# Global age-sex-specific mortality, life expectancy, and population estimates in 204 countries and territories and 811 subnational locations, 1950–2021, and the impact of the COVID-19 pandemic: a comprehensive demographic analysis for the Global Burden of Disease Study 2021

**DOI:** 10.1016/S0140-6736(24)00476-8

**Published:** 2024-05-18

**Authors:** GBD 2021 Demographics Collaborators, Austin E Schumacher, Hmwe Hmwe Kyu, Amirali Aali, Cristiana Abbafati, Jaffar Abbas, Rouzbeh Abbasgholizadeh, Madineh Akram Abbasi, Mohammadreza Abbasian, Samar Abd ElHafeez, Michael Abdelmasseh, Sherief Abd-Elsalam, Ahmed Abdelwahab, Mohammad Abdollahi, Meriem Abdoun, Auwal Abdullahi, Ame Mehadi Abdurehman, Mesfin Abebe, Aidin Abedi, Armita Abedi, Tadesse M Abegaz, Roberto Ariel Abeldaño Zuñiga, E S Abhilash, Olugbenga Olusola Abiodun, Richard Gyan Aboagye, Hassan Abolhassani, Mohamed Abouzid, Lucas Guimarães Abreu, Woldu Aberhe Abrha, Michael R M Abrigo, Dariush Abtahi, Samir Abu Rumeileh, Niveen ME Abu-Rmeileh, Salahdein Aburuz, Ahmed Abu-Zaid, Juan Manuel Acuna, Tim Adair, Isaac Yeboah Addo, Oladimeji M Adebayo, Oyelola A Adegboye, Victor Adekanmbi, Bashir Aden, Abiola Victor Adepoju, Charles Oluwaseun Adetunji, Temitayo Esther Adeyeoluwa, Olorunsola Israel Adeyomoye, Rishan Adha, Amin Adibi, Wirawan Adikusuma, Qorinah Estiningtyas Sakilah Adnani, Saryia Adra, Abel Afework, Aanuoluwapo Adeyimika Afolabi, Ali Afraz, Shadi Afyouni, Saira Afzal, Pradyumna Agasthi, Shahin Aghamiri, Antonella Agodi, Williams Agyemang-Duah, Bright Opoku Ahinkorah, Aqeel Ahmad, Danish Ahmad, Firdos Ahmad, Muayyad M Ahmad, Tauseef Ahmad, Keivan Ahmadi, Amir Mahmoud Ahmadzade, Mohadese Ahmadzade, Ayman Ahmed, Haroon Ahmed, Luai A Ahmed, Muktar Beshir Ahmed, Syed Anees Ahmed, Marjan Ajami, Budi Aji, Olufemi Ajumobi, Gizachew Taddesse Akalu, Essona Matatom Akara, Karolina Akinosoglou, Sreelatha Akkala, Samuel Akyirem, Hanadi Al Hamad, Syed Mahfuz Al Hasan, Ammar Al Homsi, Mohammad Al Qadire, Moein Ala, Timothy Olukunle Aladelusi, Tareq Mohammed Ali AL-Ahdal, Samer O Alalalmeh, Ziyad Al-Aly, Khurshid Alam, Manjurul Alam, Zufishan Alam, Rasmieh Mustafa Al-amer, Fahad Mashhour Alanezi, Turki M Alanzi, Mohammed Albashtawy, Mohammad T AlBataineh, Robert W Aldridge, Sharifullah Alemi, Ayman Al-Eyadhy, Adel Ali Saeed Al-Gheethi, Khalid F Alhabib, Fadwa Alhalaiqa Naji Alhalaiqa, Mohammed Khaled Al-Hanawi, Abid Ali, Akhtar Ali, Beriwan Abdulqadir Ali, Hassam Ali, Mohammed Usman Ali, Rafat Ali, Syed Shujait Shujait Ali, Zahid Ali, Shohreh Alian Samakkhah, Gianfranco Alicandro, Sheikh Mohammad Alif, Mohammad Aligol, Rasoul Alimi, Ahmednur Adem Aliyi, Adel Al-Jumaily, Syed Mohamed Aljunid, Wael Almahmeed, Sabah Al-Marwani, Sadeq Ali Ali Al-Maweri, Joseph Uy Almazan, Hesham M Al-Mekhlafi, Omar Almidani, Mahmoud A Alomari, Nivaldo Alonso, Jaber S Alqahtani, Ahmed Yaseen Alqutaibi, Salman Khalifah Al-Sabah, Awais Altaf, Jaffar A Al-Tawfiq, Khalid A Altirkawi, Farrukh Jawad Alvi, Hassan Alwafi, Yaser Mohammed Al-Worafi, Hany Aly, Karem H Alzoubi, Azmeraw T Amare, Edward Kwabena Ameyaw, Abebe Feyissa Amhare, Tarek Tawfik Amin, Alireza Amindarolzarbi, Javad Aminian Dehkordi, Sohrab Amiri, Hubert Amu, Dickson A Amugsi, Jimoh Amzat, Robert Ancuceanu, Deanna Anderlini, Pedro Prata Andrade, Catalina Liliana Andrei, Tudorel Andrei, Dhanalakshmi Angappan, Abhishek Anil, Afifa Anjum, Catherine M Antony, Ernoiz Antriyandarti, Iyadunni Adesola Anuoluwa, Sumadi Lukman Anwar, Anayochukwu Edward Anyasodor, Seth Christopher Yaw Appiah, Muhammad Aqeel, Jalal Arabloo, Razman Arabzadeh Bahri, Morteza Arab-Zozani, Mosab Arafat, Ana Margarida Araújo, Aleksandr Y Aravkin, Abdulfatai Aremu, Hany Ariffin, Timur Aripov, Benedetta Armocida, Mahwish Arooj, Anton A Artamonov, Kurnia Dwi Artanti, Judie Arulappan, Idowu Thomas Aruleba, Raphael Taiwo Aruleba, Ashokan Arumugam, Malke Asaad, Saeed Asgary, Mubarek Yesse Ashemo, Muhammad Ashraf, Marvellous O Asika, Seyyed Shamsadin Athari, Maha Moh'd Wahbi Atout, Alok Atreya, Sameh Attia, Avinash Aujayeb, Abolfazl Avan, Adedapo Wasiu Awotidebe, Beatriz Paulina Ayala Quintanilla, Martin Amogre Ayanore, Getnet Melaku Ayele, Jose L Ayuso-Mateos, Seyed Mohammad Ayyoubzadeh, Sina Azadnajafabad, Gulrez Shah Azhar, Shahkaar Aziz, Ahmed Y Azzam, Mina Babashahi, Abraham Samuel Babu, Muhammad Badar, Alaa Badawi, Ashish D Badiye, Soroush Baghdadi, Nasser Bagheri, Sara Bagherieh, Sulaiman Bah, Saeed Bahadorikhalili, Jianjun Bai, Ruhai Bai, Jennifer L Baker, Shankar M Bakkannavar, Abdulaziz T Bako, Senthilkumar Balakrishnan, Saliu A Balogun, Ovidiu Constantin Baltatu, Kiran Bam, Maciej Banach, Soham Bandyopadhyay, Biswajit Banik, Palash Chandra Banik, Hansi Bansal, Shirin Barati, Martina Barchitta, Mainak Bardhan, Suzanne Lyn Barker-Collo, Francesco Barone-Adesi, Hiba Jawdat Barqawi, Ronald D Barr, Lope H Barrero, Zarrin Basharat, Asma'u I J Bashir, Hameed Akande Bashiru, Pritish Baskaran, Buddha Basnyat, Quique Bassat, João Diogo Basso, Saurav Basu, Kavita Batra, Ravi Batra, Bernhard T Baune, Mohsen Bayati, Nebiyou Simegnew Bayileyegn, Thomas Beaney, Neeraj Bedi, Tahmina Begum, Emad Behboudi, Amir Hossein Behnoush, Maryam Beiranvand, Diana Fernanda Bejarano Ramirez, Uzma Iqbal Belgaumi, Michelle L Bell, Aminu K Bello, Muhammad Bashir Bello, Olorunjuwon Omolaja Bello, Luis Belo, Apostolos Beloukas, Salaheddine Bendak, Derrick A Bennett, Isabela M Bensenor, Habib Benzian, Zombor Berezvai, Adam E Berman, Amiel Nazer C Bermudez, Paulo J G Bettencourt, Habtamu B Beyene, Kebede A Beyene, Devidas S Bhagat, Akshaya Srikanth Bhagavathula, Neeraj Bhala, Ashish Bhalla, Dinesh Bhandari, Nikha Bhardwaj, Pankaj Bhardwaj, Prarthna V Bhardwaj, Ashish Bhargava, Sonu Bhaskar, Vivek Bhat, Gurjit Kaur Bhatti, Jasvinder Singh Bhatti, Manpreet S Bhatti, Rajbir Bhatti, Zulfiqar A Bhutta, Boris Bikbov, Nada Binmadi, Bagas Suryo Bintoro, Antonio Biondi, Catherine Bisignano, Francesca Bisulli, Atanu Biswas, Raaj Kishore Biswas, Saeid Bitaraf, Tone Bjørge, Archie Bleyer, Mary Sefa Boampong, Virginia Bodolica, Aadam Olalekan Bodunrin, Obasanjo Afolabi Bolarinwa, Milad Bonakdar Hashemi, Aime Bonny, Kaustubh Bora, Berrak Bora Basara, Safiya Bala Borodo, Rohan Borschmann, Alejandro Botero Carvajal, Souad Bouaoud, Sofiane Boudalia, Edward J Boyko, Nicola Luigi Bragazzi, Dejana Braithwaite, Hermann Brenner, Gabrielle Britton, Annie J Browne, Andre R Brunoni, Norma B Bulamu, Lemma N Bulto, Danilo Buonsenso, Katrin Burkart, Richard A Burns, Sharath Burugina Nagaraja, Reinhard Busse, Yasser Bustanji, Zahid A Butt, Florentino Luciano Caetano dos Santos, Tianji Cai, Daniela Calina, Luis Alberto Cámera, Luciana Aparecida Campos, Ismael R Campos-Nonato, Chao Cao, Carlos Alberto Cardenas, Rosario Cárdenas, Sinclair Carr, Giulia Carreras, Juan J Carrero, Andrea Carugno, Felix Carvalho, Márcia Carvalho, Joao Mauricio Castaldelli-Maia, Carlos A Castañeda-Orjuela, Giulio Castelpietra, Ferrán Catalá-López, Alberico L Catapano, Maria Sofia Cattaruzza, Arthur Caye, Christopher R Cederroth, Francieli Cembranel, Muthia Cenderadewi, Kelly M Cercy, Ester Cerin, Muge Cevik, Pamela R Uscamaita Chacón-Uscamaita, Yaacoub Chahine, Chiranjib Chakraborty, Jeffrey Shi Kai Chan, Chin-Kuo Chang, Periklis Charalampous, Jaykaran Charan, Vijay Kumar Chattu, Victoria Chatzimavridou-Grigoriadou, Malizgani Paul Chavula, Huzaifa Ahmad Cheema, An-Tian Chen, Haowei Chen, Lingxiao Chen, Meng Xuan Chen, Simiao Chen, Nicolas Cherbuin, Derek S Chew, Gerald Chi, Jesus Lorenzo Chirinos-Caceres, Abdulaal Chitheer, So Mi Jemma Cho, William C S Cho, Bryan Chong, Hitesh Chopra, Rahul Choudhary, Rajiv Chowdhury, Dinh-Toi Chu, Isaac Sunday Chukwu, Eric Chung, Eunice Chung, Sheng-Chia Chung, Karly I Cini, Cain C T Clark, Kaleb Coberly, Alyssa Columbus, Haley Comfort, Joao Conde, Sara Conti, Paolo Angelo Cortesi, Vera Marisa Costa, Ewerton Cousin, Richard G Cowden, Michael H Criqui, Natália Cruz-Martins, Garland T Culbreth, Patricia Cullen, Matthew Cunningham, Daniel da Silva e Silva, Sriharsha Dadana, Omid Dadras, Zhaoli Dai, Koustuv Dalal, Lachlan L Dalli, Giovanni Damiani, Emanuele D'Amico, Sara Daneshvar, Aso Mohammad Darwesh, Jai K Das, Saswati Das, Nihar Ranjan Dash, Mohsen Dashti, Claudio Alberto Dávila-Cervantes, Nicole Davis Weaver, Kairat Davletov, Diego De Leo, Aklilu Tamire Debele, Louisa Degenhardt, Reza Dehbandi, Lee Deitesfeld, Ivan Delgado-Enciso, Laura Delgado-Ortiz, Daniel Demant, Berecha Hundessa Demessa, Andreas K Demetriades, Xinlei Deng, Edgar Denova-Gutiérrez, Kebede Deribe, Nikolaos Dervenis, Don C Des Jarlais, Hardik Dineshbhai Desai, Rupak Desai, Keshab Deuba, Vinoth Gnana Chellaiyan Devanbu, Sourav Dey, Arkadeep Dhali, Kuldeep Dhama, Mandira Lamichhane Dhimal, Meghnath Dhimal, Sameer Dhingra, Diana Dias da Silva, Daniel Diaz, Adriana Dima, Delaney D Ding, M Ashworth Dirac, Abhinav Dixit, Shilpi Gupta Dixit, Thanh Chi Do, Thao Huynh Phuong Do, Camila Bruneli do Prado, Masoud Dodangeh, Klara Georgieva Dokova, Christiane Dolecek, E Ray Dorsey, Wendel Mombaque dos Santos, Rajkumar Doshi, Leila Doshmangir, Abdel Douiri, Robert Kokou Dowou, Tim Robert Driscoll, Haneil Larson Dsouza, John Dube, Samuel C Dumith, Susanna J Dunachie, Bruce B Duncan, Andre Rodrigues Duraes, Senbagam Duraisamy, Oyewole Christopher Durojaiye, Sulagna Dutta, Paulina Agnieszka Dzianach, Arkadiusz Marian Dziedzic, Oluwakemi Ebenezer, Ejemai Eboreime, Alireza Ebrahimi, Chidiebere Peter Echieh, Abdelaziz Ed-Dra, Hisham Atan Edinur, David Edvardsson, Kristina Edvardsson, Defi Efendi, Ferry Efendi, Shayan Eghdami, Terje Andreas Eikemo, Ebrahim Eini, Michael Ekholuenetale, Emmanuel Ekpor, Temitope Cyrus Ekundayo, Rabie Adel El Arab, Doaa Abdel Wahab El Morsi, Maysaa El Sayed Zaki, Maha El Tantawi, Iffat Elbarazi, Noha Mousaad Elemam, Frank J Elgar, Islam Y Elgendy, Ghada Metwally Tawfik ElGohary, Hala Rashad Elhabashy, Muhammed Elhadi, Omar Abdelsadek Abdou Elmeligy, Mohammed Elshaer, Ibrahim Elsohaby, Amir Emami Zeydi, Mehdi Emamverdi, Theophilus I Emeto, Luchuo Engelbert Bain, Ryenchindorj Erkhembayar, Tesfahun C Eshetie, Sharareh Eskandarieh, Juan Espinosa-Montero, Kara Estep, Farshid Etaee, Ugochukwu Anthony Eze, Natalia Fabin, Adewale Oluwaseun Fadaka, Adeniyi Francis Fagbamigbe, Saman Fahimi, Luca Falzone, Carla Sofia e Sá Farinha, MoezAlIslam Ezzat Mahmoud Faris, Mohsen Farjoud Kouhanjani, Andre Faro, Hossein Farrokhpour, Ali Fatehizadeh, Hamed Fattahi, Nelsensius Klau Fauk, Pooria Fazeli, Valery L Feigin, Ginenus Fekadu, Seyed-Mohammad Fereshtehnejad, Abdullah Hamid Feroze, Daniela Ferrante, Pietro Ferrara, Nuno Ferreira, Getahun Fetensa, Irina Filip, Florian Fischer, Joanne Flavel, Abraham D Flaxman, Luisa S Flor, Bobirca Teodor Florin, Morenike Oluwatoyin Folayan, Kristen Marie Foley, Artem Alekseevich Fomenkov, Lisa M Force, Carla Fornari, Behzad Foroutan, Matteo Foschi, Kate Louise Francis, Richard Charles Franklin, Alberto Freitas, Joseph Friedman, Sara D Friedman, Takeshi Fukumoto, John E Fuller, Peter Andras Gaal, Muktar A Gadanya, Santosh Gaihre, Abduzhappar Gaipov, Emmanuela Gakidou, Yaseen Galali, Nasrin Galehdar, Silvano Gallus, Quan Gan, Aravind P Gandhi, Balasankar Ganesan, Jalaj Garg, Shuo-Yan Gau, Prem Gautam, Rupesh K Gautam, Federica Gazzelloni, Miglas W Gebregergis, Mesfin Gebrehiwot, Tesfay Brhane Gebremariam, Urge Gerema, Motuma Erena Getachew, Tamirat Getachew, Peter W Gething, Mansour Ghafourifard, Sulmaz Ghahramani, Khalid Yaser Ghailan, Alireza Ghajar, Mohammad Javad Ghanbarnia, MohammadReza Ghasemi, Afsaneh Ghasemzadeh, Fariba Ghassemi, Ramy Mohamed Ghazy, Sailaja Ghimire, Asadollah Gholamian, Ali Gholamrezanezhad, Pooyan Ghorbani Vajargah, Ghozali Ghozali, Sherief Ghozy, Arun Digambarrao Ghuge, Alessandro Gialluisi, Ruth Margaret Gibson, Artyom Urievich Gil, Paramjit Singh Gill, Tiffany K Gill, Richard F Gillum, Themba G Ginindza, Alem Girmay, James C Glasbey, Elena V Gnedovskaya, Laszlo Göbölös, Amit Goel, Mohamad Goldust, Mahaveer Golechha, Pouya Goleij, Arefeh Golestanfar, Davide Golinelli, Philimon N Gona, Houman Goudarzi, Amir Hossein Goudarzian, Anmol Goyal, Scott Greenhalgh, Michal Grivna, Giovanni Guarducci, Mohammed Ibrahim Mohialdeen Gubari, Mesay Dechasa Gudeta, Avirup Guha, Stefano Guicciardi, Damitha Asanga Gunawardane, Sasidhar Gunturu, Cui Guo, Anish Kumar Gupta, Bhawna Gupta, Indarchand Ratanlal Gupta, Rajat Das Gupta, Sapna Gupta, Veer Bala Gupta, Vijai Kumar Gupta, Vivek Kumar Gupta, Reyna Alma Gutiérrez, Farrokh Habibzadeh, Parham Habibzadeh, Vladimir Hachinski, Mohammad Haddadi, Rasool Haddadi, Nils Haep, Adel Hajj Ali, Esam S Halboub, Sobia Ahsan Halim, Brian J Hall, Sebastian Haller, Rabih Halwani, Randah R Hamadeh, Kanaan Hamagharib Abdullah, Samer Hamidi, Mohammad Hamiduzzaman, Ahmad Hammoud, Nasrin Hanifi, Graeme J Hankey, Md Abdul Hannan, Md Nuruzzaman Haque, Harapan Harapan, Josep Maria Haro, Ahmed I Hasaballah, Faizul Hasan, Ikramul Hasan, M Tasdik Hasan, Hamidreza Hasani, Mohammad Hasanian, Ali Hasanpour- Dehkordi, Abbas M Hassan, Amr Hassan, Hossein Hassanian-Moghaddam, Soheil Hassanipour, Johannes Haubold, Rasmus J Havmoeller, Simon I Hay, Youssef Hbid, Jeffrey J Hebert, Omar E Hegazi, Golnaz Heidari, Mohammad Heidari, Mahsa Heidari-Foroozan, Reza Heidari-Soureshjani, Bartosz Helfer, Claudiu Herteliu, Hamed Hesami, Dineshani Hettiarachchi, Demisu Zenbaba Heyi, Kamal Hezam, Yuta Hiraike, Howard J Hoffman, Ramesh Holla, Nobuyuki Horita, Md Belal Hossain, Md Mahbub Hossain, Sahadat Hossain, Mohammad-Salar Hosseini, Hassan Hosseinzadeh, Mehdi Hosseinzadeh, Mihaela Hostiuc, Sorin Hostiuc, Mohamed Hsairi, Vivian Chia-rong Hsieh, Chengxi Hu, Junjie Huang, Md Nazmul Huda, Fernando N Hugo, Michael Hultström, Javid Hussain, Salman Hussain, Nawfal R Hussein, Le Duc Huy, Hong-Han Huynh, Bing-Fang Hwang, Segun Emmanuel Ibitoye, Oluwatope Olaniyi Idowu, Desta Ijo, Kevin S Ikuta, Mehran Ilaghi, Olayinka Stephen Ilesanmi, Irena M Ilic, Milena D Ilic, Mustapha Immurana, Leeberk Raja Inbaraj, Arnaud Iradukunda, Farideh Iravanpour, Kenneth Chukwuemeka Iregbu, Md Rabiul Islam, Mohammad Mainul Islam, Sheikh Mohammed Shariful Islam, Farhad Islami, Nahlah Elkudssiah Ismail, Gaetano Isola, Masao Iwagami, Chidozie C D Iwu, Chinwe Juliana Iwu-Jaja, Mahalaxmi Iyer, Linda Merin J, Jalil Jaafari, Louis Jacob, Kathryn H Jacobsen, Farhad Jadidi-Niaragh, Morteza Jafarinia, Khushleen Jaggi, Kasra Jahankhani, Nader Jahanmehr, Haitham Jahrami, Akhil Jain, Nityanand Jain, Ammar Abdulrahman Jairoun, Mihajlo Jakovljevic, Reza Jalilzadeh Yengejeh, Elham Jamshidi, Chinmay T Jani, Mark M Janko, Abubakar Ibrahim Jatau, Sathish Kumar Jayapal, Shubha Jayaram, Jayakumar Jeganathan, Alelign Tasew Jema, Digisie Mequanint Jemere, Wonjeong Jeong, Anil K Jha, Ravi Prakash Jha, John S Ji, Heng Jiang, Yingzhao Jin, Yinzi Jin, Olatunji Johnson, Nabi Jomehzadeh, Darwin Phan Jones, Tamas Joo, Abel Joseph, Nitin Joseph, Charity Ehimwenma Joshua, Jacek Jerzy Jozwiak, Mikk Jürisson, Billingsley Kaambwa, Ali Kabir, Hannaneh Kabir, Zubair Kabir, Vidya Kadashetti, Farima Kahe, Pradnya Vishal Kakodkar, Rizwan Kalani, Leila R Kalankesh, Feroze Kaliyadan, Sanjay Kalra, Ashwin Kamath, Arun Kamireddy, Thanigaivelan Kanagasabai, Himal Kandel, Edmund Wedam Kanmiki, Kehinde Kazeem Kanmodi, Rami S Kantar, Neeti Kapoor, Mehrdad Karajizadeh, Behzad Karami Matin, Shama D Karanth, Ibraheem M Karaye, Asima Karim, Hanie Karimi, Salah Eddin Karimi, Arman Karimi Behnagh, Samad Karkhah, Ajit K Karna, Faizan Zaffar Kashoo, Hengameh Kasraei, Nigussie Assefa Kassaw, Nicholas J Kassebaum, Molly B Kassel, Adarsh Katamreddy, Srinivasa Vittal Katikireddi, Patrick DMC Katoto, Joonas H Kauppila, Navjot Kaur, Neda Kaydi, Jeanne Françoise Kayibanda, Gbenga A Kayode, Foad Kazemi, Sina Kazemian, sara Kazeminia, Leila Keikavoosi-Arani, Cathleen Keller, John H Kempen, Jessica A Kerr, Emmanuelle Kesse-Guyot, Mohammad Keykhaei, Mohamad Mehdi Khadembashiri, Mohammad Amin Khadembashiri, Morteza Abdullatif Khafaie, Himanshu Khajuria, Mohammad Khalafi, Amirmohammad Khalaji, Nauman Khalid, Ibrahim A Khalil, Faham Khamesipour, Asaduzzaman Khan, Gulfaraz Khan, Ikramullah Khan, Imteyaz A Khan, Maseer Khan, Moien AB Khan, Taimoor Khan, Mahammed Ziauddin Khan suheb, Shaghayegh Khanmohammadi, Khaled Khatab, Fatemeh Khatami, Armin Khavandegar, Hamid Reza Khayat Kashani, Khalid A Kheirallah, Feriha Fatima Khidri, Elaheh Khodadoust, Moein Khormali, Mahmood Khosrowjerdi, Jagdish Khubchandani, Helda Khusun, Zemene Demelash Kifle, Grace Kim, Jihee Kim, Ruth W Kimokoti, Kasey E Kinzel, Girmay Tsegay Kiross, Adnan Kisa, Sezer Kisa, Juniper Boroka Kiss, Mika Kivimäki, Desmond Klu, Ann Kristin Skrindo Knudsen, Ali-Asghar Kolahi, Farzad Kompani, Gerbrand Koren, Soewarta Kosen, Karel Kostev, Ashwin Laxmikant Kotnis, Parvaiz A Koul, Sindhura Lakshmi Koulmane Laxminarayana, Ai Koyanagi, Michael A Kravchenko, Kewal Krishan, Hare Krishna, Vijay Krishnamoorthy, Yuvaraj Krishnamoorthy, Kris J Krohn, Barthelemy Kuate Defo, Connor M Kubeisy, Burcu Kucuk Bicer, Md Abdul Kuddus, Mohammed Kuddus, Ilari Kuitunen, Omar Kujan, Mukhtar Kulimbet, Vishnutheertha Kulkarni, Ashish Kumar, Harish Kumar, Nithin Kumar, Rahul Kumar, Shiv Kumar, Madhulata Kumari, Almagul Kurmanova, Om P Kurmi, Asep Kusnali, Dian Kusuma, Tezer Kutluk, Ambily Kuttikkattu, Evans F Kyei, Ilias Kyriopoulos, Carlo La Vecchia, Muhammad Awwal Ladan, Lucie Laflamme, Chandrakant Lahariya, Abdelilah Lahmar, Daphne Teck Ching Lai, Tri Laksono, Dharmesh Kumar Lal, Ratilal Lalloo, Tea Lallukka, Judit Lám, Demetris Lamnisos, Tuo Lan, Francesco Lanfranchi, Berthold Langguth, Van Charles Lansingh, Ariane Laplante-Lévesque, Bagher Larijani, Anders O Larsson, Savita Lasrado, Kamaluddin Latief, Mahrukh Latif, Kaveh Latifinaibin, Paolo Lauriola, Long Khanh Dao Le, Nhi Huu Hanh Le, Thao Thi Thu Le, Trang Diep Thanh Le, Munjae Lee, Paul H Lee, Sang-woong Lee, Seung Won Lee, Wei-Chen Lee, Yo Han Lee, Samson Mideksa Legesse, James Leigh, Jacopo Lenzi, Elvynna Leong, Temesgen L Lerango, Ming-Chieh Li, Wei Li, Xiaopan Li, Yichong Li, Zhihui Li, Massimo Libra, Virendra S Ligade, Andrew Tiyamike Makhiringa Likaka, Lee-Ling Lim, Ro-Ting Lin, Shuzhi Lin, Vasileios-Arsenios Lioutas, Stefan Listl, Jue Liu, Simin Liu, Xiaofeng Liu, Katherine M Livingstone, Erand Llanaj, Chun-Han Lo, Arianna Maever Loreche, László Lorenzovici, Mojgan Lotfi, Masoud Lotfizadeh, Rafael Lozano, Jailos Lubinda, Giancarlo Lucchetti, Alessandra Lugo, Raimundas Lunevicius, Jianing Ma, Stefan Ma, Zheng Feei Ma, Mahmoud Mabrok, Nikolaos Machairas, Monika Machoy, Christian Madsen, Javier A Magaña Gómez, Azzam A Maghazachi, Sandeep B Maharaj, Preeti Maharjan, Soleiman Mahjoub, Mansour Adam Mahmoud, Elham Mahmoudi, Morteza Mahmoudi, Omar Mohamed Makram, Jeadran N Malagón-Rojas, Elaheh Malakan Rad, Reza Malekzadeh, Armaan K Malhotra, Kashish Malhotra, Ahmad Azam Malik, Iram Malik, Lesibana Anthony Malinga, Deborah Carvalho Malta, Abdullah A Mamun, Yosef Manla, Fahmida Mannan, Yasaman Mansoori, Ali Mansour, Vahid Mansouri, Mohammad Ali Mansournia, Lorenzo Giovanni Mantovani, Bishnu P Marasini, Hamid Reza Marateb, Joemer C Maravilla, Agustina M Marconi, Parham Mardi, Mirko Marino, Abdoljalal Marjani, Carlos Alberto Marrugo Arnedo, Bernardo Alfonso Martinez-Guerra, Ramon Martinez-Piedra, Cleodice A Martins, Francisco Rogerlândio Martins-Melo, Miquel Martorell, Wolfgang Marx, Sharmeen Maryam, Roy Rillera Marzo, Kedar K V Mate, Clara N Matei, Alexander G Mathioudakis, Richard James Maude, Andrea Maugeri, Erin A May, Mahsa Mayeli, Maryam Mazaheri, Mohsen Mazidi, Antonio Mazzotti, Colm McAlinden, John J McGrath, Martin McKee, Anna Laura W McKowen, Susan A McLaughlin, Michael A McPhail, Steven M McPhail, Enkeleint A Mechili, Rishi P Mediratta, Jitendra Kumar Meena, Medhin Mehari, Max L Mehlman, Rahul Mehra, Kamran Mehrabani-Zeinabad, Entezar Mehrabi Nasab, Ravi Mehrotra, Mathewos M Mekonnen, Walter Mendoza, Ritesh G Menezes, Endalkachew Worku Mengesha, George A Mensah, Laverne G Mensah, Alexios-Fotios A Mentis, Sultan Ayoub Meo, Atte Meretoja, Tuomo J Meretoja, Abera M Mersha, Bezawit Afework Mesfin, Tomislav Mestrovic, Adquate Mhlanga, Laurette Mhlanga, Tianyue Mi, Georgia Micha, Irmina Maria Michalek, Ted R Miller, Sergey Nikolaevich Mindlin, Giada Minelli, Le Huu Nhat Minh, GK Mini, Neema W Minja, Niloofar Mirdamadi, Mojgan Mirghafourvand, Andreea Mirica, Seyed Kazem Mirinezhad, Omid Mirmosayyeb, Mizan Kiros Mirutse, Mohammad Mirza-Aghazadeh-Attari, Maryam Mirzaei, Tadesse Misgana, Sanjeev Misra, Philip B Mitchell, Prasanna Mithra, Chaitanya Mittal, Madhukar Mittal, Babak Moazen, Ahmed Ismail Mohamed, Jama Mohamed, Mouhand F H Mohamed, Nouh Saad Mohamed, Sakineh Mohammad-Alizadeh-Charandabi, Soheil Mohammadi, Abdollah Mohammadian-Hafshejani, Saeed Mohammad-pour, Marita Mohammadshahi, Mustapha Mohammed, Salahuddin Mohammed, Shafiu Mohammed, Hoda Mojiri-forushani, Ali H Mokdad, Peyman Mokhtarzadehazar, Kaveh Momenzadeh, Sara Momtazmanesh, Lorenzo Monasta, Mohammad Ali Moni, Fateme Montazeri, AmirAli Moodi Ghalibaf, Maryam Moradi, Yousef Moradi, Maziar Moradi-Lakeh, Mehdi Moradinazar, Farhad Moradpour, Paula Moraga, Lidia Morawska, Rafael Silveira Moreira, Negar Morovatdar, Shane Douglas Morrison, Jakub Morze, Reza Mosaddeghi Heris, Jonathan F Mosser, Elias Mossialos, Hakimeh Mostafavi, Amirmahdi Mostofinejad, Vincent Mougin, Simin Mouodi, Parsa Mousavi, Seyed Ehsan Mousavi, Amin Mousavi Khaneghah, Christine Mpundu-Kaambwa, Matías Mrejen, Sumaira Mubarik, Lorenzo Muccioli, Ulrich Otto Mueller, Faraz Mughal, Sumoni Mukherjee, George Duke Mukoro, Admir Mulita, Francesk Mulita, Malaisamy Muniyandi, Kavita Munjal, Fungai Musaigwa, Khaled M Musallam, Ghulam Mustafa, Sathish Muthu, Saravanan Muthupandian, Woojae Myung, Ashraf F Nabhan, Fredrick Muyia Nafukho, Ahamarshan Jayaraman Nagarajan, Mohsen Naghavi, Pirouz Naghavi, Ganesh R Naik, Gurudatta Naik, Mukhammad David Naimzada, Sanjeev Nair, Tapas Sadasivan Nair, Hastyar Hama Rashid Najmuldeen, Luigi Naldi, Vinay Nangia, Shumaila Nargus, Bruno Ramos Nascimento, Gustavo G Nascimento, Abdallah Y Naser, Mohammad Javad Nasiri, Zuhair S Natto, Javaid Nauman, Muhammad Naveed, Biswa Prakash Nayak, Vinod C Nayak, Ashish Kumar Nayyar, Athare Nazri-Panjaki, Hadush Negash, Amayu Kumesa Negero, Ionut Negoi, Ruxandra Irina Negoi, Serban Mircea Negru, Seyed Aria Nejadghaderi, Chakib Nejjari, Mohammad Hadi Nematollahi, Evangelia Nena, Samata Nepal, Olivia D Nesbit, Charles Richard James Newton, Josephine W Ngunjiri, Dang H Nguyen, Phat Tuan Nguyen, Phuong The Nguyen, Tuan Thanh Nguyen, Van Thanh Nguyen, Yeshambel T Nigatu, Taxiarchis Konstantinos Nikolouzakis, Ali Nikoobar, Amin Reza Nikpoor, Muhammad A Nizam, Shuhei Nomura, Mamoona Noreen, Nafise Noroozi, Abbas Norouzian Baghani, Bo Norrving, Jean Jacques Noubiap, Amanda Novotney, Chisom Adaobi Nri-Ezedi, George Ntaios, Mpiko Ntsekhe, Virginia Nuñez-Samudio, Dieta Nurrika, Bogdan Oancea, Kehinde O Obamiro, Ismail A Odetokun, Akinyemi O D Ofakunrin, Ropo Ebenezer Ogunsakin, James Odhiambo Oguta, In-Hwan Oh, Hassan Okati-Aliabad, Sylvester Reuben Okeke, Akinkunmi Paul Okekunle, Lawrence Okidi, Osaretin Christabel Okonji, Patrick Godwin Okwute, Andrew T Olagunju, Muideen Tunbosun Olaiya, Titilope O Olanipekun, Matthew Idowu Olatubi, Antonio Olivas-Martinez, Gláucia Maria Moraes Oliveira, Susan Oliver, Abdulhakeem Abayomi Olorukooba, Isaac Iyinoluwa Olufadewa, Bolajoko Olubukunola Olusanya, Jacob Olusegun Olusanya, Yinka Doris Oluwafemi, Gideon Olamilekan Oluwatunase, Hany A Omar, Goran Latif Omer, Sokking Ong, Obinna E Onwujekwe, Kenneth Ikenna Onyedibe, John Nelson Opio, Michal Ordak, E Roberto Orellana, Orish Ebere Orisakwe, Verner N Orish, Hans Orru, Doris V Ortega-Altamirano, Alberto Ortiz, Edgar Ortiz-Brizuela, Esteban Ortiz-Prado, Uchechukwu Levi Osuagwu, Adrian Otoiu, Nikita Otstavnov, Amel Ouyahia, Guoqing Ouyang, Mayowa O Owolabi, Ifeoluwa Temitayo Oyeyemi, Oyetunde T Oyeyemi, Yaz Ozten, Mahesh Padukudru P A, Jagadish Rao Padubidri, Mahsa Pahlavikhah Varnosfaderani, Pramod Kumar Pal, Tamás Palicz, Claudia Palladino, Raffaele Palladino, Raul Felipe Palma-Alvarez, Adrian Pana, Parsa Panahi, Ashok Pandey, Seithikurippu R Pandi-Perumal, Victoria Pando-Robles, Helena Ullyartha Pangaribuan, Georgios D Panos, Ioannis Pantazopoulos, Paraskevi Papadopoulou, Shahina Pardhan, Romil R Parikh, Seoyeon Park, Ashwaghosha Parthasarathi, Ava Pashaei, Deepak Kumar Pasupula, Jenil R Patel, Sangram Kishor Patel, Aslam Ramjan Pathan, Ashlesh Patil, Shankargouda Patil, Dimitrios Patoulias, Venkata Suresh Patthipati, Uttam Paudel, Shrikant Pawar, Hamidreza Pazoki Toroudi, Spencer A Pease, Amy E Peden, Paolo Pedersini, Minjin Peng, Umberto Pensato, Veincent Christian Filipino Pepito, Emmanuel K Peprah, Gavin Pereira, Jeevan Pereira, Marcos Pereira, Mario F P Peres, Arokiasamy Perianayagam, Norberto Perico, Ionela-Roxana Petcu, Fanny Emily Petermann-Rocha, Raffaele Pezzani, Hoang Tran Pham, Michael R Phillips, Daniela Pierannunzio, Manon Pigeolet, David M Pigott, Thomas Pilgrim, Marina Pinheiro, Michael A Piradov, Nishad Plakkal, Evgenii Plotnikov, Dimitri Poddighe, Peter Pollner, Ramesh Poluru, Constance Dimity Pond, Maarten J Postma, Govinda Raj Poudel, Lisasha Poudel, Ghazaleh Pourali, Naeimeh Pourtaheri, Sergio I Prada, Pranil Man Singh Pradhan, Vijay Kumar Prajapati, V Prakash, Chandra P Prasad, Manya Prasad, Akila Prashant, Elton Junio Sady Prates, Hery Purnobasuki, Bharathi M Purohit, Jagadeesh Puvvula, Rizwan Qaisar, Nameer Hashim Qasim, Ibrahim Qattea, Gangzhen Qian, Nguyen Khoi Quan, Amir Radfar, Venkatraman Radhakrishnan, Pourya Raee, Hadi Raeisi Shahraki, Seyedeh Niloufar Rafiei Alavi, Ibrar Rafique, Alberto Raggi, Fakher Rahim, Md Mosfequr Rahman, Mosiur Rahman, Muhammad Aziz Rahman, Tafhimur Rahman, Amir Masoud Rahmani, Shayan Rahmani, Niloufar Rahnavard, Pramila Rai, Sathish Rajaa, Ali Rajabpour-Sanati, Prashant Rajput, Prasanna Ram, Hazem Ramadan, Shakthi Kumaran Ramasamy, Sheena Ramazanu, Juwel Rana, Kritika Rana, Chhabi Lal Ranabhat, Nemanja Rancic, Smitha Rani, Shubham Ranjan, Chythra R Rao, Indu Ramachandra Rao, Mithun Rao, Sowmya J Rao, Drona Prakash Rasali, Davide Rasella, Sina Rashedi, Vahid Rashedi, Ahmed Mustafa Rashid, Ashkan Rasouli-Saravani, Prateek Rastogi, Azad Rasul, Ramin Ravangard, Nakul Ravikumar, David Laith Rawaf, Salman Rawaf, Reza Rawassizadeh, Iman Razeghian-Jahromi, Murali Mohan Rama Krishna Reddy, Elrashdy Moustafa Mohamed Redwan, Faizan Ur Rehman, Robert C Reiner Jr, Giuseppe Remuzzi, Bhageerathy Reshmi, Serge Resnikoff, Luis Felipe Reyes, Malihe Rezaee, Negar Rezaei, Nima Rezaei, Mohsen Rezaeian, Mavra A Riaz, Ana Isabel Ribeiro, Daniel Cury Ribeiro, Jennifer Rickard, Maria Jesus Rios-Blancas, Hannah Elizabeth Robinson-Oden, Mónica Rodrigues, Jefferson Antonio Buendia Rodriguez, Leonardo Roever, Ravi Rohilla, Peter Rohloff, Debby Syahru Romadlon, Luca Ronfani, Gholamreza Roshandel, Sharareh Roshanzamir, Morteza Rostamian, Bedanta Roy, Priyanka Roy, Enrico Rubagotti, Susan Fred Rumisha, Godfrey M Rwegerera, Andrzej Rynkiewicz, Manjula S, Chandan S N, Katharina S Sunnerhagen, Aly M A Saad, Michela Sabbatucci, Korosh Saber, Maha Mohamed Saber-Ayad, Simona Sacco, Basema Saddik, Adam Saddler, Bashdar Abuzed Sadee, Ehsan Sadeghi, Masoumeh Sadeghi, Saeid Sadeghian, Umar Saeed, Maryam Saeedi, Sare Safi, Rajesh Sagar, Amene Saghazadeh, Narjes Saheb Sharif-Askari, Soumya Swaroop Sahoo, Mohammad Ali Sahraian, Seyed Aidin Sajedi, Mirza Rizwan Sajid, Joseph W Sakshaug, Saina Salahi, Sarvenaz Salahi, Payman Salamati, Afeez Abolarinwa Salami, Luciane B Salaroli, Mohamed A Saleh, Sana Salehi, Marwa Rashad Salem, Mohammed Z Y Salem, Sohrab Salimi, Hossein Samadi Kafil, Sara Samadzadeh, Kamel A Samara, Saad Samargandy, Yoseph Leonardo Samodra, Vijaya Paul Samuel, Abdallah M Samy, Juan Sanabria, Nima Sanadgol, Edmond Sanganyado, Rama Krishna Sanjeev, Francesco Sanmarchi, Francesca Sanna, Ichtiarini Nurullita Santri, Milena M Santric-Milicevic, Made Ary Sarasmita, Aswini Saravanan, Babak Saravi, Yaser Sarikhani, Chinmoy Sarkar, Rodrigo Sarmiento-Suárez, Gargi Sachin Sarode, Sachin C Sarode, Arash Sarveazad, Brijesh Sathian, Thirunavukkarasu Sathish, Davide Sattin, Jennifer Saulam, Susan M Sawyer, Sonia Saxena, Ganesh Kumar Saya, Yaser Sayadi, Abu Sayeed, Md Abu Sayeed, Mete Saylan, Nikolaos Scarmeas, Benedikt Michael Schaarschmidt, Winfried Schlee, Maria Inês Schmidt, Art Schuermans, David C Schwebel, Falk Schwendicke, Mario Šekerija, Siddharthan Selvaraj, Mohammad H Semreen, Sabyasachi Senapati, Pallav Sengupta, Subramanian Senthilkumaran, Sadaf G Sepanlou, Dragos Serban, Addisu Sertsu, Yashendra Sethi, SeyedAhmad SeyedAlinaghi, Seyed Arsalan Seyedi, Amir Shafaat, Omid Shafaat, Mahan Shafie, Arman Shafiee, Nilay S Shah, Pritik A Shah, Saeed Shahabi, Ataollah Shahbandi, Izza Shahid, Samiah Shahid, Wajeehah Shahid, Moyad Jamal Shahwan, Masood Ali Shaikh, Alireza Shakeri, Husain Shakil, Sunder Sham, Muhammad Aaqib Shamim, Mehran Shams-Beyranvand, Hina Shamshad, Mohammad Ali Shamshirgaran, Mohammad Anas Shamsi, Mohd Shanawaz, Abhishek Shankar, Sadaf Sharfaei, Amin Sharifan, Mariam Shariff, Javad Sharifi-Rad, Manoj Sharma, Rajesh Sharma, Saurab Sharma, Vishal Sharma, Rajesh P Shastry, Amin Shavandi, David H Shaw, Amir Mehdi Shayan, Amr Mohamed Elsayed Shehabeldine, Aziz Sheikh, Rahim Ali Sheikhi, Jiabin Shen, Manjunath Mala Shenoy, B Suresh Kumar Shetty, Ranjitha S Shetty, Robert Adamu Shey, Amir Shiani, Kenji Shibuya, Desalegn Shiferaw, Mika Shigematsu, Jae Il Shin, Min-Jeong Shin, Rahman Shiri, Reza Shirkoohi, Aminu Shittu, Ivy Shiue, K M Shivakumar, Velizar Shivarov, Sina Shool, Sunil Shrestha, Kanwar Hamza Shuja, Kerem Shuval, Yafei Si, Migbar Mekonnen Sibhat, Emmanuel Edwar Siddig, Inga Dora Sigfusdottir, João Pedro Silva, Luís Manuel Lopes Rodrigues Silva, Soraia Silva, Jorge Piano Simões, Colin R Simpson, Anjali Singal, Abhinav Singh, Aditya Singh, Ambrish Singh, Balbir Bagicha Singh, Baljinder Singh, Mahendra Singh, Mayank Singh, Narinder Pal Singh, Paramdeep Singh, Surjit Singh, Md Shahjahan Siraj, Freddy Sitas, Shravan Sivakumar, Valentin Yurievich Skryabin, Anna Aleksandrovna Skryabina, David A Sleet, Erica Leigh N Slepak, Hanye Sohrabi, Hamidreza Soleimani, Sameh S M Soliman, Marco Solmi, Yonatan Solomon, Yimeng Song, Reed J D Sorensen, Joan B Soriano, Ireneous N Soyiri, Michael Spartalis, Chandrashekhar T Sreeramareddy, Joseph R Starnes, Vladimir I Starodubov, Antonina V Starodubova, Simona Cătălina Stefan, Dan J Stein, Fridolin Steinbeis, Paschalis Steiropoulos, Leo Stockfelt, Mark A Stokes, Stefan Stortecky, Saverio Stranges, Konstantinos Stroumpoulis, Muhammad Suleman, Rizwan Suliankatchi Abdulkader, Abida Sultana, Jing Sun, David Sunkersing, Sri Susanty, Chandan Kumar Swain, Bryan L Sykes, Lukasz Szarpak, Mindy D Szeto, Miklós Szócska, Payam Tabaee Damavandi, Ozra Tabatabaei Malazy, Seyed-Amir Tabatabaeizadeh, Shima Tabatabai, Karen M Tabb, Mohammad Tabish, Luis M Taborda-Barata, Takahiro Tabuchi, Birkneh Tilahun Tadesse, Amirmasoud Taheri, Yasaman Taheri Abkenar, Moslem Taheri Soodejani, Amir Taherkhani, Jabeen Taiba, Ardeshir Tajbakhsh, Iman M Talaat, Ashis Talukder, Jacques Lukenze Tamuzi, Ker-Kan Tan, Haosu Tang, Hong K Tang, Nathan Y Tat, Vivian Y Tat, Razieh Tavakoli Oliaee, Seyed Mohammad Tavangar, Nuno Taveira, Tsion Mulat Tebeje, Yibekal Manaye Tefera, Mojtaba Teimoori, Mohamad-Hani Temsah, Reem Mohamad Hani Temsah, Masayuki Teramoto, Solomon Hailemariam Tesfaye, Pugazhenthan Thangaraju, Kavumpurathu Raman Thankappan, Rajshree Thapa, Rekha Thapar, Nihal Thomas, Amanda G Thrift, Chern Choong Chern Thum, Jing Tian, Ales Tichopad, Jansje Henny Vera Ticoalu, Tenaw Yimer Tiruye, Seyed Abolfazl Tohidast, Marcello Tonelli, Mathilde Touvier, Marcos Roberto Tovani-Palone, Khai Hoan Tram, Nghia Minh Tran, Domenico Trico, Indang Trihandini, Samuel Joseph Tromans, Vien T Truong, Thien Tan Tri Tai Truyen, Evangelia Eirini Tsermpini, Munkhtuya Tumurkhuu, Kang Tung, Stefanos Tyrovolas, Chukwudi S Ubah, Aniefiok John Udoakang, Arit Udoh, Inam Ulhaq, Saeed Ullah, Sana Ullah, Muhammad Umair, Tungki Pratama Umar, Chukwuma David Umeokonkwo, Anushri Umesh, Brigid Unim, Bhaskaran Unnikrishnan, Era Upadhyay, Daniele Urso, Marco Vacante, Amir Mohammad Vahdani, Asokan Govindaraj Vaithinathan, Sahel Valadan Tahbaz, Rohollah Valizadeh, Jef Van den Eynde, Elena Varavikova, Orsolya Varga, Siddhartha Alluri Varma, Priya Vart, Shoban Babu Varthya, Tommi Juhani Vasankari, Lennert J Veerman, Narayanaswamy Venketasubramanian, Deneshkumar Venugopal, Nicholas Alexander Verghese, Madhur Verma, Pratibha Verma, Massimiliano Veroux, Georgios-Ioannis Verras, Dominique Vervoort, Rafael José Vieira, Jorge Hugo Villafañe, Leonardo Villani, Gabriela Ines Villanueva, Paul J Villeneuve, Francesco S Violante, Rachel Visontay, Vasily Vlassov, Bay Vo, Stein Emil Vollset, Simona Ruxandra Volovat, Victor Volovici, Avina Vongpradith, Theo Vos, Isidora S Vujcic, Rade Vukovic, Yohannes Dibaba Wado, Hatem A Wafa, Yasir Waheed, Richard G Wamai, Cong Wang, Denny Wang, Fang Wang, Shu Wang, Song Wang, Yanzhong Wang, Yuan-Pang Wang, Paul Ward, Stefanie Watson, Marcia R Weaver, Kosala Gayan Weerakoon, Daniel J Weiss, Abrha Hailay Weldemariam, Katherine M Wells, Yi Feng Wen, Andrea Werdecker, Ronny Westerman, Dakshitha Praneeth Wickramasinghe, Nuwan Darshana Wickramasinghe, Tissa Wijeratne, Shadrach Wilson, Marcin W Wojewodzic, Eve E Wool, Anthony D Woolf, Dongze Wu, Ratna Dwi Wulandari, Hong Xiao, Bin Xu, Xiaoyue Xu, Lalit Yadav, Sajad Yaghoubi, Lin Yang, Yuichiro Yano, Yao Yao, Pengpeng Ye, Gesila Endashaw Yesera, Renjulal Yesodharan, Subah Abderehim Yesuf, Arzu Yiğit, Vahit Yiğit, Paul Yip, Dong Keon Yon, Naohiro Yonemoto, Yuyi You, Mustafa Z Younis, Chuanhua Yu, Siddhesh Zadey, Vesna Zadnik, Nima Zafari, Mohammad Zahedi, Muhammad Nauman Zahid, Mazyar Zahir, Fathiah Zakham, Nazar Zaki, Josefina Zakzuk, Giulia Zamagni, Burhan Abdullah Zaman, Sojib Bin Zaman, Nelson Zamora, Ramin Zand, Milad Zandi, Ghazal G Z Zandieh, Aurora Zanghì, Iman Zare, Mikhail Sergeevich Zastrozhin, Mohammed G M Zeariya, Youjie Zeng, Chunxia Zhai, Chen Zhang, Haijun Zhang, Hongwei Zhang, Yunquan Zhang, Zhaofeng Zhang, Zhenyu Zhang, Hanqing Zhao, Yang Zhao, Yong Zhao, Peng Zheng, Chenwen Zhong, Juexiao Zhou, Bin Zhu, Zhaohua Zhu, Pardis Ziaeefar, Magdalena Zielińska, Zhiyong Zou, Alimuddin Zumla, Elric Zweck, Samer H Zyoud, Stephen S Lim, Christopher J L Murray

## Abstract

**Background:**

Estimates of demographic metrics are crucial to assess levels and trends of population health outcomes. The profound impact of the COVID-19 pandemic on populations worldwide has underscored the need for timely estimates to understand this unprecedented event within the context of long-term population health trends. The Global Burden of Diseases, Injuries, and Risk Factors Study (GBD) 2021 provides new demographic estimates for 204 countries and territories and 811 additional subnational locations from 1950 to 2021, with a particular emphasis on changes in mortality and life expectancy that occurred during the 2020–21 COVID-19 pandemic period.

**Methods:**

22 223 data sources from vital registration, sample registration, surveys, censuses, and other sources were used to estimate mortality, with a subset of these sources used exclusively to estimate excess mortality due to the COVID-19 pandemic. 2026 data sources were used for population estimation. Additional sources were used to estimate migration; the effects of the HIV epidemic; and demographic discontinuities due to conflicts, famines, natural disasters, and pandemics, which are used as inputs for estimating mortality and population. Spatiotemporal Gaussian process regression (ST-GPR) was used to generate under-5 mortality rates, which synthesised 30 763 location-years of vital registration and sample registration data, 1365 surveys and censuses, and 80 other sources. ST-GPR was also used to estimate adult mortality (between ages 15 and 59 years) based on information from 31 642 location-years of vital registration and sample registration data, 355 surveys and censuses, and 24 other sources. Estimates of child and adult mortality rates were then used to generate life tables with a relational model life table system. For countries with large HIV epidemics, life tables were adjusted using independent estimates of HIV-specific mortality generated via an epidemiological analysis of HIV prevalence surveys, antenatal clinic serosurveillance, and other data sources. Excess mortality due to the COVID-19 pandemic in 2020 and 2021 was determined by subtracting observed all-cause mortality (adjusted for late registration and mortality anomalies) from the mortality expected in the absence of the pandemic. Expected mortality was calculated based on historical trends using an ensemble of models. In location-years where all-cause mortality data were unavailable, we estimated excess mortality rates using a regression model with covariates pertaining to the pandemic. Population size was computed using a Bayesian hierarchical cohort component model. Life expectancy was calculated using age-specific mortality rates and standard demographic methods. Uncertainty intervals (UIs) were calculated for every metric using the 25th and 975th ordered values from a 1000-draw posterior distribution.

**Findings:**

Global all-cause mortality followed two distinct patterns over the study period: age-standardised mortality rates declined between 1950 and 2019 (a 62·8% [95% UI 60·5–65·1] decline), and increased during the COVID-19 pandemic period (2020–21; 5·1% [0·9–9·6] increase). In contrast with the overall reverse in mortality trends during the pandemic period, child mortality continued to decline, with 4·66 million (3·98–5·50) global deaths in children younger than 5 years in 2021 compared with 5·21 million (4·50–6·01) in 2019. An estimated 131 million (126–137) people died globally from all causes in 2020 and 2021 combined, of which 15·9 million (14·7–17·2) were due to the COVID-19 pandemic (measured by excess mortality, which includes deaths directly due to SARS-CoV-2 infection and those indirectly due to other social, economic, or behavioural changes associated with the pandemic). Excess mortality rates exceeded 150 deaths per 100 000 population during at least one year of the pandemic in 80 countries and territories, whereas 20 nations had a negative excess mortality rate in 2020 or 2021, indicating that all-cause mortality in these countries was lower during the pandemic than expected based on historical trends. Between 1950 and 2021, global life expectancy at birth increased by 22·7 years (20·8–24·8), from 49·0 years (46·7–51·3) to 71·7 years (70·9–72·5). Global life expectancy at birth declined by 1·6 years (1·0–2·2) between 2019 and 2021, reversing historical trends. An increase in life expectancy was only observed in 32 (15·7%) of 204 countries and territories between 2019 and 2021. The global population reached 7·89 billion (7·67–8·13) people in 2021, by which time 56 of 204 countries and territories had peaked and subsequently populations have declined. The largest proportion of population growth between 2020 and 2021 was in sub-Saharan Africa (39·5% [28·4–52·7]) and south Asia (26·3% [9·0–44·7]). From 2000 to 2021, the ratio of the population aged 65 years and older to the population aged younger than 15 years increased in 188 (92·2%) of 204 nations.

**Interpretation:**

Global adult mortality rates markedly increased during the COVID-19 pandemic in 2020 and 2021, reversing past decreasing trends, while child mortality rates continued to decline, albeit more slowly than in earlier years. Although COVID-19 had a substantial impact on many demographic indicators during the first 2 years of the pandemic, overall global health progress over the 72 years evaluated has been profound, with considerable improvements in mortality and life expectancy. Additionally, we observed a deceleration of global population growth since 2017, despite steady or increasing growth in lower-income countries, combined with a continued global shift of population age structures towards older ages. These demographic changes will likely present future challenges to health systems, economies, and societies. The comprehensive demographic estimates reported here will enable researchers, policy makers, health practitioners, and other key stakeholders to better understand and address the profound changes that have occurred in the global health landscape following the first 2 years of the COVID-19 pandemic, and longer-term trends beyond the pandemic.

**Funding:**

Bill & Melinda Gates Foundation.

## Introduction

Understanding mortality and population trends over time and across locations, age groups, and sexes is crucial for planning population-specific public health policies. Age-specific mortality rates can indicate the emergence of new adverse health risks in specific locations, while population counts can inform resource allocation and aid in planning future development. The COVID-19 pandemic has highlighted the importance of demography in understanding disease and injury burden^1^ and the roles health policy and infrastructure have in health and demographic outcomes.^1^, ^2^ As the COVID-19 pandemic enters an endemic phase in some locations, demographic indicators can provide important context for understanding and addressing COVID-19, long COVID-19,^3^ and the interaction between COVID-19 and other diseases and injuries. Furthermore, demographic trends in the decades before the COVID-19 pandemic and reversals in those trends during the first 2 years of the COVID-19 pandemic (2020–21) can provide insights into potential long-term effects of the pandemic. These shifts in demographic patterns, including in population growth and age distribution, can help policy makers and public health experts better understand how the pandemic has impacted different groups within society and inform strategies for future pandemic preparedness and health-care planning.

The Global Burden of Diseases, Injuries, and Risk Factors Study (GBD) is an evolving research effort that quantifies the state of global health.^4^ The scope of the study has historically included estimating key demographic metrics and comprehensive health metrics for a set of national and subnational locations that has expanded over time. Mortality has been estimated as part of GBD since the first GBD estimates were published in the 1993 World Bank World Development Report, and mortality estimates have been included in each update since GBD 2010.^5^, ^6^, ^7^, ^8^, ^9^, ^10^ A comprehensive, internally consistent modelling strategy for estimating population and fertility was introduced in GBD 2017, greatly improving the consistency of results.^11^ Previously, GBD drew on population estimates from the UN Population Division of the Department of Economic and Social Affairs (UNPD).^12^, ^13^ In GBD 2019, the demographic analysis used population, fertility, and mortality estimates to produce a typology that better helped to specify phases of demographic transition.^10^ The GBD demography framework is part of the greater GBD enterprise; thus, it differs from other demographic research initiatives by using estimates of disease and injury burden to inform population and mortality estimates, and vice versa. Attempting to estimate the effects of the pandemic is now a major focus of GBD and other demographic research efforts.^12^, ^14^, ^15^, ^16^

The GBD 2021 demographic analysis improved on GBD 2019 by using additional data sources and refined methods to generate updated estimates of mortality, life expectancy, and population size at the global, regional, national, and subnational levels for each year from 1950 to 2021. GBD 2021 is the first round to incorporate the COVID-19 pandemic into the modelling process through the estimation of excess mortality due to the pandemic, defined as the net difference between the number of deaths that occurred between 2020 and 2021 and the number of deaths that would be expected over the same period based on previous trends in all-cause mortality.^16^ The unified approach to estimate all-cause mortality and excess mortality in GBD 2021 is an innovation in current demographic research methods. This facilitates analysis of the interplay between wider demographic processes and the COVID-19 pandemic. In this iteration of the GBD demographic analysis, we aim to provide policy makers and the public with the information needed to gain a better understanding of the demographic context of disease and injury burden since 1950 and during the COVID-19 pandemic in 2020–21 specifically.

**Research in context**

**Evidence before this study**

The UN Population Division of the Department of Economic and Social Affairs (UNPD) produces estimates and projections of global, regional, and national demographic metrics that are updated biannually. Their latest findings, published in the World Population Prospects 2022 revision, incorporated WHO estimates of excess mortality due to the COVID-19 pandemic in 2020 and 2021. Estimates of excess mortality during the pandemic have also been generated by the Institute for Health Metrics and Evaluation and the World Mortality Dataset. The International Database of the US Census Bureau reports population estimates and projections for more than 200 countries and areas, of which a subset are updated every year. Organisations including WHO, the Organisation for Economic Co-operation and Development, and the European Union release demographic estimates less regularly and typically only for select metrics or locations. Some national statistics offices also produce their own demographic indicators. The Global Burden of Diseases, Injuries, and Risk Factors Study (GBD) generates regularly updated and globally comparable health metrics, including mortality, life expectancy, and population estimates for past years, and forecasts up to the year 2100. The current GBD 2021 cycle is directly preceded by GBD 2019, which reported demographic estimates for 204 countries and territories for each year from 1950 through 2019. While each of these studies represent important efforts to provide insights into demographic estimates and the COVID-19 pandemic, only GBD estimates comply with the Guidelines for Accurate and Transparent Health Estimates Reporting, which identifies best practices for reporting global health estimates.

**Added value of this study**

GBD 2021 is one of the first studies to fully evaluate demographic trends in the context of the first 2 years of the COVID-19 pandemic. The study employed a unified framework to calculate excess mortality rates due to the COVID-19 pandemic along with a comprehensive set of demographic metrics including all-cause mortality, life expectancy, and population counts for 204 countries and territories and 811 subnational locations. This allowed estimates of all-cause mortality to inform estimates of excess mortality due to the pandemic, and vice versa. In contrast, the demographic estimates published by UNPD for 2020 and 2021, although based on data available during the pandemic, did not use a unified framework for all-cause and excess mortality. Additionally, while the US Census Bureau published population estimates for 2020 and 2021, the estimates were adjusted to reflect the effects of the pandemic for only a subset of locations. GBD 2021 utilised a suite of customised and validated data processing and modelling tools, systematically analysing thousands of data sources to produce global, regional, national, and subnational demographic estimates by age, sex, and Socio-demographic Index (SDI) level for each year from 1950 to 2021. Compared with GBD 2019, GBD 2021 utilised 5296 additional data sources. Additionally, the model life table system used in GBD 2021 was improved to provide more accurate mortality estimates for older age groups. All estimates are packaged within freely accessible data-sharing and visualisation tools.

**Implications of all the available evidence**

Our study highlights the impact of the first 2 years of the COVID-19 pandemic at a novel level of granularity, demonstrating unprecedented reversals in adult mortality and life expectancy trends at the global, regional, and national levels. Furthermore, globally comparable measures of excess mortality due to the pandemic show substantial variation in the burden experienced by different countries and territories. Our comprehensive set of demographic estimates provides a rich description of evolving long-term trends in mortality and life expectancy across age groups, sexes, and SDI levels, and our population analyses reveal changing dynamics and age structures with implications for the future of health-care systems, economies, and societies. Collectively, the estimates reported here provide an integrated demographic framework for GBD and a valuable foundation for policy evaluation, development, and implementation around the world.

## Methods

### Overview

For each new GBD iteration, recently available data and improved methods are used to update the full time series of demographic estimates from 1950 to the latest year of analysis; GBD 2021 demographic estimates therefore supersede all previous estimates.

The GBD 2021 demographic methods closely followed those used in GBD 2019.^10^ Improvements for GBD 2021 centred on a single framework to estimate both all-cause mortality and excess mortality due to the COVID-19 pandemic. The analytical process for computing internally consistent demographic estimates included six main components: (1) estimating age-specific fertility rates; (2) estimating under-5 and adult (age 15–59 years) mortality rates; (3) estimating age-specific mortality rates using a relational model life table system with HIV adjustments; (4) estimating excess mortality due to the COVID-19 pandemic and adjusting all-cause mortality estimates accordingly; (5) accounting for fatal discontinuities such as wars, famines, and natural disasters; and (6) estimating population sizes. To resolve discrepancies due to the inherent interdependent nature of population, mortality, and fertility estimates, the estimation process was run twice: first to generate preliminary numbers, and second to refine all estimates and ensure internal consistency. A detailed description of all methods and analytical flowcharts for all-cause mortality, fertility, and population estimation are available in appendix 1 (sections 2–6, 8).

This study complies with the Guidelines for Accurate and Transparent Health Estimates Reporting (GATHER);^17^ a completed GATHER checklist is provided in appendix 1 (section 8). Python (version 3.8.17 and 3.10.4), Stata (version 15.1), and R (version 3.5 and 4.2) were used for statistical analysis This manuscript was produced with the GBD Collaborator Network and in accordance with the GBD Protocol.^18^ An international network of collaborators provides, reviews, and analyses the available data to generate health metrics; the 2021 GBD round drew on the expertise of more than 11 000 collaborators across more than 160 countries and territories.

### Data sources and processing

The GBD 2021 analysis used a range of data types for mortality and population estimation that were identified from a systematic search of available data from government websites, statistical annuals, demographic compendia, large-scale surveys, and collaborator input; comprehensive details on the sources of input data are available online via the GBD 2021 Sources Tool. Under-5 mortality rates (U5MRs), defined as the probability of death from birth to age 5 years, were estimated using 30 526 location-years of vital registration data (3179 new location-years for GBD 2021 compared with GBD 2019),^10^ 237 location-years of sample vital registration data, and 1445 other sources (including 57 new surveys, one new census, and ten other new sources; appendix 1 section 8). Adult mortality, defined as the probability of death before age 60 years assuming survival to age 15 years, was estimated using 30 207 location-years of vital registration data (3150 new location-years for GBD 2021 compared with GBD 2019), 1435 location-years of sample vital registration data, 75 censuses, 280 surveys (including 65 sources of household death data and 167 sources of sibling history data), and 24 other sources (appendix 1 section 8). Age-specific mortality was estimated using 43 758 empirical life tables for 1950–2021 (compared with 35 406 in GBD 2019; appendix 1 section 8). Prevalence surveys, antenatal clinic serosurveillance, and vital registration were used to adjust for the impact of the HIV epidemic due to its exceptional impact on age-specific mortality. Fatal discontinuities were accounted for using 2235 location-years from vital registration and 237 other sources (compared with 1812 from vital registration and 174 other sources in GBD 2019). Estimation of excess mortality due to the COVID-19 pandemic utilised an additional 146 139 datapoints of all-cause mortality data at either weekly or monthly intervals from vital registration and surveillance reports that were assessed for completeness of registration (compared with our previous excess mortality estimation,^16^ GBD 2021 used 1389 additional weeks or months of data).

Population estimates utilised national and subnational censuses (1277 overall; 25 new), population registries (749 location-years of data), and post-enumeration surveys (161 in total). Additionally, migration data on refugee movements from the UN High Commissioner for Refugees and datasets for select countries (primarily Gulf States and nations in the EU) were used to inform migration estimates.

### All-cause mortality estimation

GBD 2021 all-cause mortality estimation followed the analytical framework for mortality analysis used in GBD 2019.^10^ Point estimates from surveys were generated using both direct and indirect estimation methods for U5MR, while for adult mortality, they were generated from sibling history data with methods that correct for inherent biases such as zero-survivor and recall bias. Time series estimates of the completeness of adult vital registration data were generated using the same modelling process as GBD 2019, which used a combination of five death distribution methods, and point estimates were adjusted accordingly.

Time series of under-5 and adult mortality without fatal discontinuities were estimated using spatiotemporal Gaussian process regression (ST-GPR), including a bias-adjustment process for U5MR, to correct for systematic differences in the data sources and smooth results across time and location. Education, HIV, and lag-distributed income were included as covariates, along with U5MR for adult mortality. These estimates were used as inputs for the GBD relational model life table system with adjustments for older-age mortality to estimate HIV-free age-specific mortality rates. HIV mortality was modelled with a combination of ST-GPR, the Estimation and Projection Package Age-Sex Model,^19^ and Spectrum,^20^ and subsequently used to produce life tables that included HIV mortality. These abridged life tables were used to generate full life tables by single year age groups with further detailed age groups under the age of 1 year. Sex-redistributed and age-redistributed fatal discontinuities by cause were aggregated by age and sex and added to the estimated mortality from the previous step to generate the final all-cause mortality life tables by location, year, sex, and age. We recalculated abridged life tables, including fatal discontinuities for each location, year, and sex combination, and then calculated the final envelope from these abridged life tables. Detailed methods for estimating each mortality component are available in appendix 1 (section 2).

### Excess mortality due to the COVID-19 pandemic estimation

Excess mortality due to the COVID-19 pandemic in 2020 and 2021 is defined as the observed all-cause mortality minus the mortality that would be expected had the pandemic not occurred, based on historical trends. Excess deaths are those attributed to the COVID-19 pandemic as a whole, both from SARS-CoV-2 infection and from other pandemic-related factors such as deferred care seeking.^21^, ^22^ Excess mortality was calculated using similar methods as in Wang et al (2022),^16^ with several key improvements. We included yearly observed deaths from vital registration to supplement daily, weekly, and monthly observed death data. We then used five variants of the spline for weekly seasonal patterns that set the second-to-last knot at 18, 24, 36, 48, or 60 months to allow for more stable trends. To select covariates, we used Rover, a method developed at the Institute for Health Metrics and Evaluation based on Bayesian model averaging. Rover is conceptually similar to the Bayesian model averaging method, which is widely used to explore the parameter space and aggregate estimates across candidate models based on performance metrics.^23^ The main difference is that while Bayesian model averaging uses marginal likelihood, Rover focuses on out-of-sample performance. We included covariates pertaining to the COVID-19 pandemic, such as seroprevalence, and background population health metrics, such as the Healthcare Access and Quality Index.^24^ With the best model selected, we ran a prediction process using 100 draws for each covariate and 100 draws of estimated coefficients and residuals, estimated from the regressions run at the draw level using draw-level input data on both excess mortality and covariates. Mean values and 95% uncertainty intervals (UIs) were then generated at national, regional, and global levels. Out-of-sample predictive validity testing was conducted based on our final model specification. Complete excess mortality methodology is detailed in appendix 1 (section 2.8).

To determine age-specific and sex-specific excess mortality, we estimated all-cause mortality twice: once with data from during the pandemic in 2020 and 2021 included and once without. For location-years with vital registration data from during the pandemic, we computed the difference in estimated age-sex-specific mortality between the two sets of estimates. We then applied this distribution to our excess mortality estimates to calculate age-specific and sex-specific excess mortality. Due to instability in age-sex distributions and implausible patterns, we used the global age-sex distribution for locations with fewer than 75 000 excess deaths, unless otherwise noted (appendix 1 section 2.8). Other pandemic-related mortality (OPRM) was estimated by calculating the difference between excess mortality and the sum of deaths due directly to COVID-19 infection and indirect deaths due to lower respiratory infections, measles, and pertussis. For locations with a negative OPRM, we adjusted the non-pandemic mortality estimates downward accordingly. We redistributed small discrepancies that remained between the mortality estimates that used vital registration age-sex-specific data from during the pandemic and the non-pandemic mortality estimates plus age-sex-specific excess mortality to ensure that the final mortality estimates including mortality shocks were consistent with observed high-quality vital registration data.

### Population estimation

We used the Bayesian hierarchical cohort component model for population projection (BCCMP) from GBD 2019 to produce age-specific population estimates.^10^ This method used age-specific fertility estimates from GBD 2021 (appendix 1 section 3), the previously described age-specific mortality estimates, and available census and registry data as inputs. Auxiliary refugee and migration data were used to inform the prior distribution on net migration in countries with substantial migration or reliable data. The model estimates an age-specific 1950 baseline population, age-specific net migration, and age-specific population estimates that are fully consistent with the input fertility and mortality estimates. Complete population estimation methodology is in appendix 1 (section 4).

### Expected mortality based on Socio-demographic Index (SDI) estimation

We analysed the relationship between age-specific log mortality rates and SDI using MR-BRT (meta-regression-Bayesian regularised trimmed),^25^ a meta-regression programme (appendix 1 section 6.1). SDI is a composite indicator of a country's lag-distributed income per capita, average years of schooling, and the total fertility rate in females younger than age 25 years (appendix 1 section 5). MR-BRT defines a linear mixed-effects model with a B-spline specification for the relationship between outcomes of interest and SDI. We used a cubic spline with five knots between 0 and 1, with left-most and right-most spline segments enforced to be linear, and with slopes matching adjacent interior segments. To ensure that the results were not sensitive to the choice of spline knots, we used a model ensemble of over 50 cubic spline models, as described above. For each model, interior knot placement was randomly generated to be between 0·1 and 0·9, with minimum inter-knot distance of 0·1 and maximum inter-knot distance of 1·0. The final predictions were obtained using the ensemble aggregate over these 50 models. This model was performed separately for each GBD age-sex group. Expected mortality rates for each age-sex group based on SDI were used to estimate expected life expectancy. A similar analysis was done for excess mortality rates due to the COVID-19 pandemic, with the exception that two-degree splines were used.

### Geographical units, age groups, and time periods

We produced estimates for each demographic metric by age-sex-location-year for 25 age groups: early neonatal (0–6 days), late neonatal (7–27 days), 1–5 months, 6–11 months, 12–23 months, 2–4 years, 5–9 years, every 5-year age group up to 95 years, and 95 years and older (fertility estimated for 5-year age groups between ages 10 years and 54 years); for males, females, and all sexes combined; for 204 countries and territories grouped into 21 regions and seven super-regions; and for every year from 1950 to 2021. We also included subnational analyses for 21 countries and territories (Brazil, China, Ethiopia, India, Indonesia, Iran, Italy, Japan, Kenya, Mexico, New Zealand, Nigeria, Norway, Pakistan, the Philippines, Poland, Russia, South Africa, Sweden, the UK, and the USA) and estimates by SDI quintile. All countries and territories were assigned an SDI value ranging from 0 (lowest income and educational attainment and highest fertility) to 100 and then grouped into quintiles from low SDI to high SDI.

### Uncertainty analysis

Uncertainty was propagated throughout the estimation process. For under-5 and adult mortality, ST-GPR generated 1000 draws for every location, year, and sex combination; 1000 draws were also produced for the crude death rate associated with HIV estimates. The 100 draws of excess mortality due to the COVID-19 pandemic were repeated ten times to generate 1000 draws. These draw-level inputs were then used to create 1000 draws of all-cause mortality estimates and draw-level estimates of fatal discontinuities. Mean estimates and 95% UIs (the 25th and 975th ranked values from the 1000 draws) were generated for all demographic metrics using the draw-level estimates. The uncertainty associated with fertility and mortality estimates was included as inputs in the BCCMP model to produce 1000 draws of population estimates.

### Role of the funding source

The funders of this study had no role in study design, data collection, data analysis, data interpretation, or the writing of the report

## Results

This section presents global, regional, and national-level results for key demographic metrics; given space constraints, estimates at the subnational level are presented in appendix 2 and are also available in downloadable form through the GBD Results tool. All subnational locations are listed in appendix 1 (section 8).

### Civil registration and vital statistics completeness

This section presents global, regional, and national-level results for key demographic metrics; given space constraints, estimates at the subnational level are presented in appendix 2. All subnational locations are listed in appendix 1 (section 8).

**Figure 1:**
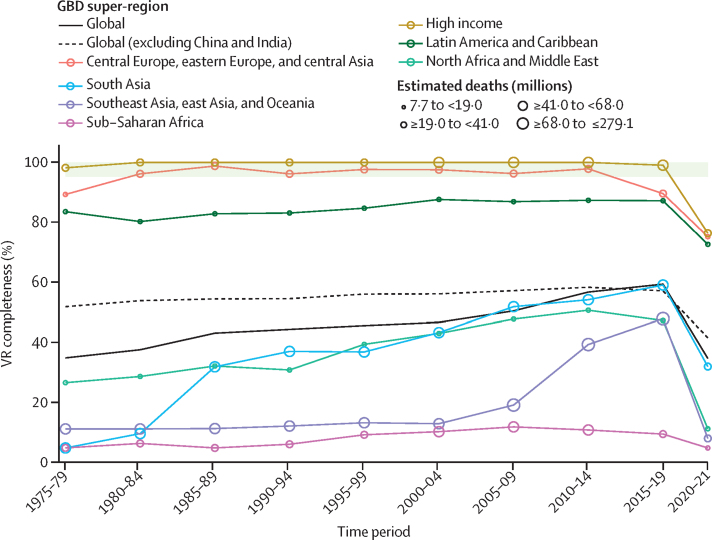
Completeness of VR systems in GBD super-regions, 1975–2021 Completeness is defined as the total number of deaths registered in all VR systems within a super-region during a 5-year period divided by the total number of estimated deaths within that super-region and period, with 100% completeness indicating that all deaths were registered. The size of the datapoints represents the number of estimated deaths. The solid black line shows the global completeness, the dashed black line indicates global completeness, excluding China and India, and other coloured lines indicate GBD super-regions. The green box indicates complete registration (defined as >95%). GBD=Global Burden of Diseases, Injuries, and Risk Factors Study. VR=vital registration.

The proportion of deaths registered in vital registration systems increased substantially at the global level during the study period, from 30·3% in 1975 to a peak of 61·1% in 2016, before declining in subsequent years due to lags in reporting (figure 1). Completeness of death registration in vital registration systems varied markedly between regions, however, most progress in completeness was observed in China (where completeness peaked at 71·2% in 2018) and India (where completeness peaked at 80·1% in 2019; appendix 2 table S1). The Indian Sample Registration System is considered complete for the sample population it covers. Outside of China and India, progress in death registration has been slow, with only a 10·3 percentage point increase observed in the rest of the world between 1975 and the peak in 2016. This increase was concentrated in north Africa and the Middle East, which improved from 20·6% completeness in 1975 to a peak of 56·0% in 2016. While registration has been complete (defined as >95%) since 1975 for nearly all countries in the high-income super-region and central Europe, eastern Europe, and central Asia, in sub-Saharan Africa peak completeness of only 8·7% was reached in 2008 and completeness has declined since then. Death registration in Latin America and the Caribbean was more variable: countries such as Costa Rica, Cuba, and Argentina have been complete for many years; registration in countries such as Peru and Ecuador has remained around 60–90% complete, and others, such as Bolivia, continue to lack registration data. At the national level, 96 countries and territories had at least 1 year of complete death registration between 2010 and 2021; 29 countries and territories without complete death registration had at least 1 year of registering more than 75% of deaths; and 47 countries and territories had no vital registration data in the GBD 2021 mortality database. Registration was incomplete or non-existent in many countries with large numbers of deaths in 2021, especially in sub-Saharan Africa, including Nigeria and Democratic Republic of Congo. In the 2020–21 period, super-regions had varying degrees of lowered completeness indicative of lags in reporting (figure 1).

### Mortality and life expectancy

**Figure 2:**
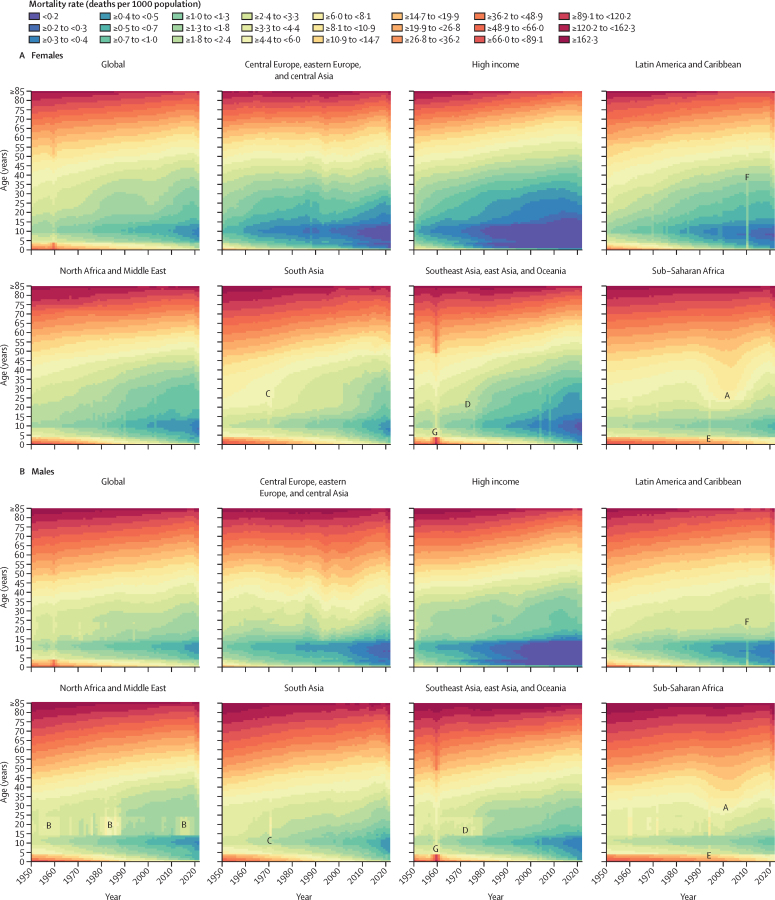
Global and GBD super-region all-cause mortality rates across the lifespan in females (A) and males (B), 1950–2021 Mortality rates are expressed as the number of deaths per 1000 population. Fatal discontinuities are indicated by the following letters: A=HIV epidemic; B=conflicts in the Middle East; C=war and genocide in India, Pakistan, and Bangladesh in 1971; D=war and genocide in Cambodia in the 1970s; E=Rwandan genocide in 1994; F=earthquake in Haiti in 2010; G=famine between 1959 and 1961. GBD=Global Burden of Diseases, Injuries, and Risk Factors Study.

Between 1950 and 2019, global age-standardised all-cause mortality rates per 100 000 population broadly declined, from 1980·5 age-standardised deaths (95% UI 1855·5–2115·0) in 1950 to 736·1 (700·1–772·8) in 2019 (appendix 2 table S3A), which equates to a 62·8% (60·5–65·1) decline in mortality during the entire period. Global all-cause mortality rates across the human lifespan for the younger than 15 years and older than 40 years age groups broadly improved for both females and males between 1950 and 2019 (figure 2). This pattern was relatively consistent across super-regions, with the exception of increased mortality in sub-Saharan Africa during the HIV epidemic and a fluctuating pattern in the central Europe, eastern Europe, and central Asia super-region. However, substantial variation in mortality levels and trends across super-regions and over time were observed in the 15–39-years age group. This age group was particularly susceptible to mortality shocks such as famine in China between 1959 and 1961; conflicts in the Middle East during multiple time periods; war in India, Pakistan, and Bangladesh and genocide in Bangladesh in 1971; war and genocide in Cambodia in the 1970s; the Rwandan genocide in 1994; and the earthquake in Haiti in 2010 (figure 2). Conflict and war had a larger impact on mortality rates in males than females. Furthermore, the HIV epidemic had an especially large impact on this age group in sub-Saharan Africa and a lesser impact in southeast Asia, east Asia, and Oceania, with a larger impact on females than males. Additionally, male mortality rates increased in Latin America and the Caribbean during the 2000s, to varying extents in countries such as El Salvador, Peru, Guatemala, Honduras, Mexico, Venezuela, and Brazil (appendix 2 figure S5). An increase in male and female mortality was observed in the high-income super-region during the late 2010s, which was most notable in the USA, Canada, and Spain (appendix 2 figure S5).

During the COVID-19 pandemic in 2020 and 2021, global age-standardised all-cause mortality rates increased by 21·9% (95% UI 13·6–31·1) for males aged 15 years and older compared with 2019 and 16·6% (10·0–23·4) for females in the same age group and time period, reversing trends in mortality observed before the pandemic (appendix 2 table S3). In contrast, during 2020 and 2021, global mortality rates for both males and females generally remained constant or further decreased for age groups younger than 15 years (figure 2). In particular, between 2019 and 2021, global U5MR decreased by 7·0% (2·3–11·1). This continued reduction in child mortality was consistent across all super-regions (figure 2).

**Figure 3:**
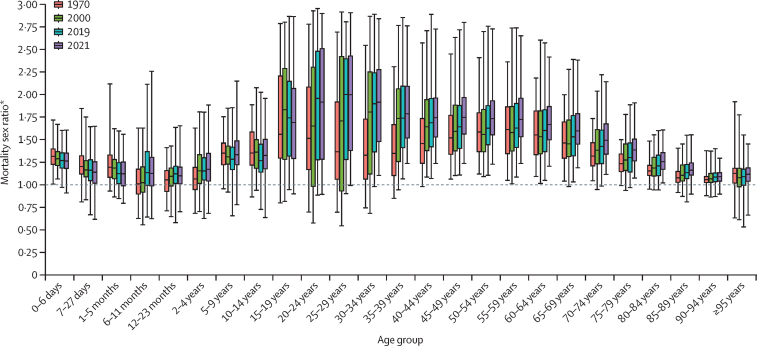
Distribution of the mortality sex ratio by age in 1970, 2000, 2019, and 2021 The distributions are for the mortality sex ratio calculated across all 204 countries and territories included in this study. The boxes represent the middle 50% of the distribution (25th and 75th percentiles), the horizontal line in boxes indicates the mean, and the whiskers show the middle 95% of the distribution (2·5th and 97·5th percentiles). *The ratio of male to female mortality rates, computed by dividing the male mortality rate by the female mortality rate for each age group and year.

All-cause mortality rates differed between sexes, and the extent of this difference varied across age groups and by location. Female mortality was generally lower than male mortality in all age groups, with substantial heterogeneity across countries and territories (figure 3). The highest variability in the ratio of male to female mortality rates across countries and territories was found in the 15–39 age groups; although little change in the mortality sex ratio has been observed between locations over time, the ratio generally increased between 1970 and 2021, indicating that the gap between male and female mortality has been increasing, generally driven by mortality rates among females decreasing at a faster rate than among males. Globally in 2021, the mortality rate for males aged 15–39 years was 65·9% (95% UI 56·8–74·7) higher than for females. The widening gap between males and females was also observed for nearly all age groups aged 40 years and older. In the neonatal age groups, the ratio of male to female mortality rates declined slightly over time towards 1, while the variability among countries and territories remained similar. Individuals aged 40 years and older had a consistent pattern of an increasing ratio of male to female mortality rates over time, with increased variability observed among those aged 65 years and older across countries and territories from 1970 to 2000, followed by little change in variability from 2000 to 2021.

**Figure 4:**
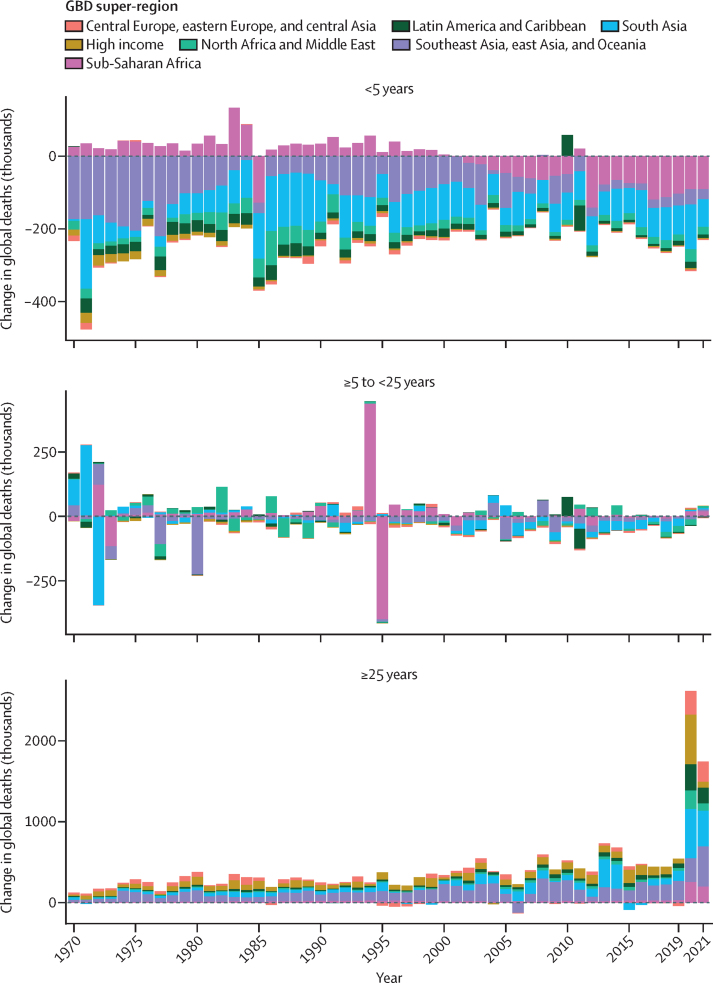
Annual change in all-cause deaths by GBD super-region across three age groups, 1970–2021 Annual change is defined as the difference between the number of deaths in the current year and the preceding year. The y-axes scales differ by age groups. The large change in the 5–24 years group between 1994 and 1995 was due to deaths during the Rwandan genocide. Different colours show GBD super-regions. GBD=Global Burden of Diseases, Injuries, and Risk Factors Study.

Despite declines in age-standardised all-cause mortality rates during the study period, the global number of deaths due to all causes combined increased from 44·0 million (95% UI 40·3–47·7) in 1950 to 50·3 million (49·3–51·4) in 2000 and 57·0 million (54·9–59·6) in 2019, largely reflecting a growing population and changing age structures. Global deaths further increased to 63·1 million (60·6–65·9) in 2020 and 67·9 million (65·0–70·8) in 2021, a notable spike attributable to the COVID-19 pandemic (table 1). Since 1970, the number of global deaths in the 25 years and older age group had increased steadily, until an unprecedented increase in 2020–21 (figure 4). This increase was observed across all GBD super-regions, with the exception of central Europe, eastern Europe, and central Asia, from 2000 to 2019. In contrast, deaths in children under 5 years declined over the entire study period, including during the COVID-19 pandemic period, with death counts of 20·0 million (17·2–23·0) in 1950, 9·21 million (8·73–9·73) in 2000, 5·21 million (4·50–6·01) in 2019, 4·89 million (4·19–5·71) in 2020, and 4·66 million (3·98–5·50) in 2021 (appendix 2 table S1). Initially, most of this decline could be attributed to declines in both U5MR and the under-5 population in southeast Asia, east Asia, and Oceania (especially China) until a tapering off around the year 2000. After this, the share of the decline attributed to sub-Saharan Africa began to increase, and this pattern continued during 2021 (figure 4). The largest number of under-5 deaths was observed in south Asia and sub-Saharan Africa during the pandemic, with south Asia accounting for 25·7% (24·1–27·2) of all deaths in children under 5 years in 2020 and 25·3% (24·0–26·6) in 2021, and sub-Saharan Africa accounting for 55·5% (53·2–57·7) in 2020 and 56·3% (54·1–58·4) in 2021. The number of global deaths in the intermediate age group (ages 5–24 years) demonstrates large yearly variability with no clear patterns, since deaths in this age group were heavily impacted by mortality shocks such as the Rwandan genocide in 1994 and natural disasters such as the earthquake in Haiti in 2010. Deaths in this age group increased slightly during 2020 and 2021 in most super-regions, but these increases were minimal compared with previous years, and in comparison to the increase observed in ages 25 years and older.

**Table 1:** Under-5 mortality rate (2021), rate of change in under-5 mortality (2000–21), probability of death between ages 15 and 59 years (2021), life expectancy at birth (2021), total number of deaths among children under-5 years, total number of deaths among all ages (2021), and excess deaths due to the COVID-19 pandemic (2020, 2021) globally and for GBD regions, super-regions, countries, and territories

		**Under-5 mortality**		**Probability of death between ages 15 and 59 years, 2021**		**Life expectancy at birth in 2021 (years)**			**Total deaths in 2021 (thousands)**	**Total deaths among children younger than 5 years in 2021 (thousands)**	**Excess deaths due to COVID-19 in 2020 (thousands)**	**Excess deaths due to COVID-19 in 2021 (thousands)**	**Excess mortality rate due to COVID-19, 2020–21 (deaths per 1000)**
		Mortality rate in 2021 (deaths per 1000)	Annualised rate of change, 2000–21	Females	Males	Females	Males	Both sexes					
**Global**		**35·7 (30·5 to 42·0)**	**−3·3% (−4·0 to −2·5)**	**0·12 (0·11 to 0·12)**	**0·19 (0·18 to 0·20)**	**74·8 (74·0 to 75·5)**	**69·0 (68·0 to 69·9)**	**71·7 (70·9 to 72·5)**	**67 900·0 (65 000·0 to 70 800·0)**	**4660·0 (3980·0 to 5500·0)**	**5890 (5480 to 6440)**	**9970 (9260 to 10 900)**	**1·04 (0·96 to 1·13)**
**Central Europe, eastern Europe, and Central Asia**		**12·0 (10·5 to 13·7)**	**−3·8% (−4·4 to −3·2)**	**0·11 (0·11 to 0·12)**	**0·25 (0·24 to 0·26)**	**75·5 (75·0 to 75·9)**	**67·4 (66·9 to 67·9)**	**71·5 (71·0 to 71·8)**	**5950·0 (5790·0 to 6130·0)**	**59·0 (51·7 to 67·6)**	**740 (681 to 801)**	**1400 (1300 to 1520)**	**2·70 (2·50 to 2·90)**
Central Asia		20·9 (17·6 to 24·6)	−4·1% (−4·8 to −3·2)	0·11 (0·10 to 0·12)	0·22 (0·21 to 0·24)	74·3 (73·3 to 75·2)	67·4 (66·4 to 68·5)	70·8 (69·8 to 71·8)	724·0 (671·0 to 779·0)	42·6 (36·0 to 50·4)	108 (80 to 133)	150 (102 to 186)	1·46 (1·06 to 1·80)
	Armenia	11·1 (9·0 to 13·8)	−4·8% (−6·0 to −3·6)	0·07 (0·06 to 0·07)	0·18 (0·16 to 0·19)	78·6 (77·8 to 79·4)	71·3 (70·3 to 72·4)	75·0 (74·1 to 76·0)	31·3 (28·9 to 33·8)	0·4 (0·3 to 0·5)	7 (5 to 9)	5 (3 to 6)	2·08 (1·43 to 2·61)
	Azerbaijan	28·6 (23·4 to 34·7)	−4·0% (−5·0 to −3·0)	0·10 (0·09 to 0·11)	0·21 (0·19 to 0·23)	73·4 (72·5 to 74·3)	67·0 (66·0 to 68·2)	70·1 (69·2 to 71·2)	89·3 (81·9 to 96·4)	3·9 (3·2 to 4·7)	21 (17 to 24)	25 (20 to 30)	2·31 (1·83 to 2·67)
	Georgia	9·7 (7·7 to 12·2)	−6·1% (−7·2 to −5·0)	0·10 (0·10 to 0·10)	0·25 (0·25 to 0·26)	75·8 (75·5 to 76·2)	67·3 (67·0 to 67·5)	71·5 (71·2 to 71·7)	59·6 (58·6 to 60·5)	0·4 (0·3 to 0·6)	6 (4 to 7)	17 (11 to 21)	3·29 (2·22 to 4·19)
	Kazakhstan	10·2 (8·4 to 12·3)	−6·1% (−7·0 to −5·1)	0·13 (0·12 to 0·14)	0·28 (0·26 to 0·30)	73·9 (73·1 to 74·7)	65·3 (64·4 to 66·2)	69·6 (68·7 to 70·4)	181·0 (169·0 to 194·0)	4·1 (3·4 to 5·0)	30 (23 to 36)	51 (41 to 60)	2·36 (1·87 to 2·76)
	Kyrgyzstan	17·0 (14·9 to 19·0)	−4·4% (−5·2 to −3·7)	0·10 (0·09 to 0·12)	0·23 (0·20 to 0·26)	76·1 (74·7 to 77·6)	68·4 (66·6 to 70·2)	72·3 (70·7 to 73·9)	38·9 (34·2 to 43·6)	2·7 (2·3 to 3·0)	7 (5 to 9)	6 (4 to 9)	1·06 (0·74 to 1·38)
	Mongolia	16·9 (14·0 to 20·5)	−5·6% (−6·6 to −4·6)	0·12 (0·10 to 0·13)	0·29 (0·26 to 0·32)	74·6 (73·5 to 75·7)	65·7 (64·3 to 67·1)	70·0 (69·1 to 71·0)	21·5 (19·9 to 23·0)	1·3 (1·1 to 1·6)	−2 (−5 to 1)	1 (−3 to 4)	−0·17 (−1·15 to 0·74)
	Tajikistan	34·5 (28·5 to 42·2)	−3·1% (−4·1 to −2·1)	0·13 (0·11 to 0·15)	0·21 (0·18 to 0·24)	72·1 (70·4 to 73·7)	66·9 (65·1 to 69·1)	69·3 (67·8 to 71·0)	59·1 (52·2 to 65·6)	9·7 (8·0 to 11·9)	12 (9 to 15)	16 (11 to 20)	1·46 (1·06 to 1·79)
	Turkmenistan	27·5 (22·2 to 33·5)	−3·7% (−4·6 to −2·6)	0·15 (0·12 to 0·19)	0·28 (0·24 to 0·34)	71·5 (69·4 to 73·7)	64·3 (62·0 to 66·8)	67·8 (65·5 to 70·1)	43·6 (36·5 to 51·2)	3·0 (2·4 to 3·7)	6 (5 to 8)	8 (6 to 10)	1·46 (1·06 to 1·79)
	Uzbekistan	21·5 (17·7 to 26·0)	−3·5% (−4·4 to −2·5)	0·10 (0·09 to 0·12)	0·18 (0·15 to 0·20)	75·1 (73·6 to 76·6)	69·9 (68·1 to 71·7)	72·5 (70·8 to 74·2)	200·0 (175·0 to 227·0)	17·0 (14·0 to 20·7)	22 (12 to 30)	21 (7 to 31)	0·69 (0·30 to 0·98)
Central Europe		5·0 (4·5 to 5·6)	−4·7% (−5·1 to −4·2)	0·08 (0·08 to 0·08)	0·18 (0·18 to 0·18)	78·3 (78·2 to 78·5)	71·3 (71·1 to 71·4)	74·7 (74·5 to 74·8)	1760·0 (1740·0 to 1780·0)	5·3 (4·8 to 5·9)	195 (140 to 243)	353 (268 to 422)	2·54 (1·89 to 3·05)
	Albania	13·1 (10·7 to 16·0)	−3·7% (−4·8 to −2·6)	0·06 (0·05 to 0·07)	0·13 (0·11 to 0·15)	78·7 (77·6 to 79·9)	73·6 (72·1 to 75·3)	76·0 (74·7 to 77·5)	30·1 (26·5 to 33·6)	0·4 (0·3 to 0·4)	5 (2 to 8)	7 (3 to 10)	2·36 (1·05 to 3·63)
	Bosnia and Herzegovina	5·2 (4·4 to 6·3)	−3·6% (−4·4 to −2·7)	0·07 (0·06 to 0·09)	0·15 (0·12 to 0·17)	78·3 (76·9 to 79·8)	72·6 (70·8 to 74·6)	75·4 (73·8 to 77·1)	46·4 (39·7 to 53·0)	0·1 (0·1 to 0·2)	5 (1 to 9)	8 (3 to 14)	2·05 (0·80 to 3·47)
	Bulgaria	6·6 (5·9 to 7·4)	−4·6% (−5·2 to −4·1)	0·13 (0·13 to 0·14)	0·26 (0·25 to 0·27)	73·7 (73·3 to 74·1)	66·4 (65·9 to 67·0)	69·9 (69·4 to 70·3)	169·0 (164·0 to 173·0)	0·4 (0·3 to 0·4)	20 (11 to 26)	47 (36 to 56)	5·21 (3·82 to 6·30)
	Croatia	4·6 (3·8 to 5·4)	−2·7% (−3·5 to −1·8)	0·06 (0·05 to 0·06)	0·13 (0·12 to 0·13)	80·3 (80·0 to 80·6)	74·1 (73·8 to 74·4)	77·2 (76·9 to 77·5)	62·4 (60·6 to 64·0)	0·2 (0·1 to 0·2)	5 (2 to 7)	10 (6 to 14)	1·84 (1·03 to 2·61)
	Czechia	2·7 (2·3 to 3·1)	−3·2% (−4·0 to −2·4)	0·06 (0·06 to 0·06)	0·12 (0·12 to 0·13)	80·9 (80·6 to 81·1)	74·4 (74·2 to 74·6)	77·6 (77·3 to 77·8)	138·0 (136·0 to 141·0)	0·3 (0·2 to 0·3)	15 (8 to 22)	23 (12 to 32)	1·88 (1·00 to 2·57)
	Hungary	4·0 (3·4 to 4·7)	−4·6% (−5·3 to −3·8)	0·09 (0·09 to 0·10)	0·19 (0·19 to 0·19)	78·0 (77·8 to 78·2)	70·9 (70·7 to 71·1)	74·5 (74·3 to 74·6)	154·0 (152·0 to 156·0)	0·4 (0·3 to 0·4)	12 (3 to 18)	26 (14 to 35)	2·02 (0·96 to 2·84)
	Montenegro	3·9 (3·2 to 4·7)	−5·5% (−6·5 to −4·5)	0·08 (0·08 to 0·09)	0·18 (0·17 to 0·19)	76·0 (75·4 to 76·6)	69·8 (69·0 to 70·5)	72·7 (72·1 to 73·3)	9·9 (9·4 to 10·4)	0·0 (0·0 to 0·0)	1 (1 to 1)	3 (3 to 3)	3·35 (2·78 to 3·90)
	North Macedonia	5·6 (4·9 to 6·3)	−4·9% (−5·5 to −4·2)	0·11 (0·09 to 0·12)	0·19 (0·17 to 0·22)	74·2 (73·2 to 75·3)	69·2 (68·0 to 70·4)	71·5 (70·4 to 72·7)	32·7 (29·3 to 36·3)	0·1 (0·1 to 0·1)	7 (5 to 8)	10 (8 to 12)	4·86 (3·79 to 5·66)
	Poland	4·4 (3·9 to 5·0)	−3·7% (−4·3 to −3·1)	0·07 (0·07 to 0·07)	0·18 (0·18 to 0·18)	79·7 (79·6 to 79·8)	71·8 (71·7 to 71·9)	75·7 (75·6 to 75·8)	517·0 (514·0 to 520·0)	1·5 (1·3 to 1·7)	65 (48 to 78)	101 (72 to 122)	2·28 (1·81 to 2·72)
	Romania	6·7 (6·1 to 7·4)	−5·7% (−6·2 to −5·3)	0·10 (0·10 to 0·10)	0·22 (0·22 to 0·22)	76·8 (76·7 to 77·0)	69·2 (69·1 to 69·4)	72·9 (72·8 to 73·0)	334·0 (332·0 to 337·0)	1·2 (1·1 to 1·3)	38 (25 to 51)	72 (49 to 90)	3·00 (2·06 to 3·85)
	Serbia	4·7 (4·2 to 5·2)	−5·4% (−6·3 to −4·6)	0·08 (0·08 to 0·09)	0·16 (0·16 to 0·16)	76·7 (76·5 to 76·9)	71·7 (71·5 to 71·8)	74·1 (74·0 to 74·3)	149·0 (147·0 to 151·0)	0·3 (0·3 to 0·4)	15 (5 to 27)	26 (6 to 44)	2·52 (0·61 to 4·24)
	Slovakia	5·8 (5·1 to 6·4)	−2·6% (−3·2 to −2·0)	0·08 (0·08 to 0·08)	0·17 (0·17 to 0·18)	78·3 (78·1 to 78·6)	71·3 (71·0 to 71·5)	74·7 (74·6 to 74·9)	72·6 (71·5 to 73·6)	0·3 (0·3 to 0·4)	5 (2 to 8)	18 (13 to 22)	2·23 (1·38 to 2·88)
	Slovenia	2·2 (2·0 to 2·5)	−4·2% (−4·8 to −3·6)	0·04 (0·04 to 0·04)	0·10 (0·09 to 0·10)	84·0 (83·4 to 84·6)	77·6 (77·2 to 78·1)	80·8 (80·4 to 81·3)	23·0 (22·0 to 23·9)	0·0 (0·0 to 0·0)	3 (1 to 4)	2 (0 to 4)	1·20 (0·31 to 1·88)
Eastern Europe		6·1 (5·6 to 6·5)	−5·2% (−5·6 to −4·8)	0·13 (0·12 to 0·14)	0·30 (0·28 to 0·32)	74·9 (74·2 to 75·5)	65·8 (65·0 to 66·6)	70·4 (69·8 to 70·9)	3470·0 (3340·0 to 3610·0)	11·1 (10·3 to 11·9)	436 (398 to 467)	899 (854 to 940)	3·33 (3·15 to 3·46)
	Belarus	4·0 (3·1 to 5·3)	−6·9% (−8·2 to −5·5)	0·11 (0·10 to 0·13)	0·29 (0·25 to 0·33)	76·0 (74·4 to 77·5)	66·0 (64·2 to 67·8)	71·0 (69·2 to 72·7)	162·0 (141·0 to 186·0)	0·3 (0·3 to 0·4)	23 (17 to 29)	42 (32 to 54)	3·67 (2·78 to 4·77)
	Estonia	2·5 (2·2 to 2·9)	−7·1% (−7·8 to −6·4)	0·07 (0·06 to 0·07)	0·17 (0·17 to 0·18)	81·2 (80·6 to 81·8)	72·4 (71·9 to 72·9)	76·9 (76·5 to 77·3)	18·6 (18·0 to 19·2)	0·0 (0·0 to 0·0)	0 (−1 to 1)	3 (2 to 5)	1·44 (0·59 to 2·33)
	Latvia	3·7 (3·2 to 4·3)	−6·1% (−6·9 to −5·4)	0·10 (0·09 to 0·10)	0·26 (0·25 to 0·27)	78·1 (77·7 to 78·5)	68·3 (67·9 to 68·7)	73·2 (73·0 to 73·5)	34·2 (33·4 to 35·0)	0·1 (0·1 to 0·1)	1 (0 to 3)	7 (5 to 9)	2·35 (1·36 to 3·41)
	Lithuania	3·5 (3·1 to 3·9)	−5·3% (−5·9 to −4·7)	0·09 (0·09 to 0·10)	0·24 (0·23 to 0·24)	78·9 (78·5 to 79·3)	69·2 (68·8 to 69·5)	74·1 (73·8 to 74·4)	47·2 (46·2 to 48·2)	0·1 (0·1 to 0·1)	5 (3 to 8)	10 (6 to 13)	2·84 (1·91 to 3·89)
	Moldova	10·9 (8·2 to 14·4)	−4·4% (−5·7 to −3·0)	0·11 (0·10 to 0·12)	0·25 (0·23 to 0·27)	76·4 (75·4 to 77·3)	67·9 (66·7 to 69·0)	72·1 (71·0 to 73·2)	50·1 (47·0 to 53·6)	0·3 (0·2 to 0·4)	5 (5 to 6)	10 (10 to 11)	2·29 (2·21 to 2·38)
	Russia	5·8 (5·5 to 6·2)	−5·6% (−5·9 to −5·2)	0·14 (0·14 to 0·14)	0·31 (0·31 to 0·31)	74·3 (74·3 to 74·4)	65·5 (65·5 to 65·6)	70·0 (69·9 to 70·0)	2410·0 (2410·0 to 2420·0)	8·1 (7·6 to 8·6)	357 (355 to 360)	690 (687 to 693)	3·70 (3·68 to 3·72)
	Ukraine	7·8 (6·2 to 9·2)	−3·3% (−4·3 to −2·4)	0·11 (0·08 to 0·15)	0·29 (0·22 to 0·37)	75·7 (72·7 to 78·6)	66·3 (62·7 to 70·1)	71·0 (68·5 to 73·6)	745·0 (614·0 to 880·0)	2·2 (1·7 to 2·6)	44 (9 to 77)	137 (96 to 179)	2·18 (1·45 to 2·93)
**High income**		**4·6 (4·2 to 5·0)**	**−2·4% (−2·8 to −2·0)**	**0·06 (0·06 to 0·06)**	**0·11 (0·11 to 0·11)**	**83·3 (83·3 to 83·4)**	**77·9 (77·8 to 78·0)**	**80·6 (80·5 to 80·7)**	**10 900·0 (10 800·0 to 10 900·0)**	**47·9 (44·0 to 52·2)**	**971 (939 to 1000)**	**947 (907 to 985)**	**0·90 (0·87 to 0·93)**
Australasia		3·3 (2·8 to 3·8)	−3·3% (−4·0 to −2·5)	0·04 (0·04 to 0·04)	0·08 (0·08 to 0·08)	85·3 (85·3 to 85·4)	81·2 (81·1 to 81·2)	83·2 (83·2 to 83·3)	210·0 (209·0 to 210·0)	1·2 (1·0 to 1·4)	−5 (−6 to −5)	4 (3 to 5)	−0·03 (−0·06 to −0·00)
	Australia	3·0 (2·5 to 3·6)	−3·6% (−4·4 to −2·7)	0·04 (0·04 to 0·04)	0·08 (0·08 to 0·08)	85·6 (85·5 to 85·7)	81·2 (81·1 to 81·3)	83·4 (83·3 to 83·5)	175·0 (174·0 to 176·0)	0·9 (0·7 to 1·0)	−3 (−4 to −3)	4 (3 to 4)	0·01 (−0·02 to 0·03)
	New Zealand	4·8 (4·3 to 5·4)	−2·3% (−2·9 to −1·6)	0·05 (0·05 to 0·05)	0·08 (0·08 to 0·08)	84·1 (83·9 to 84·3)	80·7 (80·5 to 80·9)	82·4 (82·3 to 82·6)	34·5 (34·1 to 35·0)	0·3 (0·3 to 0·3)	−2 (−2 to −2)	0 (0 to 0)	−0·21 (−0·27 to −0·15)
High-income Asia Pacific		2·2 (2·0 to 2·4)	−4·1% (−4·5 to −3·7)	0·03 (0·03 to 0·03)	0·07 (0·07 to 0·07)	87·8 (87·7 to 87·8)	81·8 (81·7 to 81·9)	84·8 (84·8 to 84·9)	1800·0 (1790·0 to 1800·0)	2·7 (2·5 to 2·9)	−27 (−32 to −22)	22 (15 to 29)	−0·01 (−0·04 to 0·01)
	Brunei	9·7 (7·7 to 12·1)	−0·3% (−1·5 to 1·0)	0·08 (0·07 to 0·10)	0·13 (0·12 to 0·15)	78·3 (77·1 to 79·3)	74·9 (73·6 to 76·0)	76·6 (75·4 to 77·7)	1·8 (1·7 to 2·0)	0·1 (0·0 to 0·1)	0 (0 to 0)	0 (0 to 0)	0·13 (−0·08 to 0·30)
	Japan	2·1 (1·9 to 2·4)	−3·5% (−4·1 to −2·9)	0·03 (0·03 to 0·03)	0·06 (0·06 to 0·06)	88·1 (88·0 to 88·2)	82·2 (82·1 to 82·2)	85·2 (85·1 to 85·2)	1440·0 (1430·0 to 1450·0)	1·8 (1·6 to 2·1)	−28 (−33 to −24)	8 (2 to 14)	−0·08 (−0·12 to −0·05)
	Singapore	1·7 (1·4 to 2·0)	−4·2% (−5·2 to −3·2)	0·03 (0·03 to 0·03)	0·05 (0·05 to 0·05)	87·7 (87·5 to 87·9)	83·6 (83·4 to 83·8)	85·7 (85·5 to 85·9)	23·7 (23·3 to 24·2)	0·1 (0·1 to 0·1)	0 (−1 to 0)	2 (1 to 2)	0·10 (0·06 to 0·15)
	South Korea	2·5 (2·0 to 2·9)	−4·9% (−5·9 to −4·0)	0·04 (0·03 to 0·04)	0·08 (0·07 to 0·08)	86·0 (85·9 to 86·2)	80·3 (80·1 to 80·5)	83·2 (83·1 to 83·4)	331·0 (326·0 to 336·0)	0·7 (0·5 to 0·8)	2 (1 to 3)	12 (12 to 14)	0·13 (0·12 to 0·15)
High-income North America		5·7 (5·2 to 6·2)	−1·7% (−2·1 to −1·3)	0·09 (0·09 to 0·09)	0·16 (0·16 to 0·16)	80·4 (80·3 to 80·6)	74·8 (74·6 to 74·9)	77·6 (77·4 to 77·7)	3780·0 (3750·0 to 3810·0)	23·1 (21·1 to 25·2)	530 (519 to 542)	560 (543 to 579)	1·53 (1·49 to 1·56)
	Canada	4·0 (3·4 to 4·8)	−1·8% (−2·6 to −0·9)	0·05 (0·05 to 0·05)	0·09 (0·09 to 0·09)	84·1 (83·9 to 84·2)	79·5 (79·4 to 79·7)	81·8 (81·7 to 82·0)	310·0 (307·0 to 314·0)	1·5 (1·2 to 1·8)	37 (35 to 39)	32 (30 to 34)	0·95 (0·90 to 0·99)
	Greenland	10·6 (9·0 to 12·3)	−3·1% (−4·1 to −2·3)	0·12 (0·11 to 0·14)	0·20 (0·17 to 0·23)	76·9 (75·7 to 77·9)	71·4 (69·7 to 72·7)	73·8 (72·4 to 75·0)	0·4 (0·4 to 0·5)	0·0 (0·0 to 0·0)	0 (0 to 0)	0 (0 to 0)	0·38 (0·08 to 0·62)
	USA	5·9 (5·4 to 6·4)	−1·7% (−2·1 to −1·2)	0·09 (0·09 to 0·09)	0·17 (0·16 to 0·17)	80·0 (79·9 to 80·2)	74·3 (74·1 to 74·4)	77·1 (77·0 to 77·2)	3470·0 (3440·0 to 3500·0)	21·6 (19·7 to 23·6)	493 (482 to 504)	528 (512 to 546)	1·59 (1·56 to 1·63)
Southern Latin America		8·5 (6·9 to 10·4)	−3·4% (−4·4 to −2·4)	0·08 (0·08 to 0·08)	0·14 (0·14 to 0·14)	79·9 (79·6 to 80·1)	73·8 (73·5 to 74·1)	76·8 (76·6 to 77·1)	553·0 (545·0 to 562·0)	6·6 (5·4 to 8·1)	41 (38 to 45)	71 (66 to 77)	0·88 (0·82 to 0·95)
	Argentina	9·7 (7·7 to 12·1)	−3·3% (−4·4 to −2·3)	0·08 (0·08 to 0·09)	0·15 (0·14 to 0·15)	79·1 (78·8 to 79·3)	73·0 (72·7 to 73·3)	76·1 (75·7 to 76·3)	378·0 (372·0 to 386·0)	5·2 (4·1 to 6·5)	30 (27 to 32)	44 (40 to 48)	0·85 (0·79 to 0·94)
	Chile	5·7 (4·9 to 6·4)	−3·5% (−4·1 to −2·8)	0·06 (0·06 to 0·06)	0·13 (0·13 to 0·13)	81·9 (81·7 to 82·1)	76·1 (76·0 to 76·3)	79·0 (78·9 to 79·2)	134·0 (133·0 to 135·0)	1·2 (1·0 to 1·3)	14 (12 to 15)	22 (21 to 23)	1·03 (0·96 to 1·10)
	Uruguay	6·8 (5·5 to 8·5)	−4·2% (−5·3 to −3·1)	0·09 (0·08 to 0·09)	0·17 (0·17 to 0·17)	79·4 (79·0 to 79·7)	72·0 (71·6 to 72·4)	75·7 (75·3 to 76·0)	40·5 (39·7 to 41·4)	0·2 (0·2 to 0·3)	−2 (−3 to −2)	5 (5 to 6)	0·49 (0·38 to 0·59)
Western Europe		3·5 (3·2 to 3·8)	−2·4% (−2·7 to −2·0)	0·04 (0·04 to 0·04)	0·08 (0·08 to 0·08)	84·2 (84·1 to 84·3)	79·4 (79·3 to 79·4)	81·8 (81·7 to 81·9)	4540·0 (4520·0 to 4560·0)	14·3 (13·3 to 15·5)	432 (411 to 448)	291 (271 to 311)	0·85 (0·80 to 0·89)
	Andorra	1·2 (0·8 to 1·5)	−5·7% (−7·4 to −4·4)	0·04 (0·03 to 0·05)	0·08 (0·06 to 0·10)	85·7 (83·5 to 87·9)	80·7 (77·9 to 83·6)	83·0 (80·5 to 85·6)	0·6 (0·5 to 0·8)	0·0 (0·0 to 0·0)	0 (0 to 0)	0 (0 to 0)	0·60 (−0·31 to 1·77)
	Austria	3·1 (2·7 to 3·5)	−2·9% (−3·5 to −2·2)	0·04 (0·04 to 0·04)	0·08 (0·08 to 0·08)	84·1 (83·9 to 84·2)	79·2 (79·1 to 79·4)	81·7 (81·5 to 81·8)	88·8 (87·7 to 89·9)	0·3 (0·2 to 0·3)	6 (5 to 7)	4 (3 to 5)	0·58 (0·44 to 0·72)
	Belgium	3·7 (3·0 to 4·4)	−2·3% (−3·3 to −1·4)	0·05 (0·05 to 0·05)	0·08 (0·08 to 0·08)	84·2 (84·0 to 84·4)	79·3 (79·1 to 79·5)	81·8 (81·6 to 81·9)	111·0 (110·0 to 112·0)	0·4 (0·3 to 0·5)	17 (16 to 18)	2 (1 to 3)	0·85 (0·76 to 0·93)
	Cyprus	2·4 (2·0 to 2·9)	−5·0% (−5·9 to −4·1)	0·04 (0·03 to 0·04)	0·07 (0·06 to 0·08)	83·2 (82·5 to 83·9)	79·2 (78·2 to 80·1)	81·2 (80·4 to 82·0)	9·2 (8·4 to 10·1)	0·0 (0·0 to 0·0)	0 (0 to 1)	1 (0 to 1)	0·30 (−0·24 to 0·76)
	Denmark	3·6 (3·2 to 4·1)	−2·1% (−2·7 to −1·4)	0·04 (0·04 to 0·05)	0·07 (0·07 to 0·07)	83·5 (83·3 to 83·7)	79·5 (79·3 to 79·7)	81·5 (81·3 to 81·7)	56·7 (55·8 to 57·7)	0·2 (0·2 to 0·3)	0 (0 to 1)	2 (2 to 3)	0·23 (0·14 to 0·34)
	Finland	2·2 (1·9 to 2·6)	−3·1% (−3·9 to −2·4)	0·04 (0·04 to 0·04)	0·09 (0·09 to 0·09)	84·9 (84·7 to 85·2)	79·5 (79·2 to 79·7)	82·2 (82·0 to 82·4)	57·1 (56·1 to 58·1)	0·1 (0·1 to 0·1)	1 (0 to 2)	2 (2 to 3)	0·30 (0·16 to 0·43)
	France	4·0 (3·6 to 4·5)	−1·4% (−1·9 to −0·9)	0·04 (0·04 to 0·04)	0·09 (0·09 to 0·09)	85·5 (85·4 to 85·6)	79·6 (79·5 to 79·7)	82·6 (82·5 to 82·7)	642·0 (639·0 to 646·0)	2·8 (2·5 to 3·1)	65 (61 to 68)	28 (24 to 32)	0·74 (0·68 to 0·79)
	Germany	3·5 (3·3 to 3·8)	−2·0% (−2·3 to −1·6)	0·05 (0·05 to 0·05)	0·09 (0·09 to 0·09)	83·4 (83·3 to 83·5)	78·5 (78·5 to 78·6)	81·0 (80·9 to 81·0)	1010·0 (1000·0 to 1010·0)	2·8 (2·6 to 3·0)	38 (34 to 44)	63 (57 to 69)	0·60 (0·54 to 0·66)
	Greece	3·9 (3·4 to 4·5)	−2·2% (−2·9 to −1·5)	0·05 (0·05 to 0·05)	0·11 (0·11 to 0·11)	82·8 (82·6 to 83·0)	77·2 (77·0 to 77·5)	80·0 (79·8 to 80·2)	144·0 (142·0 to 146·0)	0·3 (0·3 to 0·4)	5 (3 to 6)	15 (14 to 16)	0·95 (0·82 to 1·06)
	Iceland	2·4 (2·0 to 2·9)	−2·3% (−3·3 to −1·2)	0·04 (0·04 to 0·04)	0·07 (0·07 to 0·07)	84·9 (84·2 to 85·5)	82·3 (81·6 to 83·0)	83·6 (82·9 to 84·3)	2·3 (2·2 to 2·4)	0·0 (0·0 to 0·0)	0 (0 to 0)	0 (0 to 0)	−0·02 (−0·25 to 0·22)
	Ireland	3·4 (2·9 to 3·8)	−3·5% (−4·2 to −2·8)	0·04 (0·04 to 0·04)	0·07 (0·07 to 0·07)	84·5 (84·2 to 84·7)	80·8 (80·5 to 81·0)	82·6 (82·4 to 82·8)	32·2 (31·6 to 32·9)	0·2 (0·2 to 0·2)	0 (0 to 1)	1 (0 to 1)	0·12 (0·02 to 0·21)
	Israel	2·3 (2·0 to 2·7)	−5·1% (−5·8 to −4·3)	0·04 (0·03 to 0·04)	0·07 (0·07 to 0·07)	85·1 (84·9 to 85·3)	81·2 (80·9 to 81·5)	83·2 (82·9 to 83·4)	50·1 (49·0 to 51·1)	0·4 (0·4 to 0·5)	2 (2 to 3)	3 (3 to 4)	0·29 (0·24 to 0·34)
	Italy	2·9 (2·6 to 3·3)	−3·0% (−3·6 to −2·4)	0·04 (0·04 to 0·04)	0·07 (0·07 to 0·07)	84·9 (84·8 to 85·0)	80·3 (80·2 to 80·4)	82·7 (82·6 to 82·7)	699·0 (695·0 to 702·0)	1·2 (1·0 to 1·3)	98 (95 to 101)	62 (59 to 66)	1·38 (1·34 to 1·44)
	Luxembourg	3·5 (2·9 to 4·2)	−1·0% (−1·9 to −0·1)	0·04 (0·04 to 0·04)	0·07 (0·06 to 0·07)	84·9 (84·4 to 85·4)	80·4 (79·8 to 81·0)	82·6 (82·0 to 83·2)	4·5 (4·3 to 4·8)	0·0 (0·0 to 0·0)	0 (0 to 0)	0 (0 to 0)	0·31 (0·09 to 0·54)
	Malta	5·3 (4·2 to 6·6)	−1·7% (−2·9 to −0·5)	0·04 (0·04 to 0·04)	0·07 (0·07 to 0·08)	84·1 (83·4 to 84·7)	81·3 (80·6 to 82·0)	82·7 (81·9 to 83·3)	4·0 (3·8 to 4·3)	0·0 (0·0 to 0·0)	0 (0 to 0)	0 (0 to 0)	0·62 (0·32 to 0·95)
	Monaco	3·8 (3·7 to 3·9)	−1·0% (−2·2 to 0·2)	0·07 (0·05 to 0·08)	0·12 (0·10 to 0·14)	81·4 (79·8 to 83·2)	76·3 (74·7 to 77·8)	78·8 (77·2 to 80·4)	0·6 (0·5 to 0·7)	0·0 (0·0 to 0·0)	0 (0 to 0)	0 (0 to 0)	1·33 (0·51 to 2·17)
	Netherlands	3·8 (3·5 to 4·2)	−2·4% (−2·9 to −1·8)	0·05 (0·04 to 0·05)	0·06 (0·06 to 0·07)	83·2 (83·1 to 83·4)	79·8 (79·6 to 79·9)	81·5 (81·4 to 81·7)	170·0 (168·0 to 172·0)	0·7 (0·6 to 0·7)	15 (13 to 16)	15 (14 to 17)	0·92 (0·83 to 0·99)
	Norway	2·1 (1·8 to 2·4)	−3·9% (−4·6 to −3·2)	0·04 (0·04 to 0·04)	0·06 (0·06 to 0·06)	84·9 (84·7 to 85·1)	81·7 (81·5 to 81·8)	83·3 (83·1 to 83·4)	41·9 (41·3 to 42·6)	0·1 (0·1 to 0·1)	0 (−1 to 0)	1 (0 to 1)	0·06 (0·00 to 0·10)
	Portugal	2·9 (2·6 to 3·3)	−4·4% (−5·0 to −3·8)	0·04 (0·04 to 0·04)	0·10 (0·10 to 0·10)	84·4 (84·3 to 84·6)	78·5 (78·3 to 78·7)	81·5 (81·4 to 81·7)	123·0 (122·0 to 124·0)	0·2 (0·2 to 0·3)	11 (10 to 12)	10 (9 to 11)	1·05 (0·95 to 1·14)
	San Marino	1·7 (1·1 to 2·3)	−5·3% (−7·3 to −3·4)	0·03 (0·02 to 0·04)	0·06 (0·04 to 0·08)	88·1 (85·3 to 91·0)	84·4 (81·4 to 87·1)	86·2 (83·3 to 89·0)	0·3 (0·2 to 0·3)	0·0 (0·0 to 0·0)	0 (0 to 0)	0 (0 to 0)	0·78 (0·01 to 1·98)
	Spain	3·0 (2·7 to 3·3)	−2·9% (−3·3 to −2·4)	0·04 (0·04 to 0·04)	0·08 (0·07 to 0·08)	85·7 (85·6 to 85·8)	79·9 (79·8 to 80·0)	82·9 (82·8 to 82·9)	445·0 (442·0 to 448·0)	1·0 (0·9 to 1·1)	72 (69 to 74)	22 (18 to 25)	1·03 (0·97 to 1·09)
	Sweden	2·3 (2·0 to 2·5)	−2·6% (−3·2 to −2·0)	0·04 (0·03 to 0·04)	0·06 (0·05 to 0·06)	85·0 (84·1 to 85·9)	82·0 (80·9 to 83·0)	83·5 (82·8 to 84·2)	92·0 (86·0 to 98·7)	0·3 (0·2 to 0·3)	9 (8 to 9)	1 (−1 to 4)	0·50 (0·38 to 0·61)
	Switzerland	3·7 (3·3 to 4·2)	−2·4% (−3·0 to −1·7)	0·03 (0·03 to 0·03)	0·05 (0·05 to 0·05)	86·4 (86·2 to 86·6)	82·5 (82·3 to 82·7)	84·5 (84·3 to 84·7)	69·7 (68·7 to 70·7)	0·3 (0·3 to 0·4)	9 (8 to 9)	3 (2 to 4)	0·69 (0·61 to 0·76)
	UK	4·2 (3·8 to 4·6)	−2·3% (−2·9 to −1·7)	0·06 (0·06 to 0·06)	0·10 (0·10 to 0·10)	82·4 (82·3 to 82·5)	78·2 (78·1 to 78·3)	80·3 (80·2 to 80·3)	686·0 (683·0 to 690·0)	2·9 (2·6 to 3·2)	82 (80 to 85)	55 (51 to 58)	1·02 (0·99 to 1·06)
**Latin America and Caribbean**		**16·5 (13·4 to 20·2)**	**−3·5% (−4·5 to −2·5)**	**0·13 (0·12 to 0·13)**	**0·23 (0·22 to 0·24)**	**75·9 (75·2 to 76·6)**	**68·9 (68·1 to 69·7)**	**72·3 (71·5 to 73·0)**	**4980·0 (4770·0 to 5200·0)**	**155·0 (125·0 to 190·0)**	**922 (847 to 1010)**	**1390 (1280 to 1520)**	**1·99 (1·85 to 2·15)**
Andean Latin America		16·7 (13·1 to 20·8)	−4·8% (−6·0 to −3·6)	0·13 (0·11 to 0·14)	0·22 (0·20 to 0·24)	74·3 (72·9 to 75·5)	68·3 (66·9 to 69·6)	71·1 (69·8 to 72·4)	565·0 (514·0 to 621·0)	20·6 (16·2 to 25·7)	220 (209 to 231)	246 (233 to 258)	3·79 (3·59 to 3·97)
	Bolivia	27·9 (23·5 to 32·7)	−4·5% (−5·4 to −3·6)	0·19 (0·16 to 0·22)	0·28 (0·25 to 0·32)	68·8 (66·7 to 70·5)	63·8 (61·9 to 65·6)	66·2 (64·1 to 67·9)	121·0 (106·0 to 140·0)	6·8 (5·7 to 8·0)	40 (33 to 46)	53 (46 to 59)	4·19 (3·58 to 4·72)
	Ecuador	13·7 (10·5 to 17·9)	−4·3% (−5·7 to −2·9)	0·10 (0·09 to 0·12)	0·19 (0·16 to 0·22)	77·1 (75·5 to 78·7)	71·0 (69·0 to 73·1)	74·0 (72·1 to 75·7)	124·0 (107·0 to 143·0)	4·4 (3·4 to 5·8)	50 (43 to 58)	38 (28 to 46)	2·58 (2·10 to 3·02)
	Peru	14·0 (9·5 to 19·1)	−5·2% (−7·0 to −3·6)	0·12 (0·11 to 0·14)	0·21 (0·19 to 0·24)	74·9 (73·4 to 76·3)	68·8 (67·3 to 70·1)	71·6 (70·2 to 73·0)	320·0 (289·0 to 357·0)	9·4 (6·4 to 12·8)	130 (129 to 131)	155 (154 to 156)	4·27 (4·24 to 4·30)
Caribbean		40·8 (33·9 to 48·8)	−1·1% (−2·0 to −0·3)	0·15 (0·13 to 0·17)	0·23 (0·20 to 0·25)	72·5 (70·7 to 74·1)	66·9 (64·9 to 68·7)	69·6 (67·7 to 71·3)	488·0 (440·0 to 541·0)	32·5 (26·9 to 39·0)	21 (−7 to 48)	107 (60 to 155)	1·48 (0·60 to 2·32)
	Antigua and Barbuda	9·3 (8·0 to 10·7)	−1·9% (−2·8 to −0·8)	0·09 (0·09 to 0·10)	0·14 (0·13 to 0·14)	77·1 (76·7 to 77·3)	73·0 (72·7 to 73·3)	75·0 (74·8 to 75·1)	0·7 (0·7 to 0·7)	0·0 (0·0 to 0·0)	0 (0 to 0)	0 (0 to 0)	−0·12 (−0·55 to 0·28)
	The Bahamas	10·2 (7·8 to 13·5)	−2·2% (−3·5 to −0·6)	0·16 (0·14 to 0·19)	0·29 (0·25 to 0·33)	73·6 (71·7 to 75·4)	66·1 (63·7 to 68·2)	69·8 (67·5 to 71·8)	3·8 (3·3 to 4·4)	0·0 (0·0 to 0·0)	1 (0 to 1)	1 (1 to 1)	2·33 (1·56 to 2·88)
	Barbados	11·7 (8·2 to 16·3)	−1·1% (−2·6 to 0·5)	0·10 (0·08 to 0·12)	0·14 (0·11 to 0·17)	77·6 (75·5 to 79·7)	74·4 (71·8 to 76·8)	76·0 (73·7 to 78·3)	3·3 (2·8 to 3·9)	0·1 (0·1 to 0·1)	0 (−1 to 0)	0 (0 to 0)	−1·03 (−1·86 to −0·23)
	Belize	14·4 (11·9 to 17·5)	−3·5% (−4·5 to −2·4)	0·13 (0·12 to 0·14)	0·21 (0·19 to 0·23)	76·1 (74·9 to 77·3)	70·5 (69·0 to 72·3)	73·2 (71·8 to 74·7)	2·3 (2·1 to 2·6)	0·0 (0·0 to 0·0)	0 (0 to 0)	0 (0 to 1)	0·72 (0·46 to 0·96)
	Bermuda	3·8 (3·2 to 4·5)	−1·9% (−3·0 to −0·7)	0·06 (0·05 to 0·07)	0·13 (0·11 to 0·14)	83·3 (81·5 to 84·7)	75·6 (73·9 to 77·1)	79·3 (77·5 to 80·8)	0·7 (0·7 to 0·9)	0·5 (0·4 to 0·5)	0 (0 to 0)	0 (0 to 0)	1·23 (0·53 to 1·90)
	Cuba	4·6 (3·9 to 5·3)	−3·0% (−3·7 to −2·2)	0·10 (0·09 to 0·11)	0·19 (0·17 to 0·20)	77·3 (76·3 to 78·3)	70·9 (69·9 to 72·1)	73·9 (73·0 to 74·9)	165·0 (151·0 to 178·0)	0·0 (0·0 to 0·0)	1 (−4 to 7)	55 (45 to 65)	2·65 (1·96 to 3·40)
	Dominica	27·6 (20·2 to 37·1)	1·8% (0·1 to 3·3)	0·12 (0·10 to 0·15)	0·21 (0·17 to 0·26)	73·3 (70·8 to 75·5)	67·4 (64·4 to 70·3)	70·2 (67·4 to 72·7)	0·8 (0·6 to 1·0)	5·3 (4·3 to 6·4)	0 (0 to 0)	0 (0 to 0)	1·24 (0·44 to 2·38)
	Dominican Republic	24·9 (20·2 to 30·1)	−2·4% (−3·4 to −1·4)	0·10 (0·09 to 0·12)	0·20 (0·17 to 0·23)	77·3 (75·5 to 78·9)	70·5 (68·3 to 72·5)	73·7 (71·8 to 75·5)	73·0 (64·1 to 82·9)	0·0 (0·0 to 0·0)	1 (−10 to 13)	9 (−5 to 20)	0·48 (−0·62 to 1·53)
	Grenada	12·6 (10·1 to 15·6)	−1·4% (−2·3 to −0·4)	0·14 (0·12 to 0·18)	0·23 (0·19 to 0·30)	72·9 (70·5 to 74·9)	67·3 (64·1 to 69·7)	69·9 (66·9 to 72·2)	1·1 (0·9 to 1·4)	0·3 (0·3 to 0·4)	0 (0 to 0)	0 (0 to 1)	1·54 (0·58 to 3·10)
	Guyana	22·7 (17·0 to 29·7)	−2·7% (−4·2 to −1·2)	0·22 (0·17 to 0·28)	0·37 (0·29 to 0·46)	68·6 (65·0 to 72·1)	61·1 (57·0 to 65·4)	64·6 (60·6 to 68·6)	8·6 (6·4 to 11·6)	24·0 (19·9 to 28·8)	1 (0 to 2)	2 (1 to 5)	2·37 (0·77 to 4·53)
	Haiti	70·6 (59·2 to 84·1)	−1·9% (−2·9 to −1·0)	0·28 (0·23 to 0·35)	0·34 (0·26 to 0·43)	61·5 (58·2 to 64·6)	58·8 (54·9 to 62·5)	60·1 (56·5 to 63·6)	131·0 (104·0 to 166·0)	0·5 (0·4 to 0·7)	14 (5 to 27)	26 (10 to 53)	1·67 (0·65 to 3·23)
	Jamaica	15·0 (11·0 to 20·1)	−1·8% (−3·5 to 0·0)	0·12 (0·10 to 0·15)	0·16 (0·13 to 0·20)	76·4 (73·7 to 78·9)	72·0 (69·1 to 75·1)	74·1 (71·3 to 76·9)	24·2 (19·5 to 29·2)	0·1 (0·1 to 0·1)	0 (−2 to 1)	5 (3 to 7)	0·90 (0·25 to 1·61)
	Puerto Rico	6·4 (5·4 to 7·7)	−2·7% (−3·6 to −1·7)	0·06 (0·05 to 0·07)	0·16 (0·13 to 0·18)	84·5 (82·8 to 86·4)	76·6 (74·4 to 79·1)	80·6 (78·5 to 82·8)	34·1 (29·1 to 39·3)	0·0 (0·0 to 0·0)	2 (−1 to 4)	2 (−1 to 5)	0·64 (−0·21 to 1·28)
	Saint Kitts and Nevis	15·9 (12·5 to 20·4)	−1·6% (−2·9 to −0·4)	0·10 (0·09 to 0·12)	0·21 (0·18 to 0·24)	75·5 (73·9 to 77·1)	68·5 (66·7 to 70·2)	71·8 (70·1 to 73·5)	0·5 (0·5 to 0·6)	0·0 (0·0 to 0·0)	0 (0 to 0)	0 (0 to 0)	0·76 (0·30 to 1·13)
	Saint Lucia	15·6 (11·2 to 21·2)	−1·0% (−2·7 to 0·6)	0·11 (0·09 to 0·14)	0·20 (0·16 to 0·25)	76·5 (73·8 to 78·9)	69·7 (66·4 to 72·7)	72·9 (69·7 to 75·6)	1·9 (1·6 to 2·5)	0·0 (0·0 to 0·0)	0 (0 to 0)	0 (0 to 1)	1·45 (0·48 to 2·74)
	Saint Vincent and the Grenadines	13·0 (9·6 to 17·2)	−3·1% (−4·7 to −1·6)	0·14 (0·12 to 0·16)	0·22 (0·20 to 0·24)	75·2 (73·7 to 76·6)	69·7 (68·0 to 71·3)	72·2 (70·5 to 73·7)	1·2 (1·0 to 1·3)	0·2 (0·2 to 0·3)	0 (0 to 0)	0 (0 to 0)	0·62 (0·20 to 1·11)
	Suriname	24·8 (18·9 to 32·0)	−2·3% (−3·7 to −0·8)	0·14 (0·12 to 0·18)	0·25 (0·21 to 0·31)	74·2 (70·9 to 76·7)	67·5 (63·4 to 70·7)	70·8 (66·9 to 73·6)	5·4 (4·3 to 7·2)	0·0 (0·0 to 0·1)	0 (0 to 0)	1 (0 to 3)	0·79 (0·03 to 2·25)
	Trinidad and Tobago	13·6 (10·2 to 18·0)	−3·2% (−4·7 to −1·7)	0·14 (0·11 to 0·17)	0·25 (0·20 to 0·31)	75·0 (72·0 to 78·0)	67·6 (64·1 to 71·2)	71·0 (67·7 to 74·4)	16·7 (12·8 to 21·4)	0·2 (0·2 to 0·3)	1 (0 to 2)	4 (2 to 8)	2·00 (0·74 to 3·74)
	Virgin Islands	5·9 (4·8 to 7·3)	−3·1% (−3·9 to −2·2)	0·08 (0·06 to 0·10)	0·21 (0·17 to 0·26)	82·3 (79·4 to 84·6)	71·3 (67·7 to 74·5)	76·6 (73·1 to 79·5)	0·9 (0·7 to 1·2)	0·0 (0·0 to 0·0)	0 (0 to 0)	0 (0 to 0)	1·49 (0·45 to 3·33)
Central Latin America		15·4 (11·9 to 19·7)	−3·1% (−4·5 to −1·9)	0·13 (0·12 to 0·13)	0·24 (0·23 to 0·25)	75·7 (74·9 to 76·5)	68·3 (67·3 to 69·3)	71·9 (70·9 to 72·8)	2080·0 (1970·0 to 2200·0)	60·4 (46·7 to 77·3)	497 (446 to 545)	610 (538 to 688)	2·21 (2·00 to 2·43)
	Colombia	11·9 (8·6 to 16·3)	−3·8% (−5·4 to −2·1)	0·08 (0·08 to 0·10)	0·16 (0·15 to 0·18)	79·7 (78·2 to 81·2)	72·6 (70·8 to 74·5)	76·1 (74·5 to 77·8)	354·0 (314·0 to 398·0)	8·1 (5·8 to 11·0)	49 (37 to 62)	105 (78 to 127)	1·70 (1·28 to 2·08)
	Costa Rica	9·4 (8·2 to 10·7)	−1·4% (−2·0 to −0·7)	0·08 (0·08 to 0·08)	0·17 (0·17 to 0·18)	81·2 (80·8 to 81·5)	74·3 (73·9 to 74·6)	77·7 (77·3 to 78·1)	30·7 (29·9 to 31·5)	0·5 (0·5 to 0·6)	1 (0 to 3)	6 (3 to 8)	0·74 (0·30 to 1·10)
	El Salvador	9·5 (7·1 to 12·5)	−5·3% (−6·8 to −3·9)	0·12 (0·10 to 0·14)	0·28 (0·24 to 0·32)	77·2 (75·4 to 79·1)	67·9 (65·4 to 70·4)	72·7 (70·6 to 74·9)	52·0 (44·8 to 59·9)	1·1 (0·8 to 1·5)	6 (5 to 7)	11 (9 to 13)	1·40 (1·19 to 1·63)
	Guatemala	25·5 (20·0 to 32·6)	−3·2% (−4·4 to −1·9)	0·15 (0·14 to 0·17)	0·27 (0·24 to 0·29)	72·7 (71·3 to 74·1)	66·2 (64·4 to 67·9)	69·4 (67·8 to 71·0)	113·0 (102·0 to 125·0)	7·6 (6·0 to 9·8)	20 (16 to 23)	32 (27 to 37)	1·78 (1·46 to 2·06)
	Honduras	15·0 (12·2 to 18·2)	−4·1% (−5·3 to −3·1)	0·18 (0·15 to 0·22)	0·25 (0·21 to 0·30)	70·7 (68·4 to 72·6)	66·4 (64·3 to 68·2)	68·5 (66·3 to 70·3)	72·9 (64·5 to 84·7)	3·3 (2·7 to 4·0)	12 (10 to 14)	20 (16 to 26)	1·65 (1·35 to 2·06)
	Mexico	14·8 (11·6 to 18·9)	−3·2% (−4·5 to −2·0)	0·14 (0·14 to 0·14)	0·27 (0·27 to 0·27)	74·7 (74·4 to 74·9)	67·4 (67·0 to 67·7)	70·9 (70·6 to 71·2)	1120·0 (1110·0 to 1120·0)	28·1 (22·0 to 36·0)	335 (302 to 362)	341 (291 to 390)	2·61 (2·36 to 2·84)
	Nicaragua	13·8 (10·3 to 18·0)	−4·6% (−6·0 to −3·1)	0·11 (0·10 to 0·12)	0·21 (0·19 to 0·23)	76·8 (75·6 to 77·9)	69·9 (68·5 to 71·2)	73·3 (72·0 to 74·4)	38·3 (35·0 to 42·2)	1·8 (1·3 to 2·3)	14 (12 to 15)	16 (14 to 18)	2·21 (1·99 to 2·42)
	Panama	14·1 (11·0 to 17·8)	−2·3% (−3·5 to −1·0)	0·08 (0·06 to 0·09)	0·14 (0·11 to 0·16)	81·4 (79·5 to 83·5)	75·5 (73·1 to 78·2)	78·3 (76·2 to 80·8)	23·9 (19·7 to 27·9)	1·0 (0·8 to 1·3)	3 (1 to 4)	3 (1 to 5)	0·81 (0·33 to 1·20)
	Venezuela	19·7 (14·8 to 25·8)	−0·8% (−2·2 to 0·5)	0·13 (0·11 to 0·16)	0·28 (0·23 to 0·32)	74·6 (72·3 to 76·9)	65·1 (62·2 to 68·1)	69·7 (67·0 to 72·3)	276·0 (231·0 to 326·0)	8·9 (6·6 to 11·6)	58 (52 to 64)	77 (68 to 87)	2·22 (2·00 to 2·43)
Tropical Latin America		12·0 (9·9 to 14·6)	−4·8% (−5·9 to −3·7)	0·12 (0·12 to 0·12)	0·22 (0·22 to 0·23)	77·3 (77·1 to 77·6)	70·2 (69·9 to 70·4)	73·7 (73·4 to 73·9)	1850·0 (1830·0 to 1870·0)	41·4 (33·8 to 50·3)	184 (170 to 197)	426 (408 to 444)	1·35 (1·29 to 1·41)
	Brazil	11·9 (9·8 to 14·4)	−4·9% (−6·0 to −3·8)	0·12 (0·12 to 0·12)	0·22 (0·22 to 0·23)	77·4 (77·2 to 77·6)	70·2 (69·9 to 70·4)	73·7 (73·5 to 73·9)	1800·0 (1780·0 to 1810·0)	39·5 (32·4 to 47·8)	183 (169 to 197)	411 (393 to 429)	1·36 (1·29 to 1·42)
	Paraguay	14·7 (10·5 to 19·6)	−3·0% (−4·5 to −1·5)	0·11 (0·10 to 0·14)	0·21 (0·18 to 0·25)	75·9 (73·8 to 77·6)	69·0 (66·5 to 71·1)	72·2 (69·9 to 74·2)	50·7 (43·7 to 59·3)	1·9 (1·4 to 2·5)	1 (0 to 1)	15 (14 to 16)	1·11 (1·04 to 1·18)
**North Africa and Middle East**		**20·2 (17·4 to 23·3)**	**−4·8% (−5·5 to −4·1)**	**0·12 (0·11 to 0·13)**	**0·19 (0·18 to 0·21)**	**73·7 (72·6 to 74·7)**	**68·9 (67·8 to 70·1)**	**71·1 (70·0 to 72·2)**	**4050·0 (3730·0 to 4390·0)**	**243·0 (208·0 to 280·0)**	**679 (583 to 753)**	**934 (797 to 1060)**	**1·33 (1·14 to 1·49)**
	Afghanistan	48·7 (40·5 to 58·4)	−4·7% (−5·7 to −3·8)	0·33 (0·27 to 0·39)	0·42 (0·37 to 0·47)	60·7 (58·5 to 62·8)	55·9 (54·0 to 57·9)	58·2 (56·3 to 60·3)	272·0 (241·0 to 305·0)	58·0 (48·1 to 69·8)	43 (32 to 57)	50 (40 to 59)	1·01 (0·78 to 1·24)
	Algeria	16·9 (13·4 to 21·0)	−4·1% (−5·4 to −2·9)	0·10 (0·09 to 0·11)	0·15 (0·13 to 0·17)	75·4 (74·3 to 76·4)	72·1 (70·6 to 73·6)	73·6 (72·3 to 74·9)	273·0 (243·0 to 306·0)	15·5 (12·2 to 19·3)	53 (51 to 54)	79 (62 to 95)	1·56 (1·35 to 1·75)
	Bahrain	5·7 (4·8 to 6·7)	−3·5% (−4·4 to −2·7)	0·09 (0·08 to 0·10)	0·13 (0·11 to 0·14)	75·1 (74·1 to 76·0)	72·2 (71·1 to 73·3)	73·3 (72·3 to 74·4)	6·3 (5·6 to 7·0)	0·1 (0·1 to 0·1)	1 (1 to 1)	2 (1 to 2)	0·91 (0·75 to 1·03)
	Egypt	12·8 (10·5 to 15·7)	−6·0% (−7·1 to −4·8)	0·14 (0·12 to 0·17)	0·24 (0·20 to 0·27)	70·2 (68·7 to 71·6)	66·9 (65·0 to 68·7)	68·4 (66·7 to 70·0)	712·0 (612·0 to 823·0)	33·1 (27·1 to 40·7)	89 (58 to 121)	152 (98 to 196)	1·20 (0·81 to 1·55)
	Iran	5·3 (4·4 to 6·2)	−9·7% (−10·7 to −8·6)	0·09 (0·08 to 0·09)	0·17 (0·16 to 0·18)	77·2 (76·8 to 77·6)	71·9 (71·5 to 72·3)	74·4 (74·1 to 74·6)	569·0 (556·0 to 582·0)	5·6 (4·7 to 6·7)	158 (153 to 162)	205 (198 to 210)	2·12 (2·07 to 2·16)
	Iraq	18·8 (14·8 to 23·7)	−4·3% (−5·4 to −3·0)	0·13 (0·10 to 0·16)	0·21 (0·17 to 0·26)	73·5 (71·6 to 75·4)	67·5 (65·6 to 70·0)	70·2 (68·3 to 72·5)	233·0 (193·0 to 269·0)	15·7 (12·4 to 19·9)	60 (50 to 70)	50 (35 to 62)	1·65 (1·33 to 1·94)
	Jordan	11·5 (9·4 to 14·1)	−3·9% (−4·9 to −2·8)	0·08 (0·07 to 0·09)	0·13 (0·11 to 0·15)	77·6 (76·1 to 78·9)	74·1 (72·4 to 75·9)	75·7 (74·1 to 77·3)	45·5 (39·2 to 52·3)	2·5 (2·0 to 3·0)	9 (6 to 11)	15 (11 to 18)	1·01 (0·70 to 1·22)
	Kuwait	8·1 (6·6 to 9·7)	−1·7% (−2·6 to −0·7)	0·04 (0·03 to 0·04)	0·09 (0·07 to 0·10)	85·1 (84·0 to 86·2)	78·1 (76·3 to 80·0)	80·7 (79·2 to 82·3)	12·1 (10·4 to 13·9)	0·4 (0·3 to 0·5)	2 (2 to 3)	2 (1 to 3)	0·48 (0·32 to 0·62)
	Lebanon	7·7 (5·4 to 10·9)	−4·9% (−6·5 to −3·2)	0·08 (0·07 to 0·09)	0·16 (0·14 to 0·17)	78·4 (77·4 to 79·3)	72·2 (70·9 to 73·3)	75·2 (74·0 to 76·2)	49·6 (45·6 to 54·6)	0·6 (0·4 to 0·9)	8 (7 to 9)	18 (16 to 19)	2·86 (2·59 to 3·17)
	Libya	21·6 (16·9 to 27·0)	−0·7% (−1·9 to 0·5)	0·13 (0·11 to 0·16)	0·20 (0·17 to 0·24)	73·4 (70·9 to 75·4)	68·7 (66·0 to 71·1)	70·8 (68·2 to 73·1)	46·3 (38·9 to 55·7)	1·8 (1·4 to 2·2)	6 (5 to 7)	10 (8 to 12)	1·24 (0·99 to 1·48)
	Morocco	14·8 (12·1 to 17·8)	−5·9% (−6·9 to −4·8)	0·13 (0·10 to 0·16)	0·16 (0·13 to 0·19)	73·9 (72·2 to 75·8)	70·9 (69·4 to 72·9)	72·3 (70·7 to 74·3)	286·0 (241·0 to 318·0)	9·5 (7·7 to 11·4)	52 (41 to 62)	46 (36 to 57)	1·41 (1·15 to 1·68)
	Oman	9·1 (8·0 to 10·2)	−2·5% (−3·1 to −1·8)	0·09 (0·08 to 0·10)	0·16 (0·15 to 0·18)	76·3 (75·1 to 77·4)	70·5 (69·1 to 71·7)	72·7 (71·4 to 73·9)	17·0 (15·3 to 19·0)	0·7 (0·6 to 0·8)	3 (3 to 4)	6 (5 to 6)	1·05 (0·98 to 1·11)
	Palestine	10·8 (8·6 to 13·9)	−4·6% (−5·8 to −3·4)	0·08 (0·07 to 0·09)	0·15 (0·13 to 0·17)	76·2 (75·2 to 77·2)	71·5 (70·3 to 72·8)	73·8 (72·6 to 74·9)	19·5 (17·5 to 21·6)	1·3 (1·0 to 1·7)	1 (0 to 2)	4 (3 to 5)	0·50 (0·34 to 0·66)
	Qatar	3·6 (2·9 to 4·6)	−5·2% (−6·3 to −4·2)	0·05 (0·04 to 0·06)	0·09 (0·07 to 0·11)	79·2 (77·6 to 80·7)	76·1 (74·2 to 77·9)	77·2 (75·4 to 78·9)	5·1 (4·2 to 6·0)	0·1 (0·1 to 0·2)	1 (1 to 1)	1 (1 to 1)	0·31 (0·23 to 0·37)
	Saudi Arabia	4·2 (3·2 to 5·3)	−8·2% (−9·7 to −6·8)	0·14 (0·11 to 0·17)	0·19 (0·16 to 0·23)	75·1 (72·9 to 77·2)	71·8 (69·9 to 73·6)	73·1 (71·1 to 75·0)	156·0 (129·0 to 187·0)	2·0 (1·5 to 2·5)	15 (12 to 18)	12 (8 to 17)	0·38 (0·29 to 0·46)
	Sudan	36·8 (29·5 to 45·0)	−5·0% (−6·1 to −4·0)	0·16 (0·13 to 0·20)	0·22 (0·17 to 0·27)	70·1 (67·2 to 72·7)	66·3 (63·1 to 69·3)	68·0 (64·9 to 70·8)	246·0 (200·0 to 300·0)	42·5 (33·9 to 52·1)	37 (27 to 46)	48 (26 to 72)	1·08 (0·69 to 1·50)
	Syria	10·0 (8·0 to 12·4)	−2·9% (−3·9 to −1·8)	0·10 (0·08 to 0·13)	0·19 (0·15 to 0·23)	74·7 (72·5 to 76·6)	70·1 (67·5 to 72·4)	72·4 (69·9 to 74·6)	104·0 (85·4 to 128·0)	2·0 (1·6 to 2·5)	7 (5 to 8)	16 (11 to 22)	0·53 (0·38 to 0·69)
	Tunisia	10·3 (8·4 to 12·5)	−5·2% (−6·2 to −4·1)	0·09 (0·07 to 0·11)	0·17 (0·14 to 0·21)	77·1 (75·1 to 79·0)	70·8 (68·5 to 73·1)	73·7 (71·5 to 75·9)	103·0 (84·9 to 124·0)	1·7 (1·4 to 2·1)	8 (−1 to 15)	34 (26 to 42)	1·87 (1·14 to 2·54)
	Türkiye	11·1 (9·1 to 13·4)	−6·3% (−7·3 to −5·3)	0·07 (0·06 to 0·08)	0·14 (0·12 to 0·17)	78·3 (77·0 to 79·5)	72·3 (70·7 to 74·0)	75·2 (73·7 to 76·7)	654·0 (566·0 to 744·0)	11·4 (9·3 to 13·7)	111 (83 to 135)	144 (107 to 172)	1·62 (1·21 to 1·87)
	United Arab Emirates	4·8 (4·1 to 5·7)	−4·2% (−5·1 to −3·5)	0·06 (0·05 to 0·07)	0·09 (0·07 to 0·10)	71·5 (70·8 to 72·3)	77·5 (75·7 to 79·6)	75·0 (73·6 to 76·6)	20·1 (15·9 to 23·7)	0·4 (0·3 to 0·4)	−2 (−7 to 2)	4 (0 to 5)	0·21 (−0·24 to 0·61)
	Yemen	38·9 (32·0 to 46·5)	−4·1% (−5·1 to −3·2)	0·18 (0·14 to 0·23)	0·29 (0·24 to 0·35)	68·5 (65·5 to 70·9)	62·4 (59·4 to 65·2)	65·3 (62·2 to 67·9)	216·0 (181·0 to 263·0)	37·8 (30·9 to 45·3)	19 (15 to 22)	37 (15 to 65)	0·85 (0·50 to 1·29)
**South Asia**		**37·1 (31·4 to 44·2)**	**−3·6% (−4·5 to −2·7)**	**0·15 (0·14 to 0·17)**	**0·23 (0·21 to 0·25)**	**70·8 (69·8 to 71·8)**	**66·4 (65·4 to 67·4)**	**68·5 (67·6 to 69·3)**	**14 800·0 (14 000·0 to 15 600·0)**	**1180·0 (995·0 to 1410·0)**	**1610 (1500 to 1710)**	**2830 (2710 to 2960)**	**1·28 (1·24 to 1·32)**
	Bangladesh	28·0 (22·5 to 34·6)	−5·3% (−6·4 to −4·2)	0·11 (0·09 to 0·13)	0·16 (0·14 to 0·19)	74·1 (72·0 to 76·1)	70·6 (68·3 to 72·8)	72·3 (70·0 to 74·3)	1100·0 (929·0 to 1280·0)	79·2 (63·4 to 98·0)	152 (127 to 208)	180 (154 to 219)	1·07 (0·92 to 1·37)
	Bhutan	29·3 (22·8 to 36·6)	−5·2% (−6·4 to −3·9)	0·10 (0·08 to 0·13)	0·13 (0·10 to 0·16)	74·9 (72·6 to 77·3)	72·7 (70·2 to 75·2)	73·7 (71·3 to 76·2)	4·4 (3·7 to 5·2)	0·4 (0·3 to 0·5)	0 (0 to 0)	0 (0 to 0)	0·09 (0·07 to 0·11)
	India	33·1 (26·9 to 40·8)	−4·0% (−5·2 to −2·8)	0·15 (0·14 to 0·17)	0·23 (0·21 to 0·25)	71·2 (70·2 to 72·4)	66·6 (65·4 to 67·7)	68·7 (67·8 to 69·6)	11 700·0 (11 100·0 to 12 500·0)	730·0 (590·0 to 902·0)	1170 (1100 to 1240)	2270 (2160 to 2370)	1·29 (1·26 to 1·33)
	Nepal	28·4 (22·0 to 36·4)	−5·1% (−6·3 to −3·8)	0·15 (0·13 to 0·18)	0·24 (0·21 to 0·27)	70·8 (68·8 to 72·4)	66·1 (64·1 to 67·8)	68·4 (66·4 to 70·1)	252·0 (224·0 to 290·0)	18·2 (14·0 to 23·4)	29 (22 to 32)	62 (58 to 70)	1·47 (1·39 to 1·59)
	Pakistan	56·3 (46·2 to 68·0)	−2·2% (−3·2 to −1·2)	0·19 (0·15 to 0·24)	0·25 (0·20 to 0·30)	66·4 (63·8 to 68·8)	63·8 (61·3 to 66·1)	65·0 (63·1 to 66·9)	1720·0 (1520·0 to 1940·0)	353·0 (288·0 to 428·0)	254 (236 to 271)	311 (258 to 385)	1·28 (1·15 to 1·48)
**Southeast Asia, east Asia, and Oceania**		**14·6 (12·6 to 17·0)**	**−5·1% (−5·8 to −4·4)**	**0·08 (0·07 to 0·09)**	**0·15 (0·13 to 0·17)**	**78·6 (77·2 to 80·0)**	**72·5 (70·9 to 74·1)**	**75·4 (74·1 to 76·6)**	**17 800·0 (15 900·0 to 19 900·0)**	**352·0 (302·0 to 411·0)**	**165 (−39 to 534)**	**869 (424 to 1490)**	**0·24 (0·09 to 0·44)**
	East Asia	7·3 (6·2 to 8·6)	−7·9% (−8·9 to −6·9)	0·06 (0·04 to 0·07)	0·12 (0·09 to 0·15)	80·7 (78·9 to 82·5)	74·8 (72·7 to 77·0)	77·6 (76·0 to 79·1)	12 100·0 (10 400·0 to 14 000·0)	90·0 (76·2 to 107·0)	55 (−6 to 292)	12 (−14 to 72)	0·02 (−0·01 to 0·12)
	China	7·2 (6·1 to 8·6)	−7·7% (−8·5 to −6·8)	0·05 (0·04 to 0·07)	0·12 (0·09 to 0·14)	80·7 (78·9 to 82·6)	74·9 (72·7 to 77·1)	77·6 (76·0 to 79·2)	11 700·0 (9980·0 to 13 600·0)	86·1 (72·3 to 102·0)	59 (3 to 283)	11 (−2 to 55)	0·02 (0·00 to 0·12)
	North Korea	10·5 (7·8 to 13·9)	−10·9% (−15·4 to −7·3)	0·12 (0·09 to 0·15)	0·20 (0·16 to 0·25)	76·2 (73·6 to 78·5)	70·1 (67·8 to 72·5)	73·3 (70·7 to 75·7)	242·0 (202·0 to 288·0)	3·1 (2·3 to 4·1)	1 (0 to 5)	0 (0 to 1)	0·02 (0·00 to 0·12)
	Taiwan (province of China)	4·6 (4·1 to 5·2)	−2·7% (−3·4 to −2·1)	0·05 (0·05 to 0·05)	0·12 (0·12 to 0·12)	84·6 (84·4 to 84·8)	78·1 (77·9 to 78·2)	81·3 (81·1 to 81·4)	184·0 (182·0 to 186·0)	0·7 (0·7 to 0·8)	−6 (−15 to 4)	1 (−18 to 16)	−0·11 (−0·69 to 0·43)
Oceania		47·1 (38·9 to 56·1)	−1·2% (−2·2 to −0·2)	0·21 (0·18 to 0·26)	0·29 (0·24 to 0·35)	66·6 (64·2 to 69·0)	62·5 (59·4 to 65·6)	64·4 (61·6 to 67·1)	108·0 (89·4 to 131·0)	19·8 (16·3 to 23·7)	1 (0 to 3)	16 (4 to 34)	0·69 (0·17 to 1·47)
	American Samoa	12·1 (9·4 to 15·5)	−0·9% (−2·3 to 0·4)	0·16 (0·13 to 0·19)	0·23 (0·19 to 0·27)	72·8 (70·6 to 74·9)	69·3 (67·0 to 71·2)	71·0 (68·7 to 72·9)	0·4 (0·4 to 0·5)	0·0 (0·0 to 0·0)	0 (0 to 0)	0 (0 to 0)	0·00 (0·00 to 0·00)
	Cook Islands	5·4 (5·4 to 5·5)	−4·4% (−5·4 to −3·4)	0·08 (0·07 to 0·10)	0·18 (0·15 to 0·22)	79·6 (77·6 to 81·6)	72·9 (70·9 to 74·7)	76·1 (74·2 to 78·0)	0·2 (0·1 to 0·2)	0·0 (0·0 to 0·0)	0 (0 to 0)	0 (0 to 0)	0·00 (0·00 to 0·00)
	Federated States of Micronesia	15·4 (12·2 to 19·1)	−4·1% (−5·2 to −2·9)	0·21 (0·16 to 0·27)	0·32 (0·26 to 0·40)	69·7 (66·6 to 72·4)	64·5 (61·1 to 67·5)	67·0 (63·6 to 69·9)	0·8 (0·7 to 1·0)	0·0 (0·0 to 0·0)	0 (0 to 0)	0 (0 to 0)	0·00 (0·00 to 0·00)
	Fiji	19·3 (14·6 to 25·2)	−1·4% (−2·9 to 0·3)	0·21 (0·16 to 0·26)	0·31 (0·23 to 0·38)	68·8 (65·8 to 71·9)	63·8 (60·4 to 67·4)	66·1 (62·9 to 69·6)	9·4 (7·2 to 12·0)	0·3 (0·3 to 0·5)	0 (0 to 0)	2 (0 to 4)	1·08 (0·27 to 2·36)
	Guam	12·0 (9·6 to 14·9)	0·1% (−1·0 to 1·3)	0·11 (0·10 to 0·12)	0·21 (0·19 to 0·23)	82·9 (81·2 to 84·7)	73·5 (71·7 to 75·5)	77·9 (76·2 to 79·8)	1·2 (1·0 to 1·3)	0·0 (0·0 to 0·0)	0 (0 to 0)	0 (0 to 0)	1·08 (0·65 to 1·48)
	Kiribati	36·4 (29·6 to 44·7)	−2·6% (−3·6 to −1·5)	0·22 (0·17 to 0·28)	0·36 (0·30 to 0·44)	67·0 (64·1 to 69·5)	61·1 (57·8 to 64·0)	64·1 (60·9 to 66·8)	1·0 (0·8 to 1·2)	0·1 (0·1 to 0·1)	0 (0 to 0)	0 (0 to 0)	0·00 (0·00 to 0·00)
	Marshall Islands	19·9 (15·3 to 26·2)	−3·1% (−4·4 to −1·7)	0·26 (0·21 to 0·33)	0·34 (0·28 to 0·41)	66·8 (63·5 to 69·6)	63·4 (59·8 to 66·5)	65·0 (61·5 to 68·1)	0·4 (0·4 to 0·6)	0·0 (0·0 to 0·0)	0 (0 to 0)	0 (0 to 0)	0·00 (0·00 to 0·00)
	Nauru	24·5 (18·2 to 33·0)	−3·1% (−4·5 to −1·6)	0·28 (0·22 to 0·34)	0·43 (0·37 to 0·51)	65·7 (62·3 to 68·7)	59·2 (55·8 to 62·4)	62·3 (58·8 to 65·4)	0·1 (0·1 to 0·1)	0·0 (0·0 to 0·0)	0 (0 to 0)	0 (0 to 0)	0·00 (0·00 to 0·00)
	Niue	51·1 (51·0 to 52·5)	2·8% (1·8 to 3·7)	0·15 (0·12 to 0·18)	0·23 (0·19 to 0·29)	69·2 (67·6 to 71·1)	65·1 (62·9 to 66·8)	67·1 (65·1 to 69·0)	0·0 (0·0 to 0·0)	0·0 (0·0 to 0·0)	0 (0 to 0)	0 (0 to 0)	0·00 (0·00 to 0·00)
	Northern Mariana Islands	6·2 (5·0 to 7·4)	−0·7% (−1·6 to 0·1)	0·13 (0·11 to 0·15)	0·22 (0·18 to 0·25)	75·0 (73·8 to 77·1)	69·5 (68·1 to 71·9)	72·0 (70·7 to 74·2)	0·4 (0·3 to 0·4)	0·0 (0·0 to 0·0)	0 (0 to 0)	0 (0 to 0)	0·38 (−0·75 to 1·39)
	Palau	16·9 (13·9 to 20·8)	−1·5% (−2·7 to −0·4)	0·15 (0·12 to 0·19)	0·28 (0·23 to 0·33)	70·5 (68·2 to 72·6)	67·7 (64·9 to 70·5)	68·7 (66·1 to 71·1)	0·2 (0·2 to 0·2)	0·0 (0·0 to 0·0)	0 (0 to 0)	0 (0 to 0)	0·00 (0·00 to 0·00)
	Papua New Guinea	52·7 (43·5 to 62·8)	−1·4% (−2·5 to −0·4)	0·22 (0·18 to 0·27)	0·29 (0·23 to 0·37)	65·5 (62·8 to 68·3)	61·9 (58·4 to 65·4)	63·5 (60·3 to 66·7)	80·7 (65·2 to 99·6)	17·6 (14·5 to 21·1)	1 (0 to 2)	13 (3 to 29)	0·75 (0·18 to 1·62)
	Samoa	13·0 (10·1 to 16·6)	−2·4% (−3·8 to −0·9)	0·17 (0·14 to 0·21)	0·22 (0·18 to 0·27)	71·9 (69·5 to 74·2)	69·6 (67·2 to 71·5)	70·7 (68·3 to 72·8)	1·4 (1·2 to 1·6)	0·1 (0·1 to 0·1)	0 (0 to 0)	0 (0 to 0)	0·00 (0·00 to 0·00)
	Solomon Islands	19·5 (15·6 to 24·2)	−2·7% (−3·9 to −1·5)	0·23 (0·18 to 0·29)	0·33 (0·27 to 0·41)	68·4 (65·2 to 71·1)	63·7 (60·3 to 66·5)	65·9 (62·6 to 68·7)	4·6 (3·7 to 5·7)	0·4 (0·3 to 0·5)	0 (0 to 0)	0 (0 to 0)	0·00 (0·00 to 0·00)
	Tokelau	64·0 (64·0 to 64·0)	5·3% (4·1 to 6·3)	0·17 (0·14 to 0·20)	0·19 (0·15 to 0·24)	67·8 (65·6 to 70·0)	67·1 (65·1 to 69·0)	67·5 (65·3 to 69·5)	0·0 (0·0 to 0·0)	0·0 (0·0 to 0·0)	0 (0 to 0)	0 (0 to 0)	0·00 (0·00 to 0·00)
	Tonga	11·7 (9·0 to 14·9)	−2·8% (−4·2 to −1·4)	0·13 (0·10 to 0·16)	0·20 (0·16 to 0·25)	75·7 (72·9 to 78·2)	70·6 (67·9 to 73·1)	73·1 (70·4 to 75·6)	0·7 (0·6 to 0·8)	0·0 (0·0 to 0·0)	0 (0 to 0)	0 (0 to 0)	0·00 (0·00 to 0·00)
	Tuvalu	17·3 (13·2 to 22·5)	−5·4% (−6·8 to −4·0)	0·19 (0·15 to 0·24)	0·29 (0·23 to 0·35)	70·6 (67·8 to 73·2)	65·8 (62·7 to 68·7)	68·0 (65·7 to 70·1)	0·1 (0·1 to 0·1)	0·0 (0·0 to 0·0)	0 (0 to 0)	0 (0 to 0)	0·00 (0·00 to 0·00)
	Vanuatu	20·7 (16·3 to 26·6)	−2·5% (−3·8 to −1·2)	0·20 (0·17 to 0·24)	0·35 (0·30 to 0·41)	69·4 (67·3 to 71·3)	62·5 (59·9 to 64·8)	65·7 (63·3 to 67·8)	2·3 (1·9 to 2·7)	0·2 (0·1 to 0·2)	0 (0 to 0)	0 (0 to 1)	0·41 (0·10 to 0·87)
Southeast Asia		21·5 (18·2 to 25·4)	−3·9% (−4·7 to −3·1)	0·12 (0·11 to 0·14)	0·22 (0·19 to 0·25)	74·3 (72·7 to 75·8)	67·9 (66·1 to 69·7)	71·0 (69·4 to 72·5)	5510·0 (4870·0 to 6180·0)	243·0 (205·0 to 287·0)	109 (−33 to 304)	841 (428 to 1410)	0·70 (0·29 to 1·26)
	Cambodia	30·7 (25·5 to 37·4)	−5·3% (−6·2 to −4·3)	0·15 (0·12 to 0·19)	0·25 (0·20 to 0·31)	71·0 (68·2 to 73·6)	65·2 (62·3 to 68·2)	68·2 (65·3 to 71·0)	129·0 (104·0 to 156·0)	11·0 (9·1 to 13·4)	0 (0 to 0)	14 (4 to 27)	0·40 (0·12 to 0·79)
	Indonesia	24·1 (19·5 to 29·5)	−3·8% (−4·9 to −2·8)	0·14 (0·11 to 0·18)	0·21 (0·16 to 0·27)	72·0 (69·6 to 74·3)	67·3 (64·4 to 70·3)	69·5 (67·3 to 71·9)	2200·0 (1790·0 to 2630·0)	107·0 (86·1 to 130·0)	133 (47 to 271)	364 (124 to 717)	0·94 (0·32 to 1·87)
	Laos	40·2 (31·3 to 50·3)	−5·2% (−6·4 to −3·9)	0·15 (0·12 to 0·19)	0·23 (0·19 to 0·29)	70·4 (67·4 to 73·2)	65·4 (62·2 to 68·7)	67·8 (64·6 to 70·9)	51·0 (40·9 to 62·3)	7·0 (5·4 to 8·8)	0 (0 to 0)	5 (2 to 11)	0·36 (0·12 to 0·78)
	Malaysia	6·2 (5·6 to 7·0)	−1·8% (−2·4 to −1·2)	0·11 (0·11 to 0·12)	0·20 (0·19 to 0·22)	75·7 (75·2 to 76·2)	70·4 (69·5 to 71·1)	72·9 (72·1 to 73·4)	224·0 (214·0 to 240·0)	3·0 (2·7 to 3·4)	−15 (−27 to −6)	37 (19 to 52)	0·34 (−0·05 to 0·70)
	Maldives	12·5 (10·1 to 15·6)	−4·4% (−5·6 to −3·2)	0·05 (0·04 to 0·06)	0·08 (0·06 to 0·10)	81·2 (79·7 to 82·6)	78·1 (76·1 to 80·0)	79·4 (77·6 to 81·1)	1·6 (1·4 to 1·9)	0·1 (0·1 to 0·1)	0 (0 to 0)	0 (0 to 0)	0·28 (0·05 to 0·56)
	Mauritius	12·6 (10·5 to 14·3)	−1·5% (−2·4 to −0·7)	0·11 (0·10 to 0·12)	0·21 (0·19 to 0·22)	76·9 (76·1 to 78·1)	70·1 (69·1 to 71·6)	73·4 (72·5 to 74·8)	13·2 (11·9 to 14·3)	0·2 (0·1 to 0·2)	0 (−1 to 0)	2 (0 to 3)	0·44 (−0·38 to 1·04)
	Myanmar	39·2 (31·7 to 49·3)	−4·8% (−5·9 to −3·7)	0·14 (0·12 to 0·18)	0·26 (0·21 to 0·32)	71·2 (68·7 to 73·5)	64·1 (61·3 to 66·9)	67·6 (64·9 to 70·2)	511·0 (423·0 to 620·0)	42·1 (33·9 to 53·2)	17 (6 to 34)	66 (21 to 134)	0·82 (0·27 to 1·65)
	Philippines	21·0 (17·3 to 25·3)	−2·6% (−3·7 to −1·5)	0·15 (0·13 to 0·18)	0·28 (0·24 to 0·32)	72·2 (70·6 to 73·8)	64·8 (63·0 to 66·7)	68·3 (66·9 to 69·5)	880·0 (799·0 to 968·0)	47·6 (39·3 to 57·6)	−17 (−19 to −16)	229 (227 to 230)	0·94 (0·93 to 0·95)
	Seychelles	13·3 (10·8 to 16·4)	−0·0% (−1·1 to 1·1)	0·11 (0·09 to 0·12)	0·20 (0·18 to 0·21)	76·5 (75·5 to 77·4)	70·8 (69·9 to 71·7)	73·4 (72·5 to 74·3)	0·9 (0·8 to 0·9)	0·0 (0·0 to 0·0)	0 (0 to 0)	0 (0 to 0)	0·06 (−0·31 to 0·36)
	Sri Lanka	6·0 (4·6 to 7·7)	−4·9% (−6·1 to −3·6)	0·07 (0·04 to 0·09)	0·16 (0·11 to 0·21)	79·7 (76·8 to 83·1)	73·4 (69·6 to 78·1)	76·6 (73·2 to 80·5)	158·0 (110·0 to 209·0)	1·8 (1·4 to 2·3)	−10 (−54 to 23)	18 (−19 to 48)	0·17 (−1·60 to 1·58)
	Thailand	7·4 (6·5 to 8·3)	−4·2% (−5·1 to −3·2)	0·09 (0·07 to 0·11)	0·21 (0·17 to 0·25)	80·3 (77·8 to 82·6)	72·4 (69·1 to 75·8)	76·3 (73·5 to 79·1)	626·0 (499·0 to 766·0)	4·0 (3·5 to 4·5)	1 (0 to 2)	62 (20 to 117)	0·44 (0·14 to 0·83)
	Timor-Leste	35·2 (29·0 to 42·7)	−4·1% (−5·1 to −3·1)	0·16 (0·12 to 0·19)	0·21 (0·17 to 0·26)	70·5 (68·2 to 72·8)	66·9 (64·2 to 69·6)	68·6 (66·1 to 71·0)	9·5 (7·9 to 11·4)	1·4 (1·2 to 1·7)	0 (0 to 0)	1 (0 to 2)	0·45 (0·14 to 0·88)
	Viet Nam	11·1 (8·7 to 14·3)	−4·4% (−5·6 to −3·2)	0·08 (0·06 to 0·10)	0·19 (0·16 to 0·24)	78·3 (76·5 to 80·3)	69·9 (68·0 to 72·0)	74·0 (72·1 to 76·1)	701·0 (587·0 to 813·0)	17·5 (13·7 to 22·5)	1 (0 to 1)	44 (14 to 90)	0·23 (0·07 to 0·47)
**Sub-Saharan Africa**		**70·7 (59·7 to 84·0)**	**−3·5% (−4·3 to −2·7)**	**0·24 (0·22 to 0·26)**	**0·34 (0·32 to 0·37)**	**64·1 (62·4 to 65·5)**	**58·7 (56·8 to 60·3)**	**61·3 (59·5 to 62·7)**	**9430·0 (8620·0 to 10 500·0)**	**2630·0 (2210·0 to 3140·0)**	**805 (747 to 864)**	**1600 (1480 to 1720)**	**1·13 (1·05 to 1·19)**
Central sub-Saharan Africa		58·3 (49·7 to 68·9)	−4·6% (−5·4 to −3·8)	0·25 (0·22 to 0·29)	0·37 (0·33 to 0·41)	63·8 (61·5 to 66·0)	58·4 (56·1 to 60·5)	61·0 (58·7 to 63·1)	1090·0 (953·0 to 1250·0)	259·0 (220·0 to 307·0)	94 (84 to 104)	174 (150 to 202)	1·04 (0·91 to 1·17)
	Angola	54·7 (45·7 to 65·1)	−5·3% (−6·3 to −4·5)	0·27 (0·22 to 0·32)	0·37 (0·32 to 0·43)	63·7 (60·8 to 66·6)	58·4 (55·6 to 61·1)	61·0 (58·2 to 63·7)	250·0 (208·0 to 296·0)	65·3 (54·3 to 78·0)	15 (13 to 18)	40 (29 to 51)	0·92 (0·71 to 1·10)
	Central African Republic	110·0 (89·2 to 136·0)	−2·4% (−3·4 to −1·3)	0·39 (0·33 to 0·47)	0·57 (0·50 to 0·65)	55·2 (51·2 to 58·6)	48·2 (44·5 to 51·7)	51·4 (47·6 to 54·9)	73·7 (60·8 to 89·4)	20·6 (16·6 to 25·8)	9 (6 to 12)	9 (6 to 14)	1·47 (0·98 to 2·15)
	Congo (Brazzaville)	39·2 (32·4 to 47·3)	−4·6% (−5·7 to −3·6)	0·31 (0·25 to 0·37)	0·35 (0·29 to 0·42)	63·1 (60·4 to 65·6)	60·6 (58·1 to 62·9)	61·8 (59·2 to 64·2)	46·3 (39·6 to 54·4)	5·0 (4·2 to 6·1)	5 (4 to 6)	8 (5 to 10)	1·25 (0·93 to 1·49)
	Democratic Republic of the Congo	57·8 (48·3 to 71·4)	−4·6% (−5·5 to −3·6)	0·23 (0·19 to 0·28)	0·35 (0·30 to 0·40)	64·5 (62·3 to 67·0)	59·0 (56·6 to 61·4)	61·6 (59·3 to 64·1)	698·0 (595·0 to 802·0)	165·0 (137·0 to 204·0)	61 (55 to 67)	112 (96 to 135)	1·02 (0·91 to 1·16)
	Equatorial Guinea	46·3 (34·6 to 62·3)	−4·6% (−6·0 to −3·1)	0·29 (0·22 to 0·38)	0·37 (0·30 to 0·45)	63·7 (58·9 to 67·7)	59·3 (55·3 to 62·9)	61·5 (57·2 to 65·3)	10·5 (8·2 to 13·6)	1·8 (1·3 to 2·4)	1 (1 to 2)	2 (1 to 3)	1·12 (0·73 to 1·55)
	Gabon	32·5 (23·6 to 44·5)	−3·7% (−5·1 to −2·1)	0·23 (0·19 to 0·29)	0·35 (0·29 to 0·41)	67·3 (64·0 to 70·2)	60·9 (57·8 to 63·6)	63·9 (60·6 to 66·7)	15·5 (12·9 to 18·7)	1·4 (1·0 to 1·9)	2 (2 to 2)	3 (2 to 4)	1·49 (1·22 to 1·69)
Eastern sub-Saharan Africa		57·9 (47·4 to 71·6)	−4·0% (−5·0 to −3·0)	0·24 (0·22 to 0·26)	0·36 (0·33 to 0·38)	64·5 (62·9 to 66·0)	58·9 (57·2 to 60·4)	61·5 (59·8 to 63·0)	3330·0 (3040·0 to 3700·0)	787·0 (640·0 to 978·0)	282 (259 to 305)	662 (594 to 712)	1·17 (1·07 to 1·25)
	Burundi	63·9 (50·0 to 82·0)	−4·3% (−5·4 to −3·1)	0·22 (0·19 to 0·26)	0·32 (0·27 to 0·36)	64·9 (62·6 to 67·2)	60·0 (57·7 to 62·3)	62·2 (59·9 to 64·4)	97·4 (84·8 to 112·0)	29·6 (23·0 to 38·3)	4 (4 to 5)	11 (10 to 12)	0·66 (0·60 to 0·70)
	Comoros	48·0 (39·0 to 58·9)	−3·7% (−4·7 to −2·6)	0·18 (0·14 to 0·22)	0·24 (0·20 to 0·28)	68·2 (65·8 to 70·2)	64·8 (62·5 to 66·9)	66·5 (64·2 to 68·5)	5·9 (5·1 to 6·8)	0·8 (0·7 to 1·0)	0 (0 to 0)	1 (1 to 1)	0·94 (0·86 to 1·01)
	Djibouti	37·2 (30·1 to 45·6)	−4·1% (−5·1 to −3·0)	0·23 (0·18 to 0·29)	0·31 (0·26 to 0·38)	67·0 (63·4 to 70·0)	62·3 (59·0 to 65·1)	64·3 (60·9 to 67·2)	9·3 (7·5 to 11·6)	1·1 (0·9 to 1·4)	1 (1 to 2)	2 (1 to 3)	1·38 (0·98 to 1·72)
	Eritrea	45·5 (34·4 to 60·3)	−3·5% (−4·9 to −2·2)	0·25 (0·20 to 0·31)	0·38 (0·32 to 0·46)	64·8 (61·5 to 67·8)	58·7 (55·2 to 61·7)	61·7 (58·3 to 64·7)	50·8 (41·6 to 62·3)	8·8 (6·6 to 11·7)	1 (1 to 2)	7 (5 to 7)	0·52 (0·44 to 0·60)
	Ethiopia	52·2 (41·8 to 65·1)	−4·8% (−5·8 to −3·7)	0·19 (0·17 to 0·22)	0·28 (0·25 to 0·32)	67·5 (65·7 to 69·2)	62·0 (60·3 to 63·7)	64·5 (63·1 to 65·8)	737·0 (678·0 to 805·0)	180·0 (143·0 to 225·0)	72 (67 to 78)	157 (143 to 170)	1·14 (1·04 to 1·23)
	Kenya	36·6 (29·7 to 44·7)	−4·0% (−5·1 to −3·0)	0·22 (0·20 to 0·26)	0·35 (0·31 to 0·39)	67·2 (65·2 to 68·9)	61·0 (59·4 to 62·6)	63·9 (62·5 to 65·2)	357·0 (326·0 to 390·0)	43·7 (35·3 to 53·5)	56 (51 to 61)	86 (77 to 94)	1·49 (1·34 to 1·60)
	Madagascar	57·6 (46·2 to 72·4)	−3·1% (−4·2 to −2·0)	0·25 (0·20 to 0·30)	0·31 (0·27 to 0·37)	63·9 (61·7 to 66·2)	60·5 (58·2 to 63·0)	62·1 (59·9 to 64·5)	206·0 (177·0 to 237·0)	48·9 (39·0 to 62·0)	24 (22 to 26)	33 (28 to 37)	1·11 (0·97 to 1·21)
	Malawi	52·1 (43·0 to 62·7)	−5·4% (−6·4 to −4·5)	0·31 (0·27 to 0·36)	0·46 (0·41 to 0·50)	62·1 (59·5 to 64·5)	55·8 (53·7 to 57·7)	58·7 (56·7 to 60·6)	173·0 (154·0 to 196·0)	29·6 (24·3 to 35·8)	8 (7 to 9)	43 (38 to 48)	1·49 (1·31 to 1·64)
	Mozambique	62·2 (49·4 to 79·3)	−4·5% (−5·7 to −3·3)	0·33 (0·28 to 0·38)	0·50 (0·45 to 0·56)	59·9 (57·4 to 62·4)	53·4 (51·0 to 55·5)	56·4 (54·0 to 58·6)	307·0 (268·0 to 350·0)	68·5 (54·0 to 88·1)	9 (5 to 13)	54 (42 to 64)	1·11 (0·94 to 1·25)
	Rwanda	41·4 (33·7 to 49·8)	−5·9% (−6·9 to −4·9)	0·21 (0·17 to 0·24)	0·30 (0·26 to 0·34)	67·5 (65·2 to 69·7)	62·3 (60·0 to 64·3)	65·0 (62·7 to 67·1)	92·1 (79·4 to 107·0)	15·1 (12·3 to 18·3)	2 (2 to 3)	20 (16 to 22)	0·88 (0·72 to 0·97)
	Somalia	92·3 (75·9 to 112·0)	−2·6% (−3·5 to −1·6)	0·36 (0·30 to 0·43)	0·53 (0·45 to 0·61)	56·9 (53·6 to 59·9)	50·7 (47·1 to 54·0)	53·6 (50·1 to 56·9)	238·0 (197·0 to 288·0)	86·0 (70·2 to 106·0)	25 (20 to 29)	41 (30 to 54)	1·26 (0·96 to 1·57)
	South Sudan	129·0 (103·0 to 159·0)	−0·8% (−1·8 to 0·3)	0·28 (0·22 to 0·35)	0·40 (0·33 to 0·48)	58·1 (53·6 to 62·0)	52·6 (47·9 to 56·7)	55·0 (50·5 to 59·1)	115·0 (92·3 to 144·0)	47·5 (37·6 to 59·9)	10 (8 to 11)	12 (9 to 16)	0·96 (0·75 to 1·14)
	Tanzania	52·4 (42·4 to 65·6)	−4·2% (−5·2 to −3·1)	0·23 (0·19 to 0·26)	0·31 (0·28 to 0·35)	65·9 (63·8 to 67·8)	61·3 (59·2 to 63·1)	63·5 (61·4 to 65·3)	440·0 (390·0 to 498·0)	101·0 (78·6 to 131·0)	38 (35 to 42)	89 (80 to 95)	1·17 (1·07 to 1·24)
	Uganda	64·6 (50·6 to 83·0)	−3·6% (−4·8 to −2·4)	0·23 (0·19 to 0·27)	0·38 (0·32 to 0·43)	64·9 (62·2 to 67·3)	57·8 (55·3 to 60·3)	61·2 (58·7 to 63·7)	329·0 (283·0 to 382·0)	98·2 (79·1 to 123·0)	16 (11 to 18)	58 (36 to 70)	0·92 (0·67 to 1·08)
	Zambia	46·1 (36·5 to 58·1)	−5·4% (−6·5 to −4·2)	0·33 (0·28 to 0·38)	0·47 (0·40 to 0·53)	61·4 (58·4 to 64·2)	55·8 (53·0 to 58·6)	58·3 (55·4 to 61·0)	175·0 (145·0 to 207·0)	27·9 (21·9 to 35·4)	14 (13 to 16)	49 (36 to 63)	1·75 (1·35 to 2·13)
Southern sub-Saharan Africa		43·6 (36·2 to 53·2)	−2·8% (−3·7 to −1·8)	0·31 (0·30 to 0·33)	0·47 (0·45 to 0·49)	63·0 (61·8 to 63·9)	55·9 (54·7 to 57·0)	59·3 (58·2 to 60·3)	1040·0 (989·0 to 1090·0)	71·4 (59·0 to 87·7)	155 (152 to 158)	297 (281 to 311)	3·01 (2·90 to 3·10)
	Botswana	40·6 (30·3 to 53·9)	−2·8% (−4·1 to −1·4)	0·32 (0·27 to 0·36)	0·45 (0·40 to 0·51)	62·9 (60·9 to 65·0)	57·0 (55·0 to 58·9)	59·7 (58·0 to 61·6)	28·1 (24·7 to 31·3)	2·0 (1·5 to 2·6)	1 (1 to 1)	10 (7 to 12)	2·54 (1·90 to 3·06)
	Eswatini	42·1 (33·4 to 53·8)	−3·9% (−5·0 to −2·7)	0·46 (0·39 to 0·54)	0·66 (0·59 to 0·73)	56·1 (53·0 to 59·2)	49·5 (46·9 to 52·2)	52·5 (49·6 to 55·5)	17·6 (14·6 to 20·9)	1·2 (1·0 to 1·6)	2 (2 to 3)	6 (4 to 7)	3·91 (2·97 to 4·57)
	Lesotho	78·8 (64·6 to 94·5)	−1·0% (−2·0 to −0·1)	0·53 (0·46 to 0·60)	0·73 (0·67 to 0·78)	52·1 (49·7 to 54·6)	45·3 (43·5 to 47·2)	48·5 (46·5 to 50·5)	37·9 (33·0 to 42·9)	3·4 (2·7 to 4·1)	3 (3 to 3)	11 (9 to 13)	4·47 (3·79 to 5·14)
	Namibia	33·4 (26·1 to 43·0)	−3·3% (−4·4 to −2·0)	0·29 (0·25 to 0·35)	0·47 (0·41 to 0·53)	64·0 (61·3 to 66·5)	56·5 (53·8 to 58·9)	60·1 (57·4 to 62·5)	26·8 (22·9 to 31·4)	1·9 (1·5 to 2·5)	2 (2 to 2)	9 (7 to 10)	2·33 (2·00 to 2·65)
	South Africa	38·6 (31·9 to 47·1)	−3·3% (−4·2 to −2·3)	0·28 (0·27 to 0·30)	0·44 (0·42 to 0·46)	64·8 (64·0 to 65·5)	57·4 (56·6 to 58·3)	61·0 (60·3 to 61·6)	733·0 (712·0 to 754·0)	38·4 (31·6 to 47·1)	130 (130 to 130)	204 (204 to 204)	3·12 (3·12 to 3·12)
	Zimbabwe	52·7 (43·6 to 64·5)	−1·9% (−2·9 to −0·9)	0·41 (0·36 to 0·47)	0·56 (0·51 to 0·62)	58·0 (55·5 to 60·4)	52·2 (49·7 to 54·5)	55·0 (52·5 to 57·3)	193·0 (167·0 to 222·0)	24·6 (20·2 to 30·2)	16 (14 to 18)	57 (45 to 67)	2·56 (2·14 to 2·93)
Western sub-Saharan Africa		86·3 (73·5 to 101·0)	−3·2% (−3·9 to −2·5)	0·21 (0·18 to 0·23)	0·29 (0·26 to 0·32)	64·5 (62·5 to 66·3)	59·9 (57·6 to 61·9)	62·1 (59·9 to 63·8)	3970·0 (3580·0 to 4510·0)	1510·0 (1280·0 to 1780·0)	274 (248 to 299)	468 (422 to 511)	0·81 (0·75 to 0·86)
	Benin	77·3 (62·8 to 95·2)	−2·9% (−3·9 to −1·9)	0·19 (0·16 to 0·22)	0·29 (0·26 to 0·34)	65·9 (63·5 to 68·0)	60·1 (57·8 to 62·1)	62·9 (60·5 to 65·0)	105·0 (92·8 to 120·0)	39·6 (32·0 to 49·1)	4 (3 to 5)	13 (11 to 14)	0·67 (0·60 to 0·75)
	Burkina Faso	95·5 (77·9 to 117·0)	−3·0% (−4·0 to −2·0)	0·21 (0·18 to 0·25)	0·33 (0·29 to 0·37)	63·0 (60·7 to 65·1)	57·4 (54·9 to 59·6)	60·1 (57·6 to 62·3)	218·0 (192·0 to 249·0)	87·8 (71·1 to 109·0)	15 (14 to 16)	25 (19 to 28)	0·95 (0·82 to 1·04)
	Cabo Verde	15·0 (11·3 to 19·7)	−5·8% (−7·3 to −4·2)	0·08 (0·07 to 0·10)	0·20 (0·17 to 0·25)	77·8 (75·8 to 79·8)	69·0 (66·8 to 71·2)	73·2 (71·1 to 75·4)	3·7 (3·1 to 4·2)	0·1 (0·1 to 0·2)	0 (0 to 0)	0 (0 to 0)	0·41 (0·23 to 0·64)
	Cameroon	65·5 (54·3 to 77·6)	−3·2% (−4·1 to −2·3)	0·26 (0·21 to 0·31)	0·36 (0·31 to 0·42)	63·6 (60·6 to 66·1)	58·5 (55·7 to 60·8)	60·8 (58·0 to 63·2)	261·0 (225·0 to 308·0)	67·6 (55·6 to 80·4)	16 (14 to 17)	46 (39 to 51)	1·03 (0·91 to 1·14)
	Chad	112·0 (94·6 to 134·0)	−2·3% (−3·2 to −1·4)	0·25 (0·20 to 0·30)	0·33 (0·28 to 0·39)	60·5 (56·9 to 63·5)	56·5 (52·5 to 59·8)	58·3 (54·5 to 61·5)	182·0 (153·0 to 220·0)	92·9 (77·9 to 112·0)	14 (11 to 16)	12 (9 to 14)	0·80 (0·63 to 0·90)
	Côte d'Ivoire	68·5 (58·2 to 80·6)	−3·4% (−4·2 to −2·5)	0·21 (0·17 to 0·26)	0·31 (0·26 to 0·36)	65·8 (63·1 to 68·4)	60·3 (57·6 to 62·7)	62·7 (59·9 to 65·1)	209·0 (181·0 to 244·0)	64·4 (54·3 to 76·1)	19 (17 to 20)	24 (21 to 28)	0·80 (0·71 to 0·88)
	The Gambia	44·2 (35·3 to 55·4)	−4·0% (−5·1 to −2·9)	0·24 (0·19 to 0·28)	0·34 (0·29 to 0·39)	65·9 (63·4 to 68·2)	60·9 (58·5 to 63·2)	63·2 (60·9 to 65·5)	17·6 (15·2 to 20·3)	42·0 (32·3 to 53·9)	2 (2 to 3)	3 (2 to 3)	1·16 (1·01 to 1·33)
	Ghana	43·4 (33·6 to 55·5)	−4·0% (−5·2 to −2·7)	0·21 (0·18 to 0·25)	0·31 (0·27 to 0·36)	67·4 (65·0 to 69·6)	61·7 (59·5 to 63·9)	64·6 (62·3 to 66·7)	250·0 (215·0 to 289·0)	42·6 (35·3 to 51·5)	18 (16 to 20)	40 (32 to 48)	0·93 (0·80 to 1·05)
	Guinea	86·8 (72·7 to 104·0)	−3·4% (−4·3 to −2·5)	0·25 (0·20 to 0·30)	0·32 (0·27 to 0·38)	62·2 (58·9 to 65·1)	58·2 (54·6 to 61·2)	60·1 (56·6 to 63·0)	127·0 (107·0 to 152·0)	4·4 (3·6 to 5·4)	14 (12 to 17)	19 (13 to 23)	1·37 (1·07 to 1·64)
	Guinea-Bissau	61·8 (50·9 to 75·1)	−4·6% (−5·6 to −3·6)	0·31 (0·25 to 0·37)	0·45 (0·38 to 0·53)	61·3 (58·8 to 63·8)	55·1 (52·4 to 57·7)	58·1 (55·6 to 60·7)	18·4 (15·8 to 21·2)	10·9 (8·4 to 14·4)	3 (3 to 3)	3 (1 to 4)	1·45 (1·07 to 1·77)
	Liberia	66·9 (51·7 to 87·8)	−4·5% (−5·7 to −3·1)	0·23 (0·19 to 0·29)	0·28 (0·24 to 0·34)	64·1 (60·1 to 67·4)	61·6 (57·7 to 64·8)	62·7 (58·9 to 66·0)	39·5 (32·2 to 49·3)	101·0 (83·9 to 124·0)	3 (3 to 4)	4 (4 to 5)	0·88 (0·77 to 1·00)
	Mali	97·7 (81·4 to 118·0)	−3·3% (−4·1 to −2·3)	0·25 (0·22 to 0·30)	0·32 (0·28 to 0·36)	61·1 (58·8 to 63·2)	57·3 (55·1 to 59·2)	59·1 (56·8 to 61·0)	234·0 (208·0 to 265·0)	4·6 (3·8 to 5·5)	21 (18 to 23)	36 (33 to 40)	1·28 (1·17 to 1·36)
	Mauritania	33·7 (28·3 to 40·2)	−4·3% (−5·2 to −3·4)	0·17 (0·13 to 0·21)	0·19 (0·15 to 0·23)	70·1 (67·4 to 72·5)	68·4 (65·6 to 71·0)	69·2 (66·5 to 71·7)	25·0 (21·0 to 30·1)	100·0 (80·9 to 124·0)	3 (3 to 4)	3 (2 to 4)	0·82 (0·66 to 0·93)
	Niger	88·7 (72·1 to 110·0)	−4·4% (−5·3 to −3·4)	0·21 (0·17 to 0·26)	0·28 (0·23 to 0·33)	63·5 (60·0 to 66·6)	60·1 (56·3 to 63·4)	61·8 (58·1 to 65·0)	206·0 (170·0 to 253·0)	787·0 (662·0 to 938·0)	13 (12 to 15)	17 (13 to 20)	0·66 (0·56 to 0·74)
	Nigeria	96·3 (81·8 to 114·0)	−3·1% (−3·9 to −2·2)	0·19 (0·15 to 0·24)	0·25 (0·21 to 0·31)	65·0 (62·2 to 67·4)	60·7 (58·0 to 63·1)	62·8 (60·8 to 64·6)	1820·0 (1650·0 to 2030·0)	0·1 (0·1 to 0·1)	106 (96 to 116)	186 (167 to 210)	0·67 (0·62 to 0·73)
	São Tomé and Príncipe	17·8 (13·5 to 23·2)	−7·1% (−8·4 to −5·7)	0·15 (0·12 to 0·19)	0·20 (0·17 to 0·24)	72·2 (70·1 to 74·1)	68·6 (66·5 to 70·3)	70·4 (68·3 to 72·1)	1·1 (1·0 to 1·3)	19·3 (16·1 to 23·0)	0 (0 to 0)	0 (0 to 0)	0·51 (0·47 to 0·55)
	Senegal	40·5 (33·9 to 47·9)	−5·2% (−6·0 to −4·3)	0·19 (0·16 to 0·23)	0·27 (0·23 to 0·31)	68·2 (65·8 to 70·2)	63·7 (61·4 to 65·8)	65·9 (63·5 to 67·9)	111·0 (96·4 to 130·0)	28·9 (22·8 to 36·4)	12 (10 to 14)	22 (19 to 25)	1·15 (0·97 to 1·26)
	Sierra Leone	97·2 (77·3 to 121·0)	−3·9% (−5·0 to −2·8)	0·24 (0·19 to 0·29)	0·29 (0·24 to 0·34)	62·1 (58·2 to 65·5)	59·2 (54·9 to 62·8)	60·6 (56·5 to 64·1)	79·5 (65·3 to 97·7)	3·4 (2·7 to 4·2)	6 (5 to 7)	6 (5 to 7)	0·75 (0·67 to 0·83)
	Togo	56·7 (45·7 to 70·8)	−3·7% (−4·8 to −2·6)	0·21 (0·18 to 0·26)	0·33 (0·28 to 0·39)	66·0 (62·7 to 69·0)	60·2 (56·6 to 63·2)	63·1 (59·6 to 66·2)	62·8 (51·4 to 77·5)	13·8 (11·1 to 17·4)	3 (3 to 4)	8 (6 to 9)	0·72 (0·57 to 0·82)

Excess deaths due to COVID-19 include all deaths due to the pandemic. Data in parentheses are 95% uncertainty intervals.

**Figure 5:**
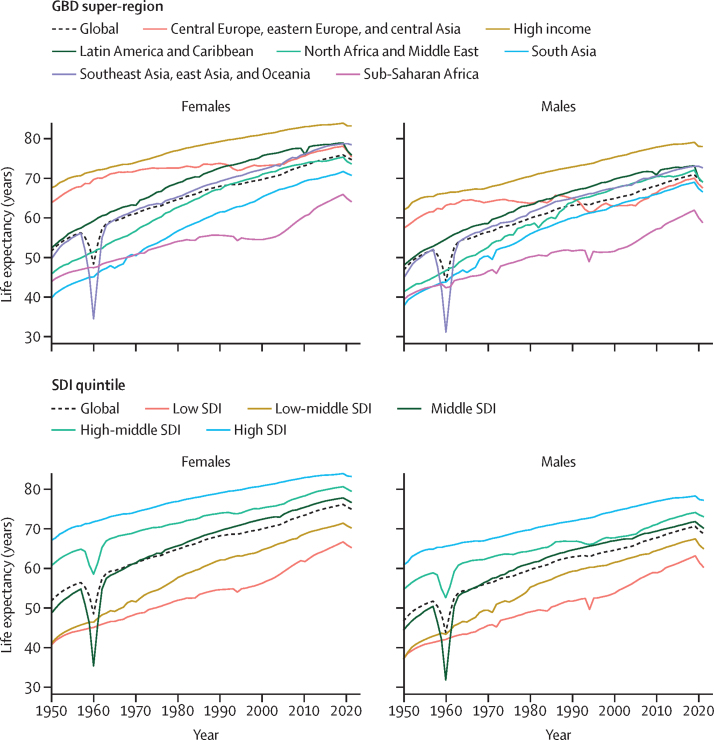
Life expectancy at birth across GBD super-regions and SDI quintiles in females and males, 1950–2021 The different colours represent GBD super-regions in the top row and SDI quintiles in the bottom row. The decline in life expectancy in 1960 for the southeast Asia, east Asia, and Oceania super-region was due to famine. GBD=Global Burden of Diseases, Injuries, and Risk Factors Study. SDI=Socio-demographic Index.

Historically, global life expectancy at birth has increased steadily; between 1950 and 2021, global life expectancy at birth increased by 22·7 years (95% UI 20·8 to 24·8), from 49·0 years (46·7 to 51·3) to 71·7 years (70·9 to 72·5; table 1; appendix 2 table S4). Life expectancy improved for females from 51·6 years (49·4 to 53·8) in 1950 to 76·0 years (75·2 to 76·7) in 2019 and for males from 46·7 years (44·3 to 49·2) in 1950 to 70·8 years (69·9 to 71·7) in 2019 (figure 5). At the super-region level, the largest increases in life expectancy occurred in south Asia and north Africa and the Middle East, while at the national level, some of the largest increases were in South Korea and Iran (appendix 2 table S4). During this time period, the smallest gains in life expectancy occurred in the central Europe, eastern Europe, and central Asia and high-income super-regions and, at the national level, in Ukraine and Lesotho. Increasing life expectancy was generally consistent across all super-regions over the entire period, with the exception of mortality shocks in several locations, stagnation in sub-Saharan Africa during the HIV/AIDS epidemic, and slow progress in central Europe, eastern Europe, and central Asia before the mid-2000s. In 2020 and 2021, however, these trends reversed. Between 2019 and 2021, global life expectancy declined by 1·6 years (1·0 to 2·2); all super-regions had decreases in life expectancy during this period, ranging from a 3·7 year (3·4 to 4·1) decline in Latin America and the Caribbean to a 0·3 year (–1·9 to 1·3) decline in southeast Asia, east Asia, and Oceania (appendix 2 table S4). An increase in life expectancy during this period was only observed in 32 (15·7%) of 204 countries and territories.

### Excess mortality due to the COVID-19 pandemic

We estimated 5·89 million (95% UI 5·48–6·44) excess deaths globally attributable to the COVID-19 pandemic in 2020 and 9·97 million (9·26–10·9) excess deaths in 2021 (table 1). The GBD super-regions with the highest all-age excess mortality rates in 2020 and 2021 combined were central Europe, eastern Europe, and central Asia (269·7 excess deaths per 100 000 population [250·0–289·6]) and Latin America and the Caribbean (199·0 [184·7–215·4]). The super-regions with the lowest all-age excess mortality rates during this time period were southeast Asia, east Asia, and Oceania (23·8 [8·9–44·1]) and high-income (90·2 [87·2–93·2]; appendix 2 figure S2). At the national level, in 2020 and 2021 combined, all-age excess mortality rates were highest in Bulgaria (520·8 [382·0–630·0]) and Lesotho (447·0 [379·3–514·0]), the highest rate in 2020 was in Peru (413·4 [410·3–416·1]), and the highest rate in 2021 was in Bulgaria (697·5 [532·4–830·5]; appendix 2 figure S2). For seven countries and territories (Taiwan [province of China], Mongolia, Japan, New Zealand, Iceland, Antigua and Barbuda, and Barbados), the all-age excess mortality rate for 2020 and 2021 combined was negative, indicating that fewer deaths occurred in these locations during the first 2 years of the pandemic than what would be expected based on past trends. In 2020, 20 countries and territories had negative excess mortality, while in 2021, only New Zealand and Barbados had negative excess mortality (table 1).

**Figure 6:**
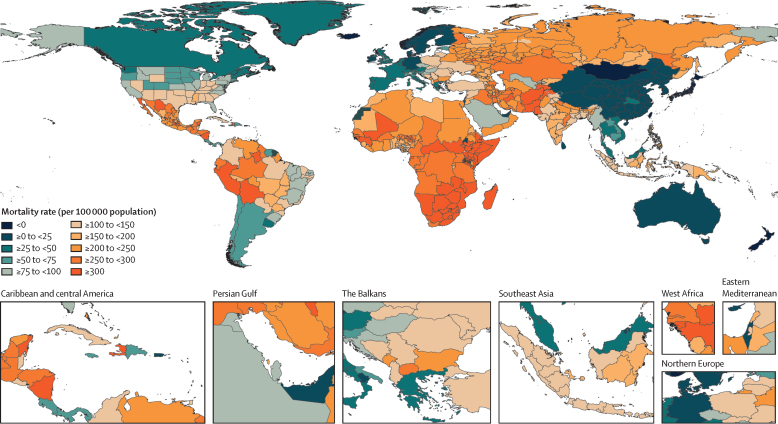
Global distribution of age-standardised excess mortality rates due to the COVID-19 pandemic, 2020 and 2021 combined Mortality rates are expressed as the number of deaths per 100 000 population. Excess mortality rates are negative in countries and territories where fewer deaths occurred than predicted.

Additionally, we computed age-standardised excess mortality rates to compare the impact of the pandemic across countries and territories while controlling for different population age structures. Age-standardised rates and all-age rates differed substantially, with the highest age-standardised excess mortality rates observed in nations in sub-Saharan Africa, Latin America, and the Middle East (figure 6). The lowest age-standardised rates were found in some countries and territories in the Caribbean, east Asia, and Oceania, and some high-income nations. There was substantial variability within all super-regions. The countries or territories with the highest age-standardised rates during 2020 and 2021 combined were Eswatini (992·5 age-standardised excess deaths per 100 000 population [95% UI 745·5 to 1173·2]), Lesotho (874·3 [734·7 to 1009·4]), and Somalia (715·6 [549·3 to 912·7]); the nations with the lowest rates were Barbados (–61·5 [–111·6 to –13·1]), Mongolia (–32·9 [–209·6 to 131·0]), and Antigua and Barbuda (–13·7 [–55·5 to 27·9]).

### Estimated mortality versus expected mortality based on SDI

**Figure 7:**
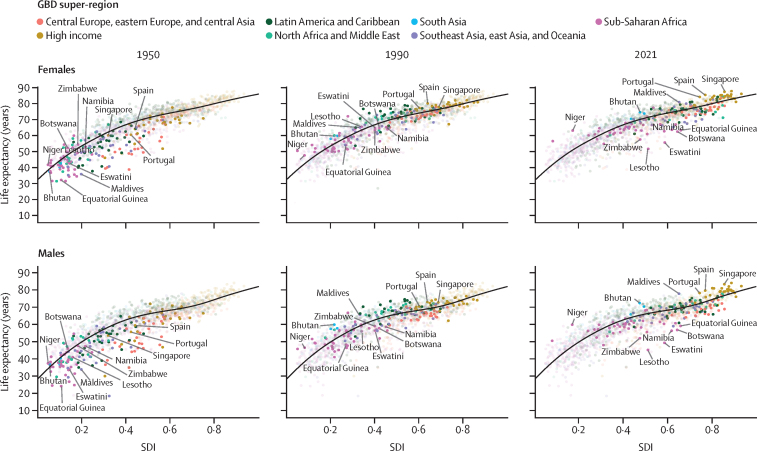
National life expectancy at birth versus SDI, and expected life expectancy based on SDI, in females and males in 1950, 1990, and 2021 Life expectancy at birth is shown for 204 countries and territories coloured by GBD super-region. Transparent points in all plots show every fifth year between 1950 and 2015, and 2021 in the first two columns. The black line represents the expected life expectancy at birth based on SDI, and the shaded area corresponds to 95% uncertainty intervals. GBD=Global Burden of Diseases, Injuries, and Risk Factors Study. SDI=Socio-demographic Index.

Between 1950 and 2021, longer life expectancies at birth were generally associated with higher SDI levels (figure 7; table 2). For females in 2021, the super-regions with the largest proportion of nations with a life expectancy higher than expected based on SDI were high-income (31 of 36 nations), south Asia (three of five nations), and Latin America and the Caribbean (16 of 33 nations), while central Europe, eastern Europe, and central Asia (23 of 29 nations), sub-Saharan Africa (35 of 46 nations), and north Africa and the Middle East (14 of 21 nations) had the highest proportion of nations with a lower life expectancy than expected based on SDI. For males in 2021, the GBD super-regions with the largest proportion of nations with a life expectancy greater than expected based on SDI were high-income (31 of 36 nations), south Asia (three of five nations), and north Africa and the Middle East (11 of 21 nations); the super-regions with the highest proportion of nations displaying a life expectancy lower than expected based on SDI were central Europe, eastern Europe, and central Asia (24 of 29 nations), sub-Saharan Africa (34 of 46 nations), and southeast Asia, east Asia, and Oceania (24 of 34 nations). Between 1950 and 2021, an increase in both life expectancy at birth and SDI was observed in all countries and territories. For females in 2021, the five countries or territories with the largest positive difference between estimated life expectancy and expected life expectancy based on SDI were Somalia (13·9 years), Niger (10·0 years), Spain (6·5 years), Portugal (6·0 years), and Singapore (5·6 years); the five countries or territories with the largest negative difference were Lesotho (–19·6 years), Eswatini (–17·9 years), Botswana (–12·8 years), Equatorial Guinea (–12·5 years), and Zimbabwe (–12·5 years; table 3). For males in 2021, the five countries or territories with the largest positive difference between estimated life expectancy and expected life expectancy based on SDI were Somalia (12·2 years), Niger (10·6 years), the Maldives (8·4 years), Bhutan (7·1 years), and Singapore (6·7 years); the five countries or territories with the largest negative difference were Lesotho (–21·2 years), Eswatini (–18·7 years), Zimbabwe (–13·4 years), South Africa (–12·8 years), and Botswana (–12·4 years; table 4).

**Table 2:** Life expectancy (estimated, expected based on SDI, and their difference), globally and by SDI quintile, for 1950, 1990, 2000, 2010, and 2021

	**1950**			**1990**			**2000**			**2010**			**2021**		
	Estimated life expectancy	Expected life expectancy	Difference	Estimated life expectancy	Expected life expectancy	Difference	Estimated life expectancy	Expected life expectancy	Difference	Estimated life expectancy	Expected life expectancy	Difference	Estimated life expectancy	Expected life expectancy	Difference
Global	49·0	63·4	−14·3	65·5	69·5	−4·0	67·2	70·7	−3·4	70·5	71·7	−1·2	71·7	72·9	−1·2
Low SDI	38·6	45·7	−7·0	53·1	54·0	−1·0	54·9	56·2	−1·2	60·2	60·2	0·1	62·6	64·9	−2·3
Low-middle SDI	38·8	50·1	−11·3	60·6	61·1	−0·5	63·0	64·1	−1·1	66·5	67·0	−0·5	67·4	69·9	−2·5
Middle SDI	46·2	55·5	−9·2	67·0	68·3	−1·3	69·6	69·9	−0·3	72·3	71·4	1·0	73·2	73·1	0·2
High-middle SDI	57·6	65·1	−7·5	70·4	71·0	−0·6	71·4	72·3	−0·9	74·7	73·9	0·8	76·2	75·7	0·5
High SDI	63·9	71·0	−7·1	75·6	75·7	−0·1	77·8	77·2	0·5	80·0	78·6	1·5	80·2	79·9	0·4

SDI=Socio-demographic Index.

**Table 3:** Female life expectancy (estimated, expected based on SDI, and their difference) for 1950, 1990, 2000, 2010, and 2021, and SDI in 2021, globally and for GBD super-regions, regions, countries, and territories

		**1950**			**1990**			**2000**			**2010**			**2021**			**SDI, 2021**
		Estimated life expectancy	Expected life expectancy	Difference	Estimated life expectancy	Expected life expectancy	Difference	Estimated life expectancy	Expected life expectancy	Difference	Estimated life expectancy	Expected life expectancy	Difference	Estimated life expectancy	Expected life expectancy	Difference	
**Global**		**51·6**	**65·6**	**−14·0**	**68·1**	**72·2**	**−4·1**	**69·8**	**73·6**	**−3·7**	**73·3**	**74·8**	**−1·6**	**74·8**	**76·2**	**−1·4**	**0·67**
**Central Europe, eastern Europe, and central Asia**		**63·8**	**72·2**	**−8·4**	**73·8**	**75·5**	**−1·7**	**73·2**	**76·6**	**−3·3**	**75·7**	**78·0**	**−2·2**	**75·5**	**79·3**	**−3·8**	**0·77**
Central Asia		51·9	68·6	−16·7	71·6	73·1	−1·5	71·0	73·9	−2·9	73·6	75·4	−1·7	74·3	76·2	−1·9	0·68
	Armenia	52·2	69·4	−17·3	73·9	72·8	1·1	74·9	73·9	1·1	77·1	75·9	1·2	78·6	77·3	1·3	0·70
	Azerbaijan	39·2	67·6	−28·4	70·6	74·3	−3·7	70·6	73·9	−3·3	73·0	75·8	−2·7	73·4	77·0	−3·6	0·69
	Georgia	57·0	73·0	−16·0	73·7	75·9	−2·2	74·0	75·2	−1·3	77·9	76·4	1·4	75·8	78·1	−2·3	0·73
	Kazakhstan	61·3	69·2	−7·9	72·6	74·1	−1·5	70·5	75·6	−5·2	73·0	76·7	−3·7	73·9	77·7	−3·8	0·73
	Kyrgyzstan	51·9	69·0	−17·1	70·9	72·0	−1·1	71·4	72·8	−1·5	73·8	73·1	0·7	76·1	74·7	1·4	0·60
	Mongolia	39·9	61·6	−21·7	65·5	70·1	−4·5	67·0	72·2	−5·2	71·1	73·7	−2·6	74·6	75·0	−0·4	0·62
	Tajikistan	40·6	62·0	−21·3	68·6	70·1	−1·4	69·0	69·6	−0·7	71·7	71·0	0·7	72·1	72·4	−0·3	0·54
	Turkmenistan	48·8	68·3	−19·6	69·3	73·4	−4·2	70·0	73·4	−3·4	73·1	75·1	−2·0	71·5	76·7	−5·2	0·68
	Uzbekistan	52·1	65·3	−13·2	72·7	71·5	1·2	71·5	73·1	−1·7	73·4	74·8	−1·4	75·1	75·6	−0·5	0·66
Central Europe		58·9	70·6	−11·8	74·6	75·4	−0·8	76·4	77·1	−0·7	79·0	78·8	0·2	78·3	80·1	−1·8	0·80
	Albania	50·2	64·4	−14·3	75·7	73·3	2·4	78·4	74·0	4·4	80·4	75·9	4·5	78·7	77·3	1·4	0·71
	Bosnia and Herzegovina	47·5	60·6	−13·2	76·2	72·7	3·5	78·0	74·5	3·5	79·8	76·6	3·3	78·3	77·8	0·4	0·72
	Bulgaria	58·9	69·9	−11·0	73·5	75·4	−1·9	73·7	76·6	−2·9	75·9	78·0	−2·1	73·7	79·3	−5·5	0·77
	Croatia	52·9	70·2	−17·4	75·7	76·3	−0·6	78·1	77·3	0·8	80·0	78·9	1·1	80·3	80·3	0·0	0·80
	Czechia	68·1	73·7	−5·6	75·6	76·6	−0·9	78·4	79·3	−0·8	80·9	80·6	0·3	80·9	81·2	−0·4	0·83
	Hungary	62·4	71·5	−9·2	73·8	75·8	−1·9	76·1	77·5	−1·5	78·5	79·1	−0·6	78·0	79·9	−2·0	0·79
	Montenegro	66·4	69·6	−3·2	78·3	76·4	1·8	76·7	76·4	0·3	77·7	78·5	−0·7	76·0	80·1	−4·1	0·80
	North Macedonia	49·3	67·6	−18·4	72·6	74·5	−2·0	73·7	75·5	−1·8	75·4	77·3	−1·9	74·2	78·6	−4·4	0·75
	Poland	59·6	71·2	−11·5	75·6	75·1	0·5	78·0	77·3	0·8	80·5	79·1	1·5	79·7	80·6	−0·9	0·81
	Romania	60·9	67·1	−6·3	73·0	75·0	−1·9	74·7	76·2	−1·5	77·5	77·8	−0·3	76·8	79·3	−2·5	0·77
	Serbia	49·9	70·4	−20·5	73·0	75·2	−2·3	73·8	76·0	−2·2	76·7	78·3	−1·6	76·7	80·1	−3·4	0·79
	Slovakia	64·4	72·2	−7·8	75·6	75·9	−0·3	77·9	78·1	−0·2	79·6	79·8	−0·1	78·3	80·6	−2·3	0·81
	Slovenia	59·5	73·3	−13·8	78·0	78·0	0·1	80·0	79·6	0·4	83·0	80·9	2·0	84·0	81·7	2·3	0·84
Eastern Europe		69·5	73·1	−3·6	74·6	76·2	−1·5	72·9	77·1	−4·2	75·1	78·8	−3·7	74·9	80·4	−5·6	0·80
	Belarus	70·6	70·6	−0·1	75·8	75·0	0·8	74·7	76·2	−1·5	76·6	78·1	−1·5	76·0	79·8	−3·8	0·78
	Estonia	70·0	73·3	−3·3	75·0	76·4	−1·4	76·2	78·3	−2·1	80·8	80·3	0·5	81·2	81·7	−0·5	0·84
	Latvia	72·0	73·6	−1·6	74·7	76·6	−1·9	76·0	78·0	−2·0	78·1	80·3	−2·1	78·1	81·2	−3·1	0·83
	Lithuania	68·7	71·5	−2·8	76·1	76·3	−0·2	77·5	77·7	−0·2	78·7	80·1	−1·4	78·9	82·2	−3·3	0·86
	Moldova	56·5	69·9	−13·4	71·5	74·5	−3·0	72·5	75·0	−2·5	74·7	76·3	−1·6	76·4	78·0	−1·6	0·73
	Russia	69·5	73·3	−3·8	74·5	76·3	−1·8	72·5	77·4	−4·9	74·8	79·1	−4·3	74·3	80·6	−6·3	0·81
	Ukraine	70·8	73·0	−2·2	74·8	75·6	−0·8	73·5	76·3	−2·8	75·4	77·7	−2·3	75·7	78·9	−3·3	0·76
**High income**		**67·7**	**74·0**	**−6·3**	**79·4**	**78·6**	**0·8**	**81·2**	**79·9**	**1·3**	**83·1**	**80·8**	**2·4**	**83·3**	**82·0**	**1·3**	**0·85**
Australasia		71·9	73·6	−1·7	79·7	78·0	1·7	82·1	79·4	2·6	84·0	80·4	3·5	85·3	81·7	3·6	0·85
	Australia	72·0	73·3	−1·3	80·0	77·8	2·1	82·3	79·3	3·1	84·2	80·4	3·8	85·6	81·7	3·9	0·84
	New Zealand	71·5	74·5	−3·0	78·4	78·6	−0·2	80·8	79·8	1·1	82·8	80·6	2·2	84·1	81·9	2·2	0·85
High-income Asia Pacific		59·6	71·5	−11·9	80·9	79·3	1·7	84·1	80·8	3·3	86·2	81·7	4·5	87·8	82·7	5·1	0·88
	Brunei	49·5	65·6	−16·1	73·1	76·2	−3·0	75·2	77·7	−2·5	77·1	79·4	−2·3	78·3	80·6	−2·3	0·81
	Japan	63·5	72·8	−9·3	82·3	79·9	2·4	85·1	81·1	4·0	86·7	81·7	5·0	88·1	82·5	5·6	0·87
	Singapore	60·5	62·6	−2·1	78·2	76·7	1·5	81·7	79·3	2·4	85·0	81·2	3·7	87·7	82·0	5·6	0·86
	South Korea	46·5	61·6	−15·1	75·9	76·8	−0·9	79·7	79·8	−0·0	84·0	81·7	2·2	86·0	83·0	3·1	0·89
High-income North America		71·1	74·8	−3·7	79·1	79·1	0·0	79·7	80·1	−0·4	81·4	81·2	0·1	80·4	82·4	−1·9	0·86
	Canada	70·9	75·0	−4·1	80·6	79·6	1·0	81·8	80·8	1·1	83·6	81·7	1·8	84·1	82·7	1·4	0·87
	Greenland	52·2	73·6	−21·3	67·5	78·0	−10·5	71·1	78·3	−7·2	74·9	80·4	−5·6	76·9	81·4	−4·5	0·83
	USA	71·2	74·8	−3·7	79·0	79·1	−0·1	79·5	80·1	−0·6	81·1	81·1	0·0	80·0	82·4	−2·3	0·86
Southern Latin America		64·0	70·2	−6·3	76·3	74·0	2·3	78·4	75·5	3·0	79·6	76·6	3·1	79·9	78·5	1·4	0·74
	Argentina	66·9	70·6	−3·7	76·0	74·0	2·0	77·9	75·5	2·4	79·0	76·3	2·7	79·1	78·1	0·9	0·72
	Chile	55·2	69·0	−13·8	76·7	74·0	2·7	79·8	75·9	3·9	81·3	77·3	4·1	81·9	79·3	2·6	0·77
	Uruguay	70·2	70·4	−0·2	76·9	73·9	3·0	78·6	75·1	3·5	80·0	76·2	3·9	79·4	77·7	1·7	0·72
Western Europe		69·2	74·0	−4·8	79·5	78·5	1·1	81·5	79·8	1·8	83·6	80·8	2·8	84·2	81·9	2·3	0·85
	Andorra	77·9	74·5	3·3	82·3	78·9	3·4	83·5	79·6	4·0	84·8	81·6	3·2	85·7	82·5	3·2	0·87
	Austria	68·6	74·4	−5·8	79·0	78·6	0·3	81·3	79·9	1·4	83·2	81·1	2·2	84·1	82·0	2·0	0·85
	Belgium	68·9	73·7	−4·9	79·3	78·3	1·0	81·0	79·6	1·4	82·8	80·8	2·0	84·2	82·0	2·2	0·85
	Cyprus	61·7	69·4	−7·7	76·3	75·8	0·5	78·1	78·5	−0·4	81·3	80·6	0·7	83·2	81·4	1·8	0·84
	Denmark	71·9	75·4	−3·4	77·9	80·3	−2·3	79·3	81·6	−2·2	81·6	82·4	−0·8	83·5	83·3	0·2	0·90
	Finland	68·1	73·4	−5·4	79·4	78·8	0·6	81·5	79·9	1·6	83·7	81·1	2·6	84·9	82·2	2·7	0·86
	France	69·8	72·7	−2·9	81·1	78·0	3·1	82·7	79·4	3·3	84·6	80·4	4·1	85·5	81·6	3·9	0·84
	Germany	70·2	75·5	−5·3	78·6	80·8	−2·2	81·2	81·9	−0·7	82·8	82·8	0·0	83·4	83·6	−0·2	0·90
	Greece	70·9	71·7	−0·9	79·4	76·4	3·0	80·8	78·1	2·7	82·7	79·4	3·3	82·8	79·9	2·9	0·79
	Iceland	74·0	73·4	0·6	80·2	79·1	1·1	82·1	80·4	1·7	83·4	81·6	1·9	84·9	82·7	2·2	0·88
	Ireland	67·2	73·9	−6·6	77·6	77·7	−0·1	79·3	79·6	−0·2	82·9	81·2	1·6	84·5	82·7	1·8	0·87
	Israel	72·7	71·7	1·0	78·8	77·4	1·4	80·6	78·6	2·0	83·4	79·4	4·0	85·1	80·6	4·5	0·81
	Italy	68·9	72·2	−3·3	80·3	77·3	3·0	82·4	78·6	3·8	84·4	79·6	4·8	84·9	80·4	4·5	0·81
	Luxembourg	68·2	75·6	−7·4	78·7	79·6	−0·8	81·4	80·9	0·4	83·4	82·0	1·4	84·9	83·0	1·9	0·88
	Malta	67·4	67·9	−0·5	78·7	75·9	2·9	81·1	77·4	3·7	83·3	78·8	4·5	84·1	80·3	3·8	0·80
	Monaco	68·1	76·8	−8·7	81·0	81·7	−0·7	81·4	82·5	−1·1	81·7	83·1	−1·4	81·4	83·7	−2·3	0·91
	Netherlands	72·9	75·8	−2·9	80·1	80·1	0·0	80·7	81·2	−0·6	82·8	82·2	0·6	83·2	83·1	0·1	0·89
	Norway	73·7	75·9	−2·2	80·1	80·1	0·0	81·6	81·7	−0·2	83·4	82·8	0·6	84·9	83·9	1·0	0·92
	Portugal	60·9	68·1	−7·2	77·6	74·4	3·2	80·1	76·0	4·1	83·1	77·3	5·9	84·4	78·5	6·0	0·74
	San Marino	76·2	75·5	0·7	82·4	80·8	1·6	84·5	82·2	2·3	87·6	82·8	4·8	88·1	83·0	5·1	0·89
	Spain	64·5	69·0	−4·5	80·4	75·4	5·1	82·9	77·0	5·9	85·0	78·3	6·7	85·7	79·3	6·5	0·77
	Sweden	72·7	75·5	−2·8	80·8	79·8	1·0	82·2	81·4	0·8	83·8	82·2	1·6	85·0	83·1	1·9	0·89
	Switzerland	71·1	78·6	−7·5	81·2	82·4	−1·2	83·1	83·0	0·2	85·0	83·7	1·3	86·4	84·4	1·9	0·93
	UK	71·3	74·7	−3·4	78·4	78·5	−0·0	80·1	79·9	0·1	82·5	80·9	1·6	82·4	82·2	0·2	0·86
	England	71·8	74·7	−2·9	78·6	78·5	0·2	80·3	79·9	0·3	82·8	80·9	1·8	82·6	82·2	0·4	0·86
	Northern Ireland	68·7	73·7	−5·1	77·2	77·8	−0·6	79·6	79·4	0·2	81·8	80·4	1·4	82·3	81·6	0·7	0·84
	Scotland	68·0	74·4	−6·4	76·7	78·5	−1·7	78·6	79·9	−1·4	80·8	80·9	−0·1	80·8	82·0	−1·2	0·85
	Wales	71·1	73·9	−2·8	78·5	77·4	1·1	79·7	78·9	0·8	81·9	79·9	2·0	81·1	81·4	−0·3	0·83
**Latin America and Caribbean**		**52·4**	**59·6**	**−7·3**	**72·7**	**71·4**	**1·4**	**75·8**	**72·8**	**3·0**	**76·1**	**74·3**	**1·8**	**75·9**	**75·6**	**0·3**	**0·65**
Andean Latin America		42·5	60·6	−18·1	70·6	71·4	−0·8	74·8	72·7	2·1	77·3	74·1	3·2	74·3	75·9	−1·6	0·65
	Bolivia	38·2	57·5	−19·3	62·4	68·3	−5·9	67·4	70·8	−3·4	71·0	72·7	−1·6	68·8	74·5	−5·8	0·60
	Ecuador	51·2	62·9	−11·7	74·2	72·0	2·1	76·6	72·8	3·7	77·5	74·1	3·4	77·1	76·2	1·0	0·66
	Peru	41·2	60·6	−19·4	72·0	71·7	0·3	76·8	73·0	3·8	79·7	74·5	5·1	74·9	76·0	−1·1	0·66
Caribbean		57·0	62·9	−5·9	69·8	72·0	−2·2	72·1	73·1	−1·0	56·4	74·5	−18·1	72·5	75·5	−3·0	0·64
	Antigua and Barbuda	60·3	62·9	−2·6	77·4	74·8	2·5	76·8	76·0	0·7	78·0	77·4	0·6	77·1	78·6	−1·6	0·75
	The Bahamas	60·1	70·4	−10·3	74·5	77·0	−2·5	74·4	78·3	−3·9	76·3	79·4	−3·1	73·6	80·4	−6·8	0·81
	Barbados	56·5	67·9	−11·4	76·1	75·9	0·2	76·7	76·6	0·2	77·2	77·5	−0·3	77·6	78·5	−0·8	0·75
	Belize	56·6	60·0	−3·4	75·6	68·3	7·3	73·3	71·4	1·9	76·2	73·3	2·9	76·1	74·7	1·4	0·61
	Bermuda	66·5	68·1	−1·6	77·4	77·0	0·4	80·8	78·1	2·6	84·9	79·9	5·0	83·3	80·9	2·4	0·82
	Cuba	68·9	65·8	3·0	76·7	73·3	3·4	79·2	73·4	5·7	80·4	74·8	5·6	77·3	76·3	1·0	0·67
	Dominica	49·7	65·8	−16·1	74·7	73·3	1·4	75·4	75·6	−0·2	75·7	77·1	−1·4	73·3	78·5	−5·1	0·75
	Dominican Republic	56·3	50·2	6·1	73·4	69·0	4·4	76·9	71·2	5·7	76·9	73·6	3·4	77·3	75·0	2·3	0·62
	Grenada	58·9	56·1	2·8	72·6	69·0	3·6	76·5	72·7	3·8	75·9	74·8	1·1	72·9	76·3	−3·3	0·67
	Guyana	52·9	60·3	−7·4	66·5	69·9	−3·3	67·8	72·4	−4·5	69·6	73·9	−4·2	68·6	75·8	−7·2	0·65
	Haiti	41·4	53·1	−11·7	54·1	62·0	−7·9	56·6	65·3	−8·6	27·6	67·6	−40·1	61·5	69·4	−7·9	0·45
	Jamaica	58·6	65·0	−6·4	76·5	72·5	4·0	76·8	74·3	2·5	79·4	75·5	3·9	76·4	76·7	−0·3	0·68
	Puerto Rico	62·9	67·1	−4·2	78·2	76·0	2·1	80·2	77·4	2·8	83·2	78·9	4·3	84·5	81·1	3·4	0·83
	Saint Kitts and Nevis	60·2	63·5	−3·3	69·2	73·9	−4·6	73·5	75·6	−2·2	75·7	77·7	−2·0	75·5	78·9	−3·4	0·75
	Saint Lucia	53·6	59·6	−6·1	72·5	71·2	1·3	76·2	73·9	2·4	79·4	75·4	4·0	76·5	76·3	0·2	0·67
	Saint Vincent and the Grenadines	53·3	58·6	−5·3	71·8	70·4	1·4	73·5	72·5	1·0	75·1	74·0	1·1	75·2	75·5	−0·2	0·64
	Suriname	61·2	59·6	1·5	71·1	71·5	−0·5	73·0	72·8	0·1	75·6	74·4	1·2	74·2	75·5	−1·2	0·63
	Trinidad and Tobago	59·2	66·6	−7·4	71·9	75·1	−3·2	73·0	76·4	−3·4	76·8	78·1	−1·3	75·0	79·3	−4·2	0·77
	Virgin Islands	64·8	69·2	−4·4	75·4	75·9	−0·5	77·0	77·4	−0·5	80·6	79·9	0·7	82·3	80·9	1·3	0·82
Central Latin America		51·0	60·0	−8·9	73·5	70·8	2·7	76·7	72·5	4·2	78·5	74·0	4·5	75·7	75·6	0·1	0·64
	Colombia	56·0	59·6	−3·7	75·0	70·8	4·1	78·4	72·4	6·0	81·2	74·0	7·2	79·7	75·9	3·8	0·66
	Costa Rica	57·4	62·0	−4·6	79·3	72·5	6·7	80·5	74·0	6·5	82·3	75·4	7·0	81·2	77·3	3·9	0·70
	El Salvador	46·2	53·5	−7·3	74·4	65·8	8·5	78·5	69·2	9·2	79·7	71·5	8·1	77·2	73·4	3·8	0·56
	Guatemala	41·8	54·3	−12·4	65·4	62·3	3·1	70·3	66·1	4·2	73·6	70·1	3·5	72·7	72·4	0·4	0·54
	Honduras	40·5	53·1	−12·6	71·0	63·2	7·8	70·7	66·6	4·1	71·8	69·6	2·1	70·7	71·9	−1·2	0·51
	Mexico	49·7	60·6	−10·9	73·2	71·5	1·7	76·4	73·3	3·1	77·7	74·4	3·3	74·7	76·2	−1·5	0·66
	Nicaragua	49·5	55·0	−5·5	77·0	64·1	12·9	80·1	67·9	12·2	79·6	70·2	9·4	76·8	72·2	4·6	0·52
	Panama	63·2	63·8	−0·6	78·9	72·8	6·1	80·9	74·1	6·8	82·0	75·1	6·9	81·4	77·3	4·1	0·71
	Venezuela	57·1	62·9	−5·8	75·2	71·9	3·3	78·7	73·4	5·2	80·1	74·4	5·7	74·6	74·8	−0·2	0·60
Tropical Latin America		55·4	57·9	−2·5	73·2	71·4	1·9	76·0	72·7	3·3	78·2	74·4	3·7	77·3	75·8	1·6	0·65
	Brazil	55·4	57·9	−2·5	73·1	71·4	1·7	76·0	72·7	3·3	78·2	74·4	3·7	77·4	75·8	1·6	0·65
	Paraguay	59·8	59·6	0·2	77·2	70·4	6·7	77·9	72·4	5·5	78·2	74·0	4·2	75·9	75·8	0·1	0·64
**North Africa and Middle East**		**45·8**	**53·5**	**−7·7**	**67·2**	**69·0**	**−1·8**	**71·1**	**72·0**	**−0·9**	**73·9**	**73·9**	**−0·0**	**73·7**	**76·0**	**−2·3**	**0·66**
	Afghanistan	38·0	45·6	−7·6	52·5	51·9	0·6	54·1	52·3	1·8	59·8	57·5	2·3	60·7	63·5	−2·8	0·34
	Algeria	44·5	49·3	−4·8	71·2	69·9	1·3	74·0	72·7	1·3	76·0	74·5	1·5	75·4	76·0	−0·6	0·66
	Bahrain	52·7	56·5	−3·8	70·5	74·0	−3·5	71·3	75·6	−4·3	75·0	77·3	−2·2	75·1	78·9	−3·9	0·75
	Egypt	45·5	56·5	−11·0	63·7	68·1	−4·4	68·7	71·5	−2·9	69·3	71·2	−1·9	70·2	74·5	−4·4	0·61
	Iran	43·7	51·9	−8·2	69·5	69·6	−0·1	75·0	73·4	1·5	78·1	75·5	2·6	77·2	77·1	0·1	0·70
	Iraq	58·6	50·2	8·4	70·3	67·4	3·0	71·8	69·9	2·0	73·8	72·2	1·6	73·5	75·9	−2·4	0·66
	Jordan	52·9	48·4	4·5	71·9	72·7	−0·8	72·2	74·1	−1·9	77·2	76·0	1·2	77·6	77·8	−0·3	0·73
	Kuwait	67·2	62·6	4·6	77·3	76·4	0·9	80·2	77·7	2·5	82·8	79·8	3·1	85·1	81·7	3·3	0·85
	Lebanon	55·8	59·3	−3·5	73·1	72·4	0·7	76·9	73·9	3·0	80·0	76·2	3·9	78·4	78·3	0·1	0·74
	Libya	43·7	50·2	−6·5	74·5	72·5	2·0	76·2	75·5	0·7	74·9	77·7	−2·8	73·4	78·1	−4·8	0·73
	Morocco	43·7	45·1	−1·4	68·3	65·0	3·3	71·3	67·9	3·4	73·1	70·4	2·7	73·9	73·3	0·6	0·56
	Oman	42·9	48·4	−5·6	72·3	68·6	3·8	75·7	74·7	1·0	77·3	77·4	−0·1	76·3	79·3	−3·0	0·77
	Palestine	46·2	49·3	−3·1	71·7	67·1	4·5	73·2	69·9	3·3	74·9	72·2	2·7	76·2	75·2	1·0	0·63
	Qatar	62·5	58·6	3·9	72·7	75·8	−3·1	73·7	77·5	−3·9	75·6	79·6	−4·0	79·2	81·7	−2·5	0·85
	Saudi Arabia	53·3	54·6	−1·3	69·4	72·7	−3·3	71·6	75·6	−4·1	73·5	78·3	−4·8	75·1	80·8	−5·7	0·82
	Sudan	47·1	48·4	−1·3	59·2	60·6	−1·4	64·1	64·1	−0·0	68·8	68·8	−0·0	70·1	72·7	−2·6	0·54
	Syria	54·6	51·1	3·5	70·7	68·6	2·1	72·8	71·5	1·2	75·6	74·3	1·3	74·7	75·1	−0·4	0·62
	Tunisia	44·0	50·2	−6·2	74·4	70·2	4·1	76·9	73·3	3·6	78·9	75·1	3·8	77·1	76·6	0·5	0·68
	Türkiye	50·0	57·2	−7·2	71·3	69·9	1·5	77·6	72·5	5·1	79·6	74·8	4·7	78·3	77·4	0·9	0·71
	United Arab Emirates	57·4	53·9	3·5	70·9	75·6	−4·7	72·5	78·9	−6·4	71·3	81·2	−10·0	71·5	81·9	−10·3	0·85
	Yemen	32·0	44·1	−12·1	60·5	55·4	5·1	64·7	61·3	3·4	69·4	66·9	2·5	68·5	69·4	−1·0	0·45
**South Asia**		**39·6**	**52·7**	**−13·1**	**61·5**	**62·6**	**−1·0**	**65·4**	**66·4**	**−1·0**	**69·4**	**69·6**	**−0·3**	**70·8**	**73·3**	**−2·5**	**0·56**
	Bangladesh	43·3	46·5	−3·3	60·2	56·5	3·7	67·1	61·3	5·8	71·1	65·6	5·6	74·1	71·2	2·9	0·49
	Bhutan	38·0	40·9	−2·9	60·2	55·4	4·8	65·6	61·3	4·3	72·5	67·1	5·4	74·9	70·4	4·5	0·47
	India	38·6	53·5	−14·9	61·7	63·2	−1·5	65·6	66·9	−1·3	69·6	70·1	−0·4	71·2	73·9	−2·6	0·58
	Nepal	40·8	45·6	−4·8	58·4	54·3	4·1	66·3	59·6	6·6	70·6	64·7	5·9	70·8	68·8	2·0	0·43
	Pakistan	46·1	50·6	−4·5	62·9	62·0	0·9	62·9	65·8	−2·9	65·7	68·8	−3·0	66·4	71·5	−5·1	0·50
Southeast Asia, east Asia, and Oceania		49·6	54·6	−5·1	69·4	70·2	−0·9	72·3	73·0	−0·7	76·2	75·2	1·0	78·6	77·0	1·6	0·70
	East Asia	50·6	53·9	−3·3	70·1	70·2	−0·2	73·3	73·3	−0·0	77·8	75·8	2·0	80·7	77·8	2·9	0·73
	China	50·7	53·1	−2·4	69·9	69·9	0·1	73·4	72·8	0·6	77·8	75·5	2·3	80·7	77·7	3·0	0·72
	North Korea	41·2	62·9	−21·7	72·4	71·2	1·2	64·8	71·2	−6·3	73·4	72·5	0·9	76·2	73·6	2·6	0·57
	Taiwan (province of China)	58·4	61·0	−2·6	77·3	76·3	1·0	79·8	78·8	1·0	83·0	81·1	1·9	84·6	82·7	1·9	0·87
Oceania		49·2	55·8	−6·6	64·5	66·6	−2·1	65·7	68·3	−2·7	66·6	69·0	−2·4	66·6	70·1	−3·4	0·47
	American Samoa	63·2	70·8	−7·6	73·8	74·8	−1·1	73·0	75·5	−2·4	72·6	76·2	−3·5	72·8	77·3	−4·4	0·72
	Cook Islands	46·7	63·5	−16·9	71·4	73·4	−2·0	75·6	75·4	0·3	78·8	77·4	1·4	79·6	79·6	0·0	0·78
	Federated States of Micronesia	45·1	56·8	−11·7	65·6	69·9	−4·3	66·8	71·7	−4·9	68·6	73·0	−4·4	69·7	74·1	−4·5	0·59
	Fiji	59·2	61·3	−2·1	69·1	72·5	−3·4	68·2	74·1	−6·0	69·2	75·0	−5·8	68·8	76·3	−7·5	0·68
	Guam	70·1	73·4	−3·3	75·8	76·6	−0·8	78·6	77·8	0·7	82·9	78·9	4·0	82·9	80·3	2·6	0·80
	Kiribati	48·0	59·3	−11·2	61·5	67·6	−6·1	63·5	69·2	−5·7	65·1	70·6	−5·5	67·0	72·2	−5·2	0·53
	Marshall Islands	53·6	56·5	−2·9	66·3	68·6	−2·3	63·9	70·2	−6·4	64·6	71·9	−7·3	66·8	73·6	−6·8	0·57
	Nauru	54·5	66·9	−12·4	64·3	72·7	−8·4	61·5	72·0	−10·6	62·0	72·7	−10·6	65·7	75·1	−9·4	0·63
	Niue	54·5	63·5	−9·0	71·9	74·0	−2·1	71·6	75·2	−3·6	72·7	76·7	−4·0	69·2	77·8	−8·6	0·73
	Northern Mariana Islands	65·4	69·2	−3·8	73·2	77·5	−4·4	75·3	78·8	−3·5	76·2	78·8	−2·5	75·0	79·6	−4·6	0·77
	Palau	50·8	68·1	−17·3	68·6	76·2	−7·5	69·7	77·3	−7·5	69·5	77·8	−8·4	70·5	78·8	−8·3	0·75
	Papua New Guinea	45·9	49·3	−3·5	62·8	62·0	0·8	64·4	64·7	−0·3	65·5	66·1	−0·6	65·5	68·1	−2·6	0·42
	Samoa	58·0	60·3	−2·3	71·1	70·8	0·3	71·7	71·9	−0·2	72·0	73·1	−1·1	71·9	74·1	−2·2	0·59
	Solomon Islands	48·6	51·9	−3·3	64·1	61·3	2·8	65·8	64·7	1·1	66·9	66·1	0·8	68·4	68·6	−0·2	0·43
	Tokelau	58·2	61·0	−2·8	68·6	72·0	−3·4	70·3	73·6	−3·3	72·2	75·2	−3·0	67·8	76·7	−8·9	0·69
	Tonga	62·9	58·9	3·9	73·1	71·0	2·1	73·9	72·8	1·1	74·6	73·9	0·8	75·7	75·2	0·5	0·63
	Tuvalu	49·2	58·6	−9·4	62·5	66·9	−4·4	63·5	70·2	−6·7	69·0	72·0	−3·1	70·6	73·7	−3·1	0·58
	Vanuatu	49·9	53·9	−4·0	67·2	64·4	2·8	68·1	66·6	1·5	69·3	68·6	0·7	69·4	70·2	−0·8	0·47
Southeast Asia		47·2	56·1	−8·9	67·9	70·1	−2·1	70·5	72·5	−2·0	73·3	74·0	−0·7	74·3	75·8	−1·5	0·65
	Cambodia	45·4	53·5	−8·1	59·6	60·6	−1·0	62·4	63·5	−1·1	69·2	67·6	1·5	71·0	70·4	0·5	0·47
	Indonesia	44·4	53·9	−9·4	65·4	69·6	−4·3	68·3	72·5	−4·2	70·8	74·0	−3·2	72·0	76·0	−4·0	0·66
	Laos	41·0	48·9	−7·9	54·6	58·9	−4·4	60·0	62·9	−2·9	67·0	67·9	−0·8	70·4	71·0	−0·6	0·49
	Malaysia	57·5	55·4	2·1	74·5	72·8	1·7	75·6	75·2	0·4	76·4	76·8	−0·5	75·7	78·3	−2·6	0·74
	Maldives	36·4	53·9	−17·5	65·4	63·2	2·2	72·8	70·6	2·2	79·3	73·9	5·4	81·2	76·0	5·2	0·65
	Mauritius	52·6	61·0	−8·4	74·1	72·8	1·2	75·5	74·5	0·9	77·8	76·0	1·8	76·9	77·7	−0·8	0·72
	Myanmar	35·8	49·3	−13·6	58·1	62·6	−4·5	61·4	65·6	−4·2	67·6	69·9	−2·2	71·2	72·4	−1·2	0·53
	Philippines	58·8	63·5	−4·7	71·8	71·7	0·1	73·8	72·8	1·0	74·0	73·6	0·4	72·2	75·9	−3·7	0·65
	Seychelles	62·9	65·6	−2·6	75·5	73·7	1·8	76·6	75·8	0·9	77·0	76·6	0·5	76·5	78·0	−1·5	0·73
	Sri Lanka	54·1	63·2	−9·1	74·1	72·0	2·1	76·5	73·9	2·6	78·2	75·4	2·9	79·7	77·1	2·6	0·70
	Thailand	53·9	56·8	−3·0	74·6	71·5	3·1	75·1	73·9	1·3	79·1	75·1	4·0	80·3	76·6	3·7	0·68
	Timor-Leste	42·7	46·1	−3·4	59·7	58·6	1·1	65·8	63·8	2·0	70·3	66·9	3·4	70·5	69·4	1·1	0·44
	Viet Nam	50·3	55·0	−4·7	73·2	67·4	5·8	76·4	71·0	5·4	77·4	73·3	4·1	78·3	75·0	3·4	0·63
**Sub-Saharan Africa**		**43·9**	**50·6**	**−6·7**	**55·6**	**61·0**	**−5·4**	**54·5**	**63·2**	**−8·7**	**60·5**	**66·4**	**−5·8**	**64·1**	**69·9**	**−5·8**	**0·46**
Central sub-Saharan Africa		44·0	50·2	−6·2	55·0	61·3	−6·3	54·6	62·6	−8·0	59·8	66·6	−6·8	63·8	70·8	−7·0	0·47
	Angola	45·3	48·4	−3·1	52·2	59·3	−7·1	55·0	62·0	−6·9	62·3	66·4	−4·1	63·7	70·6	−6·9	0·45
	Central African Republic	45·3	46·1	−0·7	50·3	55·4	−5·0	45·0	57·5	−12·5	50·4	60·0	−9·6	55·2	62·0	−6·7	0·31
	Congo (Brazzaville)	39·3	51·5	−12·2	56·9	68·1	−11·2	53·4	69·9	−16·5	60·3	71·5	−11·3	63·1	74·0	−10·9	0·58
	Democratic Republic of the Congo	44·2	49·8	−5·6	56·0	60·6	−4·6	55·3	58·9	−3·6	59·7	60·3	−0·6	64·5	66·6	−2·1	0·38
	Equatorial Guinea	32·8	46·1	−13·3	54·5	59·3	−4·8	58·6	67·6	−9·1	62·1	73·3	−11·2	63·7	76·2	−12·5	0·66
	Gabon	36·1	51·1	−15·0	64·3	69·6	−5·3	61·0	71·7	−10·7	64·7	73·1	−8·5	67·3	75·5	−8·2	0·63
Eastern sub-Saharan Africa		40·8	47·0	−6·2	53·1	56·8	−3·7	53·3	58·9	−5·7	61·7	63·2	−1·6	64·5	67·6	−3·1	0·41
	Burundi	39·5	45·6	−6·1	51·2	54·6	−3·5	48·1	55·4	−7·3	61·1	57·2	3·9	64·9	60·6	4·3	0·29
	Comoros	45·7	47·5	−1·8	59·6	60·0	−0·3	62·2	64·7	−2·5	66·7	67·9	−1·2	68·2	70·4	−2·3	0·48
	Djibouti	60·4	51·5	8·9	63·7	63·8	−0·2	62·6	65·8	−3·3	64·7	68·3	−3·6	67·0	71·2	−4·2	0·49
	Eritrea	41·4	42·5	−1·1	52·3	55·4	−3·1	58·8	62·0	−3·2	62·8	64·4	−1·6	64·8	67·4	−2·6	0·40
	Ethiopia	36·2	40·9	−4·7	49·0	50·2	−1·2	52·9	52·3	0·6	64·9	58·6	6·3	67·5	65·0	2·5	0·36
	Kenya	48·4	47·5	0·9	63·5	63·5	−0·1	56·0	66·4	−10·3	62·7	68·8	−6·0	67·2	72·2	−5·0	0·52
	Madagascar	40·4	48·4	−8·0	57·4	60·0	−2·6	60·0	60·3	−0·3	62·8	62·3	0·5	63·9	67·1	−3·2	0·40
	Malawi	38·8	48·9	−10·1	50·4	54·6	−4·2	46·3	56·5	−10·2	58·5	60·6	−2·1	62·1	66·1	−4·0	0·38
	Mozambique	42·1	44·6	−2·5	53·2	51·9	1·3	54·7	54·3	0·5	56·0	57·5	−1·6	59·9	62·9	−3·0	0·33
	Rwanda	32·1	48·0	−15·9	51·8	59·6	−7·8	52·0	60·0	−7·9	65·9	64·4	1·5	67·5	68·8	−1·3	0·44
	Somalia	45·0	41·4	3·6	50·9	40·3	10·6	53·2	40·9	12·3	53·6	42·0	11·6	56·9	43·0	13·9	0·08
	South Sudan	50·3	48·4	1·9	54·8	54·6	0·1	57·3	56·5	0·9	60·1	59·3	0·8	58·1	60·0	−1·9	0·28
	Tanzania	41·4	45·6	−4·2	56·7	58·6	−1·9	54·3	60·6	−6·4	62·2	64·7	−2·5	65·9	69·4	−3·6	0·45
	Uganda	41·5	45·1	−3·6	50·8	53·1	−2·3	51·5	56·8	−5·3	62·0	63·2	−1·2	64·9	68·3	−3·5	0·42
	Zambia	46·1	48·9	−2·7	52·5	61·6	−9·2	46·0	62·6	−16·6	59·1	66·9	−7·7	61·4	71·7	−10·3	0·51
Southern sub-Saharan Africa		52·5	61·0	−8·4	67·4	71·5	−4·1	56·3	73·3	−16·9	57·8	74·4	−16·6	63·0	75·6	−12·7	0·64
	Botswana	52·6	48·9	3·8	65·0	67·9	−2·9	50·1	71·9	−21·7	59·7	74·0	−14·3	62·9	75·6	−12·8	0·64
	Eswatini	43·2	49·3	−6·2	65·1	67·1	−2·0	50·4	70·2	−19·9	49·7	72·2	−22·5	56·1	74·0	−17·9	0·59
	Lesotho	52·9	50·6	2·2	65·9	63·8	2·1	51·2	67·1	−16·0	51·9	69·4	−17·5	52·1	71·7	−19·6	0·51
	Namibia	53·4	55·4	−2·0	65·6	69·4	−3·8	56·1	71·5	−15·5	63·6	73·1	−9·5	64·0	75·0	−10·9	0·62
	South Africa	52·4	62·9	−10·5	68·4	72·7	−4·2	59·2	74·3	−15·0	59·0	75·4	−16·3	64·8	76·6	−11·8	0·68
	Zimbabwe	54·8	52·7	2·1	63·8	67·1	−3·3	47·8	69·2	−21·4	53·5	67·9	−14·4	58·0	70·4	−12·5	0·47
Western sub-Saharan Africa		44·4	49·3	−4·9	55·7	59·6	−3·9	55·8	62·0	−6·2	60·9	65·6	−4·6	64·5	69·2	−4·7	0·45
	Benin	41·5	46·1	−4·6	57·8	55·8	2·1	59·9	58·2	1·6	63·9	61·6	2·3	65·9	65·8	0·1	0·37
	Burkina Faso	38·1	40·9	−2·8	52·4	48·4	4·0	53·8	51·9	1·9	59·8	55·8	4·1	63·0	60·3	2·7	0·29
	Cabo Verde	50·3	48·9	1·4	72·4	59·6	12·8	73·9	65·0	9·0	77·6	69·6	7·9	77·8	72·5	5·2	0·53
	Cameroon	44·2	48·9	−4·7	59·8	61·3	−1·5	55·8	64·4	−8·6	59·3	66·9	−7·6	63·6	70·6	−7·1	0·48
	Chad	43·4	40·9	2·5	54·5	47·0	7·5	53·8	49·3	4·4	58·0	53·1	4·9	60·5	56·8	3·7	0·24
	Côte d'Ivoire	47·5	47·0	0·4	58·4	60·0	−1·5	53·7	63·5	−9·8	59·4	65·0	−5·6	65·8	68·3	−2·5	0·43
	The Gambia	54·8	47·5	7·3	61·9	57·2	4·7	62·7	61·0	1·8	64·8	64·4	0·4	65·9	67·6	−1·8	0·41
	Ghana	48·7	57·2	−8·5	60·5	65·6	−5·0	60·7	68·3	−7·6	63·9	70·4	−6·5	67·4	73·3	−5·8	0·56
	Guinea	41·5	41·4	0·0	51·9	52·7	−0·9	54·8	55·4	−0·6	59·1	58·6	0·5	62·2	63·8	−1·6	0·34
	Guinea-Bissau	32·1	42·0	−9·9	52·2	54·6	−2·5	54·2	57·9	−3·7	58·8	60·6	−1·8	61·3	64·7	−3·4	0·35
	Liberia	34·8	48·9	−14·0	50·7	56·8	−6·2	55·0	56·5	−1·4	61·2	60·0	1·2	64·1	64·7	−0·6	0·35
	Mali	37·3	41·4	−4·1	50·1	48·0	2·2	53·1	51·1	2·1	59·0	54·6	4·3	61·1	59·6	1·5	0·27
	Mauritania	49·5	52·3	−2·8	60·5	63·5	−3·0	64·2	66·4	−2·2	68·3	68·1	0·2	70·1	71·2	−1·1	0·50
	Niger	42·2	40·3	1·8	48·1	44·1	4·0	52·3	46·1	6·2	61·3	49·3	12·0	63·5	53·5	10·0	0·17
	Nigeria	45·7	50·2	−4·5	55·9	61·6	−5·7	55·9	63·5	−7·7	61·2	67·6	−6·4	65·0	71·4	−6·4	0·50
	São Tomé and Príncipe	35·0	52·3	−17·3	64·7	62·0	2·7	65·0	63·2	1·8	70·0	67·1	2·8	72·2	71·5	0·7	0·51
	Senegal	46·5	46·1	0·4	60·4	56·8	3·5	61·9	60·3	1·6	67·1	63·2	3·9	68·2	67·6	0·6	0·41
	Sierra Leone	40·3	47·0	−6·7	53·1	55·0	−1·9	52·7	55·8	−3·0	56·6	59·6	−3·0	62·1	65·0	−2·8	0·36
	Togo	44·8	45·6	−0·8	59·4	59·3	0·1	58·4	61·6	−3·2	61·4	63·5	−2·1	66·0	67·6	−1·6	0·41

SDI=Socio-demographic Index. GBD=Global Burden of Diseases, Injuries, and Risk Factors Study.

**Table 4:** Male life expectancy (estimated, expected based on SDI, and their difference) for 1950, 1990, 2000, 2010, and 2021, and SDI in 2021, globally and for GBD super-regions, regions, countries, and territories

		**1950**			**1990**			**2000**			**2010**			**2021**			**SDI, 2021**
		Estimated life expectancy	Expected life expectancy	Difference	Estimated life expectancy	Expected life expectancy	Difference	Estimated life expectancy	Expected life expectancy	Difference	Estimated life expectancy	Expected life expectancy	Difference	Estimated life expectancy	Expected life expectancy	Difference	
**Global**		**46·7**	**61·4**	**−14·6**	**63·0**	**66·9**	−**3·9**	**64·8**	**67·9**	**−3·1**	**68·0**	**68·8**	**−0·8**	**69·0**	**69·9**	**−0·9**	**0·67**
**Central Europe, eastern Europe, and central Asia**		**57·3**	**66·9**	**−9·6**	**64·8**	**69·3**	−**4·5**	**62·9**	**70·3**	**−7·4**	**66·2**	**71·8**	**−5·6**	**67·4**	**73·4**	**−5·9**	**0·77**
Central Asia		45·9	64·0	−18·1	64·0	67·6	−3·6	63·2	68·1	−4·9	66·3	69·2	−2·9	67·4	69·9	−2·4	0·68
	Armenia	46·5	64·7	−18·3	67·3	67·4	−0·1	69·3	68·1	1·2	70·5	69·7	0·8	71·3	71·0	0·4	0·70
	Azerbaijan	35·1	63·2	−28·1	62·7	68·4	−5·7	64·1	68·1	−4·0	67·2	69·5	−2·3	67·0	70·7	−3·6	0·69
	Georgia	48·3	67·5	−19·2	65·2	69·7	−4·4	65·5	69·1	−3·6	67·7	70·1	−2·5	67·3	72·0	−4·7	0·73
	Kazakhstan	52·5	64·6	−12·0	63·2	68·3	−5·1	59·4	69·4	−10·0	63·1	70·4	−7·3	65·3	71·5	−6·1	0·73
	Kyrgyzstan	44·6	64·4	−19·8	62·5	66·8	−4·3	62·6	67·4	−4·8	65·2	67·6	−2·4	68·4	68·7	−0·3	0·60
	Mongolia	36·8	57·7	−20·9	59·8	65·2	−5·5	60·6	66·9	−6·3	62·6	68·0	−5·4	65·7	68·9	−3·2	0·62
	Tajikistan	39·3	58·0	−18·7	63·7	65·2	−1·6	64·7	64·9	−0·2	67·9	66·0	1·9	66·9	67·0	−0·1	0·54
	Turkmenistan	44·3	63·8	−19·6	62·6	67·8	−5·2	62·3	67·8	−5·5	65·6	69·0	−3·4	64·3	70·4	−6·1	0·68
	Uzbekistan	47·3	61·1	−13·8	66·1	66·4	−0·3	65·7	67·6	−1·9	68·1	68·8	−0·7	69·9	69·4	0·5	0·66
Central Europe		54·6	65·7	−11·1	66·9	69·2	−2·3	69·1	70·8	−1·7	71·7	72·7	−1·0	71·3	74·4	−3·2	0·80
	Albania	49·5	60·3	−10·8	69·8	67·7	2·1	71·9	68·2	3·7	75·7	69·7	6·1	73·6	71·0	2·6	0·71
	Bosnia and Herzegovina	45·6	56·7	−11·2	70·6	67·3	3·3	72·7	68·6	4·1	74·3	70·3	4·0	72·6	71·6	0·9	0·72
	Bulgaria	55·3	65·1	−9·8	66·6	69·2	−2·6	66·6	70·3	−3·7	68·7	71·8	−3·1	66·4	73·4	−6·9	0·77
	Croatia	48·9	65·4	−16·5	68·1	70·0	−1·9	70·9	71·0	−0·1	73·7	73·0	0·7	74·1	74·7	−0·5	0·80
	Czechia	63·9	68·0	−4·1	67·6	70·3	−2·7	71·7	73·4	−1·7	74·6	75·1	−0·5	74·4	75·9	−1·5	0·83
	Hungary	57·7	66·4	−8·7	65·2	69·5	−4·4	67·5	71·3	−3·8	70·8	73·2	−2·4	70·9	74·2	−3·3	0·79
	Montenegro	64·7	64·9	−0·2	71·5	70·1	1·4	71·0	70·1	0·8	72·6	72·4	0·2	69·8	74·4	−4·7	0·80
	North Macedonia	50·4	63·2	−12·8	68·3	68·6	−0·3	69·3	69·3	−0·1	71·3	71·0	0·3	69·2	72·6	−3·4	0·75
	Poland	53·1	66·1	−13·0	66·6	69·0	−2·4	69·7	71·0	−1·3	72·1	73·2	−1·0	71·8	75·1	−3·2	0·81
	Romania	57·8	62·8	−5·0	66·6	68·9	−2·3	67·7	69·9	−2·2	70·0	71·6	−1·6	69·2	73·4	−4·1	0·77
	Serbia	46·3	65·6	−19·3	67·3	69·1	−1·8	68·6	69·8	−1·2	71·7	72·2	−0·4	71·7	74·4	−2·8	0·79
	Slovakia	60·7	66·9	−6·2	66·7	69·7	−3·0	69·4	72·0	−2·6	71·9	74·0	−2·1	71·3	75·1	−3·8	0·81
	Slovenia	53·0	67·7	−14·7	70·1	71·8	−1·7	72·4	73·8	−1·4	76·3	75·5	0·7	77·6	76·5	1·1	0·84
Eastern Europe		61·7	67·6	−5·9	64·5	69·9	−5·4	60·4	70·8	−10·4	63·7	72·7	−9·0	65·8	74·9	−9·0	0·80
	Belarus	63·8	65·7	−1·9	66·3	68·9	−2·6	63·3	69·9	−6·6	64·6	72·0	−7·3	66·0	74·0	−8·0	0·78
	Estonia	62·1	67·7	−5·6	64·7	70·1	−5·4	65·6	72·2	−6·6	71·0	74·7	−3·7	72·4	76·5	−4·2	0·84
	Latvia	64·6	67·9	−3·3	64·4	70·3	−5·9	65·0	71·8	−6·8	68·0	74·7	−6·6	68·3	75·9	−7·6	0·83
	Lithuania	62·2	66·4	−4·2	66·2	70·0	−3·8	66·7	71·5	−4·8	67·5	74·4	−7·0	69·2	77·2	−8·0	0·86
	Moldova	49·2	65·1	−15·9	64·6	68·6	−4·0	65·0	68·9	−3·9	65·6	70·0	−4·4	67·9	71·8	−3·9	0·73
	Russia	60·9	67·7	−6·8	64·0	70·0	−6·0	59·3	71·1	−11·8	62·9	73·2	−10·3	65·5	75·1	−9·6	0·81
	Ukraine	64·7	67·5	−2·8	65·7	69·4	−3·8	62·3	70·0	−7·7	65·7	71·5	−5·7	66·3	73·0	−6·7	0·76
**High income**		**61·9**	**68·2**	**−6·3**	**72·7**	**72·6**	**0·2**	**75·2**	**74·2**	**1·0**	**77·7**	**75·3**	**2·4**	**77·9**	**77·0**	**0·9**	**0·85**
Australasia		67·0	67·9	−0·9	73·6	71·8	1·8	76·8	73·6	3·2	79·6	74·9	4·7	81·2	76·5	4·6	0·85
	Australia	66·9	67·7	−0·8	73·8	71·6	2·1	77·0	73·4	3·6	79·7	74·9	4·8	81·2	76·5	4·7	0·84
	New Zealand	68·6	67·6	1·0	72·6	72·6	−0·1	74·0	76·0	−2·0	75·1	79·0	−3·9	76·8	80·7	−4·0	0·85
High–income Asia Pacific		66·4	51·8	14·7	73·4	74·4	−1·0	75·3	76·9	−1·6	76·5	79·4	−2·8	77·8	81·8	−4·1	0·88
	Brunei	61·4	48·6	12·7	69·9	69·6	0·3	71·5	72·8	−1·3	73·6	74·6	−1·0	75·1	74·9	0·2	0·81
	Japan	67·4	59·9	7·5	74·2	76·2	−2·0	75·7	78·0	−2·3	76·5	79·9	−3·4	77·6	82·2	−4·6	0·87
	Singapore	58·6	53·8	4·8	70·4	73·0	−2·6	73·4	76·8	−3·4	75·9	80·3	−4·4	77·0	83·6	−6·7	0·86
	South Korea	57·7	30·1	27·5	70·5	68·0	2·5	74·0	72·6	1·4	76·5	77·2	−0·7	78·1	80·3	−2·1	0·89
High–income North America		68·8	65·5	3·3	73·2	72·3	0·9	74·4	74·4	0·0	75·9	76·6	−0·7	77·4	74·8	2·6	0·86
	Canada	68·9	66·6	2·3	73·8	74·1	−0·3	75·3	76·6	−1·3	76·5	79·2	−2·6	77·8	79·5	−1·8	0·87
	Greenland	67·9	46·9	21·0	71·8	62·4	9·4	72·2	66·5	5·7	74·9	69·5	5·3	76·1	71·4	4·7	0·83
	USA	68·8	65·5	3·3	73·2	72·1	1·1	74·4	74·2	0·3	75·7	76·3	−0·6	77·4	74·3	3·1	0·86
Southern Latin America		65·4	58·8	6·6	68·2	69·3	−1·1	69·3	71·4	−2·1	70·3	73·5	−3·2	72·4	73·8	−1·4	0·74
	Argentina	65·7	61·5	4·2	68·2	68·9	−0·7	69·3	70·5	−1·2	70·0	72·6	−2·6	72·0	73·0	−1·0	0·72
	Chile	64·4	50·6	13·8	68·2	70·3	−2·1	69·7	74·1	−4·4	71·0	75·9	−5·0	73·4	76·1	−2·8	0·77
	Uruguay	65·6	63·8	1·8	68·1	69·4	−1·3	69·0	70·9	−1·9	69·9	72·8	−2·9	71·5	72·0	−0·6	0·72
Western Europe		68·2	64·5	3·7	72·4	73·0	−0·6	74·0	75·6	−1·6	75·3	78·5	−3·2	76·8	79·4	−2·6	0·85
	Andorra	68·6	71·2	−2·6	73·0	75·8	−2·8	73·8	77·2	−3·4	76·3	79·2	−2·8	77·6	80·7	−3·1	0·87
	Austria	68·5	63·6	4·9	72·6	72·4	0·2	74·2	75·3	−1·1	75·7	77·9	−2·2	77·0	79·2	−2·3	0·85
	Belgium	68·0	63·3	4·7	72·2	72·7	−0·5	73·8	74·7	−1·0	75·3	77·5	−2·2	77·0	79·3	−2·3	0·85
	Cyprus	64·7	56·1	8·7	69·5	72·6	−3·1	72·4	74·1	−1·8	75·1	77·2	−2·1	76·1	79·2	−3·1	0·84
	Denmark	69·2	69·5	−0·2	74·7	72·3	2·3	76·3	74·7	1·7	77·4	77·4	0·0	78·5	79·5	−1·0	0·90
	Finland	67·8	60·7	7·1	72·7	71·2	1·6	74·2	74·4	−0·2	75·7	77·1	−1·4	77·2	79·5	−2·3	0·86
	France	67·3	64·5	2·8	71·8	73·0	−1·2	73·6	75·3	−1·7	74·9	78·1	−3·2	76·3	79·6	−3·2	0·84
	Germany	69·3	64·4	5·0	75·3	72·1	3·2	76·8	75·3	1·5	77·9	77·9	0·0	78·9	78·5	0·4	0·90
	Greece	66·5	67·8	−1·2	70·1	74·7	−4·6	72·0	75·9	−3·9	73·6	77·8	−4·2	74·2	77·2	−3·0	0·79
	Iceland	67·8	69·0	−1·2	73·2	75·9	−2·7	74·9	78·3	−3·4	76·3	80·0	−3·7	77·8	82·3	−4·6	0·88
	Ireland	68·1	65·0	3·1	71·5	72·2	−0·8	73·8	74·0	−0·2	75·9	78·6	−2·6	77·8	80·8	−3·0	0·87
	Israel	66·5	72·2	−5·7	71·1	75·5	−4·3	72·6	76·8	−4·2	73·6	80·1	−6·5	75·1	81·2	−6·1	0·81
	Italy	66·9	65·2	1·7	71·0	73·7	−2·7	72·6	76·5	−3·9	73·8	79·3	−5·5	74·9	80·3	−5·4	0·81
	Luxembourg	69·4	63·5	6·0	73·8	71·6	2·2	75·5	75·0	0·5	77·0	78·5	−1·5	78·1	80·4	−2·2	0·88
	Malta	63·4	64·6	−1·2	69·7	74·1	−4·4	71·1	76·3	−5·1	72·7	79·0	−6·2	74·7	81·3	−6·6	0·80
	Monaco	70·5	64·0	6·5	76·5	74·7	1·8	77·6	75·9	1·7	78·3	77·1	1·2	79·1	76·3	2·8	0·91
	Netherlands	69·5	70·6	−1·1	74·4	73·8	0·6	75·9	75·5	0·4	77·2	78·8	−1·6	78·3	79·8	−1·5	0·89
	Norway	69·7	70·6	−1·0	74·4	73·7	0·8	76·5	76·0	0·5	77·9	79·0	−1·0	79·3	81·7	−2·4	0·92
	Portugal	63·6	55·9	7·7	68·5	70·6	−2·1	69·8	73·3	−3·5	71·0	77·0	−6·0	72·4	78·5	−6·1	0·74
	San Marino	69·3	69·4	−0·1	75·3	76·6	−1·3	77·2	78·4	−1·3	77·9	80·5	−2·6	78·1	84·4	−6·2	0·89
	Spain	64·4	59·6	4·8	69·2	73·3	−4·1	70·7	75·9	−5·2	72·2	78·9	−6·8	73·4	79·9	−6·6	0·77
	Sweden	69·3	70·3	−1·0	74·0	75·0	−1·0	76·1	77·5	−1·4	77·2	79·8	−2·7	78·3	82·0	−3·6	0·89
	Switzerland	72·6	66·7	5·9	77·4	74·3	3·0	78·1	77·3	0·8	79·1	80·5	−1·4	80·0	82·5	−2·5	0·93
	UK	68·7	66·9	1·8	72·4	72·9	−0·5	74·2	75·4	−1·2	75·5	78·5	−3·0	77·2	78·2	−1·0	0·86
	England	68·7	67·1	1·6	72·4	73·1	−0·8	74·2	75·7	−1·5	75·5	78·9	−3·4	77·2	78·4	−1·2	0·86
	Northern Ireland	68·0	66·3	1·7	71·6	71·4	0·2	73·6	74·8	−1·3	74·9	77·5	−2·6	76·3	78·3	−1·9	0·84
	Scotland	68·5	65·7	2·8	72·4	71·1	1·3	74·2	73·3	0·9	75·5	76·3	−0·8	77·0	76·3	0·7	0·85
	Wales	68·1	66·3	1·8	71·1	72·9	−1·8	73·0	75·0	−2·0	74·2	77·9	−3·6	76·1	78·7	−2·6	0·83
**Latin America and Caribbean**		**55·7**	**47·9**	**7·8**	**66·3**	**66·7**	−**0·4**	**67·4**	**69·7**	−**2·3**	**68·4**	**70·7**	−**2·3**	**69·4**	**68·9**	**0·5**	**0·65**
Andean Latin America		56·7	40·4	16·3	66·3	66·6	−0·3	67·3	71·1	−3·9	68·3	73·9	−5·6	69·7	68·3	1·3	0·65
	Bolivia	53·7	36·3	17·4	63·8	60·4	3·5	65·9	65·4	0·5	67·3	69·4	−2·2	68·6	63·8	4·8	0·60
	Ecuador	58·9	49·9	9·0	66·8	69·8	−3·0	67·4	71·4	−4·0	68·3	71·9	−3·6	69·9	71·0	−1·1	0·66
	Peru	56·7	39·1	17·6	66·5	67·3	−0·7	67·5	73·1	−5·6	68·6	76·7	−8·1	69·8	68·8	1·0	0·66
Caribbean		58·9	52·8	6·1	66·8	66·0	0·8	67·6	68·2	−0·7	68·6	59·1	9·5	69·3	66·9	2·5	0·64
	Antigua and Barbuda	58·9	54·8	4·1	68·8	70·5	−1·7	69·8	72·1	−2·3	71·1	73·3	−2·2	72·6	73·0	−0·4	0·75
	The Bahamas	65·6	54·8	10·8	70·7	67·7	3·0	72·2	67·7	4·4	73·6	69·5	4·0	74·9	66·1	8·8	0·81
	Barbados	63·4	51·0	12·4	69·7	71·3	−1·7	70·3	72·4	−2·1	71·3	74·7	−3·4	72·4	74·4	−2·0	0·75
	Belize	56·1	53·3	2·8	63·8	71·7	−7·8	66·3	66·7	−0·5	67·7	71·0	−3·3	68·7	70·5	−1·8	0·61
	Bermuda	63·6	61·4	2·2	70·7	69·3	1·4	72·0	74·1	−2·1	74·2	76·6	−2·4	75·5	75·6	−0·1	0·82
	Cuba	61·6	65·0	−3·4	67·7	73·0	−5·3	67·8	74·9	−7·1	68·8	76·2	−7·4	70·0	70·9	−0·9	0·67
	Dominica	61·6	45·6	16·0	67·7	69·1	−1·4	69·4	70·1	−0·7	70·8	70·4	0·4	72·4	67·4	4·9	0·75
	Dominican Republic	46·0	53·5	−7·5	64·4	69·3	−4·9	66·1	70·6	−4·4	67·9	71·5	−3·6	68·9	70·5	−1·6	0·62
	Grenada	52·2	54·6	−2·4	64·4	67·6	−3·2	67·3	67·7	−0·4	68·8	68·2	0·6	70·0	67·3	2·7	0·67
	Guyana	56·4	49·5	6·9	65·1	60·3	4·8	67·0	62·2	4·9	68·1	63·3	4·8	69·5	61·1	8·4	0·65
	Haiti	49·1	35·2	13·9	58·0	53·2	4·8	61·1	57·2	3·9	63·2	35·4	27·8	64·7	58·8	6·0	0·45
	Jamaica	60·8	54·5	6·3	67·1	73·9	−6·7	68·4	72·7	−4·3	69·3	74·6	−5·2	70·4	72·0	−1·6	0·68
	Puerto Rico	62·8	59·7	3·1	69·8	69·8	0·0	71·1	72·6	−1·5	73·0	75·8	−2·8	75·7	76·6	−0·9	0·83
	Saint Kitts and Nevis	59·5	56·5	3·0	68·1	65·8	2·3	69·4	69·1	0·3	71·5	70·0	1·5	73·0	68·5	4·4	0·75
	Saint Lucia	55·7	50·1	5·7	66·1	67·6	−1·5	68·1	70·2	−2·1	69·2	72·3	−3·1	70·0	69·7	0·3	0·67
	Saint Vincent and the Grenadines	54·7	50·4	4·3	65·6	68·2	−2·6	67·1	68·8	−1·6	68·2	71·1	−2·9	69·3	69·7	−0·4	0·64
	Suriname	55·7	56·8	−1·1	66·4	66·3	0·1	67·4	67·0	0·3	68·5	69·2	−0·7	69·3	67·5	1·8	0·63
	Trinidad and Tobago	62·3	56·6	5·7	69·0	67·0	2·0	70·1	68·0	2·2	72·0	70·6	1·4	73·4	67·6	5·8	0·77
	Virgin Islands	64·6	58·8	5·8	69·7	69·2	0·5	71·1	70·1	1·1	74·2	71·5	2·7	75·5	71·3	4·2	0·82
Central Latin America		56·1	48·5	7·6	65·9	67·9	−2·1	67·1	70·8	−3·6	68·2	72·6	−4·4	69·4	68·3	1·1	0·64
	Colombia	55·7	53·3	2·5	65·9	68·2	−2·4	67·0	70·5	−3·4	68·2	75·0	−6·8	69·7	72·6	−3·0	0·66
	Costa Rica	58·0	55·4	2·6	67·1	74·8	−7·7	68·2	75·5	−7·3	69·2	76·7	−7·5	71·0	74·3	−3·3	0·70
	El Salvador	49·5	43·7	5·8	61·6	65·4	−3·8	64·6	68·3	−3·7	66·4	71·0	−4·5	67·8	67·9	−0·1	0·56
	Guatemala	50·3	42·4	7·9	58·3	60·1	−1·8	61·8	64·3	−2·4	65·2	67·5	−2·2	67·0	66·2	0·9	0·54
	Honduras	49·1	38·0	11·1	59·2	66·5	−7·3	62·3	69·0	−6·7	64·9	70·0	−5·1	66·7	66·4	0·2	0·51
	Mexico	56·7	46·8	9·9	66·4	68·2	−1·7	67·7	71·7	−4·0	68·5	72·4	−3·9	69·9	67·4	2·5	0·66
	Nicaragua	51·1	46·6	4·5	60·0	70·5	−10·5	63·4	73·2	−9·8	65·4	74·3	−8·9	66·9	69·9	−3·0	0·52
	Panama	59·8	59·4	0·3	67·4	74·2	−6·8	68·3	75·5	−7·2	69·0	75·5	−6·5	71·0	75·5	−4·5	0·71
	Venezuela	58·9	54·8	4·1	66·7	69·5	−2·8	67·8	69·7	−1·9	68·5	71·4	−2·9	68·8	65·1	3·7	0·60
Tropical Latin America		54·0	48·3	5·7	66·3	65·8	0·5	67·3	68·7	−1·5	68·5	70·9	−2·4	69·5	70·2	−0·6	0·65
	Brazil	54·0	48·1	5·9	66·3	65·6	0·7	67·3	68·6	−1·4	68·5	70·8	−2·3	69·5	70·2	−0·7	0·65
	Paraguay	55·7	57·7	−1·9	65·6	73·7	−8·1	67·0	73·1	−6·1	68·2	72·7	−4·5	69·5	69·0	0·6	0·64
**North Africa and Middle East**		**49·5**	**41·3**	**8·2**	**64·4**	**63·8**	**0·6**	**66·8**	**67·4**	−**0·6**	**68·1**	**70·3**	−**2·2**	**69·8**	**68·9**	**0·8**	**0·66**
	Afghanistan	41·2	38·5	2·7	47·8	52·5	−4·7	48·2	53·3	−5·0	53·7	59·1	−5·4	59·5	55·9	3·6	0·34
	Algeria	45·1	41·1	4·0	65·1	68·6	−3·5	67·3	71·2	−3·9	68·6	74·9	−6·3	69·8	72·1	−2·3	0·66
	Bahrain	52·6	50·9	1·7	68·2	67·9	0·4	69·4	68·6	0·9	71·0	73·0	−2·0	73·0	72·2	0·8	0·75
	Egypt	52·6	42·7	9·9	63·6	62·3	1·3	66·4	66·4	0·0	66·1	67·5	−1·3	68·6	66·9	1·7	0·61
	Iran	47·8	35·7	12·1	64·9	65·8	−0·9	67·8	71·2	−3·4	69·3	74·0	−4·7	70·8	71·9	−1·0	0·70
	Iraq	46·0	51·5	−5·5	63·0	64·5	−1·5	65·1	66·6	−1·6	66·9	68·5	−1·5	69·7	67·5	2·1	0·66
	Jordan	44·2	49·4	−5·2	67·3	72·1	−4·9	68·3	74·2	−5·9	69·8	77·5	−7·7	71·6	74·1	−2·5	0·73
	Kuwait	58·6	50·9	7·7	70·1	72·4	−2·3	71·5	76·0	−4·5	74·0	79·1	−5·1	76·5	78·1	−1·6	0·85
	Lebanon	55·4	51·4	4·0	67·0	65·9	1·1	68·1	74·3	−6·2	69·9	75·6	−5·8	72·2	72·2	0·0	0·74
	Libya	46·0	40·2	5·8	67·1	71·3	−4·1	69·3	72·8	−3·5	71·5	72·4	−0·9	72·0	68·7	3·3	0·73
	Morocco	40·6	38·6	2·0	60·8	65·8	−5·0	63·4	69·2	−5·8	65·6	71·5	−6·0	67·7	70·9	−3·2	0·56
	Oman	44·2	38·0	6·1	64·0	66·6	−2·6	68·7	69·4	−0·7	71·1	70·6	0·5	73·4	70·5	2·9	0·77
	Palestine	45·1	41·3	3·8	62·8	67·2	−4·5	65·1	66·8	−1·7	66·9	70·7	−3·8	69·1	71·5	−2·4	0·63
	Qatar	54·7	54·7	0·1	69·5	68·7	0·9	71·3	69·0	2·3	73·8	72·6	1·2	76·5	76·1	0·4	0·85
	Saudi Arabia	50·7	52·6	−1·9	67·3	66·6	0·7	69·4	69·1	0·3	72·2	70·3	1·9	75·3	71·8	3·5	0·82
	Sudan	44·2	47·4	−3·2	56·7	56·7	0·0	60·0	61·5	−1·5	64·2	66·4	−2·2	67·3	66·3	1·0	0·54
	Syria	46·9	52·1	−5·2	64·0	67·8	−3·8	66·4	70·4	−4·0	68·4	72·9	−4·5	69·0	70·1	−1·1	0·62
	Tunisia	46·0	39·7	6·3	65·4	70·1	−4·7	67·7	71·8	−4·1	69·0	73·9	−4·9	70·3	70·8	−0·6	0·68
	Türkiye	53·3	41·3	12·0	65·1	64·4	0·7	67·1	69·3	−2·2	68·8	73·0	−4·2	71·1	72·3	−1·1	0·71
	United Arab Emirates	49·9	53·3	−3·4	69·4	69·4	0·0	73·0	70·1	2·8	75·9	72·3	3·6	76·8	77·5	−0·8	0·85
	Yemen	39·6	29·7	9·9	51·4	57·1	−5·7	57·4	61·7	−4·4	62·5	66·3	−3·7	64·7	62·4	2·3	0·45
**South Asia**		**48·6**	**37·6**	**11·0**	**58·6**	**59·9**	−**1·3**	**62·1**	**63·0**	−**0·9**	**64·9**	**65·9**	−**0·9**	**67·7**	**66·4**	**1·3**	**0·56**
	Bangladesh	42·2	40·5	1·7	52·6	58·0	−5·4	57·4	63·6	−6·2	61·4	68·1	−6·7	66·1	70·6	−4·5	0·49
	Bhutan	36·3	37·0	−0·8	51·4	60·1	−8·7	57·4	65·5	−8·1	62·8	70·6	−7·9	65·6	72·7	−7·1	0·47
	India	49·5	36·4	13·1	59·2	60·0	−0·8	62·5	63·1	−0·5	65·2	65·9	−0·6	68·1	66·6	1·5	0·58
	Nepal	41·2	37·0	4·1	50·3	57·6	−7·3	55·7	64·2	−8·5	60·6	67·9	−7·3	64·2	66·1	−1·9	0·43
	Pakistan	46·5	47·6	−1·1	58·0	62·2	−4·2	61·6	62·3	−0·7	64·2	64·4	−0·2	66·4	63·8	2·6	0·50
**Southeast Asia, east Asia, and Oceania**		**50·7**	**44·8**	**5·9**	**65·4**	**64·9**	**0·5**	**67·5**	**67·4**	**0·1**	**69·1**	**70·4**	−**1·3**	**70·7**	**72·5**	−**1·8**	**0·70**
East Asia		49·9	46·3	3·6	65·4	65·8	−0·4	67·7	68·4	−0·7	69·5	71·9	−2·3	71·6	74·8	−3·2	0·73
	China	49·1	47·4	1·7	65·1	65·7	−0·6	67·4	68·5	−1·1	69·3	71·9	−2·6	71·5	74·9	−3·4	0·72
	North Korea	58·9	18·5	40·4	66·1	66·8	−0·7	66·1	59·3	6·9	67·1	67·6	−0·4	67·9	70·1	−2·2	0·57
	Taiwan (province of China)	57·0	55·7	1·3	70·0	72·2	−2·1	72·7	74·1	−1·4	75·7	76·9	−1·1	77·8	78·1	−0·3	0·87
Oceania		51·8	46·8	5·0	62·3	61·1	1·2	63·8	62·8	1·0	64·4	63·9	0·4	65·2	62·5	2·7	0·47
	American Samoa	65·9	60·8	5·0	68·8	67·4	1·4	69·3	68·1	1·2	69·9	68·6	1·2	71·0	69·3	1·7	0·72
	Cook Islands	59·5	46·4	13·0	67·8	66·4	1·4	69·2	69·8	−0·6	71·1	72·1	−1·0	73·8	72·9	0·9	0·78
	Federated States of Micronesia	52·9	41·5	11·4	65·1	60·5	4·6	66·5	61·6	5·0	67·5	63·4	4·1	68·3	64·5	3·8	0·59
	Fiji	57·4	58·5	−1·1	67·1	63·9	3·3	68·3	63·4	4·9	68·9	65·4	3·5	70·0	63·8	6·2	0·68
	Guam	67·8	65·9	1·9	70·3	72·1	−1·8	71·6	75·8	−4·1	73·0	76·3	−3·4	74·7	73·5	1·1	0·80
	Kiribati	55·4	44·0	11·4	63·2	56·4	6·8	64·6	57·3	7·2	65·7	59·2	6·5	66·9	61·1	5·8	0·53
	Marshall Islands	52·6	47·4	5·1	64·0	59·6	4·4	65·4	60·5	4·9	66·7	61·4	5·3	67·9	63·4	4·5	0·57
	Nauru	62·5	57·5	5·0	67·3	58·1	9·2	66·8	55·0	11·8	67·3	55·6	11·6	69·0	59·2	9·8	0·63
	Niue	59·5	51·3	8·2	68·2	65·8	2·4	69·1	65·4	3·7	70·4	66·9	3·5	71·6	65·1	6·5	0·73
	Northern Mariana Islands	64·6	62·2	2·4	71·3	70·1	1·2	72·7	71·1	1·6	72·7	71·0	1·8	73·8	69·5	4·3	0·77
	Palau	63·6	46·9	16·7	69·9	63·6	6·3	71·0	65·4	5·5	71·6	65·9	5·7	72·7	67·7	5·0	0·75
	Papua New Guinea	45·1	44·4	0·8	58·0	60·3	−2·3	60·6	62·7	−2·1	61·8	63·7	−1·9	63·6	61·9	1·7	0·42
	Samoa	56·4	55·5	0·9	65·9	65·9	−0·1	66·7	67·8	−1·1	67·6	69·3	−1·7	68·3	69·6	−1·3	0·59
	Solomon Islands	47·8	45·1	2·7	57·4	59·1	−1·7	60·6	61·1	−0·5	61·8	62·2	−0·3	64·0	63·7	0·4	0·43
	Tokelau	57·0	55·7	1·3	66·8	66·9	−0·2	67·9	68·4	−0·5	69·1	70·2	−1·1	70·4	67·1	3·3	0·69
	Tonga	55·1	59·2	−4·2	66·0	68·5	−2·5	67·4	68·1	−0·7	68·1	69·4	−1·3	69·1	70·6	−1·5	0·63
	Tuvalu	54·7	37·8	16·9	62·5	57·2	5·3	65·4	57·5	7·9	66·8	63·8	3·0	68·0	65·8	2·2	0·58
	Vanuatu	49·9	44·7	5·2	60·3	60·9	−0·6	62·3	61·7	0·6	64·0	62·8	1·2	65·4	62·5	2·9	0·47
Southeast Asia		52·2	40·8	11·4	65·2	62·8	2·4	67·1	65·4	1·7	68·2	67·8	0·4	69·5	67·9	1·6	0·65
	Cambodia	49·5	41·4	8·1	56·7	55·2	1·6	59·5	57·8	1·6	63·2	63·5	−0·3	65·6	65·2	0·3	0·47
	Indonesia	49·9	38·2	11·7	64·9	62·7	2·2	67·1	66·0	1·2	68·2	67·4	0·8	69·8	67·3	2·5	0·66
	Laos	44·6	34·8	9·8	55·1	50·6	4·4	58·9	56·1	2·8	63·4	62·4	1·0	66·0	65·4	0·6	0·49
	Malaysia	51·4	51·8	−0·3	67·4	69·9	−2·5	69·1	70·8	−1·7	70·5	72·2	−1·7	72·2	70·4	1·8	0·74
	Maldives	49·9	34·0	15·8	59·2	65·8	−6·7	65·7	72·1	−6·4	68·1	77·0	−8·9	69·8	78·1	−8·4	0·65
	Mauritius	57·0	50·8	6·3	67·4	66·3	1·1	68·6	69·0	−0·4	69·8	70·8	−1·0	71·5	70·1	1·3	0·72
	Myanmar	45·1	29·4	15·7	58·6	52·3	6·3	61·4	56·0	5·4	65·1	61·4	3·7	67·0	64·1	2·9	0·53
	Philippines	59·5	55·8	3·7	66·5	65·4	1·2	67·4	67·3	0·1	67·9	67·6	0·3	69·7	64·8	4·8	0·65
	Seychelles	61·4	57·8	3·6	68·0	66·1	1·9	69·5	68·0	1·5	70·3	69·6	0·6	71·8	70·8	1·0	0·73
	Sri Lanka	59·2	54·8	4·4	66·8	65·8	1·0	68·1	67·1	1·0	69·2	70·1	−0·8	70·8	73·4	−2·6	0·70
	Thailand	52·9	49·6	3·3	66·4	67·6	−1·2	68·1	67·7	0·4	69·0	72·6	−3·6	70·3	72·4	−2·1	0·68
	Timor–Leste	41·7	43·6	−1·9	54·7	59·0	−4·3	59·8	65·1	−5·3	62·5	68·3	−5·7	64·7	66·9	−2·1	0·44
	Viet Nam	51·1	39·6	11·5	63·0	65·4	−2·4	66·0	67·9	−1·9	67·7	68·6	−0·9	68·9	69·9	−1·0	0·63
**Sub–Saharan Africa**		**46·5**	**39·3**	**7·2**	**57·0**	**51·5**	**5·5**	**59·2**	**51·5**	**7·7**	**62·1**	**57·1**	**5·0**	**65·1**	**58·7**	**6·4**	**0·46**
Central sub–Saharan Africa		46·0	36·3	9·7	57·4	50·4	7·0	58·6	50·9	7·7	62·3	56·5	5·8	65·9	58·4	7·5	0·47
	Angola	44·2	38·7	5·5	55·4	46·5	8·9	58·0	50·1	7·9	62·1	57·9	4·2	65·7	58·4	7·3	0·45
	Central African Republic	41·7	39·0	2·7	51·4	44·4	7·0	53·7	42·4	11·3	56·1	46·2	9·9	58·0	48·2	9·8	0·31
	Congo (Brazzaville)	47·4	31·6	15·8	63·6	52·1	11·5	65·1	52·2	12·8	66·4	60·6	5·8	68·2	60·6	7·6	0·58
	Democratic Republic of the Congo	45·6	35·4	10·2	56·7	51·9	4·8	55·1	51·7	3·3	56·4	56·5	−0·1	62·3	59·0	3·3	0·38
	Equatorial Guinea	41·7	24·3	17·4	55·4	48·4	7·0	63·2	55·2	8·0	67·7	63·4	4·3	69·9	59·3	10·6	0·66
	Gabon	46·9	24·9	22·0	64·9	56·7	8·3	66·5	57·0	9·5	67·6	60·4	7·2	69·3	60·9	8·4	0·63
Eastern sub–Saharan Africa		42·7	37·3	5·4	52·9	48·9	4·0	55·1	50·3	4·8	59·2	58·0	1·2	63·2	58·9	4·3	0·41
	Burundi	41·2	35·5	5·7	50·7	47·1	3·6	51·4	42·6	8·8	53·3	58·4	−5·1	56·7	60·0	−3·3	0·29
	Comoros	43·2	42·7	0·4	56·1	56·8	−0·7	60·6	60·3	0·3	63·4	64·9	−1·5	65·6	64·8	0·8	0·48
	Djibouti	47·4	54·8	−7·4	59·8	59·1	0·7	61·6	58·9	2·7	63·8	61·7	2·2	66·1	62·3	3·8	0·49
	Eritrea	37·9	35·5	2·5	51·4	41·2	10·2	58·0	50·9	7·1	60·3	56·5	3·8	63·0	58·7	4·3	0·40
	Ethiopia	36·3	34·5	1·7	46·0	44·1	2·0	48·2	50·3	−2·1	54·7	62·0	−7·3	60·8	62·0	−1·2	0·36
	Kenya	43·2	44·6	−1·4	59·5	60·8	−1·3	62·1	53·9	8·2	64·2	59·2	5·0	66·9	61·0	5·9	0·52
	Madagascar	44·2	39·4	4·8	56·1	54·6	1·5	56·4	57·9	−1·5	58·3	60·7	−2·4	62·8	60·5	2·3	0·40
	Malawi	44·6	33·7	10·9	50·7	47·7	3·0	52·6	44·9	7·7	56·7	54·0	2·7	61·8	55·8	6·1	0·38
	Mozambique	40·1	38·0	2·1	47·8	48·5	−0·7	50·3	50·1	0·2	53·7	51·0	2·7	58·9	53·4	5·5	0·33
	Rwanda	43·7	30·7	12·9	55·7	47·8	7·9	56·1	48·8	7·3	60·3	62·0	−1·7	64·2	62·3	1·9	0·44
	Somalia	36·8	41·4	−4·6	35·7	45·9	−10·3	36·3	48·3	−12·1	37·4	48·1	−10·7	38·5	50·7	−12·2	0·08
	South Sudan	44·2	44·0	0·1	50·7	49·9	0·8	52·6	52·8	−0·2	55·4	56·2	−0·8	56·1	52·6	3·5	0·28
	Tanzania	41·2	37·2	3·9	54·7	53·4	1·3	56·7	52·2	4·5	60·6	59·8	0·8	64·7	61·3	3·5	0·45
	Uganda	40·6	36·6	4·1	49·1	46·4	2·7	52·9	47·4	5·5	59·2	56·7	2·5	63·8	57·8	6·0	0·42
	Zambia	44·6	40·5	4·1	57·7	50·3	7·4	58·6	44·6	14·0	62·5	54·6	7·9	66·5	55·8	10·8	0·51
Southern sub–Saharan Africa		57·0	46·2	10·8	66·4	60·0	6·4	67·7	51·6	16·1	68·5	53·4	15·1	69·4	55·9	13·5	0·64
	Botswana	44·6	46·0	−1·4	63·4	58·0	5·4	66·7	45·9	20·8	68·2	56·1	12·1	69·4	57·0	12·4	0·64
	Eswatini	45·1	34·0	11·1	62·8	56·6	6·1	65·4	45·6	19·8	66·9	44·3	22·6	68·2	49·5	18·7	0·59
	Lesotho	46·5	41·2	5·3	59·8	56·2	3·6	62·8	45·7	17·1	64·7	45·5	19·3	66·5	45·3	21·2	0·51
	Namibia	51·4	47·6	3·9	64·7	58·9	5·9	66·4	51·6	14·8	67·6	56·8	10·8	68·9	56·5	12·4	0·62
	South Africa	58·9	46·7	12·2	67·3	60·6	6·6	68·4	53·8	14·6	69·2	54·6	14·7	70·3	57·4	12·8	0·68
	Zimbabwe	48·6	47·7	0·9	62·8	59·1	3·6	64·6	45·9	18·7	63·4	50·4	13·0	65·6	52·2	13·4	0·47
Western sub–Saharan Africa		45·1	40·4	4·8	55·7	52·9	2·8	58·0	53·3	4·7	61·4	58·0	3·4	64·6	59·9	4·7	0·45
	Benin	41·7	36·3	5·4	51·8	53·9	−2·1	54·4	55·8	−1·4	57·7	59·3	−1·7	61·6	60·1	1·5	0·37
	Burkina Faso	36·3	35·3	0·9	44·2	49·2	−5·1	47·8	50·8	−3·0	51·8	55·5	−3·7	56·4	57·4	−1·0	0·29
	Cabo Verde	44·6	46·5	−1·9	55·7	67·0	−11·2	60·8	66·5	−5·7	64·9	70·9	−6·0	67·1	69·0	−1·8	0·53
	Cameroon	44·6	38·4	6·2	57·4	57·0	0·4	60·3	53·3	7·0	62·5	56·7	5·8	65·7	58·5	7·3	0·48
	Chad	36·3	38·4	−2·1	42·7	51·7	−9·0	45·1	50·5	−5·4	49·1	55·0	−5·9	52·9	56·5	−3·6	0·24
	Côte d'Ivoire	42·7	42·7	0·0	56·1	53·3	2·8	59·5	50·3	9·2	60·8	55·8	5·0	63·8	60·3	3·5	0·43
	The Gambia	43·2	49·1	−5·9	53·3	56·8	−3·5	57·0	58·6	−1·5	60·3	61·3	−1·0	63·2	60·9	2·3	0·41
	Ghana	53·3	43·9	9·4	61·4	57·9	3·4	63·8	58·2	5·6	65·6	59·6	6·0	67·7	61·7	6·0	0·56
	Guinea	36·8	36·8	0·1	48·6	51·9	−3·2	51·4	53·0	−1·5	54·7	56·6	−1·9	59·8	58·2	1·6	0·34
	Guinea–Bissau	37·4	24·6	12·7	50·7	45·9	4·8	54·0	48·9	5·1	56·7	54·1	2·7	60·6	55·1	5·5	0·35
	Liberia	44·6	26·9	17·8	52·9	45·4	7·6	52·6	53·8	−1·2	56·1	60·7	−4·6	60·6	61·6	−1·0	0·35
	Mali	36·8	32·8	4·1	43·7	49·5	−5·8	46·9	53·2	−6·3	50·7	57·6	−7·0	55·7	57·3	−1·5	0·27
	Mauritania	48·2	44·5	3·8	59·5	60·1	−0·6	62·1	64·4	−2·4	63·6	68·6	−5·0	66·1	68·4	−2·2	0·50
	Niger	35·7	37·5	−1·9	39·6	46·7	−7·1	41·7	51·4	−9·8	45·1	59·2	−14·1	49·5	60·1	−10·6	0·17
	Nigeria	46·0	42·6	3·4	57·7	52·9	4·8	59·5	53·3	6·2	63·2	58·4	4·8	66·3	60·7	5·5	0·50
	São Tomé and Príncipe	48·2	36·9	11·3	58·0	61·8	−3·8	59·2	64·1	−4·9	62·8	67·8	−5·1	66·4	68·6	−2·2	0·51
	Senegal	41·7	42·3	−0·6	52·9	56·6	−3·7	56·4	58·6	−2·2	59·2	64·0	−4·8	63·2	63·7	−0·5	0·41
	Sierra Leone	42·7	35·8	6·9	51·1	49·2	1·9	51·8	48·9	2·9	55·7	54·6	1·1	60·8	59·2	1·7	0·36
	Togo	41·2	38·6	2·6	55·4	56·2	−0·8	57·7	54·4	3·2	59·5	56·8	2·6	63·2	60·2	3·0	0·41

SDI=Socio-demographic Index. GBD=Global Burden of Diseases, Injuries, and Risk Factors Study.

**Figure 8:**
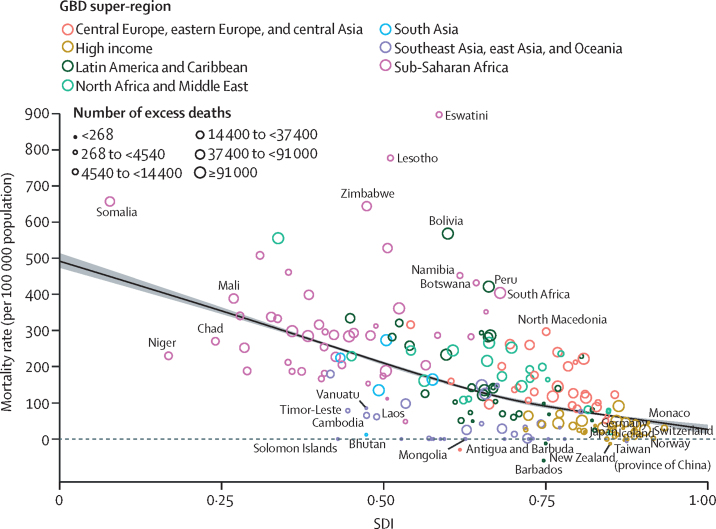
National age-standardised rates of excess mortality due to the COVID-19 pandemic versus SDI, and expected rates of excess mortality based on SDI, 2020 and 2021 combined Mortality rates are expressed as the number of deaths per 100 000 and are shown for 204 countries and territories coloured by GBD super-region. The size of the datapoints indicates the number of excess deaths. The black line represents expected age-standardised excess mortality rates based on SDI, and the shaded area indicates the 95% uncertainty intervals. GBD=Global Burden of Diseases, Injuries, and Risk Factors Study. SDI=Socio-demographic Index.

In 2020 and 2021 combined, lower age-standardised excess mortality rates due to the COVID-19 pandemic were broadly associated with higher SDI levels, but the association was not consistently strong (figure 8). The GBD super-regions with the largest proportion of countries and territories with an excess mortality rate higher than expected based on SDI were central Europe, eastern Europe, and central Asia (26 of 29 nations), Latin America and the Caribbean (21 of 33 nations), and south Asia (three of five nations); the super-regions with the largest proportion of nations with an excess mortality rate lower than expected based on SDI were southeast Asia, east Asia, and Oceania (33 of 34 nations), high-income (33 of 36 nations), and sub-Saharan Africa (27 of 46 nations). At the national level, the five countries or territories with the largest positive difference between estimated excess mortality and expected excess mortality based on SDI (ie, higher mortality than expected) were Bulgaria, North Macedonia, Lesotho, Peru, and Bolivia; the five nations with the highest negative difference between estimated excess mortality and expected excess mortality based on SDI (ie, lower mortality than expected) were Barbados, Mongolia, New Zealand, Antigua and Barbuda, and the Marshall Islands.

### Population

**Figure 9:**
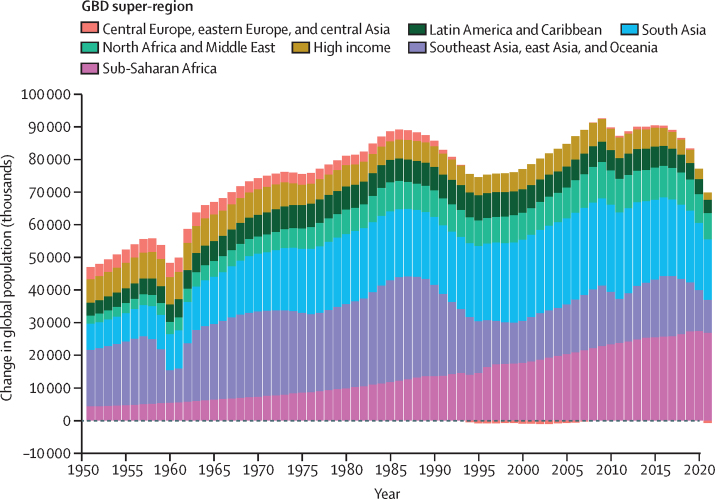
Annual change in global total population by GBD super-region, 1950–2021 Annual change is defined as the difference between the population size in the current year and the preceding year. Different colours show GBD super-regions. GBD=Global Burden of Diseases, Injuries, and Risk Factors Study.

The global total population increased annually over the study period, from 2·52 billion (95% UI 2·48–2·58) in 1950 to 6·10 billion (5·98–6·22) in 2000 and 7·89 billion (7·67–8·13) in 2021 (table 5). Annual growth in total population fluctuated over the study period, from an annual increase of 46·9 million (41·0–52·7) from 1950 to 1951 with the highest annual increase of 92·5 million (75·7–106·6) observed between 2008 and 2009 (figure 9). After 2009, population growth plateaued, and in 2017, the annual increase in population began to decline. Between 2019 and 2021, this decline accelerated, with annual gains of just 77·0 million (49·4–95·6) from 2019 to 2020 and 69·0 million (50·8–93·2) from 2020 to 2021. These reduced gains include the impact of excess deaths due to the COVID-19 pandemic, therefore the magnitude might not persist as excess mortality declines. The majority of global population growth during the study period is attributed to three GBD super-regions: sub-Saharan Africa; south Asia; and southeast Asia, east Asia, and Oceania. The population of sub-Saharan Africa grew at a steadily increasing rate throughout the study period, contributing 9·1% (7·3–11·0) of the total global population growth from 1950 to 1951, 23·3% (19·4–27·6) from 2000 to 2001, and 39·5% (28·4–52·7) from 2020 to 2021. South Asia contributed 17·1% (13·8–20·6) of the total global population growth from 1950 to 1951, rose to a peak contribution of 32·9% (28·4–37·8) from 1999 to 2000, and remained relatively constant in more recent years, with a contribution of 26·3% (9·0–44·7) from 2020 to 2021. In contrast, the annual growth of the population fluctuated in southeast Asia, east Asia, and Oceania. The contribution of this super-region to annual global population growth was relatively stable up to a peak of 37·3% (30·4–41·8) from 1956 to 1957 and then subsequently decreased, contributing 14·1% (0·0 to 30·2) from 2020 to 2021. Central Europe, eastern Europe, and central Asia contributed little to global population growth, and in fact experienced a decline in population over some periods, with growth from 1950 to 1992, a decline from 1993 to 2006, growth from 2007 to 2018, and a return to population decline in 2019. Population growth was relatively stable in Latin America and the Caribbean and north Africa and the Middle East at the super-regional level during the previous three decades, whereas population growth in the high-income super-region began to decline starting around 2015.

**Table 5:** The 2000 population and 2021 population and annualised rate of change in population (2000–21), globally and for GBD super-regions, regions, countries, and territories

		**Population in 2000 (thousands)**				**Population in 2021 (thousands)**				**Annualised rate of change in population, 2000–21**
		All ages	<15 years	15–64 years	≥65 years	All ages	<15 years	15–64 years	≥65 years	
**Global**		**6 100 000 (5 980 000 to 6 220 000)**	**1 830 000 (1 800 000 to 1 870 000)**	**3 840 000 (3 760 000 to 3 920 000)**	**423 000 (416 000 to 432 000)**	**7 890 000 (7 670 000 to 8 130 000)**	**2 010 000 (1 950 000 to 2 070 000)**	**5 110 000 (4 960 000 to 5 270 000)**	**770 000 (750 000 to 792 000)**	**1·2% (1·2 to 1·3)**
**Central Europe, eastern Europe, and central Asia**		**417 000 (404 000 to 431 000)**	**87 300 (84 500 to 90 000)**	**282 000 (272 000 to 291 000)**	**48 400 (46 600 to 50 000)**	**418 000 (393 000 to 441 000)**	**80 800 (75 900 to 85 500)**	**275 000 (259 000 to 291 000)**	**61 800 (58 100 to 65 200)**	**0·0% (−0·1 to 0·1)**
Central Asia		74 400 (70 600 to 78 100)	24 800 (23 500 to 26 100)	45 300 (43 100 to 47 600)	4310 (4120 to 4500)	95 800 (85 900 to 106 000)	27 700 (24 700 to 30 600)	62 100 (55 700 to 68 600)	6020 (5490 to 6550)	1·2% (0·9 to 1·4)
	Armenia	3320 (3070 to 3550)	849 (785 to 909)	2170 (2010 to 2320)	297 (275 to 318)	3000 (2600 to 3380)	592 (515 to 668)	2000 (1740 to 2260)	398 (346 to 449)	−0·5% (−0·8 to −0·2)
	Azerbaijan	8280 (7700 to 8890)	2580 (2400 to 2770)	5220 (4860 to 5600)	480 (447 to 515)	10 500 (9080 to 12 000)	2360 (2040 to 2700)	7440 (6440 to 8500)	699 (605 to 798)	1·1% (0·8 to 1·4)
	Georgia	4730 (4340 to 5120)	1030 (948 to 1120)	3090 (2830 to 3340)	612 (562 to 662)	3610 (3200 to 4010)	736 (653 to 817)	2300 (2040 to 2550)	572 (507 to 635)	−1·3% (−1·4 to −1·2)
	Kazakhstan	15 000 (13 900 to 16 100)	4180 (3860 to 4500)	9790 (9060 to 10 500)	1010 (934 to 1090)	19 000 (17 000 to 20 800)	5430 (4880 to 5960)	12 100 (10 900 to 13 300)	1400 (1260 to 1540)	1·1% (1·0 to 1·2)
	Kyrgyzstan	5010 (4650 to 5380)	1770 (1640 to 1900)	2970 (2750 to 3180)	279 (259 to 299)	6860 (5860 to 7900)	2270 (1940 to 2620)	4250 (3630 to 4890)	340 (290 to 391)	1·5% (1·1 to 1·8)
	Mongolia	2440 (2270 to 2610)	879 (817 to 939)	1480 (1380 to 1580)	83·6 (77·8 to 89·3)	3340 (3080 to 3580)	1090 (1000 to 1170)	2110 (1950 to 2260)	144 (134 to 155)	1·5% (1·4 to 1·5)
	Tajikistan	6360 (5950 to 6800)	2710 (2540 to 2900)	3410 (3180 to 3640)	244 (228 to 261)	10 200 (8800 to 11 600)	3580 (3110 to 4090)	6210 (5380 to 7080)	368 (319 to 420)	2·2% (1·9 to 2·5)
	Turkmenistan	4260 (3710 to 4830)	1600 (1400 to 1820)	2480 (2160 to 2810)	179 (156 to 203)	5160 (4620 to 5700)	1520 (1370 to 1680)	3350 (3000 to 3700)	284 (254 to 314)	0·9% (0·8 to 1·0)
	Uzbekistan	25 000 (21 500 to 28 700)	9150 (7880 to 10 500)	14 700 (12 700 to 16 900)	1120 (967 to 1290)	34 200 (24 500 to 43 600)	10 100 (7220 to 12 900)	22 300 (16 000 to 28 500)	1810 (1300 to 2310)	1·5% (0·6 to 2·0)
Central Europe		122 000 (118 000 to 126 000)	23 000 (22 200 to 23 700)	83 500 (80 700 to 86 200)	16 000 (15 500 to 16 500)	115 000 (110 000 to 120 000)	17 700 (16 900 to 18 500)	75 200 (71 800 to 78 500)	22 300 (21 300 to 23 300)	−0·3% (−0·4 to −0·2)
	Albania	3190 (2970 to 3430)	962 (895 to 1030)	2010 (1870 to 2160)	225 (209 to 242)	2670 (2320 to 3020)	444 (385 to 502)	1810 (1570 to 2050)	416 (361 to 471)	−0·9% (−1·2 to −0·6)
	Bosnia and Herzegovina	3980 (3490 to 4490)	806 (707 to 911)	2700 (2370 to 3060)	466 (409 to 527)	3300 (2900 to 3690)	490 (431 to 548)	2210 (1940 to 2470)	606 (532 to 677)	−0·9% (−0·9 to −0·8)
	Bulgaria	7940 (7400 to 8580)	1230 (1150 to 1330)	5390 (5030 to 5820)	1320 (1230 to 1420)	6790 (6070 to 7430)	976 (874 to 1070)	4340 (3880 to 4750)	1470 (1320 to 1610)	−0·8% (−0·9 to −0·7)
	Croatia	4570 (4250 to 4900)	794 (738 to 851)	3080 (2860 to 3310)	696 (646 to 746)	4210 (3680 to 4750)	597 (522 to 674)	2720 (2370 to 3060)	896 (783 to 1010)	−0·4% (−0·7 to −0·2)
	Czechia	10 200 (10 200 to 10 300)	1670 (1660 to 1680)	7140 (7090 to 7200)	1420 (1410 to 1430)	10 600 (9670 to 11 600)	1720 (1560 to 1870)	6710 (6100 to 7330)	2210 (2010 to 2410)	0·2% (−0·2 to 0·6)
	Hungary	10 200 (9440 to 11 000)	1720 (1590 to 1850)	6950 (6430 to 7470)	1530 (1410 to 1640)	9600 (8430 to 10 900)	1390 (1220 to 1570)	6200 (5440 to 7020)	2010 (1760 to 2280)	−0·3% (−0·5 to 0·0)
	Montenegro	637 (580 to 695)	142 (129 to 155)	425 (387 to 464)	70·1 (63·9 to 76·6)	618 (540 to 701)	111 (97·4 to 126)	413 (361 to 468)	93·7 (81·9 to 106)	−0·1% (−0·3 to 0·0)
	North Macedonia	2060 (1900 to 2230)	460 (424 to 497)	1390 (1290 to 1510)	204 (188 to 220)	2180 (1800 to 2590)	328 (270 to 390)	1540 (1270 to 1830)	308 (254 to 366)	0·2% (−0·3 to 0·7)
	Poland	38 300 (35 200 to 41 300)	7370 (6760 to 7950)	26 200 (24 100 to 28 300)	4720 (4330 to 5090)	38 200 (34 600 to 41 900)	5890 (5320 to 6450)	25 200 (22 800 to 27 600)	7170 (6480 to 7860)	0·0% (−0·1 to 0·1)
	Romania	22 400 (20 600 to 24 300)	4220 (3870 to 4570)	15 200 (14 000 to 16 500)	2960 (2720 to 3210)	18 900 (16 500 to 21 500)	3010 (2630 to 3420)	12 100 (10 600 to 13 800)	3790 (3300 to 4300)	−0·8% (−1·1 to −0·6)
	Serbia	9670 (8880 to 10 500)	1870 (1720 to 2030)	6550 (6020 to 7090)	1250 (1140 to 1350)	8920 (7750 to 10 000)	1330 (1150 to 1490)	5930 (5160 to 6670)	1660 (1440 to 1860)	−0·4% (−0·6 to −0·2)
	Slovakia	5390 (5360 to 5420)	1050 (1040 to 1050)	3720 (3700 to 3740)	624 (620 to 628)	5430 (4900 to 5960)	857 (772 to 940)	3640 (3280 to 3990)	937 (845 to 1030)	0·0% (−0·4 to 0·4)
	Slovenia	1990 (1980 to 2010)	321 (318 to 323)	1390 (1380 to 1400)	280 (278 to 282)	2070 (1890 to 2250)	312 (285 to 340)	1320 (1200 to 1440)	437 (398 to 475)	0·2% (−0·2 to 0·5)
Eastern Europe		221 000 (208 000 to 234 000)	39 600 (37 300 to 41 900)	153 000 (144 000 to 162 000)	28 100 (26 400 to 29 700)	207 000 (185 000 to 228 000)	35 400 (31 600 to 39 200)	138 000 (123 000 to 152 000)	33 500 (29 900 to 36 800)	−0·3% (−0·6 to −0·1)
	Belarus	10 200 (9460 to 11 000)	1930 (1790 to 2070)	6920 (6410 to 7440)	1360 (1260 to 1460)	9320 (8020 to 10 600)	1580 (1360 to 1800)	6250 (5380 to 7120)	1490 (1280 to 1700)	−0·4% (−0·8 to −0·2)
	Estonia	1390 (1390 to 1400)	251 (249 to 252)	936 (930 to 942)	208 (206 to 209)	1310 (1190 to 1430)	216 (196 to 236)	825 (748 to 902)	270 (244 to 295)	−0·3% (−0·7 to 0·1)
	Latvia	2380 (2210 to 2540)	431 (399 to 459)	1600 (1480 to 1700)	355 (329 to 379)	1870 (1700 to 2050)	297 (270 to 326)	1180 (1070 to 1290)	392 (356 to 430)	−1·2% (−1·3 to −1·0)
	Lithuania	3520 (3260 to 3780)	705 (653 to 756)	2330 (2160 to 2500)	483 (447 to 518)	2730 (2480 to 3010)	408 (370 to 449)	1760 (1600 to 1940)	557 (506 to 614)	−1·2% (−1·3 to −1·1)
	Moldova	4200 (3810 to 4600)	922 (836 to 1010)	2850 (2580 to 3120)	428 (388 to 469)	3590 (2970 to 4190)	522 (432 to 609)	2520 (2080 to 2940)	555 (459 to 647)	−0·8% (−1·2 to −0·4)
	Russia	149 000 (137 000 to 161 000)	26 700 (24 600 to 28 900)	104 000 (95 800 to 113 000)	18 400 (16 900 to 19 900)	145 000 (125 000 to 164 000)	26 100 (22 500 to 29 400)	96 000 (82 900 to 108 000)	22 700 (19 600 to 25 700)	−0·1% (−0·5 to 0·1)
	Ukraine	49 600 (46 000 to 53 200)	8640 (8010 to 9270)	34 100 (31 600 to 36 600)	6850 (6350 to 7350)	43 100 (34 600 to 51 400)	6350 (5100 to 7570)	29 300 (23 500 to 34 900)	7440 (5990 to 8880)	−0·7% (−1·3 to −0·2)
**High income**		**968 000 (944 000 to 990 000)**	**185 000 (180 000 to 189 000)**	**647 000 (631 000 to 661 000)**	**137 000 (134 000 to 140 000)**	**1 090 000 (1 060 000 to 1 120 000)**	**176 000 (171 000 to 181 000)**	**702 000 (682 000 to 720 000)**	**214 000 (208 000 to 219 000)**	**0·6% (0·5 to 0·6)**
Australasia		22 700 (21 300 to 24 100)	4870 (4570 to 5170)	15 100 (14 100 to 16 000)	2780 (2600 to 2950)	31 000 (29 200 to 32 700)	5730 (5400 to 6060)	20 000 (18,900 to 21,200)	5200 (4890 to 5500)	1·5% (1·4 to 1·5)
	Australia	18 900 (17 400 to 20 300)	4000 (3690 to 4290)	12 600 (11 600 to 13 500)	2330 (2150 to 2500)	25 800 (24 000 to 27 500)	4750 (4420 to 5070)	16 700 (15 500 to 17 800)	4390 (4080 to 4690)	1·5% (1·5 to 1·5)
	New Zealand	3860 (3580 to 4150)	878 (813 to 944)	2530 (2340 to 2720)	454 (421 to 488)	5170 (4720 to 5610)	982 (896 to 1060)	3380 (3080 to 3660)	810 (739 to 878)	1·4% (1·3 to 1·4)
High-income Asia Pacific		180 000 (171 000 to 190 000)	29 700 (28 200 to 31 100)	125 000 (118 000 to 131 000)	25 900 (24 300 to 27 400)	185 000 (175 000 to 196 000)	22 400 (21 200 to 23 700)	117 000 (111 000 to 123 000)	46 100 (43 300 to 49 000)	0·1% (0·1 to 0·2)
	Brunei	333 (306 to 358)	105 (96·7 to 113)	218 (201 to 235)	9·3 (8·6 to 10)	451 (394 to 510)	94·6 (82·6 to 107)	332 (290 to 375)	24·5 (21·4 to 27·7)	1·4% (1·2 to 1·7)
	Japan	129 000 (120 000 to 138 000)	18 900 (17 600 to 20 200)	87 800 (81 800 to 93 800)	22 200 (20 700 to 23 700)	128 000 (118 000 to 137 000)	15 400 (14 300 to 16 600)	75 400 (69 700 to 80 900)	36 800 (34 000 to 39 600)	0·0% (−0·1 to 0·0)
	Singapore	4030 (3740 to 4300)	754 (701 to 805)	3020 (2810 to 3220)	256 (238 to 274)	5730 (5260 to 6200)	812 (746 to 878)	4150 (3810 to 4490)	768 (706 to 831)	1·7% (1·6 to 1·7)
	South Korea	46 800 (43 500 to 49 900)	9860 (9160 to 10 500)	33 500 (31 200 to 35 800)	3390 (3150 to 3610)	51 600 (47 800 to 55 100)	6070 (5630 to 6490)	37 000 (34 300 to 39 600)	8500 (7870 to 9080)	0·5% (0·4 to 0·5)
High-income North America		311 000 (292 000 to 331 000)	66 700 (62 400 to 70 800)	206 000 (193 000 to 219 000)	38 300 (35 900 to 40 600)	370 000 (346 000 to 394 000)	65 600 (61 300 to 69 800)	240 000 (225 000 to 256 000)	64 200 (60 000 to 68 200)	0·8% (0·8 to 0·8)
	Canada	30 300 (28 100 to 32 400)	5920 (5490 to 6330)	20 600 (19 100 to 22 000)	3830 (3560 to 4100)	37 500 (35 100 to 40 200)	6170 (5770 to 6620)	24 300 (22 700 to 26 000)	7040 (6580 to 7540)	1·0% (1·0 to 1·0)
	Greenland	56·1 (55·8 to 56·5)	15·2 (15·1 to 15·3)	38·1 (37·8 to 38·3)	2·8 (2·8 to 2·8)	56·1 (50·7 to 61·1)	11·8 (10·6 to 12·8)	39·1 (35·3 to 42·6)	5·3 (4·8 to 5·8)	0·0% (−0·5 to 0·4)
	USA	281 000 (261 000 to 301 000)	60 700 (56 500 to 65 000)	186 000 (173 000 to 199 000)	34 400 (32 000 to 36 800)	333 000 (308 000 to 357 000)	59 400 (55 100 to 63 700)	216 000 (200 000 to 232 000)	57 100 (52 900 to 61 300)	0·8% (0·8 to 0·8)
Southern Latin America		55 200 (52 400 to 58 200)	15 400 (14 600 to 16 200)	34 700 (32 900 to 36 500)	5180 (4910 to 5460)	67 700 (61 400 to 74 200)	14 500 (13 100 to 15 900)	45 100 (40 900 to 49 400)	8110 (7370 to 8870)	1·0% (0·7 to 1·2)
	Argentina	36 800 (34 200 to 39 600)	10 500 (9730 to 11 300)	22 700 (21 100 to 24 500)	3590 (3340 to 3870)	45 500 (39 200 to 51 800)	10 200 (8780 to 11 600)	30 100 (25 900 to 34 300)	5250 (4530 to 5990)	1·0% (0·7 to 1·3)
	Chile	15 100 (13 900 to 16 300)	4090 (3750 to 4420)	9890 (9060 to 10 700)	1160 (1060 to 1250)	18 800 (17 100 to 20 600)	3650 (3320 to 4000)	12 800 (11 700 to 14 000)	2330 (2120 to 2550)	1·0% (1·0 to 1·1)
	Uruguay	3300 (2990 to 3600)	818 (742 to 895)	2050 (1860 to 2240)	427 (387 to 467)	3410 (2990 to 3860)	660 (578 to 748)	2210 (1940 to 2510)	531 (466 to 603)	0·1% (0·0 to 0·3)
Western Europe		398 000 (391 000 to 405 000)	68 000 (66 700 to 69 300)	266 000 (261 000 to 270 000)	64 600 (63 300 to 65 700)	437 000 (422 000 to 451 000)	68 100 (65 900 to 70 200)	279 000 (270 000 to 288 000)	90 000 (86 700 to 92 900)	0·4% (0·3 to 0·5)
	Andorra	65·6 (65·2 to 66·1)	10·1 (10 to 10·2)	47·5 (47·2 to 47·8)	8·1 (8 to 8·1)	85·6 (77·6 to 94·3)	10·2 (9·2 to 11·2)	61·7 (56 to 68)	13·7 (12·4 to 15·1)	1·3% (0·8 to 1·7)
	Austria	8020 (7450 to 8600)	1360 (1260 to 1460)	5410 (5030 to 5800)	1240 (1150 to 1330)	8980 (8090 to 9780)	1300 (1170 to 1410)	5970 (5380 to 6500)	1710 (1540 to 1870)	0·5% (0·4 to 0·6)
	Belgium	10 300 (9510 to 11 000)	1810 (1670 to 1940)	6730 (6230 to 7230)	1730 (1600 to 1860)	11 500 (10 300 to 12 600)	1910 (1720 to 2090)	7310 (6580 to 8010)	2240 (2020 to 2460)	0·5% (0·4 to 0·6)
	Cyprus	918 (851 to 983)	204 (189 to 218)	620 (575 to 664)	94·2 (87·3 to 101)	1360 (1170 to 1540)	219 (189 to 248)	941 (813 to 1070)	198 (171 to 225)	1·9% (1·5 to 2·1)
	Denmark	5330 (5290 to 5380)	982 (974 to 990)	3560 (3530 to 3590)	796 (789 to 802)	5850 (5300 to 6410)	954 (865 to 1050)	3720 (3370 to 4070)	1180 (1070 to 1290)	0·4% (0·0 to 0·8)
	Finland	5190 (5150 to 5230)	936 (929 to 942)	3470 (3440 to 3490)	784 (779 to 790)	5540 (4950 to 6060)	847 (758 to 927)	3400 (3040 to 3720)	1290 (1150 to 1410)	0·3% (−0·2 to 0·7)
	France	59 900 (55 500 to 64 400)	11 400 (10 500 to 12 200)	39 100 (36 200 to 42 000)	9440 (8740 to 10 100)	66 400 (59 500 to 73 500)	11 600 (10 400 to 12 800)	41 000 (36 800 to 45 400)	13 800 (12 300 to 15 200)	0·5% (0·3 to 0·6)
	Germany	82 300 (81 600 to 83 000)	12 800 (12 700 to 12 900)	55 800 (55 400 to 56 300)	13 700 (13 600 to 13 800)	85 400 (76 200 to 94 000)	12 000 (10 700 to 13 200)	54 900 (49 000 to 60 400)	18 600 (16 600 to 20 400)	0·2% (−0·3 to 0·6)
	Greece	11 100 (10 300 to 11 900)	1720 (1600 to 1850)	7560 (7000 to 8130)	1800 (1670 to 1940)	10 200 (8730 to 11 500)	1390 (1200 to 1580)	6470 (5550 to 7320)	2310 (1980 to 2610)	−0·4% (−0·8 to −0·2)
	Iceland	279 (277 to 282)	65 (64·5 to 65·6)	182 (180 to 183)	32·5 (32·3 to 32·8)	350 (318 to 384)	67·5 (61·3 to 74)	228 (206 to 250)	55·2 (50·1 to 60·5)	1·1% (0·7 to 1·5)
	Ireland	3870 (3560 to 4170)	849 (781 to 915)	2590 (2380 to 2790)	427 (393 to 461)	4940 (4420 to 5450)	997 (892 to 1100)	3190 (2860 to 3520)	751 (672 to 829)	1·2% (1·1 to 1·3)
	Israel	6390 (5760 to 7070)	1840 (1660 to 2040)	3940 (3550 to 4360)	614 (554 to 680)	9590 (8200 to 11 000)	2630 (2250 to 3030)	5770 (4930 to 6640)	1200 (1020 to 1380)	1·9% (1·7 to 2·1)
	Italy	56 700 (52 400 to 60 700)	8100 (7500 to 8680)	38 200 (35 300 to 40 900)	10 400 (9600 to 11 100)	59 800 (54 400 to 65 100)	7600 (6910 to 8270)	38 200 (34 700 to 41 600)	14 000 (12 700 to 15 300)	0·3% (0·2 to 0·3)
	Luxembourg	434 (401 to 466)	81·9 (75·8 to 88·1)	291 (270 to 313)	60·3 (55·8 to 64·8)	644 (589 to 703)	101 (92·5 to 110)	447 (409 to 488)	96 (87·8 to 105)	1·9% (1·8 to 1·9)
	Malta	402 (363 to 442)	80·1 (72·3 to 88·2)	272 (246 to 299)	50 (45·1 to 55)	442 (384 to 500)	64 (55·7 to 72·4)	278 (242 to 315)	100 (87 to 113)	0·4% (0·3 to 0·6)
	Monaco	33 (30·8 to 35·4)	4·3 (4 to 4·7)	20·9 (19·5 to 22·4)	7·8 (7·2 to 8·3)	37·9 (34·3 to 41·4)	5 (4·5 to 5·4)	23·2 (21 to 25·4)	9·7 (8·8 to 10·6)	0·7% (0·5 to 0·8)
	Netherlands	15 900 (15 800 to 16 000)	2950 (2930 to 2980)	10 800 (10 700 to 10 900)	2160 (2140 to 2180)	17 200 (15 600 to 18 900)	2680 (2430 to 2940)	11 100 (10 000 to 12 200)	3460 (3130 to 3800)	0·4% (−0·1 to 0·8)
	Norway	4480 (4440 to 4520)	893 (886 to 901)	2900 (2870 to 2920)	689 (684 to 695)	5420 (4930 to 5960)	924 (841 to 1020)	3520 (3210 to 3880)	972 (885 to 1070)	0·9% (0·5 to 1·3)
	Portugal	10 500 (9780 to 11 300)	1720 (1590 to 1840)	7160 (6640 to 7670)	1660 (1550 to 1780)	10 600 (9230 to 12 000)	1360 (1190 to 1550)	6830 (5940 to 7750)	2420 (2110 to 2750)	0·0% (−0·3 to 0·3)
	San Marino	27·5 (23·9 to 31)	4·3 (3·7 to 4·8)	18·6 (16·2 to 21)	4·6 (4 to 5·2)	32·7 (28·4 to 37·4)	4·4 (3·8 to 5)	21·3 (18·4 to 24·3)	7·1 (6·1 to 8·1)	0·8% (0·8 to 0·9)
	Spain	40 800 (40 500 to 41 100)	6070 (6030 to 6110)	27 900 (27 700 to 28 000)	6860 (6820 to 6900)	45 500 (41 000 to 49 900)	6480 (5830 to 7100)	29 900 (26 900 to 32 700)	9190 (8270 to 10 100)	0·5% (0·0 to 0·9)
	Sweden	8900 (8830 to 8980)	1630 (1620 to 1650)	5730 (5680 to 5770)	1540 (1530 to 1560)	10 400 (9390 to 11 400)	1820 (1650 to 2000)	6420 (5810 to 7050)	2140 (1930 to 2350)	0·7% (0·3 to 1·1)
	Switzerland	7300 (6820 to 7760)	1250 (1160 to 1330)	4930 (4600 to 5240)	1130 (1050 to 1200)	8920 (8050 to 9860)	1330 (1200 to 1470)	5890 (5310 to 6510)	1710 (1540 to 1880)	1·0% (0·8 to 1·1)
	UK	59 000 (55 400 to 62 600)	11 200 (10 500 to 11 900)	38 500 (36 100 to 40 800)	9310 (8730 to 9880)	67 800 (63 900 to 71 600)	11 800 (11 100 to 12 400)	43 600 (41 000 to 46 000)	12 500 (11 800 to 13 200)	0·7% (0·6 to 0·7)
	England	49 200 (45 600 to 52 900)	9330 (8640 to 10 000)	32 100 (29 800 to 34 500)	7780 (7210 to 8360)	57 300 (53 400 to 60 900)	10 000 (9370 to 10 700)	36 800 (34 300 to 39 100)	10 400 (9730 to 11 100)	0·7% (0·7 to 0·7)
	Northern Ireland	1700 (1570 to 1840)	384 (355 to 416)	1100 (1020 to 1190)	219 (202 to 237)	1930 (1800 to 2060)	372 (346 to 397)	1230 (1150 to 1310)	328 (305 to 350)	0·6% (0·6 to 0·6)
	Scotland	5140 (4760 to 5510)	939 (870 to 1010)	3400 (3150 to 3650)	802 (743 to 861)	5520 (4790 to 6280)	843 (732 to 960)	3590 (3120 to 4080)	1090 (943 to 1240)	0·3% (0·0 to 0·6)
	Wales	2950 (2730 to 3180)	567 (526 to 612)	1870 (1740 to 2020)	506 (468 to 546)	3150 (2940 to 3370)	524 (489 to 560)	1960 (1830 to 2100)	664 (620 to 709)	0·3% (0·3 to 0·4)
**Latin America and Caribbean**		**465 000 (450 000 to 480 000)**	**152 000 (148 000 to 157 000)**	**288 000 (278 000 to 297 000)**	**25 100 (24 200 to 25 900)**	**594 000 (560 000 to 626 000)**	**143 000 (136 000 to 150 000)**	**398 000 (374 000 to 420 000)**	**53 200 (49 800 to 56 400)**	**1·2% (1·0 to 1·3)**
Andean Latin America		46 300 (43 400 to 49 200)	16 500 (15 500 to 17 500)	27 400 (25 700 to 29 200)	2390 (2240 to 2540)	66 100 (61 400 to 70 300)	18 100 (16 800 to 19 200)	43 000 (40 000 to 45 700)	5020 (4660 to 5340)	1·7% (1·6 to 1·8)
	Bolivia	8290 (7670 to 8910)	3230 (2990 to 3470)	4690 (4340 to 5030)	373 (345 to 401)	11 800 (10 300 to 13 300)	3490 (3050 to 3930)	7560 (6620 to 8520)	750 (656 to 845)	1·7% (1·4 to 1·9)
	Ecuador	12 500 (11 600 to 13 500)	4550 (4210 to 4900)	7360 (6810 to 7930)	628 (581 to 677)	18 100 (15 500 to 20 500)	5070 (4350 to 5750)	11 600 (9930 to 13 100)	1420 (1220 to 1610)	1·7% (1·4 to 2·0)
	Peru	25 500 (22 900 to 28 200)	8690 (7820 to 9620)	15 400 (13 800 to 17 000)	1390 (1250 to 1530)	36 300 (32 900 to 39 700)	9540 (8650 to 10 400)	23 900 (21 700 to 26 100)	2850 (2580 to 3120)	1·7% (1·6 to 1·7)
Caribbean		40 100 (38 700 to 41 600)	12 100 (11 600 to 12 500)	25 200 (24 300 to 26 100)	2870 (2760 to 2970)	47 500 (44 300 to 50 900)	11 500 (10 600 to 12 500)	31 200 (29 200 to 33 500)	4750 (4470 to 5050)	0·8% (0·6 to 1·0)
	Antigua and Barbuda	76·4 (70·3 to 82·2)	21·6 (19·9 to 23·2)	49·7 (45·7 to 53·4)	5·1 (4·7 to 5·5)	89·4 (78·4 to 100)	16·9 (14·8 to 19)	63·6 (55·7 to 71·4)	8·9 (7·8 to 10)	0·7% (0·5 to 1·0)
	The Bahamas	303 (283 to 325)	85·4 (79·7 to 91·4)	202 (188 to 216)	16 (14·9 to 17·1)	388 (334 to 444)	81·2 (69·9 to 92·9)	275 (237 to 314)	31·8 (27·4 to 36·4)	1·2% (0·8 to 1·5)
	Barbados	257 (240 to 273)	56·7 (53 to 60·3)	170 (158 to 180)	30·6 (28·6 to 32·5)	299 (260 to 342)	47·1 (40·9 to 53·9)	203 (176 to 232)	49·2 (42·7 to 56·3)	0·7% (0·4 to 1·1)
	Belize	240 (223 to 256)	93·7 (87·1 to 100)	136 (126 to 145)	10·2 (9·5 to 10·9)	429 (369 to 489)	123 (106 to 140)	284 (244 to 323)	22·5 (19·3 to 25·6)	2·8% (2·4 to 3·1)
	Bermuda	63·3 (59·3 to 67·3)	12·1 (11·3 to 12·8)	44·5 (41·6 to 47·3)	6·8 (6·4 to 7·2)	63·5 (57·4 to 69·9)	8·4 (7·6 to 9·3)	42 (37·9 to 46·2)	13·1 (11·9 to 14·5)	0·0% (−0·2 to 0·2)
	Cuba	11 400 (10 500 to 12 300)	2440 (2250 to 2630)	7840 (7220 to 8450)	1120 (1030 to 1200)	11 300 (9910 to 12 700)	1780 (1560 to 2000)	7720 (6790 to 8690)	1770 (1560 to 1990)	−0·1% (−0·3 to 0·2)
	Dominica	68·6 (63·5 to 73·6)	21 (19·5 to 22·6)	41·9 (38·8 to 44·9)	5·7 (5·3 to 6·1)	67·1 (58·4 to 76·2)	13·7 (11·9 to 15·6)	46·1 (40·2 to 52·4)	7·3 (6·3 to 8·3)	−0·1% (−0·4 to 0·2)
	Dominican Republic	8600 (7900 to 9250)	2990 (2750 to 3220)	5150 (4730 to 5550)	451 (415 to 486)	11 000 (9390 to 12 600)	2940 (2510 to 3350)	7230 (6170 to 8260)	843 (719 to 963)	1·2% (0·8 to 1·5)
	Grenada	104 (95·9 to 112)	31·9 (29·4 to 34·4)	66·1 (61 to 71·2)	5·9 (5·5 to 6·4)	103 (88·9 to 116)	21·8 (18·9 to 24·6)	71·5 (61·9 to 80·5)	9·3 (8·1 to 10·5)	−0·1% (−0·4 to 0·2)
	Guyana	779 (719 to 842)	284 (262 to 307)	463 (428 to 501)	31·8 (29·3 to 34·3)	765 (670 to 859)	213 (187 to 240)	501 (439 to 563)	50 (43·7 to 56·1)	−0·1% (−0·3 to 0·1)
	Haiti	8190 (7470 to 8870)	3260 (2980 to 3540)	4610 (4210 to 5000)	314 (286 to 340)	12 900 (10 700 to 15 200)	4350 (3620 to 5140)	8010 (6660 to 9450)	506 (421 to 597)	2·1% (1·7 to 2·6)
	Jamaica	2630 (2450 to 2840)	840 (781 to 905)	1590 (1480 to 1720)	200 (186 to 215)	2800 (2450 to 3160)	584 (511 to 660)	1950 (1700 to 2200)	269 (236 to 304)	0·3% (0·0 to 0·5)
	Puerto Rico	3880 (3620 to 4130)	925 (862 to 985)	2530 (2360 to 2690)	428 (398 to 455)	3290 (3050 to 3530)	444 (411 to 477)	2120 (1970 to 2280)	725 (671 to 778)	−0·8% (−0·8 to −0·7)
	Saint Kitts and Nevis	46·4 (42·9 to 50)	13·7 (12·6 to 14·7)	29·2 (27 to 31·4)	3·6 (3·3 to 3·8)	58·6 (48·5 to 69·6)	9·8 (8·1 to 11·7)	43·4 (35·9 to 51·5)	5·4 (4·4 to 6·4)	1·1% (0·6 to 1·6)
	Saint Lucia	155 (144 to 166)	49·1 (45·4 to 52·7)	95·7 (88·6 to 103)	10·3 (9·6 to 11·1)	178 (152 to 202)	29·7 (25·4 to 33·7)	127 (109 to 144)	20·6 (17·6 to 23·4)	0·6% (0·3 to 0·9)
	Saint Vincent and the Grenadines	110 (102 to 118)	34·8 (32·3 to 37·3)	67·5 (62·7 to 72·5)	7·5 (7 to 8·1)	114 (100 to 129)	25 (21·9 to 28·2)	76·6 (67·1 to 86·6)	12·6 (11 to 14·2)	0·2% (−0·1 to 0·4)
	Suriname	449 (418 to 479)	135 (126 to 144)	287 (267 to 306)	26·9 (25 to 28·7)	579 (510 to 654)	143 (126 to 162)	384 (338 to 434)	51·8 (45·6 to 58·5)	1·2% (0·9 to 1·5)
	Trinidad and Tobago	1290 (1200 to 1380)	331 (309 to 354)	871 (812 to 930)	89·6 (83·5 to 95·6)	1390 (1210 to 1570)	272 (236 to 307)	943 (816 to 1060)	178 (154 to 200)	0·4% (0·0 to 0·6)
	Virgin Islands	111 (104 to 119)	29·7 (27·8 to 31·7)	72·5 (67·9 to 77·5)	9·1 (8·6 to 9·8)	85·9 (79·8 to 91·9)	13·4 (12·4 to 14·3)	53·9 (50 to 57·6)	18·6 (17·3 to 19·9)	−1·2% (−1·3 to −1·2)
Central Latin America		199 000 (191 000 to 208 000)	70 000 (67 400 to 73 000)	119 000 (115 000 to 125 000)	9530 (9150 to 9950)	253 000 (242 000 to 265 000)	63 500 (60 800 to 66 400)	168 000 (161 000 to 176 000)	21 200 (20 300 to 22 200)	1·1% (1·1 to 1·2)
	Colombia	39 700 (35 700 to 43 700)	13 100 (11 800 to 14 500)	24 500 (22 000 to 26 900)	2130 (1910 to 2350)	49 100 (44 500 to 53 500)	10 600 (9630 to 11 600)	33 600 (30 500 to 36 600)	4840 (4390 to 5280)	1·0% (1·0 to 1·1)
	Costa Rica	3900 (3640 to 4160)	1250 (1170 to 1340)	2440 (2270 to 2590)	214 (200 to 228)	4750 (4180 to 5340)	1020 (894 to 1140)	3250 (2860 to 3660)	481 (423 to 541)	0·9% (0·7 to 1·2)
	El Salvador	5860 (5240 to 6550)	2240 (2010 to 2510)	3280 (2930 to 3670)	336 (301 to 376)	6450 (5430 to 7380)	1820 (1530 to 2080)	4070 (3430 to 4660)	557 (469 to 637)	0·4% (0·2 to 0·6)
	Guatemala	11 100 (10 200 to 12 000)	5010 (4630 to 5420)	5680 (5250 to 6140)	388 (359 to 420)	15 800 (14 400 to 17 100)	4930 (4490 to 5360)	9910 (9030 to 10 800)	920 (838 to 1000)	1·7% (1·6 to 1·7)
	Honduras	6170 (5720 to 6660)	2630 (2440 to 2840)	3310 (3070 to 3570)	226 (210 to 244)	10 100 (8910 to 11 300)	3280 (2890 to 3660)	6330 (5580 to 7060)	508 (448 to 567)	2·3% (2·1 to 2·5)
	Mexico	101 000 (94 400 to 108 000)	34 900 (32 600 to 37 400)	61 400 (57 300 to 65 800)	4770 (4460 to 5110)	129 000 (119 000 to 139 000)	32 100 (29 600 to 34 500)	86 600 (80 000 to 93 300)	10 600 (9750 to 11 400)	1·2% (1·1 to 1·2)
	Nicaragua	4930 (4460 to 5400)	2010 (1820 to 2200)	2740 (2480 to 3000)	185 (167 to 203)	6670 (5590 to 7770)	1980 (1660 to 2310)	4300 (3600 to 5010)	391 (328 to 456)	1·4% (1·1 to 1·7)
	Panama	2910 (2730 to 3120)	927 (868 to 994)	1810 (1700 to 1940)	175 (164 to 187)	4290 (3700 to 4870)	1150 (993 to 1310)	2750 (2370 to 3120)	389 (335 to 441)	1·8% (1·4 to 2·1)
	Venezuela	23 300 (21 600 to 25 100)	7820 (7270 to 8420)	14 300 (13 300 to 15 400)	1100 (1020 to 1180)	26 600 (23 000 to 30 100)	6620 (5710 to 7480)	17 400 (15 000 to 19 700)	2580 (2220 to 2910)	0·6% (0·3 to 0·9)
Tropical Latin America		180 000 (168 000 to 192 000)	53 900 (50 300 to 57 600)	116 000 (108 000 to 124 000)	10 300 (9600 to 11 000)	228 000 (196 000 to 258 000)	50 200 (43 300 to 56 900)	155 000 (134 000 to 176 000)	22 200 (19 100 to 25 300)	1·1% (0·7 to 1·4)
	Brazil	175 000 (162 000 to 187 000)	52 000 (48 300 to 55 600)	113 000 (105 000 to 121 000)	10 000 (9340 to 10 800)	220 000 (188 000 to 251 000)	48 200 (41 100 to 54 900)	150 000 (128 000 to 171 000)	21 800 (18 600 to 24 800)	1·1% (0·7 to 1·4)
	Paraguay	5150 (4730 to 5580)	1960 (1800 to 2130)	2930 (2690 to 3180)	251 (230 to 272)	7170 (5860 to 8460)	2010 (1640 to 2370)	4680 (3830 to 5520)	481 (393 to 568)	1·6% (1·0 to 2·0)
**North Africa and Middle East**		**421 000 (407 000 to 434 000)**	**152 000 (147 000 to 157 000)**	**251 000 (243 000 to 260 000)**	**17 400 (16 800 to 18 100)**	**623 000 (600 000 to 646 000)**	**183 000 (175 000 to 191 000)**	**406 000 (390 000 to 420 000)**	**34 200 (32 900 to 35 400)**	**1·9% (1·8 to 2·0)**
	Afghanistan	15 900 (12 800 to 18 900)	7830 (6270 to 9320)	7500 (6000 to 8910)	604 (484 to 718)	31 200 (21 600 to 40 900)	14 200 (9840 to 18 600)	16 400 (11 400 to 21 500)	623 (432 to 816)	3·2% (2·5 to 3·6)
	Algeria	31 000 (28 600 to 33 500)	10 700 (9890 to 11 600)	18 900 (17 500 to 20 400)	1360 (1260 to 1470)	44 200 (37 400 to 51 000)	13 300 (11 200 to 15 300)	28 100 (23 700 to 32 300)	2840 (2400 to 3280)	1·7% (1·3 to 2·0)
	Bahrain	646 (602 to 695)	186 (173 to 200)	445 (415 to 479)	15·1 (14·1 to 16·2)	1530 (1420 to 1650)	297 (276 to 320)	1180 (1100 to 1270)	54·5 (50·7 to 58·7)	4·1% (4·1 to 4·1)
	Egypt	67 300 (61 500 to 73 000)	23 800 (21 800 to 25 900)	41 100 (37 600 to 44 600)	2290 (2090 to 2490)	106 000 (95 700 to 116 000)	36 900 (33 400 to 40 400)	64 400 (58 400 to 70 500)	4380 (3970 to 4790)	2·1% (2·1 to 2·2)
	Iran	66 200 (60 400 to 72 200)	21 900 (19 900 to 23 800)	41 300 (37 700 to 45 100)	3040 (2770 to 3310)	85 400 (76 900 to 93 900)	20 200 (18 200 to 22 200)	59 200 (53 300 to 65 100)	6010 (5410 to 6610)	1·2% (1·1 to 1·3)
	Iraq	25 100 (21 600 to 29 100)	10 200 (8790 to 11 800)	14 100 (12 100 to 16 400)	762 (654 to 881)	41 200 (29 200 to 52 100)	13 500 (9520 to 17 000)	26 100 (18 500 to 32 900)	1680 (1190 to 2120)	2·3% (1·4 to 2·8)
	Jordan	4820 (4380 to 5270)	1900 (1730 to 2080)	2780 (2530 to 3040)	134 (122 to 147)	12 300 (11 100 to 13 700)	3630 (3260 to 4030)	8180 (7340 to 9080)	512 (459 to 568)	4·5% (4·4 to 4·5)
	Kuwait	1920 (1720 to 2110)	530 (476 to 583)	1320 (1180 to 1450)	67·1 (60·2 to 73·8)	4650 (4030 to 5280)	846 (733 to 959)	3630 (3150 to 4120)	171 (148 to 194)	4·2% (4·1 to 4·4)
	Lebanon	3560 (3200 to 3970)	1110 (1000 to 1240)	2170 (1950 to 2420)	273 (245 to 304)	5540 (4670 to 6390)	1280 (1080 to 1470)	3720 (3130 to 4290)	546 (461 to 630)	2·1% (1·8 to 2·3)
	Libya	5090 (4590 to 5600)	1790 (1620 to 1970)	3100 (2800 to 3410)	199 (180 to 219)	6870 (5810 to 7980)	1490 (1260 to 1730)	5030 (4250 to 5840)	350 (296 to 406)	1·4% (1·1 to 1·7)
	Morocco	29 700 (26 800 to 32 600)	10 200 (9240 to 11 200)	18 000 (16 200 to 19 800)	1480 (1330 to 1620)	37 200 (33 100 to 41 300)	9790 (8730 to 10 900)	24 600 (22 000 to 27 400)	2740 (2440 to 3040)	1·1% (1·0 to 1·1)
	Oman	2330 (2120 to 2530)	880 (801 to 956)	1400 (1270 to 1520)	53·2 (48·4 to 57·7)	4700 (4350 to 5060)	1220 (1130 to 1320)	3370 (3120 to 3620)	115 (107 to 124)	3·3% (3·3 to 3·4)
	Palestine	3020 (2750 to 3290)	1410 (1280 to 1540)	1520 (1390 to 1660)	92 (83·8 to 100)	5140 (4660 to 5610)	1870 (1700 to 2040)	3090 (2810 to 3380)	176 (160 to 192)	2·5% (2·5 to 2·6)
	Qatar	592 (538 to 643)	159 (145 to 173)	425 (386 to 462)	7·9 (7·2 to 8·6)	2980 (2750 to 3200)	494 (456 to 531)	2450 (2260 to 2630)	37·1 (34·2 to 39·9)	7·7% (7·6 to 7·8)
	Saudi Arabia	20 800 (18 800 to 22 800)	7480 (6760 to 8210)	12 700 (11 500 to 14 000)	547 (494 to 600)	37 700 (32 600 to 43 000)	7570 (6550 to 8630)	29 100 (25 200 to 33 200)	1020 (884 to 1170)	2·8% (2·6 to 3·0)
	Sudan	26 700 (23 700 to 29 800)	11 900 (10 500 to 13 300)	13 900 (12 300 to 15 500)	922 (817 to 1030)	43 400 (37 000 to 49 700)	16 600 (14 100 to 19 000)	25 400 (21 700 to 29 100)	1390 (1180 to 1590)	2·3% (2·1 to 2·4)
	Syria	16 700 (15 100 to 18 200)	6940 (6260 to 7550)	9270 (8360 to 10 100)	519 (468 to 565)	14 000 (11 500 to 16 200)	3660 (2990 to 4240)	9350 (7640 to 10 800)	1010 (829 to 1170)	−0·9% (−1·3 to −0·5)
	Tunisia	9840 (8930 to 10 800)	2980 (2710 to 3260)	6250 (5670 to 6830)	607 (551 to 663)	11 800 (10 600 to 13 200)	2770 (2470 to 3070)	7950 (7110 to 8830)	1130 (1010 to 1260)	0·9% (0·8 to 1·0)
	Türkiye	67 100 (58 200 to 75 600)	20 100 (17 400 to 22 600)	43 100 (37 400 to 48 600)	3940 (3420 to 4450)	83 600 (77 100 to 90 000)	18 500 (17 100 to 19 900)	56 900 (52 500 to 61 200)	8170 (7530 to 8790)	1·1% (0·8 to 1·3)
	United Arab Emirates	3230 (2900 to 3550)	720 (647 to 792)	2480 (2230 to 2730)	28·5 (25·6 to 31·4)	9630 (7900 to 11 200)	1340 (1100 to 1560)	8130 (6670 to 9470)	163 (134 to 190)	5·2% (4·8 to 5·5)
	Yemen	18 600 (17 000 to 20 200)	8970 (8190 to 9730)	9160 (8370 to 9950)	490 (448 to 532)	33 600 (28 200 to 39 500)	13 800 (11 500 to 16 200)	18 800 (15 800 to 22 100)	1020 (850 to 1190)	2·8% (2·4 to 3·2)
**South Asia**		**1 330 000 (1 250 000 to 1 400 000)**	**487 000 (458 000 to 514 000)**	**781 000 (734 000 to 828 000)**	**57 400 (53 800 to 60 900)**	**1 850 000 (1 670 000 to 2 040 000)**	**507 000 (460 000 to 557 000)**	**1 220 000 (1 100 000 to 1 350 000)**	**120 000 (108 000 to 133 000)**	**1·6% (1·4 to 1·8)**
	Bangladesh	129 000 (120 000 to 139 000)	52 300 (48 400 to 56 100)	72 800 (67 400 to 78 100)	4310 (3990 to 4620)	165 000 (143 000 to 186 000)	45 800 (39 700 to 51 600)	107 000 (93 100 to 121 000)	11 600 (10 100 to 13 100)	1·1% (0·8 to 1·4)
	Bhutan	645 (582 to 712)	238 (215 to 263)	382 (344 to 421)	25·2 (22·7 to 27·8)	757 (685 to 823)	187 (169 to 204)	520 (470 to 565)	50·1 (45·3 to 54·5)	0·8% (0·7 to 0·8)
	India	1 030 000 (953 000 to 1 110 000)	366 000 (338 000 to 393 000)	620 000 (572 000 to 666 000)	47 000 (43 400 to 50 600)	1 410 000 (1 240 000 to 1 600 000)	366 000 (321 000 to 415 000)	951 000 (833 000 to 1 080 000)	97 500 (85 500 to 110 000)	1·5% (1·3 to 1·7)
	Nepal	23 900 (22 200 to 25 500)	9770 (9080 to 10 400)	13 200 (12 300 to 14 100)	904 (840 to 966)	31 100 (27 300 to 35 300)	9230 (8100 to 10 500)	20 000 (17 600 to 22 700)	1910 (1680 to 2170)	1·2% (1·0 to 1·5)
	Pakistan	139 000 (127 000 to 150 000)	58 400 (53 700 to 63 100)	75 100 (69 100 to 81 200)	5140 (4730 to 5560)	236 000 (215 000 to 257 000)	85 400 (78 100 to 93 100)	142 000 (129 000 to 154 000)	8550 (7820 to 9320)	2·5% (2·5 to 2·6)
**Southeast Asia, east Asia, and Oceania**		**1 860 000 (1 760 000 to 1 950 000)**	**483 000 (460 000 to 505 000)**	**1 250 000 (1 190 000 to 1 320 000)**	**119 000 (112 000 to 125 000)**	**2 190 000 (2 070 000 to 2 290 000)**	**445 000 (424 000 to 465 000)**	**1 490 000 (1 410 000 to 1 560 000)**	**254 000 (240 000 to 269 000)**	**0·8% (0·7 to 0·8)**
East Asia		1 300 000 (1 220 000 to 1 390 000)	305 000 (285 000 to 326 000)	907 000 (847 000 to 968 000)	92 500 (86 300 to 98 700)	1 470 000 (1 370 000 to 1 580 000)	267 000 (248 000 to 287 000)	1 000 000 (933 000 to 1 080 000)	203 000 (188 000 to 217 000)	0·6% (0·6 to 0·6)
	China	1 260 000 (1 170 000 to 1 350 000)	294 000 (274 000 to 314 000)	876 000 (816 000 to 937 000)	89 000 (82 900 to 95 200)	1 420 000 (1 320 000 to 1 530 000)	260 000 (241 000 to 279 000)	967 000 (896 000 to 1 040 000)	196 000 (182 000 to 211 000)	0·6% (0·6 to 0·6)
	North Korea	23 400 (20 900 to 26 000)	6550 (5830 to 7260)	15 300 (13 600 to 17 000)	1540 (1380 to 1710)	26 400 (22 400 to 30 300)	4770 (4040 to 5480)	18 900 (16 000 to 21 700)	2670 (2260 to 3060)	0·6% (0·3 to 0·7)
	Taiwan (province of China)	22 300 (22 100 to 22 400)	4700 (4670 to 4730)	15 600 (15 500 to 15 700)	1930 (1920 to 1940)	23 600 (21 400 to 25 900)	2950 (2670 to 3230)	16 700 (15 100 to 18 300)	4010 (3640 to 4390)	0·3% (−0·1 to 0·7)
Oceania		8350 (7950 to 8720)	3300 (3140 to 3450)	4780 (4560 to 5000)	256 (244 to 266)	13 900 (12 500 to 15 300)	5080 (4540 to 5590)	8360 (7520 to 9170)	489 (446 to 530)	2·4% (2·2 to 2·7)
	American Samoa	58·5 (54·6 to 62·6)	22·1 (20·6 to 23·6)	34·2 (31·9 to 36·6)	2·2 (2·1 to 2·4)	49·8 (45·8 to 53·2)	14·2 (13·1 to 15·2)	31·9 (29·4 to 34·1)	3·7 (3·4 to 3·9)	−0·8% (−0·8 to −0·7)
	Cook Islands	18·6 (17·1 to 20)	5·5 (5·1 to 5·9)	11·8 (10·9 to 12·7)	1·3 (1·2 to 1·4)	17·7 (16 to 19·4)	3·8 (3·4 to 4·1)	11·6 (10·5 to 12·7)	2·3 (2·1 to 2·5)	−0·2% (−0·3 to −0·1)
	Federated States of Micronesia	110 (102 to 117)	44·4 (41·3 to 47·3)	61·3 (57·1 to 65·4)	3·8 (3·5 to 4)	103 (89·5 to 116)	30·6 (26·7 to 34·7)	67·2 (58·6 to 76·2)	4·8 (4·2 to 5·5)	−0·3% (−0·6 to 0·0)
	Fiji	816 (739 to 892)	266 (241 to 290)	522 (473 to 571)	28·2 (25·5 to 30·8)	924 (839 to 1020)	272 (247 to 300)	596 (540 to 654)	56·4 (51·2 to 62)	0·6% (0·6 to 0·6)
	Guam	159 (149 to 170)	49·5 (46·2 to 52·7)	101 (94·7 to 108)	8·5 (8 to 9·1)	159 (146 to 171)	36·6 (33·7 to 39·3)	104 (95·3 to 111)	19·1 (17·6 to 20·6)	0·0% (−0·1 to 0·0)
	Kiribati	87·3 (81 to 93·8)	34·9 (32·4 to 37·5)	49·5 (45·9 to 53·1)	2·9 (2·7 to 3·1)	121 (108 to 134)	42 (37·6 to 46·6)	74·5 (66·6 to 82·7)	4·6 (4·1 to 5·1)	1·6% (1·4 to 1·7)
	Marshall Islands	52·5 (48·5 to 56·6)	21·9 (20·2 to 23·5)	29·5 (27·3 to 31·8)	1·1 (1 to 1·2)	56·3 (49·2 to 63·6)	17·5 (15·3 to 19·7)	36·5 (31·9 to 41·3)	2·3 (2 to 2·6)	0·3% (0·1 to 0·6)
	Nauru	10·8 (9·9 to 11·6)	4·2 (3·8 to 4·5)	6·3 (5·8 to 6·8)	0·3 (0·3 to 0·4)	11 (9·6 to 12·4)	4 (3·5 to 4·5)	6·6 (5·8 to 7·5)	0·4 (0·3 to 0·5)	0·1% (−0·1 to 0·3)
	Niue	1·9 (1·8 to 2·1)	0·6 (0·5 to 0·6)	1·2 (1·1 to 1·3)	0·2 (0·2 to 0·2)	1·7 (1·5 to 1·9)	0·4 (0·3 to 0·4)	1·1 (1 to 1·2)	0·2 (0·2 to 0·2)	−0·7% (−0·9 to −0·4)
	Northern Mariana Islands	72·7 (67·7 to 77·5)	17·9 (16·7 to 19·1)	53·5 (49·9 to 57·1)	1·3 (1·2 to 1·3)	48·5 (45·1 to 52·1)	11·3 (10·5 to 12·1)	33·6 (31·3 to 36·2)	3·6 (3·3 to 3·9)	−1·9% (−2·0 to −1·9)
	Palau	19·7 (18·4 to 21·1)	4·9 (4·6 to 5·2)	13·9 (13 to 14·9)	1 (0·9 to 1)	18·1 (16·2 to 20·1)	3·3 (2·9 to 3·6)	13·2 (11·8 to 14·6)	1·7 (1·5 to 1·8)	−0·4% (−0·6 to −0·2)
	Papua New Guinea	5520 (5140 to 5880)	2250 (2100 to 2400)	3110 (2900 to 3310)	156 (145 to 166)	10 500 (9100 to 11 800)	3920 (3410 to 4410)	6230 (5420 to 7020)	314 (273 to 354)	3·0% (2·7 to 3·3)
	Samoa	180 (166 to 193)	72·6 (67 to 77·6)	99·3 (91·6 to 106)	8·3 (7·6 to 8·8)	214 (193 to 236)	79·9 (72·2 to 88·1)	123 (111 to 135)	11 (10 to 12·2)	0·8% (0·7 to 1·0)
	Solomon Islands	445 (412 to 480)	190 (176 to 205)	242 (224 to 261)	13·6 (12·6 to 14·7)	684 (579 to 780)	260 (220 to 297)	401 (339 to 457)	22·6 (19·1 to 25·7)	2·0% (1·6 to 2·3)
	Tokelau	1·5 (1·4 to 1·7)	0·5 (0·5 to 0·6)	0·9 (0·8 to 0·9)	0·1 (0·1 to 0·1)	1·4 (1·2 to 1·5)	0·4 (0·4 to 0·4)	0·8 (0·8 to 0·9)	0·1 (0·1 to 0·2)	−0·6% (−0·7 to −0·5)
	Tonga	103 (93 to 113)	40·5 (36·6 to 44·3)	56·8 (51·4 to 62·2)	5·5 (5 to 6·1)	106 (96 to 117)	39 (35·2 to 42·8)	60·6 (54·7 to 66·5)	6·7 (6 to 7·3)	0·2% (0·1 to 0·2)
	Tuvalu	9·7 (8·9 to 10·5)	3·4 (3·1 to 3·7)	5·7 (5·2 to 6·2)	0·6 (0·6 to 0·7)	12·4 (10·8 to 14)	3·7 (3·3 to 4·2)	7·8 (6·8 to 8·8)	0·9 (0·8 to 1)	1·1% (0·9 to 1·3)
	Vanuatu	194 (180 to 208)	82·3 (76·3 to 88·1)	106 (98·6 to 114)	5·8 (5·4 to 6·2)	313 (291 to 336)	116 (108 to 125)	184 (171 to 198)	12·2 (11·4 to 13·1)	2·3% (2·3 to 2·3)
Southeast Asia		543 000 (513 000 to 573 000)	174 000 (165 000 to 184 000)	343 000 (323 000 to 362 000)	26 100 (24 700 to 27 500)	698 000 (670 000 to 728 000)	173 000 (166 000 to 180 000)	474 000 (456 000 to 495 000)	51 200 (49 000 to 53 300)	1·2% (1·1 to 1·3)
	Cambodia	12 500 (11 500 to 13 600)	5200 (4780 to 5640)	6910 (6350 to 7500)	430 (396 to 467)	17 000 (14 500 to 19 600)	5120 (4360 to 5890)	11 000 (9380 to 12 700)	931 (794 to 1070)	1·5% (1·1 to 1·8)
	Indonesia	212 000 (183 000 to 240 000)	66 600 (57 600 to 75 700)	135 000 (117 000 to 154 000)	9580 (8280 to 10 900)	279 000 (257 000 to 300 000)	67 300 (62 000 to 72 400)	194 000 (179 000 to 209 000)	17 500 (16 100 to 18 800)	1·3% (1·1 to 1·6)
	Laos	5390 (4850 to 5930)	2310 (2080 to 2540)	2890 (2600 to 3180)	193 (174 to 212)	7380 (6610 to 8100)	2300 (2060 to 2520)	4750 (4260 to 5220)	327 (293 to 359)	1·5% (1·5 to 1·5)
	Malaysia	23 800 (22 200 to 25 500)	7990 (7460 to 8540)	14 900 (13 900 to 15 900)	911 (851 to 974)	31 800 (27 200 to 36 000)	7610 (6510 to 8610)	21 900 (18 700 to 24 700)	2340 (2000 to 2650)	1·4% (1·0 to 1·6)
	Maldives	280 (260 to 299)	113 (105 to 121)	156 (146 to 167)	10·3 (9·6 to 11)	517 (456 to 571)	100 (88·3 to 110)	395 (348 to 436)	22·1 (19·5 to 24·4)	2·9% (2·7 to 3·1)
	Mauritius	1210 (1130 to 1300)	312 (290 to 334)	827 (769 to 887)	75·7 (70·4 to 81·1)	1270 (1100 to 1440)	207 (180 to 235)	900 (779 to 1020)	164 (142 to 186)	0·2% (−0·1 to 0·5)
	Myanmar	45 300 (38 300 to 52 300)	14 300 (12 100 to 16 500)	28 700 (24 300 to 33 100)	2300 (1950 to 2650)	56 400 (50 200 to 62 800)	15 600 (13 900 to 17 400)	37 000 (32 900 to 41 200)	3810 (3390 to 4240)	1·1% (0·9 to 1·3)
	Philippines	79 500 (73 900 to 85 100)	30 000 (27 900 to 32 100)	46 500 (43 300 to 49 800)	2940 (2740 to 3150)	113 000 (100 000 to 125 000)	34 000 (30 100 to 37 600)	73 100 (64 700 to 80 800)	6170 (5470 to 6830)	1·7% (1·5 to 1·8)
	Seychelles	81·6 (74·6 to 88)	22·3 (20·4 to 24·1)	53·2 (48·6 to 57·4)	6 (5·5 to 6·5)	105 (91·4 to 121)	23·4 (20·3 to 26·8)	73 (63·2 to 83·5)	9·1 (7·9 to 10·4)	1·2% (0·9 to 1·5)
	Sri Lanka	18 700 (16 200 to 21 200)	5090 (4390 to 5770)	12 500 (10 800 to 14 200)	1100 (954 to 1250)	22 300 (19 400 to 25 000)	5100 (4460 to 5740)	14 700 (12 800 to 16 500)	2450 (2140 to 2760)	0·8% (0·8 to 0·9)
	Thailand	62 500 (58 500 to 66 800)	15 200 (14 200 to 16 200)	43 400 (40 600 to 46 400)	3920 (3670 to 4190)	66 700 (57 500 to 75 900)	9770 (8430 to 11 100)	47 300 (40 800 to 53 800)	9640 (8320 to 11 000)	0·3% (−0·1 to 0·6)
	Timor-Leste	904 (821 to 984)	389 (353 to 423)	487 (442 to 530)	28·2 (25·6 to 30·6)	1400 (1250 to 1540)	521 (465 to 575)	803 (717 to 887)	74·4 (66·4 to 82·1)	2·1% (2·0 to 2·2)
	Viet Nam	80 200 (74 500 to 86 400)	26 300 (24 400 to 28 300)	49 400 (45 900 to 53 200)	4570 (4240 to 4920)	100 000 (92 300 to 108 000)	24 800 (22 800 to 26 600)	67 800 (62 400 to 73 000)	7670 (7060 to 8250)	1·1% (1·0 to 1·1)
**Sub-Saharan Africa**		**647 000 (629 000 to 666 000)**	**289 000 (281 000 to 297 000)**	**338 000 (329 000 to 348 000)**	**19 600 (19 000 to 20 100)**	**1 130 000 (1 090 000 to 1 180 000)**	**476 000 (457 000 to 496 000)**	**624 000 (599 000 to 650 000)**	**33 500 (32 200 to 34 800)**	**2·7% (2·6 to 2·7)**
Central sub-Saharan Africa		73 600 (65 300 to 81 300)	33 600 (29 800 to 37 200)	37 900 (33 700 to 41 800)	2020 (1780 to 2250)	137 000 (110 000 to 166 000)	58 700 (47 400 to 70 600)	74 800 (60 100 to 90 500)	3490 (2800 to 4230)	2·9% (2·5 to 3·4)
	Angola	14 700 (12 600 to 16 900)	6840 (5860 to 7850)	7560 (6480 to 8680)	323 (277 to 371)	32 700 (29 100 to 36 400)	15 200 (13 500 to 17 000)	16 700 (14 900 to 18 600)	741 (658 to 826)	3·8% (3·7 to 4·0)
	Central African Republic	3620 (3320 to 3940)	1620 (1490 to 1760)	1920 (1760 to 2080)	85·4 (78·5 to 93)	5480 (4510 to 6410)	2280 (1880 to 2670)	3080 (2530 to 3590)	125 (103 to 146)	2·0% (1·5 to 2·3)
	Congo (Brazzaville)	3150 (2790 to 3450)	1280 (1130 to 1400)	1780 (1570 to 1940)	98·1 (86·9 to 107)	5390 (4590 to 6240)	1930 (1640 to 2230)	3290 (2800 to 3810)	172 (147 to 200)	2·5% (2·3 to 2·8)
	Democratic Republic of the Congo	50 200 (41 900 to 58 100)	23 100 (19 300 to 26 700)	25 600 (21 400 to 29 700)	1450 (1210 to 1670)	90 000 (63 000 to 118 000)	38 000 (26 600 to 49 700)	49 700 (34 700 to 65 000)	2340 (1640 to 3070)	2·7% (1·9 to 3·4)
	Equatorial Guinea	654 (544 to 758)	309 (258 to 359)	328 (273 to 381)	16·3 (13·6 to 18·9)	1510 (1360 to 1680)	585 (527 to 648)	894 (805 to 990)	33·6 (30·3 to 37·3)	4·0% (3·8 to 4·3)
	Gabon	1230 (1090 to 1370)	499 (442 to 556)	675 (598 to 753)	53·2 (47·1 to 59·4)	1820 (1610 to 2020)	639 (566 to 709)	1100 (975 to 1220)	74·7 (66·1 to 82·9)	1·9% (1·8 to 1·9)
Eastern sub-Saharan Africa		250 000 (242 000 to 259 000)	117 000 (113 000 to 121 000)	127 000 (122 000 to 131 000)	6540 (6320 to 6760)	426 000 (406 000 to 447 000)	178 000 (170 000 to 187 000)	236 000 (225 000 to 247 000)	11 800 (11 300 to 12 400)	2·5% (2·5 to 2·6)
	Burundi	6390 (5610 to 7130)	3040 (2670 to 3400)	3160 (2780 to 3530)	182 (159 to 202)	13 200 (11 300 to 15 000)	5850 (5020 to 6640)	7040 (6040 to 7990)	326 (279 to 369)	3·5% (3·4 to 3·5)
	Comoros	553 (505 to 602)	233 (213 to 253)	300 (275 to 327)	19·5 (17·8 to 21·2)	744 (612 to 882)	240 (197 to 284)	467 (384 to 554)	37 (30·4 to 43·8)	1·4% (0·9% to 1·8)
	Djibouti	619 (546 to 696)	238 (210 to 268)	368 (324 to 414)	13 (11·5 to 14·7)	1260 (1080 to 1450)	413 (355 to 476)	806 (693 to 927)	39·8 (34·2 to 45·8)	3·4% (3·3 to 3·5)
	Eritrea	3980 (3370 to 4650)	1780 (1500 to 2070)	2130 (1800 to 2480)	79·7 (67·4 to 93)	6600 (4580 to 8750)	2520 (1750 to 3350)	3900 (2710 to 5180)	169 (118 to 225)	2·4% (1·5 to 3·0)
	Ethiopia	68 400 (61 800 to 75 400)	32 500 (29 400 to 35 800)	34 200 (30 900 to 37 700)	1710 (1550 to 1890)	109 000 (91 800 to 125 000)	44 400 (37 400 to 51 100)	61 400 (51 700 to 70 700)	3220 (2720 to 3710)	2·2% (1·9 to 2·4)
	Kenya	31 100 (28 800 to 33 400)	14 000 (12 900 to 15 000)	16 300 (15 100 to 17 500)	831 (768 to 892)	50 100 (46 200 to 54 000)	18 700 (17 200 to 20 100)	29 700 (27 500 to 32 100)	1650 (1530 to 1790)	2·3% (2·2 to 2·3)
	Madagascar	15 900 (14 300 to 17 500)	7270 (6530 to 8030)	8180 (7360 to 9040)	406 (365 to 448)	28 600 (26 100 to 31 000)	11 700 (10 700 to 12 700)	16 100 (14 700 to 17 500)	687 (627 to 745)	2·8% (2·7 to 2·9)
	Malawi	11 100 (10 200 to 11 900)	5080 (4660 to 5470)	5690 (5220 to 6120)	329 (302 to 354)	19 400 (17 900 to 21 000)	8120 (7460 to 8790)	10 800 (9900 to 11 700)	539 (494 to 582)	2·7% (2·7 to 2·7)
	Mozambique	17 600 (16 000 to 19 100)	8080 (7360 to 8800)	8970 (8180 to 9770)	506 (461 to 551)	31 100 (28 200 to 33 900)	14 300 (13 000 to 15 600)	16 000 (14 600 to 17 500)	767 (697 to 838)	2·7% (2·7 to 2·7)
	Rwanda	8110 (7420 to 8780)	3740 (3420 to 4050)	4180 (3820 to 4520)	197 (180 to 213)	13 300 (11 500 to 14 900)	4970 (4310 to 5600)	7850 (6810 to 8840)	451 (392 to 508)	2·3% (2·1 to 2·5)
	Somalia	10 200 (8650 to 11 700)	4780 (4070 to 5510)	5210 (4430 to 6000)	170 (144 to 195)	21 600 (15 600 to 27 000)	10 300 (7450 to 12 900)	10 900 (7850 to 13 600)	386 (279 to 484)	3·6% (2·8 to 4·0)
	South Sudan	7270 (6420 to 8090)	3300 (2920 to 3670)	3770 (3330 to 4190)	202 (178 to 225)	9670 (8120 to 11 000)	4300 (3610 to 4900)	5140 (4310 to 5860)	242 (203 to 276)	1·4% (1·1 to 1·5)
	Tanzania	34 300 (31 500 to 37 100)	15 600 (14 300 to 16 900)	17 700 (16 200 to 19 100)	1070 (985 to 1160)	58 400 (51 500 to 65 500)	24 400 (21 500 to 27 300)	32 200 (28 400 to 36 100)	1840 (1620 to 2060)	2·5% (2·3 to 2·7)
	Uganda	24 300 (22 200 to 26 300)	12 200 (11 200 to 13 300)	11 500 (10 500 to 12 400)	565 (516 to 612)	43 300 (38 700 to 48 300)	19 800 (17 700 to 22 100)	22 500 (20 000 to 25 100)	1010 (905 to 1130)	2·8% (2·6 to 2·9)
	Zambia	9930 (9220 to 10 600)	4730 (4390 to 5060)	4950 (4590 to 5290)	246 (229 to 264)	19 500 (16 800 to 22 300)	8270 (7110 to 9440)	10 800 (9270 to 12 300)	455 (391 to 519)	3·2% (2·9 to 3·5)
Southern sub-Saharan Africa		63 700 (60 000 to 67 300)	22 600 (21 300 to 23 800)	38 300 (36 100 to 40 600)	2790 (2620 to 2960)	80 300 (72 900 to 88 200)	24 100 (22 000 to 26 200)	51 700 (46 900 to 56 900)	4490 (4030 to 4970)	1·1% (0·9 to 1·3)
	Botswana	1700 (1580 to 1820)	658 (613 to 706)	978 (911 to 1050)	58·7 (54·6 to 62·9)	2390 (2080 to 2710)	698 (606 to 791)	1590 (1380 to 1800)	105 (90·8 to 118)	1·6% (1·3 to 1·9)
	Eswatini	1020 (927 to 1110)	445 (406 to 485)	546 (498 to 595)	25·8 (23·5 to 28·1)	1160 (1030 to 1260)	413 (368 to 451)	703 (626 to 767)	40 (35·7 to 43·7)	0·6% (0·5 to 0·6)
	Lesotho	1740 (1570 to 1910)	680 (617 to 748)	976 (885 to 1070)	79·7 (72·3 to 87·7)	1870 (1680 to 2070)	630 (566 to 695)	1160 (1040 to 1280)	83·9 (75·4 to 92·5)	0·4% (0·3 to 0·4)
	Namibia	1830 (1700 to 1960)	748 (695 to 800)	1020 (948 to 1090)	65·8 (61·1 to 70·4)	2430 (2090 to 2730)	825 (711 to 926)	1500 (1300 to 1690)	101 (87·2 to 114)	1·3% (1·0 to 1·6)
	South Africa	45 400 (41 800 to 48 800)	15 000 (13 800 to 16 100)	28 300 (26 000 to 30 400)	2170 (2000 to 2340)	56 900 (49 700 to 64 300)	15 200 (13 300 to 17 200)	38 000 (33 200 to 42 900)	3670 (3210 to 4140)	1·1% (0·8 to 1·3)
	Zimbabwe	12 000 (11 100 to 12 900)	5060 (4670 to 5440)	6530 (6030 to 7020)	389 (359 to 418)	15 600 (13 800 to 17 500)	6290 (5570 to 7050)	8810 (7790 to 9860)	494 (437 to 553)	1·2% (1·1 to 1·4)
Western sub-Saharan Africa		259 000 (246 000 to 273 000)	116 000 (110 000 to 122 000)	135 000 (128 000 to 142 000)	8220 (7790 to 8640)	490 000 (462 000 to 518 000)	215 000 (203 000 to 227 000)	261 000 (247 000 to 276 000)	13 700 (12 900 to 14 400)	3·0% (3·0 to 3·1)
	Benin	6720 (6170 to 7260)	3250 (2990 to 3520)	3260 (3000 to 3530)	201 (184 to 217)	13 500 (11 800 to 15 100)	6080 (5330 to 6820)	7050 (6180 to 7910)	370 (325 to 415)	3·3% (3·1 to 3·5)
	Burkina Faso	12 400 (11 300 to 13 700)	6050 (5480 to 6660)	5970 (5410 to 6560)	409 (370 to 450)	22 800 (20 900 to 24 600)	10 400 (9550 to 11 200)	11 700 (10 800 to 12 700)	690 (635 to 747)	2·9% (2·8 to 3·0)
	Cabo Verde	451 (420 to 482)	188 (176 to 201)	236 (220 to 252)	26·9 (25·1 to 28·8)	559 (487 to 634)	143 (125 to 162)	382 (333 to 434)	33·7 (29·4 to 38·2)	1·0% (0·7 to 1·3)
	Cameroon	15 100 (13 600 to 16 600)	6820 (6160 to 7530)	7780 (7020 to 8590)	453 (409 to 500)	31 800 (26 700 to 37 200)	13 500 (11 300 to 15 700)	17 500 (14 600 to 20 400)	862 (723 to 1010)	3·5% (3·2 to 3·8)
	Chad	8290 (7350 to 9220)	4130 (3660 to 4590)	3890 (3450 to 4330)	269 (238 to 299)	17 700 (15 200 to 20 300)	9010 (7720 to 10 300)	8330 (7130 to 9510)	409 (350 to 467)	3·6% (3·5 to 3·8)
	Côte d'Ivoire	16 900 (15 700 to 18 200)	7290 (6740 to 7850)	9270 (8570 to 9980)	390 (360 to 420)	27 900 (24 900 to 31 100)	11 600 (10 300 to 12 900)	15 600 (13 900 to 17 400)	728 (649 to 814)	2·4% (2·2 to 2·5)
	The Gambia	1350 (1240 to 1460)	604 (555 to 653)	706 (648 to 763)	40·6 (37·3 to 43·9)	2390 (2110 to 2680)	993 (875 to 1110)	1330 (1170 to 1490)	72·1 (63·5 to 80·9)	2·7% (2·5 to 2·9)
	Ghana	19 100 (17 800 to 20 400)	8010 (7460 to 8530)	10 500 (9770 to 11 200)	642 (598 to 683)	34 200 (29 700 to 38 900)	12 900 (11 200 to 14 600)	20 200 (17 500 to 22 900)	1200 (1040 to 1360)	2·8% (2·4 to 3·1)
	Guinea	8100 (7380 to 8800)	3750 (3420 to 4070)	3970 (3620 to 4310)	382 (348 to 415)	13 400 (12 000 to 15 000)	6050 (5380 to 6730)	6960 (6200 to 7750)	425 (379 to 474)	2·4% (2·3 to 2·5)
	Guinea-Bissau	1250 (1080 to 1410)	580 (504 to 655)	635 (552 to 717)	31·2 (27·2 to 35·3)	2060 (1780 to 2340)	898 (775 to 1020)	1120 (966 to 1270)	46·4 (40 to 52·6)	2·4% (2·4 to 2·5)
	Liberia	2850 (2520 to 3180)	1260 (1120 to 1410)	1480 (1310 to 1650)	105 (93·3 to 118)	5460 (4610 to 6310)	2190 (1840 to 2530)	3140 (2650 to 3630)	138 (117 to 160)	3·1% (2·9 to 3·3)
	Mali	11 100 (10 200 to 12 000)	5280 (4850 to 5710)	5450 (5010 to 5900)	338 (311 to 366)	24 100 (20 600 to 27 500)	11 600 (9900 to 13 200)	11 900 (10 200 to 13 600)	633 (541 to 722)	3·7% (3·4 to 4·0)
	Mauritania	2610 (2440 to 2790)	1150 (1080 to 1230)	1360 (1270 to 1450)	99·4 (92·7 to 106)	4400 (3880 to 4930)	1850 (1640 to 2080)	2370 (2100 to 2660)	169 (149 to 189)	2·5% (2·2 to 2·7)
	Niger	11 300 (10 400 to 12 100)	5560 (5130 to 5980)	5470 (5050 to 5880)	248 (229 to 267)	25 000 (21 900 to 28 000)	12 800 (11 200 to 14 300)	11 700 (10 200 to 13 100)	572 (500 to 641)	3·8% (3·5 to 4·0)
	Nigeria	123 000 (110 000 to 135 000)	53 400 (48 000 to 58 900)	65 300 (58 700 to 72 100)	3950 (3550 to 4360)	231 000 (206 000 to 258 000)	102 000 (90 400 to 113 000)	123 000 (110 000 to 138 000)	6200 (5510 to 6920)	3·0% (3·0 to 3·1)
	São Tomé and Príncipe	144 (133 to 154)	64·5 (59·7 to 69·4)	73·1 (67·7 to 78·7)	6 (5·6 to 6·5)	217 (191 to 243)	77·8 (68·6 to 87·3)	131 (116 to 147)	7·8 (6·8 to 8·7)	2·0% (1·7 to 2·2)
	Senegal	9930 (9180 to 10 700)	4390 (4060 to 4720)	5210 (4810 to 5600)	337 (312 to 362)	15 900 (14 000 to 17 600)	6360 (5620 to 7060)	8920 (7880 to 9900)	583 (515 to 647)	2·2% (2·0 to 2·4)
	Sierra Leone	4420 (4010 to 4810)	1980 (1800 to 2160)	2260 (2050 to 2450)	182 (164 to 197)	8870 (7940 to 9810)	3580 (3200 to 3960)	5010 (4490 to 5550)	276 (247 to 305)	3·3% (3·3 to 3·4)
	Togo	4850 (4270 to 5470)	2180 (1910 to 2450)	2560 (2260 to 2890)	114 (101 to 129)	8370 (7160 to 9500)	3310 (2830 to 3760)	4810 (4120 to 5460)	254 (217 to 288)	2·6% (2·5 to 2·6)

Data in parentheses are 95% uncertainty intervals. GBD=Global Burden of Diseases, Injuries, and Risk Factors Study.

**Figure 10:**
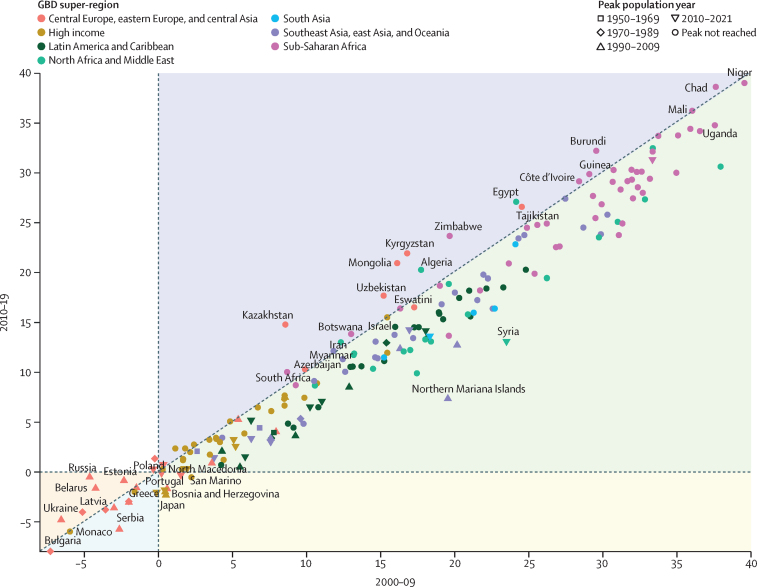
Rate of natural increase in population, 2010–19 versus 2000–09 Rate of natural increase is shown for 204 countries and territories coloured by GBD super-region. The rate of natural increase is calculated as the number of births minus the number of deaths divided by the person-years during the time period. The shape of the datapoints represents the year that peak population was reached. Purple shading indicates a higher rate of natural increase between 2010 and 2019 than between 2000 and 2009; green shading denotes a higher rate between 2000 and 2009 than between 2010 and 2019; yellow shading indicates a negative rate between 2010 and 2019 and a positive rate between 2000 and 2009; blue shading denotes a negative rate across all years that was most pronounced between 2010 and 2019; orange shading indicates a negative rate across all years that was most pronounced between 2000 and 2009; white shading denotes a negative rate between 2000 and 2009 and a positive rate between 2010 and 2019. The years 2020 and 2021 were omitted due to the impact of the COVID-19 pandemic on deaths. GBD=Global Burden of Diseases, Injuries, and Risk Factors Study.

The majority of countries and territories (154 [75·5%] of 204 countries and territories representing all seven super-regions) had a positive rate of natural increase (calculated as the number of births minus the number of deaths divided by person-years) between 2000 and 2009 followed by a smaller positive rate between 2010 and 2019 (figure 10). 26 countries and territories had a rate of natural increase that was positive during both decades and that was larger between 2010 and 2019 than between 2000 and 2009 (figure 10). Of these countries and territories, nine were in sub-Saharan Africa, eight were in central Europe, eastern Europe, and central Asia, and five were in the high-income super-region. Seven countries and territories had a positive rate of natural increase between 2000 and 2009 followed by a negative rate of natural increase between 2010 and 2019: Bosnia and Herzegovina, Greece, Japan, North Macedonia, Poland, Portugal, and San Marino (figure 10). The countries and territories of Belarus, Estonia, Latvia, Russia, and Ukraine experienced a negative rate of natural increase between 2000 and 2009 and continued to have a negative rate of natural increase between 2010 and 2019, but to a smaller extent (figure 10). The rate of natural increase was negative between 2000 and 2009 in Bulgaria, Croatia, Germany, Hungary, Italy, Lithuania, Moldova, Monaco, Romania, and Serbia, and to an even larger extent between 2010 and 2019 (figure 10). Of the 204 countries and territories, peak population was reached between 1950 and 1969 in three countries and territories, between 1970 and 1989 in eight countries and territories, between 1990 and 2009 in 23 countries and territories, between 2010 and 2021 in 22 countries and territories, and the peak population had not yet been reached as of 2021 in 148 countries and territories.

**Figure 11:**
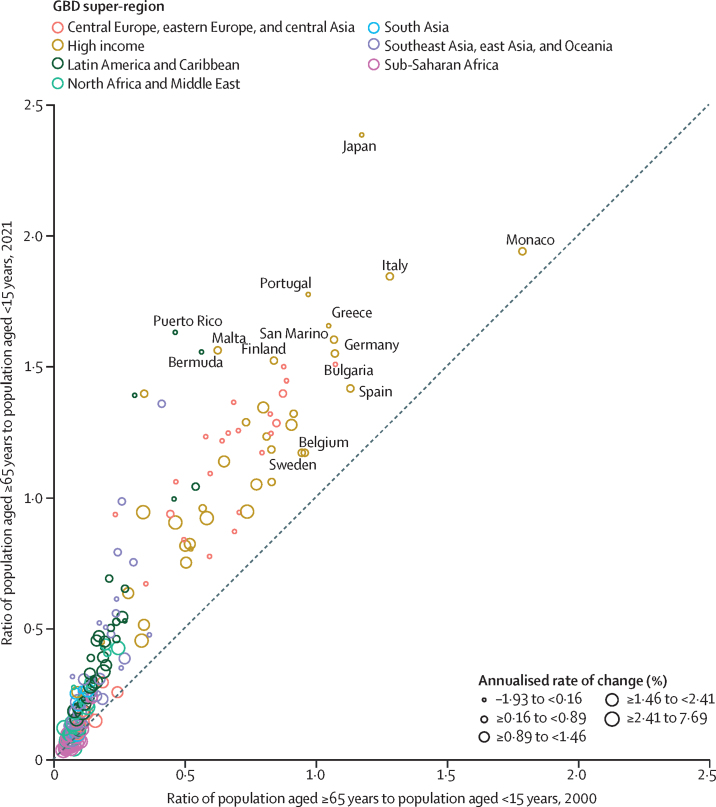
Ratio of the number of individuals older than 65 years to those younger than 15 years, 2000 versus 2021 This ratio is shown for 204 countries and territories coloured by GBD super-region. The size of the datapoints indicates the annualised rate of change in total population from 2000 to 2021, and the black dotted line represents the line of equality. GBD=Global Burden of Diseases, Injuries, and Risk Factors Study.

The age structure of populations changed substantially across the globe between 1950 and 2021, with a general shift in the distribution away from younger ages and towards older ages (table 5). From 2000 to 2021, the proportion of the population aged younger than 15 years decreased in 196 of 204 countries and territories, with some of the largest declines observed in Saudi Arabia (from 36·0% to 20·1%) and Syria (41·5% to 26·1%). The eight countries in which the proportion of the population aged younger than 15 years did not decline were Angola, Chad, Kazakhstan, Mali, Niger, Nigeria, Russia, and Somalia. During this same period, the proportion of the population aged 65 years and older increased in 175 of 204 countries and territories; some of the largest increases were observed in Japan (from 17·2% to 28·9%) and Puerto Rico (from 11·0% to 22·0%). Three of 204 countries and territories had an increase in the proportion of the population aged younger than 15 years combined with a decline in the proportion of the population aged 65 years and older; these nations (Mali, Nigeria, and Chad) are all located in sub-Saharan Africa. The ratio of the population aged 65 years and older to the population aged less than 15 years increased between 2000 and 2021 in 188 of 204 countries and territories, including all nations within the high-income; Latin America and the Caribbean; south Asia; and southeast Asia, east Asia, and Oceania super-regions (figure 11). Some of the largest increases occurred in Japan, Puerto Rico, and South Korea. The countries and territories in which this ratio did not increase were Afghanistan, Benin, Burkina Faso, Burundi, Cameroon, Chad, Democratic Republic of Congo, Guinea, Guinea-Bissau, Kyrgyzstan, Liberia, Mali, Mozambique, Nigeria, Sierra Leone, and South Sudan.

## Discussion

### Main findings

Our comprehensive set of updated demographic metrics indicate profound changes in the global health landscape during the first 2 years of the COVID-19 pandemic relative to historical trends. Long-term trends of decreasing mortality were superseded by marked increases in mortality rates in age groups older than 15 years during 2020 and 2021; in contrast, mortality in children under 5 years remained largely unaffected by the pandemic and continued to decrease globally. Global life expectancy declined sharply during 2020 and 2021, reversing the longstanding trend of life expectancy improvement. Age-standardised rates demonstrated the pandemic was disproportionately severe in countries within sub-Saharan Africa, the Middle East, south Asia, and Latin America. The COVID-19 pandemic has also highlighted the need for timely and comprehensive data collection and reporting. The development of high-quality civil registration and vital statistics systems has stagnated in many parts of the world due to multifaceted societal, financial, logistical, legislative, and political reasons, with notable exceptions including China, India, and some countries in north Africa and the Middle East. Population growth has slowed globally since 2017, although future declines might not persist at rates similar to those in 2020 and 2021 as the pandemic eases. In contrast, population growth is steady in south Asia and accelerating in sub-Saharan Africa. Increasing populations in many low-income and middle-income locations, combined with a shift in the age distribution away from younger ages and towards older ages, is likely to lead to new social, economic, and political challenges.

### Data availability and gaps

Although the proportion of registered deaths has continuously increased at the global level since 1950, we observed marked variability across GBD super-regions and individual countries and territories. Civil registration and vital statistics are particularly scarce in sub-Saharan Africa; investment in vital registration system development in these nations is recommended to improve the availability of data necessary for accurate health measurements and policy evaluation. The COVID-19 pandemic highlighted the need for accessible and up-to-date health data when trying to understand and track emerging global health events. Much uncertainty remains about the true extent of the effect of the pandemic on mortality in countries and territories with minimal to no vital registration data available, which is particularly concerning considering that these countries are potentially the most negatively impacted by the pandemic. With the exception of China, India, and some countries in north Africa and the Middle East, progress in improving the extent of global death registration has slowed—perhaps due to a focus on cheaper but less permanent and systematic data collection efforts, such as small-scale and large-scale surveys. Although surveys are an invaluable source of demographic information, investing in more expensive yet comprehensive civil registration and vital statistics systems is crucial to monitor and improve population health.^26^

Beyond creating and improving civil registration and vital statistics systems, countries and territories without data during the past decade would also benefit from collecting additional data from other sources, such as censuses and nationally representative surveys. 30 countries and territories had no available data on child mortality for the period 2015–21, and 62 countries and territories had no available data on adult mortality. 41 countries and territories had no usable census data between 2010 and 2021, but census data were available before 2000 for these countries. Furthermore, the COVID-19 pandemic interrupted many data collection efforts, such as the USAID Demographic and Health Surveys Program,^27^ and national censuses, which are now resuming.^28^ Impacts such as these must be resolved to improve future data availability.

### Impact of the COVID-19 pandemic

The COVID-19 pandemic had differential effects on mortality across the lifespan. Life expectancy decreased in every GBD super-region and 84% of countries and territories from 2019 to 2021, but younger age groups were minimally affected. This finding is a welcome contrast to early warnings about potentially devastating impacts of the pandemic on child mortality.^29^ Conversely, increases in mortality rates in populations aged 25 years and older were observed on a scale not seen in the previous 70 years.^30^ Although the burden of excess deaths and all-age excess mortality rates due to the pandemic was largest in countries in central and eastern Europe, and Latin America, our analysis of age-standardised mortality rates highlights the relative severity of the pandemic's effects on mortality in certain countries within sub-Saharan Africa, the Middle East, south Asia, and Latin America. There was a general association between higher SDI and lower excess mortality, but this association was not particularly strong, and many countries were exceptions to this association, suggesting that at the population level, SDI was not always a strong predictor of excess mortality due to the COVID-19 pandemic in 2020 and 2021. Excess mortality was particularly high in nations such as Bolivia and South Africa when compared with other countries and territories with a similar SDI, which some have argued was in part due to relaxed containment strategies and vaccine hesitancy.^31^ Conversely, excess mortality was particularly low in countries such as the Solomon Islands and Bhutan, which might be a reflection of delayed transmission in more isolated nations and of high vaccination rates.^32^ These findings emphasise that mortality outcomes during the COVID-19 pandemic were not solely determined by SDI and that vaccination efforts, public policies, and individual behaviour changes likely influenced the severity of the pandemic across countries and territories at all levels of SDI.^33^, ^34^, ^35^, ^36^, ^37^ Reports published as recently as 2023 have shown that since 2021, mortality due to the pandemic has declined,^38^, ^39^ presumably driven by vaccination efforts, public policies, individual behaviour changes, and the emergence of new SARS-CoV-2 variants with lower case-fatality ratios.^40^, ^41^ However, mortality has increased in some locations, which might be due to lifting of protective restrictions.^42^

### Long-term mortality trends

In the era of the UN Sustainable Development Goals (SDGs), there has been a decline in the global U5MR, which continued during the COVID-19 pandemic. However, progress has varied substantially between countries, and many continue to lag behind SDG targets. Based on the trajectory of U5MR between 2010 and 2021, 38 countries will not reach SDG target 3.2 of a U5MR at least as low as 25 deaths per 1000 livebirths by 2030 (appendix 2 table S2A). To eradicate preventable under-5 deaths, more equitable global strategies—intensified in regions with the highest rates—are imperative. Compared with child mortality, reductions in adult mortality have not been as consistent globally. Historically, increased adult mortality was observed in the 1990s in countries spanning eastern and southern Africa, eastern Europe, and central Asia. During the late 2010s, some high-income nations, including the USA, have had mortality spikes, particularly among the 15–39-years age group, which reflect mortality patterns associated with increased drug and alcohol misuse and mental health disorders.^43^, ^44^ The 15–39-years age group is particularly volatile globally, and is the age group most affected by fatal discontinuities such as conflict.^45^ Sex differences in mortality vary widely across the globe. The global ratio of male to female mortality has generally increased, although it has differed as a function of age. The largest variability in the ratio of male to female mortality was in the 15–39-years age group with much less variability observed in younger and older age groups. These differences go beyond biological explanations and highlight the importance of future efforts to address mortality risks to which males are particularly susceptible due to behavioural factors, war and conflict, occupational hazards, homicide, and suicide.^46^, ^47^ The substantial differences among countries show, however, that it is also important to address mortality risks that predominantly affect women, such as maternal mortality, gender-based violence, and economic disparities.^48^, ^49^ We also found that life expectancy was consistently higher in countries in the Americas, east Asia, and western Europe than countries in sub-Saharan Africa, and this effect was strongly associated with SDI. Although we did not establish causal effects, this finding is supported by many studies showing that social determinants of health are key drivers of mortality,^50^, ^51^, ^52^, ^53^, ^54^ and improving education, economic prosperity, and gender inequalities is vital for continual progress in health outcomes globally. However, notable exceptions regarding the relationship between mortality and SDI indicate that other factors are also involved.

### Population dynamics and age structures

Although the rate of global population growth has plateaued and started to decline since 2017, in lower income countries—primarily in sub-Saharan Africa—rapid population growth has continued. Thus, much of future population growth will likely occur in the poorest regions. Resource scarcity and rapid infrastructure expansion will be crucial issues to address.^55^, ^56^ These factors, and a history of colonialism, can contribute to political instability.^57^, ^58^ These challenges will require responses from governments and the global community. Furthermore, the concentration of population growth has shifted to locations with the poorest health—ie, locations with the highest child mortality rates. This might lead to challenges in continuing improvement of health outcomes.

Outside of these locations, slowing of population growth is widespread. Although most countries and territories had not reached a peak population as of 2021, in 171 of 204 countries and territories a lower rate of natural increase was observed between 2010 and 2019 than between 2000 and 2009. Furthermore, our analysis of population age structures over time indicated a prominent shift towards older ages in most regions and nations. As older populations expand and reduced younger populations reach working-age, nations could encounter economic and social challenges requiring updated policies related to health care, retirement, reproduction, childcare, and migration.^59^, ^60^, ^61^, ^62^ The shift towards a higher ratio of older people to younger people will require greater attention to be paid to labour shortages, health systems strengthening, and evaluation of government policies on retirement and health care.^61^, ^63^, ^64^ However, beneficial consequences such as the so-called second demographic dividend of greater personal wealth and investment in human capital might offset some of these challenges.^65^ Future research on these topics must seek to understand how changing population dynamics impact health outcomes and systems, and how health interventions can be tailored to address the unique challenges posed by these demographic shifts. Migration is particularly relevant to these challenges. Voluntary emigration from locations with younger adult population bulges to locations in need of more labour to support ageing populations is an open public policy discussion.^66^, ^67^ The level of migration needed to support older age populations is dynamic and is likely to change over time with technological innovations and new public policies.^68^ Furthermore, environmental constraints in some high-income countries might limit immigration possibilities. Migration of skilled workers out of lower-income countries might consequently worsen these economies.^69^, ^70^ Global cooperation is necessary, and guidelines such as the UN Global Compact for Safe, Orderly and Regular Migration^71^ can help lead this work.

### Comparisons between GBD 2021 estimates and other estimates

There are numerous differences in data processing and statistical modelling assumptions between the GBD 2021 estimates reported here and those from other demographic studies that provide important advantages. Excess mortality estimates for 2020 and 2021 have been previously reported in the GBD study and by other institutes. Our previous excess mortality estimates reported 18·2 million (95% UI 17·1–19·6) excess deaths in this study. Estimating mortality during the COVID-19 pandemic was particularly difficult due to many factors including delays in reporting, differing granularity of available data, and political will to provide accurate data. Although our earlier estimates were based on the best available data and methodology at the time, we have made data and modelling improvements that resulted in this lower estimate. We updated to more reliable data sources in some countries that corrected errors in reporting, and included more data up to the end of 2021. Methodologically, we modelled data at the yearly level, and additionally included age-specific detailed projections from our GBD mortality modelling process to inform our non-pandemic counterfactual, which generally led to higher estimates of expected non-pandemic mortality and thus lower excess mortality.

Our current estimate of global excess mortality during 2020 and 2021 is comparable to the WHO estimate of 14·9 million (95% UI 13·3–16·6) excess deaths,^15^ with our mean estimate falling within the uncertainty interval of the WHO estimate and vice versa. Our estimates tend to be higher than those of WHO for sub-Saharan Africa, with the largest differences being 233 000 more deaths in Nigeria and 177 000 more deaths in Ethiopia; and south Asia, with the largest differences being 262 000 more deaths in Pakistan and 171 000 more deaths in Bangladesh. However, our estimate for India was 1·3 million deaths lower than that of WHO, which is the largest discrepancy in this direction. We also estimated 123 000 more excess deaths in China—our results indicated positive excess, whereas WHO estimated negative excess. The largest differences occur in locations for which little or no all-cause mortality data were available for the pandemic period, and thus estimates relied on predictive models. These differences reflect different covariates used for predictions models. Additionally, WHO models and predicts all-cause mortality rates in locations without data, whereas we predict excess mortality rates directly, which leads to different assumptions and functional forms for statistical models. Differences in locations with all-cause mortality data are driven by different data processing steps and different models for expected non-pandemic mortality.

The latest estimates from UNICEF, published in 2023, reported a global U5MR of 38·1 deaths (95% UI 36·1–42·2) per 1000 livebirths in 2021,^72^ which is consistent with our estimate of 35·7 deaths (30·5–42·0) per 1000 livebirths. The mean relative difference at the national level between our 2021 U5MR estimates and those provided by UNICEF is –2·6%, ranging from –58·4% to 111·9%. Similar to our estimates, the UNICEF estimates show a continued decreasing trend in child mortality during the COVID-19 pandemic. Between 1950 and 2019, the mean relative difference between our estimates and UNICEF estimates across countries and territories was –2·0%, ranging from –64·3% to 154·6%. These differences primarily reflect differences in data inclusion, processing, and synthesis. For example, our estimate of mortality in Iran in 2021 is 58·4% lower than that of UNICEF. We included vital registration data from 2021 and our estimates closely match this observed mortality, whereas UNICEF does not include these data, leading to higher estimates. Using the most recent available data suggests our estimates are more reliable.

Adult mortality estimates at the country level from the 2022 UN World Population Prospects (WPP) report are on average 11·1% lower than our 2021 estimates,^13^ which range from 41·8% lower to 289·5% higher. Between 1950 and 2019, the mean relative difference between our adult mortality estimates and those from WPP 2022 was –4·3%, ranging from –64·0% to 229·6%. Differences between WPP 2022 estimates of national life expectancy at birth and those from GBD 2021 are primarily driven by these differences in adult mortality estimates, and variability in child mortality estimates. While location-years with complete death registration show substantial agreement between estimates, with a mean relative difference of 1·3%, our estimates for 2021 range from 7·8 years lower to 10·1 years higher, and our estimates for years before the COVID-19 pandemic range from 20·4 years lower to 38·4 years higher. The largest discrepancies were due to location-years with large fatal discontinuities or scarcity of high-quality vital registration data. Furthermore, discrepancies between 2021 estimates are highly influenced by the differences in estimation of excess mortality due to the COVID-19 pandemic. As one of the largest differences, our life expectancy estimate for Nigeria in 2021 is 10·1 years higher than the WPP estimate, driven by our estimated 41·8% lower adult mortality. Our adult mortality estimates more closely follow the bulk of the data from sibling-survival histories, and our age-specific mortality estimates rely on a database of 43 758 empirical life tables as opposed to the Coale-Demeny north model life table used by WPP 2022, which has been shown to underperform compared with other modern model life table methods.^73^, ^74^

For further comparison with WPP and as a model validation exercise, we compared estimated age-specific mortality rates and death counts from our analysis and from WPP with those calculated directly from all location-years of vital registration data deemed to have complete death registration. When comparing our results, we used our population estimates as the denominator to calculate mortality rates from vital registration; similarly, we used WPP population estimates as the denominator for that comparison. Across all location-year-age-sex mortality rates, our estimates had mean absolute error of 0·024, indicating a good fit to the data, along with root mean squared error (RMSE) of 0·52. These were lower than the respective 0·033 and 0·53 calculated for WPP. Similarly, our death count estimates had a mean absolute error of 84·8 and RMSE of 365 compared with a mean absolute error of 222 and RMSE of 1032 for WPP estimates.

Estimates of the global population from WPP 2022 are similar to that of this study, with an estimated global population of 7·91 billion in 2021, compared with our estimate of 7·89 billion (95 % UI 7·67–8·13). On average in 2021, country-level population estimates were 0·2% lower in GBD 2021 than WPP 2022 and ranged from 34·2% lower to 82·2% higher. For specific ages, differences in the younger than 15 years age group ranged from 48·0% lower to 75·3% higher, while differences in the 65 years and older age group ranged from 36·0% lower to 39·5% higher. The largest relative differences were for locations in which no recent census data were available, and those with substantial net in-migration from other countries.

### Limitations

This research has several limitations. First, estimates continue to be limited by data source availability and scope. COVID-19 showed the crucial need to create more robust vital registration systems that can highlight the differential effects of disease and injury across population subgroups in a timely manner. 93 of 204 countries and territories had no available all-cause mortality data to estimate excess mortality due to the COVID-19 pandemic, which means our estimates in these areas are solely driven by associations with covariates. These locations were largely in regions where the effects of the pandemic were most severe. Furthermore, the scarcity of high-quality civil registration and vital statistics systems to produce reliable data in many low-income and middle-income countries introduces large-scale uncertainty in all demographic estimates. Additionally, population estimates in certain countries rely on modelled projections due to no available recent censuses. Future development of reliable data sources is crucial because estimates improve as the quality of underlying data improves. Subsequent GBD cycles will provide revised estimates after additional data for recent years become available.

Second, analysis of more granular subpopulations such as subnational areas or by other population characteristics was restricted by data availability. Although our effort represents the most comprehensive global analysis of mortality and population, the estimates presented in this research mask substantial heterogeneity in smaller geographies. This limits the utility of our estimates to provide insights for more targeted interventions, for example, understanding occupational hazards in industrial regions. Improving this aspect of the research requires more comprehensive and detailed data, such as by race, ethnicity, socioeconomic status, and smaller administrative levels,^75^, ^76^, ^77^ and future work will aim to produce more comprehensive health metrics.

Third, the GBD demographics approach has not developed an encompassing model to estimate migration together with population, mortality, and fertility. Estimating migration in a model that jointly informs population, mortality, and fertility will not only improve accuracy of population estimates, but also allow assessing and improving corrections for death registration completeness and census coverage. This is crucial in locations with large migration flows, such as the United Arab Emirates and Qatar, where current methods for these corrections might not perform well.^78^, ^79^ The increased importance of migration at present and in the future, especially considering the shifting age structure in many populations, places renewed importance on producing reliable migration estimates.

Fourth, we assumed a binomial distribution when calculating data variance and did not evaluate other models of distribution. Some of our input data might be overdispersed, resulting in inaccurate estimates of data variance. However, we do not expect that changing our assumptions on the distribution would have a sizeable impact on estimates since the sampling errors on vital registration and civil registration mortality and fertility data are likely to be much smaller than non-sampling errors. In the future, we will consider testing such assumptions.

Fifth, computational resources did not permit propagation of uncertainty for all covariates throughout the analytical process. While uncertainty from model estimation was accounted for at each stage, such as U5MR, adult mortality, and age-specific mortality rates, uncertainties for some covariates such as lag-distributed income and education were not. Similarly, estimates of coefficients in the COVID-19 excess mortality prediction model did not include uncertainty. Future iterations of GBD will investigate computationally more efficient implementation of current methods and development of new methods to allow for all sources of uncertainty to be included in modelling.

### Future directions

The COVID-19 pandemic will likely continue to impact estimates of demographic trends in future years due to reporting lags and the persistent effects of the pandemic. Future research should focus on understanding the full demographic impact of the pandemic in 2022 and beyond. Methodologically, we aim to improve our incorporation of excess mortality and COVID-19 direct mortality estimates into the GBD mortality estimation process, rather than post-hoc unification of two separate modelling endeavours. We also plan to develop a standalone migration model and integrate this model into the GBD demographic estimation process. Along with this, we aim to simultaneously estimate mortality and population rather than the current sequentially iterative approach. This would allow the uncertainty in mortality estimates to inform population estimates and vice versa, helping address issues in age, period, and cohort trends that might otherwise arise.

### Conclusion

Tracking long-term health trends and evaluating the impact of the COVID-19 pandemic require accurate global, regional, and national estimates of mortality, life expectancy, and population, because these crucial demographic indicators foundationally underpin our understanding of population health. The comprehensive demographic metrics reported in this study show that marked reversals in adult mortality and life expectancy trends occurred during 2020 and 2021, leading to increased mortality and reduced life expectancy worldwide. This increased mortality did not occur in younger populations: mortality rates in children under 5 years continued to decline globally during the first 2 years of the pandemic, although more equitable and intensified investment is needed to achieve SDG targets in many locations. While global population growth is slowing, geographical distributions and age structures are undergoing fundamental shifts—low-income countries and territories continue to grow, and population structures across the globe are ageing. Nations in the post-pandemic world will need to address emerging health-care, economic, and social challenges with new policies and practices. The development, implementation, and evaluation of these health policies and practices in diverse locations around the world can be informed and guided by the GBD 2021 demographic estimates. Accurate mortality, life expectancy, and population estimates might be even more important to informing policy and practice in a post-pandemic world than in the past. Collectively, the extensive set of demographic estimates reported here represent a valuable global tool for policy evaluation, development, and implementation in diverse locations around the world.

## Data sharing

To download the data used in these analyses, please visit the GBD 2021 Sources Tool. The statistical code used in GBD 2021 is available online.

For the **Mortality Visualisation** Tool see https://vizhub.healthdata.org/mortality/

## Declaration of interests
