# Supplementary material for: Global age-sex-specific mortality, life expectancy, and population estimates in 204 countries and territories and 811 subnational locations, 1950–2021, and the impact of the COVID-19 pandemic: a comprehensive demographic analysis for the Global Burden of Disease Study 2021
Source: Lancet. 2024 May 18;403(10440):1989–2056. doi: 10.1016/S0140-6736(24)00476-8 (PMC11126395; doi:10.1016/S0140-6736(24)00476-8)
Supplement: Supplementary appendix 1 [file mmc1.pdf]

THE LANCET

Supplementary appendix 1

This appendix formed part of the original submission and has been peer reviewed. We post it as supplied by the authors.

Supplement to: GBD 2021 Demographics Collaborators. Global age-sex-specific mortality, life expectancy, and population estimates in 204 countries and territories and 811 subnational locations, 1950–2021, and the impact of the COVID-19 pandemic: a comprehensive demographic analysis for the Global Burden of Disease Study 2021. *Lancet* 2024; **403:** 1989–2056.

Appendix 1: methods appendix to "Global age-sex-specific mortality, life expectancy, and population estimates in 204 countries and territories and 811 subnational locations, 1950–2021, and the impact of the COVID-19 pandemic: a comprehensive demographic analysis for the Global Burden of Disease Study 2021"

This appendix provides further methodological detail for "Global age-sex-specific mortality, life expectancy, and population estimates in 204 countries and territories and 811 subnational locations, 1950–2021, and the impact of the COVID-19 pandemic: a comprehensive demographic analysis for the Global Burden of Disease Study 2021 ."

Preamble

This appendix provides further methodological detail for "Global age-sex-specific mortality, life expectancy, and population estimates in 204 countries and territories and 811 subnational locations, 1950–2021, and the impact of the COVID-19 pandemic: a comprehensive demographic analysis for the Global Burden of Disease Study 2021." This study complies with the Guidelines for Accurate and Transparent Health Estimates Reporting (GATHER) recommendations.[1] It includes detailed tables and information on data in an effort to maximise transparency in our estimation processes and provide a comprehensive description of analytical steps. We intend this appendix to be a living document, to be updated with each iteration of the Global Burden of Disease Study.

Portions of this appendix have been reproduced or adapted from appendices for Wang et al 2010,[2] GBD 2016 Mortality Collaborators,[3] GBD 2017 Mortality Collaborators,[4] GBD 2017 Population and Fertility Collaborators,[5] GBD 2017 DALYs and HALE Collaborators,[6] and 2019 Demographics Collaborators.[7] References are provided for reproduced sections.

Table of Contents

Preamble 2

List of appendix figures and tables 6

Appendix figures: 6

Appendix tables: 6

Section 1: GBD overview 7

Section 1.1: Global Burden of Diseases, Injuries, and Risk Factors Study 2021 7

Section 1.2: Harmonising estimation of mortality, population, and fertility 7

Section 1.3: Geographical locations of the analysis 7

Section 1.4: Time period of the analysis 7

Section 1.5: Statement of GATHER compliance 7

Section 1.6: List of abbreviations 7

Section 1.7: GBD results overview 10

Section 1.8: Data input sources overview 10

Section 1.9: Funding sources 11

Section 2: All-cause mortality, HIV mortality, and fatal discontinuities 11

Section 2.1: Overview 11

Section 2.2: Child mortality 11

Section 2.2.1: Data sources 11

Section 2.2.2: VR prioritisation 12

Section 2.2.3: VR under-enumeration for bias correction 13

Section 2.2.4: Computation of 5q0 from CBH 13

Section 2.2.5: SBH method 13

Section 2.2.6: ${}_5q_0$ data synthesis 13

Section 2.2.7: Identify and remove outliers 18

Section 2.2.8: Rake subnational estimates to national level (excluding South Africa) 18

Section 2.2.9: Review estimates for quality 19

Section 2.2.10: Under-5 age and sex pattern model estimation 19

Section 2.2.11: Identify and remove outliers from age and sex models 21

Section 2.2.12: Under-5 death number estimation 21

Section 2.3: Estimation of adult mortality rate 22

Section 2.3.1: Data sources 22

Section 2.3.2: Completeness assessment: death distribution methods and completeness estimates synthesis 22

Section 2.3.3: Sibling survival method 24

Section 2.3.4: ${}_{45}q_{15}$ data synthesis using non-linear mixed effects model and ST-GPR 27

Section 2.3.5: Identify and remove outliers 31

Section 2.3.6: Scale subnational estimates to national level (excluding South Africa) 32

Section 2.4: Model life table system 32

Section 2.4.1: Overview 32

Section 2.4.2: Building an empirical model life table database: data sources and quality review 33

Section 2.4.3: Extending age-specific mortality 34

Section 2.4.4: GBD relational model life table system with a flexible standard selection mechanism 34

Section 2.4.5: Rake subnational life tables to national level (excluding South Africa) 38

Section 2.5: HIV/AIDS estimation....38
Section 2.5.1: Age-specific mortality (with and without HIV)....38
Section 2.5.2: HIV-free survival rates (for Spectrum)....38
Section 2.5.3: EPP and Spectrum....38
Section 2.5.4: HIV mortality reckoning....39
Section 2.5.5: Envelope calculation....40
Section 2.5.6: Life table calculation....40

Section 2.6: Age-specific mortality estimation for all GBD age groups: with and without HIV....40
Section 2.6.1: HIV-deleted age-specific mortality....40
Section 2.6.2: Age-specific mortality without discontinuities (with HIV/AIDS)....40
Section 2.6.3: Add fatal discontinuities....41
Section 2.6.4: Ages-specific deaths with discontinuities and HIV/AIDS....41
Section 2.6.5: Life tables with HIV/AIDS and fatal discontinuities....41

Section 2.7: Fatal discontinuities....42
Section 2.7.1: Input data....42
Section 2.7.2: Location mapping....44
Section 2.7.3: Side splitting....45
Section 2.7.4: Prioritisation....45
Section 2.7.5: Age-sex splitting....45
Section 2.7.6: Assigning uncertainty and generating draws....45
Section 2.7.7: Changes since GBD 2019....45

Section 2.8: Accounting for the COVID-19 pandemic....46
Section 2.8.1: Excess mortality due to the COVID-19 pandemic....46
Section 2.8.2: Excess mortality age-sex splitting....47
Section 2.8.3: Balancing equation....48
Section 2.8.4: COVID-19 excess mortality harmonisation....48

Section 3: Fertility....48

Section 3.1: Overview....48
Section 3.2.1: Fertility data source types....48
Section 3.2.2: Fertility data identification and synthesis....48
Section 3.3.2: Age-specific fertility rate estimation for 15 to 49 years....49
Section 3.3.3: Data source adjustment....51
Section 3.3.4: Hyperparameter selection....51
Section 3.3.5: SBH methods....53
Section 3.3.6: Age-specific fertility rate estimation for 10- to 14-year-olds and 50- to 54-year-olds....53
Section 3.3.7: Fertility metrics....54

Section 3.4: Sex ratio at birth....54
Section 3.4.1: Overview....54
Section 3.4.2: Modelling approach....54

Section 4: Population....54

Section 4.1: Overview....54

Section 4.2: Data sources and processing....55
Section 4.2.1: Census and registry lists....55
Section 4.2.2: Data extraction....55
Section 4.2.3: Data processing....55

Section 4.3: Model inputs....58
Section 4.3.1: Migration....58
Section 4.3.2: Mortality....59

Section 4.3.3: Fertility....59
Section 4.3.4: Sex ratio at birth 59
Section 4.4: Modelling strategy 59
Section 4.4.1: CCMPP 59
This equation is for ages above 0 years, as it does not include fertility. Fertility rates are used along with populations sizes in childbearing ages to calculate the age 0 population. 59
Section 4.4.2: Bayesian demographic balancing model 59
Section 4.4.3: Model description 60
Section 4.4.4: Population uncertainty 61
Section 4.4.5: GBD world population age standard....62
Section 5: Socio-demographic Index (SDI) analysis 62
Section 5.1: Overview....62
Section 5.2: SDI calculation 62
Section 6: Additional methods....63
Section 6.1: Correlation estimation....63
Section 6.2: Annualised rates of change....63
Section 6.3: Gross domestic product (GDP) and lag-distributed income 64
Section 6.4: Educational attainment covariate....64
Section 7: References....66
Section 8: Tables and figures....71
Section 9: Authors' contributions....251

List of appendix figures and tables

See section 8 for all appendix figures and tables listed here.

Appendix figures:

Appendix Figure S1. Analytical flowchart for the estimation of all-cause mortality by age and sex, excess mortality due to COVID-19, and HIV/AIDS incidence, prevalence, and mortality for GBD 2021

Appendix Figure S2. Estimated completeness of death registration 1950−2021

Appendix Figure S3. Analytical flowchart for the GBD 2021 fertility estimation process

Appendix Figure S4. Analytical flowchart for the GBD 2021 population estimation process

Appendix Figure S5. Census and registry availability by location and year

Appendix tables:

Appendix Table S1. GBD location hierarchy with levels

Appendix Table S2. GATHER checklist

Appendix Table S3. Number of all-cause mortality data sources by type and location, 1950–2021

Appendix Table S4. Number of all-cause mortality data sources by type and year, 1950–2021

Appendix Table S5. Number of 5q0 data sources by type and location, 1950–2021

Appendix Table S6. 5q0 reference sources by source date and location, 1950–2021

Appendix Table S7. New data sources added for GBD 2021 mortality estimation

Appendix Table S8. Number of outliered datapoints for the age/sex model by location, source type, and age group

Appendix Table S9. Distribution of empirical life tables by decade and GBD super-region, 1950–2021

Appendix Table S10. Locations by HIV estimation group

Appendix Table S11. Number of sources used for the analysis of age-specific fertility by location

Appendix Table S12. Number of sources used for the analysis of age-specific fertility by year

Appendix Table S13. List of all confirmed censuses by location and year

Appendix Table S14. GBD world population age standard

Appendix Table S15. Socio-demographic Index values for all estimated GBD 2021 locations, 1950–1974

Appendix Table S16. Socio-demographic Index values for all estimated GBD 2021 locations, 1975–1999

Appendix Table S17. Socio-demographic Index values for all estimated GBD 2021 locations, 2000–2021

Section 1: GBD overview

Section 1.1: Global Burden of Diseases, Injuries, and Risk Factors Study 2021

The Global Burden of Diseases, Injuries, and Risk Factors Study (GBD) is a collaborative research effort aimed at estimating worldwide population, fertility, morbidity, and mortality. The GBD collaborator network draws on the expertise of over 11 000 contributors from around the world. For this paper, we estimated mortality and population by age, sex, and location from 1950 to 2021.

Section 1.2: Harmonising estimation of mortality, population, and fertility

The GBD demographic analytical framework is unique in that mortality and population estimates are generated using a closely interconnected modelling process that also incorporates estimation of fertility metrics. For GBD 2021, we first generated age-specific mortality and fertility estimates separately using the analytical approaches described in the sections that follow. In these steps, population was used as an input for the calculation of age-specific mortality rates and fertility rates from vital and civil registration systems. The outputs from these two processes were used to generate age-sex-specific population estimates by location and year using a Bayesian hierarchical cohort component model for population projection. Once the new population numbers were estimated, they were returned as inputs to the same mortality and fertility estimation models that were run again in a second loop. The population estimation process was then run again using the revised mortality and fertility numbers to produce the final population estimates for GBD 2021. To maintain consistency between mortality, population, and fertility in count space, we updated death numbers and birth numbers estimates using the rates estimated in the second loop and the final population estimates.

Section 1.3: Geographical locations of the analysis

We produced estimates for 204 countries and territories that were grouped into 21 regions and seven super-regions. The seven super-regions are central Europe, eastern Europe, and central Asia; high income; Latin America and the Caribbean; north Africa and the Middle East; south Asia; southeast Asia, east Asia, and Oceania; and sub-Saharan Africa. In GBD 2021 we continue to analyse at subnational levels countries that were added in previous cycles including Brazil, China, Ethiopia, India, Indonesia, Iran, Italy, Japan, Kenya, Mexico, New Zealand, Nigeria, Norway, Pakistan, Russia, the Philippines, Poland, South Africa, Sweden, the UK, and the USA. All analyses are at the first level of administrative organisation within each country except for New Zealand (by Māori ethnicity), Sweden (by Stockholm and non-Stockholm), the UK (by local government authorities), and the Philippines (by provinces). To meet data use requirements, in this publication we present subnational estimates for Brazil, Ethiopia, Indonesia, Iran, Japan, Kenya, Mexico, Norway, Pakistan, South Africa, Sweden, the UK, and the USA; given space constraints, these results are presented in Appendix 2 tables instead of the main text. Additionally, subnational estimates for China, India, Italy, Nigeria, the Philippines, and Russia are included in maps but are not reported in appendix tables. Subnational estimates for other countries will be released in separate publications. A list of locations can be found in appendix table S1 (section 8).

Section 1.4: Time period of the analysis

We estimated numbers and rates of all-cause mortality and population for the years 1950–2021.

Section 1.5: Statement of GATHER compliance

This study complies with the Guidelines for Accurate and Transparent Health Estimates Reporting (GATHER) recommendations.[1] We have documented the steps involved in our analytical procedures and detailed the data sources used. See appendix table S2 (section 8) for the GATHER checklist.

The GATHER recommendations can be found here: http://gather-statement.org/

Section 1.6: List of abbreviations

Abbreviation	Full phrase
${}_{1}q_{0}$	probability of death from birth to age 1 year
${}_{45}m_{15}$	mortality rate between age 15 years and 60 years
${}_{45}q_{15}$	probability of death between age 15 years and 60 years
${}_{4}q_{1}$	probability of death between age 1 year and 5 years
${}_{5}m_{0}$	mortality rate between birth and age 5 years

$_5q_0$	probability of death between birth and age 5 years, also commonly known as under-5 mortality rate (U5MR)
ACLED	Armed Conflict Location and Event Database
ART	antiretroviral therapy
ASFR	age-specific fertility rate
BCCMP	Bayesian hierarchical cohort component model for population projection
BMA	Bayesian model averaging
BTL	International Classification of Diseases Basic Tabulation List
CBH	complete birth history
CCMPP	cohort component method for population projection
CD4	CD4+ T lymphocyte; an indicator of immune function
CEB	children ever born
CIBA	cohort incidence bias adjustment
CIBA-SPECTRUM	cohort incidence bias adjusted spectrum output
CoD	causes of death
CODEm	Cause of Death Ensemble model
COVID-19	coronavirus disease 2019
DALYs	disability-adjusted life-years
DDM	death distribution methods
DHS	Demographic and Health Surveys
DSP	Disease Surveillance Points System
DYB	Demographic Yearbook
EDU15+	Mean years of education for those aged 15 and older
EM-DAT	Centre for Research on the Epidemiology of Disasters' International Disaster Database
enn	early neonatal
EPP	Estimation and Projection Package
GATHER	Guidelines for Accurate and Transparent Health Estimates Reporting
GBD	Global Burden of Diseases, Injuries, and Risk Factors Study
GEMS	Global Enteric Multicenter Study
GGB	generalised growth balance method
GGBSEG	combined generalised growth balance and synthetic extinct generation method

GHDx	Global Health Data Exchange
GIDEON	Global Infectious Diseases and Epidemiology Network
GK	Gakidou-King
GLMM	Gulf Labour Markets, Migration, and Population
GPR	Gaussian process regression
HDI	human development index
HFC	Human Fertility Collection
HIV	human immunodeficiency virus
HIV CDR	crude death rate due to HIV/AIDS
HMD	Human Mortality Database
IBC	Iraq Body Count
ICD	International Classification of Disease
IPUMS	Integrated Public Use Microdata Series
LDI	lag-distributed income per capita
lnn	late neonatal
LSMS	Living Standards and Measurement Surveys
MAD	median absolute deviation
MCCD	Medical Certification of Causes of Death
MCMC	Markov Chain Monte Carlo
MICS	Multiple Indicator Cluster Surveys
MPIDR	Max Planck Institute for Demographic Research
${}_{n}m_{x}$	mortality rate between age x and x+n
NCD	non-communicable disease
OPRM	other pandemic-related mortality
PCVA	Physician Certified Verbal Autopsy
PES	post-enumeration survey
pnn	post neonatal
PRIO	Peace Research Institute Oslo
${}_{n}q_{x}$	probability of death between age x and x+n
RHS	Reproductive Health Surveys
SBH	summary birth history
SCAD	Social Conflict Analysis Database

SDI	Socio-demographic Index
SEER	Surveillance, Epidemiology, and End Results Program
SEG	synthetic extinct generation
SRS	Sample Registration System
ST-GPR	spatiotemporal Gaussian process regression
TFO30	total fertility over age 30
TFR	total fertility rate
TFU25	total fertility under age 25
TMB	template model builder
U5MR	under-5 mortality rate
UCDP	Uppsala Conflict Data Program
UI	uncertainty interval
UN	United Nations
UNAIDS	Joint United Nations Programme on HIV/AIDS
UNHCR	United Nations High Commissioner for Refugees
UNICEF	United Nations International Children's Emergency Fund
UNPD	United Nations Population Division
UNSD	United Nations Statistics Division
USAID	United States Agency for International Development
VR	vital registration
WFS	World Fertility Survey
WHO	World Health Organization
YLDs	years lived with disability
YLLs	years of life lost

Section 1.7: GBD results overview

Supplementary results are available in appendix 2.

Visualisations for all results specific to demographic mortality measures for GBD 2021 are also available at https://vizhub.healthdata.org/mortality/.

Section 1.8: Data input sources overview

GBD 2021 synthesises a large and growing number of data input sources including surveys, censuses, vital statistics, and other health-related data sources. The data from these sources are used to estimate mortality and morbidity; causes of death, illness, and injury; and attributable risk for 204 countries and territories from 1990 to 2021. The input sources are accessible through an interactive citation tool available in the GHDx. While we reviewed and extracted all input sources

included in this tool, some sources were not included in our models as a result of data deduplication in the modelling process.

Citations for specific GBD components, causes and risks, and locations can be found through the GBD Sources Tool in the GHDx: https://ghdx.healthdata.org/gbd-2021/sources. This tool allows users to view and access GHDx records for input sources and export a CSV file that includes metadata, citations, and information about where the data were used in GBD. As required by GATHER, additional metadata for input sources are available through the citation tool as well.

Section 1.9: Funding sources

Research reported in this publication was supported by the Bill & Melinda Gates Foundation, the University of Melbourne, Public Health England, the Norwegian Institute of Public Health, the National Institute on Aging of the National Institutes of Health (award P30AG047845), and the National Institute of Mental Health of the National Institutes of Health (award R01MH110163). The content is solely the responsibility of the authors and does not necessarily represent the official views of the Bill & Melinda Gates Foundation or the National Institutes of Health. The funders of the study had no role in study design, data collection, data analysis, data interpretation, or writing of the report. All authors had full access to all data in the study and had final responsibility for the decision to submit for publication.

Section 2: All-cause mortality, HIV mortality, and fatal discontinuities

Section 2.1: Overview[7]

We aimed to generate the most accurate all-cause mortality estimates for all GBD age groups, by sex, for all 1055 locations in the GBD 2021 location hierarchy, for a 72-year time series from 1950 to 2021.

One methodological challenge we faced was variability in the completeness and quality of data. Calculating all-cause mortality for different age groups in different locations required drawing on a diverse set of data sources (appendix tables S3 and S4). More generally, estimating all-cause mortality was complicated by the fact that not all countries and territories have complete vital registration (VR) systems recording the event of death and periodic censuses. Several methods were used to estimate completeness and adjust VR data accordingly.

Here, we provide a detailed description of the methodology we used to estimate all-cause mortality. Due to the interdependence between HIV mortality estimation and all-cause mortality estimation, this appendix includes a partial overview of the epidemiological modelling of HIV. In what follows, we describe five major methodological tasks: estimating the probability of death between birth and age 5 years (${}_{5}q_{0}$); estimating the probability of death between age 15 years and 60 years (${}_{45}q_{15}$); estimating a complete set of age-specific mortality rates; estimating HIV mortality; and producing final estimates of age-specific mortality with HIV and fatal discontinuities incorporated. We describe primary input data and the analytical steps undertaken for each step. The appendix figure S1 flowchart shows the entire modelling process. We ran the full model twice in order to update the input mortality data with the updated population estimates to improve internal consistency among different model components in not only our mortality estimation model, but also fertility and population estimation models. Inputs that were updated for the second iteration of the model are shown in appendix figure S1 using patterned boxes.

Section 2.2: Child mortality[7]

Section 2.2.1: Data sources

Overview

For the estimation of child mortality, we used data from vital registration (VR) systems, Sample Registration Systems (SRS), Disease Surveillance Points Systems (DSP), household surveys (complete birth histories [CBH], summary birth histories [SBH]), censuses (SBHs, CBHs on rare occasions), and Demographic Surveillance Sites (DSS). It was important that data were fully representative of the given age-sex group and location-year.

VR/SRS/DSP from other sources

We endeavored to incorporate all available data from VR systems as inputs in our all-cause mortality estimation process. This included multi-country VR sources such as the WHO Mortality Database, United Nations Demographic Yearbooks, Human Mortality Database, country statistics offices, and the Organisation for Economic Co-operation and Development (OECD) databases. We updated data from these sources in our systems as new releases occurred. Wherever possible, we

also catalogued all data sources from each system for ongoing national VR systems (eg, the USA National Vital Statistics System).

When VR data were not available, we extracted data from the SRS from India and the DSP from China. Data from the Health and Demographic Surveillance System were also used.

Under-5 populations and livebirths
Livebirths and population in the under-5 age groups were produced as part of the fertility and population estimation process, described in sections 3 and 4.

CBH microdata
Where VR data were unavailable or unreliable, CBHs were the preferred method for data collection on child mortality. CBHs are surveys conducted with mothers about all livebirths they have ever had by the time of the survey regardless of the child's survival status, including birthdate, survival status, and date of death, if deceased. Since CBH data include the age at which mothers gave birth to each child, we could calculate period and age-specific mortality rates in the time period before the survey, assuming no survivor, migration-related, or recall bias. Many routine survey series, including the Demographic and Health Surveys (DHS), World Fertility Surveys (WFS), Multiple Indicator Cluster Surveys (MICS), and other national surveys, contain CBH modules. When available, we downloaded and used microdata that included individual level survey responses as opposed to the tabulated results which generally report values for three five-year periods.

CBH tabulated data
Some surveys release tabulated results before microdata, so we included these tabulated datapoints in our probability of death from birth to age 5 years (${}_5q_0$) database. However, once we were able to obtain microdata from the same survey, the tabulated report estimates were replaced in the database with point estimates from the processed microdata.

SBH microdata
SBH questionnaires are shorter and less detailed than CBHs and lack information on dates of birth and death of children. They simply ask mothers the number of livebirths they have ever had and how many of those children have died, as well as the age of the mother at the time of the interview. For this study, we collected all available SBH data with microdata or those that reported data on the proportion of a mother's children who died, by maternal age group. GBD has developed its own summary birth history method to generate under-5 mortality rate (U5MR) using the aforementioned SBH data.

Under-5 age-sex patterns for VR/SRS/DSP
In high-income countries, under-5 mortality estimates were derived primarily with data from VR systems. Often, these data were divided into several age groups: early neonatal (0-6 days), late neonatal (7-27 days), post-neonatal (28-364 days), and 1 to 4 years. Some country-years of data had less specific age groups, with the early and late neonatal age groups combined or the under-1 age groups combined. Age-sex patterns of mortality in the under-5 age groups were also available from SRS and DSP sources.

Under-5 age-sex patterns from CBH
Aside from VR/SRS/DSP, CBHs from surveys provide another important source on the distribution of mortality among under-5 age groups. These data reflect age patterns primarily from low-income countries and countries with higher levels of under-5 mortality. We only used datapoints from the 15 years leading up to the survey, so children who were born to mothers more than 15 years before the interview were not included in our analysis. We grouped observations into 5-year time periods to produce more robust input point estimates of the probability of death for each under-5 age-sex group.

Section 2.2.2: VR prioritisation
As we worked with a variety of sources of VR data, we developed a hierarchy of preferred sources. For a given location-year, our first preference was to use WHO data from GBD cause-specific mortality estimation, followed by unadjusted WHO data, then Human Mortality Database (HMD) data, and lastly UN Demographic Yearbook data. We made exceptions in some cases, however. We assessed single-country VR data sources according to whether there were inconsistencies with other data sources or VR system documentation. For instance, we used HMD VR data for Germany, Taiwan (Province of China), and Spain to estimate certain years since WHO data produced mortality rates inconsistent with established trends.

Section 2.2.3: VR under-enumeration for bias correction

For under-5 mortality, VR system completeness was estimated in the same way as in previous iterations of GBD, and as was the case in GBD 2019, most VR bias was corrected using a mixed effects non-linear model, described in section 2.2.6.

Section 2.2.4: Computation of 5q0 from CBH

We used CBH microdata from unpooled surveys to calculate biennial U5MRs. We analysed each survey separately (eg, Kenya DHS 2014; Bangladesh MICS 2012–2013) instead of pooling all DHS or MICS CBH surveys from a single country (eg, Nigeria MICS surveys from 2007, 2011, and 2016–2017) so that we could better understand and address data quality issues in specific surveys. By grouping observation points into two-year intervals to generate biennial estimates, we overcame the issues of smaller sample sizes and lower stability typically associated with the use of unpooled data. We only included biennial estimates that contained more than 10 000 person-months of data.

Processing of tabular CBH

In some instances, microdata were not available for all CBH surveys. We still added results from these surveys to our database if survey reports provided ${}_5q_0$ datapoint estimates based on CBH.

Section 2.2.5: SBH method

We used an SBH method developed and updated by Rajaratnam and colleagues to estimate ${}_5q_0$ from SBH data from censuses and surveys.[9] We found this method to be more accurate and provide more timely estimates than previous methods.

In cases where the only data available were tabular data of the number of livebirths ever born and number of children who died, aggregated by the mother's age, we applied Rajaratnam and colleagues' Maternal Age Cohort model.[9]

Section 2.2.6: ${}_5q_0$ data synthesis

Data synthesis using ST-GPR

We used a spatiotemporal Gaussian process regression (ST-GPR) to estimate U5MR.[7] We also incorporated data bias adjustment into the model based on the under-5 mortality synthesis model used in previous GBD iterations.[2,3,10–12] To adjust for bias, we ran the first-stage non-linear effects model for GBD 2021 standard locations only and then extracted predicted fixed effect coefficients from the non-linear mixed effect model and applied them to all locations. A set of fixed effects were estimated for each data source type used in our analysis. A list of source types can be found below. We also estimated a random effect for each data source within a given location. The combination of source-type-specific fixed effect and source-specific random effect was the basis for bias adjustment. When a reference source was selected, all other sources were adjusted based on the difference between the bias value from the reference source and the source of interest. We did not adjust reference sources when more than one source was used as the reference source. In this case, all other sources were adjusted based on the average bias values of all selected reference sources. Removing systematic differences in sources meant that we avoided estimating false trends caused by overlapping sources with different levels of non-sampling variance. We then synthesised the bias-adjusted child mortality data using a combination of a non-linear effects model, spatiotemporal regression, and Gaussian process regression (GPR). This process produced consistent time series estimates of mortality by location, with 95% uncertainty intervals (UIs) using the 2.5% and 97.5% percentiles of the summed draws.

Source types used in child mortality bias correction

1. CBH: AIDS Indicator Survey and Malaria Indicator Survey
2. CBH: Census
3. CBH: Demographic and Health Survey
4. CBH: Multiple Indicator Cluster Survey
5. CBH: World Fertility Survey
6. CBH: Other survey series
7. Household Death Recall: Census
8. Household Death Recall: Other survey series
9. Household Death Recall: Incomplete VR/Sample Registration/Surveillance

10. SBH: AIDS Indicator Survey and Malaria Indicator Survey
11. SBH: Census
12. SBH: Demographic and Health Survey
13. SBH: Multiple Indicator Cluster Survey
14. SBH: World Fertility Survey
15. SBH: Other survey series
16. VR/Sample Registration/Surveillance: complete
17. VR/Sample Registration/Surveillance: incomplete

A list of under-5 mortality sources is provided in appendix table S5 and a list of reference sources is in appendix table S6.

Non-linear mixed effects model

To estimate data bias and provide first stage predictions for $_5m_0$, we used a non-linear mixed effects regression model:

$$_5m_{0_{ctsi}} = \exp[\alpha_k + \gamma_{0c} + \gamma_{0cs} + (\beta_1 + \gamma_{1c}) * \log(LDI_{ct}) + (\beta_2 + \gamma_{2c}) * education_{ct}] + \beta_3 * HIV_{ct} + \varepsilon_{cysi}$$

$$\gamma_{0c} \sim N\left(0, \sigma^2_{\gamma_0}\right)$$

$$\gamma_{1c} \sim N\left(0, \sigma^2_{\gamma_1}\right)$$

$$\gamma_{2c} \sim N\left(0, \sigma^2_{\gamma_2}\right)$$

$$\gamma_{0cs} \sim N\left(0, \sigma^2_{\gamma_{0s}}\right)$$

$$\varepsilon_{cts} \sim N(0, \sigma^2_\varepsilon)$$

Where

Index c is location, t is time, s is source, k is source type of datapoint i

$_5m_0$ is U5MR

LDI is lag-distributed income per capita

education is mean years of education for women of reproductive age (15-49 years)

HIV is crude death rate due to HIV in age groups 0-4

γ_{0c} is a country random intercept

γ_{0cs} is a country-source random intercept

γ_{1c} is a country random slope for *LDI*

γ_{2c} is a country random slope for *education*

α is a fixed intercept for source

β_i is a fixed covariate coefficient

ε is the residual

$\gamma_{0c}, \gamma_{0cs}, \gamma_{1c}, \gamma_{2c}$ are all pairwise independent

Each source was categorised into one of the 17 source types (k) listed above. See sections 6.3 and 6.4 for details on how LDI and education were estimated. This regression used HIV mortality rates estimated as part of the GBD 2021 cycle (section 2.5).

We turned to country experts to identify data sources in each location that were likely to be least biased and of higher data quality to be used as the reference source or sources. When it was believed that a VR system was complete, we used it as the reference source. When data from complete VR systems were not available, we used DHS estimates from CBHs as the

reference source if available. Specific sources were selected on a location-by-location basis when neither complete VR nor CBH from DHS or other surveys were available.

The bias adjustment was applied using the equation below:

$$adjusted_5m_{0,cts} = \exp[\alpha_t + \gamma_{0c} + \gamma_{0ref,c+} (\beta_1 + \gamma_{1c}) * \log(LDI_{ct}) + (\beta_2 + \gamma_{2c}) * education_{cy}] + \beta_3 * HIV_{ct} + \varepsilon_{cts}$$

For incomplete VR data, we calculated an upward adjustment using a five-year rolling mean of the difference between incomplete VR data and a Loess regression of the already-adjusted survey data.

Spatiotemporal smoothing

To smooth the residuals between the predicted $_5q_0$ time series using the non-linear mixed effect model and the adjusted raw data, we applied a combination of spatiotemporal smoothing functions. The stage described here smoothed the residuals over time and across locations within each GBD region. The first-stage prediction of $_5q_0$ was generated using the equation below:

$$predicted_5m_{0,ct} = \exp[\alpha_t + \beta_1 * \log(LDI_{ct}) + \beta_2 * education_{ct}] + \beta_3 * HIV_{ct}$$

The predicted time series for this smoothing function only used the fixed effects from the non-linear mixed effects model; no random effects or survey-type fixed effects were included.

After calculating the predicted time series, we calculated the residuals between the predicted and the adjusted data. Next, we applied the smoothing functions to the residuals. We weighted residuals in each region for each location-year based on how close the data were to this location-year in space and time. 99% of the weight went to in-location residuals, with the remaining 1% going to out-of-location but within-GBD region residuals. The weight for time was defined using the equation below:

$$w_t = \left(1 - \left(\frac{|r_t - r_{est}|}{1 + argmax_t|r_t - r_{est}|}\right)^{\lambda}\right)^3$$

Where

r_t is the year of interest

r_{est} is the year of the residual being weighted

$argmax_t|r_t - r_{est}|$ is the maximum distance between the year of interest and a residual within the region

λ is the weighting function that dictates how quickly the weights fall off as the distance in time increases

A larger λ implies that the assigned weights will diminish more slowly over time, while a smaller λ allows the weights to diminish more rapidly with time. λ values were chosen using the parameter selection process described below. We generated one estimate of the smoothed residuals using a linear fit to this weighted data, similar to a Loess fit, and then generated a second estimate of the smoothed residuals by calculating the weighted average of the data. We then combined these two estimates of the smoothed residuals into one final estimate. We assigned more weight to the local linear fit in countries and territories with high data density and quality. Alternatively, we assigned more weight to the weighted average in countries and territories with data sparsity using the following equation:

$$final\ smoothed\ residual = k * linear\ estimate + (1 - k) * weighted\ average$$

Where

$$k = \frac{number\ of\ in\ country\ datapoints}{number\ of\ in\ country\ datapoints\ +\ number\ of\ country\ years\ with\ no\ data}$$

The pooling of two estimates of the smoothed residuals was employed to account for artificial trends in smoothed residuals from either method, especially when the year of interest was further away from the last year with empirical data. This approach also helps improve comparability among estimates for various locations that otherwise might need to employ different methods for smoothing the residuals.

Last, we incorporated the smoothed residuals back into the above predictions to produce a smoothed approximation of the adjusted data. We used this estimate as the prior for the Gaussian process regression (GPR) described in the following section.

To avoid producing space-time smoothed U5MR values less than 0 or greater than 1, both space-time smoothing and the GPR described below were done using U5MR in logit space.

GPR

We used GPR to generate a final time series of point estimates as well as UIs.

The GPR model was as follows:

$$\mu_{c,t} = f_c(t) + S_{c,t}$$

$$f_c(t) \sim GP(M, C)$$

Where

t is time, c is location

μ_t is the true $\log_{10}({}_5q_0)$

$f(t)$ is the baseline mortality risk

S_t is excess mortality due to fatal discontinuities estimated independently of $f(t)$

M is the mean function for the Gaussian process

C is the covariance function for the Gaussian process

The mean function (M) is an initial guess at our unknown function f_c. We chose this to be our second-stage prediction.

The covariance function (C) defines the covariance between any two points t_i and t_j. It controls the smoothness of realizations from the Gaussian process and the amount of deviation from the mean that is tolerated. There are some conventions regarding good choices for the covariance function in GPR, but no single function is indicated. We elected to use the Matérn covariance function:

$$C_{matern}(x_i, x_j \mid a, s, \nu) = a^2 \frac{1}{\Gamma(\nu)2^{(\nu-1)}} \left[\sqrt{2\nu}\frac{|x_i - x_j|}{s}\right]^\nu K_\nu\left(\sqrt{2\nu}\,\frac{|x_i - x_j|}{s}\right)$$

Where

a is amplitude

s is scale

ν is degree of differentiability

Γ is the gamma function

K_ν is the modified Bessel function of the second kind and order ν

To select the *amplitude* parameter, we first calculated the variance of logit-difference between the first-stage and second-stage predictions in high data-density years separately by location. Then, this location-specific variance was used as amplitude for countries with complete VR and the mean of these variances was used as amplitude for countries with incomplete VR. *Scale* was fixed by location based on data density (see table below), and degree of differentiability is fixed at 1.

In addition to our Gaussian process prior for f, we observe the value of f for some set of years o_i – these are our data. We assume the observation error is normally distributed and calculate the data variance as described in the next section. Then, the posterior distribution for f given the observations and the prior is another Gaussian process with a closed form solution for posterior $\widetilde{M}$ and $\tilde{C}$. Let V be a matrix with observation variances [v_0, … , v_{n-1}] on its diagonal and o be the vector of years for which we have observed $f(o)$. Then, the posterior distribution, $\tilde{f}$, is:

$$\tilde{f}_c \mid data \sim GP(\widetilde{M}, \tilde{C})$$

$$\widetilde{M}(t) = M(t) + C(t,o)[C(o,o) + V]^{-1}\big(f(o) - M(o)\big)$$
$$\tilde{C}(t_i, t_j) = C(t_i, t_j) - C(t_i, o)[C(o,o) + V]^{-1}\, C(o, t_j)$$

From this Gaussian process posterior distribution, we take realisations, where one realisation is a complete function from our domain of years to the outcome of interest. 1000 realisations are draws that can be collapsed to get a mean and 95% uncertainty interval for every location and year separately.

GPR was implemented using the PyMC package in Python.

Data variance

We calculated data variance for each $_5q_0$ input datapoint, which reflects sampling errors. Additional non-sampling errors were added when the datapoint was from a non-reference data source. Specifically, we calculated data variance using the following methods, depending on the type of data:

1. **Complete VR data:** We assumed there was only sampling error, and not non-sampling error, because complete VR is assumed to be unbiased. We calculated this error using the binomial distribution $p(1-p)/N$, where N is the population aged 0 to 5 years for the location and p is the U5MR, $_5m_0$. We then used the delta method[13] to transform the variance of $_5m_0$ to the variance of $\log_{10}(_5q_0)$.
2. **Incomplete VR data:** We included both sampling error and non-sampling error from uncertainty in our estimate of data completeness. We calculated the total data variance as the sum of the variances from sampling error (see calculation method for complete VR data) and non-sampling error based on the completeness estimate.
3. **CBH data:** We generated 1000 simulations of $_5q_0$, transformed these estimates into $\log_{10}$ space, and calculated the sampling variance from the transformed draws.
4. **SBH data:** We used the standard error from the mean residuals.
5. **Other:** We used the maximum standard error from non-VR points in a country or territory if we could not produce data variance for a source. When no other source was available, we set the data variance to be the maximum standard error from non-VR data sources within the GBD region the location belongs to.

Finally, for all data types other than complete VR data, we calculated the within-source-type variance of the source-specific random effect for each source type. We then converted it to $\log_{10}$ space and added it to the variance already calculated using one of the methods above.

Hyperparameter selection ST-GPR used to generate U5MR

We selected hyperparameters based on a data density score for each location, first used in GBD 2017.[4] We calculated data density scores based on the number of deaths from VR sources plus the number of unique CBH and SBH sources available for the given location, using the following computational methods for each type of data source:

1. **Complete VR data density score:** calculated based on the number of deaths reported from unbiased VR sources in a single location. We capped the number of deaths for each source-year at 500 and then divided that number by 500 to generate a score between 0 and 1 where 1 represents a complete VR system with at least 500 registered deaths and a 0 represents a complete VR system with 0 registered deaths. Final complete VR scores ranged from 0 to 78.
2. **Incomplete VR data density score:** calculated the same way as with complete VR data but using biased VR sources rather than unbiased VR sources.
3. **Total CBH sources:** the number of unique complete birth histories for a single location.
4. **Total SBH sources:** the number of unique summary birth histories for a single location.

After making these calculations, we used the following formula to compute the final data density for each location:

$$data\_density = complete\_vr\_deaths + (0.5 \times incomplete\_vr\_deaths) + (2 \times cbh\_sources) + (0.25 * sbh\_sources)$$

We then assigned hyperparameters based on final data densities, as designated in table A. Zeta determines the magnitude of space weights in the spatiotemporal smoothing. Lambda determines the magnitude of time weights. Scale is the scale parameter of the Matérn covariance function, which controls correlation over time.

Table A

Data density	Zeta	Lambda	Scale
0 to 9	0.7	0.7	15
10 to 19	0.7	0.5	15
20 to 29	0.8	0.4	15
30 to 49	0.9	0.3	10
50 plus	0.99	0.2	5

Section 2.2.7: Identify and remove outliers

We carefully reviewed child mortality data and estimates to ensure quality. First, we outliered datapoints from years in which fatal discontinuities (shocks) occurred, unless cause of death information was available for the VR datapoint such that deaths from specific causes of death identified as a fatal discontinuity could be removed. We excluded fatal discontinuity data because our objective was to identify the underlying mortality risk, which would be skewed by mortality data from large stochastic events. We incorporated fatal discontinuities back into the data later in the estimation process (see section 2.6.3). Second, we outliered data sources with quality concerns, such as the Afghanistan DHS from 2010. Collaborators from our extensive network reviewed sources in which they had expert knowledge and identified those with known quality issues.

Section 2.2.8: Rake subnational estimates to national level (excluding South Africa)

One strength of GBD is that we ensure consistency throughout our location hierarchy, meaning that subnational mortality estimates always add up to national mortality estimates. The estimation process for $_5q_0$ described above does not enforce this consistency, so the following adjustments in locations with subnational estimates after the aforementioned estimation process were made. We first population-weighted the subnational estimates to form an estimate at the national level. A scalar was then calculated by year by dividing the separately estimated national value by this weighted average of subnational estimates. We then multiplied the scalars from this calculation by all the subnational estimates to get scaled estimates for each subnational location. We chose this strategy for subnational scaling because national-level estimates are generally more reliable as they are more likely to be based on more empirical data, and the data themselves are less likely to be affected by the small number issue. The effect of scaling was minimal in locations where VR data were high-quality.

South Africa was the one country where we did not use this method. We instead aggregated provincial-level estimates from GPR to the national level. This was to ensure consistency between our estimates at the subnational level and the observed deaths from the VR system in South Africa at the national level.

Section 2.2.9: Review estimates for quality

We compared our $_5q_0$ estimates with UNICEF estimates from the 2022 revision.[14,15] We identified all differences in results and determined whether they were caused by updates to the available data, changes to the hyperparameters we set, or modifications to input covariates. If it was determined that our data or estimates needed to be updated based on this process of reviewing differences with UNICEF, necessary revisions were made. Additionally, we revised data and estimates through expert consultation with the GBD mortality collaborator network.

Section 2.2.10: Under-5 age and sex pattern model estimation

To disaggregate under-5 mortality into age- and sex-specific groups, we used a multi-stage modelling process similar to the method first published in GBD 2016.[3] The result of this process is sex-specific mortality for the early neonatal (0-6 days), late neonatal (7-27 days), 1-5 months, 6-11 months, 12-23 months, and 2-4 years age groups.

First, we estimated the ratio of male to female $_5q_0$, and then, we estimated the sex-specific age distributions of under-5 mortality. Estimating both the sex and age components relied on the following sequential processes: (1) generalised linear model, (2) spatiotemporal smoothing, and (3) GPR. Finally, the components were scaled and synthesised into age- and sex-specific mortality rates.

Data from VR, SRS, and CBHs were converted to mortality risks for each age group. Some sources included age-specific data for all four age groups, while others only had infant (early, late, and post-neonatal) and child distinctions.

The sex model first estimated the ratio of male to female $_5q_0$ in rescaled logit space for each location o in region g in year o. We excluded observed ratios below 0.8 or above 1.5 and then rescaled the ratio data from 0.8–1.5 to 0–1to place our data within the domain of the logit function. Data from non-standard locations were also excluded in the first-stage model fitting. Outliered data by location, source type, and age group is shown in appendix table S8.

With these data, we fit the following sex-ratio model:

$$logit\left(\frac{Male\ {}_5q_0\ scaled}{Female\ {}_5q_0\ \ scaled}\right)_{ijt} = \beta_0 + \delta_j + \gamma_i + S_{_5q_0} + \varepsilon_{ijt}$$

$$\delta_j \sim N\left(0, \sigma^2_{\delta_j}\right)$$

$$\gamma_i \sim N\left(0, \sigma^2_{\gamma_i}\right)$$

$$\varepsilon_{ijt} \sim N(0, \sigma^2_\varepsilon)$$

Where

i is location, j is region,

t is time

β_0 is an intercept term

$S_{_5q_0}$ is a natural spline on both-sex $_5q_0$

$\delta_j$is the region random intercept

γ_i is the location random intercept (nested in region)

ε is the residual

We then used the parameter estimates from this model, and our estimated $_5q_0$, to predict first-stage sex-ratio of under-5 mortality for all GBD location-years. Spatiotemporal smoothing and GPR were used to produce final sex-ratio estimates. We chose ST-GPR hyperparameters using the same method described in section 2.2.6.

Next, we estimated $_5q_0$ for males and females using the equation:

$$ {}_5q_0 = \left(\frac{1}{1 + r_{birth}}\right) * (female\,{}_5q_0) + \left(\frac{r_{birth}}{1 + r_{birth}}\right) * (male\,{}_5q_0) $$

Where

r_{birth} is the sex-ratio at birth

${}_5q_0$ is the direct output from the estimation process described in section 2.2.1–2.2.10

male ${}_5q_0$ can be replaced with female ${}_5q_0$ times the predicted ${}_5q_0$ sex-ratio to create a system with one unknown.

We then fit a separate age-specific model for each sex-specific age group, resulting in five models for each sex: early neonatal, late neonatal, post-neonatal, infant (neonatal, late neonatal, and post-neonatal combined), and child. The outcome modelled was the log-proportion of sex-specific under-5 deaths occurring in age group x, or in other words, the probability of death in age group x conditional on death before age 5. This outcome facilitates scaling to our estimates for ${}_5q_0$.

The first-stage model for estimating this age distribution was:

$$ log\ (Pr(death\ at\ age\ x|u5\ death)_{jits}) = \beta_0 + \gamma_{js} + \gamma_{is} + a_{1x}\beta_{1s} * HIV_{its} + a_{2x}\beta_{2s} * Mat.Ed._{it} + $$

$$ a_{3x}\beta_{3s} * Completeness_{it} + S_{{}_5q_0} + \varepsilon_{jits} $$
$$ \gamma_{js} \sim N(0, \sigma^2) $$
$$ \gamma_{is} \sim N(0, \tau^2) $$

Where

x is age, s is sex, i is location, j is region, t is time

γ_i is a country random intercept (nested in region)

$\gamma_{j[i]}$ is a region random intercept

$S_{{}_5q_0}$ is a natural spline on ${}_5q_0$

β_i is a fixed covariate coefficient

a_{1x} is an indicator for HIV covariate inclusion: 0 for enn, 1 otherwise

a_{2x} is an indicator for maternal education covariate inclusion: 1 for child, 0 otherwise

a_{3x} is an indicator for source completeness covariate inclusion: 1 for child, 0 otherwise

ε is the residual

The literature on HIV mortality in different under-5 age groups is limited, and there is no clear guidance on how to age-split under-1 deaths due to HIV.[16,17] Prior research has, however, suggested that HIV mortality risk differs between different under-5 age groups,[17,18] with essentially all HIV deaths that occur in the first year of life occurring in the post-neonatal stage. We therefore included under-5 crude death rates from HIV from GBD 2021 (see section 2.2.6) in the model for post-neonatal, infant, and child, but not the neonatal age groups. Including this covariate improved the fit and predictive validity of the model in countries with high HIV prevalence. In locations where HIV/AIDS contributes a large burden to under-5 mortality, using ${}_5q_0$ as the only predictor in this model typically leads to an overestimation of neonatal mortality.[19]

We also included a maternal education covariate and a source-specific child completeness covariate for the 1 to 4 age group only. We calculated child completeness by dividing the source-specific $_5q_0$ point estimate by the final GPR $_5q_0$ estimate. In the prediction of this model, we used a completeness value of 1, to predict from a hypothetically complete source.

After prediction of the first-stage model for all GBD location-years, ST-GPR was used to generate stage-three results by age and sex. We used the same ST-GPR process as described previously for the overall U5MR.

Finally, we ensured consistency in probability of death between the separate and collective under-5 age groups by scaling. We first scaled the infant and child conditional probabilities to sum to 1 and then scaled the early neonatal, late neonatal, and post-neonatal conditional probabilities to sum to the infant conditional probability. Next, we calculated the probability of early neonatal death by multiplying the rescaled conditional probability of early neonatal death given under-5 death by $_5q_0$ using the equation below:

$$q_{enn} = \Pr(death\ in\ enn\ |\ u5\ death) * 5q0$$

where enn is early neonatal.

To calculate the probability of late neonatal death, we multiplied the rescaled conditional probability of late neonatal death given under-5 death by $_5q_0$ and then divided that by the probability of survival to the start of the age group (7 days), using the following equation:

$$q_{lnn} = \Pr\ (death\ in\ lnn\ |\ u5\ death) * 5q0/(1 - q_{enn})$$

where lnn refers to late neonatal.

We calculated probability of death for the post-neonatal and 1–4-year age groups in the same way.

Section 2.2.11: Identify and remove outliers from age and sex models

There were several criteria for removing outliers from the under-5 age-sex pattern model.

For the sex model, we outliered non-VR datapoints from locations with high-quality VR data (based on the GBD VR quality rating system) to ensure the model followed the highest-quality data.

For the age model, we outliered the following:

1. VR data that were incomplete. We compared the nine-year rolling average of the $_5q_0$ value from the VR data to the nine-year rolling average of the $_5q_0$ estimates and then the raw data value of $_5q_0$ to the final $_5q_0$ estimate for each data-year. We outliered data for which the second comparison yielded a ratio of 0.85 or less, unless the ratio of the nine-year rolling average was above 0.85.
2. Data that were identified as outliers earlier in the $_5q_0$ analysis.
3. CBH datapoints from more than 15 years before the survey was administered.
4. CBH data in locations with both VR and CBH data, but only if the data conflicted.
5. Some datapoints were manually outliered for various reasons. For example, the definition of a livebirth included a minimum weight requirement in some eastern European countries before the 1990s, which led to inconsistencies in livebirth data over time. We therefore outliered age group data in ages that would include childbirth deaths when and where the definition of livebirth included a minimum weight.

Section 2.2.12: Under-5 death number estimation

We estimated the number of under-5 deaths by ageing birth cohorts through our estimated age-sex-specific probabilities of death. To do this, we divided the number of births for each location-year into weekly birth cohorts and moved these cohorts through our mortality estimates in weekly increments. This process produced estimates of the number of person-years and deaths in each under-5 age group.

Section 2.3: Estimation of adult mortality rate[7]

Section 2.3.1: Data sources

Adult population estimates

We used multiple data types to estimate adult mortality rate, including VR systems; surveys and censuses, from which we extracted household death recall data; the SRS in India; and the DSP system in China.

VR/SRS/DSP

Refer to section 2.2.1 for details on how we identified and prioritised VR, SRS, and DSP data.

Household recall of deaths

Household death recall modules can be found in censuses and surveys, where the information on the number of deaths that occurred in each household over a specific recall period is collected. Information on the number of usual residents in the household for the same recall period is collected as well. Such information, combined, can be used to adjust data deemed incomplete in the recall of household deaths to generate sex-specific and age-specific mortality rates.

Sibling survival histories

We also acquired sibling survival history data from surveys where participants are asked about the status of their siblings (alive or dead). Surveys were deemed usable if they asked participants to provide a full account of all siblings (children born to the same mother), including each sibling's year of death and age at death (if applicable), year of birth, and sex.

Section 2.3.2: Completeness assessment: death distribution methods and completeness estimates synthesis

As with under-5 mortality VR data, we assessed the completeness of available adult mortality VR data. For this study, we applied a suite of death distribution methods (DDMs) as well as our findings about under-5 VR data completeness to make this assessment.[20] We used the three DDMs most common in demography: generalised growth balance (GGB), synthetic extinct generation (SEG), and a combined approach (GGBSEG),[21–25] which estimate completeness by comparing the age distribution of the population between two censuses with the age distribution of deaths between those same censuses. Starting in GBD 2019, we also used two additional methods that utilise the GBD Bayesian Population Model, which only differ in whether the proportion of net migrants is also estimated in addition to completeness.

To test performance of the various methods, we simulated census and VR data while varying the level of VR completeness and factors that may bias completeness estimates, including total population size and age-structure, census completeness, net migration, and extent of age-misreporting in census and VR data. We used the Cohort Component Method of Population Projection (CCMPP) in the GBD Bayesian Population Model to estimate completeness of VR in between censuses.[20] This ages a population forward in time using CCMPP with the input mortality data and estimates of completeness in order to match the population at the second time point. For each of the five methods (new and traditional) we tested 105 age trims ranging from a starting age of 5 and an end age of 95. Table B shows the top ten age trims by method.

Table B

Rank	CCMP fixed migration		CCMP not fixed migration		GGB		GGBSEG		SEG	
	Start age trim	End age trim	Start age trim	End age trim	Start age trim	End age trim	Start age trim	End age trim	Start age trim	End age trim
1	55	95	25	95	5	70	5	70	45	85
2	65	95	25	90	15	65	10	70	40	85
3	55	90	35	95	10	65	5	75	50	95
4	65	90	30	95	20	65	15	65	45	95
5	45	95	20	95	5	85	20	65	55	85
6	60	95	15	95	5	60	15	70	50	85
7	50	95	35	90	10	85	10	60	40	90
8	45	90	45	95	10	70	10	65	60	85
9	50	90	30	90	15	85	15	60	40	95
10	60	90	20	90	10	60	10	75	35	85

From the simulation testing, we found that no single method performed significantly better than the other methods, but certain age trims for each method performed significantly better than other age trims. To account for this when producing point estimates of completeness for each pair of censuses and VR data in between censuses, we excluded the highest and lowest point estimates across the five methods and only kept the three middle estimates. For each method, we only kept the top-performing age trim as ranked in our simulation testing. These three-point estimates of completeness for each census pair were then combined to form a full time series of completeness using a spatiotemporal regression model, as described below.

Our assessment used a two-stage model. We first used estimated child completeness to predict adult completeness, followed by a spatiotemporal regression model to incorporate estimates of adult completeness from the DDMs to generate a series of estimates from 1950 to 2021 of source- and country-specific adult death registration completeness. We assume that the completeness of a system changes gradually, so within the GBD standard locations, we used DDM estimates from neighbouring years to help assess the completeness of data from any given year in the study period. We also assumed that completeness was likely to be similar between countries in the same region, so we factored in completeness estimates from nearby countries as well.

We calculated child completeness as the ratio of observed child mortality to estimated child mortality in a given source, country, and year, using the estimation process described in section 2.2. While data were only available from certain years for an individual country-source, we made a complete time series of child completeness estimates using a smoothing process. When a country-source had no more than three years of data, we assumed a constant level of child completeness that matched the mean of the available years' completeness. When a country-source had more than three years of data, we used a Loess regression to fill in gaps in the time series. In cases where we had to make out-of-sample estimates (country-sources without data from 1950 or 2021), we held child completeness constant before the first and after the last observation instead of using the Loess regression to predict completeness levels. When a country-source had a gap of more than five years between data, we filled in the gap with a linear interpolation of the two closest observations instead of using the Loess regression.

After estimating child completeness, we conducted our first-stage regression using simple linear regression of calculated adult completeness on child completeness, and predicted adult completeness using the obtained coefficients for VR, SRS, DSP, medical certification of causes of death (MCCD), and civil registration (CR) sources in log space, using the following formula:

$$\hat{\mu}(\log(Adult\ completeness)) = \hat{\beta}_0 + \hat{\beta}_1 * \log(Child\ completeness)$$

For other sources, we assumed completeness for the first stage due to lack of child mortality data. In our second-stage model, we took the residuals from the first-stage regression and applied spatiotemporal smoothing to the residuals, in order to borrow strength across the full time series and nearby locations. We then produced draw level estimates of completeness using the calculated prediction from stage 1 as the mean, and the standard deviation calculated from the smoothed residuals for stage 2 assuming a normal distribution. After setting logged completeness to 0 for estimates greater than 0, we produced final estimates and uncertainty from the anti-logged individual truncated draws.

For VR, SRS, and DSP sources, we considered country-years that had completeness estimates of 95% or higher to be 100% complete. We then scaled completeness values between 90% and 95% up to 100% as follows:

$$scaled\ completeness = 0.9 + 2\ (estimated\ completeness - 0.9).$$

We also assumed that if a country-source was complete across the full time series, its subnational locations were complete as well. Our exceptions to this assumption were Brazil and Iran, where we found that level of completeness differed across subnational locations, despite a complete and accurate time series at the national level.

Section 2.3.3: Sibling survival method

While VR systems are the preferred sources for adult mortality rates, in countries with few to no data from a working VR system, adult mortality rates can be derived from sibling survival modules, which provide much-needed information on the level and trends of adult mortality. We define a sibship as a group of offspring who have the same biological parents. Without necessary adjustment, adult mortality estimates from sibling survival modules are biased in four primary ways, resulting from the design and implementation of the method:[26]

1. Selection bias (under-representation of siblings from sibships with high mortality)
2. Zero reporter bias (sibships not represented in the survey due to sex composition and/or absence of any alive sibling of a sibship by the time of the interview)
3. Sparse data
4. Recall bias (under-reporting of deaths of siblings living in different places, having died in the distant past, or for any other reason that the respondent could not recall the death).

The technique we used to estimate adult mortality from sibling survival data and minimise bias was based on methods in Obermeyer and colleagues,[26] with the following changes to their methods:

1. Incorporated appropriate survival weights that accounted for the study design
2. Implemented a zero-survivor correction that accounted for mortality in families who were under-represented in the data because none of the siblings were alive to participate in the survey
3. Refined recall bias adjustment.

The correction to account for mortality in sibships with high mortality rates, proposed by Gakidou and King,[27] incorporates a sibship-level weight:

$$W_j = \frac{B_j}{S_j}$$

Where

j is a given sibship

B_j is the original size of sibship j

S_j is the number of siblings in sibship j who survive to the time of the survey

We used the Gakidou-King (GK) weight on observations being analysed at the sibship level to compute the weighted average of the proportions of siblings who had died, as reported by each respondent. The weight corrected for under-representation of high-mortality sibships.

Since we analyse data at the sibling level (with one observation for each sibling instead of for each sibship), we also used the following sibling-level weight:

$$W_i = \frac{1}{S_j}$$

for sibling i in sibship j. [28,29]

Previously, we applied the GK sibship-level weight to data that had been disaggregated to the sibling level, which led to inaccuracies in the estimates. Our sibling-level weight overcomes this challenge.

Eligibility criteria for different surveys also needed to be factored into the correction.[28] In GK's survivorship correction, $\frac{S_j}{B_j}$ refers to the probability that a sibling in sibship j survived and was eligible to be selected as a survey participant. For instance, only women between the ages of 15 and 49 are eligible to participate in DHSs, so for DHS data, S_j refers to the number of surviving women in sibship j who were aged 15-49 at the time of the survey. For this study, we made the S_j value consistent with the eligibility criteria for each survey.

As previously discussed, the sampled population in sibling histories did not include sibships that had no eligible siblings to participate in the surveys. As a result, these sibships are not present in the data. To account for this, we estimated the number of deaths among missing sibships by age and sibship size using a zero-survivor correction. We then added these estimated siblings to the observed sample before calculating age-specific mortality rates.

We applied the zero-survivor correction to sibships with one or two female siblings. It factored in the true number of sibships with one (or two) females as it related to the cumulative probability of dying before the time of the survey. The result of the correction was an estimate of the number of missing sibling deaths. For one-sibling sibships, we used the following formula:

$$K_{obs}^1 = K_{true}^1 * (1 - {}_aq_0^1)$$

$$K_{miss}^1 = K_{true}^1 * {}_aq_0^1$$

Where

K_{obs}^1 is the number of sibships with one sister that were observed in the sample

K_{true}^1 is the true number of sibships with one sister in the sample

K_{miss}^1 is the number of sibships with one sister that were not represented in the sample due to no surviving sibling

${}_aq_0^1$ is the probability of death between birth and age a

$(1 - {}_aq_0^1)$ is the probability that the sister survived to the time of the survey

We were able to determine that the number of sibships with only one sister that were not represented in the sampled population as a result of zero-survivor bias was:

$$K_{miss}^{1} = \frac{K_{obs}^{1}}{1 - {}_{a}q_{0}^{1}} \times {}_{a}q_{0}^{1}$$

We then multiplied the estimated number of missing sibships (K_{miss}^{1}) by the number of females in the sibship (one, in this instance) to estimate the number of females missing from each age group because they had died. We made each missing female one observation point, assigned birth and death dates based on the distribution in the sampled population, and added them to the dataset. We conducted the same process with families with two sisters using the formula below:

$$K_{obs}^{2} = K_{true}^{2} * (1 - {}_{a}q_{0}^{1} * {}_{a}q_{0}^{2})$$

$$K_{miss}^{2} = K_{true}^{2} * {}_{a}q_{0}^{1} * {}_{a}q_{0}^{2}$$

$$\therefore K_{miss}^{2} = \frac{K_{obs}^{2}}{1 - {}_{a}q_{0}^{1} * {}_{a}q_{0}^{2}} * {}_{a}q_{0}^{1} * {}_{a}q_{0}^{2}$$

Where

K_{obs}^{2} is the number of sibships with two sisters that were observed in the sampled population

K_{true}^{2} is the true number of sibships with two sisters in the population

K_{miss}^{2} is the number of sibships with two sisters that were not represented in the sampled population due to zero-survivor bias

${}_{a}q_{0}^{1}$ is the probability of death for the first sister between birth and age a

${}_{a}q_{0}^{2}$ is the probability of death for the second sister between birth and age a.

In two-sister sibships with only one 15- to 49-year-old sister, we used the following equation to account for the second sister not contributing to the probability of the sibship being observed in the sample:

$$K_{obs}^{2} = K_{true}^{2} * (1 - {}_{a}q_{0}^{1})$$

$$K_{miss}^{2} = K_{true}^{2} * {}_{a}q_{0}^{1} * {}_{a}q_{0}^{2} + K_{true}^{2} * {}_{a}q_{0}^{1} * (1 - {}_{a}q_{0}^{2})$$

$$\therefore K_{miss}^{2} = \frac{K_{obs}^{2}}{1 - {}_{a}q_{0}^{1}} * {}_{a}q_{0}^{1} * {}_{a}q_{0}^{2} * \frac{K_{obs}^{2}}{1 - {}_{a}q_{0}^{1}} * {}_{a}q_{0}^{1} * (1 - {}_{a}q_{0}^{2})$$

The original methods developed by GK and Obermeyer and colleagues used a logistic regression to account for bias in the time period before the survey.[26,27] For this study, however, we used an updated method to adjust for recall bias. Once we had estimated the probability of death between 15 and 60 years (${}_{45}q_{15}$) from each survey, we paired up estimates that overlapped the same time period. Overlaps occurred in countries and territories where at least two surveys were conducted within 15 years of one another, since we included 15 years of recall. For each year with overlapping surveys, we computed the difference in the years of recall as the number of years between surveys. We quantified the relationship between years of recall and level of mortality for each sex of sibling using the following linear regression model:

$$\Delta({}_{45}q_{15})_{i,j} = \beta \times \Delta(survey\ date)_{i,j} + \xi$$

Where

$\Delta({}_{45}q_{15})_{i,j}$ is the difference in ${}_{45}q_{15}$

$\Delta(survey\ date)_{i,j}$ is the difference in survey date

j is the survey pair

i is the country

We also calculated 95% UIs.

We adjusted the ${}_{45}q_{15}$ estimates to account for recall bias using the period coefficient.

Section 2.3.4: ${}_{45}q_{15}$ data synthesis using non-linear mixed effects model and ST-GPR

Overview of adult (${}_{45}q_{15}$) mortality estimation

We produced a time series of ${}_{45}q_{15}$ estimates for each location in the GBD hierarchy. For each location, we modelled the underlying mortality risk separately from excess mortality due to fatal discontinuities. We used a three-stage process to model underlying mortality risk. Through this process, we incorporated all available data for each country and territory that had not been outliered or excluded due to conflict or natural disaster. Broadly, we implemented the following in each stage of the model:

1. Applied a nonlinear mixed effects model that used covariates to explain variation in the mortality rate for the 15 to 59-year age group (${}_{45}m_{15}$).
2. Smoothed residuals between input data and the first-stage prediction. We then added the smoothed residuals back into the first-stage regression predictions. Through this, we generated a complete time series of prior ${}_{45}q_{15}$ for each location.
3. Produced final estimates using the prior generated from the first two steps and input data on adult mortality rates.

After completing these three stages, we modelled shocks to mortality by estimating the excess risk of mortality in country-years in which a natural disaster or conflict occurred. We then added the estimated excess mortality risk to the underlying mortality risk to generate final time series estimates of ${}_{45}q_{15}$. A full description of the stages from this modelling strategy can be found below.

First-stage nonlinear mixed effects regression

The first stage of the adult mortality model was a nonlinear mixed effects regression. This regression produced adult mortality rate estimates for each location, year, and sex. We incorporated the following covariates: lag-distributed income per capita, mean years of education in the 15 to 59-year age group, crude death rate due to HIV/AIDS in the 15 to 59-year age group, and estimated ${}_5q_0$ from our study. We also included a fixed effect on sex, which allowed us to fit the model using males and females together. The equation for the nonlinear mixed effects regression was as follows:

$$ {}_{45}m_{15}^{observed}{}_{s,c,t,i} = \exp\left(\alpha_s + \gamma_c + \beta_1 \cdot Edu_{s,c,t} + \beta_2 \cdot ln\left(LDI_{c,t}\right) + \beta_3\ {}_5q_{0_{c,t}}\right) + \beta_5 \cdot HIV + \mathcal{E}_{s,c,t,i} $$
$$ \gamma_c \sim N(0, \sigma_c^2) $$

Where

s is sex, c is location, t is time for datapoint i

Edu is the mean years of education for the age group 15 to 59

LDI is lag-distributed income

$_5q_0$ is probability of death from birth to age 5 years

α_s is a fixed intercept for sex

γ_c is a country- or territory-level random effect

HIV is the crude mortality rate from HIV for ages 15 to 59

The predictions generated through stage one of the model were based on predictions from the above equation, but without the location-level random effect. We did this to enable the modelling of spatial mortality trends in the next stage. After predictions were generated, we converted them from mortality rates to probabilities so they could be used in the second stage. When converting from mortality rate ($_nm_x$) to probability of death ($_nq_x$), we assumed a constant mortality rate within the 45-year wide age group.

Second stage spatiotemporal smoothing of residuals

We used the second stage of the regression model in part to capture more fully the pattern of variation in $_{45}q_{15}$ that the covariates could not account for. To do this, we smoothed residuals across time and among locations within the same GBD region.

We fit the local regressions for each of the 21 GBD regions. These local regressions included two variations, both of which used the same weighting scheme for incorporating spatial and temporal correlations. Both variations included conducting weighted linear regressions for each location-year of interest. A description of the weighting scheme that was used in both variations is described below, followed by a description of the two variations.

For each location-year regression, we weighted all the residuals with respect to that location and year. We first weighted residuals with respect to time using the following weighting function, which was similar to the one used in the U5MR estimation process:

$$w_t = \left(1 - \left(\frac{|r_t - r_{est}|}{1 + argmax_t|r_t - r_{est}|}\right)^{\lambda}\right)^3$$

Where

r_t is the year of interest

r_{est} is the year of the residual being weighted

$argmax_t|r_t - r_{est}|$ is the maximum distance between the year of interest and a residual within the region

The λ parameter determined how quickly the weights diminished over time, with a smaller λ reflecting a more rapidly diminishing weight over time than a larger λ.

Second, we weighted residuals with respect to space using the weighting function below, which was applied to the modified time weights. We multiplied the weight of residuals for each country of interest by a factor of:

$$\frac{\zeta \sum_{i \notin c_{est}} w_i}{(1-\zeta)\sum_{i \in c_{est}} w_i}$$

Where

c_{est} is the location of interest

w_i is the time weight

Applying this formula meant that 100·ζ% of the weight was placed on residuals in a single country or territory, with the remaining 100·(1-ζ)% placed on residuals from other countries or territories in the same region. We applied a factor of 0 to countries and territories that had no residuals (ie, those with no data), which meant they were not re-weighted. For those locations, all the weight was consequently applied to the other countries in the region. We set λ and ζ based on each location's data density.

The first local regression variation, or the linear local regression, was a weighted linear regression of the residuals by country-year. It was an indicator of the residual from the country or territory being assessed and was estimated using the following equation:

$$r_{est} = \beta_0 + \beta_1 t + \beta_2 c_{est} + \mathcal{E}$$

We dropped the indicator if it could not be estimated due to the country or territory not having any residuals.

The second variation was a simple weighted average of the residuals in the country or territory being assessed.

We combined the estimates from both variations. The local linear regression included covariates such as year, but in locations with sparse data, this led to implausible out-of-time sample predictions because the extrapolation relied so heavily on the covariate information. The second variation did not include an explicit time trend, so it did not have the same problem with out-of-time sampling, but it was less equipped to fit the data in data-rich countries and territories.

Using the equation below, we then calculated the data density (d_c) in each country and territory of interest (c_{est}) and from that, the weighted average of the predictions from the two local regression variations, with d_c% of the weight assigned to the linear local regression and the remainder to the fixed effects local regression. This gave appropriate weight to each of the variations based on completeness of VR data in each location.

$$d_c = 100 \times \left(\frac{\#\, VR\ points\ in\ c_{est}}{Maximum\ \#\, VR\ points\ in\ any\ country\ in\ the\ region} \right)$$

We logit-transformed the residuals before smoothing. We used the smoothed residuals to adjust the logit-transformed predictions from the first-stage regression, before reverse-logit transforming this estimate. Because we conducted the first two stages in logit-space, our predictions' domain was restricted to the range between 0 and 1. We called these the second-stage predictions.

Model

We used a GPR model for the third stage of our prediction model. The equation was as follows:

$$\mu_{t,c} = f_c(t) + S_{t,c}$$

$$f_c(t) \sim GP(M, C)$$

Where

t is time, c is location

μ_t is the true $\log_{10}({}_{45}q_{15})$

$f(t)$ is the baseline mortality risk

S_t captures excess mortality due to war and disasters and is estimated independently of $f(t)$

M is the Gaussian process mean function

C is the Gaussian process covariance function

Because the Dominican Republic, Peru, Madagascar, and Morocco had very different estimates from sibling histories versus VR systems and we could not determine the direction of the bias in each source, we used a slightly different model from the one above. For these countries, we instead used a model that included a bias term for each source (β_s):

$$\mu_{t,c} = f_c(t) + \beta_s + S_{t,s}$$

$$\beta_s \sim Normal(0, 0.01^2)$$

$$f_c(t) \sim GP(M, C)$$

As with ${}_5q_0$ (see section 2.2.6), the prior distribution of $f(t)$ was based on second-stage predictions as the mean prior (M) and a Matérn covariance function as the covariance prior (C). The covariance function included three parameters: scale, amplitude, and degree of differentiability. Respectively, these parameters controlled the distance over which the function was correlated; controlled the amount by which estimates from the Gaussian process distribution could deviate from the mean function; and influenced sample smoothness from the Gaussian process. The selection of these and other hyperparameters is explained in the next section. For more details about GPR and the Matérn covariance function, see section 2.2.6.

Hyperparameter and parameter selection for adult mortality rate ST-GPR

The following hyperparameters and parameters are used:

- Stage 2 (Space-time smoothing)
  - Zeta
  - Lambda
- Stage 3 (Gaussian process)
  - Scale
  - Amplitude
  - Degree of differentiability

Similar to selecting parameters for U5MR, we selected zeta, lambda, and scale hyperparameters for adult mortality based on a data density score. We calculated the data density score for each location and sex based on the number of sibling histories and number of deaths, following the steps outlined below:

1. Calculated the death number score based on the number of deaths calculated during the DDM component of the model, including unadjusted complete deaths and adjusted deaths from incomplete sources.
2. Assigned a score of 15 for each sibling history for a single location, year, and sex.
3. Added the death counts and sibling history scores together for each location, year, and sex; capped the resulting number at 1000 and then divided the number by 1000. The result was a score of 0 to 1 for each location, year, and sex.
4. Added the score for each year in the full time series to calculate the final complete VR score for each location and sex, over the study period.
5. Assigned the following parameters based on the data density for each location (table C).

Table C

Data density	Zeta	Lambda	Scale
0 to 9	0.6	0.7	25
10 to 19	0.7	0.5	20
20 to 29	0.8	0.4	15

30 to 49	0.9	0.3	10
50 plus	0.99	0.2	5

To develop the data density score system, we conducted iterative testing that accounted for the assumption that sources such as complete birth histories and complete VR systems are more susceptible to non-sampling measurement error than other sources. We tested different cutoffs for VR deaths to account for sample size. We also conducted iterative testing and refinement for weights.

To get the *amplitude* parameter for GPR, we first calculated the variance of logit-difference between the first-stage and second-stage predictions in high data-density years separately by location. Then, this location-specific variance was used as amplitude for countries with complete VR and the mean of these variances was used as amplitude for countries with incomplete VR.

The *degree of differentiability* parameter for GPR was selected as 1.

Data variance

We calculated data variance for each ${}_{45}q_{15}$ empirical observation using different methods depending on the type of data.

For sampling variance of ${}_{45}m_{15}$, we used the following binomial distribution:

$$\frac{p(1-p)}{N}$$

Where

N is the national population aged 15 to 59 years

p is the mortality rate, ${}_{45}m_{15}$

We then used the delta method to transform variance into $\log_{10}({}_{45}q_{15})$ space. For estimates that came from a combination of complete and incomplete VR data, we added the sampling variance (calculated for complete VR data) and the variance based on the level of estimated completeness from DDM synthesis (calculated as in section 2.2.6) together to get the total data variance.

For estimates that came from survey, sibling history, or census data, we calculated the mean absolute derivation (MAD) estimator of the variance by source type using the second-stage predictions. We employed the following equation:

$$\theta_s^2 = (1.4826 \cdot MAD_s)^2$$
$$= 1.4826\ x\ median\Big(\Big|\big(log_{10}\big({}_{45}q_{15}^{observed}\big) - log_{10}\big({}_{45}q_{15}^{predicted}\big)\big)$$
$$- median\ \big(log_{10}\big({}_{45}q_{15}^{observed}\big) - log_{10}\big({}_{45}q_{15}^{predicted}\big)\big)\Big|\Big)^2$$

Where s is the source type (sibling history, census, or survey).

Section 2.3.5: Identify and remove outliers

We excluded certain implausible outliers from the ST-GPR regression. We generally outliered raw datapoints that met one of the following criteria:

1. Raw input ${}_{45}q_{15}$ datapoints from years affected by natural disasters, wars, and other fatal discontinuities defined elsewhere[3] based on the analysis explained in section 2.6.3.

2. Raw datasets with poor quality data, as determined by examining the dataset. Examples include Afghanistan 2010 Mortality Survey, which was not nationally representative, and VR data from Serbia that excluded deaths from Kosovo.
3. Raw input data and time series estimates of $_{45}q_{15}$ that were unexplainably different from adjacent points from a similar source, as determined by visual inspection. Unexplained differences indicated a data reporting issue and compilation error in the direct sources.
4. Subnational-level single-year $_{45}q_{15}$ estimates from sibling survival methods that had implausibly high or low estimates due to small sample sizes.

Section 2.3.6: Scale subnational estimates to national level (excluding South Africa)

As described in section 2.2.8, a strength of GBD is that we ensure consistency throughout our location hierarchy, meaning that subnational mortality estimates always add up to national mortality estimates. National-level data were typically more robust, included more data sources, and spanned longer time periods. To eliminate any correlation between subnational and national draws that was introduced earlier in the model, we randomised the order of the 1000 draws at the subnational and national levels. Then, to ensure consistency between the aggregated subnational-level and separately estimated national-level estimates, we raked subnational $_{45}q_{15}$ to the national level using the following formula:

$$_{45}q_{15}^{s'} = 1 - e^{-45 \times {}_{45}M_{15}^{s} \times r}$$

And

$$r = \frac{\frac{ln\,(1 - {}_{45}q_{15}^{N})}{-45}}{\sum_{s=1}^{n} \frac{ln\,(1 - {}_{45}q_{15}^{N})}{-45} \times \frac{p_s}{p_N}}$$

Where

s is subnational locations within country N

p is population in age group 15 to 59

$_{45}q_{15}$ is the estimate of adult mortality rate from the ST-GPR process

$_{45}q_{15}^{s'}$ is the post-scaling $_{45}q_{15}$ for subnational locations

As with $_5q_0$, South Africa was the one country where we did not use this method, because HIV/AIDS is such a large contributor to mortality. We instead aggregated provincial-level estimates to the national level using population for the corresponding age group as weights.

Section 2.4: Model life table system[7]

Section 2.4.1: Overview

We used model life tables (ie, structured relationships between levels of age-specific mortality at different ages) to generate estimates of age-specific mortality for detailed age groups using summary $_5q_0$ and $_{45}q_{15}$. In particular, we relied on this method for location-years without VR systems as well as recent years for locations with high-quality VR systems, because it can take several years for VR data to be published after collection.

Ideally, a model life table system has the following attributes. First, it should only require a few entry parameters to generate a full life table. Second, it should accurately reflect the age patterns of mortality observed in real populations. Third, it should make reasonable estimates of age-specific mortality in countries and territories that have high levels of mortality, particularly those with large HIV/AIDS epidemics. Fourth, it should produce realistic age-specific mortality time trends. Fifth, age-specific mortality should be positively related to entry parameters such as $_5q_0$ and $_{45}q_{15}$.

For this as well as earlier GBD iterations, we used a relational model life table system akin to the one proposed by Brass and Coale.[22] They posited that the logit-transformed survival curves from two life tables can be linearly related to each other. Murray and colleagues found, however, that as mortality levels in a population moved further from the reference standard, the linearity in logit *lx* space no longer held true.[30] They instead proposed a set of bend factors to account for the deviation from linearity. This modified logit life table system also gives users the option to predict $_{45}q_{15}$ from $_{5}q_{0}$. It has been used broadly by WHO since the early 2000s.[30]

Murray and colleagues' modified logit life table system does, however, have two important limitations. First, in location-years where adult mortality was much higher than child mortality, such as with the HIV/AIDS epidemic, the mortality age patterns generated by the system did not fit well to the observed mortality age patterns. Second, when the system was applied to the $_{5}q_{0}$ and $_{45}q_{15}$ time series, a contradictory pattern emerged with adult and child mortality both declining but age-specific mortality rates increasing in certain age groups.

We extended the modified logit life table system in four distinct steps, summarised below. A full description of the modifications can be found elsewhere.[2,10]

Section 2.4.2: Building an empirical model life table database: data sources and quality review

Relational model life table systems are heavily dependent on the empirical database used to produce the standard life tables. For the latest GBD analyses of all-cause and cause-specific mortality, we compiled 65 182 abridged empirical life tables prior to outliering or smoothing using data from full VR systems and sample VR systems such as the DSP system in China and the SRS in India. We adjusted each source by estimated completeness. For ages younger than 1 and 1 to 4, we applied under-5 completeness; for ages 15 and older, we applied adult completeness; and for ages 5 to 9 and 10 to 14, we applied a combination of under-5 and adult completeness.

For each life table in each location, we sorted by year and conducted smoothing using moving averages with widths of 3, 5, and 7 years. The smoothing helped minimise drops or jumps in age-specific mortality in locations where low mortality rates contributed to high variability in mortality patterns across years.

As with previous iterations of GBD, we developed two sets of life tables that met the inclusion criteria for this study. First, there was a universal set of life tables that could be selected for standard life table calculation for all locations. Second, there was a location-specific set of life tables that were used only for the location the data originated from. New in GBD 2021, we have location-year specific life tables, which are only used in the location and year the data originated from. Life tables that were characterised by jumps in age-specific mortality or other implausible trends due to low population or low mortality numbers contributing to inconsistent estimates, old-age misreporting or heaping, or data extraction or reporting issues were excluded.

Life table categorisation was based partially on a neural network machine vision model (based in Python, using Keras from the TensorFlow package) which was trained and deployed to separate likely outliers from likely non-outliers. This machine learning was performed for GBD 2019 and the resultant outliering was carried over into GBD 2021. All life tables were formatted and saved as .jpg plots of log $_{n}q_{x}$ over age. A random set of 6000 plots were reviewed manually for implausible age patterns to create the training set. We looked for a general age pattern of mortality among the human population, which starts with relatively high probability of death in the first year of life, followed by a drop into late childhood, and then roughly log-linear increase through most of adulthood and after, assuming there is no major epidemic of any kind. Life tables with too much noise were excluded from the universal set. A two-person review system was used to verify or modify these machine vision designations. Therefore, the machine vision was not the arbiter of inclusion, but rather a tool to facilitate speedy manual review. Outliering decisions for life tables that were added since GBD 2019 were not based on machine vision and were all investigated manually for implausible trends.

Life tables that were outliered for $_{5}q_{0}$ or $_{45}q_{15}$ estimation or had estimated completeness below 50% were also excluded from the empirical life table database. Life tables with estimated completeness between 50% and 85% were included only in the location-specific category, not in the universal set.

For some locations, life tables that did not qualify upon initial review were included as location-specific life tables, in order to improve modelled envelope fit to the VR data.

Our database of life tables that met the inclusion criteria now includes 7792 universal, 33 919 location-specific, and 2047 location-year-specific life tables, for a total of 43 758 life tables (appendix table S9).

Section 2.4.3: Extending age-specific mortality

We extrapolated age-specific mortality up to age 105-109 using the recursive model below, fit to high-quality VR data from the Human Mortality Database:

$$logit\left({}_5q_{x+5}^{j,t,g}\right) - logit\left({}_5q_{x}^{j,t,g}\right) = \beta_0^g + \gamma^{j,g} + \beta^g\left[logit\left({}_5q_{x}^{j,t,g}\right) - logit\left({}_5q_{x-5}^{j,t,g}\right)\right]$$
$$\gamma^{j,g} \sim N(0, \sigma_\gamma^2)$$

Where

j refers to country

g refers to sex

t refers to time

β is a fixed covariate coefficient

γ is a random intercept

This approach was first used in GBD 2019, where a difference in ${}_nq_x$ was used to predict a difference in ${}_nq_x$. Previously, we had used the last (oldest age) observed level of ${}_nq_x$ to predict the difference to the next ${}_nq_x$ value. However, we conducted an analysis of these two extension methods and found that the newer method was a better predictor out of sample. Additionally, using the last slope to predict the next slope removed cases of sharp changes in slope in ${}_nq_x$ that were produced by the old method.

After empirical life tables were extended to age 105-109, an iterative scaling algorithm was applied to match ${}_5q_{95}$, ${}_5q_{100}$, and ${}_5q_{105}$ to the observed ${}_nm_x$ implied by VR deaths over GBD-estimated population.

Another modification, originally introduced in GBD 2019, is intended to reduce the frequency of cases where the female mortality rate becomes greater than male mortality rate in the extended ages. Life tables with male-female crossover in late adulthood were designated to the location-specific category rather than the universal life table group. The change designed to address this issue is covered in section 2.4.4.

Section 2.4.4: GBD relational model life table system with a flexible standard selection mechanism

We based our relational model life table system on the idea that, to capture the very high levels of young adult mortality that occur in locations with high HIV prevalence, we needed to first develop a model life table for a counterfactual population without HIV and then add the effects of HIV by age and sex into that life table.

Three steps were used in developing this system. First, we estimated counterfactual levels of ${}_5q_0$ and ${}_{45}q_{15}$ without HIV. Second, we generated a full set of age-specific mortality rates using the counterfactual model life table system. Third, we estimated the increase in mortality when HIV was included back in, for each age group.

Model for populations free of HIV/AIDS

Our relational model life table system was different from previous systems in several ways. First, we modelled ${}_nq_x$ rather than l_x in logit space. Modelling ${}_nq_x$ allowed us to better capture the impacts of changes to ${}_5q_0$ and ${}_{45}q_{15}$ on different age groups. We used the following equation to generate life tables for populations not affected by HIV/AIDS:

$$logit({}_nq_x^c) = logit({}_nq_x^s) + \beta_x^1 \cdot \left(logit({}_5q_0^c) - logit({}_5q_0^s)\right) + \beta_x^2 \cdot \left(logit({}_{45}q_{15}^c) - logit({}_{45}q_{15}^s)\right)$$

Where

$logit({}_{45}q_{15}^s)$ is the logit transformation of ${}_{45}q_{15}$in the standard life table

$logit({}_{45}q_{15}^c)$ is the logit transformation of ${}_{45}q_{15}$ for a country without HIV or the counterfactual level of ${}_{45}q_{15}$ in the absence of HIV in a country affected by HIV/AIDS

$logit({}_5q_0^s)$ is the logit transformation of ${}_5q_0$ in the standard population

$logit({}_5q_0^c)$ is the logit transformation of ${}_5q_0$ for a country without HIV or the counterfactual level of ${}_5q_0$ in the absence of HIV in a country affected by HIV/AIDS

$logit({}_nq_x^s)$ is the logit of the probability of death in the standard population from age x to $x + n$

$logit({}_nq_x^c)$ is the logit transformation of the probability of death from age x to $x + n$ in a country without HIV or the counterfactual level of ${}_nq_x$ in the absence of HIV in a country affected by HIV/AIDS

β_x^1 and β_x^2 are coefficients that vary by age x and measure the impact of differences in child and adult mortality rates between a target life table and the standard life table on the estimated age pattern of mortality. These coefficients determined how much the estimated age pattern of mortality deviated from the standard by age and from linearity.

The design of this equation was based on our assumption that the logit-transformed age-specific probability of death in a target life table (c) could be represented as a function of (1) the corresponding logit-transformed age-specific probability of death in a standard life table (s), (2) the difference in ${}_5q_0$ in logit scale between life tables c and s, and (3) the difference in ${}_{45}q_{15}$ in logit scale between life tables c and s. Life table c was our life table without HIV, either with counterfactual levels of probability of death without HIV/AIDS for countries and territories affected by HIV/AIDS, or as a complete life table for countries and territories not affected by HIV/AIDS. The design of the model was informed by previous observations that in the absence of an HIV/AIDS epidemic, differences in age-specific probability of death in logit scale are correlated strongly with differences in ${}_5q_0$ or ${}_{45}q_{15}$ in logit scale.

We estimated the β_x^1 and β_x^2 coefficients using the equation below:

$$logit\ {}_nq_x^c - logit\ {}_nq_x^s = \beta_x^1 \times (logit\ {}_5q_0^c - logit\ {}_5q_0^s) + \beta_x^2 \times (logit_{45}q_{15}^c - logit_{45}q_{15}^s)$$

To estimate the parameters, we used location-time-specific standards for each of the life tables in our database that were from country-years not affected by HIV/AIDS. We used country/territory-time-specific standard life tables any time there existed in our database an observed life table from the same country within a 15-year time frame. We produced region-specific standard life tables by collapsing all the life tables in our dataset without HIV from the same GBD region by sex. Next, we paired the life tables in our dataset without HIV with the region-specific life tables we had produced.

We estimated $\hat{\beta}_1$ and $\hat{\beta}_2$, the results from which are found in table D below. We restricted the effects of ${}_5q_0$ and ${}_{45}q_{15}$ to specific age groups to prevent improbable outputs in instances where ${}_5q_0$ and ${}_{45}q_{15}$ changed in opposite directions in the same population. We generated full life tables for locations without HIV/AIDS epidemics using the values in table D. We used the ${}_5q_0$ and ${}_{45}q_{15}$ values as our entry parameters for this model life table system.

Table D. Model life table coefficients

Age group	Difference in $_5q_0$ (logit scale)		Difference in $_{45}q_{15}$ (logit scale)	
	Male	Female	Male	Female
0	1.003	1.009	--	--
1-4	0.978	0.954	--	--
5-9	0.821	0.725	--	--
10-14	0.455	0.378	0.471	0.458
15-19	0.218	0.251	0.779	0.517
20-24	--	--	0.754	0.901
25-29	--	--	0.562	0.997
30-34	--	--	0.591	0.984
35-39	--	--	0.720	0.965
40-44	--	--	0.847	0.955
45-49	--	--	0.967	0.956
50-54	--	--	1.005	0.957
55-59	--	--	1.030	0.941
60-64	--	--	0.974	0.855
65-69	--	--	0.971	0.790
70-74	--	--	0.934	0.770
75-79	--	--	0.899	0.766
80-84	--	--	0.815	0.701
85-89	--	--	0.659	0.838
90-94	--	--	0.641	0.724
95-99	--	--	1.082	1.072
100-104	--	--	1.188	1.087
105-109	--	--	1.622	1.636

The process we used to compute standard life tables considered observed relationships between variances in geography, time, and mortality age patterns. To produce a standard life table, we computed the Mahalanobis distance between the target life table and all the observed life tables in our database without HIV based on $_5q_0$ and $_{45}q_{15}$ in logit scale. We used Mahalanobis distance rather than Euclidean distance because Mahalanobis distance includes the relationship between $_5q_0$ and $_{45}q_{15}$ in logit space in its calculation of distances between life table pairs. We expressed the Mahalanobis distance between two sets of $_5q_0$ and $_{45}q_{15}$ as:

$$D_M^i(Q^i) = \sqrt{(Q^i - O)^\tau S^{-1}(Q^i - O)}$$

Where

O is a multivariate vector representing entry parameters $_5q_0$ and $_{45}q_{15}$ in logit scale

$Q^i = (logit(\,_5q_0^i), logit(_{45}q_{15}^i))$ is a multivariate vector that corresponds to an empirical life table i in our life table database.

$_5q_0$ and $_{45}q_{15}$ were highly coordinated in logit space. For most super-regions, we kept the 100 life tables with the lowest Mahalanobis distance; we kept only 15 life tables for locations in the high-income super-region. We also included all the life tables in our database from the location, within 30 years. After combining all these life tables, we kept the 100 (15 for high-income locations) that were closest based on geography and distance in years.

For GBD 2017, this process was done separately by location, year, and sex. However, starting in GBD 2019, we selected life tables by location-year, but in pairs by sex. This was done to reduce unsubstantiated crossover of male and female mortality in the elderly. The result is that the life tables selected for males for a given location-year are from the same location-years as the life tables selected for females for that same location-year. For GBD 2021, to better capture the effects of the COVID-19 pandemic, data from pandemic affected years (2020-2021) were only used for estimation in the location-year of origin, and if VR was present, only the empirical life table (ELT) was used to create the standard life table. This was done to

prevent data from before the pandemic affecting the mortality trends of during-pandemic estimates and vice versa. We then calculated the weighted average of the selected life tables by applying empirical weights to each table.

Figure A. Empirical weights by lag in time and geographic region for males

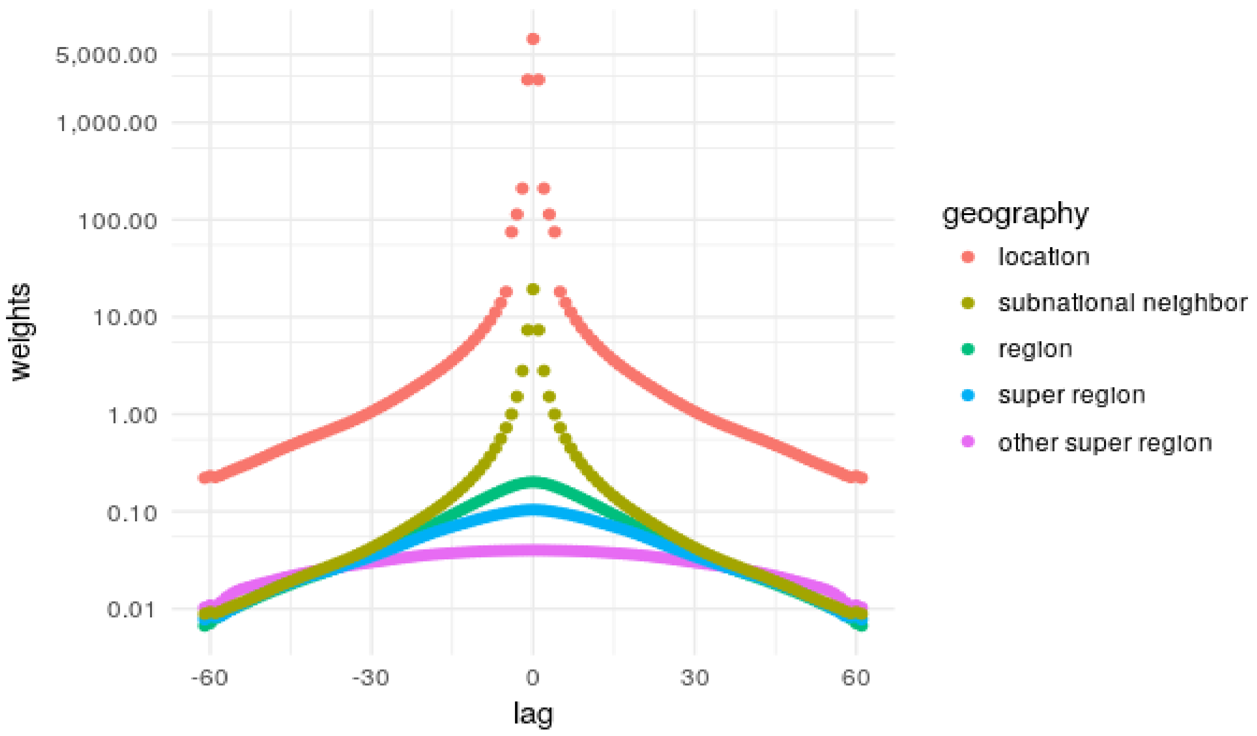

Estimating HIV/AIDS

Unlike other existing model life table systems, our system provides a solution to the challenge of incorporating deaths from HIV/AIDS. Since GBD 2016, we have incorporated an updated process to estimate the HIV counterfactual mortality age pattern (see section 2.6) using HIV counterfactual $_5q_0$ and $_{45}q_{15}$ as entry parameters. We then added excess mortality due to HIV/AIDS to the under-5 and 15–59 summary age groups as well as specific age groups by location, year, and sex to the age-specific mortality without HIV. For the age-specific step, we used the Spectrum HIV model to extract the number of deaths due to HIV/AIDS and calculated the relative risk (RR). We defined RR as the ratio of HIV-specific mortality between a specific age group and the 40 to 44 age group, using the following equation:

$$R_i = \frac{{}_nM_x^{HIV}}{{}_5M_{40}^{HIV}}$$

Where i indicates different age groups 0, 1–4, 4–9, 10–14, etc.

We ran the Estimation and Projection Package (EPP)/Spectrum at the 1000-draw level, for draws of the RR of death from HIV/AIDS by location, sex, and time.

Estimating all-cause mortality for South Africa

We followed the steps for incorporating HIV/AIDS into all-cause mortality described in the previous section for all locations, except South Africa. For estimating all-cause mortality in South Africa, we instead performed the steps in reverse order. South Africa has a near-complete VR system, so we estimated all-cause mortality with HIV using observed VR and then removed excess mortality due to HIV using the RR of death from HIV estimated in the Spectrum model.

This method allowed us to match the completeness-adjusted VR data by age, and the age pattern of HIV-specific mortality matched the age pattern from the EPP/Spectrum epidemiological model.

Uncertainty in the estimated life tables

One strength of our model is that we included uncertainty in each step of the mortality estimation process. We followed the method described in King and colleagues[31] and incorporated uncertainty from the entry parameters ($_5q_0$ and $_{45}q_{15}$) and the coefficients of each model. In short, we repeated the model life table process described in the previous sections 1000 times based on randomly paired entry parameters (ie, different standard life tables) and model coefficients. The result was wide UIs for mortality estimates in countries and territories where uncertainties in the estimated entry parameters were high.

Section 2.4.5: Rake subnational life tables to national level (excluding South Africa)

As explained in previous sections, for all countries and territories excluding South Africa, we raked subnational-level estimates to separately estimated national-level estimates. We did this because we require subnational mortality estimates to add up to national estimates, and data at the national level were generally more robust than at the subnational level.

For South Africa, we instead aggregated subnational-level estimates from our EPP-Spectrum model as our national-level estimates. As explained briefly in previous sections, we did this because the substantial heterogeneity in HIV/AIDS mortality rates at the subnational level in South Africa made it difficult to accurately estimate all-cause and HIV cause-specific mortality at the national level. We used all subnational child and adult mortality data available. The number of data sources was similar at the national and provincial levels.

Section 2.5: HIV/AIDS estimation[7]

Section 2.5.1: Age-specific mortality (with and without HIV)

We used age-specific mortality rates with-HIV and without-HIV from the model life table process as inputs to the HIV/AIDS estimation described in this section.

Section 2.5.2: HIV-free survival rates (for Spectrum)

Our inputs for Spectrum were single-year HIV-free mortality based on five-year abridged life tables, generated from the age-specific HIV-free survival probabilities produced through the process described above.

Section 2.5.3: EPP and Spectrum

For our HIV analysis, we modified two tools developed by the Joint United Nations Programme on HIV/AIDS (UNAIDS):

1. EPP and EPP-Age Sex Model (EPP-ASM):
    a. These tools generate HIV burden estimates in line with observed prevalence data. They incorporate many of the same assumptions as Spectrum. EPP fits a simpler model to HIV prevalence data from representative surveys and surveillance sites to estimate prevalence and incidence for the 15–49-year age groups. EPP-ASM incorporates the full population project of Spectrum and produces age-sex-specific prevalence, incidence, and mortality.
    b. We modified EPP-ASM by building a paediatric module that mirrored that of Spectrum. Perinatal and breastfeeding transmission was calculated as a function of prevalence among pregnant women and maternal-to-child transmission programme data. We additionally improved the fit to prevalence data by allowing flexibility in the age distribution of incidence over time. We parameterised the ratio of incidence among ages 15–24:25+ as a constant before year 2000 and a linear regression thereafter. This allowed for the shifts in the age distribution of incidence observed over the course of the HIV epidemic to be reflected in our results.
2. Spectrum natural history model:
    a. This model generates HIV incidence, prevalence, and death by age, sex, and year using time series estimates of HIV incidence, demographic inputs (such as HIV-free mortality and population), assumptions about CD4 progression rates, and assumptions about on-antiretroviral therapy (ART) and off-ART HIV mortality rates by age, sex, and CD4 rate. We used treatment coverage time series from UNAIDS.
    b. We made several modifications after creating a replica of Spectrum in Python (starting with GBD 2013). First, we sex-split incidence based on a model that was fit to the sex ratio of observed prevalence in countries and territories with nationally representative surveys. Second, our child model included CD4 progression and CD4-specific mortality rates that came from a model fit to survival data from the International epidemiology Databases to Evaluate AIDS (IeDEA), as well as ART distribution data from

IeDEA. Additionally, we scaled all input values by a uniformly sampled factor between 0.9 and 1.1 to generate estimates with realistic ranges of uncertainty.

Section 2.5.4: HIV mortality reckoning

We used the reckoning process to reconcile separate estimates of HIV mortality produced by two different estimation processes in the GBD framework. The separate estimates were those from the model life table system and from the natural history model of EPP-Spectrum. We also used space-time GPR-smoothed VR data on HIV-specific mortality in countries and territories with high-quality VR systems instead of mortality estimates from Spectrum.

We assigned all GBD 2021 locations to a modelling strategy group (appendix table S10) based on the level of HIV in the country or territory and the completeness of HIV and VR data. The group a location was assigned to determined which sources we used for HIV-specific mortality data and how we calculated final HIV and all-cause mortality estimates. The groups were as follows:

- Group 1 includes countries with HIV prevalence data from antenatal clinics or nationally- or subnationally-representative population-based seroprevalence surveys:
    - Group 1A included countries with a peak of at least 0.5% prevalence.
    - Group 1B includes countries with a peak of at least 0.25% prevalence and vital registration completeness less than 65%.
- Group 2A: locations with high-quality (4 or 5 star) VR systems, based on the GBD VR quality rating system.[32] All-cause mortality estimates in these locations used national data such as the VR system.
- Group 2B: locations with lower-quality VR systems.
- Group 2C: locations without any VR data.

For HIV-specific mortality estimates, Group 1A and 1B locations used Spectrum output, Group 2A and 2B locations used mortality output straight from the ST-GPR process, and Group 2C locations used output cohort incidence bias adjusted (CIBA) deaths caused by HIV/AIDS from the Spectrum model.[33]

The outputs from the model included the under-1 age group but not early-, late-, and post-neonatal age groups. We assumed that all HIV deaths that occurred in the first year of life occurred in the post-neonatal stage. Previous research is limited but suggests higher mortality in the post-neonatal stage.[16,17] There was no definitive guidance on how better to age-split under-1 HIV deaths.

Finally, reckoned HIV-specific mortality is determined differently by age and modelling group. For the under-5 age groups, we conducted a separate calculation using results from the under-5 age-sex process described in section 2.2.14. For Group 1A and 1B locations, we produced a scalar from HIV-specific and non-HIV deaths Spectrum results, which we then used to produce an HIV-deleted mortality envelope from the all-cause envelope. For locations in other groups, we capped HIV mortality, which was taken directly from ST-GPR or Spectrum, at 90% of the all-cause envelope, and then subtracted it from the all-cause envelope to produce an HIV-deleted envelope. We assumed that all under-1 HIV-specific deaths happened in the post-neonatal stage.

For all locations in groups other than Group 1A, we followed the same procedure described above and capped HIV mortality for ages 6-14, which was taken directly from ST-GPR or Spectrum, at 90% of the all-cause envelope, and then subtracted it from the all-cause envelope to produce an HIV-deleted envelope for that age group. For Group 1A locations, we added HIV mortality from Spectrum to the HIV-free mortality taken directly from our estimates to produce the all-cause with-HIV envelope.

For the age groups older than 15, we used one approach for Group 1A and 1B locations and another approach for Group 2A, 2B, and 2C locations. For Group 1A and 1B locations, we used HIV mortality estimated from an ensemble model. For this, we calculated the average of the implied HIV mortality from the model life table process and HIV mortality from Spectrum, which were related to one another through the age pattern of draw-level HIV-free mortality. For Group 2A, 2B, and 2C locations, we used HIV mortality taken directly from ST-GPR and CIBA-Spectrum. For all locations in groups other than Group 1A, we subtracted HIV mortality, calculated using one of the approaches just described, from the all-cause envelope to produce HIV-deleted envelope deaths. For Group 1A locations, we instead added HIV mortality, again calculated using the approach just described, to the HIV-free mortality that had been estimated through the model life table process, which produced all-cause mortality. This process allowed us to adjust all-cause mortality based on ensemble HIV estimates in

Group 1A locations and was a response to the inherent uncertainty in all-cause mortality estimates that are based primarily on sibling survival data (as was the case in Group 1A locations) and the assumptions about mortality and data that are necessary in EPP and Spectrum.

Section 2.5.5: Envelope calculation

We produced the all-cause and without-HIV envelopes by combining the results from the with-HIV and HIV-free life tables with HIV-specific mortality from ST-GPR, Spectrum output, or CIBA-Spectrum output. We determined the implied HIV mortality from the model life table system.

For the 95+ age group, we approximated the over-95 all-cause mortality rate by using our life table output and dividing l_x by T_x from the 95-99 age group values in the country-specific life tables (T_x is the life table parameter from the total person-years lived after age x). We also calculated the 95+ all-cause mortality rate using the envelope deaths/populations. Next, we developed a scalar from the approximated 95+ all-cause mortality to the 95+ all-cause mortality rate. We rescaled the with-HIV and HIV-free life tables and the implied HIV mortality rate to death counts space using this scalar. Finally, we calculated HIV-deleted and all-cause mortality using the approach described for other age groups above 15 years old.

Section 2.5.6: Life table calculation

For the most part, we calculated life tables using the same approach as for calculating the envelope. There were, however, several differences in the calculation that were related to sex and location aggregation.

For all locations in groups other than Group 1A, we divided the all-cause life table results by the ratio of HIV-free mortality to with-HIV mortality to get HIV-free life table results. For Group 1A locations, we instead multiplied the HIV-free life table results by the same ratio to get all-cause life table results. This process aligned with how we produced envelopes for different location groups.

For aggregate locations (ie, regions, super-regions, and global), we produced aggregate ${}_nm_x$ and ${}_na_x$ (defined as the average number of years lived in the age interval, from among those who died in that age interval) by weighting ${}_nm_x$ by population and ${}_na_x$ by deaths:

$${}_nm_x \times population$$

We used these death- and population-weighted ${}_nm_x$ and ${}_na_x$ values to produce life tables at the regional level.

We calculated the life tables for the under-5 age groups using a similar approach. We did, however, use national-level under-5 results instead of the aggregated ${}_nm_x$ and ${}_na_x$ values when calculating the all-cause life table for countries with subnational locations. We used this approach to preserve the under-5 mortality estimates produced through the processes described above. We did this because those estimates were not always the same as the estimates produced by the aggregated ${}_nm_x$ and ${}_na_x$ values from the model life table process. We used the standard weights for HIV-deleted life tables to aggregate subnational units.

Section 2.6: Age-specific mortality estimation for all GBD age groups: with and without HIV[7]

Section 2.6.1: HIV-deleted age-specific mortality

We used HIV-deleted age-specific mortality in our causes of death (COD) analyses to prevent the spillover effect of HIV mortality into other causes, which was a potential problem especially in locations with a high HIV burden. We calculated this as the difference between the HIV-specific mortality from the HIV reckoning process (section 2.5.4) and the with-HIV age-specific mortality without fatal discontinuities (section 2.6.3).

Section 2.6.2: Age-specific mortality without discontinuities (with HIV/AIDS)

We produced age-specific mortality rates without fatal discontinuities using the model life table system (section 2.4), the age-sex model (section 2.2.11), and the HIV reckoning process (section 2.5.4), with some methods specific to certain location groups.

For all locations, we used our age-sex model, which split U5MR into early mortality for the neonatal (enn), late neonatal (lnn), post neonatal (pnn), 12-23 months, and 2-4 years age groups, to calculate under-5 age-specific with-HIV mortality. For the age groups over 5 years, we used the GBD model life table system to calculate age-specific with-HIV mortality for

locations not in Group 1A. For Group 1A locations, we instead calculated age-specific with-HIV mortality as the sum of the HIV-specific mortality calculated in the ensemble model (section 2.5) and the HIV-free mortality calculated in the first step of the model life table system (section 2.4).

Section 2.6.3: Add fatal discontinuities

We excluded data from years with fatal discontinuities from our mortality estimation process because we did not want sudden and temporary jumps in mortality to distort long-term mortality trends in that country or territory. The following section explains how we added deaths caused by fatal discontinuities to the all-cause mortality envelope and life tables. Section 2.7 provides more information about how we estimated the fatal discontinuities mortality.

To add fatal discontinuities to the life tables, we first produced full single-year with-HIV life tables, to which the fatal discontinuity death rates would later be added. In addition to the single-year death rates, early neonatal, late neonatal, and post-neonatal age groups from the under-5 age and sex model estimates were appended to the life tables. The fatal discontinuity death rates come from 1000 draws of deaths due to fatal discontinuities, transformed by our estimates of population. We then calculated fatal discontinuity mortality rates for each single-year age by assuming the same mortality rate for each year in an age group. This preserved the original abridged fatal discontinuity deaths. We then added the 1000 draws of fatal discontinuity mortality rates to the 1000 draws of with-HIV mortality rates in the full life tables for each location, sex, and age group, creating full life tables that had both fatal discontinuities and HIV included. These full life tables were then abridged into the standard age groups produced throughout the GBD. We calculated 95% UIs in the same way as for other estimates, using the 2.5% and 97.5% percentiles of the summed draws.

In GBD 2021, we adapted our process to preserve the mortality rate from fatal discontinuities when integrated into the abridged life tables. Previously, there would often be minor discrepancies between the increase in all-cause mortality and the mortality rate of fatal discontinuities. In cases where all-cause ${}_nq_x$ would be greater than 1 if fatal discontinuities were added, we lowered the all-cause mortality rate by a corresponding amount to prevent this from happening.

Section 2.6.4: Ages-specific deaths with discontinuities and HIV/AIDS

We used the results from section 2.6.3 to produce location-sex-year-age-specific mortality numbers that included fatal discontinuities (appendix 2 figure S7).

Section 2.6.5: Life tables with HIV/AIDS and fatal discontinuities

We used the results from section 2.6.4 to produce location-sex-year-specific ${}_5q_0$, ${}_{45}q_{15}$, and life expectancy at birth estimates (appendix 2 tables S2–S4).

Section 2.7: Fatal discontinuities[7]

Figure B

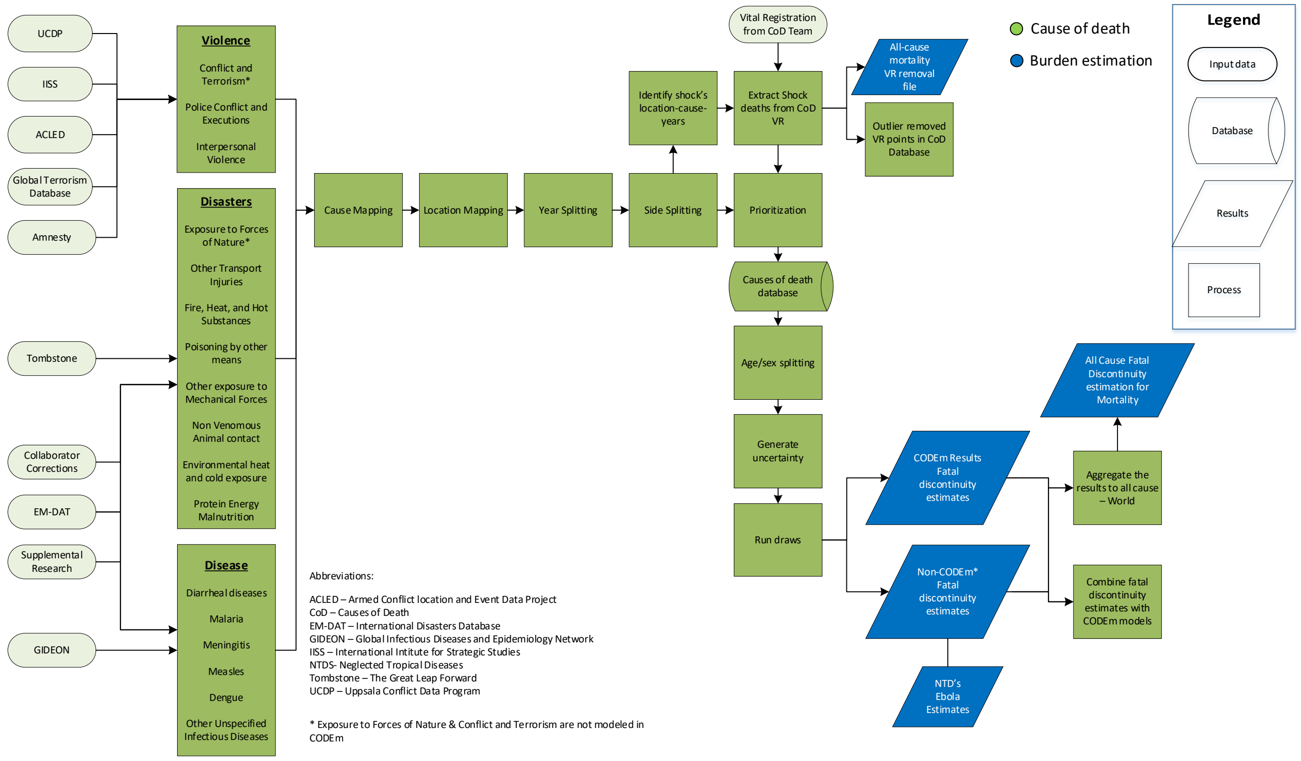

Fatal discontinuities are defined as events that are stochastic in nature and cannot be modelled because they do not have a predictable time trend. Some causes have both fatal discontinuities as well as a continuous background mortality that has a smooth time trend and can be modelled. These include police violence and executions; interpersonal violence; other transport injuries; fire, heat, and hot substances; poisoning by other means; other exposure to mechanical forces; non-venomous animal contact; environmental heat and cold exposure; protein-energy malnutrition; diarrhoeal disease; malaria; meningitis; measles; dengue; and other unspecified infectious diseases. Causes without a continuous background mortality that are exclusively estimated using the fatal discontinuity method are conflict and terrorism and exposure to forces of nature. Any other causes are not captured in fatal discontinuities.

Section 2.7.1: Input data

Overall

We collected data on fatal discontinuities from a range of sources, namely from country-level VR systems and international databases that reported several cause-specific fatal discontinuities. We also collected supplemental data that have known issues related to quality, representativeness, or time lags in reporting. We used a Twitter scraper in place of a systematic literature review as a way to identify supplemental input data for missing fatal discontinuities. We describe the different fatal discontinuity data sources, presented by fatal discontinuity sub-cause, below.

Discontinuities only (non-CODEm)

For causes not modelled in the Cause of Death Ensemble model (CODEm), all deaths captured in VR were considered to be fatal discontinuities. Deaths that were extracted from cause-specific VR were then subtracted from the all-cause VR data used in the all-cause mortality estimation process.

Conflict and terrorism

In GBD 2021, war is defined as "a state of armed conflict between states, governments, societies, and paramilitary groups." It is "generally characterised by extreme violence, aggression, destruction, and mortality, and the use of regular or irregular military forces." Terrorism is defined as "the unlawful use or threatened use of force or violence against individuals or

property in an attempt to coerce or intimidate governments or societies to achieve political, religious, or ideological objectives." We used conflict and terrorism data from the Uppsala Conflict Data Program (UCDP), International Institute for Strategic Studies (IISS), Armed Conflict Location & Event Data Project (ACLED), Global Terrorism Database (GTD), and VR systems and other supplemental data sources (table E). A case was assigned for each event using the source's cause coding and any description from available notes.

Table E. Conflict and terrorism data sources

Data source name	Date accessed	Years of data downloaded	Type of data included
**Uppsala Conflict Data Program[34]**			
Georeferenced Event Dataset, Version 19.1	8/11/2021	1989-2021	UCDP battles, non-state, and one-sided conflict deaths with the most disaggregated location information available
PRIO Battles Deaths Dataset, Version 3.1	4/7/2020	1946-2008	Armed conflict (civil wars, etc.)
**International Institute for Strategic Studies**			
Armed Conflict Dataset	11/17/2016	1997-2016	Insurgency, Inter-state, Intra-state conflict deaths
**Robert S. Strauss Center for International Security and Law**			
Armed Conflict Location and Event Dataset (ACLED)	8/10/2022	1997-2021	Actions of opposition groups, governments, and militias in selected locations in Africa, Asia, and the Middle East specifying the exact location and date of battle events, transfers of military control, headquarter establishment, civilian violence, and rioting
**University of Maryland, Global Terrorism Database**			
Global Terrorism Database (GTD)	2/27/2020	1970-2018	Attacks aimed at attaining political, economic, religious, or social goal, includes evidence of intention to coerce, action was outside precepts of International Humanitarian Law
**University of Chicago, Chicago Project on Security and Threats**			
Suicide Attack Database (CPOST SAD)	11/26/2017	1982-2016	Attacks in which an attacker kills him/herself in a deliberate attempt to kill others, includes only attacks perpetrated by non-state actors
**Amnesty International**			
Amnesty	7/20/2020	1991-2019	Police conflict and executions

Four major conflicts were identified that were not represented in these databases: genocide in Bangladesh in 1971,[35] genocide in Burundi in 1972 and 1993[36], and civil conflict in Albania in 1997[37]. In these cases, literature sources were used to account for these fatal discontinuities.

Exposure to forces of nature

In GBD 2021, exposure to forces of nature is defined as "a force that is beyond human control." The Centre for Research on the Epidemiology of Disasters' International Disaster Database (EM-DAT)[38] served as the primary non-VR source of fatal discontinuities due to exposure to forces of nature (ie, natural disasters, lightning, earthquakes, volcanic eruptions, avalanches, storms, and floods). Data from EM-DAT were last accessed August 8, 2021. Supplemental online research was conducted for events where EM-DAT and VR were not up-to-date.

Partial discontinuity (CODEm)

For causes modelled in CODEm with fatal discontinuities obscured in the time trend, a process was established to avoid duplication of fatal discontinuity deaths in CODEm and the fatal discontinuity estimates. First, location-cause-years were identified through outside non-VR sources. If these location-cause-years also had VR death estimates that were greater than the average of the immediate surrounding years, the difference between the identified year and the average of the

surrounding years was included in the relevant cause for the fatal discontinuities database. The extracted deaths for all fatal discontinuity causes from VR were then subtracted from the all-cause VR data used in the all-cause mortality estimation process.

Executions and police conflict

In GBD 2021, executions and police conflict are defined as “the lawful use or threatened use of force or violence against individuals or groups of people or property in an attempt to achieve political or socioeconomic objectives for a state.” Data for executions and police conflict came primarily from Amnesty International, but other sources such as UCDP, ACLED, and VR that reported deaths due to legal intervention were also cause-mapped to executions and police conflict.

Homicide

In GBD 2021, homicide is defined as “the use of violence against an individual or group of people in an attempt to achieve non-political, religious, or ideological objectives.” Data for homicide came from VR, IISS, GED, ACLED, and other supplements. Events were mapped to homicide where the notes found in the raw data indicated gang violence. Deaths from IISS, GED, and ACLED were then split among three homicide sub-types; physical violence by firearms, physical violence by sharp object, and physical violence by other means, based on the rates calculated from VR by country and territory if available, and by region if country VR was unavailable.

Protein-energy malnutrition (PEM)

In GBD 2021, protein-energy malnutrition is defined as “a lack of dietary protein and/or energy” and covers famines as well as severe droughts. The primary source for PEM data, other than VR, was EM-DAT. The Tombstone report was used to estimate deaths attributed to the famine during the Great Leap Forward in China in the 1960s.[39]

Other injury causes

Other injury causes include other transport injuries (eg, plane, train, and boat accidents); poisonings; fire, heat, and hot substances; and other exposure to mechanical forces (eg, building collapse). The primary data source for these events other than VR was EM-DAT. Supplemental online research was conducted for events where EM-DAT and VR were not up to date.

Meningococcal meningitis and other diseases

In GBD 2021, fatal discontinuities due to a subset of infectious diseases were estimated, including meningococcal meningitis (or meningococcal infection), diarrhoeal disease caused by cholera and dengue. These infectious diseases were first included on the fatal discontinuity cause list for GBD 2016 because (1) their current modelling strategies in CODEm did not optimally capture the potentially highly variable—or epidemic—mortality levels and trends characteristic of these causes; and (2) they can contribute to significant total fatalities in a given location-year. Other infectious diseases for which the latter is true—high death rates in the presence of an outbreak or epidemic—are currently modelled with alternative cause of death methods (eg, natural history models for measles and yellow fever), which allow for greater variation year-over-year if or when outbreaks occur.

The Global Infectious Diseases and Epidemiology Network (GIDEON) and EM-DAT served as the primary data sources for collating cholera and meningococcal meningitis or meningococcal infection death reports.[40,41] For any year that cholera or meningococcal meningitis deaths were recorded in a country or territory covered by GBD, reported deaths were directly extracted from 1950 to 2021. If GIDEON or EMDAT had reporting gaps in cholera or meningococcal meningitis deaths, and WHO reports had coverage for those years, the WHO reports were used. For the Yemen Cholera outbreak in 2016 and 2017, estimates from local collaborators were used in the absence of other data sources.

Section 2.7.2: Location mapping

Every event in the fatal discontinuities database was mapped to a GBD location using a four-step process that includes the following steps in succession: (1) manual mapping, (2) string matching, (3) GPS overlay, and (4) geocoding. If an event was manually mapped, the location was assigned without the use of any other map types. In manual mapping, events are manually assigned to locations by matching the location provided in the raw data to a GBD location. During string matching, an event’s location strings were directly compared to the GBD ASCII location names. During GPS overlay, events that have GPS coordinates provided were overlaid onto a map of GBD locations. If the event was placed over a GBD most-detailed location, the event was assigned to that location. During geocoding, the event’s location string was entered into Open Street Maps, which returns GPS coordinates. These coordinates were processed using GPS overlay to return GBD locations. This

hierarchy provides results where the results of manual mappings are considered the most reliable, followed successively by string matching, GPS coordinates, and then geocoding.

Section 2.7.3: Side splitting

Many fatal discontinuities, such as war, have deaths that are reported across multiple locations. In these instances, deaths were split between the population from both locations, unless estimates by side were provided. If the resulting locations were at the most detailed GBD location level, no further splitting was needed. If a location was not most detailed, the deaths were distributed among the child locations by population.

Section 2.7.4: Prioritisation

Where multiple sources reported shock deaths for the same location-year-cause, a cause-specific prioritisation scheme was followed that reflected the available detail in the cause-specific datasets. For example, the Georeferenced Event Dataset from UCDP was prioritised above all other non-VR sources because it included detail on how deaths were distributed between multiple actors and locations in each conflict event. In most cases, VR from 4-star or 5-star locations was used where available. In some cases, VR from 4-star or 5-star locations was not chosen if there were well-known data quality issues or discrepancies in the cause of death data reporting related to a particular event (eg, supplemental death data for Louisiana were used for Hurricane Katrina because of established data reporting issues).

Section 2.7.5: Age-sex splitting

We ran all compiled data through the causes of death age-sex splitting process, except in cases where we had substantial and reliable information about the age distribution of specific events with high mortality, such as United States mortality during the Vietnam war and Iran mortality during the Iran-Iraq conflict in the early 1980s.[32]

Section 2.7.6: Assigning uncertainty and generating draws

Uncertainty analysis

We generated UIs for deaths caused by conflict and terrorism using UCDP high and low mortality estimates, except for deaths in Iraq from 2003 to 2016. During this time period, deaths due to conflict and terrorism were estimated using a combination of supplemental sources. We used death counts from the Iraq Body Count (IBC),[42] which had the lowest number of deaths from among the sources we found, as the lower bound of the UI from 2003 to 2016. We used estimates from the Iraq Mortality Study (IMS) by Hagopian and colleagues[43] from 2003 to 2006, the deadliest years of the war, to scale deaths to produce the upper UI limits using the formula below:

$$deaths_{GBD\ 2017,high} = deaths_{IBC} \cdot \overline{\left[\frac{deaths_{IMS}}{deaths_{IBC}}\right]}_{2003-2006}$$

GBD 2021 used the average ratio between IMS and IBC reported deaths between 2003 and 2006, multiplied by the number of deaths reported by the IBC. This high estimate was carried forward through 2017 under the assumption that IBC similarly undercounts the number of deaths due to the ongoing civil war in Iraq. The final, best estimate for conflict and terrorism deaths in Iraq from 2003 to 2016 is the midpoint of the high and low estimates given above.

When high and low estimates were not included in the existing data, we applied the regional average UI to the available mortality estimate, for all fatal discontinuity causes.

We assumed a log-normal distribution using mean mortality rates and standard error based on high and low estimates. When the standard error was less than 10e-8, we set the draws equal to the mean rate. We then sampled 1000 draws from the log-normal distribution and converted the draws back to counts space. We used the count space draws to calculate final means and UIs.

Section 2.7.7: Changes since GBD 2019

The only substantive methods change since GBD 2019 was the inclusion of COVID-19 as a shock model. The calculation of COVID-19 shocks is detailed in Section 2.8 below.

Section 2.8: Accounting for the COVID-19 pandemic

Section 2.8.1: Excess mortality due to the COVID-19 pandemic

We used the methodology described in a previous publication to estimate excess mortality due to the COVID-19 pandemic[44] with some key updates. Our analysis of excess mortality due to the COVID-19 pandemic from Jan 1, 2020, to Dec 31, 2021, followed four key steps. First, we developed a database of all-cause mortality by week and month after accounting for reporting lags, anomalies such as heat waves, and under-registration of death. Second, we developed an ensemble model to predict expected deaths in the absence of the COVID-19 pandemic for years 2020 and 2021. In location and time combinations with data meeting our inclusion criteria, excess mortality was estimated as observed mortality minus expected mortality. Third, to estimate excess mortality due to COVID-19 over the entire study period for all countries and territories in the analysis, and especially for locations without weekly or monthly all-cause mortality data, we developed a statistical model to directly predict the excess mortality due to COVID-19, using covariates that pertained to both the COVID-19 pandemic and background population health-related metrics at the population level before SARS-CoV-2 emerged. Fourth, we estimated excess mortality due to COVID-19, propagating uncertainty from each step.

To estimate expected mortality, we developed six models, each fit separately by location. The first four models were based on first estimating the weekly (or monthly) seasonal pattern of mortality and then estimating the time trend in weekly or monthly mortality not explained by seasonality. We used a Bayesian spline to estimate the weekly seasonal pattern for each location using data from 2010, or the earliest year after 2010 when such data first became available, until around February 2020, when the COVID-19 pandemic started for each location. Second, using the same Bayesian spline, we estimated the time trend in the residuals. By combining the seasonal and secular trends, we generated predictions of the expected level of mortality in 2020 and 2021.

The specification of the spline can have a sizeable impact on the estimated expected mortality for a particular location. To make the results more robust to model specification, we included in our ensemble four variants according to where the second to last knot in the spline was placed: 6 months, 12 months, 18 months, and 24 months before the end of the period for the input data before the COVID-19 pandemic started for each location. We also included in the ensemble a Poisson model with fixed effects on week and year, and a model that assumed that expected mortality for 2020 and 2021 was the same as the corresponding weekly mortality observed in 2019. To derive weights for the different models in the ensemble, we assessed how each model performed in an out-of-sample predictive validity test. We fit the model to all data prior to March 1, 2019, and then evaluated how each model performed in predicting mortality between March 2019, and February 2020, compared with observed mortality in the same time period. We then weighted component models in the ensemble using 1 over the root mean squared error (RMSE) of the predictions for each component to down-weight component models with larger RMSE (and thus less accurate predictions) in the ensemble. A global weighting scheme was used for all locations. Expected mortality from the ensemble model was subtracted from observed mortality in 2020 and 2021 to estimate excess mortality due to the COVID-19 pandemic.

Based on our estimation of expected mortality, and after applying the exclusions that account for late registration and other anomalies as previously described, we generated excess mortality estimates for each location where we had input data. In addition, we added excess mortality estimates from two countries where the ensemble model could not be applied due to data constraints: South Africa (only the provinces) and India. We obtained national and province-level excess mortality rate estimates for South Africa which are regularly updated by the Medical Research Council of South Africa, and excess mortality estimates for select periods during the first and second waves of the COVID-19 epidemic for 12 states in India. Using these empirical excess mortality estimates, we developed a statistical model to predict excess mortality for all 191 national and 252 subnational locations in our analysis for the uniform period of Jan 1, 2020, to Dec 31, 2021. This model was crucial for directly estimating excess mortality in countries where reported all-cause mortality data have not been available during the pandemic.

Various studies have examined the associations between particular underlying medical conditions and increased risk of severe COVID-19. We examined all available and relevant covariates on the basis of a meta-analysis conducted by the US Centers for Disease Control and Prevention, as well as covariates directly related to the COVID-19 pandemic, including seroprevalence (lagged by 25 days), mobility (lagged by 19 days), infection detection ratio (IDR; lagged by 19 days), and reported crude death rate due to COVID-19. To help identify covariates that have sensible direction of effect on excess mortality rate, we used ROVER, a method developed at the Institute for Health Metrics and Evaluation based on Bayesian model averaging (BMA). ROVER is conceptually similar to the BMA method, which is widely used to explore the

parameter space and aggregate estimates across candidate models based on performance metrics.[45] The main difference is that while BMA uses marginal likelihood, ROVER focuses on out-of-sample performance. The implementation of ROVER used for this process can be found at https://github.com/ihmeuw-msca/modrover. Based on the outputs of ROVER, 11 covariates were included in our final log-linear model, where the dependent variable was excess mortality in logarithmic scale: lagged cumulative infection rate (seroprevalence) in log space, COVID-19 mortality rate in log space, crude death rate in log space, lagged IDR, annual inpatient admissions per capita, diabetes mortality rate in log space, HIV mortality rate in log space, , average absolute latitude, smoking prevalence, Health Access and Quality (HAQ) Index, and proportion of the population aged 75 years or older. To reduce model volatility, after fitting the initial model, data points with residuals in the top and bottom 2.5th percentiles were excluded and the model was refit. Altogether, these covariates explained 86.9% of the variation in the input data to this regression. We also calculated in-sample residuals for the locations that had directly observed excess mortality rates used in the regression (ie, India and South Africa). Regional and super-regional residuals, per the GBD regional classification system, were calculated as the mean residuals from locations included in each regional aggregate. We also calculated a country-level residual for India using the residual from the 12 states to recognise the dispersed periods and geographical regions covered by the civil registration data. To validate our modelling process, we conducted out-of-sample predictive validity testing. Given the sparsity of input data on empirical excess mortality, we validated our model by repeatedly leaving one location out of the input data, then re-estimated the model and made predictions for the left-out location. Our analysis showed that the mean relative error of predicted excess mortality rate is 10.2% and the root mean squared error of predicted excess mortality rate is 0.00066, indicating a precise prediction model that has low bias.

With this model, we predicted estimates of excess mortality for the periods of 2020, 2021, and the combined time period of Jan 1, 2020 to Dec 21, 2021. Given the broader availability of data covering the entire time period, the yearly estimates were scaled to the entire time period for locations without direct estimates of excess mortality from the first stage model. Ratios of estimated excess mortality to reported COVID-19 deaths were computed using the modelled excess mortality and the reported COVID-19 counts for each location.

As noted previously, policy makers and researchers have proposed many other causes of death that might have been affected by lockdown restrictions, unemployment, and increased poverty. To date, insufficient data are available to widely test for increases in deaths due to pandemic-related elements, such as deferred care, or for reductions in deaths, such as decreases in injury-related deaths due to reduced mobility.

Section 2.8.2: Excess mortality age-sex splitting

In order to reconcile all-ages, all-sexes excess mortality estimates with age-sex-specific all-cause mortality estimates, age-sex distributions of excess mortality were created to split the aggregate estimate. Distributions of excess were calculated by taking the difference between two all-cause envelope versions for location with VR in 2020-2021: 1) an envelope with pandemic years VR data, 2) a counterfactual envelope without pandemic years VR data. The difference between these envelope versions was the implied excess mortality and provided age- and sex-specific rates of excess. Beyond location-specific distributions of excess mortality, global distributions of positive and negative excess were created by summing the implied excess of all countries with positive and negative excess, respectively. The global positive distribution was calculated separately for 2020 and 2021 while the global negative distribution was calculated using all location-years with implied negative excess.

These distributions of excess were applied to the total COVID-19 excess mortality values and scaled such that the total excess mortality was conserved, and the implied under-5 excess remained constant. Location-years with VR largely used a location-year-specific age-sex distribution while locations without VR used either the positive or negative global distribution of excess depending on the value of excess mortality. Location-years for 2021 could also use the distribution from 2020 if VR was present in 2020, but not 2021. Other locations with VR could also be forced to use the global distribution if estimated excess mortality was below a threshold (50 000) or if the implied distribution was implausible or differed in sign from the estimated total excess mortality. All subnational locations used the same age-sex distribution as their parent locations if the sign agreed, or the corresponding global distribution if it did not.

Section 2.8.3: Balancing equation

The balancing equation formally reconciled deaths associated with COVID-19 excess mortality and cause-specific pandemic related causes. This was done by creating a measure of other pandemic related mortality (OPRM) which is equal to the difference between excess mortality and the sum of COVID-19 specific deaths, measles, lower respiratory infection (LRI), and pertussis. Measles, LRI, and pertussis were referred to as indirect COVID causes as mortality from these diseases was affected by the pandemic. OPRM captures all deaths due to the pandemic which were not specifically caused by COVID-19 or the indirect COVID causes. It can be positive or negative, though as we assert that GBD level 1 causes cannot be negative, negative OPRM is removed in the next process.

Section 2.8.4: COVID-19 excess mortality harmonisation

To create consensus between COVID-19-specific mortality, other pandemic related mortality, and all-cause mortality, we reconciled the difference between estimated and implied excess mortality, using estimates from the model life table system, First, any negative OPRM was added to the all-cause envelope and set to zero. For locations with VR data in either 2020 or 2021, the difference between implied and separately estimated excess mortality was split between the all-cause envelope, COVID-19 deaths, and other pandemic related causes. Steps were taken such that values were positive and that final COVID-19 deaths were never less than the reported COVID-19 deaths from each location. Additionally, we adjusted mortality for locations which had VR data in 2020, but not 2021, by multiplying the envelope by the ratio of the adjusted envelope in 2020 over the unadjusted envelope. Locations with no VR in either 2020 or 2021 instead use a ratio of expected deaths from the COVID EM estimation pipeline and the estimate from model life tables. This, in addition to distributing negative OPRM among the all-cause envelope and COVID mortality, allows us to have consistency between our with and without pandemic data mortality results, COVID mortality estimation, and COVID excess mortality estimation. Subnational estimates for all three indicators were scaled to the national level. After COVID-19 deaths, OPRM, indirect COVID shocks, and expected non-pandemic mortality were recalculated, we treated the results as outputs from our model life table system (section 2.4) and performed shocks addition and envelope finalization steps to attain final results. Details from these steps can be found in sections 2.5–2.7.

Section 3: Fertility[4,5,7]

Section 3.1: Overview

Prior to GBD 2016, we used United Nations World Population Prospects fertility estimates for all countries and territories.[46] Starting with GBD 2016, we estimated fertility rates within the GBD framework. We based our estimate of total fertility rate (TFR) on a systematic synthesis of all data available for all GBD 2016 locations, using the age-specific fertility pattern from World Population Prospects (WPP).[3] Starting in GBD 2017, we estimated age-specific fertility rates (ASFRs) for ages 10 to 54 years based on a systematic synthesis of all available data for all GBD locations. We calculated TFR as a function of the ASFRs.[5]. We compiled a total of 58 072 unique location-source-years of data for females aged 10 to 54 for the period between 1950 and 2021. Appendix table S11 and appendix table S12 depict the number of sources by location and by year.

Section 3.2.1: Fertility data source types

We sought to use accurate and complete accounts of livebirths reported according to the age of mothers. Complete livebirth registration reports are designed to account for all births in a single country, territory, or subnational location in a single year, which makes them the gold standard for fertility data. Most high-income countries and territories maintain high-quality VR systems with information on dates and locations for all births as well as demographic characteristics of each mother. Many lower-income countries and territories, however, rely on birth registries with incomplete data coverage or interrupted and/or delayed reporting. In these locations, we used household surveys with birth history information for women aged 15 to 49 at the time of the survey, but had to use birth registries for females aged 10 to 14 and 50 to 54 since most household surveys do not collect birth histories from those age groups.

For triangulating the level and age-pattern of fertility, we had to rely on other types of data sources, primarily household surveys and censuses in areas, where birth registration data quality and completeness were low. Household surveys and censuses contained two primary types of fertility information: CBHs and SBHs. See section 2.2.1 for more information about CBHs and SBHs.

Section 3.2.2: Fertility data identification and synthesis

We obtained VR data from the UN Demographic Yearbook (DYB) from the UN Statistics Division (UNSD),[47] the Human Fertility Collection (HFC) and Human Fertility Database (HFD) from the Max Planck Institute for Demographic Research

(MPIDR),[48,49] the WHO mortality database,[50] official publications, online data portals of national statistical offices, and international collaborators. The HFC, HFD, and DYB are compilations of registry-based fertility data from national statistical offices and research institutes. We obtained DYB data on live births by age of mother for every year available from 1950 to 2020. We obtained the complete set of age-specific empirical data from HFC up to 2019 and from HFD up to 2021 but excluded country-year-ages already accounted for by the DYB. In addition to DYB, HFC, and HFD data, we obtained data from SRSs where available, primarily in South Asian countries such as India, Pakistan, and Bangladesh. In total, at the national level, we obtained 8680 unique country-source-years of VR data, with 2441 from before 1970 and 2554 from after 2000. We also had 29 country-source-years of data from SRS.

We identified fertility data from censuses and household surveys using the Global Health Data Exchange (ger) by searching for "complete birth history," "summary birth history," and "fertility" from among the records categorised as "survey" and "census." Research team members reviewed these data to verify whether they included the necessary information for GBD analysis. We then conducted additional research to identify and fill gaps in data, primarily by data seeking on country statistical office websites and seeking recently released surveys such as DHS, MICS, WFS, and Reproductive Health Surveys (RHS). In-country collaborators also assisted in acquiring data that were not publicly available. For low-income locations (especially in sub-Saharan Africa), we sought out colonial censuses from the 1950s and 1960s with SBH data. For sources that contained microdata, we computed period ASFRs every three years over a 15-year recall using CBH data and calculated the average number of children ever born (CEB) for each year of mother's age, which we later split by cohort age patterns from the first modelling stage (see section 3.3.5), using SBH data. For sources that did not contain microdata, we extracted period ASFR or average CEB by mother's age from reports or other publications. In total, we used 735 CBH and 879 SBH sources from surveys and censuses. We were occasionally unable to identify whether a survey that contained tabulated period ASFRs was a CBH or SBH survey, but these data only accounted for 150 country-source-years from 28 sources. We have provided details on the nature and quantity of identified data sources in appendix tables S11 and S12. We then estimated fertility rates for the 10–14-year age group as a function of estimated fertility in the 15–19-year age group and for the 50–54-year age group as a function of the estimated fertility in the 45–49-year group. We provide more information about the age-specific fertility estimation process below. After estimating ASFRs, we computed summary measures of fertility including TFR, total fertility under age 25 (TFU25), and total fertility over age 30 (TFO30).

Section 3.3.2: Age-specific fertility rate estimation for 15 to 49 years

As stated above, we used ST-GPR to estimate ASFR for age groups 15–19, 20–24, 25–29, 30–34, 35–39, 40–44, and 45–49. The methods for this process have been described in full elsewhere,[7] but in short, we did the following:

(1) estimated ASFR for the 20–24 age group using age-specific data from CBH and VR sources, using mean years of education in that age group as a covariate; (2) estimated ASFR for the other age groups using age-specific data from CBH and VR sources as well as age-specific mean years of education and the 20–24 age group ASFR; (3) split SBH and other total births data by age and period using estimated location, time, and ASFR for each age group; (4) re-estimated ASFR for the 20–24 age group using CBH, VR, and period-age-split SBH data; and (5) re-estimated ASFR for the other age groups using CBH, VR, and the period-age-split SBH data.

We implemented the ST-GPR models for ASFR as explained below. The first stage of our mixed effect regression was fit in bounded logit space:

$$Logit\ (\frac{ASFR\ data - lower\ bound_{age}}{upper\ bound_{age} -\ lower\ bound_{age}})$$

We set the lower bound as the minimum fertility by age across time and location and the upper bound, after dropping implausibly high ASFRs over 0·5, as the 99·3 percentile of fertility by age across time and location. The upper bound set an implied maximum TFR of 9·35.

We used the following formula for our mixed effects regression:

$$logit_{bound}\,(ASFR_{20-24})_{c,t,s,i} = \beta_0 + \beta_1 * female\ education_{c,t} + \gamma_{cs} + \varepsilon_{c,t,s,i}$$

$$logit_{bound}\,(ASFR_{n-n+4})_{c,t,s,i} = \beta_0 + \beta_1 * fem\ edu_{c,t} + spline(ASFR_{20-24,c,t}) + \gamma_{cs} + \varepsilon_{c,t,s,i}$$

$$\gamma_{cs} \sim N(0, \sigma^2_{\gamma cs})$$

$$\varepsilon_{c,t,s,i} \sim N(0, \sigma^2_{\varepsilon})$$

Where

c is location, t is time, s is source of datapoint i

n is between 15 and 45

β_0 is the intercept

β_1 is the coefficient on female education

γ_{cs} is a location-source random intercept

ε is the residual

Female education and the 20-24 age group ASFR estimates were specific to each country or territory and year.

We only used female education as a covariate in high-income locations for the 20-24 age group, not for the other age groups. We fit separate models for the high-income, sub-Saharan Africa, central Europe, eastern Europe, and central Asia super-regions to factor in the differences in the relationships between the 20-24 age group ASFR and the ASFR of other age groups. We selected the knots in the linear spline (in logit space) by super-region and age group, as outlined in table F below.

Table F. Knots on ASFR 20–24

Region	Age	Knot
Central Europe, eastern Europe, and central Asia	15	NA
Central Europe, eastern Europe, and central Asia	25	-1·5
Central Europe, eastern Europe, and central Asia	30	-2
Central Europe, eastern Europe, and central Asia	35	-1·75
Central Europe, eastern Europe, and central Asia	40	-1·75
Central Europe, eastern Europe, and central Asia	45	-2
High-income	15	NA
High-income	25	NA
High-income	30	-2·25
High-income	35	-2
High-income	40	-2·25
High-income	45	-2·25
Others	15	NA
Others	25	-1·5
Others	30	-1·3
Others	35	-1·3
Others	40	-2
Others	45	-2·5

Sub-Saharan Africa	15	NA
Sub-Saharan Africa	25	-1·75
Sub-Saharan Africa	30	-1·25
Sub-Saharan Africa	35	-1·3
Sub-Saharan Africa	40	-1·5
Sub-Saharan Africa	45	-1·75

We outliered data that reported improbably high ASFR (ie, ASFR over 0·5); had 0 values as a result of sampling error, particularly in the 45–49-year age group; reflected an undercounting of births, when we could not adjust the data using other sources; or reported implausibly high mortality levels or trends compared to complete VR data or other more reliable sources.

Section 3.3.3: Data source adjustment

After running the mixed effects model, we adjusted data to a reference source using the random intercept on the concatenation of location and source. To get the adjustment factor, we did the following using the equation below: (1) calculated the difference between the fixed and random effects of the reference source, (2) calculated the difference between the fixed and random effects of the datapoint for the specific source, (3) added the two differences together. We then added this adjustment factor to the data to get an adjusted value.

$$Adjustment\ Factor = (Location\ Source\ RE_{ref} - Location\ Source\ RE_{data\ point})$$

where RE represents a random intercept of either a reference source or a datapoint-specific location-source.

When we had more than one reference source for a single location, we averaged the values of the location source random effects from all the reference sources and used that for the $Location\ Source\ REref$ part of the equation.

We primarily chose reference sources as those that met one of the following criteria: (1) complete VR for locations with complete VR, (2) an average of complete birth history sources for locations with one or more complete birth histories, and (3) an average of all the sources for each location for locations without complete VR or complete birth histories. We considered a location-year of VR to be complete if the estimated completeness of child death registrations was over 95% according to the previous round of GBD.[7] We also chose reference sources for some locations using expert judgement. For example, the 1950s and 1960s censuses in sub-Saharan Africa are widely viewed as an accurate reflection of depressed fertility in that region at that time, so we used those as reference sources.

Section 3.3.4: Hyperparameter selection

We used the outputs of the previous processes to implement residual smoothing and GPR. We chose hyperparameters for these steps based on a location-age-specific data density score. We calculated data density scores based on the years for which VR sources were available plus the number of unique CBH and SBH sources available for the given location, using the following computational methods for each type of data source:

1. **Complete VR sources:** calculated as the number of years for which VR data were available. If the number of births in the age group was below 100, this part of the score was down-weighted by the ratio between the number of births and 100.
2. **Incomplete VR sources:** calculated the same way as with complete VR data, but down-weighted by 0·5.
3. **Total CBH sources:** the number of unique complete birth histories for a single location.
4. **Total SBH sources:** the number of unique summary birth histories for a single location.

We calculated the data density score using the following equation:

$$DD\ Score_{loc,age} = Complete\ VR\ years_{loc,age} + (2 * Number\ CBH\ Sources_{loc,age}) + (0.25 * Number\ SBH\ sources_{loc,age}) + (0.5 * Incomplete\ VR\ years_{loc,age}) + Number\ Other\ Sources_{loc,age}$$

Where

DD is the data density

CBH is complete birth history

SBH is summary birth history

In this round of GBD, we updated our time weights to use a beta density function. We assigned hyperparameters α and β for the beta density function, generally based on final data densities, as shown in table G. However, there were exceptions where we manually assigned a different set of hyperparameters.

Table G. Hyperparameter values by data density

Data density	Alpha	Beta	Zeta	Scale
Over 50	500	500	0·99	5
Between 30 and 50	100	100	0·9	10
Between 20 and 29	20	20	0·8	15
Between 10 and 19	15	15	0·7	15
Under 10	10	10	0·6	15

Where the time weights were calculated as:

$$w_t = \frac{\frac{\Gamma(\alpha+\beta)}{\Gamma(\alpha)\Gamma(\beta)} x^{\alpha-1}(1-x)^{\beta-1}}{\frac{\Gamma(\alpha+\beta)}{\Gamma(\alpha)\Gamma(\beta)} 0.5^{\alpha-1}(1-0.5)^{\beta-1}} = \frac{x^{\alpha-1}(1-x)^{\beta-1}}{0.5^{\alpha+\beta-2}}$$

And:

$$x = \frac{(t+72)}{144}$$

In cases of incomplete VR sources, we defined data variance as the difference between the spatiotemporal prediction and the unadjusted data. Some location-ages had very little data, resulting in implausible variance. As such, for location-ages with fewer than five datapoints, we used the maximum data variance in the location's GBD region.

For complete VR sources, we assumed that non-sampling variance was 0. We calculated sampling variance for these sources using the following binomial equation:

$$Sampling\ Variance = \frac{ASFR * (1 - ASFR)}{Births}$$

We then calculated amplitude and applied it to all locations other than high-income VR-only national locations with 40+ years of VR data. For these locations, we took the mean of the location-specific variance of the difference between the data and the spatiotemporal smoothing. However, we only included national locations from 1990 to 2021.

Section 3.3.5: SBH methods

For the earlier years of the study period, CEB data were more readily available from SBHs than from CBHs. While there are numerous methods for calculating period-age-specific fertility from SBH information, the Brass Parity/Fertility ratio method is used most often. However, this method assumes a constant ASFR over time. We wanted a dynamic measure of cohort age patterns over time, so we instead used ASFR estimates from CBH and VR data from the first run-through of ST-GPR described above to split SBH into period ASFR. We used these estimates to compute implied annualised fertility for all the five-year birth cohorts represented in a given SBH from age 10 through whichever came sooner: age 54 or the year of the survey. To account for births that occurred in years when part of the initial age group had moved into the next age group, we calculated the weighted average of estimated ASFRs in the age groups on either side of the selected age group and took that average as the fertility experienced by that hypothetical cohort in that year, assuming a uniform age distribution within the age group. For example, for a cohort of women aged 20 to 24 in 1984 with ASFR F, we would compute the ASFR experienced by this cohort as women aged 21 to 25 in 1985 as

$$.8 * {}_5F_{20}^{1984} + .2 *{}_5F_{25}^{1989}$$

since 20% of the cohort had aged into the 25 to 29 age group by the following year.

We then used the implied annualised cohort ASFR to calculate cumulative cohort fertility up to the age of each cohort at the time of the survey. We compared implied cumulative fertility to observed cumulative fertility (average CEB from SBHs) by each cohort to get a scaling factor. We then applied this scaling factor to the original implied cohort age pattern, which distributed CEB back across time and age. This method only covered birth cohorts between 1940 (who began to experience ASFR 10-14 in 1950, the first year of our study period) and 2009 (who began to experience ASFR 10-14 in 2019, at the end of our study period).

Splitting of total birth and historic location aggregate data

A large portion of the data could only be obtained already aggregated by age and/or location (eg, total live births instead of births by mother's age; former USSR prior to its dissolution). For these data, we split just the CBH and VR data using the age and location proportions designated in the first ST-GPR run-through. After splitting the data, we reran the estimation process describe above using all CBH, VR, and period and age-split data as well as location and age-split miscellany. This improved the availability of past data and gave us more information about aggregate levels of fertility over time.

Section 3.3.6: Age-specific fertility rate estimation for 10- to 14-year-olds and 50- to 54-year-olds

We estimated ASFR for the 10 to 14 and 50–54-year age groups separately because data for these age groups in locations without VR systems were scarce. We used the relationship between ASFR in sequential age groups to estimate both age groups. For the 10 to 14 age group, we ran a mixed effects regression on the log of the ratio of ASFR 10-14 over ASFR 15-19, and used ASFR 15-19 and nested random intercepts by super-region, region, and location as predictors, as defined by the equation below:

$$\log\left(\frac{ASFR\ 10-14}{ASFR\ 15-19}\right) = \beta_0 + \beta_1 \log(ASFR\ 15-19) + \gamma_{k[j]} + \gamma_{jk[i]} + \gamma_{ijk}$$

$$\gamma_{k[j]} \sim N(0,\sigma^2_{\gamma_{k[j]}})$$

$$\gamma_{jk[i]} \sim N(0,\sigma^2_{\gamma_{jk[i]}})$$

$$\gamma_{ijk} \sim N(0,\sigma^2_{\gamma_{ijk}})$$

Where

i is location, j is region, k is super-region

β_i is a fixed covariate coefficient

$\gamma_{k[j]}$, $\gamma_{jk[i]}$, and γ_{ijk} are nested super-region, region, and location random intercepts

For ASFR in the 50 to 54 age group, we instead estimated a regression on the log ratio of ASFR 50–54 over ASFR 45–49 with a constant. We did this because there was not a clear relationship between this ratio and ASFR 45–49. We produced 1000 draws from the variance-covariance matrix to generate uncertainty using methods described previously.

Section 3.3.7: Fertility metrics

We calculated TFR as the time-weighted sum of the ASFRs from each age group. To do this, we added ASFR for each five-year age group and multiplied that by the five years spent in each age group. We calculated TFU25 and TFO30 in the same way, but with only the relevant age groups included. We defined livebirths as the sum of ASFR from all age groups multiplied by the 10- to 54-year-old female population from the population model described in section 4.

Section 3.4: Sex ratio at birth

Section 3.4.1: Overview

Another component of population structures and reproductive capacity is the sex ratio at birth (SRB). For the GBD 2021 analysis, we defined SRB as the ratio of total male to total female livebirths in each location in a given calendar year. The naturally occurring SRB is generally approximately 1·05 males per female, with some location-specific variations.[51] Since the introduction of ultrasound technologies and the ability to conduct sex-selective abortions, previously stable SRBs have shifted in some locations as a result of systematic sex preferences for children.[51] SRBs in recent years are particularly skewed in the Caucasus, south Asia, and east Asia. To reflect historic equilibria and recent shifts in SRB, we developed a model to estimate SRB in all GBD countries and territories, Hong Kong, and Macau from 1950 to 2021.

Section 3.4.2: Modelling approach

For GBD 2021, we updated our sex ratio at birth estimates. We implemented ST-GPR to estimate a complete time series of the sex ratio at birth for all GBD locations and years. The main differences from previous GBD rounds are additional VR and CBH data, updated outliering decisions following thorough model review, and the use of higher values of the beta hyperparameter for locations with more data. The beta hyperparameter change lowers the smoothness of the model fit, allowing the model to follow the data more closely. See the fertility section for more details on the methodology.

Section 4: Population

Section 4.1: Overview

To estimate population size over a period of time, it is necessary to estimate the initial population and how many people are entering and leaving the population through births, deaths, and migration. The demographic balancing equation expresses how population (N) changes between two points in time.

$$N(T) = N(0) + B[0,T] - D[0,T] + I[0,T] - O[0,T]$$

People can only enter a population through birth (B) or immigration (I) and exit through death (D) or emigration (O). In this analysis, immigration and emigration are collapsed into net migration (G), where net immigration is indicated by a positive value and net emigration is indicated by a negative value.

$$N(T) = N(0) + B[0,T] - D[0,T] + G[0,T]$$

The Bayesian population model used in this study reconciles data on population size obtained through population censuses and registries with GBD fertility and mortality estimates. Below we describe each data source used as an input to the model and then we describe the modelling processes used to estimate migration and population. The calculated GBD world population standard is shown in appendix table S14.

Section 4.2: Data sources and processing[7]

Section 4.2.1: Census and registry lists

Population censuses, which are generally conducted in most countries every ten years, are the primary source of data for population size by age and sex. In some countries, continuous population registries are also maintained. We synthesised population census data by first compiling a list of censuses as documented by the United Nations Statistics Division (UNSD),[52] United Nations Population Division (UNPOP),[53] United Nations Demographic Yearbook (UN DYB),[47] the Integrated Public Use Microdata Series (IPUMS),[54] and the Population Research Center at The University of Texas at Austin.[55] All censuses and registries can be found for each country in appendix figure S5. In appendix table S13, a list of all confirmed censuses can be found.

Section 4.2.2: Data extraction

We extracted age-sex-specific population census data and registry counts from the UN DYB,[47] IPUMS,[54] national statistic websites, and by searching the WorldCat catalog.[56] Occasionally, multiple sources of population counts for a given census or registry-year were available. In these instances, preference was given to registry data rather than census data, data where population counts were reported as de facto (when people are counted based on place of enumeration) over de jure (when people are counted based on place of usual residence), and data reporting population by more granular age and sex groups. In certain cases, only a portion of the total census population was reported with more granular detail for age and sex (with a sample ranging from 1% to 20% of the total census). In these instances, we assumed that the age and sex structure of the sub-sample was identical to that of the total census. Then, we scaled up the sub-sample counts using the total census population counts.

In total, data were extracted for 1277 censuses. Of the censuses extracted, 678 were de facto, 537 were de jure, and in 61 instances we were unable to determine whether the data were de facto or de jure. We were unable to extract some censuses because results were either never or have not yet released by the coordinating organisation. In total, 749 location-years of registry data was extracted for 28 locations.

Any instance of published limitations of the census was also noted, including both limitations identified by media and independent experts in addition to limitations provided by the country's statistical division upon publication. In total, data were outliered from 89 censuses because they were representative of only a subset of the population, utilised questionable methods, or were inconsistent with adjacent data. Some censuses were excluded because countries may have artificially inflated population counts or undercounted minorities for political reasons. Others were excluded because they failed to enumerate the non-white population (for example, Rwanda in 1953 and Burundi in both 1952 and 1958).

Section 4.2.3: Data processing

Population data could be inaccurate due to age misreporting, representativeness of the de facto population, and under/over-enumeration. While identifying censuses, we preferentially utilised raw census and registry population counts. Then, we corrected for the issues mentioned by applying a standardised set of methods.

The first step was to distribute counts of individuals for whom age and/or sex were unknown. The age-sex structure of the remaining data from that particular census was used to distribute the counts of this group of individuals.

The next step was to adjust for age heaping that occurs when, instead of reporting their exact age, individuals report their age as a round number (usually ending in 0 or 5). Population totals were aggregated first to the largest age interval length originally in the data, so each population count was in standardised age groups and the censuses with unusually wide age groups were collapsed to total population by sex only. For each census, the age ratio score (for males and females), sex ratio score, and joint score (JS) were calculated. These measures are explained in detail in the US Census Bureau Population Analysis System documentation.[57]

We used a combination of three previously developed age-heaping methods for this analysis. One of these, the Feeney correction, which is applicable only in census counts with single-year age groups, redistributes people reporting their age as multiples of five to the eight adjacent single-year age groups, resulting in corrected counts with proportional adjacent age groups that then form a linear progression. The corrected counts are reported in five-year age groups. The Arriaga correction takes input census counts in five-year age groups and smooths them by combining the three nearest ten-year age groups with a second-degree polynomial. The advantage of using this technique instead of other similar methods is that it also smooths the youngest and oldest age groups. The Arriaga strong method averages three consecutive ten-year age groups, which addresses cases with severe age misreporting. These three methods were used in countries outside of the high-income and central Europe, eastern Europe, and central Asia super-regions according to the age group length of the population counts and the JS, as seen below in table H.

Table H. Age heaping corrections

Age group length	Joint score (JS)	Age heaping correction
1	JS <= 20	None
1	JS > 20	Feeney
5	JS <= 20	None
5	20 < JS >= 40	Arriaga
5	JS > 40	Arriaga strong
10	JS <= 20	None
10	JS > 20	Arriaga strong
Total population	Not available	None

After performing a visual inspection, 35 censuses were identified as being incorrectly scored or poor-quality, and these data were not corrected for age heaping. This was predominantly due to sex ratio scores that were higher than normal, but still thought to be a real phenomenon, occurring among the Persian Gulf states, for example. 21 censuses were also identified as exhibiting age heaping but were not corrected due to being in either the high-income or the central Europe, eastern Europe, and central Asia super-regions. After applying age heaping corrections, we aggregated the single-year age group population counts conducted outside of the high-income and the central Europe, eastern Europe, and central Asia super-regions into five-year age groups.

For the third step, we corrected data in cases that were not representative of the de facto population of interest, either due to the coverage of geographical area, subpopulations, or a combination of both. A number of countries that exist in 2021 are combinations of two or more countries that existed in the past, such as Germany. In other countries, semi-autonomous territories have conducted separate censuses, such as Transnistria, and others still have contemporary states that are part of historical aggregates, such as the states that were previously part of Yugoslavia. Censuses for fragmented or semi-autonomous areas often occurred in different years and collected information about different age groups.

We estimated the population of the complete modern geographic unit for which we had a time series of separate censuses from all constituent historical or disputed territories. All available censuses for the constituent parts were collapsed to the most granular set of common age groups. Age-sex-specific populations were interpolated after standardisation using the age-sex-specific annual rate of change to generate annual time series spanning the available census data for each constituent. Finally, for all original census years corresponding to the largest constituent where there was also overlap with the estimated populations from the constituents that were smaller, the populations of all the constituents were summed at the age-sex level to produce a census estimate that was representative of the entire modern geography. This method was utilised in the combination of censuses from East and West Germany, Moldova and Transnistria, Cyprus and the Turkish Republic of Northern Cyprus, Serbia and Kosovo, and Malaysia Peninsular, Sarawak, and Sabah. For modern countries formerly part of Yugoslavia, only historical census data that were reported according to ethnic (rather than geographical) affiliation were able to be extracted. In these cases, the estimated age-sex proportions of the total historical aggregate population from the

previous iteration of the Bayesian demographic balancing model were applied to create modern geographies from the historic census data. These split data were used only for Serbia, as census data for the five other modern countries that were formerly part of Yugoslavia were obtained.

Singapore censuses only contained granular age and sex population counts for residents, but non-residents accounted for a significant portion of the de facto population, while registry data for Singapore residents and non-residents were only available in broad age groups (0–14, 15–64, 65+). Using available empirical death counts for both residents and non-residents, and assuming that both subpopulations have similar mortality rates, the relative age-pattern in the death counts was utilised to scale the resident population from the census data to the total population. These age-sex-specific population counts were then scaled to the broad age group data from the population registries in Singapore.

A fourth step was to correct for improper enumeration. Many censuses suffer from under-enumeration, since it is logistically very difficult to reach every single member of the population except in the smallest and most navigable countries. To address this issue, national statistical offices will often conduct post-enumeration surveys (PES) shortly after the completion of censuses to assess the degree to which individuals were either missed or double-counted. The PES quantifies the overall bias of a given census, and population totals can be adjusted using those results. We searched for PES using the UNSD's list of known or planned PES efforts in the 2000 and 2010 census rounds. We looked for PES corresponding to any known census by looking through country statistical websites, academic publications, the WorldCat catalog, and data presented by countries during UN Stats symposia on the topic of PES. 152 PES were identified that reported a net under-enumeration percentage, of which 34 reported under-enumeration percentage by age and/or sex. As most extracted census data were unadjusted and a corresponding PES was unable to be identified for many cases, a simple linear regression was used to predict percentage adjustment for all ages and both sexes on the basis of the Socio-demographic Index (SDI) (section 3).

$$totalPctAdjust = \beta_0 + \beta_1 SDI + \varepsilon$$

We used the variance-covariance matrix from this model to simulate 1000 draws of the predicted total percentage adjustment ($totalPctAdjust$) for every possible value of SDI. We incorporated the variance of the 1000 draws simulated from the model into the overall calculation of population uncertainty (section 2.3.2).

Based on observations that over- and under-enumeration varies significantly depending on age and sex, the 34 PES that reported under-enumeration by age and/or sex were used to obtain an under-enumeration global age pattern. In order to address the issue of PES reporting inconsistent age groups, we used DisMod-MR, an age-integrating Bayesian meta-regression tool widely used in GBD processes[58,59] and included SDI as a predictor in the model. The age-sex pattern was then shifted up or down to equal the predicted mean total percentage adjustment and was applied to all census counts where prior adjustment was not identified.

The Bayesian demographic balancing model and the cohort component method of population projection (CCMPP) requires a starting population as an input from which it can project populations forward in time, but most countries did not conduct a census in 1950. Therefore, the last step of data processing was to produce a prior estimate of the population in 1950 in each location in single-year age groups for each sex. In instances where post-processed censuses were available for 1950 with continuous single-year age groups up to the age group 95+, the census was used directly. In all other cases, aggregate age groups were split in the oldest census available with person-years lived (${}_nL_x$) in single-year age intervals from the estimated life table for that location in 1950.[4] Then, this age pattern was smoothed using a local first-degree polynomial with bandwidth equal to 2 if the original age groups were in age interval lengths of 1 or 5 and bandwidth equal to 5 if in larger age groups.[60] If this oldest census was not from 1950, we projected the census backward using an inverse version of CCMPP (assuming zero migration). A description of standard CCMPP can be found in section 4.4.1; backward CCMPP solves for the population at the previous time point rather than projecting to the next time point.

$${}_1N_{x-1}(t) = {}_1N_x\,(t+1) \cdot \frac{{}_1L_{x-1}(t)}{{}_1L_x\,(t)}$$

The equation for the open-ended age group is indeterminate and leaves what we call the upper missing triangle as the backward projection continues further back in time. The missing age groups are estimated using the 1950 ${}_nL_x$ age pattern found in the estimated life tables. In certain locations, as the census counts are projected backward, age misreporting in the older age groups becomes extremely evident and leads to implausible age patterns; we addressed this by collapsing the original census to a lower open-ended age group, thereby ignoring the information for the oldest age groups in the original census. 34 countries used annualised rate of change between the oldest two censuses to back-project the total population, then scaled the back-projected age-specific population to the back-projected total population. In 36 countries, the assumption of zero migration used in backward CCMPP is not appropriate, or the oldest census was not close enough temporally to 1950 to back-project a reasonable age pattern; in these countries we instead used previous GBD population estimates in 1950 as the baseline population. The 1950 populations were used as the prior for the Bayesian demographic balancing model but were input with considerable uncertainty to account for the fact that a census did not occur in 1950 in most countries.

Section 4.3: Model inputs[7]

Section 4.3.1: Migration

The demographic balancing equation has four main components: population, births, deaths, and migration. Except in high-income countries, migration was not measured as well as the other components, thus impacting CCMPP which requires either the net migration proportion or net number of migrations by age, sex, and year. In locations with frequent censuses, we set the prior for net migration to zero in the Bayesian population model (further described below). However, we recognise the model is not able to estimate migration accurately when large migration occurred between censuses or in the years after the last census was conducted. Therefore, we describe our process of replacing the zero prior in these specific types of locations by identifying and extracting migration data below.

For migration, we utilised a refugee stock dataset from the United Nations High Commissioner for Refugees (UNHCR)[61] which included end-of-year (EOY) counts of refugees residing in each country of destination (D), organised by country of origin (O), to calculate net flow of refugees in or out of countries. For example, change in the number of refugees residing in Rwanda in 1994 was calculated by taking the number of refugees in the country at the EOY in 1994 $D_{RWA}(EOY\ 1994)$ and subtracting the number of refugees at the EOY in 1993 $D_{RWA}(EOY\ 1993)$.

$$\Delta D_{RWA}[1994] = D_{RWA}(EOY\ 1994) - D_{RWA}(EOY\ 1993)$$

Likewise, change in the number of refugees originating from Rwanda who had relocated elsewhere in 1994 was calculated by taking the number of refugees originating from Rwanda at the EOY in 1994 $O_{RWA}\ (EOY\ 1994)$ and substracting the number of refugees originating from Rwanda at the EOY in 1993 $O_{RWA}(EOY\ 1993)$.

$$\Delta O_{RWA}[1994] = O_{RWA}(EOY\ 1994) - O_{RWA}(EOY\ 1993)$$

Net refugee migration for Rwanda in 1994 was calculated as:

$$\text{Net refugee migration}[1994] = (\Delta D_{RWA}[1994] - \Delta O_{RWA}[1994])$$

We converted net refugee migration to mid-year migration totals by averaging totals for adjacent years. While the UNHCR dataset also reported totals for internally displaced persons, these types of individuals were excluded from our analysis. In location-years of refugee crises, the total number of migrants were primarily made up of the large influxes or outfluxes of people, replacing the zero prior with the UNHCR data.

There are certain countries, such as Germany, Romania, the United Arab Emirates, and Bahrain, where large non-refugee migrations have taken place too recently to have been captured by the last census, so they were not accounted for in the Bayesian population model. In locations where this was the case, migration data was taken from the statistical office of the European union (EUROSTAT),[62] the Gulf Labour Markets, Migration and Population (GLMM) programme,[63] or national statistics websites.

EUROSTAT was the only source that provided age-sex-specific migration data; the other sources reported total number of migrants over specific time periods. Since age-sex-specific migration is required as an input to CCMPP,

we scaled age-sex patterns of migration to the input net migrant totals. The aggregated EUROSTAT age-sex pattern was used for most locations, but we used the Bayesian demographic balancing model's age-sex pattern for Qatar in places where the majority of migration has occurred among young adult male temporary workers (Saudi Arabia, Bahrain, United Arab Emirates, Oman, and Kuwait). Qatar was chosen because its most recent census was conducted in 2015 and captured this recent uptick in migration. Lastly, given the genocide in Rwanda in 1994, we assumed the migrant age pattern would be dissimilar to the typical migrant age pattern reported in the European Union and the age pattern of migratory workers in Qatar, so we instead used an age pattern that was uniform across age and sex for the country.

Section 4.3.2: Mortality

The GBD mortality model produced yearly complete period life tables with single-year age groups up to age 95+, as described in section 2.

Section 4.3.3: Fertility

The GBD fertility model produced single-year age group fertility rates between ages 10 and 54 as described in section 3.

Section 4.3.4: Sex ratio at birth

The GBD sex ratio at birth (SRB) model produces yearly sex ratio estimates, which we used to split total livebirths into sex-specific livebirths during CCMPP. See section 3.4 for more information.

Section 4.4: Modelling strategy[7]

Section 4.4.1: CCMPP

CCMPP allows projection of population forward in discrete time intervals by age and sex by incorporating mortality, fertility, and migration estimates. Since more information about this method is provided in Preston,[23] we only present the fundamental equation here:

$$ {}_1N_x\ (t+1) = \left[\left({}_1N_{x-1}(t) + \frac{{}_1G_{x-1}[t,t+1]}{2} \right) \cdot \frac{{}_1L_x(t)}{{}_1L_{x-1}(t)}\right] + \frac{{}_1G_{x-1}[t,t+1]}{2} $$

The survivorship ratio, $\frac{{}_1L_x}{{}_1L_{x-1}}$, which is derived from the GBD period life tables, is used to survive any age group (except the youngest and oldest age groups, which are slightly different) forward by one time interval. One key assumption made was that the number of migrants was evenly split, with half migrating during the beginning of the time interval and the other half migrating at the end of the time interval. Although G was defined by Preston as the net flow of migrants during the projection period in the age interval x - 1 to x,[23] we defined ${}_1G_{x-1}$ as the net flow of migrants during the projection period for the cohort initially between age x – 1 and x at the beginning of the projection period because we used the GBD Bayesian demographic balancing model (described below) to estimate migration. Net migration proportions were estimated by this model instead of counts. We defined these proportions as the ratio of net migrants in a cohort to the population at the beginning of the projection period.

$$ {}_1g_x = \frac{{}_1G_x}{{}_1N_x} $$

This equation is for ages above 0 years, as it does not include fertility. Fertility rates are used along with populations sizes in childbearing ages to calculate the age 0 population.

Section 4.4.2: Bayesian demographic balancing model

A Bayesian hierarchical model (popReconstruct) which reconstructs population by age and sex back in time was previously developed by Wheldon and colleagues.[64] Baseline population counts, mortality, fertility, migration, and

sex ratio at birth were simultaneously estimated by the popReconstruct model to reconcile CCMPP's population projections with recent census data.

In Wheldon's version, bias-reduced input values reported by the United Nations were used for the necessary components of population change which included net migration. In our application of the model, we used initial GBD estimates of fertility and mortality which were derived from replicable methods. Since GBD did not produce net migration estimates due to a lack of migration data and difficulties in directly modelling the variable, we modified Wheldon's popReconstruct model to estimate net migration indirectly, consistent with available census data and the input demographic estimates.

Section 4.4.3: Model description

In both popReconstruct and our demographic balancing model, CCMPP is embedded into a Bayesian hierarchical model. To keep the variables consistent with the popReconstruct model, n, g, s, f, and srb were used to symbolise population, the net migration proportion, survivorship ratio, age-specific fertility rate, and sex-ratio at birth, respectively. We indexed the variables by sex (l), single-year age groups (a) and single calendar years (t). Initial parameter values are indicated by an asterisk ($*$).

Level 1: The percentage difference between non-baseline sex-age-year-specific census counts ($n^*_{l,a,t}$) and the corresponding projected sex-age-year-specific populations counts ($n_{l,a,t}$) from CCMPP in Level 2 was modelled. We modelled percentage difference to ensure the variance ($\sigma^2_{n^*_{l,a,t}}$) associated with each census datapoint was estimated on a consistent scale across age groups and locations for a variety of population magnitudes.

$$Level\ 1\text{:}\ \frac{n^*_{l,a,t} - n_{l,a,t}}{n_{l,a,t}} \sim Normal(0, \sigma^2_{n^*_{l,a,t}})$$

Level 2: The model inputs were transformed into projected sex-age-year-specific population counts ($n_{l,a,t}$).

$$Level\ 2\text{:}\ n_{l,a,t} = CCMPP(n_{l,a,t_0}, g_{l,a,t}, s^*_{l,a,t}, f^*_{l,a,t}, srb^*_t)$$

Level 3: The net migration proportion ($g_{l,a,t}$) and baseline population in 1950 (n_{l,a,t_0}) were modelled. Like in level 1, the baseline population was modelled as the percentage difference between the estimated baseline population in 1950 and the input values. To avoid major discontinuities in the age pattern and time series, net migration proportion was modelled as an autoregressive process over age and time. Contrary to the popReconstruct model, we did not also estimate ASFR ($f^*_{l,a,t}$), survival ratios ($s^*_{l,a,t}$) and sex ratio at birth (srb^*_t) because the zero prior for migration makes it difficult to jointly estimate all components.

$$Level\ 3\text{:}\ \frac{n_{l,a,t_0} - n^*_{l,a,t_0}}{n^*_{l,a,t_0}} \sim Normal\left(0, \sigma^2_{n^*_{l,a,t_0}}\right)$$

$$g_{l,a,t} - g^*_{l,a,t} \sim AR1\text{:}\,AR1(\sigma_g, \rho_{g_a}, \rho_{g_t})$$

Level 4: Hyper-priors were defined for the prior distribution on the net migration proportion. We specified greater correlation over time than age for the net migration proportion correlation.

$$Level\ 4\text{:}\ \log(\sigma_g) \sim Normal(-5, 9)$$

$$logit(\rho_{g_a}) \sim Normal(-3, 0.01)$$

$$logit(\rho_{g_t}) \sim Normal(3, 0.01)$$

Rather than estimating one variance term for all census counts, the initial standard deviation for every age-sex-year-specific census datapoint ($\sigma_{n^*_{l,a,t}}$) was set to 0.01 for locations in the high-income super-region, 0.03 for locations in the central Europe, eastern Europe, and central Asia super-region, and 0.05 for locations in the other super-regions. Several issues were addressed by multiplying the standard deviation by scalars in order to adjust the weight of a

datapoint. These issues included: 1) We doubled the standard deviation of the 1950 baseline counts so that the estimated baseline count could change considerably from the input 1950 counts from census processing. 2) Our model was constructed to compare projected population counts to the available census age groups, whether that was single-year age groups or just total population. When a census only had less granular age groups, there were fewer datapoints included, which gave less weight to the census year as a whole. To resolve this, the standard deviation for each census datapoint was divided by the age interval's width, resulting in all censuses being assigned similar weights. 3) Age misreporting among older ages was accounted for by multiplying the standard deviation by a scalar which increased linearly from 1 among 50-year-olds to 3 among 95+-year-olds in the terminal age group. 4) Under-enumeration in the under-5 age group was addressed by multiplying standard deviation of population counts by a linear scalar that increased from 1 for age 5 to 3 for age 0. For the locations not included in the high-income or central Europe, eastern Europe, and central Asia super-regions, non-baseline under-5 population counts were not input for all censuses, making under-5 populations dependent on the fertility estimates, under-5 mortality estimates, and census counts from older age groups.

Older age misreporting seemed to be quite common, demonstrated by the unrealistically large immigration values produced by the model to compensate for the high counts of individuals among the older age groups. This problem was partly addressed by the methods we described above which gave less weight to census counts among the older age groups. In addition, we ran the model multiple times for each location with each version excluding data above a certain maximum age between 55 and 95. Upon completion of each model version for a location, we calculated: 1) mean average percentage error (MAPE) to compare input census data to the estimated population for each datapoint that was included in the model and 2) the weighted mean of the absolute value of the net migration proportions for five-year age groups between 55 and 95 by assigning a weight equal to 1 for age group 55, 2 for age group 60, and so on, capping at a weight of 9 assigned to age group 95+. Then, we selected the version with the highest maximum age that was within 5% of the minimum MAPE value from the various model versions and within 0.005 of the minimum weighted mean net migration proportion for older age groups. By using this method, we were able to balance decreasing migration among the oldest age groups and ensure a good fit to the census data we included in our model.

For each location, we separately fit the Bayesian demographic balancing model from 1950 to 2021 with single-year age groups up to the terminal age group of 95+. CCMPP was the used to derive final population estimates using GBD estimates of mortality, fertility, and sex ratio at birth as well as posterior estimates of baseline population and the net migration proportion.

Section 4.4.4: Population uncertainty

After estimating a complete time series of age-sex-specific population with CCMPP, we estimated population uncertainty using a combination of out-of-sample predictive validity and uncertainty in the adjustment for census completeness (section 2.3.1).

A major cause of uncertainty was related to the PES results and the census completeness model, leading to additional uncertainty in population estimates for all years, since censuses are used to inform population size in both census and non-census years. Using our census completeness model, we calculated the variance of the percentage adjustment for completeness ($Var_{completeness}$) that was associated with each location-year and dependent on the SDI value.

There was additional uncertainty in our population estimates for non-census years which was related to the amount of time to the closest census and GBD estimates of mortality, fertility, and migration. To measure this, we withheld census data in the 143 national locations where there were a minimum of five censuses with age-sex-specific population totals after undergoing post-census processing (section 2.3.1). For each location, the model was fit 20 times using a random subset of one to five of the location's censuses. Percentage error between the held-out census data and the projected posterior population estimates were compiled across locations by the absolute number of years from the closest census. We then fit a linear regression on the root mean squared percentage error ($RMSPE$) as a function of the number of years to the closest census ($YearsToCensus$).

$$RMSPE = \beta_0 + \beta_1 YearsToCensus + \varepsilon_i$$

For each location-year, we then had the predicted variance of the percentage adjustment for completeness ($Var_{completeness}$) based on the specific year's SDI and the predicted out-of-sample percentage error based on the distance from the closest census ($RMSPE$). We combined these sources of uncertainty in variance space.

$$Var_{pop} = Var_{completeness} + RMSPE^2$$

We used the total variance for population to take simulated draws of the population percentage error ($PopPctError$) by location-year.

$$PopPctError \sim Normal(0, Var_{pop})$$

Finally, these percentage errors were used to calculate new population draws which incorporated both PES adjustment uncertainty and also out-of-sample variance associated with estimation errors in the GBD fertility, mortality, and migration estimates and the amount of time to the closest census.

$$Pop_{draw} = Pop_{mean} + (Pop_{mean} * PopPctError_{draw})$$

Section 4.4.5: GBD world population age standard

The GBD world population age standard was used to calculate age-standardised rates presented throughout GBD. In previous GBD years, 2013, 2015, and 2016, specifically, a standard population age structure was generated by taking the non-weighted mean of the 2010 to 2035 age-specific proportional distributions for national locations reported by the UNPOP World Population Prospects 2012 revision. Beginning in GBD 2017, we used the non-weighted mean of the GBD year's age-specific proportional distributions for national locations with populations greater than 5 million in the GBD year to update the world population age standard. The final values used for the age standard can be found in appendix table S14.

Section 5: Socio-demographic Index (SDI) analysis[7]

Section 5.1: Overview

The Socio-demographic Index (SDI) is a summary indicator created to get at the background social and economic conditions that shape health outcomes in a given location. Introduced in GBD 2015,[46] and further refined in GBD 2019,[7] it represents an update to the Human Development Index (HDI), while also providing an alternative to outdated development language.

SDI includes three components. It includes an economic indicator, lag-distributed income (LDI)[7] per capita, as well as two demographic indicators, total fertility rate under the age of 25 (TFU25) and mean educational attainment for those aged 15 and older (EDU15+). TFU25 is intended to serve as an indicator of the status of women. Beginning in GBD 2017, we began using TFU25 instead of total fertility rate (TFR), because we found it to be a better proxy for women's status.[5]

To create the index, we rescaled each component to obtain a value between 0 and 1. We set this scale using selected health indicators. At the low end of this scale, 0 represents the minimum level of each covariate input past which selected health outcomes do not get worse. At the high end of this scale, 1 represents the maximum level of each covariate input past which selected health outcomes do not improve. Finally, we calculated SDI by taking the geometric mean of those rescaled values.

Section 5.2: SDI calculation

Further refinements to the method for calculating SDI have been implemented since the original development in the 2015 cycle. Beginning in GBD 2017, we decided to use TFU25 instead of the TFR component. The rationale for this was to attempt to better capture women's social status, given that it covers ages when women tend to enter the workforce and pursue further educational opportunities. It is also important that there has been a consistent decline in TFU25 over time in highly developed countries. In contrast, there have been rebounds in TFR driven by increasing fertility in older ages. The concordance correlation coefficient was 0.981 between SDI using the GBD 2016 method and the updated GBD 2017 method.[3,4]

In order to improve the stability of the interpretation of SDI over time, we switched from relative index scales to absolute index scales during GBD 2016 when we noticed the introduction of subnational units led to stretched empirical minima and maxima.[7] The minima and maxima of the scales were selected by looking at the relationships between each of the inputs and life expectancy at birth and under-5 mortality, then identifying points of limiting returns at both high and low values, if they occurred prior to theoretical limits (eg, EDU15+ of 0).

An index score of 0 therefore represents the point at which decreasing each covariate does not worsen selected health outcomes and an index score of 1 represents the level at which increasing the level of each covariate does not improve selected health outcomes. The means that a location with an SDI of 1 would have the theoretical maximum level of development relevant to these selected health outcomes. A location with an SDI of 0, on the other hand, would have the theoretical minimum level of development relevant to these selected health outcomes.

Using scales defining the upper and lower bound for each input, we computed the index scores underlying SDI as follows:

$$I_{Cly} = \frac{(C_{ly} - C_{low})}{(C_{high} - C_{low})}$$

Where I_{cly} – the index for covariate *C*, location *l*, and year *y* – is equal to the quotient of the difference between the value of that covariate in that location-year and the lower bound of the covariate and the difference between the upper and lower bounds for that covariate. If the values of input covariates fell above the upper bounds or below the lower bounds, they were set equal to the respective upper or lower bounds. The index value for TFU25 was computed as $1 - I_{TFU25ly}$, to account for the negative relationship between TFU25 and development, and thus between TFU25 and index score. For GBD 2021, we expanded the computation of SDI to 1055 national and subnational locations spanning the time period 1950 to 2021.

The composite SDI was calculated as the geometric mean of these three indices (LDI, TFU25, EDU15+) for a given location-year. The cutoff values used to determine quintiles for analysis were then computed using country-level estimates of SDI for the year 2021, excluding countries with populations less than 1 million. Annual SDI values can be found in appendix tables S15–17.

Section 6: Additional methods

Section 6.1: Correlation estimation[7]

To estimate correlation, we used the Pearson correlation coefficient, defined by the function below:[65]

$$\hat{\rho} = \frac{\sum_{i=1}^{n} w_i\,(x_i - \bar{x})(y_i - \bar{y})}{\sqrt{\sum_{i-1}^{n} w_i(x_i - \bar{x})^2}\,\sqrt{\sum_{i-1}^{n} w_i(y_i - \bar{y})^2}}$$

Where

w_i are the weights, if specified, or $w_i = 1$ if weights are not specified

$\bar{x} = (\sum w_i x_i)/(\sum w_i)$ is the mean of x

$\bar{y}$ is similarly defined

Section 6.2: Annualised rates of change[7]

We calculated annualised rates of change by taking the log of ${}_5q_0$ in the target year $(y + t)$, dividing that by the ${}_5q_0$ at the baseline year y, and dividing that by the difference in years between the target and baseline years (t), using the following equation:

$$aroc_{_5q_0} = \frac{ln\left(\frac{_5q_{0\,y+t}}{_5q_{0\,y}}\right)}{t}$$

Section 6.3: Gross domestic product (GDP) and lag-distributed income[7]

We used four gross domestic product (GDP) per capita series to calculate GDP per capita: World Bank World Development Indicators, International Monetary Fund World Economic Outlook report, Angus Maddison's research homepage at the University of Groningen Department of Economics, and the University of Pennsylvania (Penn) Center for International Comparisons of Production, Income, and Prices.[66–70] We imputed each series separately using growth regressions and then averaged the four series to make a unified GDP per capita series for GBD. We used mixed effects regression with region-specific random effects to interpolate or extrapolate any country data that were missing from all four series. These methods are described in full in by James and colleagues.[71]

Lag-distributed income (LDI) per capita is a moving average transformation of GDP per capita. We calculated LDI, a ten-year lagged average of GDP, with the formula below:

$$LDI_{pc,t} = \frac{1}{5.5}\left(GDPpc_t + \sum_{i=1}^{9} GDPpc_{t-i} \cdot \left(1 - \frac{i}{10}\right)\right)$$

where LDI at time t is a function of current GDP (at t) and the previous nine years of GDP with an inverse moving weight and a normalisation factor (the sum of all weights).

Section 6.4: Educational attainment covariate

We based our estimates of average years of education on a collection of 6319 censuses and household surveys. The approach we used to estimate educational attainment, which we describe below, has been recently published[72] and draws upon previously established methods.[23,73] Educational data sources used have information about the distribution of educational attainment and must be granular by country or territory, year, sex, and five-year or ten-year age groups. Some sources only provided education data for multi-year bins (eg, the percentage of the population with between two and five years of completed schooling), which were probabilistically split into single-year proportions using a previously published crosswalk model.[74] Data were top coded to 18 years of education.

We then adjusted for systematic biases between data providers in a regional and location-specific fashion. We used a mixed-effects regression model with random effects for data provider and nested random effects for data provider within country to adjust the quantity of interest, either proportion of the population with no education or the mean years of educational attainment. Next, we used age-cohort imputation[72] to carry educational attainment in observed cohorts forward through time, since education levels are relatively constant after age 25. To model the changes by age within cohorts, we used data from all available cohorts with multiple observations at or after age 25. For datapoints from cohorts aged 25 or older, we extrapolated the data forward and backward in time so all year-age combinations in that cohort contained that data (eg, a datapoint for a cohort aged 40–44 in 1995 was projected forward for 45–49-year-olds in 2000, 50–54-year-olds in 2005, etc. and backward for 35–39-year-olds in 1990, 30–34-year-olds in 1985, etc.). The details of both the adjustment and cohort extrapolation models are described elsewhere.[72]

After adjustment and imputation, GPR was used to ensure final model results were consistent with input data and to incorporate model and data uncertainty to produce uncertainty intervals. This allowed us to estimate a complete single-year series of educational attainment from 1950 through 2021 by age, sex, and location. GPR assumes that the trend in the underlying data follows a Gaussian process, which is defined using a mean function m(·) and a covariance function Cov(·). To define the mean function, we calculated the mean level of educational attainment of the country-age-year-specific population, $Edu_{c,a,\,s,t}$, using the following formula:

$$logit\left(\frac{Edu_{c,a,s,t}}{Edu_{max_a}}\right) = \beta_{s,r} + \delta_{s,r}\, Year + I_{s,r} + \propto_{c,a,s}$$

$$\alpha_{c,a,s} \sim N(0, \sigma_\alpha^2)$$

where:

c is location, a is age, s is sex, t is time

Edu_{max_a} is the maximum mean educational attainment for each age group, defined as 3 for ages 5-9, 8 for ages 10-14, 13 for ages 15-19, and 18 for all age groups 20-24 and up

$\beta_{s,r}$ is a sex- and region-specific intercept

$\delta_{s,r}$ captures the linear secular trend for each sex and region

$I_{s,r}$ is a natural spline on age to capture the non-linear age pattern by sex and region, with knots at 45 and 65 years of age

$\propto_{c,s}$ is a country-sex-specific random intercept.

The covariance function of the model was derived using a Matérn covariance function, consistent with prior applications of GPR:

$$M(y, y') = \sigma^2 \frac{2^{1-\nu}}{\Gamma(\nu)} \left(\frac{d(y, y')\sqrt{2\nu}}{\iota}\right)^{\nu} \kappa_\nu \left(\frac{d(y, y')\sqrt{2\nu}}{\iota}\right)$$

Where:

$d(\cdot)$ is a distance function

σ^2 is the marginal variance

ν is a smoothness hyper parameter defining the differentiability of the function

ι is a link-scale parameter approximately equivalent to the number of years at which two points are no longer correlated

κ_ν is the Bessel function

$\Gamma(\cdot)$ is the gamma function

Similar to previous applications of GPR, we approximated σ_p^2 as the super-region and sex-specific residual from the mean function, with ν set to 2 and l to 40, to reflect the inherent smoothness of educational attainment trends over time.

Lastly, we used GPR to smooth the age-period model residuals. This step allowed us to account for uncertainty in each datapoint and combine data and model uncertainty to estimate UIs. Final covariates are aggregated to the age-sex of interest.

Section 7: References

1 Stevens G, Alkema L, Black R, et al. Guidelines for Accurate and Transparent Health Estimates Reporting: the GATHER statement. *The Lancet* 2016; **388**: 19–23.

2 Wang H, Dwyer-Lindgren L, Lofgren K, et al. Age-specific and sex-specific mortality in 187 countries, 1970–2010: a systematic analysis for the Global Burden of Disease Study 2010. *The Lancet* 2012; **380**: 2071–94.

3 GBD 2016 Mortality Collaborators. Global, regional, and national under-5 mortality, adult mortality, age-specific mortality, and life expectancy, 1970–2016: a systematic analysis for the Global Burden of Disease Study 2016. *The Lancet* 2017; **390**: 1084–150.

4 GBD 2017 Mortality Collaborators. Global, regional, and national age-sex-specific mortality and life expectancy, 1950–2017: a systematic analysis for the Global Burden of Disease Study 2017. *The Lancet* 2018; **392**: 1684–735.

5 GBD 2017 Population and Fertility Collaborators. Population and fertility by age and sex for 195 countries and territories, 1950–2017: a systematic analysis for the Global Burden of Disease Study 2017. *The Lancet* 2018; **392**: 1995–2051.

6 GBD 2017 DALYs and HALE Collaborators. Global, regional, and national disability-adjusted life-years (DALYs) for 359 diseases and injuries and healthy life expectancy (HALE) for 195 countries and territories, 1990–2017: a systematic analysis for the Global Burden of Disease Study 2017. *The Lancet*; **392**: 1859–922.

7 GBD 2019 Demographics Collaborators. Global age-sex-specific fertility, mortality, healthy life expectancy (HALE), and population estimates in 204 countries and territories, 1950–2019: a comprehensive demographic analysis for the Global Burden of Disease Study 2019. *The Lancet* 2020; **396**: 1160–203.

8 Ministry of Local Government and Modernisation (Norway). Regulations for the 2019 local government elections for municipalities and counties that are subject to boundary changes that come into effect January 1st 2020. 2019; published online March 20. https://www.regjeringen.no/en/dokumenter/regulations-for-the-2019-local-government-elections-for-municipalities-and-counties-that-are-subject-to-boundary-changes-that-come-into-effect-january-1st-2020/id2637010/ (accessed Sept 1, 2023).

9 Rajaratnam J, Tran L, Lopez A, Murray C. Measuring under-five mortality: validation of new low-cost methods. *PLOS Med* 2010; **7**: e1000253.

10 Wang H, Liddell C, Coates M, et al. Global, regional, and national levels of neonatal, infant, and under-5 mortality during 1990–2013: a systematic analysis for the Global Burden of Disease Study 2013. *The Lancet* 2015; **386**: 957–79.

11 Murray C, Ezzati M, Flaxman A, et al. GBD 2010: design, definitions, and metrics. *The Lancet* 2012; **380**: 2063–6.

12 Child Mortality Collaborators. Global, regional, national, and selected subnational levels of stillbirths, neonatal, infant, and under-5 mortality, 1980–2015: a systematic analysis for the Global Burden of Disease Study 2015. *The Lancet* 2016; **388**: 1725–74.

13 Gompertz B. Gompertz B. On one uniform law of mortality from birth to extreme old age, and on the law of sickness. *J Inst Actruar Assur Mag* 1871; **16**: 329–44.

14 Sharrow D, Hug L, You D, *et al.* Global, regional, and national trends in under-5 mortality between 1990 and 2019 with scenario-based projections until 2030: a systematic analysis by the UN Inter-agency Group for Child Mortality Estimation. *Lancet Glob Health* 2022; **10**: e195–206.

15 United Nations Inter-agency Group, for Child Mortality Estimation. Levels & Trends in Child Mortality. 2022 https://childmortality.org/wp-content/uploads/2023/01/UN-IGME-Child-Mortality-Report-2022.pdf.

16 Brocklehurst P, French R. The association between maternal HIV infection and perinatal outcome: a systematic review of the literature and meta-analysis. *Br J Obstet Gynaecol* 1998; **105**: 836–48.

17 Kim H-Y, Kasonde P, Mwiya M, et al. Pregnancy loss and role of infant HIV status on perinatal mortality among HIV-infected women. *BMC Pediatr* 2012; **12**: 138.

18 Kerber K, Lawn J, Johnson L, et al. South African child deaths 1990-2011: have HIV services reversed the trend enough to meet Millennium Development Goal 4? *AIDS Lond Engl* 2013; **27**: 2637–48.

19 Bradshaw D, Dorrington R. Child mortality in South Africa - we have lost touch. *Afr Med J* 2007; **97**: 582.

20 Murray C, Rajaratnam J, Marcus J, Laakso T, Lopez A. What can we conclude from death registration? *PLOS Med* 2010; **7**: e1000262.

21 Hill K. Estimating census and death registration completeness. *Asian Pac Popul Forum* 1987; **1**: 8–13, 23–4.

22 Brass W, Coale A. Methods of analysis and estimation in mathematical demography. In: Smith D, Keyfitz N, eds. Mathematical Demography. Biomathematics, vol 6. Springer, Berlin, Heidelberg, 1977: 307–13.

23 Preston S. The changing relation between mortality and level of economic development. *Popul Stud* 1975; **29**: 231–48.

24 Preston S, Coale A, Trussell J, Weinstein M. Estimating the completeness of reporting of adult deaths in populations that are approximately stable. *Popul Index* 1980; **46**: 179–202.

25 Bennett N, Horiuchi S. Estimating the completeness of death registration in a closed population. *Popul Index* 1981; **47**: 207–11.

26 Obermeyer Z, Rajaratnam J, Park C, et al. Measuring adult mortality using sibling survival: a new analytical method and new results for 44 countries, 1974–2006. *PLOS Med* 2010; **7**: e1000260.

27 Gakidou E, King G. Death by survey: estimating adult mortality without selection bias from sibling survival data. *Demography* 2006; **43**: 569–85.

28 Masquelier B. Adult mortality from sibling survival data: a reappraisal of selection biases. *Demography* 2013; **50**: 207–28.

29 Rogers R, Crimmins E, editors. International handbook of adult mortality. Springer Netherlands, 2011.

30 Murray C, Ferguson B, Lopez A, Guillot M, Salomon J, Ahmad O. Modified logit life table system: principles, empirical validation, and application. *Popul Stud* 2003; **57**: 165–82.

31 King G, Tomz M, Wittenberg J. Making the most of statistical analyses: improving interpretation and presentation. *Am J Polit Sci* 2000; **44**: 341–55.

32 GBD 2017 Causes of Death Collaborators. Global, regional, and national age-sex-specific mortality for 282 causes of death in 195 countries and territories, 1980–2017: a systematic analysis for the Global Burden of Disease Study 2017. *The Lancet* 2018; **392**: 1736–88.

33 Wang H, Wolock T, Carter A, et al. Estimates of global, regional, and national incidence, prevalence, and mortality of HIV, 1980–2015: the Global Burden of Disease Study 2015. *Lancet HIV* 2016; **3**: e361-87.

34 Themnér L. UCDP/PRIO Armed Conflict Dataset codebook version 18.1. Uppsala Conflict Data Program (UCDP) and Center for the Study of Civil Wars - International Peace Research Institute (PRIO), 2012.

35 Obermeyer Z, Murray C, Gakidou E. Fifty years of violent war deaths from Vietnam to Bosnia: analysis of data from the world health survey programme. *BMJ* 2008; **336**: 1482–6.

36 Leitenberg M. Rwanda, 1994: international incompetence produces genocide. *Peacekeeping Int Relat* 1994; **23**.

37 Jarvis C. The rise and fall of Albania's pyramid schemes. *Finance Dev* 2000; **37**.

38 Centre for Research on the Epidemiology of Disaster (CRED). EM-DAT: The OFDA/CRED International Disaster Database. Brussels: Catholic University of Leuven.

39 Jisheng Y, Friedman E, Guo J, Mosher S. Tombstone: the great Chinese famine, 1958-1962. New York: Farrar, Straus and Giroux (Macmillan), 2012.

40 Inc G, Berger D. Bacterial meningitis: global status: 2017 edition. GIDEON Informatics Inc, 2017.

41 Inc G, Berger D. Cholera: global status: 2017 edition. GIDEON Informatics Inc, 2017.

42 Documented civilian deaths from violence. Iraq Body Count. 2003; published online 2020. https://www.iraqbodycount.org/database/ (accessed Sept 10, 2023).

43 Hagopian A, Flaxman A, Takaro T, et al. Mortality in Iraq associated with the 2003–2011 war and occupation: findings from a national cluster sample survey by the University Collaborative Iraq Mortality Study. *PLOS Med* 2013; **10**: e1001533.

44 COVID-19 Excess Mortality Collaborators. Estimating excess mortality due to the COVID-19 pandemic: a systematic analysis of COVID-19-related mortality, 2020–21. *The Lancet* 2022; **399**: 1513–36.

45 Fragoso TM, Bertoli W, Louzada F. Bayesian Model Averaging: A Systematic Review and Conceptual Classification. *Int Stat Rev* 2018; **86**: 1–28.

46 GBD 2015 Mortality and Causes of Death Collaborators. Global, regional, and national life expectancy, all-cause mortality, and cause-specific mortality for 249 causes of death, 1980–2015: a systematic analysis for the Global Burden of Disease Study 2015. *The Lancet* 2016; **388**: 1459–544.

47 United Nations Department of Economic and Social Affairs. United Nations Demographic Yearbook. New York: United Nations, 2020 https://unstats.un.org/unsd/demographic-social/products/dyb/dyb_2020/.

48 Max Planck Institute for Demographic Research (Germany), Vienna Institute of Demography (Austria). Human Fertility Collection. www.fertilitydata.org (accessed July 6, 2021).

49 Max Planck Institute for Demographic Research (Germany), Vienna Institute of Demography (Austria). Human Fertility Database. www.fertilitydata.org (accessed Sept 13, 2022).

50 World Health Organization. WHO Mortality Database. https://www.who.int/data/data-collection-tools/who-mortality-database (accessed Sept 1, 2023).

51 Preston S, Heuveline P, Guillot M. Demography: measuring and modeling population processes, 1 edition. Wiley-Blackwell, 2000.

52 United Nations Department of Economic and Social Affairs Statistics Division. Welcome to UNSD. 2020. https://unstats.un.org/home/ (accessed Sept 10, 2023).

53 United Nations Department of Economic and Social Affairs. Population. http://www.un.org/en/development/desa/population/ (accessed Sept 10, 2023).

54 IPUMS USA. U.S. census data for social, economic, and health research. 2019. https://usa.ipums.org/usa/ (accessed Sept 10, 2023).

55 The University of Texas at Austin College of Liberal Arts. Welcome to the Population Research Center (PRC). 2020. https://liberalarts.utexas.edu/prc/ (accessed Sept 10, 2023).

56 WorldCat. WorldCat. https://www.worldcat.org/ (accessed Sept 10, 2023).

57 Arriaga EE, Johnson PD, Jamison E. Population analysis with microcomputers - Volume 1 Presentation of Techniques. 1994; published online Nov. https://www2.census.gov/software/pas/documentation/pamvi-archive.pdf.

58 Flaxman AD, Vos T, Murray CJL, Kiyono P. An integrative metaregression framework for descriptive epidemiology, 1st edn. Seattle: University of Washington Press, 2015.

59 GBD 2019 Diseases and Injuries Collaborators. Global burden of 369 diseases and injuries in 204 countries and territories, 1990–2019: a systematic analysis for the Global Burden of Disease Study 2019. *The Lancet* 2020; **396**: 1204–22.

60 Wand M, Ripley B. KernSmooth: functions for Kernel Smoothing supporting Wand & Jones (1995). 2015; published online June 29. https://CRAN.R-project.org/package=KernSmooth (accessed Sept 10, 2023).

61 UNCHR. UNHCR - The UN Refugee Agency. 2001; published online 2020. http://www.unhcr.org/en-us/ (accessed Sept 10, 2023).

62 European Commission. EUROSTAT: your key to European statistics. 2020. http://ec.europa.eu/eurostat (accessed Sept 10, 2023).

63 European University Institute and Gulf Research Center. Gulf Labour Markets, Migration, and Population (GLMM) Programme. GLMM. 2020. http://gulfmigration.eu/ (accessed Sept 10, 2023).

64 Wheldon M, Raftery A, Clark S, Gerland P. Reconstructing past populations with uncertainty from fragmentary data. *J Am Stat Assoc* 2013; **108**: 96–110.

65 Stata:Corp. Stata: release 13. College Station, TX: StataCorp LP, 2013.

66 Groningen Growth and Development Centre: Faculty of Economics and Business. The Database | Penn World Table version 10.0. https://www.rug.nl/ggdc/productivity/pwt/ (accessed Feb 4, 2022).

67 The World Bank. DataBank | The World Bank. http://databank.worldbank.org/data/home.aspx (accessed Feb 4, 2022).

68 International Monetary Fund. World Economic Outlook, April 2022: War Sets Back the Global Recovery. International Monetary Fund. https://www.imf.org/en/Publications/WEO/Issues/2022/04/19/world-economic-outlook-april-2022 (accessed June 8, 2022).

69 Groningen Growth and Development Centre: Faculty of Economics and Business. Maddison Project Database 2020. https://www.rug.nl/ggdc/historicaldevelopment/maddison/releases/maddison-project-database-2020 (accessed Feb 4, 2022).

70 United Nations Statistics Division - National Health Accounts. National Accounts Main Aggregates Database. https://unstats.un.org/unsd/snaama/ (accessed Feb 4, 2022).

71 James S, Gubbins P, Murray C, Gakidou E. Developing a comprehensive time series of GDP per capita for 210 countries from 1950 to 2015. *Popul Health Metr* 2012; **10**: 12.

72 Friedman J, York H, Graetz N, *et al.* Measuring and forecasting progress towards the education-related SDG targets. *Nature* 2020; **580**: 636–9.

73 Gakidou E, Cowling K, Lozano R, Murray C. Increased educational attainment and its effect on child mortality in 175 countries between 1970 and 2009: a systematic analysis. *Lancet Lond Eng* 2010; **376**: 959–74.

74 Friedman J, Graetz N, Gakidou E. Improving the estimation of educational attainment: New methods for assessing average years of schooling from binned data. *PLOS ONE* 2018; **13**: e0208019.

Section 8: Tables and figures

Appendix Figure S1: Analytical flowchart for the estimation of all-cause mortality by age and sex, and HIV/AIDS incidence, prevalence, and mortality for GBD 2021

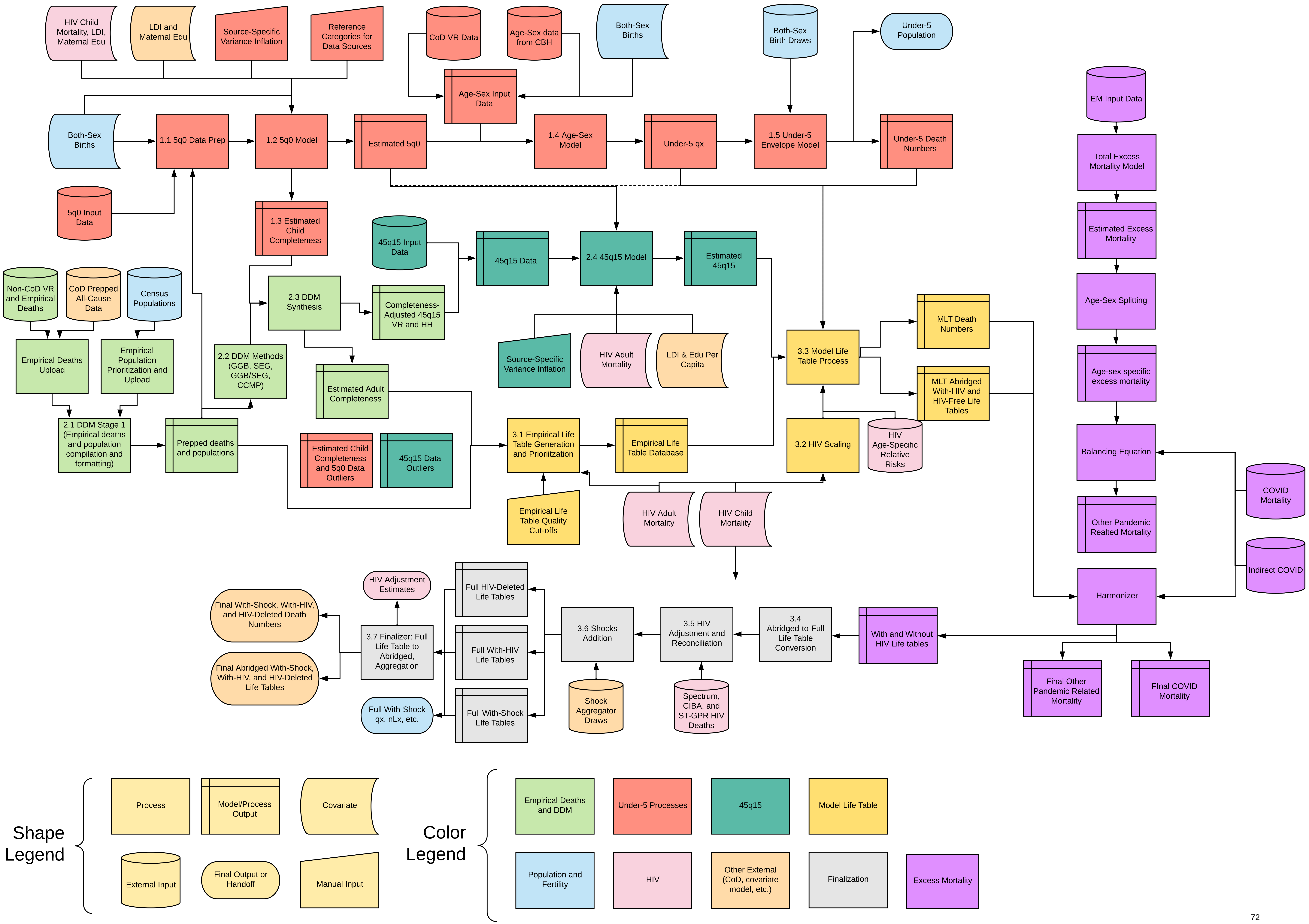

Appendix Figure S2: Estimated completeness of death registration, 1950–2021

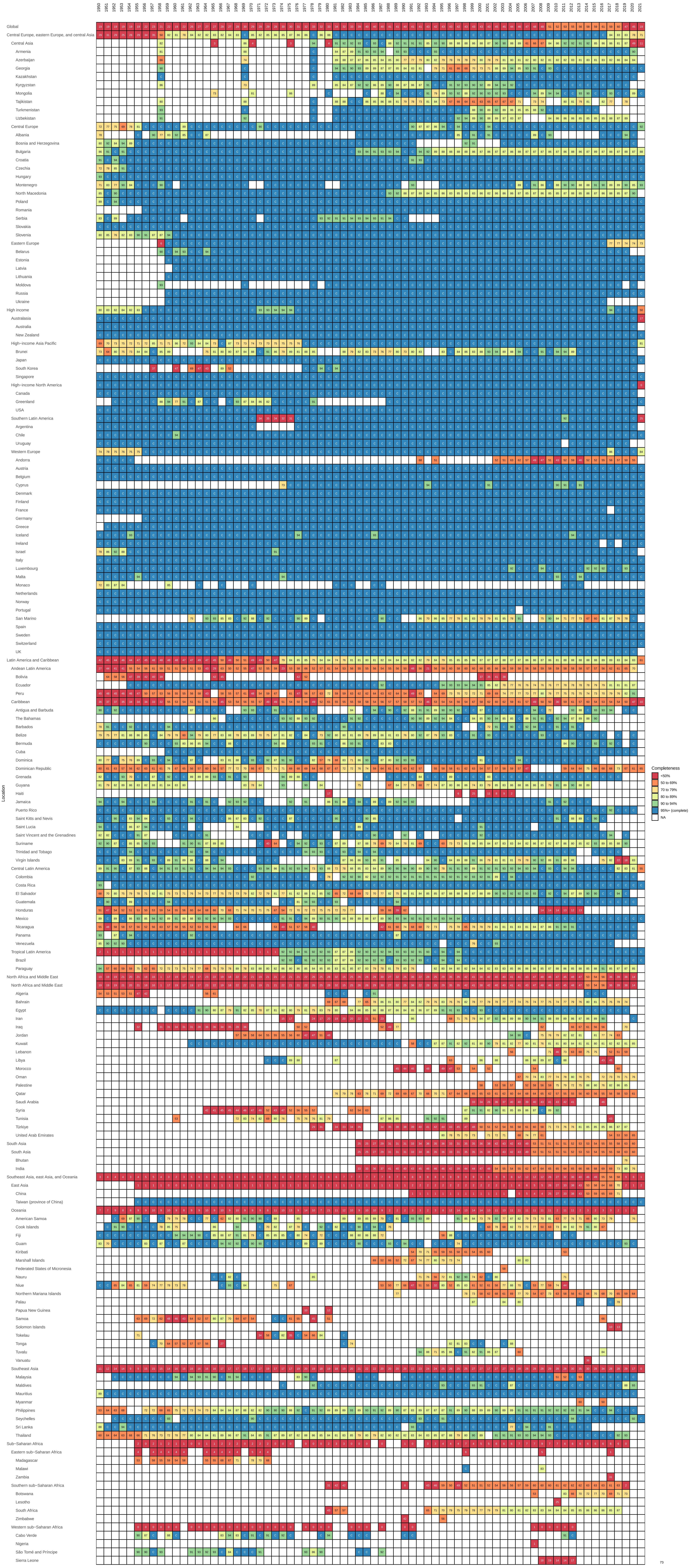

Appendix Figure S3: Analytical flowchart for the GBD 2021 fertility estimation process

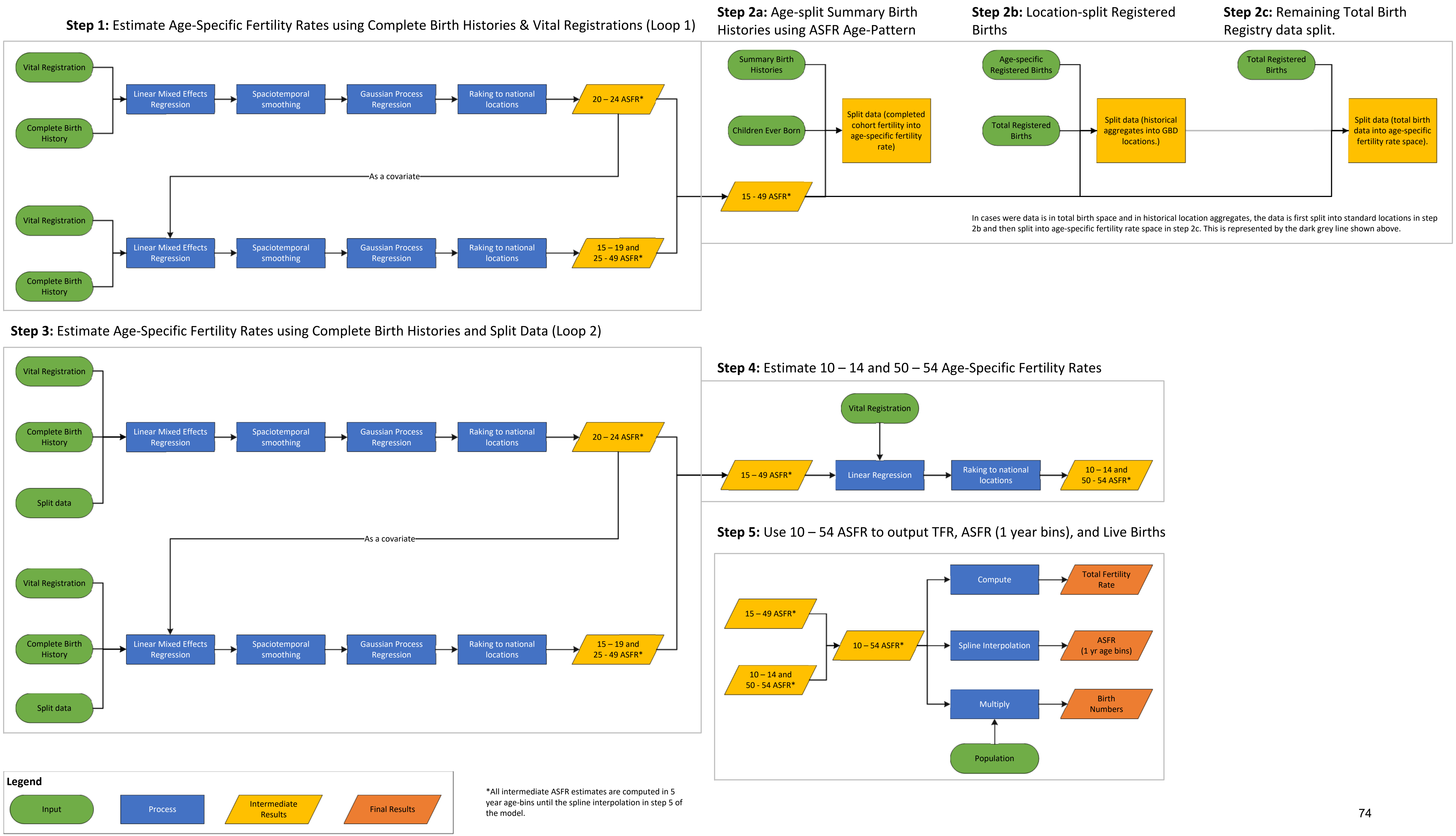

Appendix Figure S4: Analytical flowchart for the GBD 2021 population estimation process

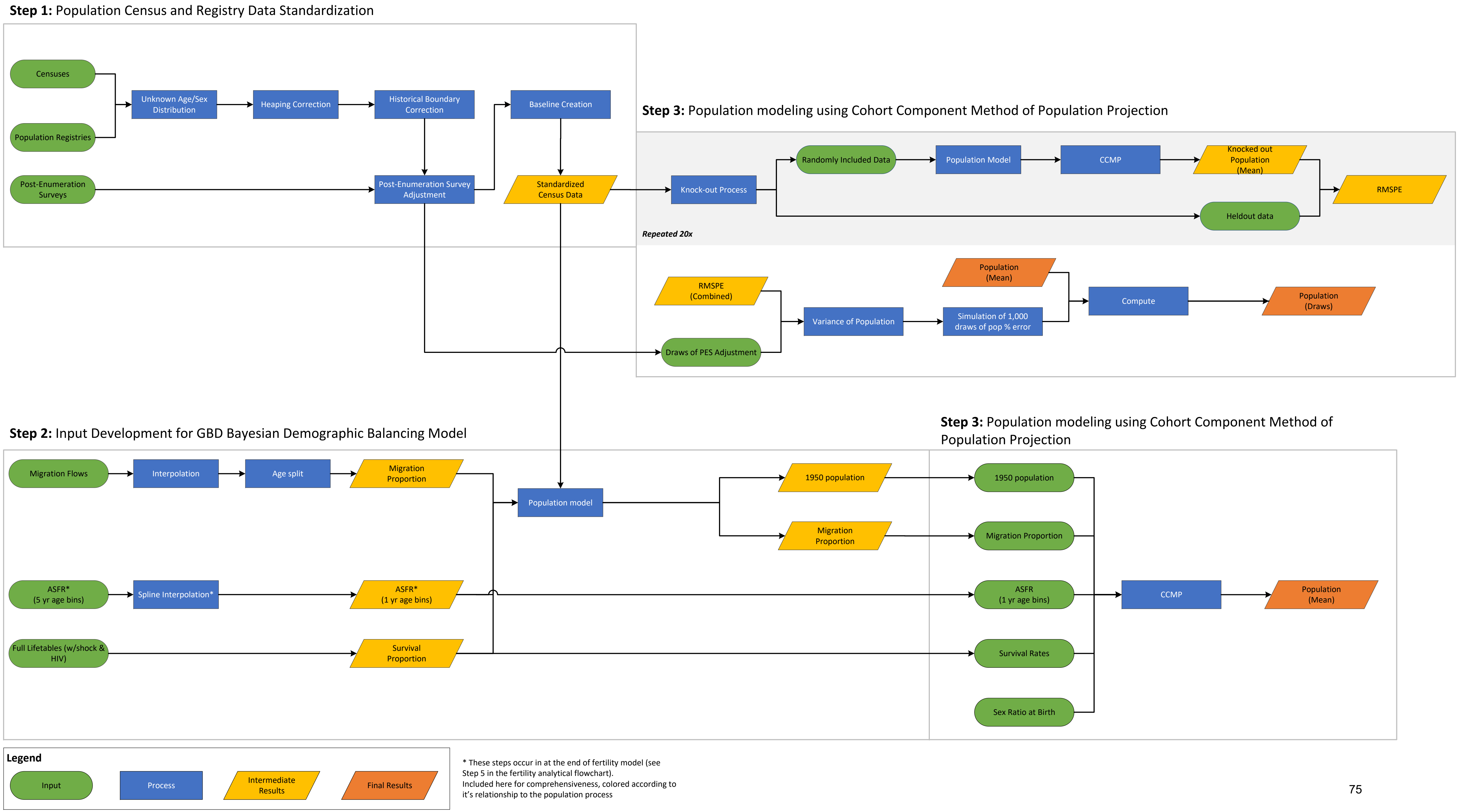

* These steps occur in at the end of fertility model (see Step 5 in the fertility analytical flowchart).
Included here for comprehensiveness, colored according to it's relationship to the population process

Appendix Figure S5: Census and registry availability by location and year
Central Europe, Eastern Europe, and Central Asia

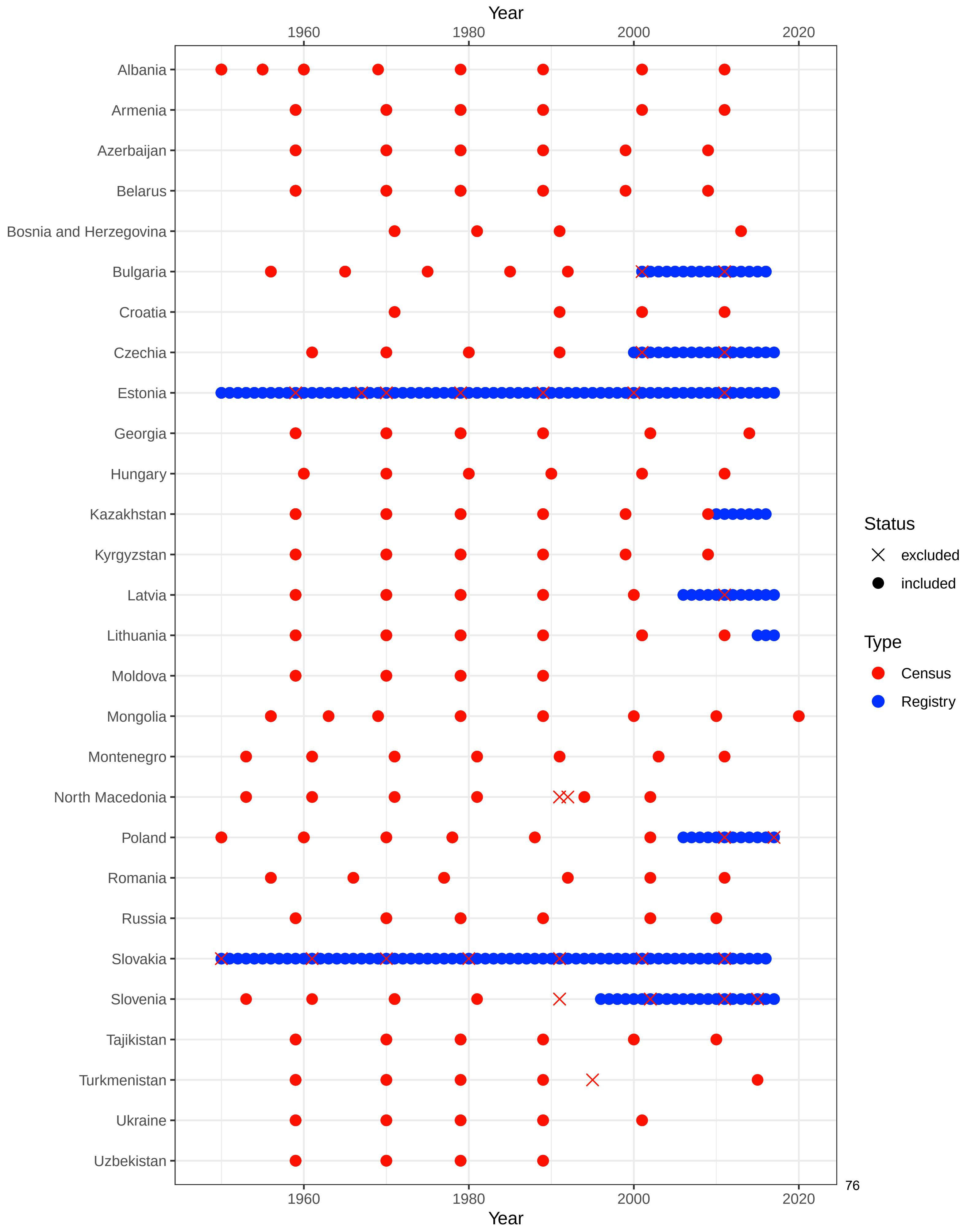

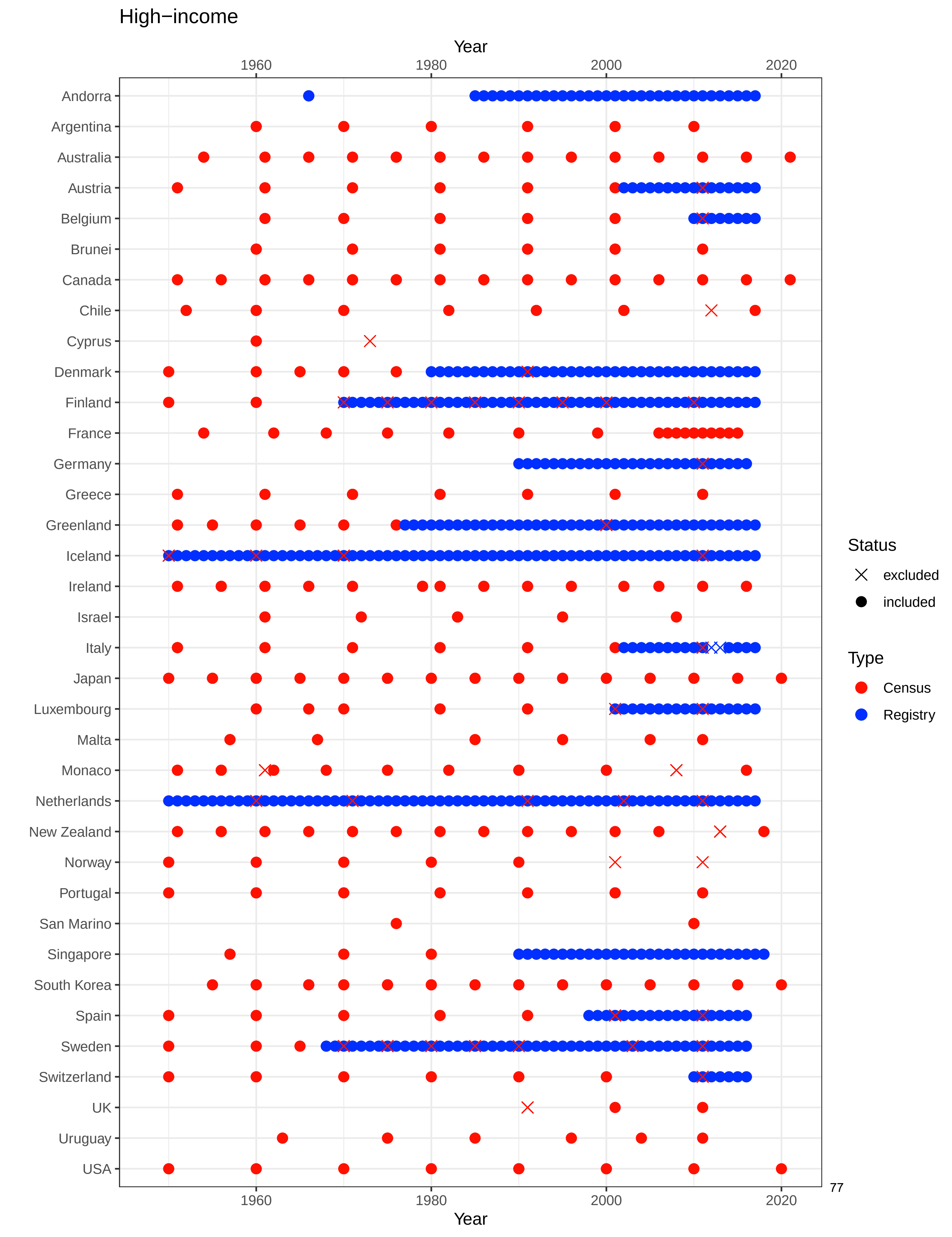
High−income
Year
1960
1980
2000
2020
Andorra
Argentina
Australia
Austria
Belgium
Brunei
Canada
Chile
Cyprus
Denmark
Finland
France
Germany
Greece
Greenland
Iceland
Ireland
Israel
Italy
Japan
Luxembourg
Malta
Monaco
Netherlands
New Zealand
Norway
Portugal
San Marino
Singapore
South Korea
Spain
Sweden
Switzerland
UK
Uruguay
USA
Status
excluded
included
Type
Census
Registry

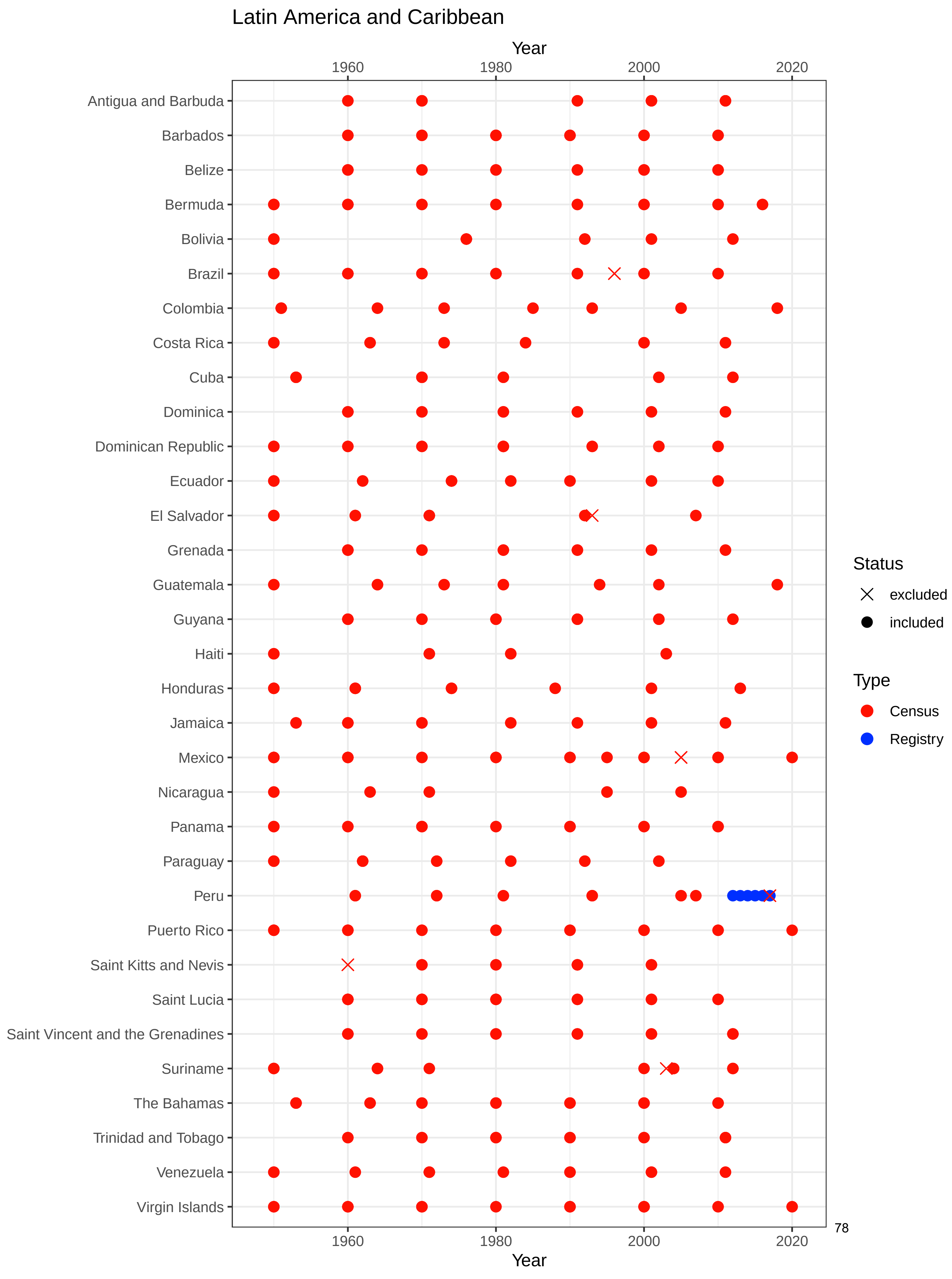

Latin America and Caribbean
Year
1960
1980
2000
2020
Antigua and Barbuda
Barbados
Belize
Bermuda
Bolivia
Brazil
Colombia
Costa Rica
Cuba
Dominica
Dominican Republic
Ecuador
El Salvador
Grenada
Guatemala
Guyana
Haiti
Honduras
Jamaica
Mexico
Nicaragua
Panama
Paraguay
Peru
Puerto Rico
Saint Kitts and Nevis
Saint Lucia
Saint Vincent and the Grenadines
Suriname
The Bahamas
Trinidad and Tobago
Venezuela
Virgin Islands
Status
excluded
included
Type
Census
Registry

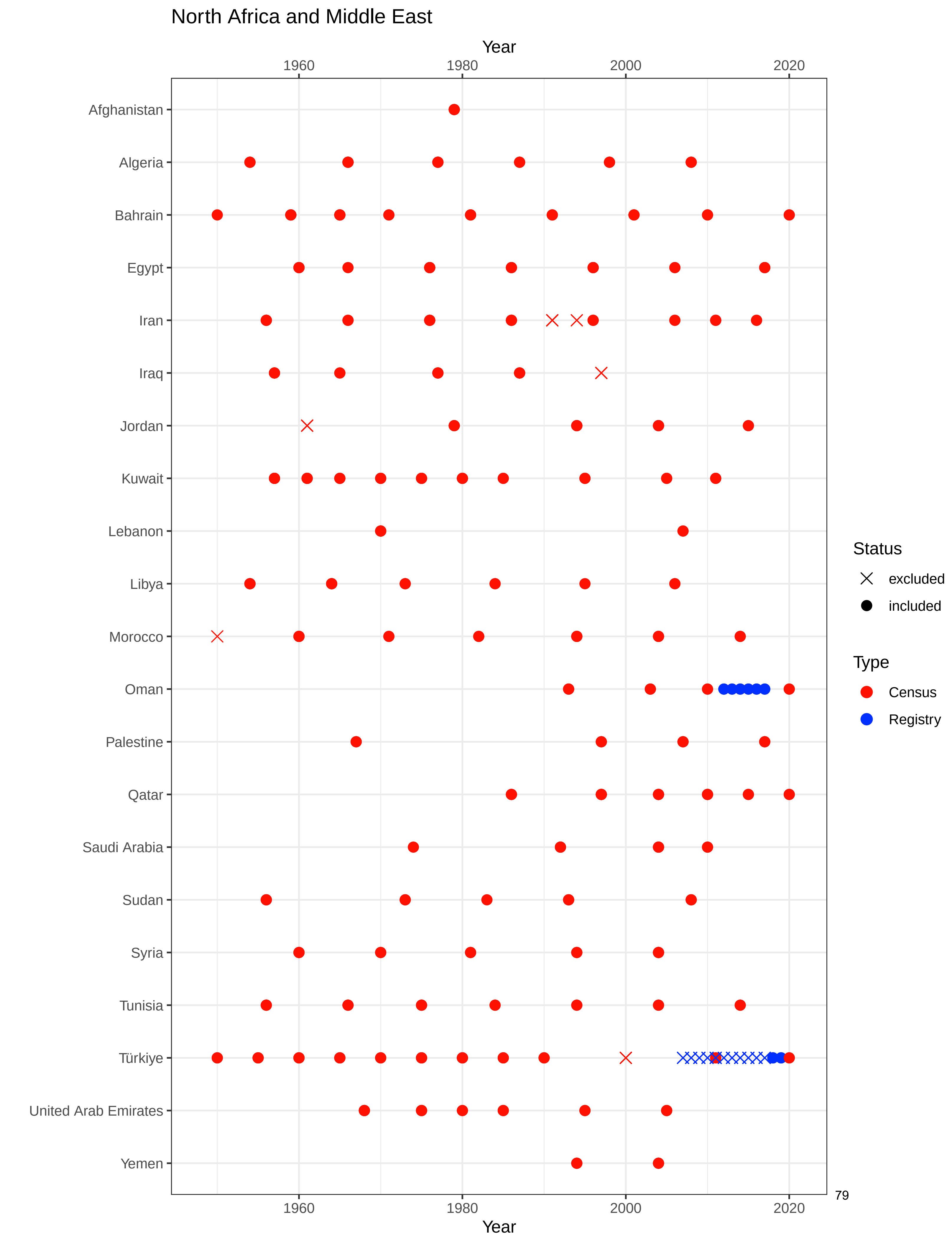

North Africa and Middle East
Year
1960
1980
2000
2020
Afghanistan
Algeria
Bahrain
Egypt
Iran
Iraq
Jordan
Kuwait
Lebanon
Libya
Morocco
Oman
Palestine
Qatar
Saudi Arabia
Sudan
Syria
Tunisia
Türkiye
United Arab Emirates
Yemen
Status
excluded
included
Type
Census
Registry

South Asia

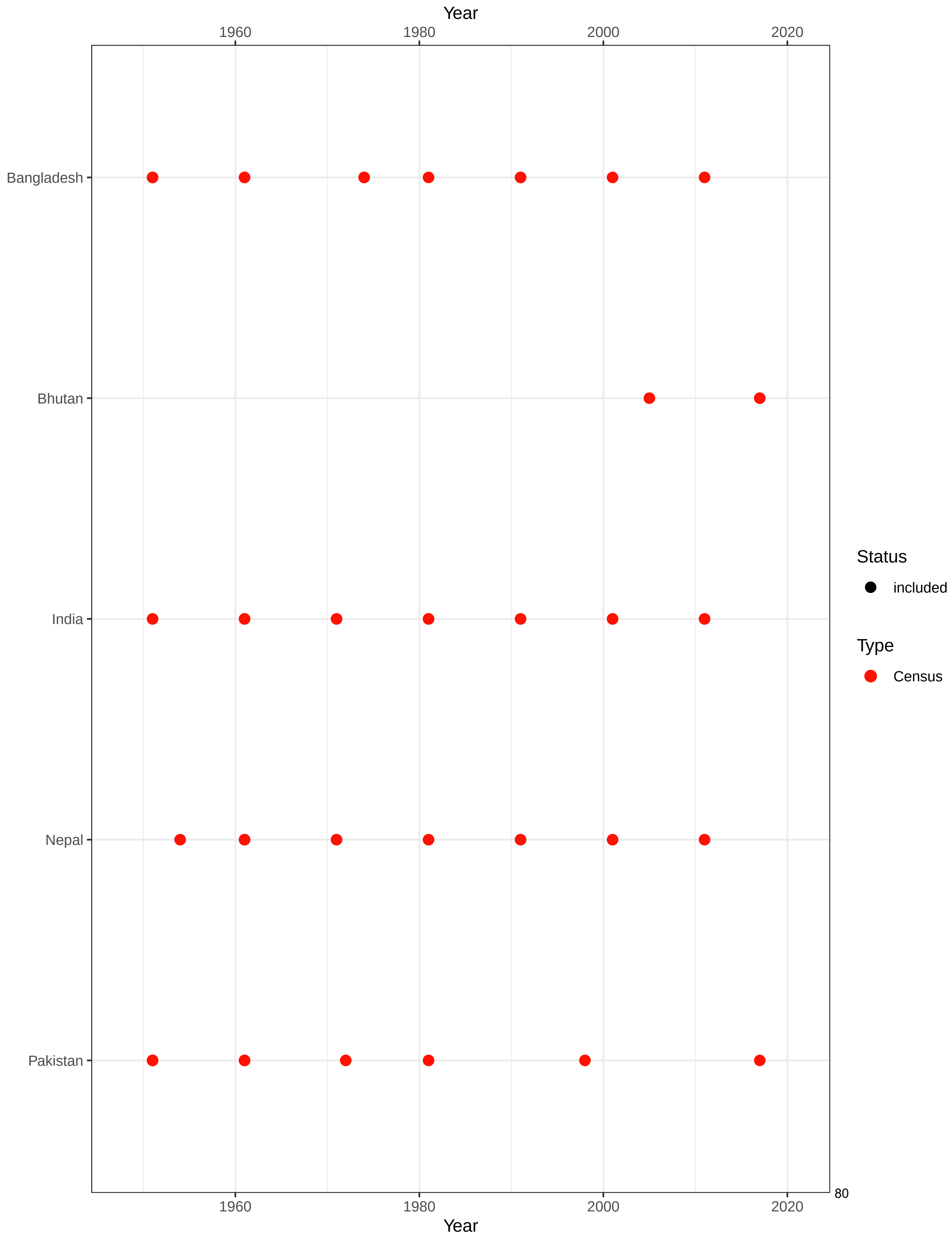

Year
1960
1980
2000
2020
Bangladesh
Bhutan
India
Nepal
Pakistan
Status
included
Type
Census
1960
1980
2000
2020
Year

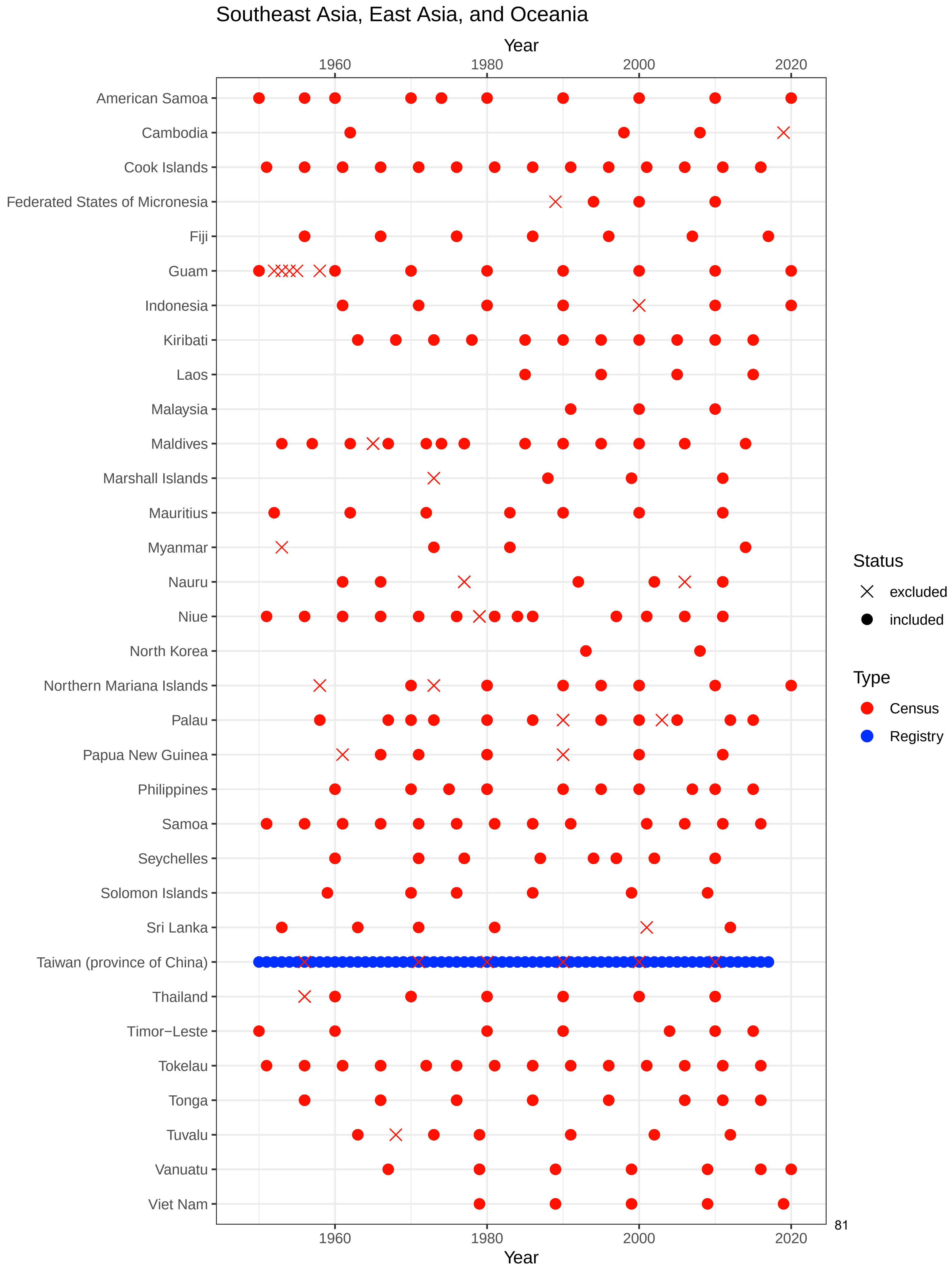
Southeast Asia, East Asia, and Oceania
Year
1960
1980
2000
2020
American Samoa
Cambodia
Cook Islands
Federated States of Micronesia
Fiji
Guam
Indonesia
Kiribati
Laos
Malaysia
Maldives
Marshall Islands
Mauritius
Myanmar
Nauru
Niue
North Korea
Northern Mariana Islands
Palau
Papua New Guinea
Philippines
Samoa
Seychelles
Solomon Islands
Sri Lanka
Taiwan (province of China)
Thailand
Timor−Leste
Tokelau
Tonga
Tuvalu
Vanuatu
Viet Nam
Status
excluded
included
Type
Census
Registry

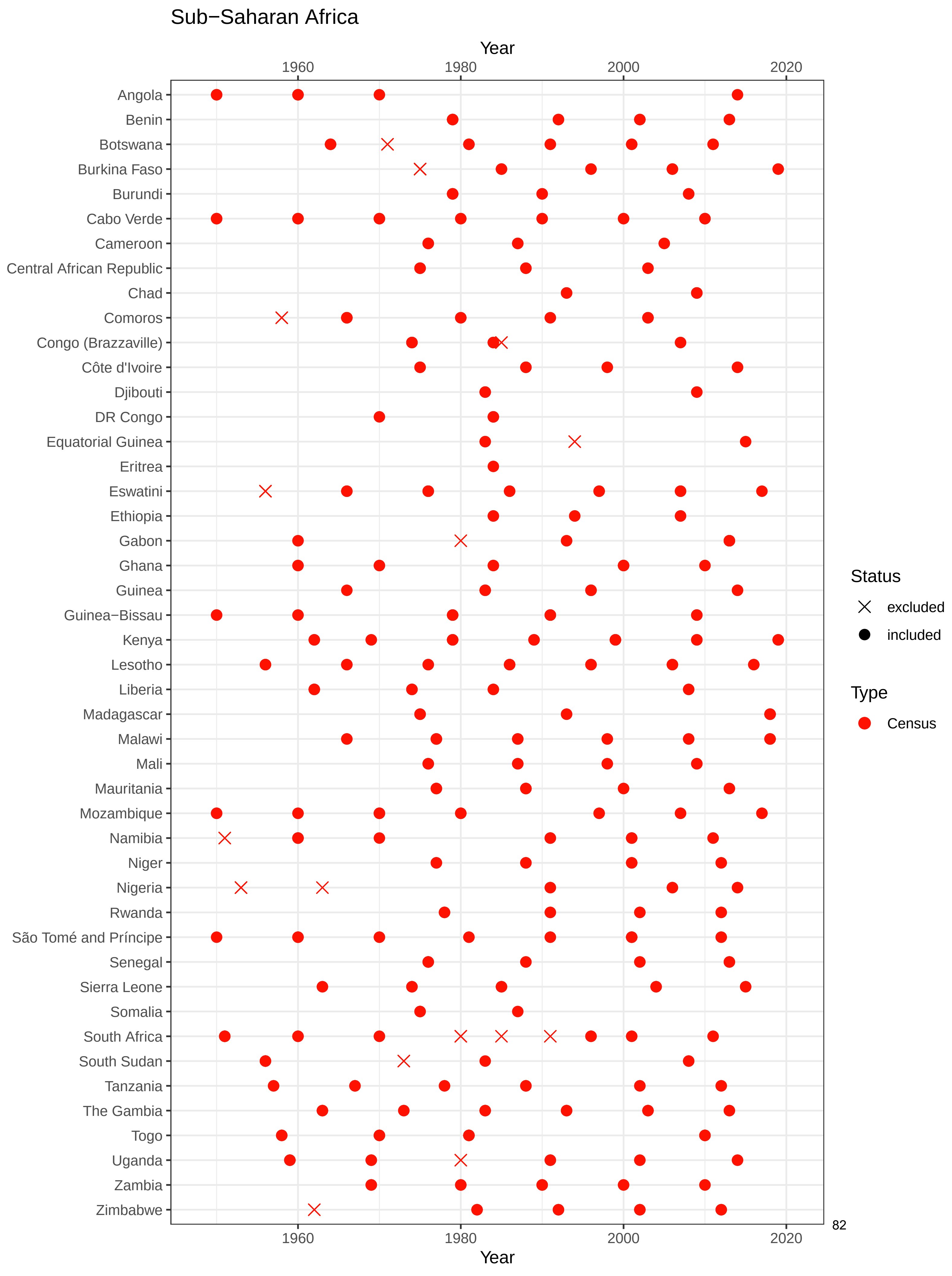
Sub−Saharan Africa
Year
1960
1980
2000
2020
Angola
Benin
Botswana
Burkina Faso
Burundi
Cabo Verde
Cameroon
Central African Republic
Chad
Comoros
Congo (Brazzaville)
Côte d'Ivoire
Djibouti
DR Congo
Equatorial Guinea
Eritrea
Eswatini
Ethiopia
Gabon
Ghana
Guinea
Guinea−Bissau
Kenya
Lesotho
Liberia
Madagascar
Malawi
Mali
Mauritania
Mozambique
Namibia
Niger
Nigeria
Rwanda
São Tomé and Príncipe
Senegal
Sierra Leone
Somalia
South Africa
South Sudan
Tanzania
The Gambia
Togo
Uganda
Zambia
Zimbabwe
Status
excluded
included
Type
Census

Appendix Table S1: GBD location hierarchy with levels	
Location	Level
Global	0
Central Europe, eastern Europe, and central Asia	1
Central Asia	2
Armenia	3
Azerbaijan	3
Georgia	3
Kazakhstan	3
Kyrgyzstan	3
Mongolia	3
Tajikistan	3
Turkmenistan	3
Uzbekistan	3
Central Europe	2
Albania	3
Bosnia and Herzegovina	3
Bulgaria	3
Croatia	3
Czechia	3
Hungary	3
Montenegro	3
North Macedonia	3
Poland	3
Romania	3
Serbia	3
Slovakia	3
Slovenia	3
Eastern Europe	2
Belarus	3
Estonia	3
Latvia	3
Lithuania	3
Moldova	3
Russia	3
Ukraine	3
High income	1
Australasia	2
Australia	3
New Zealand	3
High-income Asia Pacific	2
Brunei	3
Japan	3
Aichi	4
Akita	4
Aomori	4
Chiba	4

Ehime	4
Fukui	4
Fukuoka	4
Fukushima	4
Gifu	4
Gunma	4
Hiroshima	4
Hokkaidō	4
Hyōgo	4
Ibaraki	4
Ishikawa	4
Iwate	4
Kagawa	4
Kagoshima	4
Kanagawa	4
Kōchi	4
Kumamoto	4
Kyōto	4
Mie	4
Miyagi	4
Miyazaki	4
Nagano	4
Nagasaki	4
Nara	4
Niigata	4
Ōita	4
Okayama	4
Okinawa	4
Ōsaka	4
Saga	4
Saitama	4
Shiga	4
Shimane	4
Shizuoka	4
Tochigi	4
Tokushima	4
Tōkyō	4
Tottori	4
Toyama	4
Wakayama	4
Yamagata	4
Yamaguchi	4
Yamanashi	4
South Korea	3
Singapore	3
High-income North America	2
Canada	3

Greenland	3
USA	3
Alabama	4
Alaska	4
Arizona	4
Arkansas	4
California	4
Colorado	4
Connecticut	4
Delaware	4
Washington, DC	4
Florida	4
Georgia	4
Hawaii	4
Idaho	4
Illinois	4
Indiana	4
Iowa	4
Kansas	4
Kentucky	4
Louisiana	4
Maine	4
Maryland	4
Massachusetts	4
Michigan	4
Minnesota	4
Mississippi	4
Missouri	4
Montana	4
Nebraska	4
Nevada	4
New Hampshire	4
New Jersey	4
New Mexico	4
New York	4
North Carolina	4
North Dakota	4
Ohio	4
Oklahoma	4
Oregon	4
Pennsylvania	4
Rhode Island	4
South Carolina	4
South Dakota	4
Tennessee	4
Texas	4
Utah	4

Vermont	4
Virginia	4
Washington	4
West Virginia	4
Wisconsin	4
Wyoming	4
Southern Latin America	2
Argentina	3
Chile	3
Uruguay	3
Western Europe	2
Andorra	3
Austria	3
Belgium	3
Cyprus	3
Denmark	3
Finland	3
France	3
Germany	3
Greece	3
Iceland	3
Ireland	3
Israel	3
Italy	3
Abruzzo	4
Basilicata	4
Calabria	4
Campania	4
Emilia-Romagna	4
Friuli-Venezia Giulia	4
Lazio	4
Liguria	4
Lombardia	4
Marche	4
Molise	4
Piemonte	4
Provincia autonoma di Bolzano	4
Provincia autonoma di Trento	4
Puglia	4
Sardegna	4
Sicilia	4
Toscana	4
Umbria	4
Valle d'Aosta	4
Veneto	4
Luxembourg	3
Malta	3

Monaco	3
Netherlands	3
Norway	3
Agder	4
Innlandet	4
Møre og Romsdal	4
Nordland	4
Oslo	4
Rogaland	4
Troms og Finnmark	4
Trøndelag	4
Vestfold og Telemark	4
Vestland	4
Viken	4
Portugal	3
San Marino	3
Spain	3
Sweden	3
Stockholm	4
Sweden except Stockholm	4
Switzerland	3
UK	3
England	4
East Midlands	5
Derby	6
Derbyshire	6
Leicester	6
Leicestershire	6
Lincolnshire	6
Northamptonshire	6
Nottingham	6
Nottinghamshire	6
Rutland	6
East of England	5
Bedford	6
Cambridgeshire	6
Central Bedfordshire	6
Essex	6
Hertfordshire	6
Luton	6
Norfolk	6
Peterborough	6
Southend-on-Sea	6
Suffolk	6
Thurrock	6
Greater London	5
Barking and Dagenham	6

Barnet	6
Bexley	6
Brent	6
Bromley	6
Camden	6
Croydon	6
Ealing	6
Enfield	6
Greenwich	6
Hackney	6
Hammersmith and Fulham	6
Haringey	6
Harrow	6
Havering	6
Hillingdon	6
Hounslow	6
Islington	6
Kensington and Chelsea	6
Kingston upon Thames	6
Lambeth	6
Lewisham	6
Merton	6
Newham	6
Redbridge	6
Richmond upon Thames	6
Southwark	6
Sutton	6
Tower Hamlets	6
Waltham Forest	6
Wandsworth	6
Westminster	6
North East England	5
County Durham	6
Darlington	6
Gateshead	6
Hartlepool	6
Middlesbrough	6
Newcastle upon Tyne	6
North Tyneside	6
Northumberland	6
Redcar and Cleveland	6
South Tyneside	6
Stockton-on-Tees	6
Sunderland	6
North West England	5
Blackburn with Darwen	6
Blackpool	6

Bolton	6
Bury	6
Cheshire East	6
Cheshire West and Chester	6
Cumbria	6
Halton	6
Knowsley	6
Lancashire	6
Liverpool	6
Manchester	6
Oldham	6
Rochdale	6
Salford	6
Sefton	6
St Helens	6
Stockport	6
Tameside	6
Trafford	6
Warrington	6
Wigan	6
Wirral	6
South East England	5
Bracknell Forest	6
Brighton and Hove	6
Buckinghamshire	6
East Sussex	6
Hampshire	6
Isle of Wight	6
Kent	6
Medway	6
Milton Keynes	6
Oxfordshire	6
Portsmouth	6
Reading	6
Slough	6
Southampton	6
Surrey	6
West Berkshire	6
West Sussex	6
Windsor and Maidenhead	6
Wokingham	6
South West England	5
Bath and North East Somerset	6
Bournemouth	6
Bristol, City of	6
Cornwall	6
Devon	6

Dorset	6
Gloucestershire	6
North Somerset	6
Plymouth	6
Poole	6
Somerset	6
South Gloucestershire	6
Swindon	6
Torbay	6
Wiltshire	6
West Midlands	5
Birmingham	6
Coventry	6
Dudley	6
Herefordshire, County of	6
Sandwell	6
Shropshire	6
Solihull	6
Staffordshire	6
Stoke-on-Trent	6
Telford and Wrekin	6
Walsall	6
Warwickshire	6
Wolverhampton	6
Worcestershire	6
Yorkshire and the Humber	5
Barnsley	6
Bradford	6
Calderdale	6
Doncaster	6
East Riding of Yorkshire	6
Kingston upon Hull, City of	6
Kirklees	6
Leeds	6
North East Lincolnshire	6
North Lincolnshire	6
North Yorkshire	6
Rotherham	6
Sheffield	6
Wakefield	6
York	6
Northern Ireland	4
Scotland	4
Wales	4
Latin America and Caribbean	1
Andean Latin America	2
Bolivia	3

Ecuador	3
Peru	3
Caribbean	2
Antigua and Barbuda	3
The Bahamas	3
Barbados	3
Belize	3
Bermuda	3
Cuba	3
Dominica	3
Dominican Republic	3
Grenada	3
Guyana	3
Haiti	3
Jamaica	3
Puerto Rico	3
Saint Kitts and Nevis	3
Saint Lucia	3
Saint Vincent and the Grenadines	3
Suriname	3
Trinidad and Tobago	3
Virgin Islands	3
Central Latin America	2
Colombia	3
Costa Rica	3
El Salvador	3
Guatemala	3
Honduras	3
Mexico	3
Aguascalientes	4
Baja California	4
Baja California Sur	4
Campeche	4
Chiapas	4
Chihuahua	4
Coahuila	4
Colima	4
Durango	4
Guanajuato	4
Guerrero	4
Hidalgo	4
Jalisco	4
México	4
Mexico City	4
Michoacán de Ocampo	4
Morelos	4
Nayarit	4

Nuevo León	4
Oaxaca	4
Puebla	4
Querétaro	4
Quintana Roo	4
San Luis Potosí	4
Sinaloa	4
Sonora	4
Tabasco	4
Tamaulipas	4
Tlaxcala	4
Veracruz de Ignacio de la Llave	4
Yucatán	4
Zacatecas	4
Nicaragua	3
Panama	3
Venezuela	3
Tropical Latin America	2
Brazil	3
Acre	4
Alagoas	4
Amapá	4
Amazonas	4
Bahia	4
Ceará	4
Distrito Federal	4
Espírito Santo	4
Goiás	4
Maranhão	4
Mato Grosso	4
Mato Grosso do Sul	4
Minas Gerais	4
Pará	4
Paraíba	4
Paraná	4
Pernambuco	4
Piauí	4
Rio de Janeiro	4
Rio Grande do Norte	4
Rio Grande do Sul	4
Rondônia	4
Roraima	4
Santa Catarina	4
São Paulo	4
Sergipe	4
Tocantins	4
Paraguay	3

North Africa and Middle East	1
North Africa and Middle East	2
Afghanistan	3
Algeria	3
Bahrain	3
Egypt	3
Iran	3
Alborz	4
Ardebil	4
Bushehr	4
Chahar Mahaal and Bakhtiari	4
East Azarbayejan	4
Fars	4
Gilan	4
Golestan	4
Hamadan	4
Hormozgan	4
Ilam	4
Isfahan	4
Kerman	4
Kermanshah	4
Khorasan-e-Razavi	4
Khuzestan	4
Kohgiluyeh and Boyer-Ahmad	4
Kurdistan	4
Lorestan	4
Markazi	4
Mazandaran	4
North Khorasan	4
Qazvin	4
Qom	4
Semnan	4
Sistan and Baluchistan	4
South Khorasan	4
Tehran	4
West Azarbayejan	4
Yazd	4
Zanjan	4
Iraq	3
Jordan	3
Kuwait	3
Lebanon	3
Libya	3
Morocco	3
Oman	3
Palestine	3
Qatar	3

Saudi Arabia	3
Sudan	3
Syria	3
Tunisia	3
Türkiye	3
United Arab Emirates	3
Yemen	3
South Asia	1
South Asia	2
Bangladesh	3
Bhutan	3
India	3
Nepal	3
Pakistan	3
Azad Jammu & Kashmir	4
Balochistan	4
Gilgit-Baltistan	4
Islamabad Capital Territory	4
Khyber Pakhtunkhwa	4
Punjab	4
Sindh	4
Southeast Asia, east Asia, and Oceania	1
East Asia	2
China	3
North Korea	3
Taiwan (province of China)	3
Oceania	2
American Samoa	3
Cook Islands	3
Fiji	3
Guam	3
Kiribati	3
Marshall Islands	3
Federated States of Micronesia	3
Nauru	3
Niue	3
Northern Mariana Islands	3
Palau	3
Papua New Guinea	3
Samoa	3
Solomon Islands	3
Tokelau	3
Tonga	3
Tuvalu	3
Vanuatu	3
Southeast Asia	2
Cambodia	3

Indonesia	3
Aceh	4
Bali	4
Bangka-Belitung Islands	4
Banten	4
Bengkulu	4
Gorontalo	4
Jakarta	4
Jambi	4
West Java	4
Central Java	4
East Java	4
West Kalimantan	4
South Kalimantan	4
Central Kalimantan	4
East Kalimantan	4
North Kalimantan	4
Riau Islands	4
Lampung	4
Maluku	4
North Maluku	4
West Nusa Tenggara	4
East Nusa Tenggara	4
Papua	4
West Papua	4
Riau	4
West Sulawesi	4
South Sulawesi	4
Central Sulawesi	4
Southeast Sulawesi	4
North Sulawesi	4
West Sumatra	4
South Sumatra	4
North Sumatra	4
Yogyakarta	4
Laos	3
Malaysia	3
Maldives	3
Mauritius	3
Myanmar	3
Philippines	3
Abra	4
Agusan Del Norte	4
Agusan Del Sur	4
Aklan	4
Albay	4
Antique	4

Apayao	4
Aurora	4
Basilan	4
Bataan	4
Batanes	4
Batangas	4
Benguet	4
Biliran	4
Bohol	4
Bukidnon	4
Bulacan	4
Cagayan	4
Camarines Norte	4
Camarines Sur	4
Camiguin	4
Capiz	4
Catanduanes	4
Cavite	4
Cebu	4
Cotabato (North Cotabato)	4
Davao de Oro	4
Davao Del Norte	4
Davao Del Sur	4
Davao Occidental	4
Davao Oriental	4
Dinagat Islands	4
Eastern Samar	4
Guimaras	4
Ifugao	4
Ilocos Norte	4
Ilocos Sur	4
Iloilo	4
Isabela	4
Kalinga	4
La Union	4
Laguna	4
Lanao Del Norte	4
Lanao Del Sur	4
Leyte	4
Maguindanao	4
Marinduque	4
Masbate	4
Misamis Occidental	4
Misamis Oriental	4
Mountain Province	4
National Capital Region	4
Negros Occidental	4

Negros Oriental	4
Northern Samar	4
Nueva Ecija	4
Nueva Vizcaya	4
Occidental Mindoro	4
Oriental Mindoro	4
Palawan	4
Pampanga	4
Pangasinan	4
Quezon	4
Quirino	4
Rizal	4
Romblon	4
Samar (Western Samar)	4
Sarangani	4
Siquijor	4
Sorsogon	4
South Cotabato	4
Southern Leyte	4
Sultan Kudarat	4
Sulu	4
Surigao Del Norte	4
Surigao Del Sur	4
Tarlac	4
Tawi-Tawi	4
Zambales	4
Zamboanga Del Norte	4
Zamboanga Del Sur	4
Zamboanga Sibugay	4
Seychelles	3
Sri Lanka	3
Thailand	3
Timor-Leste	3
Viet Nam	3
Sub-Saharan Africa	1
Central sub-Saharan Africa	2
Angola	3
Central African Republic	3
Congo (Brazzaville)	3
DR Congo	3
Equatorial Guinea	3
Gabon	3
Eastern sub-Saharan Africa	2
Burundi	3
Comoros	3
Djibouti	3
Eritrea	3

Ethiopia	3
Addis Ababa	4
Afar	4
Amhara	4
Benishangul-Gumuz	4
Dire Dawa	4
Gambella	4
Harari	4
Oromia	4
Somali	4
Southern Nations, Nationalities, and Peoples	4
Tigray	4
Kenya	3
Baringo	4
Bomet	4
Bungoma	4
Busia	4
Elgeyo Marakwet	4
Embu	4
Garissa	4
Homa Bay	4
Isiolo	4
Kajiado	4
Kakamega	4
Kericho	4
Kiambu	4
Kilifi	4
Kirinyaga	4
Kisii	4
Kisumu	4
Kitui	4
Kwale	4
Laikipia	4
Lamu	4
Machakos	4
Makueni	4
Mandera	4
Marsabit	4
Meru	4
Migori	4
Mombasa	4
Murang'a	4
Nairobi	4
Nakuru	4
Nandi	4
Narok	4
Nyamira	4

Nyandarua	4
Nyeri	4
Samburu	4
Siaya	4
Taita Taveta	4
Tana River	4
Tharaka Nithi	4
Trans Nzoia	4
Turkana	4
Uasin Gishu	4
Vihiga	4
Wajir	4
West Pokot	4
Madagascar	3
Malawi	3
Mozambique	3
Rwanda	3
Somalia	3
South Sudan	3
Uganda	3
Tanzania	3
Zambia	3
Southern sub-Saharan Africa	2
Botswana	3
Eswatini	3
Lesotho	3
Namibia	3
South Africa	3
Eastern Cape	4
Free State	4
Gauteng	4
KwaZulu-Natal	4
Limpopo	4
Mpumalanga	4
North West	4
Northern Cape	4
Western Cape	4
Zimbabwe	3
Western sub-Saharan Africa	2
Benin	3
Burkina Faso	3
Cabo Verde	3
Cameroon	3
Chad	3
Côte d'Ivoire	3
The Gambia	3
Ghana	3

Guinea	3
Guinea-Bissau	3
Liberia	3
Mali	3
Mauritania	3
Niger	3
Nigeria	3
São Tomé and Príncipe	3
Senegal	3
Sierra Leone	3
Togo	3

Appendix table S2. GATHER checklist

Item #	Checklist item	Reporting location
**Objectives and funding**		
**1**	Define the indicator(s), populations (including age, sex, and geographic entities), and time period(s) for which estimates were made.	Main text (Summary—methods section); Main text (Methods section); appendix 1 (section 1.1–1.4)
**2**	List the funding sources for the work.	Main text (acknowledgement section); Primary funder given in Summary—funding; appendix 1 (section 1.9)
**Data Inputs**		
For all data inputs from multiple sources that are synthesized as part of the study:		
**3**	Describe how the data were identified and how the data were accessed.	Main text (Methods); appendix 1 (section 1.8, section 2.2.1, Section 2.3.1, section 2.7.1, section 3.2, section 4.2–4.3)
**4**	Specify the inclusion and exclusion criteria. Identify all ad-hoc exclusions.	Main text (Methods); appendix 1 (section 1.8, section 2.2.1–2.2.3, section 2.3.1–2.32, section 2.7.1–2.7.4, section 3.2, section 4.2–4.3)
**5**	Provide information on all included data sources and their main characteristics. For each data source used, report reference information or contact name/institution, population represented, data collection method, year(s) of data collection, sex and age range, diagnostic criteria or measurement method, and sample size, as relevant.	Online data citation tool: https://ghdx.healthdata.org/gbd-2021/sources
**6**	Identify and describe any categories of input data that have potentially important biases (e.g., based on characteristics listed in item 5).	Appendix 1 (section 1.8, section 2.2.1–2.2.6, section 2.3.1-2.3.4, section 2.5.4, section 2.7.1-2.7.4, section 3.2, section 4.2-4.4, section 6.4)
For data inputs that contribute to the analysis but were not synthesized as part of the study:		
**7**	Describe and give sources for any other data inputs.	Online data citation tool: https://ghdx.healthdata.org/gbd-2021/sources
For all data inputs:		
**8**	Provide all data inputs in a file format from which data can be efficiently extracted (e.g., a spreadsheet rather than a PDF), including all relevant meta-data listed in item 5. For any data inputs that cannot be shared because of ethical or legal reasons, such as third-party ownership, provide a contact name or the name of the institution that retains the right to the data.	Online data citation tool: https://ghdx.healthdata.org/gbd-2021/sources. Availability of data is dependent on data use agreements; contact information provided when not directly available.
**Data analysis**		
**9**	Provide a conceptual overview of the data analysis method. A diagram may be helpful.	Main text (Methods); appendix 1 (sections 2–5; figures S1, S3, and S4)
**10**	Provide a detailed description of all steps of the analysis, including mathematical formulae. This description should cover, as relevant, data cleaning, data pre-processing, data	Main text (Methods); appendix 1 (sections 2–5)

	adjustments and weighting of data sources, and mathematical or statistical model(s).	
**11**	Describe how candidate models were evaluated and how the final model(s) were selected.	Appendix 1 (section 2.2-2.8, section 3.3-3.4, section 4.4, section 6)
**12**	Provide the results of an evaluation of model performance, if done, as well as the results of any relevant sensitivity analysis.	Appendix 1 (section 2.2-2.8, section 3.3-3.4, section 4.4, section 6)
**13**	Describe methods for calculating uncertainty of the estimates. State which sources of uncertainty were, and were not, accounted for in the uncertainty analysis.	Appendix 1 (section 2.2.6, section 2.3.2, section 2.4.4, section 2.5.3-2.5.4, section 2.7.6, section 3.3.6, section 4.2.3, section 4.4.4, section 6.4)
**14**	State how analytic or statistical source code used to generate estimates can be accessed.	Code provided in online repository: http://ghdx.healthdata.org/gbd-2021/code
**Results and Discussion**		
**15**	Provide published estimates in a file format from which data can be efficiently extracted.	Online data/results visualisation tool: https://vizhub.healthdata.org/gbd-results/. Results can be selected from drop-down menus and downloaded as CSVs; select datasets of estimates available at http://ghdx.healthdata.org/gbd-2021
**16**	Report a quantitative measure of the uncertainty of the estimates (e.g. uncertainty intervals).	UIs given in main text and methods appendix, including in figures (as appropriate) and tables; UIs also provided in appendix 2; UIs also given for all results in our online results tool: https://vizhub.healthdata.org/gbd-results/
**17**	Interpret results in light of existing evidence. If updating a previous set of estimates, describe the reasons for changes in estimates.	Main text (Discussion)
**18**	Discuss limitations of the estimates. Include a discussion of any modelling assumptions or data limitations that affect interpretation of the estimates.	Main text (Discussion—limitations)

Appendix Table 3. Number of all-cause mortality data sources by type and location, 1950-2021						
**Location**	**SBH**	**CBH**	**HH**	**SIBS**	**VR/SRS/DSP**	**DSS**
Armenia	6	4	0	0	42	0
Azerbaijan	4	1	2	0	43	0
Georgia	3	3	1	0	43	0
Kazakhstan	8	2	0	0	43	0
Kyrgyzstan	7	4	1	0	42	0
Mongolia	9	3	2	0	40	0
Tajikistan	8	2	1	0	39	0
Turkmenistan	3	1	2	0	38	0
Uzbekistan	4	2	0	0	42	0
Albania	6	2	0	0	42	0
Bosnia and Herzegovina	0	0	0	0	65	0
Bulgaria	4	0	0	0	72	0
Croatia	0	0	0	0	72	0
Czechia	0	0	0	0	72	0
Hungary	1	0	0	0	72	0
Montenegro	1	0	0	0	63	0
North Macedonia	2	0	0	0	71	0
Poland	1	0	0	0	72	0
Romania	4	1	1	0	66	0
Serbia	4	0	1	0	65	0
Slovakia	0	0	0	0	72	0
Slovenia	0	0	0	0	72	0
Belarus	3	0	0	0	62	0
Estonia	1	0	0	0	63	0
Latvia	1	0	0	0	62	0
Lithuania	0	0	0	0	63	0
Moldova	4	2	0	0	43	0
Russia	1	0	0	0	63	0
Ukraine	6	2	0	0	58	0
Australia	0	0	0	0	71	0
New Zealand	0	0	0	0	72	0
Brunei	1	0	0	0	62	0
Japan	0	0	0	0	72	0
Aichi	0	0	0	0	43	0
Akita	0	0	0	0	43	0
Aomori	0	0	0	0	43	0
Chiba	0	0	0	0	43	0
Ehime	0	0	0	0	43	0
Fukui	0	0	0	0	43	0
Fukuoka	0	0	0	0	43	0
Fukushima	0	0	0	0	43	0
Gifu	0	0	0	0	43	0
Gunma	0	0	0	0	43	0
Hiroshima	0	0	0	0	43	0
Hokkaidō	0	0	0	0	43	0
Hyōgo	0	0	0	0	42	0
Ibaraki	0	0	0	0	43	0
Ishikawa	0	0	0	0	43	0
Iwate	0	0	0	0	42	0
Kagawa	0	0	0	0	43	0
Kagoshima	0	0	0	0	43	0
Kanagawa	0	0	0	0	43	0
Kōchi	0	0	0	0	43	0
Kumamoto	0	0	0	0	43	0
Kyōto	0	0	0	0	43	0
Mie	0	0	0	0	43	0
Miyagi	0	0	0	0	43	0
Miyazaki	0	0	0	0	43	0
Nagano	0	0	0	0	43	0
Nagasaki	0	0	0	0	43	0
Nara	0	0	0	0	43	0
Niigata	0	0	0	0	43	0
Ōita	0	0	0	0	43	0
Okayama	0	0	0	0	43	0

Appendix Table 3. Number of all-cause mortality data sources by type and location, 1950-2021						
Location	SBH	CBH	HH	SIBS	VR/SRS/DSP	DSS
Okinawa	0	0	0	0	43	0
Ōsaka	0	0	0	0	43	0
Saga	0	0	0	0	43	0
Saitama	0	0	0	0	43	0
Shiga	0	0	0	0	43	0
Shimane	0	0	0	0	43	0
Shizuoka	0	0	0	0	43	0
Tochigi	0	0	0	0	43	0
Tokushima	0	0	0	0	43	0
Tōkyō	0	0	0	0	43	0
Tottori	0	0	0	0	43	0
Toyama	0	0	0	0	43	0
Wakayama	0	0	0	0	43	0
Yamagata	0	0	0	0	43	0
Yamaguchi	0	0	0	0	43	0
Yamanashi	0	0	0	0	43	0
South Korea	6	1	0	0	45	0
Singapore	0	0	0	0	103	0
Canada	0	0	0	0	71	0
Greenland	0	0	0	0	60	0
USA	0	0	0	0	71	0
Alabama	0	0	0	0	62	0
Alaska	0	0	0	0	62	0
Arizona	0	0	0	0	62	0
Arkansas	0	0	0	0	62	0
California	0	0	0	0	62	0
Colorado	0	0	0	0	62	0
Connecticut	0	0	0	0	62	0
Delaware	0	0	0	0	62	0
Washington, DC	0	0	0	0	62	0
Florida	0	0	0	0	62	0
Georgia	0	0	0	0	62	0
Hawaii	0	0	0	0	62	0
Idaho	0	0	0	0	62	0
Illinois	0	0	0	0	62	0
Indiana	0	0	0	0	62	0
Iowa	0	0	0	0	62	0
Kansas	0	0	0	0	62	0
Kentucky	0	0	0	0	62	0
Louisiana	0	0	0	0	62	0
Maine	0	0	0	0	62	0
Maryland	0	0	0	0	62	0
Massachusetts	0	0	0	0	62	0
Michigan	0	0	0	0	62	0
Minnesota	0	0	0	0	62	0
Mississippi	0	0	0	0	62	0
Missouri	0	0	0	0	62	0
Montana	0	0	0	0	62	0
Nebraska	0	0	0	0	62	0
Nevada	0	0	0	0	62	0
New Hampshire	0	0	0	0	62	0
New Jersey	0	0	0	0	62	0
New Mexico	0	0	0	0	62	0
New York	0	0	0	0	62	0
North Carolina	0	0	0	0	62	0
North Dakota	0	0	0	0	62	0
Ohio	0	0	0	0	62	0
Oklahoma	0	0	0	0	62	0
Oregon	0	0	0	0	62	0
Pennsylvania	0	0	0	0	62	0
Rhode Island	0	0	0	0	62	0
South Carolina	0	0	0	0	62	0
South Dakota	0	0	0	0	62	0
Tennessee	0	0	0	0	62	0

Appendix Table 3. Number of all-cause mortality data sources by type and location, 1950-2021						
**Location**	**SBH**	**CBH**	**HH**	**SIBS**	**VR/SRS/DSP**	**DSS**
Texas	0	0	0	0	62	0
Utah	0	0	0	0	62	0
Vermont	0	0	0	0	62	0
Virginia	0	0	0	0	62	0
Washington	0	0	0	0	62	0
West Virginia	0	0	0	0	62	0
Wisconsin	0	0	0	0	62	0
Wyoming	0	0	0	0	62	0
Argentina	3	0	0	0	66	0
Chile	4	0	0	0	72	0
Uruguay	6	0	0	0	69	0
Andorra	0	0	0	0	21	0
Austria	0	0	0	0	72	0
Belgium	2	0	0	0	72	0
Cyprus	3	0	1	0	48	0
Denmark	0	0	0	0	72	0
Finland	0	0	0	0	72	0
France	0	0	0	0	71	0
Germany	0	0	0	0	66	0
Greece	0	0	0	0	71	0
Iceland	0	0	0	0	72	0
Ireland	0	0	1	0	70	0
Israel	0	0	0	0	71	0
Italy	0	0	0	0	72	0
Abruzzo	0	0	0	0	53	0
Basilicata	0	0	0	0	53	0
Calabria	0	0	0	0	53	0
Campania	0	0	0	0	53	0
Emilia-Romagna	0	0	0	0	53	0
Friuli-Venezia Giulia	0	0	0	0	53	0
Lazio	0	0	0	0	53	0
Liguria	0	0	0	0	53	0
Lombardia	0	0	0	0	53	0
Marche	0	0	0	0	53	0
Molise	0	0	0	0	53	0
Piemonte	0	0	0	0	53	0
Provincia autonoma di Bolzano	0	0	0	0	52	0
Provincia autonoma di Trento	0	0	0	0	52	0
Puglia	0	0	0	0	53	0
Sardegna	0	0	0	0	53	0
Sicilia	0	0	0	0	53	0
Toscana	0	0	0	0	53	0
Umbria	0	0	0	0	53	0
Valle d'Aosta	0	0	0	0	53	0
Veneto	0	0	0	0	53	0
Luxembourg	0	0	0	0	72	0
Malta	0	0	0	0	72	0
Monaco	0	0	0	0	16	0
Netherlands	0	0	0	0	72	0
Norway	0	0	0	0	72	0
Agder	0	0	0	0	70	0
Innlandet	0	0	0	0	70	0
Møre og Romsdal	0	0	0	0	70	0
Nordland	0	0	0	0	70	0
Oslo	0	0	0	0	70	0
Rogaland	0	0	0	0	70	0
Troms og Finnmark	0	0	0	0	70	0
Trøndelag	0	0	0	0	70	0
Vestfold og Telemark	0	0	0	0	70	0
Vestland	0	0	0	0	70	0
Viken	0	0	0	0	70	0
Portugal	1	1	0	0	71	0
San Marino	0	0	0	0	53	0
Spain	0	0	0	0	72	0

Appendix Table 3. Number of all-cause mortality data sources by type and location, 1950-2021						
**Location**	**SBH**	**CBH**	**HH**	**SIBS**	**VR/SRS/DSP**	**DSS**
Sweden	0	0	0	0	72	0
Stockholm	0	0	0	0	38	0
Sweden except Stockholm	0	0	0	0	39	0
Switzerland	1	0	0	0	72	0
UK	0	0	0	0	72	0
England	0	0	0	0	41	0
East Midlands	0	0	0	0	40	0
Derby	0	0	0	0	41	0
Derbyshire	0	0	0	0	41	0
Leicester	0	0	0	0	41	0
Leicestershire	0	0	0	0	41	0
Lincolnshire	0	0	0	0	41	0
Northamptonshire	0	0	0	0	40	0
Nottingham	0	0	0	0	41	0
Nottinghamshire	0	0	0	0	41	0
Rutland	0	0	0	0	41	0
East of England	0	0	0	0	40	0
Bedford	0	0	0	0	41	0
Cambridgeshire	0	0	0	0	41	0
Central Bedfordshire	0	0	0	0	41	0
Essex	0	0	0	0	41	0
Hertfordshire	0	0	0	0	41	0
Luton	0	0	0	0	41	0
Norfolk	0	0	0	0	41	0
Peterborough	0	0	0	0	41	0
Southend-on-Sea	0	0	0	0	41	0
Suffolk	0	0	0	0	41	0
Thurrock	0	0	0	0	41	0
Greater London	0	0	0	0	40	0
Barking and Dagenham	0	0	0	0	41	0
Barnet	0	0	0	0	41	0
Bexley	0	0	0	0	41	0
Brent	0	0	0	0	41	0
Bromley	0	0	0	0	41	0
Camden	0	0	0	0	41	0
Croydon	0	0	0	0	41	0
Ealing	0	0	0	0	41	0
Enfield	0	0	0	0	41	0
Greenwich	0	0	0	0	41	0
Hackney	0	0	0	0	40	0
Hammersmith and Fulham	0	0	0	0	41	0
Haringey	0	0	0	0	41	0
Harrow	0	0	0	0	41	0
Havering	0	0	0	0	41	0
Hillingdon	0	0	0	0	41	0
Hounslow	0	0	0	0	41	0
Islington	0	0	0	0	41	0
Kensington and Chelsea	0	0	0	0	41	0
Kingston upon Thames	0	0	0	0	41	0
Lambeth	0	0	0	0	41	0
Lewisham	0	0	0	0	41	0
Merton	0	0	0	0	41	0
Newham	0	0	0	0	41	0
Redbridge	0	0	0	0	41	0
Richmond upon Thames	0	0	0	0	41	0
Southwark	0	0	0	0	41	0
Sutton	0	0	0	0	41	0
Tower Hamlets	0	0	0	0	41	0
Waltham Forest	0	0	0	0	41	0
Wandsworth	0	0	0	0	41	0
Westminster	0	0	0	0	41	0
North East England	0	0	0	0	40	0
County Durham	0	0	0	0	41	0
Darlington	0	0	0	0	41	0

Appendix Table 3. Number of all-cause mortality data sources by type and location, 1950-2021						
Location	SBH	CBH	HH	SIBS	VR/SRS/DSP	DSS
Gateshead	0	0	0	0	41	0
Hartlepool	0	0	0	0	41	0
Middlesbrough	0	0	0	0	41	0
Newcastle upon Tyne	0	0	0	0	41	0
North Tyneside	0	0	0	0	41	0
Northumberland	0	0	0	0	41	0
Redcar and Cleveland	0	0	0	0	41	0
South Tyneside	0	0	0	0	41	0
Stockton-on-Tees	0	0	0	0	41	0
Sunderland	0	0	0	0	41	0
North West England	0	0	0	0	40	0
Blackburn with Darwen	0	0	0	0	41	0
Blackpool	0	0	0	0	41	0
Bolton	0	0	0	0	41	0
Bury	0	0	0	0	41	0
Cheshire East	0	0	0	0	41	0
Cheshire West and Chester	0	0	0	0	41	0
Cumbria	0	0	0	0	41	0
Halton	0	0	0	0	41	0
Knowsley	0	0	0	0	41	0
Lancashire	0	0	0	0	41	0
Liverpool	0	0	0	0	41	0
Manchester	0	0	0	0	41	0
Oldham	0	0	0	0	41	0
Rochdale	0	0	0	0	41	0
Salford	0	0	0	0	41	0
Sefton	0	0	0	0	41	0
St Helens	0	0	0	0	41	0
Stockport	0	0	0	0	41	0
Tameside	0	0	0	0	41	0
Trafford	0	0	0	0	41	0
Warrington	0	0	0	0	41	0
Wigan	0	0	0	0	41	0
Wirral	0	0	0	0	41	0
South East England	0	0	0	0	40	0
Bracknell Forest	0	0	0	0	41	0
Brighton and Hove	0	0	0	0	41	0
Buckinghamshire	0	0	0	0	41	0
East Sussex	0	0	0	0	41	0
Hampshire	0	0	0	0	41	0
Isle of Wight	0	0	0	0	41	0
Kent	0	0	0	0	41	0
Medway	0	0	0	0	41	0
Milton Keynes	0	0	0	0	41	0
Oxfordshire	0	0	0	0	41	0
Portsmouth	0	0	0	0	41	0
Reading	0	0	0	0	41	0
Slough	0	0	0	0	41	0
Southampton	0	0	0	0	41	0
Surrey	0	0	0	0	41	0
West Berkshire	0	0	0	0	41	0
West Sussex	0	0	0	0	41	0
Windsor and Maidenhead	0	0	0	0	41	0
Wokingham	0	0	0	0	41	0
South West England	0	0	0	0	40	0
Bath and North East Somerset	0	0	0	0	41	0
Bournemouth	0	0	0	0	40	0
Bristol, City of	0	0	0	0	41	0
Cornwall	0	0	0	0	41	0
Devon	0	0	0	0	41	0
Dorset	0	0	0	0	41	0
Gloucestershire	0	0	0	0	41	0
North Somerset	0	0	0	0	41	0
Plymouth	0	0	0	0	41	0

Appendix Table 3. Number of all-cause mortality data sources by type and location, 1950-2021						
**Location**	**SBH**	**CBH**	**HH**	**SIBS**	**VR/SRS/DSP**	**DSS**
Poole	0	0	0	0	40	0
Somerset	0	0	0	0	41	0
South Gloucestershire	0	0	0	0	41	0
Swindon	0	0	0	0	41	0
Torbay	0	0	0	0	41	0
Wiltshire	0	0	0	0	41	0
West Midlands	0	0	0	0	40	0
Birmingham	0	0	0	0	41	0
Coventry	0	0	0	0	41	0
Dudley	0	0	0	0	41	0
Herefordshire, County of	0	0	0	0	41	0
Sandwell	0	0	0	0	41	0
Shropshire	0	0	0	0	41	0
Solihull	0	0	0	0	41	0
Staffordshire	0	0	0	0	41	0
Stoke-on-Trent	0	0	0	0	41	0
Telford and Wrekin	0	0	0	0	41	0
Walsall	0	0	0	0	41	0
Warwickshire	0	0	0	0	41	0
Wolverhampton	0	0	0	0	41	0
Worcestershire	0	0	0	0	41	0
Yorkshire and the Humber	0	0	0	0	40	0
Barnsley	0	0	0	0	41	0
Bradford	0	0	0	0	41	0
Calderdale	0	0	0	0	41	0
Doncaster	0	0	0	0	41	0
East Riding of Yorkshire	0	0	0	0	41	0
Kingston upon Hull, City of	0	0	0	0	41	0
Kirklees	0	0	0	0	41	0
Leeds	0	0	0	0	41	0
North East Lincolnshire	0	0	0	0	41	0
North Lincolnshire	0	0	0	0	41	0
North Yorkshire	0	0	0	0	41	0
Rotherham	0	0	0	0	41	0
Sheffield	0	0	0	0	41	0
Wakefield	0	0	0	0	41	0
York	0	0	0	0	41	0
Northern Ireland	0	0	0	0	71	0
Scotland	0	0	0	0	73	0
Wales	0	0	0	0	41	0
Bolivia	26	6	1	3	12	0
Ecuador	17	7	1	0	67	0
Peru	21	14	0	5	68	0
Antigua and Barbuda	0	0	0	0	61	0
The Bahamas	0	0	0	0	50	0
Barbados	0	0	0	0	62	0
Belize	7	3	0	0	69	0
Bermuda	1	0	0	0	70	0
Cuba	1	0	0	0	62	0
Dominica	0	0	0	0	64	0
Dominican Republic	15	13	1	2	67	0
Grenada	0	0	0	0	57	0
Guyana	7	4	1	0	46	0
Haiti	9	7	3	2	1	0
Jamaica	5	1	2	0	51	0
Puerto Rico	1	1	0	0	71	0
Saint Kitts and Nevis	0	0	0	0	66	0
Saint Lucia	0	0	0	0	57	0
Saint Vincent and the Grenadines	0	0	0	0	53	0
Suriname	2	0	1	0	61	0
Trinidad and Tobago	5	2	0	0	67	0
Virgin Islands	0	0	0	0	54	0
Colombia	13	8	0	0	69	0
Costa Rica	7	3	0	0	71	0

Appendix Table 3. Number of all-cause mortality data sources by type and location, 1950-2021						
**Location**	**SBH**	**CBH**	**HH**	**SIBS**	**VR/SRS/DSP**	**DSS**
El Salvador	10	6	2	3	70	0
Guatemala	10	6	0	0	70	0
Honduras	12	5	3	1	44	0
Mexico	25	2	1	0	72	0
Aguascalientes	16	1	0	0	43	0
Baja California	16	1	0	0	43	0
Baja California Sur	16	1	0	0	43	0
Campeche	16	1	0	0	43	0
Chiapas	16	1	0	0	43	0
Chihuahua	16	1	0	0	43	0
Coahuila	16	1	0	0	43	0
Colima	16	1	0	0	43	0
Durango	16	1	0	0	43	0
Guanajuato	16	1	0	0	43	0
Guerrero	16	1	0	0	43	0
Hidalgo	14	1	0	0	43	0
Jalisco	16	1	0	0	43	0
México	16	1	0	0	43	0
Mexico City	16	1	0	0	43	0
Michoacán de Ocampo	16	1	0	0	43	0
Morelos	16	1	0	0	43	0
Nayarit	16	1	0	0	43	0
Nuevo León	16	1	0	0	43	0
Oaxaca	16	1	0	0	43	0
Puebla	16	1	0	0	43	0
Querétaro	16	1	0	0	43	0
Quintana Roo	16	1	0	0	43	0
San Luis Potosí	16	1	0	0	43	0
Sinaloa	16	1	0	0	43	0
Sonora	16	1	0	0	43	0
Tabasco	16	1	0	0	43	0
Tamaulipas	16	1	0	0	43	0
Tlaxcala	16	1	0	0	43	0
Veracruz de Ignacio de la Llave	16	1	0	0	43	0
Yucatán	16	1	0	0	43	0
Zacatecas	16	1	0	0	43	0
Nicaragua	12	5	0	0	57	0
Panama	9	1	0	0	70	0
Venezuela	4	1	1	0	67	0
Brazil	28	2	1	0	48	0
Acre	9	1	1	0	43	0
Alagoas	21	2	1	0	43	0
Amapá	9	1	1	0	43	0
Amazonas	10	1	1	0	43	0
Bahia	21	2	1	0	43	0
Ceará	21	2	1	0	43	0
Distrito Federal	21	1	1	0	43	0
Espírito Santo	21	1	1	0	43	0
Goiás	21	1	1	0	43	0
Maranhão	21	2	1	0	43	0
Mato Grosso	21	1	1	0	43	0
Mato Grosso do Sul	21	1	1	0	43	0
Minas Gerais	21	1	1	0	43	0
Pará	10	1	1	0	43	0
Paraíba	21	2	1	0	42	0
Paraná	21	1	1	0	43	0
Pernambuco	21	2	1	0	43	0
Piauí	21	2	1	0	43	0
Rio de Janeiro	21	1	1	0	43	0
Rio Grande do Norte	21	2	1	0	43	0
Rio Grande do Sul	21	1	1	0	43	0
Rondônia	9	1	1	0	43	0
Roraima	9	1	1	0	43	0
Santa Catarina	21	1	1	0	43	0

Appendix Table 3. Number of all-cause mortality data sources by type and location, 1950-2021						
**Location**	**SBH**	**CBH**	**HH**	**SIBS**	**VR/SRS/DSP**	**DSS**
São Paulo	20	1	1	0	43	0
Sergipe	21	2	1	0	43	0
Tocantins	9	1	1	0	33	0
Paraguay	15	7	0	0	71	0
Afghanistan	6	1	1	0	0	0
Algeria	5	3	1	0	25	0
Bahrain	4	0	2	0	39	0
Egypt	13	11	2	0	68	0
Iran	5	1	9	0	33	0
Alborz	3	1	6	0	16	0
Ardebil	3	1	6	0	16	0
Bushehr	3	1	6	0	16	0
Chahar Mahaal and Bakhtiari	3	1	6	0	16	0
East Azarbayejan	3	1	6	0	16	0
Fars	3	1	6	0	16	0
Gilan	3	1	6	0	16	0
Golestan	3	1	6	0	16	0
Hamadan	3	1	6	0	16	0
Hormozgan	3	1	6	0	16	0
Ilam	3	1	6	0	16	0
Isfahan	3	1	6	0	16	0
Kerman	3	1	6	0	16	0
Kermanshah	3	1	6	0	16	0
Khorasan-e-Razavi	3	1	6	0	16	0
Khuzestan	3	1	6	0	16	0
Kohgiluyeh and Boyer-Ahmad	3	1	6	0	16	0
Kurdistan	3	1	6	0	16	0
Lorestan	3	1	6	0	16	0
Markazi	3	1	6	0	16	0
Mazandaran	3	1	6	0	16	0
North Khorasan	3	1	6	0	16	0
Qazvin	3	1	6	0	16	0
Qom	3	1	6	0	16	0
Semnan	3	1	6	0	16	0
Sistan and Baluchistan	3	1	4	0	16	0
South Khorasan	3	1	6	0	16	0
Tehran	3	1	0	0	16	0
West Azarbayejan	3	1	6	0	16	0
Yazd	3	1	6	0	16	0
Zanjan	3	1	6	0	16	0
Iraq	7	5	4	0	25	1
Jordan	12	7	1	1	26	0
Kuwait	3	0	1	0	58	0
Lebanon	4	2	1	1	11	0
Libya	4	1	0	0	17	0
Morocco	9	6	2	2	11	0
Oman	4	0	2	0	16	0
Palestine	8	4	4	0	20	0
Qatar	1	0	2	0	40	0
Saudi Arabia	2	0	4	0	12	0
Sudan	9	5	2	1	0	0
Syria	6	4	1	0	31	0
Tunisia	7	5	2	0	21	0
Türkiye	14	5	3	0	39	0
United Arab Emirates	2	0	6	0	16	0
Yemen	7	5	2	0	0	0
Bangladesh	14	9	4	3	42	2
Bhutan	4	0	1	1	0	0
India	12	4	0	0	59	0
Nepal	12	7	5	2	0	0
Pakistan	14	8	9	0	23	0
Azad Jammu & Kashmir	1	0	0	0	0	0
Balochistan	5	5	0	0	0	0
Gilgit-Baltistan	2	2	0	0	0	0

Appendix Table 3. Number of all-cause mortality data sources by type and location, 1950-2021						
Location	SBH	CBH	HH	SIBS	VR/SRS/DSP	DSS
Islamabad Capital Territory	0	1	0	0	0	0
Khyber Pakhtunkhwa	4	5	1	0	0	0
Punjab	7	5	1	0	0	0
Sindh	5	5	1	0	0	0
China	5	0	31	0	23	0
North Korea	0	0	2	0	0	0
Taiwan (province of China)	0	0	0	0	67	0
American Samoa	1	0	0	0	55	0
Cook Islands	1	0	0	0	45	0
Fiji	7	1	0	0	55	0
Guam	0	0	0	0	70	0
Kiribati	7	0	2	0	12	0
Marshall Islands	2	0	1	0	14	0
Federated States of Micronesia	3	0	0	0	1	0
Nauru	2	0	2	0	17	0
Niue	0	0	0	0	43	0
Northern Mariana Islands	0	0	0	0	24	0
Palau	2	0	0	0	6	0
Papua New Guinea	8	1	3	1	2	0
Samoa	5	0	6	0	23	0
Solomon Islands	4	0	2	0	0	0
Tokelau	0	0	1	0	11	0
Tonga	3	0	3	0	18	0
Tuvalu	1	0	3	0	13	0
Vanuatu	4	0	1	0	1	0
Cambodia	11	5	1	4	0	0
Indonesia	46	15	3	5	0	0
Aceh	32	4	2	4	0	0
Bali	36	5	6	5	0	0
Bangka-Belitung Islands	25	4	5	4	0	0
Banten	33	4	4	4	0	0
Bengkulu	36	5	5	5	0	0
Gorontalo	22	4	5	5	0	0
Jakarta	40	5	4	5	0	0
Jambi	34	4	6	5	0	0
West Java	40	5	6	5	0	0
Central Java	40	5	6	5	0	0
East Java	40	5	6	5	0	0
West Kalimantan	36	5	6	5	0	0
South Kalimantan	40	5	6	5	0	0
Central Kalimantan	36	4	4	5	0	0
East Kalimantan	34	4	6	5	0	0
North Kalimantan	17	2	3	0	0	0
Riau Islands	29	3	4	5	0	0
Lampung	39	5	4	5	0	0
Maluku	33	3	0	4	0	0
North Maluku	24	3	4	3	0	0
West Nusa Tenggara	40	5	4	5	0	0
East Nusa Tenggara	34	4	6	5	0	0
Papua	30	2	3	4	0	0
West Papua	29	2	4	4	0	0
Riau	37	5	6	5	0	0
West Sulawesi	30	3	5	3	0	0
South Sulawesi	40	5	6	5	0	0
Central Sulawesi	36	5	5	5	0	0
Southeast Sulawesi	35	5	4	5	0	0
North Sulawesi	36	5	5	5	0	0
West Sumatra	40	5	4	5	0	0
South Sumatra	40	5	6	5	0	0
North Sumatra	40	5	3	5	0	0
Yogyakarta	40	5	6	5	0	0
Laos	4	2	1	1	0	0
Malaysia	3	1	0	0	58	0
Maldives	7	2	1	0	44	0

Appendix Table 3. Number of all-cause mortality data sources by type and location, 1950-2021						
**Location**	**SBH**	**CBH**	**HH**	**SIBS**	**VR/SRS/DSP**	**DSS**
Mauritius	0	0	0	0	71	0
Myanmar	6	1	2	1	2	0
Philippines	13	7	0	0	69	0
Abra	3	5	0	0	20	0
Agusan Del Norte	3	5	0	0	20	0
Agusan Del Sur	3	5	0	0	20	0
Aklan	3	5	0	0	26	0
Albay	3	5	0	0	26	0
Antique	3	5	0	0	26	0
Apayao	3	4	0	0	14	0
Aurora	3	5	0	0	14	0
Basilan	3	5	0	0	19	0
Bataan	3	5	0	0	26	0
Batanes	3	2	0	0	26	0
Batangas	3	5	0	0	26	0
Benguet	3	5	0	0	25	0
Biliran	3	4	0	0	14	0
Bohol	3	5	0	0	26	0
Bukidnon	3	5	0	0	26	0
Bulacan	3	5	0	0	26	0
Cagayan	3	5	0	0	26	0
Camarines Norte	3	5	0	0	26	0
Camarines Sur	3	5	0	0	26	0
Camiguin	3	3	0	0	25	0
Capiz	3	4	0	0	26	0
Catanduanes	3	5	0	0	26	0
Cavite	3	5	0	0	26	0
Cebu	3	5	0	0	26	0
Cotabato (North Cotabato)	3	5	0	0	26	0
Davao de Oro	2	5	0	0	14	0
Davao Del Norte	3	5	0	0	25	0
Davao Del Sur	3	5	0	0	25	0
Davao Occidental	3	1	0	0	14	0
Davao Oriental	3	5	0	0	25	0
Dinagat Islands	2	1	0	0	14	0
Eastern Samar	3	5	0	0	25	0
Guimaras	3	2	0	0	14	0
Ifugao	3	5	0	0	25	0
Ilocos Norte	3	5	0	0	26	0
Ilocos Sur	3	5	0	0	26	0
Iloilo	3	5	0	0	26	0
Isabela	2	5	0	0	26	0
Kalinga	3	5	0	0	24	0
La Union	3	5	0	0	26	0
Laguna	3	5	0	0	26	0
Lanao Del Norte	3	5	0	0	26	0
Lanao Del Sur	3	5	0	0	26	0
Leyte	3	5	0	0	26	0
Maguindanao	3	5	0	0	19	0
Marinduque	3	5	0	0	26	0
Masbate	3	5	0	0	26	0
Misamis Occidental	3	5	0	0	26	0
Misamis Oriental	3	5	0	0	26	0
Mountain Province	3	4	0	0	25	0
National Capital Region	3	5	0	0	14	0
Negros Occidental	3	5	0	0	26	0
Negros Oriental	3	2	0	0	26	0
Northern Samar	3	5	0	0	25	0
Nueva Ecija	3	5	0	0	25	0
Nueva Vizcaya	3	5	0	0	25	0
Occidental Mindoro	3	5	0	0	26	0
Oriental Mindoro	2	5	0	0	26	0
Palawan	3	5	0	0	26	0
Pampanga	2	5	0	0	26	0

Appendix Table 3. Number of all-cause mortality data sources by type and location, 1950-2021						
**Location**	**SBH**	**CBH**	**HH**	**SIBS**	**VR/SRS/DSP**	**DSS**
Pangasinan	2	5	0	0	26	0
Quezon	3	5	0	0	26	0
Quirino	3	4	0	0	20	0
Rizal	3	5	0	0	18	0
Romblon	3	5	0	0	26	0
Samar (Western Samar)	3	5	0	0	26	0
Sarangani	2	5	0	0	14	0
Siquijor	3	1	0	0	20	0
Sorsogon	3	5	0	0	26	0
South Cotabato	3	5	0	0	24	0
Southern Leyte	3	5	0	0	25	0
Sultan Kudarat	3	5	0	0	19	0
Sulu	3	5	0	0	23	0
Surigao Del Norte	3	5	0	0	26	0
Surigao Del Sur	3	5	0	0	26	0
Tarlac	3	5	0	0	26	0
Tawi-Tawi	3	2	0	0	18	0
Zambales	3	4	0	0	26	0
Zamboanga Del Norte	3	5	0	0	26	0
Zamboanga Del Sur	3	5	0	0	26	0
Zamboanga Sibugay	3	5	0	0	14	0
Seychelles	2	0	1	0	69	0
Sri Lanka	5	2	4	0	63	0
Thailand	11	2	0	0	69	0
Timor-Leste	9	6	0	4	0	0
Viet Nam	16	3	3	0	0	2
Angola	4	2	0	1	0	0
Central African Republic	5	1	1	1	0	0
Congo (Brazzaville)	5	4	1	2	0	0
DR Congo	5	2	1	2	0	0
Equatorial Guinea	1	0	1	0	0	0
Gabon	2	2	0	2	0	0
Burundi	8	3	1	2	0	0
Comoros	4	2	2	1	0	0
Djibouti	3	1	0	0	0	0
Eritrea	3	2	1	1	0	0
Ethiopia	8	5	2	4	0	5
Addis Ababa	3	4	1	2	0	0
Afar	4	5	1	2	0	0
Amhara	4	5	1	2	0	0
Benishangul-Gumuz	3	4	1	2	0	0
Dire Dawa	4	5	1	2	0	0
Gambella	4	5	1	2	0	0
Harari	4	5	1	2	0	0
Oromia	4	4	1	2	0	0
Somali	4	5	1	2	0	0
Southern Nations, Nationalities, and Peoples	4	4	1	2	0	0
Tigray	4	5	1	2	0	0
Kenya	19	7	1	4	0	4
Baringo	12	5	0	1	0	0
Bomet	9	3	1	1	0	0
Bungoma	13	7	0	1	0	0
Busia	12	6	0	0	0	0
Elgeyo Marakwet	12	6	0	1	0	0
Embu	14	7	0	0	0	0
Garissa	10	3	0	0	0	0
Homa Bay	9	4	0	1	0	0
Isiolo	11	4	0	0	0	0
Kajiado	12	5	1	1	0	0
Kakamega	12	7	0	0	0	0
Kericho	13	6	0	0	0	0
Kiambu	13	6	0	1	0	0
Kilifi	13	6	0	1	0	0
Kirinyaga	12	6	0	0	0	0

Appendix Table 3. Number of all-cause mortality data sources by type and location, 1950-2021						
Location	SBH	CBH	HH	SIBS	VR/SRS/DSP	DSS
Kisii	12	7	0	1	0	0
Kisumu	12	7	0	1	0	0
Kitui	15	7	0	1	0	0
Kwale	12	6	0	1	0	0
Laikipia	13	5	0	0	0	0
Lamu	10	4	0	0	0	0
Machakos	15	7	0	1	0	0
Makueni	11	4	0	1	0	0
Mandera	10	3	0	0	0	0
Marsabit	9	4	0	0	0	0
Meru	12	7	0	1	0	0
Migori	9	4	0	1	0	0
Mombasa	12	7	0	0	0	0
Murang'a	12	6	1	1	0	0
Nairobi	11	6	0	1	0	0
Nakuru	12	6	0	1	0	0
Nandi	13	6	0	1	0	0
Narok	11	6	0	1	0	0
Nyamira	10	4	0	1	0	0
Nyandarua	11	6	0	1	0	0
Nyeri	11	6	0	1	0	0
Samburu	8	3	0	0	0	0
Siaya	13	7	0	1	0	0
Taita Taveta	12	6	0	1	0	0
Tana River	10	3	0	1	0	0
Tharaka Nithi	10	4	0	1	0	0
Trans Nzoia	12	6	0	0	0	0
Turkana	9	4	0	1	0	0
Uasin Gishu	12	6	0	1	0	0
Vihiga	9	3	0	1	0	0
Wajir	10	3	0	0	0	0
West Pokot	12	5	0	1	0	0
Madagascar	10	4	3	4	14	0
Malawi	19	8	9	6	2	1
Mozambique	9	4	1	3	0	1
Rwanda	13	7	2	4	0	0
Somalia	2	1	0	0	0	0
South Sudan	2	1	0	0	0	0
Uganda	17	7	1	5	0	1
Tanzania	14	7	3	4	0	3
Zambia	11	5	10	5	0	0
Botswana	9	2	3	0	10	0
Eswatini	4	3	2	1	0	0
Lesotho	10	4	1	3	0	0
Namibia	5	4	2	4	0	0
South Africa	9	2	10	1	33	3
Eastern Cape	7	2	8	1	21	0
Free State	7	2	8	1	21	0
Gauteng	7	2	4	1	21	0
KwaZulu-Natal	8	2	7	1	21	0
Limpopo	7	2	8	1	21	0
Mpumalanga	7	2	6	1	21	0
North West	7	2	7	1	21	0
Northern Cape	6	2	6	1	21	0
Western Cape	7	1	7	1	21	0
Zimbabwe	9	8	4	6	2	0
Benin	9	7	2	3	0	0
Burkina Faso	12	4	6	3	0	2
Cabo Verde	3	1	1	0	24	0
Cameroon	9	6	3	3	0	0
Chad	5	3	1	3	0	0
Côte d'Ivoire	6	10	1	3	0	1
The Gambia	5	1	4	0	0	1
Ghana	20	9	3	1	0	1

Appendix Table 3. Number of all-cause mortality data sources by type and location, 1950-2021						
**Location**	**SBH**	**CBH**	**HH**	**SIBS**	**VR/SRS/DSP**	**DSS**
Guinea	4	4	4	3	0	0
Guinea-Bissau	4	1	1	1	0	0
Liberia	7	5	2	2	0	0
Mali	10	6	5	5	0	0
Mauritania	8	5	2	2	0	0
Niger	5	4	0	3	0	0
Nigeria	22	6	1	0	0	0
São Tomé and Príncipe	6	2	2	2	20	0
Senegal	13	13	3	3	0	3
Sierra Leone	6	3	2	2	5	0
Togo	8	3	3	2	0	0

Appendix Table 4. Number of all-cause mortality data sources by type and year, 1950-2021						
**Year**	**SBH**	**CBH**	**HH**	**SIBS**	**VR/SRS/DSP**	**DSS**
1950	0	0	0	0	81	0
1951	0	0	0	0	92	0
1952	0	0	0	0	98	0
1953	0	0	0	0	97	0
1954	0	0	0	0	98	0
1955	0	1	0	0	106	0
1956	2	0	0	0	105	0
1957	0	1	0	0	105	0
1958	1	0	1	0	114	0
1959	0	1	1	0	162	0
1960	23	0	2	0	161	0
1961	3	1	1	0	160	0
1962	0	0	2	0	160	0
1963	1	1	2	0	161	0
1964	1	2	3	0	163	0
1965	1	1	2	0	217	0
1966	4	4	0	0	164	0
1967	2	2	2	0	160	0
1968	1	7	0	0	229	0
1969	2	3	0	0	258	0
1970	39	10	1	0	245	0
1971	39	4	2	0	244	0
1972	3	13	1	0	246	0
1973	9	5	2	0	247	1
1974	8	22	2	0	252	0
1975	13	14	0	0	187	0
1976	14	24	2	0	260	0
1977	8	12	1	0	261	0
1978	10	29	0	0	271	0
1979	8	11	1	0	358	0
1980	114	28	1	0	312	0
1981	17	7	2	0	478	0
1982	38	27	7	0	482	0
1983	5	10	1	0	480	0
1984	8	30	23	0	485	0
1985	45	14	1	0	487	0
1986	33	46	1	0	483	0
1987	38	44	7	0	488	0
1988	48	62	3	1	534	0
1989	54	20	34	1	603	0
1990	170	53	2	1	611	4
1991	114	48	8	6	613	4
1992	144	158	15	2	611	4
1993	191	38	9	4	609	6
1994	134	49	5	33	613	7
1995	106	55	17	6	620	7
1996	111	52	50	4	653	8
1997	82	80	48	48	663	9
1998	121	112	75	3	666	9
1999	335	72	69	11	666	10
2000	291	94	50	14	668	11

Appendix Table 4. Number of all-cause mortality data sources by type and year, 1950-2021						
**Year**	**SBH**	**CBH**	**HH**	**SIBS**	**VR/SRS/DSP**	**DSS**
2001	180	44	39	2	696	11
2002	75	171	37	35	700	11
2003	275	71	7	6	667	14
2004	116	53	39	10	712	16
2005	234	110	70	7	716	18
2006	306	85	25	5	798	21
2007	221	70	10	38	834	21
2008	284	157	15	4	801	23
2009	261	48	9	10	801	26
2010	348	31	54	6	797	30
2011	390	137	55	7	767	30
2012	193	69	37	39	800	29
2013	147	87	34	42	795	24
2014	117	90	35	13	796	21
2015	221	72	32	3	797	2
2016	134	83	34	13	767	0
2017	110	88	39	2	724	0
2018	3	0	47	0	714	0
2019	0	0	31	0	670	0
2020	0	0	24	0	741	0
2021	0	0	1	0	405	0

Appendix Table 5. Number of 5q0 data sources by type and location, 1950-2021					
**Location**	**SBH**	**CBH**	**HH**	**VR/SRS/DSP**	**DSS**
Armenia	6	4	0	42	0
Azerbaijan	4	1	2	43	0
Georgia	3	3	1	43	0
Kazakhstan	8	2	0	43	0
Kyrgyzstan	7	4	1	42	0
Mongolia	10	3	2	40	0
Tajikistan	8	2	1	39	0
Turkmenistan	3	1	2	25	0
Uzbekistan	4	2	0	41	0
Albania	7	2	0	42	0
Bosnia and Herzegovina	0	0	0	65	0
Bulgaria	4	0	0	72	0
Croatia	0	0	0	71	0
Czechia	0	0	0	72	0
Hungary	1	0	0	72	0
Montenegro	2	0	0	60	0
North Macedonia	2	0	0	71	0
Poland	1	0	0	72	0
Romania	4	1	1	66	0
Serbia	5	0	1	55	0
Slovakia	0	0	0	72	0
Slovenia	0	0	0	72	0
Belarus	4	0	0	61	0
Estonia	1	0	0	63	0
Latvia	1	0	0	62	0
Lithuania	0	0	0	63	0
Moldova	4	2	0	43	0
Russia	1	0	0	63	0
Ukraine	8	2	0	58	0
Australia	0	0	0	71	0
New Zealand	0	0	0	72	0
Brunei	1	0	0	62	0
Japan	0	0	0	72	0
Aichi	0	0	0	43	0
Akita	0	0	0	43	0
Aomori	0	0	0	43	0
Chiba	0	0	0	43	0
Ehime	0	0	0	43	0
Fukui	0	0	0	43	0
Fukuoka	0	0	0	43	0
Fukushima	0	0	0	43	0
Gifu	0	0	0	43	0
Gunma	0	0	0	43	0
Hiroshima	0	0	0	43	0
Hokkaidō	0	0	0	43	0
Hyōgo	0	0	0	42	0
Ibaraki	0	0	0	43	0
Ishikawa	0	0	0	43	0
Iwate	0	0	0	42	0
Kagawa	0	0	0	43	0
Kagoshima	0	0	0	43	0
Kanagawa	0	0	0	43	0
Kōchi	0	0	0	43	0
Kumamoto	0	0	0	43	0
Kyōto	0	0	0	43	0
Mie	0	0	0	43	0
Miyagi	0	0	0	42	0

Appendix Table 5. Number of 5q0 data sources by type and location, 1950-2021					
**Location**	**SBH**	**CBH**	**HH**	**VR/SRS/DSP**	**DSS**
Miyazaki	0	0	0	43	0
Nagano	0	0	0	43	0
Nagasaki	0	0	0	43	0
Nara	0	0	0	43	0
Niigata	0	0	0	43	0
Ōita	0	0	0	43	0
Okayama	0	0	0	43	0
Okinawa	0	0	0	43	0
Ōsaka	0	0	0	43	0
Saga	0	0	0	43	0
Saitama	0	0	0	43	0
Shiga	0	0	0	43	0
Shimane	0	0	0	43	0
Shizuoka	0	0	0	43	0
Tochigi	0	0	0	43	0
Tokushima	0	0	0	43	0
Tōkyō	0	0	0	43	0
Tottori	0	0	0	43	0
Toyama	0	0	0	43	0
Wakayama	0	0	0	43	0
Yamagata	0	0	0	43	0
Yamaguchi	0	0	0	43	0
Yamanashi	0	0	0	43	0
South Korea	6	1	0	44	0
Singapore	0	0	0	72	0
Canada	0	0	0	69	0
Greenland	0	0	0	60	0
USA	0	0	0	71	0
Alabama	0	0	0	62	0
Alaska	0	0	0	62	0
Arizona	0	0	0	62	0
Arkansas	0	0	0	62	0
California	0	0	0	62	0
Colorado	0	0	0	62	0
Connecticut	0	0	0	62	0
Delaware	0	0	0	62	0
Washington, DC	0	0	0	62	0
Florida	0	0	0	62	0
Georgia	0	0	0	62	0
Hawaii	0	0	0	62	0
Idaho	0	0	0	62	0
Illinois	0	0	0	62	0
Indiana	0	0	0	62	0
Iowa	0	0	0	62	0
Kansas	0	0	0	62	0
Kentucky	0	0	0	62	0
Louisiana	0	0	0	62	0
Maine	0	0	0	62	0
Maryland	0	0	0	62	0
Massachusetts	0	0	0	62	0
Michigan	0	0	0	62	0
Minnesota	0	0	0	62	0
Mississippi	0	0	0	62	0
Missouri	0	0	0	62	0
Montana	0	0	0	62	0
Nebraska	0	0	0	62	0
Nevada	0	0	0	62	0

Appendix Table 5. Number of 5q0 data sources by type and location, 1950-2021					
**Location**	**SBH**	**CBH**	**HH**	**VR/SRS/DSP**	**DSS**
New Hampshire	0	0	0	62	0
New Jersey	0	0	0	62	0
New Mexico	0	0	0	62	0
New York	0	0	0	62	0
North Carolina	0	0	0	62	0
North Dakota	0	0	0	62	0
Ohio	0	0	0	62	0
Oklahoma	0	0	0	62	0
Oregon	0	0	0	62	0
Pennsylvania	0	0	0	62	0
Rhode Island	0	0	0	62	0
South Carolina	0	0	0	62	0
South Dakota	0	0	0	62	0
Tennessee	0	0	0	62	0
Texas	0	0	0	62	0
Utah	0	0	0	62	0
Vermont	0	0	0	62	0
Virginia	0	0	0	62	0
Washington	0	0	0	62	0
West Virginia	0	0	0	62	0
Wisconsin	0	0	0	62	0
Wyoming	0	0	0	62	0
Argentina	3	0	0	64	0
Chile	4	0	0	72	0
Uruguay	6	0	0	68	0
Andorra	0	0	0	21	0
Austria	0	0	0	72	0
Belgium	2	0	0	71	0
Cyprus	3	0	1	48	0
Denmark	0	0	0	72	0
Finland	0	0	0	72	0
France	0	0	0	71	0
Germany	0	0	0	66	0
Greece	0	0	0	71	0
Iceland	0	0	0	72	0
Ireland	0	0	0	70	0
Israel	0	0	0	71	0
Italy	0	0	0	72	0
Abruzzo	0	0	0	53	0
Basilicata	0	0	0	53	0
Calabria	0	0	0	53	0
Campania	0	0	0	53	0
Emilia-Romagna	0	0	0	53	0
Friuli-Venezia Giulia	0	0	0	53	0
Lazio	0	0	0	53	0
Liguria	0	0	0	53	0
Lombardia	0	0	0	53	0
Marche	0	0	0	53	0
Molise	0	0	0	53	0
Piemonte	0	0	0	53	0
Provincia autonoma di Bolzano	0	0	0	52	0
Provincia autonoma di Trento	0	0	0	52	0
Puglia	0	0	0	53	0
Sardegna	0	0	0	53	0
Sicilia	0	0	0	53	0
Toscana	0	0	0	53	0
Umbria	0	0	0	53	0

Appendix Table 5. Number of 5q0 data sources by type and location, 1950-2021					
Location	SBH	CBH	HH	VR/SRS/DSP	DSS
Valle d'Aosta	0	0	0	53	0
Veneto	0	0	0	53	0
Luxembourg	0	0	0	72	0
Malta	0	0	0	72	0
Monaco	0	0	0	9	0
Netherlands	0	0	0	70	0
Norway	0	0	0	72	0
Agder	0	0	0	70	0
Innlandet	0	0	0	70	0
Møre og Romsdal	0	0	0	70	0
Nordland	0	0	0	70	0
Oslo	0	0	0	70	0
Rogaland	0	0	0	70	0
Troms og Finnmark	0	0	0	70	0
Trøndelag	0	0	0	70	0
Vestfold og Telemark	0	0	0	70	0
Vestland	0	0	0	70	0
Viken	0	0	0	70	0
Portugal	1	1	0	69	0
San Marino	0	0	0	49	0
Spain	0	0	0	72	0
Sweden	0	0	0	72	0
Stockholm	0	0	0	37	0
Sweden except Stockholm	0	0	0	37	0
Switzerland	1	0	0	72	0
UK	0	0	0	70	0
England	0	0	0	41	0
East Midlands	0	0	0	38	0
Derby	0	0	0	41	0
Derbyshire	0	0	0	41	0
Leicester	0	0	0	41	0
Leicestershire	0	0	0	41	0
Lincolnshire	0	0	0	41	0
Northamptonshire	0	0	0	40	0
Nottingham	0	0	0	41	0
Nottinghamshire	0	0	0	41	0
Rutland	0	0	0	41	0
East of England	0	0	0	38	0
Bedford	0	0	0	41	0
Cambridgeshire	0	0	0	41	0
Central Bedfordshire	0	0	0	41	0
Essex	0	0	0	41	0
Hertfordshire	0	0	0	41	0
Luton	0	0	0	41	0
Norfolk	0	0	0	41	0
Peterborough	0	0	0	41	0
Southend-on-Sea	0	0	0	41	0
Suffolk	0	0	0	41	0
Thurrock	0	0	0	41	0
Greater London	0	0	0	38	0
Barking and Dagenham	0	0	0	41	0
Barnet	0	0	0	41	0
Bexley	0	0	0	41	0
Brent	0	0	0	41	0
Bromley	0	0	0	41	0
Camden	0	0	0	41	0
Croydon	0	0	0	41	0

Appendix Table 5. Number of 5q0 data sources by type and location, 1950-2021					
Location	SBH	CBH	HH	VR/SRS/DSP	DSS
Ealing	0	0	0	41	0
Enfield	0	0	0	41	0
Greenwich	0	0	0	41	0
Hackney	0	0	0	38	0
Hammersmith and Fulham	0	0	0	41	0
Haringey	0	0	0	41	0
Harrow	0	0	0	41	0
Havering	0	0	0	41	0
Hillingdon	0	0	0	41	0
Hounslow	0	0	0	41	0
Islington	0	0	0	41	0
Kensington and Chelsea	0	0	0	41	0
Kingston upon Thames	0	0	0	41	0
Lambeth	0	0	0	41	0
Lewisham	0	0	0	41	0
Merton	0	0	0	41	0
Newham	0	0	0	41	0
Redbridge	0	0	0	41	0
Richmond upon Thames	0	0	0	41	0
Southwark	0	0	0	41	0
Sutton	0	0	0	41	0
Tower Hamlets	0	0	0	41	0
Waltham Forest	0	0	0	41	0
Wandsworth	0	0	0	41	0
Westminster	0	0	0	41	0
North East England	0	0	0	38	0
County Durham	0	0	0	41	0
Darlington	0	0	0	41	0
Gateshead	0	0	0	41	0
Hartlepool	0	0	0	41	0
Middlesbrough	0	0	0	41	0
Newcastle upon Tyne	0	0	0	41	0
North Tyneside	0	0	0	41	0
Northumberland	0	0	0	41	0
Redcar and Cleveland	0	0	0	41	0
South Tyneside	0	0	0	41	0
Stockton-on-Tees	0	0	0	41	0
Sunderland	0	0	0	41	0
North West England	0	0	0	38	0
Blackburn with Darwen	0	0	0	41	0
Blackpool	0	0	0	41	0
Bolton	0	0	0	41	0
Bury	0	0	0	41	0
Cheshire East	0	0	0	41	0
Cheshire West and Chester	0	0	0	41	0
Cumbria	0	0	0	41	0
Halton	0	0	0	41	0
Knowsley	0	0	0	41	0
Lancashire	0	0	0	41	0
Liverpool	0	0	0	41	0
Manchester	0	0	0	41	0
Oldham	0	0	0	41	0
Rochdale	0	0	0	41	0
Salford	0	0	0	41	0
Sefton	0	0	0	41	0
St Helens	0	0	0	41	0
Stockport	0	0	0	41	0

Appendix Table 5. Number of 5q0 data sources by type and location, 1950-2021					
Location	SBH	CBH	HH	VR/SRS/DSP	DSS
Tameside	0	0	0	41	0
Trafford	0	0	0	41	0
Warrington	0	0	0	41	0
Wigan	0	0	0	41	0
Wirral	0	0	0	41	0
South East England	0	0	0	38	0
Bracknell Forest	0	0	0	41	0
Brighton and Hove	0	0	0	41	0
Buckinghamshire	0	0	0	41	0
East Sussex	0	0	0	41	0
Hampshire	0	0	0	41	0
Isle of Wight	0	0	0	41	0
Kent	0	0	0	41	0
Medway	0	0	0	41	0
Milton Keynes	0	0	0	41	0
Oxfordshire	0	0	0	41	0
Portsmouth	0	0	0	41	0
Reading	0	0	0	41	0
Slough	0	0	0	41	0
Southampton	0	0	0	41	0
Surrey	0	0	0	41	0
West Berkshire	0	0	0	41	0
West Sussex	0	0	0	41	0
Windsor and Maidenhead	0	0	0	41	0
Wokingham	0	0	0	41	0
South West England	0	0	0	38	0
Bath and North East Somerset	0	0	0	41	0
Bournemouth	0	0	0	40	0
Bristol, City of	0	0	0	41	0
Cornwall	0	0	0	41	0
Devon	0	0	0	41	0
Dorset	0	0	0	41	0
Gloucestershire	0	0	0	41	0
North Somerset	0	0	0	41	0
Plymouth	0	0	0	41	0
Poole	0	0	0	40	0
Somerset	0	0	0	41	0
South Gloucestershire	0	0	0	41	0
Swindon	0	0	0	41	0
Torbay	0	0	0	41	0
Wiltshire	0	0	0	41	0
West Midlands	0	0	0	38	0
Birmingham	0	0	0	41	0
Coventry	0	0	0	41	0
Dudley	0	0	0	41	0
Herefordshire, County of	0	0	0	41	0
Sandwell	0	0	0	41	0
Shropshire	0	0	0	41	0
Solihull	0	0	0	41	0
Staffordshire	0	0	0	41	0
Stoke-on-Trent	0	0	0	41	0
Telford and Wrekin	0	0	0	41	0
Walsall	0	0	0	41	0
Warwickshire	0	0	0	41	0
Wolverhampton	0	0	0	41	0
Worcestershire	0	0	0	41	0
Yorkshire and the Humber	0	0	0	38	0

Appendix Table 5. Number of 5q0 data sources by type and location, 1950-2021					
Location	SBH	CBH	HH	VR/SRS/DSP	DSS
Barnsley	0	0	0	41	0
Bradford	0	0	0	41	0
Calderdale	0	0	0	41	0
Doncaster	0	0	0	41	0
East Riding of Yorkshire	0	0	0	41	0
Kingston upon Hull, City of	0	0	0	41	0
Kirklees	0	0	0	41	0
Leeds	0	0	0	41	0
North East Lincolnshire	0	0	0	41	0
North Lincolnshire	0	0	0	41	0
North Yorkshire	0	0	0	41	0
Rotherham	0	0	0	41	0
Sheffield	0	0	0	41	0
Wakefield	0	0	0	41	0
York	0	0	0	41	0
Northern Ireland	0	0	0	70	0
Scotland	0	0	0	72	0
Wales	0	0	0	41	0
Bolivia	26	6	1	12	0
Ecuador	17	7	0	67	0
Peru	21	14	0	68	0
Antigua and Barbuda	0	0	0	61	0
The Bahamas	0	0	0	50	0
Barbados	0	0	0	62	0
Belize	7	3	0	69	0
Bermuda	1	0	0	68	0
Cuba	1	0	0	62	0
Dominica	0	0	0	64	0
Dominican Republic	15	13	1	62	0
Grenada	0	0	0	57	0
Guyana	7	4	1	46	0
Haiti	9	7	2	1	0
Jamaica	5	1	1	51	0
Puerto Rico	1	1	0	71	0
Saint Kitts and Nevis	0	0	0	66	0
Saint Lucia	0	0	0	57	0
Saint Vincent and the Grenadines	0	0	0	53	0
Suriname	2	0	1	61	0
Trinidad and Tobago	5	2	0	67	0
Virgin Islands	0	0	0	54	0
Colombia	13	8	0	69	0
Costa Rica	7	3	0	71	0
El Salvador	10	6	0	70	0
Guatemala	10	6	0	69	0
Honduras	12	5	2	44	0
Mexico	25	2	1	72	0
Aguascalientes	16	1	0	43	0
Baja California	16	1	0	43	0
Baja California Sur	16	1	0	43	0
Campeche	16	1	0	43	0
Chiapas	16	1	0	43	0
Chihuahua	16	1	0	43	0
Coahuila	16	1	0	43	0
Colima	16	1	0	43	0
Durango	16	1	0	43	0
Guanajuato	16	1	0	43	0
Guerrero	16	1	0	43	0

Appendix Table 5. Number of 5q0 data sources by type and location, 1950-2021					
**Location**	**SBH**	**CBH**	**HH**	**VR/SRS/DSP**	**DSS**
Hidalgo	14	1	0	43	0
Jalisco	16	1	0	43	0
México	16	1	0	43	0
Mexico City	16	1	0	42	0
Michoacán de Ocampo	16	1	0	43	0
Morelos	16	1	0	43	0
Nayarit	16	1	0	43	0
Nuevo León	16	1	0	43	0
Oaxaca	16	1	0	43	0
Puebla	16	1	0	43	0
Querétaro	16	1	0	43	0
Quintana Roo	16	1	0	43	0
San Luis Potosí	16	1	0	43	0
Sinaloa	16	1	0	43	0
Sonora	16	1	0	43	0
Tabasco	16	1	0	43	0
Tamaulipas	16	1	0	43	0
Tlaxcala	16	1	0	43	0
Veracruz de Ignacio de la Llave	16	1	0	43	0
Yucatán	16	1	0	43	0
Zacatecas	16	1	0	43	0
Nicaragua	12	5	0	37	0
Panama	9	1	0	70	0
Venezuela	4	1	1	66	0
Brazil	29	2	1	39	0
Acre	9	1	1	34	0
Alagoas	21	2	1	34	0
Amapá	9	1	1	34	0
Amazonas	10	1	1	34	0
Bahia	21	2	1	34	0
Ceará	21	2	1	34	0
Distrito Federal	21	1	1	34	0
Espírito Santo	21	1	1	34	0
Goiás	21	1	1	34	0
Maranhão	21	2	1	34	0
Mato Grosso	21	1	1	34	0
Mato Grosso do Sul	21	1	1	34	0
Minas Gerais	21	1	1	34	0
Pará	10	1	1	34	0
Paraíba	21	2	1	33	0
Paraná	21	1	1	34	0
Pernambuco	21	2	1	34	0
Piauí	21	2	1	34	0
Rio de Janeiro	21	1	1	34	0
Rio Grande do Norte	21	2	1	34	0
Rio Grande do Sul	21	1	1	34	0
Rondônia	9	1	1	34	0
Roraima	9	1	1	34	0
Santa Catarina	21	1	1	34	0
São Paulo	20	1	1	34	0
Sergipe	21	2	1	34	0
Tocantins	9	1	1	24	0
Paraguay	16	7	0	68	0
Afghanistan	7	1	0	0	0
Algeria	5	3	1	16	0
Bahrain	4	0	2	38	0
Egypt	14	11	2	68	0

Appendix Table 5. Number of 5q0 data sources by type and location, 1950-2021					
Location	SBH	CBH	HH	VR/SRS/DSP	DSS
Iran	5	1	3	3	0
Alborz	3	1	0	2	0
Ardebil	3	1	0	2	0
Bushehr	3	1	0	2	0
Chahar Mahaal and Bakhtiari	3	1	0	2	0
East Azarbayejan	3	1	0	2	0
Fars	3	1	0	2	0
Gilan	3	1	0	2	0
Golestan	3	1	0	2	0
Hamadan	3	1	0	2	0
Hormozgan	3	1	0	2	0
Ilam	3	1	0	2	0
Isfahan	3	1	0	2	0
Kerman	3	1	0	2	0
Kermanshah	3	1	0	2	0
Khorasan-e-Razavi	3	1	0	2	0
Khuzestan	3	1	0	2	0
Kohgiluyeh and Boyer-Ahmad	3	1	0	2	0
Kurdistan	3	1	0	2	0
Lorestan	3	1	0	2	0
Markazi	3	1	0	2	0
Mazandaran	3	1	0	2	0
North Khorasan	3	1	0	2	0
Qazvin	3	1	0	2	0
Qom	3	1	0	2	0
Semnan	3	1	0	2	0
Sistan and Baluchistan	3	1	0	2	0
South Khorasan	3	1	0	2	0
Tehran	3	1	0	2	0
West Azarbayejan	3	1	0	2	0
Yazd	3	1	0	2	0
Zanjan	3	1	0	2	0
Iraq	7	5	3	12	1
Jordan	12	7	0	26	0
Kuwait	3	0	1	58	0
Lebanon	4	2	0	8	0
Libya	4	1	0	17	0
Morocco	9	6	1	11	0
Oman	4	0	2	16	0
Palestine	9	4	4	12	0
Qatar	1	0	2	40	0
Saudi Arabia	2	0	4	2	0
Sudan	9	5	2	0	0
Syria	6	4	0	31	0
Tunisia	7	5	1	21	0
Türkiye	14	5	3	39	0
United Arab Emirates	2	0	6	16	0
Yemen	7	5	2	0	0
Bangladesh	14	9	3	42	0
Bhutan	5	0	1	0	0
India	12	4	0	33	0
Nepal	12	7	4	0	0
Pakistan	15	8	9	22	0
Azad Jammu & Kashmir	1	0	0	0	0
Balochistan	5	5	0	0	0
Gilgit-Baltistan	2	2	0	0	0
Islamabad Capital Territory	0	1	0	0	0

Appendix Table 5. Number of 5q0 data sources by type and location, 1950-2021					
Location	SBH	CBH	HH	VR/SRS/DSP	DSS
Khyber Pakhtunkhwa	4	5	1	0	0
Punjab	7	5	1	0	0
Sindh	5	5	1	0	0
China	5	0	31	23	0
North Korea	0	0	1	0	0
Taiwan (province of China)	0	0	0	67	0
American Samoa	1	0	0	55	0
Cook Islands	1	0	0	45	0
Fiji	7	1	0	55	0
Guam	0	0	0	70	0
Kiribati	7	0	2	12	0
Marshall Islands	2	0	1	14	0
Federated States of Micronesia	3	0	0	1	0
Nauru	2	0	2	17	0
Niue	0	0	0	32	0
Northern Mariana Islands	0	0	0	23	0
Palau	2	0	0	6	0
Papua New Guinea	8	1	3	2	0
Samoa	5	0	4	22	0
Solomon Islands	4	0	2	0	0
Tokelau	0	0	1	9	0
Tonga	3	0	3	16	0
Tuvalu	1	0	3	13	0
Vanuatu	5	0	1	1	0
Cambodia	11	5	1	0	0
Indonesia	46	15	3	0	0
Aceh	32	4	0	0	0
Bali	36	5	0	0	0
Bangka-Belitung Islands	25	4	2	0	0
Banten	33	4	0	0	0
Bengkulu	36	5	0	0	0
Gorontalo	22	4	1	0	0
Jakarta	40	5	0	0	0
Jambi	34	4	0	0	0
West Java	40	5	0	0	0
Central Java	40	5	0	0	0
East Java	40	5	0	0	0
West Kalimantan	36	5	0	0	0
South Kalimantan	40	5	0	0	0
Central Kalimantan	36	4	0	0	0
East Kalimantan	34	4	0	0	0
North Kalimantan	17	2	0	0	0
Riau Islands	29	3	0	0	0
Lampung	39	5	0	0	0
Maluku	33	3	0	0	0
North Maluku	24	3	0	0	0
West Nusa Tenggara	40	5	0	0	0
East Nusa Tenggara	34	4	0	0	0
Papua	30	2	1	0	0
West Papua	29	2	1	0	0
Riau	37	5	0	0	0
West Sulawesi	30	3	0	0	0
South Sulawesi	40	5	0	0	0
Central Sulawesi	36	5	0	0	0
Southeast Sulawesi	35	5	0	0	0
North Sulawesi	36	5	0	0	0
West Sumatra	40	5	0	0	0

Appendix Table 5. Number of 5q0 data sources by type and location, 1950-2021					
Location	SBH	CBH	HH	VR/SRS/DSP	DSS
South Sumatra	40	5	0	0	0
North Sumatra	40	5	0	0	0
Yogyakarta	40	5	2	0	0
Laos	4	2	1	0	0
Malaysia	3	1	0	57	0
Maldives	7	2	1	44	0
Mauritius	0	0	0	71	0
Myanmar	6	1	2	2	0
Philippines	14	7	0	69	0
Abra	3	5	0	14	0
Agusan Del Norte	3	5	0	20	0
Agusan Del Sur	3	5	0	20	0
Aklan	3	5	0	26	0
Albay	3	5	0	26	0
Antique	3	5	0	26	0
Apayao	3	4	0	14	0
Aurora	3	5	0	14	0
Basilan	3	5	0	19	0
Bataan	3	5	0	26	0
Batanes	3	2	0	26	0
Batangas	3	5	0	26	0
Benguet	3	5	0	25	0
Biliran	3	4	0	14	0
Bohol	3	5	0	26	0
Bukidnon	3	5	0	26	0
Bulacan	3	5	0	26	0
Cagayan	3	5	0	26	0
Camarines Norte	3	5	0	26	0
Camarines Sur	3	5	0	14	0
Camiguin	3	3	0	25	0
Capiz	3	4	0	26	0
Catanduanes	3	5	0	14	0
Cavite	3	5	0	26	0
Cebu	3	5	0	26	0
Cotabato (North Cotabato)	3	5	0	26	0
Davao de Oro	2	5	0	14	0
Davao Del Norte	3	5	0	25	0
Davao Del Sur	3	5	0	25	0
Davao Occidental	3	1	0	14	0
Davao Oriental	3	5	0	25	0
Dinagat Islands	2	1	0	14	0
Eastern Samar	3	5	0	25	0
Guimaras	3	2	0	14	0
Ifugao	3	5	0	25	0
Ilocos Norte	3	5	0	26	0
Ilocos Sur	3	5	0	26	0
Iloilo	3	5	0	26	0
Isabela	2	5	0	26	0
Kalinga	3	5	0	24	0
La Union	3	5	0	26	0
Laguna	3	5	0	26	0
Lanao Del Norte	3	5	0	26	0
Lanao Del Sur	3	5	0	26	0
Leyte	3	5	0	26	0
Maguindanao	3	5	0	19	0
Marinduque	3	5	0	26	0
Masbate	3	5	0	26	0

Appendix Table 5. Number of 5q0 data sources by type and location, 1950-2021					
Location	SBH	CBH	HH	VR/SRS/DSP	DSS
Misamis Occidental	3	5	0	26	0
Misamis Oriental	3	5	0	26	0
Mountain Province	3	4	0	14	0
National Capital Region	3	5	0	14	0
Negros Occidental	3	5	0	26	0
Negros Oriental	3	2	0	26	0
Northern Samar	3	5	0	25	0
Nueva Ecija	3	5	0	25	0
Nueva Vizcaya	3	5	0	25	0
Occidental Mindoro	3	5	0	26	0
Oriental Mindoro	2	5	0	26	0
Palawan	3	5	0	14	0
Pampanga	2	5	0	26	0
Pangasinan	2	5	0	26	0
Quezon	3	5	0	26	0
Quirino	3	4	0	20	0
Rizal	3	5	0	18	0
Romblon	3	5	0	26	0
Samar (Western Samar)	3	5	0	26	0
Sarangani	2	5	0	14	0
Siquijor	3	1	0	20	0
Sorsogon	3	5	0	26	0
South Cotabato	3	5	0	24	0
Southern Leyte	3	5	0	25	0
Sultan Kudarat	3	5	0	19	0
Sulu	3	5	0	23	0
Surigao Del Norte	3	5	0	26	0
Surigao Del Sur	3	5	0	26	0
Tarlac	3	5	0	26	0
Tawi-Tawi	3	2	0	17	0
Zambales	3	4	0	26	0
Zamboanga Del Norte	3	5	0	26	0
Zamboanga Del Sur	3	5	0	26	0
Zamboanga Sibugay	3	5	0	14	0
Seychelles	2	0	0	69	0
Sri Lanka	5	2	4	61	0
Thailand	11	2	0	69	0
Timor-Leste	9	6	0	0	0
Viet Nam	16	3	0	0	0
Angola	5	2	0	0	0
Central African Republic	5	1	0	0	0
Congo (Brazzaville)	5	4	0	0	0
DR Congo	5	2	1	0	0
Equatorial Guinea	1	0	1	0	0
Gabon	2	2	0	0	0
Burundi	8	3	0	0	0
Comoros	4	2	1	0	0
Djibouti	3	1	0	0	0
Eritrea	3	2	0	0	0
Ethiopia	8	5	1	0	0
Addis Ababa	3	4	1	0	0
Afar	4	5	1	0	0
Amhara	4	5	1	0	0
Benishangul-Gumuz	3	4	1	0	0
Dire Dawa	4	5	1	0	0
Gambella	4	5	1	0	0
Harari	4	5	1	0	0

Appendix Table 5. Number of 5q0 data sources by type and location, 1950-2021					
**Location**	**SBH**	**CBH**	**HH**	**VR/SRS/DSP**	**DSS**
Oromia	4	4	1	0	0
Somali	4	5	1	0	0
Southern Nations, Nationalities, and Peoples	4	4	1	0	0
Tigray	4	5	1	0	0
Kenya	19	7	0	0	0
Baringo	12	5	0	0	0
Bomet	9	3	0	0	0
Bungoma	13	7	0	0	0
Busia	12	6	0	0	0
Elgeyo Marakwet	12	6	0	0	0
Embu	14	7	0	0	0
Garissa	10	3	0	0	0
Homa Bay	9	4	0	0	0
Isiolo	11	4	0	0	0
Kajiado	12	5	0	0	0
Kakamega	12	7	0	0	0
Kericho	13	6	0	0	0
Kiambu	13	6	0	0	0
Kilifi	13	6	0	0	0
Kirinyaga	12	6	0	0	0
Kisii	12	7	0	0	0
Kisumu	12	7	0	0	0
Kitui	15	7	0	0	0
Kwale	12	6	0	0	0
Laikipia	13	5	0	0	0
Lamu	10	4	0	0	0
Machakos	15	7	0	0	0
Makueni	11	4	0	0	0
Mandera	10	3	0	0	0
Marsabit	9	4	0	0	0
Meru	12	7	0	0	0
Migori	9	4	0	0	0
Mombasa	12	7	0	0	0
Murang'a	12	6	0	0	0
Nairobi	11	6	0	0	0
Nakuru	12	6	0	0	0
Nandi	13	6	0	0	0
Narok	11	6	0	0	0
Nyamira	10	4	0	0	0
Nyandarua	11	6	0	0	0
Nyeri	11	6	0	0	0
Samburu	8	3	0	0	0
Siaya	13	7	0	0	0
Taita Taveta	12	6	0	0	0
Tana River	10	3	0	0	0
Tharaka Nithi	10	4	0	0	0
Trans Nzoia	12	6	0	0	0
Turkana	9	4	0	0	0
Uasin Gishu	12	6	0	0	0
Vihiga	9	3	0	0	0
Wajir	10	3	0	0	0
West Pokot	12	5	0	0	0
Madagascar	10	4	2	14	0
Malawi	19	8	3	0	0
Mozambique	9	4	0	0	0
Rwanda	13	7	1	0	0
Somalia	2	1	0	0	0

Appendix Table 5. Number of 5q0 data sources by type and location, 1950-2021					
**Location**	**SBH**	**CBH**	**HH**	**VR/SRS/DSP**	**DSS**
South Sudan	2	1	0	0	0
Uganda	18	7	0	0	0
Tanzania	14	7	1	0	0
Zambia	11	5	1	0	0
Botswana	9	2	1	0	0
Eswatini	4	3	0	0	0
Lesotho	10	4	1	0	0
Namibia	5	4	0	0	0
South Africa	9	2	4	32	0
Eastern Cape	7	2	7	21	0
Free State	7	2	7	21	0
Gauteng	7	2	3	21	0
KwaZulu-Natal	8	2	6	21	0
Limpopo	7	2	7	21	0
Mpumalanga	7	2	5	21	0
North West	7	2	6	21	0
Northern Cape	6	2	5	21	0
Western Cape	7	1	6	21	0
Zimbabwe	9	8	2	2	0
Benin	9	7	2	0	0
Burkina Faso	12	4	4	0	0
Cabo Verde	3	1	1	24	0
Cameroon	9	6	1	0	0
Chad	5	3	1	0	0
Côte d'Ivoire	6	10	0	0	0
The Gambia	5	1	4	0	0
Ghana	20	9	2	0	0
Guinea	4	4	2	0	0
Guinea-Bissau	4	1	1	0	0
Liberia	7	5	1	0	0
Mali	10	6	1	0	0
Mauritania	8	5	1	0	0
Niger	5	4	0	0	0
Nigeria	22	6	1	0	0
São Tomé and Príncipe	6	2	1	20	0
Senegal	13	13	2	0	0
Sierra Leone	6	3	1	5	0
Togo	8	3	3	0	0

Appendix Table S6: 5q0 reference sources by source date and location, 1950–2021

Location	Year Range of Data	Reference Source	Data Type
Armenia	1975-2015	Standard Demographic and Health Survey	CBH
Azerbaijan	1981-2006	Standard Demographic and Health Survey	CBH
Georgia	2003-2021	Vital Registration	VR/SRS
Georgia	1978-2011	Reproductive Health Survey	CBH
Georgia	1975-2004	Reproductive Health Survey	SBH
Georgia	1981-2005	Multiple Indicator Cluster Survey	SBH
Kazakhstan	2010-2020	Vital Registration	VR/SRS
Kazakhstan	1981-2010	Multiple Indicator Cluster Survey	SBH
Kyrgyzstan	2007-2019	Vital Registration	VR/SRS
Kyrgyzstan	1974-2012	Standard Demographic and Health Survey	CBH
Mongolia	1974-1999	Reproductive Health Survey	CBH
Mongolia	1986-2016	Multiple Indicator Cluster Survey	CBH
Tajikistan	1987-2017	Standard Demographic and Health Survey	CBH
Turkmenistan	1987, 1992, 1997	Standard Demographic and Health Survey	CBH
Turkmenistan	1992-2015	Multiple Indicator Cluster Survey	CBH
Uzbekistan	1973-1996	Standard Demographic and Health Survey	CBH
Albania	1983-2008	Standard Demographic and Health Survey	CBH
Bosnia and Herzegovina	1950-1991, 1998-2020	Vital Registration	VR/SRS
Bulgaria	1950-2021	Vital Registration	VR/SRS
Croatia	1950-2013, 2015-2021	Vital Registration	VR/SRS
Czechia	1950-2021	Vital Registration	VR/SRS
Hungary	1950-2021	Vital Registration	VR/SRS
Montenegro	1989-2011	Multiple Indicator Cluster Survey	SBH
North Macedonia	1950-2020	Vital Registration	VR/SRS
Poland	1950-2021	Vital Registration	VR/SRS
Romania	1956-2021	Vital Registration	VR/SRS
Serbia	1950-1952, 1954-1960, 1962-1970, 1972-1990, 1995-1997, 2008-2021	Vital Registration	VR/SRS
Slovakia	1950-2021	Vital Registration	VR/SRS
Slovenia	1950-2021	Vital Registration	VR/SRS
Belarus	1969, 1972, 1976, 1979-1980, 1982-1983, 1986, 1990, 1993, 1996, 1998	Census	SBH
Belarus	1981-2004	Multiple Indicator Cluster Survey	SBH
Estonia	1959-2021	Vital Registration	VR/SRS
Latvia	1960-2021	Vital Registration	VR/SRS
Lithuania	1959-2021	Vital Registration	VR/SRS
Moldova	1980-2005	Standard Demographic and Health Survey	CBH
Russia	1959-2021	Vital Registration	VR/SRS
Ukraine	1998-2016	Vital Registration	VR/SRS
Ukraine	1982-2007	Standard Demographic and Health Survey	CBH
Australia	1950-2020	Vital Registration	VR/SRS
New Zealand	1950-2021	Vital Registration	VR/SRS
Brunei	1950-1959, 1964-1978, 1982-1992, 1995-2020	Vital Registration	VR/SRS
Japan	1950-2021	Vital Registration	VR/SRS
Aichi	1979-2021	Vital Registration	VR/SRS
Akita	1979-2021	Vital Registration	VR/SRS
Aomori	1979-2021	Vital Registration	VR/SRS
Chiba	1979-2021	Vital Registration	VR/SRS
Ehime	1979-2021	Vital Registration	VR/SRS
Fukui	1979-2021	Vital Registration	VR/SRS
Fukuoka	1979-2021	Vital Registration	VR/SRS
Fukushima	1979-2021	Vital Registration	VR/SRS
Gifu	1979-2021	Vital Registration	VR/SRS
Gunma	1979-2021	Vital Registration	VR/SRS
Hiroshima	1979-2021	Vital Registration	VR/SRS
Hokkaidō	1979-2021	Vital Registration	VR/SRS
Hyōgo	1979-1994, 1996-2021	Vital Registration	VR/SRS
Ibaraki	1979-2021	Vital Registration	VR/SRS
Ishikawa	1979-2021	Vital Registration	VR/SRS
Iwate	1979-2010, 2012-2021	Vital Registration	VR/SRS
Kagawa	1979-2021	Vital Registration	VR/SRS
Kagoshima	1979-2021	Vital Registration	VR/SRS
Kanagawa	1979-2021	Vital Registration	VR/SRS
Kōchi	1979-2021	Vital Registration	VR/SRS
Kumamoto	1979-2021	Vital Registration	VR/SRS
Kyōto	1979-2021	Vital Registration	VR/SRS
Mie	1979-2021	Vital Registration	VR/SRS
Miyagi	1979-2010, 2012-2021	Vital Registration	VR/SRS
Miyazaki	1979-2021	Vital Registration	VR/SRS

Nagano	1979-2021	Vital Registration	VR/SRS
Nagasaki	1979-2021	Vital Registration	VR/SRS
Nara	1979-2021	Vital Registration	VR/SRS
Niigata	1979-2021	Vital Registration	VR/SRS
Ōita	1979-2021	Vital Registration	VR/SRS
Okayama	1979-2021	Vital Registration	VR/SRS
Okinawa	1979-2021	Vital Registration	VR/SRS
Ōsaka	1979-2021	Vital Registration	VR/SRS
Saga	1979-2021	Vital Registration	VR/SRS
Saitama	1979-2021	Vital Registration	VR/SRS
Shiga	1979-2021	Vital Registration	VR/SRS
Shimane	1979-2021	Vital Registration	VR/SRS
Shizuoka	1979-2021	Vital Registration	VR/SRS
Tochigi	1979-2021	Vital Registration	VR/SRS
Tokushima	1979-2021	Vital Registration	VR/SRS
Tōkyō	1979-2021	Vital Registration	VR/SRS
Tottori	1979-2021	Vital Registration	VR/SRS
Toyama	1979-2021	Vital Registration	VR/SRS
Wakayama	1979-2021	Vital Registration	VR/SRS
Yamagata	1979-2021	Vital Registration	VR/SRS
Yamaguchi	1979-2021	Vital Registration	VR/SRS
Yamanashi	1979-2021	Vital Registration	VR/SRS
South Korea	1999-2020	Vital Registration	VR/SRS
South Korea	1954-1974	World Fertility Survey	CBH
Singapore	1950-2021	Vital Registration	VR/SRS
Canada	1950-2013, 2016-2020	Vital Registration	VR/SRS
Greenland	1952-1965, 1967-1978, 1988-2021	Vital Registration	VR/SRS
USA	1950-2020	Vital Registration	VR/SRS
Alabama	1959-2020	Vital Registration	VR/SRS
Alaska	1959-2020	Vital Registration	VR/SRS
Arizona	1959-2020	Vital Registration	VR/SRS
Arkansas	1959-2020	Vital Registration	VR/SRS
California	1959-2020	Vital Registration	VR/SRS
Colorado	1959-2020	Vital Registration	VR/SRS
Connecticut	1959-2020	Vital Registration	VR/SRS
Delaware	1959-2020	Vital Registration	VR/SRS
Washington, DC	1959-2020	Vital Registration	VR/SRS
Florida	1959-2020	Vital Registration	VR/SRS
Georgia	1959-2020	Vital Registration	VR/SRS
Hawaii	1959-2020	Vital Registration	VR/SRS
Idaho	1959-2020	Vital Registration	VR/SRS
Illinois	1959-2020	Vital Registration	VR/SRS
Indiana	1959-2020	Vital Registration	VR/SRS
Iowa	1959-2020	Vital Registration	VR/SRS
Kansas	1959-2020	Vital Registration	VR/SRS
Kentucky	1959-2020	Vital Registration	VR/SRS
Louisiana	1959-2020	Vital Registration	VR/SRS
Maine	1959-2020	Vital Registration	VR/SRS
Maryland	1959-2020	Vital Registration	VR/SRS
Massachusetts	1959-2020	Vital Registration	VR/SRS
Michigan	1959-2020	Vital Registration	VR/SRS
Minnesota	1959-2020	Vital Registration	VR/SRS
Mississippi	1959-2020	Vital Registration	VR/SRS
Missouri	1959-2020	Vital Registration	VR/SRS
Montana	1959-2020	Vital Registration	VR/SRS
Nebraska	1959-2020	Vital Registration	VR/SRS
Nevada	1959-2020	Vital Registration	VR/SRS
New Hampshire	1959-2020	Vital Registration	VR/SRS
New Jersey	1959-2020	Vital Registration	VR/SRS
New Mexico	1959-2020	Vital Registration	VR/SRS
New York	1959-2020	Vital Registration	VR/SRS
North Carolina	1959-2020	Vital Registration	VR/SRS
North Dakota	1959-2020	Vital Registration	VR/SRS
Ohio	1959-2020	Vital Registration	VR/SRS
Oklahoma	1959-2020	Vital Registration	VR/SRS
Oregon	1959-2020	Vital Registration	VR/SRS
Pennsylvania	1959-2020	Vital Registration	VR/SRS
Rhode Island	1959-2020	Vital Registration	VR/SRS
South Carolina	1959-2020	Vital Registration	VR/SRS

South Dakota	1959-2020	Vital Registration	VR/SRS
Tennessee	1959-2020	Vital Registration	VR/SRS
Texas	1959-2020	Vital Registration	VR/SRS
Utah	1959-2020	Vital Registration	VR/SRS
Vermont	1959-2020	Vital Registration	VR/SRS
Virginia	1959-2020	Vital Registration	VR/SRS
Washington	1959-2020	Vital Registration	VR/SRS
West Virginia	1959-2020	Vital Registration	VR/SRS
Wisconsin	1959-2020	Vital Registration	VR/SRS
Wyoming	1959-2020	Vital Registration	VR/SRS
Argentina	1950-1956, 1958-1970, 1976-2019	Vital Registration	VR/SRS
Chile	1950-2021	Vital Registration	VR/SRS
Uruguay	1950-2009, 2012-2019	Vital Registration	VR/SRS
Andorra	1992, 1994, 2002-2020	Vital Registration	VR/SRS
Austria	1950-2021	Vital Registration	VR/SRS
Belgium	1950-2020	Vital Registration	VR/SRS
Cyprus	1974-2021	Vital Registration	VR/SRS
Denmark	1950-2021	Vital Registration	VR/SRS
Finland	1950-2021	Vital Registration	VR/SRS
France	1950-2016, 2018-2021	Vital Registration	VR/SRS
Germany	1956-2021	Vital Registration	VR/SRS
Greece	1951-2021	Vital Registration	VR/SRS
Iceland	1950-2021	Vital Registration	VR/SRS
Ireland	1950-2015, 2017, 2019-2021	Vital Registration	VR/SRS
Israel	1950-2020	Vital Registration	VR/SRS
Italy	1950-2021	Vital Registration	VR/SRS
Abruzzo	1969-2021	Vital Registration	VR/SRS
Basilicata	1969-2021	Vital Registration	VR/SRS
Calabria	1969-2021	Vital Registration	VR/SRS
Campania	1969-2021	Vital Registration	VR/SRS
Emilia-Romagna	1969-2021	Vital Registration	VR/SRS
Friuli-Venezia Giulia	1969-2021	Vital Registration	VR/SRS
Lazio	1969-2021	Vital Registration	VR/SRS
Liguria	1969-2021	Vital Registration	VR/SRS
Lombardia	1969-2021	Vital Registration	VR/SRS
Marche	1969-2021	Vital Registration	VR/SRS
Molise	1969-2021	Vital Registration	VR/SRS
Piemonte	1969-2021	Vital Registration	VR/SRS
Provincia autonoma di Bolzano	1969-2020	Vital Registration	VR/SRS
Provincia autonoma di Trento	1969-2020	Vital Registration	VR/SRS
Puglia	1969-2021	Vital Registration	VR/SRS
Sardegna	1969-2021	Vital Registration	VR/SRS
Sicilia	1969-2021	Vital Registration	VR/SRS
Toscana	1969-2021	Vital Registration	VR/SRS
Umbria	1969-2021	Vital Registration	VR/SRS
Valle d'Aosta	1969-2021	Vital Registration	VR/SRS
Veneto	1969-2021	Vital Registration	VR/SRS
Luxembourg	1950-2021	Vital Registration	VR/SRS
Malta	1950-2021	Vital Registration	VR/SRS
Monaco	1950-1953, 1959, 1963, 1966, 1970, 1983	Vital Registration	VR/SRS
Netherlands	1950-1995, 1998-2021	Vital Registration	VR/SRS
Norway	1950-2021	Vital Registration	VR/SRS
Agder	1951-2020	Vital Registration	VR/SRS
Innlandet	1951-2020	Vital Registration	VR/SRS
Møre og Romsdal	1951-2020	Vital Registration	VR/SRS
Nordland	1951-2020	Vital Registration	VR/SRS
Oslo	1951-2020	Vital Registration	VR/SRS
Rogaland	1951-2020	Vital Registration	VR/SRS
Troms og Finnmark	1951-2020	Vital Registration	VR/SRS
Trøndelag	1951-2020	Vital Registration	VR/SRS
Vestfold og Telemark	1951-2020	Vital Registration	VR/SRS
Vestland	1951-2020	Vital Registration	VR/SRS
Viken	1951-2020	Vital Registration	VR/SRS
Portugal	1950-1974, 1977-2004, 2006-2021	Vital Registration	VR/SRS
San Marino	1962, 1964-1975, 1977-1978, 1980-1989, 1992-1996, 1998-1999, 2001-2004, 2008-2020	Vital Registration	VR/SRS
Spain	1950-2021	Vital Registration	VR/SRS
Sweden	1950-2021	Vital Registration	VR/SRS
Stockholm	1980-1986, 1990-2019	Vital Registration	VR/SRS
Sweden except Stockholm	1980-1986, 1990-2019	Vital Registration	VR/SRS

Switzerland	1950-2021	Vital Registration	VR/SRS
UK	1950-1999, 2001-2020	Vital Registration	VR/SRS
England	1981-2021	Vital Registration	VR/SRS
East Midlands	1981-2017, 2019	Vital Registration	VR/SRS
Derby	1981-2021	Vital Registration	VR/SRS
Derbyshire	1981-2021	Vital Registration	VR/SRS
Leicester	1981-2021	Vital Registration	VR/SRS
Leicestershire	1981-2021	Vital Registration	VR/SRS
Lincolnshire	1981-2021	Vital Registration	VR/SRS
Northamptonshire	1981-2020	Vital Registration	VR/SRS
Nottingham	1981-2021	Vital Registration	VR/SRS
Nottinghamshire	1981-2021	Vital Registration	VR/SRS
Rutland	1981-2021	Vital Registration	VR/SRS
East of England	1981-2017, 2019	Vital Registration	VR/SRS
Bedford	1981-2021	Vital Registration	VR/SRS
Cambridgeshire	1981-2021	Vital Registration	VR/SRS
Central Bedfordshire	1981-2021	Vital Registration	VR/SRS
Essex	1981-2021	Vital Registration	VR/SRS
Hertfordshire	1981-2021	Vital Registration	VR/SRS
Luton	1981-2021	Vital Registration	VR/SRS
Norfolk	1981-2021	Vital Registration	VR/SRS
Peterborough	1981-2021	Vital Registration	VR/SRS
Southend-on-Sea	1981-2021	Vital Registration	VR/SRS
Suffolk	1981-2021	Vital Registration	VR/SRS
Thurrock	1981-2021	Vital Registration	VR/SRS
Greater London	1981-2017, 2019	Vital Registration	VR/SRS
Barking and Dagenham	1981-2021	Vital Registration	VR/SRS
Barnet	1981-2021	Vital Registration	VR/SRS
Bexley	1981-2021	Vital Registration	VR/SRS
Brent	1981-2021	Vital Registration	VR/SRS
Bromley	1981-2021	Vital Registration	VR/SRS
Camden	1981-2021	Vital Registration	VR/SRS
Croydon	1981-2021	Vital Registration	VR/SRS
Ealing	1981-2021	Vital Registration	VR/SRS
Enfield	1981-2021	Vital Registration	VR/SRS
Greenwich	1981-2021	Vital Registration	VR/SRS
Hackney	1981-2017, 2019	Vital Registration	VR/SRS
Hammersmith and Fulham	1981-2021	Vital Registration	VR/SRS
Haringey	1981-2021	Vital Registration	VR/SRS
Harrow	1981-2021	Vital Registration	VR/SRS
Havering	1981-2021	Vital Registration	VR/SRS
Hillingdon	1981-2021	Vital Registration	VR/SRS
Hounslow	1981-2021	Vital Registration	VR/SRS
Islington	1981-2021	Vital Registration	VR/SRS
Kensington and Chelsea	1981-2021	Vital Registration	VR/SRS
Kingston upon Thames	1981-2021	Vital Registration	VR/SRS
Lambeth	1981-2021	Vital Registration	VR/SRS
Lewisham	1981-2021	Vital Registration	VR/SRS
Merton	1981-2021	Vital Registration	VR/SRS
Newham	1981-2021	Vital Registration	VR/SRS
Redbridge	1981-2021	Vital Registration	VR/SRS
Richmond upon Thames	1981-2021	Vital Registration	VR/SRS
Southwark	1981-2021	Vital Registration	VR/SRS
Sutton	1981-2021	Vital Registration	VR/SRS
Tower Hamlets	1981-2021	Vital Registration	VR/SRS
Waltham Forest	1981-2021	Vital Registration	VR/SRS
Wandsworth	1981-2021	Vital Registration	VR/SRS
Westminster	1981-2021	Vital Registration	VR/SRS
North East England	1981-2017, 2019	Vital Registration	VR/SRS
County Durham	1981-2021	Vital Registration	VR/SRS
Darlington	1981-2021	Vital Registration	VR/SRS
Gateshead	1981-2021	Vital Registration	VR/SRS
Hartlepool	1981-2021	Vital Registration	VR/SRS
Middlesbrough	1981-2021	Vital Registration	VR/SRS
Newcastle upon Tyne	1981-2021	Vital Registration	VR/SRS
North Tyneside	1981-2021	Vital Registration	VR/SRS
Northumberland	1981-2021	Vital Registration	VR/SRS
Redcar and Cleveland	1981-2021	Vital Registration	VR/SRS
South Tyneside	1981-2021	Vital Registration	VR/SRS

Stockton-on-Tees	1981-2021	Vital Registration	VR/SRS
Sunderland	1981-2021	Vital Registration	VR/SRS
North West England	1981-2017, 2019	Vital Registration	VR/SRS
Blackburn with Darwen	1981-2021	Vital Registration	VR/SRS
Blackpool	1981-2021	Vital Registration	VR/SRS
Bolton	1981-2021	Vital Registration	VR/SRS
Bury	1981-2021	Vital Registration	VR/SRS
Cheshire East	1981-2021	Vital Registration	VR/SRS
Cheshire West and Chester	1981-2021	Vital Registration	VR/SRS
Cumbria	1981-2021	Vital Registration	VR/SRS
Halton	1981-2021	Vital Registration	VR/SRS
Knowsley	1981-2021	Vital Registration	VR/SRS
Lancashire	1981-2021	Vital Registration	VR/SRS
Liverpool	1981-2021	Vital Registration	VR/SRS
Manchester	1981-2021	Vital Registration	VR/SRS
Oldham	1981-2021	Vital Registration	VR/SRS
Rochdale	1981-2021	Vital Registration	VR/SRS
Salford	1981-2021	Vital Registration	VR/SRS
Sefton	1981-2021	Vital Registration	VR/SRS
St Helens	1981-2021	Vital Registration	VR/SRS
Stockport	1981-2021	Vital Registration	VR/SRS
Tameside	1981-2021	Vital Registration	VR/SRS
Trafford	1981-2021	Vital Registration	VR/SRS
Warrington	1981-2021	Vital Registration	VR/SRS
Wigan	1981-2021	Vital Registration	VR/SRS
Wirral	1981-2021	Vital Registration	VR/SRS
South East England	1981-2017, 2019	Vital Registration	VR/SRS
Bracknell Forest	1981-2021	Vital Registration	VR/SRS
Brighton and Hove	1981-2021	Vital Registration	VR/SRS
Buckinghamshire	1981-2021	Vital Registration	VR/SRS
East Sussex	1981-2021	Vital Registration	VR/SRS
Hampshire	1981-2021	Vital Registration	VR/SRS
Isle of Wight	1981-2021	Vital Registration	VR/SRS
Kent	1981-2021	Vital Registration	VR/SRS
Medway	1981-2021	Vital Registration	VR/SRS
Milton Keynes	1981-2021	Vital Registration	VR/SRS
Oxfordshire	1981-2021	Vital Registration	VR/SRS
Portsmouth	1981-2021	Vital Registration	VR/SRS
Reading	1981-2021	Vital Registration	VR/SRS
Slough	1981-2021	Vital Registration	VR/SRS
Southampton	1981-2021	Vital Registration	VR/SRS
Surrey	1981-2021	Vital Registration	VR/SRS
West Berkshire	1981-2021	Vital Registration	VR/SRS
West Sussex	1981-2021	Vital Registration	VR/SRS
Windsor and Maidenhead	1981-2021	Vital Registration	VR/SRS
Wokingham	1981-2021	Vital Registration	VR/SRS
South West England	1981-2017, 2019	Vital Registration	VR/SRS
Bath and North East Somerset	1981-2021	Vital Registration	VR/SRS
Bournemouth	1981-2020	Vital Registration	VR/SRS
Bristol, City of	1981-2021	Vital Registration	VR/SRS
Cornwall	1981-2021	Vital Registration	VR/SRS
Devon	1981-2021	Vital Registration	VR/SRS
Dorset	1981-2021	Vital Registration	VR/SRS
Gloucestershire	1981-2021	Vital Registration	VR/SRS
North Somerset	1981-2021	Vital Registration	VR/SRS
Plymouth	1981-2021	Vital Registration	VR/SRS
Poole	1981-2020	Vital Registration	VR/SRS
Somerset	1981-2021	Vital Registration	VR/SRS
South Gloucestershire	1981-2021	Vital Registration	VR/SRS
Swindon	1981-2021	Vital Registration	VR/SRS
Torbay	1981-2021	Vital Registration	VR/SRS
Wiltshire	1981-2021	Vital Registration	VR/SRS
West Midlands	1981-2017, 2019	Vital Registration	VR/SRS
Birmingham	1981-2021	Vital Registration	VR/SRS
Coventry	1981-2021	Vital Registration	VR/SRS
Dudley	1981-2021	Vital Registration	VR/SRS
Herefordshire, County of	1981-2021	Vital Registration	VR/SRS
Sandwell	1981-2021	Vital Registration	VR/SRS
Shropshire	1981-2021	Vital Registration	VR/SRS

Solihull	1981-2021	Vital Registration	VR/SRS
Staffordshire	1981-2021	Vital Registration	VR/SRS
Stoke-on-Trent	1981-2021	Vital Registration	VR/SRS
Telford and Wrekin	1981-2021	Vital Registration	VR/SRS
Walsall	1981-2021	Vital Registration	VR/SRS
Warwickshire	1981-2021	Vital Registration	VR/SRS
Wolverhampton	1981-2021	Vital Registration	VR/SRS
Worcestershire	1981-2021	Vital Registration	VR/SRS
Yorkshire and the Humber	1981-2017, 2019	Vital Registration	VR/SRS
Barnsley	1981-2021	Vital Registration	VR/SRS
Bradford	1981-2021	Vital Registration	VR/SRS
Calderdale	1981-2021	Vital Registration	VR/SRS
Doncaster	1981-2021	Vital Registration	VR/SRS
East Riding of Yorkshire	1981-2021	Vital Registration	VR/SRS
Kingston upon Hull, City of	1981-2021	Vital Registration	VR/SRS
Kirklees	1981-2021	Vital Registration	VR/SRS
Leeds	1981-2021	Vital Registration	VR/SRS
North East Lincolnshire	1981-2021	Vital Registration	VR/SRS
North Lincolnshire	1981-2021	Vital Registration	VR/SRS
North Yorkshire	1981-2021	Vital Registration	VR/SRS
Rotherham	1981-2021	Vital Registration	VR/SRS
Sheffield	1981-2021	Vital Registration	VR/SRS
Wakefield	1981-2021	Vital Registration	VR/SRS
York	1981-2021	Vital Registration	VR/SRS
Northern Ireland	1950-1978, 1980-2020	Vital Registration	VR/SRS
Scotland	1950-2021	Vital Registration	VR/SRS
Wales	1981-2021	Vital Registration	VR/SRS
Bolivia	1961-2007	Standard Demographic and Health Survey	CBH
Ecuador	1954-1994	Vital Registration	VR/SRS
Ecuador	1967-2004	Reproductive Health Survey	CBH
Peru	1961-2014	Standard Demographic and Health Survey	CBH
Antigua and Barbuda	1950-1966, 1969-1978, 1983, 1985-2009, 2012-2017, 2019-2020	Vital Registration	VR/SRS
The Bahamas	1965, 1967-2015	Vital Registration	VR/SRS
Barbados	1950-1997, 2000-2013	Vital Registration	VR/SRS
Belize	1966-1991, 1993, 1996	Census	SBH
Belize	1973-1999	Reproductive Health Survey	CBH
Belize	1966-1999	Reproductive Health Survey	SBH
Belize	1990-2015	Multiple Indicator Cluster Survey	CBH
Belize	1981-2011	Multiple Indicator Cluster Survey	SBH
Bermuda	1950-2016, 2018	Vital Registration	VR/SRS
Cuba	1959-2020	Vital Registration	VR/SRS
Dominica	1950-1963, 1966-2015	Vital Registration	VR/SRS
Dominican Republic	1959-2013	Standard Demographic and Health Survey	CBH
Grenada	1950-1969, 1974-1978, 1985, 1988-2018	Vital Registration	VR/SRS
Guyana	1982-2009	Standard Demographic and Health Survey	CBH
Haiti	1971-2016	Standard Demographic and Health Survey	CBH
Jamaica	1985, 1990, 1995	Reproductive Health Survey	CBH
Jamaica	1975-2005	Multiple Indicator Cluster Survey	SBH
Puerto Rico	1950-2020	Vital Registration	VR/SRS
Saint Kitts and Nevis	1950-1996, 1998-2016	Vital Registration	VR/SRS
Saint Lucia	1950-1961, 1963, 1968, 1972-2006, 2008-2015	Vital Registration	VR/SRS
Saint Vincent and the Grenadines	1950-1956, 1960-1964, 1970-1972, 1974, 1977-1980, 1982-1988, 1990, 1992, 1995-2017, 2019	Vital Registration	VR/SRS
Suriname	1996, 2001, 2006, 2011, 2016	Multiple Indicator Cluster Survey	CBH
Suriname	1975-2005	Multiple Indicator Cluster Survey	SBH
Trinidad and Tobago	1962-1987	Standard Demographic and Health Survey	CBH
Virgin Islands	1950-1966, 1971-1973, 1980-1987, 1989, 1993-2012, 2016-2020	Vital Registration	VR/SRS
Colombia	1961-2015	Standard Demographic and Health Survey	CBH
Costa Rica	1950, 1952-2021	Vital Registration	VR/SRS
Costa Rica	1950-1999	Census	SBH
Costa Rica	1969-1992	Reproductive Health Survey	CBH
Costa Rica	1961-1992	Reproductive Health Survey	SBH
El Salvador	1983-1984	Standard Demographic and Health Survey	CBH
Guatemala	1966-1998	Standard Demographic and Health Survey	CBH
Guatemala	1975-2008	Reproductive Health Survey	CBH
Honduras	1976-2011	Standard Demographic and Health Survey	CBH
Mexico	1960-1987	Standard Demographic and Health Survey	CBH
Aguascalientes	2010-2021	Vital Registration	VR/SRS
Baja California	1979-2021	Vital Registration	VR/SRS
Baja California Sur	1979-2021	Vital Registration	VR/SRS

Campeche	2010-2021	Vital Registration	VR/SRS
Chiapas	2010-2021	Vital Registration	VR/SRS
Chihuahua	2010-2021	Vital Registration	VR/SRS
Coahuila	2010-2021	Vital Registration	VR/SRS
Colima	2010-2021	Vital Registration	VR/SRS
Durango	2010-2021	Vital Registration	VR/SRS
Guanajuato	1979-2021	Vital Registration	VR/SRS
Guerrero	1965-2015	Census	SBH
Guerrero	1973-1992	Other Survey	CBH
Guerrero	1967-2014	Other Survey	SBH
Hidalgo	2010-2021	Vital Registration	VR/SRS
Jalisco	2010-2021	Vital Registration	VR/SRS
México	1979-2021	Vital Registration	VR/SRS
Mexico City	1979-2015, 2017-2021	Vital Registration	VR/SRS
Michoacán de Ocampo	1965-2015	Census	SBH
Michoacán de Ocampo	1971-1992	Other Survey	CBH
Michoacán de Ocampo	1967-2014	Other Survey	SBH
Morelos	2010-2021	Vital Registration	VR/SRS
Nayarit	2010-2021	Vital Registration	VR/SRS
Nuevo León	2010-2021	Vital Registration	VR/SRS
Oaxaca	1965-2015	Census	SBH
Oaxaca	1971-1992	Other Survey	CBH
Oaxaca	1967-2014	Other Survey	SBH
Puebla	2010-2021	Vital Registration	VR/SRS
Querétaro	1979-2021	Vital Registration	VR/SRS
Quintana Roo	2010-2021	Vital Registration	VR/SRS
San Luis Potosí	1965-2015	Census	SBH
San Luis Potosí	1971-1992	Other Survey	CBH
San Luis Potosí	1967-2014	Other Survey	SBH
Sinaloa	2010-2021	Vital Registration	VR/SRS
Sonora	2010-2021	Vital Registration	VR/SRS
Tabasco	2005-2021	Vital Registration	VR/SRS
Tamaulipas	2005-2021	Vital Registration	VR/SRS
Tlaxcala	1979-2021	Vital Registration	VR/SRS
Veracruz de Ignacio de la Llave	2010-2021	Vital Registration	VR/SRS
Yucatán	2010-2021	Vital Registration	VR/SRS
Zacatecas	2010-2021	Vital Registration	VR/SRS
Nicaragua	1968-2001	Standard Demographic and Health Survey	CBH
Panama	1965-2009	Census	SBH
Panama	1972-2008	Living Standards Measurement Study	SBH
Venezuela	1950-1967, 1969-1994, 1996-2017	Vital Registration	VR/SRS
Brazil	2019-2021	Vital Registration	VR/SRS
Brazil	2010-2018	Other Survey	CBH
Brazil	1972-1973, 1975-2015	Other Survey	SBH
Acre	2019-2021	Vital Registration	VR/SRS
Acre	1990-2018	Other Survey	CBH
Acre	1981-2015	Other Survey	SBH
Alagoas	2019-2021	Vital Registration	VR/SRS
Alagoas	1990-2018	Other Survey	CBH
Alagoas	1973, 1976-2015	Other Survey	SBH
Amapá	1979-2009, 2019-2021	Vital Registration	VR/SRS
Amapá	1990-2018	Other Survey	CBH
Amapá	1981-2015	Other Survey	SBH
Amazonas	2019-2021	Vital Registration	VR/SRS
Amazonas	1990-2018	Other Survey	CBH
Amazonas	1981-2015	Other Survey	SBH
Bahia	2019-2021	Vital Registration	VR/SRS
Bahia	1990-2018	Other Survey	CBH
Bahia	1973-2015	Other Survey	SBH
Ceará	2019-2021	Vital Registration	VR/SRS
Ceará	1990-2018	Other Survey	CBH
Ceará	1973-1974, 1976-2015	Other Survey	SBH
Distrito Federal	1979-2009, 2019-2021	Vital Registration	VR/SRS
Distrito Federal	1990-2018	Other Survey	CBH
Distrito Federal	1972-2015	Other Survey	SBH
Espírito Santo	1979-1999, 2019-2021	Vital Registration	VR/SRS
Espírito Santo	1990-2018	Other Survey	CBH
Espírito Santo	1971-1972, 1975-2015	Other Survey	SBH
Goiás	2019-2021	Vital Registration	VR/SRS

Goiás	1990-2018	Other Survey	CBH
Goiás	1991-2015	Other Survey	SBH
Maranhão	2019-2021	Vital Registration	VR/SRS
Maranhão	1990-2018	Other Survey	CBH
Maranhão	1973, 1975-2015	Other Survey	SBH
Mato Grosso	2019-2021	Vital Registration	VR/SRS
Mato Grosso	1990-2018	Other Survey	CBH
Mato Grosso	1972, 1974-2015	Other Survey	SBH
Mato Grosso do Sul	1979-2009, 2019-2021	Vital Registration	VR/SRS
Mato Grosso do Sul	1990-2018	Other Survey	CBH
Mato Grosso do Sul	1972-1973, 1975-2015	Other Survey	SBH
Minas Gerais	2019-2021	Vital Registration	VR/SRS
Minas Gerais	1990-2018	Other Survey	CBH
Minas Gerais	1972-1973, 1975-1976, 1978-2015	Other Survey	SBH
Pará	2019-2021	Vital Registration	VR/SRS
Pará	1990-2018	Other Survey	CBH
Pará	1981-2015	Other Survey	SBH
Paraíba	2019-2021	Vital Registration	VR/SRS
Paraíba	1990-2018	Other Survey	CBH
Paraíba	1973-1974, 1976-2015	Other Survey	SBH
Paraná	1979-2009, 2019-2021	Vital Registration	VR/SRS
Paraná	1990-2018	Other Survey	CBH
Paraná	1972-1973, 1975-2015	Other Survey	SBH
Pernambuco	2019-2021	Vital Registration	VR/SRS
Pernambuco	1990-2018	Other Survey	CBH
Pernambuco	1973, 1976-1977, 1979-2015	Other Survey	SBH
Piauí	2019-2021	Vital Registration	VR/SRS
Piauí	1950-1991, 1994, 1997	Census	SBH
Piauí	1990-2018	Other Survey	CBH
Piauí	1973-1975, 1977-2015	Other Survey	SBH
Rio de Janeiro	1979-2009, 2019-2021	Vital Registration	VR/SRS
Rio de Janeiro	1990-2018	Other Survey	CBH
Rio de Janeiro	1971-1972, 1974-2015	Other Survey	SBH
Rio Grande do Norte	2019-2021	Vital Registration	VR/SRS
Rio Grande do Norte	1990-2018	Other Survey	CBH
Rio Grande do Norte	1972, 1974, 1976-2015	Other Survey	SBH
Rio Grande do Sul	1979-2009, 2019-2021	Vital Registration	VR/SRS
Rio Grande do Sul	1990-2018	Other Survey	CBH
Rio Grande do Sul	1971-1972, 1974-2015	Other Survey	SBH
Rondônia	2019-2021	Vital Registration	VR/SRS
Rondônia	1990-2018	Other Survey	CBH
Rondônia	1981-2015	Other Survey	SBH
Roraima	2019-2021	Vital Registration	VR/SRS
Roraima	1990-2018	Other Survey	CBH
Roraima	1981-2015	Other Survey	SBH
Santa Catarina	1979-2009, 2019-2021	Vital Registration	VR/SRS
Santa Catarina	1990-2018	Other Survey	CBH
Santa Catarina	1972-1973, 1975-2015	Other Survey	SBH
São Paulo	1979-2009, 2019-2021	Vital Registration	VR/SRS
São Paulo	1990-2018	Other Survey	CBH
São Paulo	1971-1972, 1974-2015	Other Survey	SBH
Sergipe	2019-2021	Vital Registration	VR/SRS
Sergipe	1990-2018	Other Survey	CBH
Sergipe	1973-1974, 1976-2015	Other Survey	SBH
Tocantins	2019-2021	Vital Registration	VR/SRS
Tocantins	1990-2018	Other Survey	CBH
Tocantins	1981-2015	Other Survey	SBH
Paraguay	1965-1990	Standard Demographic and Health Survey	CBH
Paraguay	1970-2008	Reproductive Health Survey	CBH
Paraguay	1989-2016	Multiple Indicator Cluster Survey	CBH
Afghanistan	1984-2015	Standard Demographic and Health Survey	CBH
Afghanistan	1991-2015	Standard Demographic and Health Survey	SBH
Afghanistan	1983, 1986-2010	Multiple Indicator Cluster Survey	SBH
Afghanistan	1982-2006, 2009	Other Survey	SBH
Algeria	1980-1982, 1985-1986, 1990, 1998, 2000, 2007-2011, 2014-2016	Vital Registration	VR/SRS
Algeria	1975-2002	Pan Arab Project for Family Health	CBH
Algeria	1961-1992	Pan Arab Project for Child Development	CBH
Bahrain	1980-1982, 1984-2012, 2014-2019	Vital Registration	VR/SRS
Bahrain	1952, 1956, 1959, 1962, 1965, 1967, 1973, 1975, 1979, 1982, 1985, 1987-1988, 1992, 1995, 1998	Census	SBH

Egypt	1959-2013	Standard Demographic and Health Survey	CBH
Iran	2015-2016, 2020	Vital Registration	VR/SRS
Iran	2005, 2007	Standard Demographic and Health Survey	CBH
Iran	1967-2000	Other Survey	CBH
Iran	1975-1999	Other Survey	SBH
Alborz	2015-2016	Vital Registration	VR/SRS
Alborz	1975-2000	Standard Demographic and Health Survey	CBH
Ardebil	2015-2016	Vital Registration	VR/SRS
Ardebil	1975-2000	Standard Demographic and Health Survey	CBH
Bushehr	2015-2016	Vital Registration	VR/SRS
Bushehr	1975-2000	Standard Demographic and Health Survey	CBH
Chahar Mahaal and Bakhtiari	2015-2016	Vital Registration	VR/SRS
Chahar Mahaal and Bakhtiari	1975-2000	Standard Demographic and Health Survey	CBH
East Azarbayejan	2015-2016	Vital Registration	VR/SRS
East Azarbayejan	1975-2000	Standard Demographic and Health Survey	CBH
Fars	2015-2016	Vital Registration	VR/SRS
Fars	1975-2000	Standard Demographic and Health Survey	CBH
Gilan	2015-2016	Vital Registration	VR/SRS
Gilan	1975-2000	Standard Demographic and Health Survey	CBH
Golestan	2015-2016	Vital Registration	VR/SRS
Golestan	1975-2000	Standard Demographic and Health Survey	CBH
Hamadan	2015-2016	Vital Registration	VR/SRS
Hamadan	1973-2000	Standard Demographic and Health Survey	CBH
Hormozgan	2015-2016	Vital Registration	VR/SRS
Hormozgan	1975-2000	Standard Demographic and Health Survey	CBH
Ilam	2015-2016	Vital Registration	VR/SRS
Ilam	1973-2000	Standard Demographic and Health Survey	CBH
Isfahan	2015-2016	Vital Registration	VR/SRS
Isfahan	1975-2000	Standard Demographic and Health Survey	CBH
Kerman	2015-2016	Vital Registration	VR/SRS
Kerman	1975-2000	Standard Demographic and Health Survey	CBH
Kermanshah	2015-2016	Vital Registration	VR/SRS
Kermanshah	1975-2000	Standard Demographic and Health Survey	CBH
Khorasan-e-Razavi	2015-2016	Vital Registration	VR/SRS
Khorasan-e-Razavi	1975-2000	Standard Demographic and Health Survey	CBH
Khuzestan	2015-2016	Vital Registration	VR/SRS
Khuzestan	1973-2000	Standard Demographic and Health Survey	CBH
Kohgiluyeh and Boyer-Ahmad	2015-2016	Vital Registration	VR/SRS
Kohgiluyeh and Boyer-Ahmad	1975-2000	Standard Demographic and Health Survey	CBH
Kurdistan	2015-2016	Vital Registration	VR/SRS
Kurdistan	1975-2000	Standard Demographic and Health Survey	CBH
Lorestan	2015-2016	Vital Registration	VR/SRS
Lorestan	1973-2000	Standard Demographic and Health Survey	CBH
Markazi	2015-2016	Vital Registration	VR/SRS
Markazi	1975-2000	Standard Demographic and Health Survey	CBH
Mazandaran	2015-2016	Vital Registration	VR/SRS
Mazandaran	1975-2000	Standard Demographic and Health Survey	CBH
North Khorasan	2015-2016	Vital Registration	VR/SRS
North Khorasan	1975-2000	Standard Demographic and Health Survey	CBH
Qazvin	2015-2016	Vital Registration	VR/SRS
Qazvin	1975-2000	Standard Demographic and Health Survey	CBH
Qom	2015-2016	Vital Registration	VR/SRS
Qom	1975-2000	Standard Demographic and Health Survey	CBH
Semnan	2015-2016	Vital Registration	VR/SRS
Semnan	1977-2000	Standard Demographic and Health Survey	CBH
Sistan and Baluchistan	2015-2016	Vital Registration	VR/SRS
Sistan and Baluchistan	1975-2000	Standard Demographic and Health Survey	CBH
South Khorasan	2015-2016	Vital Registration	VR/SRS
South Khorasan	1975-2000	Standard Demographic and Health Survey	CBH
Tehran	2015-2016	Vital Registration	VR/SRS
Tehran	1981-2000	Standard Demographic and Health Survey	CBH
West Azarbayejan	2015-2016	Vital Registration	VR/SRS
West Azarbayejan	1975-2000	Standard Demographic and Health Survey	CBH
Yazd	2015-2016	Vital Registration	VR/SRS
Yazd	1975-2000	Standard Demographic and Health Survey	CBH
Zanjan	2015-2016	Vital Registration	VR/SRS
Zanjan	1975-2000	Standard Demographic and Health Survey	CBH
Iraq	1976-2017	Multiple Indicator Cluster Survey	CBH
Iraq	1981-2010	Multiple Indicator Cluster Survey	SBH

Jordan	1963-1966, 1968-2017	Standard Demographic and Health Survey	CBH
Kuwait	1962-1989, 1991-2020	Vital Registration	VR/SRS
Lebanon	1979-2004	Pan Arab Project for Family Health	CBH
Lebanon	1979-2003	Pan Arab Project for Family Health	SBH
Lebanon	1966-1995	Pan Arab Project for Child Development	CBH
Lebanon	1971-1995	Pan Arab Project for Child Development	SBH
Lebanon	1998, 2009	Multiple Indicator Cluster Survey	SBH
Libya	1972-1976, 1981, 1996, 2000, 2002, 2006-2011, 2016-2017	Vital Registration	VR/SRS
Libya	1983-2007	Pan Arab Project for Family Health	SBH
Libya	1964-1995	Pan Arab Project for Child Development	CBH
Libya	1970-1994	Pan Arab Project for Child Development	SBH
Libya	2003	Multiple Indicator Cluster Survey	SBH
Morocco	1958-2003	Standard Demographic and Health Survey	CBH
Oman	2004-2014, 2016-2020	Vital Registration	VR/SRS
Palestine	1973-1998, 2001, 2003	Census	SBH
Palestine	1992	Other Demographic and Health Survey	CBH
Palestine	1980-2004	Other Demographic and Health Survey	SBH
Palestine	2004	Pan Arab Project for Family Health	CBH
Palestine	1982-2006	Pan Arab Project for Family Health	SBH
Palestine	1977-2013, 2018	Multiple Indicator Cluster Survey	CBH
Palestine	1975-2013	Multiple Indicator Cluster Survey	SBH
Palestine	1970-2003	Other Survey	CBH
Qatar	1981-2020	Vital Registration	VR/SRS
Qatar	1979, 1984, 1989, 1994	Pan Arab Project for Family Health	CBH
Qatar	1970, 1975, 1980, 1985	Pan Arab Project for Child Development	CBH
Qatar	1973, 1975, 1980, 1985	Pan Arab Project for Child Development	SBH
Saudi Arabia	2011-2012	Vital Registration	VR/SRS
Sudan	1982-2014	Multiple Indicator Cluster Survey	CBH
Syria	2000-2010	Vital Registration	VR/SRS
Syria	1953, 1956, 1959, 1961, 1964, 1966, 1976, 1980, 1983, 1985, 1988, 1990	Census	SBH
Tunisia	1963-1988	Standard Demographic and Health Survey	CBH
Türkiye	1966-2008	Standard Demographic and Health Survey	CBH
United Arab Emirates	1995-1999, 2001-2003, 2005-2008, 2017-2020	Vital Registration	VR/SRS
United Arab Emirates	1956, 1959, 1963, 1966, 1969, 1971	Census	SBH
Yemen	1962-2013	Standard Demographic and Health Survey	CBH
Yemen	1973-2002	Pan Arab Project for Family Health	CBH
Bangladesh	1964-1970, 1972-1973, 1975-2014, 2016	Standard Demographic and Health Survey	CBH
Bangladesh	1951-1970, 1972-1973, 1975	World Fertility Survey	SBH
Bhutan	1980-2017	Census	SBH
Bhutan	1985-2009	Multiple Indicator Cluster Survey	SBH
Bhutan	1992-1993	Other Survey	CBH
Bhutan	1989-2013	Other Survey	SBH
India	1988-2020	Sample Registrations System	VR/SRS
India	1960-2014	Standard Demographic and Health Survey	CBH
Nepal	1968-2015	Standard Demographic and Health Survey	CBH
Pakistan	1969-2012, 2015	Standard Demographic and Health Survey	CBH
Azad Jammu & Kashmir	1977-2001	Other Survey	SBH
Balochistan	1975-2012	Standard Demographic and Health Survey	CBH
Gilgit-Baltistan	1993-2012	Standard Demographic and Health Survey	CBH
Islamabad Capital Territory	1995-2012	Standard Demographic and Health Survey	CBH
Khyber Pakhtunkhwa	1971-2012	Standard Demographic and Health Survey	CBH
Khyber Pakhtunkhwa	2017	Multiple Indicator Cluster Survey	CBH
Punjab	1969-2012	Standard Demographic and Health Survey	CBH
Sindh	1969-2012	Standard Demographic and Health Survey	CBH
North Korea	2005, 2010, 2015	Multiple Indicator Cluster Survey	CBH
Taiwan (province of China)	1955-2021	Vital Registration	VR/SRS
American Samoa	1952-1956, 1958-1973, 1976, 1982, 1984-1993, 1997-2017, 2020	Vital Registration	VR/SRS
Cook Islands	1951-1960, 1965, 1968, 1971-1973, 1975-1977, 1979-1988, 2000-2016	Vital Registration	VR/SRS
Fiji	1967, 1970, 1974, 1976-1977, 1980, 1982-1983, 1987-1988, 1990-1992, 1995, 1998, 2001, 2004	Census	SBH
Guam	1950-1992, 1994-2020	Vital Registration	VR/SRS
Kiribati	1987, 1989-1990, 1992, 1994-1995, 1997-1998, 2000-2002, 2004	Census	SBH
Kiribati	2007, 2012, 2017	Multiple Indicator Cluster Survey	CBH
Kiribati	1996, 2001, 2006	Other Survey	CBH
Kiribati	1991, 1994, 1997, 2001, 2004, 2006	Other Survey	SBH
Marshall Islands	1986-1997, 2005-2006	Vital Registration	VR/SRS
Marshall Islands	1981, 1984, 1987, 1990-1992, 1995, 1999, 2002, 2005, 2008	Census	SBH
Marshall Islands	1995, 2000, 2005	Standard Demographic and Health Survey	CBH
Federated States of Micronesia	2003	Vital Registration	VR/SRS
Federated States of Micronesia	1982, 1985, 1988, 1991, 1994, 1996	Census	SBH

Nauru	1965-1968, 1978, 1992-2002, 2011	Vital Registration	VR/SRS
Niue	1950-1961, 1966-1969, 1973, 1975, 1989-1991, 1993, 1995, 1997, 1999-2001, 2003-2004, 2006, 2008, 2011	Vital Registration	VR/SRS
Northern Mariana Islands	1998-2020	Vital Registration	VR/SRS
Palau	1999, 2003, 2005, 2013, 2017-2018	Vital Registration	VR/SRS
Papua New Guinea	1952, 1956, 1959, 1962-1963, 1965-1968, 1970-1971, 1974, 1976-1977, 1979, 1981, 1984, 1988, 1990, 1993, 1996, 1998	Census	SBH
Papua New Guinea	1979-2006, 2011, 2016	Standard Demographic and Health Survey	CBH
Papua New Guinea	1988, 1991, 1995, 1998, 2001, 2003, 2005	Standard Demographic and Health Survey	SBH
Papua New Guinea	1974, 1979, 1984, 1989, 1994	Other Demographic and Health Survey	CBH
Papua New Guinea	1972, 1976, 1979, 1982, 1985, 1987-1988, 1993	Other Demographic and Health Survey	SBH
Samoa	1950, 1952-1953, 1955-1958, 1960, 1962, 1965, 1968, 1970, 1972	Census	SBH
Samoa	2002, 2007, 2012	Standard Demographic and Health Survey	CBH
Solomon Islands	1952, 1955, 1958-1959, 1961, 1964, 1966-1967, 1969, 1972, 1981, 1984, 1987, 1990, 1993-1995, 1997, 2000, 2003, 2005	Census	SBH
Solomon Islands	1995, 2000, 2003, 2005, 2008, 2013	Standard Demographic and Health Survey	CBH
Tokelau	1955, 1971-1974, 1976, 1978-1979, 1982	Vital Registration	VR/SRS
Tonga	2006	Census	CBH
Tonga	1958, 1961, 1964, 1967-1968, 1970-1971, 1973-1974, 1977, 1980, 1982	Census	SBH
Tonga	2000, 2005, 2010	Other Demographic and Health Survey	CBH
Tonga	1992, 1996, 2000, 2003, 2006, 2008	Other Demographic and Health Survey	SBH
Tonga	2004, 2014	Multiple Indicator Cluster Survey	CBH
Tuvalu	1992-2002, 2005, 2016	Vital Registration	VR/SRS
Vanuatu	2014	Vital Registration	VR/SRS
Vanuatu	1952, 1956, 1959, 1961, 1963, 1991, 1994, 1998, 2001, 2003, 2006	Census	SBH
Vanuatu	2001, 2006, 2011	Other Demographic and Health Survey	CBH
Vanuatu	1994, 1997, 2001, 2004, 2006, 2009	Other Demographic and Health Survey	SBH
Vanuatu	1985-2006	Multiple Indicator Cluster Survey	SBH
Cambodia	1972-1974, 1981-2014	Standard Demographic and Health Survey	CBH
Indonesia	1958-2017	Standard Demographic and Health Survey	CBH
Aceh	1972-2017	Standard Demographic and Health Survey	CBH
Bali	1966-2015	Standard Demographic and Health Survey	CBH
Bangka-Belitung Islands	1978-2017	Standard Demographic and Health Survey	CBH
Banten	1972-2017	Standard Demographic and Health Survey	CBH
Bengkulu	1971-2015	Standard Demographic and Health Survey	CBH
Gorontalo	1976-2017	Standard Demographic and Health Survey	CBH
Jakarta	1962-2017	Standard Demographic and Health Survey	CBH
Jambi	1972-2017	Standard Demographic and Health Survey	CBH
West Java	1964-2017	Standard Demographic and Health Survey	CBH
Central Java	1964-2017	Standard Demographic and Health Survey	CBH
East Java	1962-2017	Standard Demographic and Health Survey	CBH
West Kalimantan	1968-2017	Standard Demographic and Health Survey	CBH
South Kalimantan	1966-2017	Standard Demographic and Health Survey	CBH
Central Kalimantan	1972-2017	Standard Demographic and Health Survey	CBH
East Kalimantan	1972-2017	Standard Demographic and Health Survey	CBH
North Kalimantan	1988-2017	Standard Demographic and Health Survey	CBH
Riau Islands	1981-2017	Standard Demographic and Health Survey	CBH
Lampung	1968-2017	Standard Demographic and Health Survey	CBH
Maluku	1991-2017	Standard Demographic and Health Survey	CBH
North Maluku	1990-2017	Standard Demographic and Health Survey	CBH
West Nusa Tenggara	1968-2017	Standard Demographic and Health Survey	CBH
East Nusa Tenggara	1972-2017	Standard Demographic and Health Survey	CBH
Papua	1992-2011	Standard Demographic and Health Survey	CBH
West Papua	1990-2012	Standard Demographic and Health Survey	CBH
Riau	1968-2017	Standard Demographic and Health Survey	CBH
West Sulawesi	1982-2017	Standard Demographic and Health Survey	CBH
South Sulawesi	1966-2017	Standard Demographic and Health Survey	CBH
Central Sulawesi	1970-2017	Standard Demographic and Health Survey	CBH
Southeast Sulawesi	1970-2017	Standard Demographic and Health Survey	CBH
North Sulawesi	1972-2017	Standard Demographic and Health Survey	CBH
West Sumatra	1968-2017	Standard Demographic and Health Survey	CBH
South Sumatra	1966-2015	Standard Demographic and Health Survey	CBH
North Sumatra	1966-2017	Standard Demographic and Health Survey	CBH
Yogyakarta	1950-2009	Census	SBH
Yogyakarta	1964-2017	Standard Demographic and Health Survey	CBH
Laos	1982-2017	Multiple Indicator Cluster Survey	CBH
Malaysia	1952-1972, 1976-1978, 1984-1986, 1990-2017, 2019-2020	Vital Registration	VR/SRS
Maldives	1980-2017	Standard Demographic and Health Survey	CBH
Mauritius	1950-2020	Vital Registration	VR/SRS
Myanmar	1988-2015	Standard Demographic and Health Survey	CBH
Philippines	1965-2017	Standard Demographic and Health Survey	CBH
Abra	1986-2010	Census	SBH

Abra	1987-2011	Standard Demographic and Health Survey	SBH
Agusan Del Norte	1986-2010	Census	SBH
Agusan Del Norte	1987-2011	Standard Demographic and Health Survey	SBH
Agusan Del Sur	1986-2010	Census	SBH
Agusan Del Sur	1987-2011	Standard Demographic and Health Survey	SBH
Aklan	1986-2010	Census	SBH
Aklan	1987-2011	Standard Demographic and Health Survey	SBH
Albay	1986-2010	Census	SBH
Albay	1987-2011	Standard Demographic and Health Survey	SBH
Antique	1986-2010	Census	SBH
Antique	1987-2011	Standard Demographic and Health Survey	SBH
Apayao	1986-2010	Census	SBH
Apayao	1987-2011	Standard Demographic and Health Survey	SBH
Aurora	1986-2010	Census	SBH
Aurora	1987-2011	Standard Demographic and Health Survey	SBH
Basilan	1986-2010	Census	SBH
Basilan	1987-2011	Standard Demographic and Health Survey	SBH
Bataan	1986-2010	Census	SBH
Bataan	1987-2011	Standard Demographic and Health Survey	SBH
Batanes	1986-2010	Census	SBH
Batanes	1987-2011	Standard Demographic and Health Survey	SBH
Batangas	1986-2010	Census	SBH
Batangas	1987-2011	Standard Demographic and Health Survey	SBH
Benguet	1986-2010	Census	SBH
Benguet	1987-2011	Standard Demographic and Health Survey	SBH
Biliran	1986-2010	Census	SBH
Biliran	1987-2011	Standard Demographic and Health Survey	SBH
Bohol	1986-2010	Census	SBH
Bohol	1987-2011	Standard Demographic and Health Survey	SBH
Bukidnon	1986-2010	Census	SBH
Bukidnon	1987-2011	Standard Demographic and Health Survey	SBH
Bulacan	1986-2010	Census	SBH
Bulacan	1987-2011	Standard Demographic and Health Survey	SBH
Cagayan	1986-2010	Census	SBH
Cagayan	1987-2011	Standard Demographic and Health Survey	SBH
Camarines Norte	1986-2010	Census	SBH
Camarines Norte	1987-2011	Standard Demographic and Health Survey	SBH
Camarines Sur	1986-2010	Census	SBH
Camarines Sur	1987-2011	Standard Demographic and Health Survey	SBH
Camiguin	1986-2010	Census	SBH
Camiguin	1987-2011	Standard Demographic and Health Survey	SBH
Capiz	1986-2010	Census	SBH
Capiz	1987-2011	Standard Demographic and Health Survey	SBH
Catanduanes	1986-2010	Census	SBH
Catanduanes	1987-2011	Standard Demographic and Health Survey	SBH
Cavite	1986-2010	Census	SBH
Cavite	1987-2011	Standard Demographic and Health Survey	SBH
Cebu	1986-2010	Census	SBH
Cebu	1987-2011	Standard Demographic and Health Survey	SBH
Cotabato (North Cotabato)	1986-2010	Census	SBH
Cotabato (North Cotabato)	1987-2011	Standard Demographic and Health Survey	SBH
Davao de Oro	1986-2010	Census	SBH
Davao de Oro	1987-2011	Standard Demographic and Health Survey	SBH
Davao Del Norte	1986-2010	Census	SBH
Davao Del Norte	1987-2011	Standard Demographic and Health Survey	SBH
Davao Del Sur	1986-2010	Census	SBH
Davao Del Sur	1987-2011	Standard Demographic and Health Survey	SBH
Davao Occidental	1986-2010	Census	SBH
Davao Occidental	1987-2011	Standard Demographic and Health Survey	SBH
Davao Oriental	1986-2010	Census	SBH
Davao Oriental	1987-2011	Standard Demographic and Health Survey	SBH
Dinagat Islands	1986-2010	Census	SBH
Eastern Samar	1986-2010	Census	SBH
Eastern Samar	1987-2011	Standard Demographic and Health Survey	SBH
Guimaras	1986-2010	Census	SBH
Guimaras	1987-2011	Standard Demographic and Health Survey	SBH
Ifugao	1986-2010	Census	SBH
Ifugao	1987-2011	Standard Demographic and Health Survey	SBH
Ilocos Norte	1986-2010	Census	SBH

Ilocos Norte	1987-2011	Standard Demographic and Health Survey	SBH
Ilocos Sur	1986-2010	Census	SBH
Ilocos Sur	1987-2011	Standard Demographic and Health Survey	SBH
Iloilo	1986-2010	Census	SBH
Iloilo	1987-2011	Standard Demographic and Health Survey	SBH
Isabela	1986-2010	Census	SBH
Isabela	1987-2011	Standard Demographic and Health Survey	SBH
Kalinga	1986-2010	Census	SBH
Kalinga	1987-2011	Standard Demographic and Health Survey	SBH
La Union	1986-2010	Census	SBH
La Union	1987-2011	Standard Demographic and Health Survey	SBH
Laguna	1986-2010	Census	SBH
Laguna	1987-2011	Standard Demographic and Health Survey	SBH
Lanao Del Norte	1986-2010	Census	SBH
Lanao Del Norte	1987-2011	Standard Demographic and Health Survey	SBH
Lanao Del Sur	1986-2010	Census	SBH
Lanao Del Sur	1987-2011	Standard Demographic and Health Survey	SBH
Leyte	1986-2010	Census	SBH
Leyte	1987-2011	Standard Demographic and Health Survey	SBH
Maguindanao	1986-2010	Census	SBH
Maguindanao	1987-2011	Standard Demographic and Health Survey	SBH
Marinduque	1986-2010	Census	SBH
Marinduque	1987-2011	Standard Demographic and Health Survey	SBH
Masbate	1986-2010	Census	SBH
Masbate	1987-2011	Standard Demographic and Health Survey	SBH
Misamis Occidental	1986-2010	Census	SBH
Misamis Occidental	1987-2011	Standard Demographic and Health Survey	SBH
Misamis Oriental	1986-2010	Census	SBH
Misamis Oriental	1987-2011	Standard Demographic and Health Survey	SBH
Mountain Province	1986-2010	Census	SBH
Mountain Province	1987-2011	Standard Demographic and Health Survey	SBH
National Capital Region	1986-2010	Census	SBH
National Capital Region	1987-2011	Standard Demographic and Health Survey	SBH
Negros Occidental	1986-2010	Census	SBH
Negros Occidental	1987-2011	Standard Demographic and Health Survey	SBH
Negros Oriental	1986-2010	Census	SBH
Negros Oriental	1987-2011	Standard Demographic and Health Survey	SBH
Northern Samar	1986-2010	Census	SBH
Northern Samar	1987-2011	Standard Demographic and Health Survey	SBH
Nueva Ecija	1986-2010	Census	SBH
Nueva Ecija	1987-2011	Standard Demographic and Health Survey	SBH
Nueva Vizcaya	1986-2010	Census	SBH
Nueva Vizcaya	1987-2011	Standard Demographic and Health Survey	SBH
Occidental Mindoro	1986-2010	Census	SBH
Occidental Mindoro	1987-2011	Standard Demographic and Health Survey	SBH
Oriental Mindoro	1986-2010	Census	SBH
Oriental Mindoro	1987-2011	Standard Demographic and Health Survey	SBH
Palawan	1986-2010	Census	SBH
Palawan	1987-2011	Standard Demographic and Health Survey	SBH
Pampanga	1986-2010	Census	SBH
Pampanga	1987-2011	Standard Demographic and Health Survey	SBH
Pangasinan	1986-2010	Census	SBH
Pangasinan	1987-2011	Standard Demographic and Health Survey	SBH
Quezon	1986-2010	Census	SBH
Quezon	1987-2011	Standard Demographic and Health Survey	SBH
Quirino	1986-2010	Census	SBH
Quirino	1987-2011	Standard Demographic and Health Survey	SBH
Rizal	1986-2010	Census	SBH
Rizal	1987-2011	Standard Demographic and Health Survey	SBH
Romblon	1986-2010	Census	SBH
Romblon	1987-2011	Standard Demographic and Health Survey	SBH
Samar (Western Samar)	1986-2010	Census	SBH
Samar (Western Samar)	1987-2011	Standard Demographic and Health Survey	SBH
Sarangani	1986-2010	Census	SBH
Sarangani	1987-2011	Standard Demographic and Health Survey	SBH
Siquijor	1986-2010	Census	SBH
Siquijor	1987-2011	Standard Demographic and Health Survey	SBH
Sorsogon	1986-2010	Census	SBH
Sorsogon	1987-2011	Standard Demographic and Health Survey	SBH

South Cotabato	1986-2010	Census	SBH
South Cotabato	1987-2011	Standard Demographic and Health Survey	SBH
Southern Leyte	1986-2010	Census	SBH
Southern Leyte	1987-2011	Standard Demographic and Health Survey	SBH
Sultan Kudarat	1986-2010	Census	SBH
Sultan Kudarat	1987-2011	Standard Demographic and Health Survey	SBH
Sulu	1989-2017	Standard Demographic and Health Survey	CBH
Surigao Del Norte	1986-2010	Census	SBH
Surigao Del Norte	1987-2011	Standard Demographic and Health Survey	SBH
Surigao Del Sur	1986-2010	Census	SBH
Surigao Del Sur	1987-2011	Standard Demographic and Health Survey	SBH
Tarlac	1986-2010	Census	SBH
Tarlac	1987-2011	Standard Demographic and Health Survey	SBH
Tawi-Tawi	1986-2010	Census	SBH
Tawi-Tawi	1987-2011	Standard Demographic and Health Survey	SBH
Zambales	1986-2010	Census	SBH
Zambales	1987-2011	Standard Demographic and Health Survey	SBH
Zamboanga Del Norte	1986-2010	Census	SBH
Zamboanga Del Norte	1987-2011	Standard Demographic and Health Survey	SBH
Zamboanga Del Sur	1986-2010	Census	SBH
Zamboanga Del Sur	1987-2011	Standard Demographic and Health Survey	SBH
Zamboanga Sibugay	1986-2010	Census	SBH
Zamboanga Sibugay	1987-2011	Standard Demographic and Health Survey	SBH
Seychelles	1952-1959, 1961-2021	Vital Registration	VR/SRS
Sri Lanka	1950-1989, 1991-1995, 1997-2004, 2006-2008, 2010-2014	Vital Registration	VR/SRS
Thailand	2010-2019	Vital Registration	VR/SRS
Thailand	1959-1986	Standard Demographic and Health Survey	CBH
Timor-Leste	1982-2016	Standard Demographic and Health Survey	CBH
Viet Nam	1974-2002	Standard Demographic and Health Survey	CBH
Angola	1988-2015	Standard Demographic and Health Survey	CBH
Central African Republic	1967-1994	Standard Demographic and Health Survey	CBH
Central African Republic	1970-1994	Standard Demographic and Health Survey	SBH
Central African Republic	1976-2010	Multiple Indicator Cluster Survey	SBH
Congo (Brazzaville)	1978-2011	Standard Demographic and Health Survey	CBH
DR Congo	1980-2013	Standard Demographic and Health Survey	CBH
Equatorial Guinea	2000, 2005, 2008	Standard Demographic and Health Survey	CBH
Gabon	1973-2011	Standard Demographic and Health Survey	CBH
Burundi	1964-2016	Standard Demographic and Health Survey	CBH
Comoros	1974-1995	Standard Demographic and Health Survey	CBH
Djibouti	1983-1996, 1999-2002	Pan Arab Project for Family Health	CBH
Djibouti	1977-2002	Pan Arab Project for Family Health	SBH
Djibouti	1982-2006	Multiple Indicator Cluster Survey	SBH
Djibouti	1972, 1975, 1979, 1983, 1986, 1989	Other Survey	SBH
Eritrea	1970-2001	Standard Demographic and Health Survey	CBH
Ethiopia	1970-2015, 2017	Standard Demographic and Health Survey	CBH
Addis Ababa	1975-2014	Standard Demographic and Health Survey	CBH
Afar	1975-2014	Standard Demographic and Health Survey	CBH
Amhara	1971-2014	Standard Demographic and Health Survey	CBH
Benishangul-Gumuz	1975-2014	Standard Demographic and Health Survey	CBH
Dire Dawa	1975-2014	Standard Demographic and Health Survey	CBH
Gambella	1975-2014	Standard Demographic and Health Survey	CBH
Harari	1975-2014	Standard Demographic and Health Survey	CBH
Oromia	1969-2014	Standard Demographic and Health Survey	CBH
Somali	1977-2014	Standard Demographic and Health Survey	CBH
Southern Nations, Nationalities, and Peop	1971-2014	Standard Demographic and Health Survey	CBH
Tigray	1975-2014	Standard Demographic and Health Survey	CBH
Kenya	1961-2014	Standard Demographic and Health Survey	CBH
Baringo	1984-2008	Standard Demographic and Health Survey	SBH
Bomet	1997-2014	Standard Demographic and Health Survey	CBH
Bungoma	1973-2014	Standard Demographic and Health Survey	CBH
Busia	1973-2014	Standard Demographic and Health Survey	CBH
Elgeyo Marakwet	1981-2014	Standard Demographic and Health Survey	CBH
Embu	1973-1974, 1979-1988, 1991-2014	Standard Demographic and Health Survey	CBH
Garissa	1987-2014	Standard Demographic and Health Survey	CBH
Homa Bay	1985-2014	Standard Demographic and Health Survey	CBH
Isiolo	1987-2011	Multiple Indicator Cluster Survey	SBH
Kajiado	1975-1982, 1984-1986, 1988-1989, 1992-2014	Standard Demographic and Health Survey	CBH
Kakamega	1971-2014	Standard Demographic and Health Survey	CBH
Kericho	1973-2014	Standard Demographic and Health Survey	CBH

Kiambu	1975-2014	Standard Demographic and Health Survey	CBH
Kilifi	1973-2014	Standard Demographic and Health Survey	CBH
Kirinyaga	1973-2014	Standard Demographic and Health Survey	CBH
Kisii	1971-2014	Standard Demographic and Health Survey	CBH
Kisumu	1973-2014	Standard Demographic and Health Survey	CBH
Kitui	1975-2014	Standard Demographic and Health Survey	CBH
Kwale	1971-2014	Standard Demographic and Health Survey	CBH
Laikipia	1977-1982, 1993-2014	Standard Demographic and Health Survey	CBH
Lamu	1987-1988, 1991-1992, 1997-2014	Standard Demographic and Health Survey	CBH
Machakos	1971-2014	Standard Demographic and Health Survey	CBH
Makueni	1985-1986, 1989-2014	Standard Demographic and Health Survey	CBH
Mandera	1982-2006	Multiple Indicator Cluster Survey	SBH
Marsabit	1978-2002	Standard Demographic and Health Survey	SBH
Meru	1973-2014	Standard Demographic and Health Survey	CBH
Migori	1987-2014	Standard Demographic and Health Survey	CBH
Mombasa	1973-2014	Standard Demographic and Health Survey	CBH
Murang'a	1971-2014	Standard Demographic and Health Survey	CBH
Nairobi	1973-2014	Standard Demographic and Health Survey	CBH
Nakuru	1975-2014	Standard Demographic and Health Survey	CBH
Nandi	1973-1997, 1999-2014	Standard Demographic and Health Survey	CBH
Narok	1975-1976, 1979-1980, 1983-2014	Standard Demographic and Health Survey	CBH
Nyamira	1982-2014	Standard Demographic and Health Survey	CBH
Nyandarua	1975-1994, 1996-2014	Standard Demographic and Health Survey	CBH
Nyeri	1973-2014	Standard Demographic and Health Survey	CBH
Samburu	1984-2008	Standard Demographic and Health Survey	SBH
Siaya	1973-2014	Standard Demographic and Health Survey	CBH
Taita Taveta	1975-2014	Standard Demographic and Health Survey	CBH
Tana River	1993-1996, 1999-2014	Standard Demographic and Health Survey	CBH
Tharaka Nithi	1991-1992, 1997-2014	Standard Demographic and Health Survey	CBH
Trans Nzoia	1975-2014	Standard Demographic and Health Survey	CBH
Turkana	1985-2014	Standard Demographic and Health Survey	CBH
Uasin Gishu	1973-2014	Standard Demographic and Health Survey	CBH
Vihiga	1987-2014	Standard Demographic and Health Survey	CBH
Wajir	1978-2002	Standard Demographic and Health Survey	SBH
West Pokot	1984-2008	Standard Demographic and Health Survey	SBH
Madagascar	1967-2008	Standard Demographic and Health Survey	CBH
Malawi	1967-2015	Standard Demographic and Health Survey	CBH
Mozambique	1976-2011	Standard Demographic and Health Survey	CBH
Rwanda	1967-1993, 1995-2014, 2018	Standard Demographic and Health Survey	CBH
Somalia	1983-2006	Multiple Indicator Cluster Survey	CBH
Somalia	1982-2006	Multiple Indicator Cluster Survey	SBH
South Sudan	1983-2007	Census	SBH
Uganda	1963-2016	Standard Demographic and Health Survey	CBH
Tanzania	1962-2015	Standard Demographic and Health Survey	CBH
Zambia	1964-2013, 2016	Standard Demographic and Health Survey	CBH
Botswana	1967-2011	Census	SBH
Botswana	1967-1988	Standard Demographic and Health Survey	CBH
Eswatini	1979-2006	Standard Demographic and Health Survey	CBH
Eswatini	1982-2006	Standard Demographic and Health Survey	SBH
Eswatini	1985-2014	Multiple Indicator Cluster Survey	CBH
Eswatini	1976-2014	Multiple Indicator Cluster Survey	SBH
Lesotho	1967, 1971-2006	Census	SBH
Lesotho	1979-2014	Standard Demographic and Health Survey	CBH
Lesotho	1980-2014	Standard Demographic and Health Survey	SBH
Lesotho	2006, 2011, 2016	Multiple Indicator Cluster Survey	CBH
Lesotho	1975-1999	Multiple Indicator Cluster Survey	SBH
Lesotho	1954-1977	World Fertility Survey	CBH
Lesotho	1953-1977	World Fertility Survey	SBH
Lesotho	1982, 1985, 1988, 1990, 1992, 1994-1995, 1997-1998, 2002, 2005, 2008	Other Survey	SBH
Namibia	1969-2013	Standard Demographic and Health Survey	CBH
South Africa	2003-2017	Vital Registration	VR/SRS
South Africa	1971-1998	Standard Demographic and Health Survey	CBH
Eastern Cape	1972-1996	Census	SBH
Free State	2000, 2011	Census	CBH
Free State	1972-1996	Census	SBH
Gauteng	2000, 2011	Census	CBH
Gauteng	1972-1996	Census	SBH
KwaZulu-Natal	1972-1996	Census	SBH
Limpopo	1972-1996	Census	SBH

Mpumalanga	1972-1996	Census	SBH
North West	2000, 2011	Census	CBH
North West	1972-1996	Census	SBH
Northern Cape	1972-1996	Census	SBH
Western Cape	1972-1996	Census	SBH
Zimbabwe	1963-2015	Standard Demographic and Health Survey	CBH
Benin	1969-2017	Standard Demographic and Health Survey	CBH
Burkina Faso	1967-2010	Standard Demographic and Health Survey	CBH
Cabo Verde	1955-1957, 1959-1960, 1966-1975, 1979-1980, 1983-1985, 1990-1991, 2011-2012	Vital Registration	VR/SRS
Cameroon	1968-2011, 2016	Standard Demographic and Health Survey	CBH
Chad	1969-2014	Standard Demographic and Health Survey	CBH
Côte d'Ivoire	1967-2011	Standard Demographic and Health Survey	CBH
The Gambia	1985-2013, 2018	Standard Demographic and Health Survey	CBH
Ghana	1962-2014	Standard Demographic and Health Survey	CBH
Ghana	1980-2007	Other Demographic and Health Survey	CBH
Guinea	1972-2012, 2018	Standard Demographic and Health Survey	CBH
Guinea-Bissau	1986-2013, 2017	Multiple Indicator Cluster Survey	CBH
Guinea-Bissau	1975-2013	Multiple Indicator Cluster Survey	SBH
Liberia	1961-1989, 1991-2013, 2018	Standard Demographic and Health Survey	CBH
Mali	1964-2012, 2016	Standard Demographic and Health Survey	CBH
Mauritania	1973-2000, 2007, 2012, 2017	Standard Demographic and Health Survey	CBH
Niger	1966-2011	Standard Demographic and Health Survey	CBH
Nigeria	1963-2012, 2016	Standard Demographic and Health Survey	CBH
São Tomé and Príncipe	1955-1958, 1962-1971, 1977-1979, 1984-1985, 1987	Vital Registration	VR/SRS
São Tomé and Príncipe	1973, 1976, 1980, 1982, 1984, 1987	Census	SBH
São Tomé and Príncipe	1985-2008	Standard Demographic and Health Survey	CBH
São Tomé and Príncipe	1984-2008	Standard Demographic and Health Survey	SBH
São Tomé and Príncipe	1990-2013, 2017	Multiple Indicator Cluster Survey	CBH
São Tomé and Príncipe	1976-2013	Multiple Indicator Cluster Survey	SBH
Senegal	1963-2017	Standard Demographic and Health Survey	CBH
Sierra Leone	1981-2013, 2018	Standard Demographic and Health Survey	CBH
Togo	1965-2013	Standard Demographic and Health Survey	CBH

Appendix Table S7: New data sources added for GBD 2021 mortality estimation

Location	Year	Sources
Albania	2011-2011	Albania Vital Registration - Deaths by Sex and Age Group
Algeria	2018-2019	Algeria Multiple Indicator Cluster Survey 2018-2019
American Samoa	2018-2018	American Samoa NVSS Custom Mortality Data 2018
American Samoa	2019-2019	American Samoa NVSS Custom Mortality Data 2019
Andorra	2017-2017	Andorra Deaths by Week, Sex and 5-year Age Group - Eurostat
Antigua and Barbuda	2008-2020	Antigua and Barbuda Deaths by Age Group and Sex 2008-2020
Argentina	2019-2019	Argentina Vital Statistics 2019
Argentina	2020-2020	Argentina Vital Statistics 2020
Armenia	2017-2017	Armenia Deaths by Week, Sex and 5-year Age Group - Eurostat
Australia	2019-2019	Australia Vital Registration - Deaths 2019
Austria	2017-2017	Austria Deaths by Week, Sex and 5-year Age Group - Eurostat
Azerbaijan	2020-2020	Azerbaijan Demographic Indicators 2021
Bangladesh	2017-2018	Bangladesh Demographic and Health Survey 2017-2018
Bangladesh	2019-2019	Bangladesh Multiple Indicator Cluster Survey 2019
Belarus	1990-2019	Belarus Demographic Yearbook 2019
Belarus	2000-2020	Belarus Statistical Yearbook 2020
Belgium	2000-2020	Belgium Human Mortality Database Short-term Mortality Fluctuations (STMF)
Belgium	1994-2020	Belgium Mortality Tables: In Exact Age 1994-2022
Belgium	2017-2017	Belgium Vital Registration - Causes of Deaths 2017
Belgium	2018-2018	Belgium Vital Registration - Causes of Deaths 2018
Bolivia	1990-1990	Bolivia Integrated Household Survey 1990
Bosnia and Herzegovina	2017-2017	Bosnia and Herzegovina Demography 2017
Bosnia and Herzegovina	2018-2018	Bosnia and Herzegovina Demography 2018
Bosnia and Herzegovina	2019-2019	Bosnia and Herzegovina Demography 2019
Botswana	2007-2017	Botswana Vital Statistics Report 2017
Brazil	2018-2018	Brazil Mortality Information System - Deaths 2018
Brazil	2019-2019	Brazil Mortality Information System - Deaths 2019
Brazil	2020-2020	Brazil Mortality Information System - Deaths 2020
Brazil	2021-2021	Brazil Mortality Information System - Deaths 2021 (preliminary)
Brazil	1990-2018	Brazil Mortality Rate in Children Under 5 Years 1990-2018
Brazil	2015-2015	Brazil National Household Sample Survey 2015
Brunei	2015-2020	Brunei Vital Statistics 2020
Bulgaria	2010-2020	Bulgaria Human Mortality Database Short-term Mortality Fluctuations (STMF)
Burundi	2008-2009	Burundi Population and Housing Census 2008-2009
Cameroon	2018-2019	Cameroon Demographic and Health Survey 2018-2019
Canada	1971-1971	Canada CANSIM Database - Estimates of Deaths, by Age and Sex, Annual
Central African Republic	2018-2019	Central African Republic Multiple Indicator Cluster Survey 2018-2019
Chad	2019-2019	Chad Multiple Indicator Cluster Survey 2019
Chile	2016-2020	Chile Human Mortality Database Short-term Mortality Fluctuations (STMF)
Chile	2019-2019	Chile Vital Statistics - Deaths 2019
Chile	2020-2020	Chile Vital Statistics - Deaths 2020
China	2011-2011	China Sample Survey on Population Changes 2011
China	2012-2012	China Sample Survey on Population Changes 2012
China	2019-2019	Hong Kong Vital Registration Death Data 2019
Colombia	2017-2017	Colombia Vital Statistics - Deaths 2017

Colombia	2018-2018	Colombia Vital Statistics - Deaths 2018
Colombia	2019-2019	Colombia Vital Statistics - Deaths 2019
Colombia	2020-2020	Colombia Vital Statistics - Deaths 2020
Costa Rica	2017-2017	Costa Rica Registered Deaths 2017
Costa Rica	2018-2018	Costa Rica Registered Deaths 2018
Costa Rica	2019-2019	Costa Rica Registered Deaths 2019
Costa Rica	2021-2021	Costa Rica Registered Deaths 2021 (Preliminary)
Croatia	2017-2017	Croatia Deaths by Week, Sex and 5-year Age Group - Eurostat
Croatia	2001-2020	Croatia Human Mortality Database Short-term Mortality Fluctuations (STMF)
Cuba	1774-2021	Cuba Demographic Yearbook 2021
Cuba	1960-2019	Cuba Health Statistics Yearbook 2019
Cyprus	2017-2017	Cyprus Deaths by Week, Sex and 5-year Age Group - Eurostat
Cyprus	2014-2018	Cyprus Demographic Statistics 2018
Czechia	2020-2020	Czech Republic Demographic Yearbook 2020
Czechia	2005-2020	Czech Republic Human Mortality Database Short-term Mortality Fluctuations (STMF)
North Korea	2017-2017	North Korea Multiple Indicator Cluster Survey 2017
North Korea	2014-2014	North Korea Socio-Economic, Demographic and Health Survey 2014
DR Congo	2017-2018	Democratic Republic of the Congo Multiple Indicator Cluster Survey 2017-2018
Denmark	2007-2020	Denmark Human Mortality Database Short-term Mortality Fluctuations (STMF)
Dominican Republic	2019-2019	Dominican Republic Multiple Indicator Cluster Survey 2019
Dominican Republic	2019-2019	Dominican Republic Vital Statistics Yearbook 2019
Dominican Republic	2021-2021	Dominican Republic Vital Statistics Yearbook 2021
Ecuador	2018-2018	Ecuador General Deaths 2018
Ecuador	2019-2019	Ecuador General Deaths 2019
Ecuador	2020-2020	Ecuador General Deaths 2020
El Salvador	2016-2016	El Salvador Vital Statistics - Deaths 2016
Estonia	2000-2020	Estonia Human Mortality Database Short-term Mortality Fluctuations (STMF)
Ethiopia	2019-2019	Ethiopia Interim Demographic and Health Survey 2019
Finland	1990-1990	Finland Deaths by Age (5-year), Sex and Area
Finland	1990-2020	Finland Human Mortality Database Short-term Mortality Fluctuations (STMF)
France	2018-2018	France Births, Deaths and Marriages in 2018 - Deaths
France	2019-2019	France Births, Deaths and Marriages in 2019 - Deaths
France	2020-2020	France Births, Deaths and Marriages in 2020 - Deaths
France	2000-2020	France Human Mortality Database Short-term Mortality Fluctuations (STMF)
The Gambia	2019-2020	Gambia Demographic and Health Survey 2019-2020
The Gambia	2018-2018	Gambia Multiple Indicator Cluster Survey 2018
Georgia	1990-2016	Demographic Situation in Georgia 2017
Georgia	1990-2016	Demographic Situation in Georgia 2017
Georgia	1995-2018	Demographic Situation in Georgia 2018
Georgia	1995-2018	Demographic Situation in Georgia 2018
Georgia	1996-2019	Demographic Situation in Georgia 2019
Georgia	1996-2019	Demographic Situation in Georgia 2019
Georgia	1994-2021	Georgia Deaths by Age and Sex 1994-2021
Georgia	1994-2021	Georgia Deaths by Age and Sex 1994-2021
Georgia	2014-2014	Georgia Deaths by Regions, Self-governed Units, Age and Sex 2014
Georgia	2014-2014	Georgia Deaths by Regions, Self-governed Units, Age and Sex 2014
Georgia	2020-2020	Georgia Vital Registration - Deaths 2020

Georgia	2020-2020	Georgia Vital Registration - Deaths 2020
Germany	1956-1956	Germany Deaths by Sex and Single Year Age Groups
Germany	1946-2006	Germany Natural Population Change 2006
Germany	1946-2007	Germany Natural Population Change 2007
Germany	1946-2008	Germany Natural Population Change 2008
Germany	1946-2009	Germany Natural Population Change 2009
Germany	1946-2010	Germany Natural Population Change 2010
Ghana	2017-2018	Ghana Multiple Indicator Cluster Survey 2017-2018
Greece	2019-2020	Greece Data on Vital Statistics 2020
Greece	2000-2017	Greece Deaths by Sex and Age of the Deceased 2000-2017
Greece	2016-2020	Greece Human Mortality Database Short-term Mortality Fluctuations (STMF)
Guam	2018-2018	Guam NVSS Custom Mortality Data 2018
Guam	2019-2019	Guam NVSS Custom Mortality Data 2019
Guatemala	2010-2010	Guatemala Vital Statistics 2010
Guatemala	2011-2011	Guatemala Vital Statistics 2011
Guatemala	2003-2012	Guatemala Vital Statistics 2012
Guatemala	2003-2013	Guatemala Vital Statistics 2013
Guatemala	2004-2014	Guatemala Vital Statistics 2014
Guatemala	2015-2015	Guatemala Vital Statistics 2015
Guatemala	2016-2016	Guatemala Vital Statistics 2016
Guatemala	2018-2018	Guatemala Vital Statistics 2018
Guatemala	2019-2019	Guatemala Vital Statistics 2019
Guatemala	2020-2020	Guatemala Vital Statistics 2020
Guinea	2018-2018	Guinea Demographic and Health Survey 2018
Guinea-Bissau	2018-2019	Guinea-Bissau Multiple Indicator Cluster Survey 2018-2019
Guyana	2019-2020	Guyana Multiple Indicator Cluster Survey 2019-2020
Honduras	2019-2019	Honduras Multiple Indicator Cluster Survey 2019
Hungary	2000-2020	Hungary Human Mortality Database Short-term Mortality Fluctuations (STMF)
Iceland	2019-2019	Iceland Causes of Death Register 2019
Iceland	2020-2020	Iceland Causes of Death Register 2020
Iceland	2017-2020	Iceland Human Mortality Database Short-term Mortality Fluctuations (STMF)
India	2017-2017	India - Delhi Medical Certification of Cause of Death Report 2017
India	2016-2016	India - Karnataka Medical Certification of Cause of Death Report 2016
India	2017-2017	India - Karnataka Medical Certification of Cause of Death Report 2017
India	2011-2011	India - Kerala Medical Certification of Cause of Death Report 2011
India	2012-2012	India - Kerala Medical Certification of Cause of Death Report 2012
India	2016-2016	India - Kerala Medical Certification of Cause of Death Report 2016
India	2017-2017	India - Kerala Medical Certification of Cause of Death Report 2017
India	2019-2021	India Demographic and Health Survey 2019-2020
India	2006-2018	India SRS Statistical Report 2018
India	2019-2019	India SRS Statistical Report 2019
India	1985-2020	India SRS Statistical Report 2020
Iran	2020-2020	Iran Selected Statistics of Vital Events 2020
Iraq	2019-2019	Iraq Annual Statistical Report 2019
Iraq	2018-2018	Iraq Multiple Indicator Cluster Survey 2018
Israel	1955-2017	Israel Statistical Abstract 2018
Israel	1948-2018	Israel Statistical Abstract 2019

Italy	2011-2022	Italy Daily Mortality Data 2011-2022
Italy	2017-2017	Italy Deaths by Week, Sex and 5-year Age Group - Eurostat
Italy	2015-2020	Italy Human Mortality Database Short-term Mortality Fluctuations (STMF)
Italy	2017-2017	Italy Vital Registration - Deaths 2017
Italy	2018-2018	Italy Vital Registration - Deaths 2018
Italy	2019-2019	Italy Vital Registration - Deaths 2019
Italy	2020-2020	Italy Vital Registration - Deaths 2020
Jamaica	2007-2007	Jamaica Vital Statistics Report 2007
Jamaica	2008-2008	Jamaica Vital Statistics Report 2008
Japan	1899-2004	Japan Deaths by Age Groups and Sex 1899-2004
Japan	2018-2018	Japan Deaths by Sex, Age, and Prefecture and 21 major cities 2018
Japan	2018-2018	Japan Vital Registration - Deaths 2018
Japan	2019-2019	Japan Vital Registration - Deaths 2019
Japan	2012-2022	Japan Vital Registration - Monthly Death Data 2012-2022
Japan	2010-2021	Japan Vital Registration - Weekly Causes of Death Data 2010-2021
Kiribati	2018-2019	Kiribati Multiple Indicator Cluster Survey 2018-2019
Kuwait	2020-2020	Kuwait Annual Bulletin for Vital Statistics - Births and Deaths 2020
Kuwait	1957-2013	Kuwait Annual Statistical Abstract 2013
Kyrgyzstan	2018-2018	Kyrgyzstan Multiple Indicator Cluster Survey 2018
Latvia	2000-2020	Latvia Human Mortality Database Short-term Mortality Fluctuations (STMF)
Lebanon	2007-2013	Lebanon Statistical Bulletin 2013
Lesotho	2018-2018	Lesotho Multiple Indicator Cluster Survey 2018
Liberia	2019-2020	Liberia Demographic and Health Survey 2019-2020
Lithuania	2016-2016	Lithuania Deaths by Age, Place of Residence, and Gender
Lithuania	2000-2020	Lithuania Human Mortality Database Short-term Mortality Fluctuations (STMF)
Luxembourg	1980-1980	Luxembourg Deaths by 5 Year Age Groups
Luxembourg	2000-2020	Luxembourg Human Mortality Database Short-term Mortality Fluctuations (STMF)
Madagascar	2021-2021	Madagascar Demographic and Health Survey 2021
Madagascar	2018-2018	Madagascar Multiple Indicator Cluster Survey 2018
Malawi	2019-2020	Malawi Multiple Indicator Cluster Survey 2019-2020
Malaysia	2015-2017	Malaysia Health Indicators 2018
Malaysia	2018-2018	Malaysia Vital Statistics 2019
Malaysia	2020-2020	Malaysia Vital Statistics 2021
Maldives	1985-2020	Maldives Statistical Yearbook 2020
Maldives	2010-2020	Maldives Statistical Yearbook 2021
Mali	2018-2018	Mali Demographic and Health Survey 2018
Malta	2017-2017	Malta Deaths by Week, Sex and 5-year Age Group - Eurostat
Mauritania	2019-2021	Mauritania Demographic and Health Survey 2019-2020
Mauritius	1999-2019	Mauritius Digest of Demographic Statistics 2019
Mexico	2018-2018	Mexico Vital Registration - Deaths 2018
Mexico	2019-2019	Mexico Vital Registration - Deaths 2019
Mexico	2020-2020	Mexico Vital Registration - Deaths 2020
Mexico	2021-2021	Mexico Vital Registration - Deaths 2021
Mongolia	2012-2012	Mongolia - Khuvsgul Multiple Indicator Cluster Survey 2012
Mongolia	1989-1989	Mongolia Deaths by Age and Sex
Mongolia	2018-2018	Mongolia Multiple Indicator Cluster Survey 2018
Mongolia	2018-2018	Mongolia Vital Registration - Deaths 2018

Mongolia	2019-2019	Mongolia Vital Registration - Deaths 2019
Mongolia	2020-2020	Mongolia Vital Registration - Deaths 2020
Nepal	2019-2019	Nepal Multiple Indicator Cluster Survey 2019
Netherlands	1988-1988	Netherlands Deceased by Gender, Age, Marital Status, and Region
New Zealand	2016-2016	New Zealand Mortality Collection 2016
New Zealand	2017-2017	New Zealand Mortality Collection 2017
New Zealand	2018-2018	New Zealand Mortality Collection 2018
Nicaragua	2016-2017	Nicaragua Compendium of Vital Statistics 2016-2017
Nicaragua	2019-2020	Nicaragua Compendium of Vital Statistics 2019-2020
Nigeria	1994-2007	Nigeria Report of Livebirths, Deaths and Stillbirths 1994-2007
North Macedonia	2012-2018	Macedonia Statistical Yearbook 2018
North Macedonia	2014-2019	Macedonia Statistical Yearbook 2019
Northern Mariana Islands	2018-2018	Northern Mariana Islands NVSS Custom Mortality Data 2018
Northern Mariana Islands	2019-2019	Northern Mariana Islands NVSS Custom Mortality Data 2019
Norway	2018-2018	Norway Cause of Death Registry 2018
Norway	2019-2019	Norway Cause of Death Registry 2019
Norway	2020-2020	Norway Cause of Death Registry 2020
Norway	2017-2017	Norway Deaths by Week, Sex and 5-year Age Group - Eurostat
Norway	2000-2020	Norway Human Mortality Database Short-term Mortality Fluctuations (STMF)
Pakistan	2016-2017	Pakistan - Khyber Pakhtunkhwa Multiple Indicator Cluster Survey 2016-2017
Pakistan	2018-2019	Pakistan - Sindh Multiple Indicator Cluster Survey 2018-2019
Pakistan	1992-1992	Pakistan Demographic Survey 1992
Palestine	2018-2018	Palestine - Gaza Strip and West Bank Mortality by Sex, Age, and Cause 2018
Palestine	2019-2020	Palestine Multiple Indicator Cluster Survey 2019-2020
Panama	2017-2017	Panama Vital Statistics, Volume III - Deaths 2017
Panama	2020-2020	Panama Vital Statistics, Volume III - Deaths 2020
Papua New Guinea	2016-2018	Papua New Guinea Demographic and Health Survey 2016-2018
Paraguay	2004-2004	Paraguay Vital Registration - Deaths 2004
Paraguay	2005-2005	Paraguay Vital Registration - Deaths 2005
Paraguay	2006-2006	Paraguay Vital Registration - Deaths 2006
Paraguay	2007-2007	Paraguay Vital Registration - Deaths 2007
Paraguay	2008-2008	Paraguay Vital Registration - Deaths 2008
Paraguay	2009-2009	Paraguay Vital Registration - Deaths 2009
Paraguay	2010-2010	Paraguay Vital Registration - Deaths 2010
Paraguay	2011-2011	Paraguay Vital Registration - Deaths 2011
Paraguay	2012-2012	Paraguay Vital Registration - Deaths 2012
Paraguay	2013-2013	Paraguay Vital Registration - Deaths 2013
Paraguay	2014-2014	Paraguay Vital Registration - Deaths 2014
Paraguay	2015-2015	Paraguay Vital Registration - Deaths 2015
Paraguay	2016-2016	Paraguay Vital Registration - Deaths 2016
Paraguay	2017-2017	Paraguay Vital Registration - Deaths 2017
Paraguay	2017-2017	Paraguay Vital Statistics 2017
Paraguay	2018-2018	Paraguay Vital Statistics 2018
Paraguay	2019-2019	Paraguay Vital Statistics 2019
Paraguay	2010-2020	Paraguay Vital Statistics 2020
Peru	2014-2018	Perú: Birth, Mortality and Marriage, 2018
Peru	2015-2019	Perú: Birth, Mortality and Marriage, 2019

Peru	2020-2020	Perú: Birth, Mortality and Marriage, 2020
Philippines	2016-2016	Philippines Vital Registration - Deaths 2016
Philippines	2017-2017	Philippines Vital Registration - Deaths 2017
Philippines	2018-2018	Philippines Vital Registration - Deaths 2018
Philippines	2020-2020	Philippines Vital Registration - Deaths 2020
Poland	2004-2004	Poland Deaths by Sex and Age
Poland	2000-2020	Poland Human Mortality Database Short-term Mortality Fluctuations (STMF)
Poland	1999-1999	Poland Vital Registration - Causes of Death Data 1999
Poland	2000-2000	Poland Vital Registration - Causes of Death Data 2000
Poland	2001-2001	Poland Vital Registration - Causes of Death Data 2001
Poland	2002-2002	Poland Vital Registration - Causes of Death Data 2002
Poland	2003-2003	Poland Vital Registration - Causes of Death Data 2003
Poland	2004-2004	Poland Vital Registration - Causes of Death Data 2004
Poland	2005-2005	Poland Vital Registration - Causes of Death Data 2005
Poland	2006-2006	Poland Vital Registration - Causes of Death Data 2006
Poland	2007-2007	Poland Vital Registration - Causes of Death Data 2007
Poland	2008-2008	Poland Vital Registration - Causes of Death Data 2008
Poland	2009-2009	Poland Vital Registration - Causes of Death Data 2009
Poland	2010-2010	Poland Vital Registration - Causes of Death Data 2010
Poland	2011-2011	Poland Vital Registration - Causes of Death Data 2011
Poland	2012-2012	Poland Vital Registration - Causes of Death Data 2012
Poland	2013-2013	Poland Vital Registration - Causes of Death Data 2013
Poland	2014-2014	Poland Vital Registration - Causes of Death Data 2014
Poland	2015-2015	Poland Vital Registration - Causes of Death Data 2015
Poland	2016-2016	Poland Vital Registration - Causes of Death Data 2016
Poland	2017-2017	Poland Vital Registration - Causes of Death Data 2017
Poland	2018-2018	Poland Vital Registration - Causes of Death Data 2018
Poland	2019-2019	Poland Vital Registration - Causes of Death Data 2019
Portugal	2000-2020	Portugal Human Mortality Database Short-term Mortality Fluctuations (STMF)
Puerto Rico	2018-2018	Puerto Rico NVSS Custom Mortality Data 2018
Puerto Rico	2019-2019	Puerto Rico NVSS Custom Mortality Data 2019
Qatar	2009-2018	Qatar Vital Statistics Annual Bulletin 2018
Qatar	2010-2019	Qatar Vital Statistics Annual Bulletin 2019
Romania	2017-2017	Romania Deaths by Week, Sex and 5-year Age Group - Eurostat
Romania	2019-2019	Romania Demographic Events in 2019
Romania	2020-2020	Romania Demographic Events in 2020
Russia	2015-2021	Russia Mortality Rates by Region, Age, Sex, Cause of Death, and 5-Year Age Groups 2015-2021
Russia	2001-2001	Russia Vital Registration - Deaths 2001
Russia	2002-2002	Russia Vital Registration - Deaths 2002
Russia	2003-2003	Russia Vital Registration - Deaths 2003
Rwanda	2019-2020	Rwanda Demographic and Health Survey 2019-2020
Samoa	2019-2020	Samoa Multiple Indicator Cluster Survey 2019-2020
São Tomé and Príncipe	2019-2019	Sao Tome and Principe Multiple Indicator Cluster Survey 2019
Scotland	2000-2020	Scotland Human Mortality Database Short-term Mortality Fluctuations (STMF)
Senegal	2018-2018	Senegal Continuous Demographic and Health Survey 2018
Senegal	2019-2019	Senegal Continuous Demographic and Health Survey 2019
Serbia	2017-2017	Serbia Deaths by Week, Sex and 5-year Age Group - Eurostat

Serbia	2019-2019	Serbia Multiple Indicator Cluster Survey 2019
Seychelles	1971-2018	Seychelles Statistical Bulletin: Population and Vital Statistics 2018
Seychelles	2020-2020	Seychelles Statistical Bulletin: Population and Vital Statistics 2020
Seychelles	2021-2021	Seychelles Statistical Bulletin: Population and Vital Statistics 2021
Sierra Leone	2019-2019	Sierra Leone Demographic and Health Survey 2019
Singapore	2018-2018	Singapore Causes of Death 2018
Singapore	2019-2019	Singapore Causes of Death 2019
Singapore	2020-2020	Singapore Causes of Death 2020
Singapore	1999-2018	Singapore Report on Registration of Births and Deaths 2018
Singapore	1999-2019	Singapore Report on Registration of Births and Deaths 2019
Singapore	2002-2021	Singapore Report on Registration of Births and Deaths 2021
Slovakia	2000-2020	Slovakia Human Mortality Database Short-term Mortality Fluctuations (STMF)
Slovakia	2017-2017	Slovakia Vital Registration - Deaths 2017
Slovenia	2000-2020	Slovenia Human Mortality Database Short-term Mortality Fluctuations (STMF)
South Africa	2000-2018	South Africa Rapid Mortality Surveillance Report 2018
South Africa	1997-2017	South Africa Vital Registration - Causes of Death 2017
Spain	2018-2018	Spain Cause of Death Statistics 2018
Spain	2019-2019	Spain Cause of Death Statistics 2019
Spain	2020-2020	Spain Cause of Death Statistics 2020
Spain	2017-2017	Spain Deaths by Week, Sex and 5-year Age Group - Eurostat
Spain	2000-2020	Spain Human Mortality Database Short-term Mortality Fluctuations (STMF)
Sri Lanka	2014-2014	Sri Lanka Vital Statistics - Deaths 2014
Stockholm	2018-2018	Sweden Cause of Death Register 2018
Stockholm	2019-2019	Sweden Cause of Death Register 2019
Stockholm	2020-2020	Sweden Cause of Death Register 2020
Suriname	2018-2018	Suriname Multiple Indicator Cluster Survey 2018
Sweden	2018-2018	Sweden Cause of Death Register 2018
Sweden	2019-2019	Sweden Cause of Death Register 2019
Sweden	2020-2020	Sweden Cause of Death Register 2020
Sweden	1968-1968	Sweden Deaths by Region, Age and Sex
Switzerland	2000-2020	Switzerland Human Mortality Database Short-term Mortality Fluctuations (STMF)
Taiwan (province of China)	2000-2020	Taiwan Human Mortality Database Short-term Mortality Fluctuations (STMF)
Taiwan (province of China)	1981-2017	Taiwan Statistics of Causes of Death 2017
Togo	2017-2017	Togo Multiple Indicator Cluster Survey 2017
Tonga	2019-2019	Tonga Multiple Indicator Cluster Survey 2019
Trinidad and Tobago	2015-2015	Trinidad and Tobago Vital Registration - Deaths by Sex and Age Group 2015
Trinidad and Tobago	2016-2016	Trinidad and Tobago Vital Registration - Deaths by Sex and Age Group 2016
Trinidad and Tobago	2017-2017	Trinidad and Tobago Vital Registration - Deaths by Sex and Age Group 2017
Trinidad and Tobago	2018-2018	Trinidad and Tobago Vital Registration - Deaths by Sex and Age Group 2018
Tunisia	2018-2018	Tunisia Multiple Indicator Cluster Survey 2018
Turkmenistan	2019-2019	Turkmenistan Multiple Indicator Cluster Survey 2019
Tuvalu	2019-2020	Tuvalu Multiple Indicator Cluster Survey 2019-2020
Türkiye	2017-2017	Turkey Death Statistics 2017
Türkiye	2018-2018	Turkey Death Statistics 2018
UK	2000-2020	Scotland Human Mortality Database Short-term Mortality Fluctuations (STMF)
UK	2018-2018	United Kingdom - England and Wales Mortality Statistics 2018
UK	2019-2019	United Kingdom - England and Wales Mortality Statistics 2019

UK	2020-2020	United Kingdom - England and Wales Mortality Statistics 2020
UK	2021-2021	United Kingdom - England and Wales Mortality Statistics 2021
UK	1955-2019	United Kingdom - Northern Ireland Deaths by Age 1955-2019
UK	1887-2019	United Kingdom - Northern Ireland Deaths by Sex 1887-2019
UK	2010-2020	United Kingdom - Northern Ireland Registrar General Annual Report 2020
UK	1979-1979	United Kingdom - Northern Ireland Vital Registration - Deaths 1979 ICD9
UK	1946-2018	United Kingdom - Scotland Vital Events Reference Tables 2018
UK	1946-2019	United Kingdom - Scotland Vital Events Reference Tables 2019
UK	1946-2020	United Kingdom - Scotland Vital Events Reference Tables 2020
UK	1979-1979	United Kingdom - Scotland Vital Registration - Deaths 1979 ICD9
UK	2017-2017	United Kingdom Vital Registration Deaths 2017 ICD10
UK	2018-2018	United Kingdom Vital Registration Deaths 2018
UK	2018-2018	United Kingdom Vital Registration Deaths 2018 ICD10
UK	2019-2019	United Kingdom Vital Registration Deaths 2019 ICD10
UK	2020-2020	United Kingdom Vital Registration Deaths 2020
Virgin Islands	2018-2018	United States Virgin Islands NVSS Custom Mortality Data 2018
Virgin Islands	2019-2019	United States Virgin Islands NVSS Custom Mortality Data 2019
USA	2020-2020	United States CDC Wonder Mortality Statistics 2020
USA	2015-2015	United States NVSS Custom Mortality Data 2015 and United States Military Deaths 2000-2018
USA	2016-2016	United States NVSS Custom Mortality Data 2016 and United States Military Deaths 2000-2018
USA	2017-2017	United States NVSS Custom Mortality Data 2017
USA	2017-2017	United States NVSS Custom Mortality Data 2017 and United States Military Deaths 2000-2018
USA	2018-2018	United States NVSS Custom Mortality Data 2018
USA	2018-2018	United States NVSS Custom Mortality Data 2018 and United States Military Deaths 2000-2018
USA	2019-2019	United States NVSS Custom Mortality Data 2019
USA	2020-2020	United States NVSS Custom Mortality Data 2020
Zimbabwe	2019-2019	Zimbabwe Multiple Indicator Cluster Survey 2019

Appendix Table S8: Number of outliered datapoints for the age/sex model by location, source type, and age group

Location	Source	Outliers Early Neonatal (0-7 days)	Outliers Late Neonatal (7-28 days)	Outliers Post Neonatal A (1-6 months)	Outliers Post Neonatal B (6-12 months)	Outliers Post Neonatal (1-12 months)	Outliers Infant (<1 years)	Outliers Child A (1-2 years)	Outliers Child B (2-5 years)	Outliers Child (1-5 years)
Afghanistan	DHS	21	21	21	21	21	21	21	21	21
Albania	Other	114	54	54	54	54	54	54	54	54
Albania	LSMS	15	15	15	15	15	15	15	15	15
Albania	DHS	36	21	19	21	18	18	23	23	20
Albania	RHS	12	4	3	3	3	3	3	4	3
Albania	VR	3	3	3	3	3	3	3	3	3
Algeria	Other	45	45	45	45	45	45	45	45	45
Algeria	PAPFAM	9	9	9	9	9	9	9	9	9
Algeria	MICS	9	9	9	9	9	9	9	9	9
American Samoa	Other	9	9	9	9	9	9	9	9	14
Andorra	Other	18	18	18	18	18	24	18	18	23
Angola	Other	9	9	9	9	9	9	9	9	9
Angola	DHS	9	9	9	9	9	9	9	9	9
Antigua and Barbuda	Other	44	53	39	39	42	39	39	39	44
Argentina	Other	9	9	9	9	9	9	9	9	9
Armenia	MICS	9	9	9	9	9	9	9	9	9
Armenia	DHS	27	27	27	27	27	27	33	30	28
Armenia	Other	117	117	117	117	117	117	117	117	117
Australia	Other	0	0	0	0	0	0	0	1	0
Azerbaijan	MICS	9	9	9	9	9	9	9	9	9
Azerbaijan	DHS	9	9	9	9	9	9	9	9	9
Azerbaijan	Other	111	111	111	111	111	111	111	111	111
Azerbaijan	VR	3	3	3	3	3	3	3	3	3
Bahrain	Other	3	3	3	3	3	3	3	3	3
Bangladesh	WFS	6	6	6	6	6	6	6	6	6
Bangladesh	DHS	90	90	90	90	90	90	90	90	90
Bangladesh	Other	18	18	18	18	18	18	18	18	18
Barbados	Other	3	3	3	3	3	3	3	3	8
Belarus	MICS	3	3	3	3	3	3	3	3	3
Belarus	Other	90	90	90	90	90	90	90	90	90
Belarus	VR	2	2	2	2	2	2	2	2	2
Belgium	Other	3	3	3	3	3	3	3	8	3
Belize	RHS	15	18	15	15	15	15	17	15	15
Benin	WFS	9	9	9	9	9	9	9	9	9
Benin	DHS	45	45	45	45	45	45	45	45	45
Bermuda	Other	77	93	72	72	86	77	72	72	95
Bhutan	Other	3	3	3	3	3	3	3	3	3
Bolivia	Other	66	66	66	66	66	66	66	66	66
Bolivia	DHS	54	54	45	45	45	45	45	45	45
Bosnia and Herzegovina	Other	12	12	12	12	12	12	12	13	12
Botswana	DHS	15	6	6	6	6	6	6	6	6
Botswana	Other	35	33	26	26	26	26	26	26	26
Botswana	VR	15	15	15	15	15	15	15	15	15
Brazil	DHS	24	25	24	24	24	24	25	25	25
Brazil	Other	129	129	129	129	129	129	129	129	129
Brazil - Acre	Other	123	123	123	123	123	123	123	123	123
Brazil - Acre	DHS	9	9	9	9	9	9	9	9	9
Brazil - Alagoas	DHS	17	21	12	12	12	12	17	16	14
Brazil - Alagoas	Other	129	129	129	129	129	129	129	129	129
Brazil - Amapá	Other	0	0	0	2	0	0	0	0	0
Brazil - Amapá	DHS	9	9	9	9	9	9	9	9	9
Brazil - Amazonas	DHS	12	14	9	9	12	9	11	14	11
Brazil - Bahia	DHS	21	22	21	21	21	21	22	23	21
Brazil - Bahia	Other	129	129	129	129	129	129	129	129	129
Brazil - Ceará	DHS	15	16	15	15	15	15	17	21	17
Brazil - Ceará	Other	129	129	129	129	129	129	129	129	129
Brazil - Distrito Federal	DHS	18	18	18	18	18	18	18	18	18
Brazil - Espírito Santo	DHS	21	21	21	21	21	21	21	21	21
Brazil - Espírito Santo	Other	54	54	54	54	54	54	54	54	54
Brazil - Goiás	DHS	21	21	21	21	21	21	21	21	21
Brazil - Goiás	Other	111	111	111	111	111	111	111	111	111
Brazil - Maranhão	DHS	19	17	12	12	12	12	13	18	12
Brazil - Maranhão	Other	129	129	129	129	129	129	129	129	129
Brazil - Mato Grosso	DHS	13	11	9	9	10	9	15	11	10
Brazil - Mato Grosso	Other	84	84	84	84	84	84	84	84	84
Brazil - Mato Grosso do Sul	DHS	9	13	9	9	10	9	16	16	14
Brazil - Minas Gerais	DHS	12	14	12	12	13	12	19	14	14
Brazil - Minas Gerais	Other	63	63	63	63	63	63	63	63	63
Brazil - Paraná	DHS	21	21	21	21	21	21	21	21	21
Brazil - Paraná	Other	42	42	42	42	42	42	42	42	42
Brazil - Paraíba	DHS	11	14	9	9	9	9	19	22	14
Brazil - Paraíba	Other	96	96	96	96	96	96	96	96	96
Brazil - Pará	DHS	13	13	9	9	9	9	10	12	10
Brazil - Pará	Other	120	120	120	120	120	120	120	120	120
Brazil - Pernambuco	DHS	19	18	18	18	18	18	21	22	19
Brazil - Pernambuco	Other	90	90	90	90	90	90	90	90	90
Brazil - Piauí	DHS	21	25	21	21	21	21	21	25	21
Brazil - Piauí	Other	129	129	129	129	129	129	129	129	129
Brazil - Rio Grande do Norte	DHS	18	17	12	12	12	12	16	20	14
Brazil - Rio Grande do Norte	Other	129	129	129	129	129	129	129	129	129
Brazil - Rio Grande do Sul	DHS	13	16	12	12	13	12	19	21	19
Brazil - Rio de Janeiro	DHS	13	15	12	12	13	12	19	19	17
Brazil - Rondônia	DHS	12	12	12	12	12	12	12	12	12
Brazil - Rondônia	Other	69	69	69	69	69	69	69	69	69
Brazil - Roraima	Other	21	21	21	21	21	21	21	21	21
Brazil - Roraima	DHS	9	9	9	9	9	9	9	9	9
Brazil - Santa Catarina	DHS	18	18	18	18	18	18	18	18	18
Brazil - Santa Catarina	Other	45	45	45	45	45	45	45	45	45
Brazil - Sergipe	DHS	12	15	9	9	10	9	17	19	15
Brazil - Sergipe	Other	117	117	117	117	117	117	117	117	117
Brazil - São Paulo	DHS	15	16	15	15	15	15	19	22	19
Brazil - Tocantins	DHS	9	9	9	9	9	9	9	9	9
Brazil - Tocantins	Other	96	96	96	96	96	96	96	96	96
Burkina Faso	DHS	36	36	36	36	36	36	36	36	36
Burkina Faso	Other	3	3	3	3	3	3	3	3	3
Burundi	DHS	15	15	15	15	15	15	15	15	15
Cabo Verde	RHS	6	6	6	6	6	6	6	6	6
Cambodia	DHS	54	54	54	54	54	54	54	54	54
Cameroon	WFS	9	9	9	9	9	9	9	9	9
Cameroon	DHS	39	39	39	39	39	39	39	39	39
Central African Republic	DHS	9	9	9	9	9	9	9	9	9
Chad	DHS	30	30	30	30	30	30	30	30	30
Chile	VR	0	0	0	3	0	0	0	0	0
Colombia	Other	120	120	120	120	120	120	120	120	120

Colombia	WFS	6	6	6	6	6	6	6	6	6
Colombia	DHS	57	57	57	57	57	57	57	57	57
Comoros	DHS	18	18	18	18	18	18	18	18	18
Congo (Brazzaville)	DHS	30	30	30	30	30	30	30	30	30
Congo (Brazzaville)	MICS	9	9	9	9	9	9	9	9	9
Congo (Brazzaville)	VR	3	3	3	3	3	3	3	3	3
Cook Islands	Other	33	33	33	33	33	39	33	33	57
Costa Rica	WFS	6	6	6	6	6	6	6	6	6
Costa Rica	RHS	5	5	5	5	5	5	6	6	5
Croatia	Other	3	3	3	3	3	3	3	4	3
Cyprus	Other	117	117	117	117	117	117	117	118	117
Czechia	RHS	15	15	15	15	15	15	15	15	15
Côte d'Ivoire	WFS	9	9	9	9	9	9	9	9	9
Côte d'Ivoire	DHS	24	24	24	24	24	24	24	24	24
Côte d'Ivoire	LSMS	3	3	3	3	3	3	3	3	3
Côte d'Ivoire	Other	18	18	18	18	18	18	18	18	18
Côte d'Ivoire	MICS	9	9	9	9	9	9	9	9	9
DR Congo	DHS	21	21	21	21	21	21	21	21	21
Denmark	Other	0	0	0	0	0	0	4	16	1
Djibouti	PAPFAM	15	15	15	15	15	15	15	15	15
Dominica	Other	192	192	192	192	192	192	192	192	192
Dominican Republic	Other	225	225	225	225	225	225	225	225	225
Dominican Republic	DHS	66	66	66	66	66	66	67	67	66
Dominican Republic	MICS	24	24	24	24	24	24	24	24	24
Ecuador	Other	201	90	90	90	90	90	90	90	90
Ecuador	DHS	30	12	12	12	12	12	12	12	12
Ecuador	RHS	45	27	27	27	27	27	27	27	27
Egypt	Other	192	192	192	192	192	192	192	192	192
Egypt	WFS	9	9	9	9	9	9	9	9	9
Egypt	DHS	81	81	81	81	81	81	81	81	81
El Salvador	Other	210	210	210	210	210	210	210	210	210
El Salvador	RHS	30	30	30	30	30	30	30	30	30
El Salvador	MICS	9	9	9	9	9	9	9	9	9
England - Barnet	Other	0	0	0	0	0	0	0	0	1
England - Barnsley	Other	3	3	3	3	3	3	3	3	3
England - Bath and North East Somerset	Other	1	1	0	0	1	1	0	0	3
England - Bedford	Other	3	3	3	3	3	3	3	3	6
England - Bexley	Other	0	0	0	0	0	0	0	0	2
England - Blackburn with Darwen	Other	0	0	0	0	0	0	0	0	1
England - Blackpool	Other	0	0	0	0	0	0	0	0	4
England - Bolton	Other	3	3	3	3	3	3	3	3	4
England - Bournemouth	Other	3	3	3	3	3	3	3	3	3
England - Bracknell Forest	Other	5	4	3	3	4	4	3	3	6
England - Brent	Other	3	3	3	3	3	3	3	3	3
England - Brighton and Hove	Other	0	0	0	0	0	0	0	0	3
England - Bromley	Other	0	0	0	0	0	0	0	0	1
England - Bury	Other	0	0	0	0	0	0	0	0	1
England - Calderdale	Other	3	3	3	3	3	3	3	3	4
England - Camden	Other	7	7	6	6	6	6	6	6	7
England - Central Bedfordshire	Other	3	3	3	3	3	3	3	3	5
England - Cheshire East	Other	0	0	0	0	0	0	0	0	1
England - Cornwall	Other	3	3	3	3	3	3	3	3	4
England - County Durham	Other	3	3	3	3	3	3	3	3	4
England - Coventry	Other	0	0	0	0	0	0	0	0	1
England - Croydon	Other	0	0	0	0	0	0	0	0	1
England - Cumbria	Other	0	0	0	0	0	0	0	0	1
England - Darlington	Other	1	1	0	0	0	0	0	0	6
England - Doncaster	Other	0	0	0	0	0	0	0	0	4
England - Dorset	Other	3	3	3	3	3	3	3	3	3
England - Dudley	Other	0	0	0	0	0	0	0	0	1
England - Ealing	Other	4	4	3	3	3	3	3	3	4
England - East Riding of Yorkshire	Other	0	0	0	0	0	0	0	0	2
England - East Sussex	Other	0	1	0	0	0	0	0	0	0
England - Enfield	Other	0	0	0	0	0	0	0	0	3
England - Gateshead	Other	3	3	3	3	3	3	3	3	6
England - Hackney	Other	2	2	2	2	2	2	2	2	2
England - Halton	Other	2	0	0	0	0	0	0	0	5
England - Hammersmith and Fulham	Other	4	4	3	3	4	4	3	3	5
England - Hampshire	Other	0	0	0	0	1	0	0	0	0
England - Haringey	Other	0	0	0	0	0	0	0	0	3
England - Harrow	Other	3	3	3	3	3	3	3	3	3
England - Hartlepool	Other	6	4	0	0	2	2	0	0	6
England - Havering	Other	1	0	0	0	0	0	0	0	3
England - Herefordshire, County of	Other	0	0	0	0	0	0	0	0	4
England - Hillingdon	Other	3	3	3	3	3	3	3	3	5
England - Hounslow	Other	3	3	3	3	3	3	3	3	4
England - Isle of Wight	Other	3	3	3	3	3	3	3	3	8
England - Islington	Other	1	1	0	0	1	1	0	0	3
England - Kensington and Chelsea	Other	1	0	0	0	0	0	0	0	3
England - Kingston upon Hull, City of	Other	0	0	0	0	0	0	0	0	1
England - Kingston upon Thames	Other	3	3	3	3	3	3	3	3	5
England - Knowsley	Other	0	0	0	0	0	0	0	0	2
England - Lambeth	Other	0	0	0	0	0	0	0	0	2
England - Leicester	Other	0	0	0	0	0	0	0	0	1
England - Lewisham	Other	3	3	3	3	3	3	3	3	3
England - Medway	Other	3	3	3	3	3	3	3	3	6
England - Merton	Other	0	0	0	0	0	0	0	0	3
England - Middlesbrough	Other	1	1	0	0	0	0	0	0	2
England - Milton Keynes	Other	3	3	3	3	3	3	3	3	4
England - Newcastle upon Tyne	Other	3	4	3	3	3	3	3	3	4
England - North East Lincolnshire	Other	1	1	0	0	0	0	0	0	3
England - North Lincolnshire	Other	2	1	0	0	0	0	0	0	3
England - North Somerset	Other	4	3	3	3	3	3	3	3	8
England - North Yorkshire	Other	0	0	0	0	0	0	0	0	2
England - Northumberland	Other	0	0	0	0	0	0	0	0	1
England - Nottinghamshire	Other	3	3	3	3	3	3	3	3	3
England - Oxfordshire	Other	0	0	0	0	0	0	0	0	1
England - Peterborough	Other	0	0	0	0	0	0	0	0	2
England - Plymouth	Other	0	0	0	0	0	0	0	0	2
England - Poole	Other	1	0	0	0	0	0	0	0	2
England - Portsmouth	Other	4	3	3	3	3	3	3	3	5
England - Reading	Other	0	0	0	0	0	0	0	0	5
England - Redbridge	Other	3	3	3	3	3	3	3	3	4
England - Redcar and Cleveland	Other	10	8	6	6	6	6	6	6	12
England - Richmond upon Thames	Other	3	3	3	3	3	3	3	3	6
England - Rochdale	Other	0	0	0	0	0	0	0	0	2
England - Rutland	Other	3	3	0	0	3	3	0	0	6

England - Salford	Other	0	0	0	0	0	0	0	0	1
England - Sefton	Other	3	3	3	3	3	3	3	3	3
England - Shropshire	Other	0	0	0	0	0	0	0	0	2
England - Solihull	Other	4	3	3	3	3	3	3	3	7
England - Somerset	Other	0	0	0	0	0	0	0	0	1
England - South Gloucestershire	Other	0	0	0	0	0	0	0	0	2
England - South Tyneside	Other	8	7	6	6	7	7	6	6	9
England - Southampton	Other	0	0	0	0	0	0	0	0	3
England - Southend-on-Sea	Other	0	0	0	0	0	0	0	0	2
England - Southwark	Other	3	3	3	3	3	3	3	3	4
England - St Helens	Other	1	0	0	0	0	0	0	0	2
England - Staffordshire	Other	0	0	0	0	2	0	0	0	0
England - Stockport	Other	3	3	3	3	3	3	3	3	3
England - Stockton-on-Tees	Other	6	6	6	6	6	6	6	6	8
England - Suffolk	Other	0	0	0	0	0	0	0	0	1
England - Sunderland	Other	4	3	3	3	3	3	3	3	5
England - Sutton	Other	2	1	0	0	0	0	0	0	2
England - Swindon	Other	1	0	0	0	0	0	0	0	4
England - Tameside	Other	0	0	0	0	0	0	0	0	2
England - Telford and Wrekin	Other	1	1	0	0	0	0	0	0	2
England - Thurrock	Other	3	4	3	3	3	3	3	3	3
England - Torbay	Other	4	4	3	3	4	4	3	3	8
England - Trafford	Other	6	6	6	6	6	6	6	6	9
England - Wakefield	Other	0	0	0	0	0	0	0	0	1
England - Walsall	Other	3	3	3	3	3	3	3	3	3
England - Wandsworth	Other	0	0	0	0	0	0	0	0	1
England - Warrington	Other	0	0	0	0	0	0	0	0	1
England - West Berkshire	Other	2	1	0	0	0	0	0	0	6
England - Westminster	Other	1	0	0	0	0	0	0	0	4
England - Wigan	Other	0	0	0	0	0	0	0	0	1
England - Windsor and Maidenhead	Other	4	4	3	3	3	3	3	3	7
England - Wokingham	Other	4	4	3	3	4	4	3	3	7
England - Wolverhampton	Other	3	3	3	3	3	3	3	3	4
England - York	Other	0	0	0	0	0	0	0	0	4
Eritrea	DHS	18	18	18	18	18	18	18	18	18
Estonia	MICS	3	3	3	3	3	3	3	3	3
Estonia	Other	0	0	0	0	0	0	0	2	0
Eswatini	DHS	9	9	9	9	9	9	9	9	9
Eswatini	MICS	18	18	18	18	18	18	18	18	18
Ethiopia	DHS	102	102	102	102	102	102	102	102	102
Ethiopia - Addis Ababa	DHS	30	46	37	37	34	30	38	37	32
Ethiopia - Afar	DHS	27	29	27	27	27	27	27	27	27
Ethiopia - Amhara	DHS	48	48	48	49	48	48	48	48	48
Ethiopia - Benishangul-Gumuz	DHS	24	24	25	24	24	24	24	24	24
Ethiopia - Dire Dawa	DHS	21	24	21	23	21	21	24	21	21
Ethiopia - Gambella	DHS	21	23	21	22	21	21	22	21	21
Ethiopia - Harari	DHS	24	26	24	27	24	24	24	25	24
Ethiopia - Oromia	DHS	42	42	42	42	42	42	42	42	42
Ethiopia - Somali	DHS	21	21	21	21	21	21	21	21	21
Ethiopia - Southern Nations, Nationalities, and Peoples	DHS	36	36	36	36	36	36	36	36	36
Ethiopia - Tigray	DHS	37	36	37	37	36	36	37	37	36
Fiji	Other	162	162	162	162	162	45	45	45	45
Fiji	WFS	15	15	15	15	15	6	6	6	6
Gabon	DHS	18	20	18	18	18	18	18	18	18
Georgia	MICS	6	6	6	6	6	6	6	6	6
Georgia	RHS	12	12	12	12	12	12	18	13	12
Georgia	Other	60	60	60	60	60	60	60	60	60
Germany	Other	0	0	0	0	0	0	0	1	0
Ghana	WFS	9	9	9	9	9	9	9	9	9
Ghana	DHS	75	84	75	75	75	75	75	75	75
Ghana	MICS	18	18	18	18	18	18	18	18	18
Ghana	Other	6	6	6	6	6	6	6	6	6
Ghana	VR	3	3	3	3	3	3	3	3	3
Greenland	Other	27	27	27	27	27	27	27	27	29
Greenland	VR	3	3	3	3	3	3	3	3	3
Grenada	Other	12	30	12	12	13	12	12	12	17
Guam	Other	0	3	0	0	0	0	0	0	1
Guam	VR	6	6	6	6	6	6	6	6	6
Guatemala	Other	3	3	3	3	3	3	3	3	3
Guatemala	DHS	24	24	24	24	24	24	24	24	24
Guatemala	RHS	18	18	18	18	18	18	18	18	18
Guinea	DHS	27	27	27	27	27	27	27	27	27
Guinea	MICS	9	9	9	9	9	9	9	9	9
Guinea	VR	3	3	3	3	3	3	3	3	3
Guinea-Bissau	MICS	18	18	18	18	18	18	18	18	18
Guyana	Other	156	156	156	156	156	156	156	156	156
Guyana	WFS	6	6	6	6	6	6	6	6	6
Guyana	DHS	9	9	9	9	9	9	13	10	10
Guyana	MICS	9	9	9	9	9	9	9	9	9
Haiti	WFS	6	6	6	6	6	6	6	6	6
Haiti	DHS	45	45	45	45	45	45	45	45	45
Haiti	Other	21	21	21	21	21	21	21	21	21
Honduras	Other	126	126	126	126	126	126	126	126	126
Honduras	RHS	18	18	18	18	18	18	18	18	18
Honduras	DHS	21	21	21	21	21	21	21	21	21
Hungary	Other	0	0	0	0	0	0	0	1	0
Iceland	Other	6	30	0	0	10	0	0	3	14
Iceland	VR	3	3	3	3	3	3	3	3	4
India	Other	174	174	174	174	174	174	174	174	174
India	DHS	48	48	48	48	48	48	48	48	48
Indonesia	Other	63	63	63	63	63	63	63	63	63
Indonesia	WFS	6	6	6	6	6	6	6	6	6
Indonesia	DHS	96	96	96	96	96	96	96	96	96
Indonesia - Aceh	DHS	6	6	6	8	6	6	10	7	7
Indonesia - Bali	DHS	4	9	4	12	4	4	12	8	8
Indonesia - Bangka-Belitung Islands	DHS	4	6	4	5	3	3	8	4	3
Indonesia - Banten	DHS	6	6	7	8	6	6	8	8	7
Indonesia - Bengkulu	DHS	3	7	8	5	5	3	8	7	7
Indonesia - Central Java	DHS	9	9	12	10	10	9	11	10	10
Indonesia - Central Kalimantan	DHS	3	12	5	10	5	3	6	7	4
Indonesia - Central Sulawesi	DHS	7	11	7	8	6	6	11	8	6
Indonesia - East Java	DHS	9	9	9	10	9	9	10	9	9
Indonesia - East Kalimantan	DHS	4	4	5	9	4	3	10	6	6
Indonesia - East Nusa Tenggara	DHS	6	8	6	6	6	6	7	6	6
Indonesia - Gorontalo	DHS	3	10	3	3	3	3	9	5	5
Indonesia - Jakarta	DHS	6	11	9	9	9	6	9	8	7
Indonesia - Jambi	DHS	3	5	10	8	6	3	12	10	10

Indonesia - Lampung	DHS	6	8	7	13	7	6	12	8	7
Indonesia - Maluku	DHS	6	8	6	6	6	6	7	7	6
Indonesia - North Kalimantan	DHS	4	6	3	7	3	3	8	10	8
Indonesia - North Maluku	DHS	6	8	6	6	6	6	6	7	6
Indonesia - North Sulawesi	DHS	1	5	5	4	4	0	5	7	4
Indonesia - North Sumatra	DHS	6	6	6	6	6	6	8	7	6
Indonesia - Papua	DHS	0	6	0	2	0	0	0	0	0
Indonesia - Riau	DHS	3	8	4	5	4	3	8	6	5
Indonesia - Riau Islands	DHS	4	8	10	8	8	3	8	8	6
Indonesia - South Kalimantan	DHS	3	5	7	10	7	3	7	8	4
Indonesia - South Sulawesi	DHS	6	8	7	7	6	6	11	6	6
Indonesia - South Sumatra	DHS	6	12	7	7	7	6	12	11	10
Indonesia - Southeast Sulawesi	DHS	6	6	6	7	6	6	8	6	6
Indonesia - West Java	DHS	9	9	9	9	9	9	9	9	9
Indonesia - West Kalimantan	DHS	5	7	5	10	4	3	6	6	6
Indonesia - West Nusa Tenggara	DHS	7	7	7	7	6	6	9	9	7
Indonesia - West Papua	DHS	3	1	1	5	1	0	0	2	0
Indonesia - West Sulawesi	DHS	6	7	6	6	6	6	6	6	6
Indonesia - West Sumatra	DHS	3	8	6	8	4	3	7	8	5
Indonesia - Yogyakarta	DHS	4	9	5	9	5	1	7	9	7
Iran	Other	108	108	108	108	108	108	108	108	108
Iran - Alborz	Other	42	42	42	42	42	42	42	42	42
Iran - Ardebil	Other	42	42	42	42	42	42	42	42	42
Iran - Bushehr	Other	21	21	21	21	21	21	21	21	21
Iran - Chahar Mahaal and Bakhtiari	Other	27	27	27	27	27	27	27	27	27
Iran - East Azarbayejan	Other	39	39	39	39	39	39	39	39	39
Iran - Fars	Other	36	36	36	36	36	36	36	36	36
Iran - Gilan	Other	30	30	30	30	30	30	30	30	30
Iran - Golestan	Other	42	42	42	42	42	42	42	42	42
Iran - Hamadan	Other	36	36	36	36	36	36	36	36	36
Iran - Hormozgan	Other	33	33	33	33	33	33	33	33	33
Iran - Ilam	Other	39	39	39	39	39	39	39	39	39
Iran - Isfahan	Other	39	39	39	39	39	39	39	39	39
Iran - Kerman	Other	42	42	42	42	42	42	42	42	42
Iran - Kermanshah	Other	33	33	33	33	33	33	33	33	33
Iran - Khorasan-e-Razavi	Other	36	36	36	36	36	36	36	36	36
Iran - Khuzestan	Other	42	42	42	42	42	42	42	42	42
Iran - Kohgiluyeh and Boyer-Ahmad	Other	39	39	39	39	39	39	39	39	39
Iran - Kurdistan	Other	42	42	42	42	42	42	42	42	42
Iran - Lorestan	Other	36	36	36	36	36	36	36	36	36
Iran - Markazi	Other	27	27	27	27	27	27	27	27	27
Iran - Mazandaran	Other	39	39	39	39	39	39	39	39	39
Iran - North Khorasan	Other	42	42	42	42	42	42	42	42	42
Iran - Qazvin	Other	42	42	42	42	42	42	42	42	42
Iran - Qom	Other	15	15	15	15	15	15	15	15	15
Iran - Sistan and Baluchistan	Other	42	42	42	42	42	42	42	42	42
Iran - South Khorasan	Other	33	33	33	33	33	33	33	33	33
Iran - Tehran	Other	12	12	12	12	12	12	12	12	12
Iran - West Azarbayejan	Other	39	39	39	39	39	39	39	39	39
Iran - Yazd	Other	21	21	21	21	21	21	21	21	21
Iran - Zanjan	Other	42	42	42	42	42	42	42	42	42
Iraq	Other	90	90	90	90	90	90	90	90	90
Iraq	MICS	51	51	51	51	51	51	51	51	51
Ireland	Other	3	3	3	3	3	3	3	5	3
Italy - Abruzzo	Other	0	0	0	0	0	0	0	0	3
Italy - Abruzzo	VR	0	0	0	0	0	0	0	0	1
Italy - Basilicata	Other	6	6	6	6	6	6	6	6	17
Italy - Basilicata	VR	3	3	3	3	3	3	3	3	3
Italy - Friuli-Venezia Giulia	Other	0	0	0	0	1	0	0	0	4
Italy - Liguria	Other	0	0	0	0	0	0	0	0	1
Italy - Molise	Other	6	6	6	6	6	6	6	6	20
Italy - Molise	VR	0	0	0	0	0	1	0	0	0
Italy - Provincia autonoma di Bolzano	Other	0	0	0	0	0	0	0	0	14
Italy - Provincia autonoma di Trento	Other	0	1	0	0	0	0	0	0	10
Italy - Puglia	VR	0	0	0	0	0	0	0	0	1
Italy - Umbria	Other	3	3	3	3	3	3	3	3	12
Italy - Umbria	VR	3	3	3	3	3	3	3	3	3
Italy - Valle d'Aosta	Other	21	21	21	21	21	21	21	21	61
Italy - Valle d'Aosta	VR	3	3	3	3	3	3	3	3	3
Jamaica	Other	69	69	69	69	69	69	69	69	69
Jamaica	WFS	6	6	6	6	6	6	6	6	6
Jamaica	VR	6	6	6	6	6	6	6	6	6
Japan	Other	3	3	3	3	3	3	3	5	3
Japan - Aichi	Other	0	0	0	0	0	0	0	8	0
Japan - Aomori	Other	0	0	0	0	0	0	0	6	0
Japan - Chiba	Other	0	0	0	0	0	0	0	6	0
Japan - Ehime	Other	0	0	0	0	0	0	0	1	0
Japan - Fukui	Other	0	0	0	0	0	0	0	2	0
Japan - Fukuoka	Other	0	2	0	0	0	0	0	5	0
Japan - Fukushima	Other	0	3	0	0	0	0	0	5	0
Japan - Gifu	Other	0	0	0	0	0	0	0	6	0
Japan - Gunma	Other	0	0	0	0	0	0	0	3	0
Japan - Hiroshima	Other	0	1	0	0	0	0	1	3	0
Japan - Hokkaidō	Other	0	0	0	0	0	0	0	7	0
Japan - Hyōgo	Other	3	4	3	3	3	3	3	8	3
Japan - Ibaraki	Other	0	3	0	0	0	0	3	5	0
Japan - Ishikawa	Other	0	0	0	0	0	0	0	1	0
Japan - Iwate	Other	1	1	1	1	1	1	1	4	1
Japan - Kagawa	Other	0	0	0	0	0	0	0	1	0
Japan - Kagoshima	Other	0	0	0	0	0	0	0	2	0
Japan - Kanagawa	Other	0	0	0	0	0	0	0	8	0
Japan - Kumamoto	Other	0	0	0	0	0	0	0	5	0
Japan - Kyōto	Other	0	4	0	0	0	0	1	3	0
Japan - Mie	Other	0	0	0	0	0	0	0	3	0
Japan - Miyagi	Other	3	4	3	3	3	3	3	8	3
Japan - Miyazaki	Other	0	0	0	0	0	0	0	4	0
Japan - Nagano	Other	0	2	0	0	0	0	0	1	0
Japan - Nagasaki	Other	0	0	0	0	0	0	0	2	0
Japan - Nara	Other	0	0	0	0	0	0	0	2	0
Japan - Niigata	Other	0	0	0	0	0	0	0	9	0
Japan - Okayama	Other	0	0	0	0	0	0	0	3	0
Japan - Okinawa	Other	0	0	0	0	0	0	0	5	0
Japan - Saga	Other	0	0	0	0	0	0	0	1	0
Japan - Saitama	Other	0	1	0	0	0	0	0	11	0
Japan - Shiga	Other	0	0	0	0	0	0	0	4	0
Japan - Shizuoka	Other	0	2	0	0	0	0	0	10	0
Japan - Tochigi	Other	0	0	0	0	0	0	0	6	0

Japan - Tottori	Other	3	3	3	3	3	3	3	3	3
Japan - Toyama	Other	0	0	0	0	0	0	0	3	0
Japan - Tōkyō	Other	0	0	0	0	0	0	0	6	0
Japan - Wakayama	Other	0	0	0	0	0	0	0	1	0
Japan - Yamagata	Other	0	0	0	0	0	0	0	1	0
Japan - Yamaguchi	Other	3	3	3	3	3	3	3	3	3
Japan - Ōita	Other	0	0	0	0	0	0	0	3	0
Japan - Ōsaka	Other	0	0	0	0	0	0	0	4	0
Jordan	DHS	63	63	63	63	63	63	63	63	63
Jordan	Other	75	75	75	75	75	75	75	75	75
Kazakhstan	MICS	3	3	3	3	3	3	3	3	3
Kazakhstan	DHS	21	23	21	21	21	21	22	22	21
Kazakhstan	Other	36	38	36	36	36	36	36	36	36
Kenya	WFS	9	9	9	9	9	9	9	9	9
Kenya	DHS	57	57	57	57	57	57	57	57	57
Kenya	Other	3	3	3	3	3	3	3	3	3
Kenya	VR	3	3	3	3	3	3	3	3	3
Kenya - Baringo	DHS	3	4	3	3	4	3	8	5	4
Kenya - Bomet	DHS	13	19	12	12	14	12	15	19	13
Kenya - Bungoma	DHS	14	17	6	6	7	6	11	9	6
Kenya - Busia	DHS	35	44	33	33	34	33	39	41	37
Kenya - Elgeyo Marakwet	DHS	37	46	36	36	38	36	48	47	44
Kenya - Embu	DHS	49	55	48	48	48	48	50	50	49
Kenya - Embu	MICS	6	6	6	6	6	6	6	6	6
Kenya - Garissa	DHS	1	10	0	0	4	0	8	4	2
Kenya - Homa Bay	MICS	6	6	6	6	6	6	6	6	6
Kenya - Homa Bay	DHS	8	13	6	6	6	6	7	6	6
Kenya - Isiolo	DHS	21	22	21	21	21	21	23	25	23
Kenya - Isiolo	MICS	3	4	3	3	3	3	5	4	3
Kenya - Kajiado	DHS	49	55	39	39	45	43	51	49	45
Kenya - Kakamega	DHS	14	23	9	9	9	9	10	9	9
Kenya - Kericho	DHS	20	34	18	18	19	18	43	25	23
Kenya - Kiambu	DHS	33	44	30	30	32	30	45	42	38
Kenya - Kilifi	DHS	19	32	18	18	19	18	24	25	20
Kenya - Kirinyaga	DHS	32	46	30	30	39	32	38	44	34
Kenya - Kisii	DHS	17	29	15	15	19	16	23	18	16
Kenya - Kisii	MICS	6	7	6	6	6	6	6	6	6
Kenya - Kisumu	DHS	11	23	3	3	3	3	8	7	4
Kenya - Kisumu	MICS	6	9	6	6	6	6	6	6	6
Kenya - Kitui	MICS	12	12	12	12	12	12	12	12	12
Kenya - Kitui	DHS	22	46	15	15	20	16	31	29	25
Kenya - Kwale	DHS	12	34	3	3	11	5	26	19	13
Kenya - Laikipia	DHS	48	48	48	48	49	48	52	51	51
Kenya - Lamu	DHS	24	27	23	23	23	23	27	26	24
Kenya - Machakos	DHS	30	37	30	30	32	30	44	35	34
Kenya - Machakos	MICS	6	9	6	6	7	6	7	6	6
Kenya - Makueni	DHS	25	27	24	24	25	24	29	25	25
Kenya - Makueni	MICS	6	6	6	6	6	6	8	6	6
Kenya - Mandera	DHS	11	17	3	3	5	3	19	7	7
Kenya - Marsabit	MICS	21	21	21	21	21	21	21	21	21
Kenya - Marsabit	DHS	21	24	21	21	22	21	25	24	22
Kenya - Meru	DHS	7	37	6	6	15	6	35	17	14
Kenya - Meru	MICS	9	9	9	9	9	9	10	9	9
Kenya - Migori	DHS	5	13	3	3	3	3	4	6	3
Kenya - Migori	MICS	3	4	3	3	3	3	3	3	3
Kenya - Mombasa	DHS	3	30	0	0	9	0	15	18	8
Kenya - Mombasa	MICS	3	5	3	3	3	3	5	4	3
Kenya - Murang'a	DHS	32	52	30	30	34	30	47	49	41
Kenya - Nairobi	DHS	11	25	6	6	10	6	17	11	9
Kenya - Nakuru	DHS	23	49	18	18	22	18	39	48	32
Kenya - Nandi	DHS	37	51	36	36	38	36	49	38	37
Kenya - Narok	DHS	37	47	30	30	35	33	40	41	37
Kenya - Nyamira	DHS	25	26	24	24	26	24	31	31	29
Kenya - Nyamira	MICS	6	8	6	6	6	6	10	7	6
Kenya - Nyandarua	DHS	49	45	42	42	44	43	53	52	46
Kenya - Nyeri	DHS	35	46	33	33	38	33	47	49	44
Kenya - Samburu	DHS	18	21	18	18	18	18	20	23	19
Kenya - Siaya	DHS	16	36	12	12	12	12	14	13	12
Kenya - Siaya	MICS	6	6	6	6	6	6	6	6	6
Kenya - Taita Taveta	DHS	36	43	33	33	39	34	45	42	38
Kenya - Tana River	DHS	27	29	27	27	27	27	28	27	27
Kenya - Tharaka Nithi	MICS	6	10	6	6	6	6	6	6	6
Kenya - Tharaka Nithi	DHS	14	17	14	14	17	14	18	16	15
Kenya - Trans Nzoia	DHS	40	47	30	30	33	31	41	39	36
Kenya - Turkana	DHS	14	24	12	12	13	12	14	17	12
Kenya - Uasin Gishu	DHS	27	35	24	24	27	24	35	31	26
Kenya - Vihiga	DHS	14	20	3	3	9	7	14	13	9
Kenya - Wajir	DHS	5	11	3	3	5	5	13	4	4
Kenya - West Pokot	DHS	37	42	36	36	37	36	43	43	41
Kiribati	Other	21	21	21	21	21	21	21	21	21
Kyrgyzstan	MICS	12	12	12	12	12	12	12	13	12
Kyrgyzstan	DHS	15	15	15	15	15	15	15	15	15
Kyrgyzstan	LSMS	6	6	6	6	6	6	7	6	6
Kyrgyzstan	Other	48	48	48	48	48	48	48	48	48
Laos	MICS	18	18	18	18	18	18	18	18	18
Latvia	MICS	3	3	3	3	3	3	3	3	3
Latvia	Other	0	0	0	0	0	0	0	2	0
Lebanon	Other	15	15	15	15	15	15	16	16	16
Lebanon	PAPFAM	9	9	9	9	9	9	16	12	12
Lebanon	VR	3	3	3	3	3	3	3	3	3
Lesotho	WFS	6	6	6	6	6	6	6	6	6
Lesotho	DHS	27	27	27	27	27	27	27	27	27
Liberia	DHS	30	30	30	30	30	30	30	30	30
Liberia	Other	9	9	9	9	9	9	9	9	9
Libya	Other	15	15	15	15	15	15	15	15	15
Lithuania	MICS	3	3	3	3	3	3	3	3	3
Lithuania	Other	0	0	0	0	0	0	0	2	0
Luxembourg	Other	2	18	0	0	6	0	0	1	11
Madagascar	Other	39	39	39	39	39	39	39	39	39
Madagascar	DHS	39	39	39	39	39	39	39	39	39
Malawi	DHS	39	39	39	39	39	39	39	39	39
Malawi	MICS	21	21	21	21	21	21	21	21	21
Malaysia	Other	15	15	15	15	15	15	15	15	15
Malaysia	WFS	6	6	6	6	6	6	6	6	6
Malaysia	VR	6	6	6	6	6	6	6	6	6
Maldives	Other	84	84	84	84	84	84	84	84	84
Maldives	DHS	18	18	18	19	18	18	23	18	18

Mali	DHS	45	45	45	45	45	45	45	45	45
Mali	Other	9	9	9	9	9	9	9	9	9
Malta	Other	0	6	0	0	0	0	0	2	4
Mauritania	WFS	9	9	9	9	9	9	9	9	9
Mauritania	Other	9	9	9	9	9	9	9	9	9
Mauritania	DHS	9	9	9	9	9	9	9	9	9
Mauritania	MICS	9	9	9	9	9	9	9	9	9
Mexico	Other	183	183	183	183	183	183	183	183	183
Mexico	WFS	6	6	6	6	6	6	6	6	6
Mexico	DHS	9	9	9	9	9	9	9	9	9
Mexico	VR	3	3	3	3	3	3	3	3	3
Mexico - Aguascalientes	Other	141	141	132	132	132	132	132	133	132
Mexico - Aguascalientes	DHS	9	9	9	9	9	9	9	9	9
Mexico - Baja California	Other	120	120	111	111	111	111	112	113	112
Mexico - Baja California	DHS	9	9	9	9	9	9	9	9	9
Mexico - Baja California	VR	3	3	3	3	3	3	3	3	3
Mexico - Baja California Sur	Other	111	111	102	102	102	102	106	104	103
Mexico - Baja California Sur	VR	3	3	3	3	3	3	3	3	3
Mexico - Campeche	Other	147	147	138	138	138	138	138	142	138
Mexico - Campeche	DHS	9	9	9	9	9	9	9	9	9
Mexico - Campeche	VR	3	3	3	3	3	3	3	3	3
Mexico - Chiapas	DHS	12	12	12	12	12	12	12	12	12
Mexico - Chiapas	Other	153	153	144	144	144	144	144	144	144
Mexico - Chihuahua	Other	153	153	144	144	144	144	145	145	144
Mexico - Chihuahua	DHS	9	9	9	9	9	9	9	9	9
Mexico - Coahuila	Other	150	150	141	141	141	141	142	144	141
Mexico - Coahuila	DHS	9	9	9	9	9	9	9	9	9
Mexico - Colima	Other	147	147	138	138	138	138	138	138	138
Mexico - Colima	DHS	9	9	9	9	9	9	9	9	9
Mexico - Colima	VR	3	3	3	3	3	3	3	3	3
Mexico - Durango	Other	147	147	138	138	138	138	138	138	138
Mexico - Durango	DHS	9	9	9	9	9	9	9	9	9
Mexico - Guanajuato	Other	102	102	93	93	93	93	93	95	93
Mexico - Guanajuato	DHS	9	9	9	9	9	9	9	9	9
Mexico - Guanajuato	VR	3	3	3	3	3	3	3	3	3
Mexico - Guerrero	DHS	12	12	12	12	12	12	12	12	12
Mexico - Guerrero	Other	153	153	144	144	144	144	144	144	144
Mexico - Guerrero	VR	3	3	3	3	3	3	3	3	3
Mexico - Hidalgo	Other	141	141	132	132	132	132	132	132	132
Mexico - Hidalgo	DHS	9	9	9	9	9	9	9	9	9
Mexico - Jalisco	DHS	12	12	12	12	12	12	12	12	12
Mexico - Jalisco	Other	132	132	123	123	123	123	124	125	123
Mexico - Mexico City	DHS	12	12	12	12	12	12	12	12	12
Mexico - Mexico City	Other	96	96	87	87	87	87	90	87	87
Mexico - Michoacán de Ocampo	Other	156	156	147	147	147	147	148	147	147
Mexico - Michoacán de Ocampo	DHS	9	9	9	9	9	9	9	9	9
Mexico - Michoacán de Ocampo	VR	3	3	3	3	3	3	3	3	3
Mexico - Morelos	Other	150	150	141	141	141	141	142	141	141
Mexico - Morelos	DHS	9	9	9	9	9	9	9	9	9
Mexico - Morelos	VR	3	3	3	3	3	3	3	3	3
Mexico - México	Other	108	108	99	99	99	99	99	99	99
Mexico - México	DHS	9	9	9	9	9	9	9	9	9
Mexico - México	VR	3	3	3	3	3	3	3	3	3
Mexico - Nayarit	Other	150	150	141	141	141	141	141	141	141
Mexico - Nayarit	DHS	9	9	9	9	9	9	9	9	9
Mexico - Nayarit	VR	3	3	3	3	3	3	3	3	3
Mexico - Nuevo León	DHS	12	12	12	12	12	12	12	12	12
Mexico - Nuevo León	Other	150	150	141	144	141	141	142	142	142
Mexico - Oaxaca	Other	156	156	147	147	147	147	147	147	147
Mexico - Oaxaca	DHS	9	9	9	9	9	9	9	9	9
Mexico - Oaxaca	VR	3	3	3	3	3	3	3	3	3
Mexico - Puebla	Other	141	141	132	132	132	132	132	132	132
Mexico - Puebla	DHS	9	9	9	9	9	9	9	9	9
Mexico - Puebla	VR	3	3	3	3	3	3	3	3	3
Mexico - Querétaro	DHS	12	12	12	12	12	12	12	12	12
Mexico - Querétaro	Other	114	114	105	105	105	105	105	105	105
Mexico - Quintana Roo	Other	147	147	138	138	138	138	140	138	138
Mexico - Quintana Roo	VR	3	3	3	3	3	3	3	3	3
Mexico - San Luis Potosí	Other	156	156	147	147	147	147	147	151	147
Mexico - San Luis Potosí	DHS	9	9	9	9	9	9	9	9	9
Mexico - San Luis Potosí	VR	3	3	3	3	3	3	3	3	3
Mexico - Sinaloa	Other	150	150	141	141	141	141	142	142	142
Mexico - Sinaloa	DHS	9	9	9	9	9	9	9	9	9
Mexico - Sonora	Other	150	150	141	141	141	141	143	143	142
Mexico - Sonora	DHS	9	9	9	9	9	9	9	9	9
Mexico - Sonora	VR	3	3	3	3	3	3	3	3	3
Mexico - Tabasco	Other	159	159	150	150	150	150	150	150	150
Mexico - Tabasco	DHS	9	9	9	9	9	9	9	9	9
Mexico - Tamaulipas	Other	150	150	141	141	141	141	142	144	141
Mexico - Tamaulipas	DHS	9	9	9	9	9	9	9	9	9
Mexico - Tlaxcala	Other	96	96	87	87	87	87	87	87	87
Mexico - Tlaxcala	DHS	9	9	9	9	9	9	9	9	9
Mexico - Tlaxcala	VR	3	3	3	3	3	3	3	3	3
Mexico - Veracruz de Ignacio de la Llave	DHS	12	12	12	12	12	12	12	12	12
Mexico - Veracruz de Ignacio de la Llave	Other	153	153	144	144	144	144	144	145	144
Mexico - Veracruz de Ignacio de la Llave	VR	3	3	3	3	3	3	3	3	3
Mexico - Yucatán	Other	150	150	141	141	141	141	142	142	141
Mexico - Yucatán	DHS	9	9	9	9	9	9	9	9	9
Mexico - Zacatecas	Other	150	150	141	141	141	141	143	142	142
Mexico - Zacatecas	DHS	9	9	9	9	9	9	9	9	9
Mexico - Zacatecas	VR	3	3	3	3	3	3	3	3	3
Moldova	MICS	15	19	15	15	15	15	21	21	18
Moldova	DHS	9	13	9	9	9	9	10	9	9
Moldova	Other	81	81	81	81	81	81	81	81	81
Monaco	Other	18	18	18	18	18	21	18	18	34
Mongolia	Other	9	9	9	9	9	9	9	9	9
Mongolia	MICS	9	9	9	9	9	9	9	9	9
Mongolia	VR	39	39	39	39	39	39	39	39	39
Montenegro	Other	105	105	105	105	105	105	105	109	105
Montenegro	VR	3	3	3	3	3	3	3	3	3
Morocco	WFS	9	9	9	9	9	9	9	9	9
Morocco	DHS	42	42	42	42	42	42	42	42	42
Morocco	Other	78	78	78	78	78	78	78	78	78
Morocco	PAPFAM	21	21	21	21	21	21	21	21	21
Mozambique	DHS	45	45	45	45	45	45	45	45	45
Mozambique	MICS	12	12	12	12	12	12	12	12	12
Mozambique	RHS	6	6	6	6	6	6	6	6	6

Myanmar	Other	3	3	3	3	3	3	3	3	3
Myanmar	VR	3	3	3	3	3	3	3	3	3
Namibia	DHS	36	36	36	36	36	36	36	36	36
Namibia	VR	3	3	3	3	3	3	3	3	3
Nauru	Other	3	3	3	3	3	3	3	3	7
Nauru	VR	3	3	3	3	3	3	3	3	9
Nepal	WFS	6	6	6	6	6	6	6	6	6
Nepal	DHS	36	36	36	36	36	36	36	36	36
Nepal	MICS	9	9	9	9	9	9	9	9	9
Netherlands	Other	0	0	0	0	0	0	0	1	0
New Zealand	Other	0	0	0	0	0	0	0	3	0
Nicaragua	Other	147	147	147	147	147	147	147	147	147
Nicaragua	RHS	18	18	18	18	18	18	18	18	18
Nicaragua	DHS	39	39	30	30	30	30	30	30	30
Niger	DHS	36	36	36	36	36	36	36	36	36
Nigeria	DHS	75	75	75	75	75	75	75	75	75
Nigeria	Other	21	21	21	21	21	21	21	21	21
Nigeria	MICS	12	12	12	12	12	12	12	12	12
Niue	Other	24	24	24	24	24	24	24	24	26
Niue	VR	48	48	48	48	48	61	48	48	71
North Macedonia	Other	0	0	0	0	0	0	0	3	0
Northern Mariana Islands	Other	3	7	4	6	4	3	3	3	5
Norway	Other	0	0	0	0	0	0	1	0	0
Norway - Agder	VR	24	24	24	24	24	24	24	24	35
Norway - Innlandet	VR	0	0	0	0	0	0	0	0	14
Norway - Møre og Romsdal	VR	9	9	9	9	9	9	9	9	22
Norway - Nordland	VR	9	9	9	9	9	9	9	9	18
Norway - Oslo	VR	3	3	3	3	3	3	3	3	5
Norway - Rogaland	VR	0	0	0	0	0	0	0	0	9
Norway - Troms og Finnmark	VR	0	0	0	0	0	1	0	0	14
Norway - Trøndelag	VR	6	6	6	6	6	6	6	6	12
Norway - Vestfold og Telemark	VR	9	9	9	9	9	9	9	9	17
Norway - Vestland	VR	0	0	0	0	0	0	0	0	4
Norway - Viken	VR	0	0	0	0	0	0	0	0	2
Oman	Other	12	12	12	12	12	12	12	12	12
Pakistan	WFS	6	6	6	6	6	6	6	6	6
Pakistan	DHS	66	66	66	66	66	66	66	66	66
Pakistan	Other	75	75	66	66	66	66	66	66	66
Pakistan	LSMS	18	18	12	12	12	12	12	12	12
Pakistan - Balochistan	DHS	27	27	27	27	27	27	27	27	27
Pakistan - Khyber Pakhtunkhwa	DHS	12	12	12	12	12	12	12	12	12
Pakistan - Punjab	DHS	15	15	15	15	15	15	15	15	15
Pakistan - Sindh	DHS	15	15	15	15	15	15	15	15	15
Palau	Other	3	3	3	3	3	3	3	3	3
Palestine	Other	78	78	78	78	78	78	78	78	78
Palestine	DHS	18	18	9	9	9	9	9	9	9
Palestine	MICS	21	21	21	21	21	21	21	21	21
Panama	Other	3	3	3	3	3	3	3	3	3
Panama	WFS	6	6	6	6	6	6	6	6	6
Papua New Guinea	Other	3	3	3	3	3	3	3	3	3
Paraguay	Other	156	156	156	156	156	156	156	156	156
Paraguay	DHS	9	9	9	9	9	9	9	9	9
Paraguay	RHS	27	28	27	27	27	27	27	31	27
Peru	Other	225	225	225	225	225	225	225	225	225
Peru	WFS	9	9	9	9	9	9	9	9	9
Peru	DHS	132	132	132	132	132	132	132	132	132
Peru	VR	3	3	3	3	3	3	3	3	3
Philippines	Other	204	204	204	204	204	204	204	204	204
Philippines	WFS	9	9	9	9	9	9	9	9	9
Philippines	DHS	114	114	114	114	114	114	114	114	114
Philippines - Abra	Other	36	36	36	36	36	36	36	36	36
Philippines - Abra	DHS	39	39	39	39	39	39	39	39	39
Philippines - Agusan Del Norte	Other	60	60	60	60	60	60	60	60	60
Philippines - Agusan Del Norte	DHS	42	42	42	42	42	42	42	42	42
Philippines - Agusan Del Sur	Other	48	49	48	48	48	48	48	48	48
Philippines - Agusan Del Sur	DHS	42	42	42	42	42	42	42	42	42
Philippines - Aklan	Other	39	39	39	39	39	39	39	39	39
Philippines - Aklan	DHS	39	39	39	39	39	39	39	39	39
Philippines - Albay	Other	33	33	33	33	33	33	33	33	33
Philippines - Albay	DHS	42	42	42	42	42	42	42	42	42
Philippines - Antique	Other	60	60	60	60	60	60	60	60	60
Philippines - Antique	DHS	36	36	36	36	36	36	36	36	36
Philippines - Apayao	DHS	39	39	39	39	39	39	39	39	39
Philippines - Apayao	Other	3	3	3	3	3	3	3	3	3
Philippines - Aurora	DHS	38	38	38	38	38	38	38	38	38
Philippines - Aurora	Other	27	27	27	27	27	27	27	27	27
Philippines - Basilan	Other	24	24	24	24	24	24	24	24	24
Philippines - Basilan	DHS	39	39	39	39	39	39	39	39	39
Philippines - Bataan	Other	39	39	39	39	39	39	39	39	39
Philippines - Bataan	DHS	39	39	39	39	39	39	39	39	39
Philippines - Batanes	Other	6	6	6	6	6	6	6	6	7
Philippines - Batanes	DHS	25	25	25	25	25	25	25	25	25
Philippines - Batangas	Other	42	42	42	42	42	42	42	42	42
Philippines - Batangas	DHS	42	42	42	42	42	42	42	42	42
Philippines - Benguet	Other	42	42	42	42	42	42	42	42	42
Philippines - Benguet	DHS	42	42	42	42	42	42	42	42	42
Philippines - Biliran	DHS	39	39	39	39	39	39	39	39	39
Philippines - Biliran	Other	30	30	30	31	30	30	30	30	30
Philippines - Bohol	Other	78	78	78	78	78	78	78	78	78
Philippines - Bohol	DHS	39	39	39	39	39	39	39	39	39
Philippines - Bukidnon	Other	78	78	78	78	78	78	78	78	78
Philippines - Bukidnon	DHS	42	42	42	42	42	42	42	42	42
Philippines - Bulacan	Other	42	42	42	42	42	42	42	42	42
Philippines - Bulacan	DHS	42	42	42	42	42	42	42	42	42
Philippines - Cagayan	Other	63	63	63	63	63	63	63	63	63
Philippines - Cagayan	DHS	42	42	42	42	42	42	42	42	42
Philippines - Camarines Norte	Other	42	42	42	42	42	42	42	42	42
Philippines - Camarines Norte	DHS	39	39	39	39	39	39	39	39	39
Philippines - Camarines Sur	Other	57	57	57	57	57	57	57	57	57
Philippines - Camarines Sur	DHS	42	42	42	42	42	42	42	42	42
Philippines - Camiguin	DHS	36	36	36	36	36	36	36	36	36
Philippines - Capiz	Other	78	78	78	78	78	78	78	78	78
Philippines - Capiz	DHS	42	42	42	42	42	42	42	42	42
Philippines - Catanduanes	Other	48	48	48	48	48	48	48	48	48
Philippines - Catanduanes	DHS	38	38	38	38	38	38	38	38	38
Philippines - Cavite	Other	42	42	42	42	42	42	42	42	42
Philippines - Cavite	DHS	42	42	42	42	42	42	42	42	42

Philippines - Cebu	Other	42	42	42	42	42	42	42	42	42
Philippines - Cebu	DHS	45	45	45	45	45	45	45	45	45
Philippines - Cotabato (North Cotabato)	Other	78	78	78	78	78	78	78	78	78
Philippines - Cotabato (North Cotabato)	DHS	42	42	42	42	42	42	42	42	42
Philippines - Davao Del Norte	Other	36	36	36	36	36	36	36	36	36
Philippines - Davao Del Norte	DHS	39	39	39	39	39	39	39	39	39
Philippines - Davao Del Sur	Other	42	42	42	42	42	42	42	42	42
Philippines - Davao Del Sur	DHS	45	45	45	45	45	45	45	45	45
Philippines - Davao Occidental	DHS	9	9	9	9	9	9	9	9	9
Philippines - Davao Occidental	Other	9	9	9	9	9	9	9	9	9
Philippines - Davao Oriental	Other	75	75	75	75	75	75	75	75	75
Philippines - Davao Oriental	DHS	42	42	42	42	42	42	42	42	42
Philippines - Davao de Oro	Other	45	45	45	45	45	45	45	45	45
Philippines - Davao de Oro	DHS	36	36	36	36	36	36	36	36	36
Philippines - Dinagat Islands	DHS	9	9	9	9	9	9	9	9	9
Philippines - Dinagat Islands	Other	3	3	3	3	3	3	3	3	3
Philippines - Eastern Samar	Other	51	51	51	51	51	51	51	51	51
Philippines - Eastern Samar	DHS	39	39	39	39	39	39	39	39	39
Philippines - Guimaras	DHS	35	35	35	35	35	35	35	35	35
Philippines - Ifugao	Other	48	48	48	48	48	48	48	48	48
Philippines - Ifugao	DHS	42	42	42	42	42	42	42	42	42
Philippines - Ilocos Norte	Other	42	42	42	42	42	42	42	42	42
Philippines - Ilocos Norte	DHS	39	39	39	39	39	39	39	39	39
Philippines - Ilocos Sur	Other	42	42	42	42	42	42	42	42	42
Philippines - Ilocos Sur	DHS	39	39	39	39	39	39	39	39	39
Philippines - Iloilo	Other	42	42	42	42	42	42	42	42	42
Philippines - Iloilo	DHS	42	42	42	42	42	42	42	42	42
Philippines - Isabela	Other	48	48	48	48	48	48	48	48	48
Philippines - Isabela	DHS	45	45	45	45	45	45	45	45	45
Philippines - Kalinga	Other	33	33	33	33	33	33	33	33	33
Philippines - Kalinga	DHS	39	39	39	39	39	39	39	39	39
Philippines - La Union	Other	42	42	42	42	42	42	42	42	42
Philippines - La Union	DHS	36	36	36	36	36	36	36	36	36
Philippines - Laguna	Other	42	42	42	42	42	42	42	42	42
Philippines - Laguna	DHS	42	42	42	42	42	42	42	42	42
Philippines - Lanao Del Norte	Other	78	78	78	78	78	78	78	78	78
Philippines - Lanao Del Norte	DHS	42	42	42	42	42	42	42	42	42
Philippines - Lanao Del Sur	Other	46	46	45	47	45	45	46	48	45
Philippines - Lanao Del Sur	DHS	42	42	42	42	42	42	42	42	42
Philippines - Leyte	Other	78	78	78	78	78	78	78	78	78
Philippines - Leyte	DHS	42	42	42	42	42	42	42	42	42
Philippines - Maguindanao	Other	36	36	38	39	36	36	36	37	36
Philippines - Maguindanao	DHS	42	42	42	42	42	42	42	42	42
Philippines - Marinduque	Other	39	39	39	39	39	39	39	39	39
Philippines - Marinduque	DHS	39	39	39	39	39	39	39	39	39
Philippines - Masbate	Other	78	78	78	78	78	78	78	78	78
Philippines - Masbate	DHS	39	39	39	39	39	39	39	39	39
Philippines - Misamis Occidental	Other	75	75	75	75	75	75	75	75	75
Philippines - Misamis Occidental	DHS	39	39	39	39	39	39	39	39	39
Philippines - Misamis Oriental	Other	69	69	69	69	69	69	69	69	69
Philippines - Misamis Oriental	DHS	42	42	42	42	42	42	42	42	42
Philippines - Mountain Province	Other	39	39	39	39	39	39	39	39	39
Philippines - Mountain Province	DHS	39	39	39	39	39	39	39	39	39
Philippines - National Capital Region	Other	60	60	60	60	60	60	60	60	60
Philippines - National Capital Region	DHS	67	67	67	67	67	67	67	67	67
Philippines - Negros Occidental	Other	66	66	66	66	66	66	66	66	66
Philippines - Negros Occidental	DHS	45	45	45	45	45	45	45	45	45
Philippines - Negros Oriental	Other	78	78	78	78	78	78	78	78	78
Philippines - Negros Oriental	DHS	39	39	39	39	39	39	39	39	39
Philippines - Northern Samar	Other	60	60	60	60	60	60	60	60	60
Philippines - Northern Samar	DHS	39	39	39	39	39	39	39	39	39
Philippines - Nueva Ecija	Other	42	42	42	42	42	42	42	42	42
Philippines - Nueva Ecija	DHS	42	42	42	42	42	42	42	42	42
Philippines - Nueva Vizcaya	Other	42	42	42	42	42	42	42	42	42
Philippines - Nueva Vizcaya	DHS	39	39	39	39	39	39	39	39	39
Philippines - Occidental Mindoro	Other	78	78	78	78	78	78	78	78	78
Philippines - Occidental Mindoro	DHS	39	39	39	39	39	39	39	39	39
Philippines - Oriental Mindoro	Other	72	72	72	72	72	72	72	72	72
Philippines - Oriental Mindoro	DHS	39	39	39	39	39	39	39	39	39
Philippines - Palawan	Other	78	78	78	78	78	78	78	78	78
Philippines - Palawan	DHS	41	41	41	41	41	41	41	41	41
Philippines - Pampanga	Other	75	75	75	75	75	75	75	75	75
Philippines - Pampanga	DHS	42	42	42	42	42	42	42	42	42
Philippines - Pangasinan	Other	42	42	42	42	42	42	42	42	42
Philippines - Pangasinan	DHS	45	45	45	45	45	45	45	45	45
Philippines - Quezon	Other	42	42	42	42	42	42	42	42	42
Philippines - Quezon	DHS	42	42	42	42	42	42	42	42	42
Philippines - Quirino	Other	39	39	39	39	39	39	39	39	39
Philippines - Quirino	DHS	38	38	38	38	38	38	38	38	38
Philippines - Rizal	Other	66	66	66	66	66	66	66	66	66
Philippines - Rizal	DHS	43	43	43	43	43	43	43	43	43
Philippines - Romblon	Other	78	78	78	78	78	78	78	78	78
Philippines - Romblon	DHS	41	41	41	41	41	41	41	41	41
Philippines - Samar (Western Samar)	Other	69	70	69	69	69	69	69	69	69
Philippines - Samar (Western Samar)	DHS	39	39	39	39	39	39	39	39	39
Philippines - Sarangani	DHS	42	42	42	42	42	42	42	42	42
Philippines - Sarangani	Other	12	12	12	13	12	12	12	12	12
Philippines - Siquijor	Other	21	21	21	21	21	21	21	21	21
Philippines - Siquijor	DHS	35	35	35	35	35	35	35	35	35
Philippines - Sorsogon	Other	42	42	42	42	42	42	42	42	42
Philippines - Sorsogon	DHS	39	39	39	39	39	39	39	39	39
Philippines - South Cotabato	Other	72	72	72	72	72	72	72	72	72
Philippines - South Cotabato	DHS	42	42	42	42	42	42	42	42	42
Philippines - Southern Leyte	Other	75	75	75	75	75	75	75	75	75
Philippines - Southern Leyte	DHS	39	39	39	39	39	39	39	39	39
Philippines - Sultan Kudarat	Other	51	51	51	51	51	51	51	51	51
Philippines - Sultan Kudarat	DHS	42	42	42	42	42	42	42	42	42
Philippines - Sulu	Other	42	42	42	43	42	42	42	42	42
Philippines - Sulu	DHS	42	42	42	42	42	42	42	42	42
Philippines - Surigao Del Norte	Other	72	72	72	72	72	72	72	72	72
Philippines - Surigao Del Norte	DHS	42	42	42	42	42	42	42	42	42
Philippines - Surigao Del Sur	Other	69	69	69	69	69	69	69	69	69
Philippines - Surigao Del Sur	DHS	42	42	42	42	42	42	42	42	42
Philippines - Tarlac	Other	42	42	42	42	42	42	42	42	42
Philippines - Tarlac	DHS	39	39	39	39	39	39	39	39	39
Philippines - Tawi-Tawi	Other	21	21	21	21	21	21	21	21	21
Philippines - Tawi-Tawi	DHS	42	42	42	42	42	42	42	42	42

Philippines - Zambales	Other	42	42	42	42	42	42	42	42	42
Philippines - Zambales	DHS	39	39	39	39	39	39	39	39	39
Philippines - Zamboanga Del Norte	Other	78	78	78	78	78	78	78	78	78
Philippines - Zamboanga Del Norte	DHS	42	42	42	42	42	42	42	42	42
Philippines - Zamboanga Del Sur	Other	78	78	78	78	78	78	78	78	78
Philippines - Zamboanga Del Sur	DHS	46	46	46	46	46	46	46	46	46
Philippines - Zamboanga Sibugay	DHS	39	39	39	39	39	39	39	39	39
Philippines - Zamboanga Sibugay	Other	42	42	42	42	42	42	42	42	42
Portugal	Other	12	12	12	12	12	12	12	12	12
Portugal	WFS	9	9	9	9	9	9	10	10	9
Puerto Rico	Other	0	0	0	2	0	0	0	2	0
Puerto Rico	RHS	9	9	9	9	9	9	13	13	12
Qatar	Other	9	9	9	9	9	9	9	9	9
Qatar	VR	3	3	3	3	3	3	3	3	3
Romania	LSMS	20	20	20	20	20	20	20	21	20
Rwanda	WFS	9	9	9	9	9	9	9	9	9
Rwanda	DHS	105	105	105	105	105	105	105	105	105
Saint Kitts and Nevis	Other	0	14	0	0	16	0	0	0	12
Saint Lucia	Other	3	6	3	3	3	3	3	3	9
Saint Vincent and the Grenadines	Other	24	28	24	24	25	24	24	24	30
Samoa	Other	54	54	54	54	54	54	54	54	54
San Marino	Other	41	45	39	39	43	45	39	39	87
San Marino	VR	0	0	0	0	0	3	0	0	1
Saudi Arabia	Other	36	36	36	36	36	36	36	36	36
Senegal	WFS	9	9	9	9	9	9	9	9	9
Senegal	DHS	81	81	81	81	81	81	81	81	81
Senegal	Other	9	9	9	9	9	9	9	9	9
Senegal	MICS	9	9	9	9	9	9	9	9	9
Serbia	Other	45	45	45	45	45	45	45	46	45
Seychelles	Other	15	16	15	15	16	15	15	15	26
Sierra Leone	DHS	21	21	21	21	21	21	21	21	21
Sierra Leone	MICS	9	9	9	9	9	9	9	9	9
Sierra Leone	VR	18	18	18	18	18	18	18	18	18
Singapore	Other	39	39	39	39	39	39	40	42	39
Slovakia	Other	0	0	0	0	0	0	0	1	0
Slovenia	Other	0	0	0	0	0	0	0	8	0
Solomon Islands	Other	6	6	6	6	6	6	6	6	6
Somalia	MICS	6	6	6	6	6	6	6	6	6
South Africa	DHS	18	21	18	18	18	18	18	18	18
South Africa	Other	99	99	99	99	99	99	99	99	99
South Africa - Eastern Cape	DHS	9	14	9	9	9	9	10	9	9
South Africa - Eastern Cape	Other	78	78	78	78	78	78	78	78	78
South Africa - Free State	Other	96	96	96	96	96	96	96	96	96
South Africa - Free State	DHS	9	13	8	8	7	6	11	14	8
South Africa - Gauteng	Other	117	117	117	117	117	117	117	117	117
South Africa - Gauteng	DHS	10	16	11	13	10	9	18	18	13
South Africa - KwaZulu-Natal	Other	150	150	150	150	150	150	150	150	150
South Africa - KwaZulu-Natal	DHS	9	11	9	10	9	9	14	11	11
South Africa - Limpopo	Other	132	132	132	132	132	132	132	132	132
South Africa - Limpopo	DHS	9	17	11	12	9	9	16	13	9
South Africa - Mpumalanga	Other	123	123	123	123	123	123	123	123	123
South Africa - Mpumalanga	DHS	9	14	9	9	9	9	10	10	9
South Africa - North West	Other	102	102	102	102	102	102	102	102	102
South Africa - North West	DHS	6	15	7	6	6	6	13	10	9
South Africa - Northern Cape	Other	81	81	81	81	81	81	81	81	81
South Africa - Northern Cape	DHS	9	19	13	14	12	9	15	16	12
South Africa - Western Cape	Other	114	114	114	114	114	114	114	114	114
South Africa - Western Cape	DHS	24	24	24	24	24	24	24	24	24
South Korea	WFS	6	6	6	6	6	6	6	6	6
South Korea	Other	87	87	87	87	87	87	87	87	87
South Sudan	MICS	9	9	9	9	9	9	9	9	9
Spain	Other	0	0	0	0	0	0	0	3	0
Sri Lanka	Other	9	9	9	9	9	9	9	9	9
Sri Lanka	WFS	6	6	6	6	6	6	6	6	6
Sri Lanka	DHS	9	9	9	9	9	9	9	9	9
Sudan	WFS	9	9	9	9	9	9	9	9	9
Sudan	Other	24	24	24	24	24	24	24	24	24
Sudan	DHS	9	9	9	9	9	9	9	9	9
Sudan	MICS	18	18	18	18	18	18	18	18	18
Suriname	Other	171	171	171	171	171	171	171	171	171
Sweden	Other	3	3	3	3	3	3	3	8	3
Sweden - Stockholm	Other	18	19	18	18	18	18	18	18	18
Sweden - Sweden except Stockholm	Other	12	12	12	12	12	12	12	12	12
Switzerland	Other	0	0	0	0	0	0	0	4	0
Syria	WFS	9	9	9	9	9	9	9	9	9
Syria	Other	63	63	63	63	63	63	63	63	63
Syria	PAPFAM	9	9	9	9	9	9	9	9	9
São Tomé and Principe	DHS	9	9	9	9	9	9	9	9	9
São Tomé and Principe	MICS	9	9	9	9	9	9	9	9	9
São Tomé and Principe	VR	3	3	3	3	3	3	3	3	3
Tajikistan	MICS	6	6	6	6	6	6	6	6	6
Tajikistan	LSMS	20	20	20	20	20	20	20	20	20
Tajikistan	Other	72	72	72	72	72	72	72	72	72
Tajikistan	DHS	18	18	18	18	18	18	18	18	18
Tanzania	DHS	54	54	54	54	54	54	54	54	54
Tanzania	Other	18	18	18	18	18	18	18	18	18
Thailand	Other	165	165	165	165	165	165	165	165	165
Thailand	WFS	6	6	6	6	6	6	6	6	6
Thailand	DHS	9	9	9	9	9	9	9	9	9
The Bahamas	Other	12	144	12	12	14	12	12	13	12
The Gambia	DHS	9	9	9	9	9	9	9	9	9
Timor-Leste	DHS	12	14	12	12	12	12	12	12	12
Timor-Leste	Other	6	6	6	6	6	6	6	6	6
Togo	DHS	24	24	24	24	24	24	24	24	24
Tokelau	Other	27	27	27	27	27	31	27	27	31
Tonga	Other	30	30	30	30	30	30	30	30	30
Trinidad and Tobago	Other	105	105	105	105	111	105	105	105	105
Trinidad and Tobago	WFS	9	9	9	9	9	9	9	10	9
Trinidad and Tobago	DHS	9	10	9	9	9	9	13	10	9
Trinidad and Tobago	VR	9	9	9	9	9	9	9	9	9
Tunisia	WFS	9	9	9	9	9	9	9	9	9
Tunisia	Other	81	81	81	81	81	81	81	81	81
Tunisia	DHS	9	9	9	9	9	9	9	9	9
Tunisia	PAPFAM	9	9	9	9	9	9	9	9	9
Tunisia	MICS	9	9	9	9	9	9	9	9	9
Turkmenistan	MICS	3	3	3	3	3	3	3	3	3
Turkmenistan	Other	45	45	45	45	45	45	45	45	45

Tuvalu	Other	3	3	3	3	3	4	3	3	13
Türkiye	WFS	9	9	9	9	9	9	9	9	9
Türkiye	DHS	36	36	36	36	36	36	36	36	36
Türkiye	Other	105	105	105	105	105	105	105	105	105
Türkiye	VR	6	6	6	6	6	6	6	6	6
UK	Other	30	30	30	30	30	15	15	15	15
UK - Northern Ireland	Other	27	27	27	27	27	27	27	27	27
UK - Scotland	Other	0	0	0	0	0	0	0	3	0
USA - Alabama	Other	3	3	3	3	3	3	3	3	3
USA - Alaska	Other	6	6	6	6	6	6	6	9	6
USA - Arizona	Other	3	3	3	3	3	3	3	3	3
USA - Arkansas	Other	3	3	3	4	3	3	3	3	3
USA - California	Other	3	3	3	3	3	3	3	3	3
USA - Colorado	Other	0	0	0	1	0	0	0	1	0
USA - Connecticut	Other	3	3	3	18	3	3	3	6	3
USA - Delaware	Other	0	0	0	0	0	0	0	5	0
USA - Florida	Other	3	3	3	3	3	3	3	3	3
USA - Georgia	Other	3	3	3	3	3	3	3	3	3
USA - Hawaii	Other	0	0	0	0	0	0	0	6	0
USA - Idaho	Other	0	0	0	10	0	0	0	2	0
USA - Illinois	Other	3	3	3	3	3	3	3	3	3
USA - Indiana	Other	3	3	3	3	3	3	3	3	3
USA - Iowa	Other	3	3	3	8	3	3	3	4	3
USA - Kansas	Other	3	3	3	4	3	3	3	4	3
USA - Kentucky	Other	3	3	3	4	3	3	3	3	3
USA - Louisiana	Other	3	3	3	3	3	3	3	3	3
USA - Maine	Other	3	3	3	29	3	3	3	7	3
USA - Maryland	Other	3	3	3	3	3	3	3	3	3
USA - Massachusetts	Other	3	3	3	8	3	3	4	3	3
USA - Michigan	Other	3	3	3	6	3	3	3	3	3
USA - Minnesota	Other	3	3	3	14	3	3	3	3	3
USA - Mississippi	Other	3	3	3	3	3	3	3	3	3
USA - Missouri	Other	3	3	3	3	3	3	3	3	3
USA - Montana	Other	3	3	3	9	3	3	3	5	3
USA - Nebraska	Other	0	0	0	10	0	0	0	1	0
USA - Nevada	Other	3	9	3	5	3	3	3	6	3
USA - New Hampshire	Other	0	0	0	11	0	0	0	4	0
USA - New Jersey	Other	3	3	3	5	3	3	3	3	3
USA - New Mexico	Other	3	3	3	10	3	3	3	3	3
USA - New York	Other	3	3	3	3	3	3	3	3	3
USA - North Carolina	Other	3	3	3	3	3	3	3	3	3
USA - North Dakota	Other	0	0	0	4	0	0	0	8	0
USA - Ohio	Other	3	3	3	3	3	3	3	3	3
USA - Oklahoma	Other	3	3	3	3	3	3	3	3	3
USA - Oregon	Other	3	3	3	6	3	3	3	4	3
USA - Pennsylvania	Other	3	3	3	4	3	3	3	3	3
USA - Rhode Island	Other	0	0	0	0	0	0	0	10	0
USA - South Carolina	Other	3	3	3	5	3	3	3	3	3
USA - South Dakota	Other	3	3	3	9	3	3	3	4	3
USA - Tennessee	Other	3	3	3	3	3	3	3	3	3
USA - Texas	Other	3	3	3	3	3	3	3	3	3
USA - Vermont	Other	0	0	0	12	0	0	0	1	0
USA - Virginia	Other	3	3	3	3	3	3	3	3	3
USA - Washington	Other	3	3	3	4	3	3	3	3	3
USA - Washington, DC	Other	3	3	3	3	3	3	3	3	3
USA - West Virginia	Other	3	3	3	4	3	3	3	3	3
USA - Wisconsin	Other	3	3	3	10	3	3	3	3	3
USA - Wyoming	Other	0	0	0	21	0	0	0	1	0
Uganda	DHS	45	45	45	45	45	45	45	45	45
Uganda	Other	9	9	9	9	9	9	9	9	9
Ukraine	MICS	3	3	3	3	3	3	3	3	3
Ukraine	Other	24	177	24	24	24	24	24	24	24
Ukraine	RHS	18	18	6	6	9	9	14	13	11
Ukraine	DHS	10	18	9	9	9	9	11	13	9
Uruguay	Other	3	3	3	3	3	3	3	3	3
Uzbekistan	MICS	3	3	3	3	3	3	3	3	3
Uzbekistan	DHS	15	15	15	15	15	15	15	15	15
Uzbekistan	Other	63	63	63	63	63	63	63	63	63
Viet Nam	DHS	15	15	15	15	15	15	15	15	15
Viet Nam	MICS	9	10	9	9	9	9	9	9	9
Virgin Islands	Other	6	6	7	9	6	7	6	6	14
Yemen	WFS	6	6	6	6	6	6	6	6	6
Yemen	DHS	24	24	24	24	24	24	24	24	24
Yemen	Other	21	21	21	21	21	21	21	21	21
Yemen	PAPFAM	9	9	9	9	9	9	9	9	9
Yemen	MICS	9	9	9	9	9	9	9	9	9
Zambia	DHS	60	60	60	60	60	60	60	60	60
Zambia	VR	3	3	3	3	3	3	3	3	3
Zambia	Other	3	3	3	3	3	3	3	3	3
Zimbabwe	DHS	54	54	54	54	54	54	54	54	54
Zimbabwe	MICS	18	18	18	18	18	18	18	18	18
Zimbabwe	Other	9	9	9	9	9	9	9	9	9

Appendix Table S9: Distribution of empirical life tables by decade and GBD super-region, 1950–2021										
**Location**	**Life Table Type**	**1950 - 1959**	**1960 - 1969**	**1970 - 1979**	**1980 - 1989**	**1990 - 1999**	**2000 - 2009**	**2010 - 2019**	**2020 - 2021**	**All years**
Central Europe, eastern Europe, and central Asia	Total	232	381	380	755	2311	2333	2004	446	8842
	Location specific	206	282	307	617	1812	1919	1807	0	6950
	Location-year specific	0	0	0	0	0	0	0	446	446
	Universal	26	99	73	138	499	414	197	0	1446
High income	Total	889	1905	2339	4282	4403	4237	3506	1138	22699
	Location specific	834	1735	1996	3101	2571	2935	2828	0	16000
	Location-year specific	0	0	0	0	0	0	0	1138	1138
	Universal	55	170	343	1181	1832	1302	678	0	5561
Latin America and Caribbean	Total	256	316	423	1210	1371	1251	889	273	5989
	Location specific	229	299	407	1186	1333	1219	857	0	5530
	Location-year specific	0	0	0	0	0	0	0	273	273
	Universal	27	17	16	24	38	32	32	0	186
North Africa and Middle East	Total	0	7	44	47	76	113	327	8	622
	Location specific	0	7	44	47	76	113	327	0	614
	Location-year specific	0	0	0	0	0	0	0	8	8
	Universal	0	0	0	0	0	0	0	0	0
South Asia	Total	0	0	0	9	446	968	818	33	2274
	Location specific	0	0	0	9	366	817	711	33	1936
	Location-year specific	0	0	0	0	0	0	0	0	0
	Universal	0	0	0	0	80	151	107	0	338
Southeast Asia, east Asia, and Oceania	Total	89	183	194	160	148	863	1049	184	2870
	Location specific	83	165	184	152	141	725	975	2	2427
	Location-year specific	0	0	0	0	0	0	0	182	182
	Universal	6	18	10	8	7	138	74	0	261
Sub-Saharan Africa	Total	3	3	2	20	72	200	162	0	462
	Location specific	3	3	2	20	72	200	162	0	462
	Location-year specific	0	0	0	0	0	0	0	0	0
	Universal	0	0	0	0	0	0	0	0	0
All locations	Total	1469	2795	3382	6483	8827	9965	8755	2082	43758
	Location specific	1355	2491	2940	5132	6371	7928	7667	35	33919
	Location-year specific	0	0	0	0	0	0	0	2047	2047
	Universal	114	304	442	1351	2456	2037	1088	0	7792

Appendix Table S10: Locations by HIV estimation group

Group 1	Group 2A	Group 2B	Group 2C
Angola	Antigua and Barbuda	Albania	Afghanistan
Benin	Argentina	American Samoa	Algeria
Botswana	Armenia	Azerbaijan	Andorra
Burkina Faso	Australia	Bahrain	Bangladesh
Burundi	Austria	Bosnia and Herzegovina	Bhutan
Cabo Verde	Barbados	Brunei	Bolivia
Cambodia	Belarus	China	Comoros
Cameroon	Belgium	Cyprus	Cook Islands
Central African Republic	Belize	Egypt	Federated States of Micronesia
Chad	Bermuda	El Salvador	Laos
Congo (Brazzaville)	Brazil	Fiji	Lebanon
Côte d'Ivoire	Bulgaria	Greenland	Libya
DR Congo	Canada	Guam	Marshall Islands
Djibouti	Chile	Honduras	Monaco
Dominican Republic	Colombia	Indonesia	Morocco
Equatorial Guinea	Costa Rica	Iran	Nauru
Eritrea	Croatia	Iraq	Nepal
Eswatini	Cuba	Jordan	Niue
Ethiopia	Czechia	Kiribati	North Korea
Gabon	Denmark	Malaysia	Pakistan
Ghana	Dominica	Maldives	Palau
Guinea	Ecuador	Mongolia	Saint Kitts and Nevis
Guinea-Bissau	Estonia	Montenegro	Samoa
Haiti	Finland	Nicaragua	San Marino
India	France	North Macedonia	Solomon Islands
Kenya	Georgia	Northern Mariana Islands	Timor-Leste
Lesotho	Germany	Oman	Tokelau
Liberia	Greece	Palestine	Tunisia
Madagascar	Grenada	Paraguay	Tuvalu
Malawi	Guatemala	Peru	United Arab Emirates
Mali	Guyana	Qatar	Vanuatu
Mauritania	Hungary	Saudi Arabia	Yemen
Mozambique	Iceland	Serbia	
Myanmar	Ireland	Seychelles	
Namibia	Israel	Slovakia	
Niger	Italy	Sri Lanka	
Nigeria	Jamaica	Syria	
Papua New Guinea	Japan	Tajikistan	
Rwanda	Kazakhstan	Thailand	
Senegal	Kuwait	Tonga	
Sierra Leone	Kyrgyzstan	Türkiye	
Somalia	Latvia	Viet Nam	
South Africa	Lithuania		
South Sudan	Luxembourg		

Sudan	Malta		
São Tomé and Príncipe	Mauritius		
Tanzania	Mexico		
The Gambia	Moldova		
Togo	Netherlands		
Uganda	New Zealand		
Zambia	Norway		
Zimbabwe	Panama		
	Philippines		
	Poland		
	Portugal		
	Puerto Rico		
	Romania		
	Russia		
	Saint Lucia		
	Saint Vincent and the Grenadines		
	Singapore		
	Slovenia		
	South Korea		
	Spain		
	Suriname		
	Sweden		
	Switzerland		
	Taiwan (province of China)		
	The Bahamas		
	Trinidad and Tobago		
	Turkmenistan		
	UK		
	USA		
	Ukraine		
	Uruguay		
	Uzbekistan		
	Venezuela		
	Virgin Islands		

Appendix Table S11. Number of sources used for the analysis of age-specific fertility by location

Location	Vital Registrations	Complete Birth Histories	Summary Birth Histories	Censuses	Other	Sample Registrations
Afghanistan	0	3	5	0	0	0
Albania	64	2	5	1	0	0
Algeria	61	5	0	0	0	0
American Samoa	69	0	0	2	0	0
Andorra	50	0	0	0	0	0
Angola	23	5	4	1	0	0
Antigua and Barbuda	62	0	0	1	0	0
Argentina	66	0	0	4	0	0
Armenia	61	4	4	2	0	0
Australia	71	0	0	1	0	0
Austria	71	0	0	0	25	0
Azerbaijan	66	3	2	1	0	0
Bahrain	52	1	0	2	0	0
Bangladesh	41	15	16	2	0	0
Barbados	64	0	0	3	0	0
Belarus	70	0	2	2	0	0
Belgium	69	0	0	0	0	0
Belize	69	2	5	4	0	0
Benin	2	6	7	2	0	0
Bermuda	67	0	0	2	0	0
Bhutan	5	1	3	2	0	0
Bolivia	41	7	22	4	0	0
Bosnia and Herzegovina	32	1	1	0	1	0
Botswana	20	5	6	5	0	0
Brazil	57	4	25	7	0	0
Brunei	61	0	0	0	0	0
Bulgaria	70	0	2	0	0	0
Burkina Faso	0	6	9	4	0	0
Burundi	9	6	7	1	0	0
Cabo Verde	47	1	2	1	0	0
Cambodia	1	6	8	2	0	0
Cameroon	0	8	8	1	0	0
Canada	69	0	0	1	0	0
Central African Republic	0	2	4	2	0	0
Chad	0	5	5	0	0	0
Chile	69	0	16	4	3	0
Colombia	65	9	9	4	0	0
Comoros	9	2	3	3	0	0
Congo (Brazzaville)	1	3	4	1	0	0
Cook Islands	46	0	0	3	0	0
Costa Rica	72	1	3	3	0	0
Croatia	71	0	0	2	1	0
Cuba	69	1	3	1	2	0
Cyprus	69	0	0	2	0	0
Czechia	71	0	0	2	0	0
Côte d'Ivoire	5	6	8	2	0	0
DR Congo	0	2	12	0	0	0
Denmark	72	0	0	0	0	0
Djibouti	28	3	3	0	0	0
Dominica	68	0	0	2	0	0
Dominican Republic	55	13	10	4	0	0
Ecuador	70	5	5	5	0	0

Egypt	67	13	14	1	2	0
El Salvador	60	5	6	2	0	0
Equatorial Guinea	17	1	1	0	0	0
Eritrea	0	3	2	0	0	0
Estonia	72	0	0	2	0	0
Eswatini	6	3	3	4	0	0
Ethiopia	0	10	6	2	0	0
Federated States of Micronesia	8	0	0	4	0	0
Fiji	58	2	1	5	0	0
Finland	72	0	0	2	0	0
France	71	0	0	0	0	0
Gabon	0	1	2	1	0	0
Georgia	58	1	5	0	0	0
Germany	70	0	0	0	2	0
Ghana	28	14	22	3	0	0
Greece	71	0	0	1	4	0
Greenland	72	0	0	0	0	0
Grenada	43	0	0	2	0	0
Guam	70	0	0	0	0	0
Guatemala	50	6	8	0	3	0
Guinea	2	7	5	2	0	0
Guinea-Bissau	21	2	5	0	0	0
Guyana	39	6	7	3	0	0
Haiti	0	8	6	2	0	0
Honduras	40	8	6	3	0	0
Hungary	68	0	0	2	13	0
Iceland	72	0	0	1	0	0
India	67	31	14	4	2	28
Indonesia	14	14	42	5	1	0
Iran	70	0	2	4	0	0
Iraq	43	6	5	2	0	0
Ireland	72	0	0	1	0	0
Israel	71	0	0	0	0	0
Italy	71	0	0	0	0	0
Jamaica	61	3	6	3	2	0
Japan	68	0	0	0	0	0
Jordan	52	20	7	0	0	0
Kazakhstan	58	2	6	2	0	0
Kenya	52	12	16	6	1	0
Kiribati	18	2	1	5	0	0
Kuwait	62	2	0	1	0	0
Kyrgyzstan	58	4	6	2	0	0
Laos	1	4	3	1	0	0
Latvia	71	0	0	2	0	0
Lebanon	30	2	4	0	0	0
Lesotho	8	6	6	4	0	0
Liberia	4	8	6	2	0	0
Libya	48	1	2	0	0	0
Lithuania	72	0	0	1	0	0
Luxembourg	72	0	0	1	0	0
Madagascar	24	7	9	0	0	0
Malawi	6	13	12	4	0	0
Malaysia	63	1	1	2	4	0
Maldives	46	2	2	4	0	0
Mali	2	9	8	3	0	0

Malta	71	0	0	0	0	0
Marshall Islands	26	1	0	2	0	0
Mauritania	1	7	6	1	0	0
Mauritius	71	0	0	2	0	0
Mexico	69	4	22	7	0	0
Moldova	57	2	3	1	0	0
Monaco	55	0	0	0	0	0
Mongolia	49	6	13	0	0	0
Montenegro	31	1	2	0	1	0
Morocco	27	10	7	3	0	0
Mozambique	29	6	7	5	0	0
Myanmar	10	6	2	1	0	0
Namibia	4	4	4	3	0	0
Nauru	31	1	0	2	0	0
Nepal	0	11	9	4	0	0
Netherlands	72	0	0	0	0	0
New Zealand	72	0	0	1	0	0
Nicaragua	56	5	7	3	0	0
Niger	2	5	7	1	0	0
Nigeria	2	14	22	2	0	0
Niue	68	0	0	4	0	0
North Korea	2	2	0	1	0	0
North Macedonia	36	0	2	2	1	0
Northern Mariana Islands	14	0	0	0	0	0
Norway	71	0	0	0	0	0
Oman	18	2	0	0	0	0
Pakistan	18	36	12	1	1	1
Palau	11	0	0	3	0	0
Palestine	24	4	6	1	0	0
Panama	70	1	4	5	3	0
Papua New Guinea	4	4	2	3	0	0
Paraguay	43	6	8	4	6	0
Peru	65	15	21	1	2	0
Philippines	70	18	7	3	3	0
Poland	71	0	0	0	6	0
Portugal	68	1	0	1	2	0
Puerto Rico	60	0	0	1	0	0
Qatar	51	2	0	1	0	0
Romania	70	0	0	3	17	0
Russia	72	0	0	1	0	0
Rwanda	18	11	10	3	0	0
Saint Kitts and Nevis	62	1	0	2	0	0
Saint Lucia	55	0	0	2	0	0
Saint Vincent and the Grenadines	66	0	0	2	0	0
Samoa	38	5	0	1	0	0
San Marino	57	0	0	0	0	0
Saudi Arabia	11	1	1	0	0	0
Senegal	3	16	14	3	0	0
Serbia	64	0	7	0	1	0
Seychelles	69	0	0	1	0	0
Sierra Leone	13	6	6	1	0	0
Singapore	72	0	0	1	0	0
Slovakia	72	0	1	1	0	0
Slovenia	69	0	0	2	1	0
Solomon Islands	1	2	0	4	0	0

Somalia	0	2	3	0	0	0
South Africa	30	3	13	2	0	0
South Korea	52	1	1	8	22	0
South Sudan	0	1	1	1	0	0
Spain	71	0	0	0	0	0
Sri Lanka	70	11	3	2	12	0
Sudan	4	6	5	3	0	0
Suriname	57	0	2	1	0	0
Sweden	72	0	0	0	0	0
Switzerland	71	0	0	0	0	0
Syria	56	3	6	1	0	0
São Tomé and Príncipe	44	2	4	2	0	0
Taiwan (province of China)	72	0	0	0	0	0
Tajikistan	56	3	5	1	0	0
Tanzania	1	10	12	1	1	0
Thailand	68	9	9	4	1	0
The Bahamas	68	0	0	3	0	0
The Gambia	0	4	5	3	0	0
Timor-Leste	19	5	16	0	0	0
Togo	1	4	7	1	0	0
Tokelau	18	0	0	1	0	0
Tonga	52	1	1	3	0	0
Trinidad and Tobago	67	2	5	2	2	0
Tunisia	70	6	7	0	0	0
Turkmenistan	35	3	3	0	0	0
Tuvalu	9	2	0	1	0	0
Türkiye	31	10	8	6	0	0
UK	71	0	0	0	0	0
USA	70	0	0	1	0	0
Uganda	1	8	17	2	1	0
Ukraine	71	2	4	2	0	0
United Arab Emirates	44	2	0	1	0	0
Uruguay	60	0	5	5	0	0
Uzbekistan	54	3	4	0	0	0
Vanuatu	3	1	1	4	0	0
Venezuela	65	2	0	2	0	0
Viet Nam	5	11	10	3	0	0
Virgin Islands	49	0	0	0	0	0
Yemen	9	6	6	1	0	0
Zambia	3	6	10	5	1	0
Zimbabwe	1	10	9	3	0	0

Appendix Table S12. Number of sources used for the analysis of age-specific fertility by year

Year	Vital Registrations	Complete Birth Histories	Summary Birth Histories	Censuses	Other	Sample Registrations
1950	116	0	0	4	4	0
1951	123	0	0	0	2	0
1952	122	0	0	0	1	0
1953	122	0	0	1	3	0
1954	121	0	0	1	1	0
1955	125	0	0	0	2	0
1956	120	0	0	0	2	0
1957	125	0	0	1	3	0
1958	126	0	0	0	3	0
1959	128	0	0	0	3	0
1960	113	0	0	7	11	0
1961	120	0	0	2	5	0
1962	126	0	0	1	5	0
1963	128	1	0	1	5	0
1964	130	1	0	1	7	0
1965	123	1	0	0	8	0
1966	117	1	0	4	5	0
1967	120	0	0	1	5	0
1968	119	1	0	1	6	0
1969	117	0	0	2	10	0
1970	112	2	0	10	16	0
1971	121	3	0	6	4	1
1972	118	0	0	1	10	0
1973	114	1	0	7	2	0
1974	118	5	3	6	1	0
1975	116	9	7	5	3	0
1976	115	9	5	5	1	1
1977	114	7	4	1	1	0
1978	118	10	5	1	1	0
1979	128	6	2	1	1	0
1980	120	5	3	18	2	0
1981	123	4	5	7	1	1
1982	122	2	0	6	1	0
1983	125	4	3	3	1	0
1984	124	8	1	2	1	0
1985	129	5	3	6	2	0
1986	129	8	7	9	1	1
1987	128	20	13	4	1	0
1988	125	10	14	6	1	0
1989	123	5	4	11	1	0
1990	128	10	7	28	1	0
1991	127	19	12	23	1	1
1992	127	13	17	8	1	0
1993	122	12	16	5	1	0

1994	118	11	16	5	1	0
1995	113	13	17	4	1	0
1996	112	24	19	9	0	1
1997	104	19	21	4	0	0
1998	101	18	28	4	0	0
1999	102	17	22	9	1	1
2000	109	20	63	21	0	1
2001	108	13	20	20	0	1
2002	116	12	16	13	0	1
2003	119	17	22	2	0	1
2004	114	15	21	2	0	1
2005	120	22	37	6	0	2
2006	115	33	55	6	0	1
2007	119	25	30	6	0	1
2008	121	21	27	7	0	1
2009	119	18	22	7	0	1
2010	121	22	38	11	0	1
2011	122	22	36	14	0	1
2012	144	28	39	5	0	1
2013	140	21	30	2	0	1
2014	140	35	44	1	0	1
2015	140	22	32	2	0	1
2016	145	23	37	1	0	1
2017	141	25	31	2	0	1
2018	134	19	16	1	0	1
2019	123	22	9	0	0	1
2020	107	7	0	0	0	1
2021	46	9	0	0	0	0
2022	0	2	0	0	0	0

Appendix Table S13: List of all confirmed censuses by location and year					
**Location**	**Year**	**Type**	**Age Detail**	**Enumeration Method**	**Included or Excluded from Estimation**
Afghanistan	1979	Census	5-year age groups	De facto	Included
Albania	1950	Census	5-year age groups	De facto	Included
Albania	1955	Census	5-year age groups	De facto	Included
Albania	1960	Census	non-std age groups	De facto	Included
Albania	1969	Census	all ages only	De facto	Included
Albania	1979	Census	all ages only	De jure	Included
Albania	1989	Census	all ages only	De jure	Included
Albania	2001	Census	5-year age groups	De facto	Included
Albania	2011	Census	5-year age groups	De jure	Included
Algeria	1954	Census	5-year age groups	Unknown	Included
Algeria	1966	Census	5-year age groups	De jure	Included
Algeria	1977	Census	5-year age groups	De jure	Included
Algeria	1987	Census	5-year age groups	De jure	Included
Algeria	1998	Census	5-year age groups	De jure	Included
Algeria	2008	Census	1-year age groups	De jure	Included
American Samoa	1950	Census	5-year age groups	De jure	Included
American Samoa	1956	Census	1-year age groups	De jure	Included
American Samoa	1960	Census	5-year age groups	De jure	Included
American Samoa	1970	Census	5-year age groups	De jure	Included
American Samoa	1974	Census	5-year age groups	De jure	Included
American Samoa	1980	Census	1-year age groups	De jure	Included
American Samoa	1990	Census	5-year age groups	De jure	Included
American Samoa	2000	Census	5-year age groups	De jure	Included
American Samoa	2010	Census	5-year age groups	De jure	Included
American Samoa	2020	Census	5-year age groups	De jure	Included
Andorra	1966	Registry	non-std age groups	NA	Included
Andorra	1985	Registry	1-year age groups	NA	Included
Andorra	1986	Registry	1-year age groups	NA	Included
Andorra	1987	Registry	1-year age groups	NA	Included
Andorra	1988	Registry	1-year age groups	NA	Included
Andorra	1989	Registry	1-year age groups	NA	Included
Andorra	1990	Registry	1-year age groups	NA	Included
Andorra	1991	Registry	1-year age groups	NA	Included
Andorra	1992	Registry	1-year age groups	NA	Included
Andorra	1993	Registry	1-year age groups	NA	Included
Andorra	1994	Registry	1-year age groups	NA	Included
Andorra	1995	Registry	1-year age groups	NA	Included
Andorra	1996	Registry	1-year age groups	NA	Included
Andorra	1997	Registry	1-year age groups	NA	Included
Andorra	1998	Registry	1-year age groups	NA	Included
Andorra	1999	Registry	1-year age groups	NA	Included
Andorra	2000	Registry	1-year age groups	NA	Included
Andorra	2001	Registry	1-year age groups	NA	Included
Andorra	2002	Registry	1-year age groups	NA	Included
Andorra	2003	Registry	1-year age groups	NA	Included
Andorra	2004	Registry	1-year age groups	NA	Included
Andorra	2005	Registry	1-year age groups	NA	Included
Andorra	2006	Registry	1-year age groups	NA	Included
Andorra	2007	Registry	1-year age groups	NA	Included
Andorra	2008	Registry	1-year age groups	NA	Included
Andorra	2009	Registry	1-year age groups	NA	Included
Andorra	2010	Registry	1-year age groups	NA	Included
Andorra	2011	Registry	1-year age groups	NA	Included

Andorra	2012	Registry	1-year age groups	NA	Included
Andorra	2013	Registry	1-year age groups	NA	Included
Andorra	2014	Registry	1-year age groups	NA	Included
Andorra	2015	Registry	1-year age groups	NA	Included
Andorra	2016	Registry	1-year age groups	NA	Included
Andorra	2017	Registry	1-year age groups	NA	Included
Angola	1950	Census	5-year age groups	De facto	Included
Angola	1960	Census	5-year age groups	De jure	Included
Angola	1970	Census	all ages only	De facto	Included
Angola	2014	Census	1-year age groups	De facto	Included
Antigua and Barbuda	1960	Census	1-year age groups	De facto	Included
Antigua and Barbuda	1970	Census	5-year age groups	De jure	Included
Antigua and Barbuda	1991	Census	1-year age groups	De jure	Included
Antigua and Barbuda	2001	Census	1-year age groups	De jure	Included
Antigua and Barbuda	2011	Census	5-year age groups	De jure	Included
Argentina	1960	Census	1-year age groups	De facto	Included
Argentina	1970	Census	1-year age groups	De facto	Included
Argentina	1980	Census	1-year age groups	De facto	Included
Argentina	1991	Census	1-year age groups	De facto	Included
Argentina	2001	Census	1-year age groups	De facto	Included
Argentina	2010	Census	1-year age groups	De facto	Included
Armenia	1959	Census	5-year age groups	De facto	Included
Armenia	1970	Census	5-year age groups	De facto	Included
Armenia	1979	Census	5-year age groups	De facto	Included
Armenia	1989	Census	5-year age groups	De jure	Included
Armenia	2001	Census	1-year age groups	De jure	Included
Armenia	2011	Census	1-year age groups	De jure	Included
Australia	1954	Census	5-year age groups	De facto	Included
Australia	1961	Census	1-year age groups	De facto	Included
Australia	1966	Census	5-year age groups	De facto	Included
Australia	1971	Census	1-year age groups	De facto	Included
Australia	1976	Census	5-year age groups	De facto	Included
Australia	1981	Census	5-year age groups	De facto	Included
Australia	1986	Census	1-year age groups	De facto	Included
Australia	1991	Census	1-year age groups	De facto	Included
Australia	1996	Census	1-year age groups	De facto	Included
Australia	2001	Census	1-year age groups	De jure	Included
Australia	2006	Census	1-year age groups	De jure	Included
Australia	2011	Census	1-year age groups	De jure	Included
Australia	2016	Census	5-year age groups	De jure	Excluded
Australia	2021	Census	5-year age groups	De jure	Included
Austria	1951	Census	1-year age groups	De jure	Included
Austria	1961	Census	1-year age groups	De jure	Included
Austria	1971	Census	1-year age groups	De jure	Included
Austria	1981	Census	1-year age groups	De jure	Included
Austria	1991	Census	1-year age groups	De jure	Included
Austria	2001	Census	1-year age groups	De jure	Included
Austria	2002	Registry	5-year age groups	NA	Included
Austria	2003	Registry	5-year age groups	NA	Included
Austria	2004	Registry	5-year age groups	NA	Included
Austria	2005	Registry	5-year age groups	NA	Included
Austria	2006	Registry	5-year age groups	NA	Included
Austria	2007	Registry	5-year age groups	NA	Included
Austria	2008	Registry	5-year age groups	NA	Included
Austria	2009	Registry	5-year age groups	NA	Included
Austria	2010	Registry	5-year age groups	NA	Included

Austria	2011	Registry	5-year age groups	NA	Included
Austria	2012	Registry	5-year age groups	NA	Included
Austria	2013	Registry	5-year age groups	NA	Included
Austria	2014	Registry	5-year age groups	NA	Included
Austria	2015	Registry	5-year age groups	NA	Included
Austria	2016	Registry	5-year age groups	NA	Included
Austria	2017	Registry	5-year age groups	NA	Included
Azerbaijan	1959	Census	5-year age groups	De facto	Included
Azerbaijan	1970	Census	5-year age groups	De facto	Included
Azerbaijan	1979	Census	5-year age groups	De facto	Included
Azerbaijan	1989	Census	5-year age groups	De jure	Included
Azerbaijan	1999	Census	1-year age groups	De jure	Included
Azerbaijan	2009	Census	1-year age groups	De jure	Included
Bahrain	1950	Census	all ages only	De facto	Included
Bahrain	1959	Census	non-std age groups	De facto	Included
Bahrain	1965	Census	non-std age groups	De facto	Included
Bahrain	1971	Census	1-year age groups	De facto	Included
Bahrain	1981	Census	5-year age groups	De facto	Included
Bahrain	1991	Census	5-year age groups	De jure	Excluded
Bahrain	2001	Census	1-year age groups	De jure	Included
Bahrain	2010	Census	1-year age groups	De jure	Included
Bahrain	2020	Census	5-year age groups	Unknown	Included
Bangladesh	1951	Census	non-std age groups	De jure	Included
Bangladesh	1961	Census	5-year age groups	De jure	Included
Bangladesh	1974	Census	5-year age groups	De facto	Included
Bangladesh	1981	Census	5-year age groups	De facto	Included
Bangladesh	1991	Census	1-year age groups	De facto	Included
Bangladesh	2001	Census	1-year age groups	De facto	Included
Bangladesh	2011	Census	5-year age groups	De facto	Included
Barbados	1960	Census	1-year age groups	De facto	Included
Barbados	1970	Census	1-year age groups	De facto	Included
Barbados	1980	Census	1-year age groups	De facto	Included
Barbados	1990	Census	5-year age groups	De jure	Included
Barbados	2000	Census	1-year age groups	De facto	Included
Barbados	2010	Census	5-year age groups	De jure	Included
Belarus	1959	Census	5-year age groups	De facto	Included
Belarus	1970	Census	5-year age groups	De facto	Included
Belarus	1979	Census	5-year age groups	De facto	Included
Belarus	1989	Census	1-year age groups	De jure	Included
Belarus	1999	Census	1-year age groups	De jure	Included
Belarus	2009	Census	1-year age groups	De jure	Included
Belgium	1961	Census	1-year age groups	De jure	Included
Belgium	1970	Census	1-year age groups	De jure	Included
Belgium	1981	Census	1-year age groups	De jure	Included
Belgium	1991	Census	5-year age groups	De jure	Included
Belgium	2001	Census	1-year age groups	De jure	Included
Belgium	2010	Registry	1-year age groups	NA	Included
Belgium	2011	Census	1-year age groups	De jure	Excluded
Belgium	2012	Registry	1-year age groups	NA	Included
Belgium	2013	Registry	1-year age groups	NA	Included
Belgium	2014	Registry	1-year age groups	NA	Included
Belgium	2015	Registry	1-year age groups	NA	Included
Belgium	2016	Registry	1-year age groups	NA	Included
Belgium	2017	Registry	1-year age groups	NA	Included
Belize	1960	Census	5-year age groups	De facto	Included
Belize	1970	Census	1-year age groups	De facto	Included

Belize	1980	Census	1-year age groups	De facto	Included
Belize	1991	Census	1-year age groups	De facto	Included
Belize	2000	Census	non-std age groups	De jure	Included
Belize	2010	Census	5-year age groups	De jure	Included
Benin	1979	Census	5-year age groups	De facto	Included
Benin	1992	Census	1-year age groups	De facto	Included
Benin	2002	Census	1-year age groups	De jure	Included
Benin	2013	Census	5-year age groups	De jure	Included
Bermuda	1950	Census	1-year age groups	De jure	Included
Bermuda	1960	Census	1-year age groups	De jure	Included
Bermuda	1970	Census	1-year age groups	De jure	Included
Bermuda	1980	Census	1-year age groups	De jure	Included
Bermuda	1991	Census	1-year age groups	De jure	Included
Bermuda	2000	Census	1-year age groups	De jure	Included
Bermuda	2010	Census	1-year age groups	De jure	Included
Bermuda	2016	Census	1-year age groups	De jure	Excluded
Bhutan	2005	Census	1-year age groups	De facto	Included
Bhutan	2017	Census	1-year age groups	De facto	Included
Bolivia	1950	Census	5-year age groups	De facto	Excluded
Bolivia	1976	Census	5-year age groups	De facto	Included
Bolivia	1992	Census	1-year age groups	De facto	Included
Bolivia	2001	Census	1-year age groups	De facto	Included
Bolivia	2012	Census	1-year age groups	De facto	Included
Bosnia and Herzegovina	1971	Census	5-year age groups	De jure	Excluded
Bosnia and Herzegovina	1981	Census	5-year age groups	De jure	Included
Bosnia and Herzegovina	1991	Census	1-year age groups	De jure	Included
Bosnia and Herzegovina	2013	Census	1-year age groups	De jure	Included
Botswana	1964	Census	1-year age groups	De facto	Included
Botswana	1971	Census	1-year age groups	De facto	Excluded
Botswana	1981	Census	1-year age groups	De facto	Included
Botswana	1991	Census	1-year age groups	De facto	Included
Botswana	2001	Census	5-year age groups	De facto	Included
Botswana	2011	Census	1-year age groups	De facto	Included
Brazil	1950	Census	1-year age groups	De facto	Included
Brazil	1960	Census	1-year age groups	De facto	Included
Brazil	1970	Census	1-year age groups	De facto	Included
Brazil	1980	Census	1-year age groups	De jure	Included
Brazil	1991	Census	1-year age groups	De jure	Included
Brazil	1996	Census	5-year age groups	De jure	Excluded
Brazil	2000	Census	1-year age groups	De jure	Included
Brazil	2010	Census	1-year age groups	De jure	Included
Brunei	1960	Census	5-year age groups	De facto	Included
Brunei	1971	Census	1-year age groups	De facto	Included
Brunei	1981	Census	1-year age groups	De facto	Included
Brunei	1991	Census	1-year age groups	De facto	Included
Brunei	2001	Census	5-year age groups	De facto	Included
Brunei	2011	Census	1-year age groups	De jure	Included
Bulgaria	1956	Census	5-year age groups	De facto	Included
Bulgaria	1965	Census	5-year age groups	De facto	Included
Bulgaria	1975	Census	5-year age groups	De facto	Included
Bulgaria	1985	Census	5-year age groups	De jure	Included
Bulgaria	1992	Census	5-year age groups	De jure	Included
Bulgaria	2001	Census	5-year age groups	De jure	Excluded
Bulgaria	2002	Registry	5-year age groups	NA	Included
Bulgaria	2003	Registry	5-year age groups	NA	Included
Bulgaria	2004	Registry	5-year age groups	NA	Included

Bulgaria	2005	Registry	5-year age groups	NA	Included
Bulgaria	2006	Registry	5-year age groups	NA	Included
Bulgaria	2007	Registry	5-year age groups	NA	Included
Bulgaria	2008	Registry	5-year age groups	NA	Included
Bulgaria	2009	Registry	5-year age groups	NA	Included
Bulgaria	2010	Registry	5-year age groups	NA	Included
Bulgaria	2011	Registry	5-year age groups	NA	Included
Bulgaria	2012	Registry	5-year age groups	NA	Included
Bulgaria	2013	Registry	5-year age groups	NA	Included
Bulgaria	2014	Registry	5-year age groups	NA	Included
Bulgaria	2015	Registry	5-year age groups	NA	Included
Bulgaria	2016	Registry	5-year age groups	NA	Included
Burkina Faso	1975	Census	5-year age groups	De jure	Excluded
Burkina Faso	1985	Census	1-year age groups	De jure	Included
Burkina Faso	1996	Census	1-year age groups	De jure	Included
Burkina Faso	2006	Census	1-year age groups	De jure	Included
Burkina Faso	2019	Census	5-year age groups	De jure	Included
Burundi	1979	Census	5-year age groups	De jure	Included
Burundi	1990	Census	1-year age groups	De jure	Included
Burundi	2008	Census	1-year age groups	De jure	Included
Cabo Verde	1950	Census	non-std age groups	De facto	Included
Cabo Verde	1960	Census	non-std age groups	De facto	Included
Cabo Verde	1970	Census	5-year age groups	De facto	Included
Cabo Verde	1980	Census	5-year age groups	De jure	Included
Cabo Verde	1990	Census	1-year age groups	De facto	Included
Cabo Verde	2000	Census	5-year age groups	De facto	Included
Cabo Verde	2010	Census	1-year age groups	De jure	Included
Cambodia	1962	Census	1-year age groups	De facto	Included
Cambodia	1998	Census	1-year age groups	De facto	Included
Cambodia	2008	Census	1-year age groups	De facto	Included
Cambodia	2019	Census	1-year age groups	De facto	Excluded
Cameroon	1976	Census	5-year age groups	De jure	Included
Cameroon	1987	Census	1-year age groups	Unknown	Included
Cameroon	2005	Census	1-year age groups	De jure	Included
Canada	1951	Census	5-year age groups	De jure	Included
Canada	1956	Census	1-year age groups	De jure	Included
Canada	1961	Census	5-year age groups	De jure	Included
Canada	1966	Census	5-year age groups	De jure	Included
Canada	1971	Census	5-year age groups	De jure	Included
Canada	1976	Census	5-year age groups	De jure	Included
Canada	1981	Census	1-year age groups	De jure	Included
Canada	1986	Census	5-year age groups	De jure	Included
Canada	1991	Census	1-year age groups	De jure	Included
Canada	1996	Census	1-year age groups	De jure	Included
Canada	2001	Census	1-year age groups	De jure	Included
Canada	2006	Census	1-year age groups	De jure	Included
Canada	2011	Census	1-year age groups	De jure	Included
Canada	2016	Census	1-year age groups	De jure	Included
Canada	2021	Census	1-year age groups	De jure	Included
Central African Republic	1975	Census	5-year age groups	De facto	Included
Central African Republic	1988	Census	1-year age groups	De facto	Included
Central African Republic	2003	Census	5-year age groups	De facto	Included
Chad	1993	Census	5-year age groups	De jure	Included
Chad	2009	Census	5-year age groups	De jure	Included
Chile	1952	Census	5-year age groups	De facto	Included
Chile	1960	Census	5-year age groups	De facto	Included

Chile	1970	Census	1-year age groups	De facto	Included
Chile	1982	Census	1-year age groups	De facto	Included
Chile	1992	Census	1-year age groups	De facto	Included
Chile	2002	Census	5-year age groups	De facto	Included
Chile	2012	Census	1-year age groups	De jure	Excluded
Chile	2017	Census	5-year age groups	De facto	Included
Colombia	1951	Census	5-year age groups	De facto	Included
Colombia	1964	Census	1-year age groups	De facto	Included
Colombia	1973	Census	1-year age groups	De facto	Included
Colombia	1985	Census	1-year age groups	De facto	Included
Colombia	1993	Census	1-year age groups	De facto	Included
Colombia	2005	Census	1-year age groups	De facto	Included
Colombia	2018	Census	5-year age groups	De jure	Included
Comoros	1958	Census	5-year age groups	De facto	Excluded
Comoros	1966	Census	non-std age groups	Unknown	Included
Comoros	1980	Census	1-year age groups	De facto	Included
Comoros	1991	Census	5-year age groups	De jure	Included
Comoros	2003	Census	non-std age groups	De jure	Included
Congo (Brazzaville)	1974	Census	5-year age groups	De jure	Included
Congo (Brazzaville)	1984	Census	1-year age groups	De jure	Included
Congo (Brazzaville)	1985	Census	1-year age groups	De jure	Excluded
Congo (Brazzaville)	2007	Census	1-year age groups	De facto	Included
Cook Islands	1951	Census	5-year age groups	De jure	Excluded
Cook Islands	1956	Census	5-year age groups	De facto	Included
Cook Islands	1961	Census	5-year age groups	De facto	Included
Cook Islands	1966	Census	5-year age groups	De facto	Excluded
Cook Islands	1971	Census	5-year age groups	Unknown	Included
Cook Islands	1976	Census	5-year age groups	De facto	Excluded
Cook Islands	1981	Census	1-year age groups	De facto	Included
Cook Islands	1986	Census	5-year age groups	De facto	Included
Cook Islands	1991	Census	5-year age groups	De facto	Included
Cook Islands	1996	Census	5-year age groups	De facto	Included
Cook Islands	2001	Census	5-year age groups	De facto	Included
Cook Islands	2006	Census	5-year age groups	De facto	Included
Cook Islands	2011	Census	5-year age groups	De facto	Included
Cook Islands	2016	Census	5-year age groups	De facto	Included
Costa Rica	1950	Census	1-year age groups	De jure	Included
Costa Rica	1963	Census	1-year age groups	De jure	Included
Costa Rica	1973	Census	1-year age groups	De jure	Included
Costa Rica	1984	Census	1-year age groups	De jure	Included
Costa Rica	2000	Census	5-year age groups	De jure	Included
Costa Rica	2011	Census	1-year age groups	De jure	Included
Croatia	1971	Census	1-year age groups	De facto	Included
Croatia	1991	Census	5-year age groups	Unknown	Excluded
Croatia	2001	Census	1-year age groups	De jure	Included
Croatia	2011	Census	1-year age groups	De jure	Included
Cuba	1953	Census	5-year age groups	De jure	Included
Cuba	1970	Census	5-year age groups	De jure	Included
Cuba	1981	Census	1-year age groups	De jure	Included
Cuba	2002	Census	1-year age groups	De jure	Included
Cuba	2012	Census	1-year age groups	De jure	Included
Cyprus	1960	Census	5-year age groups	De jure	Included
Cyprus	1973	Census	5-year age groups	De jure	Excluded
Czechia	1961	Census	1-year age groups	De facto	Included
Czechia	1970	Census	1-year age groups	De jure	Included
Czechia	1980	Census	1-year age groups	De jure	Included

Czechia	1991	Census	5-year age groups	De jure	Included
Czechia	2000	Registry	1-year age groups	NA	Included
Czechia	2001	Census	5-year age groups	De jure	Excluded
Czechia	2002	Registry	1-year age groups	NA	Included
Czechia	2003	Registry	1-year age groups	NA	Included
Czechia	2004	Registry	1-year age groups	NA	Included
Czechia	2005	Registry	1-year age groups	NA	Included
Czechia	2006	Registry	1-year age groups	NA	Included
Czechia	2007	Registry	1-year age groups	NA	Included
Czechia	2008	Registry	1-year age groups	NA	Included
Czechia	2009	Registry	1-year age groups	NA	Included
Czechia	2010	Registry	1-year age groups	NA	Included
Czechia	2011	Census	1-year age groups	De jure	Excluded
Czechia	2012	Registry	1-year age groups	NA	Included
Czechia	2013	Registry	1-year age groups	NA	Included
Czechia	2014	Registry	1-year age groups	NA	Included
Czechia	2015	Registry	1-year age groups	NA	Included
Czechia	2016	Registry	1-year age groups	NA	Included
Czechia	2017	Registry	1-year age groups	NA	Included
Côte d'Ivoire	1975	Census	5-year age groups	De facto	Included
Côte d'Ivoire	1988	Census	1-year age groups	De facto	Included
Côte d'Ivoire	1998	Census	5-year age groups	Unknown	Included
Côte d'Ivoire	2014	Census	1-year age groups	De jure	Included
DR Congo	1970	Census	all ages only	De jure	Included
DR Congo	1984	Census	1-year age groups	Unknown	Included
Denmark	1950	Census	1-year age groups	De jure	Included
Denmark	1960	Census	1-year age groups	De jure	Included
Denmark	1965	Census	5-year age groups	De jure	Included
Denmark	1970	Census	1-year age groups	De jure	Included
Denmark	1976	Census	1-year age groups	De jure	Included
Denmark	1980	Registry	1-year age groups	NA	Included
Denmark	1981	Registry	1-year age groups	NA	Included
Denmark	1982	Registry	1-year age groups	NA	Included
Denmark	1983	Registry	1-year age groups	NA	Included
Denmark	1984	Registry	1-year age groups	NA	Included
Denmark	1985	Registry	1-year age groups	NA	Included
Denmark	1986	Registry	1-year age groups	NA	Included
Denmark	1987	Registry	1-year age groups	NA	Included
Denmark	1988	Registry	1-year age groups	NA	Included
Denmark	1989	Registry	1-year age groups	NA	Included
Denmark	1990	Registry	1-year age groups	NA	Included
Denmark	1991	Census	1-year age groups	De jure	Excluded
Denmark	1992	Registry	1-year age groups	NA	Included
Denmark	1993	Registry	1-year age groups	NA	Included
Denmark	1994	Registry	1-year age groups	NA	Included
Denmark	1995	Registry	1-year age groups	NA	Included
Denmark	1996	Registry	1-year age groups	NA	Included
Denmark	1997	Registry	1-year age groups	NA	Included
Denmark	1998	Registry	1-year age groups	NA	Included
Denmark	1999	Registry	1-year age groups	NA	Included
Denmark	2000	Registry	1-year age groups	NA	Included
Denmark	2001	Registry	1-year age groups	NA	Included
Denmark	2002	Registry	1-year age groups	NA	Included
Denmark	2003	Registry	1-year age groups	NA	Included
Denmark	2004	Registry	1-year age groups	NA	Included
Denmark	2005	Registry	1-year age groups	NA	Included

Denmark	2006	Registry	1-year age groups	NA	Included
Denmark	2007	Registry	1-year age groups	NA	Included
Denmark	2008	Registry	1-year age groups	NA	Included
Denmark	2009	Registry	1-year age groups	NA	Included
Denmark	2010	Registry	1-year age groups	NA	Included
Denmark	2011	Registry	1-year age groups	NA	Included
Denmark	2012	Registry	1-year age groups	NA	Included
Denmark	2013	Registry	1-year age groups	NA	Included
Denmark	2014	Registry	1-year age groups	NA	Included
Denmark	2015	Registry	1-year age groups	NA	Included
Denmark	2016	Registry	1-year age groups	NA	Included
Denmark	2017	Registry	1-year age groups	NA	Included
Djibouti	1983	Census	5-year age groups	De jure	Included
Djibouti	2009	Census	5-year age groups	De facto	Excluded
Djibouti	2009	Census	5-year age groups	De jure	Included
Dominica	1960	Census	5-year age groups	De facto	Included
Dominica	1970	Census	5-year age groups	De facto	Included
Dominica	1981	Census	5-year age groups	De facto	Excluded
Dominica	1991	Census	5-year age groups	De facto	Included
Dominica	2001	Census	5-year age groups	De facto	Included
Dominica	2011	Census	5-year age groups	De facto	Included
Dominican Republic	1950	Census	1-year age groups	De jure	Included
Dominican Republic	1960	Census	1-year age groups	De jure	Included
Dominican Republic	1970	Census	1-year age groups	De jure	Included
Dominican Republic	1981	Census	5-year age groups	De jure	Included
Dominican Republic	1993	Census	1-year age groups	De jure	Included
Dominican Republic	2002	Census	1-year age groups	De jure	Included
Dominican Republic	2010	Census	1-year age groups	De jure	Included
Ecuador	1950	Census	1-year age groups	De facto	Included
Ecuador	1962	Census	1-year age groups	De facto	Included
Ecuador	1974	Census	5-year age groups	De facto	Included
Ecuador	1982	Census	1-year age groups	De facto	Included
Ecuador	1990	Census	1-year age groups	De facto	Included
Ecuador	2001	Census	1-year age groups	De facto	Included
Ecuador	2010	Census	1-year age groups	De facto	Included
Egypt	1960	Census	5-year age groups	De facto	Included
Egypt	1966	Census	all ages only	De facto	Included
Egypt	1976	Census	5-year age groups	De facto	Included
Egypt	1986	Census	5-year age groups	De facto	Included
Egypt	1996	Census	5-year age groups	De facto	Included
Egypt	2006	Census	1-year age groups	De facto	Included
Egypt	2017	Census	5-year age groups	De facto	Included
El Salvador	1950	Census	1-year age groups	De facto	Included
El Salvador	1961	Census	1-year age groups	De facto	Included
El Salvador	1971	Census	1-year age groups	De facto	Included
El Salvador	1992	Census	1-year age groups	De facto	Included
El Salvador	1993	Census	5-year age groups	Unknown	Excluded
El Salvador	2007	Census	1-year age groups	De jure	Included
Equatorial Guinea	1983	Census	5-year age groups	De facto	Included
Equatorial Guinea	1994	Census	1-year age groups	Unknown	Excluded
Equatorial Guinea	2015	Census	all ages only	Unknown	Included
Eritrea	1984	Census	all ages only	De jure	Included
Estonia	1950	Registry	5-year age groups	NA	Included
Estonia	1951	Registry	5-year age groups	NA	Included
Estonia	1952	Registry	5-year age groups	NA	Included
Estonia	1953	Registry	5-year age groups	NA	Included

Estonia	1954	Registry	5-year age groups	NA	Included
Estonia	1955	Registry	5-year age groups	NA	Included
Estonia	1956	Registry	5-year age groups	NA	Included
Estonia	1957	Registry	5-year age groups	NA	Included
Estonia	1958	Registry	5-year age groups	NA	Included
Estonia	1959	Census	5-year age groups	De facto	Excluded
Estonia	1960	Registry	5-year age groups	NA	Included
Estonia	1961	Registry	5-year age groups	NA	Included
Estonia	1962	Registry	5-year age groups	NA	Included
Estonia	1963	Registry	5-year age groups	NA	Included
Estonia	1964	Registry	5-year age groups	NA	Included
Estonia	1965	Registry	5-year age groups	NA	Included
Estonia	1966	Registry	5-year age groups	NA	Included
Estonia	1967	Census	5-year age groups	De facto	Excluded
Estonia	1968	Registry	5-year age groups	NA	Included
Estonia	1969	Registry	5-year age groups	NA	Included
Estonia	1970	Census	5-year age groups	De facto	Excluded
Estonia	1971	Registry	5-year age groups	NA	Included
Estonia	1972	Registry	5-year age groups	NA	Included
Estonia	1973	Registry	5-year age groups	NA	Included
Estonia	1974	Registry	5-year age groups	NA	Included
Estonia	1975	Registry	5-year age groups	NA	Included
Estonia	1976	Registry	5-year age groups	NA	Included
Estonia	1977	Registry	5-year age groups	NA	Included
Estonia	1978	Registry	5-year age groups	NA	Included
Estonia	1979	Census	5-year age groups	De facto	Excluded
Estonia	1980	Registry	5-year age groups	NA	Included
Estonia	1981	Registry	5-year age groups	NA	Included
Estonia	1982	Registry	5-year age groups	NA	Included
Estonia	1983	Registry	5-year age groups	NA	Included
Estonia	1984	Registry	5-year age groups	NA	Included
Estonia	1985	Registry	5-year age groups	NA	Included
Estonia	1986	Registry	5-year age groups	NA	Included
Estonia	1987	Registry	5-year age groups	NA	Included
Estonia	1988	Registry	5-year age groups	NA	Included
Estonia	1989	Census	5-year age groups	De facto	Excluded
Estonia	1990	Registry	5-year age groups	NA	Included
Estonia	1991	Registry	5-year age groups	NA	Included
Estonia	1992	Registry	5-year age groups	NA	Included
Estonia	1993	Registry	5-year age groups	NA	Included
Estonia	1994	Registry	5-year age groups	NA	Included
Estonia	1995	Registry	5-year age groups	NA	Included
Estonia	1996	Registry	5-year age groups	NA	Included
Estonia	1997	Registry	5-year age groups	NA	Included
Estonia	1998	Registry	5-year age groups	NA	Included
Estonia	1999	Registry	5-year age groups	NA	Included
Estonia	2000	Registry	5-year age groups	NA	Included
Estonia	2001	Registry	5-year age groups	NA	Included
Estonia	2002	Registry	5-year age groups	NA	Included
Estonia	2003	Registry	5-year age groups	NA	Included
Estonia	2004	Registry	5-year age groups	NA	Included
Estonia	2005	Registry	5-year age groups	NA	Included
Estonia	2006	Registry	5-year age groups	NA	Included
Estonia	2007	Registry	5-year age groups	NA	Included
Estonia	2008	Registry	5-year age groups	NA	Included
Estonia	2009	Registry	5-year age groups	NA	Included

Estonia	2010	Registry	5-year age groups	NA	Included
Estonia	2011	Registry	5-year age groups	NA	Included
Estonia	2012	Registry	5-year age groups	NA	Included
Estonia	2013	Registry	5-year age groups	NA	Included
Estonia	2014	Registry	5-year age groups	NA	Included
Estonia	2015	Registry	5-year age groups	NA	Included
Estonia	2016	Registry	5-year age groups	NA	Included
Estonia	2017	Registry	5-year age groups	NA	Included
Eswatini	1956	Census	5-year age groups	De facto	Excluded
Eswatini	1966	Census	1-year age groups	De facto	Included
Eswatini	1976	Census	5-year age groups	De facto	Included
Eswatini	1986	Census	1-year age groups	De facto	Included
Eswatini	1997	Census	1-year age groups	De facto	Included
Eswatini	2007	Census	5-year age groups	De facto	Excluded
Eswatini	2017	Census	1-year age groups	De jure	Excluded
Ethiopia	1984	Census	1-year age groups	De facto	Included
Ethiopia	1994	Census	1-year age groups	De facto	Included
Ethiopia	2007	Census	1-year age groups	De facto	Included
Federated States of Micronesia	1989	Census	all ages only	De jure	Excluded
Federated States of Micronesia	1994	Census	5-year age groups	De jure	Included
Federated States of Micronesia	2000	Census	1-year age groups	De jure	Included
Federated States of Micronesia	2010	Census	5-year age groups	De facto	Included
Fiji	1956	Census	5-year age groups	De facto	Included
Fiji	1966	Census	5-year age groups	De facto	Included
Fiji	1976	Census	5-year age groups	De facto	Included
Fiji	1986	Census	1-year age groups	De facto	Included
Fiji	1996	Census	1-year age groups	De facto	Included
Fiji	2007	Census	5-year age groups	De facto	Included
Fiji	2017	Census	5-year age groups	De jure	Included
Finland	1950	Census	1-year age groups	De jure	Included
Finland	1960	Census	5-year age groups	De jure	Excluded
Finland	1970	Census	1-year age groups	De jure	Excluded
Finland	1971	Registry	1-year age groups	NA	Included
Finland	1972	Registry	1-year age groups	NA	Included
Finland	1973	Registry	1-year age groups	NA	Included
Finland	1974	Registry	1-year age groups	NA	Included
Finland	1975	Census	5-year age groups	De jure	Excluded
Finland	1976	Registry	1-year age groups	NA	Included
Finland	1977	Registry	1-year age groups	NA	Included
Finland	1978	Registry	1-year age groups	NA	Included
Finland	1979	Registry	1-year age groups	NA	Included
Finland	1980	Registry	1-year age groups	NA	Included
Finland	1981	Registry	1-year age groups	NA	Included
Finland	1982	Registry	1-year age groups	NA	Included
Finland	1983	Registry	1-year age groups	NA	Included
Finland	1984	Registry	1-year age groups	NA	Included
Finland	1985	Census	1-year age groups	De jure	Excluded
Finland	1986	Registry	1-year age groups	NA	Included
Finland	1987	Registry	1-year age groups	NA	Included
Finland	1988	Registry	1-year age groups	NA	Included
Finland	1989	Registry	1-year age groups	NA	Included
Finland	1990	Census	1-year age groups	De jure	Excluded
Finland	1991	Registry	1-year age groups	NA	Included
Finland	1992	Registry	1-year age groups	NA	Included
Finland	1993	Registry	1-year age groups	NA	Included
Finland	1994	Registry	1-year age groups	NA	Included

Finland	1995	Registry	1-year age groups	NA	Included
Finland	1996	Registry	1-year age groups	NA	Included
Finland	1997	Registry	1-year age groups	NA	Included
Finland	1998	Registry	1-year age groups	NA	Included
Finland	1999	Registry	1-year age groups	NA	Included
Finland	2000	Census	1-year age groups	De jure	Excluded
Finland	2001	Registry	1-year age groups	NA	Included
Finland	2002	Registry	1-year age groups	NA	Included
Finland	2003	Registry	1-year age groups	NA	Included
Finland	2004	Registry	1-year age groups	NA	Included
Finland	2005	Registry	1-year age groups	NA	Included
Finland	2006	Registry	1-year age groups	NA	Included
Finland	2007	Registry	1-year age groups	NA	Included
Finland	2008	Registry	1-year age groups	NA	Included
Finland	2009	Registry	1-year age groups	NA	Included
Finland	2010	Census	1-year age groups	De jure	Excluded
Finland	2011	Registry	1-year age groups	NA	Included
Finland	2012	Registry	1-year age groups	NA	Included
Finland	2013	Registry	1-year age groups	NA	Included
Finland	2014	Registry	1-year age groups	NA	Included
Finland	2015	Registry	1-year age groups	NA	Included
Finland	2016	Registry	1-year age groups	NA	Included
Finland	2017	Registry	1-year age groups	NA	Included
France	1954	Census	5-year age groups	De jure	Included
France	1962	Census	5-year age groups	De jure	Included
France	1968	Census	5-year age groups	De jure	Included
France	1975	Census	5-year age groups	De jure	Included
France	1982	Census	5-year age groups	De jure	Included
France	1990	Census	5-year age groups	De jure	Included
France	1999	Census	5-year age groups	De jure	Included
France	2006	Census	1-year age groups	De jure	Included
France	2007	Census	1-year age groups	De jure	Included
France	2008	Census	1-year age groups	De jure	Included
France	2009	Census	1-year age groups	De jure	Included
France	2010	Census	1-year age groups	De jure	Included
France	2011	Census	1-year age groups	De jure	Included
France	2012	Census	1-year age groups	De jure	Included
France	2013	Census	1-year age groups	De jure	Included
France	2014	Census	1-year age groups	De jure	Included
France	2015	Census	5-year age groups	Unknown	Included
Gabon	1960	Census	5-year age groups	De facto	Included
Gabon	1980	Census	5-year age groups	De jure	Excluded
Gabon	1993	Census	1-year age groups	De facto	Included
Gabon	2013	Census	non-std age groups	De jure	Included
Georgia	1959	Census	5-year age groups	De facto	Included
Georgia	1970	Census	5-year age groups	De facto	Included
Georgia	1979	Census	5-year age groups	De facto	Included
Georgia	1989	Census	5-year age groups	De jure	Included
Georgia	2002	Census	1-year age groups	De jure	Included
Georgia	2014	Census	1-year age groups	De jure	Included
Germany	1990	Registry	1-year age groups	NA	Included
Germany	1991	Registry	1-year age groups	NA	Included
Germany	1992	Registry	1-year age groups	NA	Included
Germany	1993	Registry	1-year age groups	NA	Included
Germany	1994	Registry	1-year age groups	NA	Included
Germany	1995	Registry	1-year age groups	NA	Included

Germany	1996	Registry	1-year age groups	NA	Included
Germany	1997	Registry	1-year age groups	NA	Included
Germany	1998	Registry	1-year age groups	NA	Included
Germany	1999	Registry	1-year age groups	NA	Included
Germany	2000	Registry	1-year age groups	NA	Included
Germany	2001	Registry	1-year age groups	NA	Included
Germany	2002	Registry	1-year age groups	NA	Included
Germany	2003	Registry	1-year age groups	NA	Included
Germany	2004	Registry	1-year age groups	NA	Included
Germany	2005	Registry	1-year age groups	NA	Included
Germany	2006	Registry	1-year age groups	NA	Included
Germany	2007	Registry	1-year age groups	NA	Included
Germany	2008	Registry	1-year age groups	NA	Included
Germany	2009	Registry	1-year age groups	NA	Included
Germany	2010	Registry	1-year age groups	NA	Included
Germany	2011	Census	1-year age groups	De jure	Excluded
Germany	2012	Registry	1-year age groups	NA	Included
Germany	2013	Registry	1-year age groups	NA	Included
Germany	2014	Registry	1-year age groups	NA	Included
Germany	2015	Registry	1-year age groups	NA	Included
Germany	2016	Registry	1-year age groups	NA	Included
Ghana	1960	Census	5-year age groups	De facto	Excluded
Ghana	1970	Census	1-year age groups	De facto	Included
Ghana	1984	Census	1-year age groups	De facto	Included
Ghana	2000	Census	5-year age groups	De facto	Included
Ghana	2010	Census	1-year age groups	De facto	Included
Greece	1951	Census	5-year age groups	De facto	Included
Greece	1961	Census	5-year age groups	De facto	Included
Greece	1971	Census	5-year age groups	De facto	Included
Greece	1981	Census	1-year age groups	De facto	Included
Greece	1991	Census	1-year age groups	De facto	Included
Greece	2001	Census	1-year age groups	De jure	Included
Greece	2011	Census	1-year age groups	De facto	Included
Greenland	1951	Census	5-year age groups	De jure	Included
Greenland	1955	Census	5-year age groups	De jure	Included
Greenland	1960	Census	non-std age groups	De jure	Included
Greenland	1965	Census	1-year age groups	De jure	Included
Greenland	1970	Census	1-year age groups	De jure	Included
Greenland	1976	Census	5-year age groups	De jure	Included
Greenland	1977	Registry	1-year age groups	NA	Included
Greenland	1978	Registry	1-year age groups	NA	Included
Greenland	1979	Registry	1-year age groups	NA	Included
Greenland	1980	Registry	1-year age groups	NA	Included
Greenland	1981	Registry	1-year age groups	NA	Included
Greenland	1982	Registry	1-year age groups	NA	Included
Greenland	1983	Registry	1-year age groups	NA	Included
Greenland	1984	Registry	1-year age groups	NA	Included
Greenland	1985	Registry	1-year age groups	NA	Included
Greenland	1986	Registry	1-year age groups	NA	Included
Greenland	1987	Registry	1-year age groups	NA	Included
Greenland	1988	Registry	1-year age groups	NA	Included
Greenland	1989	Registry	1-year age groups	NA	Included
Greenland	1990	Registry	1-year age groups	NA	Included
Greenland	1991	Registry	1-year age groups	NA	Included
Greenland	1992	Registry	1-year age groups	NA	Included
Greenland	1993	Registry	1-year age groups	NA	Included

Greenland	1994	Registry	1-year age groups	NA	Included
Greenland	1995	Registry	1-year age groups	NA	Included
Greenland	1996	Registry	1-year age groups	NA	Included
Greenland	1997	Registry	1-year age groups	NA	Included
Greenland	1998	Registry	1-year age groups	NA	Included
Greenland	1999	Registry	1-year age groups	NA	Included
Greenland	2000	Census	5-year age groups	De jure	Excluded
Greenland	2001	Registry	1-year age groups	NA	Included
Greenland	2002	Registry	1-year age groups	NA	Included
Greenland	2003	Registry	1-year age groups	NA	Included
Greenland	2004	Registry	1-year age groups	NA	Included
Greenland	2005	Registry	1-year age groups	NA	Included
Greenland	2006	Registry	1-year age groups	NA	Included
Greenland	2007	Registry	1-year age groups	NA	Included
Greenland	2008	Registry	1-year age groups	NA	Included
Greenland	2009	Registry	1-year age groups	NA	Included
Greenland	2010	Registry	1-year age groups	NA	Included
Greenland	2011	Registry	1-year age groups	NA	Included
Greenland	2012	Registry	1-year age groups	NA	Included
Greenland	2013	Registry	1-year age groups	NA	Included
Greenland	2014	Registry	1-year age groups	NA	Included
Greenland	2015	Registry	1-year age groups	NA	Included
Greenland	2016	Registry	1-year age groups	NA	Included
Greenland	2017	Registry	1-year age groups	NA	Included
Grenada	1960	Census	5-year age groups	De facto	Included
Grenada	1970	Census	5-year age groups	De facto	Included
Grenada	1981	Census	5-year age groups	De facto	Excluded
Grenada	1991	Census	5-year age groups	De facto	Included
Grenada	2001	Census	5-year age groups	De facto	Included
Grenada	2011	Census	5-year age groups	De jure	Included
Guam	1950	Census	5-year age groups	De jure	Included
Guam	1952	Census	all ages only	Unknown	Excluded
Guam	1953	Census	all ages only	Unknown	Excluded
Guam	1954	Census	all ages only	Unknown	Excluded
Guam	1955	Census	all ages only	Unknown	Excluded
Guam	1958	Census	all ages only	Unknown	Excluded
Guam	1960	Census	non-std age groups	De jure	Included
Guam	1970	Census	5-year age groups	De jure	Included
Guam	1980	Census	1-year age groups	De jure	Included
Guam	1990	Census	1-year age groups	De jure	Included
Guam	2000	Census	1-year age groups	De jure	Included
Guam	2010	Census	5-year age groups	De jure	Included
Guam	2020	Census	5-year age groups	De jure	Included
Guatemala	1950	Census	1-year age groups	De jure	Included
Guatemala	1964	Census	1-year age groups	De jure	Included
Guatemala	1973	Census	1-year age groups	De jure	Included
Guatemala	1981	Census	1-year age groups	De jure	Included
Guatemala	1994	Census	1-year age groups	De jure	Included
Guatemala	2002	Census	all ages only	De jure	Included
Guatemala	2018	Census	5-year age groups	De jure	Included
Guinea	1966	Census	all ages only	Unknown	Included
Guinea	1983	Census	1-year age groups	Unknown	Included
Guinea	1996	Census	5-year age groups	De facto	Included
Guinea	2014	Census	1-year age groups	De jure	Included
Guinea-Bissau	1950	Census	5-year age groups	De facto	Included
Guinea-Bissau	1960	Census	5-year age groups	De facto	Included

Guinea-Bissau	1979	Census	5-year age groups	De jure	Included
Guinea-Bissau	1991	Census	non-std age groups	De facto	Included
Guinea-Bissau	2009	Census	1-year age groups	De jure	Included
Guyana	1960	Census	5-year age groups	De facto	Included
Guyana	1970	Census	1-year age groups	De facto	Included
Guyana	1980	Census	5-year age groups	Unknown	Excluded
Guyana	1991	Census	5-year age groups	De facto	Included
Guyana	2002	Census	5-year age groups	De facto	Included
Guyana	2012	Census	5-year age groups	De jure	Included
Haiti	1950	Census	1-year age groups	De jure	Included
Haiti	1971	Census	1-year age groups	De jure	Included
Haiti	1982	Census	1-year age groups	De jure	Included
Haiti	2003	Census	1-year age groups	Unknown	Included
Honduras	1950	Census	5-year age groups	De facto	Included
Honduras	1961	Census	5-year age groups	De facto	Included
Honduras	1974	Census	5-year age groups	De facto	Included
Honduras	1988	Census	1-year age groups	De facto	Included
Honduras	2001	Census	1-year age groups	De jure	Included
Honduras	2013	Census	1-year age groups	De jure	Included
Hungary	1960	Census	1-year age groups	De facto	Included
Hungary	1970	Census	1-year age groups	De facto	Included
Hungary	1980	Census	1-year age groups	De facto	Included
Hungary	1990	Census	1-year age groups	De facto	Included
Hungary	2001	Census	1-year age groups	Unknown	Included
Hungary	2011	Census	1-year age groups	De facto	Included
Iceland	1950	Census	5-year age groups	De jure	Excluded
Iceland	1951	Registry	1-year age groups	NA	Included
Iceland	1952	Registry	1-year age groups	NA	Included
Iceland	1953	Registry	1-year age groups	NA	Included
Iceland	1954	Registry	1-year age groups	NA	Included
Iceland	1955	Registry	1-year age groups	NA	Included
Iceland	1956	Registry	1-year age groups	NA	Included
Iceland	1957	Registry	1-year age groups	NA	Included
Iceland	1958	Registry	1-year age groups	NA	Included
Iceland	1959	Registry	1-year age groups	NA	Included
Iceland	1960	Census	1-year age groups	De jure	Excluded
Iceland	1961	Registry	1-year age groups	NA	Included
Iceland	1962	Registry	1-year age groups	NA	Included
Iceland	1963	Registry	1-year age groups	NA	Included
Iceland	1964	Registry	1-year age groups	NA	Included
Iceland	1965	Registry	1-year age groups	NA	Included
Iceland	1966	Registry	1-year age groups	NA	Included
Iceland	1967	Registry	1-year age groups	NA	Included
Iceland	1968	Registry	1-year age groups	NA	Included
Iceland	1969	Registry	1-year age groups	NA	Included
Iceland	1970	Census	5-year age groups	De jure	Excluded
Iceland	1971	Registry	1-year age groups	NA	Included
Iceland	1972	Registry	1-year age groups	NA	Included
Iceland	1973	Registry	1-year age groups	NA	Included
Iceland	1974	Registry	1-year age groups	NA	Included
Iceland	1975	Registry	1-year age groups	NA	Included
Iceland	1976	Registry	1-year age groups	NA	Included
Iceland	1977	Registry	1-year age groups	NA	Included
Iceland	1978	Registry	1-year age groups	NA	Included
Iceland	1979	Registry	1-year age groups	NA	Included
Iceland	1980	Registry	1-year age groups	NA	Included

Iceland	1981	Registry	1-year age groups	NA	Included
Iceland	1982	Registry	1-year age groups	NA	Included
Iceland	1983	Registry	1-year age groups	NA	Included
Iceland	1984	Registry	1-year age groups	NA	Included
Iceland	1985	Registry	1-year age groups	NA	Included
Iceland	1986	Registry	1-year age groups	NA	Included
Iceland	1987	Registry	1-year age groups	NA	Included
Iceland	1988	Registry	1-year age groups	NA	Included
Iceland	1989	Registry	1-year age groups	NA	Included
Iceland	1990	Registry	1-year age groups	NA	Included
Iceland	1991	Registry	1-year age groups	NA	Included
Iceland	1992	Registry	1-year age groups	NA	Included
Iceland	1993	Registry	1-year age groups	NA	Included
Iceland	1994	Registry	1-year age groups	NA	Included
Iceland	1995	Registry	1-year age groups	NA	Included
Iceland	1996	Registry	1-year age groups	NA	Included
Iceland	1997	Registry	1-year age groups	NA	Included
Iceland	1998	Registry	1-year age groups	NA	Included
Iceland	1999	Registry	1-year age groups	NA	Included
Iceland	2000	Registry	1-year age groups	NA	Included
Iceland	2001	Registry	1-year age groups	NA	Included
Iceland	2002	Registry	1-year age groups	NA	Included
Iceland	2003	Registry	1-year age groups	NA	Included
Iceland	2004	Registry	1-year age groups	NA	Included
Iceland	2005	Registry	1-year age groups	NA	Included
Iceland	2006	Registry	1-year age groups	NA	Included
Iceland	2007	Registry	1-year age groups	NA	Included
Iceland	2008	Registry	1-year age groups	NA	Included
Iceland	2009	Registry	1-year age groups	NA	Included
Iceland	2010	Registry	1-year age groups	NA	Included
Iceland	2011	Census	5-year age groups	De jure	Excluded
Iceland	2012	Registry	1-year age groups	NA	Included
Iceland	2013	Registry	1-year age groups	NA	Included
Iceland	2014	Registry	1-year age groups	NA	Included
Iceland	2015	Registry	1-year age groups	NA	Included
Iceland	2016	Registry	1-year age groups	NA	Included
Iceland	2017	Registry	1-year age groups	NA	Included
India	1951	Census	1-year age groups	De facto	Included
India	1961	Census	5-year age groups	De facto	Included
India	1971	Census	1-year age groups	De facto	Included
India	1981	Census	5-year age groups	De facto	Included
India	1991	Census	1-year age groups	De facto	Included
India	2001	Census	1-year age groups	De facto	Included
India	2011	Census	1-year age groups	De facto	Included
Indonesia	1961	Census	non-std age groups	De facto	Included
Indonesia	1971	Census	5-year age groups	De jure	Excluded
Indonesia	1980	Census	1-year age groups	De facto	Included
Indonesia	1990	Census	5-year age groups	De facto	Included
Indonesia	2000	Census	1-year age groups	De jure	Excluded
Indonesia	2010	Census	1-year age groups	De jure	Included
Indonesia	2020	Census	5-year age groups	De jure	Included
Iran	1956	Census	5-year age groups	De facto	Included
Iran	1966	Census	5-year age groups	De jure	Excluded
Iran	1976	Census	5-year age groups	De facto	Included
Iran	1986	Census	5-year age groups	De facto	Included
Iran	1991	Census	5-year age groups	De jure	Excluded

Iran	1994	Census	1-year age groups	De jure	Excluded
Iran	1996	Census	5-year age groups	De jure	Excluded
Iran	2006	Census	1-year age groups	De jure	Included
Iran	2011	Census	1-year age groups	De jure	Included
Iran	2016	Census	5-year age groups	De jure	Excluded
Iraq	1957	Census	5-year age groups	De facto	Included
Iraq	1965	Census	1-year age groups	De facto	Included
Iraq	1977	Census	5-year age groups	De facto	Included
Iraq	1987	Census	5-year age groups	De facto	Included
Iraq	1997	Census	5-year age groups	De facto	Excluded
Ireland	1951	Census	1-year age groups	De facto	Included
Ireland	1956	Census	5-year age groups	De jure	Included
Ireland	1961	Census	5-year age groups	De facto	Included
Ireland	1966	Census	5-year age groups	De facto	Included
Ireland	1971	Census	1-year age groups	De facto	Included
Ireland	1979	Census	5-year age groups	De facto	Included
Ireland	1981	Census	5-year age groups	De facto	Included
Ireland	1986	Census	5-year age groups	De facto	Included
Ireland	1991	Census	1-year age groups	De facto	Included
Ireland	1996	Census	1-year age groups	De facto	Included
Ireland	2002	Census	1-year age groups	De facto	Included
Ireland	2006	Census	1-year age groups	De facto	Included
Ireland	2011	Census	1-year age groups	De facto	Included
Ireland	2016	Census	1-year age groups	De jure	Included
Israel	1961	Census	1-year age groups	De jure	Included
Israel	1972	Census	5-year age groups	De jure	Included
Israel	1983	Census	5-year age groups	De jure	Included
Israel	1995	Census	1-year age groups	De facto	Included
Israel	2008	Census	1-year age groups	De facto	Included
Italy	1951	Census	5-year age groups	De jure	Included
Italy	1961	Census	1-year age groups	De jure	Included
Italy	1971	Census	1-year age groups	De jure	Included
Italy	1981	Census	1-year age groups	De facto	Included
Italy	1991	Census	1-year age groups	De facto	Included
Italy	2001	Census	1-year age groups	De jure	Included
Italy	2002	Registry	1-year age groups	NA	Included
Italy	2003	Registry	1-year age groups	NA	Included
Italy	2004	Registry	1-year age groups	NA	Included
Italy	2005	Registry	1-year age groups	NA	Included
Italy	2006	Registry	1-year age groups	NA	Included
Italy	2007	Registry	1-year age groups	NA	Included
Italy	2008	Registry	1-year age groups	NA	Included
Italy	2009	Registry	1-year age groups	NA	Included
Italy	2010	Registry	1-year age groups	NA	Included
Italy	2011	Census	5-year age groups	De jure	Excluded
Italy	2012	Registry	1-year age groups	NA	Excluded
Italy	2013	Registry	1-year age groups	NA	Excluded
Italy	2014	Registry	1-year age groups	NA	Included
Italy	2015	Registry	1-year age groups	NA	Included
Italy	2016	Registry	1-year age groups	NA	Included
Italy	2017	Registry	1-year age groups	NA	Included
Jamaica	1953	Census	5-year age groups	De jure	Included
Jamaica	1960	Census	5-year age groups	De jure	Included
Jamaica	1970	Census	5-year age groups	De jure	Included
Jamaica	1982	Census	1-year age groups	De jure	Included
Jamaica	1991	Census	1-year age groups	De jure	Included

Jamaica	2001	Census	1-year age groups	De jure	Included
Jamaica	2011	Census	1-year age groups	De jure	Included
Japan	1950	Census	1-year age groups	De facto	Included
Japan	1955	Census	5-year age groups	De facto	Included
Japan	1960	Census	5-year age groups	De facto	Included
Japan	1965	Census	1-year age groups	De facto	Included
Japan	1970	Census	1-year age groups	De facto	Included
Japan	1975	Census	5-year age groups	De facto	Included
Japan	1980	Census	1-year age groups	De facto	Included
Japan	1985	Census	5-year age groups	De facto	Included
Japan	1990	Census	1-year age groups	De facto	Included
Japan	1995	Census	1-year age groups	De facto	Included
Japan	2000	Census	1-year age groups	De jure	Included
Japan	2005	Census	1-year age groups	De jure	Included
Japan	2010	Census	1-year age groups	De jure	Included
Japan	2015	Census	5-year age groups	De jure	Excluded
Japan	2020	Census	1-year age groups	De jure	Included
Jordan	1961	Census	non-std age groups	De facto	Excluded
Jordan	1979	Census	5-year age groups	De facto	Included
Jordan	1994	Census	5-year age groups	De facto	Included
Jordan	2004	Census	5-year age groups	De facto	Included
Jordan	2015	Census	1-year age groups	De facto	Included
Kazakhstan	1959	Census	non-std age groups	De facto	Included
Kazakhstan	1970	Census	non-std age groups	De facto	Included
Kazakhstan	1979	Census	5-year age groups	De facto	Included
Kazakhstan	1989	Census	1-year age groups	De facto	Included
Kazakhstan	1999	Census	1-year age groups	De jure	Included
Kazakhstan	2009	Census	5-year age groups	De facto	Included
Kazakhstan	2010	Registry	all ages only	NA	Included
Kazakhstan	2011	Registry	all ages only	NA	Included
Kazakhstan	2012	Registry	all ages only	NA	Included
Kazakhstan	2013	Registry	all ages only	NA	Included
Kazakhstan	2014	Registry	all ages only	NA	Included
Kazakhstan	2015	Registry	all ages only	NA	Included
Kazakhstan	2016	Registry	all ages only	NA	Included
Kenya	1962	Census	5-year age groups	Unknown	Excluded
Kenya	1969	Census	1-year age groups	De facto	Included
Kenya	1979	Census	5-year age groups	De facto	Included
Kenya	1989	Census	1-year age groups	De jure	Included
Kenya	1999	Census	5-year age groups	De facto	Included
Kenya	2009	Census	1-year age groups	De facto	Included
Kenya	2019	Census	5-year age groups	De facto	Included
Kiribati	1963	Census	5-year age groups	De facto	Included
Kiribati	1968	Census	5-year age groups	De facto	Included
Kiribati	1973	Census	5-year age groups	De facto	Included
Kiribati	1978	Census	5-year age groups	De facto	Included
Kiribati	1985	Census	5-year age groups	De facto	Included
Kiribati	1990	Census	5-year age groups	De facto	Included
Kiribati	1995	Census	5-year age groups	De facto	Included
Kiribati	2000	Census	5-year age groups	De facto	Included
Kiribati	2005	Census	5-year age groups	De facto	Included
Kiribati	2010	Census	1-year age groups	De facto	Included
Kiribati	2015	Census	5-year age groups	De jure	Included
Kuwait	1957	Census	5-year age groups	De facto	Included
Kuwait	1961	Census	non-std age groups	De facto	Included
Kuwait	1965	Census	1-year age groups	De facto	Included

Kuwait	1970	Census	1-year age groups	De facto	Included
Kuwait	1975	Census	5-year age groups	De facto	Included
Kuwait	1980	Census	5-year age groups	De facto	Included
Kuwait	1985	Census	1-year age groups	De facto	Included
Kuwait	1995	Census	5-year age groups	De facto	Included
Kuwait	2005	Census	5-year age groups	De facto	Included
Kuwait	2011	Census	1-year age groups	De facto	Included
Kyrgyzstan	1959	Census	non-std age groups	De facto	Included
Kyrgyzstan	1970	Census	non-std age groups	De facto	Included
Kyrgyzstan	1979	Census	5-year age groups	De facto	Included
Kyrgyzstan	1989	Census	1-year age groups	De jure	Included
Kyrgyzstan	1999	Census	1-year age groups	De jure	Included
Kyrgyzstan	2009	Census	1-year age groups	De jure	Included
Laos	1985	Census	5-year age groups	De jure	Included
Laos	1995	Census	5-year age groups	De facto	Included
Laos	2005	Census	1-year age groups	De jure	Included
Laos	2015	Census	1-year age groups	De jure	Included
Latvia	1959	Census	non-std age groups	De facto	Included
Latvia	1970	Census	non-std age groups	De facto	Included
Latvia	1979	Census	5-year age groups	De jure	Included
Latvia	1989	Census	5-year age groups	De jure	Included
Latvia	2000	Census	5-year age groups	De jure	Included
Latvia	2006	Registry	1-year age groups	NA	Included
Latvia	2007	Registry	1-year age groups	NA	Included
Latvia	2008	Registry	1-year age groups	NA	Included
Latvia	2009	Registry	1-year age groups	NA	Included
Latvia	2010	Registry	1-year age groups	NA	Included
Latvia	2011	Census	1-year age groups	De jure	Excluded
Latvia	2012	Registry	1-year age groups	NA	Included
Latvia	2013	Registry	1-year age groups	NA	Included
Latvia	2014	Registry	1-year age groups	NA	Included
Latvia	2015	Registry	1-year age groups	NA	Included
Latvia	2016	Registry	1-year age groups	NA	Included
Latvia	2017	Registry	1-year age groups	NA	Included
Lebanon	1970	Survey	5-year age groups	De facto	Included
Lebanon	2007	Census	5-year age groups	De facto	Included
Lesotho	1956	Census	non-std age groups	De facto	Included
Lesotho	1966	Census	1-year age groups	De facto	Included
Lesotho	1976	Census	5-year age groups	De facto	Included
Lesotho	1986	Census	5-year age groups	De facto	Included
Lesotho	1996	Census	5-year age groups	De facto	Included
Lesotho	2006	Census	5-year age groups	De facto	Included
Lesotho	2016	Census	5-year age groups	De facto	Included
Liberia	1962	Census	5-year age groups	De facto	Included
Liberia	1974	Census	5-year age groups	De facto	Included
Liberia	1984	Census	1-year age groups	De facto	Included
Liberia	2008	Census	1-year age groups	De facto	Included
Libya	1954	Census	5-year age groups	De facto	Included
Libya	1964	Census	non-std age groups	De facto	Included
Libya	1973	Census	1-year age groups	De jure	Included
Libya	1984	Census	1-year age groups	De facto	Included
Libya	1995	Census	all ages only	Unknown	Included
Libya	2006	Census	5-year age groups	De facto	Included
Lithuania	1959	Census	non-std age groups	De facto	Included
Lithuania	1970	Census	non-std age groups	De facto	Included
Lithuania	1979	Census	5-year age groups	De facto	Included

Lithuania	1989	Census	1-year age groups	De jure	Included
Lithuania	2001	Census	5-year age groups	De jure	Included
Lithuania	2011	Census	1-year age groups	De jure	Included
Lithuania	2015	Registry	1-year age groups	NA	Included
Lithuania	2016	Registry	1-year age groups	NA	Included
Lithuania	2017	Registry	1-year age groups	NA	Included
Luxembourg	1960	Census	1-year age groups	De jure	Included
Luxembourg	1966	Census	5-year age groups	De jure	Included
Luxembourg	1970	Census	1-year age groups	De jure	Included
Luxembourg	1981	Census	1-year age groups	De jure	Included
Luxembourg	1991	Census	5-year age groups	De jure	Included
Luxembourg	2001	Registry	5-year age groups	NA	Included
Luxembourg	2002	Registry	5-year age groups	NA	Included
Luxembourg	2003	Registry	5-year age groups	NA	Included
Luxembourg	2004	Registry	5-year age groups	NA	Included
Luxembourg	2005	Registry	5-year age groups	NA	Included
Luxembourg	2006	Registry	5-year age groups	NA	Included
Luxembourg	2007	Registry	5-year age groups	NA	Included
Luxembourg	2008	Registry	5-year age groups	NA	Included
Luxembourg	2009	Registry	5-year age groups	NA	Included
Luxembourg	2010	Registry	5-year age groups	NA	Included
Luxembourg	2011	Registry	5-year age groups	NA	Included
Luxembourg	2012	Registry	5-year age groups	NA	Included
Luxembourg	2013	Registry	5-year age groups	NA	Included
Luxembourg	2014	Registry	5-year age groups	NA	Included
Luxembourg	2015	Registry	5-year age groups	NA	Included
Luxembourg	2016	Registry	5-year age groups	NA	Included
Luxembourg	2017	Registry	5-year age groups	NA	Included
Madagascar	1975	Census	5-year age groups	De facto	Included
Madagascar	1993	Census	5-year age groups	De facto	Included
Madagascar	2018	Census	5-year age groups	De jure	Included
Malawi	1966	Census	5-year age groups	De facto	Included
Malawi	1977	Census	5-year age groups	De facto	Included
Malawi	1987	Census	1-year age groups	De facto	Included
Malawi	1998	Census	1-year age groups	De facto	Included
Malawi	2008	Census	1-year age groups	De facto	Included
Malawi	2018	Census	5-year age groups	De jure	Included
Malaysia	1991	Census	1-year age groups	De facto	Included
Malaysia	2000	Census	5-year age groups	De jure	Included
Malaysia	2010	Census	1-year age groups	De jure	Included
Maldives	1953	Census	all ages only	De jure	Included
Maldives	1957	Census	all ages only	De jure	Included
Maldives	1962	Census	all ages only	De jure	Included
Maldives	1965	Census	non-std age groups	De facto	Excluded
Maldives	1967	Census	1-year age groups	De facto	Included
Maldives	1972	Census	all ages only	De jure	Included
Maldives	1974	Census	5-year age groups	De facto	Included
Maldives	1977	Census	5-year age groups	De facto	Included
Maldives	1985	Census	1-year age groups	De facto	Included
Maldives	1990	Census	1-year age groups	De facto	Included
Maldives	1995	Census	1-year age groups	De facto	Included
Maldives	2000	Census	1-year age groups	De facto	Included
Maldives	2006	Census	1-year age groups	De facto	Included
Maldives	2014	Census	1-year age groups	De facto	Included
Mali	1976	Census	5-year age groups	De facto	Included
Mali	1987	Census	1-year age groups	De jure	Included

Mali	1998	Census	5-year age groups	De jure	Included
Mali	2009	Census	1-year age groups	De facto	Included
Malta	1957	Census	5-year age groups	De facto	Included
Malta	1967	Census	5-year age groups	De facto	Included
Malta	1985	Census	5-year age groups	De facto	Included
Malta	1995	Census	1-year age groups	De jure	Included
Malta	2005	Census	1-year age groups	De jure	Included
Malta	2011	Census	1-year age groups	De facto	Included
Marshall Islands	1973	Census	5-year age groups	De facto	Excluded
Marshall Islands	1988	Census	1-year age groups	De facto	Included
Marshall Islands	1999	Census	1-year age groups	De facto	Included
Marshall Islands	2011	Census	1-year age groups	De facto	Excluded
Marshall Islands	2011	Census	all ages only	De jure	Included
Mauritania	1977	Census	5-year age groups	De facto	Included
Mauritania	1988	Census	5-year age groups	De facto	Included
Mauritania	2000	Census	5-year age groups	De facto	Included
Mauritania	2013	Census	5-year age groups	De jure	Included
Mauritius	1952	Census	1-year age groups	De jure	Included
Mauritius	1962	Census	1-year age groups	De facto	Included
Mauritius	1972	Census	1-year age groups	De jure	Included
Mauritius	1983	Census	all ages only	De jure	Included
Mauritius	1990	Census	1-year age groups	De facto	Included
Mauritius	2000	Census	1-year age groups	De jure	Included
Mauritius	2011	Census	1-year age groups	De jure	Included
Mexico	1950	Census	1-year age groups	De jure	Included
Mexico	1960	Census	5-year age groups	De jure	Excluded
Mexico	1970	Census	1-year age groups	De jure	Included
Mexico	1980	Census	1-year age groups	De jure	Included
Mexico	1990	Census	1-year age groups	De jure	Included
Mexico	1995	Census	5-year age groups	Unknown	Included
Mexico	2000	Census	1-year age groups	De jure	Included
Mexico	2005	Census	1-year age groups	De jure	Excluded
Mexico	2010	Census	1-year age groups	De facto	Included
Mexico	2020	Census	1-year age groups	De jure	Included
Moldova	1959	Census	non-std age groups	De facto	Included
Moldova	1970	Census	non-std age groups	De facto	Included
Moldova	1979	Census	5-year age groups	De facto	Included
Moldova	1989	Census	1-year age groups	De jure	Included
Monaco	1951	Census	5-year age groups	De jure	Included
Monaco	1956	Census	5-year age groups	De jure	Included
Monaco	1961	Census	1-year age groups	De jure	Excluded
Monaco	1962	Census	5-year age groups	De jure	Included
Monaco	1968	Census	5-year age groups	De jure	Included
Monaco	1975	Census	5-year age groups	De jure	Excluded
Monaco	1982	Census	5-year age groups	De jure	Excluded
Monaco	1990	Census	non-std age groups	De jure	Excluded
Monaco	2000	Census	5-year age groups	Unknown	Included
Monaco	2008	Census	5-year age groups	De jure	Excluded
Monaco	2016	Census	5-year age groups	De jure	Excluded
Mongolia	1956	Census	non-std age groups	De facto	Included
Mongolia	1963	Census	5-year age groups	De jure	Included
Mongolia	1969	Census	5-year age groups	De jure	Included
Mongolia	1979	Census	5-year age groups	De jure	Included
Mongolia	1989	Census	5-year age groups	De facto	Included
Mongolia	2000	Census	1-year age groups	De facto	Included
Mongolia	2010	Census	1-year age groups	De jure	Included

Mongolia	2020	Census	5-year age groups	De jure	Included
Montenegro	1953	Census	5-year age groups	De jure	Included
Montenegro	1961	Census	5-year age groups	De jure	Included
Montenegro	1971	Census	5-year age groups	De jure	Included
Montenegro	1981	Census	5-year age groups	De jure	Included
Montenegro	1991	Census	5-year age groups	De facto	Included
Montenegro	2003	Census	1-year age groups	De jure	Included
Montenegro	2011	Census	1-year age groups	De jure	Included
Morocco	1950	Census	all ages only	De jure	Excluded
Morocco	1960	Census	5-year age groups	De jure	Included
Morocco	1971	Census	1-year age groups	De jure	Included
Morocco	1982	Census	5-year age groups	De facto	Included
Morocco	1994	Census	5-year age groups	De facto	Included
Morocco	2004	Census	1-year age groups	De facto	Included
Morocco	2014	Census	5-year age groups	De jure	Included
Mozambique	1950	Census	5-year age groups	De facto	Included
Mozambique	1960	Census	5-year age groups	De jure	Included
Mozambique	1970	Census	5-year age groups	De facto	Included
Mozambique	1980	Census	1-year age groups	De facto	Included
Mozambique	1997	Census	1-year age groups	De jure	Included
Mozambique	2007	Census	1-year age groups	De facto	Included
Mozambique	2017	Census	1-year age groups	De jure	Included
Myanmar	1953	Census	all ages only	Unknown	Excluded
Myanmar	1973	Census	5-year age groups	De jure	Included
Myanmar	1983	Census	1-year age groups	De facto	Included
Myanmar	2014	Census	1-year age groups	De facto	Included
Namibia	1951	Census	5-year age groups	De facto	Excluded
Namibia	1960	Census	5-year age groups	De facto	Included
Namibia	1970	Census	all ages only	De jure	Included
Namibia	1991	Census	1-year age groups	De facto	Included
Namibia	2001	Census	1-year age groups	De facto	Included
Namibia	2011	Census	1-year age groups	De facto	Included
Nauru	1961	Census	5-year age groups	De facto	Excluded
Nauru	1966	Census	5-year age groups	De facto	Excluded
Nauru	1977	Census	5-year age groups	De facto	Excluded
Nauru	1992	Census	5-year age groups	De facto	Excluded
Nauru	2002	Census	5-year age groups	De facto	Excluded
Nauru	2006	Census	5-year age groups	Unknown	Excluded
Nauru	2011	Census	5-year age groups	Unknown	Included
Nepal	1954	Census	5-year age groups	De facto	Included
Nepal	1961	Census	5-year age groups	De facto	Included
Nepal	1971	Census	non-std age groups	De jure	Included
Nepal	1981	Census	1-year age groups	De jure	Included
Nepal	1991	Census	1-year age groups	De jure	Included
Nepal	2001	Census	1-year age groups	De jure	Included
Nepal	2011	Census	1-year age groups	De jure	Included
Netherlands	1950	Registry	1-year age groups	NA	Included
Netherlands	1951	Registry	1-year age groups	NA	Included
Netherlands	1952	Registry	1-year age groups	NA	Included
Netherlands	1953	Registry	1-year age groups	NA	Included
Netherlands	1954	Registry	1-year age groups	NA	Included
Netherlands	1955	Registry	1-year age groups	NA	Included
Netherlands	1956	Registry	1-year age groups	NA	Included
Netherlands	1957	Registry	1-year age groups	NA	Included
Netherlands	1958	Registry	1-year age groups	NA	Included
Netherlands	1959	Registry	1-year age groups	NA	Included

Netherlands	1960	Census	1-year age groups	De jure	Excluded
Netherlands	1961	Registry	1-year age groups	NA	Included
Netherlands	1962	Registry	1-year age groups	NA	Included
Netherlands	1963	Registry	1-year age groups	NA	Included
Netherlands	1964	Registry	1-year age groups	NA	Included
Netherlands	1965	Registry	1-year age groups	NA	Included
Netherlands	1966	Registry	1-year age groups	NA	Included
Netherlands	1967	Registry	1-year age groups	NA	Included
Netherlands	1968	Registry	1-year age groups	NA	Included
Netherlands	1969	Registry	1-year age groups	NA	Included
Netherlands	1970	Registry	1-year age groups	NA	Included
Netherlands	1971	Registry	1-year age groups	NA	Included
Netherlands	1971	Census	1-year age groups	Unknown	Excluded
Netherlands	1972	Registry	1-year age groups	NA	Included
Netherlands	1973	Registry	1-year age groups	NA	Included
Netherlands	1974	Registry	1-year age groups	NA	Included
Netherlands	1975	Registry	1-year age groups	NA	Included
Netherlands	1976	Registry	1-year age groups	NA	Included
Netherlands	1977	Registry	1-year age groups	NA	Included
Netherlands	1978	Registry	1-year age groups	NA	Included
Netherlands	1979	Registry	1-year age groups	NA	Included
Netherlands	1980	Registry	1-year age groups	NA	Included
Netherlands	1981	Registry	1-year age groups	NA	Included
Netherlands	1982	Registry	1-year age groups	NA	Included
Netherlands	1983	Registry	1-year age groups	NA	Included
Netherlands	1984	Registry	1-year age groups	NA	Included
Netherlands	1985	Registry	1-year age groups	NA	Included
Netherlands	1986	Registry	1-year age groups	NA	Included
Netherlands	1987	Registry	1-year age groups	NA	Included
Netherlands	1988	Registry	1-year age groups	NA	Included
Netherlands	1989	Registry	1-year age groups	NA	Included
Netherlands	1990	Registry	1-year age groups	NA	Included
Netherlands	1991	Census	5-year age groups	De jure	Excluded
Netherlands	1992	Registry	1-year age groups	NA	Included
Netherlands	1993	Registry	1-year age groups	NA	Included
Netherlands	1994	Registry	1-year age groups	NA	Included
Netherlands	1995	Registry	1-year age groups	NA	Included
Netherlands	1996	Registry	1-year age groups	NA	Included
Netherlands	1997	Registry	1-year age groups	NA	Included
Netherlands	1998	Registry	1-year age groups	NA	Included
Netherlands	1999	Registry	1-year age groups	NA	Included
Netherlands	2000	Registry	1-year age groups	NA	Included
Netherlands	2001	Registry	1-year age groups	NA	Included
Netherlands	2002	Census	1-year age groups	De jure	Excluded
Netherlands	2003	Registry	1-year age groups	NA	Included
Netherlands	2004	Registry	1-year age groups	NA	Included
Netherlands	2005	Registry	1-year age groups	NA	Included
Netherlands	2006	Registry	1-year age groups	NA	Included
Netherlands	2007	Registry	1-year age groups	NA	Included
Netherlands	2008	Registry	1-year age groups	NA	Included
Netherlands	2009	Registry	1-year age groups	NA	Included
Netherlands	2010	Registry	1-year age groups	NA	Included
Netherlands	2011	Census	1-year age groups	De jure	Excluded
Netherlands	2012	Registry	1-year age groups	NA	Included
Netherlands	2013	Registry	1-year age groups	NA	Included
Netherlands	2014	Registry	1-year age groups	NA	Included

Netherlands	2015	Registry	1-year age groups	NA	Included
Netherlands	2016	Registry	1-year age groups	NA	Included
Netherlands	2017	Registry	1-year age groups	NA	Included
New Zealand	1951	Census	1-year age groups	De facto	Included
New Zealand	1956	Census	5-year age groups	De jure	Included
New Zealand	1961	Census	1-year age groups	De jure	Included
New Zealand	1966	Census	5-year age groups	De facto	Included
New Zealand	1971	Census	5-year age groups	De facto	Included
New Zealand	1976	Census	5-year age groups	De facto	Included
New Zealand	1981	Census	1-year age groups	De jure	Included
New Zealand	1986	Census	1-year age groups	De jure	Included
New Zealand	1991	Census	1-year age groups	De facto	Included
New Zealand	1996	Census	5-year age groups	De jure	Excluded
New Zealand	2001	Census	5-year age groups	De jure	Excluded
New Zealand	2006	Census	5-year age groups	De jure	Excluded
New Zealand	2013	Census	5-year age groups	De jure	Excluded
New Zealand	2018	Census	1-year age groups	De jure	Included
Nicaragua	1950	Census	1-year age groups	De jure	Included
Nicaragua	1963	Census	1-year age groups	De jure	Included
Nicaragua	1971	Census	1-year age groups	De jure	Included
Nicaragua	1995	Census	1-year age groups	Unknown	Included
Nicaragua	2005	Census	1-year age groups	De jure	Included
Niger	1977	Census	5-year age groups	De jure	Included
Niger	1988	Census	5-year age groups	De facto	Included
Niger	2001	Census	1-year age groups	De jure	Included
Niger	2012	Census	1-year age groups	De jure	Included
Nigeria	1953	Census	non-std age groups	De facto	Excluded
Nigeria	1963	Census	1-year age groups	De facto	Excluded
Nigeria	1991	Census	1-year age groups	De facto	Included
Nigeria	2006	Census	5-year age groups	De facto	Included
Nigeria	2014	Census	5-year age groups	Micro-census	Included
Niue	1951	Census	5-year age groups	De facto	Excluded
Niue	1956	Census	1-year age groups	De facto	Included
Niue	1961	Census	1-year age groups	De facto	Included
Niue	1966	Census	5-year age groups	De jure	Included
Niue	1971	Census	1-year age groups	De facto	Excluded
Niue	1976	Census	5-year age groups	De facto	Excluded
Niue	1979	Census	5-year age groups	De facto	Excluded
Niue	1981	Census	5-year age groups	De facto	Included
Niue	1984	Census	1-year age groups	De facto	Included
Niue	1986	Census	1-year age groups	De facto	Included
Niue	1997	Census	5-year age groups	De facto	Excluded
Niue	2001	Census	5-year age groups	De facto	Excluded
Niue	2006	Census	5-year age groups	De jure	Included
Niue	2011	Census	5-year age groups	De facto	Included
North Korea	1993	Census	1-year age groups	De jure	Included
North Korea	2008	Census	1-year age groups	De jure	Included
North Macedonia	1953	Census	5-year age groups	De jure	Included
North Macedonia	1961	Census	5-year age groups	De jure	Included
North Macedonia	1971	Census	5-year age groups	De jure	Included
North Macedonia	1981	Census	5-year age groups	De jure	Included
North Macedonia	1991	Census	5-year age groups	De jure	Excluded
North Macedonia	1992	Census	5-year age groups	Unknown	Excluded
North Macedonia	1994	Census	5-year age groups	De jure	Included
North Macedonia	2002	Census	5-year age groups	De jure	Excluded
Northern Mariana Islands	1958	Census	5-year age groups	De jure	Excluded

Northern Mariana Islands	1970	Census	non-std age groups	De jure	Included
Northern Mariana Islands	1973	Census	1-year age groups	De facto	Excluded
Northern Mariana Islands	1980	Census	5-year age groups	De facto	Included
Northern Mariana Islands	1990	Census	1-year age groups	De facto	Included
Northern Mariana Islands	1995	Census	5-year age groups	De facto	Included
Northern Mariana Islands	2000	Census	non-std age groups	De jure	Included
Northern Mariana Islands	2010	Census	5-year age groups	De facto	Included
Northern Mariana Islands	2020	Census	5-year age groups	De jure	Included
Norway	1950	Census	5-year age groups	De jure	Excluded
Norway	1960	Census	5-year age groups	De jure	Excluded
Norway	1970	Census	1-year age groups	De jure	Included
Norway	1980	Census	1-year age groups	De jure	Included
Norway	1990	Census	5-year age groups	De jure	Included
Norway	2001	Census	1-year age groups	De jure	Excluded
Norway	2011	Census	1-year age groups	De jure	Excluded
Oman	1993	Census	5-year age groups	De facto	Included
Oman	2003	Census	1-year age groups	De facto	Included
Oman	2010	Census	5-year age groups	De facto	Included
Oman	2012	Registry	5-year age groups	NA	Included
Oman	2013	Registry	5-year age groups	NA	Included
Oman	2014	Registry	5-year age groups	NA	Included
Oman	2015	Registry	5-year age groups	NA	Included
Oman	2016	Registry	5-year age groups	NA	Included
Oman	2017	Registry	5-year age groups	NA	Included
Oman	2020	Census	5-year age groups	Unknown	Included
Pakistan	1951	Census	non-std age groups	De jure	Included
Pakistan	1961	Census	non-std age groups	De jure	Included
Pakistan	1972	Census	5-year age groups	De facto	Excluded
Pakistan	1981	Census	1-year age groups	De facto	Included
Pakistan	1998	Census	5-year age groups	De facto	Included
Pakistan	2017	Census	all ages only	Unknown	Included
Palau	1958	Census	5-year age groups	De facto	Included
Palau	1967	Census	5-year age groups	De facto	Included
Palau	1970	Census	5-year age groups	De jure	Excluded
Palau	1973	Census	5-year age groups	De facto	Excluded
Palau	1980	Census	5-year age groups	De facto	Excluded
Palau	1986	Census	5-year age groups	De facto	Excluded
Palau	1990	Census	5-year age groups	De jure	Excluded
Palau	1995	Census	5-year age groups	De facto	Included
Palau	2000	Census	1-year age groups	De jure	Excluded
Palau	2003	Census	non-std age groups	Unknown	Excluded
Palau	2005	Census	5-year age groups	De jure	Included
Palau	2012	Census	5-year age groups	De jure	Excluded
Palau	2015	Census	5-year age groups	De jure	Excluded
Palestine	1967	Census	5-year age groups	De jure	Included
Palestine	1997	Census	1-year age groups	De facto	Included
Palestine	2007	Census	1-year age groups	De facto	Included
Palestine	2017	Census	5-year age groups	De facto	Excluded
Panama	1950	Census	1-year age groups	De facto	Included
Panama	1960	Census	1-year age groups	De facto	Included
Panama	1970	Census	5-year age groups	De facto	Included
Panama	1980	Census	1-year age groups	De facto	Included
Panama	1990	Census	1-year age groups	De facto	Included
Panama	2000	Census	1-year age groups	De facto	Included
Panama	2010	Census	5-year age groups	De facto	Included
Papua New Guinea	1961	Census	1-year age groups	De facto	Excluded

Papua New Guinea	1966	Census	5-year age groups	De facto	Included
Papua New Guinea	1971	Census	1-year age groups	De facto	Included
Papua New Guinea	1980	Census	1-year age groups	De facto	Included
Papua New Guinea	1990	Census	5-year age groups	De facto	Excluded
Papua New Guinea	2000	Census	1-year age groups	De facto	Included
Papua New Guinea	2011	Census	5-year age groups	De facto	Included
Paraguay	1950	Census	1-year age groups	De facto	Included
Paraguay	1962	Census	1-year age groups	De facto	Included
Paraguay	1972	Census	1-year age groups	De facto	Included
Paraguay	1982	Census	1-year age groups	De facto	Included
Paraguay	1992	Census	1-year age groups	De facto	Included
Paraguay	2002	Census	1-year age groups	De facto	Included
Peru	1961	Census	1-year age groups	De facto	Included
Peru	1972	Census	1-year age groups	De facto	Included
Peru	1981	Census	1-year age groups	De facto	Included
Peru	1993	Census	1-year age groups	Unknown	Included
Peru	2005	Census	1-year age groups	De facto	Included
Peru	2007	Census	1-year age groups	De facto	Included
Peru	2012	Registry	all ages only	NA	Included
Peru	2013	Registry	all ages only	NA	Included
Peru	2014	Registry	all ages only	NA	Included
Peru	2015	Registry	all ages only	NA	Included
Peru	2016	Registry	all ages only	NA	Included
Peru	2017	Census	1-year age groups	De facto	Excluded
Peru	2017	Registry	all ages only	NA	Included
Philippines	1960	Census	1-year age groups	De jure	Included
Philippines	1970	Census	1-year age groups	De jure	Included
Philippines	1975	Census	5-year age groups	De jure	Included
Philippines	1980	Census	5-year age groups	De jure	Included
Philippines	1990	Census	1-year age groups	De jure	Included
Philippines	1995	Census	1-year age groups	De jure	Included
Philippines	2000	Census	1-year age groups	De jure	Included
Philippines	2007	Census	1-year age groups	De jure	Included
Philippines	2010	Census	5-year age groups	De jure	Included
Philippines	2015	Census	1-year age groups	De jure	Included
Poland	1950	Census	5-year age groups	De facto	Included
Poland	1960	Census	5-year age groups	De facto	Included
Poland	1970	Census	1-year age groups	De facto	Included
Poland	1978	Census	5-year age groups	De facto	Included
Poland	1988	Census	1-year age groups	De facto	Included
Poland	2002	Census	1-year age groups	De jure	Included
Poland	2006	Registry	1-year age groups	NA	Included
Poland	2007	Registry	1-year age groups	NA	Included
Poland	2008	Registry	1-year age groups	NA	Included
Poland	2009	Registry	1-year age groups	NA	Included
Poland	2010	Registry	1-year age groups	NA	Included
Poland	2011	Census	1-year age groups	De jure	Excluded
Poland	2012	Registry	1-year age groups	NA	Included
Poland	2013	Registry	1-year age groups	NA	Included
Poland	2014	Registry	1-year age groups	NA	Included
Poland	2015	Registry	1-year age groups	NA	Included
Poland	2016	Registry	1-year age groups	NA	Included
Poland	2017	Registry	1-year age groups	NA	Included
Poland	2017	Census	1-year age groups	Unknown	Excluded
Portugal	1950	Census	1-year age groups	De facto	Included
Portugal	1960	Census	5-year age groups	De facto	Included

Portugal	1970	Census	1-year age groups	De facto	Included
Portugal	1981	Census	1-year age groups	De facto	Included
Portugal	1991	Census	1-year age groups	De facto	Included
Portugal	2001	Census	5-year age groups	De facto	Included
Portugal	2011	Census	1-year age groups	De jure	Included
Puerto Rico	1950	Census	1-year age groups	De jure	Included
Puerto Rico	1960	Census	5-year age groups	De jure	Included
Puerto Rico	1970	Census	1-year age groups	De jure	Included
Puerto Rico	1980	Census	1-year age groups	De jure	Included
Puerto Rico	1990	Census	1-year age groups	De jure	Included
Puerto Rico	2000	Census	1-year age groups	De jure	Included
Puerto Rico	2010	Census	1-year age groups	De jure	Included
Puerto Rico	2020	Census	all ages only	De jure	Included
Qatar	1986	Census	1-year age groups	De facto	Included
Qatar	1997	Census	5-year age groups	De facto	Included
Qatar	2004	Census	5-year age groups	De facto	Included
Qatar	2010	Census	5-year age groups	De facto	Included
Qatar	2015	Census	5-year age groups	De jure	Excluded
Qatar	2020	Census	5-year age groups	De jure	Included
Romania	1956	Census	5-year age groups	De jure	Included
Romania	1966	Census	5-year age groups	De jure	Excluded
Romania	1977	Census	5-year age groups	De facto	Included
Romania	1992	Census	1-year age groups	De jure	Included
Romania	2002	Census	1-year age groups	De jure	Included
Romania	2011	Census	1-year age groups	De facto	Included
Russia	1959	Census	5-year age groups	De facto	Included
Russia	1970	Census	5-year age groups	De facto	Included
Russia	1979	Census	5-year age groups	De jure	Included
Russia	1989	Census	1-year age groups	De jure	Included
Russia	2002	Census	5-year age groups	De jure	Included
Russia	2010	Census	1-year age groups	De jure	Included
Rwanda	1978	Census	5-year age groups	De facto	Included
Rwanda	1991	Census	5-year age groups	De facto	Included
Rwanda	2002	Census	1-year age groups	De jure	Included
Rwanda	2012	Census	1-year age groups	De jure	Included
Saint Kitts and Nevis	1960	Census	1-year age groups	De facto	Excluded
Saint Kitts and Nevis	1970	Census	1-year age groups	De facto	Included
Saint Kitts and Nevis	1980	Census	5-year age groups	Unknown	Included
Saint Kitts and Nevis	1991	Census	5-year age groups	De facto	Excluded
Saint Kitts and Nevis	2001	Census	5-year age groups	Unknown	Included
Saint Lucia	1960	Census	5-year age groups	De facto	Included
Saint Lucia	1970	Census	5-year age groups	De facto	Included
Saint Lucia	1980	Census	1-year age groups	De facto	Included
Saint Lucia	1991	Census	1-year age groups	De facto	Included
Saint Lucia	2001	Census	1-year age groups	De facto	Included
Saint Lucia	2010	Census	5-year age groups	De jure	Included
Saint Vincent and the Grenadines	1960	Census	5-year age groups	De facto	Included
Saint Vincent and the Grenadines	1970	Census	5-year age groups	De facto	Included
Saint Vincent and the Grenadines	1980	Census	1-year age groups	De facto	Included
Saint Vincent and the Grenadines	1991	Census	1-year age groups	De facto	Included
Saint Vincent and the Grenadines	2001	Census	1-year age groups	De facto	Included
Saint Vincent and the Grenadines	2012	Census	5-year age groups	De jure	Included
Samoa	1951	Census	5-year age groups	De facto	Included
Samoa	1956	Census	1-year age groups	De facto	Included
Samoa	1961	Census	5-year age groups	De facto	Included
Samoa	1966	Census	5-year age groups	De facto	Included

Samoa	1971	Census	1-year age groups	De facto	Included
Samoa	1976	Census	5-year age groups	De facto	Included
Samoa	1981	Census	5-year age groups	De facto	Included
Samoa	1986	Census	5-year age groups	De facto	Included
Samoa	1991	Census	5-year age groups	De facto	Included
Samoa	2001	Census	1-year age groups	De facto	Included
Samoa	2006	Census	5-year age groups	De facto	Included
Samoa	2011	Census	1-year age groups	De facto	Included
Samoa	2016	Census	1-year age groups	De facto	Excluded
San Marino	1976	Census	5-year age groups	De facto	Excluded
San Marino	2010	Census	non-std age groups	De facto	Included
Saudi Arabia	1974	Census	all ages only	Unknown	Included
Saudi Arabia	1992	Census	5-year age groups	De facto	Included
Saudi Arabia	2004	Census	5-year age groups	De facto	Included
Saudi Arabia	2010	Census	5-year age groups	De facto	Included
Senegal	1976	Census	5-year age groups	De jure	Included
Senegal	1988	Census	5-year age groups	De jure	Included
Senegal	2002	Census	5-year age groups	De jure	Included
Senegal	2013	Census	5-year age groups	De facto	Included
Seychelles	1960	Census	5-year age groups	De facto	Included
Seychelles	1971	Census	1-year age groups	De jure	Included
Seychelles	1977	Census	5-year age groups	De facto	Included
Seychelles	1987	Census	1-year age groups	De facto	Included
Seychelles	1994	Census	5-year age groups	De facto	Included
Seychelles	1997	Census	1-year age groups	De facto	Included
Seychelles	2002	Census	1-year age groups	De jure	Included
Seychelles	2010	Census	1-year age groups	De facto	Included
Sierra Leone	1963	Census	5-year age groups	De facto	Included
Sierra Leone	1974	Census	5-year age groups	De facto	Included
Sierra Leone	1985	Census	1-year age groups	De facto	Included
Sierra Leone	2004	Census	1-year age groups	Unknown	Included
Sierra Leone	2015	Census	5-year age groups	De facto	Included
Singapore	1957	Census	5-year age groups	De facto	Included
Singapore	1970	Census	1-year age groups	De facto	Included
Singapore	1980	Census	1-year age groups	De facto	Included
Singapore (Residents Only)	1990	Registry	non-std age groups	NA	Included
Singapore (Residents Only)	1991	Registry	non-std age groups	NA	Included
Singapore (Residents Only)	1992	Registry	non-std age groups	NA	Included
Singapore (Residents Only)	1993	Registry	non-std age groups	NA	Included
Singapore (Residents Only)	1994	Registry	non-std age groups	NA	Included
Singapore (Residents Only)	1995	Registry	non-std age groups	NA	Included
Singapore (Residents Only)	1996	Registry	non-std age groups	NA	Included
Singapore (Residents Only)	1997	Registry	non-std age groups	NA	Included
Singapore (Residents Only)	1998	Registry	non-std age groups	NA	Included
Singapore (Residents Only)	1999	Registry	non-std age groups	NA	Included
Singapore (Residents Only)	2000	Registry	non-std age groups	NA	Included
Singapore (Residents Only)	2001	Registry	non-std age groups	NA	Included
Singapore (Residents Only)	2002	Registry	non-std age groups	NA	Included
Singapore (Residents Only)	2003	Registry	non-std age groups	NA	Included
Singapore (Residents Only)	2004	Registry	non-std age groups	NA	Included
Singapore (Residents Only)	2005	Registry	non-std age groups	NA	Included
Singapore (Residents Only)	2006	Registry	non-std age groups	NA	Included
Singapore (Residents Only)	2007	Registry	non-std age groups	NA	Included
Singapore (Residents Only)	2008	Registry	non-std age groups	NA	Included
Singapore (Residents Only)	2009	Registry	non-std age groups	NA	Included
Singapore (Residents Only)	2010	Registry	non-std age groups	NA	Included

Singapore (Residents Only)	2011	Registry	non-std age groups	NA	Included
Singapore (Residents Only)	2012	Registry	non-std age groups	NA	Included
Singapore (Residents Only)	2013	Registry	non-std age groups	NA	Included
Singapore (Residents Only)	2014	Registry	non-std age groups	NA	Included
Singapore (Residents Only)	2015	Registry	non-std age groups	NA	Included
Singapore (Residents Only)	2016	Registry	non-std age groups	NA	Included
Singapore (Residents Only)	2017	Registry	non-std age groups	NA	Included
Singapore (Residents Only)	2018	Registry	non-std age groups	NA	Included
Slovakia	1950	Census	5-year age groups	De jure	Excluded
Slovakia	1951	Registry	1-year age groups	NA	Included
Slovakia	1952	Registry	1-year age groups	NA	Included
Slovakia	1953	Registry	1-year age groups	NA	Included
Slovakia	1954	Registry	1-year age groups	NA	Included
Slovakia	1955	Registry	1-year age groups	NA	Included
Slovakia	1956	Registry	1-year age groups	NA	Included
Slovakia	1957	Registry	1-year age groups	NA	Included
Slovakia	1958	Registry	1-year age groups	NA	Included
Slovakia	1959	Registry	1-year age groups	NA	Included
Slovakia	1960	Registry	1-year age groups	NA	Included
Slovakia	1961	Census	1-year age groups	De jure	Excluded
Slovakia	1962	Registry	1-year age groups	NA	Included
Slovakia	1963	Registry	1-year age groups	NA	Included
Slovakia	1964	Registry	1-year age groups	NA	Included
Slovakia	1965	Registry	1-year age groups	NA	Included
Slovakia	1966	Registry	1-year age groups	NA	Included
Slovakia	1967	Registry	1-year age groups	NA	Included
Slovakia	1968	Registry	1-year age groups	NA	Included
Slovakia	1969	Registry	1-year age groups	NA	Included
Slovakia	1970	Registry	1-year age groups	NA	Included
Slovakia	1970	Census	all ages only	Unknown	Excluded
Slovakia	1971	Registry	1-year age groups	NA	Included
Slovakia	1972	Registry	1-year age groups	NA	Included
Slovakia	1973	Registry	1-year age groups	NA	Included
Slovakia	1974	Registry	1-year age groups	NA	Included
Slovakia	1975	Registry	1-year age groups	NA	Included
Slovakia	1976	Registry	1-year age groups	NA	Included
Slovakia	1977	Registry	1-year age groups	NA	Included
Slovakia	1978	Registry	1-year age groups	NA	Included
Slovakia	1979	Registry	1-year age groups	NA	Included
Slovakia	1980	Census	5-year age groups	De jure	Excluded
Slovakia	1981	Registry	1-year age groups	NA	Included
Slovakia	1982	Registry	1-year age groups	NA	Included
Slovakia	1983	Registry	1-year age groups	NA	Included
Slovakia	1984	Registry	1-year age groups	NA	Included
Slovakia	1985	Registry	1-year age groups	NA	Included
Slovakia	1986	Registry	1-year age groups	NA	Included
Slovakia	1987	Registry	1-year age groups	NA	Included
Slovakia	1988	Registry	1-year age groups	NA	Included
Slovakia	1989	Registry	1-year age groups	NA	Included
Slovakia	1990	Registry	1-year age groups	NA	Included
Slovakia	1991	Census	5-year age groups	De jure	Excluded
Slovakia	1992	Registry	1-year age groups	NA	Included
Slovakia	1993	Registry	1-year age groups	NA	Included
Slovakia	1994	Registry	1-year age groups	NA	Included
Slovakia	1995	Registry	1-year age groups	NA	Included
Slovakia	1996	Registry	1-year age groups	NA	Included

Slovakia	1997	Registry	1-year age groups	NA	Included
Slovakia	1998	Registry	1-year age groups	NA	Included
Slovakia	1999	Registry	1-year age groups	NA	Included
Slovakia	2000	Registry	1-year age groups	NA	Included
Slovakia	2001	Census	1-year age groups	De jure	Excluded
Slovakia	2002	Registry	1-year age groups	NA	Included
Slovakia	2003	Registry	1-year age groups	NA	Included
Slovakia	2004	Registry	1-year age groups	NA	Included
Slovakia	2005	Registry	1-year age groups	NA	Included
Slovakia	2006	Registry	1-year age groups	NA	Included
Slovakia	2007	Registry	1-year age groups	NA	Included
Slovakia	2008	Registry	1-year age groups	NA	Included
Slovakia	2009	Registry	1-year age groups	NA	Included
Slovakia	2010	Registry	1-year age groups	NA	Included
Slovakia	2011	Census	1-year age groups	De jure	Excluded
Slovakia	2012	Registry	1-year age groups	NA	Included
Slovakia	2013	Registry	1-year age groups	NA	Included
Slovakia	2014	Registry	1-year age groups	NA	Included
Slovakia	2015	Registry	1-year age groups	NA	Included
Slovakia	2016	Registry	1-year age groups	NA	Included
Slovenia	1953	Census	all ages only	Unknown	Included
Slovenia	1961	Census	all ages only	Unknown	Included
Slovenia	1971	Census	1-year age groups	De facto	Included
Slovenia	1981	Census	all ages only	Unknown	Included
Slovenia	1991	Census	1-year age groups	De jure	Excluded
Slovenia	1996	Registry	1-year age groups	NA	Included
Slovenia	1997	Registry	1-year age groups	NA	Included
Slovenia	1998	Registry	1-year age groups	NA	Included
Slovenia	1999	Registry	1-year age groups	NA	Included
Slovenia	2000	Registry	1-year age groups	NA	Included
Slovenia	2001	Registry	1-year age groups	NA	Included
Slovenia	2002	Census	1-year age groups	De jure	Excluded
Slovenia	2003	Registry	1-year age groups	NA	Included
Slovenia	2004	Registry	1-year age groups	NA	Included
Slovenia	2005	Registry	1-year age groups	NA	Included
Slovenia	2006	Registry	1-year age groups	NA	Included
Slovenia	2007	Registry	1-year age groups	NA	Included
Slovenia	2008	Registry	1-year age groups	NA	Included
Slovenia	2009	Registry	1-year age groups	NA	Included
Slovenia	2010	Registry	1-year age groups	NA	Included
Slovenia	2011	Census	1-year age groups	De jure	Excluded
Slovenia	2012	Registry	1-year age groups	NA	Included
Slovenia	2013	Registry	1-year age groups	NA	Included
Slovenia	2014	Registry	1-year age groups	NA	Included
Slovenia	2015	Census	1-year age groups	De jure	Excluded
Slovenia	2016	Registry	1-year age groups	NA	Included
Slovenia	2017	Registry	1-year age groups	NA	Included
Solomon Islands	1959	Census	5-year age groups	De facto	Included
Solomon Islands	1970	Census	5-year age groups	De facto	Included
Solomon Islands	1976	Census	5-year age groups	De facto	Included
Solomon Islands	1986	Census	5-year age groups	De facto	Included
Solomon Islands	1999	Census	5-year age groups	De facto	Included
Solomon Islands	2009	Census	5-year age groups	De facto	Included
Somalia	1975	Census	5-year age groups	De jure	Included
Somalia	1987	Census	all ages only	Unknown	Included
South Africa	1951	Census	5-year age groups	De facto	Included

South Africa	1960	Census	1-year age groups	De facto	Included
South Africa	1970	Census	1-year age groups	De facto	Included
South Africa	1980	Census	5-year age groups	De facto	Excluded
South Africa	1985	Census	1-year age groups	De facto	Excluded
South Africa	1991	Census	1-year age groups	De facto	Excluded
South Africa	1996	Census	1-year age groups	De facto	Included
South Africa	2001	Census	1-year age groups	De facto	Included
South Africa	2011	Census	1-year age groups	De facto	Included
South Korea	1955	Census	5-year age groups	De facto	Included
South Korea	1960	Census	5-year age groups	De facto	Included
South Korea	1966	Census	1-year age groups	De facto	Included
South Korea	1970	Census	1-year age groups	De facto	Included
South Korea	1975	Census	5-year age groups	De facto	Included
South Korea	1980	Census	1-year age groups	De facto	Included
South Korea	1985	Census	1-year age groups	De facto	Included
South Korea	1990	Census	1-year age groups	De facto	Included
South Korea	1995	Census	1-year age groups	De jure	Included
South Korea	2000	Census	1-year age groups	De jure	Included
South Korea	2005	Census	1-year age groups	De jure	Included
South Korea	2010	Census	5-year age groups	De jure	Included
South Korea	2015	Census	1-year age groups	De jure	Included
South Korea	2020	Census	all ages only	De jure	Included
South Sudan	1956	Census	non-std age groups	De jure	Included
South Sudan	1973	Census	5-year age groups	De facto	Excluded
South Sudan	1983	Census	all ages only	De jure	Included
South Sudan	2008	Census	1-year age groups	De facto	Included
Spain	1950	Census	1-year age groups	De facto	Included
Spain	1960	Census	1-year age groups	De jure	Included
Spain	1970	Census	5-year age groups	De jure	Included
Spain	1981	Census	5-year age groups	De jure	Included
Spain	1991	Census	1-year age groups	De jure	Included
Spain	1998	Registry	1-year age groups	NA	Included
Spain	1999	Registry	1-year age groups	NA	Included
Spain	2000	Registry	1-year age groups	NA	Included
Spain	2001	Census	1-year age groups	De facto	Excluded
Spain	2002	Registry	1-year age groups	NA	Included
Spain	2003	Registry	1-year age groups	NA	Included
Spain	2004	Registry	1-year age groups	NA	Included
Spain	2005	Registry	1-year age groups	NA	Included
Spain	2006	Registry	1-year age groups	NA	Included
Spain	2007	Registry	1-year age groups	NA	Included
Spain	2008	Registry	1-year age groups	NA	Included
Spain	2009	Registry	1-year age groups	NA	Included
Spain	2010	Registry	1-year age groups	NA	Included
Spain	2011	Census	1-year age groups	De jure	Excluded
Spain	2012	Registry	1-year age groups	NA	Included
Spain	2013	Registry	1-year age groups	NA	Included
Spain	2014	Registry	1-year age groups	NA	Included
Spain	2015	Registry	1-year age groups	NA	Included
Spain	2016	Registry	1-year age groups	NA	Included
Sri Lanka	1953	Census	5-year age groups	De facto	Included
Sri Lanka	1963	Census	1-year age groups	De facto	Included
Sri Lanka	1971	Census	1-year age groups	De facto	Included
Sri Lanka	1981	Census	1-year age groups	De facto	Included
Sri Lanka	2001	Census	1-year age groups	De facto	Excluded
Sri Lanka	2012	Census	1-year age groups	De jure	Included

Sudan	1956	Census	non-std age groups	De jure	Included
Sudan	1973	Census	5-year age groups	De facto	Included
Sudan	1983	Census	all ages only	De jure	Included
Sudan	1993	Census	5-year age groups	De jure	Included
Sudan	2008	Census	1-year age groups	Unknown	Included
Suriname	1950	Census	1-year age groups	De facto	Included
Suriname	1964	Census	5-year age groups	De facto	Included
Suriname	1971	Census	all ages only	Unknown	Included
Suriname	2000	Census	all ages only	Unknown	Included
Suriname	2003	Census	5-year age groups	De jure	Excluded
Suriname	2004	Census	1-year age groups	De jure	Included
Suriname	2012	Census	1-year age groups	De jure	Included
Sweden	1950	Census	1-year age groups	De jure	Included
Sweden	1960	Census	5-year age groups	De jure	Included
Sweden	1965	Census	5-year age groups	De jure	Included
Sweden	1968	Registry	1-year age groups	NA	Included
Sweden	1969	Registry	1-year age groups	NA	Included
Sweden	1970	Census	5-year age groups	De jure	Excluded
Sweden	1971	Registry	1-year age groups	NA	Included
Sweden	1972	Registry	1-year age groups	NA	Included
Sweden	1973	Registry	1-year age groups	NA	Included
Sweden	1974	Registry	1-year age groups	NA	Included
Sweden	1975	Census	5-year age groups	De jure	Excluded
Sweden	1976	Registry	1-year age groups	NA	Included
Sweden	1977	Registry	1-year age groups	NA	Included
Sweden	1978	Registry	1-year age groups	NA	Included
Sweden	1979	Registry	1-year age groups	NA	Included
Sweden	1980	Census	1-year age groups	De jure	Excluded
Sweden	1981	Registry	1-year age groups	NA	Included
Sweden	1982	Registry	1-year age groups	NA	Included
Sweden	1983	Registry	1-year age groups	NA	Included
Sweden	1984	Registry	1-year age groups	NA	Included
Sweden	1985	Census	5-year age groups	De jure	Excluded
Sweden	1986	Registry	1-year age groups	NA	Included
Sweden	1987	Registry	1-year age groups	NA	Included
Sweden	1988	Registry	1-year age groups	NA	Included
Sweden	1989	Registry	1-year age groups	NA	Included
Sweden	1990	Census	1-year age groups	De jure	Excluded
Sweden	1991	Registry	1-year age groups	NA	Included
Sweden	1992	Registry	1-year age groups	NA	Included
Sweden	1993	Registry	1-year age groups	NA	Included
Sweden	1994	Registry	1-year age groups	NA	Included
Sweden	1995	Registry	1-year age groups	NA	Included
Sweden	1996	Registry	1-year age groups	NA	Included
Sweden	1997	Registry	1-year age groups	NA	Included
Sweden	1998	Registry	1-year age groups	NA	Included
Sweden	1999	Registry	1-year age groups	NA	Included
Sweden	2000	Registry	1-year age groups	NA	Included
Sweden	2001	Registry	1-year age groups	NA	Included
Sweden	2002	Registry	1-year age groups	NA	Included
Sweden	2003	Census	1-year age groups	De jure	Excluded
Sweden	2004	Registry	1-year age groups	NA	Included
Sweden	2005	Registry	1-year age groups	NA	Included
Sweden	2006	Registry	1-year age groups	NA	Included
Sweden	2007	Registry	1-year age groups	NA	Included
Sweden	2008	Registry	1-year age groups	NA	Included

Sweden	2009	Registry	1-year age groups	NA	Included
Sweden	2010	Registry	1-year age groups	NA	Included
Sweden	2011	Census	1-year age groups	De jure	Excluded
Sweden	2012	Registry	1-year age groups	NA	Included
Sweden	2013	Registry	1-year age groups	NA	Included
Sweden	2014	Registry	1-year age groups	NA	Included
Sweden	2015	Registry	1-year age groups	NA	Included
Sweden	2016	Registry	1-year age groups	NA	Included
Switzerland	1950	Census	1-year age groups	De jure	Included
Switzerland	1960	Census	5-year age groups	De jure	Included
Switzerland	1970	Census	1-year age groups	De jure	Included
Switzerland	1980	Census	1-year age groups	De jure	Included
Switzerland	1990	Census	1-year age groups	De jure	Included
Switzerland	2000	Census	5-year age groups	De facto	Included
Switzerland	2010	Registry	1-year age groups	NA	Included
Switzerland	2011	Census	1-year age groups	De jure	Excluded
Switzerland	2012	Registry	1-year age groups	NA	Included
Switzerland	2013	Registry	1-year age groups	NA	Included
Switzerland	2014	Registry	1-year age groups	NA	Included
Switzerland	2015	Registry	1-year age groups	NA	Included
Switzerland	2016	Registry	1-year age groups	NA	Included
Syria	1960	Census	5-year age groups	De facto	Included
Syria	1970	Census	1-year age groups	De facto	Included
Syria	1981	Census	1-year age groups	De facto	Included
Syria	1994	Census	1-year age groups	De facto	Included
Syria	2004	Census	5-year age groups	De facto	Included
São Tomé and Príncipe	1950	Census	all ages only	Unknown	Included
São Tomé and Príncipe	1960	Census	5-year age groups	De jure	Included
São Tomé and Príncipe	1970	Census	5-year age groups	De facto	Included
São Tomé and Príncipe	1981	Census	1-year age groups	De facto	Included
São Tomé and Príncipe	1991	Census	1-year age groups	De facto	Included
São Tomé and Príncipe	2001	Census	5-year age groups	De facto	Included
São Tomé and Príncipe	2012	Census	1-year age groups	De jure	Included
Taiwan (province of China)	1950	Registry	1-year age groups	NA	Included
Taiwan (province of China)	1951	Registry	1-year age groups	NA	Included
Taiwan (province of China)	1952	Registry	1-year age groups	NA	Included
Taiwan (province of China)	1953	Registry	1-year age groups	NA	Included
Taiwan (province of China)	1954	Registry	1-year age groups	NA	Included
Taiwan (province of China)	1955	Registry	1-year age groups	NA	Included
Taiwan (province of China)	1956	Census	5-year age groups	De facto	Excluded
Taiwan (province of China)	1957	Registry	1-year age groups	NA	Included
Taiwan (province of China)	1958	Registry	1-year age groups	NA	Included
Taiwan (province of China)	1959	Registry	1-year age groups	NA	Included
Taiwan (province of China)	1960	Registry	1-year age groups	NA	Included
Taiwan (province of China)	1961	Registry	1-year age groups	NA	Included
Taiwan (province of China)	1962	Registry	1-year age groups	NA	Included
Taiwan (province of China)	1963	Registry	1-year age groups	NA	Included
Taiwan (province of China)	1964	Registry	1-year age groups	NA	Included
Taiwan (province of China)	1965	Registry	5-year age groups	NA	Included
Taiwan (province of China)	1966	Registry	5-year age groups	NA	Included
Taiwan (province of China)	1967	Registry	5-year age groups	NA	Included
Taiwan (province of China)	1968	Registry	5-year age groups	NA	Included
Taiwan (province of China)	1969	Registry	5-year age groups	NA	Included
Taiwan (province of China)	1970	Registry	1-year age groups	NA	Included
Taiwan (province of China)	1971	Census	5-year age groups	De jure	Excluded
Taiwan (province of China)	1972	Registry	1-year age groups	NA	Included

Taiwan (province of China)	1973	Registry	1-year age groups	NA	Included
Taiwan (province of China)	1974	Registry	1-year age groups	NA	Included
Taiwan (province of China)	1975	Registry	1-year age groups	NA	Included
Taiwan (province of China)	1976	Registry	1-year age groups	NA	Included
Taiwan (province of China)	1977	Registry	1-year age groups	NA	Included
Taiwan (province of China)	1978	Registry	1-year age groups	NA	Included
Taiwan (province of China)	1979	Registry	1-year age groups	NA	Included
Taiwan (province of China)	1980	Census	5-year age groups	Unknown	Excluded
Taiwan (province of China)	1981	Registry	1-year age groups	NA	Included
Taiwan (province of China)	1982	Registry	1-year age groups	NA	Included
Taiwan (province of China)	1983	Registry	1-year age groups	NA	Included
Taiwan (province of China)	1984	Registry	1-year age groups	NA	Included
Taiwan (province of China)	1985	Registry	1-year age groups	NA	Included
Taiwan (province of China)	1986	Registry	1-year age groups	NA	Included
Taiwan (province of China)	1987	Registry	1-year age groups	NA	Included
Taiwan (province of China)	1988	Registry	1-year age groups	NA	Included
Taiwan (province of China)	1989	Registry	1-year age groups	NA	Included
Taiwan (province of China)	1990	Census	1-year age groups	De facto	Excluded
Taiwan (province of China)	1991	Registry	1-year age groups	NA	Included
Taiwan (province of China)	1992	Registry	1-year age groups	NA	Included
Taiwan (province of China)	1993	Registry	1-year age groups	NA	Included
Taiwan (province of China)	1994	Registry	1-year age groups	NA	Included
Taiwan (province of China)	1995	Registry	1-year age groups	NA	Included
Taiwan (province of China)	1996	Registry	1-year age groups	NA	Included
Taiwan (province of China)	1997	Registry	1-year age groups	NA	Included
Taiwan (province of China)	1998	Registry	1-year age groups	NA	Included
Taiwan (province of China)	1999	Registry	1-year age groups	NA	Included
Taiwan (province of China)	2000	Census	1-year age groups	De facto	Excluded
Taiwan (province of China)	2001	Registry	1-year age groups	NA	Included
Taiwan (province of China)	2002	Registry	1-year age groups	NA	Included
Taiwan (province of China)	2003	Registry	1-year age groups	NA	Included
Taiwan (province of China)	2004	Registry	1-year age groups	NA	Included
Taiwan (province of China)	2005	Registry	1-year age groups	NA	Included
Taiwan (province of China)	2006	Registry	1-year age groups	NA	Included
Taiwan (province of China)	2007	Registry	1-year age groups	NA	Included
Taiwan (province of China)	2008	Registry	1-year age groups	NA	Included
Taiwan (province of China)	2009	Registry	1-year age groups	NA	Included
Taiwan (province of China)	2010	Census	5-year age groups	De facto	Excluded
Taiwan (province of China)	2011	Registry	1-year age groups	NA	Included
Taiwan (province of China)	2012	Registry	1-year age groups	NA	Included
Taiwan (province of China)	2013	Registry	1-year age groups	NA	Included
Taiwan (province of China)	2014	Registry	1-year age groups	NA	Included
Taiwan (province of China)	2015	Registry	1-year age groups	NA	Included
Taiwan (province of China)	2016	Registry	1-year age groups	NA	Included
Taiwan (province of China)	2017	Registry	1-year age groups	NA	Included
Tajikistan	1959	Census	non-std age groups	De facto	Included
Tajikistan	1970	Census	non-std age groups	De facto	Included
Tajikistan	1979	Census	5-year age groups	De facto	Included
Tajikistan	1989	Census	1-year age groups	De jure	Included
Tajikistan	2000	Census	1-year age groups	De facto	Included
Tajikistan	2010	Census	1-year age groups	De facto	Included
Tanzania	1957	Census	5-year age groups	De jure	Included
Tanzania	1967	Census	1-year age groups	De facto	Included
Tanzania	1978	Census	5-year age groups	De facto	Included
Tanzania	1988	Census	1-year age groups	De facto	Included
Tanzania	2002	Census	5-year age groups	De facto	Included

Tanzania	2012	Census	1-year age groups	De facto	Included
Thailand	1956	Census	5-year age groups	De jure	Excluded
Thailand	1960	Census	5-year age groups	De jure	Included
Thailand	1970	Census	5-year age groups	De jure	Included
Thailand	1980	Census	1-year age groups	De jure	Included
Thailand	1990	Census	5-year age groups	De jure	Included
Thailand	2000	Census	1-year age groups	De jure	Included
Thailand	2010	Census	1-year age groups	De jure	Included
The Bahamas	1953	Census	5-year age groups	De facto	Included
The Bahamas	1963	Census	non-std age groups	De facto	Included
The Bahamas	1970	Census	1-year age groups	De facto	Included
The Bahamas	1980	Census	1-year age groups	De facto	Included
The Bahamas	1990	Census	1-year age groups	De facto	Included
The Bahamas	2000	Census	1-year age groups	De facto	Included
The Bahamas	2010	Census	1-year age groups	De jure	Included
The Gambia	1963	Census	non-std age groups	De facto	Included
The Gambia	1973	Census	1-year age groups	De facto	Included
The Gambia	1983	Census	1-year age groups	De facto	Included
The Gambia	1993	Census	1-year age groups	De facto	Included
The Gambia	2003	Census	5-year age groups	De facto	Included
The Gambia	2013	Census	5-year age groups	De facto	Included
Timor-Leste	1950	Census	all ages only	Unknown	Included
Timor-Leste	1960	Census	all ages only	Unknown	Included
Timor-Leste	1980	Census	all ages only	Unknown	Included
Timor-Leste	1990	Census	1-year age groups	De jure	Included
Timor-Leste	2004	Census	5-year age groups	De facto	Included
Timor-Leste	2010	Census	1-year age groups	De facto	Included
Timor-Leste	2015	Census	5-year age groups	De facto	Included
Togo	1958	Census	1-year age groups	De facto	Included
Togo	1970	Census	5-year age groups	De jure	Included
Togo	1981	Census	5-year age groups	De facto	Included
Togo	2010	Census	5-year age groups	De jure	Included
Tokelau	1951	Census	5-year age groups	De facto	Excluded
Tokelau	1956	Census	1-year age groups	De facto	Included
Tokelau	1961	Census	5-year age groups	Unknown	Included
Tokelau	1966	Census	5-year age groups	Unknown	Included
Tokelau	1972	Census	5-year age groups	Unknown	Included
Tokelau	1976	Census	5-year age groups	De facto	Excluded
Tokelau	1981	Census	1-year age groups	Unknown	Included
Tokelau	1986	Census	5-year age groups	Unknown	Included
Tokelau	1991	Census	5-year age groups	De facto	Included
Tokelau	1996	Census	5-year age groups	De facto	Included
Tokelau	2001	Census	5-year age groups	De facto	Included
Tokelau	2006	Census	5-year age groups	De facto	Included
Tokelau	2011	Census	5-year age groups	De facto	Included
Tokelau	2016	Census	5-year age groups	De facto	Included
Tonga	1956	Census	5-year age groups	De facto	Included
Tonga	1966	Census	5-year age groups	De facto	Included
Tonga	1976	Census	5-year age groups	De jure	Included
Tonga	1986	Census	5-year age groups	De facto	Included
Tonga	1996	Census	5-year age groups	De facto	Included
Tonga	2006	Census	1-year age groups	De jure	Included
Tonga	2011	Census	non-std age groups	De jure	Included
Tonga	2016	Census	1-year age groups	De facto	Included
Trinidad and Tobago	1960	Census	1-year age groups	De jure	Included
Trinidad and Tobago	1970	Census	1-year age groups	De facto	Included

Trinidad and Tobago	1980	Census	1-year age groups	De facto	Included
Trinidad and Tobago	1990	Census	5-year age groups	De facto	Included
Trinidad and Tobago	2000	Census	5-year age groups	De facto	Included
Trinidad and Tobago	2011	Census	1-year age groups	De jure	Included
Tunisia	1956	Census	non-std age groups	De facto	Included
Tunisia	1966	Census	1-year age groups	De facto	Included
Tunisia	1975	Census	5-year age groups	De facto	Included
Tunisia	1984	Census	1-year age groups	De facto	Included
Tunisia	1994	Census	1-year age groups	De facto	Included
Tunisia	2004	Census	5-year age groups	De facto	Included
Tunisia	2014	Census	5-year age groups	De facto	Included
Turkmenistan	1959	Census	non-std age groups	De facto	Included
Turkmenistan	1970	Census	non-std age groups	De facto	Included
Turkmenistan	1979	Census	5-year age groups	De facto	Included
Turkmenistan	1989	Census	5-year age groups	De facto	Included
Turkmenistan	1995	Census	5-year age groups	De facto	Excluded
Turkmenistan	2015	Census	all ages only	Unknown	Included
Tuvalu	1963	Census	5-year age groups	De facto	Included
Tuvalu	1968	Census	5-year age groups	De facto	Excluded
Tuvalu	1973	Census	5-year age groups	De facto	Included
Tuvalu	1979	Census	5-year age groups	De facto	Included
Tuvalu	1991	Census	5-year age groups	De facto	Included
Tuvalu	2002	Census	5-year age groups	De facto	Included
Tuvalu	2012	Census	5-year age groups	De facto	Included
Türkiye	1950	Census	5-year age groups	De facto	Included
Türkiye	1955	Census	5-year age groups	De facto	Included
Türkiye	1960	Census	5-year age groups	De facto	Included
Türkiye	1965	Census	5-year age groups	De facto	Included
Türkiye	1970	Census	5-year age groups	De facto	Excluded
Türkiye	1975	Census	5-year age groups	De facto	Included
Türkiye	1980	Census	5-year age groups	De facto	Included
Türkiye	1985	Census	5-year age groups	De facto	Excluded
Türkiye	1990	Census	5-year age groups	De facto	Excluded
Türkiye	2000	Census	5-year age groups	De facto	Excluded
Türkiye	2007	Registry	5-year age groups	NA	Excluded
Türkiye	2008	Registry	5-year age groups	NA	Excluded
Türkiye	2009	Registry	5-year age groups	NA	Excluded
Türkiye	2010	Registry	5-year age groups	NA	Excluded
Türkiye	2011	Registry	5-year age groups	NA	Excluded
Türkiye	2012	Registry	5-year age groups	NA	Excluded
Türkiye	2013	Registry	5-year age groups	NA	Excluded
Türkiye	2014	Registry	5-year age groups	NA	Excluded
Türkiye	2015	Registry	5-year age groups	NA	Excluded
Türkiye	2016	Registry	5-year age groups	NA	Excluded
Türkiye	2017	Registry	5-year age groups	NA	Excluded
Türkiye	2018	Registry	5-year age groups	NA	Included
Türkiye	2019	Registry	5-year age groups	NA	Included
Türkiye	2020	Census	5-year age groups	De jure	Included
UK	1991	Census	1-year age groups	De facto	Excluded
UK	2001	Census	1-year age groups	De facto	Included
UK	2011	Census	1-year age groups	De jure	Included
United States	1950	Census	1-year age groups	De jure	Included
United States	1960	Census	5-year age groups	De jure	Excluded
United States	1970	Census	5-year age groups	De jure	Included
United States	1980	Census	5-year age groups	De jure	Included
United States	1990	Census	5-year age groups	De jure	Included

United States	2000	Census	5-year age groups	De jure	Included
United States	2010	Census	5-year age groups	De jure	Included
United States	2020	Census	all ages only	De jure	Included
Uganda	1959	Census	5-year age groups	De jure	Included
Uganda	1969	Census	1-year age groups	De facto	Included
Uganda	1980	Census	5-year age groups	De facto	Excluded
Uganda	1991	Census	1-year age groups	De facto	Included
Uganda	2002	Census	1-year age groups	De facto	Included
Uganda	2014	Census	1-year age groups	De facto	Included
Ukraine	1959	Census	non-std age groups	De facto	Included
Ukraine	1970	Census	non-std age groups	De facto	Included
Ukraine	1979	Census	5-year age groups	De facto	Included
Ukraine	1989	Census	5-year age groups	De jure	Included
Ukraine	2001	Census	1-year age groups	De facto	Included
United Arab Emirates	1968	Census	non-std age groups	De facto	Included
United Arab Emirates	1975	Census	5-year age groups	De facto	Included
United Arab Emirates	1980	Census	5-year age groups	De jure	Included
United Arab Emirates	1985	Census	5-year age groups	De facto	Included
United Arab Emirates	1995	Census	5-year age groups	De facto	Included
United Arab Emirates	2005	Census	5-year age groups	De facto	Included
Uruguay	1963	Census	1-year age groups	De facto	Included
Uruguay	1975	Census	5-year age groups	De facto	Included
Uruguay	1985	Census	1-year age groups	De facto	Included
Uruguay	1996	Census	1-year age groups	De facto	Included
Uruguay	2004	Census	1-year age groups	De facto	Included
Uruguay	2011	Census	1-year age groups	De jure	Included
Uzbekistan	1959	Census	non-std age groups	De facto	Included
Uzbekistan	1970	Census	non-std age groups	De facto	Included
Uzbekistan	1979	Census	5-year age groups	De facto	Included
Uzbekistan	1989	Census	5-year age groups	De jure	Included
Vanuatu	1967	Census	5-year age groups	De facto	Included
Vanuatu	1979	Census	5-year age groups	De facto	Included
Vanuatu	1989	Census	1-year age groups	De jure	Included
Vanuatu	1999	Census	5-year age groups	De facto	Included
Vanuatu	2009	Census	1-year age groups	De facto	Included
Vanuatu	2016	Census	5-year age groups	De facto	Excluded
Vanuatu	2020	Census	5-year age groups	De facto	Included
Venezuela	1950	Census	1-year age groups	De facto	Included
Venezuela	1961	Census	1-year age groups	De facto	Included
Venezuela	1971	Census	5-year age groups	De facto	Included
Venezuela	1981	Census	1-year age groups	De facto	Included
Venezuela	1990	Census	1-year age groups	De facto	Included
Venezuela	2001	Census	non-std age groups	De facto	Included
Venezuela	2011	Census	1-year age groups	De jure	Included
Viet Nam	1979	Census	5-year age groups	De facto	Included
Viet Nam	1989	Census	1-year age groups	De jure	Included
Viet Nam	1999	Census	1-year age groups	De jure	Included
Viet Nam	2009	Census	1-year age groups	De jure	Included
Viet Nam	2019	Census	5-year age groups	De jure	Included
Virgin Islands, U.S.	1950	Census	5-year age groups	De jure	Included
Virgin Islands, U.S.	1960	Census	5-year age groups	De jure	Included
Virgin Islands, U.S.	1970	Census	5-year age groups	De jure	Included
Virgin Islands, U.S.	1980	Census	1-year age groups	De jure	Included
Virgin Islands, U.S.	1990	Census	5-year age groups	De jure	Included
Virgin Islands, U.S.	2000	Census	5-year age groups	De jure	Included
Virgin Islands, U.S.	2010	Census	5-year age groups	De jure	Included

Virgin Islands, U.S.	2020	Census	5-year age groups	De jure	Included
Yemen	1994	Census	1-year age groups	De facto	Included
Yemen	2004	Census	5-year age groups	De facto	Included
Zambia	1969	Census	1-year age groups	De facto	Included
Zambia	1980	Census	1-year age groups	De facto	Included
Zambia	1990	Census	1-year age groups	De facto	Included
Zambia	2000	Census	1-year age groups	De facto	Included
Zambia	2010	Census	1-year age groups	De jure	Included
Zimbabwe	1962	Census	non-std age groups	De jure	Excluded
Zimbabwe	1982	Census	1-year age groups	De facto	Included
Zimbabwe	1992	Census	1-year age groups	De facto	Included
Zimbabwe	2002	Census	1-year age groups	De facto	Included
Zimbabwe	2012	Census	1-year age groups	De facto	Included

Appendix Table S14: GBD world population age standard

Age Group	Percent of Population	Rounded
Early Neonatal	0.0396232	0.04
Late Neonatal	0.117777	0.12
1 to 5 months	0.864776	0.86
6 to 11 months	1.01009	1.01
12 to 23 months	2.01613	2.02
2 to 4	5.9934	5.99
5 to 9	9.65824	9.66
10 to 14	8.99361	8.99
15 to 19	8.28913	8.29
20 to 24	7.80122	7.8
25 to 29	7.59144	7.59
30 to 34	7.32171	7.32
35 to 39	6.82805	6.83
40 to 44	6.14735	6.15
45 to 49	5.51133	5.51
50 to 54	4.91312	4.91
55 to 59	4.34586	4.35
60 to 64	3.68223	3.68
65 to 69	2.98509	2.99
70 to 74	2.26526	2.27
75 to 79	1.59758	1.6
80 to 84	1.09729	1.1
85 to 89	0.604519	0.6
90 to 94	0.246663	0.25
95 plus	0.0785092	0.08

Appendix Table S15: Socio-demographic Index values for all estimated GBD 2021 locations, 1950-1974

Location	1950	1951	1952	1953	1954	1955	1956	1957	1958	1959	1960	1961	1962	1963	1964	1965	1966	1967	1968	1969	1970	1971	1972	1973	1974
Global	0.369234923	0.37169409	0.374024861	0.376187157	0.37781724	0.37988415	0.382011933	0.384286646	0.386644114	0.389516286	0.392818134	0.396293884	0.400273251	0.40416286	0.408609845	0.412669343	0.416380305	0.420521809	0.425421196	0.431105159	0.437294762	0.443084266	0.448792233	0.454520836	0.460214675
Central Europe, eastern Europe, and central Asia	0.525915472	0.528858565	0.533114928	0.535589017	0.536475644	0.538361176	0.539946803	0.5410634	0.543174716	0.546040611	0.55050322	0.556651863	0.5636164	0.569286395	0.5746996	0.57781188	0.578634146	0.579536026	0.58191658	0.584656841	0.588059314	0.591712863	0.595248844	0.598222896	0.600640055
Central Asia	0.432762555	0.434969133	0.438246947	0.44078989	0.442226282	0.444127416	0.446516153	0.44853582	0.450660084	0.452157098	0.455129108	0.459653445	0.465536402	0.471220117	0.476825923	0.481673135	0.486650363	0.492060251	0.498137404	0.504378215	0.510658364	0.514649841	0.517629489	0.521179665	0.524905734
Armenia	0.453517664	0.454023955	0.455654447	0.45718278	0.458422736	0.460100183	0.462100229	0.464007925	0.466417405	0.468904786	0.473148786	0.478665006	0.484290673	0.489122739	0.494729882	0.500336784	0.506092577	0.511813766	0.517318684	0.522310704	0.527273689	0.531845528	0.535808199	0.539890486	0.543529017
Azerbaijan	0.412950445	0.414169179	0.417118485	0.419281938	0.420988686	0.423370706	0.426585025	0.429031213	0.432101791	0.434298002	0.438129357	0.442484643	0.446846651	0.450119449	0.455707366	0.46256794	0.470052646	0.478330886	0.487308757	0.496042875	0.50575247	0.515517841	0.524952224	0.534980795	0.54449763
Georgia	0.552054833	0.554569585	0.558398407	0.562153597	0.565724701	0.569878222	0.574293273	0.577493363	0.580011634	0.581209704	0.583301829	0.586168615	0.589752316	0.593117517	0.597335693	0.601708843	0.606084942	0.610476073	0.614713091	0.618107718	0.6204636	0.619369683	0.614671656	0.611318148	0.61167819
Kazakhstan	0.447574251	0.450007679	0.454095151	0.455830418	0.45469141	0.45378201	0.45391035	0.453835852	0.454136398	0.453408228	0.455273713	0.461774399	0.472800843	0.485299147	0.495478854	0.502721375	0.509366077	0.516094257	0.522777208	0.529353024	0.537192102	0.545595417	0.552905552	0.559061329	0.564585103
Kyrgyzstan	0.438539858	0.441459358	0.444678606	0.447573712	0.450393855	0.453243903	0.456046238	0.458608898	0.461043954	0.462941257	0.46545628	0.468671133	0.473045311	0.477517165	0.482558553	0.487881186	0.493170774	0.498798416	0.504038791	0.507629414	0.505772507	0.495040845	0.485071365	0.483971538	0.487288704
Mongolia	0.305923411	0.308394264	0.311112812	0.313947087	0.316640595	0.318702028	0.32034839	0.321400355	0.322520867	0.323635992	0.325215291	0.326921855	0.328609074	0.330153389	0.331588039	0.333008418	0.334656956	0.336249121	0.338093904	0.340534368	0.343946515	0.348153916	0.353248099	0.359112611	0.365332168
Tajikistan	0.314889706	0.316571192	0.319118283	0.321704615	0.324540316	0.327725955	0.331342147	0.334783941	0.338505298	0.341804773	0.345653865	0.349562367	0.353616498	0.357242865	0.361327413	0.365644046	0.370039881	0.37462681	0.379309802	0.383735128	0.38836781	0.392899955	0.397344198	0.4020139	0.406556299
Turkmenistan	0.425192724	0.426231529	0.42959495	0.432675154	0.435659557	0.439640258	0.444422537	0.447758863	0.451509673	0.453772976	0.457551084	0.46185661	0.465761436	0.468310795	0.472741132	0.477270763	0.481677134	0.485732055	0.489788819	0.492880811	0.4965652	0.499921512	0.502635658	0.50650986	0.51038848
Uzbekistan	0.371074328	0.373438652	0.376658285	0.379957351	0.383334853	0.387130304	0.391294378	0.395265266	0.399508308	0.403441682	0.40789952	0.412437265	0.416831727	0.420766368	0.425327297	0.429924563	0.434734471	0.439377746	0.444024231	0.448325491	0.452575107	0.45701741	0.461187121	0.465499361	0.469515559
Central Europe	0.482313114	0.486887283	0.489599688	0.491324328	0.492300228	0.494557281	0.497155374	0.500758501	0.50465848	0.509149151	0.51444649	0.520350368	0.525752815	0.531012003	0.536469595	0.540779864	0.542119272	0.543157576	0.547498133	0.553458239	0.558965662	0.563657099	0.567186407	0.568918381	0.570870821
Albania	0.349281637	0.351958678	0.353129027	0.35407423	0.354952893	0.356422772	0.358039077	0.360250073	0.362783957	0.366031771	0.370473181	0.375540016	0.381322266	0.3877382	0.394661039	0.401672409	0.408602746	0.415264551	0.422203304	0.429837359	0.4380956	0.447022444	0.456676603	0.466873486	0.477117386
Bosnia and Herzegovina	0.293421744	0.297179584	0.301110904	0.304188936	0.306774156	0.312766498	0.315680683	0.320662184	0.325803518	0.332958297	0.340760693	0.348131402	0.355004931	0.36278687	0.37117366	0.379327574	0.387895775	0.39560435	0.403417926	0.41154723	0.419486484	0.4273884	0.434834803	0.441608965	0.448371737
Bulgaria	0.462106505	0.475004104	0.478951904	0.483296316	0.484631708	0.485567314	0.486774015	0.489483349	0.49128079	0.492603037	0.496709849	0.503192275	0.509983855	0.515722565	0.522918397	0.53071273	0.534975612	0.534384085	0.534291147	0.539273055	0.545895395	0.551057085	0.551988953	0.549835052	0.55056634
Croatia	0.471246319	0.475883509	0.478223844	0.480705665	0.48346522	0.489177721	0.495553944	0.50448341	0.511320939	0.51744793	0.524389986	0.531446615	0.538323721	0.544422998	0.547224554	0.548074233	0.552025016	0.557283606	0.561767976	0.56734707	0.571307276	0.574434133	0.579190601	0.586082903	0.593926569
Czechia	0.572979218	0.576308858	0.580801049	0.584606615	0.586907891	0.587874148	0.589517857	0.593701879	0.598874002	0.602227784	0.603527075	0.603122545	0.600916114	0.600518075	0.606224163	0.614958905	0.622782257	0.628398351	0.631226934	0.632647492	0.633842015	0.632567254	0.627337993	0.620422512	0.617208623
Hungary	0.502327291	0.50667663	0.507027404	0.504027896	0.503293252	0.507380053	0.513064642	0.51971855	0.525851826	0.531788688	0.538128636	0.545698289	0.552565093	0.557558484	0.560862544	0.561733688	0.5609632	0.560870819	0.563412072	0.568071035	0.572409361	0.575679008	0.576402982	0.5723433	0.567928658
Montenegro	0.45701218	0.461607048	0.466452019	0.469318289	0.47337344	0.481914442	0.488515757	0.495989477	0.502369643	0.508763075	0.515805554	0.524347785	0.531452074	0.539084803	0.547179118	0.553843395	0.559922845	0.565545861	0.571065037	0.577064121	0.583142194	0.588849053	0.594861823	0.600714409	0.607059096
North Macedonia	0.412207488	0.415984927	0.419761691	0.423764017	0.427600219	0.433818824	0.439384406	0.445556733	0.452070037	0.458350233	0.465021115	0.471384371	0.477442646	0.484513748	0.49377737	0.500636501	0.507730037	0.51452918	0.521134315	0.527997064	0.534869721	0.541270676	0.546943357	0.552238608	0.556887948
Poland	0.494196525	0.495773827	0.496412027	0.496378253	0.495492118	0.495041072	0.494964558	0.495911018	0.499650596	0.505959452	0.513548332	0.520726972	0.526866994	0.533448791	0.540290603	0.547665619	0.555654256	0.562069102	0.566717772	0.569341353	0.57147282	0.575098522	0.579780239	0.583539281	0.586629937
Romania	0.401225454	0.414242296	0.420756634	0.426331273	0.42907061	0.434674719	0.437918347	0.443362052	0.447692862	0.453635808	0.460748641	0.468431626	0.475639785	0.482748631	0.489765496	0.489991357	0.473768829	0.457287519	0.463306128	0.480115059	0.495570931	0.506933067	0.514283104	0.516881202	0.51867515
Serbia	0.47735674	0.481182519	0.484454318	0.486847063	0.490409237	0.497607451	0.50460705	0.508386378	0.508948502	0.508242168	0.508019288	0.510192879	0.513406855	0.515796038	0.517179272	0.520336771	0.525871589	0.531166098	0.536424438	0.543058292	0.54895365	0.552875475	0.556872783	0.56088327	0.563894341
Slovakia	0.523674882	0.526183112	0.5306769	0.534787576	0.53622914	0.535869195	0.536651652	0.540662154	0.544670273	0.54684428	0.549413095	0.554687852	0.559009654	0.562927451	0.569525273	0.576740167	0.584688555	0.591905578	0.59513059	0.597639928	0.601619653	0.603826904	0.603655807	0.602276377	0.603140751
Slovenia	0.559861423	0.564426972	0.568758718	0.572964426	0.576724793	0.579989761	0.583847292	0.589905516	0.595158107	0.598251888	0.601154973	0.603179176	0.60564268	0.60843604	0.609301093	0.610465145	0.615281076	0.621503208	0.62742027	0.63053467	0.631721905	0.6343369	0.637605107	0.641929302	0.645354247
Eastern Europe	0.554107568	0.557231216	0.562886232	0.566325045	0.567958438	0.570389826	0.571623938	0.571540436	0.572877754	0.57546392	0.580101993	0.58710374	0.595422663	0.602238939	0.608647381	0.612140134	0.613218197	0.613857201	0.613986181	0.613681908	0.615060505	0.618462191	0.622835568	0.62701255	0.63018609
Belarus	0.480655583	0.486252522	0.495503233	0.500825581	0.502592051	0.505061562	0.508478423	0.51104427	0.513639638	0.517664987	0.52312584	0.529781801	0.536969972	0.542102957	0.547617643	0.55083451	0.551379973	0.552032198	0.553822314	0.553961756	0.55338061	0.557156012	0.562790166	0.568622082	0.573403852
Estonia	0.559352965	0.563931955	0.570632018	0.576770948	0.58148465	0.586518959	0.591394364	0.594473261	0.59721815	0.599677729	0.602611025	0.606809467	0.612563793	0.616603934	0.619998816	0.623923125	0.626036596	0.624758675	0.62233971	0.621295738	0.622845544	0.626799345	0.631557686	0.635264082	0.637372505
Latvia	0.56887308	0.573458017	0.579417859	0.583496107	0.58599305	0.589434704	0.592249357	0.59387401	0.596489661	0.599453261	0.602502413	0.606387472	0.612465374	0.618072901	0.62407694	0.628592237	0.63010798	0.630430949	0.630467534	0.630141614	0.631613573	0.635380953	0.639713287	0.643920335	0.647435951
Lithuania	0.502805695	0.510457115	0.519538803	0.527470287	0.533723585	0.540697743	0.547503344	0.551807982	0.555369883	0.55855114	0.562840669	0.568745363	0.57580056	0.581775397	0.587374347	0.591147557	0.593797073	0.595276607	0.595849488	0.596221895	0.59856229	0.603367582	0.610225346	0.617344204	0.622428334
Moldova	0.459020825	0.467302	0.476129036	0.483904562	0.49054233	0.496407512	0.501377824	0.505963268	0.510697063	0.516070261	0.522877983	0.530191301	0.538253575	0.54556331	0.552895091	0.559821403	0.566011959	0.571991728	0.577364648	0.581742059	0.582925267	0.577929662	0.57218538	0.572253288	0.575741954
Russia	0.560026856	0.562538648	0.567377134	0.569721696	0.570012203	0.571861371	0.573225182	0.573960691	0.576721764	0.58054399	0.585918336	0.593833863	0.603018244	0.609476623	0.614887027	0.617562852	0.618130407	0.619206124	0.62014556	0.620855747	0.623573899	0.627581854	0.631892649	0.635679801	0.638413314
Ukraine	0.549439708	0.553578936	0.560524555	0.566501119	0.571143115	0.574168535	0.573218076	0.569400594	0.566165475	0.565322412	0.567903801	0.573189869	0.579861994	0.587742458	0.596288632	0.601065745	0.602234216	0.601576597	0.599343395	0.595704089	0.593143515	0.594796281	0.600195534	0.605768502	0.610059009
High income	0.584939692	0.588131353	0.590418687	0.592486809	0.5938818	0.594998837	0.596026986	0.597448998	0.598638593	0.600277642	0.602561031	0.6054303	0.609162543	0.61358421	0.619191711	0.625971638	0.63220931	0.638189725	0.644193003	0.649118571	0.654278732	0.660840108	0.66817359	0.674908028	0.68082293
Australasia	0.570681353	0.567394689	0.564216632	0.562846351	0.561189614	0.559149827	0.557500552	0.556848503	0.557069023	0.557824477	0.558331919	0.561417921	0.568462829	0.577482646	0.587049066	0.594083171	0.59862403	0.601610573	0.604193093	0.606727133	0.607806718	0.612049383	0.622526403	0.634420158	0.645238181
Australia	0.560717179	0.557513608	0.554132353	0.552838378	0.551363563	0.549173068	0.547527731	0.547386798	0.548082281	0.548999439	0.549802897	0.553029096	0.56001043	0.568987465	0.578581434	0.58583859	0.590621112	0.593712774	0.596147608	0.598358767	0.599367788	0.603694947	0.614300658	0.626191373	0.636769113
New Zealand	0.603510389	0.600364905	0.598681683	0.597199321	0.594758812	0.593587374	0.591929646	0.588633294	0.586547168	0.586783257	0.586204231	0.58930164	0.59718487	0.606436335	0.61562357	0.621215862	0.624211225	0.626782041	0.63114902	0.636435502	0.6387495	0.643066292	0.653172466	0.664998179	0.676903103
High-income Asia Pacific	0.50585163	0.515708212	0.523797485	0.531049999	0.536633141	0.54233767	0.548600092	0.553462155	0.55720162	0.56273178	0.570204917	0.577984625	0.585676973	0.593008939	0.599389706	0.607974294	0.615223041	0.622001762	0.63263787	0.641458615	0.647304361	0.651611377	0.656713264	0.663225828	0.670579819
Brunei	0.379203403	0.376102976	0.372549892	0.367227455	0.361112114	0.356724232	0.355415278	0.353611991	0.352220567	0.35655249	0.365304897	0.378203219	0.393746028	0.406553604	0.41751878	0.428966871	0.439749782	0.449702597	0.461131229	0.473912199	0.486740101	0.500534816	0.514540477	0.527080206	0.537980006
Japan	0.543099876	0.554506236	0.563978185	0.572083525	0.57846668	0.584903312	0.592055915	0.597318217	0.601182643	0.606982144	0.614623452	0.622604022	0.630047387	0.63656464	0.641471877	0.649889202	0.656742538	0.66223274	0.672327217	0.680168129	0.684332671	0.686587057	0.690498836	0.696071482	0.703312325
Aichi	0.553003423	0.564770987	0.574377247	0.582515369	0.588769755	0.595015664	0.6026819	0.608472853	0.611758574	0.61751671	0.625767166	0.633714704	0.640791856	0.646761593	0.650577781	0.658486774	0.664659339	0.669409031	0.679866687	0.68775387	0.691387466	0.692622813	0.695839471	0.701001095	0.708190474
Akita	0.508231642	0.51950578	0.5285652	0.536150463	0.541858177	0.547535723	0.554118479	0.557948721	0.559975491	0.564487545	0.570698547	0.577162647	0.583025276	0.587867433	0.591469791	0.599991595	0.606921266	0.61235399	0.623288908	0.630545642	0.633295404	0.633525237	0.636063697	0.641103791	0.648876356
Aomori	0.482924481	0.494866231	0.504489751	0.512524643	0.518496772	0.524480399	0.531337798	0.535899927	0.538741369	0.543683018	0.549978122	0.556195574	0.562670496	0.56803641	0.571463063	0.580470119	0.587609582	0.592851695	0.603842503	0.611639575	0.614801742	0.616057873	0.619429032	0.624831374	0.633404578
Chiba	0.526312805	0.537481509	0.546650155	0.55434582	0.560303252	0.566308447	0.572327477	0.57670772	0.579936622	0.585341032	0.593076838	0.602146593	0.611058867	0.620013466	0.627190413	0.638140795	0.647395814	0.655442932	0.668231295	0.678606241	0.68413807	0.687102592	0.69167924	0.698125203	0.705603529
Ehime	0.522310044	0.5335944	0.542759759	0.550422234	0.556199906	0.561958367	0.568262616	0.572019757	0.574112349	0.578941338	0.585722894	0.592908789	0.599529627	0.60524048	0.609634469	0.618140591	0.625025765	0.630094065	0.640602543	0.648649811	0.652738968	0.65456369	0.657657258	0.66273811	0.670019943
Fukui	0.514305047	0.525882596	0.535063008	0.54271154	0.54839153	0.554048642	0.560300464	0.564186232	0.566523401	0.57119707	0.577509368	0.583933187	0.590119692	0.59529935	0.598566421	0.606970541	0.613502302	0.6181664	0.628978879	0.636633196	0.639697562	0.640780115	0.644547255	0.650589071	0.6594637
Fukuoka	0.55719677	0.567964406	0.576856609	0.584343961	0.590164007	0.595996015	0.602563218	0.607338206	0.610885859	0.615754539	0.622088592	0.628751894	0.63448248	0.638833624	0.642286788	0.649218227	0.655078986	0.660202239	0.669612976	0.677175195	0.681696901	0.684769541	0.68953582	0.696096904	0.704156799
Fukushima	0.499350015	0.51071917	0.519916774	0.527602283	0.533342895	0.539099031	0.54530107	0.549188005	0.55166308	0.556303305	0.562607303	0.56859814	0.574303477	0.579213574	0.582571575	0.590974082	0.598171696	0.603947719	0.614954577	0.62299094	0.626733804	0.628305962	0.63258184	0.639190975	0.648157514
Gifu	0.524979326	0.536039609	0.545087413	0.55270342	0.558550201	0.56444034	0.570826675	0.575043563	0.577609009	0.583291804	0.590986918	0.598647098	0.605558442	0.611399769	0.615305367	0.623374086	0.629926572	0.634953457	0.645720938	0.65394176	0.65796354	0.66017433	0.664237541	0.670291514	0.678038956
Gunma	0.517353327	0.528077121	0.536916923	0.544310814	0.55003588	0.55573384	0.561366437	0.565355089	0.568705676	0.573951539	0.581019051	0.588995514	0.597079024	0.604384345	0.610472854	0.620185304	0.628500896	0.635385997	0.646789694	0.655710783	0.660915166	0.664049263	0.668390089	0.674119332	0.681458544
Hiroshima	0.541659822	0.553062975	0.562322712	0.570086466	0.575985405	0.581895253	0.589177882	0.595057359	0.598709496	0.604273236	0.612085061	0.619955834	0.627151566	0.633250046	0.638276748	0.647365376	0.65461123	0.660466396	0.671697426	0.680320189	0.68433705	0.686157078	0.690235675	0.69623985	0.704134447
Hokkaidō	0.538429375	0.55016942	0.559704847	0.56774025	0.573923295	0.580040982	0.586054824	0.590819953	0.594059823	0.59901598	0.605388518	0.611649566	0.617232842	0.622335389	0.62558298	0.633076778	0.638935906	0.643466277	0.652767424	0.65936031	0.662278287	0.663524533	0.666986096	0.67263335	0.680492444
Hyōgo	0.570574965	0.58157474	0.590591432	0.598216185	0.604109955	0.610000522	0.616188873	0.620412653	0.623337222	0.628183688	0.634349091	0.641219914	0.647065544	0.651846865	0.655316661	0.662842164	0.668850393	0.673564042	0.682995152	0.690438886	0.694216985	0.696219658	0.699595805	0.704550013	0.711170047
Ibaraki	0.508731202	0.520071772	0.529334332	0.53709299	0.542983742	0.548909073	0.55519407	0.55991689	0.563453859	0.569378198	0.577010515	0.585804441	0.593927454	0.600132753	0.604524124	0.612789663	0.619394717	0.625112805	0.636377087	0.645069263	0.65044314	0.653703082	0.658422014	0.6649957	0.67390514
Ishikawa	0.516433295	0.528230331	0.53756119	0.545334563	0.551116769	0.556851033	0.563246843	0.567231281	0.570470677	0.576609693	0.584567185	0.591917367	0.598804553	0.604452783	0.608010076	0.616631289	0.623326146	0.628283188	0.639693281	0.647749849	0.650796105	0.651453091	0.654411523	0.659835398	0.668217304
Iwate	0.484583696	0.496502336	0.506109803	0.514162486	0.520186066	0.526193208	0.53288692	0.537148583	0.539625252	0.544804268	0.552053962	0.558946169	0.565854283	0.571876062	0.575925251	0.58516926	0.592188688	0.597321342	0.607976667	0.615457133	0.618431729	0.619358917	0.622836772	0.628902323	0.637622789
Kagawa	0.529421589	0.54088638	0.550149818	0.557891676	0.563682446	0.569449056	0.575760886	0.579685197	0.581976678	0.586382742	0.592406794	0.598844421	0.604956498	0.610244796	0.614199188	0.622993614	0.630303289	0.636521036	0.648563811	0.65754414	0.661679483	0.663541193	0.667380477	0.673355481	0.681985987
Kagoshima	0.495007372	0.50549852	0.514104013	0.521261903	0.526764945	0.532305184	0.537993949	0.541619477	0.544396875	0.548909844	0.554630031	0.560788	0.56692594	0.572685549	0.577037623	0.585705464	0.593107843	0.598980486	0.609056794	0.616736799	0.621031416	0.624292615	0.629798047	0.637047914	0.646162653
Kanagawa	0.585392014	0.596179014	0.60511544	0.61272818	0.618646955	0.624548343	0.632870086	0.638882054	0.643198677	0.649660882	0.658677207	0.668308409	0.676761414	0.684434178	0.690177128	0.698497607	0.704894011	0.709782297	0.719148949	0.726235274	0.729733214	0.731067992	0.733509989	0.73744556	0.742676384
Kōchi	0.498104604	0.509860421	0.519285559	0.527178165	0.533075997	0.538935881	0.545331855	0.549478778	0.552000402	0.557000355	0.563727581	0.570960725	0.577726999	0.583428964	0.587600752	0.596305549	0.603383566	0.608698691	0.619322234	0.627119239	0.630768192	0.632839829	0.636455942	0.642141467	0.650430694
Kumamoto	0.502968626	0.514122122	0.523283219	0.530946906	0.536800552	0.542653139	0.548242504	0.551457658	0.553847559	0.55811768	0.5638271	0.57033206	0.576540773	0.582166133	0.586909617	0.59612565	0.603821945	0.609980457	0.621183116	0.629466912	0.633873243	0.636780105	0.641853815	0.648856997	0.657773048
Kyōto	0.569164644	0.578917445	0.587126479	0.594131408	0.599741628	0.605348683	0.611750822	0.616575196	0.620390876	0.625854594	0.632949156	0.640359451	0.647639085	0.654123329	0.659244476	0.666843411	0.673362142	0.679097758	0.688754749	0.696705274	0.701643768	0.704848153	0.709395099	0.715283084	0.722124787
Mie	0.52484417	0.53648639	0.545924786	0.553825877	0.559811805	0.565757472	0.571933798	0.57568857	0.577334073	0.581824813	0.589088102	0.59695652	0.6048222	0.611220791	0.615393515	0.623662721	0.629882274	0.634405019	0.644878661	0.652523657	0.655763505	0.6567558	0.659539695	0.664770171	0.672645675
Miyagi	0.522680193	0.53359785	0.542526391	0.549991274	0.555703063	0.561379179	0.567506912	0.571518706	0.574309668	0.57939159	0.586127456	0.593121944	0.599947869	0.606088388	0.61066329	0.619628807	0.626678144	0.632230895	0.642957465	0.651137149	0.655570816	0.658378686	0.663037672	0.669552953	0.678138328
Miyazaki	0.49275502	0.504481341	0.513942709	0.521872582	0.527847606	0.533779828	0.540126167	0.543691839	0.54525665	0.549265942	0.555570601	0.561720531	0.567784484	0.573296727	0.577704059	0.58747793	0.595424761	0.601159714	0.612365835	0.620174156	0.623697357	0.62534862	0.629084444	0.635076286	0.643637149
Nagano	0.52622091	0.535788105	0.543817019	0.550626797	0.556066402	0.561585758	0.567611155	0.572451595	0.576605988	0.58276056	0.590866281	0.599199514	0.607239075	0.614498786	0.620553677	0.629461702	0.637238211	0.644028345	0.654280794	0.662856506	0.668459086	0.672573375	0.677718245	0.6841352	0.691645393
Nagasaki	0.50486327	0.515708979	0.524643482	0.532149699	0.53795986	0.543804902	0.550887632	0.556348005	0.559693943	0.564437927	0.569641749	0.574948386	0.579395012	0.583123835	0.586216134	0.593983802	0.600535896	0.605639499	0.615722271	0.623796716	0.628651516	0.632064618	0.636992155	0.643806729	0.65281054

Nara	0.553205777	0.563709356	0.572414239	0.579759417	0.585547647	0.591346753	0.597076327	0.601542943	0.605234632	0.610121397	0.616520247	0.623221949	0.629670624	0.63537769	0.640152424	0.64822561	0.655461433	0.66193599	0.67228168	0.680220657	0.684636999	0.686953102	0.690691669	0.695519083	0.701478624
Niigata	0.510307515	0.521080583	0.529846128	0.537282424	0.542935486	0.548610721	0.555134039	0.55989867	0.562898924	0.567992655	0.574607711	0.581180542	0.588017615	0.594071117	0.598716285	0.607683821	0.614952584	0.62064305	0.631228653	0.638965487	0.642672872	0.644316923	0.647751477	0.653194826	0.661382504
Ōita	0.518977941	0.529936321	0.53893256	0.54648886	0.552330847	0.558194893	0.563918528	0.568448002	0.572215638	0.577494114	0.58368827	0.589653624	0.595735416	0.600771087	0.604823784	0.613230251	0.620525532	0.626286683	0.636776802	0.645245317	0.649959538	0.652674157	0.657130712	0.663395987	0.671223676
Okayama	0.526736277	0.538168055	0.547457276	0.555239867	0.561114187	0.566987087	0.573630519	0.577722858	0.580083018	0.585017976	0.592166379	0.599972407	0.607226166	0.613699692	0.618668016	0.628071696	0.63603832	0.642577855	0.654763051	0.66424487	0.6690797	0.670933353	0.674451542	0.679920917	0.68745579
Okinawa	0.455427212	0.469564901	0.480855493	0.490262813	0.497234393	0.504119407	0.511629127	0.516414458	0.519594144	0.525194065	0.532786427	0.539817339	0.546491637	0.551541661	0.554621892	0.562921728	0.569185045	0.573608451	0.584074871	0.59098168	0.593382111	0.594392373	0.599476152	0.608396827	0.620697149
Ōsaka	0.580290437	0.591425423	0.600586254	0.608388042	0.614484104	0.620584642	0.627358484	0.632513145	0.636965165	0.643288942	0.651256147	0.659408226	0.666995872	0.673517358	0.678069707	0.686152291	0.692430197	0.697230444	0.706595572	0.71369359	0.717164079	0.718466571	0.721373087	0.726137834	0.732605143
Saga	0.512640985	0.523388018	0.532271328	0.539720322	0.545455256	0.551189458	0.556841063	0.560863188	0.563865216	0.568264467	0.574691755	0.580693751	0.586569969	0.591751047	0.596386637	0.605421245	0.613116209	0.618794121	0.629056269	0.636974494	0.641124195	0.643597661	0.648086355	0.654397211	0.662564098
Saitama	0.523857385	0.534939994	0.544091309	0.5518128	0.557819518	0.563873988	0.570430609	0.575827664	0.579742424	0.586281631	0.594831485	0.603902791	0.613026267	0.621894555	0.62863531	0.638314776	0.646352012	0.653030085	0.664335892	0.673124362	0.678069517	0.680623817	0.685003509	0.69079393	0.697604213
Shiga	0.540730672	0.551720389	0.560692666	0.56818829	0.573891269	0.57962371	0.586313629	0.590650941	0.593295085	0.598471942	0.605296016	0.611986301	0.618275143	0.623868861	0.627616713	0.635200477	0.641779924	0.647109332	0.657752211	0.666473863	0.671740785	0.675444447	0.681425613	0.689633188	0.698980156
Shimane	0.498182288	0.509207948	0.518247492	0.525822667	0.531613796	0.537319046	0.54339594	0.547448168	0.550480188	0.555702996	0.562475699	0.569078885	0.575188934	0.580080035	0.583625853	0.591868768	0.598873476	0.604102713	0.614405377	0.622061311	0.625936652	0.62819722	0.632427071	0.638980481	0.647777439
Shizuoka	0.53726799	0.548652875	0.557974367	0.565818466	0.571850954	0.577855363	0.584317622	0.589227971	0.592685464	0.598335439	0.605953683	0.613870991	0.621335003	0.627604197	0.631741953	0.639837593	0.646175379	0.650719277	0.660588296	0.668035866	0.671363522	0.672778456	0.676002865	0.681370971	0.688781692
Tochigi	0.523301752	0.534369568	0.543397272	0.550980161	0.556722389	0.562464026	0.56856453	0.572265425	0.574895939	0.579679238	0.586492834	0.593750927	0.600850922	0.607013313	0.611734311	0.620686545	0.628057126	0.633904514	0.644767255	0.653077598	0.65761033	0.660676464	0.665458671	0.672046034	0.680078043
Tokushima	0.504898894	0.516317835	0.52561129	0.533378555	0.539289356	0.545172421	0.551766355	0.55575487	0.558689813	0.564411927	0.57215504	0.579912398	0.587074044	0.593183918	0.597792888	0.606322897	0.613126179	0.618542758	0.629675809	0.638149013	0.642738594	0.645409681	0.649852837	0.65607638	0.664344217
Tōkyō	0.647961492	0.657170419	0.66508591	0.671973776	0.677580251	0.683199466	0.689661804	0.694660058	0.698436622	0.702602516	0.708276882	0.714775576	0.720761517	0.726073183	0.730455631	0.73643337	0.741448303	0.746298983	0.754445154	0.761586156	0.766272864	0.769729013	0.774412843	0.779875929	0.786003363
Tottori	0.519531734	0.530770344	0.539916411	0.547612525	0.55342321	0.559174561	0.565109801	0.568581488	0.57055574	0.57471205	0.580491773	0.586239736	0.591737794	0.596313215	0.599628312	0.607613672	0.614608849	0.620030565	0.630611961	0.638792259	0.643170848	0.645670716	0.65004719	0.656329969	0.664910488
Toyama	0.52743851	0.538873188	0.548000954	0.555715224	0.561407298	0.567073204	0.573598794	0.577884587	0.580123349	0.585212601	0.593014181	0.601497573	0.608296703	0.613514103	0.616347908	0.624609578	0.631005094	0.63561257	0.646453766	0.654177426	0.657194541	0.657764359	0.660698374	0.666232728	0.674568172
Wakayama	0.531670739	0.543826233	0.55357031	0.561715053	0.567853911	0.573917916	0.579975381	0.583855229	0.585491947	0.58975688	0.596277013	0.603107549	0.610161761	0.61649138	0.621582714	0.631181243	0.63837342	0.643595372	0.65364677	0.660610682	0.663298129	0.664110627	0.667004329	0.672235686	0.679878046
Yamagata	0.499093849	0.509669012	0.518344313	0.525630848	0.531221748	0.536929759	0.543375586	0.547865176	0.55106964	0.556258084	0.563235046	0.570042939	0.576304282	0.581750259	0.585642554	0.594223942	0.601420609	0.607079974	0.618109541	0.626057782	0.629467946	0.630992832	0.634767055	0.640931805	0.649351665
Yamaguchi	0.543696508	0.554947112	0.564126586	0.571857595	0.577763085	0.583656314	0.589278871	0.59209236	0.594051515	0.598854498	0.606501706	0.613528962	0.620004732	0.625624031	0.62978458	0.638480906	0.645465105	0.650740794	0.660924788	0.668474751	0.672036958	0.673178224	0.67604826	0.681190761	0.688645281
Yamanashi	0.522148361	0.531818887	0.539981811	0.546855035	0.552353045	0.557924147	0.564073828	0.568891961	0.572972725	0.57883006	0.586628203	0.5947328	0.602758444	0.609802	0.615638289	0.624378975	0.632168847	0.638669184	0.648134309	0.656187023	0.661786571	0.665895887	0.671690604	0.67871037	0.686395246
South Korea	0.303972464	0.30559976	0.308771314	0.316528922	0.322844058	0.328908043	0.334229059	0.340167646	0.346199802	0.352563005	0.359270917	0.366515153	0.373780419	0.381798474	0.390498688	0.399631123	0.409852504	0.420745503	0.432686663	0.445874311	0.459410895	0.472786071	0.486116063	0.500498781	0.51414546
Singapore	0.318369437	0.315367934	0.309000754	0.307812448	0.309977569	0.312827932	0.31809929	0.323831334	0.331753136	0.343341857	0.355570554	0.365636488	0.375442913	0.386054595	0.3970718	0.408970975	0.422026037	0.437406206	0.452861354	0.466753741	0.479760218	0.492046721	0.505365171	0.519609847	0.533803647
High-income North America	0.615405805	0.611227566	0.607941611	0.603376826	0.598092488	0.59259942	0.586748927	0.5848632	0.585316232	0.586459857	0.589760589	0.597211196	0.608778081	0.622198011	0.637370308	0.651323442	0.662700727	0.672446482	0.67890948	0.6831949	0.691413982	0.704363053	0.716190649	0.724142524	0.729803133
Canada	0.621815371	0.61808478	0.613632384	0.609806277	0.60724977	0.606433663	0.603861787	0.601483406	0.600804454	0.601721252	0.605129671	0.610019535	0.616790539	0.626138157	0.63922646	0.652544009	0.663270424	0.671620399	0.678478824	0.684461405	0.691758273	0.700928989	0.709245948	0.715559862	0.719995623
Greenland	0.564999971	0.564103814	0.5599585	0.552467284	0.543043839	0.534858189	0.52920606	0.523779853	0.517467732	0.510111737	0.502417073	0.495455563	0.488889923	0.483348866	0.486242537	0.50216974	0.525449914	0.548114088	0.575224968	0.608293546	0.637682618	0.659854786	0.67752582	0.69151684	0.701230038
USA	0.614249877	0.60998157	0.606821298	0.602157938	0.596532943	0.590459137	0.584201308	0.582380743	0.583007096	0.584187621	0.587468888	0.595208609	0.607390968	0.621313025	0.636729173	0.650753374	0.662202683	0.672114057	0.678514402	0.682594826	0.690945201	0.704378203	0.716626257	0.724785468	0.730619178
Alabama	0.549755387	0.546032465	0.543073584	0.537895176	0.53155665	0.525024205	0.518133641	0.516222382	0.517083084	0.518273591	0.521906968	0.530586782	0.544564772	0.560469003	0.57790238	0.593371171	0.606023144	0.61681338	0.62289093	0.624741776	0.63078292	0.645732386	0.661050525	0.670880366	0.678682044
Alaska	0.593678794	0.587693434	0.582312225	0.572835676	0.561295402	0.547541416	0.53282326	0.525586304	0.522850616	0.520765837	0.523179081	0.533219506	0.550902299	0.572084577	0.59568636	0.61601546	0.631392854	0.643992752	0.651382723	0.655553903	0.668012502	0.689266325	0.707641338	0.716868957	0.720065491
Arizona	0.605681653	0.600731934	0.596586534	0.589956151	0.581992823	0.573214192	0.564638419	0.561567586	0.561296303	0.561709062	0.564698612	0.57327017	0.587117829	0.602694291	0.620021725	0.635131406	0.646804477	0.656547851	0.662031367	0.664521385	0.670616185	0.681825076	0.693337761	0.702403762	0.710200153
Arkansas	0.544457087	0.540159055	0.536460496	0.530462385	0.523587014	0.516194133	0.508415806	0.505588608	0.505941279	0.506466336	0.509705297	0.518789082	0.533387677	0.550171822	0.56842563	0.58458529	0.597924796	0.609070807	0.61503348	0.616140904	0.621092293	0.634651317	0.647832845	0.655459782	0.662571371
California	0.649715291	0.646345488	0.644024896	0.640141678	0.635295375	0.630177437	0.625176023	0.624329688	0.625435746	0.627056266	0.630242378	0.637315187	0.648231635	0.660550504	0.674254737	0.686427513	0.696243617	0.704796871	0.710281275	0.714336484	0.721978656	0.732544215	0.741614931	0.747205711	0.750609682
Colorado	0.639437378	0.636501755	0.633984834	0.629026031	0.623257482	0.617184471	0.610930386	0.608811057	0.608678689	0.608913685	0.611677501	0.61854734	0.629938072	0.643189871	0.657955069	0.671101967	0.681341334	0.690174168	0.695743889	0.698975643	0.708106223	0.724082167	0.737867392	0.747056582	0.753947377
Connecticut	0.660094523	0.657593691	0.655721374	0.652600592	0.648534472	0.644052405	0.639500664	0.637952439	0.637802692	0.63809451	0.640411629	0.646422805	0.656147373	0.667811271	0.681262076	0.693582189	0.703877667	0.713267261	0.71988317	0.725803601	0.737510818	0.754452792	0.767923852	0.776063777	0.781861733
Delaware	0.632275645	0.626404982	0.622386456	0.617068936	0.611079366	0.605311155	0.599897616	0.5990917	0.600541024	0.602290649	0.605925197	0.614127382	0.62656618	0.640524794	0.655846109	0.669649366	0.68061485	0.690279726	0.696663518	0.701314254	0.709687096	0.721962199	0.733621546	0.742079715	0.74806395
Washington, DC	0.654295419	0.651310808	0.650148402	0.6472395	0.644204067	0.64063683	0.636990867	0.637834517	0.640798418	0.643395909	0.646655802	0.653606697	0.664412044	0.675987918	0.687931709	0.698625606	0.707613748	0.715954707	0.721311645	0.724138377	0.730395852	0.740541789	0.750548385	0.758021265	0.763889462
Florida	0.611519146	0.606326708	0.602718341	0.597327159	0.59098834	0.584129834	0.57704255	0.574414337	0.574218408	0.574313251	0.576494163	0.583398508	0.595313392	0.609105675	0.624332036	0.637981817	0.648957118	0.658444426	0.663653984	0.664848283	0.670795042	0.686214422	0.702032016	0.713287764	0.72418227
Georgia	0.547056829	0.541760777	0.537850791	0.531697936	0.524764739	0.51737111	0.510061924	0.509047954	0.511388638	0.514031511	0.518936834	0.529446708	0.545391754	0.562839966	0.581464911	0.598114071	0.611870587	0.624081241	0.632008496	0.636982248	0.645781143	0.659426492	0.672917342	0.683732206	0.692650516
Hawaii	0.612824817	0.60949547	0.607053008	0.602506373	0.596822413	0.590356472	0.583816623	0.581579864	0.581902636	0.582693045	0.586612739	0.595434867	0.6088464	0.624298168	0.641432457	0.657115982	0.669851661	0.680627471	0.687414408	0.691176155	0.69985469	0.713859309	0.725252922	0.732187306	0.736873358
Idaho	0.59758135	0.591346458	0.586515749	0.578331631	0.569023706	0.55927321	0.549566383	0.545146082	0.543958197	0.543612133	0.54612633	0.554435744	0.568565734	0.585150508	0.604100893	0.621415528	0.634433956	0.644811563	0.650400577	0.652604346	0.658359732	0.669419313	0.680446945	0.687601357	0.691408845
Illinois	0.6298194	0.625739868	0.6225865	0.618048339	0.612513181	0.60652897	0.600401042	0.598222891	0.59813528	0.5982576	0.600679895	0.607603958	0.619002654	0.632297008	0.647290909	0.66085852	0.671977222	0.68167826	0.687386372	0.68961567	0.69728706	0.71259772	0.726369981	0.734711057	0.7403342
Indiana	0.607746975	0.603758673	0.600286334	0.595192188	0.588367312	0.580895797	0.573110995	0.569733829	0.568648504	0.567988684	0.569928223	0.576804215	0.588909393	0.603138984	0.619302951	0.634072402	0.64583193	0.655818821	0.661293829	0.662848589	0.670026694	0.686172179	0.700381707	0.70888913	0.715486063
Iowa	0.626567895	0.62139474	0.617548541	0.611467163	0.605144299	0.597664783	0.58994406	0.586497733	0.585377633	0.584692817	0.586512587	0.592910556	0.603816858	0.617264688	0.633023851	0.647713029	0.659333516	0.66883617	0.675131753	0.679556843	0.690010799	0.706927319	0.721094606	0.729134308	0.733239654
Kansas	0.626431842	0.622242528	0.620147718	0.614919284	0.608957074	0.602005028	0.594626592	0.591441242	0.590921237	0.590609241	0.592879529	0.599378471	0.610603131	0.623868373	0.638971449	0.652599351	0.663271103	0.672397479	0.678554072	0.683543585	0.69379754	0.709746922	0.723336198	0.730243814	0.732224702
Kentucky	0.550919711	0.547532759	0.54470618	0.539835316	0.53393871	0.527373917	0.520147112	0.518162807	0.518971549	0.520270314	0.523909638	0.532856042	0.546937904	0.562743798	0.579802782	0.595197603	0.607732838	0.618747967	0.625275706	0.626772781	0.631798623	0.646826058	0.662270324	0.671147231	0.67632861
Louisiana	0.546053849	0.54007329	0.536018259	0.529608557	0.521893083	0.513284241	0.504450096	0.501479773	0.501568406	0.501838297	0.504747496	0.513881893	0.528839573	0.545985887	0.564953723	0.581734536	0.595145561	0.606914124	0.614620096	0.620511847	0.63234827	0.649571737	0.664514506	0.673769457	0.67881862
Maine	0.594069133	0.588805199	0.585112271	0.578942988	0.571839243	0.564325102	0.556443089	0.553198698	0.552686824	0.552790011	0.555924585	0.564172292	0.577718799	0.593532516	0.611459544	0.627546607	0.640095966	0.650474675	0.656015577	0.657795321	0.664333687	0.680560201	0.697478561	0.708435624	0.716018184
Maryland	0.632834865	0.629678015	0.627590484	0.623646513	0.618656997	0.613209502	0.607610717	0.606049355	0.606536308	0.6071957	0.60973265	0.617113626	0.628609499	0.641804961	0.656418626	0.669592914	0.680650988	0.69078697	0.697655434	0.703408596	0.71427293	0.731678346	0.747917713	0.758359864	0.765109444
Massachusetts	0.666920468	0.664099488	0.662073966	0.659175527	0.655451788	0.651667028	0.647752059	0.646565822	0.646766942	0.647672764	0.65026867	0.65642109	0.665486849	0.676226419	0.68859293	0.699951972	0.709384129	0.717865198	0.723697544	0.727765198	0.73663688	0.750931119	0.763403309	0.771748126	0.777970816
Michigan	0.61999377	0.615421615	0.612057154	0.607535109	0.601404545	0.594782514	0.587498511	0.584191314	0.582934447	0.582119619	0.583820195	0.590003654	0.600967159	0.614848935	0.63067939	0.645163337	0.656934444	0.666961997	0.673112708	0.676797821	0.686829356	0.703802639	0.718107194	0.727917935	0.736088326
Minnesota	0.634288677	0.630716163	0.627786313	0.623791994	0.619012575	0.613881076	0.608487846	0.606389701	0.606299848	0.606689576	0.609303629	0.615861377	0.626286102	0.638470678	0.652616627	0.665905079	0.676681366	0.686170492	0.692719925	0.697856092	0.709060475	0.72575861	0.739012732	0.74648588	0.750829817
Mississippi	0.518344268	0.511662227	0.506653312	0.499061444	0.490234175	0.480863306	0.47043225	0.466076621	0.465900625	0.466385925	0.469970333	0.480148397	0.497130727	0.51652755	0.537309957	0.555646707	0.570153408	0.582500747	0.589245326	0.59102624	0.597816134	0.614190255	0.631192808	0.643151137	0.652782744
Missouri	0.603936585	0.599608217	0.596241906	0.591462314	0.585525836	0.579146912	0.57244987	0.569970112	0.569950648	0.570677969	0.573554115	0.581019821	0.593243576	0.607339478	0.622915464	0.637070582	0.648450894	0.658356414	0.664709121	0.668103999	0.676399913	0.691215624	0.70473826	0.712993212	0.718873796
Montana	0.626518584	0.621053942	0.616290139	0.609709173	0.602119921	0.594238552	0.586042178	0.582039211	0.580357127	0.578972142	0.580339409	0.586539442	0.598603678	0.612963253	0.629336596	0.644218344	0.655910886	0.665315846	0.669615208	0.669118156	0.675167337	0.691552264	0.705990527	0.713317024	0.718123401
Nebraska	0.632597032	0.627351859	0.623679338	0.617827737	0.611677528	0.604523547	0.597171883	0.594285259	0.593623868	0.593203382	0.595420305	0.601856091	0.612827054	0.625969212	0.640909044	0.654666883	0.66563452	0.674606519	0.679962137	0.682838935	0.69095018	0.706347941	0.719689186	0.726706588	0.7311827
Nevada	0.615722422	0.609788395	0.606483527	0.600294021	0.593198107	0.585043278	0.575544695	0.571594253	0.570733187	0.57008683	0.572154766	0.580425176	0.59509102	0.61242079	0.631366191	0.64742582	0.6594054	0.669338866	0.675442399	0.680134631	0.691668921	0.709596355	0.725591717	0.736199659	0.743267615
New Hampshire	0.61903004	0.614605141	0.611224753	0.606426407	0.60094648	0.594839462	0.588388991	0.585990233	0.585810368	0.586474922	0.589764131	0.597591859	0.610069734	0.624621655	0.641237083	0.656190063	0.667882702	0.67790993	0.684663166	0.689048651	0.697767409	0.713714173	0.729669685	0.740726447	0.749062284
New Jersey	0.651348296	0.648593813	0.646616129	0.643288439	0.638899709	0.634048147	0.629077139	0.627278083	0.626833811	0.627050756	0.629334278	0.635266408	0.645217045	0.656982691	0.670349847	0.682711036	0.692879134	0.701924153	0.707672774	0.711230321	0.720198128	0.736182677	0.750306196	0.758978329	0.76556371
New Mexico	0.576692638	0.570704509	0.566135417	0.559249008	0.551319997	0.54234934	0.533118982	0.530194682	0.531033129	0.53237744	0.53622468	0.545757787	0.560960009	0.577877298	0.596504356	0.612884337	0.625525185	0.635739013	0.641472716	0.644770393	0.652663193	0.665273475	0.677081165	0.685264395	0.69150527
New York	0.647868806	0.643682514	0.640648456	0.636856161	0.632364478	0.6277255	0.622827936	0.621718958	0.622737083	0.624624353	0.628134808	0.635366808	0.64615488	0.658318387	0.671941523	0.684539121	0.695033731	0.704302628	0.711101396	0.716334852	0.724937233	0.736609406	0.747074537	0.754249635	0.759041655
North Carolina	0.563646789	0.558658234	0.554578415	0.549024278	0.542904125	0.536460553	0.529623235	0.527935683	0.529432805	0.531474337	0.535554597	0.54483381	0.558977149	0.574444053	0.59113544	0.606286558	0.619046422	0.630330573	0.638024943	0.643256234	0.652079175	0.665098359	0.677994187	0.688225945	0.696448749
North Dakota	0.593734307	0.586796715	0.580356281	0.573323465	0.566021693	0.558985882	0.552411168	0.550674718	0.552349506	0.554490304	0.559529101	0.568787676	0.584471504	0.600869594	0.618430518	0.635106649	0.648200242	0.658552882	0.665191999	0.669855298	0.676880434	0.687321055	0.698130989	0.708260827	0.715247129
Ohio	0.61554286	0.611159669	0.607739753	0.603083827	0.59710641	0.590644755	0.583706575	0.581344121	0.581572842	0.582668636	0.585765645	0.59328052	0.605209804	0.618825045	0.634033848	0.648039918	0.65948199	0.669383175	0.675997058	0.680593397	0.688931126	0.701287248	0.712841925	0.721036692	0.726746293
Oklahoma	0.585931138	0.580956518	0.577536586	0.572161845	0.565649622	0.558503184	0.550857702	0.548351553	0.549330069	0.550940466	0.554645174	0.563298426	0.576741673	0.591669288	0.608017616	0.622782219	0.634587503	0.644913104	0.651259586	0.655088387	0.662822601	0.6748856	0.686300221	0.69427158	0.699737983
Oregon	0.641781303	0.637681701	0.634516117	0.629737932	0.62394802	0.618154719	0.612151282	0.610025827	0.610351224	0.611416518	0.614357275	0.621270458	0.632373361	0.645275418	0.659902107	0.673354692	0.684166495	0.69343194	0.699612226	0.704439446	0.712964421	0.724556839	0.734992434	0.741941555	0.746360654
Pennsylvania	0.629577496	0.625653226	0.622707649	0.618906593	0.613955259	0.608667893	0.603509236	0.602187065	0.602819017	0.604182549	0.607396963	0.614269009	0.624840362	0.637071773	0.650676621	0.663418315	0.674004929	0.683255044	0.689751947	0.694542991	0.702568445	0.71406615	0.725048062	0.733118328	0.738908271
Rhode Island	0.623777083	0.619562124	0.616489046	0.612555768	0.607574371	0.602384422	0.59730238	0.596201111	0.597503122	0.599771632	0.603932635	0.61180741	0.623701493	0.637169941	0.652138801	0.666048564	0.677610249	0.687901212	0.695415401	0.701015089	0.70964126	0.721192549	0.732532772	0.741254202	0.747643472
South Carolina	0.547597249	0.544069805	0.540709662	0.534800806	0.52726626	0.519021396	0.510184146	0.507189617	0.50793345	0.509463681	0.513235473	0.522515581	0.537396239	0.554187919	0.572422358	0.589086825	0.602930963	0.615084974	0.623044308	0.628052679	0.636643872	0.649669874	0.662755135	0.673336738	0.682270742
South Dakota	0.602771205	0.598148456	0.592137292	0.585906865	0.578911262	0.570598196	0.562527834	0.559843982	0.559756347	0.55995784	0.563610314	0.571901304	0.585318731	0.600234116	0.616406533	0.631685558	0.643893863	0.653626081	0.659767134	0.663664235	0.670988614	0.682098765	0.6927687	0.700941892	0.705744
Tennessee	0.557305922	0.552543052	0.54907748	0.544373846	0.538833652	0.532820686	0.526422427	0.525319045	0.527312557	0.529943273	0.534268003	0.54358768	0.55760594	0.57291282	0.589373041	0.604368465	0.616955874	0.628036976	0.635594039	0.64049643	0.648823646	0.661312387	0.673449977	0.682786229	0.69003801
Texas	0.57797166	0.572483566	0.56843569	0.562100453	0.554958727	0.546991293	0.538665746	0.536027536	0.536683065	0.537784205	0.541071235	0.549705524	0.563819033	0.579766033	0.597024246	0.612609611	0.62515441	0.636144081	0.643320138	0.648462464	0.657947752	0.671390329	0.684038296	0.693319079	0.700125536

Utah	0.627797881	0.622645686	0.6179987	0.61127746	0.603183915	0.59470863	0.586033942	0.582114	0.58084358	0.580623591	0.583182708	0.590988548	0.603873175	0.619016464	0.63609343	0.651555809	0.663228637	0.672284066	0.676857098	0.678015553	0.682614403	0.691557045	0.699014635	0.701341653	0.70019957
Vermont	0.610148978	0.605973013	0.602294737	0.597399177	0.59160982	0.585326777	0.579459804	0.578261624	0.579598355	0.581953848	0.586689417	0.595639731	0.608864137	0.623604318	0.639909777	0.654972617	0.667437101	0.678268718	0.68588175	0.691307487	0.700190368	0.712567899	0.724719038	0.733939345	0.740873799
Virginia	0.603507465	0.600076142	0.597490134	0.593050404	0.587736824	0.581825705	0.575819211	0.574574827	0.575994833	0.577997398	0.581889619	0.590278635	0.603142259	0.617672012	0.63350261	0.648050922	0.660151736	0.670982113	0.678692325	0.684564899	0.693887377	0.706866899	0.719211874	0.728697673	0.736065806
Washington	0.643667478	0.639160833	0.635987678	0.631585759	0.626387284	0.620743245	0.615130003	0.613945989	0.615092218	0.61695726	0.620596483	0.628298368	0.640070303	0.653195016	0.667372254	0.68040389	0.691335325	0.701098371	0.708180431	0.714234037	0.724073047	0.736484254	0.747002871	0.753826341	0.758020862
West Virginia	0.569026094	0.563940283	0.559799079	0.553839703	0.546655815	0.539138612	0.531957662	0.529873122	0.530416419	0.531626755	0.535091967	0.543290136	0.556382265	0.571336091	0.587741921	0.602834218	0.615035439	0.625328752	0.631647738	0.634794588	0.64141982	0.652983599	0.66476948	0.673123682	0.67875769
Wisconsin	0.629425136	0.626154656	0.623224283	0.619011403	0.613728514	0.608567839	0.603394435	0.601883735	0.602509748	0.60404906	0.607447634	0.614356306	0.625106409	0.637583298	0.651878776	0.665200525	0.676074506	0.685396593	0.691969806	0.697127113	0.705524342	0.716845788	0.727079427	0.734145595	0.739025411
Wyoming	0.613429941	0.606778209	0.600639907	0.592508307	0.583270626	0.573514396	0.563961534	0.560322466	0.559900789	0.560304417	0.563615543	0.572585318	0.587367762	0.604017907	0.622427662	0.638737451	0.651031454	0.661180055	0.6670606	0.670543877	0.678790517	0.691073917	0.702208831	0.709550928	0.714302362
Southern Latin America	0.470229439	0.473016503	0.473956852	0.475152482	0.476753889	0.478111673	0.479413528	0.482539358	0.486331297	0.489127784	0.492020081	0.494001152	0.49614339	0.49873004	0.502746207	0.508293987	0.513844816	0.517934198	0.518992222	0.520620226	0.523495644	0.525975528	0.529325445	0.533448991	0.533778427
Argentina	0.479474011	0.482486206	0.483157905	0.484355541	0.486586009	0.488890106	0.490812827	0.494485358	0.498757065	0.502325319	0.506390831	0.509071167	0.511312096	0.514057492	0.518572448	0.524415317	0.529119626	0.531300199	0.529308105	0.529137463	0.532730559	0.53739124	0.542521281	0.545863041	0.541627207
Chile	0.438382509	0.440184668	0.442526612	0.444437352	0.445374708	0.444642274	0.443975042	0.446132915	0.450008788	0.45274579	0.455074052	0.456582867	0.458293902	0.460754965	0.464556943	0.469886886	0.477185978	0.485622425	0.494297043	0.500725194	0.502545904	0.500396936	0.500003854	0.506754886	0.517099415
Uruguay	0.477361517	0.481436112	0.482161313	0.48246253	0.481886668	0.482377206	0.484963753	0.487538128	0.488802871	0.487222429	0.483842664	0.482572856	0.486400712	0.489403249	0.490980772	0.495507839	0.503391641	0.510114646	0.512014232	0.512607922	0.513406479	0.514121861	0.515896827	0.517731723	0.520268153
Western Europe	0.582396	0.586146567	0.588782084	0.591627374	0.594391251	0.596431191	0.59815897	0.600115728	0.601686861	0.603100537	0.604644925	0.605883273	0.606890444	0.608045448	0.610561643	0.614306433	0.618809712	0.623985573	0.629315384	0.634631661	0.639408968	0.644891197	0.651796502	0.658470824	0.664507292
Andorra	0.608750024	0.613073552	0.617280861	0.621602199	0.625911361	0.629955029	0.633994652	0.637749135	0.641154504	0.644477008	0.648247714	0.651901807	0.65556335	0.65933922	0.663402127	0.667750629	0.672495956	0.677426945	0.682492643	0.687455379	0.692211439	0.696159216	0.700792822	0.706205231	0.712158759
Austria	0.600576811	0.604456956	0.605690107	0.606650728	0.607344174	0.606516403	0.605338862	0.60442145	0.602914298	0.603285038	0.604505952	0.605544815	0.608247685	0.612581829	0.617388815	0.619188577	0.619200938	0.620335939	0.62435109	0.631864371	0.64051539	0.648283078	0.655614213	0.661676383	0.668553879
Belgium	0.573464435	0.576075574	0.577168567	0.577909298	0.57815209	0.578422186	0.578543306	0.578791227	0.579124285	0.580260986	0.581080893	0.581685631	0.582634258	0.583972943	0.587751766	0.593103485	0.599035204	0.604874608	0.60997701	0.614162668	0.618296137	0.624310798	0.632554803	0.641501939	0.64993109
Cyprus	0.451021417	0.455536	0.461172077	0.465368805	0.467408481	0.467353282	0.467594479	0.469190507	0.472554381	0.477361842	0.480903431	0.485188011	0.490810111	0.49719066	0.502266353	0.506906658	0.510465769	0.516758223	0.525865671	0.537463979	0.547287141	0.555279656	0.56339037	0.570417477	0.573991866
Denmark	0.63415132	0.633744266	0.633048055	0.633358439	0.633694014	0.633389148	0.633923402	0.63598159	0.638766579	0.641508853	0.643194147	0.644557132	0.645638916	0.646976812	0.649749326	0.653266951	0.661847135	0.675997917	0.689340493	0.6989053	0.70386327	0.707064206	0.711731349	0.716144957	0.719492671
Finland	0.565768936	0.568974085	0.569593436	0.570130085	0.571804211	0.573610574	0.57591829	0.580583219	0.584127355	0.58624276	0.589884135	0.592838927	0.594804984	0.599328233	0.606454453	0.612956691	0.618277019	0.624872449	0.633173343	0.641927113	0.650317738	0.65887052	0.666740639	0.671903802	0.67523077
France	0.540784786	0.54559229	0.54925391	0.552073469	0.554424414	0.557020619	0.560044061	0.562983585	0.564154199	0.564830053	0.566059382	0.567221525	0.567349365	0.567289846	0.571011229	0.576603406	0.583391746	0.591131666	0.597744504	0.60270952	0.606155282	0.610924055	0.618243754	0.628221148	0.639306824
Germany	0.640368557	0.645682046	0.650650846	0.656981337	0.663156923	0.667077697	0.669769062	0.672124219	0.672986542	0.673654462	0.674859598	0.67556276	0.67690254	0.679490539	0.682325689	0.684927192	0.689282188	0.69472535	0.700933255	0.708541878	0.715861969	0.724030326	0.733818633	0.741401382	0.746605617
Greece	0.508330956	0.512960832	0.516162781	0.519773701	0.521811704	0.525431544	0.530475125	0.534749337	0.537590518	0.540407175	0.544450952	0.548404687	0.550365637	0.551567666	0.552105326	0.551905296	0.549713497	0.548574737	0.551881472	0.558703708	0.56613558	0.572343549	0.577627671	0.580644387	0.581840085
Iceland	0.563962495	0.561914373	0.558867392	0.555127828	0.552033799	0.550416771	0.55003063	0.550564256	0.552630572	0.556812778	0.561344081	0.564574005	0.567273002	0.571386962	0.578372699	0.587096122	0.598083334	0.609206401	0.618287423	0.624832594	0.628455151	0.631704013	0.637114718	0.646936556	0.657485032
Ireland	0.578381662	0.580197841	0.58307841	0.586546975	0.588718778	0.590500053	0.592106185	0.594131245	0.595269388	0.595563094	0.596083091	0.596489063	0.596769765	0.597861166	0.599751267	0.601903563	0.603971534	0.605774365	0.607160657	0.608067399	0.608405233	0.60935087	0.612532691	0.617769342	0.623941995
Israel	0.508746839	0.502561464	0.501822689	0.507824965	0.51514546	0.518869983	0.522498182	0.529308982	0.538776512	0.544814534	0.548939387	0.554679123	0.558392596	0.561271387	0.566060035	0.574385884	0.58551663	0.594719263	0.599896917	0.602591739	0.604799768	0.608979602	0.613530887	0.616401058	0.618655046
Italy	0.525606918	0.530982125	0.53446099	0.537040127	0.539461854	0.542313521	0.545335783	0.548938632	0.552106337	0.555060867	0.558362641	0.56113472	0.562664288	0.562794157	0.563732205	0.567301378	0.571956413	0.576362665	0.580501701	0.584149954	0.587531495	0.590604734	0.593447578	0.596578962	0.601348946
Abruzzo	0.52726463	0.532990758	0.536695119	0.539382731	0.541896598	0.544846333	0.547975832	0.551653826	0.554831618	0.557753781	0.561013421	0.563707362	0.565157971	0.565305661	0.566449569	0.570384891	0.57542939	0.580236107	0.584841768	0.58906664	0.593135349	0.596773292	0.600342701	0.604225626	0.609617091
Basilicata	0.477542098	0.483001402	0.486445831	0.488853743	0.491078686	0.493738994	0.496556148	0.499912907	0.502695528	0.505113939	0.507777227	0.509689529	0.510058713	0.508765594	0.508507147	0.511472882	0.515710776	0.5196083	0.523239955	0.526462263	0.529565134	0.532330628	0.535123295	0.538397771	0.543899755
Calabria	0.463910847	0.469726685	0.473150293	0.475350083	0.477295961	0.479733801	0.482278538	0.485476241	0.487964261	0.48996143	0.492272596	0.493687051	0.493124663	0.490414694	0.488866728	0.491405469	0.495684016	0.499476243	0.50287276	0.50573347	0.508483496	0.510788901	0.513117018	0.515840619	0.521279991
Campania	0.48598305	0.491891084	0.495587956	0.498187497	0.500554818	0.503339378	0.5062383	0.509696275	0.512496405	0.514831359	0.51743942	0.519274644	0.519332501	0.51745112	0.516508499	0.518998348	0.522946296	0.526335773	0.529231373	0.531418446	0.533220208	0.534466183	0.535558998	0.536849211	0.540625116
Emilia-Romagna	0.549160251	0.554537065	0.557761073	0.559898349	0.561829266	0.564249076	0.566821465	0.569971772	0.572575695	0.574839117	0.577485196	0.579439671	0.579897065	0.578756449	0.578677682	0.581744055	0.586290912	0.590446202	0.594353113	0.597749981	0.60085485	0.603577651	0.606265315	0.60924779	0.614108495
Friuli-Venezia Giulia	0.544289614	0.549436751	0.552916398	0.555668371	0.558252352	0.561205885	0.564279196	0.567882199	0.571156836	0.574337973	0.577834481	0.581040343	0.583434471	0.584921822	0.58714246	0.59124438	0.59623451	0.601055873	0.605759965	0.610190428	0.614522697	0.618536472	0.622478963	0.62670129	0.631942077
Lazio	0.556949502	0.562410699	0.566092002	0.568926578	0.571599152	0.574676958	0.577909099	0.581621472	0.584959171	0.588129459	0.591624893	0.594717884	0.596768574	0.597763418	0.599539172	0.603507785	0.608421251	0.613128201	0.617648892	0.621795217	0.625750826	0.629267273	0.632680029	0.636372977	0.64132795
Liguria	0.55742253	0.562327119	0.565861162	0.568843918	0.57169685	0.574899691	0.578217417	0.581946989	0.585448354	0.588929635	0.592734423	0.596380054	0.599399309	0.601763892	0.604554274	0.608673346	0.613447201	0.618115857	0.62271392	0.627038931	0.631262098	0.63508064	0.63880746	0.642737521	0.64741462
Lombardia	0.563270293	0.568329518	0.571819033	0.574603452	0.577297782	0.580358953	0.583548762	0.587194212	0.59053462	0.593784043	0.597330769	0.600575218	0.602986978	0.604532546	0.606744664	0.610844465	0.615774564	0.620539947	0.625179889	0.629565797	0.633835297	0.637735074	0.641590223	0.645692381	0.650753524
Marche	0.518683744	0.523961757	0.527214099	0.529482828	0.531581964	0.534134528	0.536794773	0.540038825	0.542779218	0.54517645	0.547894909	0.550003569	0.55064764	0.549756292	0.549847595	0.552974547	0.55750341	0.561761276	0.565811308	0.569385893	0.572755957	0.575694384	0.578646938	0.581937311	0.587039215
Molise	0.488454702	0.494117707	0.497604875	0.499959202	0.502088401	0.504651412	0.507353723	0.510639536	0.513275462	0.515488595	0.518017994	0.519756134	0.519776421	0.517994119	0.517404441	0.520322289	0.524723394	0.528733106	0.532551758	0.536010331	0.539192798	0.541955819	0.54477516	0.548069494	0.553511628
Piemonte	0.543344804	0.548464327	0.552056358	0.554985113	0.557760651	0.560891014	0.564197251	0.567951996	0.571397799	0.574709438	0.578333563	0.581669477	0.584173358	0.585811783	0.588078038	0.592148097	0.597060459	0.601789893	0.606401499	0.610678845	0.614801659	0.618545705	0.622228024	0.62602603	0.630838397
Provincia autonoma di Bolzano	0.544085141	0.549682657	0.553077724	0.555394425	0.557551185	0.560162397	0.562975106	0.566428288	0.569336752	0.57192419	0.574884061	0.577245173	0.57807821	0.577207403	0.577351003	0.580734589	0.585579313	0.590088763	0.594342738	0.59807264	0.601543614	0.60464575	0.60769447	0.611037594	0.61632058
Provincia autonoma di Trento	0.554482399	0.559706546	0.563136658	0.565687726	0.568088835	0.570908951	0.573918927	0.577461771	0.580640052	0.583659314	0.58702102	0.589957143	0.591783021	0.592454822	0.594036098	0.597966824	0.602947243	0.607644132	0.612186214	0.616462776	0.620514559	0.624237654	0.627961074	0.631986571	0.637335613
Puglia	0.475895281	0.481555084	0.485077701	0.487578003	0.489864703	0.4925738	0.495402612	0.49880459	0.501594662	0.503982486	0.506727984	0.508807668	0.509185451	0.507723857	0.507213171	0.510041507	0.514297413	0.518138468	0.521608903	0.524484497	0.527027844	0.529064811	0.530922601	0.533027634	0.537450504
Sardegna	0.49048865	0.495928089	0.499606573	0.502519548	0.505281506	0.508401776	0.511624571	0.515341989	0.518652211	0.521784493	0.525307624	0.528470239	0.530406858	0.531111062	0.532600778	0.536469254	0.541416869	0.546218137	0.55089274	0.555201499	0.559311064	0.562966467	0.566465993	0.570284523	0.575631833
Sicilia	0.48002454	0.486031261	0.489664364	0.492118882	0.494345461	0.49698283	0.499735963	0.503085283	0.505760413	0.507949123	0.510526012	0.512324454	0.512121371	0.509730862	0.508316599	0.510638789	0.514696653	0.51833995	0.521687131	0.524165789	0.526159802	0.527462052	0.528472785	0.529690442	0.53354839
Toscana	0.532612721	0.537681773	0.541006672	0.543515693	0.54586721	0.548610244	0.551461935	0.554864225	0.557861041	0.560692921	0.563840854	0.566611118	0.568396819	0.56912214	0.57068087	0.574480135	0.579316072	0.583969048	0.588499623	0.592735459	0.596918823	0.6007825	0.604637248	0.608838453	0.614241946
Umbria	0.526551159	0.531925993	0.535392772	0.537907877	0.540230762	0.542965809	0.545882101	0.549324263	0.552304527	0.55502262	0.558035526	0.560523046	0.561813973	0.56189075	0.562912298	0.566518981	0.571253335	0.575718595	0.58001342	0.58394148	0.587811814	0.591395521	0.595079195	0.599008402	0.604406321
Valle d'Aosta	0.554481384	0.559927338	0.563529742	0.566202313	0.568669728	0.571532269	0.574559571	0.578093448	0.581191855	0.584048104	0.587183321	0.589839057	0.591299281	0.591553569	0.592637059	0.596058343	0.600493098	0.604642581	0.608579997	0.612067652	0.61529221	0.618137515	0.620885104	0.623764028	0.628095301
Veneto	0.531221557	0.536290681	0.539784818	0.54256501	0.545169349	0.54813851	0.551235223	0.554754904	0.557957434	0.56101997	0.564404052	0.567408905	0.569571086	0.57081426	0.572723071	0.57661853	0.581348307	0.585941641	0.590456234	0.594713658	0.598924277	0.60278554	0.60664305	0.610790174	0.616121977
Luxembourg	0.643187413	0.643615206	0.644378967	0.643619918	0.642930581	0.642640614	0.642961648	0.643437453	0.643967811	0.64462593	0.645907914	0.647724449	0.64897509	0.649563791	0.650676116	0.653374859	0.657902271	0.662527492	0.667784517	0.673945093	0.68044404	0.688521054	0.697251397	0.70449265	0.710100015
Malta	0.414459413	0.420594514	0.426136679	0.43109521	0.436149328	0.440800759	0.445192134	0.448249668	0.451164555	0.454758602	0.460797446	0.468445771	0.476967289	0.486424896	0.496024876	0.504518787	0.511532098	0.517673758	0.5237294	0.529370576	0.535115375	0.540590796	0.546166425	0.551001272	0.555760529
Monaco	0.688311979	0.691976384	0.695490292	0.698999219	0.702550712	0.706188231	0.709928124	0.71376613	0.717661316	0.721660122	0.725878752	0.730002039	0.73429732	0.738663474	0.743197339	0.747798756	0.752335228	0.756834978	0.761358941	0.765919049	0.770446032	0.775147335	0.77984121	0.784658132	0.789307161
Netherlands	0.650974337	0.651465828	0.653474962	0.656373551	0.65850687	0.660448461	0.661506937	0.661798052	0.661789381	0.662676186	0.66335523	0.66283452	0.662591467	0.662996682	0.664779476	0.666019215	0.667471129	0.67181782	0.675649576	0.680101122	0.688755057	0.69802934	0.706818753	0.715313068	0.72295598
Norway	0.655818211	0.654909638	0.652454377	0.649754876	0.647104486	0.644755385	0.643763658	0.643744994	0.644762706	0.646105811	0.644820223	0.64273712	0.643116561	0.644325886	0.646215667	0.649033516	0.65192053	0.654826205	0.657881419	0.66249232	0.668495858	0.679016814	0.691738175	0.701573266	0.710465522
Agder	0.653525916	0.652836887	0.65046484	0.647781514	0.645015653	0.642433817	0.641220209	0.640852604	0.641457135	0.642404434	0.640822548	0.638399423	0.638346032	0.63914076	0.640543918	0.642755516	0.645083783	0.647493755	0.650053984	0.65423451	0.659866141	0.670206162	0.68271815	0.691805608	0.699740441
Innlandet	0.634129557	0.633206369	0.630764007	0.628052072	0.625397319	0.623044997	0.622019343	0.621922309	0.62285504	0.6241196	0.622714703	0.620471567	0.620740297	0.621829178	0.623645615	0.626432262	0.629289824	0.632218313	0.635335955	0.640110899	0.646396163	0.65724462	0.67043085	0.681080122	0.690853696
Møre og Romsdal	0.633885784	0.632640461	0.629758681	0.626617045	0.623550449	0.620861759	0.619526682	0.619146012	0.61986825	0.620975726	0.619193637	0.616513089	0.61657268	0.617497308	0.61912554	0.621761605	0.624359309	0.626936413	0.629738664	0.634320299	0.640568992	0.651871906	0.665762638	0.676626367	0.686725869
Nordland	0.621341661	0.619941674	0.616748978	0.613260375	0.609743034	0.606562159	0.605053168	0.604720395	0.605637237	0.606889977	0.604997028	0.602111807	0.602160998	0.603135569	0.604738957	0.607378442	0.610093216	0.612842379	0.615771574	0.620539243	0.627007593	0.638693302	0.65318079	0.664213103	0.674025591
Oslo	0.7128523	0.712867245	0.711742607	0.710459947	0.709248247	0.708306911	0.708369704	0.709019563	0.710284293	0.71185441	0.711859577	0.711424268	0.712736473	0.71473816	0.717341575	0.720645745	0.724078364	0.72767743	0.731454948	0.736297787	0.741933917	0.750374395	0.759781765	0.766931846	0.773205308
Rogaland	0.650037728	0.64872047	0.645721504	0.642428111	0.639107814	0.636034181	0.634500656	0.63399469	0.634652982	0.635713938	0.63373772	0.630923022	0.630874048	0.631793144	0.633324919	0.635734331	0.638286595	0.6407961	0.643528498	0.647918992	0.653985497	0.66514699	0.678749277	0.688705066	0.697273605
Troms og Finnmark	0.621521224	0.619780168	0.616291812	0.612479148	0.608742351	0.605360667	0.603602016	0.603085164	0.603900351	0.605070256	0.602671279	0.599269288	0.599036493	0.599861927	0.601471929	0.604318227	0.607239364	0.61023544	0.613569888	0.618906374	0.626085834	0.638774052	0.654463686	0.667288197	0.679583992
Trøndelag	0.638265328	0.636756458	0.633477092	0.629929647	0.626350931	0.623115884	0.621447091	0.620886406	0.621598164	0.62268599	0.620722469	0.617857969	0.617872759	0.618809415	0.620373857	0.622893273	0.625515847	0.628073627	0.630935699	0.635668189	0.642126312	0.653968177	0.668515533	0.679998314	0.690503493
Vestfold og Telemark	0.65156194	0.650659369	0.648243398	0.645521794	0.642870938	0.640545393	0.639481712	0.639278179	0.640089273	0.641234273	0.639701313	0.637254661	0.637296418	0.638123747	0.639642732	0.642079532	0.644539759	0.647054406	0.649790574	0.654299186	0.660445883	0.671464356	0.684995324	0.695618702	0.70517137
Vestland	0.650778589	0.649806831	0.647127419	0.64417146	0.641163466	0.638376887	0.637057528	0.636673733	0.637345484	0.638366537	0.636764801	0.634346065	0.634416957	0.635370912	0.636968691	0.639490313	0.642089578	0.64477201	0.647642358	0.65224699	0.65837949	0.669296644	0.682592324	0.692786259	0.701933118
Viken	0.669202575	0.668604743	0.666492538	0.664089118	0.66175472	0.659713599	0.658869638	0.658859041	0.659781426	0.661035576	0.65982542	0.657837221	0.65825458	0.659459539	0.661345432	0.664207713	0.667052434	0.669981385	0.67314767	0.67793435	0.684183813	0.694813582	0.707547258	0.717439594	0.726478526
Portugal	0.421596118	0.424737683	0.427600868	0.431131072	0.433383866	0.435097461	0.437197828	0.439088931	0.440726151	0.442444701	0.44440452	0.446673331	0.450244257	0.454246246	0.457841751	0.461697398	0.465277629	0.469772303	0.475875501	0.482238691	0.487556879	0.493101119	0.500583951	0.507423363	0.508077868
San Marino	0.643648817	0.647610198	0.651386011	0.655136412	0.658824987	0.66244086	0.665890383	0.669161832	0.672207283	0.675082236	0.677865835	0.680347218	0.682527981	0.684373903	0.685911776	0.687025946	0.688122466	0.689105328	0.690178303	0.691408343	0.693059417	0.69487428	0.697571408	0.701420591	0.706325908
Spain	0.440265751	0.443869173	0.446604757	0.448634549	0.450981734	0.452666349	0.454715073	0.457181073	0.46072885	0.464537809	0.468562399	0.472468526	0.476159959	0.479739407	0.483984728	0.489106784	0.493829272	0.497450796	0.500813464	0.505536204	0.509504484	0.512493302	0.516750339	0.521472351	0.525792323
Sweden	0.641208305	0.643372131	0.644020619	0.645700864	0.646987941	0.647643646	0.65006427	0.653746052	0.657658225	0.661682745	0.664757238	0.666232011	0.666597602	0.666100252	0.666909894	0.670256394	0.676910835	0.688668978	0.700540719	0.70684593	0.709496744	0.712346726	0.716465782	0.720970146	0.726992421
Stockholm	0.706836166	0.708825978	0.709702158	0.711303626	0.712472029	0.713066991	0.715132638	0.718103546	0.721215319	0.724415529	0.726951441	0.728435729	0.729102656	0.729134639	0.730304092	0.733471194	0.738883503	0.748024227	0.75667305	0.760045367	0.760293962	0.761854905	0.766036299	0.771833753	0.778752074
Sweden except Stockholm	0.628219139	0.630407685	0.630993664	0.632639385	0.633890374	0.63446519	0.636830513	0.640506363	0.644423932	0.648439272	0.651445063	0.652712704	0.652794978	0.651967668	0.65248783	0.655684945	0.662462949	0.674653696	0.687126892	0.694002446	0.697123448	0.700207145	0.70428542	0.708482799	0.714287671
Switzerland	0.748828775	0.751295373	0.752691141	0.754233608	0.755408871	0.756021377	0.755994707	0.756352519	0.757688865	0.758665981	0.75807533	0.756197022	0.754065944	0.753548715	0.756112228	0.760592933	0.765050357	0.769033638	0.773469999	0.778127934	0.783150436	0.789183212	0.79564575	0.801997215	0.80905276
UK	0.58808604	0.591168077	0.592040927	0.593694987	0.596388508	0.596660049	0.595026325	0.594348544	0.595054018	0.594723557	0.593440648	0.592967783	0.593786343	0.595668592	0.599880075	0.606522192	0.613566308	0.620896909	0.628820825	0.636212576	0.641901351	0.650181193	0.661345511	0.67094875	0.679086813

England	0.590307919	0.593260536	0.594096165	0.595899555	0.59879415	0.59902495	0.597393804	0.596807453	0.597659329	0.597393298	0.595747944	0.595106281	0.596122459	0.598134573	0.602310685	0.608658375	0.615806303	0.623341173	0.631165471	0.638525132	0.64436248	0.652685855	0.663816556	0.673248147	0.681208535
East Midlands	0.554358889	0.557386935	0.558136961	0.559956375	0.562974961	0.563090156	0.56116742	0.560371024	0.56113203	0.560652274	0.558637291	0.557731616	0.558658291	0.560699316	0.565173113	0.572084959	0.579967269	0.58826666	0.596878583	0.605031478	0.611632144	0.621026659	0.633321619	0.643755197	0.652534252
Derby	0.563087955	0.566087362	0.566494775	0.567982803	0.570813217	0.570138881	0.56700298	0.565257315	0.565373831	0.563859196	0.560344469	0.558246285	0.558404992	0.559747223	0.563882056	0.571160857	0.579708818	0.588689827	0.597981674	0.606666384	0.613414046	0.623593054	0.637214022	0.648563675	0.65810384
Derbyshire	0.555516841	0.558449016	0.559147802	0.560867306	0.56375316	0.563725168	0.561655262	0.560765828	0.561463103	0.560926143	0.558825179	0.557816144	0.558655904	0.560635169	0.564947467	0.571652972	0.579372743	0.587511787	0.595981932	0.604042925	0.610604141	0.619933462	0.632072805	0.642348817	0.650978668
Leicester	0.530102761	0.53282366	0.532877794	0.53401048	0.536553193	0.535673573	0.532342583	0.53025429	0.529974915	0.528153394	0.524391848	0.521884917	0.521689555	0.522727283	0.526855531	0.5342635	0.542958794	0.552225081	0.561975804	0.571122345	0.578236769	0.588883876	0.603129255	0.614880112	0.624999452
Leicestershire	0.58981736	0.593007964	0.594104577	0.596194856	0.599386534	0.600070523	0.599109608	0.599005101	0.600231997	0.600376297	0.599272428	0.599088697	0.600352866	0.602671047	0.607306668	0.613991766	0.621407561	0.629219191	0.637254576	0.644829883	0.651044811	0.659464017	0.670328511	0.67966609	0.687503314
Lincolnshire	0.555545743	0.558497727	0.55920562	0.560935838	0.563853403	0.563823195	0.56172616	0.560828366	0.561547331	0.561002314	0.558858697	0.557841633	0.558715606	0.560728901	0.565104869	0.5719308	0.579813584	0.588156834	0.596788446	0.604916534	0.611500135	0.620935086	0.633251872	0.643657954	0.652406439
Northamptonshire	0.56707858	0.570151865	0.570863988	0.572603651	0.575578854	0.575437757	0.573084184	0.572001932	0.57259537	0.571883862	0.569556378	0.56853376	0.569543976	0.571622366	0.57617423	0.583227361	0.591372795	0.599950567	0.608839216	0.617208069	0.623942686	0.633609138	0.646292566	0.656965393	0.66598133
Nottingham	0.567214918	0.570142967	0.570704199	0.572290044	0.575098921	0.574949495	0.572760752	0.571657803	0.57214617	0.571357546	0.56899005	0.567719136	0.568311127	0.570005562	0.57423164	0.581147766	0.589049533	0.597406201	0.606118565	0.614383036	0.620989773	0.630505111	0.642993998	0.653435222	0.662402237
Nottinghamshire	0.555788731	0.558813128	0.559625698	0.561457353	0.564442055	0.564546883	0.562624716	0.561862378	0.56265706	0.562227079	0.560248309	0.559339955	0.560237983	0.562258586	0.566587595	0.57327809	0.580958946	0.589058413	0.597479719	0.605486093	0.612003871	0.621223306	0.633226891	0.643396273	0.651924368
Rutland	0.605750896	0.608985173	0.610148124	0.61245182	0.615832748	0.616710231	0.615926465	0.616060686	0.617527589	0.617882501	0.616999615	0.617188125	0.619043841	0.621875488	0.62671782	0.633422217	0.640790755	0.648442371	0.656268838	0.663539862	0.669518502	0.677567393	0.687979971	0.696817777	0.704171528
East of England	0.582422005	0.585262889	0.586096108	0.587911767	0.59076275	0.590990888	0.589381998	0.588813163	0.589681292	0.589475697	0.587934171	0.587411951	0.588560197	0.590729881	0.595076836	0.60158773	0.608957791	0.616716707	0.624722567	0.632231588	0.638207462	0.646641045	0.657780919	0.667253945	0.675229496
Bedford	0.588351252	0.591253561	0.592086903	0.59397001	0.596930879	0.597035623	0.595150709	0.594411533	0.595235148	0.594875563	0.593052471	0.592396025	0.593524536	0.595730021	0.600109132	0.606662998	0.614183797	0.622089682	0.630207581	0.63778532	0.643737655	0.652329933	0.663727835	0.673308374	0.681310214
Cambridgeshire	0.623299139	0.626030203	0.62698439	0.628893861	0.631706677	0.632343659	0.631491027	0.631482549	0.632672044	0.633064662	0.632457239	0.632641359	0.63407568	0.636423212	0.640661138	0.646724763	0.65349721	0.660691328	0.668130031	0.675178645	0.680915542	0.688404819	0.698229461	0.706741554	0.713991116
Central Bedfordshire	0.586574053	0.589583238	0.590521741	0.592440052	0.595414506	0.595587761	0.593786945	0.593111301	0.593970766	0.593638092	0.591834159	0.591174894	0.592270211	0.594443935	0.598808967	0.605364152	0.612897976	0.620831808	0.628996963	0.636640358	0.642669947	0.651378329	0.662932748	0.672688264	0.680860217
Essex	0.573187088	0.576110294	0.576982806	0.578871468	0.58179783	0.582105509	0.580607304	0.580084352	0.580997302	0.580863789	0.579416343	0.578926072	0.580030273	0.582186578	0.586571601	0.593171596	0.600619231	0.608453187	0.616524086	0.624106713	0.630080619	0.638573011	0.649787725	0.659286788	0.667363015
Hertfordshire	0.628290402	0.631275254	0.632487706	0.634551674	0.637528772	0.63820264	0.637214228	0.637184505	0.638363771	0.638608144	0.637733502	0.637769293	0.639176973	0.641419775	0.645537814	0.651477858	0.658215816	0.665278423	0.672531633	0.679318809	0.684844723	0.692464671	0.702370474	0.710947985	0.718127119
Luton	0.554298739	0.557067366	0.557254255	0.558538767	0.561158766	0.560428054	0.557322985	0.555423445	0.555371007	0.553763331	0.550175874	0.547803595	0.547639103	0.548831986	0.552976166	0.560208618	0.568641457	0.577532324	0.586731614	0.595273603	0.601656963	0.611662989	0.62528013	0.636348356	0.645745756
Norfolk	0.566623432	0.569197144	0.569763679	0.571292086	0.573884603	0.573783846	0.571787481	0.570933422	0.571585749	0.571134612	0.569282044	0.568498463	0.569524033	0.571585448	0.575858964	0.582390324	0.589896243	0.597744183	0.605859645	0.613463933	0.619572527	0.628238785	0.639633924	0.649351614	0.657554778
Peterborough	0.559541142	0.562452836	0.562661816	0.563973581	0.566564529	0.565492951	0.562063269	0.559944938	0.559817731	0.558081041	0.554241262	0.551802671	0.551982711	0.553318317	0.557634715	0.565014245	0.573521665	0.582461182	0.591831216	0.600560468	0.607144593	0.617246903	0.631062513	0.64249037	0.652130734
Southend-on-Sea	0.54751174	0.55001463	0.550452043	0.55186312	0.554372731	0.554082786	0.551828308	0.550742648	0.551250467	0.550555281	0.548375774	0.547367806	0.548254475	0.55024867	0.554526672	0.561178809	0.568935651	0.577062694	0.585445524	0.593321566	0.599569222	0.608621219	0.620528579	0.630640839	0.639194023
Suffolk	0.568703808	0.571356648	0.571791371	0.573236043	0.575834648	0.575374694	0.572768166	0.571402411	0.5717603	0.57079594	0.568177999	0.566833098	0.567537245	0.569442556	0.573761004	0.580546023	0.588440488	0.596770369	0.605369937	0.613414164	0.61966685	0.628911859	0.641264994	0.651559542	0.660205282
Thurrock	0.556858105	0.559653899	0.560199014	0.561717862	0.56430893	0.563799047	0.561150526	0.55968475	0.559932021	0.558851768	0.556046467	0.554446736	0.555117218	0.556858401	0.561196971	0.568091647	0.575909964	0.584292105	0.593107808	0.601307432	0.607783246	0.617173677	0.629720742	0.640195177	0.649037187
Greater London	0.621722468	0.625404187	0.626893338	0.629376418	0.633095815	0.63418471	0.633551955	0.634133193	0.636267776	0.63740536	0.637332141	0.638304132	0.640890678	0.644376298	0.649705909	0.656830164	0.664542061	0.672481293	0.68050786	0.687972228	0.694016342	0.701959989	0.712165829	0.720871028	0.728299012
Barking and Dagenham	0.513853288	0.516942696	0.517331658	0.51888737	0.521813434	0.521511202	0.519173814	0.518122498	0.518910329	0.518395629	0.51622362	0.515382751	0.516808485	0.519307568	0.524291104	0.531869961	0.540605518	0.549618333	0.558908979	0.567666029	0.574580061	0.584504267	0.597263295	0.608174559	0.617494689
Barnet	0.609994224	0.613402474	0.614862951	0.617291748	0.620845205	0.62219452	0.622015379	0.622859477	0.624960074	0.626270856	0.62668749	0.628042054	0.630701163	0.634168127	0.639228873	0.645756127	0.653085142	0.660677955	0.668369647	0.67557642	0.681656944	0.689642952	0.69949509	0.70817247	0.715499699
Bexley	0.554120676	0.55758739	0.558926336	0.561278825	0.56479656	0.565814585	0.565096788	0.565485311	0.567366141	0.568268982	0.567990479	0.568768498	0.571171591	0.574465319	0.579638721	0.586601785	0.594385775	0.602482808	0.61071594	0.618377163	0.624682677	0.633407658	0.644297647	0.653746455	0.66176039
Brent	0.570567924	0.574012571	0.575028291	0.577160847	0.580658241	0.581178619	0.57979435	0.5796634	0.581313827	0.581757943	0.580711066	0.580892351	0.582993653	0.586119961	0.591435086	0.598900183	0.607342886	0.616145327	0.625110386	0.633422734	0.639973684	0.649337122	0.661317177	0.671466661	0.680155905
Bromley	0.599915065	0.603541064	0.605257593	0.607901921	0.611603771	0.613132341	0.613184336	0.61420219	0.616458726	0.617922468	0.618498117	0.619989388	0.622749932	0.626310291	0.631368917	0.637848108	0.645083778	0.652564421	0.660116721	0.667201849	0.67317622	0.681077909	0.690758197	0.699294033	0.706516801
Camden	0.716813381	0.720301933	0.722303742	0.725171298	0.728940165	0.731228086	0.732536481	0.734560854	0.737439897	0.73988787	0.741822889	0.744336355	0.747634193	0.751503925	0.756538292	0.762668335	0.769304151	0.7761705	0.783074554	0.789623665	0.795379389	0.801795294	0.809547959	0.816320698	0.821407096
Croydon	0.571158426	0.574833154	0.576177808	0.578587983	0.582273966	0.583193389	0.582209782	0.582449472	0.584338271	0.585122954	0.584568054	0.585170727	0.587558129	0.590904911	0.596282574	0.603622681	0.611841698	0.620399917	0.629096245	0.637149113	0.643699791	0.652864647	0.664439129	0.674374894	0.68278384
Ealing	0.592713607	0.596156443	0.59730898	0.599588135	0.603154753	0.603946906	0.602942118	0.603157618	0.604967553	0.605703771	0.605146053	0.605774183	0.608115853	0.611322952	0.616601359	0.623916223	0.632094057	0.640533915	0.649132227	0.657200899	0.663665294	0.672602522	0.683972896	0.693842308	0.702267022
Enfield	0.560162305	0.563527048	0.564646483	0.566799099	0.570206305	0.570978681	0.569875084	0.569965104	0.57161292	0.572230878	0.571592709	0.572060249	0.574305458	0.577464992	0.582666701	0.589777645	0.597779529	0.606108582	0.614607603	0.622492541	0.628957914	0.638009803	0.649396659	0.659229469	0.667579106
Greenwich	0.547538044	0.550754544	0.551585015	0.553583341	0.556879278	0.557210354	0.55564595	0.555320838	0.556749614	0.55707755	0.555948953	0.555929826	0.557764164	0.560667224	0.565819064	0.573261033	0.581709905	0.59049632	0.599420203	0.607710813	0.614271927	0.623624512	0.635606295	0.645966025	0.654835329
Hackney	0.623975483	0.627362065	0.627247362	0.628108321	0.630534819	0.62840743	0.623481966	0.620333989	0.619836273	0.61731133	0.612319371	0.609078388	0.608681411	0.609677296	0.614052011	0.621920543	0.631455122	0.641299338	0.651293124	0.660416341	0.666843	0.677639891	0.692304889	0.703990869	0.71423521
Hammersmith and Fulham	0.659274254	0.66263401	0.664275006	0.666860724	0.670490403	0.672117013	0.672422211	0.673734103	0.676230601	0.678007738	0.678973676	0.680827239	0.683888139	0.687723102	0.692979323	0.699554897	0.706855249	0.714462867	0.72218456	0.729472769	0.735762472	0.743336131	0.752632669	0.760891693	0.768001662
Haringey	0.580485376	0.584034001	0.585428169	0.587890457	0.591637781	0.592843168	0.592299719	0.592966227	0.595104912	0.596266013	0.596287497	0.597364547	0.600001018	0.603567044	0.608930301	0.616050329	0.624114983	0.632450213	0.640893776	0.648745722	0.655187785	0.663850528	0.6747583	0.684201857	0.692246838
Harrow	0.594410262	0.59777404	0.599013007	0.601287535	0.604753789	0.605695633	0.604901549	0.60531983	0.607165578	0.608152228	0.608056723	0.608912423	0.611213165	0.614445257	0.619454903	0.62624154	0.633908092	0.641708763	0.649606732	0.657056624	0.663179082	0.671603909	0.682156156	0.691398644	0.699285636
Havering	0.54977936	0.553404572	0.554831027	0.557240426	0.560815906	0.561767275	0.560916211	0.561246125	0.563061422	0.563985098	0.563737217	0.564462163	0.566767599	0.57003946	0.575169794	0.582179174	0.590079379	0.598103287	0.606247629	0.613952011	0.620270051	0.628839071	0.639515833	0.648823917	0.656748352
Hillingdon	0.609176339	0.612836613	0.614238159	0.616622851	0.620268218	0.621204367	0.620267958	0.620536493	0.622380053	0.623280771	0.622956912	0.623655614	0.625962437	0.629274764	0.634493001	0.641522326	0.649509642	0.657678405	0.665944663	0.673724937	0.68007386	0.68884353	0.699817623	0.709269795	0.717349502
Hounslow	0.596854922	0.600397155	0.601616937	0.60388597	0.607439263	0.60821275	0.607083511	0.607194799	0.608974938	0.609637111	0.608937235	0.609405133	0.611665488	0.614897276	0.620180888	0.627464331	0.635641893	0.644186563	0.652894753	0.660976611	0.667569582	0.676753894	0.688393754	0.698395643	0.706926428
Islington	0.653273829	0.656899025	0.658304362	0.660775235	0.664466713	0.665530795	0.664894356	0.665385244	0.667345906	0.668398022	0.668346392	0.669288191	0.671694368	0.675026845	0.680283385	0.687480854	0.695508768	0.703744403	0.712055093	0.719833289	0.726117126	0.734584617	0.745358152	0.754658665	0.762770404
Kensington and Chelsea	0.706921114	0.710369026	0.712663028	0.715792983	0.719644309	0.722327771	0.724186917	0.726724087	0.730010109	0.732939662	0.735456208	0.738507407	0.742247919	0.746482195	0.751728997	0.757774365	0.764372533	0.771373763	0.778518398	0.785336509	0.791558663	0.797528923	0.804600292	0.811134919	0.816683823
Kingston upon Thames	0.645601424	0.648744081	0.650411885	0.652962848	0.656347001	0.658138703	0.658832356	0.660284096	0.662667927	0.664627568	0.666028808	0.668080973	0.671032515	0.674663475	0.679479449	0.685311462	0.691675021	0.698248579	0.704922267	0.711330006	0.716987845	0.723870661	0.732112931	0.739636628	0.74605422
Lambeth	0.601520411	0.604986688	0.606161333	0.608386645	0.611959626	0.61284159	0.611847812	0.612085613	0.613880444	0.614606173	0.614074302	0.614666942	0.616915653	0.62013297	0.625309274	0.632371093	0.640466072	0.648848963	0.657331792	0.665182476	0.671526341	0.680328513	0.69150643	0.701109128	0.709245347
Lewisham	0.558488586	0.561968076	0.563251378	0.565618994	0.569231731	0.570303488	0.569644522	0.570126334	0.572102762	0.5731266	0.572987497	0.573896823	0.57646959	0.579873507	0.58520575	0.592297633	0.600135063	0.608271509	0.616521945	0.624139201	0.630349633	0.638863455	0.64956073	0.658805208	0.666549185
Merton	0.600019648	0.603383893	0.604705351	0.607037115	0.610481552	0.611579257	0.611059087	0.611596103	0.613520373	0.614559002	0.6145447	0.615528061	0.617962809	0.621221424	0.626229476	0.632869876	0.640242985	0.647951328	0.655802508	0.663111863	0.669200298	0.677503198	0.687859218	0.696909734	0.704547991
Newham	0.520097175	0.52315563	0.523199032	0.524487621	0.527374937	0.526588035	0.523381172	0.521617891	0.52195173	0.520675096	0.517359946	0.515533018	0.516318623	0.518310413	0.523271033	0.531343688	0.540794156	0.550610518	0.560709801	0.570123995	0.577262539	0.588102599	0.602404125	0.614439681	0.624700219
Redbridge	0.571329633	0.574629448	0.575820769	0.578031897	0.581416339	0.58238487	0.581635166	0.581990691	0.583765302	0.584629785	0.584399994	0.585207657	0.587588676	0.590827301	0.595955344	0.602809856	0.610454845	0.618445142	0.626608501	0.634214228	0.640538975	0.64910446	0.65979319	0.669109682	0.676990437
Richmond upon Thames	0.677994614	0.681019841	0.682753018	0.685404417	0.688826326	0.690869596	0.691955654	0.693718325	0.696250523	0.698381143	0.700089304	0.702410093	0.705488389	0.7091409	0.713847222	0.719405518	0.725444401	0.731731933	0.73808123	0.744107749	0.74946648	0.755826159	0.763376412	0.770319041	0.776162917
Southwark	0.612431223	0.615909575	0.616951872	0.619022766	0.62248574	0.62301261	0.621509389	0.621307123	0.622784248	0.623086519	0.621997056	0.622119872	0.624071483	0.627066885	0.632157049	0.63930518	0.647594438	0.65615671	0.664822245	0.672832862	0.679237563	0.688483951	0.700222533	0.710221683	0.718723159
Sutton	0.572706421	0.576170518	0.57759183	0.580015639	0.583558324	0.584732283	0.584268491	0.584868838	0.586881713	0.587999784	0.588040084	0.589079437	0.591624565	0.595018817	0.600185744	0.607014341	0.614586556	0.622470079	0.63050543	0.638045881	0.644360922	0.652923908	0.66355391	0.672869123	0.68078576
Tower Hamlets	0.582109668	0.584941978	0.58418166	0.58466026	0.58711385	0.584859411	0.579248326	0.575467042	0.574368856	0.571116495	0.565019137	0.560636014	0.559483078	0.560234458	0.564803463	0.573379932	0.584261147	0.595579898	0.607154996	0.617776058	0.625420069	0.638360522	0.655724303	0.669785724	0.681826394
Waltham Forest	0.535211738	0.538389248	0.539140479	0.540986811	0.544161408	0.544383428	0.54279028	0.542449039	0.543868866	0.54414066	0.542940365	0.542936633	0.544874616	0.547874823	0.553040288	0.560508975	0.569133383	0.578011785	0.586984081	0.59533946	0.601965494	0.611401369	0.62348917	0.633923663	0.642773234
Wandsworth	0.635639938	0.639015388	0.640540902	0.643055017	0.64659551	0.648064176	0.648083046	0.649111764	0.651362453	0.652855645	0.653444316	0.654929545	0.65772233	0.661263871	0.666402518	0.672975331	0.680166058	0.687675468	0.695320484	0.702467312	0.708575425	0.716364467	0.72601281	0.734517052	0.741719191
Westminster	0.735091086	0.738203163	0.740030913	0.74221274	0.745143485	0.746389845	0.746595012	0.747643513	0.749703837	0.751047736	0.75155064	0.752732018	0.754981091	0.75777627	0.761514001	0.766305549	0.771738655	0.777442322	0.783186517	0.788556857	0.793117759	0.798612815	0.805427011	0.811218979	0.816534218
North East England	0.546116003	0.548962502	0.549439772	0.551037195	0.553838536	0.553477948	0.550862931	0.549376256	0.54949664	0.548183649	0.545074271	0.543086419	0.543005586	0.54405884	0.547778508	0.554234742	0.561829367	0.569921855	0.578408307	0.586384797	0.592503746	0.601947722	0.614886775	0.625770258	0.634937555
County Durham	0.54531058	0.548156721	0.548656299	0.550234282	0.553002537	0.552704172	0.550195726	0.54876856	0.548873734	0.547655767	0.544745068	0.542858913	0.542770471	0.543767703	0.547363253	0.553651311	0.561044326	0.568897077	0.577113355	0.584839274	0.590696951	0.59980181	0.612285718	0.622763331	0.631685174
Darlington	0.569063658	0.57197384	0.572504042	0.574066132	0.576784287	0.576202236	0.573252329	0.571551098	0.571565282	0.570064743	0.566597307	0.564334359	0.564247626	0.565212763	0.568828665	0.575189327	0.582634596	0.590676917	0.599196553	0.60711502	0.613204987	0.622760379	0.635871257	0.646777862	0.655957162
Gateshead	0.553517611	0.556342249	0.556687396	0.55812324	0.560759564	0.559988991	0.556984226	0.555158508	0.555040723	0.553315692	0.549661201	0.547232425	0.546774342	0.547495838	0.550921565	0.557282735	0.564892674	0.573037034	0.581576801	0.589606759	0.595603246	0.605162011	0.618391164	0.629389817	0.638810788
Hartlepool	0.525456465	0.528207471	0.528427899	0.529742278	0.532308473	0.531289199	0.527663218	0.525346049	0.524896067	0.522727838	0.518290088	0.515130484	0.514415638	0.514872254	0.518312265	0.524892247	0.532728418	0.541204523	0.55021927	0.558586053	0.564897586	0.575093344	0.589254429	0.600915264	0.610745471
Middlesbrough	0.527068797	0.529867794	0.529978333	0.53117729	0.533726136	0.532696755	0.529048245	0.526631223	0.525993077	0.523628576	0.519041317	0.515611732	0.514447986	0.514608737	0.517862264	0.52451737	0.532631272	0.541316691	0.55045397	0.558984771	0.565298025	0.575806147	0.590406593	0.602262667	0.612429301
Newcastle upon Tyne	0.592004618	0.594883291	0.59576008	0.597579049	0.600443107	0.600798241	0.599465139	0.598941111	0.599709667	0.599321964	0.597567723	0.59665585	0.597135725	0.598675193	0.602503592	0.608387308	0.615099935	0.622232672	0.629623109	0.636623183	0.642239352	0.650402332	0.661265301	0.670583049	0.678455944
North Tyneside	0.561670607	0.564583164	0.565310789	0.567034008	0.569860323	0.569684972	0.567366536	0.566162915	0.566484394	0.565427513	0.56266913	0.560978487	0.561050702	0.562151775	0.56558549	0.571553952	0.578685987	0.586252329	0.594149914	0.601582835	0.607350606	0.616263387	0.628374037	0.638667314	0.64736738
Northumberland	0.559512359	0.562429775	0.563000137	0.564655339	0.567458202	0.567162387	0.564799327	0.56343801	0.563613316	0.562423637	0.55955868	0.557794729	0.557760972	0.558808375	0.562337688	0.568514862	0.575793145	0.583529655	0.59162123	0.59927761	0.605096272	0.614170893	0.626625278	0.637077992	0.645986227
Redcar and Cleveland	0.531615675	0.534459515	0.534805184	0.53623324	0.538894312	0.538055431	0.534672633	0.532580892	0.532311991	0.530388412	0.526286784	0.523444575	0.522960105	0.523625192	0.527198578	0.533800599	0.541585394	0.549977788	0.558863082	0.56708558	0.573290856	0.583253766	0.597083875	0.608482001	0.618041083
South Tyneside	0.525941693	0.528694023	0.529162754	0.530671586	0.533351041	0.532877107	0.530094456	0.528497901	0.528499338	0.52704536	0.523794602	0.521705243	0.52158481	0.522561542	0.526063469	0.532152581	0.539509921	0.547345105	0.555563935	0.563294821	0.569238796	0.578579889	0.591299954	0.601975672	0.610968432
Stockton-on-Tees	0.565542756	0.568529487	0.569038471	0.570591453	0.573342346	0.572606089	0.569355349	0.567354723	0.567164927	0.565292705	0.56127078	0.558512373	0.557970687	0.558573181	0.56198554	0.568342257	0.575874495	0.584048329	0.592685672	0.600742382	0.606813426	0.616611515	0.630249163	0.641539858	0.6510503
Sunderland	0.533962087	0.536700663	0.536875439	0.538107045	0.54068163	0.539609555	0.535851963	0.533441441	0.532886638	0.530552373	0.525966412	0.522675347	0.521725986	0.522012039	0.525317366	0.53191974	0.540074744	0.548742617	0.557849153	0.566348765	0.572691395	0.583009112	0.597359604	0.609272174	0.619370518
North West England	0.570415395	0.572994556	0.573379783	0.574777812	0.577270184	0.576859926	0.574385566	0.572991755	0.573141935	0.572035396	0.56935146	0.567776396	0.56807548	0.56951942	0.57348085	0.580008043	0.587610868	0.595710431	0.60415829	0.612093913	0.61828957	0.627432087	0.639702462	0.650034527	0.658724179

Blackburn with Darwen	0.525727257	0.52804705	0.527657846	0.528340619	0.530409894	0.528624247	0.52404079	0.520887487	0.519797422	0.516829637	0.511503445	0.50768254	0.506517983	0.506774799	0.510435508	0.517775693	0.526817015	0.536384866	0.546359317	0.555641326	0.562482003	0.573511953	0.588773164	0.601276424	0.611813493
Blackpool	0.531173547	0.533408726	0.533546211	0.534650286	0.53681576	0.536199221	0.533639143	0.531979028	0.531846135	0.530590407	0.527814511	0.526234489	0.52668056	0.528133037	0.532056481	0.538485165	0.545957084	0.553934355	0.562319499	0.5701988	0.576415726	0.585692734	0.597946062	0.608239963	0.617039783
Bolton	0.549102045	0.551498159	0.551521175	0.552565142	0.554799185	0.553733393	0.55033574	0.54816401	0.54781008	0.545974702	0.542217286	0.539688858	0.539342414	0.540384607	0.544222609	0.550982454	0.559084599	0.567714055	0.57673992	0.585293266	0.591853969	0.60183264	0.615329588	0.62651726	0.63591511
Bury	0.570927898	0.57356029	0.574116377	0.575643178	0.57820336	0.577973753	0.575770578	0.574614045	0.57495358	0.57407127	0.571703773	0.570516546	0.571096594	0.572755476	0.576698175	0.58299359	0.590381491	0.598279563	0.606467834	0.61415055	0.62015936	0.628936614	0.640667784	0.650570175	0.658849226
Cheshire East	0.630427145	0.633078813	0.633851694	0.635499022	0.638066565	0.638172545	0.63651385	0.635860712	0.636489484	0.636121314	0.634525643	0.633836539	0.634633355	0.636410292	0.6402694	0.646190521	0.652982528	0.660193274	0.667693052	0.674817059	0.680560048	0.688589097	0.699134304	0.708218582	0.715810035
Cheshire West and Chester	0.614447483	0.617210608	0.61797932	0.619656464	0.622308927	0.622304748	0.620428806	0.619567777	0.620096463	0.619455945	0.617431801	0.616516784	0.61718744	0.618832919	0.622657949	0.628799168	0.635938569	0.643516645	0.651324887	0.658621085	0.66434181	0.672609485	0.683651096	0.693074429	0.700942427
Cumbria	0.577540496	0.580123122	0.580653636	0.582139232	0.584652044	0.584395146	0.582191102	0.581074636	0.581446306	0.580635762	0.578348121	0.577125658	0.577629566	0.579246091	0.58314541	0.589420539	0.596736222	0.604478633	0.612530938	0.620170117	0.626195479	0.634879852	0.646433269	0.656262892	0.664510292
Halton	0.555353966	0.55797203	0.558058404	0.559184139	0.561515592	0.560311706	0.556578575	0.554109409	0.553530162	0.551300652	0.54694366	0.543996583	0.54355393	0.544343406	0.548200817	0.555213581	0.563460299	0.572378177	0.581800084	0.590508036	0.597171812	0.607438563	0.621516008	0.633097432	0.642839043
Knowsley	0.52931184	0.531984932	0.532062588	0.533188588	0.53558147	0.534541657	0.531015992	0.528621376	0.5279929	0.525768047	0.521542338	0.518520718	0.51769951	0.518184792	0.521810866	0.528702521	0.536925386	0.54568101	0.55484801	0.56341952	0.569846171	0.579949896	0.593790621	0.605084717	0.614675989
Lancashire	0.579221347	0.581702327	0.581947005	0.58320387	0.585565351	0.585087874	0.582609823	0.581163708	0.581185465	0.580005643	0.577363467	0.57579277	0.575995648	0.577315851	0.581163364	0.58763183	0.59516991	0.603196494	0.611570423	0.619421265	0.625474463	0.634474946	0.646618919	0.656819633	0.665515241
Liverpool	0.564128306	0.566617638	0.567117085	0.568597265	0.571122262	0.570986818	0.568877336	0.567776596	0.568095584	0.567195959	0.564754034	0.563309896	0.563793351	0.565524849	0.569747707	0.576287383	0.583638533	0.591445199	0.599640459	0.607412905	0.613731792	0.622627798	0.634323881	0.644208877	0.652553407
Manchester	0.586517416	0.588946901	0.589099867	0.590250599	0.592525918	0.591875122	0.589201639	0.58757097	0.587486367	0.586155539	0.583257916	0.581441029	0.581496772	0.582691294	0.586487997	0.593015938	0.600612706	0.608734924	0.6172535	0.625251067	0.631447935	0.640524483	0.652782381	0.663080387	0.671909513
Oldham	0.530217178	0.532618122	0.532571742	0.533642101	0.535961088	0.5348159	0.531212267	0.528869902	0.528362725	0.526286065	0.522205023	0.519522611	0.519227369	0.520227477	0.524103303	0.531035429	0.539367349	0.548241016	0.55750046	0.566095086	0.572604327	0.582734397	0.596529893	0.607884874	0.61742564
Rochdale	0.533987857	0.536517527	0.536608224	0.537712739	0.540039087	0.538981514	0.535474796	0.533233477	0.532804663	0.530842526	0.526922294	0.524227679	0.523795289	0.524725806	0.52858308	0.535413899	0.543668678	0.552476757	0.561658257	0.570237605	0.576803639	0.586929971	0.600660818	0.612041016	0.621606047
Salford	0.558782836	0.56115897	0.561060736	0.562021012	0.564208591	0.563163759	0.559850079	0.557654553	0.557167187	0.555253538	0.551511513	0.548941586	0.54850087	0.549321401	0.553049105	0.559879786	0.567944157	0.57655773	0.585543886	0.593885839	0.600226923	0.610024386	0.62331013	0.634149903	0.64331542
Sefton	0.569585795	0.572320485	0.57312224	0.574854094	0.577565306	0.577707492	0.575987435	0.575267241	0.575920378	0.575532145	0.573821992	0.573053887	0.573909081	0.575782592	0.579821584	0.585979684	0.593091546	0.600649785	0.608458801	0.615765938	0.621588159	0.629983193	0.641029152	0.650437773	0.658290198
St Helens	0.543159602	0.545802743	0.546157325	0.547533749	0.5500588	0.549439214	0.546597677	0.544922586	0.544968447	0.543650265	0.540569636	0.53868877	0.538829165	0.540233362	0.544224926	0.55090289	0.558813303	0.567242471	0.575953341	0.584049229	0.590239108	0.599575732	0.612197035	0.622690333	0.631456076
Stockport	0.603439586	0.606223614	0.607020189	0.608747612	0.611422499	0.611484833	0.609651485	0.60882472	0.609386955	0.608821562	0.606893884	0.606054951	0.606901548	0.60865211	0.612671834	0.618823318	0.625794524	0.633282789	0.641087947	0.648334539	0.654075848	0.662362439	0.673386126	0.682722044	0.690468732
Tameside	0.540065892	0.542536646	0.542687206	0.543839334	0.546157556	0.545239614	0.542030469	0.540010921	0.539774915	0.538107397	0.534578736	0.532287628	0.53214786	0.533328536	0.537212941	0.543946276	0.552020162	0.560660516	0.569631986	0.577999885	0.584397736	0.594174135	0.607395918	0.618379626	0.627637782
Trafford	0.635703074	0.63840648	0.63919086	0.640899436	0.643485765	0.643491226	0.641794113	0.641029436	0.641619633	0.641208742	0.639448695	0.638588913	0.639302841	0.640915736	0.644714674	0.650702389	0.657494031	0.664802464	0.672406891	0.679431459	0.684924592	0.692854332	0.703472471	0.712597482	0.720292882
Warrington	0.608946444	0.61172752	0.612356992	0.613928595	0.616559601	0.616244115	0.613863926	0.612585845	0.612904469	0.611968381	0.609423994	0.607994574	0.60838747	0.609957746	0.613912384	0.620320458	0.627838724	0.635862887	0.644129414	0.651792333	0.657689013	0.666508464	0.678384472	0.688302045	0.696578246
Wigan	0.540354006	0.542802984	0.542959123	0.54414619	0.546505843	0.545691608	0.542621869	0.540747686	0.540592863	0.53896181	0.535519698	0.533401708	0.533364843	0.534533901	0.538408845	0.545234946	0.553364083	0.561995268	0.570936715	0.579293109	0.585681584	0.595289414	0.608324848	0.61924835	0.628423827
Wirral	0.558790029	0.561377789	0.561800403	0.563224178	0.565733848	0.565306573	0.562800264	0.561391806	0.56157186	0.560472074	0.557754661	0.556155875	0.55642576	0.557905273	0.561824954	0.568235969	0.575784615	0.583803042	0.592148692	0.600033808	0.606179546	0.615285856	0.62745481	0.637663068	0.646173418
South East England	0.637105014	0.63997374	0.64132546	0.643489041	0.646358086	0.64734096	0.646950604	0.647206585	0.648356307	0.648711449	0.648092708	0.648034417	0.648988396	0.650603058	0.653738038	0.658390249	0.663681608	0.669373192	0.675328598	0.680965448	0.685494638	0.692012962	0.700882486	0.708588782	0.715081083
Bracknell Forest	0.650806511	0.654119865	0.655788847	0.658270522	0.661415738	0.662419942	0.661951886	0.662130817	0.663237133	0.663359566	0.66232589	0.661980566	0.662752949	0.664100093	0.667160868	0.671934697	0.6773504	0.683165737	0.68925381	0.694936522	0.699340054	0.705852902	0.714887675	0.722585309	0.729094479
Brighton and Hove	0.651188412	0.653497192	0.654762655	0.656782008	0.659340354	0.660731858	0.661234639	0.662199086	0.663718208	0.66481504	0.665421385	0.666469155	0.668152763	0.670325018	0.673565946	0.677801369	0.682491847	0.687535274	0.692798696	0.697866639	0.702259962	0.707765392	0.714867271	0.721444831	0.7270241
Buckinghamshire	0.665890041	0.668903176	0.670491652	0.672842082	0.675819621	0.677054021	0.676989037	0.677493313	0.6788143	0.679439063	0.679180735	0.67941935	0.680504026	0.682242953	0.68534629	0.689754467	0.694753584	0.700137637	0.705689416	0.710875334	0.715052978	0.72111661	0.729299525	0.736443569	0.742367883
East Sussex	0.592407456	0.594859776	0.595771628	0.597533787	0.600024433	0.600603558	0.599744615	0.599599622	0.600453634	0.600513141	0.599610047	0.599399944	0.600415827	0.602052714	0.605345741	0.610169834	0.61561951	0.621503858	0.627702197	0.633524596	0.638246435	0.645203843	0.654560451	0.662683773	0.669493916
Hampshire	0.634567837	0.63754984	0.638950237	0.641226143	0.64424148	0.645197599	0.644644943	0.644773879	0.645899335	0.646185428	0.645389873	0.645187638	0.646053734	0.647679272	0.650887434	0.655667183	0.661183187	0.667143172	0.673307888	0.679047941	0.683569943	0.690253118	0.699404	0.707263707	0.713797215
Isle of Wight	0.573773032	0.576220996	0.576933267	0.578613242	0.581090477	0.581271123	0.579900053	0.579296576	0.579867376	0.579555702	0.578020505	0.577137337	0.577639956	0.578880781	0.582022758	0.587072206	0.592891225	0.5991433	0.60570104	0.611778635	0.616460146	0.623754447	0.633842788	0.642550552	0.649927472
Kent	0.600515763	0.603350027	0.6045142	0.606520594	0.609334846	0.610010197	0.609096561	0.608974391	0.60989872	0.609926177	0.608823205	0.608353956	0.6091021	0.610634699	0.613891692	0.618928624	0.624776379	0.630960851	0.637426841	0.643565499	0.648451816	0.655666473	0.665460002	0.673905735	0.681045293
Medway	0.568535144	0.571449982	0.572445808	0.574365681	0.577218673	0.577515551	0.575937358	0.57523679	0.575833454	0.575271631	0.57322986	0.571950681	0.572150706	0.573294722	0.576515917	0.581949569	0.588376085	0.595225483	0.602397987	0.60919436	0.614436166	0.622418433	0.633512462	0.642921186	0.650853567
Milton Keynes	0.62823551	0.631281762	0.632109965	0.633868731	0.636544205	0.636399715	0.634375582	0.633241761	0.633492518	0.632479771	0.629870804	0.628236358	0.628251415	0.629124337	0.632236401	0.637638358	0.644174151	0.651081748	0.658453858	0.665440167	0.670717435	0.679114641	0.690980016	0.701064461	0.709645684
Oxfordshire	0.665986618	0.668928635	0.670479125	0.672844084	0.675836264	0.677242945	0.677496738	0.678225347	0.679662656	0.6804752	0.680548063	0.681055179	0.682250128	0.684004119	0.687119768	0.691552479	0.696543569	0.701944293	0.70754619	0.712819536	0.717057726	0.722622852	0.730277736	0.737013993	0.742675055
Portsmouth	0.611750215	0.614352173	0.615196583	0.61691274	0.619479194	0.620014964	0.619086865	0.618840599	0.619604523	0.61950742	0.618350091	0.617830774	0.61853746	0.620015993	0.62331613	0.628486431	0.634390628	0.640755603	0.647471904	0.653850621	0.658935845	0.666273236	0.676297654	0.68499106	0.692504487
Reading	0.665750953	0.668415387	0.669561203	0.671553734	0.674264725	0.674937359	0.674132717	0.6740424	0.674918058	0.674943642	0.673873184	0.673394487	0.674019971	0.675419905	0.678414661	0.682998595	0.688391018	0.694285219	0.700451631	0.706238431	0.710871571	0.717527517	0.726732051	0.734685293	0.741465669
Slough	0.626753911	0.629834378	0.630498539	0.632093639	0.634703779	0.634149584	0.63143954	0.62971113	0.629523954	0.627844238	0.624321525	0.621881863	0.621276626	0.621592256	0.624406277	0.629745265	0.636367614	0.643380027	0.650844192	0.657860102	0.662912748	0.671389151	0.683572058	0.693726604	0.702342786
Southampton	0.617320647	0.620075623	0.621185585	0.623130805	0.625849349	0.626453811	0.625517384	0.625337624	0.626208971	0.626217871	0.625101314	0.624603095	0.625337972	0.626894327	0.630136448	0.635054399	0.640743605	0.646855127	0.65328375	0.659393564	0.664342144	0.671345049	0.680833777	0.689016429	0.695927638
Surrey	0.680445914	0.683217746	0.684697131	0.686930144	0.689712536	0.69106102	0.691363558	0.692128946	0.693490151	0.694276784	0.694474123	0.695126342	0.696418586	0.698198674	0.701234044	0.705408339	0.710037325	0.715034552	0.720241295	0.725190028	0.729275027	0.734844572	0.742237203	0.748850616	0.754424879
West Berkshire	0.669591402	0.672622466	0.673924242	0.675976839	0.678738677	0.679296343	0.678423638	0.678212297	0.679049863	0.678994421	0.677754685	0.677181349	0.677873285	0.679192914	0.682234478	0.687173276	0.692896815	0.698982905	0.705290259	0.711159753	0.715787683	0.722741375	0.732367783	0.740650523	0.747612954
West Sussex	0.622821581	0.625544547	0.626741855	0.62874001	0.631465771	0.632267296	0.631663202	0.631778541	0.632849595	0.633126571	0.632421979	0.632307217	0.633267201	0.634969381	0.638203032	0.642996747	0.648501873	0.654348998	0.660446109	0.666271082	0.670994149	0.677858224	0.687026839	0.695048883	0.70180407
Windsor and Maidenhead	0.688232647	0.691119229	0.692653007	0.694920311	0.697731473	0.698923451	0.698896689	0.699409758	0.700646167	0.701172418	0.700874574	0.701131703	0.702259601	0.703920765	0.706962921	0.711257929	0.716053404	0.721328884	0.726884462	0.732087075	0.736365701	0.742316473	0.750333229	0.757343856	0.763178823
Wokingham	0.699538167	0.702422653	0.704122957	0.706556872	0.709468576	0.710995631	0.711539717	0.712497335	0.714027917	0.714981683	0.715279278	0.716039853	0.717428856	0.719243354	0.72229236	0.726464156	0.731052905	0.736013152	0.741165924	0.745998412	0.749953905	0.755210325	0.762257378	0.76850273	0.773683081
South West England	0.606946781	0.609553658	0.610379379	0.612076464	0.614605488	0.614992325	0.613802446	0.61334945	0.613925206	0.613585665	0.612184871	0.611492934	0.612042166	0.613431367	0.616685281	0.621761029	0.627554164	0.633816017	0.640563568	0.646984379	0.652152941	0.659663045	0.669727262	0.678423176	0.685730649
Bath and North East Somer	0.653758079	0.656242543	0.657436256	0.659388209	0.661925016	0.663185617	0.663457469	0.664148288	0.665361317	0.666096325	0.666331233	0.666972899	0.668239192	0.670108007	0.673231969	0.677492707	0.682191786	0.687244988	0.692614297	0.697801759	0.702240828	0.708026302	0.715449926	0.722167367	0.727852998
Bournemouth	0.609994677	0.612244066	0.613131793	0.614794795	0.617119238	0.617813366	0.617280383	0.617392613	0.618305002	0.618591043	0.618114908	0.618164798	0.619268626	0.621128573	0.624431013	0.629083147	0.634285432	0.639895929	0.645928603	0.651796717	0.656808064	0.663572369	0.672240264	0.679961339	0.686509455
Bristol, City of	0.639805833	0.642429538	0.643343918	0.645078168	0.647576453	0.64808154	0.647136238	0.646831552	0.647451118	0.647282997	0.646174826	0.645656064	0.646217218	0.647598031	0.650735564	0.655616762	0.661200614	0.6672364	0.673753308	0.679987218	0.685036513	0.692103335	0.701562074	0.709811655	0.716865961
Cornwall	0.588252547	0.590676801	0.591220525	0.592627368	0.594886628	0.594768323	0.592864456	0.591789797	0.591906035	0.590920135	0.588648826	0.587222133	0.587288353	0.58827024	0.591309585	0.596355878	0.602236439	0.608692279	0.615699664	0.622299893	0.627560931	0.63562624	0.646545663	0.655864133	0.663661128
Devon	0.607324818	0.609793443	0.610548424	0.612144973	0.614557516	0.615093922	0.614203969	0.613944925	0.614566311	0.614416373	0.613396344	0.612986739	0.613630672	0.6150686	0.618260779	0.62313327	0.628692278	0.634644785	0.641030647	0.647137398	0.652053725	0.659143758	0.668556209	0.676774816	0.683738457
Dorset	0.607794577	0.610365743	0.611209814	0.612879297	0.615333881	0.615636261	0.614407174	0.613928264	0.614485102	0.614098229	0.612646812	0.611952868	0.612538099	0.613846536	0.617031342	0.622027852	0.627715859	0.633836151	0.64040071	0.646611478	0.651569686	0.658933365	0.668767505	0.677246322	0.684391676
Gloucestershire	0.623925681	0.626687851	0.627601567	0.629365365	0.631961873	0.632400947	0.631225518	0.63073748	0.631234081	0.630844731	0.629426224	0.628652857	0.629037315	0.630266564	0.633406031	0.638422784	0.644147299	0.650309839	0.656919423	0.663184583	0.668113644	0.67543704	0.685319855	0.693819263	0.700992211
North Somerset	0.602147846	0.604787038	0.605624595	0.607296926	0.609826033	0.61010068	0.608708958	0.608116035	0.608629394	0.608119211	0.606476395	0.605658936	0.606174847	0.607587907	0.610824278	0.615872556	0.621809522	0.628225115	0.635069092	0.641521914	0.646707768	0.654462605	0.664837103	0.673743691	0.681188959
Plymouth	0.582868106	0.585386922	0.585867204	0.587251737	0.589604181	0.589307266	0.587066047	0.58577645	0.585804476	0.584686464	0.582092148	0.580271855	0.580113015	0.581071267	0.584223788	0.589571038	0.595950689	0.602788892	0.610258211	0.617418119	0.62309373	0.631637292	0.64319269	0.653000554	0.661280601
Poole	0.616592546	0.619120811	0.619871812	0.621411309	0.623799998	0.623941102	0.62238013	0.62164027	0.621988564	0.621311786	0.619495664	0.618445098	0.618809048	0.620025713	0.623125838	0.628090397	0.633856865	0.640172661	0.646957093	0.653368041	0.658577809	0.66638116	0.676800409	0.685760792	0.693303552
Somerset	0.59486328	0.597542665	0.598326166	0.599947226	0.602465114	0.60258005	0.600925094	0.600111498	0.600475032	0.599723037	0.597729085	0.596607336	0.59692508	0.598183447	0.601361538	0.606474695	0.612539251	0.619103324	0.626124009	0.632733468	0.637987573	0.645940916	0.656656131	0.66578266	0.673419772
South Gloucestershire	0.638381505	0.641403454	0.642626109	0.644681654	0.647514362	0.648193838	0.647392946	0.647174467	0.647899267	0.647693136	0.646478844	0.646004732	0.646580373	0.647980634	0.651180475	0.656162142	0.661860146	0.667946042	0.67443074	0.680593828	0.685464583	0.692608476	0.702226685	0.710508028	0.717517173
Swindon	0.618863452	0.621895778	0.622751585	0.624447676	0.627104129	0.62690563	0.624595596	0.623255941	0.623325014	0.621989758	0.619015088	0.617032647	0.616815802	0.617604581	0.620711803	0.626150837	0.632520778	0.639500675	0.647044405	0.654038362	0.659422203	0.667952444	0.679723359	0.689535514	0.69777053
Torbay	0.570953223	0.573061368	0.573419361	0.574622218	0.576629766	0.576419002	0.574654899	0.573668735	0.573784442	0.572942427	0.570972554	0.569810279	0.57008835	0.571154072	0.57416993	0.579163563	0.584991563	0.591288268	0.598132682	0.604673361	0.60997725	0.617944094	0.628554759	0.637792473	0.645747765
Wiltshire	0.614315405	0.617137595	0.618039775	0.619811328	0.622435241	0.622609012	0.621067836	0.620352606	0.620811326	0.620184347	0.618231047	0.617053771	0.617318433	0.618412204	0.621589811	0.626871191	0.632907714	0.63945122	0.646493449	0.653072301	0.658187484	0.665859166	0.676352416	0.685234662	0.692685654
West Midlands	0.549988216	0.552702939	0.552920696	0.554156081	0.556672626	0.556116684	0.553365571	0.55181509	0.551982463	0.550810839	0.547943255	0.546318617	0.546759859	0.548442185	0.552836918	0.559992196	0.568284216	0.577064825	0.586195656	0.59478216	0.601531344	0.611395147	0.624472636	0.635471381	0.64475772
Birmingham	0.547425735	0.549967644	0.549796903	0.550686765	0.552953383	0.551848467	0.548270265	0.545984294	0.545600876	0.543606592	0.539539125	0.536738362	0.53649645	0.537738209	0.542060893	0.549419931	0.558013605	0.567150535	0.576741619	0.585777129	0.592827966	0.603324141	0.617281301	0.628788764	0.638493962
Coventry	0.571879934	0.57457296	0.574733348	0.575849878	0.578254645	0.577570196	0.574696889	0.572991098	0.573008332	0.571690702	0.568661725	0.566835492	0.566956727	0.568328945	0.572424624	0.579414638	0.587670762	0.596467757	0.605569951	0.614088189	0.62068474	0.630447858	0.643340809	0.65401898	0.663058533
Dudley	0.541767103	0.544537966	0.544783128	0.546021031	0.548523354	0.547842378	0.544945337	0.543316399	0.543422258	0.542060493	0.538952657	0.537196858	0.537455729	0.538910711	0.54309516	0.550199045	0.55858283	0.567478517	0.576700554	0.585365564	0.592020457	0.601899169	0.615030452	0.626051349	0.635413464
Herefordshire, County of	0.570717081	0.573500126	0.573896545	0.575281744	0.577811947	0.577433045	0.5751827	0.574071531	0.574579458	0.573888308	0.571667942	0.570696018	0.571735129	0.573705854	0.578112185	0.585001168	0.592891666	0.601235248	0.609947597	0.618135452	0.624671437	0.633902333	0.645995458	0.65635872	0.665042769
Sandwell	0.514488464	0.516888887	0.516515988	0.517076285	0.519088111	0.51736956	0.512906087	0.509910223	0.508940963	0.506178986	0.501161445	0.497722501	0.496959637	0.497617032	0.501448244	0.508707046	0.517772651	0.527402272	0.537478033	0.546821147	0.553876112	0.564955927	0.579906216	0.592154539	0.602551519
Shropshire	0.57684514	0.579543237	0.58010189	0.581555995	0.584157568	0.584069871	0.582042883	0.581188548	0.581852028	0.581394074	0.579587537	0.578974457	0.580118193	0.582277762	0.586685735	0.593298928	0.601038201	0.60920766	0.617650444	0.625560646	0.631943213	0.640924675	0.652563901	0.662496175	0.67079573
Solihull	0.609891956	0.612893701	0.613680402	0.61536977	0.618179302	0.618130118	0.616069594	0.615147181	0.615792883	0.615285136	0.61330333	0.612430494	0.613279908	0.615277583	0.619562887	0.626165197	0.633927065	0.642185882	0.650683715	0.65859325	0.664862703	0.673708661	0.6852199	0.694927173	0.702988923
Staffordshire	0.566887274	0.569756093	0.570273447	0.571762823	0.574449139	0.57427121	0.572074718	0.570977515	0.571472278	0.570791931	0.568608826	0.567541277	0.568226388	0.570068705	0.574424635	0.581398944	0.589490377	0.598054141	0.606911609	0.615263243	0.62182443	0.631196086	0.643507099	0.653934304	0.662778501
Stoke-on-Trent	0.519334691	0.521813587	0.521618683	0.522378592	0.524483906	0.523053404	0.51905319	0.516446359	0.51583435	0.513599129	0.509224359	0.506344236	0.506089509	0.507096375	0.511214183	0.518553636	0.527308386	0.536692467	0.546582614	0.555748845	0.562854671	0.573599997	0.587968497	0.599849069	0.609863629
Telford and Wrekin	0.558438482	0.561094742	0.56104022	0.56197244	0.564280896	0.563008027	0.559152284	0.556737442	0.556353108	0.554340529	0.550186731	0.547541241	0.547487651	0.548693436	0.553020758	0.560559714	0.56946566	0.579011205	0.589038195	0.598298923	0.605484581	0.616272847	0.63074637	0.642707732	0.652741329

Walsall	0.523199469	0.525929177	0.525955129	0.526964375	0.529387857	0.528448411	0.52501217	0.522827669	0.522470539	0.520593145	0.516742217	0.51416477	0.513846763	0.514812874	0.518864554	0.526181584	0.534899244	0.544159074	0.553794864	0.562763921	0.569589524	0.580089484	0.594110085	0.605632056	0.615388813
Warwickshire	0.598158944	0.601008827	0.601582607	0.603083687	0.605749779	0.605730617	0.603819417	0.602959137	0.603595338	0.603130102	0.601287406	0.600530571	0.601417121	0.603399392	0.607737	0.614521439	0.622351019	0.630675128	0.639235707	0.64723958	0.653598229	0.662626129	0.674342436	0.684185823	0.692474288
Wolverhampton	0.534096193	0.536753628	0.536662572	0.537571645	0.539866427	0.538675896	0.535002574	0.532596862	0.53211796	0.530075926	0.526013529	0.523310828	0.522882631	0.523826652	0.527840276	0.535173486	0.543976007	0.553327744	0.563049109	0.572134229	0.578978277	0.589479434	0.603599982	0.615272022	0.625211791
Worcestershire	0.57620441	0.579047102	0.579623816	0.581150857	0.58382026	0.58374587	0.58185121	0.580960032	0.581556909	0.581016014	0.579116692	0.578415992	0.579367679	0.58136808	0.58571532	0.592500363	0.600376832	0.608718504	0.617347479	0.625493559	0.632004505	0.641165968	0.653114909	0.663315333	0.671977602
Yorkshire and the Humber	0.553470972	0.556579982	0.557267813	0.559023113	0.562054069	0.562137421	0.560148562	0.559331406	0.560133373	0.559687039	0.557689339	0.556831773	0.557843222	0.559959336	0.564502047	0.571474175	0.57940016	0.587724052	0.596314263	0.604349713	0.61065829	0.619774204	0.631898155	0.642160858	0.650806254
Barnsley	0.516494878	0.519431613	0.519795004	0.52124476	0.524049959	0.523533144	0.520648855	0.519044034	0.5192529	0.517957806	0.514811862	0.513079319	0.513562466	0.515275279	0.519648226	0.526828763	0.53530383	0.544265448	0.553486571	0.562032729	0.568601189	0.578412028	0.591509708	0.602409244	0.611557341
Bradford	0.527565432	0.530715588	0.530923589	0.532246064	0.535046261	0.534135369	0.530731518	0.528584148	0.528435103	0.526620177	0.522694005	0.520145644	0.519943152	0.521116561	0.525440739	0.533155729	0.54226238	0.551854642	0.561760575	0.570927866	0.577742692	0.588276699	0.602638378	0.614425332	0.624467008
Calderdale	0.555930196	0.558987743	0.559501757	0.561015598	0.563860189	0.563495754	0.560828437	0.559471614	0.559862578	0.558845519	0.556115117	0.554714728	0.5554444	0.557329846	0.561811571	0.568910692	0.577263968	0.586076204	0.595161542	0.603586829	0.610135201	0.619854272	0.632742503	0.643530737	0.652589581
Doncaster	0.519446004	0.522528016	0.522894511	0.524300183	0.527099199	0.526415581	0.5232395	0.521470291	0.521582704	0.520155789	0.516704631	0.514506331	0.514632248	0.516063279	0.520360753	0.527708701	0.536356727	0.545383229	0.554761271	0.563531028	0.570272208	0.5803157	0.593787087	0.604998398	0.614409581
East Riding of Yorkshire	0.574029768	0.577272153	0.578269393	0.580262477	0.58340255	0.583834664	0.582535065	0.582261165	0.583461395	0.583497533	0.582269258	0.582140254	0.583495738	0.585756264	0.590353554	0.597076631	0.604647394	0.612602571	0.620749088	0.628415929	0.634518229	0.643037275	0.654165757	0.663773665	0.671801463
Kingston upon Hull, City o	0.518994035	0.521937958	0.522197523	0.523472638	0.526134536	0.52533309	0.522046446	0.520101912	0.520034407	0.518388334	0.514734632	0.512488476	0.512681827	0.514153572	0.518468821	0.525807197	0.534514094	0.543753148	0.553372968	0.562298178	0.569201946	0.579516462	0.593323196	0.604780131	0.614477572
Kirklees	0.545296916	0.548399005	0.548877224	0.550416054	0.553308993	0.553101889	0.550713035	0.549482718	0.549958314	0.549075485	0.546509652	0.545135282	0.545726431	0.5475025	0.551955093	0.559236104	0.567617791	0.57645444	0.585549575	0.594004292	0.600495114	0.610143835	0.622986359	0.633653768	0.642692151
Leeds	0.587893355	0.591126163	0.592111154	0.594076674	0.597175986	0.59762685	0.596254241	0.595878122	0.596929053	0.596931572	0.59564434	0.595333608	0.59656563	0.598794274	0.603228765	0.609927548	0.617478323	0.625438949	0.633623957	0.641296653	0.647352427	0.655759999	0.666792621	0.676213264	0.684235337
North East Lincolnshire	0.520481392	0.523484042	0.523611918	0.524860003	0.527638964	0.526626419	0.522849659	0.520541572	0.520289158	0.518273679	0.514042877	0.511310098	0.511214857	0.512466842	0.516869446	0.524563367	0.53373833	0.543451627	0.553488598	0.562734397	0.56969293	0.58046831	0.595069074	0.607021145	0.6170504
North Lincolnshire	0.566127695	0.56935538	0.569856339	0.571442002	0.574437381	0.573894121	0.570857094	0.569196252	0.569417762	0.568084658	0.564793218	0.56291591	0.563384713	0.565085882	0.569532856	0.576867301	0.585482363	0.594564498	0.603931163	0.612535615	0.619116291	0.629021933	0.642309883	0.653266605	0.662443836
North Yorkshire	0.59031343	0.59352931	0.594645698	0.596707052	0.599874764	0.600483701	0.599303091	0.599201547	0.600526049	0.600817463	0.599819956	0.599811959	0.601359788	0.603919512	0.608482703	0.615025011	0.622423516	0.630179465	0.638137093	0.645637116	0.65169474	0.660017393	0.670772623	0.679988929	0.687682439
Rotherham	0.527581411	0.530606956	0.531052283	0.532579927	0.535480831	0.535054173	0.532221154	0.530707344	0.530986446	0.529791868	0.526780937	0.525123424	0.525673093	0.527419013	0.531882543	0.539091197	0.547580095	0.556519071	0.565728833	0.574254326	0.580825761	0.590589829	0.603611959	0.614440473	0.623503699
Sheffield	0.582593823	0.585599698	0.586684938	0.588712375	0.591784977	0.592628322	0.591808284	0.591941115	0.593299933	0.593715418	0.592964338	0.593066771	0.59470822	0.597363932	0.602041866	0.608495793	0.615510788	0.622838296	0.630391208	0.637502937	0.643418203	0.651286554	0.66132472	0.669992616	0.677253794
Wakefield	0.532276866	0.53533545	0.535764677	0.537215364	0.540035605	0.53947285	0.536509048	0.53487939	0.535153152	0.533948288	0.53080707	0.528936465	0.529295211	0.530992977	0.535427659	0.542731614	0.55134815	0.560439108	0.569787671	0.578478082	0.585153187	0.595131108	0.608462535	0.619538516	0.62886795
York	0.628200457	0.631353388	0.632650068	0.634848063	0.6379931	0.639133469	0.638845536	0.639357296	0.640897967	0.64166839	0.641535887	0.642157596	0.643944783	0.646532284	0.65087502	0.656915424	0.663594814	0.67064203	0.677867435	0.684676196	0.690293481	0.697668593	0.707070716	0.715255512	0.722191453
Northern Ireland	0.488105781	0.493008039	0.495716886	0.50066953	0.506721997	0.510604561	0.512044554	0.513631628	0.515778778	0.516194966	0.521469942	0.524787445	0.522255305	0.522472127	0.529701254	0.543528529	0.55468943	0.562607193	0.571664763	0.581277945	0.588636359	0.596965144	0.609212192	0.628441482	0.645264135
Scotland	0.578740331	0.581943785	0.582980822	0.583438133	0.584503874	0.584774181	0.58324598	0.582589688	0.583610164	0.584162181	0.585224119	0.586158414	0.587018167	0.588852592	0.593211432	0.600989313	0.606840051	0.612595158	0.620565873	0.628347058	0.633234455	0.640982577	0.651624179	0.659542018	0.666026147
Wales	0.561013743	0.563266824	0.563583525	0.564788689	0.5669366	0.566767063	0.56477525	0.563426091	0.56329217	0.562007906	0.559472716	0.557965635	0.557873507	0.558844577	0.561908353	0.567640235	0.574062565	0.581243941	0.588774463	0.595941315	0.601455066	0.609883785	0.621649971	0.631555206	0.640018189
Latin America and Caribbean	0.278069555	0.281450394	0.284564058	0.287260465	0.29030217	0.29374457	0.297464628	0.301438018	0.305188532	0.308851612	0.312706911	0.316792299	0.321395835	0.326487575	0.332141418	0.338214487	0.344566673	0.350995616	0.357352736	0.363517666	0.369739739	0.375907427	0.382599238	0.38984351	0.396960925
Andean Latin America	0.289483418	0.293317926	0.296869181	0.300132308	0.303400473	0.306598046	0.309652349	0.312783866	0.315646115	0.318438814	0.321728253	0.325228306	0.329324186	0.333890679	0.339050992	0.344667513	0.351011131	0.357693133	0.363929388	0.369673813	0.375321001	0.381012919	0.387270955	0.394601279	0.402694431
Bolivia	0.247141415	0.250911192	0.253836369	0.255590096	0.257761454	0.260455313	0.26273702	0.264898196	0.267213169	0.269617935	0.272303472	0.275203221	0.278567609	0.282466577	0.286714873	0.291286654	0.296372948	0.30167101	0.306914268	0.312201171	0.317606372	0.323084695	0.328668573	0.334369537	0.340145294
Ecuador	0.321070493	0.324568431	0.328740617	0.332613922	0.336122543	0.338325107	0.339905371	0.341253321	0.34218702	0.342607989	0.343073819	0.343909024	0.345914038	0.349242369	0.35410033	0.360185131	0.368003215	0.376690724	0.383900724	0.389127635	0.393322381	0.397547198	0.403537083	0.41246235	0.423389498
Peru	0.289569533	0.293188664	0.296501383	0.299912497	0.303411118	0.307150601	0.310993602	0.315045342	0.318989421	0.322949987	0.327522041	0.332245035	0.337222016	0.342233444	0.34748848	0.353022146	0.358888914	0.364877475	0.370752872	0.376750437	0.383050515	0.389451606	0.396010479	0.402961208	0.41025865
Caribbean	0.323185331	0.328031489	0.331208181	0.332423062	0.332674112	0.333173747	0.334945864	0.337278754	0.338267339	0.337794907	0.336782098	0.336049257	0.337550599	0.341340062	0.347316009	0.356119508	0.36650268	0.375846952	0.382012545	0.385820529	0.388692714	0.392386863	0.399128417	0.408428996	0.417874709
Antigua and Barbuda	0.331150382	0.333590435	0.33445288	0.333731314	0.332094502	0.331833365	0.33558559	0.341674621	0.348752979	0.356987146	0.366510563	0.376075119	0.384914588	0.393796363	0.403752778	0.414143	0.424371686	0.435206858	0.447167903	0.458707983	0.469010458	0.479637316	0.49005921	0.498396773	0.503781161
The Bahamas	0.47868588	0.47962534	0.478718821	0.47821729	0.484302216	0.493577335	0.503110777	0.513456305	0.520682013	0.52351318	0.523025483	0.521194233	0.514950336	0.509108651	0.513937906	0.524651491	0.537511271	0.550553462	0.561216549	0.570800274	0.579270983	0.587274857	0.594984439	0.602269695	0.605857316
Barbados	0.414855871	0.409530283	0.404636181	0.400413755	0.398958116	0.404712198	0.412608655	0.419139517	0.42414908	0.427765391	0.436951596	0.449834879	0.459508357	0.466970052	0.472304314	0.477213201	0.486110719	0.497252311	0.50721403	0.517124941	0.525111346	0.531718137	0.540028701	0.551133896	0.563091512
Belize	0.284624064	0.286027726	0.286628059	0.286505909	0.285447948	0.283872897	0.282196115	0.280433266	0.278553009	0.276787402	0.275136925	0.273538332	0.272570726	0.272860394	0.274474545	0.27689707	0.280058266	0.283878947	0.287904445	0.291509006	0.294447516	0.297603734	0.300545474	0.303731427	0.307895117
Bermuda	0.423249931	0.428151294	0.432121064	0.435381277	0.438103364	0.440694741	0.443702281	0.447189479	0.450691301	0.455056724	0.461067101	0.468754626	0.477885248	0.488009805	0.499226257	0.510571495	0.522332542	0.533704444	0.543560144	0.55221435	0.560878129	0.570540222	0.581154083	0.591612986	0.601236996
Cuba	0.375371138	0.382969181	0.38809249	0.388430012	0.387648127	0.387187385	0.38806919	0.389262786	0.38687513	0.380207387	0.370470689	0.36095365	0.356293179	0.357127974	0.362415438	0.37284331	0.386246571	0.397570612	0.400997802	0.398387009	0.393082527	0.390623518	0.39698838	0.411379141	0.426811839
Dominica	0.379561382	0.378844682	0.375844009	0.368119588	0.358778191	0.352926339	0.349334218	0.344317045	0.341109619	0.343569126	0.350096765	0.356448788	0.36119444	0.359716524	0.353257133	0.348401977	0.349740772	0.351473982	0.353036036	0.357439438	0.36270996	0.371070495	0.389338181	0.419530431	0.452220347
Dominican Republic	0.161988768	0.166551226	0.170460188	0.174147185	0.178312776	0.182511896	0.187153703	0.192105959	0.197073452	0.202107464	0.20754042	0.212892755	0.219355893	0.226399667	0.233980131	0.240949541	0.248577158	0.25635442	0.264182252	0.27249415	0.281473728	0.290718938	0.300379117	0.310438183	0.320461196
Grenada	0.230028379	0.228274225	0.225890495	0.223418242	0.221315136	0.220345735	0.22100139	0.223763154	0.22916169	0.237040894	0.246884966	0.257546967	0.268328726	0.278783364	0.288798476	0.298082565	0.306687311	0.314489034	0.321835696	0.328784566	0.33550683	0.342218118	0.349440909	0.357063651	0.36500584
Guyana	0.290248945	0.290448793	0.290237875	0.289929067	0.289886254	0.290364251	0.291580907	0.29365037	0.29650832	0.300373523	0.305589013	0.311665213	0.318264168	0.3247682	0.332718445	0.342094269	0.352516437	0.363552691	0.374649126	0.385781029	0.396491548	0.406900669	0.416666626	0.426188057	0.435147223
Haiti	0.185039023	0.186820534	0.18864627	0.190548363	0.192588605	0.194704257	0.196878849	0.199118551	0.201389035	0.203823256	0.206461166	0.208852521	0.211667599	0.214493768	0.217151228	0.219715745	0.222361203	0.2247703	0.227228446	0.229846587	0.232536175	0.235383705	0.238312436	0.241485845	0.244938211
Jamaica	0.359081004	0.361653474	0.362993938	0.363963138	0.363853783	0.362899771	0.36118016	0.359492976	0.357050499	0.354498395	0.352161946	0.349847884	0.347839283	0.346716804	0.346899348	0.349079885	0.354023204	0.360472377	0.367311961	0.374458498	0.38270347	0.392469292	0.404073369	0.416134293	0.428238359
Puerto Rico	0.397437103	0.405243523	0.414434372	0.41952506	0.41904236	0.417688699	0.421230405	0.427951033	0.434825216	0.442376211	0.450019517	0.456912559	0.462771463	0.469274954	0.478310217	0.493738541	0.512419886	0.52862932	0.540888762	0.549043343	0.55400065	0.559068107	0.565225662	0.571249209	0.577219697
Saint Kitts and Nevis	0.337799346	0.330385502	0.316769547	0.296543865	0.270346507	0.240390699	0.210410157	0.181154165	0.152960434	0.138418979	0.148747264	0.168531109	0.187203027	0.20723462	0.231595794	0.261737199	0.297005892	0.331656405	0.360811092	0.381728583	0.395018833	0.403426761	0.409719068	0.415873744	0.423787573
Saint Lucia	0.279792998	0.280254757	0.280052406	0.279036673	0.277383479	0.275351862	0.272976968	0.270468559	0.268087103	0.265929532	0.26397854	0.261897869	0.259724647	0.257593276	0.25556256	0.253902239	0.253013659	0.253303119	0.254774334	0.257867832	0.264221571	0.273866355	0.284922441	0.296724192	0.308685092
Saint Vincent and the Grenadines	0.255203357	0.249887717	0.241101158	0.229112812	0.214461987	0.199575629	0.187408667	0.181294882	0.181658105	0.186170591	0.19365283	0.201749127	0.208555371	0.212288594	0.215336965	0.22106516	0.231781599	0.245468816	0.257904681	0.264902469	0.268155382	0.273845872	0.287055377	0.302876558	0.317840463
Suriname	0.279715462	0.281092095	0.28203398	0.283014817	0.283993831	0.284948191	0.285938815	0.286936078	0.288072184	0.289893304	0.292621513	0.296505456	0.301961361	0.308735771	0.316404486	0.325209707	0.335371597	0.346147206	0.357071088	0.367972931	0.378689408	0.389416291	0.39942533	0.408082906	0.414572557
Trinidad and Tobago	0.393243186	0.395692625	0.394695284	0.391527034	0.388559883	0.388085657	0.389414222	0.390779257	0.392416783	0.394562835	0.398183855	0.403793799	0.411290494	0.42189311	0.434801667	0.448603279	0.462787921	0.476474823	0.489680437	0.50142852	0.510907726	0.518404492	0.525643933	0.532994604	0.540534364
Virgin Islands	0.443557824	0.443091353	0.441281688	0.437993933	0.433464889	0.428484796	0.423785607	0.420327605	0.418351254	0.418425385	0.420465313	0.42343555	0.427323533	0.432035935	0.437929004	0.445179127	0.453921315	0.464220132	0.475555115	0.487695154	0.501187501	0.515845095	0.531438425	0.546299339	0.559555874
Central Latin America	0.278621301	0.281250054	0.283462847	0.285204286	0.287526561	0.290292306	0.29308539	0.295892667	0.298335769	0.300932166	0.304022264	0.307640729	0.311795649	0.316621576	0.32210259	0.327648996	0.333129893	0.338911581	0.345073279	0.351527248	0.35827912	0.364599377	0.371322779	0.378536969	0.38595064
Colombia	0.272653816	0.274717924	0.27709384	0.279636113	0.282263377	0.28450148	0.286600921	0.28871735	0.290898943	0.293715596	0.297077858	0.30128476	0.306333453	0.312044426	0.318624028	0.325739606	0.333469921	0.341486859	0.349775347	0.35809364	0.366465206	0.374790191	0.38326855	0.39168277	0.399718129
Costa Rica	0.311276237	0.312195446	0.316046172	0.31868935	0.317736203	0.318088831	0.318634502	0.317454677	0.312509729	0.310451341	0.31542784	0.323461698	0.333722611	0.346818336	0.361332184	0.375270679	0.388682977	0.402183738	0.415681911	0.428144516	0.439420783	0.448414774	0.455915616	0.461303244	0.464035672
El Salvador	0.191343921	0.193761798	0.196381953	0.199044036	0.201494867	0.203937104	0.206579041	0.209721619	0.212956439	0.216154228	0.219337003	0.222542443	0.226197806	0.22994198	0.234086842	0.23840785	0.243061374	0.24763528	0.252102216	0.256168731	0.259914376	0.263495685	0.267200465	0.271273001	0.275551748
Guatemala	0.203053031	0.205230594	0.207577319	0.210027588	0.213774023	0.217946419	0.220818179	0.222556354	0.223308573	0.223868333	0.22527322	0.228262181	0.232140891	0.236387209	0.241723457	0.247372552	0.252446444	0.256578481	0.259795687	0.261977148	0.26267232	0.262791984	0.263903472	0.265078613	0.266555229
Honduras	0.182498396	0.184850758	0.187212883	0.189971672	0.192082645	0.194300239	0.197049014	0.199789121	0.202579639	0.205357475	0.208009719	0.210462433	0.212976734	0.215506343	0.218176567	0.220860215	0.223636911	0.226384276	0.229257219	0.231819423	0.234364078	0.236938275	0.239798367	0.243081436	0.246513924
Mexico	0.28681193	0.290001467	0.292508665	0.294132678	0.296272333	0.298857904	0.301810131	0.305244235	0.308780345	0.312200996	0.31565197	0.318802711	0.322006672	0.325756411	0.329869975	0.333757295	0.337763907	0.34216761	0.347074531	0.35204841	0.356848552	0.36138932	0.367120069	0.374380358	0.382690148
Aguascalientes	0.278383395	0.281130984	0.283106227	0.284097431	0.285565335	0.287464344	0.289813331	0.29271513	0.295805454	0.298864416	0.301971924	0.304826078	0.307792495	0.311486354	0.315631084	0.319719967	0.324088669	0.329247462	0.335247988	0.341442798	0.347608731	0.353650105	0.361344395	0.370809589	0.381281332
Baja California	0.337416328	0.340803641	0.343335783	0.344861098	0.346819352	0.34926051	0.352095227	0.355443809	0.358862933	0.36211284	0.365426515	0.368443776	0.371521429	0.375053626	0.378747506	0.382229551	0.385864303	0.390689274	0.397015605	0.404031383	0.410794101	0.417214533	0.424900771	0.433930018	0.443706392
Baja California Sur	0.309167578	0.312254085	0.314490062	0.31568066	0.317370836	0.319524855	0.322046972	0.32512839	0.328282414	0.331301446	0.334345009	0.337079869	0.339848332	0.343099226	0.346631452	0.350086734	0.353859093	0.358435734	0.363817147	0.369337877	0.374628508	0.379575354	0.386022098	0.39449492	0.404423038
Campeche	0.280702117	0.283447774	0.285503354	0.286702622	0.288463575	0.29065479	0.293174231	0.296115652	0.299129585	0.302006917	0.304829714	0.307293456	0.309705093	0.312385684	0.315199273	0.317803662	0.320514874	0.323698015	0.327176377	0.330436105	0.333547808	0.336381752	0.340211809	0.345083744	0.350595227
Chiapas	0.234506943	0.236564856	0.238068496	0.238820591	0.239993567	0.241543765	0.243326117	0.245491014	0.247753868	0.249881903	0.251990772	0.253741433	0.25545084	0.257537958	0.259861455	0.262018375	0.263974108	0.266158136	0.268648681	0.271102027	0.273323769	0.275020719	0.277444871	0.281107923	0.285648063
Chihuahua	0.301119369	0.303862309	0.305793538	0.306708996	0.308043336	0.309831533	0.312010491	0.314718948	0.317531537	0.320252419	0.322996339	0.325471115	0.328061946	0.331364614	0.335141312	0.338747937	0.342297713	0.346498595	0.351503091	0.356804502	0.361920079	0.366737099	0.373043883	0.381348752	0.39108104
Coahuila	0.303359896	0.306327762	0.308485408	0.30961806	0.311195373	0.313227287	0.315639417	0.318562546	0.321566923	0.324425198	0.327341101	0.329936408	0.33264163	0.336115775	0.340106507	0.343960007	0.347812965	0.352114514	0.356898692	0.361681937	0.366301367	0.370665858	0.376541578	0.384467635	0.393831243
Colima	0.267101418	0.270286539	0.272698311	0.274121621	0.276064785	0.278642973	0.281749389	0.285477802	0.289421829	0.293399828	0.297437039	0.301095462	0.304897085	0.309454827	0.314462605	0.319491286	0.324912901	0.33106837	0.33789941	0.344899612	0.351943584	0.358767245	0.366981525	0.376797357	0.387459545
Durango	0.257687219	0.260186051	0.261865251	0.262490762	0.263527797	0.265014247	0.266872375	0.269238235	0.271697209	0.274048394	0.276370997	0.278358066	0.280412121	0.283314567	0.286829222	0.290291537	0.293855448	0.297873799	0.302339473	0.306840907	0.311341259	0.315613555	0.321337714	0.328818836	0.337496451
Guanajuato	0.232545993	0.235020983	0.236863692	0.237928448	0.239469061	0.24144093	0.243764417	0.24656155	0.249489918	0.252316732	0.255155101	0.257683015	0.260201705	0.263192471	0.26648222	0.269501534	0.272566106	0.276157881	0.280529806	0.285264341	0.290021233	0.29467891	0.300701715	0.308260127	0.316819178
Guerrero	0.213574617	0.215532185	0.216973087	0.21772336	0.218892706	0.220449737	0.22233266	0.224687114	0.22716278	0.229593319	0.232026115	0.234205064	0.236446092	0.23921169	0.24225523	0.245088722	0.247851616	0.250887356	0.254206961	0.257489054	0.26054666	0.263158622	0.266850719	0.272121318	0.278592194
Hidalgo	0.235084521	0.237719626	0.239764324	0.241037946	0.24283528	0.245071217	0.247657976	0.250711629	0.253881872	0.256997086	0.260154212	0.262963181	0.265750879	0.268791834	0.271950221	0.274870671	0.277836174	0.280970382	0.284256549	0.287464903	0.290600334	0.293468289	0.297487886	0.303071447	0.309861622
Jalisco	0.291170149	0.294185611	0.296504851	0.2979404	0.299850242	0.302142304	0.30482731	0.307961992	0.311208251	0.314370192	0.317695279	0.320819446	0.32406573	0.32796384	0.332313159	0.336668933	0.341155039	0.346051161	0.351219546	0.356191754	0.361081188	0.36581219	0.371826862	0.3793732	0.388045642
México	0.281023021	0.284071318	0.28647713	0.288015956	0.290040409	0.292529697	0.295451745	0.298980033	0.302683042	0.306363285	0.310129788	0.313662223	0.317279394	0.321542667	0.326241858	0.330692596	0.335239434	0.340262508	0.34567116	0.351018501	0.3561863	0.361087649	0.367576259	0.375808604	0.384983394
Mexico City	0.400009653	0.404001205	0.407131602	0.409213105	0.411931541	0.415142206	0.418693332	0.422731581	0.426895402	0.430952035	0.43523075	0.439337067	0.443588793	0.448409332	0.453620276	0.458579164	0.463484263	0.46883785	0.475094492	0.481719162	0.488051895	0.493944321	0.500824535	0.508916535	0.517701294

Michoacán de Ocampo	0.23494096	0.237415891	0.239299989	0.240393551	0.241907062	0.243793792	0.246038039	0.248711629	0.251500597	0.254219543	0.256969193	0.259458941	0.26204868	0.265298543	0.269080474	0.272727327	0.276316316	0.280174328	0.284361329	0.288489269	0.292517275	0.296272589	0.301137479	0.307489833	0.314917819
Morelos	0.286792185	0.289999382	0.292581639	0.29438129	0.296695651	0.29948575	0.302676542	0.306377806	0.310234586	0.314013403	0.317696729	0.321078021	0.32453474	0.328494052	0.332742861	0.336708558	0.340682901	0.345205232	0.35031897	0.355494077	0.360366672	0.364885608	0.370745435	0.378172911	0.386551234
Nayarit	0.23980089	0.242134209	0.243781807	0.244545134	0.245744578	0.247431616	0.249567529	0.252291841	0.255201819	0.258103761	0.261047539	0.263766456	0.266566894	0.269947148	0.273666606	0.277246262	0.280978731	0.285285887	0.290168387	0.295216789	0.300261688	0.305164977	0.311696694	0.32020001	0.330040732
Nuevo León	0.321979685	0.325322645	0.327791515	0.329204589	0.331141653	0.333599954	0.336461085	0.33992776	0.343605305	0.34725058	0.35100487	0.354563103	0.358445583	0.363419307	0.369299725	0.375204512	0.381232267	0.387868195	0.39509323	0.402300197	0.409247267	0.415830994	0.423790478	0.43339054	0.444024471
Oaxaca	0.23170455	0.233878898	0.235580913	0.236638429	0.238193456	0.240125733	0.242362594	0.244996242	0.247704249	0.250306812	0.252949026	0.255284946	0.257591734	0.260253993	0.263086707	0.265687201	0.268194255	0.270877674	0.273740731	0.276496858	0.27910663	0.281469587	0.284818593	0.289482526	0.295100753
Puebla	0.253349702	0.25587441	0.257746066	0.258772528	0.260379969	0.26240397	0.264716323	0.267456489	0.270240047	0.272870701	0.275454277	0.277654891	0.279793056	0.28232562	0.285050739	0.28743798	0.289679733	0.292258697	0.295489219	0.298947325	0.302248136	0.305165925	0.30923079	0.314867454	0.321673804
Querétaro	0.257773016	0.260944799	0.263544286	0.265405411	0.267795178	0.270633776	0.273944806	0.277834682	0.281914009	0.285962296	0.290066007	0.293933591	0.297877169	0.302386331	0.30723659	0.311757331	0.316508195	0.321921397	0.32810973	0.334626935	0.34113756	0.347420967	0.354945222	0.363734603	0.373186523
Quintana Roo	0.255445354	0.25788789	0.259566308	0.260305028	0.261533894	0.263359754	0.265615283	0.268395481	0.271373531	0.274286453	0.277106865	0.279469016	0.281839159	0.284806523	0.288060783	0.290990701	0.293801177	0.297217325	0.301615665	0.306419429	0.311046206	0.315250637	0.320779862	0.327978012	0.336271846
San Luis Potosí	0.248630425	0.251185389	0.253124159	0.254259219	0.255927811	0.258020416	0.260421965	0.26327562	0.266210127	0.26900021	0.271784701	0.274212694	0.276531131	0.278959662	0.281383769	0.283480854	0.28565182	0.288411772	0.29176732	0.295135421	0.298488215	0.301701919	0.30631988	0.312575407	0.320053991
Sinaloa	0.26568203	0.268117737	0.269813425	0.270586341	0.271809798	0.273455979	0.275556778	0.278184184	0.280941914	0.283637032	0.286399374	0.288880884	0.291508768	0.294946577	0.298956886	0.302938505	0.307123435	0.311760765	0.316857586	0.322032939	0.327271907	0.332489402	0.339488377	0.348589654	0.359119931
Sonora	0.310868942	0.31425037	0.316822423	0.318385274	0.320417829	0.322888686	0.32582125	0.329294535	0.332923863	0.336528923	0.340466011	0.344303231	0.348191714	0.352506178	0.357117611	0.361844166	0.36694046	0.372489867	0.378406353	0.384248805	0.389999995	0.39542278	0.402052599	0.410243291	0.419636463
Tabasco	0.2395824	0.242065989	0.243768155	0.244519398	0.245698047	0.247348875	0.249415143	0.25204079	0.254839873	0.257561955	0.260201362	0.26244842	0.264850126	0.26815844	0.272088214	0.275943175	0.279757898	0.284091359	0.288808576	0.293430227	0.297663035	0.301373489	0.306174046	0.312434727	0.31969526
Tamaulipas	0.305630068	0.308755719	0.311103863	0.312483568	0.31438089	0.316782015	0.319594225	0.322927057	0.326360986	0.32967222	0.332992221	0.335989641	0.339052288	0.342669522	0.34667022	0.350340431	0.354024095	0.35841274	0.363638819	0.369174551	0.374556788	0.379637912	0.385904259	0.393735231	0.402628965
Tlaxcala	0.234268681	0.23666685	0.238404372	0.239247105	0.240657972	0.242563842	0.244861998	0.247638416	0.250519847	0.253312967	0.256172592	0.258769904	0.261425437	0.264641488	0.268193162	0.271475659	0.274817169	0.278646594	0.283146163	0.28788776	0.292585826	0.296982974	0.302677247	0.310182456	0.319034224
Veracruz de Ignacio de la Llave	0.269606117	0.272509639	0.274791107	0.276278266	0.2782438	0.280640999	0.283388121	0.286576011	0.289853585	0.292997258	0.296041582	0.298692734	0.301367688	0.304513599	0.308010582	0.31124091	0.314349669	0.317561167	0.320848522	0.323988604	0.326957166	0.329657481	0.333336295	0.338413995	0.34469108
Yucatán	0.295988291	0.298686583	0.300690594	0.301823852	0.303472937	0.305489448	0.307745191	0.31034736	0.312968396	0.31533961	0.317573986	0.319304283	0.320973915	0.32304877	0.325397273	0.327288871	0.328902653	0.330910509	0.333571536	0.336357546	0.338830154	0.340951006	0.344622762	0.350116286	0.356700901
Zacatecas	0.239760425	0.241964178	0.243458104	0.244022723	0.244988459	0.246424277	0.248220928	0.250518689	0.252920069	0.255202813	0.257498302	0.259470796	0.261430955	0.263909212	0.266671213	0.269353813	0.272198564	0.275524152	0.27932675	0.283257904	0.287330339	0.291307342	0.29694585	0.304830245	0.314356144
Nicaragua	0.21197197	0.214098279	0.216432216	0.217825487	0.218878588	0.219860775	0.220367132	0.22091106	0.221092673	0.221315927	0.221404653	0.221609971	0.222021327	0.222766679	0.223839252	0.225127109	0.226351962	0.228011699	0.230256559	0.233116043	0.236123047	0.239280795	0.242557962	0.245892948	0.24918873
Panama	0.340534201	0.33244354	0.32130165	0.312089965	0.30786483	0.308233394	0.310582141	0.313956895	0.316670184	0.318154537	0.3165629	0.315029413	0.318452361	0.327584953	0.342054272	0.352797039	0.357436674	0.363849056	0.371098698	0.379210326	0.389105421	0.400932899	0.414491261	0.426782148	0.439185888
Venezuela	0.322694559	0.323825559	0.323997405	0.324115127	0.326354791	0.330050499	0.332591039	0.332501934	0.328959009	0.32605393	0.327438809	0.33342367	0.341373766	0.350023203	0.359367041	0.368657077	0.376999852	0.385357965	0.394041753	0.404649242	0.418174574	0.430010054	0.439262065	0.446521322	0.452886555
Tropical Latin America	0.253906836	0.257468883	0.261502901	0.265548106	0.270059178	0.274998412	0.280104696	0.285544288	0.291198197	0.296901683	0.302699323	0.308591893	0.314534962	0.320301472	0.326092494	0.332042762	0.338252276	0.344569175	0.351186984	0.357810146	0.364621227	0.37162953	0.378934524	0.386498923	0.39340026
Brazil	0.252898989	0.256453133	0.260498008	0.264550294	0.269072182	0.27402219	0.279142791	0.28460689	0.290285684	0.296020161	0.301852622	0.30777823	0.313758096	0.319560247	0.325393333	0.331390346	0.337652553	0.344023501	0.350698295	0.357374369	0.364232306	0.371287192	0.378643302	0.386254198	0.393188146
Acre	0.152603938	0.154310928	0.15652263	0.158931731	0.161823496	0.165163554	0.16878935	0.172755936	0.176949015	0.181163013	0.185343929	0.189403881	0.193830692	0.198035723	0.202452008	0.206951335	0.211443041	0.216046512	0.220889713	0.225749513	0.230948199	0.23672123	0.242472169	0.245933954	0.248219815
Alagoas	0.154370437	0.156249386	0.158638715	0.161073397	0.163968337	0.167328894	0.170902118	0.174833927	0.179074698	0.183389161	0.187796553	0.192386838	0.197116919	0.201832899	0.20648732	0.210973146	0.215300957	0.219841298	0.224516745	0.229012676	0.233732414	0.238810035	0.244189778	0.249718071	0.254701588
Amapá	0.21000777	0.21236063	0.215394118	0.218612014	0.222448532	0.226746306	0.231280604	0.236173317	0.241328904	0.246407417	0.25141698	0.256233759	0.261434294	0.266271971	0.271261252	0.276290985	0.281253795	0.287002428	0.293738993	0.300299065	0.307021988	0.314175229	0.321073281	0.324814059	0.327376674
Amazonas	0.211220104	0.213885705	0.217081783	0.220410673	0.224282267	0.228614596	0.23325079	0.238260847	0.243541784	0.248842074	0.254163668	0.260070536	0.26458623	0.268475427	0.2724635	0.27661391	0.28069387	0.285026057	0.289686158	0.294474759	0.299619343	0.305429439	0.311965174	0.319048874	0.325207462
Bahia	0.174727352	0.176832645	0.179386737	0.181928198	0.184903267	0.188312399	0.191909351	0.195842156	0.200043365	0.204309907	0.208628708	0.212228775	0.215859691	0.21918614	0.223343022	0.227954159	0.232747478	0.237575579	0.242510697	0.247348111	0.252487863	0.257993643	0.263901611	0.270224821	0.275941867
Ceará	0.175741015	0.178089794	0.180945747	0.183735609	0.186963467	0.190532058	0.194189951	0.198195388	0.202479566	0.206895261	0.211491917	0.216099269	0.22151931	0.226661212	0.232080286	0.237449871	0.242173727	0.246924394	0.250839532	0.253718619	0.256919651	0.260725029	0.265121234	0.269747072	0.274216827
Distrito Federal	0.345173226	0.350054625	0.355452782	0.360991411	0.366980868	0.373426739	0.380142856	0.387198417	0.394386433	0.401500738	0.408469519	0.415424413	0.422750756	0.429347548	0.435637642	0.441185373	0.446079688	0.451294862	0.456580416	0.461462214	0.46641723	0.471910057	0.478006603	0.48329373	0.488042159
Espírito Santo	0.219409297	0.222412379	0.225923821	0.229440091	0.233441439	0.237861882	0.242426341	0.24734212	0.252603586	0.258057748	0.26388331	0.268965905	0.274611025	0.280596978	0.288600779	0.295885572	0.30328421	0.310680164	0.318275745	0.325692513	0.333012196	0.340418375	0.347987777	0.355011145	0.361116755
Goiás	0.170459075	0.172982166	0.176255582	0.179393786	0.183148193	0.187444967	0.191817966	0.196641155	0.201813527	0.207129267	0.212692222	0.219564553	0.227927297	0.235544772	0.243030787	0.249744203	0.255731108	0.261911344	0.27172167	0.285008711	0.296877652	0.30775137	0.318044628	0.327442801	0.335576729
Maranhão	0.136569102	0.138063147	0.1400426	0.141959284	0.144290384	0.146975209	0.149669295	0.152685525	0.155912322	0.159187916	0.162532229	0.165704522	0.16950284	0.17262975	0.1756885	0.178836819	0.182148023	0.185485492	0.188359755	0.190601782	0.193321988	0.196725563	0.200680477	0.204592139	0.207962042
Mato Grosso	0.242101734	0.244483416	0.24726481	0.250029894	0.253148006	0.256611275	0.260220854	0.26413131	0.268211027	0.272197752	0.275821061	0.279927332	0.284174749	0.287128895	0.290542858	0.293975193	0.296826375	0.300299044	0.304662102	0.309510062	0.31457907	0.319989466	0.325767708	0.329873155	0.333263497
Mato Grosso do Sul	0.231146312	0.234093679	0.237577286	0.241033542	0.244995029	0.24932999	0.25374192	0.258547415	0.263589597	0.268666296	0.273834376	0.280131225	0.286736579	0.291645002	0.296880525	0.301998031	0.305989396	0.310388745	0.315424046	0.320728807	0.326354771	0.332376592	0.339269891	0.349809637	0.359246217
Minas Gerais	0.234501653	0.237611623	0.241363686	0.245085791	0.249307749	0.253993214	0.258847623	0.264085282	0.269592938	0.275274384	0.2812089	0.287329152	0.29385272	0.299730555	0.306326985	0.313133998	0.320284585	0.327601635	0.334688399	0.340993465	0.347055789	0.35306279	0.359528359	0.367196768	0.374497626
Pará	0.202682747	0.205214403	0.208289949	0.211450866	0.215100897	0.219244736	0.223603215	0.228307435	0.233259957	0.238224996	0.243237999	0.248503487	0.253068961	0.257417512	0.261590403	0.266124141	0.270963873	0.275893601	0.28017765	0.28356812	0.287127629	0.291250811	0.295749709	0.299748969	0.303035452
Paraíba	0.177268025	0.179625499	0.182434722	0.185214559	0.188422999	0.192075552	0.195825384	0.199943181	0.204352278	0.208880734	0.213570346	0.218109612	0.223643228	0.228558852	0.233560555	0.238598329	0.242363877	0.246361484	0.249617771	0.251877541	0.25442003	0.257483192	0.261418137	0.266701013	0.272240294
Paraná	0.248624646	0.251605076	0.255185875	0.25884136	0.263011001	0.267656081	0.272601369	0.277943585	0.283554163	0.289238936	0.294978805	0.300193187	0.305632652	0.310160634	0.314859027	0.320422667	0.32595162	0.331867666	0.33788843	0.343626016	0.349848437	0.35704662	0.365342216	0.376040446	0.386088772
Pernambuco	0.183310705	0.185749262	0.188674534	0.191699567	0.19526485	0.199368363	0.203738788	0.208511995	0.213534639	0.218587807	0.223695483	0.228896013	0.234540654	0.240421337	0.246219359	0.251961193	0.25756473	0.263312995	0.268779592	0.273689042	0.27878079	0.284262522	0.290204506	0.29646479	0.302227928
Piauí	0.133518359	0.135363266	0.137779222	0.140036291	0.142764913	0.145851074	0.148926465	0.152335918	0.156053617	0.159938375	0.164090739	0.169464563	0.175903588	0.180302475	0.185310439	0.190166174	0.194724527	0.19920509	0.202963234	0.205787923	0.208992249	0.212847309	0.21748695	0.222958904	0.228210382
Rio de Janeiro	0.348857748	0.353737294	0.359243556	0.364712149	0.370643095	0.37691891	0.383293724	0.389885141	0.396646256	0.403473432	0.41038455	0.417335377	0.423857037	0.430705261	0.437326709	0.443653474	0.44992307	0.456171167	0.462801264	0.469507142	0.476409244	0.483832887	0.49154003	0.498894323	0.505559715
Rio Grande do Norte	0.180049175	0.182572172	0.185585295	0.188640645	0.192183445	0.196230435	0.200472876	0.205168659	0.210173572	0.215312923	0.220601118	0.225770396	0.231620132	0.237392292	0.242955796	0.248620094	0.254272859	0.25999019	0.264377823	0.267282627	0.270373047	0.273850242	0.278062047	0.28385671	0.289600266
Rio Grande do Sul	0.303732435	0.3080207	0.312952955	0.317858479	0.323298032	0.329277745	0.335447588	0.341978844	0.348720622	0.355518255	0.362432987	0.369324985	0.37655143	0.383567337	0.390561407	0.397542171	0.404621951	0.411670681	0.418940279	0.42596391	0.43270349	0.439398769	0.446494625	0.454184318	0.461003002
Rondônia	0.21396004	0.21617512	0.218891432	0.221645869	0.224802421	0.228313774	0.232018416	0.236063039	0.240340866	0.244635255	0.248994868	0.253404369	0.258191121	0.262664277	0.267373925	0.272202309	0.277254454	0.283118774	0.290114812	0.29719307	0.304517533	0.312112073	0.319565711	0.324952225	0.329450807
Roraima	0.234817353	0.237384279	0.240456312	0.243451961	0.246910048	0.25063543	0.254440354	0.258568056	0.262938692	0.267377473	0.271908959	0.276524044	0.281469944	0.285936377	0.290532399	0.295053389	0.2993408	0.303736119	0.308516259	0.313555199	0.319320489	0.325803653	0.332415436	0.338375123	0.343813526
Santa Catarina	0.273699276	0.277454766	0.281735751	0.285942417	0.290549923	0.295513899	0.300577695	0.306024658	0.311763683	0.317660776	0.323983384	0.330411646	0.336370331	0.342066289	0.347844165	0.353438452	0.360187113	0.367153007	0.374919561	0.383081306	0.391234207	0.399444818	0.407926523	0.417096538	0.425488993
São Paulo	0.325975987	0.330158203	0.334875768	0.339603268	0.344765022	0.350300067	0.355954316	0.361886732	0.367989931	0.374131147	0.380361262	0.386673395	0.392917861	0.39916927	0.405286092	0.411572315	0.418218297	0.424804866	0.431991764	0.439487682	0.447035554	0.454611784	0.461781851	0.468536123	0.474424714
Sergipe	0.18165468	0.183860958	0.186593639	0.189298982	0.19245165	0.196023717	0.199725244	0.203829925	0.208267371	0.212853134	0.217608007	0.222911361	0.229231617	0.234858575	0.240081028	0.245571107	0.251554721	0.257599528	0.263386963	0.268591437	0.274042131	0.27978109	0.285954361	0.292349166	0.298450338
Tocantins	0.157010618	0.158626572	0.16088938	0.162913031	0.165452396	0.168318514	0.171122086	0.174272043	0.177742593	0.181285514	0.184972524	0.189638346	0.19555792	0.200875323	0.206067157	0.21053018	0.214350445	0.218208088	0.225028033	0.234892085	0.244134174	0.252958373	0.26137202	0.26915297	0.275919065
Paraguay	0.292716473	0.296722924	0.30041227	0.304190091	0.308198701	0.312615627	0.31703661	0.321323678	0.325713909	0.329883806	0.33392163	0.338043356	0.342104923	0.34605684	0.349788701	0.35347424	0.356988288	0.360672505	0.364358659	0.368256029	0.372617475	0.377218376	0.382028555	0.387074578	0.39233767
North Africa and Middle East	0.191330356	0.195771221	0.200315434	0.205062706	0.209722612	0.214293635	0.218965053	0.223644133	0.22819607	0.232619839	0.236852141	0.240406416	0.243240251	0.245402036	0.247871504	0.252271357	0.25879351	0.266094089	0.273379469	0.280319173	0.287453976	0.294570824	0.301759638	0.308438353	0.315027515
North Africa and Middle East	0.191330356	0.195771221	0.200315434	0.205062706	0.209722612	0.214293635	0.218965053	0.223644133	0.22819607	0.232619839	0.236852141	0.240406416	0.243240251	0.245402036	0.247871504	0.252271357	0.25879351	0.266094089	0.273379469	0.280319173	0.287453976	0.294570824	0.301759638	0.308438353	0.315027515
Afghanistan	0.113209591	0.114962171	0.116750475	0.118729012	0.120632391	0.122484165	0.124369418	0.126124107	0.127932744	0.12969735	0.131696251	0.133614057	0.135391617	0.136979487	0.138262986	0.139601928	0.141362837	0.143139537	0.14494882	0.14671002	0.148594098	0.150104207	0.150914473	0.151652679	0.152558731
Algeria	0.141578713	0.14447119	0.147796193	0.151113881	0.154884396	0.158738668	0.163018874	0.167836298	0.172994673	0.178764359	0.184680466	0.189981965	0.194699239	0.200406124	0.206522932	0.212948307	0.219226606	0.225882762	0.232744036	0.239740447	0.247295794	0.254548912	0.262761261	0.271205277	0.280506335
Bahrain	0.23153911	0.23706227	0.242760405	0.248714882	0.254903805	0.261217499	0.267667682	0.27448138	0.281613958	0.289101445	0.296863953	0.304899505	0.313234841	0.321869376	0.331021595	0.340799671	0.349610261	0.358957526	0.368891128	0.379453296	0.390770341	0.402667415	0.415124025	0.428694126	0.441753798
Egypt	0.227207604	0.232047502	0.236534617	0.241140008	0.245746093	0.250267365	0.254580399	0.258389853	0.26143127	0.263666376	0.264912289	0.264083495	0.260410927	0.254671754	0.249377925	0.247054126	0.249781738	0.256970169	0.266340733	0.275942341	0.28440791	0.290974843	0.295863505	0.299667394	0.30311317
Iran	0.168392913	0.171542336	0.17492501	0.178432242	0.182095124	0.185948289	0.190295532	0.195256578	0.200584738	0.20602654	0.211254247	0.215977547	0.219381072	0.220237992	0.222125637	0.231871635	0.247323258	0.26159456	0.271455249	0.278530529	0.286144233	0.296600153	0.307274674	0.315019461	0.320986431
Alborz	0.227121454	0.230879349	0.234836919	0.238838209	0.242907307	0.247128933	0.251841985	0.25719708	0.262925926	0.268773315	0.274433297	0.279629666	0.283453156	0.284917435	0.287764827	0.298675216	0.314762902	0.329957348	0.340988077	0.349444125	0.358578006	0.370557418	0.382826401	0.392197599	0.399746417
Ardebil	0.120229706	0.122033134	0.124026022	0.126108853	0.128322096	0.130704307	0.133496785	0.136757501	0.140296486	0.143900732	0.147241415	0.150087251	0.151498647	0.15004344	0.14950499	0.157934239	0.172166236	0.185088013	0.193212705	0.198368949	0.20408329	0.213309088	0.222719841	0.228826372	0.232770072
Bushehr	0.16453501	0.16616696	0.167953489	0.169849484	0.171860814	0.174032191	0.176617161	0.17977777	0.183292184	0.186998769	0.190601196	0.193883384	0.195978191	0.196032956	0.197177164	0.205320858	0.218846469	0.231708444	0.240927056	0.247875661	0.25556249	0.266023733	0.277037506	0.285611501	0.292661527
Chahar Mahaal and Bakhtiari	0.141998288	0.144082785	0.146336779	0.148662453	0.151069579	0.153601944	0.156521499	0.15994378	0.163670136	0.167518256	0.171158724	0.1743696	0.176244146	0.175667945	0.176093564	0.183826834	0.196692316	0.208647251	0.216620829	0.222040813	0.228009888	0.236835426	0.246062541	0.252678285	0.257626941
East Azarbayejan	0.156948333	0.159309851	0.161850339	0.164472369	0.167241178	0.170184301	0.173576786	0.177496332	0.181705643	0.185988526	0.18998815	0.193393361	0.195228034	0.194391777	0.194623019	0.203403981	0.217838144	0.230985636	0.239427246	0.244921478	0.251001724	0.260244505	0.269711766	0.276090048	0.280524842
Fars	0.178775115	0.181606337	0.184642398	0.187794547	0.191074158	0.194518823	0.198459811	0.203044692	0.207889443	0.212886802	0.217762786	0.222324804	0.22564455	0.226798485	0.22912332	0.238639094	0.253314949	0.26708231	0.276908045	0.284249145	0.292128047	0.302644249	0.31339891	0.321504825	0.327915046
Gilan	0.17302109	0.17533375	0.177839562	0.180451951	0.183184819	0.186109351	0.189540035	0.193624526	0.198004914	0.202633005	0.207200077	0.211473397	0.214826297	0.216251846	0.218769373	0.228156163	0.243341291	0.257828389	0.268815847	0.277621219	0.287176392	0.299125	0.311647188	0.321836754	0.330734721
Golestan	0.131122226	0.133241018	0.135609751	0.138074766	0.140717291	0.143580446	0.14692686	0.150810522	0.15503821	0.159369112	0.163499952	0.167122214	0.169237132	0.168218411	0.168129624	0.177383222	0.193052821	0.207391406	0.216642671	0.222700505	0.22938465	0.239585062	0.250011752	0.256916151	0.261524976
Hamadan	0.137508765	0.139543622	0.141784267	0.144121733	0.146573999	0.149191278	0.152239343	0.15583059	0.159772379	0.163851508	0.167730665	0.171215151	0.173365272	0.172844255	0.173243162	0.181767821	0.195746477	0.208592419	0.217035855	0.222742435	0.22902929	0.238505624	0.248231743	0.254977335	0.25986195
Hormozgan	0.11843365	0.120265509	0.122273577	0.124399357	0.126664205	0.129065749	0.131847972	0.135184013	0.138866751	0.142757843	0.146644441	0.150330342	0.152978884	0.153616185	0.155039246	0.162998245	0.176489704	0.189084392	0.197952046	0.204451346	0.211553256	0.221037676	0.230963215	0.238268073	0.243807795
Ilam	0.117100463	0.11895361	0.120960559	0.123092796	0.12541302	0.127948751	0.130920408	0.134548255	0.138465347	0.142329018	0.145828967	0.148748474	0.150402344	0.149948673	0.150773179	0.15943471	0.172995952	0.185399411	0.193884704	0.19974001	0.205699211	0.214616361	0.224078324	0.231308279	0.237158661
Isfahan	0.190146558	0.19268217	0.195474574	0.198419266	0.201493848	0.20472092	0.208411789	0.212759773	0.21754625	0.222568527	0.227534172	0.232269687	0.23596278	0.237866342	0.241003254	0.250700275	0.264904562	0.278602139	0.289001047	0.297260366	0.306073874	0.317303862	0.32887374	0.338048465	0.345818842
Kerman	0.147861341	0.149955517	0.152243958	0.154640669	0.157169073	0.15987507	0.163035948	0.166763559	0.170822673	0.17501727	0.178989754	0.182495807	0.184718752	0.184361287	0.185090572	0.193907504	0.208616578	0.222228632	0.231474787	0.237917297	0.244924821	0.254937792	0.265320415	0.272726696	0.278272355

Kermanshah	0.141566871	0.143639914	0.145922189	0.148314921	0.150828703	0.153509707	0.156620301	0.160334939	0.164403071	0.168616474	0.172698904	0.176466116	0.179110132	0.179736632	0.181142031	0.189360062	0.20241381	0.21461404	0.223152749	0.229349707	0.236090071	0.24556305	0.255349856	0.262648983	0.268462215
Khorasan-e-Razavi	0.154486616	0.156340912	0.15842494	0.160626295	0.162985854	0.165512607	0.168523496	0.172150494	0.176118849	0.180090614	0.183694549	0.186626435	0.187941514	0.18611805	0.185427121	0.194447199	0.210066074	0.224139049	0.23298062	0.238550137	0.244690631	0.25426379	0.264110942	0.270528456	0.274760625
Khuzestan	0.137887781	0.139844043	0.142028814	0.144353893	0.146839382	0.149510798	0.152609236	0.156225808	0.160173134	0.16424398	0.168087398	0.171409486	0.173335308	0.172180598	0.171981097	0.181079651	0.196850057	0.211234562	0.22061512	0.226881326	0.233878159	0.244341791	0.255118455	0.262533861	0.26779682
Kohgiluyeh and Boyer-Ahmad	0.118825124	0.120167129	0.121669223	0.123262981	0.124956636	0.12674996	0.128883641	0.131515752	0.134435267	0.137460844	0.140338762	0.142885506	0.144404625	0.143803996	0.144215907	0.152017417	0.165299155	0.177498319	0.185892884	0.191927757	0.198633188	0.20824548	0.218413532	0.226041789	0.232022061
Kurdistan	0.121904796	0.123596005	0.125448038	0.12737048	0.12938607	0.131502287	0.133979071	0.136932847	0.140179853	0.143522119	0.146734907	0.149690653	0.151656851	0.151992856	0.15322472	0.160090695	0.170488442	0.180337271	0.187344369	0.192529901	0.197987068	0.205859814	0.213993207	0.220111707	0.225002023
Lorestan	0.122441379	0.124289478	0.126322961	0.12844603	0.130683595	0.13307019	0.135862125	0.139152804	0.142736148	0.146400245	0.149813917	0.152746041	0.154406438	0.153509453	0.153462421	0.161400919	0.174927949	0.187218811	0.195143744	0.200338052	0.206079819	0.214900797	0.224067276	0.230339584	0.234733926
Markazi	0.155027048	0.156926943	0.158964039	0.161050188	0.163244752	0.165583753	0.168371629	0.171685332	0.175392074	0.179333602	0.183281941	0.187060435	0.190074314	0.191411951	0.193681447	0.201577984	0.213896235	0.225850575	0.235111729	0.242703231	0.251021653	0.261715859	0.272933758	0.282115818	0.290022844
Mazandaran	0.17596479	0.178901106	0.182100941	0.18545738	0.188985172	0.19274282	0.197010994	0.201922811	0.207286083	0.212635835	0.218014409	0.223197374	0.227320889	0.229594095	0.232965746	0.242970787	0.257907845	0.2722308	0.283035868	0.291533665	0.300560398	0.312082084	0.323948393	0.333292939	0.341116981
North Khorasan	0.136963889	0.138046056	0.139128887	0.140115032	0.141068135	0.141951298	0.143076222	0.144516075	0.146063226	0.147528598	0.148641822	0.149195917	0.148495841	0.145313976	0.143099783	0.149012299	0.160639384	0.171221791	0.177627897	0.181515513	0.186228193	0.194185209	0.202635723	0.20830478	0.212088659
Qazvin	0.159587127	0.161391799	0.163306585	0.165208769	0.167161229	0.169205464	0.171512582	0.174309363	0.177418655	0.180635565	0.18372845	0.186405726	0.188144025	0.187851556	0.188391933	0.195620605	0.207973888	0.21956943	0.227661885	0.23361149	0.240282009	0.249618833	0.25932291	0.266541611	0.272256514
Qom	0.17983877	0.182372577	0.185032732	0.187737167	0.190512038	0.193384989	0.196706585	0.200523903	0.204679087	0.208919987	0.212987119	0.21669323	0.219369354	0.22007944	0.221796038	0.23006691	0.243452358	0.256244521	0.265689187	0.273099534	0.281333541	0.292223464	0.303662426	0.312668578	0.32007658
Semnan	0.177577491	0.180102786	0.182886178	0.185834925	0.188964878	0.192320098	0.196202644	0.200595874	0.205499239	0.210741102	0.216080132	0.221356415	0.225729016	0.228480985	0.232504989	0.243067017	0.258088103	0.272748223	0.284301921	0.29377199	0.303839298	0.316474253	0.329552237	0.340348336	0.349780548
Sistan and Baluchistan	0.112021915	0.113321586	0.114741574	0.116198293	0.117726574	0.119346013	0.121290073	0.123630024	0.126195492	0.128789985	0.131173259	0.133093395	0.133843305	0.132372758	0.131708475	0.138311212	0.150026603	0.160847627	0.16781043	0.172164092	0.176941586	0.184507183	0.192417901	0.197678285	0.20119656
South Khorasan	0.130716276	0.131779004	0.132967203	0.134197368	0.135497302	0.136896745	0.138622184	0.140851983	0.143385088	0.146022558	0.148501826	0.150647858	0.15180526	0.151103804	0.151243695	0.157552447	0.168220082	0.178102693	0.184731069	0.189322956	0.194514452	0.202188807	0.210279598	0.216151497	0.22060967
Tehran	0.305052544	0.309594601	0.314115862	0.318451997	0.322679668	0.326834754	0.331362127	0.336444227	0.341836259	0.347248738	0.35231078	0.356798913	0.359826599	0.36044378	0.362570181	0.37279244	0.388540084	0.403274603	0.413872333	0.42201607	0.430921912	0.442554207	0.454495715	0.463763766	0.471480347
West Azarbayejan	0.137745409	0.140011246	0.142455048	0.145001805	0.147662825	0.15047027	0.153694816	0.15741078	0.161441322	0.16557982	0.169569571	0.173223379	0.175667518	0.175997981	0.177237351	0.185173985	0.197614881	0.209243513	0.217194984	0.222846979	0.229042232	0.237986291	0.24719021	0.253869703	0.258974642
Yazd	0.154689132	0.15671483	0.158951897	0.161291852	0.163770936	0.166456102	0.169595223	0.173319717	0.177448944	0.181689957	0.185720022	0.189373695	0.191974206	0.19240113	0.19387825	0.202682905	0.217969693	0.232348215	0.242714876	0.250523107	0.258963696	0.270011935	0.281553924	0.290382177	0.297540765
Zanjan	0.155539325	0.157278415	0.159173475	0.161074654	0.163051704	0.165155721	0.167616621	0.170462547	0.173518325	0.176540763	0.179244767	0.181255663	0.181855302	0.179541037	0.178172462	0.185564068	0.19902182	0.211119951	0.218493319	0.223053202	0.228348085	0.237001028	0.245902897	0.251711412	0.255596498
Iraq	0.15870381	0.162480892	0.166522961	0.172806444	0.179011621	0.183867683	0.188602044	0.192971903	0.197494306	0.201968156	0.206797849	0.211927695	0.217019245	0.22135964	0.225997307	0.230863014	0.236316037	0.241470297	0.247260777	0.253074549	0.258912887	0.264898075	0.270641893	0.276956265	0.283611904
Jordan	0.115944182	0.121085435	0.126453274	0.132005408	0.13781945	0.143620733	0.150492845	0.157437922	0.164830102	0.172543233	0.180657964	0.189799876	0.199266588	0.20960912	0.221856687	0.23543094	0.250568241	0.266323979	0.281272544	0.295543333	0.308443193	0.320746193	0.333044239	0.345023809	0.35680545
Kuwait	0.319760648	0.327139953	0.334686447	0.340960274	0.346920349	0.352202892	0.357124869	0.357193401	0.350981995	0.337855489	0.323416688	0.312543526	0.309994469	0.313583617	0.313844374	0.311654803	0.311854021	0.319436247	0.339876098	0.372040224	0.403648304	0.422540425	0.433765942	0.440227271	0.447443904
Lebanon	0.272311277	0.284146158	0.293800129	0.30372259	0.313750398	0.323478641	0.332373698	0.340961294	0.348423002	0.356020768	0.363506415	0.370905415	0.37819536	0.385182743	0.3922797	0.39981633	0.407896505	0.415474156	0.423688546	0.431784548	0.440113528	0.448860946	0.460228581	0.472961104	0.48297272
Libya	0.152541634	0.156384219	0.160329916	0.164198584	0.168054009	0.172931222	0.17844878	0.183779323	0.189304144	0.194980194	0.201532287	0.208161229	0.215589025	0.223771975	0.232936329	0.241940967	0.251550221	0.260987855	0.271283051	0.281726766	0.291455624	0.30035485	0.309796057	0.319896476	0.331954797
Morocco	0.098941338	0.101938917	0.105028715	0.108420438	0.11202693	0.115701893	0.119485758	0.123291102	0.127400311	0.131647215	0.136016938	0.140630263	0.145846827	0.151269673	0.156844365	0.162103468	0.168102996	0.174401747	0.181016187	0.18802707	0.195314122	0.202648924	0.210267978	0.218078186	0.226275718
Oman	0.130316682	0.13307961	0.136008212	0.139036366	0.142394307	0.14600993	0.149632724	0.153454564	0.157695853	0.161949791	0.166232949	0.171291323	0.176231271	0.18128171	0.186205494	0.191730269	0.197921076	0.206685444	0.218488968	0.230126554	0.240494925	0.249553309	0.258023874	0.265122061	0.272661344
Palestine	0.142587436	0.146398148	0.150400151	0.154873712	0.159476553	0.16427554	0.169161574	0.174227711	0.179943852	0.186014405	0.192564973	0.198816109	0.204777632	0.212065708	0.219220787	0.226451321	0.234461335	0.242202146	0.249140435	0.256562701	0.264442932	0.273051856	0.28245602	0.290723756	0.300085327
Qatar	0.261957144	0.269160535	0.27672783	0.284515657	0.292664291	0.301641957	0.310631518	0.320011631	0.330194289	0.34084272	0.35128714	0.362999547	0.374915632	0.387528699	0.400534182	0.412489902	0.42526512	0.438424984	0.451854191	0.465433768	0.478843115	0.492254658	0.505394747	0.51805446	0.530179792
Saudi Arabia	0.205572639	0.209905926	0.214391016	0.219194619	0.22434357	0.229493503	0.234848374	0.240368451	0.246035859	0.252043675	0.25851818	0.265381938	0.272619954	0.28004373	0.287661187	0.295935738	0.303195846	0.310832505	0.318784458	0.326957758	0.336291182	0.346262939	0.35650237	0.367149757	0.378483207
Sudan	0.132435328	0.135837009	0.139381872	0.143019568	0.146721903	0.150465209	0.15440726	0.157918549	0.161383087	0.165108226	0.168618704	0.171918247	0.175348252	0.1783891	0.181049283	0.184051429	0.186916604	0.189609343	0.192469417	0.195540744	0.198602734	0.201957948	0.20529431	0.208486703	0.212342371
Syria	0.156562742	0.15944426	0.164852905	0.170311851	0.175993404	0.180557385	0.185894522	0.191379265	0.195954975	0.200714925	0.205475316	0.210410956	0.216413122	0.221865214	0.227844831	0.233728446	0.238978807	0.244674815	0.250533756	0.257381593	0.263961774	0.270934681	0.27921793	0.286635253	0.295518684
Tunisia	0.15253053	0.155953676	0.160512228	0.164984759	0.169448935	0.173538702	0.178280332	0.182768959	0.187967792	0.193036224	0.198869814	0.205218633	0.211595862	0.218714755	0.226018809	0.233868942	0.241808369	0.249800697	0.258221662	0.266687659	0.275726405	0.28526783	0.295833364	0.305846463	0.316096718
Türkiye	0.239594884	0.246350882	0.252623378	0.258831603	0.263862412	0.268989512	0.27384015	0.279186959	0.284633775	0.289845937	0.295059498	0.30005799	0.305325497	0.310793929	0.31598687	0.320577971	0.325892429	0.330762657	0.335333829	0.339462756	0.343103857	0.346133399	0.349009396	0.351608045	0.354327062
United Arab Emirates	0.195075326	0.200453129	0.205763354	0.211038446	0.216253494	0.221518929	0.22678481	0.232095944	0.237690431	0.243488581	0.24947214	0.256002625	0.262932552	0.270376593	0.278387789	0.287102821	0.296190364	0.305920396	0.316301998	0.327340199	0.33883625	0.350977314	0.363050096	0.375043817	0.387148718
Yemen	0.085084064	0.086183663	0.087375129	0.088656045	0.089976767	0.091299006	0.09265047	0.094031251	0.095432106	0.096837769	0.098376262	0.099939895	0.101515709	0.103122864	0.104647817	0.106456514	0.108146238	0.109926615	0.111785869	0.113791054	0.116489012	0.119777832	0.123181464	0.126934426	0.130697654
South Asia	0.182001764	0.184245538	0.186537671	0.189192771	0.191899987	0.194409096	0.197167707	0.199538413	0.202224469	0.204910556	0.207910648	0.210787804	0.213690235	0.216780791	0.220230214	0.223282209	0.226614244	0.230306292	0.234130924	0.238333734	0.242455216	0.246134893	0.249564267	0.252825442	0.255575054
South Asia	0.182001764	0.184245538	0.186537671	0.189192771	0.191899987	0.194409096	0.197167707	0.199538413	0.202224469	0.204910556	0.207910648	0.210787804	0.213690235	0.216780791	0.220230214	0.223282209	0.226614244	0.230306292	0.234130924	0.238333734	0.242455216	0.246134893	0.249564267	0.252825442	0.255575054
Bangladesh	0.103047763	0.103695803	0.104586369	0.10549928	0.106344679	0.107025892	0.108301718	0.109387531	0.110225025	0.110358244	0.108101071	0.101757325	0.091356291	0.078050255	0.06191319	0.05290196	0.053360883	0.053971729	0.075143126	0.094701727	0.111067672	0.12525482	0.137091088	0.14623109	0.153107653
Bhutan	0.05302094	0.054353671	0.055660525	0.057068781	0.058578803	0.060177984	0.061873189	0.063652048	0.065497855	0.067508756	0.069799133	0.072172634	0.074657752	0.077233102	0.080032366	0.082983422	0.086110434	0.089391958	0.092872056	0.096549732	0.100463118	0.104234708	0.107926511	0.111610116	0.115439398
India	0.189821934	0.192258752	0.194669826	0.197467231	0.200332887	0.203068618	0.205992401	0.208529376	0.211532784	0.214519365	0.217946466	0.22133314	0.224757059	0.22840267	0.232493748	0.236044022	0.239505523	0.243442147	0.247376983	0.251714197	0.25587181	0.259613226	0.263145092	0.266554095	0.26932174
Nepal	0.09920641	0.100391138	0.10169819	0.103342942	0.104899267	0.106401457	0.108110149	0.10972909	0.111632863	0.113626414	0.115710639	0.117729235	0.119726109	0.121591602	0.123605457	0.125335025	0.127365618	0.129022813	0.130380887	0.131786034	0.133229247	0.134372756	0.135581746	0.13670416	0.138271958
Pakistan	0.15340427	0.15480295	0.156256217	0.158302012	0.160405293	0.162384323	0.164543432	0.166819406	0.169163234	0.171740373	0.174492496	0.177613604	0.181109058	0.184952466	0.18925144	0.193952861	0.198919414	0.20374749	0.208645902	0.213494415	0.218239673	0.222208278	0.225543082	0.228546617	0.231045815
Azad Jammu & Kashmir	0.129182152	0.130923182	0.132686106	0.134918504	0.137322792	0.13966674	0.14216162	0.144807117	0.147676711	0.150739989	0.154051997	0.157763123	0.161915215	0.166490505	0.171555812	0.176946861	0.18296284	0.188933517	0.194990268	0.20105224	0.207001578	0.212228097	0.216863377	0.221145339	0.224987788
Balochistan	0.13949383	0.140357819	0.141253072	0.142608138	0.143996744	0.14526032	0.146666406	0.148087676	0.149549482	0.151130409	0.152843394	0.154913667	0.157303397	0.160073007	0.163180324	0.16662371	0.170413723	0.174112266	0.177872747	0.181643226	0.185369214	0.188513021	0.191161791	0.193541224	0.195437958
Gilgit-Baltistan	0.088230229	0.088903109	0.089571748	0.09089343	0.092202793	0.093400688	0.094787082	0.096267788	0.097890095	0.099746384	0.101769537	0.104386762	0.107240347	0.11024768	0.113963686	0.118341981	0.121875663	0.125298826	0.128905715	0.132530454	0.135987062	0.139616367	0.142761705	0.145743736	0.148507337
Islamabad Capital Territory	0.250760914	0.254248952	0.257615955	0.261630779	0.265672914	0.26951988	0.273561449	0.277673372	0.281882162	0.286318967	0.290997807	0.296206875	0.301702573	0.307834499	0.314474758	0.321588776	0.328687541	0.335625895	0.342611387	0.349569854	0.356540149	0.36238914	0.367419139	0.372188014	0.376631312
Khyber Pakhtunkhwa	0.128890546	0.129942146	0.131057328	0.132831017	0.134612917	0.136248654	0.13808525	0.139952462	0.141974448	0.144218901	0.146590533	0.149510339	0.152711369	0.156085297	0.159923928	0.163975542	0.168202985	0.172165995	0.176266827	0.180478154	0.184732317	0.188280627	0.191234231	0.193975011	0.196295474
Punjab	0.154929922	0.156332502	0.157800779	0.159867258	0.161997189	0.163993077	0.166160565	0.168464527	0.170791063	0.173347354	0.176083899	0.179158956	0.182645752	0.186574753	0.190866212	0.195578416	0.200660449	0.205636479	0.210670726	0.215634676	0.220535371	0.224599564	0.228048721	0.231146965	0.233791378
Sindh	0.169280806	0.170694543	0.172140447	0.174078477	0.176086017	0.177980166	0.180036732	0.182245424	0.184579843	0.18710503	0.189809829	0.192912575	0.196331809	0.20014557	0.20447129	0.209173092	0.214251427	0.219179036	0.224101826	0.229039781	0.233802697	0.237635925	0.240797584	0.243577686	0.245839982
Southeast Asia, east Asia, and Oceania	0.205729404	0.211494367	0.216773458	0.222167392	0.226170656	0.230761149	0.235527311	0.240455794	0.24509712	0.249831048	0.254552762	0.258425377	0.262772154	0.267724964	0.273695705	0.278711202	0.283703718	0.288161995	0.293348827	0.30200906	0.311766396	0.321053039	0.330709493	0.341226434	0.351997672
East Asia	0.193972364	0.201738111	0.208495292	0.215250906	0.219469654	0.22453378	0.229869896	0.235210212	0.240124559	0.244643318	0.248943106	0.251423165	0.254378358	0.25857644	0.264365689	0.269406105	0.274268978	0.27862899	0.283454724	0.293286534	0.304498383	0.315247067	0.326238488	0.338241342	0.350425352
China	0.184143547	0.193051286	0.200374731	0.207565879	0.211673034	0.216733027	0.222084536	0.227378202	0.232162359	0.236401029	0.240284561	0.2419707	0.244161425	0.247755593	0.253190073	0.258055616	0.262737111	0.266847937	0.271257674	0.280891057	0.292101462	0.302829123	0.313590402	0.325385168	0.33745371
North Korea	0.323069256	0.329208848	0.335472699	0.341849007	0.348163416	0.354551883	0.360884587	0.367134368	0.373247651	0.379188798	0.385152563	0.390971787	0.396622393	0.402091527	0.40763929	0.412944004	0.418252887	0.42337254	0.428352343	0.433220159	0.438087218	0.442826232	0.447394496	0.451880603	0.456208661
Taiwan (province of China)	0.295925327	0.297780719	0.30485235	0.31259191	0.319246496	0.325991814	0.333404291	0.340538394	0.34614441	0.351148628	0.35669276	0.36233286	0.368279385	0.374598022	0.380777859	0.386484889	0.394394589	0.404820147	0.417617608	0.431975305	0.446639743	0.461192996	0.474546156	0.486096207	0.494350309
Oceania	0.220001956	0.222463448	0.224725014	0.22700273	0.229349896	0.231798653	0.234653182	0.237534954	0.240532014	0.243682778	0.24706343	0.25077545	0.254654727	0.258949453	0.263937024	0.269335372	0.274615804	0.279992964	0.285432008	0.290936586	0.296652249	0.302247223	0.307841481	0.313553747	0.319170484
American Samoa	0.48489396	0.490904299	0.497417258	0.504192433	0.510752999	0.516605357	0.521417663	0.525028771	0.527208026	0.528356228	0.528327132	0.526791242	0.524101243	0.521475999	0.52009455	0.520685348	0.523394619	0.527697141	0.53354301	0.540669865	0.547834523	0.555243331	0.562851136	0.570614358	0.578142826
Cook Islands	0.337651274	0.34119051	0.343695711	0.345193673	0.345602067	0.344753732	0.342820466	0.339948358	0.336396624	0.332640796	0.329616655	0.327489067	0.326576767	0.327252182	0.329956787	0.334498891	0.340629382	0.348288448	0.357864325	0.369456936	0.382739913	0.395498179	0.40755532	0.420152747	0.432592446
Fiji	0.298635685	0.302840275	0.307037215	0.311383485	0.315901654	0.320570943	0.325427145	0.330555426	0.335947109	0.341787626	0.348296652	0.354981597	0.362058434	0.370215321	0.379112143	0.386740988	0.394308783	0.402125302	0.410102103	0.417581236	0.425115221	0.432462395	0.439661085	0.447007453	0.454416728
Guam	0.56570105	0.559390746	0.550162708	0.53899944	0.524149229	0.505586929	0.489687194	0.482181443	0.481051877	0.479881791	0.479418839	0.484606	0.498155113	0.517862854	0.537379639	0.55377475	0.568339853	0.57872945	0.582768118	0.583244065	0.581156491	0.579247417	0.582649163	0.594623163	0.612836249
Kiribati	0.276751223	0.279640557	0.282162744	0.284572468	0.287030688	0.289713911	0.292623658	0.295587061	0.298568759	0.301650042	0.305089181	0.308984352	0.313415924	0.318284946	0.323544495	0.328810944	0.334173704	0.339481111	0.34475786	0.349916981	0.354865454	0.359104527	0.363898412	0.370239317	0.380152342
Marshall Islands	0.237158263	0.241967415	0.246699586	0.251585967	0.25656693	0.261684462	0.26678536	0.27191911	0.27694196	0.28207037	0.287337274	0.292635575	0.297966001	0.303230601	0.308551385	0.313705576	0.318886565	0.323921963	0.328858023	0.333817974	0.338871446	0.343526383	0.347575852	0.35382879	0.360792089
Federated States of Micronesia	0.235773344	0.238742152	0.241737277	0.244938898	0.248317916	0.251973556	0.255784928	0.259821772	0.263968925	0.268384243	0.273224597	0.278347603	0.283779015	0.289407263	0.295384612	0.301056104	0.307885326	0.314707519	0.321540323	0.328452676	0.335360432	0.34251719	0.349675789	0.356984286	0.364493109
Nauru	0.401252441	0.404308722	0.406623898	0.408534091	0.410429019	0.412621487	0.415301718	0.418420054	0.42184566	0.425808534	0.430429538	0.435767123	0.441670149	0.447548285	0.453259838	0.45884393	0.464452911	0.469961123	0.475342878	0.480610073	0.485750308	0.4909482	0.496048738	0.501065258	0.506051031
Niue	0.339378972	0.343714131	0.347744768	0.351591861	0.355187497	0.358679451	0.362141559	0.365716224	0.369459927	0.373587394	0.378139666	0.382828169	0.387893665	0.393519111	0.400032039	0.406957831	0.414385128	0.422082573	0.430075524	0.438557235	0.447415271	0.456445776	0.465336167	0.47382858	0.481832885
Northern Mariana Islands	0.446763718	0.453066068	0.459672034	0.466449687	0.473597652	0.481086556	0.488858874	0.496741115	0.504858345	0.513077139	0.521435328	0.529859509	0.538381077	0.546974479	0.555629659	0.564350692	0.572505942	0.580382229	0.587967101	0.595436336	0.60295852	0.609778733	0.616206398	0.622426752	0.628433435
Palau	0.421918527	0.42845101	0.435174128	0.442249416	0.449534443	0.45692617	0.464302991	0.471722949	0.479310465	0.487080122	0.494903378	0.502388825	0.509785501	0.517395025	0.525586737	0.533792456	0.541290469	0.548585488	0.555840196	0.563339858	0.570842783	0.577215269	0.583070317	0.588503536	0.593686652
Papua New Guinea	0.138627688	0.140389623	0.142236276	0.144204604	0.146304457	0.148550905	0.150842339	0.153320897	0.155904104	0.158633842	0.161534054	0.164776855	0.168263305	0.171873666	0.17583642	0.180196541	0.184923624	0.189557373	0.1943644	0.199583229	0.205356977	0.211210098	0.217002419	0.222783212	0.228426503
Samoa	0.284366516	0.287950355	0.291429947	0.295016462	0.298707836	0.302518302	0.306444005	0.310486906	0.314638633	0.318994604	0.323611746	0.328351877	0.333106237	0.337882924	0.342978901	0.348141125	0.353430644	0.358751577	0.364188517	0.369711568	0.375470113	0.382125067	0.388617031	0.395415189	0.401008386
Solomon Islands	0.168911699	0.170671283	0.172431623	0.174317499	0.176316833	0.178465685	0.180704237	0.183023593	0.185340937	0.187742329	0.190299634	0.193042149	0.195810579	0.198617841	0.201551995	0.204481705	0.207614797	0.210777282	0.214097438	0.217456012	0.220832909	0.224202631	0.227316151	0.230685651	0.235061307
Tokelau	0.295961347	0.299993422	0.304036252	0.308322188	0.312801929	0.317525559	0.322472253	0.327730094	0.333060124	0.33847212	0.344085452	0.349660714	0.355095112	0.360506453	0.366035955	0.371496872	0.377066697	0.382737384	0.388671083	0.3948854	0.40152042	0.408898856	0.416191874	0.422821743	0.429040019
Tonga	0.266900015	0.271533007	0.276006424	0.280749307	0.285751673	0.290908296	0.296104223	0.301240526	0.30618984	0.311103799	0.316233533	0.321198692	0.32602889	0.330557094	0.335071388	0.339309292	0.343312347	0.347034834	0.350686086	0.354374742	0.358228073	0.361823813	0.365412153	0.368899429	0.372463985

Tuvalu	0.264210883	0.26706284	0.269840981	0.272716209	0.275693982	0.278728017	0.281809676	0.284896025	0.287943762	0.291095188	0.294434881	0.297740113	0.301102333	0.304445205	0.307926721	0.311367903	0.315004864	0.31861685	0.322347047	0.326149597	0.330172743	0.333904546	0.337427884	0.340800327	0.344074842
Vanuatu	0.196423511	0.198838081	0.201204137	0.203610421	0.206114892	0.20871589	0.211462452	0.214287101	0.217157632	0.220168119	0.223472605	0.226950313	0.230550332	0.234303674	0.238370823	0.242471936	0.246680827	0.250782106	0.254889467	0.259140891	0.263530106	0.267826267	0.271952829	0.277035138	0.284212867
Southeast Asia	0.224047997	0.227247285	0.230335523	0.233648191	0.237178955	0.240779603	0.244335415	0.248210552	0.251961384	0.256179095	0.260630413	0.265377225	0.270533709	0.275843194	0.281177776	0.286217826	0.291821386	0.297258359	0.302969022	0.308887313	0.315238047	0.32147148	0.327859474	0.334584811	0.341470846
Cambodia	0.188150847	0.190000127	0.192336351	0.194286669	0.197265447	0.199597942	0.202870065	0.206318862	0.209833887	0.213867653	0.218085283	0.221826747	0.225877673	0.230334277	0.233979868	0.237601584	0.241067117	0.244565954	0.248004328	0.251231811	0.253501367	0.255353774	0.25667296	0.258106822	0.258662188
Indonesia	0.197775374	0.200947244	0.20377711	0.206753205	0.209948159	0.213076208	0.216146768	0.219553953	0.22254183	0.225827264	0.229295266	0.233061262	0.236781744	0.240265885	0.244060083	0.24801791	0.252163827	0.25660637	0.26190722	0.267968514	0.274928401	0.282402984	0.2902632	0.298487541	0.307195224
Aceh	0.221005106	0.224023336	0.226717067	0.229528396	0.232573197	0.235604987	0.238621758	0.241932759	0.244908183	0.248161974	0.251521627	0.255138278	0.258666828	0.262106182	0.265859664	0.269717214	0.273743369	0.277975206	0.282838828	0.288407317	0.294744988	0.301696662	0.308970467	0.316418345	0.324433866
Bali	0.176582688	0.179048578	0.181256114	0.183672558	0.18635554	0.189028759	0.19174393	0.194795499	0.197558657	0.200653508	0.203969326	0.207717488	0.211552238	0.215327373	0.219411661	0.223804507	0.22859911	0.233871528	0.240007886	0.246931885	0.254741629	0.263096399	0.272006678	0.281400344	0.291366126
Bangka-Belitung Islands	0.230148138	0.233510332	0.236517453	0.239619028	0.242917105	0.246066771	0.249140831	0.252430415	0.255290516	0.2583784	0.261589083	0.265028484	0.268324952	0.271263546	0.274382979	0.277507444	0.280649827	0.283893276	0.287910201	0.292711953	0.298404922	0.304647163	0.311258581	0.318154819	0.325340533
Banten	0.222974533	0.226412603	0.229436341	0.232514115	0.235744486	0.238768518	0.241629616	0.244783089	0.24732732	0.250123339	0.253048563	0.256311213	0.259609271	0.262560103	0.265704988	0.268911512	0.272171059	0.275587074	0.279771489	0.284526175	0.290010843	0.295957207	0.302262392	0.308991509	0.316195247
Bengkulu	0.160869896	0.163498775	0.165744366	0.16808342	0.170572628	0.172918072	0.175136737	0.177706644	0.179756264	0.182073843	0.184515183	0.187232578	0.18996605	0.192479109	0.195323512	0.198360205	0.201829672	0.205757993	0.210653566	0.216397714	0.223177971	0.230599826	0.2385846	0.247020151	0.25603762
Gorontalo	0.17125938	0.175746959	0.179669335	0.183612386	0.18780138	0.191687252	0.195282109	0.199305697	0.20247176	0.206009236	0.209783109	0.213837732	0.217898574	0.221357046	0.224964801	0.228428537	0.231653656	0.234761794	0.238857188	0.243729336	0.249690728	0.256185509	0.263481984	0.271701473	0.280361937
Jakarta	0.305996381	0.310895149	0.315467438	0.320282684	0.325332132	0.330324295	0.335255907	0.340474323	0.345355487	0.350580145	0.355980627	0.361708962	0.367282811	0.372574657	0.378122725	0.383689631	0.389263603	0.395116938	0.401712444	0.409022974	0.416995671	0.425184477	0.433457635	0.441708364	0.450454294
Jambi	0.175120683	0.178100376	0.180687344	0.183410333	0.186353942	0.189132464	0.191785181	0.194850174	0.197435131	0.200235526	0.203171977	0.206432456	0.209761694	0.212763253	0.216019184	0.219342992	0.222640176	0.226019205	0.230275752	0.235286963	0.24111879	0.247311703	0.253813282	0.26079474	0.268308705
West Java	0.197230241	0.200179389	0.202807618	0.205607669	0.208662537	0.211632786	0.214480546	0.217674364	0.220442703	0.223503795	0.226729875	0.230288933	0.233839632	0.237160332	0.240788933	0.244574504	0.24856443	0.252876155	0.258030703	0.263976532	0.270791596	0.278082985	0.285591044	0.293216927	0.301289559
Central Java	0.178760696	0.181756805	0.18436568	0.187088644	0.190010932	0.19285606	0.195636959	0.198790358	0.201436082	0.204328157	0.20742337	0.210760863	0.214018464	0.216976897	0.22019343	0.223553797	0.227183432	0.231114988	0.235976739	0.24159941	0.248120887	0.255194217	0.262707503	0.270733956	0.279256645
East Java	0.186144194	0.189181138	0.191863489	0.194677178	0.197729541	0.200755402	0.203711109	0.20701536	0.209909781	0.213129609	0.216562482	0.220203173	0.223747872	0.227013616	0.230584109	0.234325155	0.238168411	0.242233421	0.247130093	0.252734542	0.259326616	0.266451542	0.274104987	0.282305985	0.290977378
West Kalimantan	0.149746973	0.151895977	0.153725401	0.155701321	0.157853063	0.159945741	0.162028891	0.164406338	0.166459226	0.168807947	0.171285615	0.174122995	0.176984743	0.179658122	0.182646209	0.185769321	0.189153203	0.192891583	0.19739664	0.202652749	0.208686967	0.215297645	0.222251224	0.229479146	0.237183556
South Kalimantan	0.18157697	0.184374053	0.186838797	0.189467505	0.192293786	0.195049984	0.197704024	0.200655753	0.203222666	0.206095176	0.209013544	0.212173239	0.215209875	0.217987641	0.221098276	0.224382258	0.227836742	0.231740365	0.236658134	0.242453707	0.249191042	0.256668414	0.264706922	0.273286857	0.282549114
Central Kalimantan	0.214929455	0.218407207	0.221544713	0.224846688	0.228349981	0.231723876	0.234976301	0.238585277	0.241727989	0.245112228	0.248591505	0.252244442	0.255755095	0.258900521	0.262297136	0.265810233	0.269419027	0.273154418	0.277722557	0.28304214	0.28919397	0.295787147	0.30272711	0.310003744	0.317911611
East Kalimantan	0.245344925	0.2486046	0.251653315	0.255017118	0.258632459	0.262316311	0.266214021	0.270413103	0.274533203	0.278902874	0.283304043	0.288062454	0.292761722	0.297383193	0.302301851	0.307310408	0.312595784	0.318287245	0.324611955	0.331740878	0.339856033	0.348918762	0.358564658	0.368564359	0.379224204
North Kalimantan	0.324212951	0.328226064	0.331936323	0.335830582	0.339822483	0.343752875	0.34766911	0.351719142	0.355318055	0.359091155	0.362960231	0.367154685	0.371295626	0.375184602	0.379314353	0.383519934	0.387956284	0.392513944	0.397583537	0.403141377	0.409309088	0.415860952	0.422723169	0.429663012	0.436877705
Riau Islands	0.311419591	0.315831931	0.319875984	0.324077702	0.328474541	0.332758655	0.336999913	0.341469614	0.345482844	0.349737948	0.354070717	0.358701682	0.36319243	0.367336352	0.371666511	0.376058771	0.380382993	0.384669073	0.389465188	0.39476587	0.400815467	0.407420174	0.414546684	0.422136459	0.430224889
Lampung	0.158054958	0.160822381	0.16313448	0.165566683	0.16822869	0.170737371	0.173078641	0.175804429	0.177987915	0.180531815	0.183249741	0.186183837	0.189062268	0.191520048	0.19421096	0.19690156	0.199603724	0.202450748	0.206244604	0.210891895	0.216505654	0.222849589	0.229829592	0.237465205	0.245672397
Maluku	0.238091823	0.242529077	0.246593908	0.25078766	0.255169243	0.259404207	0.263576926	0.268100951	0.272100361	0.276378548	0.280720976	0.285428385	0.290094091	0.294341266	0.298797027	0.303291664	0.307907775	0.312693024	0.318218163	0.32431548	0.331050788	0.338161206	0.345385396	0.352755772	0.360412316
North Maluku	0.159962714	0.16430179	0.167869256	0.171498118	0.175448017	0.179033869	0.182264293	0.186108086	0.188868198	0.192020546	0.19550364	0.199282537	0.202832008	0.205455403	0.208188858	0.210708209	0.212930804	0.214982167	0.218283072	0.222367938	0.227601003	0.233351616	0.239920765	0.247434597	0.255345334
West Nusa Tenggara	0.115878841	0.118304037	0.120268058	0.122326176	0.124612065	0.126731057	0.12872552	0.131113078	0.13292532	0.135076959	0.137446714	0.140111065	0.142716669	0.144928734	0.147397512	0.149918239	0.15244413	0.155138919	0.158737302	0.163106726	0.168423833	0.174306748	0.180754085	0.187839846	0.195489186
East Nusa Tenggara	0.1422902	0.146286268	0.149639545	0.153076945	0.156816067	0.16027839	0.16346525	0.167233813	0.170045302	0.173194686	0.176655923	0.180418745	0.183996236	0.186786195	0.189732357	0.192566054	0.195250243	0.197923873	0.20187953	0.206747729	0.212844025	0.219646957	0.227454521	0.236395884	0.245903001
Papua	0.252098871	0.256083699	0.259790742	0.2636191	0.267552736	0.27132695	0.274929985	0.278799133	0.282208122	0.285776126	0.289282898	0.292970112	0.296623213	0.299968394	0.303435368	0.306783973	0.310122866	0.313649983	0.317833126	0.322592396	0.327907812	0.333595189	0.33949516	0.345654012	0.352263253
West Papua	0.249346603	0.252797985	0.255853132	0.258975031	0.262212117	0.265284685	0.268217761	0.271413135	0.274066086	0.276983722	0.279984258	0.283217252	0.286302055	0.289099091	0.2921221	0.295190252	0.298251527	0.301386462	0.305340526	0.310033414	0.315589616	0.321614409	0.327898888	0.334434207	0.341372788
Riau	0.238538586	0.241478154	0.244204011	0.247137884	0.250290772	0.253446897	0.256664644	0.26016044	0.263355858	0.266725153	0.270211585	0.274046163	0.278028797	0.282213634	0.286893901	0.291893907	0.297363351	0.30341102	0.310172786	0.317637076	0.325807439	0.334494162	0.343346349	0.352075791	0.361163033
West Sulawesi	0.181466232	0.184921219	0.187913576	0.190991352	0.194276698	0.197331519	0.200220177	0.2034682	0.206100566	0.209003767	0.212049766	0.215317865	0.218444088	0.221031424	0.223751906	0.226395304	0.228941733	0.231376019	0.23459697	0.238403874	0.243140457	0.248535065	0.254509097	0.261078478	0.268113572
South Sulawesi	0.157348392	0.159981088	0.162291177	0.164776274	0.167501587	0.170174621	0.172849716	0.175927501	0.178620774	0.181647296	0.184890409	0.188494373	0.192046009	0.195328376	0.198966413	0.202805656	0.206790568	0.211091669	0.216283055	0.222292408	0.229214099	0.236733958	0.244757678	0.25329872	0.262317563
Central Sulawesi	0.165641135	0.169008852	0.171907215	0.174941515	0.178260859	0.18142476	0.18441419	0.187795969	0.190590399	0.19369069	0.197022798	0.200726967	0.204374168	0.207531946	0.210915296	0.214340711	0.21796138	0.221861391	0.226804397	0.232589524	0.239388727	0.246850129	0.254937763	0.263709593	0.272970519
Southeast Sulawesi	0.132526106	0.134828473	0.136747425	0.138794109	0.141030598	0.143182535	0.145331516	0.147852381	0.14996675	0.152398435	0.155015675	0.158038793	0.161096408	0.163924884	0.167114532	0.170538125	0.174245104	0.178390499	0.183418683	0.18914769	0.195738436	0.202958125	0.210803333	0.219274145	0.228509026
North Sulawesi	0.20826993	0.212361179	0.216068135	0.219937905	0.224022402	0.227960171	0.231782072	0.236023543	0.239723189	0.243733148	0.247990407	0.252708659	0.257390751	0.261708731	0.266339286	0.271170012	0.276308347	0.28205246	0.289053672	0.296894636	0.305620877	0.31485308	0.324355565	0.334227969	0.344409374
West Sumatra	0.206245268	0.209850315	0.213063365	0.21638941	0.219900112	0.223226026	0.2263941	0.229913746	0.232871669	0.23616154	0.239593055	0.243310397	0.246982576	0.25041444	0.254161412	0.257990552	0.261991164	0.266060798	0.271006369	0.276692984	0.283327497	0.290555398	0.298229935	0.306381829	0.315062306
South Sumatra	0.208422091	0.211537731	0.21431567	0.217242834	0.220337258	0.223289671	0.22614412	0.229324205	0.232140674	0.23530839	0.238597213	0.242165554	0.245679833	0.2490307	0.252707468	0.256527596	0.260569392	0.264904491	0.270038929	0.275836947	0.282387967	0.289322221	0.296420175	0.303627909	0.311339296
North Sumatra	0.211737126	0.215141387	0.218185195	0.221375548	0.224777059	0.228043549	0.231214807	0.234732344	0.23778366	0.24113952	0.24466686	0.248615399	0.25259683	0.256363269	0.260539845	0.264955223	0.269635837	0.274565078	0.280336813	0.286857069	0.294219181	0.302094511	0.310238673	0.318612089	0.327489439
Yogyakarta	0.174459668	0.177752247	0.180683539	0.183746096	0.187089562	0.19038213	0.193689742	0.197469368	0.200763566	0.204448788	0.208384484	0.212784758	0.217333929	0.221687679	0.226451375	0.2314843	0.237132817	0.243010119	0.249903008	0.257591499	0.266251713	0.275449451	0.285174636	0.295559563	0.306434309
Laos	0.136230788	0.137243895	0.13828806	0.139348943	0.140479002	0.141706143	0.143067057	0.144539209	0.146130291	0.147808256	0.149596904	0.151638672	0.153819152	0.156104778	0.158402023	0.160718167	0.163379669	0.166105988	0.168919725	0.171821919	0.17466846	0.177897692	0.181280787	0.185184046	0.189086719
Malaysia	0.214102682	0.215775912	0.21818809	0.220644982	0.223554669	0.226625261	0.23011193	0.234975036	0.241171424	0.248655932	0.256965083	0.267839595	0.281595789	0.295868254	0.308301891	0.318813071	0.329659661	0.340785849	0.351626351	0.36111786	0.369386312	0.378867664	0.389228539	0.399695466	0.40967634
Maldives	0.196661027	0.199264454	0.201715159	0.204250245	0.20692188	0.209609964	0.212319017	0.214970943	0.217494802	0.220072274	0.222887292	0.225488361	0.227882331	0.22959184	0.230715777	0.231683054	0.232464374	0.232847847	0.233104108	0.233407229	0.234098037	0.235034095	0.236164234	0.23740706	0.238871519
Mauritius	0.292283123	0.299651549	0.304094441	0.306833962	0.307643713	0.305276848	0.306338595	0.312147178	0.31968163	0.323480962	0.323194373	0.322906493	0.325209288	0.333274977	0.344687721	0.356418209	0.370451695	0.384414848	0.39653424	0.408925049	0.419722196	0.429666705	0.438429985	0.443774574	0.449011606
Myanmar	0.137807234	0.143116661	0.145907156	0.148246744	0.149096422	0.151696932	0.154744578	0.158073317	0.160675738	0.164771526	0.168857814	0.172608012	0.176714486	0.181151693	0.184823484	0.188345557	0.191459416	0.193949995	0.196849149	0.199800404	0.203087132	0.206364192	0.209459197	0.212499016	0.216023098
Philippines	0.336157457	0.340666317	0.344903632	0.349763549	0.354535322	0.359295945	0.363994637	0.368647307	0.37305548	0.377699718	0.382182369	0.386538716	0.390915719	0.395367072	0.39950021	0.403638702	0.407734501	0.411871454	0.416168007	0.420429547	0.424659192	0.428961118	0.433423765	0.438418133	0.44324379
Abra	0.314755525	0.318431485	0.321850245	0.325777143	0.329680605	0.333605727	0.337518358	0.34141542	0.345098591	0.349027439	0.352988216	0.35689676	0.36090009	0.364905821	0.368584726	0.372276066	0.375984304	0.379684091	0.383395396	0.38694105	0.390277312	0.393775751	0.397429452	0.401675403	0.405987687
Agusan Del Norte	0.310043171	0.314963914	0.319572311	0.324783001	0.329891284	0.334970837	0.340010226	0.345006191	0.349728554	0.354735076	0.359593073	0.364309072	0.369007525	0.37376323	0.378069246	0.382308718	0.386339302	0.390289869	0.394227236	0.398001691	0.401570114	0.405039618	0.408558655	0.412721154	0.416874498
Agusan Del Sur	0.237234497	0.240431709	0.243368248	0.246911731	0.250418765	0.253913838	0.257373686	0.260808431	0.264016739	0.267506074	0.27093081	0.274194803	0.277534398	0.280974096	0.284062934	0.287156623	0.290106658	0.293042807	0.296050955	0.298874115	0.301585091	0.304422989	0.307510024	0.311273223	0.315108785
Aklan	0.339669684	0.343881237	0.347815649	0.352474359	0.357059072	0.361733694	0.366386196	0.37099585	0.375357517	0.380037648	0.384623144	0.389066274	0.39351226	0.398032829	0.402213358	0.40635801	0.41048885	0.414660353	0.418961412	0.423221937	0.427392565	0.431610511	0.435858186	0.440582947	0.44499657
Albay	0.328175285	0.332526746	0.33659853	0.341342845	0.346001325	0.350699833	0.355357857	0.359973666	0.364279288	0.368854569	0.373316516	0.377648534	0.381996231	0.386368661	0.390354585	0.39429296	0.398143132	0.402024114	0.40599326	0.409835611	0.413511447	0.417155419	0.420912533	0.425267982	0.429483904
Antique	0.247658603	0.250953036	0.253984746	0.257628873	0.26123212	0.2648266	0.268432354	0.271984043	0.27522342	0.278733285	0.282187888	0.285418702	0.288636028	0.291871053	0.294640601	0.29732989	0.299829044	0.302227799	0.304563952	0.306621313	0.308403507	0.310157326	0.312051027	0.314620406	0.317270024
Apayao	0.251895569	0.255345261	0.258555451	0.262385794	0.266188624	0.270039518	0.273853668	0.277608401	0.281136838	0.284910499	0.288596748	0.292172268	0.295876049	0.299689214	0.303257481	0.30686366	0.310482361	0.314196042	0.317995449	0.321773671	0.325572949	0.32959765	0.333921022	0.339043833	0.34425266
Aurora	0.281842873	0.285446876	0.288767766	0.292750486	0.296700918	0.300656728	0.304624863	0.308504569	0.312142356	0.316071486	0.319906895	0.323582211	0.32732664	0.331160326	0.334663016	0.338195174	0.341677931	0.345251639	0.348939646	0.35256835	0.35609117	0.359739685	0.363526909	0.367975494	0.37240444
Basilan	0.198432619	0.200502667	0.202330932	0.20470589	0.207056038	0.209432352	0.211802797	0.214138946	0.216250678	0.21859039	0.220857162	0.222992992	0.225253915	0.227605705	0.229669287	0.23173589	0.233724169	0.23579476	0.237933845	0.239993733	0.24202393	0.244287265	0.246909576	0.250334129	0.2540134
Bataan	0.3588602	0.36288674	0.366601468	0.370964017	0.375241264	0.379546479	0.383810924	0.388057205	0.392088236	0.396420688	0.400698299	0.404927303	0.409276483	0.413564433	0.417523079	0.421529895	0.425579069	0.429707072	0.433856273	0.437844907	0.44163568	0.445418174	0.449235233	0.453593261	0.457739232
Batanes	0.347632494	0.352692606	0.357328984	0.362842805	0.368206837	0.373565352	0.378872052	0.38417241	0.389194809	0.394605493	0.399779724	0.404825723	0.409806996	0.414800454	0.419276267	0.423696253	0.428053808	0.432423637	0.436858003	0.441051175	0.444945109	0.448789233	0.452579907	0.456995625	0.461175506
Batangas	0.368466351	0.373026704	0.377326748	0.382209203	0.387050665	0.391911189	0.396780807	0.401680452	0.406364904	0.411347073	0.416270917	0.421195873	0.426203151	0.431227128	0.435948483	0.440704215	0.445400598	0.450155316	0.455000089	0.459730782	0.464282847	0.468735574	0.473252044	0.478255983	0.48305325
Benguet	0.407110329	0.412222602	0.416983031	0.422360027	0.427597849	0.432782484	0.437862078	0.442886919	0.447744922	0.452923312	0.458006584	0.462963945	0.467891069	0.472827248	0.477428578	0.482028989	0.486589788	0.491131066	0.495711161	0.500128453	0.504403815	0.508663754	0.512929566	0.517641284	0.522174344
Biliran	0.312561376	0.316381921	0.31993918	0.324020291	0.328073501	0.332134888	0.336175542	0.340180646	0.343988295	0.348032655	0.351985746	0.355809621	0.359688321	0.363609775	0.367248263	0.370852083	0.374410048	0.377990854	0.381651087	0.385230599	0.388676843	0.392238325	0.396031556	0.400478144	0.404963618
Bohol	0.277496961	0.281367633	0.28496236	0.289198366	0.293382616	0.297608537	0.301835058	0.306031461	0.309972193	0.314176726	0.318254193	0.322197547	0.326157396	0.330146821	0.333774863	0.337324174	0.340770362	0.344228378	0.347725155	0.351137623	0.354487553	0.357912395	0.361487951	0.36571551	0.3698938
Bukidnon	0.248661767	0.251928964	0.254937163	0.25856232	0.262131394	0.265709207	0.269269136	0.272773174	0.276023942	0.279548496	0.283028794	0.286343583	0.289749953	0.293212842	0.296307681	0.299358348	0.302308453	0.305207323	0.308112533	0.31082431	0.313360137	0.315966432	0.318781943	0.322219166	0.325696303
Bulacan	0.389557844	0.394396876	0.398947527	0.404057232	0.409079415	0.414113957	0.419120719	0.424098734	0.428829696	0.433805692	0.438680405	0.443487683	0.448276484	0.453054181	0.457522448	0.462037076	0.466442806	0.470877594	0.475358912	0.479734923	0.48397853	0.488121083	0.492310564	0.496880601	0.501170022
Cagayan	0.312646071	0.316542422	0.320150958	0.324407714	0.328562771	0.332723163	0.336850922	0.340956263	0.344829237	0.348942977	0.352965655	0.356861032	0.360792568	0.364824638	0.368536833	0.372250903	0.375839525	0.37947211	0.383238216	0.386875747	0.390402637	0.393998972	0.397691142	0.401943748	0.406077505
Camarines Norte	0.317369043	0.321575027	0.325455271	0.330046254	0.334504857	0.338955913	0.343358798	0.347726714	0.351815621	0.356152593	0.36027751	0.364258542	0.368209846	0.372162862	0.375692583	0.379112875	0.382316998	0.385477241	0.388663438	0.391605172	0.394335423	0.396970427	0.399663254	0.402881636	0.405831427
Camarines Sur	0.315785232	0.320149991	0.324239382	0.3289776	0.33362018	0.338279572	0.34288022	0.347448068	0.351755276	0.356282005	0.360657419	0.364844293	0.36902003	0.37324002	0.377055097	0.380760632	0.384287642	0.387846168	0.39147473	0.394959942	0.398293501	0.401655825	0.405138289	0.409230176	0.413274822
Camiguin	0.328193804	0.333018076	0.337471519	0.342726461	0.347822185	0.352918754	0.357919709	0.362883971	0.367521067	0.372481339	0.377190992	0.381729187	0.386221031	0.390728404	0.394845048	0.39899258	0.402989779	0.406927043	0.410881547	0.414646946	0.41821685	0.421707917	0.425258052	0.429510089	0.43369754
Capiz	0.262324021	0.265977441	0.269358125	0.273370005	0.277298716	0.28120761	0.285100695	0.288968733	0.292588101	0.296500315	0.30027121	0.303873423	0.307509808	0.311244462	0.314637324	0.31804946	0.321272832	0.324520052	0.327840592	0.33105252	0.334163685	0.337338449	0.340685708	0.344695301	0.34872269
Catanduanes	0.312145292	0.316076536	0.319757373	0.324047878	0.328281806	0.33255241	0.336761255	0.340882181	0.344693662	0.348719713	0.352657999	0.356399916	0.36013153	0.363804189	0.367079725	0.37028931	0.37328544	0.376262591	0.3792311	0.382044655	0.384692653	0.387360775	0.390178082	0.393621841	0.396974423
Cavite	0.428764082	0.434242749	0.439407015	0.445177783	0.450794908	0.456385119	0.461925499	0.467460408	0.472761828	0.478300568	0.483708673	0.489027668	0.494324565	0.499589313	0.50449097	0.509336084	0.513989259	0.518677169	0.52346005	0.528095133	0.53256642	0.53689677	0.541179328	0.545937323	0.550513246
Cebu	0.362861695	0.36784282	0.372544066	0.377889213	0.383098673	0.388324353	0.393482091	0.398561531	0.403346807	0.408387209	0.413300159	0.418014885	0.422691014	0.427430338	0.431854823	0.43626138	0.440468863	0.444660511	0.448950796	0.453140599	0.457240434	0.461279992	0.465383381	0.470033787	0.474570383

Cotabato (North Cotabato)	0.261584336	0.265352225	0.268835065	0.273040069	0.277187916	0.281338546	0.285489227	0.289630799	0.293523009	0.29773994	0.301860558	0.305850627	0.309888873	0.313968087	0.317657305	0.321301459	0.324854426	0.328375502	0.3319158	0.335257306	0.338446919	0.341694732	0.345107015	0.349179002	0.353207313
Davao de Oro	0.237672208	0.241143832	0.244300751	0.248162946	0.251944713	0.255677948	0.259368811	0.263009119	0.266381165	0.270088483	0.273612186	0.276958624	0.280352322	0.283821169	0.286938893	0.290067499	0.293090749	0.296082235	0.29913035	0.302058021	0.304888584	0.307710966	0.310738073	0.314545339	0.318430241
Davao Del Norte	0.296878457	0.300691897	0.304225591	0.308355795	0.312437727	0.316531511	0.320608891	0.324665818	0.328545024	0.332719227	0.33680588	0.340786505	0.344865611	0.349071581	0.352964379	0.356876527	0.360682207	0.364529132	0.368476903	0.372359057	0.37616383	0.38001977	0.384066552	0.38874351	0.393437972
Davao Del Sur	0.327152754	0.331909161	0.336457516	0.341565321	0.346613939	0.351654897	0.356682361	0.361664382	0.366427849	0.371501266	0.376435407	0.38123466	0.386088359	0.391005179	0.395541719	0.400061483	0.404395639	0.408695951	0.413021696	0.417132227	0.420961328	0.424613548	0.42835856	0.432728568	0.437061892
Davao Occidental	0.265485314	0.26789766	0.27009734	0.272542967	0.274917876	0.27729413	0.279657112	0.281930652	0.283981203	0.286133443	0.288248683	0.290294476	0.292437225	0.294589152	0.296530704	0.298489986	0.300375532	0.30224654	0.304137289	0.305857006	0.307484134	0.30922247	0.311170554	0.313600088	0.316179609
Davao Oriental	0.232270173	0.235593455	0.238630215	0.242378322	0.246040518	0.249689238	0.253293898	0.256848954	0.260163932	0.263734616	0.267184492	0.270516532	0.273941917	0.277449746	0.280556599	0.283619337	0.286618652	0.289618356	0.292636566	0.295516286	0.298283641	0.301132904	0.304209623	0.308048461	0.31188785
Dinagat Islands	0.282534428	0.285622188	0.288514389	0.291875642	0.295246518	0.298646865	0.302135187	0.305611053	0.308906924	0.312409413	0.315901397	0.319305461	0.322823007	0.326420234	0.329743734	0.333078545	0.336395079	0.339780713	0.34324332	0.346580041	0.3498291	0.353215411	0.356857804	0.36111082	0.3655003
Eastern Samar	0.230556646	0.233785989	0.236693084	0.24026574	0.243766954	0.247259179	0.250682967	0.254017389	0.257032816	0.260333067	0.26348414	0.266414386	0.269391528	0.272402955	0.274980909	0.277424387	0.279637523	0.281816162	0.284029853	0.286035167	0.287818566	0.289648022	0.29162234	0.294306334	0.297066759
Guimaras	0.285950539	0.288744742	0.291233536	0.294384446	0.297478776	0.300607326	0.303748205	0.306879589	0.309829027	0.313081782	0.316269454	0.319436498	0.322781091	0.326256633	0.329470304	0.332709321	0.336026223	0.339366669	0.342788089	0.346123941	0.349385398	0.352831297	0.356548524	0.361004525	0.365470778
Ifugao	0.280005043	0.282686285	0.285130964	0.288096879	0.291010741	0.293938928	0.296852686	0.299737285	0.30245487	0.305412883	0.308349421	0.311224717	0.314232833	0.317308719	0.320144858	0.323022452	0.325959677	0.328932006	0.331974219	0.334978556	0.33796365	0.341163085	0.344662302	0.348796156	0.353056866
Ilocos Norte	0.373461503	0.378058071	0.382410119	0.387390308	0.392292492	0.397279143	0.402242842	0.407206588	0.412009418	0.417115821	0.422132731	0.427083997	0.432047642	0.437122095	0.441850931	0.446554118	0.451185855	0.455800572	0.460498987	0.46510185	0.469583449	0.47406271	0.478631906	0.483685793	0.488553338
Ilocos Sur	0.356200325	0.36010521	0.363763849	0.367984578	0.372139886	0.376358372	0.380582832	0.384774627	0.388705902	0.39284281	0.396930007	0.400885359	0.404861093	0.408947625	0.412750856	0.416524769	0.42033334	0.424247385	0.428334334	0.432406587	0.436467599	0.440688745	0.445082395	0.449979727	0.454645181
Iloilo	0.34069459	0.345204671	0.349424952	0.354250085	0.359020803	0.36380823	0.368624982	0.373457982	0.378075645	0.383029119	0.387877767	0.392677599	0.397565838	0.40250191	0.407113549	0.411694441	0.416156511	0.420587885	0.425031178	0.429299173	0.433382009	0.437405751	0.441453037	0.446059461	0.450626467
Isabela	0.322446266	0.326465057	0.330205885	0.334549137	0.338830953	0.343088316	0.347307106	0.351488585	0.355451368	0.359654263	0.363740267	0.367715137	0.371727259	0.375775863	0.379506316	0.383264477	0.386891991	0.390599881	0.394425106	0.398161336	0.401815269	0.405527831	0.409406433	0.413820395	0.418065889
Kalinga	0.243899538	0.246708669	0.249205219	0.252385504	0.255473576	0.258537129	0.261526037	0.264470702	0.267169214	0.270131767	0.272958436	0.275609446	0.278333313	0.281150304	0.28365034	0.286193487	0.288672031	0.29119	0.293757506	0.29626195	0.298733043	0.301422859	0.304389789	0.308099079	0.311861106
La Union	0.365975488	0.3704422	0.374610947	0.379417958	0.384121114	0.388840478	0.393544445	0.398254272	0.402737597	0.407471201	0.412126756	0.416697092	0.421310049	0.425941096	0.430230371	0.434458619	0.438615811	0.442813792	0.447103992	0.451229347	0.455205683	0.45920877	0.463239275	0.467771579	0.472086897
Laguna	0.405151709	0.41027653	0.415094561	0.4204839	0.42578813	0.431100861	0.43636235	0.441603107	0.446620951	0.451909919	0.457112796	0.462208877	0.467262322	0.472307537	0.477026738	0.481798759	0.486437919	0.491081019	0.495734869	0.500241126	0.504560979	0.508775797	0.51303879	0.517739173	0.522256541
Lanao Del Norte	0.291818523	0.29594554	0.299811334	0.304251715	0.30862835	0.313020802	0.317424304	0.321814317	0.325997625	0.330466507	0.334888318	0.339258639	0.343728359	0.348237338	0.352380714	0.356523067	0.360528906	0.364515435	0.368547593	0.372372125	0.376022911	0.379694846	0.383462588	0.387863009	0.392283758
Lanao Del Sur	0.24354173	0.245657235	0.247566426	0.250080577	0.252592569	0.255135035	0.257728671	0.260364073	0.262814596	0.265580288	0.268351983	0.271070073	0.27400749	0.277127547	0.280038444	0.283033165	0.28583311	0.288656915	0.291594136	0.294443893	0.297233287	0.30016083	0.303397463	0.30739113	0.311470334
Leyte	0.284513753	0.288315231	0.291853202	0.296008879	0.300127822	0.304268861	0.308367956	0.312433354	0.316278339	0.320354989	0.324281508	0.328065303	0.331905841	0.335809569	0.339373101	0.342929023	0.346374102	0.349875341	0.353448377	0.356917223	0.360298468	0.363780134	0.36745498	0.371737036	0.375992973
Maguindanao	0.201119246	0.203300905	0.205222006	0.207770046	0.210285874	0.212805769	0.215303709	0.217757393	0.219979151	0.222444255	0.224789666	0.226998201	0.229332161	0.231797296	0.23396749	0.236130244	0.238127231	0.240111857	0.242184411	0.244146059	0.246086355	0.248223233	0.250667149	0.253834472	0.257115746
Marinduque	0.274274209	0.277510346	0.280436397	0.283986159	0.287465662	0.29095873	0.294425958	0.297848553	0.30097793	0.304298164	0.307503747	0.310586432	0.313736175	0.316900078	0.319708031	0.322479696	0.325207057	0.327960465	0.330728708	0.333338693	0.335848092	0.338500063	0.341387648	0.344911256	0.348382701
Masbate	0.237800774	0.241302866	0.244497151	0.24836582	0.25213697	0.255884473	0.259549441	0.263149365	0.266455618	0.27003715	0.27341861	0.27657078	0.279699138	0.282873563	0.285631569	0.288276774	0.290668283	0.292956487	0.295272047	0.297350192	0.299202747	0.30103877	0.302954228	0.305477181	0.307949783
Misamis Occidental	0.309250772	0.314147046	0.318757677	0.323956355	0.329078083	0.334203725	0.339279723	0.344320393	0.349128348	0.354222223	0.359162768	0.363925382	0.368649193	0.373459826	0.377796137	0.381995272	0.386046634	0.390018175	0.393982204	0.397774372	0.401340905	0.404867153	0.408409461	0.412526505	0.416563409
Misamis Oriental	0.358667079	0.36418679	0.369407041	0.375257443	0.381000478	0.386720748	0.392387658	0.397985767	0.403336777	0.40900276	0.414559482	0.419927643	0.425232862	0.430504012	0.435352321	0.44009856	0.444712054	0.449248223	0.45377954	0.458071299	0.462154016	0.466199449	0.47030599	0.474951648	0.47946872
Mountain Province	0.233704033	0.236023406	0.238079439	0.240697147	0.243295078	0.245878701	0.248472477	0.251028934	0.253366033	0.255979377	0.258626011	0.26117326	0.263826664	0.266551592	0.26892026	0.271279087	0.273690271	0.276095453	0.278490462	0.280676889	0.282750633	0.284919759	0.28744801	0.290760934	0.294370629
National Capital Region	0.472158923	0.47840546	0.48421356	0.490501071	0.496550365	0.502446216	0.508154832	0.513735806	0.519077851	0.524659447	0.529940811	0.534922853	0.539725483	0.544465249	0.54868864	0.552759134	0.556592044	0.560261975	0.563980758	0.567490378	0.570690864	0.573626531	0.576462365	0.579781584	0.582976802
Negros Occidental	0.322150922	0.326847059	0.331256574	0.336310546	0.34127672	0.346227416	0.351136893	0.355982113	0.360560982	0.365400995	0.370088836	0.374590544	0.37902606	0.383498512	0.387604571	0.391672457	0.395599834	0.399507859	0.403503547	0.407371518	0.411153899	0.414978543	0.418921398	0.423428461	0.427825207
Negros Oriental	0.277547121	0.281655769	0.285516288	0.290036685	0.294485159	0.298944706	0.303365412	0.307726063	0.311828529	0.316197374	0.320445971	0.324523504	0.328538505	0.332593725	0.33631251	0.340017875	0.343611483	0.34720212	0.350848624	0.354373502	0.35785149	0.361376691	0.365008014	0.369171974	0.373248591
Northern Samar	0.231834136	0.235062102	0.237991993	0.24156999	0.245069929	0.248554594	0.252022925	0.255449833	0.258570608	0.261977453	0.265235427	0.268323417	0.271446883	0.274625209	0.27739973	0.280088093	0.282606072	0.285114319	0.287704205	0.290142446	0.292447245	0.294891303	0.297528902	0.300856479	0.304225449
Nueva Ecija	0.366139213	0.370792669	0.375175672	0.380193351	0.385103381	0.390043196	0.394941496	0.399831023	0.404518084	0.409481126	0.414314793	0.419066195	0.423817846	0.428596529	0.433039488	0.437429109	0.44169485	0.445973012	0.450339892	0.4545451	0.458616044	0.462663754	0.466725467	0.471279836	0.47567308
Nueva Vizcaya	0.305156746	0.308927531	0.312453501	0.316501764	0.320559008	0.32462558	0.328727771	0.332812857	0.336650243	0.340751845	0.344870766	0.348909562	0.353041386	0.357102148	0.360804697	0.364459035	0.368132437	0.371788917	0.375423796	0.378825452	0.382030688	0.385257011	0.388666287	0.392671827	0.396787766
Occidental Mindoro	0.21517283	0.21751622	0.219607878	0.222168582	0.224752387	0.227316612	0.229978691	0.232589048	0.234848016	0.237365128	0.239948669	0.242367979	0.244917375	0.247398743	0.249353542	0.251174102	0.252814605	0.25429602	0.255632529	0.256482298	0.256894079	0.257286638	0.258010591	0.259466112	0.261301352
Oriental Mindoro	0.268748126	0.272045048	0.275086669	0.278668581	0.282219686	0.285782661	0.28933041	0.292820221	0.296055784	0.299510801	0.302974973	0.306270791	0.309662792	0.313102875	0.31620953	0.319294215	0.322290465	0.325361867	0.328503715	0.331506592	0.334393422	0.33738336	0.340655561	0.344659666	0.348788453
Palawan	0.236761187	0.24019281	0.243325034	0.247151613	0.250917929	0.254688668	0.258413829	0.262105322	0.26557147	0.269390057	0.273090256	0.276596977	0.280186955	0.283889312	0.287197611	0.290464554	0.293515747	0.296507755	0.299539797	0.302331377	0.30487986	0.307485731	0.310220047	0.31366033	0.317167016
Pampanga	0.373586249	0.378405239	0.382937609	0.388036314	0.393066873	0.398092637	0.403099827	0.408082063	0.412834773	0.417844442	0.422788854	0.427703932	0.432669614	0.437593823	0.442167957	0.446722971	0.451177742	0.455666052	0.460190753	0.464575717	0.468686013	0.472704188	0.476797206	0.481378773	0.485802215
Pangasinan	0.369327095	0.374004393	0.378388729	0.383415803	0.388337756	0.393268617	0.398165345	0.403073094	0.407768868	0.412705666	0.417519956	0.422211282	0.426917406	0.431569291	0.435837758	0.440041988	0.44407325	0.448123633	0.452261956	0.45623216	0.46005037	0.463854922	0.467677901	0.472029124	0.476181606
Quezon	0.33341631	0.337928969	0.342150244	0.346970217	0.351737426	0.356516912	0.361258853	0.365907179	0.370279613	0.374898003	0.379432653	0.383814617	0.388178657	0.392546601	0.396534239	0.400416535	0.404161462	0.407912878	0.411705998	0.415332841	0.418770563	0.42218294	0.425668729	0.429702406	0.433669326
Quirino	0.258993847	0.262157215	0.265061628	0.268533726	0.27196944	0.275412147	0.278854965	0.282258474	0.285406021	0.288763422	0.292049099	0.295205055	0.298454168	0.301731046	0.304675776	0.307597112	0.310383956	0.313198048	0.316072327	0.318768149	0.32134782	0.324038211	0.327011179	0.330685486	0.334419804
Rizal	0.41017577	0.410751821	0.410906866	0.411551242	0.412090518	0.412609082	0.41308757	0.413724189	0.414667681	0.416503243	0.419041909	0.423720971	0.430879357	0.440477518	0.451429352	0.46360583	0.476262232	0.489181048	0.502058374	0.514103318	0.524886139	0.533886792	0.54128644	0.547660072	0.552349792
Romblon	0.238311843	0.241409475	0.244183777	0.247714473	0.251167284	0.25464277	0.258047569	0.261381662	0.2644089	0.267684115	0.270786139	0.273692022	0.276659543	0.279671479	0.282282252	0.284853422	0.287287549	0.289685717	0.292087713	0.294337316	0.296466004	0.298727982	0.301199841	0.304374796	0.307502441
Samar (Western Samar)	0.24518897	0.248803757	0.252135044	0.256141674	0.260076385	0.264010956	0.267880174	0.271694548	0.275230042	0.279040029	0.282721765	0.286249524	0.289804035	0.293415812	0.296736658	0.299969186	0.303076676	0.306188167	0.309394508	0.312479234	0.315453712	0.318525342	0.32179598	0.325737988	0.329714821
Sarangani	0.246178447	0.249859897	0.253262461	0.257390302	0.26148327	0.265611776	0.269740036	0.273856935	0.277723724	0.281895166	0.285914765	0.289851503	0.293827152	0.297857928	0.301569462	0.305270965	0.308888318	0.312517341	0.316161621	0.319724036	0.323173244	0.326675585	0.330304469	0.334567195	0.338787811
Siquijor	0.272094758	0.275918198	0.279477265	0.283833732	0.288131871	0.292456001	0.296757977	0.301009924	0.304986724	0.309223058	0.31323016	0.317137433	0.32112371	0.325259433	0.329120786	0.333137126	0.336973504	0.340924999	0.344945169	0.348792993	0.352425966	0.355983411	0.359548964	0.363599437	0.367371358
Sorsogon	0.304613587	0.308699365	0.312490907	0.317041357	0.321485962	0.32597912	0.330407885	0.33476336	0.338810504	0.343139667	0.347324196	0.351287808	0.355234575	0.359192872	0.362747483	0.366223439	0.369534256	0.372807375	0.376130072	0.379304634	0.382344339	0.385373754	0.388504414	0.392217937	0.395804843
South Cotabato	0.320311941	0.324430012	0.328282935	0.332799928	0.337270332	0.341774062	0.346333138	0.350874371	0.355215379	0.359894882	0.364467075	0.368972803	0.373542567	0.378140078	0.382363424	0.386556531	0.39062055	0.394651029	0.398681311	0.402520472	0.406156416	0.409728154	0.413425219	0.417714209	0.421944223
Southern Leyte	0.291830906	0.295464066	0.298789698	0.302717851	0.306566977	0.310445298	0.314319063	0.318138004	0.321719079	0.325555703	0.329318564	0.332989933	0.336734014	0.340556747	0.344052753	0.347544212	0.350922367	0.35424182	0.357574555	0.3607171	0.363704793	0.366720038	0.369856795	0.37356515	0.377137729
Sultan Kudarat	0.236439973	0.239339425	0.241979894	0.245216908	0.248428298	0.251635404	0.254851798	0.258046933	0.260992297	0.264219828	0.267383342	0.270384092	0.273512658	0.276727416	0.279575069	0.282411885	0.285049101	0.287674577	0.29032372	0.29273839	0.295004841	0.297365459	0.299994766	0.303413562	0.307018939
Sulu	0.16487517	0.167211352	0.169243746	0.172045247	0.17482083	0.177657138	0.180436407	0.183111632	0.185432279	0.187896214	0.190034674	0.191989886	0.194048902	0.196344627	0.198500423	0.20079178	0.203001412	0.205027792	0.206890793	0.208442026	0.209763936	0.211306999	0.213240357	0.216106108	0.219189286
Surigao Del Norte	0.300549053	0.304617703	0.308424092	0.312934862	0.317394335	0.321890129	0.326343736	0.330795974	0.335031468	0.339556558	0.343939004	0.348135603	0.352366921	0.356735355	0.360772003	0.364819058	0.368720578	0.372626918	0.37661432	0.380494525	0.384248895	0.388005909	0.391848136	0.396242998	0.400532894
Surigao Del Sur	0.270512381	0.274332598	0.277889281	0.282060858	0.286181452	0.290293506	0.294415034	0.298509955	0.302307393	0.306406473	0.310470122	0.314362731	0.318312476	0.322252383	0.325789176	0.329264955	0.332610392	0.33593355	0.339231364	0.342316969	0.345215204	0.348196643	0.351404633	0.35528526	0.35918521
Tarlac	0.358801899	0.363179074	0.367270505	0.37195229	0.376577211	0.381203895	0.385818821	0.390437329	0.394819589	0.399475517	0.4040465	0.40855886	0.41311169	0.41765773	0.421837275	0.425942732	0.429970122	0.434036596	0.438169112	0.442163724	0.445946006	0.449656525	0.4534291	0.457695708	0.461744295
Tawi-Tawi	0.22647579	0.229373498	0.23194602	0.235214609	0.23837598	0.241521905	0.244591477	0.247575328	0.250275329	0.253234872	0.256001818	0.258598518	0.261197316	0.263829278	0.266019896	0.268129773	0.270151762	0.272103623	0.274036816	0.275799708	0.277407271	0.279107531	0.281029087	0.283662355	0.286412322
Zambales	0.389611196	0.394779234	0.399596768	0.405051189	0.410365418	0.415677838	0.420899949	0.426003124	0.430792261	0.435877951	0.440808244	0.445571227	0.450274834	0.454921803	0.459149143	0.463314603	0.467242569	0.471083272	0.47489884	0.478561752	0.482030388	0.485364288	0.488678518	0.492462893	0.496008822
Zamboanga Del Norte	0.245977158	0.249680895	0.253075468	0.257165886	0.261147485	0.265121222	0.269058881	0.272958946	0.276575948	0.28049334	0.284227352	0.287851334	0.291493405	0.295179883	0.298448937	0.301671975	0.304789332	0.30788231	0.311021302	0.314056559	0.316988393	0.320001171	0.323160973	0.327023874	0.330887406
Zamboanga Del Sur	0.294335573	0.298149348	0.301687335	0.305831308	0.309929715	0.31404447	0.318156816	0.322253247	0.326130207	0.330315117	0.334413031	0.338429691	0.342561372	0.346714537	0.350581815	0.354464063	0.358324582	0.362256245	0.366258633	0.3702034	0.374047888	0.377989595	0.382152156	0.386978255	0.391834991
Zamboanga Sibugay	0.231275189	0.234592219	0.237610786	0.241353949	0.245033246	0.248716255	0.252350898	0.255939204	0.25926493	0.262858185	0.266272665	0.269528751	0.27285338	0.276240699	0.279237481	0.282239236	0.285155214	0.288081042	0.29106231	0.293956609	0.296740833	0.299648239	0.302781885	0.306646106	0.310556253
Seychelles	0.371025606	0.373939673	0.375293215	0.375513102	0.37522191	0.374195838	0.372890847	0.372018006	0.371265235	0.370321303	0.369047772	0.36675591	0.365217719	0.365249424	0.367080463	0.369623424	0.372537596	0.375571088	0.379066131	0.381857761	0.385405305	0.390476813	0.397162009	0.404987424	0.413705802
Sri Lanka	0.328662531	0.333450682	0.336907935	0.341192602	0.346265614	0.351379644	0.355533339	0.359702898	0.36417793	0.368557288	0.372268741	0.375071547	0.378726392	0.383261334	0.389091324	0.395446792	0.40200519	0.408687616	0.415456141	0.421518709	0.426312003	0.429112248	0.430484773	0.431270793	0.432311393
Thailand	0.235012245	0.238122963	0.24113305	0.244391295	0.247618788	0.251015526	0.25449248	0.258271551	0.262227459	0.266951364	0.272590294	0.278685584	0.285346755	0.292347652	0.29946285	0.306619822	0.314025627	0.320999381	0.327633397	0.334052799	0.341224093	0.348290121	0.355253937	0.362557942	0.36964658
Timor-Leste	0.103452589	0.103970085	0.10450757	0.105178901	0.106005743	0.107022391	0.108212588	0.109495242	0.110823016	0.112264012	0.113642174	0.115461233	0.117460576	0.119595021	0.121919179	0.124335196	0.12697123	0.129813923	0.133054089	0.136827033	0.14015419	0.144181727	0.148495838	0.152972624	0.157603042
Viet Nam	0.208889388	0.211818896	0.214894462	0.218148703	0.221642019	0.225303251	0.229115688	0.233111162	0.237283066	0.241658291	0.246251453	0.251049282	0.256816791	0.262568536	0.26849832	0.273718816	0.279491124	0.28363869	0.287434648	0.291849218	0.296176006	0.300847394	0.305948068	0.311307998	0.31637379
Sub-Saharan Africa	0.157558733	0.159593141	0.161379893	0.163161162	0.165131839	0.167118024	0.16910603	0.171075725	0.173167708	0.175454313	0.178075511	0.180734213	0.183521902	0.18653603	0.189611353	0.192548746	0.195828738	0.199134932	0.202490921	0.206226712	0.210319781	0.214353613	0.218404449	0.222660001	0.227293264
Central sub-Saharan Africa	0.154393232	0.156506742	0.158438311	0.160443417	0.162243229	0.164178157	0.166039125	0.16800144	0.169994102	0.172037052	0.174272122	0.176433766	0.179010262	0.181723355	0.184558604	0.187265457	0.190900796	0.194626842	0.198570734	0.202919811	0.207494969	0.212277209	0.217055939	0.222317044	0.227895632
Angola	0.134403694	0.135915072	0.137398724	0.138869386	0.140068457	0.14155641	0.142920178	0.144563186	0.146335324	0.148135335	0.149964856	0.152260011	0.154388775	0.156654283	0.159320382	0.162630163	0.166048995	0.169684689	0.173272468	0.177121635	0.181071965	0.185282503	0.189551998	0.194144029	0.198765181
Central African Republic	0.10686999	0.108530716	0.11023895	0.112033402	0.11390405	0.115834721	0.117864391	0.119985137	0.122161665	0.124452417	0.126740776	0.129101777	0.131298968	0.13332634	0.135678779	0.138039483	0.140092192	0.142049457	0.144430616	0.147053695	0.149996468	0.152852035	0.155375788	0.158184686	0.161090466
Congo (Brazzaville)	0.167245078	0.169682517	0.172224516	0.174793186	0.177487301	0.180546858	0.183735356	0.187095052	0.19057857	0.194234847	0.198020484	0.20211642	0.206373484	0.210983091	0.21534577	0.219002255	0.223739367	0.228747751	0.234043418	0.239805263	0.246165307	0.252876146	0.259958283	0.267746326	0.275646315
DR Congo	0.150145905	0.153044538	0.155475122	0.158055152	0.160428869	0.162754112	0.165014722	0.167156465	0.169205651	0.171325501	0.173646948	0.175380775	0.178066255	0.180827283	0.18338575	0.185850906	0.18943291	0.192895661	0.196767555	0.201266897	0.205808545	0.210475903	0.21492365	0.219997228	0.225425667
Equatorial Guinea	0.10607384	0.108354054	0.110560526	0.11284247	0.115305773	0.117816435	0.120403474	0.123018464	0.125693912	0.128651428	0.132084324	0.135647971	0.139452986	0.144478692	0.149309839	0.15380272	0.158926733	0.164034953	0.169006348	0.173499039	0.176825024	0.180219935	0.183451484	0.187080438	0.191084043
Gabon	0.165303001	0.167644566	0.170160213	0.172797994	0.175675904	0.178784065	0.18202468	0.185493451	0.189065508	0.192968297	0.197175526	0.201977725	0.207288142	0.213155864	0.218688503	0.224483594	0.232621711	0.241008154	0.249466762	0.258534476	0.268118573	0.277754807	0.287798344	0.298426519	0.310764166

Eastern sub-Saharan Africa	0.118171039	0.12025662	0.121811588	0.123199257	0.124835323	0.126510703	0.128265125	0.130059105	0.131847984	0.133889493	0.136161187	0.138354729	0.140693761	0.143168027	0.145845018	0.148609856	0.151707235	0.154961832	0.158322311	0.161771636	0.165146402	0.16843057	0.171850774	0.175289507	0.178774247
Burundi	0.100491601	0.102754615	0.104696179	0.106715086	0.109027346	0.111277363	0.113595613	0.116003117	0.118438819	0.121084058	0.123776098	0.125150773	0.127066088	0.12913928	0.131305465	0.133354534	0.135166579	0.137771706	0.140176642	0.142107684	0.145751518	0.149281093	0.151700921	0.15446796	0.156966291
Comoros	0.121469026	0.123080339	0.12435819	0.12552086	0.126636339	0.127472578	0.128077235	0.128461377	0.128628209	0.128652602	0.128784079	0.128833391	0.129331252	0.130146189	0.130997062	0.131582138	0.132749288	0.13419329	0.135793744	0.137592995	0.140039989	0.142719657	0.145796161	0.149242213	0.152916076
Djibouti	0.163063165	0.166458793	0.169891563	0.173502372	0.177371024	0.181391594	0.185539197	0.189826893	0.19419623	0.198659469	0.203222997	0.207806481	0.212345568	0.21695456	0.221760928	0.225588074	0.230097087	0.234553953	0.238940896	0.243279937	0.248751683	0.254061846	0.259295901	0.264531566	0.269428553
Eritrea	0.069217837	0.070610911	0.072088797	0.073786642	0.075653687	0.077669938	0.079885761	0.082221642	0.084614833	0.087177265	0.090018749	0.09284129	0.095773172	0.098758651	0.101941109	0.105332917	0.108973221	0.112711585	0.116620887	0.120718144	0.124891904	0.128997927	0.133139035	0.137415285	0.141780876
Ethiopia	0.056066525	0.058178579	0.059518604	0.060934369	0.062062405	0.063124683	0.064340038	0.065459957	0.066564735	0.06792514	0.069487978	0.071083945	0.072733918	0.074464124	0.076400376	0.078438537	0.080646679	0.082863584	0.085163683	0.087679964	0.09018517	0.092572538	0.095090167	0.097683302	0.100272527
Addis Ababa	0.194662834	0.197987138	0.200798378	0.203806588	0.206640215	0.20943435	0.212531239	0.215686235	0.219000804	0.222621729	0.226575715	0.230633563	0.234873234	0.239443454	0.24433011	0.249302641	0.254906353	0.260680354	0.266583059	0.272708729	0.27915083	0.28582235	0.292799425	0.300067586	0.307678597
Afar	0.035167123	0.037787707	0.03891631	0.040010031	0.040894627	0.041927556	0.043080445	0.044030901	0.044931169	0.046135613	0.047564882	0.048985664	0.050391939	0.051762265	0.053278337	0.054852568	0.05666379	0.058399263	0.060199449	0.062198286	0.064028795	0.065637882	0.067331188	0.069042953	0.070701898
Amhara	0.034822681	0.037817496	0.039002273	0.040110069	0.040608418	0.04094711	0.041412609	0.041627483	0.041743654	0.042142289	0.042681639	0.043184235	0.044230601	0.045395298	0.046779283	0.04824823	0.049884791	0.051446577	0.053052167	0.054860343	0.056578046	0.058155393	0.059825284	0.061539476	0.063200138
Benishangul-Gumuz	0.027019971	0.031463647	0.033139535	0.034622643	0.035309767	0.035778633	0.036377748	0.036815403	0.037486727	0.038494269	0.039710581	0.040897083	0.042084248	0.043263369	0.044685776	0.046206334	0.047872577	0.049426501	0.051040561	0.052908477	0.054676158	0.056210809	0.05787255	0.059593055	0.061274065
Dire Dawa	0.132814322	0.135417354	0.137536229	0.139802717	0.141863644	0.143862627	0.146059841	0.148280314	0.150586336	0.15316351	0.155994877	0.158902216	0.161945522	0.165098618	0.168489073	0.172126505	0.175796172	0.179540612	0.183372306	0.187467491	0.191538016	0.195873006	0.200409031	0.205195459	0.210128355
Gambella	0.060271776	0.06230702	0.063490108	0.064696264	0.065560385	0.066311669	0.067137512	0.067780581	0.068306802	0.069009293	0.069834961	0.070650346	0.071475226	0.072286025	0.073193634	0.07413442	0.075426907	0.076711488	0.078078187	0.079721208	0.081433533	0.08295887	0.08472622	0.086689927	0.088755263
Harari	0.131834225	0.134559325	0.136895857	0.139500737	0.141946725	0.144298045	0.146931163	0.149616751	0.15237384	0.155452032	0.158738842	0.162023071	0.165379233	0.168864844	0.172543845	0.176379119	0.180430296	0.184561494	0.188790603	0.193255607	0.197740152	0.202303585	0.207028969	0.211975963	0.217070642
Oromia	0.043593468	0.045305914	0.045982108	0.046953068	0.047992373	0.04894581	0.050045183	0.051016751	0.051960008	0.053185383	0.054630461	0.056111172	0.057644625	0.059240606	0.061041079	0.062900718	0.065131439	0.067356222	0.0696469	0.072152821	0.07464706	0.076887345	0.079257504	0.081695837	0.084096564
Somali	0.035537127	0.037429025	0.038175256	0.038908128	0.039221054	0.039427802	0.039736973	0.040107639	0.040824056	0.041800356	0.042979863	0.04416129	0.04536977	0.046569441	0.047916617	0.049384616	0.050967347	0.052501867	0.054105538	0.055886939	0.057543131	0.05909412	0.060744878	0.062448955	0.064130306
Southern Nations, Nationalities, and P	0.041692248	0.044598001	0.04612356	0.047669266	0.048732556	0.049676286	0.050792454	0.05171402	0.052580802	0.053766647	0.055169351	0.056604544	0.058091008	0.059659249	0.061533889	0.063529773	0.065759105	0.067948069	0.070244324	0.072821783	0.075350307	0.077768198	0.080346421	0.082993355	0.085603362
Tigray	0.053765134	0.054970618	0.055415528	0.055858743	0.055992104	0.05617572	0.057126367	0.057991269	0.058848716	0.059917721	0.061178751	0.062464772	0.06381306	0.065260873	0.066919861	0.068589776	0.070647056	0.072712148	0.074816204	0.077085646	0.079435258	0.081526672	0.083731326	0.086013468	0.088348455
Kenya	0.125031917	0.128129491	0.129201313	0.130192307	0.131955394	0.1340218	0.136224274	0.138611575	0.141138829	0.143679475	0.146564044	0.149323934	0.152418922	0.155821068	0.159594099	0.163575597	0.168270986	0.173211469	0.178428514	0.183968494	0.18949852	0.195434142	0.201653561	0.208022658	0.21475058
Baringo	0.100216189	0.101990576	0.10213515	0.102351339	0.103223938	0.104338895	0.105632493	0.107110256	0.108728584	0.110435996	0.112430142	0.114355189	0.11648818	0.118823346	0.121256997	0.123490788	0.126654567	0.129828439	0.132926181	0.136093253	0.139035166	0.142190569	0.14550825	0.148967397	0.152714641
Bomet	0.078039451	0.079014741	0.078586151	0.07807884	0.077954978	0.077994887	0.078261091	0.078811285	0.079797549	0.080982205	0.083048462	0.085637022	0.088845939	0.092966518	0.097573331	0.10257359	0.109640976	0.117259085	0.125542066	0.134193084	0.142529021	0.150797993	0.158660682	0.166113631	0.173256827
Bungoma	0.061363784	0.067141805	0.071091903	0.074458652	0.07810426	0.081796715	0.085408358	0.089181238	0.093128028	0.097029712	0.101450935	0.105790085	0.110387405	0.115200612	0.120390822	0.125592363	0.131420316	0.137394198	0.14347677	0.14985433	0.155641103	0.161318711	0.167043425	0.172547457	0.178146115
Busia	0.10159745	0.102843847	0.102481119	0.10220787	0.102596823	0.103299277	0.104178378	0.105269734	0.106659279	0.108182845	0.11001102	0.111862737	0.114039402	0.116481927	0.119223477	0.121695319	0.125270263	0.128821773	0.132316367	0.135927632	0.139397898	0.142576759	0.145769088	0.148822244	0.151844659
Elgeyo Marakwet	0.076523776	0.077211969	0.076426665	0.075697571	0.075557384	0.075753161	0.07623612	0.076971489	0.078053019	0.079343603	0.08103438	0.082853336	0.084992659	0.087547322	0.090323861	0.093023272	0.097525353	0.102225356	0.107345965	0.112855373	0.118323616	0.123792364	0.129517447	0.135395484	0.141668124
Embu	0.131148973	0.134938404	0.136736475	0.138558297	0.141285099	0.144373425	0.147665324	0.151231234	0.154979842	0.158699632	0.162781411	0.166715431	0.171015366	0.175595279	0.180526778	0.185314751	0.191281467	0.197206214	0.203050091	0.208900457	0.214449233	0.2204726	0.226775345	0.233220249	0.240292085
Garissa	0.060310233	0.062142857	0.062892349	0.06364527	0.064789557	0.066088304	0.067427631	0.068852006	0.070331895	0.071859975	0.07351721	0.075075295	0.07681976	0.078696165	0.080781887	0.083046908	0.085504366	0.088089304	0.090826125	0.093692695	0.096419987	0.099494926	0.102707733	0.1059691	0.109260626
Homa Bay	0.094902098	0.099230006	0.101575469	0.103411759	0.105644595	0.107913731	0.110101238	0.11222427	0.114302021	0.11611702	0.118128927	0.119791159	0.121545116	0.123412423	0.125450371	0.12741775	0.130111766	0.132850876	0.135582071	0.138328744	0.140973187	0.143460186	0.145888935	0.148194017	0.150686826
Isiolo	0.09029721	0.092475643	0.09295731	0.093447404	0.094632157	0.096116378	0.097770679	0.099589746	0.101530696	0.103516107	0.105791082	0.107946437	0.110378945	0.11298623	0.115854434	0.119000617	0.122416224	0.12593527	0.129583502	0.133370742	0.136706234	0.140693962	0.144771319	0.148757291	0.152823132
Kajiado	0.086185035	0.087690281	0.087930593	0.088193684	0.088999398	0.090022486	0.091341747	0.092933178	0.094822745	0.09683855	0.099299966	0.101879446	0.10478603	0.108074003	0.111444787	0.114950289	0.119772906	0.124872916	0.130372885	0.136190707	0.141866439	0.147852768	0.153944336	0.160213951	0.166972332
Kakamega	0.07643576	0.078988712	0.080178764	0.081197987	0.082836304	0.084754705	0.086822463	0.089121613	0.091732158	0.094514826	0.097890991	0.101285129	0.105073391	0.109405998	0.11442217	0.119748087	0.125922973	0.13236404	0.138984515	0.145891195	0.152543515	0.159226737	0.166091629	0.173143218	0.180636815
Kericho	0.100019085	0.101110564	0.100495865	0.099829252	0.099670678	0.09965147	0.099725473	0.09996136	0.100357465	0.100771629	0.101743131	0.10289687	0.104385296	0.106485243	0.108962272	0.111917848	0.116195945	0.12087399	0.126227169	0.13206714	0.137629413	0.143860067	0.150293201	0.156963446	0.16428123
Kiambu	0.162365397	0.164955641	0.165224418	0.165497723	0.166758893	0.168508697	0.170518725	0.172920712	0.175539686	0.178288853	0.181545598	0.184699348	0.188298099	0.192204834	0.196578136	0.201257984	0.206915411	0.212930582	0.219345917	0.225854353	0.232152537	0.239452923	0.247014452	0.254812622	0.263067206
Kilifi	0.11222977	0.113881557	0.113740693	0.11347923	0.113764583	0.114228102	0.114731474	0.11528925	0.115916444	0.116488453	0.117245537	0.117776592	0.118497374	0.119447153	0.120867133	0.122087466	0.124683119	0.127567291	0.130820819	0.134922741	0.13828586	0.142669604	0.147357302	0.152124425	0.156867212
Kirinyaga	0.117213338	0.11961837	0.1201436	0.120732331	0.122133734	0.123965645	0.126054227	0.128449255	0.13118547	0.134156928	0.137665856	0.141250801	0.145522678	0.150391589	0.155967188	0.161570611	0.168741967	0.176459083	0.184600158	0.193189835	0.201807173	0.211493189	0.221589604	0.231932124	0.242741556
Kisii	0.045164744	0.046355138	0.046768685	0.047156775	0.047822245	0.048573676	0.049353871	0.055675978	0.069869971	0.080147331	0.089181082	0.0970177	0.104336196	0.111433106	0.118584038	0.125825324	0.132975245	0.139990505	0.146958492	0.153800575	0.16018799	0.166552566	0.172891341	0.179161227	0.18566937
Kisumu	0.10015269	0.104315886	0.106470977	0.108340675	0.110738026	0.113245496	0.115735966	0.118318599	0.120988255	0.123564549	0.126353721	0.128966132	0.131704903	0.13461505	0.137762425	0.140910838	0.144948553	0.149111728	0.153372645	0.157755344	0.162029458	0.166182187	0.170376047	0.174456824	0.178838152
Kitui	0.085629292	0.087986679	0.088882062	0.089845487	0.091520015	0.093588347	0.095914459	0.098472597	0.101208559	0.104017606	0.107150441	0.110206628	0.113595428	0.117296661	0.121321562	0.124589308	0.129759879	0.134891893	0.140059533	0.145352779	0.150571137	0.155731759	0.161082098	0.166450725	0.172122483
Kwale	0.100641319	0.102162839	0.10211891	0.102086132	0.102676785	0.103536628	0.104532448	0.1056295	0.106909393	0.108244651	0.109877456	0.111413086	0.113226718	0.115358343	0.11796373	0.120440529	0.124304658	0.128482494	0.133020521	0.138177794	0.142908617	0.14847349	0.154362652	0.160327436	0.166391014
Laikipia	0.127648199	0.130736177	0.132173609	0.133729795	0.136051844	0.138692017	0.141613305	0.144836014	0.148340888	0.151967304	0.156168044	0.160472647	0.165090115	0.169996496	0.175192554	0.180294025	0.186715731	0.19337957	0.200303422	0.207478313	0.214490375	0.222047637	0.229759013	0.237543171	0.245804255
Lamu	0.105124286	0.106753468	0.106796736	0.106796918	0.107422702	0.108298899	0.109264826	0.110366512	0.111631574	0.112940074	0.114569296	0.116186196	0.118103498	0.120332365	0.123015068	0.125496243	0.129752808	0.134391383	0.139478152	0.145251909	0.15103406	0.157381276	0.16423384	0.17135666	0.178798735
Machakos	0.146171594	0.148793304	0.149201434	0.14964013	0.151004534	0.152760981	0.154734833	0.157031933	0.159519145	0.161993988	0.164909482	0.167734284	0.171015288	0.174691447	0.178783738	0.182999684	0.188436985	0.194080233	0.199978552	0.20608006	0.211918758	0.218425972	0.225100722	0.231825068	0.238825313
Makueni	0.092049576	0.095052694	0.096397282	0.097737363	0.099767825	0.102153233	0.104753059	0.107551424	0.110532307	0.113548145	0.11696486	0.120264888	0.123937234	0.127843409	0.131985252	0.13570411	0.141299789	0.146853743	0.152483877	0.158182274	0.163622885	0.169214919	0.174933013	0.180663544	0.18655238
Mandera	0.060863262	0.062593706	0.063102044	0.063565925	0.06440717	0.065364148	0.066304803	0.0672427	0.068156964	0.069069842	0.07007507	0.070921616	0.071923063	0.072991087	0.074196856	0.075445449	0.076803119	0.078167091	0.079582978	0.081036366	0.082291245	0.083748217	0.085230109	0.086646827	0.087981814
Marsabit	0.091372909	0.09327186	0.093477906	0.093677553	0.094530966	0.095642487	0.096864717	0.098217068	0.09967324	0.101168152	0.102941864	0.104590149	0.106482439	0.108471917	0.110670659	0.11294876	0.115670166	0.118504794	0.121385827	0.124323068	0.126942564	0.129926652	0.133008931	0.136051616	0.139198311
Meru	0.111958851	0.114232023	0.114752271	0.115310201	0.116714354	0.11853571	0.120603674	0.123003251	0.125629016	0.1283655	0.131508037	0.134602385	0.138079544	0.141930189	0.146216854	0.150332984	0.155877741	0.16148862	0.167289745	0.173220465	0.17893522	0.18495555	0.191061365	0.197019797	0.2031446
Migori	0.147057031	0.149208745	0.148947254	0.148477563	0.148831452	0.149369357	0.149889223	0.150414763	0.150933853	0.151382938	0.151930593	0.152158938	0.152562629	0.153070904	0.153828048	0.154295628	0.155582606	0.156807018	0.15792518	0.159072225	0.160058333	0.160852028	0.161578878	0.162180157	0.16281908
Mombasa	0.180233532	0.184416437	0.185751865	0.187036761	0.189445145	0.192324455	0.195399637	0.198728476	0.20219985	0.205758414	0.209839604	0.213655689	0.217845028	0.222331929	0.227301731	0.232520026	0.238645187	0.24497612	0.251337519	0.257762902	0.263436456	0.27118392	0.279097613	0.287150829	0.295729128
Murang'a	0.140276309	0.14384471	0.145308156	0.146634758	0.148749855	0.151171724	0.153679777	0.156372717	0.15907774	0.161811944	0.164785625	0.167554577	0.170627242	0.173845269	0.177227522	0.180302091	0.184867754	0.189538778	0.194463918	0.19963744	0.204777549	0.210966298	0.217449188	0.224140839	0.231387078
Nairobi	0.250245423	0.256539571	0.259709927	0.262674286	0.266746233	0.271305942	0.276163349	0.281378975	0.286858342	0.292378191	0.298430675	0.304447229	0.311003945	0.318035551	0.325626139	0.333628787	0.342430912	0.351434952	0.360366935	0.369062513	0.377555057	0.386806168	0.395789095	0.404416186	0.412905442
Nakuru	0.137996946	0.140743253	0.141108859	0.141415827	0.142652073	0.144269963	0.146064755	0.148084749	0.150283922	0.152574265	0.155359936	0.158043065	0.161008419	0.164318425	0.167855531	0.171518662	0.176381067	0.181446218	0.186798255	0.192370896	0.197708241	0.203756943	0.20995502	0.216215539	0.222880817
Nandi	0.092713086	0.094252069	0.09440943	0.094605358	0.09534718	0.096292987	0.097451484	0.098826812	0.100435401	0.102004798	0.103912493	0.105903526	0.108033915	0.110453815	0.113009581	0.115106867	0.118776339	0.122565263	0.126710341	0.131139534	0.135460675	0.139963746	0.144580459	0.149480058	0.154944472
Narok	0.091600616	0.092301732	0.091546742	0.090859701	0.090712237	0.090751792	0.090946576	0.091279022	0.091770311	0.092234478	0.093037675	0.093870959	0.094848392	0.096019112	0.09724923	0.09834659	0.100748485	0.103282434	0.106045564	0.10908984	0.111931732	0.114779439	0.117624566	0.120458619	0.123448047
Nyamira	0.12650398	0.13080169	0.133030415	0.135061592	0.137788553	0.140830851	0.143924072	0.147154906	0.15039255	0.153671837	0.157317037	0.160815597	0.164619598	0.168723039	0.173165836	0.177297784	0.182693873	0.18815857	0.193734537	0.199411358	0.205096036	0.210704916	0.216539422	0.222497874	0.228694697
Nyandarua	0.08116973	0.088265953	0.093886255	0.099305027	0.105149388	0.111095469	0.117053844	0.123149856	0.129422521	0.135548064	0.141923025	0.147962907	0.154260598	0.160745253	0.167553225	0.174541754	0.181805152	0.189144327	0.196526725	0.203705995	0.210348566	0.21784325	0.225308833	0.232681564	0.240205776
Nyeri	0.145876193	0.149808874	0.151330106	0.152884841	0.155465089	0.158473745	0.161695575	0.165232708	0.168944949	0.172664651	0.176714382	0.180760947	0.185368605	0.19045371	0.196160217	0.201522724	0.208389725	0.215407175	0.22261102	0.230062319	0.237211994	0.245219496	0.253437797	0.261604263	0.270053811
Samburu	0.09515128	0.097112659	0.096936869	0.096685344	0.097200678	0.09799197	0.098861794	0.099833118	0.10088557	0.101953979	0.103297912	0.104523961	0.105979249	0.107576927	0.10932087	0.110941449	0.113213655	0.115511354	0.117814667	0.120222545	0.122339559	0.124457197	0.126638893	0.128855611	0.13121858
Siaya	0.052155878	0.063431198	0.070833804	0.076478666	0.081748977	0.086634162	0.091168549	0.095416138	0.099453791	0.103117914	0.10678079	0.110022042	0.113258717	0.116441489	0.119691606	0.122698407	0.126240911	0.129715016	0.133108566	0.136451225	0.139510337	0.142404317	0.145108304	0.147604531	0.150261808
Taita Taveta	0.125900606	0.128346893	0.128672591	0.128996137	0.13013911	0.131570719	0.133148635	0.134950118	0.136969146	0.139066962	0.141593109	0.144111586	0.147032907	0.150420777	0.154443836	0.15856021	0.164112465	0.170124442	0.17651232	0.183501919	0.190193467	0.198063503	0.206249008	0.214470795	0.222939225
Tana River	0.07012098	0.071884516	0.072119722	0.072290567	0.073037408	0.073991842	0.075018895	0.076164913	0.07744954	0.078785554	0.080342028	0.081813743	0.083598261	0.085675736	0.088204786	0.090658602	0.094471141	0.098629542	0.103190275	0.108358041	0.113380504	0.118998783	0.124999393	0.131127526	0.137339341
Tharaka Nithi	0.10456205	0.107096281	0.108010049	0.108947513	0.110595201	0.112606588	0.114800736	0.117197673	0.119805129	0.122454306	0.125536715	0.128613988	0.132067793	0.13568174	0.13945393	0.14288844	0.148267492	0.1537471	0.159457212	0.165469416	0.171356763	0.177401439	0.183619569	0.189872623	0.196347195
Trans Nzoia	0.142629915	0.144284421	0.143780792	0.143226204	0.143420593	0.143855218	0.144407609	0.145054638	0.145761826	0.1463499	0.147322722	0.148170954	0.149237597	0.150689171	0.15232441	0.154074846	0.156997303	0.160254591	0.163985519	0.168177899	0.172269229	0.177106899	0.18215007	0.18737432	0.193028372
Turkana	0.073399571	0.074946524	0.075145392	0.075300078	0.075965511	0.076764914	0.077614849	0.078519827	0.079460317	0.080434978	0.081611556	0.082713547	0.083993004	0.085405139	0.086995786	0.088706957	0.09085007	0.093132706	0.095576138	0.098234206	0.100744487	0.103709785	0.106872961	0.110163099	0.113629355
Uasin Gishu	0.128535792	0.131602896	0.132832596	0.134045253	0.135934542	0.138117882	0.140453691	0.142987354	0.145721569	0.148522258	0.15171735	0.154837844	0.158196003	0.161851586	0.165736545	0.169634074	0.174544603	0.179553793	0.184647193	0.189947045	0.194841453	0.200324805	0.205867774	0.211506012	0.217456742
Vihiga	0.128542755	0.131205854	0.131423063	0.131513437	0.132456229	0.133739639	0.1351462	0.136610009	0.138163621	0.139716583	0.141699981	0.143603658	0.145800463	0.148185747	0.150833807	0.153316684	0.157076327	0.160875657	0.164693195	0.168625932	0.172379535	0.176369536	0.18068048	0.185083519	0.189690333
Wajir	0.062860064	0.064720314	0.065211579	0.065643005	0.066494822	0.067455911	0.068390978	0.069345719	0.070262443	0.071151909	0.072144017	0.072954843	0.073901883	0.074890015	0.075985072	0.077115247	0.078433221	0.079745532	0.081055268	0.082360881	0.083376096	0.084545204	0.085757645	0.086976726	0.088167281
West Pokot	0.080053767	0.082036732	0.08258951	0.083214041	0.084472837	0.085986087	0.087637757	0.089450648	0.091411914	0.093408648	0.095675845	0.097904104	0.100345043	0.102947696	0.105627834	0.108040036	0.111447709	0.114808012	0.118122277	0.121475533	0.124564806	0.127494531	0.130424141	0.133234327	0.136047577
Madagascar	0.137958583	0.139505393	0.141163878	0.143008443	0.145072718	0.147344562	0.149779971	0.152454095	0.155278381	0.158191344	0.161155978	0.16430342	0.167691579	0.171124208	0.174658967	0.178159046	0.181949977	0.186005059	0.190417357	0.195034092	0.199930908	0.204962145	0.209933365	0.214750111	0.219540402
Malawi	0.13847763	0.140130893	0.141625048	0.143185882	0.144853711	0.146714107	0.149206036	0.151984582	0.15472309	0.157229246	0.160120195	0.162985913	0.16543152	0.167358572	0.168691415	0.170820524	0.173331984	0.175348463	0.176152985	0.176337947	0.175226909	0.17386218	0.171899814	0.168957921	0.166034822
Mozambique	0.092737426	0.094071645	0.095546798	0.097132262	0.098700046	0.100479501	0.102073866	0.103723436	0.105531883	0.10752072	0.109535282	0.111554568	0.113765496	0.115659048	0.11763209	0.119526063	0.121598195	0.123977274	0.126748946	0.129976398	0.133275054	0.13663352	0.13976905	0.142863739	0.145457553
Rwanda	0.125752406	0.128583101	0.130969477	0.133168448	0.135466363	0.13743881	0.138828926	0.139587149	0.140274685	0.141672159	0.143688565	0.145790126	0.148885545	0.151316351	0.153102985	0.155306486	0.158264865	0.161432999	0.164656053	0.168064232	0.171497913	0.174540367	0.177876521	0.18201403	0.186578572
Somalia	0.060459262	0.061033972	0.061591029	0.062240085	0.062971518	0.063752859	0.064556777	0.065321198	0.065949516	0.066537385	0.067180982	0.067826139	0.068513164	0.069186163	0.070130047	0.071075596	0.072049316	0.072895169	0.073815667	0.074790324	0.076021794	0.077561916	0.081631437	0.082931392	0.083761899

South Sudan	0.13025669	0.131979709	0.13367535	0.135379192	0.137078104	0.138768056	0.140409847	0.142064109	0.143656044	0.14525887	0.146872971	0.148451042	0.149985642	0.151478537	0.15309743	0.154674381	0.156380993	0.158138986	0.159964966	0.161839667	0.163808442	0.165804099	0.167853697	0.169941679	0.172114705
Uganda	0.100429346	0.102054187	0.103238494	0.103922601	0.104888324	0.106076546	0.107387912	0.10904016	0.110570639	0.11234179	0.114136206	0.115849889	0.117782235	0.120109675	0.122866445	0.125320492	0.12825585	0.131076521	0.133970995	0.137152381	0.140563614	0.143757683	0.147129741	0.150444493	0.153582784
Tanzania	0.104125026	0.106698332	0.108547865	0.109869833	0.111735736	0.113586944	0.115448708	0.117415184	0.119398748	0.121652887	0.124111941	0.126558781	0.129543474	0.132897678	0.136594042	0.140266524	0.144586113	0.149033261	0.153724954	0.158491821	0.163596234	0.168540376	0.17357502	0.178740815	0.183974501
Zambia	0.14048678	0.13952882	0.138695489	0.138015462	0.137503484	0.136884412	0.136692571	0.136502278	0.136432647	0.137281595	0.138514515	0.140123562	0.142065829	0.144518629	0.147908686	0.152336321	0.157602331	0.163694105	0.170240113	0.177362872	0.184819413	0.192265752	0.200338875	0.208657715	0.217100578
Southern sub-Saharan Africa	0.294116446	0.297643525	0.300632588	0.304361393	0.308250641	0.312113763	0.31613122	0.320088623	0.324098908	0.328212481	0.332559866	0.337263837	0.342129624	0.346963264	0.351918485	0.356972338	0.362033133	0.367450358	0.372782471	0.378111567	0.383589635	0.388921039	0.394032766	0.399271827	0.404652724
Botswana	0.135694371	0.137445634	0.139426441	0.141547839	0.143740521	0.146065636	0.148527742	0.151114238	0.153810468	0.156667429	0.159645893	0.16294302	0.166556404	0.170484168	0.174830993	0.178919979	0.183637084	0.188888896	0.194724268	0.200855466	0.207796748	0.215912441	0.225235835	0.235174439	0.244621411
Eswatini	0.142694293	0.144961214	0.147082892	0.149315245	0.151713821	0.154124896	0.15665543	0.159246142	0.161950823	0.164896147	0.168632956	0.173078231	0.17863962	0.184177574	0.190289475	0.196961392	0.203415564	0.210149158	0.216131133	0.222000927	0.229339257	0.237095741	0.245314565	0.253188051	0.26132221
Lesotho	0.15805657	0.161286951	0.164078673	0.167027554	0.170283569	0.173425669	0.176642485	0.180032973	0.183561837	0.187130033	0.191226013	0.195006849	0.199968166	0.205518183	0.211402136	0.216732075	0.221416666	0.226499498	0.230747082	0.234398534	0.237416073	0.238909163	0.241637903	0.246336188	0.250800856
Namibia	0.215669268	0.218841175	0.222177191	0.22579144	0.229643212	0.233515933	0.237575115	0.241870336	0.246292513	0.251029854	0.256241529	0.261346753	0.267030509	0.272997485	0.279584722	0.286300601	0.293264862	0.299748323	0.306108389	0.312591667	0.319107275	0.325945548	0.332973935	0.340254253	0.3479103
South Africa	0.32413852	0.327933629	0.331300019	0.33530168	0.339536274	0.343790408	0.348189358	0.352641023	0.356979703	0.36140171	0.366114551	0.370931697	0.375839135	0.380860252	0.38612398	0.391565013	0.397013791	0.402679156	0.408368326	0.414078752	0.419846807	0.425638162	0.431231368	0.436840719	0.442300428
Eastern Cape	0.270349949	0.27323126	0.275565595	0.278502405	0.281619058	0.284852926	0.288317531	0.291854718	0.295331151	0.299023875	0.303074901	0.30743496	0.312072672	0.316989935	0.322263785	0.327827685	0.334152883	0.34081883	0.347566333	0.354308123	0.361060599	0.367805411	0.37418841	0.38048038	0.386495035
Free State	0.308342487	0.312130101	0.315391517	0.31921429	0.323239552	0.327350998	0.331693427	0.336134715	0.340497316	0.345050888	0.349901281	0.354972494	0.360229544	0.365687428	0.371444459	0.377354637	0.383512106	0.389915526	0.396371321	0.402739584	0.408935602	0.415084535	0.420965066	0.426845377	0.432570608
Gauteng	0.445983903	0.45084065	0.454888298	0.459493982	0.464298674	0.468834675	0.473344826	0.477909325	0.482337886	0.48677019	0.491572794	0.496500814	0.501472006	0.506531104	0.511874518	0.517399886	0.522940052	0.528467778	0.533721301	0.538915137	0.544170997	0.549422579	0.55436046	0.559157673	0.563616798
KwaZulu-Natal	0.276265737	0.279688973	0.282711423	0.286364719	0.290188042	0.294082111	0.298144838	0.302332178	0.306444074	0.310690412	0.315310923	0.320240161	0.325320018	0.330626658	0.336284828	0.342222552	0.348449494	0.354992972	0.361708943	0.368452443	0.375186375	0.382128477	0.388914901	0.395725428	0.402457419
Limpopo	0.195386002	0.197371377	0.199037772	0.201219007	0.203701942	0.206359269	0.209307699	0.212425834	0.215561428	0.218903433	0.222616738	0.226643206	0.230779044	0.235218037	0.240062024	0.245278988	0.251593365	0.258242852	0.265047952	0.271938619	0.278957943	0.285871601	0.292788463	0.299837382	0.306887867
Mpumalanga	0.216849869	0.219393061	0.221738344	0.224598002	0.227775552	0.231090951	0.234683882	0.238434162	0.24225961	0.246287991	0.250811911	0.255497931	0.260440047	0.265717668	0.271460016	0.277594009	0.284137323	0.291132548	0.298326645	0.305602197	0.312790853	0.320190736	0.32765764	0.335370433	0.343051296
North West	0.270386895	0.274069027	0.277332524	0.281177874	0.285265379	0.289457908	0.293902894	0.298466287	0.302990452	0.30775774	0.312839738	0.318168745	0.323731143	0.329533727	0.335621864	0.341932852	0.348476326	0.355326677	0.362273566	0.369351818	0.376614221	0.384055779	0.391345387	0.39877057	0.406140406
Northern Cape	0.324812676	0.328741816	0.332072217	0.335862923	0.339739083	0.34356644	0.347481949	0.351371593	0.35509017	0.358950089	0.363008088	0.367148222	0.371424243	0.375840954	0.380452293	0.385196195	0.390103264	0.395308876	0.400628825	0.406012012	0.411429601	0.41698349	0.422478896	0.428186898	0.433810957
Western Cape	0.461318617	0.465846473	0.469362249	0.473365455	0.477425651	0.481106703	0.484649698	0.48811439	0.491350888	0.494544943	0.498075028	0.501673116	0.505261249	0.508931167	0.512827178	0.516898992	0.521064268	0.525309414	0.529378382	0.533537717	0.537972902	0.542721029	0.547371114	0.552180368	0.556916577
Zimbabwe	0.182130351	0.184706319	0.187040187	0.189860422	0.192647246	0.195527012	0.198878518	0.202392501	0.205629614	0.209250825	0.213065317	0.216981021	0.220623314	0.224149706	0.227404276	0.230706304	0.233901166	0.237232868	0.240354826	0.244261407	0.249417249	0.254889876	0.260660878	0.266401786	0.272445224
Western sub-Saharan Africa	0.139948263	0.142193858	0.144290465	0.146197478	0.148296663	0.150369292	0.152277114	0.154134279	0.156188577	0.158488489	0.160985557	0.163407033	0.165858566	0.168594302	0.171281015	0.174181886	0.176943259	0.179375534	0.181886758	0.18505349	0.18857391	0.192588032	0.196668757	0.200864203	0.205613791
Benin	0.107444054	0.108744576	0.110064543	0.111514978	0.112902408	0.114400391	0.115884878	0.117392052	0.119128966	0.121091139	0.123068374	0.125071409	0.126763738	0.129059736	0.131339934	0.133302462	0.13603734	0.138770275	0.141466672	0.144306264	0.147497779	0.150552508	0.153796544	0.157237403	0.160896883
Burkina Faso	0.056640768	0.056763165	0.056874778	0.056960652	0.057049361	0.057318402	0.05848246	0.059739834	0.061069253	0.062459768	0.064005578	0.065410879	0.067072162	0.068540709	0.069993539	0.071458073	0.07287009	0.074360353	0.075914876	0.077521117	0.079000184	0.080543912	0.082376539	0.084007892	0.085720491
Cabo Verde	0.13526356	0.135189471	0.135061297	0.135035376	0.135154112	0.135767179	0.136678521	0.137414711	0.138309924	0.14014046	0.14247901	0.145085554	0.146992885	0.148640608	0.15080645	0.153527508	0.157768512	0.162898121	0.168952922	0.175526654	0.182399637	0.189054913	0.195281021	0.200791143	0.205454574
Cameroon	0.136048225	0.138337357	0.140571921	0.143001962	0.145516528	0.148080339	0.150535906	0.153109593	0.155765604	0.158574575	0.16165404	0.164757461	0.167517008	0.17057563	0.173457633	0.176208433	0.178995521	0.181321835	0.183510971	0.185934766	0.188458978	0.191027613	0.193844497	0.19686644	0.200149354
Chad	0.057413946	0.058263937	0.059143118	0.060023423	0.060951701	0.06206143	0.063227965	0.064478951	0.065812039	0.067360477	0.068760011	0.070208577	0.071770418	0.073090947	0.074296054	0.075440836	0.076699344	0.077900551	0.079064893	0.080164287	0.081535498	0.082723495	0.083712202	0.084484609	0.085571915
Côte d'Ivoire	0.117305699	0.11888832	0.120496059	0.122170625	0.123946739	0.125856562	0.127836192	0.12993196	0.132149716	0.134530566	0.137153289	0.139906951	0.142743498	0.145869388	0.149584867	0.152835146	0.156129641	0.159653207	0.16355948	0.167621265	0.171920732	0.176457391	0.180978114	0.185551105	0.190259641
The Gambia	0.119641808	0.121437138	0.123070245	0.124683282	0.126368179	0.127969619	0.129567611	0.131154507	0.132677915	0.1342238	0.135797721	0.137281752	0.138513076	0.139541193	0.140560697	0.141770446	0.142951283	0.143989611	0.145010623	0.146008047	0.147138833	0.148341575	0.149678813	0.151504903	0.153759992
Ghana	0.244808112	0.246424008	0.247043376	0.248687936	0.250569454	0.251204052	0.251768822	0.252199769	0.252904825	0.254404072	0.256415944	0.258267395	0.2603182	0.26263173	0.264964129	0.267054646	0.268866788	0.270426544	0.271870938	0.273626366	0.276258976	0.279272961	0.28190699	0.285276958	0.289670131
Guinea	0.058843135	0.060476997	0.062202185	0.064153972	0.066452169	0.069019097	0.071829111	0.074820179	0.077946659	0.081297878	0.084906956	0.088618555	0.092468115	0.096384599	0.099991496	0.103691717	0.107436969	0.110920609	0.114288207	0.117525121	0.120121841	0.122301859	0.125035901	0.128325882	0.131379947
Guinea-Bissau	0.065085757	0.067102461	0.069266781	0.071691513	0.075202011	0.078085527	0.081652303	0.085438465	0.089351374	0.093336848	0.097646809	0.102171032	0.10679537	0.111654534	0.116771994	0.121969203	0.127119948	0.132468254	0.137684956	0.143026264	0.14828282	0.152547215	0.156641647	0.160225823	0.163431377
Liberia	0.141993144	0.144715701	0.147303768	0.149702923	0.151891815	0.154471696	0.15710882	0.159753153	0.162006492	0.164724467	0.167337474	0.169662775	0.171770448	0.174308887	0.176317084	0.177689663	0.180149013	0.182586822	0.184684378	0.187195826	0.189677658	0.192011418	0.194122604	0.196448674	0.19872841
Mali	0.058974306	0.059775074	0.060622377	0.061494273	0.062467232	0.063538204	0.064669588	0.065886552	0.067197334	0.068577792	0.070071666	0.071551894	0.073020847	0.074620524	0.076149819	0.077741449	0.079497388	0.08130687	0.083309257	0.085268433	0.087482254	0.089691069	0.091792613	0.093693133	0.095358471
Mauritania	0.179851331	0.182057773	0.18433452	0.186528657	0.188816612	0.191061608	0.193652912	0.196294775	0.198573579	0.201147859	0.203989137	0.206610151	0.208941964	0.210899404	0.214551472	0.218684877	0.222185087	0.225610034	0.229230541	0.232668281	0.236576739	0.240287223	0.243892482	0.247338075	0.251494262
Niger	0.046479795	0.046500404	0.046506912	0.046539859	0.046589196	0.04663238	0.046641856	0.046619391	0.046692778	0.047564148	0.048531395	0.049539195	0.050566659	0.051734315	0.052788293	0.053931156	0.054831363	0.055684741	0.056430541	0.057005058	0.057452644	0.058191151	0.058735199	0.058976698	0.05944712
Nigeria	0.151738725	0.15424946	0.156954708	0.15924235	0.161810736	0.164386653	0.166696831	0.169126876	0.171681049	0.174549819	0.177722355	0.180827614	0.183991162	0.187380385	0.190849646	0.194785516	0.198298708	0.201188713	0.204181049	0.208229909	0.212778148	0.218024903	0.223405538	0.229095102	0.235308337
São Tomé and Príncipe	0.176227804	0.17961261	0.182200596	0.18351159	0.183839494	0.183900593	0.184027099	0.184067432	0.184152654	0.184911972	0.186554266	0.188380574	0.19014371	0.191776285	0.19370802	0.196115441	0.19912807	0.202825271	0.207276142	0.21245847	0.21831647	0.224591444	0.231105966	0.237796	0.244286809
Senegal	0.104813737	0.105897155	0.107080044	0.108244641	0.109527127	0.110847741	0.112203821	0.113683395	0.115147555	0.116662206	0.118077367	0.119355213	0.120183185	0.120988884	0.122055196	0.123487085	0.125200937	0.127711222	0.131007268	0.134085078	0.137177381	0.140165981	0.143283478	0.145993564	0.14851364
Sierra Leone	0.115354819	0.117369623	0.119131591	0.120857539	0.122726408	0.124514448	0.126381476	0.128248652	0.130081352	0.132097131	0.134205627	0.136329253	0.138455337	0.140425885	0.142717128	0.145227424	0.147651766	0.149780298	0.15224173	0.155021347	0.158104105	0.161205953	0.164248869	0.16731421	0.170417684
Togo	0.1038512	0.1056322	0.107489841	0.10938831	0.11136955	0.113411103	0.11558842	0.117871815	0.120298284	0.122820779	0.125731924	0.128946491	0.131816435	0.134806209	0.139379197	0.144815652	0.150042872	0.155235077	0.160480301	0.16580834	0.170795233	0.176068033	0.181219526	0.185960798	0.191377016

Appendix Table S16: Socio-demographic Index values for all estimated GBD 2021 locations, 1975-1999

Location	1975	1976	1977	1978	1979	1980	1981	1982	1983	1984	1985	1986	1987	1988	1989	1990	1991	1992	1993	1994	1995	1996	1997	1998	1999
Global	0.465606096	0.470957347	0.475713762	0.479876314	0.48338419	0.487328502	0.491661156	0.495111541	0.497997959	0.501048526	0.504679017	0.508919816	0.513156151	0.517366219	0.521265908	0.525528856	0.530272485	0.534636544	0.538598319	0.542389433	0.546327999	0.550703413	0.555139426	0.559380567	0.563738017
Central Europe, eastern Europe, and central Asia	0.603262199	0.606743531	0.610059175	0.61276742	0.615311483	0.618098778	0.61987509	0.620292085	0.620985113	0.62255254	0.623780776	0.624865428	0.626637989	0.629437243	0.633341443	0.638316821	0.644260725	0.650300228	0.654984112	0.658896401	0.663230612	0.6673985	0.671061614	0.674441709	0.678044379
Central Asia	0.528454544	0.532797288	0.536815093	0.540599036	0.543672385	0.545839227	0.547222721	0.548119008	0.548650545	0.54900101	0.549028287	0.549205307	0.549772042	0.551028647	0.552200525	0.553361946	0.555147774	0.557269541	0.560082892	0.562699531	0.565585602	0.568760584	0.571766483	0.574659386	0.578120701
Armenia	0.546297084	0.548481046	0.550002932	0.551209488	0.551802009	0.551896425	0.551319847	0.549864573	0.547969509	0.54581102	0.54368769	0.542147301	0.540787582	0.540289143	0.542086913	0.544414535	0.547561863	0.548974724	0.551214674	0.553783844	0.556815513	0.560537778	0.564627462	0.569556663	0.574323064
Azerbaijan	0.552952483	0.561006271	0.568327513	0.574805161	0.580160089	0.584475269	0.587931128	0.590950266	0.593480733	0.595198523	0.596132828	0.596548507	0.597146128	0.597706836	0.597270004	0.595986033	0.595807712	0.594651335	0.593542676	0.592088785	0.589441457	0.586315946	0.583221813	0.580392893	0.579192506
Georgia	0.614089728	0.617292992	0.620547947	0.623799565	0.626667538	0.629195534	0.631285551	0.632891489	0.634258651	0.635635388	0.637247097	0.639454895	0.64234741	0.646470033	0.651101623	0.656136044	0.662113186	0.665021428	0.663466862	0.658134139	0.651363604	0.64455809	0.638525631	0.633347931	0.630277946
Kazakhstan	0.569739555	0.574859312	0.579872459	0.584858355	0.588795462	0.591232799	0.592663276	0.593348489	0.593307363	0.593180709	0.591925213	0.590898296	0.590374887	0.589966465	0.589463464	0.589435804	0.590967832	0.596127664	0.603138328	0.6105911	0.618573582	0.626013138	0.631166626	0.635388624	0.640180485
Kyrgyzstan	0.49113686	0.494876419	0.498414483	0.501443384	0.504083977	0.506103725	0.507346585	0.508099337	0.508632337	0.509461266	0.51045871	0.510979352	0.51137384	0.513085108	0.515695875	0.519407652	0.523746714	0.527901552	0.532431008	0.53623298	0.53905282	0.541628927	0.54334115	0.543258546	0.542867282
Mongolia	0.371754111	0.377960679	0.384495008	0.391086682	0.397710301	0.404545046	0.410939449	0.417731432	0.424202004	0.430573436	0.436600742	0.442690293	0.448616012	0.45430497	0.459899126	0.466550118	0.472199014	0.477586554	0.482726727	0.488276683	0.495006781	0.501703718	0.508558268	0.514793383	0.520781189
Tajikistan	0.410812332	0.414884738	0.418839952	0.422513868	0.425792098	0.428722412	0.431252233	0.43369313	0.436126158	0.438737776	0.441547338	0.444838719	0.448702418	0.454405219	0.460008462	0.466155413	0.472342173	0.475745195	0.478238813	0.478726011	0.477586868	0.474346027	0.470274796	0.465341742	0.460505616
Turkmenistan	0.513853251	0.517824962	0.521740605	0.525753376	0.529341537	0.532581854	0.535554261	0.538533692	0.541703204	0.544868161	0.547878907	0.550862762	0.55403316	0.557985535	0.560749007	0.563126887	0.564939386	0.565587505	0.567174449	0.567288976	0.567198635	0.567061261	0.565623084	0.564405264	0.564716006
Uzbekistan	0.473106197	0.476852536	0.48026472	0.483447718	0.486227356	0.488470809	0.489968129	0.491153234	0.492209369	0.493122022	0.494008	0.494828119	0.495902398	0.497772556	0.499284578	0.500241735	0.501544609	0.502905194	0.506556615	0.511018525	0.516986264	0.52439291	0.532686328	0.541279069	0.549739391
Central Europe	0.574219489	0.578528759	0.582971826	0.586768876	0.590839925	0.595664901	0.599532514	0.602183211	0.604750942	0.607523737	0.610965569	0.614893623	0.619185181	0.624749993	0.631219883	0.637272174	0.643082622	0.648772145	0.654469912	0.661219388	0.667954947	0.674121167	0.680037255	0.685968374	0.692056184
Albania	0.487017799	0.496393844	0.505192357	0.513256991	0.520471621	0.526911652	0.532539571	0.53753882	0.542113587	0.545973442	0.5490642	0.551751438	0.553739175	0.555221294	0.556930698	0.5577733	0.555629412	0.553107897	0.552453328	0.553763669	0.557040889	0.562248886	0.566802804	0.572534102	0.578295462
Bosnia and Herzegovina	0.454739566	0.460687455	0.467284694	0.474362999	0.48198725	0.489540301	0.496922256	0.503953804	0.510377483	0.516451151	0.522034045	0.527385165	0.532006243	0.536021638	0.539589525	0.54113254	0.541346251	0.539303227	0.536031882	0.53385339	0.534025536	0.545549215	0.563565415	0.581829515	0.597997469
Bulgaria	0.553740824	0.556821082	0.560671202	0.564694705	0.569955861	0.575937776	0.580520556	0.584777646	0.589695496	0.595052684	0.598647009	0.60165957	0.605925367	0.612756563	0.622760792	0.633446498	0.643069488	0.651186876	0.659161569	0.66743046	0.673999977	0.678862584	0.680221466	0.678496877	0.677446543
Croatia	0.600863736	0.606713063	0.611585629	0.616115666	0.621040028	0.625948373	0.629943115	0.633390704	0.636376071	0.640353445	0.645275978	0.649991038	0.65371905	0.657906386	0.662982715	0.668906358	0.6747246	0.678093133	0.678221047	0.677548133	0.677029465	0.679507318	0.68536501	0.691365519	0.696636411
Czechia	0.618669848	0.622186903	0.625581533	0.630475092	0.638878762	0.647827578	0.6527152	0.655394548	0.657695961	0.660040934	0.663932424	0.668319716	0.671715419	0.675089913	0.678338143	0.681848021	0.688002859	0.697577809	0.710853416	0.725969686	0.737981722	0.74604138	0.751680344	0.757033755	0.76242888
Hungary	0.569274165	0.574671134	0.581527319	0.588630694	0.596932604	0.605767823	0.61409279	0.621303847	0.625488474	0.627315354	0.629570361	0.633829912	0.6386332	0.643156103	0.646529744	0.649419991	0.653707155	0.660382905	0.667543556	0.675051406	0.683186412	0.690883334	0.697935514	0.70420272	0.709980627
Montenegro	0.613645258	0.620309771	0.627323535	0.634035814	0.639741987	0.646788091	0.652311905	0.657165602	0.661003539	0.664274806	0.666768495	0.669096613	0.670708025	0.672100351	0.673607764	0.67422572	0.674305723	0.672664772	0.668665719	0.665011931	0.662765437	0.663414769	0.665542032	0.669024771	0.672096103
North Macedonia	0.56089041	0.564209289	0.567231231	0.570571655	0.574319507	0.577998064	0.581777716	0.585418603	0.588666257	0.591950256	0.594830427	0.598023776	0.600786721	0.6033673	0.606470185	0.609026094	0.611337453	0.612708856	0.613195099	0.614362905	0.616994878	0.620944017	0.626223811	0.63205941	0.636687259
Poland	0.589355625	0.592943292	0.596669198	0.598165652	0.598484046	0.599200556	0.598253205	0.595953796	0.596630111	0.599943077	0.605133922	0.610534284	0.615122522	0.619639073	0.623877348	0.627227888	0.632671471	0.640661793	0.6489505	0.658010514	0.666678867	0.674378829	0.682587612	0.690805671	0.698536056
Romania	0.523194328	0.528660277	0.533748103	0.539190108	0.544903688	0.552054381	0.561068826	0.569537029	0.573686682	0.574256374	0.574272451	0.576329801	0.582058622	0.59245852	0.606508237	0.619298862	0.626795627	0.630475082	0.633801422	0.638371932	0.643718879	0.648104195	0.651225576	0.654927769	0.659544846
Serbia	0.566454555	0.570456598	0.574901248	0.57884846	0.58296455	0.588951845	0.593128795	0.594741824	0.597153095	0.600743456	0.605642189	0.609559826	0.612865003	0.618454979	0.624548816	0.63051102	0.638016934	0.641971994	0.642096034	0.642223221	0.642739671	0.644846417	0.647162094	0.650245989	0.655534174
Slovakia	0.605865969	0.608552674	0.610702504	0.612007859	0.616853841	0.623251057	0.626307321	0.627187135	0.628411049	0.629678025	0.633509659	0.639201414	0.643959336	0.648472501	0.651630331	0.653853505	0.658731402	0.665830111	0.675967937	0.688136477	0.697476536	0.704405003	0.71151321	0.719058766	0.726144173
Slovenia	0.647488438	0.650853815	0.654123481	0.657329841	0.662635026	0.669228099	0.675173683	0.681520934	0.688441383	0.694364649	0.69895274	0.703277437	0.708290188	0.714666779	0.721529582	0.727463928	0.732666986	0.737391552	0.741774172	0.746234861	0.750994975	0.756348987	0.762275292	0.767728526	0.773236192
Eastern Europe	0.633069214	0.636422518	0.63950606	0.641877365	0.644050261	0.646546266	0.647983331	0.647751211	0.648235209	0.65030993	0.651549107	0.652063357	0.653315833	0.655336287	0.6587192	0.664250165	0.671452375	0.678752887	0.683359195	0.686011586	0.689459344	0.692791353	0.695202953	0.696939833	0.69881365
Belarus	0.577409341	0.581455817	0.584873966	0.587825526	0.590715025	0.594203365	0.59777516	0.598781653	0.599932923	0.603531332	0.606135245	0.607857717	0.610020181	0.612566985	0.617359513	0.622446576	0.627038267	0.632891737	0.639394169	0.644652781	0.648959504	0.652855221	0.655627296	0.657246467	0.66017101
Estonia	0.63894581	0.640775891	0.643108325	0.645622867	0.647175602	0.648095702	0.65006535	0.651489648	0.652979616	0.65582452	0.657762741	0.656973907	0.656293863	0.658796325	0.665688248	0.674967632	0.685223089	0.695586674	0.70369315	0.708456181	0.713172725	0.718813024	0.72465244	0.729027035	0.733250616
Latvia	0.650871143	0.654798782	0.657691865	0.659136587	0.659635732	0.660981945	0.661526384	0.659864738	0.659553615	0.661420296	0.662219798	0.663127677	0.665579196	0.669401816	0.674080647	0.680193638	0.688117297	0.696157036	0.705249855	0.712531353	0.717639459	0.721139538	0.72374658	0.725427336	0.727219685
Lithuania	0.626492074	0.630688067	0.635069002	0.639479505	0.644870116	0.652086539	0.658576338	0.662416834	0.664536226	0.665775177	0.666941599	0.667912488	0.667703869	0.667400584	0.667327204	0.668503938	0.673234121	0.68099288	0.687480621	0.691151653	0.69473438	0.698591301	0.70284095	0.707468182	0.712834171
Moldova	0.579709054	0.583609988	0.587021483	0.589793187	0.591650316	0.592754089	0.593357432	0.593533348	0.593766823	0.594424438	0.595500995	0.59723372	0.598543988	0.599579008	0.601535526	0.604251762	0.607270774	0.609664558	0.61415292	0.617603767	0.620951321	0.623113984	0.624370105	0.624116761	0.622834529
Russia	0.641013274	0.644156539	0.647253516	0.649767757	0.652067441	0.654514677	0.655805177	0.655713903	0.656541006	0.658654626	0.659893562	0.66024988	0.661117906	0.662526769	0.6653755	0.671600578	0.680099211	0.688068022	0.692036194	0.694144966	0.697854214	0.701468231	0.703752867	0.705198518	0.706863538
Ukraine	0.613810274	0.61780909	0.620842083	0.622641434	0.624350662	0.626953869	0.628428893	0.627487991	0.6269903	0.629075522	0.630419621	0.631335391	0.633947392	0.637977027	0.643160354	0.647461466	0.651950021	0.657600333	0.662867405	0.665506375	0.666950167	0.668104744	0.669207846	0.670322301	0.670642905
High income	0.6860049	0.690821853	0.695537231	0.699778981	0.703501141	0.707686161	0.712444468	0.717459907	0.722716264	0.727750825	0.732457021	0.736822637	0.740653953	0.74422797	0.7477962	0.751589646	0.755897938	0.760450188	0.764678286	0.768692619	0.772362673	0.775646213	0.778714489	0.781567859	0.78449535
Australasia	0.654930309	0.66308771	0.670343538	0.676845542	0.682062728	0.685858294	0.689918626	0.694455516	0.699975724	0.70527836	0.709999077	0.714961182	0.719655998	0.724079539	0.727781089	0.731234397	0.734915293	0.739005622	0.743426014	0.747726041	0.752015072	0.756387302	0.760990093	0.765299502	0.769521272
Australia	0.646254065	0.654266895	0.661374595	0.667951862	0.67333341	0.677126776	0.681031207	0.685483038	0.691304069	0.696940181	0.702090783	0.707764355	0.713214724	0.718230863	0.722307649	0.725982519	0.729759859	0.733901404	0.738374743	0.742846572	0.747426824	0.751991736	0.756633901	0.761133265	0.765697506
New Zealand	0.687587653	0.696562291	0.704806777	0.711323747	0.716202413	0.72054969	0.725916658	0.731371317	0.735928745	0.740065504	0.743069454	0.744940586	0.746183723	0.74784186	0.749834018	0.752321655	0.755616091	0.759461372	0.763634604	0.767118658	0.769998036	0.773575283	0.778205776	0.781730757	0.784428638
High-income Asia Pacific	0.677844313	0.684424905	0.690417226	0.695597198	0.700694836	0.7055453	0.710232484	0.715416918	0.721560239	0.728809716	0.735995085	0.74253355	0.749100887	0.755710432	0.762006233	0.767803786	0.773332908	0.778834454	0.783967344	0.789028457	0.794099242	0.798862943	0.803127763	0.806831345	0.810468966
Brunei	0.54808298	0.557183679	0.565267057	0.573398716	0.582230162	0.591269088	0.600324124	0.609195876	0.617785135	0.625425407	0.632386792	0.639321917	0.646528698	0.65366606	0.660202399	0.666081917	0.671233831	0.676702526	0.68217544	0.687394093	0.692471156	0.697602233	0.702944386	0.708630119	0.714851237
Japan	0.711220194	0.718592436	0.725361948	0.731552146	0.737416864	0.74263454	0.747135291	0.750890396	0.754768577	0.759704294	0.764629107	0.769316577	0.774436507	0.779852068	0.785320012	0.790253516	0.794616189	0.799156225	0.802972994	0.806689483	0.810510771	0.813870746	0.816675782	0.819016202	0.821066637
Aichi	0.716378843	0.724172772	0.731207163	0.737521907	0.743464659	0.748871755	0.753757865	0.757859586	0.761985611	0.767249395	0.772583265	0.777654569	0.783328661	0.789335943	0.795394569	0.800854834	0.805713722	0.810525696	0.814428732	0.818156687	0.821991858	0.825359323	0.827980287	0.830020885	0.831752637
Akita	0.657694581	0.665984396	0.674187251	0.681551483	0.68872598	0.695405251	0.700762585	0.705230077	0.709649503	0.715161589	0.720496333	0.725450973	0.730363539	0.735070484	0.739707006	0.743856278	0.747574745	0.751582445	0.755273102	0.759436294	0.764014469	0.768510645	0.772345859	0.775568933	0.778279035
Aomori	0.643634968	0.65299641	0.661785848	0.669817693	0.677329081	0.683574939	0.688686782	0.69297673	0.69724597	0.702996809	0.708598524	0.713765224	0.719251935	0.724772747	0.73024966	0.734632276	0.738220585	0.742503691	0.746331387	0.750745944	0.755390883	0.759330321	0.762402008	0.765051749	0.767594613
Chiba	0.713316385	0.720424974	0.726566115	0.732087704	0.737482501	0.742039997	0.746047397	0.749374171	0.752855238	0.757492143	0.762466334	0.767212092	0.772345581	0.777871815	0.783514555	0.788716613	0.793422788	0.798192042	0.80219287	0.806285916	0.810242865	0.813860435	0.816764764	0.818997279	0.820582065
Ehime	0.678611183	0.686932832	0.693940085	0.700362101	0.706756635	0.711826398	0.716235501	0.720104443	0.723872347	0.728720073	0.733168765	0.737308483	0.741790976	0.7465509	0.751577796	0.756008354	0.759776393	0.764179312	0.768344579	0.772700377	0.777227284	0.781698624	0.785531592	0.789125021	0.792193405
Fukui	0.66932111	0.678520143	0.686741275	0.69390998	0.700940241	0.707944895	0.714217713	0.719482352	0.724730034	0.731042886	0.737228797	0.742858103	0.748980005	0.755257252	0.761332543	0.766481771	0.770995682	0.775919886	0.780107783	0.784392079	0.788687729	0.792784195	0.796523142	0.799770058	0.802613732
Fukuoka	0.712448467	0.719834014	0.726517297	0.73260308	0.738668133	0.743839701	0.747892972	0.750827101	0.75368912	0.757558573	0.761401525	0.765085791	0.76923232	0.773623867	0.777967997	0.781940943	0.785543342	0.789628637	0.793282003	0.797043517	0.800880098	0.804436744	0.807588525	0.810293078	0.81268464
Fukushima	0.65822213	0.667373255	0.676007967	0.683960344	0.691382019	0.697787058	0.702921264	0.706966963	0.711126533	0.716783971	0.722318528	0.727841048	0.733617724	0.739265822	0.744748891	0.749067309	0.752895552	0.757341189	0.760912537	0.764659856	0.768845833	0.772560203	0.775574524	0.777881776	0.780010634
Gifu	0.6862101	0.69385663	0.700750406	0.706990671	0.713013807	0.718929922	0.724085865	0.72840373	0.732855126	0.738295143	0.743531311	0.748554975	0.754016597	0.759772737	0.765524277	0.77061016	0.775167156	0.779921097	0.784063798	0.787935465	0.791899237	0.795713773	0.798845458	0.801413024	0.803599335
Gunma	0.689688751	0.697583686	0.704735405	0.711143216	0.717271848	0.722875349	0.727841205	0.731787768	0.736035094	0.741739704	0.74702428	0.751714642	0.756848484	0.762458719	0.767946767	0.772669278	0.776841517	0.781302495	0.78512725	0.789110353	0.793289401	0.796752555	0.799746577	0.801983844	0.803736657
Hiroshima	0.713192895	0.721387128	0.728526579	0.734852719	0.740638689	0.745518819	0.749565752	0.752755498	0.756120709	0.760702379	0.765124867	0.769343797	0.773946762	0.778904486	0.784047586	0.788739945	0.792996904	0.797721054	0.801685783	0.805563645	0.809687101	0.81343513	0.816491478	0.818883202	0.820972318
Hokkaidō	0.689132181	0.697503203	0.705361208	0.712354526	0.718923838	0.724642774	0.729234199	0.732975074	0.736434767	0.740816717	0.745071406	0.749043445	0.753398262	0.757897526	0.762501265	0.766618759	0.770545049	0.775002204	0.778997897	0.783027038	0.787104355	0.790624396	0.793472978	0.795841261	0.797971991
Hyōgo	0.718752125	0.72638271	0.733315369	0.739608419	0.745643653	0.750450015	0.754404255	0.757556874	0.760917614	0.765437351	0.769746264	0.773643633	0.778205579	0.783337531	0.788546103	0.793422254	0.797854129	0.8025584	0.806739758	0.810591501	0.815021468	0.818935508	0.821963838	0.824275794	0.826204856
Ibaraki	0.683752885	0.692735927	0.700647307	0.707442272	0.713798828	0.719849385	0.724673347	0.728728075	0.732813306	0.738508752	0.74450505	0.750145906	0.755601032	0.76133802	0.766937556	0.771923199	0.776240273	0.780828611	0.784864357	0.789197702	0.7934051	0.796815911	0.799620314	0.80165805	0.803334713
Ishikawa	0.67746643	0.685942055	0.693789233	0.700804379	0.707478179	0.713763992	0.719411182	0.724137457	0.729159383	0.735356064	0.741318018	0.746815938	0.752906903	0.75939058	0.766008328	0.772257224	0.777743697	0.783048924	0.787478366	0.791675216	0.796129464	0.800359655	0.803859554	0.807072346	0.810199083
Iwate	0.647667195	0.656681171	0.665202283	0.673069108	0.680355525	0.686329033	0.691184636	0.695095323	0.699361998	0.705299801	0.710919836	0.716091945	0.721544911	0.726671536	0.731849437	0.736184911	0.740022447	0.744750245	0.748974983	0.753613781	0.758465624	0.762700621	0.766128626	0.768943899	0.77169128
Kagawa	0.691300872	0.699812679	0.707463814	0.713896098	0.720053117	0.725606706	0.729925156	0.733298652	0.736859459	0.741701353	0.746350577	0.750801025	0.755437917	0.760445881	0.765968105	0.771183472	0.776049865	0.781312969	0.785691208	0.789987341	0.794422443	0.797920663	0.800638876	0.803051941	0.805151605
Kagoshima	0.655727776	0.66475667	0.673040417	0.680368607	0.686996609	0.693119523	0.698346672	0.702936694	0.707740222	0.7135625	0.719023983	0.723926626	0.728762438	0.733781038	0.738837004	0.743332441	0.747341162	0.751621404	0.755297035	0.759208018	0.763288767	0.766955466	0.770187831	0.773109002	0.775891103
Kanagawa	0.748751057	0.754713928	0.760183036	0.765279333	0.770287738	0.774962216	0.779216897	0.782701773	0.786223359	0.790341151	0.794552777	0.798493194	0.802982832	0.807646574	0.812414897	0.817021862	0.821082745	0.825155986	0.828639824	0.832064556	0.835578487	0.839006037	0.841826124	0.843998149	0.845619122
Kōchi	0.659811295	0.668641371	0.677055974	0.684773988	0.691997019	0.697857016	0.702257062	0.705872827	0.709581721	0.714579359	0.719507187	0.724177608	0.72902114	0.733720252	0.738273194	0.74200445	0.745306112	0.749418836	0.753381034	0.757898646	0.762671737	0.767032245	0.770866113	0.774272806	0.77745194
Kumamoto	0.667546079	0.676600846	0.684753103	0.691927879	0.698759497	0.70476974	0.709777695	0.71382029	0.717875197	0.723355024	0.728813343	0.733949685	0.739191679	0.7443413	0.74936445	0.753731159	0.757620049	0.761919289	0.765363874	0.768826777	0.77248233	0.775352054	0.777632614	0.779395773	0.781053455
Kyōto	0.729049789	0.735505423	0.741681009	0.747603074	0.752980554	0.757954063	0.762192258	0.765341315	0.768371669	0.772328047	0.776789016	0.781017557	0.785724478	0.790679798	0.795497229	0.799903927	0.804110731	0.808471829	0.812385279	0.816066282	0.8196826	0.8229264	0.825615659	0.828035273	0.83024396
Mie	0.681087861	0.688864702	0.695794139	0.701773124	0.707704239	0.713147704	0.717578946	0.721096775	0.724739647	0.729780762	0.734880914	0.739902987	0.745610186	0.751811991	0.75816	0.763830036	0.768821888	0.77394322	0.778119696	0.782166127	0.786285717	0.790482619	0.793990014	0.79686977	0.799521316
Miyagi	0.687283877	0.69548467	0.703180643	0.710203357	0.716704373	0.722375122	0.727305485	0.731486605	0.735703642	0.741063831	0.746420268	0.751547871	0.756999743	0.762336922	0.767714246	0.77250193	0.777005693	0.781843374	0.785881585	0.789943637	0.793938841	0.797152556	0.799749629	0.801736292	0.80353098
Miyazaki	0.652849785	0.661253282	0.669314911	0.676767491	0.683556197	0.689525702	0.694534837	0.698886822	0.70315303	0.708545062	0.713671194	0.71876397	0.72423548	0.729607575	0.734840447	0.73910863	0.742497639	0.74629663	0.749475469	0.752936057	0.75675515	0.760702676	0.764435684	0.767829809	0.77098375
Nagano	0.69962284	0.707070985	0.714257275	0.720801166	0.726884454	0.732466181	0.737320564	0.741079072	0.744961082	0.749599025	0.753990963	0.758166311	0.762587745	0.767274102	0.772068801	0.776599842	0.780650743	0.784828945	0.78830185	0.791792619	0.795782568	0.799540002	0.802747372	0.805406739	0.80787798
Nagasaki	0.662415194	0.671181125	0.678815335	0.685322936	0.691457706	0.697004365	0.70169091	0.705500715	0.709475433	0.714507831	0.71931181	0.72370468	0.728261783	0.732745686	0.737606563	0.741965825	0.745796116	0.750451731	0.754563164	0.758831053	0.763247037	0.766907912	0.769883826	0.772443732	0.774924835

Nara	0.708079763	0.714431081	0.720302332	0.725960558	0.731201366	0.735206452	0.738377733	0.741236793	0.744604084	0.749021127	0.753600696	0.757710102	0.762473794	0.767706747	0.773187763	0.77849492	0.783115286	0.78771718	0.79188812	0.796021231	0.800103269	0.804190177	0.807484441	0.810068329	0.812418526
Niigata	0.670581505	0.679045892	0.68713775	0.694498769	0.701140525	0.707346485	0.712524202	0.716661195	0.720772354	0.726063609	0.731415553	0.736535539	0.741671773	0.746642286	0.751528867	0.755942624	0.760144111	0.764757048	0.768690995	0.772862924	0.77731692	0.781102782	0.784394068	0.787313486	0.790064429
Ōita	0.679662923	0.68762299	0.695469069	0.702837074	0.709752622	0.715998745	0.720986326	0.724975093	0.729159865	0.734437825	0.739215337	0.743697028	0.748455849	0.753567682	0.759170266	0.763945301	0.768203854	0.772857222	0.776865072	0.780927437	0.785177869	0.788782354	0.792177023	0.794961335	0.797469451
Okayama	0.696099995	0.704169198	0.711313617	0.717590592	0.723451151	0.728342839	0.732262555	0.73544616	0.73917599	0.744679276	0.750104137	0.755322252	0.760894845	0.766712348	0.772394784	0.777378183	0.781781398	0.786620382	0.790512314	0.794237375	0.798187391	0.801324812	0.803913327	0.806408327	0.808773094
Okinawa	0.633858401	0.644802646	0.654044327	0.662210896	0.670077112	0.676751631	0.682559786	0.687450376	0.692308914	0.698622973	0.704952407	0.71148536	0.718372822	0.724817988	0.730922539	0.735453216	0.739066472	0.743244	0.746807885	0.75059463	0.754516933	0.757191367	0.759079599	0.760650243	0.762281886
Ōsaka	0.739420417	0.745953394	0.751973018	0.757215747	0.761842677	0.76604371	0.769455223	0.772177234	0.774897193	0.778553737	0.782207596	0.78568708	0.789903885	0.794796638	0.799716953	0.804615792	0.809253996	0.814292929	0.818462434	0.822327463	0.826243329	0.829397081	0.831927222	0.834077574	0.835937911
Saga	0.671526067	0.679784235	0.687431499	0.69435853	0.700675778	0.706368992	0.71108761	0.714997989	0.718725158	0.72336709	0.728106512	0.732889919	0.737775292	0.742547272	0.7471603	0.75102028	0.754552146	0.758988687	0.763078633	0.767195485	0.771356713	0.774823474	0.777808464	0.780496425	0.783006033
Saitama	0.705414172	0.712638747	0.719007109	0.724875622	0.730622651	0.735790248	0.740298596	0.743842694	0.747514532	0.752342256	0.757217341	0.761674789	0.766650857	0.772042904	0.777483279	0.782473382	0.787026452	0.7919327	0.796062996	0.799950232	0.803740545	0.807022733	0.809773681	0.811852464	0.813468055
Shiga	0.707585882	0.714533629	0.721465666	0.728223112	0.73478716	0.740459956	0.744566806	0.74825351	0.752159063	0.757311401	0.762656041	0.767629366	0.773689345	0.780477816	0.787179343	0.792940745	0.798110131	0.80365653	0.808228355	0.812630828	0.816938757	0.820598571	0.823777408	0.826135886	0.828136198
Shimane	0.65702213	0.665571235	0.673146671	0.679676724	0.685902863	0.691877608	0.696883155	0.700604278	0.704467812	0.710332741	0.715054019	0.719470211	0.724638849	0.730012168	0.735107266	0.739553491	0.743602718	0.748040781	0.751888094	0.756163362	0.760864658	0.765509795	0.769777421	0.773586608	0.776878236
Shizuoka	0.696947698	0.704698054	0.71187935	0.718416514	0.724677616	0.72998394	0.734554427	0.738399302	0.742479272	0.747803959	0.753145122	0.758342781	0.763803178	0.769519847	0.775266118	0.780199834	0.784630605	0.789107265	0.792731175	0.796358359	0.800079048	0.803573974	0.806491752	0.808828856	0.810842339
Tochigi	0.6889634	0.697517007	0.705122423	0.712166654	0.719121494	0.724957892	0.729355782	0.732721886	0.736272733	0.740945622	0.745511277	0.750201127	0.755507687	0.761259844	0.767142037	0.772234384	0.776753976	0.781631541	0.785867332	0.789941714	0.794094128	0.797501856	0.800171508	0.802235255	0.80389371
Tokushima	0.673506764	0.68218869	0.689369883	0.695756808	0.701606422	0.706523869	0.710522617	0.713785615	0.717822531	0.723326233	0.728228747	0.732753019	0.737641506	0.742931245	0.748694662	0.7538988	0.758620934	0.763702302	0.767994545	0.772177487	0.776639766	0.780695318	0.784247331	0.787488054	0.790802494
Tōkyō	0.792699383	0.798984216	0.804733964	0.809997444	0.814929429	0.819277621	0.823457574	0.827203284	0.831041498	0.835602033	0.840263701	0.844809809	0.84985257	0.855330541	0.861024383	0.866280939	0.870778848	0.875074043	0.878657556	0.881894869	0.885104598	0.887853602	0.890256549	0.892409102	0.894271154
Tottori	0.674548793	0.683591338	0.691465492	0.698329477	0.704811047	0.710286392	0.714650867	0.717982743	0.721657889	0.72703001	0.732309094	0.737522168	0.743001075	0.748295483	0.753501903	0.757802249	0.761596984	0.766011272	0.769773137	0.773845827	0.778242264	0.782119283	0.785438087	0.788207078	0.790690858
Toyama	0.683932807	0.692611657	0.700749994	0.708471841	0.716130771	0.722720836	0.728001412	0.732374899	0.736990219	0.742442595	0.747831884	0.753218642	0.758934821	0.765015508	0.771156348	0.776809557	0.781820272	0.786683387	0.790791202	0.794681022	0.798741596	0.802895812	0.806275928	0.809217162	0.811903678
Wakayama	0.687508635	0.694095165	0.699464603	0.703386952	0.706053424	0.708431199	0.710870604	0.713489858	0.71684246	0.722582968	0.728546246	0.733724483	0.738986506	0.744192739	0.749270987	0.753739111	0.757745147	0.76215474	0.766116135	0.769938364	0.773914914	0.777687657	0.780840732	0.78355184	0.786085234
Yamagata	0.658870489	0.667442201	0.67570305	0.683342592	0.690619745	0.697278588	0.702898944	0.707589168	0.711816175	0.716776189	0.72160017	0.72647451	0.731688724	0.73685149	0.741908775	0.746246131	0.750074468	0.754225012	0.757638499	0.761162835	0.764952999	0.768540479	0.771656499	0.774287768	0.776609157
Yamaguchi	0.69683159	0.704230552	0.710629332	0.716297842	0.721760329	0.726588812	0.730240567	0.733144499	0.736721569	0.741542662	0.746285638	0.750673191	0.755768576	0.761141669	0.766303409	0.770512746	0.774213385	0.778637263	0.782122399	0.785735172	0.789678824	0.793129836	0.796171204	0.798722798	0.800975841
Yamanashi	0.694382027	0.701782465	0.708337646	0.714701842	0.720720145	0.726350121	0.731480735	0.735634157	0.740028622	0.74547308	0.751127302	0.755967889	0.761221458	0.766749119	0.772432158	0.777354522	0.781500795	0.785662771	0.78921375	0.792929522	0.796882169	0.800258994	0.803108456	0.805065438	0.806818886
South Korea	0.526364442	0.537586595	0.547742533	0.5571267	0.565689135	0.572827314	0.580983556	0.591168672	0.604314543	0.618504904	0.632336641	0.645791816	0.658455429	0.670795768	0.681912046	0.692329307	0.702631128	0.712440842	0.72228674	0.732197906	0.74194003	0.75155324	0.760591683	0.768281885	0.776773436
Singapore	0.546755378	0.559005074	0.570841486	0.58126999	0.590943375	0.600567381	0.610427856	0.619969723	0.629425137	0.638533137	0.646777566	0.654596187	0.661834567	0.669834801	0.677845829	0.686404444	0.695055558	0.703427834	0.712315204	0.721256797	0.729982231	0.739028235	0.747571898	0.754873204	0.760831136
High-income North America	0.734293506	0.737481983	0.740407477	0.742904143	0.744602274	0.746935982	0.750221832	0.753566815	0.757135201	0.760126177	0.76280236	0.765284022	0.766354872	0.765796236	0.765001791	0.765659862	0.768049984	0.771549819	0.774977626	0.778206755	0.781348237	0.784104747	0.78634761	0.788569028	0.791196294
Canada	0.724080285	0.729451626	0.735113681	0.740482364	0.744939549	0.749141443	0.753062643	0.756617165	0.760756701	0.765338487	0.769863434	0.773932138	0.777196654	0.77911309	0.780462416	0.781977864	0.78415936	0.787030537	0.789716593	0.792598518	0.796521166	0.80106497	0.804833356	0.808135441	0.81226594
Greenland	0.706849497	0.711145974	0.716291499	0.721014176	0.724031534	0.72731353	0.731386856	0.735285514	0.738431157	0.739474072	0.740241425	0.740031413	0.738041563	0.735671014	0.73447558	0.732258252	0.730626532	0.729781103	0.729894842	0.730875896	0.730831638	0.730216225	0.730551087	0.733242588	0.73655713
USA	0.735185665	0.738141015	0.740757435	0.742914886	0.744290928	0.746410428	0.749633105	0.752971768	0.756490765	0.759316396	0.761797203	0.764115508	0.764950695	0.764128282	0.763095783	0.76364769	0.766044295	0.769595492	0.773103517	0.776368588	0.779410254	0.78193477	0.783979278	0.786076332	0.788544535
Alabama	0.685422482	0.689205485	0.693337141	0.69755218	0.700320002	0.704975409	0.711057026	0.715938946	0.719504043	0.721789883	0.724170197	0.727067502	0.727866966	0.725948555	0.723861687	0.723971524	0.726751726	0.731172405	0.735422776	0.739157219	0.742448805	0.744779226	0.745964171	0.747298417	0.749782983
Alaska	0.725798612	0.732627174	0.73838161	0.742831553	0.74387355	0.741629113	0.736201251	0.730787074	0.730246707	0.73298464	0.739983059	0.74930527	0.755083256	0.754668953	0.749386336	0.745456697	0.745077011	0.748832169	0.755756001	0.762254073	0.767586469	0.771153384	0.773369618	0.774814237	0.776839421
Arizona	0.717089075	0.722216369	0.726033912	0.728740677	0.730174346	0.730997078	0.731963668	0.732662828	0.733613027	0.734124692	0.73478737	0.736253829	0.736908767	0.736377049	0.735810141	0.736537113	0.738789693	0.741489192	0.743897802	0.745861893	0.74756578	0.749188043	0.750627388	0.752124723	0.753689992
Arkansas	0.670364248	0.674964868	0.679699468	0.683630464	0.684664706	0.688194532	0.693927594	0.697383487	0.699565703	0.701361687	0.704071743	0.707384241	0.707462749	0.704769415	0.701921143	0.702645719	0.707897114	0.714654406	0.718950616	0.721219502	0.722524935	0.724044374	0.726629462	0.729878243	0.733090647
California	0.753205373	0.755155798	0.757001498	0.758875239	0.760353373	0.761824818	0.763594057	0.765145602	0.76672237	0.767544089	0.767740528	0.767470596	0.765512715	0.761654283	0.757484935	0.75549857	0.756354327	0.759310592	0.763141736	0.767219317	0.771536824	0.77579064	0.77980965	0.78378332	0.787586289
Colorado	0.759439627	0.763397577	0.766158555	0.767519398	0.766801557	0.765649412	0.765075142	0.766882787	0.77115991	0.774439862	0.776890974	0.780438421	0.783637883	0.784807783	0.784387672	0.783966537	0.784800899	0.788062706	0.792444753	0.7964655	0.799673811	0.801839991	0.802452207	0.80204853	0.801964458
Connecticut	0.78584845	0.787695749	0.789269525	0.79103456	0.793148408	0.795886657	0.7993033	0.802895613	0.807009877	0.810906655	0.814136172	0.817093884	0.819010153	0.820235614	0.82167974	0.824312178	0.827833018	0.831569583	0.83447056	0.836957596	0.839146647	0.840550575	0.841612081	0.843664579	0.847149752
Delaware	0.752609507	0.755878791	0.758509868	0.760890324	0.762821856	0.764805486	0.767199468	0.769502492	0.772052872	0.774342098	0.776467069	0.778593486	0.779941418	0.780585007	0.781564031	0.783519768	0.786707633	0.790250993	0.793239859	0.795656336	0.797818794	0.799505987	0.800487658	0.801527954	0.803179514
Washington, DC	0.768546063	0.771962826	0.775013064	0.777412231	0.778588942	0.779251115	0.78016315	0.781274301	0.782799181	0.784225733	0.785491567	0.786538266	0.786401771	0.78498512	0.783923839	0.785119619	0.788995157	0.794617144	0.801002677	0.807851272	0.814989188	0.821998365	0.828325838	0.834034202	0.839474011
Florida	0.733776577	0.739021495	0.742621596	0.744482038	0.743736411	0.743549605	0.745518079	0.748075529	0.751326002	0.753831897	0.75593019	0.75783203	0.757557482	0.755416293	0.753727687	0.755299508	0.759577357	0.764214506	0.768635499	0.772756742	0.776116633	0.77817577	0.779535058	0.781221493	0.783279839
Georgia	0.699770125	0.704866332	0.709145114	0.712876943	0.71571039	0.718178001	0.721054251	0.723896972	0.727027668	0.729797335	0.732288511	0.734912718	0.736347956	0.736660319	0.737241545	0.739511622	0.743490724	0.748286271	0.752772815	0.756508865	0.75943308	0.761496988	0.76281708	0.764068933	0.765608241
Hawaii	0.741504824	0.746426848	0.751219229	0.754492168	0.754974464	0.755654801	0.755971275	0.755464206	0.75806387	0.763421352	0.767779774	0.770040173	0.770585452	0.76926104	0.76612565	0.765363789	0.767370787	0.770565923	0.774531808	0.780201178	0.785663235	0.790617446	0.794722364	0.795831504	0.797273841
Idaho	0.694443231	0.697188211	0.699742492	0.702613629	0.704897939	0.708188105	0.712903511	0.718110693	0.724446805	0.729859622	0.734389592	0.738087371	0.739547707	0.739122775	0.737633931	0.736462409	0.73732233	0.740411992	0.744371806	0.74781858	0.751005264	0.754557185	0.757603191	0.760381495	0.763630894
Illinois	0.744471966	0.746718399	0.749133499	0.750237475	0.749643505	0.751872777	0.757004989	0.761837348	0.766070134	0.769574076	0.772643272	0.77475693	0.77461829	0.772428904	0.769665756	0.769244886	0.771253878	0.774669165	0.778294843	0.782376993	0.787158475	0.791538331	0.79467225	0.797249579	0.799893083
Indiana	0.720585276	0.722888967	0.725414505	0.727567438	0.72896073	0.733085666	0.738670228	0.743360703	0.747512775	0.750238684	0.752726727	0.755189836	0.755081778	0.752631268	0.750544317	0.750328787	0.752783713	0.756627315	0.760335613	0.763370222	0.765609249	0.767485194	0.768930907	0.77055891	0.77291208
Iowa	0.736423036	0.737716806	0.739047322	0.740774566	0.741462316	0.744730051	0.750649421	0.755789193	0.760285152	0.764694522	0.769288525	0.773334495	0.774954694	0.774019768	0.772781542	0.77326663	0.775424525	0.778950883	0.782341469	0.78534145	0.787707685	0.790466653	0.793014723	0.795066897	0.797782149
Kansas	0.733634739	0.735578352	0.737727867	0.738861889	0.737907918	0.738129021	0.741213561	0.74577691	0.750948222	0.755559724	0.759028721	0.762758836	0.765391915	0.765746969	0.765096042	0.765782681	0.767582978	0.770095478	0.773377613	0.776860843	0.780048096	0.781992392	0.782827972	0.783895322	0.78541963
Kentucky	0.680319055	0.683077462	0.686897725	0.6909346	0.693296119	0.697267477	0.702761436	0.708276612	0.713781812	0.718075018	0.721625878	0.724835709	0.726431449	0.724789944	0.721830789	0.721698825	0.72457641	0.729310325	0.733694156	0.737528023	0.740997266	0.743590395	0.745736402	0.747721967	0.749786034
Louisiana	0.682408559	0.685382657	0.688229892	0.690451967	0.690668036	0.691285595	0.693096033	0.696464204	0.701697097	0.706206078	0.711138619	0.717003699	0.72006801	0.719769725	0.718271619	0.717356733	0.718644553	0.722292552	0.727389573	0.733052482	0.73756337	0.739839768	0.740648793	0.742007099	0.744576037
Maine	0.720966486	0.723113987	0.726134847	0.730304299	0.733253773	0.736608977	0.740629211	0.744571109	0.748792806	0.75262954	0.756271192	0.759888249	0.762233034	0.763144918	0.765024596	0.76873693	0.774261462	0.780753171	0.786406603	0.790840643	0.794018772	0.795956749	0.796972597	0.798548665	0.801118613
Maryland	0.769439092	0.771930449	0.774619541	0.777557325	0.780283915	0.782777742	0.784903591	0.787425747	0.790446582	0.792017295	0.79311482	0.794896779	0.795493351	0.794816828	0.794523907	0.796023679	0.800123865	0.805640343	0.810165128	0.813717476	0.816936095	0.819304762	0.820462715	0.821113793	0.822317266
Massachusetts	0.782321075	0.784995815	0.787416621	0.789559534	0.791216002	0.793586171	0.797294361	0.801454853	0.805506556	0.809154875	0.812921563	0.816520794	0.818830361	0.820133576	0.822067356	0.825701409	0.829794176	0.833579189	0.83688148	0.840107349	0.843307106	0.845791623	0.847710767	0.850036784	0.853151393
Michigan	0.741699731	0.74393054	0.745741033	0.747285261	0.749595945	0.75468868	0.760467145	0.764976863	0.768185783	0.769899135	0.771479866	0.772964252	0.77315874	0.770580977	0.767012162	0.766908044	0.770918039	0.776702532	0.781831965	0.78626406	0.790168022	0.792991452	0.795174389	0.79715201	0.799259746
Minnesota	0.754386763	0.756315785	0.757906339	0.759394661	0.760332702	0.762925369	0.767664766	0.773638899	0.778942987	0.783010296	0.787528469	0.792248814	0.795215169	0.796372554	0.797524021	0.799549491	0.802800135	0.806657992	0.809858578	0.812711516	0.815039875	0.816853863	0.818606518	0.820740585	0.823131666
Mississippi	0.66065119	0.665687967	0.670074921	0.673150715	0.67383464	0.67644421	0.680991181	0.685730151	0.690744363	0.694877201	0.699161485	0.70295425	0.703267214	0.700512118	0.697901508	0.697709614	0.700217175	0.704872701	0.709860449	0.714793277	0.719703472	0.722782499	0.723481129	0.724910651	0.727549666
Missouri	0.723692864	0.72557927	0.726864248	0.727634032	0.727686106	0.7304803	0.735431556	0.740002021	0.744459396	0.747262851	0.749855772	0.753228412	0.754547612	0.7533793	0.751439287	0.751221706	0.754144786	0.759033459	0.763528036	0.767727876	0.770676976	0.772616132	0.774189081	0.775833232	0.778570909
Montana	0.723424667	0.727253321	0.730500255	0.733557217	0.734598507	0.735789663	0.737654103	0.739364571	0.741804293	0.745365629	0.750319406	0.755154562	0.758548607	0.759069611	0.757853163	0.756650394	0.757937467	0.761274633	0.765982268	0.770667046	0.775171489	0.779342863	0.782092826	0.785168355	0.788348735
Nebraska	0.735913882	0.738984492	0.741990905	0.744000537	0.743346857	0.743673289	0.747296002	0.751826847	0.756848423	0.762121092	0.76722358	0.771746901	0.774056528	0.774215311	0.773918281	0.775521295	0.778575121	0.782198328	0.785008349	0.78729054	0.790375571	0.793533526	0.795744941	0.797403922	0.798826658
Nevada	0.749036578	0.753540819	0.755425272	0.754704676	0.752661235	0.752268145	0.754526144	0.759374042	0.764216897	0.766369373	0.766614467	0.765433707	0.76166955	0.755084755	0.747592962	0.744408991	0.747795607	0.75322181	0.756591811	0.758586729	0.760947269	0.763339269	0.764626561	0.766305068	0.76947594
New Hampshire	0.755485533	0.758906731	0.761675331	0.764838566	0.767436623	0.771614165	0.777235636	0.782910281	0.788149374	0.790859618	0.793055019	0.795621489	0.797763302	0.800589718	0.80526684	0.811328701	0.81700015	0.821128436	0.823177296	0.824703999	0.826108506	0.827137369	0.829009623	0.832449006	0.835975626
New Jersey	0.770558022	0.77342963	0.77601025	0.778326513	0.780608957	0.784060883	0.787929722	0.791471568	0.795551756	0.799234978	0.802204684	0.804904942	0.806365893	0.807121592	0.808642481	0.811232625	0.814871765	0.81932112	0.822530721	0.825335771	0.828572215	0.830945354	0.83222171	0.833528425	0.835453315
New Mexico	0.696992978	0.700875381	0.703493946	0.704966275	0.705226906	0.705712318	0.707134703	0.709156838	0.712001561	0.714382063	0.716853844	0.719317437	0.720085218	0.718667076	0.716537963	0.715668063	0.7165368	0.719172257	0.722824713	0.726743025	0.730670777	0.734164709	0.737302352	0.740407672	0.743575257
New York	0.762777185	0.765750228	0.768641842	0.771330569	0.77355461	0.775636054	0.778103508	0.780741197	0.783759981	0.786567643	0.7890121	0.791371868	0.793142395	0.794227044	0.795398117	0.79741955	0.800179201	0.803449532	0.806504513	0.809459648	0.812489092	0.815392499	0.818000166	0.820494984	0.823397251
North Carolina	0.703381448	0.708999654	0.714141222	0.718882043	0.722826766	0.726419639	0.730110853	0.733290385	0.736192504	0.738335056	0.739941394	0.741507942	0.742298315	0.742228022	0.742433698	0.743977619	0.747181909	0.75142845	0.755446059	0.758702636	0.761241318	0.763040923	0.764300516	0.765614356	0.767476174
North Dakota	0.720514006	0.724034371	0.726832799	0.730067248	0.732000916	0.733345567	0.736468916	0.740031088	0.744309959	0.749226765	0.75451497	0.759743945	0.763846433	0.766102297	0.768517795	0.771430284	0.774178767	0.777204987	0.780101317	0.783372445	0.786797633	0.790927725	0.794867929	0.799168564	0.802966297
Ohio	0.730998976	0.733974397	0.736552931	0.738747617	0.740690365	0.743176702	0.746619074	0.750201646	0.753805387	0.756697101	0.759010799	0.760875361	0.761642462	0.761175217	0.760691056	0.761347777	0.763467823	0.766720356	0.769983371	0.773023773	0.775826089	0.778193383	0.780258958	0.782408296	0.784924235
Oklahoma	0.704053401	0.706663404	0.708033085	0.707760221	0.705853399	0.70384166	0.703616914	0.706342593	0.711984349	0.718243432	0.724508148	0.730199736	0.733399987	0.733755512	0.733120629	0.733039141	0.734196414	0.736747722	0.739537192	0.741859464	0.743721209	0.744911312	0.745878648	0.747298975	0.749183419
Oregon	0.749590419	0.75162703	0.75311167	0.754439817	0.755593171	0.757593424	0.760891328	0.764171159	0.767468335	0.76959628	0.770761418	0.771380559	0.770942021	0.769614746	0.768515656	0.769007728	0.771098606	0.774222179	0.777396013	0.780262258	0.783117654	0.786069235	0.789017842	0.79214028	0.795603641
Pennsylvania	0.74347327	0.747053937	0.750305225	0.753189155	0.755685925	0.758187588	0.761383587	0.764708072	0.76815789	0.771022054	0.773398594	0.775619074	0.77715558	0.778115688	0.779230673	0.781295559	0.784392033	0.788268353	0.792143491	0.795839015	0.799306425	0.802375058	0.804980941	0.807457013	0.810210115
Rhode Island	0.752563607	0.756283878	0.759562079	0.762428999	0.764864816	0.767460491	0.770584178	0.773586971	0.776777892	0.779761335	0.782548861	0.78544129	0.788068272	0.790294347	0.792544652	0.795258398	0.798390144	0.801855402	0.805080215	0.807904471	0.810442142	0.812506454	0.814513325	0.816822887	0.819318879
South Carolina	0.689994219	0.696278972	0.701857997	0.706768726	0.710814846	0.714648739	0.71875936	0.722479926	0.725935674	0.728303387	0.72990502	0.731264386	0.731479106	0.730479978	0.729895324	0.731363176	0.735334588	0.740915117	0.746431495	0.751163651	0.754990851	0.757648952	0.759363511	0.760937156	0.763031619
South Dakota	0.709828561	0.712736553	0.715609792	0.718375944	0.720699547	0.722793449	0.725889697	0.728997183	0.732629731	0.736557046	0.740439641	0.744301287	0.746966094	0.748316049	0.749376501	0.751220476	0.753878059	0.757773382	0.762220786	0.766643094	0.770649708	0.77477888	0.77838801	0.781724628	0.784752668
Tennessee	0.695952108	0.700424492	0.70440607	0.708203667	0.711654541	0.715237218	0.719245055	0.723006369	0.726516836	0.729018917	0.730699824	0.732032177	0.732165517	0.73094373	0.729762262	0.73014798	0.732744426	0.736784404	0.740865341	0.74467146	0.748223265	0.751039118	0.753174397	0.755385064	0.75781867
Texas	0.7056081	0.709245821	0.71149006	0.71220961	0.711423434	0.710815809	0.711902661	0.714132381	0.717690949	0.721001184	0.724427201	0.727903715	0.729774642	0.729416514	0.728316311	0.728266043	0.729424929	0.731642933	0.733962991	0.736074692	0.738228462	0.740187744	0.741934487	0.743758711	0.745750934

Utah	0.699049456	0.698146331	0.697587233	0.697421415	0.697676697	0.700024437	0.70550426	0.712928164	0.720915185	0.727437664	0.732956687	0.737803388	0.740806623	0.742055943	0.742768131	0.744547789	0.747423518	0.75088115	0.754103143	0.756690593	0.759085491	0.761724198	0.764769513	0.768070193	0.771590884
Vermont	0.746389474	0.75053381	0.753905609	0.756759082	0.758991726	0.761407296	0.764704938	0.768141021	0.771847709	0.775454233	0.779098588	0.782985121	0.786535428	0.789426943	0.792410483	0.795613598	0.799050645	0.802863714	0.80640126	0.809688005	0.812681885	0.815343961	0.818030649	0.821075338	0.824411585
Virginia	0.741993555	0.746594401	0.751138391	0.755579659	0.759381242	0.762917246	0.766813952	0.770375018	0.773860897	0.776642368	0.778898533	0.781059621	0.782551382	0.78333634	0.783997177	0.785556276	0.788297264	0.791798398	0.795226744	0.798412739	0.801287236	0.803612093	0.805400288	0.806999575	0.809009858
Washington	0.76100259	0.762803256	0.7640359	0.764991793	0.765636291	0.767013539	0.769779631	0.772899332	0.776269922	0.778749955	0.780611289	0.782150662	0.782518503	0.781580522	0.78064173	0.781101794	0.783192157	0.786443145	0.789887398	0.793226943	0.796465958	0.799622268	0.80278059	0.806271493	0.810109375
West Virginia	0.683374	0.687071532	0.690823999	0.694516404	0.697931067	0.701721081	0.706415392	0.711113857	0.715742633	0.7195075	0.722583956	0.725171486	0.726292848	0.726024886	0.725420074	0.725935457	0.72805707	0.7316111	0.735513184	0.739303585	0.742828588	0.745902371	0.748529084	0.750992443	0.753469156
Wisconsin	0.74308983	0.746146043	0.748905681	0.751344004	0.753444144	0.755983337	0.759362524	0.762786574	0.766630725	0.770261809	0.773694203	0.777046869	0.779588939	0.78104645	0.782304261	0.784207391	0.786860748	0.790207111	0.793541709	0.796764779	0.799800129	0.802553387	0.80497178	0.80727955	0.809696788
Wyoming	0.717549822	0.718624641	0.718384438	0.71679784	0.714092638	0.7128697	0.714646487	0.719161257	0.725477409	0.731684807	0.738158426	0.744512642	0.748536522	0.749605779	0.749792825	0.750880744	0.753215731	0.756800183	0.76077982	0.764501193	0.768054327	0.771234146	0.77449507	0.778014938	0.781583393
Southern Latin America	0.533806069	0.538370065	0.543225952	0.546447899	0.5493445	0.553201292	0.558394509	0.564058064	0.568978486	0.572725882	0.57443356	0.57650784	0.579113237	0.58196439	0.584606109	0.587308121	0.592118694	0.598079761	0.603588571	0.609392016	0.614372849	0.619260048	0.624756487	0.630349086	0.635748461
Argentina	0.536965534	0.539815276	0.5435016	0.544645985	0.546884347	0.552400631	0.558916031	0.564851269	0.569574921	0.572225178	0.572163855	0.574142754	0.577756688	0.582052419	0.585439617	0.587397284	0.59170754	0.597996324	0.603739312	0.60978733	0.614473706	0.618942624	0.623934409	0.628723436	0.633175129
Chile	0.525652937	0.533087358	0.539703619	0.546194858	0.549547236	0.550115782	0.553382262	0.558920487	0.564352854	0.570251166	0.575354192	0.577525532	0.578532383	0.579413176	0.581611855	0.5864951	0.59300658	0.599106083	0.604828509	0.610673933	0.617234677	0.62369446	0.630540085	0.638082758	0.645928084
Uruguay	0.524639102	0.529121603	0.533577643	0.53899033	0.545208089	0.551479599	0.557381236	0.561755782	0.565484048	0.568647813	0.571101489	0.573217512	0.574873803	0.576342898	0.578639256	0.581921855	0.586156118	0.589822355	0.592295025	0.595188424	0.596825409	0.599775104	0.605665051	0.612700815	0.619009271
Western Europe	0.669763605	0.674844759	0.680148781	0.685206826	0.689824149	0.69507567	0.700832027	0.706714423	0.712557215	0.717896023	0.722949433	0.727633622	0.731912102	0.736519785	0.741404562	0.746400479	0.751670034	0.757021934	0.762056783	0.766693114	0.770621721	0.774079855	0.777577691	0.780866892	0.784019197
Andorra	0.718279796	0.724282352	0.729540015	0.733708551	0.736416963	0.738815508	0.741319265	0.744283107	0.747356279	0.750005159	0.75212911	0.754002594	0.755458398	0.757205009	0.75862722	0.76146388	0.764709071	0.767188282	0.768953828	0.770023765	0.770988683	0.772403471	0.774580971	0.776972578	0.779629634
Austria	0.677857272	0.68679832	0.693343893	0.697734945	0.700034756	0.701620096	0.705109239	0.711670355	0.719134757	0.725394544	0.730944236	0.73582757	0.739966477	0.743791612	0.747462378	0.749853693	0.751652734	0.754375286	0.758804554	0.764704364	0.769827471	0.774446565	0.779232856	0.783521016	0.787451584
Belgium	0.656372856	0.662369337	0.667605667	0.672342138	0.67743155	0.682734369	0.688936074	0.696040391	0.702653151	0.70912336	0.714872268	0.719892127	0.724432921	0.728941539	0.733357194	0.737390656	0.742173074	0.748012299	0.753685087	0.758903638	0.763170401	0.766826075	0.770813953	0.774228268	0.77704104
Cyprus	0.57390996	0.575065674	0.577265862	0.578048003	0.579824682	0.583990097	0.58814941	0.591708517	0.596886849	0.604342555	0.611705753	0.618083195	0.624610738	0.632398398	0.640628796	0.648230872	0.655726169	0.666011849	0.677547477	0.688829361	0.699923898	0.709839946	0.718864244	0.727964382	0.736846135
Denmark	0.725802818	0.734171642	0.740408741	0.746206583	0.752661419	0.75966374	0.766156502	0.772032138	0.776941511	0.780906391	0.784786786	0.789145249	0.792974064	0.795708305	0.79814824	0.801154655	0.80454035	0.808164434	0.811585713	0.815138614	0.819104612	0.823375243	0.827679979	0.831683237	0.835381543
Finland	0.679662363	0.685405281	0.691840171	0.697867	0.703371478	0.708541698	0.71268043	0.717142537	0.722865945	0.729382405	0.735939171	0.741678026	0.74600091	0.74951717	0.753136896	0.756221509	0.758410687	0.7608041	0.763526604	0.767101901	0.771163742	0.774809059	0.778814215	0.782305059	0.785773988
France	0.647932415	0.653253546	0.658177739	0.663509085	0.66751874	0.6719179	0.677651846	0.684840458	0.691444591	0.696935057	0.702518332	0.708588261	0.714424006	0.719875374	0.725431659	0.730747466	0.736452382	0.742871973	0.748582327	0.753176132	0.757125789	0.760899579	0.764650672	0.767852561	0.770304363
Germany	0.75061305	0.754461604	0.759130658	0.76390049	0.767348303	0.771005943	0.775961971	0.781613565	0.787763874	0.793034142	0.797126793	0.800206572	0.803027232	0.806785723	0.811260443	0.817077666	0.823339898	0.828323503	0.832286809	0.835561963	0.837643152	0.839188129	0.841385903	0.843854978	0.846232398
Greece	0.583673252	0.586455077	0.589450067	0.591644872	0.594682542	0.600661907	0.608028502	0.615060968	0.622684623	0.631595553	0.640378447	0.647976203	0.654551165	0.66159025	0.668375864	0.674186465	0.68031648	0.687055389	0.693328926	0.699153022	0.704862873	0.710139336	0.715687268	0.721593982	0.727529455
Iceland	0.666882989	0.676073459	0.683795757	0.689410088	0.695250327	0.70252077	0.710222059	0.718538808	0.727059047	0.735482152	0.742113907	0.746820979	0.750994908	0.755120164	0.759390606	0.764212517	0.769027393	0.772706415	0.776389423	0.779967961	0.782740057	0.78535642	0.788528335	0.792626368	0.797368808
Ireland	0.63057433	0.636343593	0.641909093	0.646681826	0.650941344	0.65679637	0.664272464	0.671957071	0.679201592	0.686269237	0.692591194	0.69854397	0.704817435	0.710720093	0.715587162	0.719891819	0.725195019	0.731918691	0.738384074	0.744011728	0.748921575	0.754010124	0.759970384	0.766488721	0.773538834
Israel	0.621834745	0.628058262	0.636285474	0.643527178	0.649409065	0.654847084	0.659279406	0.663848278	0.669996704	0.676720861	0.683525001	0.689598893	0.695146347	0.700178033	0.704800003	0.709178347	0.713371633	0.717897923	0.722250176	0.726654736	0.731127859	0.734714026	0.738015277	0.74192719	0.745443338
Italy	0.607323677	0.614723689	0.622531132	0.630272116	0.638219991	0.646004252	0.652339956	0.658295982	0.664875447	0.671520338	0.678220405	0.68440542	0.689687493	0.695121101	0.700834576	0.706255224	0.711457349	0.716649746	0.721720992	0.726870913	0.731644082	0.73605087	0.739972336	0.743101596	0.746074084
Abruzzo	0.615929286	0.623501805	0.631244776	0.638921638	0.646751938	0.654417079	0.660697538	0.666625476	0.673094212	0.679599137	0.686090007	0.69216168	0.697509779	0.703047231	0.708928829	0.714650756	0.720314474	0.726036648	0.731441857	0.736853274	0.742135794	0.747013718	0.75138555	0.754985849	0.758414771
Basilicata	0.551095813	0.560083918	0.569492933	0.578865949	0.58837163	0.597648175	0.605087451	0.612011632	0.619677953	0.627487524	0.635040246	0.641912553	0.647829235	0.653922208	0.660294214	0.666507396	0.672611491	0.678846405	0.684857076	0.690931633	0.696773911	0.702437784	0.707815571	0.712597373	0.717439425
Calabria	0.529067984	0.539080826	0.549746388	0.560235853	0.570844286	0.58095398	0.589202585	0.596844802	0.605536423	0.614101721	0.622750424	0.630460136	0.636811229	0.643347567	0.650374927	0.656864369	0.663315571	0.669924506	0.676566868	0.683266057	0.689455482	0.695133568	0.700346283	0.704675627	0.70898274
Campania	0.546710656	0.555033946	0.564201081	0.5733159	0.582672251	0.591746557	0.598835771	0.605722552	0.613787543	0.621936099	0.63016775	0.637391782	0.643197727	0.649188787	0.655628281	0.661702253	0.667520742	0.673477318	0.679435188	0.685497259	0.690950704	0.695804806	0.700110353	0.703349843	0.706425625
Emilia-Romagna	0.620486702	0.628455996	0.636805829	0.645068213	0.653417499	0.66149498	0.667984262	0.673952855	0.680600984	0.687302263	0.694029324	0.700217621	0.705393359	0.710783306	0.71653717	0.721948871	0.727184143	0.732437186	0.737706301	0.743180402	0.748223375	0.753051528	0.757340662	0.760781386	0.764140844
Friuli-Venezia Giulia	0.637580059	0.644258499	0.651023022	0.657693689	0.664588986	0.67129433	0.676936855	0.682200425	0.687667701	0.693217476	0.698775748	0.704149706	0.709032927	0.714142915	0.719592696	0.724866124	0.72994147	0.734873099	0.739543768	0.74431686	0.74912879	0.753536933	0.757480159	0.760771763	0.763955725
Lazio	0.647012931	0.653797502	0.660746621	0.667661055	0.674749812	0.681748824	0.687570764	0.693036221	0.699017774	0.705081362	0.711167698	0.716903729	0.722002318	0.727147069	0.732488239	0.737620712	0.742562604	0.747476738	0.75217172	0.756881007	0.761258174	0.765200121	0.768876599	0.772108974	0.77514763
Liguria	0.652225453	0.657851664	0.663533067	0.669208596	0.675144182	0.681024754	0.686007553	0.690653841	0.69553761	0.700687339	0.705935794	0.710967844	0.715581949	0.720310183	0.725320266	0.730184642	0.734884027	0.739396416	0.743578301	0.747799606	0.751953443	0.75585102	0.759456466	0.762584744	0.765514591
Lombardia	0.656309236	0.662803363	0.669410163	0.675989479	0.682739754	0.689384568	0.694997915	0.700213901	0.705740941	0.711307865	0.716950878	0.722342613	0.727187305	0.73221193	0.737420402	0.742347949	0.747008975	0.751487308	0.755700771	0.759947659	0.763880108	0.767494336	0.770699542	0.773316188	0.77567219
Marche	0.593590396	0.601635261	0.610118738	0.618506521	0.626977908	0.63525514	0.641840058	0.647984135	0.654820218	0.661629623	0.668474225	0.67476553	0.680133135	0.685645386	0.691487941	0.697071608	0.702424493	0.70784338	0.713228463	0.718742438	0.723813645	0.728647889	0.733058156	0.736742308	0.740419601
Molise	0.560766926	0.569841959	0.579319131	0.588669432	0.598075293	0.607214357	0.614582285	0.621477822	0.629132197	0.63680315	0.644403388	0.651367136	0.657414562	0.66375792	0.67037206	0.676593732	0.682668668	0.688908551	0.694994766	0.701219137	0.707208041	0.712892946	0.718413588	0.723261661	0.727778774
Piemonte	0.636207013	0.642564969	0.649074072	0.655513144	0.662148294	0.668633711	0.674023711	0.679077889	0.684463009	0.689942142	0.695501409	0.700762918	0.705495386	0.71039444	0.715515911	0.720360415	0.724925882	0.729438003	0.733675332	0.738002739	0.742133738	0.745882478	0.749299394	0.752084872	0.754728908
Provincia autonoma di Bolzano	0.623143189	0.631677974	0.640539232	0.649296214	0.658194267	0.666804325	0.673767657	0.680343739	0.687638669	0.695003016	0.702351284	0.709070301	0.71472158	0.720558878	0.726640359	0.732400638	0.737857914	0.743168764	0.748346382	0.753629005	0.758853531	0.76380399	0.767954752	0.771384014	0.774456235
Provincia autonoma di Trento	0.643601395	0.651061172	0.658701978	0.666295395	0.674010802	0.681598143	0.68781824	0.693625661	0.699832157	0.706067532	0.712206479	0.717854836	0.722815446	0.727960671	0.733226041	0.73819977	0.74282506	0.747253469	0.751445442	0.755646179	0.759487795	0.763090364	0.76618372	0.768732353	0.77128382
Puglia	0.54393763	0.552525195	0.561821685	0.570948495	0.580234921	0.589229375	0.596254702	0.602869199	0.610485849	0.618135932	0.625854883	0.632841679	0.638692924	0.64476074	0.651215211	0.657350626	0.663352452	0.669409029	0.675292038	0.681240093	0.686760664	0.691821949	0.69621578	0.69972705	0.703207369
Sardegna	0.582012405	0.589769273	0.597802837	0.605678929	0.613638158	0.621417667	0.627780226	0.633728516	0.640125672	0.646628725	0.652976689	0.658848616	0.663978412	0.669202232	0.6746603	0.679922875	0.685123182	0.690290443	0.695245687	0.70019795	0.704781418	0.708967469	0.712966475	0.716299229	0.719548668
Sicilia	0.539895183	0.548806912	0.558515164	0.56798161	0.577599343	0.586868927	0.594025468	0.600917199	0.609056803	0.61732495	0.625649133	0.632962616	0.638725685	0.644621939	0.650972929	0.657025838	0.662897641	0.668946236	0.675082629	0.681367885	0.687160758	0.692495615	0.696977061	0.700008669	0.702797087
Toscana	0.620324574	0.627501142	0.634875608	0.642142589	0.649583404	0.656835552	0.663012664	0.668841535	0.674889255	0.680879853	0.686937538	0.692623889	0.697691139	0.702852618	0.708149388	0.713229274	0.718119176	0.722962647	0.727582851	0.732172134	0.736513094	0.740530304	0.744217322	0.747383239	0.750402163
Umbria	0.610875718	0.618560588	0.626452794	0.63420337	0.642018736	0.649625818	0.655845119	0.661791041	0.668069025	0.674187453	0.680308461	0.685966893	0.690907459	0.696069204	0.701479784	0.706686715	0.711703294	0.716750971	0.721590593	0.726422295	0.731104304	0.735293537	0.739163012	0.742302398	0.745232881
Valle d'Aosta	0.633547114	0.640337132	0.647382059	0.654431266	0.661727621	0.668876579	0.674760945	0.680379472	0.686667804	0.693093411	0.699650097	0.70572732	0.710906902	0.716121601	0.721580445	0.726784665	0.731705275	0.736548149	0.741102591	0.745640193	0.750137728	0.754142816	0.757432036	0.759929736	0.762095115
Veneto	0.622099286	0.629126794	0.636269379	0.643386167	0.650671479	0.657885011	0.664038229	0.66977004	0.675810854	0.681893817	0.687969629	0.693771277	0.69894006	0.70424098	0.709738591	0.714979957	0.719998279	0.724834198	0.729421168	0.733951591	0.738130376	0.742059383	0.745613004	0.748588806	0.751361817
Luxembourg	0.714224102	0.719091421	0.723460693	0.72747478	0.731584918	0.735787019	0.740135702	0.745207797	0.750302203	0.75489344	0.759241136	0.763977065	0.767610045	0.771024971	0.775585298	0.781051609	0.785961442	0.789053364	0.792843836	0.797824008	0.80268247	0.806516138	0.810235288	0.813889026	0.817570995
Malta	0.563151355	0.572343338	0.581544041	0.590403211	0.598990711	0.607091235	0.614284743	0.620940154	0.626785701	0.631948768	0.636401587	0.640141232	0.643913648	0.648028093	0.652206403	0.656504584	0.661112691	0.666324648	0.672120257	0.677847314	0.682617704	0.686623261	0.691756406	0.698095287	0.704734545
Monaco	0.793297355	0.79730867	0.801183477	0.805054201	0.808848004	0.812478976	0.815777155	0.818979258	0.822039742	0.825075248	0.828073773	0.831174877	0.834447433	0.837988755	0.841735508	0.845495153	0.84830369	0.85086394	0.853264992	0.855626927	0.857986147	0.860245432	0.86244672	0.864716513	0.866842394
Netherlands	0.729466722	0.735645559	0.741276462	0.745923826	0.750018746	0.7544748	0.75949739	0.763775043	0.767324006	0.771072819	0.775217477	0.779448391	0.783359608	0.786985545	0.790554139	0.794612123	0.799285437	0.803965978	0.808278967	0.812530216	0.816662769	0.81998422	0.82288177	0.825882234	0.828693827
Norway	0.71989992	0.728513194	0.735408914	0.741061372	0.746933367	0.753025534	0.758790929	0.764821711	0.770759723	0.775711301	0.779883364	0.783565504	0.786106321	0.788284411	0.791636504	0.795887277	0.800468886	0.805437473	0.810565689	0.815414668	0.820223522	0.825701668	0.83146877	0.836562809	0.841130237
Agder	0.70855138	0.716906845	0.723592912	0.728954891	0.734853408	0.741010537	0.746938111	0.753309743	0.759618622	0.764984923	0.769379191	0.772988597	0.7751956	0.776939678	0.779887442	0.783912098	0.788332788	0.79314313	0.798179607	0.802898816	0.807571929	0.81314131	0.819160991	0.824508265	0.829429423
Innlandet	0.701081462	0.71049614	0.718120251	0.724287423	0.730471819	0.736659443	0.742483997	0.748557189	0.754631809	0.75971283	0.76377283	0.767248516	0.769842614	0.772179842	0.775611216	0.78002909	0.784849736	0.789925941	0.795040066	0.800016866	0.804874963	0.81013378	0.815499936	0.820122385	0.824149118
Møre og Romsdal	0.697312739	0.706608561	0.713575353	0.719020357	0.724876671	0.731102444	0.737126054	0.743686509	0.750441297	0.756230084	0.761237346	0.765682812	0.76854207	0.770810091	0.774429353	0.779159162	0.784374841	0.789929274	0.7956521	0.800960694	0.805986693	0.811592672	0.817417706	0.822556658	0.827212892
Nordland	0.684125831	0.693459942	0.700828212	0.706770971	0.713082674	0.719647109	0.726180308	0.733161835	0.739957538	0.745379715	0.749680708	0.753343899	0.75550686	0.757037846	0.76010844	0.764634665	0.769725265	0.775292839	0.780791239	0.785865667	0.790924709	0.796892447	0.802989116	0.808160059	0.81237422
Oslo	0.779765685	0.785851302	0.791036006	0.795652204	0.800453524	0.805506903	0.810179635	0.814855781	0.819279598	0.823079072	0.826568639	0.829703897	0.831960887	0.833820722	0.836396334	0.839440851	0.842718294	0.846455149	0.850400684	0.854382432	0.858623102	0.863699754	0.869486225	0.874881254	0.879981632
Rogaland	0.706487836	0.714885155	0.72137047	0.726710104	0.732951473	0.740500896	0.747824263	0.755540822	0.762894019	0.76883842	0.77360119	0.777639063	0.780270794	0.782547803	0.785905624	0.790232798	0.795102916	0.800853825	0.806845242	0.812333764	0.817488085	0.823108012	0.829018909	0.834332203	0.839124112
Troms og Finnmark	0.69218593	0.703596001	0.712551713	0.719417616	0.725807421	0.732122165	0.738033826	0.744345242	0.75091319	0.75640027	0.760960155	0.764808291	0.767135395	0.768916539	0.771883887	0.77581152	0.780265229	0.7853149	0.790702564	0.796009998	0.80143653	0.807533278	0.813683372	0.818758455	0.822973826
Trøndelag	0.701569855	0.711706365	0.719722052	0.726054451	0.73232055	0.738796447	0.745073125	0.751582106	0.758094392	0.763340442	0.76766836	0.771551776	0.77413911	0.776343304	0.780121591	0.784941155	0.790034372	0.795463405	0.800889108	0.805914648	0.810700901	0.816224863	0.822159304	0.827407436	0.8320705
Vestfold og Telemark	0.715111802	0.723992315	0.73113366	0.736919147	0.742744555	0.748559323	0.753803466	0.759222017	0.764678699	0.769265244	0.773183084	0.776740185	0.779272264	0.78169124	0.785447812	0.790051833	0.794780111	0.79954847	0.804272723	0.808580434	0.812995348	0.818342689	0.824152521	0.829269348	0.833812082
Vestland	0.711571794	0.720349262	0.727120564	0.732548258	0.738329309	0.744263692	0.749933659	0.75608256	0.762287336	0.767514754	0.771952638	0.775942657	0.778790094	0.781318	0.785026694	0.789791575	0.79490379	0.800378133	0.806037267	0.811382054	0.816491018	0.822167287	0.827889616	0.832915531	0.837348232
Viken	0.736178284	0.745055158	0.752267602	0.758336369	0.764524985	0.770657595	0.776260652	0.781958779	0.787424268	0.791998282	0.795873016	0.799368691	0.801908891	0.804208246	0.807641274	0.811900165	0.816358577	0.821078772	0.825920984	0.830499396	0.835018647	0.840030512	0.845170776	0.849684467	0.853773523
Portugal	0.502809991	0.502032338	0.508734792	0.51768046	0.525579342	0.532679647	0.5388883	0.54456932	0.550374925	0.55711943	0.564394116	0.571127845	0.577772297	0.584730784	0.59214894	0.599777757	0.607269449	0.61480808	0.622101177	0.629306387	0.635498761	0.640864526	0.646192904	0.651335994	0.656120667
San Marino	0.711581055	0.717834789	0.724644949	0.731937758	0.739824288	0.74779501	0.755520709	0.762886002	0.769944163	0.776835815	0.783475427	0.78980863	0.795940409	0.801963138	0.80778051	0.813244888	0.818282513	0.822782758	0.827382324	0.831970983	0.836762726	0.841389495	0.845864865	0.850474771	0.855338613
Spain	0.529215224	0.5328777	0.539114952	0.546819851	0.554658351	0.56315025	0.571583803	0.579623946	0.587122931	0.594106964	0.601082551	0.607725658	0.614449062	0.621557695	0.629045388	0.636673166	0.644130745	0.651597597	0.658637569	0.665321549	0.671576034	0.677088943	0.682429497	0.687490717	0.692245197
Sweden	0.734739217	0.741766037	0.747487309	0.752151563	0.755846774	0.759853355	0.764635241	0.769355404	0.77348516	0.776733238	0.778878816	0.780705714	0.781792173	0.782292178	0.783005271	0.785535792	0.790096441	0.795431458	0.801009522	0.806700264	0.812244654	0.817227025	0.821694059	0.825731995	0.829487099
Stockholm	0.785803675	0.791807709	0.796433585	0.799604935	0.802407112	0.806562656	0.811756988	0.816517645	0.820044958	0.82235027	0.823326317	0.823948425	0.824632933	0.825236848	0.826148521	0.828649292	0.832525028	0.837056407	0.841511909	0.846125387	0.850654749	0.854868259	0.858962185	0.862507239	0.865644835
Sweden except Stockholm	0.722159149	0.729369425	0.735275532	0.740213861	0.74406123	0.74797357	0.75259259	0.757225246	0.761437752	0.764838296	0.767215886	0.769291797	0.770455226	0.770922736	0.77159612	0.774127452	0.778841201	0.784342898	0.790150522	0.796045895	0.801770911	0.806862729	0.811330613	0.815395871	0.819215685
Switzerland	0.816023855	0.821728235	0.82633781	0.829254233	0.830842309	0.83254267	0.834887724	0.838018452	0.841885504	0.846201236	0.850356529	0.854095961	0.856907455	0.859134224	0.86113925	0.862766844	0.864796867	0.867736079	0.870863558	0.873140871	0.874835331	0.876640557	0.878485545	0.880035152	0.882045586
UK	0.686952064	0.69390845	0.698120207	0.69967736	0.701529849	0.70590333	0.711531234	0.716156075	0.72013148	0.723132802	0.726041413	0.728923926	0.731698346	0.73579531	0.740221427	0.744334126	0.749148231	0.754556689	0.76015435	0.7651646	0.768656864	0.771576484	0.775050122	0.779057165	0.784036475

England	0.688960513	0.695845661	0.700091682	0.701653233	0.703573559	0.708282549	0.714178173	0.718854507	0.722765925	0.725615477	0.728270902	0.73089416	0.733327714	0.737015191	0.741184908	0.745220814	0.750010695	0.755362083	0.760861507	0.765852166	0.769445553	0.772571526	0.776369611	0.780575801	0.785737637
East Midlands	0.66102298	0.668482362	0.6732384	0.675324608	0.677784438	0.682976498	0.689307202	0.694342208	0.69845713	0.701438349	0.704125992	0.70663315	0.708804583	0.712286054	0.716411927	0.720595588	0.725382189	0.730646772	0.736028016	0.740970402	0.744731977	0.748029077	0.751865275	0.756017311	0.761092911
Derby	0.667357881	0.675244829	0.679804922	0.681076645	0.682776978	0.687972343	0.694853943	0.700580407	0.705772986	0.709677893	0.713117754	0.716428278	0.718991205	0.722253106	0.725645176	0.729286457	0.734133474	0.740362913	0.747124171	0.753028474	0.757013791	0.759883575	0.763064357	0.766716021	0.771726951
Derbyshire	0.659306813	0.666558993	0.671161189	0.673137741	0.675438739	0.680421464	0.686512619	0.691283402	0.695122571	0.697872077	0.700226339	0.702379712	0.704129081	0.706900403	0.71005267	0.713327582	0.717621039	0.722732128	0.727957391	0.732604274	0.735976543	0.738909926	0.742314924	0.74595707	0.750737546
Leicester	0.634886977	0.643574617	0.648870792	0.650764486	0.653083737	0.658942582	0.666501206	0.67272832	0.677901783	0.681986336	0.686116873	0.69029811	0.694086562	0.698838491	0.703969103	0.709100479	0.714837923	0.720499844	0.725856916	0.731189577	0.735508537	0.739395071	0.743846264	0.748201117	0.753065154
Leicestershire	0.69508693	0.701769853	0.706155789	0.708297754	0.710849703	0.715585412	0.721226124	0.725773148	0.729439581	0.732155014	0.734663987	0.736711612	0.738463924	0.741650362	0.745404062	0.749605138	0.754437304	0.759361182	0.764242331	0.76853066	0.771947325	0.775105495	0.778598236	0.782224664	0.786641392
Lincolnshire	0.660847176	0.668165338	0.67276943	0.674672954	0.676873734	0.681780901	0.68779255	0.692338334	0.695797448	0.698145337	0.700096853	0.701597941	0.702550914	0.705270808	0.709350176	0.713279193	0.717375606	0.721714956	0.726455465	0.731051075	0.734487907	0.73750007	0.741067708	0.744968286	0.749813215
Northamptonshire	0.674786199	0.682421917	0.687191498	0.689076374	0.691273736	0.696377481	0.702818985	0.708019033	0.712106036	0.714578988	0.716443086	0.718094488	0.719353225	0.722288776	0.726518145	0.731115469	0.736430778	0.742413863	0.748219029	0.753135116	0.756541719	0.759175237	0.76205477	0.764865474	0.768415453
Nottingham	0.671064212	0.678729731	0.683632295	0.685814006	0.688418662	0.693890082	0.700581249	0.70604558	0.710732939	0.714563489	0.718242003	0.721921782	0.725544495	0.730265993	0.735145301	0.739549358	0.744347228	0.749752031	0.755675822	0.761270252	0.765718479	0.769870011	0.774833163	0.780210006	0.785997331
Nottinghamshire	0.66014475	0.667292561	0.671829248	0.673783332	0.676062919	0.680975339	0.686948792	0.691550779	0.695235961	0.69777903	0.700088857	0.702412869	0.7044673	0.707755005	0.711552482	0.715311951	0.71968933	0.724606164	0.729613454	0.734297939	0.737770308	0.740783285	0.74447891	0.748548904	0.753457256
Rutland	0.71127601	0.717514307	0.721365047	0.722814829	0.724435723	0.728398867	0.73350636	0.737816778	0.74166892	0.744842738	0.747908696	0.751094626	0.754062181	0.758046283	0.762388796	0.766192523	0.769723391	0.773297051	0.777004638	0.780391624	0.783018585	0.785485478	0.788347669	0.790914093	0.793736539
East of England	0.68297451	0.689787912	0.694049312	0.695724901	0.697828318	0.702791846	0.709232634	0.714397744	0.718424832	0.721292897	0.723840098	0.726172073	0.728377501	0.732261046	0.736966261	0.741478228	0.746266375	0.751345668	0.756660707	0.761739487	0.765640975	0.768889275	0.772612162	0.776766244	0.781857965
Bedford	0.689167758	0.69597323	0.700125332	0.701474043	0.703216938	0.708145736	0.714961351	0.720960529	0.725896545	0.729300464	0.73239268	0.735153967	0.737513109	0.741462474	0.746546958	0.751540717	0.756436515	0.761124416	0.766028513	0.770676544	0.774226978	0.77774583	0.78174237	0.785784479	0.79042273
Cambridgeshire	0.720956556	0.727059127	0.731068767	0.73298772	0.73534954	0.740077469	0.746001168	0.750849064	0.754666322	0.757406793	0.760166297	0.762957164	0.765527779	0.769513492	0.774308746	0.779009799	0.783985181	0.78906669	0.794080165	0.798821016	0.802652426	0.805838099	0.809458806	0.813666046	0.818714151
Central Bedfordshire	0.68887446	0.695840758	0.700155141	0.701669072	0.703547457	0.708498847	0.715118584	0.720476437	0.724516775	0.727260583	0.730053159	0.732960801	0.735761801	0.74012669	0.745238403	0.749851975	0.754455551	0.759106474	0.764070857	0.769062023	0.77297579	0.775964356	0.779029842	0.782147226	0.786078181
Essex	0.675151222	0.682012835	0.686303824	0.688028922	0.690200046	0.695179773	0.701547527	0.70657322	0.710508132	0.713414509	0.715914139	0.717939897	0.719759298	0.723340025	0.727949656	0.732582426	0.737593163	0.742876315	0.748384483	0.753574414	0.757518885	0.760760768	0.764430182	0.768444949	0.773439012
Hertfordshire	0.725075553	0.731156324	0.735083072	0.736857625	0.739053756	0.743696238	0.749613669	0.754493158	0.758563717	0.76164428	0.764270085	0.766664878	0.768993472	0.772830462	0.777333859	0.781615003	0.786069739	0.790941758	0.796102505	0.801069367	0.804952951	0.808196939	0.811891827	0.816073697	0.821142625
Luton	0.654962932	0.662957314	0.667531487	0.668580905	0.670229242	0.675856736	0.683661774	0.690042674	0.695047822	0.698719316	0.701417341	0.703692321	0.706055193	0.710084192	0.715353074	0.720456369	0.725553255	0.730739857	0.736365577	0.741805998	0.746002485	0.74981492	0.753976308	0.757898739	0.76232555
Norfolk	0.665562785	0.672532869	0.676900282	0.678647522	0.680809654	0.685810344	0.692251287	0.697242242	0.701063073	0.703678029	0.705877657	0.707934603	0.709937863	0.713757774	0.718291366	0.722519771	0.726932383	0.731763446	0.736990424	0.742026703	0.745919726	0.749228008	0.752979265	0.757142663	0.762415377
Peterborough	0.661596828	0.669886182	0.67460479	0.675642024	0.677095659	0.682540553	0.690197058	0.696272384	0.700630727	0.703170619	0.705455443	0.707915984	0.709881893	0.713574798	0.717621734	0.720906113	0.724486723	0.728629234	0.733591239	0.738835596	0.742688022	0.74577596	0.749385711	0.753029136	0.75782605
Southend-on-Sea	0.6475814	0.654901499	0.659511885	0.661360489	0.663662799	0.669091941	0.676180983	0.682163328	0.68662899	0.689205962	0.691366634	0.694275617	0.697939365	0.702700577	0.70721961	0.710768644	0.714455755	0.718963861	0.724101622	0.729301332	0.733065945	0.735870203	0.739467426	0.74388285	0.749656346
Suffolk	0.668740592	0.676108554	0.680542789	0.681868553	0.683566067	0.688666386	0.695625231	0.701140563	0.705237462	0.70815129	0.710903377	0.713191384	0.715156353	0.71903496	0.723913544	0.728635207	0.734038687	0.73963033	0.74494139	0.749561717	0.752729101	0.755145422	0.758265898	0.76219203	0.767158916
Thurrock	0.657739131	0.665382741	0.67006771	0.671779384	0.673851722	0.679131461	0.685946408	0.690863179	0.693984217	0.695839246	0.697889158	0.700078132	0.701938322	0.705709409	0.710219368	0.714583085	0.71931345	0.724233006	0.72995803	0.7359403	0.740257075	0.743684038	0.747559204	0.75183529	0.756916493
Greater London	0.73550707	0.741837948	0.745885665	0.747578579	0.749583079	0.754076472	0.759717636	0.764468834	0.768663992	0.77188902	0.774975307	0.778139783	0.78126931	0.78559774	0.790367391	0.79481868	0.799834703	0.805204307	0.810571153	0.815466559	0.819126417	0.822454571	0.826380411	0.830469675	0.835217016
Barking and Dagenham	0.626504542	0.633868923	0.638590564	0.640581589	0.642974088	0.648252934	0.654611802	0.659628442	0.663548602	0.665995815	0.668316385	0.67086136	0.67310099	0.67664716	0.680469734	0.684001119	0.688516583	0.694051852	0.700523075	0.707012651	0.711712654	0.715453534	0.720077271	0.725050347	0.730521788
Barnet	0.722473201	0.728174415	0.732135008	0.734274754	0.73675998	0.741247991	0.746627243	0.751306255	0.755534133	0.758757372	0.761601776	0.764552657	0.767943826	0.772574715	0.777426508	0.781612853	0.786012382	0.790506684	0.794961341	0.799470411	0.803264526	0.806863184	0.811078659	0.815559927	0.820742881
Bexley	0.669455974	0.675838175	0.680197766	0.682494234	0.685127253	0.689958656	0.69547236	0.69980442	0.703262408	0.705723199	0.708134269	0.710414428	0.71264459	0.716390969	0.720982626	0.725753639	0.731228465	0.736819341	0.742315968	0.74726382	0.750917475	0.754069305	0.757639668	0.761348499	0.766013793
Brent	0.688540649	0.695367871	0.699695095	0.701448675	0.703632273	0.708728133	0.715125755	0.720606241	0.725545373	0.729206687	0.732769664	0.736714187	0.740561434	0.745485241	0.750465758	0.754591719	0.758736052	0.763433359	0.768771548	0.773904287	0.777701948	0.781248313	0.785590309	0.789871932	0.794789662
Bromley	0.713378284	0.719070333	0.723116403	0.72544812	0.728096529	0.73257372	0.737718749	0.741990056	0.745646827	0.748197908	0.750479213	0.752788415	0.755266081	0.759238209	0.763992764	0.768353433	0.77275209	0.777301364	0.782008337	0.786466597	0.789948728	0.793321613	0.797411783	0.801741687	0.806731006
Camden	0.826427218	0.830292423	0.832871426	0.834094814	0.83560187	0.838711837	0.84289712	0.846622902	0.850065476	0.852686672	0.854739085	0.856928378	0.8589265	0.861285535	0.863992199	0.866757584	0.870073842	0.873487908	0.876929449	0.880212413	0.882802077	0.885330458	0.888101161	0.890852059	0.893707053
Croydon	0.69087916	0.697446717	0.701690095	0.703505481	0.705657881	0.71044315	0.716290299	0.72123425	0.725490074	0.728407284	0.731107499	0.733914364	0.736523831	0.740250706	0.744676131	0.749157558	0.754470413	0.760135211	0.765609579	0.770455557	0.773919422	0.776817337	0.780426701	0.784393556	0.78928507
Ealing	0.710261193	0.716755796	0.720937455	0.722715238	0.724884898	0.729700731	0.735677964	0.740771566	0.74556633	0.749626218	0.753761398	0.758094202	0.762326443	0.767440036	0.772324933	0.776440874	0.780781212	0.785687945	0.790925528	0.795884412	0.79991058	0.803736232	0.808046513	0.812508974	0.81758411
Enfield	0.675634114	0.682250985	0.686659886	0.688766989	0.691204989	0.696072915	0.701765647	0.706190617	0.709740406	0.712413913	0.715194949	0.718017742	0.720945273	0.725329903	0.72993644	0.734015063	0.7388007	0.744266862	0.749820519	0.754511903	0.757858488	0.761079071	0.765104673	0.769544589	0.774731301
Greenwich	0.66329042	0.670174653	0.674617594	0.676535795	0.678851988	0.683884827	0.689971961	0.694852242	0.698996437	0.701785496	0.704379746	0.707364447	0.710324338	0.714772309	0.719892935	0.724794432	0.730568978	0.737056996	0.743585009	0.749387751	0.753646856	0.757402944	0.761515247	0.765732658	0.770821244
Hackney	0.724575034	0.732223771	0.735924198	0.735290349	0.734983265	0.739116648	0.74568143	0.750886656	0.755234118	0.758331501	0.760298086	0.760727471	0.760719955	0.763479046	0.767868573	0.772855945	0.780149291	0.788048608	0.794849376	0.799756997	0.801617428	0.802540605	0.804391122	0.806670726	0.810459046
Hammersmith and Fulham	0.774842679	0.78044629	0.784534238	0.787105039	0.79009818	0.794966933	0.800843202	0.806179681	0.81115145	0.815417628	0.819798962	0.824327874	0.82877516	0.834074776	0.839668047	0.84480445	0.849996132	0.855195257	0.860183244	0.864779843	0.868625952	0.8722719	0.876150721	0.880063755	0.884634222
Haringey	0.699999752	0.706239593	0.710384307	0.712313727	0.71463567	0.719462445	0.725486451	0.730690118	0.73543147	0.739019239	0.742320529	0.745578579	0.748809939	0.75333307	0.75795613	0.762031142	0.766734225	0.771662106	0.776384587	0.78046201	0.783315684	0.786091474	0.789701982	0.793422832	0.797741588
Harrow	0.70687213	0.713061206	0.717223823	0.719274417	0.721682113	0.726319259	0.731810984	0.736246652	0.739967598	0.742668295	0.74510995	0.7476234	0.750659432	0.755547302	0.761064879	0.76590342	0.770692636	0.775646349	0.780600528	0.785093902	0.788395101	0.79148835	0.795565116	0.800355253	0.805905431
Havering	0.664399406	0.670656734	0.674879238	0.677038181	0.679565634	0.684237081	0.689704766	0.694060703	0.697596516	0.700018478	0.702057619	0.703639069	0.705156328	0.708672532	0.713406768	0.718170603	0.723397767	0.728622374	0.733407867	0.737906412	0.741479928	0.744618622	0.748109635	0.752005979	0.757329069
Hillingdon	0.725197925	0.731563154	0.735834007	0.737869039	0.740248765	0.745012331	0.750820522	0.755711096	0.759975355	0.763198446	0.766235125	0.768892591	0.771043921	0.774807721	0.779407447	0.783602665	0.78858177	0.794126231	0.799479633	0.804346255	0.807977966	0.811165388	0.815091125	0.819769381	0.825370586
Hounslow	0.715180194	0.721875813	0.726264956	0.728243474	0.730570899	0.735513105	0.741481022	0.746370421	0.750331542	0.753152615	0.756512184	0.760282304	0.763738413	0.768226067	0.773119159	0.777744991	0.782777713	0.78797048	0.793088964	0.797766026	0.801045775	0.803720178	0.807413818	0.811981973	0.817880504
Islington	0.770585831	0.776989636	0.781321178	0.783484625	0.786062172	0.791055427	0.797121961	0.802138702	0.806748753	0.810716629	0.813786346	0.817075509	0.819864102	0.823011571	0.826301659	0.829730557	0.834538971	0.840074285	0.845991632	0.851344046	0.855202575	0.858405045	0.861427577	0.864283728	0.867619926
Kensington and Chelsea	0.821944967	0.826145185	0.829465898	0.832027959	0.835010709	0.839095381	0.843616468	0.847370017	0.85066972	0.853472363	0.856352852	0.859462034	0.862723575	0.866821791	0.871248119	0.87554948	0.879909108	0.884016873	0.887919316	0.891609781	0.894876949	0.89819944	0.901878354	0.90481233	0.906671066
Kingston upon Thames	0.752143962	0.757363217	0.761399827	0.764246655	0.767376811	0.771755919	0.776505054	0.780424175	0.784023555	0.787133271	0.790251795	0.793703847	0.797510244	0.802191238	0.807066597	0.811664993	0.816506309	0.821409292	0.825913128	0.830163346	0.833946915	0.837507878	0.841469241	0.845810942	0.850801759
Lambeth	0.717116093	0.723383572	0.727458658	0.729219455	0.731489958	0.736517525	0.742891831	0.748570681	0.753910078	0.758251633	0.762407024	0.766503612	0.770448358	0.775627878	0.781725234	0.787597726	0.793585528	0.799554012	0.805240529	0.810384225	0.814263416	0.817846569	0.822277641	0.826880899	0.832010865
Lewisham	0.673977732	0.680070177	0.68409387	0.686001027	0.68837183	0.693315737	0.699469743	0.705287522	0.710717544	0.714824569	0.718658923	0.722530942	0.726498948	0.731594302	0.736799157	0.741501455	0.746637661	0.75223091	0.757916286	0.763039852	0.766982601	0.770342927	0.774216109	0.778468843	0.783543336
Merton	0.711825165	0.717820127	0.721927595	0.724062382	0.726554111	0.731250419	0.736769586	0.741356367	0.745382019	0.748462141	0.751494962	0.754913273	0.758802126	0.763926509	0.769227349	0.774133503	0.779264091	0.784517026	0.789931319	0.795075297	0.799208115	0.802983981	0.807597715	0.812524816	0.81785755
Newham	0.634723953	0.642764565	0.647574796	0.648997163	0.65091178	0.656504829	0.663657876	0.669347941	0.674167824	0.67788663	0.681505843	0.685336605	0.688866633	0.693371998	0.697883475	0.701795321	0.706435607	0.711724521	0.717248995	0.722188557	0.725423043	0.728685786	0.733076299	0.737392944	0.742819967
Redbridge	0.68456216	0.690806705	0.695079497	0.697322712	0.699920099	0.704721583	0.710304943	0.71490024	0.718907508	0.721913288	0.724647114	0.727169772	0.729685776	0.733426406	0.737406044	0.741118203	0.745625644	0.750744848	0.756022404	0.760918122	0.764646431	0.768051696	0.772273	0.776802421	0.781831216
Richmond upon Thames	0.781620844	0.786298991	0.789942483	0.792575315	0.795507208	0.799600225	0.804110804	0.808020818	0.811636685	0.814628747	0.81759509	0.82070112	0.823974347	0.828168467	0.83274575	0.837032814	0.841391747	0.84569853	0.84989642	0.853761379	0.857003294	0.860178395	0.863807321	0.867759476	0.872359082
Southwark	0.726961929	0.733548131	0.737808653	0.7395855	0.741824976	0.746968697	0.753568115	0.759470343	0.765082004	0.769651916	0.773815776	0.777880501	0.781875242	0.786874734	0.792422615	0.797907534	0.803926439	0.810137544	0.816098289	0.821544009	0.825849944	0.829764819	0.834456408	0.839649071	0.845546146
Sutton	0.688357377	0.694654681	0.699025707	0.701391586	0.704049197	0.708798373	0.714214378	0.71853657	0.722116451	0.724609666	0.726862025	0.7292262	0.731880311	0.736230362	0.741150014	0.745539692	0.750322856	0.755413986	0.760752139	0.76603767	0.770410677	0.774311397	0.778686753	0.783260471	0.788775107
Tower Hamlets	0.693846597	0.703069881	0.708178091	0.708281195	0.708192647	0.713203598	0.721021623	0.727548737	0.733299188	0.738213266	0.742875143	0.746905391	0.750162506	0.754332929	0.759653841	0.765109358	0.771711626	0.778966354	0.786159164	0.792573377	0.797321441	0.802062544	0.807837588	0.813677375	0.81971193
Waltham Forest	0.651270218	0.658133222	0.662551507	0.664391308	0.666599312	0.671674021	0.677946289	0.683101236	0.687700421	0.691374733	0.6948878	0.698521207	0.702280236	0.707086942	0.712274527	0.716668274	0.72145392	0.727050373	0.733124582	0.738929149	0.743403588	0.747381584	0.751791267	0.756269456	0.76142493
Wandsworth	0.748590027	0.754235311	0.758208073	0.760490373	0.763191042	0.767945188	0.773620607	0.778773655	0.783931793	0.788737503	0.79384068	0.799276531	0.804620569	0.810360965	0.815920909	0.820916201	0.825894317	0.831010381	0.836144422	0.84099764	0.845261428	0.849505433	0.85423898	0.858944733	0.86399287
Westminster	0.821799649	0.825884684	0.828678513	0.830083384	0.831681435	0.834798645	0.838785418	0.841861447	0.844255817	0.846109323	0.847941108	0.850208338	0.852602907	0.855443494	0.858266653	0.860957245	0.864667843	0.86896405	0.873169724	0.876962898	0.879974952	0.882941481	0.885943119	0.888570465	0.891199613
North East England	0.643949896	0.651863555	0.656695001	0.658412052	0.660610532	0.666057592	0.672740161	0.677863752	0.681955814	0.684643122	0.687408501	0.690785546	0.694179867	0.698567922	0.702518588	0.705613776	0.70969006	0.715108428	0.721157918	0.726624022	0.730569673	0.73413026	0.738518133	0.743378297	0.749166653
County Durham	0.640401976	0.648035824	0.652613652	0.654141382	0.656158488	0.661391813	0.667988883	0.673423683	0.678143894	0.681408471	0.684401627	0.687744097	0.690717327	0.694085307	0.696747718	0.698702667	0.702094932	0.707437931	0.713686849	0.718932791	0.722237487	0.72504864	0.728821377	0.733442582	0.739289883
Darlington	0.665043613	0.672979567	0.67769031	0.679087716	0.680791057	0.685883831	0.692236425	0.69683141	0.700476769	0.703018576	0.705469475	0.707603286	0.708681017	0.710457436	0.712115802	0.713740668	0.717208445	0.722525017	0.728664363	0.734149787	0.737707345	0.740342727	0.743345895	0.74677718	0.752013583
Gateshead	0.64807241	0.656052531	0.660789089	0.662241531	0.664187633	0.669562319	0.676328465	0.681619334	0.685420495	0.687534959	0.690167272	0.693489082	0.696542241	0.700523922	0.704486325	0.707597434	0.711674996	0.716966528	0.722399178	0.72717075	0.730390263	0.733155036	0.737095049	0.741590115	0.746754291
Hartlepool	0.620540243	0.629054851	0.634036334	0.635400981	0.637121712	0.642561945	0.649393913	0.654125609	0.657384887	0.659480383	0.661502557	0.663672585	0.665940697	0.669807792	0.673587677	0.676173399	0.679522189	0.683978451	0.688914886	0.693795603	0.697925929	0.701718428	0.705386794	0.708784926	0.71319269
Middlesbrough	0.622450493	0.631147978	0.636172501	0.637425931	0.63917708	0.644688247	0.651509168	0.655950998	0.659734328	0.662788123	0.665612224	0.668656612	0.671788009	0.677053204	0.682496584	0.686457603	0.690513802	0.695091863	0.700220906	0.705304231	0.709086957	0.712340786	0.716402145	0.720571197	0.725772294
Newcastle upon Tyne	0.686230418	0.693108904	0.697684814	0.699948487	0.702775824	0.708027105	0.714203472	0.719208231	0.723319039	0.726059052	0.728831141	0.73258195	0.737232222	0.743027527	0.747909747	0.751971871	0.756852693	0.762151849	0.767520106	0.77259528	0.776904285	0.781153688	0.786083077	0.791241968	0.796583502
North Tyneside	0.655904762	0.663331324	0.6679129	0.669641138	0.671778882	0.676872262	0.683053013	0.687693108	0.691061542	0.692872004	0.694885104	0.697511439	0.700394871	0.704550742	0.708326415	0.711450488	0.715545308	0.721175663	0.727445092	0.732544195	0.735868226	0.738895127	0.742604096	0.746971489	0.752173036
Northumberland	0.65469366	0.662227227	0.666733289	0.668119681	0.66993712	0.6749815	0.681368847	0.686463465	0.690732765	0.693750226	0.696795594	0.700041435	0.702772314	0.706163795	0.709374181	0.711789114	0.714907396	0.71927485	0.724587736	0.729679695	0.733250929	0.73626221	0.739826495	0.743823392	0.749123568
Redcar and Cleveland	0.627534647	0.63577773	0.64054351	0.641741875	0.643297749	0.648488355	0.655083668	0.659717217	0.663280458	0.665389014	0.667257477	0.669996404	0.673089562	0.677331639	0.680929066	0.683036341	0.685800422	0.690393576	0.696219709	0.701839301	0.705764239	0.709095171	0.713212033	0.71757085	0.722753167
South Tyneside	0.619885816	0.627679145	0.632537642	0.634360951	0.636563733	0.641862206	0.648256327	0.652823055	0.655417354	0.655604039	0.65602303	0.658662087	0.662404939	0.66708685	0.671286691	0.674543573	0.678543978	0.683716248	0.689358644	0.694396754	0.698025126	0.701471242	0.705756197	0.710404589	0.716041346
Stockton-on-Tees	0.660507175	0.668718921	0.673549367	0.674869226	0.67653035	0.681794943	0.688420527	0.693044531	0.696169877	0.698337918	0.701033899	0.703931681	0.706455646	0.709911422	0.71319571	0.716320949	0.721109506	0.727228349	0.734102101	0.740372526	0.744452093	0.747815414	0.751862566	0.75599085	0.760936797
Sunderland	0.629409883	0.637993383	0.642925998	0.644161511	0.64586324	0.651415853	0.65859983	0.664090447	0.668572407	0.671527458	0.674700378	0.678609016	0.682075953	0.686442115	0.690361213	0.693436907	0.697931364	0.704371173	0.711319643	0.717271466	0.721360113	0.724995257	0.729357509	0.734016404	0.739538447
North West England	0.667194323	0.674596629	0.679084594	0.680578328	0.682432783	0.687297604	0.693314653	0.697892471	0.701802756	0.7046943	0.707549476	0.710480947	0.712924685	0.716417867	0.720155269	0.723853024	0.728909506	0.734960051	0.741129046	0.746459648	0.74992915	0.752791702	0.756477484	0.760650507	0.76598297

Blackburn with Darwen	0.622289576	0.631165574	0.63598704	0.636579188	0.637544831	0.642822739	0.650141003	0.656127161	0.66109824	0.664390121	0.667513359	0.670447875	0.672918874	0.676829485	0.680108255	0.68238976	0.685731636	0.690008092	0.695005459	0.699698108	0.702632539	0.7053888	0.70887123	0.712786846	0.718095097
Blackpool	0.625674726	0.633473723	0.638465704	0.640630049	0.643064139	0.648278491	0.654368643	0.658683492	0.661926228	0.663392097	0.663565754	0.663750169	0.664586912	0.667946091	0.671813741	0.675252664	0.679927587	0.68518121	0.690174524	0.694593438	0.697103334	0.69907228	0.70250716	0.706827137	0.712501767
Bolton	0.645162631	0.653102366	0.657733718	0.658877519	0.660288439	0.665122189	0.67129069	0.675652248	0.679353869	0.682518138	0.686049718	0.689295292	0.691400371	0.694485084	0.697918118	0.701717327	0.707123313	0.713437318	0.719670447	0.724604349	0.727095634	0.728802704	0.731464409	0.734662473	0.739275432
Bury	0.666945776	0.673962965	0.678235305	0.679652125	0.681344384	0.685846107	0.691407896	0.695465437	0.698635188	0.700609475	0.702695786	0.704906643	0.70649462	0.709223363	0.71294179	0.717064481	0.722315648	0.728541718	0.734837387	0.740145419	0.743550793	0.74612714	0.749191337	0.752662934	0.757184989
Cheshire East	0.723144145	0.729516984	0.733474633	0.734978575	0.736777622	0.740975561	0.746037247	0.749799301	0.752951495	0.75521995	0.757509715	0.759879738	0.761792121	0.764650991	0.768018427	0.771674646	0.776171884	0.781368716	0.786590269	0.791325813	0.794808609	0.797632724	0.800895963	0.804325731	0.808972433
Cheshire West and Chester	0.708560027	0.715122846	0.719072412	0.720360954	0.72196777	0.726236076	0.731496372	0.735373993	0.738813479	0.741274903	0.743674075	0.74610457	0.747776408	0.750796996	0.754838943	0.759102313	0.764448654	0.770695945	0.776715784	0.781653165	0.784738117	0.787267683	0.790772656	0.794815905	0.799793841
Cumbria	0.672553861	0.67950906	0.683733287	0.685167875	0.686911079	0.691425188	0.696975982	0.701103219	0.70460335	0.707372733	0.71048972	0.713503528	0.715650697	0.718516205	0.721115255	0.723302241	0.726945661	0.732305222	0.738372222	0.743815552	0.747052396	0.749318145	0.752561756	0.756270732	0.760885712
Halton	0.652469602	0.660767741	0.665472351	0.666450614	0.667658279	0.67252298	0.678793664	0.683253435	0.686524127	0.688089853	0.689232935	0.690605601	0.692068462	0.695714127	0.700176283	0.704162024	0.709000642	0.715316123	0.722218377	0.7283098	0.732239076	0.734714285	0.736727017	0.739207013	0.743894948
Knowsley	0.624102727	0.632259501	0.636934501	0.638011111	0.639493787	0.644563199	0.651078915	0.65598094	0.660820537	0.664653592	0.667852655	0.671473248	0.674295911	0.677240136	0.680203498	0.68322195	0.687997471	0.693783342	0.698891684	0.703117436	0.706171182	0.709475536	0.713689691	0.717494486	0.722184627
Lancashire	0.673872551	0.6811868	0.685590055	0.686971387	0.688678895	0.693337228	0.699089634	0.703333564	0.707022174	0.709950092	0.712732459	0.715362428	0.717356892	0.720325905	0.723927276	0.727956978	0.733591254	0.740115399	0.746543407	0.751957131	0.755326739	0.757928218	0.761319536	0.765075881	0.769850615
Liverpool	0.660790207	0.668005731	0.672552469	0.674450237	0.676858878	0.681922416	0.687906554	0.692465733	0.696357071	0.699529231	0.703097414	0.707234734	0.711187186	0.716011892	0.720688613	0.725467323	0.731475935	0.737600847	0.743305292	0.748057395	0.751380354	0.754931797	0.75970248	0.765176947	0.77130966
Manchester	0.680469453	0.688004378	0.692687966	0.694470465	0.696717861	0.702056037	0.708722823	0.714296612	0.719012566	0.722470693	0.726085928	0.730244118	0.734296135	0.738879935	0.742927951	0.746647615	0.751705623	0.757925193	0.764651684	0.77085982	0.775441989	0.77948319	0.784412505	0.790043864	0.796729323
Oldham	0.626873004	0.635017917	0.639681128	0.640704358	0.642000631	0.646912064	0.653311188	0.658013787	0.66187441	0.664490157	0.667144767	0.669930905	0.671844949	0.674620741	0.677734074	0.680659819	0.684881077	0.690479928	0.696535421	0.70102578	0.703103672	0.704684602	0.707004718	0.709396487	0.713174671
Rochdale	0.631042934	0.639131038	0.643844233	0.644979869	0.646382508	0.651285938	0.657651642	0.662417826	0.666177037	0.668600503	0.670859864	0.672940704	0.674442904	0.677161622	0.679946088	0.682495148	0.686914997	0.692740297	0.69886136	0.70449136	0.70786881	0.710105757	0.713178503	0.716891	0.722227937
Salford	0.652243216	0.660012919	0.664498388	0.665634937	0.667223187	0.672240313	0.678601933	0.68325404	0.687244294	0.689932745	0.692676526	0.695921869	0.698079446	0.701322922	0.70485491	0.707740126	0.712145618	0.718390732	0.725411036	0.731641191	0.735455032	0.737696	0.740745454	0.745601195	0.75253971
Sefton	0.665938628	0.672660922	0.676921916	0.678616262	0.680590262	0.685103163	0.690530815	0.694600118	0.69811698	0.70087405	0.70357637	0.706277387	0.7083442	0.711101022	0.714596406	0.718750428	0.724685208	0.73131607	0.737480071	0.742595906	0.745922692	0.748511595	0.751985561	0.756073253	0.760803623
St Helens	0.640089871	0.647526905	0.651889368	0.65303823	0.654509323	0.659306018	0.665643235	0.671069232	0.67580035	0.678577518	0.680982607	0.683682402	0.685819436	0.688869918	0.69176922	0.694233681	0.69856037	0.704405114	0.710740518	0.71592096	0.718794057	0.721045314	0.723957516	0.727473651	0.732620088
Stockport	0.698009536	0.704634776	0.708709095	0.710152897	0.711883779	0.716306198	0.721755571	0.726077088	0.729782911	0.7323715	0.7345752	0.736609765	0.738857765	0.743008454	0.747425913	0.751620973	0.757002826	0.763161137	0.769049941	0.773894448	0.777004091	0.779429439	0.782650547	0.786493222	0.791417208
Tameside	0.636791853	0.644707824	0.64943368	0.650828412	0.652514483	0.657501878	0.663772557	0.668363093	0.672100052	0.674751796	0.677386543	0.679793595	0.681501131	0.684128613	0.687094973	0.690169822	0.694965954	0.701117587	0.707610216	0.713230143	0.716889481	0.720061883	0.723720255	0.727376926	0.732192994
Trafford	0.727642531	0.73417047	0.738178732	0.739679198	0.741493421	0.74587759	0.751234671	0.755351117	0.758872074	0.76156352	0.764288238	0.767201919	0.769870956	0.773783062	0.77796702	0.781976839	0.78671279	0.792185092	0.797857675	0.802838184	0.806303374	0.80907728	0.812217293	0.815846012	0.82081479
Warrington	0.704677446	0.71164746	0.715749155	0.716843443	0.718216734	0.722596247	0.728191997	0.732342466	0.735730447	0.738466098	0.741165293	0.743592428	0.745894199	0.74955784	0.753665102	0.757425219	0.762524405	0.768492764	0.774199848	0.778864593	0.781605683	0.783694627	0.786230134	0.78928744	0.794297245
Wigan	0.637422241	0.645123185	0.649597156	0.650791452	0.652347952	0.657206081	0.66337558	0.667979065	0.672045997	0.67536852	0.678604635	0.681661625	0.684074921	0.687316814	0.690657985	0.69385144	0.698265316	0.703972367	0.710030694	0.7151698	0.718306821	0.720969646	0.724216723	0.727230537	0.731261727
Wirral	0.654477293	0.661653783	0.665938763	0.667178622	0.668696606	0.673261996	0.679073669	0.683575877	0.687373508	0.689808418	0.691954809	0.694083699	0.695817699	0.699120945	0.702917033	0.706506429	0.711299157	0.71684603	0.722583903	0.727889248	0.731471658	0.734278981	0.737502235	0.740843839	0.745220385
South East England	0.721427058	0.727069672	0.730606254	0.731944157	0.733671145	0.737924372	0.743549611	0.748192363	0.751900207	0.754476819	0.756769053	0.758912706	0.76092911	0.764415286	0.768630707	0.772790602	0.777244962	0.781939055	0.786728675	0.791151604	0.794546578	0.797511116	0.801016407	0.804897274	0.809591967
Bracknell Forest	0.735496194	0.741147601	0.744413549	0.745325631	0.74669339	0.750870415	0.756683098	0.761685124	0.766180798	0.769761488	0.772781875	0.775286615	0.777221854	0.780432183	0.784403207	0.78835599	0.792376918	0.796362939	0.800201995	0.80372606	0.806494029	0.809234574	0.812806613	0.817085183	0.822449514
Brighton and Hove	0.73243881	0.737388185	0.741085823	0.743554451	0.746437054	0.750889589	0.756082488	0.760577225	0.764517265	0.767782076	0.771031528	0.774375301	0.777907968	0.782579994	0.787872125	0.792979183	0.797690676	0.802289163	0.806994316	0.811592102	0.815718978	0.819632815	0.823822985	0.82813446	0.832806187
Buckinghamshire	0.748151934	0.75328534	0.756567152	0.757864501	0.75951169	0.763476809	0.768745777	0.773275077	0.777027081	0.779696669	0.782193729	0.784516279	0.786389935	0.789009604	0.792242059	0.795667014	0.79972445	0.804153468	0.808566331	0.812712818	0.816047897	0.819147253	0.822745824	0.826370169	0.830647683
East Sussex	0.676190321	0.682252478	0.686169488	0.687815767	0.689794697	0.694361249	0.700221026	0.704945592	0.708603984	0.711220184	0.713607145	0.715561429	0.717281137	0.720726938	0.725110334	0.729563972	0.734239331	0.739055297	0.743947209	0.748758441	0.752523436	0.755398663	0.758712415	0.762538853	0.767349635
Hampshire	0.72021626	0.725819522	0.729232636	0.730294118	0.731724242	0.735834013	0.741420111	0.74593626	0.749578187	0.752157091	0.754382391	0.756466555	0.758531116	0.762006742	0.766264559	0.770575146	0.775101568	0.779529051	0.78406633	0.788214791	0.791303029	0.794093757	0.797339118	0.800995234	0.805765068
Isle of Wight	0.657090164	0.663548182	0.667440723	0.668672903	0.670263165	0.674827662	0.680936194	0.685698412	0.689162787	0.691009179	0.692375104	0.694114258	0.695940485	0.699551845	0.70411063	0.708723981	0.71354308	0.718447987	0.723447718	0.728367559	0.732199272	0.735867966	0.740313162	0.744732602	0.749753512
Kent	0.688069789	0.694219603	0.697988923	0.699290959	0.701010078	0.705507935	0.711496781	0.716335882	0.719999051	0.722120097	0.723705744	0.725166308	0.726613977	0.729869827	0.733868876	0.737842246	0.742361735	0.747373795	0.752527314	0.757224036	0.760513289	0.763050684	0.766333125	0.77029105	0.775213379
Medway	0.65873076	0.665501805	0.669418633	0.670366717	0.671756699	0.676439118	0.682847953	0.687613653	0.691106747	0.693425335	0.695737189	0.697954927	0.699623296	0.703218473	0.707813477	0.712321819	0.717281118	0.722438665	0.72800876	0.733302788	0.73683364	0.739660696	0.743128307	0.746490245	0.750536591
Milton Keynes	0.718088795	0.725547622	0.729790125	0.730583768	0.731676735	0.736404992	0.743108404	0.74812465	0.75149573	0.753547584	0.75558151	0.757740183	0.759710864	0.763693961	0.76863977	0.77322075	0.778331804	0.783797352	0.789245102	0.794136978	0.79737607	0.799996722	0.803147662	0.806334838	0.810432989
Oxfordshire	0.748137726	0.752921226	0.755943067	0.757174249	0.758876948	0.762829491	0.768049364	0.772672102	0.776696782	0.779761779	0.782608514	0.785149698	0.787252783	0.790371555	0.794141832	0.797879483	0.801991449	0.806535965	0.811259773	0.815680507	0.819112355	0.821896669	0.825133939	0.828850108	0.833407475
Portsmouth	0.699814062	0.706334153	0.710535999	0.712318572	0.714563631	0.719500114	0.725701547	0.730375842	0.733835586	0.736120651	0.738421589	0.741096847	0.744165865	0.749270366	0.754935064	0.759838477	0.764590616	0.769683769	0.775121398	0.780223929	0.784492061	0.788624308	0.793325128	0.797954383	0.80271548
Reading	0.748238274	0.754158204	0.757924566	0.759404832	0.761257397	0.765709592	0.771541794	0.776043241	0.779632795	0.782523722	0.785516629	0.789006245	0.792921287	0.798080467	0.803456444	0.808190771	0.812818	0.817601328	0.822252547	0.826413735	0.829859222	0.833249724	0.837373085	0.841883449	0.847268994
Slough	0.710923497	0.718424221	0.72256972	0.72316777	0.724262611	0.729373754	0.736772977	0.742767315	0.747054243	0.749837132	0.752673272	0.755619513	0.758262883	0.762693573	0.767829963	0.772614527	0.777852952	0.783348505	0.789105341	0.79477329	0.799107234	0.803009866	0.807531049	0.811901035	0.815957412
Southampton	0.702755092	0.708736202	0.712554065	0.714081001	0.716030605	0.720673866	0.726824078	0.731978766	0.736264061	0.739319244	0.741954839	0.744289496	0.746172671	0.74976095	0.754374562	0.759131491	0.764184185	0.769582897	0.775357415	0.780772518	0.785228459	0.789500375	0.794431093	0.799486349	0.804825417
Surrey	0.759758625	0.764609792	0.76789668	0.769613892	0.771727645	0.775713553	0.780645896	0.784891365	0.788342127	0.790854542	0.793238376	0.79547011	0.797667786	0.801156657	0.805295183	0.809241765	0.813218803	0.81740927	0.821597713	0.825347581	0.828574383	0.831625334	0.835056831	0.839083767	0.844177461
West Berkshire	0.754438843	0.760443653	0.764037916	0.765053921	0.766356178	0.770546401	0.77634614	0.780976244	0.78433781	0.786385904	0.788229284	0.79020517	0.792452744	0.796194543	0.800888057	0.805550058	0.810452711	0.815744156	0.820928839	0.825327085	0.828370424	0.830796334	0.833740327	0.837243331	0.841790163
West Sussex	0.708429479	0.714312144	0.718051348	0.719574218	0.721480856	0.725917942	0.731737387	0.736656239	0.740857703	0.743943682	0.746445971	0.748392176	0.750174534	0.75370288	0.757884207	0.762071928	0.766742633	0.771673315	0.776482782	0.780726888	0.783815722	0.786408682	0.789594608	0.793359433	0.798066328
Windsor and Maidenhead	0.768857709	0.77393041	0.777199038	0.778608734	0.78033828	0.78424769	0.78931283	0.793580163	0.796950944	0.799490689	0.802248931	0.805282705	0.808068639	0.811311933	0.814767088	0.818140871	0.82161379	0.825335136	0.829300668	0.833100456	0.83615734	0.838814994	0.842056621	0.845764989	0.850320792
Wokingham	0.778623792	0.783110618	0.786083445	0.787539866	0.789334754	0.792936821	0.797409505	0.800874844	0.803335436	0.804939764	0.806788492	0.809087485	0.811710475	0.815803347	0.820418927	0.82484241	0.829020445	0.83302523	0.836971846	0.840701088	0.843846097	0.846792561	0.850285993	0.85408238	0.858443204
South West England	0.692804927	0.699082842	0.703053813	0.704729227	0.70679342	0.711387734	0.717074903	0.721744346	0.725565227	0.728221882	0.730560566	0.732809578	0.735019022	0.73869875	0.743269024	0.747943254	0.752830357	0.757839338	0.762930652	0.767653174	0.77118217	0.77424567	0.778030724	0.782430638	0.787870311
Bath and North East Somer	0.733314326	0.738327645	0.742022565	0.744456026	0.747247529	0.751485636	0.756209831	0.760201748	0.763530868	0.766227097	0.769045952	0.771876555	0.774753917	0.778517426	0.782941254	0.787410378	0.791679434	0.796062083	0.800686236	0.80515302	0.808856243	0.812099586	0.815971076	0.820464266	0.825483228
Bournemouth	0.692866308	0.698657711	0.702865741	0.705524158	0.708555654	0.713413147	0.71899038	0.723823961	0.727709147	0.730615118	0.733846435	0.73736742	0.740855612	0.745273113	0.750127947	0.755273408	0.760904777	0.76639109	0.771589882	0.776447775	0.780418675	0.78395412	0.788175663	0.793129783	0.798834399
Bristol, City of	0.723666071	0.729692466	0.73364564	0.735545291	0.737904478	0.74257173	0.748270068	0.753072713	0.756968944	0.759762067	0.762707946	0.766147804	0.769721135	0.774421634	0.779649141	0.784610852	0.789563536	0.794631999	0.799891908	0.804923182	0.809005773	0.812692564	0.817008436	0.8216647	0.826973496
Cornwall	0.671289382	0.67796759	0.682095397	0.683613677	0.685433624	0.690066842	0.695765894	0.699874614	0.702911425	0.705038107	0.706843938	0.708204291	0.709599018	0.71318012	0.718133707	0.723353527	0.728807695	0.734489376	0.740317544	0.745287422	0.74856818	0.751176734	0.754545903	0.758684459	0.764192088
Devon	0.690463029	0.696455017	0.700375234	0.702213022	0.704426667	0.708932293	0.714458304	0.719207585	0.72327782	0.725920555	0.727767477	0.729567191	0.731656269	0.735159925	0.739539267	0.744138186	0.749037326	0.753944293	0.758875878	0.763286888	0.766307015	0.768956137	0.772504244	0.776764326	0.782179207
Dorset	0.691321278	0.697436389	0.701174539	0.702629922	0.704481694	0.708851054	0.714283095	0.718631195	0.72191478	0.723919309	0.725595861	0.727334185	0.729162039	0.732511698	0.736671927	0.740994535	0.745585307	0.750206833	0.754874337	0.759291606	0.762327758	0.765060227	0.768865932	0.773326451	0.778577111
Gloucestershire	0.707888947	0.71397213	0.717710973	0.719099753	0.720915036	0.725313172	0.730920275	0.735718698	0.739857092	0.742768858	0.745221091	0.747381425	0.749279808	0.752496541	0.756654331	0.76125096	0.766177348	0.771016768	0.775795147	0.780262872	0.783751041	0.786736732	0.790009743	0.793687427	0.798643084
North Somerset	0.688443519	0.694758127	0.698689352	0.700173974	0.701983707	0.706479323	0.71226028	0.717193851	0.721355817	0.724128745	0.726234615	0.727842387	0.729389354	0.732935153	0.737945774	0.74308392	0.748176981	0.753257523	0.758151374	0.762368074	0.76532543	0.767841052	0.770787331	0.774183006	0.778905521
Plymouth	0.669432575	0.676492942	0.680844995	0.682416513	0.684373568	0.68944716	0.696043137	0.701620933	0.706465309	0.710248012	0.71394912	0.717379586	0.720224047	0.724416906	0.729344846	0.73401315	0.73892965	0.744467059	0.750099797	0.755131717	0.758732071	0.76162238	0.765366246	0.769783922	0.775008813
Poole	0.700664573	0.707135054	0.71126884	0.713021502	0.715091335	0.719658199	0.725130246	0.728906084	0.731520173	0.733326058	0.735357597	0.7378981	0.740498749	0.744342458	0.748972799	0.753452228	0.75793914	0.762633126	0.767637869	0.772653461	0.776587663	0.779892358	0.783655076	0.787872761	0.793233602
Somerset	0.680892999	0.68735765	0.691311078	0.692675001	0.694360033	0.698862174	0.704686722	0.709467637	0.713319336	0.715906238	0.717799143	0.718918954	0.719583741	0.721862527	0.725578077	0.729813379	0.734663347	0.739871994	0.745266885	0.750237632	0.753664979	0.756291819	0.759357155	0.763015065	0.767910485
South Gloucestershire	0.724268581	0.730144457	0.733825589	0.735290201	0.737145865	0.741350718	0.746503528	0.750537143	0.753642794	0.75579177	0.757980621	0.760258654	0.762616987	0.766436632	0.7706774	0.77431501	0.778082843	0.782047764	0.786109976	0.789994211	0.793285599	0.796854367	0.801304921	0.805966637	0.811302141
Swindon	0.705921225	0.712935616	0.717041553	0.718183613	0.719723541	0.724641252	0.731123834	0.736439603	0.740526436	0.742948427	0.744632404	0.746370278	0.748506367	0.752835827	0.758151614	0.763337469	0.768908604	0.774454839	0.779791493	0.784518226	0.787462164	0.78949609	0.792243574	0.795621411	0.8002938
Torbay	0.653525002	0.660513301	0.665031574	0.667184912	0.669676872	0.674620419	0.680324876	0.684207431	0.686835333	0.688288902	0.689636807	0.691798822	0.694287375	0.698534572	0.703537608	0.708212902	0.712970993	0.718010472	0.723061826	0.727404081	0.730273039	0.732910662	0.736375272	0.74010756	0.745153112
Wiltshire	0.699923755	0.706160147	0.709633549	0.710465205	0.711734687	0.716002872	0.721705826	0.726410204	0.73041563	0.733296387	0.735774413	0.738067885	0.739944551	0.743038396	0.7469491	0.751135505	0.755451157	0.759884685	0.764427612	0.768893382	0.77224644	0.774881797	0.778147472	0.782047535	0.787235972
West Midlands	0.653783571	0.661680498	0.666581029	0.668454055	0.670661728	0.675755675	0.681590926	0.685968893	0.689932514	0.692987339	0.695874479	0.698596065	0.701025711	0.704496589	0.708362168	0.712229751	0.717294817	0.722991462	0.728771674	0.73384891	0.737082996	0.73977625	0.743457914	0.747776577	0.753376821
Birmingham	0.648085705	0.656363631	0.661277703	0.662801433	0.664820264	0.670036233	0.676245447	0.681007957	0.685381	0.689019564	0.692818992	0.696684055	0.700261363	0.704551719	0.708482322	0.711918055	0.716941572	0.722790385	0.728461133	0.733522363	0.736635931	0.739095684	0.742751257	0.74712647	0.752647744
Coventry	0.671798763	0.679368883	0.684032075	0.685710257	0.68774696	0.692719917	0.69848234	0.702704008	0.706262362	0.70884663	0.711502829	0.714318232	0.71742645	0.721461637	0.725931404	0.730835783	0.73669449	0.742633422	0.748709332	0.754362863	0.758228958	0.761683748	0.765997002	0.770440984	0.775696268
Dudley	0.644449909	0.652271589	0.65703272	0.658729107	0.660732817	0.665654842	0.671262967	0.675279791	0.678846018	0.681563954	0.684043429	0.686613396	0.689123012	0.692199019	0.695488772	0.698687632	0.702693024	0.707436032	0.712484459	0.716784504	0.719634967	0.722330173	0.725895686	0.72971007	0.734414914
Herefordshire, County of	0.673366593	0.680684732	0.685260354	0.687037806	0.688994426	0.693602952	0.698887758	0.702844044	0.706498518	0.709083372	0.711009613	0.712464719	0.713443573	0.715943156	0.71918065	0.722860527	0.728012127	0.733900249	0.739954651	0.745296614	0.748918535	0.751922712	0.755598086	0.75991016	0.765623471
Sandwell	0.61285576	0.621652836	0.626905906	0.628494574	0.630355588	0.635837349	0.642411373	0.64739892	0.651852461	0.655171609	0.658221384	0.661254755	0.664204031	0.66806084	0.671783655	0.674777822	0.678886945	0.684257203	0.690250227	0.69529774	0.698067871	0.700465198	0.703999554	0.707805314	0.712879546
Shropshire	0.678836068	0.685791398	0.690261984	0.692152583	0.694233362	0.698742543	0.703848854	0.707600795	0.710782067	0.712838659	0.714797963	0.716993467	0.718845537	0.721671671	0.725267247	0.72909223	0.733978943	0.739324288	0.7444821	0.749057252	0.751921113	0.754203631	0.757586414	0.761759383	0.767237234
Solihull	0.710766784	0.717445793	0.721635199	0.723256642	0.725092191	0.729418406	0.73439608	0.738040648	0.740966915	0.742571557	0.743804226	0.7449756	0.746249765	0.749071927	0.752920356	0.75697799	0.762336153	0.768332551	0.773961556	0.778524879	0.781575749	0.784494296	0.788254026	0.792430764	0.797823903
Staffordshire	0.671257323	0.678614716	0.68320062	0.685011252	0.687110577	0.69182994	0.697161984	0.701050637	0.70453898	0.70712211	0.709427894	0.711357756	0.712815541	0.715614152	0.719381017	0.723512602	0.728904975	0.734918802	0.740985181	0.74579796	0.748529298	0.750866348	0.754092905	0.75817765	0.763618739
Stoke-on-Trent	0.619765399	0.628333483	0.633540123	0.635330721	0.637376988	0.642861701	0.649324396	0.654384397	0.659055901	0.662970978	0.66656333	0.669287346	0.671088218	0.674198317	0.678299055	0.682717867	0.688223883	0.6937868	0.699181327	0.704248652	0.707532771	0.710120289	0.713765953	0.717625826	0.722274839
Telford and Wrekin	0.662597422	0.67102687	0.675951112	0.67725523	0.678698198	0.683582037	0.689404153	0.693418569	0.697159652	0.700351547	0.703167837	0.705421323	0.706989643	0.709682335	0.713011919	0.716348041	0.721031078	0.726515931	0.732272613	0.737756079	0.741501812	0.744657839	0.748356888	0.752034519	0.757024051

Walsall	0.62488714	0.63308885	0.637967313	0.639457063	0.641272813	0.646293116	0.652103016	0.656141645	0.659781689	0.66256738	0.665576143	0.668719444	0.671091216	0.673945018	0.67654204	0.67868011	0.681778926	0.686454909	0.692208265	0.696833385	0.698847612	0.699530532	0.70106263	0.704017822	0.708980276
Warwickshire	0.700395589	0.707294027	0.711651488	0.713413755	0.715494821	0.720101154	0.725387707	0.729453595	0.733227523	0.735957437	0.738098336	0.739928867	0.741869868	0.744974809	0.748713938	0.753085339	0.758705831	0.764235811	0.769224656	0.773814492	0.777157593	0.78000855	0.783515693	0.787519487	0.792720704
Wolverhampton	0.634883512	0.643189204	0.648093071	0.649564833	0.65138712	0.65658727	0.662806583	0.667705324	0.672107604	0.675421373	0.678553267	0.681129035	0.682862647	0.685676409	0.688963415	0.692133664	0.696834259	0.702632522	0.709165938	0.715134949	0.718299619	0.720305573	0.7235514	0.727692618	0.73356159
Worcestershire	0.680282608	0.68743242	0.691973385	0.693855234	0.695992309	0.700602294	0.705786557	0.709658596	0.713270984	0.715996559	0.718193873	0.719999311	0.721402379	0.724143416	0.727860609	0.731756994	0.736468614	0.741498891	0.746667055	0.751165511	0.754157486	0.756841299	0.76053245	0.76511163	0.771225385
Yorkshire and the Humber	0.659229649	0.666639352	0.671253038	0.673017088	0.675173315	0.680273137	0.686449891	0.691169558	0.695197866	0.698287659	0.701127136	0.703891066	0.706538596	0.710190867	0.713993411	0.71744891	0.721942348	0.727224023	0.732715226	0.7376591	0.741090229	0.744213656	0.748039192	0.752248852	0.757564663
Barnsley	0.620549502	0.628326198	0.633034438	0.634553122	0.636396046	0.641518521	0.647921585	0.652748548	0.656765489	0.660015489	0.662979696	0.66560477	0.668004251	0.670971645	0.67322365	0.674409277	0.677297607	0.682143092	0.687701817	0.692117147	0.694451745	0.697069072	0.701108238	0.70552609	0.710642371
Bradford	0.634297734	0.642670653	0.647401422	0.648324566	0.649639113	0.654861109	0.661719678	0.667002675	0.671483114	0.675005653	0.678807249	0.682550153	0.685491537	0.689159611	0.692828636	0.695777168	0.699723241	0.704427991	0.709368942	0.714104834	0.717070903	0.719157139	0.721203086	0.723017633	0.726441976
Calderdale	0.661465644	0.669165508	0.67384972	0.675339955	0.677103409	0.682075315	0.688284352	0.692750852	0.696128489	0.698746471	0.701674257	0.704340814	0.70653281	0.710082193	0.713994537	0.717583821	0.72220176	0.727794375	0.733498025	0.738242957	0.740888394	0.743001819	0.746067255	0.749534048	0.754305043
Doncaster	0.623626048	0.631512198	0.636063702	0.637219662	0.638739909	0.643690061	0.649965522	0.654484218	0.658158295	0.660682714	0.662815707	0.665084613	0.667535624	0.670812828	0.673265643	0.67510562	0.678434292	0.682996246	0.688022272	0.692463822	0.694956323	0.696786009	0.699423852	0.703136631	0.708291833
East Riding of Yorkshire	0.679561103	0.686366344	0.690713992	0.692584342	0.694791902	0.699483358	0.705007175	0.709205469	0.7126438	0.715024616	0.717040481	0.718845628	0.720592174	0.723422251	0.726517608	0.729479586	0.734058197	0.739801277	0.745398719	0.750075423	0.753204147	0.755850404	0.759027005	0.76253921	0.767140291
Kingston upon Hull, City o	0.624034002	0.632292156	0.637286655	0.638904169	0.640842828	0.646225137	0.652924104	0.65772897	0.661430535	0.663679192	0.665653915	0.668118977	0.670958609	0.675071442	0.678803473	0.681942142	0.686303885	0.691360749	0.696855984	0.702058029	0.705435171	0.708841245	0.713685639	0.719310797	0.725860694
Kirklees	0.65143351	0.659085795	0.663704817	0.665203278	0.667108138	0.672205001	0.678478264	0.68316737	0.687395957	0.691018322	0.694187779	0.697207027	0.70035972	0.704183266	0.70760555	0.710984285	0.715952904	0.721728673	0.727298233	0.731498351	0.733768462	0.73592606	0.739005644	0.742545969	0.747270177
Leeds	0.6919331	0.698718063	0.703059822	0.704968988	0.70730039	0.712224075	0.718133781	0.722965722	0.72743283	0.730946778	0.733858525	0.73677986	0.739757693	0.743848184	0.748651027	0.753340826	0.758704729	0.764464312	0.770128491	0.775218036	0.779274871	0.783217875	0.787712447	0.792454193	0.79795548
North East Lincolnshire	0.626952595	0.635403592	0.640198085	0.641134949	0.642359703	0.647451981	0.654133989	0.658907866	0.6623228	0.664098415	0.665776334	0.667410571	0.668698537	0.671786636	0.675729662	0.679517747	0.685012163	0.691269153	0.697182622	0.702389449	0.705504761	0.707733487	0.710493044	0.713452048	0.717885014
North Lincolnshire	0.671441453	0.679148906	0.683579929	0.684581229	0.685849574	0.690585956	0.696740654	0.701192761	0.704534659	0.706846915	0.709273747	0.711474854	0.713000616	0.715495573	0.718647306	0.721431975	0.724986018	0.729588766	0.734674199	0.739194201	0.74239997	0.745328254	0.748478341	0.7514857	0.754696264
North Yorkshire	0.695095858	0.701576056	0.705770574	0.70756697	0.709643976	0.714190562	0.719672232	0.72400205	0.727701747	0.73046908	0.732927683	0.734939503	0.736578435	0.739328477	0.742653356	0.746089107	0.750306404	0.755072311	0.759882813	0.763926127	0.766510062	0.768917453	0.772331385	0.77626258	0.781084123
Rotherham	0.632401962	0.64006706	0.644667525	0.646065966	0.647777351	0.652733301	0.65894172	0.663455408	0.667151256	0.669815713	0.672332261	0.674729601	0.67643149	0.678953696	0.681671068	0.684048953	0.688005253	0.693073788	0.698683561	0.703887797	0.706812985	0.709061667	0.712089621	0.715385087	0.719984009
Sheffield	0.684314212	0.690592923	0.694817141	0.697053844	0.69976635	0.704543165	0.709878349	0.713883602	0.7173748	0.720379368	0.723111343	0.725910611	0.729020227	0.733092509	0.737276286	0.740870442	0.744939332	0.749592409	0.754683289	0.759741945	0.763734483	0.767149838	0.770746987	0.774813454	0.779963093
Wakefield	0.638052704	0.645986158	0.650779505	0.652301656	0.654142917	0.659334712	0.665854311	0.67077565	0.674818463	0.677579024	0.680040285	0.682320619	0.684115151	0.686780113	0.689723832	0.692436467	0.69653247	0.701823136	0.707800514	0.713152829	0.716363594	0.719061388	0.722368367	0.725655502	0.730279377
York	0.728791465	0.734709865	0.738778205	0.741009663	0.743613407	0.748141593	0.753303075	0.757511335	0.761324295	0.764541979	0.767578241	0.770539318	0.773673717	0.777873025	0.782495084	0.787154023	0.792373666	0.797581364	0.802250845	0.806402138	0.809928483	0.813354821	0.817286745	0.821463734	0.826269566
Northern Ireland	0.653098705	0.658238832	0.661547301	0.662705423	0.664823028	0.670119087	0.676181736	0.681143041	0.684928039	0.688559072	0.692440326	0.696583257	0.702287329	0.70988532	0.717200619	0.722723259	0.728469763	0.735161603	0.742021784	0.74762993	0.751320924	0.754622495	0.758643468	0.762872168	0.768131943
Scotland	0.674062217	0.68222803	0.687151073	0.690330222	0.693625344	0.697866375	0.703098264	0.708457223	0.713610632	0.717759561	0.722247589	0.726160912	0.730088219	0.735496458	0.74015662	0.743972492	0.748220021	0.753417051	0.759543792	0.764809282	0.76863789	0.771395327	0.774650905	0.779539941	0.785256752
Wales	0.648275763	0.655541021	0.660064788	0.66159301	0.66359397	0.668767905	0.675256149	0.680523115	0.684767563	0.687937256	0.690924537	0.693808619	0.696634754	0.700843334	0.705665935	0.710140734	0.715478036	0.721407686	0.727533596	0.733105415	0.73695851	0.74048567	0.744775328	0.7493989	0.755031994
Latin America and Caribbean	0.40383881	0.411017814	0.418340727	0.425829887	0.433388462	0.440743884	0.447621896	0.454274731	0.460548904	0.466731188	0.472761295	0.478548545	0.484067874	0.489101581	0.493628183	0.497738092	0.501730505	0.505674834	0.509875876	0.51433915	0.518546498	0.522877323	0.527604	0.532559673	0.537509233
Andean Latin America	0.410742754	0.418557012	0.426284844	0.433908539	0.442070054	0.450259122	0.457862167	0.464925657	0.470919908	0.476431604	0.48172427	0.487435915	0.492942412	0.496878965	0.498686893	0.50001149	0.501652202	0.50365586	0.506388431	0.510305574	0.514896344	0.519376447	0.52403649	0.528443909	0.532743717
Bolivia	0.345873606	0.351765135	0.357739647	0.363489399	0.369190547	0.374807433	0.380507944	0.385954156	0.390946082	0.395947743	0.400770903	0.405084336	0.409535493	0.413982576	0.418755154	0.423917961	0.429397795	0.434835542	0.440601646	0.4468225	0.453479243	0.460220669	0.466982897	0.473796201	0.48019431
Ecuador	0.433668943	0.443142907	0.45171698	0.459909681	0.469274417	0.478717584	0.486143206	0.492879092	0.499006123	0.503656832	0.508317601	0.514307967	0.518982985	0.520983619	0.520092625	0.518430614	0.517679421	0.51837907	0.520707291	0.524908291	0.529380752	0.532691399	0.535349652	0.53719633	0.539032308
Peru	0.417704545	0.425219727	0.432991227	0.440808087	0.44891626	0.457002154	0.465017271	0.472474999	0.478506764	0.484411304	0.489959176	0.495693497	0.501711501	0.506331065	0.508666775	0.510419852	0.512161525	0.513821552	0.515981506	0.51930608	0.523640527	0.528312042	0.533576491	0.538721717	0.543853377
Caribbean	0.426671613	0.435939967	0.445261908	0.453975835	0.461868791	0.468547315	0.474107305	0.479174357	0.484564107	0.490223429	0.495771101	0.500812841	0.50479768	0.508616834	0.513050909	0.518111381	0.522788586	0.526984192	0.530506786	0.533584311	0.536650593	0.539775932	0.543302886	0.547365066	0.552060172
Antigua and Barbuda	0.506683804	0.509416268	0.513944636	0.519259299	0.526509586	0.534534369	0.542770621	0.551210415	0.559292499	0.567122296	0.575214632	0.58380438	0.591665364	0.598827628	0.60535671	0.612104591	0.618817129	0.624695818	0.630075854	0.634768955	0.638008629	0.641647192	0.645895591	0.650614287	0.655534994
The Bahamas	0.605786239	0.605926221	0.609080786	0.61218986	0.616023611	0.62055622	0.626249094	0.634072041	0.644065706	0.655951118	0.669295723	0.680824877	0.68800996	0.691915792	0.694160731	0.693509268	0.689126784	0.687343838	0.693816501	0.706819892	0.719381607	0.728478844	0.734781215	0.739281293	0.741622848
Barbados	0.575192808	0.58496775	0.591249017	0.596160993	0.601694021	0.605284484	0.606978102	0.609455834	0.615001674	0.622921377	0.631699992	0.640193523	0.646222036	0.649159747	0.65144169	0.653582517	0.656654073	0.662446635	0.668815012	0.673409365	0.676012247	0.677193054	0.678644826	0.679827167	0.680398459
Belize	0.311969143	0.315571159	0.319994497	0.325362732	0.331895865	0.340243433	0.348207477	0.355945264	0.363914715	0.372257946	0.380429709	0.388156432	0.396025885	0.404159089	0.413349679	0.423726992	0.433834392	0.444198507	0.455258577	0.465729308	0.475278511	0.482702165	0.488020129	0.491819335	0.495070196
Bermuda	0.610163014	0.618814786	0.626963365	0.634301664	0.641051672	0.647305219	0.652584969	0.657304338	0.662132484	0.667034845	0.672256576	0.677461193	0.682481741	0.687336357	0.692050702	0.696451196	0.700276851	0.703984095	0.707678222	0.711068998	0.714468828	0.717831671	0.721642813	0.725853528	0.730539823
Cuba	0.442200101	0.458765879	0.474979281	0.488832297	0.499663022	0.50664608	0.5107356	0.513632044	0.517985644	0.523937032	0.530819721	0.537019094	0.540874356	0.544980657	0.550867987	0.558019071	0.563413638	0.566169006	0.565441047	0.563186313	0.560765902	0.559346329	0.558924568	0.55936322	0.561820953
Dominica	0.476263074	0.491697195	0.502597541	0.512382497	0.51596844	0.518029362	0.521576269	0.524660861	0.529604309	0.535461459	0.539657995	0.543554662	0.547298265	0.552282225	0.558704478	0.56360259	0.565237843	0.569570684	0.578811811	0.590119147	0.601986932	0.61438205	0.625057353	0.633017994	0.639453915
Dominican Republic	0.330387332	0.339759179	0.348936529	0.357728194	0.36653058	0.375443006	0.384039941	0.392527796	0.400996069	0.408901363	0.415678973	0.42158459	0.427388536	0.432993125	0.438427858	0.442654076	0.446144578	0.449953081	0.454229297	0.458620614	0.463313869	0.468516938	0.474504389	0.481170939	0.488178541
Grenada	0.373174086	0.381484845	0.389030819	0.39601518	0.402246472	0.406722075	0.409427129	0.410875857	0.411516873	0.412120221	0.413131292	0.414720217	0.417866935	0.422412583	0.428718553	0.436734419	0.445887953	0.455910058	0.46599489	0.476410341	0.486558999	0.496589808	0.506217602	0.516590019	0.527333411
Guyana	0.443163512	0.44959075	0.454547082	0.458292951	0.460988506	0.463302962	0.465540542	0.466879148	0.466779867	0.465388112	0.463452604	0.461846951	0.460913182	0.46016878	0.459920818	0.460430129	0.462677069	0.466753989	0.472174229	0.478992924	0.486573457	0.495216036	0.504362779	0.513068898	0.521595978
Haiti	0.248386712	0.252475726	0.256343189	0.260294053	0.264495416	0.26894684	0.27299178	0.27666252	0.280273306	0.283944889	0.287810297	0.291855834	0.2961609	0.300838102	0.305478428	0.31033463	0.315455441	0.320389979	0.324895851	0.328399456	0.332999152	0.338229396	0.343880049	0.349698073	0.355980743
Jamaica	0.440544335	0.452136996	0.462838574	0.47231965	0.480460735	0.487085773	0.493195843	0.498857508	0.50447731	0.509870611	0.514381507	0.518851494	0.523211717	0.526812112	0.530472797	0.534781234	0.53959448	0.545088753	0.551218492	0.557643773	0.564315038	0.570940131	0.577212091	0.583048035	0.588846467
Puerto Rico	0.580710548	0.582270072	0.586069444	0.593113208	0.601249406	0.609278849	0.618127556	0.627114352	0.635041177	0.641041656	0.645030874	0.648127841	0.650641859	0.65256843	0.654689425	0.658758146	0.66326172	0.667272192	0.671024105	0.67545081	0.68067395	0.685865893	0.691274382	0.697040445	0.702850671
Saint Kitts and Nevis	0.430746482	0.437562743	0.445446639	0.453719485	0.462131712	0.471284207	0.481552022	0.49317654	0.505474827	0.518538446	0.530935948	0.542647742	0.55298204	0.562140599	0.571065375	0.580685877	0.59025495	0.59918719	0.607235892	0.614514658	0.620716498	0.62620741	0.63127474	0.635539988	0.639713739
Saint Lucia	0.320493113	0.333527527	0.346838999	0.36022238	0.373004939	0.384405225	0.395032522	0.405322381	0.415622879	0.426234001	0.437450031	0.449506564	0.461366756	0.473609108	0.485225832	0.49629657	0.505975555	0.515368261	0.524234949	0.532731083	0.541479794	0.550242412	0.558293461	0.566138703	0.573428555
Saint Vincent and the Grenadines	0.330451607	0.34224299	0.351367195	0.358862071	0.365017944	0.370977312	0.378476746	0.388187633	0.399772134	0.412076455	0.423884516	0.435208528	0.446178253	0.458283419	0.46852922	0.475930186	0.481075658	0.485977871	0.490998791	0.496379132	0.50298609	0.509527158	0.515482619	0.521209679	0.527099552
Suriname	0.418952155	0.422936402	0.428111752	0.433035999	0.436658993	0.438875195	0.442183286	0.446602958	0.452299131	0.459276563	0.467643349	0.475902962	0.483000564	0.489922413	0.49641918	0.502054305	0.506655795	0.510959772	0.514419437	0.517650245	0.521002622	0.525059318	0.529750812	0.534599696	0.538932791
Trinidad and Tobago	0.546505354	0.551641642	0.55582113	0.559490823	0.563302955	0.567738526	0.572276271	0.577082801	0.582027707	0.587710514	0.594023565	0.600880178	0.607644674	0.613674512	0.618963308	0.62397015	0.628758918	0.634039552	0.639191914	0.64424291	0.649220055	0.654169455	0.659293633	0.664507714	0.670244574
Virgin Islands	0.57083364	0.580356724	0.588634477	0.595684637	0.602349903	0.608464169	0.614080344	0.61841838	0.622971683	0.627437766	0.631021877	0.634140962	0.637291325	0.640039052	0.642991817	0.655160856	0.664018659	0.671081871	0.676872745	0.681982223	0.686730211	0.691247699	0.695598204	0.699768686	0.703949342
Central Latin America	0.39356845	0.401580858	0.409929782	0.418702592	0.427261473	0.435234794	0.442673657	0.449682372	0.456024327	0.461746878	0.466752708	0.471058847	0.47534372	0.479172906	0.482436591	0.48578741	0.489558534	0.494110188	0.499417966	0.505060641	0.509874246	0.514693467	0.520170671	0.526131813	0.531807023
Colombia	0.407281661	0.414608736	0.421704657	0.428873084	0.435705631	0.442424437	0.448564645	0.454103394	0.459131488	0.463698319	0.467575029	0.470990334	0.473946282	0.476554517	0.478770437	0.480720054	0.482339894	0.484572359	0.488229373	0.493278182	0.499102757	0.504986969	0.511079502	0.517022505	0.52243594
Costa Rica	0.467740789	0.471317542	0.474475623	0.478531214	0.483842575	0.489665239	0.495410483	0.499538744	0.500858402	0.499795309	0.501156822	0.507647597	0.514812279	0.520928424	0.52763543	0.534125181	0.539342164	0.544624238	0.549946626	0.55517125	0.560547086	0.565180862	0.570128417	0.576067051	0.582229492
El Salvador	0.280302097	0.285810596	0.292638731	0.30059813	0.309511621	0.31876506	0.327910736	0.336585627	0.344549321	0.351377289	0.356975746	0.361399821	0.365142047	0.368300362	0.370887473	0.373057975	0.374855325	0.377076643	0.380307326	0.385149094	0.391780266	0.400178468	0.410455826	0.42142119	0.432733148
Guatemala	0.267606032	0.266585585	0.265298816	0.264782141	0.264812901	0.268375177	0.275578072	0.283960779	0.292128738	0.299225072	0.305226098	0.309049845	0.310632995	0.310797305	0.310572703	0.311792455	0.314329484	0.319165481	0.326840819	0.3334691	0.339642101	0.346961212	0.355995963	0.366461193	0.374455316
Honduras	0.24989992	0.254018014	0.258863833	0.264338822	0.270259013	0.276490304	0.282697225	0.288810507	0.294620913	0.300298927	0.305858338	0.310970395	0.316124414	0.321395291	0.326839918	0.332042889	0.337034649	0.342305657	0.347838446	0.353164852	0.358787268	0.364328424	0.369983894	0.375950111	0.381752316
Mexico	0.392089624	0.402293915	0.41317937	0.424671769	0.435537624	0.444787901	0.452807183	0.460289992	0.467076321	0.473368659	0.479276141	0.484803029	0.490700444	0.496063271	0.500707846	0.504996083	0.50951067	0.514638057	0.520493167	0.526749241	0.531554141	0.536530827	0.541988171	0.547842121	0.55381337
Aguascalientes	0.392605475	0.404573069	0.416956206	0.429933751	0.442362935	0.453166566	0.462684482	0.471617212	0.479842655	0.487563414	0.494897326	0.501677026	0.50857726	0.514695469	0.519945874	0.524645983	0.529086308	0.533396884	0.538117209	0.543268306	0.547452751	0.552198621	0.55740598	0.563132208	0.56946169
Baja California	0.454529015	0.466324932	0.478411708	0.490532895	0.501603855	0.511059156	0.519398254	0.52703311	0.533593873	0.539423611	0.544589432	0.548868101	0.552657592	0.555253773	0.556697804	0.557776897	0.559365698	0.561957605	0.565692125	0.570004399	0.573086528	0.576674209	0.581389375	0.587181672	0.593318034
Baja California Sur	0.415699204	0.427945576	0.440784284	0.454163456	0.466795091	0.477605932	0.486952688	0.495632708	0.503463592	0.510486652	0.51689328	0.522877748	0.529403819	0.535924607	0.542069415	0.547365468	0.552228229	0.55700866	0.562550597	0.568593089	0.573090747	0.577591103	0.58235863	0.587326778	0.59177257
Campeche	0.357737386	0.366565184	0.376539375	0.387261937	0.39742956	0.40619762	0.413954355	0.421435283	0.428210406	0.434332544	0.439968487	0.445467781	0.451742931	0.4576992	0.462961401	0.467809577	0.473008376	0.479048708	0.486466285	0.494683355	0.501700286	0.509180613	0.516994964	0.525105941	0.533195202
Chiapas	0.29119164	0.297968162	0.305699606	0.314484534	0.322998632	0.330033195	0.336201334	0.342454592	0.348581115	0.354476655	0.359725729	0.364458909	0.369265774	0.373284345	0.376578959	0.380122713	0.385087423	0.391658899	0.399667728	0.408412838	0.415031544	0.420853035	0.42670943	0.432571889	0.438500296
Chihuahua	0.402134722	0.414250913	0.427014104	0.440282597	0.45275542	0.463471198	0.472821379	0.481462518	0.489213783	0.496287572	0.502846974	0.508827162	0.514688495	0.519582247	0.523353165	0.526085567	0.528369742	0.530621214	0.533088262	0.535463962	0.536770448	0.538957206	0.542141191	0.546449106	0.551381625
Coahuila	0.404189699	0.415455562	0.427406567	0.43989489	0.451506083	0.461091722	0.469277042	0.476889832	0.483646855	0.489841903	0.495750868	0.501519996	0.50788063	0.513893932	0.519252608	0.524303567	0.529617755	0.535356954	0.541523107	0.547873874	0.552441837	0.557008538	0.562049036	0.567506083	0.573174326
Colima	0.399067675	0.411390192	0.424157918	0.437389712	0.449904567	0.460870156	0.47076412	0.480079341	0.488540798	0.496291317	0.503969088	0.511649227	0.519982297	0.527732769	0.534754687	0.541152933	0.547263727	0.553181144	0.559053374	0.564887752	0.569709808	0.575167134	0.580692342	0.586060065	0.591773151
Durango	0.347607579	0.359073227	0.371303363	0.384108984	0.396229946	0.406744537	0.416178005	0.425146127	0.433253016	0.440701276	0.447340909	0.4531517	0.459052304	0.464192696	0.468333729	0.471994856	0.475753101	0.480095953	0.484867553	0.489882342	0.494135503	0.499273794	0.505544681	0.512813591	0.519955161
Guanajuato	0.326621704	0.337655319	0.349287841	0.361391852	0.372945701	0.383222115	0.39255167	0.401382012	0.40954639	0.417289079	0.425075977	0.43298142	0.441394291	0.449210208	0.456238683	0.463111395	0.470188385	0.477361521	0.484588445	0.491856735	0.498138011	0.505096921	0.512397226	0.520005532	0.52749219
Guerrero	0.286300833	0.295324186	0.305365295	0.316383736	0.326981655	0.336145587	0.344446847	0.352761356	0.360714941	0.368162881	0.375129558	0.381776666	0.388840397	0.395139646	0.400324543	0.404457237	0.408255466	0.412470402	0.417130337	0.422254534	0.42746483	0.433915892	0.440722245	0.447597172	0.454441561
Hidalgo	0.318097154	0.327646935	0.337916078	0.348871996	0.359344831	0.368078196	0.37543944	0.382318409	0.388647404	0.394860057	0.401146816	0.407461795	0.414654925	0.421622339	0.427900136	0.433772282	0.439869628	0.446651297	0.454131297	0.462109622	0.469017512	0.476188075	0.483745892	0.491469785	0.498988995
Jalisco	0.397871353	0.408837303	0.420349317	0.43223578	0.443374464	0.452976299	0.461462682	0.46923026	0.476088284	0.482464255	0.488969266	0.49553968	0.502634725	0.508958019	0.514446995	0.519722519	0.525127651	0.530888564	0.537022622	0.543348927	0.548287987	0.553519239	0.559297302	0.565395705	0.571402201
México	0.395390877	0.406861583	0.419460521	0.432895679	0.445713251	0.4568822	0.466916405	0.476836436	0.486220696	0.494873283	0.502621623	0.509437167	0.516159084	0.521873907	0.52647631	0.530648662	0.535123213	0.540586547	0.547051784	0.5538444	0.558051963	0.561445872	0.564846598	0.568399018	0.572593908
Mexico City	0.526813689	0.536152449	0.545568928	0.555314461	0.564530164	0.572406629	0.579105011	0.584830752	0.589599385	0.593858457	0.597770805	0.601324099	0.605557305	0.609878483	0.61413282	0.618325598	0.622830107	0.628087498	0.634543876	0.64174362	0.646990861	0.652117183	0.657804747	0.663818173	0.669722375

Michoacán de Ocampo	0.323413506	0.333057269	0.343489336	0.354722381	0.365649326	0.375176448	0.383844388	0.39227894	0.400316601	0.408036702	0.415573293	0.423082729	0.431400455	0.439568803	0.447191642	0.454293326	0.461317384	0.468512261	0.475423283	0.481909927	0.487128237	0.492489666	0.498260802	0.504338873	0.510458735
Morelos	0.395947932	0.406271323	0.417080039	0.428313581	0.438772864	0.447464767	0.454951981	0.462283507	0.469463024	0.476622361	0.483956653	0.491456059	0.499696531	0.507418412	0.51453232	0.520991486	0.527159101	0.533231346	0.539467741	0.545786953	0.550933604	0.556407268	0.56197648	0.567471342	0.573149825
Nayarit	0.341242587	0.353645215	0.366886503	0.380943008	0.394401474	0.405995587	0.41594581	0.424981641	0.432924879	0.439981349	0.446166386	0.451771536	0.457801302	0.463596409	0.468863135	0.47346391	0.477936384	0.482866787	0.48830129	0.494012839	0.499079268	0.505010842	0.511456927	0.518532192	0.526147165
Nuevo León	0.455435684	0.4673658	0.479743862	0.492604814	0.504793179	0.515463501	0.524976476	0.533923053	0.542042478	0.549552745	0.556442705	0.562349889	0.568135074	0.573252447	0.577658705	0.581722309	0.585732591	0.589944071	0.594231065	0.598487076	0.601406582	0.60454057	0.608331656	0.612985238	0.61816602
Oaxaca	0.301933064	0.31019872	0.31948217	0.329611923	0.339403006	0.348130665	0.356412266	0.364691392	0.372566561	0.380076679	0.387136111	0.393796381	0.4004924	0.406153508	0.410527386	0.414302017	0.418255379	0.422765462	0.42802641	0.433694002	0.43843657	0.443780236	0.449239643	0.454492855	0.459714435
Puebla	0.329592582	0.338745107	0.348824078	0.359753384	0.370251701	0.379309835	0.387414715	0.395233518	0.402470288	0.409305234	0.415890257	0.42230252	0.429545931	0.436662205	0.443151664	0.449529952	0.456266656	0.463574103	0.471067188	0.478342588	0.483552566	0.488206112	0.493198426	0.498537683	0.504066375
Querétaro	0.38355983	0.394882022	0.406762912	0.419036337	0.4305566	0.440463751	0.4491896	0.457380354	0.464546683	0.470845249	0.476714253	0.482536359	0.489381337	0.496077848	0.502161548	0.507320191	0.512180108	0.517465864	0.523870787	0.530888522	0.536820255	0.5430477	0.550031272	0.557462457	0.565822742
Quintana Roo	0.345948068	0.357017469	0.369238927	0.382635865	0.395606998	0.406711371	0.416578559	0.426209211	0.435370818	0.443910943	0.452001824	0.459757862	0.468073834	0.476036253	0.483569469	0.491221072	0.499658554	0.508849401	0.518966035	0.529275714	0.538097916	0.547338794	0.55607698	0.564034086	0.571284805
San Luis Potosí	0.328966028	0.339272101	0.350645182	0.362763683	0.37417791	0.383865327	0.392308106	0.400459157	0.408177778	0.415643776	0.422494313	0.428450195	0.434550159	0.440273325	0.445323873	0.450145657	0.455178109	0.460865887	0.467886019	0.475908899	0.482813693	0.490262913	0.498007231	0.505981191	0.513788587
Sinaloa	0.371084311	0.384298933	0.398377856	0.413184517	0.427336619	0.439764881	0.450832878	0.461096476	0.470072413	0.477981127	0.48521253	0.491884555	0.4986511	0.504371056	0.508811519	0.51239837	0.515724538	0.519110064	0.523493293	0.529171679	0.533824543	0.539021242	0.545017962	0.551864493	0.558729337
Sonora	0.43044312	0.44242958	0.45491834	0.467824109	0.479924481	0.49020023	0.499081041	0.507238111	0.514439317	0.520994777	0.527168292	0.532756424	0.53795544	0.541749605	0.544516427	0.546866387	0.549324775	0.552173274	0.55580514	0.560149323	0.563537758	0.567396802	0.572244411	0.578181096	0.584797789
Tabasco	0.328228057	0.338201916	0.349201212	0.361195591	0.372654835	0.382540796	0.391519929	0.400582192	0.409594178	0.418366892	0.426778133	0.434796249	0.443065919	0.450609223	0.457237724	0.463410559	0.469836195	0.476814745	0.483912394	0.490924045	0.497132636	0.504137673	0.511622806	0.519274266	0.526820229
Tamaulipas	0.412671477	0.423523914	0.434773751	0.4465676	0.457766992	0.467333665	0.475483414	0.482786067	0.489173736	0.494987806	0.501180399	0.508070612	0.516366623	0.524701371	0.53245622	0.538947104	0.544638461	0.549815236	0.555307892	0.560934086	0.566102025	0.572559594	0.578748304	0.584483131	0.588908393
Tlaxcala	0.329075806	0.340190702	0.351875631	0.364041638	0.375504326	0.385657584	0.395027143	0.403994675	0.412010591	0.419284365	0.426666938	0.434472649	0.44341739	0.452121529	0.460219558	0.467929212	0.475615886	0.483461998	0.491556131	0.499593393	0.505981625	0.512412878	0.519057294	0.525890662	0.532981824
Veracruz de Ignacio de la Llave	0.352462466	0.361663052	0.371844434	0.382783936	0.393072986	0.401581038	0.408723784	0.415499352	0.422050278	0.428527163	0.434581925	0.439953062	0.445404096	0.450150911	0.454006897	0.457310627	0.460899618	0.46549269	0.47103541	0.477123296	0.481811683	0.486716706	0.492450681	0.498808516	0.504983565
Yucatán	0.364316299	0.372916326	0.382442622	0.392700949	0.402439135	0.410685289	0.417949658	0.425080876	0.431909992	0.43848449	0.444472059	0.449680643	0.455010299	0.460004211	0.464484883	0.469101155	0.474451956	0.480864076	0.488652617	0.497235252	0.504223762	0.511318755	0.518418074	0.525292033	0.531390302
Zacatecas	0.325461692	0.338069664	0.351611549	0.366000307	0.379852753	0.392211099	0.403696966	0.415001311	0.425747311	0.435943792	0.445485984	0.454189656	0.46261388	0.469936231	0.476285586	0.482045818	0.48758905	0.49321387	0.498885367	0.504545124	0.508949999	0.51353475	0.518745791	0.524468712	0.530542762
Nicaragua	0.252051026	0.255543118	0.259668832	0.263430042	0.266387363	0.271363409	0.278431376	0.286565396	0.295418156	0.304184338	0.312795906	0.320980002	0.328678848	0.334696178	0.340453631	0.346035235	0.351740622	0.357712704	0.363562069	0.369727968	0.376625345	0.384087266	0.391775893	0.399807535	0.408085965
Panama	0.450575377	0.458541469	0.464546329	0.471174268	0.47902401	0.487356756	0.495835402	0.503997318	0.510315895	0.515684203	0.522012073	0.528991887	0.534913355	0.538787075	0.542037901	0.546048123	0.550484218	0.554972755	0.559168666	0.561714534	0.563353023	0.56531007	0.569395939	0.574697318	0.580432842
Venezuela	0.458251611	0.463483403	0.468876331	0.474387006	0.480227056	0.486449674	0.49293421	0.499350353	0.50552503	0.510704494	0.513177048	0.513329709	0.513547236	0.514229083	0.514372787	0.516891218	0.522272296	0.529517991	0.536279978	0.541866058	0.546348234	0.548649226	0.552364437	0.557243117	0.560277999
Tropical Latin America	0.399625598	0.406081332	0.412417776	0.41874208	0.425362369	0.432188928	0.438599937	0.44510488	0.451500771	0.458342168	0.465637664	0.473239141	0.480567964	0.487575205	0.49418597	0.499587794	0.504290478	0.508056827	0.511622219	0.515330113	0.519290875	0.523498539	0.527841481	0.532164921	0.536658546
Brazil	0.399436877	0.405912711	0.412265442	0.41860778	0.425253325	0.4321045	0.438535858	0.445077694	0.451525258	0.458430447	0.465799036	0.473488093	0.480898326	0.487975515	0.494640751	0.500070509	0.504784798	0.508539943	0.512081793	0.515760842	0.519685963	0.523863329	0.528177889	0.532481169	0.536972035
Acre	0.250165899	0.252930516	0.256431062	0.262741671	0.270142988	0.278016002	0.285833758	0.294175421	0.303340661	0.313454382	0.324311483	0.335686097	0.34597837	0.355756409	0.364709096	0.372460174	0.378980241	0.384504394	0.38967272	0.395394211	0.401619627	0.407548372	0.412889884	0.418094805	0.423294984
Alagoas	0.259362654	0.2647279	0.2703422	0.276580857	0.283350429	0.29042807	0.297304045	0.304850771	0.314139662	0.324424866	0.335368671	0.345944889	0.355972196	0.363743089	0.369721487	0.375345821	0.38023745	0.384027164	0.386951623	0.389896012	0.392646954	0.395593369	0.399214316	0.403276779	0.407308246
Amapá	0.3297457	0.332872489	0.33668864	0.342840896	0.350428329	0.358851286	0.367381496	0.376812864	0.388392655	0.400603142	0.413184066	0.424991076	0.435763137	0.446571992	0.458107841	0.466489972	0.473046435	0.478145562	0.482182483	0.486829328	0.492740827	0.49860984	0.504140235	0.508931047	0.513368306
Amazonas	0.330819384	0.336996664	0.343434763	0.352247156	0.361589559	0.370927512	0.37997468	0.38989871	0.401906422	0.414856413	0.428276594	0.442464765	0.455444358	0.466920434	0.476692348	0.484414577	0.490171921	0.4941568	0.498171213	0.500865898	0.502034694	0.502823745	0.503017744	0.503369852	0.504117595
Bahia	0.281308409	0.28723781	0.293543555	0.301200286	0.309463401	0.318107578	0.326737782	0.336098665	0.34691226	0.358595923	0.370786977	0.382625637	0.393082664	0.402645134	0.411087294	0.41801382	0.423900821	0.428644816	0.432921354	0.436994935	0.440272212	0.443807533	0.447529476	0.451064095	0.454853367
Ceará	0.278738058	0.284174778	0.290226384	0.298344849	0.307141693	0.316107822	0.32449157	0.333125884	0.342405119	0.351897332	0.361457777	0.370944747	0.378997929	0.386256266	0.392695326	0.398353718	0.40427565	0.409294965	0.414032839	0.419088629	0.424445663	0.430080828	0.435248358	0.43991082	0.444360723
Distrito Federal	0.492673114	0.497961612	0.503674431	0.511122887	0.519545948	0.528432473	0.536807209	0.544610536	0.549939206	0.555256767	0.560062164	0.564661219	0.568829083	0.573227432	0.578511834	0.583730192	0.591082949	0.595897693	0.599876679	0.604819954	0.618006745	0.630290826	0.640613076	0.649632422	0.657487179
Espírito Santo	0.366594034	0.372289065	0.378415252	0.387389912	0.396756843	0.4060653	0.414555237	0.423035163	0.432176634	0.441816923	0.451529638	0.460959402	0.468520939	0.475942096	0.483642809	0.489800826	0.495332665	0.500161475	0.504654971	0.509780209	0.515480061	0.520844987	0.526081348	0.53127897	0.536847495
Goiás	0.342660156	0.349784427	0.356616259	0.363843025	0.371262941	0.378536337	0.384832534	0.390880778	0.397411192	0.404800304	0.413061686	0.422812895	0.431358821	0.439277037	0.446422118	0.453438148	0.460179009	0.465097122	0.46976741	0.474631888	0.479858862	0.48553627	0.491472893	0.497707742	0.504092618
Maranhão	0.211089243	0.214977009	0.219352821	0.224992603	0.231279201	0.237865931	0.244222118	0.250776873	0.256932503	0.264028241	0.272160586	0.281284058	0.289496675	0.297912226	0.306065659	0.313023543	0.319224358	0.324157618	0.328661244	0.333630525	0.339376001	0.345669569	0.351066591	0.355270891	0.359166843
Mato Grosso	0.336583578	0.34082945	0.345605048	0.351787724	0.35861621	0.366015878	0.373654427	0.382141794	0.39075727	0.399992153	0.409950182	0.421216817	0.431609611	0.441549911	0.450326724	0.457312691	0.463967763	0.469716305	0.475569609	0.481304068	0.486368479	0.491495218	0.497158911	0.502799965	0.509001634
Mato Grosso do Sul	0.367218918	0.374837695	0.381944995	0.390576611	0.398984383	0.406953546	0.414622021	0.423065353	0.431089397	0.439440814	0.447301111	0.455405794	0.462576379	0.469310215	0.475677031	0.480886355	0.485393804	0.488630055	0.491891739	0.495863954	0.49877929	0.502012889	0.505864168	0.510425772	0.515585159
Minas Gerais	0.38122242	0.388356374	0.39566315	0.404124412	0.412851794	0.421327756	0.428806946	0.435993449	0.442975304	0.450389012	0.458218421	0.466084512	0.473761599	0.481159423	0.488316779	0.494231041	0.499747029	0.504362287	0.508531841	0.51311003	0.516926011	0.520857254	0.524825805	0.528604659	0.532472401
Pará	0.306129397	0.310140292	0.314913774	0.322584537	0.331143156	0.339936219	0.348307929	0.356851803	0.365168272	0.374294396	0.384118052	0.394934226	0.405161448	0.414614388	0.424852581	0.433188565	0.439782409	0.443914048	0.449390902	0.454416572	0.456197802	0.457380857	0.458097603	0.458675175	0.45933038
Paraíba	0.27801642	0.284756786	0.291756757	0.298948539	0.306505827	0.31431974	0.321614299	0.32906562	0.337214094	0.34589328	0.355101761	0.365086648	0.374072672	0.381848606	0.389253835	0.396846309	0.403849551	0.408899007	0.413799628	0.419115289	0.423480248	0.427852546	0.43197639	0.435780989	0.439453252
Paraná	0.395272482	0.404626731	0.413676345	0.422336743	0.431086038	0.43982474	0.447770523	0.455263625	0.462679642	0.470388396	0.477819122	0.485100853	0.49202677	0.498414315	0.504658964	0.509650881	0.513544215	0.516665469	0.519940801	0.5240295	0.528442393	0.533466096	0.538918647	0.544828513	0.551061592
Pernambuco	0.307611597	0.313598281	0.319743371	0.326143052	0.333164352	0.340539017	0.347745094	0.355313997	0.363433807	0.372388865	0.382080922	0.392600286	0.402358588	0.410962753	0.418652546	0.425212065	0.431196773	0.435294585	0.438625264	0.441795382	0.444270997	0.447065377	0.450063197	0.453262352	0.456550562
Piauí	0.233327299	0.239229627	0.245379175	0.252178429	0.25939616	0.266786435	0.2735907	0.280622777	0.288018873	0.296102987	0.304773473	0.314354272	0.322816921	0.330081176	0.33700328	0.344034125	0.350490702	0.355144925	0.360301859	0.366061245	0.372743273	0.379283202	0.384930973	0.38973714	0.394110991
Rio de Janeiro	0.511555161	0.517509563	0.523006498	0.527697527	0.532445495	0.537565032	0.542447592	0.546927052	0.55023749	0.553798972	0.557976594	0.56216022	0.566219677	0.570346183	0.574056962	0.577165929	0.580732921	0.583569006	0.586183932	0.588730546	0.59157667	0.594989694	0.598729205	0.602983575	0.607692053
Rio Grande do Norte	0.295160522	0.301396313	0.308090055	0.316090999	0.324740892	0.333471107	0.34150129	0.349639379	0.358864948	0.368696825	0.378930029	0.388685601	0.397448788	0.405026906	0.412532584	0.41829153	0.423517752	0.426995884	0.43064853	0.434084359	0.437251325	0.441177128	0.445355343	0.449355275	0.453643052
Rio Grande do Sul	0.466955246	0.473152028	0.479277191	0.485345712	0.491653444	0.497885768	0.503243045	0.5084363	0.513596375	0.519452404	0.526155831	0.533521179	0.540234683	0.546457037	0.552334436	0.556901852	0.560585518	0.564129687	0.567855382	0.571433991	0.57367372	0.576171806	0.579124158	0.582577123	0.586737395
Rondônia	0.333614295	0.338267823	0.343055806	0.349348984	0.356146763	0.36323639	0.370020162	0.37754512	0.386458146	0.396159376	0.406415392	0.417249761	0.426742679	0.43522742	0.443567335	0.449843647	0.454054902	0.456571687	0.45884913	0.460785842	0.462871321	0.465668136	0.469333872	0.4735258	0.478398792
Roraima	0.349082468	0.35485716	0.360770213	0.366960051	0.373912434	0.381623386	0.389430226	0.397877602	0.407409406	0.417627812	0.428289429	0.439802347	0.450184874	0.459525073	0.468730861	0.47622098	0.481078873	0.483531055	0.483848404	0.483918678	0.489355873	0.495096223	0.500238417	0.505062325	0.510358709
Santa Catarina	0.433092512	0.440793017	0.448409896	0.456932779	0.465525435	0.473951489	0.481363733	0.488642139	0.495601158	0.502741721	0.510003214	0.5177102	0.525047834	0.532639922	0.541309372	0.547671627	0.552450808	0.556656648	0.560199304	0.56401257	0.567741304	0.57195993	0.576439366	0.580941199	0.585807915
São Paulo	0.479624456	0.484862109	0.489789983	0.494138384	0.498892436	0.504303636	0.50978033	0.515845005	0.521726306	0.527513369	0.53344811	0.539916029	0.54680771	0.553564569	0.559802773	0.564758448	0.568849951	0.5721516	0.575334285	0.578935322	0.583745835	0.588962422	0.594491028	0.599875602	0.605392837
Sergipe	0.304367454	0.310995456	0.317877733	0.32494134	0.332512152	0.340496026	0.348172262	0.356692645	0.371489723	0.386352641	0.40052775	0.413174961	0.423051742	0.431552285	0.438581754	0.444644766	0.450327693	0.454549905	0.45810966	0.460630267	0.462694986	0.465478785	0.468966292	0.472533866	0.476130944
Tocantins	0.282136868	0.289063342	0.296136345	0.303330267	0.310856931	0.318579628	0.325944776	0.333522673	0.341029322	0.34899483	0.357312677	0.36619072	0.373585236	0.379909905	0.383551658	0.385988031	0.388290427	0.389253254	0.390525148	0.392989216	0.398731675	0.404750399	0.411020436	0.417674795	0.424378506
Paraguay	0.397514309	0.402824444	0.408470827	0.414263514	0.420039643	0.426169264	0.432298238	0.437481668	0.441713706	0.445680968	0.449565943	0.45297599	0.456493047	0.460414067	0.464841982	0.469527785	0.474352286	0.479314169	0.4846142	0.490226935	0.496336876	0.502322804	0.508286948	0.513819896	0.518720583
North Africa and Middle East	0.321037626	0.327692032	0.33391326	0.339075728	0.343774011	0.349320733	0.356646526	0.365075438	0.374014514	0.382928316	0.391621546	0.400665958	0.410114919	0.419275786	0.428310235	0.437420668	0.445920934	0.453971432	0.462102156	0.470393819	0.478553399	0.486462858	0.493980049	0.501445054	0.508955784
North Africa and Middle East	0.321037626	0.327692032	0.33391326	0.339075728	0.343774011	0.349320733	0.356646526	0.365075438	0.374014514	0.382928316	0.391621546	0.400665958	0.410114919	0.419275786	0.428310235	0.437420668	0.445920934	0.453971432	0.462102156	0.470393819	0.478553399	0.486462858	0.493980049	0.501445054	0.508955784
Afghanistan	0.15365569	0.155085054	0.156289933	0.157264469	0.156458646	0.154615606	0.154736532	0.1571246	0.160275239	0.16300982	0.165154865	0.167450008	0.169758659	0.171559467	0.172480174	0.173832165	0.17647255	0.179633724	0.180183713	0.17851866	0.178279128	0.178085404	0.177884124	0.177513403	0.177075239
Algeria	0.28982945	0.299497649	0.30955721	0.320320836	0.331913601	0.343998921	0.356583056	0.369653233	0.383037288	0.396500207	0.409941706	0.422108115	0.433052236	0.44285155	0.452032068	0.460486906	0.468319197	0.475950937	0.483255591	0.49045066	0.497957543	0.505990967	0.514056566	0.522382419	0.530785583
Bahrain	0.450597401	0.460850848	0.473139414	0.48362128	0.492791626	0.502275119	0.511669507	0.521255102	0.532455321	0.543727769	0.553132353	0.561240607	0.567611013	0.57260731	0.57794461	0.584578852	0.590596897	0.596040229	0.602165622	0.608133725	0.613850722	0.619485376	0.624596062	0.630738301	0.638083187
Egypt	0.306967813	0.311503392	0.315824463	0.319961812	0.324739854	0.331685947	0.340321237	0.349689666	0.359011983	0.367202322	0.375093911	0.383330311	0.392095866	0.400810898	0.40904001	0.417182742	0.426909452	0.437744555	0.448490053	0.458727004	0.468035368	0.476116627	0.482970886	0.489184071	0.495502619
Iran	0.326233539	0.332791653	0.336996966	0.33219596	0.316875615	0.300519562	0.297261251	0.305839639	0.321248257	0.33868607	0.354526813	0.373670326	0.39513065	0.414787892	0.434767997	0.453799944	0.468982858	0.480626697	0.492277025	0.505072857	0.517531494	0.52860072	0.537727779	0.546366017	0.555624332
Alborz	0.406388003	0.41431643	0.419941288	0.417591379	0.406869803	0.39592933	0.395405668	0.404366523	0.419268997	0.436015192	0.451934252	0.470036194	0.489676155	0.507363312	0.524662871	0.540408689	0.552446156	0.561720763	0.571360408	0.581991853	0.59235619	0.601919501	0.609791771	0.617160272	0.624754093
Ardebil	0.23588118	0.240531012	0.242397353	0.231951165	0.202548194	0.162656015	0.146699775	0.161232828	0.187594124	0.214373126	0.235417704	0.261390992	0.289324224	0.313709968	0.337949776	0.360726369	0.378799381	0.393000243	0.407753646	0.424543993	0.44117041	0.456014466	0.468500206	0.480594273	0.493810004
Bushehr	0.298991889	0.306580444	0.311980404	0.308590576	0.294525438	0.278644654	0.275881709	0.286024439	0.303905135	0.324234544	0.343158813	0.365367462	0.389632753	0.411717932	0.433615168	0.453839596	0.469448794	0.481662416	0.494246564	0.508013522	0.521385121	0.533890659	0.544378403	0.554069826	0.563930667
Chahar Mahaal and Bakhtiari	0.261831291	0.267499305	0.270890611	0.26446418	0.244410939	0.21938776	0.209636008	0.21742996	0.235812372	0.257469679	0.27676859	0.300748382	0.327194563	0.350830972	0.374236303	0.396177428	0.413752843	0.428041937	0.442933803	0.459408764	0.475501709	0.490244611	0.502691384	0.514474966	0.526414669
East Azarbayejan	0.284223069	0.289336049	0.291918675	0.284116747	0.262868214	0.238643767	0.231610775	0.240800257	0.258755919	0.279073092	0.29694495	0.319138626	0.343890146	0.366397205	0.389261396	0.41107167	0.428565789	0.441380504	0.453432793	0.466432141	0.478877163	0.489608139	0.498394773	0.506906517	0.516334524
Fars	0.333494973	0.340283596	0.34480849	0.341204482	0.328513626	0.314838309	0.311993061	0.319478088	0.333345111	0.349218208	0.363911863	0.381566245	0.401452525	0.419948745	0.439196397	0.458096531	0.473693688	0.486439073	0.499443128	0.51335231	0.526754556	0.538837966	0.549504903	0.559797448	0.57048609
Gilan	0.339053445	0.348800219	0.35652602	0.356859046	0.349470563	0.34218072	0.344564859	0.355636398	0.371759592	0.388936593	0.404572168	0.421455707	0.439285679	0.455230192	0.471418793	0.487126643	0.499460221	0.508700148	0.518056868	0.528092897	0.537867504	0.546866678	0.554629344	0.562175507	0.570205493
Golestan	0.265327676	0.270770906	0.273246109	0.262734646	0.233028621	0.194042865	0.178801866	0.192389966	0.218977799	0.247174717	0.270227334	0.298478831	0.32902486	0.355938572	0.382528734	0.407352738	0.426926755	0.441059963	0.454286526	0.468554203	0.482081553	0.49346702	0.502532992	0.511246358	0.520758265
Hamadan	0.263977026	0.269531112	0.272538391	0.265317751	0.244674001	0.221057823	0.214925628	0.225223388	0.243732978	0.264149634	0.281857583	0.303682704	0.327857188	0.349713324	0.3718437	0.393037826	0.410220293	0.423912725	0.437800504	0.452938893	0.467590975	0.480586561	0.491692757	0.502193297	0.513311656
Hormozgan	0.24855541	0.254521451	0.258038939	0.251652784	0.231631819	0.206921409	0.197730336	0.205847135	0.223752258	0.244445834	0.262654894	0.285577308	0.311403866	0.334949598	0.358647986	0.380909867	0.398548745	0.412525116	0.426860879	0.442821254	0.458529988	0.472603368	0.484619542	0.496242767	0.508746293
Ilam	0.242510816	0.249308219	0.253952445	0.249394805	0.232419034	0.211841435	0.206808353	0.217976419	0.238368255	0.261427476	0.282505072	0.307758255	0.335441555	0.360862158	0.386150146	0.409636699	0.428514542	0.443906192	0.459715963	0.47669329	0.492956965	0.507819049	0.520575019	0.532693066	0.545783519
Isfahan	0.352749997	0.360856741	0.366945138	0.36600127	0.357624861	0.348381808	0.347509041	0.355187799	0.368719509	0.384249723	0.399071075	0.416047079	0.434785512	0.45221869	0.470285884	0.487959498	0.502499113	0.513759556	0.524654244	0.535936687	0.546644571	0.556328697	0.564527037	0.572314491	0.580505822
Kerman	0.283096537	0.28948002	0.293447473	0.287386551	0.268216833	0.246072116	0.240393569	0.251108571	0.27084409	0.293082997	0.312977467	0.336761172	0.362732793	0.386101035	0.409089582	0.430231243	0.446669591	0.459613095	0.472939055	0.487557248	0.50177046	0.514550658	0.525228903	0.535080841	0.545405976

Kermanshah	0.273564993	0.280029988	0.284308154	0.279676294	0.264002663	0.246382552	0.242390989	0.251304192	0.267473442	0.28578619	0.30226832	0.322251259	0.344487164	0.364725119	0.385043512	0.404063329	0.418944664	0.430677775	0.442969461	0.456671649	0.470165926	0.482910245	0.493319538	0.502877113	0.512764657
Khorasan-e-Razavi	0.278115859	0.283144639	0.285442601	0.276447973	0.252250958	0.22385901	0.215300983	0.226300605	0.247113945	0.269825754	0.289289675	0.312721695	0.338210052	0.360624414	0.382670827	0.40318232	0.419388051	0.431379703	0.443165339	0.456264538	0.468991438	0.48000785	0.488685804	0.496687726	0.505420726
Khuzestan	0.272325618	0.278312713	0.281409574	0.272840893	0.24826907	0.219048678	0.210761227	0.222876014	0.244604137	0.268194305	0.288139735	0.312590054	0.339601415	0.363920718	0.388443598	0.411717104	0.430329418	0.444092399	0.457051262	0.471014342	0.484259839	0.495041354	0.503511676	0.511585072	0.520772892
Kohgiluyeh and Boyer-Ahmad	0.237289248	0.243860034	0.247957478	0.24165266	0.220288307	0.192515236	0.182187455	0.192428408	0.213763497	0.237812453	0.258717512	0.284422352	0.312617712	0.337835593	0.362657643	0.385444625	0.403033901	0.416642534	0.430717139	0.446547889	0.462229473	0.47612742	0.487763929	0.499036207	0.511072379
Kurdistan	0.229293839	0.234621095	0.238194385	0.234652119	0.222290277	0.207900464	0.203834493	0.210133745	0.222803648	0.237740799	0.251535811	0.26893988	0.288836503	0.307496119	0.327039396	0.34632778	0.362447943	0.375440307	0.38862839	0.402963883	0.416962406	0.429685212	0.440474448	0.450905653	0.46191712
Lorestan	0.238362116	0.243457531	0.246029608	0.237741005	0.213674693	0.183168616	0.172267744	0.183332035	0.204798452	0.228289793	0.248155637	0.272892125	0.300199586	0.324718101	0.349296601	0.372692648	0.391835021	0.407133613	0.422519576	0.439322885	0.455649233	0.470104029	0.482000472	0.493415816	0.505665767
Markazi	0.297174638	0.305186996	0.310981544	0.309498059	0.299886213	0.289134231	0.287769886	0.295446133	0.309042452	0.324556909	0.339227552	0.356541267	0.375854356	0.393897798	0.412690417	0.431126436	0.446318425	0.458666642	0.471251666	0.484631548	0.497579881	0.509690596	0.520238798	0.530425095	0.540916614
Mazandaran	0.348223725	0.356638352	0.363044116	0.361892797	0.35239617	0.342072791	0.341763318	0.35131684	0.36738982	0.385627242	0.402788162	0.422419541	0.443860411	0.463850072	0.484410206	0.504227331	0.520307133	0.532162754	0.543172259	0.554438776	0.564985363	0.574153477	0.581401075	0.587722361	0.594308132
North Khorasan	0.215206206	0.219874482	0.221991799	0.212426534	0.183948393	0.141768587	0.120561228	0.135406217	0.164560662	0.193533321	0.216281115	0.244113357	0.27387687	0.29965405	0.324724417	0.347842053	0.365863242	0.37902068	0.391915395	0.406447733	0.420753657	0.43325316	0.4435523	0.453444139	0.464441351
Qazvin	0.277368524	0.283715583	0.287673451	0.283038493	0.267780452	0.250712864	0.247196394	0.256198326	0.272544699	0.291277075	0.308605923	0.329465021	0.3524539	0.373265103	0.393985581	0.413374606	0.42875875	0.439910721	0.450246346	0.461099117	0.471356931	0.480631073	0.488819062	0.497443183	0.507680985
Qom	0.326652117	0.334306124	0.339418989	0.336144031	0.323195603	0.308695661	0.305210552	0.312611009	0.326967818	0.343575641	0.359011659	0.377445555	0.397973178	0.416694319	0.435390785	0.452628907	0.465722037	0.475802913	0.486425484	0.498440084	0.510319504	0.521096027	0.530026839	0.53828674	0.546971243
Semnan	0.358501474	0.368412412	0.37634748	0.37759925	0.372385518	0.367201216	0.369893386	0.379552597	0.393534193	0.408297309	0.422047523	0.437238067	0.453609547	0.468309518	0.482906681	0.496089451	0.505579479	0.512703331	0.520769634	0.530215318	0.539840308	0.549412651	0.557994613	0.566637746	0.575727814
Sistan and Baluchistan	0.20397385	0.207959639	0.209289378	0.19931685	0.170785168	0.126912813	0.098775032	0.11047068	0.138376046	0.165274574	0.185040674	0.210675068	0.238704775	0.262864306	0.286682311	0.308840734	0.326078692	0.338899321	0.351732463	0.366584876	0.381150014	0.393055912	0.401954761	0.410016208	0.419505526
South Khorasan	0.224407616	0.229484422	0.232540176	0.226950622	0.209363353	0.187033666	0.177925026	0.184782278	0.201498423	0.221195604	0.238914597	0.260854581	0.285255746	0.30728272	0.329445475	0.350446131	0.367440589	0.38062956	0.394002213	0.408852797	0.423488664	0.436553451	0.447312873	0.457270791	0.46779298
Tehran	0.478419914	0.486730262	0.49295921	0.492303794	0.485253454	0.478243092	0.478516578	0.485426168	0.496622869	0.508897831	0.520463334	0.533567089	0.548103674	0.561356535	0.574843226	0.587612685	0.597405602	0.605000433	0.613073986	0.622138665	0.631118522	0.639506955	0.646425935	0.652979878	0.659879899
West Azarbayejan	0.263387809	0.268945656	0.272306282	0.266971083	0.250658	0.231988957	0.226578814	0.234050089	0.248867683	0.265938	0.28119905	0.30028072	0.321764958	0.341306068	0.361121307	0.37997815	0.394902017	0.406170759	0.417408542	0.429901147	0.442058721	0.452581184	0.460986464	0.469157741	0.47852181
Yazd	0.304000259	0.311862337	0.317343081	0.313250772	0.297441052	0.279516925	0.275575592	0.285542336	0.303930598	0.324809217	0.343815886	0.366186008	0.390783099	0.412909792	0.434595614	0.454227587	0.468809771	0.479870146	0.491575953	0.505026011	0.518510439	0.530704079	0.540959313	0.550735949	0.561282626
Zanjan	0.258860257	0.263649665	0.265937161	0.257192251	0.232713179	0.203183205	0.194118009	0.205762584	0.226817131	0.249798876	0.26945853	0.294041631	0.321108026	0.34519094	0.368955117	0.391316094	0.4088901	0.420937843	0.432099645	0.444346897	0.456107387	0.465850585	0.473262332	0.47968483	0.486947981
Iraq	0.290943585	0.299145775	0.307206393	0.316015085	0.325935421	0.335781385	0.344435451	0.353194598	0.361306377	0.369251553	0.37720537	0.38514542	0.393527968	0.400968304	0.406189665	0.412044173	0.415814116	0.420353594	0.424834933	0.428751731	0.432075545	0.436818479	0.443104027	0.452384092	0.462736441
Jordan	0.368409008	0.380691529	0.393138975	0.406745609	0.420794485	0.435902805	0.450856751	0.465009081	0.478172806	0.490773667	0.502054229	0.512393706	0.521588209	0.529372691	0.534899991	0.539147468	0.542819411	0.547458846	0.55218235	0.557254335	0.562571536	0.567529655	0.572581508	0.57764357	0.582759199
Kuwait	0.458767819	0.474013994	0.492787283	0.511050991	0.528670099	0.545114971	0.560005405	0.575133534	0.589115985	0.603255637	0.615050297	0.623032781	0.630905625	0.639118442	0.6524387	0.664517904	0.668809621	0.67015911	0.670036252	0.671393213	0.677215523	0.686093304	0.695789389	0.704383736	0.711841328
Lebanon	0.488412241	0.488025883	0.486763849	0.487714414	0.491599174	0.495578721	0.499407051	0.500169325	0.502686687	0.509159818	0.518005501	0.525136146	0.532911349	0.536798112	0.536694513	0.536718969	0.539197492	0.541793516	0.544581306	0.547684033	0.55147172	0.556436159	0.562120818	0.56905907	0.576078528
Libya	0.344091404	0.357001316	0.370356463	0.38388977	0.397882454	0.41182861	0.424397682	0.436831252	0.448981015	0.460583478	0.47259186	0.483869528	0.494361829	0.504923151	0.515822934	0.527998168	0.541432399	0.554468227	0.566798415	0.578651416	0.589578634	0.600350044	0.610620315	0.619973984	0.628600604
Morocco	0.2347727	0.243714071	0.252724902	0.261661251	0.27066064	0.279790018	0.288215687	0.296840972	0.305017744	0.312984655	0.321029983	0.329104564	0.336448516	0.344036155	0.351183359	0.35807287	0.364984629	0.371187194	0.376778977	0.382849451	0.38816411	0.394150096	0.399504009	0.405038792	0.410303191
Oman	0.280854555	0.289399891	0.298182206	0.306456584	0.314868361	0.323278201	0.33241893	0.342490872	0.352772069	0.363117844	0.373788109	0.383804749	0.39461055	0.405806975	0.417140511	0.429270949	0.442268122	0.457737972	0.474999033	0.493513107	0.513194359	0.533246394	0.554315537	0.574474902	0.59239847
Palestine	0.308656452	0.317867145	0.326190701	0.334710612	0.342289372	0.350274806	0.356831105	0.363546472	0.369069886	0.374353743	0.379008353	0.384601878	0.389278644	0.393137249	0.396847157	0.40179221	0.40572715	0.411146869	0.416779649	0.423136673	0.429307027	0.434731371	0.440901776	0.447782415	0.454909692
Qatar	0.541776023	0.55256233	0.5626787	0.571912588	0.580422582	0.588609449	0.596089855	0.603026431	0.609408466	0.615408025	0.621415895	0.62728062	0.633120694	0.638936185	0.645008178	0.651208376	0.655338767	0.660673763	0.666194399	0.672025384	0.678320339	0.684835919	0.693109654	0.701409064	0.708880981
Saudi Arabia	0.388905955	0.399881524	0.410888103	0.421664137	0.432640025	0.443730453	0.455183831	0.465693507	0.475433073	0.484775213	0.493541152	0.502704195	0.511448471	0.520470469	0.529331214	0.538954515	0.549907717	0.561144266	0.572186913	0.582878871	0.593251423	0.603648418	0.613749116	0.623518214	0.632796447
Sudan	0.217013081	0.222818506	0.229336371	0.235337848	0.240596264	0.245903129	0.251297433	0.257120602	0.262697366	0.267620101	0.271504609	0.275213291	0.279230127	0.283638065	0.288166353	0.292178643	0.296473446	0.300863677	0.305409326	0.31009643	0.315208259	0.320680229	0.326761462	0.333087761	0.339754884
Syria	0.305427355	0.315107399	0.32394027	0.332914494	0.341622471	0.350668957	0.359872992	0.368737701	0.377347757	0.385221672	0.393178804	0.40060321	0.40801172	0.416137665	0.423245609	0.430492643	0.437907493	0.445904236	0.45414786	0.462437298	0.470692043	0.478743085	0.48623235	0.493707992	0.500474869
Tunisia	0.326258196	0.336282914	0.346213176	0.356360852	0.366907982	0.377601353	0.38818539	0.398258906	0.408204742	0.417938788	0.427627678	0.436567505	0.445631478	0.45428266	0.462667146	0.471138521	0.479543057	0.488285281	0.496852039	0.505564882	0.51419694	0.523294274	0.532064044	0.540498161	0.548724563
Türkiye	0.357515307	0.361752702	0.366757646	0.372433792	0.378796563	0.385762839	0.393136765	0.400490401	0.407867554	0.415047098	0.422412708	0.429715847	0.437703584	0.445704541	0.453439208	0.461606984	0.469244066	0.476814569	0.48465697	0.49161995	0.49882484	0.506238546	0.513933356	0.521509898	0.528512416
United Arab Emirates	0.399434437	0.412277456	0.424740893	0.436115657	0.447865738	0.461559504	0.478517598	0.498864976	0.521984398	0.545306146	0.566370978	0.584312419	0.599362919	0.61336513	0.628285219	0.644412271	0.660739899	0.675926211	0.689463801	0.701656792	0.713138288	0.724181976	0.734723175	0.744476623	0.753253916
Yemen	0.134530687	0.138796841	0.143286666	0.147914703	0.152658223	0.157279709	0.162155058	0.167129757	0.172438813	0.17801423	0.183625231	0.18942127	0.195506738	0.20192585	0.208649589	0.215664586	0.222885409	0.230415046	0.238025246	0.245879426	0.253962572	0.262278613	0.270782536	0.279500507	0.288261301
South Asia	0.25846839	0.260922925	0.26329615	0.265808662	0.268003285	0.270685034	0.27363955	0.276786514	0.280606571	0.284967828	0.289905894	0.295369424	0.300997053	0.307111453	0.313419397	0.319796517	0.325854134	0.331940961	0.338013384	0.344322901	0.350726453	0.357286569	0.363719215	0.370287576	0.376979322
South Asia	0.25846839	0.260922925	0.26329615	0.265808662	0.268003285	0.270685034	0.27363955	0.276786514	0.280606571	0.284967828	0.289905894	0.295369424	0.300997053	0.307111453	0.313419397	0.319796517	0.325854134	0.331940961	0.338013384	0.344322901	0.350726453	0.357286569	0.363719215	0.370287576	0.376979322
Bangladesh	0.157317834	0.160164793	0.162134301	0.163770281	0.165976981	0.168540022	0.171890929	0.175979109	0.18106345	0.186578302	0.192222233	0.198408668	0.205248148	0.212446856	0.22014772	0.228548934	0.237207413	0.245247956	0.25209072	0.258997294	0.265293918	0.271177781	0.27777328	0.28451594	0.290951431
Bhutan	0.119126516	0.123174481	0.127546567	0.132261112	0.137216383	0.142358518	0.148255671	0.154512641	0.161352375	0.168363124	0.175478177	0.182805401	0.190958929	0.199102088	0.20735349	0.21503985	0.22131244	0.228148272	0.236004687	0.244331246	0.253291957	0.262362746	0.271487422	0.280440384	0.289510488
India	0.272416268	0.275017652	0.27751157	0.280059937	0.282034986	0.284534635	0.287271254	0.290117187	0.293657196	0.297752691	0.302461918	0.307814138	0.313374897	0.319592963	0.326068915	0.332593603	0.338591779	0.344591727	0.350685397	0.35699603	0.363418501	0.370018644	0.376358763	0.382817848	0.389453889
Nepal	0.140078248	0.142244119	0.144773542	0.147742466	0.151019556	0.15415285	0.157864464	0.161819522	0.165410418	0.169544707	0.17396327	0.178518205	0.183114547	0.188304622	0.193831366	0.199560654	0.205687443	0.21211309	0.218843431	0.22607306	0.233253154	0.240700616	0.248382797	0.256227179	0.26435918
Pakistan	0.233287781	0.235711095	0.238315543	0.241526051	0.24513888	0.249452723	0.2542854	0.259694735	0.265786661	0.272195243	0.278914046	0.285511563	0.291998501	0.298455306	0.304611346	0.310467621	0.316265667	0.322248051	0.328219252	0.334433128	0.340926296	0.347656203	0.354218627	0.360875336	0.367641397
Azad Jammu & Kashmir	0.228560683	0.232312028	0.236281924	0.240830749	0.245835302	0.25154516	0.257774925	0.264543797	0.271899218	0.279526615	0.287433889	0.295281899	0.303106306	0.31091997	0.3185368	0.325857101	0.333077208	0.340429071	0.347704205	0.355188695	0.36290103	0.370811847	0.378509273	0.386226886	0.39394301
Balochistan	0.196989521	0.198529691	0.200016213	0.201704648	0.20344345	0.205624691	0.208225762	0.211350471	0.21514899	0.21936468	0.223987895	0.228749407	0.23366456	0.238779195	0.243832882	0.248830294	0.253867226	0.259137459	0.264445518	0.270032258	0.275808956	0.281657954	0.28728496	0.292936773	0.298615917
Gilgit-Baltistan	0.151274892	0.153600284	0.156026535	0.158983373	0.162266088	0.165966014	0.17051346	0.175445768	0.180897541	0.186540046	0.192385192	0.198544256	0.204672499	0.210800093	0.216671019	0.222103674	0.227640973	0.233261906	0.238770234	0.244414316	0.250163095	0.256324108	0.262331705	0.268435004	0.274619029
Islamabad Capital Territory	0.380755527	0.384930874	0.389407212	0.394638876	0.400491191	0.40714335	0.414423106	0.422170465	0.430375842	0.438656582	0.447147464	0.455648222	0.46395827	0.472125962	0.479895424	0.487150872	0.494111387	0.501070598	0.50780845	0.514676352	0.521761529	0.529113389	0.536212659	0.543302326	0.550405879
Khyber Pakhtunkhwa	0.198399845	0.200634584	0.202976651	0.205922164	0.209327203	0.213468129	0.218074472	0.223086386	0.228643373	0.234376407	0.240370408	0.246243299	0.252048221	0.257736423	0.263045307	0.267901742	0.272521311	0.277278922	0.282095856	0.287208184	0.292664619	0.298363471	0.303939494	0.309610949	0.315386855
Punjab	0.236264425	0.238956883	0.241824598	0.245298052	0.249125554	0.253625359	0.258615211	0.264173259	0.27038314	0.276892768	0.283708382	0.29038815	0.296967112	0.303573433	0.309918059	0.316049943	0.322229572	0.328631987	0.335042017	0.341726759	0.348740259	0.356039491	0.363112926	0.370279528	0.377573896
Sindh	0.247725226	0.249771817	0.252130624	0.25520188	0.258807705	0.263196241	0.268185382	0.273881621	0.280403952	0.287302867	0.294527377	0.301603736	0.308466816	0.31511828	0.32133154	0.327061045	0.332559514	0.338170748	0.343712065	0.349423746	0.355314165	0.361355173	0.367341984	0.373442645	0.3796483
Southeast Asia, east Asia, and Oceania	0.362642075	0.3722482	0.380935925	0.389051136	0.395944664	0.403542902	0.411933655	0.418450795	0.423222012	0.42824036	0.434505761	0.442074373	0.449760298	0.457339948	0.463918161	0.471036942	0.479318189	0.487345622	0.49528092	0.502971282	0.511167456	0.520513218	0.529602631	0.53725808	0.54471189
East Asia	0.362549331	0.372789681	0.381665669	0.389981084	0.396625378	0.404018172	0.41223236	0.418110807	0.421556366	0.425716375	0.432082425	0.440406586	0.448974806	0.457273657	0.463961582	0.471179162	0.479935584	0.488431396	0.496859473	0.504885881	0.513530275	0.5237577	0.533815513	0.542652548	0.551367028
China	0.349604513	0.359497348	0.368005968	0.376158968	0.382701576	0.390010001	0.398095091	0.404047927	0.407582709	0.412004231	0.418845612	0.427533623	0.436360758	0.444881967	0.451570027	0.458668935	0.46730197	0.475784031	0.484353831	0.492578645	0.501500908	0.512033996	0.522326866	0.531379484	0.540333688
North Korea	0.460058425	0.463817961	0.467441426	0.471005361	0.474296497	0.477353608	0.480067944	0.48237968	0.48442177	0.486384165	0.488343322	0.490211204	0.492018583	0.493961259	0.495904808	0.497780128	0.499120512	0.499629841	0.499700646	0.49953134	0.499080529	0.498429014	0.497136869	0.49615921	0.496484754
Taiwan (province of China)	0.500109025	0.507353793	0.517007555	0.52731473	0.538198101	0.549866346	0.561935998	0.574847062	0.58856227	0.602646172	0.616044226	0.628119226	0.638880321	0.648859954	0.658477778	0.667633854	0.676537237	0.684803636	0.69292413	0.70107875	0.709626821	0.719128415	0.729145497	0.738125787	0.745338399
Oceania	0.324338236	0.329550689	0.334447166	0.339446421	0.344518258	0.349187431	0.353691723	0.357937098	0.362192252	0.366573185	0.370948098	0.37541561	0.379670255	0.38375425	0.387649926	0.391195082	0.394418822	0.397572264	0.401113099	0.404892361	0.408507251	0.412437065	0.415893127	0.419224844	0.422520527
American Samoa	0.585416832	0.592601515	0.598801654	0.603703944	0.607022491	0.609108848	0.610109377	0.610099127	0.609160066	0.607901289	0.607331017	0.607575415	0.608515401	0.609926737	0.611599963	0.613633924	0.615847612	0.618583654	0.622078598	0.626053241	0.629814054	0.632706983	0.634926685	0.636597976	0.638322193
Cook Islands	0.444263944	0.454264467	0.465272976	0.476579811	0.487465067	0.49759977	0.506281766	0.514238284	0.520993817	0.528205896	0.535148116	0.54204534	0.547882889	0.55334404	0.558808216	0.564514854	0.570622776	0.577123468	0.584059175	0.591540868	0.598789968	0.606414151	0.613991564	0.621202228	0.62802903
Fiji	0.461969615	0.468637165	0.474709495	0.480064234	0.485703114	0.490412472	0.494706345	0.498515777	0.502076312	0.506560248	0.51109269	0.516112761	0.520549868	0.524909606	0.529700607	0.534648908	0.53877826	0.543188552	0.547776474	0.55284902	0.558530972	0.565041879	0.571187753	0.577172354	0.583573942
Guam	0.633050573	0.652759275	0.668536716	0.678982081	0.684925249	0.688121296	0.689967134	0.691174671	0.691953907	0.692786266	0.693373422	0.693442503	0.692039304	0.688683588	0.682976126	0.676220305	0.669974292	0.66635634	0.667318181	0.672111712	0.678120215	0.684173735	0.691684871	0.701819289	0.713641593
Kiribati	0.389314019	0.394584452	0.399097793	0.403345394	0.406262956	0.406547397	0.406354928	0.405785509	0.404842573	0.404022804	0.403251727	0.403406033	0.403902437	0.405856549	0.407960647	0.410389821	0.412884199	0.415577609	0.418481661	0.421716217	0.425024354	0.428300682	0.431641941	0.435741904	0.439692923
Marshall Islands	0.366988046	0.372522967	0.37756879	0.382583579	0.387365332	0.390909147	0.394384697	0.397302307	0.400612738	0.40372678	0.405948072	0.410308684	0.415245816	0.420686198	0.42573995	0.430839288	0.435571412	0.440491018	0.445479084	0.450848221	0.456859674	0.46120943	0.464752728	0.468133032	0.471292727
Federated States of Micronesia	0.371802272	0.379182821	0.386538091	0.393732828	0.400788384	0.407599926	0.413873766	0.419771212	0.425343153	0.430949836	0.436518419	0.441809809	0.446926307	0.451991792	0.457210422	0.462511617	0.468144831	0.473792936	0.479939595	0.485300006	0.491015185	0.495570308	0.499110106	0.502861564	0.506663431
Nauru	0.510908879	0.515335525	0.519280219	0.522902157	0.52612838	0.528918229	0.531219753	0.533026317	0.534405739	0.535529124	0.536435428	0.537195208	0.537841858	0.538358485	0.538779782	0.539145981	0.538433557	0.537038893	0.535059742	0.533001097	0.530890925	0.528544474	0.52624159	0.523885302	0.521894324
Niue	0.48888836	0.495590843	0.501995082	0.508145796	0.514240047	0.520303045	0.526460377	0.532787574	0.539491289	0.546318579	0.553127327	0.55996122	0.566867746	0.573897144	0.580822259	0.587532984	0.593882057	0.599881243	0.605514779	0.610650175	0.615374609	0.619734555	0.623913848	0.627578934	0.630857172
Northern Mariana Islands	0.634332929	0.640114344	0.645772599	0.651364822	0.656803467	0.661896783	0.66686141	0.671725666	0.676543405	0.681340174	0.686092063	0.69096019	0.695843183	0.70056405	0.70484855	0.708593838	0.712223642	0.715787607	0.719179396	0.722510494	0.726219053	0.730074086	0.733795366	0.737423127	0.740934815
Palau	0.59866261	0.603119536	0.607544019	0.611957256	0.616286153	0.620548078	0.624713482	0.62850873	0.63208686	0.635719985	0.639699067	0.644039045	0.648590228	0.653263499	0.657958653	0.66290951	0.668230731	0.673302462	0.676648715	0.680195775	0.68483214	0.690095673	0.695122123	0.69965291	0.703115322
Papua New Guinea	0.23370729	0.238935559	0.243780543	0.249126475	0.254643221	0.259975116	0.265281599	0.270395306	0.275722683	0.280938515	0.286204957	0.291559108	0.296844805	0.301949303	0.306603454	0.310668629	0.314967305	0.319530259	0.325005984	0.330664468	0.335421285	0.340348698	0.344258325	0.347801408	0.351250621
Samoa	0.406172637	0.412049519	0.417804458	0.423995339	0.431185438	0.437535409	0.442836484	0.44789373	0.452839869	0.457854963	0.463073422	0.468591712	0.473873293	0.478653977	0.483465947	0.487491428	0.490987146	0.494027448	0.497079264	0.499484836	0.502369735	0.505778026	0.509096382	0.512367566	0.51540905
Solomon Islands	0.238495024	0.242517336	0.247062284	0.251918106	0.257827301	0.262731229	0.267184117	0.271257558	0.275222958	0.279017666	0.282508217	0.285823197	0.289547085	0.293202854	0.297070697	0.301217167	0.305830668	0.311431697	0.317151092	0.323300247	0.329968564	0.336208053	0.341835627	0.34713506	0.351867191
Tokelau	0.434882297	0.441021583	0.447668217	0.454634726	0.461777857	0.468692965	0.47512885	0.480946699	0.486454299	0.491937784	0.497259699	0.502243682	0.50709941	0.512012909	0.517001954	0.521942386	0.526628473	0.53130785	0.535935005	0.540523665	0.545295634	0.550282652	0.555631326	0.561047611	0.566415406
Tonga	0.37626617	0.380318847	0.385148034	0.390093517	0.395077594	0.401559508	0.409340958	0.4179958	0.426513216	0.438447276	0.449826079	0.460788363	0.470482745	0.478488441	0.485448771	0.49180684	0.498166205	0.503659848	0.50890445	0.514207835	0.52021502	0.525808135	0.531009092	0.536172314	0.54131702

Tuvalu	0.347139687	0.34997101	0.352241994	0.354094108	0.355776177	0.35732842	0.359218586	0.3598763	0.361524915	0.364280369	0.367239079	0.372817787	0.37991215	0.389002233	0.397107188	0.406247566	0.414819937	0.42290198	0.43051826	0.438563051	0.444962242	0.44980498	0.455368428	0.462454678	0.468845802
Vanuatu	0.290145532	0.295705395	0.300789106	0.306668437	0.312254418	0.316338465	0.320341658	0.324123109	0.32794428	0.332196287	0.336213349	0.339919887	0.34328249	0.346182993	0.349176127	0.353100252	0.357005379	0.360893296	0.364444155	0.368501002	0.372479126	0.376460885	0.380667171	0.384690196	0.388300526
Southeast Asia	0.348333333	0.355788962	0.36341272	0.370925143	0.37859758	0.386548048	0.394799035	0.402832515	0.410937444	0.419094094	0.426805521	0.434239758	0.441559965	0.448933427	0.456496702	0.46410364	0.471749871	0.479490406	0.487349418	0.495348107	0.50335114	0.511232528	0.518664155	0.52443938	0.529667781
Cambodia	0.258549547	0.258678627	0.258816057	0.259894899	0.261050804	0.262846114	0.264971426	0.267223445	0.269691645	0.272414814	0.275294876	0.277979108	0.280689992	0.283636313	0.286510019	0.289075059	0.2924792	0.296900955	0.301627202	0.305722592	0.310678881	0.31563097	0.32050375	0.324974299	0.330224246
Indonesia	0.315568117	0.324100654	0.332659493	0.340843543	0.349585997	0.359261592	0.369504192	0.379197745	0.38898835	0.399257925	0.409291949	0.419202424	0.428883378	0.438314124	0.447787846	0.457134954	0.466505583	0.475753157	0.484775241	0.4936071	0.502008381	0.510108146	0.518102189	0.524045604	0.52917633
Aceh	0.332323221	0.34044383	0.348524292	0.356118591	0.36431562	0.373521264	0.383474378	0.393061292	0.403062504	0.413997387	0.425076525	0.43651001	0.448067521	0.459715023	0.471360713	0.482673083	0.493504261	0.503725238	0.513293532	0.522194442	0.530244852	0.537785887	0.545269986	0.550976463	0.555867589
Bali	0.301166437	0.311217094	0.321388301	0.331217975	0.341516835	0.352467246	0.36354755	0.373884473	0.384189083	0.394998728	0.405695972	0.416417168	0.427116449	0.437720353	0.448293203	0.458655011	0.468848044	0.478844524	0.488700458	0.498232752	0.507060762	0.515231093	0.522993626	0.528355586	0.532622854
Bangka-Belitung Islands	0.331930988	0.33856247	0.345038917	0.351022297	0.357393319	0.364785011	0.372879421	0.380520657	0.388327684	0.396818569	0.405328062	0.414252704	0.423497744	0.432956189	0.442772547	0.452646767	0.462591923	0.472391479	0.481888282	0.491048594	0.499481814	0.507367902	0.514946326	0.520543825	0.525260596
Banten	0.322993957	0.329948457	0.336965887	0.343633918	0.350940814	0.35932125	0.368503459	0.377046071	0.385697502	0.394865338	0.403904871	0.413196986	0.422608315	0.43193669	0.441443871	0.450965928	0.460784951	0.470637488	0.48025382	0.489594952	0.498356376	0.506821488	0.514934415	0.520591505	0.525083292
Bengkulu	0.264650575	0.27320642	0.281572191	0.289392754	0.29776622	0.307120038	0.317116097	0.326458338	0.335971023	0.346333816	0.356775037	0.367458272	0.378243527	0.389133694	0.399996415	0.410618592	0.421338809	0.43188088	0.441945224	0.451774972	0.4609698	0.46969509	0.478202963	0.484290714	0.489268721
Gorontalo	0.288173313	0.295838585	0.303338575	0.310082817	0.31703164	0.324617828	0.332445507	0.339059624	0.345406024	0.352014744	0.358185183	0.364384754	0.37053853	0.376664404	0.383129629	0.389888128	0.397140957	0.404527932	0.411806004	0.418943452	0.425658298	0.43220747	0.438480857	0.44209189	0.44455879
Jakarta	0.458853352	0.467389708	0.475950628	0.484109026	0.492890758	0.502724191	0.513244166	0.523295138	0.533533141	0.544256055	0.554842021	0.565287577	0.575343086	0.584930465	0.594354638	0.603337382	0.612305583	0.621480363	0.631010736	0.64050786	0.649534732	0.658244406	0.666732384	0.672972623	0.678467268
Jambi	0.275521202	0.283033271	0.290885412	0.298629084	0.30708201	0.31662195	0.326979069	0.337123055	0.34779819	0.359236989	0.370503473	0.381718722	0.39270254	0.403367094	0.413861708	0.423986255	0.434086314	0.443835819	0.453051079	0.461985935	0.470551523	0.478941705	0.487365789	0.493585119	0.498877558
West Java	0.308984808	0.316820591	0.324732402	0.332346259	0.340637477	0.350076224	0.360378827	0.370393745	0.380788795	0.39194348	0.402987095	0.413798986	0.424174209	0.43407296	0.443828829	0.453362331	0.462900417	0.472227416	0.481260302	0.490077931	0.498420828	0.50635713	0.51402432	0.519463845	0.523829587
Central Java	0.287416724	0.295747633	0.304065631	0.311938239	0.320245254	0.329351069	0.338916924	0.347866496	0.356794694	0.366106612	0.375102566	0.383920025	0.392565727	0.401053271	0.409729409	0.418426869	0.427270317	0.436058992	0.444525024	0.452776675	0.460755364	0.468489217	0.476047616	0.481393227	0.48599492
East Java	0.299353001	0.308007915	0.316795453	0.325348257	0.33450918	0.344578966	0.355149032	0.365131572	0.375081001	0.385232595	0.394935918	0.404299322	0.413324329	0.422034978	0.430753929	0.439345376	0.448002535	0.456568647	0.464981405	0.473362709	0.481554707	0.489625168	0.497734151	0.503899208	0.509461652
West Kalimantan	0.244664347	0.252347074	0.260369955	0.268296807	0.276867886	0.28636671	0.296385722	0.306090584	0.316272257	0.327304335	0.338371005	0.349364424	0.360099787	0.370681712	0.381525894	0.392420939	0.403546934	0.414559711	0.425181012	0.435596831	0.445592807	0.455135464	0.464363134	0.471289924	0.476975088
South Kalimantan	0.291592988	0.300874946	0.310121348	0.318963931	0.328438771	0.338835909	0.349883782	0.360363133	0.370844859	0.381649025	0.392059854	0.402254338	0.412163539	0.421889367	0.431597187	0.4410948	0.450659333	0.460186271	0.469587689	0.478715406	0.487260019	0.495360632	0.50319087	0.50876176	0.513397467
Central Kalimantan	0.325620885	0.333465534	0.341196047	0.348331788	0.355886934	0.364317685	0.373313685	0.381898675	0.390909589	0.400725033	0.410676982	0.420774231	0.430638215	0.440089434	0.449476762	0.458816473	0.468694807	0.478852254	0.489080158	0.499159777	0.508785407	0.517859927	0.526160727	0.531642782	0.535617829
East Kalimantan	0.389745054	0.400386931	0.411232008	0.42215952	0.43400079	0.447026932	0.460914127	0.47461461	0.488343529	0.502199882	0.515544716	0.528235772	0.539785408	0.550431471	0.560691841	0.570464049	0.579709067	0.588155672	0.59575493	0.603144549	0.610012928	0.61667543	0.623547959	0.629222386	0.634267668
North Kalimantan	0.443762507	0.450660913	0.457388652	0.463628861	0.470218607	0.477609204	0.485449551	0.492658424	0.499829258	0.50747387	0.515109282	0.523007829	0.53100256	0.538998696	0.547131276	0.555308193	0.563444647	0.571335245	0.578925818	0.586396151	0.593391674	0.60026322	0.607352228	0.613417455	0.619052162
Riau Islands	0.438121497	0.446076725	0.454064101	0.461688022	0.469851629	0.478922428	0.488565867	0.497538283	0.506570643	0.516173498	0.52560243	0.535183932	0.544744445	0.554145033	0.56368694	0.573195374	0.582604149	0.591698026	0.600398522	0.608909874	0.617005694	0.624868761	0.632527177	0.638224392	0.642955195
Lampung	0.253441061	0.261407197	0.269545695	0.27734394	0.285727108	0.295049237	0.304859328	0.313964135	0.323192429	0.333016086	0.34267837	0.352402983	0.362100546	0.371711024	0.38159731	0.391631433	0.401893655	0.412229167	0.422372896	0.432420699	0.442266267	0.45188518	0.461299065	0.468325456	0.474384663
Maluku	0.367450177	0.37440182	0.381023396	0.387075002	0.393592706	0.400909346	0.408639275	0.415514228	0.422351476	0.429737544	0.436943051	0.444443087	0.452121035	0.459916203	0.468071258	0.476402589	0.484988378	0.493485215	0.50141601	0.50877279	0.515041977	0.520424846	0.524928074	0.526587821	0.525959227
North Maluku	0.262394429	0.26958038	0.27688229	0.283624882	0.290757842	0.298585957	0.306713539	0.313554777	0.320315991	0.327619047	0.334595893	0.341754806	0.349082843	0.356533101	0.364544976	0.372965609	0.382162104	0.391831112	0.40169747	0.411834386	0.421759355	0.431455905	0.44083814	0.447156939	0.45223301
West Nusa Tenggara	0.202735621	0.21016827	0.217673604	0.224778175	0.232445633	0.241071692	0.250202031	0.258623019	0.26726109	0.27670195	0.286243935	0.296075177	0.306015453	0.316072112	0.326521854	0.337279768	0.348596437	0.360281417	0.3721195	0.383887897	0.395203377	0.406173953	0.416987393	0.425463443	0.433193111
East Nusa Tenggara	0.254583059	0.263275096	0.271868273	0.279695763	0.287706815	0.296102542	0.304390005	0.311373036	0.318069271	0.32505525	0.331699621	0.33830881	0.344837563	0.351314406	0.358260401	0.365588037	0.373390941	0.38136121	0.389191357	0.396974847	0.404505209	0.411801645	0.418800405	0.422702411	0.425734701
Papua	0.358479206	0.364575165	0.370494637	0.375953729	0.381847934	0.388549688	0.395830761	0.402600428	0.40937063	0.41658137	0.423762792	0.431265605	0.439008218	0.446753851	0.454545017	0.462138557	0.469889521	0.477720022	0.485722916	0.493730909	0.501559539	0.509246938	0.516897106	0.523062066	0.528222317
West Papua	0.347852455	0.354456581	0.360944595	0.366973145	0.373448873	0.380731021	0.388468829	0.39539962	0.402285222	0.409709595	0.417005501	0.424708416	0.432617031	0.440579509	0.448859654	0.457306637	0.466233925	0.475295762	0.484221157	0.492996739	0.501069789	0.508700871	0.516041171	0.521316627	0.525568147
Riau	0.370010941	0.378865057	0.387474481	0.395393136	0.403919554	0.413498726	0.42382017	0.433860257	0.444458513	0.456088983	0.467974676	0.480206084	0.492401891	0.504340168	0.516250084	0.527812917	0.539003159	0.549370911	0.558666293	0.567224066	0.574760449	0.581775474	0.588952942	0.594783332	0.600042462
West Sulawesi	0.274594108	0.281258898	0.287936366	0.294117592	0.30084295	0.30853347	0.316838864	0.324415568	0.33207608	0.340253606	0.348189568	0.356232321	0.364116261	0.371907812	0.379977836	0.388169169	0.396456145	0.404603634	0.412332862	0.419839087	0.426883335	0.433684845	0.44045657	0.444978373	0.448429021
South Sulawesi	0.271044544	0.279913985	0.288829096	0.297301362	0.306315847	0.316235565	0.326622854	0.336254244	0.345900174	0.356066732	0.365923618	0.375660427	0.385196829	0.394538094	0.403931863	0.41327701	0.422717823	0.432096037	0.441230921	0.450205126	0.458776699	0.467112157	0.475422193	0.481601972	0.486907342
Central Sulawesi	0.281553632	0.289974562	0.298099321	0.305669111	0.313774771	0.322939947	0.332682113	0.341701359	0.350724901	0.360150367	0.369121656	0.377773314	0.386152278	0.394479327	0.403135726	0.411857501	0.420823131	0.42982927	0.438825231	0.447877725	0.456371897	0.4643591	0.472248668	0.478015632	0.483022386
Southeast Sulawesi	0.237568006	0.246838772	0.256168159	0.265046459	0.27452357	0.285094151	0.296229339	0.306520564	0.316462014	0.32652746	0.336322764	0.346224998	0.356191508	0.366261833	0.376554535	0.386931522	0.397433053	0.407674437	0.417535343	0.427348765	0.43685672	0.446111545	0.455278285	0.462217344	0.468035766
North Sulawesi	0.35386482	0.363249302	0.372480825	0.381149186	0.390126842	0.399815533	0.409645332	0.418366368	0.426708135	0.435220131	0.443261697	0.450934859	0.458244147	0.465292957	0.472482441	0.479692906	0.487089939	0.494600444	0.502300884	0.510088618	0.517712785	0.525358743	0.532788289	0.537612742	0.541079774
West Sumatra	0.323300377	0.331679441	0.340009029	0.347894384	0.356360192	0.36592952	0.376133032	0.38574479	0.39556386	0.406039428	0.416354371	0.426638219	0.436637255	0.446300852	0.455945463	0.465210234	0.474324214	0.483333039	0.492277723	0.501058181	0.509330998	0.517298811	0.525176518	0.530886205	0.535735015
South Sumatra	0.318813917	0.326423104	0.33399718	0.341140494	0.348919158	0.357778225	0.367365715	0.376499545	0.385907789	0.396046768	0.406107664	0.41632102	0.426443413	0.436414309	0.446431963	0.456248761	0.465829716	0.474790255	0.48295831	0.490696948	0.497941212	0.505024074	0.512306214	0.517875345	0.522473583
North Sumatra	0.335877234	0.344328588	0.352538899	0.360060629	0.36805504	0.377056167	0.38663116	0.3955098	0.404474339	0.414106862	0.423607026	0.433173008	0.442525811	0.451607781	0.460808236	0.469947965	0.479159906	0.488431968	0.497510076	0.506390271	0.514469837	0.522109974	0.529810711	0.535438327	0.540252562
Yogyakarta	0.316848685	0.327248137	0.337554814	0.34725376	0.357197924	0.367741716	0.378319872	0.387911315	0.397313668	0.407016191	0.41633466	0.425558545	0.434863507	0.444170661	0.453827144	0.463588443	0.473680778	0.484262987	0.495513713	0.506926164	0.517913177	0.528175512	0.537648885	0.544056497	0.549395041
Laos	0.193279408	0.197469498	0.201328218	0.205368668	0.209334804	0.213787254	0.21926064	0.224797626	0.230187743	0.235540832	0.241043245	0.246410558	0.251008488	0.255026114	0.259670101	0.264283164	0.268787618	0.273435312	0.278140418	0.28327023	0.288795341	0.294838372	0.301477967	0.308402545	0.315972615
Malaysia	0.418769126	0.428775462	0.43896002	0.448377002	0.457105256	0.465305839	0.473697717	0.482659671	0.491547853	0.500426112	0.509060201	0.517455154	0.525539959	0.533244457	0.540010354	0.545799405	0.551683548	0.558186752	0.565845656	0.574380446	0.583441103	0.593152135	0.603418105	0.612645412	0.621724901
Maldives	0.239845807	0.240833313	0.242472115	0.244524987	0.247083582	0.251210031	0.255552339	0.260488265	0.265333323	0.271544324	0.279398671	0.288418239	0.298262995	0.30866601	0.319832941	0.331601544	0.344398607	0.35825234	0.372806999	0.388081604	0.403945583	0.420519128	0.437436026	0.453973361	0.469338066
Mauritius	0.45558873	0.462850414	0.46877004	0.47473134	0.482359057	0.491156497	0.502078129	0.513167087	0.522123322	0.528459449	0.532473311	0.535069506	0.537567295	0.539889698	0.541830556	0.544586533	0.548209121	0.55345463	0.56068864	0.569092171	0.57731939	0.584797061	0.591497735	0.597363039	0.602034234
Myanmar	0.21991358	0.224523499	0.229757309	0.235633104	0.242085505	0.24956624	0.25757056	0.266088986	0.275066295	0.284269698	0.29307277	0.300872888	0.307350544	0.311640301	0.315581023	0.319219724	0.322034719	0.325628884	0.329707434	0.33429012	0.339590976	0.345191641	0.350892883	0.356696902	0.363037732
Philippines	0.448022144	0.45299842	0.457921243	0.462968888	0.468146205	0.473509584	0.478711545	0.483796927	0.488618517	0.492093993	0.494388185	0.496645329	0.49909606	0.502206758	0.505979517	0.510011796	0.513707183	0.517109502	0.520600378	0.524613607	0.528827907	0.532938849	0.536589868	0.53949647	0.542257968
Abra	0.410435365	0.415336201	0.420511824	0.426215833	0.432386185	0.438926063	0.445372873	0.451871479	0.458431388	0.463796563	0.468168	0.472448387	0.476814652	0.48180236	0.487402273	0.493114157	0.498240114	0.502877528	0.507468037	0.512240724	0.516914091	0.521143513	0.524627936	0.527188491	0.529350873
Agusan Del Norte	0.421261164	0.42623924	0.431492699	0.437212361	0.443257503	0.44950764	0.455511679	0.46131149	0.466761749	0.470677278	0.473205321	0.475616367	0.478074206	0.481131138	0.48473654	0.488478852	0.491758668	0.494592713	0.497382002	0.500520542	0.503695088	0.506641442	0.508979151	0.510384405	0.511557489
Agusan Del Sur	0.319132171	0.3236215	0.328343605	0.333569703	0.339308787	0.345475789	0.351653923	0.357826785	0.363909993	0.368710095	0.372375098	0.37607269	0.379919489	0.38430766	0.389261883	0.394358429	0.398943598	0.403099123	0.407256794	0.411808217	0.416351049	0.420605615	0.424133121	0.426757	0.429158978
Aklan	0.44933943	0.453928505	0.458487119	0.463158528	0.467909678	0.472800823	0.47736745	0.481674842	0.485610987	0.488097808	0.489352662	0.490592872	0.49208809	0.49422928	0.497035373	0.500143976	0.503005165	0.505672601	0.508519903	0.511977488	0.515774545	0.519592613	0.522999766	0.525531724	0.527903028
Albay	0.433853053	0.438625186	0.443548241	0.448713971	0.454050856	0.459549768	0.464669612	0.469487785	0.473807267	0.476557863	0.477906019	0.479141303	0.480541847	0.482671687	0.485502706	0.488673629	0.491577906	0.494292548	0.497167734	0.500767412	0.50478604	0.50882488	0.512453496	0.515364353	0.518140187
Antique	0.32015694	0.323519497	0.327225492	0.331712559	0.336840054	0.342627015	0.348551311	0.354863155	0.36148227	0.367129336	0.37193246	0.376851948	0.382094413	0.388129816	0.394873795	0.401776103	0.408036697	0.413740711	0.419361308	0.425361161	0.431335493	0.436817936	0.441544273	0.445299125	0.44869387
Apayao	0.34962455	0.355421365	0.361466415	0.367955099	0.374809669	0.382069363	0.389210594	0.396285417	0.403107535	0.408525568	0.412633057	0.416567874	0.420611033	0.425197579	0.430345332	0.435660953	0.440534223	0.44499167	0.449480294	0.454497768	0.459735846	0.464886627	0.469586845	0.473454544	0.477184743
Aurora	0.376918554	0.381825713	0.386912571	0.392373964	0.398196131	0.404314582	0.410320967	0.416239407	0.42194832	0.426308433	0.429529095	0.432832034	0.43644422	0.440868102	0.446147856	0.451738519	0.456950748	0.46184658	0.466781985	0.472254264	0.477906092	0.483338571	0.488149519	0.492013942	0.49558613
Basilan	0.257935787	0.262267705	0.266899911	0.272221118	0.278181778	0.2848098	0.291635493	0.298768446	0.306117848	0.312520003	0.318014711	0.323570973	0.329342662	0.335801633	0.342940357	0.350254265	0.357039364	0.363322389	0.369543406	0.37621656	0.383008064	0.389521453	0.395442204	0.400600427	0.405563315
Bataan	0.461887582	0.46629021	0.470771183	0.475538065	0.480432345	0.485492386	0.490198409	0.494637353	0.498813791	0.501638883	0.503401029	0.505183868	0.507234194	0.510028922	0.513538965	0.517309034	0.520735717	0.523928236	0.527231496	0.531170753	0.535383608	0.539494776	0.54317543	0.546147216	0.549052443
Batanes	0.46549706	0.470411809	0.475524789	0.480974333	0.486640969	0.492484704	0.497971198	0.503182784	0.508058921	0.511249288	0.512966487	0.514744575	0.516887227	0.519929399	0.523809354	0.528049634	0.531939891	0.535585737	0.539339097	0.54393731	0.54910787	0.554562617	0.559594854	0.5634099	0.566946327
Batangas	0.487952052	0.493188899	0.498559891	0.504178752	0.509944932	0.515882462	0.52155873	0.526958294	0.532006591	0.535617885	0.537993749	0.540223224	0.542495318	0.545358567	0.548793456	0.552429857	0.555717665	0.558729844	0.561799595	0.565363594	0.569134324	0.572820149	0.576062009	0.578537285	0.580780854
Benguet	0.52676574	0.531783225	0.536918007	0.54223172	0.547703564	0.553250442	0.558464594	0.563355736	0.567797037	0.570745946	0.572395197	0.574041166	0.575899778	0.578334935	0.581317521	0.584464349	0.58722893	0.589732125	0.59234214	0.59546542	0.598804337	0.602104872	0.604939372	0.606964205	0.608896272
Biliran	0.409617445	0.414661503	0.419950767	0.425678603	0.431793314	0.438260683	0.444591859	0.450889841	0.457023224	0.461943088	0.465790529	0.469665351	0.473762367	0.478370372	0.483569082	0.488957278	0.493982806	0.498632759	0.503306257	0.508460646	0.51375464	0.518918048	0.523558344	0.52737613	0.530937113
Bohol	0.374173764	0.378802223	0.383594249	0.388711984	0.39422347	0.400095572	0.405900597	0.411698574	0.417324084	0.421601402	0.424720299	0.427753623	0.430906853	0.434665699	0.439155619	0.443903869	0.448302517	0.452348692	0.456438737	0.461137897	0.466146757	0.471126903	0.475564943	0.479107722	0.482418945
Bukidnon	0.329270793	0.33321662	0.337419249	0.342119602	0.347329559	0.353030996	0.35876578	0.364588918	0.370392227	0.374928735	0.378323664	0.381671925	0.385159172	0.389367945	0.394273799	0.399380416	0.404023114	0.408275929	0.412588651	0.417425495	0.42236631	0.427035384	0.431084392	0.434323197	0.437297738
Bulacan	0.505408761	0.509851369	0.514367388	0.519067294	0.523962937	0.529065036	0.534002158	0.538807329	0.543299993	0.546463327	0.548500235	0.55045195	0.552608199	0.555468283	0.558992305	0.562840071	0.566428461	0.569766048	0.57318035	0.577094385	0.581283004	0.585510602	0.589436448	0.592693193	0.595756173
Cagayan	0.410273012	0.414818475	0.419505837	0.424501021	0.429823968	0.435461479	0.440941026	0.446340548	0.451499045	0.455353818	0.458059133	0.46084832	0.463862024	0.467519389	0.471872796	0.476523863	0.480897844	0.484963897	0.489117194	0.493926938	0.498971508	0.50394762	0.508414834	0.512001765	0.515321235
Camarines Norte	0.408720008	0.411859447	0.415072233	0.418593772	0.422463239	0.426706103	0.430841941	0.434883634	0.438713653	0.441129015	0.442346221	0.443629076	0.445237979	0.447596648	0.450777679	0.454389757	0.457840474	0.461063575	0.464462618	0.468428777	0.472583487	0.476532628	0.47984856	0.482216625	0.484312886
Camarines Sur	0.417402471	0.421919203	0.426640454	0.431767396	0.43724925	0.4430776	0.448779187	0.454384785	0.459769571	0.463798099	0.46662499	0.469478861	0.472582612	0.476291545	0.480618044	0.485164875	0.489361424	0.493200974	0.49705719	0.501506153	0.506143495	0.510733672	0.514853108	0.518063872	0.52104005
Camiguin	0.438142103	0.443219279	0.448542889	0.454056515	0.459645338	0.465347308	0.470681696	0.475664985	0.480122568	0.482893251	0.48418786	0.485469042	0.487001011	0.489241223	0.492130297	0.495305819	0.498206943	0.500951424	0.503858005	0.507181188	0.510702709	0.514173609	0.517129638	0.519112757	0.520852399
Capiz	0.352855172	0.3574687	0.362307669	0.36767071	0.373469011	0.379682693	0.385880944	0.392107261	0.398189232	0.402874269	0.406289705	0.409646954	0.413102876	0.417182603	0.42180589	0.42655316	0.430796418	0.434569814	0.438338288	0.442581245	0.446866816	0.450868951	0.454242389	0.456737284	0.458974499
Catanduanes	0.400358147	0.404021249	0.407814761	0.412017259	0.416579943	0.421558957	0.426570595	0.431653593	0.436678247	0.440481362	0.44331894	0.446197281	0.449313012	0.453124318	0.457710438	0.462551509	0.467032831	0.471102942	0.475205722	0.479818552	0.484521052	0.489002939	0.492805339	0.495766235	0.498440936
Cavite	0.555210287	0.560297412	0.565479431	0.57084511	0.576315192	0.581847388	0.587016496	0.59183009	0.596138995	0.598914564	0.600338272	0.601707895	0.603190843	0.60517402	0.607626579	0.610211825	0.612466496	0.614431865	0.616456474	0.619049671	0.621937059	0.624878303	0.627476237	0.629373258	0.631148984
Cebu	0.479186407	0.484201184	0.489302415	0.494525813	0.499772806	0.5050335	0.510006578	0.514672812	0.518839997	0.521484103	0.522795419	0.52402804	0.525451488	0.527473837	0.53000716	0.532735498	0.535184099	0.537409336	0.539696072	0.542495726	0.545542569	0.54856922	0.551193724	0.553063284	0.554743529

Cotabato (North Cotabato)	0.357309374	0.361905987	0.366695444	0.371866176	0.377427081	0.38329108	0.389026585	0.394660302	0.400095896	0.404085928	0.406795524	0.409506685	0.412388778	0.415899768	0.420041124	0.424363132	0.42818722	0.431601429	0.435025749	0.438957556	0.443011906	0.446847905	0.450027437	0.45227877	0.454300441
Davao de Oro	0.322504273	0.327052218	0.331812532	0.337026181	0.342674908	0.348716075	0.354738521	0.360800997	0.366747426	0.371246162	0.374460123	0.377585917	0.380842061	0.384798286	0.389390211	0.394188933	0.398475716	0.402272643	0.406034811	0.410264612	0.414613669	0.418697157	0.422188964	0.42477907	0.427079438
Davao Del Norte	0.398256162	0.403532403	0.408983989	0.414852585	0.421026341	0.427522423	0.433921751	0.44026345	0.446378916	0.451157363	0.454755008	0.458396122	0.462241551	0.466750714	0.471878871	0.47725283	0.482257954	0.486909844	0.491538247	0.4966396	0.501958362	0.507110136	0.511742636	0.515444042	0.518837267
Davao Del Sur	0.441601689	0.446553643	0.451637071	0.457014755	0.462629083	0.4684376	0.474078154	0.479667826	0.48495193	0.488899757	0.49165912	0.49437574	0.497210582	0.500676079	0.50479512	0.509118509	0.513016088	0.516571489	0.52016433	0.524170872	0.528321223	0.532319316	0.535822319	0.538557143	0.541112922
Davao Occidental	0.318888906	0.321891997	0.325169315	0.32897166	0.333304584	0.338183496	0.34322381	0.348596582	0.354263805	0.359237308	0.363573797	0.367888861	0.372322494	0.377308938	0.382966842	0.388923824	0.394538632	0.399788939	0.405090332	0.410838851	0.416692939	0.422258121	0.42732412	0.431901298	0.436420405
Davao Oriental	0.315880582	0.320297312	0.324950954	0.330074718	0.335587616	0.341509674	0.347320021	0.353072815	0.358636714	0.362878841	0.365894755	0.368876951	0.372062906	0.375912551	0.380455384	0.385245835	0.389594617	0.393582355	0.397643446	0.402508439	0.407752423	0.413024333	0.417851716	0.421871716	0.425709117
Dinagat Islands	0.370014902	0.374893694	0.380006924	0.385658405	0.391882762	0.398629045	0.405409974	0.412359099	0.419307205	0.425157454	0.430026644	0.434822044	0.439748502	0.445258082	0.451283989	0.45740369	0.463025617	0.468196986	0.473345622	0.478953085	0.484672292	0.490109225	0.494991968	0.499196657	0.503243733
Eastern Samar	0.299888097	0.303101407	0.30654052	0.310527211	0.31500936	0.320018209	0.324952304	0.329942743	0.334887501	0.338490806	0.34101713	0.343586764	0.346324103	0.349826026	0.354178275	0.358787376	0.362878035	0.36639932	0.369924619	0.373965159	0.378004916	0.38158973	0.384331237	0.38619929	0.387758011
Guimaras	0.370115451	0.375163988	0.380463668	0.386235939	0.392399348	0.398956341	0.40537451	0.411793562	0.418047151	0.422940327	0.426586899	0.430119631	0.43386812	0.43821473	0.443211546	0.448414677	0.453165677	0.457533785	0.46197678	0.466960044	0.472143782	0.477133844	0.481523946	0.484957624	0.488114925
Ifugao	0.357504217	0.362377895	0.367587905	0.373312544	0.379539332	0.386241056	0.392890802	0.399652127	0.406354794	0.411942513	0.416516753	0.420952633	0.425565681	0.430776644	0.436594903	0.442548221	0.447995267	0.45305081	0.458133786	0.463600232	0.46907812	0.474208503	0.478664686	0.482238057	0.485578325
Ilocos Norte	0.493487141	0.498735363	0.504088339	0.50957863	0.515173445	0.520880315	0.526291116	0.531507752	0.536403529	0.539918823	0.542216123	0.54437054	0.546651791	0.549418921	0.552701805	0.556207974	0.559411424	0.562402595	0.565460193	0.568922794	0.572547553	0.576090887	0.579200076	0.58153885	0.583742928
Ilocos Sur	0.45922358	0.463928134	0.468617767	0.473512931	0.47864967	0.48402581	0.48925689	0.494462136	0.499517971	0.50333779	0.506116885	0.508881673	0.511885487	0.515524773	0.519838931	0.524452639	0.528775256	0.532831024	0.536976835	0.541527269	0.546162984	0.550553538	0.554366487	0.557282695	0.559956615
Iloilo	0.455360885	0.46056385	0.465904984	0.471602193	0.477573463	0.483827141	0.489841567	0.495675798	0.501262334	0.505542916	0.508586886	0.511581179	0.514738988	0.518501929	0.522867694	0.527433705	0.531643384	0.535534166	0.539448689	0.543682832	0.548062125	0.552240901	0.555844502	0.558512792	0.560955746
Isabela	0.422379654	0.427011256	0.431767209	0.436800031	0.442106279	0.447668476	0.452985845	0.458151295	0.463034441	0.466608819	0.46910089	0.471685607	0.474476001	0.478002149	0.482305497	0.486994063	0.49145654	0.495622618	0.49979648	0.504431543	0.509272084	0.513958911	0.518081911	0.521359963	0.524337691
Kalinga	0.315765271	0.320121774	0.324741773	0.329926751	0.335628417	0.34188145	0.348200573	0.354710731	0.361265529	0.3665967	0.370826419	0.375064457	0.379546955	0.384676057	0.390507417	0.396586905	0.402252916	0.407611127	0.413029176	0.418959489	0.425070996	0.431054064	0.436504759	0.441050314	0.445317027
La Union	0.476449956	0.481121233	0.485860102	0.490830952	0.496021355	0.501444211	0.506585448	0.511480507	0.516000832	0.519061358	0.520893807	0.522670001	0.524537339	0.526903527	0.529890759	0.533148639	0.536071317	0.538675071	0.541342804	0.544516874	0.547894539	0.551145072	0.553907373	0.555905488	0.557709025
Laguna	0.526849954	0.531851106	0.537043938	0.54246415	0.548026556	0.553637368	0.55887314	0.56375243	0.568075047	0.570870137	0.572397632	0.57376555	0.575278397	0.577432657	0.580156839	0.583112351	0.585723601	0.587962551	0.590211801	0.592919678	0.595829433	0.598658227	0.601091405	0.60282229	0.604381112
Lanao Del Norte	0.396869869	0.401934339	0.407170822	0.412823446	0.418776137	0.424929223	0.430777888	0.436383176	0.441741681	0.445662978	0.448279393	0.450767997	0.453322049	0.456418965	0.460115962	0.46393371	0.467302053	0.470247295	0.473227993	0.476695249	0.480238009	0.48352204	0.486144581	0.48790355	0.489433351
Lanao Del Sur	0.315720949	0.320386726	0.325298094	0.330774503	0.336737447	0.343174246	0.349557454	0.355944861	0.36223439	0.367230075	0.371047981	0.374731598	0.378468032	0.382798459	0.387850316	0.393071715	0.397833358	0.402118499	0.406409448	0.410865852	0.415142323	0.418885318	0.42184144	0.42389968	0.425612163
Leyte	0.380335427	0.385113926	0.390124895	0.395509624	0.401223487	0.40728494	0.41324228	0.41912706	0.424832203	0.429242988	0.432521702	0.43576054	0.43915348	0.443164781	0.447858365	0.452754461	0.457255664	0.461338765	0.46545871	0.470031788	0.474745284	0.47934116	0.483398128	0.486532868	0.489419634
Maguindanao	0.260591141	0.264391895	0.268449869	0.273080985	0.278236737	0.283977171	0.289879058	0.296005641	0.302229778	0.307353212	0.311543103	0.315863188	0.320439671	0.325748325	0.331783206	0.338054564	0.343896149	0.349301553	0.354721946	0.360538202	0.366364899	0.371911775	0.376854923	0.380888447	0.384578933
Marinduque	0.351875429	0.355681722	0.359679994	0.364137887	0.369059602	0.37449034	0.379950718	0.385435268	0.39087548	0.394994622	0.39794197	0.400759994	0.403709631	0.407239448	0.411454986	0.415902044	0.419914225	0.423549257	0.427238105	0.431340635	0.435469671	0.43925705	0.442293083	0.44439647	0.446224249
Masbate	0.310552129	0.313467189	0.316508217	0.319943636	0.323813081	0.328049674	0.332204855	0.336365856	0.3404732	0.343235687	0.344833601	0.34640423	0.348170263	0.350642455	0.35382128	0.357228929	0.360130301	0.362581514	0.365084303	0.367976114	0.370850194	0.37327649	0.374858543	0.375489568	0.375839335
Misamis Occidental	0.420744671	0.425388076	0.430186269	0.435315435	0.440688262	0.446224857	0.451466894	0.456480246	0.461128792	0.464290837	0.466156038	0.46799789	0.46996978	0.472555694	0.475743432	0.479137772	0.482130921	0.484755605	0.487375966	0.490228026	0.493005319	0.49542158	0.497170318	0.497955155	0.498451251
Misamis Oriental	0.48411022	0.489290044	0.494650303	0.500290501	0.506146151	0.512122423	0.517787671	0.523140888	0.528059053	0.531428497	0.533428066	0.535338304	0.537353186	0.53989214	0.542945676	0.546148333	0.548962943	0.551466481	0.553998532	0.55705062	0.5603096	0.56349272	0.566164354	0.567962378	0.569569385
Mountain Province	0.298294744	0.302729494	0.307502586	0.313002927	0.319057941	0.325681732	0.332253798	0.339017671	0.345854208	0.351550288	0.356303028	0.361057217	0.365909848	0.371476401	0.377750087	0.384142978	0.389890999	0.394983191	0.399825221	0.404880792	0.409762101	0.414070159	0.417486337	0.419958369	0.421967521
National Capital Region	0.586319472	0.590223265	0.594372353	0.598771386	0.603318058	0.607985787	0.612366237	0.616478324	0.620135125	0.622375477	0.623368668	0.624237274	0.625276642	0.626867315	0.629088258	0.631604081	0.633853859	0.635862419	0.637940062	0.640545157	0.643394295	0.646241426	0.648794579	0.650709013	0.652618625
Negros Occidental	0.432289904	0.43716663	0.442163415	0.447371371	0.452795471	0.458377979	0.463745514	0.468879662	0.473637482	0.476932791	0.478915524	0.480889608	0.48300431	0.485627829	0.488794684	0.492135423	0.495108113	0.497767506	0.500450479	0.50352918	0.506708844	0.509752136	0.512205671	0.513725278	0.514994993
Negros Oriental	0.377356926	0.381785403	0.386345339	0.391178826	0.396270999	0.401595338	0.406773972	0.411872172	0.41667369	0.420133723	0.422444571	0.424785653	0.427339742	0.430501214	0.434234068	0.438173264	0.44179132	0.445143902	0.448556065	0.452496588	0.456665244	0.460740328	0.464351625	0.467150815	0.469714873
Northern Samar	0.307765735	0.311764875	0.315953632	0.320647224	0.325748489	0.33133074	0.336915597	0.342574564	0.34826053	0.352678797	0.356030065	0.359431403	0.363048028	0.367350246	0.372362158	0.377649131	0.382447777	0.386814676	0.391203467	0.395976421	0.400712917	0.405101783	0.408750108	0.411410092	0.413746228
Nueva Ecija	0.480082812	0.484803067	0.489590937	0.494550616	0.499603766	0.504744704	0.50953792	0.514012503	0.518019279	0.520532604	0.521751427	0.522962038	0.524341988	0.526247882	0.528695265	0.531381357	0.533786251	0.535898211	0.538114883	0.540863167	0.543851476	0.546716691	0.549103589	0.550740588	0.55222002
Nueva Vizcaya	0.401094357	0.405840397	0.410840002	0.416457831	0.42268185	0.429365478	0.435927127	0.442480509	0.448936926	0.454155981	0.458313092	0.462364056	0.466512104	0.47129561	0.476712177	0.482176184	0.486871208	0.490908008	0.494807479	0.499088297	0.503269888	0.506990304	0.50997028	0.512084895	0.513901027
Occidental Mindoro	0.263350133	0.265759745	0.268431788	0.272106174	0.276782522	0.282210075	0.287701222	0.293666719	0.300066389	0.305507253	0.310193292	0.314969526	0.319889545	0.325661997	0.332261883	0.339037642	0.344967049	0.350049168	0.354918039	0.359983643	0.364635755	0.368377774	0.370958902	0.37274986	0.374176824
Oriental Mindoro	0.353104884	0.357879176	0.363005121	0.368767343	0.375020145	0.381737128	0.388470101	0.395266527	0.402066844	0.407648463	0.41217922	0.416618469	0.42120517	0.42640049	0.432168991	0.438054195	0.443436909	0.448351613	0.453247272	0.458485922	0.463696843	0.468584791	0.472821106	0.476154401	0.479238124
Palawan	0.320886982	0.325114842	0.329553763	0.334541712	0.339997309	0.345892264	0.351719553	0.357555433	0.363367161	0.367800485	0.370980922	0.374215422	0.37761946	0.381668334	0.386342859	0.391125531	0.395348366	0.399044743	0.402657344	0.406857344	0.411162737	0.415200162	0.418529354	0.42092942	0.423082913
Pampanga	0.490364347	0.4954146	0.500638789	0.50621198	0.511985975	0.517973115	0.523717785	0.529253292	0.534484203	0.538319735	0.540990925	0.543578461	0.546302277	0.54965365	0.553597978	0.557746428	0.56147988	0.564849035	0.56814459	0.571902747	0.5759152	0.579865413	0.583400558	0.586245314	0.588925177
Pangasinan	0.480351452	0.484785466	0.489260063	0.493987273	0.498914664	0.504044689	0.508946761	0.513661336	0.518066102	0.521061809	0.522807421	0.524575314	0.5265044	0.529019745	0.532166597	0.535583582	0.538726455	0.541605051	0.544599252	0.548122585	0.551787063	0.555310788	0.558284381	0.560421018	0.562357038
Quezon	0.437781419	0.442271899	0.446957364	0.451919852	0.457076992	0.462432965	0.467480591	0.472304362	0.476753741	0.479758717	0.481530015	0.483317972	0.485326497	0.487981517	0.491250983	0.494755975	0.497904923	0.500708955	0.503549027	0.506778012	0.51003994	0.513068701	0.515543543	0.517203748	0.518652522
Quirino	0.338297696	0.342558982	0.34710205	0.352284807	0.358058078	0.364352292	0.370763679	0.377289977	0.383826362	0.389140184	0.393343203	0.397487773	0.401728587	0.406562764	0.412038142	0.417716701	0.423038166	0.428007276	0.433081114	0.438683669	0.444393929	0.449863614	0.45469782	0.458600441	0.462218691
Rizal	0.555592439	0.557835676	0.559086436	0.559758065	0.559782167	0.559314873	0.558461229	0.557593883	0.556837037	0.555300696	0.553112679	0.551436379	0.550649947	0.551374146	0.553480054	0.556537796	0.559979648	0.563691384	0.567884345	0.573027924	0.578714588	0.584603287	0.590210396	0.59504784	0.599613195
Romblon	0.310593407	0.314003886	0.317571	0.321615793	0.326191141	0.331395155	0.336643865	0.341953444	0.347258982	0.351234513	0.354084705	0.356944478	0.360075524	0.363927062	0.368564323	0.373491683	0.377969909	0.381990022	0.386059667	0.390773318	0.395689695	0.400402555	0.404387302	0.407427651	0.410250436
Samar (Western Samar)	0.333819494	0.338342841	0.343041585	0.348126835	0.353553734	0.359406246	0.365219626	0.370988784	0.376590741	0.380790729	0.383812979	0.386797774	0.389892216	0.393540701	0.397753805	0.402122967	0.406060274	0.40956694	0.41303879	0.416847929	0.420653889	0.424128116	0.426910148	0.428750932	0.430251405
Sarangani	0.343179923	0.348007492	0.353056231	0.358486884	0.364180465	0.370155669	0.375988961	0.381734868	0.387200442	0.391323663	0.394271791	0.397215537	0.400350786	0.404136034	0.408560598	0.413238597	0.41756627	0.421676191	0.425924586	0.430727485	0.435847042	0.441004866	0.44584224	0.449861615	0.453705551
Siquijor	0.371219888	0.375658796	0.380450487	0.38564324	0.391010339	0.39648361	0.401734662	0.406863059	0.411774194	0.415216179	0.417472008	0.420052177	0.42311877	0.427072089	0.431707066	0.436579156	0.441220798	0.445638191	0.450172774	0.455378212	0.460941425	0.466650478	0.471978037	0.476360385	0.480491999
Sorsogon	0.399457437	0.403543395	0.40773249	0.412166153	0.416771098	0.421577235	0.42620952	0.430670003	0.434775396	0.437377803	0.438735627	0.440140189	0.441881064	0.444431259	0.447684247	0.451186555	0.454349618	0.457268478	0.460329264	0.464040471	0.468062619	0.47202674	0.475468521	0.478059933	0.480413304
South Cotabato	0.426277002	0.431042185	0.4359823	0.441295526	0.446948596	0.452878283	0.458591746	0.464192409	0.469552996	0.473523714	0.476300521	0.478953797	0.481740351	0.48512843	0.489136658	0.493304671	0.49706363	0.500426958	0.503817861	0.507708152	0.511725939	0.515581972	0.518903425	0.521396419	0.523662082
Southern Leyte	0.3807562	0.384698254	0.38884245	0.39338246	0.398320429	0.403649147	0.408936774	0.414207438	0.419343331	0.423187182	0.425938887	0.428717071	0.431755861	0.435485597	0.439960231	0.444659979	0.448982679	0.452956869	0.456982715	0.461473383	0.466026471	0.470339646	0.474005831	0.4767812	0.479322292
Sultan Kudarat	0.310851032	0.315205077	0.319831852	0.325162486	0.33103693	0.337393513	0.343726302	0.350123595	0.356585025	0.361784016	0.365832849	0.369864603	0.373996541	0.378764463	0.384211972	0.389772512	0.394785842	0.399217783	0.40355312	0.408228945	0.412786795	0.416883858	0.42011876	0.422402672	0.424357512
Sulu	0.222556401	0.226342832	0.230258103	0.23434246	0.238650282	0.243297942	0.248174696	0.25345244	0.258991514	0.26358154	0.267199326	0.270991889	0.275085244	0.279876247	0.285340503	0.291025666	0.29649175	0.301892531	0.30759409	0.313756704	0.32001469	0.326065953	0.33159151	0.336166694	0.340449304
Surigao Del Norte	0.404900788	0.40965756	0.41452462	0.419635731	0.424974434	0.430568838	0.435979195	0.441239817	0.446220528	0.44988996	0.452406415	0.454810401	0.457437709	0.460731129	0.464717188	0.468963284	0.472884636	0.47654864	0.480356046	0.48489205	0.489801918	0.494734744	0.49930364	0.503123817	0.506793805
Surigao Del Sur	0.363276847	0.367868602	0.372697114	0.378063996	0.383940102	0.390220184	0.396417958	0.402620219	0.408703143	0.413473804	0.417140759	0.420843888	0.424696029	0.429252273	0.434525965	0.44007541	0.445207018	0.449961915	0.454740389	0.459897717	0.46504644	0.469852059	0.473986797	0.477235737	0.480264831
Tarlac	0.465840518	0.470196896	0.474599746	0.479183391	0.483884468	0.488717658	0.493148896	0.49731916	0.50111435	0.503517382	0.504779825	0.506079206	0.507643336	0.509990054	0.513071086	0.516577193	0.519922204	0.523110165	0.526450561	0.53036813	0.534556241	0.538590973	0.542085881	0.544809162	0.547277083
Tawi-Tawi	0.289342529	0.292766621	0.296408095	0.300582991	0.305383869	0.310766673	0.316233308	0.321988482	0.327862456	0.332617209	0.336426612	0.340411199	0.344756201	0.349978873	0.355978189	0.362263287	0.36809861	0.373558566	0.379019491	0.384748407	0.390361297	0.395535287	0.39997237	0.403411219	0.406466383
Zambales	0.499608714	0.503566022	0.507570837	0.511765442	0.516062245	0.520425517	0.524430182	0.528111985	0.531288963	0.532948781	0.533392088	0.533818633	0.534437988	0.535699949	0.537563203	0.53965418	0.541446109	0.543004867	0.544689636	0.546872467	0.549227103	0.551467688	0.553240691	0.554230586	0.555102245
Zamboanga Del Norte	0.334902582	0.339396181	0.344080887	0.349150277	0.354525084	0.360172131	0.365655264	0.371054985	0.376234372	0.379951718	0.382371632	0.384733063	0.387259144	0.390492334	0.394364123	0.398464758	0.402127833	0.405412583	0.408717262	0.4125698	0.416593958	0.420464873	0.423802083	0.426194965	0.428362735
Zamboanga Del Sur	0.396866061	0.402406183	0.408152316	0.414249517	0.420604882	0.427284263	0.433734541	0.440108226	0.446296709	0.451097827	0.454630325	0.458031847	0.461552239	0.465657898	0.470334568	0.475176508	0.479541486	0.483501746	0.487530367	0.492059641	0.496769323	0.50135883	0.505476055	0.50877701	0.511864566
Zamboanga Sibugay	0.314586107	0.319036789	0.323748271	0.328895091	0.334465522	0.340485871	0.346468792	0.352440826	0.35828369	0.362737893	0.366015251	0.369297663	0.372771754	0.376973444	0.381898147	0.387055592	0.391744715	0.396017619	0.400336691	0.405210618	0.410316488	0.41527972	0.419610867	0.423007552	0.426101387
Seychelles	0.423539942	0.434840635	0.446694035	0.4594739	0.473605655	0.486886858	0.499135932	0.509683846	0.518951245	0.527813447	0.536709926	0.544823087	0.552748799	0.560367755	0.567974696	0.575526496	0.582698331	0.590052919	0.598044194	0.606041268	0.613853281	0.62213517	0.630697732	0.638438882	0.645339666
Sri Lanka	0.435507766	0.441051338	0.446872679	0.452358842	0.45666911	0.460615686	0.465131104	0.470146672	0.475836759	0.482454881	0.489603035	0.49697926	0.503943432	0.510587395	0.516837237	0.522622553	0.528239203	0.533345535	0.538891102	0.545124551	0.551744058	0.558938888	0.566128422	0.572326187	0.577630048
Thailand	0.377166383	0.38554919	0.394339195	0.403341437	0.41208232	0.420320904	0.428661694	0.437263786	0.445865998	0.454644845	0.463192975	0.471217831	0.479387776	0.488095394	0.497255863	0.506644481	0.515911466	0.525161869	0.534303709	0.543325547	0.552452209	0.561302487	0.568449492	0.573817902	0.578854168
Timor-Leste	0.162346545	0.167469327	0.172925047	0.178772331	0.185000799	0.190575744	0.196926731	0.203463701	0.21018687	0.21718423	0.223865337	0.231231067	0.238862622	0.246760449	0.254842162	0.262468083	0.270943349	0.27991676	0.289266629	0.298763189	0.308018684	0.31758875	0.326705374	0.334894497	0.33925214
Viet Nam	0.320952309	0.326930934	0.333655088	0.339942523	0.346056494	0.351206034	0.358056809	0.36381168	0.370218675	0.37670852	0.382656564	0.387905939	0.392607305	0.397443235	0.402478517	0.407630048	0.41321725	0.419781657	0.427072185	0.435106272	0.443858785	0.45310241	0.462853518	0.472478892	0.481445774
Sub-Saharan Africa	0.231771346	0.236373453	0.241114798	0.245813958	0.25049216	0.255143394	0.259686318	0.264199391	0.268635435	0.273130199	0.277759505	0.281742287	0.285602625	0.289540486	0.29343912	0.297147968	0.30063245	0.303873882	0.306848923	0.309705038	0.312728678	0.316139229	0.319699114	0.323309973	0.327028574
Central sub-Saharan Africa	0.232787007	0.23758921	0.242379451	0.246837575	0.251534147	0.256465874	0.26125575	0.265948412	0.270673031	0.275940939	0.281087763	0.285677481	0.290074335	0.2944483	0.298709908	0.302374953	0.305220591	0.307803569	0.309082037	0.309897978	0.311059245	0.312674396	0.314501685	0.316506276	0.318568997
Angola	0.20281399	0.206614511	0.210571291	0.214714103	0.219078744	0.223575748	0.227972195	0.232476613	0.236992077	0.24194387	0.246968876	0.251587013	0.256510204	0.261329764	0.266090018	0.270736935	0.275160625	0.2792315	0.281867703	0.284487594	0.287447813	0.291421279	0.295433993	0.299553353	0.303720042
Central African Republic	0.164099184	0.167339334	0.170973575	0.174712601	0.178232604	0.181356083	0.185086953	0.188790396	0.192118909	0.19564704	0.199008068	0.202526043	0.205792217	0.20933149	0.213149898	0.216825191	0.220191656	0.223007541	0.225451125	0.228487724	0.231696792	0.233989591	0.236671999	0.239743529	0.24298853
Congo (Brazzaville)	0.283507592	0.291531723	0.299146338	0.307074777	0.315842485	0.325844159	0.33643967	0.347698589	0.359066095	0.370546962	0.381395709	0.390385922	0.398711172	0.406534779	0.413916454	0.420654655	0.426734374	0.431987662	0.436606594	0.440168347	0.443744125	0.447566642	0.451026961	0.454445045	0.45717654
DR Congo	0.229833639	0.233862484	0.237998123	0.241645221	0.245685162	0.249963154	0.254070969	0.258047305	0.262085386	0.267108835	0.271770303	0.27604442	0.280060613	0.284050328	0.28761916	0.28984321	0.290431567	0.290959242	0.28962879	0.286859146	0.283967532	0.28041316	0.276336503	0.271813823	0.267367662
Equatorial Guinea	0.196254783	0.20154355	0.206998601	0.211173168	0.214774437	0.217947662	0.220978029	0.224089974	0.228472362	0.232920045	0.238231249	0.2430807	0.248761536	0.255118938	0.26204985	0.268783629	0.275296992	0.283487764	0.292236032	0.30068634	0.310443551	0.324601221	0.350550963	0.371237163	0.391128023
Gabon	0.323388183	0.336870863	0.348958267	0.359700131	0.370389425	0.380971717	0.390411012	0.399381844	0.408049405	0.416487895	0.424458813	0.431316748	0.437186947	0.442911008	0.449161213	0.455421187	0.461563098	0.467024048	0.472394463	0.478061947	0.483826218	0.48946146	0.495074162	0.50037255	0.50480635

Eastern sub-Saharan Africa	0.1821715	0.185810887	0.189484923	0.193214565	0.196828716	0.200281218	0.203594321	0.207001074	0.210559615	0.214107943	0.217642016	0.221008234	0.224324533	0.22761531	0.230659376	0.233622123	0.236340461	0.238615291	0.240816973	0.242901814	0.245423542	0.24858486	0.252144544	0.255890509	0.259957122
Burundi	0.159273072	0.162315635	0.166031542	0.169400786	0.172622423	0.175229027	0.178452622	0.181298481	0.184114532	0.18673467	0.1901267	0.193575788	0.196778882	0.200096007	0.203060966	0.20586736	0.20849572	0.210864619	0.213249386	0.215222973	0.215631676	0.215526325	0.21576182	0.216402603	0.216855767
Comoros	0.156990492	0.161343468	0.166013945	0.171094425	0.176862412	0.183368413	0.190265538	0.197566045	0.205342677	0.213546023	0.222307323	0.231754843	0.241358131	0.251098548	0.260524198	0.270048116	0.279145649	0.288506271	0.297607417	0.305706587	0.314220341	0.322249069	0.330148125	0.337517914	0.344774694
Djibouti	0.274612886	0.280495589	0.286312118	0.291864769	0.297047093	0.300981569	0.303511503	0.306233738	0.308995526	0.31165697	0.315063847	0.318534579	0.322421589	0.327992479	0.333883603	0.337781789	0.338770011	0.340986061	0.344904394	0.347981404	0.351245633	0.354385634	0.358096401	0.361596764	0.365305211
Eritrea	0.145971922	0.150347514	0.154835934	0.159424933	0.164093099	0.168603771	0.172977742	0.177283062	0.181640729	0.186173536	0.190749505	0.195413787	0.20025618	0.205359622	0.210698757	0.216028239	0.222574737	0.230296066	0.239385041	0.250752541	0.261498485	0.272527055	0.283551721	0.293764828	0.30314234
Ethiopia	0.102827582	0.105494053	0.108109568	0.110637005	0.113382558	0.116816831	0.120210373	0.123489071	0.127240555	0.130620338	0.13350026	0.136552289	0.140019974	0.142885158	0.145481708	0.148033885	0.14959971	0.150174386	0.151582364	0.152982637	0.155085605	0.158127229	0.161874743	0.16507771	0.168903545
Addis Ababa	0.315306701	0.32317527	0.331364515	0.339875403	0.349355741	0.360737397	0.372648995	0.384788555	0.397933506	0.411252313	0.4244142	0.43784439	0.451655228	0.4643373	0.476103176	0.487060041	0.495700289	0.502057376	0.508601978	0.514369793	0.520299676	0.526683615	0.533187064	0.538390746	0.543738777
Afar	0.072320697	0.074148279	0.075925326	0.077607283	0.07942445	0.081736289	0.084016252	0.086300706	0.089133385	0.091611	0.09346685	0.09539253	0.097760105	0.099575049	0.101094883	0.102500069	0.102923831	0.102304887	0.102424632	0.102491546	0.103319651	0.105128034	0.107613013	0.109385397	0.111748498
Amhara	0.064778755	0.066542935	0.068267857	0.069931218	0.071743351	0.074098889	0.076444859	0.078745373	0.081572297	0.084025812	0.085954444	0.088033871	0.090573102	0.092619268	0.094461217	0.096349458	0.097325773	0.097289302	0.098146296	0.098993032	0.100681932	0.103542465	0.107321049	0.110521867	0.114484273
Benishangul-Gumuz	0.062915703	0.064753274	0.066518147	0.068223847	0.070072664	0.07248303	0.074869012	0.077221362	0.080139227	0.082565239	0.084302918	0.08613711	0.088460472	0.090159597	0.091554043	0.0929276	0.093242299	0.092373199	0.092442514	0.092501201	0.093497947	0.095699051	0.098780885	0.101087265	0.104172732
Dire Dawa	0.215095	0.220277974	0.225491744	0.230693922	0.236350187	0.243033079	0.249885906	0.256656269	0.26401774	0.271231985	0.278030951	0.285151416	0.292675525	0.299460697	0.305909509	0.312205934	0.317408907	0.32136416	0.325969914	0.330416077	0.335292053	0.340748931	0.346489448	0.351424683	0.356676908
Gambella	0.09090095	0.093403277	0.096113031	0.099025519	0.102478814	0.10694519	0.111645388	0.116687261	0.122646717	0.128777028	0.134876607	0.141388573	0.148626572	0.155469411	0.162107981	0.168610253	0.17373969	0.177538991	0.18211209	0.186359835	0.190956687	0.196128721	0.201735096	0.206360155	0.211526595
Harari	0.222257431	0.227622673	0.233066	0.238417926	0.244216562	0.251084737	0.258103036	0.265136045	0.272852157	0.280371246	0.287529796	0.29500617	0.302921846	0.310066366	0.316661292	0.322842077	0.327439623	0.330356931	0.333740959	0.336843341	0.340420403	0.344672091	0.349115288	0.352605147	0.356454396
Oromia	0.086443392	0.088884222	0.091232458	0.093467358	0.095899245	0.098987601	0.101996787	0.104904351	0.108326898	0.111324465	0.113710437	0.116234253	0.119204645	0.121521608	0.123553461	0.125544882	0.126509567	0.126441794	0.127249568	0.128025155	0.129534961	0.132016399	0.135189515	0.137689748	0.140860153
Somali	0.065778272	0.067617634	0.069405171	0.07110859	0.072901074	0.075109628	0.07729279	0.079378294	0.081864655	0.083992433	0.08557422	0.087294811	0.089407126	0.090991451	0.092310531	0.093590078	0.094118884	0.093758812	0.094108373	0.094380873	0.095260047	0.096930178	0.099120064	0.100596636	0.102567257
Southern Nations, Nationalities, and P	0.088170135	0.090803806	0.09336322	0.09582366	0.098500442	0.101891653	0.105292305	0.108603573	0.112556993	0.115979993	0.118705082	0.121654381	0.125125146	0.127872405	0.130214404	0.132426296	0.13344635	0.133144737	0.133851279	0.134522203	0.136105734	0.138925428	0.14251578	0.145163238	0.148526634
Tigray	0.090652226	0.093164871	0.095671127	0.098099862	0.10066209	0.103811987	0.106859747	0.10973915	0.113059521	0.116128407	0.118776697	0.12156054	0.124778	0.127494831	0.130083906	0.132771026	0.134682067	0.135957655	0.138249694	0.140704188	0.143957874	0.148141461	0.153040465	0.157343997	0.162224438
Kenya	0.221349852	0.228065548	0.235329709	0.242827683	0.250330792	0.258087041	0.265769908	0.273523081	0.281128869	0.288676671	0.296231311	0.304003274	0.311721058	0.319427343	0.326811725	0.333850293	0.34047622	0.346298636	0.351611769	0.356779194	0.361535807	0.366203076	0.370436465	0.374581401	0.378630009
Baringo	0.156926932	0.160753006	0.165362223	0.170578269	0.176478002	0.183415948	0.19091793	0.199123407	0.20778492	0.216826568	0.226397213	0.236435863	0.246781445	0.25722455	0.267418801	0.277332089	0.286878009	0.295822039	0.304372219	0.312974314	0.321200334	0.32941504	0.337292157	0.345077278	0.352742706
Bomet	0.180186206	0.185838872	0.191688587	0.197518322	0.203455153	0.209904488	0.216408123	0.22326482	0.230133083	0.237111194	0.244296277	0.251730247	0.25932241	0.267002672	0.2746064	0.281945483	0.288978204	0.295293783	0.301345026	0.30755692	0.313625026	0.319692092	0.325478983	0.331270626	0.337193122
Bungoma	0.183966058	0.188976532	0.194331561	0.19989697	0.205729152	0.212233349	0.219077921	0.226297901	0.233611589	0.241051753	0.248877396	0.257122533	0.265410728	0.273662552	0.281599445	0.289281609	0.296687682	0.303245026	0.30916962	0.314806558	0.319902117	0.324822574	0.329336651	0.333755213	0.338064073
Busia	0.15509054	0.157598127	0.160309177	0.163135937	0.166212024	0.169795477	0.173853835	0.178485443	0.183388644	0.188590075	0.194406496	0.200772431	0.207431483	0.214420289	0.221769875	0.229493355	0.237501538	0.245283572	0.253022094	0.260764321	0.268296617	0.275677969	0.282650691	0.289517931	0.296358333
Elgeyo Marakwet	0.148453577	0.154677342	0.161491456	0.16851283	0.175711487	0.183496886	0.191283079	0.199552673	0.207927733	0.216350295	0.225091087	0.23410945	0.243498766	0.253282104	0.263045406	0.272798674	0.282228145	0.290881506	0.2990872	0.307222665	0.315020211	0.322700147	0.330033053	0.337356882	0.344705996
Embu	0.247623894	0.254725354	0.26282842	0.271669522	0.280782404	0.290301355	0.299741997	0.309233202	0.31850306	0.327419341	0.335940876	0.344285675	0.352114896	0.359559153	0.366323376	0.372525502	0.378069856	0.382859621	0.387355614	0.39192078	0.396238366	0.400489568	0.404374282	0.408165465	0.411846168
Garissa	0.112443915	0.115487621	0.118779518	0.122079064	0.125258174	0.12834887	0.13137681	0.13434826	0.137183805	0.14004957	0.142895758	0.145907273	0.148867444	0.152005725	0.155249281	0.158613685	0.162075617	0.165442493	0.16880049	0.172383212	0.176036287	0.179871369	0.183679778	0.187553513	0.191494598
Homa Bay	0.15340213	0.155532102	0.157993462	0.160834996	0.164081657	0.168075831	0.172604795	0.177734483	0.18324038	0.189105132	0.195361712	0.202121549	0.209273259	0.216528155	0.22378737	0.231035423	0.238208688	0.24496969	0.25161778	0.257939844	0.263911807	0.269616851	0.274818633	0.280234259	0.285549547
Isiolo	0.156953518	0.160699796	0.164843556	0.169165474	0.173458017	0.178035261	0.182802512	0.187682537	0.192600079	0.19770441	0.202974916	0.208825474	0.21482591	0.221111145	0.227492433	0.233789194	0.240136919	0.246029388	0.251700393	0.257510266	0.263085837	0.268767424	0.27424321	0.279685288	0.28506706
Kajiado	0.174150902	0.180609734	0.1876964	0.195207233	0.203126202	0.211762717	0.220696153	0.229889685	0.239006933	0.248023986	0.256991987	0.266024642	0.274902365	0.283546157	0.291765726	0.299327765	0.306299633	0.312299085	0.317767754	0.323185612	0.328127219	0.333007381	0.337434417	0.341698631	0.34578703
Kakamega	0.188406989	0.195567018	0.203177159	0.21100054	0.218894502	0.22711843	0.235429846	0.243853059	0.252181498	0.260419133	0.268702334	0.27712109	0.285559385	0.293894269	0.301744877	0.309166492	0.316082197	0.322142025	0.327535645	0.332549378	0.3369821	0.341248814	0.345135014	0.349037071	0.352837825
Kericho	0.172186207	0.179265135	0.186938121	0.195086113	0.203666051	0.212919113	0.222478376	0.232160063	0.241646827	0.250837826	0.25988287	0.269120461	0.278183899	0.286859759	0.294820936	0.30207479	0.308864481	0.314698953	0.319936672	0.325052651	0.329781843	0.334690188	0.339360909	0.344219039	0.349340188
Kiambu	0.271410553	0.279151411	0.287633754	0.296815005	0.306328694	0.31638509	0.326358843	0.33647719	0.346341954	0.356191801	0.365904212	0.375631037	0.385035958	0.394272917	0.402763587	0.410469356	0.417320566	0.422914458	0.427542947	0.43178245	0.435489619	0.439143408	0.442421965	0.445771814	0.449176001
Kilifi	0.163009109	0.167489091	0.172482288	0.177853152	0.183637355	0.189921981	0.196255583	0.20275536	0.209211165	0.215751634	0.222592408	0.229801457	0.23710253	0.24435425	0.251346061	0.258065918	0.264638143	0.270643202	0.276129217	0.281366995	0.286267669	0.29116174	0.295678144	0.299911897	0.303960339
Kirinyaga	0.253551455	0.26399321	0.275209771	0.286819711	0.29852601	0.310597491	0.32233492	0.333969272	0.345168409	0.355805401	0.365849228	0.37535115	0.384165083	0.392382789	0.399793479	0.406480966	0.412135271	0.416486334	0.419920108	0.42311659	0.425837437	0.428359335	0.430413807	0.432451874	0.434515702
Kisii	0.192334474	0.198578393	0.205253999	0.212356721	0.219766789	0.227818236	0.236341256	0.245319976	0.254611058	0.264410247	0.274527168	0.285131262	0.295698624	0.306058069	0.315843761	0.325030301	0.333612106	0.341175248	0.347928262	0.354020159	0.359278054	0.36421181	0.368447746	0.372632803	0.376657104
Kisumu	0.183451624	0.187619196	0.192366207	0.197742195	0.203700039	0.210614717	0.218202813	0.22645006	0.235120671	0.244269329	0.254030598	0.264323115	0.274879052	0.285585696	0.296147159	0.306497721	0.316416331	0.325436172	0.333735869	0.341396111	0.348410666	0.354855748	0.360556843	0.366052267	0.371220317
Kitui	0.178075475	0.183641839	0.189732851	0.196071468	0.202511671	0.209587812	0.21657336	0.223701002	0.230817236	0.237988867	0.245292072	0.252683238	0.260057574	0.2674577	0.274621277	0.281505982	0.287870185	0.293429062	0.298578876	0.303634717	0.30832271	0.312815604	0.316995208	0.321138962	0.325151708
Kwale	0.173289396	0.178990585	0.185235521	0.191663041	0.198196809	0.204977972	0.211753982	0.218607907	0.225449793	0.232351547	0.23945964	0.246767511	0.253972602	0.261138168	0.267960035	0.27463023	0.281139161	0.287040655	0.29245678	0.297643945	0.302320604	0.306849191	0.310865037	0.314636206	0.318185739
Laikipia	0.254005196	0.261503774	0.269780296	0.278503097	0.287427552	0.29674231	0.305947139	0.315119747	0.323936213	0.332373553	0.340604787	0.348656147	0.35621787	0.363280933	0.3696249	0.375311824	0.380289937	0.384354573	0.387921611	0.391472315	0.394764889	0.39812225	0.401319836	0.404635168	0.408182913
Lamu	0.187007053	0.19414701	0.201933134	0.209999507	0.218168258	0.226704108	0.234905144	0.243285082	0.251570949	0.259907312	0.268372765	0.276780561	0.285014996	0.293151479	0.301040321	0.308688861	0.315858991	0.32230194	0.328268032	0.334149911	0.339731428	0.345165422	0.350113364	0.354774515	0.359235424
Machakos	0.24592089	0.252472139	0.259633199	0.267173924	0.274878058	0.283022954	0.291234314	0.299555246	0.307747818	0.315817368	0.323713929	0.331695765	0.339366789	0.346921675	0.354025134	0.36059665	0.366629546	0.371698098	0.376123795	0.380260963	0.383958354	0.387674486	0.391111035	0.394660229	0.398261804
Makueni	0.192577937	0.198000294	0.20394572	0.210219624	0.216573147	0.223399656	0.230103486	0.236951599	0.243770313	0.250769435	0.257920778	0.265316079	0.272710956	0.280299207	0.287869082	0.295311685	0.302407578	0.308792118	0.31480265	0.320786566	0.326346147	0.331794835	0.336949064	0.342080128	0.347085149
Mandera	0.089137525	0.090202804	0.091553311	0.093036361	0.094543744	0.096072984	0.097545777	0.099010014	0.100443519	0.101929524	0.10351639	0.105272757	0.107022402	0.108902733	0.110820447	0.112796963	0.114710494	0.116499203	0.118206025	0.120081361	0.12206423	0.124250297	0.126571695	0.129153551	0.131879035
Marsabit	0.142338741	0.145296613	0.148697318	0.152331596	0.155988805	0.159929416	0.163920008	0.167991884	0.171998543	0.17608228	0.180277027	0.184817004	0.189424395	0.194265763	0.1992139	0.204167587	0.209181919	0.213861832	0.218392609	0.223077631	0.22765943	0.232422125	0.237073522	0.241817875	0.246536497
Meru	0.209399585	0.215108986	0.221294907	0.227753129	0.23433083	0.241294302	0.248142175	0.255171428	0.262121307	0.269159233	0.276371391	0.283939234	0.291623119	0.299436952	0.306883664	0.313881707	0.32028248	0.325855052	0.330852571	0.335717435	0.340262064	0.344674977	0.348596344	0.35240216	0.356104582
Migori	0.16351009	0.163611745	0.163850392	0.164174217	0.164682375	0.165733695	0.166964251	0.168644063	0.170608313	0.172961301	0.17593511	0.179628702	0.184118941	0.189040117	0.194255493	0.199865536	0.205883867	0.211993161	0.218439856	0.224884551	0.231356707	0.237868705	0.244064712	0.25054333	0.257141704
Mombasa	0.304050493	0.310865206	0.318460539	0.326247202	0.333925899	0.341247048	0.348735053	0.355944232	0.36265609	0.369132049	0.375262981	0.382012034	0.38860328	0.395288753	0.401781793	0.407873141	0.413952754	0.419216297	0.423935689	0.428567486	0.43261654	0.436912702	0.440749575	0.444374099	0.447876932
Murang'a	0.238904792	0.245801808	0.253359333	0.261154785	0.269182034	0.27790802	0.286654529	0.295792354	0.30502728	0.314455807	0.323857526	0.333301676	0.342526199	0.351610785	0.360113199	0.367814565	0.374654381	0.380424549	0.385632142	0.390711094	0.395411575	0.399939801	0.404054967	0.408210411	0.412402098
Nairobi	0.420196905	0.426523817	0.433874454	0.441755447	0.449571536	0.457437148	0.465384429	0.473213237	0.480595093	0.487522277	0.494005989	0.500590704	0.506722207	0.512556629	0.517878489	0.522778723	0.527295829	0.530936286	0.534057641	0.537299975	0.540339994	0.543621476	0.546664333	0.549828568	0.553306654
Nakuru	0.229482289	0.235456755	0.242215448	0.249544183	0.257183794	0.265388492	0.273946621	0.28297469	0.292161707	0.30149479	0.310987503	0.320762573	0.330303293	0.339587866	0.348047252	0.355830725	0.362978684	0.36906454	0.374535834	0.379914658	0.384788103	0.38972562	0.394251801	0.398727016	0.403222967
Nandi	0.161190714	0.166670648	0.172820158	0.179444288	0.186664618	0.194876825	0.203490024	0.212550563	0.221701565	0.231019915	0.240787547	0.250827789	0.260995077	0.271054296	0.280734474	0.289951784	0.298532129	0.30628018	0.313513689	0.320512487	0.327070198	0.333285169	0.338996734	0.344421211	0.349670131
Narok	0.126970116	0.129888638	0.133182111	0.136822473	0.14093859	0.145924473	0.151375103	0.157316811	0.163431522	0.169780678	0.17658345	0.18366651	0.191084128	0.198602392	0.205935266	0.213001059	0.219832858	0.226263942	0.232615568	0.23909751	0.245397357	0.251663101	0.257634254	0.263614734	0.269653794
Nyamira	0.234855834	0.240592483	0.24690659	0.25366573	0.260733589	0.268587831	0.276409158	0.284603445	0.292840779	0.301185382	0.309853265	0.318664864	0.327577727	0.336526857	0.345262475	0.353838595	0.361933874	0.369320218	0.376132473	0.382747587	0.388888308	0.394719451	0.399922482	0.40508713	0.410234682
Nyandarua	0.247899354	0.254966448	0.262658687	0.270806101	0.27924605	0.288322398	0.297598109	0.307055364	0.316416501	0.325683679	0.334730192	0.343686144	0.352033917	0.35999466	0.367359942	0.374020323	0.380029956	0.384965144	0.389294058	0.393686702	0.397903926	0.402347268	0.406838521	0.411643022	0.416676136
Nyeri	0.278329142	0.286052435	0.294625503	0.303648566	0.31287664	0.322643467	0.332182607	0.341843909	0.351300511	0.360525007	0.36952597	0.378315877	0.386595329	0.394602642	0.402145388	0.409157218	0.41528834	0.420285295	0.424671172	0.428909275	0.432821312	0.436575649	0.439918068	0.44321536	0.446406337
Samburu	0.133640094	0.136014677	0.138989162	0.142340323	0.145998958	0.150294813	0.154852935	0.159891977	0.165094918	0.170519784	0.17610954	0.181870308	0.187600441	0.193284976	0.198754862	0.203841145	0.208472034	0.212419411	0.216068502	0.219785184	0.223259402	0.226828881	0.230249271	0.233745767	0.237228318
Siaya	0.153057026	0.155213327	0.157668475	0.160470611	0.163614265	0.167487797	0.171861031	0.176841416	0.182192285	0.187961113	0.194154516	0.200866775	0.207909631	0.215233254	0.222584636	0.229809712	0.236939692	0.243607305	0.250120001	0.25651642	0.262582972	0.26846617	0.273755658	0.279084929	0.284349415
Taita Taveta	0.231723575	0.238917332	0.246846509	0.25499766	0.263054627	0.271085595	0.278938765	0.286783245	0.294354161	0.301672219	0.30885465	0.316178974	0.323300556	0.330344993	0.337120889	0.343602499	0.349733192	0.355083819	0.359999178	0.364840536	0.369378824	0.374013622	0.378267827	0.382338517	0.386231377
Tana River	0.144069302	0.149806503	0.156038557	0.162400833	0.168693915	0.175138509	0.181102877	0.187082025	0.192875301	0.19858754	0.204464829	0.210335021	0.216001761	0.221514491	0.226808385	0.231789736	0.236304822	0.240055184	0.24317251	0.246068031	0.248472631	0.250663384	0.252335202	0.253672818	0.254820981
Tharaka Nithi	0.203028869	0.20928023	0.21605867	0.22311847	0.230197757	0.23768833	0.244893023	0.252168184	0.259369288	0.26658971	0.274000809	0.281525243	0.289015826	0.296570579	0.303993784	0.311294385	0.318161516	0.324406452	0.330220761	0.336010206	0.341489728	0.346834873	0.351980976	0.357078214	0.362093357
Trans Nzoia	0.199189767	0.204668503	0.21083984	0.217713794	0.225345135	0.233721204	0.242622495	0.251835264	0.261012526	0.270173693	0.279378921	0.289042217	0.298861293	0.308639353	0.317779548	0.326157148	0.334040233	0.341036141	0.347356539	0.353497308	0.359018313	0.364477075	0.369464613	0.374460727	0.379496401
Turkana	0.11715434	0.120508122	0.124207914	0.12801663	0.13182525	0.13575086	0.139757471	0.143862565	0.147898008	0.151968486	0.156135941	0.160644336	0.165279478	0.170006889	0.174763237	0.17963154	0.184595301	0.189320069	0.194012228	0.198911833	0.203689386	0.208625628	0.213444575	0.218359032	0.223219564
Uasin Gishu	0.223698765	0.229293629	0.23572238	0.242843231	0.250592241	0.259156457	0.268211173	0.277730844	0.287286896	0.296679081	0.305913212	0.315379704	0.324827762	0.334116164	0.342895404	0.351121368	0.358988709	0.365838113	0.371976508	0.378017822	0.38359007	0.389104903	0.394281318	0.399377787	0.404554879
Vihiga	0.194222883	0.198288066	0.203038674	0.208286058	0.213835536	0.219984353	0.226352083	0.233134674	0.239942444	0.246803837	0.253985319	0.261459724	0.269070502	0.276888599	0.284792132	0.292847673	0.300805202	0.308182414	0.315385132	0.322616471	0.329540915	0.336348736	0.342660257	0.348901362	0.355161749
Wajir	0.089263315	0.09012498	0.091307773	0.092558026	0.093840928	0.095155211	0.09652116	0.097980484	0.099507489	0.10120275	0.103018545	0.105072456	0.107176078	0.109513048	0.112029517	0.114632781	0.117329999	0.120087561	0.122832208	0.125758988	0.128681816	0.131747473	0.134866156	0.138041871	0.141228778
West Pokot	0.138942685	0.141529775	0.14449126	0.147617644	0.150970196	0.154953273	0.159231591	0.164066749	0.169214462	0.174725424	0.180815556	0.187386652	0.194322905	0.201518969	0.208629827	0.215643183	0.22239318	0.228657488	0.234625908	0.240556788	0.24610384	0.251641623	0.256799193	0.261983743	0.267220236
Madagascar	0.224364376	0.229138085	0.233984192	0.238554886	0.243625659	0.248740284	0.253199335	0.25733887	0.261389632	0.265181337	0.268729859	0.272245373	0.27493821	0.276894373	0.278469868	0.279889465	0.280429589	0.280501283	0.280204299	0.279615149	0.279164003	0.279066493	0.27942619	0.280317671	0.281571072
Malawi	0.163521183	0.161866794	0.16141968	0.16240672	0.164406043	0.167332577	0.170559716	0.174250485	0.178326605	0.182866386	0.188013131	0.192737822	0.196782403	0.19976217	0.202176537	0.204010245	0.206043786	0.206732359	0.208091205	0.209144566	0.211756232	0.215264475	0.219416252	0.22312233	0.227068368
Mozambique	0.147310977	0.148938409	0.150514167	0.152049243	0.153658119	0.155480821	0.15750112	0.159426011	0.160764746	0.162321076	0.163467878	0.164421754	0.165677828	0.167687994	0.170385624	0.173064715	0.175550435	0.177285188	0.179851807	0.182236861	0.183427982	0.185705869	0.188650436	0.191998629	0.195435288
Rwanda	0.190882409	0.196180607	0.201193029	0.206106935	0.211456221	0.216856479	0.222519039	0.228211303	0.23489809	0.241708418	0.24876074	0.255813078	0.262222198	0.267799955	0.272186501	0.27509719	0.276194698	0.277178171	0.276980133	0.272331258	0.271102227	0.271097922	0.272455861	0.274582551	0.277014387
Somalia	0.085933189	0.086085228	0.086754222	0.086712477	0.083293455	0.07729974	0.071284131	0.065264853	0.052358579	0.045635023	0.046329677	0.046955609	0.047526878	0.048075003	0.048487257	0.048848564	0.049422382	0.050115531	0.050797999	0.051471696	0.052148606	0.052840548	0.053570967	0.054321527	0.055071838

South Sudan	0.17416594	0.176247424	0.178355506	0.180584835	0.182848586	0.18499318	0.187086568	0.189151775	0.191186739	0.193248801	0.195339107	0.197455978
Uganda	0.156667288	0.159955496	0.163162729	0.165810519	0.167545891	0.168939194	0.170220792	0.17197761	0.174114877	0.175779494	0.177277786	0.178704665
Tanzania	0.189416143	0.195222066	0.20092684	0.206860651	0.212946957	0.218793687	0.223822893	0.228577205	0.233472452	0.238394237	0.243390113	0.246973661
Zambia	0.225132847	0.23339648	0.241021409	0.248330434	0.255397912	0.262146724	0.268445634	0.273754157	0.278707803	0.283449248	0.287974975	0.291668283
Southern sub-Saharan Africa	0.410064413	0.415428545	0.420670714	0.426039805	0.431643183	0.437878768	0.444376459	0.450832332	0.457289296	0.464337594	0.471528002	0.47876299
Botswana	0.253754384	0.262901794	0.272010106	0.281306853	0.291007345	0.301238382	0.311653552	0.322403679	0.333497633	0.344932802	0.356544997	0.368358538
Eswatini	0.270673543	0.280126898	0.288898816	0.29701118	0.304938423	0.313046076	0.321494815	0.32963802	0.33766489	0.345593484	0.35331633	0.362157318
Lesotho	0.254183149	0.258592265	0.26496141	0.27331445	0.279600436	0.285263691	0.290866075	0.296302355	0.30124452	0.306288132	0.311268468	0.316191879
Namibia	0.355673184	0.363398527	0.370812125	0.37817338	0.385518333	0.393267047	0.400604532	0.407761406	0.414409125	0.420864298	0.427145532	0.432864673
South Africa	0.447607469	0.45280142	0.457869286	0.463068315	0.468534307	0.474595547	0.480811757	0.486891496	0.492903948	0.499575429	0.506276765	0.513035041
Eastern Cape	0.392262317	0.397753151	0.403222403	0.408884084	0.414822434	0.421251948	0.427597794	0.433743386	0.439848083	0.446684351	0.453511261	0.460116823
Free State	0.43813168	0.443589544	0.449223914	0.455191321	0.46160804	0.468611896	0.475876105	0.483256329	0.490774734	0.499008051	0.50721678	0.515385339
Gauteng	0.567665159	0.571469747	0.575107948	0.578913779	0.582904305	0.587522324	0.592364341	0.596934541	0.601223861	0.605802051	0.61028279	0.6148696
KwaZulu-Natal	0.408976052	0.41508104	0.420844995	0.426636204	0.432655621	0.439259327	0.44611958	0.452818288	0.459474646	0.466800583	0.474080705	0.481537168
Limpopo	0.314124959	0.321322894	0.328470565	0.335741056	0.343436975	0.351872589	0.360323524	0.368784093	0.377293617	0.386779123	0.396506659	0.406146899
Mpumalanga	0.350870518	0.35865684	0.366484154	0.374549603	0.382850932	0.39171601	0.400731252	0.409591628	0.418323356	0.427686484	0.437314688	0.447223304
North West	0.413384999	0.42032217	0.426914195	0.433174193	0.439406314	0.44599619	0.45260817	0.45892953	0.465041659	0.471693274	0.478251676	0.484792636
Northern Cape	0.439276342	0.444543248	0.449792444	0.455259892	0.461169385	0.467625332	0.474212273	0.480528822	0.486509629	0.492676465	0.498458412	0.504112385
Western Cape	0.56138495	0.565653229	0.569796632	0.574181533	0.578795541	0.584087463	0.58938858	0.594314233	0.59886572	0.603756888	0.608423096	0.612888711
Zimbabwe	0.278660062	0.284986278	0.290473906	0.295849558	0.301229991	0.307586744	0.31549716	0.323969234	0.333184161	0.342525996	0.353062717	0.363804263
Western sub-Saharan Africa	0.210238435	0.214964816	0.220045325	0.225011911	0.229920409	0.234605172	0.239142507	0.243652901	0.247983094	0.252146412	0.256639907	0.260019835
Benin	0.164057652	0.167511029	0.171022328	0.174298691	0.177894107	0.182073262	0.18613468	0.189903259	0.193306598	0.197043535	0.201241584	0.205009596
Burkina Faso	0.087391264	0.089244883	0.091220197	0.093615271	0.096248548	0.098939757	0.101922491	0.1047398	0.107267216	0.109781399	0.112819662	0.116362838
Cabo Verde	0.208107033	0.210519141	0.21259042	0.215359345	0.218148297	0.221190507	0.224441268	0.22752595	0.231413695	0.235909377	0.241388352	0.247513927
Cameroon	0.203793674	0.207467405	0.211927787	0.21707417	0.222499129	0.228182409	0.234558379	0.241358816	0.248550257	0.25632889	0.26442889	0.272644284
Chad	0.087196718	0.088826606	0.090297844	0.091743729	0.092512392	0.093231451	0.093570212	0.094182397	0.095722338	0.097566097	0.099944643	0.102492739
Côte d'Ivoire	0.194954818	0.200111668	0.205511067	0.211015954	0.216170001	0.220707978	0.225604629	0.230580398	0.235554303	0.240561345	0.245928769	0.251723777
The Gambia	0.156508915	0.159478849	0.162966681	0.167174471	0.171685579	0.176349554	0.181480154	0.187276053	0.193338953	0.199514073	0.205952582	0.212594339
Ghana	0.293782369	0.29814996	0.303505936	0.309240556	0.314862471	0.320540172	0.326042847	0.330983697	0.335422922	0.340080442	0.345138376	0.350321836
Guinea	0.135044771	0.139990419	0.144068569	0.147319789	0.150239704	0.153039529	0.155521643	0.157862847	0.160001275	0.162090516	0.164245337	0.166563994
Guinea-Bissau	0.166415868	0.169131934	0.170882408	0.173219277	0.175590916	0.177267328	0.178788779	0.180760779	0.182806508	0.185380478	0.188091224	0.191165051
Liberia	0.200597006	0.202671462	0.20474926	0.207070689	0.209735257	0.212220835	0.214308455	0.216560025	0.218998805	0.221952339	0.225281944	0.228638523
Mali	0.097407465	0.099870306	0.102366134	0.104579212	0.107316074	0.109480843	0.111469771	0.113064451	0.114592341	0.11603076	0.117667881	0.11939356
Mauritania	0.256142667	0.261351661	0.266558394	0.271200337	0.276413585	0.281708634	0.287388842	0.292931607	0.298524537	0.30402263	0.309437661	0.314858148
Niger	0.059853476	0.060273959	0.060873872	0.061712737	0.062948488	0.064292405	0.065781826	0.067408308	0.069064019	0.070379493	0.071859445	0.073575556
Nigeria	0.241147042	0.247164144	0.253471946	0.259372952	0.265077574	0.270477524	0.275558016	0.280520775	0.285218633	0.289577847	0.29422275	0.296770098
São Tomé and Príncipe	0.250451427	0.256276983	0.262732297	0.268998104	0.275509189	0.28089326	0.28535751	0.289950539	0.293890622	0.297036006	0.300003717	0.302125129
Senegal	0.151085341	0.1537951	0.157069167	0.161090175	0.166094169	0.17138167	0.177045386	0.183613079	0.190316373	0.196660447	0.203335964	0.210380872
Sierra Leone	0.173267164	0.176005419	0.178697693	0.1814183	0.184194891	0.186617996	0.189205058	0.191924712	0.19435178	0.196951233	0.199207385	0.201507763
Togo	0.1963377	0.201326882	0.207012904	0.213363712	0.219537913	0.22542915	0.230636914	0.235457234	0.239670271	0.243802881	0.247976713	0.252098084

South Sudan	0.199655711	0.201950711	0.204299398	0.2066565	0.208932716	0.211199401	0.213416471	0.215612089	0.217920463	0.220399437	0.223037171	0.225788869	0.228648585
Uganda	0.180527084	0.182563402	0.18477309	0.187001096	0.188924156	0.191003433	0.193557456	0.197293193	0.201835423	0.207080731	0.213091696	0.219712442	0.226916945
Tanzania	0.249728841	0.252959039	0.256009594	0.259306074	0.262416238	0.264989448	0.267254915	0.269417204	0.272078202	0.275507909	0.279259184	0.28315261	0.287381679
Zambia	0.295400772	0.299090218	0.301726863	0.304008549	0.30584236	0.307316277	0.308780208	0.309223449	0.309995813	0.311326691	0.31308733	0.314926435	0.317396437
Southern sub-Saharan Africa	0.486127133	0.493648609	0.500850123	0.506946633	0.512377786	0.517569473	0.522772503	0.528063317	0.533379781	0.538780402	0.544134279	0.549141665	0.553884659
Botswana	0.38035963	0.392833119	0.405752857	0.418077748	0.429752286	0.440490843	0.450523614	0.459954967	0.469266513	0.478588352	0.487767857	0.496193622	0.504978826
Eswatini	0.371573631	0.380768275	0.389954206	0.399420955	0.408269969	0.416683379	0.424802995	0.432503472	0.440127047	0.447399238	0.454089854	0.460136984	0.465990845
Lesotho	0.321010757	0.326632174	0.332783906	0.339155125	0.345496571	0.35216963	0.358780457	0.365310801	0.371265315	0.377221202	0.383096648	0.388406478	0.393495001
Namibia	0.437887002	0.442267806	0.446320825	0.450040233	0.45453444	0.460021314	0.465734135	0.471873217	0.477794329	0.483116378	0.488012938	0.492812548	0.497613862
South Africa	0.520198788	0.527869022	0.535352269	0.541571435	0.546921136	0.552066899	0.557198439	0.562448636	0.567905304	0.573453988	0.579099278	0.584437313	0.589407349
Eastern Cape	0.46703156	0.474378483	0.481422094	0.487156629	0.491945768	0.496558751	0.501227233	0.505989096	0.51093299	0.516048444	0.521361921	0.526467367	0.531267891
Free State	0.523883998	0.532653336	0.54083705	0.547462374	0.552883824	0.557829772	0.562617174	0.567353036	0.572049629	0.576614774	0.581126929	0.585306812	0.58910135
Gauteng	0.619842516	0.625331393	0.630677378	0.634850535	0.638354675	0.641818295	0.64537204	0.649149055	0.653144945	0.657292104	0.661432136	0.6651077	0.668250714
KwaZulu-Natal	0.489474205	0.497972497	0.506410146	0.513597109	0.519883128	0.525872525	0.531685065	0.537429182	0.54332559	0.549247727	0.555189823	0.560854845	0.566177928
Limpopo	0.416070449	0.426366667	0.436395992	0.445061873	0.452611513	0.459856043	0.467197201	0.474783033	0.482535902	0.490389071	0.498458945	0.506234473	0.513697542
Mpumalanga	0.457648449	0.468632807	0.479294211	0.48855668	0.497037138	0.505426137	0.513684302	0.521975611	0.53017023	0.537754564	0.544821961	0.551184002	0.556967812
North West	0.491593642	0.498774937	0.505784734	0.511582018	0.516519186	0.521134664	0.525462575	0.529596926	0.533738699	0.537988641	0.542500563	0.546909151	0.551071976
Northern Cape	0.51010346	0.516622896	0.523012152	0.528126699	0.532204689	0.535917002	0.539584307	0.543479865	0.547881717	0.552713272	0.558067867	0.563489877	0.568810429
Western Cape	0.617548244	0.622537018	0.627208488	0.630621788	0.633124877	0.635137853	0.636892409	0.638716432	0.640890698	0.643456189	0.646366031	0.649109334	0.651688918
Zimbabwe	0.373574249	0.38270234	0.39108167	0.398559341	0.406052877	0.412083826	0.4179742	0.424051564	0.42892904	0.434187192	0.438141988	0.441093135	0.443246604
Western sub-Saharan Africa	0.263174796	0.266492683	0.270006039	0.273700004	0.277410732	0.281024681	0.284431723	0.287699583	0.2909832	0.294618466	0.298302443	0.302025797	0.305809779
Benin	0.208478229	0.21192808	0.21520582	0.218907154	0.222539019	0.226323465	0.230312594	0.234032036	0.238009696	0.241895323	0.245731534	0.249376451	0.253215341
Burkina Faso	0.119648728	0.123151322	0.12649486	0.129695615	0.133299128	0.136594798	0.139825465	0.142848973	0.146115673	0.150041686	0.154228401	0.158707048	0.163468067
Cabo Verde	0.254448606	0.261898837	0.269643143	0.276723367	0.282931722	0.288460371	0.294517286	0.302216794	0.310675634	0.319736229	0.329049585	0.338786069	0.349296918
Cameroon	0.280776182	0.288503108	0.295986728	0.303055334	0.309623458	0.315601269	0.320831654	0.325410123	0.329539318	0.333425982	0.337346001	0.34119709	0.344955364
Chad	0.105188296	0.108321744	0.11144706	0.114638829	0.117954634	0.121132832	0.123600072	0.126080572	0.128289492	0.130169484	0.132136021	0.134090304	0.135825261
Côte d'Ivoire	0.257841066	0.264707492	0.272091406	0.279320353	0.286492681	0.293451293	0.299743215	0.305210653	0.310105508	0.314965385	0.319843979	0.324712873	0.329201009
The Gambia	0.219257207	0.225801621	0.232259101	0.238714846	0.245010164	0.251087863	0.256996141	0.262424792	0.267696507	0.27295019	0.278274122	0.283946056	0.290070621
Ghana	0.355633384	0.361390606	0.367212438	0.373112005	0.379239256	0.384856767	0.389968494	0.394953663	0.399930204	0.405003047	0.410038073	0.414888532	0.419755496
Guinea	0.169153216	0.171971433	0.175059715	0.178295421	0.181459391	0.184485016	0.187686242	0.191128837	0.194573245	0.198633755	0.203280449	0.208003383	0.212412705
Guinea-Bissau	0.194698741	0.198634825	0.202947615	0.207614839	0.212505984	0.217026088	0.221678978	0.226434933	0.231223526	0.236106374	0.241360584	0.244371797	0.248217317
Liberia	0.232421172	0.234779324	0.234657513	0.235296847	0.238212218	0.238283313	0.235984406	0.232656774	0.228424478	0.222682591	0.219672765	0.219053855	0.222212867
Mali	0.120964591	0.12244413	0.124371032	0.126526428	0.12912815	0.131537449	0.134177054	0.136937829	0.139866225	0.143078916	0.146414326	0.14998673	0.153737336
Mauritania	0.320254175	0.325337242	0.33051367	0.335780942	0.341135175	0.346531288	0.352176572	0.357585912	0.363037695	0.368508328	0.373021339	0.37708025	0.381112297
Niger	0.075272959	0.077138716	0.079005944	0.08086848	0.082848208	0.084771578	0.08669649	0.088608733	0.090473708	0.092346359	0.094219115	0.096545153	0.098813686
Nigeria	0.298775472	0.300866912	0.303207394	0.305868047	0.308489225	0.311234052	0.31398306	0.316747447	0.319637439	0.323137685	0.326690553	0.330263851	0.3339365
São Tomé and Príncipe	0.304114405	0.306225211	0.308205942	0.309542852	0.310601028	0.311515447	0.312743558	0.314261087	0.31584392	0.317729288	0.31979567	0.322519219	0.325651916
Senegal	0.217430721	0.224495629	0.231316215	0.238047613	0.244482178	0.250416532	0.255767964	0.260379997	0.264438773	0.268318965	0.272072875	0.275995656	0.280111918
Sierra Leone	0.204165566	0.206994948	0.209611451	0.211569335	0.213013567	0.21422304	0.216089201	0.218277411	0.219822604	0.21996927	0.219089791	0.218584313	0.217943042
Togo	0.25571614	0.260086689	0.264564939	0.269692273	0.274530568	0.27878931	0.281224631	0.284653387	0.288536692	0.292606677	0.296947835	0.300022333	0.303020232

Appendix Table S17: Socio-demographic Index values for all estimated GBD 2021 locations, 2000-2021

Location	2000	2001	2002	2003	2004	2005	2006	2007	2008	2009	2010	2011	2012	2013	2014	2015	2016	2017	2018	2019	2020	2021
Global	0.56800316	0.57177753	0.57568796	0.57990708	0.58437859	0.58897852	0.59375662	0.59864137	0.6033701	0.60775944	0.6129525	0.61775922	0.62184498	0.62634328	0.63098237	0.63544149	0.64015359	0.6455643	0.65104847	0.6565759	0.66134354	0.66582097
Central Europe, eastern Europe, and central Asia	0.68204328	0.68616546	0.69041062	0.69548048	0.70141441	0.70707179	0.71222406	0.71722196	0.72255514	0.72727958	0.73207766	0.73607829	0.73967451	0.74328837	0.74665736	0.74987717	0.75335999	0.75709847	0.76076037	0.76422712	0.76683705	0.76946635
Central Asia	0.58215554	0.5866458	0.59145711	0.59652977	0.60173672	0.60729382	0.61307828	0.61910458	0.62493038	0.63010289	0.6352956	0.64031356	0.64467352	0.64888814	0.65299988	0.65681783	0.66031242	0.66357678	0.66666645	0.6698373	0.67248671	0.67516398
Armenia	0.57943757	0.5854193	0.59271061	0.60080917	0.60903177	0.61775963	0.62668834	0.63587098	0.64443159	0.64971484	0.65481153	0.65981809	0.66483314	0.6695345	0.67393219	0.67805463	0.6817464	0.68598027	0.69027687	0.69476596	0.6982689	0.70183319
Azerbaijan	0.58002057	0.58219371	0.58571614	0.59003871	0.59464608	0.60248519	0.6137711	0.62611093	0.63690111	0.64516334	0.65205651	0.65797163	0.662897	0.66765878	0.67216274	0.67624491	0.67980764	0.6832632	0.68656109	0.68983471	0.69260519	0.69485127
Georgia	0.63026599	0.6333883	0.63837011	0.64406558	0.64892098	0.653616	0.65840337	0.66369797	0.6684425	0.67244938	0.67703489	0.68156704	0.68551922	0.68860327	0.69215521	0.69731613	0.70373149	0.71087472	0.71799365	0.72453646	0.72912589	0.7324736
Kazakhstan	0.64468962	0.64839726	0.65152934	0.65483593	0.65873307	0.66294394	0.66704886	0.67136721	0.67635287	0.68160684	0.68699664	0.69175895	0.69531277	0.69905359	0.70351192	0.70822974	0.71256904	0.71602669	0.718532	0.7207976	0.72278877	0.7251445
Kyrgyzstan	0.54303649	0.54380555	0.54437467	0.54564926	0.54726234	0.54826045	0.54938359	0.55114619	0.55320638	0.55512387	0.55641654	0.55892333	0.56154157	0.56577677	0.57056936	0.57546323	0.58038855	0.5857661	0.59123713	0.59650164	0.60049955	0.60397933
Mongolia	0.52665034	0.5326202	0.53842483	0.54397165	0.5495515	0.55468028	0.55956114	0.5642368	0.5685006	0.57134169	0.57440667	0.57860066	0.58325589	0.58808081	0.59286735	0.59685965	0.60026077	0.60372196	0.60750183	0.61125068	0.61462964	0.61762156
Tajikistan	0.45706133	0.45625651	0.45880778	0.46332971	0.46915421	0.47463779	0.47965183	0.48421116	0.48844114	0.49206404	0.49589396	0.4998698	0.50395388	0.50797068	0.51198952	0.51578324	0.519897	0.52449261	0.52915392	0.53376995	0.53769053	0.54151119
Turkmenistan	0.56742103	0.57104382	0.57559617	0.58082013	0.58671235	0.59338889	0.59989607	0.6060422	0.61192331	0.61765291	0.62323459	0.62923372	0.63526318	0.64136189	0.64754242	0.65335279	0.65894561	0.66428469	0.6694336	0.67430354	0.67829818	0.68216078
Uzbekistan	0.55752231	0.56480338	0.57169014	0.57800654	0.58394299	0.58953078	0.59449806	0.59962805	0.60488916	0.61035175	0.61609791	0.62134462	0.62620213	0.6307964	0.63525705	0.63980564	0.6442306	0.64811935	0.65191814	0.65583696	0.6591242	0.66262169
Central Europe	0.69877971	0.70565678	0.7120006	0.71782364	0.72349425	0.72867409	0.73347644	0.73807667	0.74314784	0.74876572	0.75491949	0.76034929	0.76480327	0.76856771	0.77175547	0.77484401	0.77781725	0.78135932	0.78547633	0.78974898	0.79311112	0.79624445
Albania	0.58466419	0.59241295	0.59986598	0.60697117	0.61386425	0.62067961	0.62748562	0.63416797	0.64076613	0.64681613	0.6526542	0.65819677	0.66355678	0.66873956	0.67411299	0.6796686	0.68503665	0.6903792	0.69556688	0.70028786	0.70379025	0.70684979
Bosnia and Herzegovina	0.61095068	0.62198946	0.63207552	0.6406351	0.64779373	0.65385914	0.65981366	0.66557874	0.67110081	0.6760329	0.6808242	0.68539926	0.68950657	0.69350871	0.69703189	0.70044605	0.70407934	0.70805343	0.7123473	0.71685712	0.72020206	0.72307789
Bulgaria	0.68100452	0.68648737	0.69151167	0.69556596	0.69988195	0.70447121	0.70886829	0.7131924	0.71767245	0.72367663	0.73070174	0.73574928	0.73917752	0.74213595	0.74537264	0.74897906	0.7522592	0.75566586	0.75914846	0.76235979	0.76508971	0.76815094
Croatia	0.7030399	0.70953684	0.71538342	0.72113038	0.72632765	0.73215414	0.73806688	0.74314878	0.7484696	0.75406561	0.75947225	0.76368638	0.76740534	0.77115188	0.77447924	0.77786589	0.78144361	0.78507454	0.78867141	0.79247542	0.79546227	0.79834103
Czechia	0.76776616	0.77216724	0.77692793	0.78244808	0.7871264	0.79133395	0.79492278	0.79825412	0.80176485	0.80496222	0.80874733	0.81205478	0.8142304	0.81561536	0.81622726	0.81656468	0.81715303	0.81906768	0.82203449	0.82478447	0.82662663	0.82845043
Hungary	0.71580528	0.72209906	0.7283846	0.73445066	0.74009997	0.74535681	0.75051538	0.75494294	0.75931747	0.76356369	0.76732865	0.76970194	0.77078425	0.7715617	0.77257505	0.77404738	0.77615031	0.77911642	0.78208366	0.78491031	0.78762039	0.79075477
Montenegro	0.67730685	0.68343345	0.68993948	0.69651751	0.70299828	0.70906546	0.71545907	0.72250394	0.72998176	0.73653893	0.74334787	0.75008033	0.75557838	0.76088691	0.76577653	0.77056128	0.77520077	0.77976463	0.78440897	0.7890269	0.79255439	0.79580058
North Macedonia	0.64165124	0.64804968	0.65539879	0.66340889	0.67123752	0.67804727	0.68404434	0.68972948	0.69537696	0.70045305	0.70558932	0.71059252	0.71517315	0.71967115	0.72408959	0.72860377	0.73317362	0.73733996	0.74133437	0.74519357	0.7478499	0.7506297
Poland	0.70666205	0.7148313	0.72200946	0.72815525	0.73367319	0.73842877	0.74236371	0.74600882	0.75052143	0.75642738	0.76318497	0.76940716	0.77468077	0.77943426	0.78368237	0.78744534	0.79065101	0.79453034	0.79947898	0.80479133	0.80879532	0.81204281
Romania	0.66529435	0.67202017	0.67829742	0.68407493	0.69041705	0.69499895	0.69949077	0.70452373	0.71075713	0.71744512	0.72389135	0.72901827	0.73389248	0.73788047	0.74039547	0.74347353	0.74705871	0.75111737	0.75553333	0.76028397	0.76427644	0.76845386
Serbia	0.6607528	0.66540733	0.67151107	0.67833493	0.68681984	0.69609757	0.70494502	0.7132423	0.72150531	0.7299656	0.74009131	0.75007276	0.75710508	0.76217091	0.76604309	0.76984222	0.77365408	0.77746038	0.78149497	0.7857683	0.78918333	0.79241629
Slovakia	0.73404624	0.74139255	0.74679114	0.75116727	0.75583669	0.76172419	0.76767304	0.77291415	0.7771974	0.78105226	0.78625442	0.79146538	0.79545622	0.79790372	0.7996188	0.80039418	0.80087821	0.80227732	0.8043573	0.80644846	0.80830597	0.81061053
Slovenia	0.77957801	0.78624078	0.79180557	0.79637153	0.80096224	0.80560125	0.8097086	0.81300632	0.81634725	0.81887856	0.82117849	0.82302356	0.82433656	0.82582628	0.82779925	0.82983707	0.83136792	0.83303814	0.83547248	0.83826678	0.84037385	0.84243073
Eastern Europe	0.70101118	0.70350622	0.70672714	0.71202517	0.71921544	0.72626571	0.73261745	0.73878686	0.74545042	0.75107016	0.75660648	0.7610253	0.76542275	0.77033829	0.7750917	0.77966348	0.78480469	0.79001487	0.79448558	0.79813838	0.80050506	0.80285101
Belarus	0.66470582	0.67133327	0.67812178	0.68486856	0.69250214	0.69898548	0.7051191	0.71149656	0.71912087	0.72729278	0.73495463	0.73950433	0.74346884	0.74852932	0.75351148	0.75820574	0.76432822	0.77113147	0.77656896	0.78050802	0.78238156	0.78448471
Estonia	0.73971315	0.74660221	0.7526299	0.75781568	0.76322373	0.76911218	0.77445184	0.78107444	0.78859887	0.79472821	0.80082972	0.80663308	0.81110936	0.81488718	0.81877073	0.82322128	0.82736436	0.83097531	0.8345663	0.83857351	0.84181714	0.84491779
Latvia	0.73095145	0.7362942	0.74187044	0.74816816	0.75526321	0.76192943	0.76845993	0.77648196	0.78632457	0.79450443	0.79817159	0.79908078	0.80102647	0.80266049	0.80372149	0.80698599	0.81146359	0.81692312	0.82141981	0.82478178	0.82790045	0.83066352
Lithuania	0.71989696	0.72713358	0.7337934	0.7412296	0.74894976	0.75635236	0.76305912	0.77022964	0.77827371	0.78568974	0.79336468	0.80092499	0.80782703	0.81321972	0.81788638	0.82311263	0.8293037	0.83587174	0.84191891	0.84790634	0.85275239	0.85648405
Moldova	0.62200493	0.62293641	0.62587484	0.62998504	0.63539482	0.64153097	0.64799626	0.65421585	0.6610466	0.66628439	0.67198962	0.67800414	0.6829798	0.68851917	0.69410456	0.69935517	0.70514454	0.71120664	0.71724443	0.72301165	0.72771054	0.73221488
Russia	0.70864365	0.71057036	0.71346096	0.71887512	0.72630532	0.73359278	0.74006466	0.74619151	0.75262324	0.7579604	0.76332012	0.76763359	0.77192038	0.77652698	0.78061752	0.78491377	0.79033015	0.79566948	0.80010814	0.8036582	0.80601147	0.808536
Ukraine	0.671095	0.67246782	0.67454322	0.67861464	0.68538094	0.6920233	0.69809338	0.70420231	0.71098018	0.71618547	0.72101987	0.72489196	0.7288172	0.73403357	0.73988356	0.74379086	0.74685799	0.75059332	0.75445173	0.75798008	0.75992987	0.76077391
High income	0.78791261	0.79147794	0.79480055	0.7975774	0.80008676	0.80217744	0.80403794	0.80641939	0.80949267	0.8128359	0.81652603	0.820039	0.82331217	0.8264454	0.82954203	0.83297246	0.83654703	0.84019794	0.84388377	0.84753265	0.8498679	0.85188481
Australasia	0.77379116	0.77807392	0.78248002	0.78628529	0.78942762	0.79172173	0.79285655	0.79394958	0.79640196	0.79993153	0.80411233	0.80816063	0.81246356	0.81734922	0.82164728	0.82561103	0.82945687	0.83292442	0.83661889	0.84058158	0.84333343	0.84551406
Australia	0.77022384	0.77466279	0.77936094	0.78360018	0.78701654	0.78961552	0.79115569	0.79253268	0.7952929	0.79917437	0.80356154	0.80752022	0.81164446	0.81645366	0.82084085	0.82486072	0.82855994	0.83179827	0.83535462	0.83931743	0.84205131	0.84425281
New Zealand	0.78765956	0.79142608	0.79451959	0.79628688	0.79817748	0.79910729	0.79845687	0.79844762	0.79966951	0.80172753	0.80513318	0.80976035	0.81495756	0.82014599	0.82390894	0.82746249	0.83184397	0.83630179	0.84059786	0.84453321	0.84739874	0.8494425
High-income Asia Pacific	0.81408902	0.81754476	0.82111049	0.82463581	0.82807376	0.83106214	0.83376633	0.83654611	0.83927964	0.84177063	0.84468204	0.84772196	0.85069345	0.85367141	0.85661156	0.85960261	0.86273318	0.86587024	0.8690726	0.87221884	0.87443725	0.876767
Brunei	0.72155249	0.72853507	0.73534392	0.74157809	0.74731171	0.75248179	0.75731866	0.76164313	0.76571426	0.76960168	0.77321922	0.77663189	0.78021655	0.78401704	0.78773329	0.79136757	0.79498077	0.79830994	0.80143655	0.80449939	0.80742409	0.81023437
Japan	0.822939	0.82472324	0.82693677	0.82939222	0.83203738	0.83428869	0.83622226	0.83829416	0.84030122	0.84213366	0.84436181	0.84675027	0.84906149	0.85135699	0.85369275	0.8560975	0.85869817	0.86139331	0.86423714	0.86714818	0.86910688	0.87124181
Aichi	0.8332455	0.83490721	0.83708961	0.8394057	0.84194956	0.84427199	0.84666045	0.84920441	0.85111704	0.85277674	0.8546368	0.85683271	0.85932579	0.86194822	0.86460482	0.86723678	0.87003729	0.87290738	0.87590019	0.8788931	0.88091865	0.88304884
Akita	0.78054579	0.78279736	0.78541866	0.78816362	0.79094398	0.7931404	0.79441252	0.79573206	0.79717433	0.79884151	0.80086617	0.80326262	0.80585363	0.80861305	0.81154735	0.8144146	0.81764909	0.82098122	0.82435531	0.82767269	0.82984444	0.83224902
Aomori	0.76987495	0.77227942	0.77527108	0.77851107	0.78187887	0.78447708	0.78680775	0.78906722	0.79106378	0.79324665	0.79589154	0.79885447	0.80177429	0.8043424	0.80705578	0.80993558	0.81318129	0.81656442	0.82007856	0.8235253	0.8258641	0.82834421
Chiba	0.82190151	0.82296686	0.82447662	0.82641886	0.82857765	0.83034976	0.83172731	0.83338772	0.83513779	0.83673563	0.83861528	0.84053708	0.84235991	0.84447398	0.84657607	0.8486335	0.85084245	0.85309259	0.85544962	0.85794583	0.85947215	0.86129264
Ehime	0.79488205	0.79715003	0.79948284	0.8020362	0.80478449	0.80663557	0.80764538	0.80872204	0.80990072	0.81141894	0.81355548	0.81615461	0.81822375	0.82040592	0.82277036	0.82550713	0.82868215	0.83195796	0.83543283	0.83887631	0.84113997	0.84350171
Fukui	0.80520343	0.80742616	0.8098834	0.81242651	0.81496141	0.81723055	0.81944211	0.82197871	0.82462179	0.82713517	0.82991351	0.83265551	0.8347411	0.83667757	0.83847613	0.84057593	0.84311726	0.84585546	0.84878473	0.85184471	0.85383607	0.85614258
Fukuoka	0.81482105	0.81667403	0.81896676	0.82151248	0.82408526	0.82607366	0.82711703	0.82834918	0.82969873	0.83135498	0.83363064	0.83594027	0.83803372	0.83992485	0.84181947	0.84386213	0.84614641	0.84860519	0.85138688	0.85430094	0.85623286	0.85834771
Fukushima	0.78182647	0.78360994	0.78646247	0.78964778	0.79327859	0.79625177	0.79860764	0.80083603	0.80285161	0.8048784	0.80708522	0.80894285	0.81102141	0.81340328	0.81636905	0.81945357	0.82303381	0.82699751	0.83125774	0.83539288	0.83816268	0.84094147
Gifu	0.8055828	0.80757215	0.80983054	0.81238671	0.815011	0.81723705	0.81911886	0.82105667	0.82296334	0.82466643	0.8267801	0.8291073	0.83144978	0.83361806	0.83587588	0.83819813	0.84075883	0.84340128	0.8462117	0.84913588	0.85112348	0.85334719
Gunma	0.80504171	0.80644285	0.80891736	0.81196848	0.81493971	0.8172735	0.81911682	0.82122607	0.82330426	0.82547856	0.82826356	0.83123679	0.83399606	0.83686271	0.83994849	0.84315203	0.84645534	0.84973033	0.85313864	0.85656404	0.85880568	0.86111136
Hiroshima	0.82297162	0.82467757	0.82664335	0.82894115	0.83136415	0.83335881	0.83457326	0.8360478	0.83730996	0.83866117	0.8405394	0.8429427	0.84510631	0.84740061	0.8500611	0.85299511	0.85609248	0.85915007	0.86231082	0.86549114	0.86765875	0.8699086
Hokkaidō	0.799858	0.80178408	0.80427382	0.80697344	0.80974588	0.81163841	0.81267846	0.81377925	0.8149429	0.81626318	0.8179953	0.8199283	0.82188928	0.82389077	0.82607466	0.82841474	0.83100338	0.83370273	0.83654063	0.83953173	0.84155411	0.84374398
Hyōgo	0.82789823	0.82919088	0.83063265	0.83210027	0.83380631	0.83513412	0.83627039	0.83752276	0.838957	0.84023015	0.84229974	0.84448463	0.84658012	0.84878255	0.85117382	0.85366344	0.85625707	0.85889748	0.86159612	0.86434714	0.8661734	0.86817016
Ibaraki	0.80498668	0.80652655	0.80886007	0.81155625	0.81427517	0.81643648	0.81877336	0.82163186	0.82438561	0.82686706	0.829813	0.83285232	0.83559506	0.83793715	0.84032202	0.84291606	0.84584205	0.84894424	0.85222168	0.85547838	0.857592	0.85980457
Ishikawa	0.81315169	0.81580031	0.81838035	0.8209175	0.82355549	0.82573252	0.82689538	0.82802182	0.82927008	0.83031172	0.83167657	0.8331578	0.83485392	0.83699127	0.8393891	0.84207206	0.84509037	0.84817826	0.85146605	0.85474863	0.85697088	0.85937894
Iwate	0.774133	0.77606918	0.77871648	0.78171599	0.78509849	0.78775726	0.78932272	0.79099356	0.79280502	0.79477593	0.7970759	0.79988635	0.80294938	0.80620657	0.80977659	0.81336791	0.81736283	0.82153995	0.82569305	0.82961876	0.83230429	0.83497969
Kagawa	0.80713888	0.80924832	0.81188091	0.81461316	0.81759622	0.81969154	0.8208718	0.8221698	0.82375216	0.82577386	0.82807738	0.83093591	0.83367028	0.83603564	0.83852418	0.84112252	0.84413236	0.84715498	0.85026935	0.85340351	0.85555665	0.85781836
Kagoshima	0.7784486	0.78087689	0.78373284	0.78661203	0.78943215	0.79164939	0.79327096	0.79535829	0.79737271	0.79924869	0.80152326	0.80401512	0.80652573	0.80899375	0.81157205	0.81436806	0.81737057	0.82038433	0.82366461	0.82713188	0.82953918	0.83204731
Kanagawa	0.84694841	0.84806167	0.84948108	0.85124523	0.85315852	0.85483109	0.85659608	0.85864149	0.86061805	0.8619109	0.8636736	0.86550684	0.86713614	0.86864031	0.87001606	0.87161307	0.87338986	0.87530079	0.87742255	0.87967672	0.88103038	0.88265439

Kōchi	0.78045311	0.78341967	0.786654	0.78961915	0.79221878	0.79404857	0.79541345	0.79680627	0.79841258	0.80023214	0.80257547	0.80524945	0.80774471	0.81030636	0.81302166	0.81595017	0.81925189	0.82272476	0.82632776	0.82989924	0.832364	0.83490616
Kumamoto	0.78293029	0.78506212	0.7878304	0.79091605	0.79398795	0.79632154	0.79754144	0.79891207	0.80041557	0.80189814	0.80396303	0.80650876	0.80927457	0.81208558	0.81493941	0.8176735	0.82066318	0.82364727	0.82677894	0.8300365	0.83222153	0.83453659
Kyōto	0.83257268	0.83446341	0.83660877	0.83902629	0.8416575	0.84395744	0.84570039	0.84752644	0.84908256	0.85045396	0.85227442	0.85413512	0.85590821	0.85762926	0.8596532	0.86206125	0.86452426	0.86698786	0.86957116	0.8722428	0.87404097	0.87601835
Mie	0.80211902	0.8046487	0.80764589	0.81079547	0.8142186	0.81711858	0.81946188	0.82195069	0.82390435	0.82599892	0.82861707	0.83114417	0.8337827	0.83666254	0.83962612	0.8427325	0.84601697	0.84924362	0.85253608	0.85582009	0.85812473	0.86056119
Miyagi	0.80523442	0.80695671	0.80927991	0.81199468	0.81489873	0.81738953	0.81927682	0.82094772	0.82257561	0.82420999	0.8261855	0.8284245	0.83139288	0.83452577	0.83789656	0.84134574	0.84497345	0.84854135	0.85199609	0.85535643	0.85759114	0.85988657
Miyazaki	0.77361448	0.77604665	0.77908425	0.78222887	0.78535599	0.7874564	0.78823714	0.78939737	0.790878	0.79254962	0.79461097	0.7972054	0.79990634	0.80274632	0.805668	0.80854478	0.81162327	0.81466352	0.81794356	0.82146135	0.82397392	0.82658986
Nagano	0.81026469	0.81234308	0.81448684	0.8167925	0.81933682	0.8216002	0.82351302	0.82558167	0.82750706	0.82924148	0.83131053	0.83351826	0.83539514	0.83724094	0.83932896	0.84186063	0.84471469	0.84768055	0.85078159	0.85396811	0.85619146	0.85857784
Nagasaki	0.77718146	0.7792525	0.78166337	0.78414904	0.7868197	0.78909626	0.79025376	0.79147035	0.7928075	0.79446748	0.7968678	0.79978117	0.80265729	0.80533637	0.807847	0.81074883	0.81396269	0.81740776	0.82095367	0.82439151	0.82674305	0.82921388
Nara	0.81465722	0.81672094	0.81892478	0.82114727	0.8233192	0.82502828	0.82607449	0.82719269	0.8284619	0.82958941	0.83127913	0.83279357	0.83436246	0.83615687	0.83794932	0.83973697	0.84173331	0.84378632	0.84606016	0.84844766	0.8499352	0.8516522
Niigata	0.79270107	0.79536472	0.79837768	0.80136145	0.80442238	0.80697612	0.80856016	0.81027861	0.81194731	0.81366644	0.81595803	0.81846309	0.82109167	0.82387172	0.82660024	0.82918937	0.83193627	0.83476685	0.83769159	0.84074048	0.84285295	0.8451521
Ōita	0.80007512	0.80245469	0.80536363	0.80858375	0.81173714	0.81398563	0.81528513	0.81650498	0.81780326	0.81893576	0.82088887	0.8233097	0.8256154	0.82769824	0.82994155	0.83239437	0.8352293	0.8382653	0.8414539	0.84471141	0.84693883	0.84928244
Okayama	0.81068495	0.81281515	0.81547384	0.8181863	0.82098671	0.82343597	0.8257387	0.82809923	0.83024672	0.8322331	0.83446952	0.83699498	0.83923484	0.84150667	0.84377162	0.84627974	0.84896787	0.85182062	0.85489701	0.85807399	0.86027156	0.86256022
Okinawa	0.76401725	0.76644775	0.77006847	0.77378759	0.77766314	0.78078458	0.78279709	0.78447154	0.78614472	0.78818894	0.7903229	0.79259381	0.79462211	0.79684744	0.79950476	0.80230924	0.80553625	0.80906299	0.81286285	0.81669732	0.8192232	0.82180305
Ōsaka	0.8374334	0.83874731	0.8403772	0.84214541	0.84411331	0.84567855	0.84682826	0.84810578	0.8494596	0.85077574	0.85258357	0.8546753	0.85666015	0.85852076	0.86051359	0.86260459	0.8649027	0.8673414	0.86993601	0.8726364	0.87443418	0.87640925
Saga	0.78523171	0.78749215	0.79047822	0.79362705	0.79678813	0.79925514	0.8005295	0.80216197	0.80391131	0.80541167	0.80748932	0.80954996	0.81136767	0.81317367	0.81523093	0.81760275	0.82035528	0.82336197	0.82668387	0.83011189	0.83245558	0.8349259
Saitama	0.814842	0.81604769	0.81767503	0.81975178	0.82218282	0.82436765	0.82602183	0.82767181	0.82943717	0.83108422	0.83309052	0.8352491	0.83719716	0.83924866	0.84128281	0.84335055	0.84560362	0.8479581	0.85046102	0.85300708	0.85459453	0.85637105
Shiga	0.82986369	0.83156427	0.83427903	0.83720508	0.84008554	0.84221347	0.84351544	0.84523639	0.84689308	0.84839055	0.85033482	0.85220357	0.85417981	0.85621791	0.85827185	0.86042497	0.86271728	0.86536673	0.86808768	0.87074143	0.87242284	0.87432337
Shimane	0.7796426	0.78232731	0.78556393	0.78911113	0.79279072	0.79552484	0.79748084	0.7996164	0.80143878	0.80306571	0.80532182	0.80832427	0.8114897	0.81450981	0.81719972	0.81995576	0.82325668	0.82653441	0.83002969	0.8336211	0.83608512	0.83867226
Shizuoka	0.81292247	0.81477053	0.81741221	0.82035273	0.82338208	0.82598914	0.82816662	0.83038886	0.83253433	0.83441062	0.83666622	0.83912754	0.8415696	0.84412845	0.84662065	0.84925416	0.85215925	0.85512197	0.85814927	0.86120135	0.86325076	0.86545045
Tochigi	0.80540865	0.80715178	0.8096336	0.81245611	0.81556709	0.81824367	0.82039122	0.82263	0.82476673	0.82701468	0.82970196	0.83231255	0.8347045	0.83713149	0.83990111	0.84308497	0.84643456	0.84970327	0.85313389	0.85652734	0.85879538	0.8611865
Tokushima	0.79397171	0.79725871	0.80114346	0.80546339	0.80963532	0.81277519	0.81507553	0.81712433	0.81927235	0.82144075	0.82423053	0.82720682	0.82999467	0.83275736	0.83565784	0.83864297	0.84195162	0.84547443	0.84917384	0.85272355	0.85506056	0.85748626
Tōkyō	0.8960117	0.8976953	0.89948479	0.90125465	0.90300785	0.90451959	0.90585958	0.90724926	0.90879511	0.91061338	0.912389	0.91424523	0.91590124	0.91740228	0.91888298	0.92027589	0.92171952	0.92310058	0.92457188	0.92606561	0.9274607	0.92881011
Tottori	0.7927611	0.79476359	0.7970453	0.79943445	0.8020004	0.80388624	0.80530368	0.80667932	0.80797056	0.80919693	0.810385	0.81188448	0.81345937	0.81516252	0.81712678	0.81933739	0.82183984	0.82465627	0.82792754	0.83130918	0.83357647	0.83595696
Toyama	0.8145953	0.81715206	0.81999204	0.82317001	0.82639689	0.82912046	0.83097413	0.8328277	0.83471494	0.83622232	0.83836985	0.84062285	0.84267203	0.84483545	0.84712486	0.84954174	0.85221074	0.85493825	0.85785099	0.86084067	0.86288001	0.86518113
Wakayama	0.78860826	0.79118409	0.7940618	0.79710686	0.80036864	0.80339604	0.80590873	0.80822047	0.81048014	0.81255037	0.81535727	0.81868589	0.82200534	0.82512279	0.82811175	0.83091736	0.8338982	0.8367232	0.83965402	0.84273187	0.84476128	0.84703748
Yamagata	0.77863534	0.78059314	0.78336657	0.78646429	0.78985381	0.79282314	0.7953713	0.79815611	0.80046852	0.802631	0.80530324	0.80805942	0.8107854	0.81366333	0.81652403	0.81960797	0.82298556	0.82634022	0.82975998	0.83316026	0.83545631	0.83793315
Yamaguchi	0.8031616	0.80535395	0.80828079	0.81121021	0.81415528	0.81655968	0.81818226	0.82006813	0.82208683	0.824121	0.82683188	0.82979826	0.83254721	0.83524197	0.83777225	0.83989353	0.8424799	0.84538757	0.84859169	0.85179793	0.85396251	0.85625589
Yamanashi	0.80863842	0.8104224	0.81257207	0.81505088	0.81792044	0.82047173	0.82319808	0.82601919	0.8285141	0.83019439	0.83272313	0.83546504	0.83777821	0.83984945	0.84196589	0.84419901	0.84649973	0.84875021	0.85117713	0.85388208	0.85570176	0.85778285
South Korea	0.78542882	0.79349628	0.8010243	0.80768779	0.81372093	0.8190934	0.82417982	0.82912795	0.83389463	0.83810833	0.84251143	0.84691323	0.85114026	0.855441	0.85966557	0.86390741	0.86831782	0.87261776	0.87682332	0.88073151	0.88371757	0.88667527
Singapore	0.76771737	0.77429466	0.77973577	0.78416556	0.78985277	0.79645575	0.80334269	0.8106253	0.81812416	0.82425152	0.83022216	0.83382961	0.83739181	0.84042018	0.84289454	0.84540186	0.847597	0.84905152	0.85076273	0.85249734	0.85429601	0.85609777
High-income North America	0.79499703	0.79927482	0.80296133	0.80582125	0.80799119	0.80888669	0.80972116	0.81248611	0.81708393	0.82247001	0.82781414	0.83206088	0.83566894	0.83905868	0.84234095	0.8458767	0.84942337	0.85296011	0.85631618	0.8597494	0.86187425	0.86346547
Canada	0.81668506	0.82069625	0.82440227	0.8278011	0.83092811	0.83328829	0.83491566	0.8366251	0.83891368	0.84158367	0.84485349	0.84801783	0.85097805	0.85384495	0.85675993	0.8595329	0.86217423	0.86505383	0.86790554	0.87055448	0.87199263	0.87317068
Greenland	0.74085701	0.74628961	0.75287837	0.75944415	0.76540157	0.77209958	0.77914141	0.78532192	0.79144126	0.79702575	0.80300313	0.80810519	0.81168456	0.81436831	0.81652706	0.81771919	0.8189619	0.8197811	0.82081551	0.82234	0.82435903	0.82621034
USA	0.79231665	0.79667679	0.80040332	0.80322802	0.80530469	0.80603308	0.80678358	0.80968805	0.8145771	0.82029457	0.82588466	0.83026639	0.83395819	0.83741299	0.84074653	0.84437034	0.84801552	0.8516303	0.85504919	0.85857806	0.86079277	0.86244835
Alabama	0.75562221	0.7634569	0.76967632	0.77372193	0.77544902	0.7737957	0.77140728	0.77285401	0.77897235	0.78761845	0.79555018	0.8008662	0.8045349	0.80709759	0.80885174	0.81000381	0.81118185	0.81358806	0.81642184	0.82052655	0.82377652	0.82560514
Alaska	0.78083077	0.78521972	0.7889819	0.79222383	0.79491632	0.79540335	0.79443709	0.79555489	0.79964399	0.80458663	0.80994445	0.81596775	0.82090836	0.82457178	0.82889147	0.83450064	0.84015487	0.84488933	0.84859956	0.85323225	0.85613572	0.8575246
Arizona	0.75636002	0.75953736	0.76246112	0.76501479	0.7674259	0.76937805	0.77264764	0.77881287	0.78717604	0.79577678	0.80338079	0.80908547	0.81356378	0.81774779	0.82165123	0.82564794	0.82971346	0.8340636	0.83821615	0.84226178	0.84475283	0.84673287
Arkansas	0.73748229	0.74320166	0.74775529	0.75153552	0.75372879	0.75302192	0.7514672	0.75412153	0.76043973	0.76857519	0.77587478	0.78133396	0.78603757	0.78957499	0.79265214	0.79556586	0.79882242	0.8027062	0.80635235	0.81171342	0.8156793	0.81770902
California	0.79193486	0.79616917	0.79984861	0.80304853	0.80590143	0.8079627	0.81048972	0.81448132	0.81964069	0.82507426	0.83048851	0.83516311	0.83936823	0.84342579	0.84738175	0.85153146	0.85567482	0.8599178	0.86388959	0.86717602	0.86897947	0.87021505
Colorado	0.80418719	0.80819566	0.81279125	0.81694526	0.81988706	0.82109149	0.82171493	0.82398986	0.82833782	0.83413826	0.84036149	0.84514076	0.8485599	0.85151037	0.85496469	0.86041387	0.86588934	0.87024427	0.87330682	0.87475059	0.875278	0.87665477
Connecticut	0.85175425	0.85669719	0.86069919	0.86256747	0.86334374	0.86325105	0.86340962	0.86495183	0.86811132	0.8723255	0.87642813	0.8796926	0.88265225	0.88567728	0.88874147	0.89176531	0.89437426	0.89643387	0.89825006	0.90042212	0.90214477	0.90310756
Delaware	0.80628035	0.81003888	0.8131746	0.81552757	0.81744644	0.81845518	0.81992419	0.82298905	0.82741328	0.83262738	0.83771046	0.84208282	0.84554445	0.84850874	0.85080132	0.85294475	0.85513302	0.85761662	0.86022942	0.86307653	0.86480183	0.86636623
Washington, DC	0.84538819	0.85135363	0.85648338	0.85952174	0.86200405	0.8638492	0.8658439	0.86874988	0.87235519	0.87629923	0.88019268	0.88368874	0.88667212	0.88931788	0.89176552	0.8942426	0.89684475	0.89943496	0.90160674	0.90346778	0.90487397	0.90596476
Florida	0.78706618	0.79234156	0.7965943	0.79874702	0.79966403	0.79950852	0.80016046	0.805009	0.81281767	0.82085509	0.82752137	0.83194251	0.83518066	0.83764876	0.83990453	0.84241877	0.84525239	0.84895038	0.85290197	0.85735427	0.86028435	0.86197928
Georgia	0.76834849	0.77184119	0.7748813	0.77719682	0.77900131	0.77995581	0.7817221	0.7858995	0.79204157	0.79908012	0.8058816	0.81150306	0.81598273	0.82006035	0.82373031	0.82736398	0.83101053	0.83485388	0.83852415	0.84223495	0.84459493	0.84656427
Hawaii	0.80052211	0.80419143	0.80717508	0.81010875	0.81356265	0.81489632	0.8141772	0.81594575	0.82045392	0.82543604	0.82968069	0.83403066	0.83817902	0.8425203	0.84641972	0.84950336	0.85219162	0.85544707	0.85915421	0.86422252	0.86787809	0.86954692
Idaho	0.7676966	0.77187798	0.77524921	0.77750502	0.77896201	0.77886097	0.77874014	0.78135431	0.7868806	0.79382761	0.80052621	0.80524672	0.80861984	0.81168413	0.8142482	0.81711009	0.82053129	0.8246588	0.82859735	0.83217305	0.83442742	0.83651792
Illinois	0.80421124	0.80999749	0.81550795	0.81969255	0.8227012	0.82363116	0.82380506	0.82643505	0.83132047	0.83697893	0.84250671	0.84664728	0.85013995	0.85340074	0.85624513	0.85927826	0.86237907	0.86593665	0.87003437	0.8746681	0.87758765	0.8791878
Indiana	0.77761538	0.78309917	0.78675308	0.78833451	0.78895133	0.78780042	0.78661414	0.78833222	0.79326555	0.79995392	0.80632191	0.8102658	0.81307024	0.81593646	0.81917592	0.8234687	0.82749875	0.83068857	0.83390058	0.83808164	0.84104285	0.84285983
Iowa	0.80196891	0.80658782	0.80977591	0.81121385	0.8112952	0.80994721	0.80908545	0.81119664	0.81593675	0.82209863	0.82805597	0.83220459	0.83512885	0.83795847	0.84120798	0.84602567	0.85118322	0.85520558	0.85820268	0.86106504	0.8627406	0.86405652
Kansas	0.78938978	0.79410424	0.79756874	0.79995935	0.8006555	0.79961479	0.79850218	0.79928582	0.80271151	0.80829952	0.81486303	0.82015747	0.82515781	0.82992241	0.8336819	0.83909081	0.84489567	0.84882384	0.85189158	0.85568546	0.85811325	0.8593838
Kentucky	0.75420833	0.76000231	0.7650894	0.76902435	0.7707573	0.76929998	0.76689502	0.76742391	0.77170995	0.77828183	0.78458599	0.78858841	0.79172061	0.79502482	0.79821007	0.80161768	0.80487866	0.80837217	0.81203222	0.81652405	0.81947102	0.82132538
Louisiana	0.74980683	0.75602604	0.76109762	0.76553255	0.7706367	0.77222979	0.77020122	0.77128654	0.77606806	0.78261679	0.78935779	0.79348297	0.79613755	0.79868449	0.801748	0.80655191	0.81153375	0.81566598	0.8189813	0.82202013	0.82386216	0.82534646
Maine	0.80478314	0.80919862	0.81319548	0.81615377	0.81817186	0.81857963	0.81895572	0.82131027	0.82551619	0.83054852	0.83562734	0.83953726	0.84241783	0.84487464	0.84723843	0.8494598	0.85206084	0.85539736	0.85908815	0.86254016	0.86429199	0.8657542
Maryland	0.82571292	0.83077548	0.83635915	0.84126649	0.84435198	0.84477433	0.84452601	0.84619351	0.85022895	0.85575781	0.86155986	0.86624088	0.87018057	0.87322171	0.8755142	0.87750082	0.87928748	0.88135015	0.883316	0.88590752	0.8881701	0.88922557
Massachusetts	0.85738343	0.86187639	0.86574216	0.8686235	0.87068959	0.87146087	0.87208267	0.87407476	0.87735192	0.88089729	0.88434169	0.88709265	0.88981969	0.89234999	0.89463298	0.89726832	0.89979076	0.90190062	0.90349056	0.90474149	0.90580568	0.90662136
Michigan	0.80337512	0.80849511	0.81240646	0.81477789	0.81641922	0.81680786	0.81702579	0.81890976	0.82218168	0.82572813	0.82932005	0.8321474	0.8345947	0.83717766	0.84036805	0.84464767	0.84921515	0.85350714	0.85719813	0.86039602	0.86229482	0.86397113
Minnesota	0.82630704	0.82986937	0.83301535	0.83560568	0.83763177	0.83803366	0.83795736	0.84012973	0.84460103	0.84998694	0.85501805	0.85837635	0.86116456	0.86410928	0.86735216	0.8711792	0.8749	0.8784915	0.88153238	0.88423098	0.88574722	0.88701394
Mississippi	0.73300925	0.73981595	0.74419718	0.74613448	0.74634436	0.74294979	0.73785966	0.73885629	0.74712518	0.758399	0.76796198	0.7745266	0.77966675	0.78326701	0.78670274	0.79102178	0.79498021	0.79840411	0.80154719	0.80562982	0.80865305	0.81047168
Missouri	0.78301074	0.78769155	0.79081752	0.7925965	0.79337314	0.79232988	0.79135331	0.79339483	0.79897713	0.80608923	0.8124277	0.81666516	0.82016489	0.82327026	0.8261325	0.82968185	0.83324877	0.8361164	0.83920406	0.84360312	0.84654037	0.84816914
Montana	0.79165948	0.79471095	0.79732911	0.79948287	0.80167168	0.80231763	0.80216067	0.80360098	0.80747296	0.81286301	0.81802596	0.821628	0.82410869	0.82682805	0.83079225	0.83698502	0.8438016	0.84935036	0.85362466	0.85640132	0.85767995	0.85910771
Nebraska	0.80074384	0.80284267	0.80547258	0.80843384	0.81044858	0.81049013	0.80975643	0.81121977	0.81465532	0.81995089	0.82627361	0.83120772	0.83431704	0.83701273	0.84065182	0.84676417	0.8524637	0.85630276	0.85938856	0.86223712	0.8639163	0.86512865

Nevada	0.77461151	0.78005427	0.78373812	0.78586148	0.78656206	0.7854999	0.7856779	0.7907889	0.79990851	0.80957506	0.81742273	0.82207684	0.82500747	0.82696897	0.82767345	0.82752292	0.8278382	0.83048291	0.83515676	0.84159686	0.84571734	0.84768278
New Hampshire	0.84029615	0.84544038	0.84970579	0.85248253	0.85443341	0.85516249	0.85570297	0.85740274	0.86065025	0.86473947	0.86873586	0.87242888	0.87575614	0.8785022	0.88140241	0.8852025	0.88880547	0.89179398	0.89440537	0.89650406	0.89742575	0.89826368
New Jersey	0.83848901	0.84240382	0.84660477	0.85003077	0.85246915	0.85330488	0.85375873	0.85558551	0.85896792	0.86301163	0.86666814	0.86978633	0.87315008	0.87628333	0.87902439	0.88159339	0.88383729	0.8855329	0.88704083	0.88916574	0.89095974	0.89192244
New Mexico	0.74758474	0.75190787	0.75553552	0.75837515	0.76050242	0.76140853	0.76245308	0.76599787	0.77194502	0.77932585	0.78677938	0.79288974	0.79800391	0.80278881	0.80720041	0.81139341	0.81550741	0.81964264	0.82352103	0.82749269	0.82994177	0.83171658
New York	0.82716208	0.83122233	0.83486238	0.83795089	0.8404858	0.84207842	0.84362318	0.84623957	0.84944106	0.85309236	0.85699376	0.86048824	0.86382948	0.86721026	0.87037887	0.87317968	0.87567647	0.87796616	0.88003638	0.88222699	0.88398769	0.88517687
North Carolina	0.77077581	0.77480776	0.77830555	0.78103261	0.78319075	0.78422871	0.78556983	0.78913744	0.79472081	0.80127194	0.80764867	0.8127426	0.81697851	0.82058114	0.82370885	0.82684578	0.83009444	0.83346043	0.83679007	0.84032264	0.84258991	0.84444531
North Dakota	0.80659318	0.80925588	0.81089045	0.81204049	0.81248854	0.8124327	0.81248085	0.81435885	0.81775702	0.82128937	0.82529299	0.82907567	0.83370259	0.83918961	0.84572032	0.85272723	0.85873152	0.86332878	0.86677772	0.86946411	0.8714634	0.87303178
Ohio	0.7886042	0.79263071	0.79603592	0.79870679	0.80065882	0.80130891	0.80190295	0.80408154	0.80777078	0.81238808	0.81708476	0.82086955	0.82400733	0.82714939	0.83015518	0.83327117	0.83655872	0.83998301	0.84319001	0.84650352	0.84864434	0.85051343
Oklahoma	0.75247452	0.75642876	0.76012639	0.76330169	0.76566904	0.76645306	0.7672088	0.76995141	0.77497592	0.78074761	0.78676629	0.79175017	0.79623219	0.80102825	0.80569204	0.80992102	0.81395989	0.81779762	0.82113423	0.82460971	0.82685731	0.82866324
Oregon	0.80004149	0.80454559	0.80836245	0.81147765	0.81383084	0.81497578	0.81617291	0.81890423	0.82313795	0.82804832	0.8328378	0.83671981	0.84001866	0.84333901	0.84688273	0.85094112	0.8552195	0.85929779	0.86294828	0.8663105	0.86836459	0.87017769
Pennsylvania	0.81375928	0.81734631	0.82040861	0.82292176	0.8249734	0.82604657	0.82706427	0.82941159	0.83286198	0.83694557	0.84125132	0.8449341	0.8482014	0.85128821	0.85408598	0.85693113	0.85982866	0.86281186	0.86577408	0.86888627	0.87079815	0.87241675
Rhode Island	0.82233186	0.82559926	0.8287903	0.83178457	0.83460619	0.83663359	0.83868594	0.84186213	0.84585046	0.85014421	0.85468085	0.85848081	0.86180964	0.8648276	0.86752102	0.87025444	0.87296488	0.87566678	0.87836046	0.88099217	0.88247115	0.88389348
South Carolina	0.76652614	0.77076555	0.77450325	0.77729273	0.77920729	0.77960739	0.78010407	0.78291056	0.78800914	0.79443254	0.80091042	0.80618206	0.81048096	0.81424638	0.8175081	0.82066857	0.82388592	0.82714201	0.83027907	0.83361293	0.83580625	0.83771865
South Dakota	0.78803856	0.79077123	0.79233892	0.79345023	0.79423175	0.79421848	0.79435773	0.79685798	0.80112718	0.80615925	0.81131194	0.8155374	0.81913629	0.82321513	0.82770429	0.83301613	0.83866908	0.84375463	0.84799196	0.85183974	0.85405383	0.85572087
Tennessee	0.76107311	0.76432077	0.76649212	0.76761658	0.76807324	0.76753847	0.76767102	0.77057345	0.77597665	0.78274085	0.7892576	0.79424059	0.79836996	0.80248224	0.80665125	0.81149604	0.81647233	0.82085239	0.82464378	0.8284145	0.83091173	0.83303324
Texas	0.74903484	0.75299572	0.75643618	0.75911811	0.76117221	0.76203418	0.7632655	0.76692595	0.77287204	0.77970558	0.78669844	0.79242585	0.79736383	0.80233401	0.80747158	0.81284725	0.81801207	0.8228579	0.82687354	0.83058669	0.83288269	0.83478328
Utah	0.77590353	0.78009663	0.78341518	0.78583595	0.78742259	0.78794973	0.7888842	0.79235853	0.79807673	0.80480725	0.81132807	0.81656466	0.82078805	0.82476785	0.82866223	0.83307259	0.83765989	0.84209526	0.84621962	0.8501301	0.85256905	0.85461679
Vermont	0.82817222	0.83198085	0.83551304	0.83878558	0.84189602	0.84401485	0.84594287	0.8486519	0.85197197	0.85531919	0.85872895	0.86189018	0.86485876	0.86798383	0.87125207	0.87481964	0.87833683	0.8815877	0.88452235	0.88722315	0.88867724	0.8899999
Virginia	0.81219476	0.81590062	0.81933188	0.82250774	0.8253964	0.82758542	0.82995382	0.83381356	0.83871469	0.84410621	0.84938871	0.85368094	0.85720844	0.86024216	0.86282264	0.8653471	0.86802926	0.87085374	0.87369732	0.87680399	0.8786606	0.8801199
Washington	0.81463575	0.81898821	0.82260236	0.82537505	0.82758068	0.8284537	0.82931957	0.83170398	0.83531685	0.83943885	0.84369776	0.84731836	0.85078179	0.85426781	0.85775008	0.8613466	0.86494843	0.86857319	0.87188454	0.87493693	0.87649116	0.87758484
West Virginia	0.75672341	0.75994047	0.76229799	0.76373938	0.76447246	0.76404071	0.76366599	0.76496691	0.76801167	0.77239549	0.77702527	0.78069254	0.78381686	0.78743297	0.79146958	0.79591759	0.80041998	0.80464921	0.80861081	0.81284408	0.81563762	0.81771351
Wisconsin	0.81282335	0.81619951	0.81920468	0.82161328	0.8234171	0.82428381	0.82515004	0.82724699	0.83049166	0.83452608	0.83875451	0.8424053	0.84566819	0.84895952	0.8522444	0.85567348	0.85909193	0.86235081	0.86537871	0.86843457	0.87023746	0.8717261
Wyoming	0.78565335	0.789883	0.79369659	0.79716501	0.80001754	0.80182422	0.80402247	0.80785235	0.81331096	0.81874135	0.82428207	0.82878864	0.83251057	0.83601344	0.83964154	0.84343901	0.84726434	0.85105917	0.85469332	0.85833834	0.86052084	0.86214872
Southern Latin America	0.64123743	0.64604037	0.64982584	0.6525524	0.65571888	0.66051913	0.66438624	0.6673925	0.67068862	0.67415239	0.67876122	0.68413428	0.68842791	0.69171599	0.69603955	0.70328723	0.71078773	0.71754842	0.72495216	0.73113382	0.73396336	0.73598472
Argentina	0.63819447	0.6421757	0.64447251	0.64590658	0.64829683	0.65336883	0.6574459	0.66018758	0.66307924	0.66591194	0.66992588	0.6752427	0.67921824	0.68163321	0.68497451	0.69171013	0.69861803	0.70513553	0.71288869	0.71900284	0.7212943	0.72312297
Chile	0.65298903	0.66026551	0.66788841	0.67344593	0.67825821	0.68258919	0.68589275	0.68893032	0.69235979	0.69660214	0.7023047	0.70859794	0.71474348	0.72044878	0.72727593	0.73609808	0.74497222	0.75205808	0.75862571	0.76512032	0.76921368	0.77151472
Uruguay	0.62320829	0.62579607	0.6282858	0.63143777	0.63421691	0.63697577	0.6405907	0.64633171	0.65352552	0.66032113	0.66609914	0.66707973	0.66580219	0.66822836	0.67333426	0.68059551	0.68981673	0.69918946	0.70690493	0.71253659	0.71616977	0.71928344
Western Europe	0.78740001	0.7909044	0.79416518	0.79696151	0.79971572	0.80241404	0.80506685	0.80772333	0.81048438	0.81311022	0.81614217	0.81937524	0.82263373	0.8257869	0.82860951	0.8313218	0.83421602	0.83752217	0.84083355	0.84421211	0.84655036	0.84872632
Andorra	0.78197651	0.78541703	0.79015876	0.7972789	0.80473198	0.81221398	0.81978726	0.82661725	0.83182171	0.83592989	0.83948997	0.8427948	0.84581083	0.84832991	0.85101049	0.85359122	0.85625705	0.85947703	0.86258895	0.86562102	0.86744917	0.86944411
Austria	0.79184768	0.79577012	0.79915412	0.80204325	0.80488914	0.80841919	0.81206461	0.81548052	0.81879366	0.82154117	0.82450011	0.828002	0.83111331	0.83367978	0.83583858	0.8375799	0.83988379	0.84317333	0.84661447	0.84986251	0.85202039	0.853837
Belgium	0.78039633	0.78471304	0.78883605	0.79201438	0.79522494	0.79849724	0.80157276	0.80461266	0.80775794	0.81105583	0.81503021	0.81947036	0.82422602	0.82861983	0.83242505	0.8358784	0.83946294	0.84321204	0.84661763	0.84949839	0.85134665	0.85365402
Cyprus	0.7451993	0.75359472	0.76150247	0.76789622	0.77376477	0.77903136	0.78456096	0.79206838	0.79945409	0.80511209	0.80984019	0.81372613	0.81697853	0.8193806	0.8211843	0.82273285	0.82428362	0.82613988	0.82834324	0.83098073	0.83305952	0.83563055
Denmark	0.8391843	0.84317775	0.84708636	0.85044133	0.8533041	0.85569073	0.85803321	0.85978173	0.86178206	0.86394805	0.86676708	0.86977868	0.87251751	0.87493297	0.87681493	0.87854394	0.88058978	0.88368849	0.88752985	0.89162457	0.8943683	0.8964242
Finland	0.78998523	0.79445383	0.79877027	0.80232543	0.80569423	0.80893679	0.81208148	0.81559761	0.81887554	0.82145759	0.82484498	0.82827058	0.83143448	0.8344254	0.83743187	0.84087848	0.84462234	0.84858613	0.85220294	0.85536518	0.85765555	0.85983137
France	0.77279203	0.77622516	0.78008887	0.78317876	0.78608465	0.78904209	0.79232192	0.79555765	0.79817743	0.80050867	0.80324025	0.80637874	0.80961539	0.81298402	0.81655145	0.82023839	0.82373093	0.82709844	0.83044859	0.8337728	0.83604944	0.83836488
Germany	0.84892576	0.85207041	0.85506679	0.85767579	0.86024033	0.86289418	0.86587371	0.86914044	0.87255285	0.87508791	0.87794671	0.88102751	0.88365641	0.88592695	0.88765161	0.88886785	0.89089637	0.89405047	0.89697555	0.89970316	0.90143861	0.90295709
Greece	0.73271054	0.73715401	0.74194522	0.74718596	0.75241861	0.7563844	0.7602097	0.76392717	0.76752766	0.77124736	0.77515581	0.77852587	0.78078948	0.78194905	0.78244201	0.78248952	0.78279844	0.78410859	0.78599975	0.78813814	0.78963222	0.79185441
Iceland	0.80303033	0.80896579	0.81340039	0.81659707	0.81952809	0.82276654	0.82624474	0.83028989	0.83462268	0.83812101	0.84138464	0.84461993	0.84729944	0.85017395	0.8535587	0.85772498	0.86184447	0.86570289	0.86921982	0.87242558	0.87432315	0.87636168
Ireland	0.78019066	0.78635374	0.79301061	0.80000587	0.80685314	0.81171382	0.81450548	0.81693103	0.81993213	0.82392967	0.82810385	0.83180479	0.83532536	0.83864443	0.84208211	0.84743789	0.8526425	0.85781616	0.86392528	0.8694125	0.87195882	0.87375385
Israel	0.74918408	0.7528343	0.75598808	0.75887541	0.76221329	0.76554346	0.7687339	0.77075614	0.77201161	0.77330391	0.77525953	0.77764967	0.78040341	0.78326717	0.78615108	0.78887446	0.79177245	0.79517344	0.79912499	0.80323299	0.80635159	0.80901165
Italy	0.74967599	0.75355484	0.75719148	0.76009374	0.76278775	0.76568991	0.76840241	0.77069838	0.77306847	0.77549926	0.77810921	0.78070347	0.78309098	0.78535688	0.78753396	0.78990212	0.79247724	0.79531119	0.79836269	0.80153002	0.80363568	0.80577353
Abruzzo	0.76230951	0.76627143	0.76981078	0.77259129	0.7749208	0.77748367	0.78003411	0.7823351	0.78475363	0.7870674	0.78959427	0.79238875	0.79503352	0.79742888	0.79964069	0.80191169	0.8043285	0.80702798	0.8099339	0.81294168	0.81485374	0.81684436
Basilicata	0.72247405	0.72738238	0.7318562	0.73532332	0.73845318	0.74157218	0.74473821	0.74771626	0.75056069	0.75297114	0.75530103	0.75779697	0.76012226	0.76253445	0.76456645	0.7672315	0.76993871	0.77299233	0.77619881	0.77947954	0.78170773	0.78401643
Calabria	0.71376972	0.71876051	0.72334746	0.72711597	0.73064081	0.73425293	0.73768069	0.74069277	0.74375746	0.74681285	0.74979242	0.75258591	0.7550263	0.75710955	0.75894233	0.76088193	0.76314053	0.76578207	0.7686683	0.77172208	0.77366768	0.77571305
Campania	0.710384	0.71483251	0.71912469	0.72245743	0.72551192	0.72881216	0.73179638	0.73429793	0.73680287	0.73939939	0.7419047	0.74417748	0.74630984	0.74826609	0.75010838	0.75218558	0.75464023	0.75724465	0.76009759	0.76308919	0.76501158	0.76694337
Emilia-Romagna	0.76827105	0.77253873	0.77628209	0.77901961	0.78142918	0.78412452	0.7867322	0.78893364	0.79133854	0.7939658	0.79692883	0.8000847	0.80307826	0.8060609	0.80895656	0.81196621	0.81513592	0.81840568	0.82182386	0.82524279	0.82749918	0.82972471
Friuli-Venezia Giulia	0.7676116	0.77141897	0.77482504	0.7773821	0.77965972	0.78219666	0.78473396	0.78691235	0.78898627	0.79097991	0.7932738	0.79563655	0.79771559	0.7998653	0.80196795	0.80437676	0.80702286	0.80984508	0.81287264	0.8160362	0.81812693	0.82030206
Lazio	0.77856881	0.78217168	0.78561692	0.78842547	0.79113289	0.79392525	0.79643317	0.79854493	0.80055006	0.80261321	0.80480954	0.80696679	0.80882967	0.81055663	0.81216286	0.81395194	0.8159524	0.8182954	0.82089297	0.82365327	0.82544852	0.82733885
Liguria	0.76891022	0.77253027	0.77570885	0.77832143	0.78071815	0.78324859	0.7856375	0.78779409	0.79010402	0.79236739	0.79470308	0.79712919	0.79937259	0.80154651	0.80375766	0.80618761	0.80880576	0.81158151	0.81458299	0.81775469	0.81990495	0.82213339
Lombardia	0.77846409	0.78151123	0.78440318	0.78665847	0.7886867	0.7909113	0.79294652	0.7946035	0.79657114	0.79868819	0.801242	0.80384378	0.80622528	0.80849255	0.81074986	0.81319289	0.81583541	0.81875497	0.8218674	0.82509769	0.82724976	0.82942992
Marche	0.74471793	0.74912098	0.75326634	0.75668577	0.75982501	0.76309227	0.76622111	0.76899491	0.77171339	0.77445264	0.77720901	0.77984978	0.78222301	0.7844159	0.78654715	0.78879484	0.79118125	0.79372013	0.79655013	0.79950756	0.80146643	0.80347318
Molise	0.73262645	0.73749377	0.74196354	0.74561357	0.74901584	0.75235123	0.75582293	0.75910818	0.76205899	0.7647674	0.76724005	0.76957705	0.77155013	0.77292281	0.77407393	0.77544044	0.77727982	0.77940558	0.78188174	0.78460817	0.78636097	0.7883506
Piemonte	0.75780246	0.76104798	0.76406478	0.76647121	0.76868012	0.77106543	0.77334562	0.77524675	0.77714958	0.77897951	0.78114853	0.78340101	0.78542205	0.78743869	0.78941068	0.79161836	0.79404043	0.79674922	0.79970669	0.8028134	0.80490063	0.80703906
Provincia autonoma di Bolzano	0.77819388	0.78213178	0.78557187	0.78819775	0.79059636	0.79319121	0.79577814	0.79798146	0.80049298	0.80329674	0.80635564	0.80950749	0.81268678	0.81577868	0.81868233	0.82180111	0.82508984	0.8283592	0.83180397	0.83522364	0.83753358	0.83971134
Provincia autonoma di Trento	0.77445619	0.7779654	0.78124091	0.78386558	0.78621061	0.78874659	0.79105893	0.79301487	0.79519578	0.79760891	0.80025814	0.80291827	0.80544577	0.80816631	0.8107183	0.81329985	0.81595375	0.81881509	0.82189684	0.82509021	0.82722537	0.82938492
Puglia	0.70743213	0.71187185	0.71597439	0.71924634	0.72219891	0.72525553	0.72809395	0.73048468	0.7327421	0.73502167	0.73741543	0.73975685	0.74204733	0.74412625	0.74611999	0.74836044	0.75072908	0.75359937	0.75666901	0.75981903	0.7618825	0.76398208
Sardegna	0.72321647	0.72709317	0.73064696	0.7337018	0.736569	0.73939613	0.74211495	0.74451652	0.74693411	0.74917971	0.7514295	0.75358639	0.75558526	0.75719746	0.75867488	0.76046161	0.76236154	0.76453972	0.76692095	0.76946266	0.77106593	0.77279343
Sicilia	0.70656829	0.71083671	0.71488561	0.71801036	0.72091595	0.72432638	0.72745396	0.72993636	0.73245307	0.73520871	0.73794983	0.74040256	0.74271317	0.74480941	0.74663498	0.74869127	0.75092322	0.75345291	0.75627166	0.75928364	0.76118761	0.76308681
Toscana	0.75387827	0.75750944	0.76092276	0.76374256	0.76638726	0.76919584	0.77191678	0.77429999	0.77680198	0.77942872	0.78219827	0.78497973	0.7875831	0.79006865	0.79250611	0.79511923	0.79791767	0.80082984	0.80395294	0.80719229	0.80940387	0.81165411
Umbria	0.74866176	0.75221567	0.75544479	0.75805393	0.76049987	0.76315973	0.76579504	0.76811451	0.77058336	0.77290828	0.77541458	0.77783289	0.77998695	0.7819531	0.78360069	0.78553629	0.78755754	0.78986584	0.79249864	0.7952744	0.79713307	0.79910132
Valle d'Aosta	0.76461864	0.76754572	0.77028118	0.77254796	0.77456816	0.77681635	0.77896987	0.78069425	0.78265594	0.78470077	0.78718271	0.78981185	0.79242449	0.79469401	0.79675235	0.79884438	0.80108053	0.80343824	0.80598988	0.80868573	0.81044976	0.81230429
Veneto	0.75464995	0.75809357	0.76116472	0.76377475	0.76627952	0.76894777	0.77151022	0.77364916	0.77581791	0.77803542	0.78042181	0.78293418	0.78522309	0.78745176	0.78963029	0.79200151	0.79464011	0.79760854	0.80078415	0.80404827	0.80626835	0.80853444

Luxembourg	0.82200657	0.82531282	0.82822349	0.83098395	0.83367257	0.83667526	0.84057445	0.84470701	0.84797888	0.85068873	0.85325828	0.85621683	0.85978903	0.86336879	0.86669356	0.87011294	0.87342088	0.87634768	0.8786379	0.8805939	0.88249532	0.88442896
Malta	0.71180525	0.71780304	0.72279027	0.72751113	0.73193385	0.73618875	0.74014018	0.74423781	0.7484271	0.75217106	0.7562505	0.76031055	0.76453788	0.76861614	0.77272874	0.77727691	0.78167341	0.78641018	0.79096562	0.79519785	0.79837517	0.80158503
Monaco	0.86897358	0.87109475	0.87318277	0.87519765	0.87723155	0.87928809	0.88127415	0.88326229	0.88533139	0.88732828	0.88926284	0.89123759	0.89307382	0.89486447	0.89654787	0.89835634	0.9000513	0.90175271	0.90338869	0.90503099	0.90668544	0.90826283
Netherlands	0.83191398	0.83551815	0.83867608	0.84163948	0.84480735	0.84793419	0.85079881	0.85359539	0.85636847	0.85860046	0.8612553	0.86424668	0.86695063	0.86940298	0.87184891	0.87440284	0.87678405	0.87930015	0.88190022	0.88464384	0.88655857	0.88846426
Norway	0.84647545	0.85211194	0.85675789	0.86030258	0.86346891	0.86621749	0.86858843	0.87092967	0.87374512	0.87658282	0.88037838	0.88435167	0.88816048	0.89198962	0.89549955	0.89891264	0.90258419	0.90604405	0.90926178	0.91227876	0.91452992	0.91613281
Agder	0.835206	0.84113718	0.84578887	0.84886127	0.85123076	0.85324717	0.8551314	0.85711177	0.85964656	0.863013	0.86777517	0.8728041	0.87766051	0.88240259	0.88646454	0.89023755	0.89391665	0.89749111	0.90098392	0.90436101	0.90618338	0.90748954
Innlandet	0.82887656	0.83407175	0.83855199	0.84213011	0.84545935	0.84817672	0.85025528	0.85198146	0.85389993	0.85647485	0.86025388	0.86429668	0.86850093	0.87274681	0.87668321	0.88069691	0.88472309	0.88869096	0.89239856	0.89598152	0.89808057	0.89975406
Møre og Romsdal	0.83271586	0.83838194	0.84312554	0.84689381	0.85036074	0.85329532	0.85573521	0.85788948	0.86034628	0.86376497	0.86864624	0.87373355	0.87786255	0.88199174	0.88583973	0.88949011	0.89335062	0.89693686	0.90026227	0.90339563	0.9057401	0.90742629
Nordland	0.81744423	0.82300433	0.82759556	0.83086046	0.83356025	0.83570012	0.83729213	0.8385198	0.84015423	0.84295699	0.84755707	0.85268038	0.85783537	0.86292616	0.8676087	0.87260291	0.87791292	0.8830385	0.88784245	0.8922541	0.89505198	0.89694228
Oslo	0.88602844	0.89230445	0.89618757	0.89909056	0.90174914	0.90422249	0.90644455	0.90866384	0.91121623	0.91441107	0.91810535	0.92160992	0.92485806	0.92795307	0.93076681	0.9334734	0.93625252	0.93878562	0.94101721	0.94300674	0.94451283	0.94569179
Rogaland	0.84475928	0.85055916	0.85498429	0.85824602	0.86126299	0.86390076	0.86634491	0.86740461	0.86848793	0.87109461	0.87519366	0.87975868	0.88423191	0.88871555	0.89270768	0.89646973	0.90046203	0.90422208	0.90784815	0.91113922	0.91350965	0.91508373
Troms og Finnmark	0.82812721	0.83378226	0.83857985	0.84214319	0.84513613	0.84721552	0.84880157	0.85022512	0.85222337	0.8556188	0.8609487	0.8667806	0.87234976	0.87734784	0.88147376	0.8853693	0.88954817	0.89336986	0.89697248	0.90055047	0.90326508	0.9051632
Trøndelag	0.83751572	0.8434422	0.84849593	0.85243307	0.85574463	0.85854685	0.86096613	0.86338902	0.86648419	0.87047319	0.87559483	0.88072764	0.8855232	0.89017439	0.89398584	0.89755853	0.90132784	0.90493847	0.90832641	0.9117067	0.91423553	0.91597759
Vestfold og Telemark	0.83906515	0.8446608	0.84933615	0.85273725	0.85557901	0.85791226	0.85977761	0.86148978	0.8636156	0.86641602	0.87024156	0.87417044	0.87811767	0.88205685	0.8857214	0.88943605	0.89319456	0.8969139	0.90049229	0.90394682	0.90587119	0.90730742
Vestland	0.84276313	0.8486332	0.85347442	0.85716964	0.86051206	0.86339923	0.86592234	0.8684169	0.87168977	0.87581105	0.8800681	0.88446835	0.88859936	0.89264388	0.89625125	0.89984226	0.90370657	0.9073585	0.91064885	0.91367553	0.91595896	0.91759629
Viken	0.85847148	0.86327925	0.86714811	0.87006156	0.8726784	0.87484938	0.8764248	0.87673685	0.87764793	0.8798049	0.8831166	0.88654101	0.88986353	0.89322663	0.89630876	0.89929186	0.90253907	0.9056458	0.90854788	0.91122676	0.91323434	0.91469261
Portugal	0.66161085	0.66724715	0.67258394	0.67766341	0.68235293	0.6868871	0.6911588	0.69497485	0.6986046	0.7022234	0.70656355	0.71126289	0.71580408	0.71983699	0.72302128	0.72575622	0.72830085	0.73122553	0.73440295	0.73809048	0.74103738	0.74415185
San Marino	0.85969536	0.86374776	0.86568059	0.86752201	0.86928713	0.8709796	0.87265215	0.87430725	0.87599188	0.87770523	0.87944081	0.88119752	0.88292453	0.88459263	0.88535369	0.88469337	0.88447766	0.88455761	0.88513352	0.88620066	0.88686794	0.88800547
Spain	0.69705603	0.70172607	0.70630965	0.71067858	0.71488623	0.71871729	0.72197013	0.72521144	0.72941104	0.73395605	0.73835206	0.74209645	0.74518187	0.74765198	0.74980093	0.75226982	0.75475794	0.75766328	0.76065957	0.76394292	0.76650603	0.7692837
Sweden	0.83302811	0.8359564	0.83874153	0.84166314	0.84450688	0.84687634	0.84911587	0.85142085	0.85370585	0.85570275	0.85872794	0.86189238	0.86470667	0.86738377	0.86992333	0.87238851	0.87466165	0.87712061	0.87988891	0.88298412	0.88506201	0.8868803
Stockholm	0.86874746	0.87169989	0.87428166	0.87670657	0.8792564	0.88162691	0.88385143	0.88624949	0.88886385	0.89115289	0.8943043	0.89749399	0.90023896	0.9023454	0.90437172	0.90635999	0.90830839	0.91004256	0.9119315	0.91388968	0.91544818	0.91662271
Sweden except Stockholm	0.82278713	0.82565124	0.82844554	0.83145624	0.83433632	0.83666605	0.83885815	0.84106732	0.84317932	0.84498859	0.84785759	0.85089033	0.85358927	0.85616454	0.85858364	0.86086365	0.86292915	0.86530307	0.86803189	0.87118114	0.87330347	0.87515115
Switzerland	0.88524353	0.88844595	0.89090281	0.89278081	0.89490981	0.89727263	0.8999623	0.90291341	0.90577845	0.90805	0.91053052	0.91327657	0.91571489	0.91795501	0.91999418	0.92183299	0.9239306	0.92620229	0.92855059	0.9306821	0.93202764	0.93305911
UK	0.78921896	0.79351317	0.79665556	0.79918611	0.80195087	0.80448919	0.80681705	0.80935277	0.81203929	0.81479364	0.81803399	0.821719	0.82645375	0.83150867	0.83551664	0.83895192	0.84246023	0.84626362	0.84996979	0.85413057	0.85692029	0.85900018
England	0.79121817	0.79572874	0.79893122	0.80142212	0.80422916	0.80686705	0.80929741	0.81187828	0.8145103	0.81714915	0.82024807	0.82388806	0.82881473	0.83417598	0.83828785	0.8417623	0.84530113	0.84919211	0.8530158	0.85723951	0.85984675	0.86143731
East Midlands	0.76650495	0.77091617	0.77397647	0.7763029	0.77888155	0.78127626	0.78346778	0.78581163	0.78826944	0.79074877	0.79372244	0.79731194	0.80229019	0.8076191	0.81163509	0.81514094	0.8188665	0.82302766	0.82710738	0.83156984	0.83424814	0.83585605
Derby	0.77727355	0.78168517	0.78481718	0.78724388	0.78985701	0.79167985	0.79300453	0.79467539	0.79702261	0.80003206	0.8032148	0.80666595	0.81201513	0.81803361	0.82281919	0.82710299	0.83102614	0.83472001	0.83797194	0.84175987	0.84430589	0.84601068
Derbyshire	0.75576888	0.75958277	0.76190732	0.76326194	0.76493949	0.76673709	0.76862781	0.7704933	0.7720735	0.77373934	0.77647066	0.78049547	0.78658728	0.79317459	0.79811537	0.80233342	0.80655966	0.81107255	0.81535806	0.81982696	0.82255145	0.8242381
Leicester	0.75814382	0.76226968	0.76531609	0.76805804	0.77149691	0.77486092	0.77783426	0.78095923	0.78457021	0.78835353	0.79265757	0.79717113	0.80216128	0.80690263	0.81011027	0.81260416	0.81521784	0.81815553	0.8212361	0.82503059	0.82748417	0.8290671
Leicestershire	0.79142005	0.79524965	0.79788962	0.80013468	0.80299005	0.80592575	0.80876558	0.81154376	0.81400821	0.81584611	0.81780299	0.82019958	0.82408761	0.82853589	0.83164571	0.83422482	0.83734304	0.84095748	0.84444094	0.84821489	0.85046112	0.85188895
Lincolnshire	0.75489142	0.75899655	0.76164522	0.76338967	0.7651122	0.76660914	0.76770246	0.76860633	0.76969957	0.77149597	0.77416751	0.777765	0.78314061	0.78899952	0.79364411	0.79785562	0.80214629	0.80683445	0.81131396	0.81623843	0.81944532	0.82136376
Northamptonshire	0.77258434	0.77623332	0.77860274	0.78020194	0.78186061	0.78325157	0.78452215	0.78612642	0.78786248	0.78985085	0.79273203	0.79666712	0.8024164	0.80860972	0.81311018	0.81686085	0.82082775	0.82538893	0.82991887	0.83481513	0.83788898	0.83963536
Nottingham	0.79161737	0.79628495	0.79981123	0.80292014	0.80603745	0.80887743	0.81175174	0.8152604	0.81938004	0.82305858	0.82612397	0.82906958	0.83250605	0.83595844	0.83871921	0.84121217	0.84415762	0.8475535	0.85104215	0.85501249	0.85740749	0.85887136
Nottinghamshire	0.75850171	0.76236891	0.76487914	0.76661287	0.76879528	0.77084519	0.77256148	0.77435406	0.77595881	0.77744296	0.77962092	0.78274283	0.78752169	0.79283125	0.79688645	0.80049784	0.80445653	0.80905635	0.8136382	0.81843411	0.82131702	0.82300786
Rutland	0.79691224	0.79962497	0.80161127	0.8032843	0.80524775	0.80723545	0.80935123	0.81166631	0.81374273	0.8158297	0.81864541	0.82176025	0.82565923	0.82991478	0.83340509	0.83643048	0.83943789	0.84265611	0.84578287	0.84937926	0.8514898	0.85273375
East of England	0.78730323	0.79188721	0.79524007	0.79792144	0.80087581	0.80356092	0.80588557	0.80824658	0.81069862	0.8131753	0.81602737	0.81922047	0.8235436	0.82846015	0.83243345	0.83592015	0.83956349	0.84362321	0.8476727	0.85213271	0.85481968	0.85640792
Bedford	0.79524673	0.79924353	0.80228154	0.80485986	0.80756347	0.81013504	0.81235424	0.81404365	0.81552582	0.81756501	0.82027151	0.82355576	0.8279465	0.83263739	0.83637132	0.83976089	0.84320546	0.84698617	0.85060917	0.85442102	0.85666666	0.85809366
Cambridgeshire	0.82400557	0.82859093	0.83209721	0.83513843	0.83817458	0.84090443	0.84337313	0.84577472	0.84830744	0.85060155	0.85294263	0.85562805	0.85935958	0.86354669	0.86697755	0.87016403	0.87358348	0.87728827	0.88088434	0.88467525	0.88677226	0.88802758
Central Bedfordshire	0.79070657	0.79479039	0.7978689	0.80010626	0.80233225	0.80430673	0.80596054	0.80757382	0.80928668	0.81112697	0.81341284	0.81640747	0.82064807	0.82581232	0.83024547	0.83383725	0.8373315	0.84104542	0.84464343	0.84859672	0.85088571	0.85225452
Essex	0.77883546	0.78351507	0.78708095	0.79006216	0.79320559	0.79608436	0.7986483	0.80124219	0.80369954	0.80585803	0.80821737	0.81088406	0.81473743	0.81916366	0.82268804	0.82574806	0.82889356	0.8325995	0.83656553	0.8410991	0.84386727	0.84549145
Hertfordshire	0.82676247	0.83186779	0.83583109	0.8389243	0.84205508	0.84460373	0.84659708	0.8483645	0.85003062	0.85160974	0.85379003	0.8564126	0.85998709	0.86411927	0.86754888	0.87067495	0.8739574	0.87745039	0.88076523	0.88429238	0.88629333	0.88751072
Luton	0.76686617	0.7704984	0.77365374	0.77712462	0.78104911	0.7847005	0.78789807	0.79141464	0.79524194	0.79916157	0.80325852	0.80737134	0.81225534	0.81703972	0.82010678	0.82261018	0.8256479	0.82887828	0.83174543	0.83551944	0.83808561	0.83971713
Norfolk	0.76826306	0.77303409	0.77616366	0.7783889	0.78107126	0.78355972	0.78582226	0.78822315	0.79081153	0.79331269	0.79579735	0.79834074	0.80211798	0.80673339	0.81057724	0.81409921	0.81808582	0.82273758	0.82736907	0.83239679	0.83550271	0.83730968
Peterborough	0.76296228	0.76688204	0.76934652	0.7708413	0.77255259	0.77391286	0.77465406	0.77549251	0.7765108	0.7784663	0.78167115	0.7853315	0.79081991	0.79777602	0.80350336	0.8084507	0.81372439	0.81960737	0.82528317	0.83148331	0.83561547	0.83787447
Southend-on-Sea	0.75576757	0.76087273	0.76507208	0.76848403	0.7717417	0.77401582	0.77553406	0.77742574	0.77966746	0.78249494	0.78575933	0.78870666	0.79247893	0.79723967	0.8012837	0.80452588	0.80786245	0.8119344	0.81627964	0.82116516	0.82413703	0.82580139
Suffolk	0.7723517	0.77642029	0.77887141	0.78054771	0.78298291	0.78536666	0.7873079	0.78917872	0.79120836	0.79353145	0.79642961	0.79999049	0.80496748	0.81059607	0.81499207	0.8187038	0.82246061	0.82671827	0.83114002	0.83616767	0.83932516	0.84115103
Thurrock	0.7617984	0.76508055	0.76692578	0.76808568	0.76955836	0.77078143	0.77156193	0.77244608	0.77419036	0.77687307	0.78015505	0.78349176	0.78748853	0.79209418	0.79558483	0.79834589	0.80148926	0.80543057	0.80971971	0.81464239	0.81776351	0.81959772
Greater London	0.84030883	0.84470211	0.84808838	0.85094349	0.85408771	0.85702182	0.8598156	0.86280693	0.86555189	0.86835084	0.87160102	0.87543247	0.88013157	0.8848343	0.8881433	0.89057307	0.89296303	0.89553814	0.89802884	0.9008461	0.90328554	0.90486476
Barking and Dagenham	0.73572423	0.73971404	0.74214235	0.74335222	0.74435237	0.74487122	0.7453979	0.74677634	0.74881659	0.75143482	0.75443637	0.75814817	0.76424208	0.77143218	0.77682347	0.78108087	0.78561971	0.79100111	0.79622478	0.80168939	0.80525367	0.80732495
Barnet	0.82620045	0.83078806	0.83413865	0.83676581	0.83930163	0.84162286	0.84382766	0.84604543	0.84810945	0.85015497	0.85299744	0.85662641	0.86126516	0.86594832	0.86928202	0.8718356	0.87426116	0.87697559	0.87976099	0.88298601	0.8848172	0.88597606
Bexley	0.77138492	0.7762055	0.77995794	0.78293343	0.78604034	0.78878367	0.79118262	0.79371234	0.7962597	0.79854463	0.80093545	0.80412363	0.80903412	0.81463145	0.81944244	0.82388089	0.82819052	0.83243853	0.83639173	0.84046774	0.84293186	0.84454763
Brent	0.80006207	0.80431918	0.80731606	0.80980032	0.81252935	0.81474281	0.81656274	0.81819565	0.81939383	0.82072269	0.82331879	0.82693645	0.83211444	0.83775859	0.84167643	0.84433317	0.84667456	0.84928146	0.8521231	0.85596076	0.85856847	0.86016434
Bromley	0.81208574	0.81672076	0.82012981	0.82304021	0.82609268	0.82874628	0.83103164	0.8332419	0.83510496	0.83640616	0.8375565	0.83945508	0.84286939	0.8468273	0.8501393	0.85303116	0.85586052	0.85911733	0.86254855	0.8661867	0.86822346	0.86954533
Camden	0.89650609	0.89896685	0.90098518	0.90281368	0.90495797	0.90704259	0.90909842	0.91143591	0.91404634	0.91661255	0.91892516	0.92148464	0.92419541	0.92661573	0.92829456	0.92934758	0.93040276	0.9316126	0.93290834	0.93428657	0.93549264	0.93647483
Croydon	0.7948106	0.79944393	0.80265198	0.80493859	0.807574	0.80979112	0.81143552	0.81321221	0.81484408	0.8162574	0.81820851	0.82072449	0.82441292	0.82849933	0.83128042	0.83355712	0.83645535	0.84012104	0.84384323	0.84805561	0.85062101	0.85206634
Ealing	0.82289891	0.8273953	0.83078645	0.83324708	0.83559593	0.83738075	0.83869019	0.83989127	0.84093462	0.8419383	0.8437711	0.84693741	0.85188405	0.8574838	0.86198393	0.86574523	0.8692633	0.87281969	0.87611372	0.8795415	0.88149643	0.88272888
Enfield	0.78013717	0.78439149	0.78740562	0.78976075	0.79218572	0.79435668	0.79635243	0.79851456	0.80092507	0.80349802	0.80689946	0.8109965	0.81622114	0.82170996	0.8255965	0.82864724	0.83185869	0.8354004	0.83882577	0.84265274	0.84500303	0.846461
Greenwich	0.77631967	0.78094286	0.78427594	0.78659955	0.78891732	0.79098356	0.79330042	0.79637723	0.79950247	0.80218477	0.8050603	0.80888263	0.81436808	0.82024314	0.8244898	0.82784916	0.83134099	0.83509882	0.83871121	0.84254074	0.84475097	0.84608019
Hackney	0.81471258	0.8181534	0.82082866	0.82326581	0.82683393	0.83082455	0.83530327	0.84042971	0.84497184	0.84974099	0.85541438	0.86139314	0.86770897	0.87339639	0.87671631	0.87877768	0.88063149	0.88268841	0.8848806	0.88806219	0.89105735	0.89294628
Hammersmith and Fulham	0.88902354	0.89150181	0.89348866	0.89540412	0.89754032	0.89971064	0.90205138	0.90452785	0.90695206	0.90956889	0.91239215	0.91529824	0.9183679	0.92145253	0.92378413	0.92559759	0.92732396	0.92906332	0.93074204	0.93255592	0.93408915	0.93515317
Haringey	0.8026294	0.80732689	0.81104019	0.81404371	0.81731621	0.82032251	0.82282993	0.82525506	0.82736214	0.82926629	0.83224998	0.83617217	0.84079127	0.84556738	0.84921561	0.85238221	0.85596973	0.86014756	0.86423234	0.86845276	0.87078061	0.87208455
Harrow	0.81145076	0.81584836	0.81886292	0.82138838	0.82467462	0.82768907	0.83020534	0.83242872	0.83427321	0.83562057	0.83741689	0.83986909	0.84324814	0.84676932	0.84885397	0.84999922	0.85083322	0.85201227	0.85371187	0.85660861	0.8586377	0.86001527

Havering	0.76311541	0.76796775	0.77155728	0.77422303	0.77764239	0.78130919	0.78450011	0.78730519	0.79005801	0.79238045	0.79480725	0.79793974	0.80220709	0.8067285	0.81023208	0.81353735	0.81715744	0.82119701	0.82536884	0.82993939	0.83279668	0.83464387
Hillingdon	0.83113758	0.83593895	0.83958379	0.84203799	0.84417612	0.8459004	0.8473667	0.84901091	0.85104679	0.8538497	0.85691681	0.86043264	0.86496569	0.87020307	0.87423136	0.87692208	0.87948897	0.88235649	0.8852682	0.88848906	0.89126898	0.89313813
Hounslow	0.82420358	0.8291261	0.83268461	0.83526961	0.83797114	0.84048106	0.84263564	0.84484453	0.84698829	0.84887354	0.85138265	0.85509044	0.86064099	0.86695427	0.87206989	0.87641897	0.88066009	0.88493488	0.88885189	0.89300646	0.89553523	0.89699458
Islington	0.87150706	0.87508324	0.87763267	0.87984139	0.88237152	0.8849225	0.8876219	0.89057345	0.89372454	0.89718682	0.90071609	0.90419345	0.90750645	0.91031388	0.91232004	0.91404133	0.91575754	0.91763015	0.91961203	0.9217552	0.92352931	0.92474007
Kensington and Chelsea	0.90843189	0.91006559	0.91150955	0.91296531	0.9147092	0.91641701	0.91834677	0.9204801	0.92272821	0.92509751	0.92750215	0.93009406	0.93264645	0.93489989	0.93658587	0.93796648	0.93952173	0.94116257	0.94266851	0.94420575	0.94550614	0.9464767
Kingston upon Thames	0.85603353	0.86055739	0.86411828	0.86704239	0.87006145	0.87296388	0.87577009	0.8787043	0.88166979	0.88396299	0.88611596	0.88829931	0.89081257	0.893262	0.89490464	0.89636244	0.89828529	0.90080218	0.90347627	0.90637444	0.90780389	0.90869828
Lambeth	0.83752619	0.84236157	0.84614767	0.84958141	0.85362941	0.85770494	0.86183536	0.86616036	0.87025638	0.87409945	0.87835678	0.88282194	0.88775428	0.89275294	0.8967257	0.90014163	0.9034326	0.90685971	0.91016639	0.91356358	0.91535783	0.91638082
Lewisham	0.7892805	0.79422735	0.79790394	0.80066658	0.80332108	0.80553478	0.80769716	0.81006311	0.81249982	0.8149258	0.81793909	0.82158935	0.82660821	0.8321343	0.83639314	0.83983936	0.8432392	0.8468051	0.85023211	0.85395556	0.85607039	0.85730894
Merton	0.82330884	0.82786023	0.83118703	0.83348895	0.83576605	0.83767292	0.83947687	0.84182137	0.84448647	0.84673501	0.8492308	0.85250906	0.85734091	0.86259802	0.86664475	0.87005724	0.8733615	0.87673744	0.88013501	0.88413348	0.88650159	0.88773396
Newham	0.74938282	0.75550038	0.76051169	0.7649267	0.76947164	0.77330625	0.77643043	0.7797725	0.78301783	0.78655411	0.79145262	0.79744619	0.80471522	0.81192357	0.81712044	0.82111356	0.82500948	0.82916684	0.83304735	0.83732911	0.84006801	0.84168813
Redbridge	0.78723136	0.79189485	0.79547287	0.79841522	0.801404	0.80383152	0.80618249	0.80887257	0.81103299	0.81247961	0.81454633	0.81772521	0.82253196	0.82777521	0.83155791	0.83436611	0.8371229	0.84019408	0.84320787	0.84679322	0.84896208	0.85026508
Richmond upon Thames	0.87717294	0.88124833	0.88434753	0.88689502	0.88938374	0.89154436	0.89359517	0.89581427	0.89795278	0.89959898	0.90163792	0.9043754	0.90773199	0.91139663	0.91465004	0.91772744	0.92077201	0.9239145	0.92695264	0.92995653	0.93132447	0.93213255
Southwark	0.85185331	0.85723068	0.8612693	0.8647362	0.86753676	0.86990066	0.87252757	0.87568665	0.87926389	0.88306436	0.8870974	0.8916206	0.89642638	0.9007784	0.90380911	0.90606481	0.90835988	0.9109518	0.91345716	0.91605289	0.91816468	0.91950983
Sutton	0.79482141	0.79972559	0.80326486	0.80604838	0.80871934	0.81083275	0.8126601	0.81467805	0.8167627	0.81875456	0.82118182	0.82433217	0.82895974	0.83413145	0.83836008	0.84190393	0.84524533	0.84853633	0.85157254	0.85503627	0.85711221	0.85842158
Tower Hamlets	0.82560101	0.83049202	0.83477564	0.83914333	0.84429237	0.84934343	0.85398229	0.85841338	0.86236273	0.86667264	0.87145478	0.87624438	0.88124895	0.88603276	0.88944824	0.89195975	0.89427139	0.89666401	0.89879702	0.90105103	0.90308201	0.90448814
Waltham Forest	0.76692302	0.77158819	0.77497571	0.77763364	0.78052014	0.78297295	0.78490751	0.78678602	0.78857743	0.79042113	0.79305762	0.79682894	0.80251966	0.80910434	0.81450239	0.81900025	0.82330467	0.82778592	0.83204346	0.83644284	0.83902216	0.8405096
Wandsworth	0.86930362	0.87407677	0.87806733	0.88146394	0.88472489	0.88765211	0.89051156	0.89344408	0.89620639	0.8983738	0.90052498	0.90286992	0.90572514	0.9088197	0.91129499	0.91346733	0.9157198	0.91806224	0.92033556	0.922739	0.92370388	0.92424149
Westminster	0.89395024	0.89655897	0.8986167	0.90046019	0.90251471	0.90464551	0.90703524	0.90966241	0.91235581	0.91472829	0.9164292	0.91839146	0.92113793	0.92391496	0.92595501	0.92756193	0.92927966	0.93114166	0.93286599	0.93462227	0.93611797	0.93719863
North East England	0.75517656	0.76000092	0.76344209	0.76610925	0.76900843	0.77181052	0.77460176	0.77761649	0.78049179	0.78306961	0.78615205	0.79003667	0.79540193	0.80107439	0.80511943	0.80829669	0.81151855	0.81514928	0.8188021	0.82310774	0.82571259	0.82727303
County Durham	0.74518594	0.74970548	0.75271731	0.75490689	0.75746944	0.76012681	0.76250071	0.76518332	0.76809846	0.77067482	0.77361282	0.77744124	0.78260244	0.78797616	0.79160399	0.79445489	0.79755543	0.80101293	0.80429708	0.80820503	0.81071906	0.81227652
Darlington	0.75775031	0.76164668	0.76418989	0.76635394	0.76952012	0.77273096	0.77560942	0.77801142	0.77963178	0.78159551	0.7852023	0.79002344	0.79688458	0.80457829	0.8103757	0.81514191	0.81963678	0.82406911	0.82807476	0.83254584	0.83546751	0.83720409
Gateshead	0.75234321	0.75731376	0.76122134	0.76445465	0.76791895	0.77124027	0.77429699	0.77713292	0.77945133	0.78248103	0.78714876	0.79238245	0.79833724	0.80419905	0.80822169	0.8114731	0.81513915	0.8192299	0.82258115	0.82623236	0.82866343	0.83024418
Hartlepool	0.71845461	0.72282349	0.72562898	0.72730131	0.72916009	0.73082238	0.73313271	0.73627497	0.73904738	0.7413062	0.74413561	0.74873543	0.75654915	0.76493273	0.77058336	0.77476087	0.77898656	0.78363469	0.78841263	0.79367659	0.796959	0.79887777
Middlesbrough	0.73196823	0.73688981	0.73963252	0.74149081	0.74415086	0.74749801	0.75101106	0.754776	0.75789713	0.75984869	0.76201042	0.76535148	0.77078309	0.77641283	0.78004115	0.78249349	0.78498498	0.78812686	0.7915783	0.79618331	0.79943482	0.801471
Newcastle upon Tyne	0.80202396	0.80722356	0.81229027	0.8171593	0.82195979	0.82625526	0.8305273	0.835104	0.83961735	0.84325483	0.84616954	0.84904908	0.85248202	0.85585328	0.85836593	0.86022435	0.86188351	0.86398528	0.86641485	0.86947394	0.87130709	0.87252066
North Tyneside	0.757655	0.76226785	0.76571699	0.76863718	0.77204743	0.77565546	0.77932008	0.78244648	0.7849722	0.78743786	0.79083703	0.79534912	0.80150615	0.80783385	0.81251038	0.8164165	0.82009684	0.82406908	0.82804409	0.83229832	0.83483439	0.8364006
Northumberland	0.754331	0.7583374	0.76108912	0.76318611	0.76580619	0.76865151	0.7714102	0.77387572	0.77586278	0.77786207	0.78077744	0.78431666	0.78967859	0.79558811	0.79963404	0.80313107	0.8067701	0.81060907	0.8144867	0.81902426	0.82177669	0.82331701
Redcar and Cleveland	0.72772366	0.73125509	0.73339543	0.73446566	0.736414	0.73919644	0.74231865	0.74511908	0.74720774	0.7486004	0.75059426	0.75444991	0.76139509	0.76923425	0.77451559	0.77772875	0.78075988	0.78427063	0.78810445	0.79310696	0.79644544	0.79836962
South Tyneside	0.72199177	0.72691563	0.73052272	0.73358975	0.73708044	0.74023518	0.74276091	0.74551789	0.7484208	0.75083025	0.75371171	0.75781144	0.76391028	0.77075899	0.77581579	0.77984432	0.78380594	0.78798301	0.79201587	0.79652902	0.79932541	0.80107303
Stockton-on-Tees	0.76591902	0.76933421	0.77137674	0.77284083	0.77461364	0.77620877	0.77816682	0.78018326	0.78180059	0.78396922	0.78735343	0.79176717	0.79783644	0.80411822	0.80850829	0.81170065	0.8149351	0.81842686	0.82187762	0.8263084	0.82931055	0.83098489
Sunderland	0.74535827	0.74997041	0.75329407	0.75609381	0.75904144	0.76134892	0.76372052	0.76661445	0.76961527	0.77241229	0.77550064	0.77947828	0.78497747	0.7907802	0.79506434	0.79839481	0.80167355	0.80548224	0.80953853	0.81426814	0.81730961	0.81916413
North West England	0.7718241	0.77664303	0.77984929	0.78202154	0.7846043	0.78724829	0.78980914	0.79258226	0.79540342	0.79826012	0.80162241	0.8055136	0.81076052	0.81647096	0.82080593	0.82440582	0.82800597	0.83195251	0.83582963	0.84018255	0.84284778	0.84444382
Blackburn with Darwen	0.7238112	0.72831272	0.73147637	0.73371769	0.7364082	0.73904631	0.74185751	0.74468719	0.74684093	0.74945207	0.75378134	0.75912251	0.76671182	0.77546956	0.78201727	0.78691731	0.79123695	0.79548674	0.80005974	0.80572532	0.80942581	0.81150374
Blackpool	0.71846253	0.7224849	0.7237447	0.72343032	0.72460302	0.72670251	0.72885299	0.73089932	0.73281453	0.73507899	0.73802993	0.74142686	0.74684695	0.75326273	0.75816527	0.76236711	0.76695876	0.7723135	0.77759512	0.78355735	0.78758585	0.78992589
Bolton	0.74433186	0.74837426	0.75101369	0.75247887	0.75429027	0.7560104	0.75773083	0.75934402	0.76056701	0.76243759	0.76605381	0.77079774	0.77736255	0.78433391	0.78911719	0.79270227	0.79629417	0.80056448	0.80471298	0.80930634	0.81234076	0.81417076
Bury	0.76184501	0.76538935	0.7675506	0.76897756	0.77117702	0.77371005	0.77584538	0.77782938	0.77943043	0.78091871	0.78349505	0.78759983	0.79381521	0.80071533	0.80604363	0.81029901	0.81420225	0.81815505	0.82186126	0.82601912	0.8285613	0.83008916
Cheshire East	0.81418307	0.81855068	0.82158838	0.82352578	0.82576388	0.82778333	0.82983807	0.83213362	0.83433343	0.83635945	0.83892111	0.84231857	0.8475738	0.85365725	0.85839506	0.86253848	0.86663733	0.87119795	0.87582037	0.88048343	0.88319117	0.88475463
Cheshire West and Chester	0.80497692	0.80935167	0.81256748	0.81471177	0.8171595	0.81989868	0.8227027	0.82579129	0.82895026	0.83141617	0.83380251	0.83677423	0.84138353	0.84658544	0.85069508	0.85413584	0.85743423	0.86079497	0.86387828	0.86750888	0.8697802	0.87117793
Cumbria	0.76598984	0.77009799	0.77249706	0.77399634	0.77645186	0.77891592	0.78125837	0.7838395	0.78657279	0.78952738	0.79311674	0.79736644	0.80368699	0.81079256	0.81632317	0.82112445	0.82576807	0.83045631	0.8347251	0.83908273	0.84177636	0.8434669
Halton	0.7494757	0.75427543	0.757266	0.75848716	0.76002317	0.76228671	0.7652617	0.76862377	0.77195658	0.77531719	0.77925367	0.78347154	0.78922992	0.79624839	0.80225792	0.80746561	0.81277136	0.81855164	0.82414361	0.8302224	0.83409953	0.83618135
Knowsley	0.72871711	0.7351277	0.73911406	0.74111117	0.7432513	0.74633275	0.74965489	0.75322783	0.75728608	0.76104959	0.76431571	0.76828803	0.77423718	0.78060947	0.78545745	0.78948257	0.79340886	0.79768751	0.80211415	0.80747793	0.81132351	0.81367171
Lancashire	0.77491484	0.77909261	0.78159083	0.78300964	0.78514659	0.78768313	0.79007049	0.79255696	0.79530341	0.79841727	0.80204457	0.80607239	0.81109734	0.81618565	0.81983333	0.82299312	0.82627369	0.82984765	0.83332213	0.83721137	0.83961368	0.84112554
Liverpool	0.77769986	0.78344546	0.78812525	0.79246909	0.79693049	0.8010914	0.80546792	0.81013324	0.81468745	0.81830189	0.8210977	0.82391341	0.82742119	0.83094411	0.8333797	0.83509089	0.83692164	0.83912808	0.84144421	0.84478329	0.84703646	0.84843764
Manchester	0.80363785	0.80955397	0.81403795	0.81762344	0.82152632	0.82561957	0.82988392	0.83485281	0.84025763	0.8451014	0.84898639	0.85230152	0.85607922	0.86006397	0.86321152	0.86598442	0.86882049	0.87190682	0.87483461	0.8781406	0.88015764	0.88142914
Oldham	0.71780555	0.7214547	0.72403929	0.725877	0.72826509	0.73060863	0.73277793	0.7350019	0.73623757	0.73819361	0.7428259	0.74898586	0.75669133	0.76466026	0.77025821	0.77412929	0.77767871	0.78214086	0.78694605	0.7925242	0.79625616	0.79840829
Rochdale	0.72799553	0.73223349	0.73485662	0.73671113	0.73893372	0.74103171	0.74251341	0.74411545	0.74570559	0.74777554	0.75157152	0.75679946	0.76405722	0.77178826	0.77720887	0.78100561	0.78440606	0.7881374	0.79199254	0.79681444	0.80011008	0.80206932
Salford	0.75973452	0.76548316	0.76987678	0.77320434	0.77643396	0.77950527	0.78257535	0.78569202	0.78908628	0.79257324	0.79591883	0.79940812	0.80447952	0.8101548	0.81395997	0.8169183	0.82054222	0.82478679	0.82891253	0.83367174	0.83684926	0.83869917
Sefton	0.76577516	0.769984	0.7730009	0.77556717	0.77886283	0.78178457	0.78411247	0.7862983	0.78800487	0.78942336	0.79119608	0.79306048	0.79621548	0.80046564	0.80399812	0.80709585	0.81013867	0.81368371	0.81753595	0.82186386	0.82438642	0.8258419
St Helens	0.73858007	0.74341145	0.74625448	0.74828049	0.75107331	0.75341307	0.75551871	0.75813763	0.76087717	0.7639785	0.7676737	0.7717539	0.7772925	0.78357098	0.78804636	0.7911898	0.79421081	0.79810586	0.80250587	0.80740478	0.81042007	0.8122146
Stockport	0.79690384	0.80148098	0.80477528	0.80704286	0.80934283	0.81152974	0.8135672	0.81570169	0.81761666	0.81944177	0.82200484	0.82523717	0.83001618	0.83560134	0.83997996	0.84362522	0.84733211	0.85117696	0.85477669	0.85849273	0.86052225	0.86171971
Tameside	0.73750282	0.74192775	0.74498667	0.7468947	0.74909784	0.75065637	0.75143437	0.75236238	0.75311745	0.75440437	0.75689691	0.76046529	0.76633805	0.77303027	0.77791402	0.7815795	0.78478989	0.78811133	0.79136903	0.79573372	0.79874919	0.80054669
Trafford	0.82635424	0.83102853	0.83470571	0.8377655	0.84100298	0.84381435	0.84621897	0.84832994	0.84982075	0.85138938	0.85409001	0.85750281	0.86222785	0.86778532	0.87254436	0.87687609	0.88101739	0.88520163	0.88929246	0.89332238	0.89550613	0.89675378
Warrington	0.80042762	0.80552801	0.80924165	0.81229377	0.81576474	0.81900113	0.82121982	0.82299003	0.8246727	0.82680404	0.83044779	0.83511681	0.84105702	0.84739354	0.85249578	0.85701058	0.86150674	0.86594896	0.87009959	0.87459779	0.87725742	0.87875621
Wigan	0.73656244	0.74105777	0.7435692	0.74472141	0.74655518	0.74906725	0.75146393	0.75306437	0.75426927	0.75639054	0.76019588	0.76518481	0.77141251	0.77745707	0.78178146	0.78549911	0.78944013	0.79391114	0.79819132	0.80256961	0.80520008	0.80685214
Wirral	0.75033765	0.75447106	0.75693217	0.75855582	0.76094516	0.76333047	0.76548729	0.76751363	0.76941677	0.77131613	0.77392955	0.77743861	0.78305632	0.78956171	0.79458561	0.79886987	0.80294669	0.80722854	0.81120829	0.81532945	0.81778247	0.81931629
South East England	0.81453592	0.81866875	0.82173457	0.82417183	0.82676548	0.82899217	0.83094146	0.83305352	0.83517101	0.83719356	0.8396068	0.84249881	0.84653195	0.85107907	0.85477468	0.85812498	0.86158042	0.8652482	0.86877208	0.8725336	0.87472821	0.8760666
Bracknell Forest	0.82807357	0.83272796	0.8361016	0.83862188	0.8412608	0.84347634	0.8451873	0.84672589	0.84814235	0.84972147	0.8520067	0.85497518	0.85932558	0.86439815	0.8686428	0.87262318	0.87661109	0.88053256	0.8842129	0.88784011	0.88981581	0.89100266
Brighton and Hove	0.83775655	0.8424023	0.84643095	0.85007394	0.85368981	0.8569527	0.86005652	0.86318119	0.8662899	0.86900759	0.87172474	0.87446877	0.87750885	0.88060818	0.88320914	0.88556575	0.88804263	0.89074714	0.89337716	0.89605158	0.89732773	0.8982572
Buckinghamshire	0.8352728	0.83909176	0.84187946	0.84408786	0.84643126	0.84829935	0.84960667	0.85079262	0.85178781	0.85267416	0.85417882	0.85667491	0.86062679	0.86508993	0.86866959	0.87183366	0.87501242	0.87851956	0.88207819	0.88571458	0.88762548	0.88878524
East Sussex	0.77251839	0.77674968	0.77982055	0.78224736	0.78489792	0.78706102	0.78873477	0.79040214	0.79190036	0.79330811	0.79559561	0.79867364	0.80327563	0.80874454	0.81334036	0.81759223	0.82199993	0.82652316	0.83077296	0.83531134	0.83800984	0.83959479
Hampshire	0.81089733	0.81509293	0.81801107	0.82011123	0.82238382	0.82439782	0.8261282	0.82804709	0.82987976	0.83163608	0.83398175	0.83709505	0.84157957	0.8465606	0.85056054	0.85437533	0.85834057	0.86227937	0.86579977	0.86946715	0.87155772	0.87285
Isle of Wight	0.7551791	0.75993144	0.76322741	0.76519787	0.76719031	0.76904815	0.77124374	0.77385158	0.77629632	0.77820529	0.78018829	0.78277279	0.78683895	0.79178496	0.79608654	0.80041738	0.80519924	0.81067578	0.81603557	0.82130478	0.82451604	0.82639963

Kent	0.78031289	0.7844356	0.78744966	0.78971696	0.79214864	0.79413323	0.79595689	0.7979821	0.79995811	0.80200709	0.80478083	0.80819853	0.81286395	0.8179832	0.82194814	0.82530204	0.8287368	0.83266675	0.83663029	0.84102153	0.84372411	0.84534539
Medway	0.75510823	0.75877207	0.76162445	0.76420479	0.76713692	0.76942453	0.77118967	0.77297525	0.77468939	0.7766228	0.77942113	0.78273483	0.78741044	0.79255667	0.79636819	0.79958832	0.8030539	0.8073482	0.81167293	0.81618517	0.81893955	0.82066784
Milton Keynes	0.8150882	0.81897639	0.8218732	0.82421027	0.82689155	0.8289722	0.83033809	0.83142131	0.83226545	0.83339458	0.83547402	0.83881144	0.84444555	0.85141317	0.85757335	0.86320655	0.86875047	0.87415256	0.87832676	0.88221577	0.88530394	0.88717934
Oxfordshire	0.83835575	0.84271664	0.84599586	0.84859294	0.8511909	0.8535381	0.85566033	0.85804485	0.86054188	0.8627231	0.8649828	0.86756895	0.87109535	0.87512378	0.87863759	0.88218828	0.88591533	0.88953937	0.89279149	0.89632717	0.89831888	0.89947593
Portsmouth	0.8076178	0.81199062	0.81542911	0.81822759	0.82090657	0.82322772	0.82545209	0.82805508	0.83115236	0.83416247	0.83671976	0.83909559	0.84233073	0.84606216	0.84911677	0.85179364	0.8544725	0.8573527	0.86001186	0.86292961	0.86467472	0.86587593
Reading	0.85293744	0.85784299	0.8618166	0.8650803	0.86823015	0.87057453	0.87185217	0.87366865	0.87611377	0.87822923	0.87904761	0.87957825	0.88140175	0.88456014	0.88765942	0.89049271	0.89330058	0.89621678	0.89897063	0.90190157	0.90431908	0.90587359
Slough	0.81900265	0.8214746	0.82330758	0.82451084	0.8257586	0.82621675	0.82598375	0.82585896	0.82615659	0.82804461	0.83135486	0.83583971	0.84213206	0.84874124	0.85385335	0.85803702	0.86202653	0.86594187	0.86947605	0.87328253	0.8765373	0.87855725
Southampton	0.81015023	0.81475129	0.81851083	0.82164415	0.82455951	0.82693701	0.82906817	0.83136861	0.83384975	0.83565154	0.83688952	0.83828507	0.84058256	0.84330949	0.84534903	0.84725151	0.84961047	0.85243428	0.85517765	0.85828484	0.86010019	0.86134908
Surrey	0.84950138	0.85398569	0.85728936	0.85994151	0.8624662	0.86445316	0.86607386	0.86788482	0.86970367	0.87132598	0.8732674	0.87548292	0.87877828	0.88260538	0.88588635	0.88904663	0.89228143	0.89553774	0.89874817	0.9020841	0.90382965	0.9048594
West Berkshire	0.84681987	0.85087726	0.85347602	0.85414895	0.85477897	0.85482124	0.85439969	0.85404108	0.85425028	0.85578691	0.85834418	0.86188037	0.86685645	0.87226468	0.87666497	0.88025107	0.88359419	0.88702536	0.89021858	0.89345061	0.89608614	0.89775701
West Sussex	0.80303899	0.80727905	0.81031956	0.81248303	0.81496096	0.81707191	0.81883466	0.82058725	0.82211726	0.82358414	0.82590089	0.82901353	0.83347447	0.83865143	0.84291021	0.84659433	0.8501431	0.85380912	0.85733381	0.86114164	0.86339007	0.86479215
Windsor and Maidenhead	0.85526134	0.85947554	0.86298159	0.8660002	0.86886598	0.87117198	0.87301075	0.87488871	0.87664683	0.87802149	0.87989865	0.88232926	0.88603994	0.89085791	0.89511708	0.89881061	0.90235087	0.9059027	0.90929175	0.91284889	0.91473804	0.91581805
Wokingham	0.86306258	0.86710861	0.8704076	0.87311457	0.87589429	0.87834707	0.88028486	0.88213974	0.88400656	0.88525218	0.88610988	0.88727031	0.88997221	0.8936533	0.89665973	0.89922274	0.90173139	0.90424761	0.90651357	0.90918875	0.91072748	0.91165875
South West England	0.79343697	0.79786834	0.80104634	0.80368179	0.80652576	0.80900425	0.8111514	0.81334646	0.81563201	0.81778862	0.82044909	0.82367897	0.82820951	0.83331304	0.83751707	0.84135103	0.8452322	0.84936962	0.85337881	0.85759435	0.86000988	0.86146702
Bath and North East Somerset	0.83075085	0.83584502	0.84050575	0.84482246	0.8487305	0.85221763	0.85568901	0.85938525	0.86296555	0.86555203	0.86777709	0.87001823	0.8727443	0.87575204	0.87828145	0.88090449	0.88409246	0.88745563	0.89031457	0.8930694	0.89445098	0.89543536
Bournemouth	0.8048075	0.81011008	0.8145398	0.81852419	0.82241845	0.82560447	0.82841321	0.8313547	0.83434427	0.83682931	0.83898966	0.84123649	0.84459133	0.84861968	0.85211305	0.85519666	0.85822726	0.86143265	0.86447808	0.86761169	0.86931921	0.87046477
Bristol, City of	0.83222633	0.83658094	0.8398284	0.84242176	0.84513932	0.8478601	0.8506721	0.85393064	0.85767376	0.86085072	0.86339527	0.8658109	0.86912093	0.87306573	0.87672518	0.88024725	0.88375121	0.8873875	0.89072045	0.89406699	0.89587235	0.89704187
Cornwall	0.76994659	0.77429251	0.77761354	0.78045262	0.78339497	0.78573839	0.78780296	0.78975369	0.79148465	0.79322605	0.7954772	0.79843508	0.80332577	0.80902662	0.81370439	0.81775645	0.82192046	0.82638101	0.83073446	0.83539397	0.8381743	0.83976856
Devon	0.7877467	0.79236557	0.79589158	0.79892481	0.80224409	0.80541911	0.80820232	0.81077568	0.81318568	0.81530465	0.81780667	0.8208979	0.82539621	0.83043518	0.83442479	0.83778135	0.84089371	0.84426645	0.8477182	0.85162009	0.85396499	0.8553761
Dorset	0.7835336	0.78734836	0.79016348	0.79235565	0.79467044	0.79649652	0.79779012	0.79882781	0.79984405	0.80141766	0.8044968	0.808423	0.81357242	0.81921679	0.82387957	0.82816077	0.83261981	0.83748523	0.84228551	0.84711044	0.84993704	0.85161514
Gloucestershire	0.80408717	0.80862767	0.81196667	0.8150688	0.81841569	0.82094651	0.82260538	0.8241468	0.82582094	0.82759457	0.83056038	0.83437906	0.83915456	0.84429117	0.84859362	0.8526156	0.85648445	0.86041398	0.86417148	0.86810169	0.87037831	0.8718063
North Somerset	0.78395343	0.78812337	0.7913713	0.79407923	0.79690982	0.79935442	0.8012024	0.80330976	0.80574388	0.80823865	0.81152651	0.81562505	0.82109059	0.82696856	0.83172396	0.8360364	0.84056734	0.84552716	0.85013781	0.85473285	0.85742559	0.85901767
Plymouth	0.78027017	0.78432143	0.78720446	0.78957262	0.7922817	0.79456638	0.79694807	0.80028357	0.80441718	0.80773762	0.81000155	0.8120936	0.81574968	0.82019961	0.82356052	0.8264205	0.82930036	0.83241611	0.83570451	0.83954392	0.84182482	0.84330754
Poole	0.79869131	0.80269517	0.80521256	0.80721052	0.80983995	0.81231308	0.81401556	0.81550983	0.81678664	0.81801996	0.82049631	0.82436683	0.82995814	0.83599646	0.8408079	0.84482	0.84865368	0.85248022	0.85598662	0.859739	0.86203606	0.8634943
Somerset	0.77303053	0.77698019	0.77940974	0.78145855	0.78400478	0.78625955	0.78807283	0.78949123	0.79052535	0.79190708	0.79472084	0.79856494	0.8038229	0.80964748	0.81434725	0.81878724	0.82340885	0.82842183	0.83335523	0.83846511	0.84156352	0.84340431
South Gloucestershire	0.81687563	0.82137858	0.82449811	0.82722374	0.83025257	0.83326002	0.8360384	0.83866626	0.84133347	0.84359673	0.84584925	0.84861734	0.85315551	0.85859109	0.86302103	0.86698568	0.87066636	0.87417854	0.87753267	0.88130736	0.8836038	0.88498476
Swindon	0.80526529	0.80913587	0.81160586	0.81326735	0.81487751	0.81593085	0.81649947	0.81731366	0.81855129	0.82002005	0.82294159	0.8271647	0.8329984	0.83908782	0.84340154	0.84689113	0.85066236	0.85496726	0.85914119	0.8634704	0.86605834	0.86754591
Torbay	0.75030851	0.75338146	0.75438811	0.754903	0.75646469	0.75815326	0.75962425	0.76116012	0.76302188	0.76474152	0.76640881	0.76847408	0.7725493	0.77785163	0.78236604	0.78666901	0.79130529	0.79652416	0.80181322	0.80739957	0.8109517	0.81303848
Wiltshire	0.79261431	0.79672143	0.79958959	0.80197267	0.8047506	0.80677253	0.8079798	0.80848882	0.80853481	0.80925605	0.81188872	0.81586941	0.8213935	0.82725146	0.83199469	0.83652255	0.84123202	0.84609662	0.85059802	0.85515896	0.85786894	0.85946624
West Midlands	0.75940199	0.76419493	0.76739212	0.76990253	0.77296591	0.77602218	0.77876534	0.78158843	0.78460552	0.78762594	0.79093321	0.79473515	0.80022585	0.80627967	0.81090038	0.81466979	0.81833681	0.82245982	0.82655635	0.83119289	0.8341226	0.83586514
Birmingham	0.75847432	0.76318415	0.7664411	0.76927616	0.77298803	0.7766293	0.77985239	0.78339913	0.78761596	0.79191728	0.79568782	0.79949509	0.80504462	0.81099026	0.81533317	0.81856957	0.82169385	0.82547098	0.82928301	0.83360171	0.8363727	0.8380669
Coventry	0.78134072	0.78585288	0.7890003	0.79146355	0.79441863	0.79685247	0.79863054	0.80071697	0.80362602	0.80689408	0.81012859	0.81365593	0.81832839	0.8231046	0.82667426	0.82985796	0.8332547	0.83682862	0.84027758	0.84430573	0.846954	0.84861807
Dudley	0.73979363	0.74427842	0.74729128	0.74936771	0.75152197	0.75396139	0.75620269	0.7580014	0.75919646	0.76011798	0.76160445	0.76384667	0.7688098	0.77512062	0.77979086	0.78316793	0.78637504	0.79038813	0.79458623	0.79924052	0.80229133	0.80416452
Herefordshire, County of	0.77159358	0.77626011	0.77935754	0.78182235	0.78456624	0.78703024	0.78902465	0.79093782	0.792409	0.79360618	0.79585412	0.79905394	0.804075	0.81024118	0.81570086	0.82069788	0.82570314	0.83106166	0.83617974	0.84137324	0.84451711	0.84631047
Sandwell	0.71857945	0.72283216	0.72545606	0.72756304	0.73041819	0.7333331	0.73538172	0.73642892	0.73729346	0.73898715	0.7418434	0.74609279	0.75330892	0.76150626	0.76752487	0.77225644	0.77634958	0.78060815	0.78485671	0.78995172	0.79354727	0.7957223
Shropshire	0.77301822	0.77746284	0.78066583	0.78391262	0.78757885	0.79078434	0.79328886	0.79522814	0.79663556	0.79833618	0.80141039	0.80520777	0.81027226	0.81576026	0.81969129	0.82297794	0.82657257	0.83065305	0.83468107	0.83906667	0.84168606	0.84321876
Solihull	0.80360557	0.80827892	0.81137497	0.81380002	0.81646701	0.81882621	0.82106282	0.82343308	0.82558809	0.82715904	0.82862908	0.83099922	0.83534223	0.84059777	0.84515986	0.84933905	0.85328059	0.85760922	0.86220782	0.86719634	0.87026072	0.87207923
Staffordshire	0.76907437	0.7732957	0.77582975	0.77754142	0.77990074	0.78247342	0.78465858	0.78686557	0.78936718	0.79166767	0.7942002	0.79715621	0.80145485	0.80613642	0.80949591	0.81215811	0.81474739	0.81785817	0.82112974	0.82517526	0.82776957	0.82932024
Stoke-on-Trent	0.72714976	0.73027224	0.73156998	0.73185777	0.73252905	0.73342029	0.73404156	0.73489991	0.7372284	0.74076156	0.74467652	0.7489263	0.75520015	0.76256378	0.76850354	0.77337781	0.77786649	0.78239312	0.78680682	0.79227991	0.79608339	0.79824252
Telford and Wrekin	0.76241506	0.76602649	0.7679446	0.76862517	0.76962126	0.77069061	0.77158447	0.77271811	0.77365602	0.77452161	0.77645308	0.77990406	0.78621931	0.79337891	0.79871453	0.80263221	0.80638653	0.81091226	0.81586187	0.82163784	0.82535598	0.82734156
Walsall	0.71460718	0.71886749	0.72126627	0.72305864	0.72548179	0.72792705	0.73059278	0.73319739	0.73508391	0.73755521	0.74138152	0.745819	0.75194796	0.75862015	0.76366623	0.76777489	0.77161849	0.77601823	0.78061959	0.78627209	0.79021275	0.79252944
Warwickshire	0.79830751	0.80270591	0.80567369	0.80770336	0.81001716	0.81227052	0.81455719	0.81714308	0.81986034	0.82256539	0.82578936	0.82959047	0.83444076	0.83970743	0.84378855	0.84723101	0.85097732	0.85493102	0.85854707	0.86257368	0.86512987	0.86667266
Wolverhampton	0.73990862	0.74486558	0.74770409	0.74969265	0.75237235	0.7549415	0.75717898	0.75971752	0.76222535	0.7641851	0.76655327	0.76997031	0.77522982	0.78094124	0.78544814	0.78948146	0.79360241	0.79831587	0.80276611	0.80748807	0.81054331	0.81238984
Worcestershire	0.7776588	0.78260173	0.78565652	0.78788183	0.79043604	0.79306715	0.79546466	0.79761486	0.79925241	0.80033585	0.80217175	0.80494064	0.80937894	0.81463709	0.81890503	0.82268224	0.82643128	0.83070607	0.83489777	0.83932159	0.84201697	0.8436113
Yorkshire and the Humber	0.7632249	0.76782316	0.77092397	0.77316885	0.77585492	0.77852448	0.78108279	0.78377648	0.78647	0.78942334	0.793	0.79715448	0.80273245	0.80873012	0.81300926	0.81641848	0.8199989	0.82412766	0.82823204	0.83292531	0.83578028	0.83745988
Barnsley	0.71580215	0.71939295	0.7212779	0.72254548	0.72447191	0.72614909	0.72784122	0.72996402	0.73143156	0.7332235	0.73632621	0.74085492	0.74768513	0.75503387	0.75979321	0.76331118	0.76738425	0.77228768	0.77724381	0.7831531	0.78704739	0.78926182
Bradford	0.73074201	0.73449237	0.73735089	0.73968877	0.74245972	0.74526746	0.74804615	0.75042709	0.75214499	0.75484221	0.75935368	0.7651499	0.77307222	0.78146074	0.78744802	0.79192952	0.79610027	0.80078437	0.80549308	0.81077249	0.8140866	0.8159859
Calderdale	0.75936439	0.76307062	0.76503626	0.76611469	0.76814065	0.77065512	0.77299568	0.77493226	0.77632046	0.77826625	0.78181311	0.78700259	0.79460396	0.8028525	0.80879226	0.81317984	0.81741625	0.82242643	0.82754697	0.83290612	0.8360443	0.83786393
Doncaster	0.71366349	0.71796464	0.72074742	0.72231981	0.72469089	0.72730461	0.72996197	0.7320148	0.73296918	0.73508999	0.7403241	0.74723035	0.75546636	0.7634393	0.7685089	0.77235445	0.77655094	0.78103803	0.78508206	0.79005164	0.7934974	0.79561402
East Riding of Yorkshire	0.77190792	0.77541984	0.77784142	0.77983664	0.78254281	0.78494296	0.78677659	0.78887476	0.7906523	0.79251856	0.79525519	0.79827886	0.802608	0.807537	0.81120261	0.81447687	0.81818708	0.82262412	0.82699617	0.8315954	0.83420933	0.83573194
Kingston upon Hull, City of	0.73235985	0.73746846	0.74056624	0.7423618	0.7446305	0.74708158	0.75004301	0.75388144	0.75809192	0.76192186	0.76485484	0.76791287	0.77243865	0.77748853	0.78028336	0.78180226	0.78360443	0.78627627	0.78943781	0.79430767	0.7978394	0.79993173
Kirklees	0.7518841	0.75527989	0.75751735	0.75945609	0.76230224	0.7649492	0.767068	0.76923034	0.77148322	0.77402084	0.77707879	0.78097817	0.78678323	0.79334374	0.79804513	0.8017518	0.80570369	0.81014066	0.81454694	0.81950305	0.82251833	0.82420765
Leeds	0.80360137	0.80853145	0.81256029	0.8160545	0.81962674	0.82311888	0.82664365	0.83033843	0.83416856	0.83744808	0.83988924	0.84211762	0.84523085	0.84877432	0.85134105	0.85342879	0.85566212	0.85836533	0.86129905	0.86502661	0.86733998	0.86876125
North East Lincolnshire	0.72312402	0.7267298	0.72790829	0.72769286	0.72856609	0.73075318	0.73366541	0.73679164	0.73916289	0.74188384	0.74587937	0.7508597	0.75815562	0.76636165	0.77223551	0.77695238	0.78176291	0.78697509	0.79201602	0.7981714	0.80242105	0.80483785
North Lincolnshire	0.75826371	0.76155487	0.7636934	0.76474285	0.76581921	0.76708404	0.76874501	0.77039975	0.77132587	0.77398941	0.77932018	0.78539781	0.79260005	0.79967865	0.80377813	0.80644455	0.80953287	0.813241	0.8169412	0.82141363	0.82424309	0.82590487
North Yorkshire	0.78635729	0.79060775	0.79354854	0.79585924	0.7987629	0.80141609	0.80357594	0.80507233	0.80621353	0.80807882	0.81118381	0.81539244	0.82126436	0.82717479	0.83104951	0.83419753	0.83777266	0.84240359	0.8471695	0.85190497	0.85461769	0.85619466
Rotherham	0.72528019	0.72943114	0.73208845	0.73415469	0.73697094	0.73948253	0.74122531	0.74298449	0.74503668	0.74759725	0.75118263	0.7552126	0.76091717	0.76809633	0.77342731	0.77770324	0.78242769	0.78782923	0.79301919	0.79862188	0.80215957	0.80422881
Sheffield	0.7856316	0.79071623	0.79450324	0.79751226	0.8011134	0.80470991	0.80794172	0.81160302	0.81580425	0.81959563	0.82283648	0.82613627	0.83025416	0.83426294	0.83707787	0.83945781	0.8421767	0.84527023	0.84819258	0.8515876	0.85360997	0.85489097
Wakefield	0.73579856	0.73988621	0.74183011	0.74291679	0.74498837	0.74700719	0.74842609	0.74943653	0.75002797	0.75165293	0.75497844	0.75973113	0.7670525	0.77524882	0.78087355	0.7846803	0.78832109	0.79254754	0.7965832	0.80144121	0.80474471	0.80681149
York	0.83125939	0.83585085	0.83970369	0.84293237	0.84627296	0.84947327	0.85252994	0.85559102	0.85859821	0.86126199	0.86379114	0.86637336	0.8694626	0.872595	0.87495675	0.87715855	0.87947393	0.8818522	0.88410163	0.8865909	0.88781128	0.88873893
Northern Ireland	0.77384799	0.77883952	0.78350445	0.78767388	0.79111671	0.79359254	0.79596234	0.79834743	0.80111937	0.80382392	0.80626793	0.80926674	0.81311377	0.81679016	0.82006248	0.82378622	0.82777576	0.83096932	0.83280699	0.8352507	0.83704932	0.83853194
Scotland	0.7904048	0.79483002	0.7984167	0.80117767	0.80371686	0.80595463	0.80752952	0.80918775	0.81205983	0.81565915	0.81936935	0.82244997	0.82485759	0.82758968	0.83094453	0.83410999	0.83743176	0.84114064	0.84476207	0.84863859	0.85078715	0.85206142

Wales	0.76099681	0.76584341	0.76943011	0.77209612	0.77506018	0.7777931	0.78019275	0.78269139	0.78514879	0.78762431	0.79065666	0.79419495	0.79911822	0.80492191	0.80941694	0.81315138	0.81681818	0.8205084	0.82461706	0.8297892	0.8327143	0.83427854
Latin America and Caribbean	0.54272237	0.54788854	0.55297248	0.55781592	0.56284923	0.56790262	0.57305602	0.57835546	0.58376018	0.58847432	0.59355664	0.5990377	0.60455091	0.61016338	0.61554288	0.62047013	0.62510555	0.62980657	0.63453797	0.63917311	0.64298253	0.64654128
Andean Latin America	0.53756051	0.54257235	0.54791107	0.55310898	0.55823544	0.56309152	0.5679874	0.57299897	0.57856419	0.58399894	0.59020118	0.59694462	0.60383923	0.61070517	0.61705999	0.62276075	0.62816126	0.6335379	0.63886016	0.64392132	0.64780682	0.65160246
Bolivia	0.4863279	0.49203076	0.49734884	0.5023658	0.50727304	0.51216649	0.51714619	0.52194613	0.52694375	0.53179244	0.53700907	0.54268074	0.54861548	0.55478822	0.56093458	0.5670009	0.57308032	0.57924128	0.58518411	0.59069204	0.59485444	0.5990108
Ecuador	0.54304478	0.54855215	0.55489733	0.56095548	0.56629029	0.56992734	0.57251627	0.5747047	0.57812504	0.58248426	0.58876306	0.59672908	0.60545432	0.61408027	0.62202743	0.62854671	0.63428114	0.64001122	0.64588852	0.65178799	0.65671446	0.66101705
Peru	0.5489522	0.55377545	0.55885326	0.56387107	0.56906302	0.574517	0.58050336	0.58695849	0.59375081	0.59995499	0.60652093	0.61305122	0.61935526	0.62551902	0.63111435	0.63627827	0.64128528	0.64618085	0.65097732	0.65543373	0.65867224	0.66205404
Caribbean	0.55736894	0.56311049	0.56906859	0.57488862	0.58053331	0.58575799	0.59060234	0.59470961	0.59829691	0.60165483	0.60548008	0.60952505	0.61325472	0.61687685	0.62044265	0.62407467	0.62758207	0.63078807	0.63397217	0.63732123	0.63970853	0.64200305
Antigua and Barbuda	0.66081906	0.66562063	0.6703608	0.67531149	0.68041243	0.68548644	0.69082602	0.69636004	0.70217536	0.70712856	0.71143225	0.71548999	0.71950932	0.72286498	0.72558598	0.72837276	0.73159894	0.73496371	0.73887911	0.74305296	0.74634533	0.74988689
The Bahamas	0.74251795	0.74359288	0.74625032	0.74991671	0.75375964	0.75678266	0.75918594	0.761991	0.7655905	0.76956167	0.77411981	0.77828189	0.78183864	0.78462391	0.78725349	0.78995615	0.7927387	0.79534718	0.79817903	0.80103307	0.80294802	0.80502067
Barbados	0.6813042	0.68332939	0.68780758	0.69323596	0.6980297	0.70151005	0.70385619	0.70587165	0.7085145	0.71214336	0.71620855	0.72006069	0.72416246	0.72782617	0.73038329	0.73260299	0.73491253	0.73715726	0.73953144	0.74223931	0.74436665	0.74674876
Belize	0.49864373	0.50239943	0.50687166	0.51267946	0.51969733	0.52723106	0.53488962	0.54190069	0.54809322	0.55346587	0.55854689	0.56344036	0.56845624	0.57323646	0.57809133	0.58327909	0.58827591	0.59333729	0.59851201	0.6033516	0.60706005	0.610229
Bermuda	0.7359965	0.74191891	0.7475445	0.75330516	0.75900888	0.7646909	0.77059677	0.77611426	0.78035833	0.78475911	0.78918147	0.79338052	0.79748487	0.8013882	0.80492341	0.80799879	0.81055084	0.81280723	0.8148928	0.81700034	0.81920342	0.82136542
Cuba	0.5666769	0.57275386	0.57996829	0.58764776	0.59434872	0.5998638	0.60497057	0.6076181	0.60940092	0.61177564	0.61526126	0.62038669	0.62615602	0.6318398	0.63730413	0.64281896	0.64790787	0.65230929	0.65685865	0.66167472	0.66521075	0.66872986
Dominica	0.64583094	0.65236857	0.65842355	0.66367887	0.66895213	0.67454908	0.68034248	0.68541592	0.69019752	0.69446187	0.69848919	0.70246326	0.70687061	0.71221048	0.71845556	0.72478554	0.7317608	0.73606247	0.73893595	0.74213207	0.74441994	0.74696718
Dominican Republic	0.49546859	0.50277542	0.51029802	0.51739557	0.52454327	0.53222865	0.54022269	0.54818702	0.55537131	0.56178375	0.56813058	0.57383481	0.57896638	0.58362583	0.58823674	0.59292416	0.59761961	0.60214029	0.60696084	0.61191302	0.61563563	0.6193882
Grenada	0.53838812	0.54905897	0.55947313	0.56971804	0.57848746	0.58715888	0.59405452	0.60076634	0.60687928	0.61180526	0.616487	0.62097852	0.62497751	0.62904834	0.6337347	0.63893608	0.64433523	0.64983152	0.65537277	0.66080851	0.66508635	0.66899303
Guyana	0.52869399	0.53486981	0.54051427	0.54551647	0.55032637	0.5548448	0.55946561	0.56454114	0.56959521	0.57476993	0.58008391	0.58568827	0.59133658	0.59682629	0.60213651	0.60760549	0.61391436	0.62060261	0.62716704	0.63364035	0.64228464	0.65081234
Haiti	0.36257221	0.36864351	0.37447332	0.38006725	0.38506151	0.38984542	0.394322	0.39897344	0.40328587	0.40794442	0.41184011	0.41617719	0.42002153	0.42398555	0.4279409	0.43175676	0.43528872	0.438541	0.44167893	0.44432885	0.44639061	0.44827828
Jamaica	0.59459292	0.60019202	0.60559175	0.61087742	0.61583628	0.62040006	0.62501886	0.62954947	0.63396785	0.63811084	0.64212939	0.64639583	0.65058376	0.65457362	0.65850534	0.66246581	0.66646928	0.67029105	0.67407531	0.67777854	0.68056718	0.68326306
Puerto Rico	0.71003565	0.71895586	0.72595686	0.73047514	0.73421955	0.73774227	0.74162943	0.74588381	0.75092217	0.75636169	0.76176325	0.76678488	0.7723118	0.77846116	0.78512034	0.79180017	0.79908408	0.80664896	0.81290607	0.81884319	0.82291844	0.82552585
Saint Kitts and Nevis	0.64435338	0.64922665	0.65459341	0.65992531	0.66756813	0.67914546	0.69007867	0.69772763	0.70414149	0.7094995	0.71446042	0.71932438	0.72343615	0.72670659	0.72953022	0.73222067	0.73556614	0.73947068	0.74373646	0.74815207	0.75156841	0.75498705
Saint Lucia	0.5797889	0.58508756	0.59016758	0.59567515	0.60162548	0.60723987	0.6127553	0.61799471	0.623273	0.62816985	0.6327829	0.63743944	0.64156713	0.64515308	0.64874351	0.65215748	0.65565334	0.65934135	0.66308668	0.66684036	0.66972049	0.67250974
Saint Vincent and the Grenadines	0.53317329	0.53945571	0.54606362	0.55269372	0.55866354	0.56358812	0.56822175	0.57281905	0.57761114	0.58254911	0.58719038	0.5902591	0.59215391	0.59461407	0.59837867	0.60375413	0.61005921	0.61643847	0.62263494	0.62832599	0.6328609	0.63719596
Suriname	0.54366002	0.54868352	0.55387943	0.55941837	0.56510971	0.57046219	0.57564705	0.58065457	0.58564483	0.5905128	0.59546975	0.60035634	0.60474187	0.60891061	0.61270442	0.61609732	0.61886809	0.62168418	0.62470801	0.62789678	0.63068371	0.63366574
Trinidad and Tobago	0.67668384	0.68276349	0.68887227	0.69582834	0.70261081	0.70852098	0.71460021	0.72019642	0.7257207	0.72953889	0.73323708	0.7368024	0.73999907	0.74359396	0.74733896	0.75151068	0.75495335	0.75822699	0.76124199	0.7641696	0.76642208	0.76876325
Virgin Islands	0.70843656	0.71375111	0.72439827	0.73460985	0.7446136	0.75426756	0.7634475	0.77218059	0.78034262	0.78705515	0.7932197	0.7980717	0.80109285	0.80340579	0.80564138	0.80788925	0.81019904	0.81240517	0.81482544	0.81742901	0.81960171	0.82183085
Central Latin America	0.53730037	0.54243984	0.54713435	0.5514461	0.5560115	0.56074953	0.56564869	0.57070727	0.57583933	0.57994006	0.58425317	0.58930755	0.59482717	0.60073102	0.60650929	0.61181533	0.61708609	0.62244243	0.62776464	0.63284707	0.63699589	0.6406851
Colombia	0.52783909	0.5330765	0.53818934	0.54326513	0.54860183	0.55408919	0.56018273	0.56682264	0.57349465	0.57987647	0.58640811	0.59334048	0.60025424	0.60725064	0.61415192	0.62088923	0.6275545	0.63413036	0.64025507	0.64603885	0.65085535	0.65544291
Costa Rica	0.58824319	0.59488911	0.60089443	0.60576569	0.6103157	0.61441262	0.61785016	0.62120057	0.62570391	0.63129138	0.63659543	0.6412013	0.64657409	0.65194581	0.65689046	0.66203014	0.66743009	0.67362139	0.68129615	0.69004909	0.69619461	0.70034048
El Salvador	0.44396267	0.45417766	0.46296886	0.47039741	0.47653666	0.48204172	0.486826	0.49080975	0.49507774	0.49948971	0.50417134	0.50896924	0.51339687	0.51812191	0.5234689	0.52938695	0.53565027	0.54199896	0.54814317	0.55403165	0.55891989	0.56377519
Guatemala	0.3798773	0.38692769	0.39689964	0.40823416	0.41977706	0.43032753	0.43885638	0.44724596	0.45654187	0.46400308	0.47047736	0.47690968	0.48186285	0.48728669	0.49402719	0.50065561	0.50714979	0.51373568	0.52099547	0.52863047	0.53457141	0.53997242
Honduras	0.38798805	0.39417401	0.4003718	0.40666838	0.41306182	0.41961233	0.426353	0.43324031	0.43999816	0.44615105	0.45238223	0.45870033	0.46492515	0.47076683	0.4765878	0.48229418	0.48770593	0.49313069	0.49865723	0.50415651	0.50866925	0.51303725
Mexico	0.55953255	0.56477418	0.57011353	0.57569821	0.58098498	0.58522408	0.58854323	0.59144741	0.59445456	0.59668436	0.59952703	0.6036461	0.60891429	0.61508999	0.6218574	0.62889672	0.63592535	0.6426672	0.64905332	0.65509534	0.66011906	0.6645753
Aguascalientes	0.57602722	0.58238199	0.5889208	0.59528251	0.60098395	0.6052851	0.60866303	0.61151629	0.61424432	0.61621885	0.61901915	0.62293883	0.62772639	0.63339809	0.63976965	0.64638692	0.6529246	0.65932105	0.66550502	0.67148323	0.67656818	0.68115928
Baja California	0.59933828	0.60536455	0.61185852	0.61819065	0.6233876	0.62712069	0.6298734	0.6322404	0.63481885	0.63658599	0.63888763	0.64266689	0.64772087	0.65351265	0.65971276	0.66640432	0.67356449	0.68055944	0.68732717	0.69378576	0.69923592	0.70408197
Baja California Sur	0.59584449	0.60006956	0.60486462	0.61048615	0.61622124	0.62103964	0.62487862	0.62826975	0.6318338	0.63507846	0.63924246	0.64472575	0.65111728	0.65803001	0.66517874	0.67275445	0.68022975	0.68730215	0.6939065	0.70006349	0.70516978	0.70969428
Campeche	0.54081983	0.54766425	0.55399832	0.56010839	0.56568472	0.57021152	0.57369097	0.57695541	0.58046699	0.58358722	0.58795187	0.59396891	0.6012821	0.60945014	0.61820947	0.62666517	0.63461986	0.64212714	0.6490849	0.65552325	0.66079487	0.66536343
Chiapas	0.44413082	0.44941525	0.45517242	0.46203366	0.46912156	0.47529399	0.48064481	0.48513561	0.48943204	0.49313887	0.49767803	0.50360146	0.51055297	0.51827703	0.52633506	0.53433331	0.54174283	0.54860507	0.55484592	0.56063971	0.56553923	0.56994091
Chihuahua	0.55667892	0.56253594	0.56932625	0.57637622	0.5828403	0.58806159	0.59205091	0.59546385	0.59911245	0.60119468	0.60315739	0.60632246	0.61056631	0.6163539	0.62315116	0.63106406	0.63913046	0.64693782	0.65440955	0.66158134	0.66781309	0.67338657
Coahuila	0.57883433	0.5840259	0.5890641	0.5944839	0.59954588	0.60333064	0.60590202	0.60765195	0.60931691	0.60973611	0.61041472	0.61244547	0.61575958	0.62103424	0.62794617	0.63548626	0.64341066	0.6513131	0.65889094	0.66610472	0.67219156	0.67756288
Colima	0.59772777	0.60285289	0.60751331	0.61204098	0.61615131	0.61976899	0.62281415	0.62561546	0.62862318	0.63102798	0.63432309	0.63871612	0.64401238	0.65030157	0.65720227	0.66443733	0.6715459	0.67817972	0.68432488	0.69002339	0.69479277	0.69911677
Durango	0.52647474	0.53278507	0.53938985	0.54636552	0.55270206	0.55723541	0.5603506	0.56274673	0.5651476	0.56687741	0.56933197	0.57313736	0.57834545	0.58478399	0.59198057	0.59964337	0.60736431	0.61475571	0.62176943	0.62839404	0.63414276	0.63932484
Guanajuato	0.53458618	0.54100819	0.54720638	0.55323782	0.55873953	0.56315616	0.56669951	0.56968314	0.57274518	0.57518014	0.57837607	0.58297839	0.58883586	0.59514959	0.60177357	0.60879	0.61629616	0.62342171	0.63017593	0.63652515	0.64178294	0.64637431
Guerrero	0.46093254	0.46720119	0.47372739	0.48043549	0.48664537	0.49140142	0.49528328	0.49907592	0.50348019	0.50750852	0.51248156	0.5187392	0.52611801	0.53432833	0.54284332	0.55090221	0.55826047	0.56494497	0.57089293	0.57631346	0.58083578	0.58485047
Hidalgo	0.50586657	0.51211415	0.51828996	0.52481297	0.53118129	0.53641315	0.54068698	0.54463751	0.54886168	0.55265827	0.55741998	0.56348608	0.57072053	0.57876194	0.5872382	0.59585308	0.60387871	0.61131371	0.61810621	0.62427846	0.62919666	0.63347845
Jalisco	0.57701229	0.58202038	0.58694671	0.59202047	0.59691328	0.60072816	0.60388805	0.60674647	0.60984851	0.6119959	0.61466819	0.6184028	0.6231613	0.62878405	0.63501843	0.64149564	0.64800707	0.65435007	0.6604767	0.66641179	0.67142597	0.67599837
México	0.57691942	0.58106381	0.58582736	0.59120498	0.59686804	0.60177977	0.60567699	0.60910883	0.61242263	0.61495132	0.61801981	0.62217966	0.62735517	0.6333249	0.63982252	0.64641531	0.65315396	0.65977775	0.66614588	0.67220359	0.67717449	0.68158761
Mexico City	0.67514284	0.67961917	0.68392976	0.68818145	0.69202952	0.69492232	0.69665424	0.69788087	0.69910929	0.69977615	0.70105554	0.70359496	0.70757164	0.71272226	0.7187518	0.72509475	0.73171036	0.73834093	0.74480794	0.75091056	0.75573933	0.75984841
Michoacán de Ocampo	0.51653161	0.52210021	0.5274816	0.53296124	0.53789494	0.54188616	0.54540142	0.54837031	0.55118386	0.55309694	0.55560424	0.55941862	0.56425136	0.5697645	0.57574863	0.58200157	0.58799894	0.59374718	0.59921892	0.60446897	0.60894406	0.61308202
Morelos	0.57915846	0.58428072	0.58878902	0.59302338	0.5964867	0.59876495	0.60044167	0.60198986	0.60392155	0.60521237	0.60739619	0.61082863	0.61539009	0.62113935	0.6277245	0.63476951	0.64183492	0.64865498	0.65501762	0.66090471	0.66575519	0.67007405
Nayarit	0.53361802	0.54084317	0.54829926	0.55618327	0.56372443	0.56997449	0.57504674	0.57852923	0.58130463	0.58358488	0.58699834	0.5919435	0.59832169	0.60563177	0.61348113	0.62147646	0.62901613	0.63620787	0.6428643	0.64890895	0.65383828	0.65817891
Nuevo León	0.62340677	0.62844154	0.63362729	0.6385176	0.64273628	0.64574276	0.6479221	0.64969342	0.65138713	0.65225715	0.65370374	0.65651089	0.66056596	0.66545621	0.67097312	0.67685353	0.68283157	0.68869497	0.69450271	0.70033179	0.7054379	0.71014182
Oaxaca	0.4647321	0.47001697	0.47596674	0.48289772	0.48994688	0.49571783	0.50049736	0.50547992	0.51104287	0.51590312	0.52153162	0.52772142	0.53427677	0.54121777	0.54829387	0.55548763	0.562415	0.56882615	0.57475406	0.58032331	0.5849127	0.5890419
Puebla	0.50946911	0.51476248	0.5204563	0.52662078	0.53246478	0.53734586	0.54150447	0.54515706	0.54877225	0.55173609	0.55548434	0.56038968	0.56637828	0.57313399	0.58022349	0.58729009	0.59416603	0.60068456	0.60680583	0.61261265	0.61754268	0.62203183
Querétaro	0.57471192	0.58262614	0.59020629	0.5969834	0.60286326	0.60763528	0.61150265	0.61493464	0.61829291	0.62065493	0.62337074	0.62740736	0.63267686	0.63847015	0.64455963	0.65067975	0.65690943	0.66285294	0.66847775	0.67385834	0.67840425	0.68255812
Quintana Roo	0.57777696	0.583471	0.58886607	0.59427902	0.59922215	0.60306565	0.60619519	0.60928534	0.6127802	0.61582787	0.6197026	0.62455861	0.63012531	0.63622861	0.64252622	0.64896632	0.65546411	0.66152672	0.6671612	0.67247292	0.6769749	0.68106867
San Luis Potosí	0.52106938	0.52781326	0.53446477	0.54137996	0.54808194	0.55416128	0.55964176	0.56486215	0.57023152	0.57481416	0.58010304	0.58599604	0.59232433	0.59920789	0.6064493	0.61351907	0.62014295	0.62633442	0.63214448	0.63767532	0.64234352	0.64665151
Sinaloa	0.56506574	0.5706942	0.57621237	0.58228089	0.58823541	0.59291479	0.59635723	0.59943278	0.60286843	0.60536385	0.60845589	0.61299726	0.61868781	0.6254509	0.63289691	0.64051841	0.64764677	0.65448631	0.66103819	0.66730947	0.67256473	0.67716497
Sonora	0.59178041	0.59785695	0.60344527	0.6092954	0.61458273	0.61897369	0.62255647	0.6253938	0.62814627	0.62963753	0.63155817	0.63550766	0.64130954	0.64865557	0.65705101	0.66606179	0.67505335	0.6836726	0.69186868	0.69948653	0.70560693	0.7106796
Tabasco	0.5338914	0.53980678	0.54523273	0.55086714	0.55619872	0.5609007	0.56483121	0.56845656	0.57224247	0.57543617	0.57954117	0.58503691	0.59166737	0.59880351	0.60636409	0.61399416	0.62114236	0.62785981	0.6341084	0.63985315	0.64460154	0.64872802
Tamaulipas	0.59220815	0.59502879	0.59778925	0.60077571	0.60345225	0.60547266	0.60721869	0.60929697	0.61200372	0.61373213	0.61576083	0.6193195	0.62410571	0.63000066	0.63670817	0.64390979	0.65124145	0.65831479	0.66513256	0.67169784	0.67723621	0.68208611
Tlaxcala	0.54001133	0.54645412	0.55282779	0.55926087	0.56543135	0.57055635	0.57485642	0.57876288	0.58275381	0.58589762	0.58962131	0.59407332	0.59916092	0.604873	0.61091633	0.61710525	0.62316194	0.62889651	0.63427565	0.63937401	0.64374855	0.64782243

Veracruz de Ignacio de la Llave	0.51022218	0.51476074	0.51949377	0.52506646	0.53078548	0.5355783	0.53959946	0.54351481	0.54783522	0.55154502	0.55591504	0.56146529	0.56811892	0.5754009	0.58309252	0.5910758	0.59882257	0.60607728	0.61278797	0.61895227	0.62388476	0.62813696
Yucatán	0.53669085	0.54134838	0.54573208	0.55111079	0.55712125	0.56270542	0.56789218	0.57301505	0.57831159	0.58267134	0.58748259	0.59310568	0.59933071	0.60603672	0.61308507	0.62008059	0.62649344	0.63256604	0.63835718	0.64393674	0.64867162	0.65305081
Zacatecas	0.53671219	0.54257064	0.54849739	0.55423957	0.55947384	0.56341252	0.56656109	0.56944972	0.57257197	0.57447126	0.57658921	0.57995006	0.58436328	0.5897267	0.59565048	0.6019342	0.60826097	0.61430989	0.62003687	0.6255434	0.63033879	0.63477366
Nicaragua	0.41610836	0.4237903	0.43081375	0.43724815	0.44327451	0.44884474	0.4539505	0.4590428	0.4638377	0.46796184	0.472172	0.47659347	0.4812969	0.48610547	0.49120735	0.49639166	0.50185334	0.50748202	0.51233823	0.51659705	0.52029267	0.52395847
Panama	0.58737839	0.5945357	0.60013868	0.60404336	0.60687703	0.60949412	0.61224971	0.61528542	0.61895891	0.62240281	0.62511632	0.62863394	0.63426344	0.64132435	0.64910817	0.65796205	0.66727676	0.6767835	0.68671992	0.69667068	0.70378849	0.70886483
Venezuela	0.56350626	0.56545838	0.56260446	0.55521222	0.55097226	0.55394168	0.56333419	0.57536717	0.58618489	0.59328842	0.59912792	0.60482348	0.61018747	0.61444102	0.61468681	0.61067536	0.60699321	0.60516013	0.60406825	0.60231389	0.60003478	0.59651306
Tropical Latin America	0.54171565	0.54693151	0.55236462	0.55769144	0.56326866	0.56889732	0.57472031	0.58091535	0.58735364	0.59330312	0.599646	0.60595697	0.61178731	0.61740172	0.62264612	0.62739963	0.63153336	0.63576518	0.6401799	0.64469363	0.64860285	0.65244239
Brazil	0.5420515	0.54730443	0.55277603	0.55814571	0.56377291	0.56945812	0.57533877	0.58158876	0.58807782	0.5940733	0.60044585	0.60677729	0.61261934	0.61821824	0.62343381	0.6281498	0.63223511	0.63642219	0.64080297	0.64529801	0.64920157	0.65304389
Acre	0.42910086	0.4352422	0.44171553	0.44810708	0.45499678	0.4622089	0.46954104	0.47766215	0.48632357	0.49449839	0.50267316	0.51015135	0.51717238	0.52383613	0.53006515	0.5356433	0.54055077	0.54550663	0.55066535	0.55603086	0.56087343	0.56585153
Alagoas	0.4119514	0.41679849	0.42219061	0.42769309	0.43363762	0.43978004	0.44625282	0.45313153	0.46010413	0.46685006	0.47413253	0.48187333	0.48887141	0.49558388	0.50181441	0.50753087	0.51247415	0.51742963	0.52241993	0.52738526	0.5315926	0.53565998
Amapá	0.51824111	0.52356672	0.52916824	0.53394388	0.53867638	0.54376686	0.54992141	0.5567305	0.56379686	0.5703049	0.57661695	0.58263976	0.58882984	0.59478011	0.60037187	0.60532117	0.60951412	0.61382742	0.61842977	0.62329167	0.62765185	0.63217964
Amazonas	0.50606222	0.5083553	0.51161263	0.51529552	0.52014233	0.52558709	0.53183699	0.5382475	0.54462383	0.55048988	0.55676855	0.56268609	0.56758545	0.57235744	0.57702076	0.58139865	0.58537281	0.589701	0.59452521	0.59967702	0.60452735	0.60948447
Bahia	0.45926884	0.46395652	0.46935003	0.47474591	0.48082891	0.48749493	0.49424201	0.50147269	0.50889675	0.51622681	0.52363228	0.53042636	0.53657321	0.54246878	0.5479409	0.55278909	0.55691537	0.56103638	0.56529991	0.56967424	0.57350195	0.57739425
Ceará	0.44905546	0.45376096	0.45907005	0.46430322	0.46989832	0.47575904	0.48218972	0.48899228	0.49650801	0.50377829	0.51166156	0.51960503	0.52649963	0.5329569	0.53883263	0.54403997	0.54843112	0.5527994	0.55713287	0.56146657	0.56509036	0.5686657
Distrito Federal	0.66441614	0.67097978	0.6771953	0.68278788	0.68818668	0.6934783	0.69910631	0.70523097	0.71186188	0.71828101	0.72484482	0.73120969	0.73692571	0.74230807	0.74721747	0.7515915	0.75540551	0.75937914	0.76355805	0.76790887	0.7719088	0.77601664
Espírito Santo	0.54356148	0.55028861	0.55685332	0.56311935	0.5703109	0.57792774	0.5856401	0.59346908	0.60121699	0.60738254	0.61422973	0.62154727	0.62832847	0.63451315	0.63999595	0.64483223	0.64898961	0.65318995	0.65761295	0.66217932	0.66617627	0.67019259
Goiás	0.51143262	0.51912453	0.52749671	0.53541233	0.54316138	0.55036251	0.55758046	0.56505097	0.57259058	0.57975964	0.58687107	0.59396246	0.60064445	0.60670179	0.61209319	0.61682493	0.62077903	0.62478839	0.6289701	0.63327306	0.63708137	0.64093387
Maranhão	0.36358774	0.36818221	0.37328639	0.37876805	0.38490452	0.39180998	0.3992711	0.40732112	0.4162305	0.42420826	0.4322344	0.44044871	0.44837228	0.45559983	0.46205531	0.4678107	0.47286503	0.47798381	0.48330632	0.48883121	0.4938582	0.49892987
Mato Grosso	0.51622992	0.52354486	0.53205088	0.5415599	0.55233966	0.56168368	0.56946023	0.57784383	0.58689801	0.59482991	0.60169954	0.60844833	0.61456396	0.6198616	0.62450143	0.62859601	0.63205244	0.63562973	0.63947142	0.64361388	0.64739224	0.65118576
Mato Grosso do Sul	0.5214238	0.52776671	0.5343377	0.5412101	0.54783577	0.55405041	0.56047904	0.56723467	0.57417317	0.58067343	0.58769634	0.59473936	0.60139531	0.60765836	0.61356757	0.61898809	0.62369262	0.62852876	0.63358374	0.63874298	0.64321738	0.64753332
Minas Gerais	0.5371023	0.54220372	0.54796245	0.55392298	0.5604885	0.56697721	0.57366948	0.58057011	0.5876665	0.59364851	0.60026782	0.60687262	0.61287949	0.61862861	0.62396609	0.62876546	0.63279528	0.63682282	0.64098257	0.64523321	0.64878904	0.65229325
Pará	0.46088399	0.46326273	0.46652025	0.47027497	0.47537039	0.48131073	0.4880692	0.49540936	0.50332024	0.51029576	0.51874817	0.5272334	0.53455948	0.54135179	0.54753813	0.55306489	0.55785553	0.56272867	0.56781093	0.57302851	0.57757771	0.5821344
Paraíba	0.44356385	0.44828987	0.45357463	0.45878929	0.46396904	0.4696234	0.47627149	0.48337298	0.49096978	0.49830088	0.50571183	0.5131686	0.52023424	0.52685659	0.53296335	0.53834627	0.54285845	0.54731419	0.55182047	0.55632748	0.56013944	0.56386815
Paraná	0.55764124	0.56430073	0.57125354	0.57848667	0.58556012	0.59191819	0.59816094	0.60494367	0.61151613	0.61731326	0.62318032	0.62898059	0.63439992	0.63969304	0.64480177	0.64956588	0.6537573	0.65808635	0.66266957	0.6673212	0.67118223	0.67485653
Pernambuco	0.46036538	0.46463093	0.46970161	0.47484008	0.48033601	0.48638652	0.49287223	0.49985444	0.50714301	0.51431826	0.52227143	0.53013497	0.53780983	0.54507415	0.55178047	0.55775813	0.56292157	0.56802278	0.57314208	0.5781638	0.58251064	0.58676422
Piauí	0.39882112	0.4033661	0.40839295	0.41363987	0.41928292	0.42540848	0.43225262	0.43962404	0.44780789	0.45596594	0.46446946	0.47285306	0.48032714	0.48729461	0.49362619	0.49922648	0.50392286	0.50851113	0.51309203	0.51764091	0.52147543	0.52524312
Rio de Janeiro	0.61300501	0.61835324	0.62369883	0.62844558	0.63356798	0.63859363	0.64363218	0.64869694	0.65412966	0.65890302	0.6640695	0.66959267	0.6749798	0.68021835	0.68514694	0.68975329	0.69393831	0.69841903	0.70321155	0.70811781	0.71223305	0.71609384
Rio Grande do Norte	0.45873082	0.46428238	0.47050124	0.47640276	0.48275443	0.48958579	0.49701859	0.50477026	0.51260986	0.52007218	0.52790584	0.53575974	0.54318056	0.55013798	0.55654885	0.56226331	0.56716165	0.57201278	0.57691059	0.58176686	0.58587668	0.58985482
Rio Grande do Sul	0.59177442	0.59721093	0.60286137	0.60844358	0.61397434	0.61918651	0.62451543	0.63026995	0.63616683	0.64153856	0.64721481	0.65241174	0.65712977	0.66170394	0.66604768	0.67015455	0.67381145	0.67766695	0.68176946	0.68604738	0.68972093	0.69333753
Rondônia	0.48414852	0.49035524	0.4974075	0.50509925	0.51331333	0.52168498	0.52935747	0.53721999	0.54554468	0.55363802	0.56199134	0.57060824	0.57832751	0.58536475	0.59173397	0.59738986	0.60224486	0.60706357	0.6119503	0.6167603	0.62087528	0.62485467
Roraima	0.51631806	0.5226118	0.5289284	0.53490412	0.54009252	0.54515053	0.55076404	0.55686254	0.56331634	0.56946522	0.57550591	0.58117384	0.58602361	0.59065211	0.59495541	0.59875076	0.60192626	0.60532266	0.60900961	0.61316143	0.61700942	0.62100557
Santa Catarina	0.59174644	0.59792221	0.60428927	0.61066548	0.61721484	0.6234999	0.62967057	0.63612891	0.64293118	0.64899146	0.65541699	0.66170105	0.66721635	0.67226091	0.67671186	0.68056338	0.68374263	0.68712054	0.69078787	0.69466464	0.69801177	0.7013407
São Paulo	0.61146726	0.61742224	0.62305869	0.62826422	0.63342469	0.63856599	0.64372872	0.64914092	0.6545714	0.65967818	0.66517002	0.67062106	0.67567899	0.68071911	0.68550619	0.68983514	0.69359848	0.69753702	0.70174714	0.70614456	0.70995406	0.7137276
Sergipe	0.48022488	0.48525471	0.49100615	0.49676668	0.50282255	0.50910864	0.51578591	0.52283265	0.53017827	0.53658309	0.54358193	0.55045065	0.55674883	0.56264961	0.56800955	0.57273835	0.57671048	0.5807074	0.58481942	0.58899082	0.59243863	0.59584018
Tocantins	0.43220813	0.4414859	0.45121971	0.46155584	0.4718882	0.48187005	0.49162077	0.50186296	0.51250914	0.522696	0.53302898	0.54252897	0.55132071	0.55961728	0.56732128	0.57440259	0.58065846	0.58681648	0.59287864	0.5988406	0.60386448	0.60858768
Paraguay	0.52300779	0.52691676	0.53117736	0.53536013	0.53955666	0.5436227	0.5479433	0.55275868	0.5580169	0.56290006	0.56884656	0.57495446	0.58065019	0.58724704	0.59395315	0.60048413	0.60701814	0.61358159	0.62003236	0.62607014	0.63105769	0.6357181
North Africa and Middle East	0.51682421	0.52409127	0.53114945	0.53829213	0.54567816	0.55304739	0.56008896	0.5665161	0.57233926	0.57706216	0.58184904	0.58759308	0.5941117	0.60112707	0.60841869	0.61577742	0.62314232	0.63056077	0.63791791	0.64509479	0.65167349	0.65822472
North Africa and Middle East	0.51682421	0.52409127	0.53114945	0.53829213	0.54567816	0.55304739	0.56008896	0.5665161	0.57233926	0.57706216	0.58184904	0.58759308	0.5941117	0.60112707	0.60841869	0.61577742	0.62314232	0.63056077	0.63791791	0.64509479	0.65167349	0.65822472
Afghanistan	0.17702577	0.17777314	0.18380843	0.19051899	0.19690354	0.20392813	0.21107419	0.21962761	0.22808917	0.23790082	0.24775995	0.25704168	0.26648441	0.27563656	0.28403035	0.2918495	0.2996307	0.30742462	0.31486609	0.32245405	0.32983007	0.3372
Algeria	0.53948151	0.54752928	0.55517222	0.56253696	0.56935285	0.57600196	0.58212636	0.58779054	0.59319097	0.59787038	0.60282353	0.60782553	0.61270353	0.6174222	0.62208729	0.62674557	0.63171074	0.63697318	0.64250004	0.64821078	0.65365147	0.65950092
Bahrain	0.6467507	0.65719606	0.6649012	0.66956411	0.67414778	0.6797906	0.68614429	0.69304515	0.69980639	0.70488584	0.70790262	0.70895874	0.71036242	0.71334316	0.71676806	0.7199266	0.7236113	0.7293231	0.73619258	0.7428478	0.74810308	0.7530432
Egypt	0.50216989	0.50871682	0.51503813	0.5206234	0.52518168	0.52866548	0.53039581	0.52945759	0.52474383	0.51625583	0.50771548	0.50459404	0.50915707	0.51933446	0.53174981	0.54437015	0.55601247	0.56691463	0.57744279	0.58773688	0.59736334	0.60678709
Iran	0.56547456	0.57461821	0.58409727	0.59396301	0.60371745	0.61329106	0.6212681	0.62774944	0.63286223	0.63739838	0.64255917	0.64789909	0.65150059	0.65447529	0.65796151	0.66205799	0.66765245	0.67434346	0.68090962	0.68674166	0.69191876	0.6972074
Alborz	0.63279463	0.64006476	0.64754683	0.65527469	0.66295954	0.67055544	0.6770396	0.6825559	0.68702035	0.6910409	0.69581766	0.70090292	0.70454243	0.70798813	0.71207689	0.71648139	0.72214212	0.72849718	0.73437934	0.73937839	0.74376241	0.74830202
Ardebil	0.50770823	0.52056048	0.53345248	0.54654539	0.55914828	0.57122281	0.58096928	0.58852021	0.59446442	0.59981496	0.60585685	0.61209234	0.61637548	0.61966722	0.62313981	0.6268815	0.63178915	0.63780159	0.64382993	0.64916853	0.65381615	0.65863977
Bushehr	0.57396415	0.58308205	0.59244592	0.60232832	0.61204096	0.62173823	0.63002304	0.63680753	0.64211431	0.64697555	0.65255811	0.65844479	0.66239921	0.66555968	0.66914772	0.67329909	0.6789001	0.68568407	0.69235058	0.69821121	0.70330496	0.70841871
Chahar Mahaal and Bakhtiari	0.53854403	0.54949036	0.56030347	0.57137699	0.58221791	0.59270157	0.60137153	0.60831844	0.61355128	0.61809149	0.62319591	0.62849679	0.63172724	0.63414164	0.63714608	0.64097387	0.64662885	0.65353781	0.66048041	0.66674984	0.67234977	0.6779886
East Azarbayejan	0.52643573	0.53582323	0.54556048	0.55555939	0.56532694	0.57470787	0.58229499	0.58822174	0.59281479	0.59702743	0.6020572	0.60751365	0.61166103	0.61544217	0.620065	0.62567214	0.63288757	0.64099987	0.64881945	0.65576851	0.6619758	0.66819323
Fars	0.58115914	0.59118372	0.60153883	0.61180187	0.62155638	0.63081941	0.63833705	0.64498069	0.65053186	0.65562818	0.66152584	0.66779732	0.67239882	0.67613073	0.68008035	0.68425814	0.6895716	0.69564725	0.70134214	0.70620755	0.71040186	0.71483077
Gilan	0.57887202	0.58701381	0.59569803	0.60481486	0.61396167	0.62314844	0.63152725	0.63926951	0.64585756	0.65180483	0.65813963	0.6643904	0.66883051	0.67267945	0.6768535	0.68124253	0.68686625	0.69308583	0.69878458	0.70363634	0.70788343	0.71231945
Golestan	0.53108678	0.5406348	0.55058163	0.56092596	0.57104193	0.58110741	0.58933407	0.59519379	0.59936676	0.6029337	0.60729219	0.61191122	0.61449407	0.61605468	0.61810864	0.62100968	0.62574684	0.63212231	0.63885318	0.64501391	0.65048709	0.65607753
Hamadan	0.52490645	0.5357609	0.54697637	0.55872245	0.57020542	0.58137325	0.59086598	0.59886027	0.60532807	0.61110702	0.61713502	0.62300822	0.62675037	0.62948069	0.63253792	0.63593216	0.64069585	0.64657251	0.6523723	0.65746758	0.66197252	0.66673482
Hormozgan	0.52171885	0.53348297	0.54516827	0.5572524	0.56951993	0.58145571	0.59111459	0.5982347	0.6032555	0.60744938	0.612072	0.61714823	0.62047072	0.62305076	0.62649556	0.63092541	0.63691823	0.64425644	0.65167453	0.6584113	0.66439043	0.67033137
Ilam	0.55953875	0.57259365	0.58594631	0.59960845	0.61244793	0.6244424	0.63492378	0.64446234	0.65269259	0.66003048	0.66714449	0.67356111	0.67740557	0.68004545	0.68245549	0.68473158	0.68837089	0.69282091	0.69689833	0.70022852	0.70302049	0.70619493
Isfahan	0.58908825	0.59704574	0.60532413	0.61395121	0.62256599	0.63124642	0.63909362	0.64623614	0.65227017	0.65777781	0.66359148	0.66930034	0.67319066	0.67640926	0.67978134	0.68315896	0.68767327	0.69295059	0.69786856	0.70206375	0.70572271	0.70971684
Kerman	0.55617564	0.56588313	0.57569564	0.58614764	0.59604588	0.60509009	0.61200228	0.61701861	0.6203251	0.62293311	0.62613935	0.62968143	0.63148626	0.63263676	0.6344102	0.63701352	0.64126343	0.64698714	0.6529345	0.65836188	0.66327078	0.66843927
Kermanshah	0.52332682	0.53336772	0.54406293	0.55564768	0.56743563	0.5790679	0.58910471	0.59778718	0.60498501	0.61158977	0.61865164	0.6257156	0.63073523	0.63475457	0.63877166	0.64288124	0.64803394	0.65412878	0.66001774	0.66516019	0.66970802	0.67446647
Khorasan-e-Razavi	0.51490729	0.5238786	0.5336534	0.54438878	0.55547648	0.56681338	0.57643632	0.58438061	0.59086725	0.5966024	0.60302374	0.6095685	0.6140323	0.61774605	0.62207644	0.62715662	0.63377622	0.64167964	0.64959811	0.65681776	0.66334337	0.66982664
Khuzestan	0.53104134	0.540748	0.55094375	0.56138294	0.57169178	0.58179572	0.58968845	0.59541384	0.59979113	0.60378766	0.60865116	0.61396507	0.61764073	0.6207112	0.62442376	0.62905901	0.63524247	0.64272055	0.65029543	0.65719997	0.66339721	0.66958791
Kohgiluyeh and Boyer-Ahmad	0.52355661	0.5353544	0.54776883	0.56086145	0.57385919	0.58642175	0.5966095	0.60489609	0.61149933	0.61765013	0.624879	0.63251482	0.63842728	0.64381055	0.64959119	0.65560125	0.66262008	0.6703172	0.67747087	0.68356697	0.68865258	0.6937062
Kurdistan	0.47371049	0.48522519	0.49764178	0.51107844	0.52453113	0.53764009	0.54886023	0.55845825	0.56649055	0.57375338	0.58125951	0.58865669	0.59407374	0.59857926	0.60324242	0.60802492	0.61391165	0.62056984	0.6269015	0.63241361	0.63723346	0.64226169
Lorestan	0.51879755	0.53142939	0.54443517	0.55766933	0.57051034	0.58266223	0.59258997	0.60040075	0.60660969	0.61209201	0.61775946	0.62330088	0.62683319	0.62950842	0.63256128	0.63628853	0.64145836	0.64770928	0.65385361	0.659311	0.664169	0.66911829

Markazi	0.55165623	0.56158775	0.57175965	0.58231232	0.59263299	0.60243907	0.61079517	0.61792865	0.62362005	0.62872574	0.63421755	0.63974771	0.64343683	0.64636712	0.64958868	0.65315602	0.6580912	0.66391587	0.66941126	0.6741577	0.67837742	0.68284382
Mazandaran	0.60136911	0.60818109	0.61615703	0.62565229	0.63572296	0.64607724	0.6553376	0.66354985	0.67026376	0.67607989	0.68194593	0.68754549	0.6912232	0.69416052	0.69731276	0.70078164	0.70577577	0.71153246	0.71691625	0.72153492	0.72560047	0.7299299
North Khorasan	0.47646473	0.48808215	0.50063279	0.5141521	0.52795859	0.54177227	0.55362013	0.56325539	0.57115498	0.57803034	0.58535378	0.59267629	0.59772944	0.60177242	0.60606771	0.61091752	0.61724259	0.62480784	0.63240584	0.63931158	0.64549311	0.6516102
Qazvin	0.5195944	0.53207715	0.54603257	0.56101473	0.57585077	0.59022113	0.60256638	0.61284177	0.62093951	0.62778342	0.63474628	0.64134344	0.64578637	0.64917743	0.65259076	0.65626529	0.661262	0.6672753	0.67316865	0.67833935	0.6829032	0.6876536
Qom	0.5560084	0.56442097	0.573137	0.5822297	0.59142963	0.60085109	0.60906901	0.61559525	0.62091434	0.62596655	0.63193756	0.63831677	0.64310698	0.64734331	0.6520977	0.65700564	0.66322942	0.67039789	0.67729151	0.6833538	0.68869247	0.69407591
Semnan	0.58526817	0.59426707	0.60359812	0.61339484	0.6230342	0.63250122	0.64080173	0.64805461	0.65418495	0.65985729	0.66614538	0.67269035	0.67749802	0.68199998	0.68687585	0.69177677	0.6977284	0.70432963	0.71030264	0.71528784	0.71953728	0.72392638
Sistan and Baluchistan	0.4298579	0.43874689	0.44740568	0.45628781	0.4653299	0.47454565	0.48059877	0.4829184	0.48320659	0.48305692	0.48456532	0.48722436	0.48829234	0.48878132	0.49069476	0.49500313	0.50203843	0.51167765	0.52247451	0.53282763	0.54220697	0.55130598
South Khorasan	0.47900871	0.48962953	0.5010653	0.51342883	0.52606123	0.5388346	0.54973374	0.55879773	0.56611706	0.57261367	0.57981909	0.58729861	0.59279322	0.59761989	0.60304895	0.60898831	0.61634647	0.62476791	0.63297067	0.64027843	0.64672361	0.65312538
Tehran	0.66735854	0.67430059	0.68149363	0.68901755	0.69649539	0.70389952	0.7103273	0.71589513	0.72041864	0.72443014	0.72898465	0.7336284	0.73690465	0.73985661	0.7432248	0.74690488	0.7518639	0.75762071	0.76300458	0.76764971	0.77174329	0.77603245
West Azarbayejan	0.48878611	0.49836255	0.50842601	0.51912335	0.53000796	0.54087308	0.54972823	0.55624435	0.56090299	0.56483796	0.56947461	0.57442046	0.57758425	0.58009813	0.58325168	0.58733977	0.59304459	0.60020635	0.60762044	0.61448487	0.62073248	0.62711246
Yazd	0.57230228	0.5821624	0.59202505	0.60198352	0.61180759	0.62171034	0.63017657	0.63719027	0.64266609	0.64757973	0.65319099	0.65912493	0.66313631	0.66646806	0.67028166	0.67485775	0.68107005	0.68846204	0.6956733	0.70205677	0.7076976	0.71334428
Zanjan	0.49513564	0.50343129	0.51348507	0.52535976	0.53835734	0.55220776	0.56492451	0.57577594	0.58434419	0.59152648	0.59889183	0.60624799	0.61159146	0.61603449	0.62063635	0.62547478	0.63161215	0.63866159	0.6454688	0.65140858	0.65653951	0.66172913
Iraq	0.4723764	0.48145832	0.48866474	0.49217444	0.49893388	0.50471943	0.51091805	0.51769897	0.52524291	0.53320091	0.54234328	0.55304856	0.56474547	0.576969	0.58831765	0.59949401	0.61150865	0.62267453	0.63323211	0.64363532	0.65330038	0.66262623
Jordan	0.58790939	0.5931828	0.59900038	0.60494595	0.6119344	0.61990923	0.62780381	0.63567028	0.64389269	0.65219632	0.65994949	0.66739668	0.67456892	0.68111996	0.68713404	0.69295203	0.6984733	0.70392383	0.70941288	0.71499895	0.72006824	0.72530723
Kuwait	0.71835756	0.72284063	0.72681698	0.73177868	0.73815371	0.74638491	0.75492849	0.76241148	0.77000261	0.77754655	0.78487393	0.79181207	0.79835699	0.80458383	0.81074323	0.81699841	0.82302653	0.82888699	0.83437146	0.83968831	0.84345981	0.84665105
Lebanon	0.58269828	0.5891855	0.59597879	0.60296724	0.61084576	0.61939136	0.62779122	0.63693823	0.64666766	0.65743319	0.66880452	0.68092062	0.69391098	0.704811	0.71242387	0.71869729	0.72458335	0.72996329	0.73474624	0.73864401	0.74200972	0.74474635
Libya	0.63704793	0.64468513	0.65166915	0.65934512	0.66691393	0.67532227	0.683702	0.69185513	0.69887325	0.70499847	0.7113625	0.71146666	0.71670069	0.71766525	0.71604221	0.71365137	0.71076014	0.71066626	0.71272044	0.71615724	0.72027039	0.7257714
Morocco	0.41534403	0.42059513	0.42577575	0.43121781	0.43682235	0.44253134	0.44867616	0.45503632	0.46180355	0.46892989	0.4762698	0.48392215	0.49160339	0.49952861	0.50744327	0.51558289	0.52351859	0.53161086	0.53972967	0.54783744	0.55524607	0.5626983
Oman	0.60842154	0.62216063	0.63516213	0.64724909	0.65762612	0.66640123	0.67535389	0.68505558	0.69495226	0.70309857	0.71068869	0.71826453	0.72645007	0.73343249	0.73874377	0.74359718	0.74879719	0.75378606	0.75917951	0.76452786	0.76885422	0.7733916
Palestine	0.4606815	0.4652991	0.46860026	0.47288173	0.47852732	0.48532059	0.49189041	0.49897843	0.50684948	0.51581584	0.52571191	0.53677763	0.54829543	0.55916348	0.56923988	0.57920426	0.58949277	0.59915719	0.60812486	0.61646684	0.62381002	0.63101167
Qatar	0.71483161	0.72083106	0.72711217	0.7335366	0.74022321	0.74718936	0.75412303	0.76096013	0.76765923	0.77427685	0.78080707	0.78721245	0.7934949	0.79961155	0.80572571	0.81187285	0.81797984	0.82401687	0.82996213	0.83580528	0.8414674	0.84686058
Saudi Arabia	0.64231177	0.65150209	0.66035686	0.66983958	0.67991786	0.69052085	0.70115261	0.71144214	0.7219602	0.73115849	0.74030067	0.74995945	0.75905494	0.76738786	0.77517813	0.7823008	0.78886788	0.79488629	0.80065698	0.80608784	0.81054186	0.81514349
Sudan	0.34687581	0.35421551	0.36172824	0.36969365	0.37792066	0.38668571	0.39615137	0.40607999	0.4160876	0.42597592	0.4361711	0.44723944	0.45679026	0.466436	0.47636213	0.48654944	0.49684822	0.50688353	0.51621409	0.52500912	0.53345542	0.54194974
Syria	0.50714369	0.51385011	0.52130974	0.52893576	0.53808447	0.54975045	0.56048476	0.57002812	0.57899049	0.58778912	0.59596776	0.60152446	0.6028726	0.60241013	0.60223576	0.60228291	0.60291118	0.60489637	0.60850716	0.61333684	0.61772759	0.62300407
Tunisia	0.55667265	0.56442101	0.57171953	0.57888267	0.58603259	0.59293776	0.59975851	0.60669678	0.61352932	0.62010683	0.62643277	0.63201984	0.63757834	0.64300851	0.6482974	0.65341354	0.65835352	0.66322285	0.668219	0.67317055	0.67761515	0.68243222
Türkiye	0.53586633	0.54235913	0.54902364	0.55610253	0.564024	0.57249589	0.58138318	0.59044239	0.59921206	0.60710462	0.61559723	0.62478721	0.63409709	0.64372382	0.65338883	0.66297174	0.67194815	0.68109627	0.68989587	0.69826377	0.70579974	0.71269267
United Arab Emirates	0.76096909	0.76785879	0.77434642	0.78114399	0.78852595	0.79642809	0.80553702	0.81487088	0.82279344	0.82830417	0.83128425	0.8329128	0.83358611	0.83380382	0.83385873	0.8341755	0.83522071	0.83721355	0.83990014	0.84298533	0.84615198	0.84931773
Yemen	0.29714516	0.30611242	0.31506502	0.32408169	0.33337129	0.34346256	0.35347295	0.36330122	0.37314029	0.38276225	0.39293664	0.4014718	0.40956135	0.4176424	0.42518967	0.43019779	0.43423298	0.43754916	0.44072799	0.4440014	0.44689307	0.45037638
South Asia	0.38338846	0.38953334	0.39519407	0.40098342	0.40718055	0.41398666	0.42136736	0.42926185	0.43701186	0.44519735	0.45407579	0.46333927	0.47308056	0.48343427	0.49408436	0.50489146	0.515311	0.52512844	0.53447741	0.5432457	0.55058557	0.55786466
South Asia	0.38338846	0.38953334	0.39519407	0.40098342	0.40718055	0.41398666	0.42136736	0.42926185	0.43701186	0.44519735	0.45407579	0.46333927	0.47308056	0.48343427	0.49408436	0.50489146	0.515311	0.52512844	0.53447741	0.5432457	0.55058557	0.55786466
Bangladesh	0.29725198	0.30318855	0.30864009	0.31436004	0.32092285	0.32809051	0.3356906	0.34393255	0.35252003	0.36148151	0.37059896	0.38012489	0.39062355	0.40170648	0.41326317	0.42548212	0.43770991	0.44975241	0.46171775	0.473265	0.48307917	0.49242088
Bhutan	0.29880487	0.30820065	0.31794354	0.32767042	0.33728649	0.34699858	0.35682421	0.3675605	0.37823354	0.38897716	0.39975194	0.41014925	0.41944322	0.42779067	0.43496927	0.44181075	0.44840185	0.4542805	0.45962471	0.46463335	0.46871448	0.47306238
India	0.39575233	0.40183747	0.40741141	0.41311578	0.41918496	0.42591306	0.43332156	0.44135146	0.44923585	0.45767233	0.467019	0.47683291	0.48721088	0.49831798	0.5097221	0.52124272	0.53222002	0.54238851	0.55193033	0.56080993	0.56813815	0.57540165
Nepal	0.27271911	0.28128747	0.28951361	0.2977726	0.30619653	0.3146618	0.32305635	0.33144561	0.34015071	0.34870189	0.3568681	0.3646426	0.37243759	0.38002233	0.38768909	0.39502115	0.40162878	0.40846387	0.41523668	0.42200725	0.42741709	0.43317463
Pakistan	0.37431913	0.38059393	0.38662999	0.39271926	0.39908417	0.40566744	0.41223483	0.41851976	0.4243482	0.43001983	0.43564066	0.44145559	0.4470966	0.4528231	0.45878142	0.46479267	0.47102217	0.47754414	0.48432613	0.49102298	0.49736441	0.50402869
Azad Jammu & Kashmir	0.40149838	0.40860326	0.41545557	0.42235537	0.42958201	0.43704644	0.44466119	0.45231842	0.45978972	0.46714764	0.47426164	0.48132564	0.48792453	0.49423462	0.50053617	0.50665208	0.51270136	0.51879497	0.52499004	0.53093598	0.53639063	0.54211877
Balochistan	0.3041235	0.30936329	0.31448679	0.31976754	0.32540818	0.33135828	0.33731296	0.34294716	0.34816028	0.35325682	0.35839244	0.36376712	0.36895028	0.37414089	0.37949329	0.38469183	0.38988999	0.39534393	0.4010568	0.40675045	0.41216125	0.4179715
Gilgit-Baltistan	0.2806372	0.28644967	0.29209912	0.29789534	0.30407231	0.31063787	0.31745107	0.32422086	0.33074442	0.33714888	0.34333231	0.34956674	0.35526468	0.3605803	0.36562052	0.37025268	0.37486591	0.37949799	0.38426318	0.38884361	0.39301978	0.39754999
Islamabad Capital Territory	0.55742471	0.56408607	0.5705217	0.57701613	0.58382639	0.59105605	0.5985292	0.60613854	0.61359788	0.62095494	0.62811281	0.63527361	0.64200777	0.64843124	0.65479537	0.66093533	0.6670449	0.67316109	0.67934485	0.68524364	0.69059481	0.69619537
Khyber Pakhtunkhwa	0.32101648	0.32620678	0.33115461	0.336239	0.34175944	0.34772621	0.35399984	0.36051255	0.36699385	0.37353807	0.3799994	0.38656624	0.39283876	0.39899187	0.40528866	0.41149302	0.41772104	0.42407868	0.43070554	0.43734857	0.44373658	0.45058115
Punjab	0.38480276	0.39161443	0.39816643	0.40472799	0.41152918	0.41853623	0.42554756	0.43229033	0.43853633	0.44455107	0.45044463	0.45646969	0.46227533	0.46818561	0.47427629	0.48043288	0.48684477	0.49355939	0.50047832	0.50724092	0.51358412	0.52020049
Sindh	0.38578503	0.39154363	0.3970977	0.40275713	0.40870602	0.41484554	0.42084535	0.42634357	0.43126977	0.43606369	0.44097512	0.44624365	0.4515419	0.45706338	0.46304727	0.4692352	0.47578641	0.48279361	0.49024318	0.49775152	0.50498481	0.51257496
Southeast Asia, east Asia, and Oceania	0.55128675	0.55646847	0.56247943	0.56954356	0.57706574	0.58501175	0.59352872	0.60222627	0.61040808	0.61839042	0.62814353	0.63636052	0.64172537	0.64735119	0.6527504	0.65681903	0.6616056	0.66891988	0.67660333	0.68470468	0.69221505	0.6988499
East Asia	0.55881567	0.56423475	0.57079838	0.57890473	0.58768267	0.59709617	0.60727342	0.61752384	0.6269558	0.63627115	0.64798501	0.65750207	0.66292987	0.66868191	0.67406275	0.67730686	0.68152833	0.68974757	0.69862583	0.70832781	0.71770931	0.7257049
China	0.54798919	0.55368037	0.56054294	0.56901883	0.57817809	0.58805219	0.59875817	0.60952025	0.61945483	0.62930773	0.64152144	0.65144798	0.65714429	0.66310301	0.66864085	0.67194011	0.67621844	0.68461966	0.693718	0.70368682	0.71336458	0.72162976
North Korea	0.49746199	0.49967404	0.50263357	0.50616524	0.51022913	0.51509776	0.51974144	0.52406697	0.5288135	0.53311441	0.53719292	0.54121227	0.54533171	0.5494833	0.55360356	0.55732127	0.56074356	0.56338829	0.56516148	0.56676571	0.56818855	0.56985463
Taiwan (province of China)	0.75292684	0.76082981	0.76900898	0.7769558	0.78467798	0.79244513	0.80017467	0.80813769	0.81540069	0.82143228	0.82722675	0.83206407	0.83667766	0.84128046	0.84569556	0.85005527	0.85446604	0.85888802	0.86315836	0.86741185	0.87124795	0.87474705
Oceania	0.42506966	0.42700403	0.42853491	0.43005967	0.43158133	0.43315184	0.4345841	0.43610175	0.43739244	0.43880798	0.44075187	0.44261777	0.4445244	0.44659351	0.44936352	0.45239726	0.45545381	0.45837779	0.4608181	0.46333393	0.46553464	0.46744513
American Samoa	0.64060955	0.64321442	0.64600206	0.64895196	0.65192114	0.65506101	0.65772572	0.66078118	0.66402576	0.66730053	0.67124892	0.67597929	0.68086154	0.68583306	0.69134314	0.69716291	0.7027264	0.70764791	0.71280059	0.71730813	0.7208385	0.72372753
Cook Islands	0.63536561	0.64442144	0.65279049	0.66146504	0.66876968	0.6740041	0.680644	0.68750519	0.69559207	0.70377698	0.71186161	0.71944467	0.72696339	0.73331702	0.7395957	0.7465503	0.75235309	0.75823357	0.76404118	0.76979591	0.77470058	0.77910995
Fiji	0.58919663	0.59449742	0.59932203	0.60348962	0.60754489	0.61081294	0.61393277	0.61658292	0.61895881	0.62088347	0.62330879	0.62643825	0.62988133	0.63427811	0.63939007	0.64502635	0.65074268	0.65646401	0.66199145	0.66706098	0.67143149	0.67505163
Guam	0.72480236	0.73426738	0.74128425	0.74591153	0.74901652	0.75086587	0.75117625	0.75262547	0.75515241	0.7582523	0.76148432	0.76398739	0.76684598	0.77022496	0.77344168	0.77672361	0.78051164	0.78555492	0.79129477	0.79677545	0.80096947	0.8039822
Kiribati	0.44443634	0.4487823	0.4534623	0.45820837	0.46233621	0.46672228	0.4705572	0.47422745	0.47720193	0.4800356	0.48244769	0.48502821	0.48817586	0.49188077	0.49531724	0.5000597	0.50520784	0.51001924	0.51481038	0.51944132	0.52345411	0.52718658
Marshall Islands	0.47456254	0.47858832	0.48277626	0.48624862	0.48957308	0.49327139	0.49745617	0.50225004	0.50635959	0.51079454	0.51608514	0.52117709	0.52604202	0.5314463	0.53664013	0.54179317	0.54678294	0.55206515	0.55759928	0.56342875	0.56883751	0.57409113
Federated States of Micronesia	0.51100423	0.51531681	0.51954567	0.5238296	0.52761096	0.53170332	0.53571868	0.53943146	0.54276635	0.5462755	0.55012255	0.55424041	0.55796585	0.56108282	0.56384003	0.56715134	0.5704581	0.57403983	0.57755508	0.58119525	0.58452723	0.58753497
Nauru	0.52025489	0.51923542	0.51843871	0.51845999	0.51880954	0.51989286	0.52267318	0.52320135	0.5260841	0.53022109	0.53568999	0.54234274	0.54989062	0.56011291	0.57142436	0.58128323	0.59010299	0.59761908	0.60531188	0.61269725	0.61933008	0.62517783
Niue	0.63406895	0.63736663	0.64112202	0.64577954	0.65061483	0.65728126	0.66416298	0.6701993	0.67639686	0.68257917	0.68628063	0.69031558	0.69415095	0.69722108	0.70054592	0.70423298	0.7074761	0.711221	0.71510823	0.71907889	0.72279025	0.72622205
Northern Mariana Islands	0.74449287	0.74717061	0.74875519	0.75017153	0.75152884	0.75182669	0.75179781	0.75160842	0.75080719	0.74859717	0.74661726	0.74441191	0.74293735	0.74228436	0.74260545	0.7437974	0.74845547	0.75649548	0.7612554	0.76577065	0.76942766	0.77153521
Palau	0.70519694	0.70740239	0.70958764	0.71100022	0.71254381	0.71433983	0.71635343	0.71880954	0.72080662	0.72228827	0.72406596	0.72673345	0.7296129	0.73195347	0.7347683	0.73816643	0.74136191	0.74422477	0.74776125	0.75071531	0.75278093	0.75404693
Papua New Guinea	0.35413579	0.35658899	0.35857209	0.36073682	0.36282754	0.36529496	0.36782676	0.37079415	0.37342621	0.37652828	0.38028678	0.38358754	0.38678103	0.38992644	0.39400457	0.39828586	0.40240727	0.40615344	0.40916508	0.41231115	0.41518688	0.41779744
Samoa	0.51890233	0.52297163	0.52724267	0.53160374	0.53600328	0.54055791	0.54481207	0.54911075	0.5531646	0.55647402	0.55962291	0.56289157	0.56532039	0.56744854	0.56949366	0.57199898	0.57540205	0.57892544	0.58232066	0.5862567	0.5900753	0.59339277
Solomon Islands	0.35479914	0.35662675	0.35791012	0.35942436	0.36129056	0.36358002	0.3664437	0.36982795	0.37376488	0.37782103	0.38271759	0.38805534	0.39300464	0.39790625	0.40224474	0.4062339	0.41040696	0.41467639	0.41888922	0.42273639	0.42611243	0.42936032

Tokelau	0.57181437	0.5769156	0.58185832	0.58685435	0.59234244	0.59817612	0.60442939	0.61116324	0.61794025	0.62407008	0.63017554	0.63601652	0.64161199	0.64796615	0.653232	0.65879899	0.66397432	0.66905413	0.67417951	0.67924784	0.68293738	0.68642562
Tonga	0.5463032	0.55105425	0.55600835	0.56073607	0.56480638	0.56844917	0.57161987	0.57435151	0.57730542	0.57917075	0.5806573	0.58265672	0.58477088	0.58735114	0.59082685	0.59496575	0.60034367	0.60624359	0.61195394	0.61732406	0.62210446	0.62634994
Tuvalu	0.47495682	0.48085089	0.48725225	0.49275471	0.49769215	0.50185131	0.50597541	0.5105043	0.5156116	0.51987057	0.52345551	0.52774424	0.53120323	0.53516623	0.53912224	0.54404788	0.54917393	0.55446648	0.5598594	0.56604549	0.5716661	0.57662053
Vanuatu	0.39215835	0.39532506	0.39772378	0.40039646	0.4032261	0.40648857	0.41050173	0.41491558	0.41985324	0.42485465	0.42965805	0.43428304	0.438662	0.44272018	0.4466873	0.45032986	0.45407276	0.45801683	0.46208022	0.46627616	0.47003839	0.47310071
Southeast Asia	0.53456661	0.53901043	0.54347855	0.54805101	0.55270742	0.5573913	0.56230217	0.56765426	0.57320839	0.57849739	0.58426847	0.59041462	0.59678029	0.60314492	0.60943868	0.61568907	0.62183986	0.62797813	0.63409588	0.64010676	0.64506966	0.6497773
Cambodia	0.33607503	0.34250791	0.34946571	0.35692966	0.36485464	0.37333181	0.38169817	0.38987623	0.39767952	0.40422093	0.41021112	0.41596441	0.42176975	0.42771506	0.43359775	0.43948736	0.44535817	0.45122174	0.45734149	0.46364343	0.46887605	0.47362149
Indonesia	0.53378959	0.53797798	0.54224568	0.54655026	0.55090231	0.55542491	0.56026592	0.5658119	0.57222527	0.5788669	0.58598242	0.59365298	0.6011857	0.6085099	0.61549989	0.62222247	0.62866662	0.63489574	0.64094875	0.64686546	0.65192665	0.65686834
Aceh	0.56026794	0.56438388	0.56872189	0.57307457	0.57772252	0.58230388	0.58672602	0.59150689	0.59674327	0.60181305	0.60705953	0.61268272	0.61854653	0.62454873	0.63050526	0.63648236	0.642395	0.6482508	0.65405877	0.65986518	0.66475876	0.66955164
Bali	0.53616426	0.53927741	0.54257347	0.54593923	0.54936586	0.55303279	0.55703291	0.56190502	0.56780069	0.57417724	0.58114054	0.58857033	0.59582696	0.60287149	0.60965942	0.61620184	0.62245407	0.62847275	0.63430388	0.64000539	0.64485235	0.64958588
Bangka-Belitung Islands	0.52892114	0.53212382	0.53552897	0.53917523	0.54298703	0.54698955	0.5512765	0.55631699	0.56225432	0.56835578	0.5747897	0.58178671	0.58868338	0.59537815	0.60187021	0.60823522	0.61460851	0.62102515	0.62738145	0.63359559	0.638989	0.64425964
Banten	0.52859153	0.53158023	0.53463584	0.53775383	0.54095116	0.54436088	0.54818718	0.55279114	0.55837624	0.56437318	0.57102114	0.57857249	0.5859379	0.59310912	0.59998839	0.60662567	0.61303665	0.61918089	0.62514768	0.63096052	0.63587866	0.64071299
Bengkulu	0.49336859	0.49684776	0.50031399	0.50391788	0.50767793	0.51176383	0.51629332	0.52173677	0.52817261	0.53479748	0.54181862	0.54940364	0.55681278	0.56409049	0.57103184	0.5777767	0.5843805	0.59086907	0.5972185	0.60341936	0.60863108	0.61367065
Gorontalo	0.44602471	0.44716892	0.44876276	0.45080182	0.45329135	0.45642028	0.4604025	0.46566364	0.4724715	0.48008009	0.48850748	0.49763464	0.50656442	0.51519052	0.52338898	0.53119496	0.53859668	0.5456919	0.55250868	0.55910806	0.56463912	0.56999021
Jakarta	0.68357742	0.68836419	0.6932305	0.69805222	0.70277371	0.70751587	0.71242858	0.71786297	0.72395734	0.73023891	0.73689221	0.74393246	0.75070975	0.75717729	0.76334451	0.76925722	0.77492035	0.78034984	0.78558506	0.79069715	0.79508951	0.79940944
Jambi	0.50341566	0.50758427	0.51194615	0.51647752	0.52114362	0.5258922	0.53111719	0.5374601	0.54511489	0.55317563	0.56192132	0.57129382	0.58031825	0.58885735	0.59684259	0.60427083	0.61117963	0.61763925	0.62378203	0.62971761	0.63470231	0.63956368
West Java	0.52755512	0.53072386	0.5339323	0.53721964	0.54068547	0.54455824	0.54898783	0.55426903	0.56051638	0.56700756	0.57393897	0.58144324	0.58878356	0.5959741	0.60283135	0.6094301	0.61576965	0.62195319	0.62798418	0.6338634	0.63884136	0.64367663
Central Java	0.49003761	0.49375055	0.49769922	0.50190521	0.50636632	0.51119198	0.51647028	0.52242419	0.52913658	0.53597076	0.54318954	0.5508159	0.55824886	0.56549478	0.57238966	0.57903147	0.58540672	0.5915727	0.59758761	0.60349025	0.60850881	0.61338882
East Java	0.51482937	0.51994422	0.52515545	0.53039257	0.53563368	0.54100965	0.54661489	0.55280696	0.55971581	0.5667625	0.57426857	0.58226911	0.59008313	0.59756583	0.60465166	0.61140792	0.61783986	0.62401929	0.62998262	0.63578677	0.64072987	0.64551738
West Kalimantan	0.48143807	0.48499739	0.48839009	0.49165766	0.49474646	0.4975574	0.5004363	0.50407497	0.50861467	0.5136176	0.5193251	0.52577988	0.53252363	0.53933342	0.54594146	0.55237456	0.55860712	0.56466654	0.57058178	0.57636235	0.58128935	0.58612167
South Kalimantan	0.51724759	0.5204995	0.52380167	0.52709316	0.53026868	0.53339914	0.5366955	0.5406522	0.54545082	0.55062024	0.55642207	0.56299804	0.56966811	0.57633518	0.5828793	0.58924978	0.59547153	0.6015648	0.60751384	0.61334189	0.61831046	0.62320608
Central Kalimantan	0.53838456	0.54053222	0.5429412	0.5454602	0.54791457	0.55040024	0.55309563	0.55639235	0.56077621	0.56569527	0.57137421	0.57788061	0.58453086	0.59117447	0.59765603	0.60395753	0.61019142	0.61640037	0.62255626	0.62864572	0.63386662	0.63899383
East Kalimantan	0.63879647	0.64279042	0.64685469	0.6511169	0.65555905	0.66020366	0.66508645	0.67062866	0.67711686	0.68365906	0.69066649	0.6984318	0.70607231	0.71336138	0.72020158	0.72672101	0.73287396	0.73872114	0.74436852	0.74989106	0.75463949	0.75929387
North Kalimantan	0.62429697	0.6292073	0.63436734	0.63974222	0.64531857	0.65115801	0.65717162	0.66355161	0.67050898	0.6771463	0.68391938	0.69114793	0.69826096	0.70517295	0.71188818	0.71842697	0.7247458	0.73089841	0.73687353	0.74271922	0.74790202	0.75298676
Riau Islands	0.64675899	0.64991663	0.65299036	0.65606106	0.65911176	0.66231984	0.6659116	0.6702922	0.6755603	0.68112742	0.68712948	0.69358947	0.69999368	0.70623153	0.7121994	0.71794241	0.72348517	0.72885742	0.73407381	0.73915486	0.7434696	0.74775431
Lampung	0.47950514	0.4838987	0.48810632	0.49217245	0.49620385	0.50041727	0.50517671	0.51099373	0.51802019	0.52564643	0.53407416	0.54289371	0.5513703	0.55939671	0.56688711	0.57397078	0.58064169	0.58699908	0.59308111	0.598979	0.60397338	0.60887136
Maluku	0.52298834	0.51852653	0.51349154	0.50800674	0.5022141	0.49670039	0.49251133	0.49083345	0.49229133	0.49595364	0.50170832	0.50914016	0.51704502	0.52509196	0.5329444	0.54067919	0.54813291	0.55534118	0.56233884	0.56918695	0.57482534	0.58032188
North Maluku	0.45598959	0.45832082	0.45989066	0.46091262	0.46163489	0.46238837	0.46358432	0.46605875	0.47046924	0.47616491	0.48333748	0.49176511	0.50026361	0.50862948	0.51668894	0.524421	0.5318067	0.53888887	0.5457041	0.55229142	0.55764156	0.56291087
West Nusa Tenggara	0.44070553	0.44798776	0.4552512	0.46240815	0.46962199	0.47675744	0.48383361	0.491206	0.49889746	0.50653697	0.51404228	0.52193726	0.52966433	0.53717467	0.54441446	0.5513934	0.55809279	0.56453903	0.57078447	0.5768659	0.5820152	0.58703462
East Nusa Tenggara	0.42845105	0.43095467	0.4338165	0.43696531	0.44029416	0.44383243	0.44776538	0.4525351	0.45847722	0.46489578	0.47209961	0.48008645	0.48801472	0.49578864	0.50327052	0.51053667	0.51761217	0.52461615	0.53149789	0.53824536	0.54391325	0.54945119
Papua	0.53252213	0.53609896	0.53928483	0.5420573	0.54444202	0.54693318	0.54958513	0.55306955	0.55766566	0.56313014	0.56958178	0.57705575	0.58465497	0.59219642	0.59954624	0.60674908	0.61376989	0.62068688	0.62747226	0.63412061	0.64001264	0.64580288
West Papua	0.52885419	0.53153173	0.53433132	0.53743263	0.54079912	0.54472282	0.54952541	0.55578751	0.56394076	0.57329506	0.58421778	0.59648716	0.60772107	0.61803843	0.62740989	0.6359972	0.64387731	0.65115933	0.65795906	0.6644003	0.66982939	0.67512564
Riau	0.60519772	0.6100163	0.61472702	0.61896732	0.62276314	0.62626858	0.62992535	0.63440792	0.64000295	0.6459983	0.65260316	0.65996766	0.66750217	0.67490295	0.68198684	0.68878372	0.69518683	0.70131759	0.70725774	0.7130141	0.71796346	0.72278452
West Sulawesi	0.45086369	0.45264887	0.4545774	0.45667968	0.45898469	0.46174285	0.46529875	0.47021593	0.47685496	0.48430645	0.49271753	0.50194275	0.51109162	0.51991839	0.5283045	0.53627554	0.54378197	0.55095746	0.55786177	0.56453058	0.57018471	0.57565685
South Sulawesi	0.49163905	0.49594103	0.50036845	0.50482569	0.50923435	0.51364434	0.51836497	0.52395152	0.53070743	0.5380618	0.5461011	0.55461525	0.56283476	0.57071405	0.57822434	0.58540084	0.59222524	0.59873435	0.60497768	0.61102327	0.61614532	0.62113541
Central Sulawesi	0.48736392	0.49125871	0.49536197	0.49950147	0.50362144	0.50768352	0.51201061	0.51720467	0.52358948	0.53057415	0.53837511	0.54702868	0.55550698	0.56367404	0.57144215	0.5788657	0.58590289	0.59265171	0.59916342	0.60551197	0.61094788	0.61625384
Southeast Sulawesi	0.47301416	0.47752407	0.4823181	0.48736429	0.49261118	0.49832063	0.50458344	0.51183548	0.52030102	0.52903955	0.53805543	0.54740529	0.55647391	0.56510272	0.57318621	0.58073303	0.5875894	0.59394341	0.59997204	0.60579632	0.61061776	0.61532587
North Sulawesi	0.54363949	0.54556523	0.54751292	0.54952838	0.55169202	0.55419005	0.5571767	0.56115905	0.56642855	0.57237058	0.57907107	0.58648729	0.59387226	0.60110657	0.60806176	0.61478816	0.62130855	0.62767902	0.63391322	0.64002776	0.64529661	0.65046831
West Sumatra	0.53988665	0.54357323	0.54745489	0.55149686	0.55570819	0.56023794	0.56525197	0.5710871	0.57780879	0.5847555	0.59220161	0.60018689	0.60806795	0.6157333	0.62300956	0.63003218	0.63670136	0.64314623	0.64936973	0.65548106	0.66065894	0.66576366
South Sumatra	0.526408	0.52982247	0.53328289	0.53690512	0.54067667	0.54476334	0.549304	0.55464109	0.56085431	0.56721212	0.57404763	0.58148689	0.58895007	0.596305	0.6033241	0.61015675	0.61668165	0.62300434	0.629152	0.63521332	0.64041033	0.64553676
North Sumatra	0.5441669	0.54752018	0.55114495	0.55502277	0.55907586	0.56327535	0.56783008	0.57316613	0.5794509	0.58607463	0.59332584	0.60125332	0.60901473	0.61657934	0.62379014	0.63068585	0.63739153	0.64387346	0.65020385	0.65640729	0.66174552	0.66702565
Yogyakarta	0.55391417	0.55777964	0.56164756	0.56558187	0.56967347	0.57403804	0.57888155	0.58456988	0.59111386	0.59792884	0.60523456	0.61307587	0.62074417	0.62816785	0.63521167	0.64195219	0.64831678	0.65444207	0.66036427	0.66613281	0.67086108	0.67542992
Laos	0.32394736	0.33258041	0.34166727	0.35065818	0.35968176	0.36872868	0.37812228	0.38772365	0.3970113	0.40613902	0.41486557	0.42335452	0.43114936	0.43869104	0.44586942	0.4527496	0.459584	0.46616889	0.4726181	0.47881137	0.48419296	0.48913609
Malaysia	0.63052354	0.63825748	0.64504425	0.65088568	0.65624333	0.66124224	0.66595629	0.67084373	0.67639991	0.68207065	0.68825862	0.6940834	0.69966473	0.70493072	0.70995376	0.71465987	0.71922342	0.72423541	0.72938808	0.73458775	0.73874399	0.74252383
Maldives	0.48349567	0.4959237	0.50740486	0.51892686	0.52991019	0.53825333	0.54795041	0.55752925	0.56683963	0.57444206	0.58198099	0.58968446	0.59694852	0.60423852	0.61157807	0.61845439	0.62487595	0.63108329	0.63720566	0.64308279	0.64760859	0.65088663
Mauritius	0.6070879	0.61224082	0.61719001	0.62276453	0.62903019	0.63476658	0.63954518	0.64435866	0.64963085	0.6550003	0.6604698	0.66649763	0.67312765	0.68011402	0.6866165	0.69223973	0.69702056	0.70161814	0.70626665	0.71093504	0.7146298	0.71826045
Myanmar	0.37036002	0.37817198	0.38634363	0.39521877	0.40469236	0.41466208	0.42470507	0.43453693	0.44359553	0.45195488	0.46018043	0.46785087	0.47533642	0.48282186	0.49025515	0.49737416	0.50411598	0.51082343	0.51755747	0.52410897	0.5294347	0.53390084
Philippines	0.54499772	0.54740943	0.54952115	0.55156797	0.55370429	0.5556641	0.55797627	0.56082202	0.56397472	0.56735582	0.57176589	0.57724028	0.58376763	0.59083755	0.59838452	0.60621562	0.61422496	0.62231503	0.63046428	0.63854738	0.64486723	0.65121933
Abra	0.53128071	0.53273718	0.53376801	0.53478051	0.53592088	0.53697419	0.53856713	0.54110627	0.54455748	0.5487971	0.55428983	0.56096209	0.56878773	0.57723633	0.58624882	0.59554948	0.60492779	0.61425622	0.62341822	0.63236923	0.63953061	0.64654691
Agusan Del Norte	0.51259648	0.51320465	0.5134108	0.51351357	0.51379208	0.51394635	0.51462695	0.51615474	0.51849637	0.52144351	0.52559722	0.53097806	0.53761675	0.54493819	0.55285983	0.56118542	0.56979452	0.57849767	0.58715901	0.59578107	0.60257288	0.60936062
Agusan Del Sur	0.43140784	0.43320888	0.43462048	0.43597421	0.43742142	0.43874005	0.4405241	0.44308084	0.44631035	0.45008245	0.45502525	0.4610141	0.46809602	0.47573261	0.48389936	0.49238263	0.50112963	0.50998689	0.51893209	0.52794931	0.53518578	0.54241367
Aklan	0.53022348	0.53217388	0.53385301	0.53563188	0.53776595	0.53993197	0.54263086	0.54623701	0.55059763	0.55522387	0.5607583	0.56692079	0.57386867	0.5812155	0.58886003	0.59662837	0.60450352	0.61235292	0.62016064	0.6279543	0.63393133	0.64007481
Albay	0.52093014	0.52339654	0.52560853	0.52788142	0.53042267	0.53292506	0.5359961	0.53986962	0.5444071	0.54930352	0.55513605	0.56166922	0.56904064	0.57679863	0.58491181	0.59324439	0.60166376	0.61006834	0.61845767	0.62675705	0.63327635	0.6398634
Antique	0.45178835	0.45429256	0.45623893	0.45810206	0.45999414	0.46163724	0.4636722	0.46651766	0.47020268	0.47458869	0.48023859	0.48688442	0.49467046	0.50309332	0.51202966	0.52134594	0.53080936	0.54032422	0.54978241	0.55918367	0.56678776	0.57433252
Apayao	0.48086003	0.48416371	0.48716531	0.49018527	0.4935853	0.4970595	0.5010587	0.50583892	0.51126283	0.51696672	0.52362411	0.53091238	0.53899792	0.54752406	0.55619904	0.56486221	0.57345629	0.58194008	0.59027874	0.59844973	0.60475473	0.61105972
Aurora	0.49900479	0.50198737	0.50456618	0.50710023	0.50968107	0.5120625	0.51477039	0.51816251	0.5220613	0.52633573	0.53163879	0.53772448	0.54485065	0.55266036	0.56078751	0.56896931	0.57717183	0.58537656	0.5935932	0.60179478	0.60814536	0.61450607
Basilan	0.41037568	0.41474166	0.41870216	0.42251806	0.42622531	0.42958818	0.43327269	0.43752808	0.44231082	0.44751166	0.4537452	0.46091357	0.4689508	0.47743764	0.48631662	0.49541035	0.50464591	0.51393515	0.52320306	0.53235515	0.53964471	0.54682572
Bataan	0.5519477	0.55448765	0.55672351	0.55889357	0.56108759	0.56305373	0.56533508	0.56820748	0.57158244	0.57517505	0.57969749	0.58498552	0.59112679	0.59780381	0.60497966	0.61250585	0.62027546	0.62816166	0.63619725	0.64426059	0.65061044	0.65690151
Batanes	0.57059109	0.5740181	0.57728226	0.58051613	0.58398298	0.58749535	0.59165252	0.59664492	0.60211166	0.60739106	0.6131907	0.61895739	0.6251476	0.63171027	0.63845433	0.64519718	0.65190275	0.65863143	0.66539208	0.67219288	0.67707236	0.68230486
Batangas	0.58297807	0.58484085	0.58645468	0.58810538	0.58989451	0.59153416	0.59355417	0.59622005	0.59943515	0.60302307	0.60760594	0.61299383	0.61927929	0.62608892	0.63337662	0.64097125	0.64874973	0.65658235	0.66444634	0.67220937	0.67827952	0.68441127
Benguet	0.61088718	0.61265109	0.6142778	0.6160018	0.61799382	0.61996378	0.62242933	0.62566677	0.62953794	0.63371516	0.63876873	0.64445207	0.65085035	0.657592	0.66465272	0.67201387	0.67962665	0.68728025	0.69483144	0.70223486	0.70783292	0.71350365
Biliran	0.53436303	0.53739152	0.54004356	0.54268936	0.54542497	0.54798481	0.5508268	0.55430762	0.5584497	0.56277987	0.5677837	0.57325657	0.57939771	0.58595626	0.5929906	0.60035125	0.60789209	0.61555329	0.62337909	0.63130052	0.63764281	0.64405335
Bohol	0.48559586	0.4882872	0.49060598	0.49281291	0.49518294	0.49745455	0.50017767	0.50369931	0.50794585	0.51261214	0.51825989	0.52473747	0.532207	0.5402093	0.54850296	0.55694141	0.56546884	0.57392992	0.58229391	0.59059041	0.5971473	0.60378059

Bukidnon	0.44007909	0.44233074	0.44411197	0.44578894	0.44744354	0.44882422	0.45049022	0.45275917	0.45564395	0.45905516	0.4636238	0.46930827	0.47618836	0.48368451	0.49175506	0.50024768	0.50900102	0.51791313	0.5269522	0.53596388	0.5432682	0.55051685
Bulacan	0.59879603	0.60152106	0.6040156	0.60653658	0.60925856	0.61188916	0.61489421	0.61850779	0.62258289	0.62685917	0.63197556	0.63774941	0.64430353	0.65124647	0.65846391	0.66586807	0.67339665	0.68093464	0.68845606	0.69588286	0.70165408	0.70752202
Cagayan	0.51849835	0.52120883	0.52350087	0.52569649	0.52794402	0.53001031	0.53246614	0.53570295	0.53962958	0.54397672	0.54938061	0.5556774	0.56297305	0.57088411	0.57924451	0.58780075	0.59650305	0.6051487	0.61370978	0.6220833	0.62860478	0.63506809
Camarines Norte	0.48628166	0.48777093	0.48886074	0.48987763	0.49124432	0.49261278	0.49456887	0.49740224	0.50092203	0.50481171	0.50969936	0.51547939	0.52234025	0.52981666	0.53781991	0.54612331	0.55462317	0.56323271	0.57186191	0.58049559	0.58738351	0.59428649
Camarines Sur	0.52388328	0.52629929	0.52836034	0.53035073	0.5323433	0.53412759	0.53627212	0.53913326	0.54268436	0.54657535	0.55142964	0.5570564	0.56363917	0.57079921	0.57846816	0.58641965	0.59457897	0.60282115	0.61107904	0.61929616	0.62586626	0.63255464
Camiguin	0.52257327	0.5241482	0.52556588	0.52698641	0.52906275	0.53142481	0.5345077	0.53852345	0.54321424	0.54787944	0.55338061	0.55942911	0.56627343	0.57367671	0.581441	0.58933593	0.59733393	0.60531239	0.61318479	0.62090381	0.62657239	0.6324379
Capiz	0.46102131	0.46249156	0.46348009	0.4644371	0.46564337	0.46669233	0.46822757	0.47060478	0.47382466	0.47749504	0.4822294	0.48802417	0.49494841	0.50238271	0.51040366	0.51882531	0.52752021	0.53634523	0.54517963	0.55396279	0.56102037	0.56808796
Catanduanes	0.50091321	0.50289905	0.50440134	0.50578591	0.50712867	0.50809942	0.50927829	0.51096617	0.51320001	0.51608385	0.52020463	0.525721	0.53265367	0.54034958	0.54878823	0.55761777	0.56667033	0.57585695	0.58513414	0.59443609	0.60201223	0.60947869
Cavite	0.63294777	0.63448097	0.63585756	0.63735987	0.63907192	0.64077745	0.6429148	0.64573383	0.64908738	0.65266111	0.65713881	0.66226784	0.66809263	0.67431285	0.68087015	0.68761723	0.69457625	0.70160767	0.70870624	0.71579043	0.72123057	0.72678437
Cebu	0.55642898	0.55783064	0.55904088	0.56034274	0.56183098	0.56327943	0.56518138	0.56786317	0.57124477	0.57495432	0.57963133	0.58504329	0.59131123	0.59806098	0.60527238	0.61281872	0.62062354	0.62851333	0.63637842	0.64408431	0.65001485	0.65607636
Cotabato (North Cotabato)	0.45617086	0.4575233	0.45841112	0.45918173	0.46004843	0.46065059	0.46165937	0.46333898	0.46559733	0.46834209	0.47224272	0.47727292	0.48351726	0.49046199	0.49798351	0.50597266	0.51424832	0.52273164	0.53134207	0.53999565	0.54690644	0.55377082
Davao de Oro	0.42921786	0.43083905	0.43201518	0.4330585	0.43417484	0.43514979	0.43663131	0.43889782	0.44175208	0.44512159	0.4496941	0.45536184	0.46218962	0.46966269	0.47761347	0.48584193	0.49431108	0.50298274	0.51174337	0.52052061	0.52746244	0.53441776
Davao Del Norte	0.52212084	0.52505647	0.52769764	0.5303122	0.53308924	0.53575812	0.53881707	0.54248804	0.54659841	0.55097192	0.55618365	0.56207274	0.56883134	0.57603195	0.58364044	0.5915541	0.59976688	0.60810743	0.61643135	0.62474035	0.63133764	0.63810495
Davao Del Sur	0.54363175	0.5457578	0.54757006	0.54942521	0.55143046	0.55334759	0.55572699	0.55888083	0.56266338	0.56688136	0.57218286	0.57839167	0.58562196	0.59348122	0.60187632	0.61064279	0.61964858	0.62867747	0.63761483	0.64631332	0.65316974	0.66000147
Davao Occidental	0.44086023	0.44490754	0.44856808	0.45211209	0.45553339	0.4586042	0.46190319	0.46566822	0.4699581	0.47488221	0.48087414	0.48819166	0.49678071	0.50589737	0.51561995	0.52561758	0.53566598	0.54566161	0.55547244	0.5651286	0.57338474	0.5814625
Davao Oriental	0.42947631	0.432804	0.43568961	0.43849756	0.4412418	0.44359074	0.44616016	0.44930632	0.45292752	0.45694815	0.46194415	0.46785605	0.47478946	0.48230605	0.49033515	0.49867918	0.50717729	0.51575039	0.52437516	0.53298609	0.53985027	0.54667344
Dinagat Islands	0.50715463	0.51070535	0.51380372	0.51672098	0.51959612	0.52218647	0.52500842	0.52826576	0.53176201	0.53548164	0.54012138	0.54571947	0.5523491	0.55945463	0.56702533	0.57490673	0.58298046	0.59118251	0.59934323	0.60750915	0.61416855	0.62078136
Eastern Samar	0.38903916	0.38967202	0.38964507	0.38937881	0.38922443	0.3887952	0.38886177	0.38983075	0.39169623	0.39439736	0.39851924	0.40441011	0.41208072	0.42047546	0.42967434	0.43935852	0.44929564	0.45942445	0.46963707	0.47979713	0.48796383	0.49567523
Guimaras	0.49114348	0.49370461	0.49585227	0.49798168	0.50056942	0.50323377	0.50645176	0.51053953	0.51534873	0.52028262	0.52601927	0.53240545	0.53963773	0.54731477	0.55528574	0.56341961	0.57161359	0.57977664	0.58797511	0.59620624	0.60268828	0.60924067
Ifugao	0.48872139	0.4913429	0.49345894	0.49540788	0.49742238	0.49921543	0.50131215	0.50400927	0.50719384	0.5107684	0.51541552	0.52116326	0.52797046	0.53524472	0.54299618	0.55103808	0.55922049	0.56752477	0.57579771	0.58406663	0.59062678	0.59712822
Ilocos Norte	0.58592588	0.58778312	0.5893994	0.59100477	0.59275388	0.59441944	0.59655341	0.59943024	0.602955	0.60683779	0.61166485	0.61727603	0.62372521	0.63059125	0.6377458	0.64510911	0.65258588	0.66009901	0.66761516	0.67507917	0.68093679	0.68692159
Ilocos Sur	0.56252015	0.56464872	0.56645519	0.56822488	0.57022958	0.57222931	0.57470133	0.57793142	0.58183559	0.58606239	0.59125395	0.59725136	0.6041791	0.61156802	0.61934029	0.62730858	0.63543429	0.64352496	0.65156554	0.65954628	0.66589429	0.67227776
Iloilo	0.56335959	0.5653781	0.5671425	0.56892978	0.57108015	0.57327554	0.57599799	0.57963491	0.58399969	0.58863906	0.59420191	0.60035015	0.60725643	0.61459278	0.62225255	0.63002535	0.63791008	0.64576382	0.65349013	0.66111503	0.66693733	0.67291782
Isabela	0.52716772	0.52954407	0.53154412	0.53351323	0.53560178	0.53752363	0.53984775	0.54286959	0.5464968	0.55053601	0.55560088	0.56153599	0.5684623	0.57594551	0.58384658	0.59196152	0.60022421	0.60847328	0.61669159	0.62477231	0.63104261	0.63731965
Kalinga	0.44945356	0.45307383	0.45628207	0.45949121	0.4630008	0.46646004	0.470394	0.47513579	0.48055517	0.4863254	0.49299647	0.50017572	0.50808955	0.5163918	0.52481059	0.53317686	0.54148945	0.54972099	0.55786137	0.56590304	0.57200029	0.57819828
La Union	0.55944715	0.56079972	0.56181975	0.56281121	0.56390595	0.56486544	0.56624375	0.56840593	0.57124369	0.57457037	0.57905343	0.58454445	0.59117864	0.59849276	0.60641322	0.61462279	0.62302889	0.63144707	0.63982786	0.64806169	0.65451761	0.66096366
Laguna	0.60593034	0.60716869	0.60818691	0.60927961	0.610613	0.61188147	0.61362688	0.61611386	0.61922123	0.62267607	0.62702988	0.63215151	0.63814732	0.64460403	0.6515515	0.65882266	0.66633926	0.67391037	0.68153834	0.68909832	0.69499712	0.70092466
Lanao Del Norte	0.49081105	0.49166191	0.49199088	0.49216804	0.49235395	0.49223712	0.49246926	0.49335385	0.49493157	0.49709516	0.50042609	0.50507825	0.5110446	0.5177011	0.52514132	0.5331415	0.54157525	0.55027829	0.55915655	0.56804898	0.57530936	0.58250552
Lanao Del Sur	0.4270541	0.42796934	0.42834406	0.42863386	0.42914705	0.42953723	0.43047656	0.43224423	0.43479719	0.4380233	0.44249021	0.44817248	0.45507745	0.46267006	0.4709311	0.47958677	0.48849782	0.49755357	0.50672528	0.51590961	0.52329783	0.53070277
Leyte	0.49216187	0.49451497	0.49651534	0.49847772	0.50066122	0.50277523	0.5053147	0.50858904	0.51252135	0.51695936	0.52254593	0.52905307	0.53666957	0.54494778	0.55365192	0.56248618	0.57140471	0.58023973	0.58889864	0.5974381	0.60416111	0.61094256
Maguindanao	0.3880739	0.39102055	0.39347961	0.39579569	0.39824316	0.40062091	0.40344862	0.40702858	0.41118786	0.41568944	0.42128118	0.42786739	0.43549019	0.44362905	0.45217335	0.46098824	0.46993436	0.47895129	0.48799597	0.49699725	0.5041702	0.51129551
Marinduque	0.44780766	0.44883518	0.44938912	0.44984263	0.45049874	0.45092478	0.45178308	0.45340269	0.45583086	0.45897958	0.46338761	0.46916505	0.47638621	0.4842276	0.4927244	0.50158883	0.51051274	0.51945399	0.5284135	0.53740949	0.54465332	0.55186124
Masbate	0.37592122	0.37541368	0.37428736	0.37296442	0.37150644	0.36959719	0.36788461	0.36683888	0.36655854	0.36726094	0.36942342	0.37347963	0.37934986	0.38599463	0.39366559	0.40206283	0.41091618	0.42009045	0.42946856	0.43896097	0.44664412	0.45410809
Misamis Occidental	0.49877893	0.49865014	0.49814921	0.49755264	0.49727484	0.49699739	0.49728091	0.4983823	0.50021534	0.50260102	0.50617145	0.51100583	0.51714556	0.5239952	0.5314557	0.53933911	0.54751223	0.55582144	0.56412563	0.57247424	0.57908862	0.5857211
Misamis Oriental	0.57111426	0.57223796	0.5730367	0.57382276	0.57471239	0.5754302	0.57656557	0.57831873	0.58056242	0.5831553	0.58674428	0.59127318	0.59683424	0.60302059	0.60979533	0.61704272	0.62460115	0.63232768	0.64012107	0.64791731	0.65403382	0.66020852
Mountain Province	0.42357614	0.42441576	0.42454524	0.42438025	0.42406488	0.42328321	0.4227177	0.42292593	0.42413562	0.42630628	0.42974588	0.4345494	0.4407264	0.44768079	0.4557423	0.46462027	0.47396115	0.48380356	0.49393712	0.50417424	0.51254507	0.52052229
National Capital Region	0.65460432	0.65633226	0.6579052	0.65951263	0.66133099	0.66314196	0.66546212	0.66855547	0.67221608	0.6761265	0.68094521	0.68641653	0.69257274	0.69909269	0.70589905	0.71288225	0.72002705	0.72718223	0.73426032	0.74120819	0.74642606	0.75174222
Negros Occidental	0.51616173	0.51691183	0.51731886	0.51766022	0.51814239	0.51848851	0.51929894	0.52083332	0.52300414	0.52562109	0.52929347	0.53391943	0.53958533	0.54585822	0.55269499	0.55997196	0.56754244	0.57525008	0.58299961	0.59077908	0.59691778	0.60317817
Negros Oriental	0.47216998	0.47422025	0.47593512	0.47761205	0.47929826	0.48076246	0.48254915	0.48498773	0.48805853	0.49160108	0.49618221	0.50156702	0.50789908	0.51488165	0.52241419	0.53026019	0.53836243	0.54654158	0.55479699	0.56301219	0.5695307	0.57608402
Northern Samar	0.41583515	0.41730969	0.41822878	0.41897252	0.41992488	0.42082439	0.42226388	0.42458261	0.42771499	0.43129026	0.43588865	0.44151493	0.44827083	0.45562729	0.46356622	0.4719156	0.48057928	0.4894836	0.49855788	0.50769037	0.51501261	0.52220911
Nueva Ecija	0.55366637	0.55480685	0.55570562	0.55666473	0.55780036	0.55887801	0.56040271	0.5626609	0.56559324	0.56893863	0.5732195	0.57829571	0.5842918	0.59086093	0.59799809	0.6055098	0.61332977	0.62127825	0.62931526	0.63734588	0.6437457	0.6501666
Nueva Vizcaya	0.51549017	0.51651509	0.51713657	0.51763804	0.5182423	0.51866244	0.51945415	0.52090488	0.52309082	0.52603599	0.53017195	0.53563317	0.54236745	0.54962416	0.55755534	0.56595879	0.57467543	0.58359332	0.59262937	0.60170588	0.60904706	0.61619295
Occidental Mindoro	0.37516621	0.37535431	0.37464914	0.37351343	0.37183662	0.36944013	0.36714958	0.36547909	0.36472746	0.36531598	0.36746442	0.37175191	0.37796597	0.38495383	0.39349826	0.40317466	0.41360415	0.42464139	0.43608394	0.44773088	0.45755516	0.46683504
Oriental Mindoro	0.48214392	0.48463363	0.48675639	0.48881242	0.49099868	0.49304322	0.49556269	0.49889006	0.50300885	0.5076352	0.51339077	0.5200893	0.5277632	0.53591926	0.54453945	0.55339106	0.56237847	0.57133617	0.58022105	0.58907426	0.59616333	0.60323574
Palawan	0.42505142	0.42646715	0.42737038	0.42812927	0.42886414	0.42925955	0.43002779	0.43149819	0.43370491	0.43654124	0.44065406	0.44615281	0.45303761	0.46060718	0.4688514	0.47756987	0.48667678	0.4960264	0.5054682	0.5149248	0.52253468	0.53000513
Pampanga	0.59161387	0.59400007	0.59614123	0.59830123	0.60050269	0.60247068	0.60475493	0.60760769	0.61094089	0.61458145	0.61917099	0.62455238	0.63084502	0.63765863	0.64493023	0.65251482	0.66029655	0.66811679	0.6759948	0.68377512	0.68986456	0.69599928
Pangasinan	0.5642316	0.5657424	0.56694724	0.56815525	0.56952375	0.57082225	0.57258064	0.57508422	0.57826383	0.58187691	0.58654922	0.5921009	0.59862753	0.60576604	0.61336556	0.62119627	0.6292134	0.63720241	0.64519851	0.65310651	0.65929418	0.66557535
Quezon	0.52004659	0.52118402	0.52212822	0.52315832	0.52445697	0.52578499	0.52766585	0.53041695	0.53395268	0.53808912	0.5433584	0.5494748	0.55657661	0.564322	0.57258105	0.58107409	0.58972957	0.59837124	0.60695527	0.61537239	0.62199063	0.62856238
Quirino	0.46556972	0.46836819	0.47061397	0.47264702	0.47463839	0.47629671	0.47826004	0.48083002	0.48397154	0.48742756	0.49180455	0.49717358	0.50362741	0.51057238	0.51802951	0.5257954	0.53383301	0.54194224	0.55015005	0.55837508	0.56494078	0.57149884
Rizal	0.60397361	0.60779266	0.61114182	0.61433544	0.61742208	0.62012651	0.62297682	0.62624142	0.62979738	0.6334617	0.63794313	0.64300619	0.64879787	0.65501257	0.6616108	0.66848546	0.6756337	0.6829413	0.69032797	0.69772185	0.70349531	0.7094079
Romblon	0.41286539	0.41488296	0.41639351	0.41781538	0.41933921	0.42054717	0.42210838	0.4244441	0.42765163	0.43161897	0.43687056	0.44355362	0.45172336	0.46052892	0.46986101	0.47937041	0.48892931	0.49842319	0.5078754	0.5173138	0.52489829	0.53239065
Samar (Western Samar)	0.43153768	0.43227449	0.43252622	0.43266752	0.43314898	0.4336472	0.43471247	0.43662302	0.43927735	0.44229819	0.44633676	0.45145341	0.45773953	0.46464227	0.47205886	0.47978456	0.48775288	0.49588352	0.50416108	0.51245123	0.51901364	0.5255082
Sarangani	0.45758851	0.46112008	0.46443457	0.4678022	0.47139606	0.47491678	0.47884591	0.48344688	0.48840828	0.49361508	0.49983808	0.50663018	0.51420378	0.52230177	0.53047681	0.53864512	0.54684426	0.55502382	0.56316235	0.57121446	0.5774474	0.58386943
Siquijor	0.48456544	0.48836387	0.49190731	0.49549428	0.498892	0.50182594	0.50494068	0.5085996	0.51265358	0.51698715	0.52235974	0.52834551	0.53514931	0.54251028	0.55013087	0.55782956	0.56563678	0.57346837	0.58125928	0.5889612	0.59472782	0.6007397
Sorsogon	0.48269207	0.48461039	0.4862521	0.487954	0.48995273	0.49193837	0.49446113	0.49789712	0.50217657	0.50683034	0.51250641	0.51902351	0.52648866	0.53441031	0.54272952	0.55128515	0.56002191	0.56882099	0.57764814	0.58640728	0.59330996	0.60025457
South Cotabato	0.5258649	0.52764294	0.52902999	0.5304012	0.53192992	0.53328942	0.5350743	0.53759213	0.54068989	0.54424253	0.5489041	0.5545683	0.56135752	0.56877584	0.57673543	0.58503259	0.5935306	0.60208225	0.61056141	0.61893074	0.62549704	0.63208137
Southern Leyte	0.48171349	0.48372561	0.48541075	0.48713178	0.4891427	0.49104828	0.49350235	0.49676617	0.50064954	0.505049	0.5105676	0.51719274	0.52494954	0.53333255	0.54211491	0.55103816	0.56003126	0.56894778	0.57775058	0.58646596	0.59333487	0.60021125
Sultan Kudarat	0.42603049	0.42710189	0.42755292	0.42774562	0.42787117	0.42760143	0.42767816	0.42841617	0.42984594	0.43192742	0.43524464	0.43998786	0.44614033	0.45297282	0.46062176	0.46884483	0.47754768	0.48662115	0.49596074	0.50539423	0.513117	0.52067608
Sulu	0.34463609	0.34847542	0.35204626	0.3556816	0.35944538	0.36301171	0.366933	0.37151761	0.37654503	0.38202734	0.38877923	0.3964133	0.40501488	0.41425509	0.42366848	0.43307547	0.44246982	0.45174437	0.46089166	0.46985252	0.47661869	0.48353585
Surigao Del Norte	0.51047132	0.51378696	0.51683805	0.51991578	0.52303817	0.52596493	0.52927105	0.53315197	0.53740327	0.54182556	0.54712347	0.55304533	0.55981052	0.5670046	0.57460873	0.58242857	0.59042628	0.59850541	0.60659591	0.61465793	0.62097748	0.6274452
Surigao Del Sur	0.48312167	0.48546311	0.48735736	0.48915469	0.49099669	0.49265909	0.49469545	0.49741893	0.50071438	0.50435405	0.5090011	0.514462	0.520834	0.52769563	0.53506364	0.5427527	0.55072028	0.558849	0.56709674	0.57547677	0.58210371	0.58874071
Tarlac	0.54963501	0.55154068	0.55307732	0.55456012	0.55611742	0.55744202	0.55906925	0.56130022	0.5640823	0.56729701	0.5715885	0.57688769	0.58322587	0.5900956	0.59749217	0.60527788	0.61332015	0.62144073	0.62961204	0.6377408	0.6441904	0.65064553

Tawi-Tawi	0.40929984	0.41157424	0.41341358	0.41516651	0.41741326	0.41987784	0.42311573	0.42740368	0.43242302	0.43773004	0.44409331	0.45122105	0.45920788	0.46764062	0.47641084	0.48541296	0.49452887	0.503662	0.51277203	0.5218073	0.52879969	0.53581204
Zambales	0.55597225	0.5565431	0.55686696	0.55729537	0.55793563	0.55849005	0.55952704	0.56136711	0.5638859	0.56693501	0.57114626	0.57629563	0.5824457	0.58924208	0.59668824	0.60453675	0.61268794	0.62098829	0.62934206	0.63767085	0.64426807	0.6508406
Zamboanga Del Norte	0.43045888	0.43208335	0.43328012	0.43439178	0.43571821	0.4369501	0.43863676	0.44107476	0.44419401	0.44776552	0.45248399	0.45824558	0.46512348	0.47261233	0.48056852	0.48883426	0.49731943	0.5059329	0.51465801	0.52334977	0.53029097	0.53718454
Zamboanga Del Sur	0.51488974	0.51750465	0.51983278	0.52216686	0.52474886	0.52730215	0.53031595	0.53407927	0.53841831	0.54300328	0.54853417	0.55477975	0.56181956	0.56936313	0.5772568	0.58539659	0.59366976	0.60194062	0.61015045	0.61826857	0.62455206	0.63090878
Zamboanga Sibugay	0.429073	0.431565	0.43365052	0.43562788	0.43789889	0.44008967	0.44280836	0.44640623	0.45067228	0.45528606	0.46094767	0.46752674	0.47518019	0.48339369	0.49195798	0.50070771	0.5095203	0.51831286	0.52707055	0.53581192	0.54274036	0.54969602
Seychelles	0.65155199	0.65649067	0.66090779	0.66416203	0.66646135	0.66926474	0.67214991	0.67507744	0.67659095	0.67784703	0.68016364	0.68368149	0.68762939	0.69252573	0.69711459	0.70172282	0.70618362	0.71102728	0.71640782	0.72207368	0.72666377	0.73015077
Sri Lanka	0.58294264	0.5875566	0.59235913	0.59741028	0.60225006	0.60690666	0.61180708	0.61697927	0.62241432	0.62814963	0.63426312	0.64079957	0.64804982	0.65543584	0.66278243	0.66976029	0.67627744	0.68240527	0.68812586	0.69351606	0.69764854	0.70153494
Thailand	0.58350092	0.58766156	0.5917591	0.59604892	0.60052067	0.60504078	0.61035668	0.61636859	0.62106754	0.62432907	0.62858944	0.63322673	0.63841097	0.64367292	0.64877206	0.65401562	0.6593693	0.66480413	0.67022559	0.6752739	0.67911999	0.68254793
Timor-Leste	0.34379365	0.34949032	0.35416202	0.35844003	0.362688	0.36691358	0.37065409	0.37539077	0.38122753	0.38794482	0.39488702	0.40187236	0.4089103	0.41521788	0.42115554	0.42638362	0.43120727	0.43451973	0.43701866	0.44019585	0.44234934	0.44466762
Viet Nam	0.48980205	0.49763035	0.50545345	0.51336751	0.52122939	0.52895633	0.53630065	0.54334265	0.55012712	0.55645594	0.56276857	0.56894546	0.57507867	0.58102921	0.58691381	0.59284957	0.59884949	0.60501942	0.61135141	0.61773382	0.62309032	0.62793372
Sub-Saharan Africa	0.33090196	0.33496328	0.33935347	0.34403438	0.34924842	0.35502181	0.36107474	0.36756161	0.37410063	0.38055519	0.38731002	0.39412176	0.40091964	0.40775813	0.41467049	0.42143301	0.42788695	0.43425414	0.4406728	0.44717187	0.45311872	0.4585873
Central sub-Saharan Africa	0.32084148	0.32374919	0.3275669	0.33193465	0.33742002	0.34409927	0.35145974	0.35956551	0.36848818	0.37666929	0.38538095	0.39469176	0.40423388	0.41352221	0.42257865	0.43098753	0.43874592	0.44617858	0.45324977	0.46015956	0.46645637	0.47225565
Angola	0.30760331	0.31169753	0.31670657	0.32177736	0.32739527	0.33378519	0.34101262	0.34884684	0.35708201	0.36440774	0.37203354	0.38000661	0.38789186	0.39579633	0.40372009	0.41137203	0.41882206	0.42617708	0.43335501	0.4404546	0.44728399	0.45372195
Central African Republic	0.24602113	0.24924674	0.25252437	0.25510152	0.25777878	0.26045192	0.26371245	0.26733219	0.27115812	0.27533852	0.27998138	0.28509725	0.29046002	0.29010493	0.2899995	0.29082787	0.2924304	0.29475224	0.29801122	0.30195306	0.3054263	0.30916769
Congo (Brazzaville)	0.46076481	0.46375168	0.46703727	0.47013437	0.47361244	0.47813763	0.48335582	0.48764074	0.49282285	0.49829997	0.50554285	0.51381578	0.52292828	0.53155168	0.54059449	0.54841403	0.55510861	0.5618341	0.56779397	0.57337367	0.57845155	0.58307524
DR Congo	0.26245675	0.2578575	0.25413636	0.25233388	0.25247092	0.25416102	0.25735512	0.26227227	0.26810387	0.27436114	0.28222375	0.29087295	0.30045478	0.31083126	0.32162293	0.33242131	0.34198879	0.35110204	0.36028768	0.36934068	0.37671227	0.38317985
Equatorial Guinea	0.41138345	0.43359006	0.451939	0.46784683	0.48387961	0.49868372	0.51157983	0.52407646	0.53701899	0.54851468	0.55910938	0.57027742	0.5818339	0.59280061	0.60347317	0.61313462	0.62185816	0.63023454	0.63802282	0.64530309	0.6521249	0.65785746
Gabon	0.50913309	0.51304571	0.51691762	0.52105141	0.52547907	0.53048857	0.53479772	0.53944142	0.54411089	0.54859132	0.55402271	0.56023208	0.56684296	0.57380813	0.58091566	0.588365	0.5960623	0.60381625	0.61172438	0.61990473	0.62760965	0.63469139
Eastern sub-Saharan Africa	0.26416531	0.26877226	0.27348445	0.27843367	0.28393661	0.28997039	0.29645526	0.30357591	0.3109185	0.31845677	0.32629597	0.3342534	0.34162808	0.3492073	0.35700952	0.36490704	0.37275883	0.38049999	0.38835511	0.39634466	0.40348021	0.40972098
Burundi	0.21686986	0.21698873	0.21778248	0.21896154	0.22091871	0.22308437	0.22582189	0.22899682	0.23285355	0.23694304	0.24144779	0.24627728	0.2515723	0.25719136	0.26279472	0.26732794	0.27158785	0.2758405	0.27973329	0.28344251	0.286496	0.28937436
Comoros	0.35187551	0.359212	0.36648613	0.37341057	0.38003598	0.38689184	0.39352143	0.39905109	0.40464976	0.41025358	0.41554338	0.42109297	0.42672996	0.43278993	0.43884451	0.44471239	0.45050698	0.45628084	0.46195134	0.46738801	0.47200313	0.47597869
Djibouti	0.36901509	0.37285316	0.37665	0.38068662	0.38498771	0.38956871	0.39503747	0.40061855	0.4066583	0.41196933	0.41699935	0.42231614	0.42771144	0.43336044	0.43945572	0.44585528	0.45344285	0.46044015	0.46770567	0.47526324	0.48187995	0.48795837
Eritrea	0.31024816	0.31724799	0.32356658	0.32859358	0.33287938	0.33668251	0.33983582	0.34284317	0.3434278	0.34514774	0.34814618	0.35416315	0.36011275	0.36434365	0.37252759	0.37693516	0.38217167	0.38593603	0.39115488	0.39633056	0.40045941	0.40386394
Ethiopia	0.17341853	0.17897297	0.18413801	0.18869481	0.1950307	0.20286824	0.21152423	0.22181993	0.23287219	0.24426582	0.25608146	0.26788086	0.27900696	0.28988527	0.30048455	0.31073642	0.32043187	0.32946924	0.33805007	0.34642266	0.35321635	0.3588233
Addis Ababa	0.54939268	0.55542161	0.56043045	0.56431784	0.56943151	0.5755078	0.58179341	0.58915693	0.59692114	0.60488966	0.61329088	0.62176334	0.62952179	0.63741593	0.64555244	0.65380755	0.66176964	0.66940403	0.67681666	0.68417585	0.68996669	0.6947296
Afar	0.11480189	0.11905238	0.12278243	0.12576246	0.13060772	0.13697002	0.14420382	0.15310516	0.16278367	0.17290444	0.18356445	0.19430056	0.2046402	0.21494064	0.22521527	0.23547254	0.24550483	0.25517861	0.26462211	0.27398706	0.28194644	0.28877341
Amhara	0.11932173	0.12553943	0.13127708	0.13619058	0.14324911	0.15213541	0.16202923	0.17382063	0.18639961	0.19927525	0.2126035	0.22578478	0.23798506	0.24961776	0.26090417	0.27172065	0.28187013	0.29117117	0.29993476	0.30840144	0.31505898	0.32045319
Benishangul-Gumuz	0.10818158	0.11363627	0.11849202	0.12246567	0.12882057	0.137216	0.14690757	0.15884862	0.17184181	0.18538265	0.19953234	0.21347031	0.22643598	0.23921067	0.25166825	0.26378196	0.27530219	0.28602094	0.29611152	0.30580875	0.31370507	0.32022848
Dire Dawa	0.36228323	0.3682846	0.37345748	0.3777916	0.38340132	0.39013703	0.39752568	0.40621972	0.41557533	0.42532002	0.43556487	0.4458478	0.45562	0.46568671	0.47595852	0.48634401	0.49651842	0.50638749	0.51601433	0.52556768	0.53366763	0.54066865
Gambella	0.21745483	0.22461352	0.23119634	0.23711931	0.24538736	0.2555678	0.26698988	0.28069613	0.2955801	0.31103477	0.32707721	0.34285349	0.35748561	0.37176026	0.38576422	0.39934786	0.41203614	0.4234045	0.43390134	0.44395416	0.4518667	0.45822008
Harari	0.36060537	0.36532529	0.36946047	0.3729444	0.37798011	0.3843402	0.39140154	0.39987955	0.40909223	0.41866488	0.42869506	0.43878276	0.44832266	0.45822213	0.46856024	0.47925971	0.49003538	0.50059889	0.51105614	0.5214891	0.53044876	0.53816876
Oromia	0.14476873	0.14986609	0.15453324	0.15856473	0.16460966	0.17231514	0.18092932	0.19136077	0.20263326	0.21427913	0.22638323	0.23858833	0.25029045	0.26190747	0.27326951	0.28418588	0.29455197	0.30422946	0.313416	0.32234769	0.32969397	0.33581502
Somali	0.10517341	0.10876118	0.1119455	0.11450819	0.1187302	0.12439919	0.13091725	0.1390476	0.14804035	0.15757025	0.1676664	0.17788091	0.18780065	0.19775066	0.20767007	0.21752944	0.2270536	0.23620899	0.24514346	0.25402577	0.26165225	0.26825381
Southern Nations, Nationalities, and Peop	0.15273481	0.15831281	0.16324994	0.16728194	0.17364111	0.1819693	0.19136959	0.20285632	0.21523697	0.22804416	0.24140239	0.25472511	0.2672688	0.27940218	0.29102727	0.30235607	0.31306423	0.32310623	0.33260584	0.3418741	0.34933889	0.35552153
Tigray	0.16787049	0.17469034	0.18138198	0.18763219	0.19577268	0.20558104	0.2164032	0.22904742	0.24256363	0.25637622	0.27021671	0.28342429	0.295752	0.3077623	0.31943002	0.33072634	0.34133269	0.3511351	0.36033055	0.36923235	0.37634669	0.38218351
Kenya	0.38239562	0.38618841	0.38988626	0.39368048	0.39774365	0.40244401	0.40774576	0.41373623	0.41973815	0.42612389	0.43330584	0.44089383	0.4486664	0.45666694	0.4647243	0.47308405	0.48170907	0.49029662	0.49910127	0.50800421	0.51613657	0.52376808
Baringo	0.36026177	0.3677072	0.37496099	0.38210732	0.38895144	0.39587713	0.40284885	0.40994404	0.41653543	0.42322585	0.43042939	0.43777825	0.44523747	0.45286559	0.46046064	0.46832563	0.47637004	0.48432326	0.49242459	0.50052297	0.50783667	0.51488321
Bomet	0.34323324	0.34934892	0.35546306	0.36177148	0.36834362	0.37558909	0.38328393	0.3915904	0.40000859	0.4088541	0.41837562	0.42814142	0.43815825	0.44836723	0.45863924	0.46914511	0.47968712	0.49000683	0.50038282	0.51072064	0.52036646	0.52936785
Bungoma	0.34201606	0.34596829	0.34983724	0.35388879	0.35830797	0.3635242	0.3694102	0.37593137	0.38237783	0.38928534	0.39713017	0.40544736	0.41394024	0.4227508	0.43167406	0.44081965	0.45027186	0.45978388	0.46954151	0.47933502	0.48823412	0.49672518
Busia	0.30278151	0.30904917	0.31520972	0.32128512	0.32740101	0.33406055	0.34113386	0.34876109	0.35623582	0.36404012	0.37258396	0.38140168	0.39034518	0.39962196	0.40893397	0.41852651	0.42842547	0.43836816	0.4485658	0.4588495	0.46846162	0.47766502
Elgeyo Marakwet	0.35191274	0.35903312	0.36593207	0.37276681	0.3797114	0.38719587	0.39510572	0.40358442	0.41184969	0.42018957	0.42915307	0.43829199	0.44745659	0.45665741	0.46575298	0.47495817	0.48413876	0.49304217	0.50192682	0.51078433	0.51880107	0.52629704
Embu	0.41528903	0.41884967	0.42235551	0.42595306	0.42982265	0.43422769	0.4391254	0.44462982	0.45009413	0.45605514	0.46276491	0.46990422	0.47713466	0.48460733	0.49219877	0.50006134	0.50815116	0.51615204	0.5243762	0.53268266	0.54029862	0.5475554
Garissa	0.19529926	0.19935953	0.20350887	0.2078281	0.212455	0.21753118	0.22314372	0.22929606	0.2354951	0.24187021	0.24873366	0.25582027	0.26289237	0.26980041	0.27650687	0.28318003	0.28979082	0.29619717	0.30269397	0.30923082	0.31529842	0.32106827
Homa Bay	0.29059477	0.29554062	0.30017308	0.3049778	0.31024252	0.31645244	0.32342622	0.33135032	0.33957262	0.34864525	0.35879107	0.36957989	0.38094713	0.3929373	0.40514106	0.41777086	0.43074586	0.44375587	0.45697261	0.47018344	0.48259887	0.49387181
Isiolo	0.2902204	0.29538253	0.30044752	0.30553593	0.31049802	0.31571355	0.32120826	0.32686212	0.33216512	0.33791517	0.34450006	0.35170062	0.35906996	0.36685988	0.37473934	0.38296336	0.39158026	0.4001813	0.40898882	0.4179019	0.42612666	0.43396683
Kajiado	0.34982514	0.35388005	0.35791287	0.36201337	0.36634563	0.37149759	0.37725651	0.38376645	0.39051628	0.39771444	0.40562934	0.41384177	0.42223328	0.43102159	0.43998136	0.44933957	0.45898524	0.46861712	0.47843969	0.48840649	0.49800022	0.50696971
Kakamega	0.35637908	0.35982513	0.36310493	0.36643328	0.37019984	0.37474499	0.3800939	0.38640671	0.392941	0.40007452	0.40821719	0.41688159	0.42572593	0.4349214	0.4442112	0.4536624	0.46330091	0.47283502	0.48247028	0.49208871	0.50101962	0.509529
Kericho	0.35459767	0.36015215	0.36587346	0.37178928	0.37784211	0.38445833	0.39139443	0.39886043	0.4063073	0.41412668	0.42268709	0.43151634	0.44047099	0.44953552	0.45872171	0.46813571	0.47767996	0.48705026	0.49650186	0.5060331	0.51507995	0.52357336
Kiambu	0.4524213	0.45573397	0.45902724	0.46260647	0.46667551	0.47155409	0.47715126	0.48352829	0.48998317	0.49674967	0.50424438	0.51206453	0.51986702	0.52781749	0.53571055	0.543913	0.55237716	0.56074853	0.56934094	0.57798717	0.58578667	0.59311015
Kilifi	0.30776104	0.31144386	0.31512961	0.31879326	0.32260586	0.32721863	0.33272644	0.33929043	0.34646581	0.35427583	0.363203	0.3728217	0.38298144	0.39355055	0.40442284	0.41584185	0.42760697	0.43943563	0.45138702	0.46343468	0.47510409	0.48600916
Kirinyaga	0.43636437	0.43832667	0.44020221	0.44220373	0.44435849	0.44702609	0.45030034	0.45447253	0.45866836	0.46331757	0.46882596	0.47469599	0.48066269	0.48689365	0.49323641	0.50005561	0.50722261	0.51438214	0.52178596	0.52935903	0.53631716	0.54301445
Kisii	0.38037322	0.38425154	0.38803172	0.39191847	0.39630802	0.4015104	0.40755891	0.41450806	0.42167082	0.42947671	0.43834573	0.44780142	0.4575415	0.46745597	0.47747863	0.48752835	0.49770319	0.50760074	0.51753212	0.52732535	0.53629584	0.54460484
Kisumu	0.37590796	0.38034198	0.38454179	0.38873661	0.39336326	0.39883663	0.4051727	0.41255596	0.42026899	0.42845044	0.43746431	0.44687155	0.45645513	0.46614566	0.47583221	0.48576018	0.49593723	0.50598977	0.51617172	0.52638188	0.5357574	0.54440932
Kitui	0.32895175	0.33260177	0.33629652	0.34014204	0.34433206	0.34927059	0.35472603	0.3607764	0.36675813	0.37324773	0.38067412	0.38858268	0.39666831	0.40510681	0.41365085	0.4224483	0.43142387	0.44027473	0.4492419	0.45827618	0.46673185	0.47474946
Kwale	0.32134295	0.32435135	0.32718985	0.33006997	0.33310789	0.3366711	0.34102117	0.34636993	0.3521558	0.35854506	0.36598907	0.37418784	0.38300052	0.39234386	0.40200224	0.41220808	0.42276399	0.43337097	0.44402927	0.45478114	0.46520963	0.47513821
Laikipia	0.41189389	0.41603417	0.42050785	0.42545425	0.43087243	0.43714841	0.44411975	0.45185932	0.45983744	0.46796876	0.47655284	0.4851985	0.49394184	0.50279173	0.51165431	0.52079312	0.53015216	0.53939312	0.54878653	0.55827591	0.56720183	0.57571177
Lamu	0.36335844	0.36741006	0.37141826	0.37538164	0.37941555	0.38391899	0.38905098	0.39479026	0.40051306	0.40653145	0.41333928	0.42057444	0.42808183	0.43582573	0.44361782	0.45191862	0.46054325	0.46921016	0.47816096	0.48724627	0.49569401	0.50378649
Machakos	0.40165728	0.40531003	0.40908603	0.41314001	0.41753381	0.42254273	0.42828797	0.4347004	0.44101769	0.4477737	0.45529974	0.46328831	0.47138925	0.47967766	0.48792392	0.4964443	0.50517693	0.51387265	0.52278938	0.53179865	0.54009577	0.54803579
Makueni	0.35185986	0.35656801	0.36123336	0.36607408	0.37123528	0.37717237	0.38371751	0.39092963	0.39804248	0.40557284	0.41400964	0.42282974	0.43165202	0.44070173	0.44967929	0.4588639	0.46819627	0.47735896	0.48666166	0.49599583	0.50447246	0.51256894
Mandera	0.13460548	0.1375094	0.14070298	0.1441618	0.14793749	0.15200491	0.1565108	0.16153015	0.16669031	0.17220618	0.1781146	0.18412552	0.19002917	0.19582674	0.20162299	0.20734981	0.21288621	0.2181795	0.22341598	0.2285393	0.23343644	0.23826383
Marsabit	0.25106416	0.25558303	0.26014092	0.26479248	0.26959364	0.27487534	0.28060571	0.28673229	0.29279861	0.2993658	0.30671286	0.31457732	0.32255832	0.33086408	0.33922532	0.34785963	0.35677731	0.36561218	0.3745712	0.38360518	0.39207979	0.40018158
Meru	0.35959025	0.36316546	0.36672433	0.37051689	0.37474811	0.3796408	0.38518795	0.39147474	0.39781287	0.40455772	0.41207553	0.42002137	0.42812692	0.43645421	0.44493826	0.4538223	0.46297858	0.47206584	0.48136409	0.49080099	0.49971654	0.50821615

Migori	0.2635732	0.26982946	0.27590578	0.28214766	0.28865359	0.29585831	0.30346298	0.31174422	0.32031054	0.32946746	0.33942096	0.3498345	0.36078118	0.37213835	0.38367253	0.39571105	0.40839196	0.42127645	0.43443386	0.44776645	0.46078865	0.47280301
Mombasa	0.45100524	0.45442917	0.45770631	0.46099166	0.46472932	0.46926463	0.47486824	0.48158893	0.48845504	0.49565237	0.50364394	0.51213761	0.52080746	0.52964475	0.53831094	0.54705859	0.55588246	0.56440592	0.57302215	0.58157873	0.58916859	0.59638778
Murang'a	0.41638265	0.4203242	0.42420926	0.42818837	0.43238445	0.43706301	0.44220975	0.44799978	0.45371495	0.45978679	0.46661773	0.47378175	0.48096035	0.48830174	0.49563739	0.50333779	0.51132457	0.51930252	0.52756449	0.53594549	0.54357788	0.55086949
Nairobi	0.55674507	0.56066175	0.56458962	0.56869147	0.57302695	0.57790573	0.58340574	0.58956907	0.59554139	0.60163438	0.60837269	0.61533231	0.62218961	0.62896797	0.6355324	0.64233846	0.64942335	0.65644035	0.66370308	0.67099893	0.67750179	0.68372244
Nakuru	0.40762374	0.41232169	0.41693325	0.42163269	0.42699353	0.43325419	0.44054664	0.44879448	0.45699148	0.46538676	0.47440038	0.48366594	0.49293477	0.50219343	0.51125128	0.52028537	0.52929916	0.53792534	0.54658946	0.55516073	0.56279661	0.5700454
Nandi	0.35473974	0.35965211	0.36450596	0.36924546	0.37408417	0.37943795	0.38518209	0.3917347	0.39854001	0.40581572	0.41379656	0.42209894	0.43072787	0.43971352	0.44895729	0.45857811	0.46833688	0.47796967	0.48774354	0.49768713	0.50725822	0.51627971
Narok	0.27584801	0.28205725	0.28840901	0.29478531	0.30108875	0.30787153	0.31485739	0.32228601	0.32995789	0.33814823	0.34707429	0.35633481	0.36586487	0.37565561	0.38574137	0.39618848	0.40679733	0.4173734	0.42805452	0.43882838	0.44929615	0.45910307
Nyamira	0.41530409	0.42038402	0.42531827	0.43048923	0.43613874	0.44249102	0.44933411	0.4569359	0.46445611	0.47263268	0.48183352	0.4914672	0.50138743	0.51158593	0.52189969	0.53227555	0.54259666	0.55257876	0.56251727	0.57232096	0.58118467	0.58926708
Nyandarua	0.42171721	0.4270748	0.43250056	0.43811961	0.4438892	0.45013407	0.45703348	0.46449859	0.47175062	0.47923949	0.48725052	0.49545696	0.50355318	0.51173563	0.51983166	0.52815573	0.53657032	0.54478966	0.55310811	0.56141338	0.56890929	0.57610986
Nyeri	0.44935217	0.45246709	0.45554338	0.45871321	0.46239312	0.46694089	0.4722988	0.47860757	0.48490753	0.49146844	0.49873753	0.50623899	0.51363901	0.52107548	0.52842088	0.53589522	0.54344866	0.55077546	0.5582799	0.5658029	0.57243095	0.57878748
Samburu	0.24070063	0.24433472	0.24798385	0.25169334	0.2555988	0.26005399	0.26492789	0.27031853	0.2757737	0.28167267	0.28823706	0.29515973	0.30237877	0.30988598	0.31759035	0.32583984	0.3344649	0.3430872	0.35205085	0.3612377	0.37000448	0.3783124
Siaya	0.28932821	0.29424968	0.29897473	0.30383257	0.30918423	0.31546086	0.32247835	0.33029095	0.3382463	0.34683951	0.35642434	0.36663269	0.37725414	0.38813675	0.39915091	0.41029717	0.42171914	0.43306226	0.44451682	0.45591235	0.46663229	0.4765244
Taita Taveta	0.38979897	0.3935252	0.39721597	0.40092075	0.40492704	0.40959221	0.41515821	0.42170599	0.42840066	0.43544198	0.44333336	0.45173013	0.4603975	0.46926755	0.47814447	0.48726442	0.49656209	0.50571664	0.51498696	0.52426924	0.53276871	0.54098098
Tana River	0.25568915	0.25649831	0.25728435	0.25805622	0.25922065	0.26126131	0.26432605	0.26838592	0.27277766	0.27783552	0.28408346	0.2910988	0.29868733	0.3069085	0.3154006	0.32450322	0.33402448	0.34367519	0.35352843	0.36355944	0.37345301	0.38284535
Tharaka Nithi	0.36689183	0.37162648	0.37651194	0.38163932	0.38708279	0.39324083	0.40003186	0.4074344	0.41482035	0.42269232	0.4314317	0.44045676	0.44955571	0.45878203	0.46793069	0.47715169	0.48638513	0.4953951	0.50444461	0.51349274	0.52178997	0.52968011
Trans Nzoia	0.3844205	0.38957093	0.39475898	0.4002202	0.40589697	0.41198674	0.41846072	0.42554918	0.43267935	0.44011034	0.44818471	0.45662743	0.46527019	0.47408703	0.48301679	0.49231628	0.50189977	0.51136886	0.52104663	0.53079554	0.53986969	0.54850455
Turkana	0.22786553	0.23258178	0.23728846	0.24207306	0.24684801	0.2519513	0.25732239	0.26312162	0.26895789	0.27534796	0.28253537	0.2901888	0.29809817	0.30628331	0.31471543	0.32341465	0.33228026	0.34097037	0.34958168	0.35797862	0.36591471	0.37343424
Uasin Gishu	0.40964379	0.41485127	0.4201437	0.4256286	0.43129544	0.43744848	0.44400576	0.4510338	0.45793381	0.46514626	0.47296452	0.48106746	0.48936919	0.49784053	0.50638642	0.51517421	0.52411778	0.53296834	0.54195286	0.55096959	0.55934577	0.56734559
Vihiga	0.3611816	0.36723014	0.37308259	0.3788441	0.38468248	0.39112676	0.39806668	0.40559811	0.4128218	0.42027675	0.42848862	0.43693298	0.44545206	0.45414737	0.46268723	0.47145079	0.48042863	0.48925912	0.49820909	0.50720557	0.51532506	0.52308979
Wajir	0.14435906	0.14763667	0.15106587	0.15465826	0.15847489	0.16259706	0.16714514	0.17210837	0.17713011	0.18244053	0.18821	0.19427243	0.20030946	0.20634144	0.21229647	0.21821194	0.2240721	0.22976103	0.23551673	0.24129824	0.24676802	0.25205011
West Pokot	0.27246151	0.27780292	0.28321097	0.28880577	0.29463593	0.30113574	0.30798146	0.31536315	0.32274818	0.3305135	0.33896688	0.34782901	0.35706252	0.36657439	0.37624551	0.3861947	0.39625639	0.40613952	0.41605866	0.42600268	0.43532573	0.4441209
Madagascar	0.28319072	0.28566818	0.28715315	0.28967546	0.29262988	0.29591933	0.29972339	0.30402624	0.30899026	0.31345089	0.31835318	0.32380104	0.32994754	0.33651923	0.34335261	0.3504436	0.35802634	0.36623559	0.37496964	0.3842092	0.39277871	0.40024694
Malawi	0.23090081	0.23402051	0.23760216	0.24155923	0.24643454	0.25167232	0.25759565	0.26522693	0.27368986	0.28229651	0.29117962	0.30044851	0.30919762	0.31793621	0.32704243	0.33614014	0.34473154	0.35312107	0.36147041	0.37011461	0.37787902	0.38455363
Mozambique	0.1985344	0.20224192	0.20639006	0.21053449	0.21497246	0.21969237	0.22475809	0.23015823	0.23579017	0.24136066	0.2467515	0.25223247	0.25831556	0.26528046	0.27289281	0.28103269	0.28915242	0.29716477	0.30522677	0.31317451	0.32029118	0.32646261
Rwanda	0.28098345	0.28608205	0.29295543	0.29967692	0.30694392	0.31421958	0.32199852	0.32961659	0.33703923	0.34449463	0.35165942	0.35894034	0.36641182	0.37376968	0.38153318	0.38989541	0.39828796	0.40641147	0.41465791	0.42308673	0.4298966	0.43558871
Somalia	0.05584206	0.05664316	0.05745761	0.05827215	0.05907695	0.05989854	0.06074148	0.06159815	0.06247539	0.06338223	0.06432213	0.06530681	0.06635488	0.06745949	0.0686093	0.06981152	0.07106405	0.07233478	0.07364225	0.07497981	0.07636337	0.07768811
South Sudan	0.23173034	0.23501413	0.23834119	0.24179719	0.2454625	0.2492809	0.25330051	0.25749176	0.26174046	0.26617923	0.27057262	0.27471914	0.27506614	0.27581394	0.27631146	0.27583363	0.27445549	0.27438786	0.27498785	0.2757062	0.27678792	0.27837113
Uganda	0.23432323	0.24218639	0.25055239	0.25916488	0.26800461	0.27736919	0.28703058	0.29674042	0.30674884	0.31673115	0.32654366	0.33621772	0.34536452	0.35438162	0.3634309	0.37253799	0.38131223	0.39008226	0.39911778	0.40843156	0.41684608	0.42326118
Tanzania	0.29182726	0.29671565	0.301984	0.30761549	0.31377298	0.32043734	0.327234	0.33431324	0.34135263	0.34837803	0.35556756	0.36303632	0.37041249	0.37796317	0.38597458	0.39427776	0.40287931	0.41182497	0.42098536	0.43022538	0.43874724	0.44656827
Zambia	0.32062532	0.32440137	0.32868012	0.3337214	0.33948892	0.34604886	0.35354071	0.36245097	0.3716885	0.38166712	0.39247099	0.40347563	0.4145745	0.4254923	0.43624401	0.44674472	0.4571226	0.46753263	0.47788771	0.48791152	0.49748556	0.50594895
Southern sub-Saharan Africa	0.55849062	0.56251127	0.56604984	0.56921519	0.5723897	0.57611569	0.58068483	0.585673	0.59040532	0.59458507	0.59883555	0.60334685	0.60803777	0.61284381	0.61745259	0.62183758	0.62591906	0.6297615	0.63341066	0.63694019	0.63988183	0.64220028
Botswana	0.51349822	0.52137018	0.52925296	0.53700018	0.54434922	0.55170697	0.55922162	0.5667025	0.57388381	0.57969641	0.58582415	0.59198546	0.59773955	0.60406473	0.61008605	0.61541112	0.62079757	0.62570495	0.63060684	0.63532409	0.6392753	0.64272163
Eswatini	0.47185284	0.47723337	0.48247442	0.48760883	0.49266916	0.49804385	0.50365829	0.50926022	0.5147309	0.5204274	0.5262458	0.53191391	0.53779802	0.54383642	0.54961385	0.55537374	0.56106119	0.56669849	0.57215481	0.57720631	0.58165307	0.58545971
Lesotho	0.3986358	0.40399013	0.40902166	0.41398257	0.41867092	0.42321123	0.42780673	0.43274504	0.43834057	0.44358013	0.44896648	0.4545301	0.46041969	0.46669226	0.47318816	0.47975446	0.48614483	0.49197815	0.49738179	0.50245732	0.50674665	0.51039307
Namibia	0.50240696	0.50671903	0.51081827	0.51457465	0.5191036	0.52388174	0.52935012	0.53506213	0.54089699	0.54651648	0.55274157	0.5596189	0.5669787	0.57459034	0.58232142	0.58982104	0.59628306	0.60175464	0.6066184	0.61092109	0.61443589	0.61756487
South Africa	0.59409368	0.59788417	0.60092168	0.6035156	0.60626251	0.60998998	0.61501469	0.62060815	0.62594803	0.63064563	0.63526854	0.63999172	0.64471566	0.64943055	0.65396449	0.65841776	0.66264249	0.66662261	0.67033537	0.67404104	0.6771661	0.6796266
Eastern Cape	0.53587488	0.53946484	0.54227296	0.5448034	0.54760921	0.55139461	0.55641342	0.56194656	0.56694482	0.57110398	0.57515226	0.5792332	0.58339892	0.58767183	0.59183366	0.59599022	0.59997241	0.60386294	0.60756497	0.61135182	0.61463222	0.6172047
Free State	0.59266132	0.59559149	0.59809989	0.60023778	0.60247516	0.60569103	0.61041265	0.61594219	0.62140353	0.62640535	0.63153084	0.63683378	0.64221275	0.647592	0.65273241	0.65756921	0.66189221	0.66580999	0.66939803	0.67288493	0.6757477	0.6779086
Gauteng	0.67101892	0.67293906	0.67416507	0.6749922	0.67604683	0.67807479	0.68139232	0.68528898	0.6890725	0.69246862	0.69595935	0.69969533	0.70354202	0.7075252	0.7114802	0.71551531	0.71941139	0.72314472	0.72667679	0.73020676	0.73314709	0.73542663
KwaZulu-Natal	0.57121523	0.5753755	0.57878692	0.58173547	0.58480061	0.58896077	0.59451299	0.60066207	0.60656677	0.61163366	0.61642504	0.62123685	0.62600658	0.63069576	0.63517323	0.63958765	0.64377135	0.64773975	0.65141775	0.6550703	0.65812815	0.6604837
Limpopo	0.52094561	0.52718196	0.53241909	0.53689973	0.5412736	0.54631837	0.55238608	0.5589643	0.56522629	0.57071318	0.57600195	0.58110199	0.58591085	0.59050406	0.59461867	0.59829389	0.60148795	0.60413435	0.60646107	0.60889869	0.61084494	0.61228784
Mpumalanga	0.56228324	0.56662508	0.56993159	0.57236109	0.57467005	0.578043	0.58284765	0.58837325	0.59384114	0.59889605	0.60391027	0.60897037	0.61382709	0.61847148	0.62285777	0.62709222	0.63111764	0.63480553	0.63810419	0.64128793	0.64395427	0.64605747
North West	0.5550737	0.55826226	0.56072338	0.56280564	0.56517049	0.5688307	0.57418797	0.58047243	0.58670005	0.59231345	0.59794054	0.60388036	0.60983482	0.61576094	0.62148756	0.62720277	0.63267377	0.63758414	0.64194456	0.64616033	0.64971093	0.65247643
Northern Cape	0.57398248	0.57837268	0.58197434	0.58498589	0.58780972	0.59119745	0.59557452	0.60024316	0.60447754	0.60794924	0.61148962	0.61557621	0.62031935	0.625579	0.63103997	0.63666516	0.64221084	0.64748292	0.65240921	0.65714213	0.66107246	0.66414062
Western Cape	0.6541842	0.65612973	0.65774184	0.65929781	0.66126574	0.6640782	0.66788147	0.67202766	0.67591549	0.67938032	0.68294892	0.68670705	0.69046072	0.69416794	0.69767622	0.70107381	0.7043196	0.70754415	0.7106979	0.713881	0.71651464	0.7185329
Zimbabwe	0.4448414	0.44635468	0.4469709	0.44600218	0.4436964	0.43965166	0.43440576	0.42916514	0.42244897	0.41833061	0.41730536	0.41972969	0.42574852	0.43288189	0.43999723	0.44655287	0.45247304	0.45842498	0.46437926	0.46838444	0.47157317	0.47381949
Western sub-Saharan Africa	0.30978096	0.31402809	0.318881	0.32423289	0.33022827	0.33677971	0.34324817	0.35004017	0.35671646	0.36349154	0.37060875	0.37760914	0.38475339	0.39189044	0.39916209	0.4062382	0.41292729	0.41961502	0.42643735	0.43338389	0.43982233	0.44602298
Benin	0.25738068	0.26138801	0.26541746	0.26954428	0.27394114	0.27840675	0.28293394	0.28767305	0.29262745	0.29770223	0.30280901	0.30792554	0.31337359	0.31931869	0.32572727	0.33221635	0.33876392	0.34560676	0.3526527	0.36003116	0.36696449	0.37348657
Burkina Faso	0.16808434	0.1729964	0.17798199	0.18325354	0.188565	0.19408613	0.19957195	0.20489109	0.21022329	0.21542337	0.22100103	0.22669663	0.23258913	0.23849749	0.2443789	0.25020826	0.25608177	0.26203301	0.26810207	0.27428709	0.27987132	0.2851184
Cabo Verde	0.35989902	0.37007028	0.37984667	0.38941979	0.39883873	0.40800922	0.41743379	0.42737979	0.43737213	0.44669151	0.45564757	0.46447397	0.47285777	0.48079648	0.4882599	0.49522949	0.50185542	0.50843579	0.51528589	0.52248801	0.52846159	0.53353454
Cameroon	0.34881479	0.35263187	0.35651182	0.36066858	0.36510003	0.36954576	0.37435588	0.37949035	0.3848383	0.39028864	0.39606715	0.4020928	0.40858012	0.41560796	0.42303356	0.43083028	0.43887895	0.4471851	0.45568785	0.46424647	0.47228585	0.47969122
Chad	0.13741237	0.13966369	0.1420417	0.14534805	0.15091087	0.15703304	0.1625764	0.16816106	0.17339678	0.17833536	0.18411062	0.18959161	0.19526275	0.20095288	0.20665884	0.2124287	0.21753218	0.22220695	0.22691388	0.23170475	0.23610308	0.24043602
Côte d'Ivoire	0.33301539	0.33654551	0.33997764	0.34334893	0.34637541	0.34872791	0.35071594	0.35272748	0.35508764	0.35793875	0.36120519	0.36408365	0.36783169	0.37235379	0.37757544	0.38349138	0.38982519	0.39669397	0.40409193	0.41172558	0.4190401	0.42594188
The Gambia	0.29635428	0.30254326	0.30771293	0.31293092	0.31861503	0.32356835	0.32815143	0.33264015	0.33731525	0.34238961	0.34799783	0.35286924	0.35837365	0.36425588	0.3699013	0.37584048	0.38156922	0.38727364	0.39315568	0.39918978	0.40452419	0.40971416
Ghana	0.42472521	0.42941185	0.43410413	0.43911821	0.44423698	0.44936002	0.45469274	0.46060625	0.46725907	0.47384805	0.48077789	0.48902097	0.49751393	0.50600465	0.51411906	0.5215392	0.52862959	0.53616994	0.54384054	0.55163203	0.55846217	0.56493039
Guinea	0.21664979	0.22109094	0.22589878	0.23052259	0.23513497	0.23969185	0.24401013	0.24847169	0.25316769	0.25755279	0.26240595	0.2676246	0.27342832	0.27960044	0.28596126	0.29236361	0.29911241	0.30688146	0.31483959	0.32283985	0.329878	0.33640129
Guinea-Bissau	0.25202652	0.25578677	0.25932182	0.26274772	0.26627174	0.27005613	0.27380399	0.27780502	0.2821515	0.28642335	0.29114164	0.29668297	0.30156234	0.30660201	0.31160291	0.31709001	0.32297967	0.32932649	0.33566783	0.34215611	0.347799	0.35310962
Liberia	0.23117336	0.23959439	0.24831197	0.25159546	0.25517961	0.25896799	0.26289726	0.26723957	0.2718536	0.27680411	0.28223575	0.28801514	0.29485432	0.30298566	0.31095544	0.31848517	0.32508517	0.33152779	0.33778458	0.34340326	0.34813389	0.35244245
Mali	0.15719003	0.16134445	0.1654207	0.1697335	0.17377955	0.17802984	0.18245247	0.18691299	0.19153675	0.19653765	0.20180354	0.20709722	0.21246506	0.21799387	0.22372998	0.22982626	0.23618623	0.24272613	0.24941973	0.25625075	0.26262922	0.26857994
Mauritania	0.38420538	0.38648742	0.38856448	0.39110488	0.39401293	0.3974991	0.40315947	0.40837712	0.41323362	0.41791061	0.42320244	0.42886729	0.43472438	0.44106891	0.44765388	0.45452349	0.46126954	0.46845237	0.47583951	0.48376774	0.4913656	0.4989451
Niger	0.10088695	0.10327178	0.10574111	0.10832289	0.1107645	0.11345202	0.11624707	0.11897163	0.12195779	0.12482938	0.127953	0.13095807	0.13446972	0.13797817	0.14173174	0.1454405	0.14920011	0.15295917	0.1568752	0.16088948	0.16453926	0.16807277
Nigeria	0.33820174	0.34302975	0.34909253	0.35591139	0.36378081	0.37258355	0.38102604	0.38977965	0.39803771	0.40635331	0.41499775	0.42335191	0.43178038	0.44012466	0.44876682	0.45724099	0.46518378	0.47297287	0.48084441	0.48878736	0.49620474	0.50339083

São Tomé and Príncipe	0.32946011	0.33448479	0.34030284	0.34685198	0.35372238	0.36110588	0.36941301	0.37781352	0.38668894	0.39582124	0.40553067	0.41578373	0.42572131	0.43559246	0.44514624	0.45405147	0.46323008	0.47217093	0.4810982	0.48983823	0.49786141	0.50541375
Senegal	0.2837849	0.2874519	0.29104629	0.29488717	0.29898452	0.30325721	0.30763391	0.3125703	0.31789409	0.32365773	0.32980407	0.3360599	0.34297711	0.350134	0.35718157	0.3641445	0.37111453	0.37835148	0.38607967	0.39401232	0.40119752	0.40805419
Sierra Leone	0.21849284	0.22039123	0.22458583	0.2292359	0.23413167	0.23954994	0.24564789	0.25252308	0.25978156	0.26705838	0.27427027	0.28167357	0.29033851	0.30073669	0.31034887	0.31699865	0.32409156	0.33121343	0.33834156	0.34577002	0.35241202	0.35866588
Togo	0.30547296	0.30782826	0.31046991	0.31379206	0.31668794	0.31902558	0.32165939	0.3243825	0.32774314	0.33155854	0.33589958	0.34077726	0.3463205	0.3522786	0.35866144	0.36560017	0.37291003	0.38033623	0.38792123	0.39566727	0.40235692	0.4085337

Section 9: Authors' Contributions

Managing the overall research enterprise

Simon I Hay, Kasey E Kinzel, Christopher J L Murray, and Austin E Schumacher.

Writing the first draft of the manuscript

Catherine Bisignano, Simon I Hay, Max L Mehlman, Susan A McLaughlin, Christopher J L Murray, and Austin E Schumacher.

Primary responsibility for applying analytical methods to produce estimates

Haley Comfort, Darwin Phan Jones, Erin A May, and Spencer A Pease.

Primary responsibility for seeking, cataloguing, extracting, or cleaning data; designing or coding figures and tables

Haley Comfort, John E Fuller, Yaz Ozten, Hannah Elizabeth Robinson-Oden, Nicholas Alexander Verghese, Denny Wang, and Stephanie Watson.

Providing data or critical feedback on data sources

Cristiana Abbafati, Jaffar Abbas, Madineh Akram Abbasi, Ahmed Abdelwahab, Meriem Abdoun, Auwal Abdullahi, Ame Mehadi Abdurehman, Aidin Abedi, Armita Abedi, E S Abhilash, Richard Gyan Aboagye, Hassan Abolhassani, Lucas Guimarães Abreu, Niveen ME Abu-Rmeileh, Ahmed Abu-Zaid, Tim Adair, Oladimeji M Adebayo, Victor Adekanmbi, Rishan Adha, Qorinah Estiningtyas Sakilah Adnani, Saira Afzal, Pradyumna Agasthi, Antonella Agodi, Bright Opoku Ahinkorah, Danish Ahmad, Muayyad M Ahmad, Tauseef Ahmad, Keivan Ahmadi, Ayman Ahmed, Haroon Ahmed, Muktar Beshir Ahmed, Budi Aji, Sreelatha Akkala, Samuel Akyirem, Hanadi Al Hamad, Ammar Al Homsi, Mohammad Al Qadire, Khurshid Alam, Fahad Mashhour Alanezi, Turki M Alanzi, Mohammed Albashtawy, Mohammad T AlBataineh, Khalid F Alhabib, Abid Ali, Beriwan Abdulqadir Ali, Hassam Ali, Rafat Ali, Syed Shujait Shujait Ali, Sheikh Mohammad Alif, Syed Mohamed Aljunid, Wael Almahmeed, Joseph Uy Almazan, Omar Almidani, Mahmoud A Alomari, Salman Khalifah Al-Sabah, Awais Altaf, Farrukh Jawad Alvi, Hassan Alwafi, Hany wq, Azmeraw T Amare, Edward Kwabena Ameyaw, Alireza Amindarolzarbi, Hubert Amu, Deanna Anderlini, Pedro Prata Andrade, Tudorel Andrei, Dhanalakshmi Angappan, Sumadi Lukman Anwar, Seth Christopher Yaw Appiah, Muhammad Aqeel, Jalal Arabloo, Hany Ariffin, Timur Aripov, Benedetta Armocida, Anton A Artamonov, Judie Arulappan, Marvellous O Asika, Seyyed Shamsadin Athari, Alok Atreya, Sameh Attia, Beatriz Paulina Ayala Quintanilla, Getnet Melaku Ayele, Jose L Ayuso-Mateos, Gulrez Shah Azhar, Ahmed Y Azzam, Mina Babashahi, Ashish D Badiye, Soroush Baghdadi, Sara Bagherieh, Abdulaziz T Bako, Senthilkumar Balakrishnan, Ovidiu Constantin Baltatu, Maciej Banach, Palash Chandra Banik, Hansi Bansal, Martina Barchitta, Mainak Bardhan, Hiba Jawdat Barqawi, Ronald D Barr, Zarrin Basharat, Pritish Baskaran, Nebiyou Simegnew Bayileyegn, Emad Behboudi, Diana Fernanda Bejarano Ramirez, Uzma Iqbal Belgaumi, Michelle L Bell, Aminu K Bello, Olorunjuwon Omolaja Bello, Apostolos Beloukas, Salaheddine Bendak, Zombor Berezvai, Adam E Berman, Habtamu B Beyene, Akshaya Srikanth Bhagavathula, Dinesh Bhandari, Sonu Bhaskar, Vivek Bhat, Gurjit Kaur Bhatti, Jasvinder Singh Bhatti, Zulfiqar A Bhutta, Boris Bikbov, Bagas Suryo Bintoro, Francesca Bisulli, Aadam Olalekan Bodunrin, Obasanjo Afolabi Bolarinwa, Milad Bonakdar Hashemi, Berrak Bora Basara, Souad Bouaoud, Nicola Luigi Bragazzi, Dejana Braithwaite, Lemma N Bulto, Danilo Buonsenso, Reinhard Busse, Florentino Luciano Caetano dos Santos, Daniela Calina, Chao Cao, Juan J Carrero, Joao Mauricio Castaldelli-Maia, Carlos A Castañeda-Orjuela, Ferrán Catalá-López, Alberico L Catapano, Christopher R Cederroth, Francieli Cembranel, Pamela R Uscamaita Chacón-Uscamaita, Chiranjib Chakraborty, Vijay Kumar Chattu, Malizgani Paul Chavula, Derek S Chew, Abdulaal Chitheer, William C S Cho, Bryan Chong, Hitesh Chopra, Rajiv Chowdhury, Dinh-Toi Chu, Eric Chung, Alyssa Columbus, Haley Comfort, Joao Conde, Paolo Angelo Cortesi, Michael H Criqui, Natália Cruz-Martins, Matthew Cunningham, Giovanni Damiani, Aso Mohammad Darwesh, Saswati Das, Claudio Alberto Dávila-Cervantes, Aklilu Tamire Debele, Louisa Degenhardt, Lee Deitesfeld, Ivan Delgado-Enciso, Berecha Hundessa Demessa, Andreas K Demetriades, Nikolaos Dervenis, Hardik Dineshbhai Desai, Rupak Desai, Vinoth Gnana Chellaiyan Devanbu, Sameer Dhingra, Diana Dias da Silva, Daniel Diaz, M Ashworth Dirac, Abhinav Dixit, Shilpi Gupta Dixit, Thanh Chi Do, Thao Huynh Phuong Do, Masoud Dodangeh, Klara Georgieva Dokova, Christiane Dolecek, E Ray Dorsey, Wendel Mombaque dos Santos, Rajkumar Doshi, Leila Doshmangir, Abdel Douiri, Haneil Larson Dsouza, Susanna J Dunachie, Bruce B Duncan, Andre Rodrigues Duraes, Senbagam Duraisamy, Paulina Agnieszka Dzianach, Ebrahim Eini, Michael Ekholuenetale, Temitope Cyrus Ekundayo, Rabie

Adel El Arab, Doaa Abdel Wahab El Morsi, Maysaa El Sayed Zaki, Frank J Elgar, Ryenchindorj Erkhembayar, Tesfahun C Eshetie, Sharareh Eskandarieh, Ugochukwu Anthony Eze, Adewale Oluwaseun Fadaka, Adeniyi Francis Fagbamigbe, Andre Faro, Hossein Farrokhpour, Ali Fatehizadeh, Valery L Feigin, Seyed-Mohammad Fereshtehnejad, Luisa S Flor, Artem Alekseevich Fomenkov, Richard Charles Franklin, Sara D Friedman, Takeshi Fukumoto, John E Fuller, Peter Andras Gaal, Muktar A Gadanya, Santosh Gaihre, Yaseen Galali, Jalaj Garg, Peter W Gething, Mansour Ghafourifard, Asadollah Gholamian, Pooyan Ghorbani Vajargah, Sherief Ghozy, Ruth Margaret Gibson, Alem Girmay, Laszlo Göbölös, Amit Goel, Mahaveer Golechha, Pouya Goleij, Houman Goudarzi, Mesay Dechasa Gudeta, Bhawna Gupta, Rajat Das Gupta, Sapna Gupta, Vijai Kumar Gupta, Vladimir Hachinski, Rasool Haddadi, Nils Haep, Adel Hajj Ali, Sobia Ahsan Halim, Brian J Hall, Rabih Halwani, Kanaan Hamagharib Abdullah, Mohammad Hamiduzzaman, Nasrin Hanifi, Josep Maria Haro, Faizul Hasan, M Tasdik Hasan, Abbas M Hassan, Soheil Hassanipour, Johannes Haubold, Rasmus J Havmoeller, Simon I Hay, Mahsa Heidari-Foroozan, Reza Heidari-Soureshjani, Claudiu Herteliu, Demisu Zenbaba Heyi, Md Mahbub Hossain, Mehdi Hosseinzadeh, Chengxi Hu, Md Nazmul Huda, Michael Hultström, Salman Hussain, Nawfal R Hussein, Le Duc Huy, Hong-Han Huynh, Segun Emmanuel Ibitoye, Desta Ijo, Olayinka Stephen Ilesanmi, Arnaud Iradukunda, Farideh Iravanpour, Kenneth Chukwuemeka Iregbu, Mohammad Mainul Islam, Sheikh Mohammed Shariful Islam, Nahlah Elkudssiah Ismail, Gaetano Isola, Mahalaxmi Iyer, Linda Merin J, Jalil Jaafari, Kathryn H Jacobsen, Morteza Jafarinia, Khushleen Jaggi, Nader Jahanmehr, Haitham Jahrami, Nityanand Jain, Mihajlo Jakovljevic, Sathish Kumar Jayapal, Shubha Jayaram, Digisie Mequanint Jemere, John S Ji, Yinzi Jin, Nabi Jomehzadeh, Darwin Phan Jones, Tamas Joo, Abel Joseph, Charity Ehimwenma Joshua, Jacek Jerzy Jozwiak, Mikk Jürisson, Billingsley Kaambwa, Zubair Kabir, Vidya Kadashetti, Pradnya Vishal Kakodkar, Leila R Kalankesh, Himal Kandel, Rami S Kantar, Neeti Kapoor, Mehrdad Karajizadeh, Samad Karkhah, Ajit K Karna, Faizan Zaffar Kashoo, Nicholas J Kassebaum, Adarsh Katamreddy, Srinivasa Vittal Katikireddi, Patrick DMC Katoto, Neda Kaydi, Gbenga A Kayode, Cathleen Keller, Morteza Abdullatif Khafaie, Himanshu Khajuria, Nauman Khalid, Ibrahim A Khalil, Imteyaz A Khan, Maseer Khan, Moien AB Khan, Mahammed Ziauddin Khan suheb, Khaled Khatab, Feriha Fatima Khidri, Jagdish Khubchandani, Grace Kim, Jihee Kim, Adnan Kisa, Sezer Kisa, Juniper Boroka Kiss, Ann Kristin Skrindo Knudsen, Gerbrand Koren, Soewarta Kosen, Sindhura Lakshmi Koulmane Laxminarayana, Kewal Krishan, Hare Krishna, Vijay Krishnamoorthy, Yuvaraj Krishnamoorthy, Barthelemy Kuate Defo, Mukhtar Kulimbet, Vishnutheertha Kulkarni, Asep Kusnali, Dian Kusuma, Ilias Kyriopoulos, Hmwe Hmwe Kyu, Muhammad Awwal Ladan, Chandrakant Lahariya, Daphne Teck Ching Lai, Tri Laksono, Dharmesh Kumar Lal, Judit Lám, Anders O Larsson, Savita Lasrado, Kamaluddin Latief, Kaveh Latifinaibin, Nhi Huu Hanh Le, Thao Thi Thu Le, Trang Diep Thanh Le, Munjae Lee, Sang-woong Lee, Seung Won Lee, James Leigh, Yichong Li, Lee-Ling Lim, Stephen S Lim, Stefan Listl, Jue Liu, Erand Llanaj, László Lorenzovici, Rafael Lozano, Jianing Ma, Zheng Feei Ma, Monika Machoy, Azzam A Maghazachi, Elham Mahmoudi, Jeadran N Malagón-Rojas, Elaheh Malakan Rad, Kashish Malhotra, Deborah Carvalho Malta, Abdullah A Mamun, Yasaman Mansoori, Mohammad Ali Mansournia, Lorenzo Giovanni Mantovani, Joemer C Maravilla, Agustina M Marconi, Parham Mardi, Abdoljalal Marjani, Carlos Alberto Marrugo Arnedo, Bernardo Alfonso Martinez-Guerra, Francisco Rogerlândio Martins-Melo, Sharmeen Maryam, Roy Rillera Marzo, Clara N Matei, Andrea Maugeri, Erin A May, Maryam Mazaheri, Michael A McPhail, Enkeleint A Mechili, Jitendra Kumar Meena, Entezar Mehrabi Nasab, Walter Mendoza, Ritesh G Menezes, Endalkachew Worku Mengesha, Atte Meretoja, Tuomo J Meretoja, Irmina Maria Michalek, Ted R Miller, Giada Minelli, Le Huu Nhat Minh, Babak Moazen, Soheil Mohammadi, Abdollah Mohammadian-Hafshejani, Mustapha Mohammed, Salahuddin Mohammed, Shafiu Mohammed, Ali H Mokdad, Peyman Mokhtarzadehazar, Sara Momtazmanesh, Lorenzo Monasta, Mohammad Ali Moni, Maryam Moradi, Yousef Moradi, Maziar Moradi-Lakeh, Mehdi Moradinazar, Shane Douglas Morrison, Jakub Morze, Jonathan F Mosser, Vincent Mougin, Sumaira Mubarik, Ulrich Otto Mueller, Faraz Mughal, Sumoni Mukherjee, George Duke Mukoro, Admir Mulita, Francesk Mulita, Malaisamy Muniyandi, Christopher J L Murray, Fungai Musaigwa, Ghulam Mustafa, Saravanan Muthupandian, Ahamarshan Jayaraman Nagarajan, Mohsen Naghavi, Ganesh R Naik, Mukhammad David Naimzada, Sanjeev Nair, Tapas Sadasivan Nair, Luigi Naldi, Shumaila Nargus, Bruno Ramos Nascimento, Gustavo G Nascimento, Mohammad Javad Nasiri, Zuhair S Natto, Muhammad Naveed, Biswa Prakash Nayak, Amayu Kumesa Negero, Ionut Negoi, Ruxandra Irina Negoi, Evangelia Nena, Charles Richard James Newton, Josephine W Ngunjiri, Dang H Nguyen, Phat Tuan Nguyen, Van Thanh Nguyen, Taxiarchis Konstantinos Nikolouzakis, Amin Reza Nikpoor, Muhammad A Nizam, Shuhei Nomura, Mamoona Noreen, Bo Norrving, Jean Jacques Noubiap, Dieta Nurrika, Bogdan Oancea, Kehinde O Obamiro, Ismail A Odetokun, Akinkunmi Paul Okekunle, Osaretin Christabel Okonji, Andrew T Olagunju, Bolajoko Olubukunola Olusanya, Jacob Olusegun Olusanya, Hany A Omar, Sokking Ong, Obinna E Onwujekwe, Orish Ebere Orisakwe, Edgar Ortiz-Brizuela, Uchechukwu Levi Osuagwu, Amel Ouyahia, Mayowa O Owolabi, Mahesh Padukudru P A, Jagadish Rao Padubidri, Raffaele Palladino, Ioannis Pantazopoulos, Paraskevi Papadopoulou, Shahina Pardhan, Romil R Parikh, Ashwaghosha Parthasarathi, Deepak Kumar Pasupula,

Sangram Kishor Patel, Aslam Ramjan Pathan, Shankargouda Patil, Venkata Suresh Patthipati, Uttam Paudel, Shrikant Pawar, Paolo Pedersini, Veincent Christian Filipino Pepito, Gavin Pereira, Jeevan Pereira, Mario F P Peres, Arokiasamy Perianayagam, Hoang Tran Pham, Daniela Pierannunzio, David M Pigott, Peter Pollner, Ramesh Poluru, Maarten J Postma, Naeimeh Pourtaheri, Sergio I Prada, Vijay Kumar Prajapati, Elton Junio Sady Prates, Nguyen Khoi Quan, Fakher Rahim, Tafhimur Rahman, Amir Masoud Rahmani, Sathish Rajaa, Prasanna Ram, Shakthi Kumaran Ramasamy, Sheena Ramazanu, Juwel Rana, Kritika Rana, Chhabi Lal Ranabhat, Nemanja Rancic, Chythra R Rao, Sowmya J Rao, Sina Rashedi, Vahid Rashedi, Ahmed Mustafa Rashid, Prateek Rastogi, Nakul Ravikumar, Salman Rawaf, Reza Rawassizadeh, Bhageerathy Reshmi, Serge Resnikoff, Luis Felipe Reyes, Nima Rezaei, Maria Jesus Rios-Blancas, Hannah Elizabeth Robinson-Oden, Mónica Rodrigues, Jefferson Antonio Buendia Rodriguez, Leonardo Roever, Peter Rohloff, Debby Syahru Romadlon, Luca Ronfani, Priyanka Roy, Enrico Rubagotti, Aly M A Saad, Korosh Saber, Basema Saddik, Adam Saddler, Bashdar Abuzed Sadee, Ehsan Sadeghi, Umar Saeed, Maryam Saeedi, Rajesh Sagar, Narjes Saheb Sharif-Askari, Mohammad Ali Sahraian, Mirza Rizwan Sajid, Joseph W Sakshaug, Afeez Abolarinwa Salami, Marwa Rashad Salem, Sara Samadzadeh, Vijaya Paul Samuel, Abdallah M Samy, Juan Sanabria, Nima Sanadgol, Francesca Sanna, Milena M Santric-Milicevic, Babak Saravi, Arash Sarveazad, Brijesh Sathian, Sonia Saxena, Yaser Sayadi, Md Abu Sayeed, Mete Saylan, Maria Inês Schmidt, David C Schwebel, Mario Šekerija, Subramanian Senthilkumaran, Yashendra Sethi, Amir Shafaat, Pritik A Shah, Saeed Shahabi, Masood Ali Shaikh, Husain Shakil, Sunder Sham, Mehran Shams-Beyranvand, Mohammad Anas Shamsi, Abhishek Shankar, Javad Sharifi-Rad, Rajesh Sharma, Vishal Sharma, Rajesh P Shastry, David H Shaw, Jiabin Shen, B Suresh Kumar Shetty, Kenji Shibuya, Jae Il Shin, Aminu Shittu, K M Shivakumar, Sunil Shrestha, Inga Dora Sigfusdottir, Luís Manuel Lopes Rodrigues Silva, Abhinav Singh, Aditya Singh, Ambrish Singh, Baljinder Singh, Narinder Pal Singh, Paramdeep Singh, Valentin Yurievich Skryabin, Anna Aleksandrovna Skryabina, Erica Leigh N Slepak, Sameh S M Soliman, Yonatan Solomon, Ireneous N Soyiri, Michael Spartalis, Chandrashekhar T Sreeramareddy, Fridolin Steinbeis, Muhammad Suleman, Rizwan Suliankatchi Abdulkader, Abida Sultana, Chandan Kumar Swain, Bryan L Sykes, Mindy D Szeto, Miklós Szócska, Payam Tabaee Damavandi, Shima Tabatabai, Karen M Tabb, Mohammad Tabish, Takahiro Tabuchi, Amirmasoud Taheri, Yasaman Taheri Abkenar, Amir Taherkhani, Jabeen Taiba, Ker-Kan Tan, Nuno Taveira, Yibekal Manaye Tefera, Pugazhenthan Thangaraju, Nihal Thomas, Amanda G Thrift, Chern Choong Chern Thum, Marcello Tonelli, Mathilde Touvier, Marcos Roberto Tovani-Palone, Domenico Trico, Indang Trihandini, Samuel Joseph Tromans, Kang Tung, Sana Ullah, Muhammad Umair, Tungki Pratama Umar, Bhaskaran Unnikrishnan, Era Upadhyay, Sahel Valadan Tahbaz, Jef Van den Eynde, Shoban Babu Varthya, Tommi Juhani Vasankari, Narayanaswamy Venketasubramanian, Nicholas Alexander Verghese, Pratibha Verma, Massimiliano Veroux, Georgios-Ioannis Verras, Vasily Vlassov, Bay Vo, Simona Ruxandra Volovat, Victor Volovici, Avina Vongpradith, Theo Vos, Yohannes Dibaba Wado, Yasir Waheed, Denny Wang, Shu Wang, Stefanie Watson, Kosala Gayan Weerakoon, Daniel J Weiss, Andrea Werdecker, Ronny Westerman, Dakshitha Praneeth Wickramasinghe, Tissa Wijeratne, Sajad Yaghoubi, Yuichiro Yano, Yao Yao, Pengpeng Ye, Gesila Endashaw Yesera, Renjulal Yesodharan, Paul Yip, Dong Keon Yon, Naohiro Yonemoto, Mustafa Z Younis, Chuanhua Yu, Vesna Zadnik, Mohammad Zahedi, Nazar Zaki, Josefina Zakzuk, Ghazal G Z Zandieh, Iman Zare, Mikhail Sergeevich Zastrozhin, Mohammed G M Zeariya, Chunxia Zhai, Zhaohua Zhu, and Magdalena Zielińska.

Developing methods or computational machinery

Cristiana Abbafati, Rouzbeh Abbasgholizadeh, Rishan Adha, Qorinah Estiningtyas Sakilah Adnani, Saira Afzal, Muktar Beshir Ahmed, Tareq Mohammed Ali AL-Ahdal, Mohammed Albashtawy, Robert W Aldridge, Hubert Amu, Aleksandr Y Aravkin, Judie Arulappan, Ahmed Y Azzam, Emad Behboudi, Michelle L Bell, Akshaya Srikanth Bhagavathula, Aadam Olalekan Bodunrin, Souad Bouaoud, Kelly M Cercy, William C S Cho, Eunice Chung, Kaleb Coberly, Haley Comfort, Garland T Culbreth, Matthew Cunningham, Aso Mohammad Darwesh, Hardik Dineshbhai Desai, Thanh Chi Do, Paulina Agnieszka Dzianach, Michael Ekholuenetale, Maysaa El Sayed Zaki, Mehdi Emamverdi, Ali Fatehizadeh, Abraham D Flaxman, Jalaj Garg, Peter W Gething, Pooyan Ghorbani Vajargah, Sherief Ghozy, Alem Girmay, Sobia Ahsan Halim, Kanaan Hamagharib Abdullah, Mohammad Hasanian, Simon I Hay, Mohammad Heidari, Hamed Hesami, Mehdi Hosseinzadeh, Md Nazmul Huda, Javid Hussain, Hong-Han Huynh, Kevin S Ikuta, Farideh Iravanpour, Mohammad Mainul Islam, Gaetano Isola, Linda Merin J, Morteza Jafarinia, Sathish Kumar Jayapal, Wonjeong Jeong, Nabi Jomehzadeh, Darwin Phan Jones, Charity Ehimwenma Joshua, Samad Karkhah, Faizan Zaffar Kashoo, Nicholas J Kassebaum, Ikramullah Khan, Adnan Kisa, Juniper Boroka Kiss, Chandrakant Lahariya, Nhi Huu Hanh Le, Thao Thi Thu Le, Sang-woong Lee, Erand Llanaj, Erin A May, Michael A McPhail, Le Huu Nhat Minh, Seyed Kazem Mirinezhad, Abdollah Mohammadian-Hafshejani, Saeed Mohammad-pour, Hoda Mojiri-forushani, Ali H Mokdad, Mohammad Ali Moni, Yousef Moradi, Admir Mulita, Francesk Mulita, Christopher J L Murray, Mohsen Naghavi, Josephine W Ngunjiri, Phat Tuan Nguyen, Van

Thanh Nguyen, Andrew T Olagunju, Michal Ordak, Yaz Ozten, Jagadish Rao Padubidri, Adrian Pana, Shrikant Pawar, Spencer A Pease, Gavin Pereira, Hoang Tran Pham, Govinda Raj Poudel, Hadi Raeisi Shahraki, Amir Masoud Rahmani, Chhabi Lal Ranabhat, Reza Rawassizadeh, Robert C Reiner Jr, Mónica Rodrigues, Enrico Rubagotti, Susan Fred Rumisha, Korosh Saber, Adam Saddler, Umar Saeed, Abdallah M Samy, Ganesh Kumar Saya, Yaser Sayadi, Austin E Schumacher, Amir Shafaat, Mohammad Anas Shamsi, Javad Sharifi-Rad, David H Shaw, Reed J D Sorensen, Michael Spartalis, Muhammad Suleman, Chandan Kumar Swain, Razieh Tavakoli Oliaee, Chern Choong Chern Thum, Muhammad Umair, Shoban Babu Varthya, Bay Vo, Stein Emil Vollset, Theo Vos, Daniel J Weiss, Ronny Westerman, Ghazal G Z Zandieh, Mohammed G M Zeariya, Yang Zhao, and Peng Zheng.

Providing critical feedback on methods or results

Amirali Aali, Cristiana Abbafati, Jaffar Abbas, Rouzbeh Abbasgholizadeh, Madineh Akram Abbasi, Mohammadreza Abbasian, Samar Abd ElHafeez, Michael Abdelmasseh, Sherief Abd-Elsalam, Ahmed Abdelwahab, Mohammad Abdollahi, Meriem Abdoun, Auwal Abdullahi, Ame Mehadi Abdurehman, Mesfin Abebe, Aidin Abedi, Armita Abedi, Tadesse M Abegaz, Roberto Ariel Abeldaño Zuñiga, E S Abhilash, Olugbenga Olusola Abiodun, Richard Gyan Aboagye, Hassan Abolhassani, Mohamed Abouzid, Lucas Guimarães Abreu, Woldu Aberhe Abrha, Michael R M Abrigo, Dariush Abtahi, Samir Abu Rumeileh, Niveen ME Abu-Rmeileh, Salahdein Aburuz, Ahmed Abu-Zaid, Juan Manuel Acuna, Tim Adair, Isaac Yeboah Addo, Oladimeji M Adebayo, Oyelola A Adegboye, Victor Adekanmbi, Bashir Aden, Abiola Victor Adepoju, Charles Oluwaseun Adetunji, Temitayo Esther Adeyeoluwa, Olorunsola Israel Adeyomoye, Rishan Adha, Amin Adibi, Wirawan Adikusuma, Qorinah Estiningtyas Sakilah Adnani, Saryia Adra, Abel Afework, Aanuoluwapo Adeyimika Afolabi, Shadi Afyouni, Saira Afzal, Pradyumna Agasthi, Shahin Aghamiri, Antonella Agodi, Williams Agyemang-Duah, Bright Opoku Ahinkorah, Aqeel Ahmad, Danish Ahmad, Firdos Ahmad, Muayyad M Ahmad, Tauseef Ahmad, Amir Mahmoud Ahmadzade, Mohadese Ahmadzade, Ayman Ahmed, Haroon Ahmed, Luai A Ahmed, Muktar Beshir Ahmed, Syed Anees Ahmed, Budi Aji, Olufemi Ajumobi, Gizachew Taddesse Akalu, Essona Matatom Akara, Karolina Akinosoglou, Sreelatha Akkala, Samuel Akyirem, Hanadi Al Hamad, Syed Mahfuz Al Hasan, Ammar Al Homsi, Mohammad Al Qadire, Moein Ala, Timothy Olukunle Aladelusi, Samer O Alalalmeh, Ziyad Al-Aly, Khurshid Alam, Manjurul Alam, Zufishan Alam, Rasmieh Mustafa Al-amer, Fahad Mashhour Alanezi, Turki M Alanzi, Mohammed Albashtawy, Mohammad T AlBataineh, Sharifullah Alemi, Adel Ali Saeed Al-Gheethi, Khalid F Alhabib, Mohammed Khaled Al-Hanawi, Abid Ali, Akhtar Ali, Hassam Ali, Mohammed Usman Ali, Rafat Ali, Syed Shujait Shujait Ali, Zahid Ali, Shohreh Alian Samakkhah, Gianfranco Alicandro, Mohammad Aligol, Rasoul Alimi, Ahmednur Adem Aliyi, Syed Mohamed Aljunid, Wael Almahmeed, Sabah Al-Marwani, Sadeq Ali Ali Al-Maweri, Joseph Uy Almazan, Hesham M Al-Mekhlafi, Omar Almidani, Mahmoud A Alomari, Jaber S Alqahtani, Ahmed Yaseen Alqutaibi, Salman Khalifah Al-Sabah, Awais Altaf, Jaffar A Al-Tawfiq, Khalid A Altirkawi, Farrukh Jawad Alvi, Hassan Alwafi, Yaser Mohammed Al-Worafi, Hany wq, Karem H Alzoubi, Azmeraw T Amare, Edward Kwabena Ameyaw, Abebe Feyissa Amhare, Tarek Tawfik Amin, Alireza Amindarolzarbi, Javad Aminian Dehkordi, Sohrab Amiri, Hubert Amu, Dickson A Amugsi, Jimoh Amzat, Deanna Anderlini, Pedro Prata Andrade, Catalina Liliana Andrei, Tudorel Andrei, Dhanalakshmi Angappan, Afifa Anjum, Ernoiz Antriyandarti, Iyadunni Adesola Anuoluwa, Sumadi Lukman Anwar, Anayochukwu Edward Anyasodor, Seth Christopher Yaw Appiah, Jalal Arabloo, Morteza Arab-Zozani, Mosab Arafat, Abdulfatai Aremu, Hany Ariffin, Timur Aripov, Benedetta Armocida, Mahwish Arooj, Anton A Artamonov, Kurnia Dwi Artanti, Judie Arulappan, Idowu Thomas Aruleba, Raphael Taiwo Aruleba, Ashokan Arumugam, Saeed Asgary, Mubarek Yesse Ashemo, Muhammad Ashraf, Marvellous O Asika, Seyyed Shamsadin Athari, Maha Moh'd Wahbi Atout, Alok Atreya, Sameh Attia, Avinash Aujayeb, Abolfazl Avan, Adedapo Wasiu Awotidebe, Beatriz Paulina Ayala Quintanilla, Martin Amogre Ayanore, Getnet Melaku Ayele, Sina Azadnajafabad, Gulrez Shah Azhar, Shahkaar Aziz, Ahmed Y Azzam, Mina Babashahi, Abraham Samuel Babu, Muhammad Badar, Alaa Badawi, Ashish D Badiye, Soroush Baghdadi, Nasser Bagheri, Sara Bagherieh, Sulaiman Bah, Saeed Bahadorikhalili, Jianjun Bai, Ruhai Bai, Jennifer L Baker, Shankar M Bakkannavar, Abdulaziz T Bako, Senthilkumar Balakrishnan, Saliu A Balogun, Ovidiu Constantin Baltatu, Maciej Banach, Soham Bandyopadhyay, Biswajit Banik, Palash Chandra Banik, Hansi Bansal, Shirin Barati, Martina Barchitta, Mainak Bardhan, Hiba Jawdat Barqawi, Lope H Barrero, Zarrin Basharat, Asma'u I J Bashir, Hameed Akande Bashiru, Pritish Baskaran, Quique Bassat, João Diogo Basso, Saurav Basu, Kavita Batra, Ravi Batra, Bernhard T Baune, Mohsen Bayati, Nebiyou Simegnew Bayileyegn, Tahmina Begum, Emad Behboudi, Amir Hossein Behnoush, Diana Fernanda Bejarano Ramirez, Uzma Iqbal Belgaumi, Michelle L Bell, Aminu K Bello, Olorunjuwon Omolaja Bello, Apostolos Beloukas, Salaheddine Bendak, Derrick A Bennett, Isabela M Bensenor, Habib Benzian, Adam E Berman, Amiel Nazer C Bermudez, Habtamu B Beyene, Devidas S Bhagat, Akshaya Srikanth Bhagavathula, Neeraj Bhala, Ashish Bhalla, Dinesh Bhandari, Nikha Bhardwaj, Pankaj Bhardwaj, Prarthna

V Bhardwaj, Ashish Bhargava, Sonu Bhaskar, Vivek Bhat, Gurjit Kaur Bhatti, Jasvinder Singh Bhatti, Manpreet S Bhatti, Rajbir Bhatti, Zulfiqar A Bhutta, Boris Bikbov, Bagas Suryo Bintoro, Francesca Bisulli, Atanu Biswas, Raaj Kishore Biswas, Archie Bleyer, Virginia Bodolica, Aadam Olalekan Bodunrin, Obasanjo Afolabi Bolarinwa, Milad Bonakdar Hashemi, Aime Bonny, Kaustubh Bora, Safiya Bala Borodo, Alejandro Botero Carvajal, Souad Bouaoud, Sofiane Boudalia, Edward J Boyko, Nicola Luigi Bragazzi, Dejana Braithwaite, Hermann Brenner, Gabrielle Britton, Annie J Browne, Andre R Brunoni, Norma B Bulamu, Lemma N Bulto, Danilo Buonsenso, Katrin Burkart, Sharath Burugina Nagaraja, Yasser Bustanji, Zahid A Butt, Florentino Luciano Caetano dos Santos, Tianji Cai, Ismael R Campos-Nonato, Chao Cao, Carlos Alberto Cardenas, Rosario Cárdenas, Joao Mauricio Castaldelli-Maia, Carlos A Castañeda-Orjuela, Giulio Castelpietra, Ferrán Catalá-López, Alberico L Catapano, Christopher R Cederroth, Francieli Cembranel, Muthia Cenderadewi, Ester Cerin, Muge Cevik, Pamela R Uscamaita Chacón-Uscamaita, Yaacoub Chahine, Chiranjib Chakraborty, Chin-Kuo Chang, Periklis Charalampous, Jaykaran Charan, Vijay Kumar Chattu, Victoria Chatzimavridou-Grigoriadou, Malizgani Paul Chavula, Huzaifa Ahmad Cheema, An-Tian Chen, Haowei Chen, Lingxiao Chen, Meng Xuan Chen, Nicolas Cherbuin, Derek S Chew, Jesus Lorenzo Chirinos-Caceres, William C S Cho, Bryan Chong, Hitesh Chopra, Rajiv Chowdhury, Dinh-Toi Chu, Isaac Sunday Chukwu, Eric Chung, Eunice Chung, Sheng-Chia Chung, Karly I Cini, Cain C T Clark, Alyssa Columbus, Haley Comfort, Joao Conde, Sara Conti, Paolo Angelo Cortesi, Vera Marisa Costa, Ewerton Cousin, Richard G Cowden, Michael H Criqui, Natália Cruz-Martins, Garland T Culbreth, Patricia Cullen, Matthew Cunningham, Daniel da Silva e Silva, Omid Dadras, Zhaoli Dai, Koustuv Dalal, Lachlan L Dalli, Giovanni Damiani, Emanuele D'Amico, Sara Daneshvar, Aso Mohammad Darwesh, Jai K Das, Saswati Das, Nihar Ranjan Dash, Mohsen Dashti, Claudio Alberto Dávila-Cervantes, Kairat Davletov, Aklilu Tamire Debele, Louisa Degenhardt, Reza Dehbandi, Ivan Delgado-Enciso, Daniel Demant, Berecha Hundessa Demessa, Andreas K Demetriades, Xinlei Deng, Kebede Deribe, Nikolaos Dervenis, Don C Des Jarlais, Hardik Dineshbhai Desai, Rupak Desai, Keshab Deuba, Vinoth Gnana Chellaiyan Devanbu, Sourav Dey, Arkadeep Dhali, Kuldeep Dhama, Mandira Lamichhane Dhimal, Meghnath Dhimal, Sameer Dhingra, Diana Dias da Silva, Daniel Diaz, Adriana Dima, Delaney D Ding, M Ashworth Dirac, Abhinav Dixit, Shilpi Gupta Dixit, Thanh Chi Do, Thao Huynh Phuong Do, Camila Bruneli do Prado, Masoud Dodangeh, Klara Georgieva Dokova, Christiane Dolecek, E Ray Dorsey, Wendel Mombaque dos Santos, Rajkumar Doshi, Leila Doshmangir, Abdel Douiri, Robert Kokou Dowou, Tim Robert Driscoll, Haneil Larson Dsouza, John Dube, Susanna J Dunachie, Senbagam Duraisamy, Oyewole Christopher Durojaiye, Sulagna Dutta, Arkadiusz Marian Dziedzic, Oluwakemi Ebenezer, Ejemai Eboreime, Alireza Ebrahimi, Abdelaziz Ed-Dra, Hisham Atan Edinur, David Edvardsson, Kristina Edvardsson, Defi Efendi, Ferry Efendi, Shayan Eghdami, Terje Andreas Eikemo, Ebrahim Eini, Michael Ekholuenetale, Emmanuel Ekpor, Temitope Cyrus Ekundayo, Rabie Adel El Arab, Doaa Abdel Wahab El Morsi, Maysaa El Sayed Zaki, Noha Mousaad Elemam, Frank J Elgar, Islam Y Elgendy, Ghada Metwally Tawfik ElGohary, Muhammed Elhadi, Omar Abdelsadek Abdou Elmeligy, Mohammed Elshaer, Ibrahim Elsohaby, Amir Emami Zeydi, Mehdi Emamverdi, Theophilus I Emeto, Tesfahun C Eshetie, Sharareh Eskandarieh, Juan Espinosa-Montero, Farshid Etaee, Ugochukwu Anthony Eze, Natalia Fabin, Adewale Oluwaseun Fadaka, Adeniyi Francis Fagbamigbe, Saman Fahimi, Luca Falzone, Carla Sofia e Sá Farinha, MoezAlIslam Ezzat Mahmoud Faris, Andre Faro, Hossein Farrokhpour, Ali Fatehizadeh, Hamed Fattahi, Pooria Fazeli, Ginenus Fekadu, Seyed-Mohammad Fereshtehnejad, Abdullah Hamid Feroze, Daniela Ferrante, Pietro Ferrara, Getahun Fetensa, Irina Filip, Florian Fischer, Joanne Flavel, Luisa S Flor, Morenike Oluwatoyin Folayan, Kristen Marie Foley, Artem Alekseevich Fomenkov, Lisa M Force, Carla Fornari, Matteo Foschi, Kate Louise Francis, Richard Charles Franklin, Alberto Freitas, Joseph Friedman, Sara D Friedman, Takeshi Fukumoto, Peter Andras Gaal, Muktar A Gadanya, Santosh Gaihre, Abduzhappar Gaipov, Emmanuela Gakidou, Yaseen Galali, Quan Gan, Aravind P Gandhi, Balasankar Ganesan, Jalaj Garg, Shuo-Yan Gau, Prem Gautam, Rupesh K Gautam, Federica Gazzelloni, Miglas W Gebregergis, Mesfin Gebrehiwot, Tesfay Brhane Gebremariam, Urge Gerema, Motuma Erena Getachew, Tamirat Getachew, Peter W Gething, Sulmaz Ghahramani, Khalid Yaser Ghailan, Alireza Ghajar, Mohammad Javad Ghanbarnia, MohammadReza Ghasemi, Afsaneh Ghasemzadeh, Fariba Ghassemi, Ramy Mohamed Ghazy, Sailaja Ghimire, Asadollah Gholamian, Ali Gholamrezanezhad, Pooyan Ghorbani Vajargah, Sherief Ghozy, Arun Digambarrao Ghuge, Alessandro Gialluisi, Ruth Margaret Gibson, Artyom Urievich Gil, Paramjit Singh Gill, Tiffany K Gill, Themba G Ginindza, Alem Girmay, James C Glasbey, Elena V Gnedovskaya, Laszlo Göbölös, Mohamad Goldust, Mahaveer Golechha, Arefeh Golestanfar, Davide Golinelli, Philimon N Gona, Amir Hossein Goudarzian, Scott Greenhalgh, Michal Grivna, Giovanni Guarducci, Mohammed Ibrahim Mohialdeen Gubari, Mesay Dechasa Gudeta, Avirup Guha, Stefano Guicciardi, Damitha Asanga Gunawardane, Sasidhar Gunturu, Cui Guo, Anish Kumar Gupta, Bhawna Gupta, Indarchand Ratanlal Gupta, Rajat Das Gupta, Sapna Gupta, Veer Bala Gupta, Vijai Kumar Gupta, Vivek Kumar Gupta, Reyna Alma Gutiérrez, Farrokh Habibzadeh, Parham Habibzadeh, Vladimir Hachinski, Mohammad Haddadi, Rasool Haddadi, Nils Haep, Adel Hajj Ali, Esam S Halboub, Sobia Ahsan Halim, Brian J Hall, Sebastian Haller, Rabih Halwani, Randah R Hamadeh, Kanaan

Hamagharib Abdullah, Samer Hamidi, Mohammad Hamiduzzaman, Ahmad Hammoud, Nasrin Hanifi, Md Nuruzzaman Haque, Harapan Harapan, Josep Maria Haro, Ahmed I Hasaballah, Faizul Hasan, Ikramul Hasan, M Tasdik Hasan, Hamidreza Hasani, Ali Hasanpour- Dehkordi, Abbas M Hassan, Hossein Hassanian-Moghaddam, Soheil Hassanipour, Johannes Haubold, Rasmus J Havmoeller, Simon I Hay, Youssef Hbid, Jeffrey J Hebert, Omar E Hegazi, Golnaz Heidari, Mohammad Heidari, Mahsa Heidari-Foroozan, Bartosz Helfer, Claudiu Herteliu, Hamed Hesami, Dineshani Hettiarachchi, Demisu Zenbaba Heyi, Kamal Hezam, Yuta Hiraike, Howard J Hoffman, Ramesh Holla, Nobuyuki Horita, Md Mahbub Hossain, Md Belal Hossain, Sahadat Hossain, Mohammad-Salar Hosseini, Hassan Hosseinzadeh, Mehdi Hosseinzadeh, Mihaela Hostiuc, Mohamed Hsairi, Chengxi Hu, Md Nazmul Huda, Fernando N Hugo, Michael Hultström, Salman Hussain, Nawfal R Hussein, Le Duc Huy, Hong-Han Huynh, Bing-Fang Hwang, Segun Emmanuel Ibitoye, Oluwatope Olaniyi Idowu, Desta Ijo, Kevin S Ikuta, Mehran Ilaghi, Irena M Ilic, Milena D Ilic, Mustapha Immurana, Arnaud Iradukunda, Farideh Iravanpour, Kenneth Chukwuemeka Iregbu, Md Rabiul Islam, Mohammad Mainul Islam, Sheikh Mohammed Shariful Islam, Farhad Islami, Nahlah Elkudssiah Ismail, Gaetano Isola, Masao Iwagami, Chidozie C D Iwu, Chinwe Juliana Iwu-Jaja, Mahalaxmi Iyer, Linda Merin J, Jalil Jaafari, Louis Jacob, Kathryn H Jacobsen, Farhad Jadidi-Niaragh, Morteza Jafarinia, Khushleen Jaggi, Haitham Jahrami, Nityanand Jain, Ammar Abdulrahman Jairoun, Mihajlo Jakovljevic, Elham Jamshidi, Chinmay T Jani, Mark M Janko, Abubakar Ibrahim Jatau, Sathish Kumar Jayapal, Shubha Jayaram, Jayakumar Jeganathan, Alelign Tasew Jema, Digisie Mequanint Jemere, Anil K Jha, Ravi Prakash Jha, John S Ji, Heng Jiang, Yingzhao Jin, Yinzi Jin, Olatunji Johnson, Nabi Jomehzadeh, Darwin Phan Jones, Tamas Joo, Abel Joseph, Nitin Joseph, Charity Ehimwenma Joshua, Jacek Jerzy Jozwiak, Mikk Jürisson, Billingsley Kaambwa, Ali Kabir, Hannaneh Kabir, Zubair Kabir, Vidya Kadashetti, Farima Kahe, Pradnya Vishal Kakodkar, Rizwan Kalani, Leila R Kalankesh, Sanjay Kalra, Ashwin Kamath, Thanigaivelan Kanagasabai, Himal Kandel, Kehinde Kazeem Kanmodi, Rami S Kantar, Neeti Kapoor, Mehrdad Karajizadeh, Behzad Karami Matin, Ibraheem M Karaye, Asima Karim, Hanie Karimi, Salah Eddin Karimi, Arman Karimi Behnagh, Samad Karkhah, Ajit K Karna, Faizan Zaffar Kashoo, Nigussie Assefa Kassaw, Nicholas J Kassebaum, Adarsh Katamreddy, Srinivasa Vittal Katikireddi, Patrick DMC Katoto, Joonas H Kauppila, Navjot Kaur, Neda Kaydi, Gbenga A Kayode, Foad Kazemi, Sina Kazemian, sara Kazeminia, Leila Keikavoosi-Arani, Cathleen Keller, John H Kempen, Jessica A Kerr, Mohammad Keykhaei, Mohamad Mehdi Khadembashiri, Mohammad Amin Khadembashiri, Morteza Abdullatif Khafaie, Himanshu Khajuria, Mohammad Khalafi, Amirmohammad Khalaji, Nauman Khalid, Faham Khamesipour, Asaduzzaman Khan, Ikramullah Khan, Imteyaz A Khan, Maseer Khan, Moien AB Khan, Taimoor Khan, Mahammed Ziauddin Khan suheb, Shaghayegh Khanmohammadi, Khaled Khatab, Fatemeh Khatami, Armin Khavandegar, khalid a kheirallah, Feriha Fatima Khidri, Moein Khormali, Jagdish Khubchandani, Helda Khusun, Zemene Demelash Kifle, Grace Kim, Jihee Kim, Ruth W Kimokoti, Girmay Tsegay Kiross, Adnan Kisa, Sezer Kisa, Juniper Boroka Kiss, Mika Kivimäki, Desmond Klu, Ann Kristin Skrindo Knudsen, Ali-Asghar Kolahi, Farzad Kompani, Gerbrand Koren, Karel Kostev, Ashwin Laxmikant Kotnis, Sindhura Lakshmi Koulmane Laxminarayana, Ai Koyanagi, Michael A Kravchenko, Kewal Krishan, Hare Krishna, Vijay Krishnamoorthy, Yuvaraj Krishnamoorthy, Barthelemy Kuate Defo, Burcu Kucuk Bicer, Md Abdul Kuddus, Mohammed Kuddus, Ilari Kuitunen, Omar Kujan, Vishnutheertha Kulkarni, Ashish Kumar, Harish Kumar, Nithin Kumar, Rahul Kumar, Shiv Kumar, Almagul Kurmanova, Om P Kurmi, Asep Kusnali, Dian Kusuma, Tezer Kutluk, Ambily Kuttikkattu, Evans F Kyei, Ilias Kyriopoulos, Hmwe Hmwe Kyu, Carlo La Vecchia, Muhammad Awwal Ladan, Chandrakant Lahariya, Abdelilah Lahmar, Daphne Teck Ching Lai, Tri Laksono, Dharmesh Kumar Lal, Tea Lallukka, Judit Lám, Demetris Lamnisos, Tuo Lan, Francesco Lanfranchi, Berthold Langguth, Van Charles Lansingh, Ariane Laplante-Lévesque, Bagher Larijani, Savita Lasrado, Kamaluddin Latief, Mahrukh Latif, Kaveh Latifinaibin, Paolo Lauriola, Long Khanh Dao Le, Nhi Huu Hanh Le, Thao Thi Thu Le, Trang Diep Thanh Le, Munjae Lee, Sang-woong Lee, Seung Won Lee, Wei-Chen Lee, Yo Han Lee, Samson Mideksa Legesse, James Leigh, Jacopo Lenzi, Elvynna Leong, Temesgen L Lerango, Ming-Chieh Li, Wei Li, Xiaopan Li, Zhihui Li, Massimo Libra, Virendra S Ligade, Andrew Tiyamike Makhiringa Likaka, Lee-Ling Lim, Stephen S Lim, Ro-Ting Lin, Vasileios-Arsenios Lioutas, Stefan Listl, Jue Liu, Simin Liu, Xiaofeng Liu, Katherine M Livingstone, Erand Llanaj, Chun-Han Lo, Arianna Maever Loreche, László Lorenzovici, Mojgan Lotfi, Masoud Lotfizadeh, Rafael Lozano, Jailos Lubinda, Giancarlo Lucchetti, Stefan Ma, Zheng Feei Ma, Mahmoud Mabrok, Nikolaos Machairas, Monika Machoy, Javier A Magaña Gómez, Azzam A Maghazachi, Sandeep B Maharaj, Preeti Maharjan, Soleiman Mahjoub, Mansour Adam Mahmoud, Elham Mahmoudi, Morteza Mahmoudi, Omar Mohamed Makram, Jeadran N Malagón-Rojas, Elaheh Malakan Rad, Reza Malekzadeh, Armaan K Malhotra, Kashish Malhotra, Ahmad Azam Malik, Iram Malik, Lesibana Anthony Malinga, Deborah Carvalho Malta, Abdullah A Mamun, Yosef Manla, Fahmida Mannan, Yasaman Mansoori, Ali Mansour, Vahid Mansouri, Mohammad Ali Mansournia, Hamid Reza Marateb, Joemer C Maravilla, Agustina M Marconi, Parham Mardi, Mirko Marino, Abdoljalal Marjani, Bernardo Alfonso Martinez-Guerra, Ramon Martinez-Piedra, Francisco Rogerlândio Martins-Melo, Miquel Martorell, Wolfgang Marx, Sharmeen Maryam, Roy Rillera Marzo,

Kedar K V Mate, Clara N Matei, Alexander G Mathioudakis, Richard James Maude, Andrea Maugeri, Erin A May, Mahsa Mayeli, Mohsen Mazidi, Colm McAlinden, John J McGrath, Martin McKee, Anna Laura Wensel McKowen, Michael A McPhail, Steven M McPhail, Enkeleint A Mechili, Rishi P Mediratta, Jitendra Kumar Meena, Medhin Mehari, Rahul Mehra, Kamran Mehrabani-Zeinabad, Entezar Mehrabi Nasab, Ravi Mehrotra, Mathewos M Mekonnen, Walter Mendoza, Ritesh G Menezes, Endalkachew Worku Mengesha, Alexios-Fotios A Mentis, Sultan Ayoub Meo, Atte Meretoja, Tuomo J Meretoja, Abera M Mersha, Bezawit Afework Mesfin, Tomislav Mestrovic, Adquate Mhlanga, Laurette Mhlanga, Tianyue Mi, Georgia Micha, Irmina Maria Michalek, Ted R Miller, Sergey Nikolaevich Mindlin, Le Huu Nhat Minh, GK Mini, Neema W Minja, Niloofar Mirdamadi, Andreea Mirica, Omid Mirmosayyeb, Mizan Kiros Mirutse, Mohammad Mirza-Aghazadeh-Attari, Maryam Mirzaei, Tadesse Misgana, Sanjeev Misra, Philip B Mitchell, Prasanna Mithra, Chaitanya Mittal, Madhukar Mittal, Babak Moazen, Ahmed Ismail Mohamed, Jama Mohamed, Mouhand F H Mohamed, Nouh Saad Mohamed, Sakineh Mohammad-Alizadeh-Charandabi, Soheil Mohammadi, Abdollah Mohammadian-Hafshejani, Saeed Mohammad-pour, Marita Mohammadshahi, Mustapha Mohammed, Salahuddin Mohammed, Shafiu Mohammed, Hoda Mojiri-forushani, Ali H Mokdad, Peyman Mokhtarzadehazar, Kaveh Momenzadeh, Sara Momtazmanesh, Mohammad Ali Moni, Fateme Montazeri, AmirAli Moodi Ghalibaf, Maryam Moradi, Yousef Moradi, Maziar Moradi-Lakeh, Mehdi Moradinazar, Farhad Moradpour, Paula Moraga, Rafael Silveira Moreira, Negar Morovatdar, Reza Mosaddeghi Heris, Jonathan F Mosser, Elias Mossialos, Hakimeh Mostafavi, Amirmahdi Mostofinejad, Simin Mouodi, Seyed Ehsan Mousavi, Amin Mousavi Khaneghah, Christine Mpundu-Kaambwa, Sumaira Mubarik, Lorenzo Muccioli, Ulrich Otto Mueller, Faraz Mughal, Sumoni Mukherjee, George Duke Mukoro, Admir Mulita, Francesk Mulita, Malaisamy Muniyandi, Kavita Munjal, Christopher J L Murray, Fungai Musaigwa, Khaled M Musallam, Ghulam Mustafa, Sathish Muthu, Saravanan Muthupandian, Woojae Myung, Ashraf F Nabhan, Fredrick Muyia Nafukho, Ahamarshan Jayaraman Nagarajan, Mohsen Naghavi, Pirouz Naghavi, Ganesh R Naik, Gurudatta Naik, Mukhammad David Naimzada, Sanjeev Nair, Tapas Sadasivan Nair, Hastyar Hama Rashid Najmuldeen, Vinay Nangia, Shumaila Nargus, Bruno Ramos Nascimento, Abdallah Y Naser, Mohammad Javad Nasiri, Zuhair S Natto, Javaid Nauman, Muhammad Naveed, Biswa Prakash Nayak, Vinod C Nayak, Athare Nazri-Panjaki, Hadush Negash, Ionut Negoi, Ruxandra Irina Negoi, Seyed Aria Nejadghaderi, Chakib Nejjari, Mohammad Hadi Nematollahi, Olivia D Nesbit, Charles Richard James Newton, Josephine W Ngunjiri, Dang H Nguyen, Phat Tuan Nguyen, Phuong The Nguyen, Van Thanh Nguyen, Yeshambel T Nigatu, Taxiarchis Konstantinos Nikolouzakis, Ali Nikoobar, Amin Reza Nikpoor, Muhammad A Nizam, Shuhei Nomura, Mamoona Noreen, Nafise Noroozi, Bo Norrving, Chisom Adaobi Nri-Ezedi, George Ntaios, Mpiko Ntsekhe, Dieta Nurrika, Bogdan Oancea, Kehinde O Obamiro, Ismail A Odetokun, Akinyemi O D Ofakunrin, Ropo Ebenezer Ogunsakin, James Odhiambo Oguta, Hassan Okati-Aliabad, Sylvester Reuben Okeke, Akinkunmi Paul Okekunle, Lawrence Okidi, Osaretin Christabel Okonji, Andrew T Olagunju, Muideen Tunbosun Olaiya, Titilope O Olanipekun, Matthew Idowu Olatubi, Antonio Olivas-Martinez, Gláucia Maria Moraes Oliveira, Abdulhakeem Abayomi Olorukooba, Isaac Iyinoluwa Olufadewa, Bolajoko Olubukunola Olusanya, Jacob Olusegun Olusanya, Yinka Doris Oluwafemi, Gideon Olamilekan Oluwatunase, Hany A Omar, Goran Latif Omer, Obinna E Onwujekwe, Kenneth Ikenna Onyedibe, John Nelson Opio, Michal Ordak, E Roberto Orellana, Orish Ebere Orisakwe, Verner N Orish, Hans Orru, Doris V Ortega-Altamirano, Alberto Ortiz, Uchechukwu Levi Osuagwu, Adrian Otoiu, Nikita Otstavnov, Amel Ouyahia, Mayowa O Owolabi, Ifeoluwa Temitayo Oyeyemi, Oyetunde T Oyeyemi, Mahesh Padukudru P A, Jagadish Rao Padubidri, Mahsa Pahlavikhah Varnosfaderani, Pramod Kumar Pal, Tamás Palicz, Raffaele Palladino, Adrian Pana, Parsa Panahi, Ashok Pandey, Seithikurippu R Pandi-Perumal, Victoria Pando-Robles, Helena Ullyartha Pangaribuan, Georgios D Panos, Paraskevi Papadopoulou, Shahina Pardhan, Romil R Parikh, Seoyeon Park, Ashwaghosha Parthasarathi, Ava Pashaei, Deepak Kumar Pasupula, Jenil R Patel, Sangram Kishor Patel, Aslam Ramjan Pathan, Ashlesh Patil, Shankargouda Patil, Dimitrios Patoulias, Venkata Suresh Patthipati, Uttam Paudel, Shrikant Pawar, Spencer A Pease, Amy E Peden, Paolo Pedersini, Veincent Christian Filipino Pepito, Emmanuel K Peprah, Gavin Pereira, Jeevan Pereira, Marcos Pereira, Mario F P Peres, Arokiasamy Perianayagam, Ionela-Roxana Petcu, Fanny Emily Petermann-Rocha, Raffaele Pezzani, Hoang Tran Pham, Michael R Phillips, Manon Pigeolet, David M Pigott, Michael A Piradov, Nishad Plakkal, Evgenii Plotnikov, Dimitri Poddighe, Peter Pollner, Ramesh Poluru, Constance Dimity Pond, Maarten J Postma, Govinda Raj Poudel, Lisasha Poudel, Ghazaleh Pourali, Sergio I Prada, Pranil Man Singh Pradhan, Vijay Kumar Prajapati, V Prakash, Chandra P Prasad, Manya Prasad, Akila Prashant, Elton Junio Sady Prates, Hery Purnobasuki, Bharathi M Purohit, Jagadeesh Puvvula, Rizwan Qaisar, Nameer Hashim Qasim, Ibrahim Qattea, Gangzhen Qian, Nguyen Khoi Quan, Amir Radfar, Venkatraman Radhakrishnan, Pourya Raee, Hadi Raeisi Shahraki, Seyedeh Niloufar Rafiei Alavi, Fakher Rahim, Md Mosfequr Rahman, Mosiur Rahman, Muhammad Aziz Rahman, Tafhimur Rahman, Amir Masoud Rahmani, Niloufar Rahnavard, Pramila Rai, Sathish Rajaa, Ali Rajabpour-Sanati, Prashant Rajput, Prasanna Ram, Hazem Ramadan, Shakthi Kumaran Ramasamy, Sheena Ramazanu, Juwel Rana, Kritika Rana, Chhabi Lal Ranabhat, Nemanja Rancic, Smitha Rani, Shubham

Ranjan, Chythra R Rao, Indu Ramachandra Rao, Sowmya J Rao, Drona Prakash Rasali, Sina Rashedi, Vahid Rashedi, Ahmed Mustafa Rashid, Ashkan Rasouli-Saravani, Prateek Rastogi, Azad Rasul, Ramin Ravangard, Nakul Ravikumar, David Laith Rawaf, Salman Rawaf, Reza Rawassizadeh, Iman Razeghian-Jahromi, Murali Mohan Rama Krishna Reddy, Elrashdy Moustafa Mohamed Redwan, Faizan Ur Rehman, Robert C Reiner Jr, Bhageerathy Reshmi, Serge Resnikoff, Luis Felipe Reyes, Malihe Rezaee, Negar Rezaei, Nima Rezaei, Mohsen Rezaeian, Mavra A Riaz, Ana Isabel Ribeiro, Jennifer Rickard, Maria Jesus Rios-Blancas, Mónica Rodrigues, Jefferson Antonio Buendia Rodriguez, Leonardo Roever, Peter Rohloff, Debby Syahru Romadlon, Gholamreza Roshandel, Sharareh Roshanzamir, Priyanka Roy, Enrico Rubagotti, Godfrey M Rwegerera, Chandan S N, Katharina S Sunnerhagen, Aly M A Saad, Michela Sabbatucci, Korosh Saber, Maha Mohamed Saber-Ayad, Simona Sacco, Basema Saddik, Bashdar Abuzed Sadee, Ehsan Sadeghi, Saeid Sadeghian, Umar Saeed, Maryam Saeedi, Sare Safi, Rajesh Sagar, Amene Saghazadeh, Narjes Saheb Sharif-Askari, Soumya Swaroop Sahoo, Mohammad Ali Sahraian, Seyed Aidin Sajedi, Mirza Rizwan Sajid, Joseph W Sakshaug, Payman Salamati, Afeez Abolarinwa Salami, Luciane B Salaroli, Mohamed A Saleh, Sana Salehi, Marwa Rashad Salem, Hossein Samadi Kafil, Sara Samadzadeh, Kamel A Samara, Saad Samargandy, Yoseph Leonardo Samodra, Vijaya Paul Samuel, Abdallah M Samy, Juan Sanabria, Nima Sanadgol, Edmond Sanganyado, Rama Krishna Sanjeev, Francesco Sanmarchi, Ichtiarini Nurullita Santri, Milena M Santric-Milicevic, Babak Saravi, Yaser Sarikhani, Chinmoy Sarkar, Rodrigo Sarmiento-Suárez, Gargi Sachin Sarode, Sachin C Sarode, Arash Sarveazad, Brijesh Sathian, Thirunavukkarasu Sathish, Davide Sattin, Jennifer Saulam, Susan M Sawyer, Sonia Saxena, Ganesh Kumar Saya, Yaser Sayadi, Abu Sayeed, Md Abu Sayeed, Mete Saylan, Nikolaos Scarmeas, Winfried Schlee, Art Schuermans, David C Schwebel, Falk Schwendicke, Mario Šekerija, Mohammad H Semreen, Sabyasachi Senapati, Pallav Sengupta, Subramanian Senthilkumaran, Sadaf G Sepanlou, Dragos Serban, Addisu Sertsu, Yashendra Sethi, SeyedAhmad SeyedAlinaghi, Seyed Arsalan Seyedi, Amir Shafaat, Omid Shafaat, Mahan Shafie, Arman Shafiee, Nilay S Shah, Pritik A Shah, Ataollah Shahbandi, Izza Shahid, Samiah Shahid, Wajeehah Shahid, Moyad Jamal Shahwan, Masood Ali Shaikh, Alireza Shakeri, Husain Shakil, Muhammad Aaqib Shamim, Mehran Shams-Beyranvand, Hina Shamshad, Mohammad Ali Shamshirgaran, Mohammad Anas Shamsi, Mohd Shanawaz, Abhishek Shankar, Sadaf Sharfaei, Amin Sharifan, Mariam Shariff, Javad Sharifi-Rad, Saurab Sharma, Vishal Sharma, Rajesh P Shastry, Amin Shavandi, Amir Mehdi Shayan, Amr Mohamed Elsayed Shehabeldine, Aziz Sheikh, Rahim Ali Sheikhi, Jiabin Shen, Ranjitha S Shetty, Robert Adamu Shey, Amir Shiani, Kenji Shibuya, Desalegn Shiferaw, Mika Shigematsu, Jae Il Shin, Min-Jeong Shin, Rahman Shiri, Aminu Shittu, Ivy Shiue, K M Shivakumar, Sina Shool, Sunil Shrestha, Kanwar Hamza Shuja, Kerem Shuval, Migbar Mekonnen Sibhat, Emmanuel Edwar Siddig, Inga Dora Sigfusdottir, João Pedro Silva, Luís Manuel Lopes Rodrigues Silva, Soraia Silva, Jorge Piano Simões, Anjali Singal, Abhinav Singh, Aditya Singh, Ambrish Singh, Balbir Bagicha Singh, Baljinder Singh, Mahendra Singh, Mayank Singh, Narinder Pal Singh, Paramdeep Singh, Md Shahjahan Siraj, Freddy Sitas, Shravan Sivakumar, Valentin Yurievich Skryabin, Anna Aleksandrovna Skryabina, David A Sleet, Hamidreza Soleimani, Sameh S M Soliman, Marco Solmi, Yonatan Solomon, Yimeng Song, Reed J D Sorensen, Joan B Soriano, Ireneous N Soyiri, Michael Spartalis, Chandrashekhar T Sreeramareddy, Joseph R Starnes, Antonina V Starodubova, Simona Cătălina Stefan, Fridolin Steinbeis, Paschalis Steiropoulos, Stefan Stortecky, Muhammad Suleman, Rizwan Suliankatchi Abdulkader, Abida Sultana, Jing Sun, David Sunkersing, Chandan Kumar Swain, Bryan L Sykes, Lukasz Szarpak, Mindy D Szeto, Miklós Szócska, Payam Tabaee Damavandi, Ozra Tabatabaei Malazy, Seyed-Amir Tabatabaeizadeh, Shima Tabatabai, Karen M Tabb, Mohammad Tabish, Luis M Taborda-Barata, Birkneh Tilahun Tadesse, Amirmasoud Taheri, Yasaman Taheri Abkenar, Moslem Taheri Soodejani, Jabeen Taiba, Iman M Talaat, Jacques Lukenze Tamuzi, Ker-Kan Tan, Haosu Tang, Hong K Tang, Razieh Tavakoli Oliaee, Seyed Mohammad Tavangar, Nuno Taveira, Tsion Mulat Tebeje, Yibekal Manaye Tefera, Mohamad-Hani Temsah, Reem Mohamad Hani Temsah, Masayuki Teramoto, Solomon Hailemariam Tesfaye, Pugazhenthan Thangaraju, Kavumpurathu Raman Thankappan, Rajshree Thapa, Rekha Thapar, Nihal Thomas, Chern Choong Chern Thum, Jing Tian, Jansje Henny Vera Ticoalu, Tenaw Yimer Tiruye, Seyed Abolfazl Tohidast, Marcello Tonelli, Mathilde Touvier, Marcos Roberto Tovani-Palone, Khai Hoan Tram, Nghia Minh Tran, Domenico Trico, Indang Trihandini, Samuel Joseph Tromans, Vien T Truong, Thien Tan Tri Tai Truyen, Evangelia Eirini Tsermpini, Munkhtuya Tumurkhuu, Kang Tung, Stefanos Tyrovolas, Chukwudi S Ubah, Aniefiok John Udoakang, Arit Udoh, Inam Ulhaq, Saeed Ullah, Sana Ullah, Muhammad Umair, Chukwuma David Umeokonkwo, Anushri Umesh, Bhaskaran Unnikrishnan, Era Upadhyay, Amir Mohammad Vahdani, Sahel Valadan Tahbaz, Rohollah Valizadeh, Jef Van den Eynde, Orsolya Varga, Siddhartha Alluri Varma, Priya Vart, Shoban Babu Varthya, Lennert J Veerman, Narayanaswamy Venketasubramanian, Deneshkumar Venugopal, Nicholas Alexander Verghese, Madhur Verma, Pratibha Verma, Massimiliano Veroux, Georgios-Ioannis Verras, Dominique Vervoort, Rafael José Vieira, Jorge Hugo Villafañe, Gabriela Ines Villanueva, Paul J Villeneuve, Bay Vo, Stein Emil Vollset, Simona Ruxandra Volovat, Victor Volovici, Theo Vos, Isidora S Vujcic, Yohannes Dibaba Wado, Hatem A Wafa, Yasir Waheed, Richard G Wamai, Cong Wang, Fang Wang, Shu Wang, Song Wang, Yanzhong Wang, Yuan-Pang

Wang, Paul Ward, Stefanie Watson, Kosala Gayan Weerakoon, Daniel J Weiss, Abrha Hailay Weldemariam, Yi Feng Wen, Andrea Werdecker, Ronny Westerman, Dakshitha Praneeth Wickramasinghe, Nuwan Darshana Wickramasinghe, Tissa Wijeratne, Shadrach Wilson, Eve E Wool, Anthony D Woolf, Dongze Wu, Ratna Dwi Wulandari, Hong Xiao, Bin Xu, Xiaoyue Xu, Lalit Yadav, Yao Yao, Pengpeng Ye, Gesila Endashaw Yesera, Renjulal Yesodharan, Subah Abderehim Yesuf, Arzu Yiğit, Vahit Yiğit, Dong Keon Yon, Naohiro Yonemoto, Yuyi You, Mustafa Z Younis, Chuanhua Yu, Siddhesh Zadey, Vesna Zadnik, Nima Zafari, Mohammad Zahedi, Muhammad Nauman Zahid, Mazyar Zahir, Fathiah Zakham, Nazar Zaki, Giulia Zamagni, Burhan Abdullah Zaman, Sojib Bin Zaman, Milad Zandi, Ghazal G Z Zandieh, Mikhail Sergeevich Zastrozhin, Mohammed G M Zeariya, Youjie Zeng, Chunxia Zhai, Chen Zhang, Haijun Zhang, Hongwei Zhang, Yunquan Zhang, Zhenyu Zhang, Hanqing Zhao, Yang Zhao, Chenwen Zhong, Juexiao Zhou, Bin Zhu, Zhaohua Zhu, Magdalena Zielińska, Zhiyong Zou, Alimuddin Zumla, Elric Zweck, and Samer H Zyoud.

Drafting the work or revising it critically for important intellectual content

Amirali Aali, Cristiana Abbafati, Jaffar Abbas, Samar Abd ElHafeez, Michael Abdelmasseh, Sherief Abd-Elsalam, Ahmed Abdelwahab, Meriem Abdoun, Auwal Abdullahi, Ame Mehadi Abdurehman, Aidin Abedi, Armita Abedi, Tadesse M Abegaz, Roberto Ariel Abeldaño Zuñiga, Olugbenga Olusola Abiodun, Hassan Abolhassani, Mohamed Abouzid, Lucas Guimarães Abreu, Samir Abu Rumeileh, Salahdein Aburuz, Ahmed Abu-Zaid, Juan Manuel Acuna, Isaac Yeboah Addo, Oladimeji M Adebayo, Oyelola A Adegboye, Victor Adekanmbi, Abiola Victor Adepoju, Charles Oluwaseun Adetunji, Olorunsola Israel Adeyomoye, Amin Adibi, Qorinah Estiningtyas Sakilah Adnani, Saryia Adra, Aanuoluwapo Adeyimika Afolabi, Ali Afraz, Saira Afzal, Pradyumna Agasthi, Antonella Agodi, Bright Opoku Ahinkorah, Danish Ahmad, Firdos Ahmad, Muayyad M Ahmad, Ayman Ahmed, Haroon Ahmed, Luai A Ahmed, Muktar Beshir Ahmed, Syed Anees Ahmed, Marjan Ajami, Olufemi Ajumobi, Essona Matatom Akara, Karolina Akinosoglou, Ammar Al Homsi, Mohammad Al Qadire, Samer O Alalalmeh, Khurshid Alam, Rasmieh Mustafa Al-amer, Mohammed Albashtawy, Mohammad T AlBataineh, Ayman Al-Eyadhy, Fadwa Alhalaiqa Naji Alhalaiqa, Mohammed Khaled Al-Hanawi, Abid Ali, Akhtar Ali, Hassam Ali, Mohammed Usman Ali, Syed Shujait Shujait Ali, Gianfranco Alicandro, Sheikh Mohammad Alif, Ahmednur Adem Aliyi, Sadeq Ali Ali Al-Maweri, Joseph Uy Almazan, Omar Almidani, Mahmoud A Alomari, Nivaldo Alonso, Jaber S Alqahtani, Salman Khalifah Al-Sabah, Jaffar A Al-Tawfiq, Hassan Alwafi, Yaser Mohammed Al-Worafi, Hany wq, Karem H Alzoubi, Azmeraw T Amare, Abebe Feyissa Amhare, Tarek Tawfik Amin, Alireza Amindarolzarbi, Sohrab Amiri, Hubert Amu, Dickson A Amugsi, Jimoh Amzat, Robert Ancuceanu, Deanna Anderlini, Pedro Prata Andrade, Catalina Liliana Andrei, Tudorel Andrei, Dhanalakshmi Angappan, Abhishek Anil, Afifa Anjum, Catherine M Antony, Ernoiz Antriyandarti, Iyadunni Adesola Anuoluwa, Anayochukwu Edward Anyasodor, Seth Christopher Yaw Appiah, Muhammad Aqeel, Jalal Arabloo, Razman Arabzadeh Bahri, Morteza Arab-Zozani, Ana Margarida Araújo, Abdulfatai Aremu, Hany Ariffin, Timur Aripov, Benedetta Armocida, Mahwish Arooj, Kurnia Dwi Artanti, Judie Arulappan, Idowu Thomas Aruleba, Raphael Taiwo Aruleba, Ashokan Arumugam, Malke Asaad, Saeed Asgary, Muhammad Ashraf, Marvellous O Asika, Seyyed Shamsadin Athari, Maha Moh'd Wahbi Atout, Alok Atreya, Avinash Aujayeb, Abolfazl Avan, Beatriz Paulina Ayala Quintanilla, Martin Amogre Ayanore, Getnet Melaku Ayele, Seyed Mohammad Ayyoubzadeh, Sina Azadnajafabad, Ahmed Y Azzam, Abraham Samuel Babu, Muhammad Badar, Alaa Badawi, Ashish D Badiye, Soroush Baghdadi, Sara Bagherieh, Sulaiman Bah, Jianjun Bai, Abdulaziz T Bako, Senthilkumar Balakrishnan, Saliu A Balogun, Ovidiu Constantin Baltatu, Kiran Bam, Maciej Banach, Soham Bandyopadhyay, Hansi Bansal, Martina Barchitta, Mainak Bardhan, Suzanne Lyn Barker-Collo, Francesco Barone-Adesi, Hiba Jawdat Barqawi, Ronald D Barr, Lope H Barrero, Asma'u I J Bashir, Hameed Akande Bashiru, Pritish Baskaran, Buddha Basnyat, Quique Bassat, João Diogo Basso, Bernhard T Baune, Thomas Beaney, Neeraj Bedi, Emad Behboudi, Amir Hossein Behnoush, Maryam Beiranvand, Diana Fernanda Bejarano Ramirez, Uzma Iqbal Belgaumi, Michelle L Bell, Aminu K Bello, Muhammad Bashir Bello, Olorunjuwon Omolaja Bello, Luis Belo, Apostolos Beloukas, Derrick A Bennett, Isabela M Bensenor, Habib Benzian, Zombor Berezvai, Paulo J G Bettencourt, Habtamu B Beyene, Kebede A Beyene, Akshaya Srikanth Bhagavathula, Neeraj Bhala, Dinesh Bhandari, Prarthna V Bhardwaj, Ashish Bhargava, Sonu Bhaskar, Vivek Bhat, Gurjit Kaur Bhatti, Jasvinder Singh Bhatti, Manpreet S Bhatti, Rajbir Bhatti, Boris Bikbov, Nada Binmadi, Antonio Biondi, Catherine Bisignano, Francesca Bisulli, Atanu Biswas, Saeid Bitaraf, Tone Bjørge, Archie Bleyer, Mary Sefa Boampong, Virginia Bodolica, Aadam Olalekan Bodunrin, Milad Bonakdar Hashemi, Aime Bonny, Kaustubh Bora, Safiya Bala Borodo, Rohan Borschmann, Alejandro Botero Carvajal, Souad Bouaoud, Sofiane Boudalia, Edward J Boyko, Nicola Luigi Bragazzi, Dejana Braithwaite, Hermann Brenner, Norma B Bulamu, Danilo Buonsenso, Richard A Burns, Sharath Burugina Nagaraja, Reinhard Busse, Yasser Bustanji, Florentino Luciano Caetano dos Santos, Luis Alberto Cámera, Luciana Aparecida Campos, Ismael R Campos-Nonato, Chao Cao, Sinclair Carr, Giulia Carreras, Juan J Carrero,

Andrea Carugno, Felix Carvalho, Márcia Carvalho, Joao Mauricio Castaldelli-Maia, Carlos A Castañeda-Orjuela, Giulio Castelpietra, Ferrán Catalá-López, Alberico L Catapano, Maria Sofia Cattaruzza, Arthur Caye, Christopher R Cederroth, Francieli Cembranel, Muthia Cenderadewi, Ester Cerin, Muge Cevik, Pamela R Uscamaita Chacón-Uscamaita, Yaacoub Chahine, Chiranjib Chakraborty, Jeffrey Shi Kai Chan, Chin-Kuo Chang, Vijay Kumar Chattu, Victoria Chatzimavridou-Grigoriadou, Malizgani Paul Chavula, Huzaifa Ahmad Cheema, An-Tian Chen, Haowei Chen, Lingxiao Chen, Meng Xuan Chen, Simiao Chen, Nicolas Cherbuin, Gerald Chi, Jesus Lorenzo Chirinos-Caceres, So Mi Jemma Cho, William C S Cho, Bryan Chong, Hitesh Chopra, Rahul Choudhary, Rajiv Chowdhury, Dinh-Toi Chu, Eric Chung, Eunice Chung, Karly I Cini, Cain C T Clark, Alyssa Columbus, Haley Comfort, Joao Conde, Sara Conti, Paolo Angelo Cortesi, Vera Marisa Costa, Ewerton Cousin, Richard G Cowden, Michael H Criqui, Natália Cruz-Martins, Patricia Cullen, Daniel da Silva e Silva, Sriharsha Dadana, Zhaoli Dai, Koustuv Dalal, Giovanni Damiani, Emanuele D'Amico, Sara Daneshvar, Nihar Ranjan Dash, Claudio Alberto Dávila-Cervantes, Nicole Davis Weaver, Diego De Leo, Aklilu Tamire Debele, Louisa Degenhardt, Ivan Delgado-Enciso, Laura Delgado-Ortiz, Berecha Hundessa Demessa, Andreas K Demetriades, Edgar Denova-Gutiérrez, Kebede Deribe, Nikolaos Dervenis, Don C Des Jarlais, Hardik Dineshbhai Desai, Rupak Desai, Keshab Deuba, Sourav Dey, Mandira Lamichhane Dhimal, Meghnath Dhimal, Sameer Dhingra, Diana Dias da Silva, Daniel Diaz, Adriana Dima, Delaney D Ding, M Ashworth Dirac, Abhinav Dixit, Shilpi Gupta Dixit, Thanh Chi Do, Camila Bruneli do Prado, Masoud Dodangeh, Christiane Dolecek, E Ray Dorsey, Wendel Mombaque dos Santos, Rajkumar Doshi, Leila Doshmangir, Abdel Douiri, Robert Kokou Dowou, Tim Robert Driscoll, Haneil Larson Dsouza, John Dube, Samuel C Dumith, Bruce B Duncan, Oyewole Christopher Durojaiye, Sulagna Dutta, Arkadiusz Marian Dziedzic, Chidiebere Peter Echieh, David Edvardsson, Defi Efendi, Shayan Eghdami, Ebrahim Eini, Michael Ekholuenetale, Rabie Adel El Arab, Maysaa El Sayed Zaki, Maha El Tantawi, Iffat Elbarazi, Noha Mousaad Elemam, Frank J Elgar, Islam Y Elgendy, Ghada Metwally Tawfik ElGohary, Hala Rashad Elhabashy, Muhammed Elhadi, Omar Abdelsadek Abdou Elmeligy, Ibrahim Elsohaby, Mehdi Emamverdi, Theophilus I Emeto, Luchuo Engelbert Bain, Ryenchindorj Erkhembayar, Tesfahun C Eshetie, Sharareh Eskandarieh, Juan Espinosa-Montero, Farshid Etaee, Ugochukwu Anthony Eze, Natalia Fabin, Adeniyi Francis Fagbamigbe, Saman Fahimi, Luca Falzone, Mohsen Farjoud Kouhanjani, Andre Faro, Ali Fatehizadeh, Nelsensius Klau Fauk, Valery L Feigin, Seyed-Mohammad Fereshtehnejad, Abdullah Hamid Feroze, Pietro Ferrara, Nuno Ferreira, Getahun Fetensa, Irina Filip, Florian Fischer, Joanne Flavel, Bobirca Teodor Florin, Morenike Oluwatoyin Folayan, Kristen Marie Foley, Behzad Foroutan, Matteo Foschi, Kate Louise Francis, Richard Charles Franklin, Alberto Freitas, Joseph Friedman, Sara D Friedman, Takeshi Fukumoto, Peter Andras Gaal, Muktar A Gadanya, Santosh Gaihre, Yaseen Galali, Nasrin Galehdar, Silvano Gallus, Aravind P Gandhi, Balasankar Ganesan, Jalaj Garg, Prem Gautam, Rupesh K Gautam, Federica Gazzelloni, Miglas W Gebregergis, Mesfin Gebrehiwot, Urge Gerema, Motuma Erena Getachew, Tamirat Getachew, Mansour Ghafourifard, Sulmaz Ghahramani, Khalid Yaser Ghailan, Alireza Ghajar, MohammadReza Ghasemi, Fariba Ghassemi, Ramy Mohamed Ghazy, Sailaja Ghimire, Ghozali Ghozali, Sherief Ghozy, Alessandro Gialluisi, Ruth Margaret Gibson, Tiffany K Gill, Richard F Gillum, Themba G Ginindza, Alem Girmay, James C Glasbey, Elena V Gnedovskaya, Laszlo Göbölös, Arefeh Golestanfar, Davide Golinelli, Philimon N Gona, Anmol Goyal, Scott Greenhalgh, Michal Grivna, Giovanni Guarducci, Mesay Dechasa Gudeta, Avirup Guha, Stefano Guicciardi, Sasidhar Gunturu, Cui Guo, Bhawna Gupta, Rajat Das Gupta, Sapna Gupta, Veer Bala Gupta, Vivek Kumar Gupta, Reyna Alma Gutiérrez, Farrokh Habibzadeh, Parham Habibzadeh, Vladimir Hachinski, Mohammad Haddadi, Nils Haep, Adel Hajj Ali, Esam S Halboub, Sobia Ahsan Halim, Brian J Hall, Rabih Halwani, Randah R Hamadeh, Mohammad Hamiduzzaman, Ahmad Hammoud, Nasrin Hanifi, Graeme J Hankey, Md Abdul Hannan, Harapan Harapan, Josep Maria Haro, Ahmed I Hasaballah, Faizul Hasan, M Tasdik Hasan, Hamidreza Hasani, Abbas M Hassan, Amr Hassan, Johannes Haubold, Rasmus J Havmoeller, Simon I Hay, Youssef Hbid, Jeffrey J Hebert, Omar E Hegazi, Golnaz Heidari, Reza Heidari-Soureshjani, Bartosz Helfer, Claudiu Herteliu, Hamed Hesami, Dineshani Hettiarachchi, Demisu Zenbaba Heyi, Kamal Hezam, Yuta Hiraike, Howard J Hoffman, Ramesh Holla, Nobuyuki Horita, Md Mahbub Hossain, Sahadat Hossain, Mohammad-Salar Hosseini, Sorin Hostiuc, Vivian Chia-rong Hsieh, Junjie Huang, Md Nazmul Huda, Fernando N Hugo, Michael Hultström, Javid Hussain, Salman Hussain, Hong-Han Huynh, Segun Emmanuel Ibitoye, Oluwatope Olaniyi Idowu, Desta Ijo, Mehran Ilaghi, Olayinka Stephen Ilesanmi, Irena M Ilic, Milena D Ilic, Mustapha Immurana, Leeberk Raja Inbaraj, Farideh Iravanpour, Kenneth Chukwuemeka Iregbu, Md Rabiul Islam, Mohammad Mainul Islam, Sheikh Mohammed Shariful Islam, Farhad Islami, Nahlah Elkudssiah Ismail, Gaetano Isola, Chidozie C D Iwu, Chinwe Juliana Iwu-Jaja, Mahalaxmi Iyer, Linda Merin J, Louis Jacob, Kathryn H Jacobsen, Morteza Jafarinia, Khushleen Jaggi, Kasra Jahankhani, Nader Jahanmehr, Haitham Jahrami, Akhil Jain, Mihajlo Jakovljevic, Reza Jalilzadeh Yengejeh, Chinmay T Jani, Mark M Janko, Abubakar Ibrahim Jatau, Sathish Kumar Jayapal, Shubha Jayaram, Alelign Tasew Jema, Digisie Mequanint Jemere, Wonjeong Jeong, Ravi Prakash Jha, Nabi Jomehzadeh, Darwin Phan Jones, Tamas Joo, Abel Joseph, Nitin Joseph, Charity Ehimwenma Joshua, Jacek Jerzy Jozwiak, Mikk Jürisson, Ali Kabir,

Hannaneh Kabir, Vidya Kadashetti, Farima Kahe, Rizwan Kalani, Feroze Kaliyadan, Sanjay Kalra, Arun Kamireddy, Thanigaivelan Kanagasabai, Himal Kandel, Edmund Wedam Kanmiki, Kehinde Kazeem Kanmodi, Rami S Kantar, Neeti Kapoor, Mehrdad Karajizadeh, Shama D Karanth, Asima Karim, Hanie Karimi, Faizan Zaffar Kashoo, Hengameh Kasraei, Nigussie Assefa Kassaw, Nicholas J Kassebaum, Adarsh Katamreddy, Srinivasa Vittal Katikireddi, Patrick DMC Katoto, Joonas H Kauppila, Navjot Kaur, Jeanne Françoise Kayibanda, Gbenga A Kayode, Foad Kazemi, Sina Kazemian, sara Kazeminia, John H Kempen, Jessica A Kerr, Emmanuelle Kesse-Guyot, Mohamad Mehdi Khadembashiri, Himanshu Khajuria, Mohammad Khalafi, Amirmohammad Khalaji, Nauman Khalid, Gulfaraz Khan, Ikramullah Khan, Maseer Khan, Moien AB Khan, Taimoor Khan, Mahammed Ziauddin Khan suheb, Shaghayegh Khanmohammadi, Khaled Khatab, Armin Khavandegar, Hamid Reza Khayat Kashani, khalid a kheirallah, Feriha Fatima Khidri, Elaheh Khodadoust, Mahmood Khosrowjerdi, Jagdish Khubchandani, Grace Kim, Adnan Kisa, Sezer Kisa, Mika Kivimäki, Ann Kristin Skrindo Knudsen, Farzad Kompani, Ashwin Laxmikant Kotnis, Parvaiz A Koul, Sindhura Lakshmi Koulmane Laxminarayana, Ai Koyanagi, Michael A Kravchenko, Kewal Krishan, Hare Krishna, Kris J Krohn, Barthelemy Kuate Defo, Connor M Kubeisy, Md Abdul Kuddus, Mohammed Kuddus, Ilari Kuitunen, Mukhtar Kulimbet, Vishnutheertha Kulkarni, Harish Kumar, Madhulata Kumari, Om P Kurmi, Asep Kusnali, Dian Kusuma, Tezer Kutluk, Evans F Kyei, Carlo La Vecchia, Muhammad Awwal Ladan, Lucie Laflamme, Chandrakant Lahariya, Abdelilah Lahmar, Ratilal Lalloo, Tea Lallukka, Berthold Langguth, Van Charles Lansingh, Ariane Laplante-Lévesque, Anders O Larsson, Savita Lasrado, Kamaluddin Latief, Mahrukh Latif, Kaveh Latifinaibin, Nhi Huu Hanh Le, Thao Thi Thu Le, Paul H Lee, Samson Mideksa Legesse, Jacopo Lenzi, Elvynna Leong, Temesgen L Lerango, Wei Li, Zhihui Li, Massimo Libra, Andrew Tiyamike Makhiringa Likaka, Lee-Ling Lim, Stefan Listl, Jue Liu, Katherine M Livingstone, Erand Llanaj, Chun-Han Lo, László Lorenzovici, Giancarlo Lucchetti, Alessandra Lugo, Raimundas Lunevicius, Zheng Feei Ma, Mahmoud Mabrok, Nikolaos Machairas, Monika Machoy, Christian Madsen, Javier A Magaña Gómez, Preeti Maharjan, Soleiman Mahjoub, Mansour Adam Mahmoud, Elham Mahmoudi, Omar Mohamed Makram, Elaheh Malakan Rad, Reza Malekzadeh, Armaan K Malhotra, Kashish Malhotra, Ahmad Azam Malik, Deborah Carvalho Malta, Abdullah A Mamun, Fahmida Mannan, Yasaman Mansoori, Vahid Mansouri, Lorenzo Giovanni Mantovani, Bishnu P Marasini, Hamid Reza Marateb, Parham Mardi, Bernardo Alfonso Martinez-Guerra, Ramon Martinez-Piedra, Cleodice A Martins, Francisco Rogerlândio Martins-Melo, Miquel Martorell, Wolfgang Marx, Sharmeen Maryam, Roy Rillera Marzo, Clara N Matei, Alexander G Mathioudakis, Andrea Maugeri, Erin A May, Mahsa Mayeli, Mohsen Mazidi, Antonio Mazzotti, Colm McAlinden, John J McGrath, Anna Laura Wensel McKowen, Susan A McLaughlin, Steven M McPhail, Enkeleint A Mechili, Rishi P Mediratta, Jitendra Kumar Meena, Max L Mehlman, Entezar Mehrabi Nasab, Mathewos M Mekonnen, Walter Mendoza, Ritesh G Menezes, George A Mensah, Laverne G Mensah, Alexios-Fotios A Mentis, Sultan Ayoub Meo, Atte Meretoja, Tuomo J Meretoja, Tomislav Mestrovic, Georgia Micha, Irmina Maria Michalek, Ted R Miller, Sergey Nikolaevich Mindlin, Giada Minelli, Le Huu Nhat Minh, GK Mini, Neema W Minja, Mojgan Mirghafourvand, Andreea Mirica, Omid Mirmosayyeb, Philip B Mitchell, Prasanna Mithra, Chaitanya Mittal, Madhukar Mittal, Babak Moazen, Mouhand F H Mohamed, Nouh Saad Mohamed, Sakineh Mohammad-Alizadeh-Charandabi, Soheil Mohammadi, Mustapha Mohammed, Salahuddin Mohammed, Shafiu Mohammed, Ali H Mokdad, Kaveh Momenzadeh, Sara Momtazmanesh, Lorenzo Monasta, Mohammad Ali Moni, Fateme Montazeri, AmirAli Moodi Ghalibaf, Maryam Moradi, Yousef Moradi, Maziar Moradi-Lakeh, Paula Moraga, Lidia Morawska, Rafael Silveira Moreira, Shane Douglas Morrison, Jakub Morze, Reza Mosaddeghi Heris, Jonathan F Mosser, Amirmahdi Mostofinejad, Simin Mouodi, Parsa Mousavi, Amin Mousavi Khaneghah, Matías Mrejen, Faraz Mughal, George Duke Mukoro, Malaisamy Muniyandi, Christopher J L Murray, Fungai Musaigwa, Khaled M Musallam, Ghulam Mustafa, Sathish Muthu, Ashraf F Nabhan, Ahamarshan Jayaraman Nagarajan, Mohsen Naghavi, Mukhammad David Naimzada, Sanjeev Nair, Hastyar Hama Rashid Najmuldeen, Shumaila Nargus, Bruno Ramos Nascimento, Gustavo G Nascimento, Abdallah Y Naser, Zuhair S Natto, Javaid Nauman, Biswa Prakash Nayak, Vinod C Nayak, Ashish Kumar Nayyar, Hadush Negash, Ionut Negoi, Ruxandra Irina Negoi, Serban Mircea Negru, Seyed Aria Nejadghaderi, Samata Nepal, Charles Richard James Newton, Josephine W Ngunjiri, Dang H Nguyen, Phat Tuan Nguyen, Phuong The Nguyen, Tuan Thanh Nguyen, Van Thanh Nguyen, Taxiarchis Konstantinos Nikolouzakis, Amin Reza Nikpoor, Muhammad A Nizam, Mamoona Noreen, Abbas Norouzian Baghani, Bo Norrving, Jean Jacques Noubiap, Chisom Adaobi Nri-Ezedi, George Ntaios, Mpiko Ntsekhe, Virginia Nuñez-Samudio, Dieta Nurrika, Bogdan Oancea, Kehinde O Obamiro, Ismail A Odetokun, Akinyemi O D Ofakunrin, In-Hwan Oh, Sylvester Reuben Okeke, Osaretin Christabel Okonji, Patrick Godwin Okwute, Andrew T Olagunju, Titilope O Olanipekun, Matthew Idowu Olatubi, Antonio Olivas-Martinez, Gláucia Maria Moraes Oliveira, Susan Oliver, Abdulhakeem Abayomi Olorukooba, Bolajoko Olubukunola Olusanya, Jacob Olusegun Olusanya, Hany A Omar, Obinna E Onwujekwe, Kenneth Ikenna Onyedibe, Michal Ordak, Verner N Orish, Hans Orru, Doris V Ortega-Altamirano, Alberto Ortiz, Edgar Ortiz-Brizuela, Esteban Ortiz-Prado, Uchechukwu Levi Osuagwu, Adrian Otoiu,

Nikita Otstavnov, Guoqing Ouyang, Mayowa O Owolabi, Oyetunde T Oyeyemi, Yaz Ozten, Mahesh Padukudru P A, Jagadish Rao Padubidri, Mahsa Pahlavikhah Varnosfaderani, Tamás Palicz, Claudia Palladino, Raul Felipe Palma-Alvarez, Seithikurippu R Pandi-Perumal, Ioannis Pantazopoulos, Paraskevi Papadopoulou, Shahina Pardhan, Romil R Parikh, Seoyeon Park, Ashwaghosha Parthasarathi, Ava Pashaei, Jenil R Patel, Ashlesh Patil, Shankargouda Patil, Dimitrios Patoulias, Venkata Suresh Patthipati, Uttam Paudel, Shrikant Pawar, Hamidreza Pazoki Toroudi, Spencer A Pease, Amy E Peden, Paolo Pedersini, Minjin Peng, Umberto Pensato, Veincent Christian Filipino Pepito, Gavin Pereira, Jeevan Pereira, Marcos Pereira, Mario F P Peres, Arokiasamy Perianayagam, Norberto Perico, Ionela-Roxana Petcu, Fanny Emily Petermann-Rocha, Hoang Tran Pham, Daniela Pierannunzio, Thomas Pilgrim, Marina Pinheiro, Michael A Piradov, Nishad Plakkal, Dimitri Poddighe, Peter Pollner, Maarten J Postma, Govinda Raj Poudel, Lisasha Poudel, Ghazaleh Pourali, Naeimeh Pourtaheri, Sergio I Prada, Pranil Man Singh Pradhan, Vijay Kumar Prajapati, Chandra P Prasad, Manya Prasad, Akila Prashant, Elton Junio Sady Prates, Rizwan Qaisar, Nameer Hashim Qasim, Ibrahim Qattea, Nguyen Khoi Quan, Amir Radfar, Venkatraman Radhakrishnan, Hadi Raeisi Shahraki, Seyedeh Niloufar Rafiei Alavi, Ibrar Rafique, Alberto Raggi, Fakher Rahim, Shayan Rahmani, Niloufar Rahnavard, Pramila Rai, Sathish Rajaa, Ali Rajabpour-Sanati, Prashant Rajput, Prasanna Ram, Hazem Ramadan, Shakthi Kumaran Ramasamy, Sheena Ramazanu, Kritika Rana, Chhabi Lal Ranabhat, Nemanja Rancic, Smitha Rani, Shubham Ranjan, Chythra R Rao, Mithun Rao, Sowmya J Rao, Davide Rasella, Vahid Rashedi, Ahmed Mustafa Rashid, Ashkan Rasouli-Saravani, Ramin Ravangard, David Laith Rawaf, Salman Rawaf, Iman Razeghian-Jahromi, Elrashdy Moustafa Mohamed Redwan, Faizan Ur Rehman, Giuseppe Remuzzi, Bhageerathy Reshmi, Serge Resnikoff, Luis Felipe Reyes, Malihe Rezaee, Nima Rezaei, Mavra A Riaz, Ana Isabel Ribeiro, Daniel Cury Ribeiro, Jennifer Rickard, Mónica Rodrigues, Jefferson Antonio Buendia Rodriguez, Leonardo Roever, Ravi Rohilla, Peter Rohloff, Debby Syahru Romadlon, Luca Ronfani, Gholamreza Roshandel, Morteza Rostamian, Bedanta Roy, Enrico Rubagotti, Susan Fred Rumisha, Godfrey M Rwegerera, Andrzej Rynkiewicz, Manjula S, Chandan S N, Katharina S Sunnerhagen, Aly M A Saad, Korosh Saber, Maha Mohamed Saber-Ayad, Simona Sacco, Basema Saddik, Bashdar Abuzed Sadee, Ehsan Sadeghi, Masoumeh Sadeghi, Umar Saeed, Maryam Saeedi, Rajesh Sagar, Soumya Swaroop Sahoo, Mohammad Ali Sahraian, Seyed Aidin Sajedi, Mirza Rizwan Sajid, Joseph W Sakshaug, Saina Salahi, Sarvenaz Salahi, Afeez Abolarinwa Salami, Marwa Rashad Salem, Mohammed Z Y Salem, Sohrab Salimi, Hossein Samadi Kafil, Sara Samadzadeh, Kamel A Samara, Saad Samargandy, Vijaya Paul Samuel, Abdallah M Samy, Juan Sanabria, Edmond Sanganyado, Rama Krishna Sanjeev, Francesco Sanmarchi, Milena M Santric-Milicevic, Made Ary Sarasmita, Aswini Saravanan, Babak Saravi, Yaser Sarikhani, Chinmoy Sarkar, Rodrigo Sarmiento-Suárez, Gargi Sachin Sarode, Sachin C Sarode, Thirunavukkarasu Sathish, Susan M Sawyer, Sonia Saxena, Ganesh Kumar Saya, Yaser Sayadi, Abu Sayeed, Md Abu Sayeed, Mete Saylan, Nikolaos Scarmeas, Benedikt Michael Schaarschmidt, Maria Inês Schmidt, Art Schuermans, Austin E Schumacher, David C Schwebel, Falk Schwendicke, Mario Šekerija, Siddharthan Selvaraj, Mohammad H Semreen, Sabyasachi Senapati, Pallav Sengupta, Sadaf G Sepanlou, Dragos Serban, Addisu Sertsu, Yashendra Sethi, Amir Shafaat, Mahan Shafie, Arman Shafiee, Nilay S Shah, Saeed Shahabi, Izza Shahid, Samiah Shahid, Moyad Jamal Shahwan, Alireza Shakeri, Husain Shakil, Muhammad Aaqib Shamim, Mehran Shams-Beyranvand, Hina Shamshad, Mohammad Ali Shamshirgaran, Mohammad Anas Shamsi, Mohd Shanawaz, Abhishek Shankar, Amin Sharifan, Javad Sharifi-Rad, Manoj Sharma, Saurab Sharma, Rajesh P Shastry, David H Shaw, Amr Mohamed Elsayed Shehabeldine, Manjunath Mala Shenoy, Kenji Shibuya, Mika Shigematsu, Reza Shirkoohi, Aminu Shittu, K M Shivakumar, Velizar Shivarov, Sina Shool, Sunil Shrestha, Kerem Shuval, Yafei Si, Emmanuel Edwar Siddig, João Pedro Silva, Soraia Silva, Jorge Piano Simões, Colin R Simpson, Anjali Singal, Abhinav Singh, Aditya Singh, Mahendra Singh, Narinder Pal Singh, Paramdeep Singh, Surjit Singh, Shravan Sivakumar, Valentin Yurievich Skryabin, Anna Aleksandrovna Skryabina, Hanye Sohrabi, Hamidreza Soleimani, Marco Solmi, Yonatan Solomon, Joan B Soriano, Ireneous N Soyiri, Michael Spartalis, Chandrashekhar T Sreeramareddy, Joseph R Starnes, Vladimir I Starodubov, Antonina V Starodubova, Simona Cătălina Stefan, Dan J Stein, Fridolin Steinbeis, Leo Stockfelt, Mark A Stokes, Stefan Stortecky, Saverio Stranges, Konstantinos Stroumpoulis, Muhammad Suleman, Abida Sultana, David Sunkersing, Sri Susanty, Chandan Kumar Swain, Bryan L Sykes, Lukasz Szarpak, Miklós Szócska, Payam Tabaee Damavandi, Seyed-Amir Tabatabaeizadeh, Karen M Tabb, Mohammad Tabish, Takahiro Tabuchi, Amirmasoud Taheri, Yasaman Taheri Abkenar, Ardeshir Tajbakhsh, Iman M Talaat, Ashis Talukder, Jacques Lukenze Tamuzi, Ker-Kan Tan, Nathan Y Tat, Vivian Y Tat, Razieh Tavakoli Oliaee, Seyed Mohammad Tavangar, Nuno Taveira, Yibekal Manaye Tefera, Mojtaba Teimoori, Mohamad-Hani Temsah, Reem Mohamad Hani Temsah, Masayuki Teramoto, Solomon Hailemariam Tesfaye, Pugazhenthan Thangaraju, Nihal Thomas, Amanda G Thrift, Chern Choong Chern Thum, Jing Tian, Ales Tichopad, Tenaw Yimer Tiruye, Seyed Abolfazl Tohidast, Marcello Tonelli, Mathilde Touvier, Marcos Roberto Tovani-Palone, Nghia Minh Tran, Domenico Trico, Samuel Joseph Tromans, Vien T Truong, Thien Tan Tri Tai Truyen, Evangelia Eirini Tsermpini, Stefanos Tyrovolas, Chukwudi S Ubah, Aniefiok John Udoakang, Arit Udoh, Inam

Ulhaq, Muhammad Umair, Tungki Pratama Umar, Chukwuma David Umeokonkwo, Brigid Unim, Bhaskaran Unnikrishnan, Era Upadhyay, Daniele Urso, Marco Vacante, Amir Mohammad Vahdani, Asokan Govindaraj Vaithinathan, Sahel Valadan Tahbaz, Jef Van den Eynde, Elena Varavikova, Orsolya Varga, Siddhartha Alluri Varma, Priya Vart, Shoban Babu Varthya, Tommi Juhani Vasankari, Narayanaswamy Venketasubramanian, Nicholas Alexander Verghese, Madhur Verma, Georgios-Ioannis Verras, Dominique Vervoort, Rafael José Vieira, Jorge Hugo Villafañe, Leonardo Villani, Gabriela Ines Villanueva, Francesco S Violante, Rachel Visontay, Vasily Vlassov, Stein Emil Vollset, Simona Ruxandra Volovat, Victor Volovici, Isidora S Vujcic, Rade Vukovic, Yasir Waheed, Cong Wang, Denny Wang, Shu Wang, Yanzhong Wang, Yuan-Pang Wang, Paul Ward, Stefanie Watson, Marcia R Weaver, Andrea Werdecker, Ronny Westerman, Dakshitha Praneeth Wickramasinghe, Nuwan Darshana Wickramasinghe, Marcin W Wojewodzic, Dongze Wu, Lalit Yadav, Sajad Yaghoubi, Lin Yang, Yao Yao, Gesila Endashaw Yesera, Subah Abderehim Yesuf, Arzu Yiğit, Vahit Yiğit, Dong Keon Yon, Naohiro Yonemoto, Siddhesh Zadey, Vesna Zadnik, Nima Zafari, Mohammad Zahedi, Mazyar Zahir, Josefina Zakzuk, Giulia Zamagni, Burhan Abdullah Zaman, Sojib Bin Zaman, Nelson Zamora, Ramin Zand, Ghazal G Z Zandieh, Aurora Zanghì, Iman Zare, Mikhail Sergeevich Zastrozhin, Mohammed G M Zeariya, Youjie Zeng, Chunxia Zhai, Chen Zhang, Haijun Zhang, Zhaofeng Zhang, Yang Zhao, Yong Zhao, Chenwen Zhong, Zhaohua Zhu, Pardis Ziaeefar, Magdalena Zielińska, Zhiyong Zou, Alimuddin Zumla, and Samer H Zyoud.

Managing the estimation or publications process

Catherine M Antony, Catherine Bisignano, Haley Comfort, Kara Estep, Simon I Hay, Nicholas J Kassebaum, Molly B Kassel, Kasey E Kinzel, Anna Laura W McKowen, Ali H Mokdad, Christopher J L Murray, Mohsen Naghavi, Amanda Novotney, David M Pigott, Nicholas Alexander Verghese, Stein Emil Vollset, Katherine M Wells, and Eve E Wool.
