# Supplementary material for: Global age-sex-specific mortality, life expectancy, and population estimates in 204 countries and territories and 811 subnational locations, 1950–2021, and the impact of the COVID-19 pandemic: a comprehensive demographic analysis for the Global Burden of Disease Study 2021
Source: Lancet. 2024 May 18;403(10440):1989–2056. doi: 10.1016/S0140-6736(24)00476-8 (PMC11126395; doi:10.1016/S0140-6736(24)00476-8)
Supplement: Supplementary appendix 3 [file mmc3.pdf]

THE LANCET

Supplementary appendix 3

This appendix formed part of the original submission and has been peer reviewed. We post it as supplied by the authors.

Supplement to: GBD 2021 Demographics Collaborators. Global age-sex-specific mortality, life expectancy, and population estimates in 204 countries and territories and 811 subnational locations, 1950–2021, and the impact of the COVID-19 pandemic: a comprehensive demographic analysis for the Global Burden of Disease Study 2021. *Lancet* 2024; **403:** 1989–2056.

Appendix 3: Authorship appendix to "Global age-sex-specific mortality, life expectancy, and population estimates in 204 countries and territories and 811 subnational locations, 1950–2021, and the impact of the COVID-19 pandemic: a comprehensive demographic analysis for the Global Burden of Disease Study 2021"

This appendix provides further authorship detail for "Global age-sex-specific mortality, life expectancy, and population estimates in 204 countries and territories and 811 subnational locations, 1950–2021, and the impact of the COVID-19 pandemic: a comprehensive demographic analysis for the Global Burden of Disease Study 2021"

Table of Contents

GBD 2021 Demographics Collaborators 2

Affiliations 10

Authors' Contributions 53

- Managing the overall research enterprise 53
- Writing the first draft of the manuscript 53
- Primary responsibility for applying analytical methods to produce estimates 53
- Primary responsibility for seeking, cataloguing, extracting, or cleaning data; designing or coding figures and tables 53
- Providing data or critical feedback on data sources 53
- Developing methods or computational machinery 56
- Providing critical feedback on methods or results 57
- Drafting the work or revising it critically for important intellectual content 64
- Managing the estimation or publications process 69

GBD 2021 Demographics Collaborators

Austin E Schumacher*, Hmwe Hmwe Kyu*, Amirali Aali, Cristiana Abbafati, Jaffar Abbas, Rouzbeh Abbasgholizadeh, Madineh Akram Abbasi, Mohammadreza Abbasian, Samar Abd ElHafeez, Michael Abdelmasseh, Sherief Abd-Elsalam, Ahmed Abdelwahab, Mohammad Abdollahi, Meriem Abdoun, Auwal Abdullahi, Ame Mehadi Abdurehman, Mesfin Abebe, Aidin Abedi, Armita Abedi, Tadesse M Abegaz, Roberto Ariel Abeldaño Zuñiga, E S Abhilash, Olugbenga Olusola Abiodun, Richard Gyan Aboagye, Hassan Abolhassani, Mohamed Abouzid, Lucas Guimarães Abreu, Woldu Aberhe Abrha, Michael R M Abrigo, Dariush Abtahi, Samir Abu Rumeileh, Niveen ME Abu-Rmeileh, Salahdein Aburuz, Ahmed Abu-Zaid, Juan Manuel Acuna, Tim Adair, Isaac Yeboah Addo, Oladimeji M Adebayo, Oyelola A Adegboye, Victor Adekanmbi, Bashir Aden, Abiola Victor Adepoju, Charles Oluwaseun Adetunji, Temitayo Esther Adeyeoluwa, Olorunsola Israel Adeyomoye, Rishan Adha, Amin Adibi, Wirawan Adikusuma, Qorinah Estiningtyas Sakilah Adnani, Saryia Adra, Abel Afework, Aanuoluwapo Adeyimika Afolabi, Ali Afraz, Shadi Afyouni, Saira Afzal, Pradyumna Agasthi, Shahin Aghamiri, Antonella Agodi, Williams Agyemang-Duah, Bright Opoku Ahinkorah, Aqeel Ahmad, Danish Ahmad, Firdos Ahmad, Muayyad M Ahmad, Tauseef Ahmad, Keivan Ahmadi, Amir Mahmoud Ahmadzade, Mohadese Ahmadzade, Ayman Ahmed, Haroon Ahmed, Luai A Ahmed, Muktar Beshir Ahmed, Syed Anees Ahmed, Marjan Ajami, Budi Aji, Olufemi Ajumobi, Gizachew Taddesse Akalu, Essona Matatom Akara, Karolina Akinosoglou, Sreelatha Akkala, Samuel Akyirem, Hanadi Al Hamad, Syed Mahfuz Al Hasan, Ammar Al Homsi, Mohammad Al Qadire, Moein Ala, Timothy Olukunle Aladelusi, Tareq Mohammed Ali AL-Ahdal, Samer O Alalalmeh, Ziyad Al-Aly, Khurshid Alam, Manjurul Alam, Zufishan Alam, Rasmieh Mustafa Al-amer, Fahad Mashhour Alanezi, Turki M Alanzi, Mohammed Albashtawy, Mohammad T AlBataineh, Robert W Aldridge, Sharifullah Alemi, Ayman Al-Eyadhy, Adel Ali Saeed Al-Gheethi, Khalid F Alhabib, Fadwa Alhalaiqa Naji Alhalaiqa, Mohammed Khaled Al-Hanawi, Abid Ali, Akhtar Ali, Beriwan Abdulqadir Ali, Hassam Ali, Mohammed Usman Ali, Rafat Ali, Syed Shujait Shujait Ali, Zahid Ali, Shohreh Alian Samakkhah, Gianfranco Alicandro, Sheikh Mohammad Alif, Mohammad Aligol, Rasoul Alimi, Ahmednur Adem Aliyi, Adel Al-Jumaily, Syed Mohamed Aljunid, Wael Almahmeed, Sabah Al-Marwani, Sadeq Ali Ali Al-Maweri, Joseph Uy Almazan, Hesham M Al-Mekhlafi, Omar Almidani, Mahmoud A Alomari, Nivaldo Alonso, Jaber S Alqahtani, Ahmed Yaseen Alqutaibi, Salman Khalifah Al-Sabah, Awais Altaf, Jaffar A Al-Tawfiq, Khalid A Altirkawi, Farrukh Jawad Alvi, Hassan Alwafi, Yaser Mohammed Al-Worafi, Hany Aly, Karem H Alzoubi, Azmeraw T Amare, Edward Kwabena Ameyaw, Abebe Feyissa Amhare, Tarek Tawfik Amin, Alireza Amindarolzarbi, Javad Aminian Dehkordi, Sohrab Amiri, Hubert Amu, Dickson A Amugsi, Jimoh Amzat, Robert Ancuceanu, Deanna Anderlini, Pedro Prata Andrade, Catalina Liliana Andrei, Tudorel Andrei, Dhanalakshmi Angappan, Abhishek Anil, Afifa Anjum, Catherine M Antony, Ernoiz Antriyandarti, Iyadunni Adesola Anuoluwa, Sumadi Lukman Anwar, Anayochukwu Edward Anyasodor, Seth Christopher Yaw Appiah, Muhammad Aqeel, Jalal Arabloo, Razman Arabzadeh Bahri, Morteza Arab-Zozani, Mosab Arafat, Ana Margarida Araújo, Aleksandr Y Aravkin, Abdulfatai Aremu, Hany Ariffin, Timur Aripov, Benedetta Armocida, Mahwish Arooj, Anton A Artamonov, Kurnia Dwi Artanti, Judie Arulappan, Idowu Thomas Aruleba, Raphael Taiwo Aruleba, Ashokan Arumugam, Malke Asaad, Saeed Asgary, Mubarek Yesse Ashemo, Muhammad Ashraf, Marvellous O Asika, Seyyed Shamsadin Athari, Maha Moh'd Wahbi Atout, Alok Atreya, Sameh Attia, Avinash Aujayeb, Abolfazl Avan, Adedapo Wasiu Awotidebe, Beatriz Paulina Ayala Quintanilla, Martin Amogre Ayanore, Getnet Melaku Ayele, Jose L Ayuso-Mateos, Seyed Mohammad Ayyoubzadeh, Sina Azadnajafabad, Gulrez Shah Azhar, Shahkaar Aziz, Ahmed Y Azzam, Mina Babashahi, Abraham Samuel Babu, Muhammad Badar, Alaa Badawi, Ashish D Badiye, Soroush Baghdadi, Nasser Bagheri, Sara Bagherieh, Sulaiman Bah, Saeed Bahadorikhalili, Jianjun Bai, Ruhai Bai,

Jennifer L Baker, Shankar M Bakkannavar, Abdulaziz T Bako, Senthilkumar Balakrishnan, Saliu A Balogun, Ovidiu Constantin Baltatu, Kiran Bam, Maciej Banach, Soham Bandyopadhyay, Biswajit Banik, Palash Chandra Banik, Hansi Bansal, Shirin Barati, Martina Barchitta, Mainak Bardhan, Suzanne Lyn Barker-Collo, Francesco Barone-Adesi, Hiba Jawdat Barqawi, Ronald D Barr, Lope H Barrero, Zarrin Basharat, Asma'u I J Bashir, Hameed Akande Bashiru, Pritish Baskaran, Buddha Basnyat, Quique Bassat, João Diogo Basso, Saurav Basu, Kavita Batra, Ravi Batra, Bernhard T Baune, Mohsen Bayati, Nebiyou Simegnew Bayileyegn, Thomas Beaney, Neeraj Bedi, Tahmina Begum, Emad Behboudi, Amir Hossein Behnoush, Maryam Beiranvand, Diana Fernanda Bejarano Ramirez, Uzma Iqbal Belgaumi, Michelle L Bell, Aminu K Bello, Muhammad Bashir Bello, Olorunjuwon Omolaja Bello, Luis Belo, Apostolos Beloukas, Salaheddine Bendak, Derrick A Bennett, Isabela M Bensenor, Habib Benzian, Zombor Berezvai, Adam E Berman, Amiel Nazer C Bermudez, Paulo J G Bettencourt, Habtamu B Beyene, Kebede A Beyene, Devidas S Bhagat, Akshaya Srikanth Bhagavathula, Neeraj Bhala, Ashish Bhalla, Dinesh Bhandari, Nikha Bhardwaj, Pankaj Bhardwaj, Prarthna V Bhardwaj, Ashish Bhargava, Sonu Bhaskar, Vivek Bhat, Gurjit Kaur Bhatti, Jasvinder Singh Bhatti, Manpreet S Bhatti, Rajbir Bhatti, Zulfiqar A Bhutta, Boris Bikbov, Nada Binmadi, Bagas Suryo Bintoro, Antonio Biondi, Catherine Bisignano, Francesca Bisulli, Atanu Biswas, Raaj Kishore Biswas, Saeid Bitaraf, Tone Bjørge, Archie Bleyer, Mary Sefa Boampong, Virginia Bodolica, Aadam Olalekan Bodunrin, Obasanjo Afolabi Bolarinwa, Milad Bonakdar Hashemi, Aime Bonny, Kaustubh Bora, Berrak Bora Basara, Safiya Bala Borodo, Rohan Borschmann, Alejandro Botero Carvajal, Souad Bouaoud, Sofiane Boudalia, Edward J Boyko, Nicola Luigi Bragazzi, Dejana Braithwaite, Hermann Brenner, Gabrielle Britton, Annie J Browne, Andre R Brunoni, Norma B Bulamu, Lemma N Bulto, Danilo Buonsenso, Katrin Burkart, Richard A Burns, Sharath Burugina Nagaraja, Reinhard Busse, Yasser Bustanji, Zahid A Butt, Florentino Luciano Caetano dos Santos, Tianji Cai, Daniela Calina, Luis Alberto Cámera, Luciana Aparecida Campos, Ismael R Campos-Nonato, Chao Cao, Carlos Alberto Cardenas, Rosario Cárdenas, Sinclair Carr, Giulia Carreras, Juan J Carrero, Andrea Carugno, Felix Carvalho, Márcia Carvalho, Joao Mauricio Castaldelli-Maia, Carlos A Castañeda-Orjuela, Giulio Castelpietra, Ferrán Catalá-López, Alberico L Catapano, Maria Sofia Cattaruzza, Arthur Caye, Christopher R Cederroth, Francieli Cembranel, Muthia Cenderadewi, Kelly M Cercy, Ester Cerin, Muge Cevik, Pamela R Chacón-Uscamaita, Yaacoub Chahine, Chiranjib Chakraborty, Jeffrey Shi Kai Chan, Chin-Kuo Chang, Periklis Charalampous, Jaykaran Charan, Vijay Kumar Chattu, Victoria Chatzimavridou-Grigoriadou, Malizgani Paul Chavula, Huzaifa Ahmad Cheema, An-Tian Chen, Haowei Chen, Lingxiao Chen, Meng Xuan Chen, Simiao Chen, Nicolas Cherbuin, Derek S Chew, Gerald Chi, Jesus Lorenzo Chirinos-Caceres, Abdulaal Chitheer, So Mi Jemma Cho, William C S Cho, Bryan Chong, Hitesh Chopra, Rahul Choudhary, Rajiv Chowdhury, Dinh-Toi Chu, Isaac Sunday Chukwu, Eric Chung, Eunice Chung, Sheng-Chia Chung, Karly I Cini, Cain C T Clark, Kaleb Coberly, Alyssa Columbus, Haley Comfort, Joao Conde, Sara Conti, Paolo Angelo Cortesi, Vera Marisa Costa, Ewerton Cousin, Richard G Cowden, Michael H Criqui, Natália Cruz-Martins, Garland T Culbreth, Patricia Cullen, Matthew Cunningham, Daniel da Silva e Silva, Sriharsha Dadana, Omid Dadras, Zhaoli Dai, Koustuv Dalal, Lachlan L Dalli, Giovanni Damiani, Emanuele D'Amico, Sara Daneshvar, Aso Mohammad Darwesh, Jai K Das, Saswati Das, Nihar Ranjan Dash, Mohsen Dashti, Claudio Alberto Dávila-Cervantes, Nicole Davis Weaver, Kairat Davletov, Diego De Leo, Aklilu Tamire Debele, Louisa Degenhardt, Reza Dehbandi, Lee Deitesfeld, Ivan Delgado-Enciso, Laura Delgado-Ortiz, Daniel Demant, Berecha Hundessa Demessa, Andreas K Demetriades, Xinlei Deng, Edgar Denova-Gutiérrez, Kebede Deribe, Nikolaos Dervenis, Don C Des Jarlais, Hardik Dineshbhai Desai, Rupak Desai, Keshab Deuba, Vinoth Gnana Chellaiyan Devanbu, Sourav Dey, Arkadeep Dhali, Kuldeep Dhama, Mandira Lamichhane Dhimal, Meghnath Dhimal, Sameer Dhingra, Diana Dias da Silva, Daniel Diaz, Adriana Dima, Delaney D Ding, M Ashworth Dirac, Abhinav

Dixit, Shilpi Gupta Dixit, Thanh Chi Do, Thao Huynh Phuong Do, Camila Bruneli do Prado, Masoud Dodangeh, Klara Georgieva Dokova, Christiane Dolecek, E Ray Dorsey, Wendel Mombaque dos Santos, Rajkumar Doshi, Leila Doshmangir, Abdel Douiri, Robert Kokou Dowou, Tim Robert Driscoll, Haneil Larson Dsouza, John Dube, Samuel C Dumith, Susanna J Dunachie, Bruce B Duncan, Andre Rodrigues Duraes, Senbagam Duraisamy, Oyewole Christopher Durojaiye, Sulagna Dutta, Paulina Agnieszka Dzianach, Arkadiusz Marian Dziedzic, Oluwakemi Ebenezer, Ejemai Eboreime, Alireza Ebrahimi, Chidiebere Peter Echieh, Abdelaziz Ed-Dra, Hisham Atan Edinur, David Edvardsson, Kristina Edvardsson, Defi Efendi, Ferry Efendi, Shayan Eghdami, Terje Andreas Eikemo, Ebrahim Eini, Michael Ekholuenetale, Emmanuel Ekpor, Temitope Cyrus Ekundayo, Rabie Adel El Arab, Doaa Abdel Wahab El Morsi, Maysaa El Sayed Zaki, Maha El Tantawi, Iffat Elbarazi, Noha Mousaad Elemam, Frank J Elgar, Islam Y Elgendy, Ghada Metwally Tawfik ElGohary, Hala Rashad Elhabashy, Muhammed Elhadi, Omar Abdelsadek Abdou Elmeligy, Mohammed Elshaer, Ibrahim Elsohaby, Amir Emami Zeydi, Mehdi Emamverdi, Theophilus I Emeto, Luchuo Engelbert Bain, Ryenchindorj Erkhembayar, Tesfahun C Eshetie, Sharareh Eskandarieh, Juan Espinosa-Montero, Kara Estep, Farshid Etaee, Ugochukwu Anthony Eze, Natalia Fabin, Adewale Oluwaseun Fadaka, Adeniyi Francis Fagbamigbe, Saman Fahimi, Luca Falzone, Carla Sofia e Sá Farinha, MoezAlIslam Ezzat Mahmoud Faris, Mohsen Farjoud Kouhanjani, Andre Faro, Hossein Farrokhpour, Ali Fatehizadeh, Hamed Fattahi, Nelsensius Klau Fauk, Pooria Fazeli, Valery L Feigin, Ginenus Fekadu, Seyed-Mohammad Fereshtehnejad, Abdullah Hamid Feroze, Daniela Ferrante, Pietro Ferrara, Nuno Ferreira, Getahun Fetensa, Irina Filip, Florian Fischer, Joanne Flavel, Abraham D Flaxman, Luisa S Flor, Bobirca Teodor Florin, Morenike Oluwatoyin Folayan, Kristen Marie Foley, Artem Alekseevich Fomenkov, Lisa M Force, Carla Fornari, Behzad Foroutan, Matteo Foschi, Kate Louise Francis, Richard Charles Franklin, Alberto Freitas, Joseph Friedman, Sara D Friedman, Takeshi Fukumoto, John E Fuller, Peter Andras Gaal, Muktar A Gadanya, Santosh Gaihre, Abduzhappar Gaipov, Emmanuela Gakidou, Yaseen Galali, Nasrin Galehdar, Silvano Gallus, Quan Gan, Aravind P Gandhi, Balasankar Ganesan, Jalaj Garg, Shuo-Yan Gau, Prem Gautam, Rupesh K Gautam, Federica Gazzelloni, Miglas W Gebregergis, Mesfin Gebrehiwot, Tesfay Brhane Gebremariam, Urge Gerema, Motuma Erena Getachew, Tamirat Getachew, Peter W Gething, Mansour Ghafourifard, Sulmaz Ghahramani, Khalid Yaser Ghailan, Alireza Ghajar, Mohammad Javad Ghanbarnia, MohammadReza Ghasemi, Afsaneh Ghasemzadeh, Fariba Ghassemi, Ramy Mohamed Ghazy, Sailaja Ghimire, Asadollah Gholamian, Ali Gholamrezanezhad, Pooyan Ghorbani Vajargah, Ghozali Ghozali, Sherief Ghozy, Arun Digambarrao Ghuge, Alessandro Gialluisi, Ruth Margaret Gibson, Artyom Urievich Gil, Paramjit Singh Gill, Tiffany K Gill, Richard F Gillum, Themba G Ginindza, Alem Girmay, James C Glasbey, Elena V Gnedovskaya, Laszlo Göbölös, Amit Goel, Mohamad Goldust, Mahaveer Golechha, Pouya Goleij, Arefeh Golestanfar, Davide Golinelli, Philimon N Gona, Houman Goudarzi, Amir Hossein Goudarzian, Anmol Goyal, Scott Greenhalgh, Michal Grivna, Giovanni Guarducci, Mohammed Ibrahim Mohialdeen Gubari, Mesay Dechasa Gudeta, Avirup Guha, Stefano Guicciardi, Damitha Asanga Gunawardane, Sasidhar Gunturu, Cui Guo, Anish Kumar Gupta, Bhawna Gupta, Indarchand Ratanlal Gupta, Rajat Das Gupta, Sapna Gupta, Veer Bala Gupta, Vijai Kumar Gupta, Vivek Kumar Gupta, Reyna Alma Gutiérrez, Farrokh Habibzadeh, Parham Habibzadeh, Vladimir Hachinski, Mohammad Haddadi, Rasool Haddadi, Nils Haep, Adel Hajj Ali, Esam S Halboub, Sobia Ahsan Halim, Brian J Hall, Sebastian Haller, Rabih Halwani, Randah R Hamadeh, Kanaan Hamagharib Abdullah, Samer Hamidi, Mohammad Hamiduzzaman, Ahmad Hammoud, Nasrin Hanifi, Graeme J Hankey, Md Abdul Hannan, Md Nuruzzaman Haque, Harapan Harapan, Josep Maria Haro, Ahmed I Hasaballah, Faizul Hasan, Ikramul Hasan, M Tasdik Hasan, Hamidreza Hasani, Mohammad Hasanian, Ali Hasanpour-Dehkordi, Abbas M Hassan, Amr Hassan, Hossein Hassanian-Moghaddam, Soheil Hassanipour, Johannes

Haubold, Rasmus J Havmoeller, Simon I Hay, Youssef Hbid, Jeffrey J Hebert, Omar E Hegazi, Golnaz Heidari, Mohammad Heidari, Mahsa Heidari-Foroozan, Reza Heidari-Soureshjani, Bartosz Helfer, Claudiu Herteliu, Hamed Hesami, Dineshani Hettiarachchi, Demisu Zenbaba Heyi, Kamal Hezam, Yuta Hiraike, Howard J Hoffman, Ramesh Holla, Nobuyuki Horita, Md Belal Hossain, Md Mahbub Hossain, Sahadat Hossain, Mohammad-Salar Hosseini, Hassan Hosseinzadeh, Mehdi Hosseinzadeh, Mihaela Hostiuc, Sorin Hostiuc, Mohamed Hsairi, Vivian Chia-rong Hsieh, Chengxi Hu, Junjie Huang, Md Nazmul Huda, Fernando N Hugo, Michael Hultström, Javid Hussain, Salman Hussain, Nawfal R Hussein, Le Duc Huy, Hong-Han Huynh, Bing-Fang Hwang, Segun Emmanuel Ibitoye, Oluwatope Olaniyi Idowu, Desta Ijo, Kevin S Ikuta, Mehran Ilaghi, Olayinka Stephen Ilesanmi, Irena M Ilic, Milena D Ilic, Mustapha Immurana, Leeberk Raja Inbaraj, Arnaud Iradukunda, Farideh Iravanpour, Kenneth Chukwuemeka Iregbu, Md Rabiul Islam, Mohammad Mainul Islam, Sheikh Mohammed Shariful Islam, Farhad Islami, Nahlah Elkudssiah Ismail, Gaetano Isola, Masao Iwagami, Chidozie C D Iwu, Chinwe Juliana Iwu-Jaja, Mahalaxmi Iyer, Linda Merin J, Jalil Jaafari, Louis Jacob, Kathryn H Jacobsen, Farhad Jadidi-Niaragh, Morteza Jafarinia, Khushleen Jaggi, Kasra Jahankhani, Nader Jahanmehr, Haitham Jahrami, Akhil Jain, Nityanand Jain, Ammar Abdulrahman Jairoun, Mihajlo Jakovljevic, Reza Jalilzadeh Yengejeh, Elham Jamshidi, Chinmay T Jani, Mark M Janko, Abubakar Ibrahim Jatau, Sathish Kumar Jayapal, Shubha Jayaram, Jayakumar Jeganathan, Alelign Tasew Jema, Digisie Mequanint Jemere, Wonjeong Jeong, Anil K Jha, Ravi Prakash Jha, John S Ji, Heng Jiang, Yingzhao Jin, Yinzi Jin, Olatunji Johnson, Nabi Jomehzadeh, Darwin Phan Jones, Tamas Joo, Abel Joseph, Nitin Joseph, Charity Ehimwenma Joshua, Jacek Jerzy Jozwiak, Mikk Jürisson, Billingsley Kaambwa, Ali Kabir, Hannaneh Kabir, Zubair Kabir, Vidya Kadashetti, Farima Kahe, Pradnya Vishal Kakodkar, Rizwan Kalani, Leila R Kalankesh, Feroze Kaliyadan, Sanjay Kalra, Ashwin Kamath, Arun Kamireddy, Thanigaivelan Kanagasabai, Himal Kandel, Edmund Wedam Kanmiki, Kehinde Kazeem Kanmodi, Rami S Kantar, Neeti Kapoor, Mehrdad Karajizadeh, Behzad Karami Matin, Shama D Karanth, Ibraheem M Karaye, Asima Karim, Hanie Karimi, Salah Eddin Karimi, Arman Karimi Behnagh, Samad Karkhah, Ajit K Karna, Faizan Zaffar Kashoo, Hengameh Kasraei, Nigussie Assefa Kassaw, Nicholas J Kassebaum, Molly B Kassel, Adarsh Katamreddy, Srinivasa Vittal Katikireddi, Patrick DMC Katoto, Joonas H Kauppila, Navjot Kaur, Neda Kaydi, Jeanne Françoise Kayibanda, Gbenga A Kayode, Foad Kazemi, Sina Kazemian, sara Kazeminia, Leila Keikavoosi-Arani, Cathleen Keller, John H Kempen, Jessica A Kerr, Emmanuelle Kesse-Guyot, Mohammad Keykhaei, Mohamad Mehdi Khadembashiri, Mohammad Amin Khadembashiri, Morteza Abdullatif Khafaie, Himanshu Khajuria, Mohammad Khalafi, Amirmohammad Khalaji, Nauman Khalid, Ibrahim A Khalil, Faham Khamesipour, Asaduzzaman Khan, Gulfaraz Khan, Ikramullah Khan, Imteyaz A Khan, Maseer Khan, Moien AB Khan, Taimoor Khan, Mahammed Ziauddin Khan suheb, Shaghayegh Khanmohammadi, Khaled Khatab, Fatemeh Khatami, Armin Khavandegar, Hamid Reza Khayat Kashani, Khalid A Kheirallah, Feriha Fatima Khidri, Elaheh Khodadoust, Moein Khormali, Mahmood Khosrowjerdi, Jagdish Khubchandani, Helda Khusun, Zemene Demelash Kifle, Grace Kim, Jihee Kim, Ruth W Kimokoti, Kasey E Kinzel, Girmay Tsegay Kiross, Adnan Kisa, Sezer Kisa, Juniper Boroka Kiss, Mika Kivimäki, Desmond Klu, Ann Kristin Skrindo Knudsen, Ali-Asghar Kolahi, Farzad Kompani, Gerbrand Koren, Soewarta Kosen, Karel Kostev, Ashwin Laxmikant Kotnis, Parvaiz A Koul, Sindhura Lakshmi Koulmane Laxminarayana, Ai Koyanagi, Michael A Kravchenko, Kewal Krishan, Hare Krishna, Vijay Krishnamoorthy, Yuvaraj Krishnamoorthy, Kris J Krohn, Barthelemy Kuate Defo, Connor M Kubeisy, Burcu Kucuk Bicer, Md Abdul Kuddus, Mohammed Kuddus, Ilari Kuitunen, Omar Kujan, Mukhtar Kulimbet, Vishnutheertha Kulkarni, Ashish Kumar, Harish Kumar, Nithin Kumar, Rahul Kumar, Shiv Kumar, Madhulata Kumari, Almagul Kurmanova, Om P Kurmi, Asep Kusnali, Dian Kusuma, Tezer Kutluk, Ambily Kuttikkattu, Evans F Kyei, Ilias Kyriopoulos, Carlo La Vecchia, Muhammad Awwal Ladan,

Lucie Laflamme, Chandrakant Lahariya, Abdelilah Lahmar, Daphne Teck Ching Lai, Tri Laksono, Dharmesh Kumar Lal, Ratilal Lalloo, Tea Lallukka, Judit Lám, Demetris Lamnisos, Tuo Lan, Francesco Lanfranchi, Berthold Langguth, Van Charles Lansingh, Ariane Laplante-Lévesque, Bagher Larijani, Anders O Larsson, Savita Lasrado, Kamaluddin Latief, Mahrukh Latif, Kaveh Latifinaibin, Paolo Lauriola, Long Khanh Dao Le, Nhi Huu Hanh Le, Thao Thi Thu Le, Trang Diep Thanh Le, Munjae Lee, Paul H Lee, Sang-woong Lee, Seung Won Lee, Wei-Chen Lee, Yo Han Lee, Samson Mideksa Legesse, James Leigh, Jacopo Lenzi, Elvynna Leong, Temesgen L Lerango, Ming-Chieh Li, Wei Li, Xiaopan Li, Yichong Li, Zhihui Li, Massimo Libra, Virendra S Ligade, Andrew Tiyamike Makhiringa Likaka, Lee-Ling Lim, Ro-Ting Lin, Shuzhi Lin, Vasileios-Arsenios Lioutas, Stefan Listl, Jue Liu, Simin Liu, Xiaofeng Liu, Katherine M Livingstone, Erand Llanaj, Chun-Han Lo, Arianna Maever Loreche, László Lorenzovici, Mojgan Lotfi, Masoud Lotfizadeh, Rafael Lozano, Jailos Lubinda, Giancarlo Lucchetti, Alessandra Lugo, Raimundas Lunevicius, Jianing Ma, Stefan Ma, Zheng Feei Ma, Mahmoud Mabrok, Nikolaos Machairas, Monika Machoy, Christian Madsen, Javier A Magaña Gómez, Azzam A Maghazachi, Sandeep B Maharaj, Preeti Maharjan, Soleiman Mahjoub, Mansour Adam Mahmoud, Elham Mahmoudi, Morteza Mahmoudi, Omar Mohamed Makram, Jeadran N Malagón-Rojas, Elaheh Malakan Rad, Reza Malekzadeh, Armaan K Malhotra, Kashish Malhotra, Ahmad Azam Malik, Iram Malik, Lesibana Anthony Malinga, Deborah Carvalho Malta, Abdullah A Mamun, Yosef Manla, Fahmida Mannan, Yasaman Mansoori, Ali Mansour, Vahid Mansouri, Mohammad Ali Mansournia, Lorenzo Giovanni Mantovani, Bishnu P Marasini, Hamid Reza Marateb, Joemer C Maravilla, Agustina M Marconi, Parham Mardi, Mirko Marino, Abdoljalal Marjani, Carlos Alberto Marrugo Arnedo, Bernardo Alfonso Martinez-Guerra, Ramon Martinez-Piedra, Cleodice A Martins, Francisco Rogerlândio Martins-Melo, Miquel Martorell, Wolfgang Marx, Sharmeen Maryam, Roy Rillera Marzo, Kedar K V Mate, Clara N Matei, Alexander G Mathioudakis, Richard James Maude, Andrea Maugeri, Erin A May, Mahsa Mayeli, Maryam Mazaheri, Mohsen Mazidi, Antonio Mazzotti, Colm McAlinden, John J McGrath, Martin McKee, Anna Laura W McKowen, Susan A McLaughlin, Michael A McPhail, Steven M McPhail, Enkeleint A Mechili, Rishi P Mediratta, Jitendra Kumar Meena, Medhin Mehari, Max L Mehlman, Rahul Mehra, Kamran Mehrabani-Zeinabad, Entezar Mehrabi Nasab, Ravi Mehrotra, Mathewos M Mekonnen, Walter Mendoza, Ritesh G Menezes, Endalkachew Worku Mengesha, George A Mensah, Laverne G Mensah, Alexios-Fotios A Mentis, Sultan Ayoub Meo, Atte Meretoja, Tuomo J Meretoja, Abera M Mersha, Bezawit Afework Mesfin, Tomislav Mestrovic, Adquate Mhlanga, Laurette Mhlanga, Tianyue Mi, Georgia Micha, Irmina Maria Michalek, Ted R Miller, Sergey Nikolaevich Mindlin, Giada Minelli, Le Huu Nhat Minh, GK Mini, Neema W Minja, Niloofar Mirdamadi, Mojgan Mirghafourvand, Andreea Mirica, Seyed Kazem Mirinezhad, Omid Mirmosayyeb, Mizan Kiros Mirutse, Mohammad Mirza-Aghazadeh-Attari, Maryam Mirzaei, Tadesse Misgana, Sanjeev Misra, Philip B Mitchell, Prasanna Mithra, Chaitanya Mittal, Madhukar Mittal, Babak Moazen, Ahmed Ismail Mohamed, Jama Mohamed, Mouhand F H Mohamed, Nouh Saad Mohamed, Sakineh Mohammad-Alizadeh-Charandabi, Soheil Mohammadi, Abdollah Mohammadian-Hafshejani, Saeed Mohammad-pour, Marita Mohammadshahi, Mustapha Mohammed, Salahuddin Mohammed, Shafiu Mohammed, Hoda Mojiri-forushani, Ali H Mokdad, Peyman Mokhtarzadehazar, Kaveh Momenzadeh, Sara Momtazmanesh, Lorenzo Monasta, Mohammad Ali Moni, Fateme Montazeri, AmirAli Moodi Ghalibaf, Maryam Moradi, Yousef Moradi, Maziar Moradi-Lakeh, Mehdi Moradinazar, Farhad Moradpour, Paula Moraga, Lidia Morawska, Rafael Silveira Moreira, Negar Morovatdar, Shane Douglas Morrison, Jakub Morze, Reza Mosaddeghi Heris, Jonathan F Mosser, Elias Mossialos, Hakimeh Mostafavi, Amirmahdi Mostofinejad, Vincent Mougin, Simin Mouodi, Parsa Mousavi, Seyed Ehsan Mousavi, Amin Mousavi Khaneghah, Christine Mpundu-Kaambwa, Matías Mrejen, Sumaira Mubarik, Lorenzo Muccioli, Ulrich

Otto Mueller, Faraz Mughal, Sumoni Mukherjee, George Duke Mukoro, Admir Mulita, Francesk Mulita, Malaisamy Muniyandi, Kavita Munjal, Fungai Musaigwa, Khaled M Musallam, Ghulam Mustafa, Sathish Muthu, Saravanan Muthupandian, Woojae Myung, Ashraf F Nabhan, Fredrick Muyia Nafukho, Ahamarshan Jayaraman Nagarajan, Mohsen Naghavi, Pirouz Naghavi, Ganesh R Naik, Gurudatta Naik, Mukhammad David Naimzada, Sanjeev Nair, Tapas Sadasivan Nair, Hastyar Hama Rashid Najmuldeen, Luigi Naldi, Vinay Nangia, Shumaila Nargus, Bruno Ramos Nascimento, Gustavo G Nascimento, Abdallah Y Naser, Mohammad Javad Nasiri, Zuhair S Natto, Javaid Nauman, Muhammad Naveed, Biswa Prakash Nayak, Vinod C Nayak, Ashish Kumar Nayyar, Athare Nazri-Panjaki, Hadush Negash, Amayu Kumesa Negero, Ionut Negoi, Ruxandra Irina Negoi, Serban Mircea Negru, Seyed Aria Nejadghaderi, Chakib Nejjari, Mohammad Hadi Nematollahi, Evangelia Nena, Samata Nepal, Olivia D Nesbit, Charles Richard James Newton, Josephine W Ngunjiri, Dang H Nguyen, Phat Tuan Nguyen, Phuong The Nguyen, Tuan Thanh Nguyen, Van Thanh Nguyen, Yeshambel T Nigatu, Taxiarchis Konstantinos Nikolouzakis, Ali Nikoobar, Amin Reza Nikpoor, Muhammad A Nizam, Shuhei Nomura, Mamoona Noreen, Nafise Noroozi, Abbas Norouzian Baghani, Bo Norrving, Jean Jacques Noubiap, Amanda Novotney, Chisom Adaobi Nri-Ezedi, George Ntaios, Mpiko Ntsekhe, Virginia Nuñez-Samudio, Dieta Nurrika, Bogdan Oancea, Kehinde O Obamiro, Ismail A Odetokun, Akinyemi O D Ofakunrin, Ropo Ebenezer Ogunsakin, James Odhiambo Oguta, In-Hwan Oh, Hassan Okati-Aliabad, Sylvester Reuben Okeke, Akinkunmi Paul Okekunle, Lawrence Okidi, Osaretin Christabel Okonji, Patrick Godwin Okwute, Andrew T Olagunju, Muideen Tunbosun Olaiya, Titilope O Olanipekun, Matthew Idowu Olatubi, Antonio Olivas-Martinez, Gláucia Maria Moraes Oliveira, Susan Oliver, Abdulhakeem Abayomi Olorukooba, Isaac Iyinoluwa Olufadewa, Bolajoko Olubukunola Olusanya, Jacob Olusegun Olusanya, Yinka Doris Oluwafemi, Gideon Olamilekan Oluwatunase, Hany A Omar, Goran Latif Omer, Sokking Ong, Obinna E Onwujekwe, Kenneth Ikenna Onyedibe, John Nelson Opio, Michal Ordak, E Roberto Orellana, Orish Ebere Orisakwe, Verner N Orish, Hans Orru, Doris V Ortega-Altamirano, Alberto Ortiz, Edgar Ortiz-Brizuela, Esteban Ortiz-Prado, Uchechukwu Levi Osuagwu, Adrian Otoiu, Nikita Otstavnov, Amel Ouyahia, Guoqing Ouyang, Mayowa O Owolabi, Ifeoluwa Temitayo Oyeyemi, Oyetunde T Oyeyemi, Yaz Ozten, Mahesh Padukudru P A, Jagadish Rao Padubidri, Mahsa Pahlavikhah Varnosfaderani, Pramod Kumar Pal, Tamás Palicz, Claudia Palladino, Raffaele Palladino, Raul Felipe Palma-Alvarez, Adrian Pana, Parsa Panahi, Ashok Pandey, Seithikurippu R Pandi-Perumal, Victoria Pando-Robles, Helena Ullyartha Pangaribuan, Georgios D Panos, Ioannis Pantazopoulos, Paraskevi Papadopoulou, Shahina Pardhan, Romil R Parikh, Seoyeon Park, Ashwaghosha Parthasarathi, Ava Pashaei, Deepak Kumar Pasupula, Jenil R Patel, Sangram Kishor Patel, Aslam Ramjan Pathan, Ashlesh Patil, Shankargouda Patil, Dimitrios Patoulias, Venkata Suresh Patthipati, Uttam Paudel, Shrikant Pawar, Hamidreza Pazoki Toroudi, Spencer A Pease, Amy E Peden, Paolo Pedersini, Minjin Peng, Umberto Pensato, Veincent Christian Filipino Pepito, Emmanuel K Peprah, Gavin Pereira, Jeevan Pereira, Marcos Pereira, Mario F P Peres, Arokiasamy Perianayagam, Norberto Perico, Ionela-Roxana Petcu, Fanny Emily Petermann-Rocha, Raffaele Pezzani, Hoang Tran Pham, Michael R Phillips, Daniela Pierannunzio, Manon Pigeolet, David M Pigott, Thomas Pilgrim, Marina Pinheiro, Michael A Piradov, Nishad Plakkal, Evgenii Plotnikov, Dimitri Poddighe, Peter Pollner, Ramesh Poluru, Constance Dimity Pond, Maarten J Postma, Govinda Raj Poudel, Lisasha Poudel, Ghazaleh Pourali, Naeimeh Pourtaheri, Sergio I Prada, Pranil Man Singh Pradhan, Vijay Kumar Prajapati, V Prakash, Chandra P Prasad, Manya Prasad, Akila Prashant, Elton Junio Sady Prates, Hery Purnobasuki, Bharathi M Purohit, Jagadeesh Puvvula, Rizwan Qaisar, Nameer Hashim Qasim, Ibrahim Qattea, Gangzhen Qian, Nguyen Khoi Quan, Amir Radfar, Venkatraman Radhakrishnan, Pourya Raee, Hadi Raeisi Shahraki, Seyedeh Niloufar Rafiei Alavi, Ibrar Rafique, Alberto Raggi, Fakher Rahim, Md Mosfequr Rahman, Mosiur

Rahman, Muhammad Aziz Rahman, Tafhimur Rahman, Amir Masoud Rahmani, Shayan Rahmani, Niloufar Rahnavard, Pramila Rai, Sathish Rajaa, Ali Rajabpour-Sanati, Prashant Rajput, Prasanna Ram, Hazem Ramadan, Shakthi Kumaran Ramasamy, Sheena Ramazanu, Juwel Rana, Kritika Rana, Chhabi Lal Ranabhat, Nemanja Rancic, Smitha Rani, Shubham Ranjan, Chythra R Rao, Indu Ramachandra Rao, Mithun Rao, Sowmya J Rao, Drona Prakash Rasali, Davide Rasella, Sina Rashedi, Vahid Rashedi, Ahmed Mustafa Rashid, Ashkan Rasouli-Saravani, Prateek Rastogi, Azad Rasul, Ramin Ravangard, Nakul Ravikumar, David Laith Rawaf, Salman Rawaf, Reza Rawassizadeh, Iman Razeghian-Jahromi, Murali Mohan Rama Krishna Reddy, Elrashdy Moustafa Mohamed Redwan, Faizan Ur Rehman, Robert C Reiner Jr, Giuseppe Remuzzi, Bhageerathy Reshmi, Serge Resnikoff, Luis Felipe Reyes, Malihe Rezaee, Negar Rezaei, Nima Rezaei, Mohsen Rezaeian, Mavra A Riaz, Ana Isabel Ribeiro, Daniel Cury Ribeiro, Jennifer Rickard, Maria Jesus Rios-Blancas, Hannah Elizabeth Robinson-Oden, Mónica Rodrigues, Jefferson Antonio Buendia Rodriguez, Leonardo Roever, Ravi Rohilla, Peter Rohloff, Debby Syahru Romadlon, Luca Ronfani, Gholamreza Roshandel, Sharareh Roshanzamir, Morteza Rostamian, Bedanta Roy, Priyanka Roy, Enrico Rubagotti, Susan Fred Rumisha, Godfrey M Rwegerera, Andrzej Rynkiewicz, Manjula S, Chandan S N, Katharina S Sunnerhagen, Aly M A Saad, Michela Sabbatucci, Korosh Saber, Maha Mohamed Saber-Ayad, Simona Sacco, Basema Saddik, Adam Saddler, Bashdar Abuzed Sadee, Ehsan Sadeghi, Masoumeh Sadeghi, Saeid Sadeghian, Umar Saeed, Maryam Saeedi, Sare Safi, Rajesh Sagar, Amene Saghazadeh, Narjes Saheb Sharif-Askari, Soumya Swaroop Sahoo, Mohammad Ali Sahraian, Seyed Aidin Sajedi, Mirza Rizwan Sajid, Joseph W Sakshaug, Saina Salahi, Sarvenaz Salahi, Payman Salamati, Afeez Abolarinwa Salami, Luciane B Salaroli, Mohamed A Saleh, Sana Salehi, Marwa Rashad Salem, Mohammed Z Y Salem, Sohrab Salimi, Hossein Samadi Kafil, Sara Samadzadeh, Kamel A Samara, Saad Samargandy, Yoseph Leonardo Samodra, Vijaya Paul Samuel, Abdallah M Samy, Juan Sanabria, Nima Sanadgol, Edmond Sanganyado, Rama Krishna Sanjeev, Francesco Sanmarchi, Francesca Sanna, Ichtiarini Nurullita Santri, Milena M Santric-Milicevic, Made Ary Sarasmita, Aswini Saravanan, Babak Saravi, Yaser Sarikhani, Chinmoy Sarkar, Rodrigo Sarmiento-Suárez, Gargi Sachin Sarode, Sachin C Sarode, Arash Sarveazad, Brijesh Sathian, Thirunavukkarasu Sathish, Davide Sattin, Jennifer Saulam, Susan M Sawyer, Sonia Saxena, Ganesh Kumar Saya, Yaser Sayadi, Abu Sayeed, Md Abu Sayeed, Mete Saylan, Nikolaos Scarmeas, Benedikt Michael Schaarschmidt, Winfried Schlee, Maria Inês Schmidt, Art Schuermans, David C Schwebel, Falk Schwendicke, Mario Šekerija, Siddharthan Selvaraj, Mohammad H Semreen, Sabyasachi Senapati, Pallav Sengupta, Subramanian Senthilkumaran, Sadaf G Sepanlou, Dragos Serban, Addisu Sertsu, Yashendra Sethi, SeyedAhmad SeyedAlinaghi, Seyed Arsalan Seyedi, Amir Shafaat, Omid Shafaat, Mahan Shafie, Arman Shafiee, Nilay S Shah, Pritik A Shah, Saeed Shahabi, Ataollah Shahbandi, Izza Shahid, Samiah Shahid, Wajeehah Shahid, Moyad Jamal Shahwan, Masood Ali Shaikh, Alireza Shakeri, Husain Shakil, Sunder Sham, Muhammad Aaqib Shamim, Mehran Shams-Beyranvand, Hina Shamshad, Mohammad Ali Shamshirgaran, Mohammad Anas Shamsi, Mohd Shanawaz, Abhishek Shankar, Sadaf Sharfaei, Amin Sharifan, Mariam Shariff, Javad Sharifi-Rad, Manoj Sharma, Rajesh Sharma, Saurab Sharma, Vishal Sharma, Rajesh P Shastry, Amin Shavandi, David H Shaw, Amir Mehdi Shayan, Amr Mohamed Elsayed Shehabeldine, Aziz Sheikh, Rahim Ali Sheikhi, Jiabin Shen, Manjunath Mala Shenoy, B Suresh Kumar Shetty, Ranjitha S Shetty, Robert Adamu Shey, Amir Shiani, Kenji Shibuya, Desalegn Shiferaw, Mika Shigematsu, Jae Il Shin, Min-Jeong Shin, Rahman Shiri, Reza Shirkoohi, Aminu Shittu, Ivy Shiue, K M Shivakumar, Velizar Shivarov, Sina Shool, Sunil Shrestha, Kanwar Hamza Shuja, Kerem Shuval, Yafei Si, Migbar Mekonnen Sibhat, Emmanuel Edwar Siddig, Inga Dora Sigfusdottir, João Pedro Silva, Luís Manuel Lopes Rodrigues Silva, Soraia Silva, Jorge Piano Simões, Colin R Simpson, Anjali Singal, Abhinav Singh, Aditya Singh, Ambrish Singh, Balbir Bagicha Singh, Baljinder

Singh, Mahendra Singh, Mayank Singh, Narinder Pal Singh, Paramdeep Singh, Surjit Singh, Md Shahjahan Siraj, Freddy Sitas, Shravan Sivakumar, Valentin Yurievich Skryabin, Anna Aleksandrovna Skryabina, David A Sleet, Erica Leigh N Slepak, Hanye Sohrabi, Hamidreza Soleimani, Sameh S M Soliman, Marco Solmi, Yonatan Solomon, Yimeng Song, Reed J D Sorensen, Joan B Soriano, Ireneous N Soyiri, Michael Spartalis, Chandrashekhar T Sreeramareddy, Joseph R Starnes, Vladimir I Starodubov, Antonina V Starodubova, Simona Cătălina Stefan, Dan J Stein, Fridolin Steinbeis, Paschalis Steiropoulos, Leo Stockfelt, Mark A Stokes, Stefan Stortecky, Saverio Stranges, Konstantinos Stroumpoulis, Muhammad Suleman, Rizwan Suliankatchi Abdulkader, Abida Sultana, Jing Sun, David Sunkersing, Sri Susanty, Chandan Kumar Swain, Bryan L Sykes, Lukasz Szarpak, Mindy D Szeto, Miklós Szócska, Payam Tabaee Damavandi, Ozra Tabatabaei Malazy, Seyed-Amir Tabatabaeizadeh, Shima Tabatabai, Karen M Tabb, Mohammad Tabish, Luis M Taborda-Barata, Takahiro Tabuchi, Birkneh Tilahun Tadesse, Amirmasoud Taheri, Yasaman Taheri Abkenar, Moslem Taheri Soodejani, Amir Taherkhani, Jabeen Taiba, Ardeshir Tajbakhsh, Iman M Talaat, Ashis Talukder, Jacques Lukenze Tamuzi, Ker-Kan Tan, Haosu Tang, Hong K Tang, Nathan Y Tat, Vivian Y Tat, Razieh Tavakoli Oliaee, Seyed Mohammad Tavangar, Nuno Taveira, Tsion Mulat Tebeje, Yibekal Manaye Tefera, Mojtaba Teimoori, Mohamad-Hani Temsah, Reem Mohamad Hani Temsah, Masayuki Teramoto, Solomon Hailemariam Tesfaye, Pugazhenthan Thangaraju, Kavumpurathu Raman Thankappan, Rajshree Thapa, Rekha Thapar, Nihal Thomas, Amanda G Thrift, Chern Choong Chern Thum, Jing Tian, Ales Tichopad, Jansje Henny Vera Ticoalu, Tenaw Yimer Tiruye, Seyed Abolfazl Tohidast, Marcello Tonelli, Mathilde Touvier, Marcos Roberto Tovani-Palone, Khai Hoan Tram, Nghia Minh Tran, Domenico Trico, Indang Trihandini, Samuel Joseph Tromans, Vien T Truong, Thien Tan Tri Tai Truyen, Evangelia Eirini Tsermpini, Munkhtuya Tumurkhuu, Kang Tung, Stefanos Tyrovolas, Chukwudi S Ubah, Aniefiok John Udoakang, Arit Udoh, Inam Ulhaq, Saeed Ullah, Sana Ullah, Muhammad Umair, Tungki Pratama Umar, Chukwuma David Umeokonkwo, Anushri Umesh, Brigid Unim, Bhaskaran Unnikrishnan, Era Upadhyay, Daniele Urso, Marco Vacante, Amir Mohammad Vahdani, Asokan Govindaraj Vaithinathan, Sahel Valadan Tahbaz, Rohollah Valizadeh, Jef Van den Eynde, Elena Varavikova, Orsolya Varga, Siddhartha Alluri Varma, Priya Vart, Shoban Babu Varthya, Tommi Juhani Vasankari, Lennert J Veerman, Narayanaswamy Venketasubramanian, Deneshkumar Venugopal, Nicholas Alexander Verghese, Madhur Verma, Pratibha Verma, Massimiliano Veroux, Georgios-Ioannis Verras, Dominique Vervoort, Rafael José Vieira, Jorge Hugo Villafañe, Leonardo Villani, Gabriela Ines Villanueva, Paul J Villeneuve, Francesco S Violante, Rachel Visontay, Vasily Vlassov, Bay Vo, Stein Emil Vollset, Simona Ruxandra Volovat, Victor Volovici, Avina Vongpradith, Theo Vos, Isidora S Vujcic, Rade Vukovic, Yohannes Dibaba Wado, Hatem A Wafa, Yasir Waheed, Richard G Wamai, Cong Wang, Denny Wang, Fang Wang, Shu Wang, Song Wang, Yanzhong Wang, Yuan-Pang Wang, Paul Ward, Stefanie Watson, Marcia R Weaver, Kosala Gayan Weerakoon, Daniel J Weiss, Abrha Hailay Weldemariam, Katherine M Wells, Yi Feng Wen, Andrea Werdecker, Ronny Westerman, Dakshitha Praneeth Wickramasinghe, Nuwan Darshana Wickramasinghe, Tissa Wijeratne, Shadrach Wilson, Marcin W Wojewodzic, Eve E Wool, Anthony D Woolf, Dongze Wu, Ratna Dwi Wulandari, Hong Xiao, Bin Xu, Xiaoyue Xu, Lalit Yadav, Sajad Yaghoubi, Lin Yang, Yuichiro Yano, Yao Yao, Pengpeng Ye, Gesila Endashaw Yesera, Renjulal Yesodharan, Subah Abderehim Yesuf, Arzu Yiğit, Vahit Yiğit, Paul Yip, Dong Keon Yon, Naohiro Yonemoto, Yuyi You, Mustafa Z Younis, Chuanhua Yu, Siddhesh Zadey, Vesna Zadnik, Nima Zafari, Mohammad Zahedi, Muhammad Nauman Zahid, Mazyar Zahir, Fathiah Zakham, Nazar Zaki, Josefina Zakzuk, Giulia Zamagni, Burhan Abdullah Zaman, Sojib Bin Zaman, Nelson Zamora, Ramin Zand, Milad Zandi, Ghazal G Z Zandieh, Aurora Zanghì, Iman Zare, Mikhail Sergeevich Zastrozhin, Mohammed G M Zeariya, Youjie Zeng, Chunxia Zhai, Chen Zhang, Haijun Zhang, Hongwei Zhang, Yunquan Zhang,

Zhaofeng Zhang, Zhenyu Zhang, Hanqing Zhao, Yang Zhao, Yong Zhao, Peng Zheng, Chenwen Zhong, Juexiao Zhou, Bin Zhu, Zhaohua Zhu, Pardis Ziaeefar, Magdalena Zielińska, Zhiyong Zou, Alimuddin Zumla, Elric Zweck, Samer H Zyoud, Stephen S Lim# , and Christopher J L Murray#,

*Joint first authors

#Joint senior authors

Affiliations

Institute for Health Metrics and Evaluation (A E Schumacher PhD, H H Kyu PhD, C M Antony MA, A Y Aravkin PhD, G S Azhar PhD, C Bisignano MPH, K Burkart PhD, K M Cercy BS, E Chung MSc, K Coberly BS, H Comfort MPH, E Cousin PhD, G T Culbreth PhD, M Cunningham MSc, N Davis Weaver MPH, Prof L Degenhardt PhD, L Deitesfeld MA, M A Dirac MD, K Estep MPA, Prof V L Feigin PhD, A D Flaxman PhD, L S Flor MPH, L M Force MD, J E Fuller MLIS, Prof E Gakidou PhD, Prof S I Hay FMedSci, K S Ikuta MD, D P Jones BS, N J Kassebaum MD, M B Kassel BA, C Keller MPH, K E Kinzel MSPH, K J Krohn MPH, Prof R Lozano MD, E A May MS, A W McKowen MA, S A McLaughlin PhD, M L Mehlman PhD, T Mestrovic PhD, A H Mokdad PhD, J F Mosser MD, V Mougin BA, Prof M Naghavi PhD, O D Nesbit MA, A Novotney MPH, Y Ozten MS, S A Pease BS, D M Pigott PhD, R C Reiner Jr PhD, H E Robinson-Oden MLIS, D H Shaw BA, E N Slepak MLIS, R J D Sorensen PhD, N A Verghese BA, Prof S Vollset DrPH, A Vongpradith BA, Prof T Vos PhD, D Wang BA, S Watson MS, Prof M R Weaver PhD, K M Wells BA, S Wilson BS, E E Wool MPH, P Zheng PhD, Prof S S Lim PhD, Prof C J L Murray DPhil), Department of Health Metrics Sciences, School of Medicine (H H Kyu PhD, A Y Aravkin PhD, K Burkart PhD, E Cousin PhD, M A Dirac MD, A D Flaxman PhD, L S Flor MPH, L M Force MD, Prof E Gakidou PhD, Prof S I Hay FMedSci, N J Kassebaum MD, Prof R Lozano MD, A H Mokdad PhD, Prof M Naghavi PhD, D M Pigott PhD, R C Reiner Jr PhD, Prof S Vollset DrPH, Prof T Vos PhD, Prof M R Weaver PhD, P Zheng PhD, Prof S S Lim PhD, Prof C J L Murray DPhil), Department of Applied Mathematics (A Y Aravkin PhD), School of Medicine (Prof E J Boyko MD), Department of Internal Medicine (Y Chahine MD), Department of Cardiology (Y Chahine MD), Department of Family Medicine (M A Dirac MD), Division of Pediatric Hematology-Oncology (L M Force MD), Department of Neurology (R Kalani MD), Department of Anesthesiology & Pain Medicine (N J Kassebaum MD, V Krishnamoorthy MD), Department of Global Health (I A Khalil MD, N W Minja MD, R J D Sorensen PhD), Division of Plastic and Reconstructive Surgery (S D Morrison MD), Foster School of Business (F M Nafukho PhD), Department of Biostatistics (A Olivas-Martinez MD), School of Social Work (E Orellana PhD), Division of Allergy and Infectious Diseases (K Tram MD), University of Washington, Seattle, WA, USA; Faculty of Medicine (A Aali MD, N Rahnavard MD), Department of Neuroscience (A Ahmadzade MD), Emam-Reza Hospital (S Mohammad-pour PhD), Clinical Research Development Unit (N Morovatdar MD), Metabolic Syndrome Research Center (G Pourali MD), International UNESCO Center for Health-related Basic Sciences and Human Nutrition (G Pourali MD), Department of Medical Genetics (N Zafari MD), Mashhad University of Medical Sciences, Mashhad, Iran; Department of Juridical and Economic Studies (C Abbafati PhD), Department of Public Health and Infectious Diseases (M S Cattaruzza PhD), La Sapienza University, Rome, Italy; Antai College of Economics (J Abbas PhD), Shanghai Mental Health Center (Prof M R Phillips MD), Shanghai Jiao Tong University, Shanghai, China; Doheny Eye Institute (R Abbasgholizadeh MD), Department of Ophthalmology (M Emamverdi MD), Center for Social Medicine (J Friedman PhD), University of California Los Angeles, Los Angeles, CA, USA; Infectious and Tropical Research Center (M A Abbasi PhD), Tuberculosis and Lung Diseases Research Center (S Daneshvar MD), Department of Radiology (M Dashti MD, A Ghasemzadeh MD, M Mirza-Aghazadeh-

Attari MD), Department of Health Policy and Management (L Doshmangir PhD), Department of Medical Surgical Nursing (M Ghafourifard PhD, M Lotfi PhD), Research Center for Evidence-Based Medicine (M Hosseini MD), Department of Immunology (F Jadidi-Niaragh PhD), School of Management and Medical Informatics (L R Kalankesh PhD), Social Determinants of Health Research Center (S Karimi PhD, Prof S Mohammad-Alizadeh-Charandabi PhD), Radiology Research Committee (M Khalafi MD), Medical Education Research Center (M Lotfi PhD), Midwifery Department (Prof M Mirghafourvand PhD, Prof S Mohammad-Alizadeh-Charandabi PhD), Liver and Gastrointestinal Disease Research Center (S Mirinezhad PhD), Neurosciences Research Center (NSRC) (R Mosaddeghi Heris MD), Student Research Committee (R Mosaddeghi Heris MD), Department of Community Medicine (S Mousavi MD), Drug Applied Research Center (H Samadi Kafil PhD), Tabriz University of Medical Sciences, Tabriz, Iran; Department of Orthopedic Surgery (M Abbasian MD, K Momenzadeh MD), Harvard Business School (F Caetano dos Santos PhD), Department of Epidemiology (S Carr MS), Division of Cardiovascular Medicine (G Chi MD), Division of Cardiology (I Y Elgendy MD), Department of Neurological Surgery at Brigham and Women's Hospital (A H Feroze MD), Department of Ophthalmology (Prof J H Kempen MD), Department of Health Policy and Management (C M Kubeisy BA), Department of Global Health and Population (Z Li PhD, P Rohloff MD), Radiology and Data Science Department (X Liu PhD), Department of Health Policy and Oral Epidemiology (Z S Natto DrPH), Department of Pulmonary and Critical Care (T O Olanipekun MD), Department of Global Health and Social Medicine (M Pigeolet MD), Harvard T.H. Chan School of Public Health (P M S Pradhan MD, E Zweck MD), Beth Israel Deaconess Medical Center (S Sharfaei MD), Division of General Internal Medicine (Prof A Sheikh MD), Harvard University, Boston, MA, USA; Department of Orthopaedic Surgery (M Abbasian MD), Department of Anesthesiology (D Abtahi MD, S Salimi MD, A Shakeri MD, A Tajbakhsh MD, A Tajbakhsh MD), Department of Biotechnology (S Aghamiri PhD), Urology Department (M Ahmadzade MD, M Bonakdar Hashemi MD), National Nutrition and Food Technology Research Institute (M Ajami PhD), Research Institute of Dental Sciences (Prof S Asgary MSc), Department of Medical Genetics (M Ghasemi PhD), Center for Comprehensive Genetic Services (M Ghasemi PhD), Social Determinants of Health Research Center (Prof H Hassanian-Moghaddam MD, A Kolahi MD, A Nikoobar DipSc), School of Medicine (M Heidari-Foroozan BSc, F Montazeri MD, S Nejadghaderi MD, S Rahmani MD, P Ziaeefar MD), Urology and Nephrology Research Center (H Hesami MD, M Zahir MD, P Ziaeefar MD), Ophtalmic Reserch Centre (H Hesami MD), Department of Immunology (K Jahankhani MSc, A Rasouli-Saravani PhD), Department of Health Policy and Management (N Jahanmehr PhD), Safety Promotion and Injury Prevention Research Center (N Jahanmehr PhD), Department of Neurosurgery (H Khayat Kashani MD), Department of Microbiology and Infectious Diseases (M Nasiri PhD), Department of Biology and Anatomical Sciences (P Raee PhD), Department of Pharmacology (M Rezaee MD), Ophthalmic Research Center (S Safi PhD), Emergency Department (S Shool MD), Department of Medical Education (S Tabatabai PhD), Shahid Beheshti University of Medical Sciences, Tehran, Iran; Epidemiology Department (S Abd ElHafeez DrPH), Pediatric Dentistry and Dental Public Health Department (Prof M El Tantawi PhD, Prof O A A Elmeligy PhD), Tropical Health Department (R M Ghazy PhD), Pathology Department (Prof I M Talaat PhD), Alexandria University, Alexandria, Egypt; Department of Surgery (M Abdelmasseh MD, Prof J Sanabria MD), Marshall University, Huntington, WV, USA; Tropical Medicine Department (S Abd-Elsalam PhD), Tanta University, Tanta, Egypt; Department of Internal Medicine (A Abdelwahab MD), Baylor College of Medicine, Houston, TX, USA; The Institute of Pharmaceutical Sciences (TIPS) (Prof M Abdollahi PhD), School of Pharmacy (Prof M Abdollahi PhD), Research Center for Immunodeficiencies (H Abolhassani PhD, Prof N Rezaei PhD, A Saghazadeh MD), Department of Pharmacology (M Ala MD, N Noroozi DVM), Urology Research Center (R Arabzadeh Bahri

MD, Prof F Khatami PhD), Department of Health Information Management (S Ayyoubzadeh PhD), Non-communicable Diseases Research Center (S Azadnajafabad MD, M Keykhaei MD, S Momtazmanesh MD, F Montazeri MD, P Mousavi MD, S Rahmani MD, N Rezaei PhD), School of Medicine (A Behnoush BS, H Farrokhpour MD, H Karimi MD, A Khalaji BS, S Khanmohammadi MD, M Mayeli MD, S Mohammadi MD, S Momtazmanesh MD), Multiple Sclerosis Research Center (S Eskandarieh PhD, Prof M Sahraian MD), Digestive Diseases Research Institute (S Fahimi MD, Prof R Malekzadeh MD, V Mansouri MD, S G Sepanlou MD), Ophthalmology Department (Prof F Ghassemi MD), Vali-E-Asr Reproductive Health Research Center (M Haddadi MD), Cardiac Primary Prevention Research Center (S Kazemian MD), Department of Cardiac Electrophysiology (S Kazemian MD), Students' Scientific Research Center (SSRC) (M Keykhaei MD, M Khadembashiri MD), Center for Research and Training in Skin Diseases and Leprosy (F Khamesipour PhD), Sina Trauma and Surgery Research Center (A Khavandegar MD, M Khormali MD, Prof P Salamati MD, S Shool MD), Children's Medical Center (F Kompani MD), Endocrinology and Metabolism Research Institute (Prof B Larijani FACE, N Mirdamadi MD, N Rezaei PhD, S Seyedi MD, O Tabatabaei Malazy PhD), Department of Cardiology (E Mahmoudi MD, S Rashedi MD), Department of Pediatric Cardiology (Prof E Malakan Rad MD), Department of Epidemiology and Biostatistics (M Mansournia PhD), Tehran Heart Center (E Mehrabi Nasab MD, M Rezaee MD), Sports and Exercise Medicine Research Center (N Mirdamadi MD), National Institute for Health Research (M Mohammadshahi PhD), Health Equity Research Center (H Mostafavi PhD), Iranian Research Center for HIV/AIDS (S SeyedAlinaghi PhD), Department of Neurology (M Shafie MD), Department of Medicine (A Shahbandi MD, A M Vahdani MD), Department of Pharmaceutical Care (A Sharifan PharmD), Research Center for Rational Use of Drugs (A Sharifan PharmD), Cancer Research Center (R Shirkoohi PhD), Cancer Biology Research Center (R Shirkoohi PhD), Faculty of Medicine (H Sohrabi MD), Department of Pathology (Prof S Tavangar MD), Tehran University of Medical Sciences, Tehran, Iran (R Heidari-Soureshjani MSc, M M Khadembashiri MD); Department of Medicine (Prof M Abdoun BMedSc), University of Setif Algeria, Sétif, Algeria; Department of Physiotherapy (A Abdullahi PhD, A W Awotidebe PhD), Department of Pharmacology and Therapeutics (S B Borodo MSc), Community Medicine Department (Prof M A Gadanya FMCPH), Department of Nursing Science (M Ladan PhD), Bayero University Kano, Kano, Nigeria; Department of Rehabilitation Sciences (A Abdullahi PhD, M U Ali MSc), School of Nursing (S Tyrovolas PhD), Hong Kong Polytechnic University, Hong Kong, China; Department of Emergency and Critical Care Nursing (A M Abdurehman MSc), Department of Health Policy and Management (A T Debele MSc), School of Nursing and Midwifery (T Getachew MSc), Department of Clinical Pharmacy (M D Gudeta MSc), Department of Psychiatry (T Misgana MSc), Department of Nursing (A Sertsu MSc), Haramaya University, Harar, Ethiopia; Department of Midwifery (M Abebe MSc, G M Ayele MSc), Infection Prevention and Control Department (A Afework MPH), Department of Public Health (T L Lerango MPH, T M Tebeje MPH), Department of Pediatrics and Child Health Nursing (M M Sibhat MSc), School of Public Health (S H Tesfaye PhD), Dilla University, Dilla, Ethiopia; Department of Neurosurgery (A Abedi MD), Keck School of Medicine (A Abedi MD), Department of Radiology (A Gholamrezanezhad MD), Mark and Mary Stevens Neuroimaging and Informatics Institute (S Salehi MD), University of Southern California, Los Angeles, CA, USA; Department of Emergency Medicine (A Abedi MD), Department of Immunology (S Athari PhD), Department of Critical Care and Emergency Nursing (N Hanifi PhD), Zanjan University of Medical Sciences, Zanjan, Iran; Department of Clinical Pharmacy (T M Abegaz MS), Department of Pharmacology (Z D Kifle MSc), University of Gondar, Gondar, Ethiopia; College of Pharmacy and Pharmaceutical Sciences (T M Abegaz MS), Florida A&M University, Tallahassee, FL, USA; Postgraduate Department (Prof R A Abeldaño Zuñiga PhD), University of Sierra Sur,

Miahuatlan de Porfirio Diaz, Mexico; National Research Council of Mexico, Mexico City, Mexico (Prof R A Abeldaño Zuñiga PhD); Department of Botany (E S Abhilash PhD), Sree Narayana Guru College Chelannur, Kozhikode, India; Department of Internal Medicine (O O Abiodun FWACP), Federal Medical Centre, Abuja, Nigeria; Department of Family and Community Health (R G Aboagye MPH), Department of Population and Behavioural Sciences (H Amu PhD), Department of Health Policy Planning and Management (M A Ayanore PhD), Department of Epidemiology and Biostatistics (R K Dowou MPhil), Institute of Health Research (M Immurana PhD, D Klu PhD), Department of Microbiology and Immunology (V N Orish PhD), University of Health and Allied Sciences, Ho, Ghana; Department of Medical Biochemistry and Biophysics (H Abolhassani PhD), Department of Medical Epidemiology and Biostatistics (Prof J J Carrero PhD), Department of Physiology and Pharmacology (C R Cederroth PhD), Department of Global Public Health (K Deuba DrPH, Prof L Laflamme PhD), Department of Neurobiology, Care Sciences, and Society (S Fereshtehnejad PhD), Department of Molecular Medicine and Surgery (Prof J H Kauppila MD), Karolinska Institute, Stockholm, Sweden; Department of Physical Pharmacy and Pharmacokinetics (M Abouzid PharmD), Poznan University of Medical Sciences, Poznan, Poland; Department of Pediatric Dentistry (Prof L G Abreu PhD), Department of Maternal and Child Nursing and Public Health (Prof D C Malta PhD, E J S Prates BS), Department of Clinical Medicine (Prof B R Nascimento PhD), Clinical Hospital (Prof B R Nascimento PhD), Federal University of Minas Gerais, Belo Horizonte, Brazil; Department of Adult Health Nursing (W A Abrha MSc, A H Weldemariam MSc), Department of Nursing (A Girmay MSc), Aksum University, Aksum, Ethiopia; Department of Research (M R M Abrigo PhD), Philippine Institute for Development Studies, Quezon City, Philippines; Department of Neurology (S Abu Rumeileh MD), Martin Luther University Halle-Wittenberg, Halle (Saale), Germany; Institute of Community and Public Health (Prof N M Abu-Rmeileh PhD), Birzeit University, Ramallah, Palestine; Department of Therapeutics (Prof S Aburuz PhD), Institute of Public Health (L A Ahmed PhD, Z Alam PhD, I Elbarazi DrPH), College of Medicine and Health Sciences (Prof M Grivna PhD, J Nauman PhD), Department of Medical Microbiology & Immunology (Prof G Khan PhD), Family Medicine Department (M A Khan MSc), Department of Computer Science and Software Engineering (Prof N Zaki PhD), United Arab Emirates University, Al Ain, United Arab Emirates; College of Pharmacy (Prof S Aburuz PhD), Department of Clinical Nursing (Prof M M Ahmad PhD), University of Jordan, Amman, Jordan; Department of Surgery (A Abu-Zaid MD), College of Pharmacy (R M H Temsah PharmD), Alfaisal University, Riyadh, Saudi Arabia; College of Graduate Health Sciences (A Abu-Zaid MD), Department of Neurology (R Zand MD), University of Tennessee, Memphis, TN, USA; Clinical Medicine Department (Prof J M Acuna MD), American University of Antigua, Osbourn, Antigua and Barbuda; FIU Robert Stempel College of Public Health & Social Work (Prof J M Acuna MD), Department of Epidemiology (P Gautam MPH), Florida International University, Miami, FL, USA (Prof R Chowdhury PhD); Melbourne School of Population and Global Health (T Adair PhD, H Jiang PhD), Department of Medicine (A S Babu PhD), Justice Health Unit (R Borschmann PhD), School of Health Sciences (A Meretoja MD), Department of Neurology (Prof T Wijeratne MD), University of Melbourne, Melbourne, VIC, Australia; Centre for Social Research in Health (I Y Addo PhD, S R Okeke PhD), School of Population Health (Z Dai PhD, X Xu PhD), National Drug and Alcohol Research Centre (Prof L Degenhardt PhD), School of Clinical Medicine (M Huda PhD), School of Psychiatry (Prof P B Mitchell MD), School of Public Health and Community Medicine (A E Peden PhD), School of Optometry and Vision Science (Prof S Resnikoff MD), Faculty of Medicine and Health (S Sharma PhD), School of Risk and Actuarial Studies (Y Si PhD), Centre for Primary Health Care and Equity (CPHCE) (F Sitas PhD), The George Institute for Global Health (P Ye MPH), University of New South Wales, Sydney, NSW, Australia; Quality and Systems Performance Unit (I Y Addo

PhD), Cancer Institute NSW, Sydney, NSW, Australia; College of Medicine (O M Adebayo MD), Department of Oral and Maxillofacial Surgery (T O Aladelusi FWACS, A A Salami BDS), Department of Community Medicine (O S Ilesanmi PhD), Department of Medicine (Prof M O Owolabi DrM), University College Hospital, Ibadan, Ibadan, Nigeria; Menzies School of Health Research (O A Adegboye PhD), Charles Darwin University, Darwin, NT, Australia; Department of Obstetrics and Gynecology (V Adekanmbi PhD), University of Texas Medical Branch, Galveston, TX, USA; Department of Molecular Biology and Genetics (Prof M T AlBataineh PhD), College of Medicine and Health Sciences Academic Programs (Prof W Almahmeed MD), Khalifa University, Abu Dhabi, United Arab Emirates (B Aden PhD); Institute of Public Health (B Aden PhD), Walden University, Al Ain, United Arab Emirates; HIV and Infectious Diseases Department (A V Adepoju MD), Jhpiego, Abuja, Nigeria; Department of Adolescent Research and Care (A V Adepoju MD), Adolescent Friendly Research Initiative and Care, Ado Ekiti, Nigeria; Department of Microbiology (Prof C O Adetunji PhD), Edo State University Uzairue, Iyamho, Nigeria; Department of Biosciences and Biotechnology (T E Adeyeoluwa PhD, I T Oyeyemi PhD, O T Oyeyemi PhD, A J Udoakang PhD), Department of Physiology (O I Adeyomoye PhD), Department of Microbiology (I A Anuoluwa PhD, O O Bello PhD, Y D Oluwafemi PhD), Department of Biological Sciences (T C Ekundayo PhD), Department of Chemistry (O O Idowu MSc), Department of Anatomy (G O Oluwatunase MSc), University of Medical Sciences, Ondo, Ondo, Nigeria; Department of Veterinary Medicine (T E Adeyeoluwa PhD), Department of Community Medicine (A A Afolabi MPH, O S Ilesanmi PhD), Oral and Maxillofacial Surgery (T O Aladelusi FWACS), Department of Epidemiology and Medical Statistics (M Ekholuenetale MSc, A F Fagbamigbe PhD), Faculty of Public Health (M Ekholuenetale MSc, I I Olufadewa MHS), Department of Health Promotion and Education (S E Ibitoye MPH), College of Medicine (A P Okekunle PhD), Department of Medicine (Prof M O Owolabi DrM), University of Ibadan, Ibadan, Nigeria; Department of Business Administration (R Adha PhD), Department of Pharmacy (W Adikusuma PhD), Muhammadiyah University of Mataram, Mataram, Indonesia; Department of Pharmaceutical Sciences (A Adibi MSc), Department of Oral Biological and Medical Sciences (M Chen BDS), School of Population and Public Health (M Hossain MSc, D P Rasali PhD), School of Nursing (A Pashaei MSc), University of British Columbia, Vancouver, BC, Canada; Faculty of Medicine (Q E S Adnani PhD), Center of Excellence in Higher Education for Pharmaceutical Care Innovation (Prof M J Postma PhD), Universitas Padjadjaran (Padjadjaran University), Bandung, Indonesia; Clinical Sciences Department (S Adra MD, H J Barqawi MPhil, N R Dash MD, Prof R Halwani PhD, Prof A A Maghazachi PhD, M M Saber-Ayad MD, N Saheb Sharif-Askari PhD, Prof I M Talaat PhD), College of Medicine (F Ahmad PhD, Prof R Halwani PhD, Prof B Saddik PhD, M A Saleh PhD), Department of Pharmacy Practice and Pharmacotherapeutics (Prof K H Alzoubi PhD, Prof H A Omar PhD), Department of Physiotherapy (A Arumugam PhD), Department of Basic Biomedical Sciences (Y Bustanji PhD), Sharjah Institute for Medical Research (N M Elemam PhD), Department of Clinical Nutrition and Dietetics (M E M Faris PhD), Department of Basic Medical Sciences (A Karim PhD, R Qaisar PhD), College of Pharmacy (Prof M H Semreen PhD), Research Institute of Medical & Health Sciences (Prof M H Semreen PhD), Department of Medicinal Chemistry (S S M Soliman PhD), University of Sharjah, Sharjah, United Arab Emirates (K A Altirkawi MD); Department of Medical Information Sciences (A Afraz MSc), Neurology Research Center (M Ilaghi MD), Kerman Neuroscience Research Center (M Ilaghi MD), Research Center for Hydatid Disease (F Khamesipour PhD), Department of Clinical Biochemistry (M Nematollahi PhD), Kerman University of Medical Sciences, Kerman, Iran; Department of Radiology (S Afyouni PhD, A Amindarolzarbi MD, G G Z Zandieh MD), Department of Biostatistics (A Columbus MS), Russell H. Morgan Department of Radiology and Radiological Science (A Kamireddy MD, O Shafaat MD), Department of Neurosurgery (F

Kazemi MD), Department of Health Policy and Management (D Vervoort MD), Department of International Health (H Zhang MS), Johns Hopkins University, Baltimore, MD, USA (E Jamshidi PharmD); Department of Community Medicine (Prof S Afzal PhD), King Edward Memorial Hospital, Lahore, Pakistan; Department of Public Health (Prof S Afzal PhD), Public Health Institute, Lahore, Pakistan; Department of Cardiovascular Medicine (P Agasthi MD), Mayo Clinic, Scottsdale, AZ, USA; Department of Medical and Surgical Sciences and Advanced Technologies "GF Ingrassia" (Prof A Agodi PhD, M Barchitta PhD, E D'Amico MD, A Maugeri PhD, Prof M Veroux PhD), Department of General Surgery and Medical-Surgical Specialties (Prof A Biondi PhD, Prof G Isola PhD, M Vacante PhD), Department of Biomedical and Biotechnological Sciences (L Falzone PhD, Prof M Libra PhD), University of Catania, Catania, Italy; Department of Geography and Planning (W Agyemang-Duah MSc), Department of Biomedical and Molecular Sciences (A Nikpoor PhD), Queen's University, Kingston, ON, Canada; School of Public Health (B O Ahinkorah MPhil, D Demant PhD), University of Technology Sydney, Sydney, NSW, Australia; Department of Medical Biochemistry (A Ahmad PhD), Department of Pediatrics (Prof G Mustafa MD), Department of Pharmacology (A R Pathan PhD, M Tabish MPharm), Shaqra University, Shaqra, Saudi Arabia; School of Medicine and Psychology (D Ahmad PhD), Research School of Population Health (N Bagheri PhD, R A Burns PhD, Prof N Cherbuin PhD), Australian National University, Canberra, ACT, Australia; Public Health Foundation of India, Gandhinagar, India (D Ahmad PhD); Department of Epidemiology and Health Statistics (T Ahmad MS), Southeast University, Nanjing, China; School of Public Health (K Ahmadi PhD, Prof S Saxena MD), Department of Primary Care and Public Health (T Beaney MSc, R Palladino MD, Prof S Rawaf MD), Department of Surgery and Cancer (Prof E Mossialos PhD), WHO Collaborating Centre for Public Health Education and Training (D L Rawaf MRCS), Imperial College London, London, UK; Institute of Endemic Diseases (A Ahmed MSc), Unit of Basic Medical Sciences (E E Siddig MD), University of Khartoum, Khartoum, Sudan; Swiss Tropical and Public Health Institute (A Ahmed MSc), University of Basel, Basel, Switzerland; Department of Biosciences (H Ahmed PhD), COMSATS Institute of Information Technology, Islamabad, Pakistan; Department of Epidemiology (M B Ahmed MPH, D Shiferaw MPH), Department of Public Health (M Y Ashemo PhD, U Gerema MSc, M E Getachew MPH), Department of Surgery (N S Bayileyegn MD), Institute of Health Science (A I Mohamed MSc), Jimma University, Jimma, Ethiopia; Australian Center for Precision Health (M B Ahmed MPH), UniSA Clinical and Health Sciences (T C Eshetie PhD), Department of Allied Health and Human Performance (T Y Tiruye PhD), University of South Australia, Adelaide, SA, Australia; Brody School of Medicine (S Ahmed PhD), Department of Internal Medicine (H Ali MD), Department of Computer Science (A O Bodunrin MSc), Department of Physiology (M Tumurkhuu PhD), Diabetes & Obesity Institute and Physiology (K Tung PhD), Department of Public Health (C S Ubah MPH), East Carolina University, Greenville, NC, USA (R T Aruleba PhD); Department of Food and Nutrition Policy and Planning Research (M Ajami PhD), National Institute of Nutrition, Tehran, Iran; Faculty of Medicine and Public Health (B Aji DrPH), Jenderal Soedirman University, Purwokerto, Indonesia; School of Community Health Sciences (O Ajumobi MPH), University of Nevada Reno, Reno, NV, USA; National Malaria Elimination Program (O Ajumobi MPH), Federal Ministry of Health, Abuja, Nigeria; Microbiology, Immunology and Parasitology Department (G T Akalu MSc), St. Paul's Hospital Millennium Medical College, Addis Ababa, Ethiopia; Microbial, Cellular and Molecular Biology Department (G T Akalu MSc), Department of Microbiology (H B Beyene PhD), School of Public Health (K Deribe PhD), Emergency Department (D Ijo MSc), Department of Health Management Information Systems (D Ijo MSc), Department of Reproductive, Family and Population Health (N A Kassaw MPH), Addis Ababa University, Addis Ababa, Ethiopia; Moyen Mono Health District (E Akara MD), Ministry of health, Tohoun, Togo; Department of Internal Medicine (K

Akinosoglou PhD), University of Patras, Greece, Patras, Greece; Department of Internal Medicine and Infectious Diseases (K Akinosoglou PhD), University General Hospital of Patras, Patras, Greece; Department of Management, Policy, and Community Health (S Akkala MPH), Department of Plastic Surgery (M Asaad MD, A M Hassan MD), McGovern Medical School (A Bleyer MD), University of Texas, Houston, TX, USA; Yale School of Nursing (S Akyirem Mres), School of the Environment (Prof M L Bell PhD, Y Song PhD), Department of Internal Medicine (F Etaee MD), Department of Dermatology (M Goldust MD), Department of Psychiatry (W Li PhD), Department of Genetics (S Pawar PhD), Yale University, New Haven, CT, USA; Geriatric and Long Term Care Department (H Al Hamad MD, B Sathian PhD), Rumailah Hospital (H Al Hamad MD), Hamad Medical Corporation, Doha, Qatar; Division of Public Health Sciences (S Al Hasan PhD), Washington University School of Medicine, St Louis, MO, USA; Department of Urology (A Al Homsi MD, O Almidani MSc), Department of Cardiology, Heart, Vascular, and Thoracic Institute (Prof W Almahmeed MD), Department of Cardiac Surgery (L Göbölös PhD), Cleveland Clinic Abu Dhabi, Abu Dhabi, United Arab Emirates; Department of Nursing (Prof M Al Qadire PhD), Al Al-Bayt University, Mafraq, Jordan; Heidelberg Institute of Global Health (HIGH) (T M A AL-Ahdal MPH, S Chen DSc, B Moazen MSc), Heidelberg University, Heidelberg, Germany; Department of Clinical Sciences (S O Alalalmeh BPharm, O E Hegazi BPharm), Center for Medical and Bio-Allied Health Sciences Research (Prof M J Shahwan PhD, M A Shamsi PhD, S H Zyoud PhD), Ajman University, Ajman, United Arab Emirates; John T. Milliken Department of Internal Medicine (Z Al-Aly MD), Brown School (C Wang MPH), Department of Surgery (C Wang MPH), Washington University in St. Louis, St. Louis, MO, USA; Clinical Epidemiology Center (Z Al-Aly MD), US Department of Veterans Affairs (VA), St Louis, MO, USA; Murdoch Business School (K Alam PhD), Murdoch University, Perth, WA, Australia; Department of Bioengineering (M Alam PhD), Department of Nutrition and Food Studies (S Tyrovolas PhD), George Mason University, Fairfax, VA, USA; School of Nursing (R M Al-amer PhD), Yarmouk University, Irbid, Jordan; School of Nursing and Midwifery (R M Al-amer PhD), Department of Engineering (G R Naik PhD), Western Sydney University, Sydney, NSW, Australia; Health Information Management and Technology Department (T M Alanzi PhD), Department of Public Health (Prof S Bah PhD), Forensic Medicine Division (Prof R G Menezes MD), Imam Abdulrahman Bin Faisal University, Dammam, Saudi Arabia (F M Alanezi PhD); Community and Mental Health Department (Prof M Albashtawy PhD), Al al-Bayt University, Mafraq, Jordan; Institute of Health Informatics (R W Aldridge PhD), Department of Health Informatics (S Chung PhD), Department of Behavioural Science and Health (S Hossain MS), Institute of Epidemiology and Health Care (J Kim MSc), Department of Epidemiology and Public Health (Prof M Kivimäki PhD), Department of Population Health Sciences (D Sunkersing PhD), Division of Surgery and Interventional Science (T Umar MD), Department of Infection (Prof A Zumla PhD), University College London, London, UK; Global Health Entrepreneurship (S Alemi PhD), Tokyo Medical and Dental University, Tokyo, Japan; Pediatric Intensive Care Unit (A Al-Eyadhy MD, M Temsah MD), Department of Cardiac Sciences (Prof K F Alhabib MD), Section of Adult Hematology (Prof G M T ElGohary MD), Department of Physiology (Prof S A Meo PhD), King Saud University, Riyadh, Saudi Arabia; Global Centre for Environmental Remediation (A A S Al-Gheethi PhD), Department of Women's Health (G T Kiross MPH), University of Newcastle, Newcastle, NSW, Australia; Cooperative Research Centre for Contamination Assessment and Remediation of the Environment, Newcastle, NSW, Australia (A A S Al-Gheethi PhD); College of Nursing (Prof F A N Alhalaiqa PhD), College of Dental Medicine (S A A Al-Maweri PhD), Department of Physical Education (Prof M A Alomari PhD), QU Health (M Mohammed PhD), Qatar University, Doha, Qatar; Psychological Sciences Association, Amman, Jordan (Prof F A N Alhalaiqa PhD); Department of Health Services and Hospital Administration (M K Al-Hanawi PhD), Health Economics Research Group (M K Al-

Hanawi PhD), Department of Oral Diagnostic Sciences (N Binmadi PhD), Pediatric Dentistry Department (Prof O A A Elmeligy PhD), Rabigh Faculty of Medicine (A A Malik PhD), Department of Dental Public Health (Z S Natto DrPH), Department of Community Medicine (S Samargandy PhD), King Abdulaziz University, Jeddah, Saudi Arabia; Department of Zoology (A Ali PhD), Department of Botany (Prof I Khan PhD), Abdul Wali Khan University Mardan, Mardan, Pakistan; School of Agriculture, Food and Ecosystem Sciences (A Ali PhD), Department of Paediatrics (Prof S M Sawyer MD), University of Melbourne, Parkville, VIC, Australia; Erbil Technical Health College (B A Ali PhD), Erbil Polytechnic University, Erbil, Iraq; School of Pharmacy (B A Ali PhD), Tishk International University, Erbil, Iraq; Department of Internal Medicine (H Ali MD), Brody School of Medicine, Greenville, NC, USA; Department of Medical Rehabilitation (Physiotherapy) (M U Ali MSc), University of Maiduguri, Maiduguri, Nigeria; Department of Biosciences (R Ali MPhil), Centre For Interdisciplinary Research In Basic Sciences (CIRBSc) (M A Shamsi PhD), Jamia Millia Islamia, New Delhi, India; Center for Biotechnology and Microbiology (S S Ali PhD), University of Swat, Swat, Pakistan; Department of Pharmacy (Z Ali PharmD), University of Peshawar, Peshawar, Pakistan; Department of Food Hygiene (S Alian Samakkhah PhD), Amol University of Special Modern Technologies, Amol, Iran; Department of Pathophysiology and Transplantation (G Alicandro PhD), Università degli Studi di Milano (University of Milan), Milan, Italy; Cystic Fibrosis Center (G Alicandro PhD), Fondazione IRCCS Ospedale Maggiore Policlinico (IRCCS "Ca' Granda Maggiore Policlinico" Hospital Foundation), Milan, Italy; School of Public Health and Preventive Medicine (S M Alif PhD, P Maharjan MPH, P Rai MPH), Department of Human Centered Computing (M Hasan MSc), Eastern Health Clinical School (R Thapa PhD), Department of Medicine (Prof A G Thrift PhD), Monash University, Melbourne, VIC, Australia; Department of Public Health (M Aligol PhD), Qom University of Medical Sciences, Qom, Iran; Department of Epidemiology and Biostatistics (R Alimi PhD), Torbat Heydariyeh University of Medical Sciences, Torbat Heydariyeh, Iran; Department of Public Health (A A Aliyi MPH, A Jema MPH), Madda Walabu University, Goba, Ethiopia; School of Physics, Mathematics and Computing (Prof A Al-Jumaily PhD), Centre for Neuromuscular and Neurological Disorders (Prof G J Hankey MD), Dental School (O Kujan PhD), The University of Western Australia, Perth, WA, Australia; Information and Communication Sciences and Technologies Pole, Mathematics, Algorithms and Decision Team (Prof A Al-Jumaily PhD), STIC (Prof A Mansour PhD), ENSTA Bretagne, Brest, France; Department of Health Policy and Management (Prof S M Aljunid PhD), Department of Surgery (S K Al-Sabah MD), Kuwait University, Kuwait, Kuwait; International Centre for Casemix and Clinical Coding (Prof S M Aljunid PhD), National University of Malaysia, Bandar Tun Razak, Malaysia; Department of Dentistry (S Al-Marwani MSc), Independent Consultant, Sana'a, Yemen; Public Health and Community Medicine (S Al-Marwani MSc), Independent Consultant, Irbid, Jordan; Department of Medicine (J U Almazan PhD, Prof D Poddighe PhD), Nazarbayev University, Astana, Kazakhstan; Department of Parasitology (Prof H M Al-Mekhlafi PhD), Department of Paediatrics (Prof H Ariffin MD), University of Malaya Medical Centre (Prof H Ariffin MD), Department of Medicine (L Lim MRCP), University of Malaya, Kuala Lumpur, Malaysia; Department of Parasitology (Prof H M Al-Mekhlafi PhD), Sana'a University, Sana'a, Yemen; Nuffield Department of Surgical Sciences (O Almidani MSc, S Bandyopadhyay BA), Nuffield Department of Medicine (B Basnyat MD, Prof R J Maude PhD), Nuffield Department of Population Health (D A Bennett PhD), Big Data Institute (A J Browne MPH), Oxford Centre for Global Health Research (C Dolecek PhD), Centre for Tropical Medicine and Global Health (S J Dunachie PhD), Department of Psychiatry (Prof C R J Newton MD), Health Economics Research Centre (Prof J A B Rodriguez PhD), University of Oxford, Oxford, UK; Department of Rehabilitation Sciences and Physical Therapy (Prof M A Alomari PhD), Department of Clinical Pharmacy (Prof K H Alzoubi PhD), Department of Public health (Prof K A Kheirallah PhD), Jordan

University of Science and Technology, Irbid, Jordan; Department of Surgery (N Alonso MD), Department of Internal Medicine (I M Bensenor PhD, Prof A R Brunoni PhD), Department of Psychiatry (Prof A R Brunoni PhD, Prof J Castaldelli-Maia PhD, Prof M F P Peres MD, Y Wang PhD), University of São Paulo, São Paulo, Brazil; Department of Respiratory Care (J S Alqahtani PhD), Prince Sultan Military College of Health Sciences, Dammam, Saudi Arabia; Department of Prosthodontics and Implant Dentistry (A Alqutaibi PhD), Taibah University, Medinah, Saudi Arabia; Department of Prosthodontics (A Alqutaibi PhD), Ibb University, Ibb, Yemen; Jaber Al Ahmad Al Sabah Hospital (S K Al-Sabah MD), Ministry of Health, Kuwait, Kuwait; Institute of Molecular Biology and Biotechnology (A Altaf PhD), University Institute of Public Health (F J Alvi MPH, A A Malik PhD, S Nargus PhD, S Nargus PhD), University College of Medicine & Dentistry (Prof M Arooj PhD), Radiological Sciences and Medical Imaging Technology (M Latif PhD), Institute of Molecular Biology and Biotechnology (IMBB) (S Shahid PhD), Research Centre for Health Sciences (RCHS) (S Shahid PhD), Department of Physics (W Shahid PhD), The University of Lahore, Lahore, Pakistan (Prof M Ashraf PhD, M A Riaz Mcom); Department of Specialty Internal Medicine (Prof J A Al-Tawfiq MD), Johns Hopkins Aramco Healthcare, Dhahran, Saudi Arabia; Medicine Department (Prof J A Al-Tawfiq MD), Indiana University School of Medicine, Indianapolis, IN, USA; Department of Clinical Pharmacology and Toxicology (H Alwafi PhD), Institute of Center and Research Studies (F Rehman PhD), Umm Al-Qura University, Makkah, Saudi Arabia; Department of Medical Sciences (Prof Y M Al-Worafi PhD), Azal University for Human Development, Sana'a, Yemen; Department of Clinical Sciences (Prof Y M Al-Worafi PhD), University of Science and Technology of Fujairah, Fujairah, United Arab Emirates; Department of Pediatrics (Prof H Aly MD), Heart, Vascular, and Thoracic Institute (A Hajj Ali MD), Cleveland Clinic, Cleveland, OH, USA; School of Medicine (A Amare PhD), Adelaide Medical School (T K Gill PhD, L Yadav PhD), Centre for Heart Rhythm Disorders (J Noubiap MD), Joanna Briggs Institute (J Opio MPH), University of Adelaide, Adelaide, SA, Australia; College of Medicine and Health Science (A Amare PhD), Department of Reproductive Health and Population Studies (E W Mengesha MPH), Bahir Dar University, Bahir Dar, Ethiopia; School of Graduate Studies (E K Ameyaw MPhil), Lingnan University, Hong Kong, China; Department of Public Health (A Amhare MSc), Salale University, Fitche, Ethiopia; School of Public Health (A Amhare MSc), The Center for Drug Safety and Policy Research (S Lin MS), Xi'an Jiaotong University, Xi'an, China; Public Heath Department (Prof T T Amin MD), Neurophysiology Department (Prof H R Elhabashy MD), Department of Neurology (A Hassan MD), Cairo University, Cairo, Egypt; Applied Science and Technology (J Aminian Dehkordi PhD), Department of Bioengineering (H Kabir MSc), University of California Berkeley, Berkeley, CA, USA; Chemical Engineering Department (J Aminian Dehkordi PhD), Tarbiat Modares University, Tehran, Iran; Medicine, Quran and Hadith Research Center (S Amiri PhD), Baqiyatallah University of Medical Sciences, Tehran, Iran; Department of Maternal and Child Wellbeing (D A Amugsi PhD), Department of Population Dynamics and Sexual and Reproductive Health (Y D Wado PhD), African Population and Health Research Center, Nairobi, Kenya; Department of Sociology (Prof J Amzat PhD), Department of Veterinary Microbiology (M B Bello PhD), Department of Veterinary Public Health and Preventive Medicine (A Shittu MSc), Usmanu Danfodiyo University, Sokoto, Sokoto, Nigeria; Department of Sociology (Prof J Amzat PhD), Electrical and Electronics Engineering Science (I T Aruleba MSc), University of Johannesburg, Johannesburg, South Africa; Faculty of Pharmacy (Prof R Ancuceanu PhD), Cardiology Department (C Andrei PhD), Department of General Surgery (B T Florin PhD, I Negoi PhD, D Serban PhD), Internal Medicine Department (M Hostiuc PhD), Department of Legal Medicine and Bioethics (S Hostiuc PhD), Department of Dermatology (C N Matei PhD), Department of Anatomy and Embryology (R I Negoi PhD), Carol Davila University of Medicine and Pharmacy, Bucharest, Romania; Centre for Sensorimotor Performance (D

Anderlini MD), Center of Research Excellence in Stillbirth (T Begum MPH), Department of Urology (Prof E Chung MD), Institute for Social Science Research (E Kanmiki MPH, J C Maravilla PhD), School of Health and Rehabilitation Sciences (A Khan PhD, M Moni PhD), School of Dentistry (R Lalloo PhD), Queensland Brain Institute (Prof J J McGrath MD), School of Public Health (Prof L J Veerman PhD), The University of Queensland, Brisbane, QLD, Australia; Neurology Department (D Anderlini MD), Royal Brisbane and Women's Hospital, Brisbane, QLD, Australia; Department of Health Care Management (P P Andrade MD, Prof R Busse PhD, S Mohammed PhD), Technical University of Berlin, Berlin, Germany; European University, Lisbon, Portugal (P P Andrade MD); Department of Statistics and Econometrics (Prof T Andrei PhD, Prof C Herteliu PhD, A Mirica PhD, A Otoiu PhD, I Petcu PhD), Management Department (A Dima PhD, S Stefan PhD), Bucharest University of Economic Studies, Bucharest, Romania; Department of Child Neurology (D Angappan MD), Department of Radiation Medicine (A Bleyer MD), Oregon Health and Science University, Portland, OR, USA; Department of Pharmacology (A Anil MD, J Charan MD, M Shamim MBBS, S Singh MD, S B Varthya MD), Department of Community Medicine and Family Medicine (P Baskaran MD, P Bhardwaj MD), Department of Anatomy (Prof N Bhardwaj MD, Prof S G Dixit MD, H Krishna MD, A K Nayyar MD), School of Public Health (P Bhardwaj MD), Department of Cardiology (R Choudhary MD), Department of Physiology (Prof A Dixit MD), Department of Surgical Oncology (Prof S Misra MCh), Department of Endocrinology & Metabolism (Prof M Mittal MD), Department of Pharmacology and Research (A Saravanan MD), Department of Urology and Kidney Transplant (M Singh MCh Urology), All India Institute of Medical Sciences, Jodhpur, India; Department of Urology (P Ram MS), All India Institute of Medical Sciences, Bhubaneswar, India (A Anil MD); Department of Psychiatry (A Anjum BHlthSci), University of Cambridge, Cambridge, UK; Agribusiness Study Program (E Antriyandarti DrAgrSc), Sebelas Maret University, Surakarta, Indonesia; Department of Surgery (S Anwar PhD), Gadjah Mada University, Yogyakarta, Indonesia; School of Dentistry and Medical Sciences (A E Anyasodor PhD), Charles Sturt University, Orange, NSW, Australia; Department of Sociology and Social Work (S Appiah PhD, M S Boampong PhD), Kwame Nkrumah University of Science and Technology, Kumasi, Ghana; Center for International Health (S Appiah PhD), Ludwig Maximilians University, Munich, Germany; Department of Psychology (M Aqeel PhD, M Aqeel PhD), Foundation University Islamabad, Rawalpandi, Pakistan; Health Management and Economics Research Center (J Arabloo PhD), School of Medicine (S Eghdami MD, P Panahi MD), Minimally Invasive Surgery Research Center (A Kabir MD, S Salahi MD), Endocrine Research Center (A Karimi Behnagh MD), Department of Echocardiography (A Karimi Behnagh MD), Eye Research Center (H Kasraei MD), Neuro Musculoskeletal Research Center (M Khadembashiri MD), Educational Development Center (E Khodadoust MD), Department of Anesthesiology (K Latifinaibin MD), Gastrointestinal and Liver Diseases Research Center (M Moradi-Lakeh MD), Preventive Medicine and Public Health Research Center (M Moradi-Lakeh MD), Department of Physiology (H Pazoki Toroudi PhD), Physiology Research Center (H Pazoki Toroudi PhD), Colorectal Research Center (A Sarveazad PhD), Department of Medical Biotechnology (M Zahedi MSc), Student Research Committee (M Zahedi MSc), Iran University of Medical Sciences, Tehran, Iran (M M Khadembashiri MD, M Moradi MD); Social Determinants of Health Research Center (M Arab-Zozani PhD), Faculty of Medicine (A Moodi Ghalibaf MD, A Rajabpour-Sanati MD), Birjand University of Medical Sciences, Birjand, Iran; College of Pharmacy (M Arafat PhD), Al Ain University, Abu Dhabi, United Arab Emirates; Associated Laboratory for Green Chemistry (LAQV) (A M Araújo PhD), Biological Sciences Department (L Belo PhD), Research Unit on Applied Molecular Biosciences (UCIBIO) (L Belo PhD, Prof F Carvalho PhD, V M Costa PhD, Prof D Dias da Silva PhD, J P Silva PhD), LAQV-REQUIMTE (M Carvalho PhD), Institute for Research and Innovation in Health (Prof N Cruz-Martins PhD), Department of

Community Medicine, Information and Health Decision Sciences (A Freitas PhD, R J Vieira MD), Department of Chemistry (M Pinheiro PhD), Epidemiology Research Unit (EPIUnit) (A Ribeiro PhD), University of Porto, Porto, Portugal; Department of Veterinary Pharmacology and Toxicology (A Aremu PhD), Department of Veterinary Public Health and Preventive Medicine (I A Odetokun PhD), University of Ilorin, Ilorin, Nigeria; Public Health and Healthcare Management (T Aripov PhD), Tashkent Institute of Postgraduate Medical Education, Tashkent, Uzbekistan; Boston Children's Hospital, Boston, MA, USA (T Aripov PhD); Department of Cardiovascular, Endocrine-metabolic Diseases and Aging (B Armocida MSc, B Unim PhD), Department of Infectious Diseases (M Sabbatucci PhD), National Institute of Health, Rome, Italy; Division of Tropical and Humanitarian Medicine (B Armocida MSc), University of Geneva, Geneva, Switzerland; Department of Biophysics (A A Artamonov PhD), Russian Academy of Sciences, Moscow, Russia; Department of Epidemiology (K D Artanti MSc), Department of Community Health Nursing (F Efendi PhD), Department of Biology (Prof H Purnobasuki PhD), Department of Health Policy and Administration (R D Wulandari DrPH), Universitas Airlangga (Airlangga University), Surabaya, Indonesia; Department of Maternal and Child Health (J Arulappan DSc), Sultan Qaboos University, Muscat, Oman; Department of Community Medicine and Rehabilitation (A Arumugam PhD), Department of Epidemiology and Global Health (M P Chavula MPH), Department of Nursing (Prof D Edvardsson PhD), Section of Sustainable Health (H Orru PhD), Umeå University, Umea, Sweden; National Agency for Strategic Research in Medical Education (NASRME) (Prof S Asgary MSc), Centre for Primary Health Care Network Management (H Fattahi PhD), Ministry of Health and Medical Education, Tehran, Iran; Department of Public Health (M Y Ashemo PhD), Wachemo University, Hossana, Ethiopia; Department of Medical Laboratory Sciences (M O Asika BMLS), Department of Pharmacology and Therapeutics (Prof O E Onwujekwe PhD), University of Nigeria Nsukka, Enugu, Nigeria; Telemedicine Department (M O Asika BMLS), Society For Disease Prevention, Inc., Hummelstown, PA, USA; Faculty of Nursing (M M W Atout PhD), Philadelphia University, Amman, Jordan; Department of Forensic Medicine (A Atreya MD), Lumbini Medical College, Palpa, Nepal; Department of Oral and Maxillofacial Surgery (S Attia MSc), Justus Liebig University of Giessen, Giessen, Germany; Northumbria HealthCare NHS Foundation Trust, Newcastle upon Tyne, UK (A Aujayeb MBBS); Robarts Research Institute (A Avan MD), Clinical Neurological Sciences (Prof V Hachinski MD), Department of Epidemiology & Biostatistics (Prof S Stranges MD), The University of Western Ontario, London, ON, Canada; School of Nursing and Public Health (A W Awotidebe PhD), Discipline of Public Health Medicine (O A Bolarinwa MSc, T G Ginindza PhD, R E Ogunsakin PhD), University of KwaZulu-Natal, Durban, South Africa; The Judith Lumley Centre (B Ayala Quintanilla PhD), School of Nursing and Midwifery (Prof D Edvardsson PhD, F Efendi PhD, M Rahman PhD), Department of Public Health (H Jiang PhD), La Trobe University, Melbourne, VIC, Australia; San Martin de Porres University, Lima, Peru (B Ayala Quintanilla PhD); Department of Health Economics (M A Ayanore PhD), Centre for Health Policy Advocacy Innovation & Research in Africa (CHPAIR-Africa), Accra, Ghana; Department of Psychiatry (Prof J L Ayuso-Mateos PhD), Department of Medicine (Prof A Ortiz MD), Hospital Universitario de La Princesa (Princess University Hospital) (Prof J B Soriano MD), Universidad Autónoma de Madrid (Autonomous University of Madrid), Madrid, Spain; Biomedical Research Networking Center for Mental Health Network (CIBERSAM) (Prof J L Ayuso-Mateos PhD), National School of Public Health (F Catalá-López PhD), Institute of Health Carlos III, Madrid, Spain; Badan Pusat Statistik (BPS) (Central Bureau of Statistics) (G S Azhar PhD), The RAND Corporation, Santa Monica, CA, USA; Institute of Biotechnology and Genetic Engineering (S Aziz MS), The University of Agriculture, Peshawar, Pakistan; Department of Neurovascular Research (A Y Azzam MBBCh), Nested Knowledge, Inc., Saint Paul, MN, USA; Faculty of Medicine (A Y Azzam MBBCh), October 6 University, 6th

of October City, Egypt; Nutrition Research Center (M Babashahi PhD), Health Human Resources Research Center (M Bayati PhD), Medical School (M Farjoud Kouhanjani MD), Epilepsy Research Center (M Farjoud Kouhanjani MD), Trauma Research Center (P Fazeli MSc, M Karajizadeh PhD), Department of Medical Immunology (P Fazeli MSc), Health Policy Research Center (S Ghahramani MD, H Kasraei MD, Y Sarikhani PhD, S Shahabi PhD), Basic Science Laboratory (F Iravanpour PhD), Shiraz Neuroscience Research Center (M Jafarinia PhD, R Tavakoli Oliaee PhD), Non-communicable Disease Research Center (Prof R Malekzadeh MD, S G Sepanlou MD), Department of Health Services Management (R Ravangard PhD), Cardiovascular Research Center (I Razeghian-Jahromi PhD), Department of Physical Medicine and Rehabilitation (S Roshanzamir MD), Burn and Wound Healing Research Center (S Roshanzamir MD), Shiraz University of Medical Sciences, Shiraz, Iran (Y Mansoori MD); Department of Physiotherapy (A S Babu PhD), Department of Forensic Medicine and Toxicology (S M Bakkannavar MD, Prof V C Nayak MD), Kasturba Medical College, Mangalore (R Holla MD, A Kamath MD, M Rao MD), Department of Pharmacy Management (V S Ligade PhD), Department of Community Medicine (C R Rao MD, R S Shetty MD), Department of Nephrology (I Rao DM), Department of Health Information Management (B Reshmi PhD), Manipal Academy of Higher Education, Manipal, India (B Reshmi PhD); Gomal Center of Biochemistry and Biotechnology (M Badar PhD), Gomal University, Dera Ismail Khan, Pakistan; Public Health Risk Sciences Division (A Badawi PhD), Public Health Agency of Canada, Toronto, ON, Canada; Department of Nutritional Sciences (A Badawi PhD), Centre for Global Child Health (Prof Z A Bhutta PhD), Temerty Faculty of Medicine (V Chattu MD), Division of Neurology (S Fereshtehnejad PhD), Department of Neurosurgery (A K Malhotra MD), Mechanical and Industrial Engineering (A Mostofinejad MSc), Institute of Health Policy, Management, and Evaluation (H Shakil MD), Division of Neurosurgery (H Shakil MD), University of Toronto, Toronto, ON, Canada; Department of Forensic Science (A D Badiye PhD, H Bansal MSc, N Kapoor PhD), Government Institute of Forensic Science, Nagpur, India; Division of Orthopaedics (S Baghdadi MD), Children's Hospital of Philadelphia, Philadelphia, PA, USA; Health Research Institute (N Bagheri PhD), University of Canberra, Canberra, ACT, Australia; School of Medicine (S Bagherieh BSc), Department of Environmental Health Engineering (A Fatehizadeh PhD), Cardiac Rehabilitation Research Center (K Mehrabani-Zeinabad PhD, Prof M Sadeghi MD), Department of Neurology (O Mirmosayyeb MD), Department of Medical Physics (K Saber PhD), Musculoskeletal Research Center (A Shafaat MS), Department of Radiology and Interventional Neuroradiology (O Shafaat MD), Isfahan University of Medical Sciences, Isfahan, Iran; NanoElectronics and Photonics Systems (MEPHOS) (S Bahadorikhalili PhD), Universitat Rovira i Virgili, Tarragona, Spain; Department of Epidemiology and Biostatistics (J Bai BA, Prof C Yu PhD), Wuhan University, Wuhan, China; School of Public Affairs (R Bai MD), Nanjing University of Science and Technology, Nanjing, China; Center for Clinical Research and Prevention (J L Baker PhD), Bispebjerg University Hospital, Frederiksberg, Denmark; Department of Neurosurgery (A T Bako PhD), Center for Health & Nature (O M Makram MD), Houston Methodist Hospital, Houston, TX, USA; Division of Biological Sciences (S Balakrishnan PhD), Tamil Nadu State Council for Science and Technology, Tamil Nadu, India; Menzies Institute for Medical Research (S A Balogun PhD, A Singh MTech, J Tian PhD), School of Pharmacy and Pharmacology (A Jatau PhD), University of Tasmania, Hobart, TAS, Australia; Center of Innovation, Technology and Education (CITE) (Prof O C Baltatu PhD), Center of Innovation, Technology and Education (CITE)/ Institute of Biomedical Engineering (Prof L A Campos PhD), Anhembi Morumbi University, Sao Jose dos Campos, Brazil; Department of Medicine (K Bam MPH, M T Olaiya PhD), School of Nursing and Midwifery (D Bhandari PhD), Stroke and Ageing Research, Victorian Heart Institute (L L Dalli PhD), Monash University, Clayton, VIC, Australia; Department of Hypertension (Prof M Banach PhD), Medical University of Lodz,

Lodz, Poland; Polish Mothers' Memorial Hospital Research Institute, Lodz, Poland (Prof M Banach PhD); Department of Neurosurgery (S Bandyopadhyay BA), University of Southampton, Southampton, UK; Institute of Health and Wellbeing (IHW) (B Banik PhD), Federation University Australia, Melbourne, VIC, Australia; Manna Institute (B Banik PhD), University of New England, Armidale, NSW, Australia; Department of Non-communicable Diseases (P C Banik MPhil), Bangladesh University of Health Sciences, Dhaka, Bangladesh; Department of Anatomy (S Barati PhD), Nursing and Midwifery Department (M Saeedi PhD), Saveh University of Medical Sciences, Saveh, Iran; Miami Cancer Institute (M Bardhan MD), Baptist Health South Florida, Miami, FL, USA; School of Psychology (Prof S L Barker-Collo PhD), School of Pharmacy (K A Beyene PhD), University of Auckland, Auckland, New Zealand; Department of Translational Medicine (F Barone-Adesi PhD), University of Eastern Piedmont, Novara, Italy; Department of Pediatrics (Prof R D Barr MD), Department of Medicine (O P Kurmi PhD), Population Health Research Institute (PHRI) (F Mannan MD), Department of Psychiatry and Behavioural Neurosciences (A T Olagunju MD), McMaster University, Hamilton, ON, Canada; Department of Industrial Engineering (Prof L H Barrero DSc), Pontifical Javeriana University, Bogota, Colombia; Alpha Genomics, Islamabad, Pakistan (Z Basharat PhD); Department of Pharmacology and Toxicology (A I J Bashir PhD), Kaduna State University, Kaduna, Nigeria; Department of Animal Sciences (H A Bashiru MSc), Department of Child Dental Health (Prof M O Folayan FWACS), Obafemi Awolowo University, Ile-Ife, Nigeria; Tuberculosis Department (B Basnyat MD), Birat Nepal Medical Trust, Kathmandu, Nepal; Barcelona Institute for Global Health (Prof Q Bassat MD), Universitat de Barcelona (University of Barcelona), Barcelona, Spain; Catalan Institution for Research and Advanced Studies (ICREA), Barcelona, Spain (Prof Q Bassat MD); Faculty of Pharmacy (J D Basso PharmD, S Silva MSc), Coimbra Chemistry Centre (J D Basso PharmD), Department of Geography and Demography (M Rodrigues PhD), Coimbra Institute for Biomedical Imaging and Translational Research (S Silva MSc), University of Coimbra, Coimbra, Portugal; Department of Academics (S Basu MD), Indian Institute of Public Health, Gurgaon, India; Department of Medical Education (K Batra PhD), School of Public Health (R Batra MS), Department of Social and Behavioral Health (Prof M Sharma PhD), University of Nevada Las Vegas, Las Vegas, NV, USA; IT Department (R Batra MS), Coforge, Georgia, GA, USA; Department of Psychiatry (Prof B T Baune PhD), University of Münster, Münster, Germany; Department of Psychiatry (Prof B T Baune PhD), Melbourne Medical School, Melbourne, VIC, Australia; School of Public Health (Prof N Bedi MD), Dr. D. Y. Patil University, Mumbai, India; Department of Epidemiology (K Y Ghailan PhD, M Khan MD), Department of Maxillofacial Surgery and Diagnostic Sciences (E S Halboub PhD), Department of Health Education and Promotion (M Shanawaz MD), Jazan University, Jazan, Saudi Arabia (Prof N Bedi MD); Health System and Population Studies Division (T Begum MPH), Maternal and Child Health Division (A Sayeed MSc, M Siraj MSc), International Centre for Diarrhoeal Disease Research, Bangladesh, Dhaka, Bangladesh; Department of Basic Sciences (E Behboudi PhD), Khoy University of Medical Sciences, Khoy, Iran; Endocrinology and Metabolism Research Institute (H Farrokhpour MD), Department of Epidemiology (M Heidari-Foroozan BSc, S Khanmohammadi MD, S Nejadghaderi MD, S Rashedi MD, H Sohrabi MD, H Soleimani MD), Non-Communicable Diseases Research Center (NCDRC), Tehran, Iran (A Behnoush BS, A Khalaji BS); Division of Pulmonary, Critical Care, and Sleep (M Beiranvand PhD), University of Florida, Jacksonville, FL, USA; Department of Medicine (D F Bejarano Ramirez BN), Faculty of Medicine (J N Malagón-Rojas MSc), El Bosque University, Bogota, Colombia; Transplant Service (D F Bejarano Ramirez BN), University Hospital Foundation Santa Fe de Bogotá, Bogota, Colombia; Department of Oral Pathology and Microbiology (U I Belgaumi MD), Krishna Vishwa Vidyapeeth Deemed to be University, Karad, India; Department of Medicine (A K Bello PhD), Department of Physics (O Ebenezer PhD), Department of Psychiatry (E Eboreime PhD), University

of Alberta, Edmonton, AB, Canada; Infectious Disease Research Department (M B Bello PhD), Medical Genomics Research Department (M Umair PhD), King Abdullah International Medical Research Center, Riyadh, Saudi Arabia; Department of Biomedical Sciences (Prof A Beloukas PhD), University of West Attica, Athens, Greece; Institute of Infection and Global Health (Prof A Beloukas PhD), Department of Surgery (Prof R Lunevicius DSc), University of Liverpool, Liverpool, UK; Department of Industrial Engineering (S Bendak PhD), Haliç University, Istanbul, Türkiye; Department of Epidemiology and Health Promotion (Prof H Benzian PhD), School of Global Public Health (S D Friedman BA, E K Peprah PhD), New York University, New York, NY, USA; Institute of Marketing (Z Berezvai PhD), Corvinus University of Budapest, Budapest, Hungary; Competition Economics and Market Research Section (Z Berezvai PhD), Hungarian Competition Authority, Budapest, Hungary; Department of Medicine (A E Berman MD), Medical College of Georgia at Augusta University, Augusta, GA, USA; Department of Epidemiology and Biostatistics (A C Bermudez MD), National Institutes of Health (A Loreche BS), University of the Philippines Manila, Manila, Philippines; Department of Epidemiology (A C Bermudez MD, S Liu MD), Department of Internal Medicine (M F H Mohamed MSc), Brown University, Providence, RI, USA; Faculty of Medicine (P J G Bettencourt PhD), Catholic University of Portugal, Rio de Mouro, Portugal; Metabolomics Laboratory (H B Beyene PhD), Baker Heart and Diabetes Institute, Melbourne, VIC, Australia; Department of Pharmaceutical and Administrative Sciences (K A Beyene PhD), University of Health Sciences and Pharmacy in St. Louis, St Louis, MO, USA; Department of Forensic Chemistry (D S Bhagat PhD), Government Institute of Forensic Science, Aurangabad, Aurangabad, India; Department of Public Health (A S Bhagavathula PhD), North Dakota State University, Fargo, ND, USA; Institutes of Applied Health Research and Translational Medicine (N Bhala PhD), Queen Elizabeth Hospital Birmingham, Birmingham, UK; Institute of Applied Health Research (N Bhala PhD), School of Geography, Earth and Environmental Sciences (R Dehbandi PhD), NIHR Global Health Research Unit on Global Surgery (J C Glasbey MSc), University of Birmingham, Birmingham, UK; Department of Internal Medicine (Prof A Bhalla MD), Post Graduate Institute of Medical Education and Research, Chandigarh, India; Public Health Research Laboratory (D Bhandari PhD), Department of Biotechnology, National College (B P Marasini PhD), Faculty of Humanities and Social Sciences (U Paudel PhD), Central Department of Public Health (L Poudel MPH), Department of Community Medicine (P M S Pradhan MD), Tribhuvan University, Kathmandu, Nepal; Department of Hematology Oncology (P V Bhardwaj MD), University of Massachusetts Medical School, Springfield, MA, USA; Department of Internal Medicine (A Bhargava MD, F Kahe MD), Wayne State University, Detroit, MI, USA; Global Health Neurology Lab (S Bhaskar PhD), NSW Brain Clot Bank, Sydney, NSW, Australia; Department of Neurology and Neurophysiology (S Bhaskar PhD), South West Sydney Local Heath District and Liverpool Hospital, Sydney, NSW, Australia; Department of Internal Medicine (V Bhat MBBS), St. John's National Academy of Health Sciences, Bangalore, India; Medical Lab Technology (G K Bhatti PhD), University Centre for Research and Development (S Kalra DM), Chandigarh University, Mohali, India; Department of Human Genetics and Molecular Medicine (Prof J S Bhatti PhD, S Senapati PhD), Central University of Punjab, Bathinda, India; Department of Botanical and Environmental Sciences (Prof M S Bhatti PhD), Department of Pharmaceutical Sciences (R Bhatti PhD), Guru Nanak Dev University, Amritsar, India; Centre of Excellence in Women & Child Health (Prof Z A Bhutta PhD), Division of Women and Child Health (J K Das MD), Aga Khan University, Karachi, Pakistan; Scientific-Tools.org, Bergamo, Italy (B Bikbov MD); Department of Health Behaviour, Environment and Social Medicine (B Bintoro MD), Center of Health and Behavior and Promotion (B Bintoro MD), Universitas Gadjah Mada (Gadjah Mada University), Sleman, Indonesia; Department of Biomedical and NeuroMotor Sciences (Prof F Bisulli PhD),

Department of Biomedical and Neuromotor Sciences (D Golinelli MD, S Guicciardi MD, J Lenzi PhD, A Mazzotti PhD, L Muccioli MD, F Sanmarchi MD), Department of Medical and Surgical Sciences (Prof F S Violante MD), University of Bologna, Bologna, Italy; UOC Clinica Neurologica (Prof F Bisulli PhD), IRCCS Istituto delle Scienze Neurologiche di Bologna (Institute of Neurological Sciences of Bologna), Bologna, Italy; Department of Neurology (Prof A Biswas DM), Department of GI Surgery (A Dhali MBBS), Institute of Post-Graduate Medical Education and Research and Seth Sukhlal Karnani Memorial Hospital, Kolkata, India; Charles Perkins Centre (R Biswas PhD), Institute of Bone and Joint Research (L Chen MD), School of Pharmacy and Charles Perkins Centre (Z Dai PhD), School of Public Health (Prof T R Driscoll PhD, H K Tang PhD), Chapter of Addiction Medicine (Prof H Hassanian-Moghaddam MD), Sydney Medical School (S Islam PhD), Save Sight Institute (H Kandel PhD, Y You PhD), Asbestos Diseases Research Institute (J Leigh MD), School of Veterinary Science (B B Singh PhD), Menzies Centre for Health Policy (F Sitas PhD), The Matilda Centre for Research in Mental Health and Substance Use (R Visontay BA), University of Sydney, Sydney, NSW, Australia; Clinical Research Centre (R Biswas PhD), Sydney Local Health District, Sydney, NSW, Australia; Department of Biostatistics and Epidemiology (Prof S Bitaraf PhD), Environmental Technologies Research Center (R Dehbandi PhD, N Kaydi PhD), Department of Orthodontics (E Eini DDS), Department of Public Health (M A Khafaie PhD), Department of Pediatric Neurology (S Sadeghian MD), Ahvaz Jundishapur University of Medical Sciences, Ahvaz, Iran; Department of Global Public Health and Primary Care (Prof T Bjørge PhD, O Dadras DrPH), University of Bergen, Bergen, Norway; Department of Research (M W Wojewodzic PhD), Cancer Registry of Norway, Oslo, Norway (Prof T Bjørge PhD); School of Business Administration (Prof V Bodolica PhD), American University of Sharjah, Sharjah, United Arab Emirates; Faculty of Medicine and Pharmaceutical Sciences (A Bonny MD), University of Douala, Douala, Cameroon; Department of Cardiology (A Bonny MD), Centre Hospitalier Montfermeil (Montfermeil Hospital Center), Montfermeil, France; Regional Medical Research Centre, North East Region (K Bora MD), Indian Council of Medical Research, Dibrugarh, India; General Directorate of Health Information Systems (B Bora Basara PhD), Ministry of Health, Ankara, Türkiye; Centre for Adolescent Health (R Borschmann PhD, K L Francis MBiostat, J A Kerr PhD), Murdoch Childrens Research Institute, Melbourne, VIC, Australia; Facultad de Salud (Faculty of Health) (Prof A Botero Carvajal MSc), Universidad Santiago de Cali (Santiago de Cali University), Cali, Colombia; Facultad de Salud (Faculty of Health) (Prof A Botero Carvajal MSc), Fundación Universitaria San Martín (San Martin University), Cali, Colombia; Department of Medicine (Prof S Bouaoud MD), Faculty of Medicine (Prof A Ouyahia PhD), University Ferhat Abbas of Setif, Setif, Algeria; Department of Epidemiology and Preventive Medicine (Prof S Bouaoud MD), University Hospital Saadna Abdenour, Setif, Algeria; Faculty of Natural Sciences and Life Sciences (S Boudalia PhD), Guelma University, Guelma, Algeria; General Medicine Service (Prof E J Boyko MD), Department of Veterans Affairs, Seattle, WA, USA; Department of Health Sciences (DISSAL) (F Lanfranchi MD), University of Genoa, Genoa, Italy (N L Bragazzi PhD); Department of Epidemiology (D Braithwaite PhD, D D Ding BS), UF Health Cancer Center (S D Karanth PhD), Department of Computer and Information Science and Engineering (P Naghavi MSc), University of Florida, Gainesville, FL, USA; Cancer Population Sciences Program (D Braithwaite PhD), University of Florida Health Cancer Center, Gainesville, FL, USA; Division of Clinical Epidemiology and Aging Research (Prof H Brenner MD), German Cancer Research Center, Heidelberg, Germany; Department of Neuroscience (G Britton PhD), University of Panama, Ancon, Panama; Infectious Diseases Department (G Britton PhD), Gorgas Memorial Institute for Health Studies, Panama City, Panama; Flinders Health and Medical Research Institute (N B Bulamu PhD), College of Nursing and Health Sciences (L N Bulto PhD, K M Foley MPH), Caring Futures Institute (D Jemere MBA), Health Economics Unit (B Kaambwa PhD),

College of Medicine and Public Health (B Kaambwa PhD, G R Naik PhD), Health and Social Care Economics Group (C Mpundu-Kaambwa PhD), Flinders University, Adelaide, SA, Australia; Department of Woman and Child Health and Public Health (D Buonsenso MD), Fondazione Policlinico Universitario A. Gemelli IRCCS (Agostino Gemelli University Polyclinic IRCCS), Rome, Italy; Global Health Research Institute (D Buonsenso MD), Department of Health Science and Public Health (L Villani DrPH), Università Cattolica del Sacro Cuore (Catholic University of Sacred Heart), Rome, Italy; Department of Community Medicine (Prof S Burugina Nagaraja MD), Employee State Insurance Post Graduate Institute of Medical Sciences and Research, Bangalore, India; Department of Biopharmaceutics and Clinical Pharmacy (Y Bustanji PhD), The University of Jordan, Amman, Jordan; School of Public Health and Health Systems (Z A Butt PhD), University of Waterloo, Waterloo, ON, Canada; Al Shifa School of Public Health (Z A Butt PhD), Al Shifa Trust Eye Hospital, Rawalpindi, Pakistan; Department of Sociology (Prof T Cai PhD), University of Macau, Macau, China; Department of Clinical Pharmacy (Prof D Calina PhD), University of Medicine and Pharmacy of Craiova, Craiova, Romania; Internal Medicine Department (Prof L A Cámera MD), Hospital Italiano de Buenos Aires (Italian Hospital of Buenos Aires), Buenos Aires, Argentina; Board of Directors (Prof L A Cámera MD), Argentine Society of Medicine, Buenos Aires, Argentina; College of Health Sciences (Prof L A Campos PhD), Abu Dhabi University, Abu Dhabi, United Arab Emirates; Center for Nutrition and Health Research (I R Campos-Nonato PhD, E Denova-Gutiérrez DSc), Center for Health Systems Research (D V Ortega-Altamirano DrPH, M Rios-Blancas DSc), Infectious Disease Research Center (Prof V Pando-Robles PhD), National Institute of Public Health, Cuernavaca, Mexico; Dana-Farber Cancer Institute, Boston, MA, USA (C Cao MPH); Foundation for Research and Sciences (C A Cardenas MD), Independent Consultant, Los Angeles, CA, USA; Department of Health Care (Prof R Cárdenas DSc), Metropolitan Autonomous University, Mexico City, Mexico; Institute for Cancer Research, Prevention and Clinical Network, Florence, Italy (G Carreras PhD); Dermatology Unit (A Carugno MD), Territorial Healthcare Company Pope John XXIII (Azienda Socio Sanitaria Territoriale Papa Giovanni XXIII), Bergamo, Italy; Instituto de Investigação, Inovação e Desenvolvimento (Institute of Research Innovation and Development) (M Carvalho PhD), University Fernando Pessoa, Porto, Portugal; Colombian National Health Observatory (C A Castañeda-Orjuela MD), Department of Public Health Research (J N Malagón-Rojas MSc), National Institute of Health, Bogota, Colombia; Epidemiology and Public Health Evaluation Group (C A Castañeda-Orjuela MD), National University of Colombia, Bogota, Colombia; Department of Medicine (G Castelpietra PhD), University of Udine, Udine, Italy; Department of Mental Health (G Castelpietra PhD), Healthcare Agency "Friuli Occidentale", Pordenone, Italy; Clinical Epidemiology Program (F Catalá-López PhD), Ottawa Hospital Research Institute, Ottawa, ON, Canada; Department of Pharmacological and Biomolecular Sciences (Prof A L Catapano PhD), IRCCS Istituto Ortopedico Galeazzi (Galeazzi Orthopedic Institute IRCCS) (G Damiani MD), Department of Clinical Sciences and Community Health (Prof C La Vecchia MD), University of Milan, Milan, Italy; MultiMedica (Prof A L Catapano PhD), IRCCS, Sesto S. Giovanni, Italy; Department of Psychiatry (A Caye PhD), Postgraduate Program in Epidemiology (Prof B B Duncan MD, Prof M I Schmidt MD), Department of Preventive and Social Dentistry (F N Hugo PhD), Federal University of Rio Grande do Sul, Porto Alegre, Brazil; Department of Otolaryngology, Head and Neck Surgery (C R Cederroth PhD), University of Tübingen, Tübingen, Germany; Department of Nutrition (Prof F Cembranel DSc), Federal University of Santa Catarina, Florianópolis, Brazil; College of Public Health, Medical and Veterinary Sciences (M Cenderadewi MPHTM), Department of Public Health and Tropical Medicine (T I Emeto PhD), College of Public Health, Medical, and Veterinary Sciences (A E Peden PhD), James Cook University, Townsville, QLD, Australia (K O Obamiro PhD); Public Health Department (M Cenderadewi MPHTM), University of Mataram,

Mataram, Indonesia; Mary MacKillop Institute for Health Research (Prof E Cerin PhD), Faculty of Health Sciences (G R Poudel PhD), Australian Catholic University, Melbourne, VIC, Australia; School of Public Health (Prof E Cerin PhD), Department of Urban Planning and Design (C Guo PhD, C Sarkar PhD), Centre for Suicide Research and Prevention (Prof P Yip PhD), Department of Social Work and Social Administration (Prof P Yip PhD), University of Hong Kong, Hong Kong, China; Infection and Global Health Research (M Cevik MD), University of St Andrews, St Andrews, UK; Regional Infectious Diseases Unit (M Cevik MD), NHS National Services Scotland, Edinburgh, UK; Carolina Health Informatics Program (P R Chacón-Uscamaita DDS), University of North Caroline Chapel Hill, Chapel Hill, NC, USA; Department of Public Health, Administration, and Social Sciences (J L Chirinos-Caceres DrPH), Cayetano Heredia University, Lima, Peru; Department of Biotechnology (Prof C Chakraborty PhD), Adamas University, Kolkata, India; Skeletal Aging & Orthopedic Surgery (Prof C Chakraborty PhD), Hallym University, Chuncheon, South Korea; Heart Failure and Structural Heart Disease Unit (J Chan MBChB), Cardiovascular Analytics Group, Hong Kong, China; Institute of Epidemiology and Preventive Medicine (C Chang PhD), National Taiwan University, Taipei City, Taiwan; Department of Psychological Medicine (C Chang PhD), School of Population Health and Environmental Sciences (A Douiri PhD, H A Wafa MPH, Y Wang PhD), School of Life Course & Population Sciences (Y Hbid PhD), Department of Twin Research and Genetic Epidemiology (M Mazidi PhD), Institute of Psychiatry, Psychology & Neuroscience (D Urso MD), King's College London, London, UK; Department of Public Health (P Charalampous PhD), Department of Neurosurgery (V Volovici PhD), Erasmus University Medical Center, Rotterdam, Netherlands; Department of Community Medicine (V Chattu MD), Datta Meghe Institute of Medical Sciences, Sawangi, India; Department of Endocrinology (V Chatzimavridou-Grigoriadou MD), Department of Mathematics (O Johnson PhD), Department of Cardiovascular Science (F Mannan MD), Division of Immunology, Immunity to Infection and Respiratory Medicine (A G Mathioudakis PhD), Division of Psychology and Mental Health (F Mughal FRCGP), University of Manchester, Manchester, UK; Department of Endocrinology (V Chatzimavridou-Grigoriadou MD), Christie Hospital NHS Foundation Trust, Manchester, UK; School of Public Health (M P Chavula MPH), University of Zambia, Lusaka, Zambia; Department of Medicine (H A Cheema MB), Department of Community Medicine and Public Health (H A Cheema MB), King Edward Medical University, Lahore, Pakistan; Fuwai Hospital (A Chen PhD), Chinese Academy of Medical Sciences & Peking Union Medical College, Beijing, China; Department of Computer Science (A Chen PhD), University of Texas Austin, Austin, TX, USA; Clinical Research Center (H Chen MB), Zhujiang Hospital (Z Zhu PhD), Southern Medical University, Guangzhou, China; Department of Cardiac Sciences (D S Chew MD), Department of Medicine (Prof M Tonelli MD), Department of Oncology (L Yang PhD), University of Calgary, Calgary, AB, Canada; Iraq Field Epidemiology Training Program (I-FETP) (A Chitheer MD), Ministry of Health, Baghdad, Iraq; Program in Medical and Population Genetics (S J Cho PhD), Broad Institute of MIT and Harvard, Cambridge, MA, USA; Cardiovascular Research Center (S J Cho PhD), Department of Orthopaedic Surgery (A Ebrahimi MD), Division of Cardiology (I Y Elgendy MD, D H Nguyen BS), Department of Radiology (X Liu PhD), Massachusetts General Hospital, Boston, MA, USA; Department of Clinical Oncology (W C S Cho PhD), Queen Elizabeth Hospital, Hong Kong, China; Department of Medicine (B Chong MBBS), Saw Swee Hock School of Public Health (S Ma PhD), Leadership Institute for Global Health Transformation (LIGHT) (S Ramazanu PhD), Department of Surgery (K Tan PhD), Yong Loo Lin School of Medicine (Prof N Venketasubramanian MBBS), National University of Singapore, Singapore, Singapore; Department of Biosciences (H Chopra PhD), Department of Public Health Dentistry (Prof G Mini PhD), Saveetha Dental College and Hospitals (M R Tovani-Palone PhD), Saveetha Institute of Medical and Technical Sciences,

Chennai, India; Department of Epidemiology (Prof R Chowdhury PhD), Department of Emergency Medicine (I Pantazopoulos PhD), Department of Cardiology (T Pilgrim MD, S Stortecky MD), University of Bern, Bern, Switzerland; Center for Biomedicine and Community Health (D Chu PhD), VNU-International School, Hanoi, Viet Nam; Department of Paediatric Surgery (I S Chukwu BMedSc), Federal Medical Centre, Umuahia, Nigeria; Department of AndroUrology (Prof E Chung MD), AndroUrology Centre, Brisbane, QLD, Australia; Health Data Research UK, London, UK (S Chung PhD); Centre for Adolescent Health (K I Cini MClinEpi, Prof S M Sawyer MD), Murdoch Childrens Research Institute, Parkville, VIC, Australia; Global Adolescent Health Group (K I Cini MClinEpi), Burnet Institute, Melbourne, VIC, Australia; College of Life Sciences (C C T Clark PhD), Birmingham City University, Birmingham, UK; Nova Medical School (J Conde PhD), Nova University of Lisbon, Lisbon, Portugal; School of Medicine and Surgery (S Conti PhD, P A Cortesi PhD, C Fornari PhD, Prof L G Mantovani DSc), Research Center on Public Health (P Ferrara MD), University of Milan Bicocca, Monza, Italy; Department of Psychology (R G Cowden PhD), University of the Free State, Park West, South Africa; Department of Family Medicine and Public Health (Prof M H Criqui MD), University of California San Diego, La Jolla, CA, USA; Therapeutic and Diagnostic Technologies (Prof N Cruz-Martins PhD), Cooperativa de Ensino Superior Politécnico e Universitário (Polytechnic and University Higher Education Cooperative), Gandra, Portugal; School of Population Health (P Cullen PhD), Centre for Healthy Brain Ageing (R Visontay BA), University of New South Wales, Kensington, NSW, Australia; Global Women's Health Program (P Cullen PhD), The George Institute for Global Health, Newtown, NSW, Australia; Department of Neuroscience (D da Silva e Silva PhD), Department of Psychiatry (Prof D C Des Jarlais PhD, S Gunturu MD), Icahn School of Medicine at Mount Sinai, New York, NY, USA; Department of Internal Medicine (S Dadana MD), Cheyenne Regional Medical Center, Cheyenne, WY, USA; Department of Addiction Medicine (O Dadras DrPH), Haukland University Hospital, Bergen, Norway; Division of Public Health Science (Prof K Dalal PhD), Mid Sweden University, Sundsvall, Sweden; Higher School of Public Health (Prof K Dalal PhD), Department of Clinical Subjects (A Kurmanova MD), Al Farabi Kazakh National University, Almaty, Kazakhstan; Department of Dermatology (G Damiani MD), Lerner College of Medicine (L Göbölös PhD), Harrington Heart and Vascular Institute (A Guha MD), Department of Neonatology (I Qattea MD), Department of Nutrition and Preventive Medicine (Prof J Sanabria MD), Case Western Reserve University, Cleveland, OH, USA; Department of Information Technology (A M Darwesh PhD), Department of Computer Science (Prof M Hosseinzadeh PhD), University of Human Development, Sulaymaniyah, Iraq; Department of Biochemistry (S Das MD), Ministry of Health and Welfare, New Delhi, India; Department of Population and Development (C A Dávila-Cervantes PhD), Latin American Faculty of Social Sciences Mexico, Mexico City, Mexico; Health Research Institute (K Davletov PhD), Asfendiyarov Kazakh National Medical University, Almaty, Kazakhstan; Australian Institute for Suicide Research and Prevention (Prof D De Leo DSc), Griffith University, Mount Gravatt, QLD, Australia; School of Medicine (I Delgado-Enciso DSc), University of Colima, Colima, Mexico; Department of Research (I Delgado-Enciso DSc), Colima State Health Services, Colima, Mexico; NCDs and Environment Programme (L Delgado-Ortiz MSc), ISGlobal Instituto de Salud Global de Barcelona (Barcelona Institute for Global Health), Barcelona, Spain; Department of Experimental and Health Sciences (L Delgado-Ortiz MSc), Pompeu Fabra University, Barcelona, Spain; School of Public Health and Social Work (D Demant PhD), International Laboratory for Air Quality and Health (Prof L Morawska PhD), Queensland University of Technology, Brisbane, QLD, Australia; USAID-JSI (B H Demessa MPH), Jimma University, Addis Ababa, Ethiopia; Department of Neurosurgery (A K Demetriades MD), Postgraduate School (U A Eze MD), Centre for Medical Informatics (Prof A Sheikh MD), Usher Institute (Prof C R Simpson PhD), College of Medicine and Veterinary

Medicine (G Verras MD), University of Edinburgh, Edinburgh, UK; Department of Neurosurgery (A K Demetriades MD), National Health Service (NHS) Scotland, Edinburgh, UK; Epidemiology Branch (X Deng PhD), National Institute of Health, Durham, NC, USA; Wellcome Trust Brighton and Sussex Centre for Global Health Research (K Deribe PhD), Brighton and Sussex Medical School, Brighton, UK; St Paul's Eye Unit (N Dervenis MD), Royal Liverpool University Hospital, Liverpool, UK; Department of Ophthalmology (N Dervenis MD), Second Department of Cardiology (D Patoulias PhD), Aristotle University of Thessaloniki, Thessaloniki, Greece; Graduate Medical Education (H D Desai MD), Gujarat Adani Institute of Medical Sciences, Bhuj, India; Division of Cardiology (R Desai MBBS), Atlanta Veterans Affairs Medical Center, Decatur, GA, USA; National Centre for AIDS and STD Control (K Deuba DrPH), Save the Children, Kathmandu, Nepal; Department of Community Medicine (V G C Devanbu MD), Chettinad Academy of Research and Education, Chennai, India; Department of Biostatistics and Epidemiology (S Dey Mphil), Department of Development Studies (Prof A Perianayagam PhD), International Institute for Population Sciences, Mumbai, India; Division of Pathology (K Dhama PhD), ICAR-Indian Veterinary Research Institute, Bareilly, India; Research Department (C L Ranabhat PhD), Policy Research Institute, Kathmandu, Nepal (M L Dhimal PhD); Global Institute for Interdisciplinary Studies, Kathmandu, Nepal (M L Dhimal PhD); Research Department (M Dhimal PhD, S Ghimire MPH, B P Marasini PhD, A Pandey MPH, U Paudel PhD), Nepal Health Research Council, Kathmandu, Nepal; Department of Pharmacy Practice (S Dhingra PhD), National Institute of Pharmaceutical Education and Research, Hajipur, India; Toxicology Research Unit (TOXRUN) (Prof D Dias da Silva PhD), Cooperativa de Ensino Superior Politécnico e Universitário (University Polytechnic Higher Education Cooperative (CESP)), Gandra, Portugal; Faculty of Science (Prof D Diaz PhD), School of Medicine (Prof R Lozano MD), National Autonomous University of Mexico, Mexico City, Mexico; Department of Medicine (T C Do MD), Medical School (H Pham MD), Department of Epidemiology (H K Tang PhD), Pham Ngoc Thach University of Medicine, Ho Chi Minh City, Viet Nam; Department of Medicine (T H Do MD), Can Tho University of Medicine and Pharmacy, Can Tho, Viet Nam; Center for Health Sciences (C B do Prado MSc), Federal University of Espírito Santo, Vitória, Brazil; Department of Biostatistics (M Dodangeh Mcom), Independent Consultant, Tehran, Iran; Department of Social Medicine and Health Care Organisation (K G Dokova PhD), Medical University "Prof. Dr. Paraskev Stoyanov", Varna, Bulgaria; Mahidol Oxford Tropical Medicine Research Unit (C Dolecek PhD), Mahidol University, Bangkok, Thailand; University of Rochester, Rochester, NY, USA (E Dorsey MD); Departamento de Responsabilidade Social (Department of Social Responsibility) (W M dos Santos PhD), Oswaldo Cruz German Hospital, São Paulo, Brazil; Brazilian Centre for Evidence-based Healthcare (W M dos Santos PhD), Joanna Briggs Institute, São Paulo, Brazil; Department of Cardiology (R Doshi MD), St. Joseph's University Medical Center, Paterson, NJ, USA; Department of Forensic Medicine and Toxicology (H L Dsouza MD, Prof P Rastogi MD, Prof B K Shetty MD), Department of General Medicine (J Jeganathan MD), Department of Community Medicine (N Joseph MD, N Kumar MD, P Mithra MD, R Thapar MD), Department of Internal Medicine (M M R Reddy MD), Kasturba Medical College (Prof B Unnikrishnan MD), Manipal Academy of Higher Education, Mangalore, India; Forensic Medicine and Toxicology Department (H L Dsouza MD), Kasturba Medical College Mangalore, Mangalore, India; Office of Institutional Analysis (J Dube MA), University of Windsor, Windsor, ON, Canada; Post-graduate Program in Health Sciences (S C Dumith PhD), Federal University of Rio Grande, Rio Grande, Brazil; Epidemiology Department (Prof R J Maude PhD), Mahidol Oxford Tropical Medicine Research Unit, Bangkok, Thailand (S J Dunachie PhD); School of Medicine (Prof A R Duraes PhD), Institute of Collective Health (Prof M Pereira PhD, Prof D Rasella PhD), Federal University of Bahia, Salvador, Brazil; Department of Internal Medicine (Prof A R Duraes PhD), Escola Bahiana de Medicina e Saúde

Pública (Bahiana School of Medicine and Public Health), Salvador, Brazil; Department of Biotechnology (S Duraisamy PhD), SRM College of Pharmacy (M R Tovani-Palone PhD), SRM Institute of Science and Technology (SRMIST), Chennai, India; Department of Infection and Tropical Medicine (O C Durojaiye MPH), School of Health and Related Research (J O Oguta MSc), University of Sheffield, Sheffield, UK; School of Life Sciences (S Dutta PhD), Manipal Academy of Higher Education, Dubai, United Arab Emirates; Child Health Analytics Research Program (P A Dzianach PhD, Prof P W Gething PhD, F Sanna PhD, D J Weiss PhD), The Malaria Atlas Project (J Kiss MRes, M A McPhail PhD, S F Rumisha PhD), Geospatial Health and Development Team (J Lubinda PhD, A Saddler PhD), Telethon Kids Institute, Perth, WA, Australia; Department of Conservative Dentistry with Endodontics (A M Dziedzic DSc), Medical University of Silesia, Katowice, Poland; Department of Psychiatry (E Eboreime PhD, E Tsermpini PhD), Dalhousie University, Halifax, NS, Canada; Division of Cardiothoracic Vascular Surgery (C P Echieh FWACS), University of Calabar, Calabar, Nigeria; Division of Cardiothoracic Surgery (C P Echieh FWACS), University of Arizona, Tucson, AZ, USA; Higher School of Technology (Prof A Ed-Dra PhD), Sultan Moulay Slimane University, Beni Mellal, Morocco; School of Health Sciences (H A Edinur PhD), Universiti Sains Malaysia (University of Science Malaysia), Kubang Kerian, Malaysia; College of Science, Health and Engineering (K Edvardsson PhD), La Trobe University, Bundoora, VIC, Australia; Department Pediatric Nursing (D Efendi MN), Faculty of Public Health (D Kusuma DSc, Prof I Trihandini PhD), Centre for Family Welfare (K Latief Mepi), University of Indonesia, Depok, Indonesia; Neonatal Intensive Care Unit (D Efendi MN), University of Indonesia Hospital, Depok, Indonesia; Centre for Global Health Inequalities Research (CHAIN) (Prof T Eikemo PhD), Department of Circulation and Medical Imaging (J Nauman PhD), Norwegian University of Science and Technology, Trondheim, Norway; Nursing Department (E Ekpor BSN), St. Martin de Porres Hospital, Eikwe, Ghana; Nursing Department (E Ekpor BSN), Christian Health Association of Ghana, Accra, Ghana; Al Ghad International Medical Sciences Colleges, Dammam, Saudi Arabia (R A El Arab MSc); Department of Forensic Medicine and Clinical Toxicology (Prof D A El Morsi MD), Clinical Pathology Department (Prof M El Sayed Zaki PhD, M Elshaer MD), Hygiene and Zoonoses Department (H Ramadan PhD), Faculty of Pharmacy (M A Saleh PhD), Mansoura University, Mansoura, Egypt; Head of Medical Education Department (Prof D A El Morsi MD), Delta University for Science and Technology, Mansoura, Egypt; School of Population and Global Health (Prof F J Elgar PhD), Department of Family Medicine (K K V Mate PhD), Department of Epidemiology, Biostatistics and Occupational Health (E Ortiz-Brizuela MSc, J Rana MPH), McGill University, Montreal, QC, Canada; Department of Internal Medicine and Hematology Unit (Prof G M T ElGohary MD), Department of Obstetrics and Gynecology (Prof A F Nabhan PhD), Department of Entomology (A M Samy PhD), Medical Ain Shams Research Institute (MASRI) (A M Samy PhD), Ain Shams University, Cairo, Egypt; Faculty of Medicine (M Elhadi MD), University of Tripoli, Tripoli, Libya; Department of Infectious Diseases and Public Health (I Elsohaby PhD), City University of Hong Kong, Hong Kong, China; Department of Animal Medicine (I Elsohaby PhD), Cardiovascular Department (Prof A M A Saad MD), Zagazig University, Zagazig, Egypt; Department of Medical-surgical Nursing (A Emami Zeydi PhD), Faculty of Nursing and Midwifery (A Goudarzian MSc), Department of Infectious Disease (A Taheri MD), Department of Dermatology (A Taheri MD), Mazandaran University of Medical Sciences, Sari, Iran; Lincoln International Institute for Rural Health (L Engelbert Bain PhD), University of Lincoln, Lincoln, UK; Department of International Cyber Education (R Erkhembayar MD), Mongolian National University of Medical Sciences, Ulaanbaatar, Mongolia; Registry of Senior Australians (ROSA) (T C Eshetie PhD), South Australian Health and Medical Research Institute, Adelaide, SA, Australia; Department of Obesity, Diabetes and Cardiovascular Risk (Prof J Espinosa-Montero PhD), National Institute of Public Health Mexico, Cuernavaca, Mexico;

Department of Ophthalmology (U A Eze MD), Federal Medical Centre, Asaba, Nigeria; Independent Consultant, Bologna, Italy (N Fabin MD); Department of Anesthesia (A O Fadaka PhD), Cincinnati Children's Hospital Medical Center, Cincinnati, OH, USA; Department of Biotechnology (A O Fadaka PhD), School of Pharmacy (O C Okonji MSc), University of the Western Cape, Cape Town, South Africa; Research Centre for Healthcare and Community (A F Fagbamigbe PhD), Faculty of Health and Life Sciences (O P Kurmi PhD), Coventry University, Coventry, UK; Epidemiology and Biostatistics Unit IRCCS Pascale (L Falzone PhD), IRCCS, Naples, Italy; Dissemination Division (C S e Farinha MSc), National Institute of Statistics, Lisbon, Portugal; Activity Planning and Control Unit (C S e Farinha MSc), Directorate-General of Health (DGS), Lisbon, Portugal; Department of Psychology (Prof A Faro PhD), Federal University of Sergipe, São Cristóvão, Brazil; Department of Public Health, Equity, and Human Flourishing (K M Foley MPH), Centre for Health Policy Research (Prof P Ward PhD), Torrens University Australia, Adelaide, SA, Australia (N K Fauk MSc); Institute of Resource Governance and Social Change, Kupang, Indonesia (N K Fauk MSc); National Institute for Stroke and Applied Neurosciences (Prof V L Feigin PhD), Auckland University of Technology, Auckland, New Zealand; Third Department of Neurology (E V Gnedovskaya PhD), Research Center of Neurology, Moscow, Russia (Prof V L Feigin PhD, M A Kravchenko PhD, Prof M A Piradov DSc); School of Pharmacy (G Fekadu MSc), Jockey Club School of Public Health and Primary Care (J Huang MD, C Zhong MD), Department of Medicine & Therapeutics (Y Jin MD), Department of Medicine and Therapeutics (L Lim MRCP), The Chinese University of Hong Kong, Hong Kong, China; Department of Pharmacy (G Fekadu MSc), Department of Nursing (G Fetensa MSc), Department of Public Health (M E Getachew MPH), Wollega University, Nekemte, Ethiopia; Department of Translational Medicine (D Ferrante PhD), University of Piemonte Orientale, Italy, Novara, Italy; Department of Social Sciences (Prof N Ferreira PhD), University of Nicosia, Nicosia, Cyprus; Psychiatry Department (I Filip MD), Kaiser Permanente, Fontana, CA, USA; School of Health Sciences (I Filip MD), A.T. Still University, Mesa, AZ, USA; Institute of Public Health (F Fischer PhD), Department of Infectious Diseases and Respiratory Medicine (F Steinbeis MD), Charité Universitätsmedizin Berlin (Charité Medical University Berlin), Berlin, Germany; School of Social Sciences (J Flavel PhD), Stretton Health Equity, Adelaide, SA, Australia; Clinical Science Department (Prof M O Folayan FWACS), Nigerian Institute of Medical Research, Yaba, Nigeria; Department of Cell Biology and Biotechnology (A A Fomenkov PhD), K.A. Timiryazev Institute of Plant Physiology, Moscow, Russia; Department of Pharmacology (Prof B Foroutan PhD), Iranshahr University of Medical Sciences, Iranshahr, Iran; Department of Biotechnological and Applied Clinical Sciences (DISCAB) (M Foschi MD), Department of Neurology (Prof S Sacco MD), University of L'Aquila, L'Aquila, Italy; Department of Neuroscience (M Foschi MD), Hospital Santa Maria delle Croci, Ravenna, Italy; School of Public Health, Medical, and Veterinary Sciences (Prof R C Franklin PhD), James Cook University, Douglas, QLD, Australia; Evidence-Based Decision Making, Research Synthesis and Health Technology Assessment (R J Vieira MD), Center for Health Technology and Services Research (CINTESIS), Porto, Portugal (A Freitas PhD); Department of Dermatology (T Fukumoto PhD), Kobe University, Kobe, Japan; Health Services Management Training Centre (P A Gaal PhD, T Joo PhD, J Lám PhD, T Palicz MD), Institute of Digital Health Sciences (P Pollner PhD), Faculty of Health and Public Administration (M Szócska PhD), Semmelweis University, Budapest, Hungary; Department of Applied Social Sciences (P A Gaal PhD), Sapientia Hungarian University of Transylvania, Târgu-Mureş, Romania; Department of Community Medicine (Prof M A Gadanya FMCPH), Aminu Kano Teaching Hospital, Kano, Nigeria; Institute of Applied Health Sciences (S Gaihre PhD), University of Aberdeen, Aberdeen, UK; Department of Medicine (A Gaipov PhD), Nazarbayev University School of Medicine, Astana, Kazakhstan; Food Technology Department (Y Galali ResM, B A Sadee PhD), Salahaddin

University-Erbil, Erbil, Iraq; Department of Nutrition and Dietetics (Y Galali ResM, B A Sadee PhD), Cihan University-Erbil, Erbil, Iraq; Faculty of Paramedicine (N Galehdar PhD), Environmental Health Research Center (A Norouzian Baghani PhD), Hepatitis Research Center (M Zandi PhD), Lorestan University of Medical Sciences, Khorramabad, Iran; Department of Environmental Health Sciences (S Gallus DSc, A Lugo PhD), Mario Negri Institute for Pharmacological Research, Milan, Italy; Nutrition and Metabolism Branch (Q Gan MPH), International Agency for Research on Cancer, Lyon, France; Department of Community Medicine and Family Medicine (A P Gandhi MD), Department of Physiology (A Patil MD), All India Institute of Medical Sciences, Nagpur, India; Institute of Health and Wellbeing (B Ganesan PhD), Federation University, Churchill, VIC, Australia; Division of Cardiovascular Medicine (J Garg MD), Medical College of Wisconsin, Milwaukee, WI, USA; School of Medicine (S Gau MD), Chung Shan Medical University, Taichung, Taiwan; Department of Pharmacology (Prof R K Gautam PhD), Indore Institute of Pharmacy, Indore, India; Institute and Faculty of Actuaries, London, UK (F Gazzelloni BSc); Department of Midwifery (M W Gebregergis MSc), Department of Epidemiology (M Mehari MPH), Department of Medical Laboratory Sciences (H Negash MSc), Adigrat University, Adigrat, Ethiopia; Department of Environmental Health (M Gebrehiwot DSc), Wollo University, Dessie, Ethiopia; Department of Public Health (T B Gebremariam MPH), Debre Berhan University, Debre Berhan, Ethiopia; Public Health (T B Gebremariam MPH), Independent Consultant, Addis Ababa, Ethiopia (S A Yesuf MSc); School of Population Health (Prof P W Gething PhD), School of Public Health (T R Miller PhD), Curtin School of Population Health (D J Weiss PhD), Curtin University, Perth, WA, Australia; Center of Health Management (K Y Ghailan PhD), Aden University, Aden, Yemen; Mount Auburn Hospital (A Ghajar MD), Harvard Medical School, Cambridge, MA, USA; Department of Ophthalmology (M Ghanbarnia MD), Cellular and Molecular Biology Research Center (Prof S Mahjoub PhD), Department of Clinical Biochemistry (Prof S Mahjoub PhD), Social Determinants of Health Research Center (S Mouodi PhD), Babol University of Medical Sciences, Babol, Iran; Young Researchers and Elite Club (A Gholamian MSc), Islamic Azad University, Rasht, Iran; Department of Biology (A Gholamian MSc), Department of Microbiology (S Valadan Tahbaz PhD), Islamic Azad University, Tehran, Iran; Department of Medical-Surgical Nursing (P Ghorbani Vajargah MSc, S Karkhah MSc), Gastrointestinal and Liver Diseases Research Center (S Hassanipour PhD), Caspian Digestive Disease Research Center (S Hassanipour PhD), Department of Environmental Health Engineering (J Jaafari PhD), Guilan University of Medical Sciences, Rasht, Iran; Department of Public Health (G Ghozali PhD), University of Muhammadiyah Kalimantan Timur, Samarinda, Indonesia; Department of Radiology (S Ghozy MD), Division of Nephrology and Hypertension (s Kazeminia MD), Mayo Clinic, Rochester, MN, USA; Department of Forensic Biology (A D Ghuge MPhil), Government Institute of Forensic Science, Aurangabad, India; Department of Clinical Research 1 (A D Ghuge MPhil), National Institute For Research In Reproductive and Child Health, Mumbai, India; Department of Epidemiology and Prevention (A Gialluisi PhD), IRCCS Neuromed, Pozzilli, Italy; Department of Medicine (R M Gibson PhD), Blood and Marrow Transplantation and Cellular Therapy Program (A Goyal MD), Division of Pediatric Hospital Medicine (R P Mediratta MD), Stanford University, Palo Alto, CA, USA; NCD Surveillance Unit (A U Gil PhD), World Health Organization (WHO), Moscow, Russia; Institute for Leadership and Health Management (A U Gil PhD), Moscow Medical Academy, Moscow, Russia; Warwick Medical School (Prof P S Gill DM), University of Warwick, Coventry, UK (J W Sakshaug PhD); Division of General Internal Medicine (R F Gillum MD), Department of Community and Family Medicine (R F Gillum MD), Howard University, Washington, DC, USA; Department of Hepatology (Prof A Goel DM), Sanjay Gandhi Postgraduate Institute of Medical Sciences, Lucknow, India; Health Systems and Policy Research Department (M Golechha PhD), Indian Institute of

Public Health, Gandhinagar, India; Department of Genetics (P Goleij MSc), Sana Institute of Higher Education, Sari, Iran; Universal Scientific Education and Research Network (USERN) (P Goleij MSc), Research Center for Environmental Determinants of Health (Prof B Karami Matin PhD, Prof E Sadeghi PhD), Department of Rehabilitation and Sports Medicine (M Mirzaei MSc), Department of Epidemiology (M Moradinazar PhD), Substance Abuse Prevention Research Center (Y Sayadi PhD), Department of Speech Therapy (A Shiani PhD), Kermanshah University of Medical Sciences, Kermanshah, Iran; Department of Theriogenology (A Golestanfar PhD), University of Tehran, Tehran, Iran; Department of Exercise and Health Sciences (P N Gona PhD), Department of Nursing (E F Kyei MSc), University of Massachusetts Boston, Boston, MA, USA; Department of Respiratory Medicine (H Goudarzi PhD), Center for Environmental and Health Sciences (H Goudarzi PhD), Hokkaido University, Sapporo, Japan; Department of Mathematics (S Greenhalgh PhD), Siena College, Loudonville, NY, USA; Department of Public Health and Preventive Medicine (Prof M Grivna PhD), Charles University, Prague, Czech Republic; Post Graduate School of Public Health (G Guarducci MD), University of Siena, Siena, Italy; Department of Family and Community Medicine (M I M Gubari PhD), University Of Sulaimani, Sulaimani, Iraq; Division of Cardiovascular Medicine (A Guha MD), Center for Biostatistics (J Ma MS), Ohio State University, Columbus, OH, USA; Health Directorate (S Guicciardi MD), Local Health Authority of Bologna, Bologna, Italy; Department of Community Medicine (D A Gunawardane MD), University of Peradeniya, Kandy, Sri Lanka; Department of Psychiatry (S Gunturu MD), Bronxcare Health System, Bronx, NY, USA; Department of Internal Medicine (A K Gupta PharmD), Faculty of Medicine and Health Sciences (Prof N P Singh MD), Shree Guru Gobind Singh Tricentenary University, Gurugram, India; Non-communicable Division (NCD) (A K Gupta PharmD), Indian Council of Medical Research, Delhi, India; Department of Public Health (B Gupta PhD), Torrens University Australia, Melbourne, VIC, Australia; Department of Biotechnology (I R Gupta PhD), Government Institute of Science, Aurangabad, India; Department of Biotechnology (I R Gupta PhD), Sant Gadge Baba Amravati University, Amravati, India; Department of Epidemiology and Biostatistics (R Gupta MPH), Department of Health Promotion, Education, and Behavior (T Mi PhD), University of South Carolina, Columbia, SC, USA; Centre for Noncommunicable Diseases and Nutrition (R Gupta MPH), James P Grant School of Public Health (M Hossain MSc), BRAC University, Dhaka, Bangladesh; Toxicology Department (S Gupta MSc), Shriram Institute for Industrial Research, Delhi, India; School of Medicine (V Gupta PhD), Institute for Mental and Physical Health and Clinical Translation (IMPACT) (W Marx PhD), Deakin University, Geelong, VIC, Australia; School of Biotechnology (V Gupta PhD), Dublin City University, Glasnevin, Ireland; Faculty of Medicine Health and Human Sciences (Prof V K Gupta PhD), Macquarie Medical School (Y You PhD), Macquarie University, Sydney, NSW, Australia; Department of Epidemiology and Psychosocial Research (R A Gutiérrez PhD), Ramón de la Fuente Muñiz National Institute of Psychiatry, Mexico City, Mexico; Global Virus Network, Middle East Region, Shiraz, Iran (F Habibzadeh MD); School of Medicine (P Habibzadeh MD), University of Maryland, Baltimore, MD, USA; Lawson Health Research Institute, London, ON, Canada (Prof V Hachinski MD); Department of Pharmacology and Toxicology (R Haddadi PhD), Research Center for Molecular Medicine (A Taherkhani PhD), Hamadan University of Medical Sciences, Hamadan, Iran; Departement of Surgery (N Haep MD), Department of Neurology (S Samadzadeh MD), Charité University Medical Center Berlin, Berlin, Germany; Clinician Scientist Program (N Haep MD), Berlin Institute of Health, Berlin, Germany; Natural and Medical Sciences Research Center (S A Halim PhD), University of Nizwa, Nizwa, Oman; NYU Shanghai, Shanghai, China (B J Hall PhD); Department of Infectious Disease Epidemiology (S Haller MD), Robert Koch Institute, Berlin, Germany; Department of Public Health (S Haller MD), Charité Institute of Public Health, Berlin, Germany; Department of Family and Community

Medicine (Prof R R Hamadeh PhD), College of Medicine and Medical Sciences (H Jahrami PhD), Arabian Gulf University, Manama, Bahrain; College of Law and Political Science (K Hamagharib Abdullah PhD), University of Human Development, Sulaimaniyah, Iraq; School of Health and Environmental Studies (Prof S Hamidi DrPH), Hamdan Bin Mohammed Smart University, Dubai, United Arab Emirates; Faculty of Health (M Hamiduzzaman PhD), Southern Cross University, Bilinga, QLD, Australia; Department of Medical and Technical Information Technology (A Hammoud MSc), Bauman Moscow State Technical University, Moscow, Russia; Perron Institute for Neurological and Translational Science, Perth, WA, Australia (Prof G J Hankey MD); Department of Biochemistry and Molecular Biology (Prof M Hannan PhD), Bangladesh Agricultural University, Mymensingh, Bangladesh; Department of Anatomy (Prof M Hannan PhD), Dongguk University, Gyeongju, South Korea; Department of Population Science and Human Resource Development (Prof M Haque PhD, Prof M Rahman PhD, M Rahman DrPH), Department of Mathematics (M Kuddus PhD), University of Rajshahi, Rajshahi, Bangladesh; Medical Research Unit (H Harapan PhD), Universitas Syiah Kuala (Syiah Kuala University), Banda Aceh, Indonesia; Research Unit (J M Haro MD), University of Barcelona, Barcelona, Spain; Biomedical Research Networking Center for Mental Health Network (CiberSAM), Barcelona, Spain (J M Haro MD); Department of Zoology and Entomology (A I Hasaballah PhD), Al Azhar University, Cairo, Egypt; Department of Nursing (F Hasan MSc), School of Health Care Administration (L D Huy MBA), International Master Program for Translational Science (H Huynh BS), Department of Global Health and Health Security (K Latief Mepi), International Ph.D. Program in Medicine (L Minh MD), Research Center for Artificial Intelligence in Medicine (L Minh MD), School of Public Health (Y L Samodra MPH, Y L Samodra MPH), Department of Clinical Pharmacy (M A Sarasmita PharmD), School of Nursing (S Susanty PhD), Taipei Medical University, Taipei, Taiwan; Department of Pharmaceutical Technology (I Hasan MPharm), Department of Population Sciences (Prof M Islam PhD), University of Dhaka, Dhaka, Bangladesh; Department of Ophthalmology (H Hasani MD), Iran University of Medical Sciences, Karaj, Iran; Department of Radiology (M Hasanian MD), Arak University of Medical Sciences, Arak, Iran; Department of Medical Surgical (Prof A Hasanpour-Dehkordi PhD), Shahroud University of Medical Sciences, Shahrekord, Iran; Department of Diagnostic and Interventional Radiology and Neuroradiology (J Haubold MD, Prof B M Schaarschmidt MD), Institute of Artificial Intelligence in Medicine (J Haubold MD), University Hospital Essen, Essen, Germany; Skaane University Hospital (R J Havmoeller PhD), Skaane County Council, Malmoe, Sweden; Faculty of Kinesiology (Prof J J Hebert PhD), University of New Brunswick, Fredericton, NB, Canada; School of Allied Health (Prof J J Hebert PhD), Murdoch University, Murdoch, WA, Australia; Independent Consultant, Santa Clara, CA, USA (G Heidari MD); Community-Oriented Nursing Midwifery Research Center (M Heidari PhD), Department of Community Health (M Lotfizadeh PhD), Social Determinants of Health Research Center (M Lotfizadeh PhD), Department of Epidemiology and Biostatistics (A Mohammadian-Hafshejani PhD), Department of Health in Disasters and Emergencies (R Sheikhi BHlthSci), Shahrekord University of Medical Sciences, Shahrekord, Iran; Institute of Psychology (B Helfer PhD), University of Wroclaw, Wroclaw, Poland; Meta Research Centre (B Helfer PhD), University of Wrocław, Wroclaw, Poland; School of Business (Prof C Herteliu PhD), London South Bank University, London, UK; Department of Anatomy Genetics and Biomedical Informatics (D Hettiarachchi PhD), Department of Surgery (D P Wickramasinghe MD), University of Colombo, Colombo, Sri Lanka; Department of Public Health (D Z Heyi MPH), Madda Walabu University, Robe, Ethiopia; Department of Microbiology (K Hezam PhD), Taiz University, Taiz, Yemen; School of Medicine (K Hezam PhD), Nankai University, Tianjin, China; Division for Health Service Promotion (Y Hiraike PhD), Department of Global Health Policy (S Nomura PhD), University of Tokyo, Tokyo, Japan; National Institute on Deafness and Other

Communication Disorders (H J Hoffman MA), Eunice Kennedy Shriver National Institute of Child Health and Human Development (L G Mensah MD), National Institute of Health, Bethesda, MD, USA; Division of Scientific Programs (H J Hoffman MA), National Human Genome Research Institute (NHGRI) (N Horita PhD), Center for Translation Research and Implementation Science (G A Mensah MD), National Institutes of Health, Bethesda, MD, USA; Department of Pulmonology (N Horita PhD), Yokohama City University, Yokohama, Japan; Social and Environmental Health Research (M Hossain MPH), Nature Study Society of Bangladesh, Khulna, Bangladesh; Department of Health Promotion and Community Health Sciences (M Hossain MPH), Texas A&M University, College Station, TX, USA; Department of Public Health and Informatics (S Hossain MS), Jahangirnagar University, Dhaka, Bangladesh; School of Health and Society (H Hosseinzadeh PhD), University of Wollongong, Wollongong, NSW, Australia; Institute of Research and Development (Prof M Hosseinzadeh PhD), Duy Tan University, Da Nang, Viet Nam; Clinical Legal Medicine Department (S Hostiuc PhD), National Institute of Legal Medicine Mina Minovici, Bucharest, Romania; Faculty of Medicine of Tunis (Prof M Hsairi MPH), University Tunis El Manar, Tunis, Tunisia; Department of Health Services Administration (V Hsieh PhD), Department of Occupational Safety and Health (Prof B Hwang PhD), College of Public Health (R Lin PhD), China Medical University, Taichung, Taiwan; Department of Psychology (C Hu PhD), Vanke School of Public Health (J S Ji DSc), Tsinghua Vanke School of Public Health (Z Li PhD), Tsinghua University, Beijing, China; Research Division (M Huda PhD), ARCED Foundation, Dhaka, Bangladesh; Department of Surgical Sciences (M Hultström PhD), Department of Medical Cell Biology (M Hultström PhD), Department of Medical Sciences (Prof A O Larsson PhD), Uppsala University, Uppsala, Sweden; Department of Biological Sciences and Chemistry (Prof J Hussain PhD), Natural and Medical Sciences Research Center (S Ullah MSc), University of Nizwa Oman, Nizwa, Oman; Czech National Centre for Evidence-Based Healthcare and Knowledge Translation (S Hussain PhD), Institute of Biostatistics and Analyses (S Hussain PhD), Masaryk University, Brno, Czech Republic; Department of Biomolecular Sciences (N R Hussein PhD), University of Zakho, Zakho, Iraq; College of Health Sciences (L D Huy MBA, N Quan MD), VinUniversity, Hanoi, Viet Nam; Department of Occupational Therapy (Prof B Hwang PhD), Asia University, Taiwan, Taichung, Taiwan; Division of Infectious Diseases (K S Ikuta MD), Veterans Affairs Greater Los Angeles, Los Angeles, CA, USA; Faculty of Medicine (I M Ilic PhD, Prof M M Santric-Milicevic PhD, I S Vujcic PhD), School of Public Health and Health Management (Prof M M Santric-Milicevic PhD), School of Medicine (R Vukovic PhD), University of Belgrade, Belgrade, Serbia; Department of Epidemiology (Prof M D Ilic PhD), University of Kragujevac, Kragujevac, Serbia; Department of Health Research (L R Inbaraj MD), ICMR National Institute for Research in Tuberculosis, Chennai, India; Department of Medicine (A Iradukunda MD), University of Burundi, Bujumbura, Burundi; Research Department (A Iradukunda MD), ARNECH Research and Consulting Office, Bujumbura, Burundi; Department of Medical Microbiology (K C Iregbu MD), University of Abuja, Abuja, Nigeria; Department of Medical Microbiology (K C Iregbu MD), National Hospital, Abuja, Nigeria; Department of Pharmacy (M R Islam PhD), University of Asia Pacific, Dhaka, Bangladesh; Institute for Physical Activity and Nutrition (S Islam PhD, K M Livingstone PhD), Department of Psychology (M A Stokes PhD), Deakin University, Burwood, VIC, Australia; Surveillance and Health Services Research Department (F Islami PhD), American Cancer Society, Atlanta, GA, USA; Department of Clinical Pharmacy & Pharmacy Practice (Prof N Ismail PhD), Asian Institute of Medicine, Science and Technology, Kedah, Malaysia; Malaysian Academy of Pharmacy, Puchong, Malaysia (Prof N Ismail PhD); Department of Health Services Research (M Iwagami PhD), University of Tsukuba, Tsukuba, Japan; Department of Non-Communicable Disease Epidemiology (M Iwagami PhD), Department of Health Services Research and Policy (Prof M McKee DSc), London School of Hygiene & Tropical Medicine,

London, UK; School of Health Systems and Public Health (C C D Iwu MPH), Department of Medical Microbiology (L A Malinga PhD), University of Pretoria, Pretoria, South Africa; Department of Global Health (C J Iwu-Jaja PhD), Risk and Resilience in Mental Disorders Unit (Prof D J Stein MD), South African Medical Research Council, Cape Town, South Africa; Department of Global Health (C J Iwu-Jaja PhD, P D Katoto PhD), South African Centre for Epidemiological Modelling and Analysis (SACEMA) (L Mhlanga PhD), Department of Epidemiology (J L Tamuzi MSc), Stellenbosch University, Cape Town, South Africa; Department of Biotechnology (M Iyer PhD, S Muthu MS), Karpagam Academy of Higher Education (Deemed to be University), Coimbatore, India; Department of Orthodontics & Dentofacial Orthopedics (L J BDS), Department of Oral Pathology and Microbiology (Prof G S Sarode PhD, Prof S C Sarode PhD), Dr. D. Y. Patil University, Pune, India; Research and Development Unit (L Jacob MD), Biomedical Research Networking Center for Mental Health Network (CiberSAM), Sant Boi de Llobregat, Spain; Faculty of Medicine (L Jacob MD), University of Versailles Saint-Quentin-en-Yvelines, Montigny-le-Bretonneux, France; Department of Health Studies (K H Jacobsen PhD), University of Richmond, Richmond, VA, USA; Department of Nephrology (K Jaggi MD), San Mateo Medical Center, San Mateo, CA, USA; Department of Nephrology (K Jaggi MD), Mills Peninsula Medical Center, Burlingame, CA, USA; Ministry of Health, Manama, Bahrain (H Jahrami PhD); Department of Leukemia (A Jain MD), The University of MD Anderson Cancer Center, Houston, TX, USA; Statistics Unit (N Jain MD), Riga Stradins University, Riga, Latvia; Health and Safety Department (A A Jairoun PhD), Dubai Municipality, Dubai, United Arab Emirates; The World Academy of Sciences UNESCO, Trieste, Italy (Prof M Jakovljevic PhD); Shaanxi University of Technology, Hanzhong, China (Prof M Jakovljevic PhD); Department of Environmental Health Engineering (R Jalilzadeh Yengejeh PhD), Islamic Azad University, Ahvaz, Iran; Department of Internal Medicine (C T Jani MD), Harvard University, Cambridge, MA, USA; Duke Global Health Institute (M M Janko PhD), Department of Anesthesiology (V Krishnamoorthy MD), Center for the Study of Aging and Human Development (Y Yao MD), Duke University, Durham, NC, USA; Centre of Studies and Research (S Jayapal PhD), Ministry of Health, Muscat, Oman; Department of Biochemistry (Prof S Jayaram MD), Government Medical College, Mysuru, India; Department of Public Health (W Jeong PhD), Yonsei University, Seoul, South Korea; Department of Cardiovascular Medicine (A K Jha MD), Saint Vincent Hospital, Worcester, MA, USA; Department of Community Medicine (R P Jha MSc), Dr. Baba Saheb Ambedkar Medical College & Hospital, Delhi, India; Department of Community Medicine (R P Jha MSc), Department of Geography (A Singh PhD), Banaras Hindu University, Varanasi, India; Department of Global Health (Y Jin PhD, Prof Z Zhang PhD), Department of Epidemiology and Biostatistics (Prof J Liu PhD), China Center for Health Development Studies (Y Yao MD), School of Public Health (H Zhang MS), Department of Nutrition and Food Hygiene (Z Zhang PhD), Institute of Child and Adolescent Health (Z Zou MD), Peking University, Beijing, China; Department of Microbiology (N Jomehzadeh PhD), Department of Pharmacology (H Mojiri-forushani PhD), Abadan School of Medical Sciences, Abadan, Iran; Hungarian Health Management Association (T Palicz MD), Hungarian Health Management Association, Budapest, Hungary (T Joo PhD); Department of Gastroenterology and Hepatology (A Joseph MD), Department of Biomedical Data Science (S Park MD), Department of Radiology (S Ramasamy MD), Stanford University, Stanford, CA, USA; Department of Economics (C E Joshua BSc), National Open University, Benin City, Nigeria; Department of Family Medicine and Public Health (J J Jozwiak PhD), University of Opole, Opole, Poland; Institute of Family Medicine and Public Health (M Jürisson PhD, H Orru PhD), University of Tartu, Tartu, Estonia; School of Public Health (Z Kabir PhD), University College Cork, Cork, Ireland; Department of Oral and Maxillofacial Pathology (V Kadashetti MDS), Department of Public Health Dentistry (Prof K M Shivakumar PhD), Department of Periodontology (S A Varma MDS),

Krishna Vishwa Vidyapeeth (Deemed to be University), Karad, India; Independent Consultant, Pune, India (P V Kakodkar MDS); Dermatology Department (F Kaliyadan MD), King Faisal University, Hofuf, Saudi Arabia; Department of Endocrinology (S Kalra DM), Bharti Hospital Karnal, Karnal, India; School of Graduate Studies (T Kanagasabai PhD), Meharry Medical College, Nashville, TN, USA; Sydney Eye Hospital (H Kandel PhD), South Eastern Sydney Local Health District, Sydney, NSW, Australia; Regional Institute for Population Studies (E Kanmiki MPH), University of Ghana, Accra, Ghana; Faculty of Dentistry (K K Kanmodi MPH), University of Puthisastra, Phnom Penh, Cambodia; Office of the Executive Director (K K Kanmodi MPH), Campaign for Health and Neck Cancer Education (CHANCE) Programme (A A Salami BDS), Cephas Health Research Initiative Inc, Ibadan, Nigeria; The Hansjörg Wyss Department of Plastic and Reconstructive Surgery (R S Kantar MD), Nab'a Al-Hayat Foundation for Medical Sciences and Health Care, New York, NY, USA; Cleft Lip and Palate Surgery Division (R S Kantar MD), Global Smile Foundation, Norwood, MA, USA; School of Health Professions and Human Services (I M Karaye MD), Hofstra University, Hempstead, NY, USA; Department of Anesthesiology (I M Karaye MD), Montefiore Medical Center, Bronx, NY, USA; Department of Biology (A K Karna PhD), Department of Public Health (Prof J Khubchandani PhD), New Mexico State University, Las Cruces, NM, USA; Department of Physical Therapy and Health Rehabilitation (F Z Kashoo MSc), Majmaah University, Majmaah, Saudi Arabia; Department of Medicine (A Katamreddy MD), Jacobi Medical Center, New York, NY, USA; MRC/CSO Social and Public Health Sciences Unit (S V Katikireddi PhD), School of Cardiovascular and Metabolic Health (F E Petermann-Rocha PhD), University of Glasgow, Glasgow, UK; Centre for Tropical Diseases and Global Health (P D Katoto PhD), Catholic University of Bukavu, Bukavu, Democratic Republic of the Congo; Surgery Research Unit (Prof J H Kauppila MD), Center for Environmental and Respiratory Health Research (I Shiue PhD), Martti Ahtisaari Institute (I Shiue PhD), University of Oulu, Oulu, Finland; Department of ENT (N Kaur MS), Dr. B. R. Ambedkar State Institute of Medical Sciences (AIMS), Mohali, India; LUPUS Gatineau, Gatineau, QC, Canada (J Kayibanda PhD); International Research Center of Excellence (G A Kayode PhD), Institute of Human Virology Nigeria, Abuja, Nigeria; Julius Centre for Health Sciences and Primary Care (G A Kayode PhD), Copernicus Institute of Sustainable Development (G Koren PhD), Utrecht University, Utrecht, Netherlands; Department of Healthcare Services Management (L Keikavoosi-Arani PhD), Non-communicable Diseases Research Center (P Mardi MD, A Shafiee MD), School of Medicine (M Shams-Beyranvand MSc), Alborz University of Medical Sciences, Karaj, Iran; Eye Unit (Prof J H Kempen MD), MyungSung Medical College, Addis Ababa, Ethiopia; Department of Psychological Medicine (J A Kerr PhD), University of Otago, Christchurch, New Zealand; Department of Human Nutrition (E Kesse-Guyot PhD), National Research Institute for Agriculture, Food and Environment, Jouy-en-Josas, France; University Sorbonne Paris Nord (E Kesse-Guyot PhD), Department of Health, Medicine and Human Biology (M Touvier PhD), Sorbonne Paris Nord University, Bobigny, France; Amity Institute of Forensic Sciences (H Khajuria PhD, B P Nayak PhD), Amity Institute of Pharmacy (K Munjal PhD), Amity University, Noida, India; College of Health Sciences (N Khalid PhD), Abu Dhabi University, Adu Dhabi, United Arab Emirates; Department of Pediatrics (I A Khan MD), Center for Pharmacoepidemiology and Treatment Science (A Parthasarathi MD), Rutgers University, New Brunswick, NJ, USA; Primary Care Department (M A Khan MSc), NHS North West London, London, UK; Department of Radiation Oncology (T Khan PhD), Department of Epidemiology and Biostatistics (M Teramoto MD), Department of Bioengineering and Therapeutic Sciences (Prof M S Zastrozhin PhD), University of California San Francisco, San Francisco, CA, USA; Department of Critical Care Medicine (M Z Khan suheb MD), St. Luke's Aurora Medical Center, Milwaukee, WI, USA; College of Health, Wellbeing and Life Sciences (Prof K Khatab PhD), Sheffield Hallam University, Sheffield, UK; College of Arts and

Sciences (Prof K Khatab PhD), Ohio University, Zanesville, OH, USA; Department of Biochemistry (F Khidri PhD), Liaquat University Of Medical and Health Sciences, Jamshoro, Pakistan; Research Department (M Khosrowjerdi PhD), Inland Norway University of Applied Sciences, Elverum, Norway; Faculty of Health Sciences (H Khusun PhD), University of Muhammadiyah Prof. Dr. HAMKA, Jakarta, Indonesia; Program Division (H Khusun PhD), SEAMEO Regional Center for Food and Nutrition, Jakarta, Indonesia; Department of Pediatrics (G Kim MD), Case Western Reserve University School of Medicine, Cleveland, OH, USA; Division of Pediatric Hospital Medicine (G Kim MD), UH Rainbow Babies and Children's Hospital, Cleveland, OH, USA; Millennium Prevention, Westwood, MA, USA (R W Kimokoti MD); Department of Public Health (G T Kiross MPH), Debre Markos University, East Gojjam, Ethiopia; School of Health Sciences (Prof A Kisa PhD), Kristiania University College, Oslo, Norway; Department of International Health and Sustainable Development (Prof A Kisa PhD), Tulane University, New Orleans, LA, USA; Department of Nursing and Health Promotion (S Kisa PhD), Oslo Metropolitan University, Oslo, Norway; Department of Public Health (Prof M Kivimäki PhD, Prof T Lallukka PhD), Department of Virology (F Zakham PhD), University of Helsinki, Helsinki, Finland (T J Meretoja MD); Department of Disease Burden (A S Knudsen PhD, C Madsen PhD), Norwegian Institute of Public Health, Bergen, Norway; Independent Consultant, Jakarta, Indonesia (S Kosen MD); Department of Epidemiology (Prof K Kostev PhD), IQVIA, Frankfurt, Germany; Department of Gynecology (Prof K Kostev PhD), Philipps-Universität Marburg, Marburg, Germany; Department of Biochemistry (A L Kotnis PhD), Department of Dentistry (A Singh MD), All India Institute of Medical Sciences, Bhopal, India; Department of Internal and Pulmonary Medicine (Prof P A Koul MD), Sheri Kashmir Institute of Medical Sciences, Srinagar, India; Kasturba Medical College (S Koulmane Laxminarayana MD), Manipal College of Nursing (R Yesodharan MSc), Manipal Academy of Higher Education, Udupi, India; San Juan de Dios Sanitary Park, Barcelona, Spain (A Koyanagi MD); Department of Anthropology (Prof K Krishan PhD), Department of Community Medicine (R Rohilla MD), Institute of Forensic Science & Criminology (V Sharma PhD), Panjab University, Chandigarh, India; Department of Community Medicine (Y Krishnamoorthy MD, S Rajaa MD), Employees' State Insurance Model Hospital, Chennai, India; Department of Demography (Prof B Kuate Defo PhD), Department of Social and Preventive Medicine (Prof B Kuate Defo PhD), University of Montreal, Montreal, QC, Canada; Foundation for Drug Policy Solutions, Washington, DC, USA (C M Kubeisy BA); Faculty of Medicine (B Kucuk Bicer PhD), Gazi University, Ankara, Türkiye; Department of Biochemistry (Prof M Kuddus PhD), Department of Public Health (M G M Zeariya PhD), University of Hail, Hail, Saudi Arabia; Pediatrics Department (I Kuitunen PhD), Kuopio University Hospital, Kuopio, Finland; Institute of Clinical Medicine (I Kuitunen PhD), University of Eastern Finland, Kuopio, Finland; Department of Health Research (M Kulimbet MSc), Atchabarov Scientific Research Institute of Fundamental and Applied Medicine (M Kulimbet MSc), Kazakh National Medical University, Almaty, Kazakhstan; Department of Medicine (V Kulkarni MS), Digital Health and Informatics Directorate (Prof S M McPhail PhD), Queensland Health, Brisbane, QLD, Australia; Department of Internal Medicine (A Kumar MD), Cabrini Institute, Akron, OH, USA; Department of Food Technology (Prof H Kumar PhD), Shri Vishwakarma Skill University, Palwal, India; Department of Biotechnology (Prof H Kumar PhD), Amity Institute of Biotechnology (M Kumari PhD, E Upadhyay PhD), Amity University Rajasthan, Jaipur, India; Department of Chemistry (R Kumar PhD), Indian Institute of Technology Madras, Chennai, India; Department of Food Science and Technology (S Kumar PhD, R Mehra PhD), Maharishi Markandeshwar (Deemed to be University), Ambala, India; National Research and Innovation Agency of the Republic of Indonesia, Jakarta, Indonesia (A Kusnali LLB); Department of Health Services Research and Management (D Kusuma DSc), City University of London, London, UK; Department of Pediatric Oncology (Prof T Kutluk

MD), Hacettepe University, Ankara, Türkiye; Department of Nephrology (A Kuttikkattu MD), Pushpagiri Institute of Medical Sciences and Research Centre, Thiruvalla, India; Department of Health Policy (I Kyriopoulos PhD, Prof E Mossialos PhD), London School of Economics and Political Science, London, UK; Institute for Social and Health Sciences (Prof L Laflamme PhD), University of South Africa, Pretoria, South Africa; Department of Health Policy and Strategy (Prof C Lahariya MD), Foundation for People-centric Health Systems, New Delhi, India; SD Gupta School of Public Health (Prof C Lahariya MD), Indian Institute of Health Management Research University, Jaipur, India; Department of Family Medicine (A Lahmar MD), University of Medicine, Oujda, Morocco; School of Digital Science (D T C Lai PhD), Universiti Brunei Darussalam (University of Brunei Darussalam), Gadong, Brunei; Institute of Applied Data Analytics (D T C Lai PhD), (University of Brunei Darussalam) (Universiti Brunei Darussalam), Gadong, Brunei; Department of Physiotherapy (T Laksono MS), Universitas Aisyiyah Yogyakarta, Yogyakarta, Indonesia; Institute of Allied Health Sciences (T Laksono MS), National Cheng Kung University, Tainan, Taiwan; India Cancer Research Consortium (Prof R Mehrotra DPhil), Indian Council of Medical Research, New Delhi, India (D K Lal MD); NEVES Society for Patient Safety, Budapest, Hungary (J Lám PhD); Department of Health Sciences (D Lamnisos PhD), European University Cyprus, Nicosia, Cyprus; Department of surgery (T Lan PhD), Washington University in St. Louis, Saint Louis, MO, USA; Department of Psychiatry and Psychotherapy (B Langguth PhD, W Schlee PhD), University of Regensburg, Regensburg, Germany; Chief Medical Office (Prof V C Lansingh PhD), HelpMeSee, New York, NY, USA; Mexican Institute of Ophthalmology, Queretaro, Mexico (Prof V C Lansingh PhD); Department of Behavioural Sciences and Learning (A Laplante-Lévesque PhD), Linköping University, Linköping, Sweden; Department of Clinical Chemistry and Pharmacology (Prof A O Larsson PhD), Uppsala University Hospital, Uppsala, Sweden; Department of Otorhinolaryngology (S Lasrado MS), Father Muller Medical College, Mangalore, India; International Society Doctors for the Environment, Arezzo, Italy (P Lauriola MD); Health Economics Division (L K D Le PhD), Monash University, Burwood, VIC, Australia; Faculty of Medicine (N Le MD), Department of Medicine (T Nguyen MD), Department of General Medicine (V T Nguyen MD), University of Medicine and Pharmacy at Ho Chi Minh City, Ho Chi Minh City, Viet Nam (T T Le MD, T D T Le MD); Cardiovascular Research Department (N Le MD), Methodist Hospital, Merrillville, IN, USA; Independent Consultant, Ho Chi Minh City, Viet Nam (T D T Le MD); Department of Medical Science (M Lee PhD), Ajou University School of Medicine, Suwon, South Korea; Department of Health Sciences (P H Lee PhD, S J Tromans PhD), University of Leicester, Leicester, UK; Pattern Recognition and Machine Learning Lab (Prof S Lee PhD), Gachon University, Seongnam, South Korea; Department of Precision Medicine (Prof S W Lee MD), Sungkyunkwan University, Suwon-si, South Korea; Department of Family Medicine (W Lee PhD), Department of Pathology (V Y Tat BS), University of Texas, Galveston, TX, USA; Department of Preventive Medicine (Prof Y Lee PhD), Korea University, Seoul, South Korea (Prof M Shin PhD); Knowledge Translation Directorate (S M Legesse PhD), Ethiopian Public Health Institute, Addis Ababa, Ethiopia; Faculty of Science (E Leong PhD), Universiti Brunei Darussalam (University of Brunei Darussalam), Bandar Seri Begawan, Brunei; Department of Health Promotion and Health Education (M Li PhD), National Taiwan Normal University, Taipei, Taiwan; Department of Health Management Center (X Li PhD), Fudan University, Shanghai, China; National Clinical Research Center for Cardiovascular Diseases (Y Li PhD), Chinese Academy of Medical Sciences, Shenzhen, China; Directorate of Quality Management and Digital Health (A T M Likaka MPH), Ministry of Health, Lilongwe, Malawi; Asbestos Diseases Research Institute, Concord, NSW, Australia (R Lin PhD); Department of Neurology (V Lioutas MD), Beth Israel Deaconess Medical Center, Harvard Medical School, Boston, MA, USA; Department of Neurology (V Lioutas MD), Framingham Heart Study, Framingham, MA, USA; Department of Dentistry- Quality and

Safety of Oral Health Care (Prof S Listl PhD), Radboud University, Nijmegen, Netherlands; Department of Translational Health Economics (Prof S Listl PhD), Heidelberg University Hospital, Heidelberg, Germany; Department of Molecular Epidemiology (E Llanaj PhD), German Institute of Human Nutrition Potsdam-Rehbrücke, Potsdam, Germany; German Center for Diabetes Research (DZD), München-Neuherberg, Germany (E Llanaj PhD); Department of Internal Medicine (C Lo MD), Kirk Kerkorian School of Medicine at UNLV, Las Vegas, NV, USA; School of Medicine and Public Health (A Loreche BS), Center for Research and Innovation (V F Pepito MSc), Ateneo De Manila University, Pasig City, Philippines; Department of Health Economics (L Lorenzovici MSc), Syreon Research Romania, Targu Mures, Romania; Department of Doctoral Studies (L Lorenzovici MSc), George Emil Palade University of Medicine, Pharmacy, Science, and Technology of Targu Mures, Targu Mures, Romania; School of Medicine (Prof G Lucchetti PhD), Federal University of Juiz de Fora, Juiz de Fora, Brazil; Department of General Surgery (Prof R Lunevicius DSc), Liverpool University Hospitals NHS Foundation Trust, Liverpool, UK; Epidemiology & Disease Control Department (S Ma PhD), Ministry of Health, Singapore, Singapore; Centre for Public Health and Wellbeing (Z Ma PhD), University of the West of England, Bristol, UK; Faculty of Veterinary Microbiology (M Mabrok PhD), Suez Canal University, Ismailia, Egypt; Department of Microbiology and Parasitology (M Mabrok PhD), King Salman International University, South of Sinai, Egypt; 2nd Department of Propaedeutic Surgery (N Machairas PhD), Department of Biophysics (Prof P Papadopoulou PhD), 3rd Department of Cardiology (M Spartalis PhD), University of Athens, Athens, Greece; Periodontal Department (Prof M Machoy PhD), Pomeranian Medical University, Szczecin, Poland; Department of Human Nutrition Research (J A Magaña Gómez PhD), Autonomous University of Sinaloa, Culiacán, Mexico; School of Pharmacy (S B Maharaj DBA), University of the West Indies, St. Augustine, Trinidad and Tobago; Planetary Health Alliance, Boston, MA, USA (S B Maharaj DBA); Department of Clinical and Hospital Pharmacy (M A Mahmoud PhD), Taibah University, Al-Madinah Al-Munawarrah, Saudi Arabia; Radiology and Precision Health Program (M Mahmoudi PhD), Michigan State University, East Lansing, MI, USA; Department of Cardiology (O M Makram MD), October 6 University, Cairo, Egypt; Department of Internal Medicine (K Malhotra MBBS), Dayanand Medical College and Hospital, Ludhiana, India; Electrical Engineering Department (I Malik PhD), Prince Sattam bin Abdulaziz University, Al Kharj, Saudi Arabia; Department of Health Research (L A Malinga PhD), Ministry of Health, Pretoria, South Africa; Institute for Social Science Research (A A Mamun PhD), The University of Queensland, Indooroopilly, QLD, Australia; Smidt Heart Institute (Y Manla MD), Cedars-Sinai Medical Center, Los Angeles, CA, USA; Security, Intelligence and Integrity of Information Team (SI3) (Prof A Mansour PhD), Laboratoire des Sciences et Techniques de l'Information de la Communication et de la Connaissance (LABSTICC), Brest, France; Laboratory of Public Health (Prof L G Mantovani DSc), Instituto Auxologico Italiano IRCCS (Italian Auxological Institute), Milan, Italy; Biomedical Engineering Research Center (CREB) (H Marateb PhD), Universitat Politècnica de Catalunya (Barcelona Tech - UPC), Barcelona, Spain; Biomedical Engineering (H Marateb PhD), University of Isfahan, Isfahan, Iran; University Health Services (A M Marconi MD), University of Wisconsin- Madison, Madison, WI, USA; Centro de Estudio e Investigación para la prevención y el tratamiento de las adicciones (Center for the Study and Investigation of Addiction Prevention and Treatment) (A M Marconi MD), Universidad de Buenos Aires (University of Buenos Aires), Buenos Aires, Argentina; Department of Food, Environmental and Nutritional Sciences (M Marino PhD), University of Milan, Milano, Italy; Department of Biochemistry (A Marjani PhD), Golestan Research Center of Gastroenterology and Hepatology (G Roshandel PhD), Neurology Department (S Sajedi MD), Golestan University of Medical Sciences, Gorgan, Iran; Department of Health Economics (Prof C A Marrugo Arnedo MSc), Mayor University, Cartagena, Colombia; Research Group in Health Economics

(Prof C A Marrugo Arnedo MSc), Institute for Immunological Research (Prof J Zakzuk PhD), University of Cartagena, Cartagena, Colombia; Department of Infectious Diseases (B A Martinez-Guerra MSc, E Ortiz-Brizuela MSc), Department of Medicine (A Olivas-Martinez MD), Instituto Nacional de Nutrición Salvador Zubirán (Salvador Zubiran National Institute of Medical Sciences and Nutrition), Mexico City, Mexico; Noncommunicable Diseases and Mental Health Department (R Martinez-Piedra BSc), Pan American Health Organization, Washington, DC, USA; Centre for Health Sciences (C A Martins MSc), Federal University of Espirito Santo, Vitoria, Brazil; Campus Fortaleza (F R Martins-Melo PhD), Federal Institute of Education, Science and Technology of Ceará, Fortaleza, Brazil; Department of Nutrition and Dietetics (M Martorell PhD), University of Concepcion, Concepción, Chile; Centre for Healthy Living (M Martorell PhD), University of Concepción, Concepción, Chile; Department of Pharmacy (S Maryam PharmD), Bahauddin Zakariya University, Multan, Pakistan; Faculty of Humanities and Health Sciences (Prof R R Marzo MD), Curtin University, Malaysia, Sarawak, Malaysia; Jeffrey Cheah School of Medicine and Health Sciences (Prof R R Marzo MD), Monash University, Subang Jaya, Malaysia; Department of Orthopaedic Surgery (K K V Mate PhD), Mayo Clinic, Phoenix, AZ, USA; Board of Directors (C N Matei PhD), Association of Resident Physicians, Bucharest, Romania; North West Lung Centre (A G Mathioudakis PhD), Manchester University NHS Foundation Trust, Manchester, UK; Department of Social Medicine and Family (M Mazaheri PhD), Dezful University of Medical Sciences, Dezful, Iran; Orthopedic Trauma Pathology Department (A Mazzotti PhD), IRCCS, Bologna, Italy; Department of Ophthalmology (C McAlinden PhD), Princess of Wales Hospital, Wales, UK; School of Optometry and Vision Sciences (C McAlinden PhD), Cardiff University, Cardiff, UK; National Centre for Register-based Research (Prof J J McGrath MD), Aarhus University, Aarhus, Denmark; Australian Centre for Health Services Innovation (Prof S M McPhail PhD), Queensland University of Technology, Kelvin Grove, QLD, Australia; Department of Healthcare (E A Mechili PhD), University of Vlora, Vlora City, Albania; Clinic of Social and Family Medicine (E A Mechili PhD), Laboratory of Toxicology (T K Nikolouzakis PhD), University of Crete, Heraklion, Greece; Department of Preventive Oncology (J K Meena MD), Medical Oncology Lab (C P Prasad PhD, M Singh PhD), Centre for Dental Education and Research (B M Purohit MDS), Department of Psychiatry (Prof R Sagar MD), Department of Radiation Oncology (A Shankar MD), All India Institute of Medical Sciences, New Delhi, India; Department of Nursing (M M Mekonnen MSc), Salale University, Fiche, Ethiopia; Peru Country Office (W Mendoza MD), United Nations Population Fund (UNFPA), Lima, Peru; Department of Medicine (G A Mensah MD), Division of Cardiology (Prof M Ntsekhe PhD), University of Cape Town, Cape Town, South Africa; International Dx Department (A A Mentis MD), BGI Genomics, Copenhagen, Denmark; Neurology Unit (A Meretoja MD), Breast Surgery Unit (T J Meretoja MD), Helsinki University Hospital, Helsinki, Finland; Department of Nursing (A M Mersha MSc), Department of Clinical Midwifery (B A Mesfin BMedSc), School of Nursing (G E Yesera MSc), Arba Minch University, Arba Minch, Ethiopia; University Centre Varazdin (T Mestrovic PhD), University North, Varazdin, Croatia; Stritch School of Medicine (A Mhlanga PhD), Loyola University Chicago, Chicago, IL, USA; Department of Preventive Medicine (L Mhlanga PhD), Department of Medicine (Cardiology) (N S Shah MD), Northwestern University, Chicago, IL, USA; Anaesthesiology Department (G Micha PhD), "Helena Venizelou" General and Maternity Hospital, Athens, Greece; Department of Epidemiology (I Michalek PhD), National Cancer Registry (I Michalek PhD), Maria Sklodowska-Curie National Research Institute of Oncology, Warsaw, Poland; Pacific Institute for Research & Evaluation, Calverton, MD, USA (T R Miller PhD); Department of Otolaryngology (S N Mindlin MD), Addiction Psychiatry Department (V Y Skryabin MD), Addictology Department (Prof M S Zastrozhin PhD), Russian Medical Academy of Continuous Professional Education, Moscow, Russia; Unit of Statistics

(G Minelli PhD), Istituto Superiore di Sanità, Rome, Italy; Global Institute of Public Health (Prof G Mini PhD), Ananthapuri Hospitals and Research Institute, Trivandrum, India; Clinical Research Department (N W Minja MD), Kilimanjaro Clinical Research Centre (KCRI), Moshi, Tanzania; Department of Neurology (O Mirmosayyeb MD), State University of New York, Buffalo, NY, USA; Office of the Minster (M K Mirutse MPH), Federal Ministry of Health, Addis Ababa, Ethiopia; Social Determinants of Health Center (M Mirza-Aghazadeh-Attari MD), School of Medicine (P Mokhtarzadehazar MD), Urmia University of Medical Sciences, Urmia, Iran (R Valizadeh PhD); Department of Forensic Medicine and Toxicology (C Mittal MD), Dr. B. C. Roy Multi-Specialty Medical Research Centre, Kharagpur, India; Institute of Addiction Research (ISFF) (B Moazen MSc), Frankfurt University of Applied Sciences, Frankfurt, Germany; College of Health Science (A I Mohamed MSc), College of Applied and Natural Science (J Mohamed MSc), University of Hargeisa, Hargeisa, Somalia; Molecular Biology Unit (N S Mohamed MSc), Bio-Statistical and Molecular Biology Department (N S Mohamed MSc), Sirius Training and Research Centre, Khartoum, Sudan; Health Economics Division (S Mohammad-pour PhD), Ministry of Health and Medical Education, Mashhad, Iran; Department of Pharmaceutical Sciences (S Mohammed PhD), Notre Dame of Maryland University, Baltimore, MD, USA; Department of Pharmacy (S Mohammed PhD), Mizan-Tepi University, Mizan, Ethiopia; Health Systems and Policy Research Unit (S Mohammed PhD), Department of Community Medicine (A A Olorukooba MSc), Ahmadu Bello University, Zaria, Nigeria; Clinical Epidemiology and Public Health Research Unit (L Monasta DSc, L Ronfani PhD, G Zamagni MSc), Burlo Garofolo Institute for Maternal and Child Health, Trieste, Italy; Department of Epidemiology and Biostatistics (Y Moradi PhD), Social Determinants of Health Research Center (F Moradpour PhD), Kurdistan University of Medical Sciences, Sanandaj, Iran; Computer, Electrical, and Mathematical Sciences and Engineering Division (P Moraga PhD), King Abdullah University of Science and Technology, Thuwal, Saudi Arabia; Department of Public Health (Prof R S Moreira PhD), Oswaldo Cruz Foundation, Recife, Brazil; Department of Public Health (Prof R S Moreira PhD), Federal University of Pernambuco, Recife, Brazil; Department of Biology and Biological Engineering (J Morze PhD), Chalmers University of Technology, Gothenburg, Sweden; College of Medical Sciences (J Morze PhD), SGMK Copernicus University, Warsaw, Poland; Epidemiology Department (S Mousavi MD), Aging Research Institute, Tabriz, Iran; Department of Fruit and Vegetable Product Technology (Prof A Mousavi Khaneghah PhD), Prof. Wacław Dąbrowski Institute of Agricultural and Food Biotechnology State Research Institute, Warsaw, Poland; Research Department (M Mrejen PhD), Instituto de Estudos para Políticas de Saúde (IEPS), São Paulo, Brazil; Unit of Pharmacotherapy, Epidemiology and Economy (S Mubarik MS), University Medical Center Groningen (Prof M J Postma PhD), Department of Internal Medicine (P Vart PhD), University of Groningen, Groningen, Netherlands; Demographic Change and Aging Research Area (A Werdecker PhD), Competence Center of Mortality-Follow-Up of the German National Cohort (R Westerman DSc), Federal Institute for Population Research, Wiesbaden, Germany (Prof U O Mueller MD); Center for Population and Health, Wiesbaden, Germany (Prof U O Mueller MD); School of Medicine (F Mughal FRCGP), Keele University, Keele, UK; Knowledge Management Department (S Mukherjee PhD), Prahlad Omkarwati Foundation (POF), Mumbai, India; Independent Consultant, New Delhi, India (S Mukherjee PhD); Department of Surgery (G D Mukoro MD), Ahmadu Bello University Teaching Hospital, Zaria, Nigeria; Department of Medicine (A Mulita PhD, E Nena MD, P Steiropoulos MD), Democritus University of Thrace, Alexandroupolis, Greece; Department of Surgery (F Mulita PhD, G Verras MD), General University Hospital of Patras, Patras, Greece; Faculty of Medicine (F Mulita PhD), Department of Internal Medicine (G Ntaios PhD), Department of Emergency Medicine (I Pantazopoulos PhD), University of Thessaly, Larissa, Greece; Department of Health Economics (M Muniyandi PhD), National Institute for Research in Tuberculosis, Chennai, India; School of

Veterinary Medicine (F Musaigwa PhD), Department of Biostatistics Epidemiology and Informatics (J Puvvula PhD), University of Pennsylvania, Philadelphia, PA, USA; Research & Innovation Department (Prof K M Musallam MD), Burjeel Medical City, Abu Dhabi, United Arab Emirates; Department of Pediatrics & Pediatric Pulmonology (Prof G Mustafa MD), Institute of Mother & Child Care, Multan, Pakistan; Department of Research Methodology (S Muthu MS), Orthopaedic Research Group, Coimbatore, India; Department of Medical Microbiology and Immunology (S Muthupandian PhD), Mekelle University, Mekelle, Ethiopia; Saveetha Dental College (S Muthupandian PhD), Saveetha Institute of Medical and Technical Sciences (SIMATS), Chennai, India; Department of Neuropsychiatry (W Myung PhD), Seoul National University, Seongnam-si, South Korea; Knowledge Translation and Utilization (Prof A F Nabhan PhD), Egyptian Center for Evidence Based Medicine, Cairo, Egypt; Research and Analytics Department (A J Nagarajan MTech), Initiative for Financing Health and Human Development, Chennai, India; Department of Research and Analytics (A J Nagarajan MTech), Bioinsilico Technologies, Chennai, India; Comprehensive Cancer Center (G Naik MPH), Department of Psychology (D C Schwebel PhD), University of Alabama at Birmingham, Birmingham, AL, USA; Laboratory of Public Health Indicators Analysis and Health Digitalization (M Naimzada MD, N Otstavnov BA), Moscow Institute of Physics and Technology, Dolgoprudny, Russia; Experimental Surgery and Oncology Laboratory (M Naimzada MD), Kursk State Medical University, Kursk, Russia; Department of Pulmonary Medicine (S Nair MD), Government Medical College Trivandrum, Trivandrum, India; Health Action by People, Trivandrum, India (S Nair MD); Department of Community Medicine (T S Nair MD), MOSC Medical College, Kolenchery, India; Medical Laboratory Analysis Department (H H Najmuldeen PhD), Cihan University-Sulaimaniya, Sulaimaniya, Iraq; Department of Dermatology (Prof L Naldi MD), San Bortolo Hospital, Vicenza, Italy; GISED Study Center, Bergamo, Italy (Prof L Naldi MD); Suraj Eye Institute, Nagpur, India (V Nangia MD); National Dental Research Institute Singapore (G G Nascimento PhD), Duke-NUS Medical School, Singapore, Singapore; Department of Applied Pharmaceutical Sciences and Clinical Pharmacy (A Y Naser PhD), Isra University, Amman, Jordan; Department of Biotechnology (M Naveed PhD), University of Central Punjab, Lahore, Pakistan; Department of Health Promotion (A Nazri-Panjaki MSc), Health Promotion Research Center (H Okati-Aliabad PhD), Zahedan University of Medical Sciences, Zahedan, Iran; Diseases Prevention and Control Team (A K Negero MPH), Public Health Department, Gedo, Ethiopia; Department of General Surgery (I Negoi PhD), Fourth Department of General Surgery (D Serban PhD), Emergency University Hospital Bucharest, Bucharest, Romania; Department of Cardiology (R I Negoi PhD), Cardio-Aid, Bucharest, Romania; Department of Oncology (S Negru MD), Victor Babes University of Medicine and Pharmacy, Timisoara, Romania; Faculty of Medicine (Prof C Nejjari PhD), Euromed University of Fes, Fez, Morocco; Faculty of Medicine (Prof C Nejjari PhD), University Sidi Mohammed Ben Abdellah, Fez, Morocco; Department of Community Medicine (S Nepal MD), Kathmandu University, Palpa, Nepal; Department of Neurosciences (Prof C R J Newton MD), Kenya Medical Research Institute/Wellcome Trust Research Programme, Kilifi, Kenya; Department of Biological Sciences (J W Ngunjiri DrPH), University of Embu, Embu, Kenya; Department of Medical Engineering (D H Nguyen BS), University of South Florida, Tampa, FL, USA; Department of Surgery (P T Nguyen MD), Danang Family Hospital, Danang, Viet Nam; Institute for Cancer Control (P T Nguyen MPH), National Cancer Center, Tokyo, Japan; Graduate School of Public Health (P T Nguyen MPH), St. Luke's International University, Chuo-ku, Japan; Department of Urology (T Nguyen MD), Department of Radiology (S Rafiei Alavi MD), University of California Irvine, Irvine, CA, USA; Institute for Mental Health and Policy (Y T Nigatu PhD), Centre for Addiction and Mental Health, Toronto, ON, Canada; Department of General Surgery (T K Nikolouzakis PhD), University Hospital of Heraklion, Heraklion, Crete, Greece;

Department of Internal Medicine (M A Nizam MD), Ziauddin University, Karachi, Pakistan; Department of Health Policy and Management (S Nomura PhD), Keio University, Tokyo, Japan; Department of Microbiology and Molecular Genetics (M Noreen PhD), The Women University Multan, Multan, Pakistan; Department of Clinical Sciences (Prof B Norrving PhD), Lund University, Lund, Sweden; Department of Paediatrics (C A Nri-Ezedi MD), Nnamdi Azikiwe University, Awka, Nigeria; The Cardiac Clinic (Prof M Ntsekhe PhD), Groote Schuur Hospital, Cape Town, South Africa; Unit of Microbiology and Public Health (V Nuñez-Samudio PhD), Institute of Medical Sciences, Las Tablas, Panama; Department of Public Health (V Nuñez-Samudio PhD), Ministry of Health, Herrera, Panama; Public Health Department (D Nurrika PhD), Banten School of Health Science, South Tangerang, Indonesia; Ministry of Research, Technology and Higher Education (D Nurrika PhD), Higher Education Service Institutions (LL-DIKTI) Region IV, Bandung, Indonesia; Department of Applied Economics and Quantitative Analysis (Prof B Oancea PhD), University of Bucharest, Bucharest, Romania; Department of Pediatrics (A O D Ofakunrin MSc), University of Jos, Jos, Nigeria; Department of Pediatrics (A O D Ofakunrin MSc), Jos University Teaching Hospital, Jos, Nigeria; Department of Preventive Medicine (I Oh PhD), Department of Pediatrics (Prof D Yon MD), Kyung Hee University, Seoul, South Korea; Independent Consultant, Sydney, NSW, Australia (S R Okeke PhD); Department of Food and Nutrition (A P Okekunle PhD), Department of Orthopedic Surgery (B Xu MD), Seoul National University, Seoul, South Korea; Department of Food Science and Postharvest Technology (L Okidi MSc), Gulu University, Gulu, Uganda; Department of Medical Physiology (P G Okwute MSc), Babcock University, Ilisan-Remo, Nigeria; Department of Medical Physiology (P G Okwute MSc), Department of Psychiatry (A T Olagunju MD), University of Lagos, Lagos, Nigeria; Department of Nursing Science (M I Olatubi PhD), Bowen University, Iwo, Nigeria; Cardiology Department (G M M Oliveira PhD), Federal University of Rio de Janeiro, Rio de Janeiro, Brazil; Department of Literature, Film, and Theatre Studies (Prof S Oliver PhD), University of Essex, Colchester, UK; Slum and Rural Health Initiative Research Academy (I I Olufadewa MHS), Slum and Rural Health Initiative, Ibadan, Nigeria; Centre for Healthy Start Initiative, Lagos, Nigeria (B O Olusanya PhD, J O Olusanya MBA); Department of Anatomy (G O Oluwatunase MSc), Olabisi Onabanjo University, Sagamu, Nigeria; Department of Pharmacology and Toxicology (Prof H A Omar PhD), Beni-Suef University, Beni-Suef, Egypt; Surgery Department (G L Omer MD), Sulaimani University, Sulaimani, Iraq; ENT Department (G L Omer MD), Tor Vergata University of Rome, Rome, Italy; Non-communicable Disease Prevention Unit (S Ong FAMS), Ministry of Health, Bandar Seri Begawan, Brunei; Early Detection & Cancer Prevention Services (S Ong FAMS), Pantai Jerudong Specialist Centre, Bandar Seri Begawan, Brunei; Department of Biomedical Sciences (K I Onyedibe PhD), Mercer University School of Medicine, Macon, GA, USA; Department of Health (J Opio MPH), Lira District Local Government, Lira, Uganda; Department of Pharmacotherapy and Pharmaceutical Care (M Ordak PhD), Department of Biochemistry and Pharmacogenomics (M Zielińska MPharm), Medical University of Warsaw, Warsaw, Poland; Research Department (E Orellana PhD), Asociacion IDEI Guatemala, Quetzaltenango, Guatemala; University of Port Harcourt, Port Harcourt, Nigeria (Prof O E Orisakwe PhD); Sickle Cell Unit (V N Orish PhD), Ho Teaching Hospital, Ho Municipality, Ghana; Department of Nephrology and Hypertension (Prof A Ortiz MD), The Institute for Health Research Foundation Jiménez Díaz University Hospital, Madrid, Spain; One Health Global Research Group (Prof E Ortiz-Prado PhD), Universidad de las Americas (University of the Americas), Quito, Ecuador; School of Medicine (U L Osuagwu PhD), Translation Health Research Institute (K Rana PhD), Western Sydney University, Campbelltown, NSW, Australia; Department of Optometry and Vision Science (U L Osuagwu PhD), University of KwaZulu-Natal, KwaZulu-Natal, South Africa; Division of Infectious Diseases (Prof A Ouyahia PhD), University Hospital of Setif, Setif, Algeria;

Department of General Surgery (G Ouyang MD), Central South University, ChangSha, China; Department of Respiratory Medicine (Prof M P P A DNB), Department of Forensic Medicine and Toxicology (S Rani MD), Department of Oral and Maxillofacial Surgery (M S MDS, C S N PhD), Jagadguru Sri Shivarathreeswara University, Mysore, India; Department of Forensic Medicine and Toxicology (J Padubidri MD), Kasturba Medical College, Mangalore, Mangalore, India; Systems and Information Engineering (M Pahlavikhah Varnosfaderani MSc), University of Virginia, Charlottesville, VA, USA; Department of Neurology (Prof P K Pal DM), National Institute of Mental Health and Neurosciences, Bengalore, India; Research Institute for Medicines-FFUL (iMed.ULisboa) (C Palladino PhD), Research Institute for Medicines (Prof N Taveira PhD), Universidade de Lisboa (University of Lisbon), Lisbon, Portugal; Department of Public Health (R Palladino MD), University of Naples Federico II, Naples, Italy; Department of Mental Health (R F Palma-Alvarez PhD), Hospital Universitari Vall d'Hebron (Vall d'Hebron University Hospital), Barcelona, Spain; Department of Psychiatry, Mental Health and Addictions (R F Palma-Alvarez PhD), Vall d'Hebron Institut de Recerca (Vall d'Hebron Research Institute), Barcelona, Spain; Department of Public Health (A Pana PhD), Babes Bolyai University, Cluj Napoca, Romania; Department of Health Metrics (A Pana PhD), Center for Health Outcomes & Evaluation, Bucharest, Romania; Research Department (A Pandey MPH), Public Health Research Society Nepal, Kathmandu, Nepal; Saveetha Medical College and Hospitals (S R Pandi-Perumal MSc), Centre of Molecular Medicine and Diagnostics (COMManD) (Prof S Patil PhD), Saveetha University, Chennai, India; Division of Research and Development (S R Pandi-Perumal MSc), Lovely Professional University, Phagwara, India; National Research and Innovation Agency, Jakarta, Indonesia (H U Pangaribuan MSc); Department of Ophthalmology (G D Panos PhD), Nottingham University Hospitals QMC Campus, Nottingham, UK; Division of Ophthalmology & Visual Sciences (G D Panos PhD), University of Nottingham, Nottingham, UK; Department of Science and Mathematics (Prof P Papadopoulou PhD), Deree-The American College of Greece, Athens, Greece; Vision and Eye Research Institute (Prof S Pardhan PhD), Anglia Ruskin University, Cambridge, UK; Department of Epidemiology and Community Health (R R Parikh MD), University of Minnesota School of Public Health, Minneapolis, MN, USA; Research Center (A Parthasarathi MD), Allergy Asthma and Chest Center, Mysore, India; Cardiology Department (D Pasupula MD), MercyOne North Iowa Medical Center, Mason City, IA, USA; Department of Epidemiology, Human Genetics and Environmental Sciences (J R Patel PhD), The University of Texas Health Science Center at Houston, Dallas, TX, USA; Department of Epidemiology (J R Patel PhD), University of Arkansas for Medical Sciences, Little Rock, AR, USA; Department of Poverty, Gender and Youth (S K Patel PhD), Population Council, New Delhi, India; Research Consultancy (A R Pathan PhD), Author Gate Publications, Malegaon, India; College of Dental Medicine (Prof S Patil PhD), Roseman University of Health Sciences, South Jordan, UT, USA; Second Department of Internal Medicine (D Patoulias PhD), European Interbalkan Medical Center, Thessaloniki, Greece; Department of Internal Medicine (V Patthipati MD), Advent Health, Palm Coast, FL, USA; Department of Hospital Medicine (V Patthipati MD), Sound Physicians, Palm Coast, FL, USA; Clinical Research Department (P Pedersini MSc, J H Villafañe PhD), IRCCS Fondazione Don Carlo Gnocchi, Milan, Italy; Department of Outpatient (M Peng MPH), Taihe Hospital, Shiyan, China; The First Clinical College (M Peng MPH), Hubei University of Medicine, Shiyan, China; Department of Neurology (U Pensato MD), IRCCS Humanitas Research Hospital, Milan, Italy; Curtin School of Population Health (Prof G Pereira PhD), Curtin University, Bentley, WA, Australia; Centre for Fertility and Health (Prof G Pereira PhD), Department of Chemical Toxicology (M W Wojewodzic PhD), Norwegian Institute of Public Health, Oslo, Norway; Department of Orthopedics (J Pereira MS), Department of Dermatology, Venereology and Leprosy (Prof M M Shenoy MD), Yenepoya

Medical College, Mangalore, India; International Institute for Educational Planning (IIEP) (Prof M F P Peres MD), Albert Einstein Hospital, São Paulo, Brazil; Mario Negri Institute for Pharmacological Research, Bergamo, Italy (N Perico MD, Prof G Remuzzi MD); Facultad de Medicina (Faculty of Medicine) (F E Petermann-Rocha PhD), Universidad Diego Portales (Diego Portales University), Santiago, Chile; Department of Medicine, Endocrinology Unit (R Pezzani PhD), University of Padova, Padova, Italy; AIROB (Associazione Italiana Ricerca Oncologica di Base), Padova, Italy (R Pezzani PhD); Department of Psychiatry (Prof M R Phillips MD), Department of Neurology (Prof N Scarmeas PhD), Department of Health and Behavior Studies (Prof I D Sigfusdottir PhD), Columbia University, New York, NY, USA; National Centre for Disease Prevention and Health Promotion (D Pierannunzio PhD), National Institute of Health, Roma, Italy; Department of Pediatric Orthopedic Surgery (M Pigeolet MD), Hôpital Necker - Enfants Malades, Paris, France; Department of Neonatology (N Plakkal MD), Department of Preventive and Social Medicine (G Saya MD), Jawaharlal Institute of Postgraduate Medical Education and Research, Puducherry, India; Research School of Chemistry and Applied Biomedical Sciences (E Plotnikov PhD), Tomsk Polytechnic University, Tomsk, Russia; Mental Health Research Institute (E Plotnikov PhD), Tomsk National Research Medical Center of the Russian Academy of Sciences, Tomsk, Russia; Clinical Academic Department of Pediatrics (Prof D Poddighe PhD), University Medical Center (UMC), Astana, Kazakhstan; Data Driven Health Division (P Pollner PhD), Hungarian Healthcare Management Association, Budapest, Hungary; Department of Data Management and Analysis (R Poluru PhD), The INCLEN Trust International, New Delhi, India; Discipline of General Practice (Prof C D Pond PhD), University of Newcastle, Callaghan, NSW, Australia; Non-communicable Diseases Research Center (N Pourtaheri PhD), Bam University of Medical Sciences, Bam, Iran; Centro de Investigaciones Clinicas (Clinical Research Center) (S I Prada PhD), Fundación Valle del Lili (Valle del Lili Foundation), Cali, Colombia; Centro de Estudios en Protección Social y Economía de la Salud (PROESA) (Research Center for Social and Health Economics) (S I Prada PhD), Universidad ICESI (ICESI University), Cali, Colombia; Department of Biochemistry (V K Prajapati PhD), Central University of Rajasthan, Ajmer, India; Askok & Rita Patel Institute of Physiotherapy (V Prakash PhD), Charotar University of Science and Technology, Anand, India; Department of Clinical Research and Epidemiology (M Prasad MD), Institute of Liver and Biliary Sciences, New Delhi, New Delhi, India; Department of Biochemistry (Prof A Prashant PhD), Jagadguru Sri Shivarathreeswara University, Mysuru, India; Department of Computer Science (N H Qasim PhD), Cihan University-Sulaymaniyah, Sulaymaniyah, Iraq; Department of Cardiology (G Qian MS), Third Military Medical University, Chongqing, China; College of Medicine (A Radfar MD), University of Central Florida, Orlando, FL, USA; Department of Medical Oncology (Prof V Radhakrishnan MD), Cancer Institute (W.I.A), Chennai, India; Department of Epidemiology and Biostatistics (H Raeisi Shahraki PhD), Shiraz University of Medical Sciences, Shahrekord, Iran; Research and Development Coordination (I Rafique PhD), National Institutes of Health, Islamabad, Pakistan; UO Neurologia, Salute Pubblica e Disabilità (Neurology, Public Health and Disability Unit) (A Raggi PhD), Fondazione IRCCS Istituto Neurologico Carlo Besta (Carlo Besta Neurological Institute), Milan, Italy; Department of Health Sciences (Prof F Rahim PhD), Cihan University-Sulaimaniya, Sulaymaniyah, Iraq; Cihan University Sulaimaniya Research Center (CUSRC), Sulaymaniyah, Iraq (Prof F Rahim PhD); Institute of Health and Wellbeing (M Rahman PhD), Federation University Australia, Berwick, VIC, Australia; Communication & Information Sciences (T Rahman Mcom), Independent Consultant, Dhaka, Bangladesh; Future Technology Research Center (A Rahmani PhD), National Yunlin University of Science and Technology, Yunlin, Taiwan; Centre for Chronic Disease Control, New Delhi, India (P Rajput PhD); Research and Innovation Division (J Rana MPH), South Asian Institute for Social Transformation (SAIST), Dhaka, Bangladesh; Health and Public Policy Department (C L

Ranabhat PhD), Global Center for Research and Development, Kathmandu, Nepal; Centre for Clinical Pharmacology (N Rancic PhD), University of Defence in Belgrade, Belgrade, Serbia; Centre for Clinical Pharmacology (N Rancic PhD), Medical College of Georgia at Augusta University, Belgrade, Serbia; School of Humanities and Social Sciences (S Ranjan MA), Indian Institute of Technology Mandi, Mandi, India; Department of Oral Pathology (S Rao MDS), Sharavathi Dental College and Hospital, Shimogga, India; Data Analytic Services (D P Rasali PhD), British Columbia Centre for Disease Control, Vancouver, BC, Canada; University of Social Welfare and Rehabilitation Sciences, Tehran, Iran (V Rashedi PhD); Department of Medicine (A M Rashid MD), Jinnah Sindh Medical University, Karachi, Pakistan; Department of Geography (A Rasul PhD), Soran University, Soran, Iraq; Section of Pulmonary and Critical Care Medicine (N Ravikumar MD), University of Chicago, Chicago, IL, USA; Inovus Medical, St Helens, UK (D L Rawaf MRCS); Academic Public Health England (Prof S Rawaf MD), Public Health England, London, UK; Department of Computer Science (R Rawassizadeh PhD), Boston University, Boston, MA, USA; Department Biological Sciences (Prof E M M Redwan PhD), King Abdulaziz University, Jeddah, Egypt; Department of Protein Research (Prof E M M Redwan PhD), Research and Academic Institution, Alexandria, Egypt; Grenoble Computer Science Laboratory (LIG) (F Rehman PhD), University of Grenoble Alpes, Grenoble, France; Brien Holden Vision Institute, Sydney, NSW, Australia (Prof S Resnikoff MD); Unisabana Center for Translational Science (L F Reyes PhD), Universidad de La Sabana (Savannah University), Chia, Colombia; Critical Care Department (L F Reyes PhD), Clinica Universidad De La Sabana (Savannah University Clinic), Chia, Colombia; Network of Immunity in Infection, Malignancy and Autoimmunity (NIIMA) (Prof N Rezaei PhD), Universal Scientific Education and Research Network (USERN), Tehran, Iran; Department of Epidemiology and Biostatistics (Prof M Rezaeian PhD), Rafsanjan University of Medical Sciences, Rafsanjan, Iran; School of Physiotherapy (D C Ribeiro PhD), University of Otago, Dunedin, New Zealand; Department of Surgery (J Rickard MD), University of Minnesota, Minneapolis, MN, USA; Department of Surgery (J Rickard MD), University Teaching Hospital of Kigali, Kigali, Rwanda; Carlos Slim Foundation, Mexico City, Mexico (M Rios-Blancas DSc); Departamento de Farmacologia y toxicologia (Department of Pharmacology and Toxicology) (Prof J A B Rodriguez PhD), Universidad de Antioquia (University of Antioquia), Medellin, Colombia; Department of Clinical Research (L Roever PhD), Federal University of Uberlândia, Uberlândia, Brazil; Gilbert and Rose-Marie Chagoury School of Medicine (L Roever PhD), Lebanese American University, Beirut, Lebanon; Center for Indigenous Health Research (P Rohloff MD), Wuqu' Kawoq Maya Health Alliance, Tecpan, Guatemala; Faculty of Nursing (D S Romadlon PhD), Chulalongkorn University, Bangkok, Thailand; School of Medicine (M Rostamian PhD), Gonabad University of Medical Sciences, Gonabad, Iran; Faculty of Medicine (B Roy PhD), Quest International University Perak, Ipoh, Malaysia; Department of Labour (P Roy PhD), Directorate of Factories, Government of West Bengal, Kolkata, India; Centro de Investigación Palmira (Palmira Research Center) (E Rubagotti PhD), Corporación Colombiana de Investigación Agropecuaria AGROSAVIA (Colombian Agricultural Research Corporation), Bogota, Colombia; Department of Health Statistics (S F Rumisha PhD), National Institute for Medical Research, Dar es Salaam, Tanzania; Department of Internal Medicine (G M Rwegerera MD), University of Botswana, Gaborone, Botswana; Department of Cardiology and Internal Medicine (Prof A Rynkiewicz PhD), University of Warmia and Mazury, Olsztyn, Poland; Institute of Neuroscience and Physiology (Prof K S Sunnerhagen PhD), Occupational and Environmental Medicine Department (L Stockfelt PhD), University of Gothenburg, Gothenburg, Sweden; Department of Neurocare (Prof K S Sunnerhagen PhD), Sabzevar University of Medical Sciences, Gothenburg, Sweden; Directorate General Health Prevention, Communicable Diseases and International Prophylaxis (M Sabbatucci PhD), Ministry of Health, Rome,

Italy; Department of Medical Pharmacology (M M Saber-Ayad MD), Public Health and Community Medicine Department (M R Salem MD), Cairo University, Giza, Egypt; Multidisciplinary Laboratory Foundation University School of Health Sciences (FUSH) (Prof U Saeed PhD), Foundation University, Islamabad, Pakistan; International Center of Medical Sciences Research (ICMSR), Islamabad, Pakistan (Prof U Saeed PhD); Department of Community Medicine and Family Medicine (S S Sahoo MD, M Verma MD), Department of Anatomy (A Singal PhD), Department of Radiodiagnosis (P Singh MD), All India Institute of Medical Sciences, Bathinda, India; Department of Statistics (M R Sajid PhD), University of Gujrat, Pakistan, Gujrat, Pakistan; Institute for Employment Research, Nuremberg, Germany (J W Sakshaug PhD); Medical Laboratory (S Salahi BMedSc), Azad University of Medical Sciences, Tehran, Iran; Advanced Therapy Medicinal Products Department (S Salahi MD), Royan Institution, Tehran, Iran; Department of Integrated Health Education (Prof L B Salaroli PhD), Federal University of Espirito Santo, Vitória, Brazil; Technology Management Department (Prof M Z Y Salem PhD), University College of Applied Sciences, Gaza, Palestine; School of Economics and Management (Prof M Z Y Salem PhD), University of Kassel, Kassel, Germany; Department of Neurology (S Samadzadeh MD), University of Southern Denmark, Odense, Denmark; Department of Urology (K A Samara MD), The University of Texas Health Science Center at San Antonio, San Antonio, TX, USA; Department of Anatomy (Prof V P Samuel PhD), Ras Al Khaimah Medical and Health Sciences University, Ras Al Khaimah, United Arab Emirates; Institute of Neuroanatomy (N Sanadgol PhD), Uniklinik Rhine-Westphalia Technical University of Aachen, Aachen, Germany; Department of Applied Sciences (E Sanganyado PhD), Northumbria University, Newcastle upon Tyne, UK; Science Policy Division (E Sanganyado PhD), Zimbabwe Young Academy of Sciences, Gwanda, Zimbabwe; Department of Pediatrics (R K Sanjeev MD), Pravara Institute of Medical Sciences, Loni, India; College of Public Health (I N Santri PhD), University of Ahmad Dahlan, Yogyakarta, Indonesia; Pharmacy Study Program (M A Sarasmita PharmD), Udayana University, Badung, Indonesia; Indira Gandhi Medical College and Research Institute, Puducherry, India (A Saravanan MD); Department of Orthpaedics and Trauma Surgery (B Saravi PhD), University of Freiburg, Freiburg, Germany; Department of Orthopaedics (B Saravi PhD), Loretto Hospital Freiburg, Freiburg, Germany; Department of Public Health (Y Sarikhani PhD), Jahrom University of Medical Sciences, Jahrom, Iran; Department of Health and Society (Prof R Sarmiento-Suárez MPH), University of Applied and Environmental Sciences, Bogota, Colombia; National School of Public Health (Prof R Sarmiento-Suárez MPH), Carlos III Health Institute, Madrid, Spain; Faculty of Health & Social Sciences (B Sathian PhD), Bournemouth University, Bournemouth, UK; Department of Family and Preventive Medicine (T Sathish PhD), Rollins School of Public Health (Prof D A Sleet PhD), Emory University, Atlanta, GA, USA; IRCCS Istituti Clinici Scientifici Maugeri (IRCCS Maugeri Scientific Clinical Institute), Milan, Italy (D Sattin PsyD); Department of Medical Informatics (J Saulam MSc), Kagawa University, Miki-cho, Japan; Food Processing and Nutrition (J Saulam MSc), Karnataka State Akkamahadevi Women's University, Vijayapura, India; Department of Educational Sciences (Y Sayadi PhD), Farhangian University, Kermanshah, Iran; Department of Post-Harvest Technology and Marketing (A Sayeed MSc), Patuakhali Science and Technology University, Patuakhali, Bangladesh; National Centre for Epidemiology and Population Health (M Sayeed MS, A Talukder MSc), Australian National University, Acton, ACT, Australia; Market Access Division (M Saylan MD), Bayer, Istanbul, Türkiye; Department of Neurology (Prof N Scarmeas PhD), National and Kapodistrian University of Athens, Athens, Greece; Cardiovascular Research Center (A Schuermans BSc), Massachusetts General Hospital, Cambridge, MA, USA; Department of Cardiovascular Sciences (A Schuermans BSc, J Van den Eynde BSc), Katholieke Universiteit Leuven, Leuven, Belgium; Clinic for Conservative Dentistry and Periodontology (Prof F Schwendicke PhD), University Hospital of the Ludwig-Maximilians-University

Munich, Munich, Germany; Department of Medical Statistics (M Šekerija PhD), University of Zagreb, Zagreb, Croatia; Department of Epidemiology and Prevention of Chronic Noncommunicable Diseases (M Šekerija PhD), Croatian Institute of Public Health, Zagreb, Croatia; Faculty of Dentistry (S Selvaraj PhD), AIMST University, Bedong, Malaysia; Department of Biomedical Sciences (P Sengupta PhD), Gulf Medical University, Ajman, United Arab Emirates; Emergency Department (S Senthilkumaran MD), Manian Medical Centre, Erode, India; Department of Medicine and Surgery (Y Sethi MBBS), Government Doon Medical College, Dehradun, India; Department of Infectious Diseases and Microbiology (P A Shah MBBS), Rajiv Gandhi University of Health Sciences, Bangalore, India; HepatoPancreatoBiliary Surgery and Liver Transplant Department (P A Shah MBBS), Healthcare Global Limited Cancer Care Hospital, Bangalore, India; Division of Preventive Cardiology (I Shahid MBBS), Houston Methodist Academic Institute, Houston, TX, USA; Independent Consultant, Karachi, Pakistan (M A Shaikh MD); Department of Pathology and Laboratory Medicine (S Sham MD), Northwell Health, New York, NY, USA; Research Institute of Pharmaceutical Sciences (H Shamshad PhD), International Center for Chemical and Biological Sciences (S Ullah MSc), University of Karachi, Karachi, Pakistan; Department of Pathobiology (M Shamshirgaran PhD), Shahid Bahonar University of Kerman, Kerman, Iran; Department of Clinical Review and Safety (S Sharfaei MD), Baim Institute for Clinical Research, Boston, MA, USA; Department of General Surgery (M Shariff MD), Mayo Clinic Foundation for Medical Education and Research, Rochester, MN, USA; Faculty of Medicine (J Sharifi-Rad PhD), Facultad de Medicina, Universidad del Azuay (University of Azuay), Cuenca, Ecuador; University School of Management and Entrepreneurship (R Sharma PhD), Delhi Technological University, Delhi, India; Department of Physiotherapy (S Sharma PhD), Kathmandu University, Dhulikhel, Nepal; Department of Microbiology (R P Shastry PhD), Yenepoya University, Mangalore, India; Department of Engineering (A Shavandi PhD), Department of Molecular Biology (R Shey PhD), Free University of Brussels, Brussels, Belgium; Bioengineering Department (A Shayan BS), Clemson University, Clemson, SC, USA; Botany and Microbiology Department (A M E Shehabeldine PhD), Al-Azhar University, Cairo, Egypt; Psychology Department (J Shen PhD), University of Massachusetts Lowell, Boston, MA, USA; Department of Biochemistry and Molecular Biology (R Shey PhD), University of Buea, Buea, Cameroon; Tokyo Foundation for Policy Research, Tokyo, Japan (Prof K Shibuya MD); Department of Public Health (D Shiferaw MPH), Dambi Dollo University, Dembi Dollo, Ethiopia; National Institute of Infectious Diseases, Tokyo, Japan (M Shigematsu PhD); Department of Pediatrics (Prof J Shin MD), Yonsei University College of Medicine, Seoul, South Korea; Finnish Institute of Occupational Health, Helsinki, Finland (R Shiri PhD); Department of Clinical Immunology and Hematology (V Shivarov PhD), Sofiamed University Hospital, Sofia, Bulgaria; Department of Genetics (V Shivarov PhD), Sofia University "St. Kliment Ohridiski", Sofia, Bulgaria; School of Pharmacy (S Shrestha PharmD), Monash University, Selangor Darul Ehsan, Malaysia; National Institute of Psychology (K Shuja MS), Quaid-i-Azam University, Islamabad, Pakistan; The Cooper Institute, Dallas, TX, USA (K Shuval PhD); Global Health Research Center (Y Si PhD), Duke Kunshan University, Kunshan, China; Department of Medical Microbiology and Infectious Diseases (E E Siddig MD), Erasmus University, Rotterdam, Netherlands; Department of Psychology (Prof I D Sigfusdottir PhD), Reykjavik University, Reykjavik, Iceland; Center of Potential and Innovation of Natural Resources (Prof L M R Silva PhD), Polytechnic Institute of Guarda, Guarda, Portugal; Health Sciences Research Centre (Prof L M R Silva PhD), University of Beira Interior, Covilhã, Portugal; Faculty of Behavioural, Management and Social Sciences (BMS) (J P Simões PhD), University of Twente, Enschede, Netherlands; School of Health (Prof C R Simpson PhD), Victoria University of Wellington, Wellington, New Zealand; School of Public Health & Zoonoses (B B Singh PhD), Guru Angad Dev Veterinary & Animal Sciences University, Ludhiana, India; Department of

Biochemistry (B Singh PhD), Central University of Punbjab, Bathinda, India; Department of Neurology (S Sivakumar MD), University of Massachusetts Medical School, Worcester, MA, USA; Clinical Branch (V Y Skryabin MD), Moscow Research and Practical Centre on Addictions, Moscow, Russia; Department of Infectious Diseases and Epidemiology (A A Skryabina MD), Department of Internal Disease (A V Starodubova DSc), Pirogov Russian National Research Medical University, Moscow, Russia; Division of Injury Prevention (Prof D A Sleet PhD), The Bizzell Group, Atlanta, GA, USA; Department of Neuroscience (M Solmi MD), University of Ottawa, Ottawa, ON, Canada; Department of Nursing (Y Solomon MSc), Department of Public Health (Y M Tefera MPH), Dire Dawa University, Dire Dawa, Ethiopia; Centro de Investigación Biomédica en Red Enfermedades Respiratorias (Center for Biomedical Research in Respiratory Diseases Network), Madrid, Spain (Prof J B Soriano MD); Hull York Medical School (I N Soyiri PhD), University of Hull, Hull City, UK; Division of Community Medicine (C T Sreeramareddy MD), International Medical University, Kuala Lumpur, Malaysia; Department of Pediatric Cardiology (J R Starnes MD), Vanderbilt University Medical Center, Nashville, TN, USA; Department of Research and Learning (J R Starnes MD), Lwala Community Alliance, Rongo, Kenya; Central Research Institute of Cytology and Genetics (E Varavikova PhD), Federal Research Institute for Health Organization and Informatics of the Ministry of Health (FRIHOI), Moscow, Russia (Prof V I Starodubov DSc); Nutrition and Dietetics Department (A V Starodubova DSc), Federal Research Institute of Nutrition, Biotechnology and Food Safety, Moscow, Russia; Department of Population Health (Prof S Stranges MD), Luxembourg Institute of Health, Strassen, Luxembourg; Department of Pathophysiology (K Stroumpoulis PhD), European University Cyprus Medical School, Engomi, Cyprus; Center for Biotechnology and Microbiology (M Suleman PhD), University of Swat, Mingora, Pakistan; School of Life Sciences (M Suleman PhD), Xiamen University, China, Xiamen, China; National Institute of Epidemiology (R Suliankatchi Abdulkader MD), Indian Council of Medical Research, Chennai, India; Mental Health Research (A Sultana MD), Independent Consultant, Khulna, Bangladesh; Division of Global Mental Health (A Sultana MD), EviSyn Health, Khulna, Bangladesh; Rural Health Research Institute (Prof J Sun PhD), Charles Sturt University, Bathurst, NSW, Australia; Institute of Integrated Intelligence and Systems (Prof J Sun PhD), Griffith University, QLD, Australia; Nursing Professional Education Study Program (S Susanty PhD), University Halu Oleo, Kendari, Indonesia; Department of Analytical and Applied Economics (C K Swain MPhil), Utkal University, Bhubaneswar, India; Department of Sociology (Prof B L Sykes PhD), Cornell University, Ithaca, NY, USA; Department of Clinical Outcomes (Prof L Szarpak PhD), Maria Sklodowska-Curie Medical Academy, Warsaw, Poland; Department of Clinical Research and Development (Prof L Szarpak PhD), LUXMED Group, Warsaw, Poland; Department of Dermatology (M D Szeto BS), University of Colorado, Aurora, CO, USA; Department of Neurology (P Tabaee Damavandi MD), Neurocenter of Southern Switzerland (NSI), Lugano, Switzerland; Department of Basic Medical Sciences (S Tabatabaeizadeh PhD), Department of Internal Medicine (S Tabatabaeizadeh PhD), Islamic Azad University, Mashhad, Iran; School of Social Work (Prof K M Tabb PhD), University of Illinois, Urbana, IL, USA; CICS-UBI - Health Sciences Research Centre (Prof L M Taborda-Barata PhD), University of Beira Interior, Covilha, Portugal; Department of Immunoallergology (Prof L M Taborda-Barata PhD), Cova da Beira University Hospital Center, Covilha, Portugal; Cancer Control Center (T Tabuchi MD), Osaka International Cancer Institute, Osaka, Japan; Department of Pediatrics (B T Tadesse MD), Hawassa University, Hawassa, Ethiopia; International Vaccine Institute, Seoul, South Korea (B T Tadesse MD); Living Systems Institute (Y Taheri Abkenar PharmD), Department of Health and Community Sciences (A Udoh PhD), University of Exeter, Exeter, UK; Department of Biostatistics and Epidemiology (M Taheri Soodejani PhD), Shahid Sadoughi University of Medical Sciences, Yazd, Iran; Department of Environmental, Agricultural and Occupational

Health (J Taiba MPH), University of Nebraska Medical Center, Omaha, NE, USA; Statistics Discipline (A Talukder MSc), Khulna University, Khulna, Bangladesh; Department of Medicine (J L Tamuzi MSc), Northlands Medical Group, Omuthiya, Namibia; State Key Laboratory of Numerical Modeling for Atmospheric Sciences and Geophysical Fluid Dynamics (LASG) (H Tang PhD), Chinese Academy of Sciences, Beijing, China; Department of Economics (N Y Tat MS), Rice University, Houston, TX, USA; Department of Research and Innovation (N Y Tat MS), Enventure Medical Innovation, Houston, TX, USA; University Institute "Egas Moniz", Monte da Caparica, Portugal (Prof N Taveira PhD); Department of Urology (M Teimoori MD), Sabzevar University of Medical Sciences, Sabzevar, Iran; Department of Pharmacology (P Thangaraju MD), All India Institute of Medical Sciences, Raipur, India; Public Health Department (Prof K R Thankappan MD), Amrita Institute of Medical Sciences, Kochi, India; Department of Endocrinology, Diabetes and Metabolism (Prof N Thomas PhD), Christian Medical College and Hospital (CMC), Vellore, India; Department of Psychiatry (C C Thum MB BCh BAO), Hospital Sultan Abdul Aziz Shah Universiti Putra Malaysia, Serdang, Malaysia; Faculty of Biomedical Engineering (A Tichopad PhD), Czech Technical University, Prague, Czech Republic; Faculty of Public Health (J H V Ticoalu MPH), Universitas Sam Ratulangi, Manado, Indonesia; Public Health Department (T Y Tiruye PhD), Debre Markos University, Debre Markos, Ethiopia; Neuromuscular Rehabilitation Research Center (S Tohidast PhD), Semnan University of Medical Sciences, Semnan, Iran; Nutritional Epidemiology Research Team (EREN) (M Touvier PhD), National Institute for Health and Medical Research (INSERM), Paris, France; Department of Health (N M Tran MD), Children's Hospital 1, Ho Chi Minh City, Viet Nam; Department of Surgical, Medical, Molecular Pathology and Critical Care Medicine (D Trico MD), University of Pisa, Pisa, Italy; Adult Learning Disability Service (S J Tromans PhD), Leicestershire Partnership National Health Service Trust, Leicester, UK; Internal Medicine Department (V T Truong MD), Nazareth Hospital, Philadelphia, PA, USA; School of Medicine (T T Truyen MD), Nam Can Tho University, Can Tho, Viet Nam; College of Public Health (C S Ubah MPH), Temple University, Philadelphia, PA, USA; Health Department (I Ulhaq PhD), Ministry of Health, Peshawar, Pakistan; Health Department (I Ulhaq PhD), Directorate General of Health Services, Peshawar, Pakistan; Department of Zoology (S Ullah PhD), Division of Science and Technology (S Ullah PhD), University of Education, Lahore, Lahore, Pakistan; Department of Life Sciences (M Umair PhD), University of Management and Technology, Lahore, Pakistan; Department of Community Medicine (C D Umeokonkwo MPH), Alex Ekwueme Federal University Teaching Hospital Abakaliki, Abakaliki, Nigeria; Department of Genomic Research on Complex Diseases (A Umesh MTech), Centre for Cellular and Molecular Biology, Hyderabad, India; Center for Neurodegenerative Diseases and the Aging Brain (D Urso MD), University of Bari, Tricase, Italy; College of Health and Sport Sciences (A G Vaithinathan MSc), University of Bahrain, Salmanya, Bahrain; Clinical Cancer Research Center (S Valadan Tahbaz PhD), Milad General Hospital, Tehran, Iran; Department of Public Health and Epidemiology (O Varga PhD), University of Debrecen, Debrecen, Hungary; UKK Institute, Tampere, Finland (Prof T J Vasankari MD); Faculty of Medicine and Health Technology (Prof T J Vasankari MD), Tampere University, Tampere, Finland; School of Medicine (Prof L J Veerman PhD), Griffith University, Gold Coast, QLD, Australia; Raffles Neuroscience Centre (Prof N Venketasubramanian MBBS), Raffles Hospital, Singapore, Singapore; Department of Statistics (D Venugopal PhD), Manonmaniam Sundaranar University, Tirunelveil, India; Department of Gamete Immunobiology (P Verma PhD), ICMR - National Institute for Research in Reproductive Health, Mumbai, India; Drug Combination Department (P Verma PhD), Innoplexus, Pune, India; Department Pediatric Hematology and Oncology (G I Villanueva MD), Hospital de Clinicas Jose de San Martin, Cuidad Autonoma de Buenos Aires, Argentina; School of Mathematics and Statistics (Prof P J Villeneuve PhD), Carleton University, Ottawa, ON, Canada; Occupational Health

Unit (Prof F S Violante MD), Sant'Orsola Malpighi Hospital, Bologna, Italy; Department of Health Care Administration and Economics (Prof V Vlassov MD), National Research University Higher School of Economics, Moscow, Russia; Faculty of Information Technology (B Vo PhD), HUTECH University, Ho Chi Minh City, Viet Nam; Department of Medical Oncology (S R Volovat PhD), University of Medicine and Pharmacy "Grigore T Popa" Iasi, Iaşi, Romania; Department of Medical Oncology (S R Volovat PhD), Regional Institute of Oncology, Iaşi, Romania; Center for Experimental Microsurgery (V Volovici PhD), Iuliu Hațieganu University of Medicine and Pharmacy, Cluj-Napoca, Romania; Department of Pediatric Endocrinology (R Vukovic PhD), Mother and Child Healthcare Institute of Serbia "Dr Vukan Cupic", Belgrade, Serbia; Office of Research, Innovation, and Commercialization (ORIC) (Prof Y Waheed PhD), Shaheed Zulfiqar Ali Bhutto Medical University (SZABMU), Islamabad, Pakistan; Gilbert and Rose-Marie Chagoury School of Medicine (Prof Y Waheed PhD), Lebanese American University, Byblos, Lebanon; Department of Cultures, Societies and Global Studies (R G Wamai PhD), Northeastern University, Boston, MA, USA; School of Public Health (R G Wamai PhD), University of Nairobi, Nairobi, Kenya; School of Public Health (F Wang PhD), Xuzhou Medical University, Xuzhou, China; Department of Neurosurgery (S Wang MD), Department of Neurology (C Zhang MD), National Center for Neurological Diseases (C Zhang MD), Capital Medical University, Beijing, China; Department of Gastroenterology (S Wang PhD), Shanghai Jiaotong University Affiliated Sixth People's Hospital, Shanghai, China; Department of Parasitology (Prof K G Weerakoon PhD), Department of Community Medicine (N D Wickramasinghe MD), Rajarata University of Sri Lanka, Anuradhapura, Sri Lanka; Key Laboratory of Shaanxi Province for Craniofacial Precision Medicine Research (Y Wen PhD), Stomatological Hospital (College) of Xi'an Jiaotong University, Xi'an, China; Department of Medicine (Prof T Wijeratne MD), University of Rajarata, Saliyapura Anuradhapuraya, Sri Lanka; Bone and Joint Research Group (Prof A D Woolf MBBS), Royal Cornwall Hospital, Truro, UK; Global Alliance for Musculoskeletal Health, Truro, UK (Prof A D Woolf MBBS); Department of Rheumatology and Immunology (D Wu PhD), Sichuan Provincial People's Hospital, Chengdu, China; School of Public Health (H Xiao PhD), Zhejiang University, Zhejiang, China; Department of Public Health Science (H Xiao PhD), Fred Hutchinson Cancer Research Center, Seattle, WA, USA; Department of Orthopedic Surgery (B Xu MD), Tianjin Hospital, Tianjin, China; Cardiovascular Program (X Xu PhD), The George Institute for Global Health, Sydney, NSW, Australia; Australian Institute of Health Innovation (L Yadav PhD), Macquarie University, Macquarie Park, NSW, Australia; Department of Basic Medical Sciences (S Yaghoubi PhD), Neyshabur University of Medical Sciences, Neyshabur, Iran; Department of Cancer Epidemiology and Prevention Research (L Yang PhD), Alberta Health Services, Calgary, AB, Canada; Faculty of Medicine (Y Yano MD), Department of Public Health (N Yonemoto PhD), Juntendo University, Tokyo, Japan; National Center for Chronic and Noncommunicable Disease Control and Prevention (P Ye MPH), Chinese Center for Disease Control and Prevention, Beijing, China; Department of Family Medicine (S A Yesuf MSc), St. Peter's Specialized Hospital, Addis Ababa, Ethiopia; Department of Health Management (A Yiğit PhD, V Yiğit PhD), Süleyman Demirel Üniversitesi (Süleyman Demirel University), Isparta, Türkiye; Department of Neuropsychopharmacology (N Yonemoto PhD), National Center of Neurology and Psychiatry, Kodaira, Japan; Department of Health Policy and Management (Prof M Z Younis PhD), Jackson State University, Jackson, MS, USA; School of Business & Economics (Prof M Z Younis PhD), Universiti Putra Malaysia (University of Putra Malaysia), Kuala Lumpur, Malaysia; Association for Socially Applicable Research (ASAR), Pune, India (S Zadey MS); Department of Emergency Medicine (S Zadey MS), Global Emergency Medicine Innovation and Implementation (GEMINI) Research Center, Durham, NC, USA; Epidemiology and Cancer Registry Sector (Prof V Zadnik PhD), Institute of Oncology Ljubljana, Ljubljana, Slovenia; Department of Biology (M N

Zahid PhD), University of Bahrain, Zallaq, Bahrain; Faculty of Medicine and Health Sciences (F Zakham PhD), Hodeidah University, Hodeidah, Yemen; Department of Pharmacology (B A Zaman MSc), University of Duhok, Duhok, Iraq; Department of Health Sciences (S Zaman MSc), James Madison University, Harrisonburg, VA, USA; Hospital San Juan de Dios, Tarija, Bolivia (N Zamora MD); Department of Neuroscience (R Zand MD), Geisinger Health System, Danville, PA, USA; Sant'Elia Hospital (A Zanghì MD), University of Catania, Caltanissetta, Italy; Research and Development Department (I Zare BSc), Sina Medical Biochemistry Technologies, Shiraz, Iran; Department of Zoology and Entomology (M G M Zeariya PhD), Al-Azhar University, Cairo, Egypt; Department of Anesthesiology (Y Zeng MD), Third Xiangya Hospital of Central South University, Changsha, China; Department of Epidemiology and Biostatistics (C Zhai MD), Anhui Medical University, Hefei, China; Cardiovascular Diseases Centre (H Zhang MD), China Academy of Chinese Medical Sciences, Beijing, China; School of Public Health (Y Zhang PhD), Hubei Province Key Laboratory of Occupational Hazard Identification and Control (Y Zhang PhD), Wuhan University of Science and Technology, Wuhan, China; College of Traditional Chinese Medicine (H Zhao MD), Hebei University, Baoding, China; Department of Basic Medicine (Y Zhao BS), Army Medical University, Chongqing, China; School of Public Health and Management (Prof Y Zhao MSc), Chongqing Medical University, Chongqing, China; Computational Bioscience Research Center, CEMSE (J Zhou PhD), King Abdullah University of Science and Technology (KAUST), Jeddah, Saudi Arabia; School of Public Health and Emergency Management (B Zhu PhD), Southern University of Science and Technology, Shenzhen, China; NIHR-Biomedical Research Centre (NIHR-BRC) (Prof A Zumla PhD), University College London Hospitals, London, UK; Department of Cardiology, Pulmonology, and Vascular Medicine (E Zweck MD), Heinrich-Heine-University, Duesseldorf, Germany; School of Physics (S H Zyoud PhD), Universiti Sains Malaysia (University of Science Malaysia), Penang, Malaysia;

Authors' Contributions

Managing the overall research enterprise

Simon I Hay, Kasey E Kinzel, Christopher J L Murray, and Austin E Schumacher.

Writing the first draft of the manuscript

Catherine Bisignano, Simon I Hay, Max L Mehlman, Susan A McLaughlin, Christopher J L Murray, and Austin E Schumacher.

Primary responsibility for applying analytical methods to produce estimates

Haley Comfort, Darwin Phan Jones, Erin A May, and Spencer A Pease.

Primary responsibility for seeking, cataloguing, extracting, or cleaning data; designing or coding figures and tables

Haley Comfort, John E Fuller, Yaz Ozten, Hannah Elizabeth Robinson-Oden, Nicholas Alexander Verghese, Denny Wang, and Stephanie Watson.

Providing data or critical feedback on data sources

Cristiana Abbafati, Jaffar Abbas, Madineh Akram Abbasi, Ahmed Abdelwahab, Meriem Abdoun, Auwal Abdullahi, Ame Mehadi Abdurehman, Aidin Abedi, Armita Abedi, E S Abhilash, Richard Gyan Aboagye, Hassan Abolhassani, Lucas Guimarães Abreu, Niveen ME Abu-Rmeileh, Ahmed Abu-Zaid, Tim Adair, Oladimeji M Adebayo, Victor Adekanmbi, Rishan Adha, Qorinah Estiningtyas Sakilah Adnani, Saira Afzal, Pradyumna Agasthi, Antonella Agodi, Bright Opoku Ahinkorah, Danish Ahmad, Muayyad M Ahmad, Tauseef Ahmad, Keivan Ahmadi, Ayman Ahmed, Haroon Ahmed, Muktar Beshir Ahmed, Budi Aji, Sreelatha Akkala, Samuel Akyirem, Hanadi Al Hamad, Ammar Al Homsi, Mohammad Al Qadire, Khurshid Alam, Fahad Mashhour Alanezi, Turki M Alanzi, Mohammed Albashtawy, Mohammad T AlBataineh, Khalid F Alhabib, Abid Ali, Beriwan Abdulqadir Ali, Hassam Ali, Rafat Ali, Syed Shujait Shujait Ali, Sheikh Mohammad Alif, Syed Mohamed Aljunid, Wael Almahmeed, Joseph Uy Almazan, Omar Almidani, Mahmoud A Alomari, Salman Khalifah Al-Sabah, Awais Altaf, Farrukh Jawad Alvi, Hassan Alwafi, Hany wq, Azmeraw T Amare, Edward Kwabena Ameyaw, Alireza Amindarolzarbi, Hubert Amu, Deanna Anderlini, Pedro Prata Andrade, Tudorel Andrei, Dhanalakshmi Angappan, Sumadi Lukman Anwar, Seth Christopher Yaw Appiah, Muhammad Aqeel, Jalal Arabloo, Hany Ariffin, Timur Aripov, Benedetta Armocida, Anton A Artamonov, Judie Arulappan, Marvellous O Asika, Seyyed Shamsadin Athari, Alok Atreya, Sameh Attia, Beatriz Paulina Ayala Quintanilla, Getnet Melaku Ayele, Jose L Ayuso-Mateos, Gulrez Shah Azhar, Ahmed Y Azzam, Mina Babashahi, Ashish D Badiye, Soroush Baghdadi, Sara Bagherieh, Abdulaziz T Bako, Senthilkumar Balakrishnan, Ovidiu Constantin Baltatu, Maciej Banach, Palash Chandra Banik, Hansi Bansal, Martina Barchitta, Mainak Bardhan, Hiba Jawdat Barqawi, Ronald D Barr, Zarrin Basharat, Pritish Baskaran, Nebiyou Simegnew Bayileyegn, Emad Behboudi, Diana Fernanda Bejarano Ramirez, Uzma Iqbal Belgaumi, Michelle L Bell, Aminu K Bello, Olorunjuwon Omolaja Bello, Apostolos Beloukas, Salaheddine Bendak, Zombor Berezvai, Adam E Berman, Habtamu B Beyene, Akshaya Srikanth Bhagavathula, Dinesh Bhandari, Sonu Bhaskar, Vivek Bhat, Gurjit Kaur Bhatti, Jasvinder Singh Bhatti, Zulfiqar A Bhutta, Boris Bikbov, Bagas Suryo Bintoro, Francesca Bisulli, Aadam Olalekan Bodunrin, Obasanjo Afolabi Bolarinwa, Milad Bonakdar Hashemi, Berrak Bora Basara, Souad Bouaoud, Nicola Luigi Bragazzi, Dejana Braithwaite, Lemma N Bulto, Danilo Buonsenso, Reinhard Busse, Florentino Luciano Caetano dos Santos, Daniela Calina, Chao Cao, Juan J Carrero, Joao Mauricio Castaldelli-Maia, Carlos A Castañeda-Orjuela, Ferrán Catalá-López, Alberico L Catapano, Christopher R Cederroth, Francieli

Cembranel, Pamela R Chacón-Uscamaita, Chiranjib Chakraborty, Vijay Kumar Chattu, Malizgani Paul Chavula, Derek S Chew, Abdulaal Chitheer, William C S Cho, Bryan Chong, Hitesh Chopra, Rajiv Chowdhury, Dinh-Toi Chu, Eric Chung, Alyssa Columbus, Haley Comfort, Joao Conde, Paolo Angelo Cortesi, Michael H Criqui, Natália Cruz-Martins, Matthew Cunningham, Giovanni Damiani, Aso Mohammad Darwesh, Saswati Das, Claudio Alberto Dávila-Cervantes, Aklilu Tamire Debele, Louisa Degenhardt, Lee Deitesfeld, Ivan Delgado-Enciso, Berecha Hundessa Demessa, Andreas K Demetriades, Nikolaos Dervenis, Hardik Dineshbhai Desai, Rupak Desai, Vinoth Gnana Chellaiyan Devanbu, Sameer Dhingra, Diana Dias da Silva, Daniel Diaz, M Ashworth Dirac, Abhinav Dixit, Shilpi Gupta Dixit, Thanh Chi Do, Thao Huynh Phuong Do, Masoud Dodangeh, Klara Georgieva Dokova, Christiane Dolecek, E Ray Dorsey, Wendel Mombaque dos Santos, Rajkumar Doshi, Leila Doshmangir, Abdel Douiri, Haneil Larson Dsouza, Susanna J Dunachie, Bruce B Duncan, Andre Rodrigues Duraes, Senbagam Duraisamy, Paulina Agnieszka Dzianach, Ebrahim Eini, Michael Ekholuenetale, Temitope Cyrus Ekundayo, Rabie Adel El Arab, Doaa Abdel Wahab El Morsi, Maysaa El Sayed Zaki, Frank J Elgar, Ryenchindorj Erkhembayar, Tesfahun C Eshetie, Sharareh Eskandarieh, Ugochukwu Anthony Eze, Adewale Oluwaseun Fadaka, Adeniyi Francis Fagbamigbe, Andre Faro, Hossein Farrokhpour, Ali Fatehizadeh, Valery L Feigin, Seyed-Mohammad Fereshtehnejad, Luisa S Flor, Artem Alekseevich Fomenkov, Richard Charles Franklin, Sara D Friedman, Takeshi Fukumoto, John E Fuller, Peter Andras Gaal, Muktar A Gadanya, Santosh Gaihre, Yaseen Galali, Jalaj Garg, Peter W Gething, Mansour Ghafourifard, Asadollah Gholamian, Pooyan Ghorbani Vajargah, Sherief Ghozy, Ruth Margaret Gibson, Alem Girmay, Laszlo Göbölös, Amit Goel, Mahaveer Golechha, Pouya Goleij, Houman Goudarzi, Mesay Dechasa Gudeta, Bhawna Gupta, Rajat Das Gupta, Sapna Gupta, Vijai Kumar Gupta, Vladimir Hachinski, Rasool Haddadi, Nils Haep, Adel Hajj Ali, Sobia Ahsan Halim, Brian J Hall, Rabih Halwani, Kanaan Hamagharib Abdullah, Mohammad Hamiduzzaman, Nasrin Hanifi, Josep Maria Haro, Faizul Hasan, M Tasdik Hasan, Abbas M Hassan, Soheil Hassanipour, Johannes Haubold, Rasmus J Havmoeller, Simon I Hay, Mahsa Heidari-Foroozan, Reza Heidari-Soureshjani, Claudiu Herteliu, Demisu Zenbaba Heyi, Md Mahbub Hossain, Mehdi Hosseinzadeh, Chengxi Hu, Md Nazmul Huda, Michael Hultström, Salman Hussain, Nawfal R Hussein, Le Duc Huy, Hong-Han Huynh, Segun Emmanuel Ibitoye, Desta Ijo, Olayinka Stephen Ilesanmi, Arnaud Iradukunda, Farideh Iravanpour, Kenneth Chukwuemeka Iregbu, Mohammad Mainul Islam, Sheikh Mohammed Shariful Islam, Nahlah Elkudssiah Ismail, Gaetano Isola, Mahalaxmi Iyer, Linda Merin J, Jalil Jaafari, Kathryn H Jacobsen, Morteza Jafarinia, Khushleen Jaggi, Nader Jahanmehr, Haitham Jahrami, Nityanand Jain, Mihajlo Jakovljevic, Sathish Kumar Jayapal, Shubha Jayaram, Digisie Mequanint Jemere, John S Ji, Yinzi Jin, Nabi Jomehzadeh, Darwin Phan Jones, Tamas Joo, Abel Joseph, Charity Ehimwenma Joshua, Jacek Jerzy Jozwiak, Mikk Jürisson, Billingsley Kaambwa, Zubair Kabir, Vidya Kadashetti, Pradnya Vishal Kakodkar, Leila R Kalankesh, Himal Kandel, Rami S Kantar, Neeti Kapoor, Mehrdad Karajizadeh, Samad Karkhah, Ajit K Karna, Faizan Zaffar Kashoo, Nicholas J Kassebaum, Adarsh Katamreddy, Srinivasa Vittal Katikireddi, Patrick DMC Katoto, Neda Kaydi, Gbenga A Kayode, Cathleen Keller, Morteza Abdullatif Khafaie, Himanshu Khajuria, Nauman Khalid, Ibrahim A Khalil, Imteyaz A Khan, Maseer Khan, Moien AB Khan, Mahammed Ziauddin Khan suheb, Khaled Khatab, Feriha Fatima Khidri, Jagdish Khubchandani, Grace Kim, Jihee Kim, Adnan Kisa, Sezer Kisa, Juniper Boroka Kiss, Ann Kristin Skrindo Knudsen, Gerbrand Koren, Soewarta Kosen, Sindhura Lakshmi Koulmane Laxminarayana, Kewal Krishan, Hare Krishna, Vijay Krishnamoorthy, Yuvaraj Krishnamoorthy, Barthelemy Kuate Defo, Mukhtar Kulimbet, Vishnutheertha Kulkarni, Asep Kusnali, Dian Kusuma, Ilias Kyriopoulos, Hmwe Hmwe Kyu, Muhammad Awwal Ladan, Chandrakant Lahariya, Daphne Teck Ching Lai, Tri Laksono, Dharmesh Kumar Lal, Judit Lám, Anders O Larsson, Savita Lasrado, Kamaluddin Latief, Kaveh Latifinaibin, Nhi Huu Hanh Le, Thao Thi Thu Le, Trang Diep Thanh Le, Munjae

Lee, Sang-woong Lee, Seung Won Lee, James Leigh, Yichong Li, Lee-Ling Lim, Stephen S Lim, Stefan Listl, Jue Liu, Erand Llanaj, László Lorenzovici, Rafael Lozano, Jianing Ma, Zheng Feei Ma, Monika Machoy, Azzam A Maghazachi, Elham Mahmoudi, Jeadran N Malagón-Rojas, Elaheh Malakan Rad, Kashish Malhotra, Deborah Carvalho Malta, Abdullah A Mamun, Yasaman Mansoori, Mohammad Ali Mansournia, Lorenzo Giovanni Mantovani, Joemer C Maravilla, Agustina M Marconi, Parham Mardi, Abdoljalal Marjani, Carlos Alberto Marrugo Arnedo, Bernardo Alfonso Martinez-Guerra, Francisco Rogerlândio Martins-Melo, Sharmeen Maryam, Roy Rillera Marzo, Clara N Matei, Andrea Maugeri, Erin A May, Maryam Mazaheri, Michael A McPhail, Enkeleint A Mechili, Jitendra Kumar Meena, Entezar Mehrabi Nasab, Walter Mendoza, Ritesh G Menezes, Endalkachew Worku Mengesha, Atte Meretoja, Tuomo J Meretoja, Irmina Maria Michalek, Ted R Miller, Giada Minelli, Le Huu Nhat Minh, Babak Moazen, Soheil Mohammadi, Abdollah Mohammadian-Hafshejani, Mustapha Mohammed, Salahuddin Mohammed, Shafiu Mohammed, Ali H Mokdad, Peyman Mokhtarzadehazar, Sara Momtazmanesh, Lorenzo Monasta, Mohammad Ali Moni, Maryam Moradi, Yousef Moradi, Maziar Moradi-Lakeh, Mehdi Moradinazar, Shane Douglas Morrison, Jakub Morze, Jonathan F Mosser, Vincent Mougin, Sumaira Mubarik, Ulrich Otto Mueller, Faraz Mughal, Sumoni Mukherjee, George Duke Mukoro, Admir Mulita, Francesk Mulita, Malaisamy Muniyandi, Christopher J L Murray, Fungai Musaigwa, Ghulam Mustafa, Saravanan Muthupandian, Ahamarshan Jayaraman Nagarajan, Mohsen Naghavi, Ganesh R Naik, Mukhammad David Naimzada, Sanjeev Nair, Tapas Sadasivan Nair, Luigi Naldi, Shumaila Nargus, Bruno Ramos Nascimento, Gustavo G Nascimento, Mohammad Javad Nasiri, Zuhair S Natto, Muhammad Naveed, Biswa Prakash Nayak, Amayu Kumesa Negero, Ionut Negoi, Ruxandra Irina Negoi, Evangelia Nena, Charles Richard James Newton, Josephine W Ngunjiri, Dang H Nguyen, Phat Tuan Nguyen, Van Thanh Nguyen, Taxiarchis Konstantinos Nikolouzakis, Amin Reza Nikpoor, Muhammad A Nizam, Shuhei Nomura, Mamoona Noreen, Bo Norrving, Jean Jacques Noubiap, Dieta Nurrika, Bogdan Oancea, Kehinde O Obamiro, Ismail A Odetokun, Akinkunmi Paul Okekunle, Osaretin Christabel Okonji, Andrew T Olagunju, Bolajoko Olubukunola Olusanya, Jacob Olusegun Olusanya, Hany A Omar, Sokking Ong, Obinna E Onwujekwe, Orish Ebere Orisakwe, Edgar Ortiz-Brizuela, Uchechukwu Levi Osuagwu, Amel Ouyahia, Mayowa O Owolabi, Mahesh Padukudru P A, Jagadish Rao Padubidri, Raffaele Palladino, Ioannis Pantazopoulos, Paraskevi Papadopoulou, Shahina Pardhan, Romil R Parikh, Ashwaghosha Parthasarathi, Deepak Kumar Pasupula, Sangram Kishor Patel, Aslam Ramjan Pathan, Shankargouda Patil, Venkata Suresh Patthipati, Uttam Paudel, Shrikant Pawar, Paolo Pedersini, Veincent Christian Filipino Pepito, Gavin Pereira, Jeevan Pereira, Mario F P Peres, Arokiasamy Perianayagam, Hoang Tran Pham, Daniela Pierannunzio, David M Pigott, Peter Pollner, Ramesh Poluru, Maarten J Postma, Naeimeh Pourtaheri, Sergio I Prada, Vijay Kumar Prajapati, Elton Junio Sady Prates, Nguyen Khoi Quan, Fakher Rahim, Tafhimur Rahman, Amir Masoud Rahmani, Sathish Rajaa, Prasanna Ram, Shakthi Kumaran Ramasamy, Sheena Ramazanu, Juwel Rana, Kritika Rana, Chhabi Lal Ranabhat, Nemanja Rancic, Chythra R Rao, Sowmya J Rao, Sina Rashedi, Vahid Rashedi, Ahmed Mustafa Rashid, Prateek Rastogi, Nakul Ravikumar, Salman Rawaf, Reza Rawassizadeh, Bhageerathy Reshmi, Serge Resnikoff, Luis Felipe Reyes, Nima Rezaei, Maria Jesus Rios-Blancas, Hannah Elizabeth Robinson-Oden, Mónica Rodrigues, Jefferson Antonio Buendia Rodriguez, Leonardo Roever, Peter Rohloff, Debby Syahru Romadlon, Luca Ronfani, Priyanka Roy, Enrico Rubagotti, Aly M A Saad, Korosh Saber, Basema Saddik, Adam Saddler, Bashdar Abuzed Sadee, Ehsan Sadeghi, Umar Saeed, Maryam Saeedi, Rajesh Sagar, Narjes Saheb Sharif-Askari, Mohammad Ali Sahraian, Mirza Rizwan Sajid, Joseph W Sakshaug, Afeez Abolarinwa Salami, Marwa Rashad Salem, Sara Samadzadeh, Vijaya Paul Samuel, Abdallah M Samy, Juan Sanabria, Nima Sanadgol, Francesca Sanna, Milena M Santric-Milicevic, Babak Saravi, Arash Sarveazad, Brijesh Sathian, Sonia

Saxena, Yaser Sayadi, Md Abu Sayeed, Mete Saylan, Maria Inês Schmidt, David C Schwebel, Mario Šekerija, Subramanian Senthilkumaran, Yashendra Sethi, Amir Shafaat, Pritik A Shah, Saeed Shahabi, Masood Ali Shaikh, Husain Shakil, Sunder Sham, Mehran Shams-Beyranvand, Mohammad Anas Shamsi, Abhishek Shankar, Javad Sharifi-Rad, Rajesh Sharma, Vishal Sharma, Rajesh P Shastry, David H Shaw, Jiabin Shen, B Suresh Kumar Shetty, Kenji Shibuya, Jae Il Shin, Aminu Shittu, K M Shivakumar, Sunil Shrestha, Inga Dora Sigfusdottir, Luís Manuel Lopes Rodrigues Silva, Abhinav Singh, Aditya Singh, Ambrish Singh, Baljinder Singh, Narinder Pal Singh, Paramdeep Singh, Valentin Yurievich Skryabin, Anna Aleksandrovna Skryabina, Erica Leigh N Slepak, Sameh S M Soliman, Yonatan Solomon, Ireneous N Soyiri, Michael Spartalis, Chandrashekhar T Sreeramareddy, Fridolin Steinbeis, Muhammad Suleman, Rizwan Suliankatchi Abdulkader, Abida Sultana, Chandan Kumar Swain, Bryan L Sykes, Mindy D Szeto, Miklós Szócska, Payam Tabaee Damavandi, Shima Tabatabai, Karen M Tabb, Mohammad Tabish, Takahiro Tabuchi, Amirmasoud Taheri, Yasaman Taheri Abkenar, Amir Taherkhani, Jabeen Taiba, Ker-Kan Tan, Nuno Taveira, Yibekal Manaye Tefera, Pugazhenthan Thangaraju, Nihal Thomas, Amanda G Thrift, Chern Choong Chern Thum, Marcello Tonelli, Mathilde Touvier, Marcos Roberto Tovani-Palone, Domenico Trico, Indang Trihandini, Samuel Joseph Tromans, Kang Tung, Sana Ullah, Muhammad Umair, Tungki Pratama Umar, Bhaskaran Unnikrishnan, Era Upadhyay, Sahel Valadan Tahbaz, Jef Van den Eynde, Shoban Babu Varthya, Tommi Juhani Vasankari, Narayanaswamy Venketasubramanian, Nicholas Alexander Verghese, Pratibha Verma, Massimiliano Veroux, Georgios-Ioannis Verras, Vasily Vlassov, Bay Vo, Simona Ruxandra Volovat, Victor Volovici, Avina Vongpradith, Theo Vos, Yohannes Dibaba Wado, Yasir Waheed, Denny Wang, Shu Wang, Stefanie Watson, Kosala Gayan Weerakoon, Daniel J Weiss, Andrea Werdecker, Ronny Westerman, Dakshitha Praneeth Wickramasinghe, Tissa Wijeratne, Sajad Yaghoubi, Yuichiro Yano, Yao Yao, Pengpeng Ye, Gesila Endashaw Yesera, Renjulal Yesodharan, Paul Yip, Dong Keon Yon, Naohiro Yonemoto, Mustafa Z Younis, Chuanhua Yu, Vesna Zadnik, Mohammad Zahedi, Nazar Zaki, Josefina Zakzuk, Ghazal G Z Zandieh, Iman Zare, Mikhail Sergeevich Zastrozhin, Mohammed G M Zeariya, Chunxia Zhai, Zhaohua Zhu, and Magdalena Zielińska.

Developing methods or computational machinery

Cristiana Abbafati, Rouzbeh Abbasgholizadeh, Rishan Adha, Qorinah Estiningtyas Sakilah Adnani, Saira Afzal, Muktar Beshir Ahmed, Tareq Mohammed Ali AL-Ahdal, Mohammed Albashtawy, Robert W Aldridge, Hubert Amu, Aleksandr Y Aravkin, Judie Arulappan, Ahmed Y Azzam, Emad Behboudi, Michelle L Bell, Akshaya Srikanth Bhagavathula, Aadam Olalekan Bodunrin, Souad Bouaoud, Kelly M Cercy, William C S Cho, Eunice Chung, Kaleb Coberly, Haley Comfort, Garland T Culbreth, Matthew Cunningham, Aso Mohammad Darwesh, Hardik Dineshbhai Desai, Thanh Chi Do, Paulina Agnieszka Dzianach, Michael Ekholuenetale, Maysaa El Sayed Zaki, Mehdi Emamverdi, Ali Fatehizadeh, Abraham D Flaxman, Jalaj Garg, Peter W Gething, Pooyan Ghorbani Vajargah, Sherief Ghozy, Alem Girmay, Sobia Ahsan Halim, Kanaan Hamagharib Abdullah, Mohammad Hasanian, Simon I Hay, Mohammad Heidari, Hamed Hesami, Mehdi Hosseinzadeh, Md Nazmul Huda, Javid Hussain, Hong-Han Huynh, Kevin S Ikuta, Farideh Iravanpour, Mohammad Mainul Islam, Gaetano Isola, Linda Merin J, Morteza Jafarinia, Sathish Kumar Jayapal, Wonjeong Jeong, Nabi Jomehzadeh, Darwin Phan Jones, Charity Ehimwenma Joshua, Samad Karkhah, Faizan Zaffar Kashoo, Nicholas J Kassebaum, Ikramullah Khan, Adnan Kisa, Juniper Boroka Kiss, Chandrakant Lahariya, Nhi Huu Hanh Le, Thao Thi Thu Le, Sang-woong Lee, Erand Llanaj, Erin A May, Michael A McPhail, Le Huu Nhat Minh, Seyed Kazem Mirinezhad, Abdollah Mohammadian-Hafshejani, Saeed Mohammad-pour, Hoda Mojiri-forushani, Ali H Mokdad, Mohammad Ali Moni, Yousef Moradi, Admir Mulita, Francesk Mulita, Christopher J L Murray, Mohsen Naghavi, Josephine W Ngunjiri, Phat Tuan Nguyen, Van Thanh Nguyen, Andrew T Olagunju, Michal Ordak, Yaz Ozten, Jagadish Rao

Padubidri, Adrian Pana, Shrikant Pawar, Spencer A Pease, Gavin Pereira, Hoang Tran Pham, Govinda Raj Poudel, Hadi Raeisi Shahraki, Amir Masoud Rahmani, Chhabi Lal Ranabhat, Reza Rawassizadeh, Robert C Reiner Jr, Mónica Rodrigues, Enrico Rubagotti, Susan Fred Rumisha, Korosh Saber, Adam Saddler, Umar Saeed, Abdallah M Samy, Ganesh Kumar Saya, Yaser Sayadi, Austin E Schumacher, Amir Shafaat, Mohammad Anas Shamsi, Javad Sharifi-Rad, David H Shaw, Reed J D Sorensen, Michael Spartalis, Muhammad Suleman, Chandan Kumar Swain, Razieh Tavakoli Oliaee, Chern Choong Chern Thum, Muhammad Umair, Shoban Babu Varthya, Bay Vo, Stein Emil Vollset, Theo Vos, Daniel J Weiss, Ronny Westerman, Ghazal G Z Zandieh, Mohammed G M Zeariya, Yang Zhao, and Peng Zheng.

Providing critical feedback on methods or results

Amirali Aali, Cristiana Abbafati, Jaffar Abbas, Rouzbeh Abbasgholizadeh, Madineh Akram Abbasi, Mohammadreza Abbasian, Samar Abd ElHafeez, Michael Abdelmasseh, Sherief Abd-Elsalam, Ahmed Abdelwahab, Mohammad Abdollahi, Meriem Abdoun, Auwal Abdullahi, Ame Mehadi Abdurehman, Mesfin Abebe, Aidin Abedi, Armita Abedi, Tadesse M Abegaz, Roberto Ariel Abeldaño Zuñiga, E S Abhilash, Olugbenga Olusola Abiodun, Richard Gyan Aboagye, Hassan Abolhassani, Mohamed Abouzid, Lucas Guimarães Abreu, Woldu Aberhe Abrha, Michael R M Abrigo, Dariush Abtahi, Samir Abu Rumeileh, Niveen ME Abu-Rmeileh, Salahdein Aburuz, Ahmed Abu-Zaid, Juan Manuel Acuna, Tim Adair, Isaac Yeboah Addo, Oladimeji M Adebayo, Oyelola A Adegboye, Victor Adekanmbi, Bashir Aden, Abiola Victor Adepoju, Charles Oluwaseun Adetunji, Temitayo Esther Adeyeoluwa, Olorunsola Israel Adeyomoye, Rishan Adha, Amin Adibi, Wirawan Adikusuma, Qorinah Estiningtyas Sakilah Adnani, Saryia Adra, Abel Afework, Aanuoluwapo Adeyimika Afolabi, Shadi Afyouni, Saira Afzal, Pradyumna Agasthi, Shahin Aghamiri, Antonella Agodi, Williams Agyemang-Duah, Bright Opoku Ahinkorah, Aqeel Ahmad, Danish Ahmad, Firdos Ahmad, Muayyad M Ahmad, Tauseef Ahmad, Amir Mahmoud Ahmadzade, Mohadese Ahmadzade, Ayman Ahmed, Haroon Ahmed, Luai A Ahmed, Muktar Beshir Ahmed, Syed Anees Ahmed, Budi Aji, Olufemi Ajumobi, Gizachew Taddesse Akalu, Essona Matatom Akara, Karolina Akinosoglou, Sreelatha Akkala, Samuel Akyirem, Hanadi Al Hamad, Syed Mahfuz Al Hasan, Ammar Al Homsi, Mohammad Al Qadire, Moein Ala, Timothy Olukunle Aladelusi, Samer O Alalalmeh, Ziyad Al-Aly, Khurshid Alam, Manjurul Alam, Zufishan Alam, Rasmieh Mustafa Al-amer, Fahad Mashhour Alanezi, Turki M Alanzi, Mohammed Albashtawy, Mohammad T AlBataineh, Sharifullah Alemi, Adel Ali Saeed Al-Gheethi, Khalid F Alhabib, Mohammed Khaled Al-Hanawi, Abid Ali, Akhtar Ali, Hassam Ali, Mohammed Usman Ali, Rafat Ali, Syed Shujait Shujait Ali, Zahid Ali, Shohreh Alian Samakkhah, Gianfranco Alicandro, Mohammad Aligol, Rasoul Alimi, Ahmednur Adem Aliyi, Syed Mohamed Aljunid, Wael Almahmeed, Sabah Al-Marwani, Sadeq Ali Ali Al-Maweri, Joseph Uy Almazan, Hesham M Al-Mekhlafi, Omar Almidani, Mahmoud A Alomari, Jaber S Alqahtani, Ahmed Yaseen Alqutaibi, Salman Khalifah Al-Sabah, Awais Altaf, Jaffar A Al-Tawfiq, Khalid A Altirkawi, Farrukh Jawad Alvi, Hassan Alwafi, Yaser Mohammed Al-Worafi, Hany wq, Karem H Alzoubi, Azmeraw T Amare, Edward Kwabena Ameyaw, Abebe Feyissa Amhare, Tarek Tawfik Amin, Alireza Amindarolzarbi, Javad Aminian Dehkordi, Sohrab Amiri, Hubert Amu, Dickson A Amugsi, Jimoh Amzat, Deanna Anderlini, Pedro Prata Andrade, Catalina Liliana Andrei, Tudorel Andrei, Dhanalakshmi Angappan, Afifa Anjum, Ernoiz Antriyandarti, Iyadunni Adesola Anuoluwa, Sumadi Lukman Anwar, Anayochukwu Edward Anyasodor, Seth Christopher Yaw Appiah, Jalal Arabloo, Morteza Arab-Zozani, Mosab Arafat, Abdulfatai Aremu, Hany Ariffin, Timur Aripov, Benedetta Armocida, Mahwish Arooj, Anton A Artamonov, Kurnia Dwi Artanti, Judie Arulappan, Idowu Thomas Aruleba, Raphael Taiwo Aruleba, Ashokan Arumugam, Saeed Asgary, Mubarek Yesse Ashemo, Muhammad Ashraf, Marvellous O Asika, Seyyed Shamsadin Athari, Maha Moh'd Wahbi Atout, Alok Atreya, Sameh Attia, Avinash Aujayeb, Abolfazl Avan, Adedapo Wasiu Awotidebe, Beatriz Paulina Ayala Quintanilla,

Martin Amogre Ayanore, Getnet Melaku Ayele, Sina Azadnajafabad, Gulrez Shah Azhar, Shahkaar Aziz, Ahmed Y Azzam, Mina Babashahi, Abraham Samuel Babu, Muhammad Badar, Alaa Badawi, Ashish D Badiye, Soroush Baghdadi, Nasser Bagheri, Sara Bagherieh, Sulaiman Bah, Saeed Bahadorikhalili, Jianjun Bai, Ruhai Bai, Jennifer L Baker, Shankar M Bakkannavar, Abdulaziz T Bako, Senthilkumar Balakrishnan, Saliu A Balogun, Ovidiu Constantin Baltatu, Maciej Banach, Soham Bandyopadhyay, Biswajit Banik, Palash Chandra Banik, Hansi Bansal, Shirin Barati, Martina Barchitta, Mainak Bardhan, Hiba Jawdat Barqawi, Lope H Barrero, Zarrin Basharat, Asma'u I J Bashir, Hameed Akande Bashiru, Pritish Baskaran, Quique Bassat, João Diogo Basso, Saurav Basu, Kavita Batra, Ravi Batra, Bernhard T Baune, Mohsen Bayati, Nebiyou Simegnew Bayileyegn, Tahmina Begum, Emad Behboudi, Amir Hossein Behnoush, Diana Fernanda Bejarano Ramirez, Uzma Iqbal Belgaumi, Michelle L Bell, Aminu K Bello, Olorunjuwon Omolaja Bello, Apostolos Beloukas, Salaheddine Bendak, Derrick A Bennett, Isabela M Bensenor, Habib Benzian, Adam E Berman, Amiel Nazer C Bermudez, Habtamu B Beyene, Devidas S Bhagat, Akshaya Srikanth Bhagavathula, Neeraj Bhala, Ashish Bhalla, Dinesh Bhandari, Nikha Bhardwaj, Pankaj Bhardwaj, Prarthna V Bhardwaj, Ashish Bhargava, Sonu Bhaskar, Vivek Bhat, Gurjit Kaur Bhatti, Jasvinder Singh Bhatti, Manpreet S Bhatti, Rajbir Bhatti, Zulfiqar A Bhutta, Boris Bikbov, Bagas Suryo Bintoro, Francesca Bisulli, Atanu Biswas, Raaj Kishore Biswas, Archie Bleyer, Virginia Bodolica, Aadam Olalekan Bodunrin, Obasanjo Afolabi Bolarinwa, Milad Bonakdar Hashemi, Aime Bonny, Kaustubh Bora, Safiya Bala Borodo, Alejandro Botero Carvajal, Souad Bouaoud, Sofiane Boudalia, Edward J Boyko, Nicola Luigi Bragazzi, Dejana Braithwaite, Hermann Brenner, Gabrielle Britton, Annie J Browne, Andre R Brunoni, Norma B Bulamu, Lemma N Bulto, Danilo Buonsenso, Katrin Burkart, Sharath Burugina Nagaraja, Yasser Bustanji, Zahid A Butt, Florentino Luciano Caetano dos Santos, Tianji Cai, Ismael R Campos-Nonato, Chao Cao, Carlos Alberto Cardenas, Rosario Cárdenas, Joao Mauricio Castaldelli-Maia, Carlos A Castañeda-Orjuela, Giulio Castelpietra, Ferrán Catalá-López, Alberico L Catapano, Christopher R Cederroth, Francieli Cembranel, Muthia Cenderadewi, Ester Cerin, Muge Cevik, Pamela R Chacón-Uscamaita, Yaacoub Chahine, Chiranjib Chakraborty, Chin-Kuo Chang, Periklis Charalampous, Jaykaran Charan, Vijay Kumar Chattu, Victoria Chatzimavridou-Grigoriadou, Malizgani Paul Chavula, Huzaifa Ahmad Cheema, An-Tian Chen, Haowei Chen, Lingxiao Chen, Meng Xuan Chen, Nicolas Cherbuin, Derek S Chew, Jesus Lorenzo Chirinos-Caceres, William C S Cho, Bryan Chong, Hitesh Chopra, Rajiv Chowdhury, Dinh-Toi Chu, Isaac Sunday Chukwu, Eric Chung, Eunice Chung, Sheng-Chia Chung, Karly I Cini, Cain C T Clark, Alyssa Columbus, Haley Comfort, Joao Conde, Sara Conti, Paolo Angelo Cortesi, Vera Marisa Costa, Ewerton Cousin, Richard G Cowden, Michael H Criqui, Natália Cruz-Martins, Garland T Culbreth, Patricia Cullen, Matthew Cunningham, Daniel da Silva e Silva, Omid Dadras, Zhaoli Dai, Koustuv Dalal, Lachlan L Dalli, Giovanni Damiani, Emanuele D'Amico, Sara Daneshvar, Aso Mohammad Darwesh, Jai K Das, Saswati Das, Nihar Ranjan Dash, Mohsen Dashti, Claudio Alberto Dávila-Cervantes, Kairat Davletov, Aklilu Tamire Debele, Louisa Degenhardt, Reza Dehbandi, Ivan Delgado-Enciso, Daniel Demant, Berecha Hundessa Demessa, Andreas K Demetriades, Xinlei Deng, Kebede Deribe, Nikolaos Dervenis, Don C Des Jarlais, Hardik Dineshbhai Desai, Rupak Desai, Keshab Deuba, Vinoth Gnana Chellaiyan Devanbu, Sourav Dey, Arkadeep Dhali, Kuldeep Dhama, Mandira Lamichhane Dhimal, Meghnath Dhimal, Sameer Dhingra, Diana Dias da Silva, Daniel Diaz, Adriana Dima, Delaney D Ding, M Ashworth Dirac, Abhinav Dixit, Shilpi Gupta Dixit, Thanh Chi Do, Thao Huynh Phuong Do, Camila Bruneli do Prado, Masoud Dodangeh, Klara Georgieva Dokova, Christiane Dolecek, E Ray Dorsey, Wendel Mombaque dos Santos, Rajkumar Doshi, Leila Doshmangir, Abdel Douiri, Robert Kokou Dowou, Tim Robert Driscoll, Haneil Larson Dsouza, John Dube, Susanna J Dunachie, Senbagam Duraisamy, Oyewole Christopher Durojaiye, Sulagna Dutta, Arkadiusz Marian Dziedzic, Oluwakemi Ebenezer, Ejemai Eboreime, Alireza Ebrahimi, Abdelaziz Ed-Dra, Hisham Atan

Edinur, David Edvardsson, Kristina Edvardsson, Defi Efendi, Ferry Efendi, Shayan Eghdami, Terje Andreas Eikemo, Ebrahim Eini, Michael Ekholuenetale, Emmanuel Ekpor, Temitope Cyrus Ekundayo, Rabie Adel El Arab, Doaa Abdel Wahab El Morsi, Maysaa El Sayed Zaki, Noha Mousaad Elemam, Frank J Elgar, Islam Y Elgendy, Ghada Metwally Tawfik ElGohary, Muhammed Elhadi, Omar Abdelsadek Abdou Elmeligy, Mohammed Elshaer, Ibrahim Elsohaby, Amir Emami Zeydi, Mehdi Emamverdi, Theophilus I Emeto, Tesfahun C Eshetie, Sharareh Eskandarieh, Juan Espinosa-Montero, Farshid Etaee, Ugochukwu Anthony Eze, Natalia Fabin, Adewale Oluwaseun Fadaka, Adeniyi Francis Fagbamigbe, Saman Fahimi, Luca Falzone, Carla Sofia e Sá Farinha, MoezAlIslam Ezzat Mahmoud Faris, Andre Faro, Hossein Farrokhpour, Ali Fatehizadeh, Hamed Fattahi, Pooria Fazeli, Ginenus Fekadu, Seyed-Mohammad Fereshtehnejad, Abdullah Hamid Feroze, Daniela Ferrante, Pietro Ferrara, Getahun Fetensa, Irina Filip, Florian Fischer, Joanne Flavel, Luisa S Flor, Morenike Oluwatoyin Folayan, Kristen Marie Foley, Artem Alekseevich Fomenkov, Lisa M Force, Carla Fornari, Matteo Foschi, Kate Louise Francis, Richard Charles Franklin, Alberto Freitas, Joseph Friedman, Sara D Friedman, Takeshi Fukumoto, Peter Andras Gaal, Muktar A Gadanya, Santosh Gaihre, Abduzhappar Gaipov, Emmanuela Gakidou, Yaseen Galali, Quan Gan, Aravind P Gandhi, Balasankar Ganesan, Jalaj Garg, Shuo-Yan Gau, Prem Gautam, Rupesh K Gautam, Federica Gazzelloni, Miglas W Gebregergis, Mesfin Gebrehiwot, Tesfay Brhane Gebremariam, Urge Gerema, Motuma Erena Getachew, Tamirat Getachew, Peter W Gething, Sulmaz Ghahramani, Khalid Yaser Ghailan, Alireza Ghajar, Mohammad Javad Ghanbarnia, MohammadReza Ghasemi, Afsaneh Ghasemzadeh, Fariba Ghassemi, Ramy Mohamed Ghazy, Sailaja Ghimire, Asadollah Gholamian, Ali Gholamrezanezhad, Pooyan Ghorbani Vajargah, Sherief Ghozy, Arun Digambarrao Ghuge, Alessandro Gialluisi, Ruth Margaret Gibson, Artyom Urievich Gil, Paramjit Singh Gill, Tiffany K Gill, Themba G Ginindza, Alem Girmay, James C Glasbey, Elena V Gnedovskaya, Laszlo Göbölös, Mohamad Goldust, Mahaveer Golechha, Arefeh Golestanfar, Davide Golinelli, Philimon N Gona, Amir Hossein Goudarzian, Scott Greenhalgh, Michal Grivna, Giovanni Guarducci, Mohammed Ibrahim Mohialdeen Gubari, Mesay Dechasa Gudeta, Avirup Guha, Stefano Guicciardi, Damitha Asanga Gunawardane, Sasidhar Gunturu, Cui Guo, Anish Kumar Gupta, Bhawna Gupta, Indarchand Ratanlal Gupta, Rajat Das Gupta, Sapna Gupta, Veer Bala Gupta, Vijai Kumar Gupta, Vivek Kumar Gupta, Reyna Alma Gutiérrez, Farrokh Habibzadeh, Parham Habibzadeh, Vladimir Hachinski, Mohammad Haddadi, Rasool Haddadi, Nils Haep, Adel Hajj Ali, Esam S Halboub, Sobia Ahsan Halim, Brian J Hall, Sebastian Haller, Rabih Halwani, Randah R Hamadeh, Kanaan Hamagharib Abdullah, Samer Hamidi, Mohammad Hamiduzzaman, Ahmad Hammoud, Nasrin Hanifi, Md Nuruzzaman Haque, Harapan Harapan, Josep Maria Haro, Ahmed I Hasaballah, Faizul Hasan, Ikramul Hasan, M Tasdik Hasan, Hamidreza Hasani, Ali Hasanpour- Dehkordi, Abbas M Hassan, Hossein Hassanian-Moghaddam, Soheil Hassanipour, Johannes Haubold, Rasmus J Havmoeller, Simon I Hay, Youssef Hbid, Jeffrey J Hebert, Omar E Hegazi, Golnaz Heidari, Mohammad Heidari, Mahsa Heidari-Foroozan, Bartosz Helfer, Claudiu Herteliu, Hamed Hesami, Dineshani Hettiarachchi, Demisu Zenbaba Heyi, Kamal Hezam, Yuta Hiraike, Howard J Hoffman, Ramesh Holla, Nobuyuki Horita, Md Mahbub Hossain, Md Belal Hossain, Sahadat Hossain, Mohammad-Salar Hosseini, Hassan Hosseinzadeh, Mehdi Hosseinzadeh, Mihaela Hostiuc, Mohamed Hsairi, Chengxi Hu, Md Nazmul Huda, Fernando N Hugo, Michael Hultström, Salman Hussain, Nawfal R Hussein, Le Duc Huy, Hong-Han Huynh, Bing-Fang Hwang, Segun Emmanuel Ibitoye, Oluwatope Olaniyi Idowu, Desta Ijo, Kevin S Ikuta, Mehran Ilaghi, Irena M Ilic, Milena D Ilic, Mustapha Immurana, Arnaud Iradukunda, Farideh Iravanpour, Kenneth Chukwuemeka Iregbu, Md Rabiul Islam, Mohammad Mainul Islam, Sheikh Mohammed Shariful Islam, Farhad Islami, Nahlah Elkudssiah Ismail, Gaetano Isola, Masao Iwagami, Chidozie C D Iwu, Chinwe Juliana Iwu-Jaja, Mahalaxmi Iyer, Linda Merin J, Jalil Jaafari, Louis Jacob, Kathryn H Jacobsen, Farhad Jadidi-Niaragh,

Morteza Jafarinia, Khushleen Jaggi, Haitham Jahrami, Nityanand Jain, Ammar Abdulrahman Jairoun, Mihajlo Jakovljevic, Elham Jamshidi, Chinmay T Jani, Mark M Janko, Abubakar Ibrahim Jatau, Sathish Kumar Jayapal, Shubha Jayaram, Jayakumar Jeganathan, Alelign Tasew Jema, Digisie Mequanint Jemere, Anil K Jha, Ravi Prakash Jha, John S Ji, Heng Jiang, Yingzhao Jin, Yinzi Jin, Olatunji Johnson, Nabi Jomehzadeh, Darwin Phan Jones, Tamas Joo, Abel Joseph, Nitin Joseph, Charity Ehimwenma Joshua, Jacek Jerzy Jozwiak, Mikk Jürisson, Billingsley Kaambwa, Ali Kabir, Hannaneh Kabir, Zubair Kabir, Vidya Kadashetti, Farima Kahe, Pradnya Vishal Kakodkar, Rizwan Kalani, Leila R Kalankesh, Sanjay Kalra, Ashwin Kamath, Thanigaivelan Kanagasabai, Himal Kandel, Kehinde Kazeem Kanmodi, Rami S Kantar, Neeti Kapoor, Mehrdad Karajizadeh, Behzad Karami Matin, Ibraheem M Karaye, Asima Karim, Hanie Karimi, Salah Eddin Karimi, Arman Karimi Behnagh, Samad Karkhah, Ajit K Karna, Faizan Zaffar Kashoo, Nigussie Assefa Kassaw, Nicholas J Kassebaum, Adarsh Katamreddy, Srinivasa Vittal Katikireddi, Patrick DMC Katoto, Joonas H Kauppila, Navjot Kaur, Neda Kaydi, Gbenga A Kayode, Foad Kazemi, Sina Kazemian, sara Kazeminia, Leila Keikavoosi-Arani, Cathleen Keller, John H Kempen, Jessica A Kerr, Mohammad Keykhaei, Mohamad Mehdi Khadembashiri, Mohammad Amin Khadembashiri, Morteza Abdullatif Khafaie, Himanshu Khajuria, Mohammad Khalafi, Amirmohammad Khalaji, Nauman Khalid, Faham Khamesipour, Asaduzzaman Khan, Ikramullah Khan, Imteyaz A Khan, Maseer Khan, Moien AB Khan, Taimoor Khan, Mahammed Ziauddin Khan suheb, Shaghayegh Khanmohammadi, Khaled Khatab, Fatemeh Khatami, Armin Khavandegar, khalid a kheirallah, Feriha Fatima Khidri, Moein Khormali, Jagdish Khubchandani, Helda Khusun, Zemene Demelash Kifle, Grace Kim, Jihee Kim, Ruth W Kimokoti, Girmay Tsegay Kiross, Adnan Kisa, Sezer Kisa, Juniper Boroka Kiss, Mika Kivimäki, Desmond Klu, Ann Kristin Skrindo Knudsen, Ali-Asghar Kolahi, Farzad Kompani, Gerbrand Koren, Karel Kostev, Ashwin Laxmikant Kotnis, Sindhura Lakshmi Koulmane Laxminarayana, Ai Koyanagi, Michael A Kravchenko, Kewal Krishan, Hare Krishna, Vijay Krishnamoorthy, Yuvaraj Krishnamoorthy, Barthelemy Kuate Defo, Burcu Kucuk Bicer, Md Abdul Kuddus, Mohammed Kuddus, Ilari Kuitunen, Omar Kujan, Vishnutheertha Kulkarni, Ashish Kumar, Harish Kumar, Nithin Kumar, Rahul Kumar, Shiv Kumar, Almagul Kurmanova, Om P Kurmi, Asep Kusnali, Dian Kusuma, Tezer Kutluk, Ambily Kuttikkattu, Evans F Kyei, Ilias Kyriopoulos, Hmwe Hmwe Kyu, Carlo La Vecchia, Muhammad Awwal Ladan, Chandrakant Lahariya, Abdelilah Lahmar, Daphne Teck Ching Lai, Tri Laksono, Dharmesh Kumar Lal, Tea Lallukka, Judit Lám, Demetris Lamnisos, Tuo Lan, Francesco Lanfranchi, Berthold Langguth, Van Charles Lansingh, Ariane Laplante-Lévesque, Bagher Larijani, Savita Lasrado, Kamaluddin Latief, Mahrukh Latif, Kaveh Latifinaibin, Paolo Lauriola, Long Khanh Dao Le, Nhi Huu Hanh Le, Thao Thi Thu Le, Trang Diep Thanh Le, Munjae Lee, Sang-woong Lee, Seung Won Lee, Wei-Chen Lee, Yo Han Lee, Samson Mideksa Legesse, James Leigh, Jacopo Lenzi, Elvynna Leong, Temesgen L Lerango, Ming-Chieh Li, Wei Li, Xiaopan Li, Zhihui Li, Massimo Libra, Virendra S Ligade, Andrew Tiyamike Makhiringa Likaka, Lee-Ling Lim, Stephen S Lim, Ro-Ting Lin, Vasileios-Arsenios Lioutas, Stefan Listl, Jue Liu, Simin Liu, Xiaofeng Liu, Katherine M Livingstone, Erand Llanaj, Chun-Han Lo, Arianna Maever Loreche, László Lorenzovici, Mojgan Lotfi, Masoud Lotfizadeh, Rafael Lozano, Jailos Lubinda, Giancarlo Lucchetti, Stefan Ma, Zheng Feei Ma, Mahmoud Mabrok, Nikolaos Machairas, Monika Machoy, Javier A Magaña Gómez, Azzam A Maghazachi, Sandeep B Maharaj, Preeti Maharjan, Soleiman Mahjoub, Mansour Adam Mahmoud, Elham Mahmoudi, Morteza Mahmoudi, Omar Mohamed Makram, Jeadran N Malagón-Rojas, Elaheh Malakan Rad, Reza Malekzadeh, Armaan K Malhotra, Kashish Malhotra, Ahmad Azam Malik, Iram Malik, Lesibana Anthony Malinga, Deborah Carvalho Malta, Abdullah A Mamun, Yosef Manla, Fahmida Mannan, Yasaman Mansoori, Ali Mansour, Vahid Mansouri, Mohammad Ali Mansournia, Hamid Reza Marateb, Joemer C Maravilla, Agustina M Marconi, Parham Mardi, Mirko Marino, Abdoljalal Marjani, Bernardo Alfonso Martinez-Guerra, Ramon

Martinez-Piedra, Francisco Rogerlândio Martins-Melo, Miquel Martorell, Wolfgang Marx, Sharmeen Maryam, Roy Rillera Marzo, Kedar K V Mate, Clara N Matei, Alexander G Mathioudakis, Richard James Maude, Andrea Maugeri, Erin A May, Mahsa Mayeli, Mohsen Mazidi, Colm McAlinden, John J McGrath, Martin McKee, Anna Laura Wensel McKowen, Michael A McPhail, Steven M McPhail, Enkeleint A Mechili, Rishi P Mediratta, Jitendra Kumar Meena, Medhin Mehari, Rahul Mehra, Kamran Mehrabani-Zeinabad, Entezar Mehrabi Nasab, Ravi Mehrotra, Mathewos M Mekonnen, Walter Mendoza, Ritesh G Menezes, Endalkachew Worku Mengesha, Alexios-Fotios A Mentis, Sultan Ayoub Meo, Atte Meretoja, Tuomo J Meretoja, Abera M Mersha, Bezawit Afework Mesfin, Tomislav Mestrovic, Adquate Mhlanga, Laurette Mhlanga, Tianyue Mi, Georgia Micha, Irmina Maria Michalek, Ted R Miller, Sergey Nikolaevich Mindlin, Le Huu Nhat Minh, GK Mini, Neema W Minja, Niloofar Mirdamadi, Andreea Mirica, Omid Mirmosayyeb, Mizan Kiros Mirutse, Mohammad Mirza-Aghazadeh-Attari, Maryam Mirzaei, Tadesse Misgana, Sanjeev Misra, Philip B Mitchell, Prasanna Mithra, Chaitanya Mittal, Madhukar Mittal, Babak Moazen, Ahmed Ismail Mohamed, Jama Mohamed, Mouhand F H Mohamed, Nouh Saad Mohamed, Sakineh Mohammad-Alizadeh-Charandabi, Soheil Mohammadi, Abdollah Mohammadian-Hafshejani, Saeed Mohammad-pour, Marita Mohammadshahi, Mustapha Mohammed, Salahuddin Mohammed, Shafiu Mohammed, Hoda Mojiri-forushani, Ali H Mokdad, Peyman Mokhtarzadehazar, Kaveh Momenzadeh, Sara Momtazmanesh, Mohammad Ali Moni, Fateme Montazeri, AmirAli Moodi Ghalibaf, Maryam Moradi, Yousef Moradi, Maziar Moradi-Lakeh, Mehdi Moradinazar, Farhad Moradpour, Paula Moraga, Rafael Silveira Moreira, Negar Morovatdar, Reza Mosaddeghi Heris, Jonathan F Mosser, Elias Mossialos, Hakimeh Mostafavi, Amirmahdi Mostofinejad, Simin Mouodi, Seyed Ehsan Mousavi, Amin Mousavi Khaneghah, Christine Mpundu-Kaambwa, Sumaira Mubarik, Lorenzo Muccioli, Ulrich Otto Mueller, Faraz Mughal, Sumoni Mukherjee, George Duke Mukoro, Admir Mulita, Francesk Mulita, Malaisamy Muniyandi, Kavita Munjal, Christopher J L Murray, Fungai Musaigwa, Khaled M Musallam, Ghulam Mustafa, Sathish Muthu, Saravanan Muthupandian, Woojae Myung, Ashraf F Nabhan, Fredrick Muyia Nafukho, Ahamarshan Jayaraman Nagarajan, Mohsen Naghavi, Pirouz Naghavi, Ganesh R Naik, Gurudatta Naik, Mukhammad David Naimzada, Sanjeev Nair, Tapas Sadasivan Nair, Hastyar Hama Rashid Najmuldeen, Vinay Nangia, Shumaila Nargus, Bruno Ramos Nascimento, Abdallah Y Naser, Mohammad Javad Nasiri, Zuhair S Natto, Javaid Nauman, Muhammad Naveed, Biswa Prakash Nayak, Vinod C Nayak, Athare Nazri-Panjaki, Hadush Negash, Ionut Negoi, Ruxandra Irina Negoi, Seyed Aria Nejadghaderi, Chakib Nejjari, Mohammad Hadi Nematollahi, Olivia D Nesbit, Charles Richard James Newton, Josephine W Ngunjiri, Dang H Nguyen, Phat Tuan Nguyen, Phuong The Nguyen, Van Thanh Nguyen, Yeshambel T Nigatu, Taxiarchis Konstantinos Nikolouzakis, Ali Nikoobar, Amin Reza Nikpoor, Muhammad A Nizam, Shuhei Nomura, Mamoona Noreen, Nafise Noroozi, Bo Norrving, Chisom Adaobi Nri-Ezedi, George Ntaios, Mpiko Ntsekhe, Dieta Nurrika, Bogdan Oancea, Kehinde O Obamiro, Ismail A Odetokun, Akinyemi O D Ofakunrin, Ropo Ebenezer Ogunsakin, James Odhiambo Oguta, Hassan Okati-Aliabad, Sylvester Reuben Okeke, Akinkunmi Paul Okekunle, Lawrence Okidi, Osaretin Christabel Okonji, Andrew T Olagunju, Muideen Tunbosun Olaiya, Titilope O Olanipekun, Matthew Idowu Olatubi, Antonio Olivas-Martinez, Gláucia Maria Moraes Oliveira, Abdulhakeem Abayomi Olorukooba, Isaac Iyinoluwa Olufadewa, Bolajoko Olubukunola Olusanya, Jacob Olusegun Olusanya, Yinka Doris Oluwafemi, Gideon Olamilekan Oluwatunase, Hany A Omar, Goran Latif Omer, Obinna E Onwujekwe, Kenneth Ikenna Onyedibe, John Nelson Opio, Michal Ordak, E Roberto Orellana, Orish Ebere Orisakwe, Verner N Orish, Hans Orru, Doris V Ortega-Altamirano, Alberto Ortiz, Uchechukwu Levi Osuagwu, Adrian Otoiu, Nikita Otstavnov, Amel Ouyahia, Mayowa O Owolabi, Ifeoluwa Temitayo Oyeyemi, Oyetunde T Oyeyemi, Mahesh Padukudru P A, Jagadish Rao Padubidri, Mahsa Pahlavikhah Varnosfaderani, Pramod Kumar Pal,

Tamás Palicz, Raffaele Palladino, Adrian Pana, Parsa Panahi, Ashok Pandey, Seithikurippu R Pandi-Perumal, Victoria Pando-Robles, Helena Ullyartha Pangaribuan, Georgios D Panos, Paraskevi Papadopoulou, Shahina Pardhan, Romil R Parikh, Seoyeon Park, Ashwaghosha Parthasarathi, Ava Pashaei, Deepak Kumar Pasupula, Jenil R Patel, Sangram Kishor Patel, Aslam Ramjan Pathan, Ashlesh Patil, Shankargouda Patil, Dimitrios Patoulias, Venkata Suresh Patthipati, Uttam Paudel, Shrikant Pawar, Spencer A Pease, Amy E Peden, Paolo Pedersini, Veincent Christian Filipino Pepito, Emmanuel K Peprah, Gavin Pereira, Jeevan Pereira, Marcos Pereira, Mario F P Peres, Arokiasamy Perianayagam, Ionela-Roxana Petcu, Fanny Emily Petermann-Rocha, Raffaele Pezzani, Hoang Tran Pham, Michael R Phillips, Manon Pigeolet, David M Pigott, Michael A Piradov, Nishad Plakkal, Evgenii Plotnikov, Dimitri Poddighe, Peter Pollner, Ramesh Poluru, Constance Dimity Pond, Maarten J Postma, Govinda Raj Poudel, Lisasha Poudel, Ghazaleh Pourali, Sergio I Prada, Pranil Man Singh Pradhan, Vijay Kumar Prajapati, V Prakash, Chandra P Prasad, Manya Prasad, Akila Prashant, Elton Junio Sady Prates, Hery Purnobasuki, Bharathi M Purohit, Jagadeesh Puvvula, Rizwan Qaisar, Nameer Hashim Qasim, Ibrahim Qattea, Gangzhen Qian, Nguyen Khoi Quan, Amir Radfar, Venkatraman Radhakrishnan, Pourya Raee, Hadi Raeisi Shahraki, Seyedeh Niloufar Rafiei Alavi, Fakher Rahim, Md Mosfequr Rahman, Mosiur Rahman, Muhammad Aziz Rahman, Tafhimur Rahman, Amir Masoud Rahmani, Niloufar Rahnavard, Pramila Rai, Sathish Rajaa, Ali Rajabpour-Sanati, Prashant Rajput, Prasanna Ram, Hazem Ramadan, Shakthi Kumaran Ramasamy, Sheena Ramazanu, Juwel Rana, Kritika Rana, Chhabi Lal Ranabhat, Nemanja Rancic, Smitha Rani, Shubham Ranjan, Chythra R Rao, Indu Ramachandra Rao, Sowmya J Rao, Drona Prakash Rasali, Sina Rashedi, Vahid Rashedi, Ahmed Mustafa Rashid, Ashkan Rasouli-Saravani, Prateek Rastogi, Azad Rasul, Ramin Ravangard, Nakul Ravikumar, David Laith Rawaf, Salman Rawaf, Reza Rawassizadeh, Iman Razeghian-Jahromi, Murali Mohan Rama Krishna Reddy, Elrashdy Moustafa Mohamed Redwan, Faizan Ur Rehman, Robert C Reiner Jr, Bhageerathy Reshmi, Serge Resnikoff, Luis Felipe Reyes, Malihe Rezaee, Negar Rezaei, Nima Rezaei, Mohsen Rezaeian, Mavra A Riaz, Ana Isabel Ribeiro, Jennifer Rickard, Maria Jesus Rios-Blancas, Mónica Rodrigues, Jefferson Antonio Buendia Rodriguez, Leonardo Roever, Peter Rohloff, Debby Syahru Romadlon, Gholamreza Roshandel, Sharareh Roshanzamir, Priyanka Roy, Enrico Rubagotti, Godfrey M Rwegerera, Chandan S N, Katharina S Sunnerhagen, Aly M A Saad, Michela Sabbatucci, Korosh Saber, Maha Mohamed Saber-Ayad, Simona Sacco, Basema Saddik, Bashdar Abuzed Sadee, Ehsan Sadeghi, Saeid Sadeghian, Umar Saeed, Maryam Saeedi, Sare Safi, Rajesh Sagar, Amene Saghazadeh, Narjes Saheb Sharif-Askari, Soumya Swaroop Sahoo, Mohammad Ali Sahraian, Seyed Aidin Sajedi, Mirza Rizwan Sajid, Joseph W Sakshaug, Payman Salamati, Afeez Abolarinwa Salami, Luciane B Salaroli, Mohamed A Saleh, Sana Salehi, Marwa Rashad Salem, Hossein Samadi Kafil, Sara Samadzadeh, Kamel A Samara, Saad Samargandy, Yoseph Leonardo Samodra, Vijaya Paul Samuel, Abdallah M Samy, Juan Sanabria, Nima Sanadgol, Edmond Sanganyado, Rama Krishna Sanjeev, Francesco Sanmarchi, Ichtiarini Nurullita Santri, Milena M Santric-Milicevic, Babak Saravi, Yaser Sarikhani, Chinmoy Sarkar, Rodrigo Sarmiento-Suárez, Gargi Sachin Sarode, Sachin C Sarode, Arash Sarveazad, Brijesh Sathian, Thirunavukkarasu Sathish, Davide Sattin, Jennifer Saulam, Susan M Sawyer, Sonia Saxena, Ganesh Kumar Saya, Yaser Sayadi, Abu Sayeed, Md Abu Sayeed, Mete Saylan, Nikolaos Scarmeas, Winfried Schlee, Art Schuermans, David C Schwebel, Falk Schwendicke, Mario Šekerija, Mohammad H Semreen, Sabyasachi Senapati, Pallav Sengupta, Subramanian Senthilkumaran, Sadaf G Sepanlou, Dragos Serban, Addisu Sertsu, Yashendra Sethi, SeyedAhmad SeyedAlinaghi, Seyed Arsalan Seyedi, Amir Shafaat, Omid Shafaat, Mahan Shafie, Arman Shafiee, Nilay S Shah, Pritik A Shah, Ataollah Shahbandi, Izza Shahid, Samiah Shahid, Wajeehah Shahid, Moyad Jamal Shahwan, Masood Ali Shaikh, Alireza Shakeri, Husain Shakil, Muhammad Aaqib Shamim, Mehran Shams-Beyranvand, Hina Shamshad, Mohammad Ali

Shamshirgaran, Mohammad Anas Shamsi, Mohd Shanawaz, Abhishek Shankar, Sadaf Sharfaei, Amin Sharifan, Mariam Shariff, Javad Sharifi-Rad, Saurab Sharma, Vishal Sharma, Rajesh P Shastry, Amin Shavandi, Amir Mehdi Shayan, Amr Mohamed Elsayed Shehabeldine, Aziz Sheikh, Rahim Ali Sheikhi, Jiabin Shen, Ranjitha S Shetty, Robert Adamu Shey, Amir Shiani, Kenji Shibuya, Desalegn Shiferaw, Mika Shigematsu, Jae Il Shin, Min-Jeong Shin, Rahman Shiri, Aminu Shittu, Ivy Shiue, K M Shivakumar, Sina Shool, Sunil Shrestha, Kanwar Hamza Shuja, Kerem Shuval, Migbar Mekonnen Sibhat, Emmanuel Edwar Siddig, Inga Dora Sigfusdottir, João Pedro Silva, Luís Manuel Lopes Rodrigues Silva, Soraia Silva, Jorge Piano Simões, Anjali Singal, Abhinav Singh, Aditya Singh, Ambrish Singh, Balbir Bagicha Singh, Baljinder Singh, Mahendra Singh, Mayank Singh, Narinder Pal Singh, Paramdeep Singh, Md Shahjahan Siraj, Freddy Sitas, Shravan Sivakumar, Valentin Yurievich Skryabin, Anna Aleksandrovna Skryabina, David A Sleet, Hamidreza Soleimani, Sameh S M Soliman, Marco Solmi, Yonatan Solomon, Yimeng Song, Reed J D Sorensen, Joan B Soriano, Ireneous N Soyiri, Michael Spartalis, Chandrashekhar T Sreeramareddy, Joseph R Starnes, Antonina V Starodubova, Simona Cătălina Stefan, Fridolin Steinbeis, Paschalis Steiropoulos, Stefan Stortecky, Muhammad Suleman, Rizwan Suliankatchi Abdulkader, Abida Sultana, Jing Sun, David Sunkersing, Chandan Kumar Swain, Bryan L Sykes, Lukasz Szarpak, Mindy D Szeto, Miklós Szócska, Payam Tabaee Damavandi, Ozra Tabatabaei Malazy, Seyed-Amir Tabatabaeizadeh, Shima Tabatabai, Karen M Tabb, Mohammad Tabish, Luis M Taborda-Barata, Birkneh Tilahun Tadesse, Amirmasoud Taheri, Yasaman Taheri Abkenar, Moslem Taheri Soodejani, Jabeen Taiba, Iman M Talaat, Jacques Lukenze Tamuzi, Ker-Kan Tan, Haosu Tang, Hong K Tang, Razieh Tavakoli Oliaee, Seyed Mohammad Tavangar, Nuno Taveira, Tsion Mulat Tebeje, Yibekal Manaye Tefera, Mohamad-Hani Temsah, Reem Mohamad Hani Temsah, Masayuki Teramoto, Solomon Hailemariam Tesfaye, Pugazhenthan Thangaraju, Kavumpurathu Raman Thankappan, Rajshree Thapa, Rekha Thapar, Nihal Thomas, Chern Choong Chern Thum, Jing Tian, Jansje Henny Vera Ticoalu, Tenaw Yimer Tiruye, Seyed Abolfazl Tohidast, Marcello Tonelli, Mathilde Touvier, Marcos Roberto Tovani-Palone, Khai Hoan Tram, Nghia Minh Tran, Domenico Trico, Indang Trihandini, Samuel Joseph Tromans, Vien T Truong, Thien Tan Tri Tai Truyen, Evangelia Eirini Tsermpini, Munkhtuya Tumurkhuu, Kang Tung, Stefanos Tyrovolas, Chukwudi S Ubah, Aniefiok John Udoakang, Arit Udoh, Inam Ulhaq, Saeed Ullah, Sana Ullah, Muhammad Umair, Chukwuma David Umeokonkwo, Anushri Umesh, Bhaskaran Unnikrishnan, Era Upadhyay, Amir Mohammad Vahdani, Sahel Valadan Tahbaz, Rohollah Valizadeh, Jef Van den Eynde, Orsolya Varga, Siddhartha Alluri Varma, Priya Vart, Shoban Babu Varthya, Lennert J Veerman, Narayanaswamy Venketasubramanian, Deneshkumar Venugopal, Nicholas Alexander Verghese, Madhur Verma, Pratibha Verma, Massimiliano Veroux, Georgios-Ioannis Verras, Dominique Vervoort, Rafael José Vieira, Jorge Hugo Villafañe, Gabriela Ines Villanueva, Paul J Villeneuve, Bay Vo, Stein Emil Vollset, Simona Ruxandra Volovat, Victor Volovici, Theo Vos, Isidora S Vujcic, Yohannes Dibaba Wado, Hatem A Wafa, Yasir Waheed, Richard G Wamai, Cong Wang, Fang Wang, Shu Wang, Song Wang, Yanzhong Wang, Yuan-Pang Wang, Paul Ward, Stefanie Watson, Kosala Gayan Weerakoon, Daniel J Weiss, Abrha Hailay Weldemariam, Yi Feng Wen, Andrea Werdecker, Ronny Westerman, Dakshitha Praneeth Wickramasinghe, Nuwan Darshana Wickramasinghe, Tissa Wijeratne, Shadrach Wilson, Eve E Wool, Anthony D Woolf, Dongze Wu, Ratna Dwi Wulandari, Hong Xiao, Bin Xu, Xiaoyue Xu, Lalit Yadav, Yao Yao, Pengpeng Ye, Gesila Endashaw Yesera, Renjulal Yesodharan, Subah Abderehim Yesuf, Arzu Yiğit, Vahit Yiğit, Dong Keon Yon, Naohiro Yonemoto, Yuyi You, Mustafa Z Younis, Chuanhua Yu, Siddhesh Zadey, Vesna Zadnik, Nima Zafari, Mohammad Zahedi, Muhammad Nauman Zahid, Mazyar Zahir, Fathiah Zakham, Nazar Zaki, Giulia Zamagni, Burhan Abdullah Zaman, Sojib Bin Zaman, Milad Zandi, Ghazal G Z Zandieh, Mikhail Sergeevich Zastrozhin, Mohammed G M Zeariya, Youjie Zeng, Chunxia Zhai,

Chen Zhang, Haijun Zhang, Hongwei Zhang, Yunquan Zhang, Zhenyu Zhang, Hanqing Zhao, Yang Zhao, Chenwen Zhong, Juexiao Zhou, Bin Zhu, Zhaohua Zhu, Magdalena Zielińska, Zhiyong Zou, Alimuddin Zumla, Elric Zweck, and Samer H Zyoud.

Drafting the work or revising it critically for important intellectual content

Amirali Aali, Cristiana Abbafati, Jaffar Abbas, Samar Abd ElHafeez, Michael Abdelmasseh, Sherief Abd-Elsalam, Ahmed Abdelwahab, Meriem Abdoun, Auwal Abdullahi, Ame Mehadi Abdurehman, Aidin Abedi, Armita Abedi, Tadesse M Abegaz, Roberto Ariel Abeldaño Zuñiga, Olugbenga Olusola Abiodun, Hassan Abolhassani, Mohamed Abouzid, Lucas Guimarães Abreu, Samir Abu Rumeileh, Salahdein Aburuz, Ahmed Abu-Zaid, Juan Manuel Acuna, Isaac Yeboah Addo, Oladimeji M Adebayo, Oyelola A Adegboye, Victor Adekanmbi, Abiola Victor Adepoju, Charles Oluwaseun Adetunji, Olorunsola Israel Adeyomoye, Amin Adibi, Qorinah Estiningtyas Sakilah Adnani, Saryia Adra, Aanuoluwapo Adeyimika Afolabi, Ali Afraz, Saira Afzal, Pradyumna Agasthi, Antonella Agodi, Bright Opoku Ahinkorah, Danish Ahmad, Firdos Ahmad, Muayyad M Ahmad, Ayman Ahmed, Haroon Ahmed, Luai A Ahmed, Muktar Beshir Ahmed, Syed Anees Ahmed, Marjan Ajami, Olufemi Ajumobi, Essona Matatom Akara, Karolina Akinosoglou, Ammar Al Homsi, Mohammad Al Qadire, Samer O Alalalmeh, Khurshid Alam, Rasmieh Mustafa Al-amer, Mohammed Albashtawy, Mohammad T AlBataineh, Ayman Al-Eyadhy, Fadwa Alhalaiqa Naji Alhalaiqa, Mohammed Khaled Al-Hanawi, Abid Ali, Akhtar Ali, Hassam Ali, Mohammed Usman Ali, Syed Shujait Shujait Ali, Gianfranco Alicandro, Sheikh Mohammad Alif, Ahmednur Adem Aliyi, Sadeq Ali Ali Al-Maweri, Joseph Uy Almazan, Omar Almidani, Mahmoud A Alomari, Nivaldo Alonso, Jaber S Alqahtani, Salman Khalifah Al-Sabah, Jaffar A Al-Tawfiq, Hassan Alwafi, Yaser Mohammed Al-Worafi, Hany wq, Karem H Alzoubi, Azmeraw T Amare, Abebe Feyissa Amhare, Tarek Tawfik Amin, Alireza Amindarolzarbi, Sohrab Amiri, Hubert Amu, Dickson A Amugsi, Jimoh Amzat, Robert Ancuceanu, Deanna Anderlini, Pedro Prata Andrade, Catalina Liliana Andrei, Tudorel Andrei, Dhanalakshmi Angappan, Abhishek Anil, Afifa Anjum, Catherine M Antony, Ernoiz Antriyandarti, Iyadunni Adesola Anuoluwa, Anayochukwu Edward Anyasodor, Seth Christopher Yaw Appiah, Muhammad Aqeel, Jalal Arabloo, Razman Arabzadeh Bahri, Morteza Arab-Zozani, Ana Margarida Araújo, Abdulfatai Aremu, Hany Ariffin, Timur Aripov, Benedetta Armocida, Mahwish Arooj, Kurnia Dwi Artanti, Judie Arulappan, Idowu Thomas Aruleba, Raphael Taiwo Aruleba, Ashokan Arumugam, Malke Asaad, Saeed Asgary, Muhammad Ashraf, Marvellous O Asika, Seyyed Shamsadin Athari, Maha Moh'd Wahbi Atout, Alok Atreya, Avinash Aujayeb, Abolfazl Avan, Beatriz Paulina Ayala Quintanilla, Martin Amogre Ayanore, Getnet Melaku Ayele, Seyed Mohammad Ayyoubzadeh, Sina Azadnajafabad, Ahmed Y Azzam, Abraham Samuel Babu, Muhammad Badar, Alaa Badawi, Ashish D Badiye, Soroush Baghdadi, Sara Bagherieh, Sulaiman Bah, Jianjun Bai, Abdulaziz T Bako, Senthilkumar Balakrishnan, Saliu A Balogun, Ovidiu Constantin Baltatu, Kiran Bam, Maciej Banach, Soham Bandyopadhyay, Hansi Bansal, Martina Barchitta, Mainak Bardhan, Suzanne Lyn Barker-Collo, Francesco Barone-Adesi, Hiba Jawdat Barqawi, Ronald D Barr, Lope H Barrero, Asma'u I J Bashir, Hameed Akande Bashiru, Pritish Baskaran, Buddha Basnyat, Quique Bassat, João Diogo Basso, Bernhard T Baune, Thomas Beaney, Neeraj Bedi, Emad Behboudi, Amir Hossein Behnoush, Maryam Beiranvand, Diana Fernanda Bejarano Ramirez, Uzma Iqbal Belgaumi, Michelle L Bell, Aminu K Bello, Muhammad Bashir Bello, Olorunjuwon Omolaja Bello, Luis Belo, Apostolos Beloukas, Derrick A Bennett, Isabela M Bensenor, Habib Benzian, Zombor Berezvai, Paulo J G Bettencourt, Habtamu B Beyene, Kebede A Beyene, Akshaya Srikanth Bhagavathula, Neeraj Bhala, Dinesh Bhandari, Prarthna V Bhardwaj, Ashish Bhargava, Sonu Bhaskar, Vivek Bhat, Gurjit Kaur Bhatti, Jasvinder Singh Bhatti, Manpreet S Bhatti,

Rajbir Bhatti, Boris Bikbov, Nada Binmadi, Antonio Biondi, Catherine Bisignano, Francesca Bisulli, Atanu Biswas, Saeid Bitaraf, Tone Bjørge, Archie Bleyer, Mary Sefa Boampong, Virginia Bodolica, Aadam Olalekan Bodunrin, Milad Bonakdar Hashemi, Aime Bonny, Kaustubh Bora, Safiya Bala Borodo, Rohan Borschmann, Alejandro Botero Carvajal, Souad Bouaoud, Sofiane Boudalia, Edward J Boyko, Nicola Luigi Bragazzi, Dejana Braithwaite, Hermann Brenner, Norma B Bulamu, Danilo Buonsenso, Richard A Burns, Sharath Burugina Nagaraja, Reinhard Busse, Yasser Bustanji, Florentino Luciano Caetano dos Santos, Luis Alberto Cámera, Luciana Aparecida Campos, Ismael R Campos-Nonato, Chao Cao, Sinclair Carr, Giulia Carreras, Juan J Carrero, Andrea Carugno, Felix Carvalho, Márcia Carvalho, Joao Mauricio Castaldelli-Maia, Carlos A Castañeda-Orjuela, Giulio Castelpietra, Ferrán Catalá-López, Alberico L Catapano, Maria Sofia Cattaruzza, Arthur Caye, Christopher R Cederroth, Francieli Cembranel, Muthia Cenderadewi, Ester Cerin, Muge Cevik, Pamela R Chacón-Uscamaita, Yaacoub Chahine, Chiranjib Chakraborty, Jeffrey Shi Kai Chan, Chin-Kuo Chang, Vijay Kumar Chattu, Victoria Chatzimavridou-Grigoriadou, Malizgani Paul Chavula, Huzaifa Ahmad Cheema, An-Tian Chen, Haowei Chen, Lingxiao Chen, Meng Xuan Chen, Simiao Chen, Nicolas Cherbuin, Gerald Chi, Jesus Lorenzo Chirinos-Caceres, So Mi Jemma Cho, William C S Cho, Bryan Chong, Hitesh Chopra, Rahul Choudhary, Rajiv Chowdhury, Dinh-Toi Chu, Eric Chung, Eunice Chung, Karly I Cini, Cain C T Clark, Alyssa Columbus, Haley Comfort, Joao Conde, Sara Conti, Paolo Angelo Cortesi, Vera Marisa Costa, Ewerton Cousin, Richard G Cowden, Michael H Criqui, Natália Cruz-Martins, Patricia Cullen, Daniel da Silva e Silva, Sriharsha Dadana, Zhaoli Dai, Koustuv Dalal, Giovanni Damiani, Emanuele D'Amico, Sara Daneshvar, Nihar Ranjan Dash, Claudio Alberto Dávila-Cervantes, Nicole Davis Weaver, Diego De Leo, Aklilu Tamire Debele, Louisa Degenhardt, Ivan Delgado-Enciso, Laura Delgado-Ortiz, Berecha Hundessa Demessa, Andreas K Demetriades, Edgar Denova-Gutiérrez, Kebede Deribe, Nikolaos Dervenis, Don C Des Jarlais, Hardik Dineshbhai Desai, Rupak Desai, Keshab Deuba, Sourav Dey, Mandira Lamichhane Dhimal, Meghnath Dhimal, Sameer Dhingra, Diana Dias da Silva, Daniel Diaz, Adriana Dima, Delaney D Ding, M Ashworth Dirac, Abhinav Dixit, Shilpi Gupta Dixit, Thanh Chi Do, Camila Bruneli do Prado, Masoud Dodangeh, Christiane Dolecek, E Ray Dorsey, Wendel Mombaque dos Santos, Rajkumar Doshi, Leila Doshmangir, Abdel Douiri, Robert Kokou Dowou, Tim Robert Driscoll, Haneil Larson Dsouza, John Dube, Samuel C Dumith, Bruce B Duncan, Oyewole Christopher Durojaiye, Sulagna Dutta, Arkadiusz Marian Dziedzic, Chidiebere Peter Echieh, David Edvardsson, Defi Efendi, Shayan Eghdami, Ebrahim Eini, Michael Ekholuenetale, Rabie Adel El Arab, Maysaa El Sayed Zaki, Maha El Tantawi, Iffat Elbarazi, Noha Mousaad Elemam, Frank J Elgar, Islam Y Elgendy, Ghada Metwally Tawfik ElGohary, Hala Rashad Elhabashy, Muhammed Elhadi, Omar Abdelsadek Abdou Elmeligy, Ibrahim Elsohaby, Mehdi Emamverdi, Theophilus I Emeto, Luchuo Engelbert Bain, Ryenchindorj Erkhembayar, Tesfahun C Eshetie, Sharareh Eskandarieh, Juan Espinosa-Montero, Farshid Etaee, Ugochukwu Anthony Eze, Natalia Fabin, Adeniyi Francis Fagbamigbe, Saman Fahimi, Luca Falzone, Mohsen Farjoud Kouhanjani, Andre Faro, Ali Fatehizadeh, Nelsensius Klau Fauk, Valery L Feigin, Seyed-Mohammad Fereshtehnejad, Abdullah Hamid Feroze, Pietro Ferrara, Nuno Ferreira, Getahun Fetensa, Irina Filip, Florian Fischer, Joanne Flavel, Bobirca Teodor Florin, Morenike Oluwatoyin Folayan, Kristen Marie Foley, Behzad Foroutan, Matteo Foschi, Kate Louise Francis, Richard Charles Franklin, Alberto Freitas, Joseph Friedman, Sara D Friedman, Takeshi Fukumoto, Peter Andras Gaal, Muktar A Gadanya, Santosh Gaihre, Yaseen Galali, Nasrin Galehdar, Silvano Gallus, Aravind P Gandhi, Balasankar Ganesan, Jalaj Garg, Prem Gautam, Rupesh K Gautam, Federica Gazzelloni, Miglas W Gebregergis, Mesfin Gebrehiwot, Urge Gerema, Motuma Erena Getachew, Tamirat Getachew, Mansour Ghafourifard, Sulmaz Ghahramani, Khalid Yaser Ghailan, Alireza Ghajar, MohammadReza Ghasemi, Fariba Ghassemi, Ramy Mohamed Ghazy, Sailaja Ghimire, Ghozali Ghozali, Sherief Ghozy, Alessandro Gialluisi, Ruth Margaret Gibson,

Tiffany K Gill, Richard F Gillum, Themba G Ginindza, Alem Girmay, James C Glasbey, Elena V Gnedovskaya, Laszlo Göbölös, Arefeh Golestanfar, Davide Golinelli, Philimon N Gona, Anmol Goyal, Scott Greenhalgh, Michal Grivna, Giovanni Guarducci, Mesay Dechasa Gudeta, Avirup Guha, Stefano Guicciardi, Sasidhar Gunturu, Cui Guo, Bhawna Gupta, Rajat Das Gupta, Sapna Gupta, Veer Bala Gupta, Vivek Kumar Gupta, Reyna Alma Gutiérrez, Farrokh Habibzadeh, Parham Habibzadeh, Vladimir Hachinski, Mohammad Haddadi, Nils Haep, Adel Hajj Ali, Esam S Halboub, Sobia Ahsan Halim, Brian J Hall, Rabih Halwani, Randah R Hamadeh, Mohammad Hamiduzzaman, Ahmad Hammoud, Nasrin Hanifi, Graeme J Hankey, Md Abdul Hannan, Harapan Harapan, Josep Maria Haro, Ahmed I Hasaballah, Faizul Hasan, M Tasdik Hasan, Hamidreza Hasani, Abbas M Hassan, Amr Hassan, Johannes Haubold, Rasmus J Havmoeller, Simon I Hay, Youssef Hbid, Jeffrey J Hebert, Omar E Hegazi, Golnaz Heidari, Reza Heidari-Soureshjani, Bartosz Helfer, Claudiu Herteliu, Hamed Hesami, Dineshani Hettiarachchi, Demisu Zenbaba Heyi, Kamal Hezam, Yuta Hiraike, Howard J Hoffman, Ramesh Holla, Nobuyuki Horita, Md Mahbub Hossain, Sahadat Hossain, Mohammad-Salar Hosseini, Sorin Hostiuc, Vivian Chia-rong Hsieh, Junjie Huang, Md Nazmul Huda, Fernando N Hugo, Michael Hultström, Javid Hussain, Salman Hussain, Hong-Han Huynh, Segun Emmanuel Ibitoye, Oluwatope Olaniyi Idowu, Desta Ijo, Mehran Ilaghi, Olayinka Stephen Ilesanmi, Irena M Ilic, Milena D Ilic, Mustapha Immurana, Leeberk Raja Inbaraj, Farideh Iravanpour, Kenneth Chukwuemeka Iregbu, Md Rabiul Islam, Mohammad Mainul Islam, Sheikh Mohammed Shariful Islam, Farhad Islami, Nahlah Elkudssiah Ismail, Gaetano Isola, Chidozie C D Iwu, Chinwe Juliana Iwu-Jaja, Mahalaxmi Iyer, Linda Merin J, Louis Jacob, Kathryn H Jacobsen, Morteza Jafarinia, Khushleen Jaggi, Kasra Jahankhani, Nader Jahanmehr, Haitham Jahrami, Akhil Jain, Mihajlo Jakovljevic, Reza Jalilzadeh Yengejeh, Chinmay T Jani, Mark M Janko, Abubakar Ibrahim Jatau, Sathish Kumar Jayapal, Shubha Jayaram, Alelign Tasew Jema, Digisie Mequanint Jemere, Wonjeong Jeong, Ravi Prakash Jha, Nabi Jomehzadeh, Darwin Phan Jones, Tamas Joo, Abel Joseph, Nitin Joseph, Charity Ehimwenma Joshua, Jacek Jerzy Jozwiak, Mikk Jürisson, Ali Kabir, Hannaneh Kabir, Vidya Kadashetti, Farima Kahe, Rizwan Kalani, Feroze Kaliyadan, Sanjay Kalra, Arun Kamireddy, Thanigaivelan Kanagasabai, Himal Kandel, Edmund Wedam Kanmiki, Kehinde Kazeem Kanmodi, Rami S Kantar, Neeti Kapoor, Mehrdad Karajizadeh, Shama D Karanth, Asima Karim, Hanie Karimi, Faizan Zaffar Kashoo, Hengameh Kasraei, Nigussie Assefa Kassaw, Nicholas J Kassebaum, Adarsh Katamreddy, Srinivasa Vittal Katikireddi, Patrick DMC Katoto, Joonas H Kauppila, Navjot Kaur, Jeanne Françoise Kayibanda, Gbenga A Kayode, Foad Kazemi, Sina Kazemian, sara Kazeminia, John H Kempen, Jessica A Kerr, Emmanuelle Kesse-Guyot, Mohamad Mehdi Khadembashiri, Himanshu Khajuria, Mohammad Khalafi, Amirmohammad Khalaji, Nauman Khalid, Gulfaraz Khan, Ikramullah Khan, Maseer Khan, Moien AB Khan, Taimoor Khan, Mahammed Ziauddin Khan suheb, Shaghayegh Khanmohammadi, Khaled Khatab, Armin Khavandegar, Hamid Reza Khayat Kashani, khalid a kheirallah, Feriha Fatima Khidri, Elaheh Khodadoust, Mahmood Khosrowjerdi, Jagdish Khubchandani, Grace Kim, Adnan Kisa, Sezer Kisa, Mika Kivimäki, Ann Kristin Skrindo Knudsen, Farzad Kompani, Ashwin Laxmikant Kotnis, Parvaiz A Koul, Sindhura Lakshmi Koulmane Laxminarayana, Ai Koyanagi, Michael A Kravchenko, Kewal Krishan, Hare Krishna, Kris J Krohn, Barthelemy Kuate Defo, Connor M Kubeisy, Md Abdul Kuddus, Mohammed Kuddus, Ilari Kuitunen, Mukhtar Kulimbet, Vishnutheertha Kulkarni, Harish Kumar, Madhulata Kumari, Om P Kurmi, Asep Kusnali, Dian Kusuma, Tezer Kutluk, Evans F Kyei, Carlo La Vecchia, Muhammad Awwal Ladan, Lucie Laflamme, Chandrakant Lahariya, Abdelilah Lahmar, Ratilal Lalloo, Tea Lallukka, Berthold Langguth, Van Charles Lansingh, Ariane Laplante-Lévesque, Anders O Larsson, Savita Lasrado, Kamaluddin Latief, Mahrukh Latif, Kaveh Latifinaibin, Nhi Huu Hanh Le, Thao Thi Thu Le, Paul H Lee, Samson Mideksa Legesse, Jacopo Lenzi, Elvynna Leong, Temesgen L Lerango, Wei Li, Zhihui Li, Massimo Libra, Andrew

Tiyamike Makhiringa Likaka, Lee-Ling Lim, Stefan Listl, Jue Liu, Katherine M Livingstone, Erand Llanaj, Chun-Han Lo, László Lorenzovici, Giancarlo Lucchetti, Alessandra Lugo, Raimundas Lunevicius, Zheng Feei Ma, Mahmoud Mabrok, Nikolaos Machairas, Monika Machoy, Christian Madsen, Javier A Magaña Gómez, Preeti Maharjan, Soleiman Mahjoub, Mansour Adam Mahmoud, Elham Mahmoudi, Omar Mohamed Makram, Elaheh Malakan Rad, Reza Malekzadeh, Armaan K Malhotra, Kashish Malhotra, Ahmad Azam Malik, Deborah Carvalho Malta, Abdullah A Mamun, Fahmida Mannan, Yasaman Mansoori, Vahid Mansouri, Lorenzo Giovanni Mantovani, Bishnu P Marasini, Hamid Reza Marateb, Parham Mardi, Bernardo Alfonso Martinez-Guerra, Ramon Martinez-Piedra, Cleodice A Martins, Francisco Rogerlândio Martins-Melo, Miquel Martorell, Wolfgang Marx, Sharmeen Maryam, Roy Rillera Marzo, Clara N Matei, Alexander G Mathioudakis, Andrea Maugeri, Erin A May, Mahsa Mayeli, Mohsen Mazidi, Antonio Mazzotti, Colm McAlinden, John J McGrath, Anna Laura Wensel McKowen, Susan A McLaughlin, Steven M McPhail, Enkeleint A Mechili, Rishi P Mediratta, Jitendra Kumar Meena, Max L Mehlman, Entezar Mehrabi Nasab, Mathewos M Mekonnen, Walter Mendoza, Ritesh G Menezes, George A Mensah, Laverne G Mensah, Alexios-Fotios A Mentis, Sultan Ayoub Meo, Atte Meretoja, Tuomo J Meretoja, Tomislav Mestrovic, Georgia Micha, Irmina Maria Michalek, Ted R Miller, Sergey Nikolaevich Mindlin, Giada Minelli, Le Huu Nhat Minh, GK Mini, Neema W Minja, Mojgan Mirghafourvand, Andreea Mirica, Omid Mirmosayyeb, Philip B Mitchell, Prasanna Mithra, Chaitanya Mittal, Madhukar Mittal, Babak Moazen, Mouhand F H Mohamed, Nouh Saad Mohamed, Sakineh Mohammad-Alizadeh-Charandabi, Soheil Mohammadi, Mustapha Mohammed, Salahuddin Mohammed, Shafiu Mohammed, Ali H Mokdad, Kaveh Momenzadeh, Sara Momtazmanesh, Lorenzo Monasta, Mohammad Ali Moni, Fateme Montazeri, AmirAli Moodi Ghalibaf, Maryam Moradi, Yousef Moradi, Maziar Moradi-Lakeh, Paula Moraga, Lidia Morawska, Rafael Silveira Moreira, Shane Douglas Morrison, Jakub Morze, Reza Mosaddeghi Heris, Jonathan F Mosser, Amirmahdi Mostofinejad, Simin Mouodi, Parsa Mousavi, Amin Mousavi Khaneghah, Matías Mrejen, Faraz Mughal, George Duke Mukoro, Malaisamy Muniyandi, Christopher J L Murray, Fungai Musaigwa, Khaled M Musallam, Ghulam Mustafa, Sathish Muthu, Ashraf F Nabhan, Ahamarshan Jayaraman Nagarajan, Mohsen Naghavi, Mukhammad David Naimzada, Sanjeev Nair, Hastyar Hama Rashid Najmuldeen, Shumaila Nargus, Bruno Ramos Nascimento, Gustavo G Nascimento, Abdallah Y Naser, Zuhair S Natto, Javaid Nauman, Biswa Prakash Nayak, Vinod C Nayak, Ashish Kumar Nayyar, Hadush Negash, Ionut Negoi, Ruxandra Irina Negoi, Serban Mircea Negru, Seyed Aria Nejadghaderi, Samata Nepal, Charles Richard James Newton, Josephine W Ngunjiri, Dang H Nguyen, Phat Tuan Nguyen, Phuong The Nguyen, Tuan Thanh Nguyen, Van Thanh Nguyen, Taxiarchis Konstantinos Nikolouzakis, Amin Reza Nikpoor, Muhammad A Nizam, Mamoona Noreen, Abbas Norouzian Baghani, Bo Norrving, Jean Jacques Noubiap, Chisom Adaobi Nri-Ezedi, George Ntaios, Mpiko Ntsekhe, Virginia Nuñez-Samudio, Dieta Nurrika, Bogdan Oancea, Kehinde O Obamiro, Ismail A Odetokun, Akinyemi O D Ofakunrin, In-Hwan Oh, Sylvester Reuben Okeke, Osaretin Christabel Okonji, Patrick Godwin Okwute, Andrew T Olagunju, Titilope O Olanipekun, Matthew Idowu Olatubi, Antonio Olivas-Martinez, Gláucia Maria Moraes Oliveira, Susan Oliver, Abdulhakeem Abayomi Olorukooba, Bolajoko Olubukunola Olusanya, Jacob Olusegun Olusanya, Hany A Omar, Obinna E Onwujekwe, Kenneth Ikenna Onyedibe, Michal Ordak, Verner N Orish, Hans Orru, Doris V Ortega-Altamirano, Alberto Ortiz, Edgar Ortiz-Brizuela, Esteban Ortiz-Prado, Uchechukwu Levi Osuagwu, Adrian Otoiu, Nikita Otstavnov, Guoqing Ouyang, Mayowa O Owolabi, Oyetunde T Oyeyemi, Yaz Ozten, Mahesh Padukudru P A, Jagadish Rao Padubidri, Mahsa Pahlavikhah Varnosfaderani, Tamás Palicz, Claudia Palladino, Raul Felipe Palma-Alvarez, Seithikurippu R Pandi-Perumal, Ioannis Pantazopoulos, Paraskevi Papadopoulou, Shahina Pardhan, Romil R Parikh, Seoyeon Park, Ashwaghosha Parthasarathi, Ava

Pashaei, Jenil R Patel, Ashlesh Patil, Shankargouda Patil, Dimitrios Patoulias, Venkata Suresh Patthipati, Uttam Paudel, Shrikant Pawar, Hamidreza Pazoki Toroudi, Spencer A Pease, Amy E Peden, Paolo Pedersini, Minjin Peng, Umberto Pensato, Veincent Christian Filipino Pepito, Gavin Pereira, Jeevan Pereira, Marcos Pereira, Mario F P Peres, Arokiasamy Perianayagam, Norberto Perico, Ionela-Roxana Petcu, Fanny Emily Petermann-Rocha, Hoang Tran Pham, Daniela Pierannunzio, Thomas Pilgrim, Marina Pinheiro, Michael A Piradov, Nishad Plakkal, Dimitri Poddighe, Peter Pollner, Maarten J Postma, Govinda Raj Poudel, Lisasha Poudel, Ghazaleh Pourali, Naeimeh Pourtaheri, Sergio I Prada, Pranil Man Singh Pradhan, Vijay Kumar Prajapati, Chandra P Prasad, Manya Prasad, Akila Prashant, Elton Junio Sady Prates, Rizwan Qaisar, Nameer Hashim Qasim, Ibrahim Qattea, Nguyen Khoi Quan, Amir Radfar, Venkatraman Radhakrishnan, Hadi Raeisi Shahraki, Seyedeh Niloufar Rafiei Alavi, Ibrar Rafique, Alberto Raggi, Fakher Rahim, Shayan Rahmani, Niloufar Rahnavard, Pramila Rai, Sathish Rajaa, Ali Rajabpour-Sanati, Prashant Rajput, Prasanna Ram, Hazem Ramadan, Shakthi Kumaran Ramasamy, Sheena Ramazanu, Kritika Rana, Chhabi Lal Ranabhat, Nemanja Rancic, Smitha Rani, Shubham Ranjan, Chythra R Rao, Mithun Rao, Sowmya J Rao, Davide Rasella, Vahid Rashedi, Ahmed Mustafa Rashid, Ashkan Rasouli-Saravani, Ramin Ravangard, David Laith Rawaf, Salman Rawaf, Iman Razeghian-Jahromi, Elrashdy Moustafa Mohamed Redwan, Faizan Ur Rehman, Giuseppe Remuzzi, Bhageerathy Reshmi, Serge Resnikoff, Luis Felipe Reyes, Malihe Rezaee, Nima Rezaei, Mavra A Riaz, Ana Isabel Ribeiro, Daniel Cury Ribeiro, Jennifer Rickard, Mónica Rodrigues, Jefferson Antonio Buendia Rodriguez, Leonardo Roever, Ravi Rohilla, Peter Rohloff, Debby Syahru Romadlon, Luca Ronfani, Gholamreza Roshandel, Morteza Rostamian, Bedanta Roy, Enrico Rubagotti, Susan Fred Rumisha, Godfrey M Rwegerera, Andrzej Rynkiewicz, Manjula S, Chandan S N, Katharina S Sunnerhagen, Aly M A Saad, Korosh Saber, Maha Mohamed Saber-Ayad, Simona Sacco, Basema Saddik, Bashdar Abuzed Sadee, Ehsan Sadeghi, Masoumeh Sadeghi, Umar Saeed, Maryam Saeedi, Rajesh Sagar, Soumya Swaroop Sahoo, Mohammad Ali Sahraian, Seyed Aidin Sajedi, Mirza Rizwan Sajid, Joseph W Sakshaug, Saina Salahi, Sarvenaz Salahi, Afeez Abolarinwa Salami, Marwa Rashad Salem, Mohammed Z Y Salem, Sohrab Salimi, Hossein Samadi Kafil, Sara Samadzadeh, Kamel A Samara, Saad Samargandy, Vijaya Paul Samuel, Abdallah M Samy, Juan Sanabria, Edmond Sanganyado, Rama Krishna Sanjeev, Francesco Sanmarchi, Milena M Santric-Milicevic, Made Ary Sarasmita, Aswini Saravanan, Babak Saravi, Yaser Sarikhani, Chinmoy Sarkar, Rodrigo Sarmiento-Suárez, Gargi Sachin Sarode, Sachin C Sarode, Thirunavukkarasu Sathish, Susan M Sawyer, Sonia Saxena, Ganesh Kumar Saya, Yaser Sayadi, Abu Sayeed, Md Abu Sayeed, Mete Saylan, Nikolaos Scarmeas, Benedikt Michael Schaarschmidt, Maria Inês Schmidt, Art Schuermans, Austin E Schumacher, David C Schwebel, Falk Schwendicke, Mario Šekerija, Siddharthan Selvaraj, Mohammad H Semreen, Sabyasachi Senapati, Pallav Sengupta, Sadaf G Sepanlou, Dragos Serban, Addisu Sertsu, Yashendra Sethi, Amir Shafaat, Mahan Shafie, Arman Shafiee, Nilay S Shah, Saeed Shahabi, Izza Shahid, Samiah Shahid, Moyad Jamal Shahwan, Alireza Shakeri, Husain Shakil, Muhammad Aaqib Shamim, Mehran Shams-Beyranvand, Hina Shamshad, Mohammad Ali Shamshirgaran, Mohammad Anas Shamsi, Mohd Shanawaz, Abhishek Shankar, Amin Sharifan, Javad Sharifi-Rad, Manoj Sharma, Saurab Sharma, Rajesh P Shastry, David H Shaw, Amr Mohamed Elsayed Shehabeldine, Manjunath Mala Shenoy, Kenji Shibuya, Mika Shigematsu, Reza Shirkoohi, Aminu Shittu, K M Shivakumar, Velizar Shivarov, Sina Shool, Sunil Shrestha, Kerem Shuval, Yafei Si, Emmanuel Edwar Siddig, João Pedro Silva, Soraia Silva, Jorge Piano Simões, Colin R Simpson, Anjali Singal, Abhinav Singh, Aditya Singh, Mahendra Singh, Narinder Pal Singh, Paramdeep Singh, Surjit Singh, Shravan Sivakumar, Valentin Yurievich Skryabin, Anna Aleksandrovna Skryabina, Hanye Sohrabi, Hamidreza Soleimani, Marco Solmi, Yonatan Solomon, Joan B Soriano, Ireneous N Soyiri, Michael Spartalis, Chandrashekhar T Sreeramareddy, Joseph R Starnes, Vladimir I

Starodubov, Antonina V Starodubova, Simona Cătălina Stefan, Dan J Stein, Fridolin Steinbeis, Leo Stockfelt, Mark A Stokes, Stefan Stortecky, Saverio Stranges, Konstantinos Stroumpoulis, Muhammad Suleman, Abida Sultana, David Sunkersing, Sri Susanty, Chandan Kumar Swain, Bryan L Sykes, Lukasz Szarpak, Miklós Szócska, Payam Tabaee Damavandi, Seyed-Amir Tabatabaeizadeh, Karen M Tabb, Mohammad Tabish, Takahiro Tabuchi, Amirmasoud Taheri, Yasaman Taheri Abkenar, Ardeshir Tajbakhsh, Iman M Talaat, Ashis Talukder, Jacques Lukenze Tamuzi, Ker-Kan Tan, Nathan Y Tat, Vivian Y Tat, Razieh Tavakoli Oliaee, Seyed Mohammad Tavangar, Nuno Taveira, Yibekal Manaye Tefera, Mojtaba Teimoori, Mohamad-Hani Temsah, Reem Mohamad Hani Temsah, Masayuki Teramoto, Solomon Hailemariam Tesfaye, Pugazhenthan Thangaraju, Nihal Thomas, Amanda G Thrift, Chern Choong Chern Thum, Jing Tian, Ales Tichopad, Tenaw Yimer Tiruye, Seyed Abolfazl Tohidast, Marcello Tonelli, Mathilde Touvier, Marcos Roberto Tovani-Palone, Nghia Minh Tran, Domenico Trico, Samuel Joseph Tromans, Vien T Truong, Thien Tan Tri Tai Truyen, Evangelia Eirini Tsermpini, Stefanos Tyrovolas, Chukwudi S Ubah, Aniefiok John Udoakang, Arit Udoh, Inam Ulhaq, Muhammad Umair, Tungki Pratama Umar, Chukwuma David Umeokonkwo, Brigid Unim, Bhaskaran Unnikrishnan, Era Upadhyay, Daniele Urso, Marco Vacante, Amir Mohammad Vahdani, Asokan Govindaraj Vaithinathan, Sahel Valadan Tahbaz, Jef Van den Eynde, Elena Varavikova, Orsolya Varga, Siddhartha Alluri Varma, Priya Vart, Shoban Babu Varthya, Tommi Juhani Vasankari, Narayanaswamy Venketasubramanian, Nicholas Alexander Verghese, Madhur Verma, Georgios-Ioannis Verras, Dominique Vervoort, Rafael José Vieira, Jorge Hugo Villafañe, Leonardo Villani, Gabriela Ines Villanueva, Francesco S Violante, Rachel Visontay, Vasily Vlassov, Stein Emil Vollset, Simona Ruxandra Volovat, Victor Volovici, Isidora S Vujcic, Rade Vukovic, Yasir Waheed, Cong Wang, Denny Wang, Shu Wang, Yanzhong Wang, Yuan-Pang Wang, Paul Ward, Stefanie Watson, Marcia R Weaver, Andrea Werdecker, Ronny Westerman, Dakshitha Praneeth Wickramasinghe, Nuwan Darshana Wickramasinghe, Marcin W Wojewodzic, Dongze Wu, Lalit Yadav, Sajad Yaghoubi, Lin Yang, Yao Yao, Gesila Endashaw Yesera, Subah Abderehim Yesuf, Arzu Yiğit, Vahit Yiğit, Dong Keon Yon, Naohiro Yonemoto, Siddhesh Zadey, Vesna Zadnik, Nima Zafari, Mohammad Zahedi, Mazyar Zahir, Josefina Zakzuk, Giulia Zamagni, Burhan Abdullah Zaman, Sojib Bin Zaman, Nelson Zamora, Ramin Zand, Ghazal G Z Zandieh, Aurora Zanghì, Iman Zare, Mikhail Sergeevich Zastrozhin, Mohammed G M Zeariya, Youjie Zeng, Chunxia Zhai, Chen Zhang, Haijun Zhang, Zhaofeng Zhang, Yang Zhao, Yong Zhao, Chenwen Zhong, Zhaohua Zhu, Pardis Ziaeefar, Magdalena Zielińska, Zhiyong Zou, Alimuddin Zumla, and Samer H Zyoud.

Managing the estimation or publications process

Catherine M Antony, Catherine Bisignano, Haley Comfort, Kara Estep, Simon I Hay, Nicholas J Kassebaum, Molly B Kassel, Kasey E Kinzel, Anna Laura W McKowen, , Ali H Mokdad, Christopher J L Murray, Mohsen Naghavi, Amanda Novotney, David M Pigott, Nicholas Alexander Verghese, Stein Emil Vollset, Katherine M Wells, Eve E Wool, and
