# Supplementary material for: Global age-sex-specific mortality, life expectancy, and population estimates in 204 countries and territories and 811 subnational locations, 1950–2021, and the impact of the COVID-19 pandemic: a comprehensive demographic analysis for the Global Burden of Disease Study 2021
Source: Lancet. 2024 May 18;403(10440):1989–2056. doi: 10.1016/S0140-6736(24)00476-8 (PMC11126395; doi:10.1016/S0140-6736(24)00476-8)
Supplement: Supplementary appendix 2 [file mmc2.pdf]

THE LANCET

Supplementary appendix 2

This appendix formed part of the original submission and has been peer reviewed. We post it as supplied by the authors.

Supplement to: GBD 2021 Demographics Collaborators. Global age-sex-specific mortality, life expectancy, and population estimates in 204 countries and territories and 811 subnational locations, 1950–2021, and the impact of the COVID-19 pandemic: a comprehensive demographic analysis for the Global Burden of Disease Study 2021. *Lancet* 2024; published online March 11. https://doi.org/10.1016/S0140-6736(24)00476-8.

Appendix 2: supplementary results appendix to "Global age-sex-specific mortality, life expectancy, and population estimates in 204 countries and territories and 811 subnational locations, 1950–2021, and the impact of the COVID-19 pandemic: a comprehensive demographic analysis for the Global Burden of Disease Study 2021"

This supplement provides additional figures and tables containing more detailed results for "Global age-sex-specific mortality, life expectancy, and population estimates in 204 countries and territories and 811 subnational locations, 1950–2021, and the impact of the COVID-19 pandemic: a comprehensive demographic analysis for the Global Burden of Disease Study 2021."

Visualisations for all results specific to demographic mortality measures for GBD 2021 are also available at https://vizhub.healthdata.org/mortality/.

Figures

Appendix 2 Figure S1a: Most recent year of available vital registration, by country 4

Appendix 2 Figure S1b: Completeness of vital registration systems for the latest year of reporting, by country 5

Appendix 2 Figure S2: Crude excess mortality rate, 2020-2021 6

Appendix 2 Figure S3a: Number of global deaths over time by age group, both sexes combined, 1950-2021 7

Appendix 2 Figure S3b: Number of global deaths over time by age group, females, 1950-2021 8

Appendix 2 Figure S3c: Number of global deaths over time by age group, males, 1950-2021 8

Appendix 2 Figure S4: Death structure by SDI in 2021 9

Appendix 2 Figure S5: National mortality rates across the lifespan in females and males, 1950-2021..... 10

Appendix 2 Figure S6: Correlation of baseline nqx and the annualized rate of change in every age group by decade 214

Appendix 2 Figure S7: National estimated - expected life expectancy based on SDI, by sex, 1950-2021… 215

Appendix 2 Figure S8: Population structure by SDI 216

Appendix 2 Figure S9: Population pyramids for females and males, by age and by location, for 1950, 1975, 2000, and 2021 218

Appendix 2 Figure S10: Comparison of COVID excess mortality estimates between GBD 2021 and WHO, by location 422

Appendix 2 Figure S11: Comparison of under 5 mortality estimates for 2021 between GBD 2021 and UNICEF 2022, by location 423

Appendix 2 Figure S12: Percent difference between GBD 2021 and WPP 2022 adult mortality estimates, by location and sex, for 1950, 1975, 2000, 2015, 2021 424

Appendix 2 Figure S13: Comparison of life expectancy at birth estimates for 2021 between GBD 2021 and WPP 2022, by sex and location 425

Appendix 2 Figure S14: Comparison of life expectancy at birth estimates for countries in GBD 2021 and WPP 2022, both sexes combined, for every five years from 1950 to 2020 and 2021 426

Appendix 2 Figure S15a: Percent difference between GBD 2021 and WPP 2022 population estimates, by location and sex, all ages, for 1950, 1975, 2000, 2015, 2021 442

Appendix 2 Figure S15b: Percent difference between GBD 2021 and WPP 2022 population estimates, by location and sex, ages <15 and >65 for 2021 443

Tables

Appendix 2 Table 1: Under-5 death numbers (in thousands) with 95% UIs by location, both sexes combined, 1950, 1960, 1970, 1980, 1990, 2000, 2010, 2019, 2020, 2021 444

Appendix 2 Table 2a: Under-5 mortality rates with 95% UIs by location, both sexes combined, 1950, 1960, 1970, 1980, 1990, 2000, 2010, 2019, 2020, 2021 456

Appendix 2 Table 2b: Under-5 mortality rates with 95% UIs by location, females, 1950, 1960, 1970, 1980, 1990, 2000, 2010, 2019, 2020, 2021 468

Appendix 2 Table 2c: Under-5 mortality rates with 95% UIs by location, males, 1950, 1960, 1970, 1980, 1990, 2000, 2010, 2019, 2020, 2021 480

Appendix 2 Table 3a: Age-standardised mortality rates (per 100 000) with 95% UIs by location, both sexes combined, 1950, 1960, 1970, 1980, 1990, 2000, 2010, 2019, 2020, 2021 492

Appendix 2 Table 3b: Age-standardised mortality rates (per 100 000) with 95% UIs by location, females, 1950, 1960, 1970, 1980, 1990, 2000, 2010, 2019, 2020, 2021 504

Appendix 2 Table 3c: Age-standardised mortality rates (per 100 000) with 95% UIs by location, males, 1950, 1960, 1970, 1980, 1990, 2000, 2010, 2019, 2020, 2021 516

Appendix 2 Table 4a: Life expectancy at birth with 95% UIs by location, both sexes combined, 1950, 1960, 1970, 1980, 1990, 2000, 2010, 2019, 2020, 2021 528

Appendix 2 Table 4b: Life expectancy at birth with 95% UIs by location, females, 1950, 1960, 1970, 1980, 1990, 2000, 2010, 2019, 2020, 2021 540

Appendix 2 Table 4c: Life expectancy at birth with 95% UIs by location, males, 1950, 1960, 1970, 1980, 1990, 2000, 2010, 2019, 2020, 2021 552

Appendix 2 Table 5a: Population (in thousands) with 95% UIs by location, both sexes combined, 1950, 1960, 1970, 1980, 1990, 2000, 2010, 2019, 2020, 2021.......... 564

Appendix 2 Table 5b: Population (in thousands) with 95% UIs by location, females, 1950, 1960, 1970, 1980, 1990, 2000, 2010, 2019, 2020, 2021.......... 576

Appendix 2 Table 5c: Population (in thousands) with 95% UIs by location, males, 1950, 1960, 1970, 1980, 1990, 2000, 2010, 2019, 2020, 2021.......... 588

Appendix 2 Figure S1a: Most recent year of available vital registration, by country

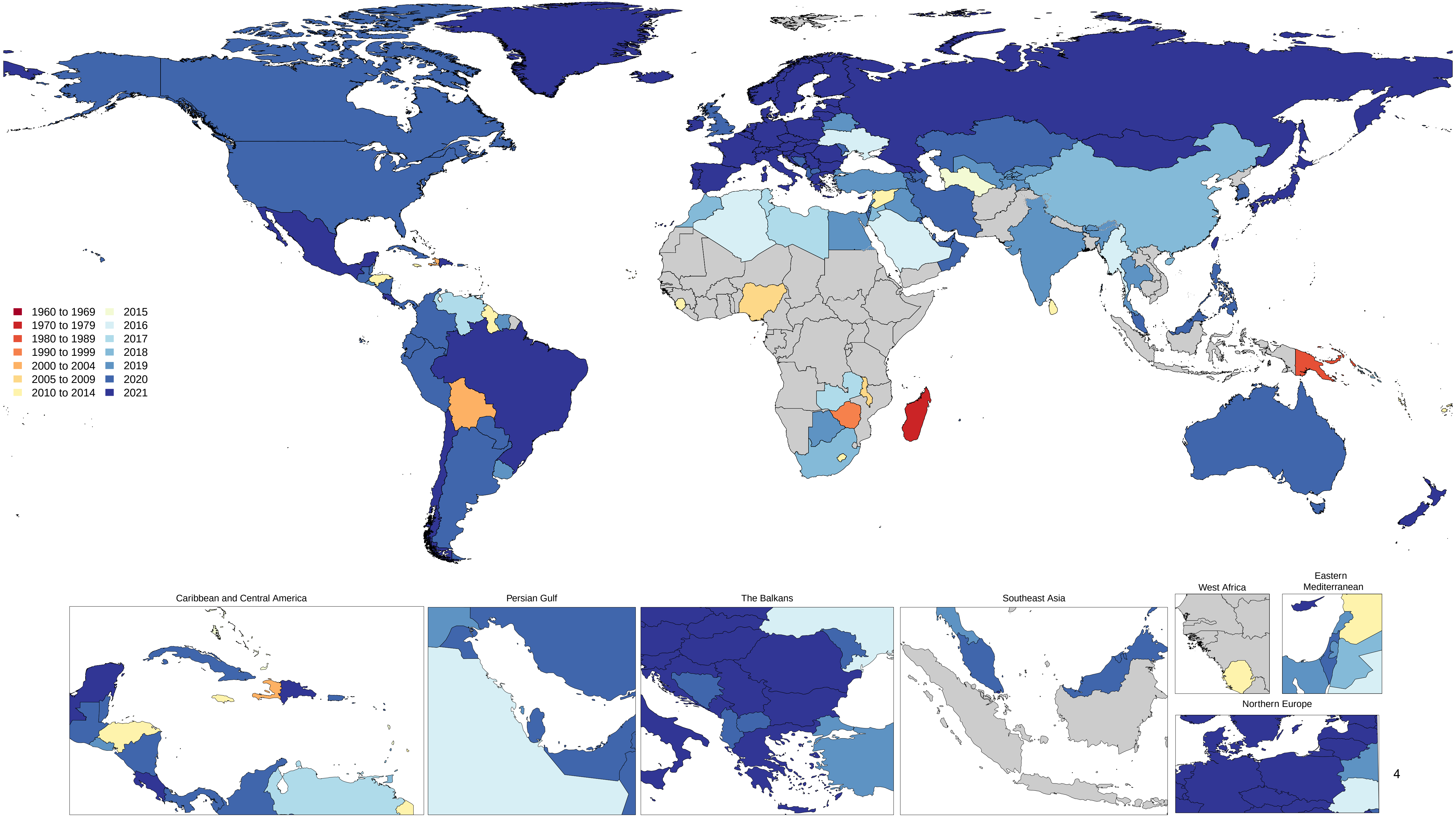

Appendix 2 Figure S1b: Completeness of vital registration systems for the latest year of reporting, by country

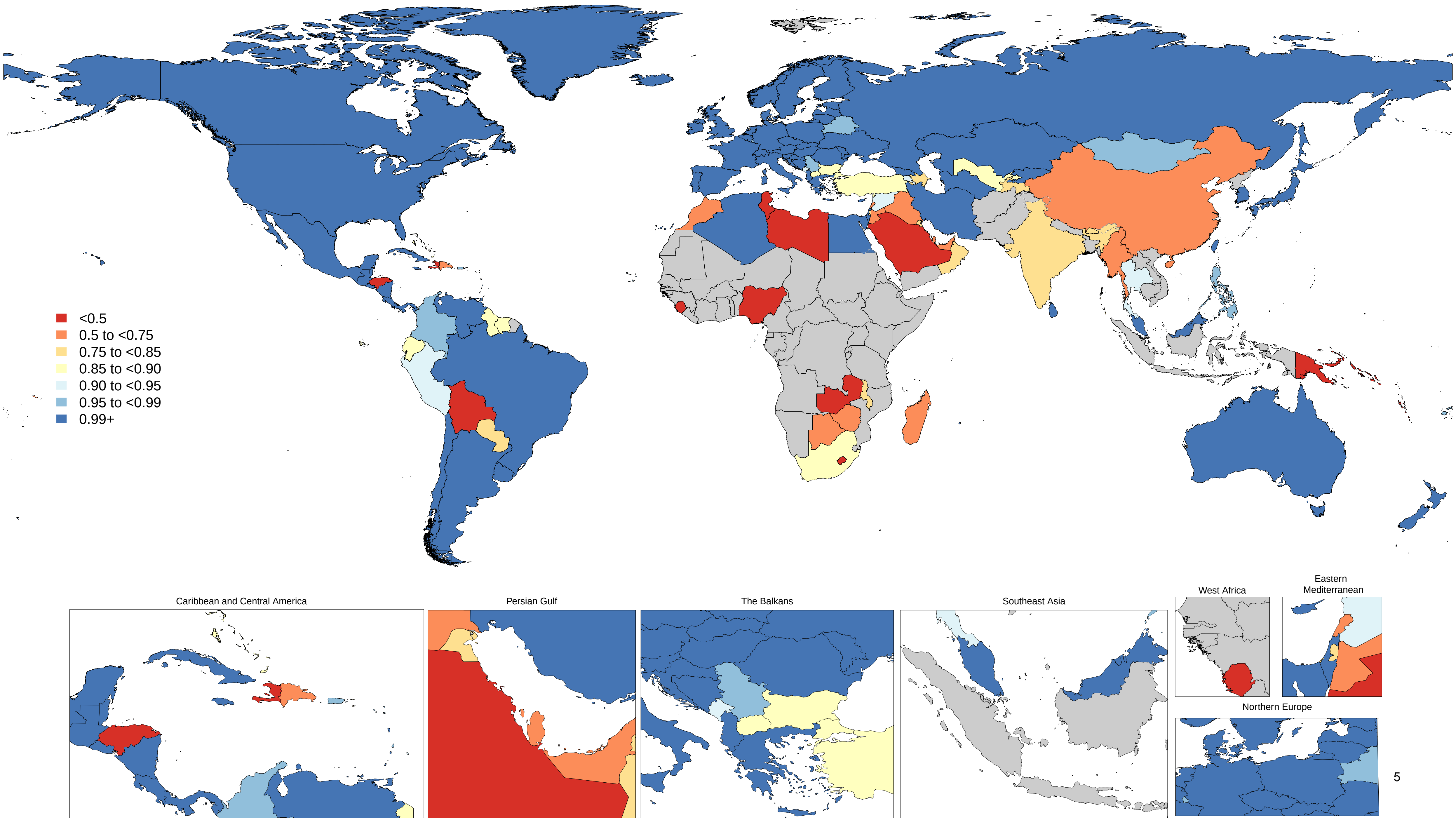

Appendix 2 Figure S2: Crude excess mortality rate, 2020–2021

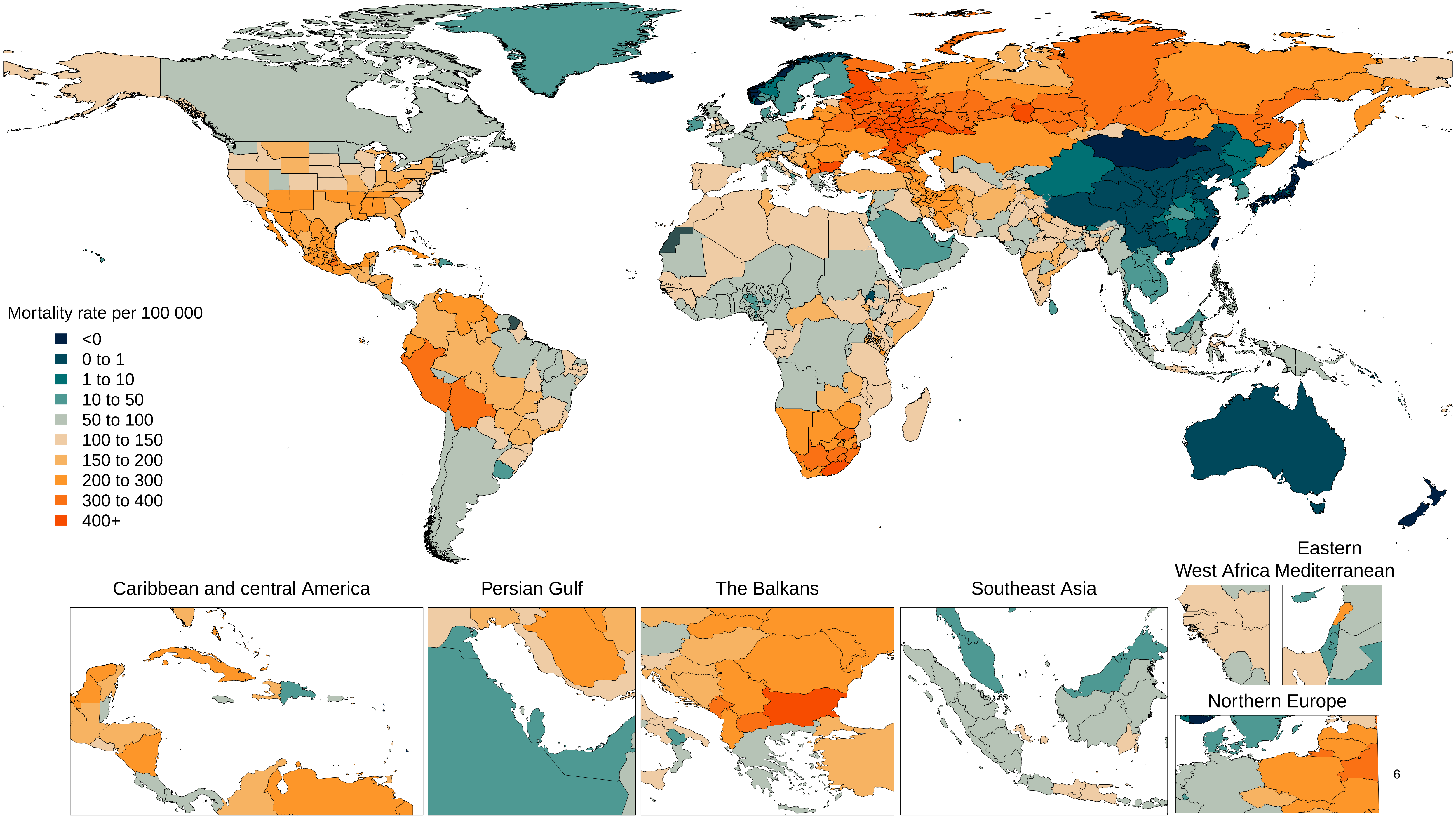

Appendix 2 Figure S3a: Number of global deaths over time by age group, both sexes combined, 1950−2021

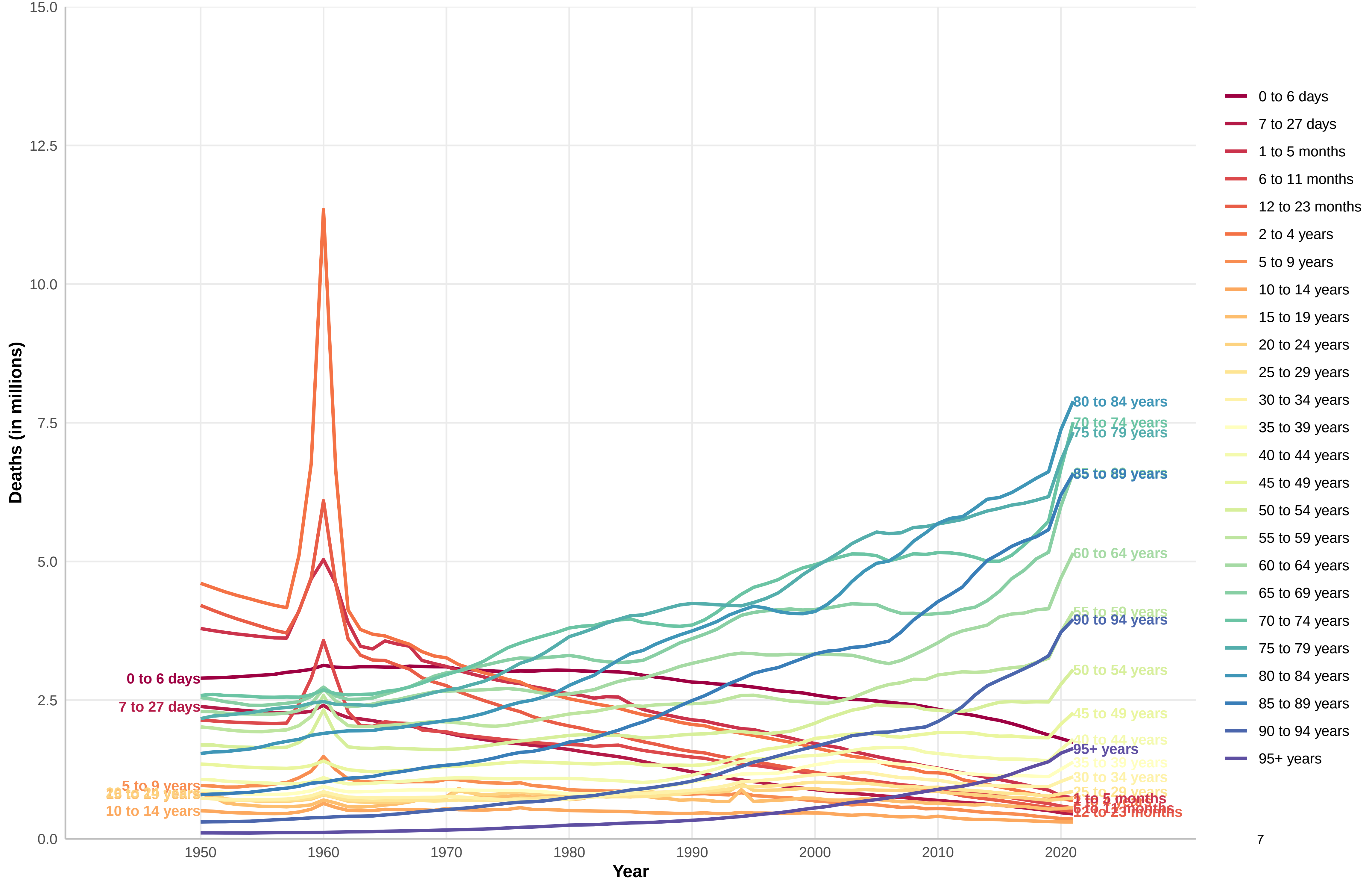

7

Appendix 2 Figure S3b: Number of global deaths over time by age group, females, 1950−2021

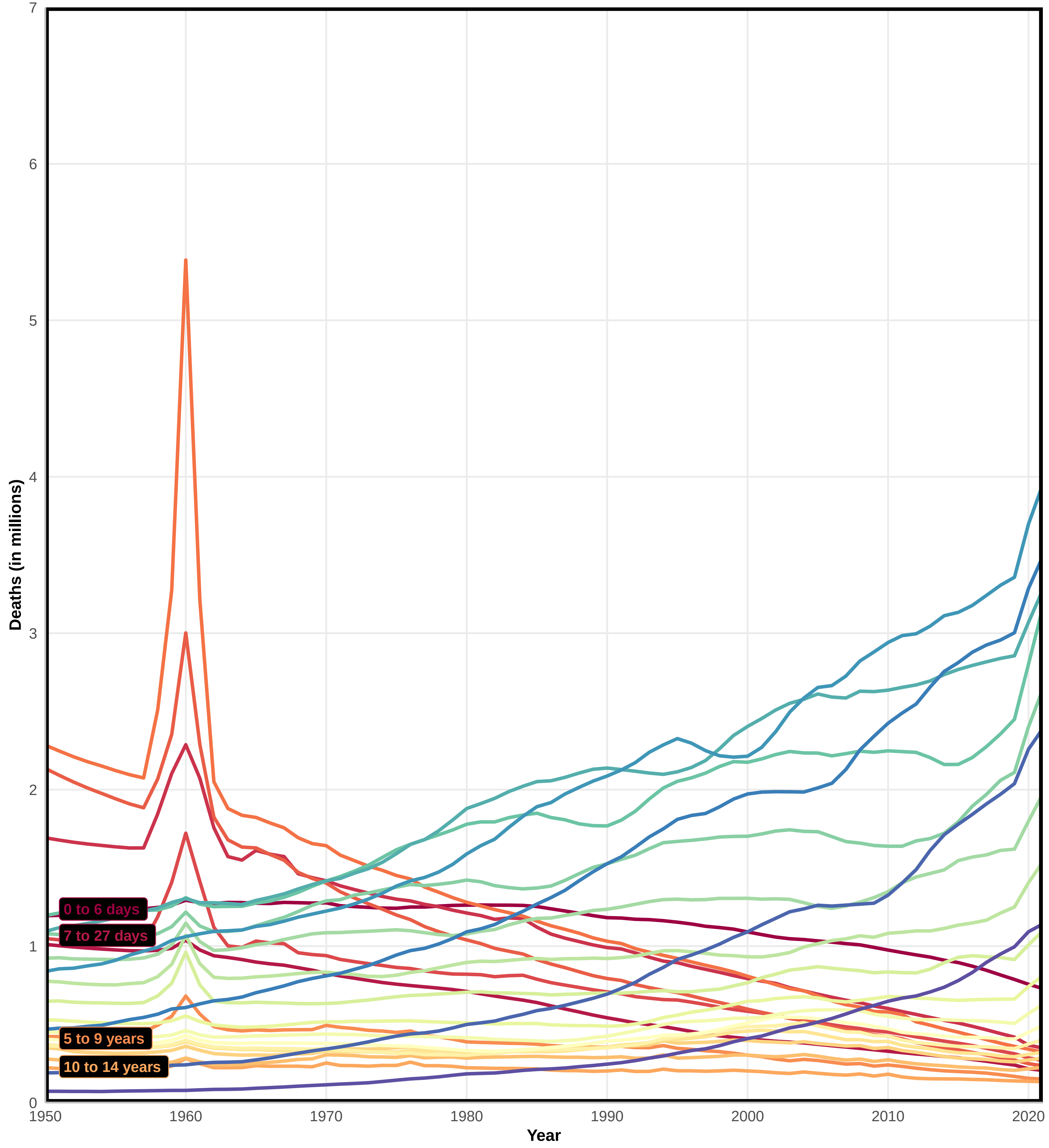

Appendix 2 Figure S3c: Number of global deaths over time by age group, males, 1950−2021

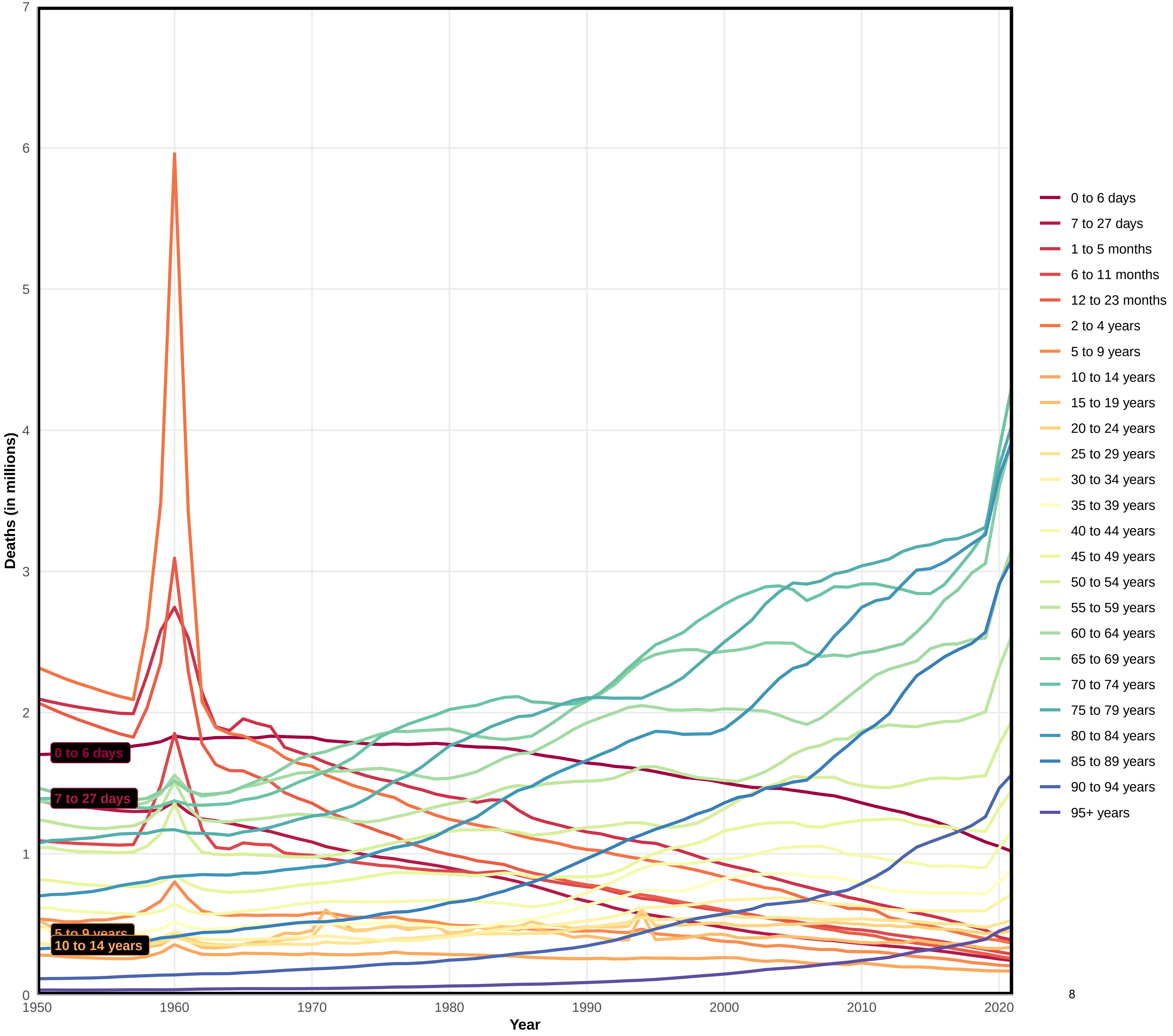

Appendix 2 Figure S4: Death Structure by SDI in 2021

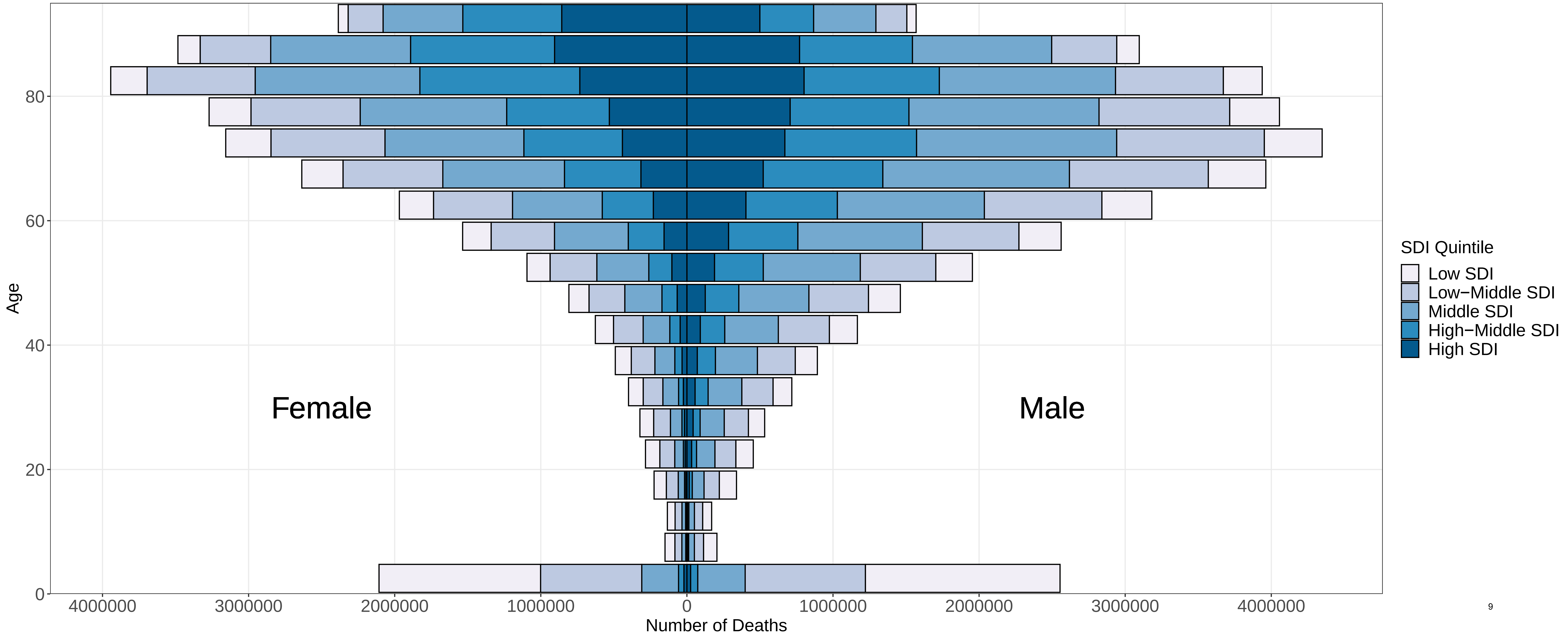

Appendix 2 Figure S5: Armenia mortality rates, 1950−2021

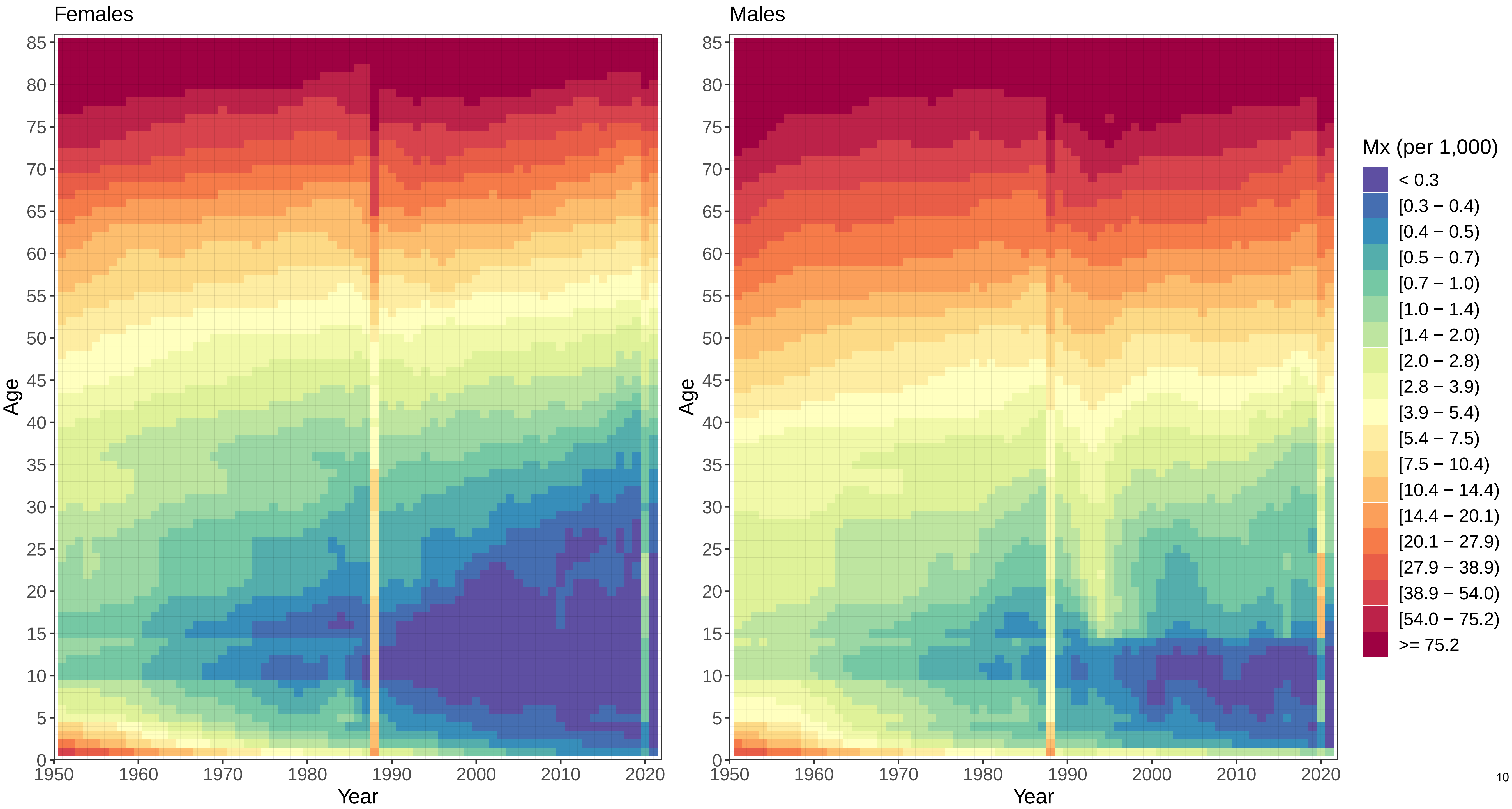

Appendix 2 Figure S5: Azerbaijan mortality rates, 1950−2021

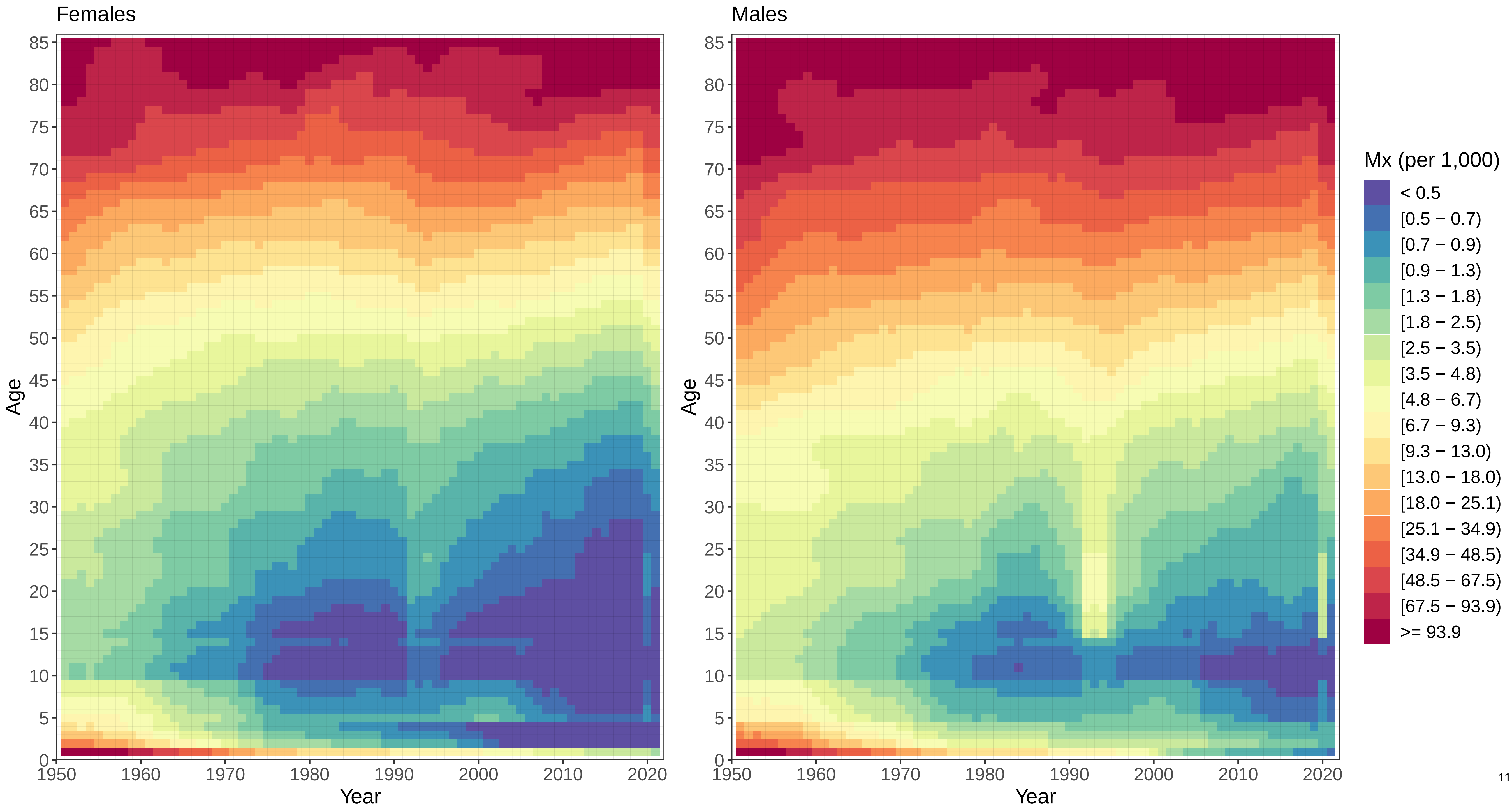

Appendix 2 Figure S5: Georgia mortality rates, 1950−2021

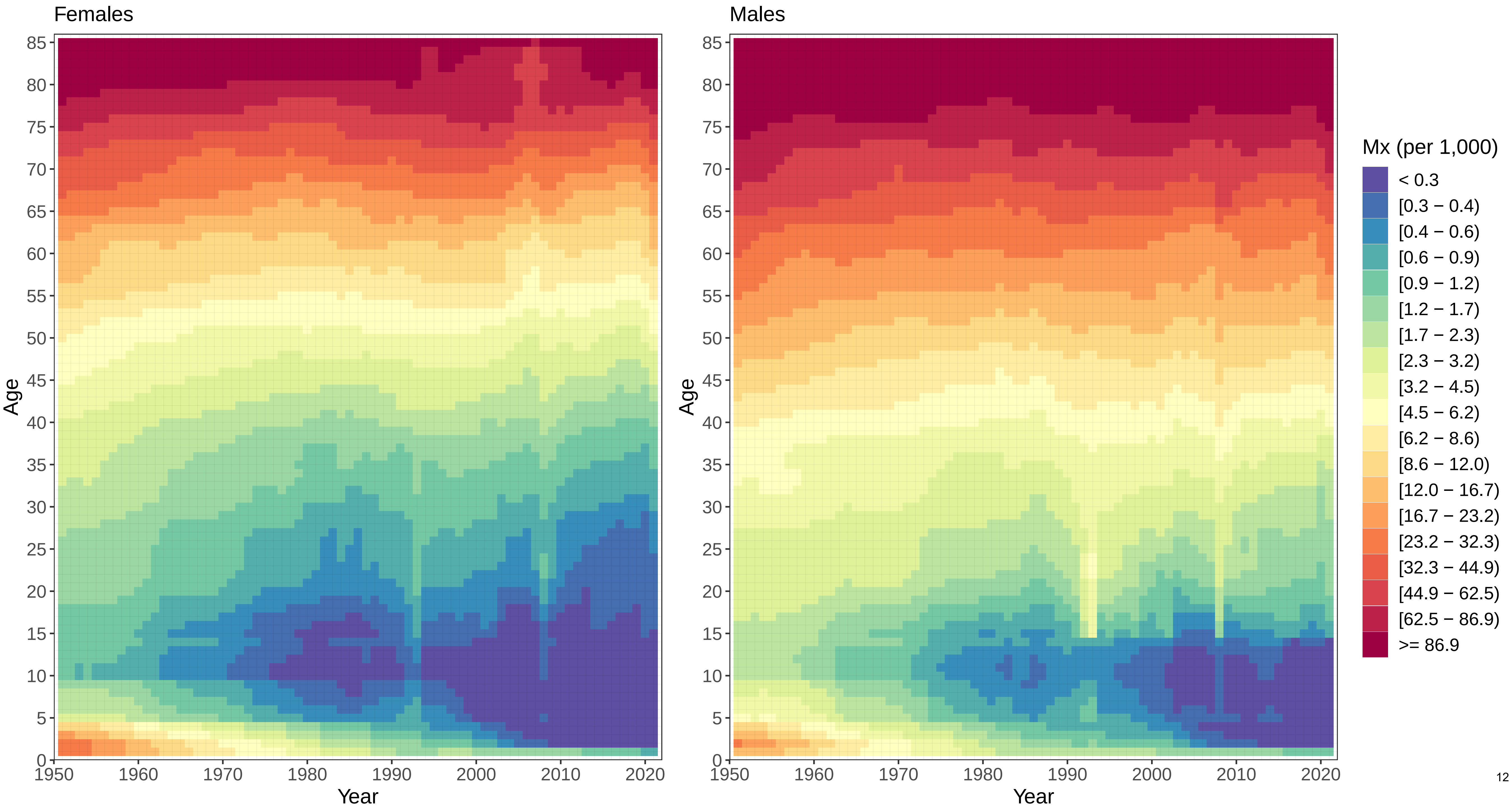

Appendix 2 Figure S5: Kazakhstan mortality rates, 1950−2021

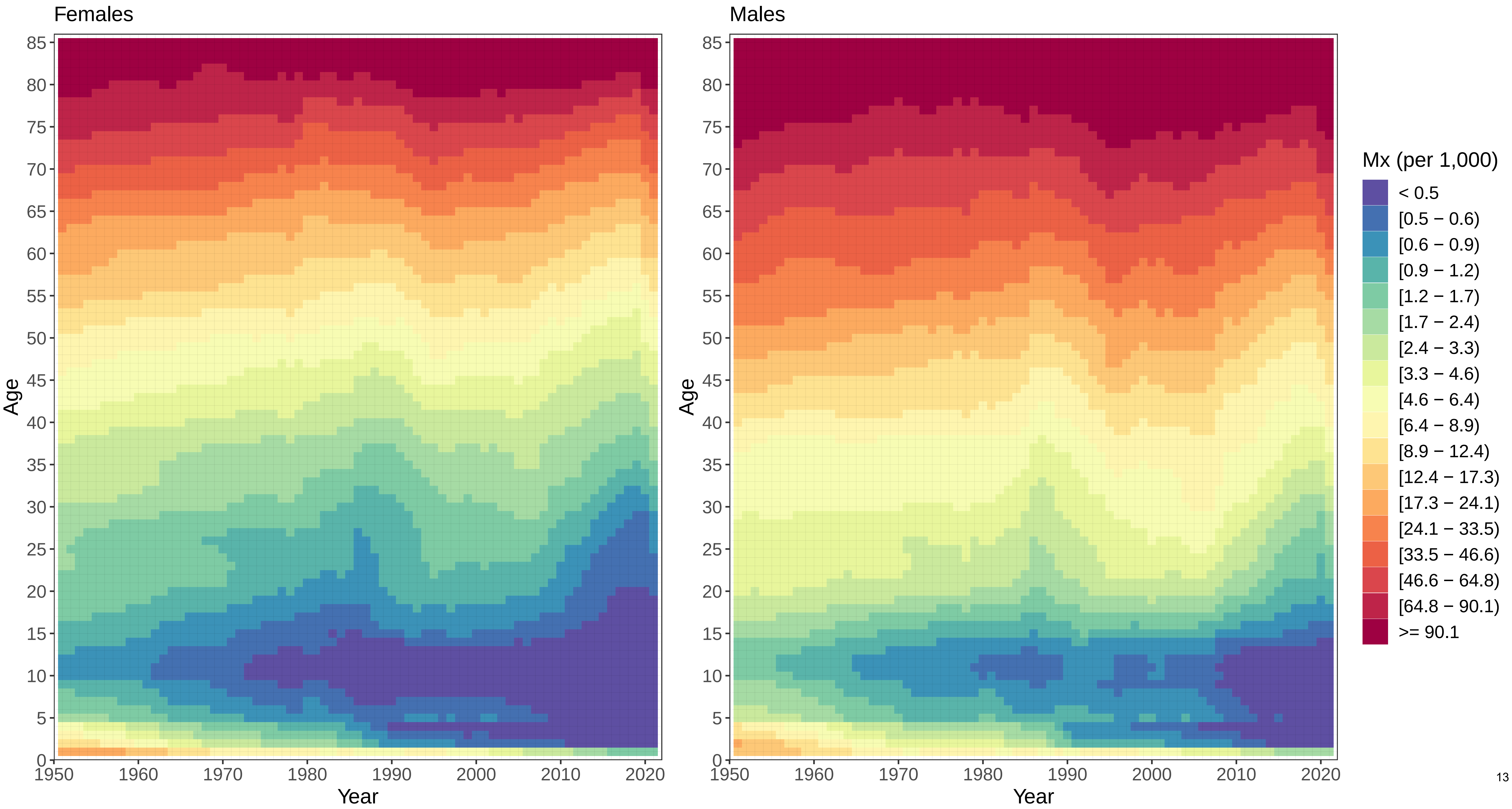

Appendix 2 Figure S5: Kyrgyzstan mortality rates, 1950−2021

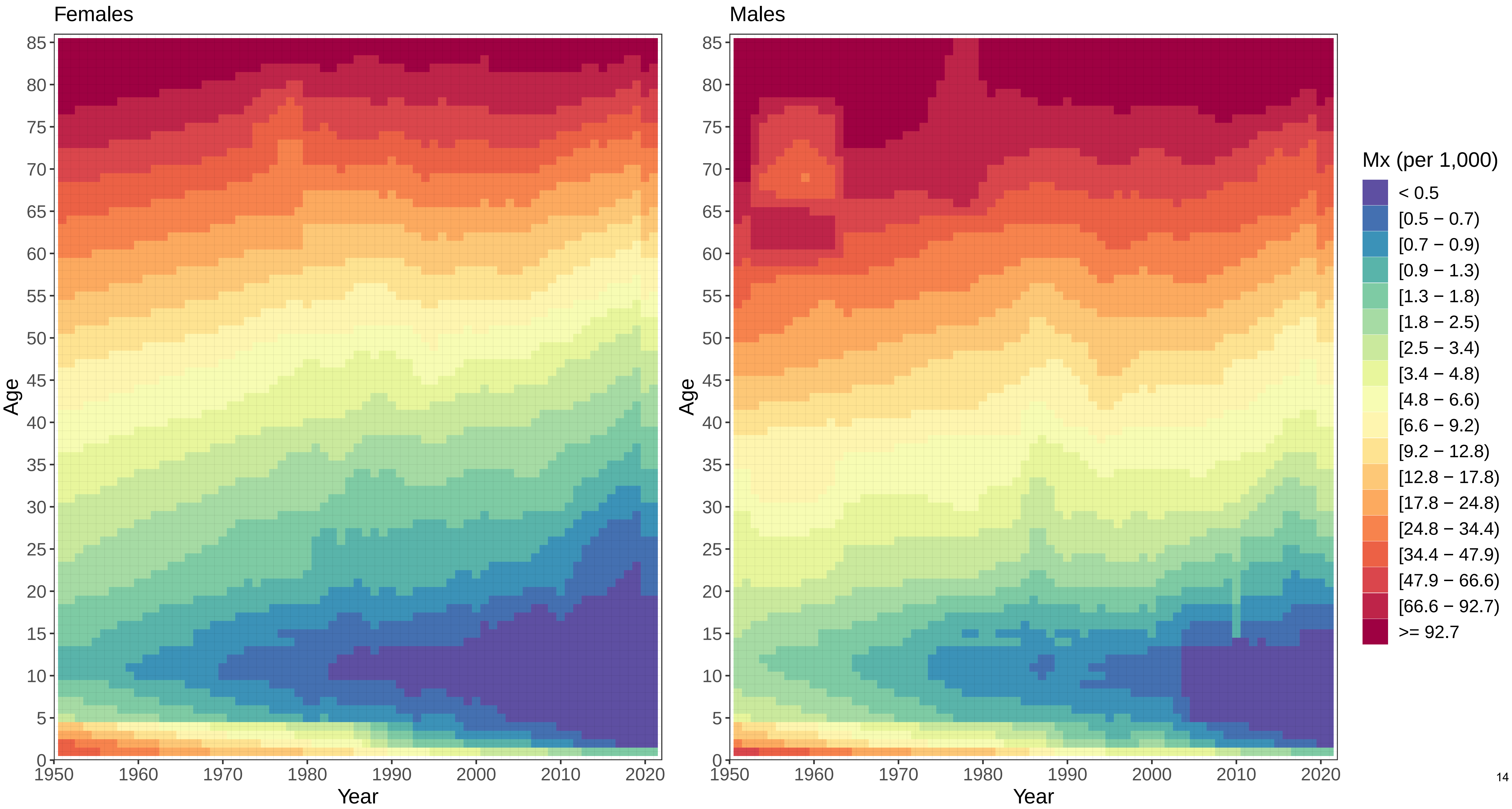

Appendix 2 Figure S5: Mongolia mortality rates, 1950−2021

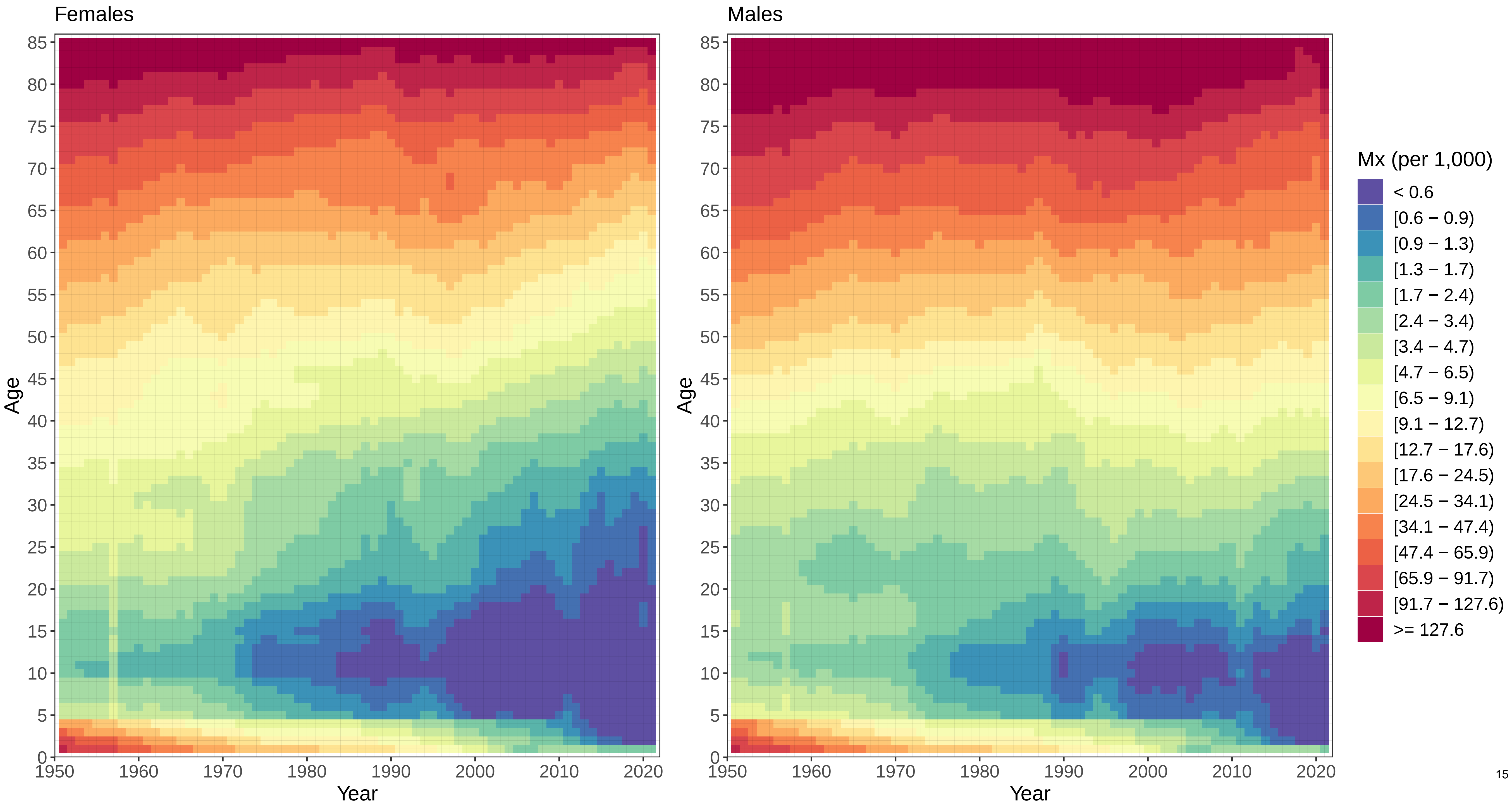

Appendix 2 Figure S5: Tajikistan mortality rates, 1950−2021

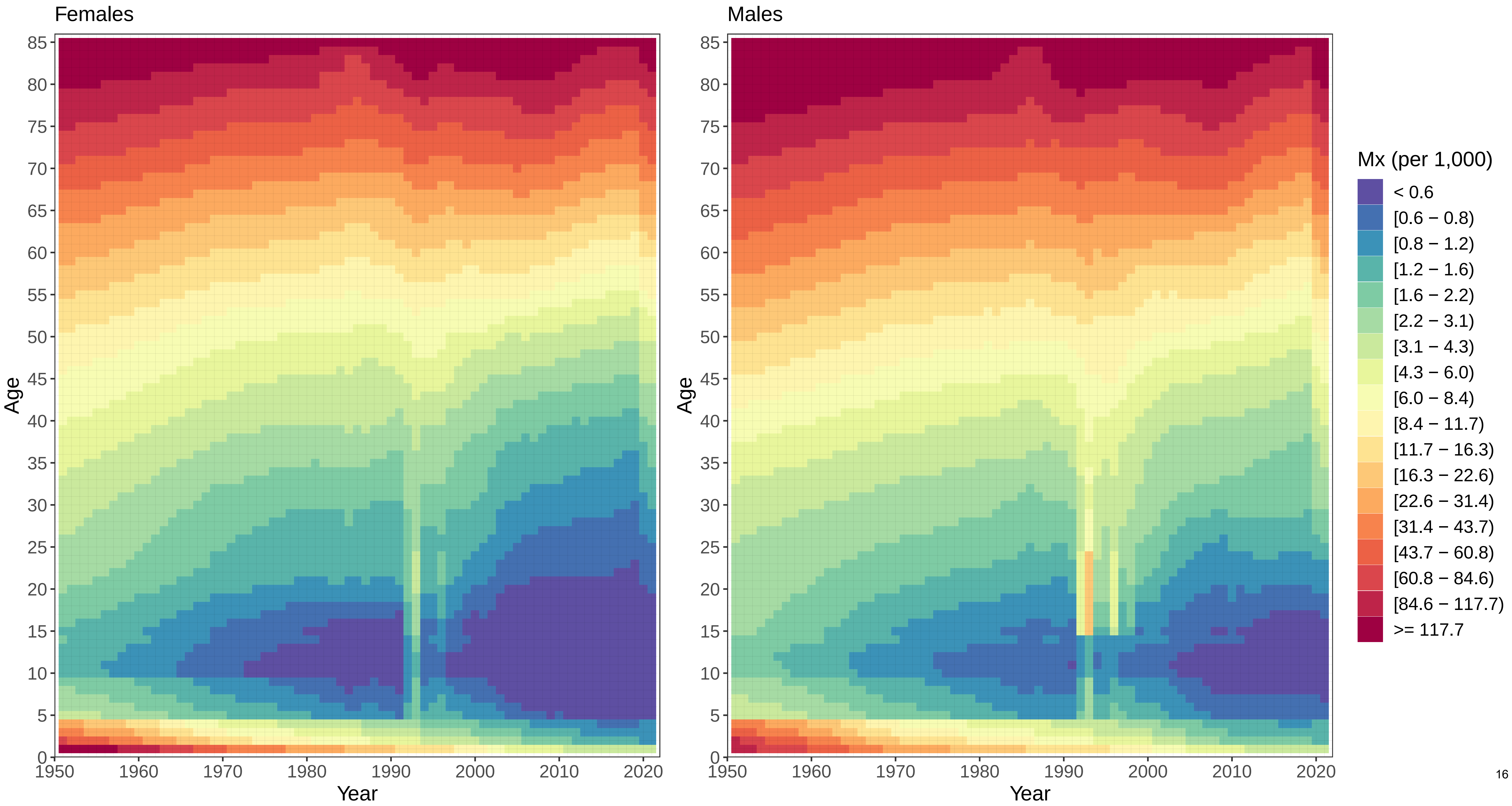

Appendix 2 Figure S5: Turkmenistan mortality rates, 1950−2021

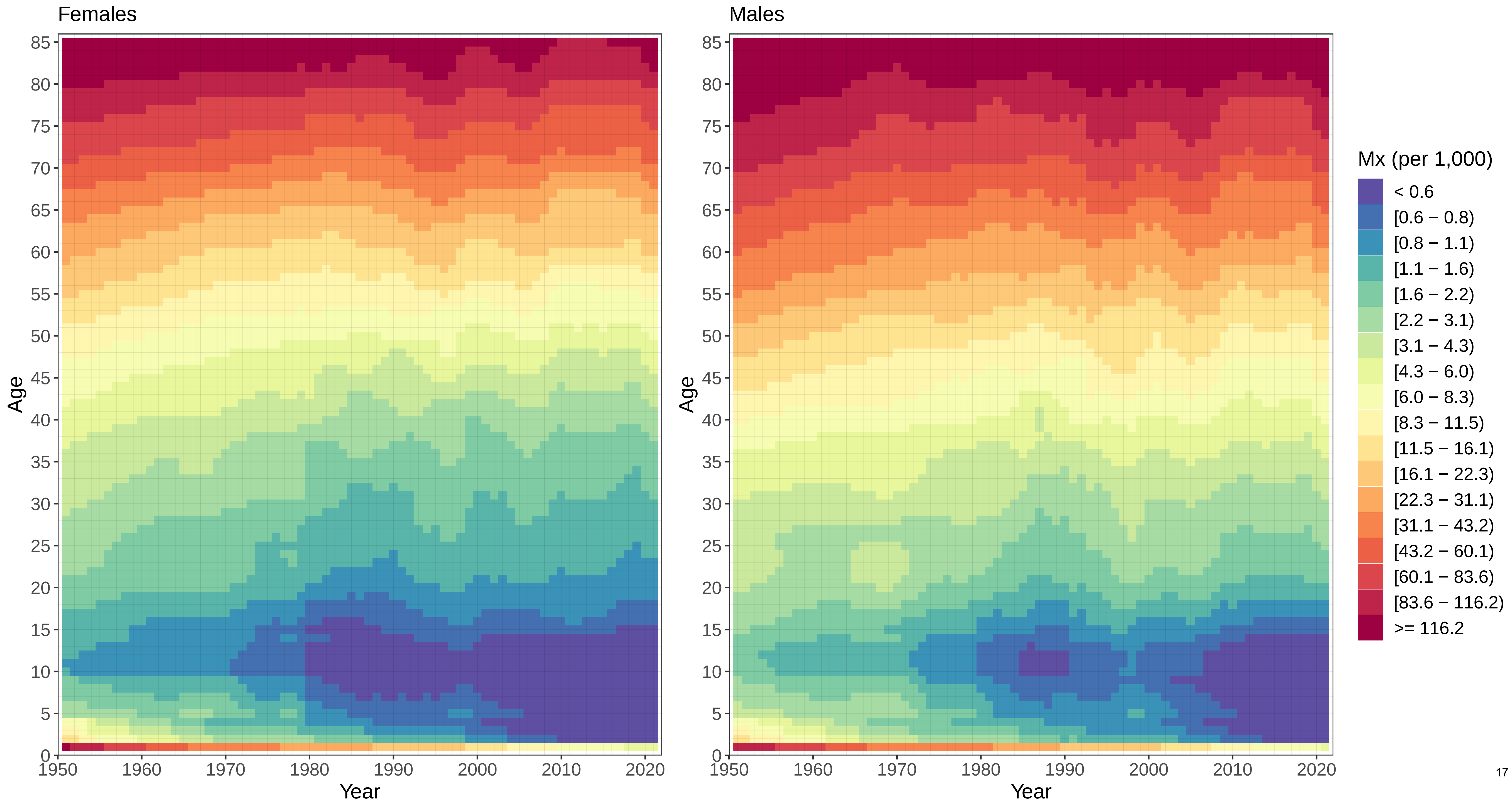

Appendix 2 Figure S5: Uzbekistan mortality rates, 1950−2021

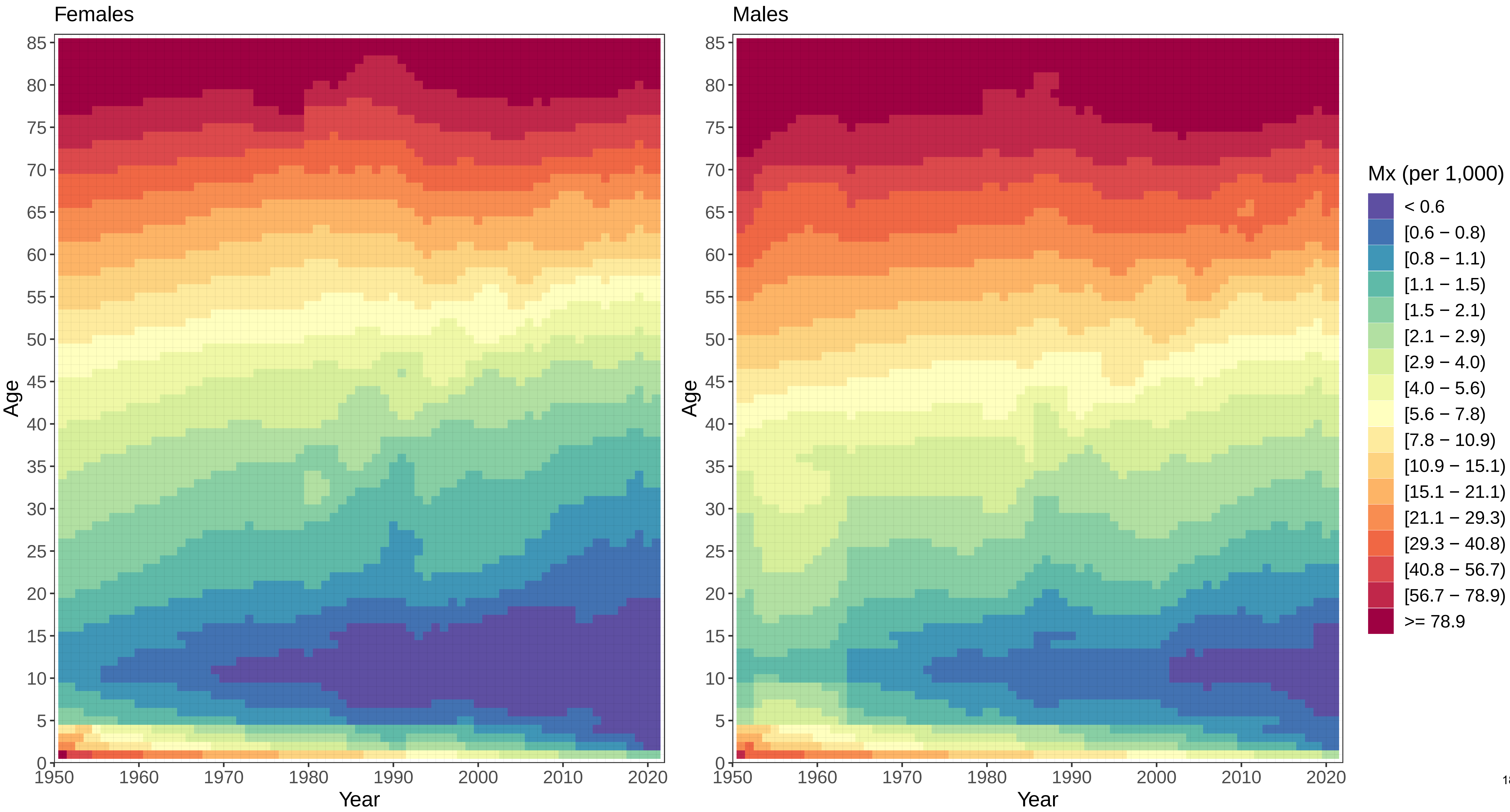

Appendix 2 Figure S5: Albania mortality rates, 1950−2021

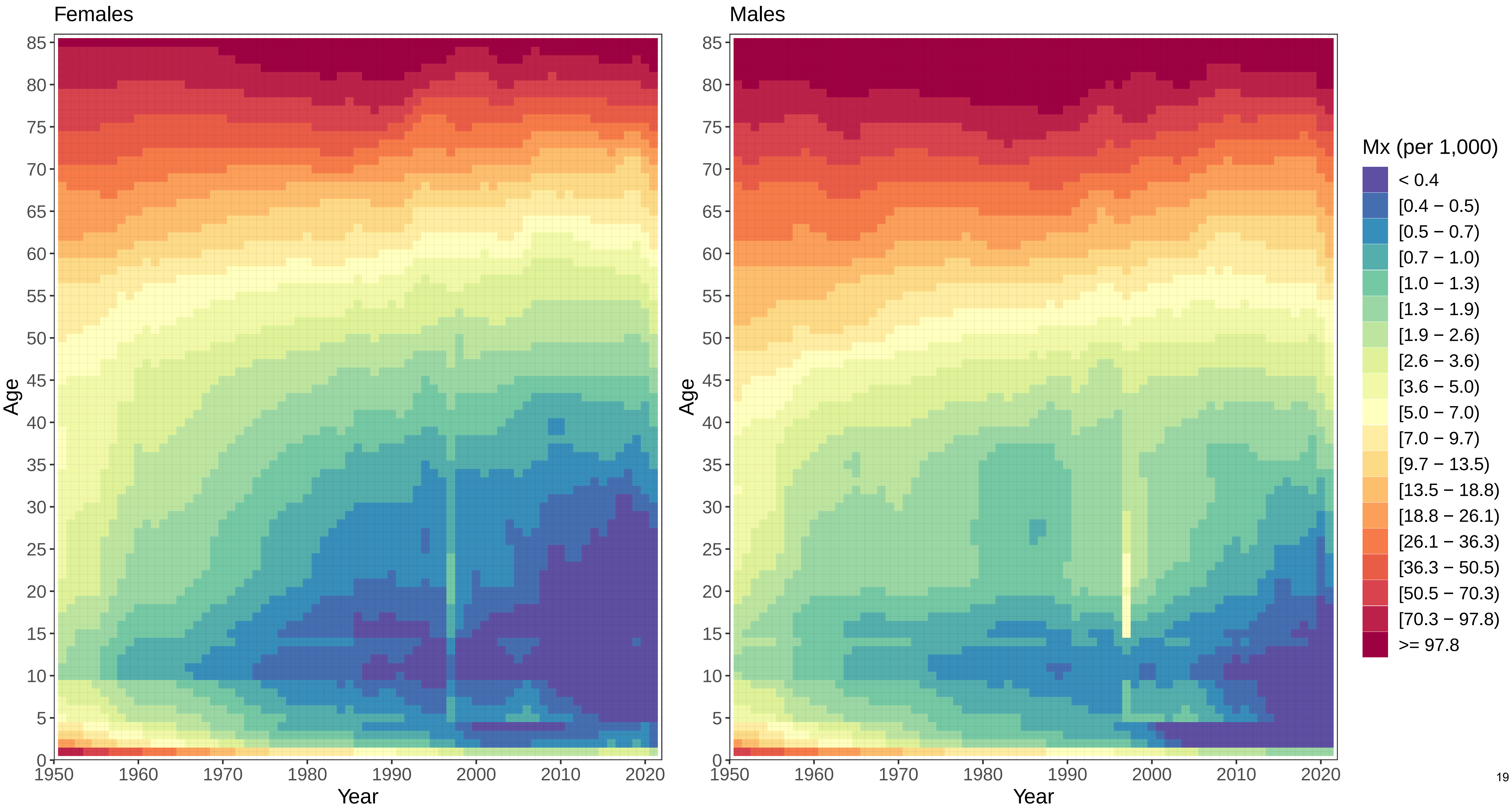

Appendix 2 Figure S5: Bosnia and Herzegovina mortality rates, 1950−2021

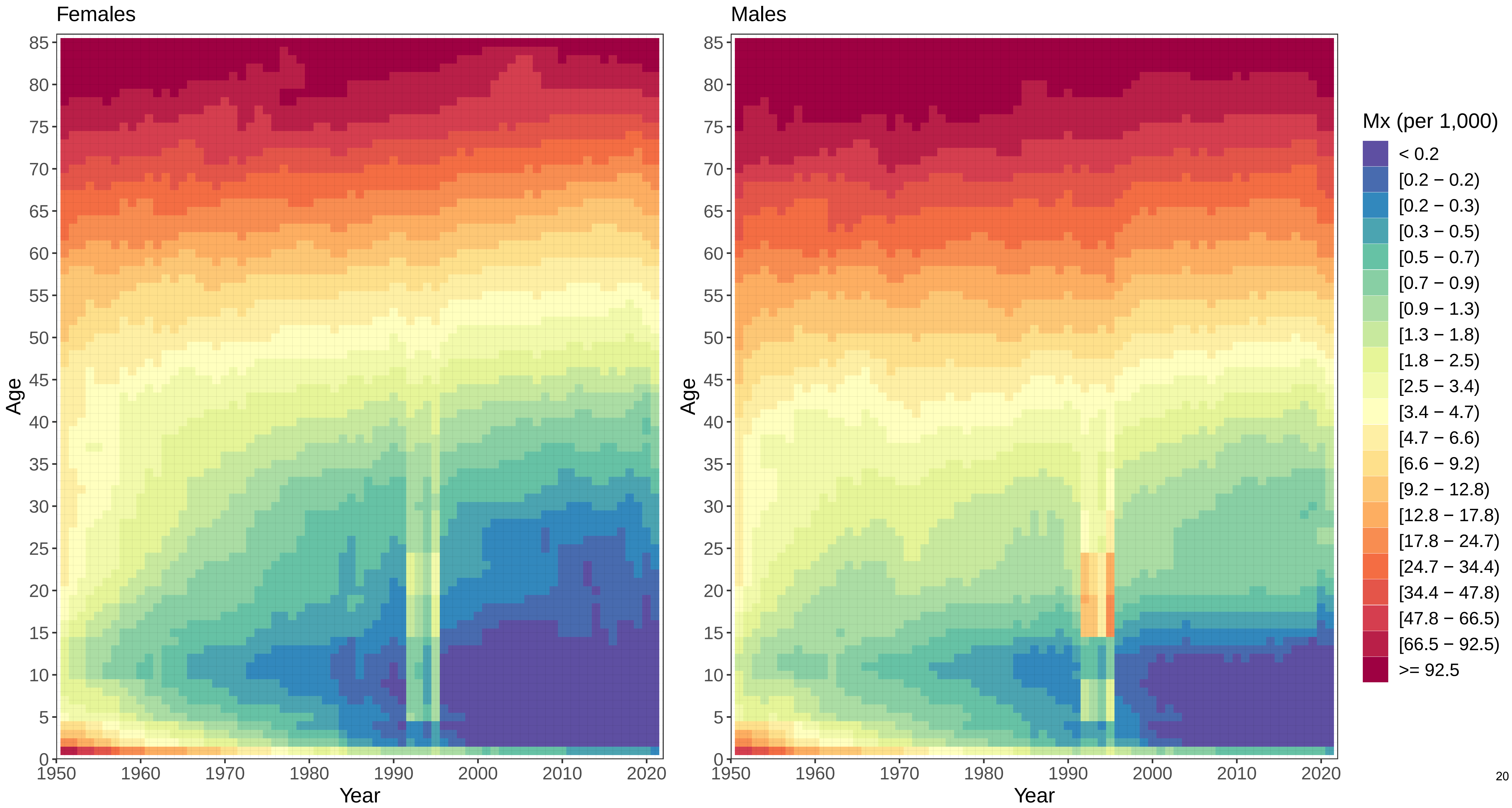

Appendix 2 Figure S5: Bulgaria mortality rates, 1950−2021

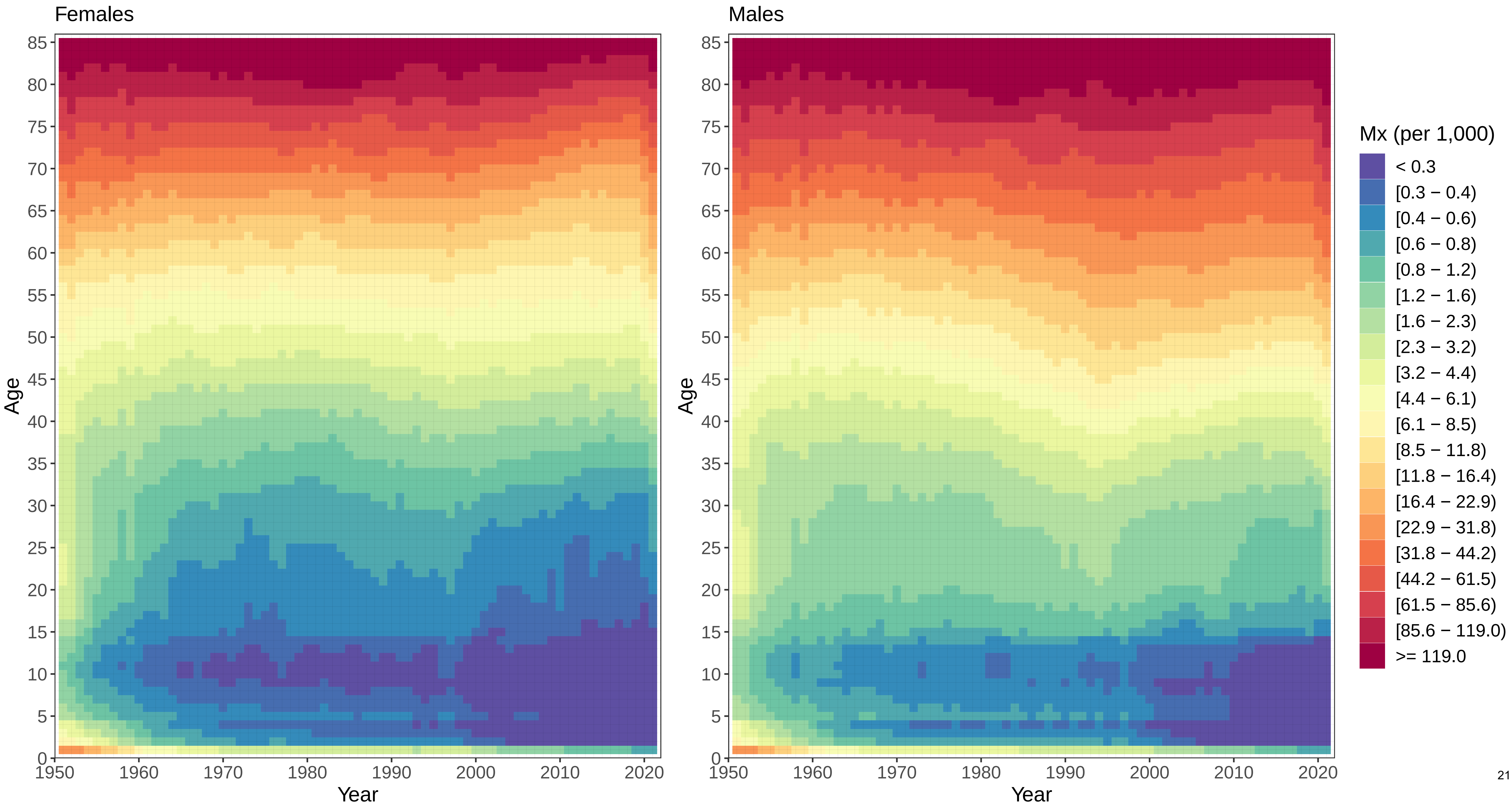

Appendix 2 Figure S5: Croatia mortality rates, 1950−2021

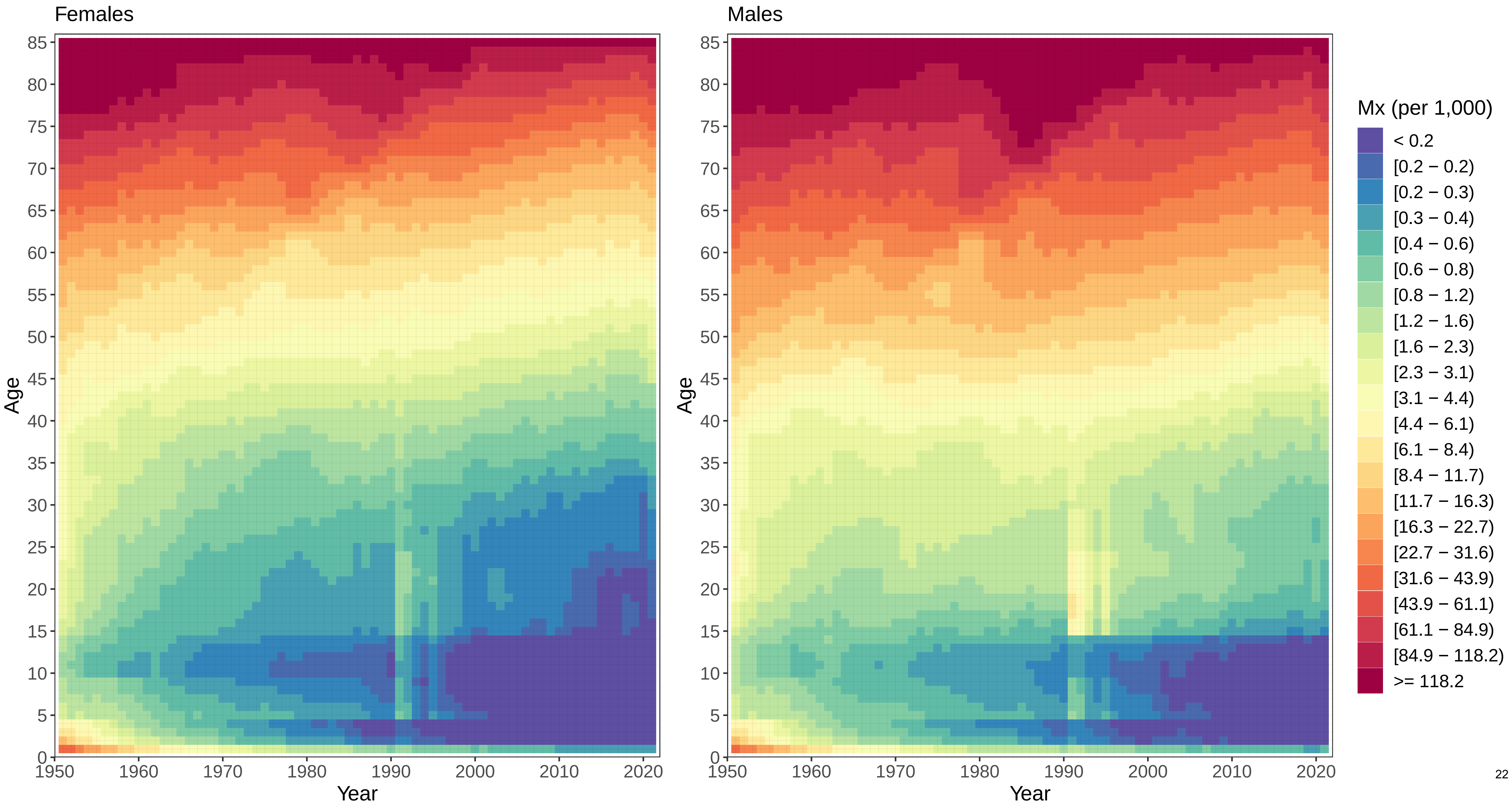

Appendix 2 Figure S5: Czechia mortality rates, 1950−2021

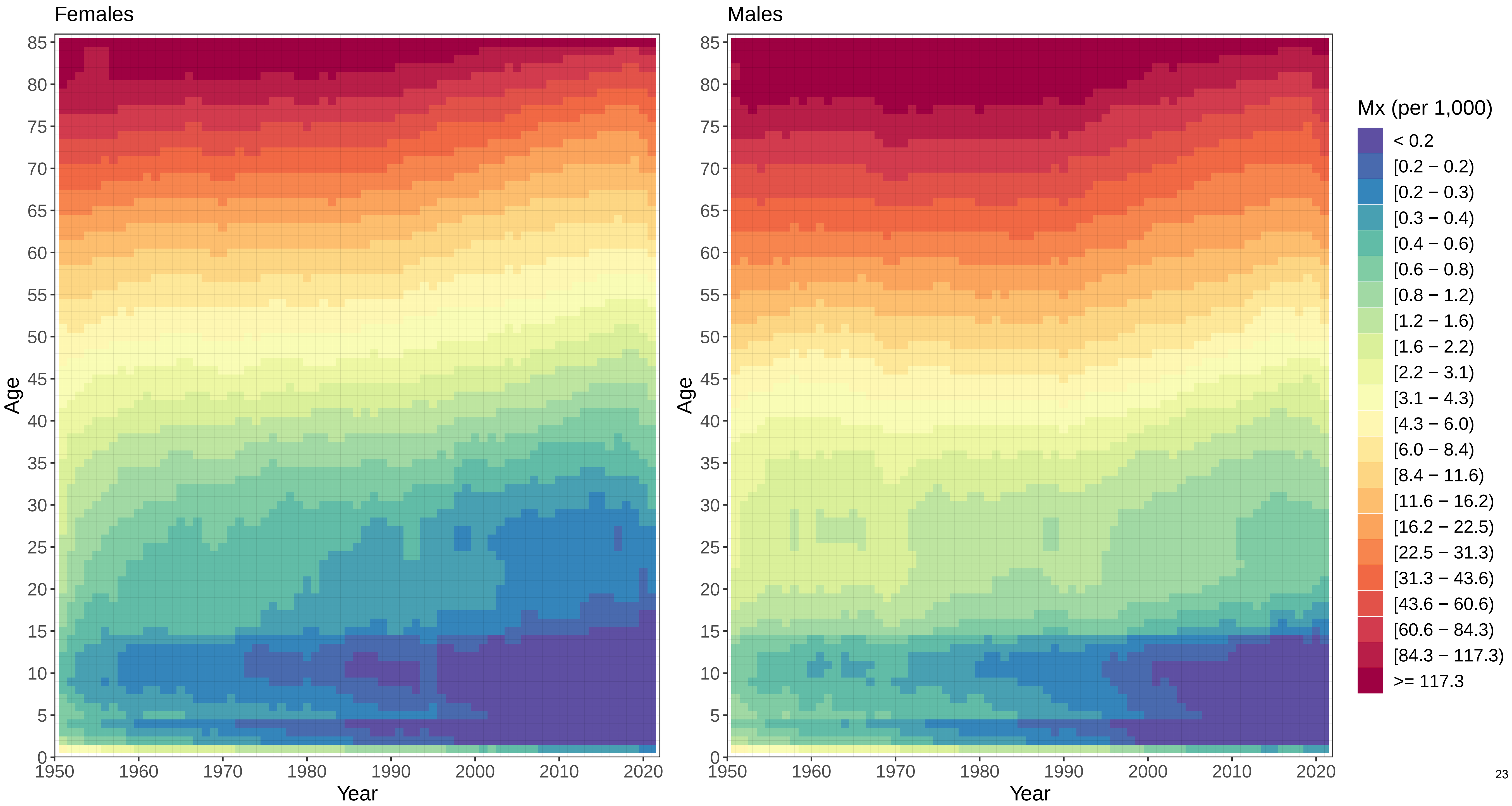

Appendix 2 Figure S5: Hungary mortality rates, 1950−2021

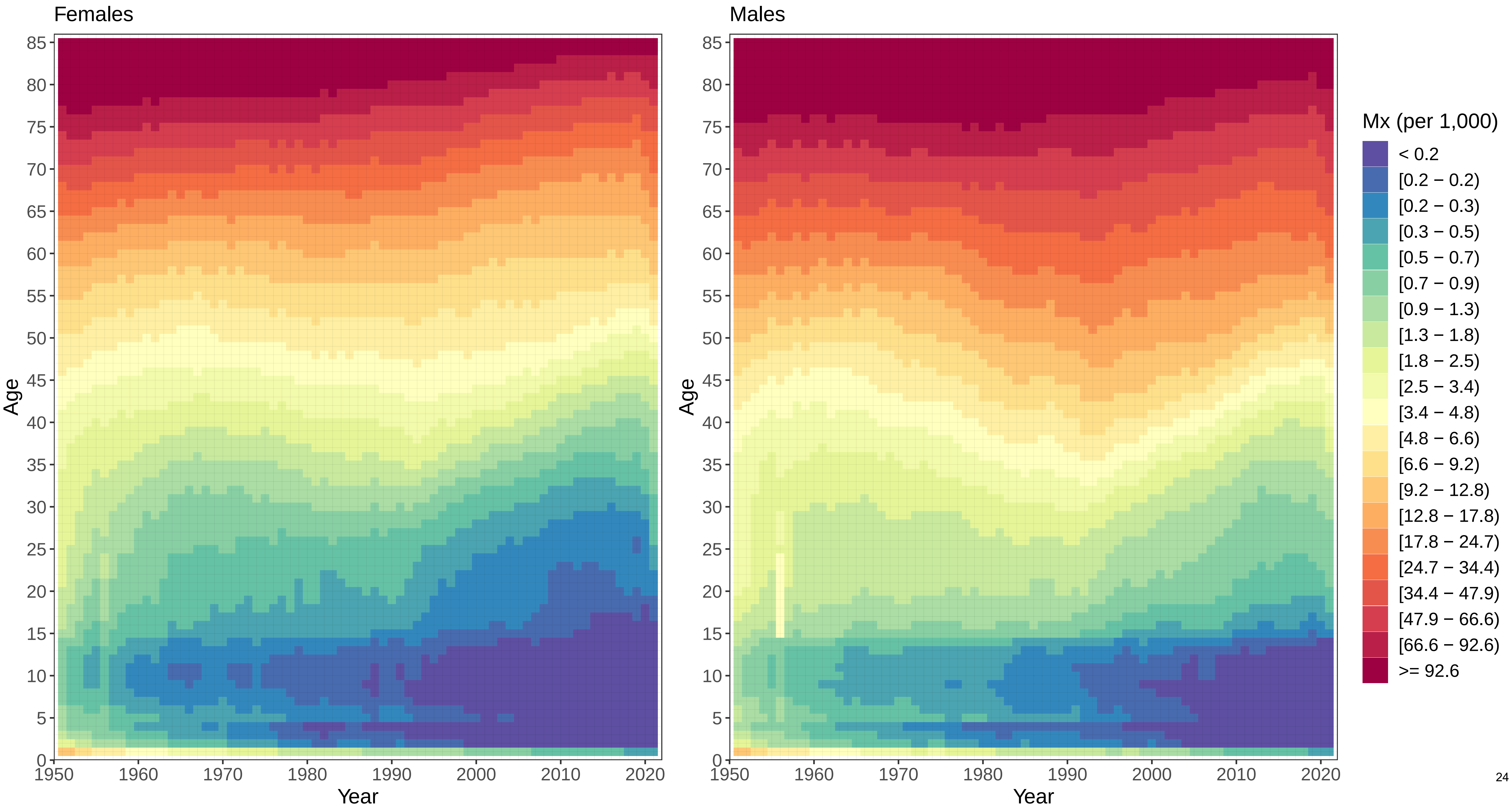

Appendix 2 Figure S5: Montenegro mortality rates, 1950−2021

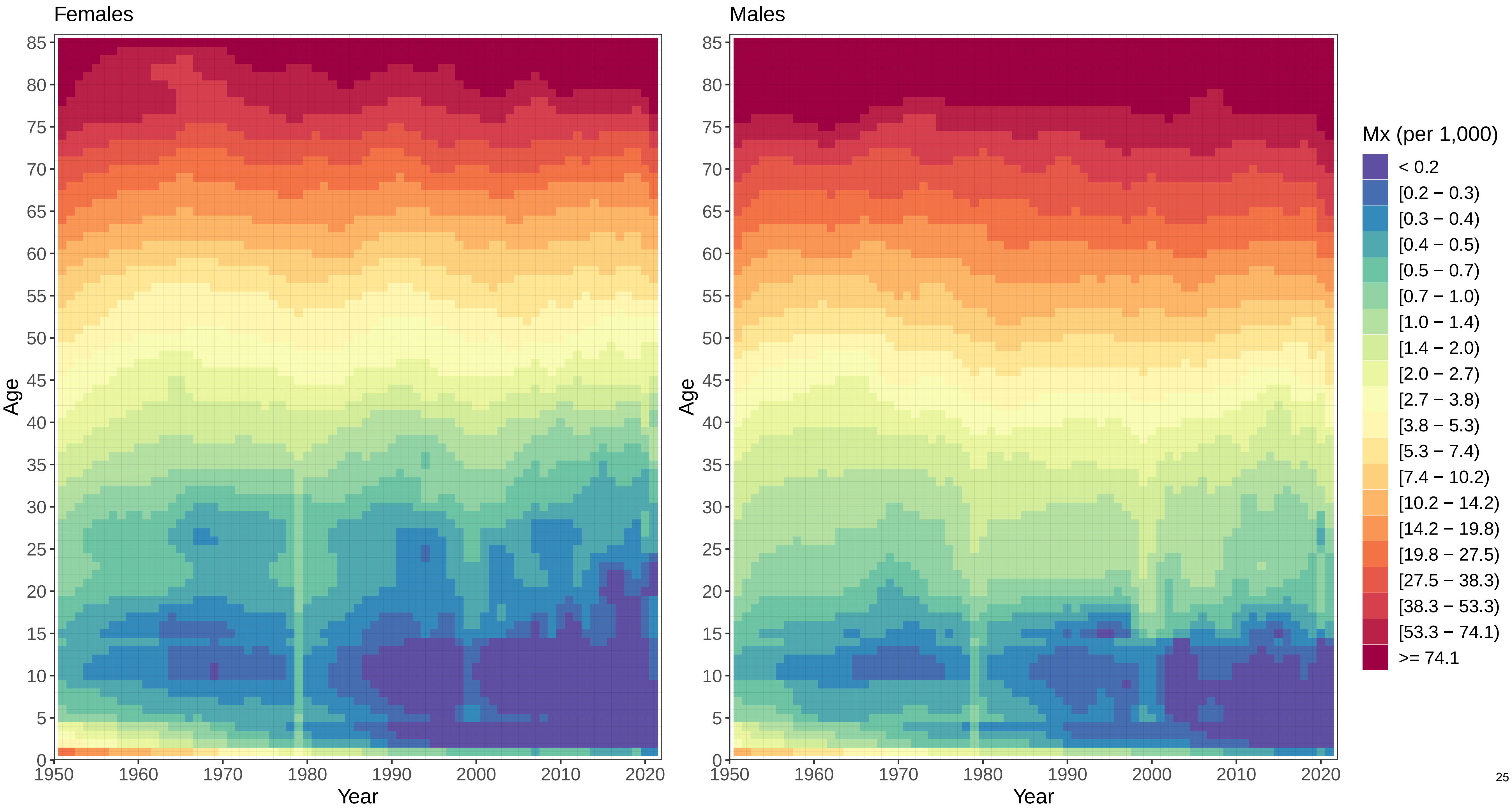

Appendix 2 Figure S5: North Macedonia mortality rates, 1950−2021

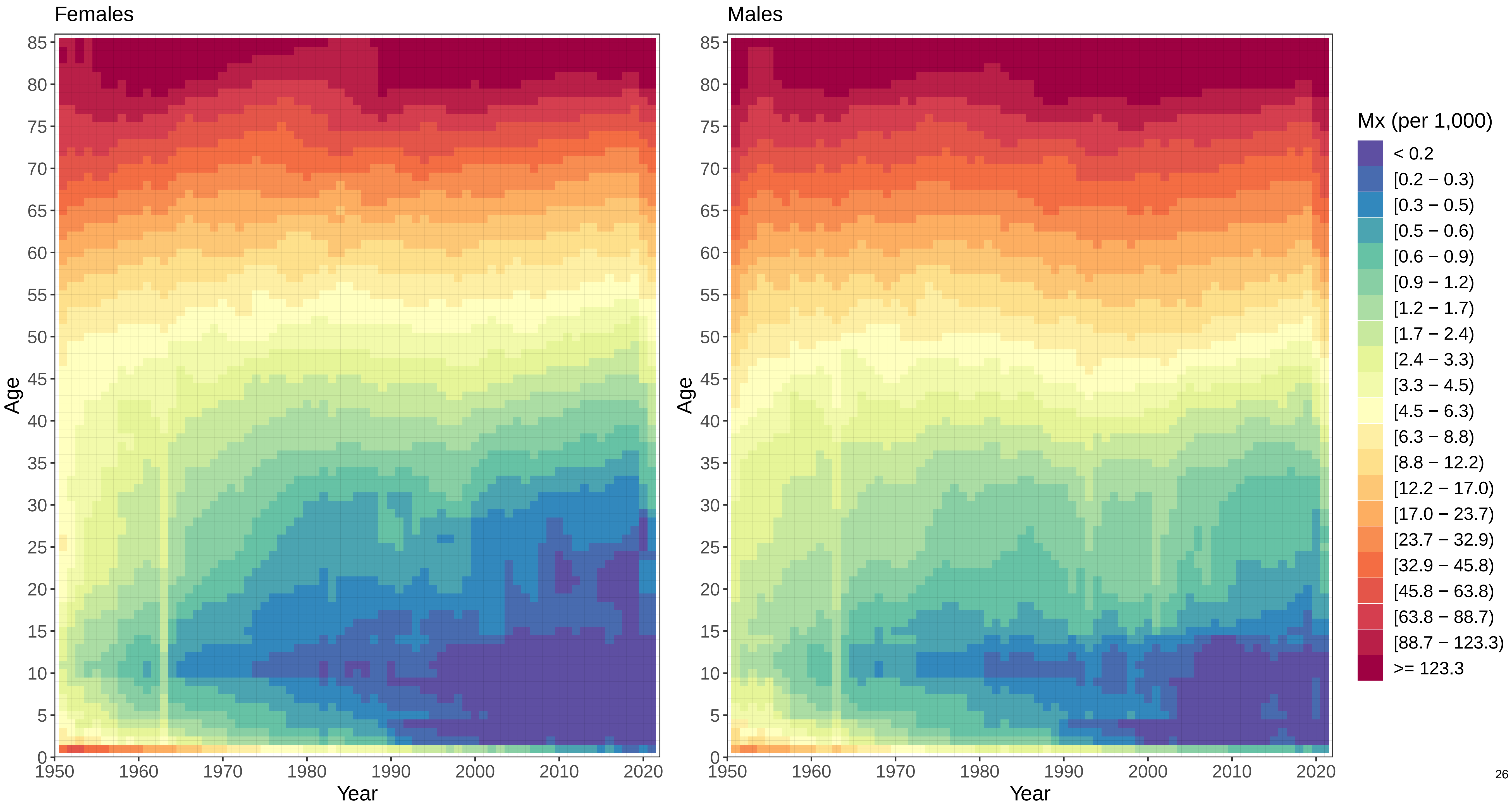

Appendix 2 Figure S5: Poland mortality rates, 1950−2021

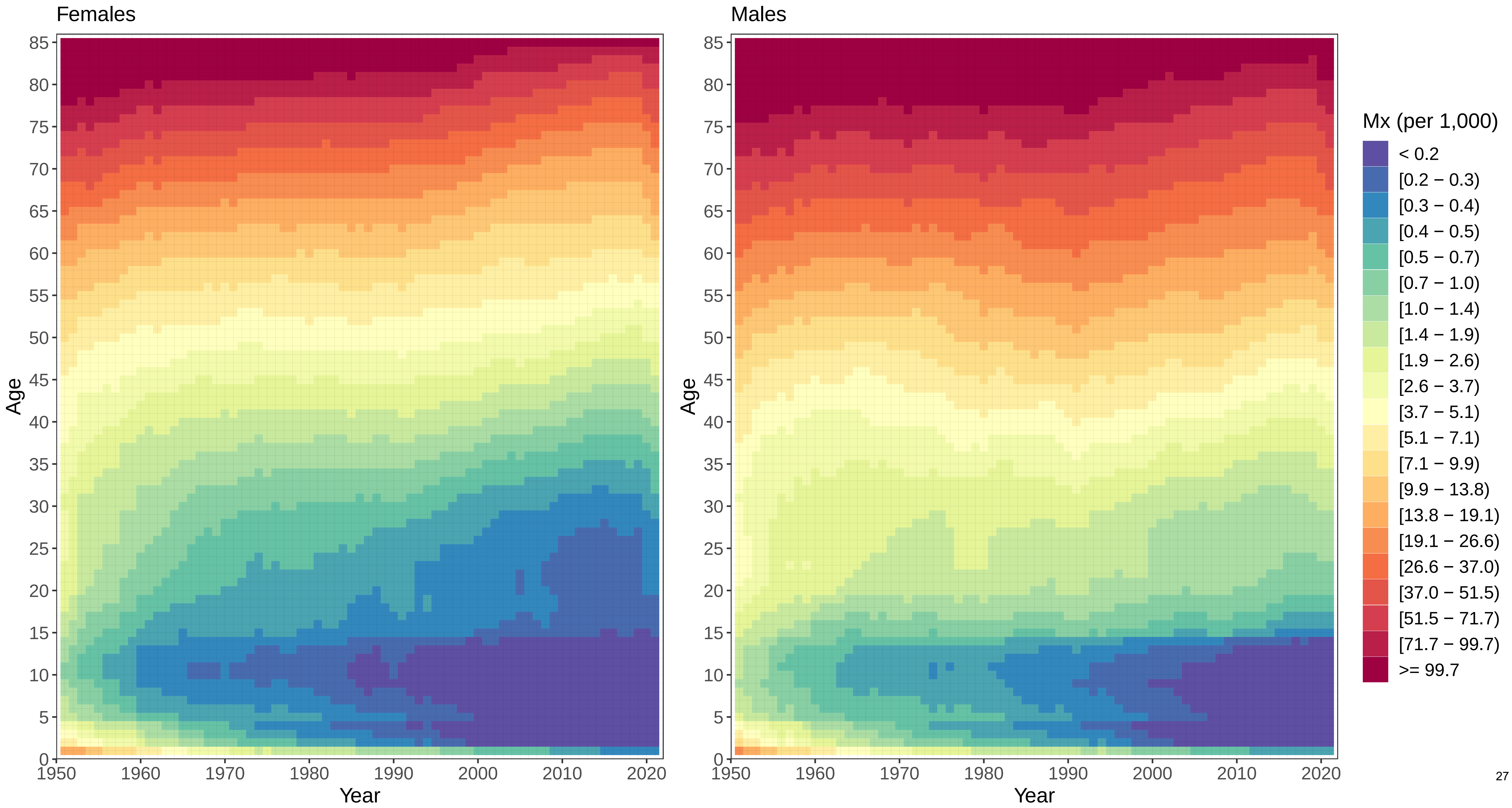

Appendix 2 Figure S5: Romania mortality rates, 1950−2021

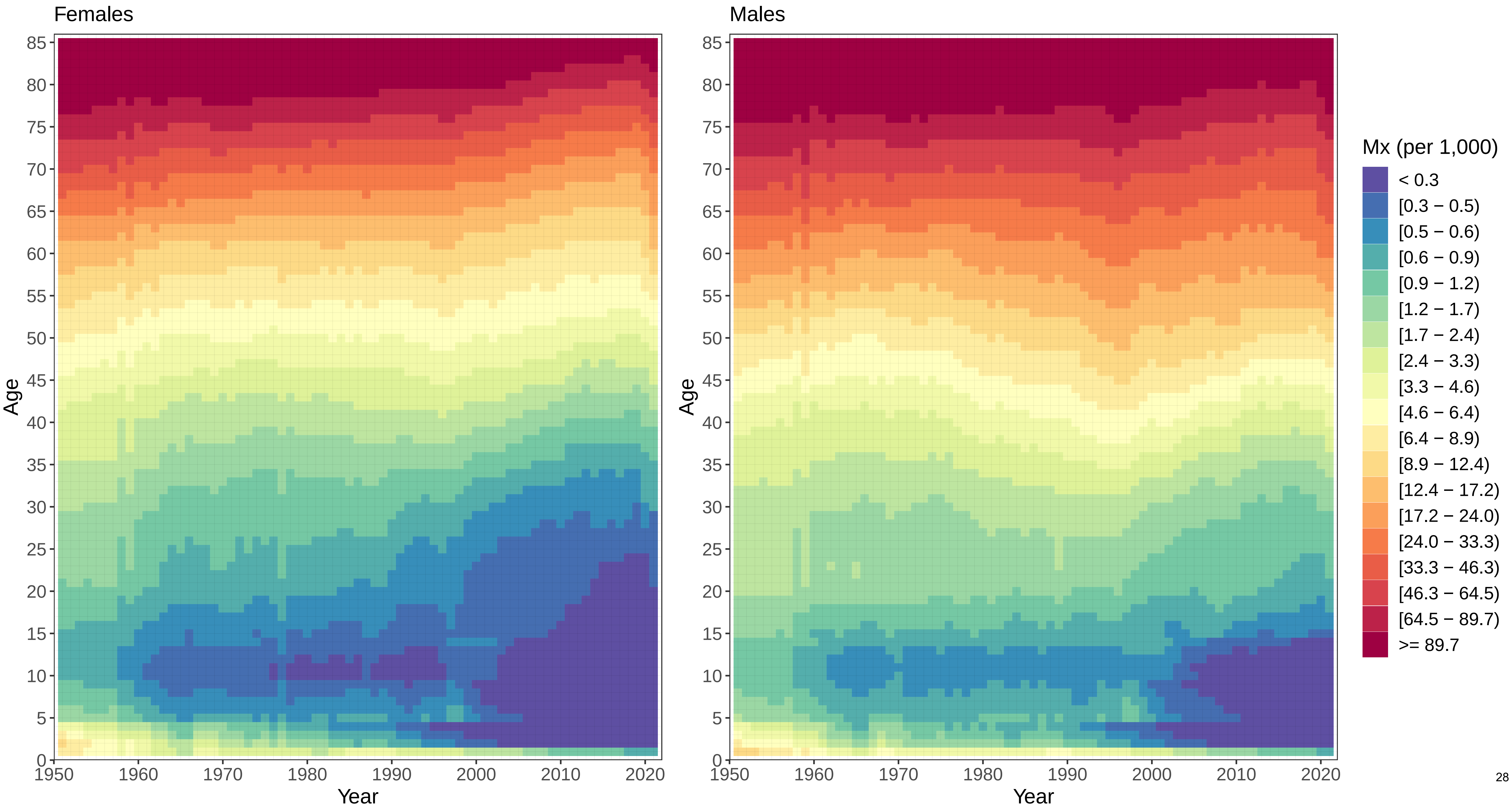

Appendix 2 Figure S5: Serbia mortality rates, 1950−2021

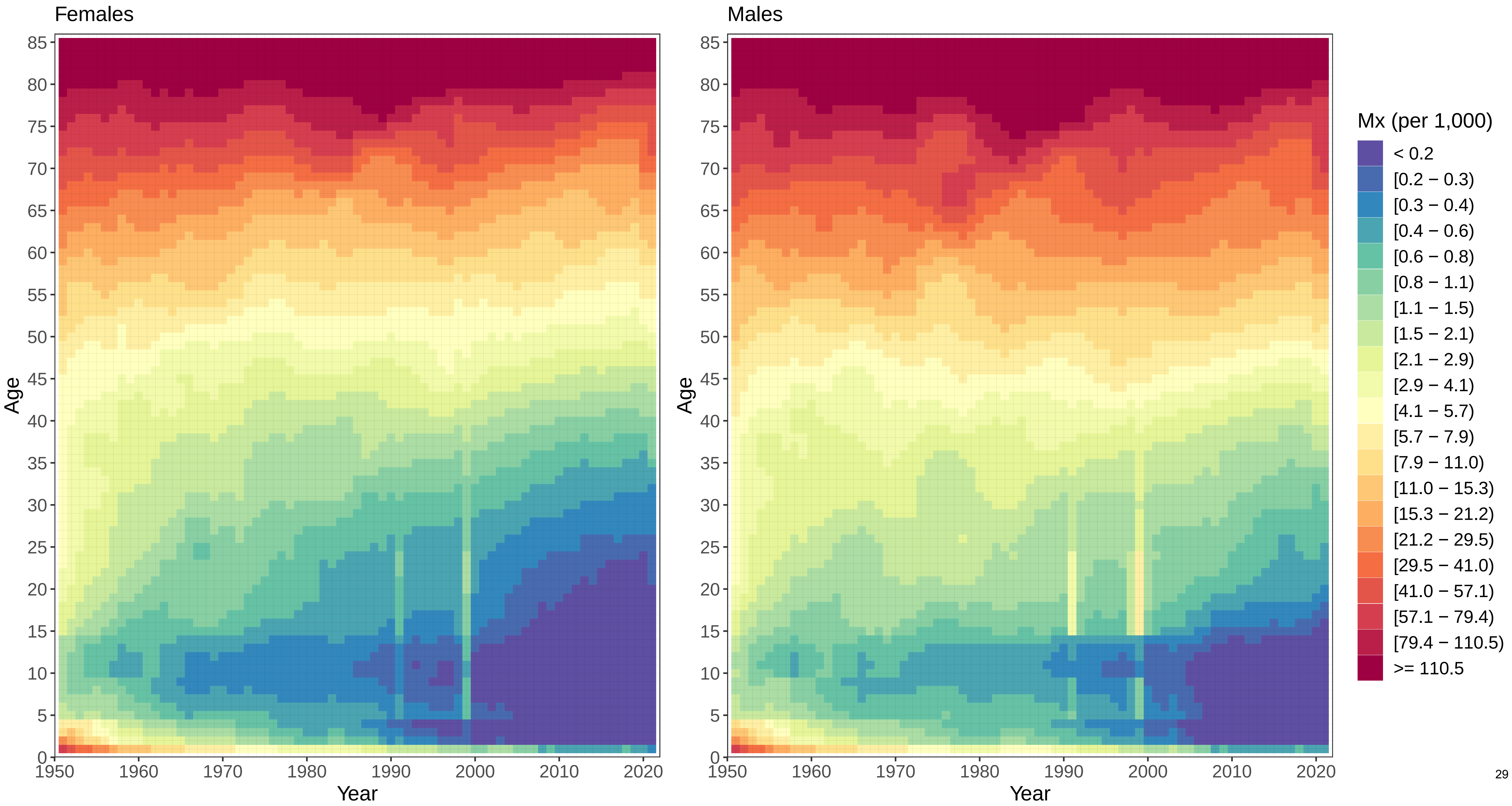

Appendix 2 Figure S5: Slovakia mortality rates, 1950−2021

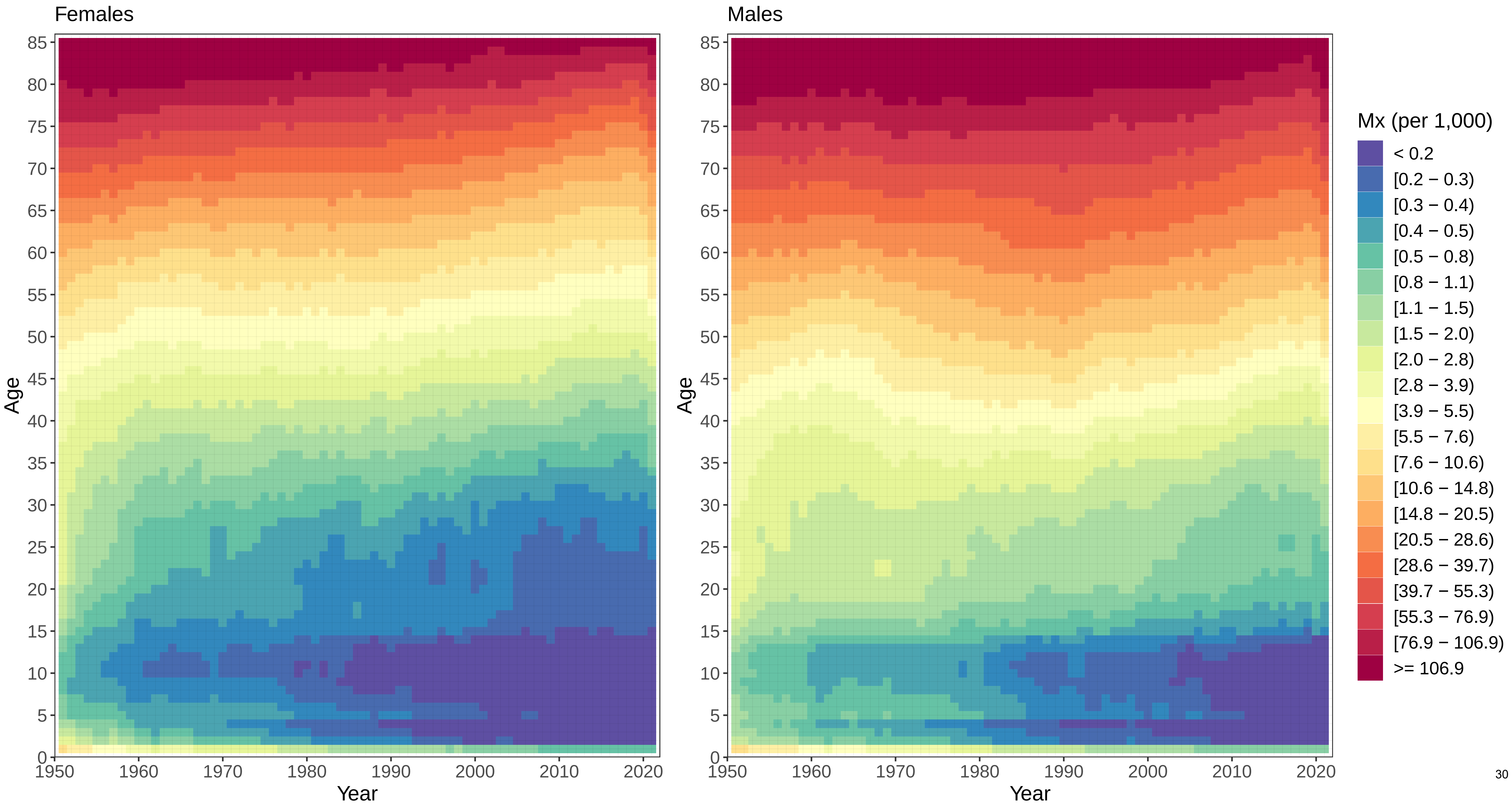

Appendix 2 Figure S5: Slovenia mortality rates, 1950−2021

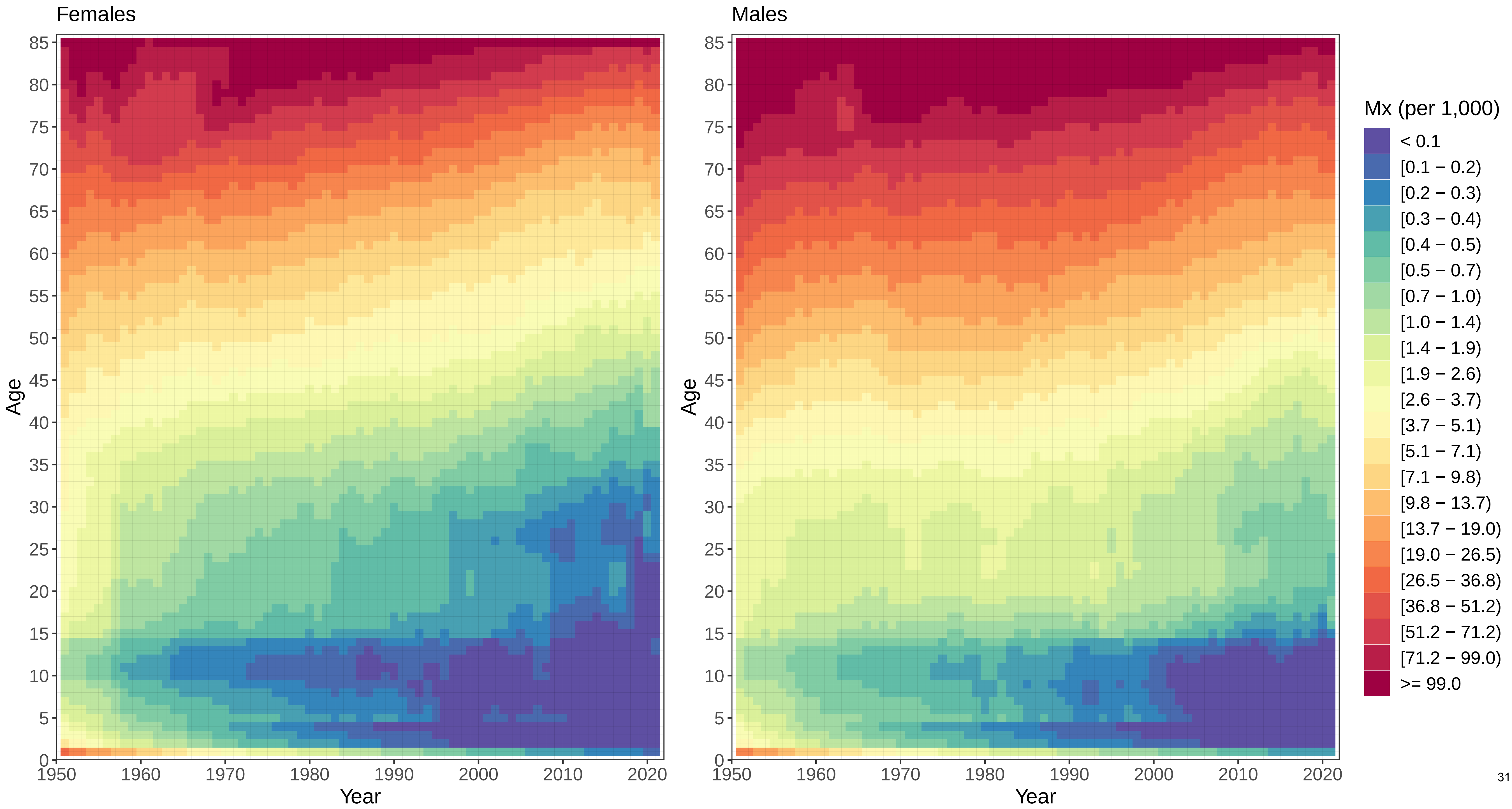

Appendix 2 Figure S5: Belarus mortality rates, 1950−2021

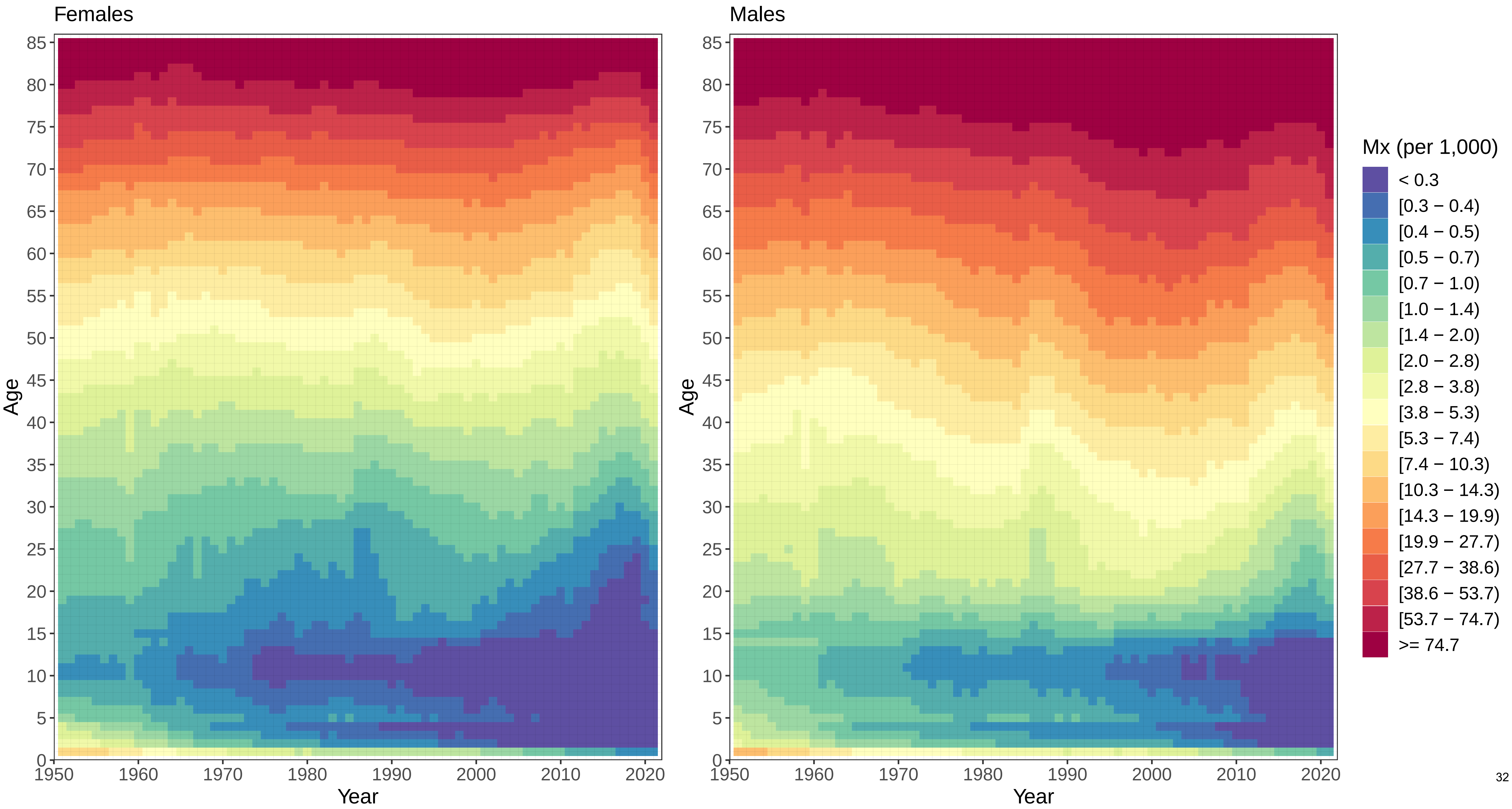

Appendix 2 Figure S5: Estonia mortality rates, 1950−2021

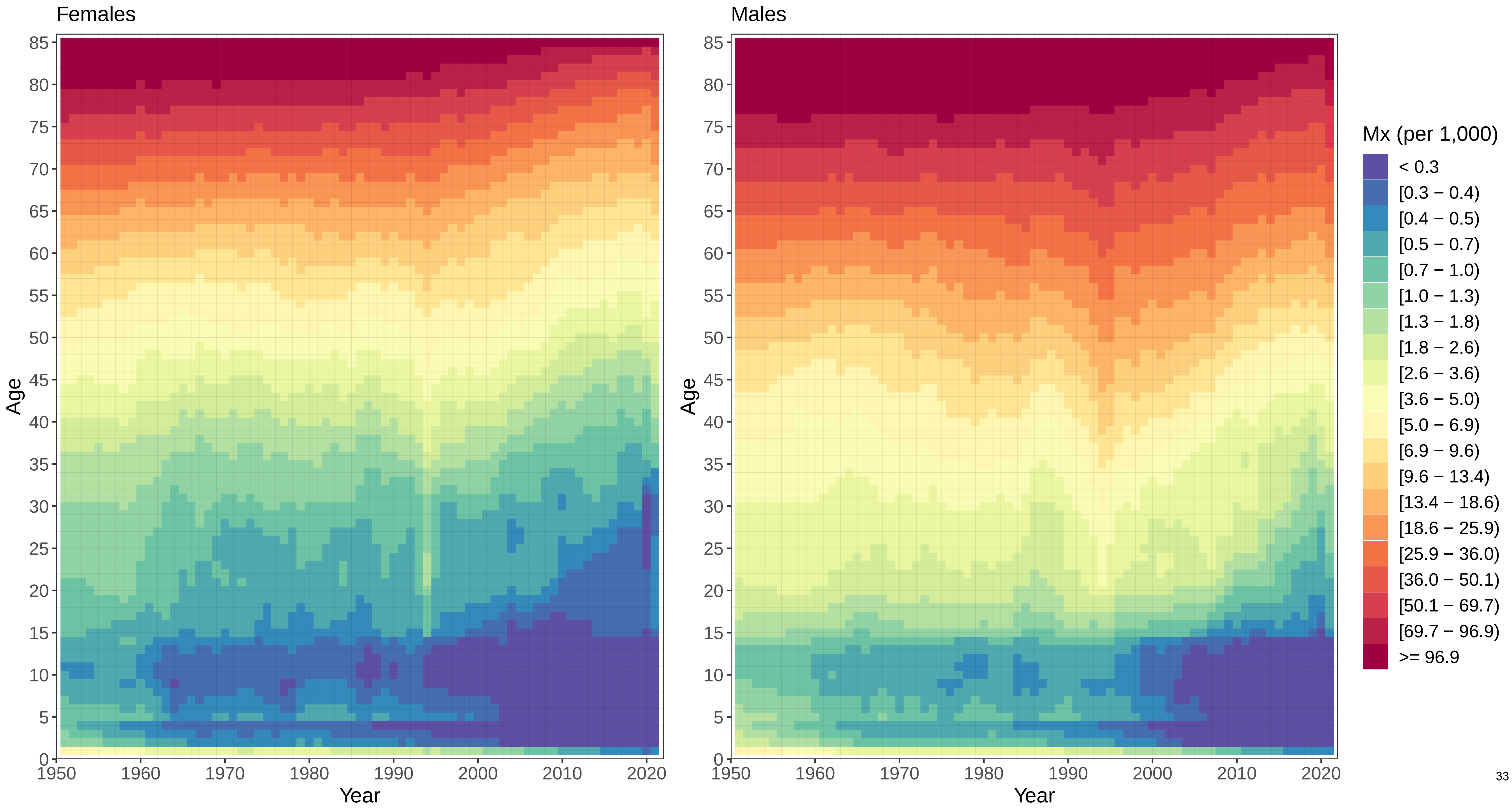

Appendix 2 Figure S5: Latvia mortality rates, 1950−2021

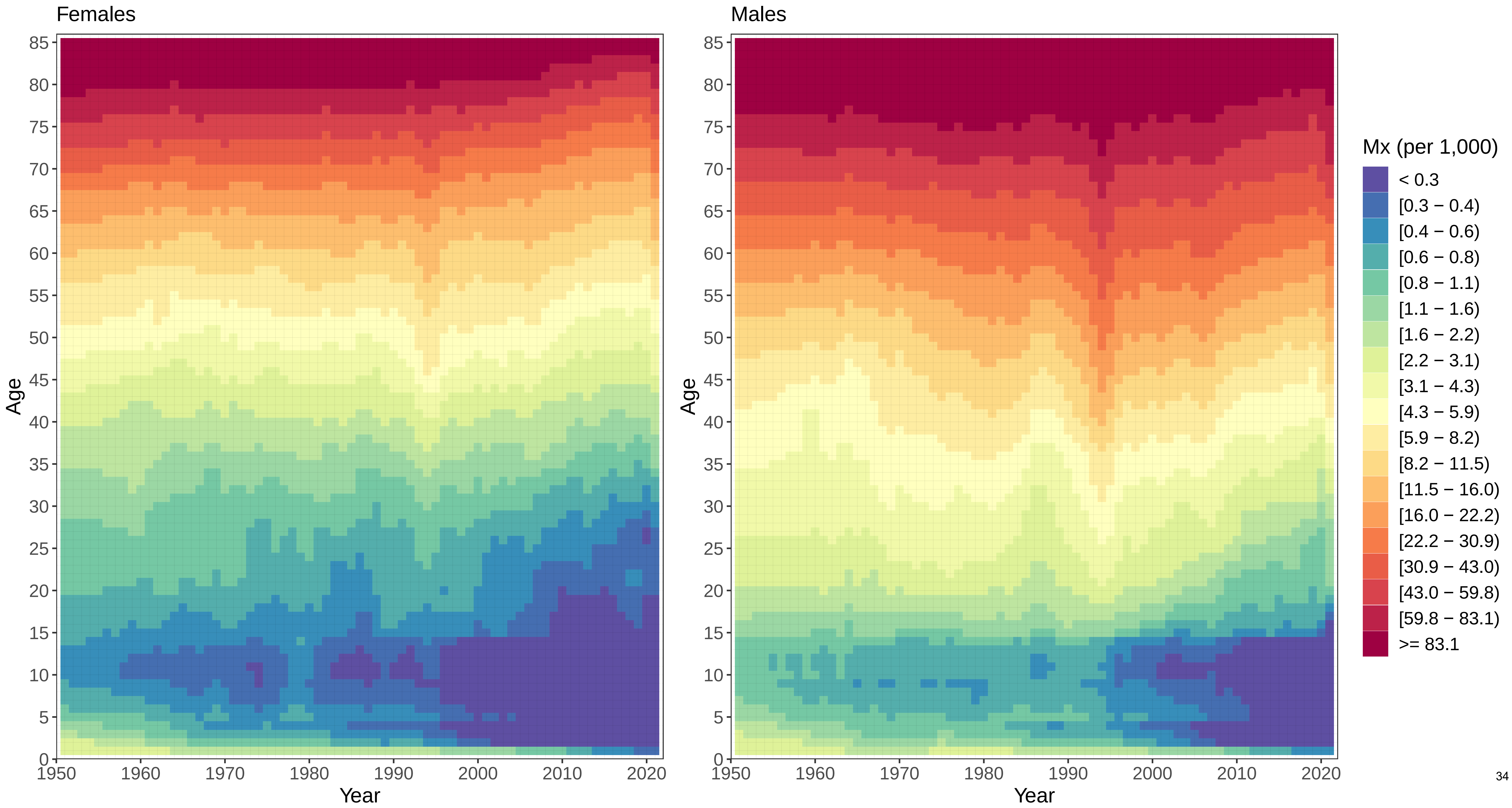

Appendix 2 Figure S5: Lithuania mortality rates, 1950−2021

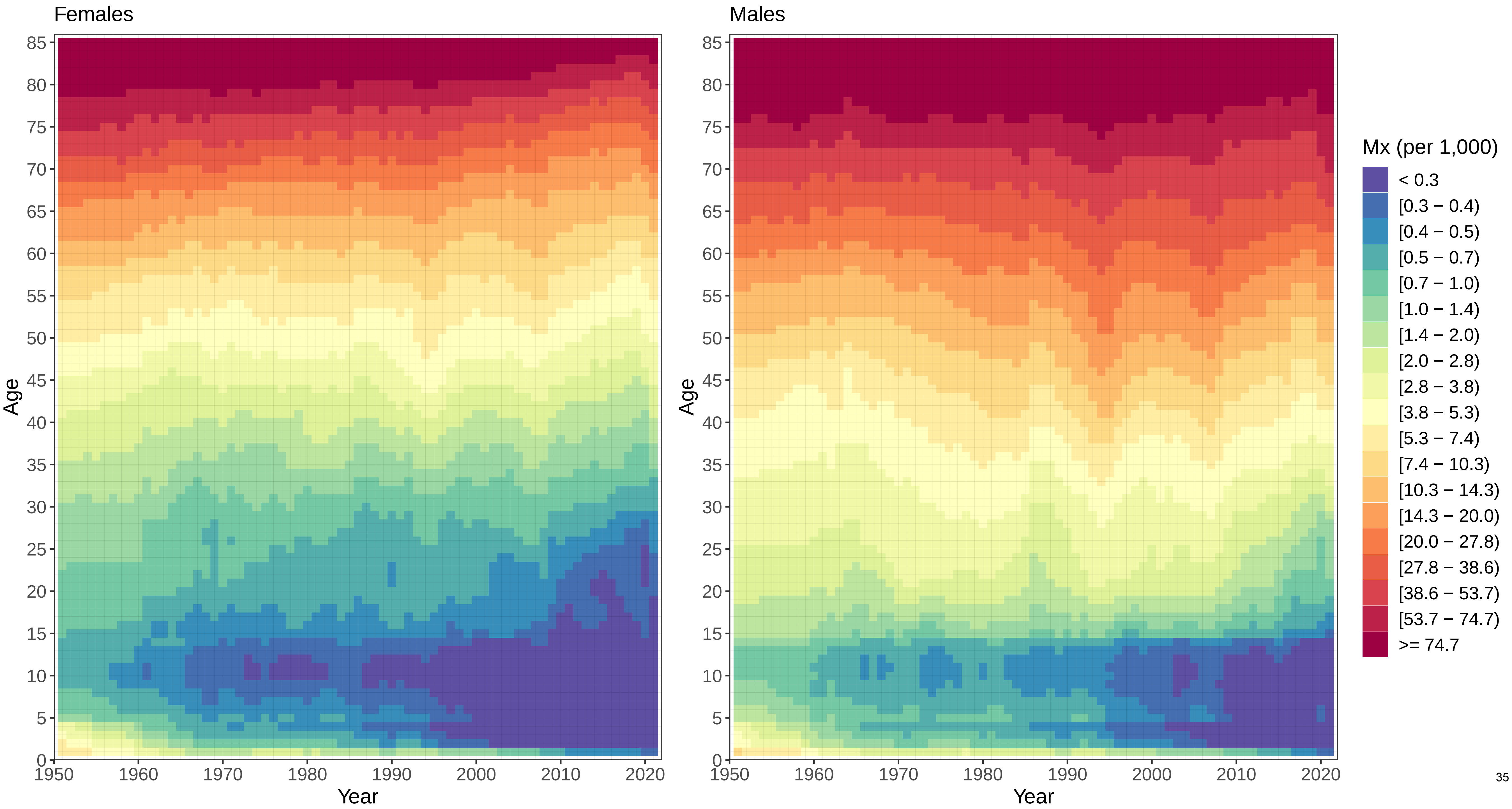

Appendix 2 Figure S5: Moldova mortality rates, 1950−2021

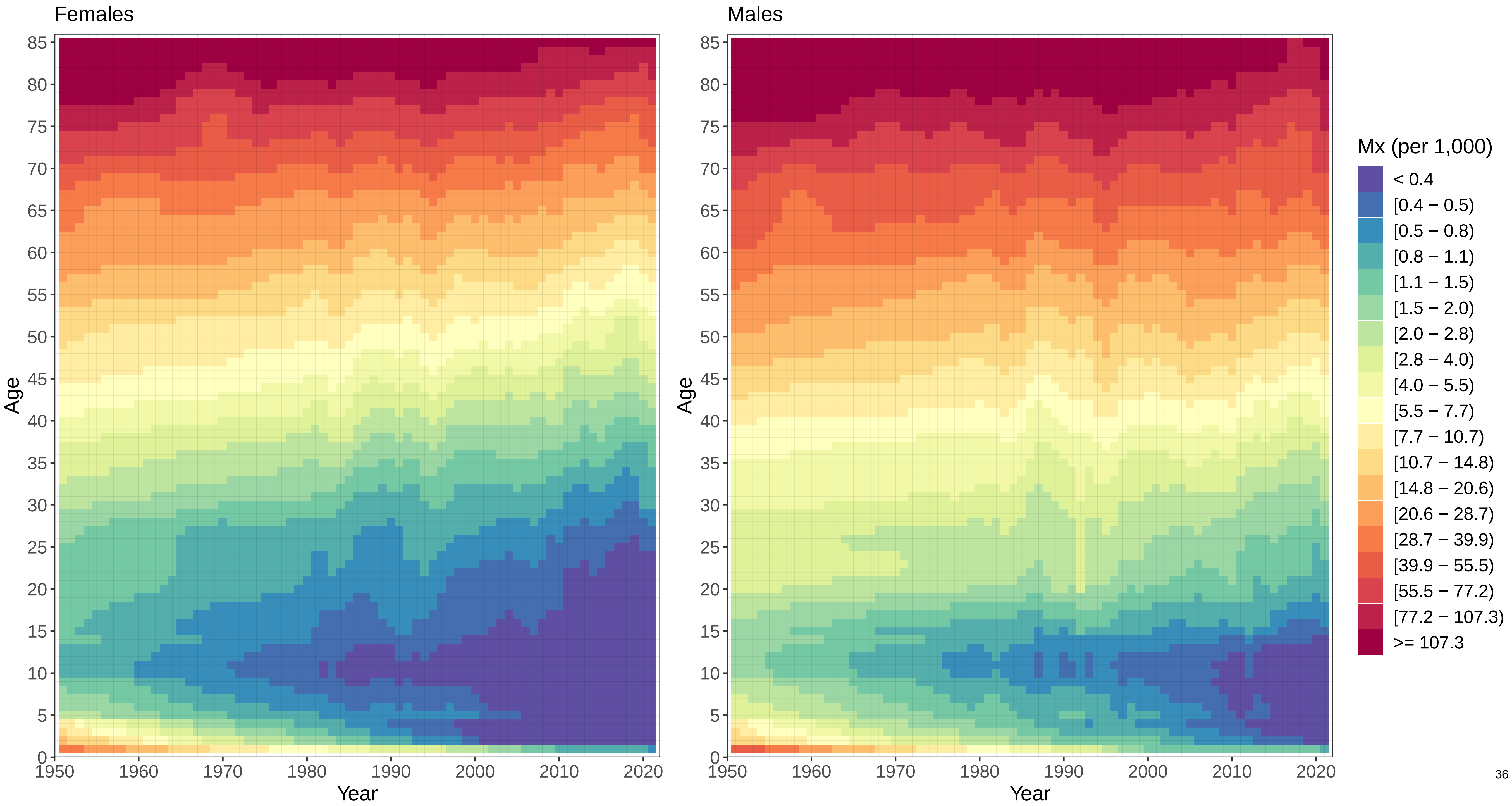

Appendix 2 Figure S5: Russia mortality rates, 1950−2021

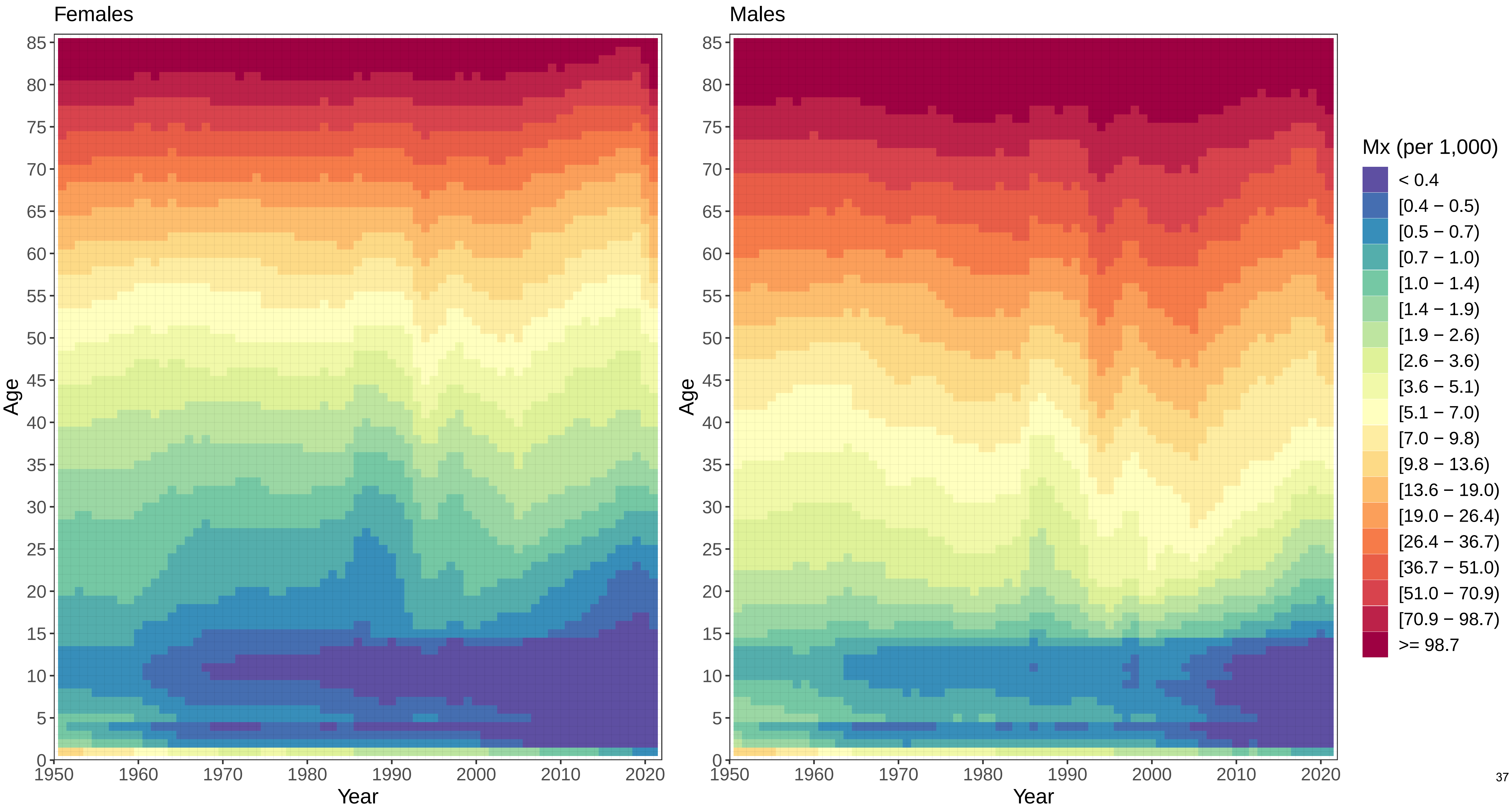

Appendix 2 Figure S5: Ukraine mortality rates, 1950−2021

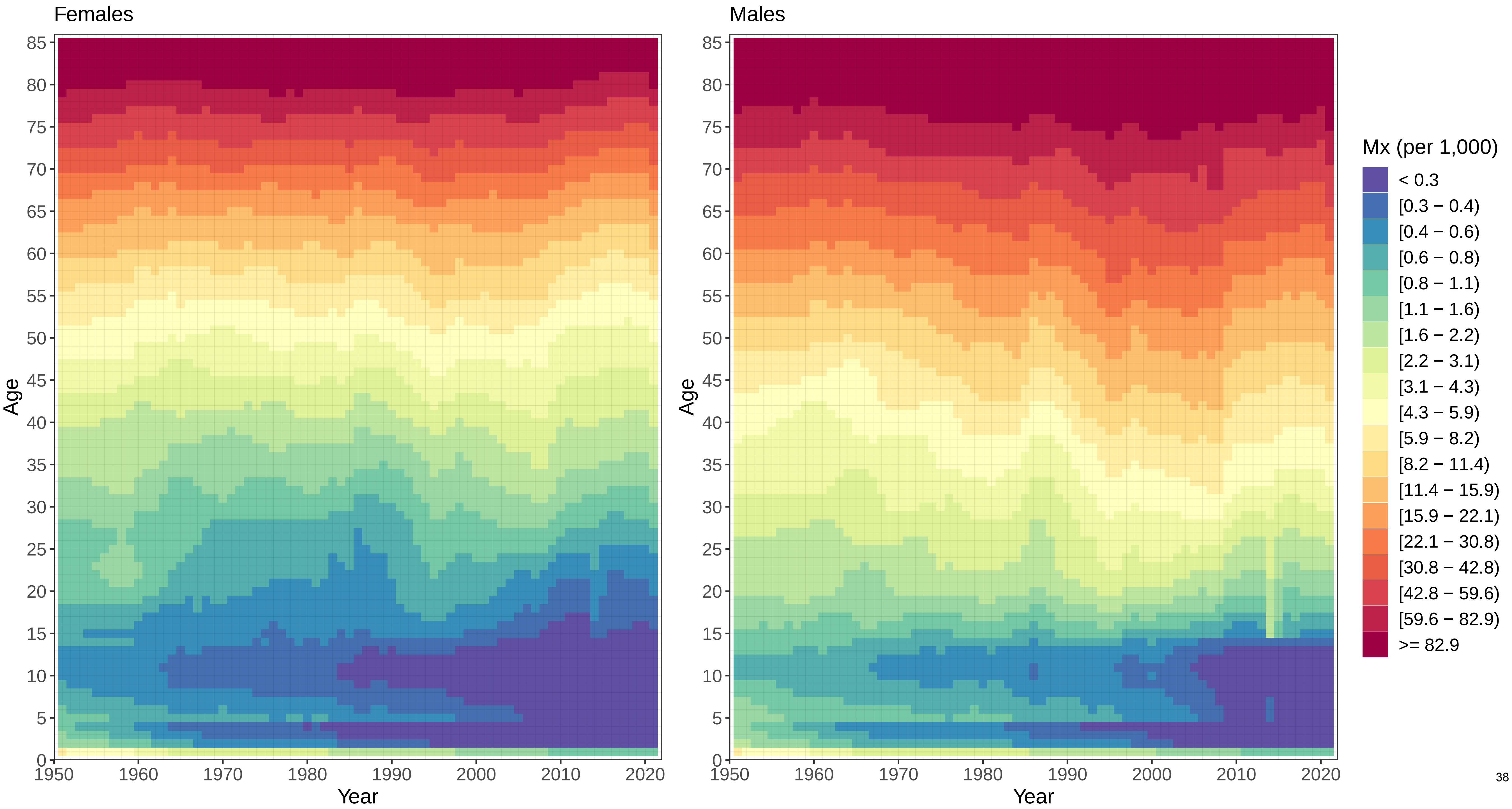

Appendix 2 Figure S5: Australia mortality rates, 1950−2021

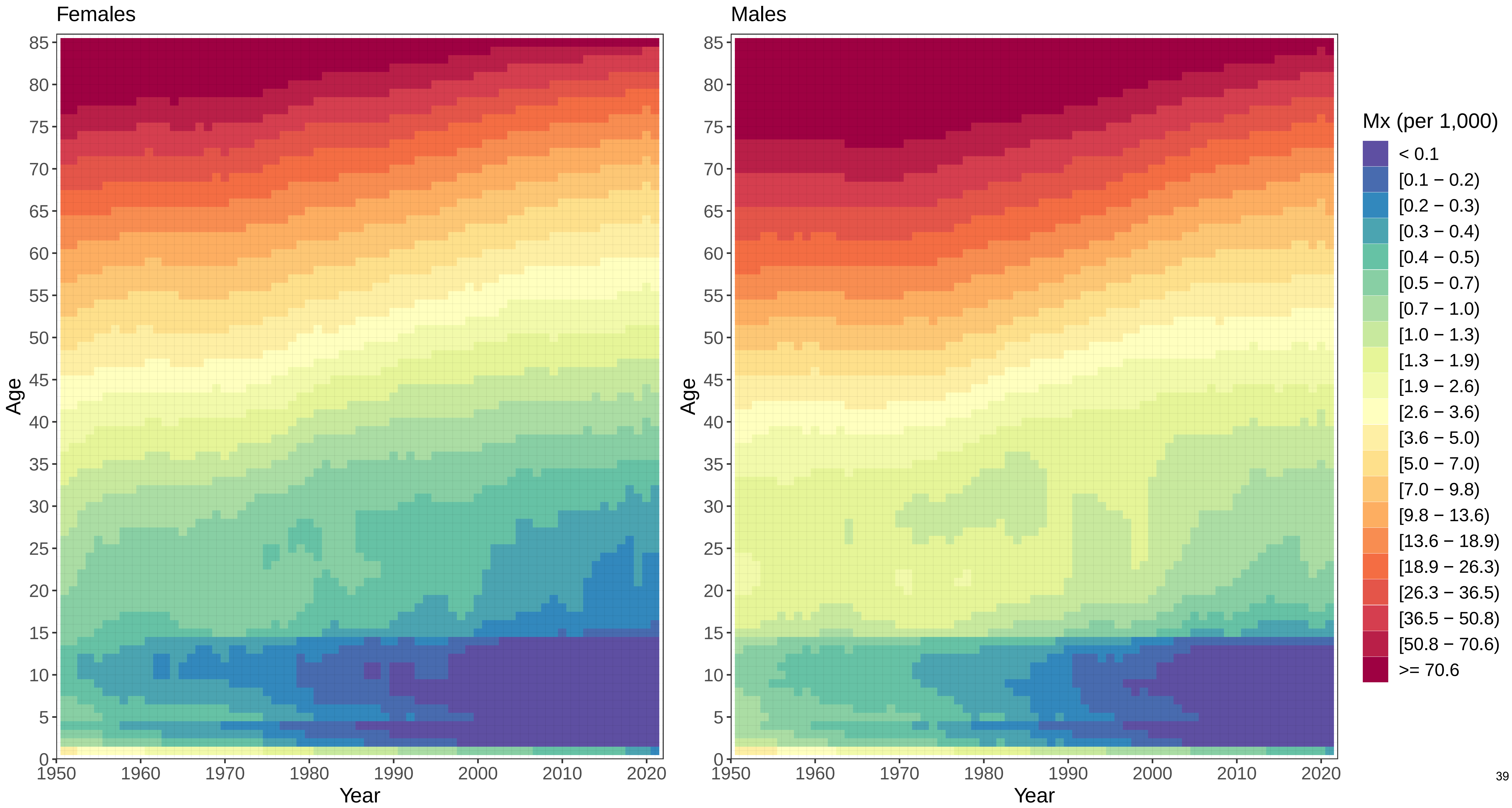

Appendix 2 Figure S5: New Zealand mortality rates, 1950−2021

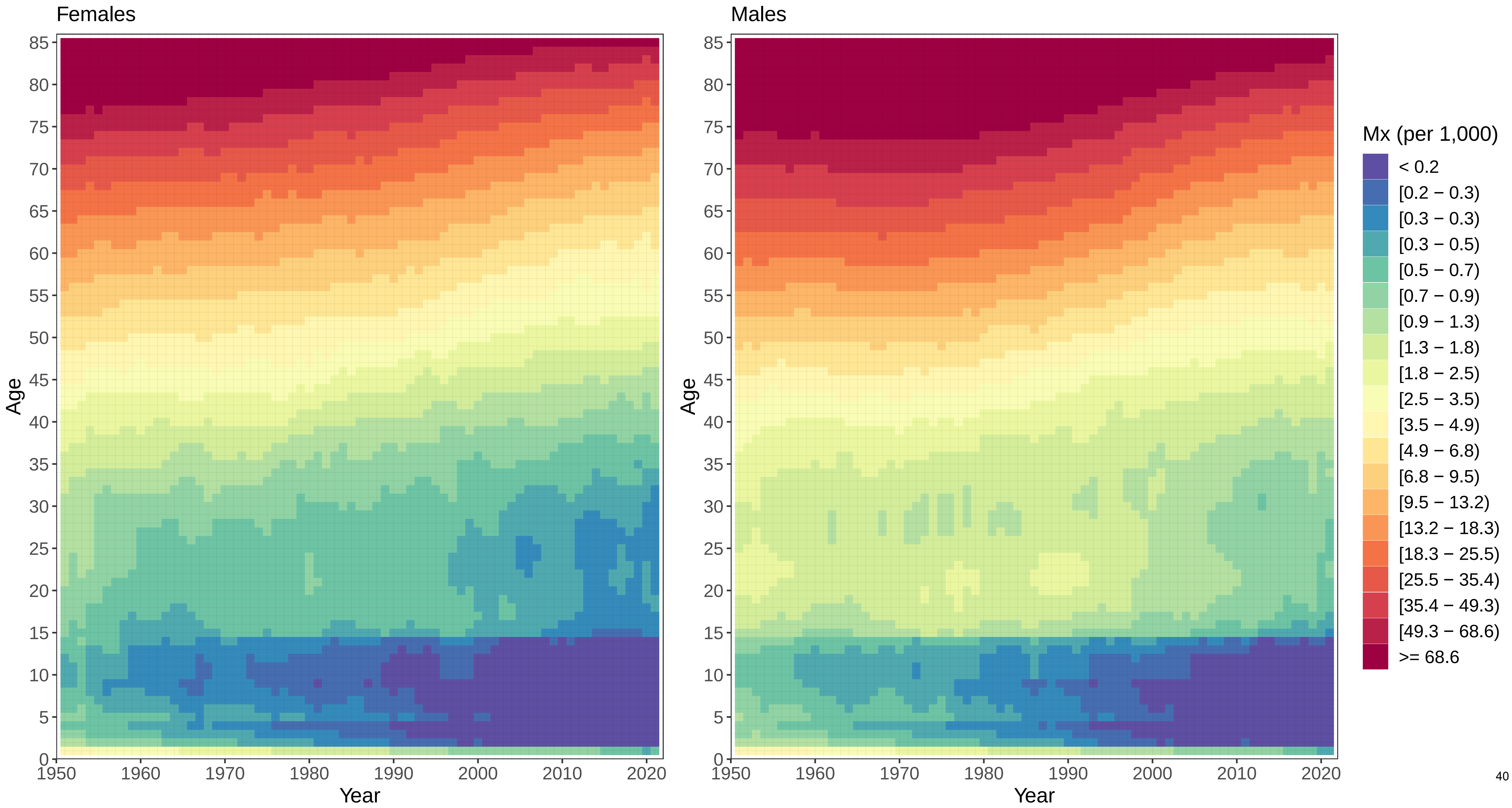

Appendix 2 Figure S5: Brunei mortality rates, 1950−2021

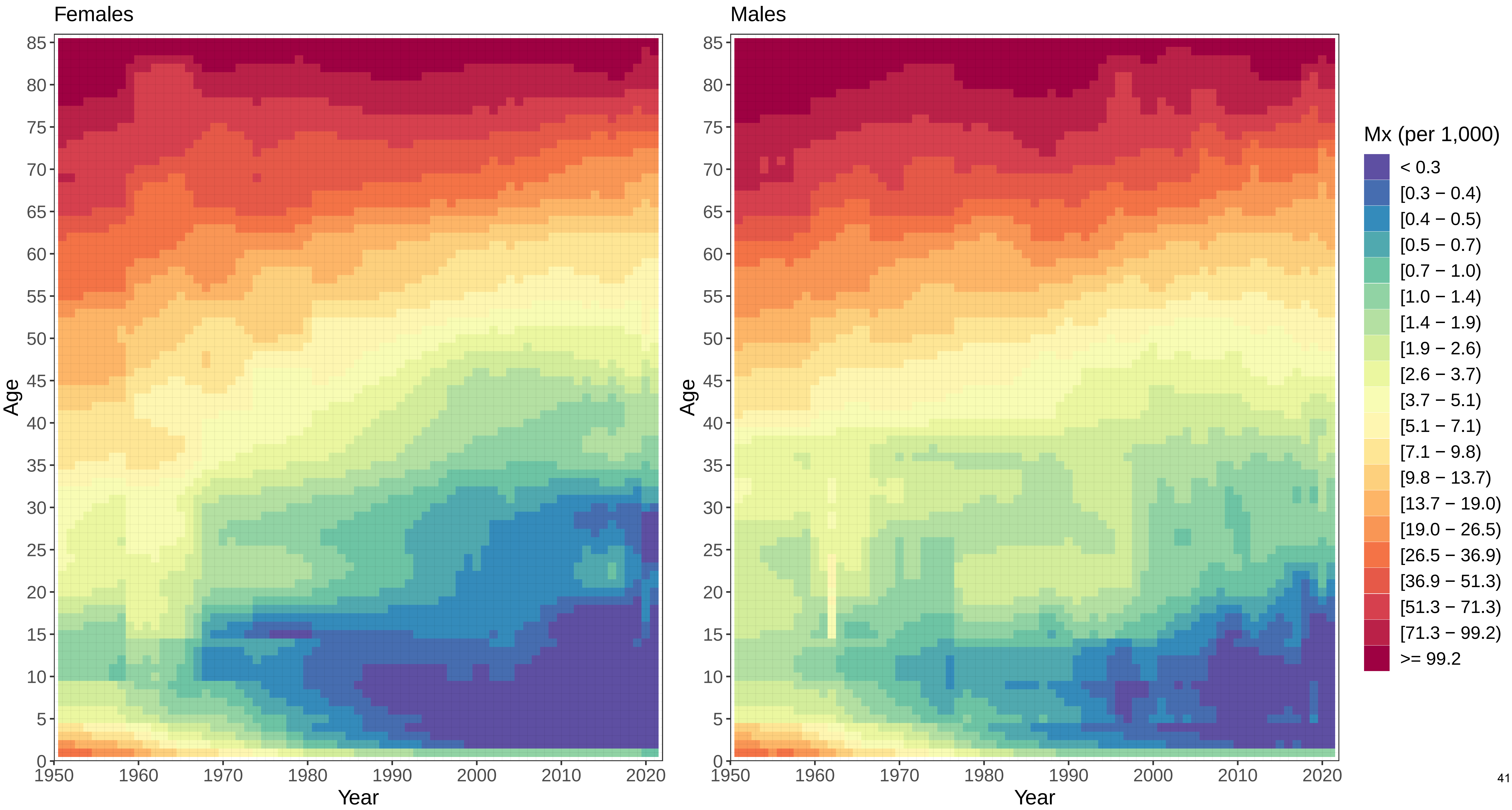

Appendix 2 Figure S5: Japan mortality rates, 1950−2021

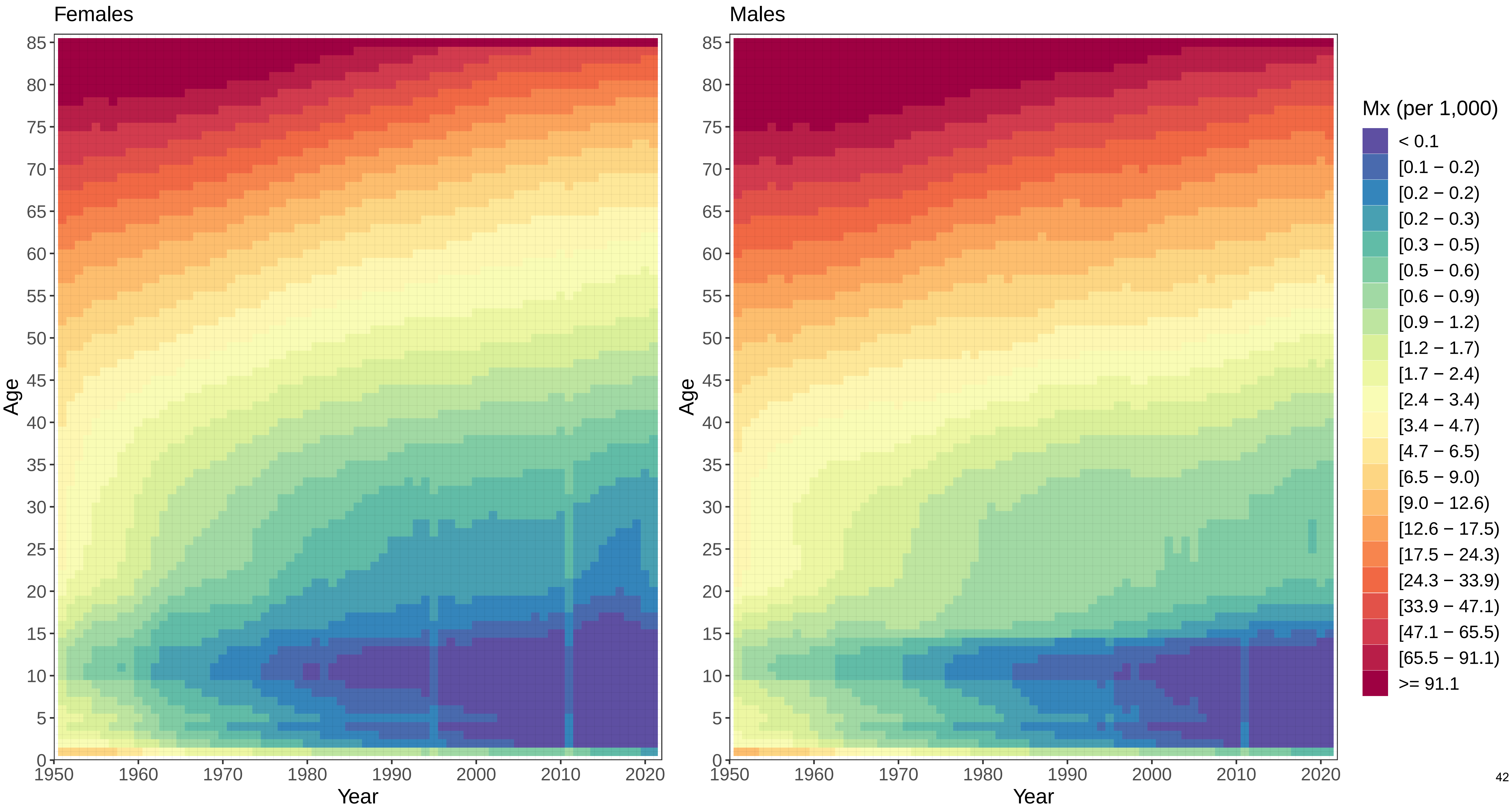

Appendix 2 Figure S5: South Korea mortality rates, 1950−2021

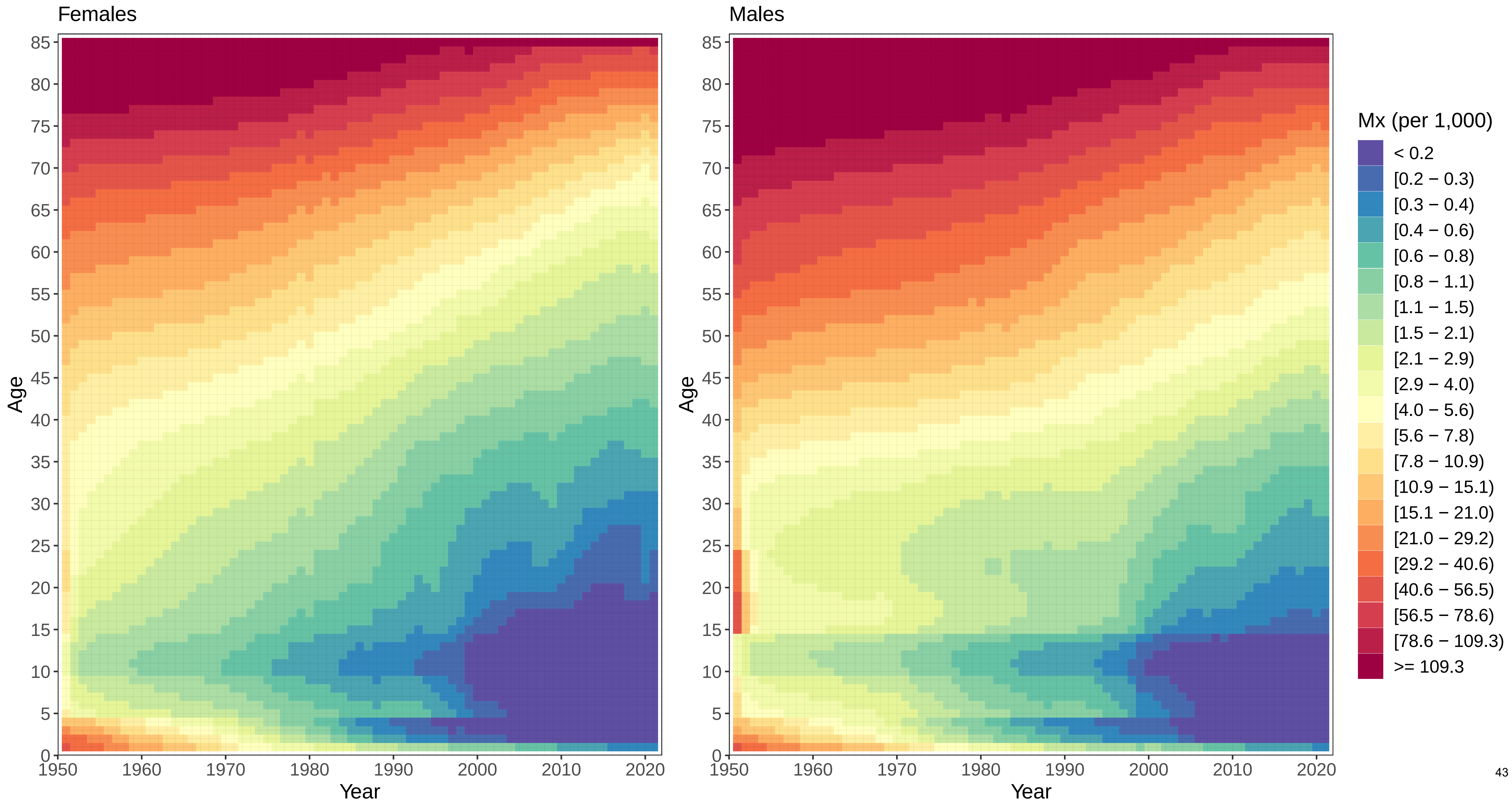

Appendix 2 Figure S5: Singapore mortality rates, 1950−2021

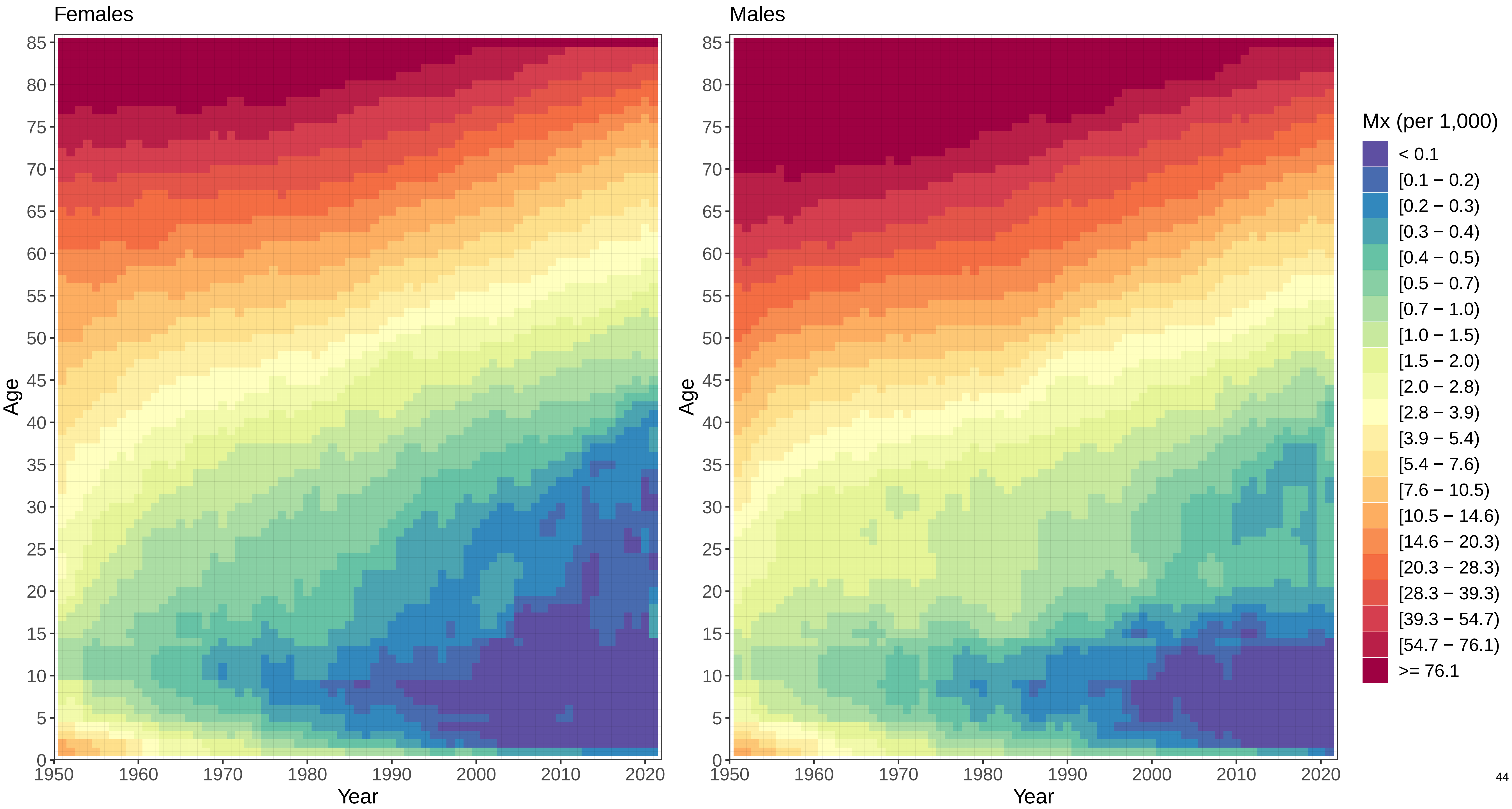

Appendix 2 Figure S5: Canada mortality rates, 1950−2021

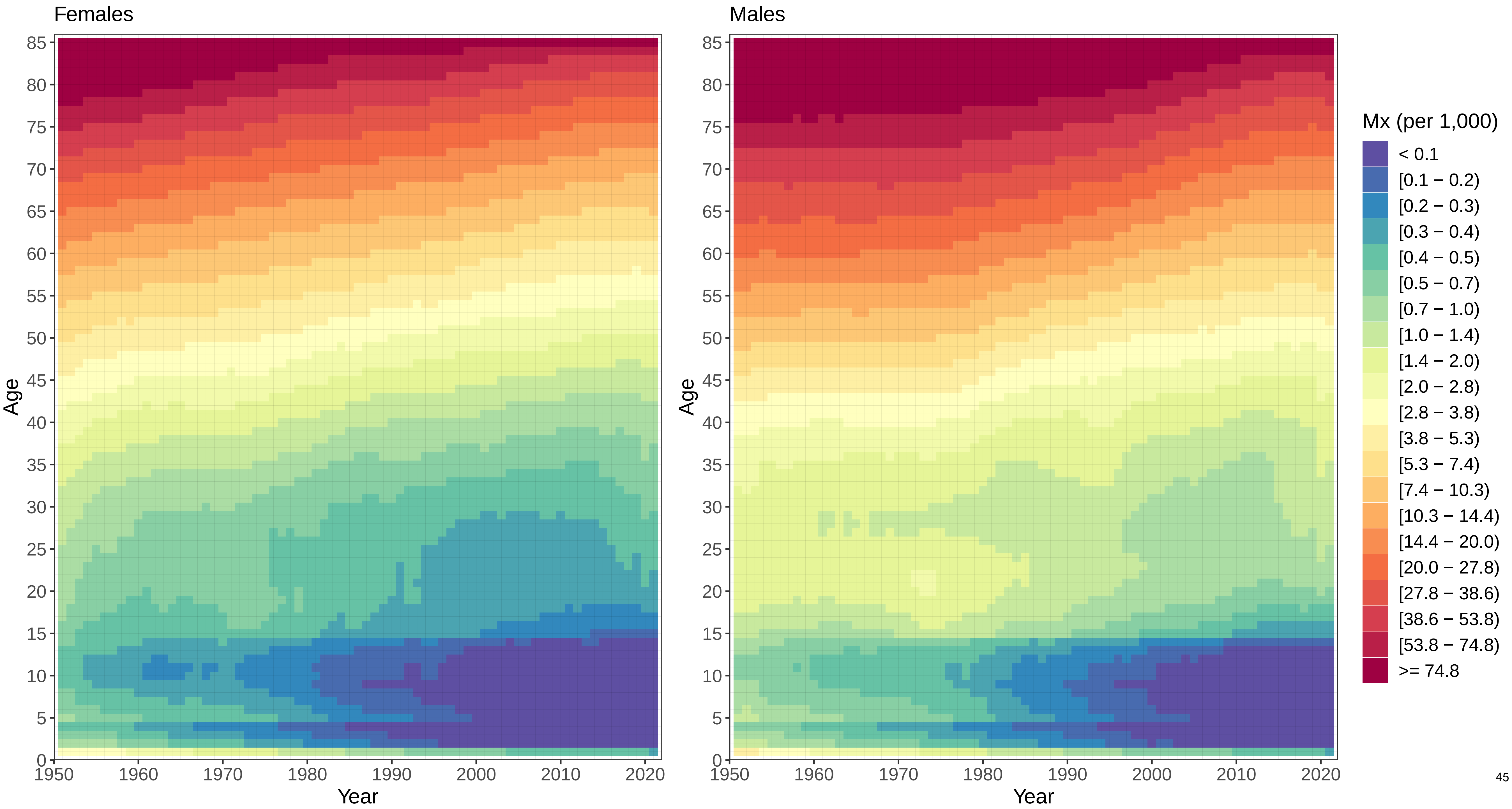

Appendix 2 Figure S5: Greenland mortality rates, 1950−2021

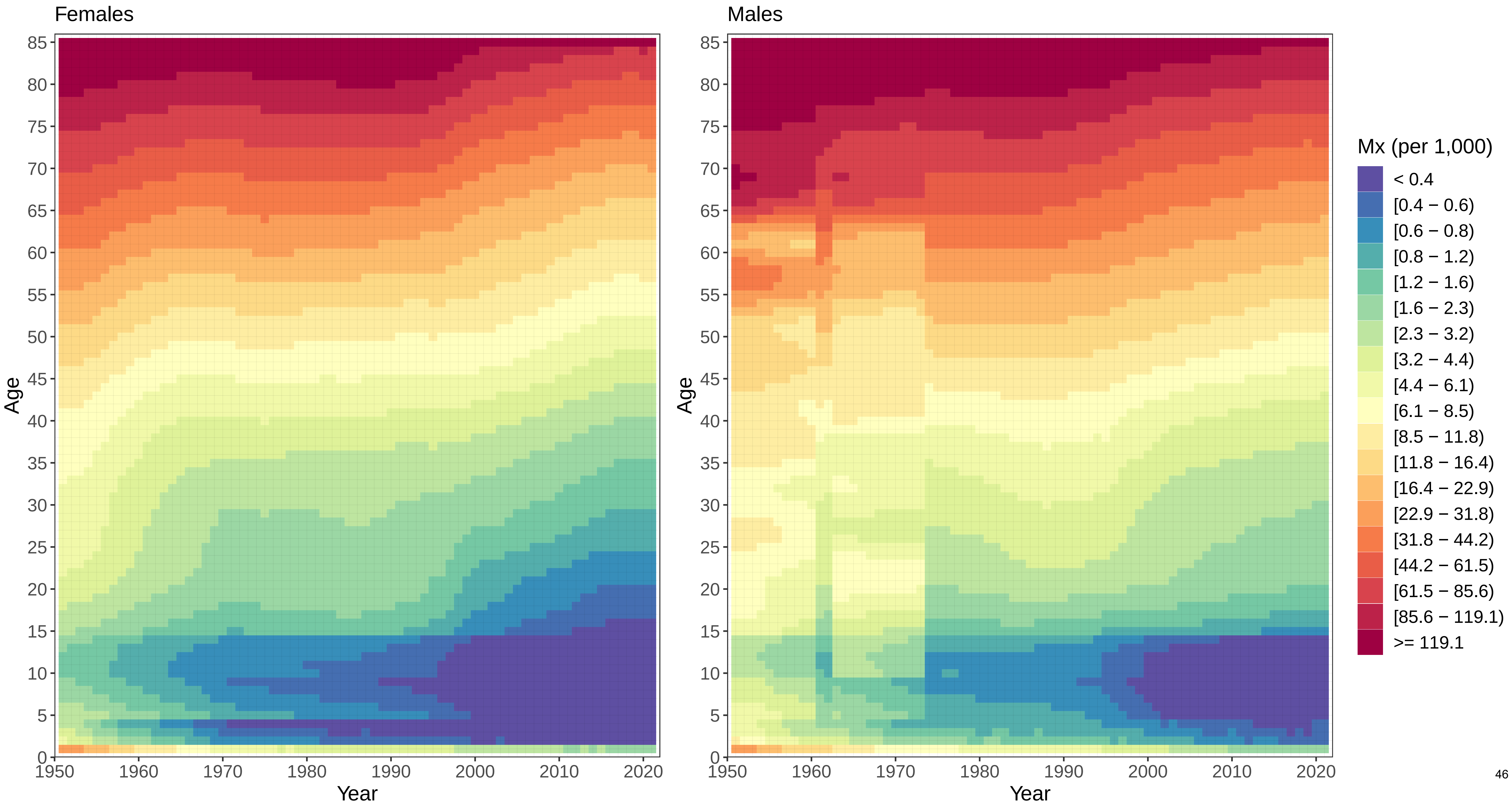

Appendix 2 Figure S5: USA mortality rates, 1950−2021

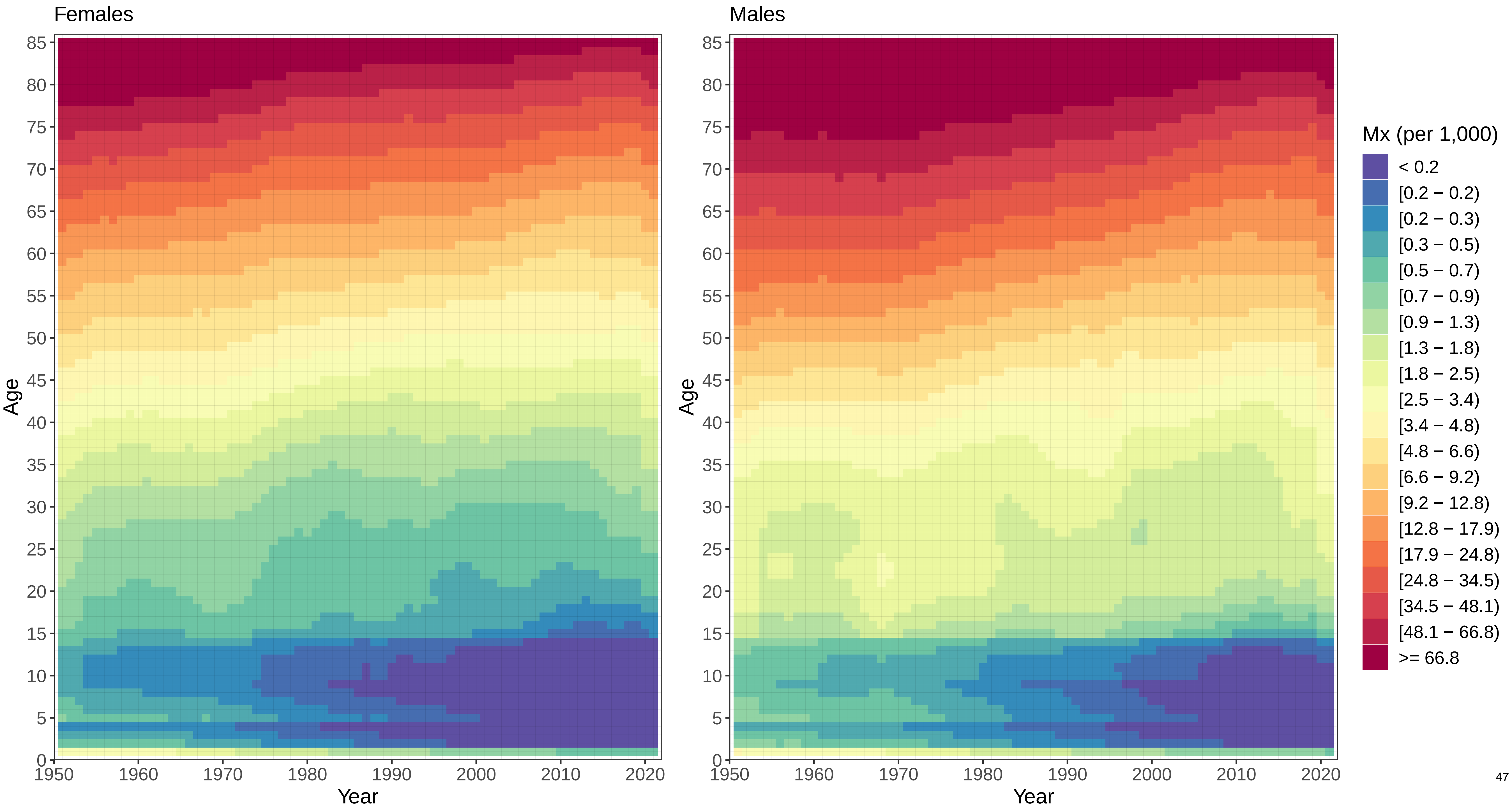

Appendix 2 Figure S5: Argentina mortality rates, 1950−2021

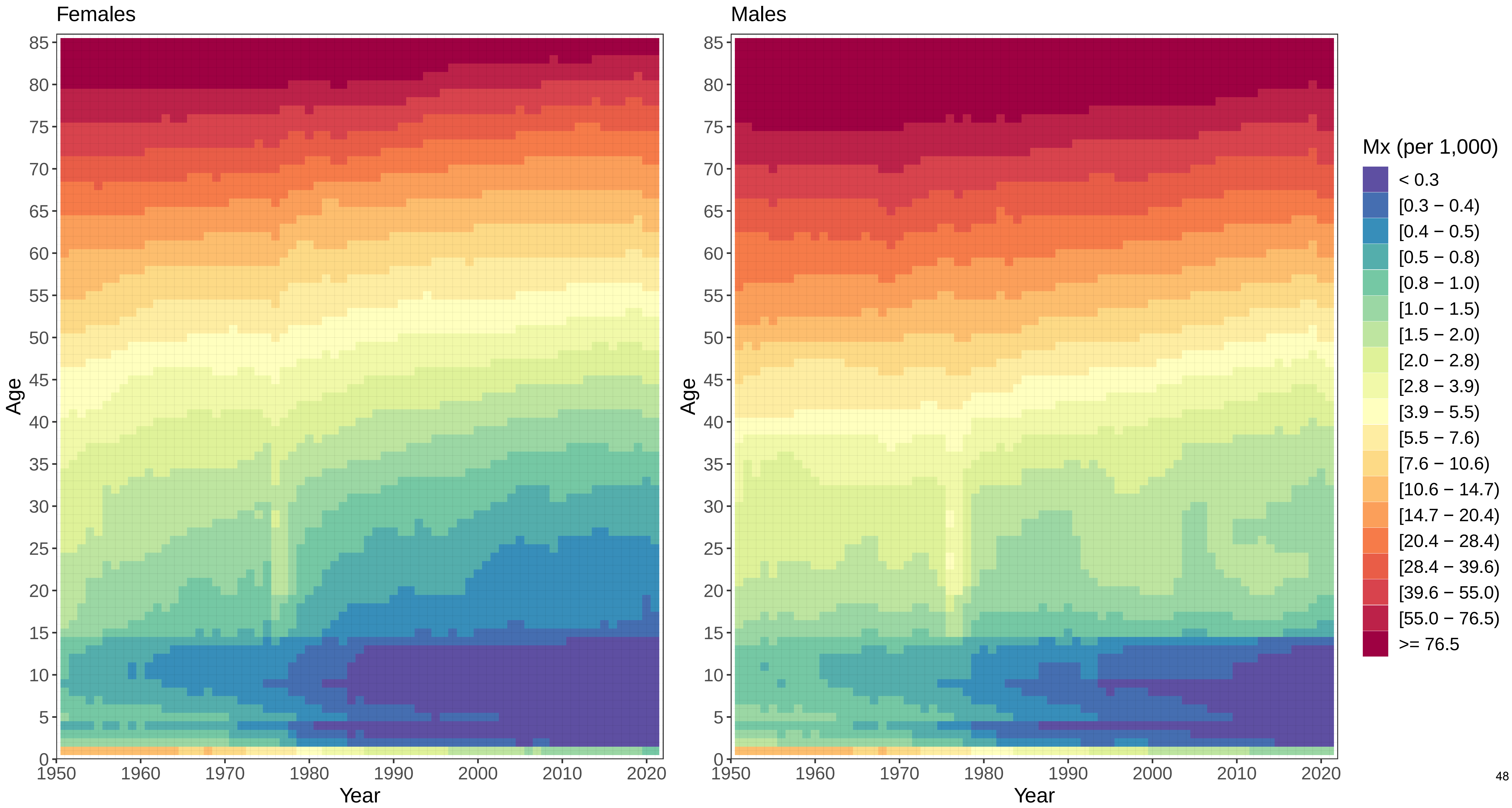

Appendix 2 Figure S5: Chile mortality rates, 1950−2021

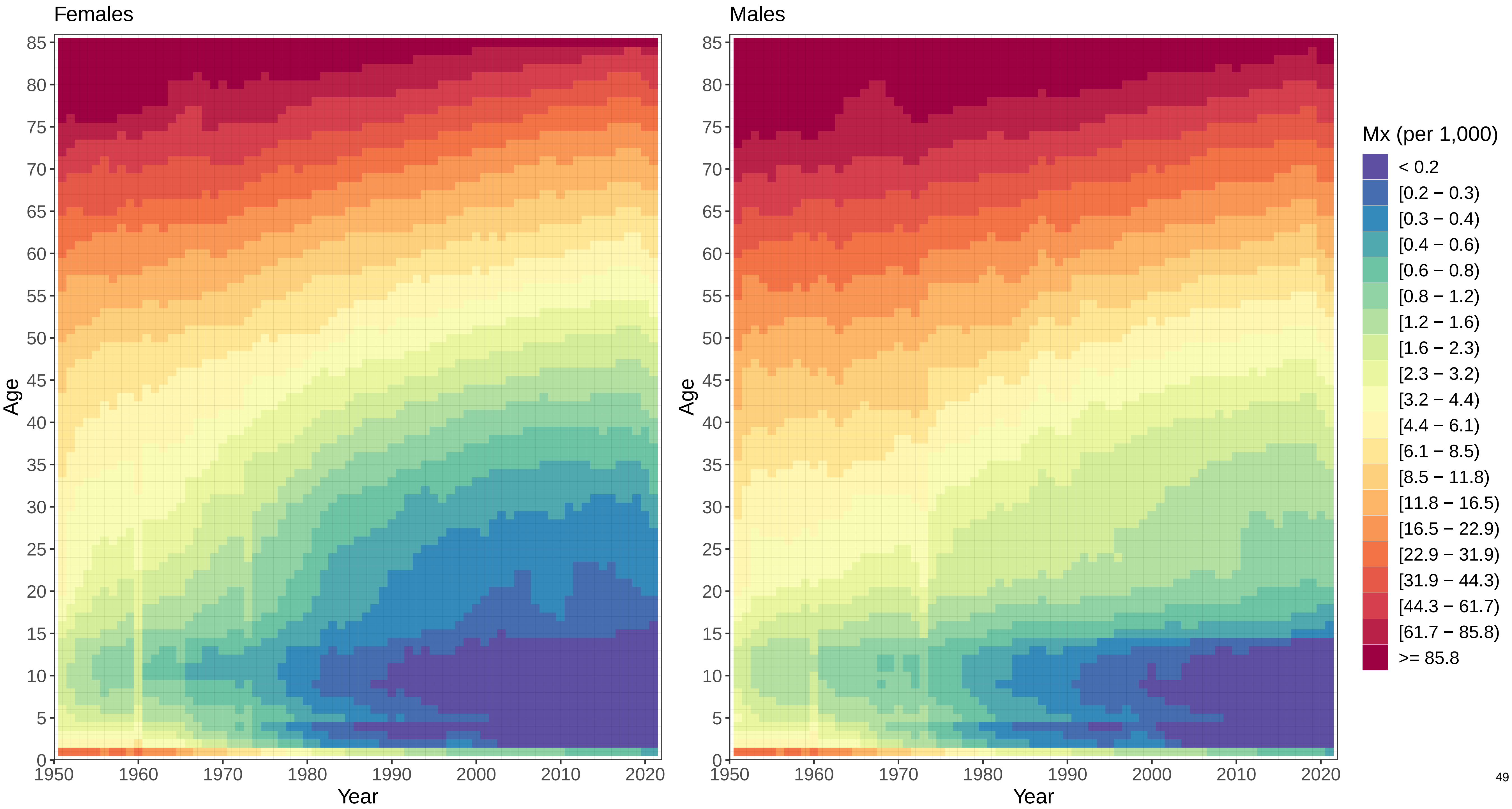

Appendix 2 Figure S5: Uruguay mortality rates, 1950−2021

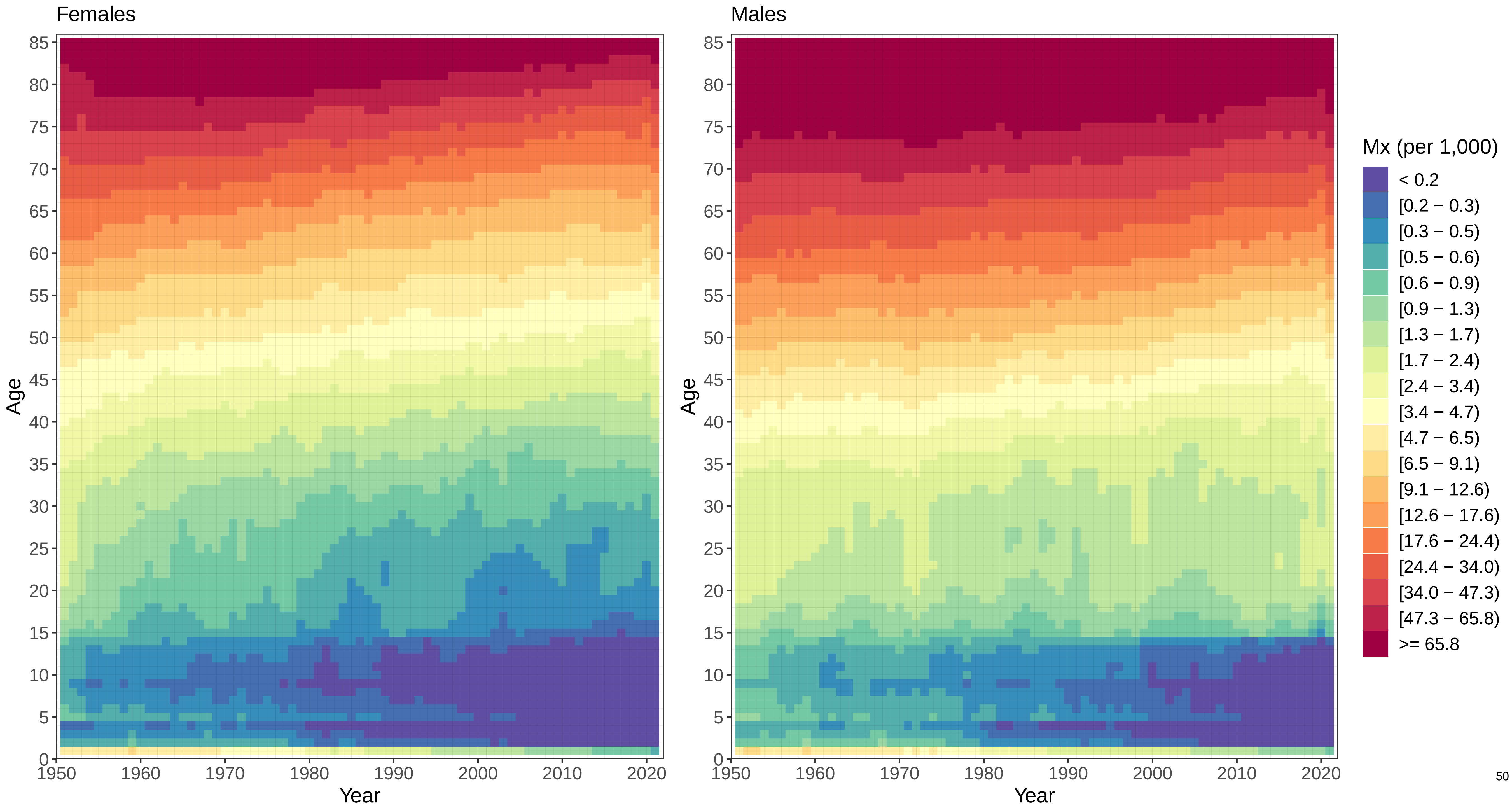

Appendix 2 Figure S5: Andorra mortality rates, 1950−2021

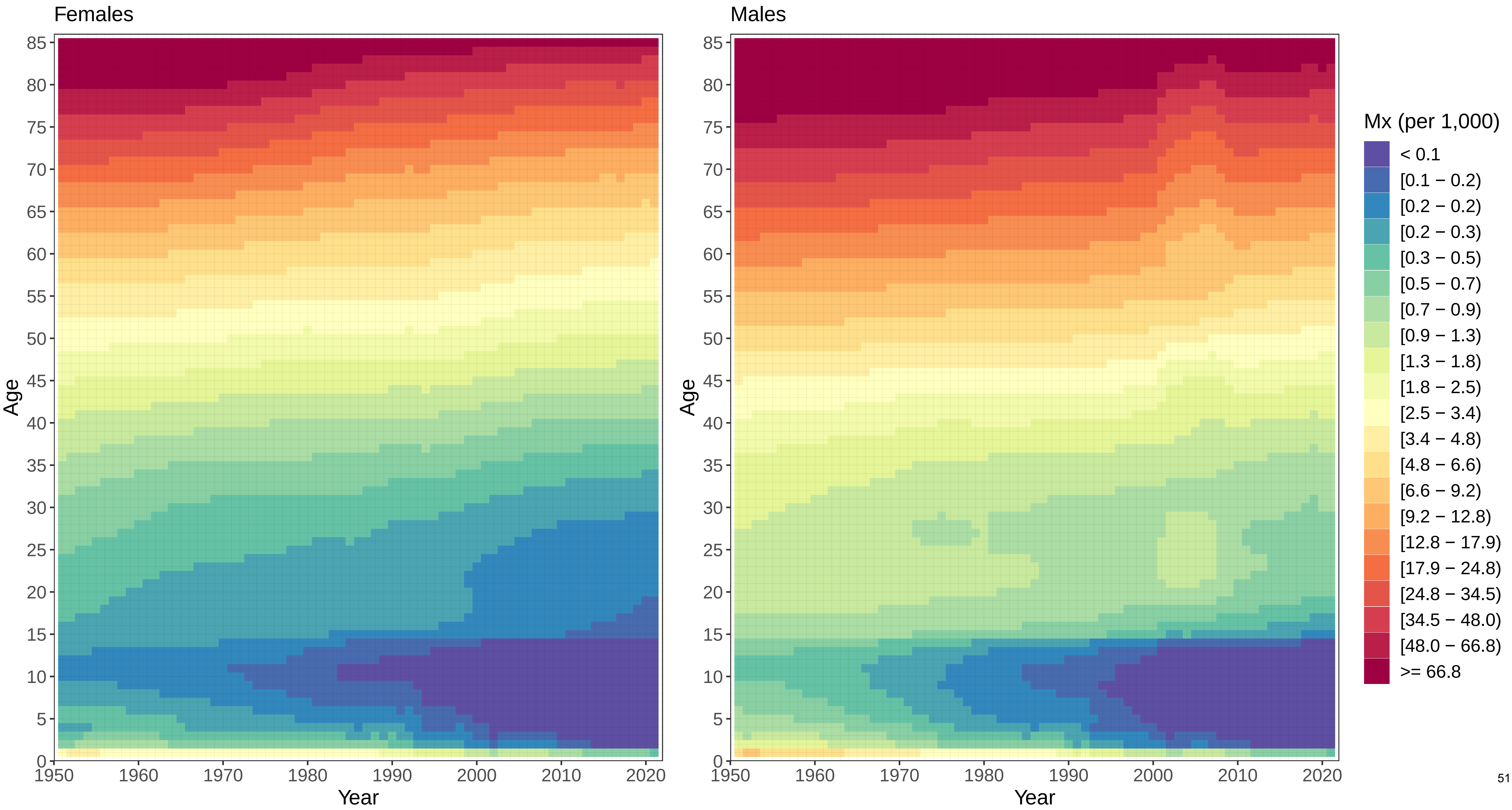

Appendix 2 Figure S5: Austria mortality rates, 1950−2021

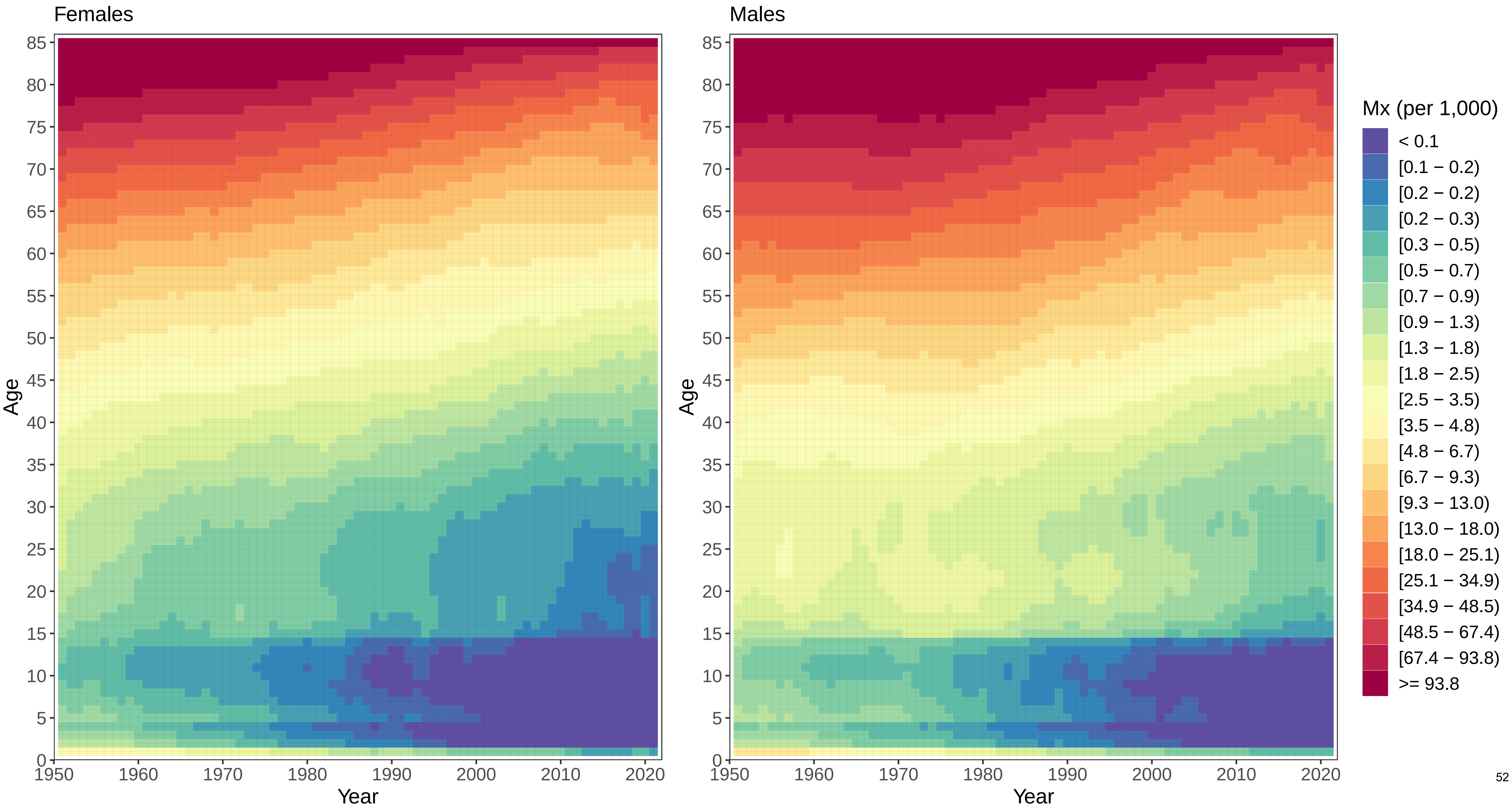

Appendix 2 Figure S5: Belgium mortality rates, 1950−2021

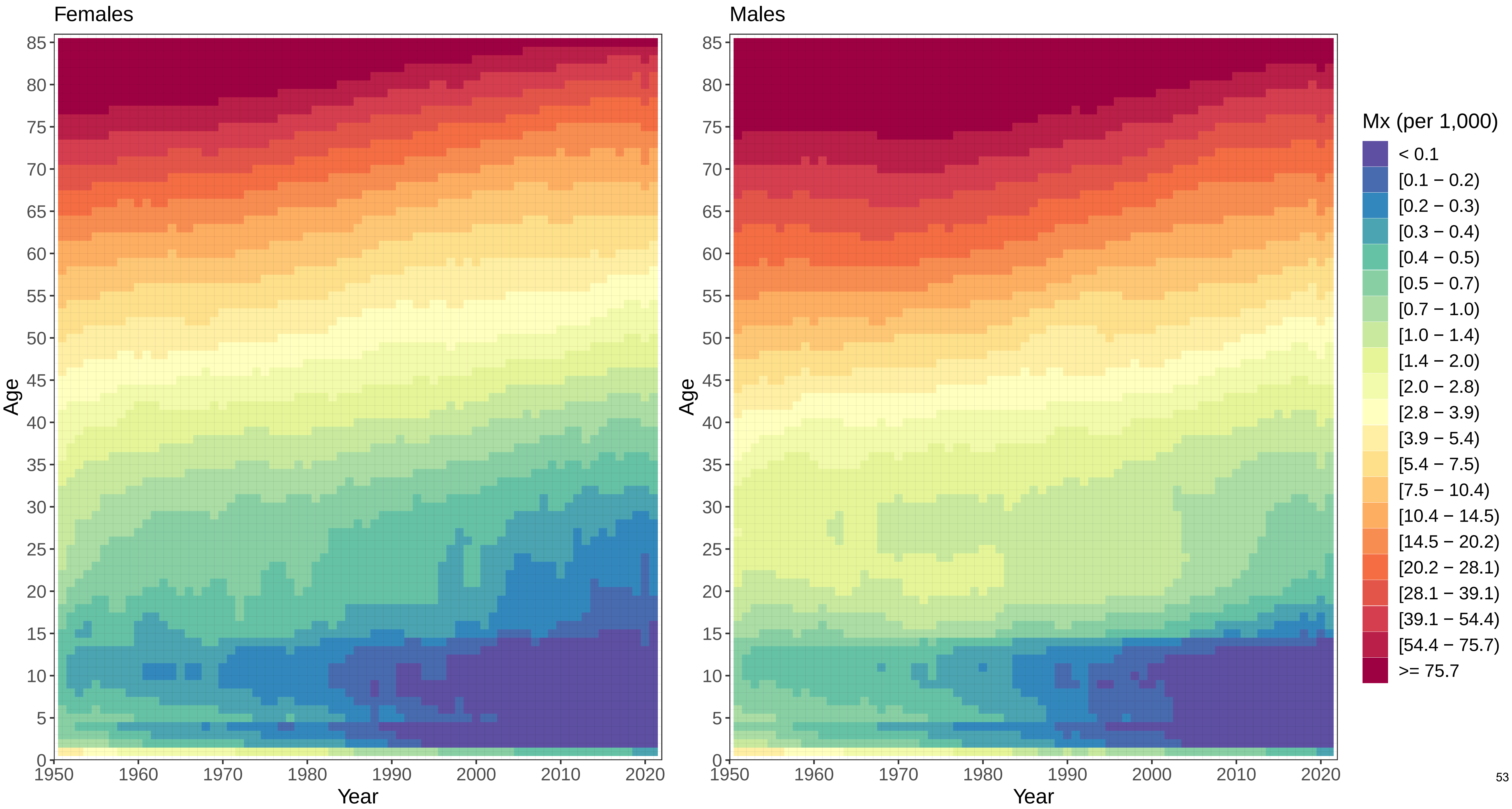

Appendix 2 Figure S5: Cyprus mortality rates, 1950−2021

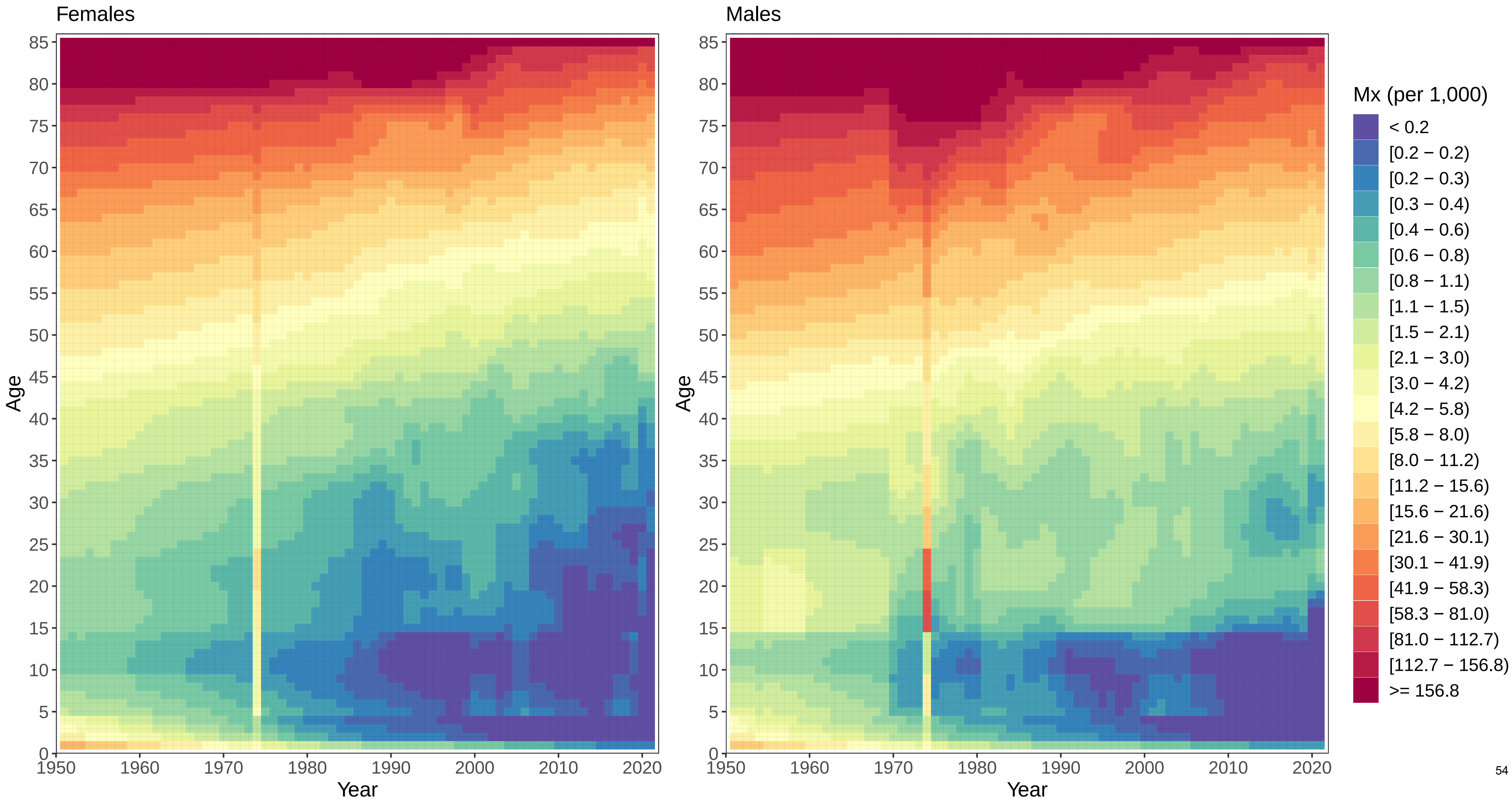

Appendix 2 Figure S5: Denmark mortality rates, 1950−2021

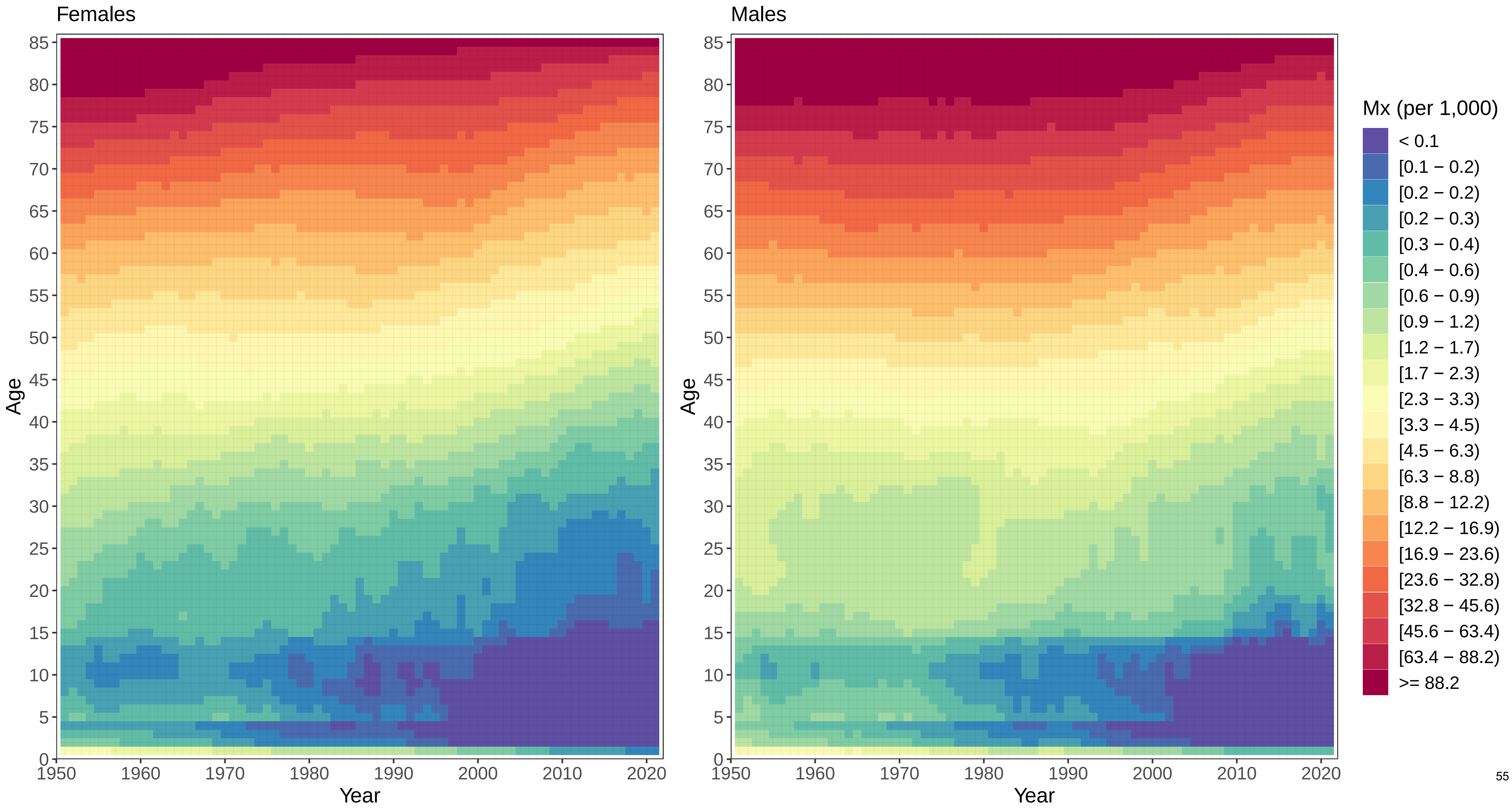

Appendix 2 Figure S5: Finland mortality rates, 1950−2021

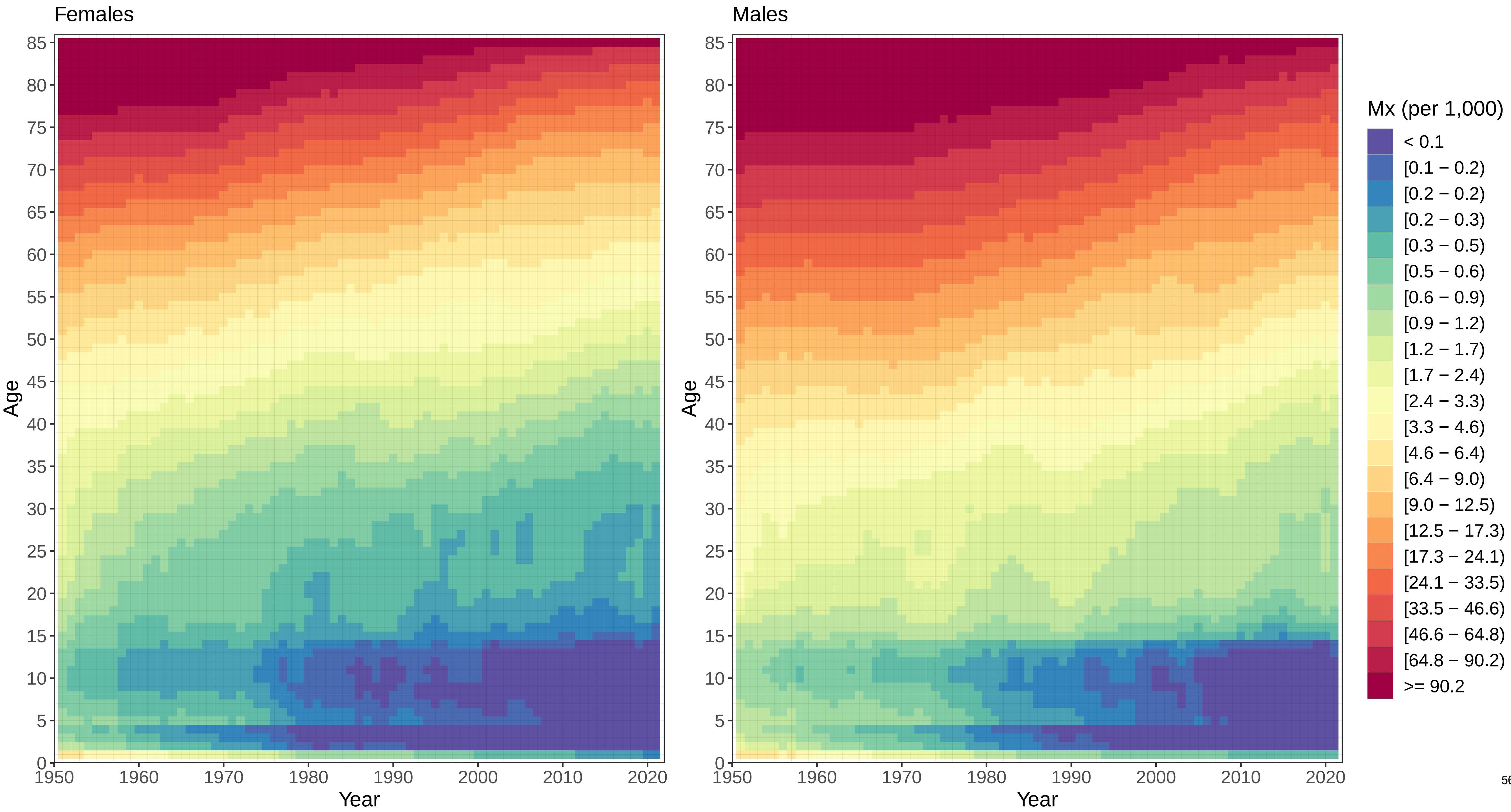

Appendix 2 Figure S5: France mortality rates, 1950−2021

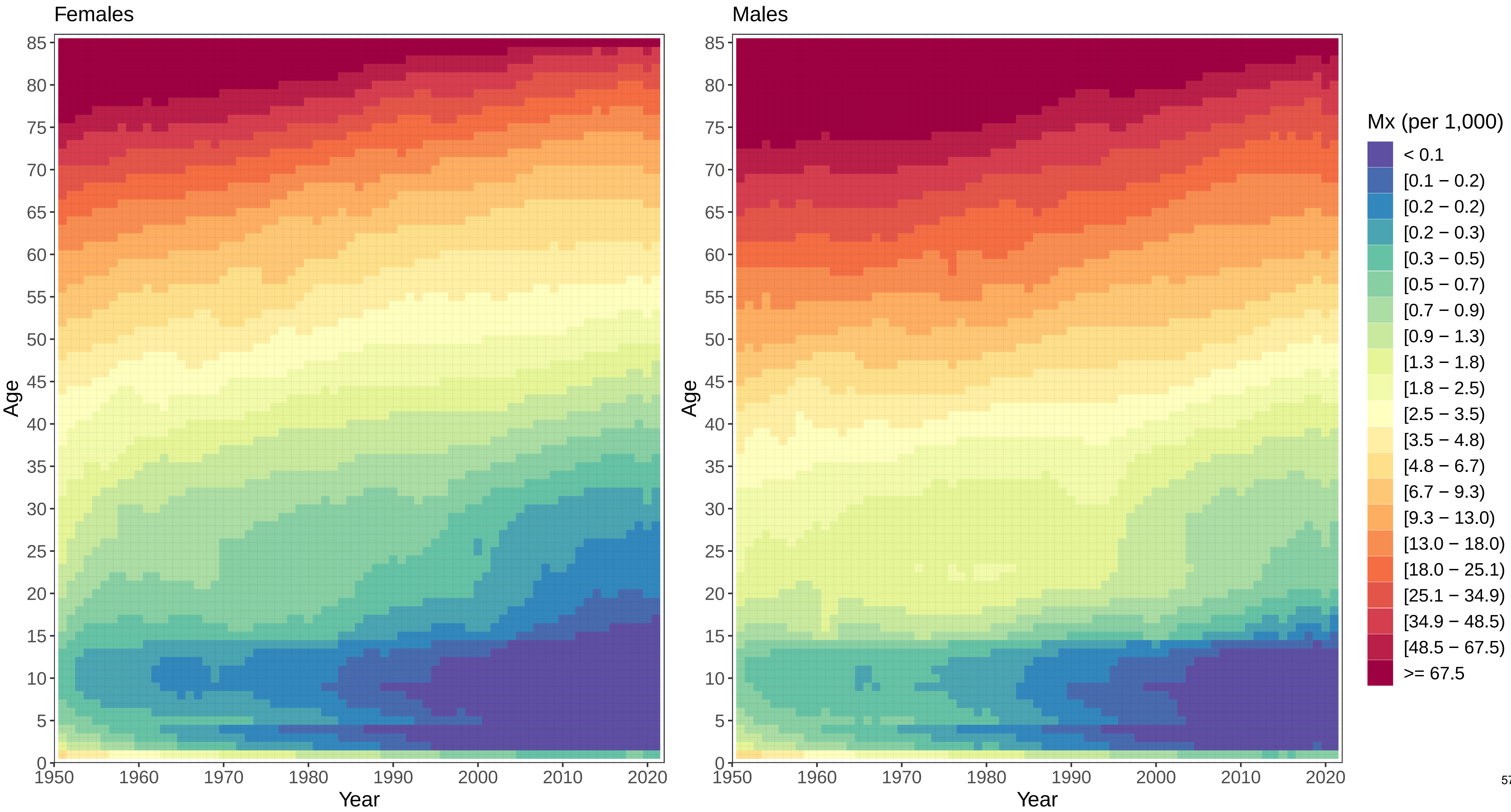

Appendix 2 Figure S5: Germany mortality rates, 1950−2021

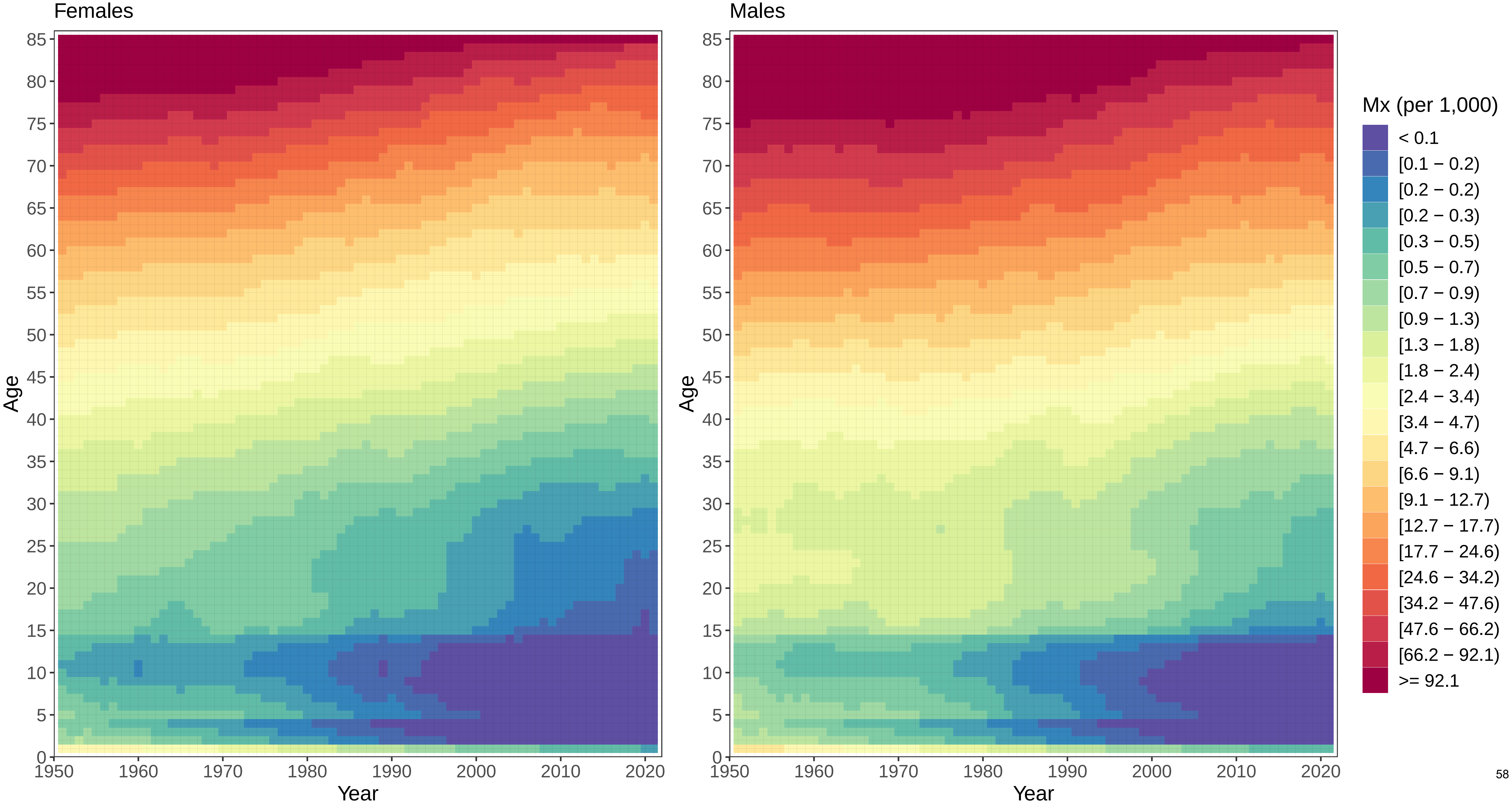

Appendix 2 Figure S5: Greece mortality rates, 1950−2021

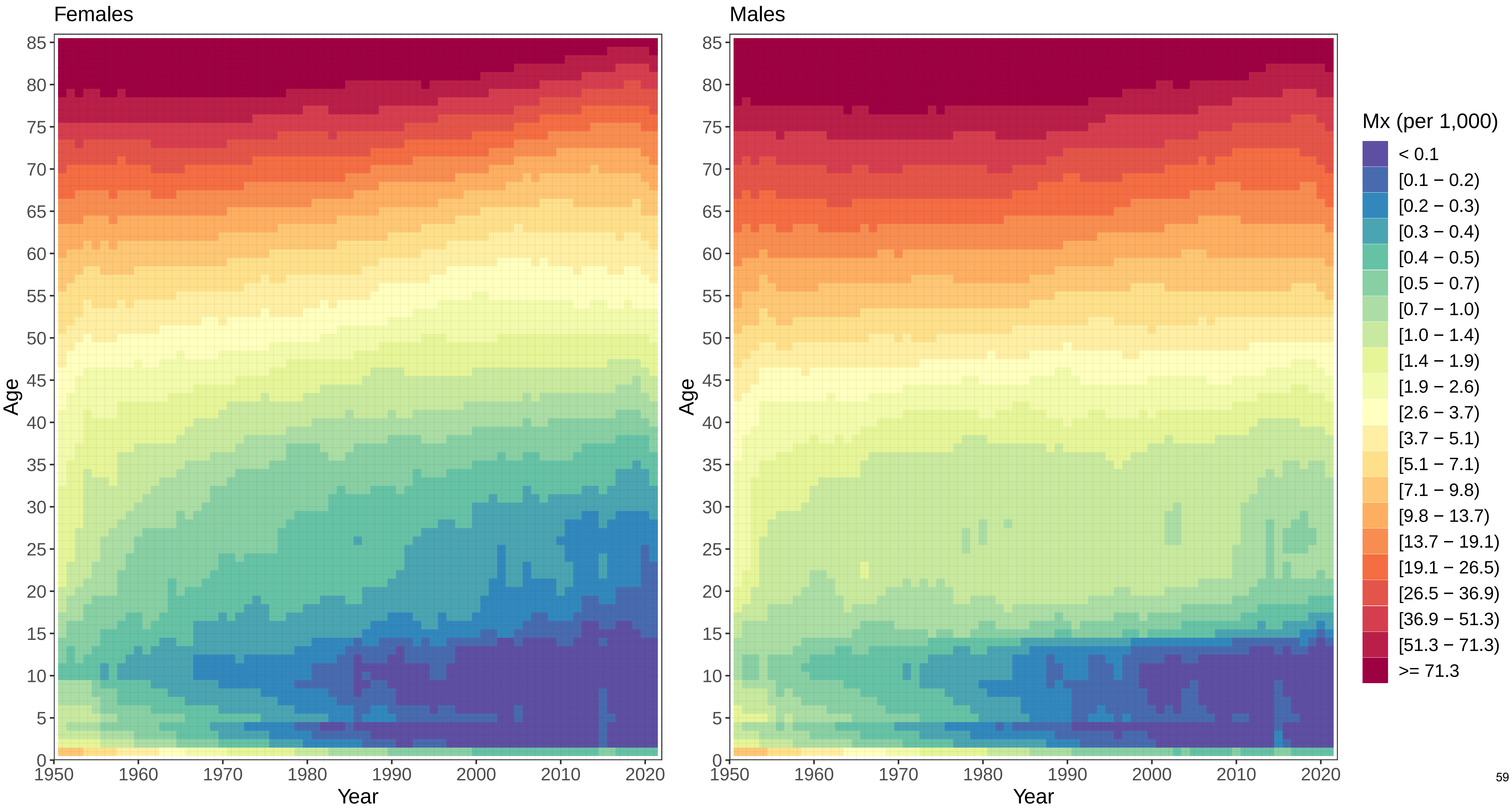

Appendix 2 Figure S5: Iceland mortality rates, 1950−2021

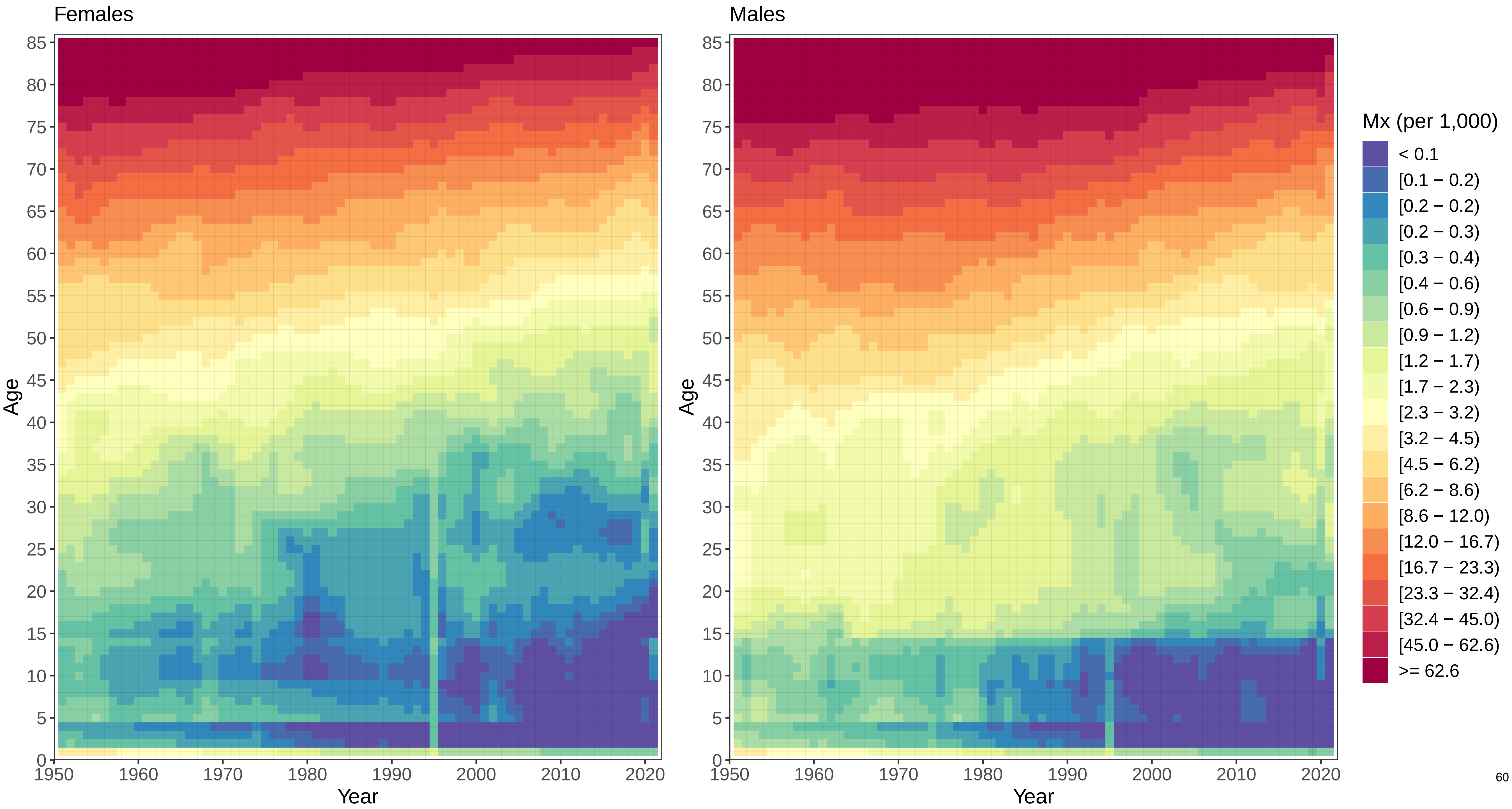

Appendix 2 Figure S5: Ireland mortality rates, 1950−2021

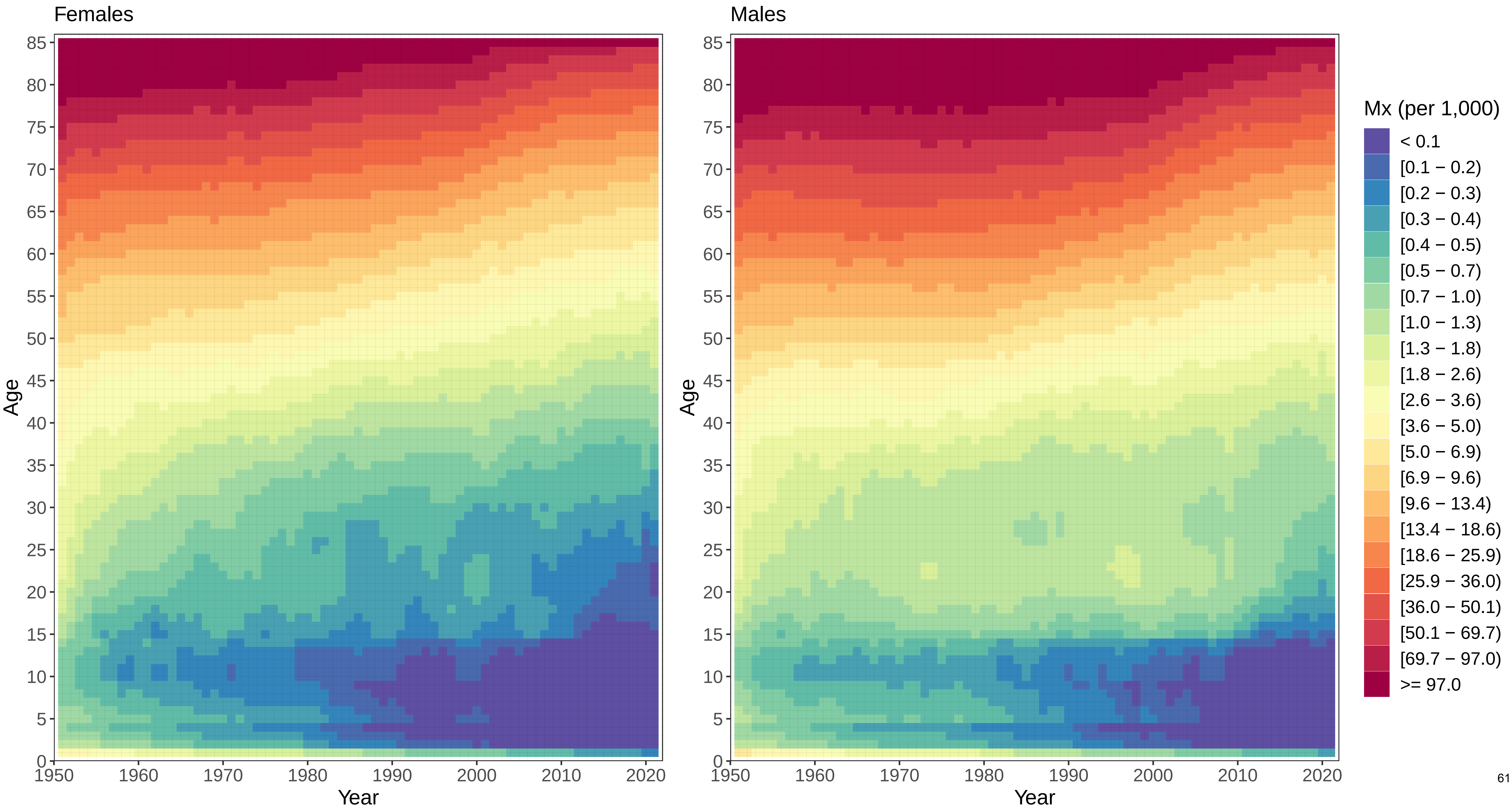

Appendix 2 Figure S5: Israel mortality rates, 1950−2021

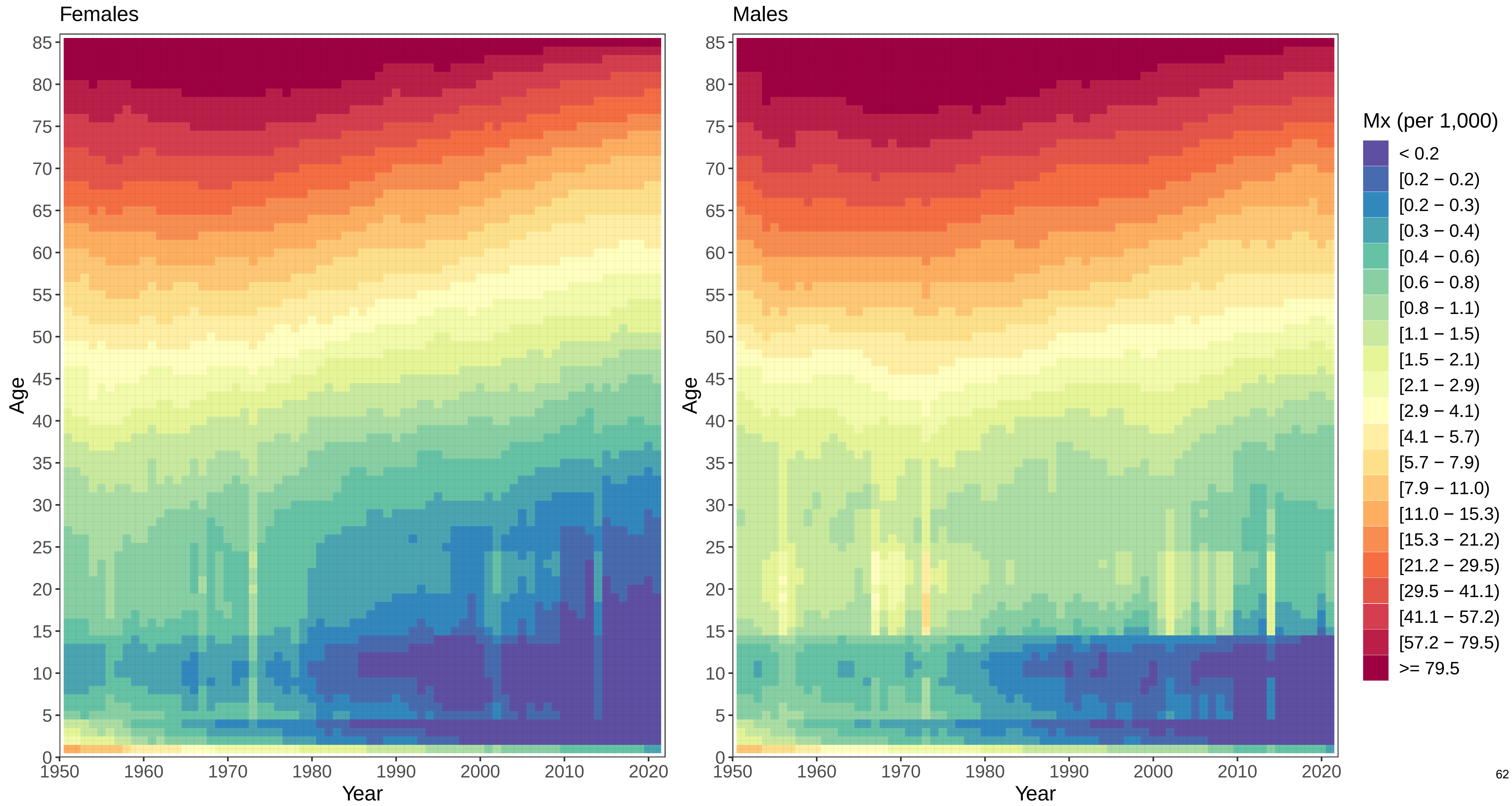

Appendix 2 Figure S5: Italy mortality rates, 1950−2021

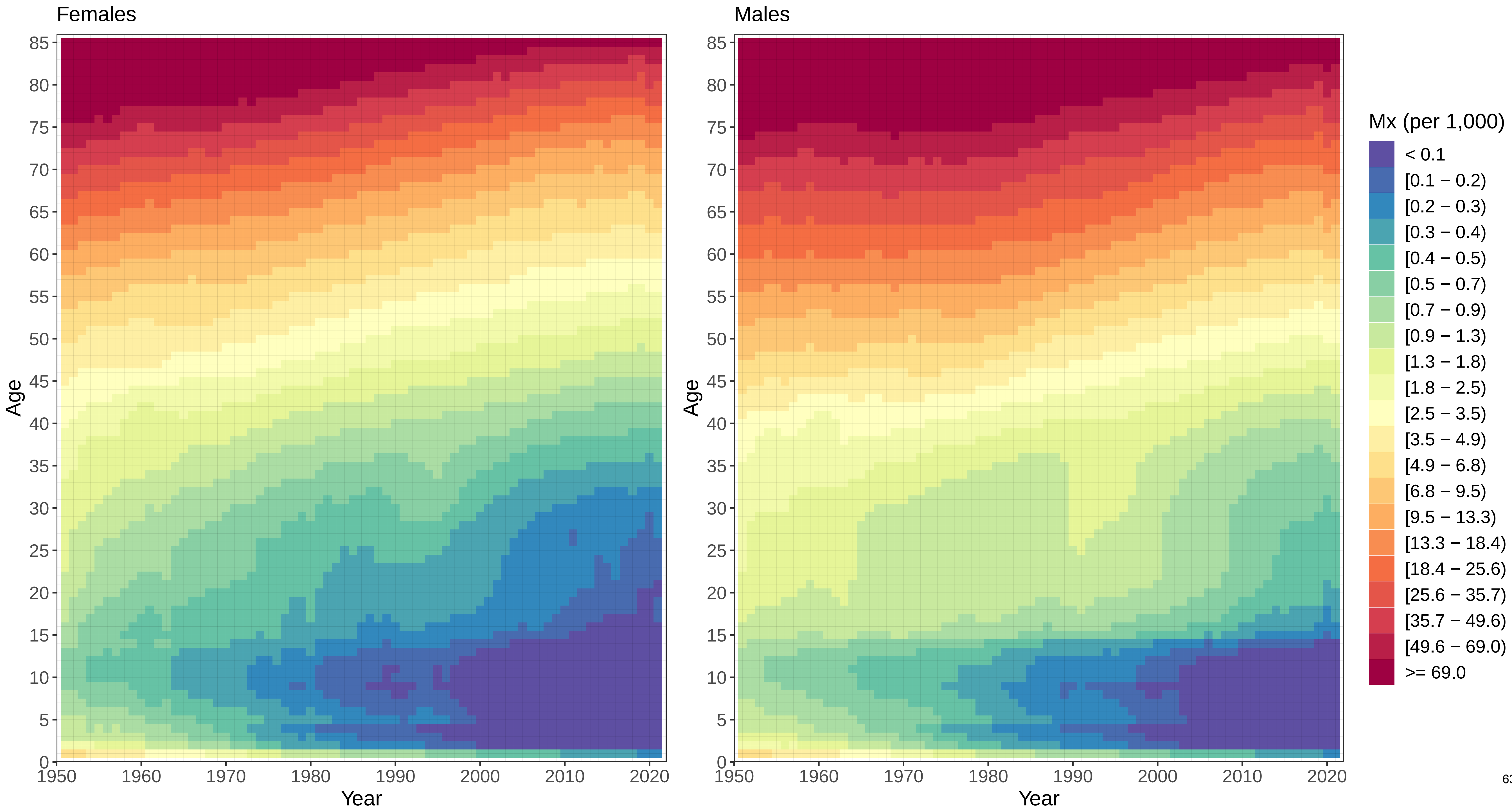

Appendix 2 Figure S5: Luxembourg mortality rates, 1950−2021

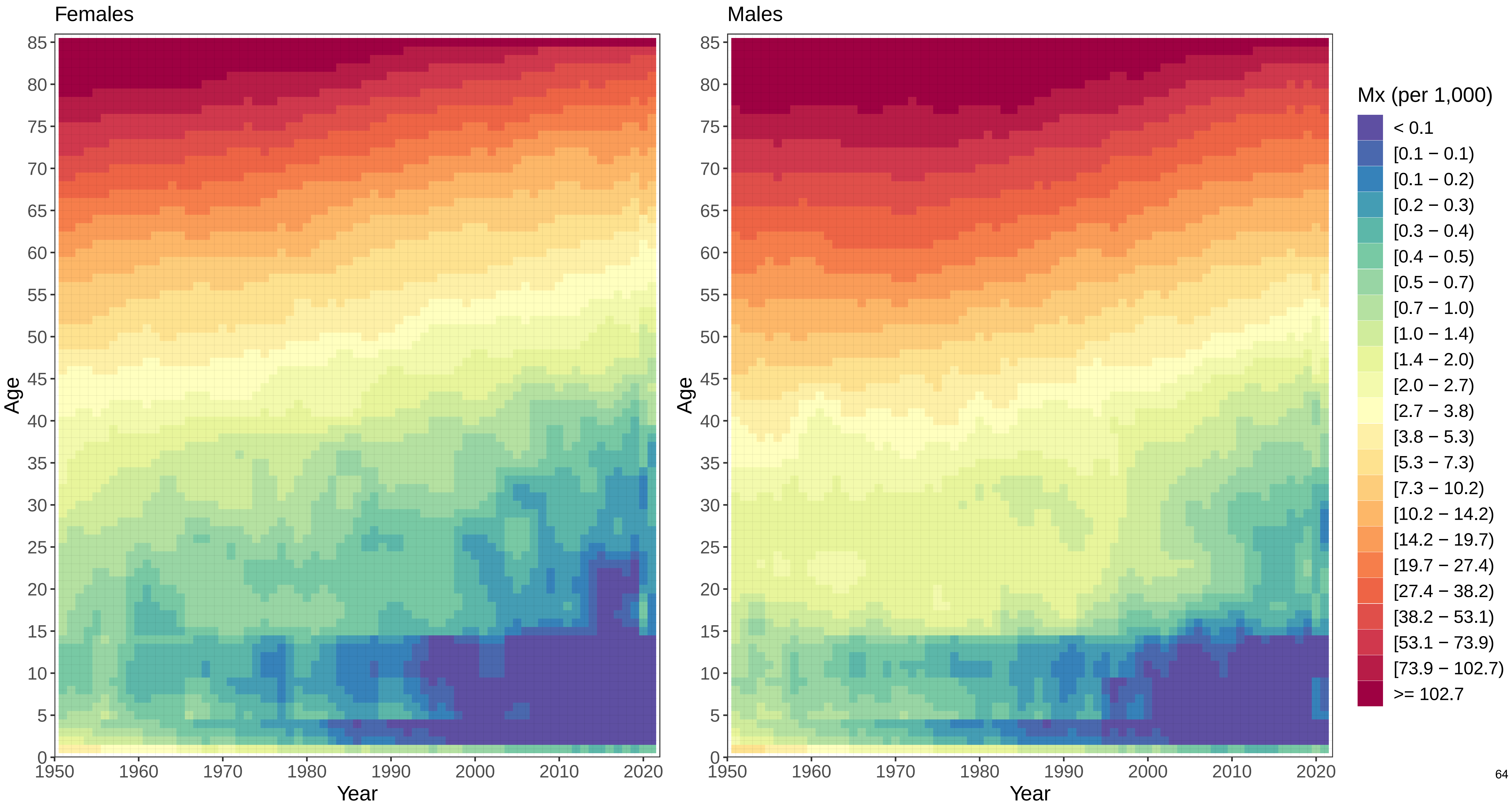

Appendix 2 Figure S5: Malta mortality rates, 1950−2021

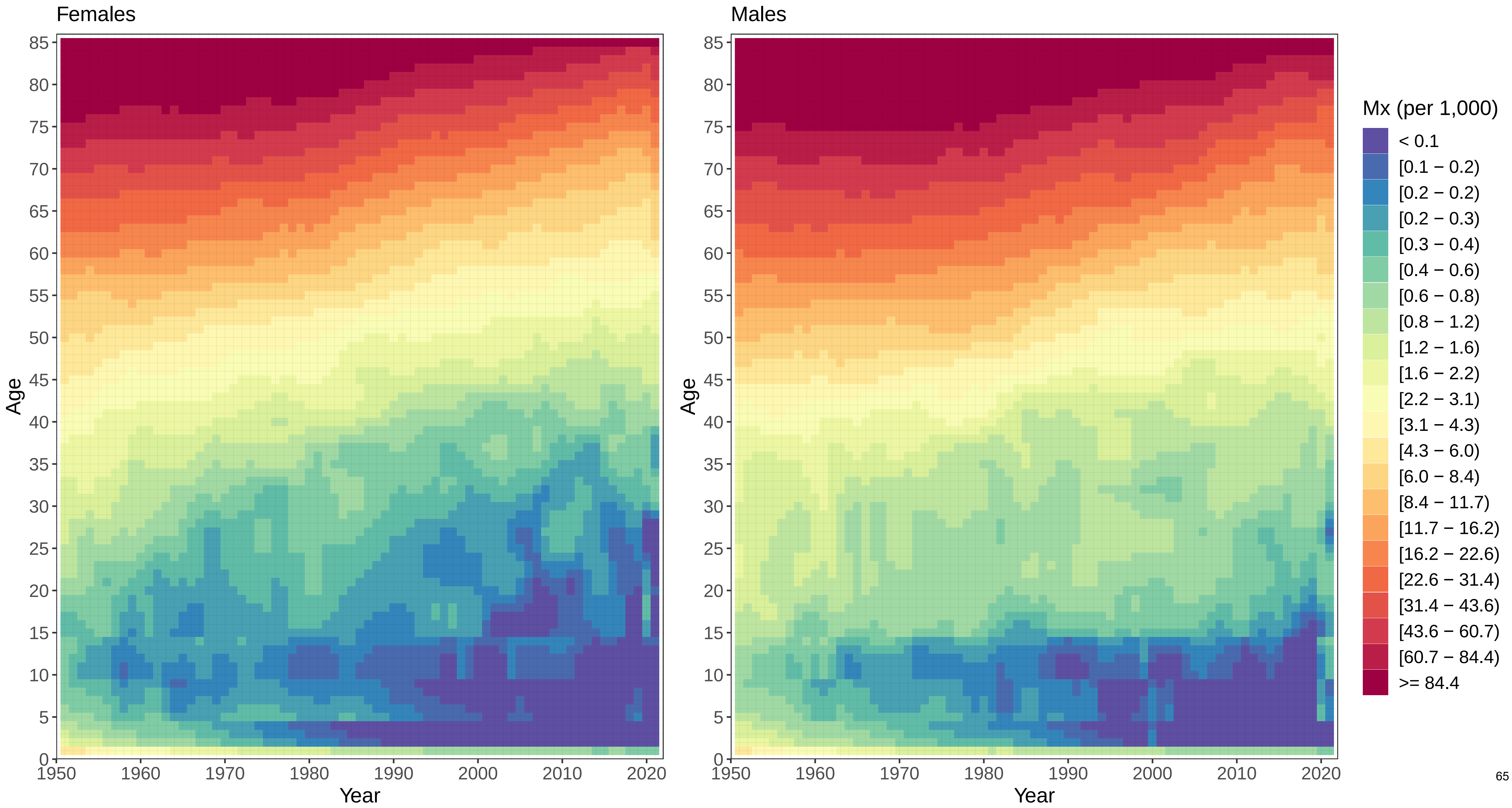

Appendix 2 Figure S5: Monaco mortality rates, 1950−2021

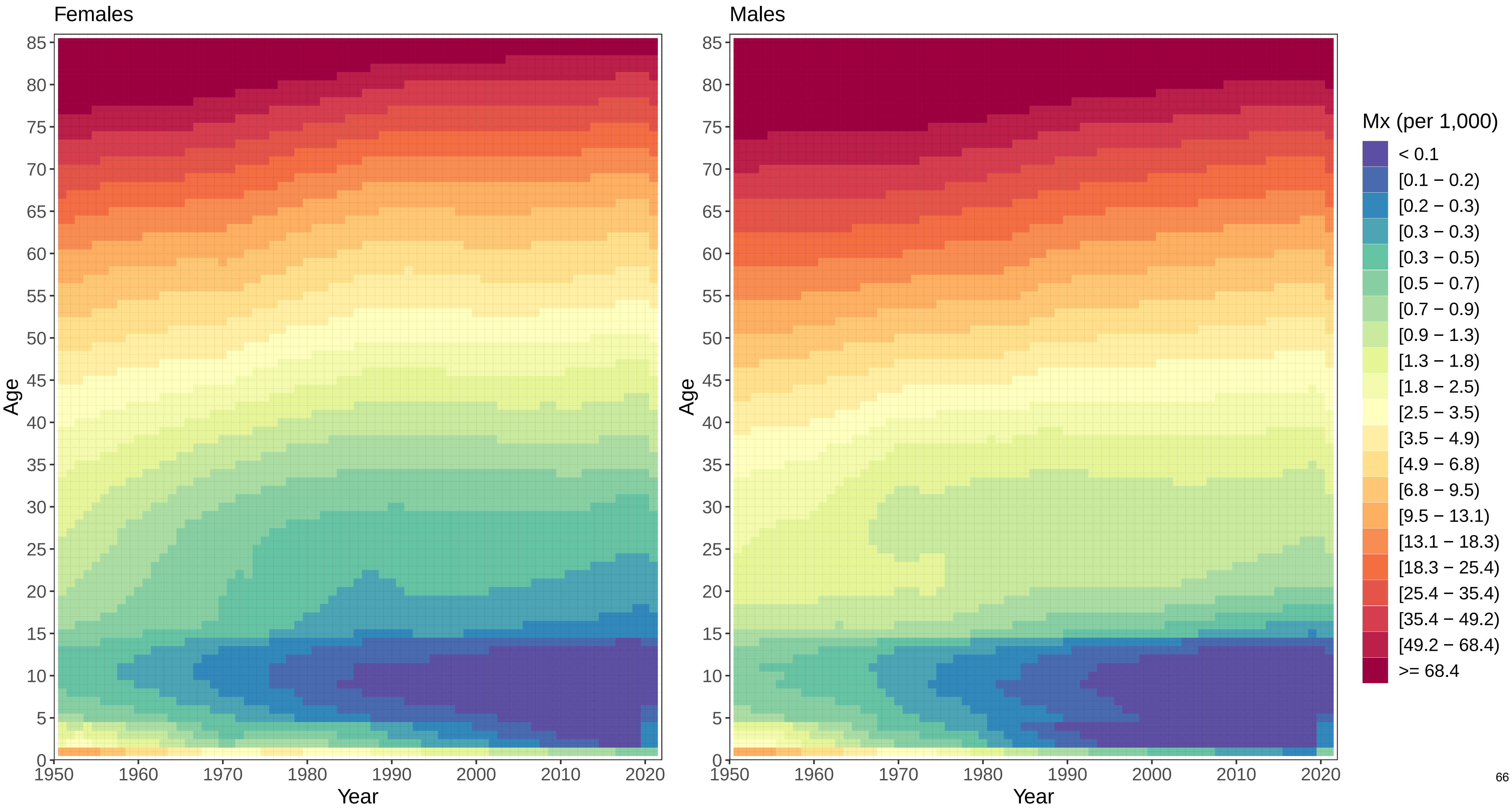

Appendix 2 Figure S5: Netherlands mortality rates, 1950−2021

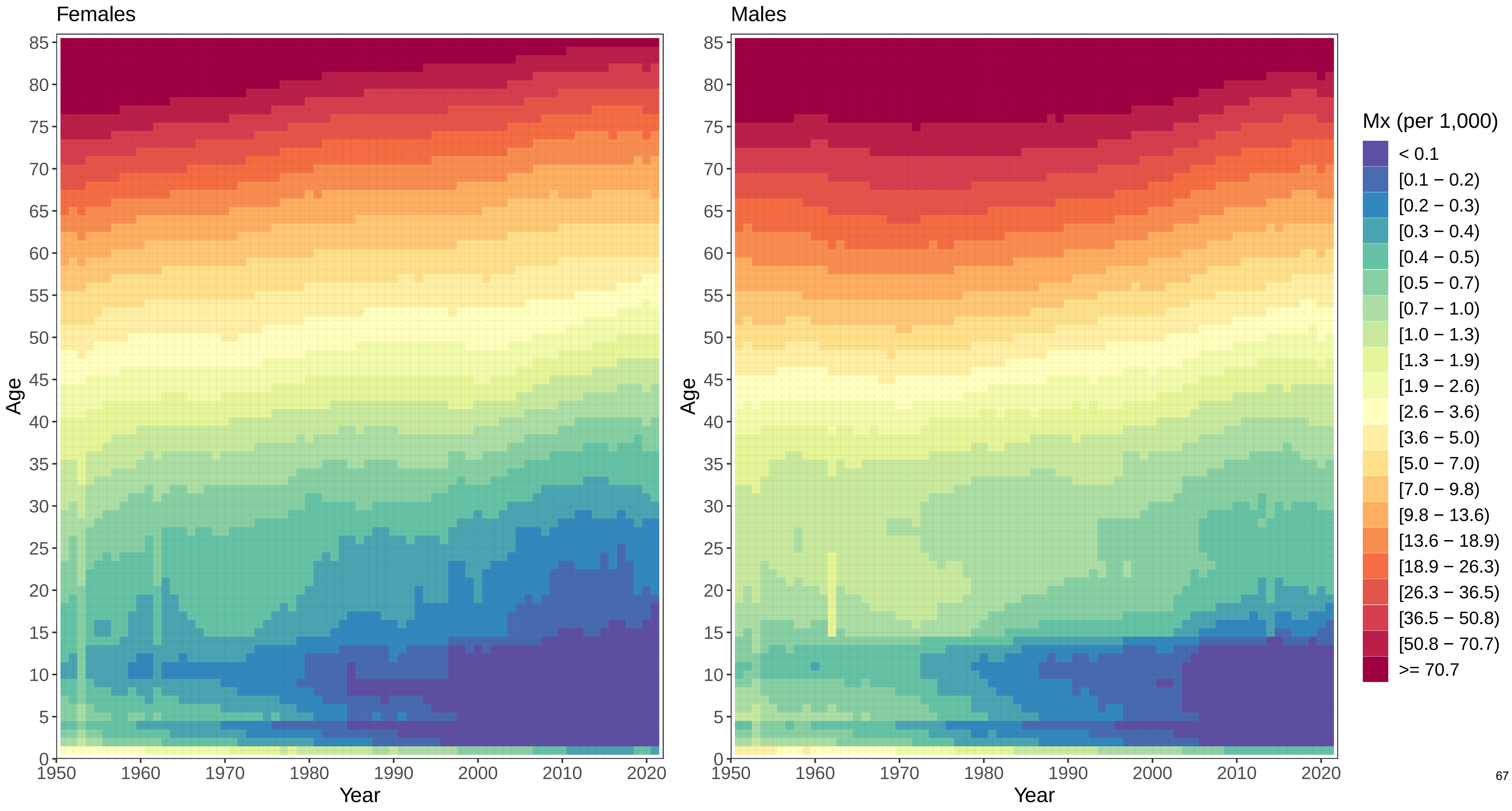

Appendix 2 Figure S5: Norway mortality rates, 1950−2021

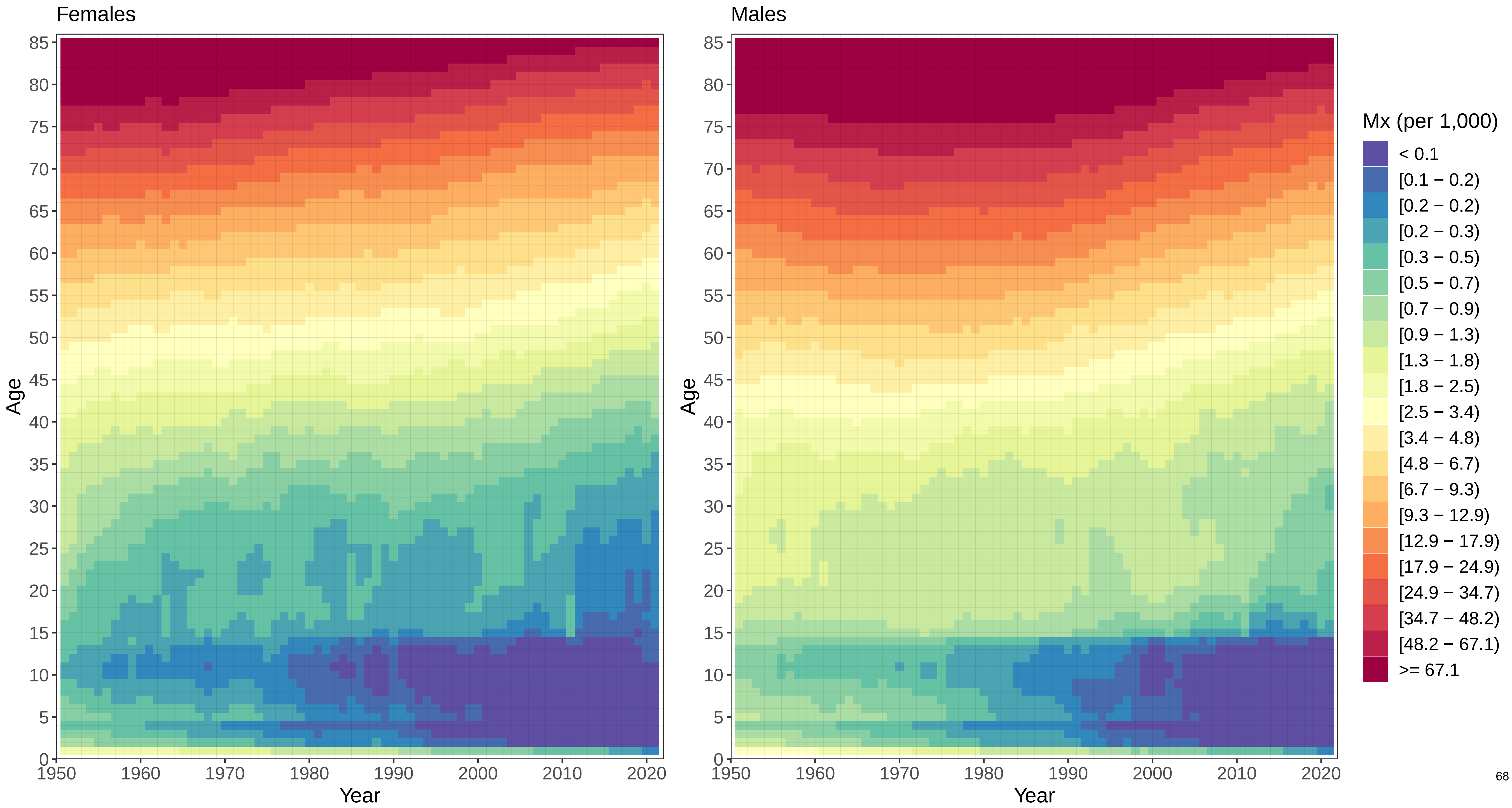

Appendix 2 Figure S5: Portugal mortality rates, 1950−2021

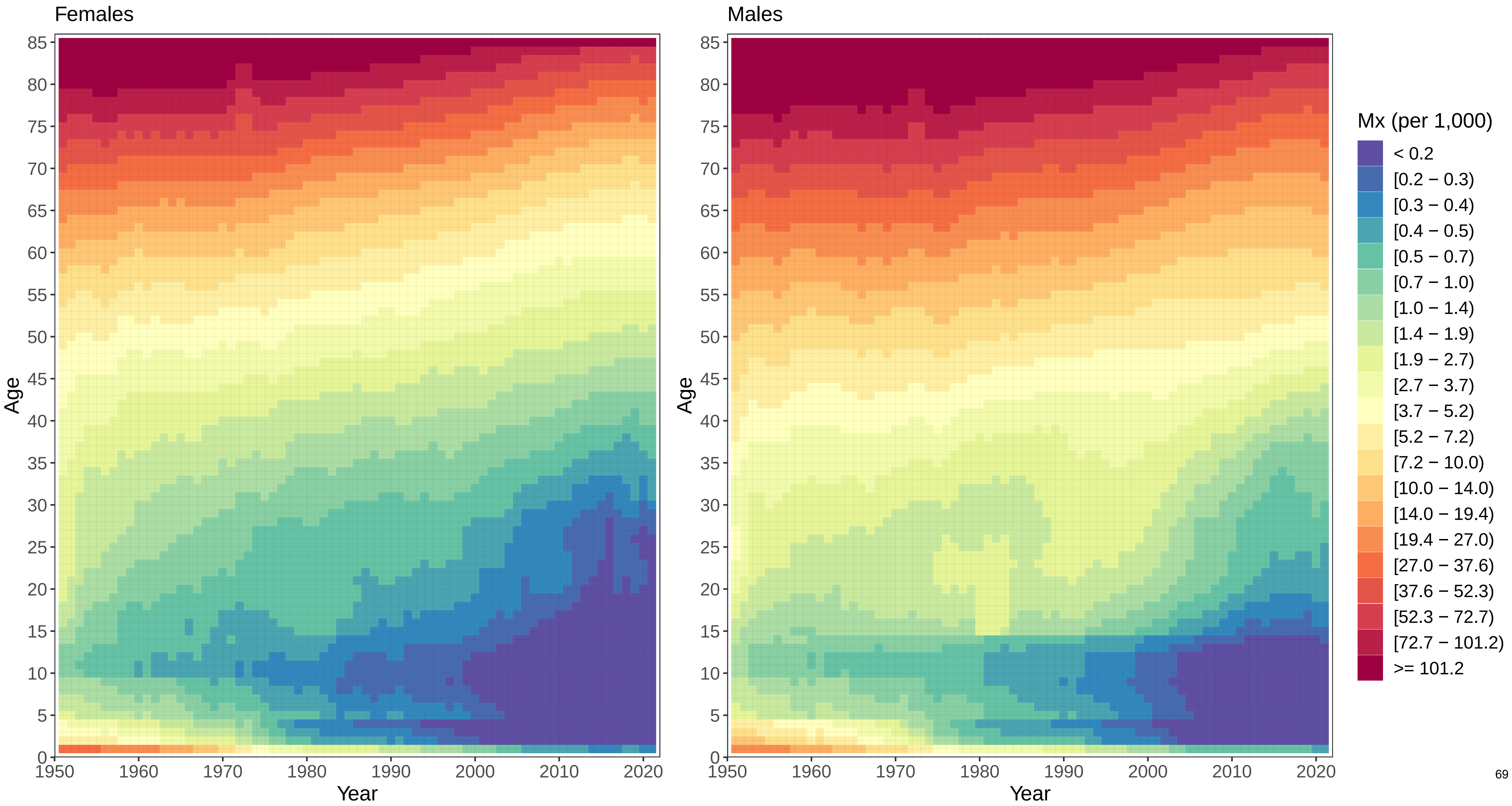

Appendix 2 Figure S5: San Marino mortality rates, 1950−2021

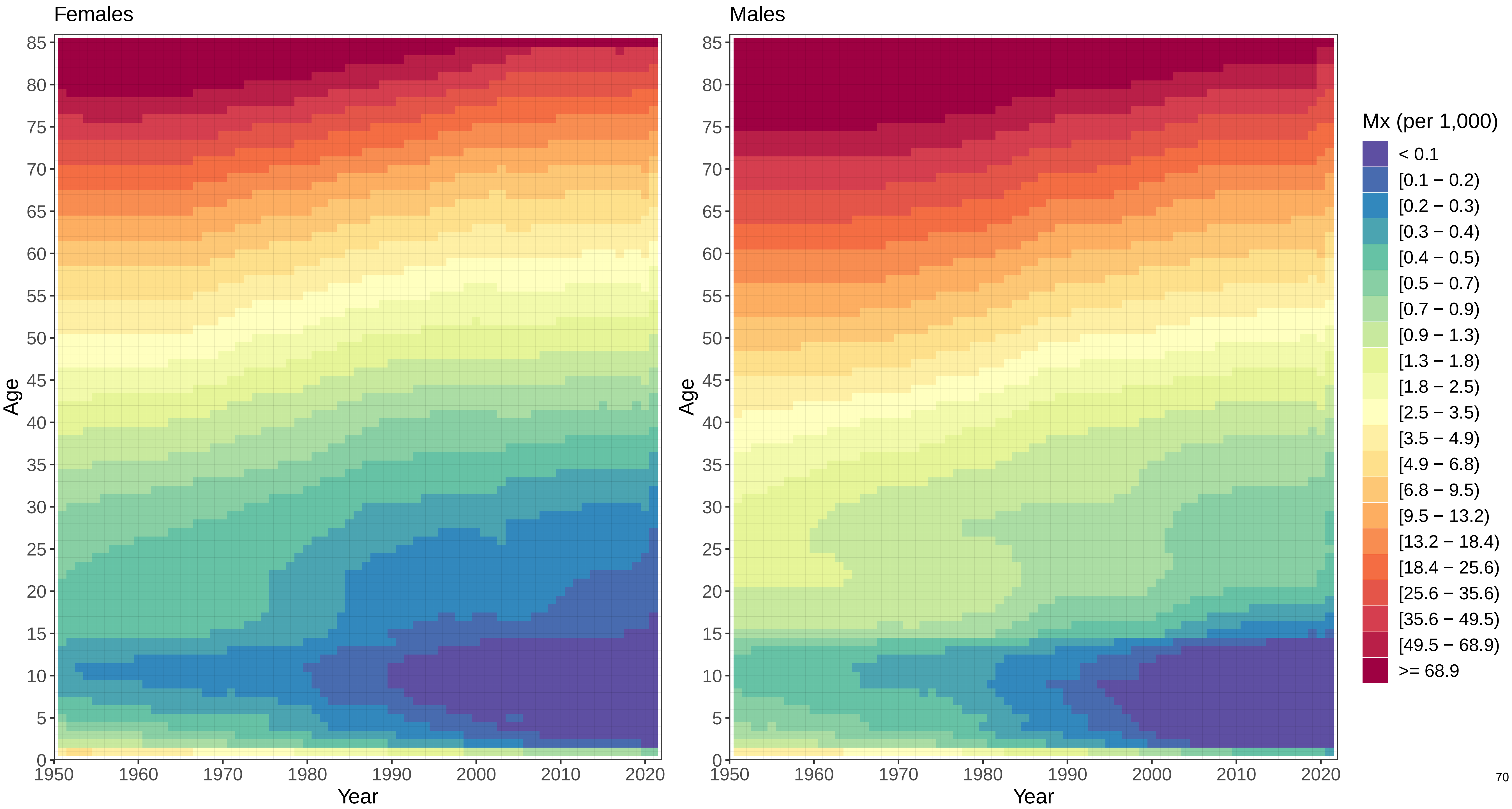

Appendix 2 Figure S5: Spain mortality rates, 1950−2021

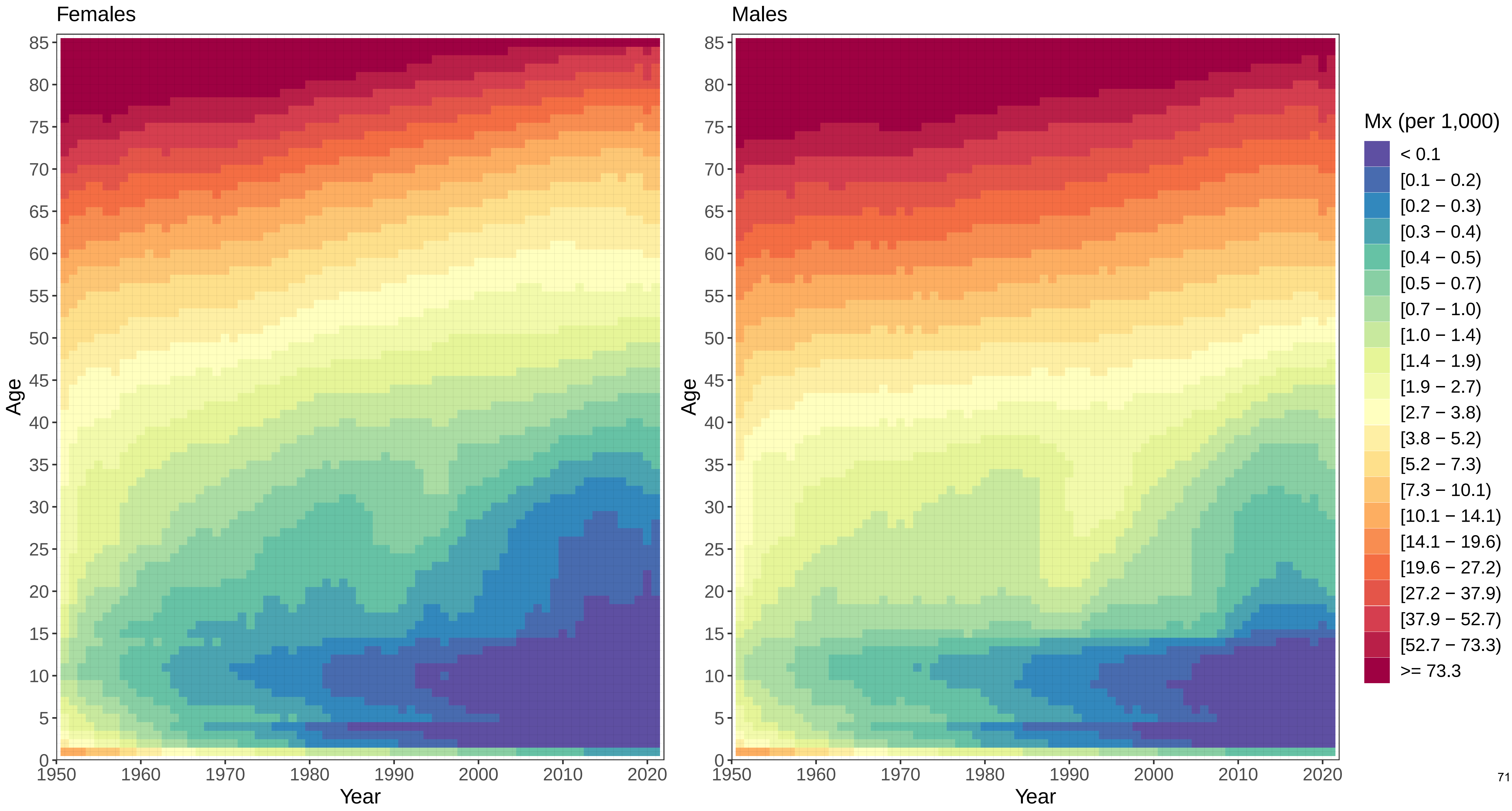

Appendix 2 Figure S5: Sweden mortality rates, 1950−2021

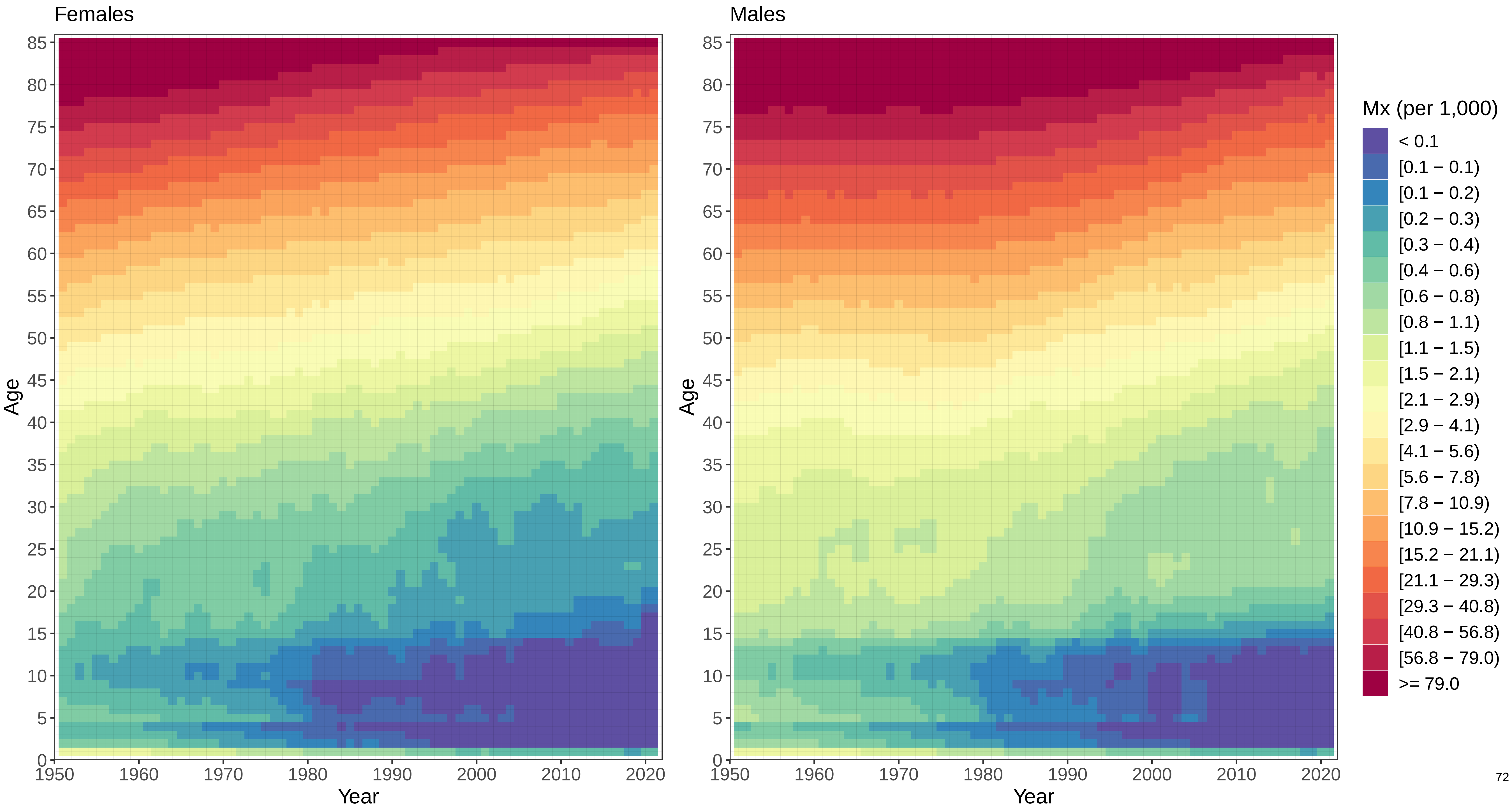

Appendix 2 Figure S5: Switzerland mortality rates, 1950−2021

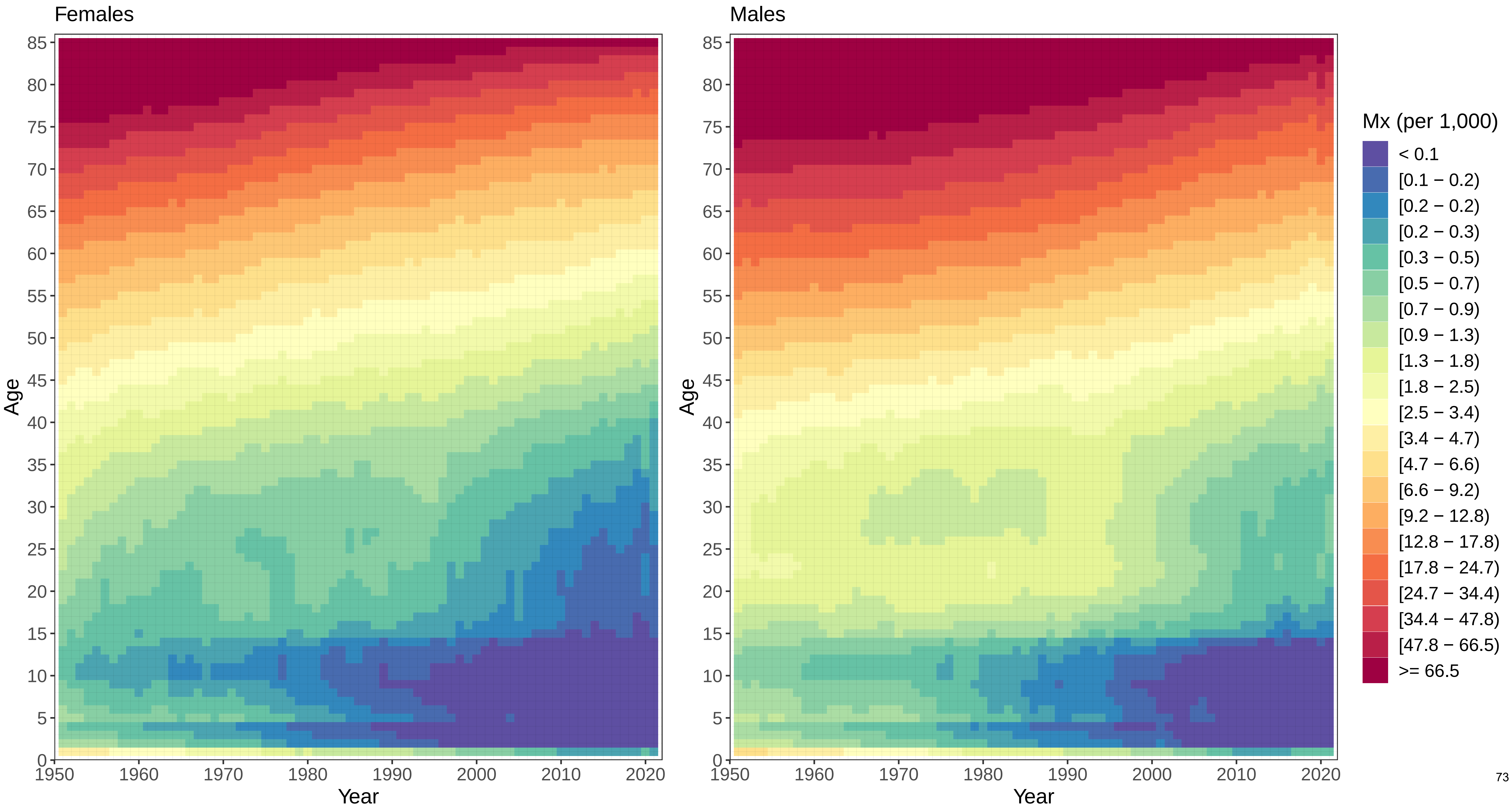

Appendix 2 Figure S5: UK mortality rates, 1950−2021

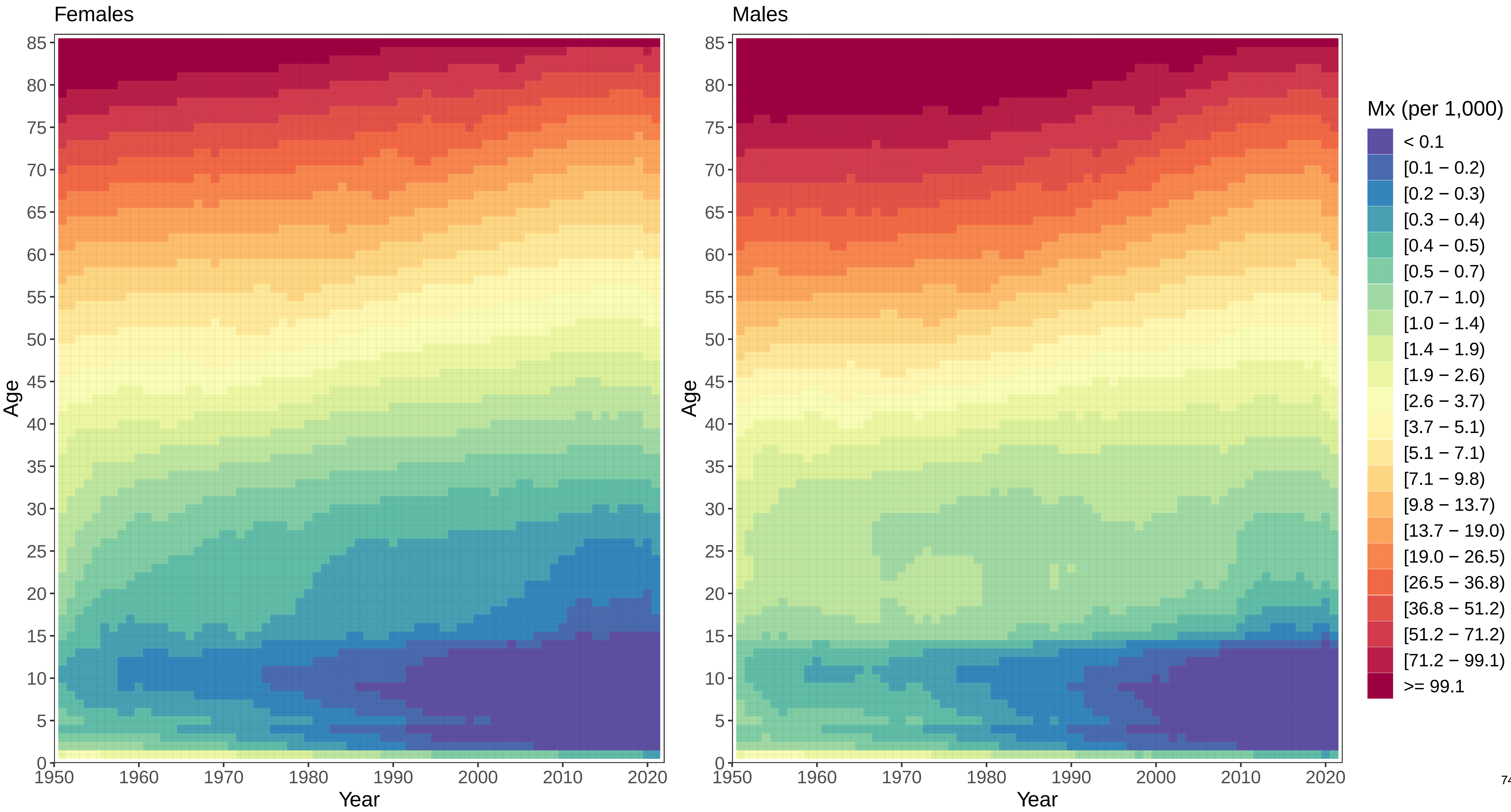

Appendix 2 Figure S5: Bolivia mortality rates, 1950−2021

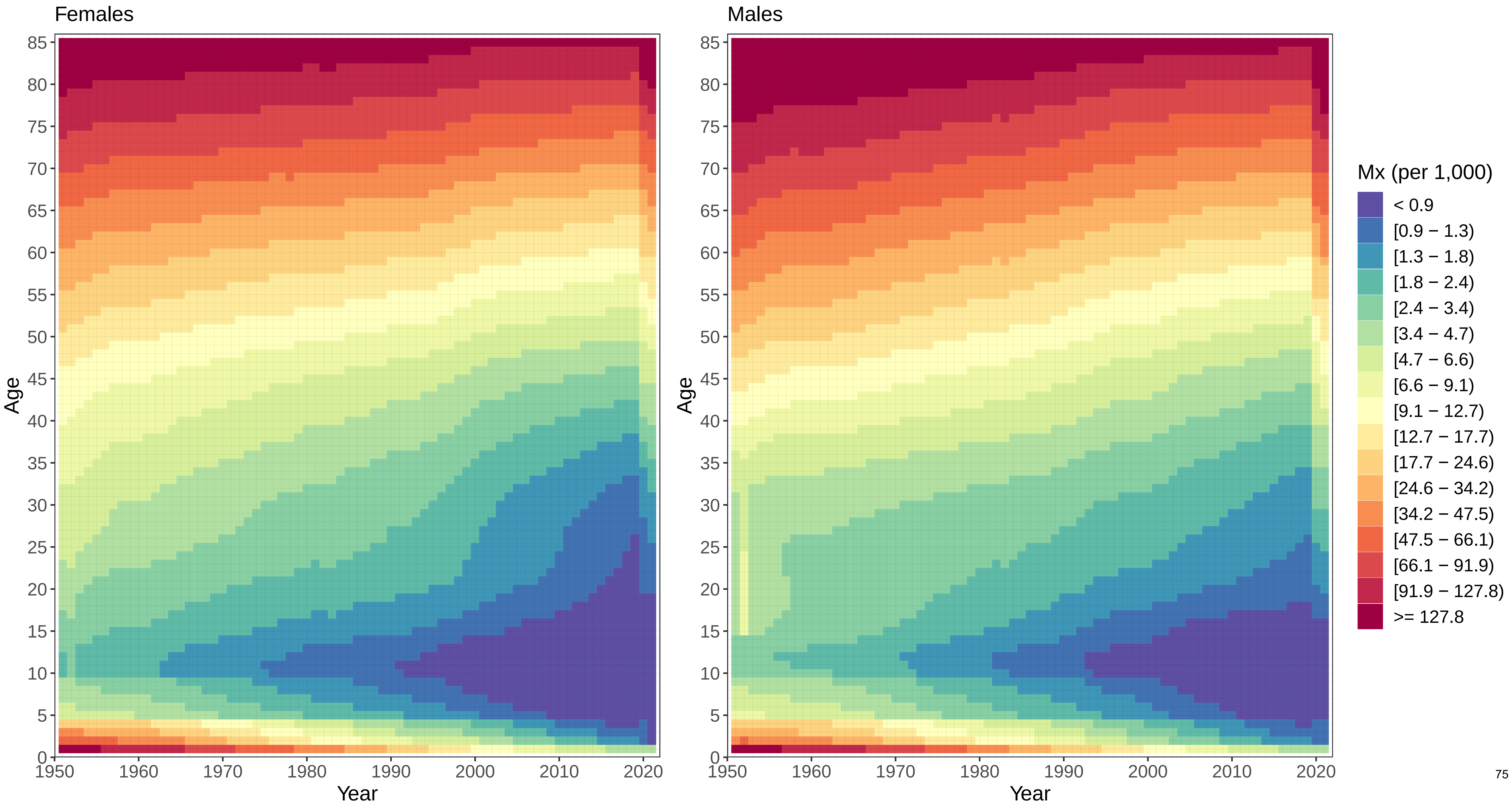

Appendix 2 Figure S5: Ecuador mortality rates, 1950−2021

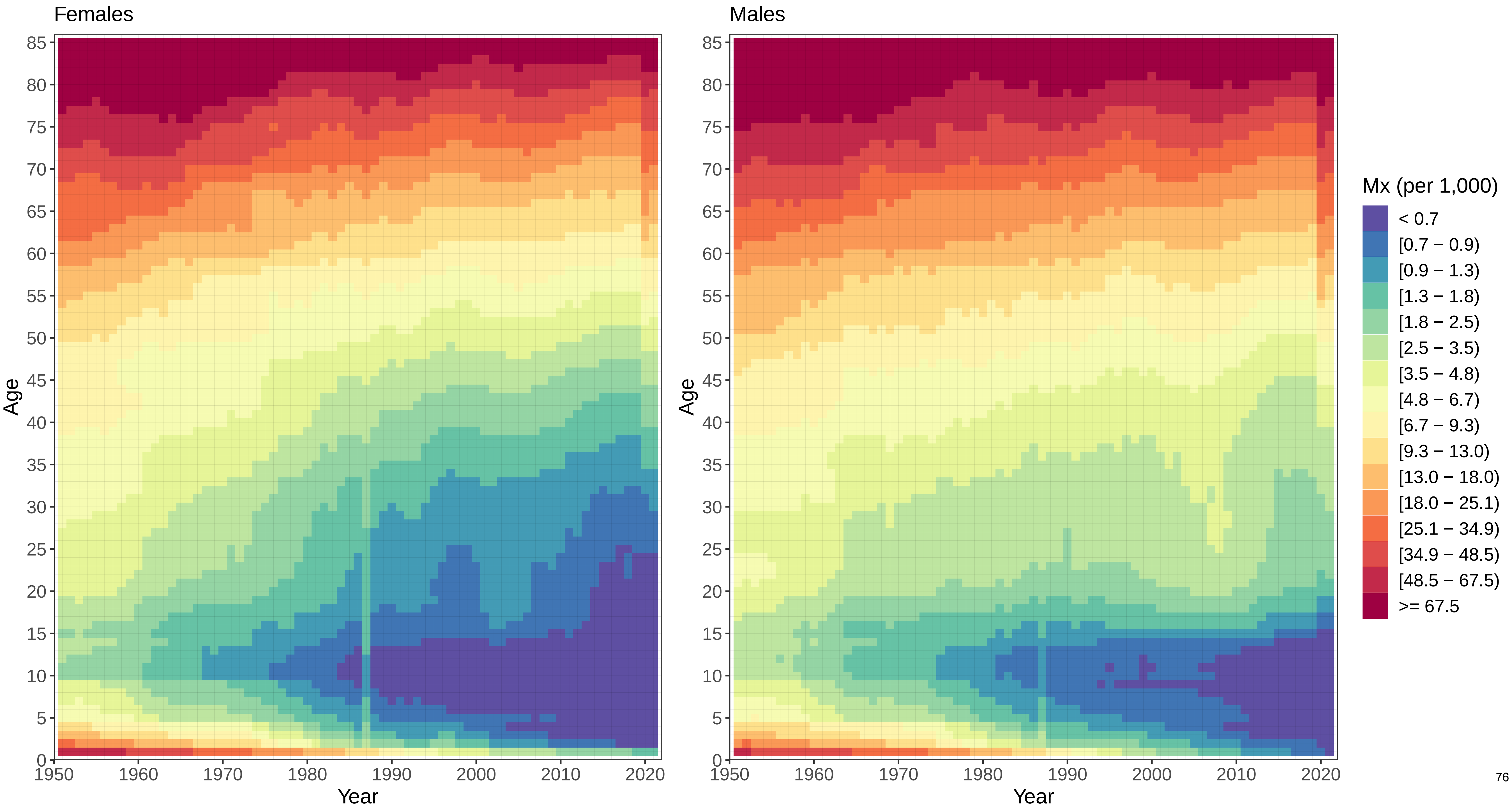

Appendix 2 Figure S5: Peru mortality rates, 1950−2021

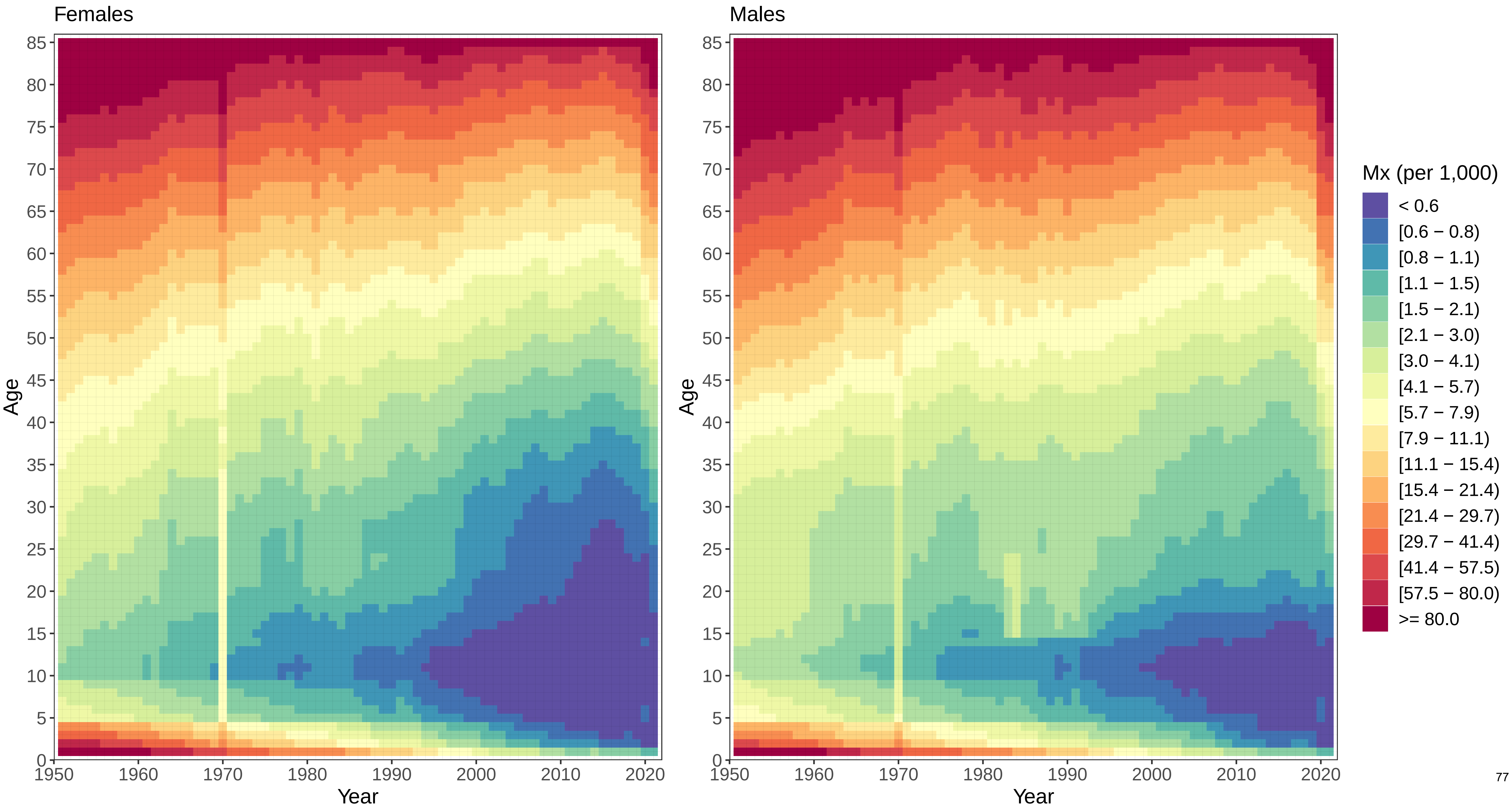

Appendix 2 Figure S5: Antigua and Barbuda mortality rates, 1950−2021

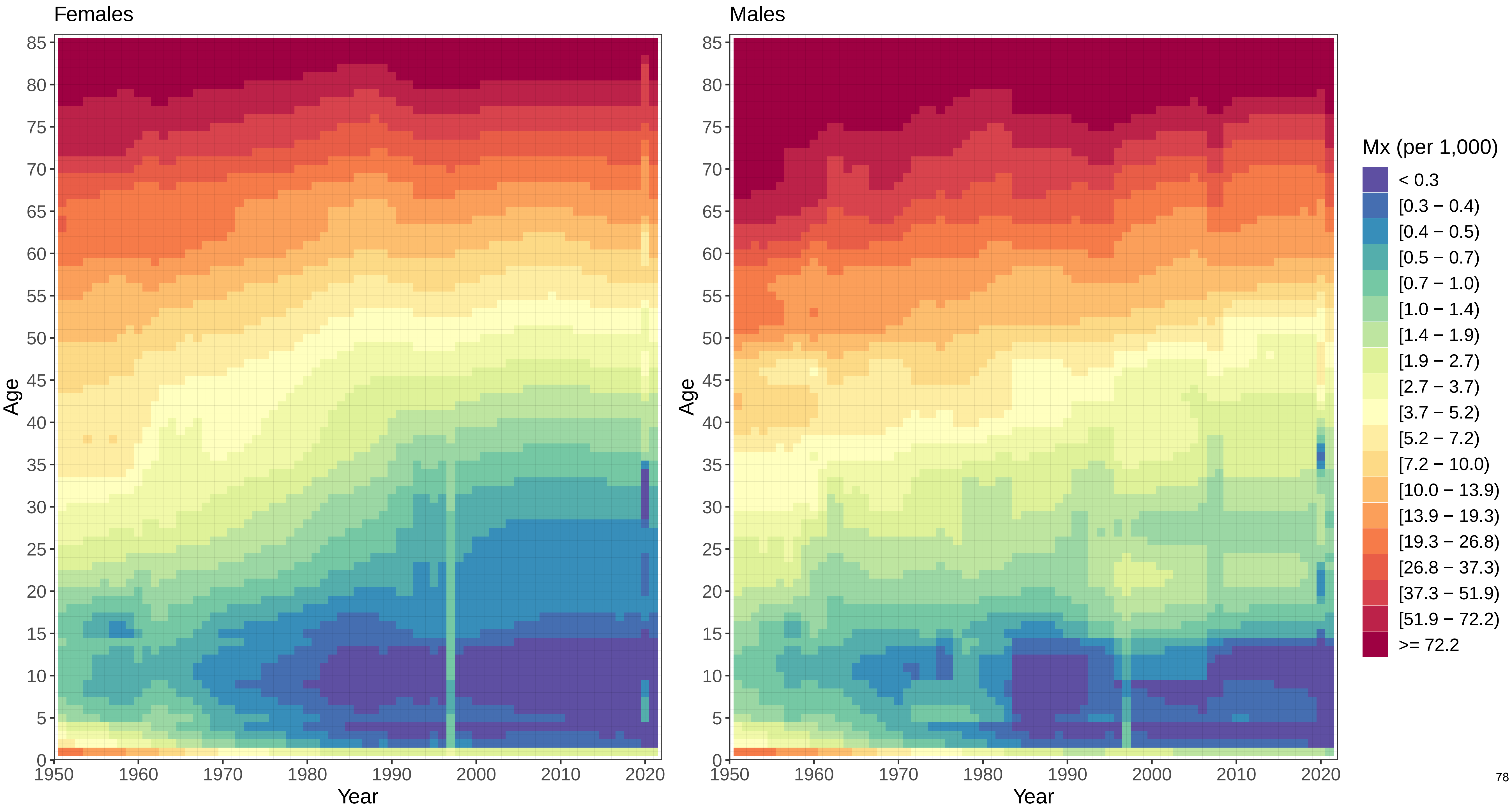

Appendix 2 Figure S5: The Bahamas mortality rates, 1950−2021

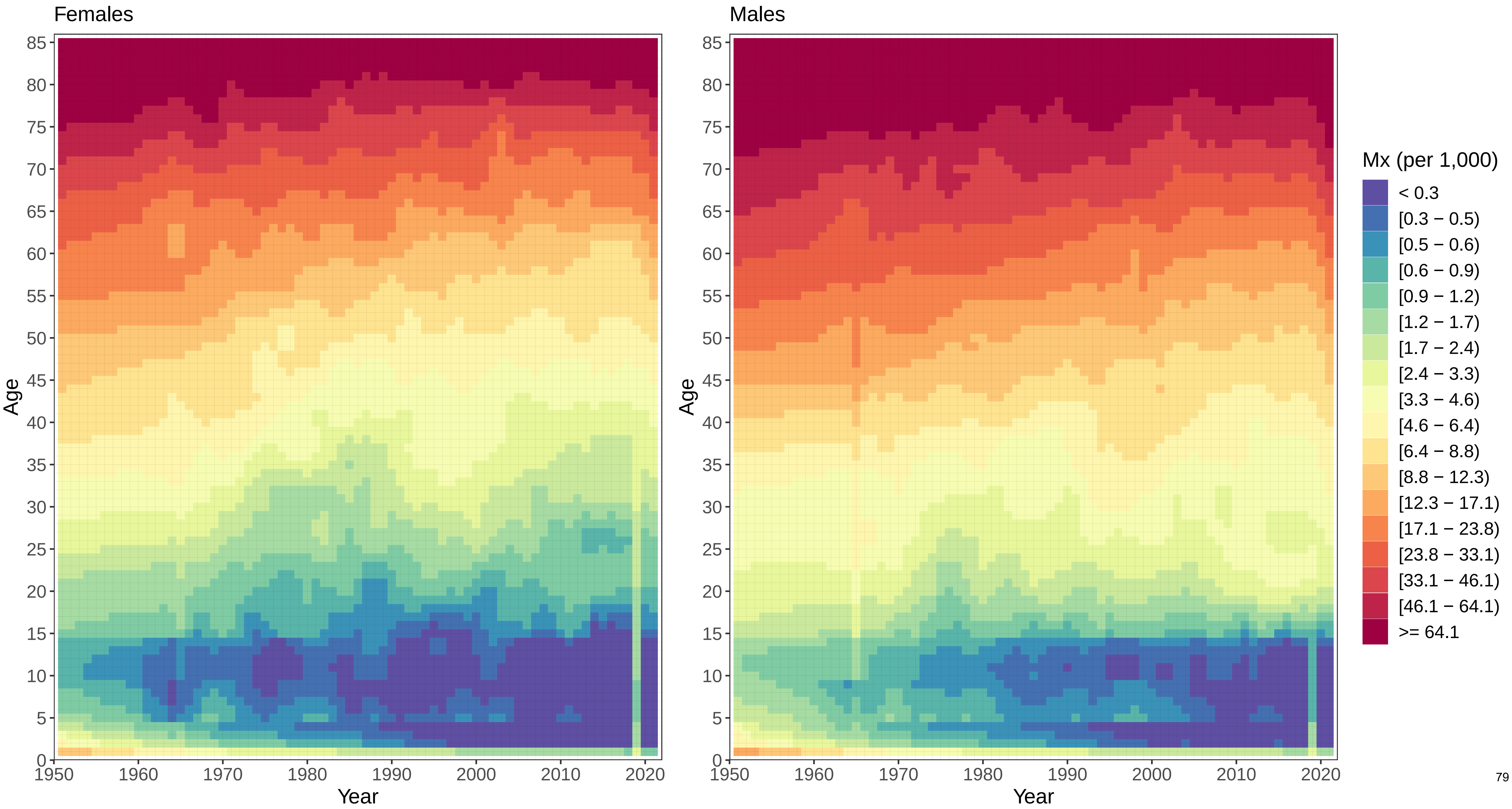

Appendix 2 Figure S5: Barbados mortality rates, 1950−2021

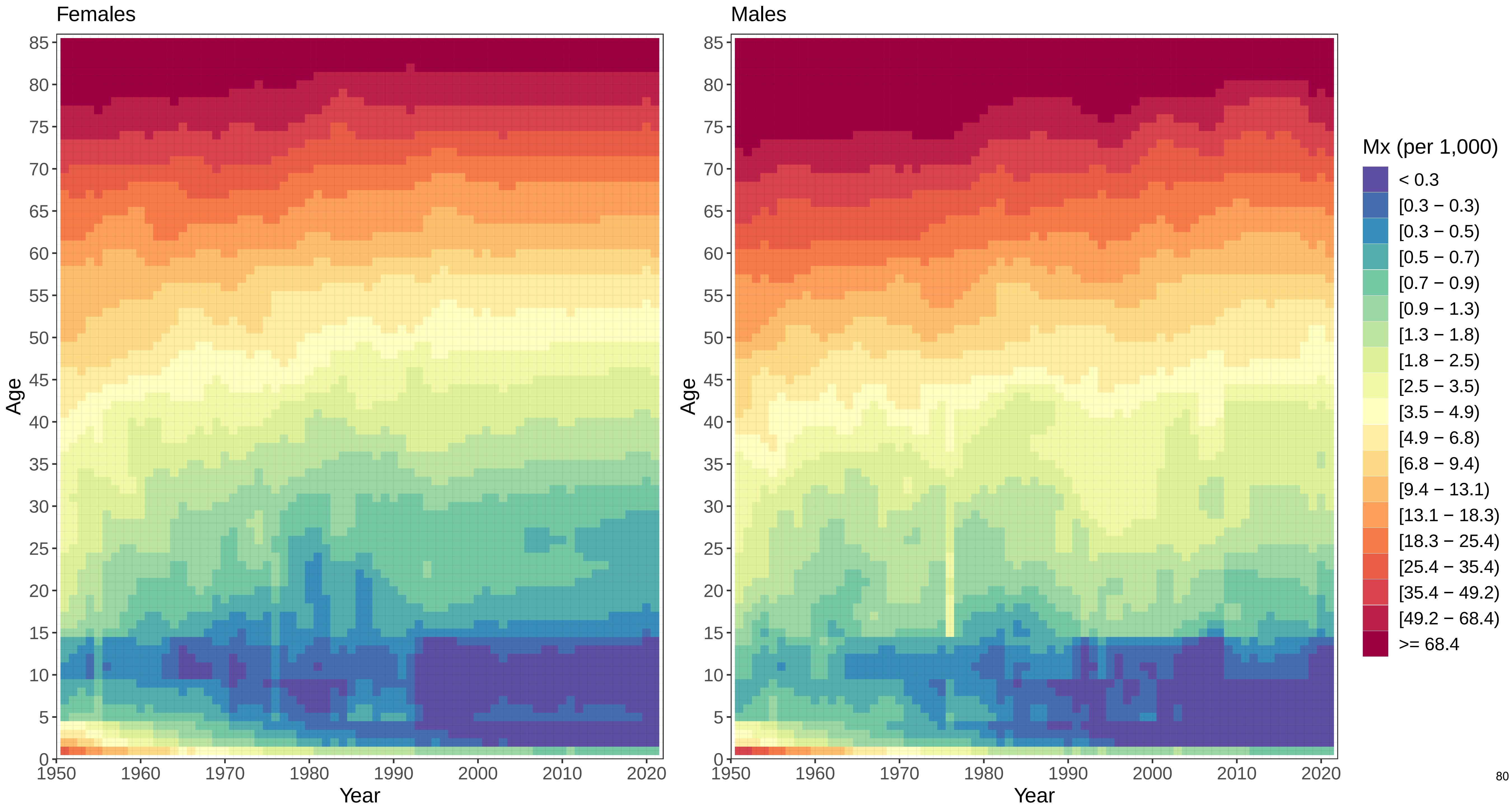

Appendix 2 Figure S5: Belize mortality rates, 1950−2021

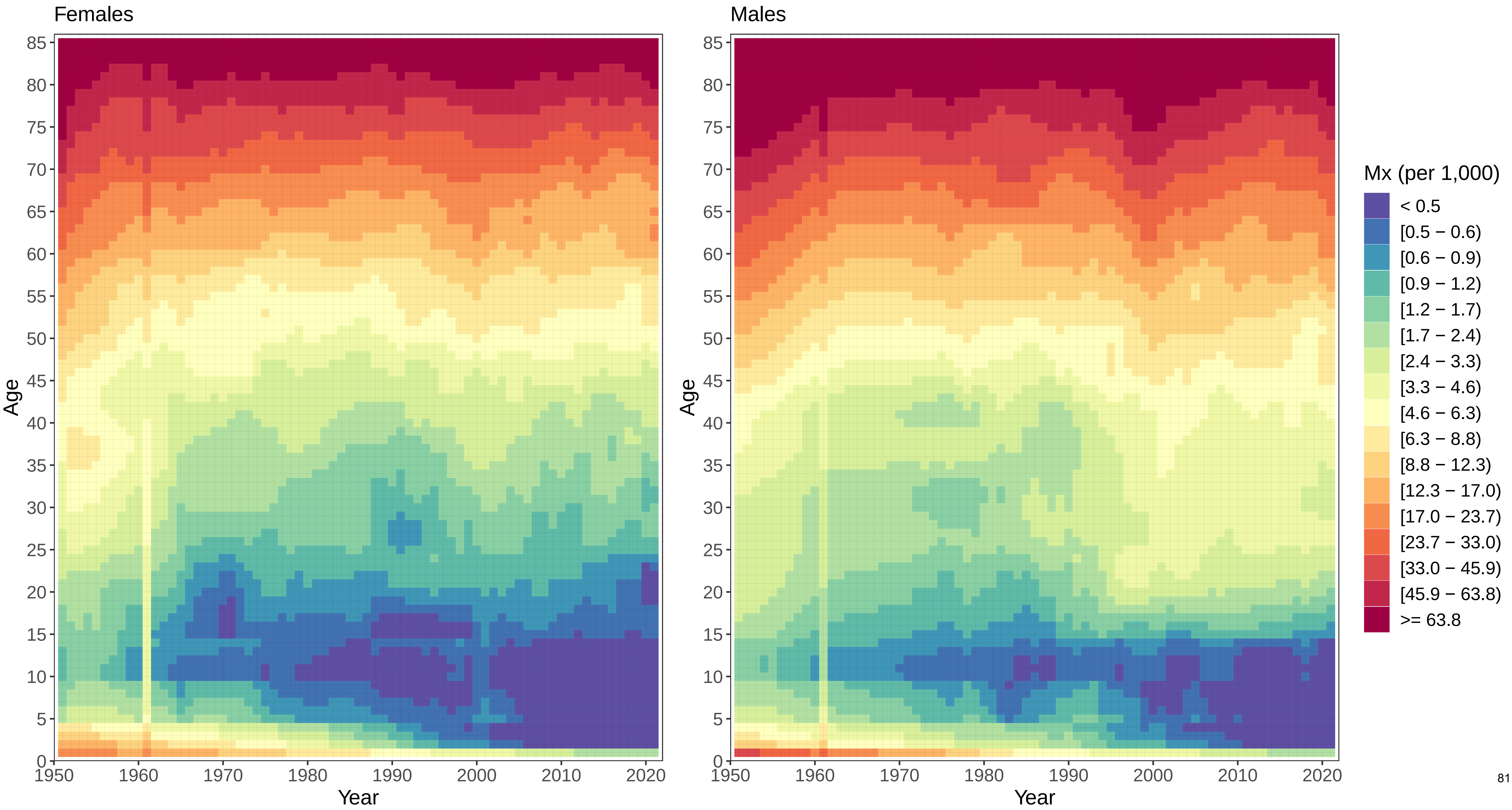

Appendix 2 Figure S5: Bermuda mortality rates, 1950−2021

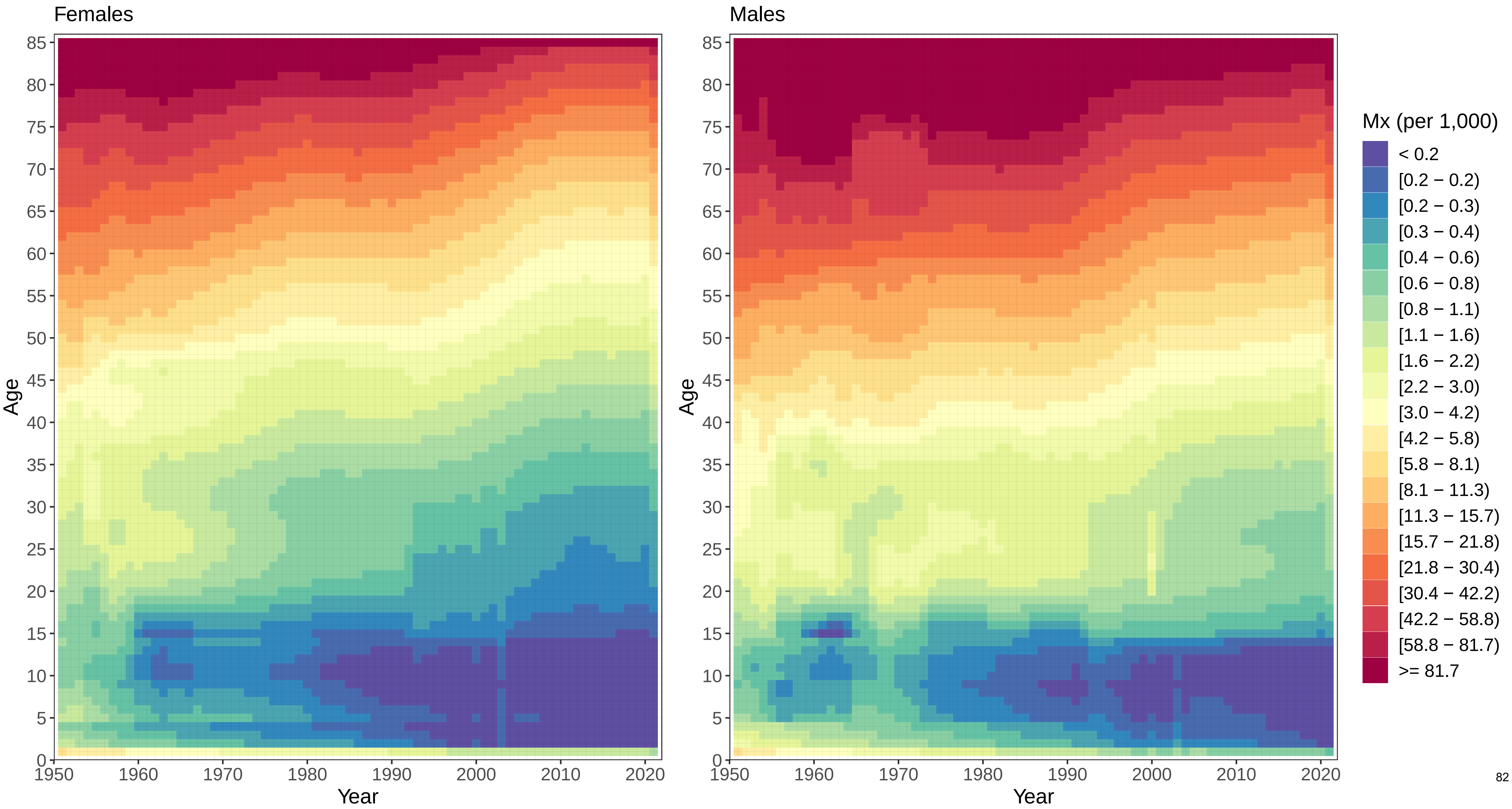

Appendix 2 Figure S5: Cuba mortality rates, 1950−2021

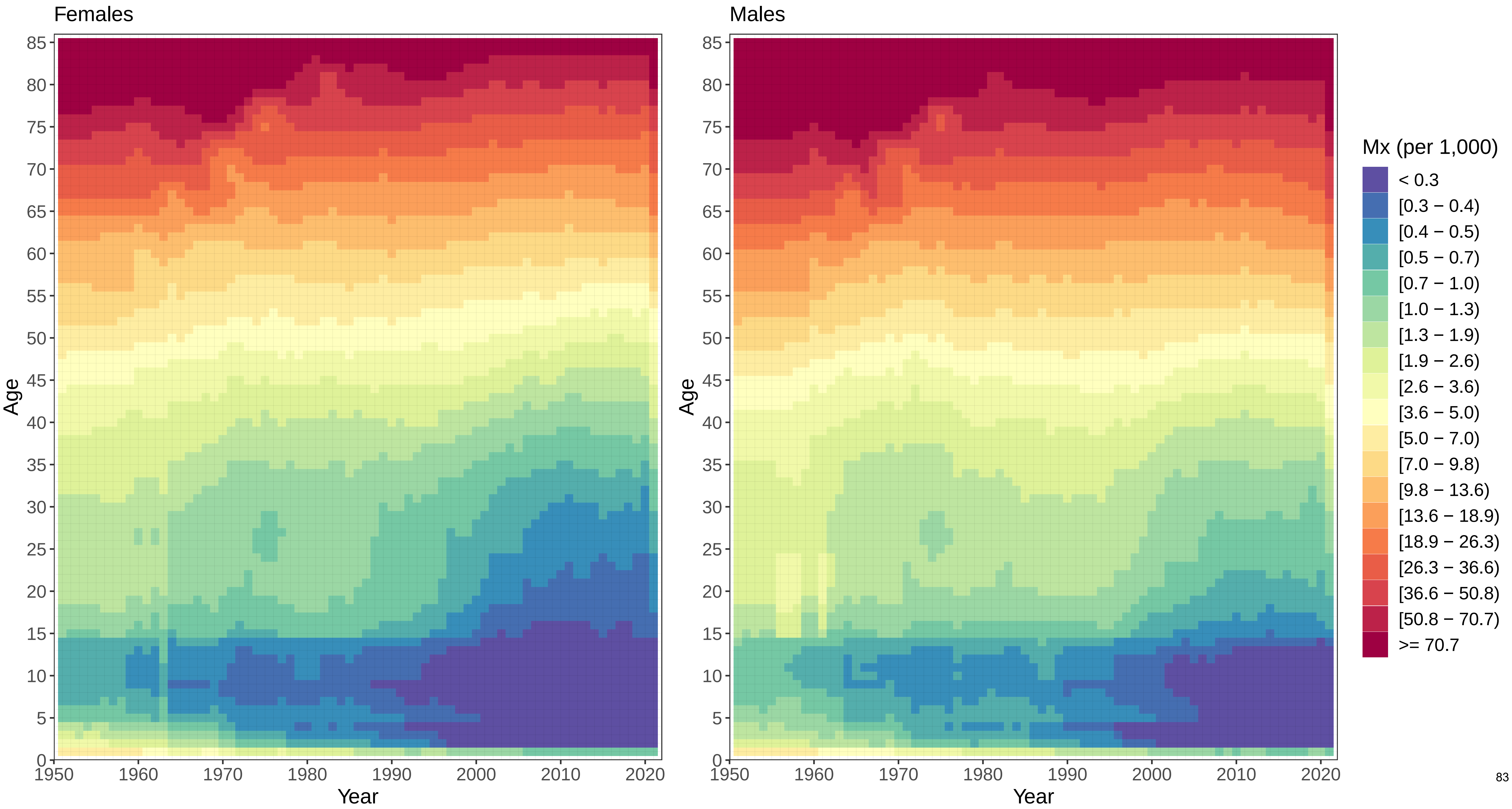

Appendix 2 Figure S5: Dominica mortality rates, 1950−2021

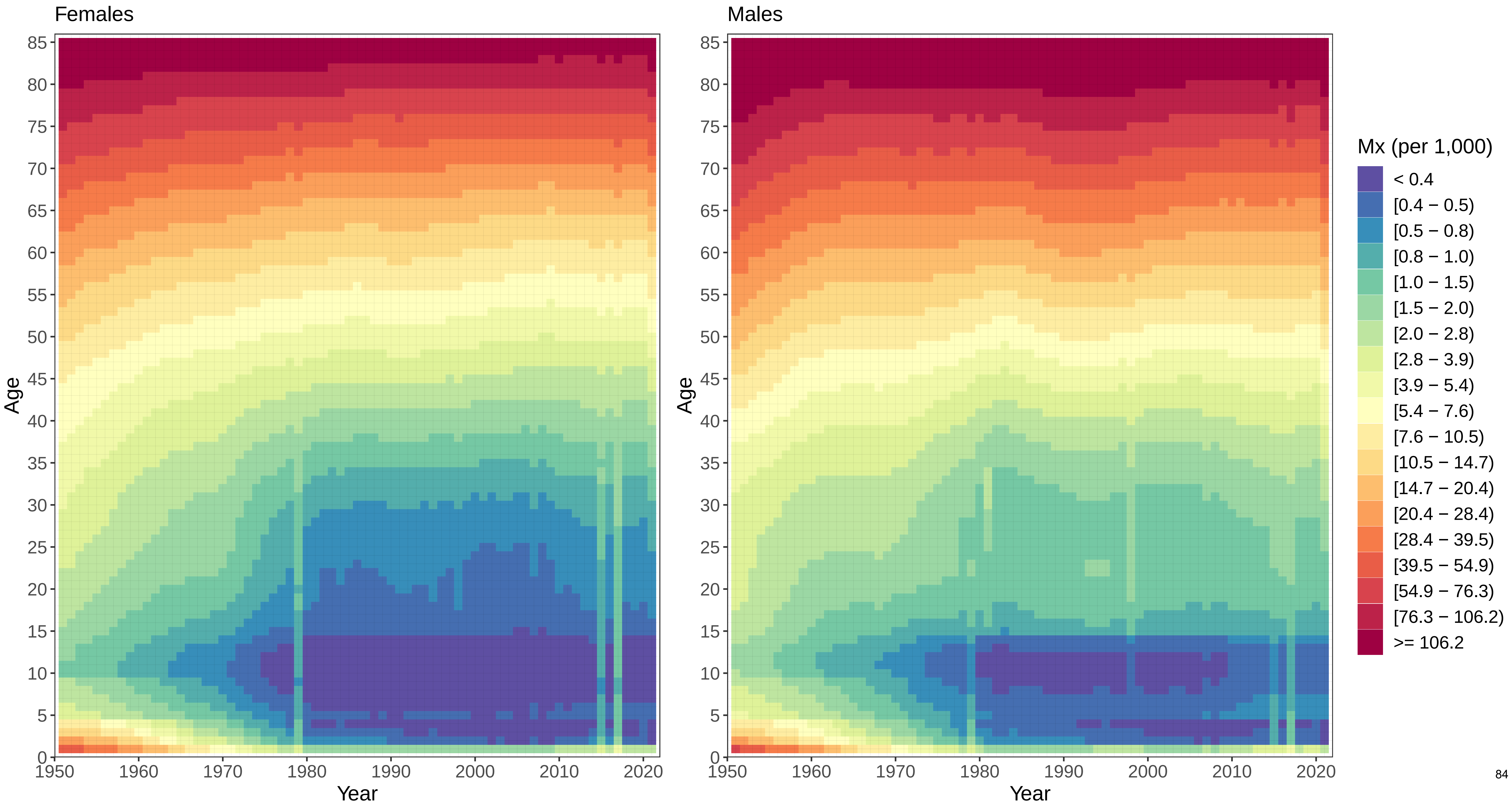

Appendix 2 Figure S5: Dominican Republic mortality rates, 1950−2021

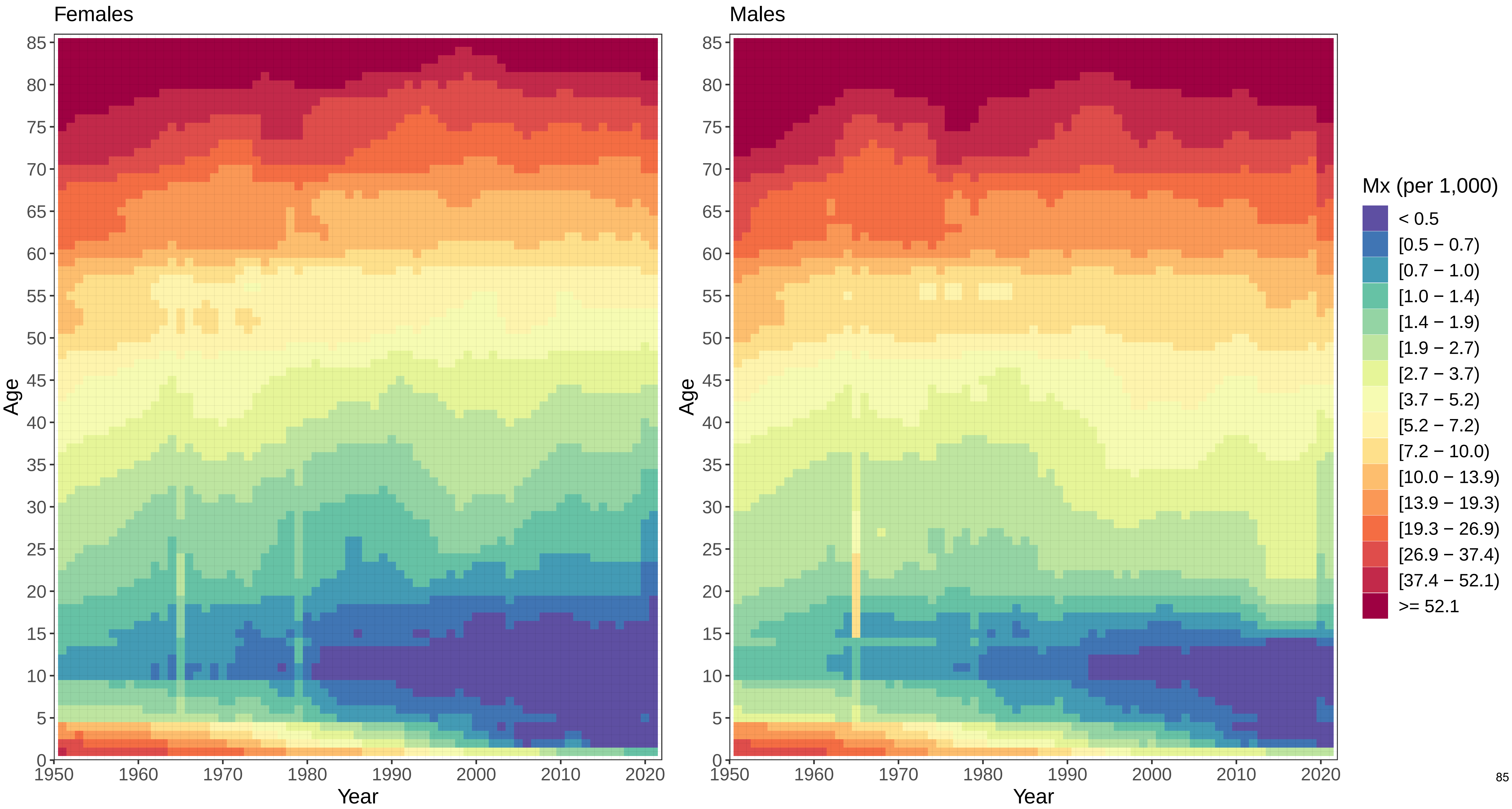

Appendix 2 Figure S5: Grenada mortality rates, 1950−2021

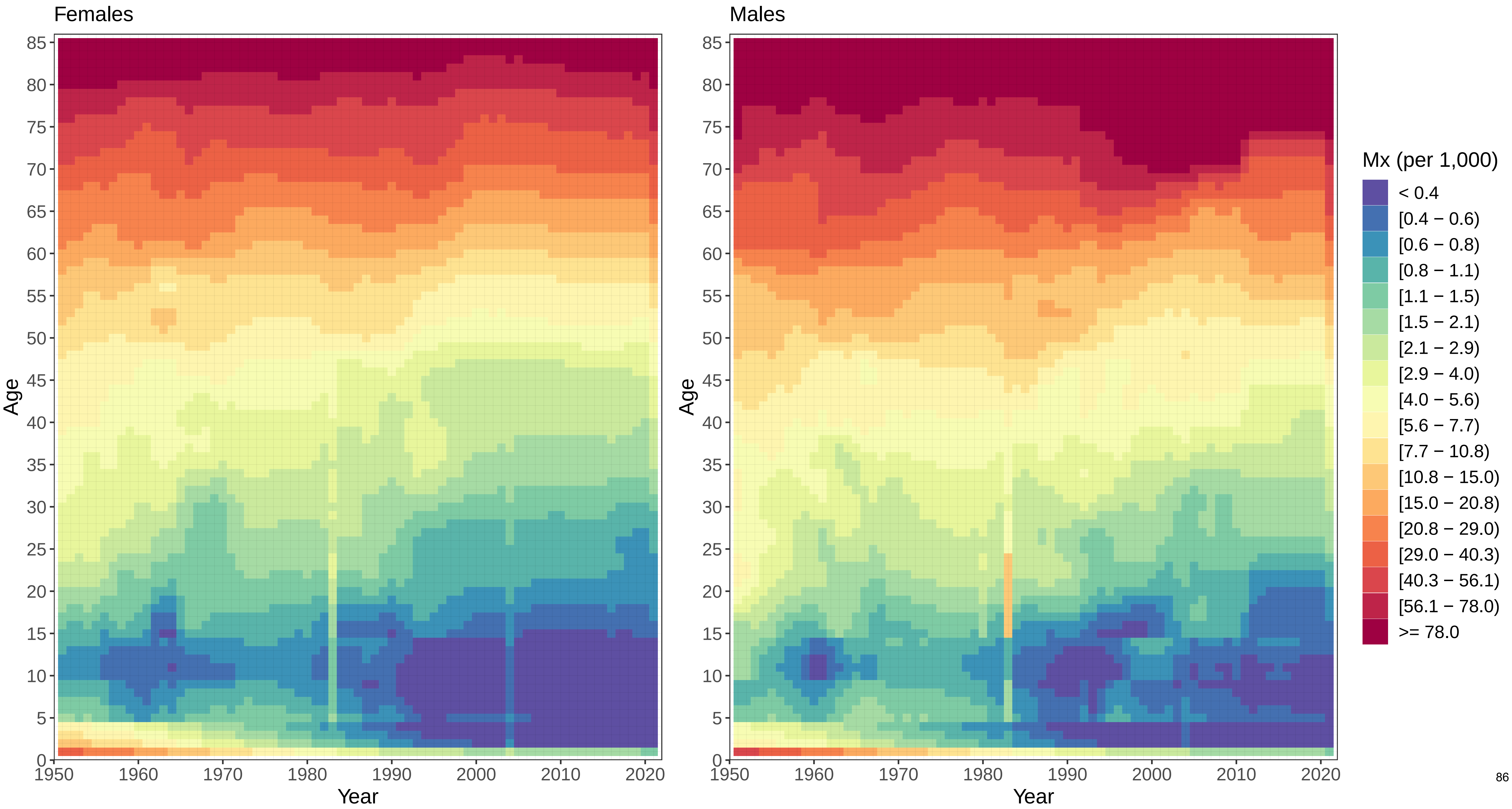

Appendix 2 Figure S5: Guyana mortality rates, 1950−2021

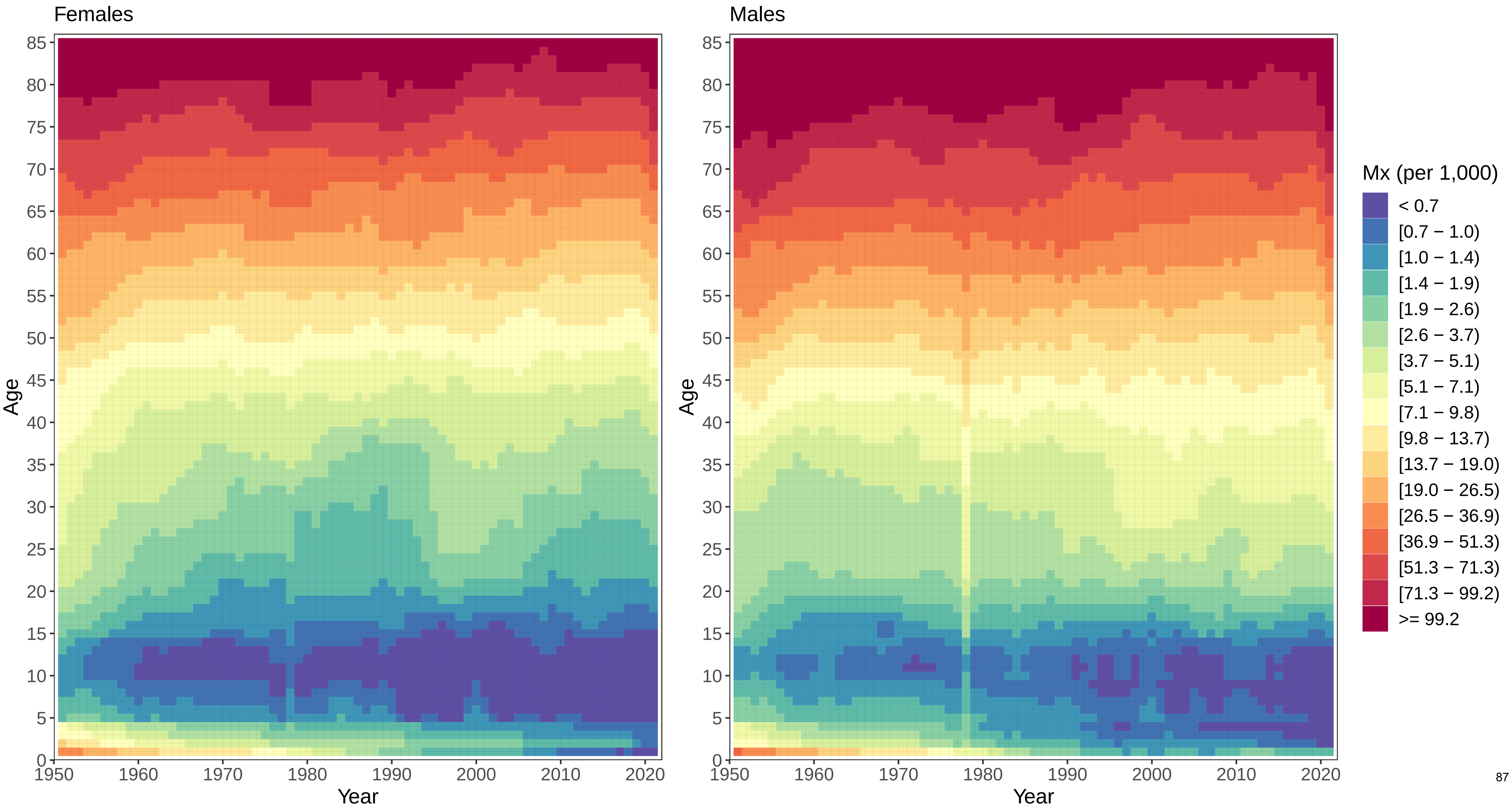

Appendix 2 Figure S5: Haiti mortality rates, 1950−2021

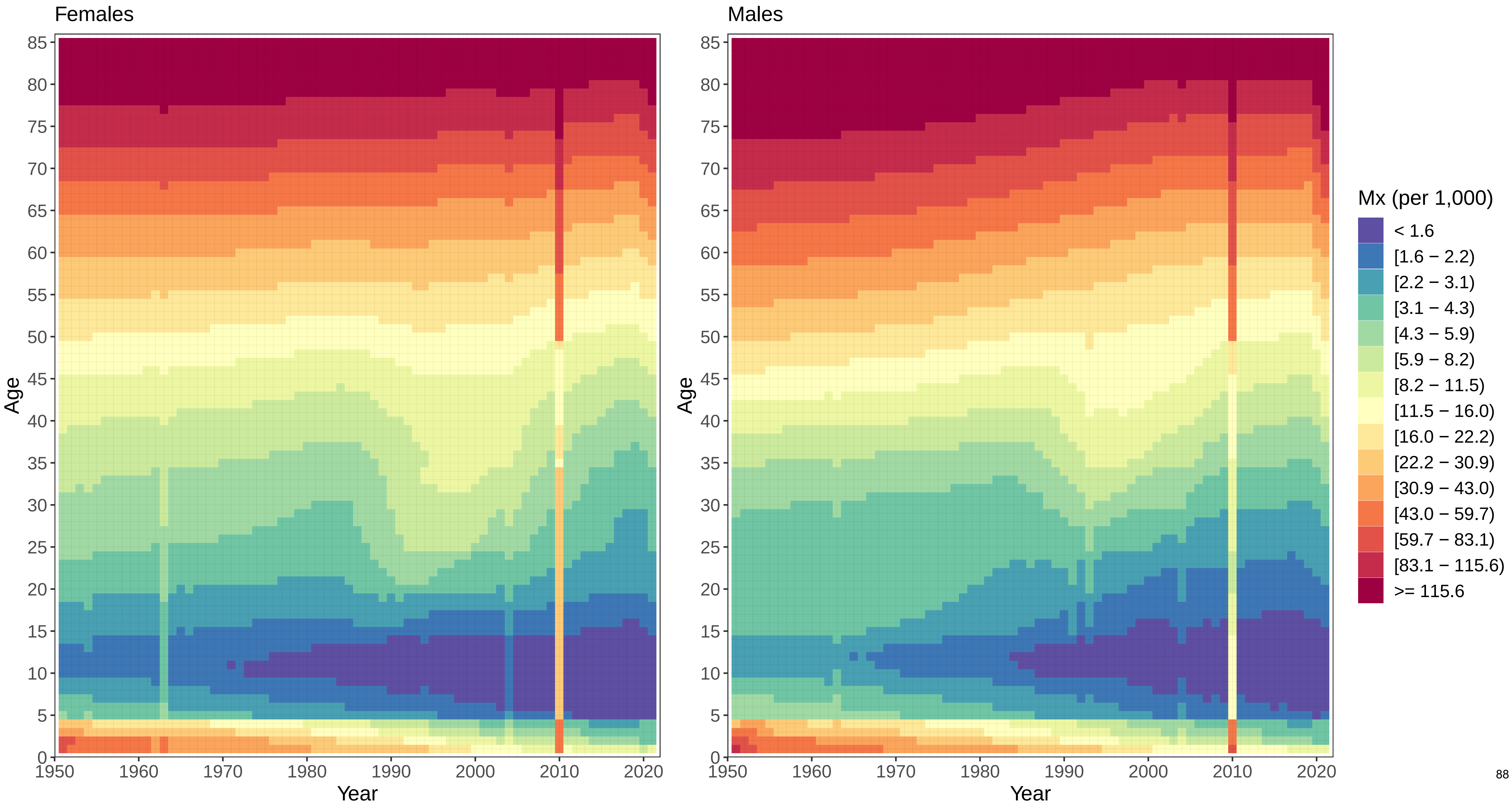

Appendix 2 Figure S5: Jamaica mortality rates, 1950−2021

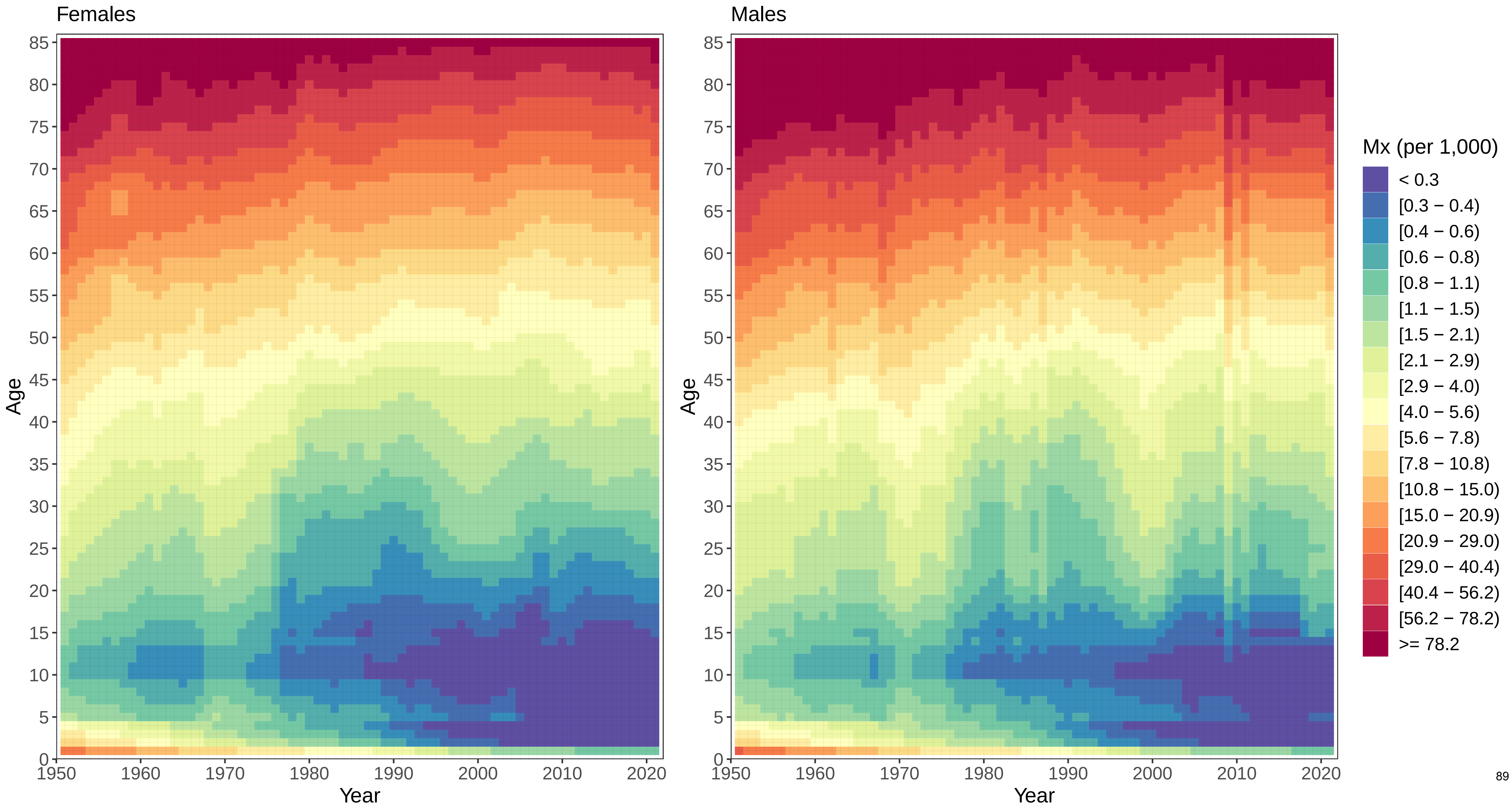

Appendix 2 Figure S5: Puerto Rico mortality rates, 1950−2021

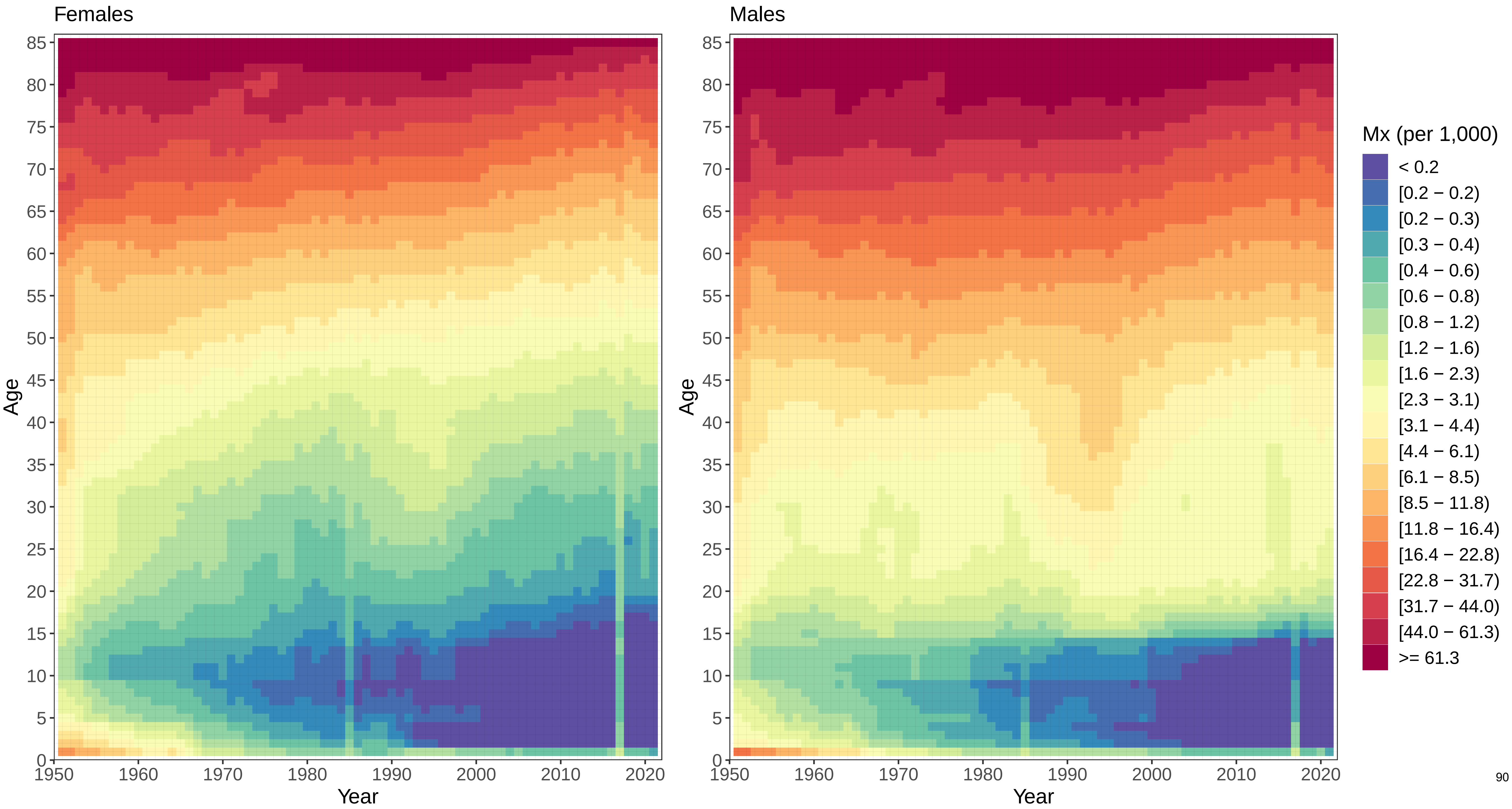

Appendix 2 Figure S5: Saint Kitts and Nevis mortality rates, 1950−2021

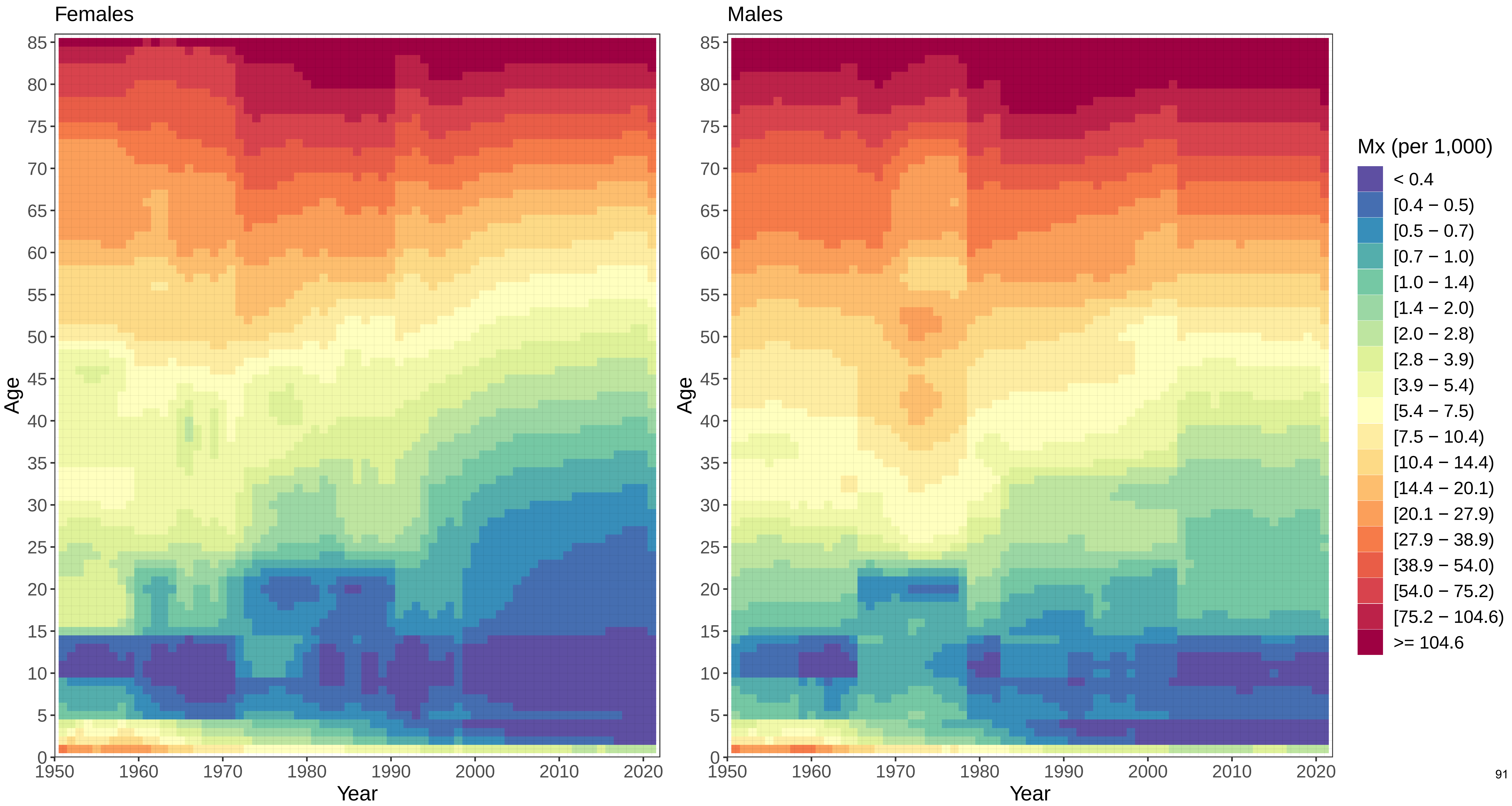

Appendix 2 Figure S5: Saint Lucia mortality rates, 1950−2021

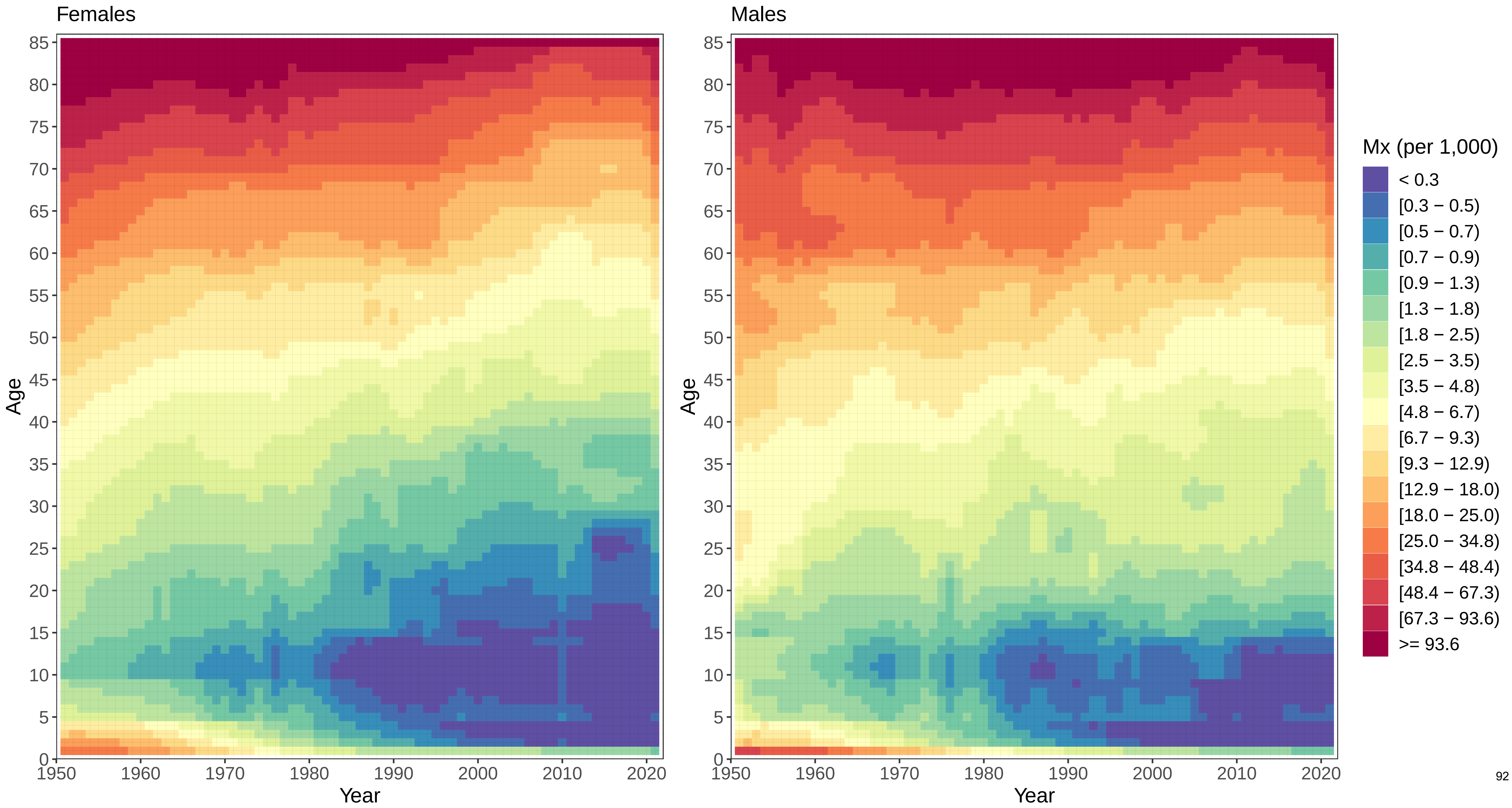

Appendix 2 Figure S5: Saint Vincent and the Grenadines mortality rates, 1950−2021

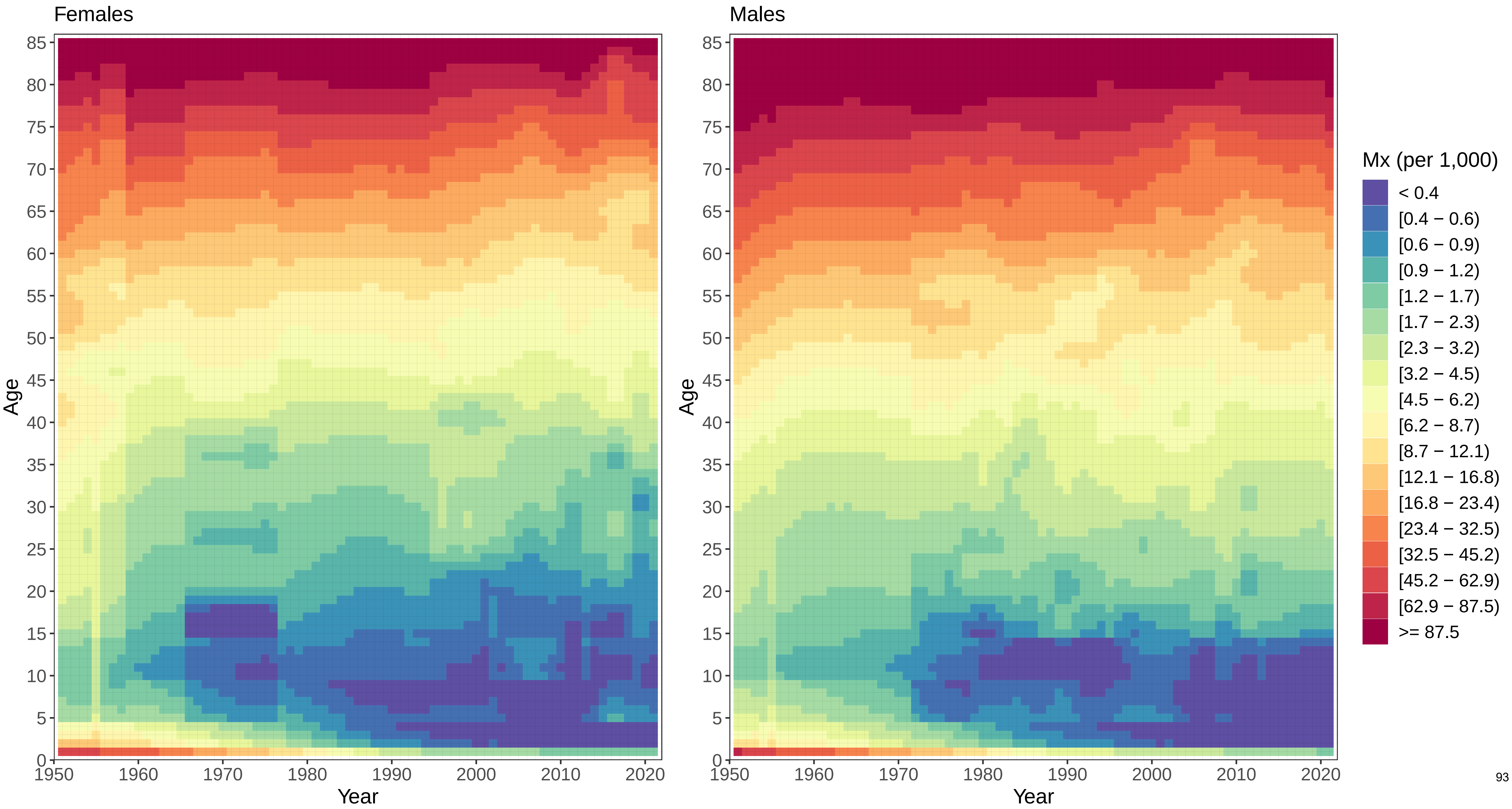

Appendix 2 Figure S5: Suriname mortality rates, 1950−2021

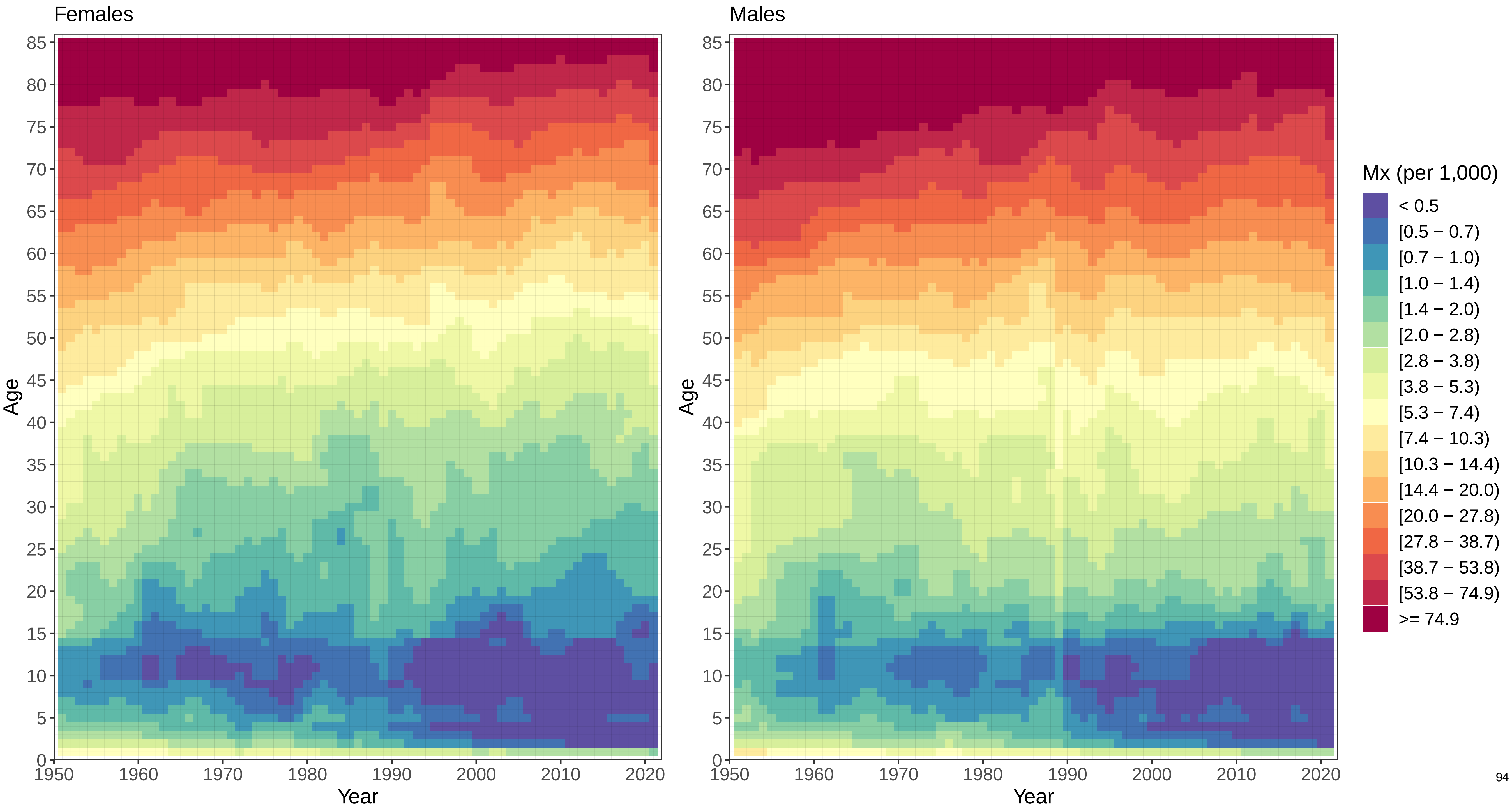

Appendix 2 Figure S5: Trinidad and Tobago mortality rates, 1950−2021

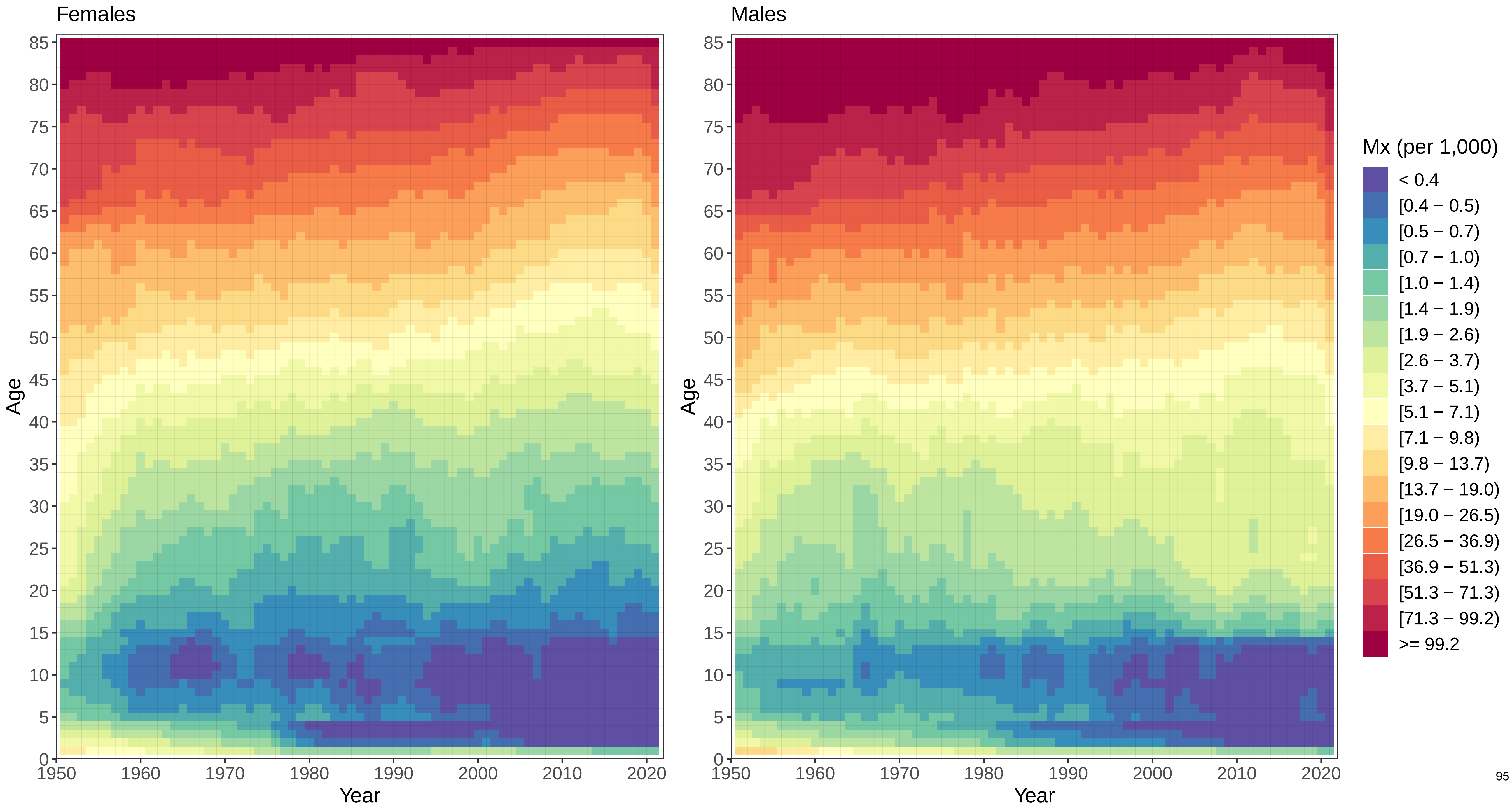

Appendix 2 Figure S5: Virgin Islands mortality rates, 1950−2021

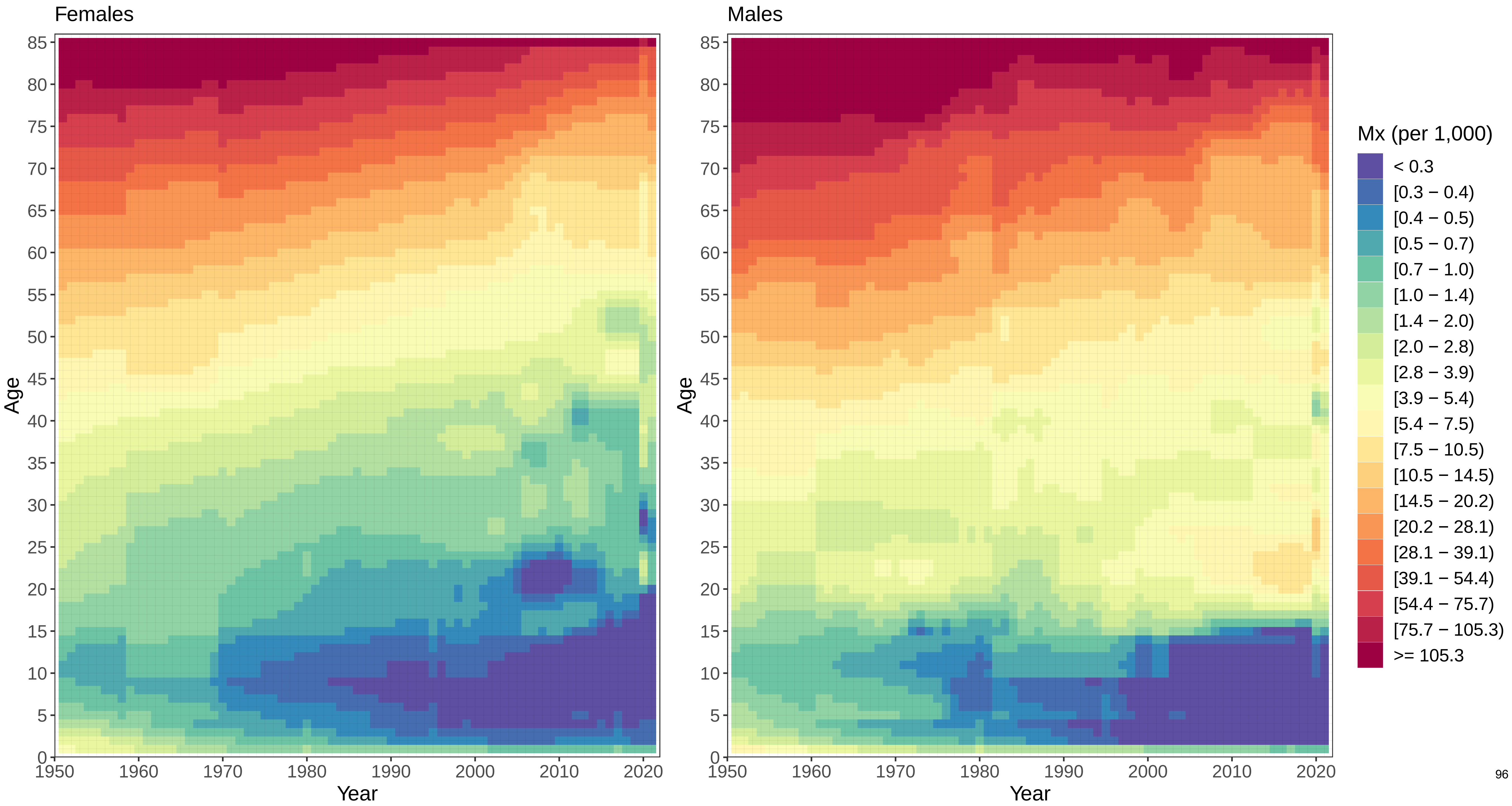

Appendix 2 Figure S5: Colombia mortality rates, 1950−2021

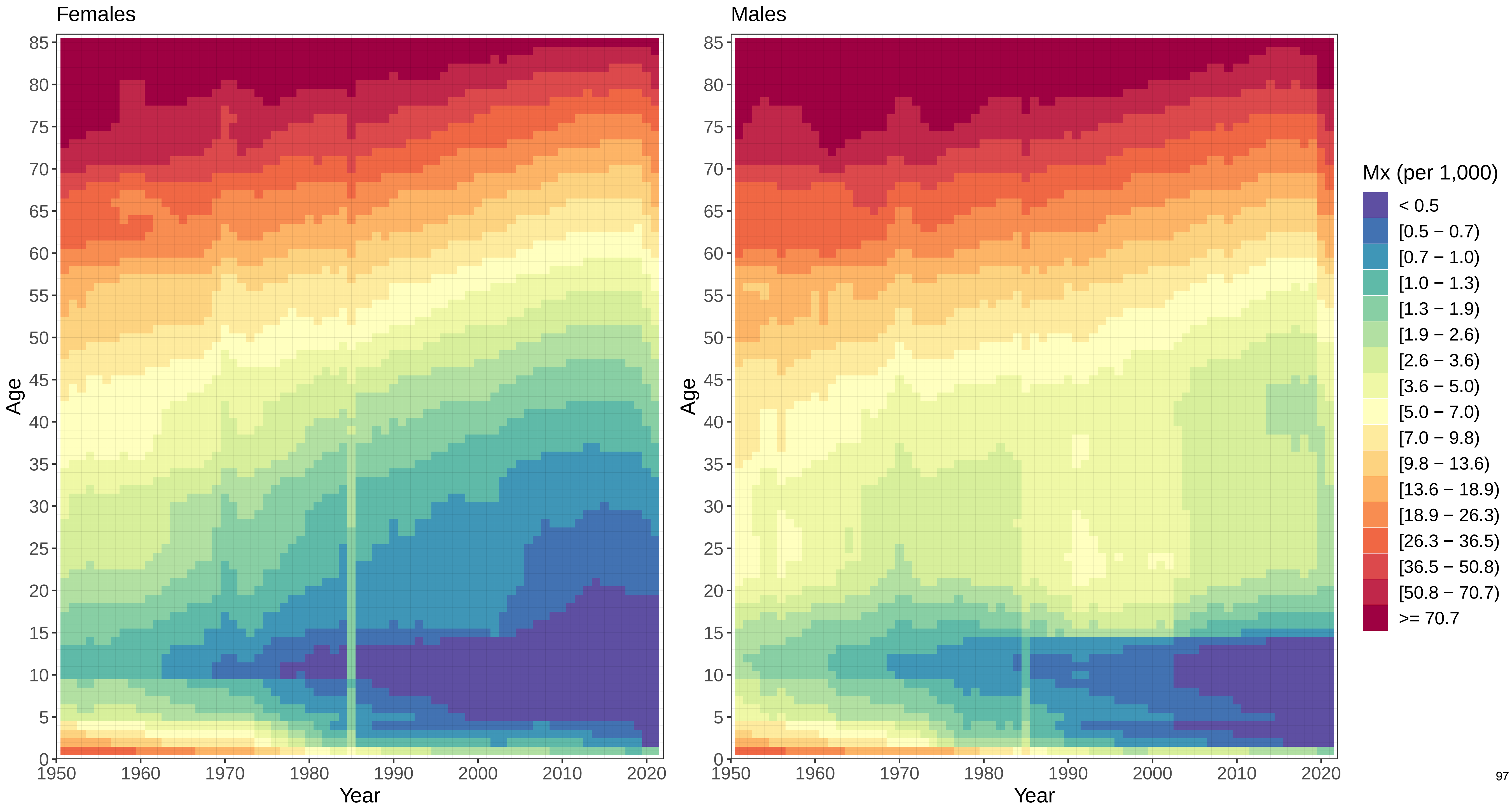

Appendix 2 Figure S5: Costa Rica mortality rates, 1950−2021

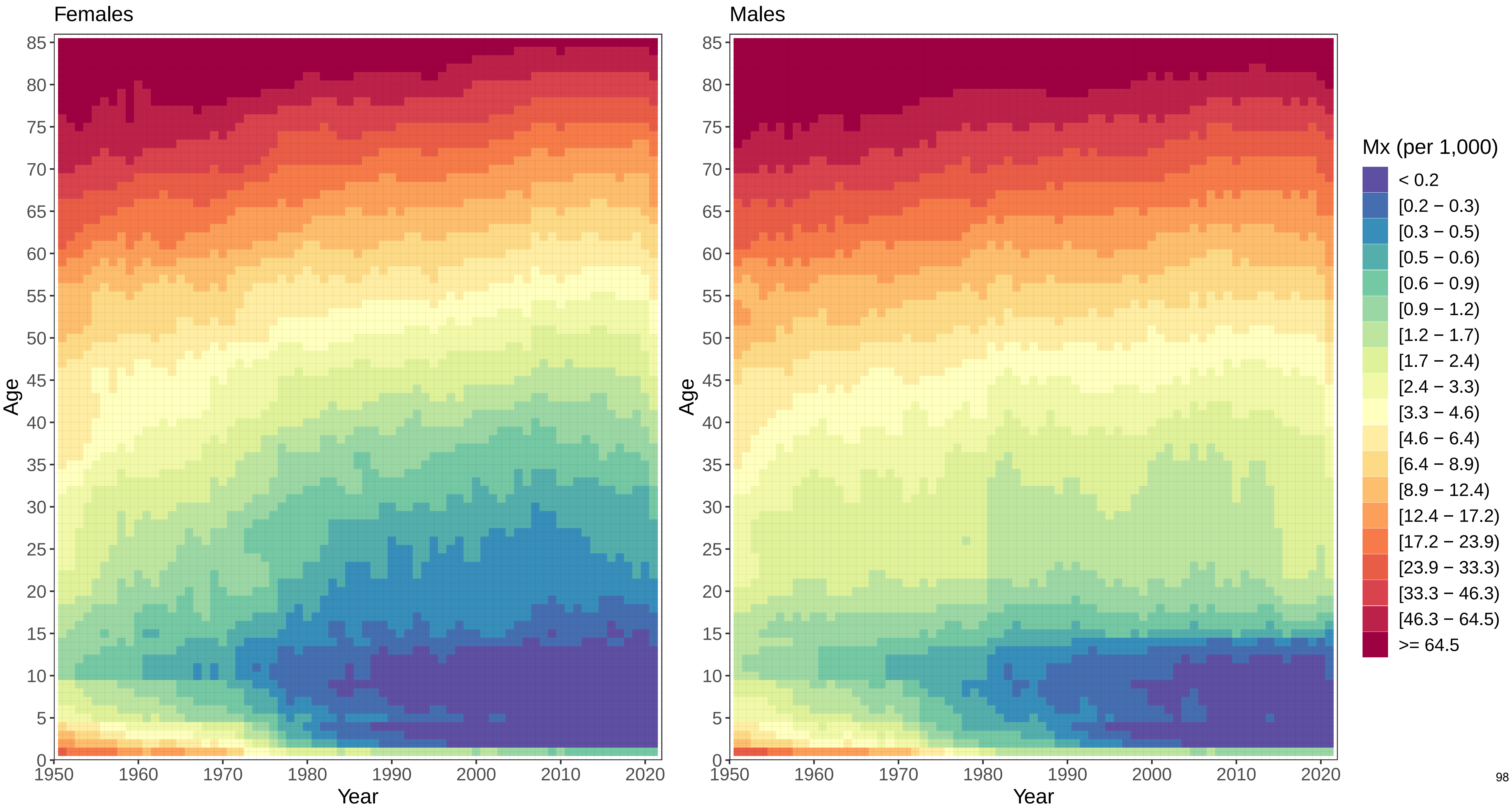

Appendix 2 Figure S5: El Salvador mortality rates, 1950−2021

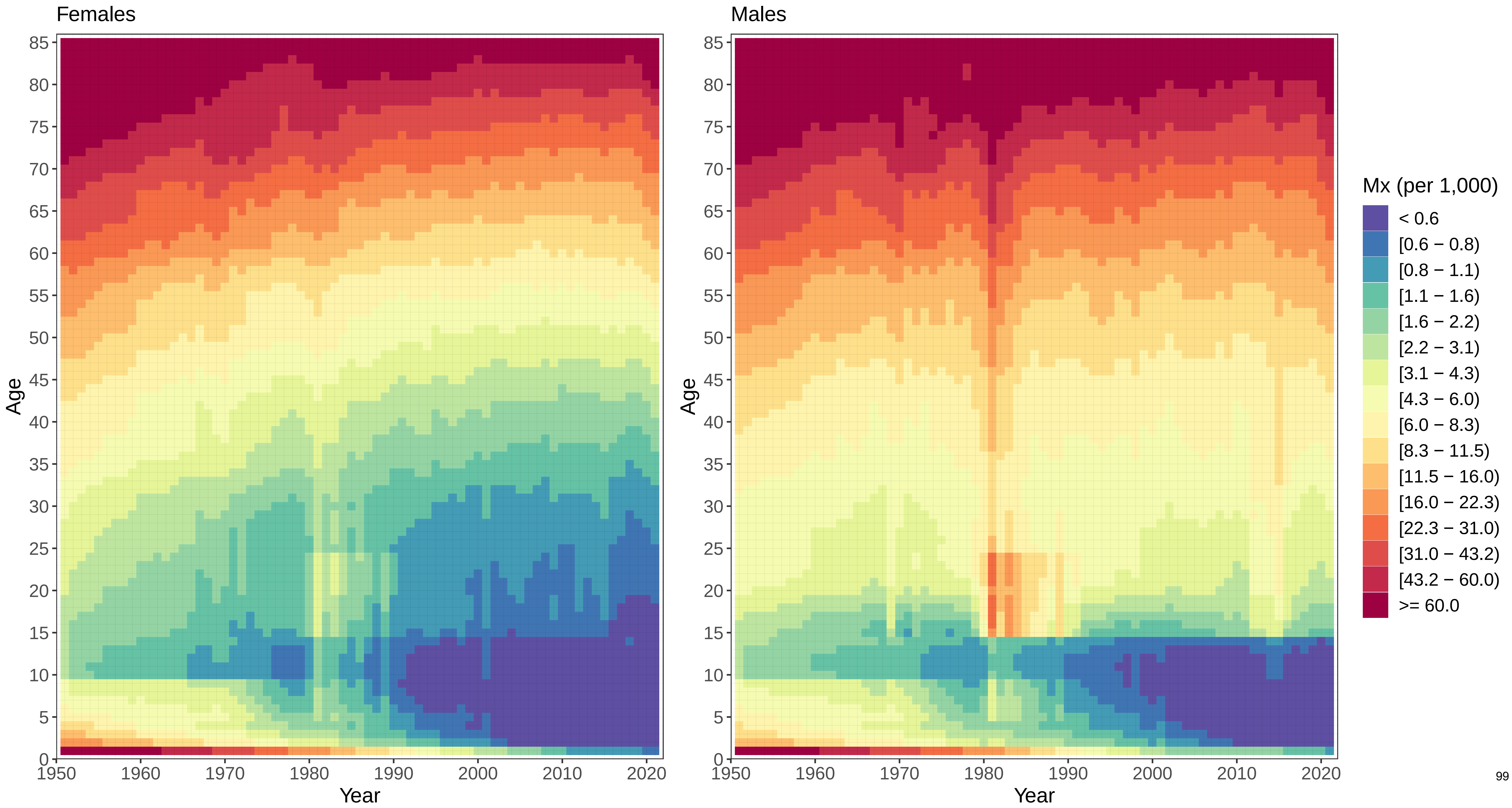

Appendix 2 Figure S5: Guatemala mortality rates, 1950−2021

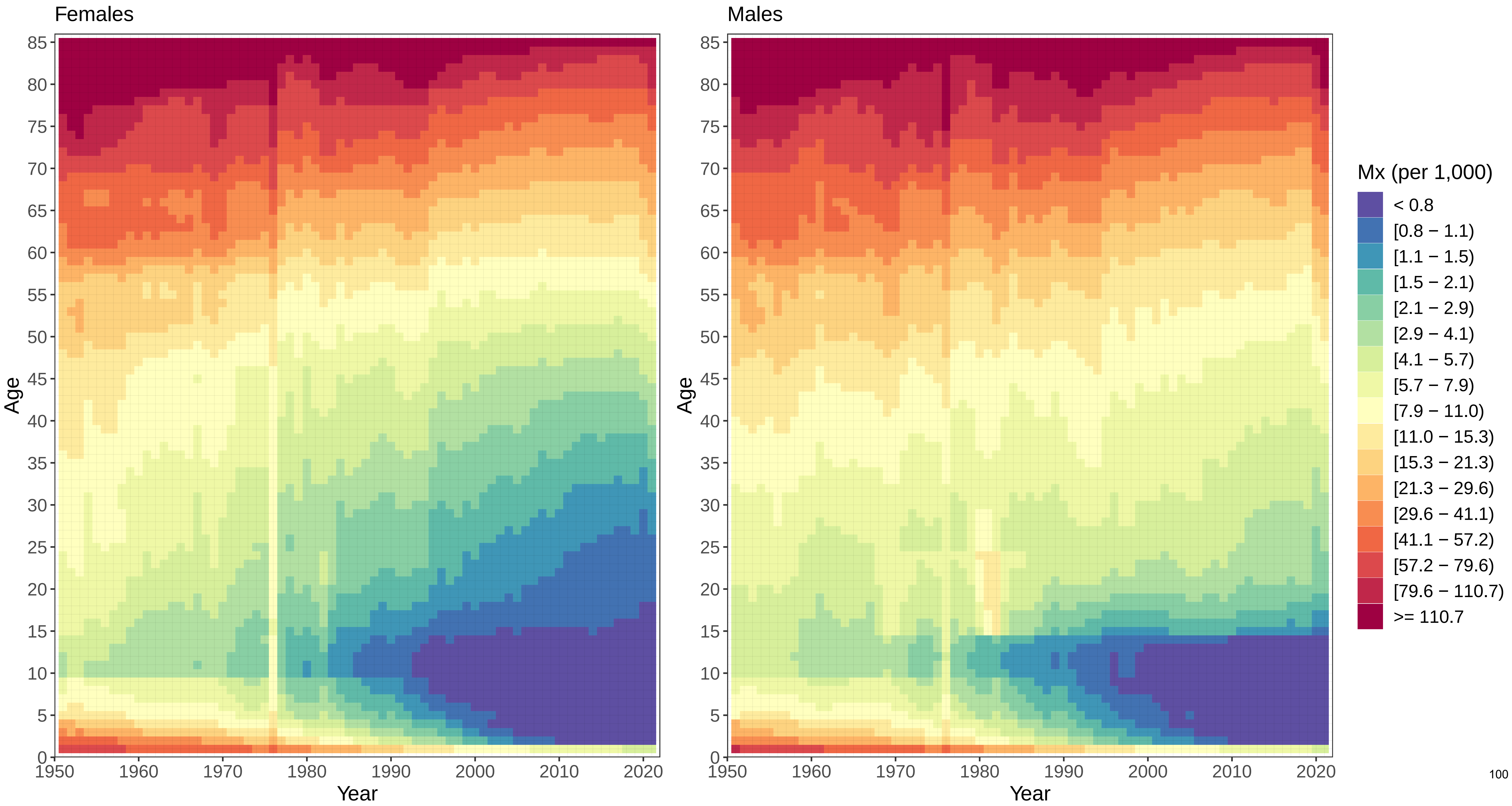

Appendix 2 Figure S5: Honduras mortality rates, 1950−2021

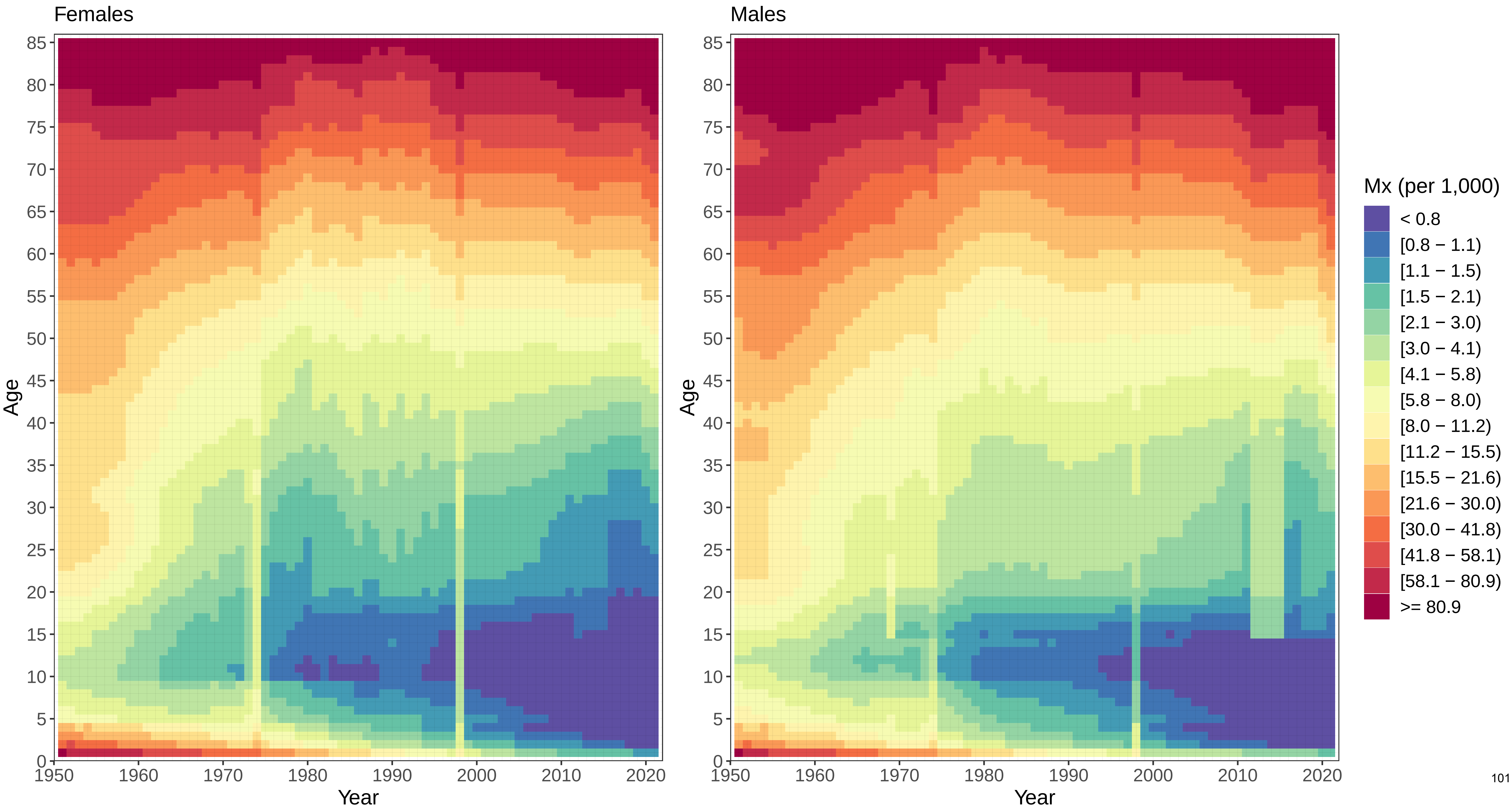

Appendix 2 Figure S5: Mexico mortality rates, 1950−2021

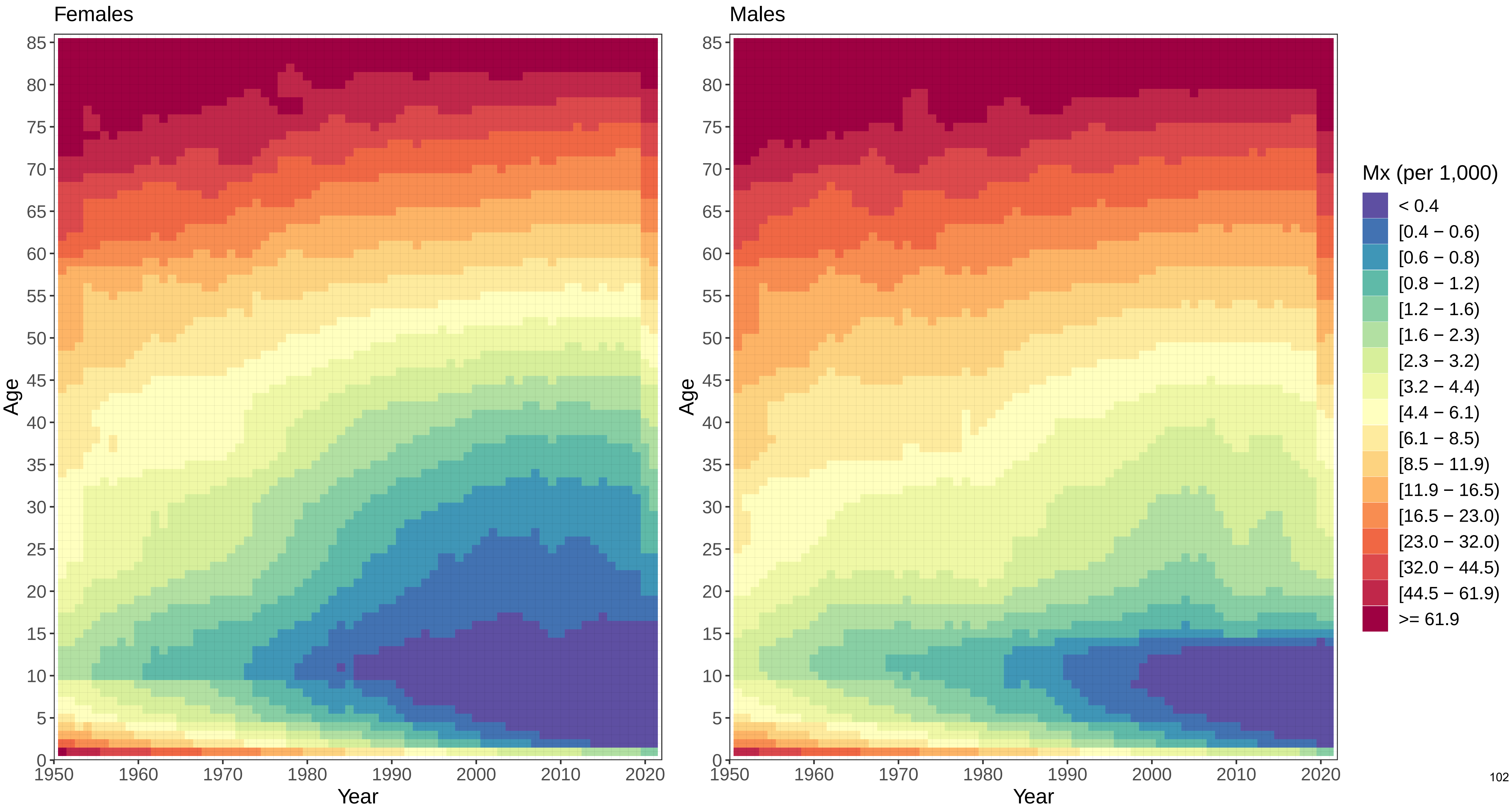

Appendix 2 Figure S5: Nicaragua mortality rates, 1950−2021

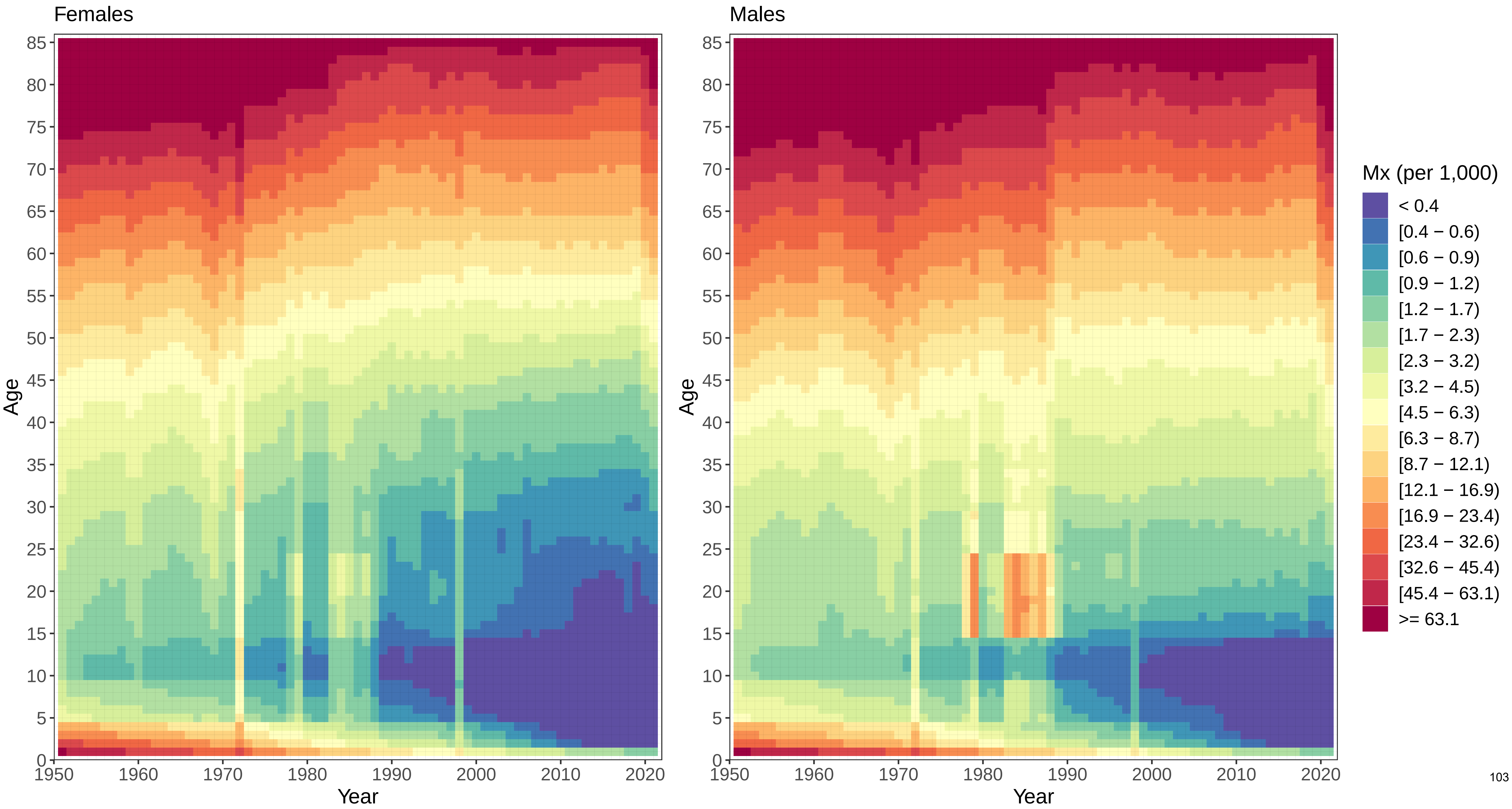

Appendix 2 Figure S5: Panama mortality rates, 1950−2021

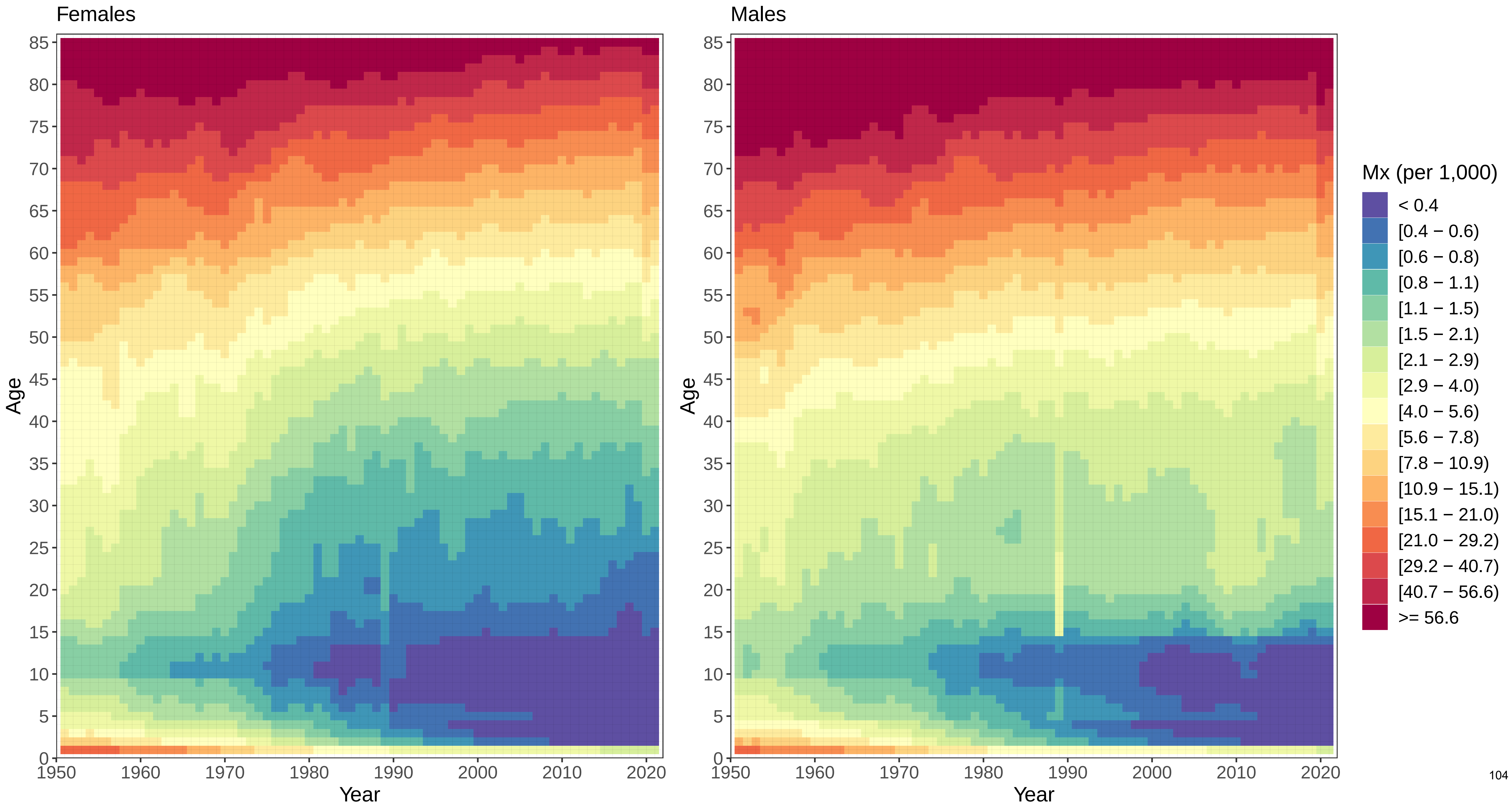

Appendix 2 Figure S5: Venezuela mortality rates, 1950−2021

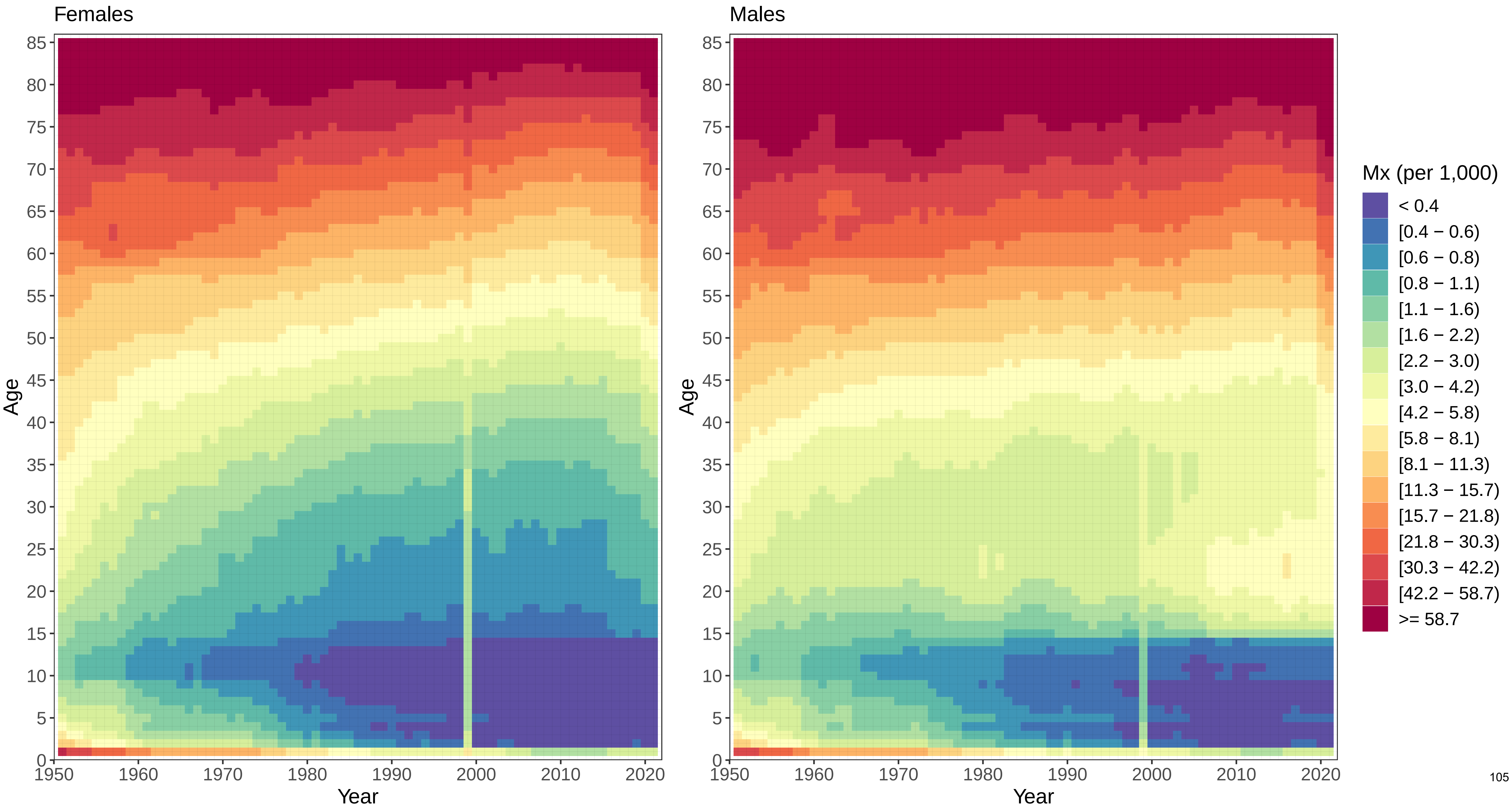

Appendix 2 Figure S5: Brazil mortality rates, 1950−2021

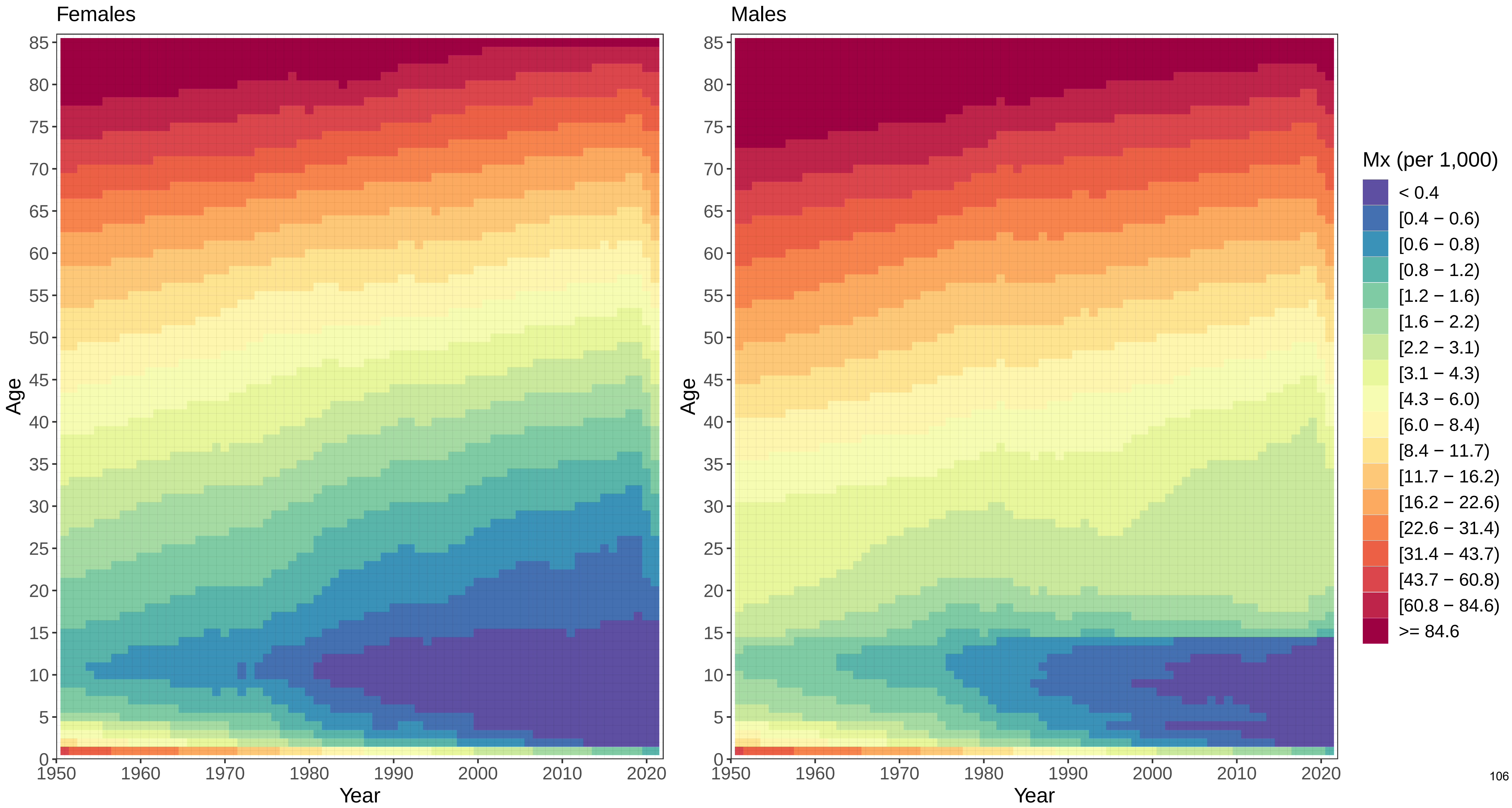

Appendix 2 Figure S5: Paraguay mortality rates, 1950−2021

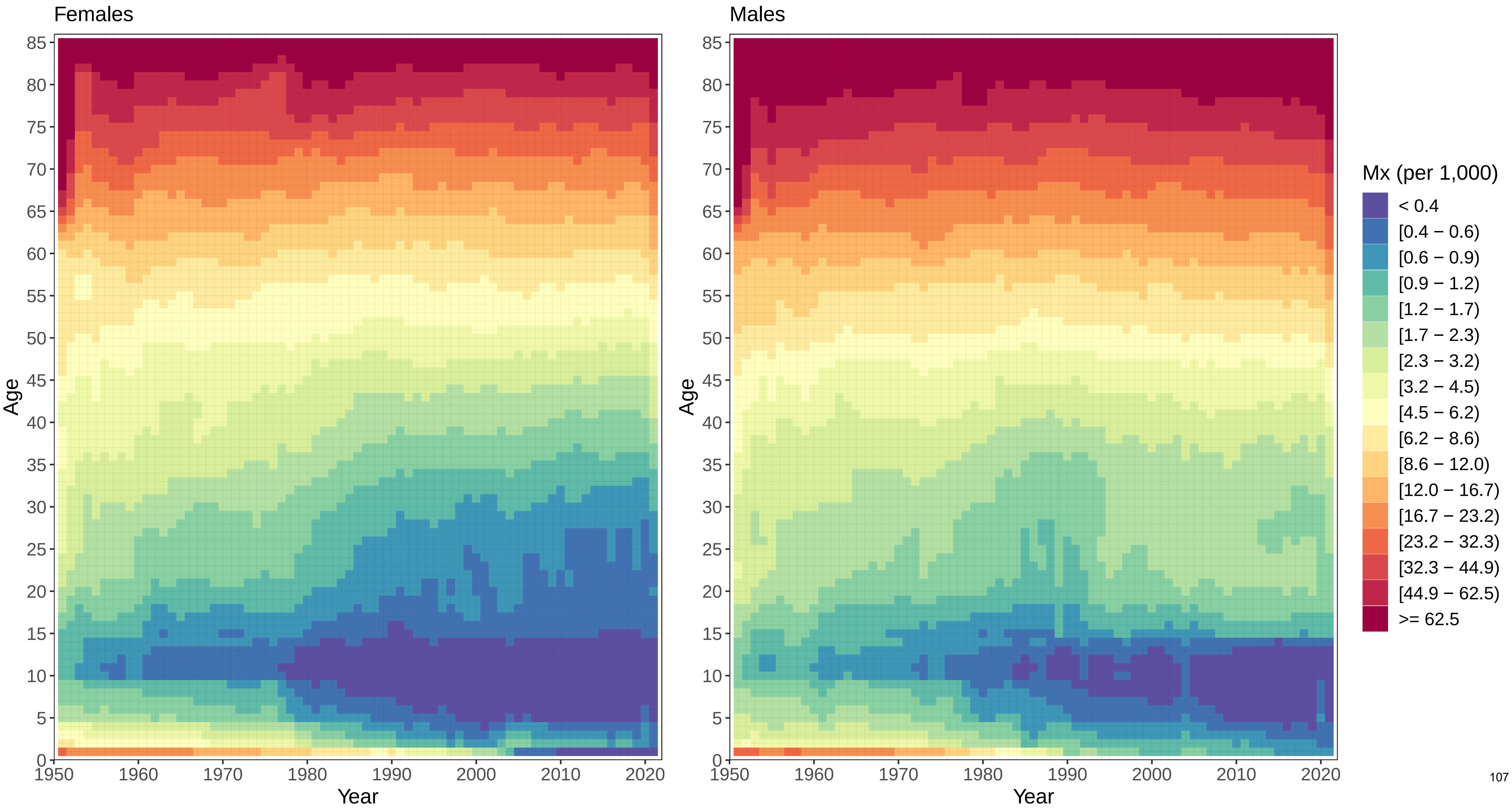

Appendix 2 Figure S5: Afghanistan mortality rates, 1950−2021

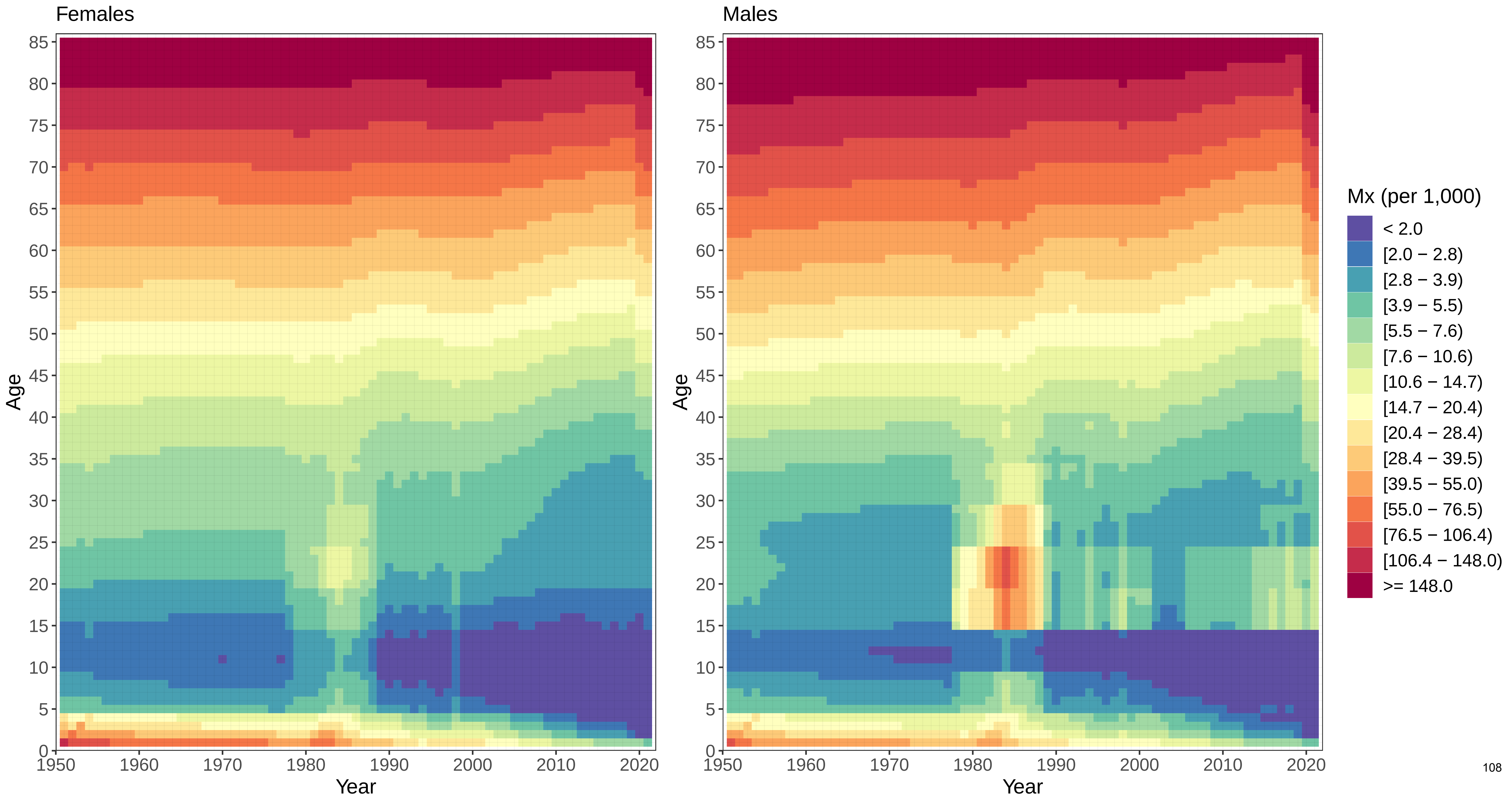

Appendix 2 Figure S5: Algeria mortality rates, 1950−2021

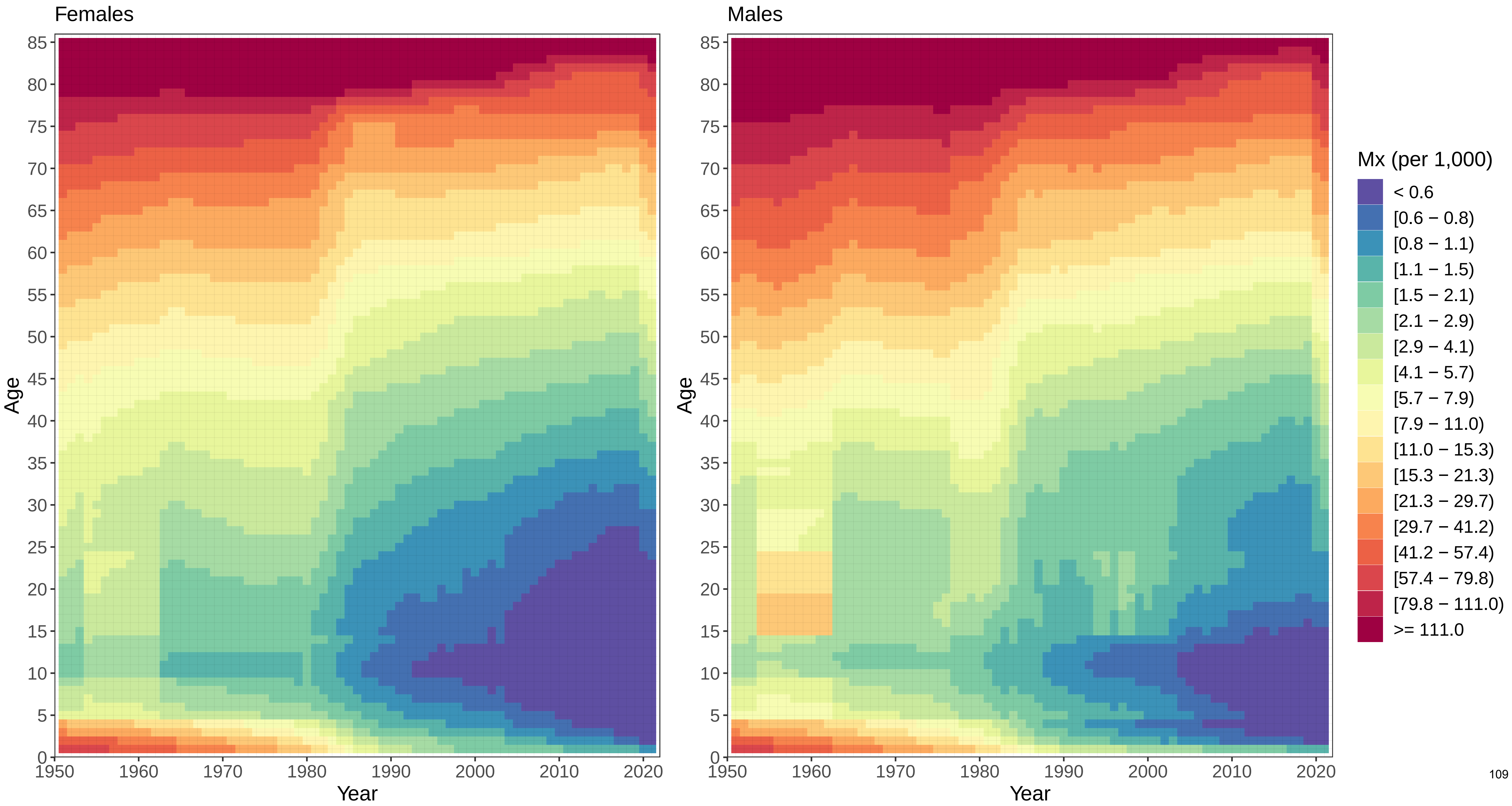

Appendix 2 Figure S5: Bahrain mortality rates, 1950−2021

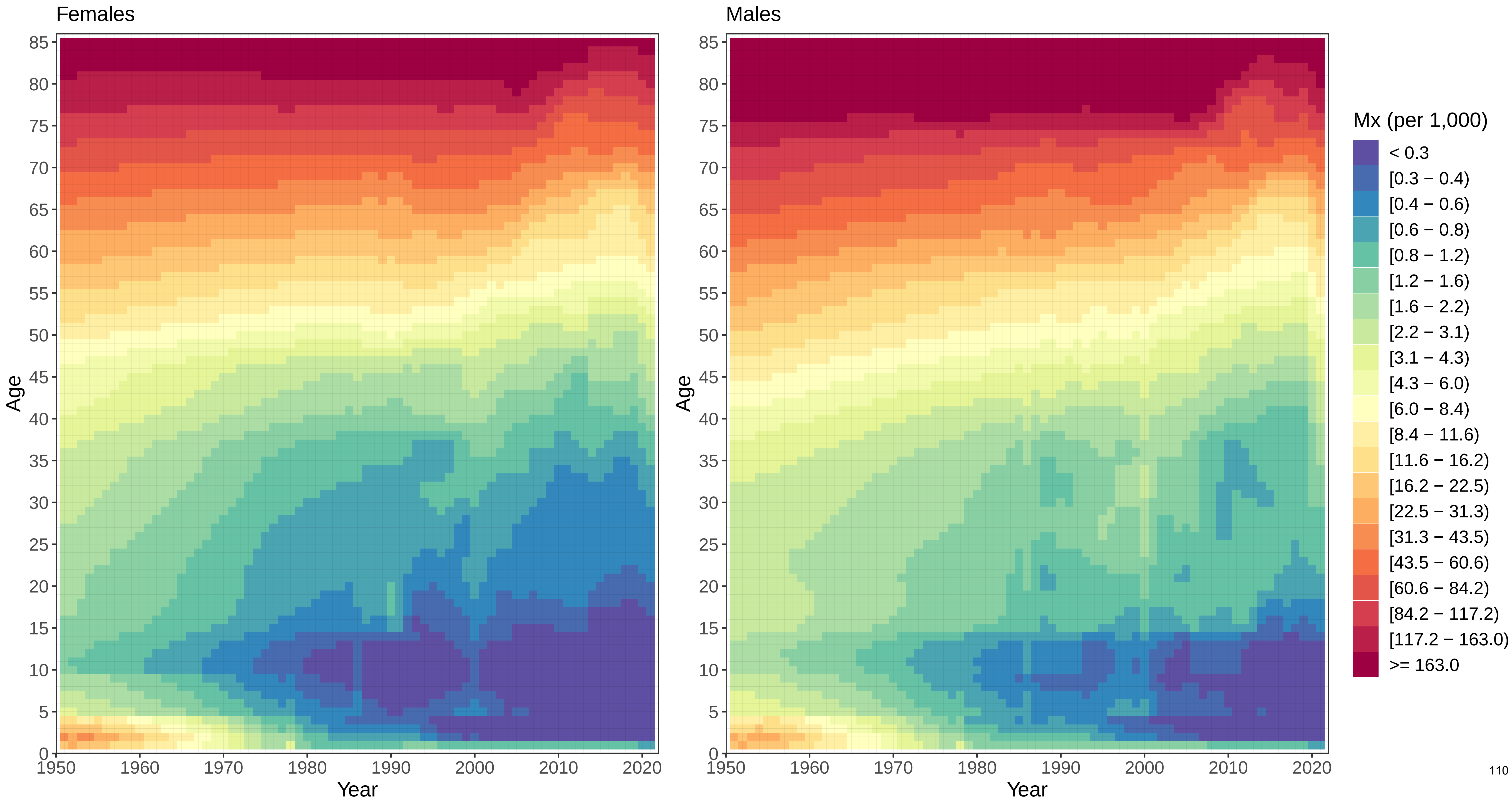

Appendix 2 Figure S5: Egypt mortality rates, 1950−2021

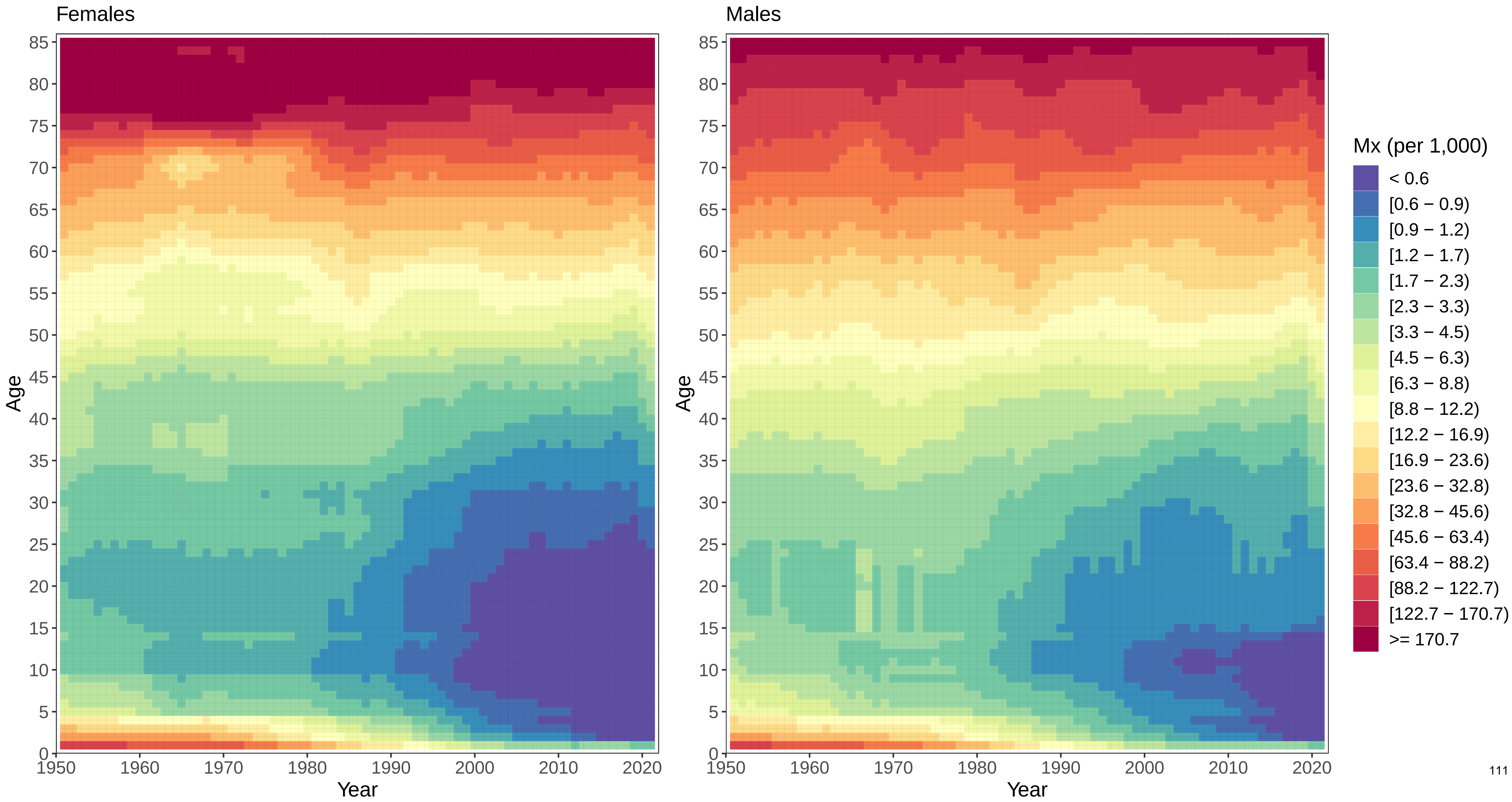

Appendix 2 Figure S5: Iran mortality rates, 1950−2021

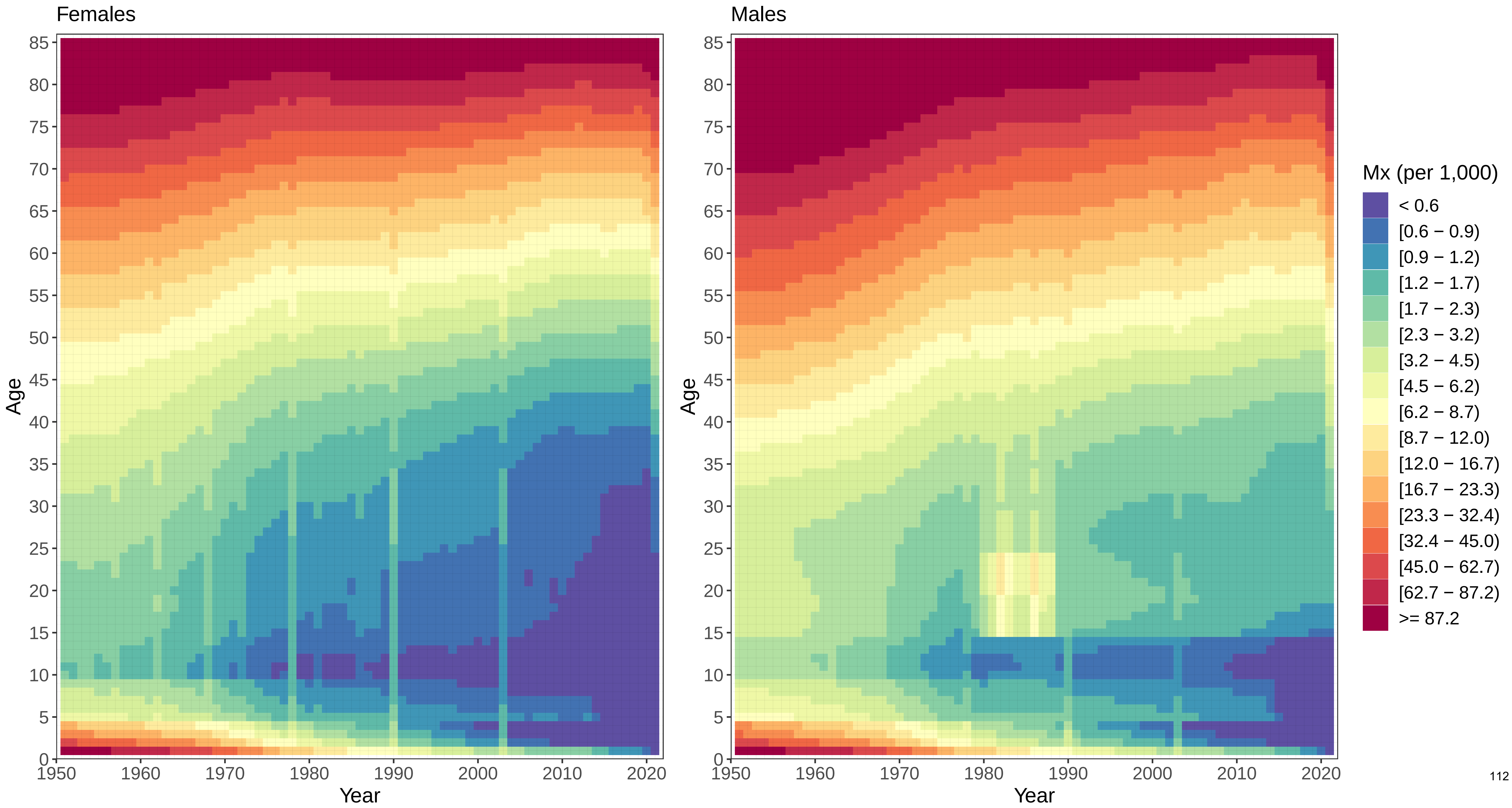

Appendix 2 Figure S5: Iraq mortality rates, 1950−2021

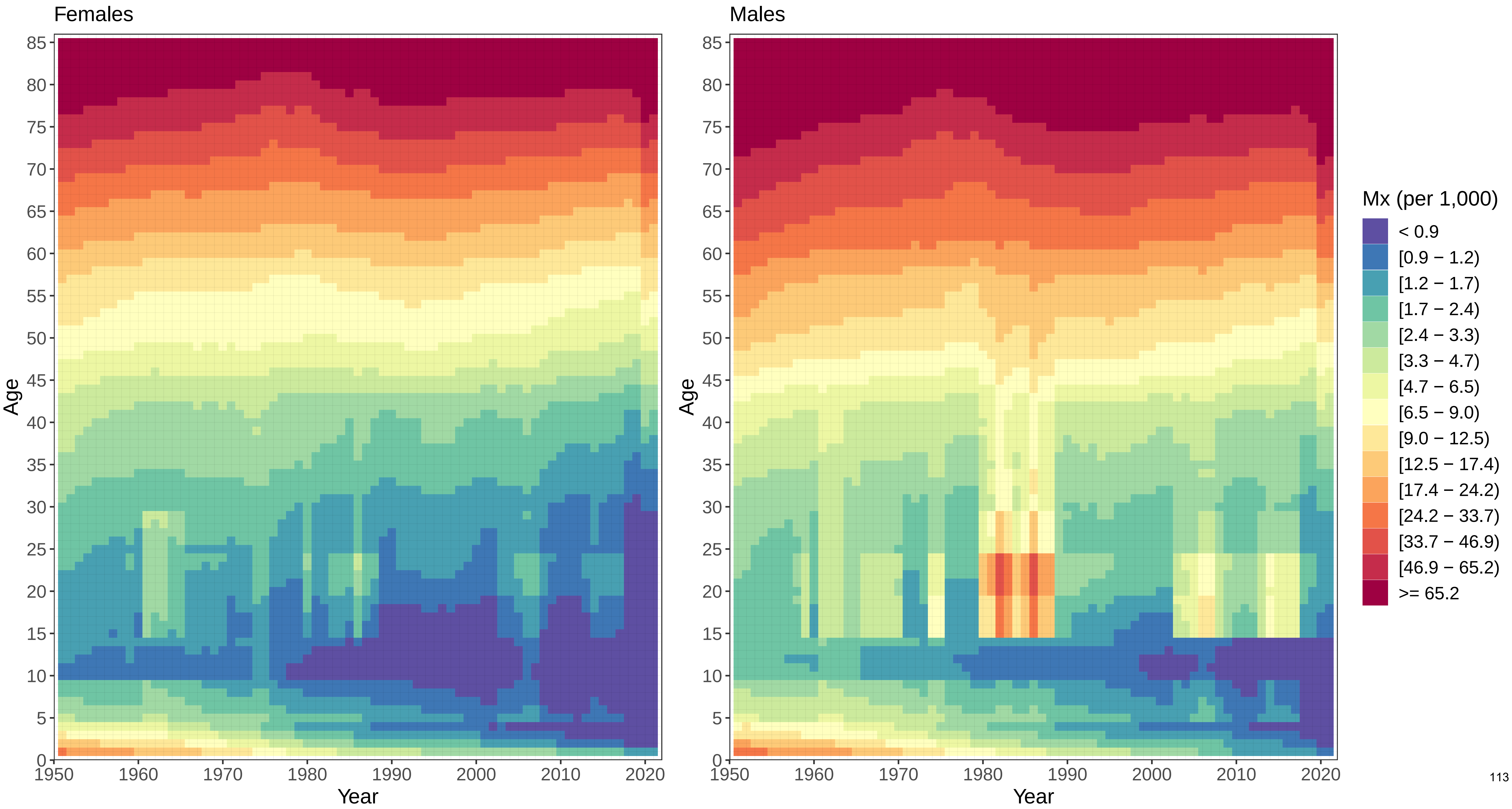

Appendix 2 Figure S5: Jordan mortality rates, 1950−2021

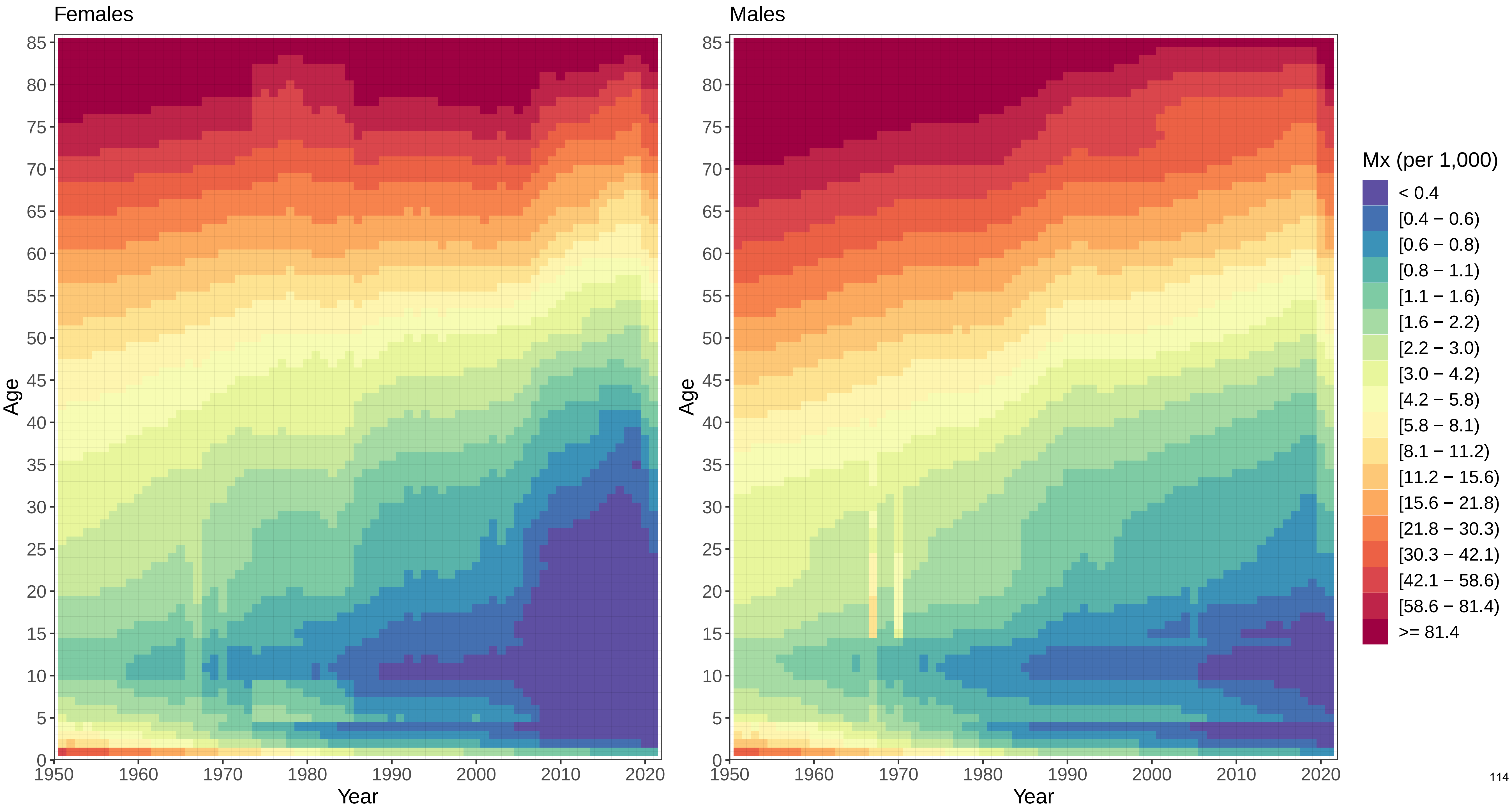

Appendix 2 Figure S5: Kuwait mortality rates, 1950−2021

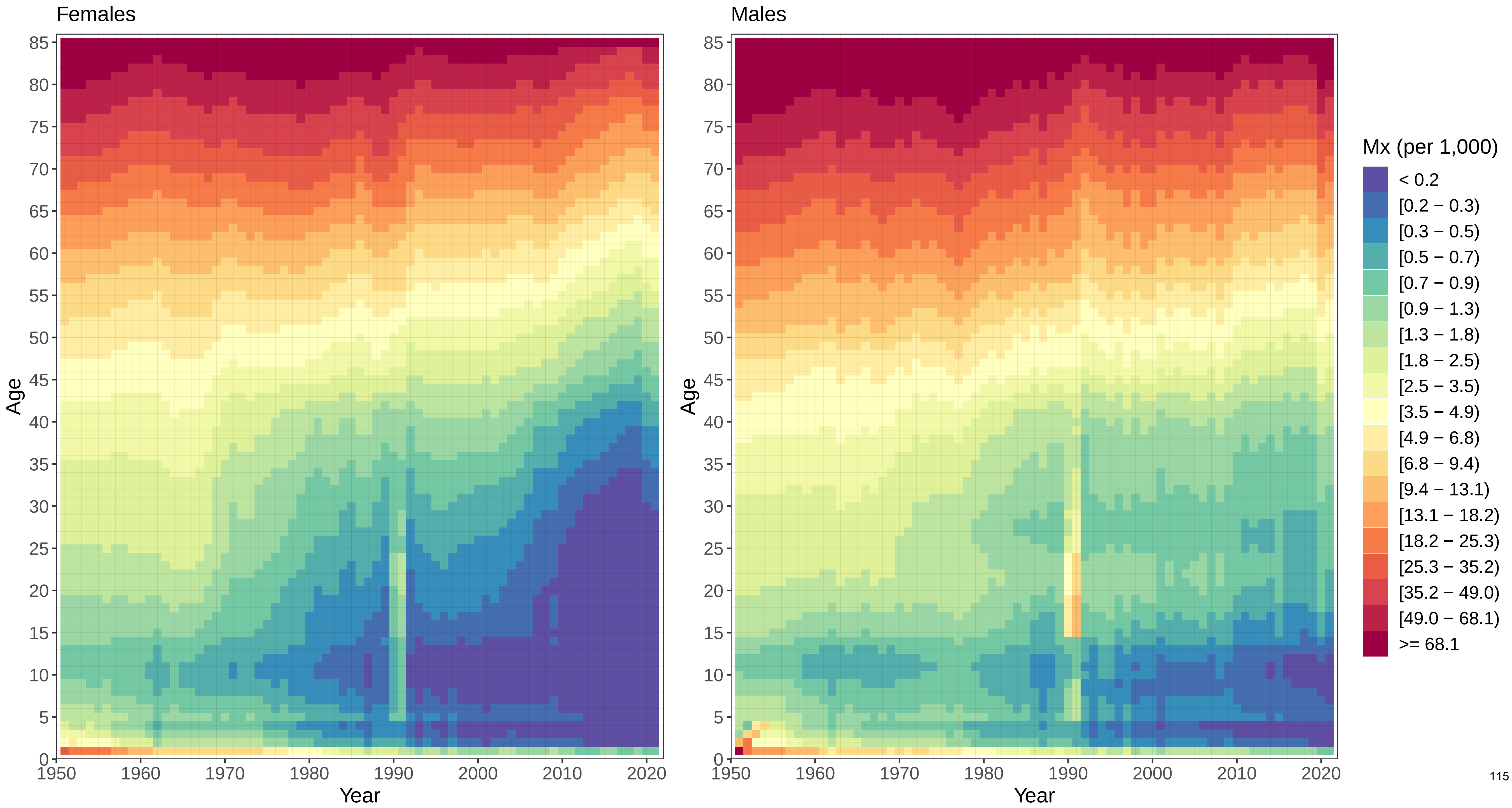

Appendix 2 Figure S5: Lebanon mortality rates, 1950−2021

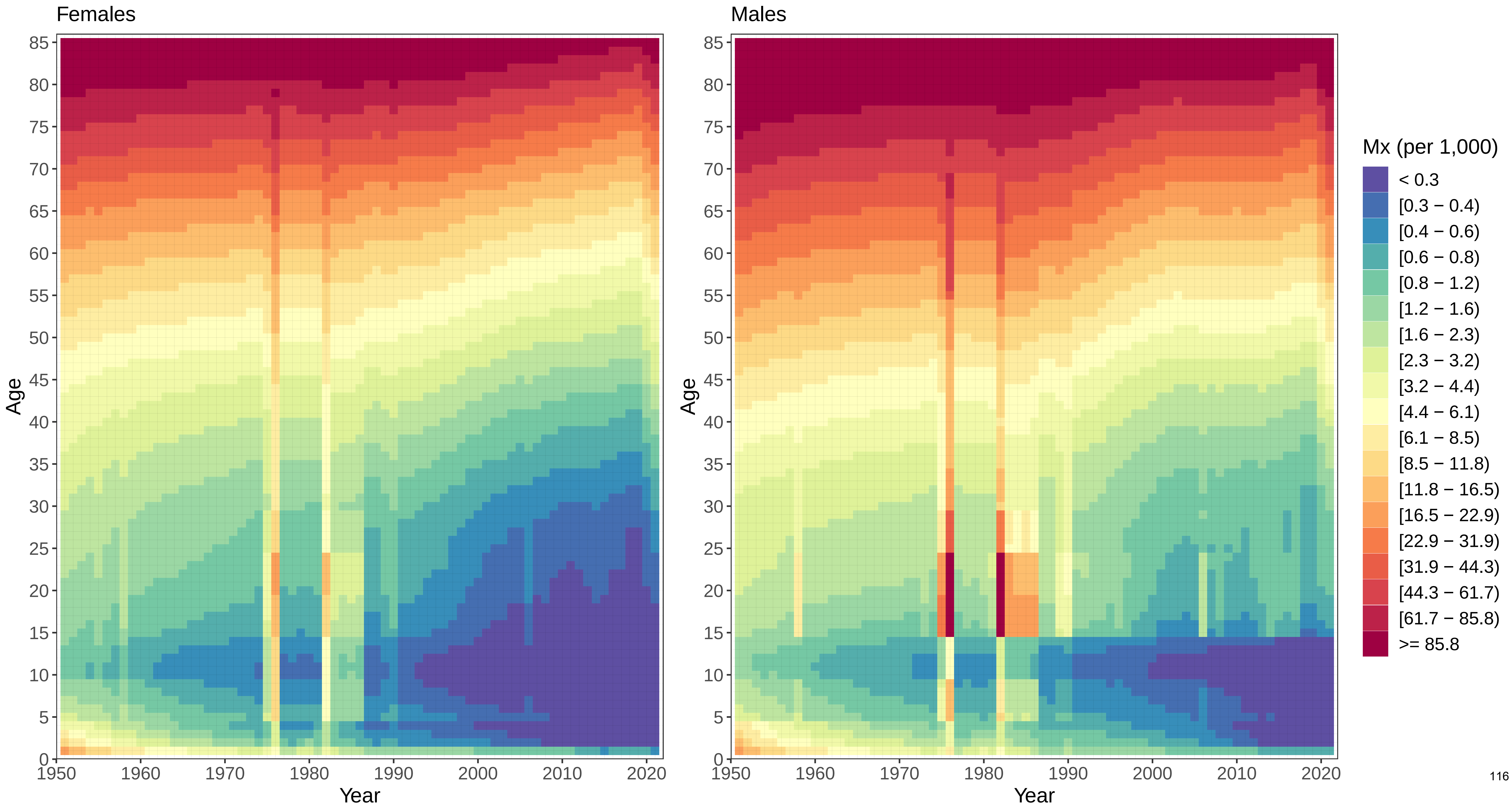

Appendix 2 Figure S5: Libya mortality rates, 1950−2021

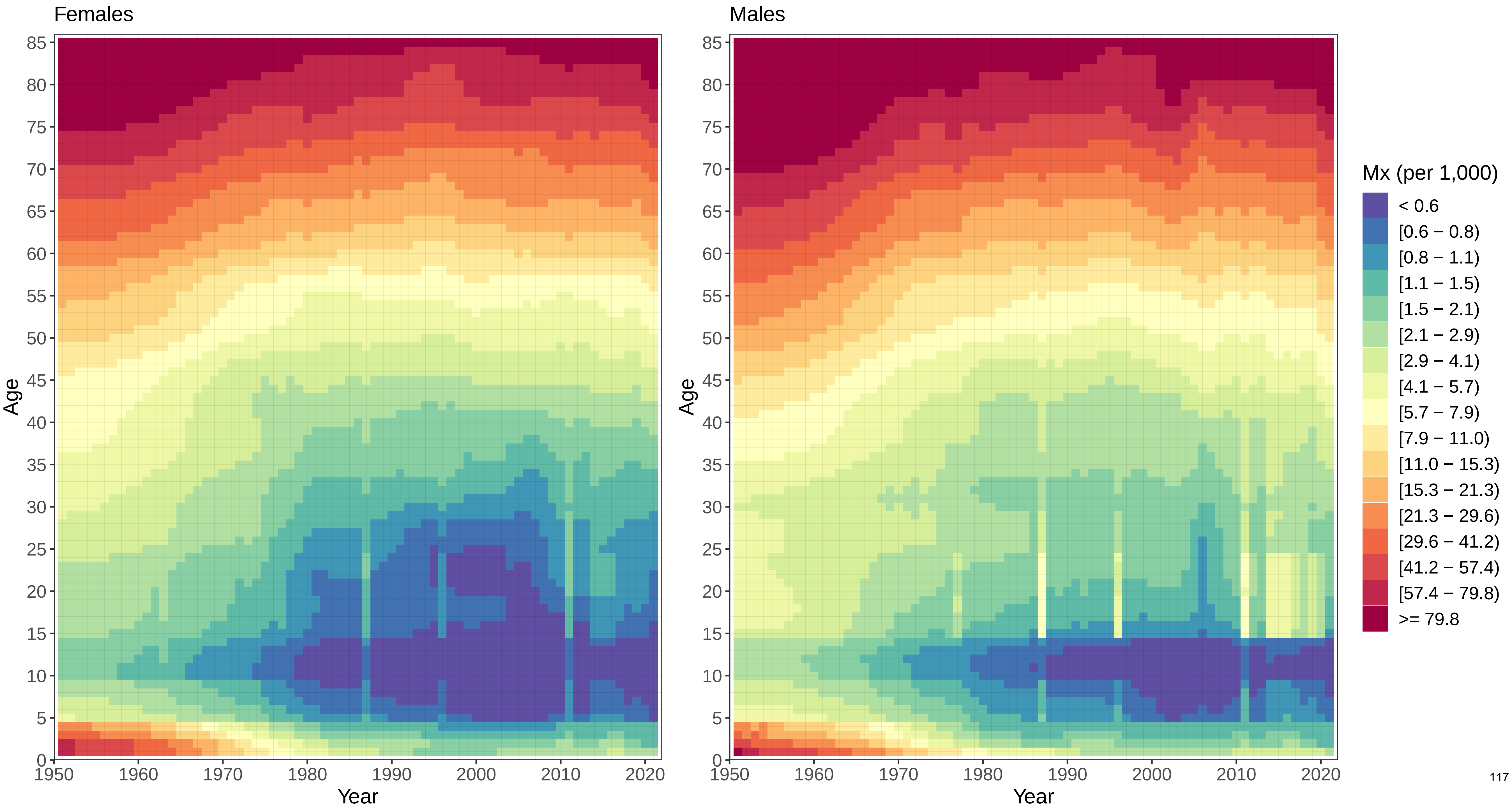

Appendix 2 Figure S5: Morocco mortality rates, 1950−2021

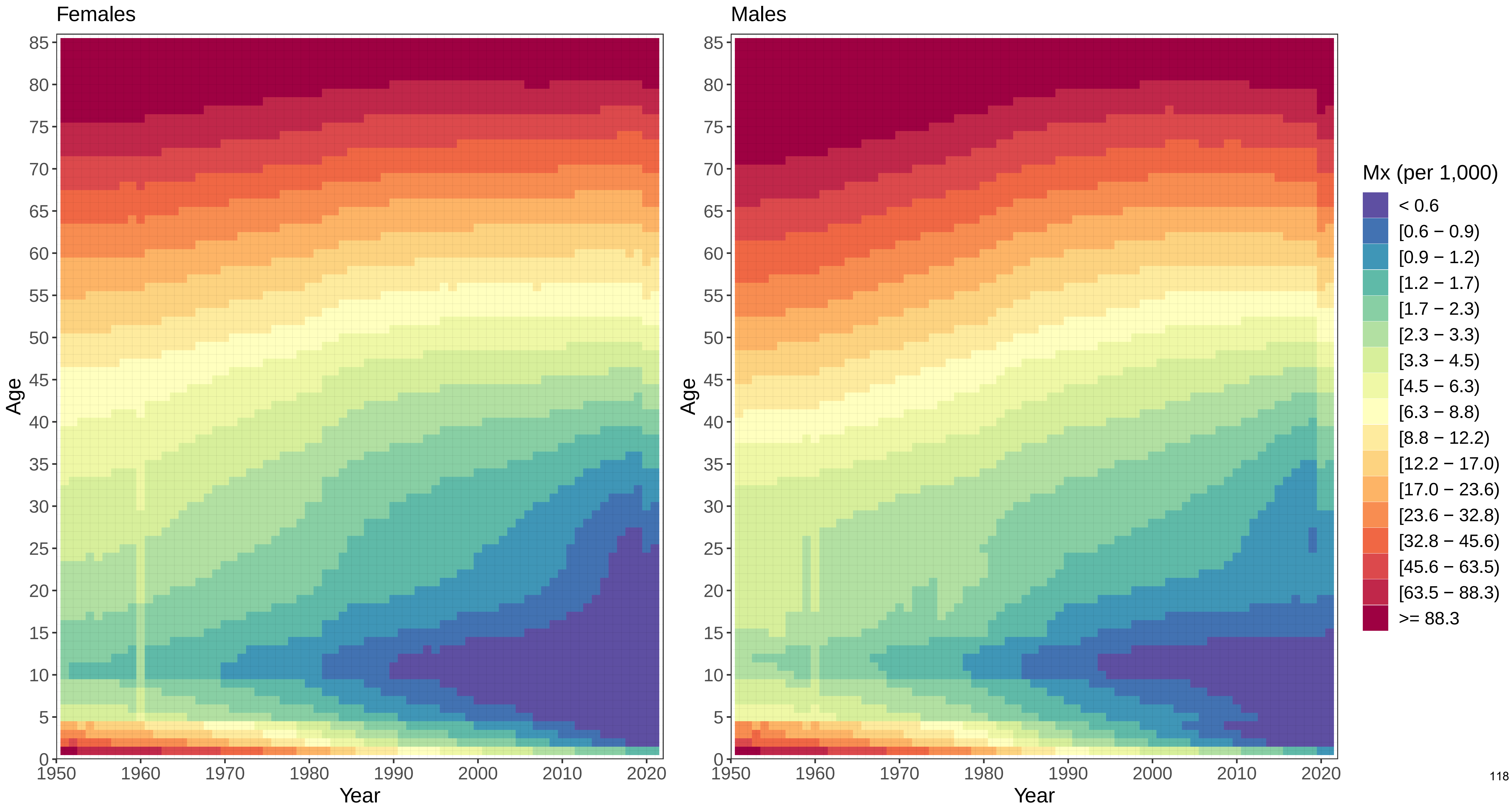

Appendix 2 Figure S5: Oman mortality rates, 1950−2021

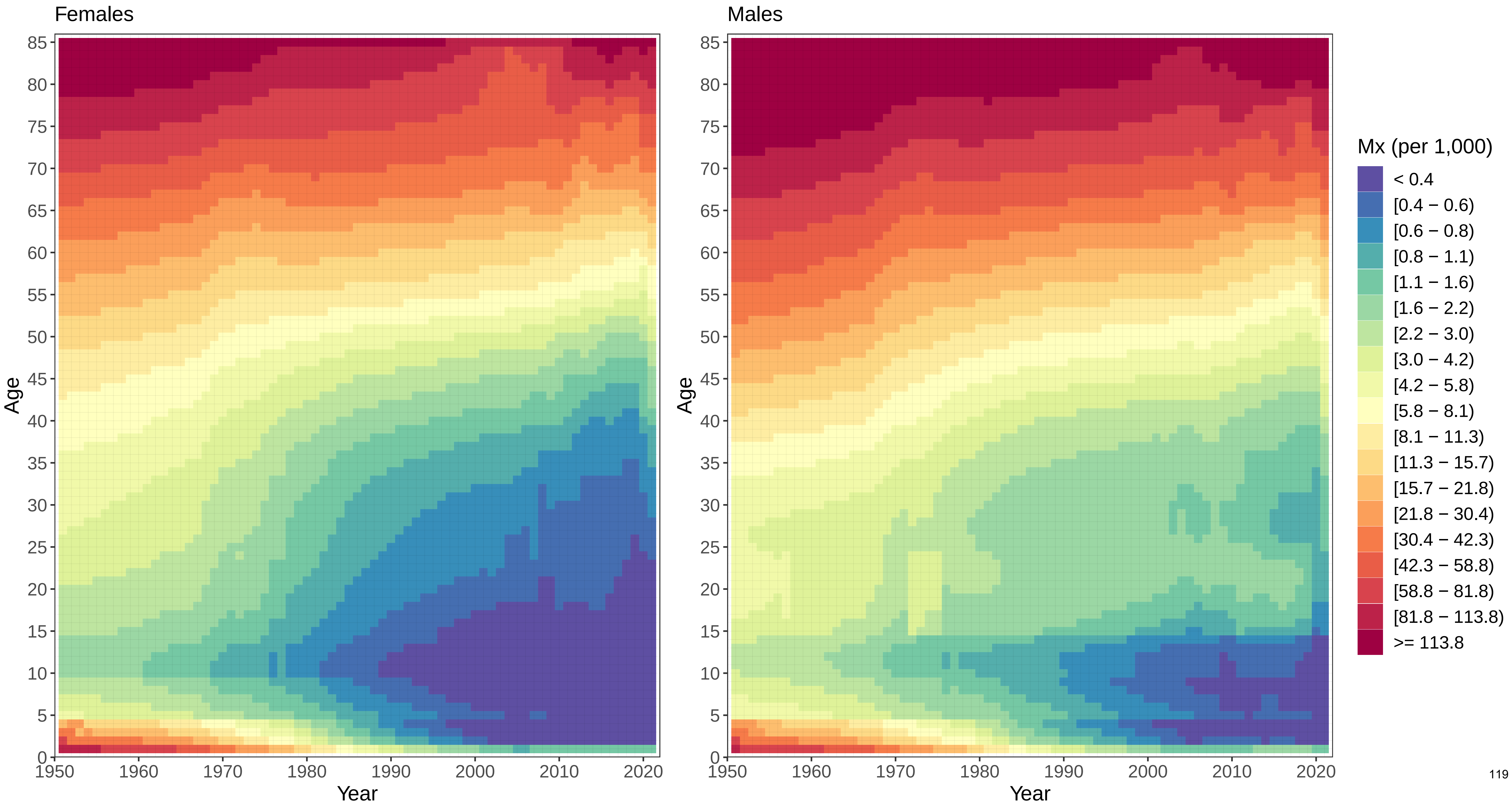

Appendix 2 Figure S5: Palestine mortality rates, 1950−2021

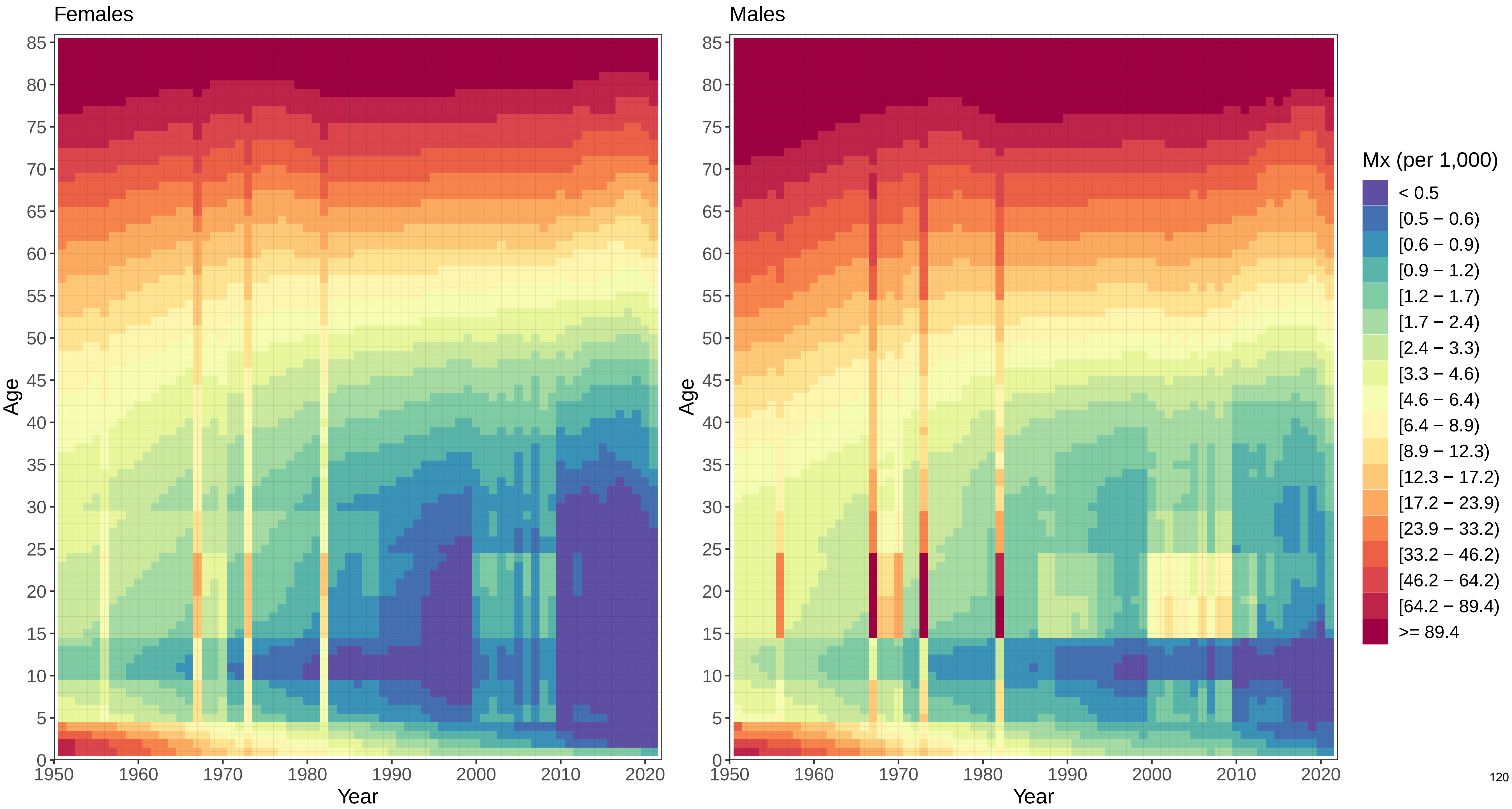

Appendix 2 Figure S5: Qatar mortality rates, 1950−2021

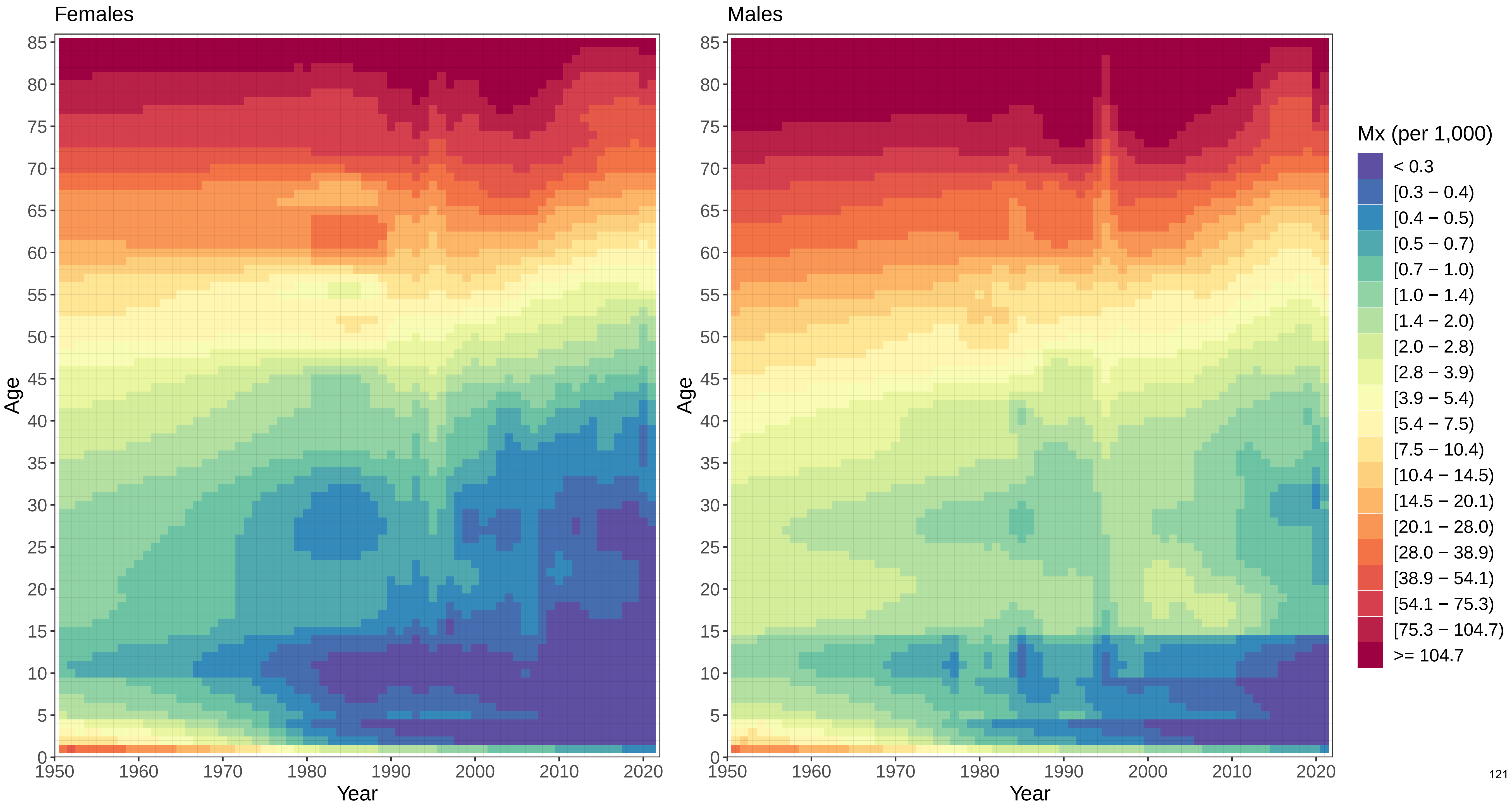

Appendix 2 Figure S5: Saudi Arabia mortality rates, 1950−2021

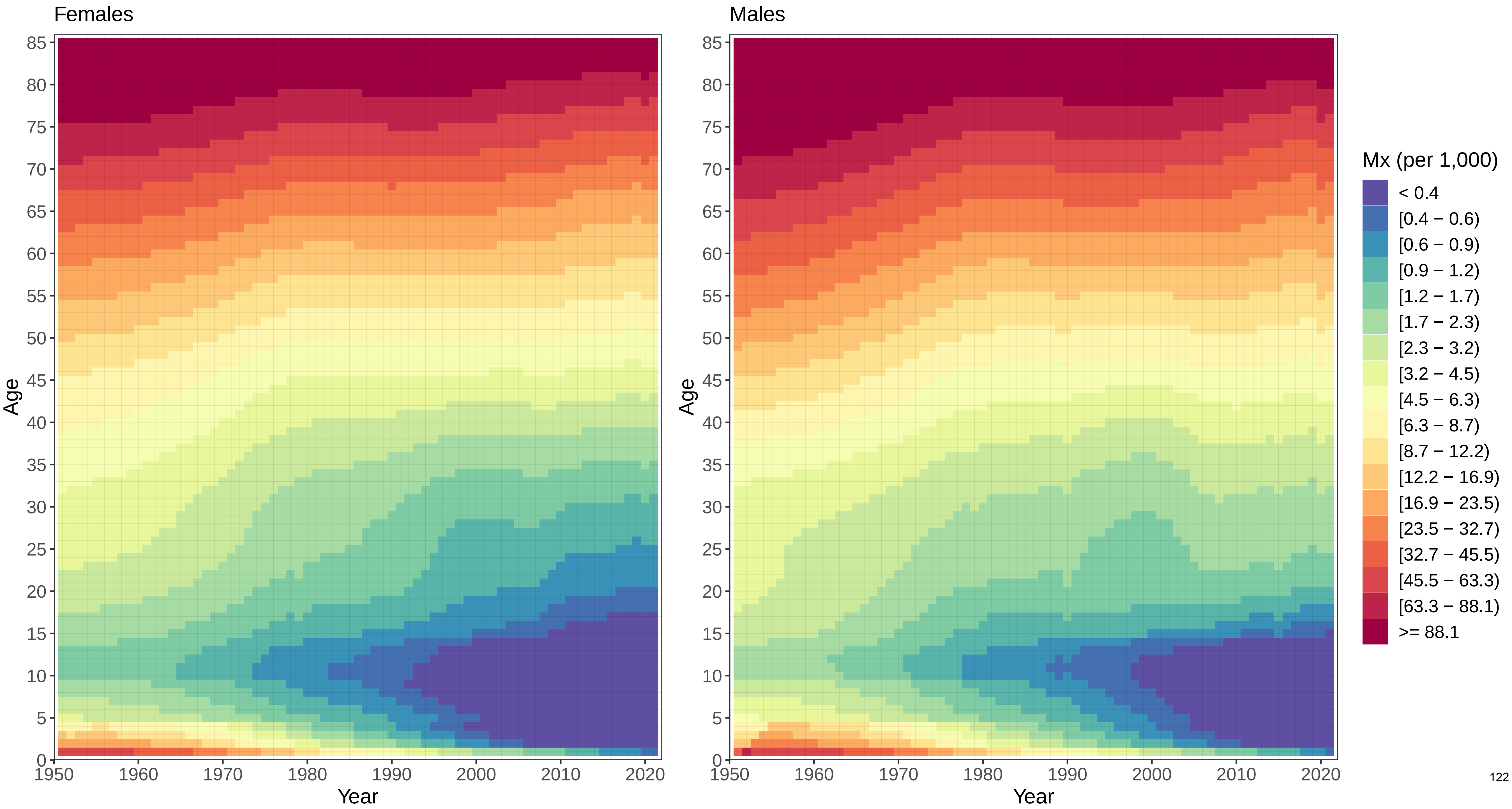

Appendix 2 Figure S5: Sudan mortality rates, 1950−2021

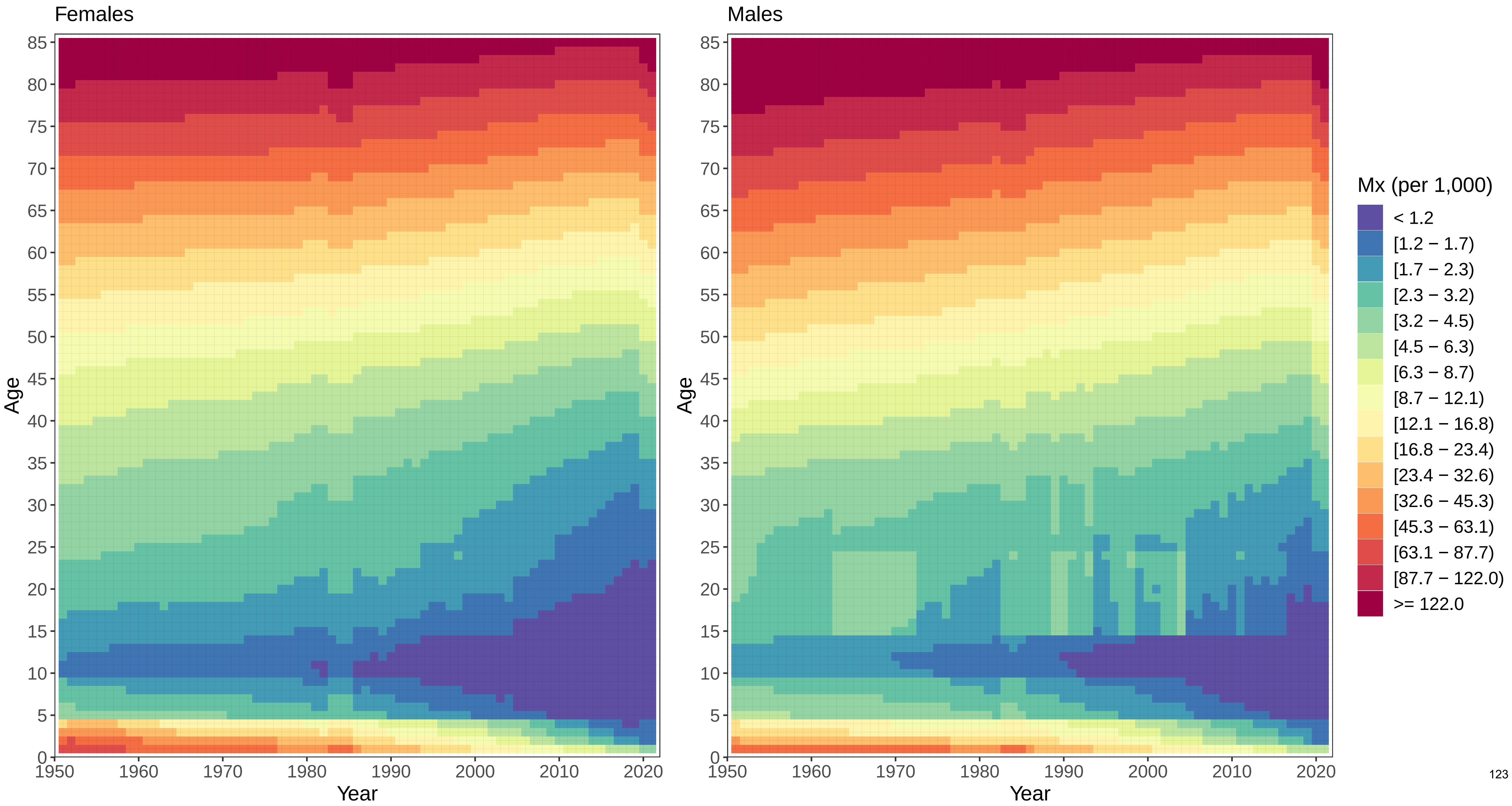

Appendix 2 Figure S5: Syria mortality rates, 1950−2021

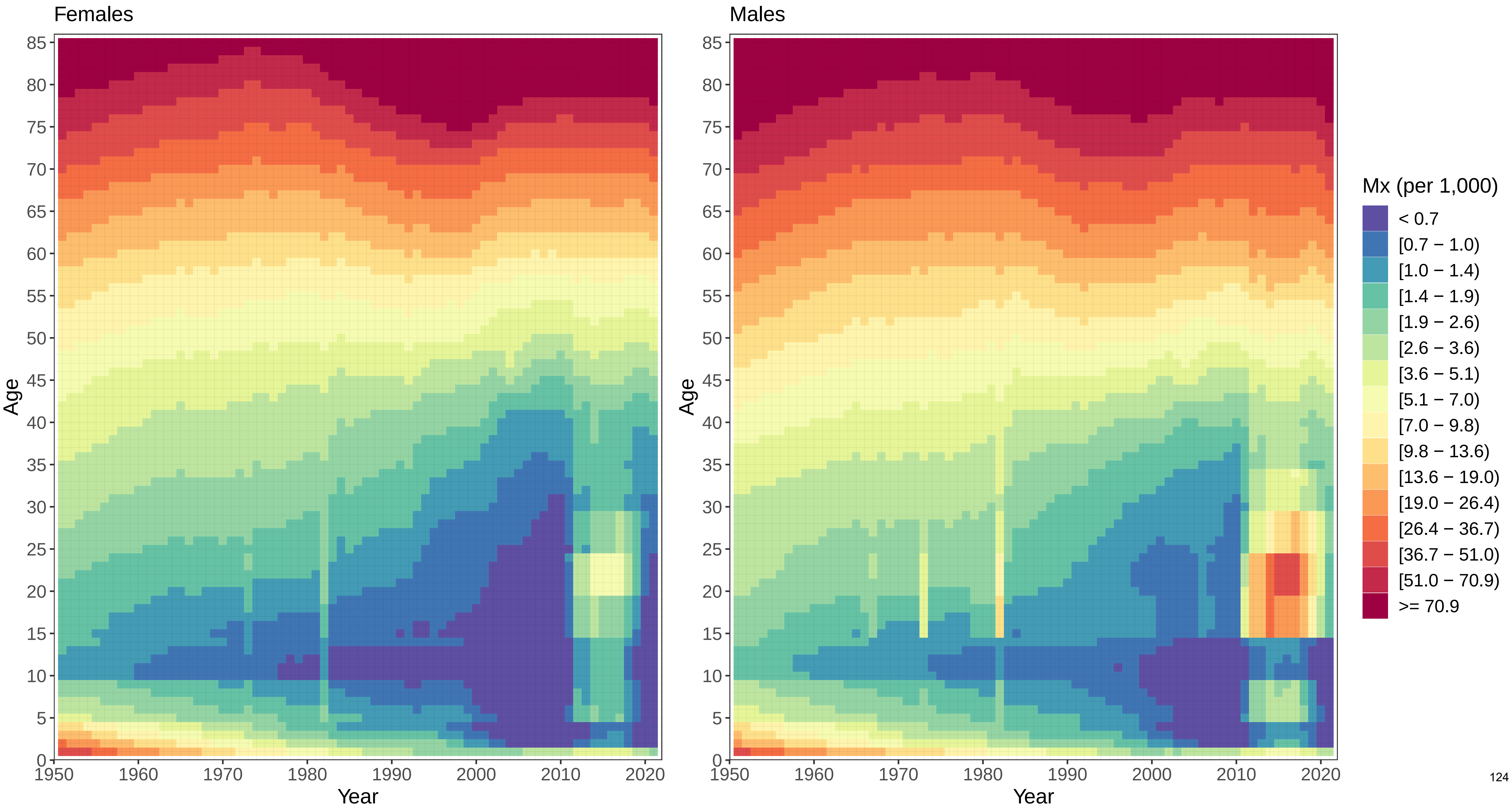

Appendix 2 Figure S5: Tunisia mortality rates, 1950−2021

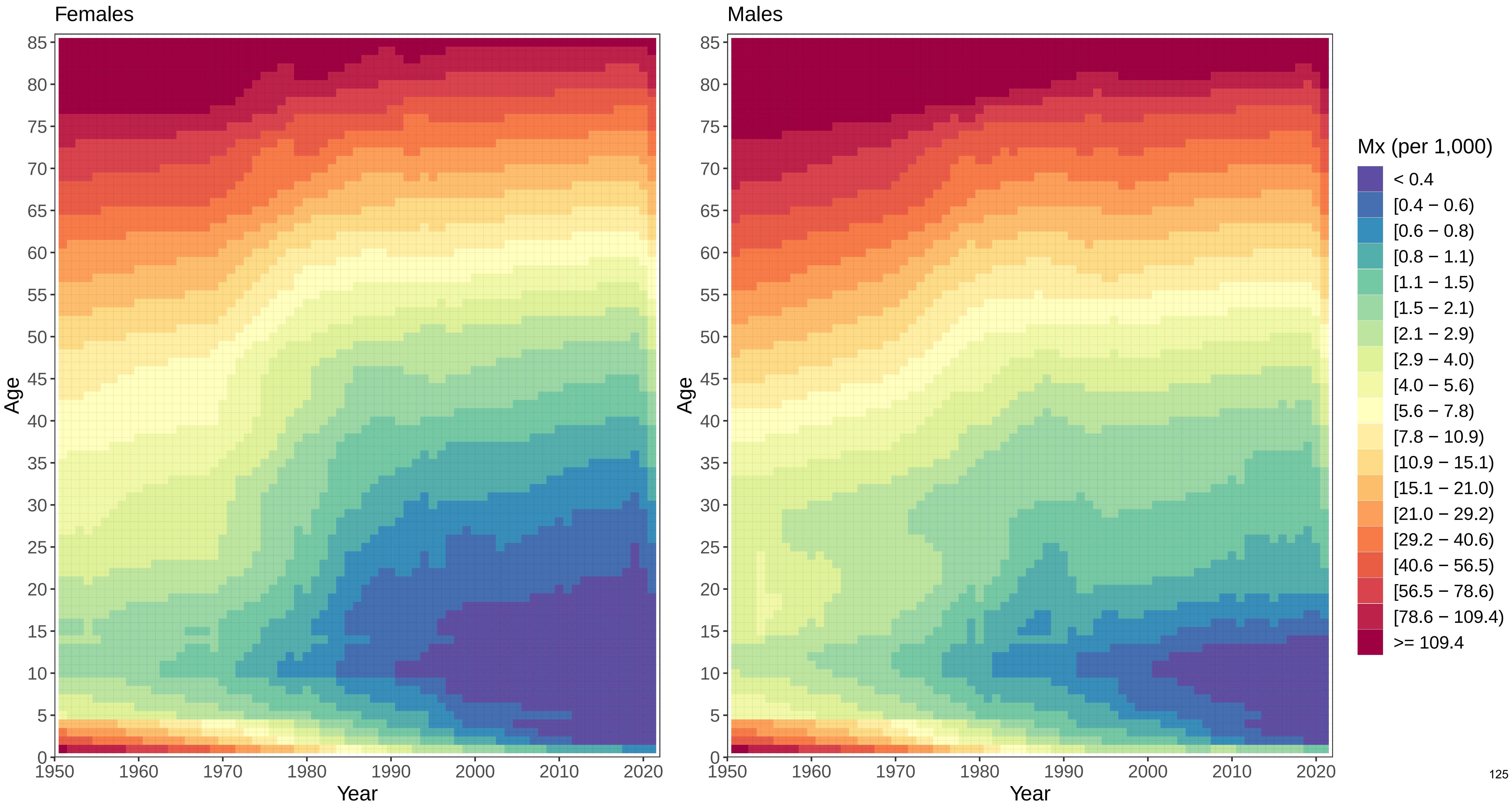

Appendix 2 Figure S5: Türkiye mortality rates, 1950−2021

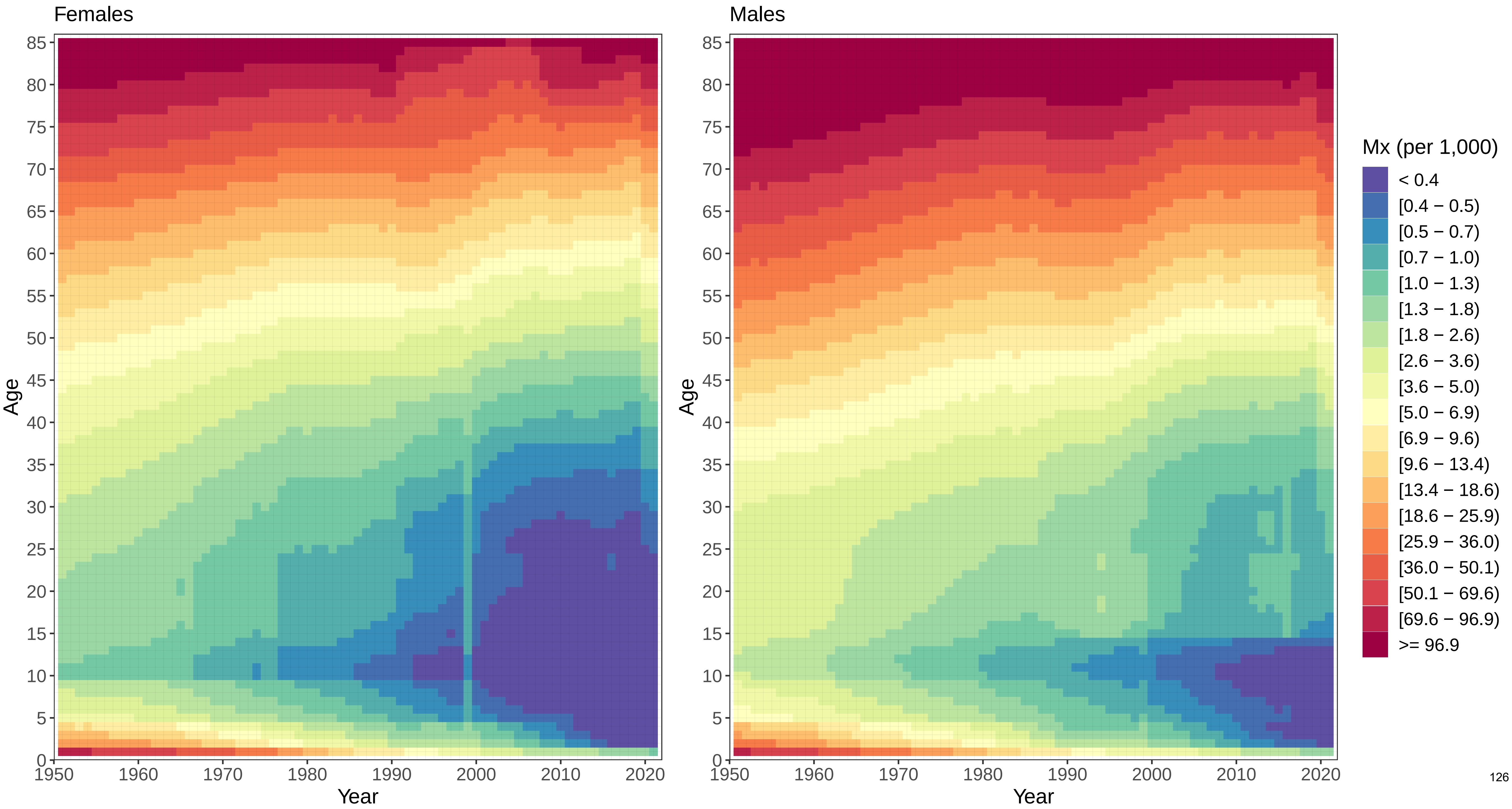

Appendix 2 Figure S5: United Arab Emirates mortality rates, 1950−2021

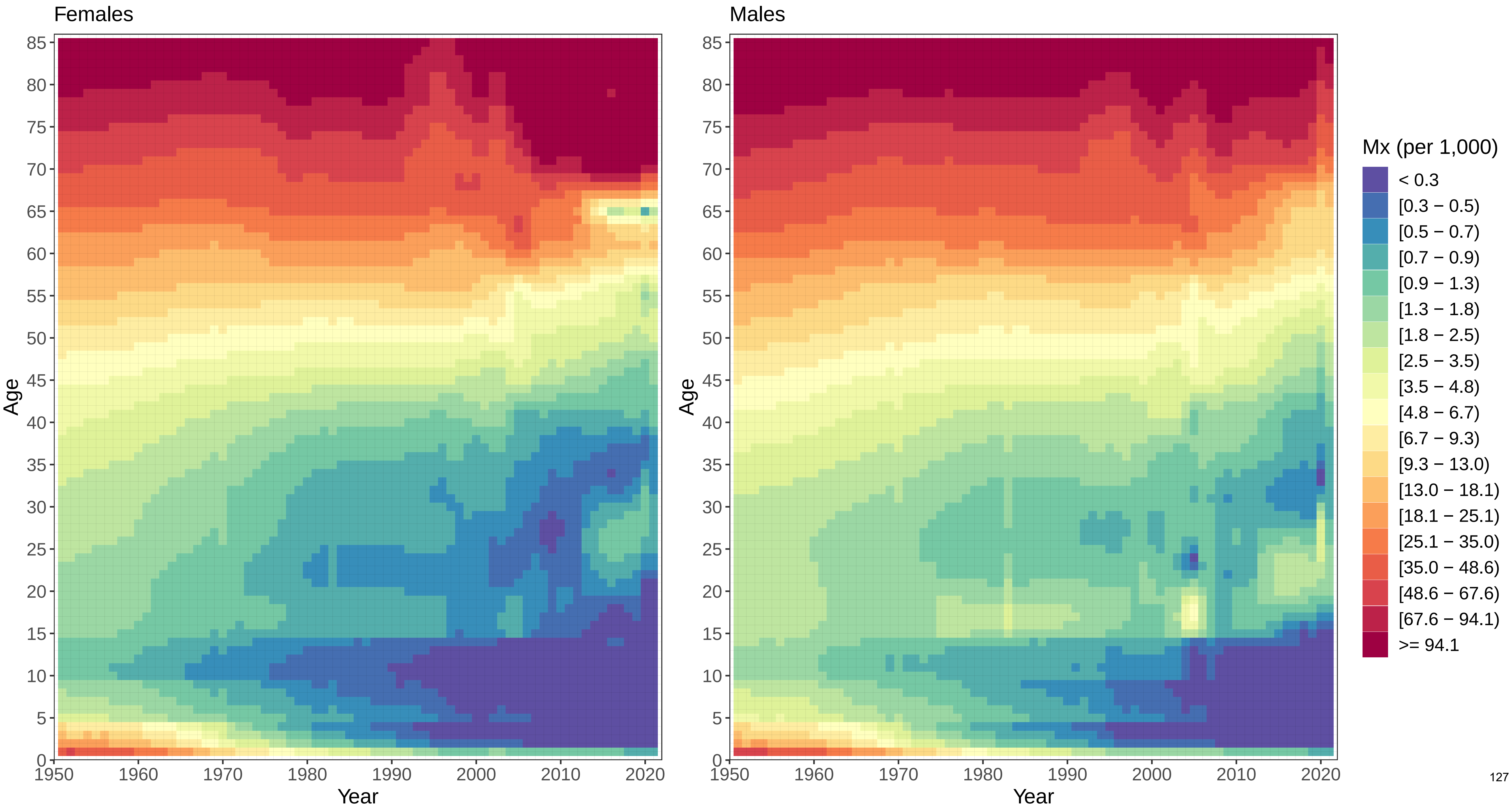

Appendix 2 Figure S5: Yemen mortality rates, 1950−2021

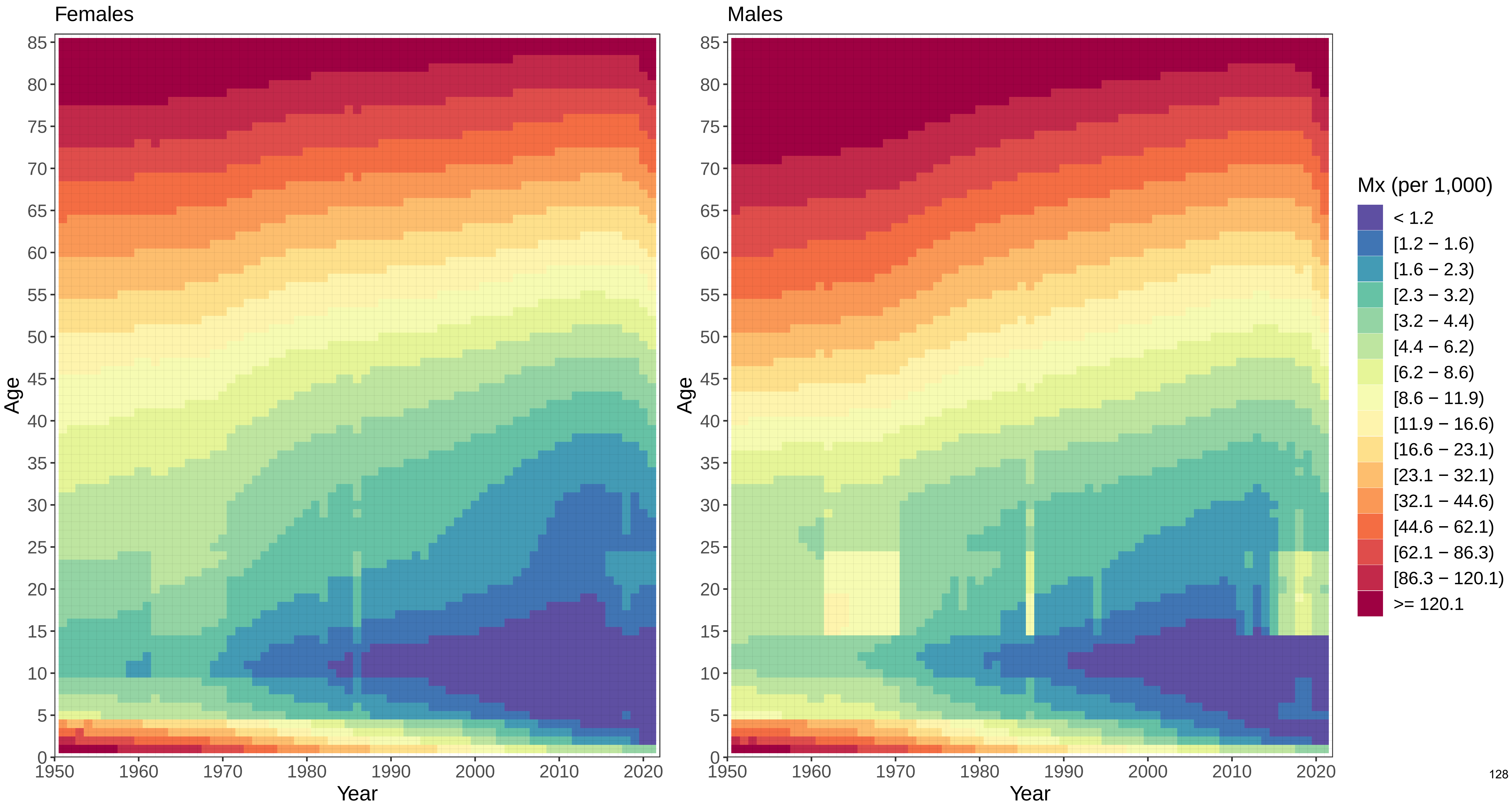

Appendix 2 Figure S5: Bangladesh mortality rates, 1950−2021

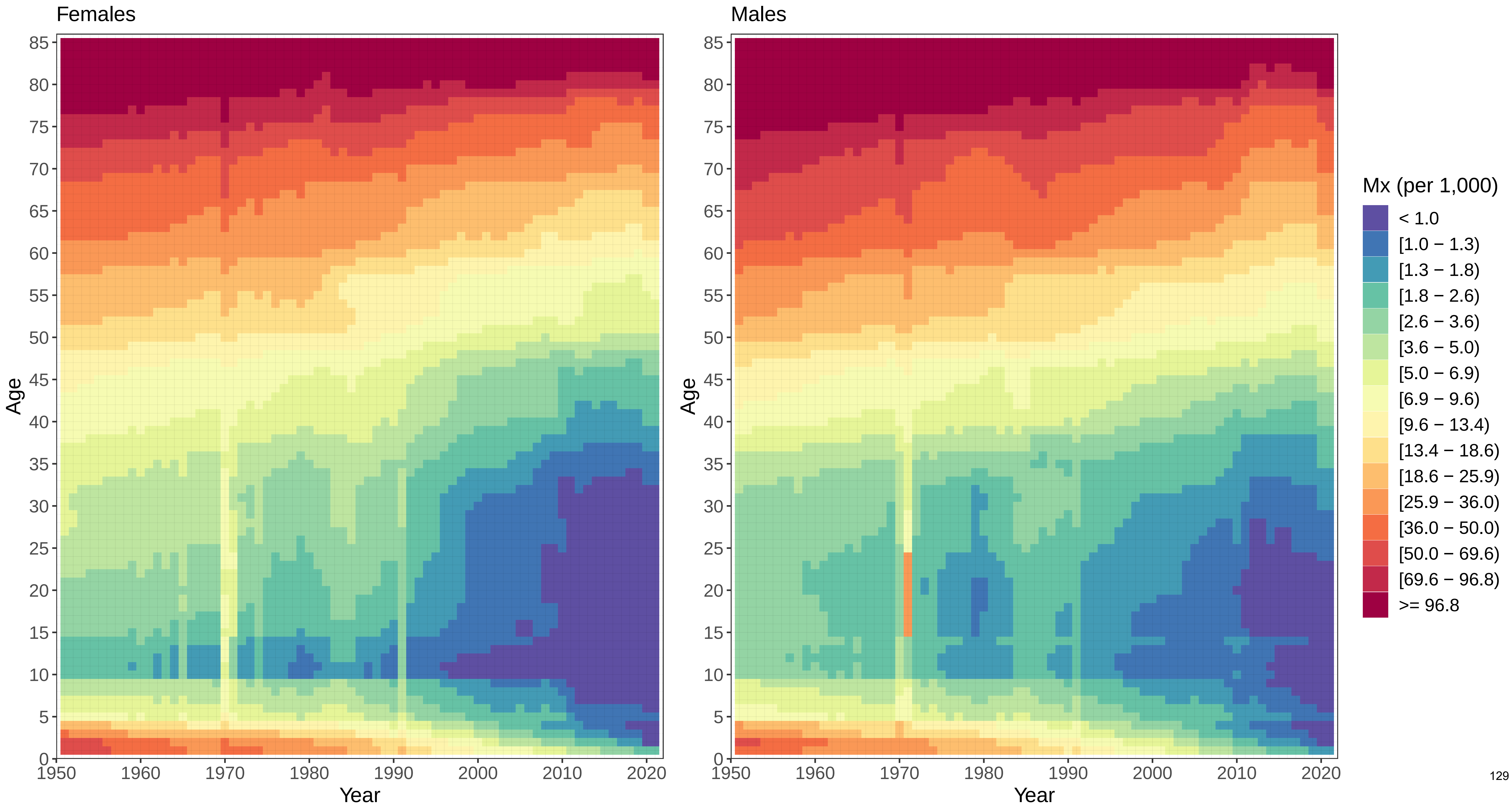

Appendix 2 Figure S5: Bhutan mortality rates, 1950−2021

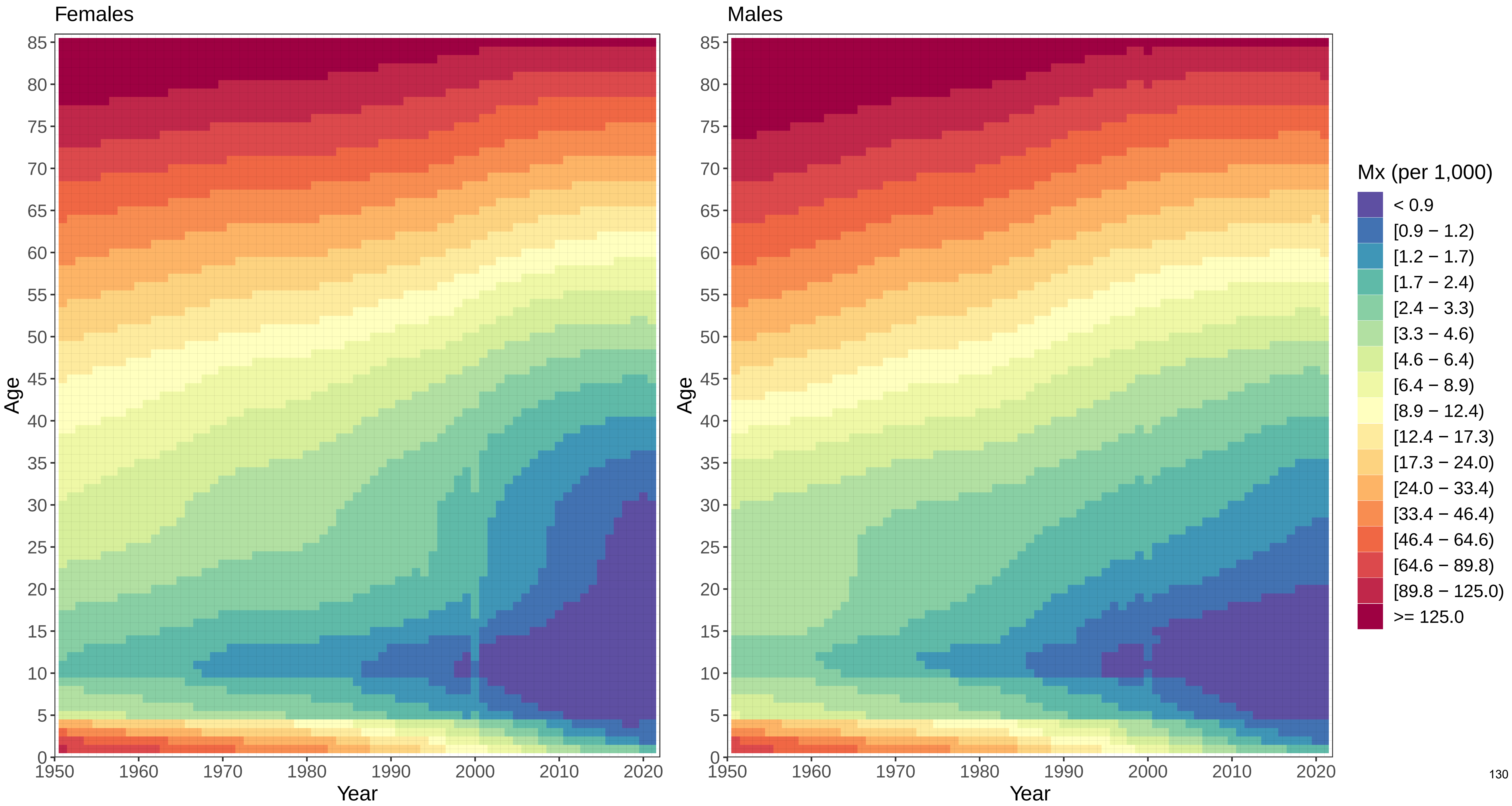

Appendix 2 Figure S5: India mortality rates, 1950−2021

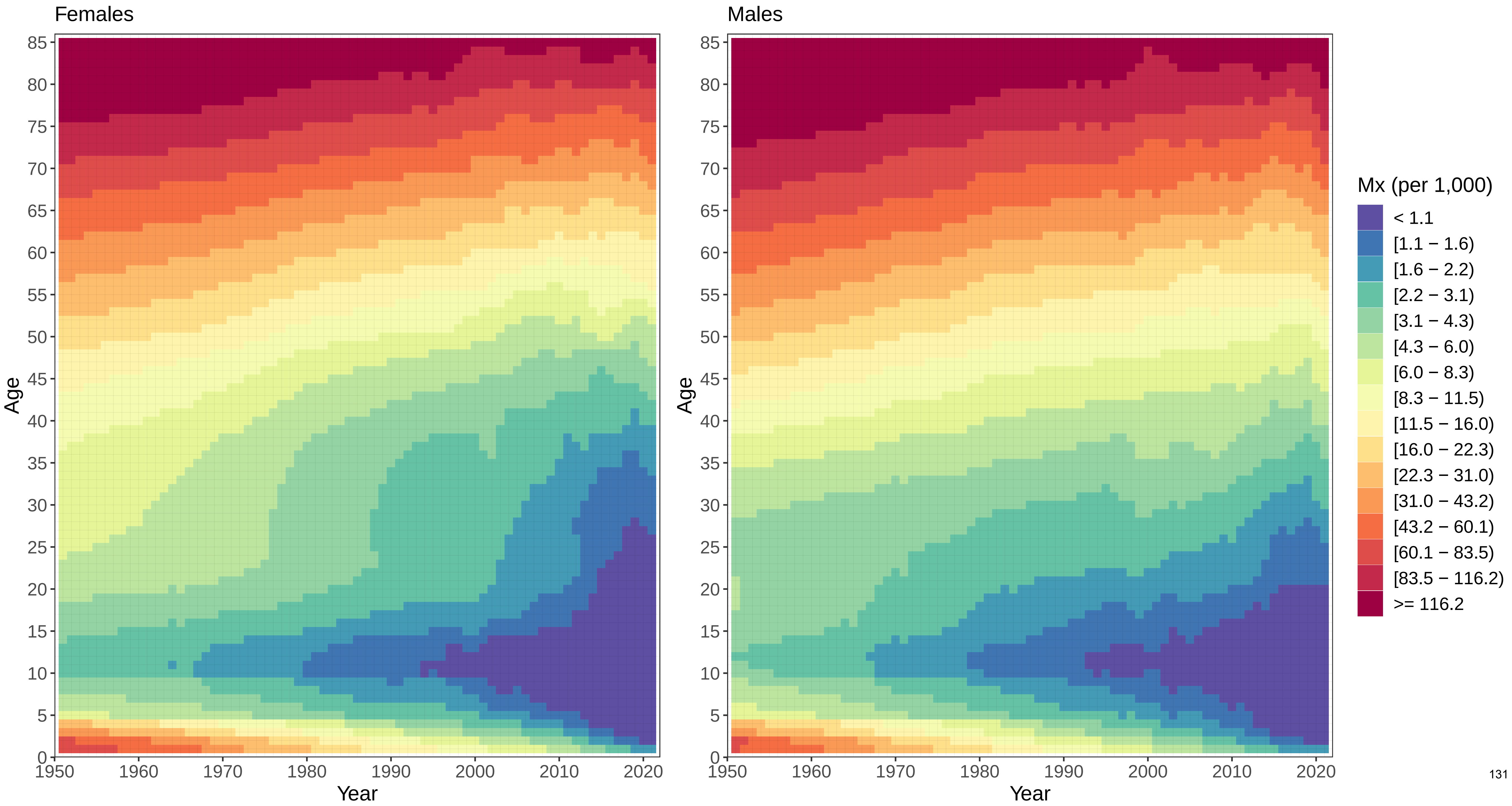

Appendix 2 Figure S5: Nepal mortality rates, 1950−2021

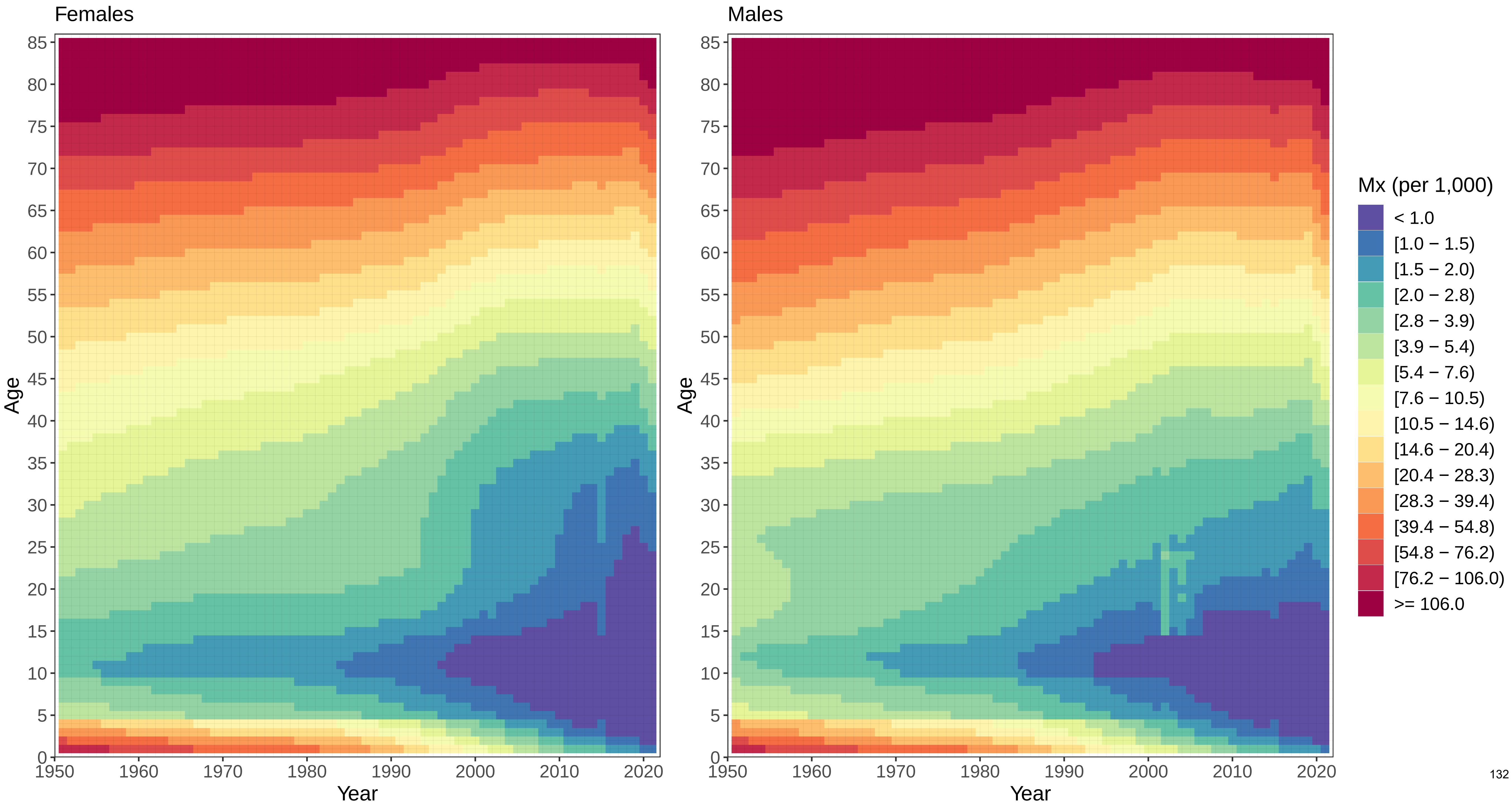

Appendix 2 Figure S5: Pakistan mortality rates, 1950−2021

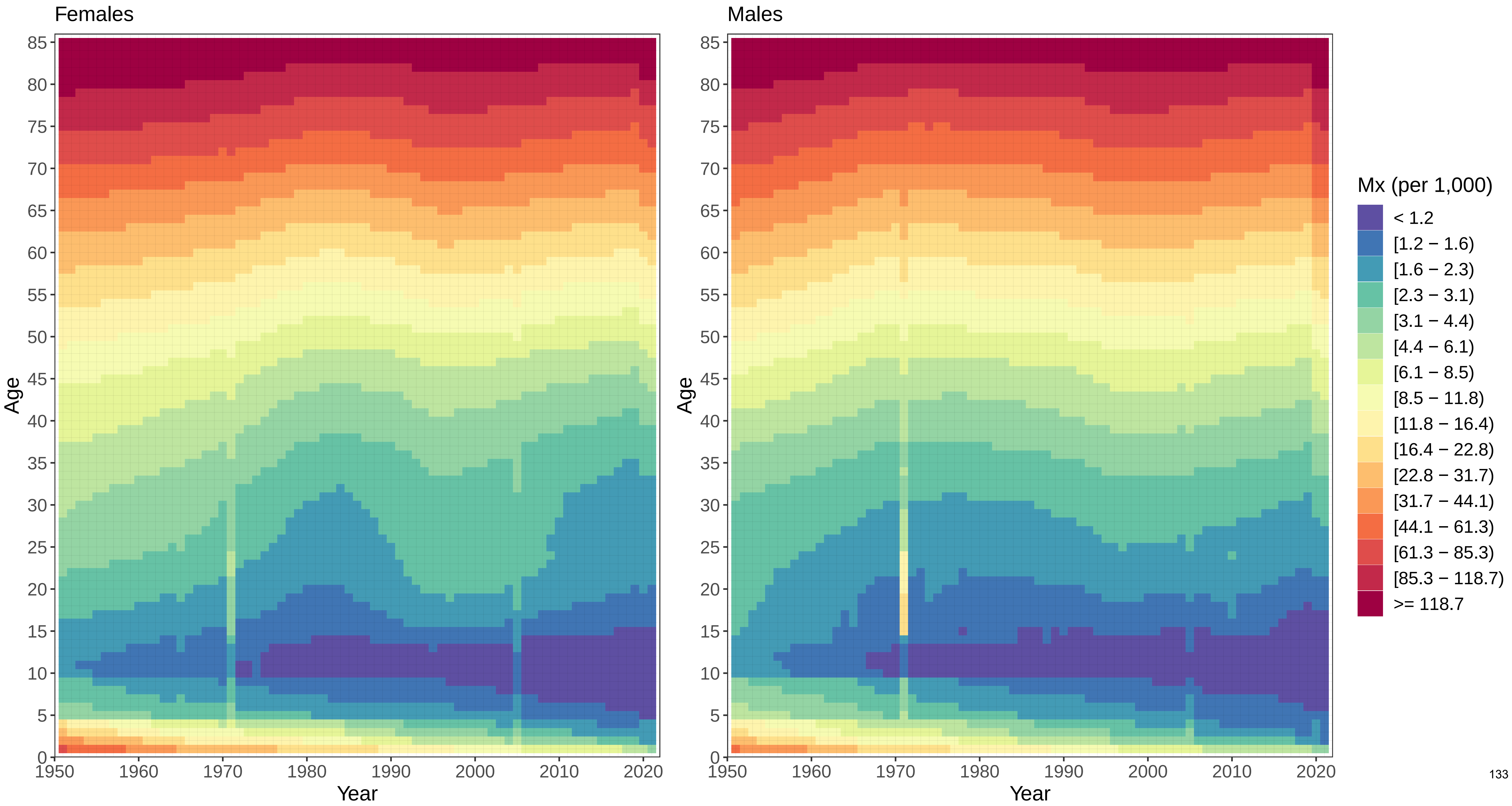

Appendix 2 Figure S5: China (without Hong Kong and Macao) mortality rates, 1950−2021

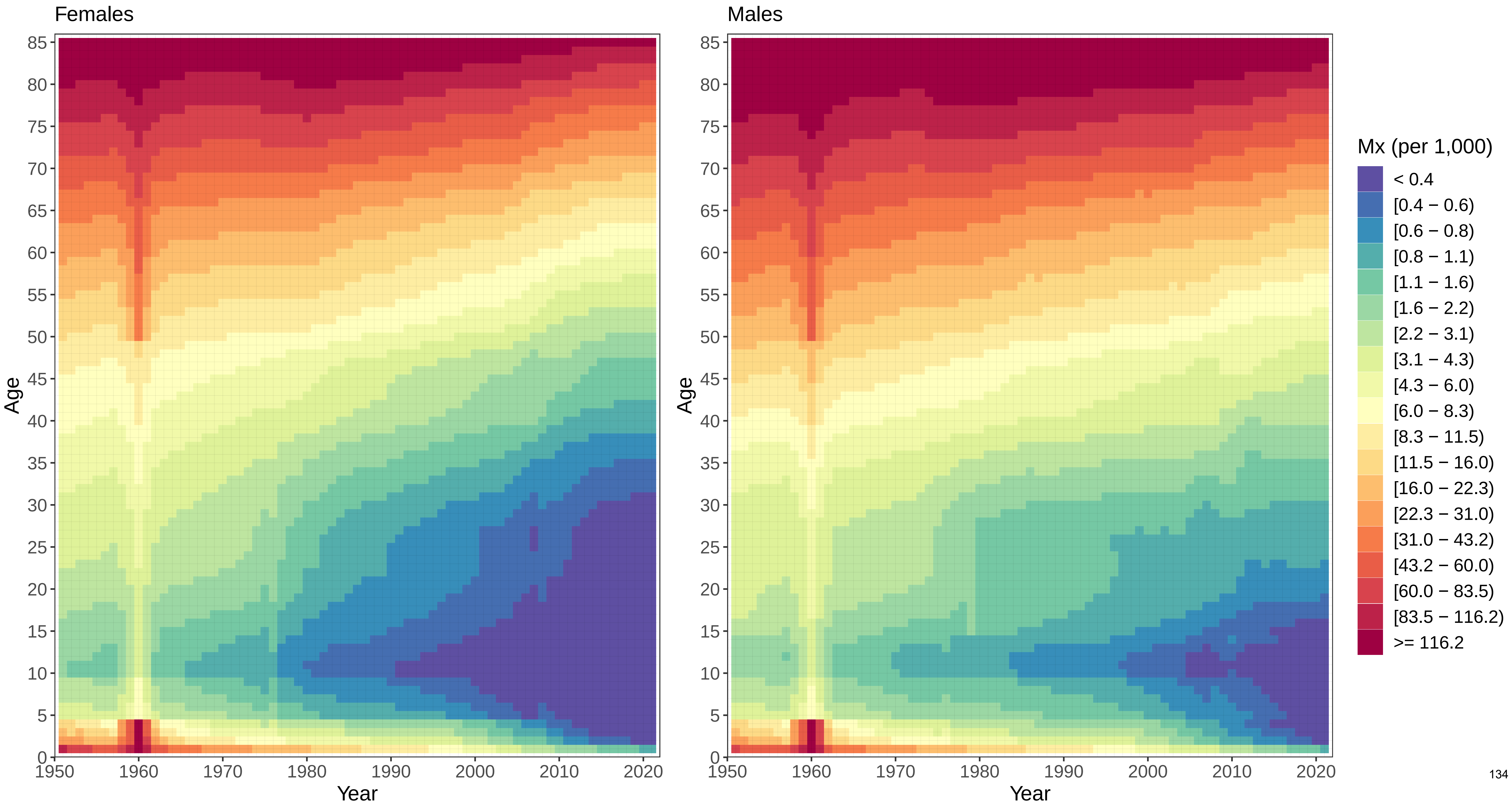

Appendix 2 Figure S5: North Korea mortality rates, 1950−2021

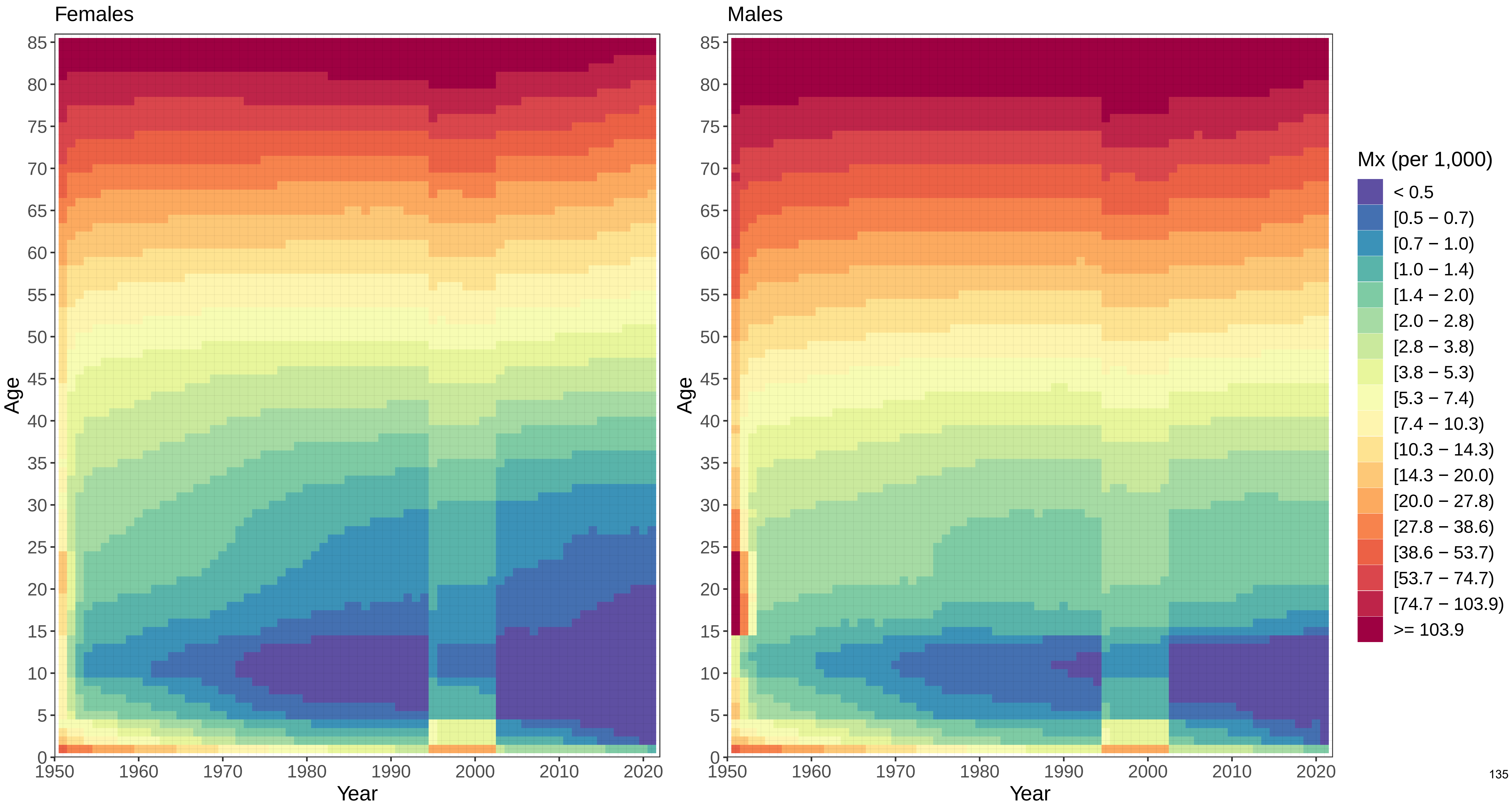

Appendix 2 Figure S5: Taiwan (province of China) mortality rates, 1950−2021

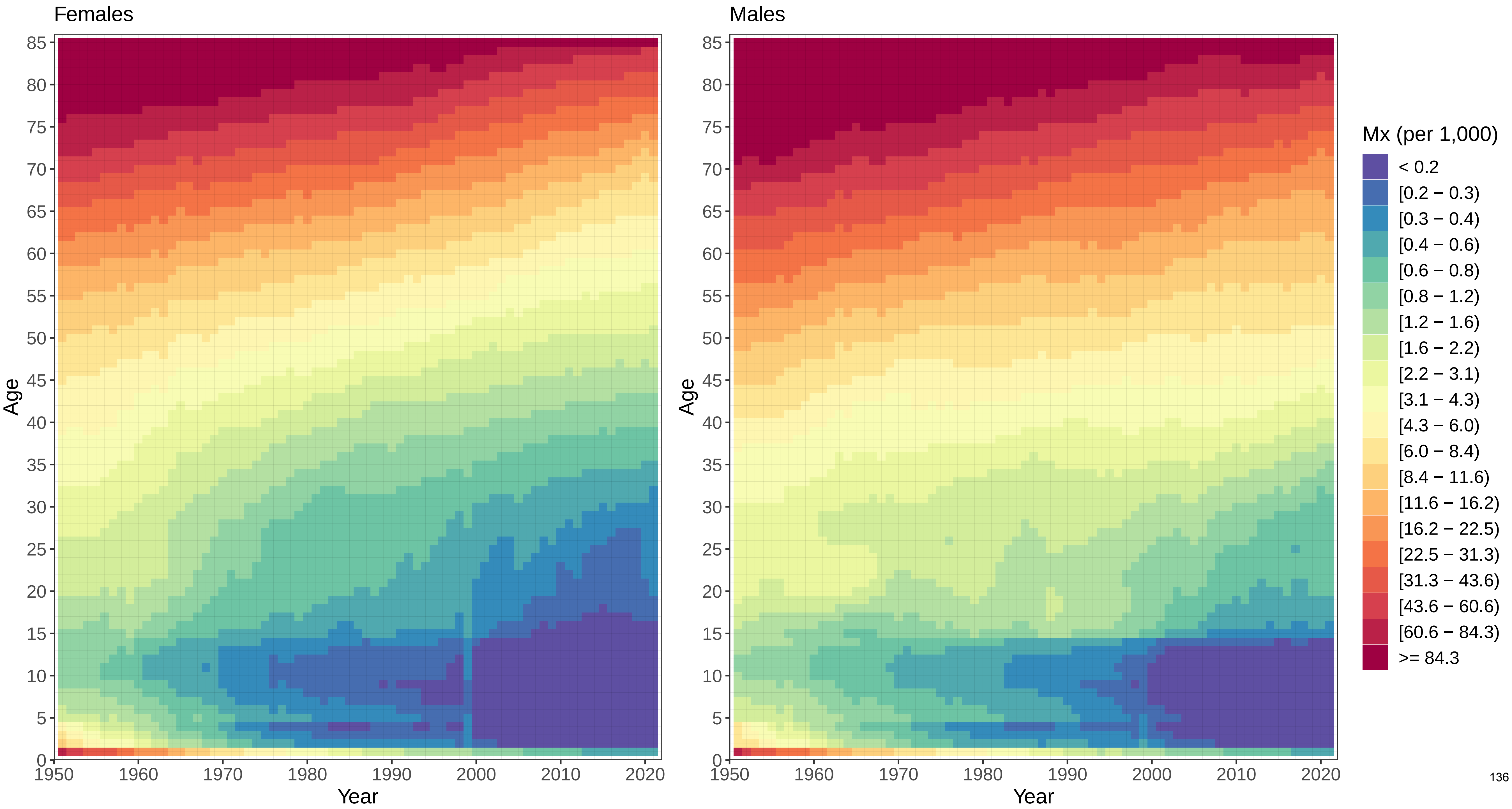

Appendix 2 Figure S5: American Samoa mortality rates, 1950−2021

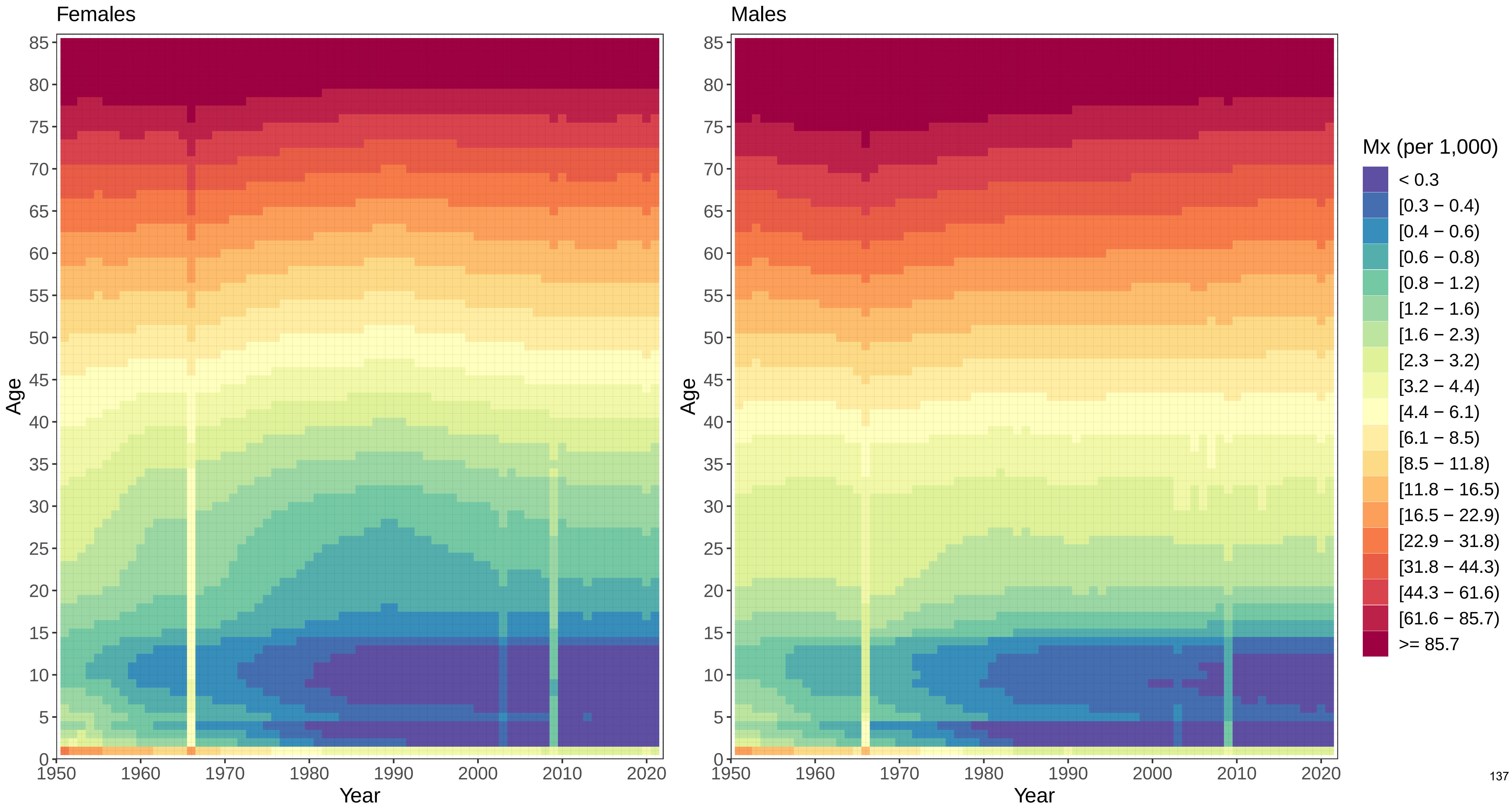

Appendix 2 Figure S5: Cook Islands mortality rates, 1950−2021

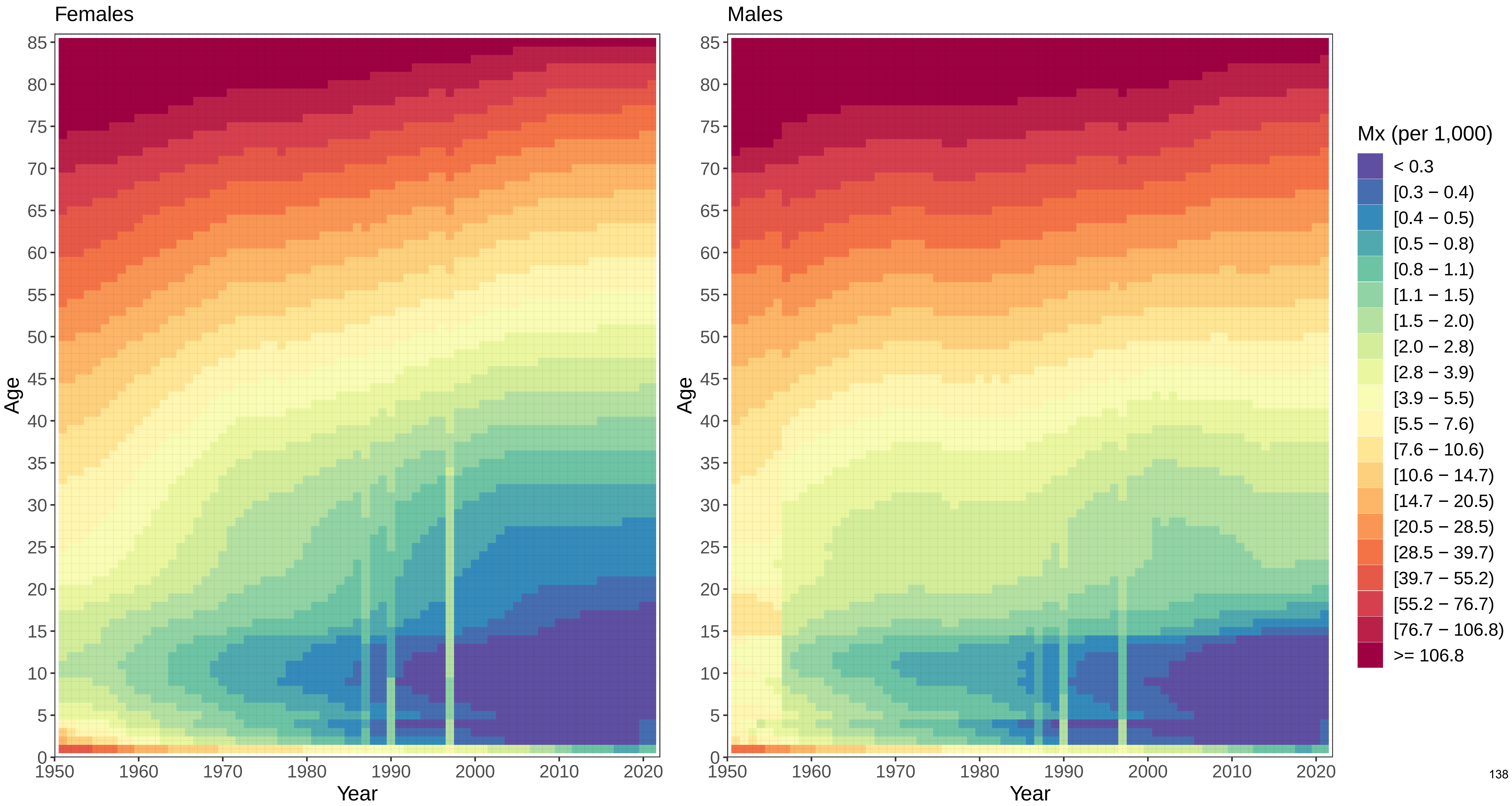

Appendix 2 Figure S5: Fiji mortality rates, 1950−2021

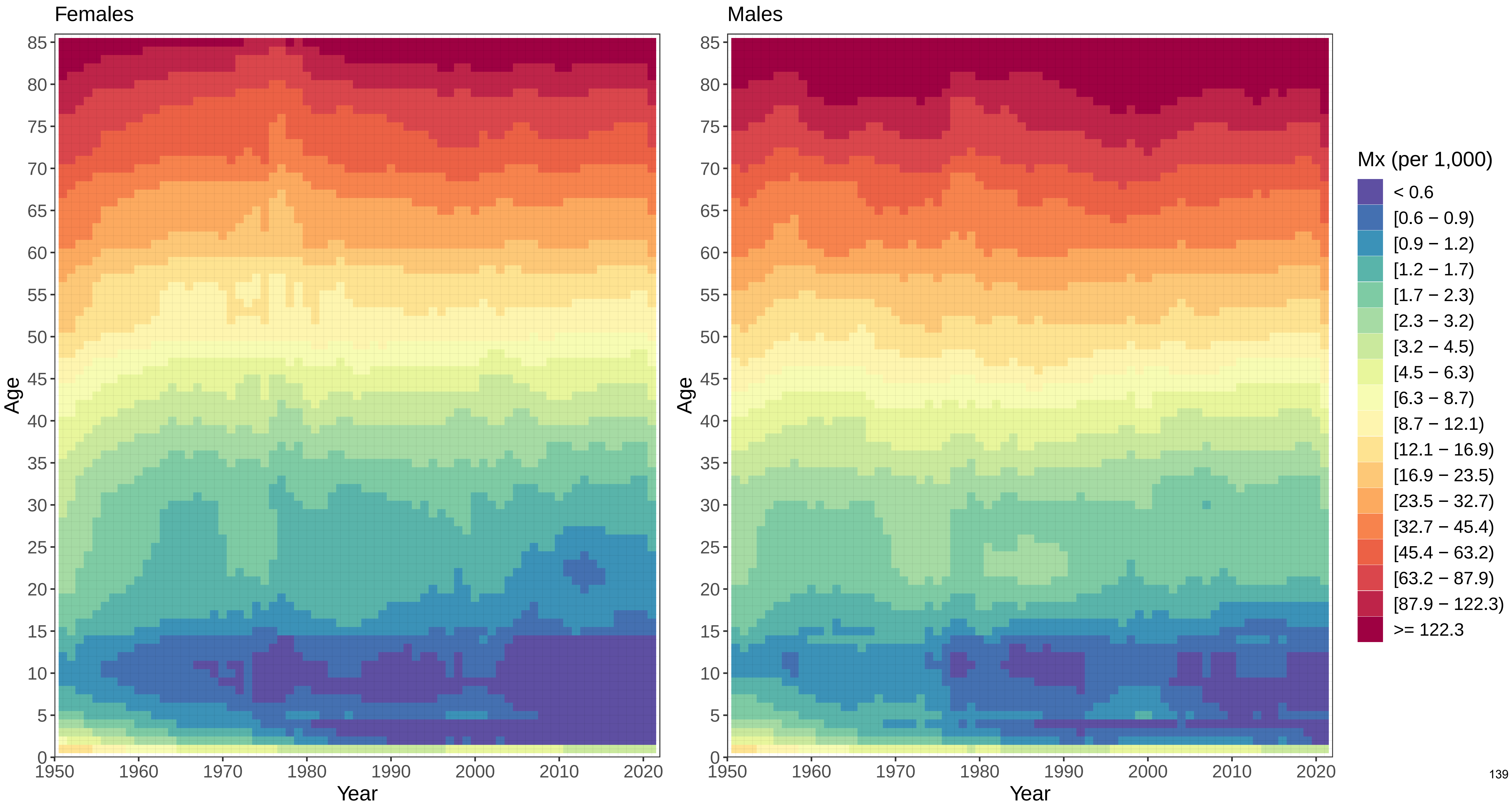

Appendix 2 Figure S5: Guam mortality rates, 1950−2021

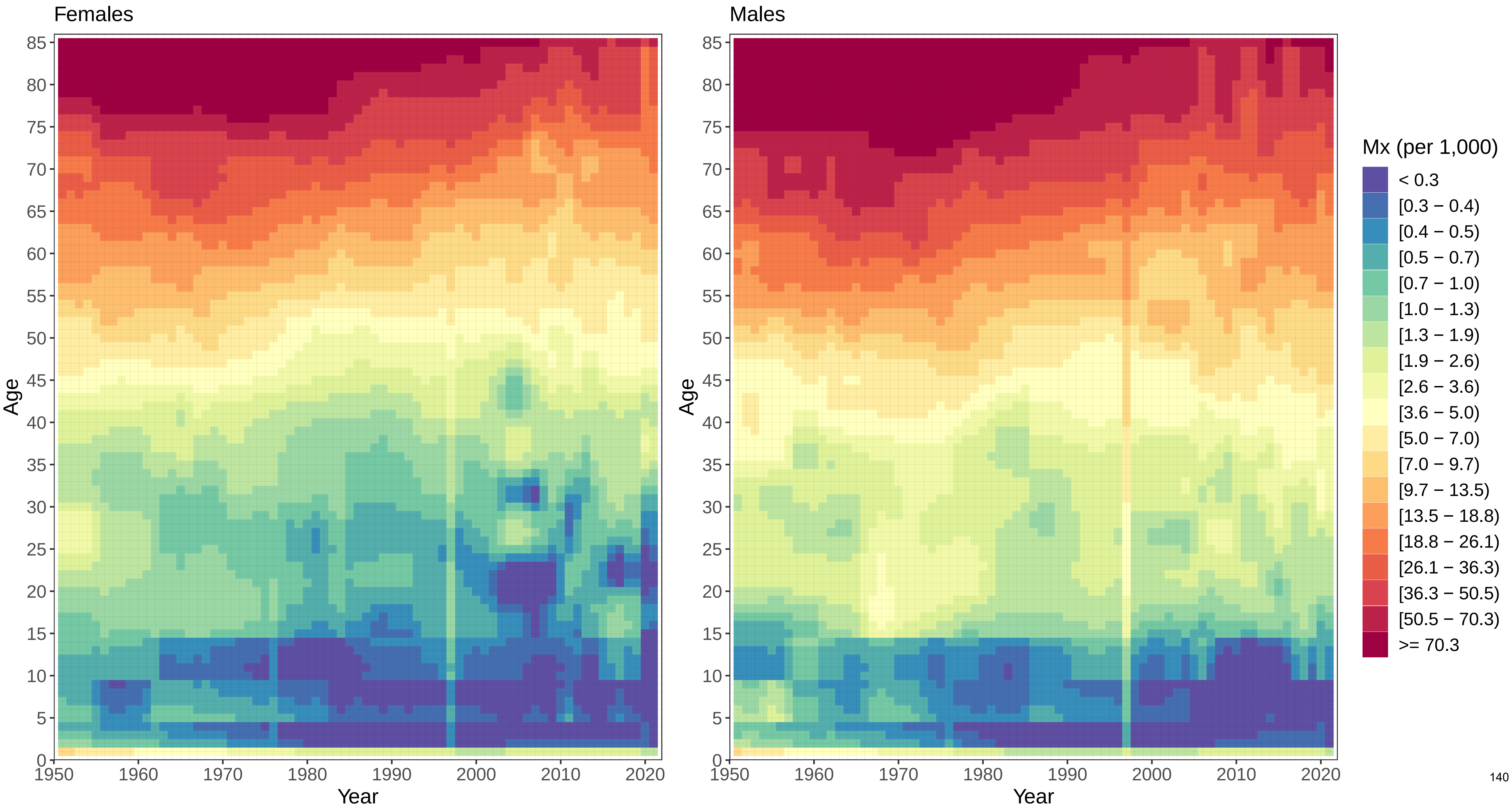

Appendix 2 Figure S5: Kiribati mortality rates, 1950−2021

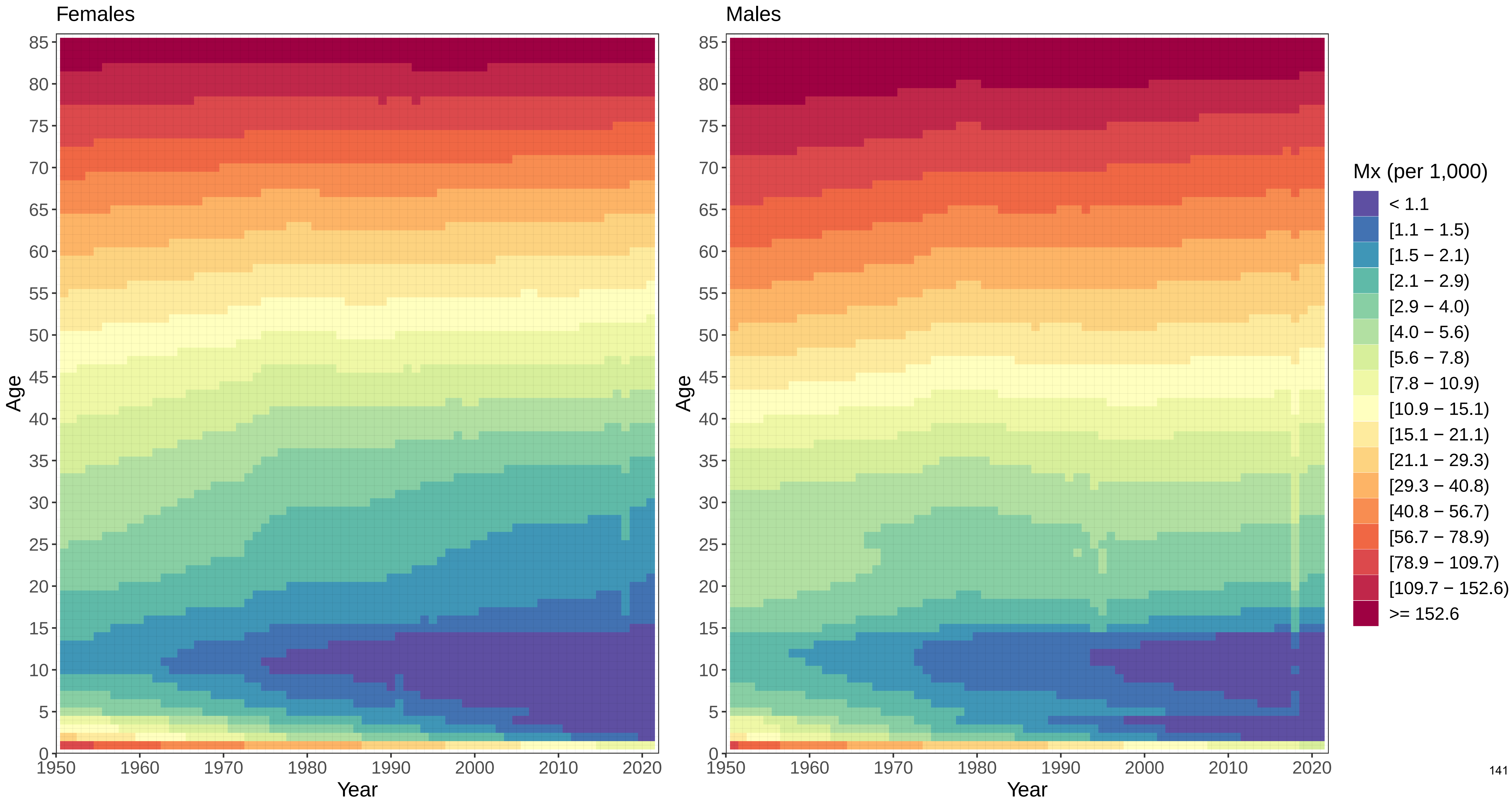

Appendix 2 Figure S5: Marshall Islands mortality rates, 1950−2021

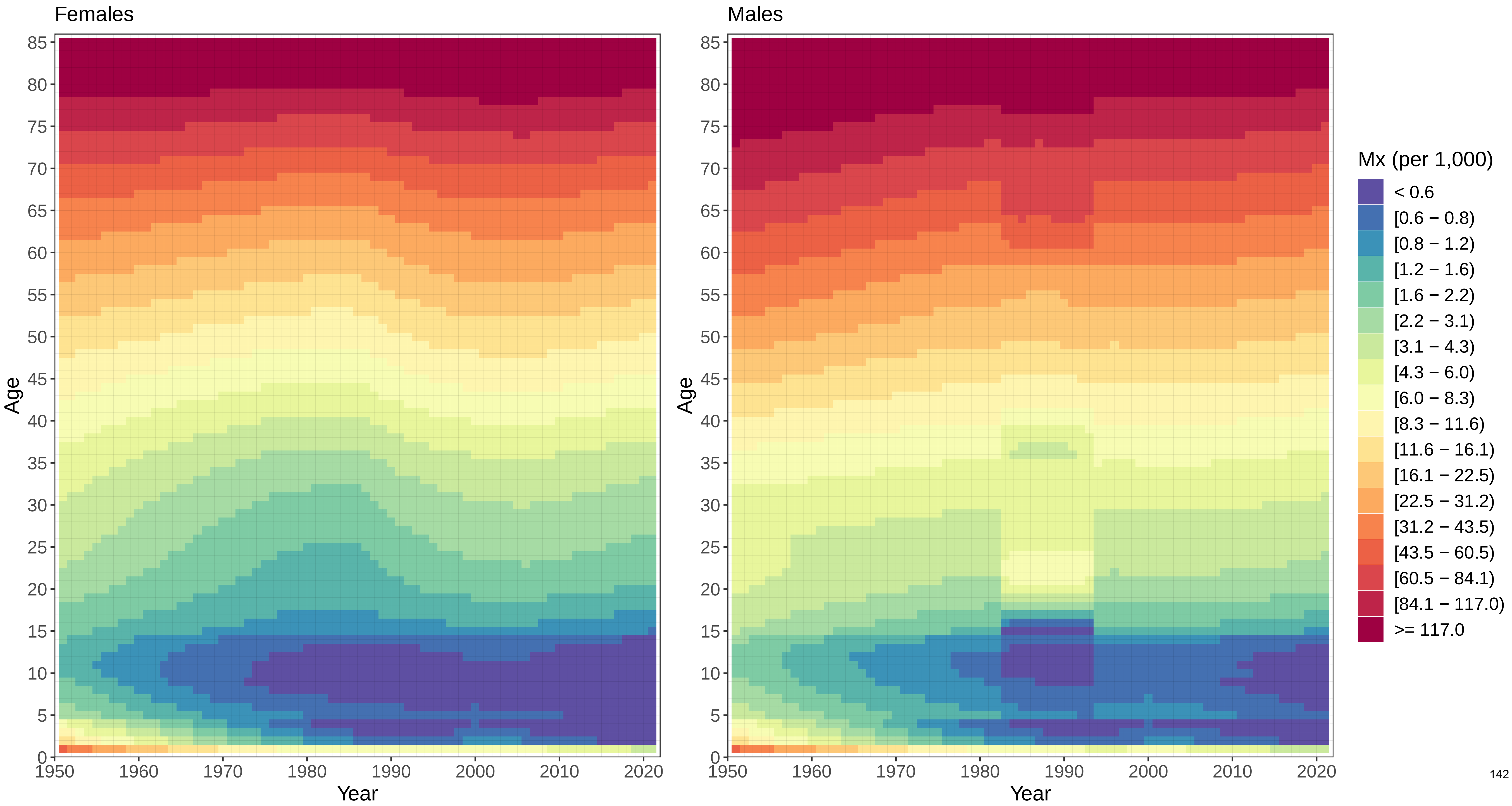

Appendix 2 Figure S5: Federated States of Micronesia mortality rates, 1950−2021

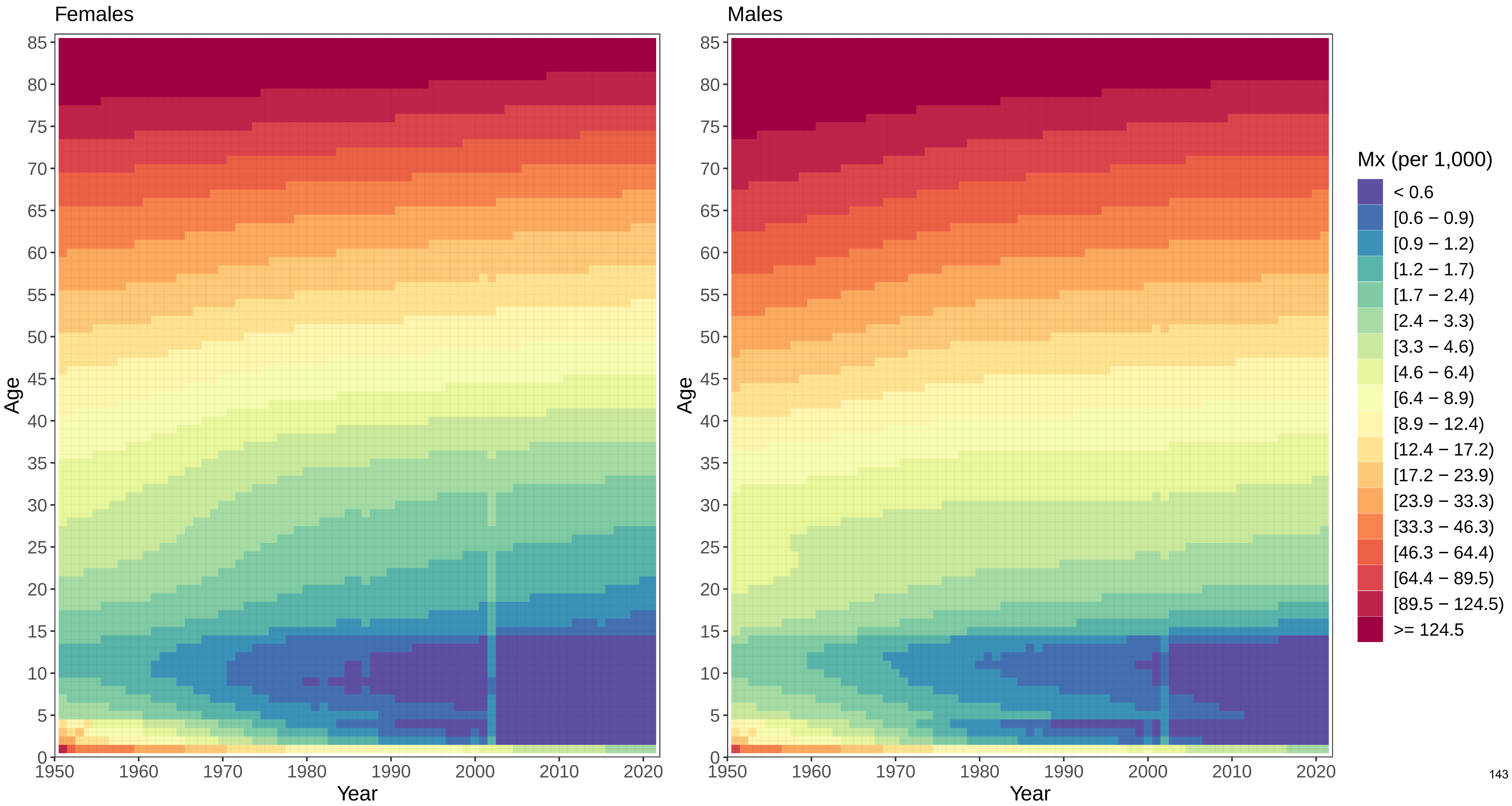

Appendix 2 Figure S5: Nauru mortality rates, 1950−2021

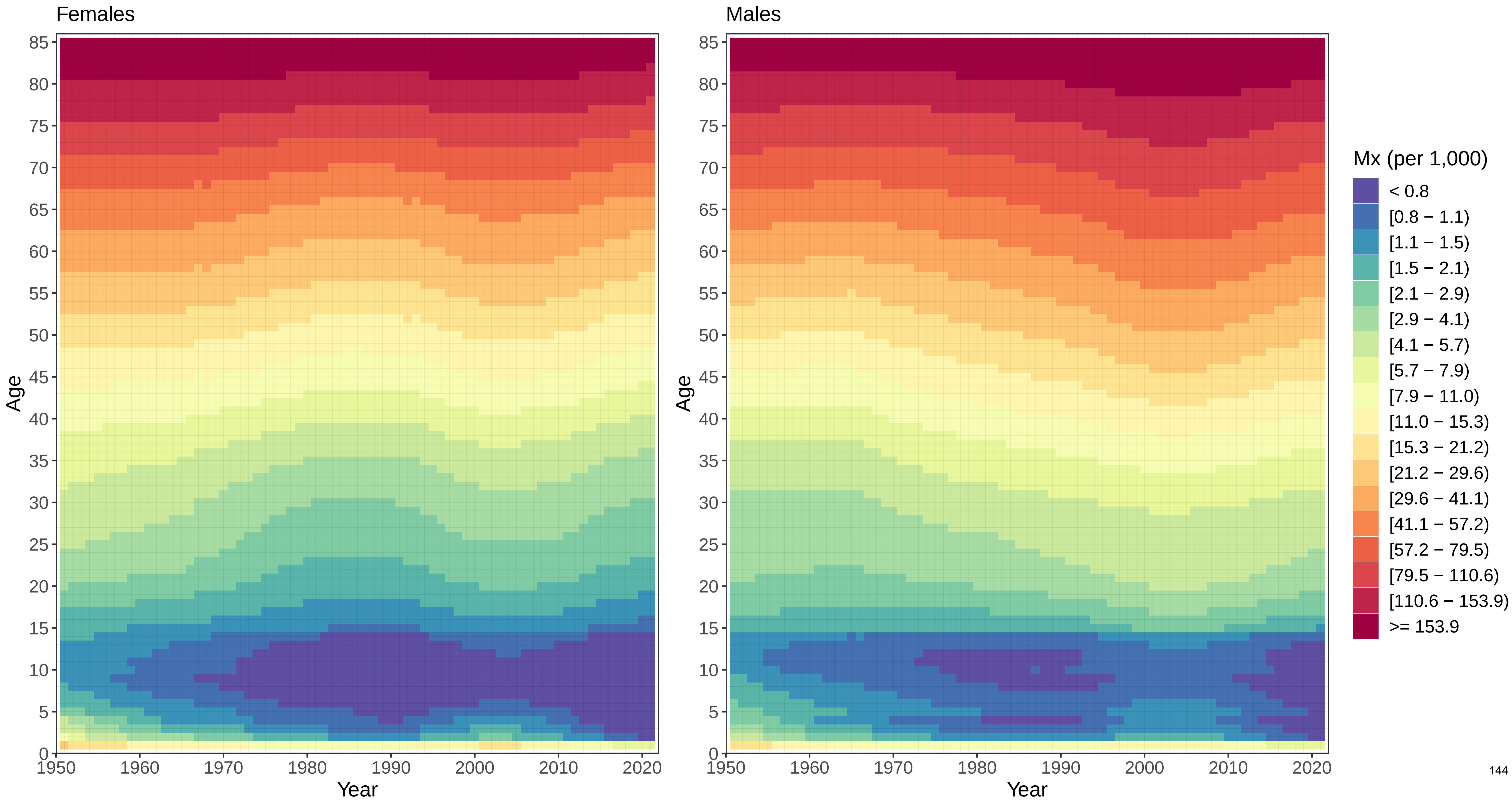

Appendix 2 Figure S5: Niue mortality rates, 1950−2021

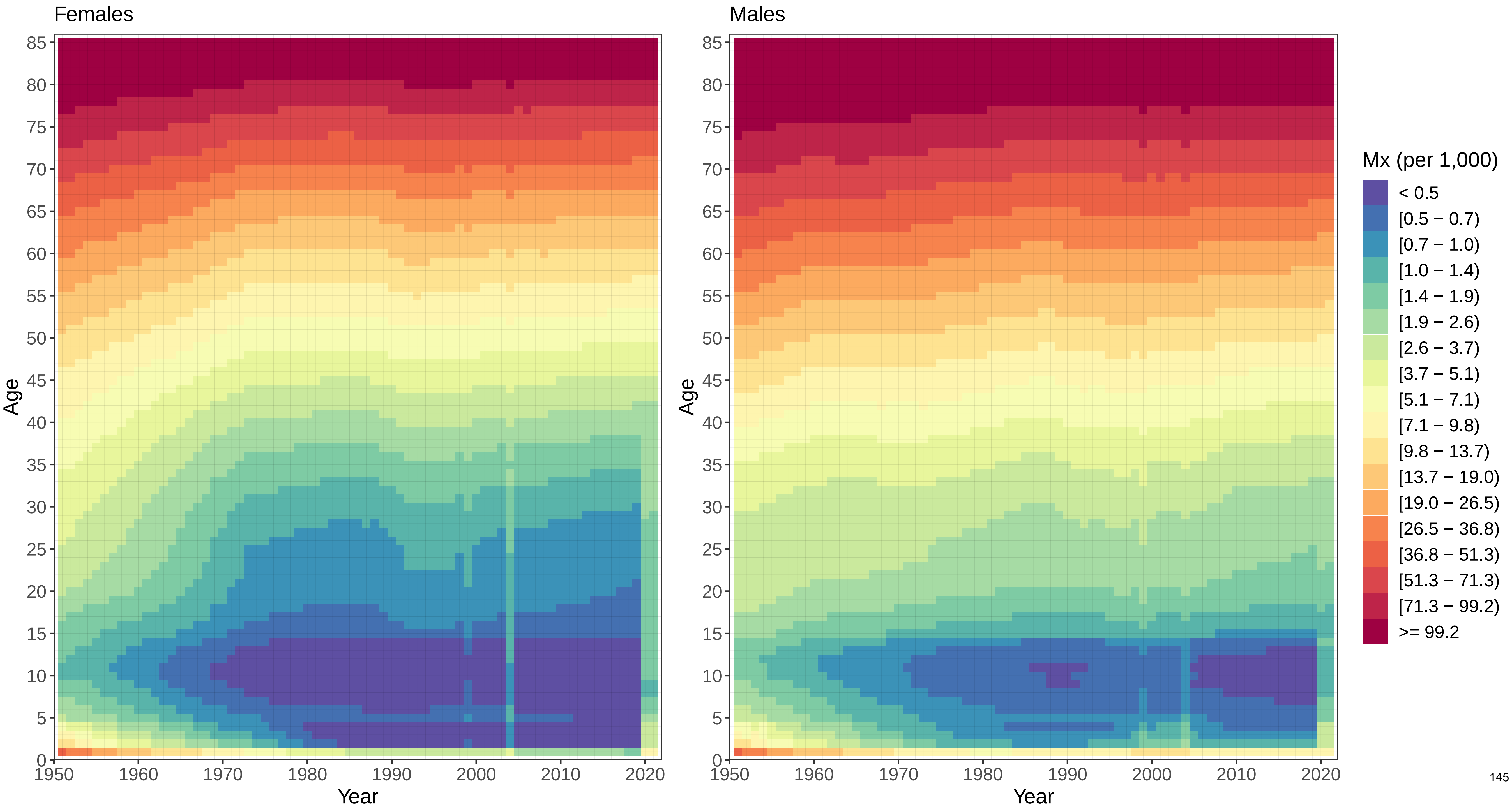

Appendix 2 Figure S5: Northern Mariana Islands mortality rates, 1950−2021

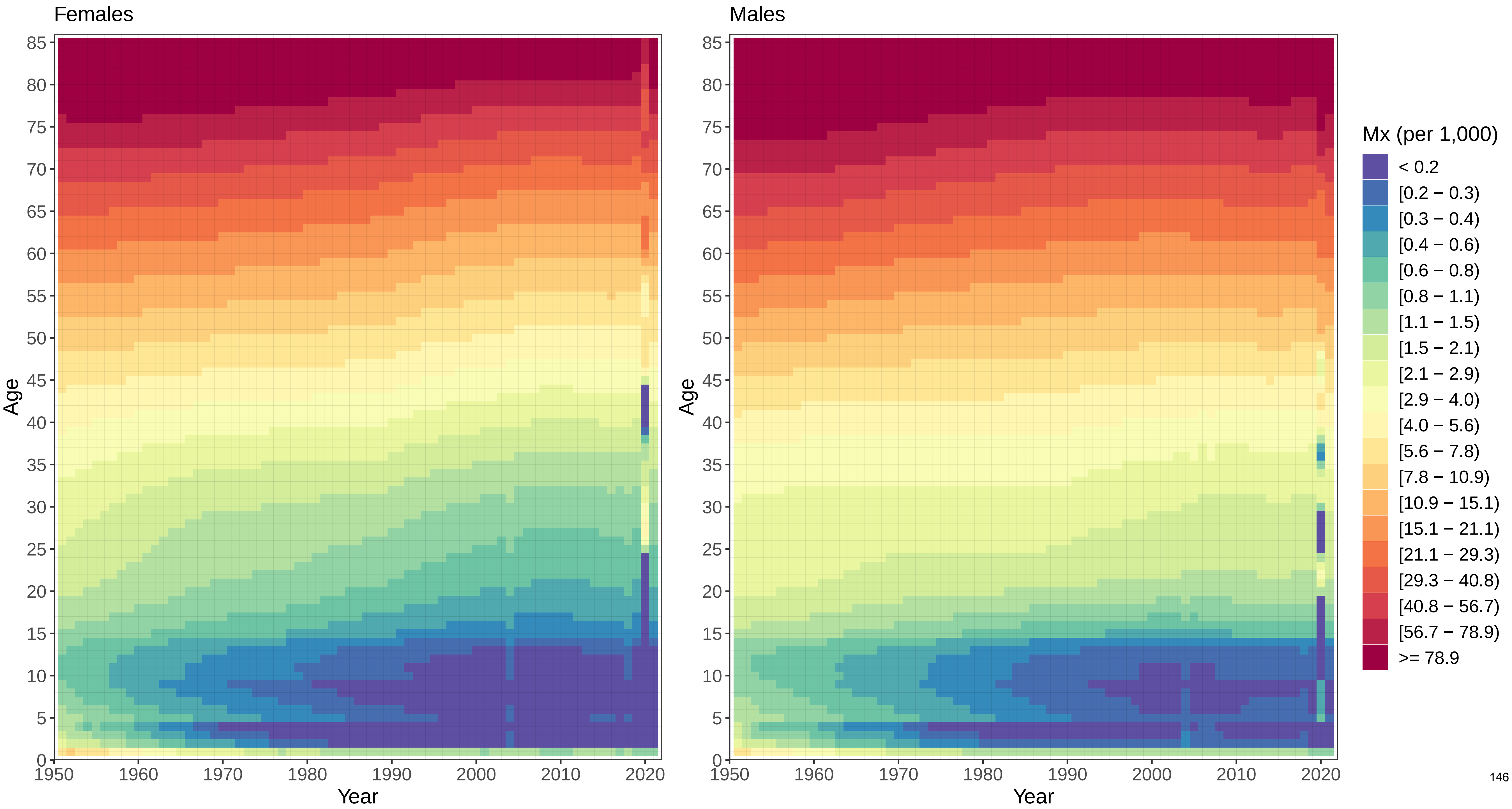

Appendix 2 Figure S5: Palau mortality rates, 1950−2021

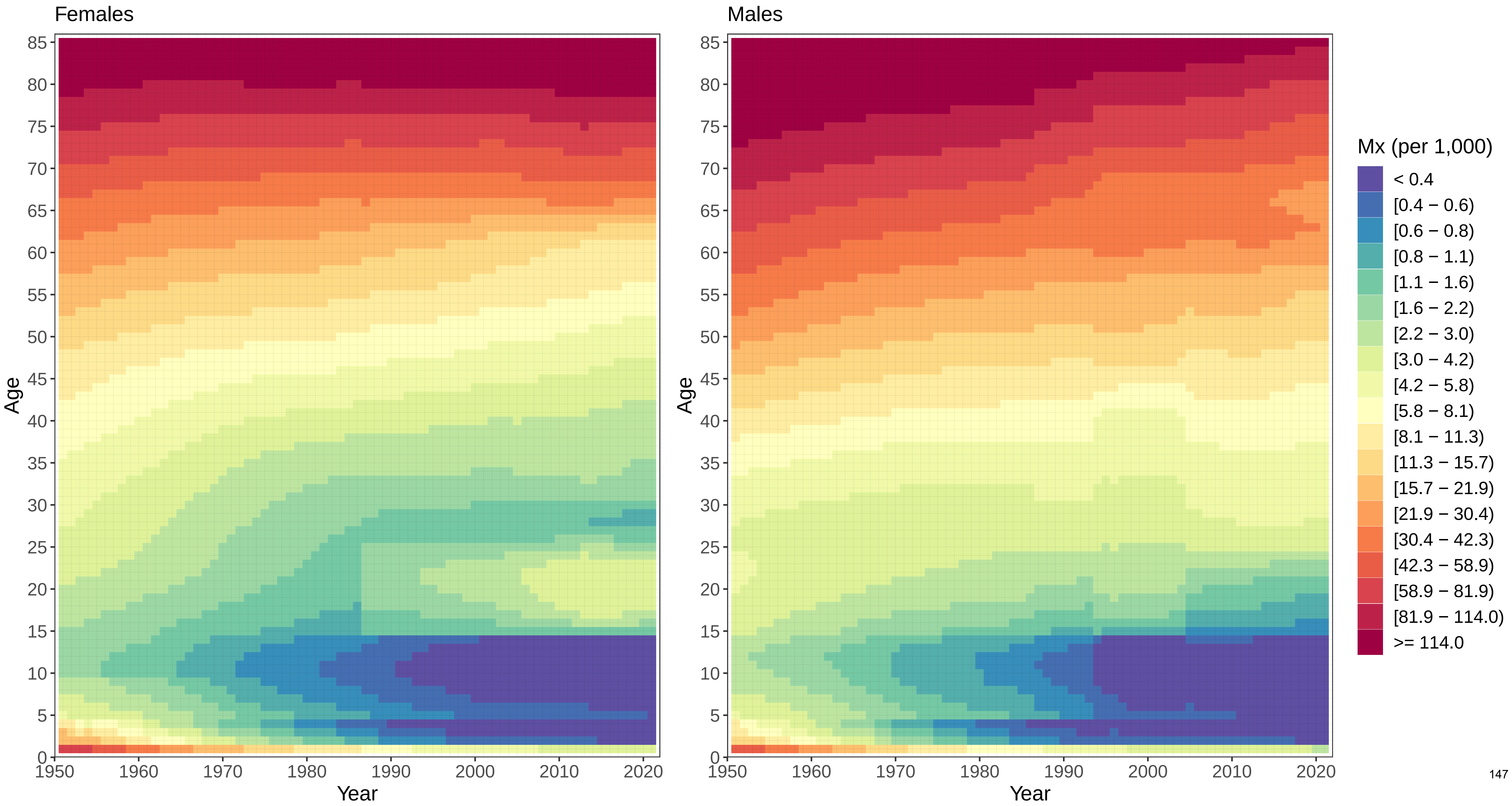

Appendix 2 Figure S5: Papua New Guinea mortality rates, 1950−2021

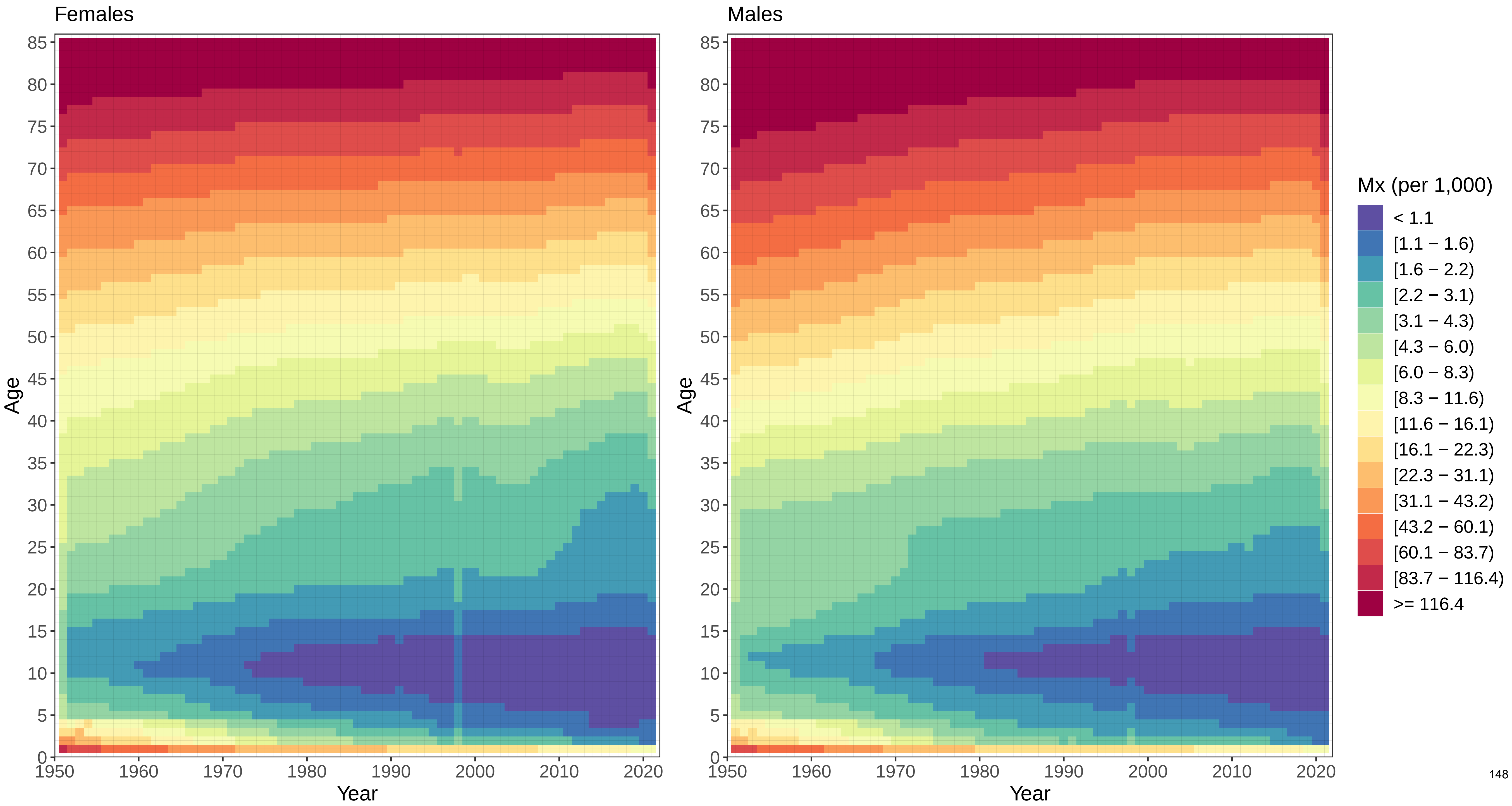

Appendix 2 Figure S5: Samoa mortality rates, 1950−2021

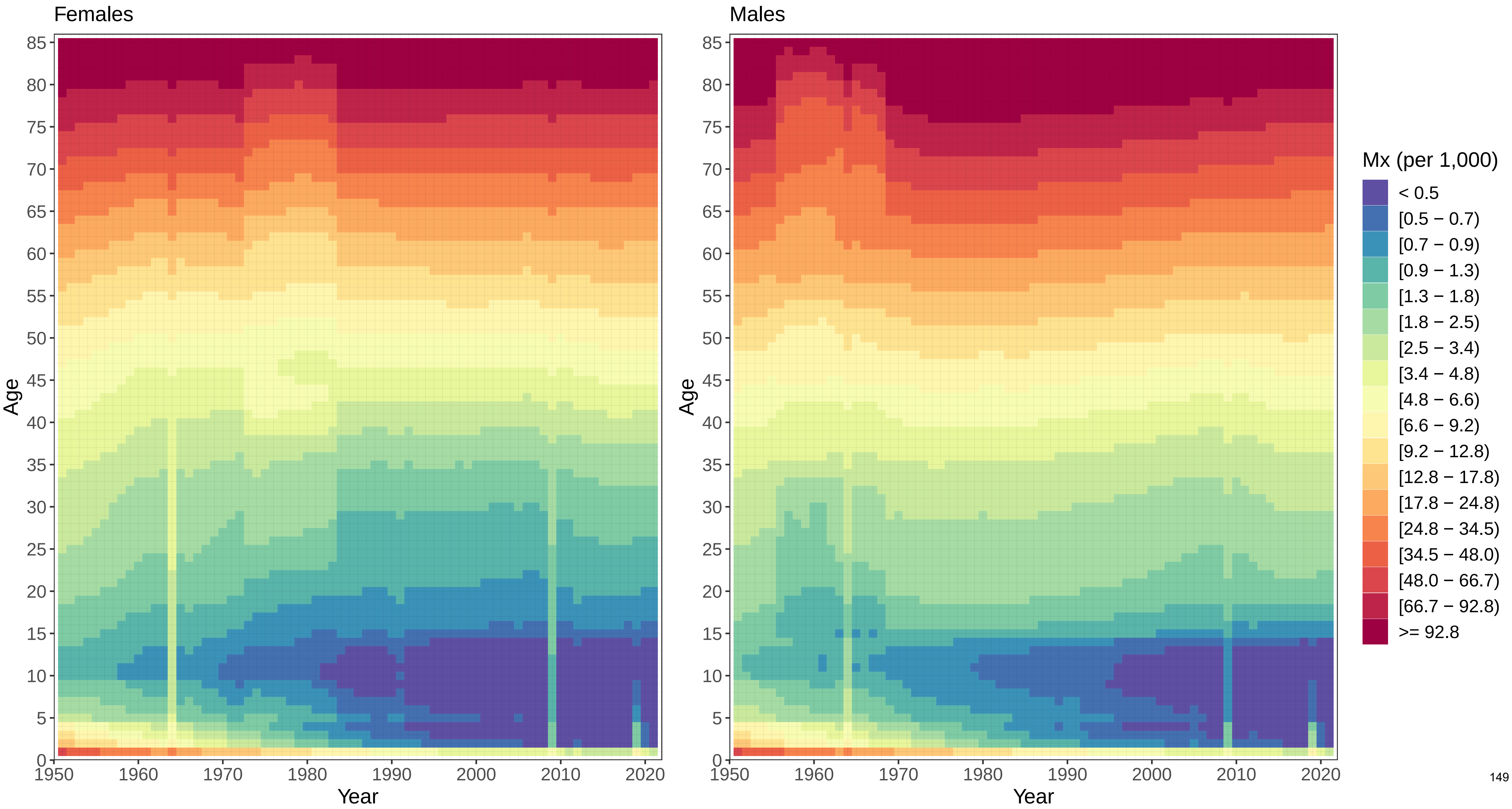

Appendix 2 Figure S5: Solomon Islands mortality rates, 1950−2021

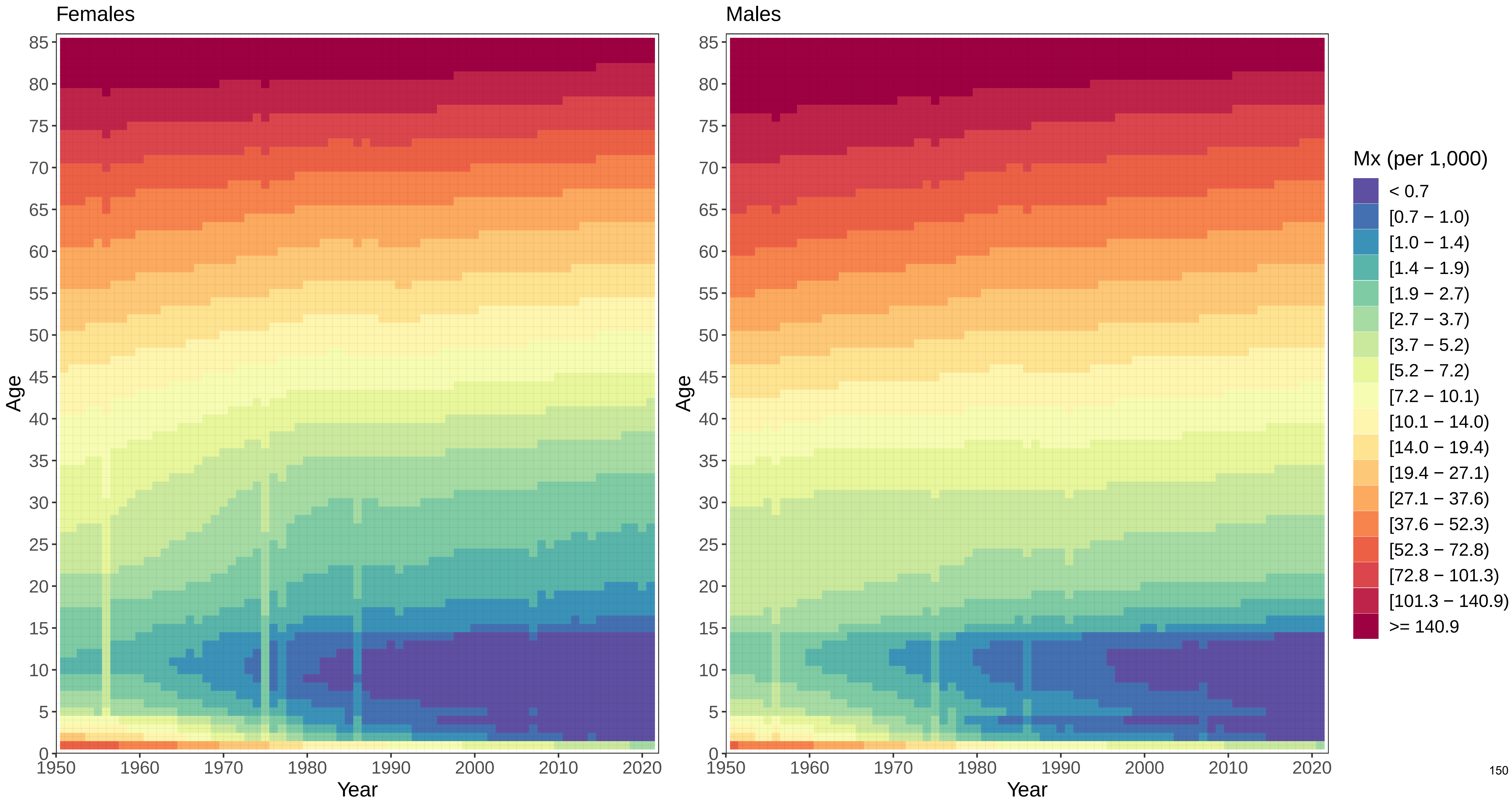

Appendix 2 Figure S5: Tokelau mortality rates, 1950−2021

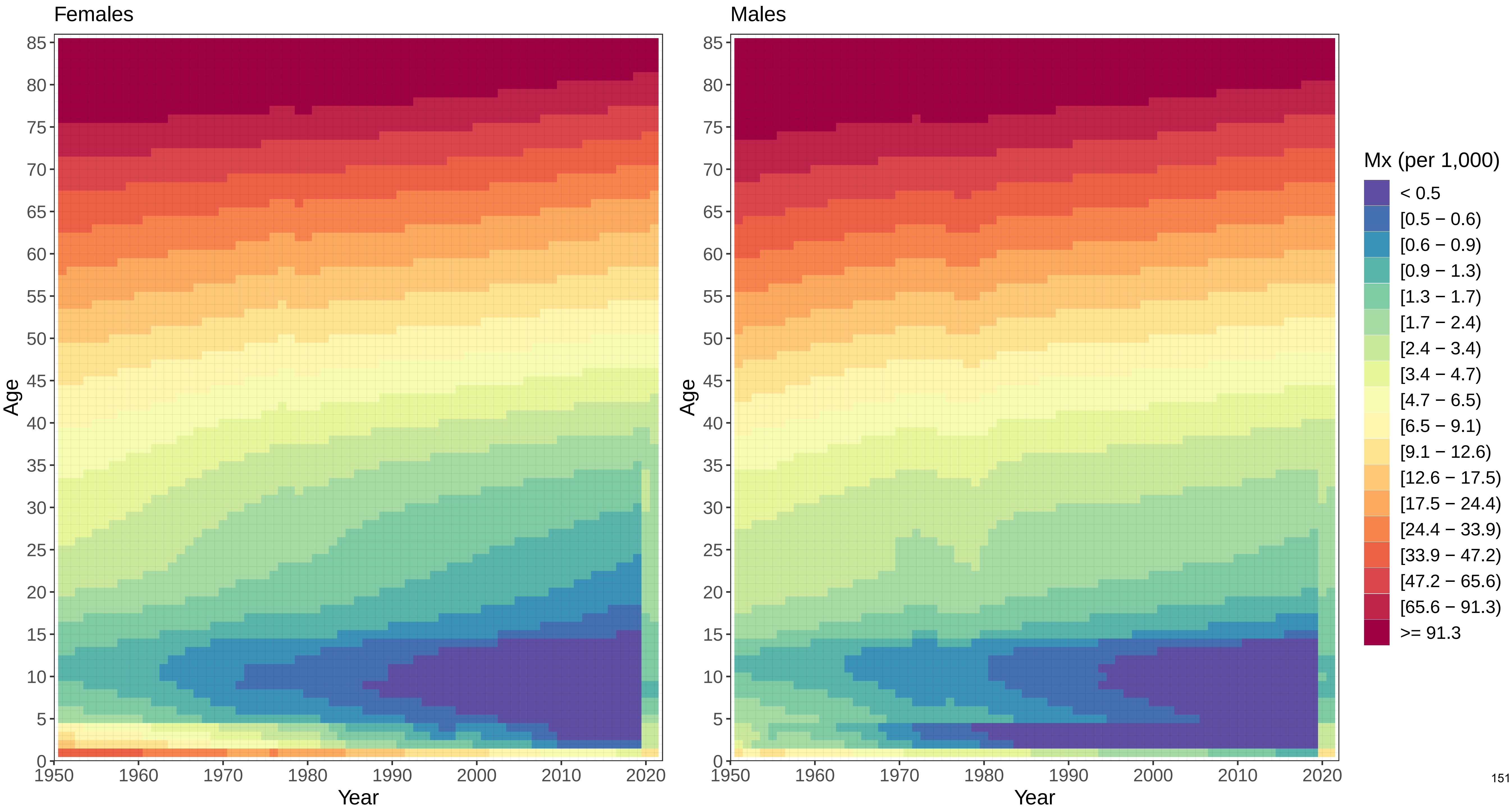

Appendix 2 Figure S5: Tonga mortality rates, 1950−2021

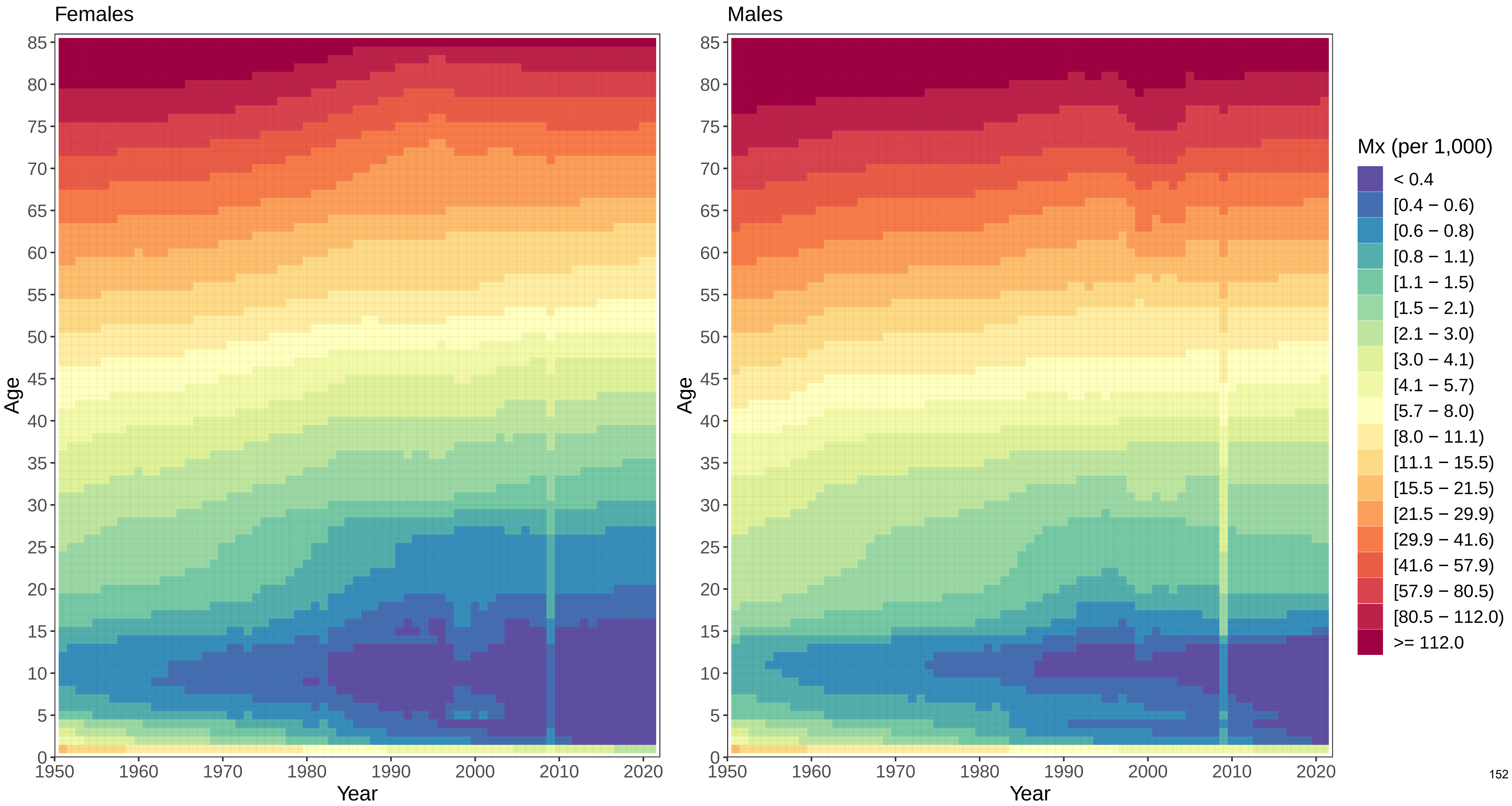

Appendix 2 Figure S5: Tuvalu mortality rates, 1950−2021

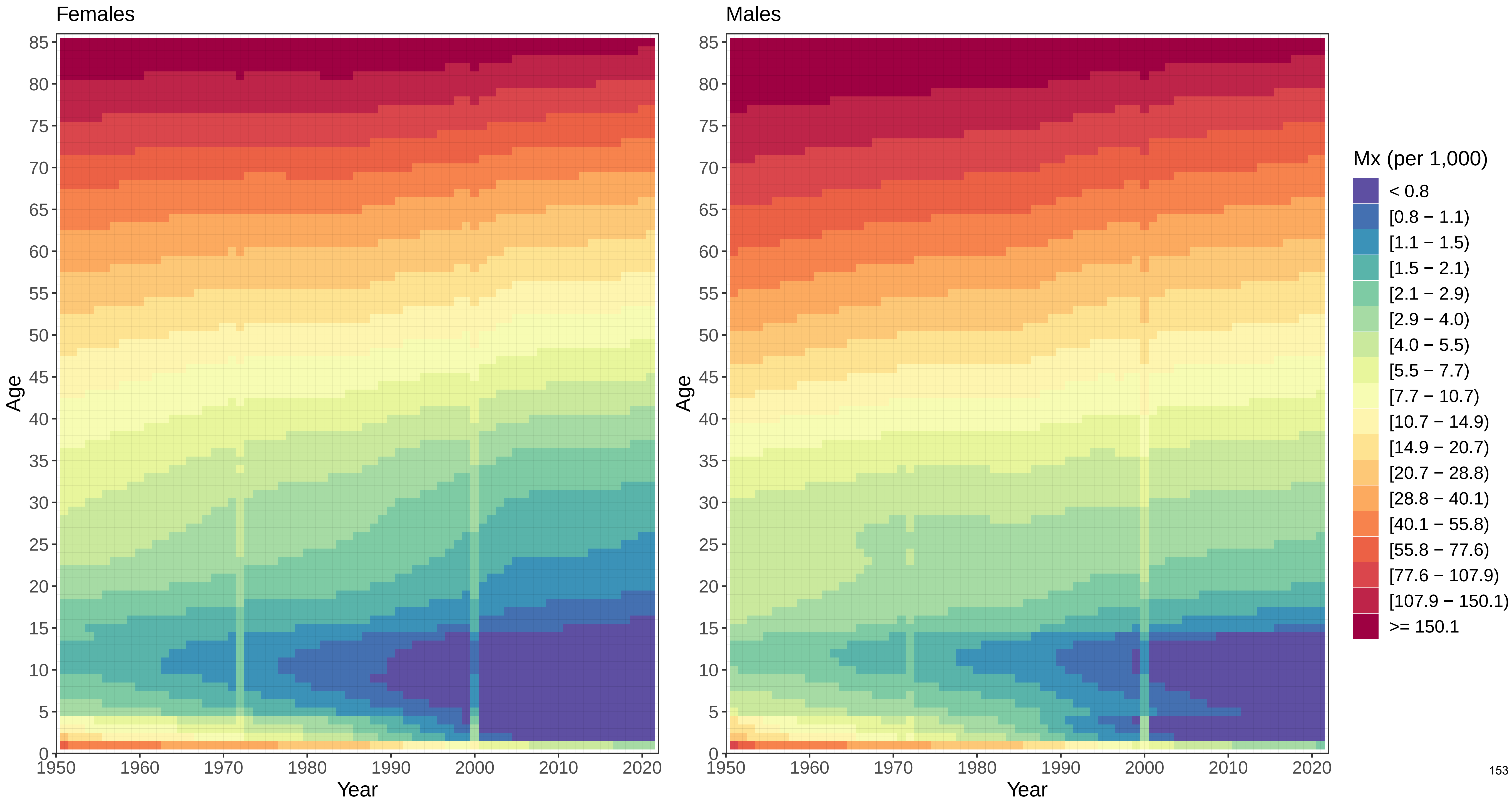

Appendix 2 Figure S5: Vanuatu mortality rates, 1950−2021

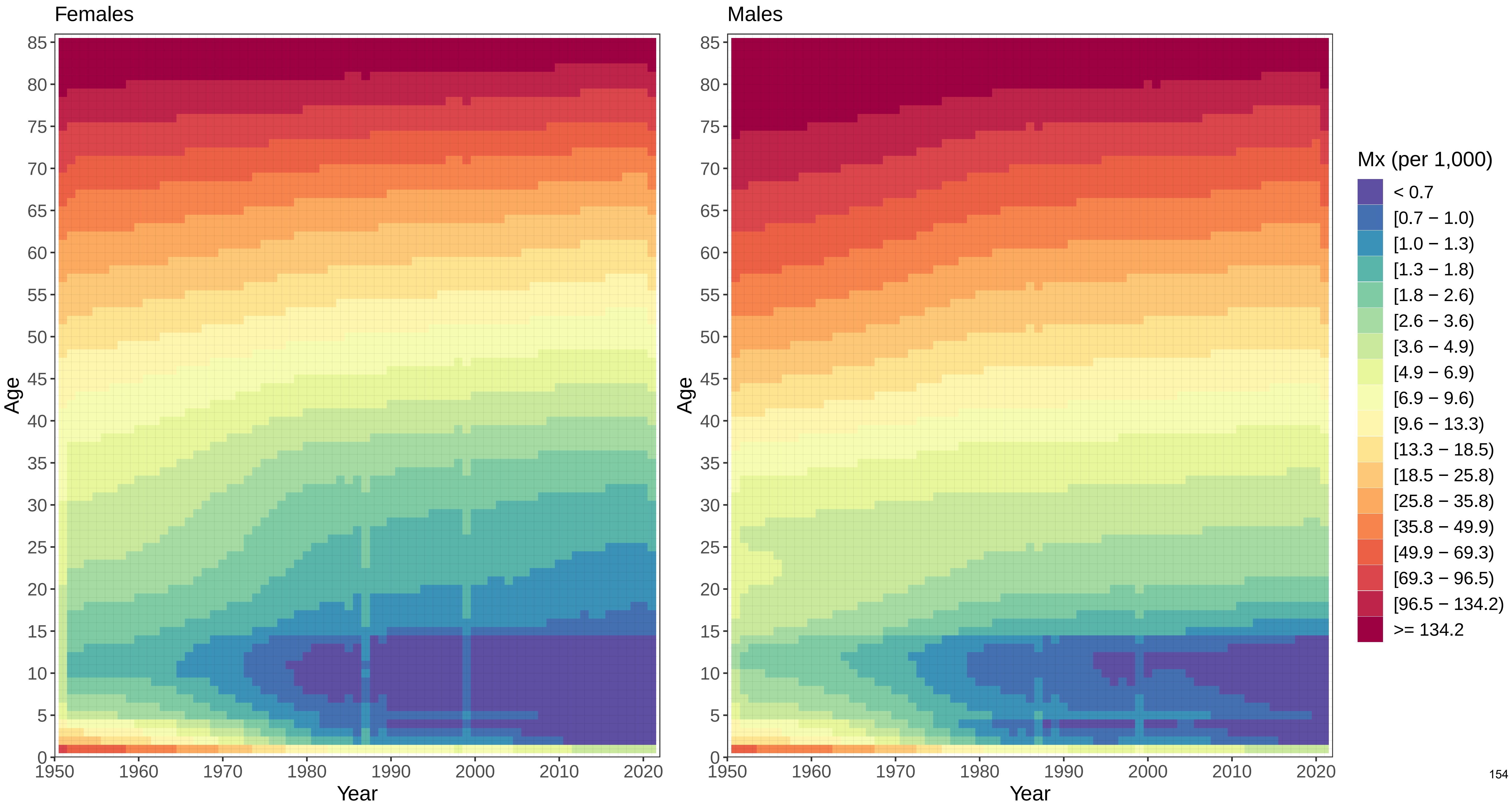

Appendix 2 Figure S5: Cambodia mortality rates, 1950−2021

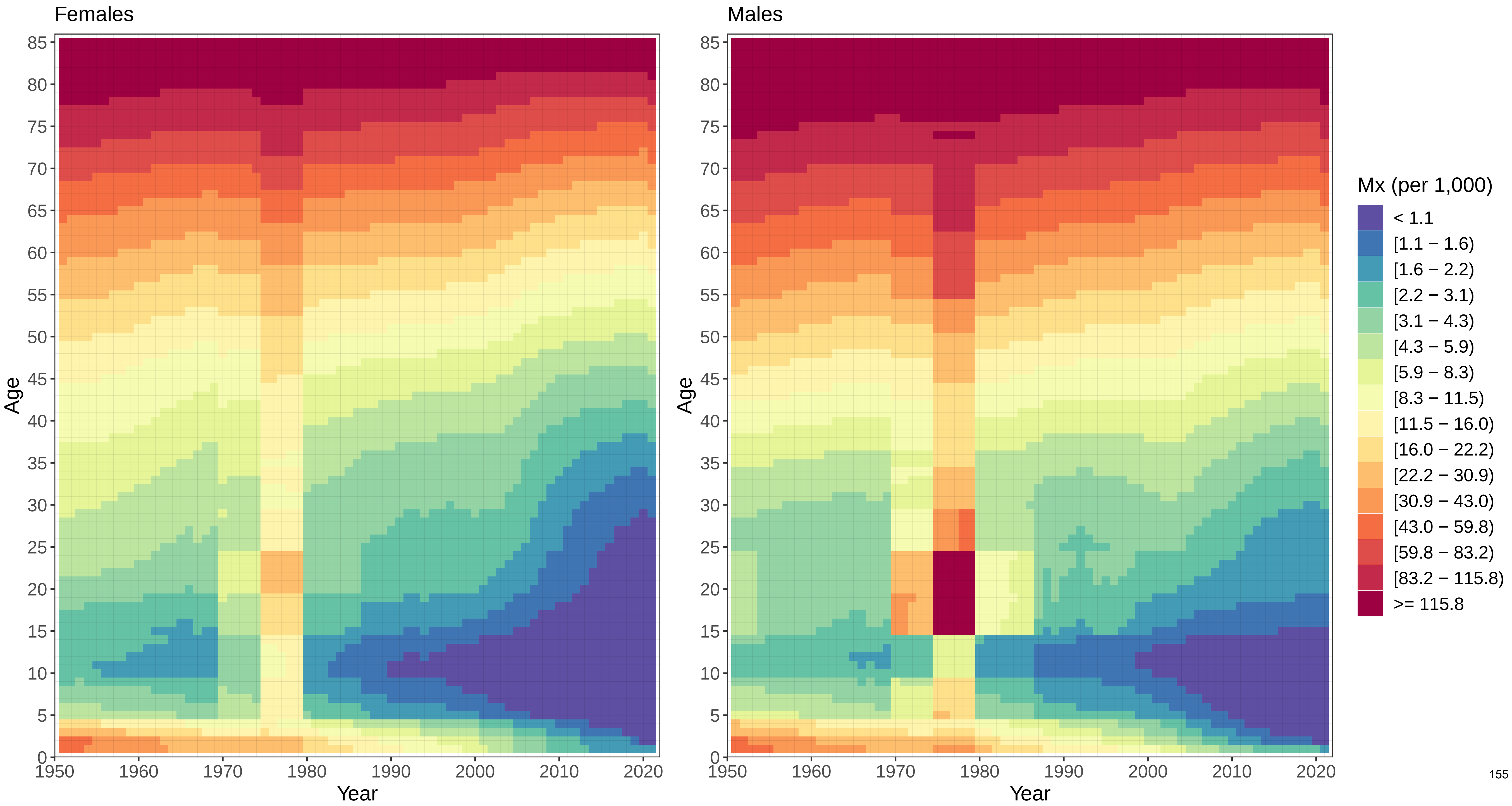

Appendix 2 Figure S5: Indonesia mortality rates, 1950−2021

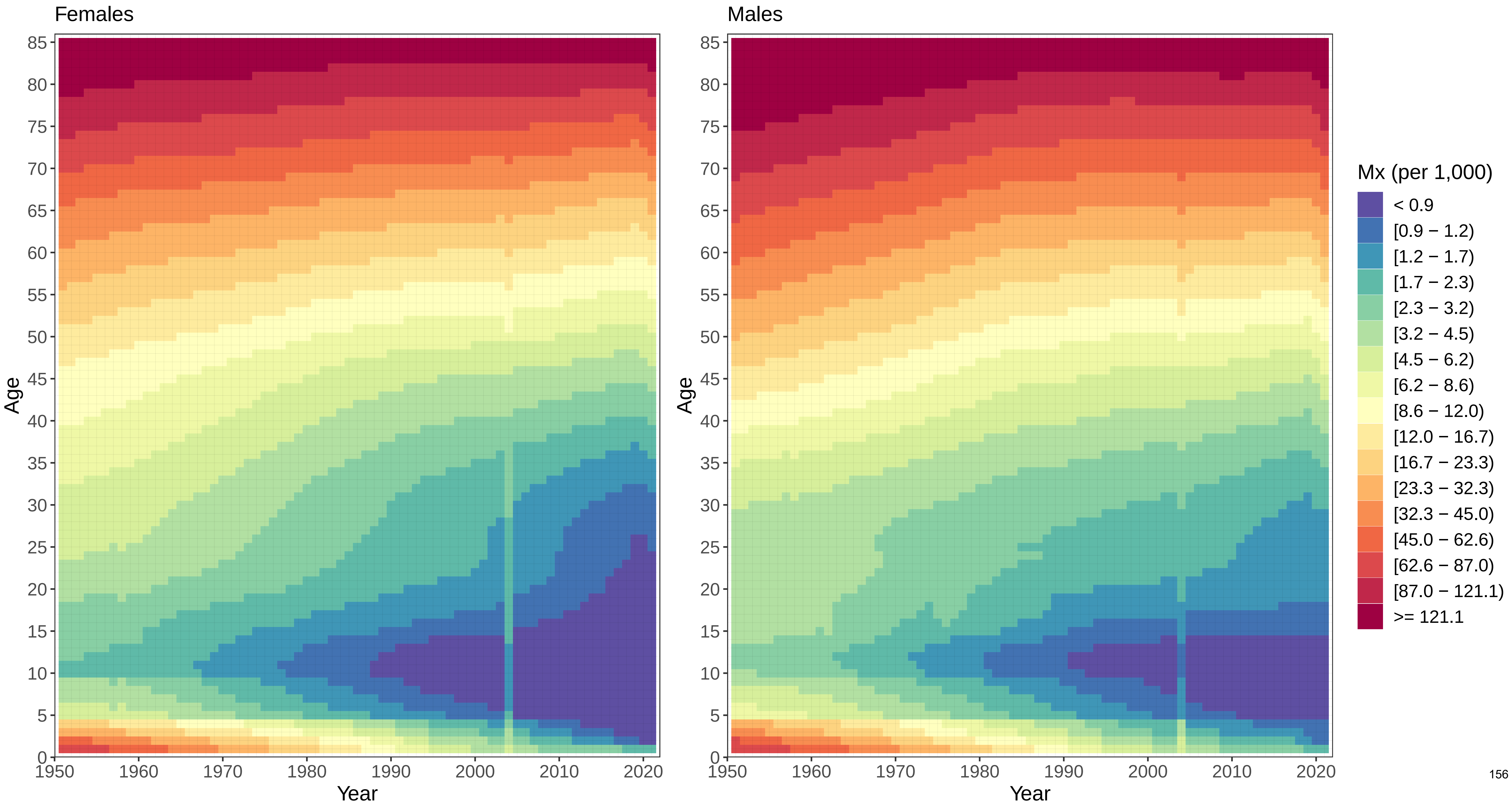

Appendix 2 Figure S5: Laos mortality rates, 1950−2021

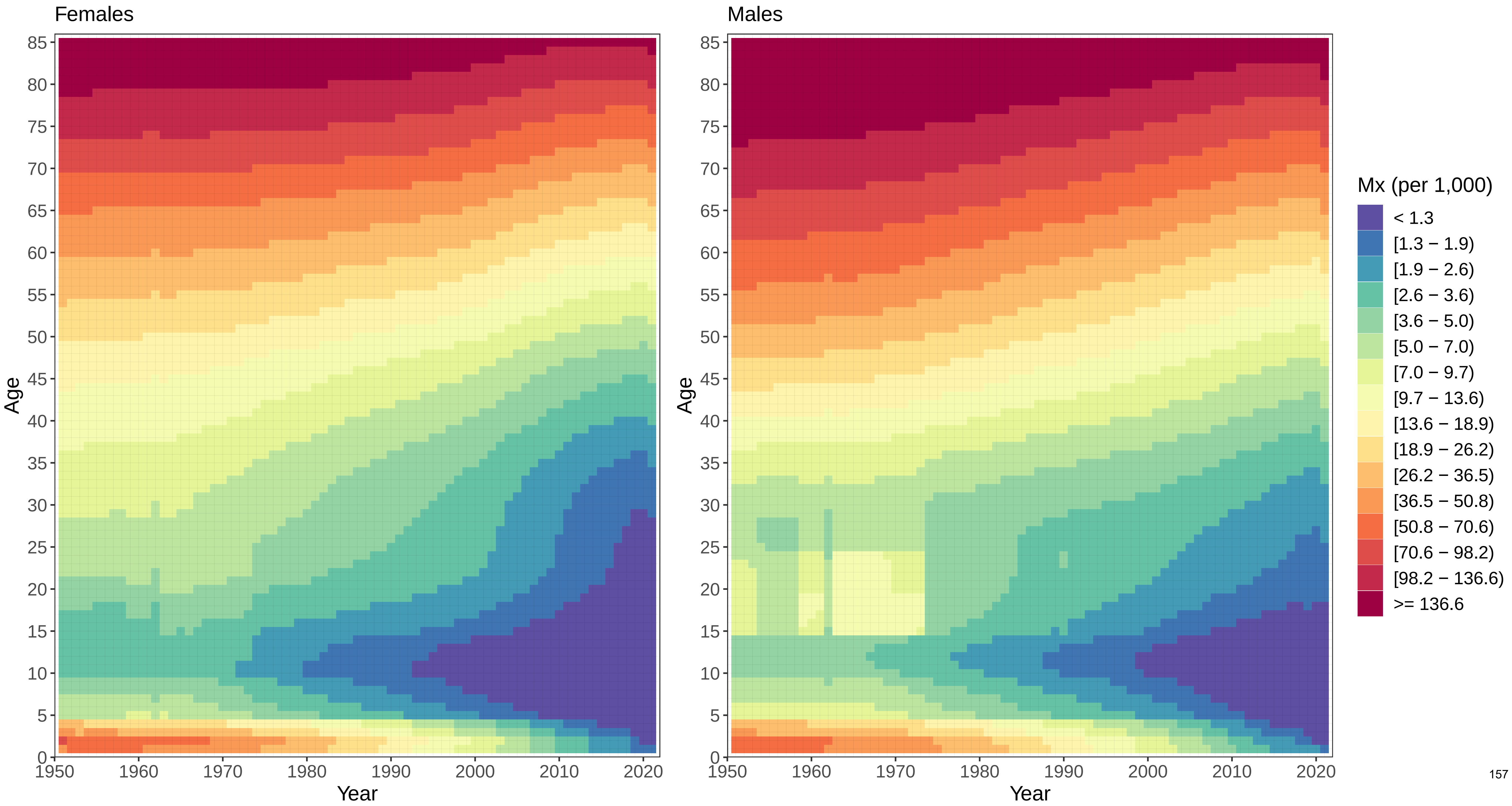

Appendix 2 Figure S5: Malaysia mortality rates, 1950−2021

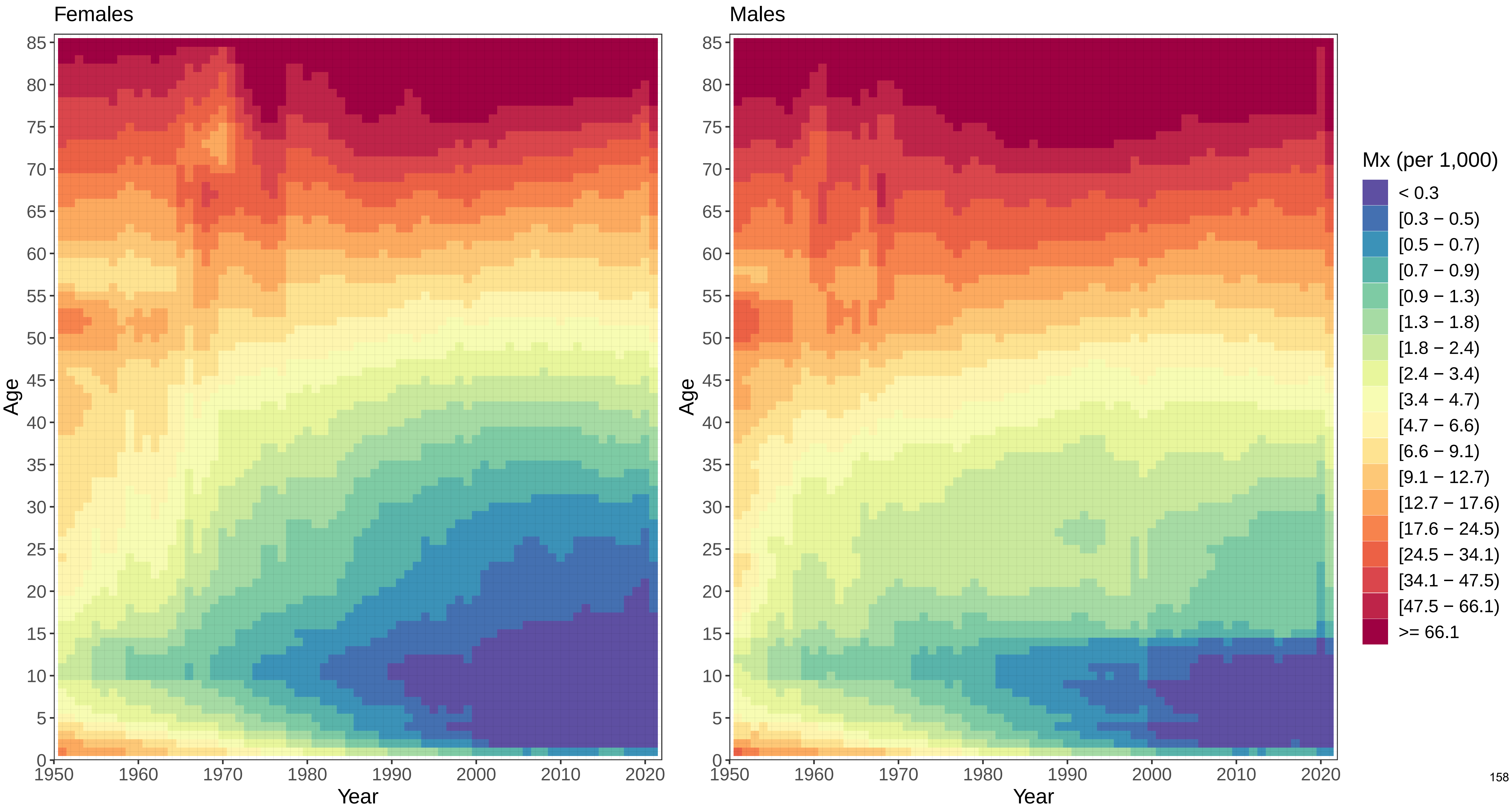

Appendix 2 Figure S5: Maldives mortality rates, 1950−2021

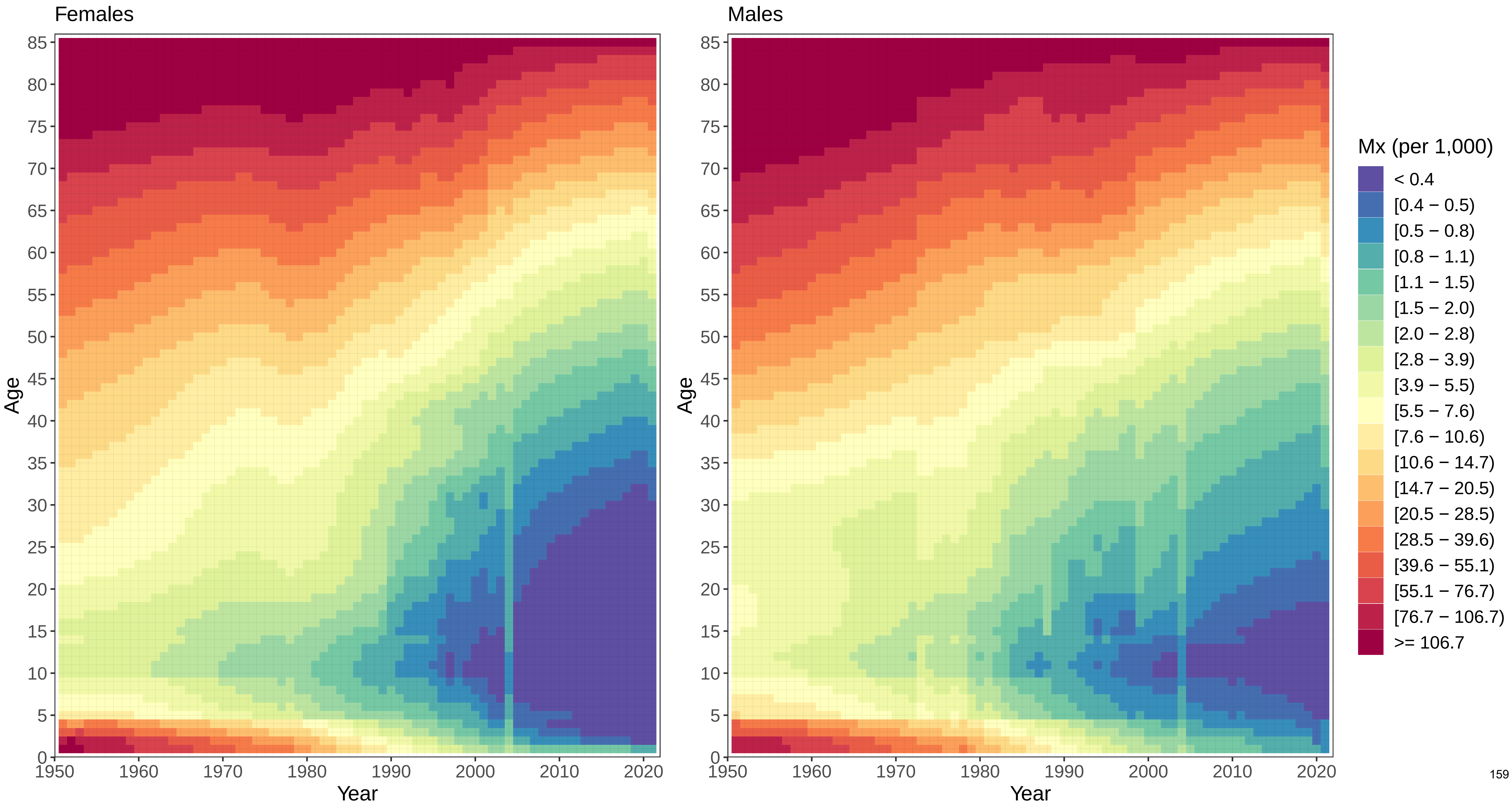

Appendix 2 Figure S5: Mauritius mortality rates, 1950−2021

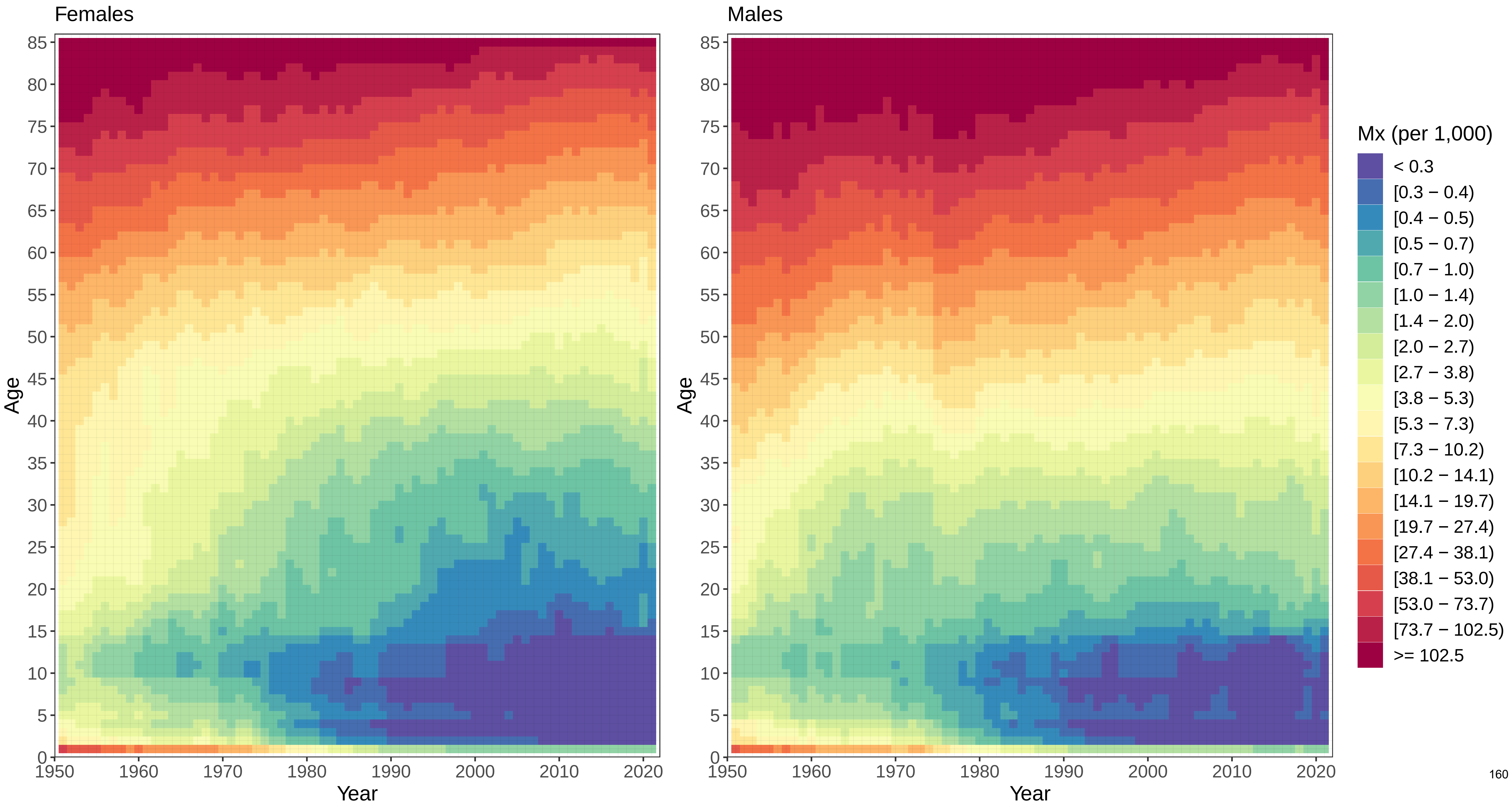

Appendix 2 Figure S5: Myanmar mortality rates, 1950−2021

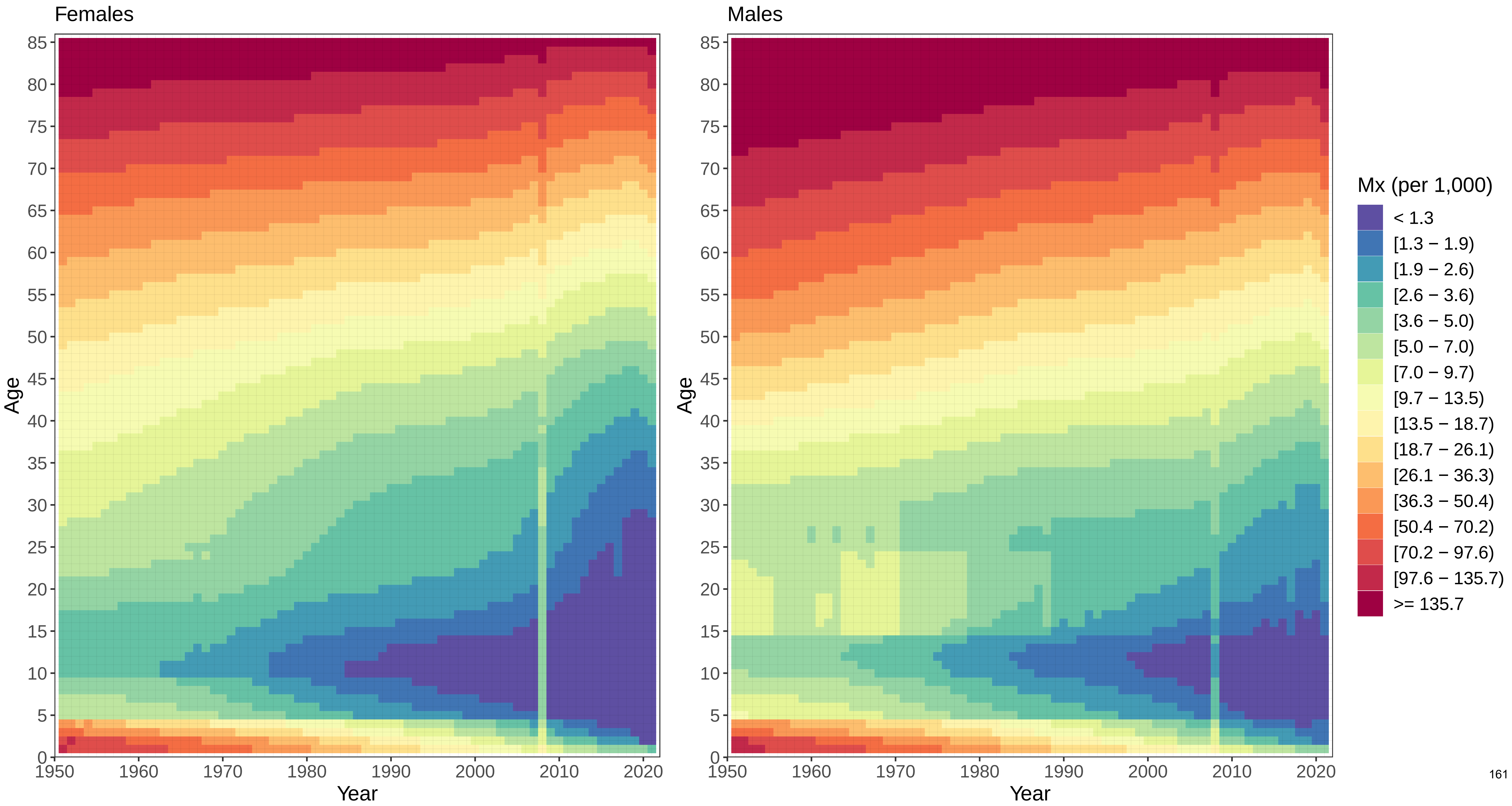

Appendix 2 Figure S5: Philippines mortality rates, 1950−2021

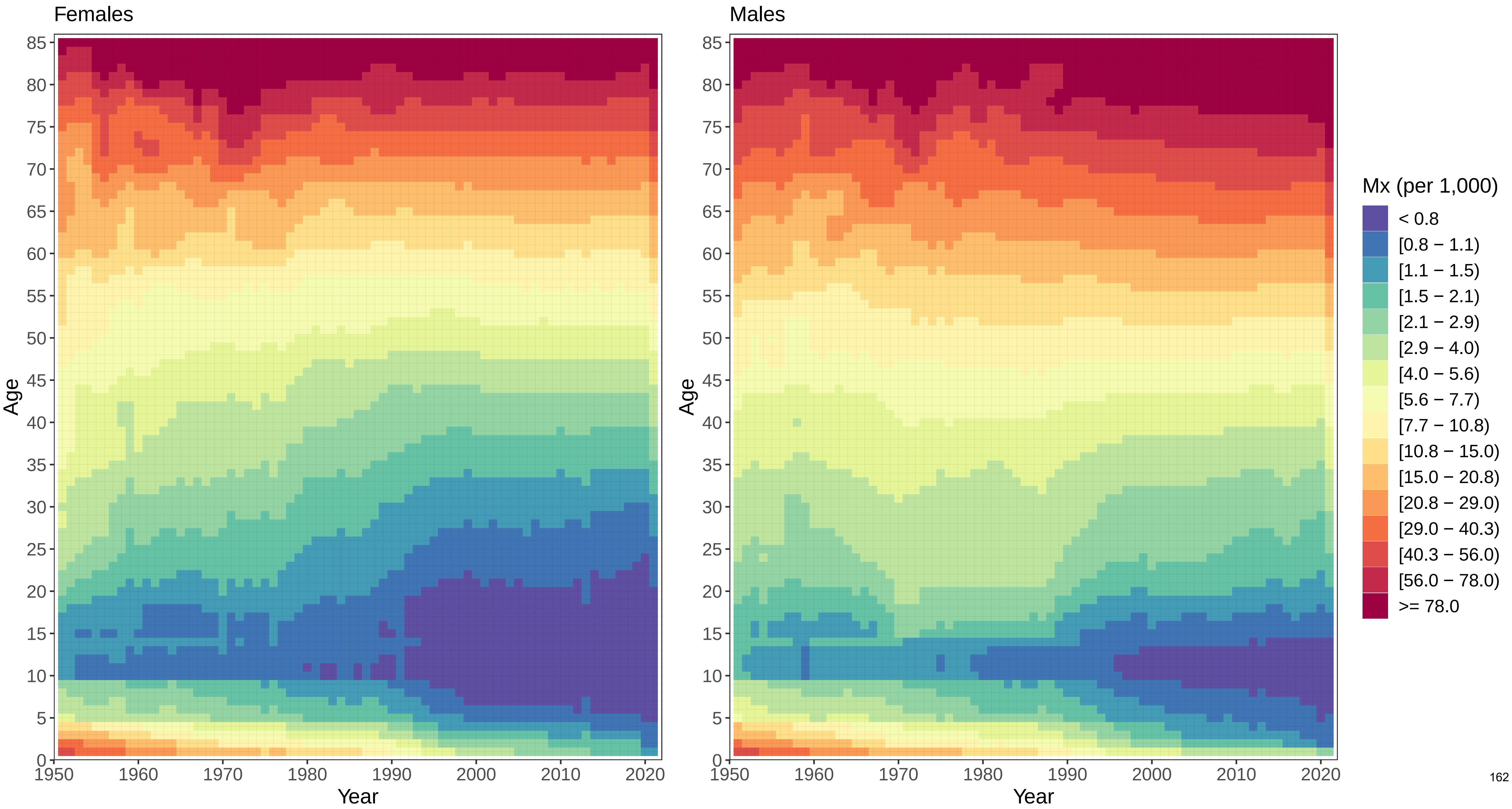

Appendix 2 Figure S5: Seychelles mortality rates, 1950−2021

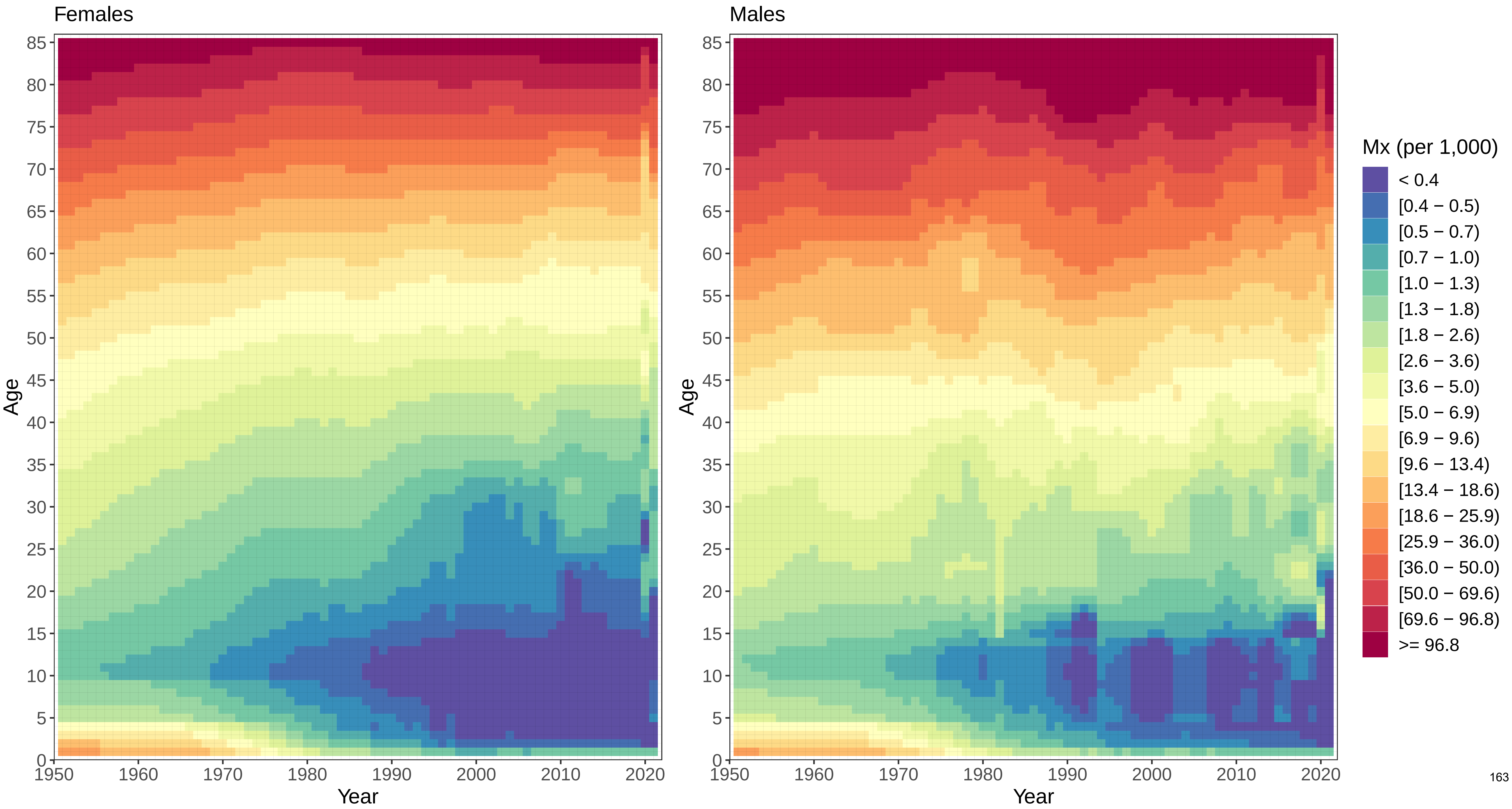

Appendix 2 Figure S5: Sri Lanka mortality rates, 1950−2021

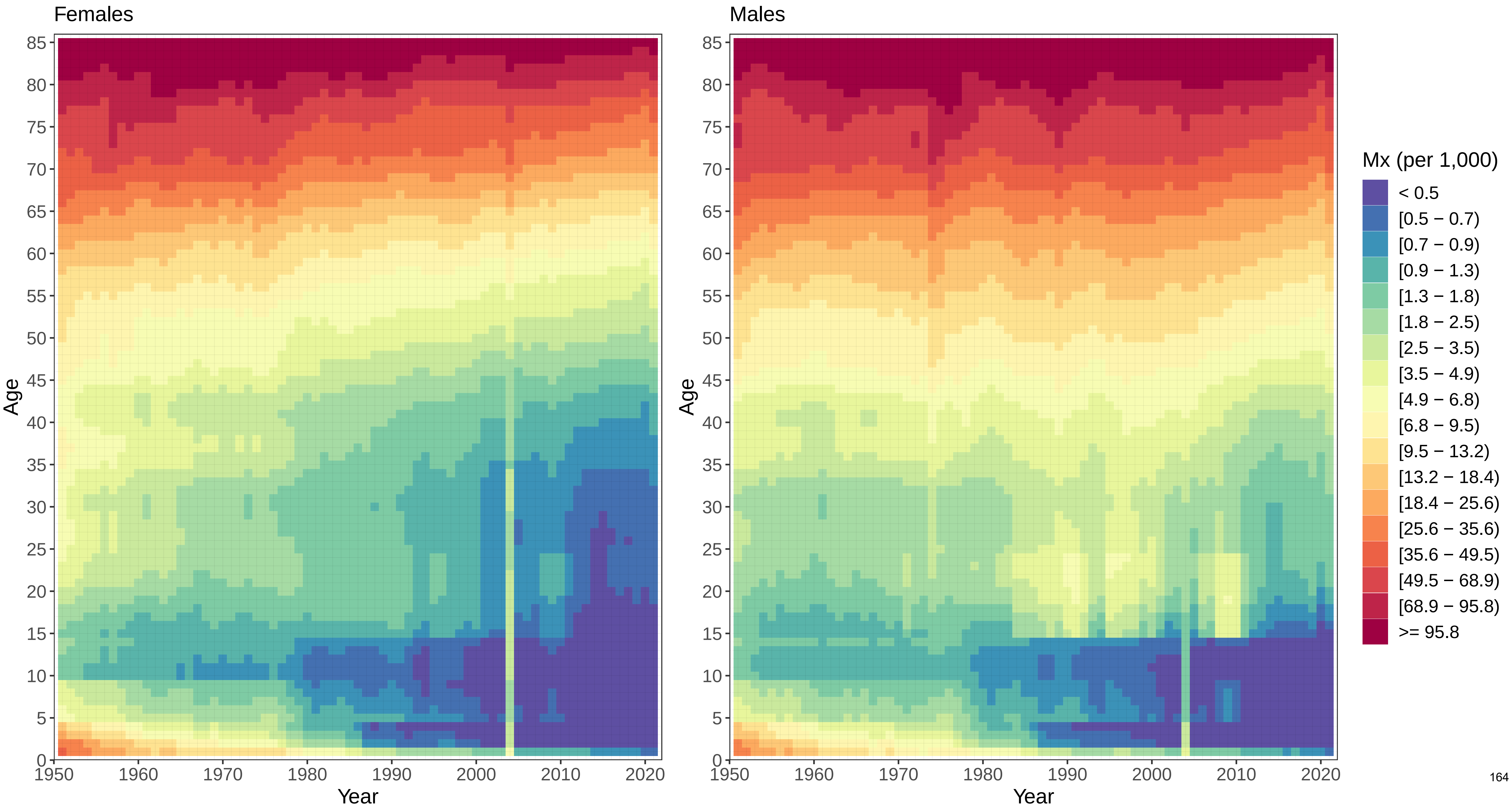

Appendix 2 Figure S5: Thailand mortality rates, 1950−2021

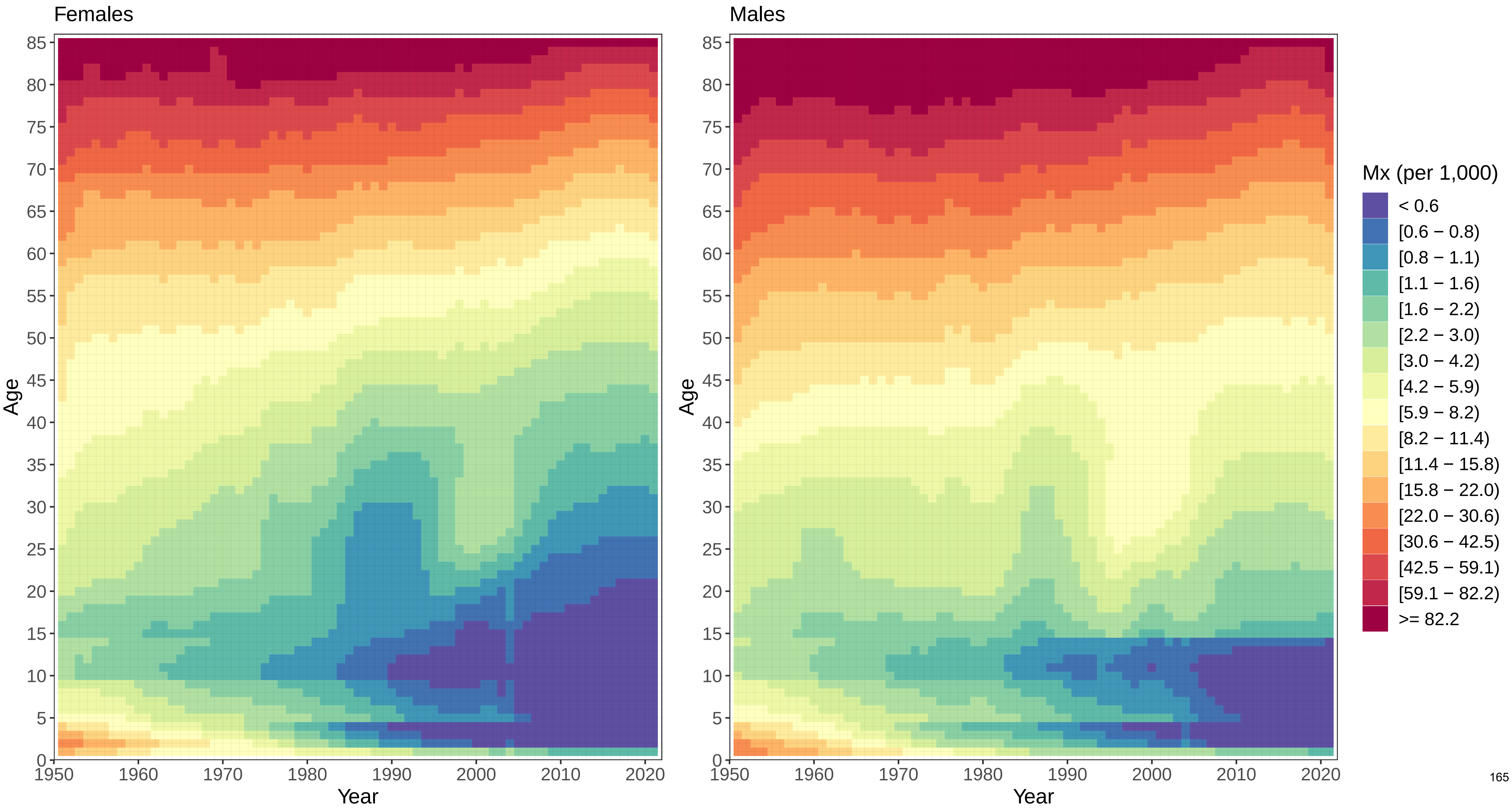

Appendix 2 Figure S5: Timor−Leste mortality rates, 1950−2021

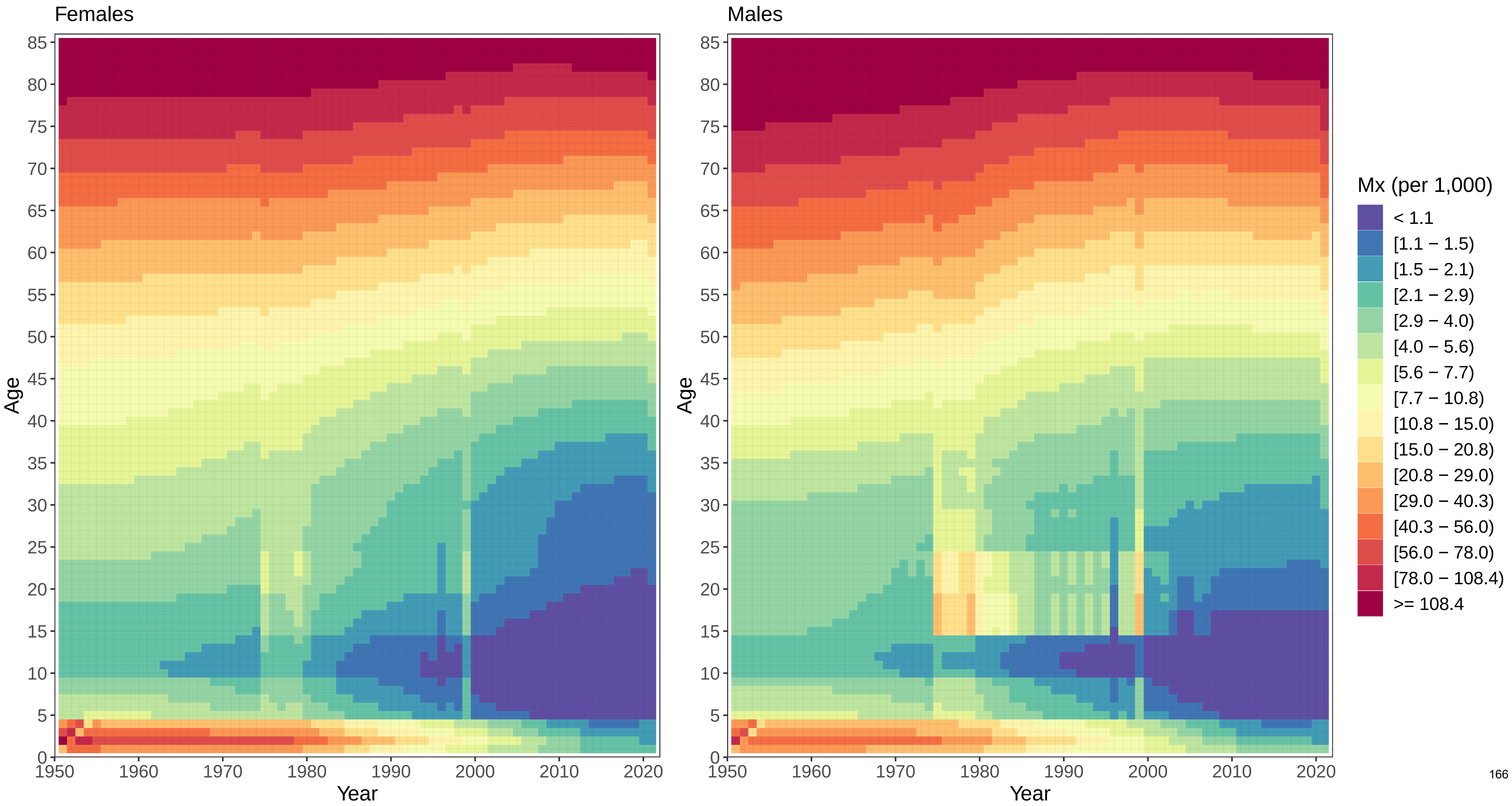

Appendix 2 Figure S5: Viet Nam mortality rates, 1950−2021

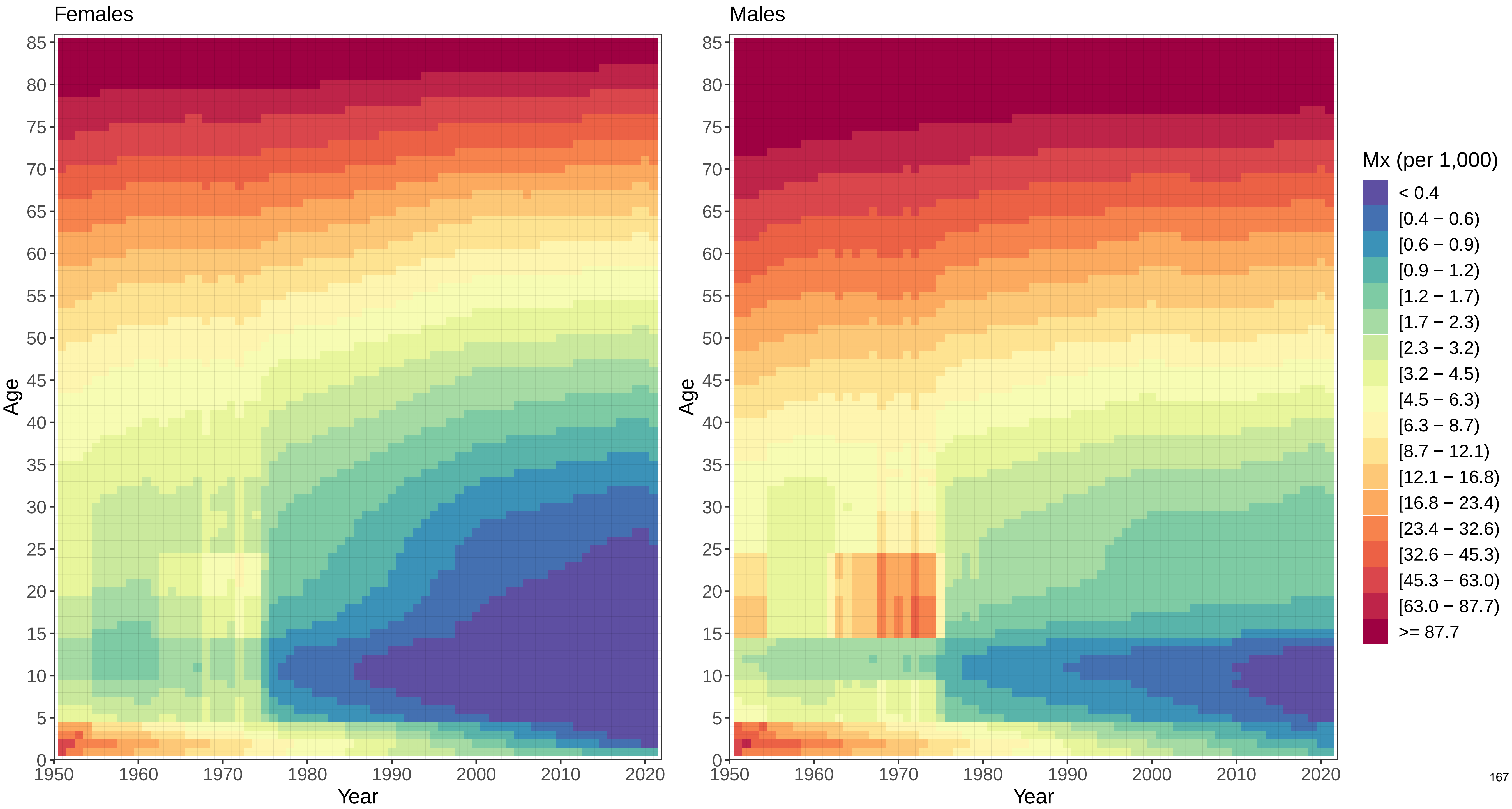

Appendix 2 Figure S5: Angola mortality rates, 1950−2021

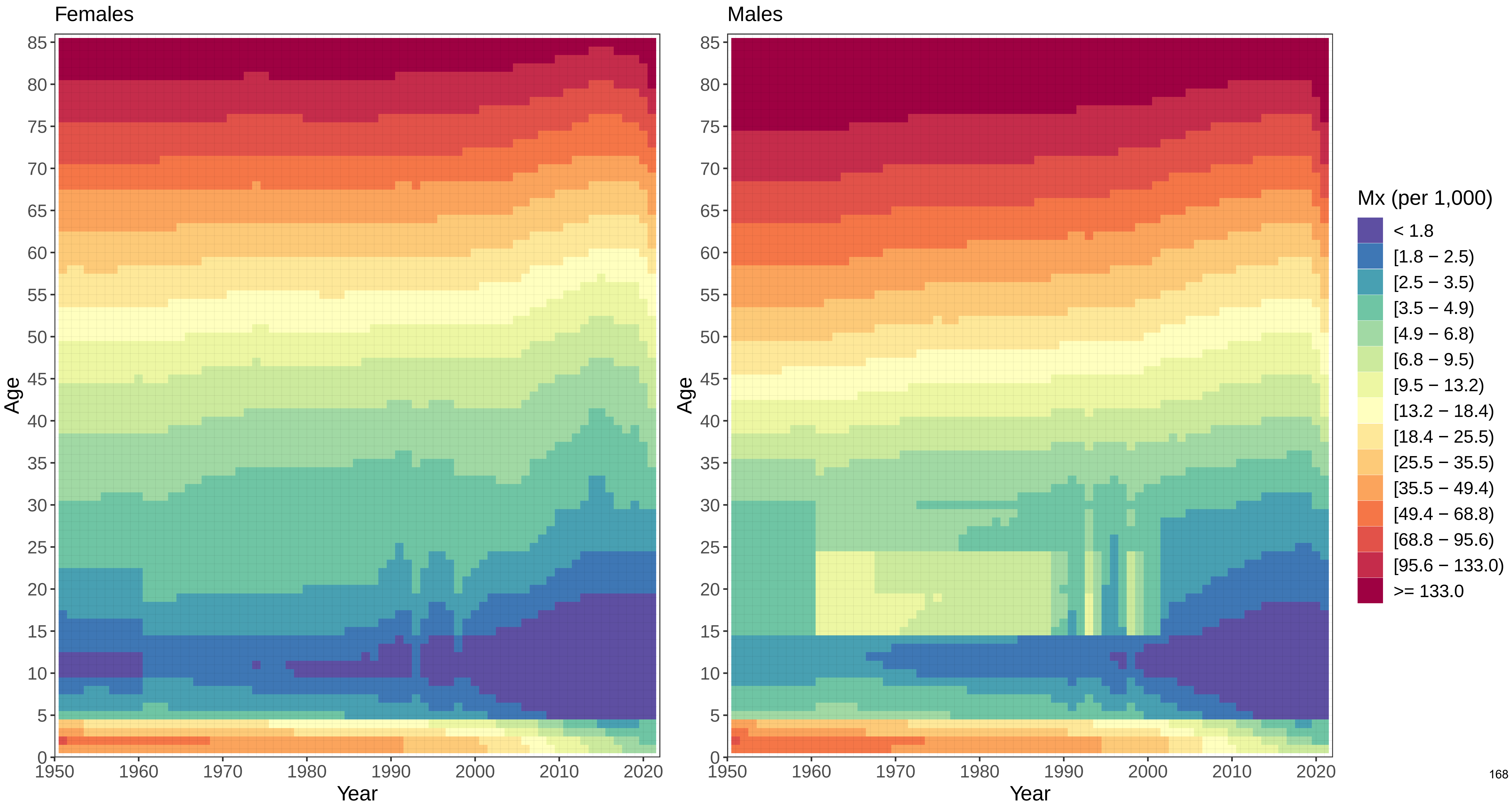

Appendix 2 Figure S5: Central African Republic mortality rates, 1950−2021

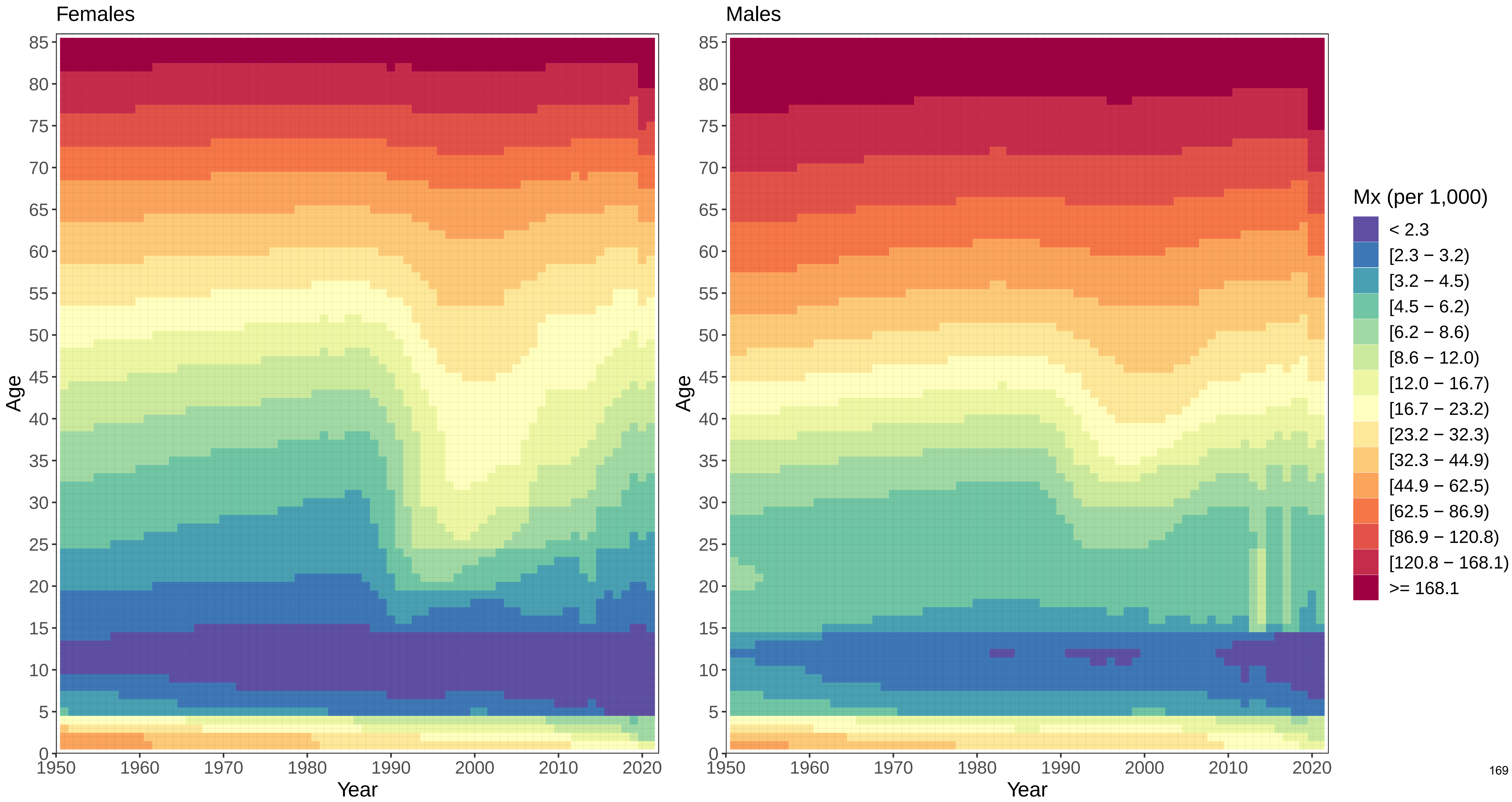

Appendix 2 Figure S5: Congo (Brazzaville) mortality rates, 1950−2021

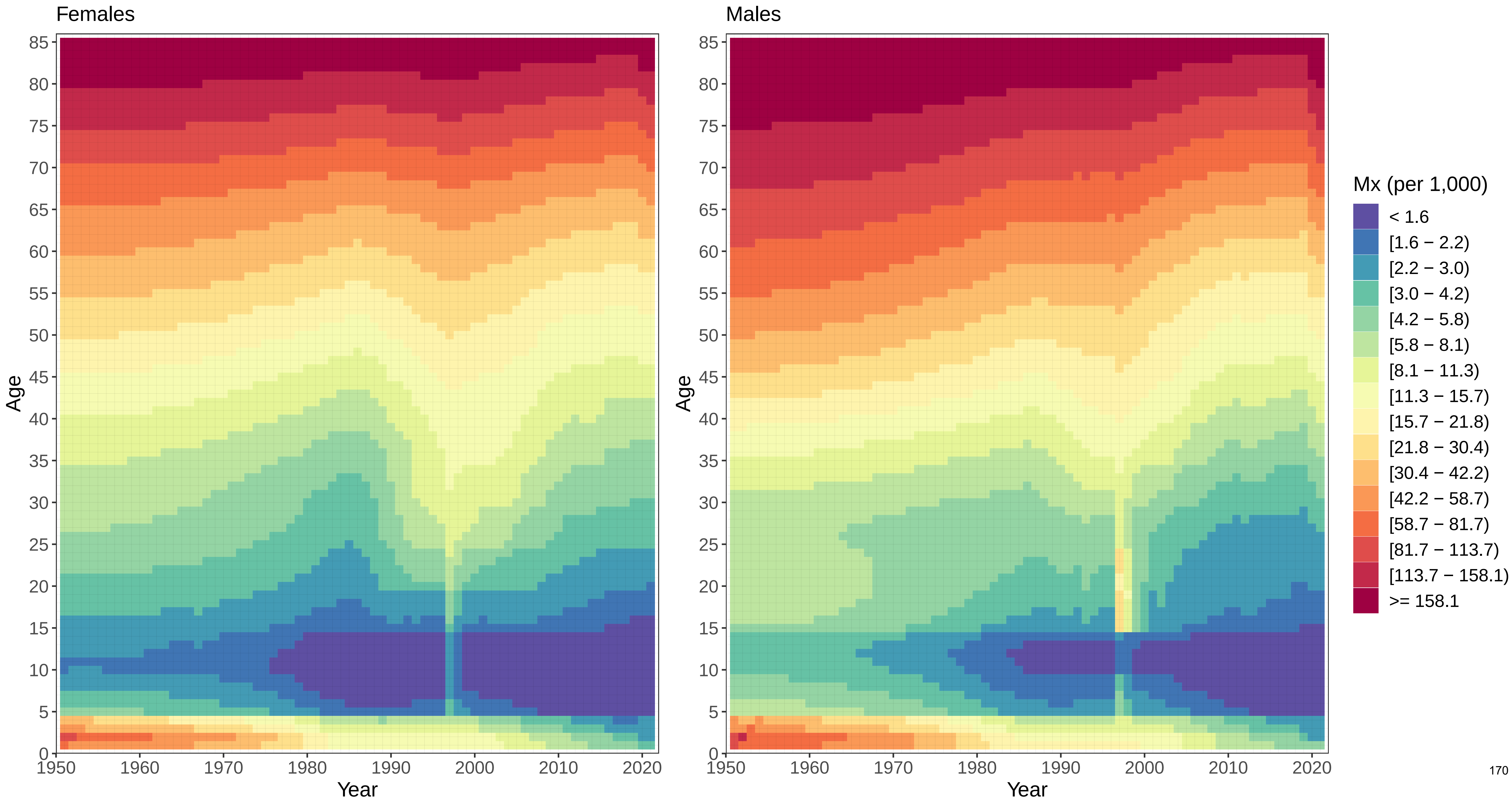

Appendix 2 Figure S5: DR Congo mortality rates, 1950−2021

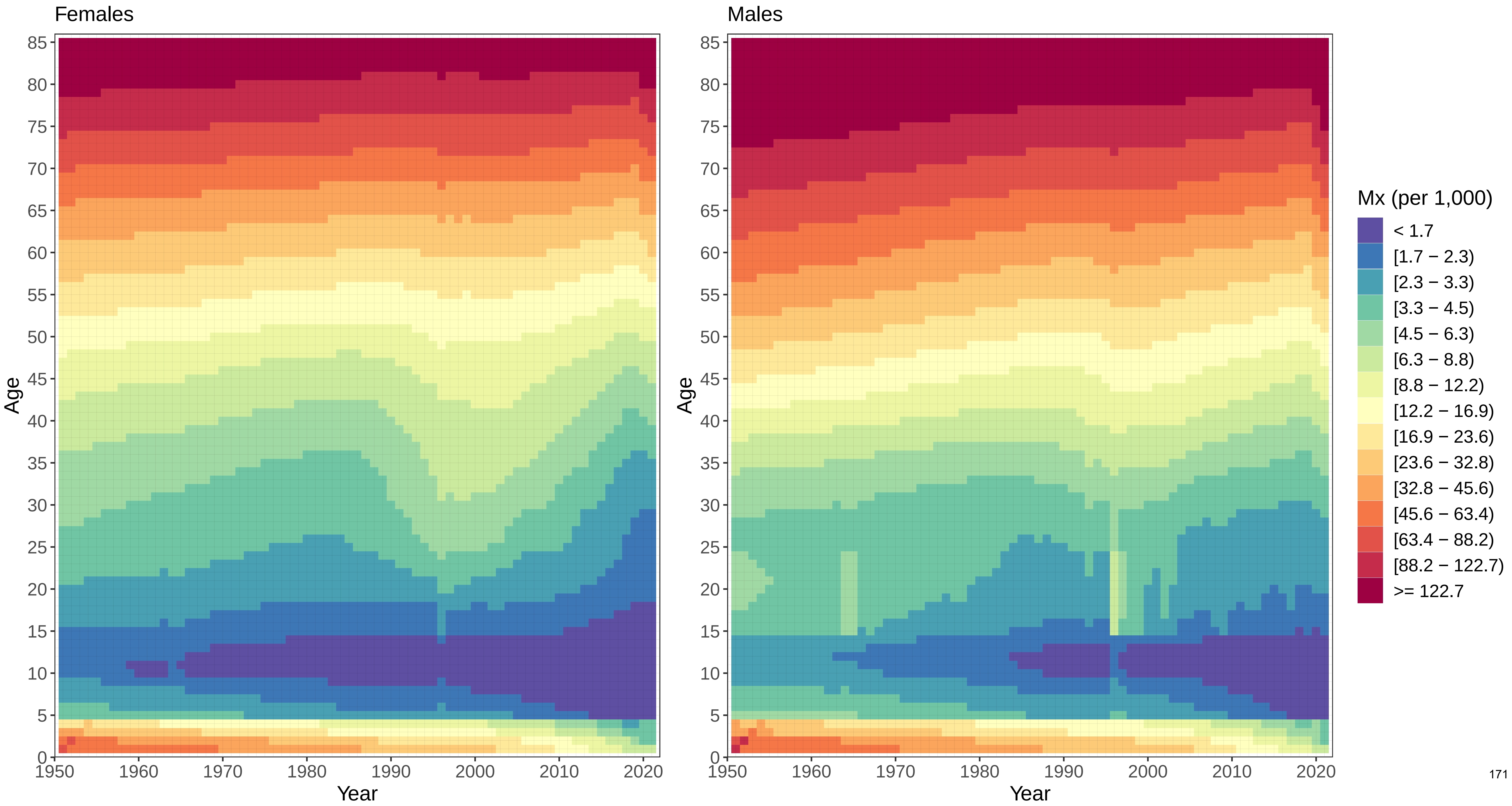

Appendix 2 Figure S5: Equatorial Guinea mortality rates, 1950−2021

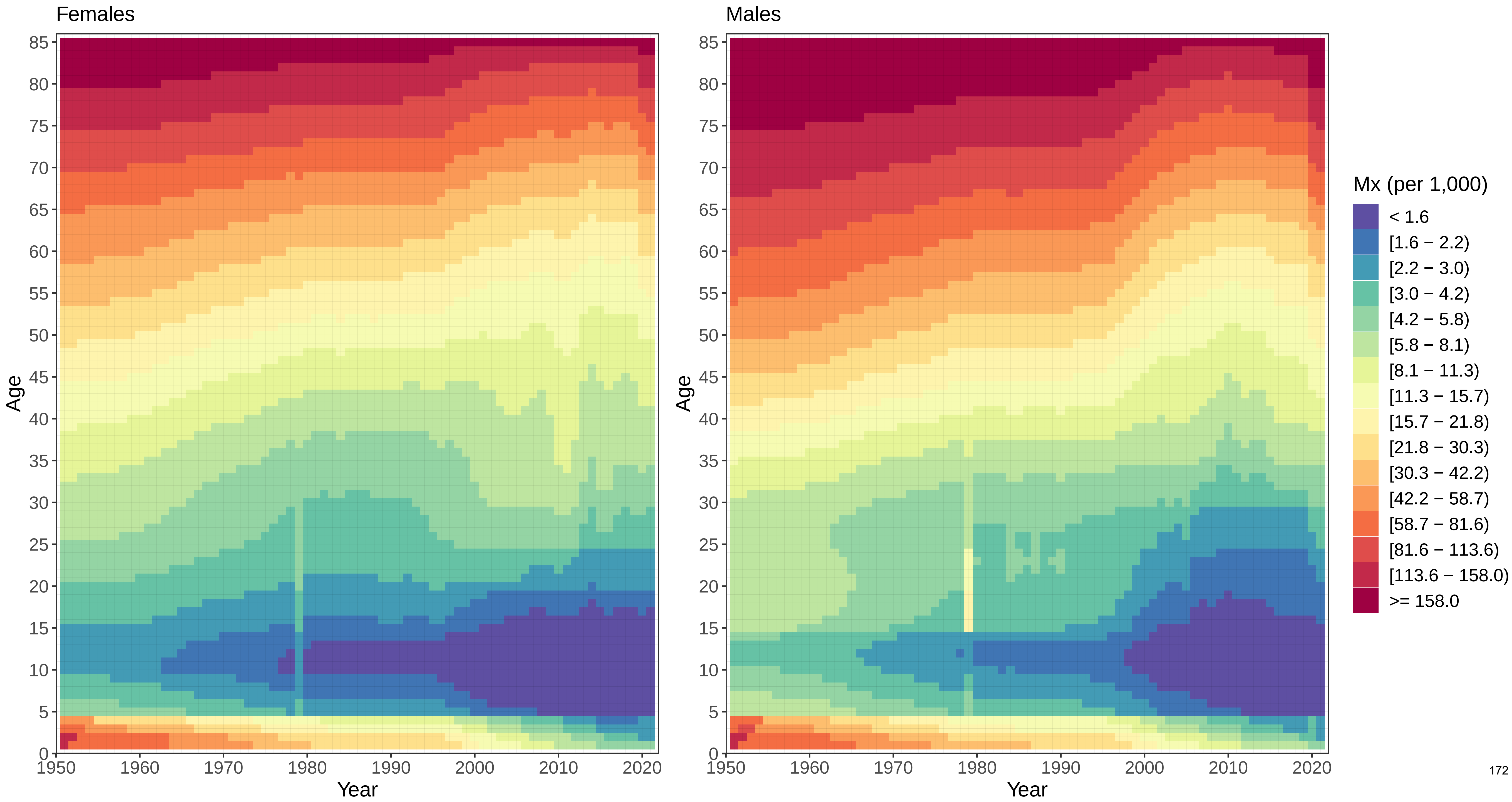

Appendix 2 Figure S5: Gabon mortality rates, 1950−2021

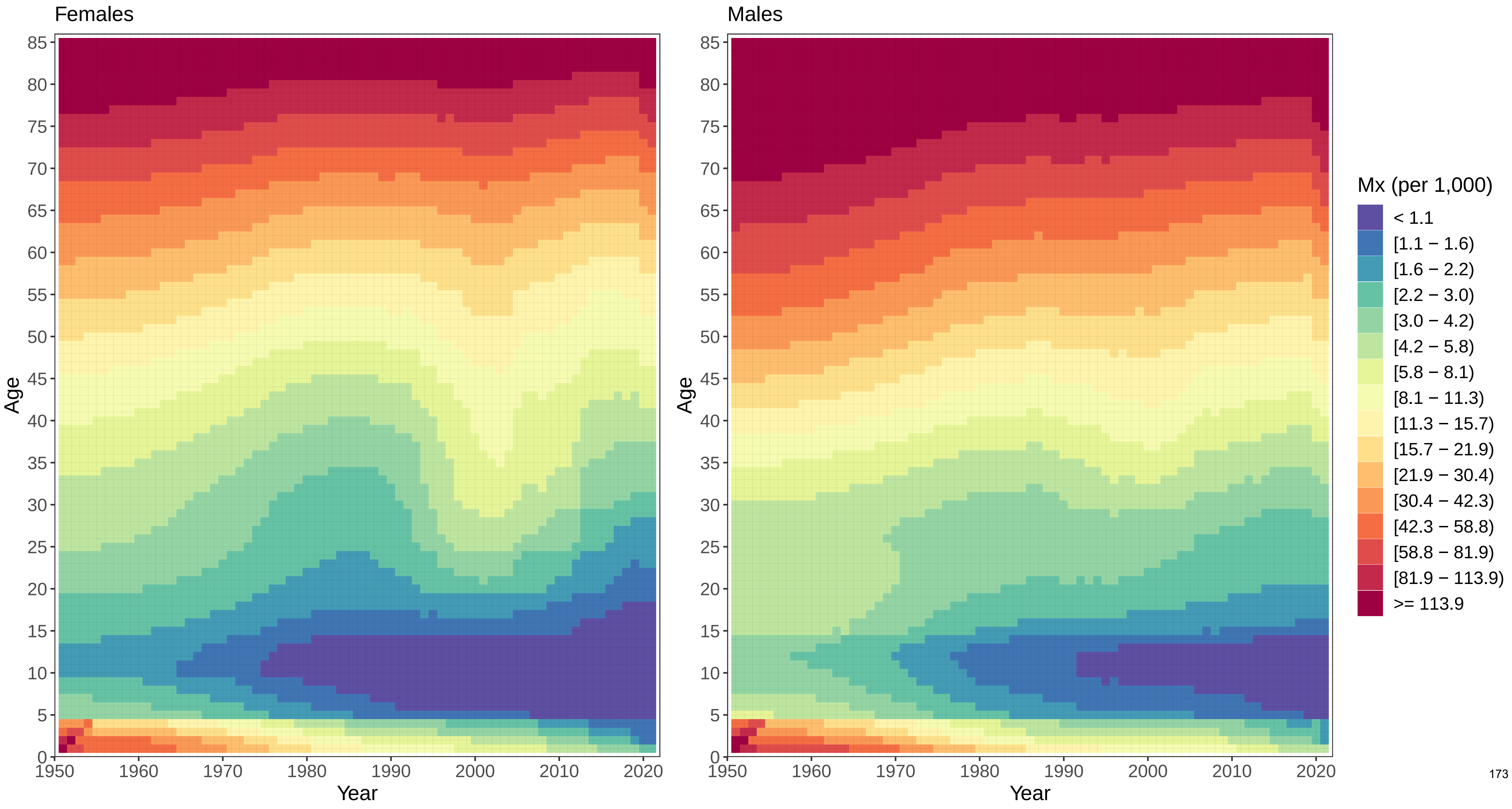

Appendix 2 Figure S5: Burundi mortality rates, 1950−2021

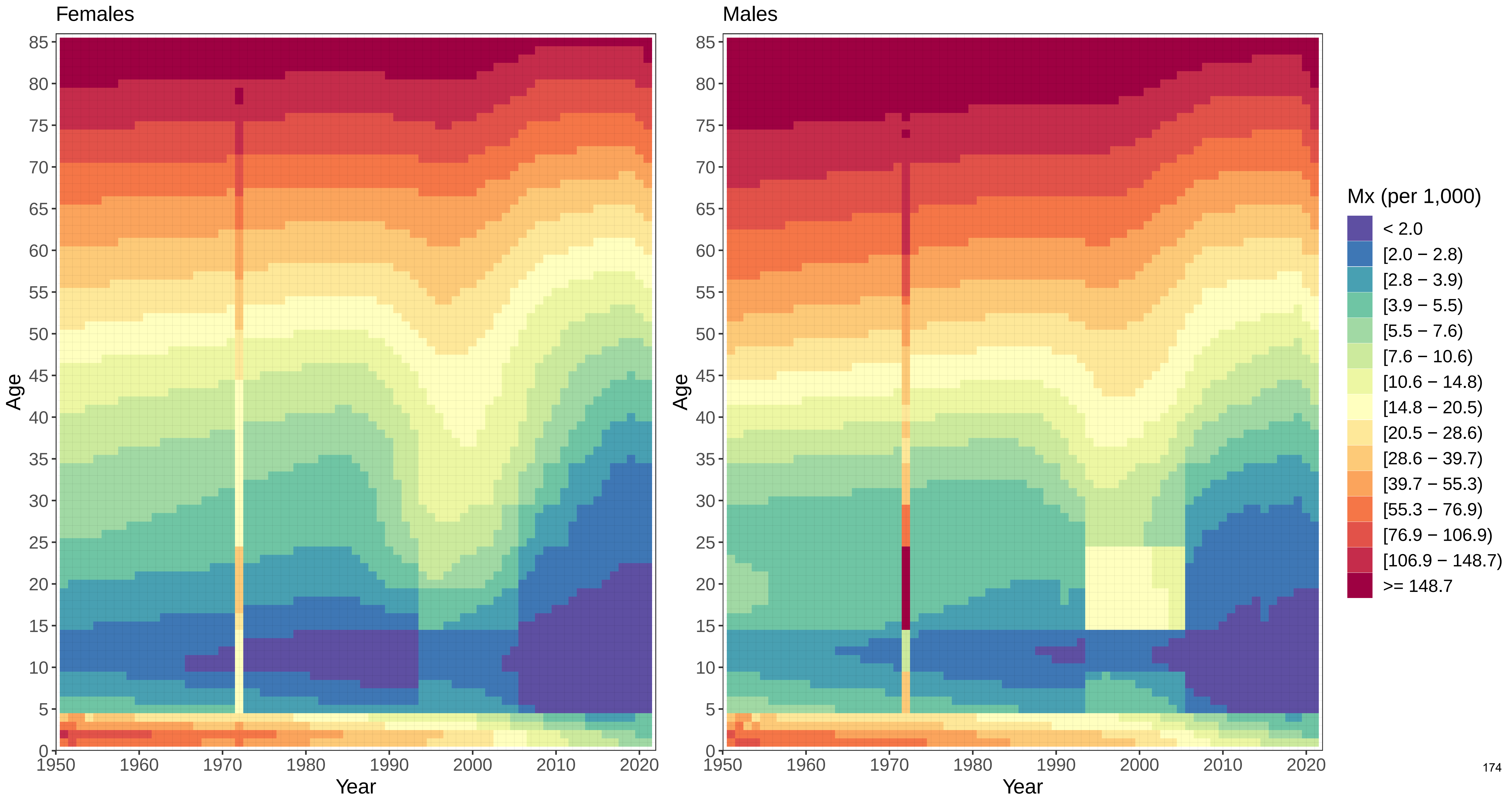

Appendix 2 Figure S5: Comoros mortality rates, 1950−2021

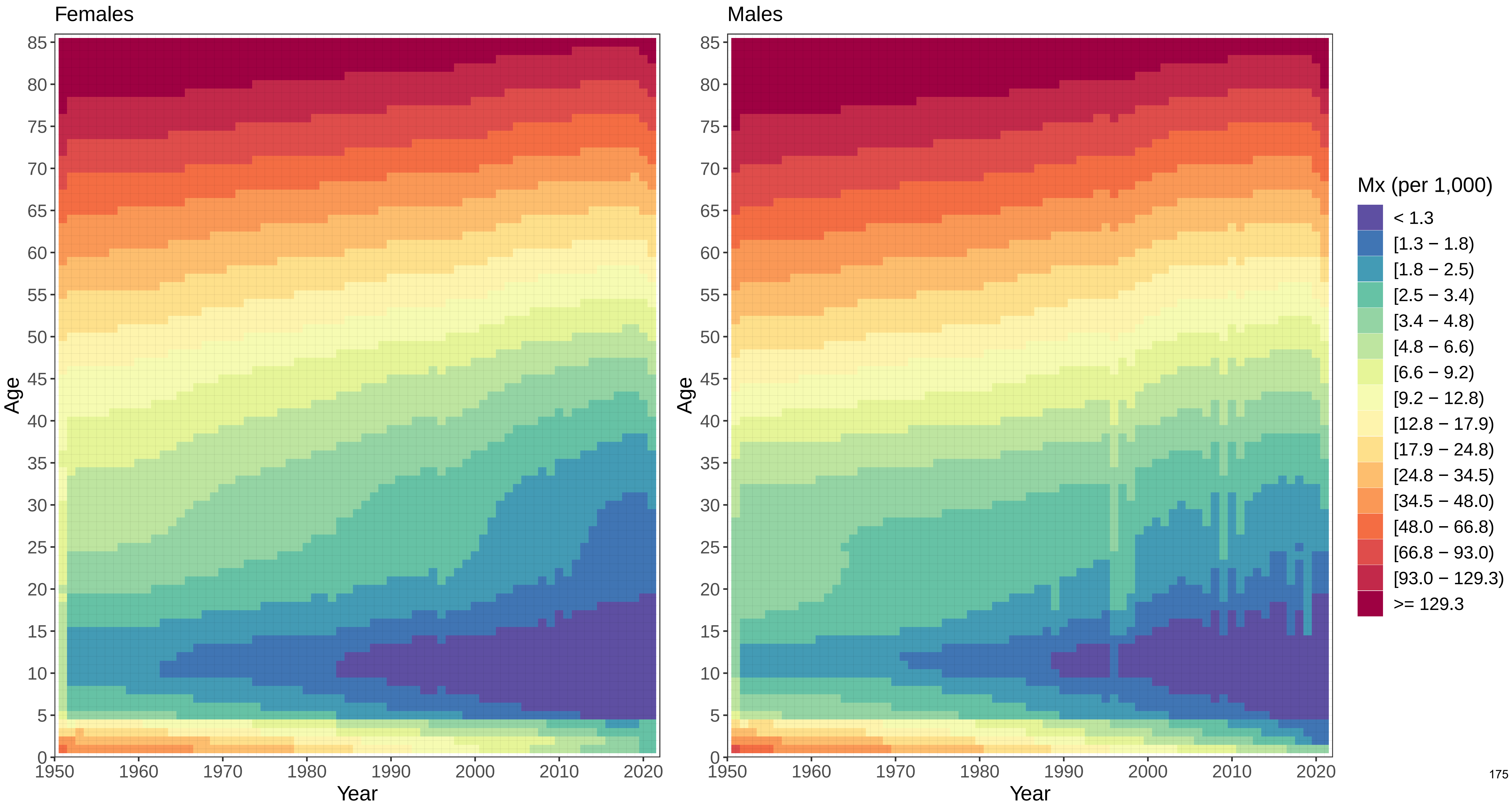

Appendix 2 Figure S5: Djibouti mortality rates, 1950−2021

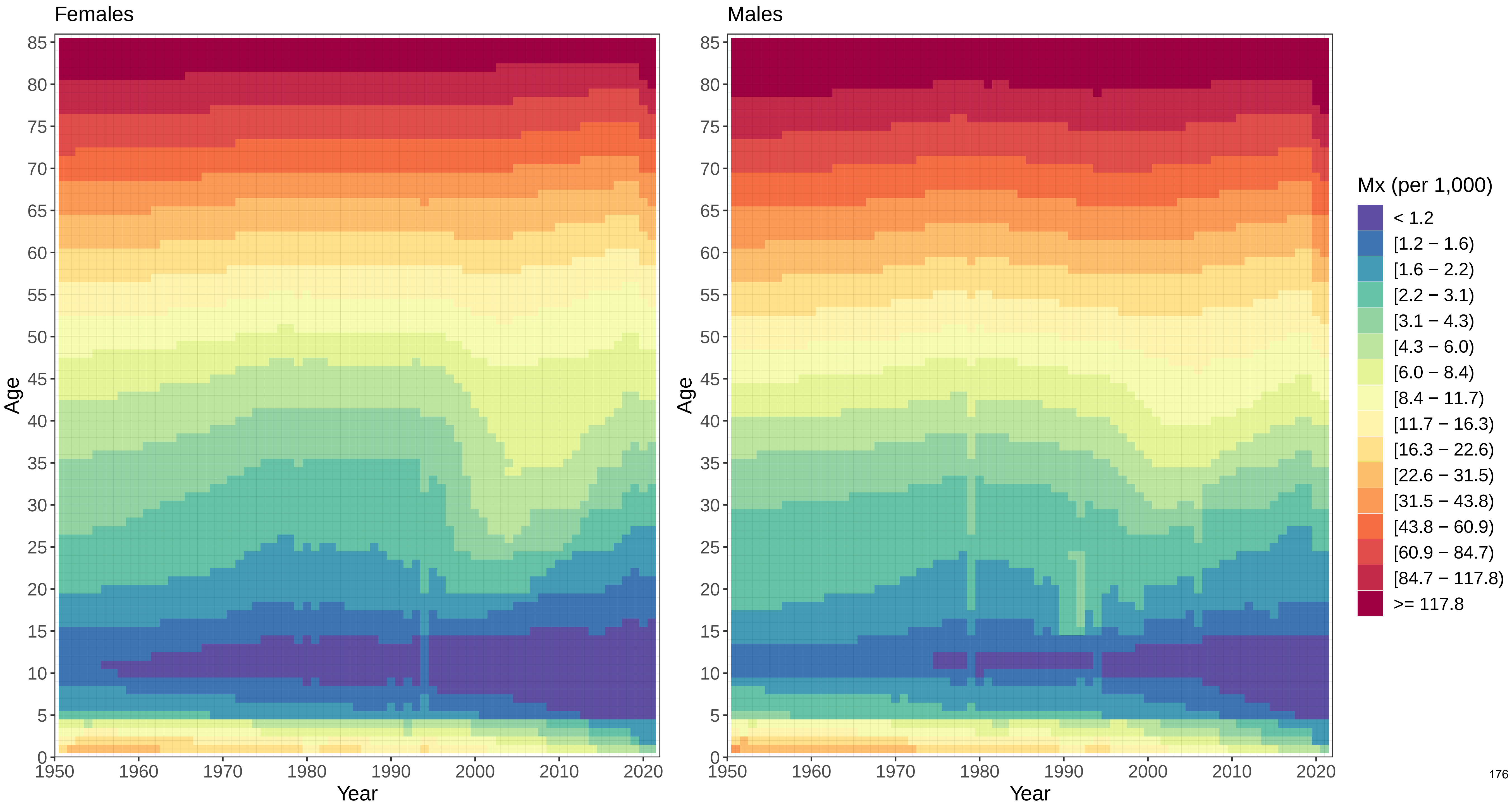

Appendix 2 Figure S5: Eritrea mortality rates, 1950−2021

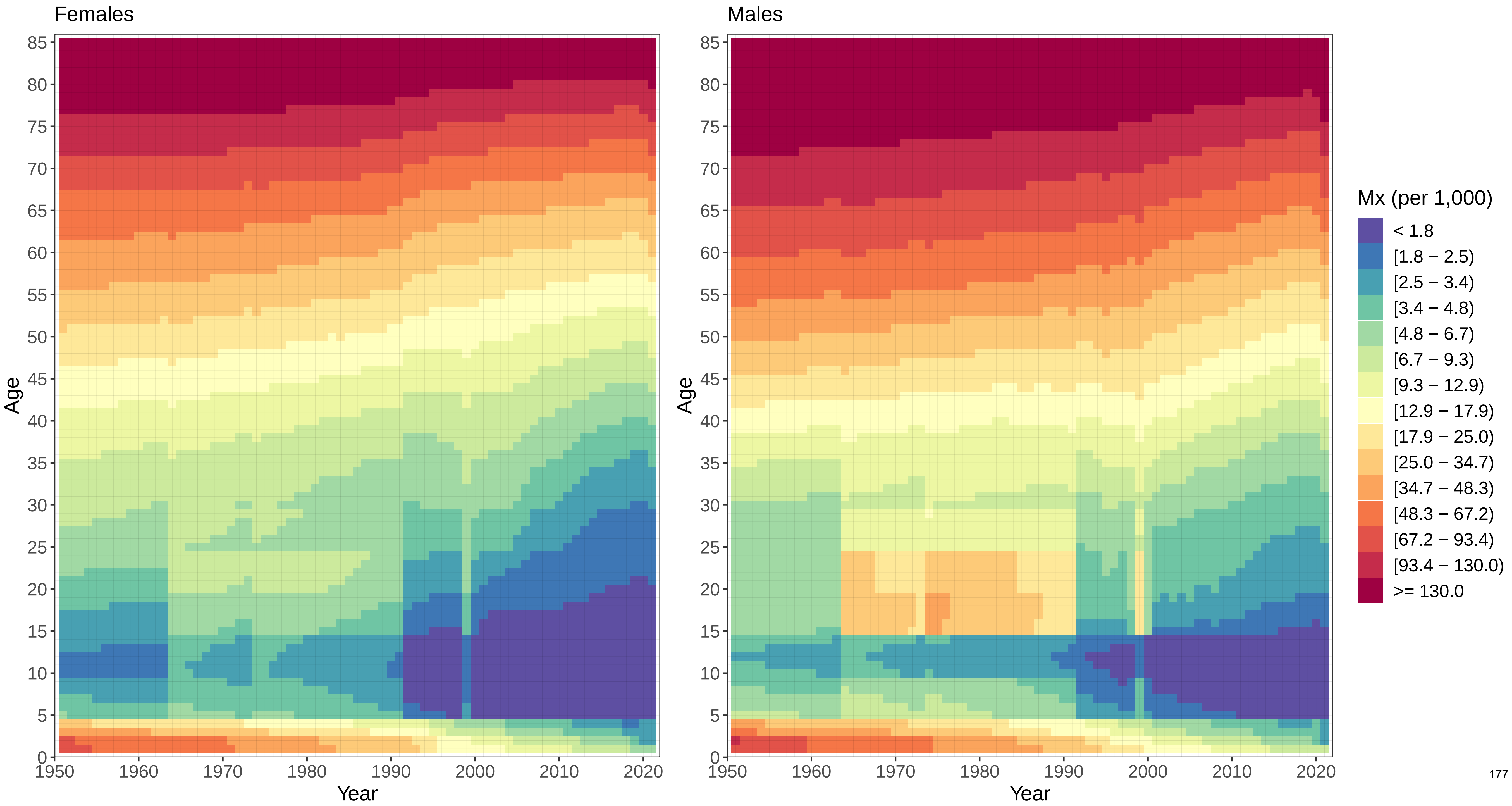

Appendix 2 Figure S5: Ethiopia mortality rates, 1950−2021

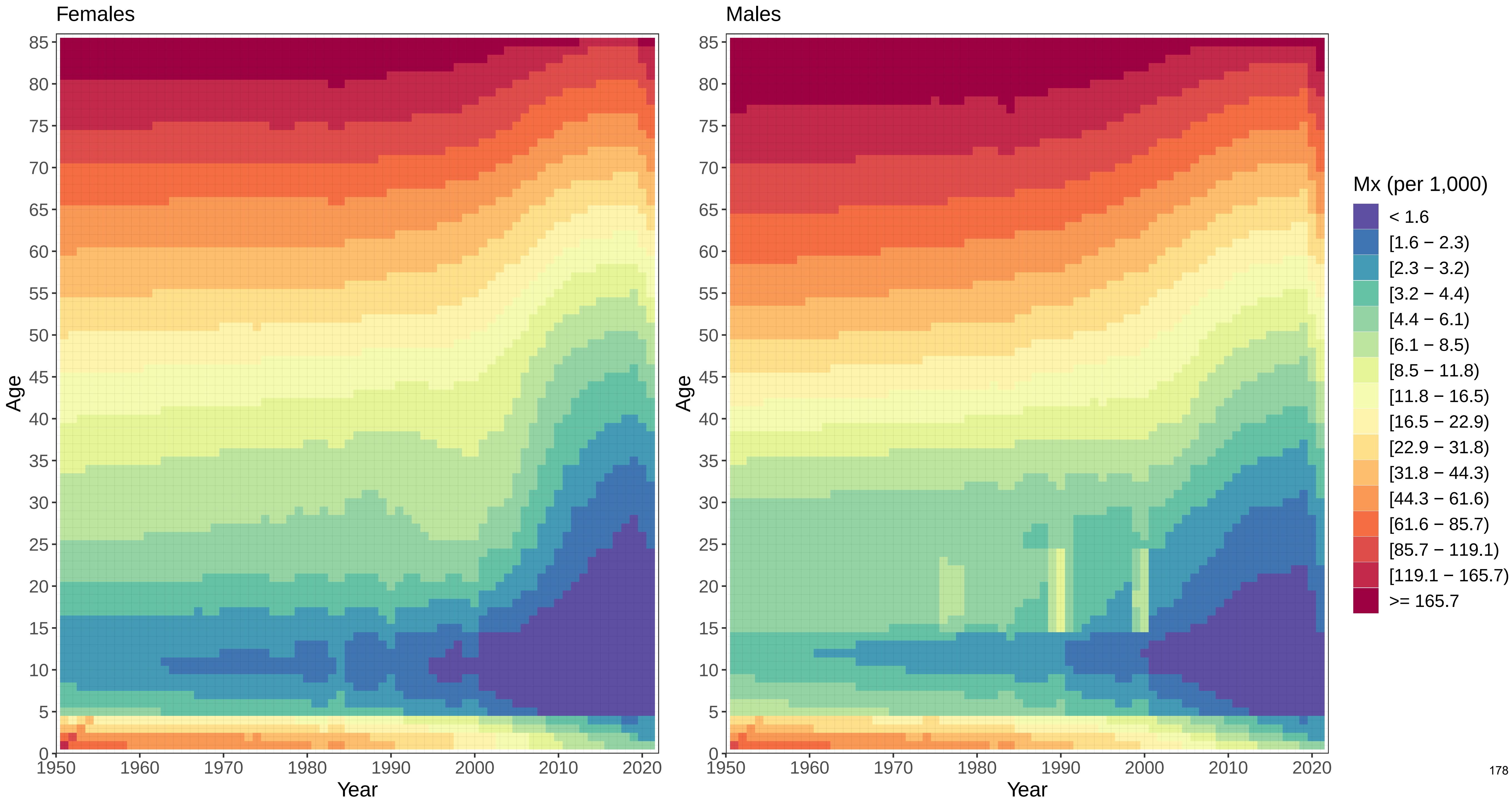

Appendix 2 Figure S5: Kenya mortality rates, 1950−2021

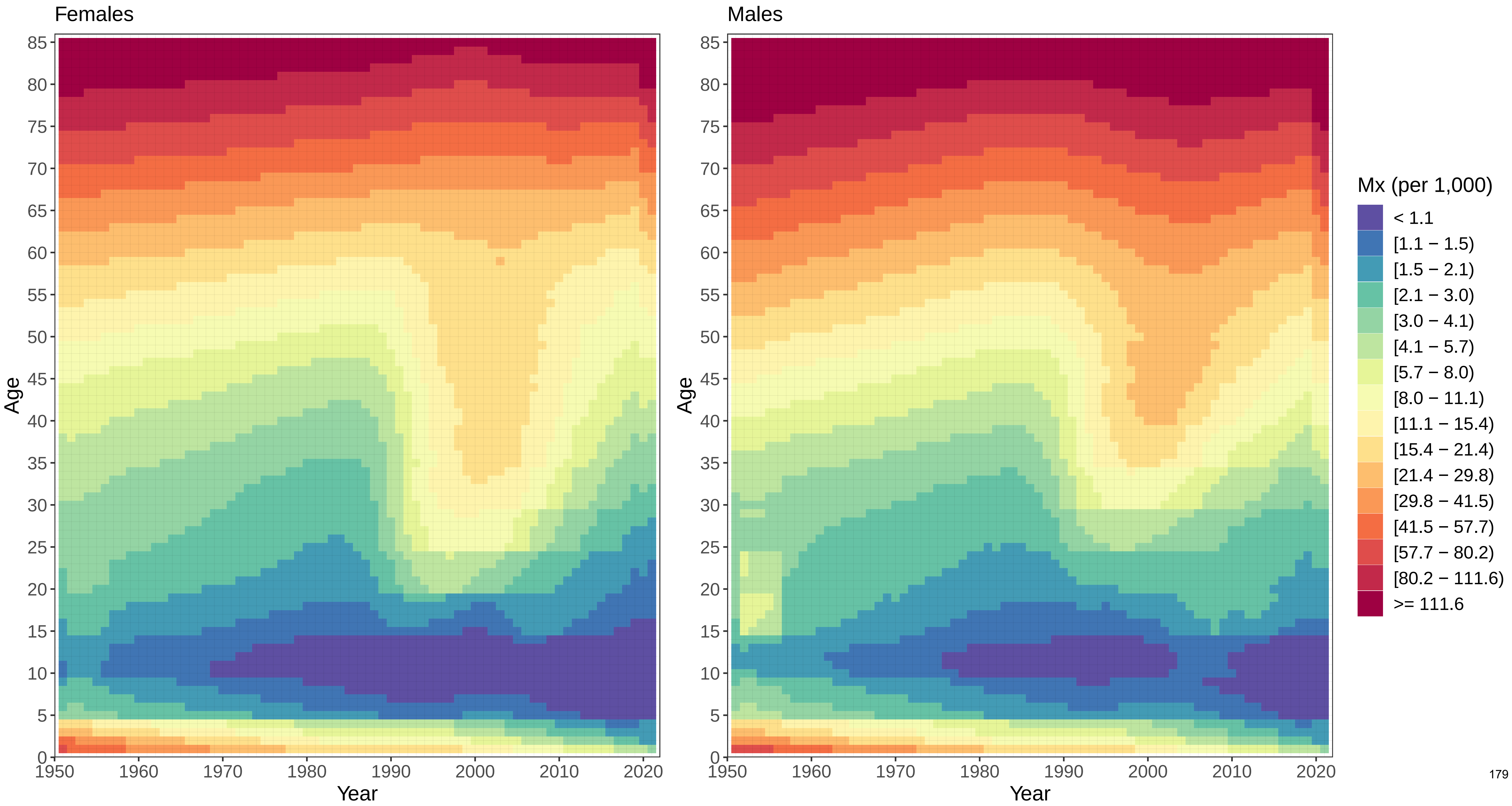

Appendix 2 Figure S5: Madagascar mortality rates, 1950−2021

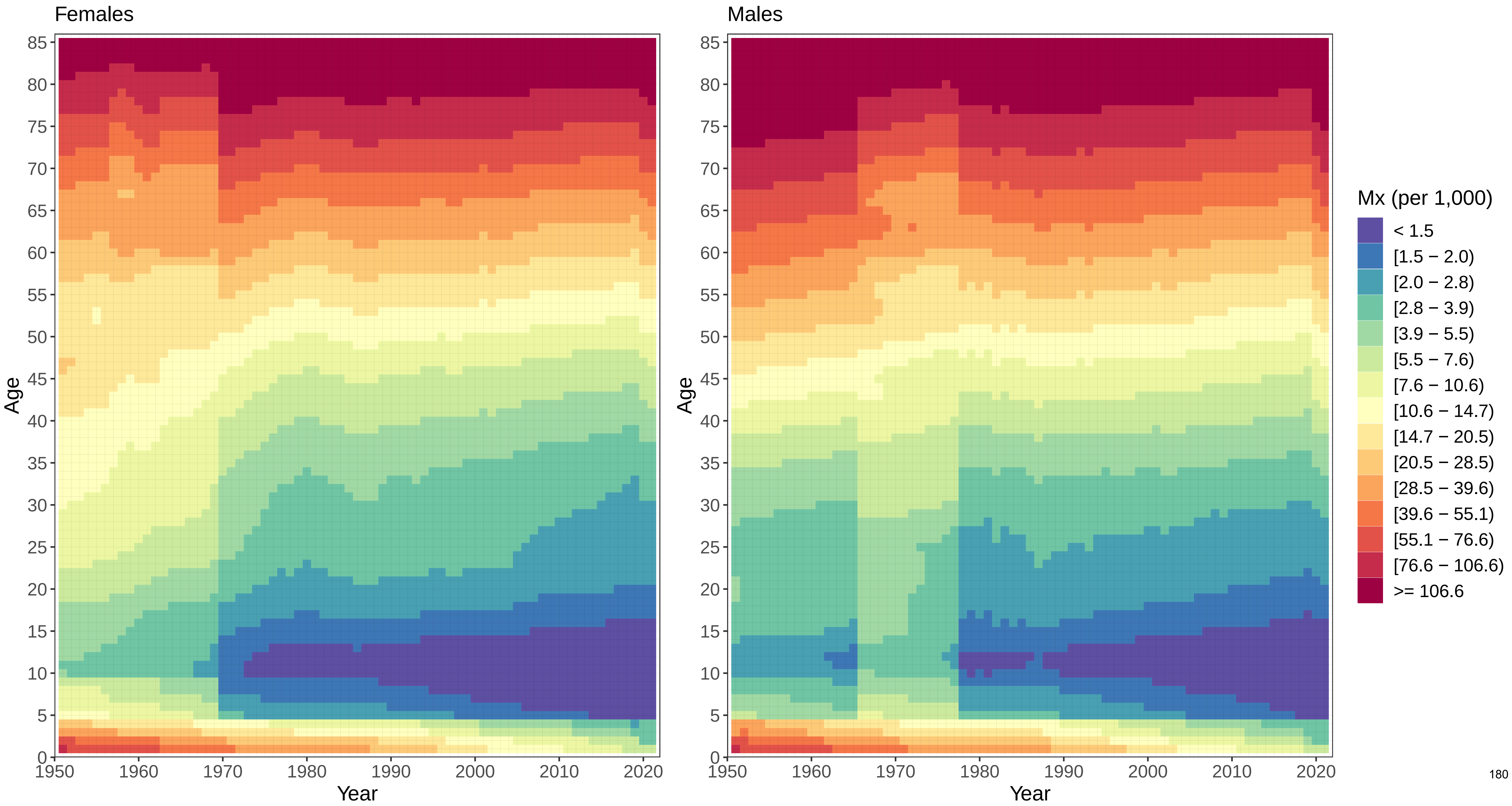

Appendix 2 Figure S5: Malawi mortality rates, 1950−2021

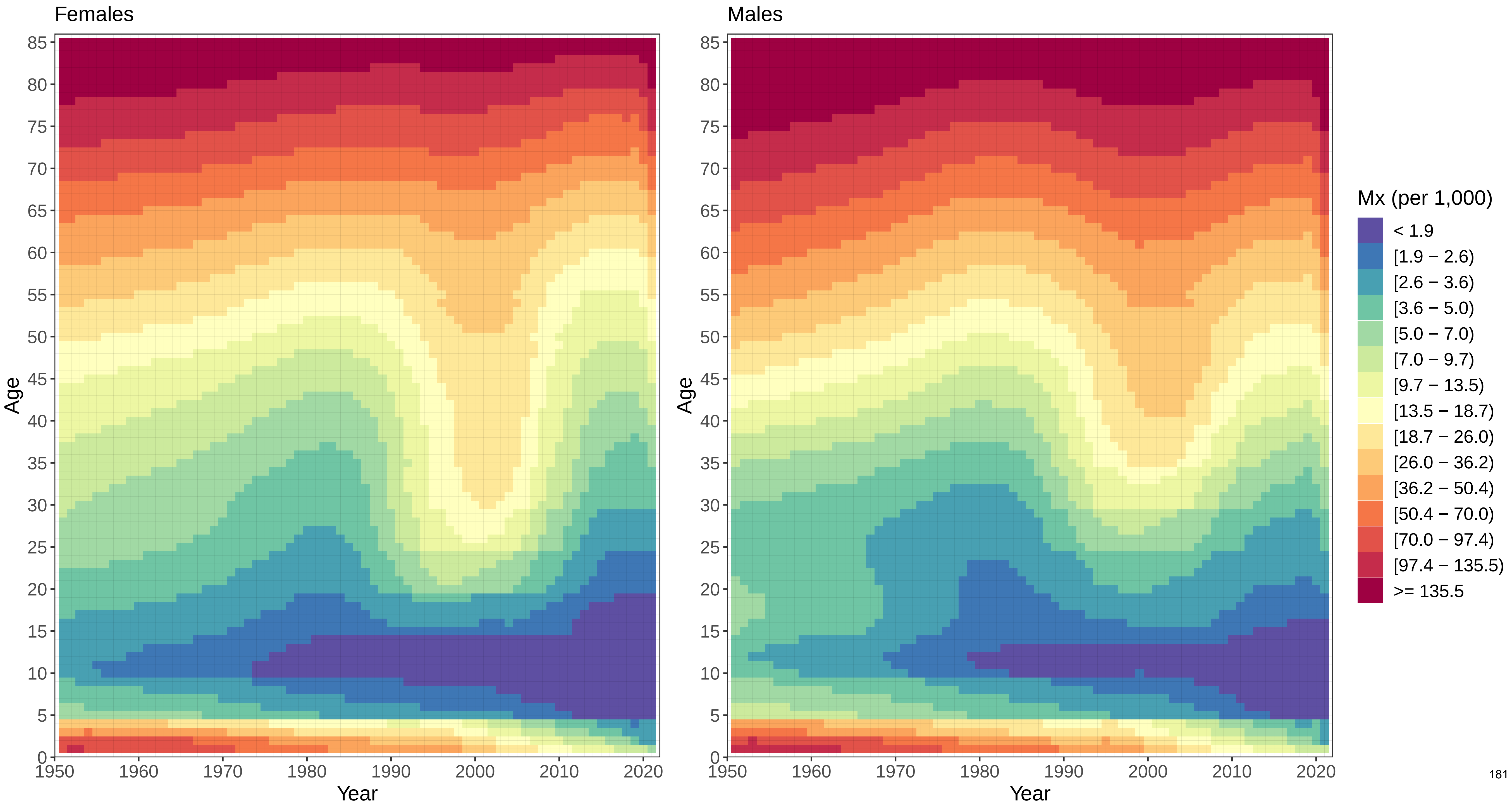

Appendix 2 Figure S5: Mozambique mortality rates, 1950−2021

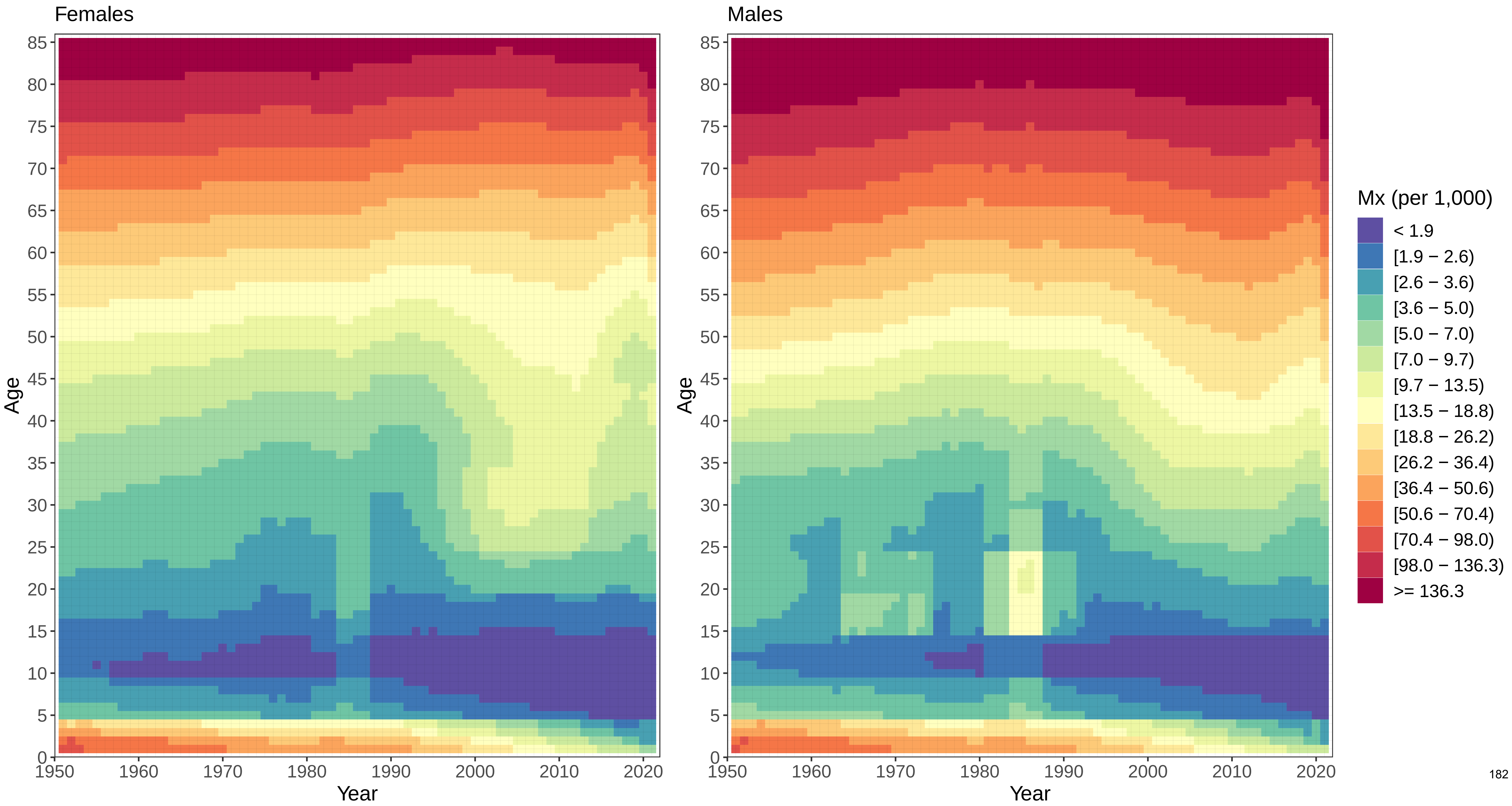

Appendix 2 Figure S5: Rwanda mortality rates, 1950−2021

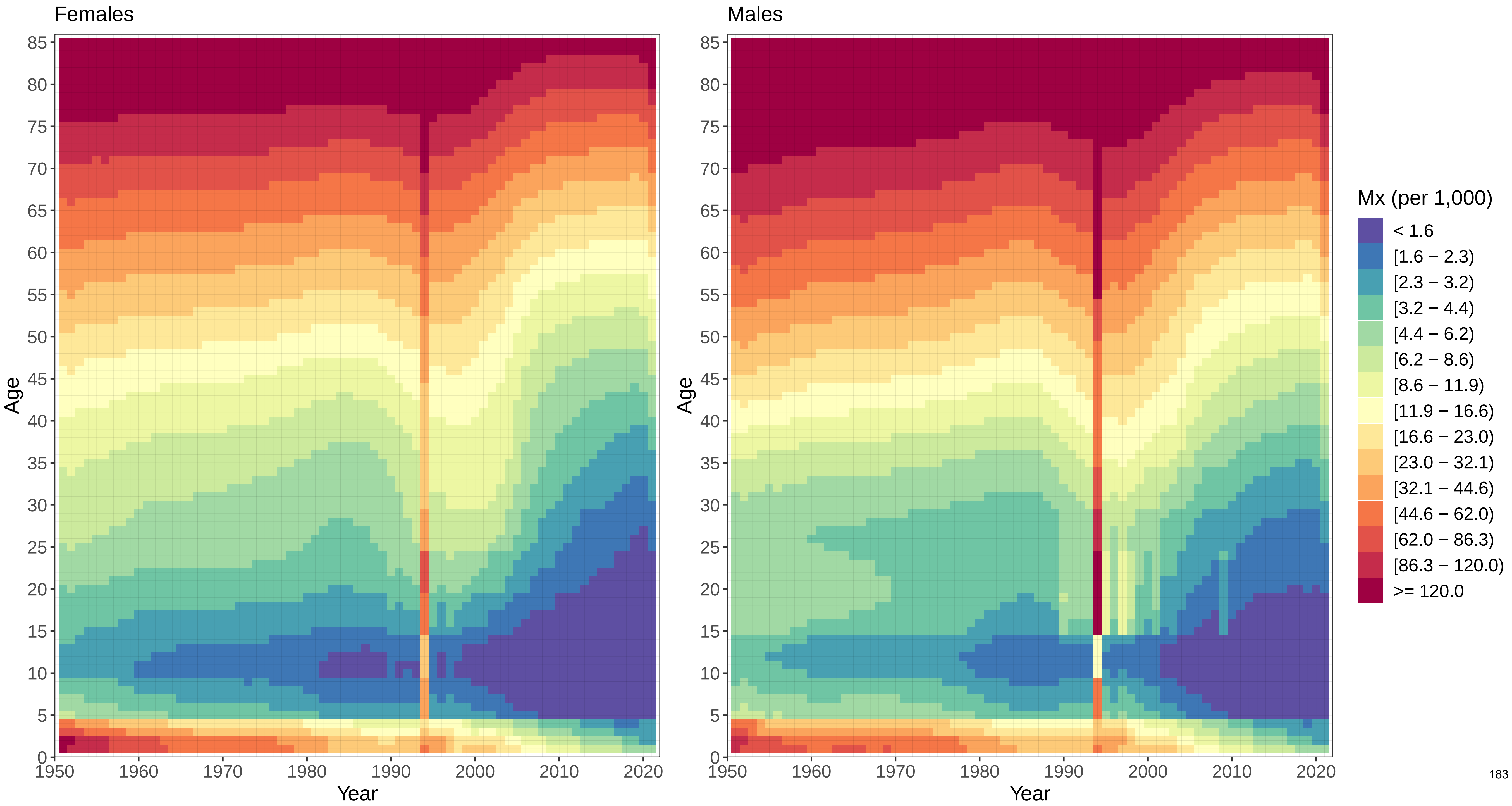

Appendix 2 Figure S5: Somalia mortality rates, 1950−2021

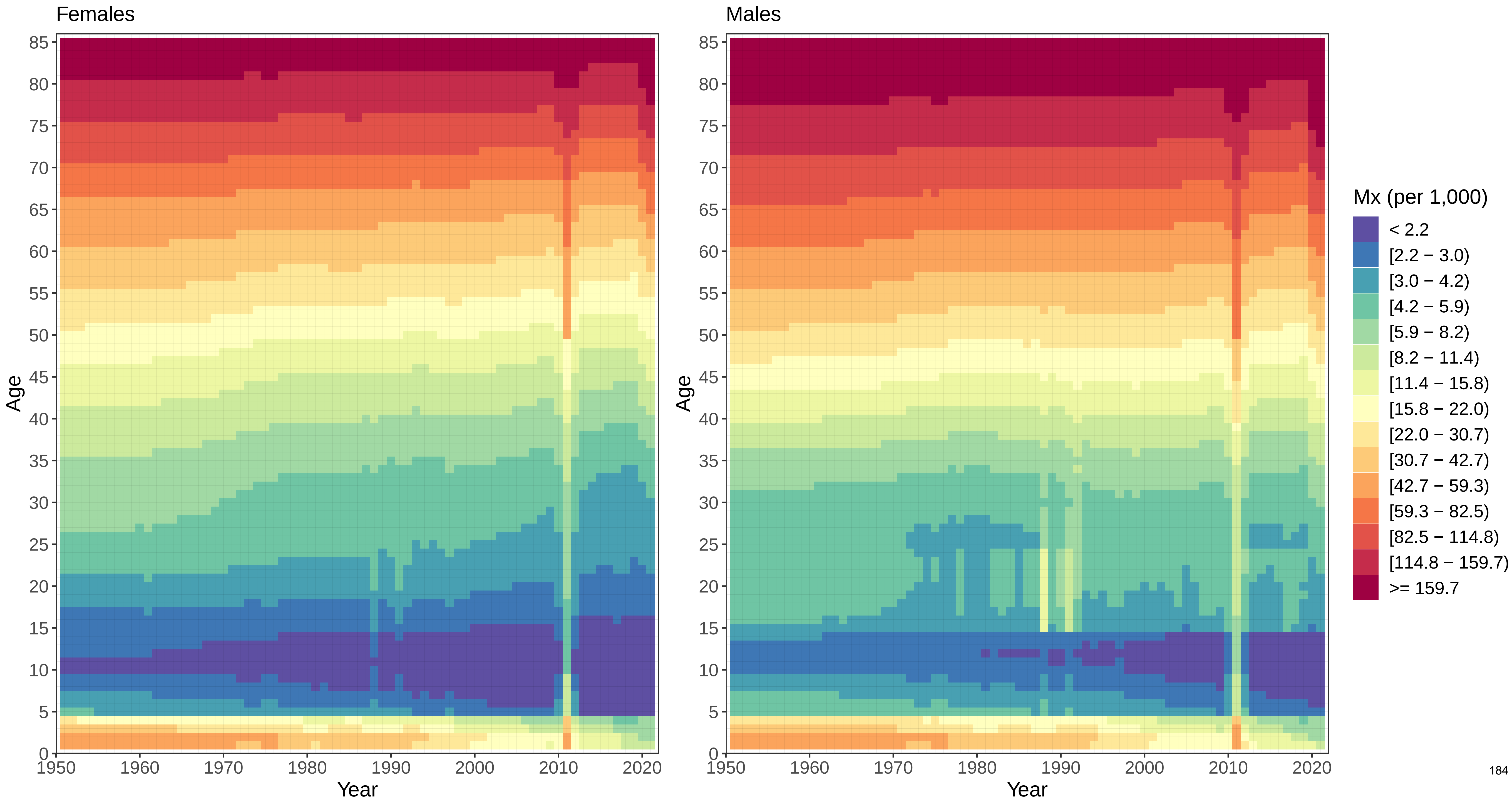

Appendix 2 Figure S5: South Sudan mortality rates, 1950−2021

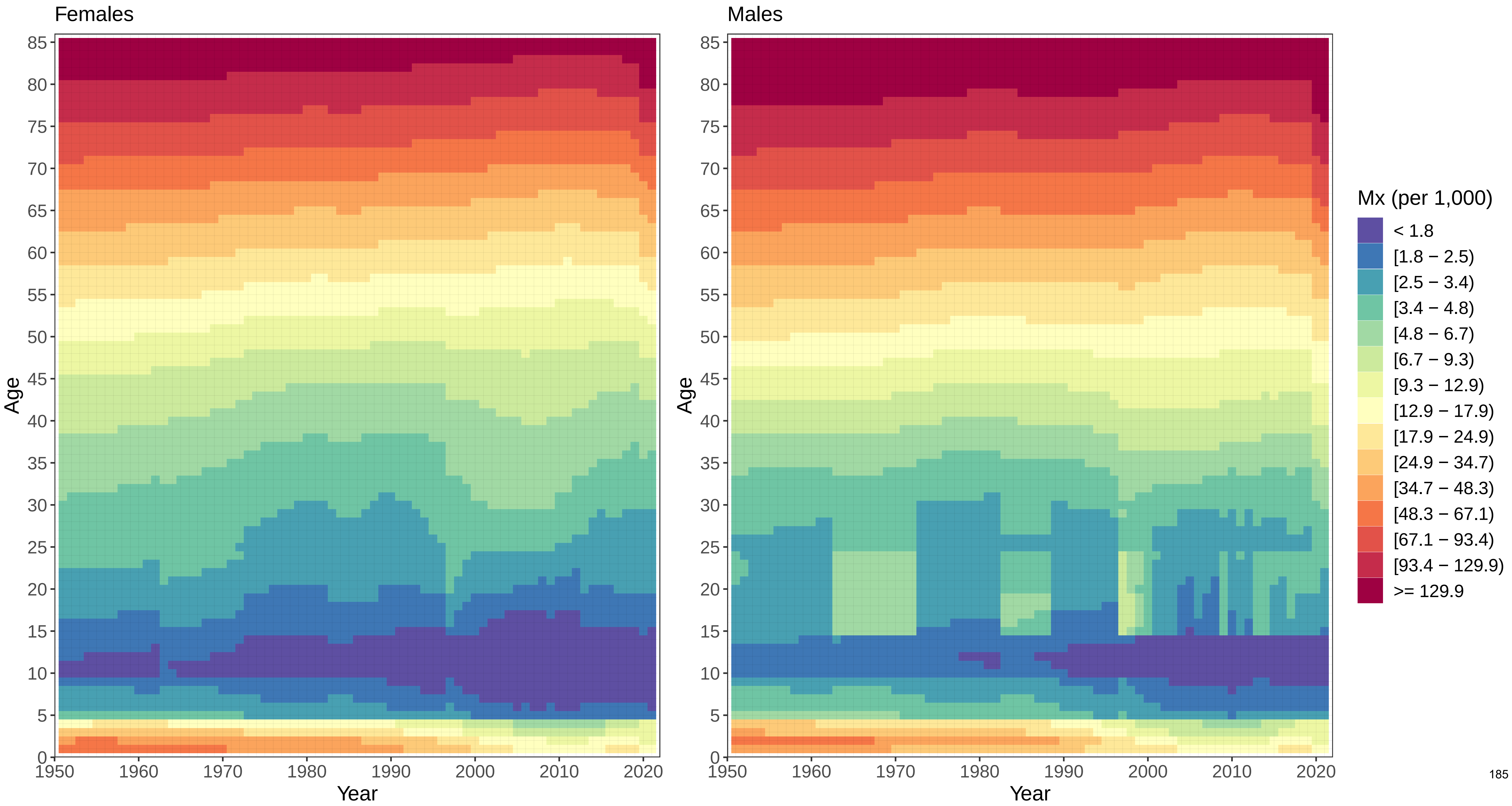

Appendix 2 Figure S5: Uganda mortality rates, 1950−2021

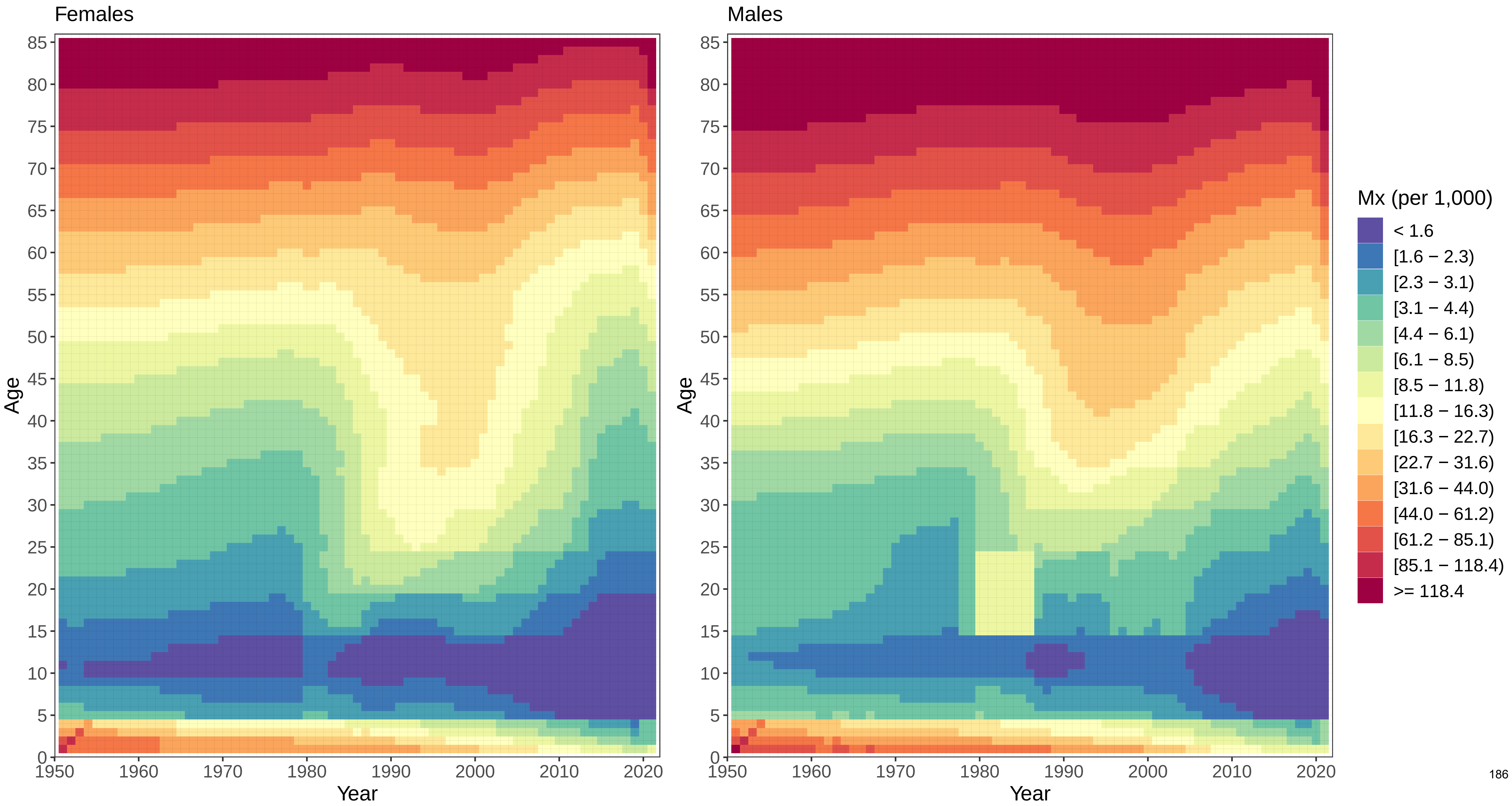

Appendix 2 Figure S5: Tanzania mortality rates, 1950−2021

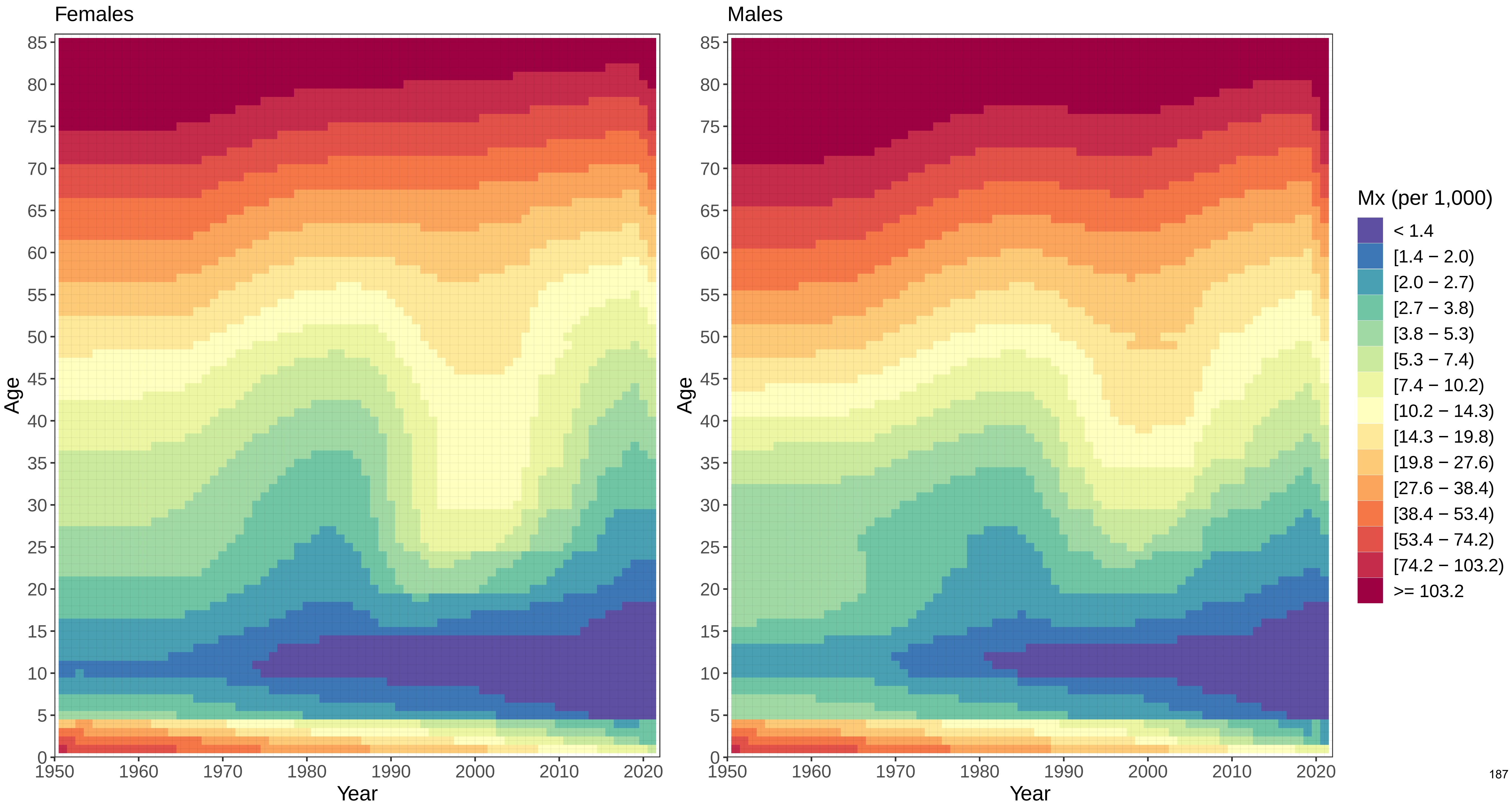

Appendix 2 Figure S5: Zambia mortality rates, 1950−2021

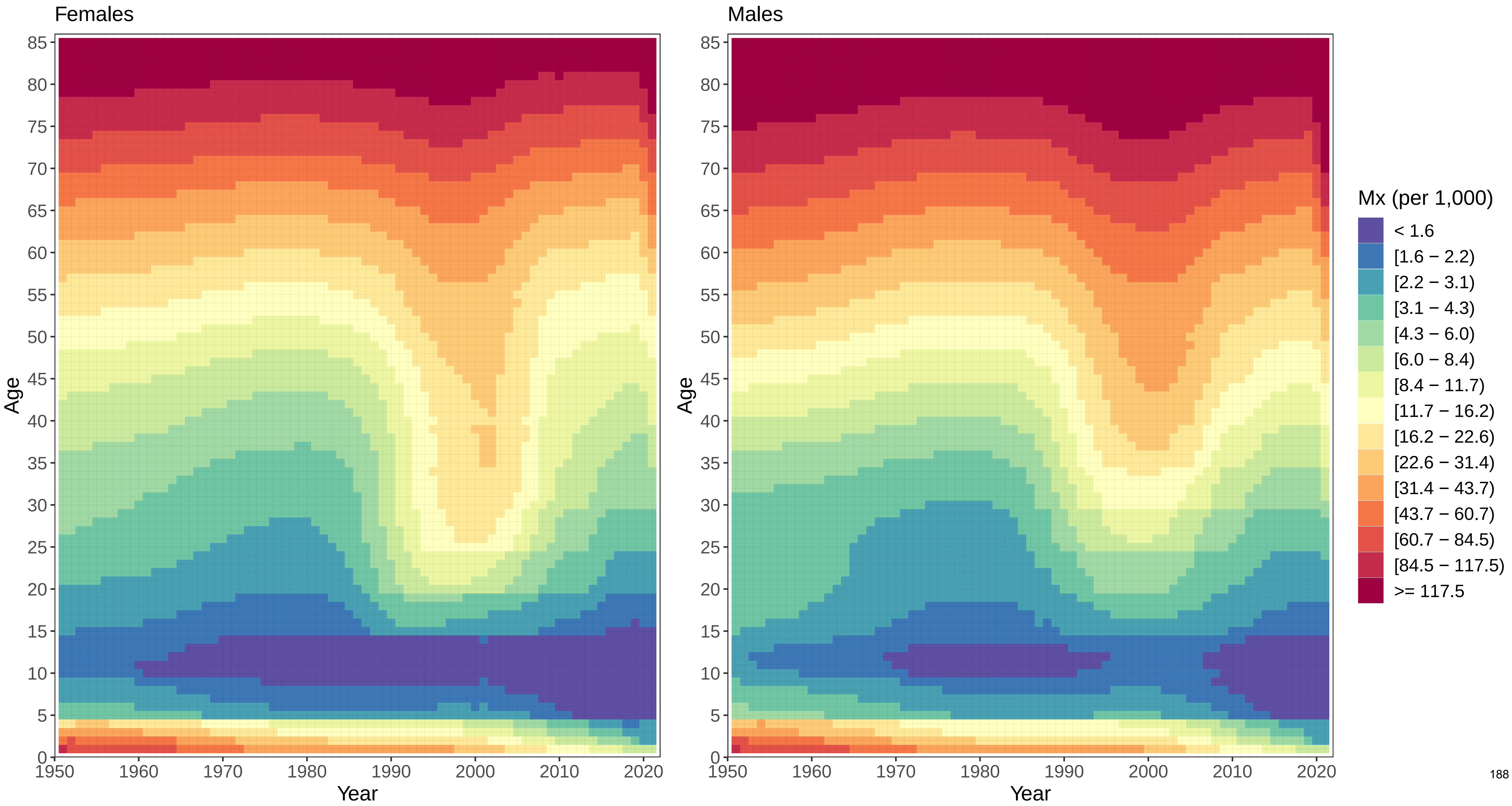

Appendix 2 Figure S5: Botswana mortality rates, 1950−2021

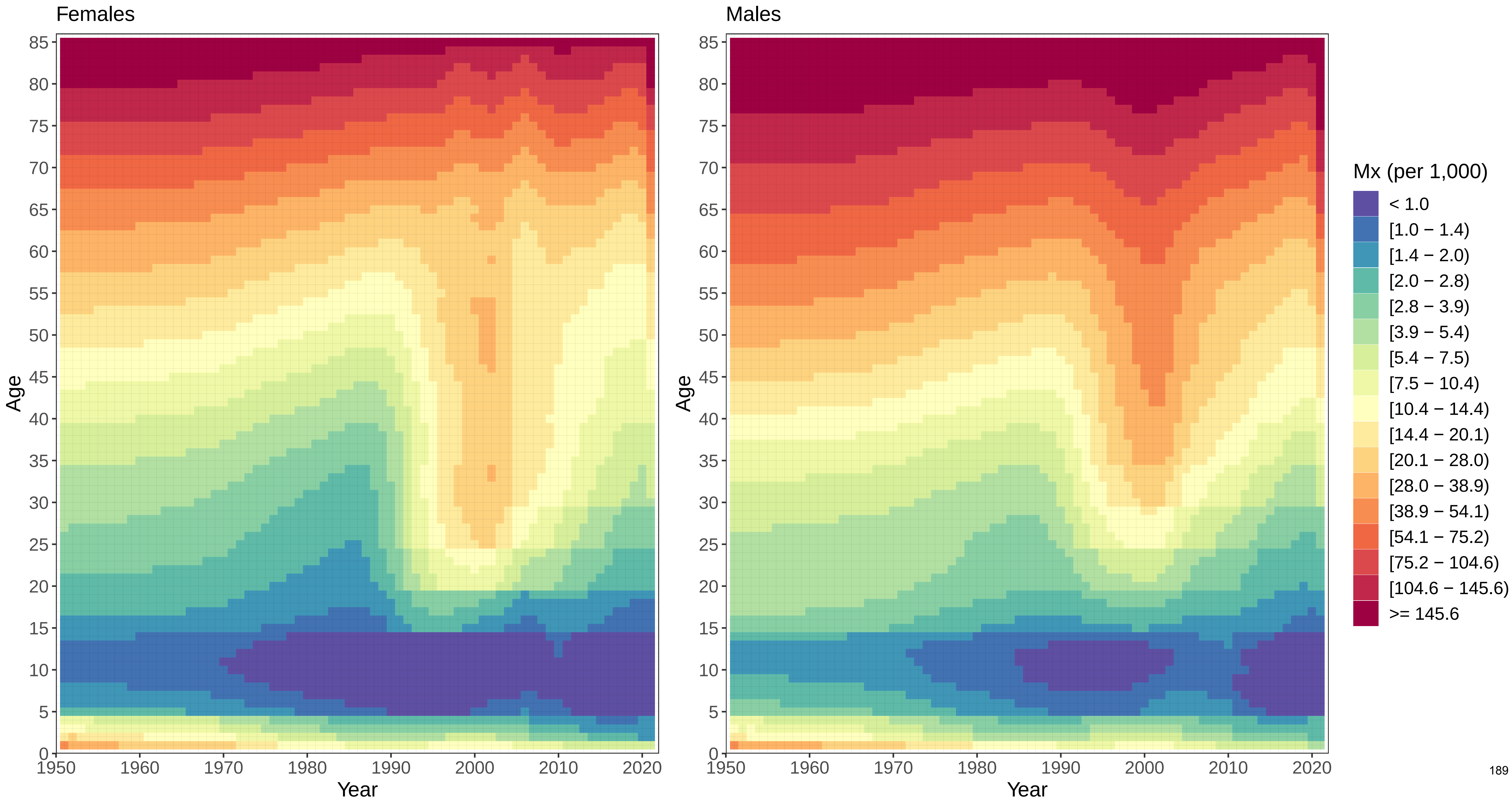

Appendix 2 Figure S5: Eswatini mortality rates, 1950−2021

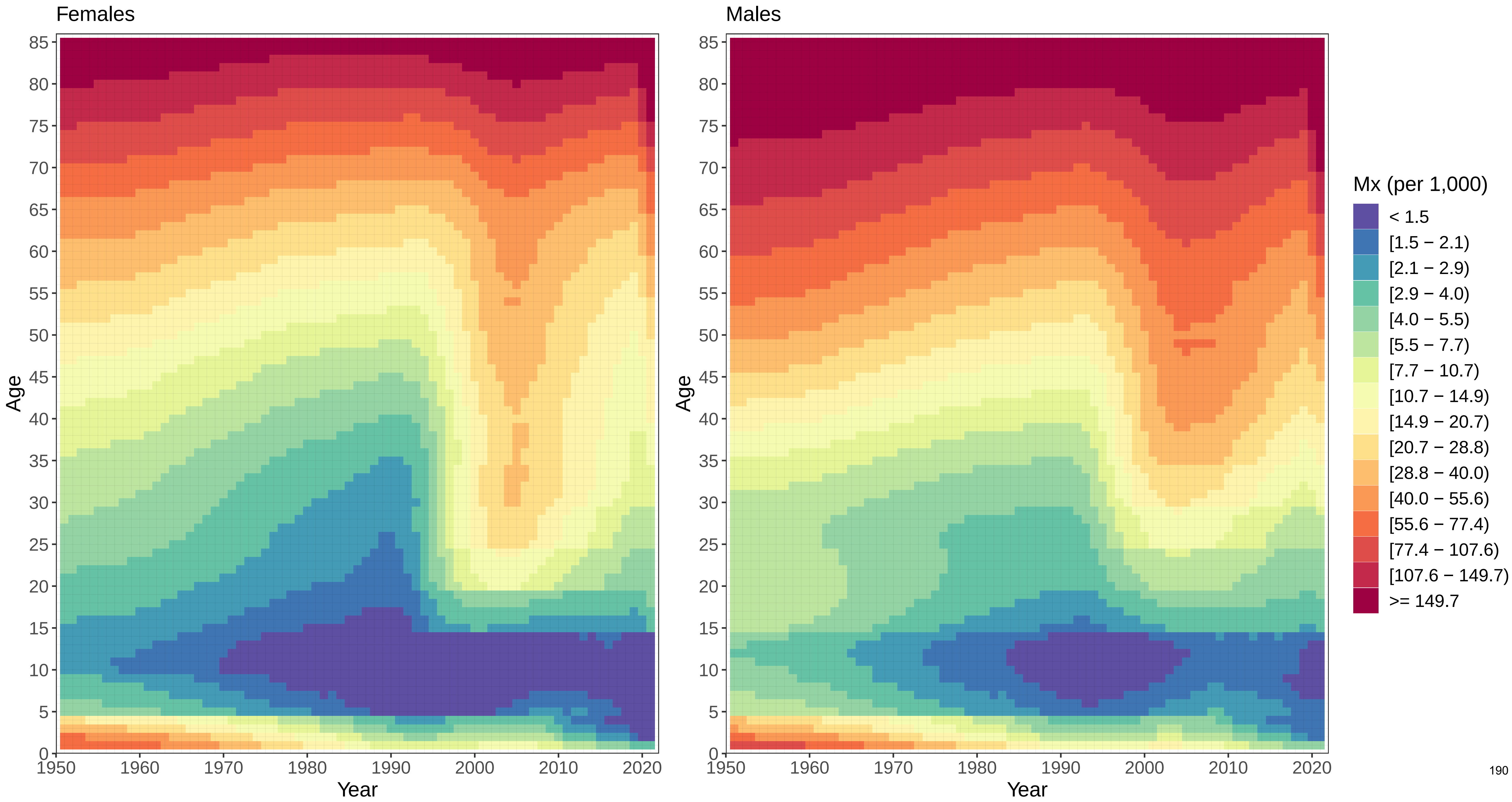

Appendix 2 Figure S5: Lesotho mortality rates, 1950−2021

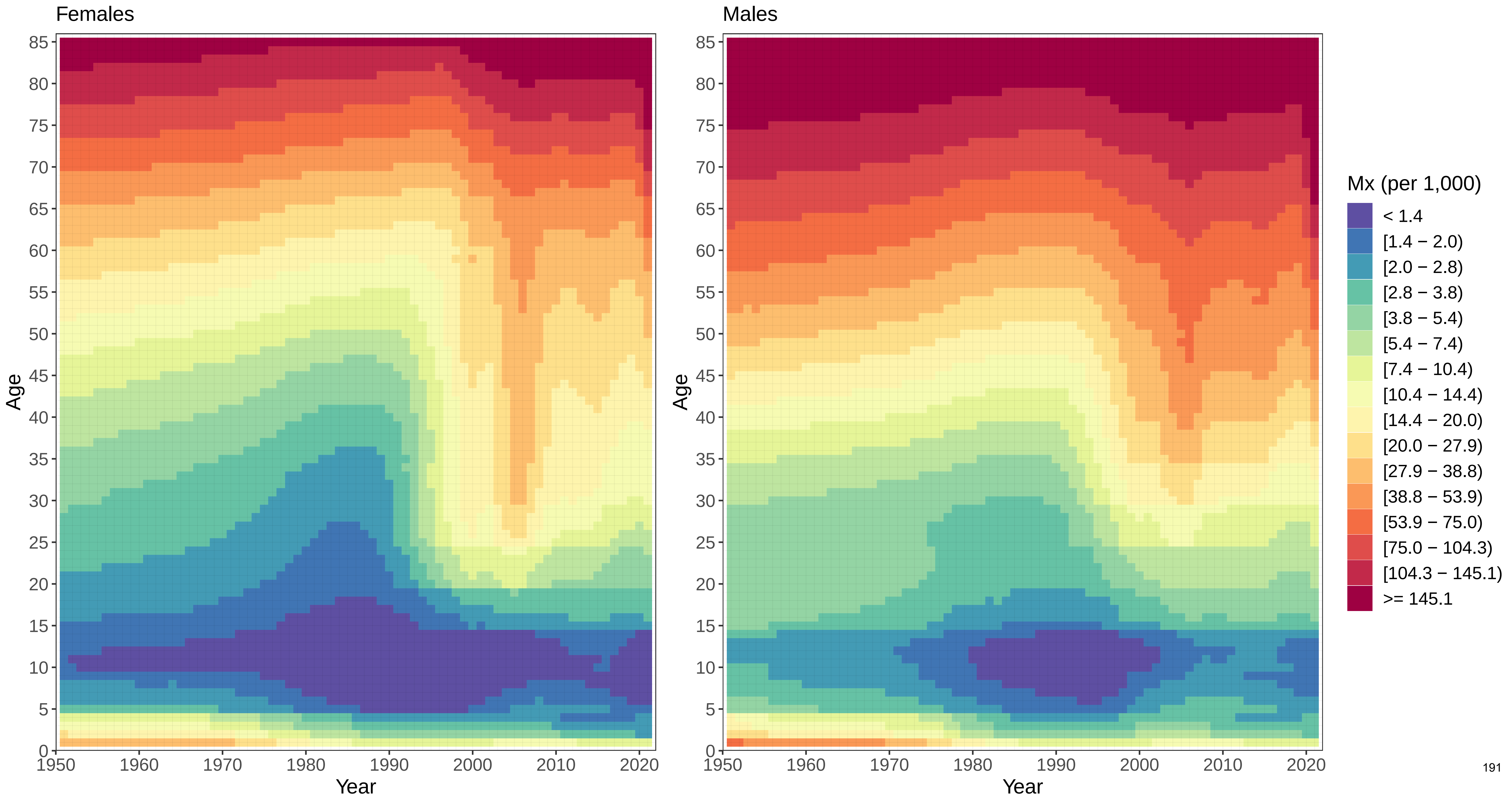

Appendix 2 Figure S5: Namibia mortality rates, 1950−2021

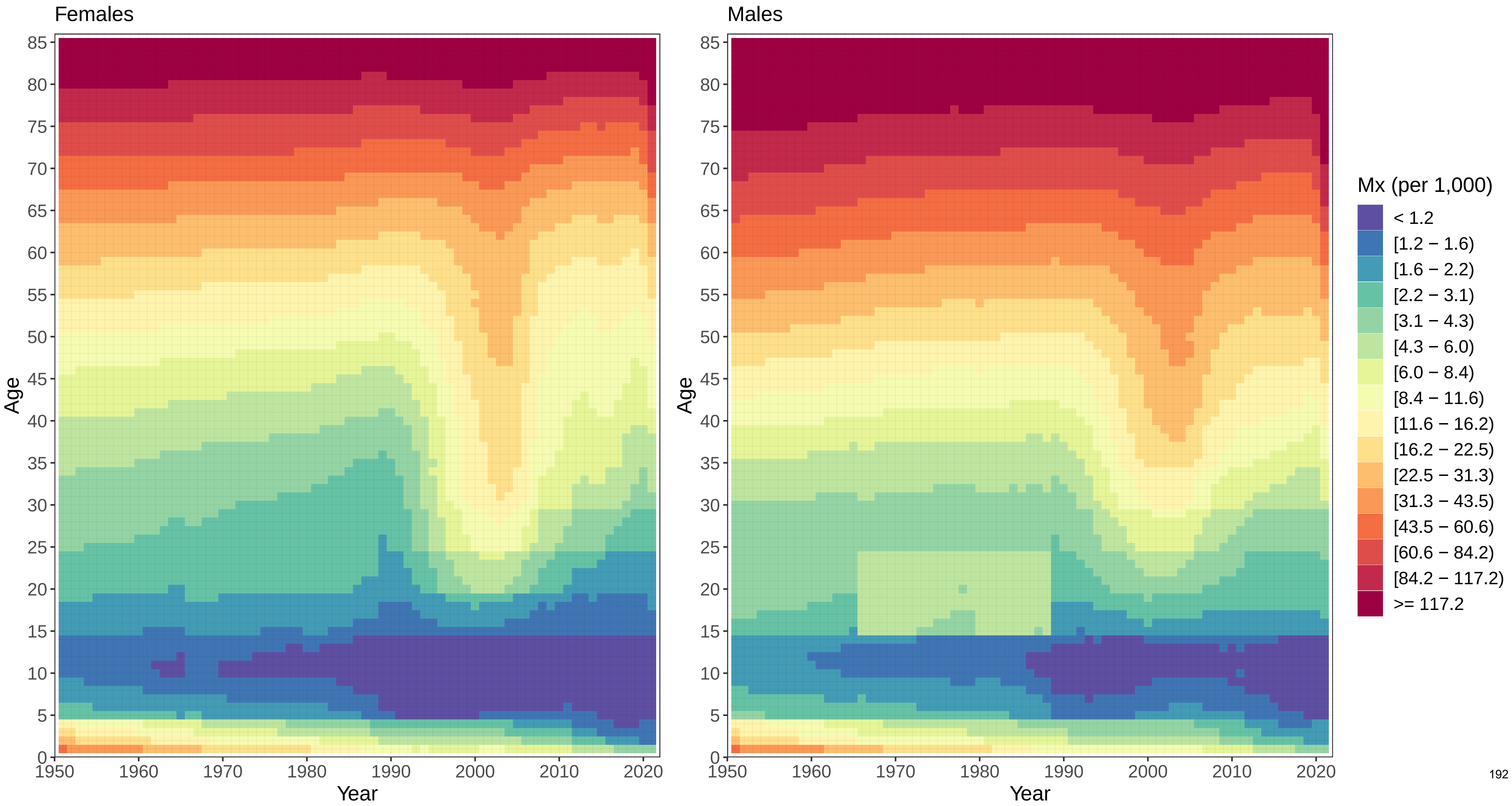

Appendix 2 Figure S5: South Africa mortality rates, 1950−2021

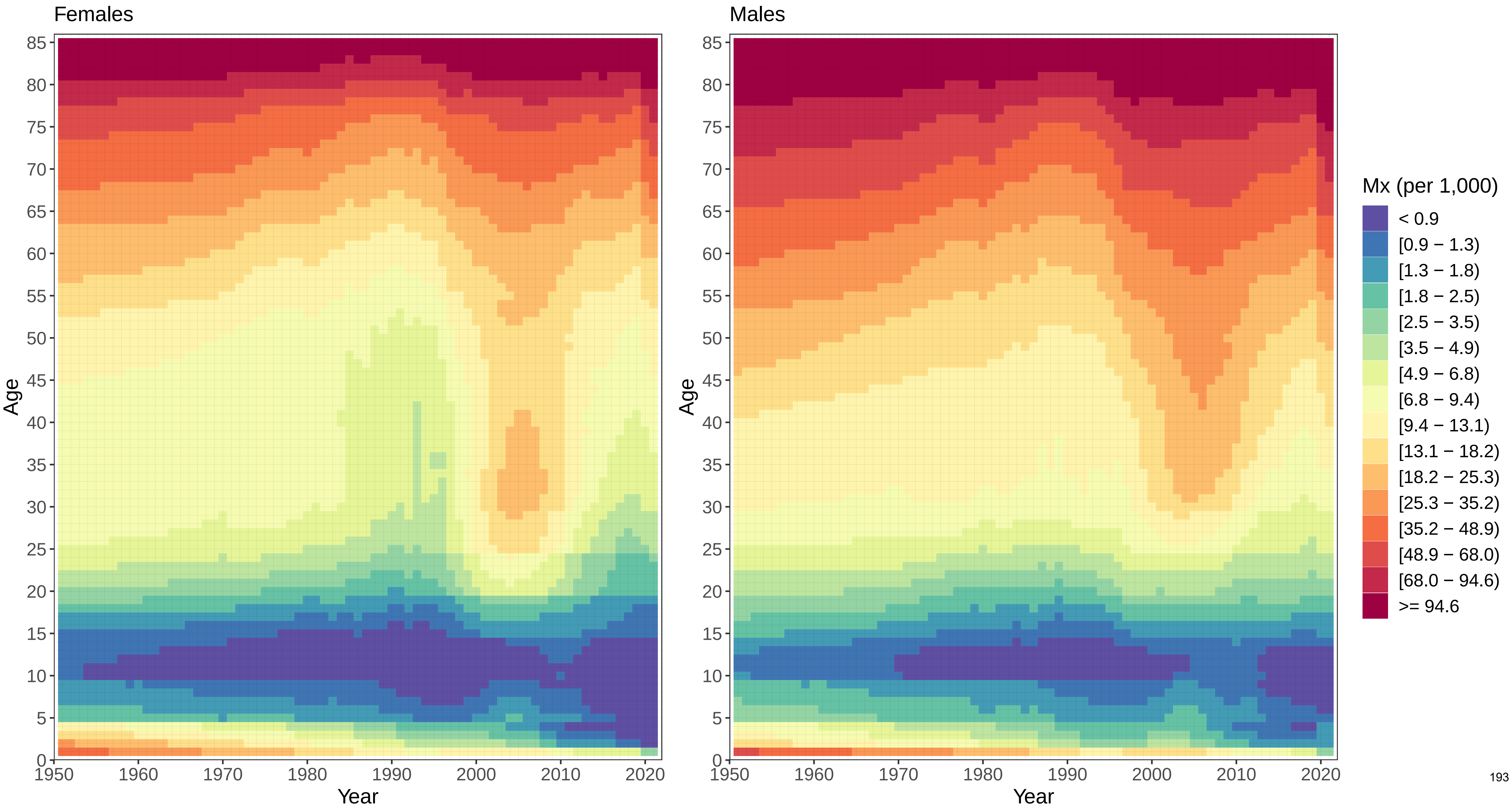

Appendix 2 Figure S5: Zimbabwe mortality rates, 1950−2021

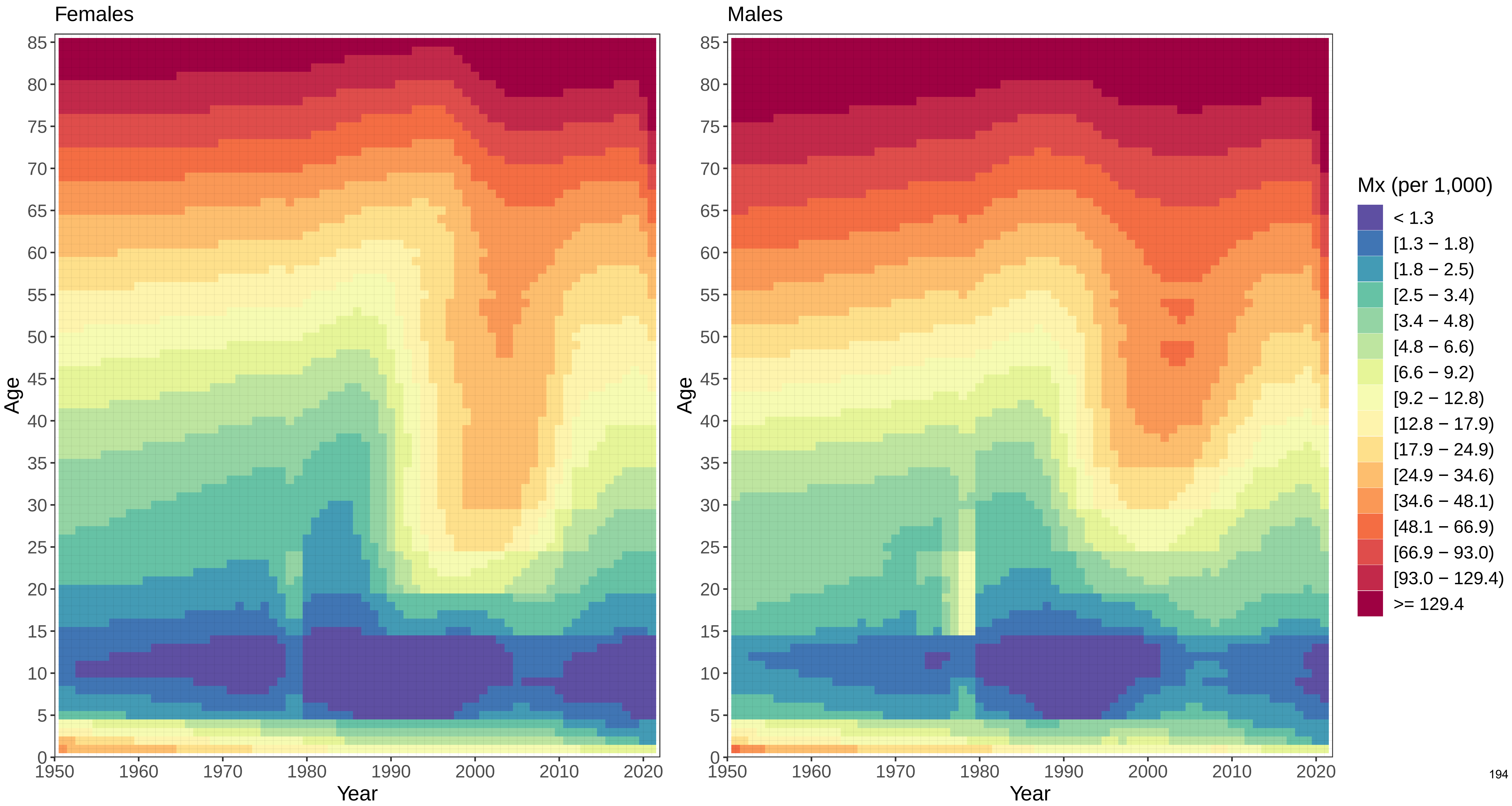

Appendix 2 Figure S5: Benin mortality rates, 1950−2021

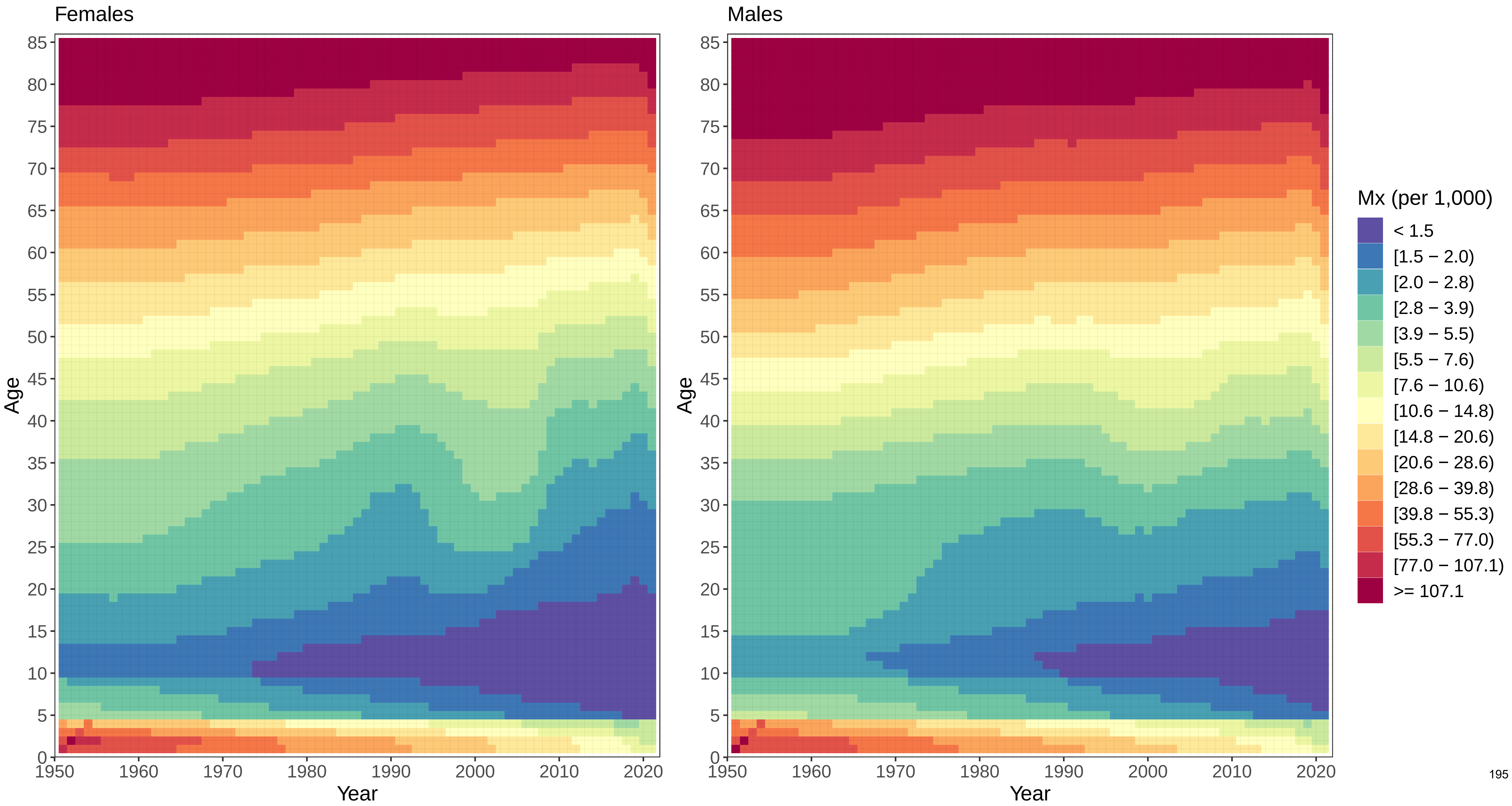

Appendix 2 Figure S5: Burkina Faso mortality rates, 1950−2021

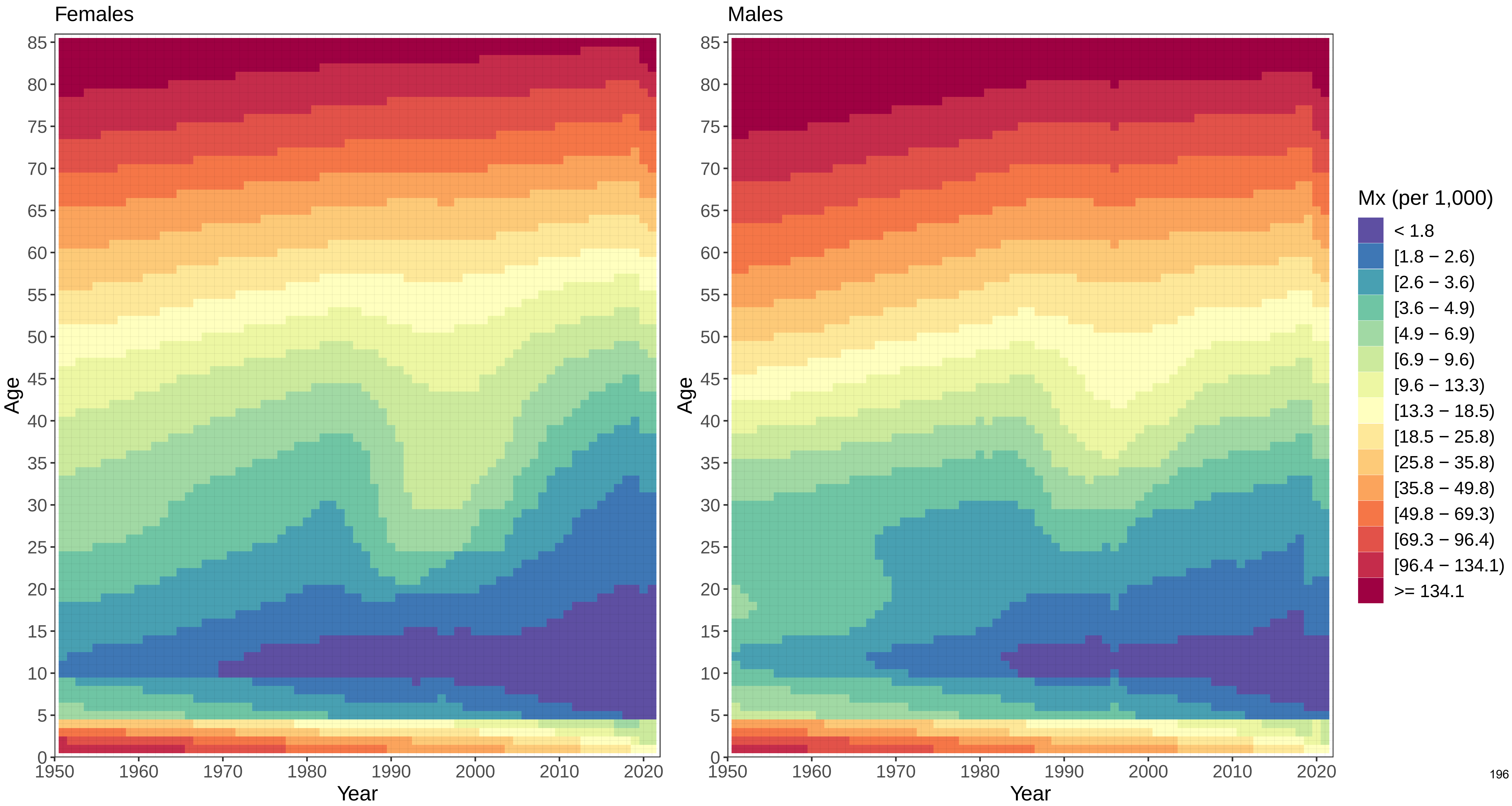

Appendix 2 Figure S5: Cabo Verde mortality rates, 1950−2021

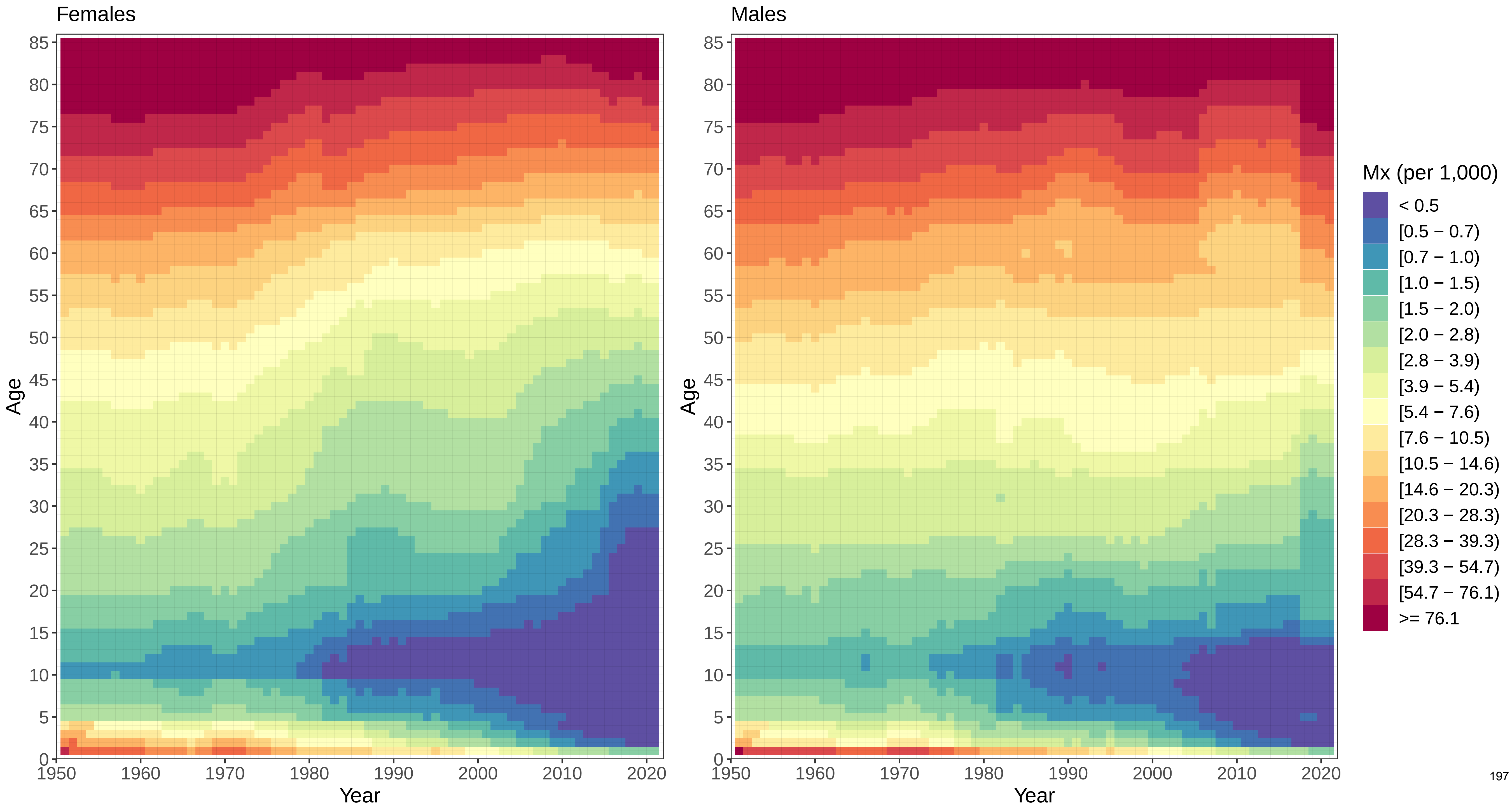

Appendix 2 Figure S5: Cameroon mortality rates, 1950−2021

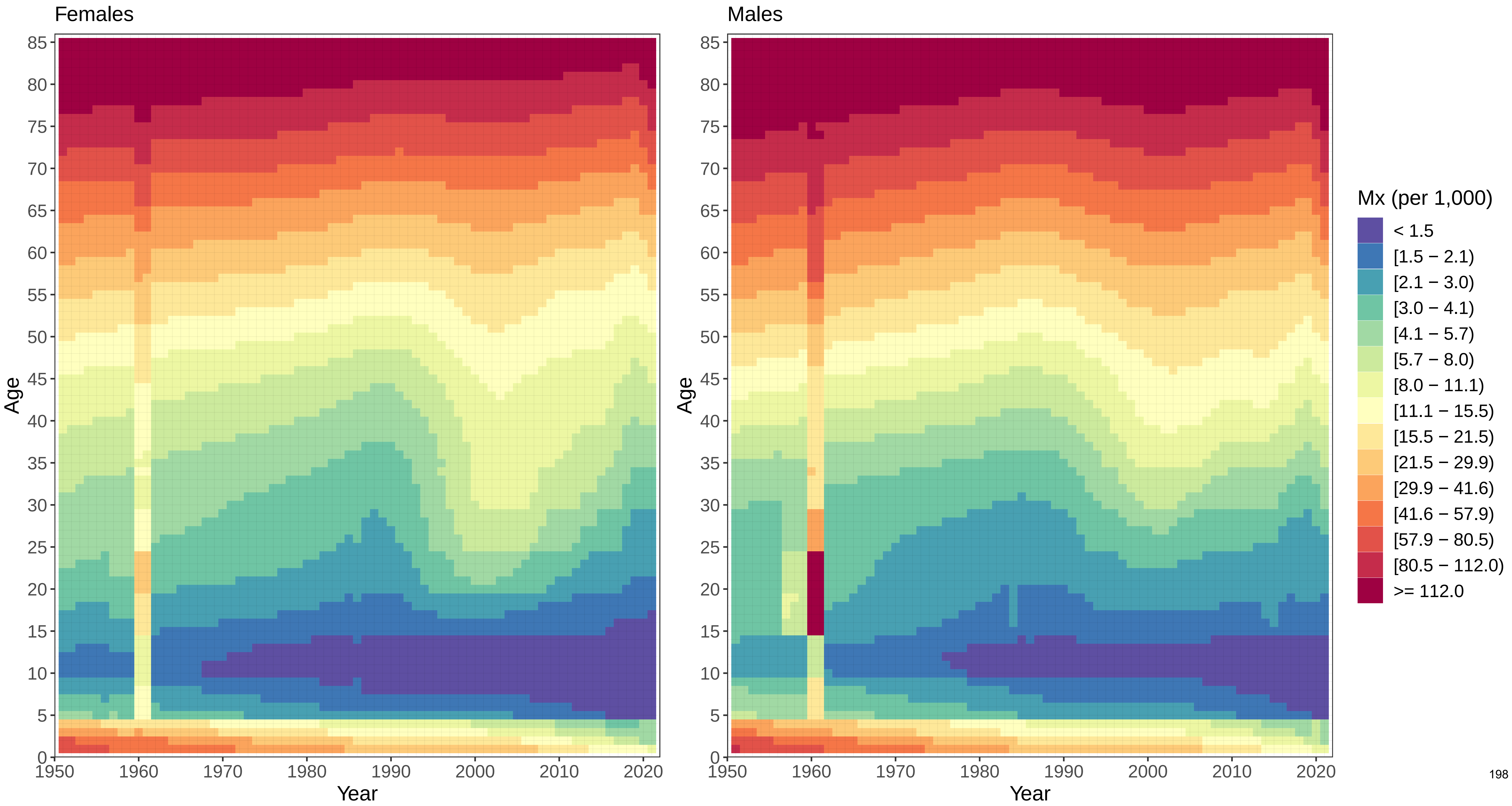

Appendix 2 Figure S5: Chad mortality rates, 1950−2021

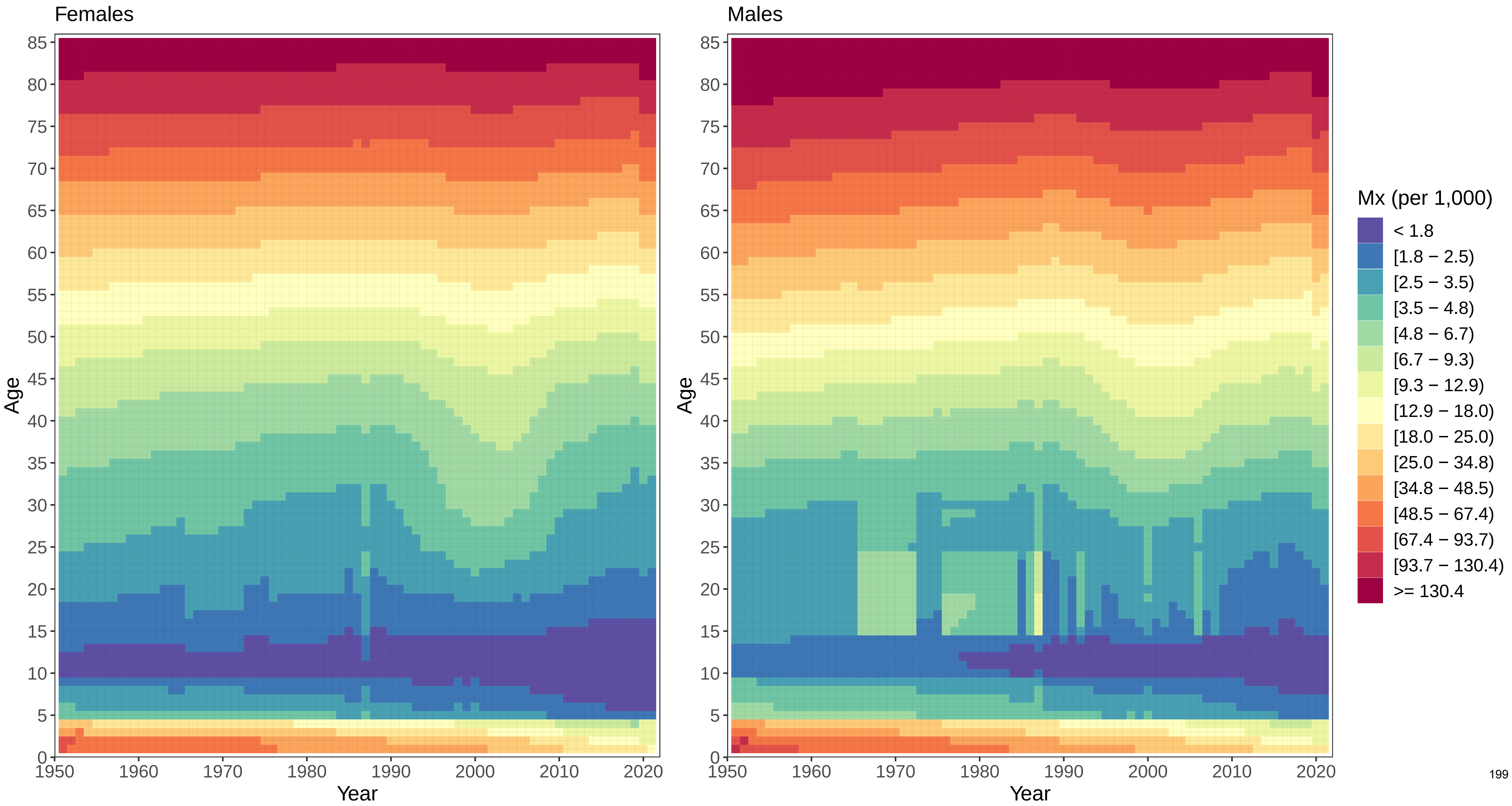

Appendix 2 Figure S5: Côte d'Ivoire mortality rates, 1950−2021

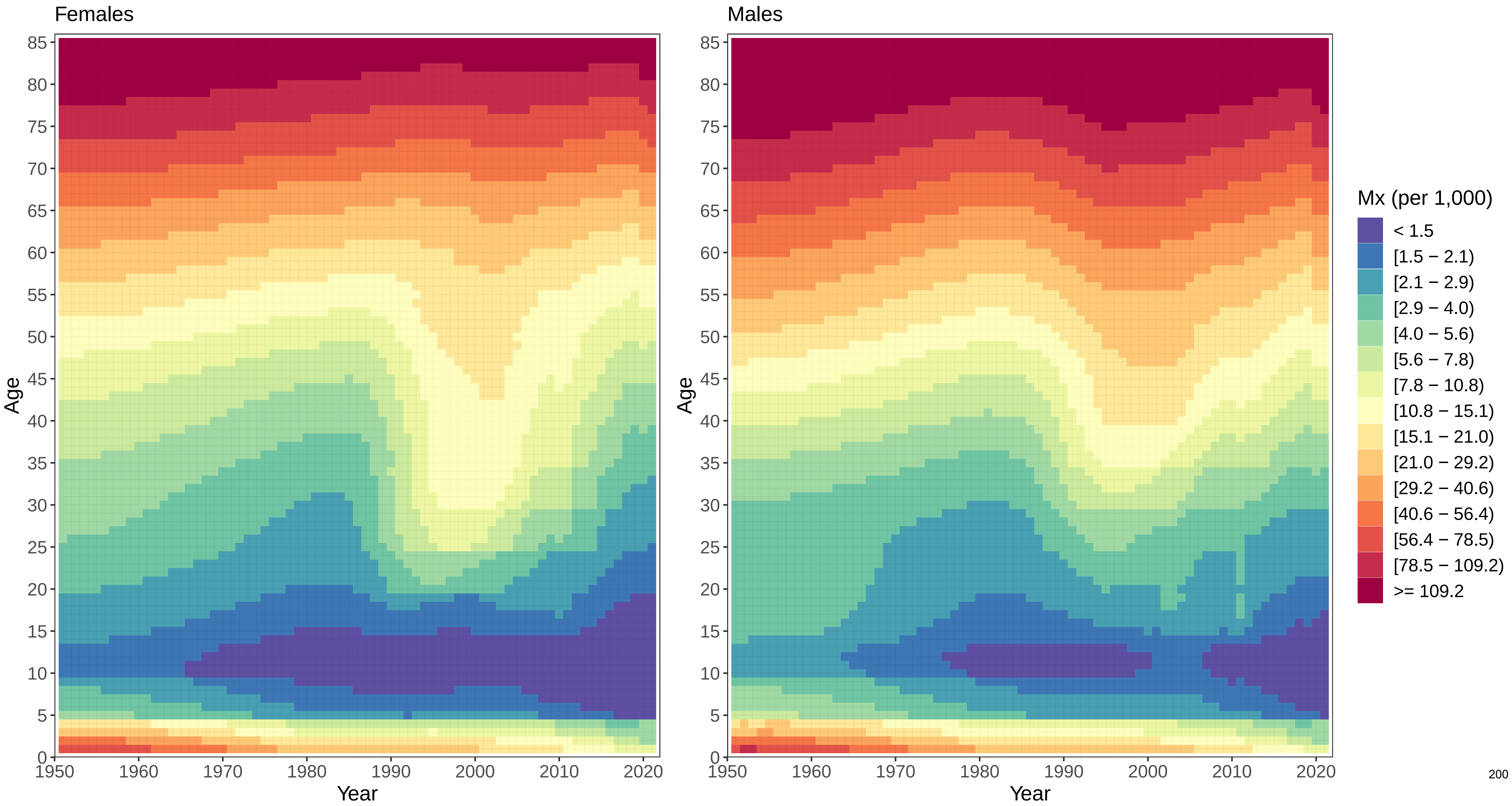

Appendix 2 Figure S5: The Gambia mortality rates, 1950−2021

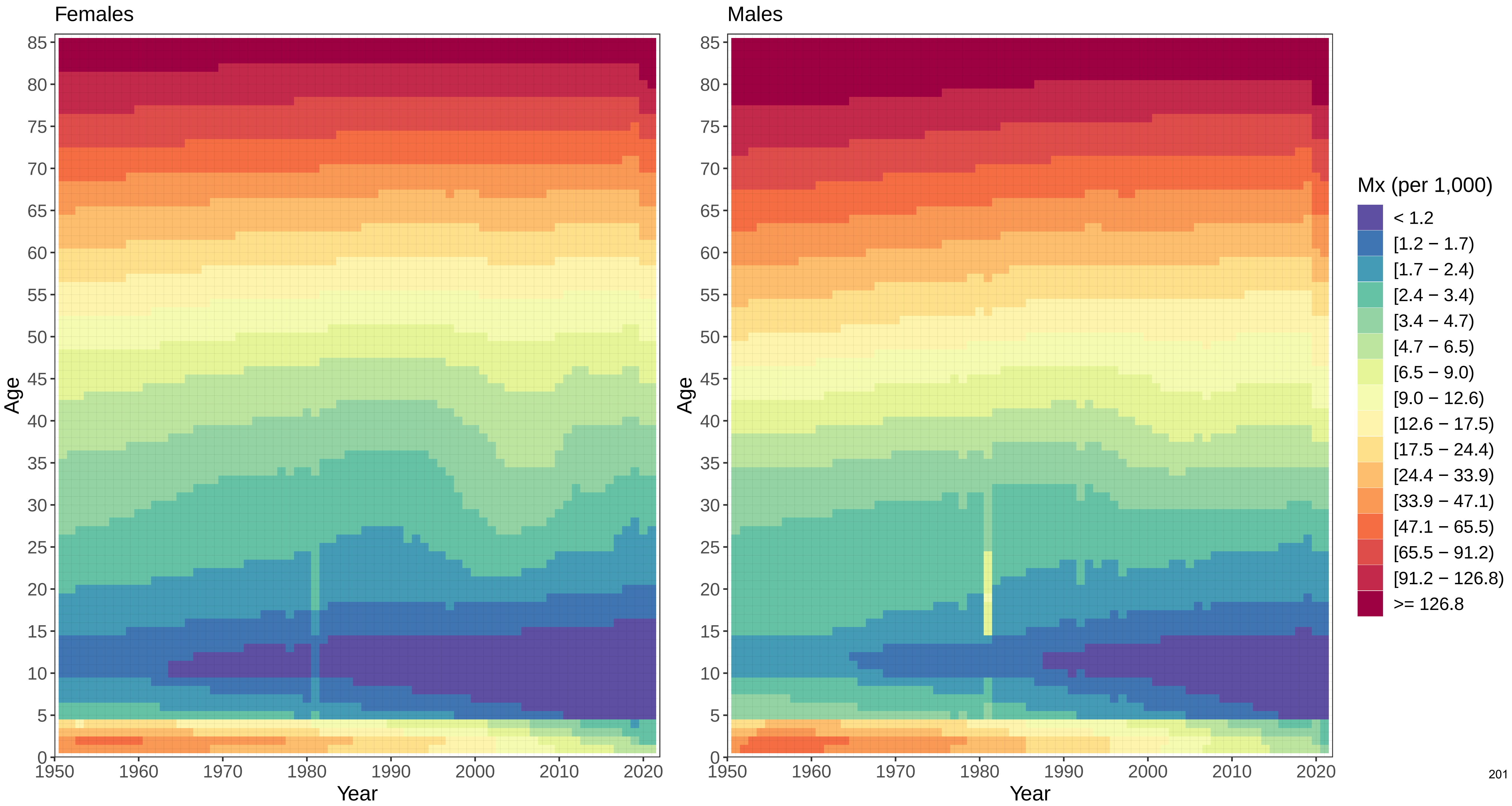

Appendix 2 Figure S5: Ghana mortality rates, 1950−2021

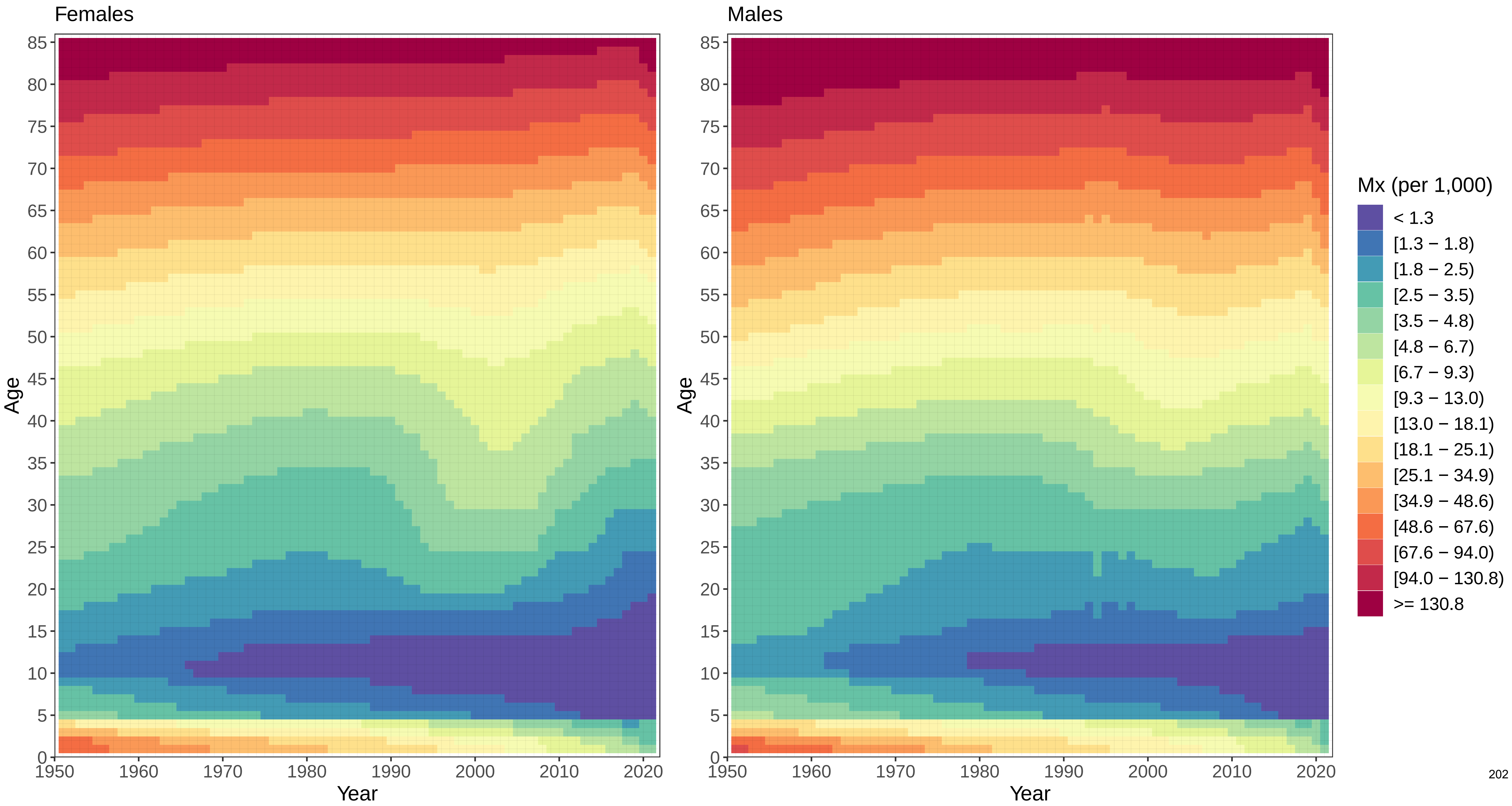

Appendix 2 Figure S5: Guinea mortality rates, 1950−2021

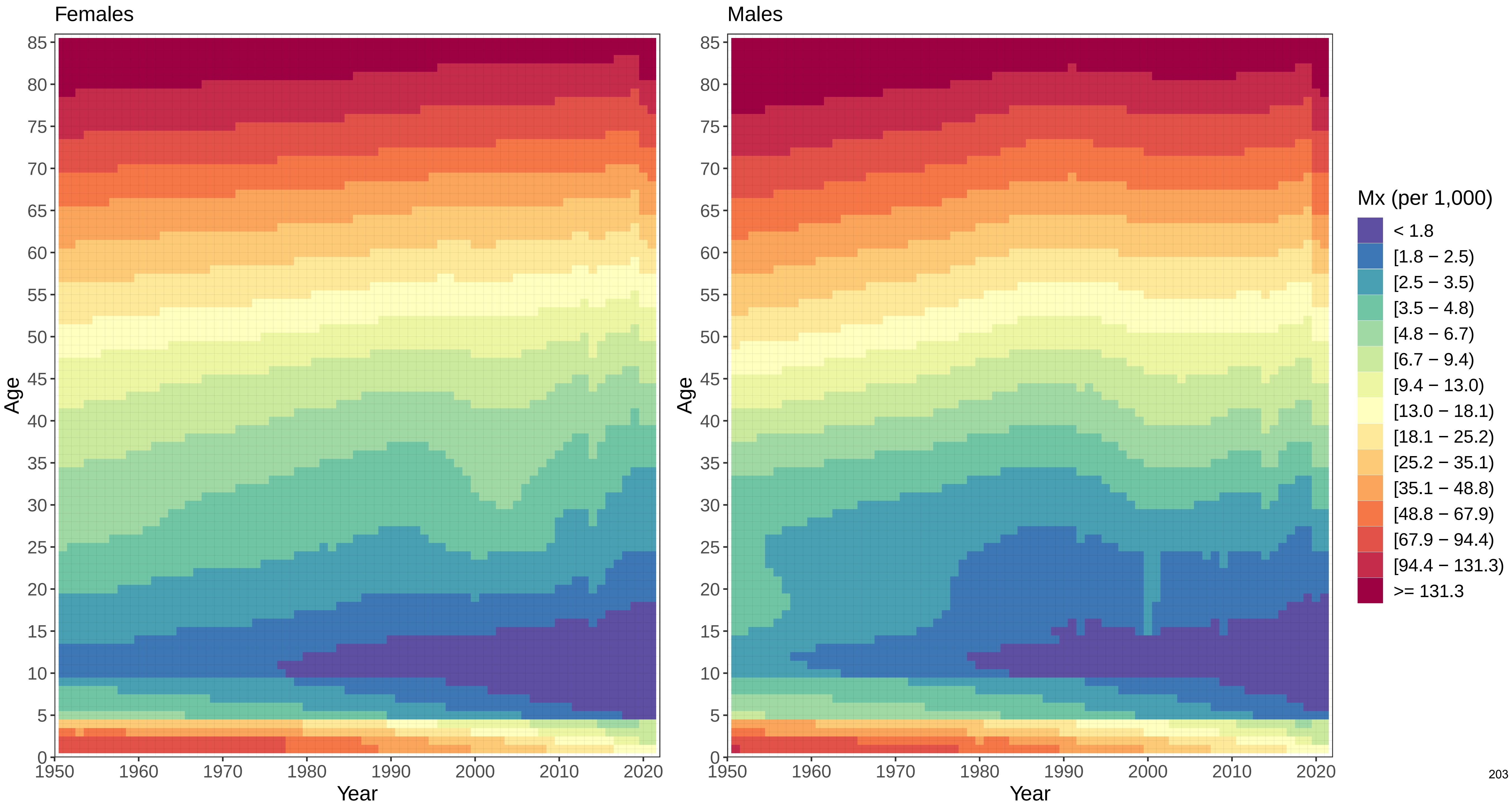

Appendix 2 Figure S5: Guinea−Bissau mortality rates, 1950−2021

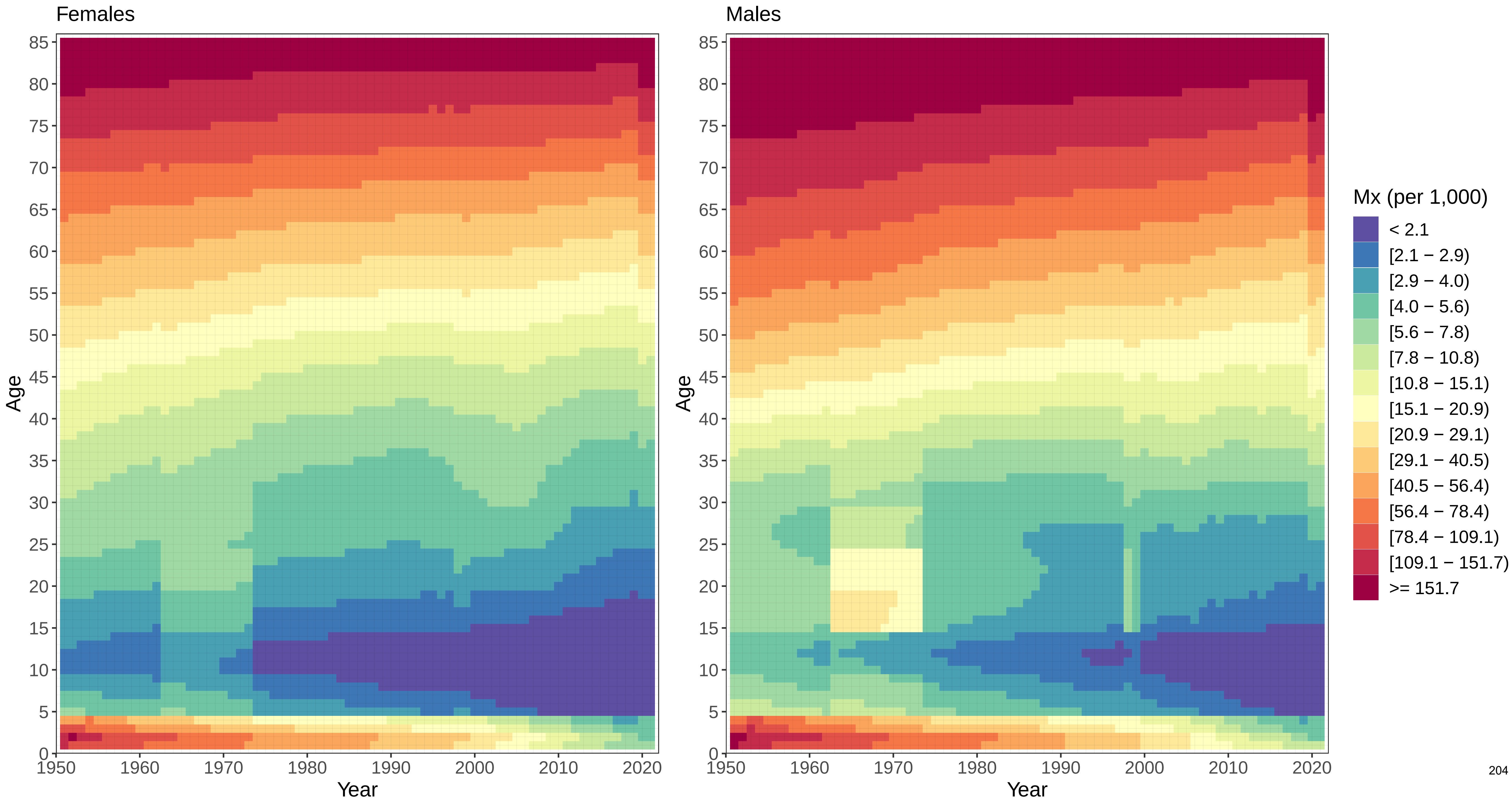

Appendix 2 Figure S5: Liberia mortality rates, 1950−2021

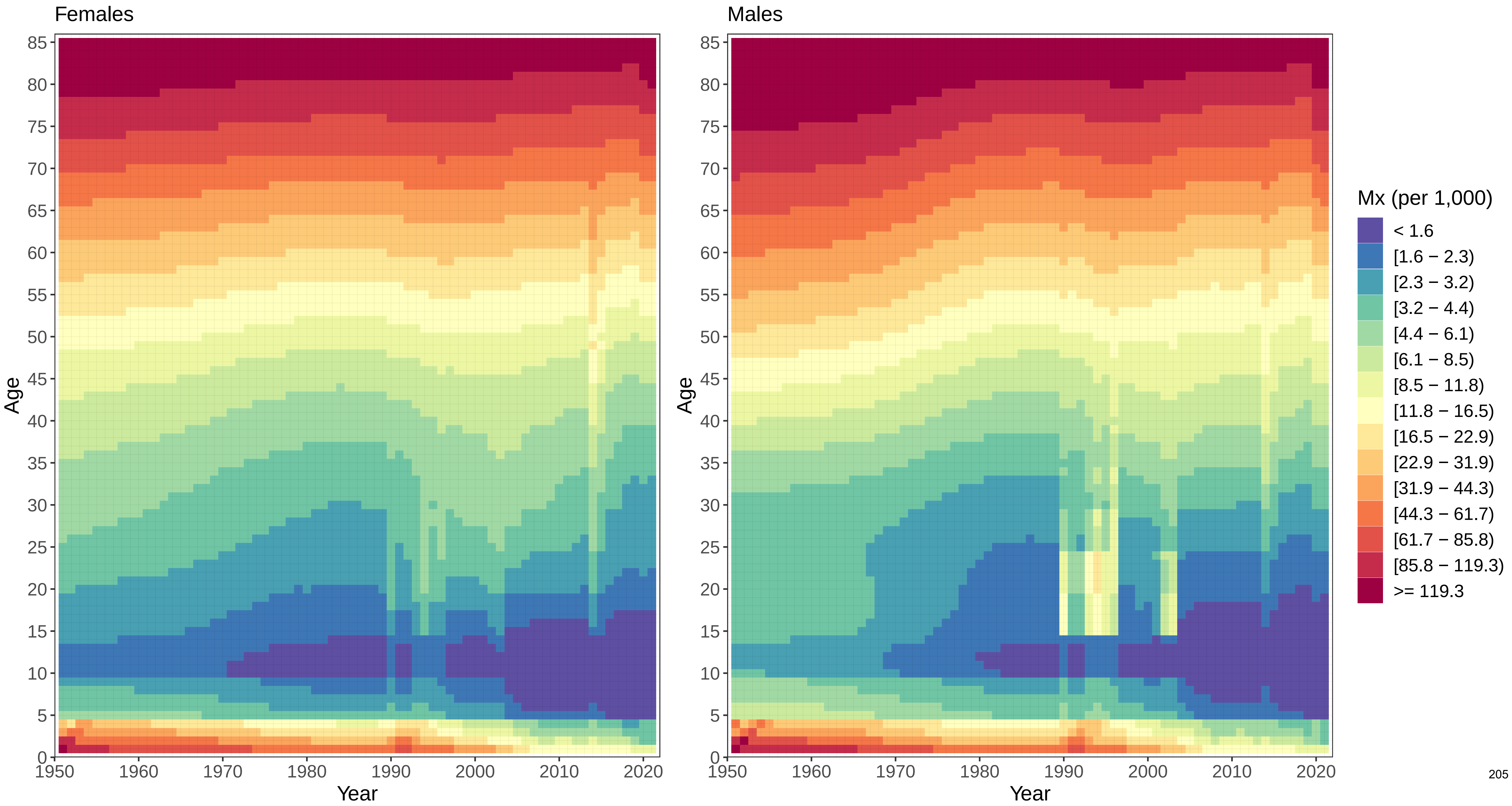

Appendix 2 Figure S5: Mali mortality rates, 1950−2021

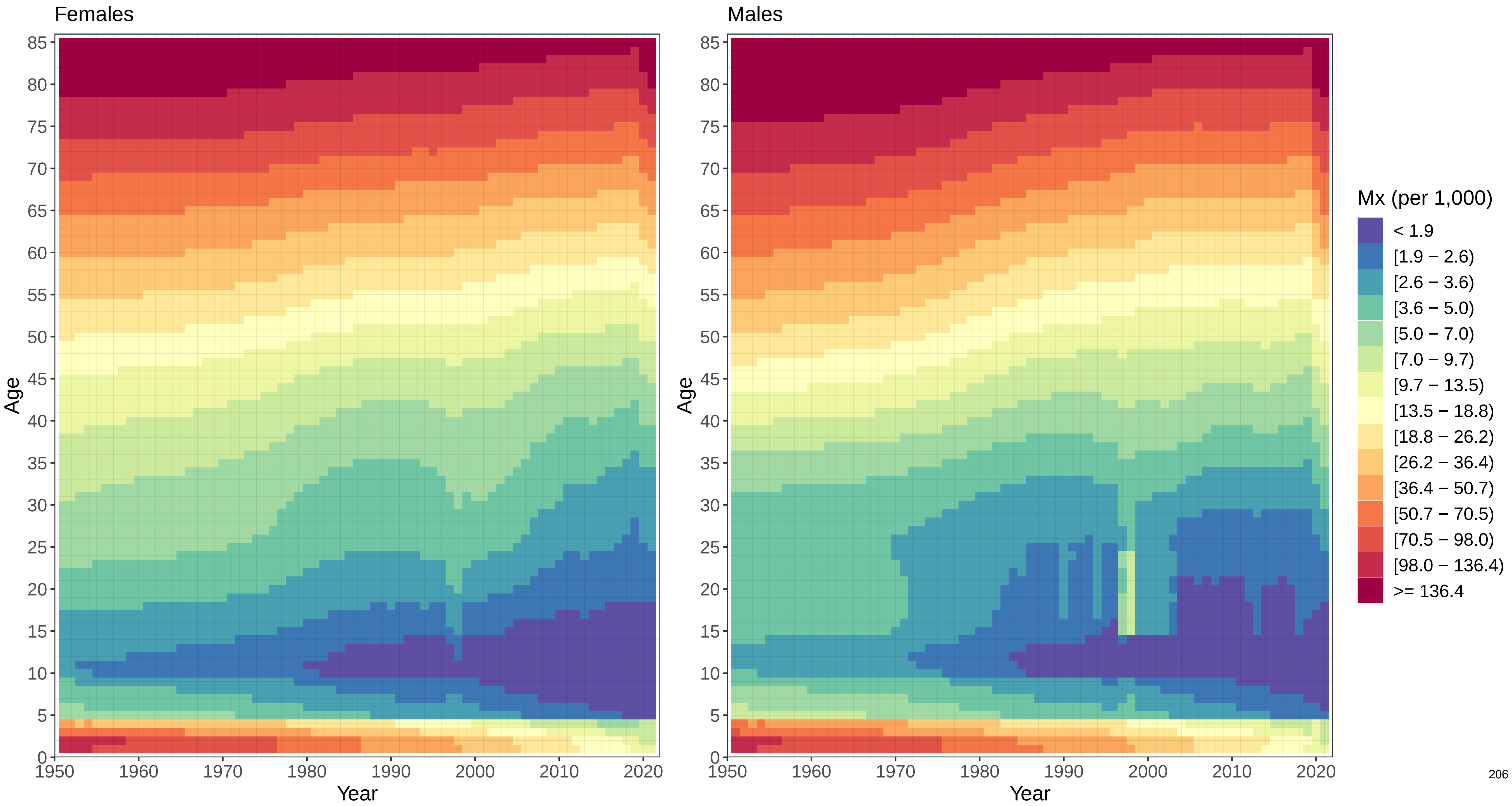

Appendix 2 Figure S5: Mauritania mortality rates, 1950−2021

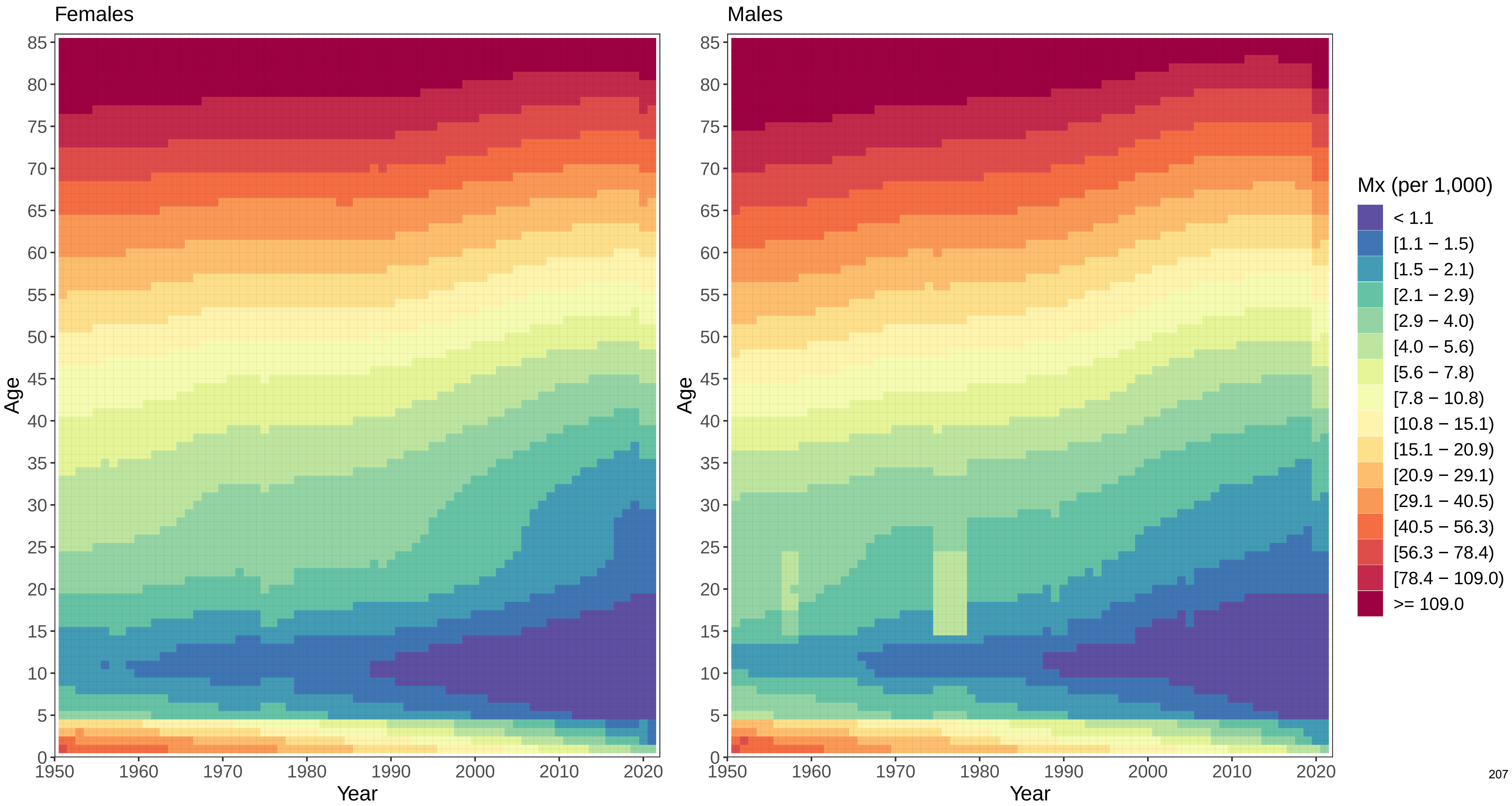

Appendix 2 Figure S5: Niger mortality rates, 1950−2021

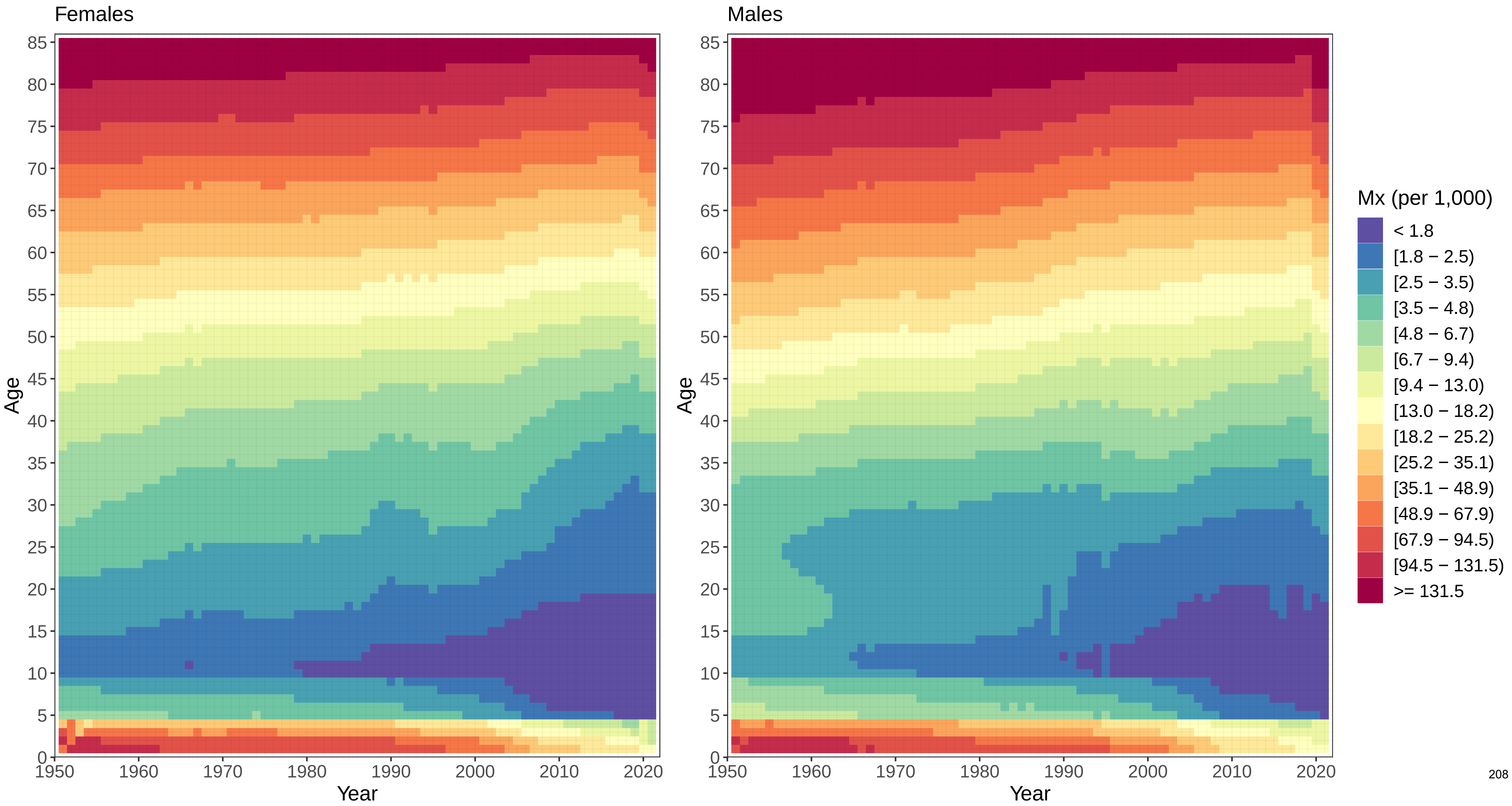

Appendix 2 Figure S5: Nigeria mortality rates, 1950−2021

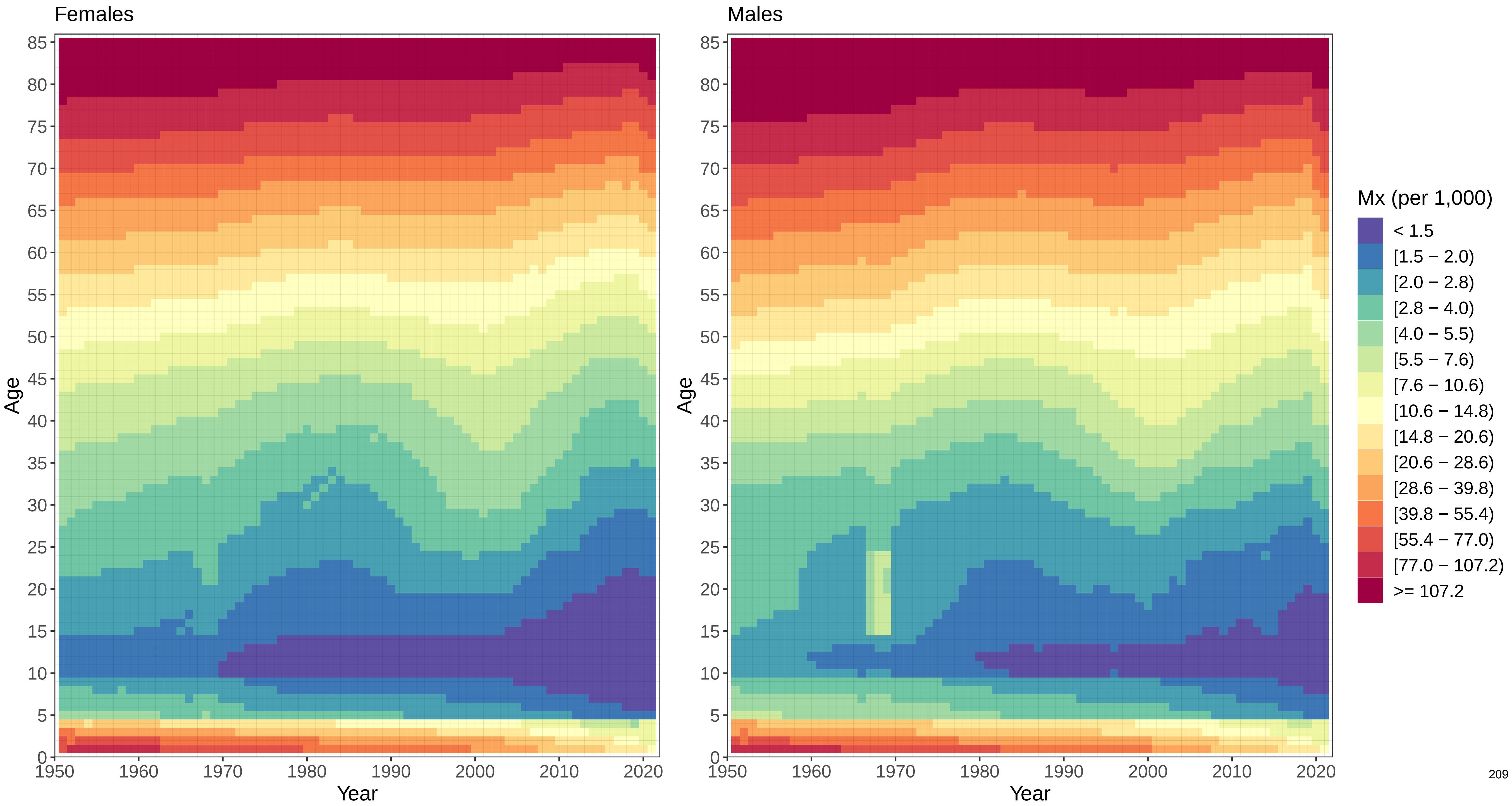

Appendix 2 Figure S5: São Tomé and Príncipe mortality rates, 1950−2021

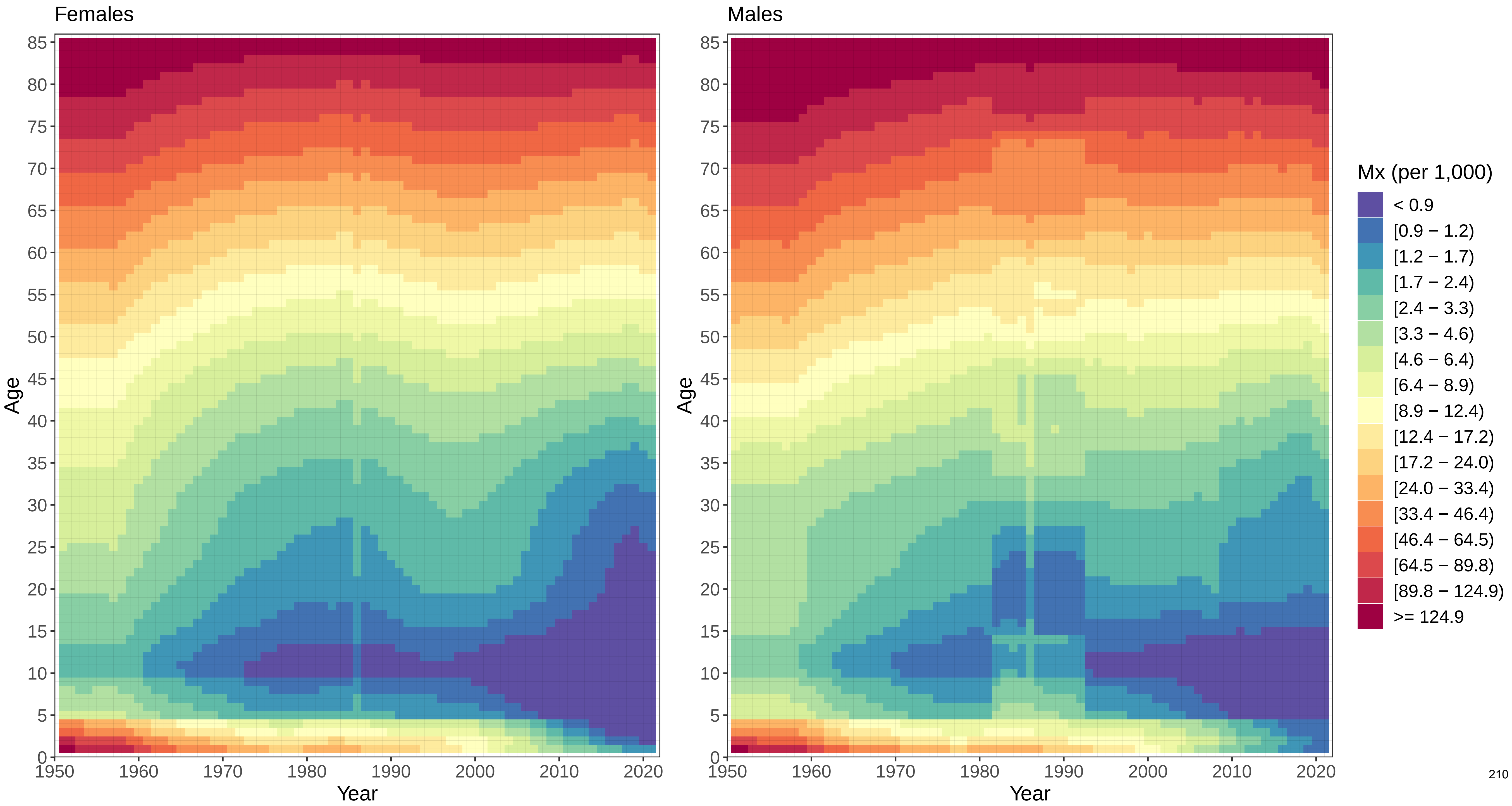

Appendix 2 Figure S5: Senegal mortality rates, 1950−2021

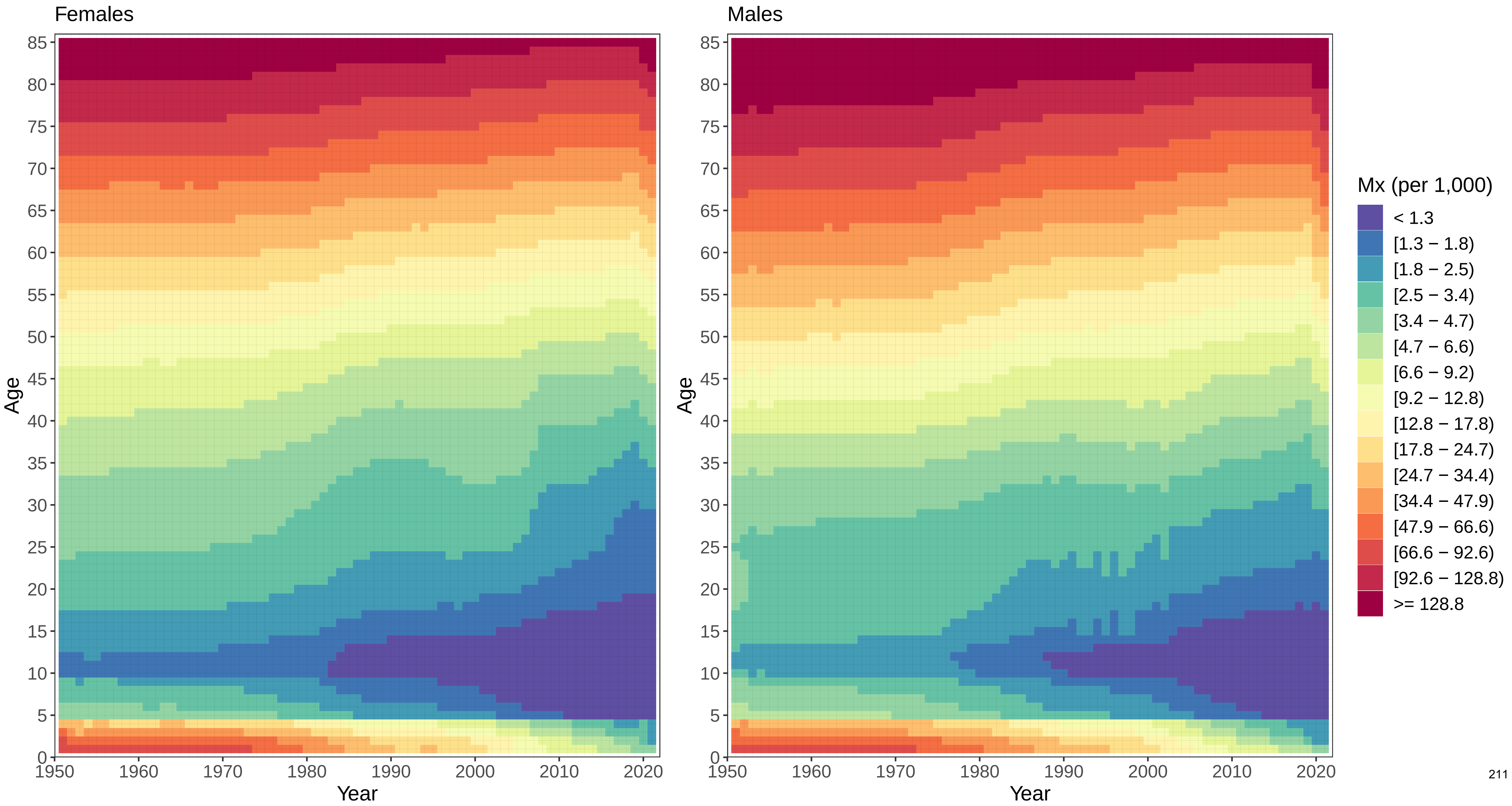

Appendix 2 Figure S5: Sierra Leone mortality rates, 1950−2021

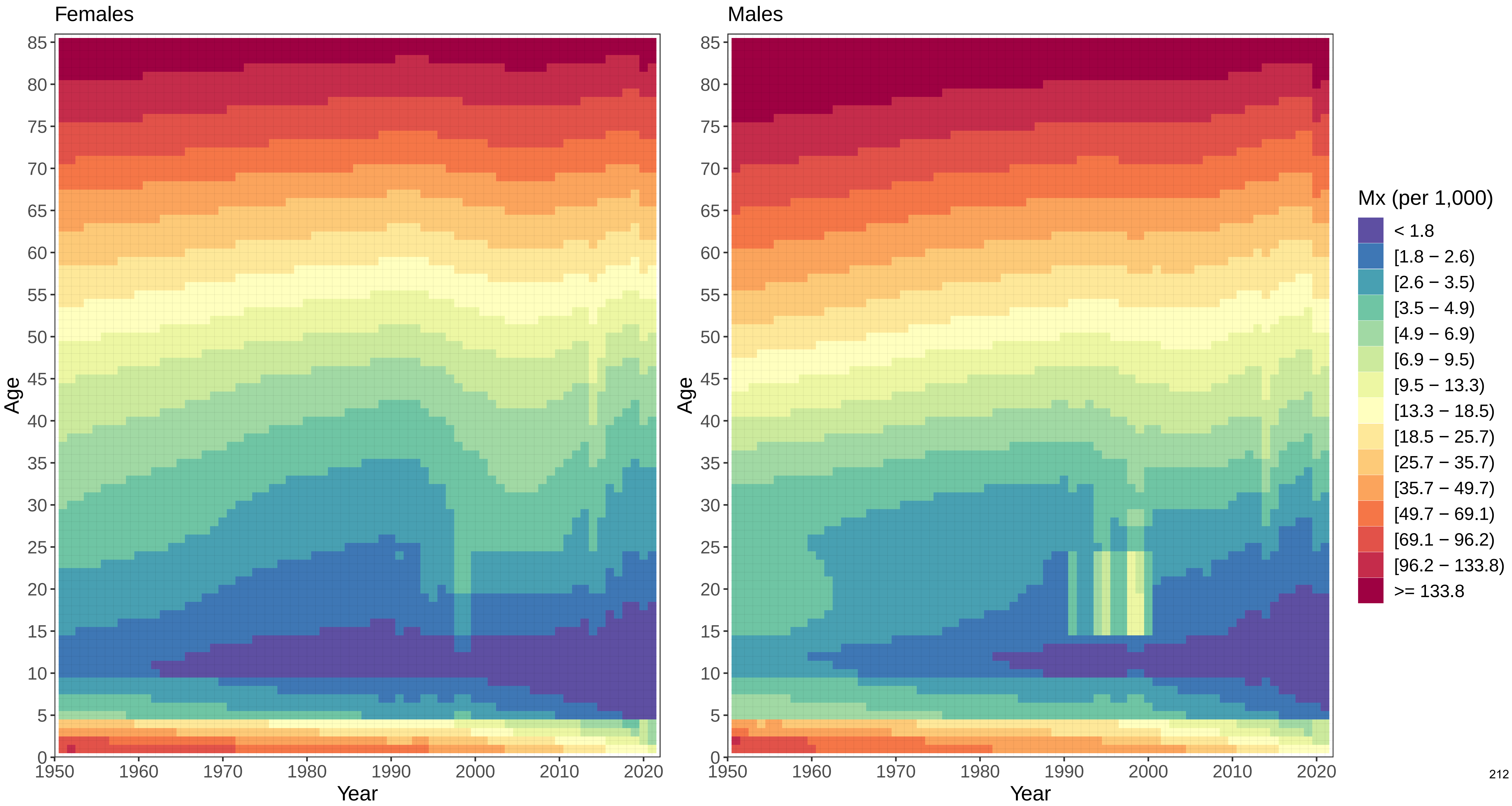

Appendix 2 Figure S5: Togo mortality rates, 1950−2021

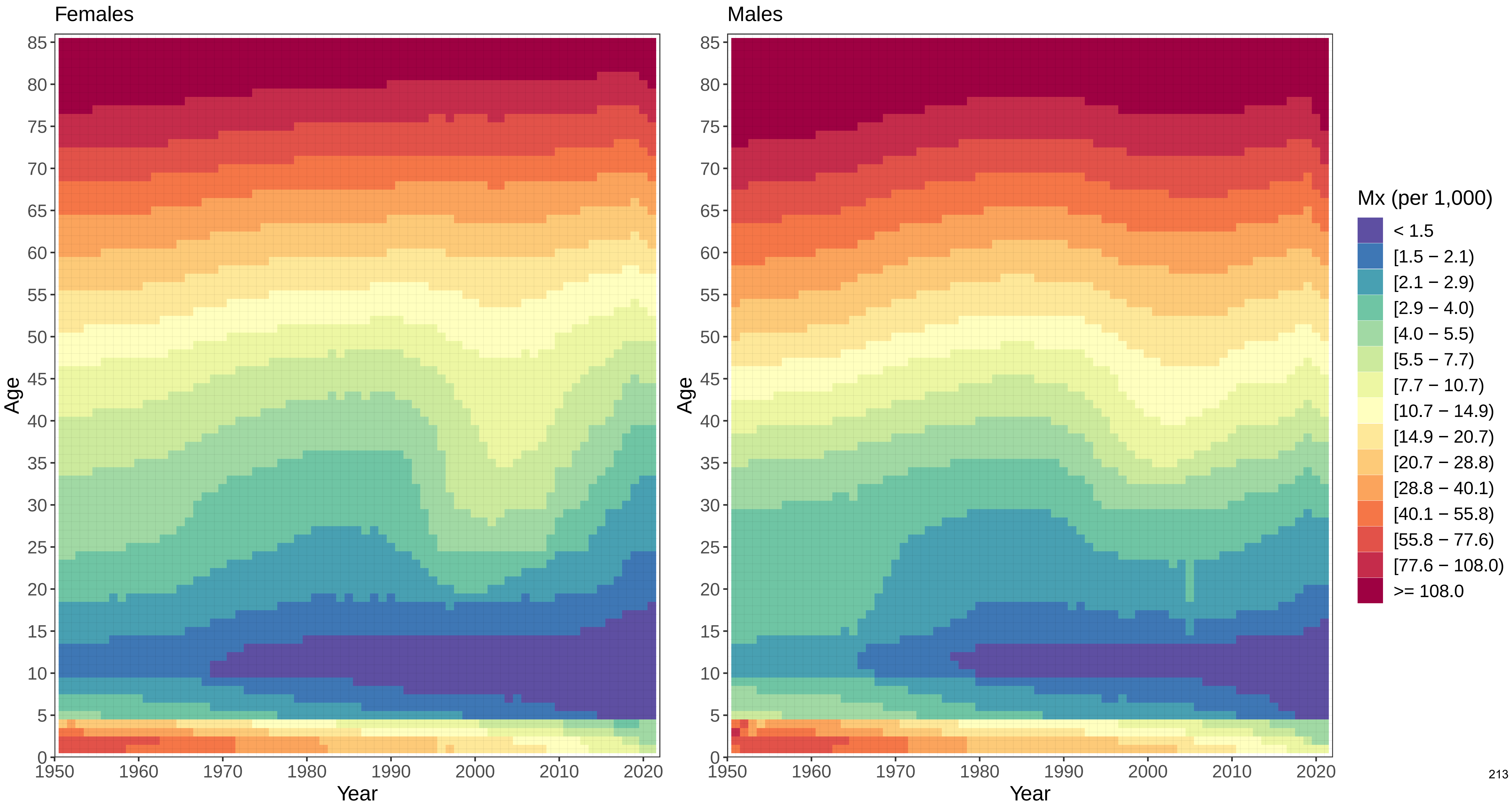

Appendix 2 Figure S6: Correlation of baseline nqx and the ARC in every decade per age group, by decade

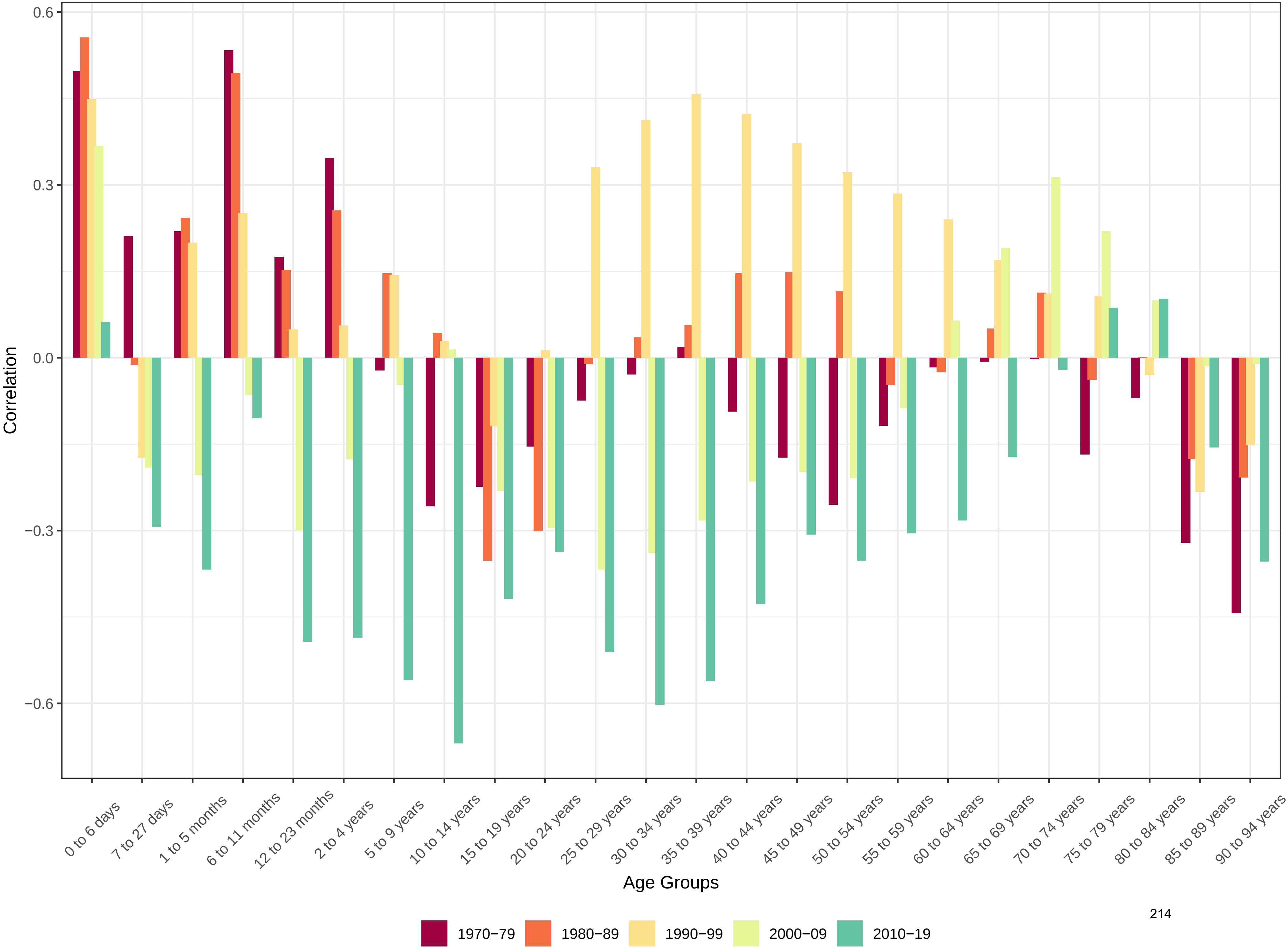

Appendix 2 Figure S7: National Estimated − Expected Life Expectancy based on SDI, by Sex, 1950−2021

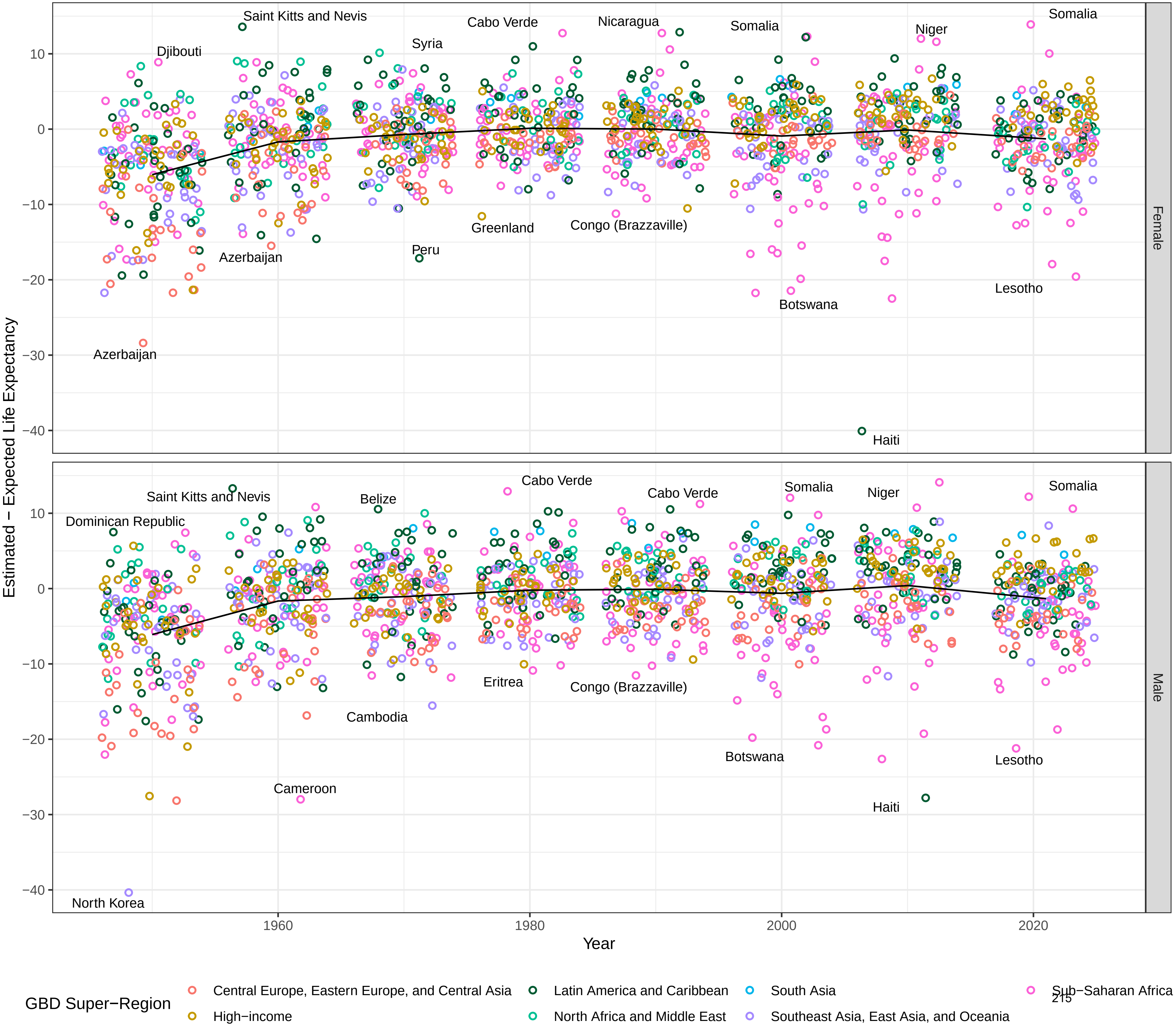

Appendix 2 Figure S8: Population Structure by SDI in 2021

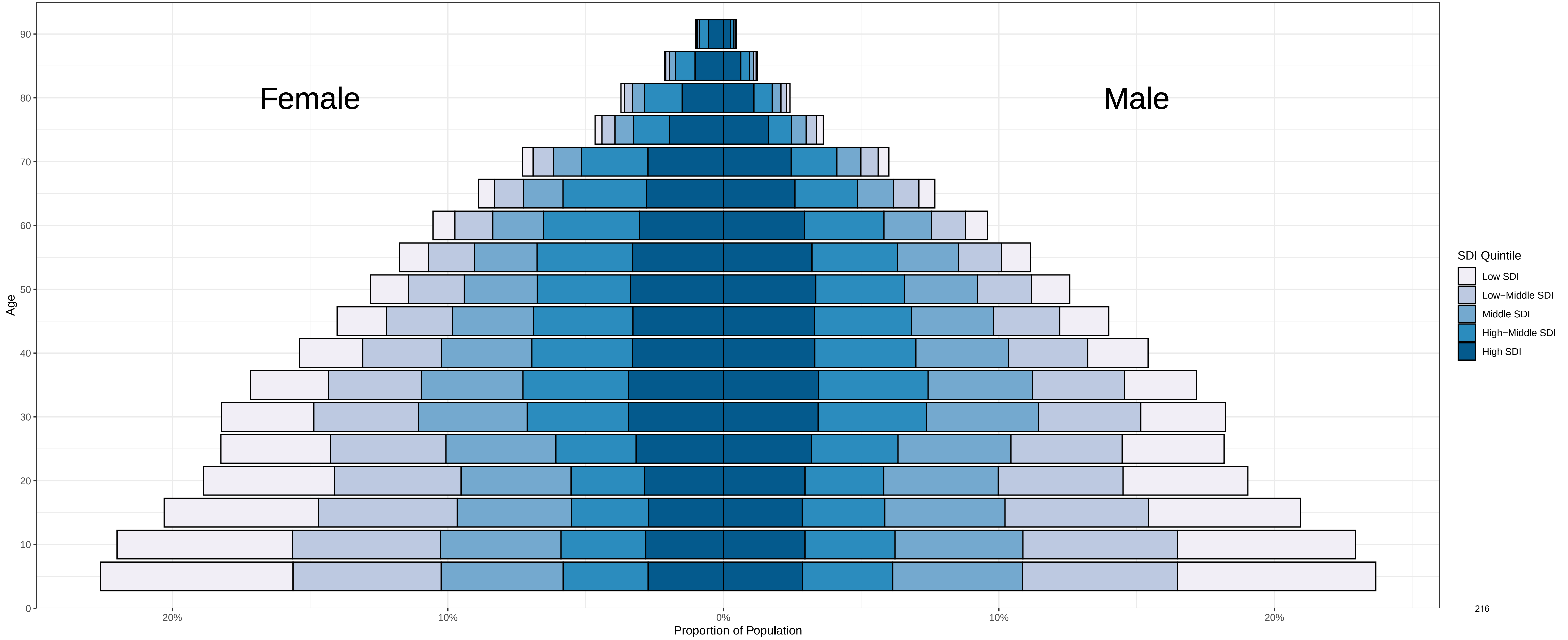

Appendix 2 Figure S9: Afghanistan population pyramids, by sex, for 1950, 1975, 2000, and 2021

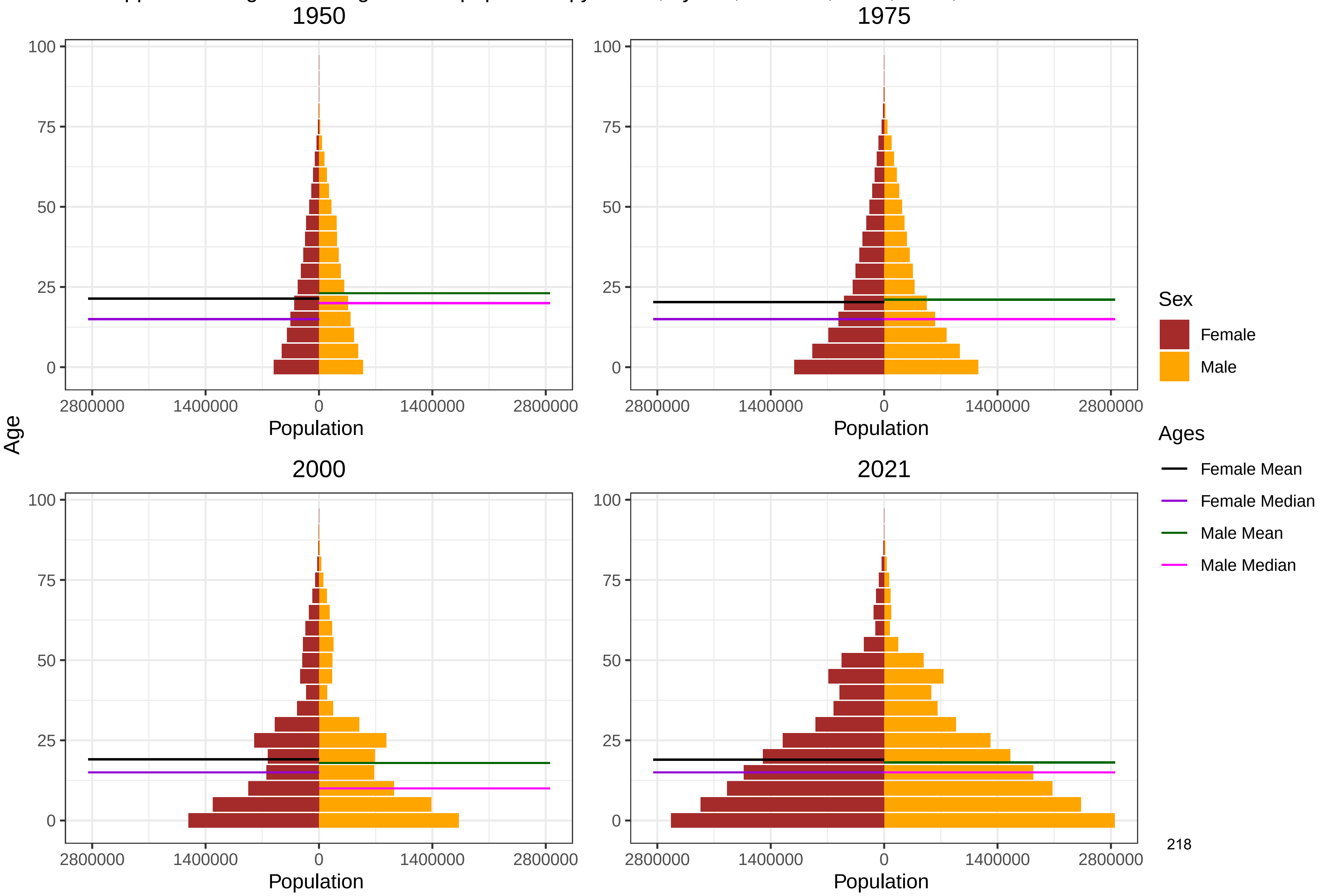

Appendix 2 Figure S9: Albania population pyramids, by sex, for 1950, 1975, 2000, and 2021

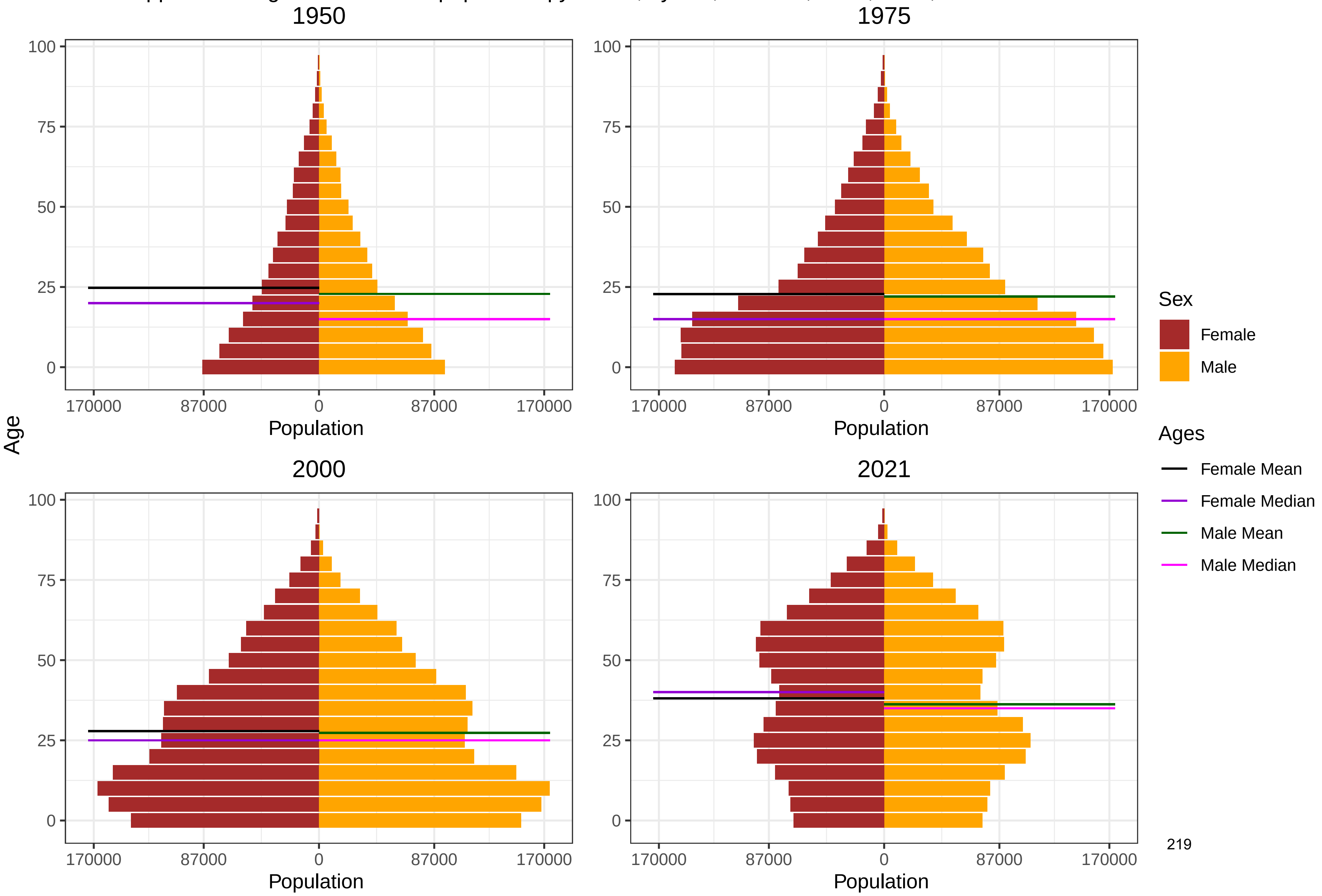

Appendix 2 Figure S9: Algeria population pyramids, by sex, for 1950, 1975, 2000, and 2021

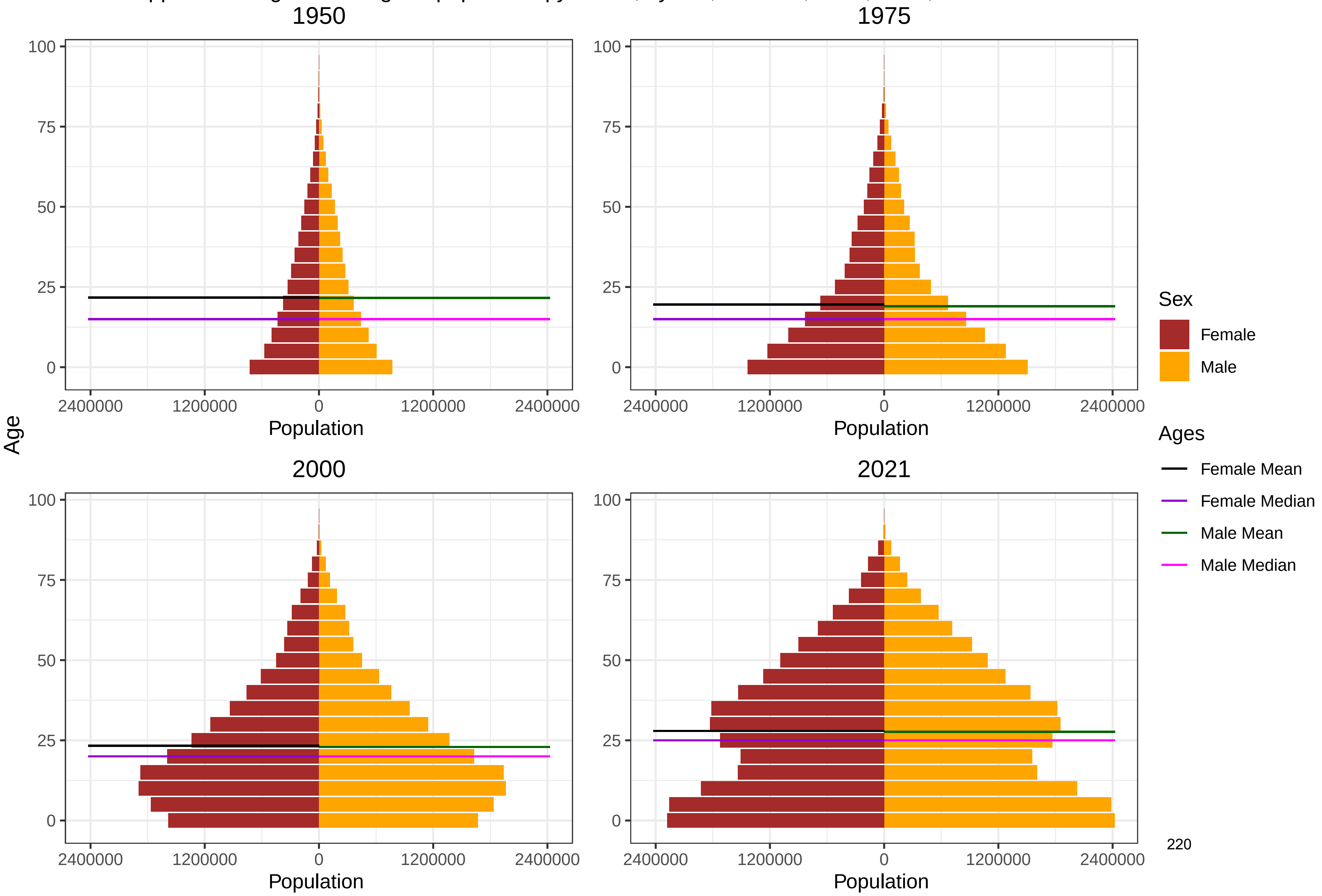

Appendix 2 Figure S9: American Samoa population pyramids, by sex, for 1950, 1975, 2000, and 2021

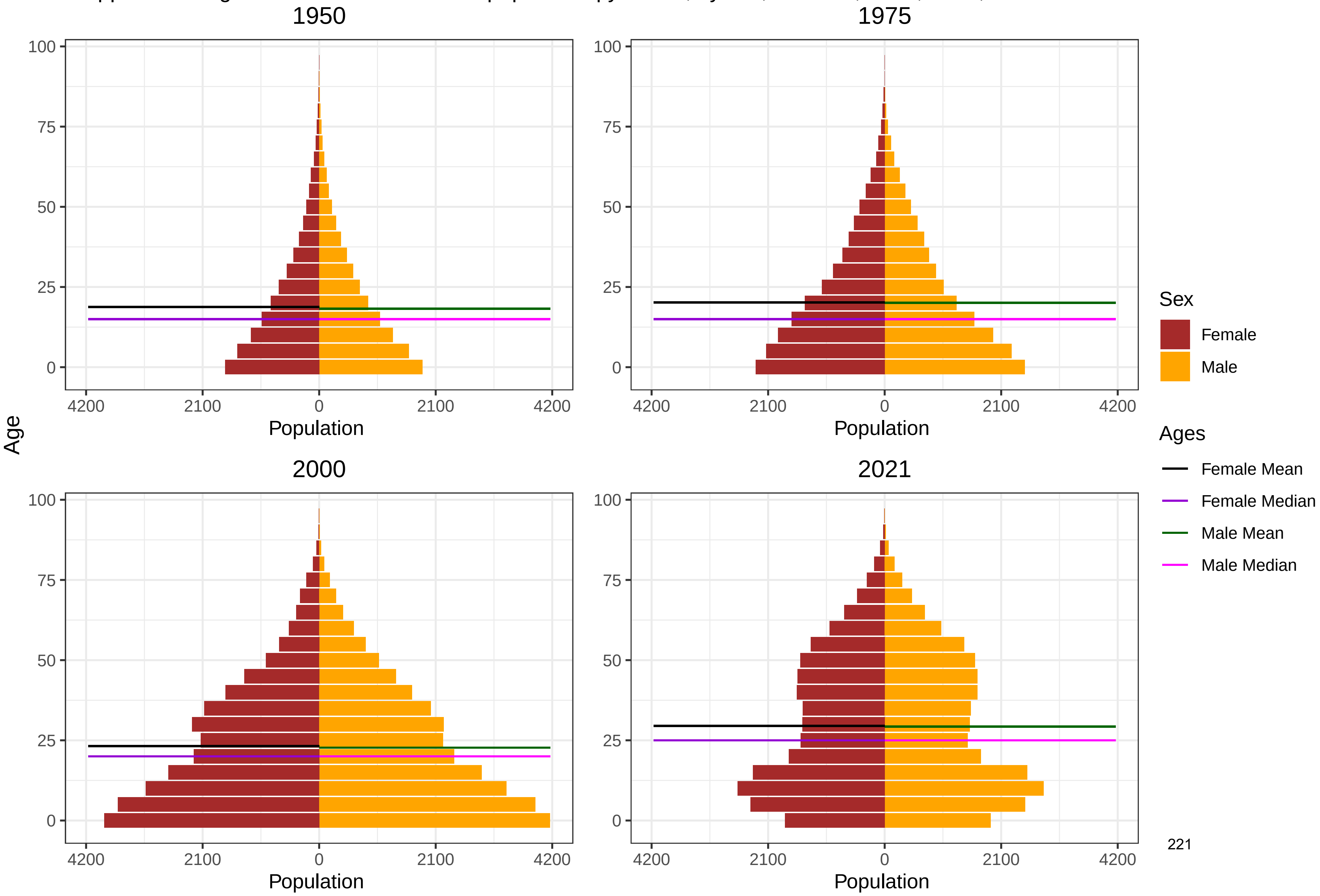

Appendix 2 Figure S9: Andorra population pyramids, by sex, for 1950, 1975, 2000, and 2021

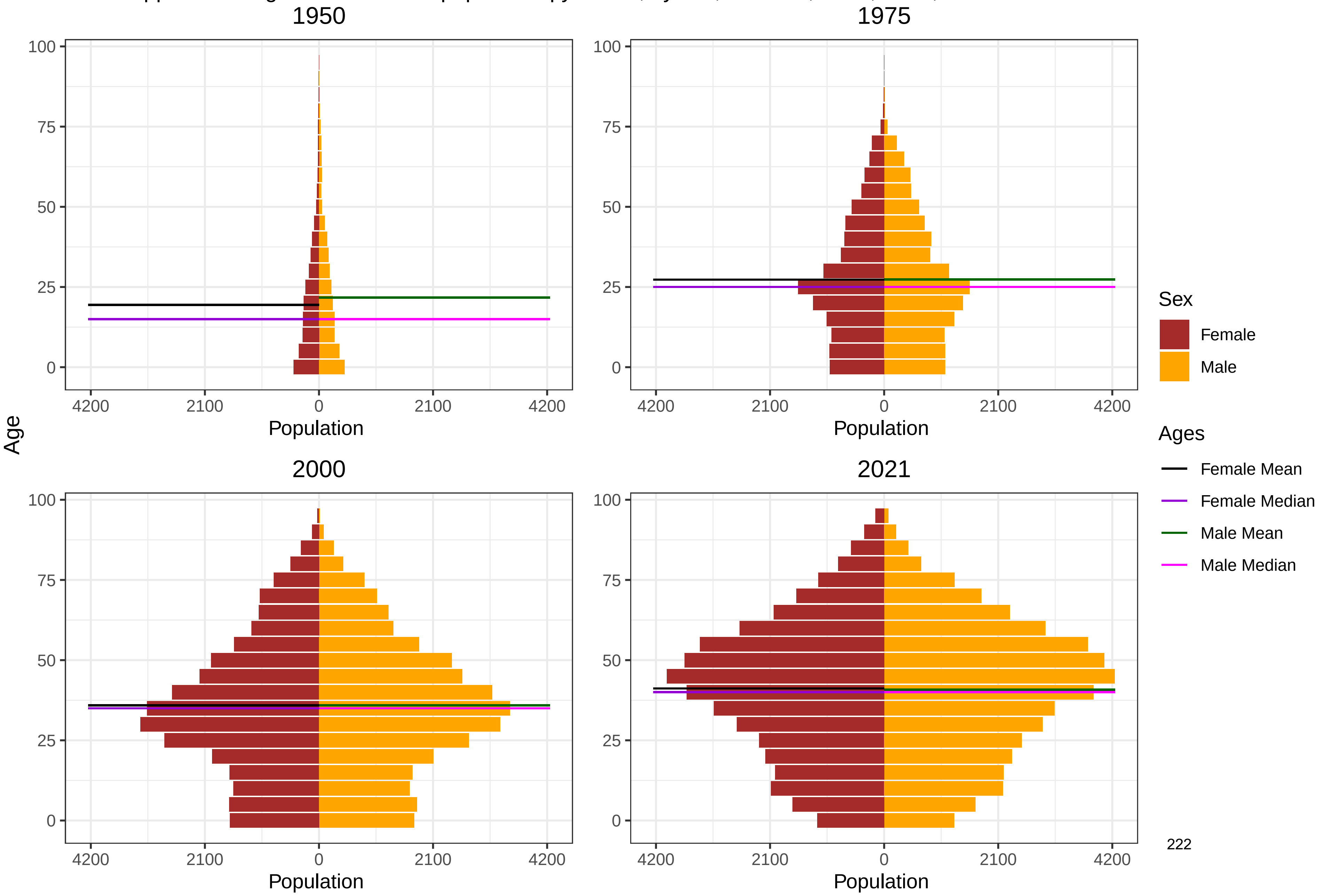

Appendix 2 Figure S9: Angola population pyramids, by sex, for 1950, 1975, 2000, and 2021

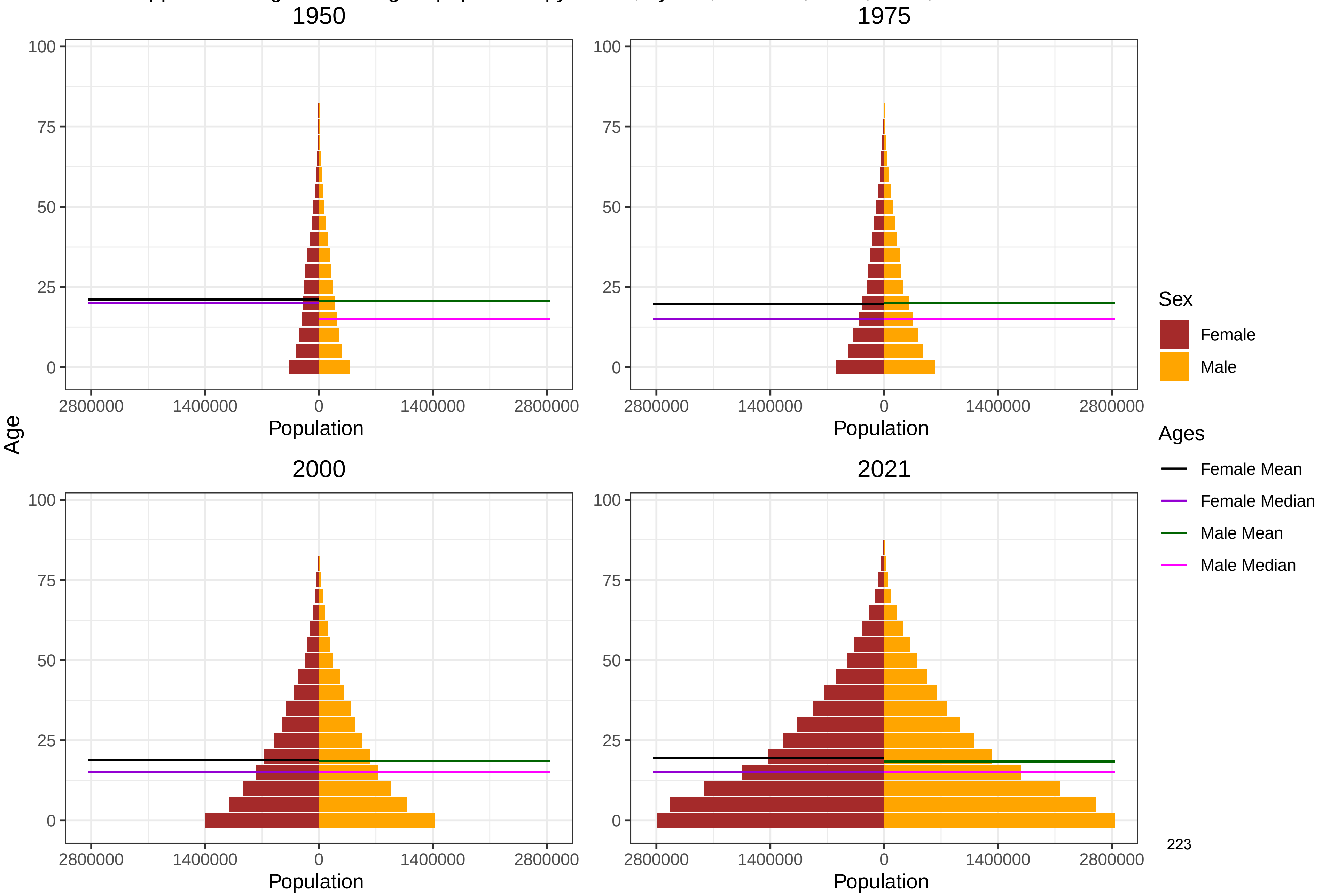

Appendix 2 Figure S9: Antigua and Barbuda population pyramids, by sex, for 1950, 1975, 2000, and 2021

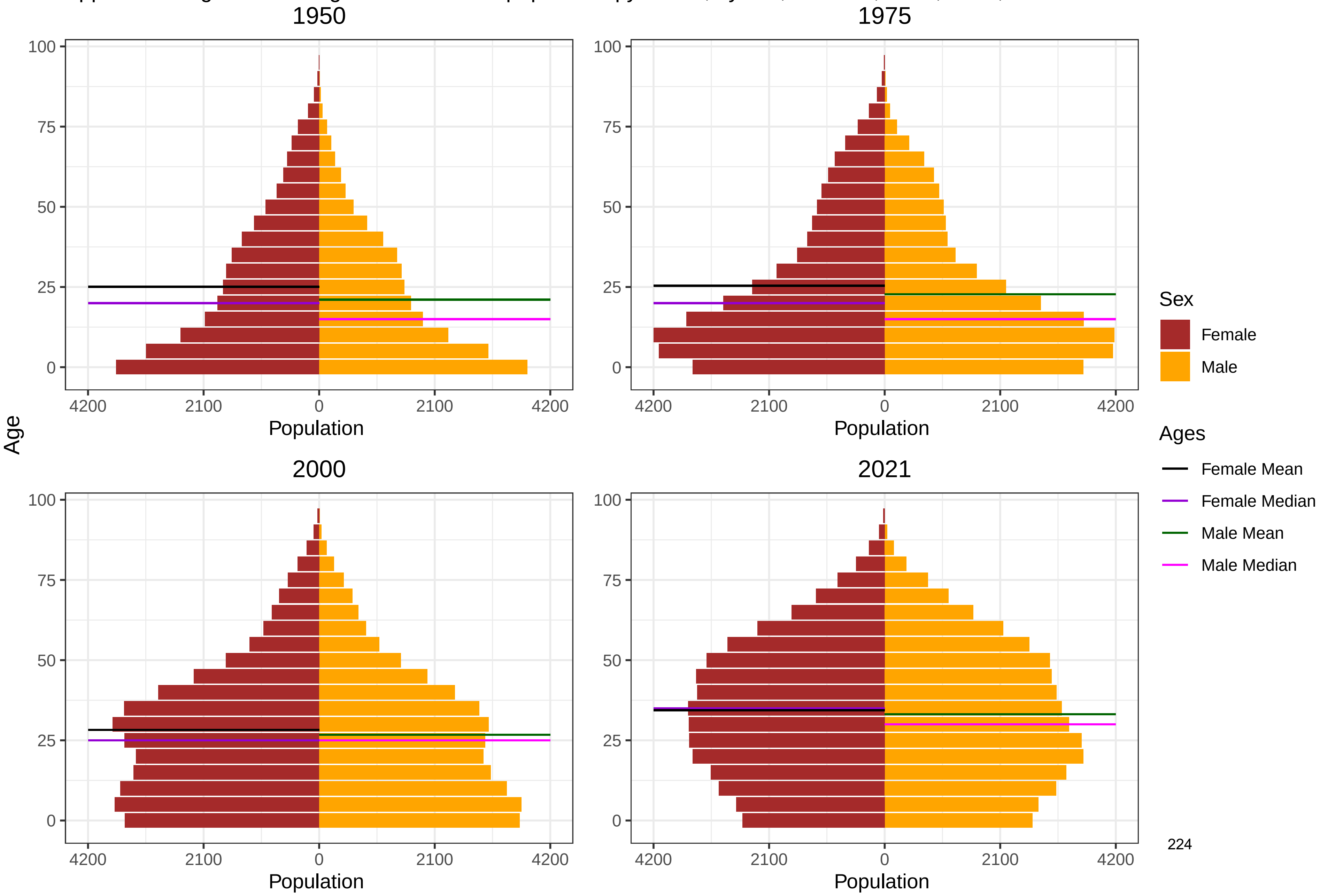

Appendix 2 Figure S9: Argentina population pyramids, by sex, for 1950, 1975, 2000, and 2021

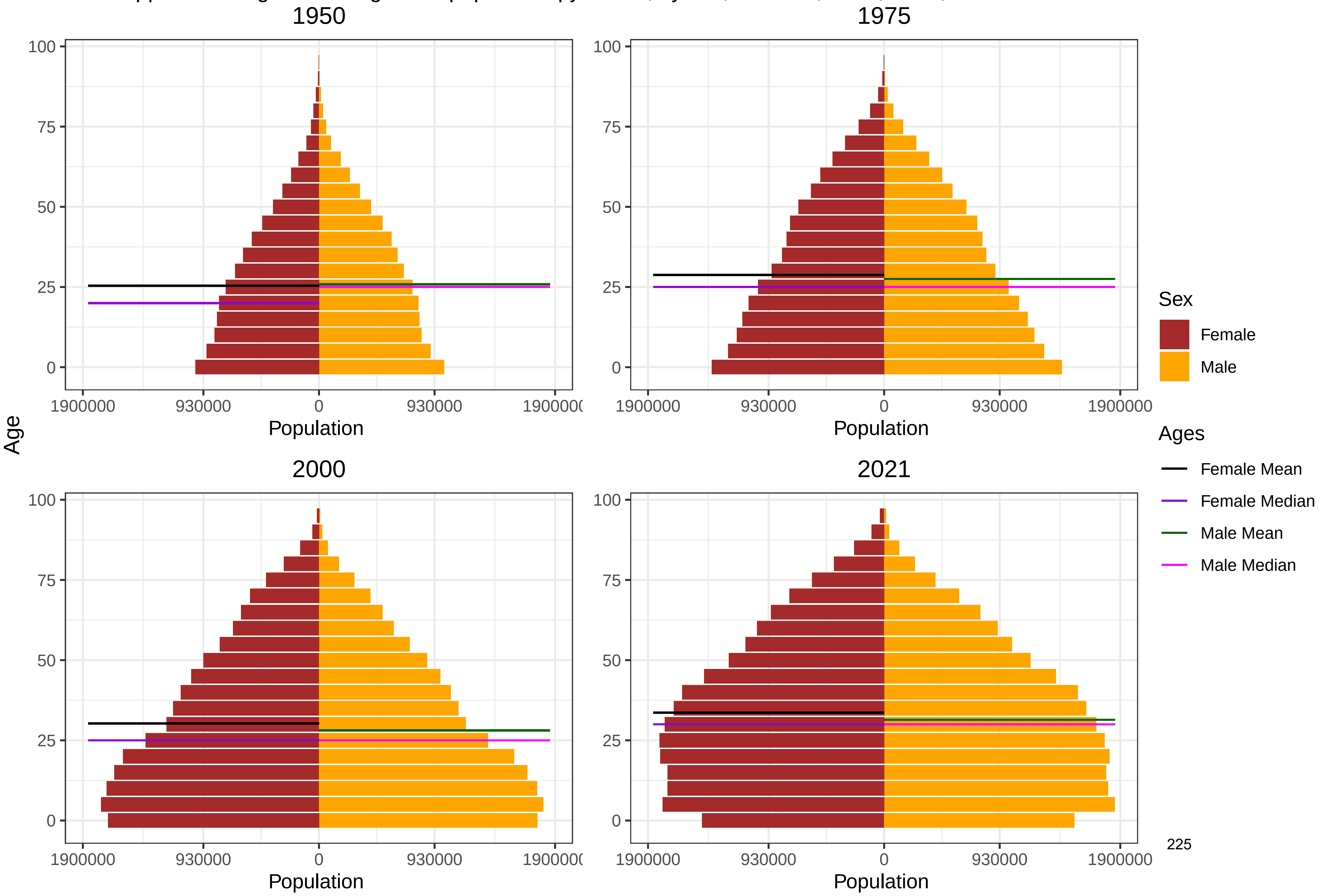

Appendix 2 Figure S9: Armenia population pyramids, by sex, for 1950, 1975, 2000, and 2021

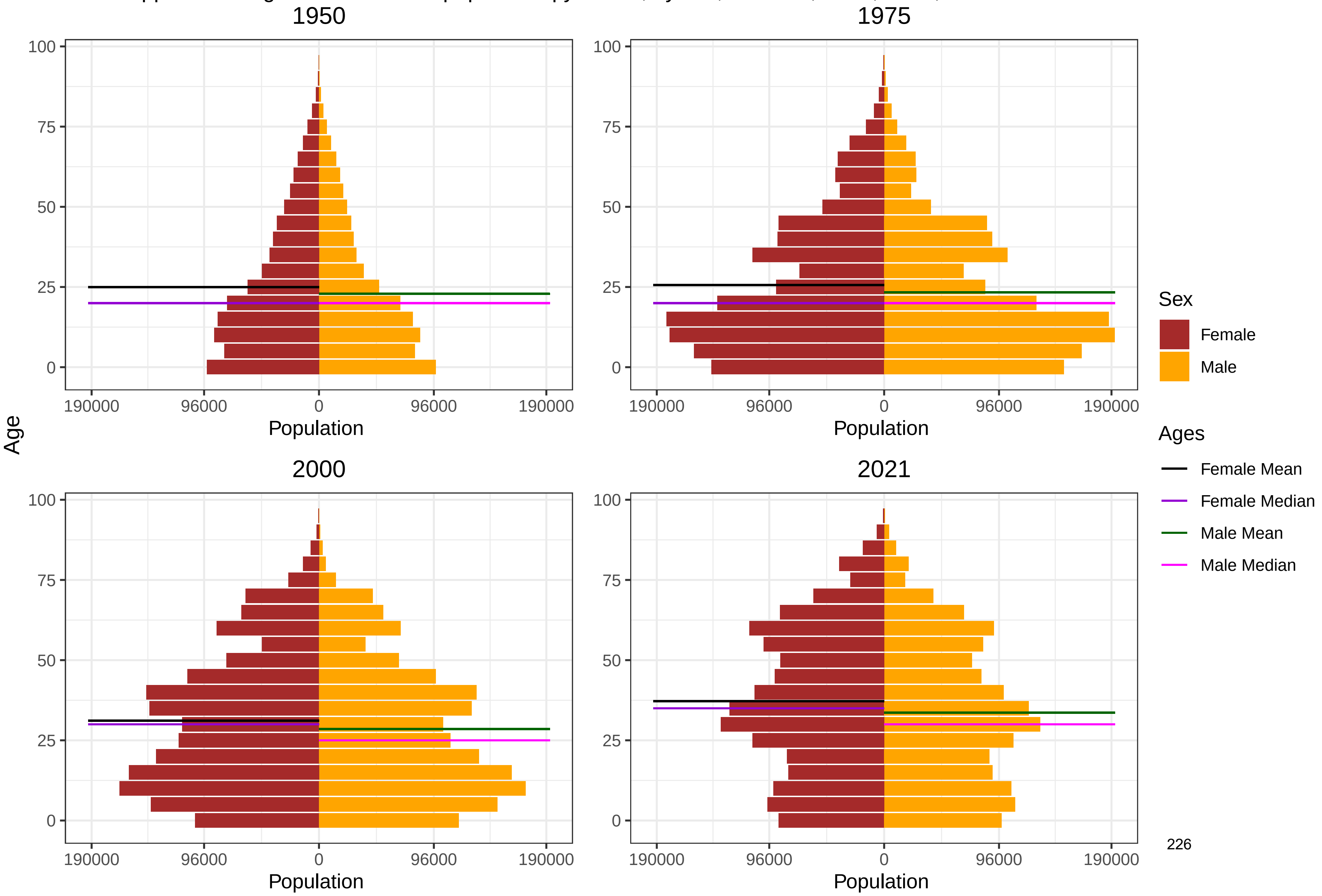

Appendix 2 Figure S9: Australia population pyramids, by sex, for 1950, 1975, 2000, and 2021

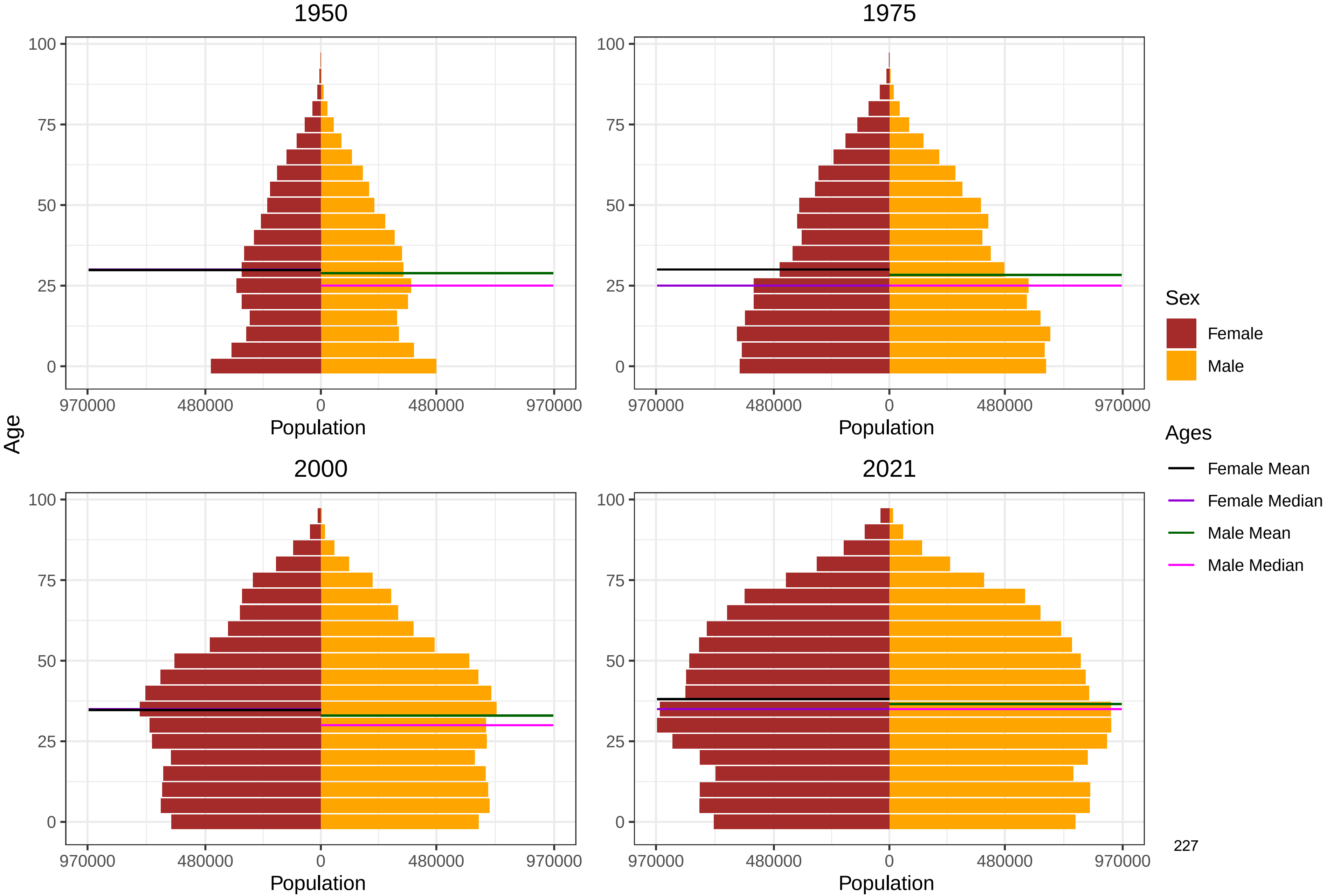

Appendix 2 Figure S9: Austria population pyramids, by sex, for 1950, 1975, 2000, and 2021

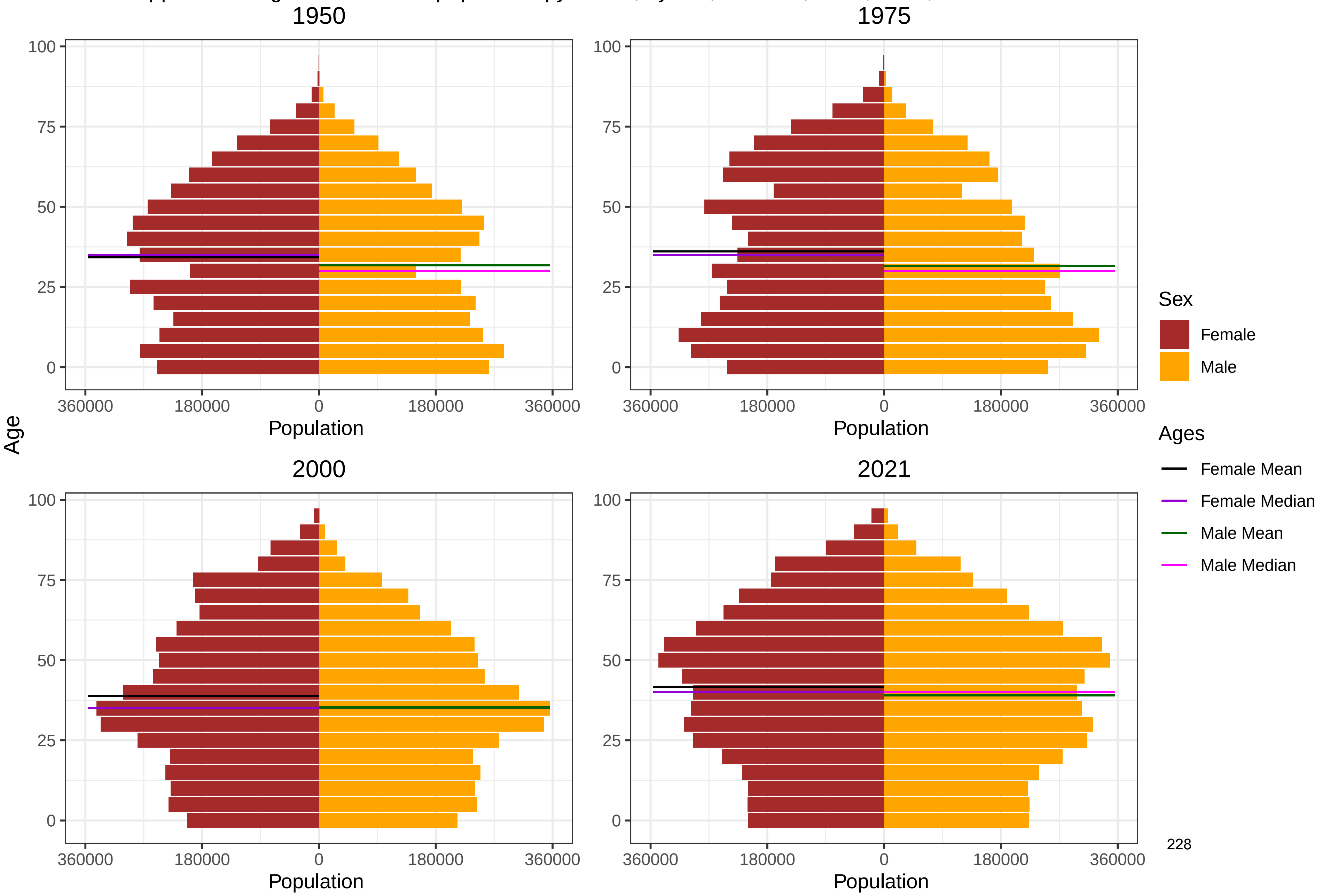

Appendix 2 Figure S9: Azerbaijan population pyramids, by sex, for 1950, 1975, 2000, and 2021

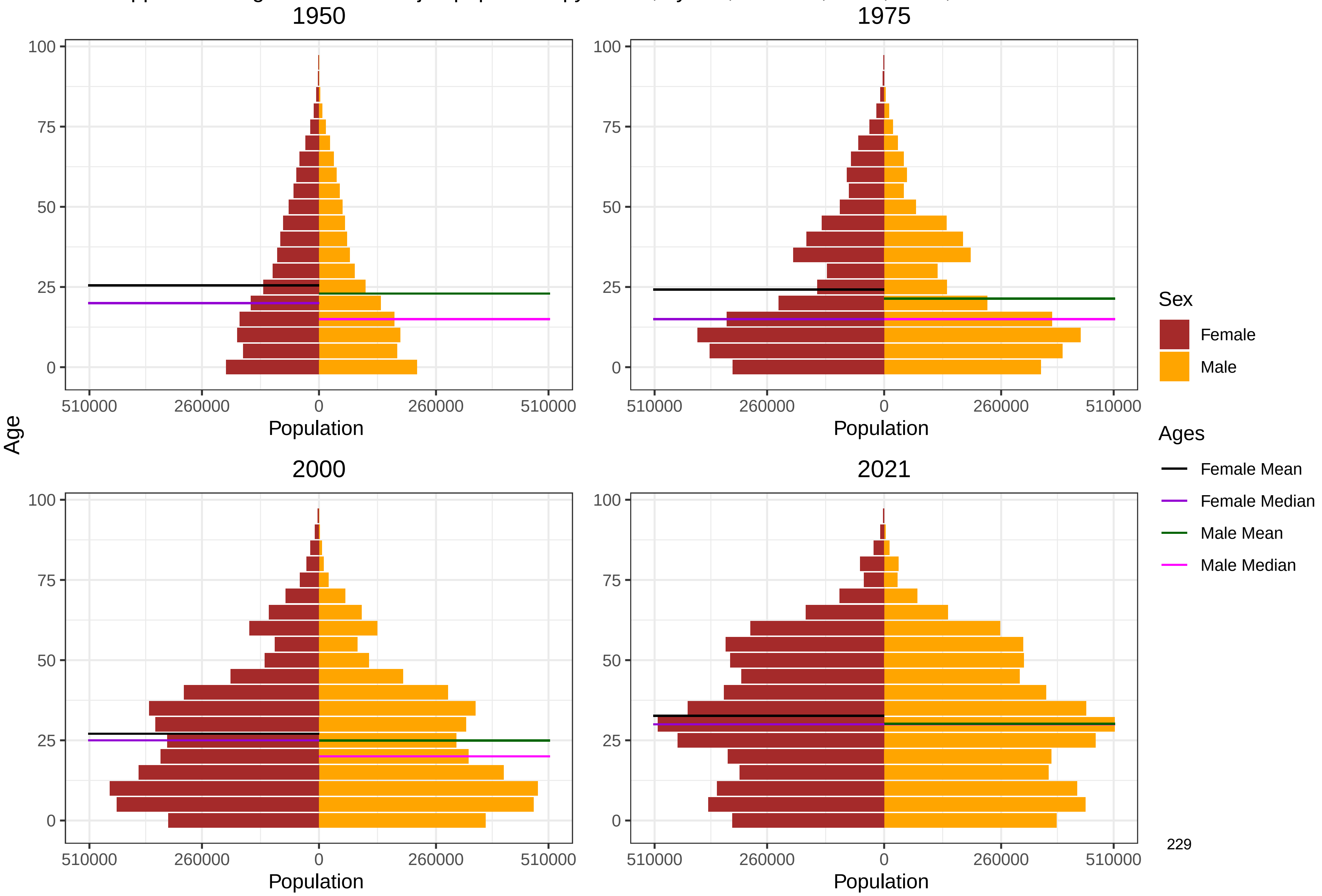

Appendix 2 Figure S9: Bahrain population pyramids, by sex, for 1950, 1975, 2000, and 2021

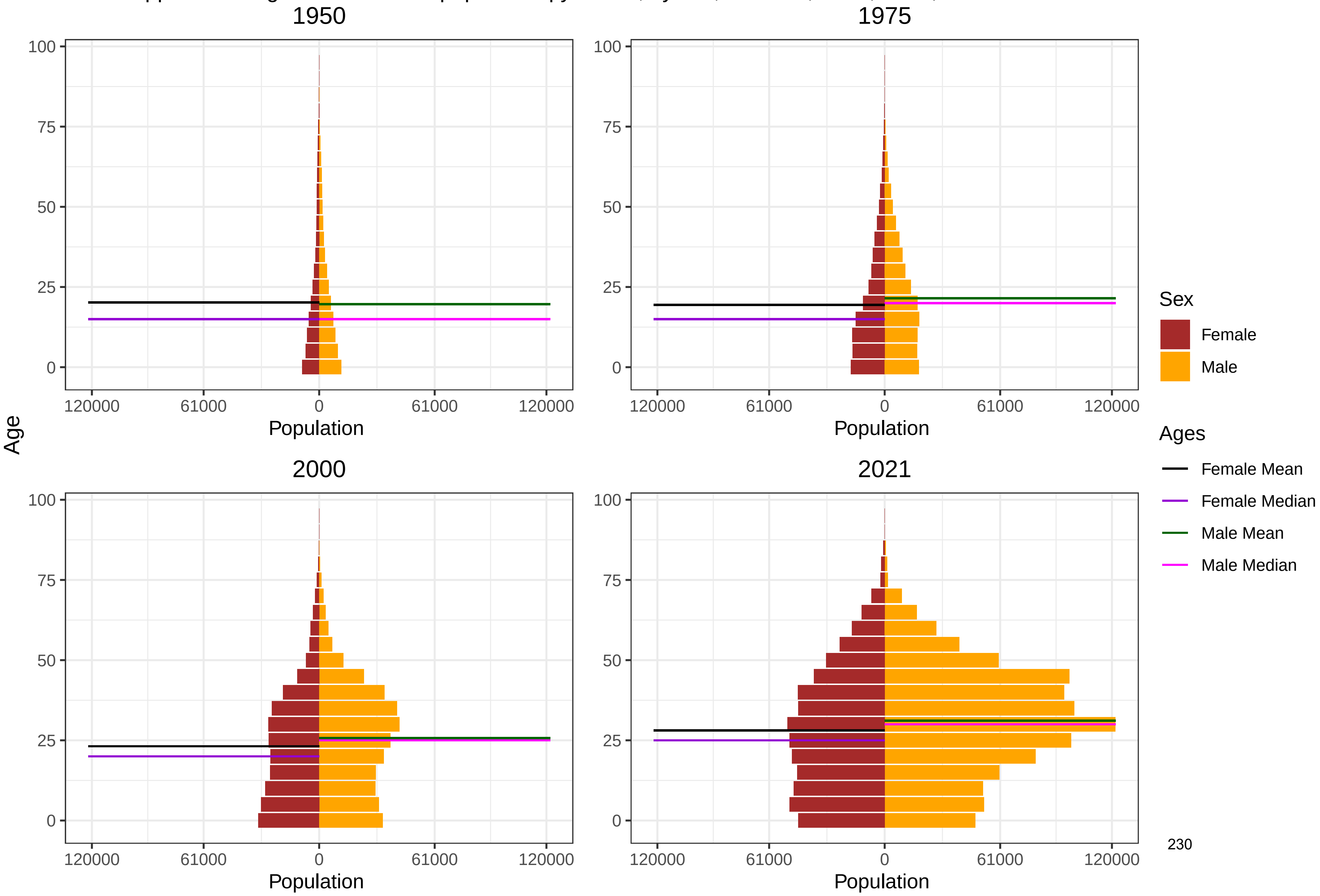

Appendix 2 Figure S9: Bangladesh population pyramids, by sex, for 1950, 1975, 2000, and 2021

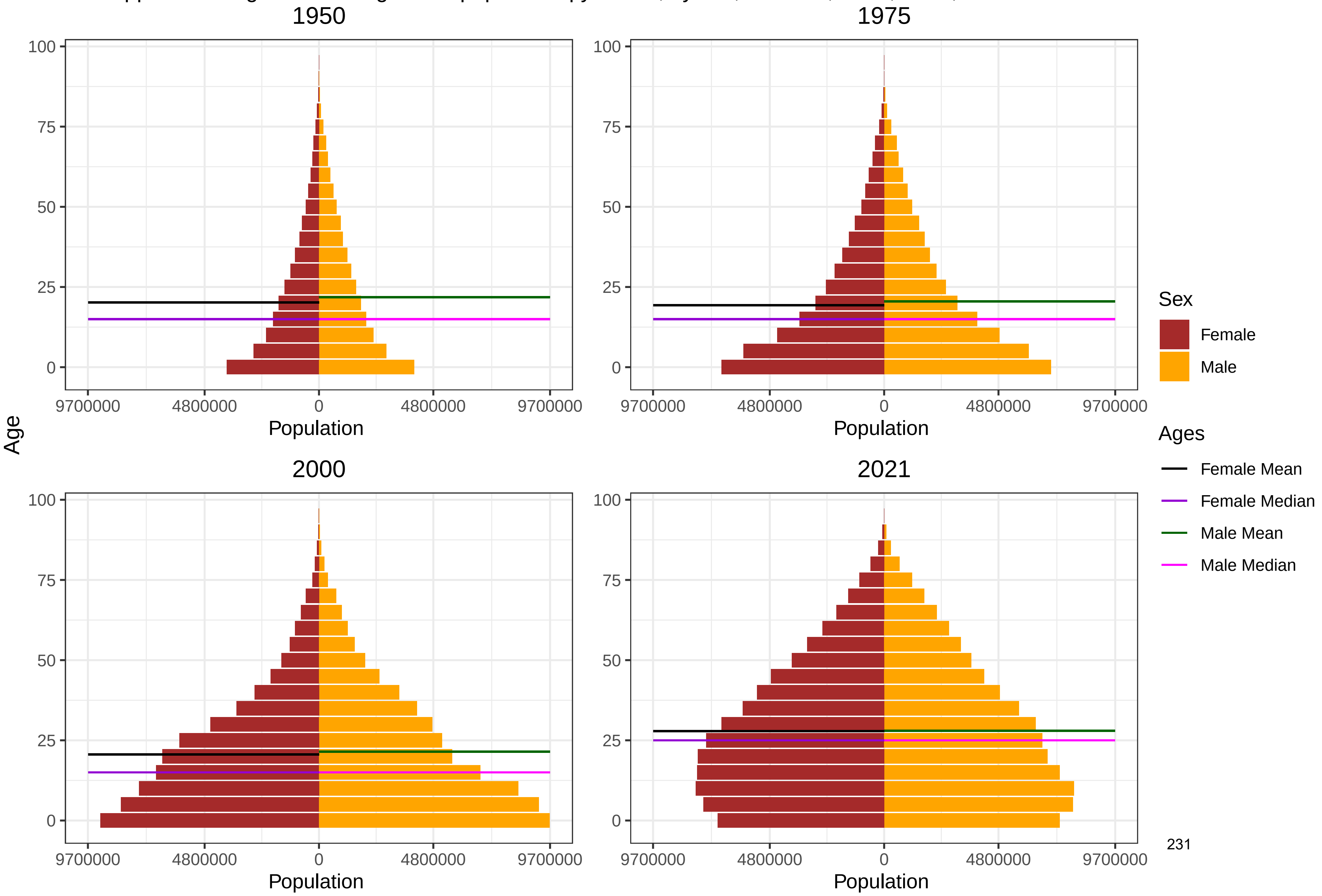

Appendix 2 Figure S9: Barbados population pyramids, by sex, for 1950, 1975, 2000, and 2021

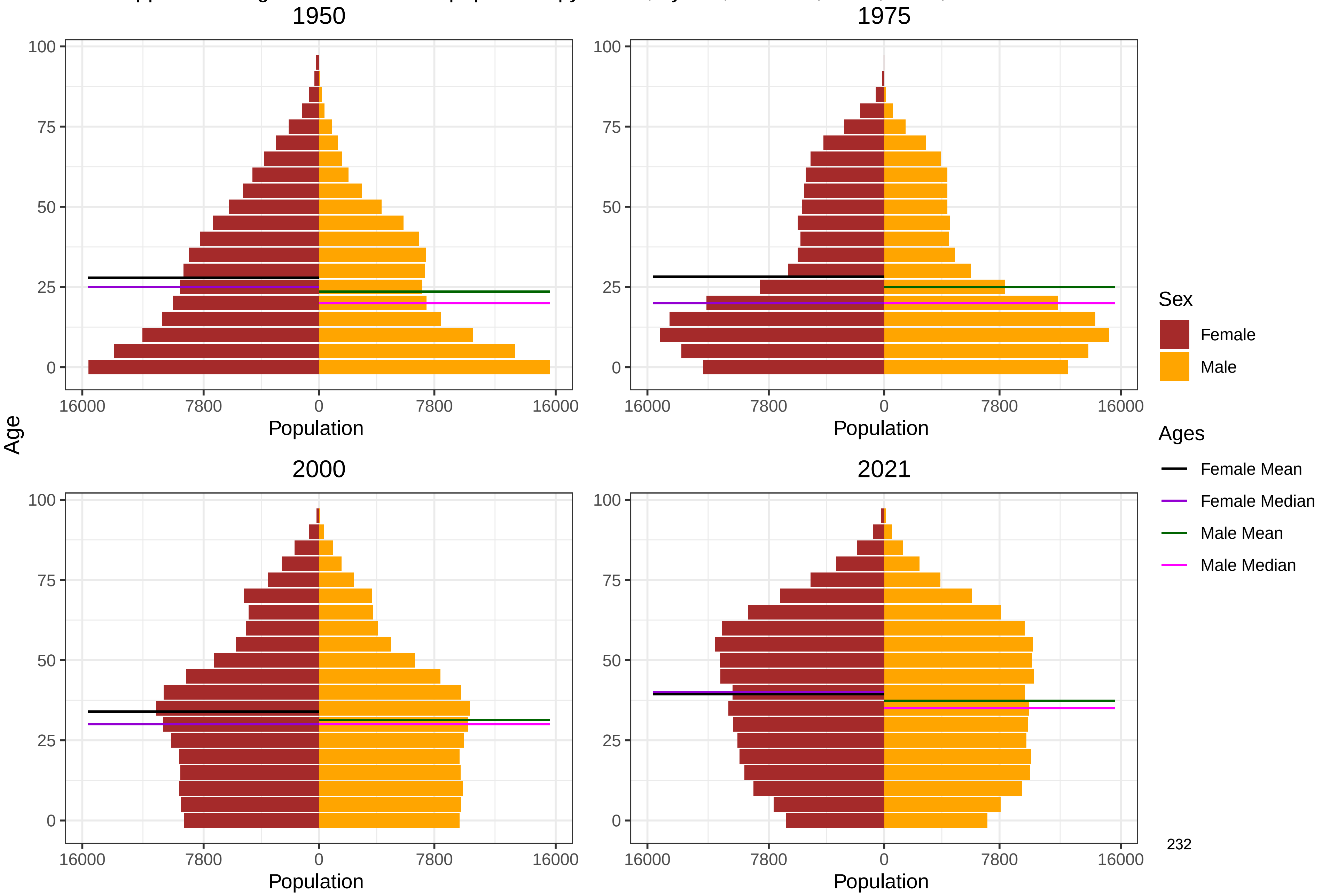

Appendix 2 Figure S9: Belarus population pyramids, by sex, for 1950, 1975, 2000, and 2021

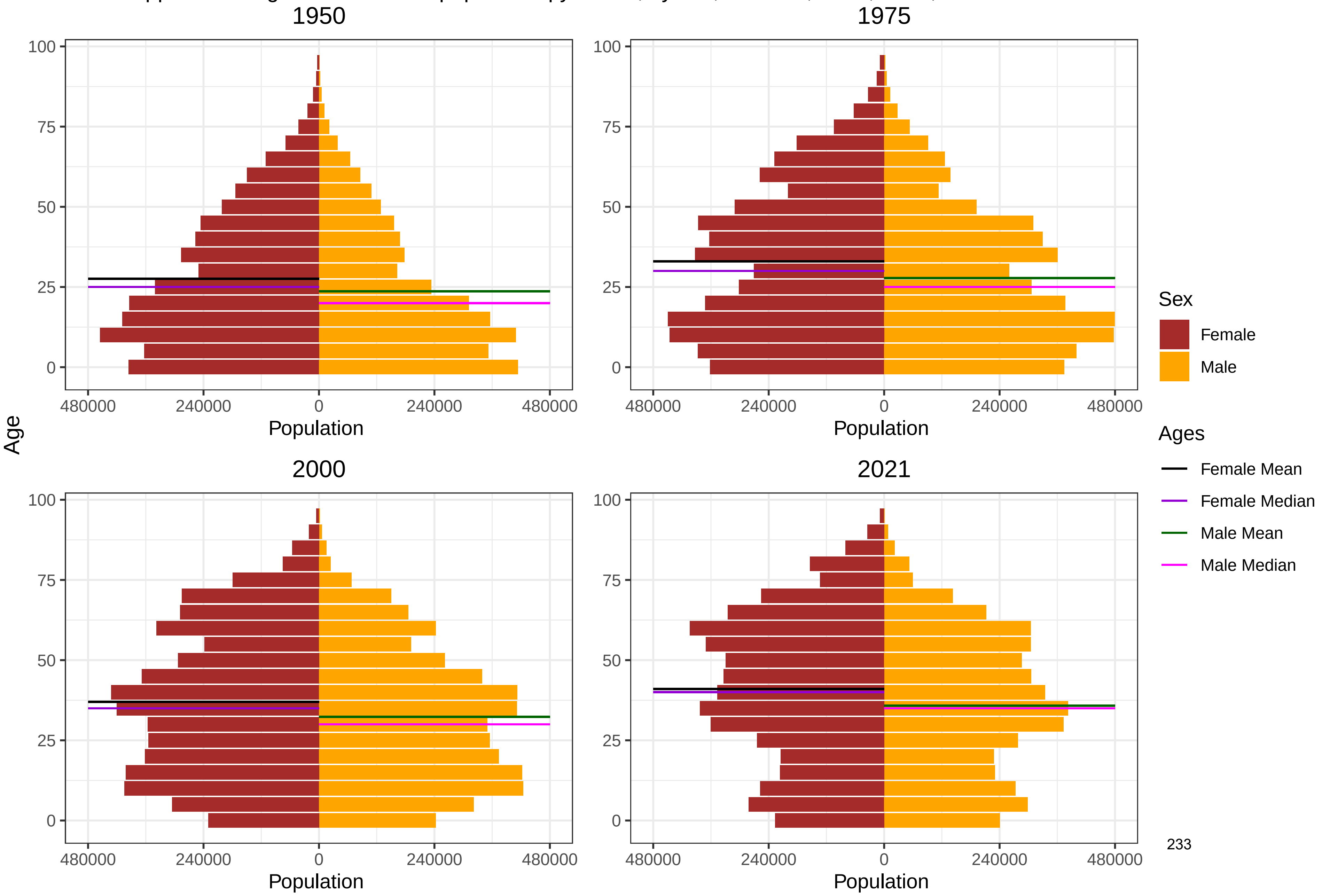

Appendix 2 Figure S9: Belgium population pyramids, by sex, for 1950, 1975, 2000, and 2021

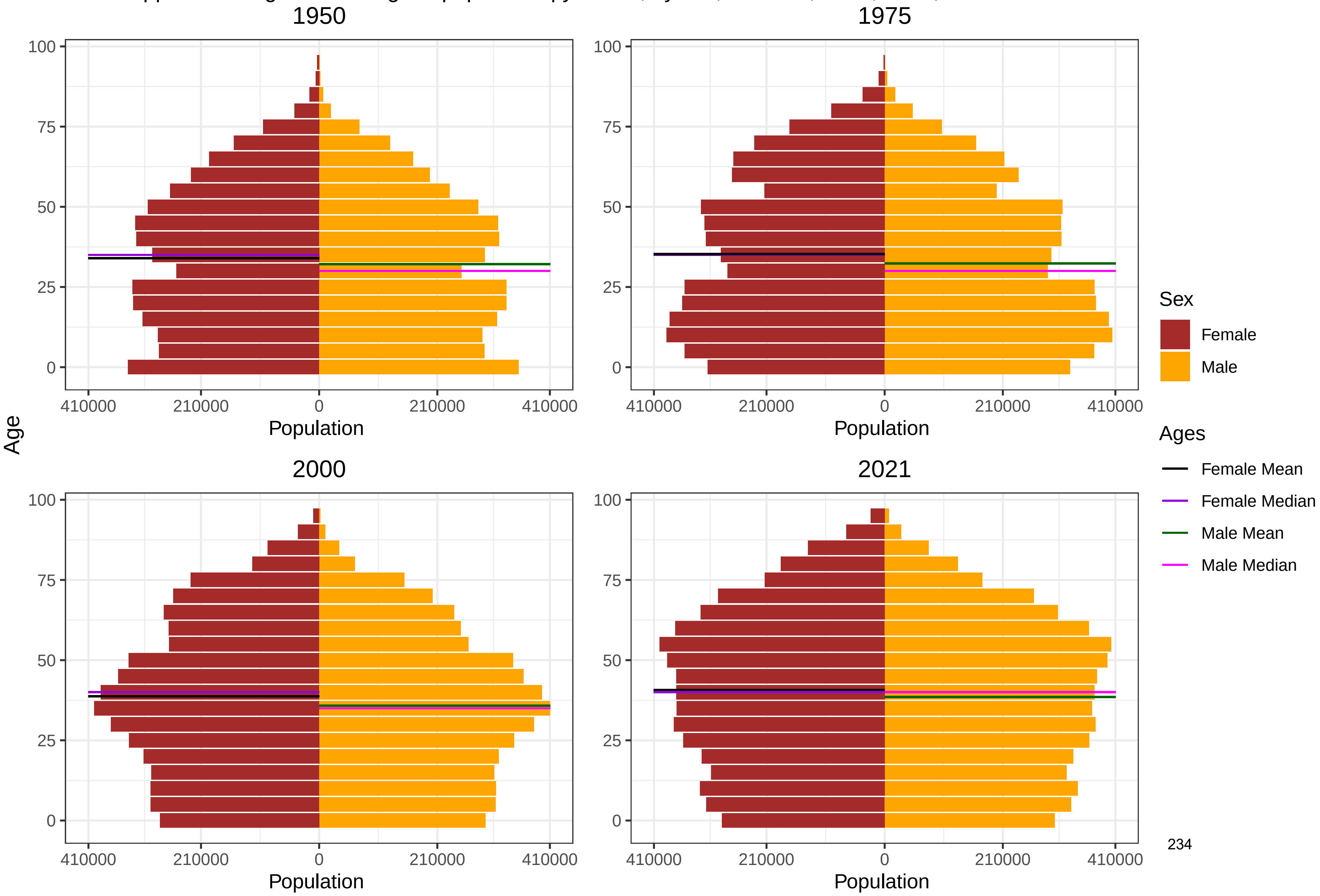

Appendix 2 Figure S9: Belize population pyramids, by sex, for 1950, 1975, 2000, and 2021

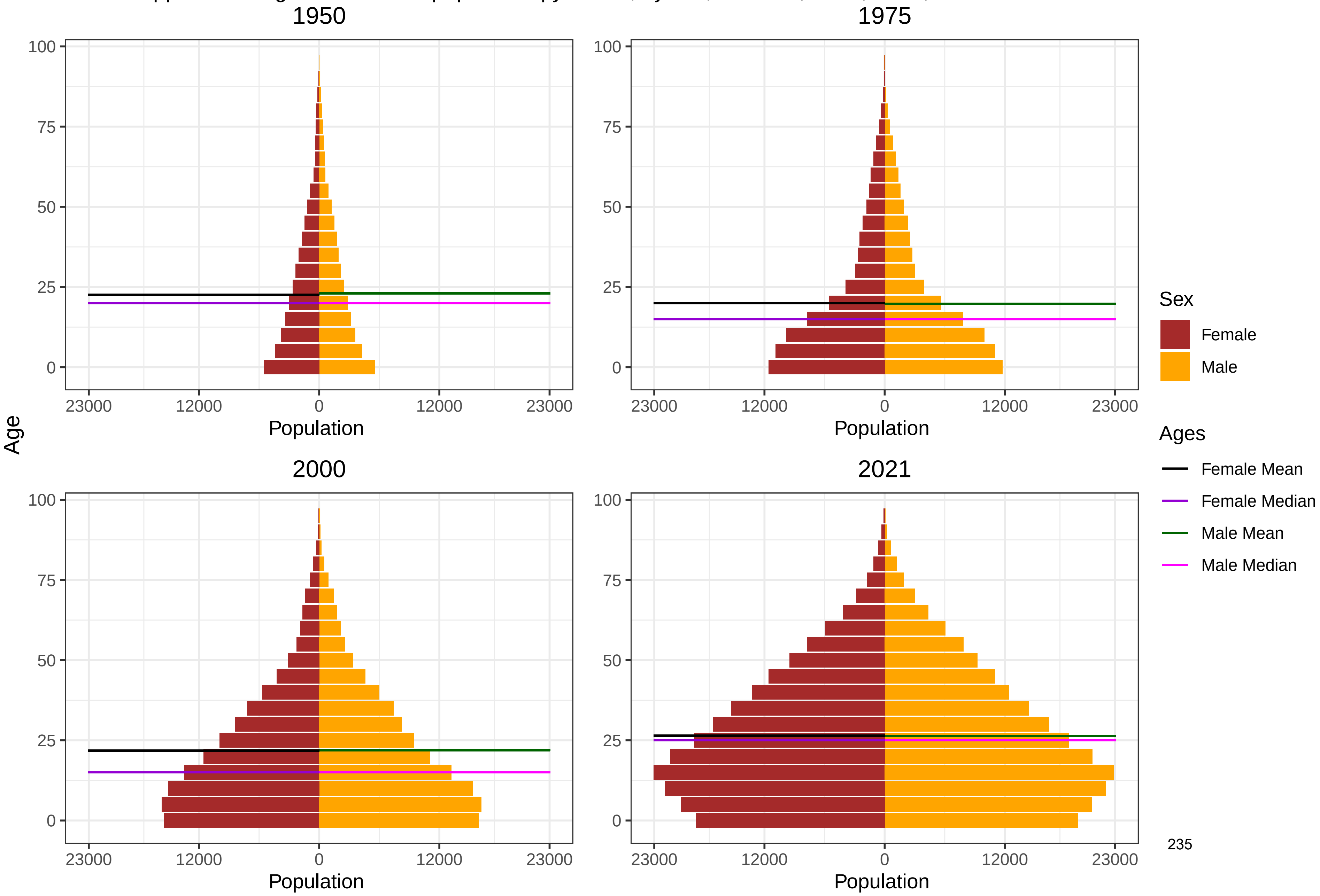

Appendix 2 Figure S9: Benin population pyramids, by sex, for 1950, 1975, 2000, and 2021

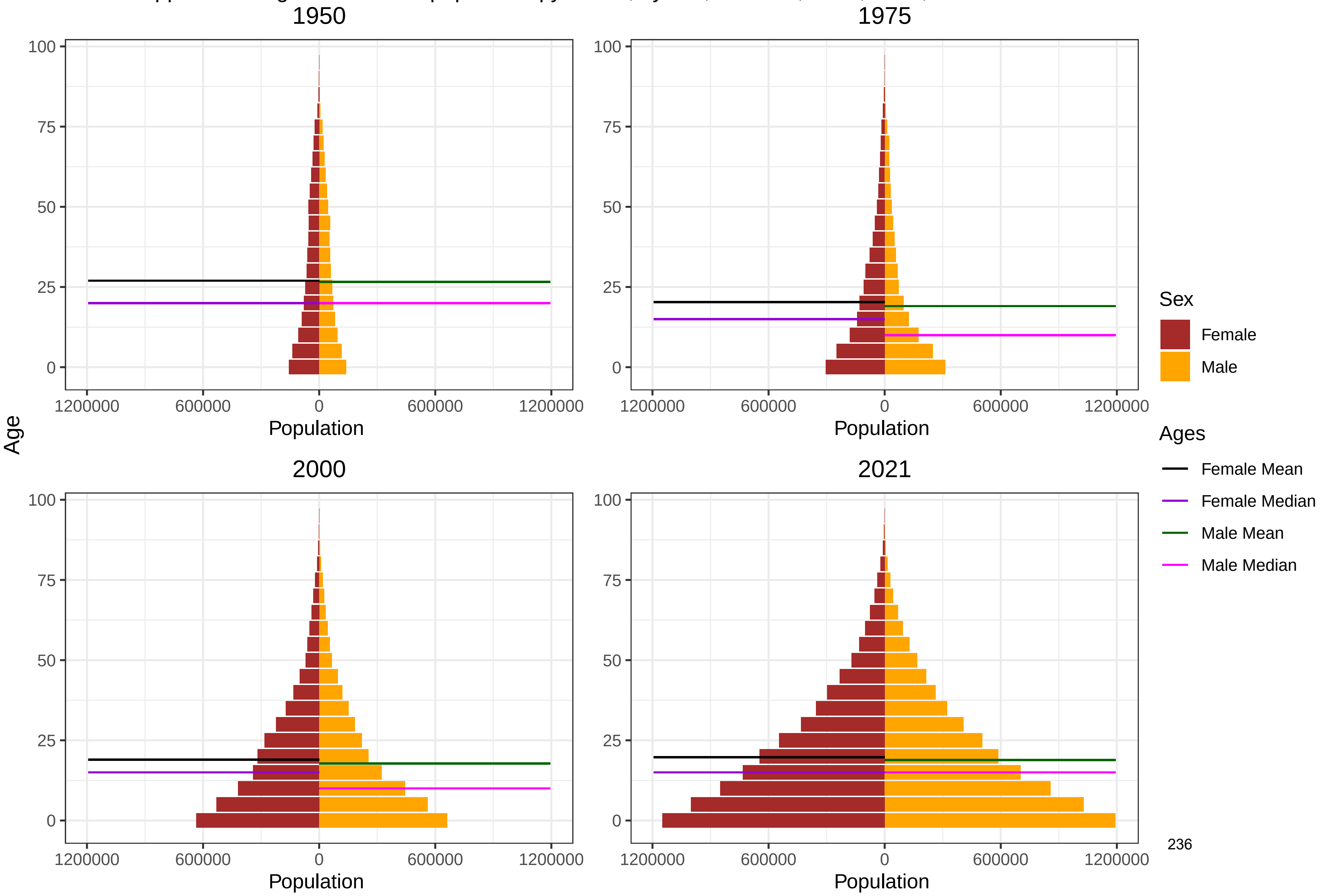

Appendix 2 Figure S9: Bermuda population pyramids, by sex, for 1950, 1975, 2000, and 2021

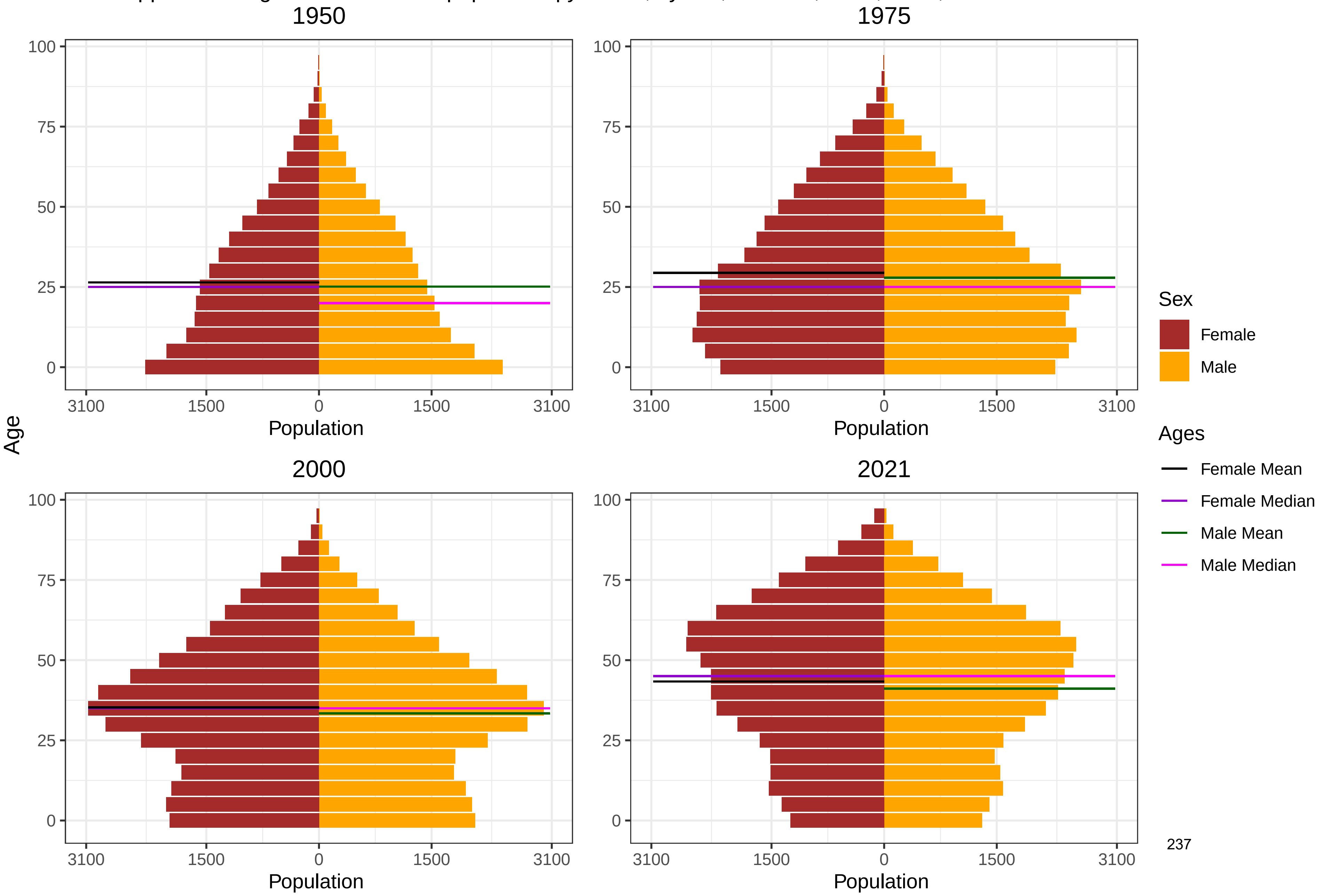

Appendix 2 Figure S9: Bhutan population pyramids, by sex, for 1950, 1975, 2000, and 2021

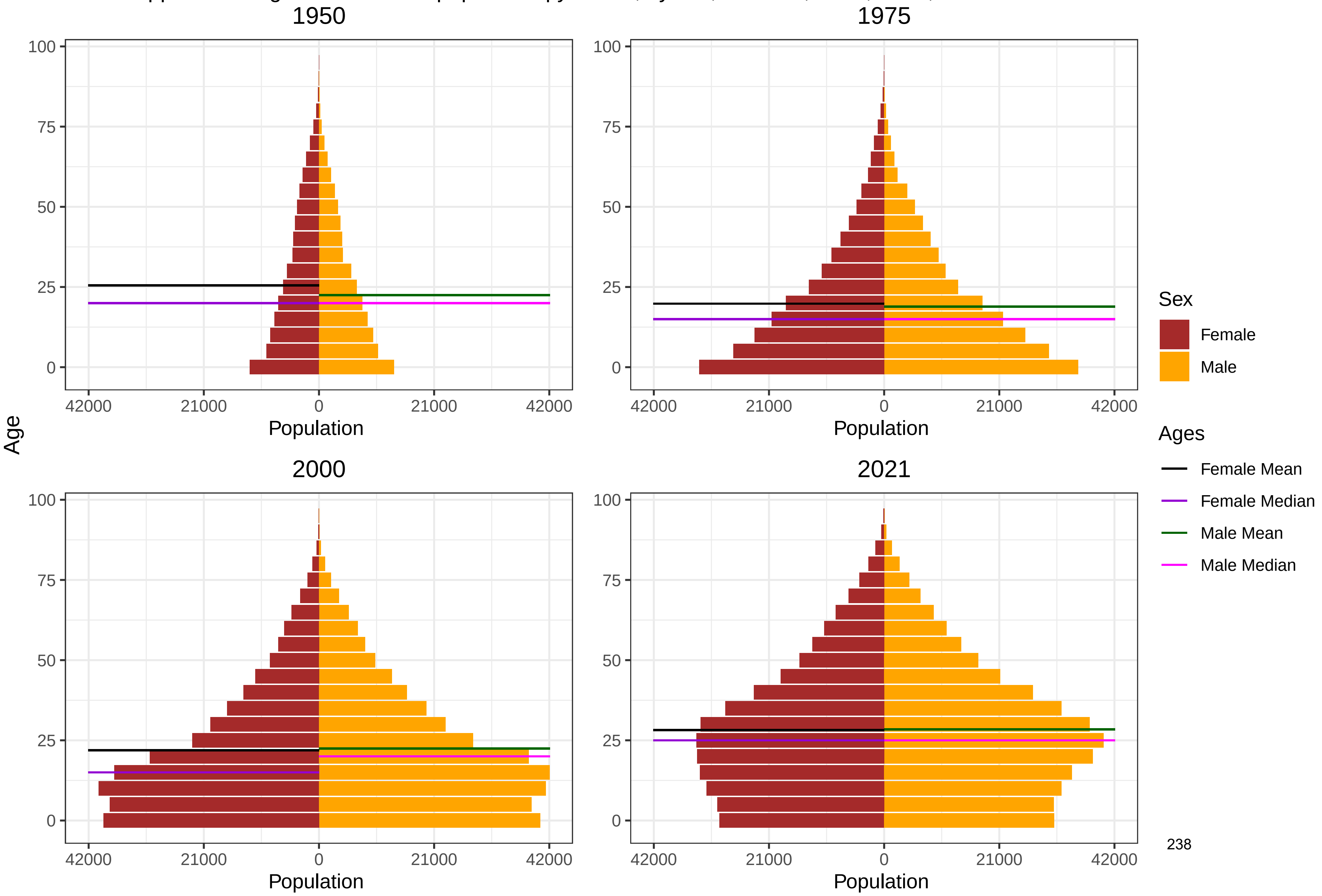

Appendix 2 Figure S9: Bolivia population pyramids, by sex, for 1950, 1975, 2000, and 2021

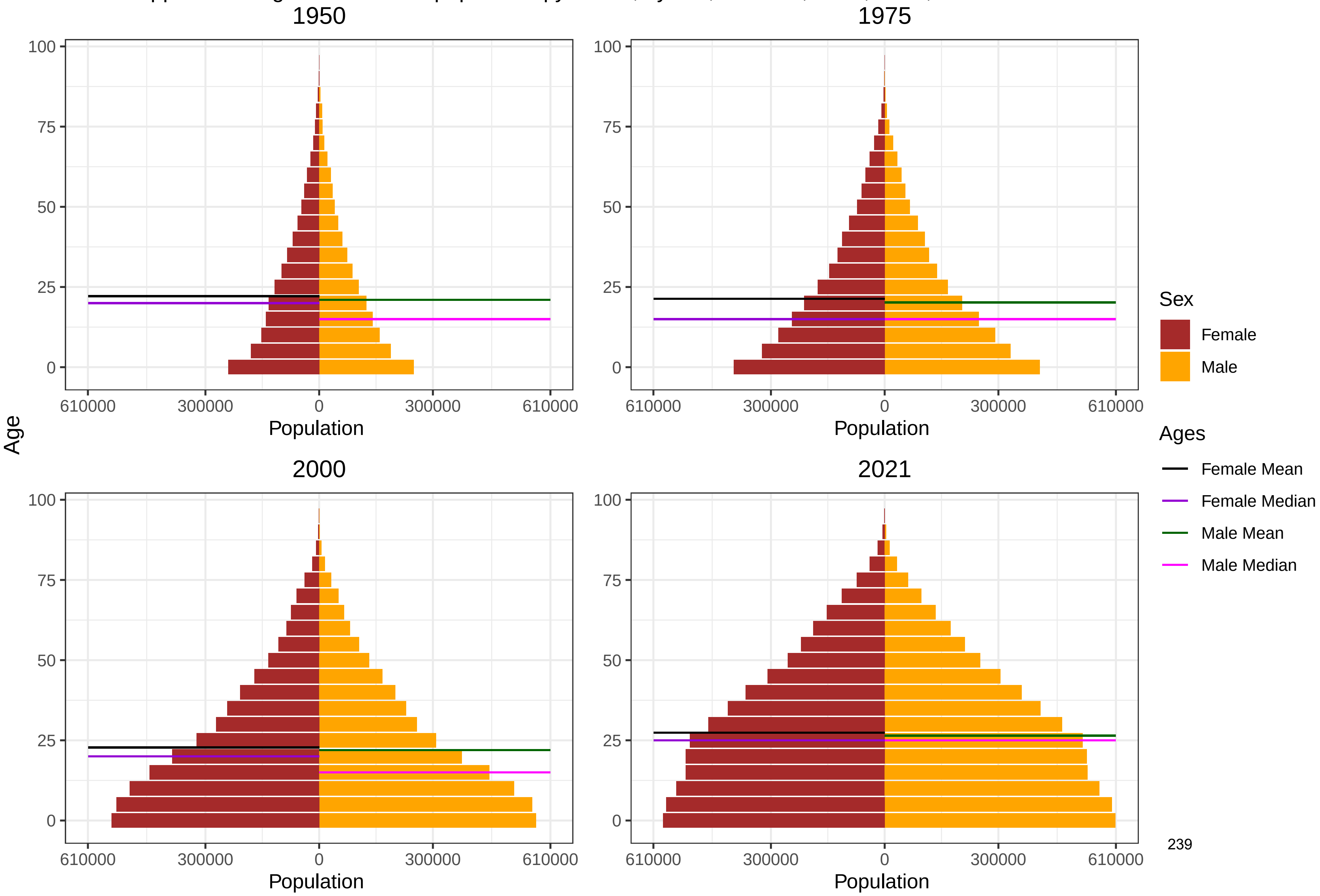

Appendix 2 Figure S9: Bosnia and Herzegovina population pyramids, by sex, for 1950, 1975, 2000, and 2021

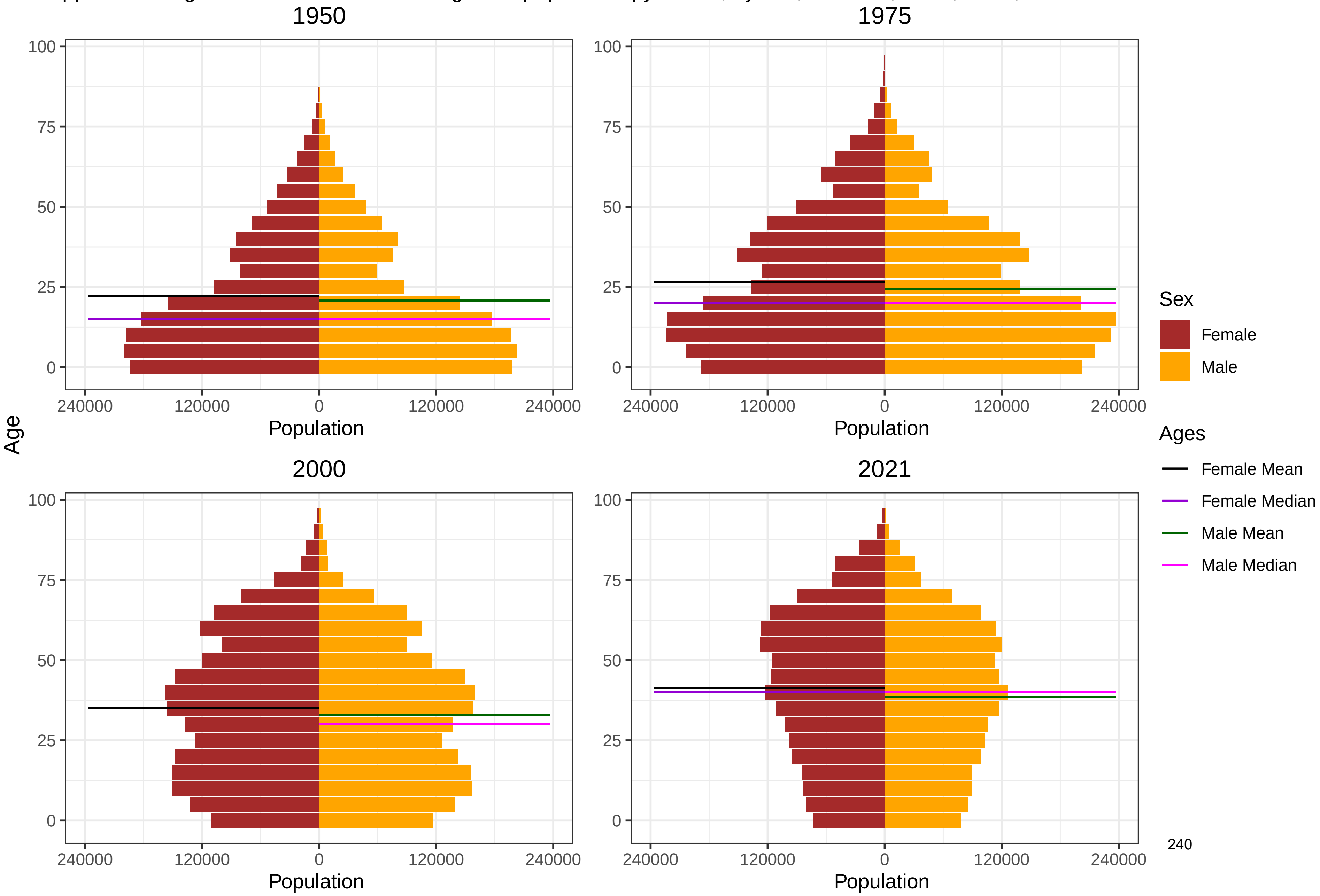

Appendix 2 Figure S9: Botswana population pyramids, by sex, for 1950, 1975, 2000, and 2021

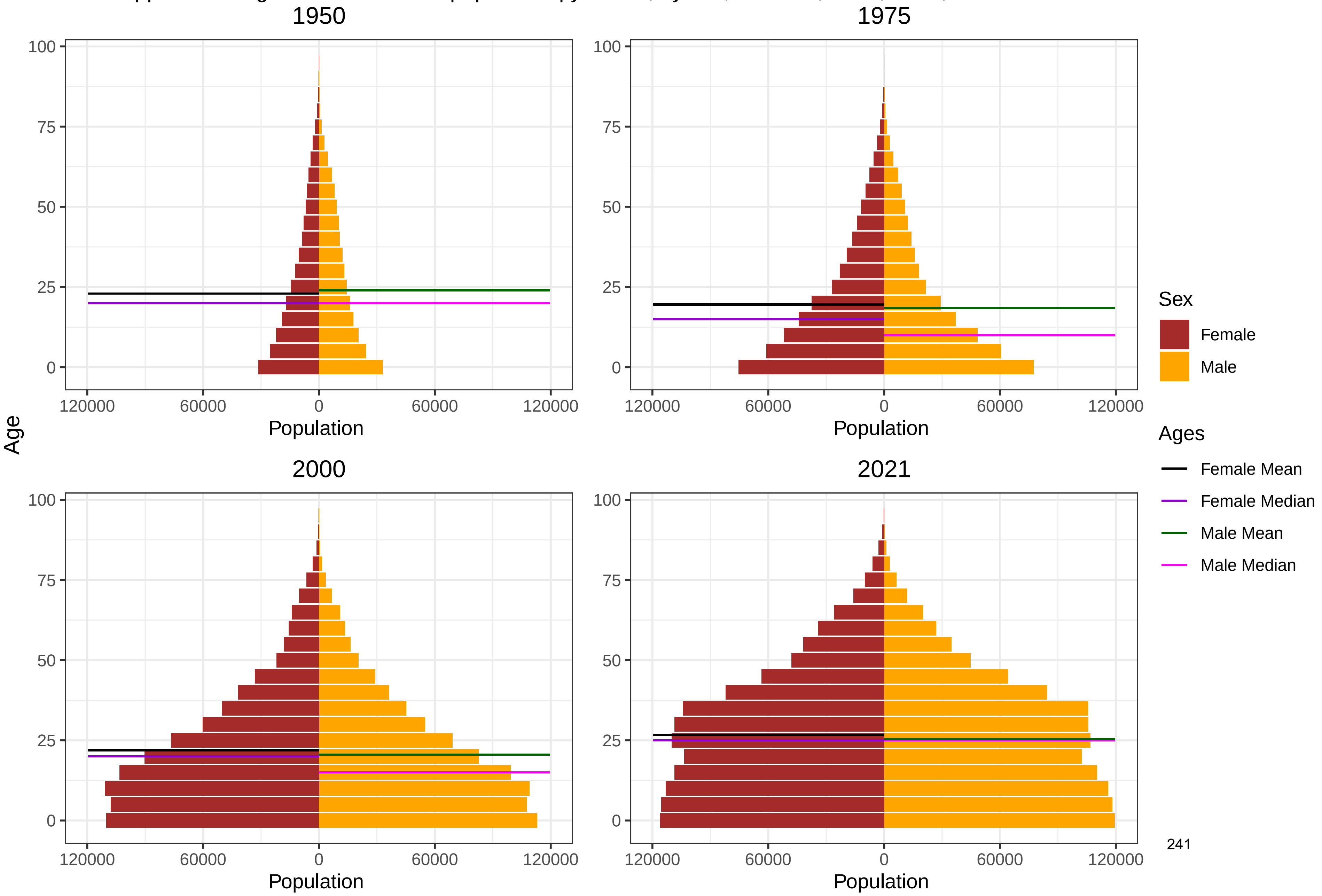

Appendix 2 Figure S9: Brazil population pyramids, by sex, for 1950, 1975, 2000, and 2021

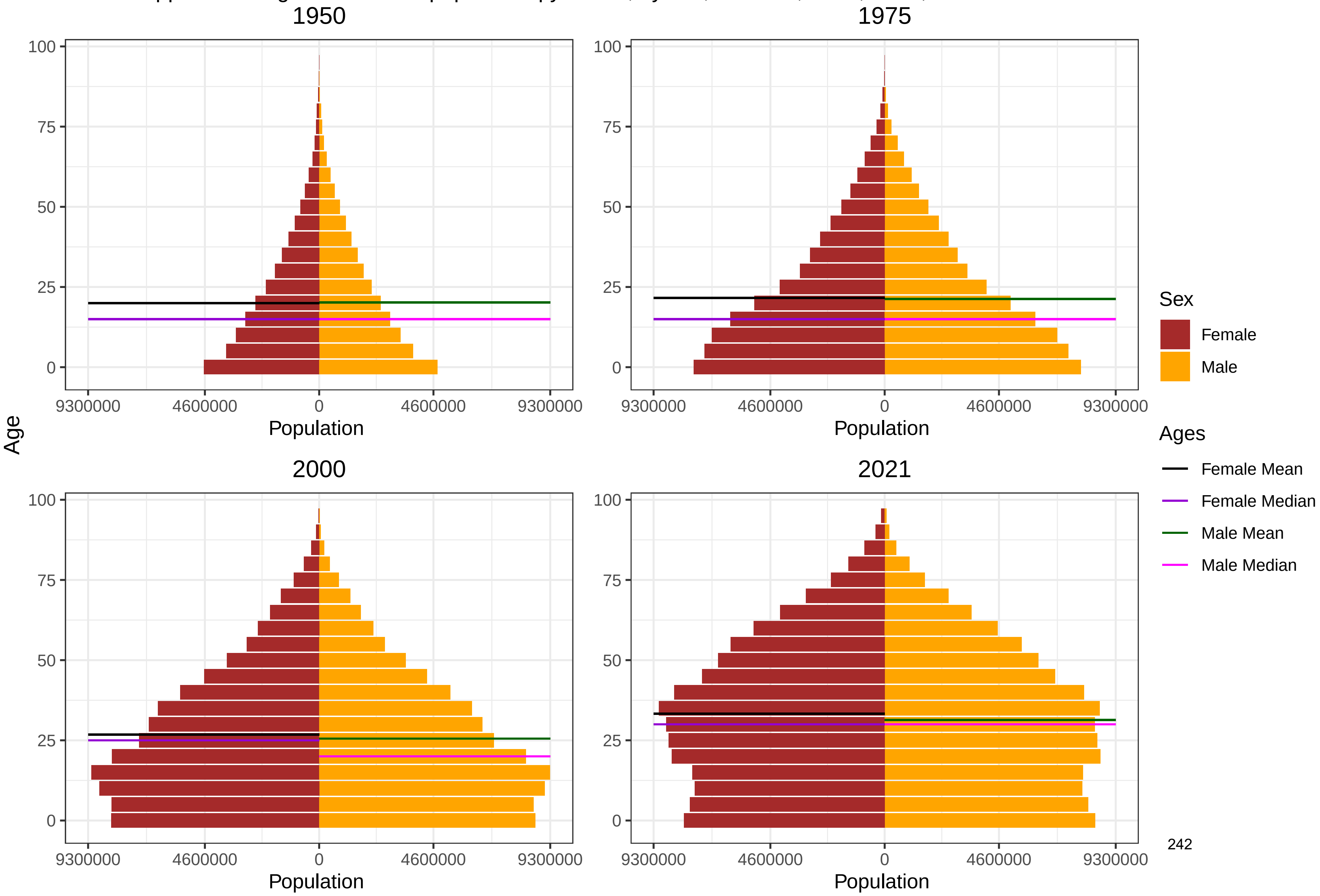

Appendix 2 Figure S9: Brunei population pyramids, by sex, for 1950, 1975, 2000, and 2021

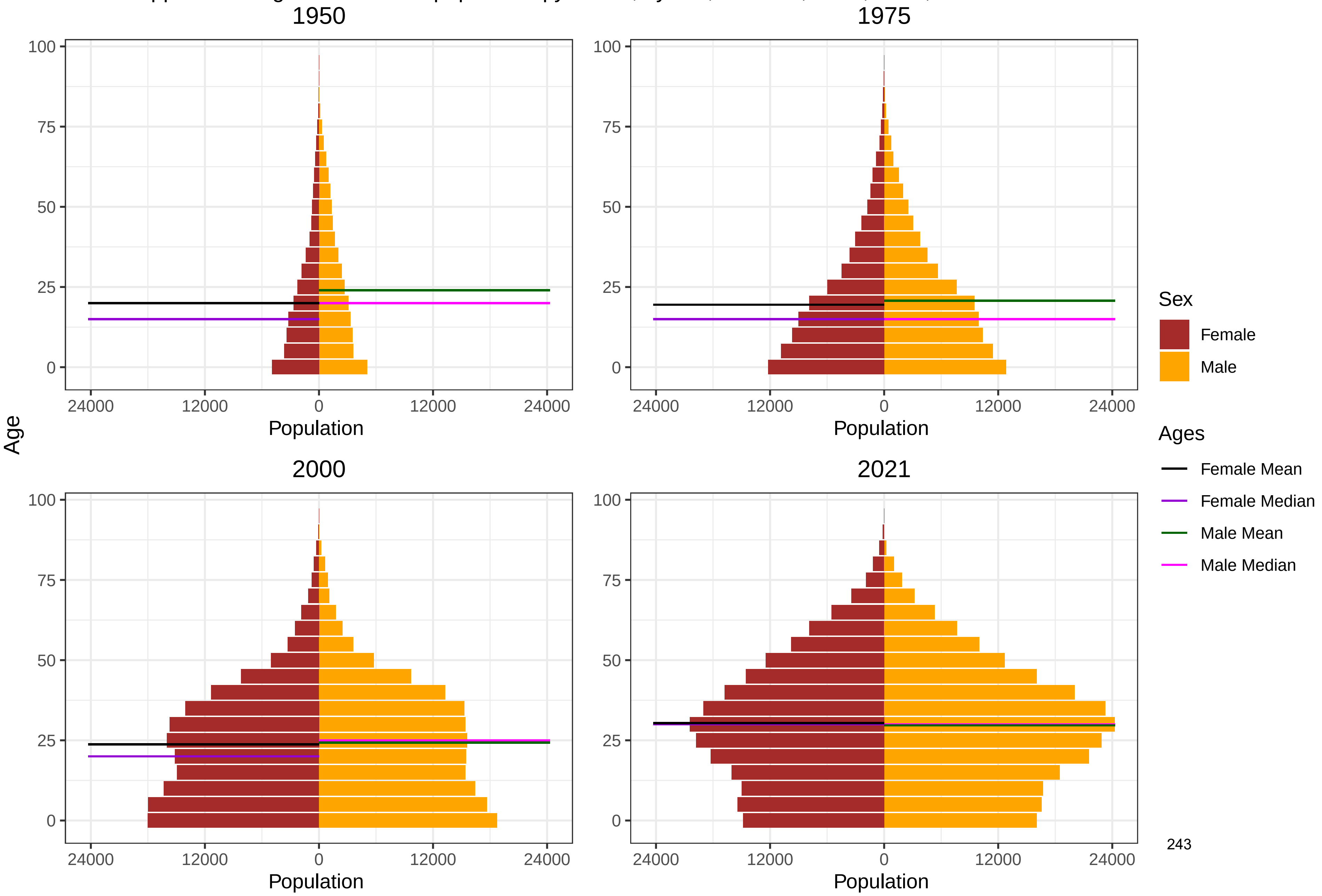

Appendix 2 Figure S9: Bulgaria population pyramids, by sex, for 1950, 1975, 2000, and 2021

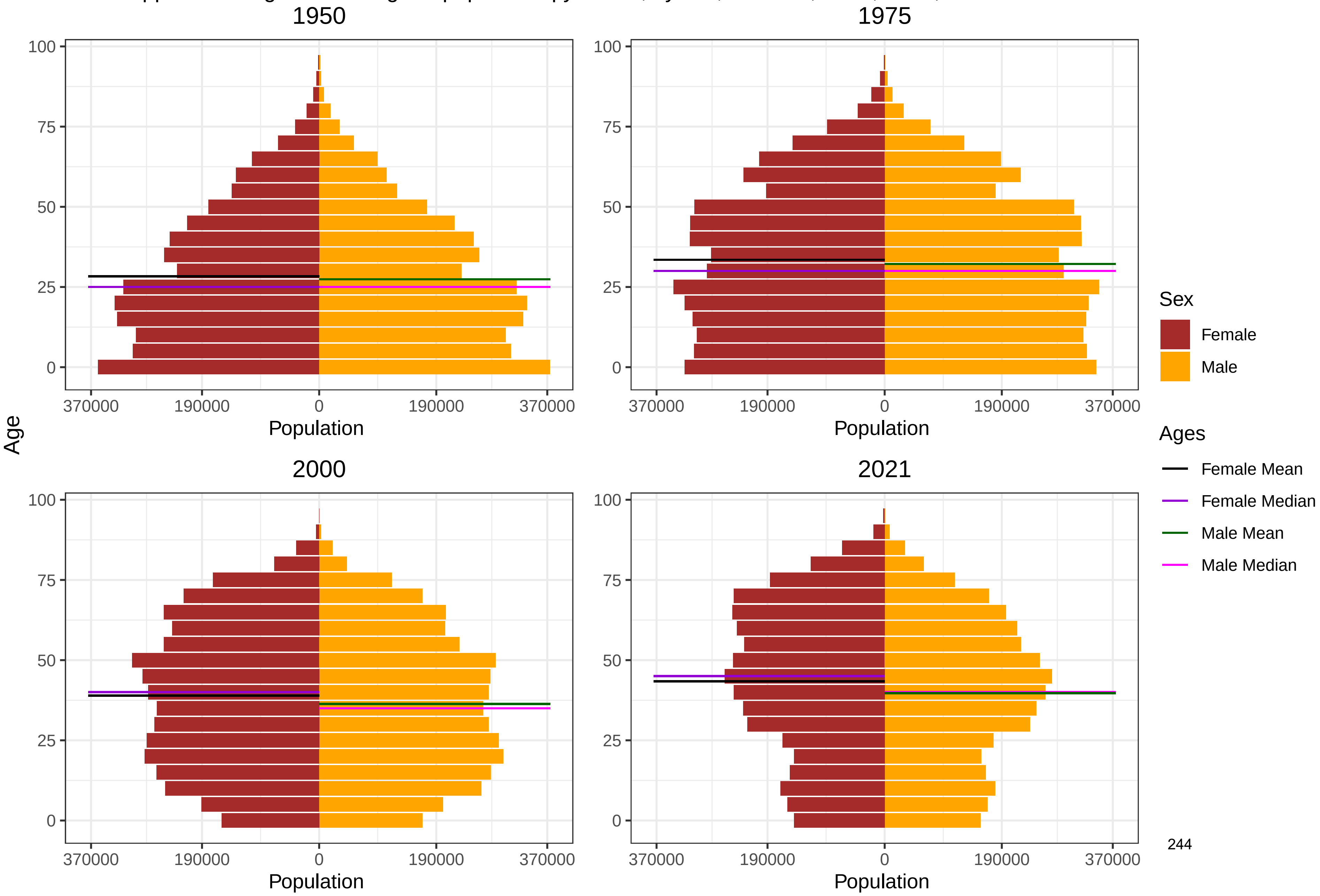

Appendix 2 Figure S9: Burkina Faso population pyramids, by sex, for 1950, 1975, 2000, and 2021

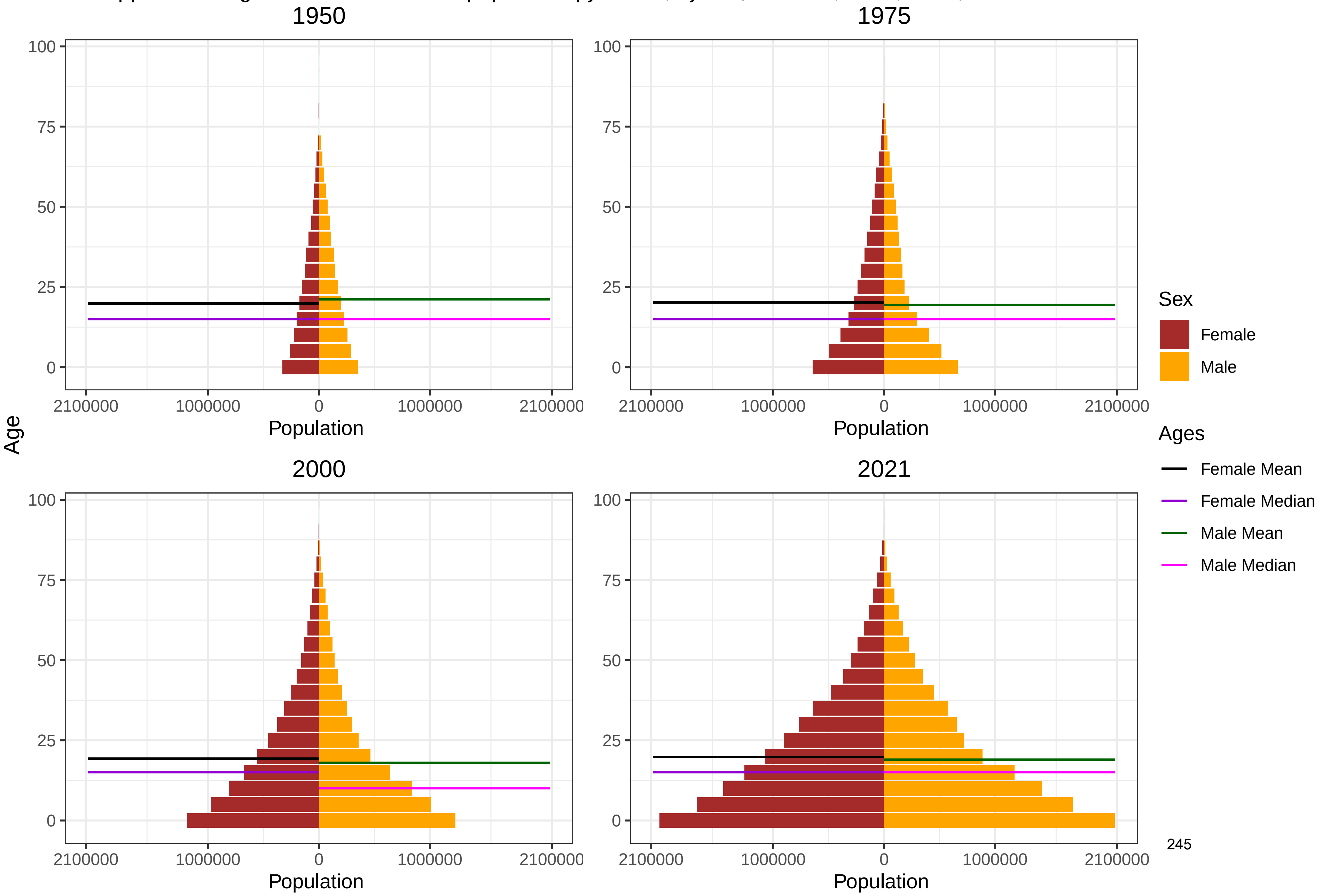

Appendix 2 Figure S9: Burundi population pyramids, by sex, for 1950, 1975, 2000, and 2021

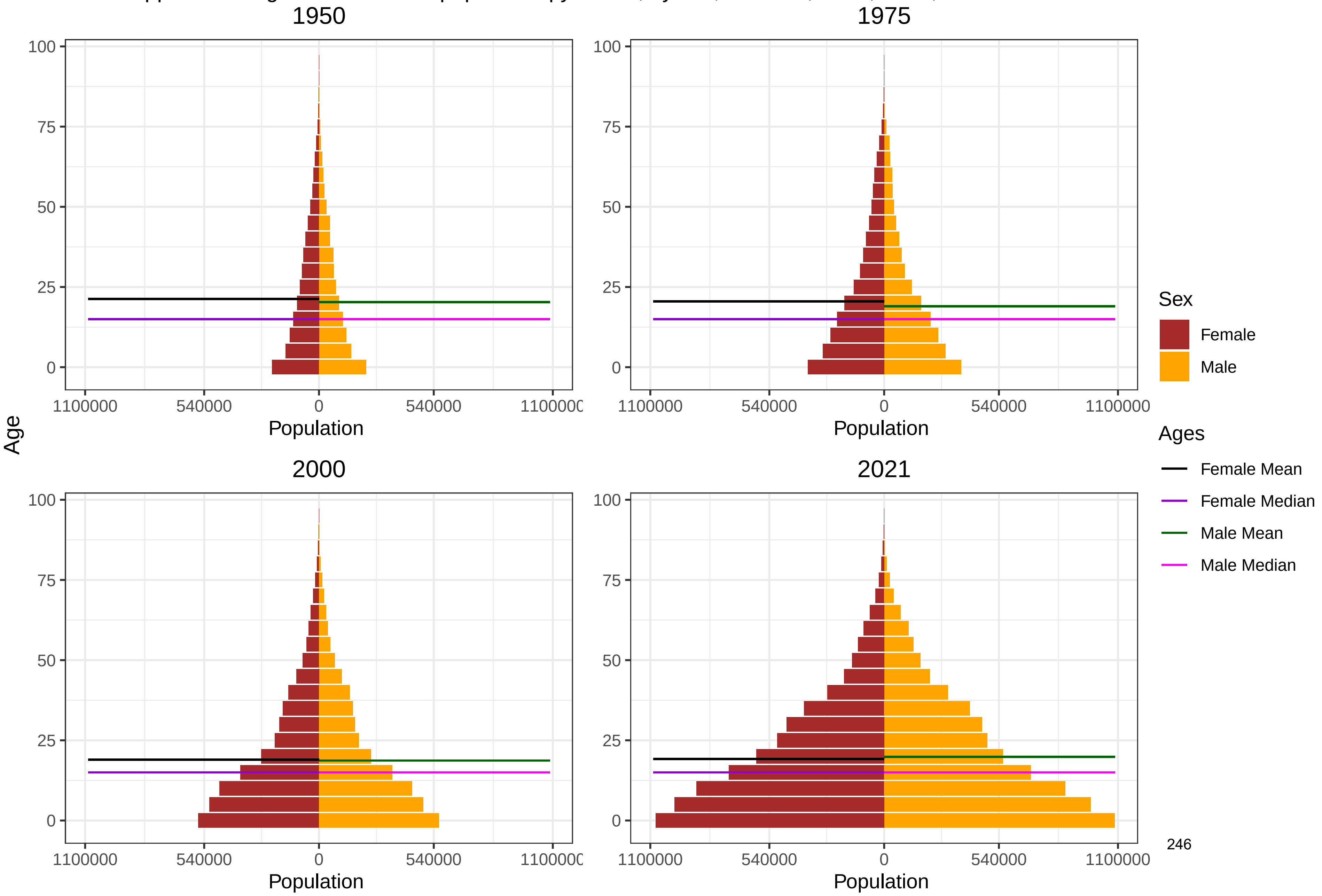

Appendix 2 Figure S9: Cabo Verde population pyramids, by sex, for 1950, 1975, 2000, and 2021

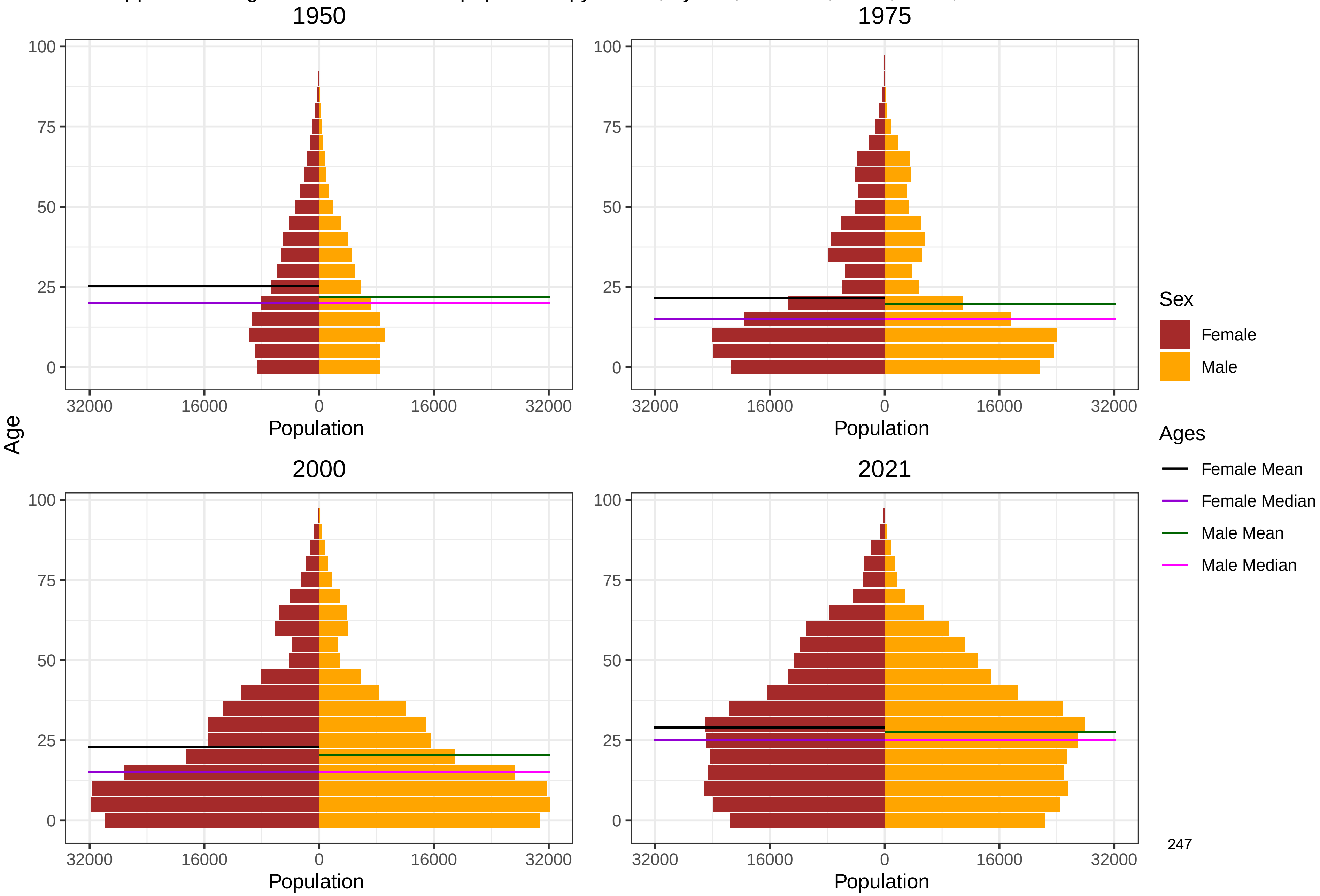

Appendix 2 Figure S9: Cambodia population pyramids, by sex, for 1950, 1975, 2000, and 2021

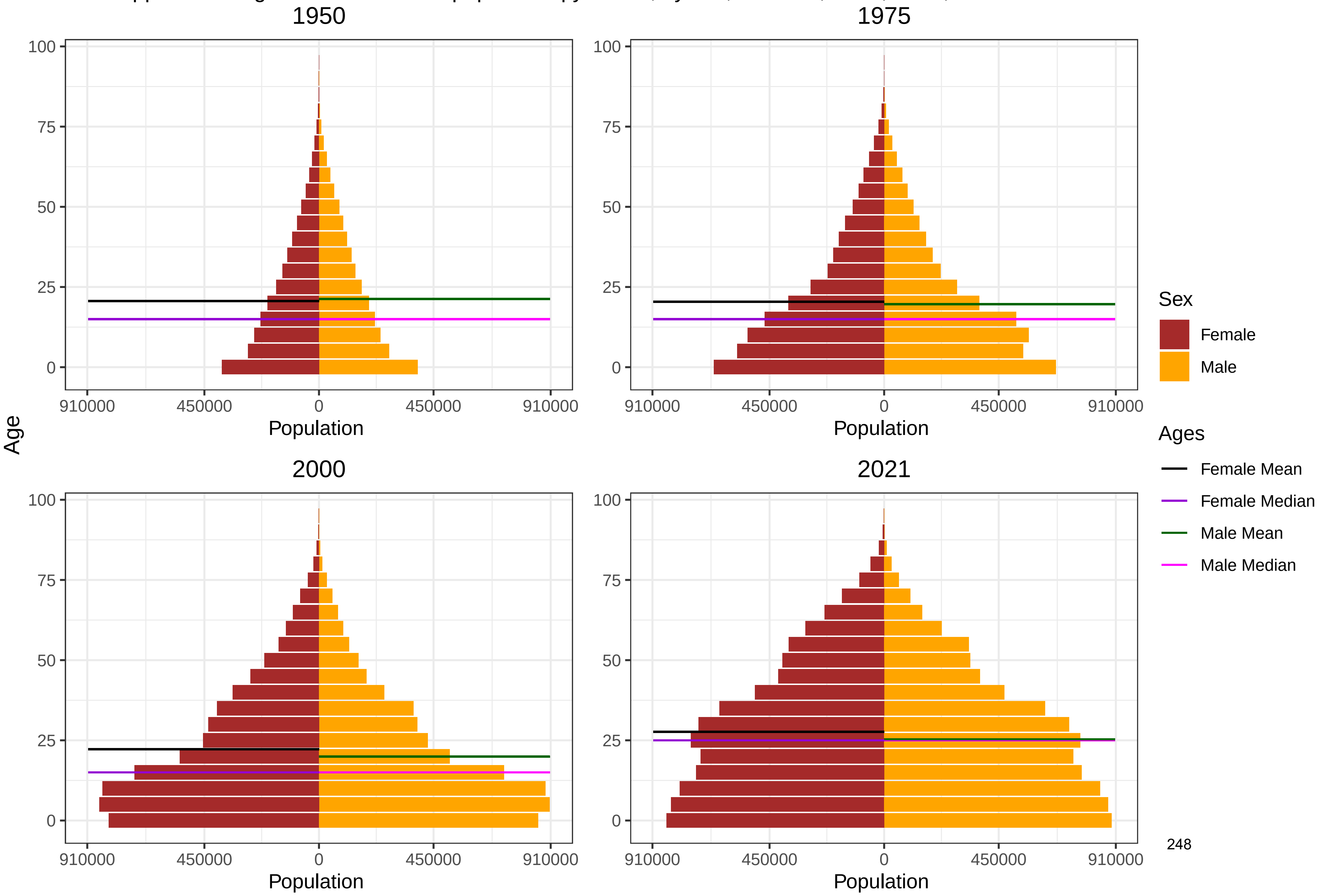

Appendix 2 Figure S9: Cameroon population pyramids, by sex, for 1950, 1975, 2000, and 2021

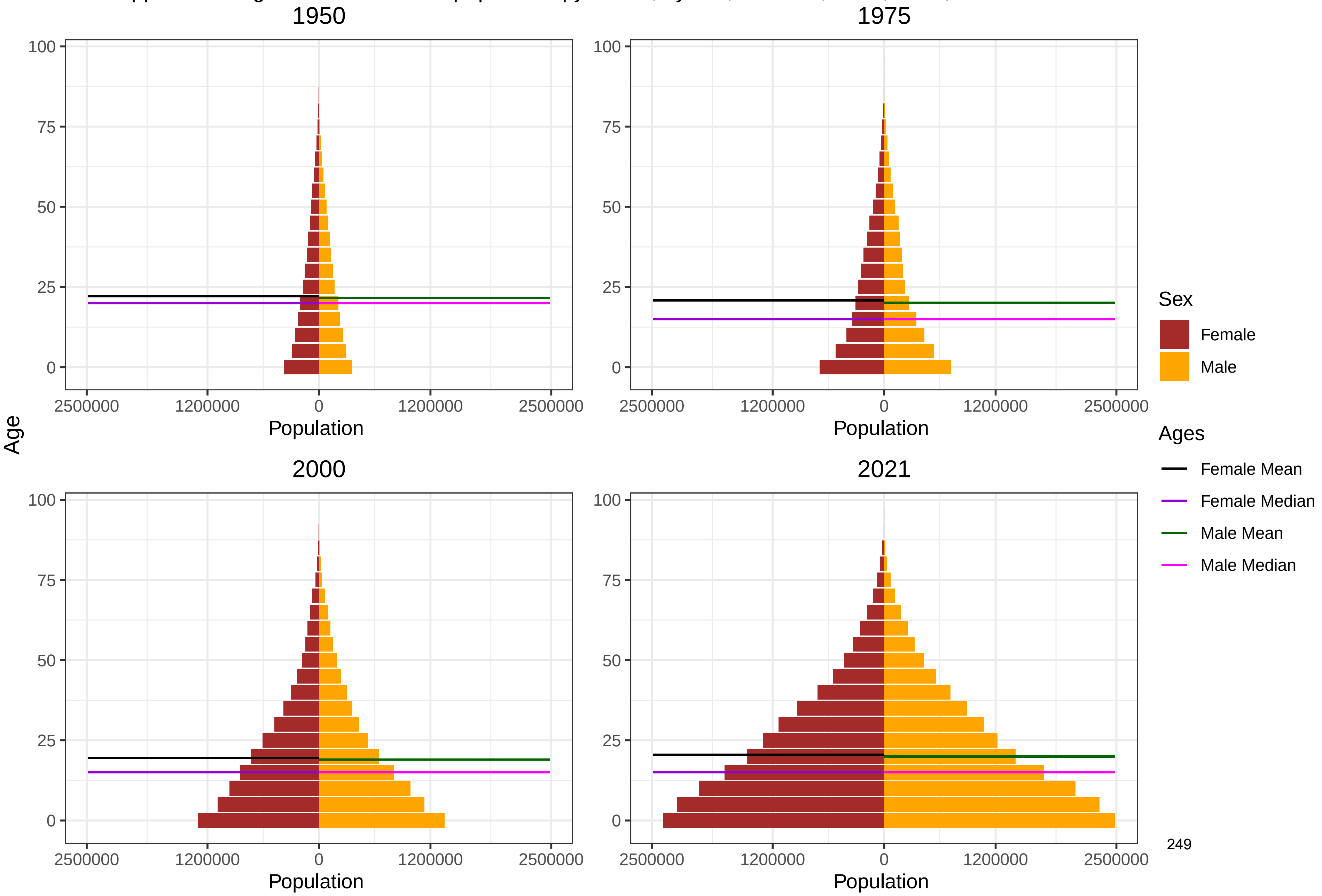

Appendix 2 Figure S9: Canada population pyramids, by sex, for 1950, 1975, 2000, and 2021

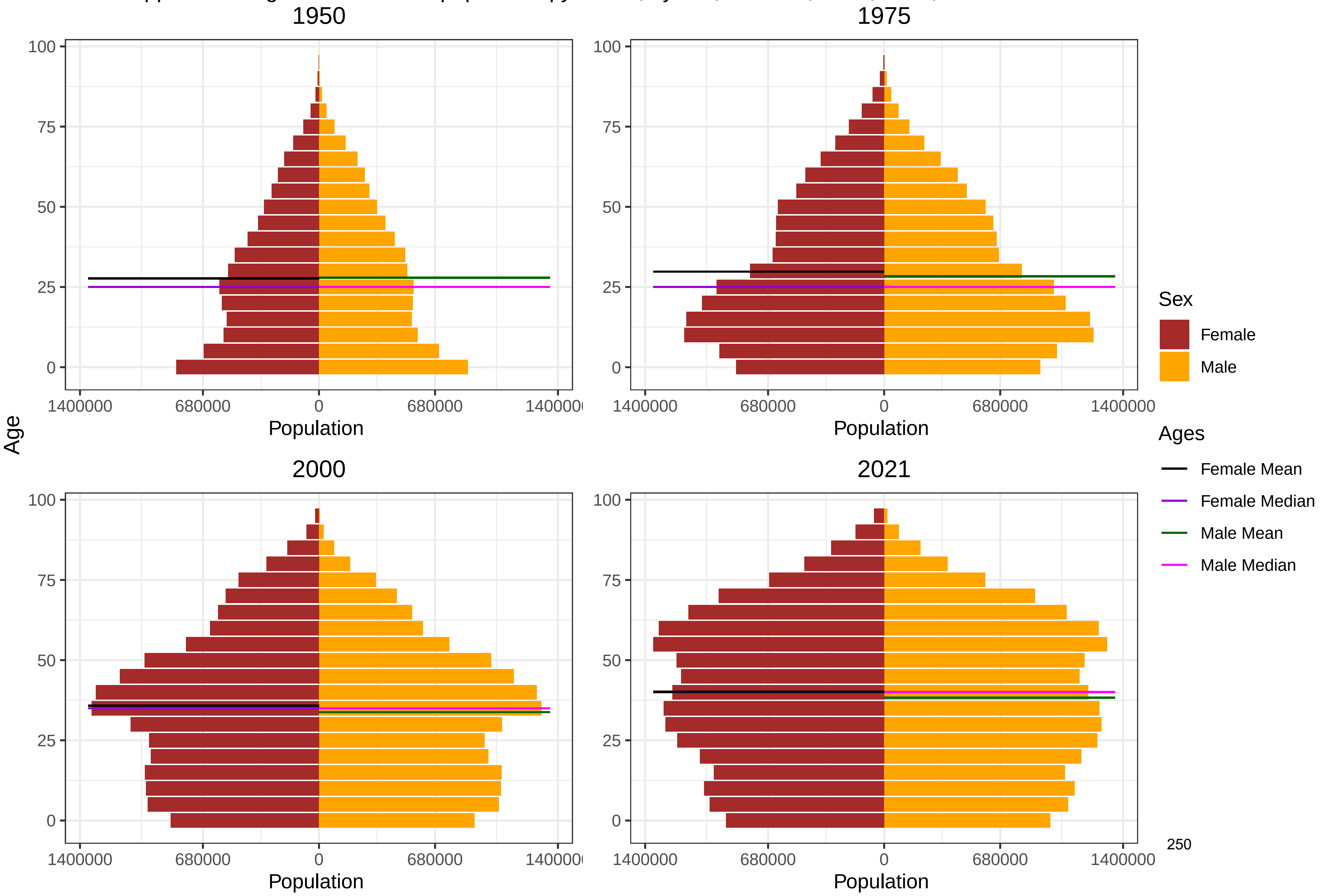

Appendix 2 Figure S9: Central African Republic population pyramids, by sex, for 1950, 1975, 2000, and 2021

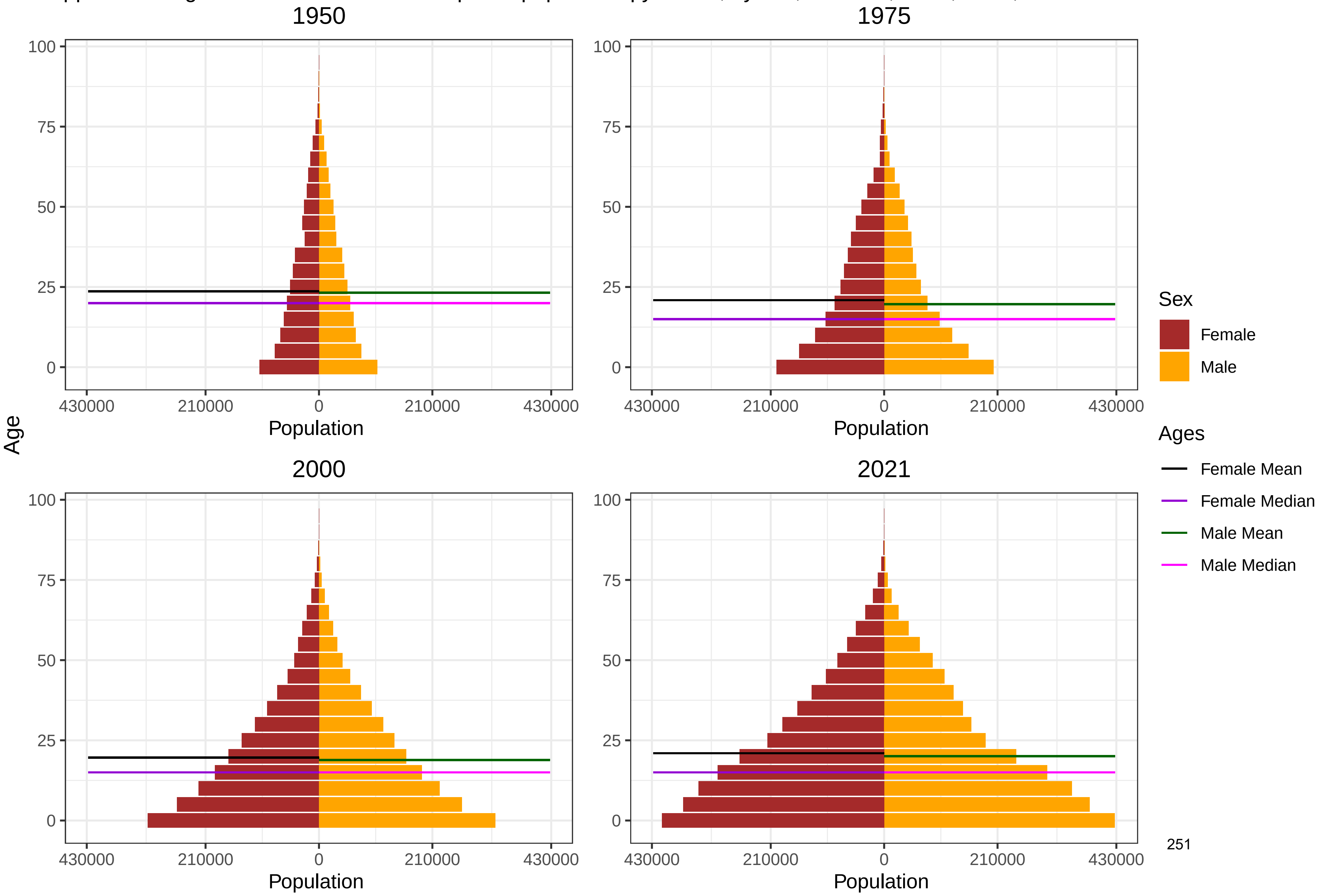

Appendix 2 Figure S9: Chad population pyramids, by sex, for 1950, 1975, 2000, and 2021

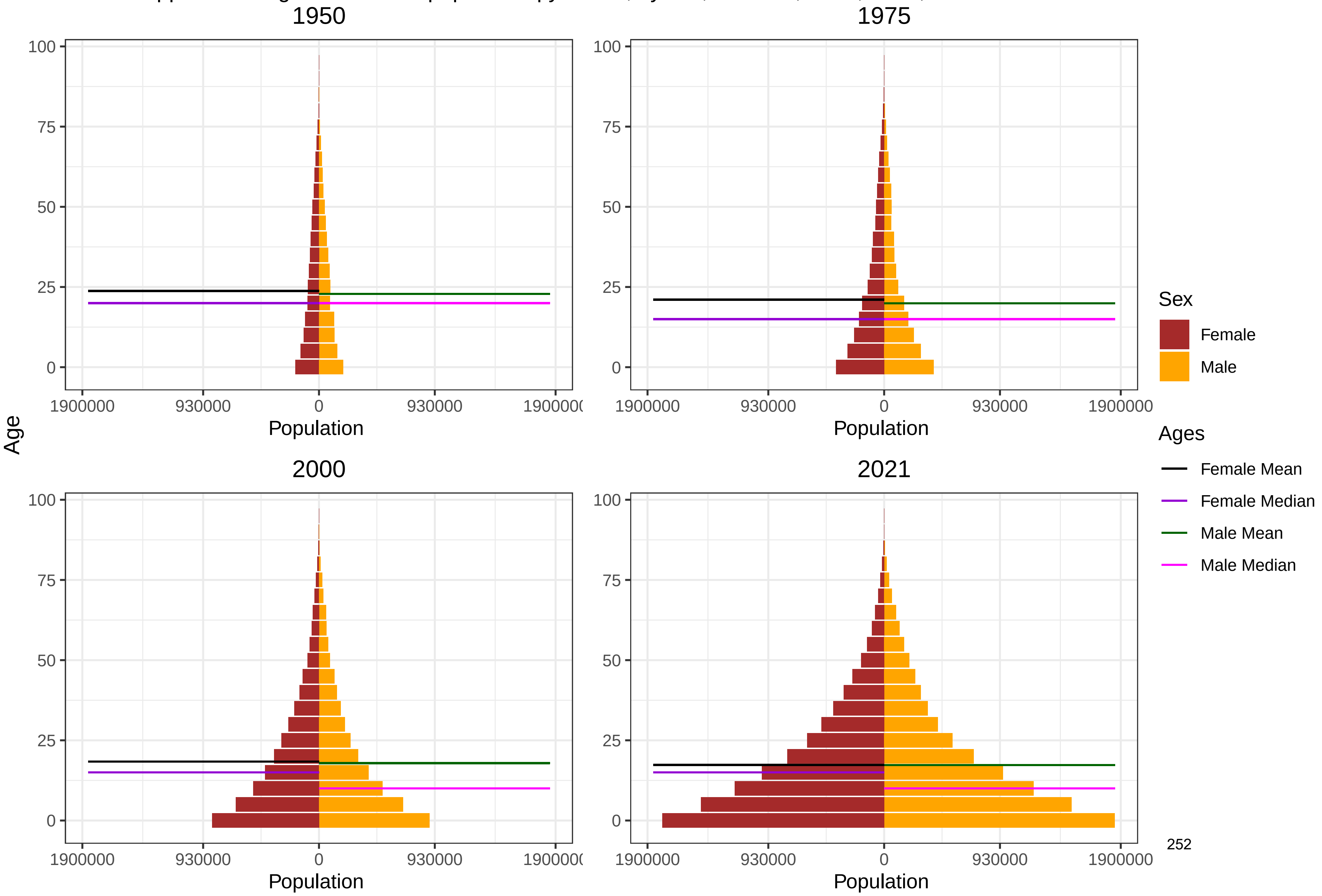

Appendix 2 Figure S9: Chile population pyramids, by sex, for 1950, 1975, 2000, and 2021

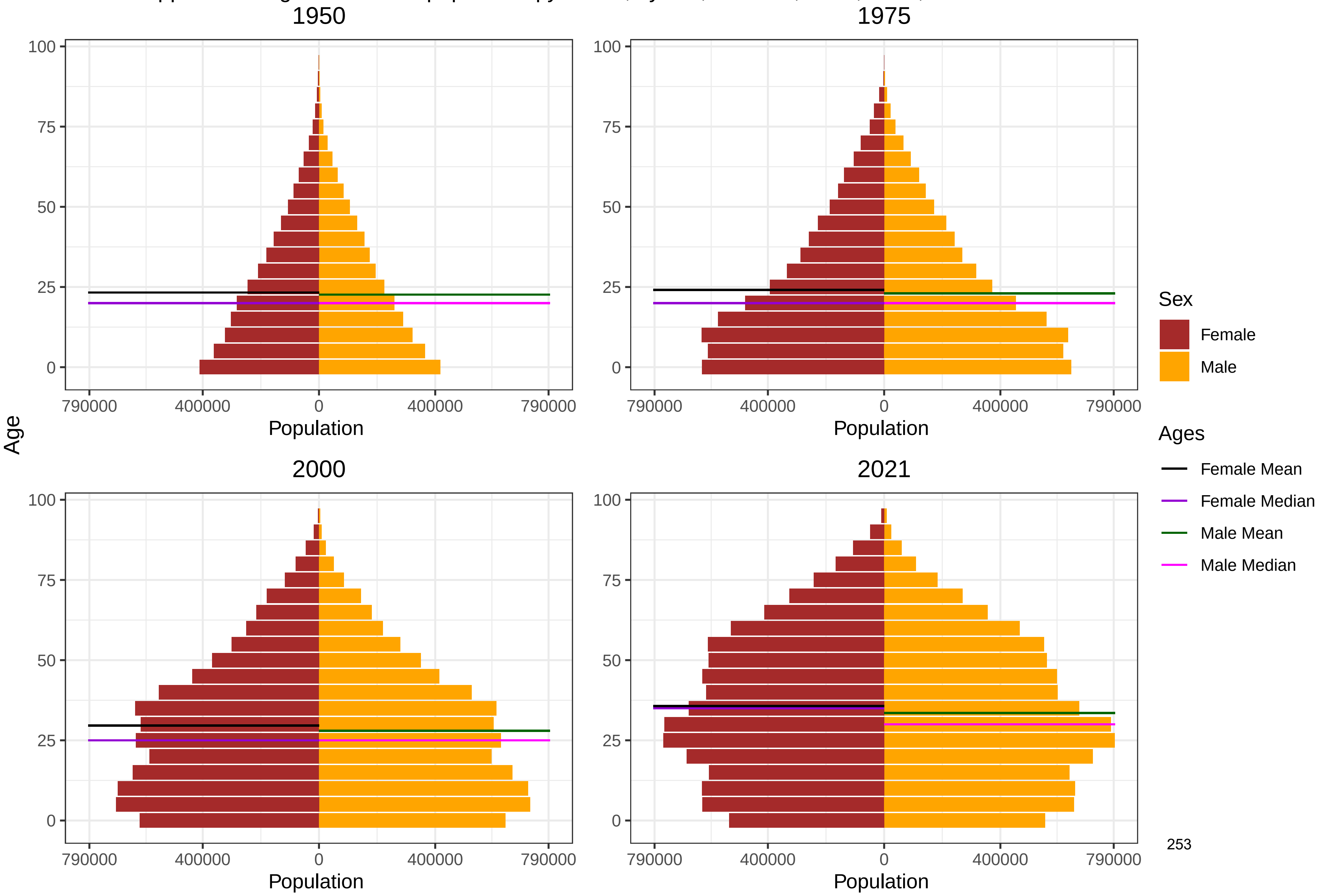

Appendix 2 Figure S9: China population pyramids, by sex, for 1950, 1975, 2000, and 2021

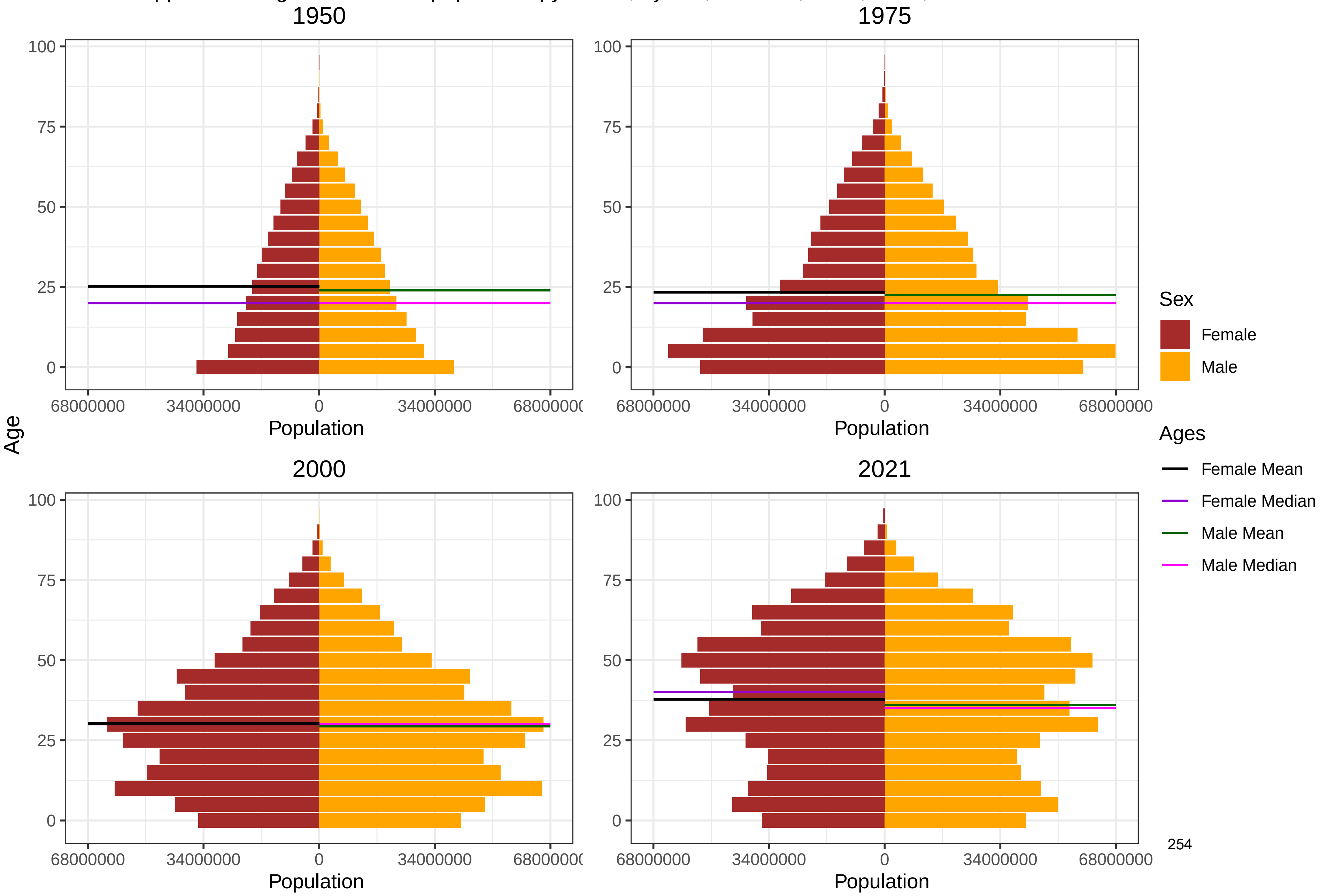

Appendix 2 Figure S9: Colombia population pyramids, by sex, for 1950, 1975, 2000, and 2021

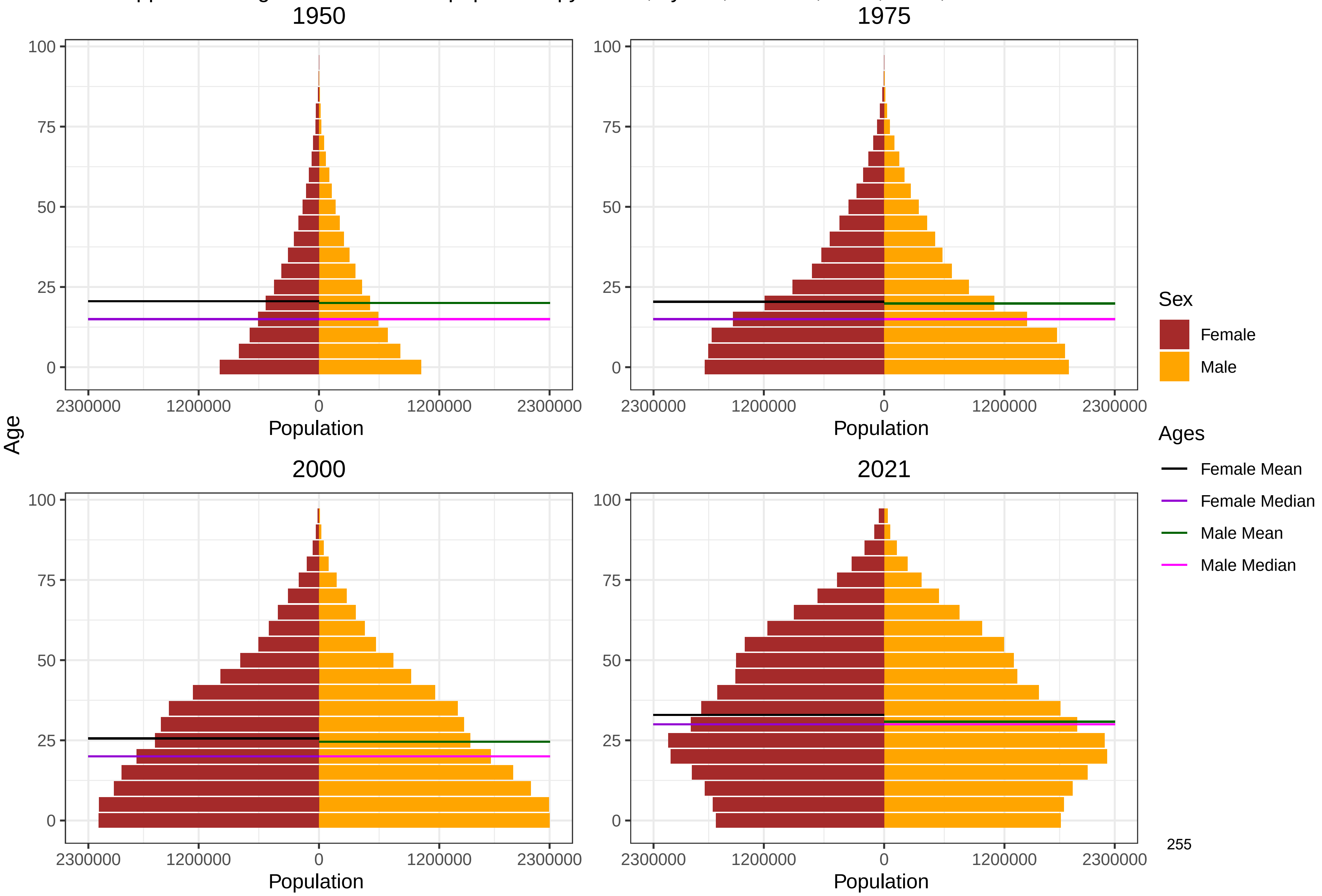

Appendix 2 Figure S9: Comoros population pyramids, by sex, for 1950, 1975, 2000, and 2021

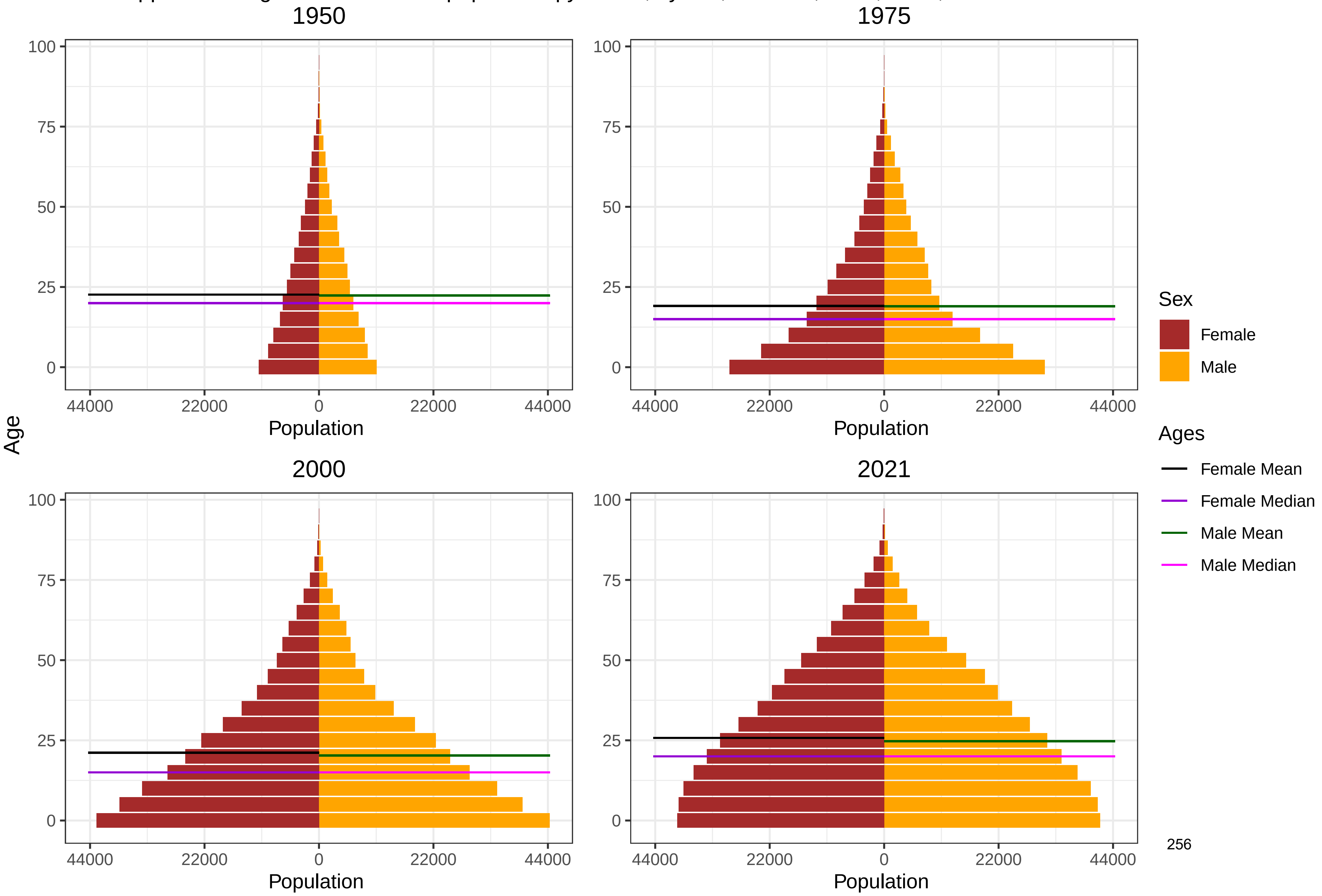

Appendix 2 Figure S9: Congo (Brazzaville) population pyramids, by sex, for 1950, 1975, 2000, and 2021

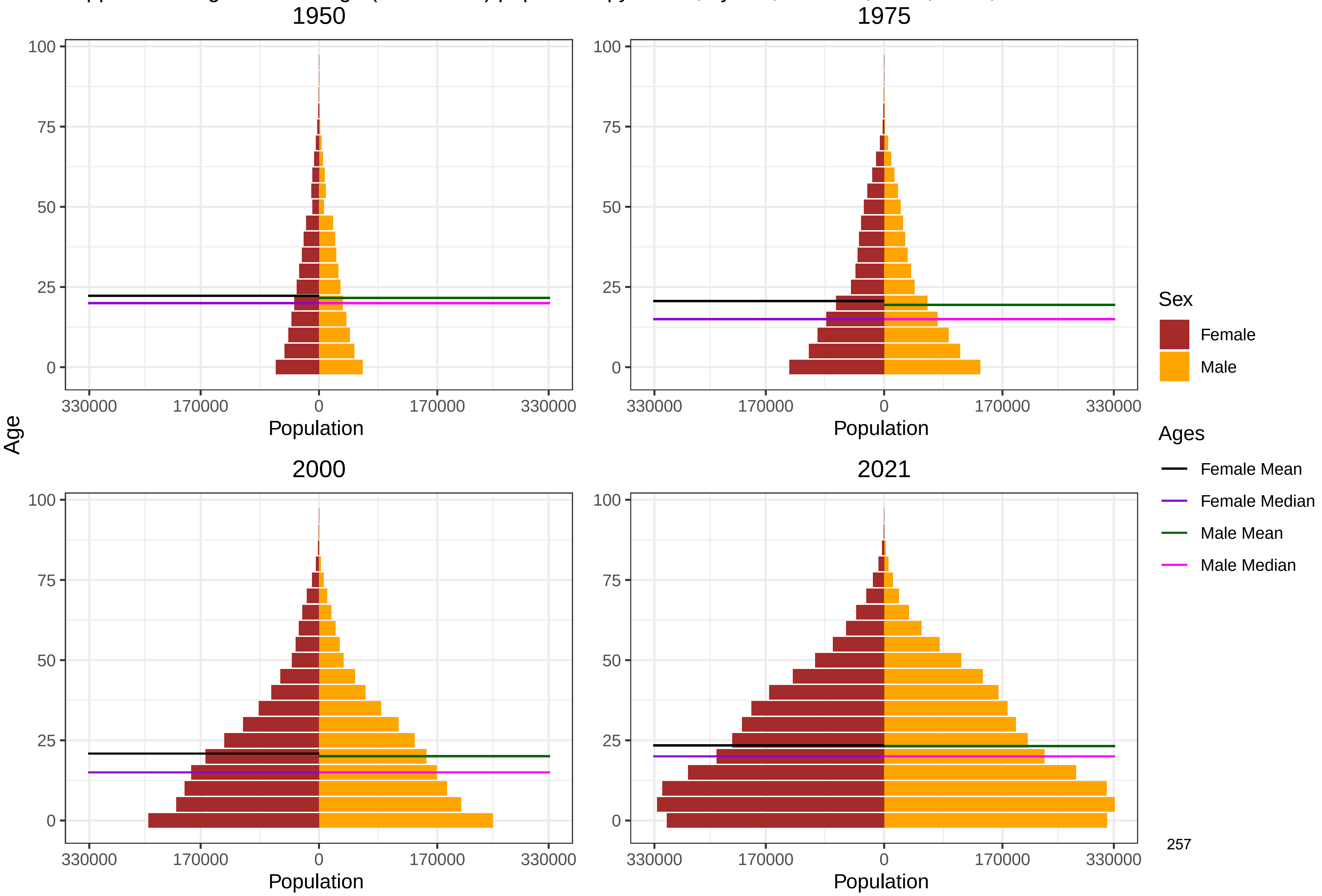

Appendix 2 Figure S9: Cook Islands population pyramids, by sex, for 1950, 1975, 2000, and 2021

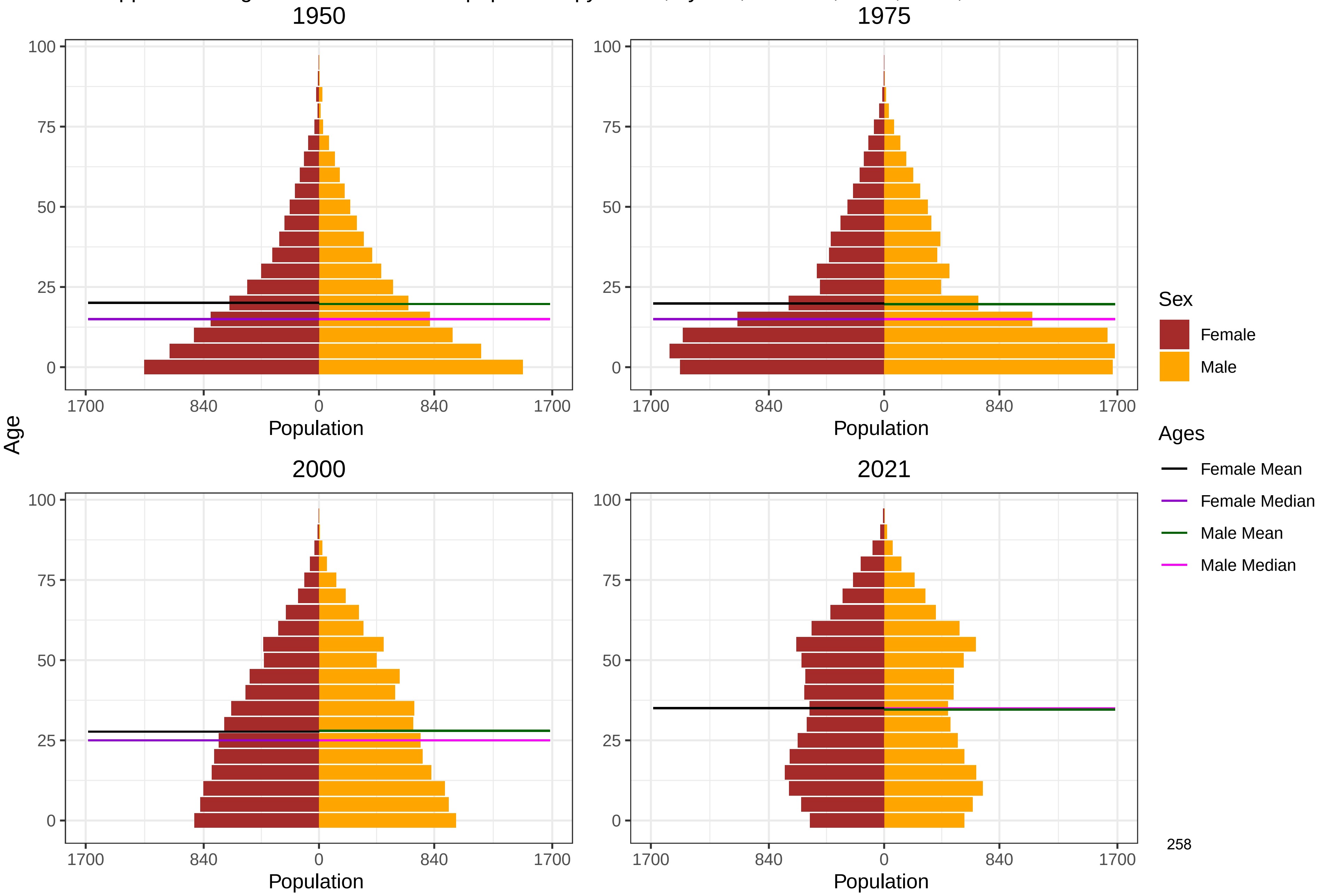

Appendix 2 Figure S9: Costa Rica population pyramids, by sex, for 1950, 1975, 2000, and 2021

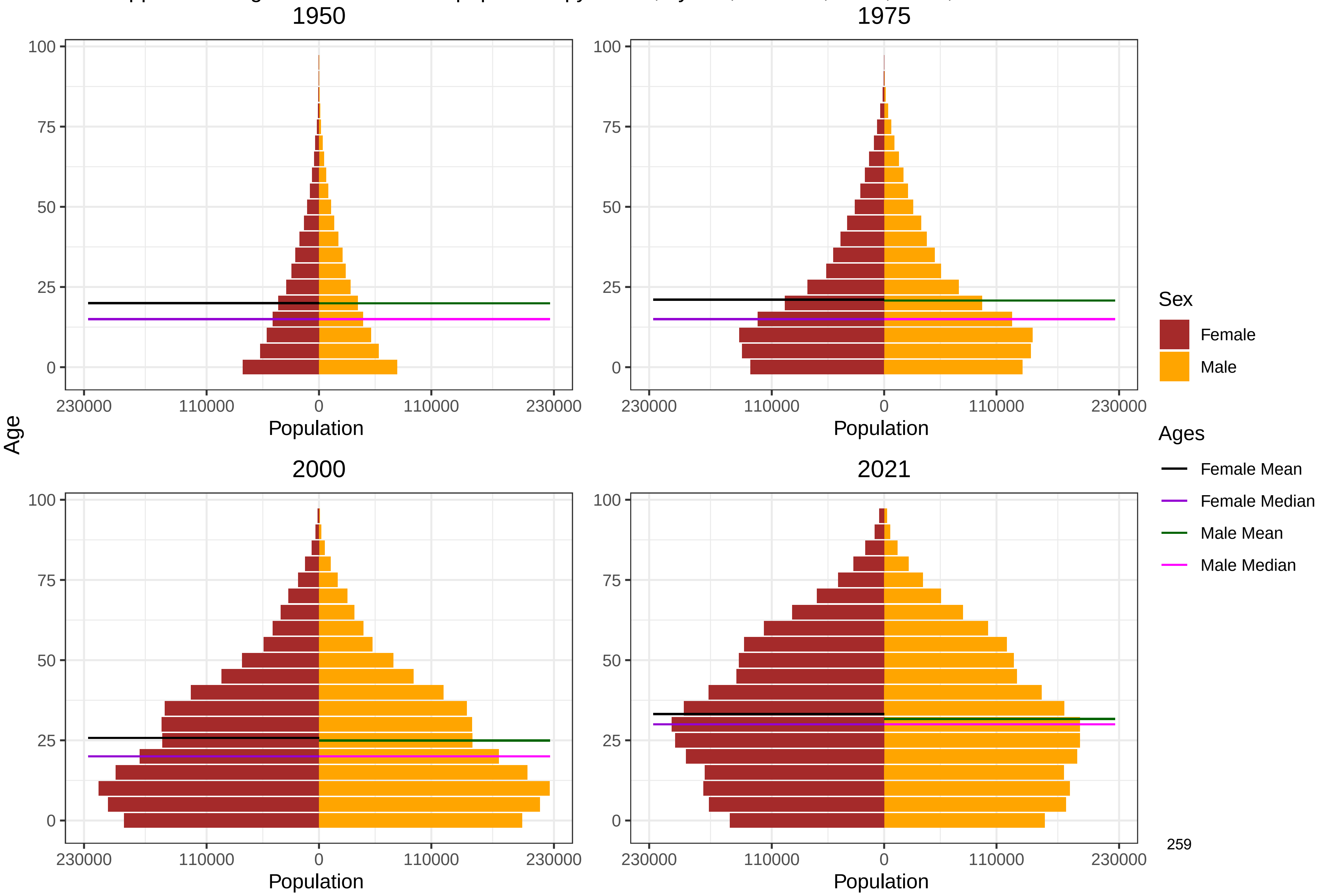

Appendix 2 Figure S9: Croatia population pyramids, by sex, for 1950, 1975, 2000, and 2021

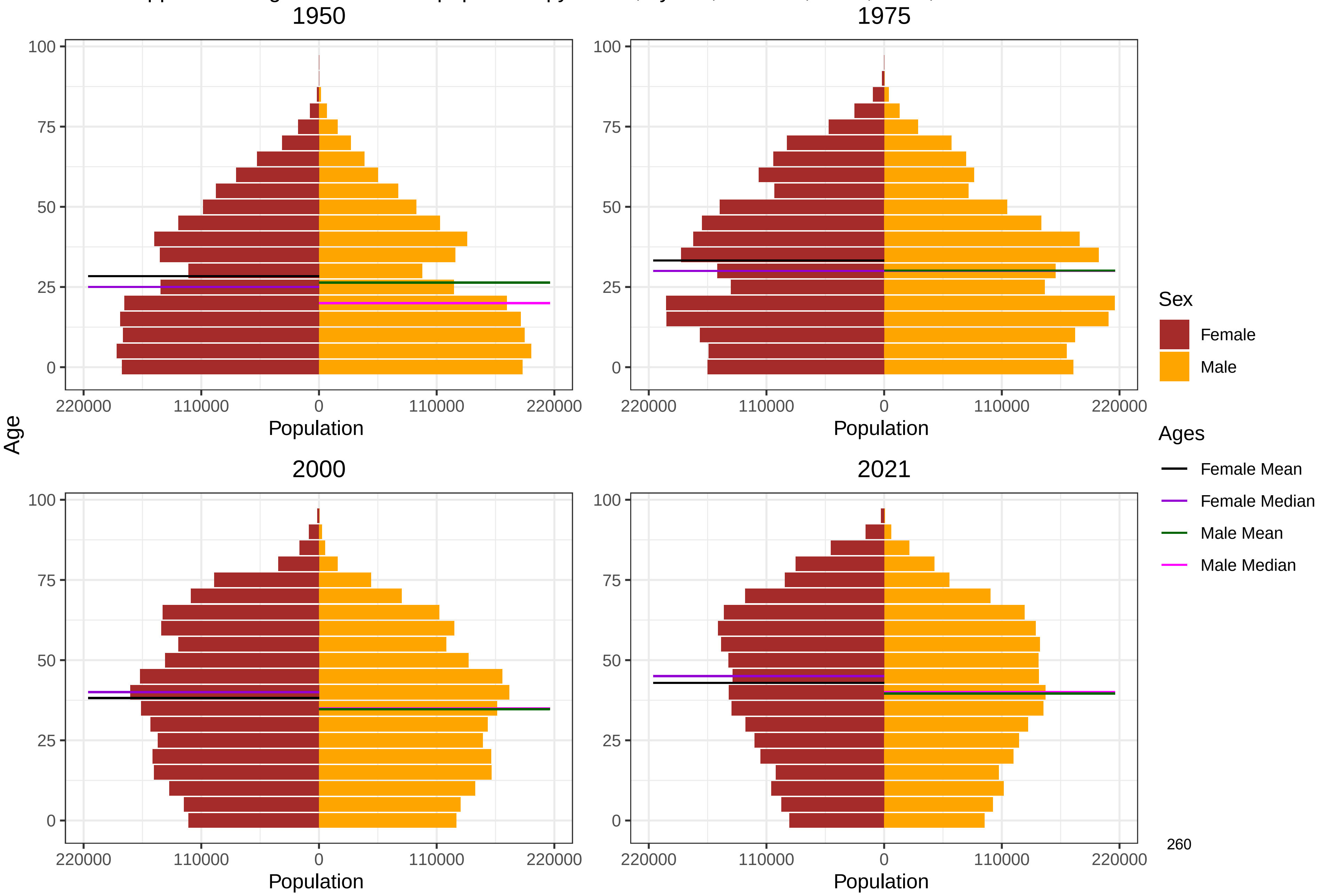

Appendix 2 Figure S9: Cuba population pyramids, by sex, for 1950, 1975, 2000, and 2021

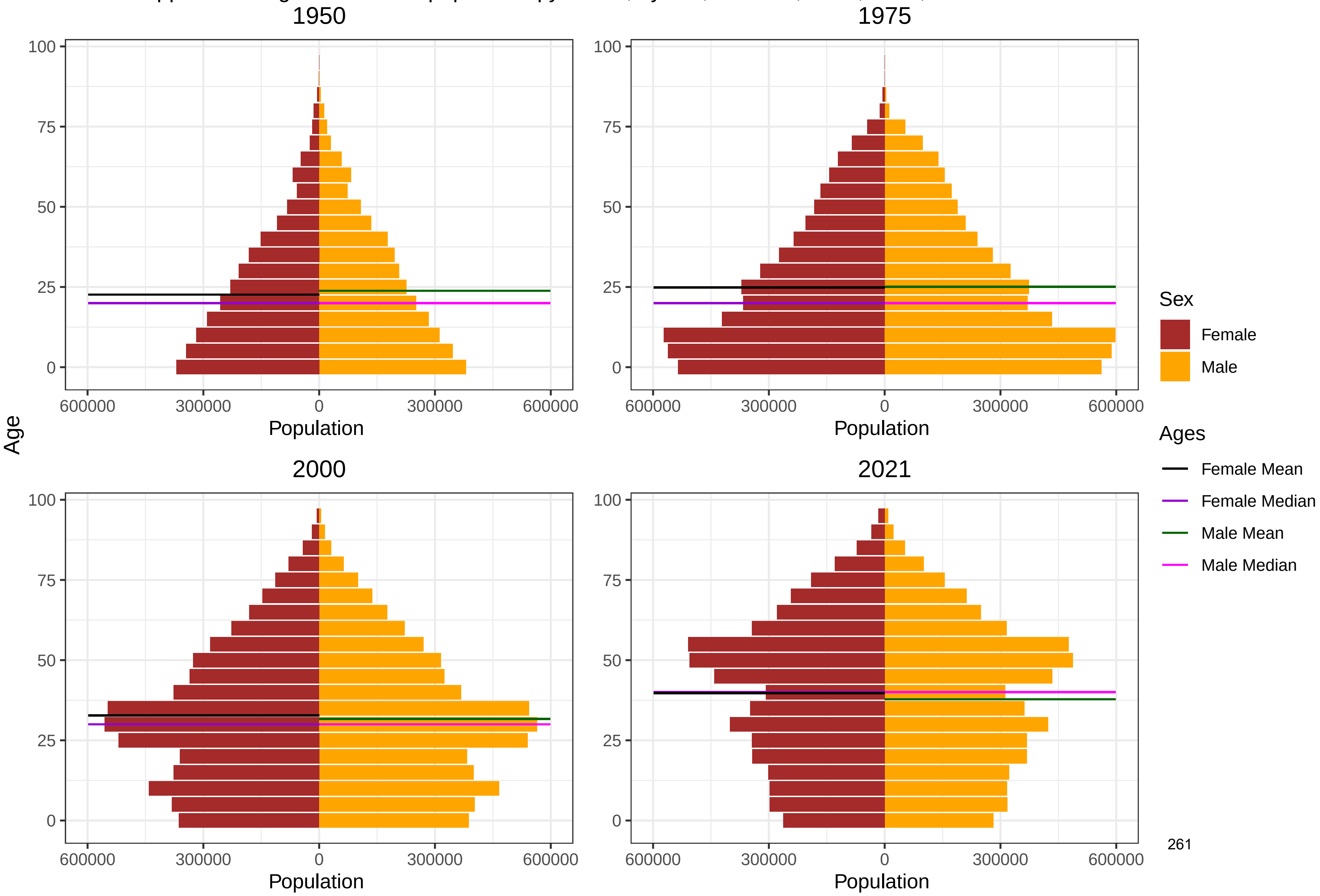

Appendix 2 Figure S9: Cyprus population pyramids, by sex, for 1950, 1975, 2000, and 2021

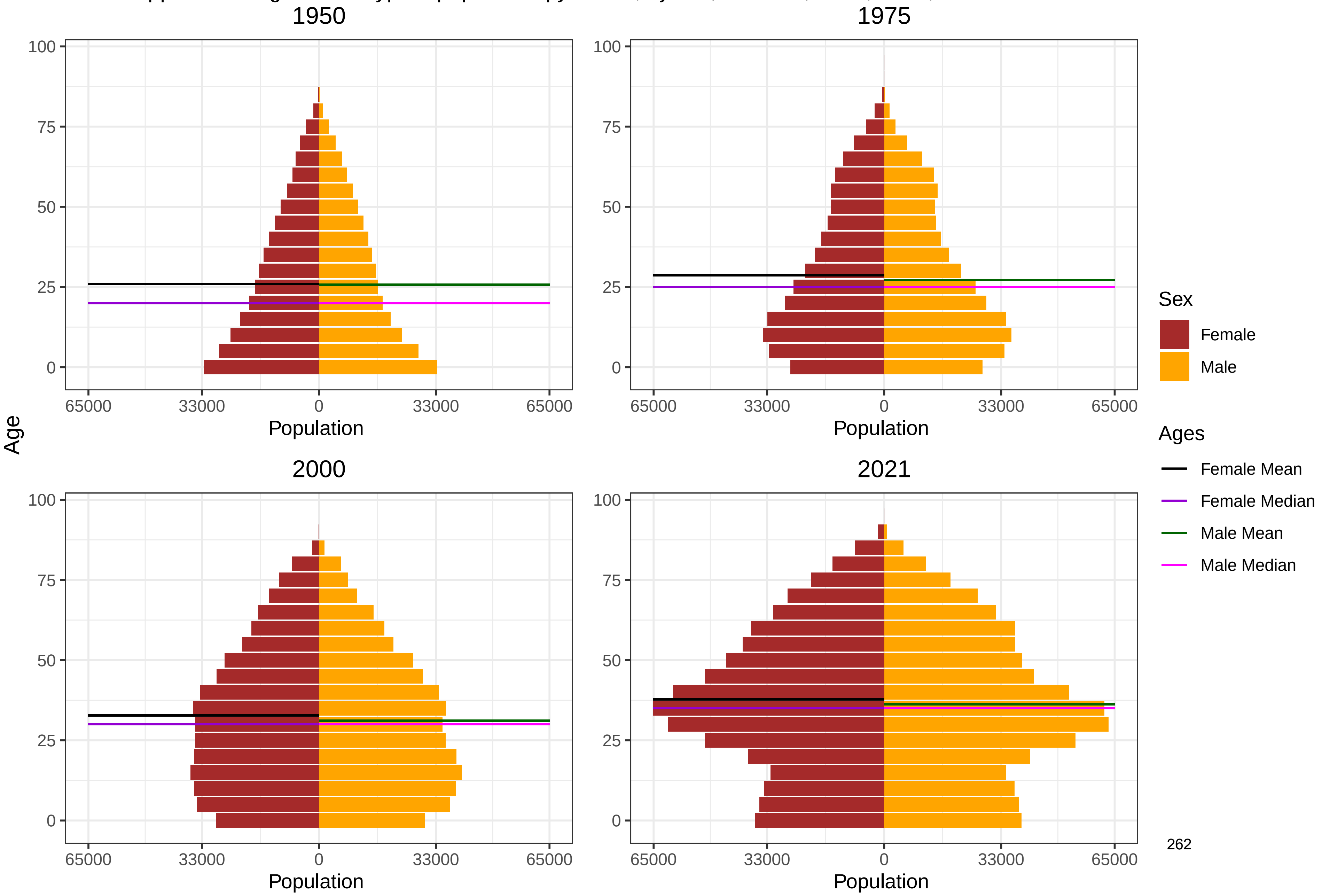

Appendix 2 Figure S9: Czechia population pyramids, by sex, for 1950, 1975, 2000, and 2021

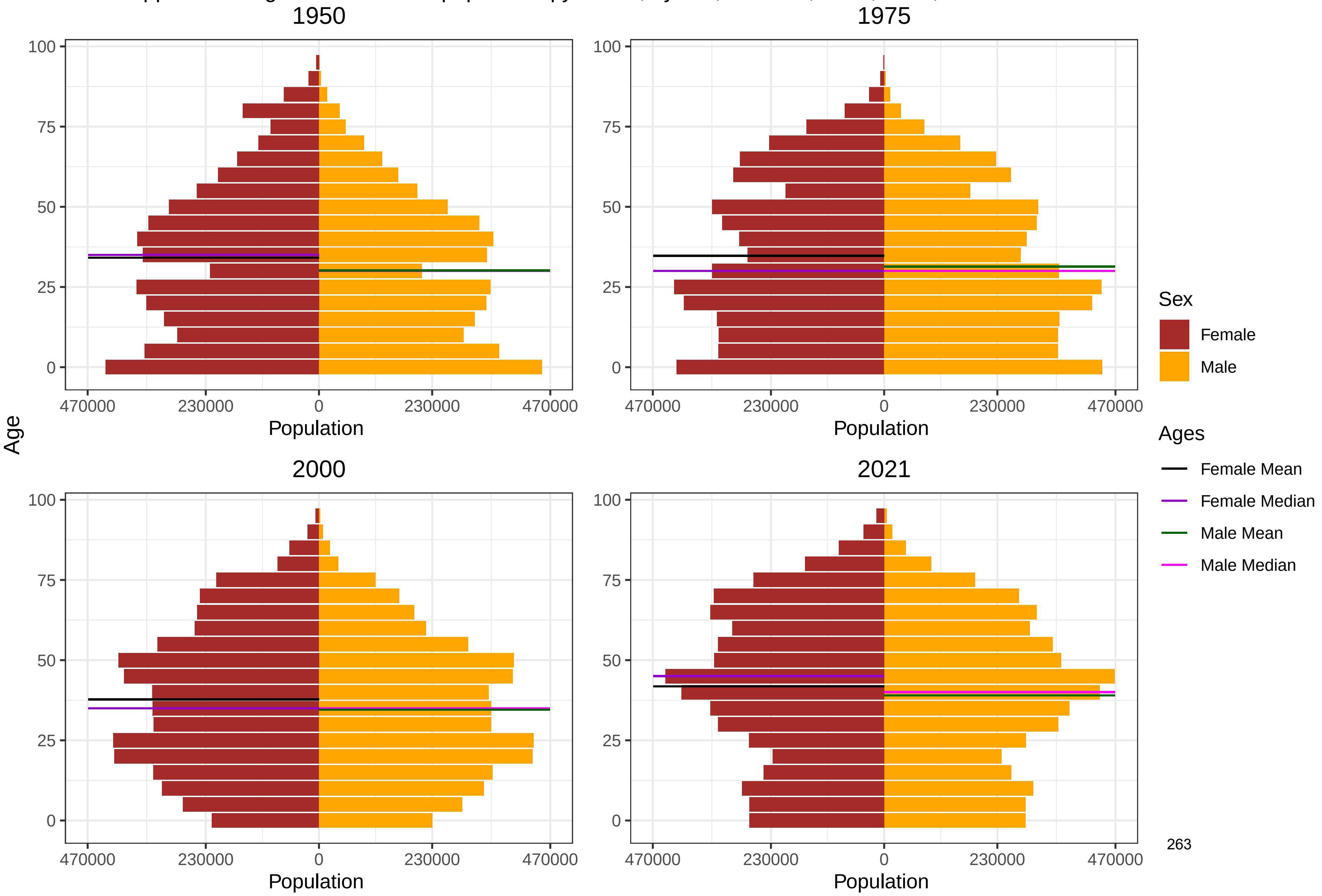

Appendix 2 Figure S9: Côte d'Ivoire population pyramids, by sex, for 1950, 1975, 2000, and 2021

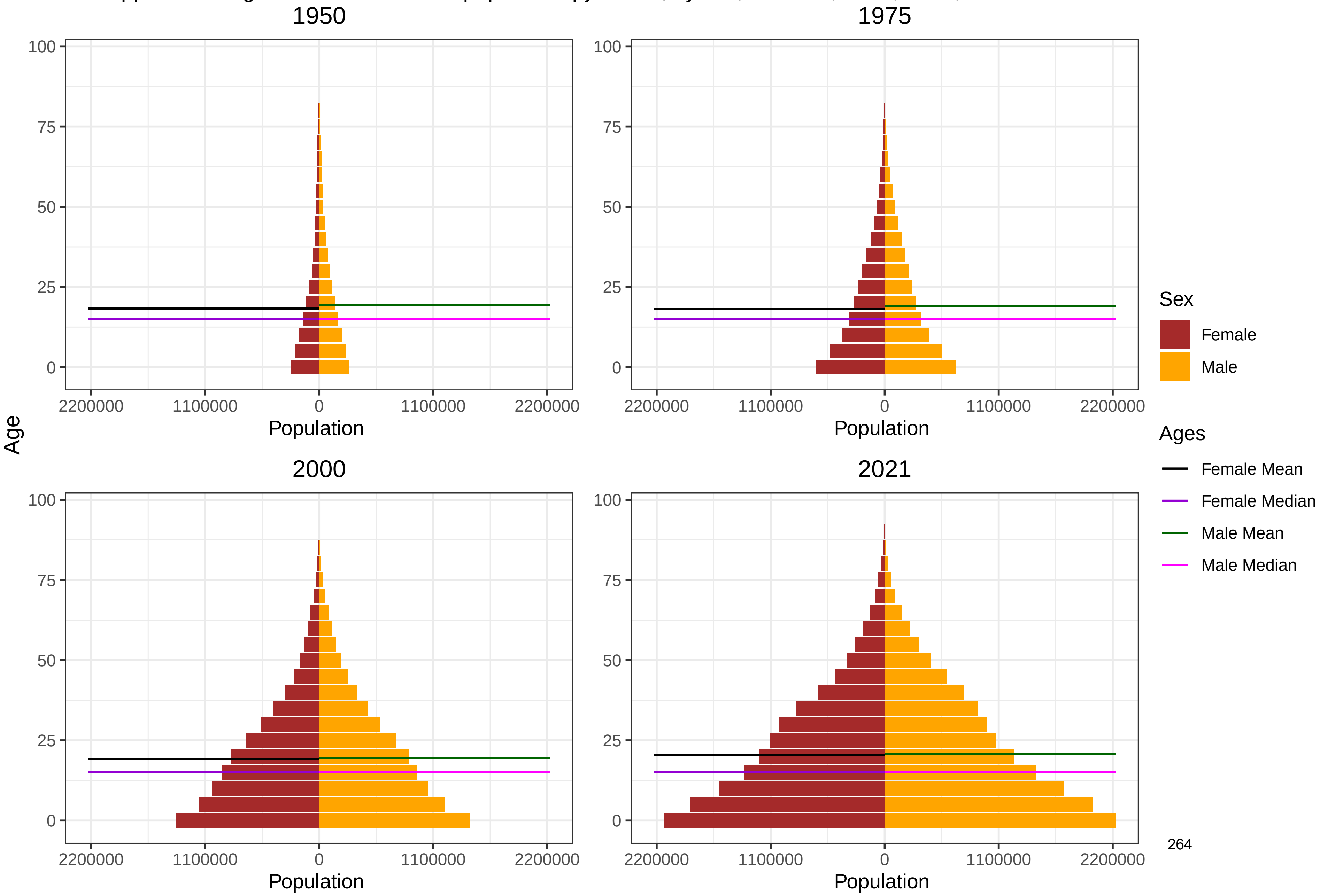

Appendix 2 Figure S9: DR Congo population pyramids, by sex, for 1950, 1975, 2000, and 2021

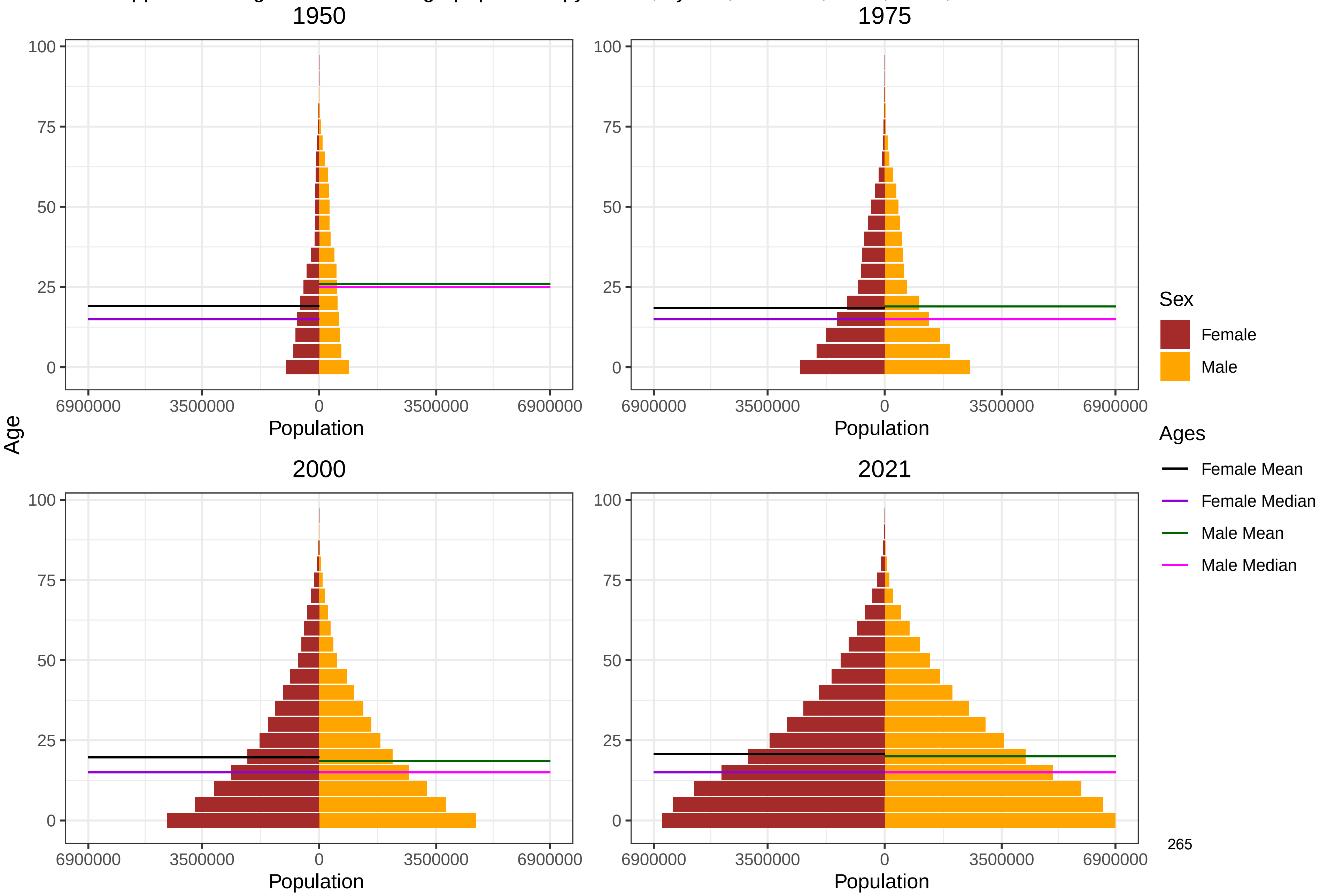

Appendix 2 Figure S9: Denmark population pyramids, by sex, for 1950, 1975, 2000, and 2021

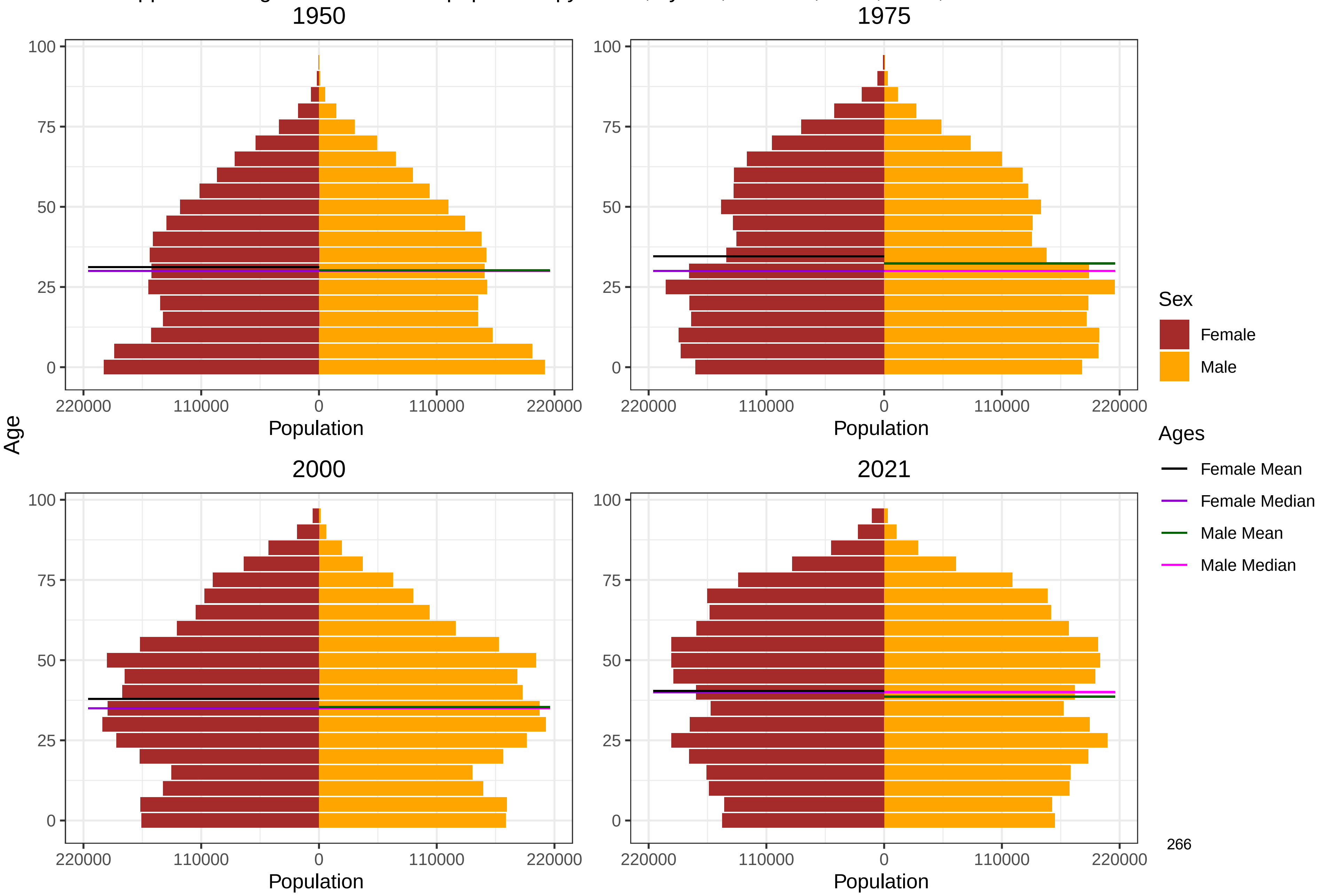

Appendix 2 Figure S9: Djibouti population pyramids, by sex, for 1950, 1975, 2000, and 2021

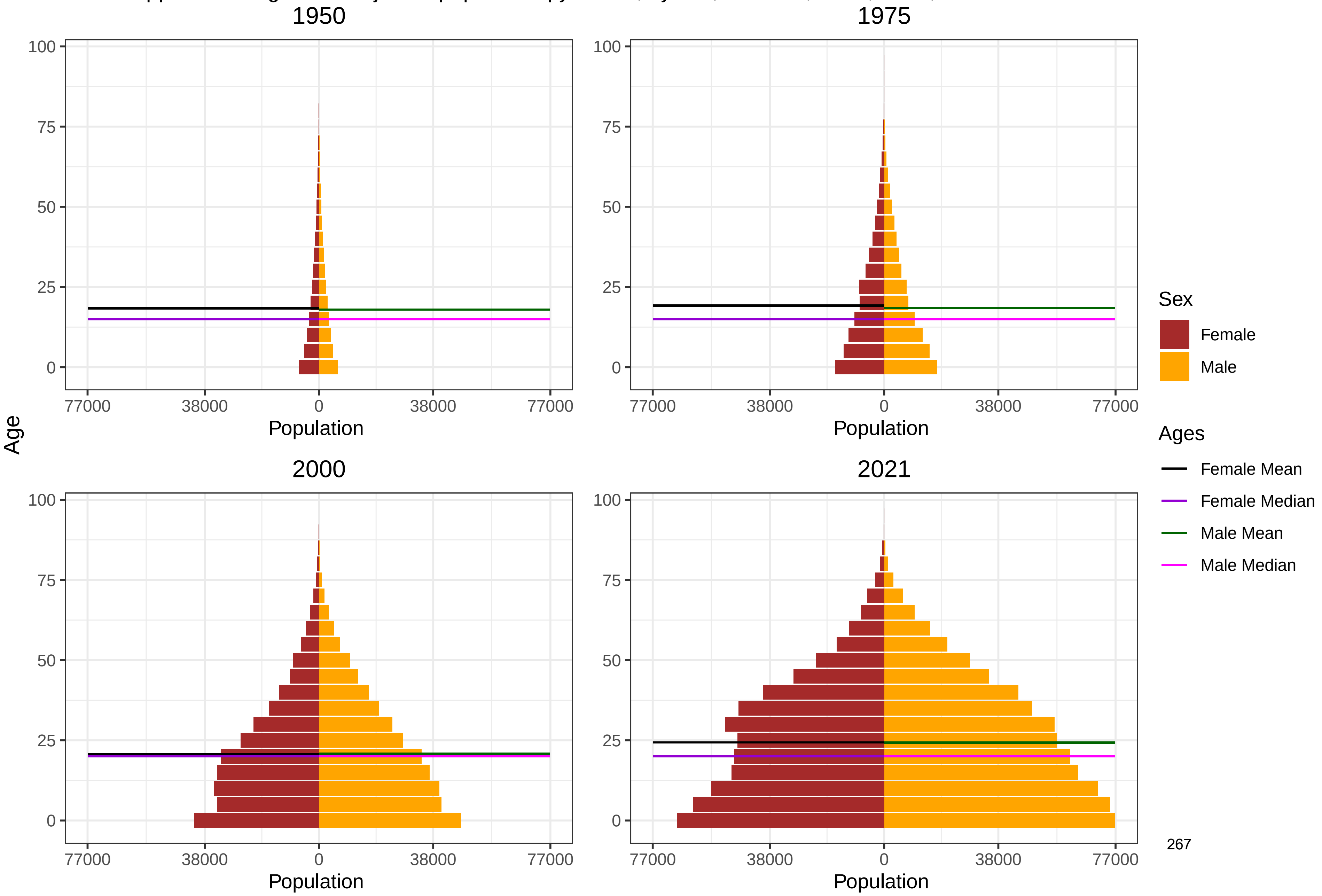

Appendix 2 Figure S9: Dominica population pyramids, by sex, for 1950, 1975, 2000, and 2021

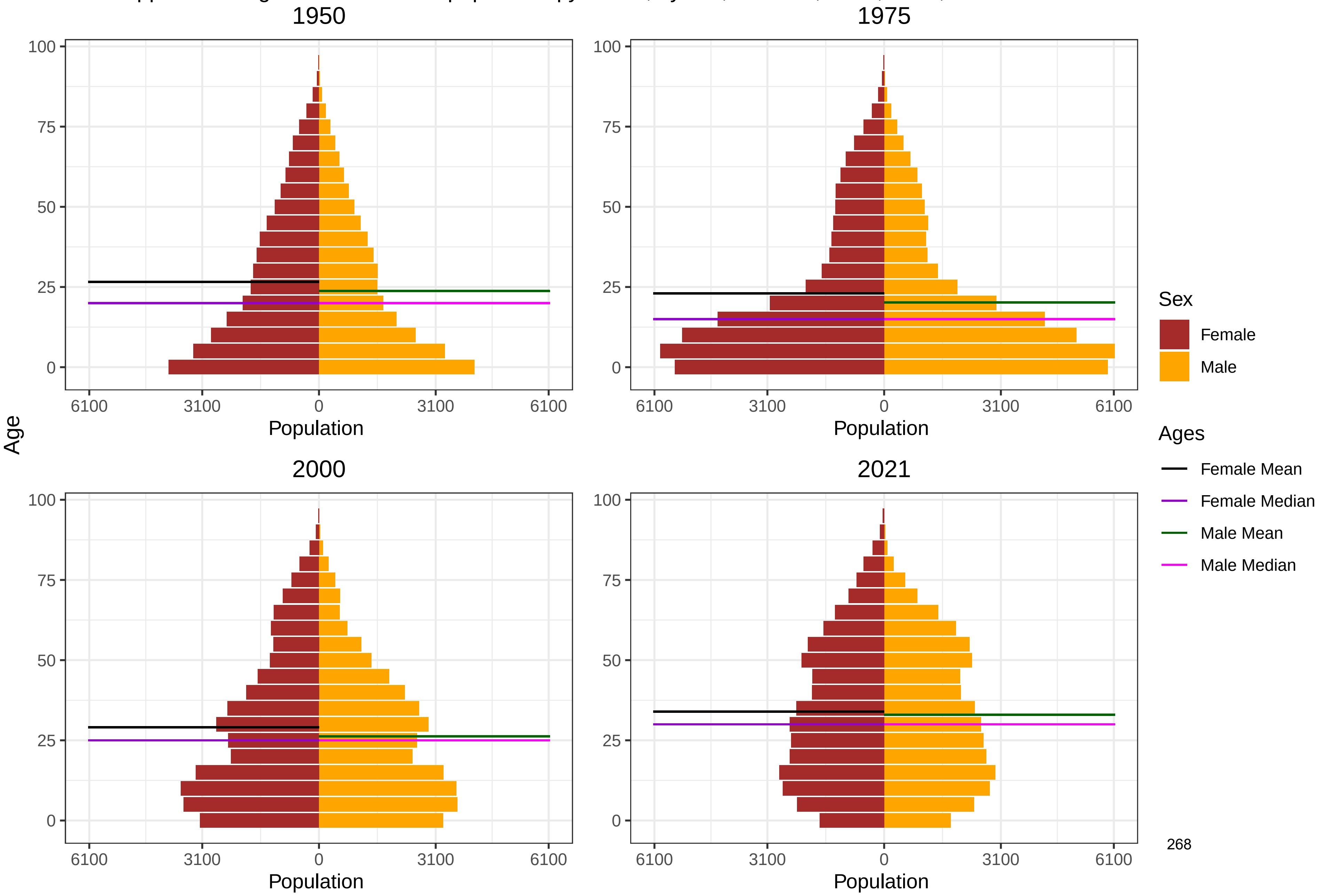

Appendix 2 Figure S9: Dominican Republic population pyramids, by sex, for 1950, 1975, 2000, and 2021

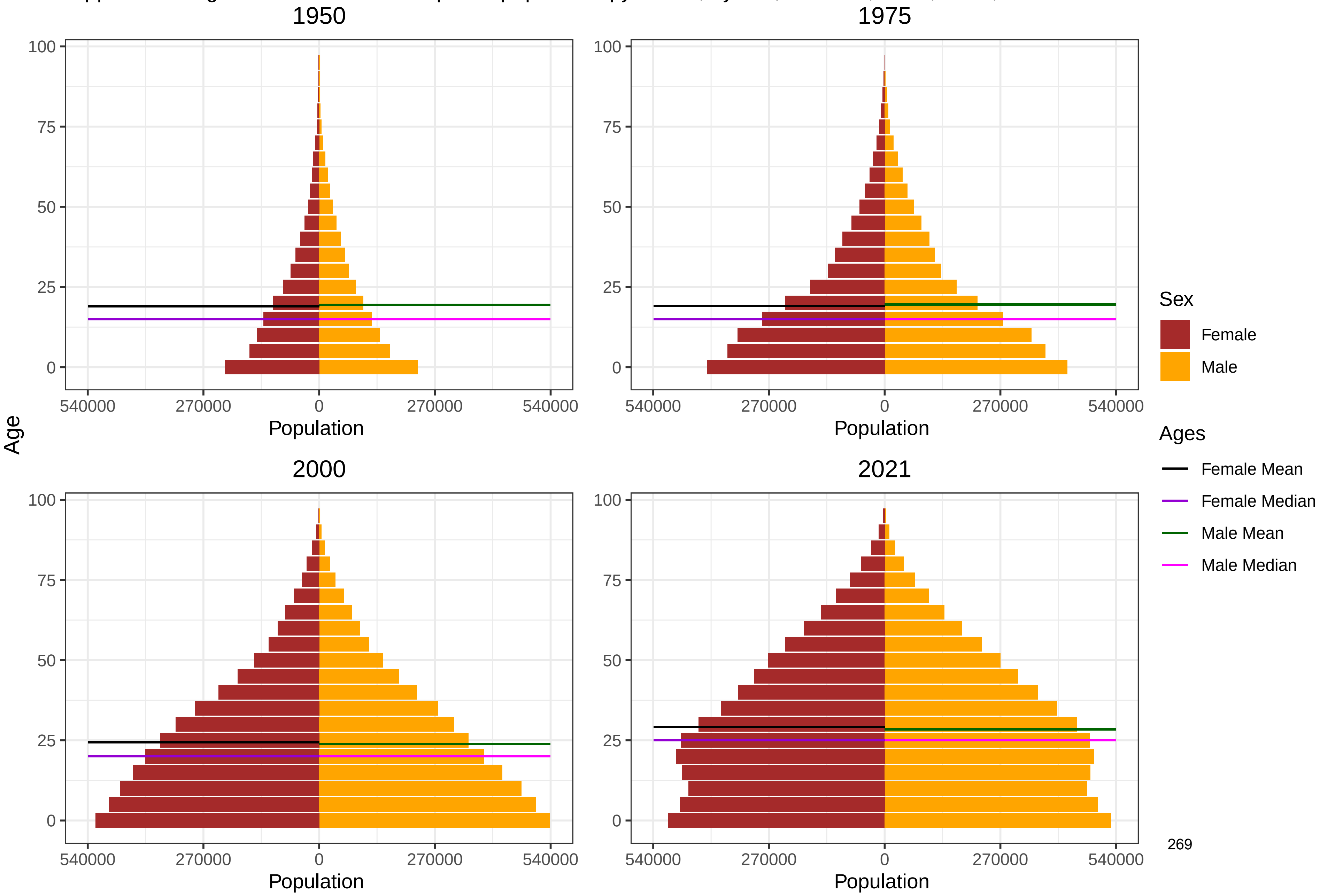

Appendix 2 Figure S9: Ecuador population pyramids, by sex, for 1950, 1975, 2000, and 2021

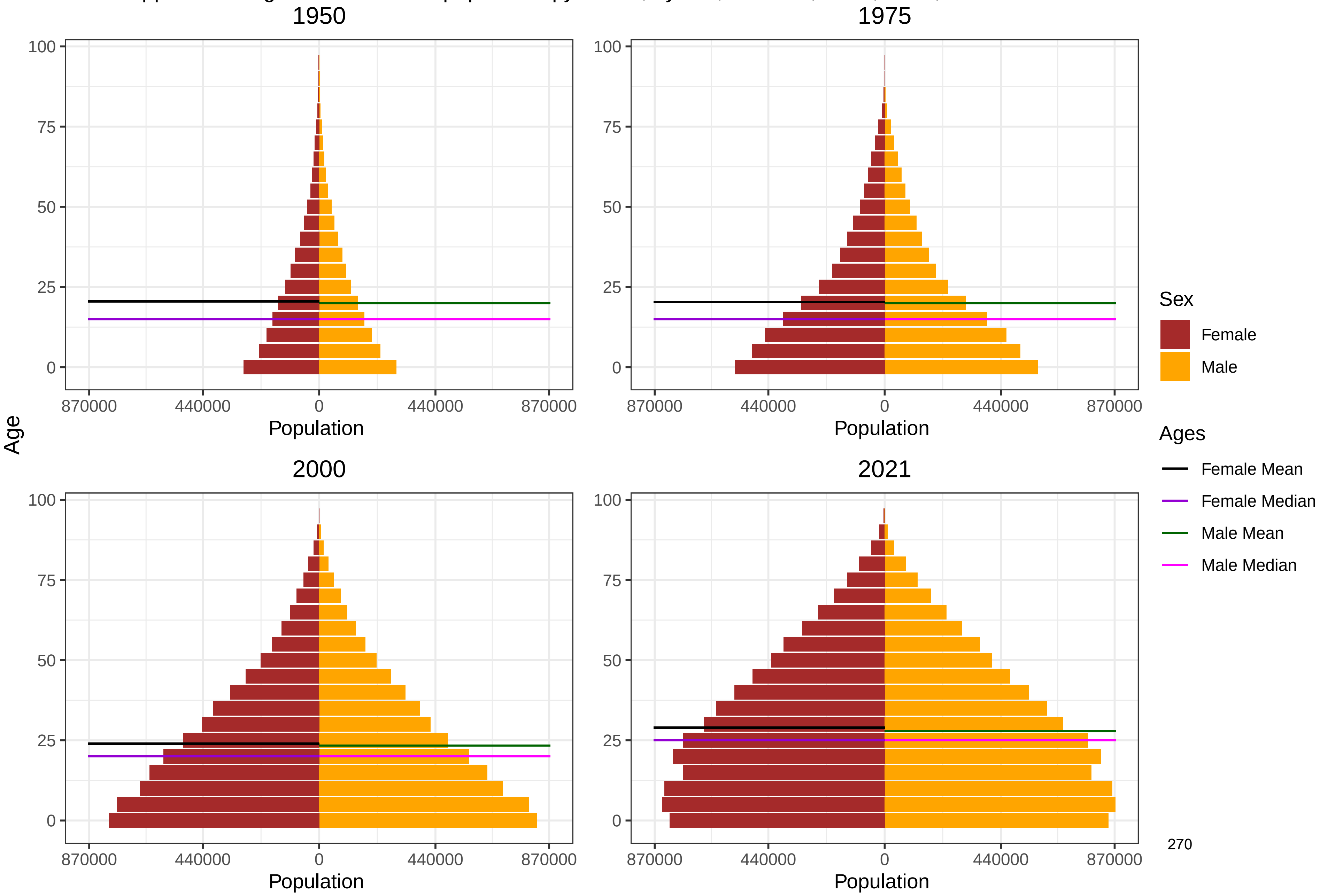

Appendix 2 Figure S9: Egypt population pyramids, by sex, for 1950, 1975, 2000, and 2021

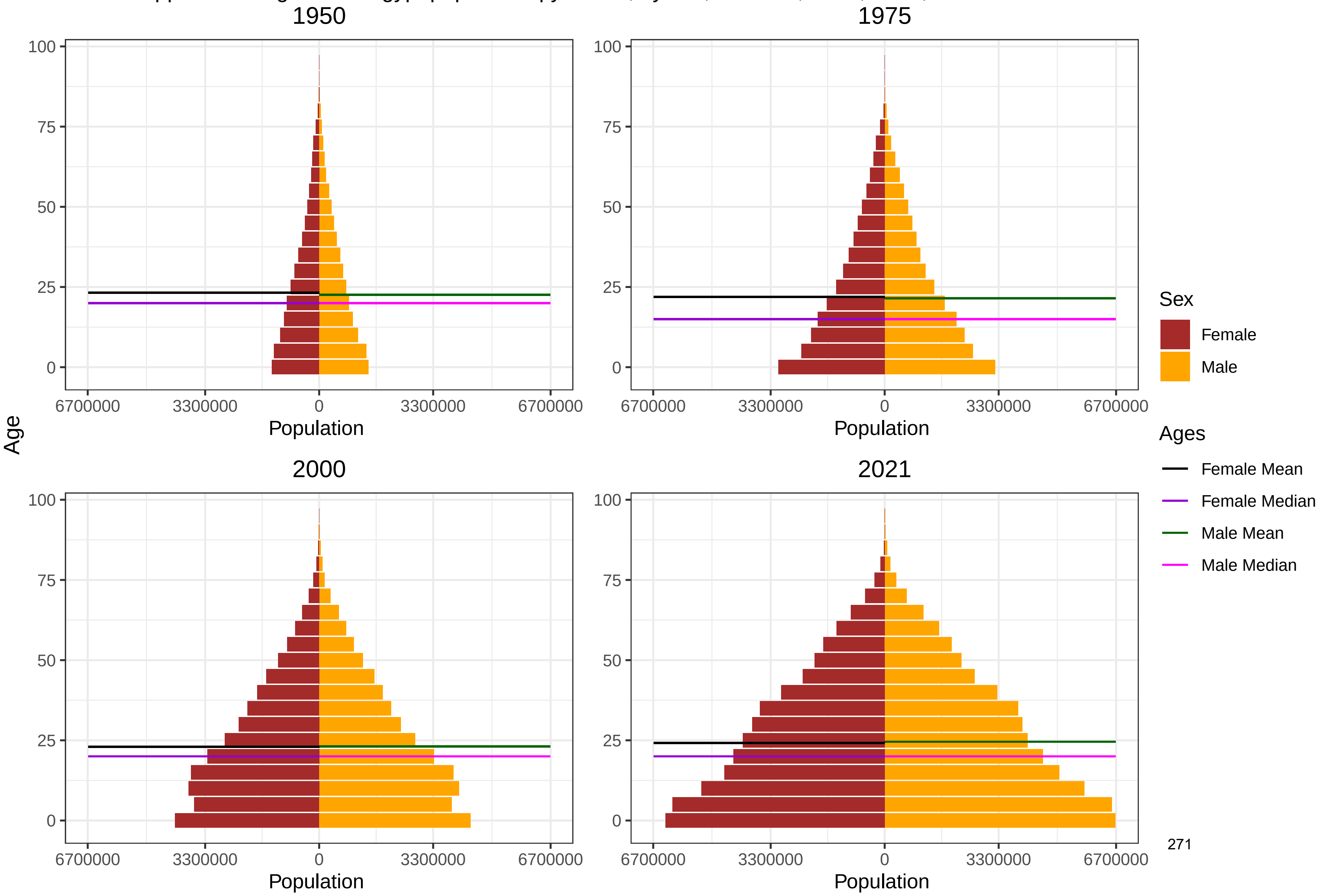

Appendix 2 Figure S9: El Salvador population pyramids, by sex, for 1950, 1975, 2000, and 2021

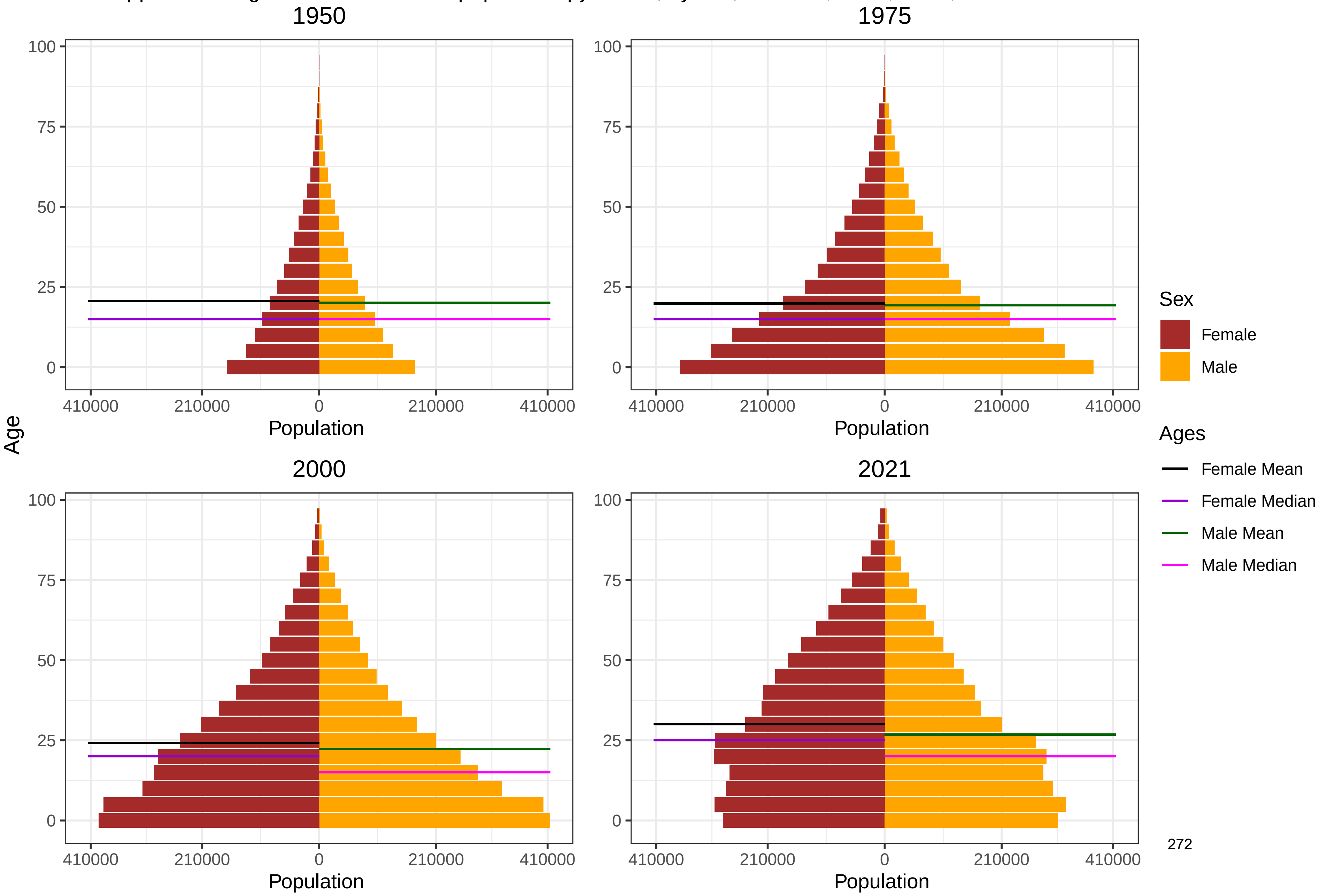

Appendix 2 Figure S9: Equatorial Guinea population pyramids, by sex, for 1950, 1975, 2000, and 2021

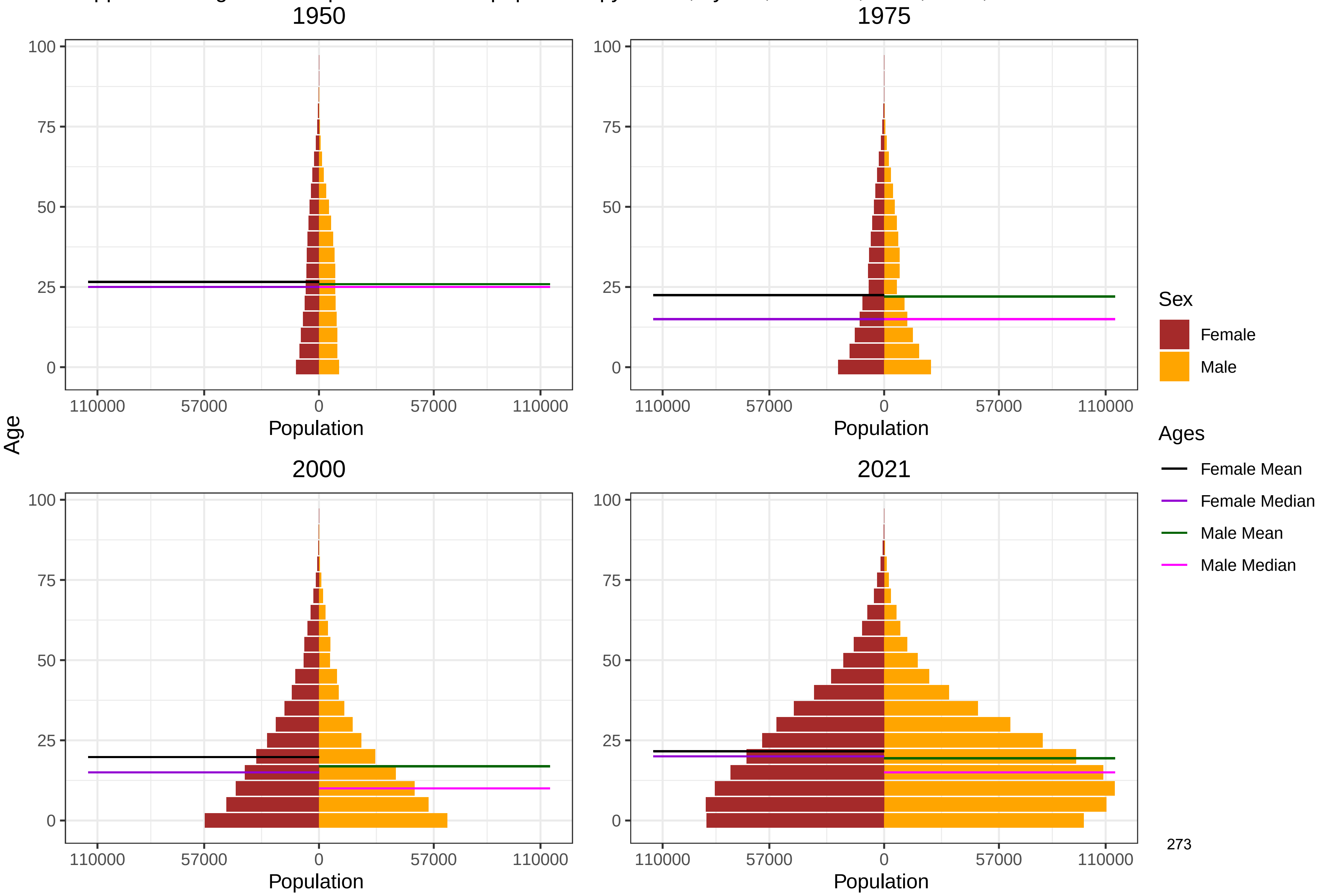

Appendix 2 Figure S9: Eritrea population pyramids, by sex, for 1950, 1975, 2000, and 2021

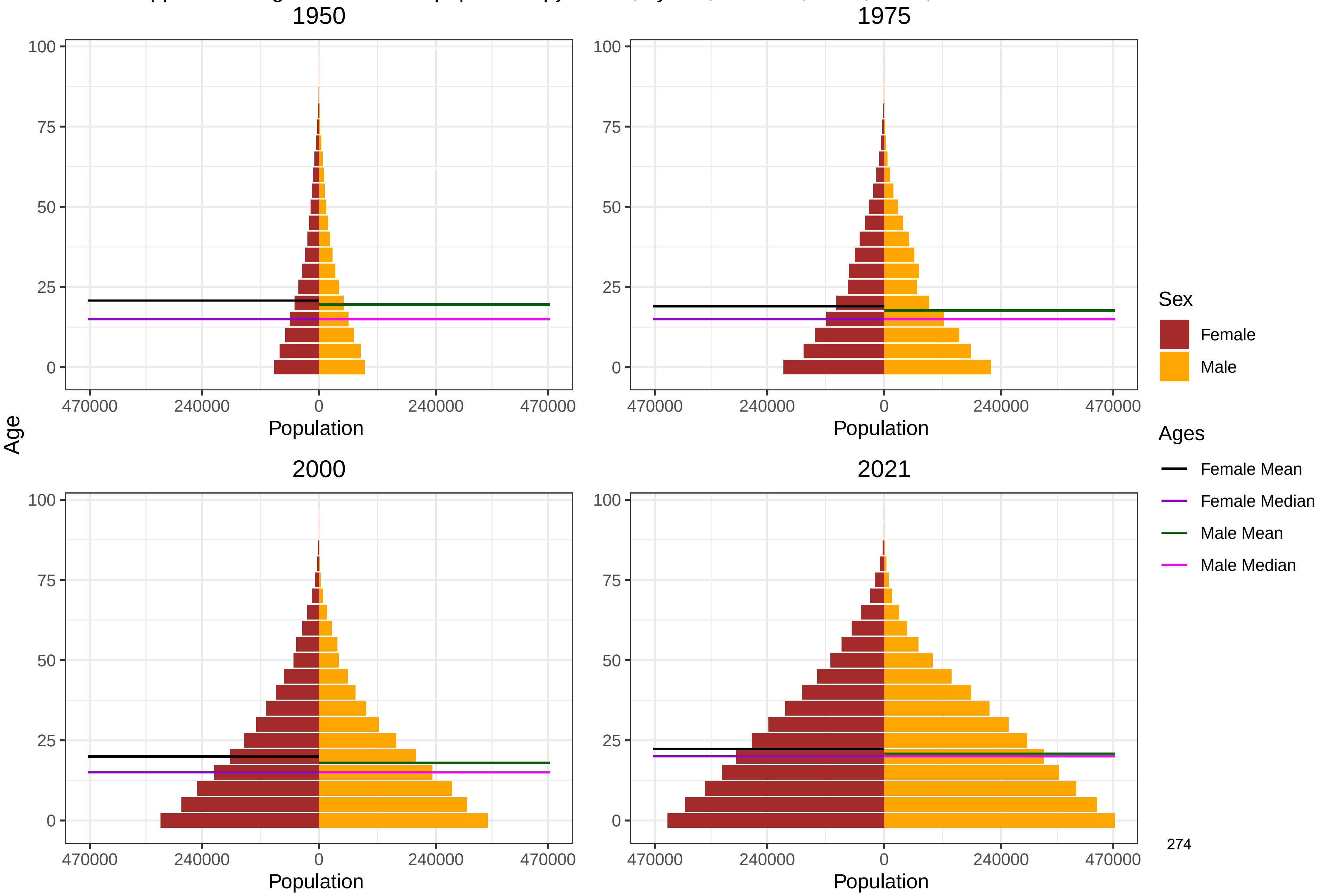

Appendix 2 Figure S9: Estonia population pyramids, by sex, for 1950, 1975, 2000, and 2021

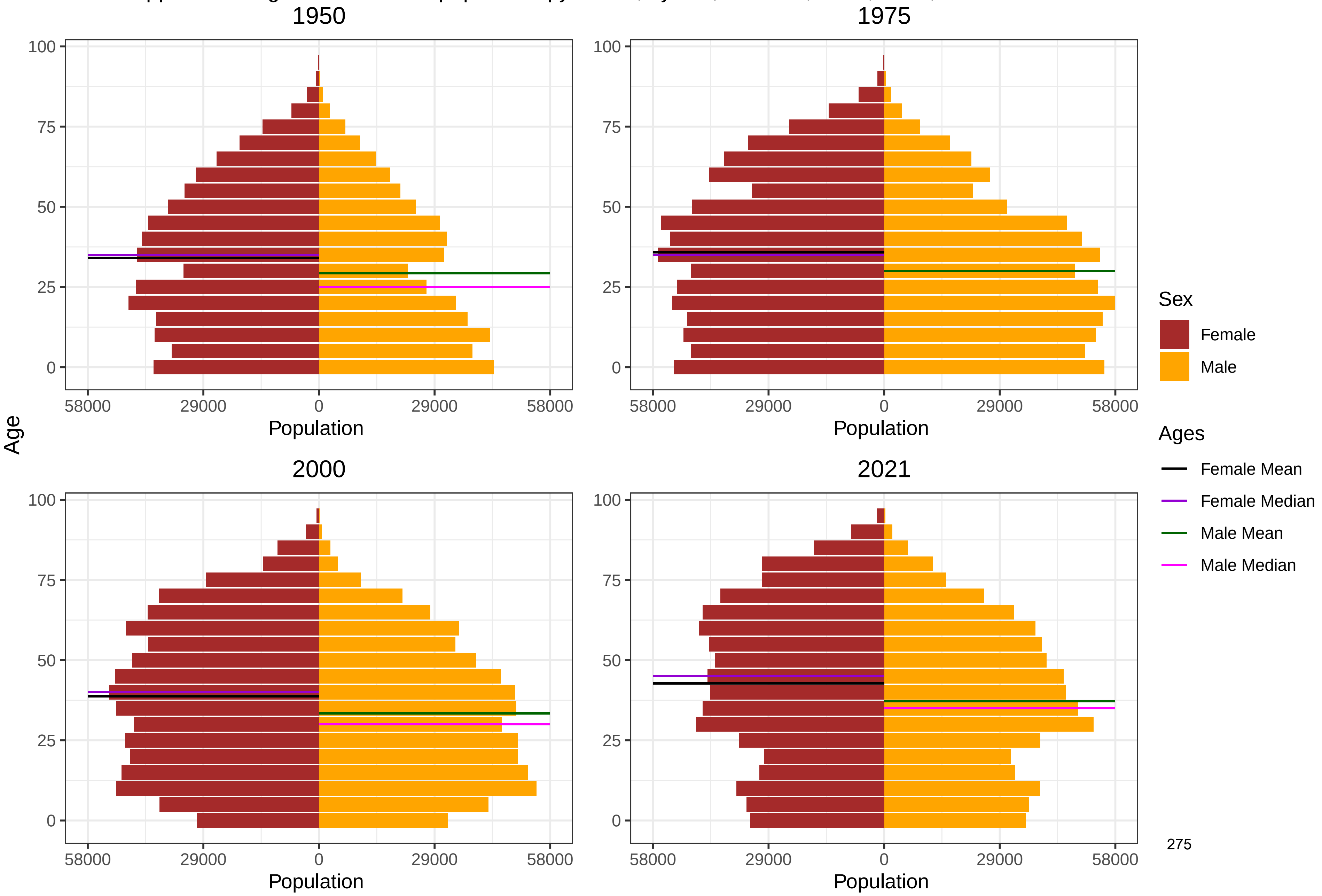

Appendix 2 Figure S9: Eswatini population pyramids, by sex, for 1950, 1975, 2000, and 2021

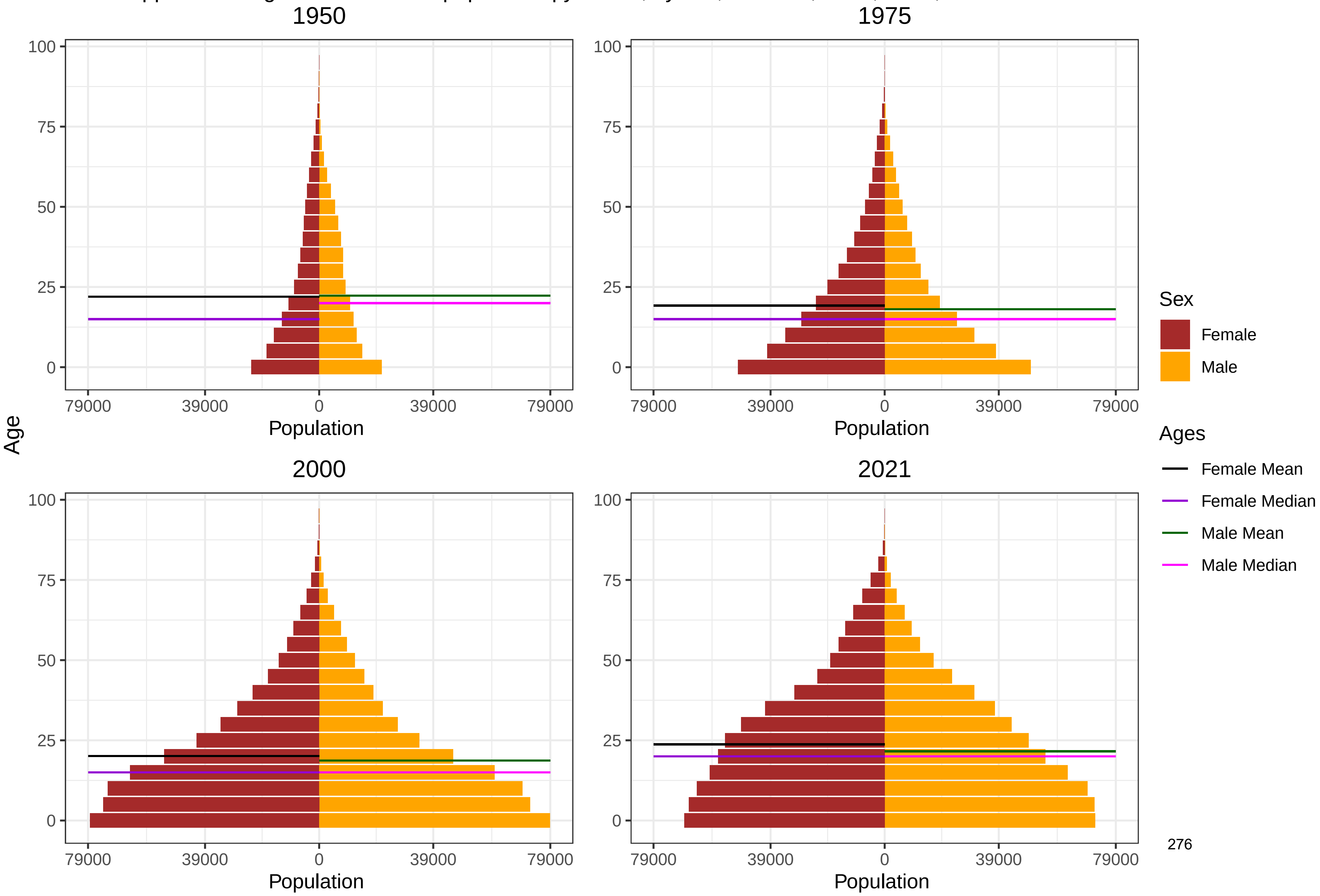

Appendix 2 Figure S9: Ethiopia population pyramids, by sex, for 1950, 1975, 2000, and 2021

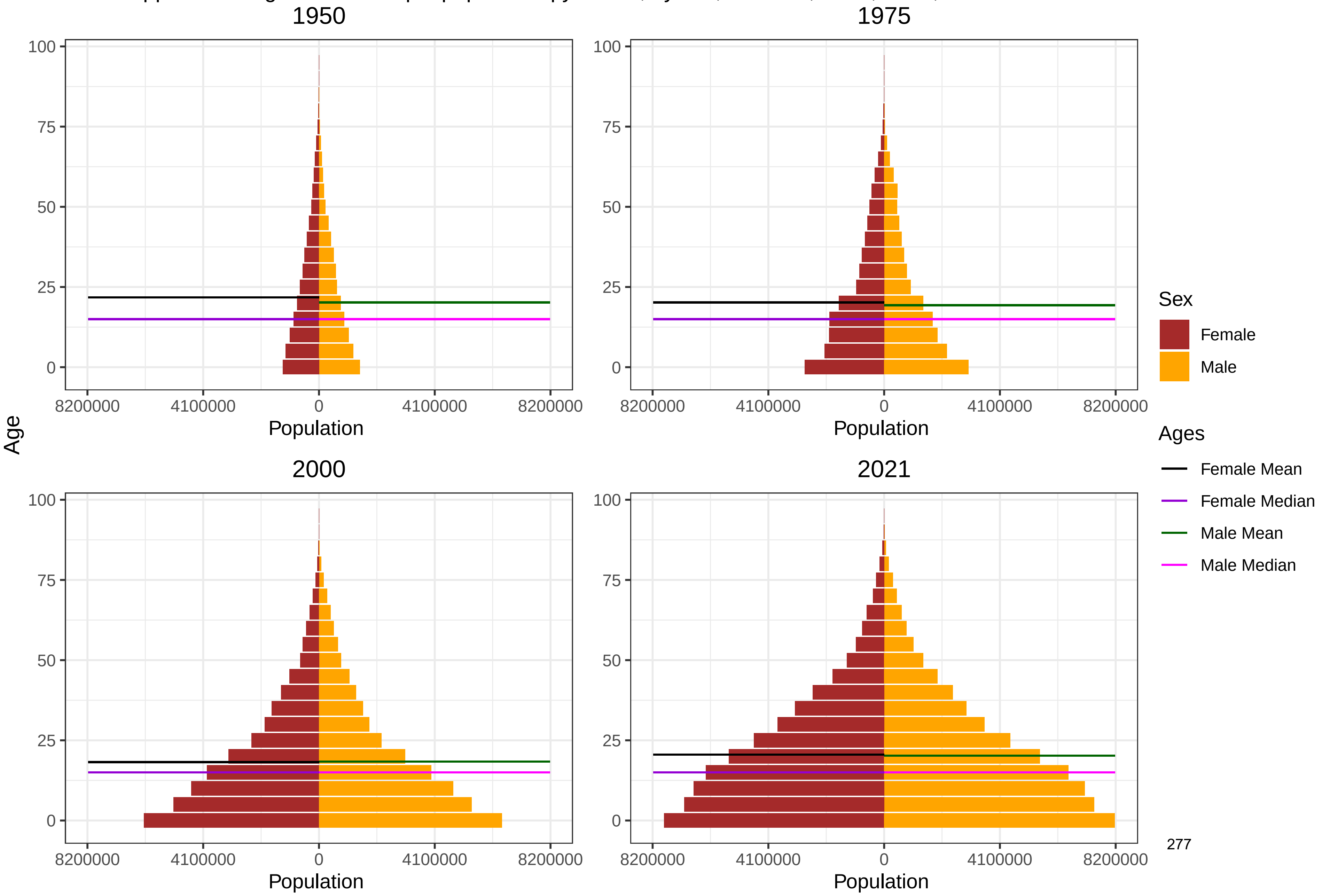

Appendix 2 Figure S9: Federated States of Micronesia population pyramids, by sex, for 1950, 1975, 2000, and 2021

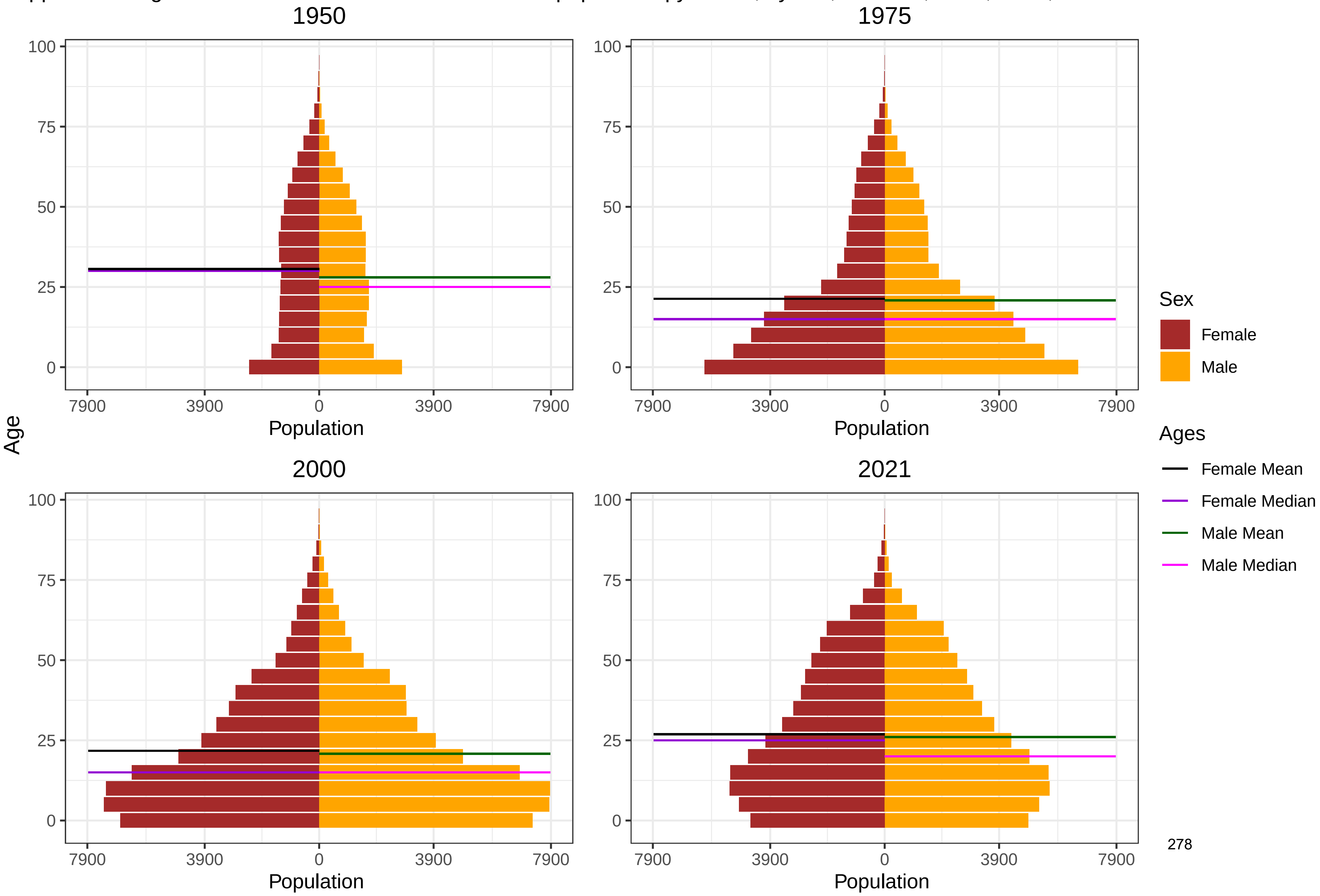

Appendix 2 Figure S9: Fiji population pyramids, by sex, for 1950, 1975, 2000, and 2021

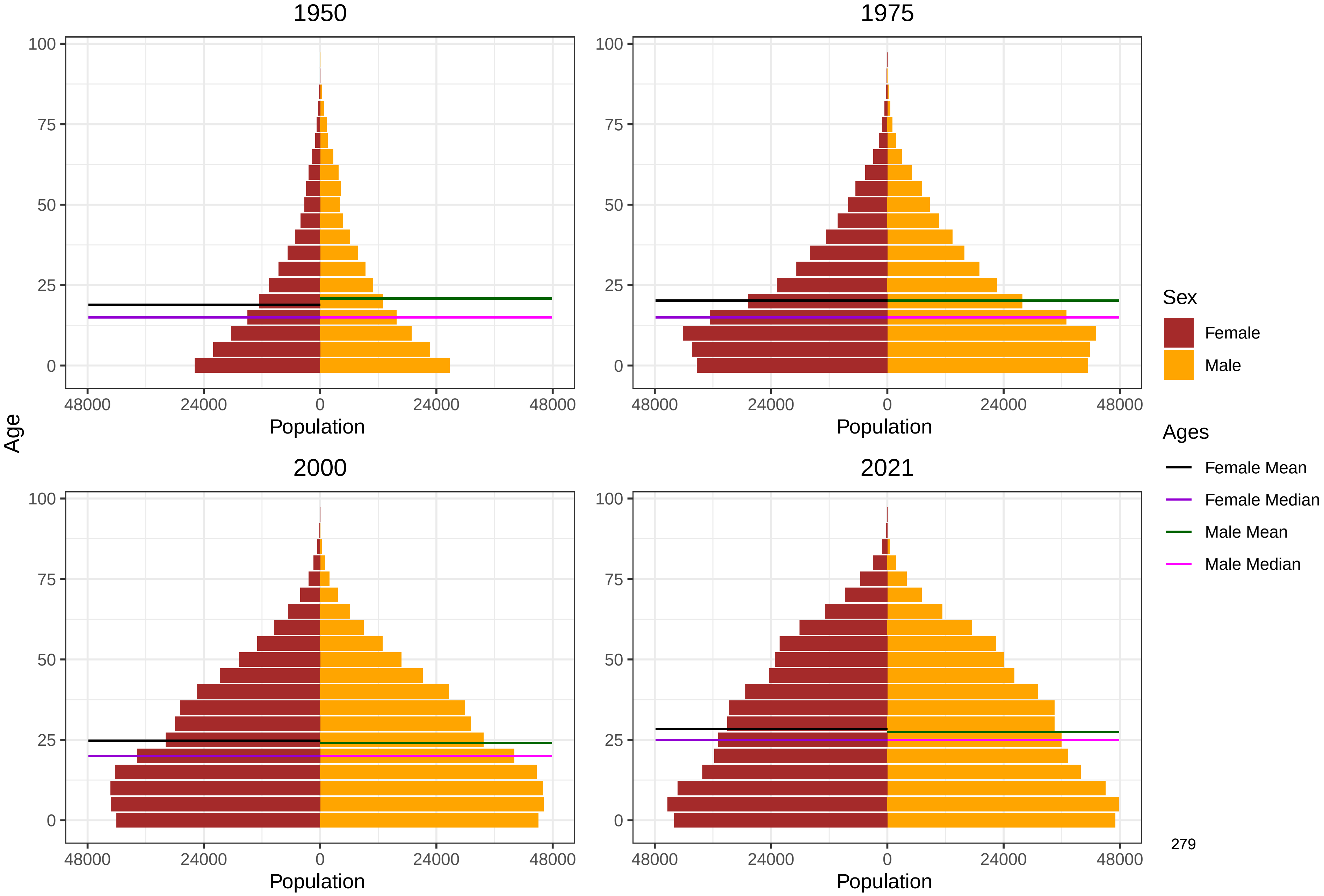

Appendix 2 Figure S9: Finland population pyramids, by sex, for 1950, 1975, 2000, and 2021

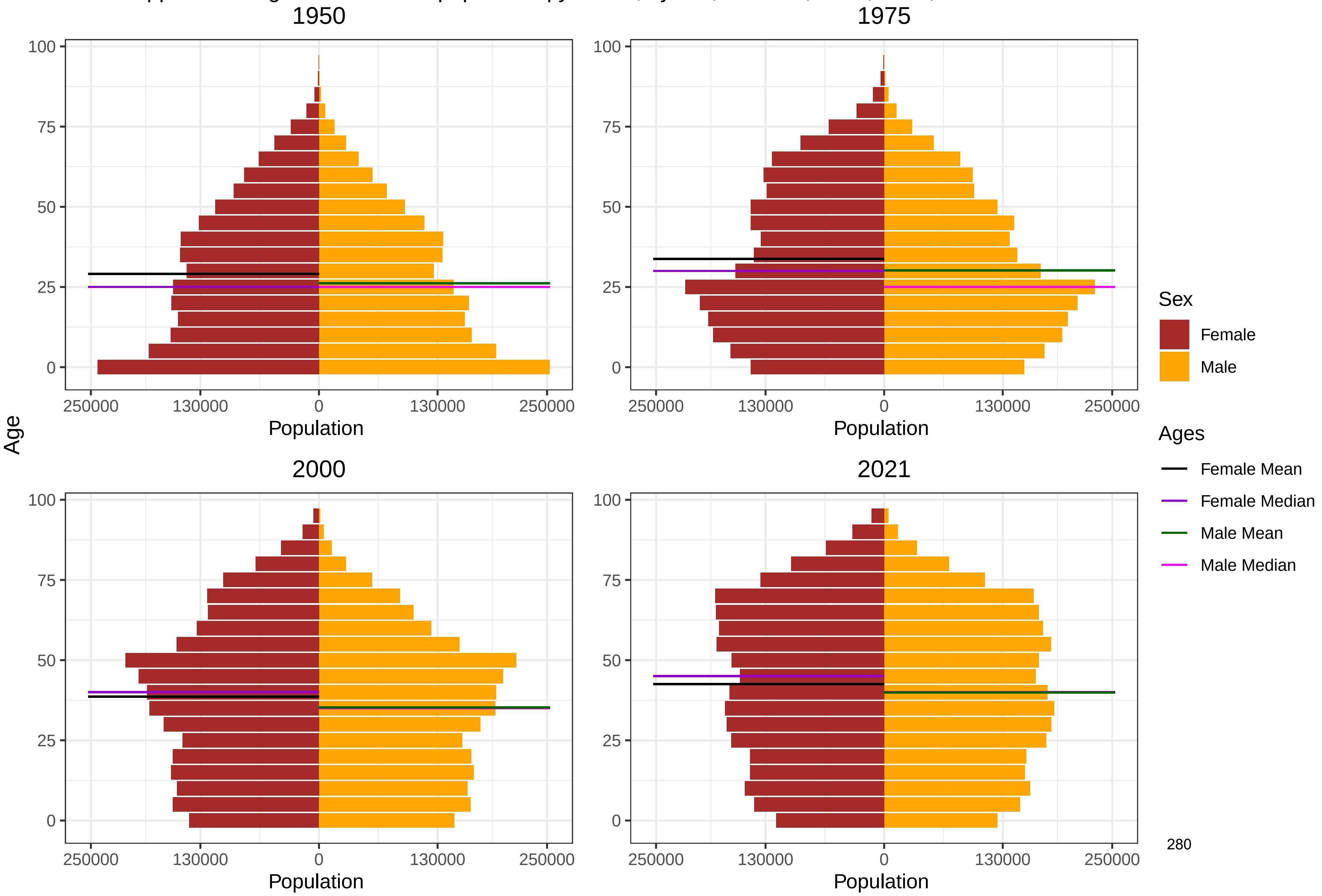

Appendix 2 Figure S9: France population pyramids, by sex, for 1950, 1975, 2000, and 2021

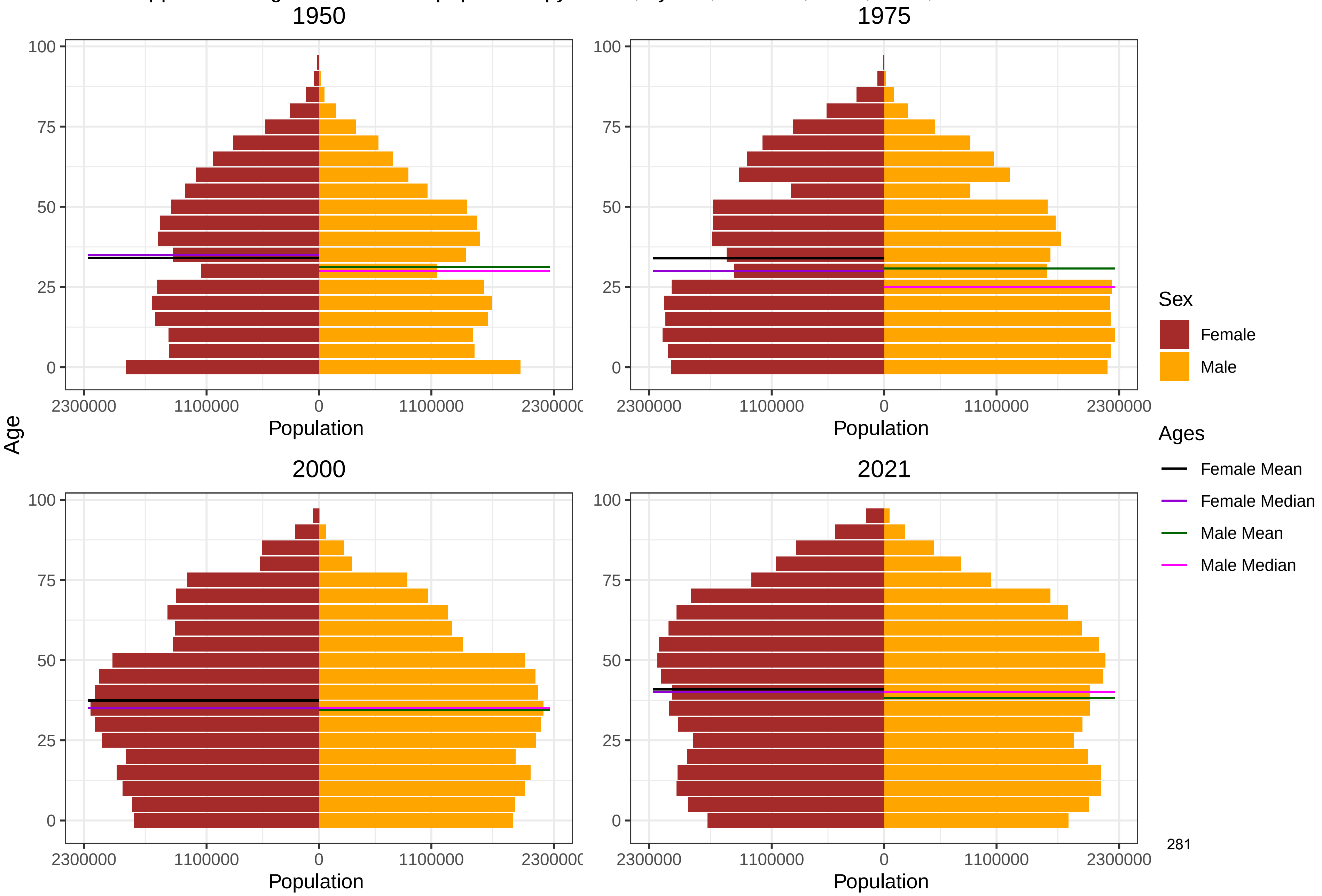

Appendix 2 Figure S9: Gabon population pyramids, by sex, for 1950, 1975, 2000, and 2021

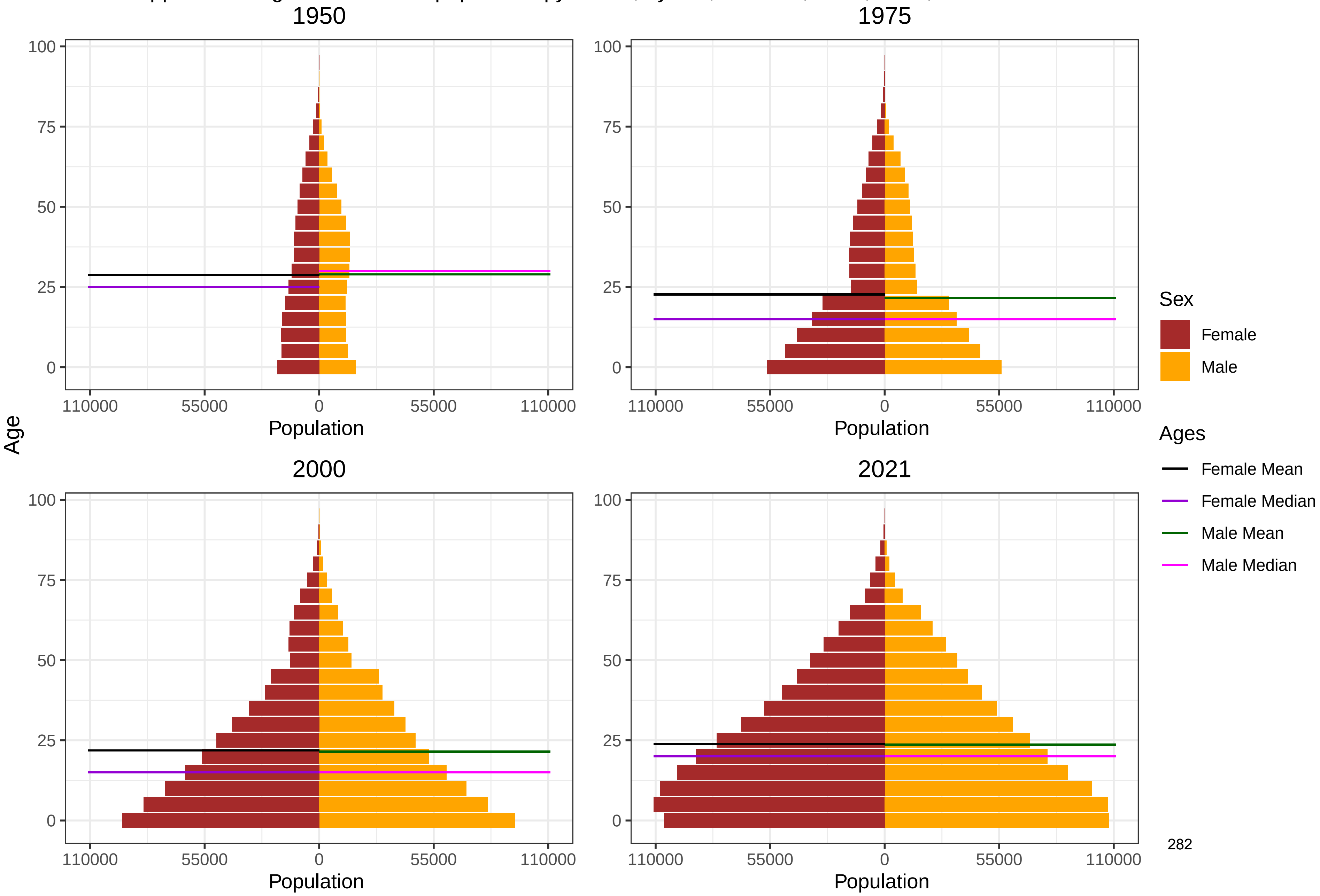

Appendix 2 Figure S9: Georgia population pyramids, by sex, for 1950, 1975, 2000, and 2021

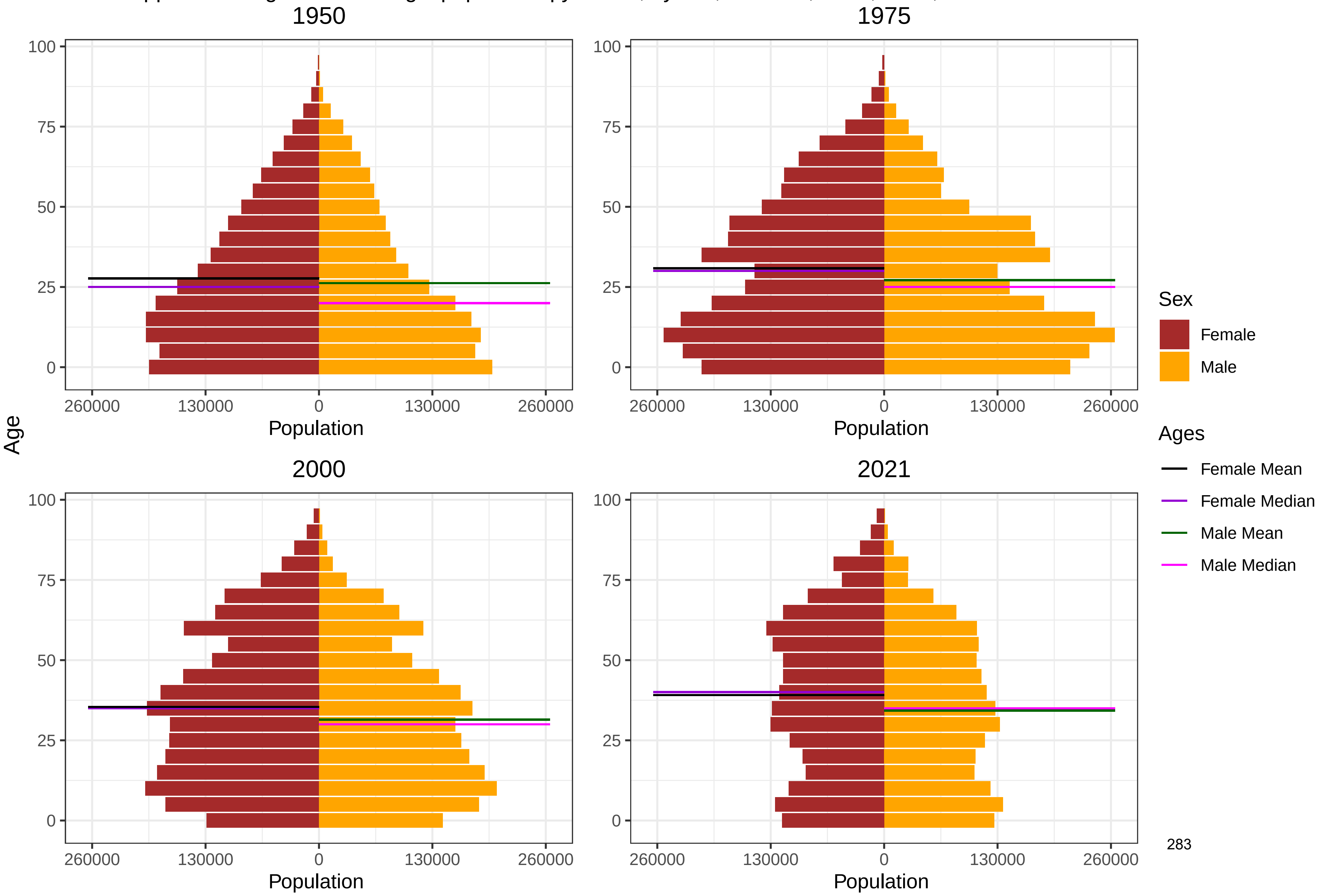

Appendix 2 Figure S9: Germany population pyramids, by sex, for 1950, 1975, 2000, and 2021

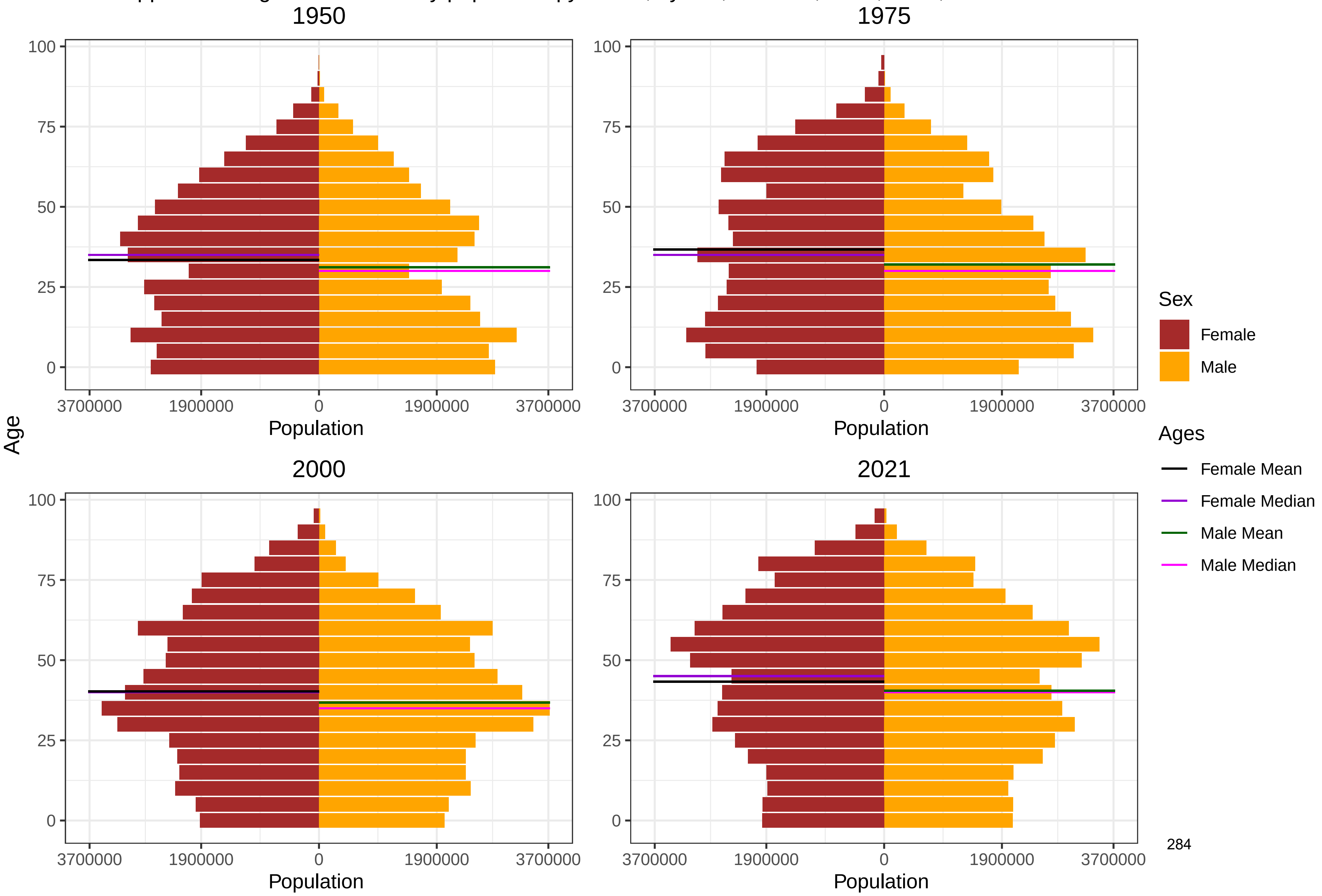

Appendix 2 Figure S9: Ghana population pyramids, by sex, for 1950, 1975, 2000, and 2021

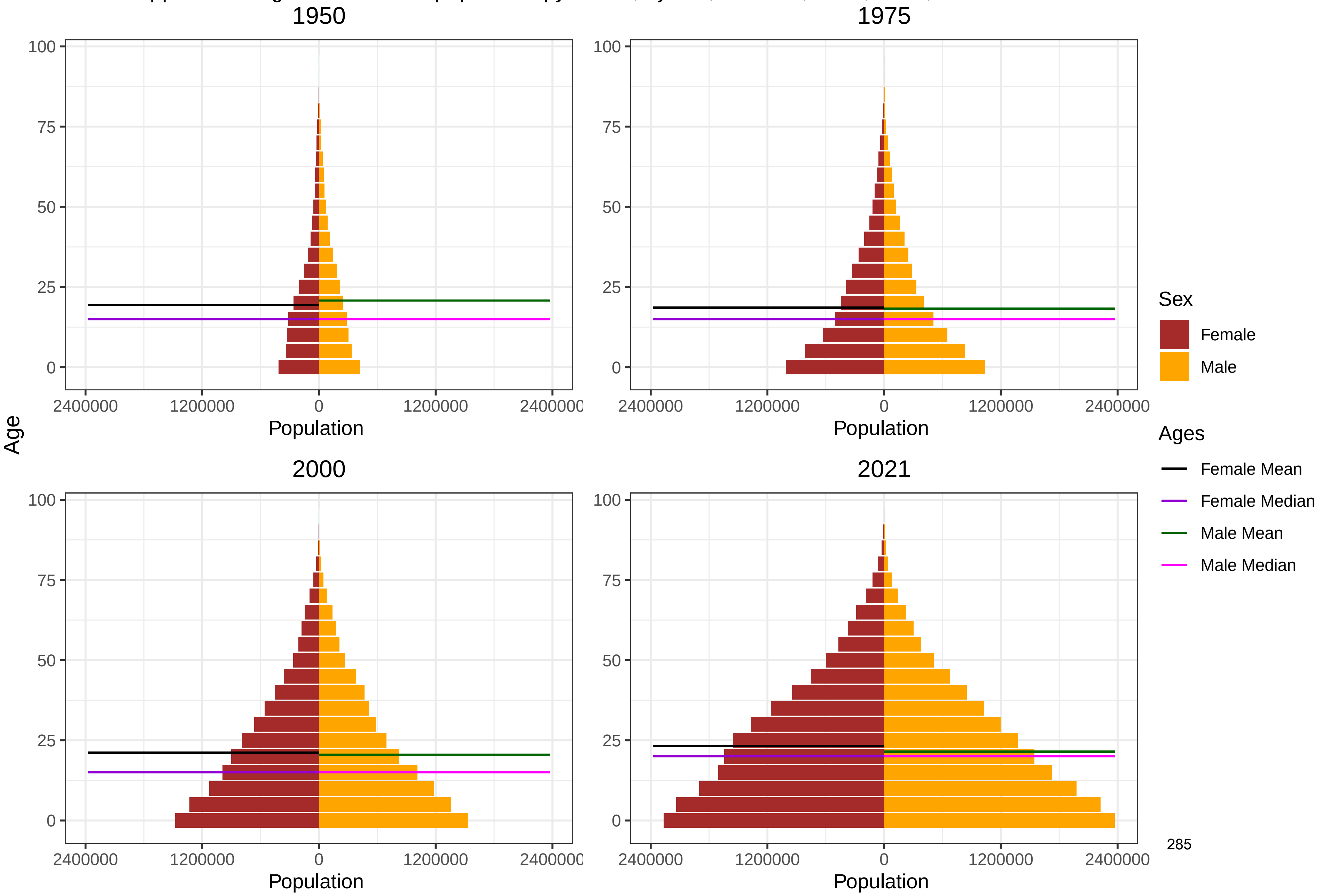

Appendix 2 Figure S9: Greece population pyramids, by sex, for 1950, 1975, 2000, and 2021

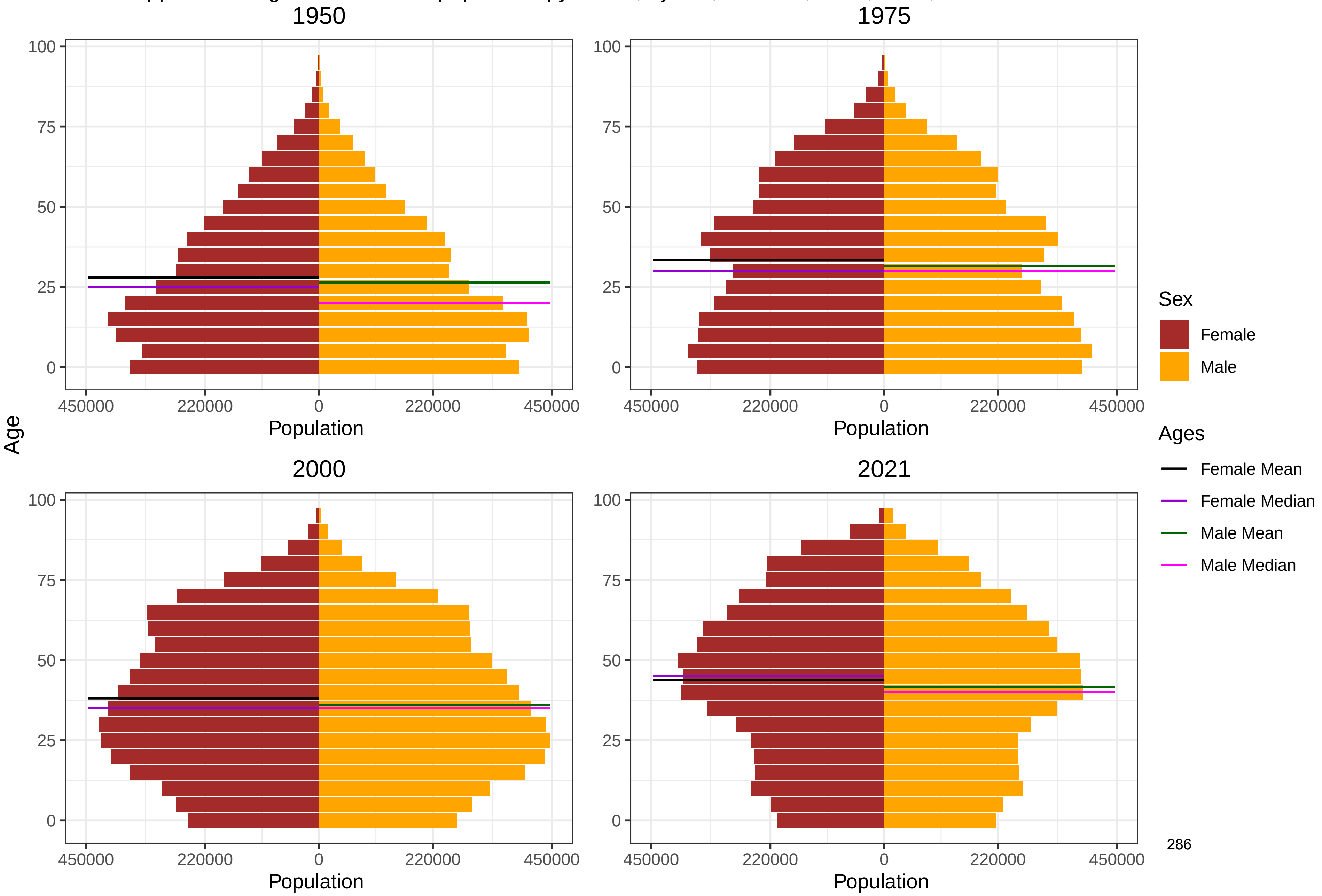

Appendix 2 Figure S9: Greenland population pyramids, by sex, for 1950, 1975, 2000, and 2021

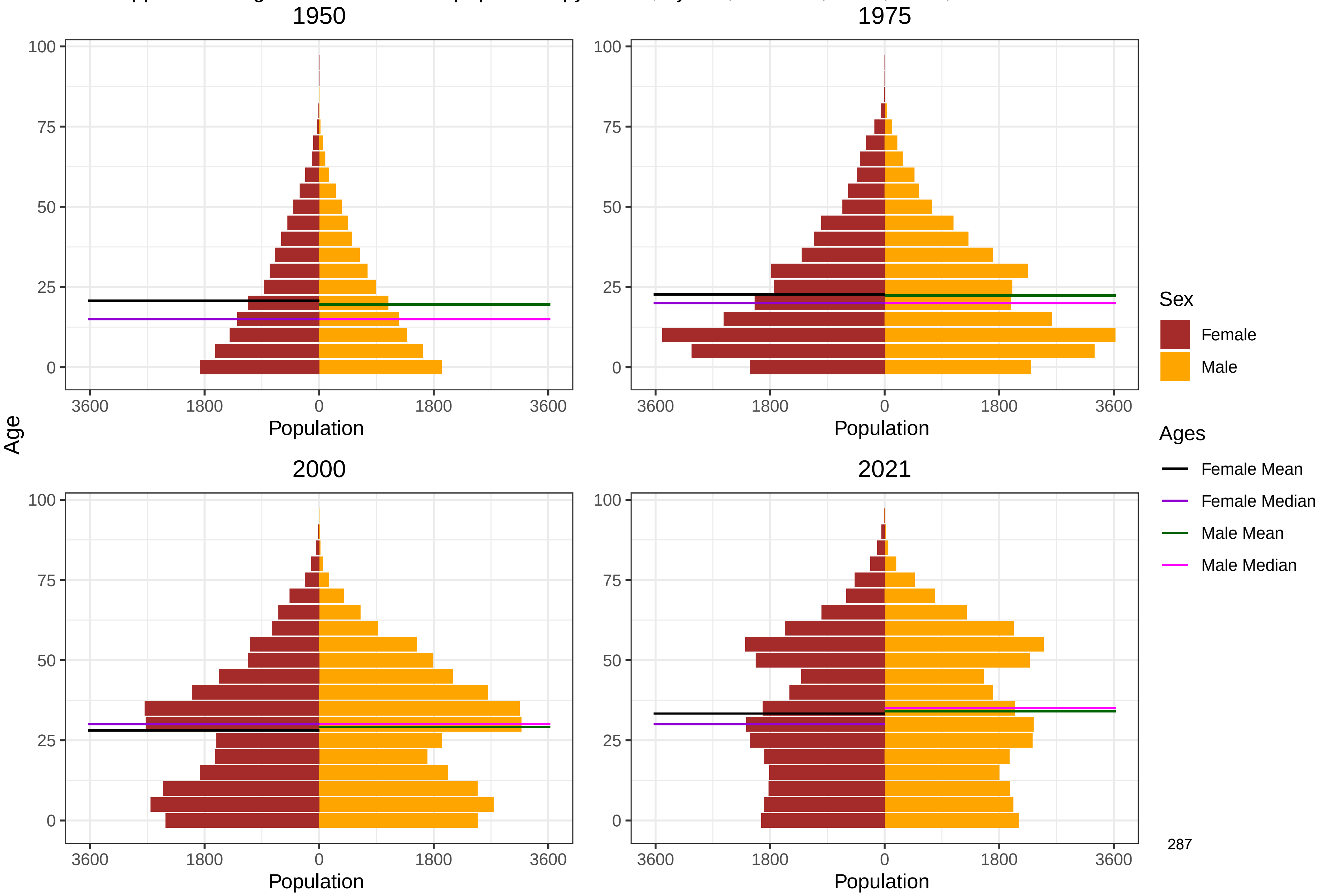

Appendix 2 Figure S9: Grenada population pyramids, by sex, for 1950, 1975, 2000, and 2021

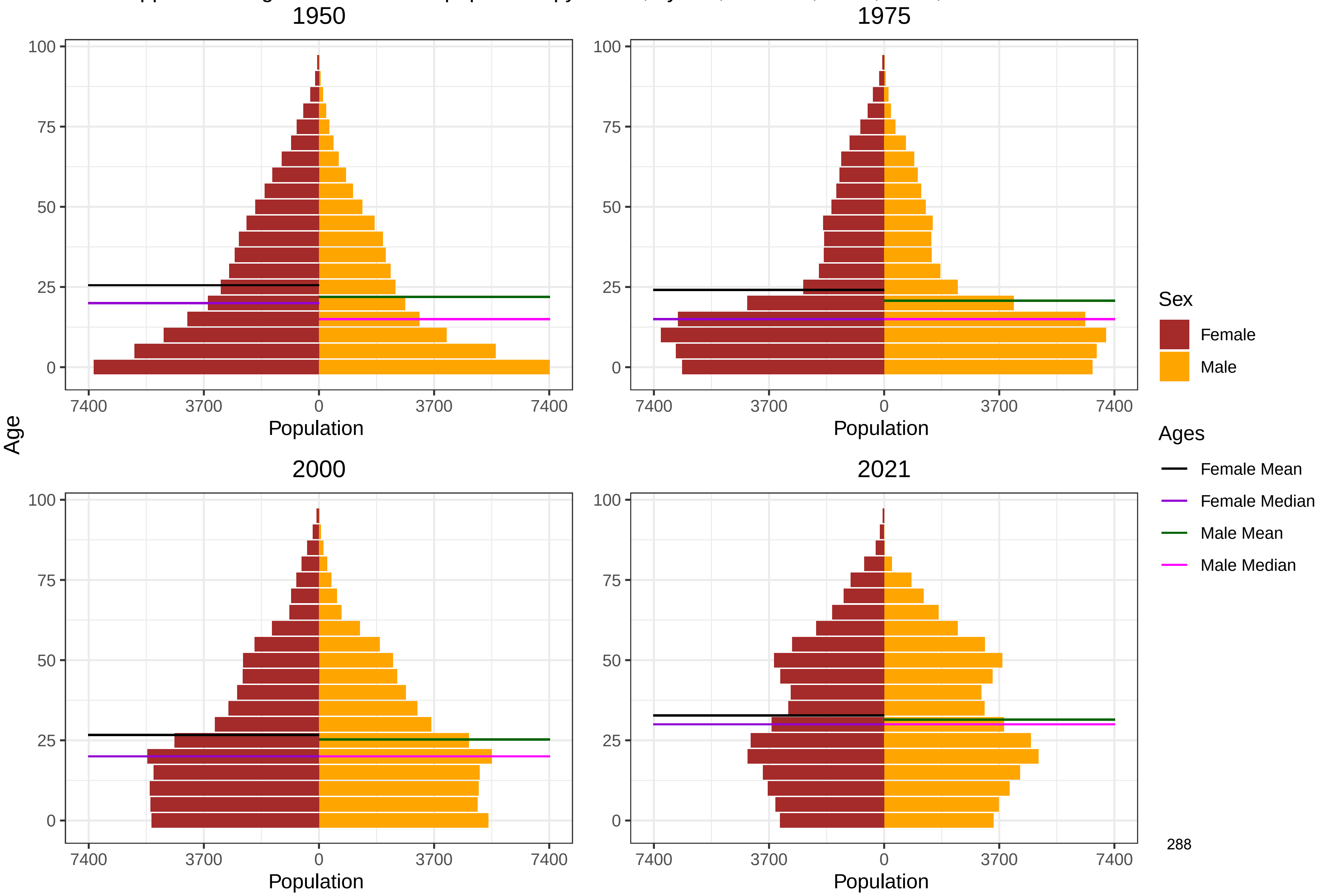

Appendix 2 Figure S9: Guam population pyramids, by sex, for 1950, 1975, 2000, and 2021

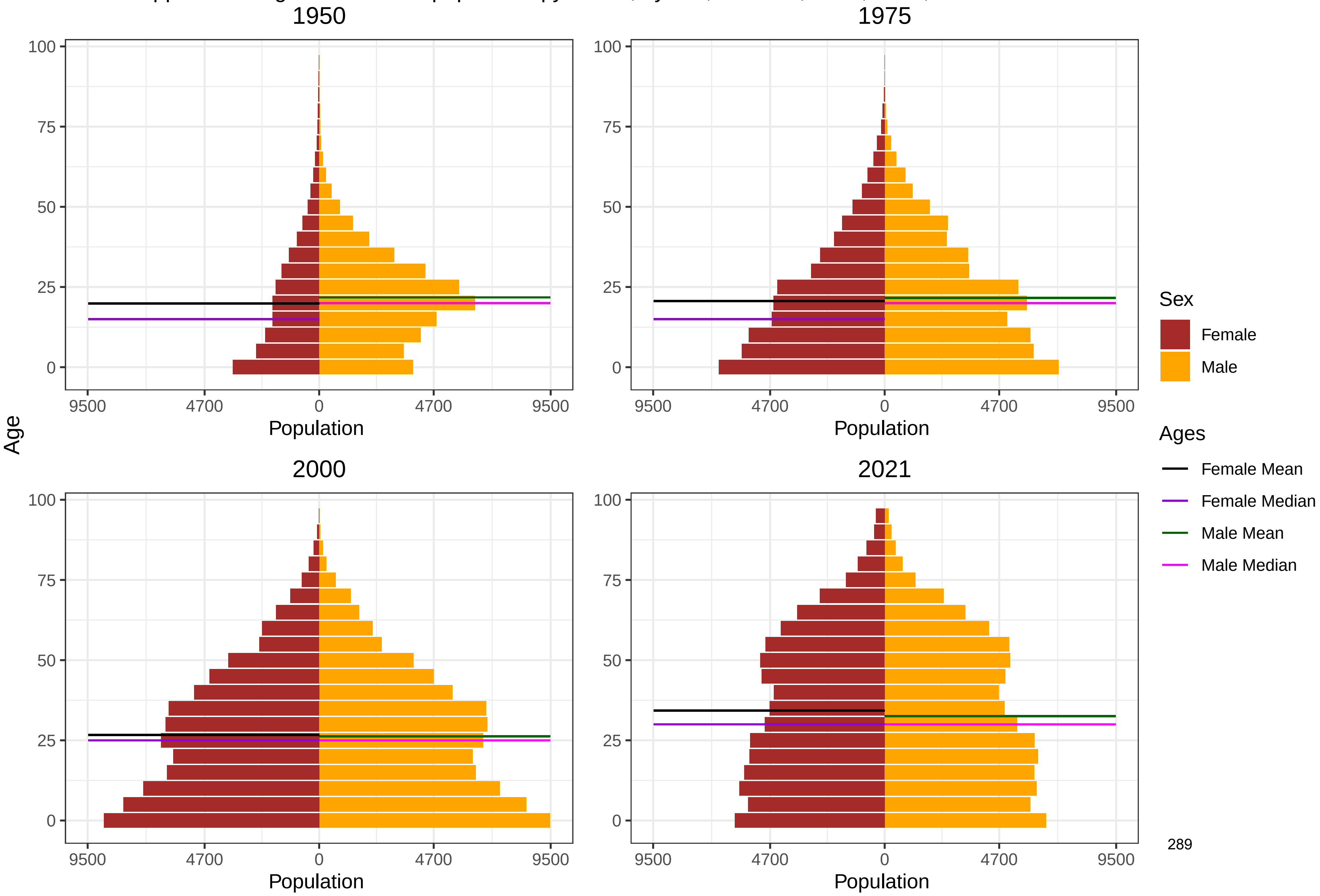

Appendix 2 Figure S9: Guatemala population pyramids, by sex, for 1950, 1975, 2000, and 2021

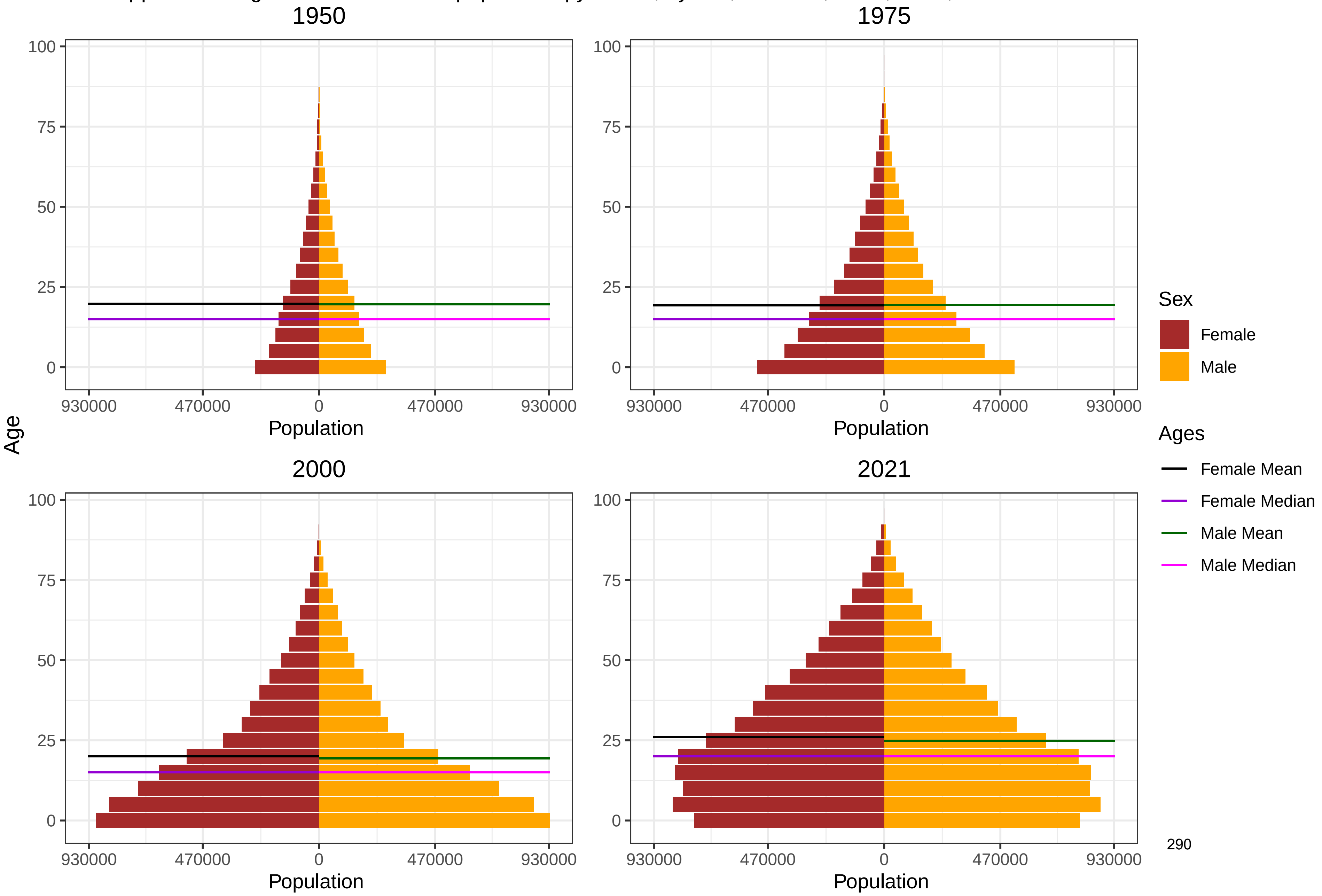

Appendix 2 Figure S9: Guinea population pyramids, by sex, for 1950, 1975, 2000, and 2021

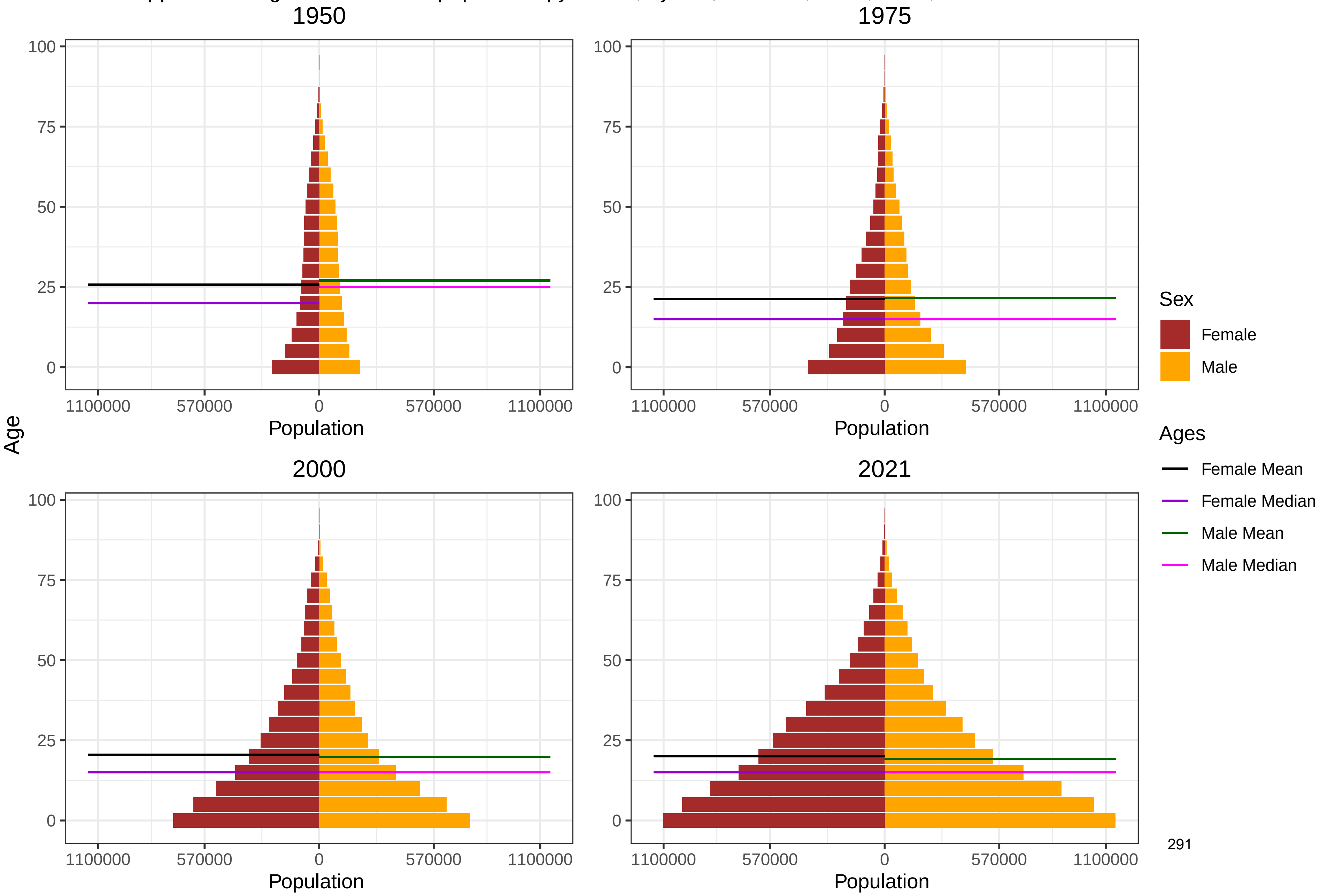

Appendix 2 Figure S9: Guinea−Bissau population pyramids, by sex, for 1950, 1975, 2000, and 2021

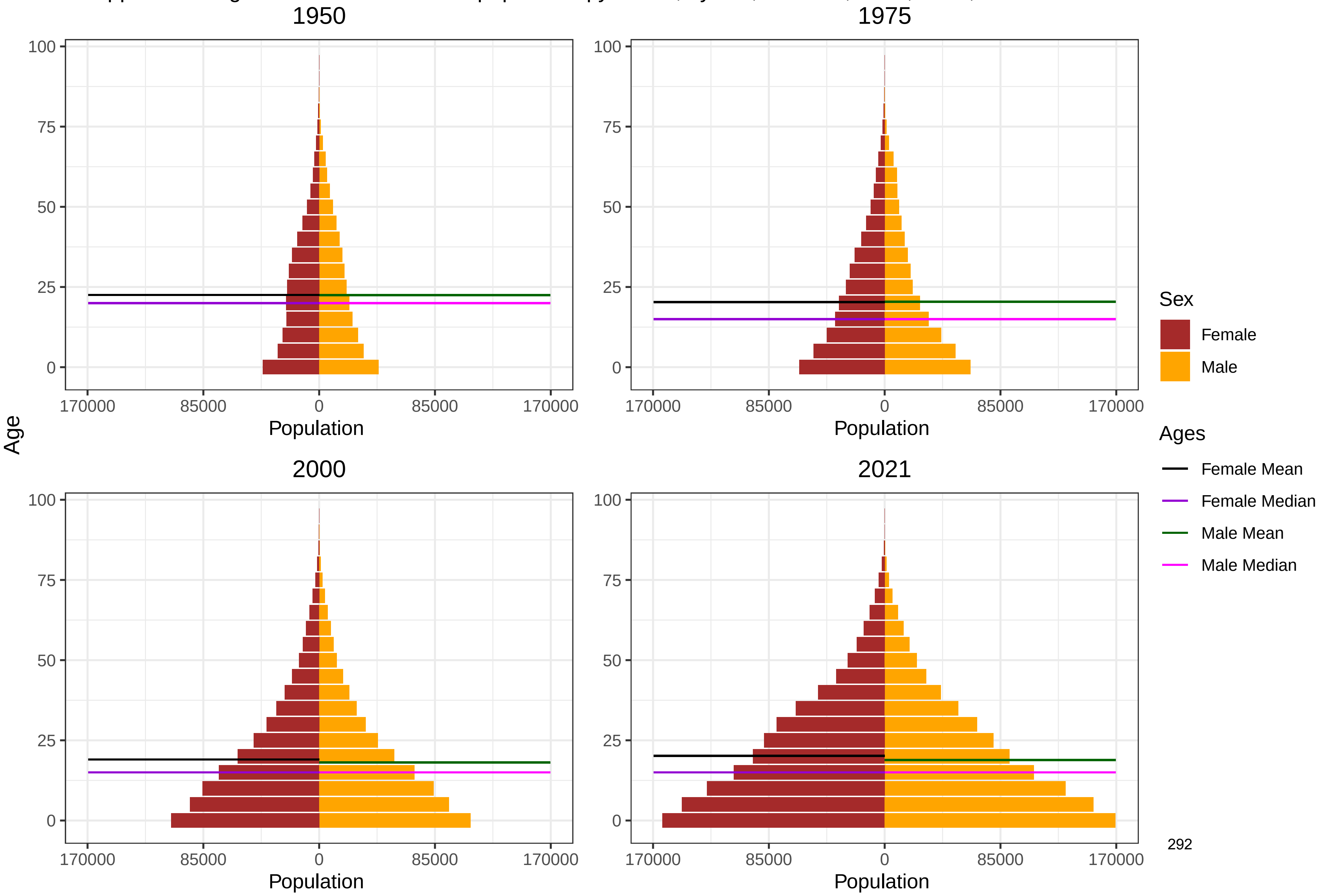

Appendix 2 Figure S9: Guyana population pyramids, by sex, for 1950, 1975, 2000, and 2021

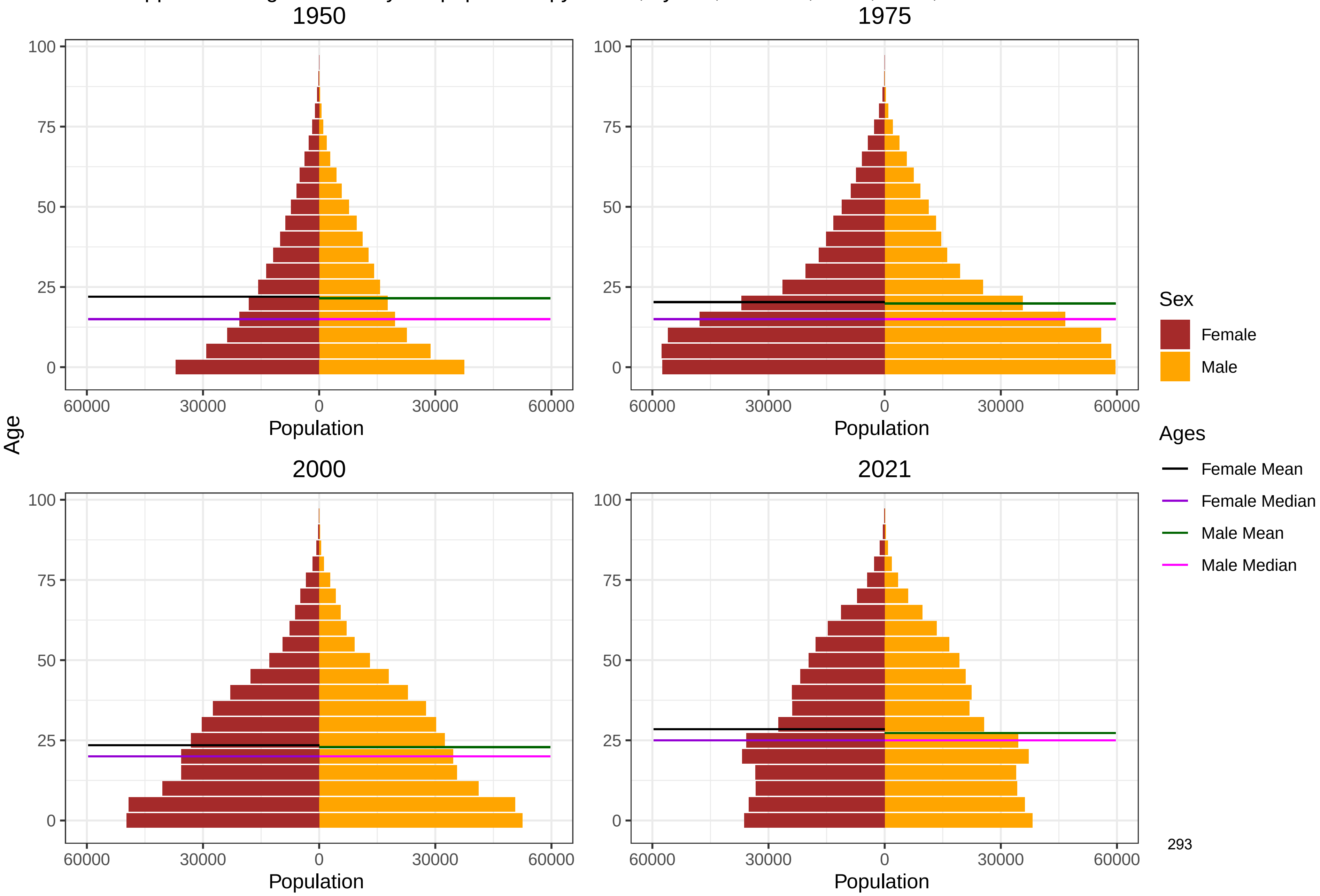

Appendix 2 Figure S9: Haiti population pyramids, by sex, for 1950, 1975, 2000, and 2021

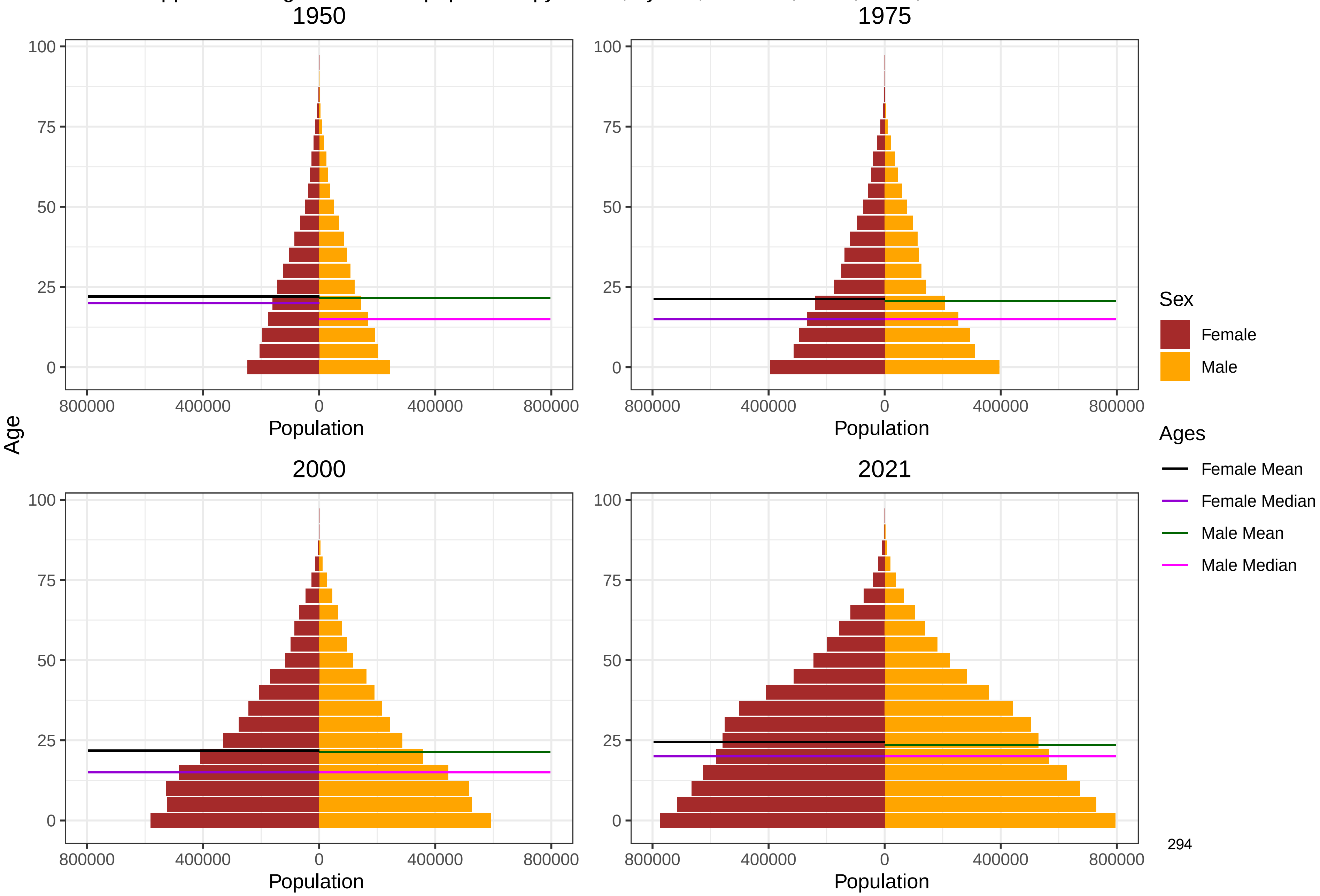

Appendix 2 Figure S9: Honduras population pyramids, by sex, for 1950, 1975, 2000, and 2021

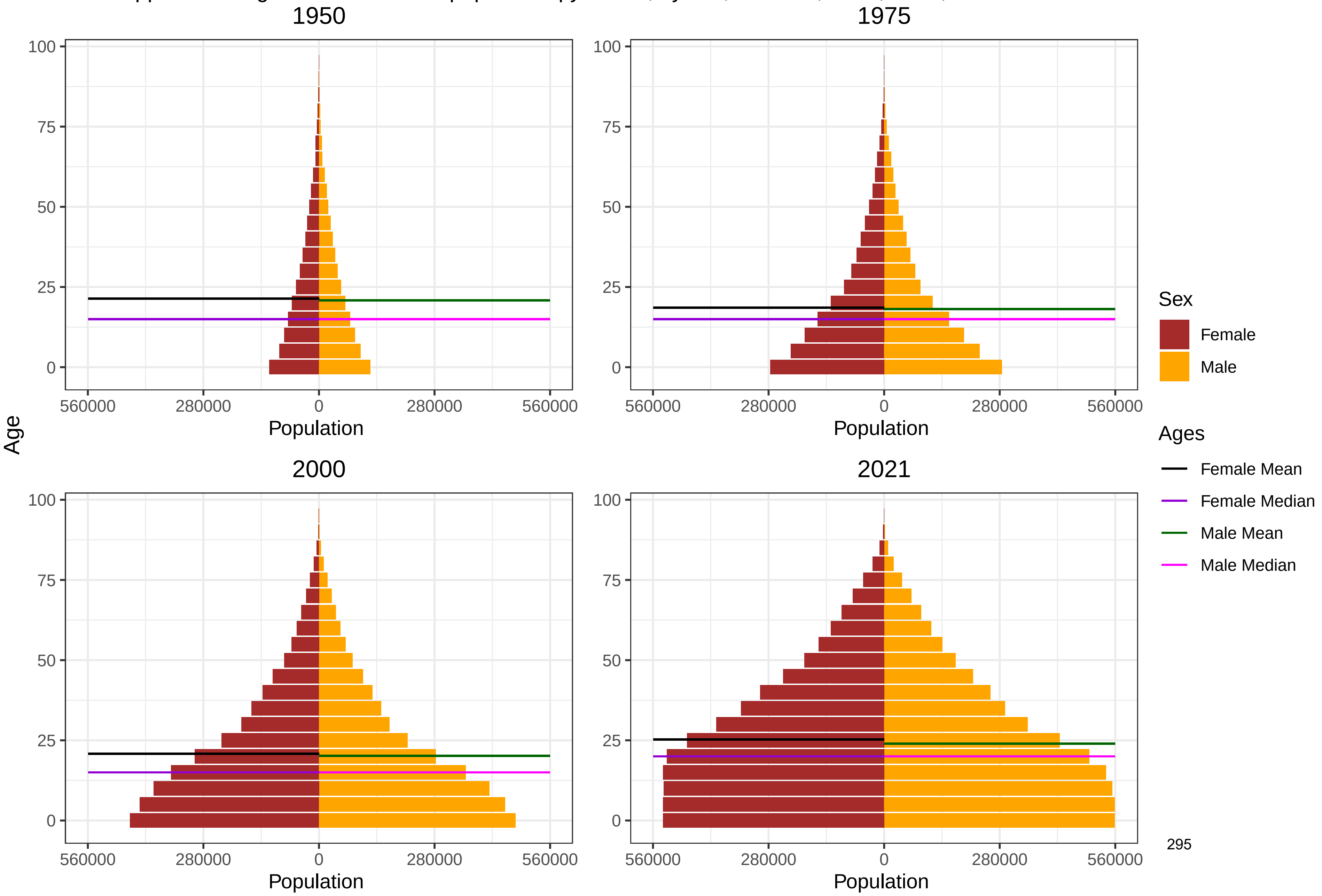

Appendix 2 Figure S9: Hungary population pyramids, by sex, for 1950, 1975, 2000, and 2021

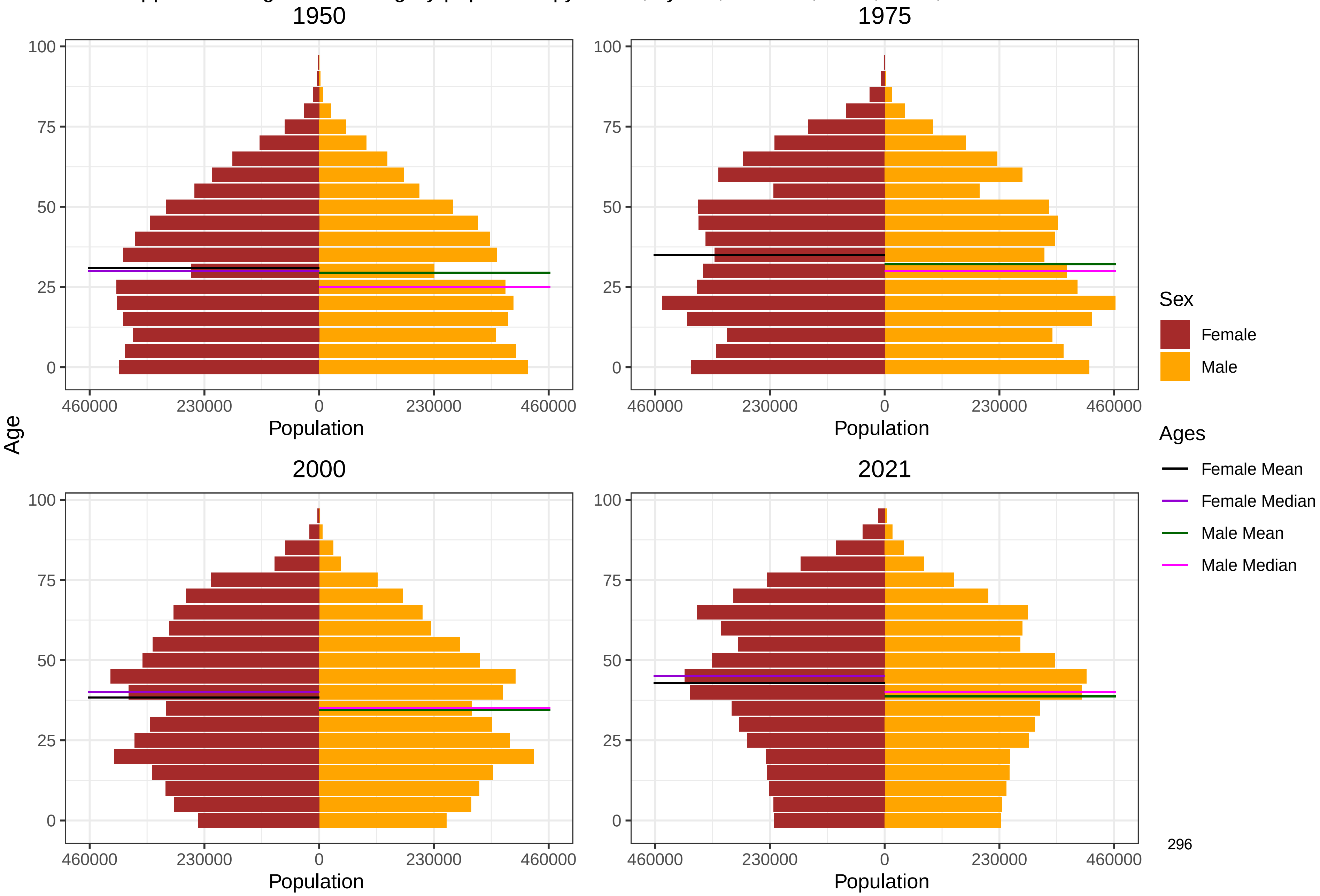

Appendix 2 Figure S9: Iceland population pyramids, by sex, for 1950, 1975, 2000, and 2021

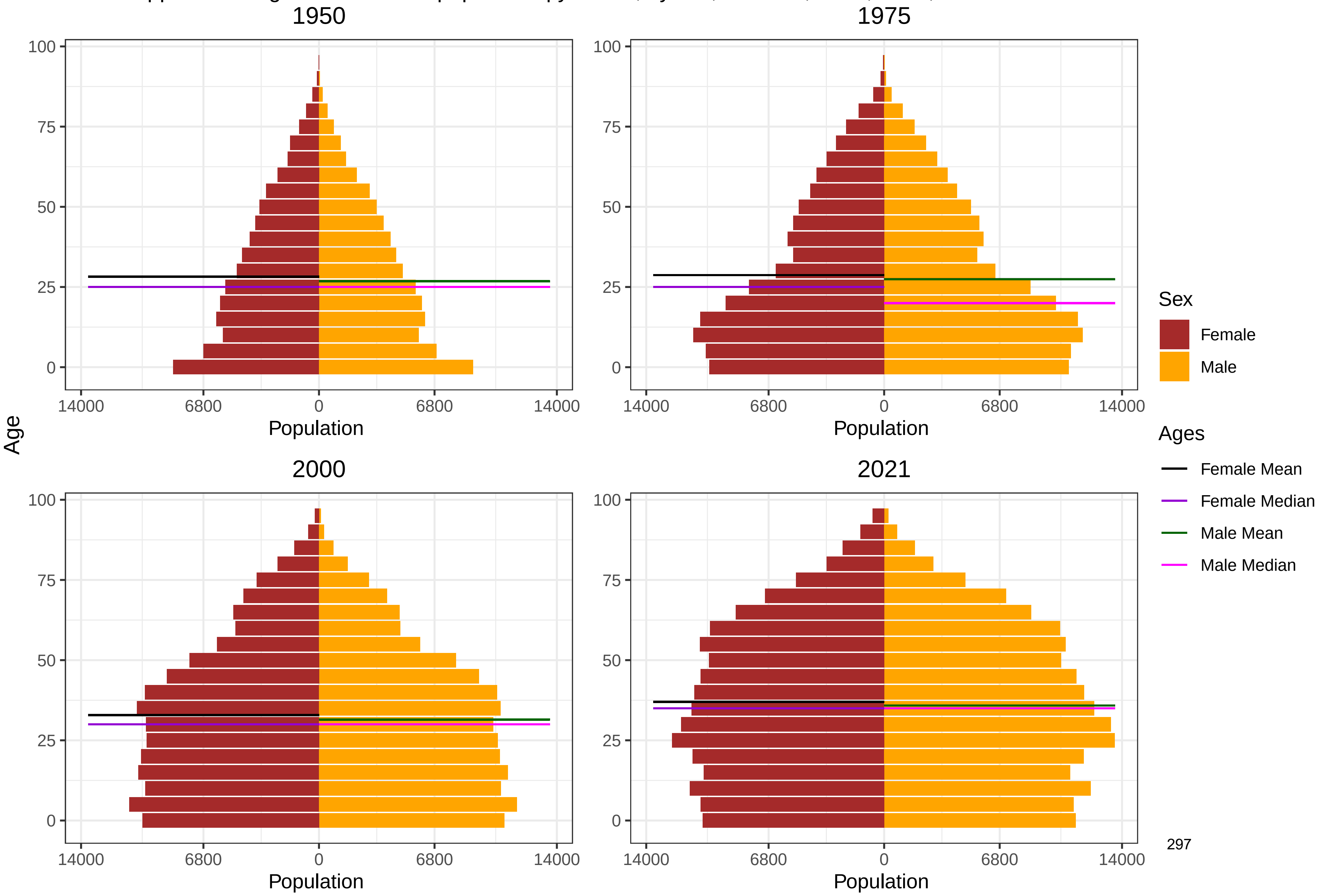

Appendix 2 Figure S9: India population pyramids, by sex, for 1950, 1975, 2000, and 2021

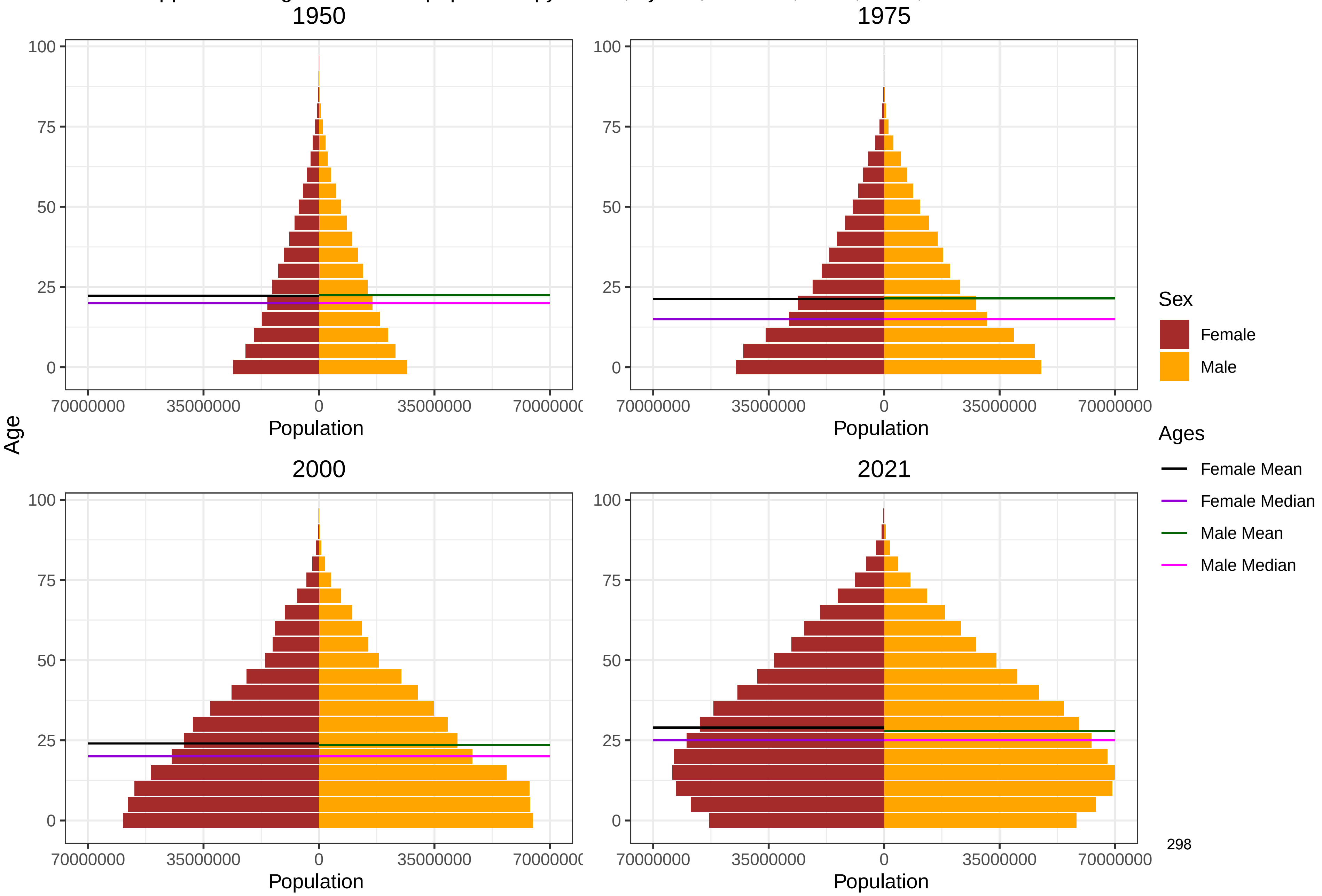

Appendix 2 Figure S9: Indonesia population pyramids, by sex, for 1950, 1975, 2000, and 2021

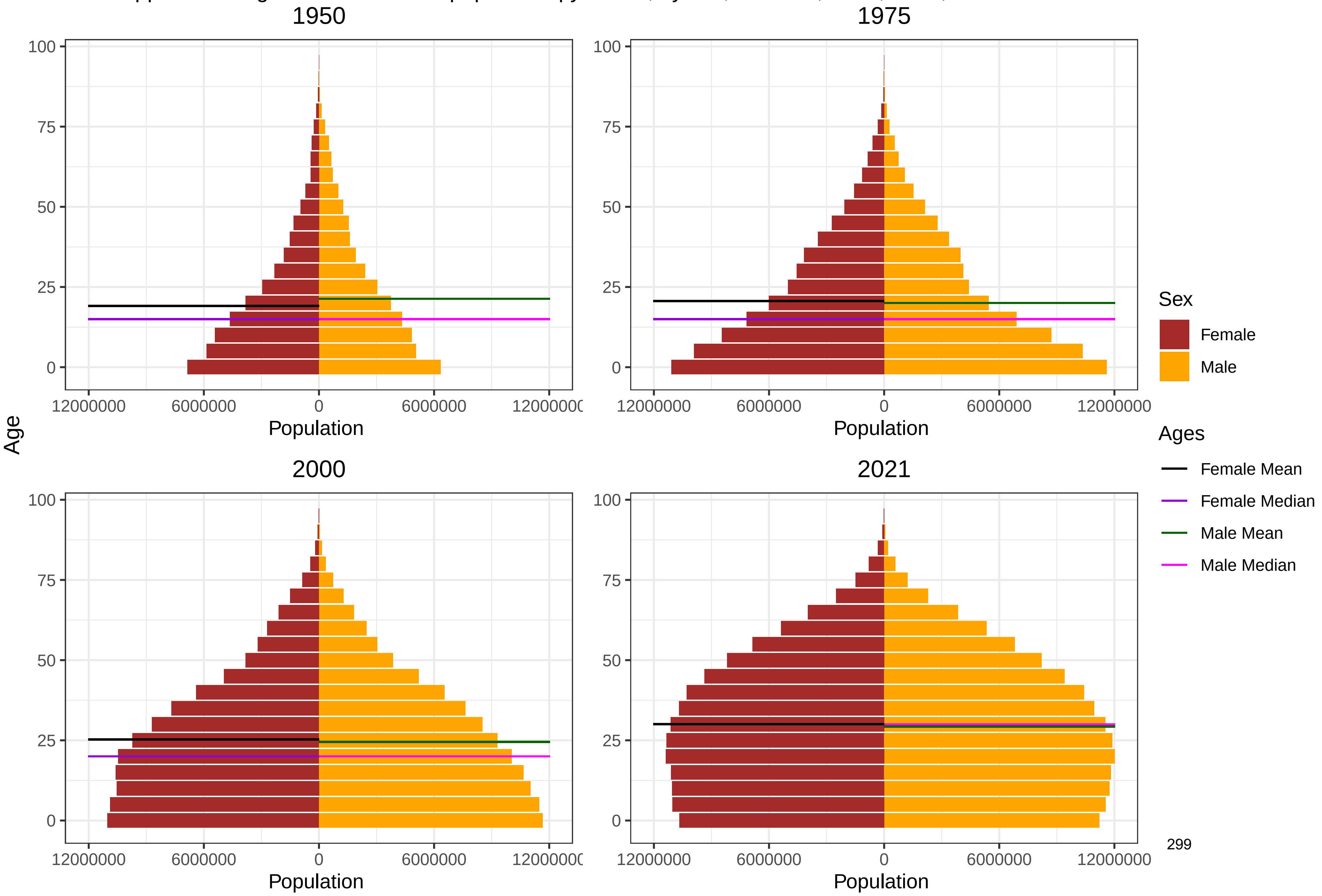

Appendix 2 Figure S9: Iran population pyramids, by sex, for 1950, 1975, 2000, and 2021

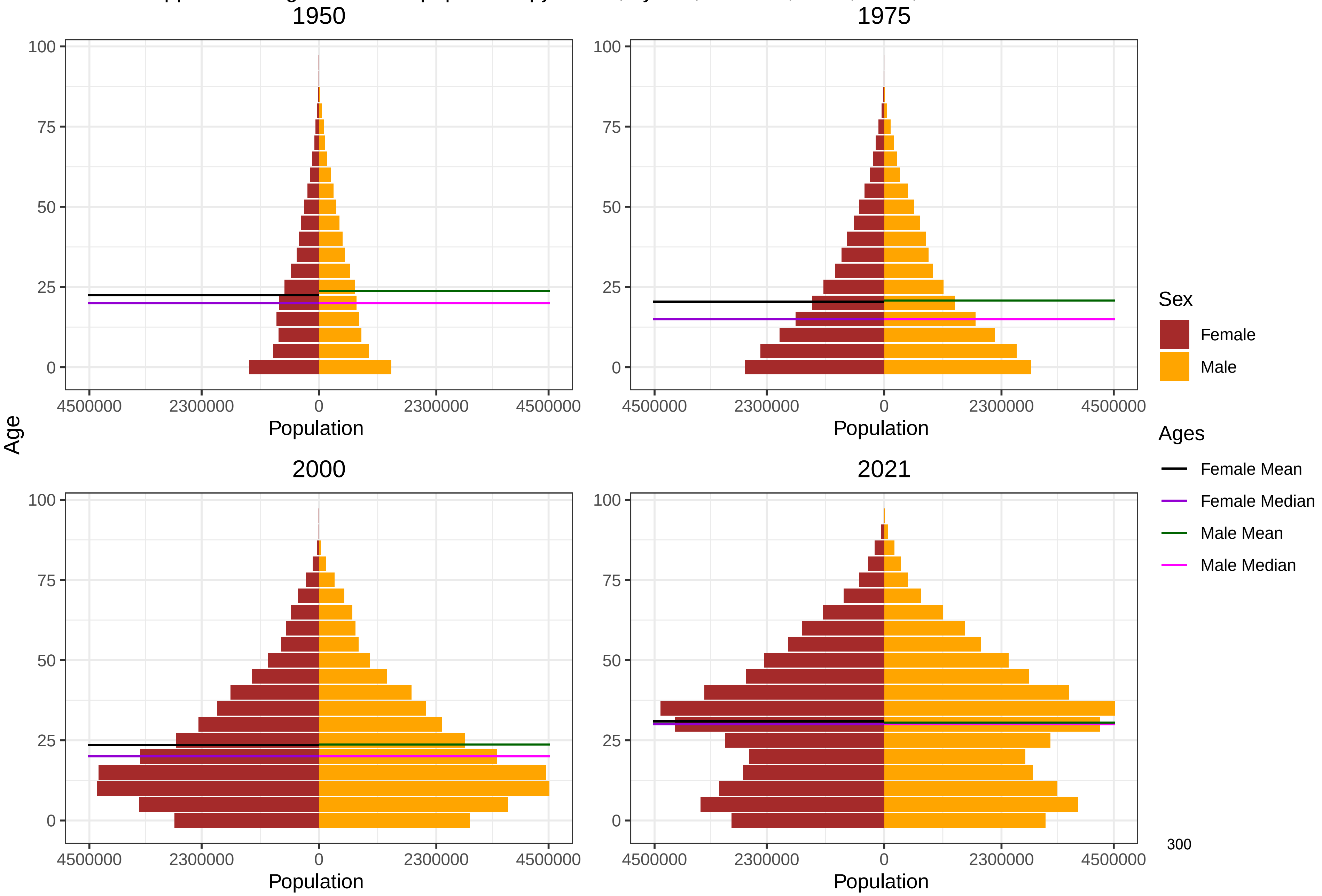

Appendix 2 Figure S9: Iraq population pyramids, by sex, for 1950, 1975, 2000, and 2021

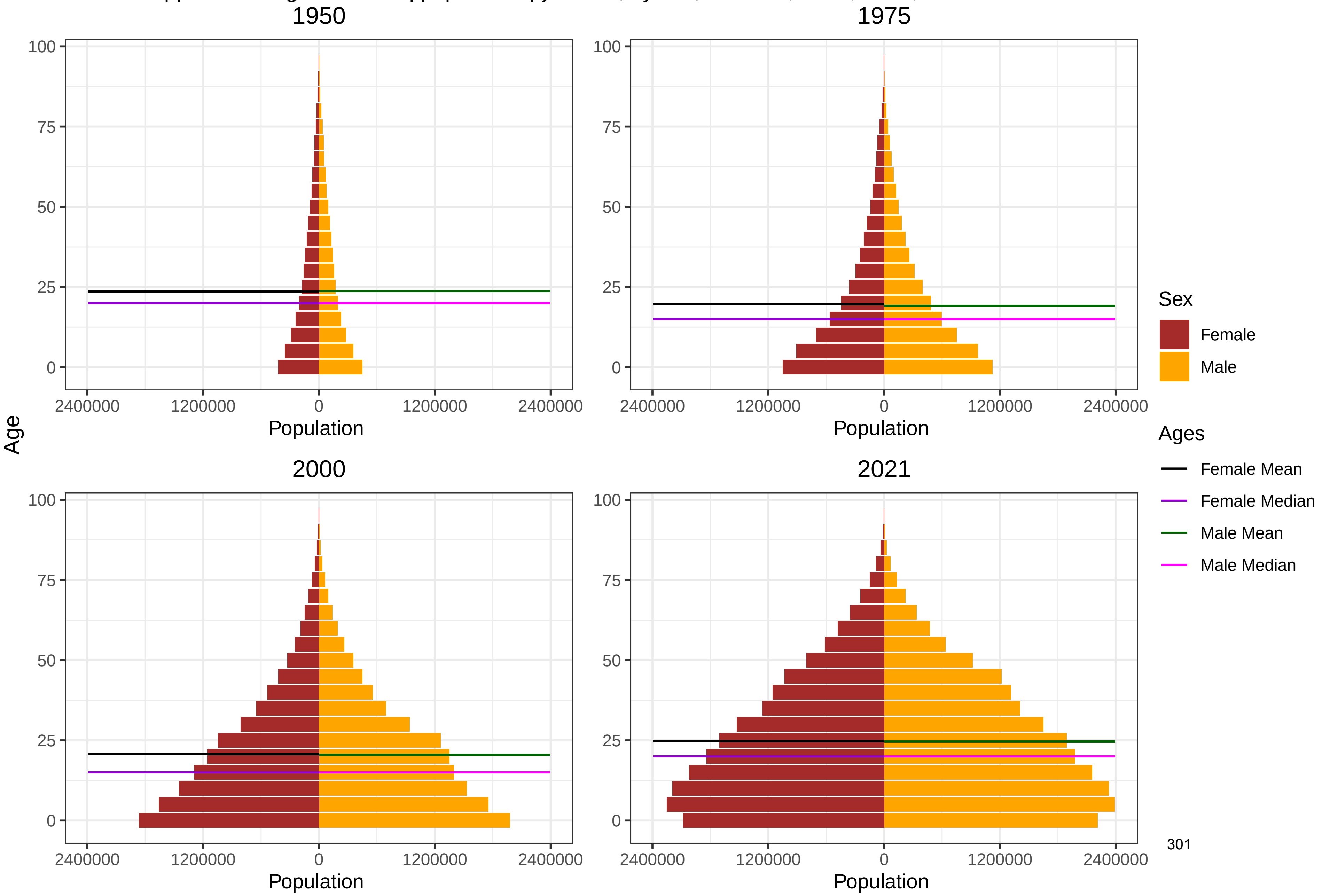

Appendix 2 Figure S9: Ireland population pyramids, by sex, for 1950, 1975, 2000, and 2021

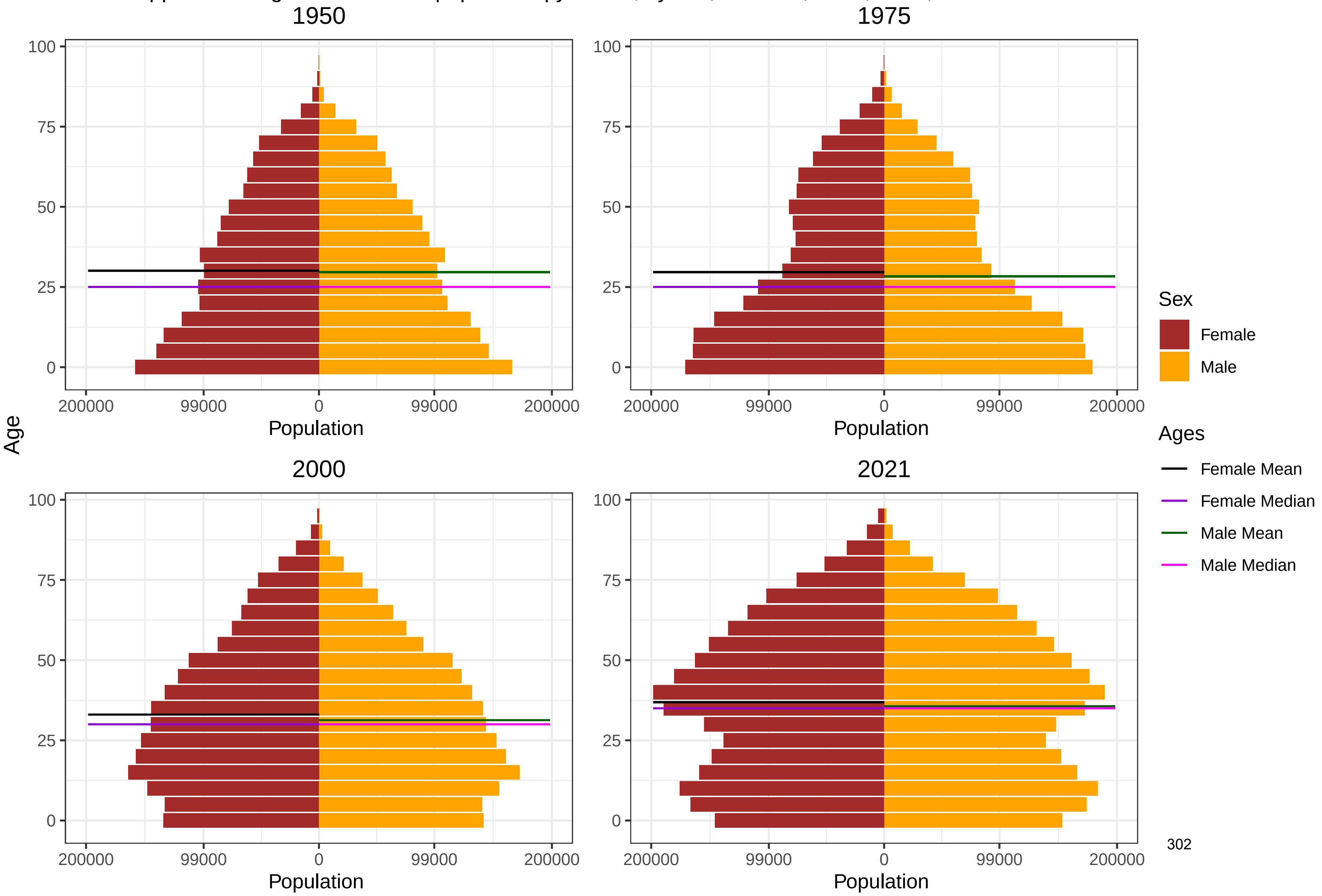

Appendix 2 Figure S9: Israel population pyramids, by sex, for 1950, 1975, 2000, and 2021

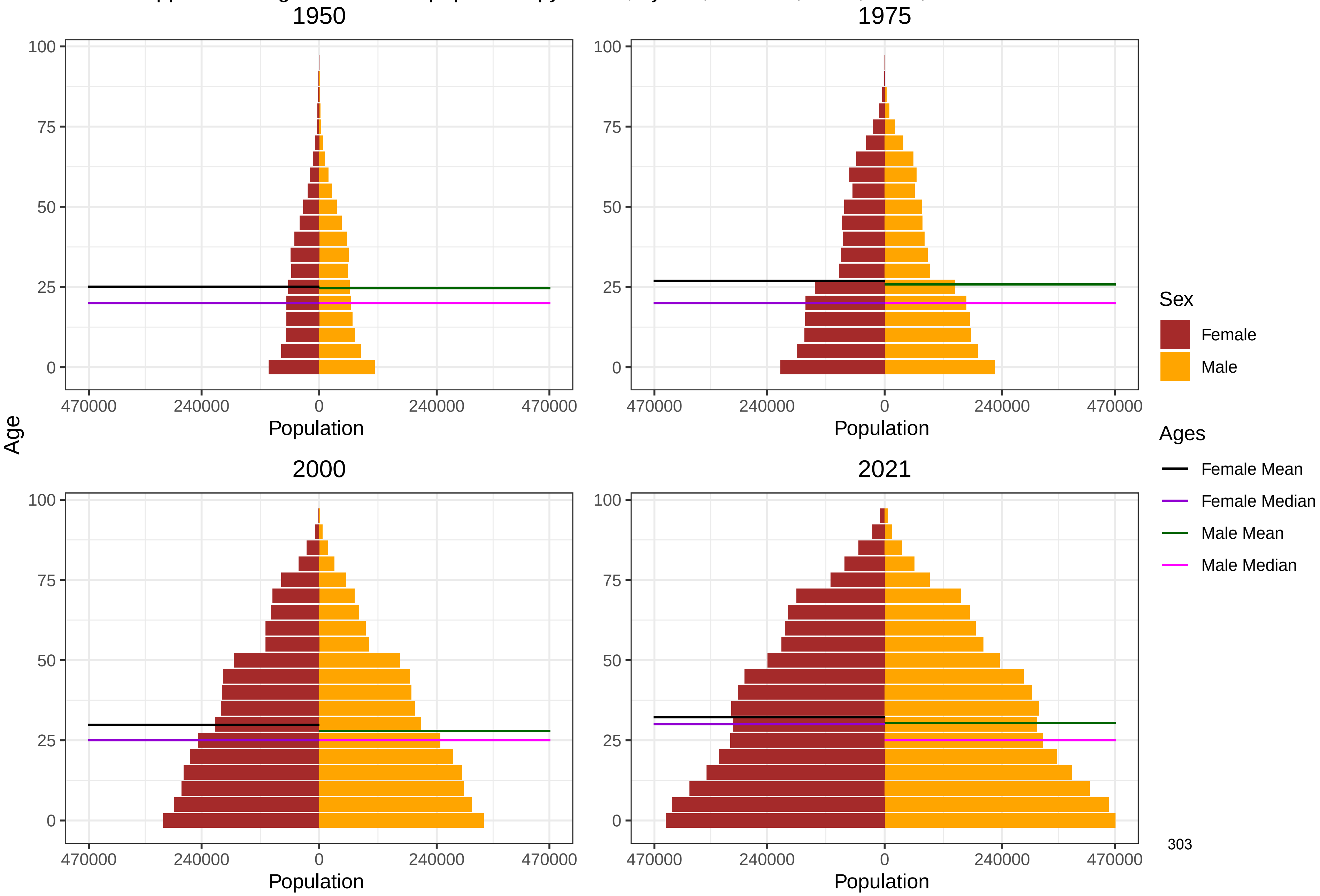

Appendix 2 Figure S9: Italy population pyramids, by sex, for 1950, 1975, 2000, and 2021

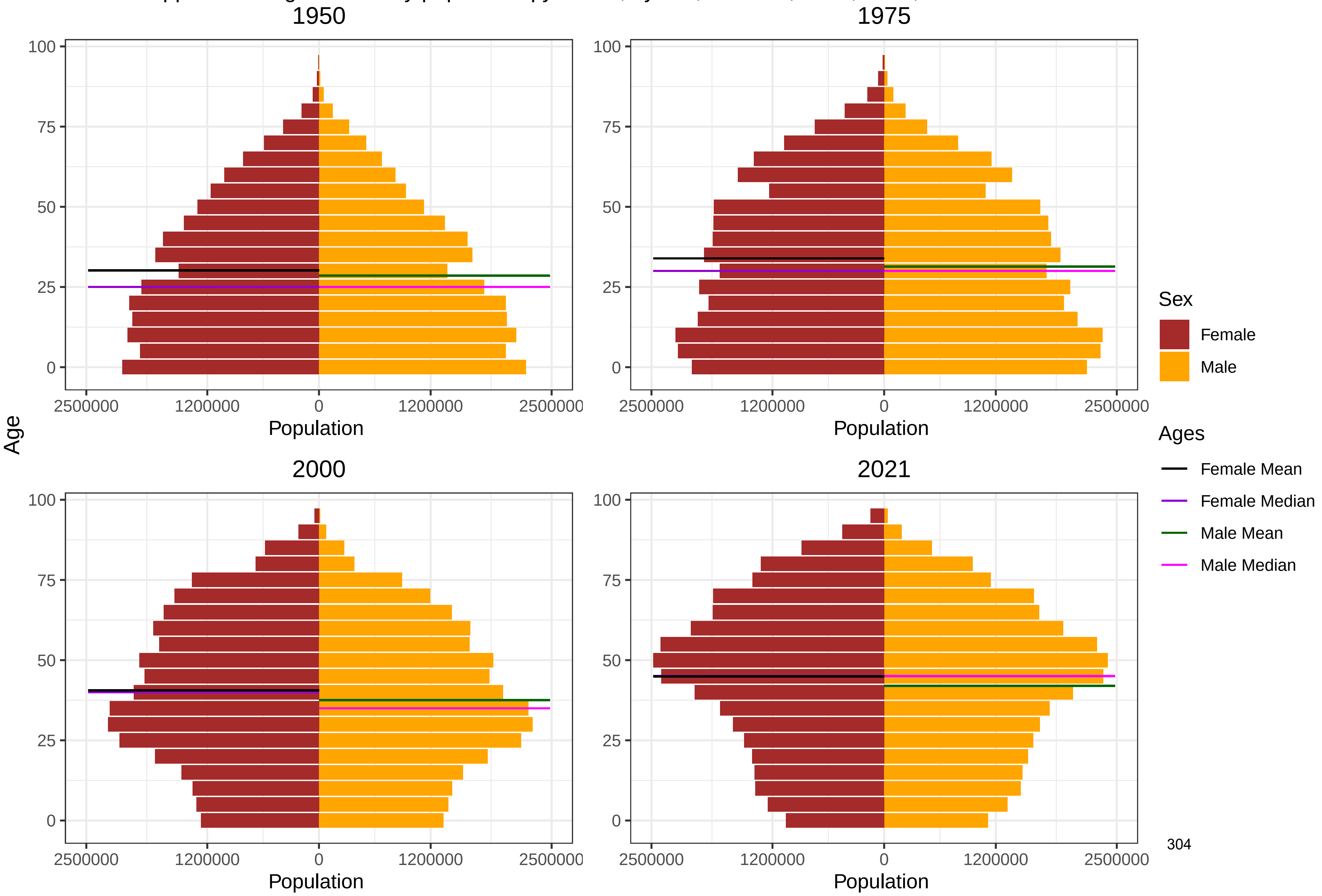

Appendix 2 Figure S9: Jamaica population pyramids, by sex, for 1950, 1975, 2000, and 2021

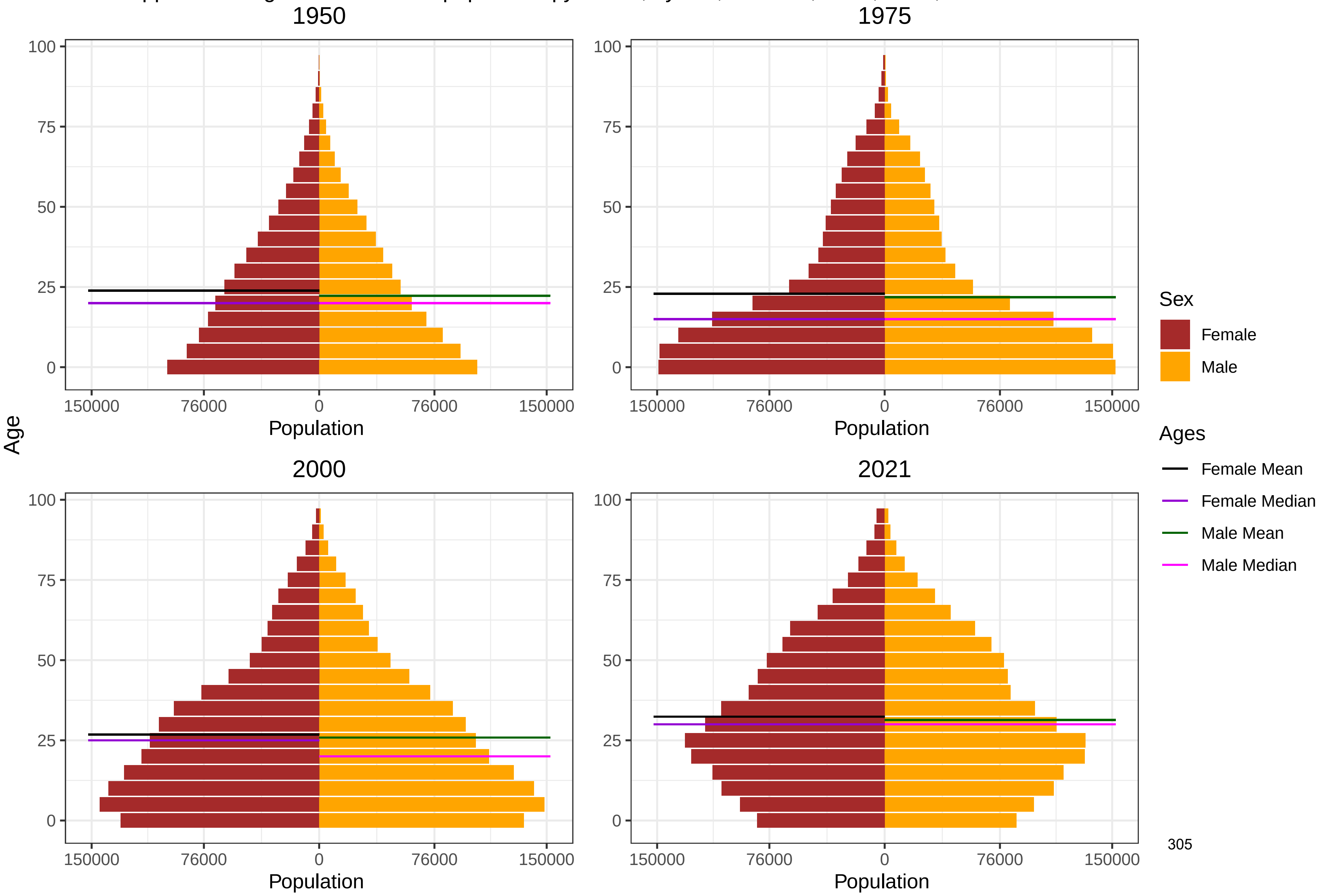

Appendix 2 Figure S9: Japan population pyramids, by sex, for 1950, 1975, 2000, and 2021

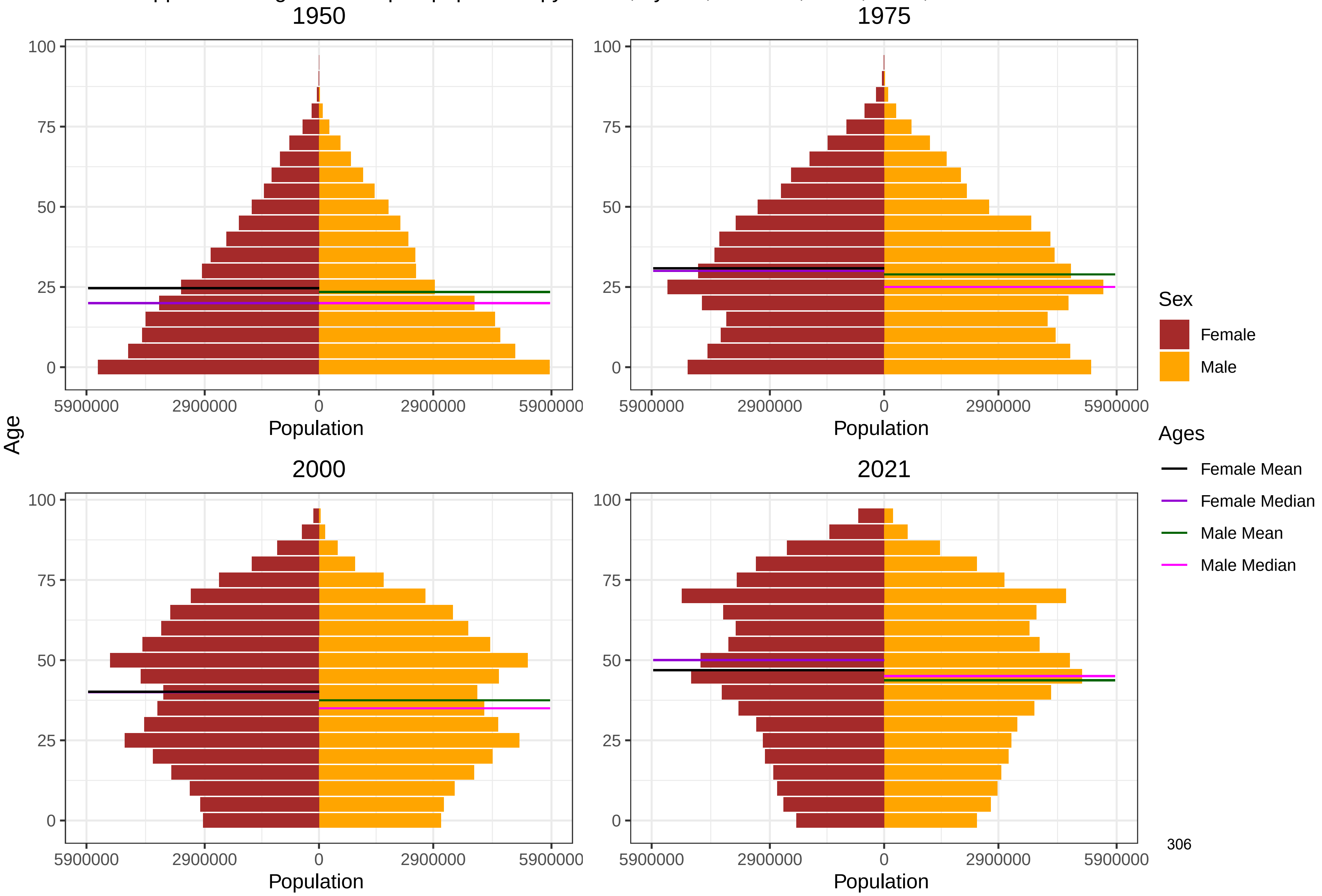

Appendix 2 Figure S9: Jordan population pyramids, by sex, for 1950, 1975, 2000, and 2021

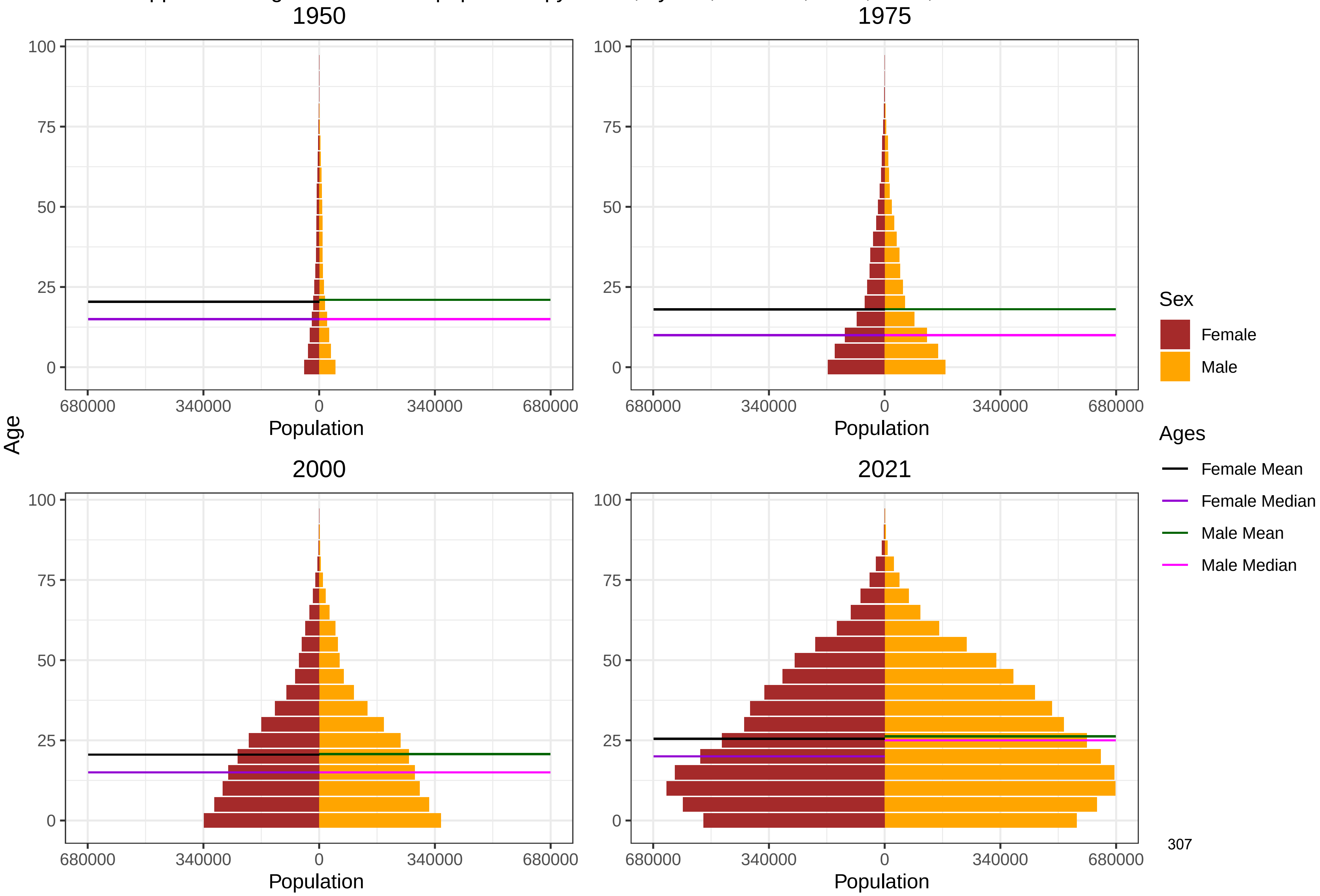

Appendix 2 Figure S9: Kazakhstan population pyramids, by sex, for 1950, 1975, 2000, and 2021

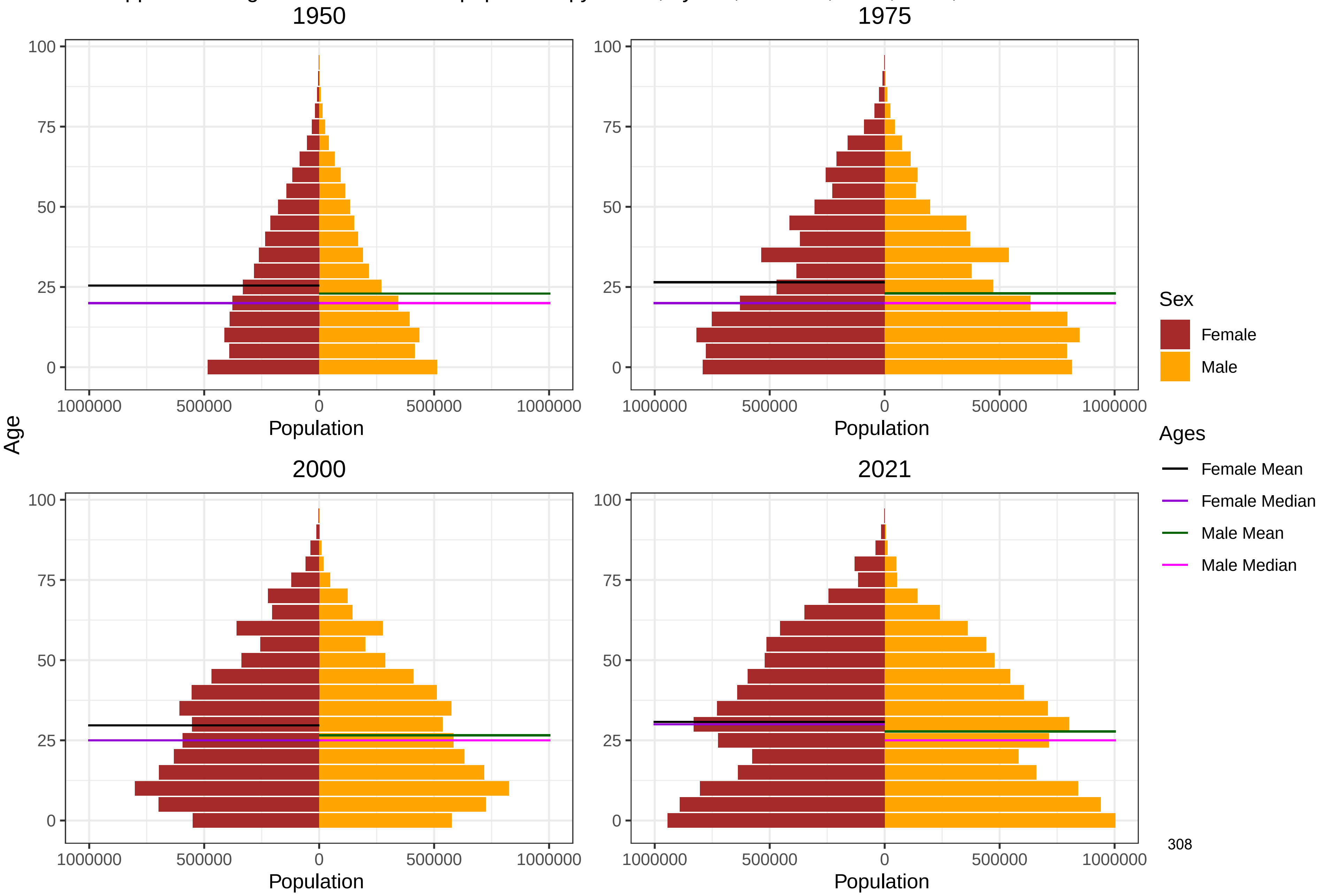

Appendix 2 Figure S9: Kenya population pyramids, by sex, for 1950, 1975, 2000, and 2021

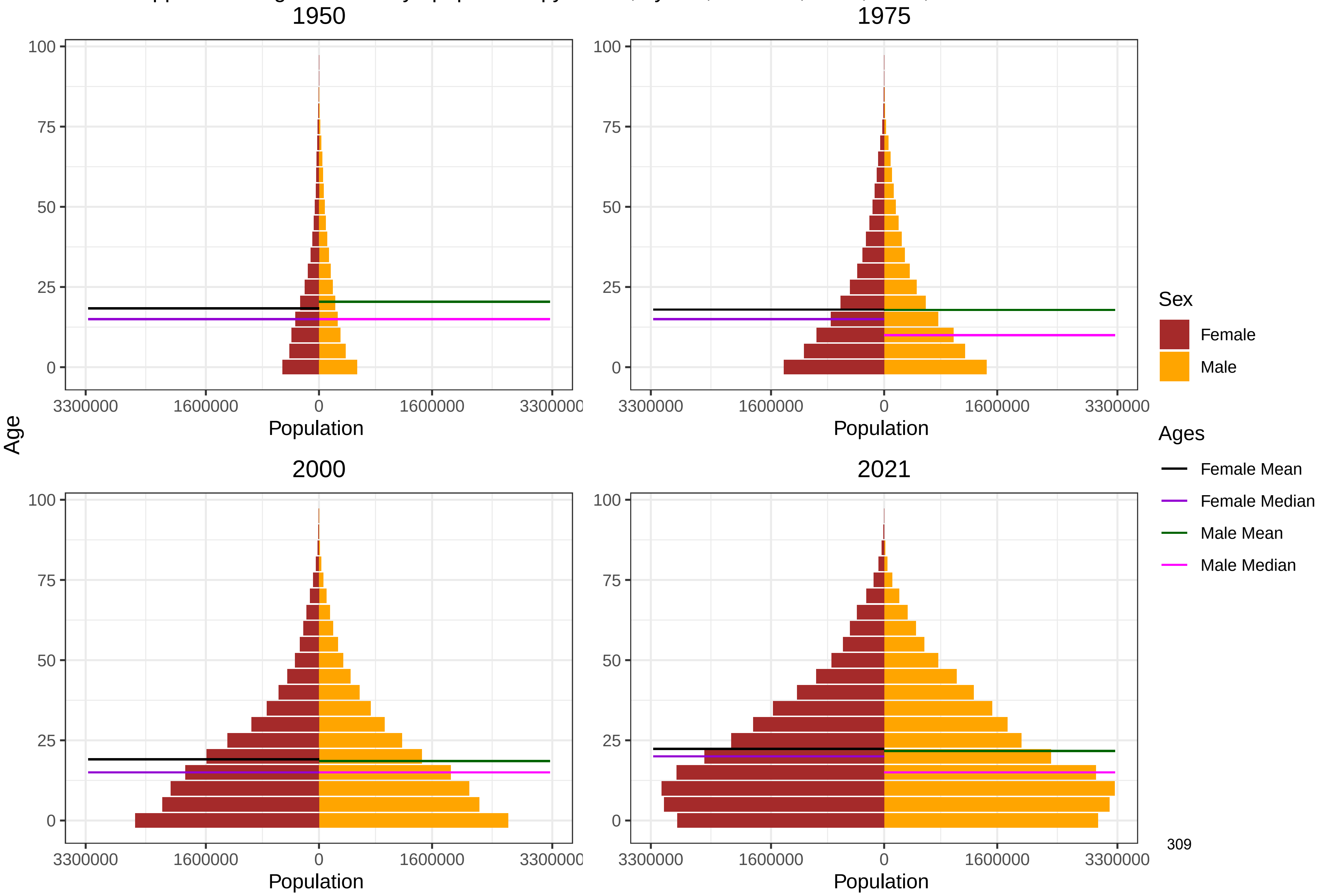

Appendix 2 Figure S9: Kiribati population pyramids, by sex, for 1950, 1975, 2000, and 2021

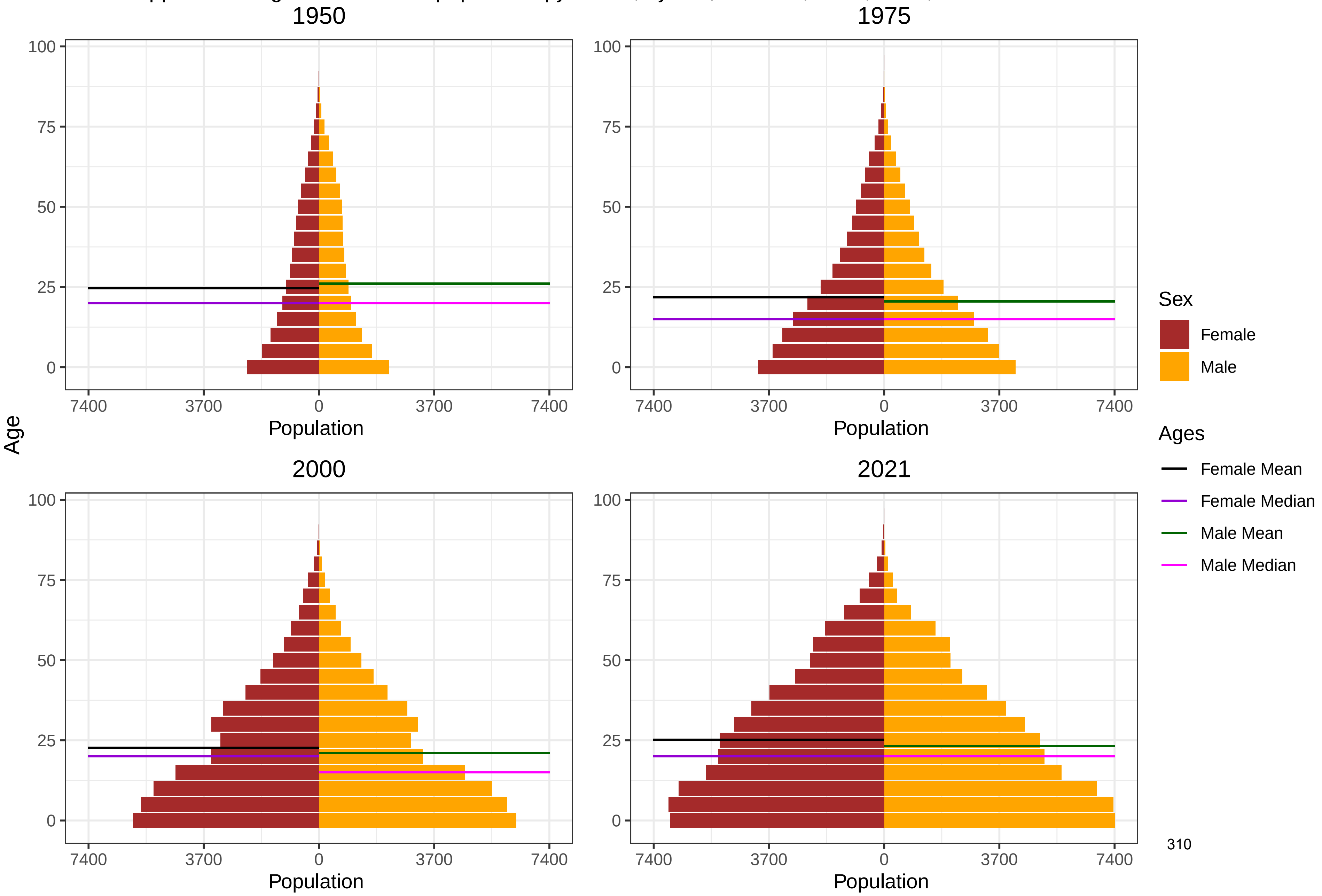

Appendix 2 Figure S9: Kuwait population pyramids, by sex, for 1950, 1975, 2000, and 2021

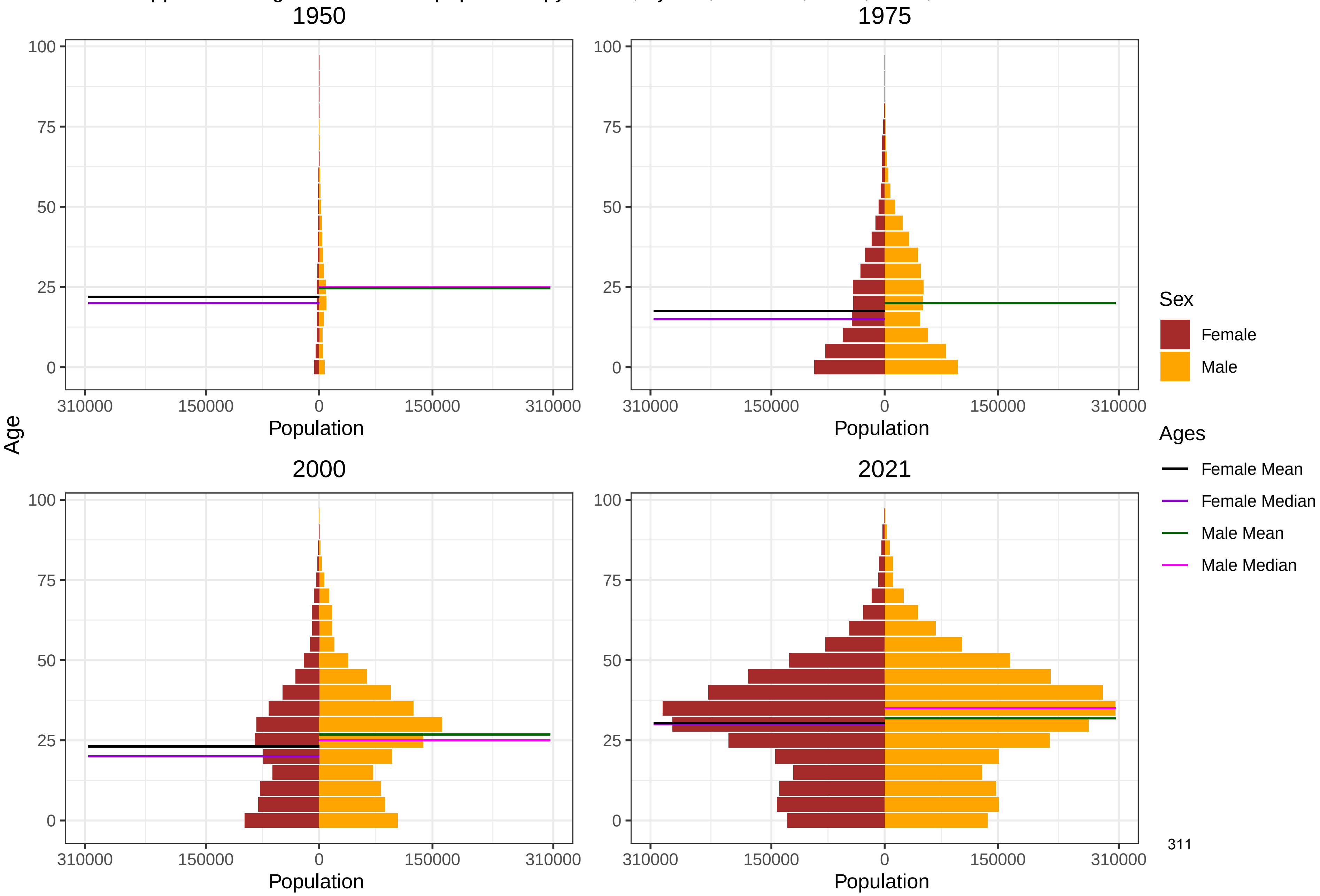

Appendix 2 Figure S9: Kyrgyzstan population pyramids, by sex, for 1950, 1975, 2000, and 2021

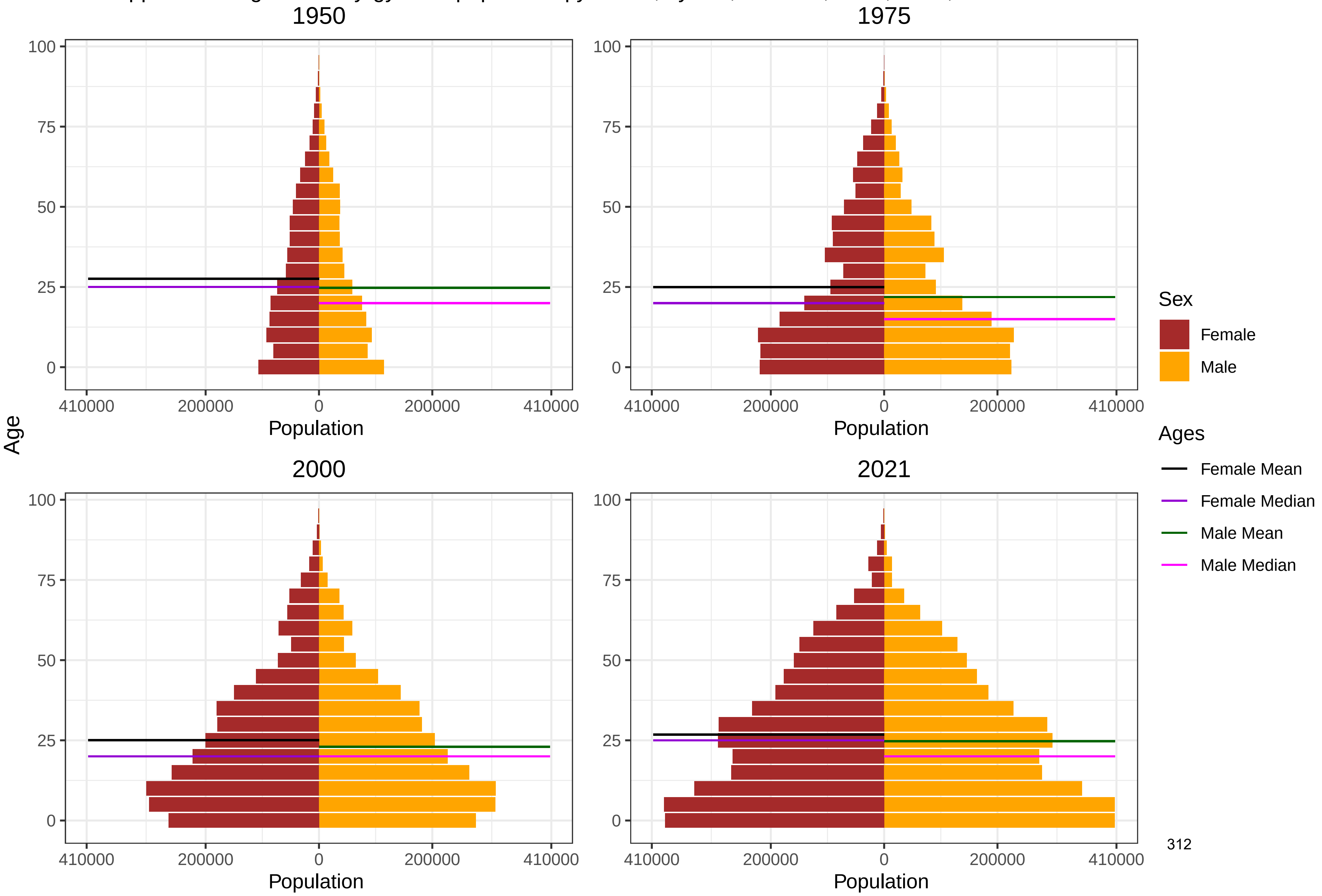

Appendix 2 Figure S9: Laos population pyramids, by sex, for 1950, 1975, 2000, and 2021

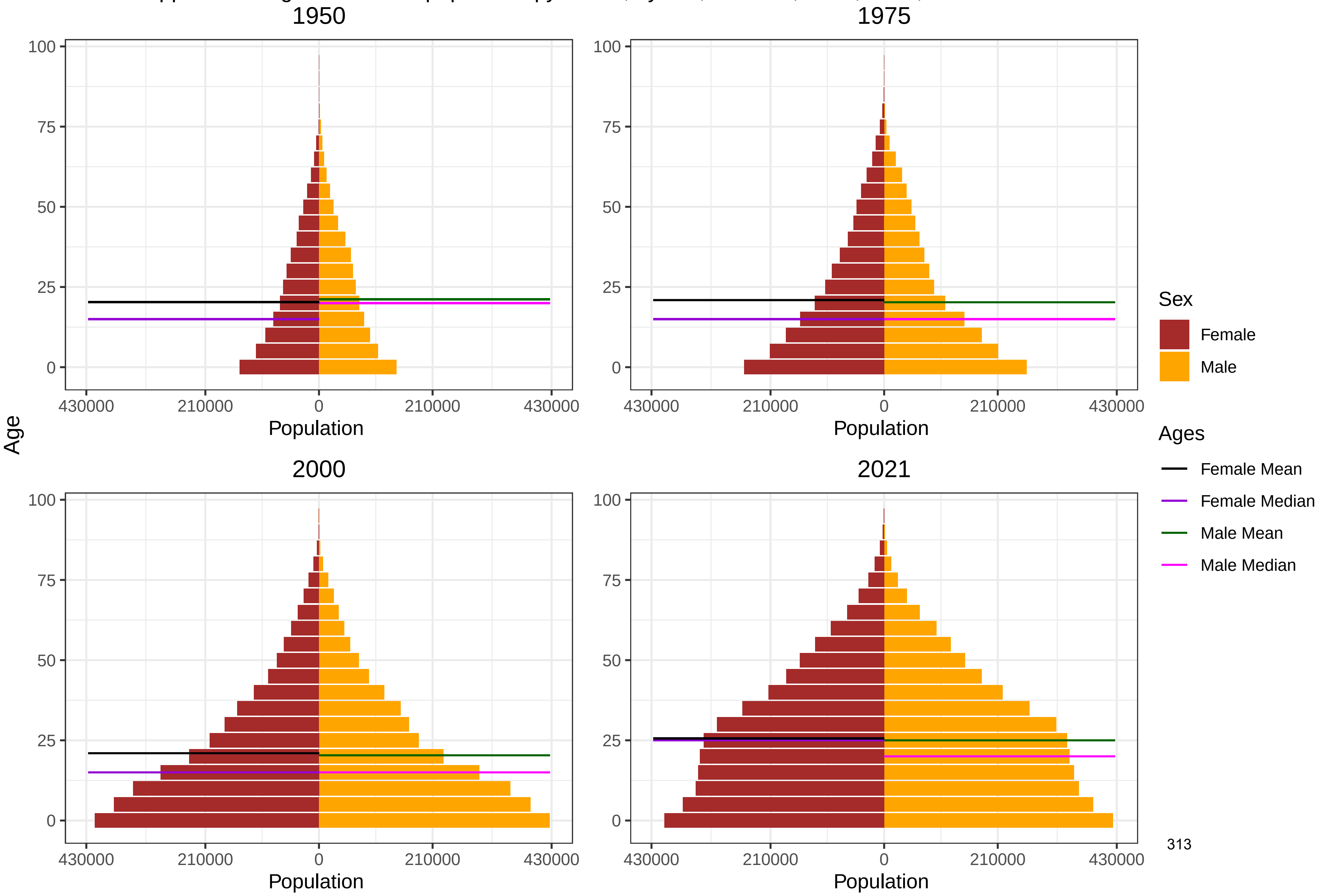

Appendix 2 Figure S9: Latvia population pyramids, by sex, for 1950, 1975, 2000, and 2021

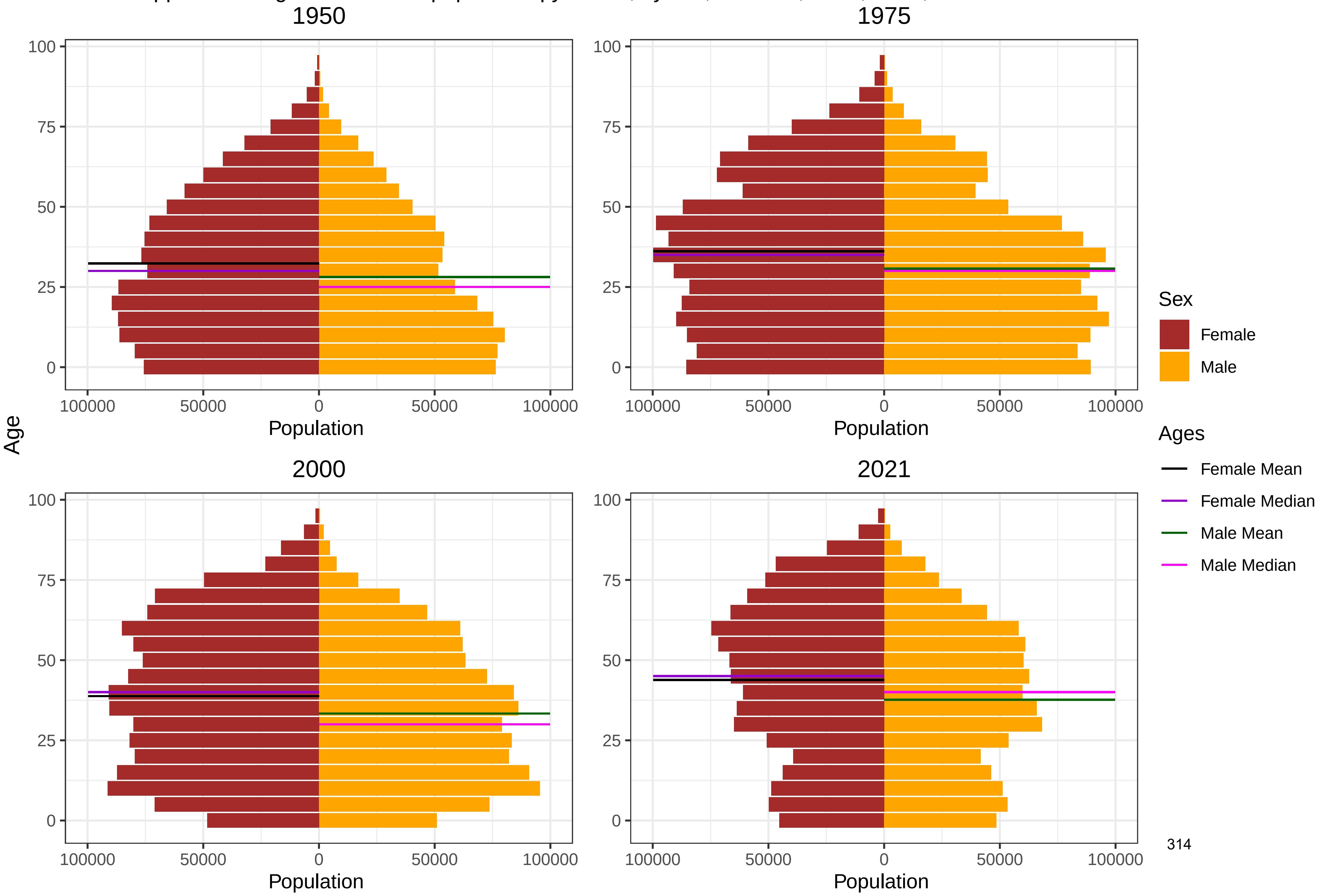

Appendix 2 Figure S9: Lebanon population pyramids, by sex, for 1950, 1975, 2000, and 2021

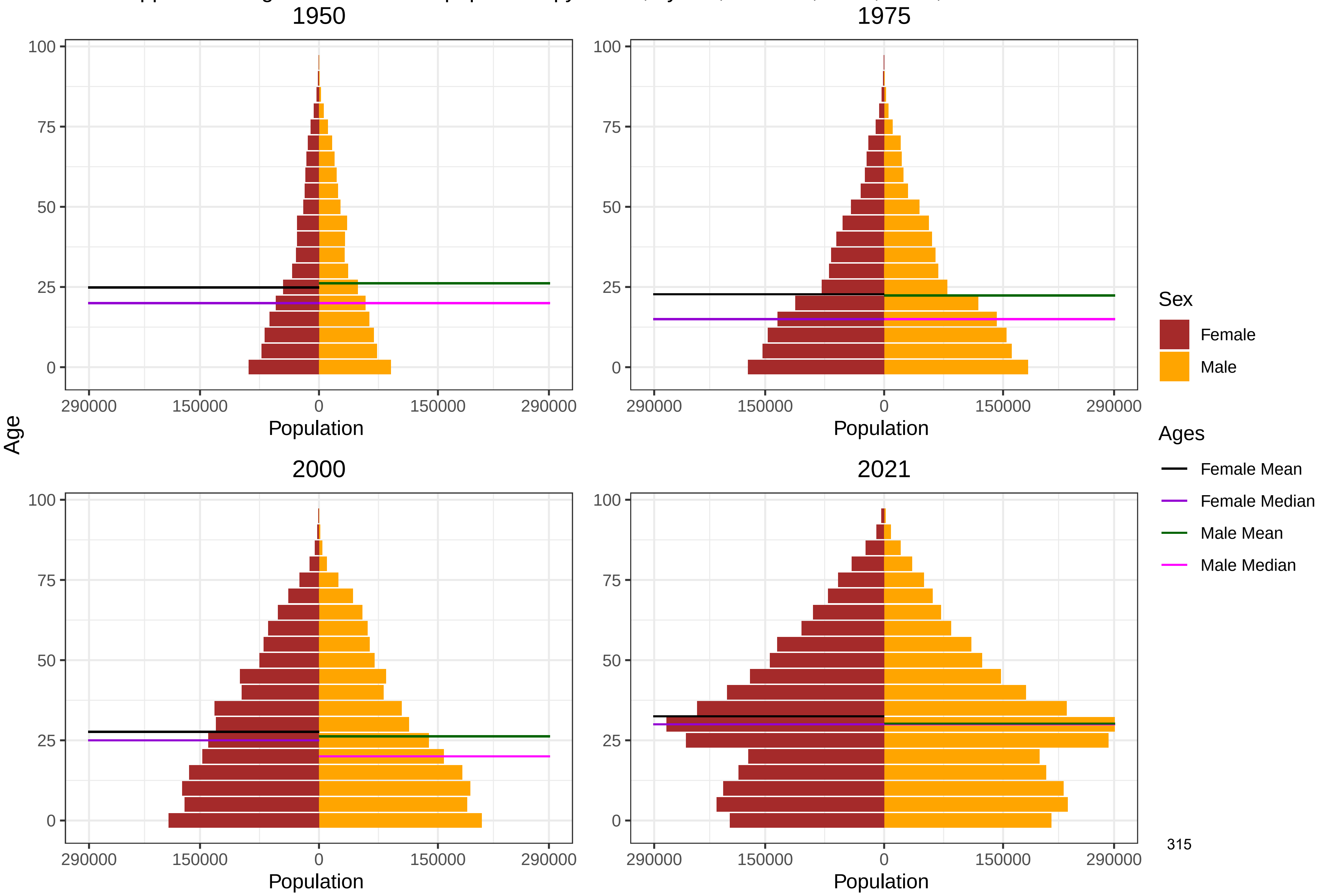

Appendix 2 Figure S9: Lesotho population pyramids, by sex, for 1950, 1975, 2000, and 2021

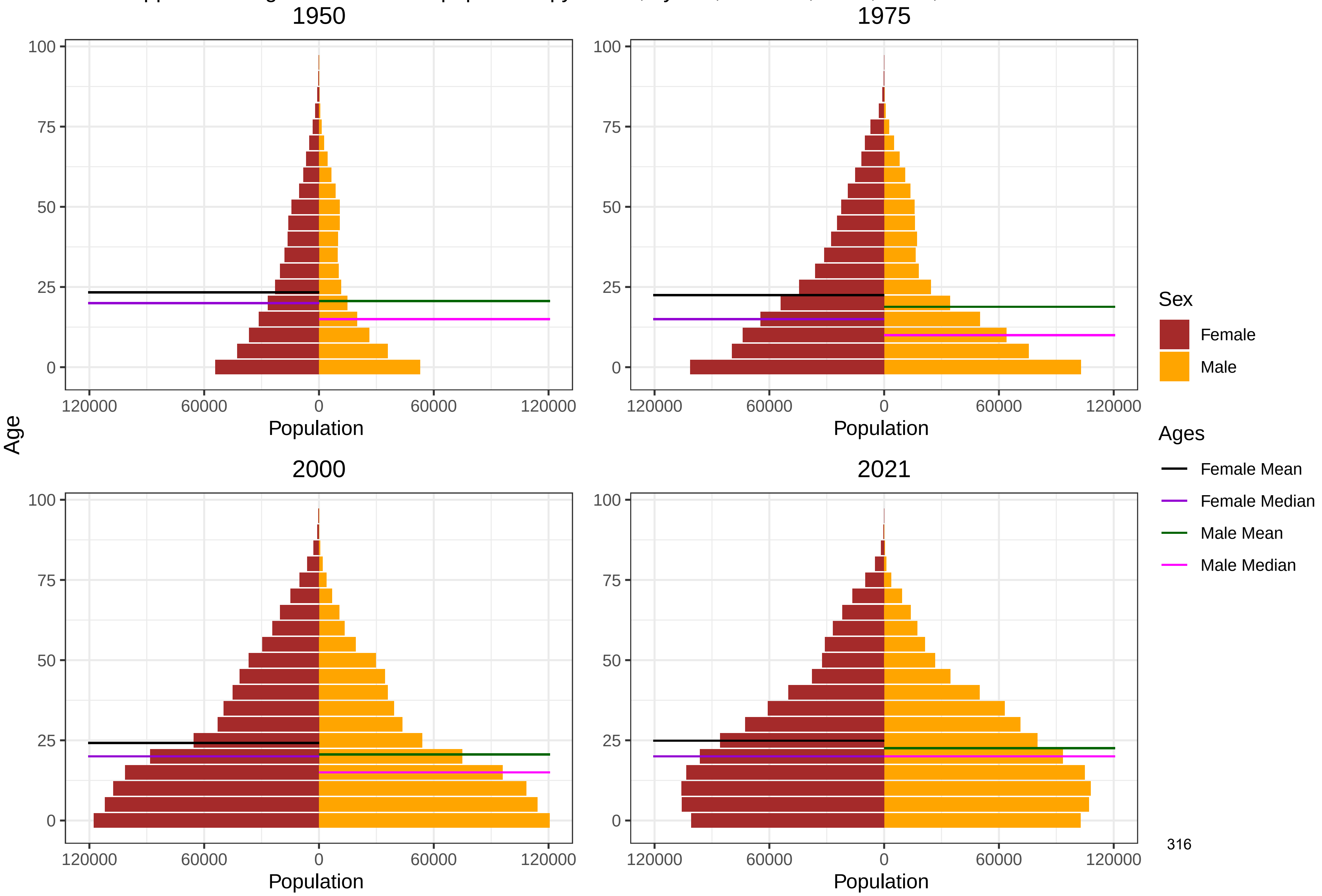

Appendix 2 Figure S9: Liberia population pyramids, by sex, for 1950, 1975, 2000, and 2021

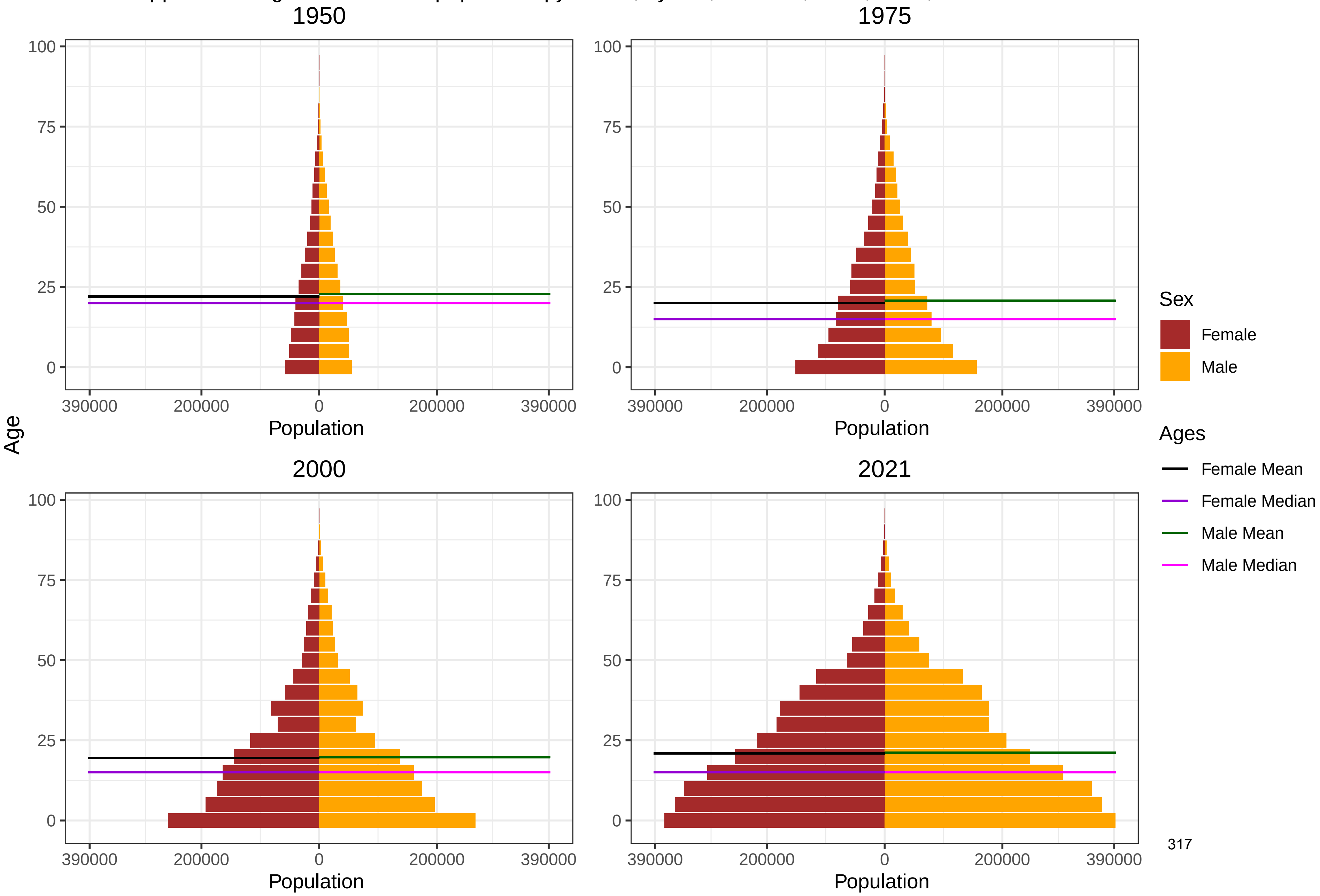

Appendix 2 Figure S9: Libya population pyramids, by sex, for 1950, 1975, 2000, and 2021

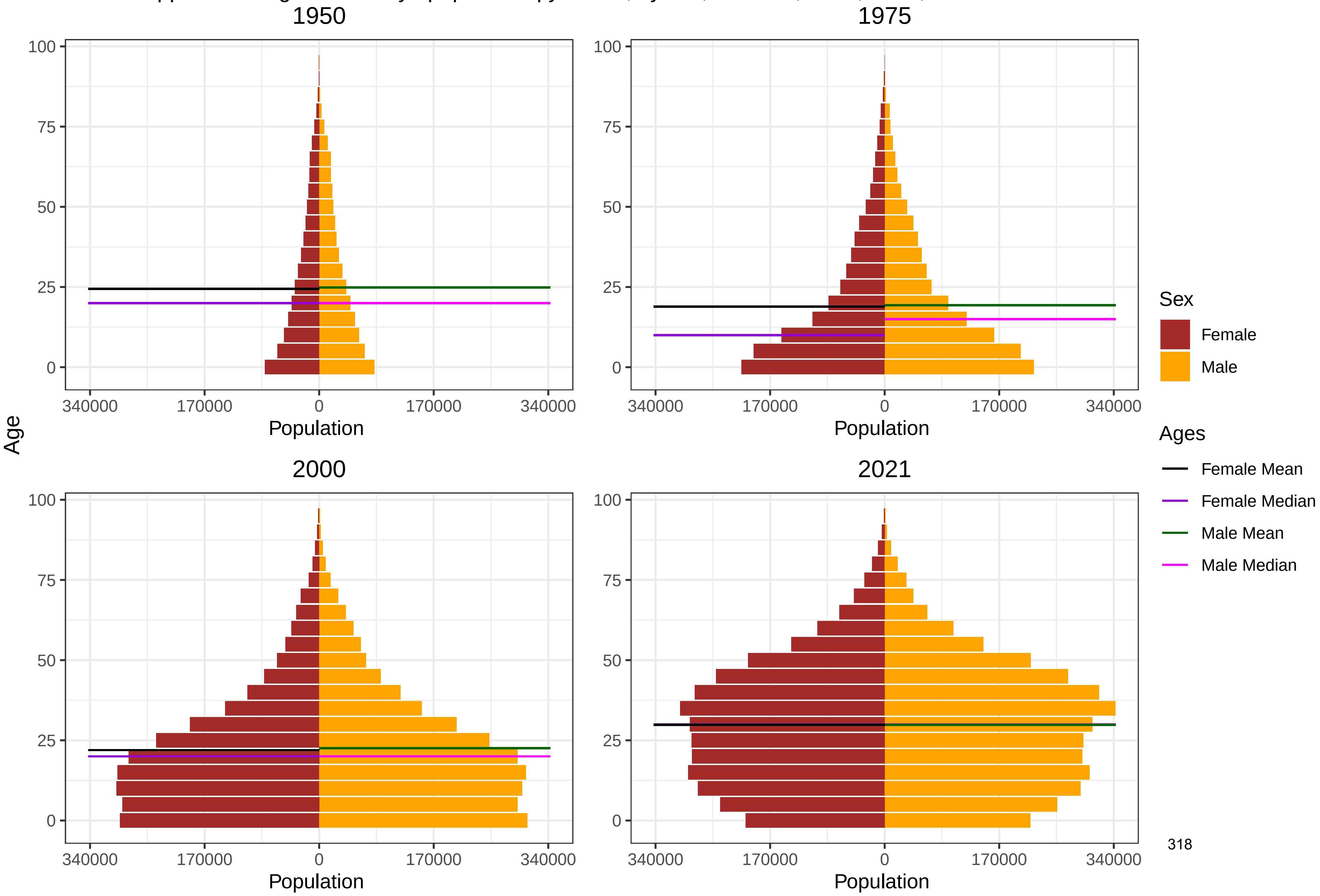

Appendix 2 Figure S9: Lithuania population pyramids, by sex, for 1950, 1975, 2000, and 2021

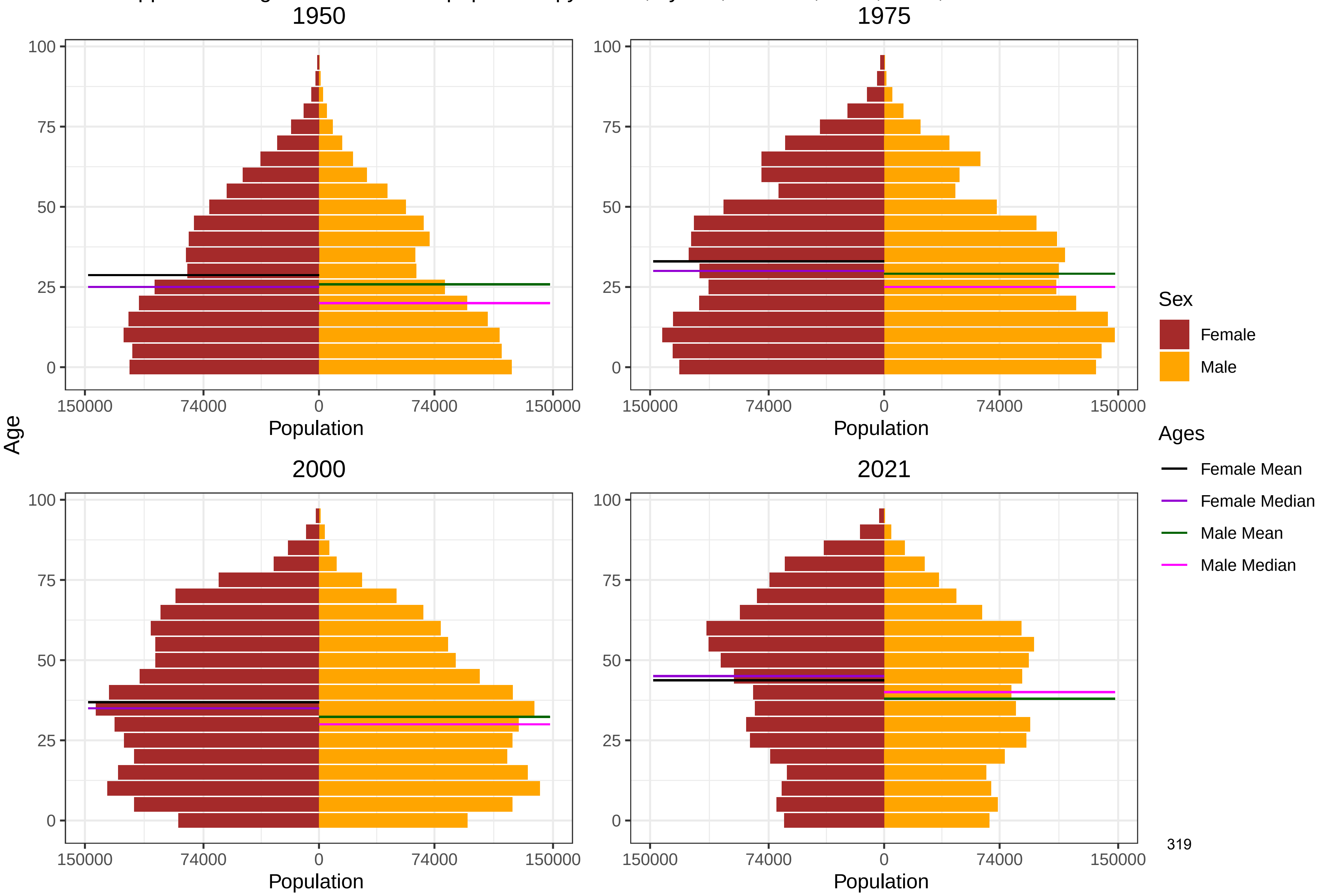

Appendix 2 Figure S9: Luxembourg population pyramids, by sex, for 1950, 1975, 2000, and 2021

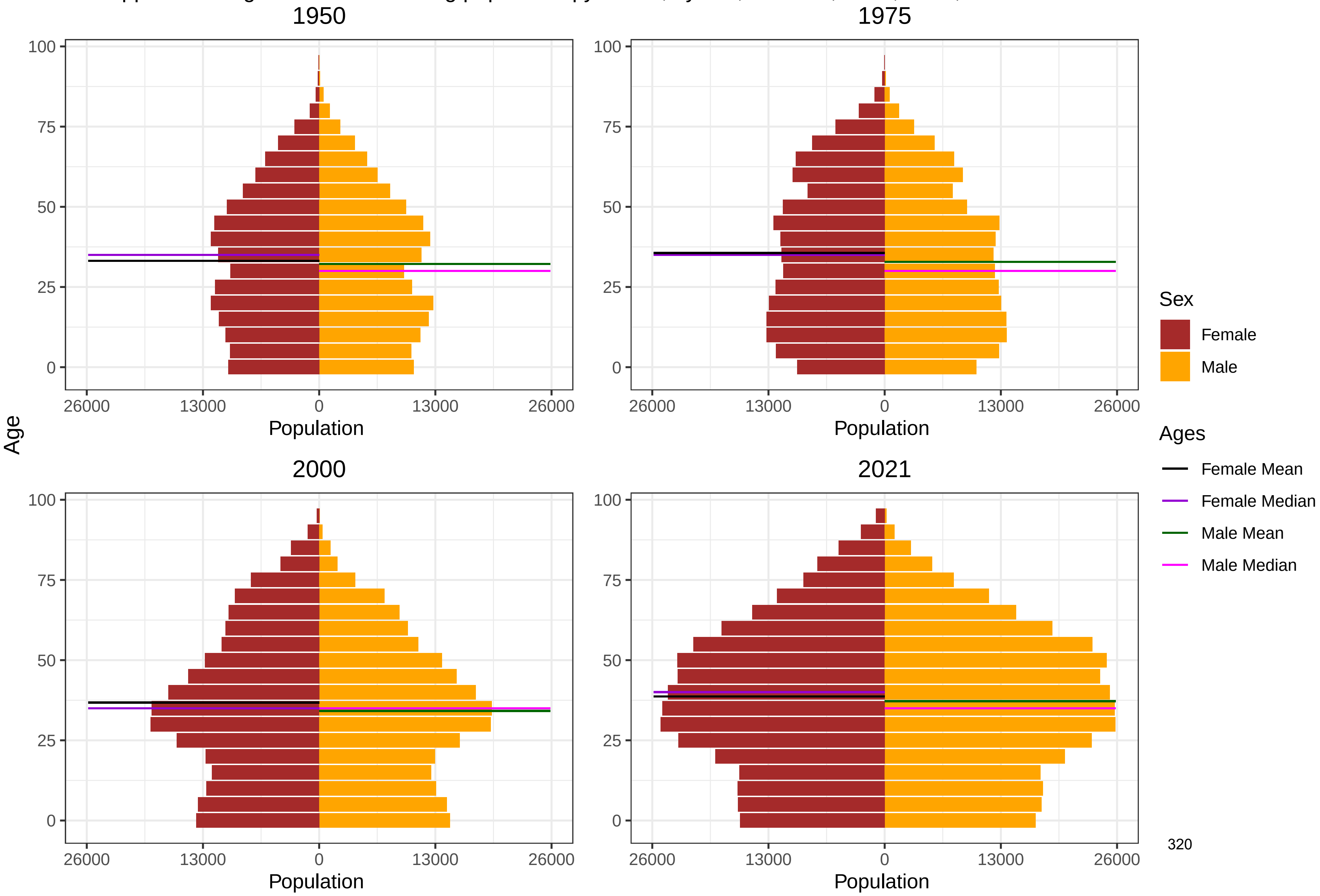

Appendix 2 Figure S9: Madagascar population pyramids, by sex, for 1950, 1975, 2000, and 2021

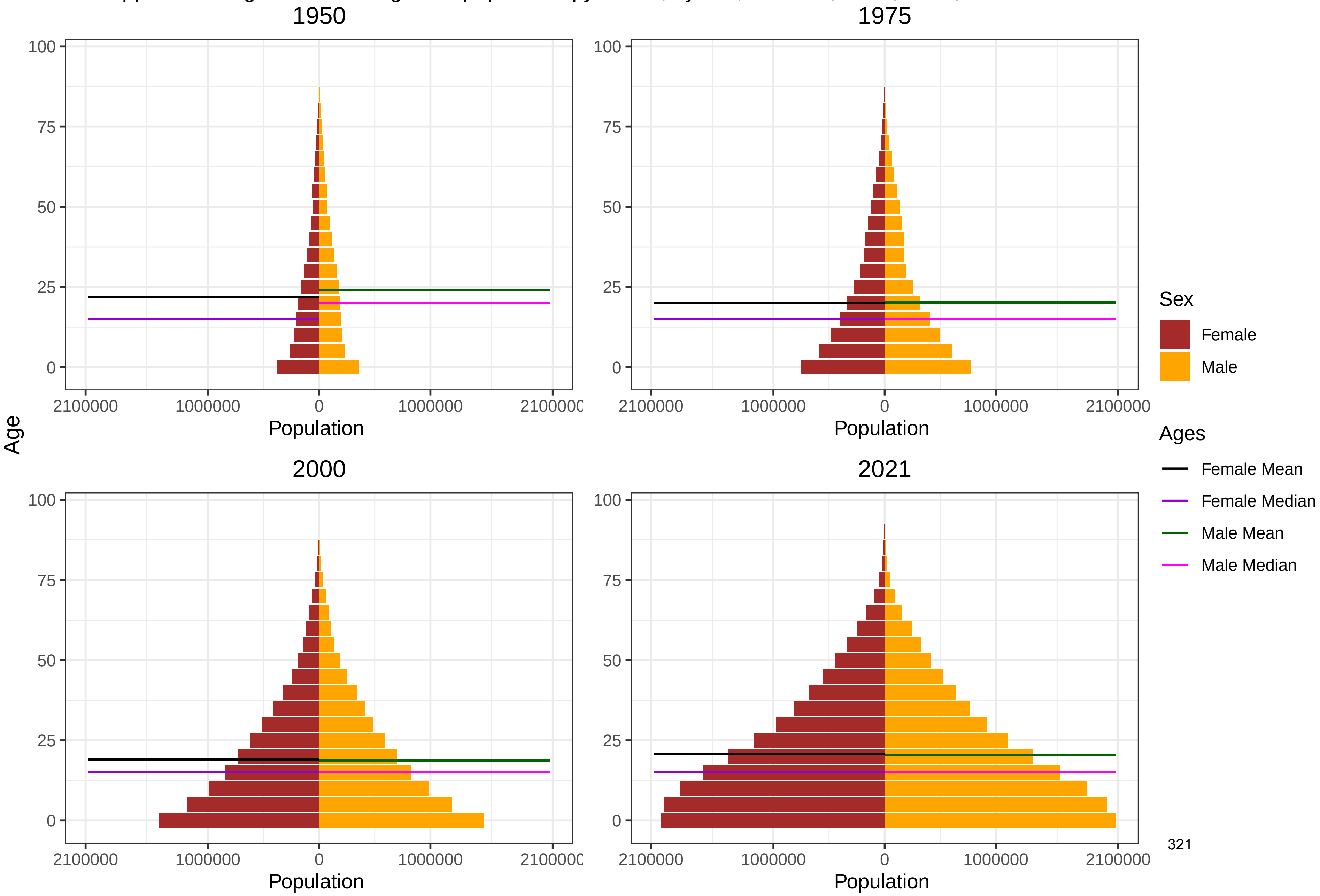

Appendix 2 Figure S9: Malawi population pyramids, by sex, for 1950, 1975, 2000, and 2021

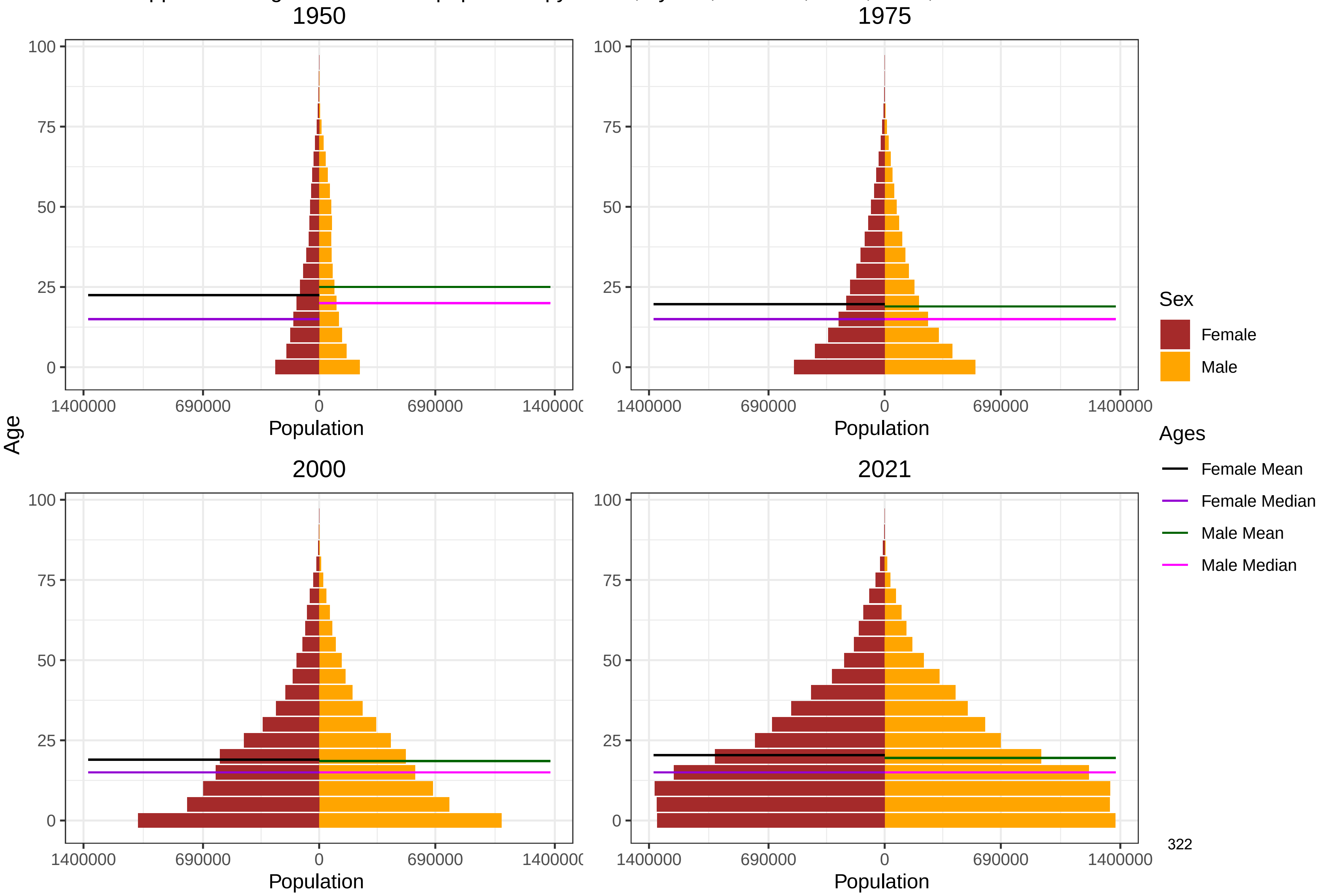

Appendix 2 Figure S9: Malaysia population pyramids, by sex, for 1950, 1975, 2000, and 2021

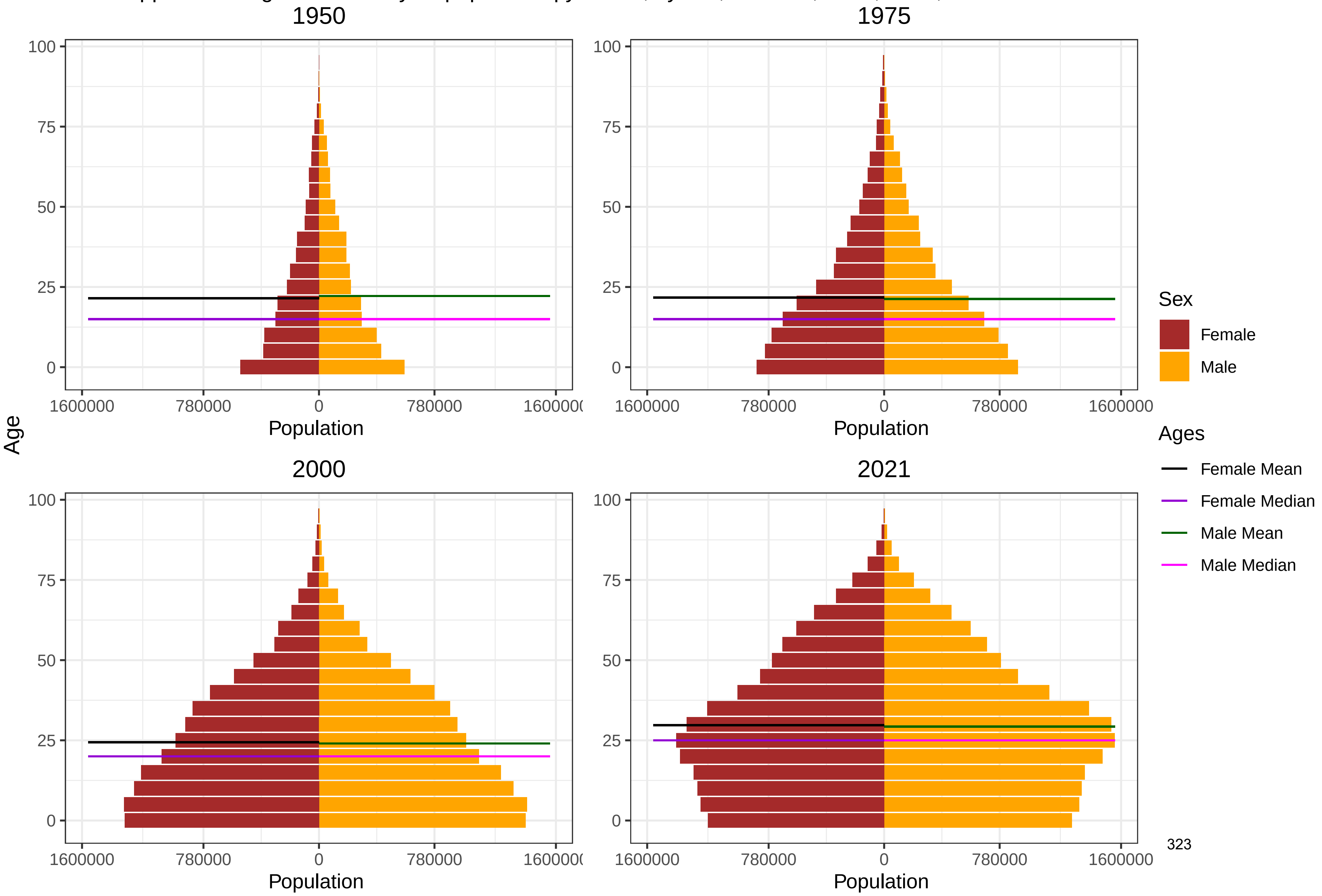

Appendix 2 Figure S9: Maldives population pyramids, by sex, for 1950, 1975, 2000, and 2021

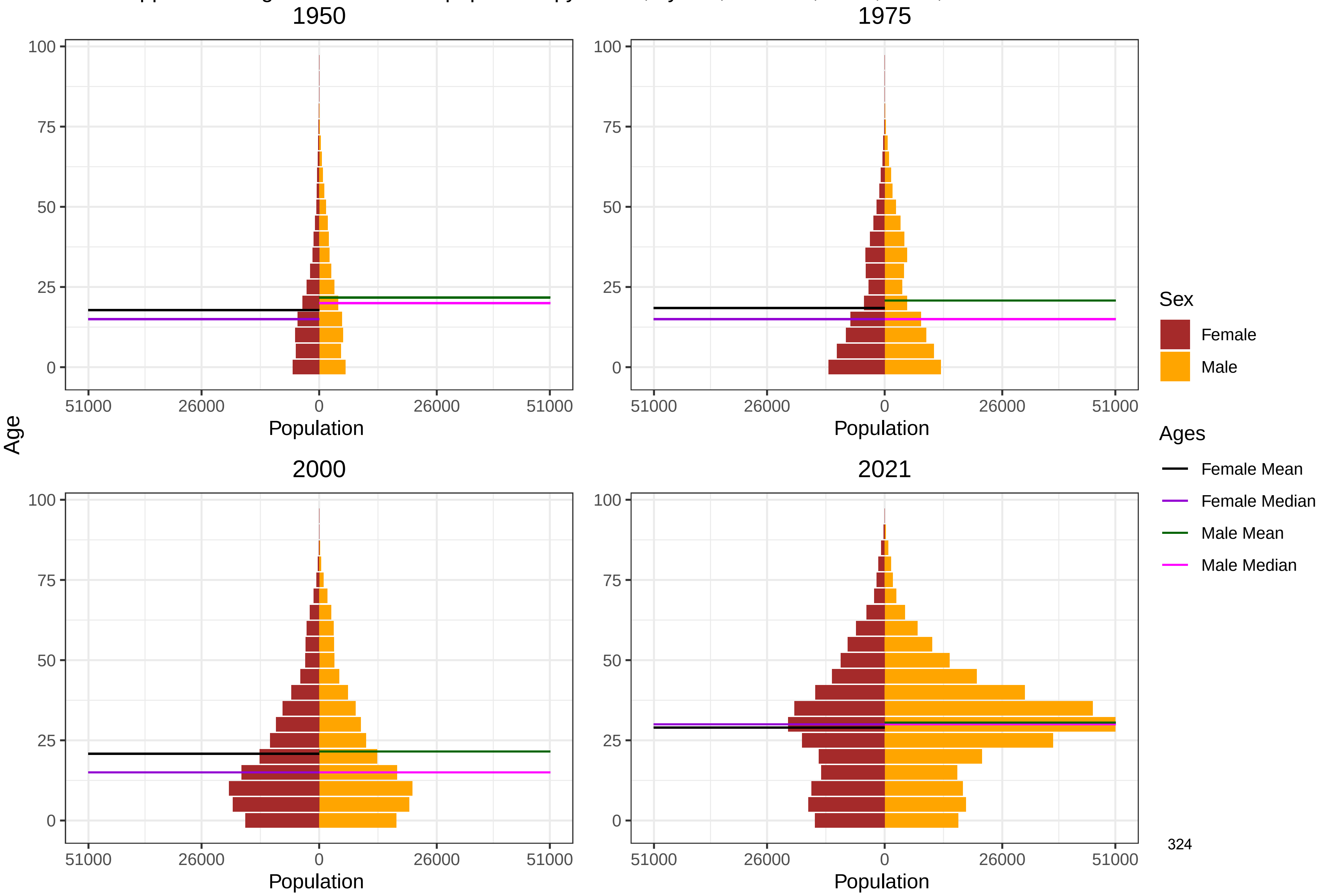

Appendix 2 Figure S9: Mali population pyramids, by sex, for 1950, 1975, 2000, and 2021

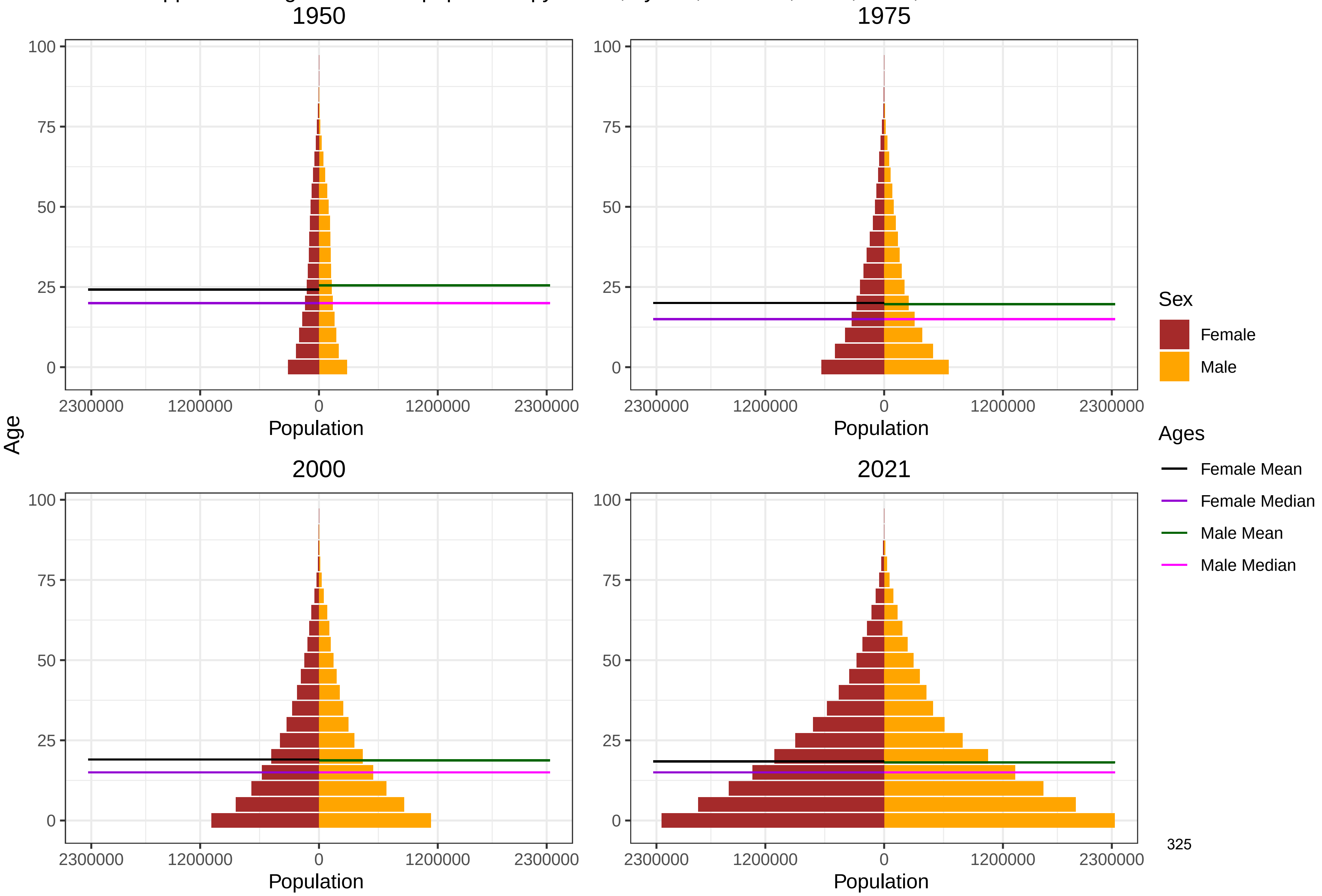

Appendix 2 Figure S9: Malta population pyramids, by sex, for 1950, 1975, 2000, and 2021

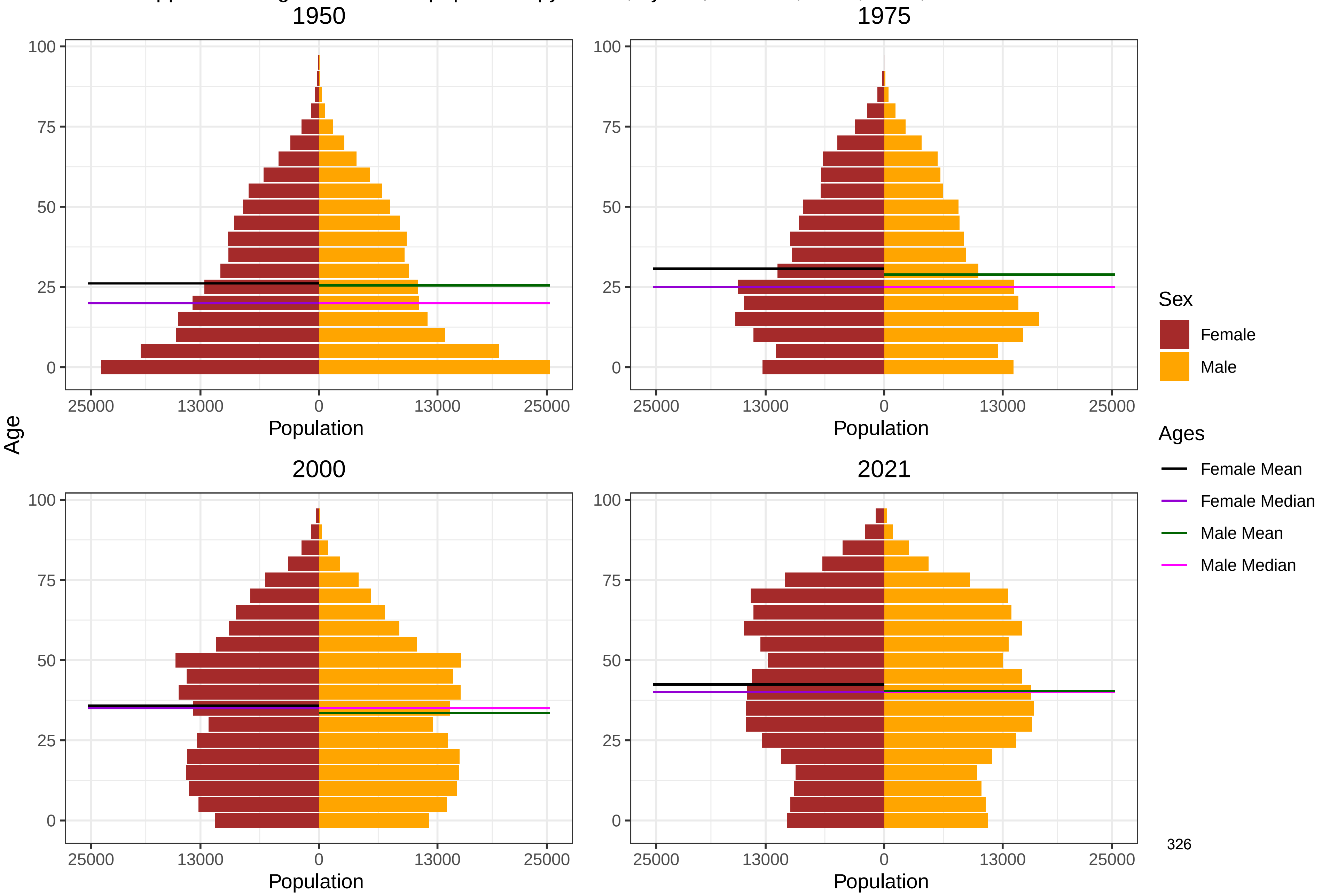

Appendix 2 Figure S9: Marshall Islands population pyramids, by sex, for 1950, 1975, 2000, and 2021

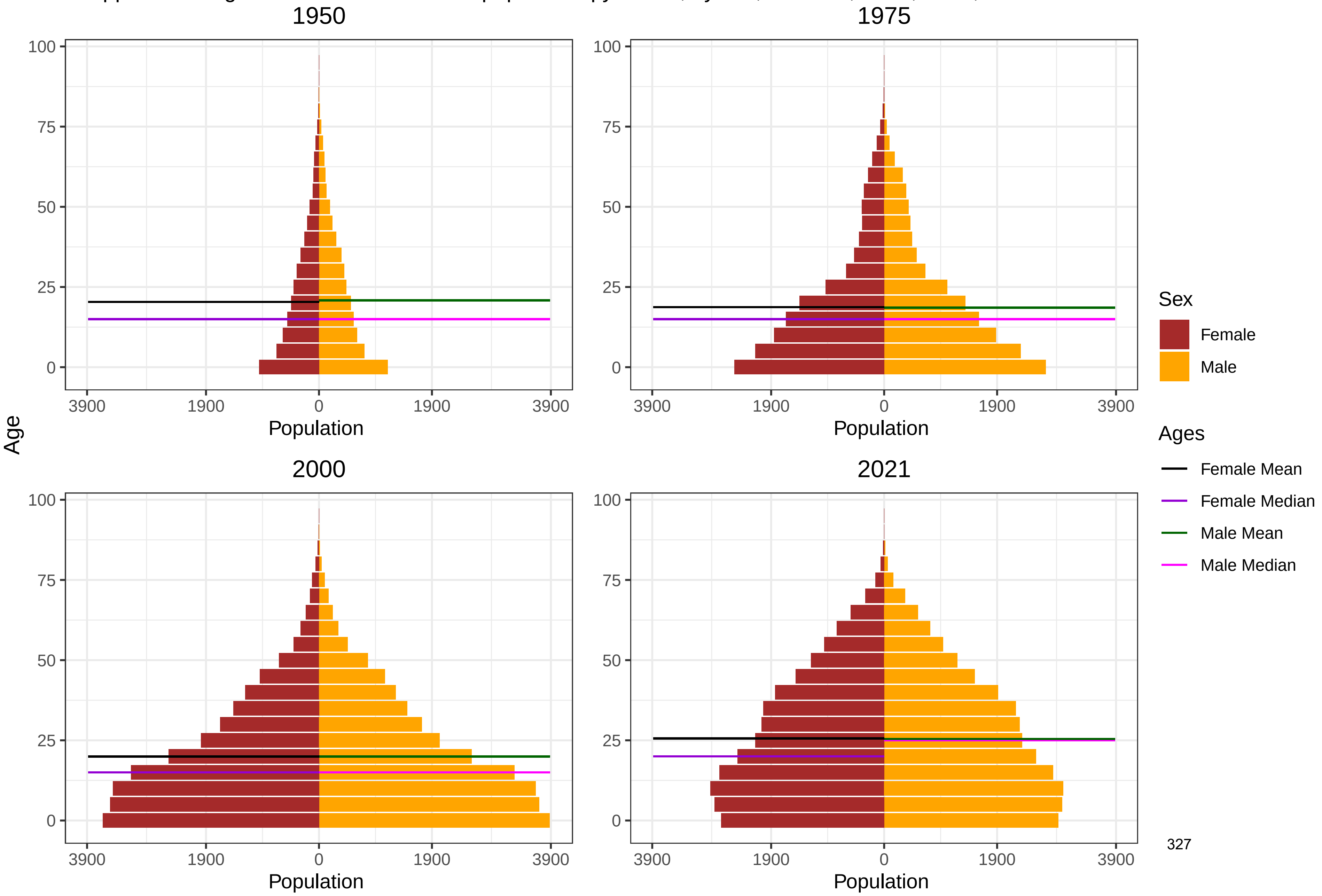

Appendix 2 Figure S9: Mauritania population pyramids, by sex, for 1950, 1975, 2000, and 2021

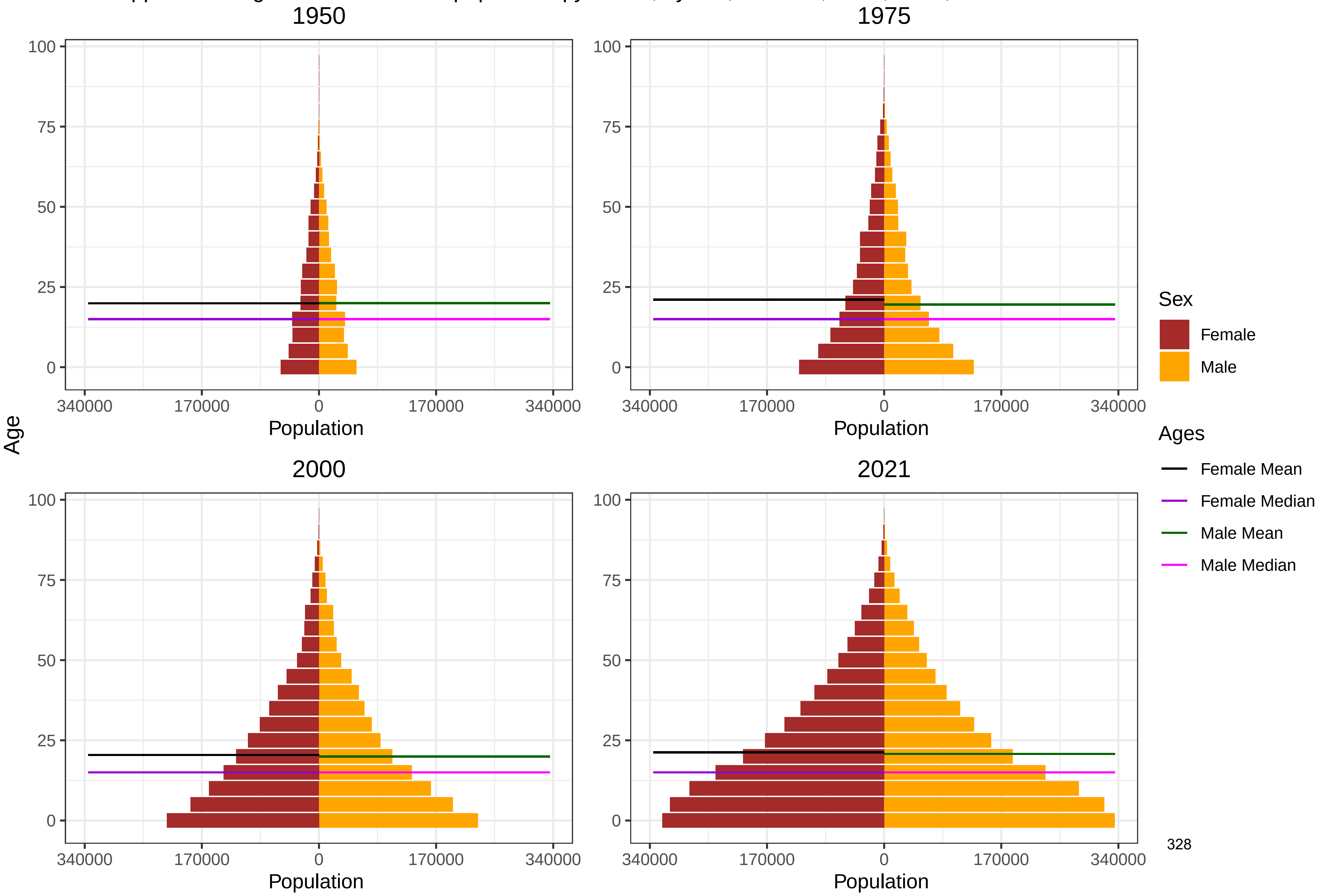

Appendix 2 Figure S9: Mauritius population pyramids, by sex, for 1950, 1975, 2000, and 2021

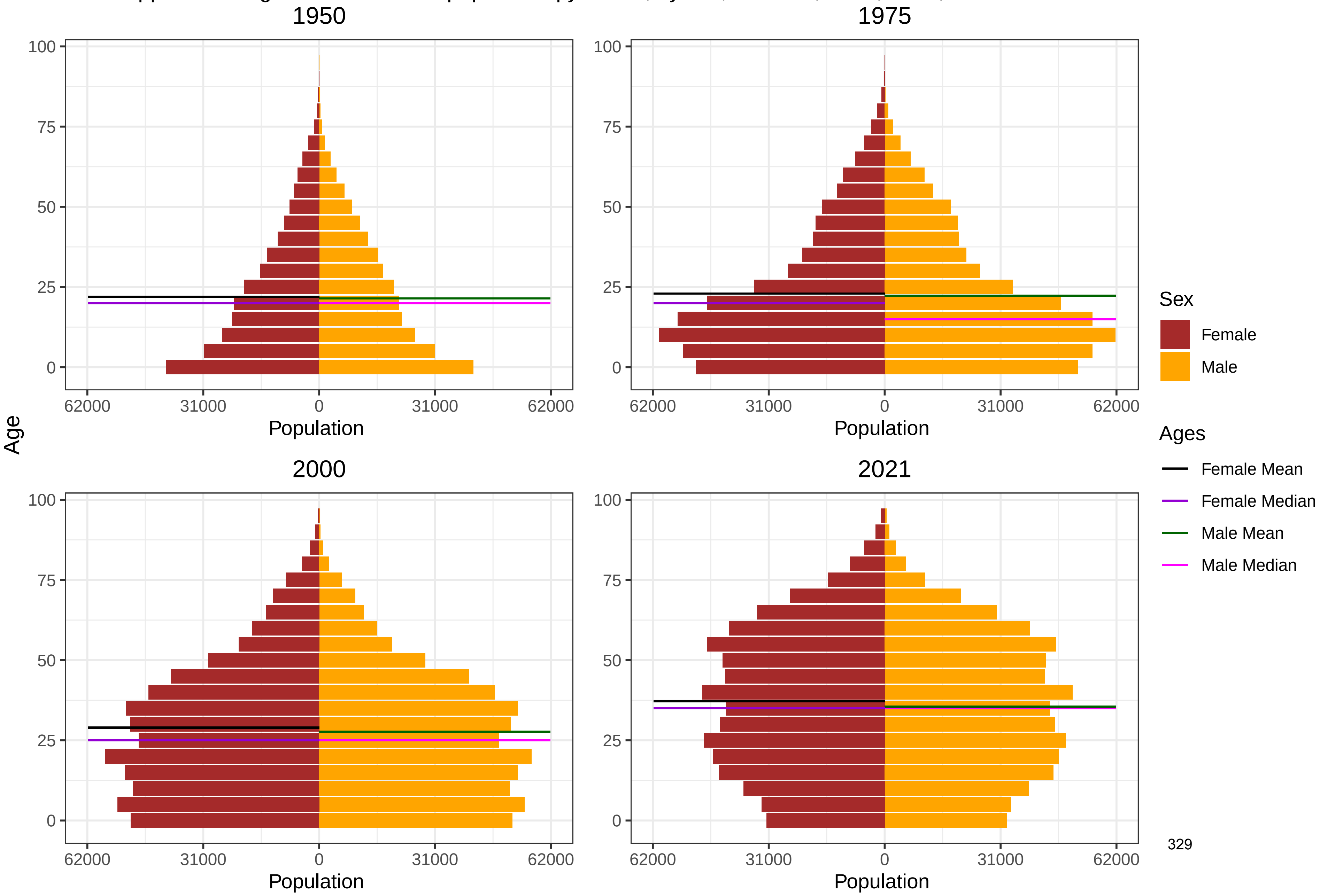

Appendix 2 Figure S9: Mexico population pyramids, by sex, for 1950, 1975, 2000, and 2021

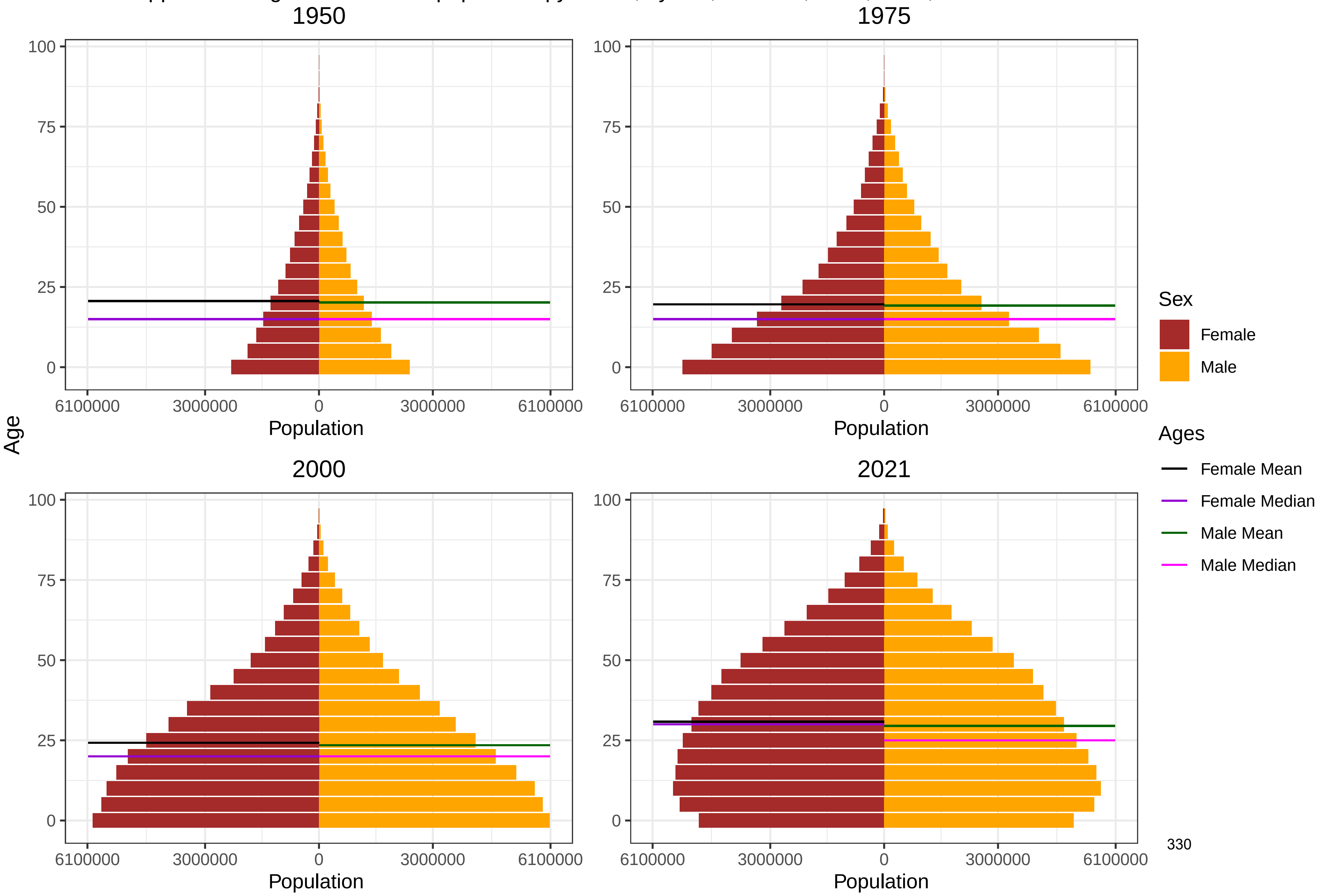

Appendix 2 Figure S9: Moldova population pyramids, by sex, for 1950, 1975, 2000, and 2021

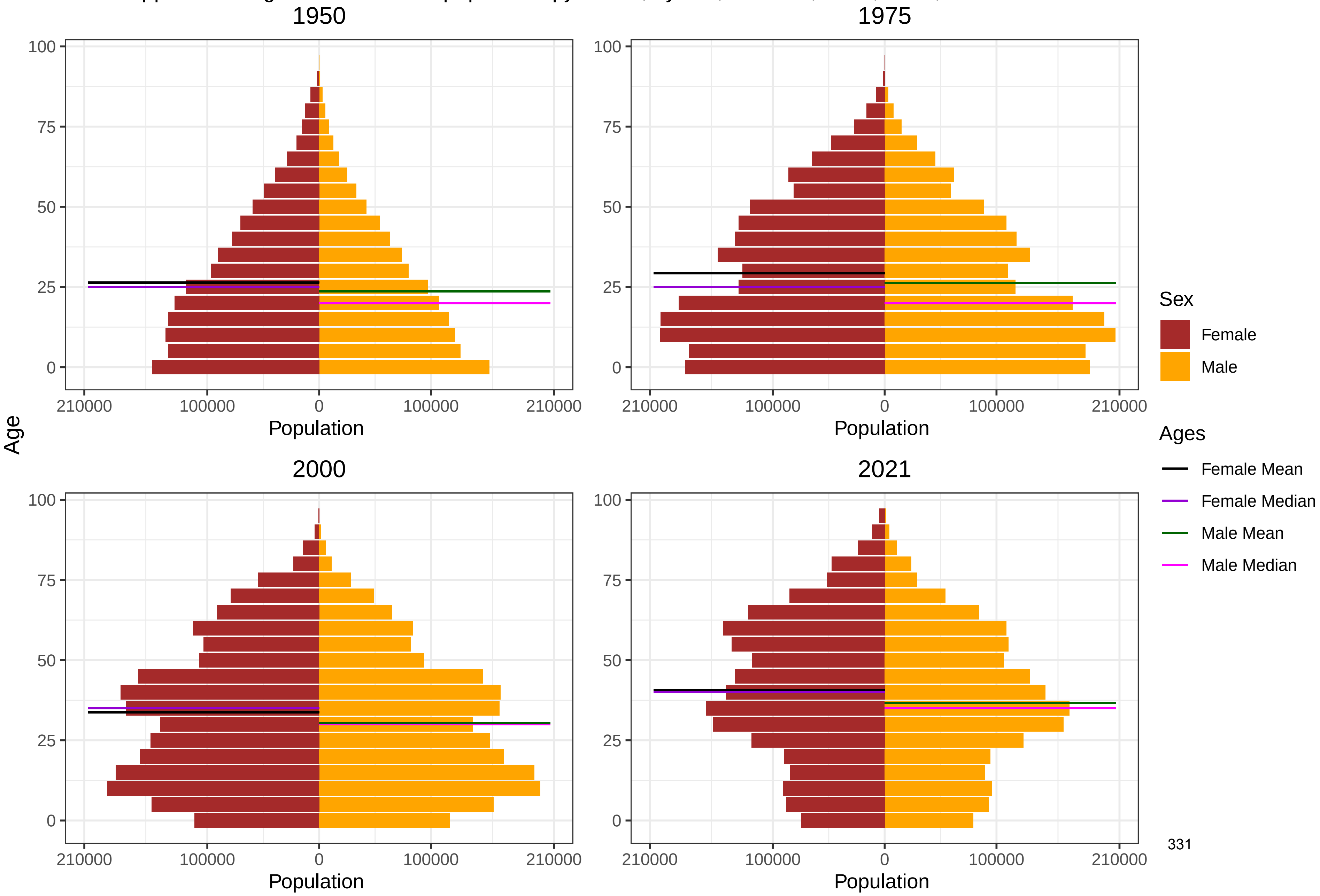

Appendix 2 Figure S9: Monaco population pyramids, by sex, for 1950, 1975, 2000, and 2021

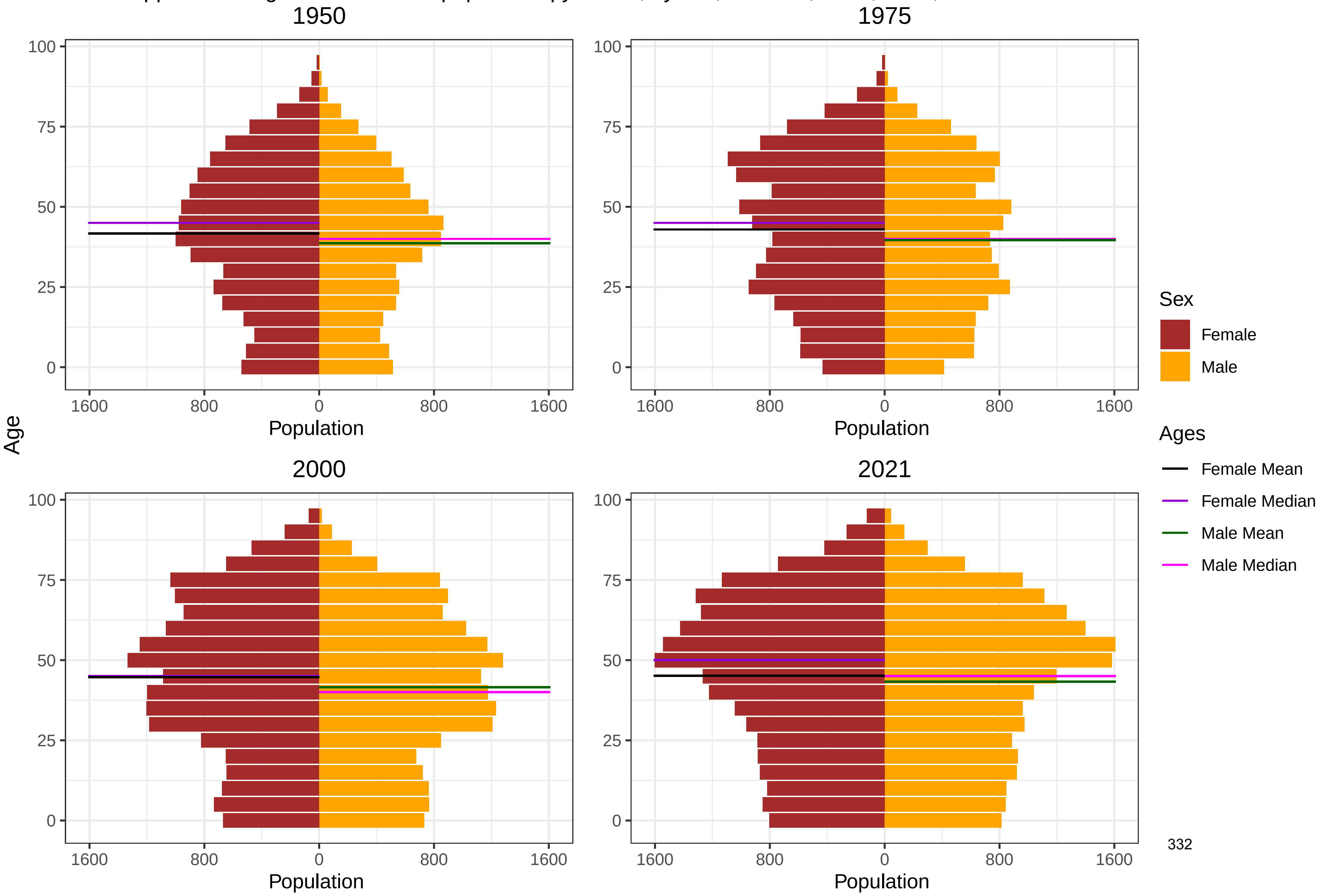

Appendix 2 Figure S9: Mongolia population pyramids, by sex, for 1950, 1975, 2000, and 2021

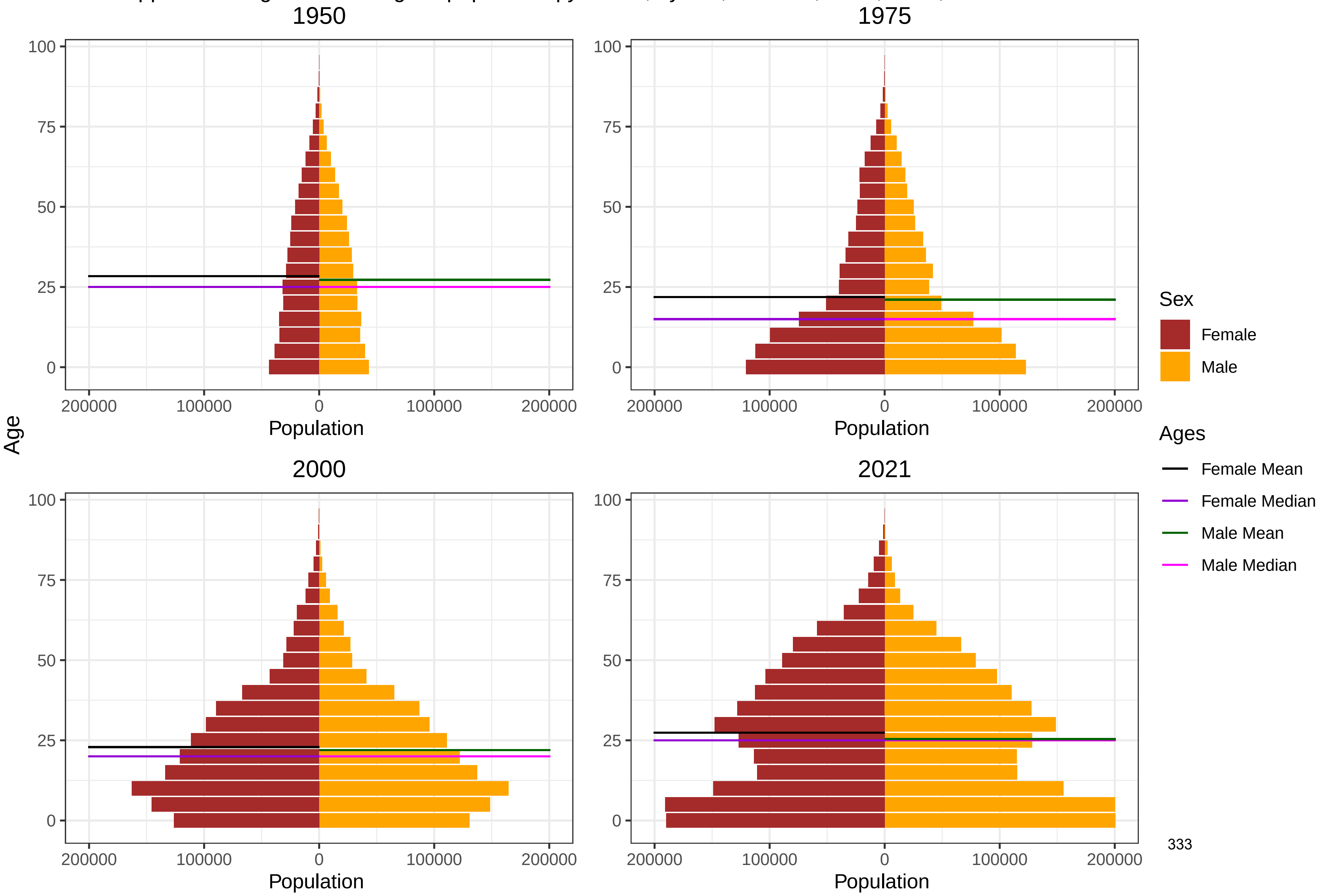

Appendix 2 Figure S9: Montenegro population pyramids, by sex, for 1950, 1975, 2000, and 2021

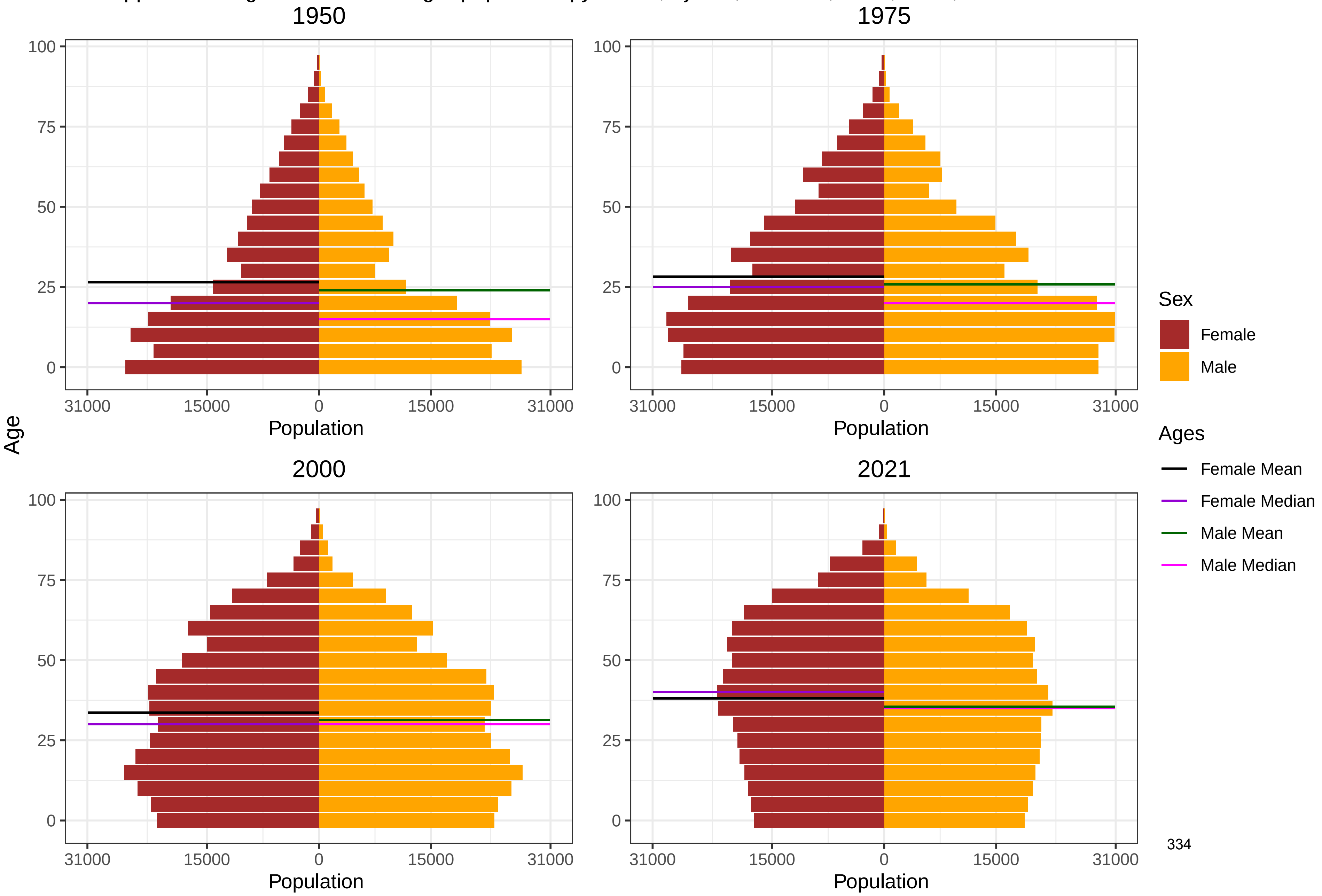

Appendix 2 Figure S9: Morocco population pyramids, by sex, for 1950, 1975, 2000, and 2021

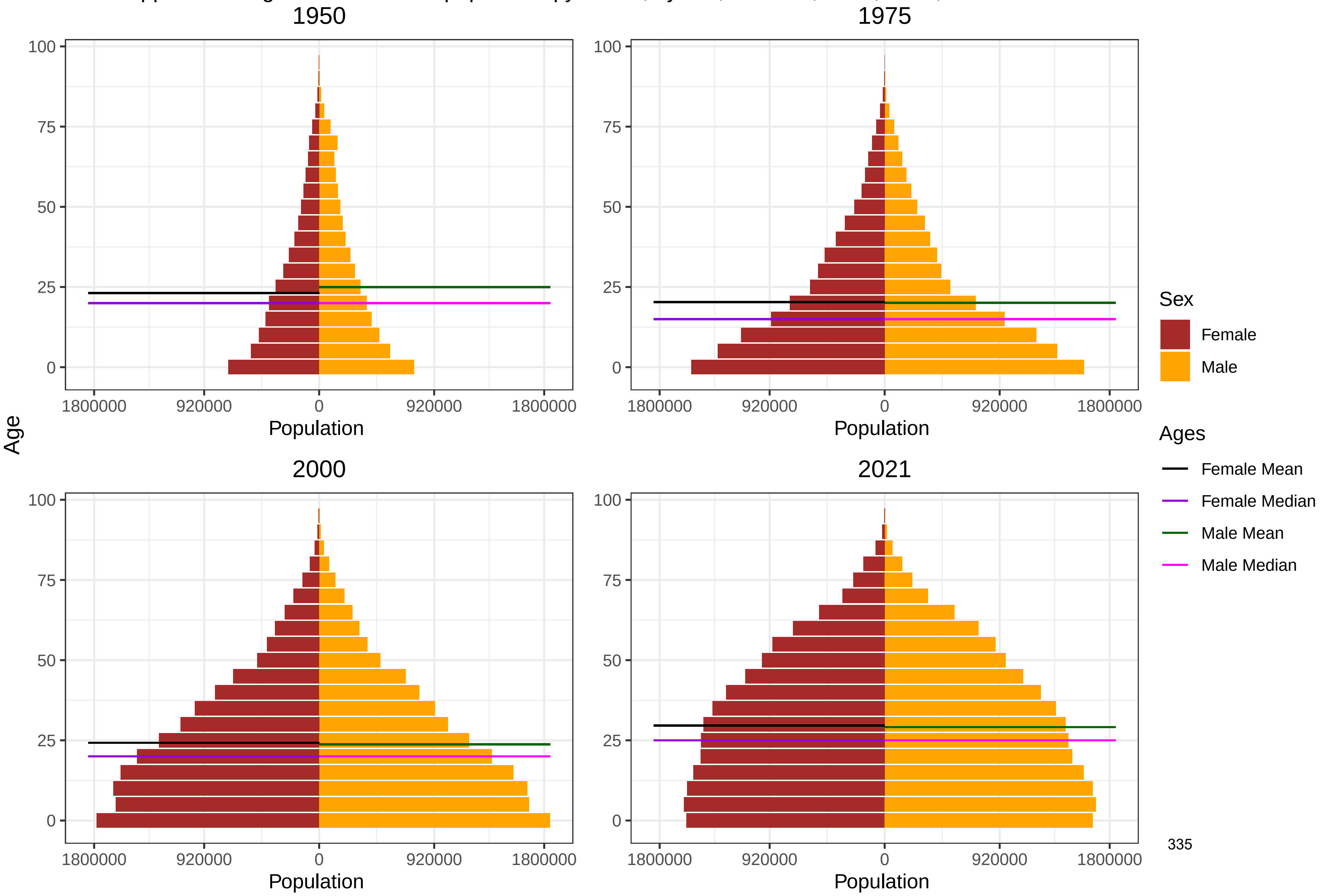

Appendix 2 Figure S9: Mozambique population pyramids, by sex, for 1950, 1975, 2000, and 2021

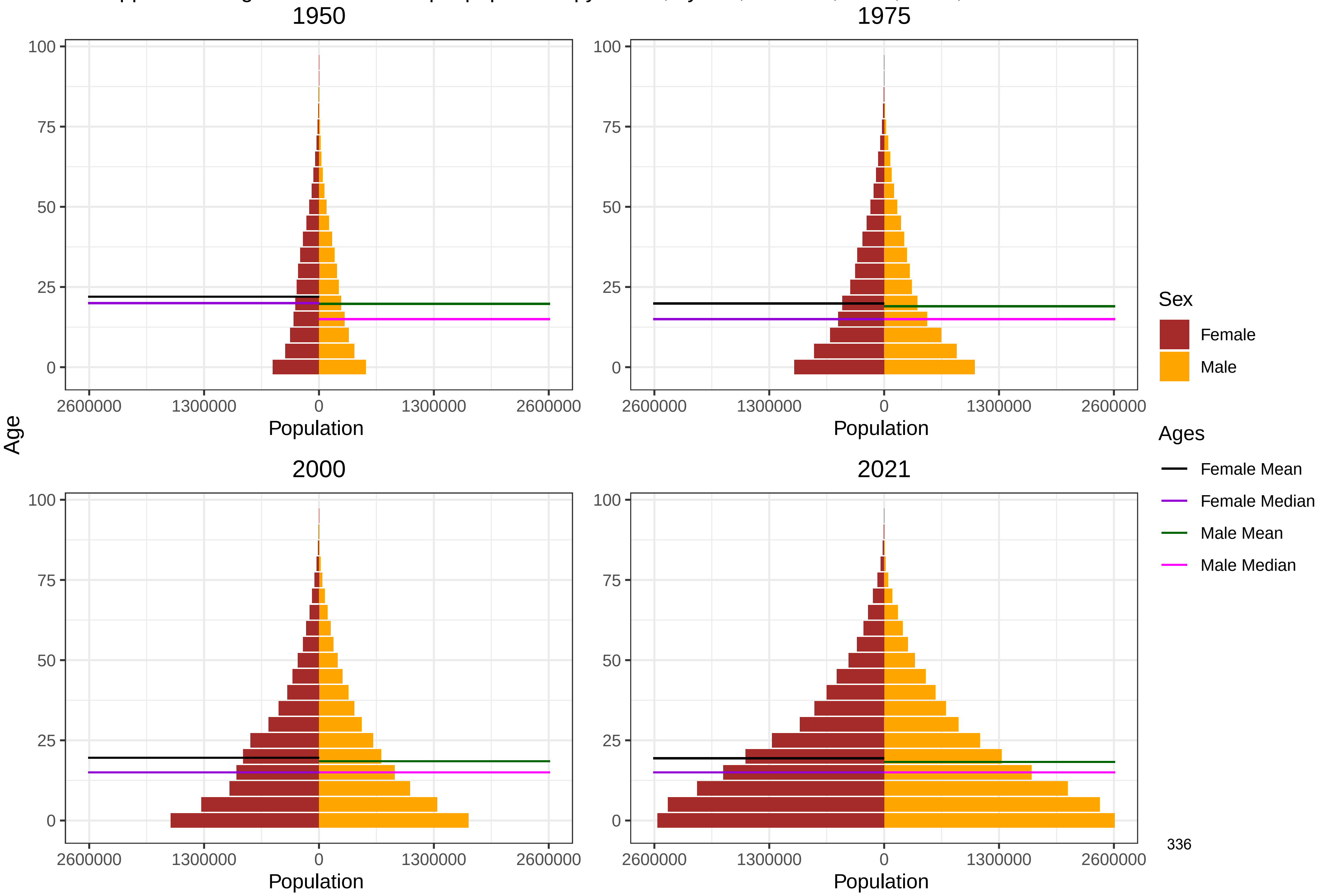

Appendix 2 Figure S9: Myanmar population pyramids, by sex, for 1950, 1975, 2000, and 2021

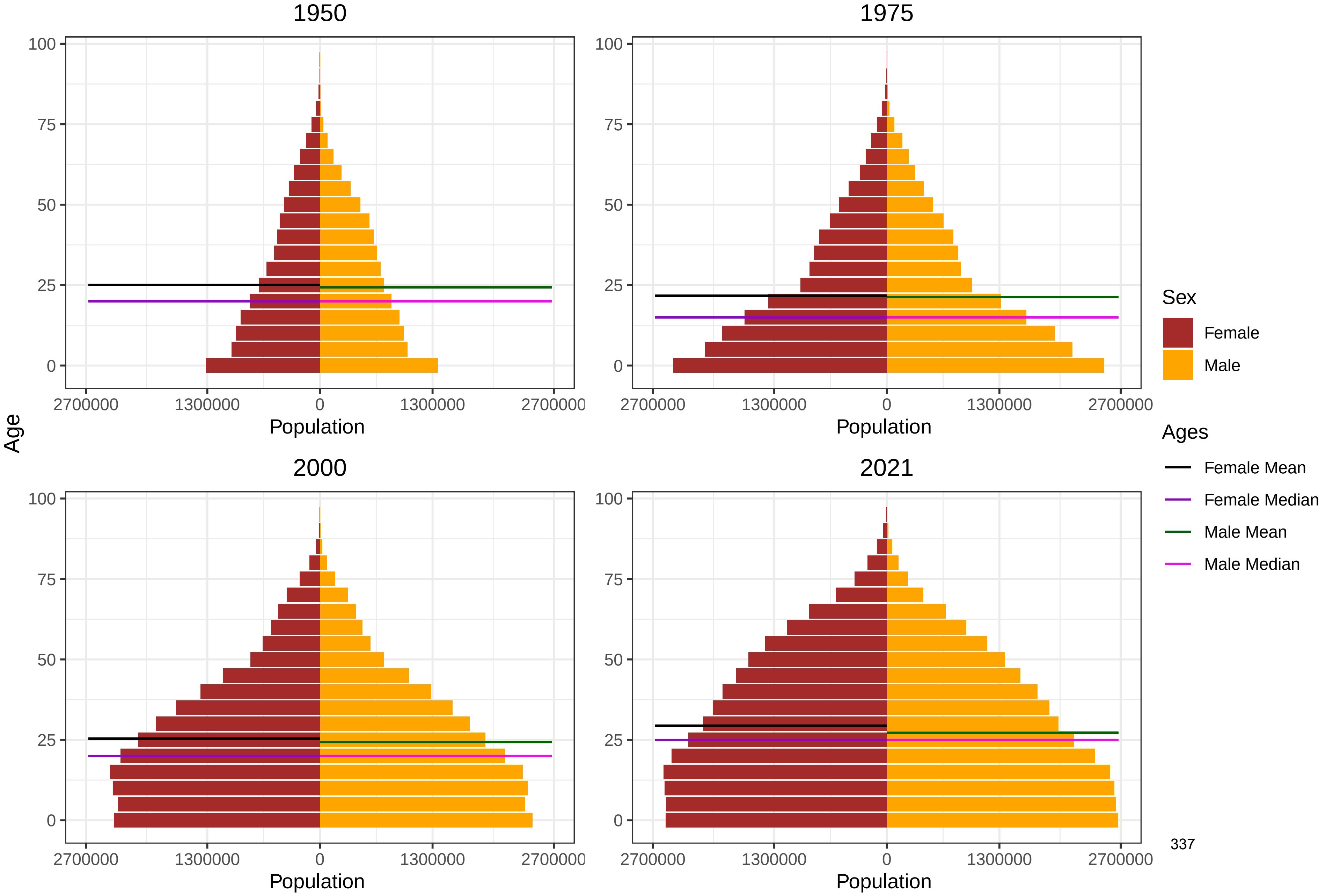

Appendix 2 Figure S9: Namibia population pyramids, by sex, for 1950, 1975, 2000, and 2021

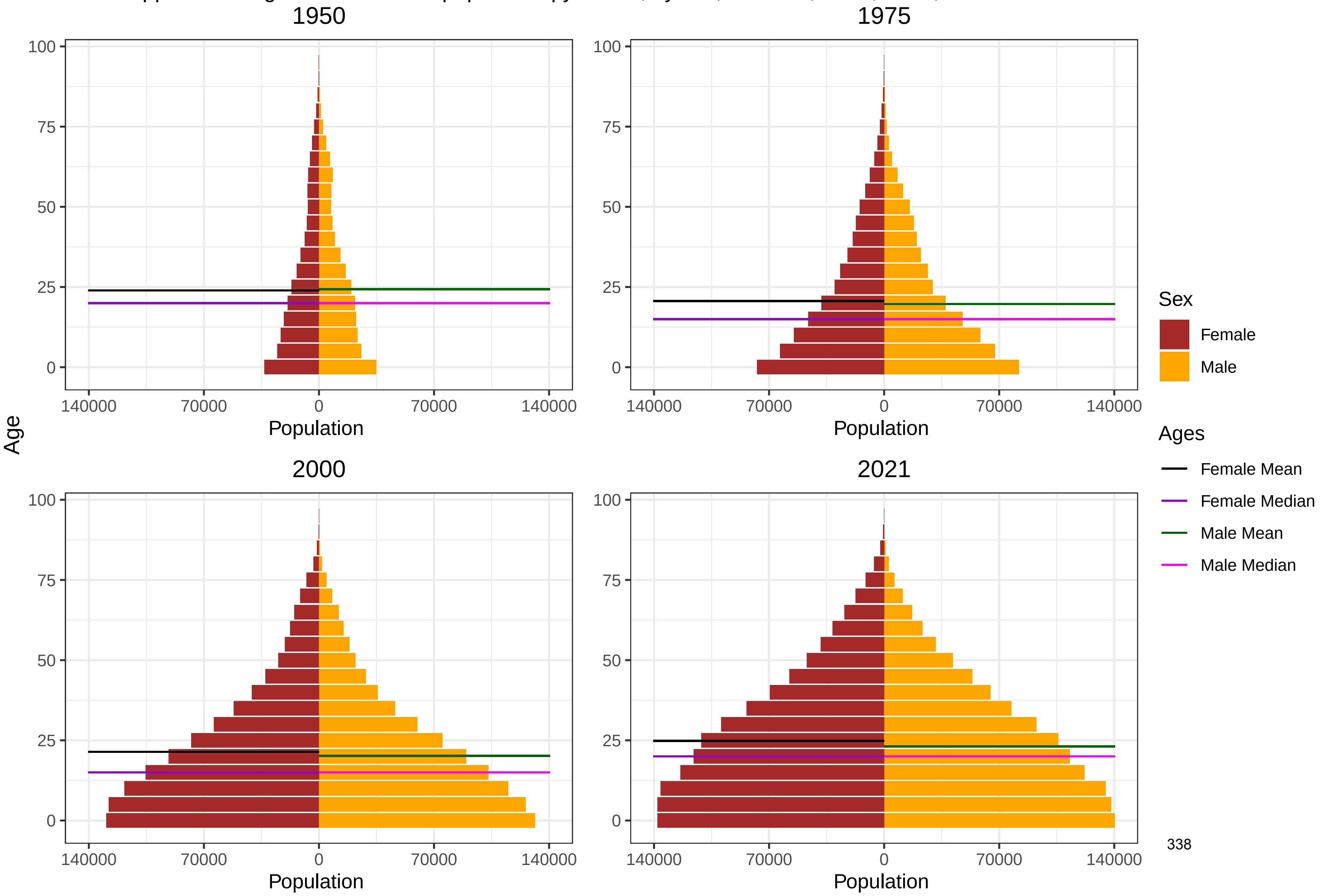

Appendix 2 Figure S9: Nauru population pyramids, by sex, for 1950, 1975, 2000, and 2021

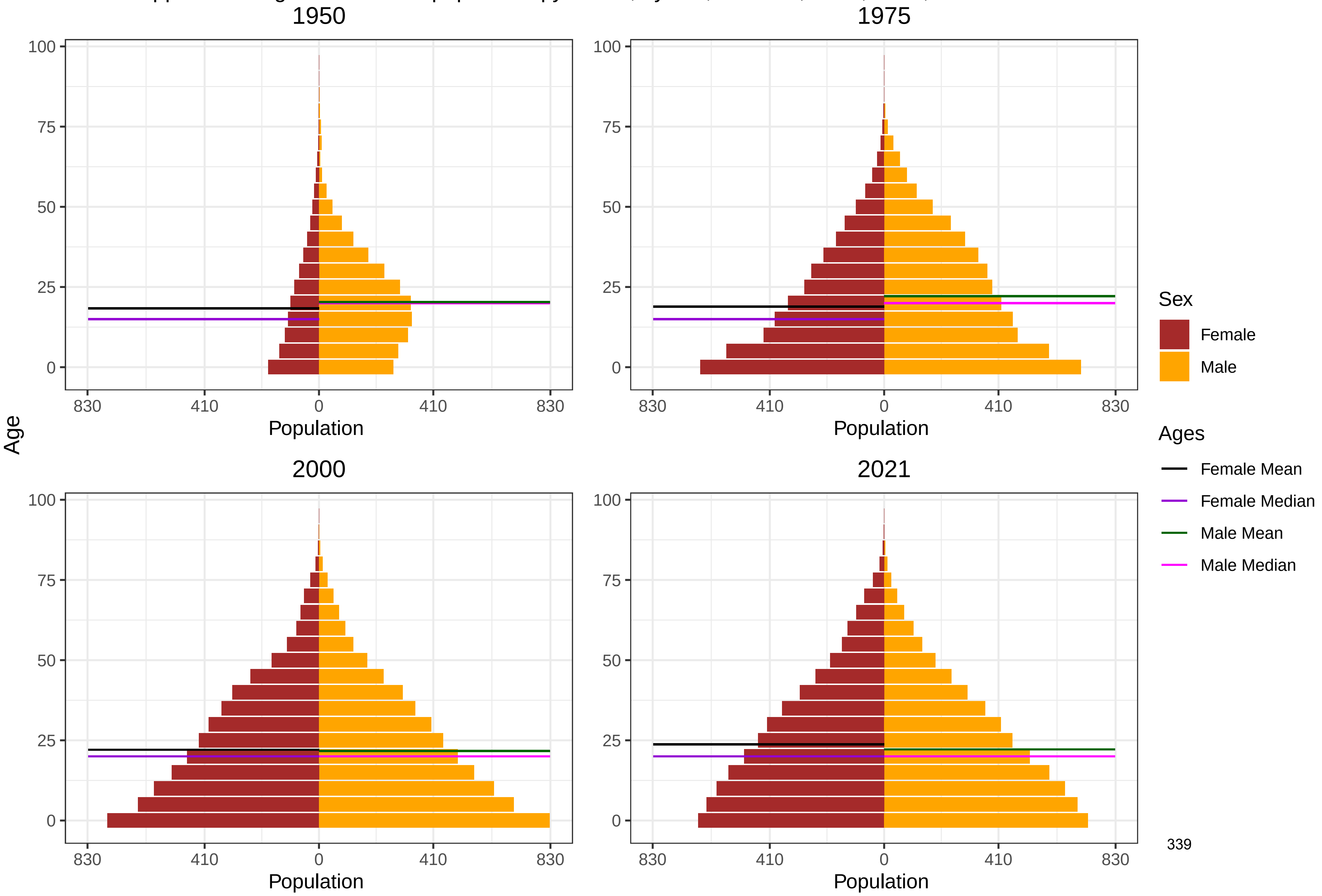

Appendix 2 Figure S9: Nepal population pyramids, by sex, for 1950, 1975, 2000, and 2021

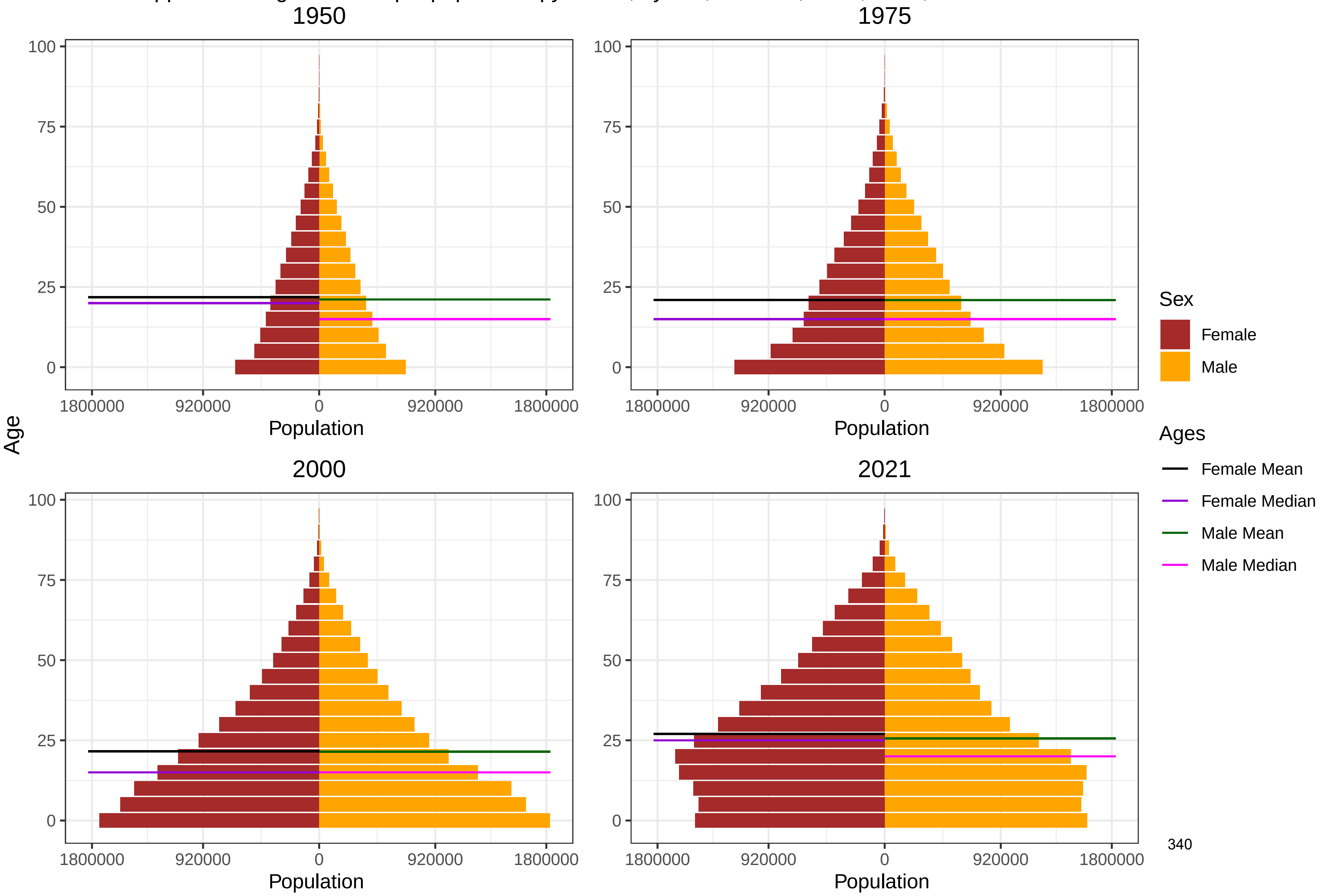

Appendix 2 Figure S9: Netherlands population pyramids, by sex, for 1950, 1975, 2000, and 2021

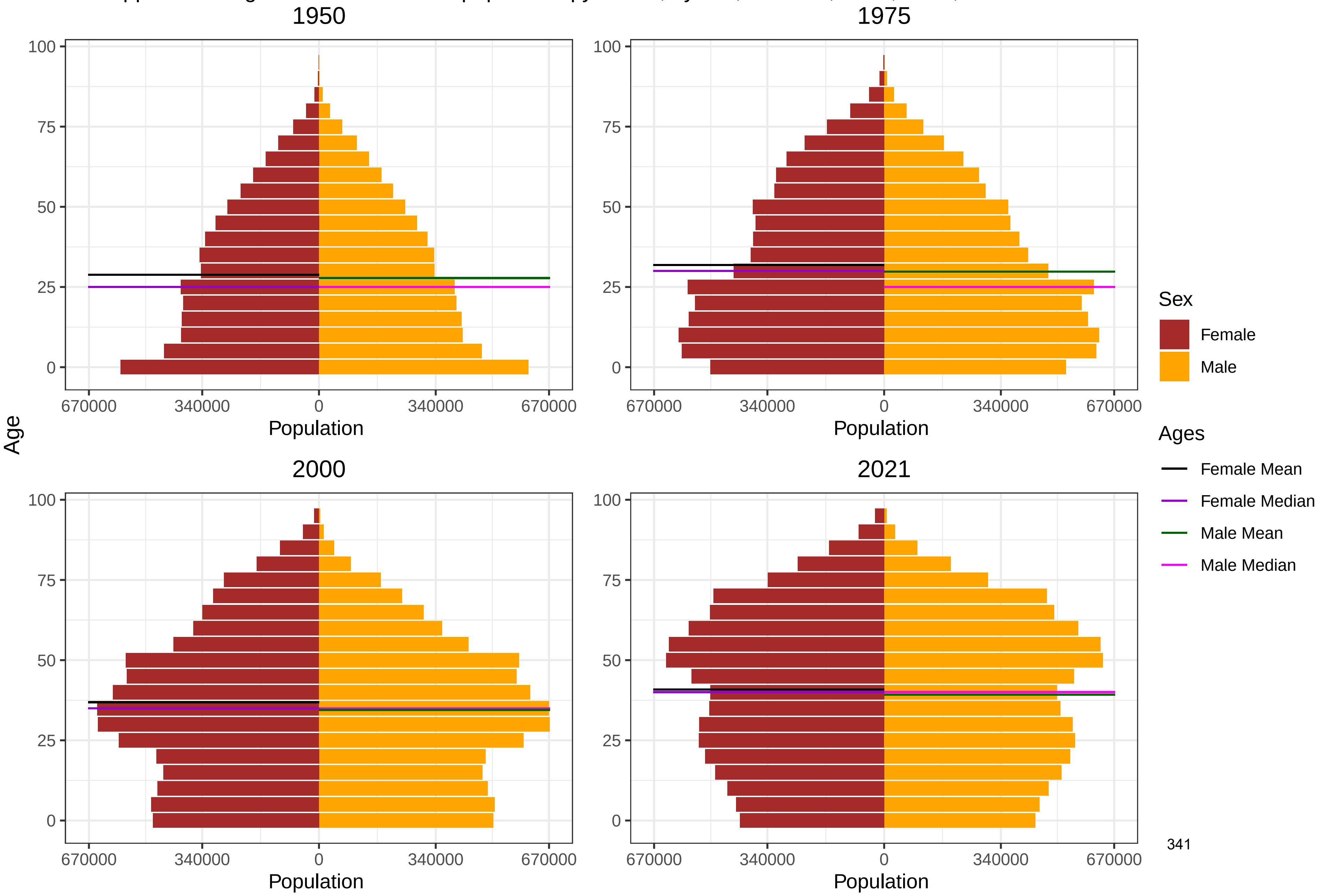

Appendix 2 Figure S9: New Zealand population pyramids, by sex, for 1950, 1975, 2000, and 2021

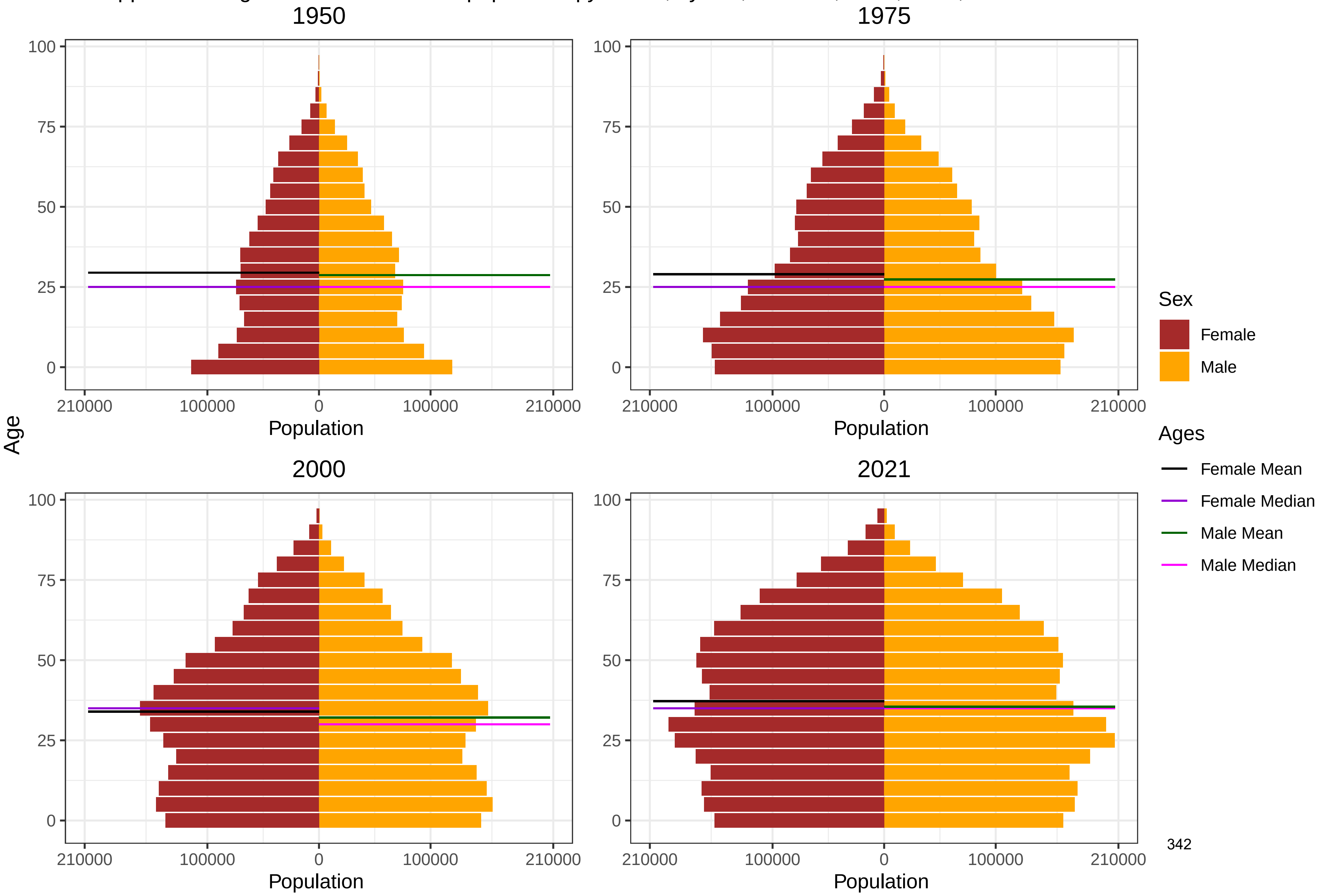

Appendix 2 Figure S9: Nicaragua population pyramids, by sex, for 1950, 1975, 2000, and 2021

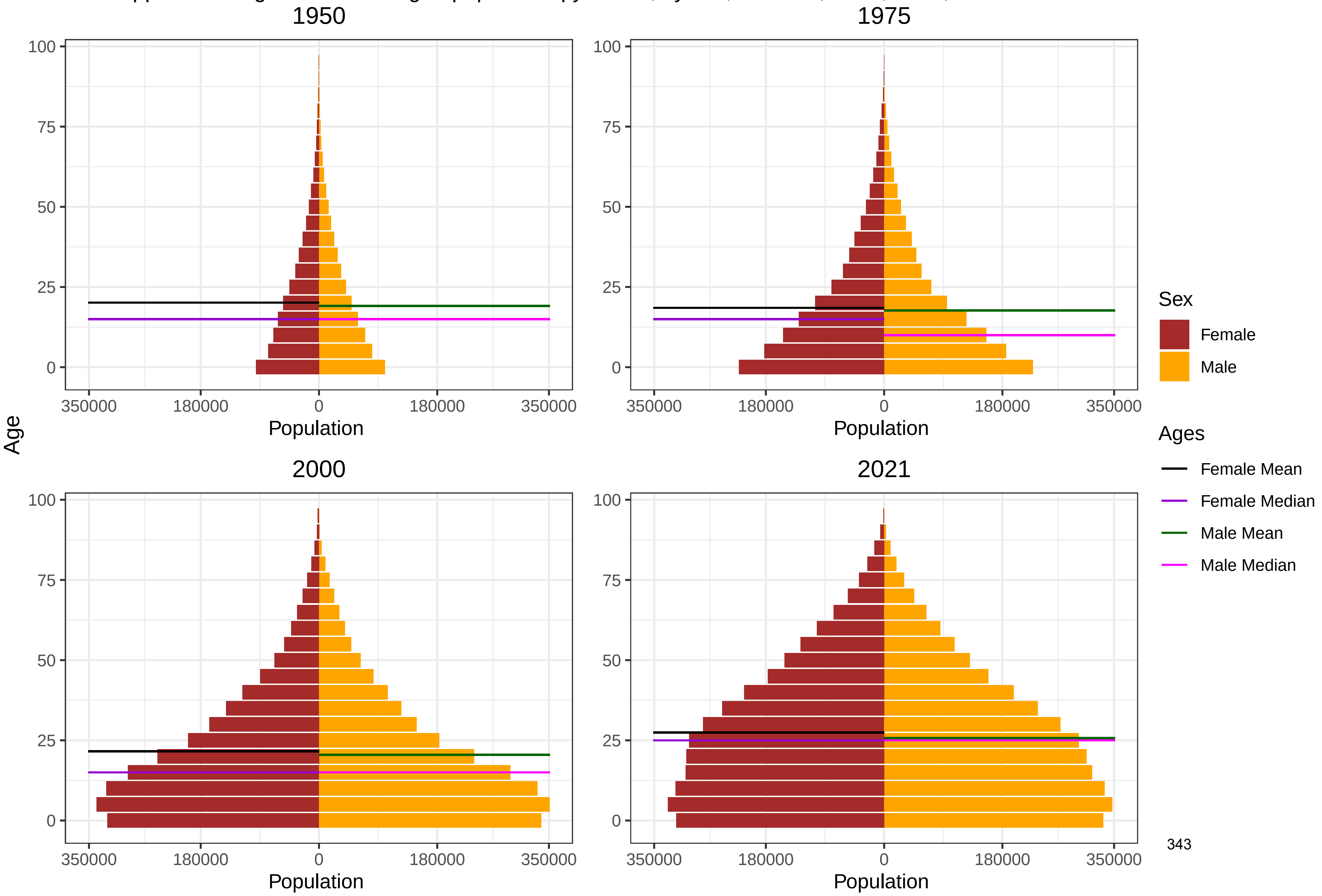

Appendix 2 Figure S9: Niger population pyramids, by sex, for 1950, 1975, 2000, and 2021

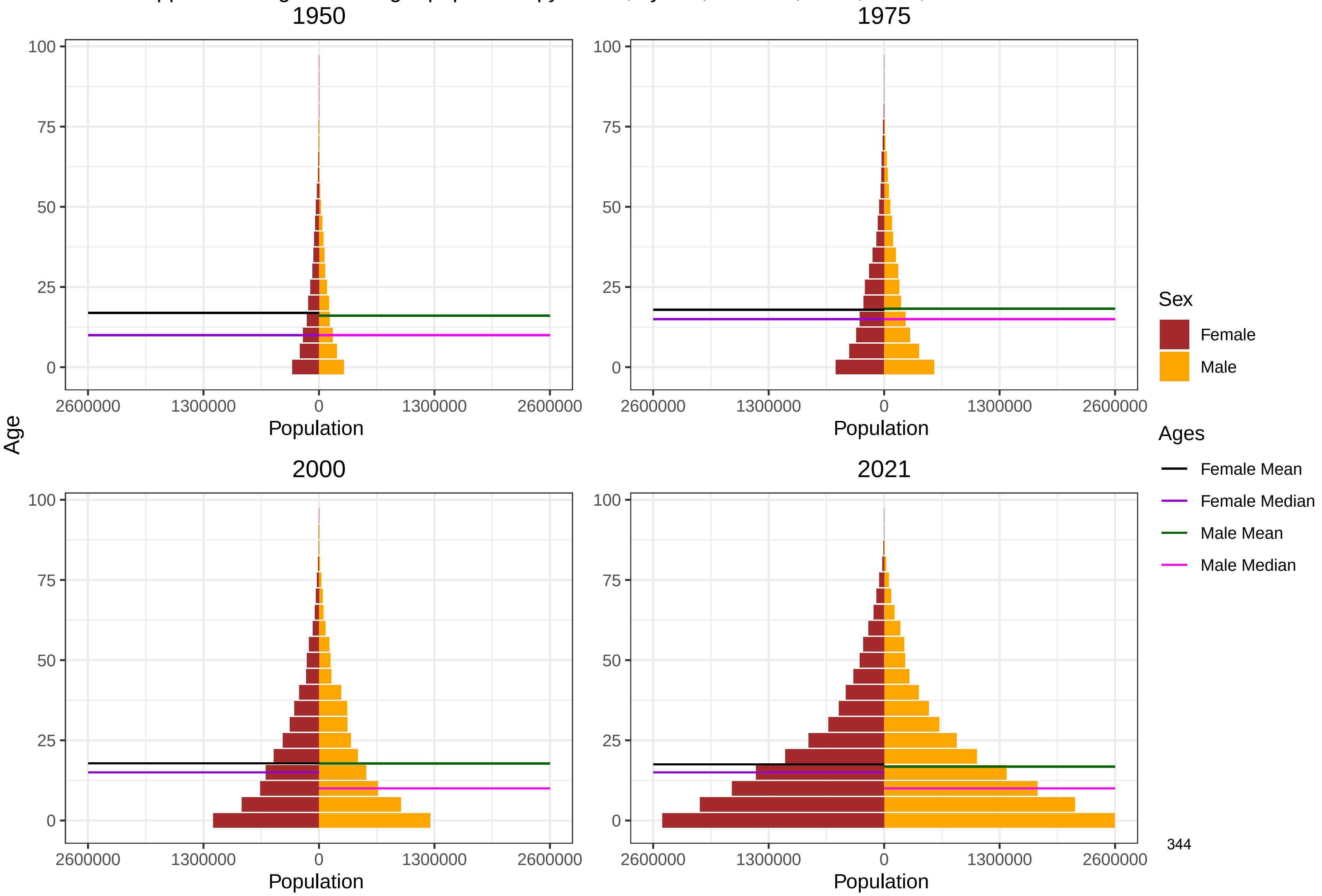

Appendix 2 Figure S9: Nigeria population pyramids, by sex, for 1950, 1975, 2000, and 2021

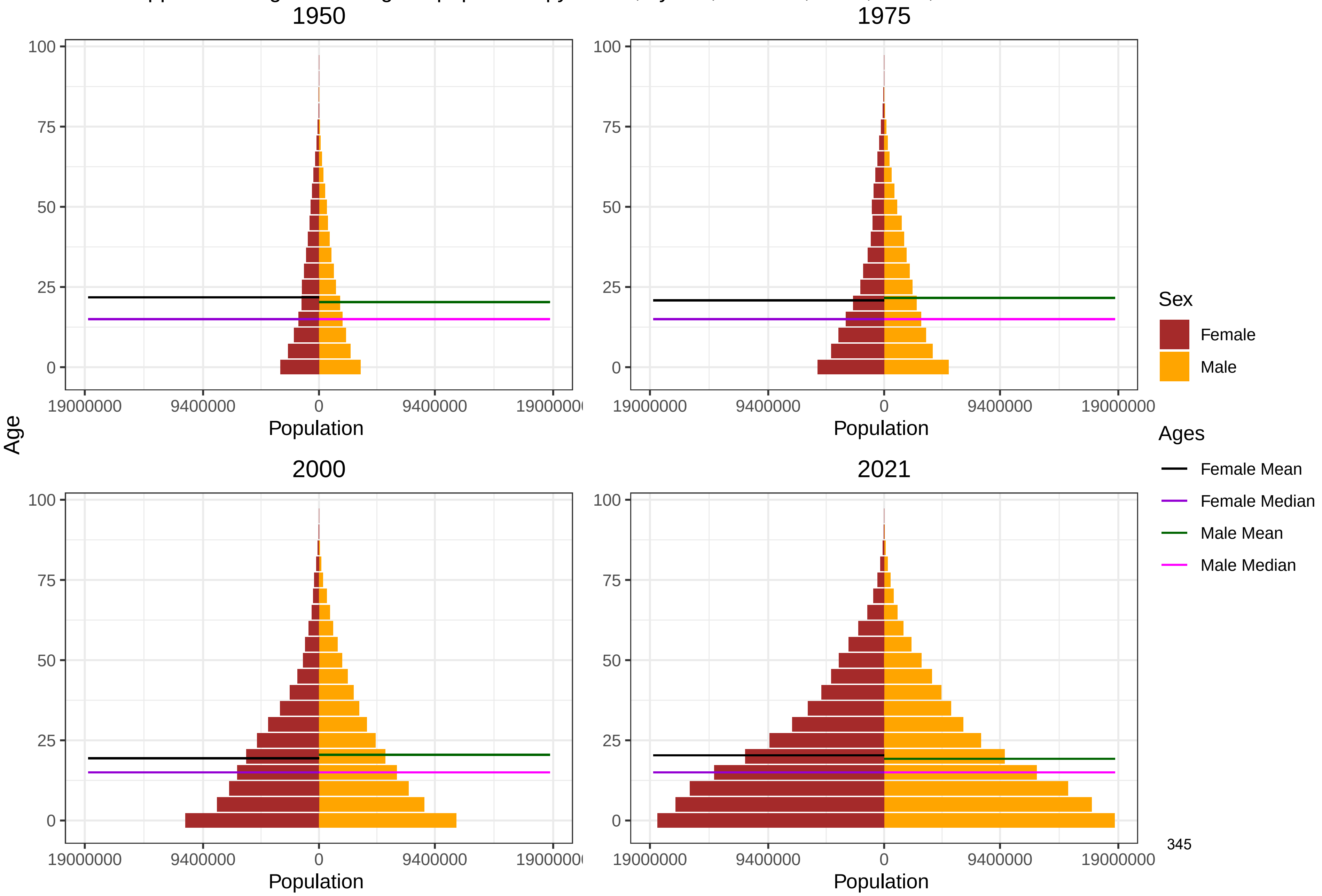

Appendix 2 Figure S9: Niue population pyramids, by sex, for 1950, 1975, 2000, and 2021

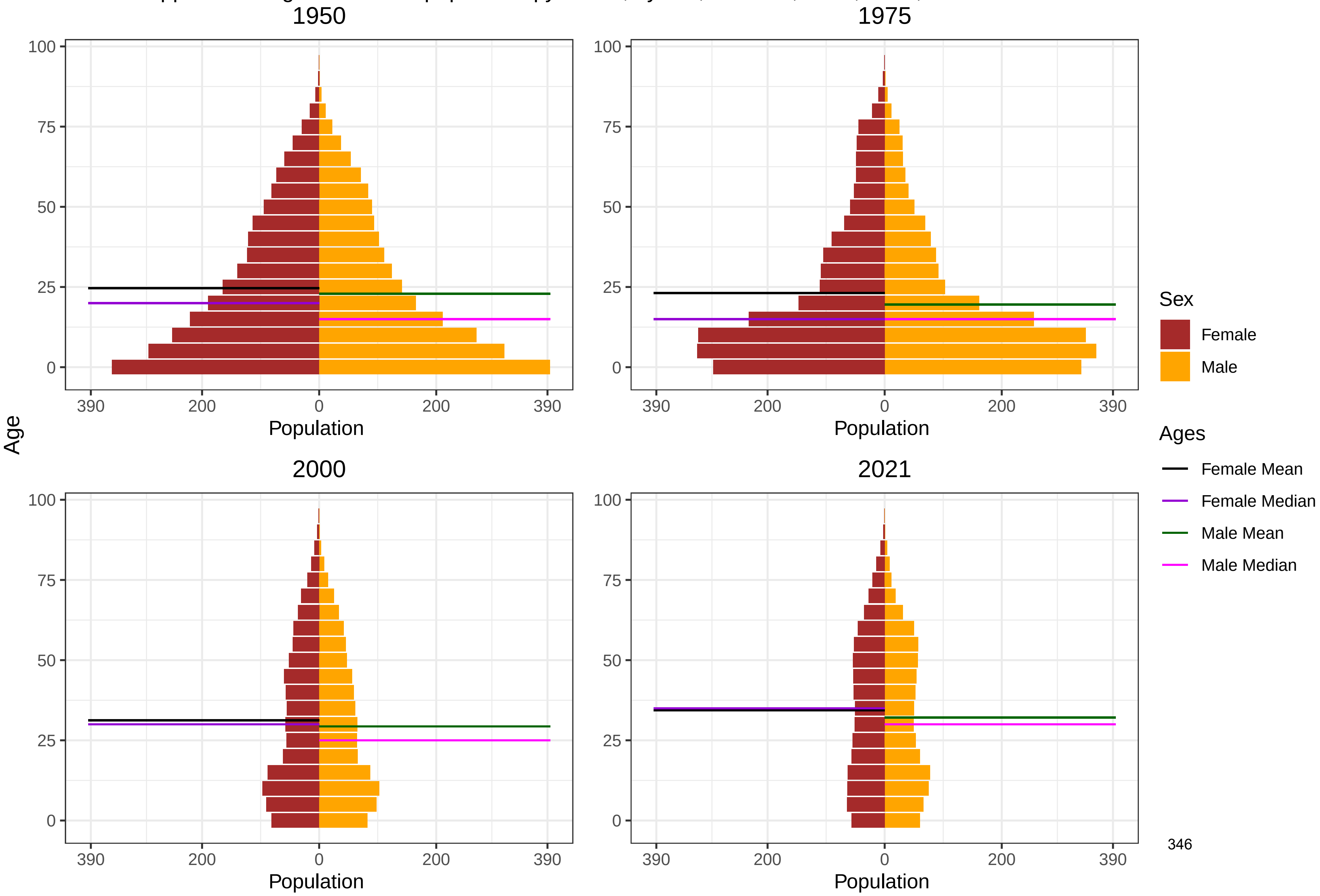

Appendix 2 Figure S9: North Korea population pyramids, by sex, for 1950, 1975, 2000, and 2021

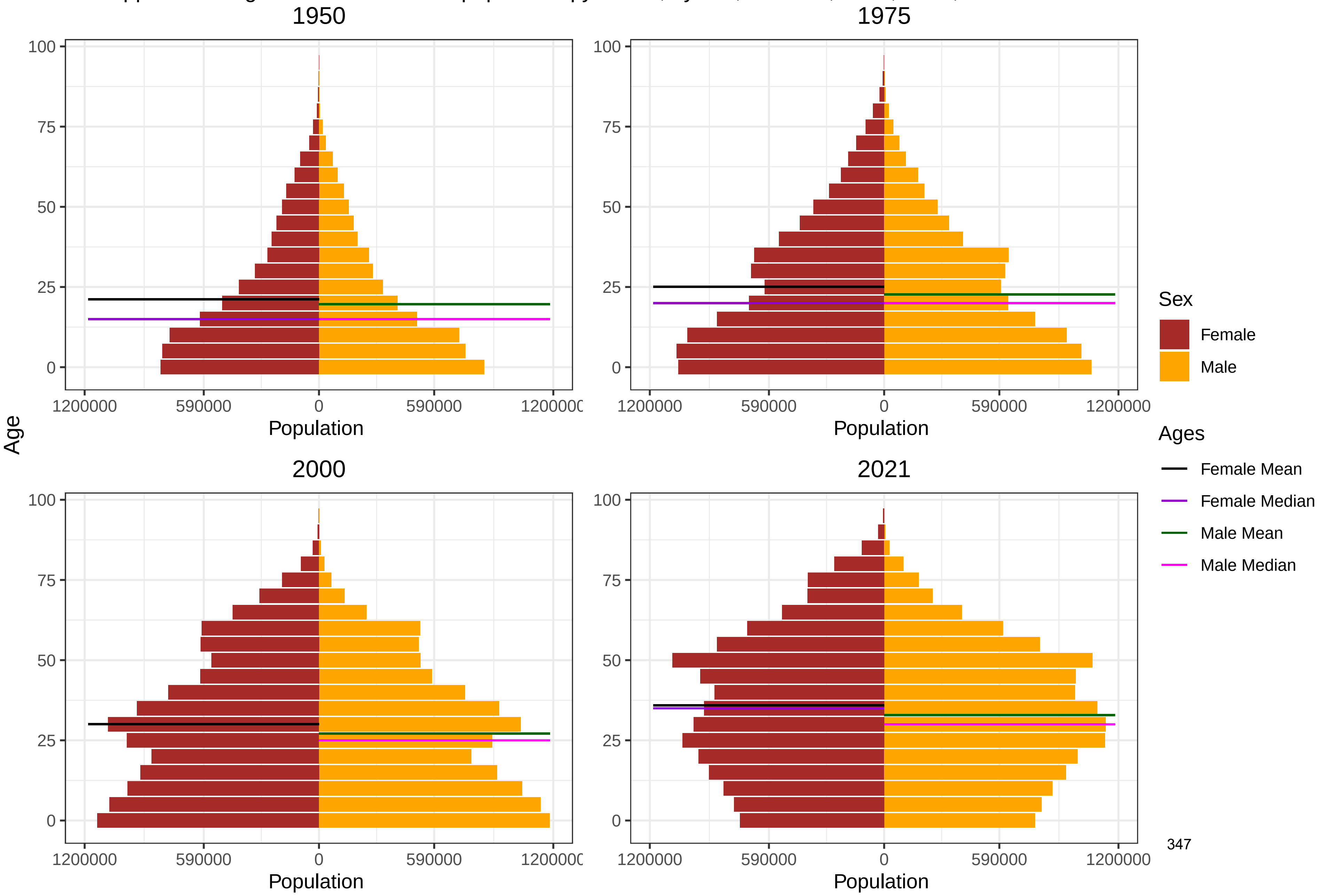

Appendix 2 Figure S9: North Macedonia population pyramids, by sex, for 1950, 1975, 2000, and 2021

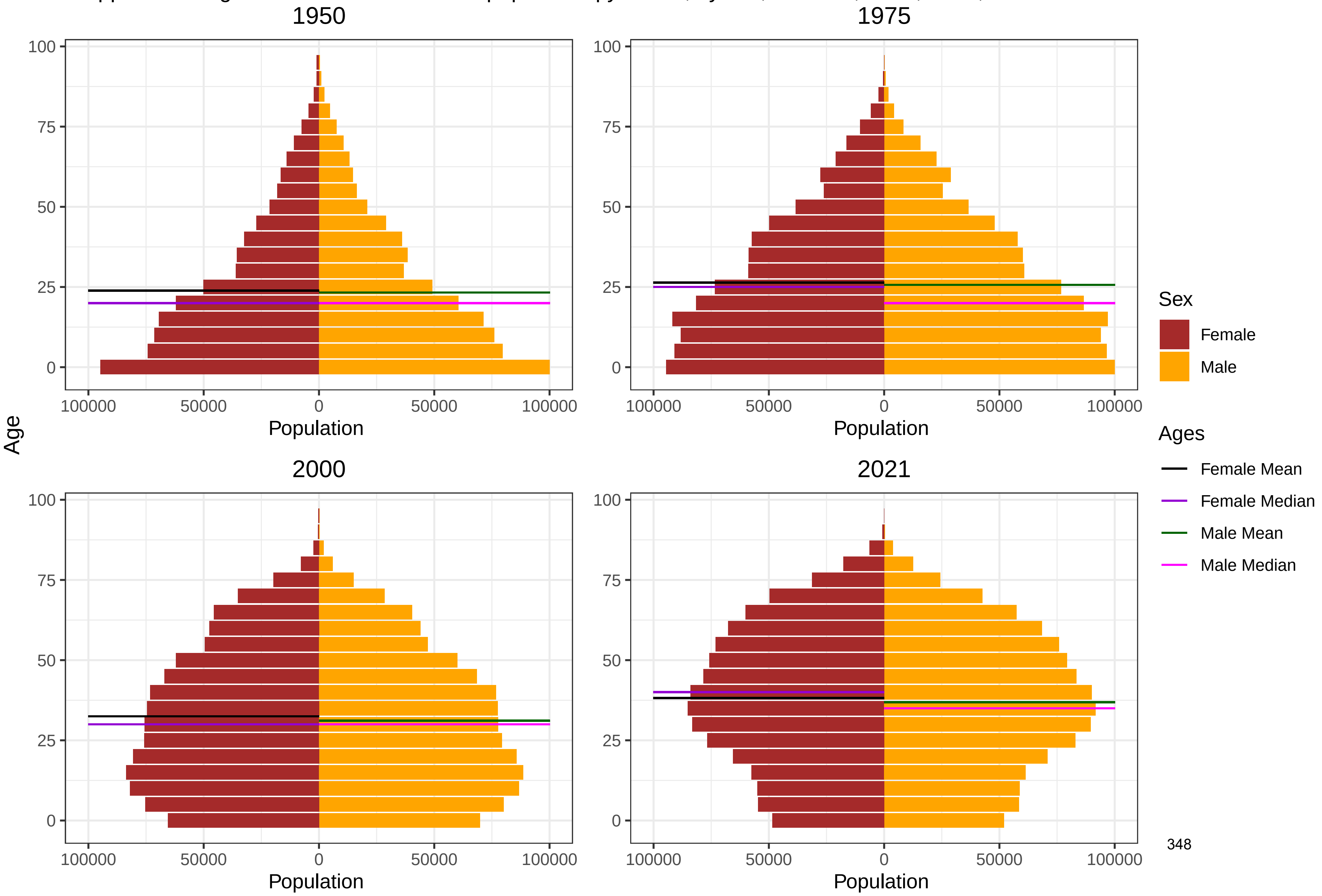

Appendix 2 Figure S9: Northern Mariana Islands population pyramids, by sex, for 1950, 1975, 2000, and 2021

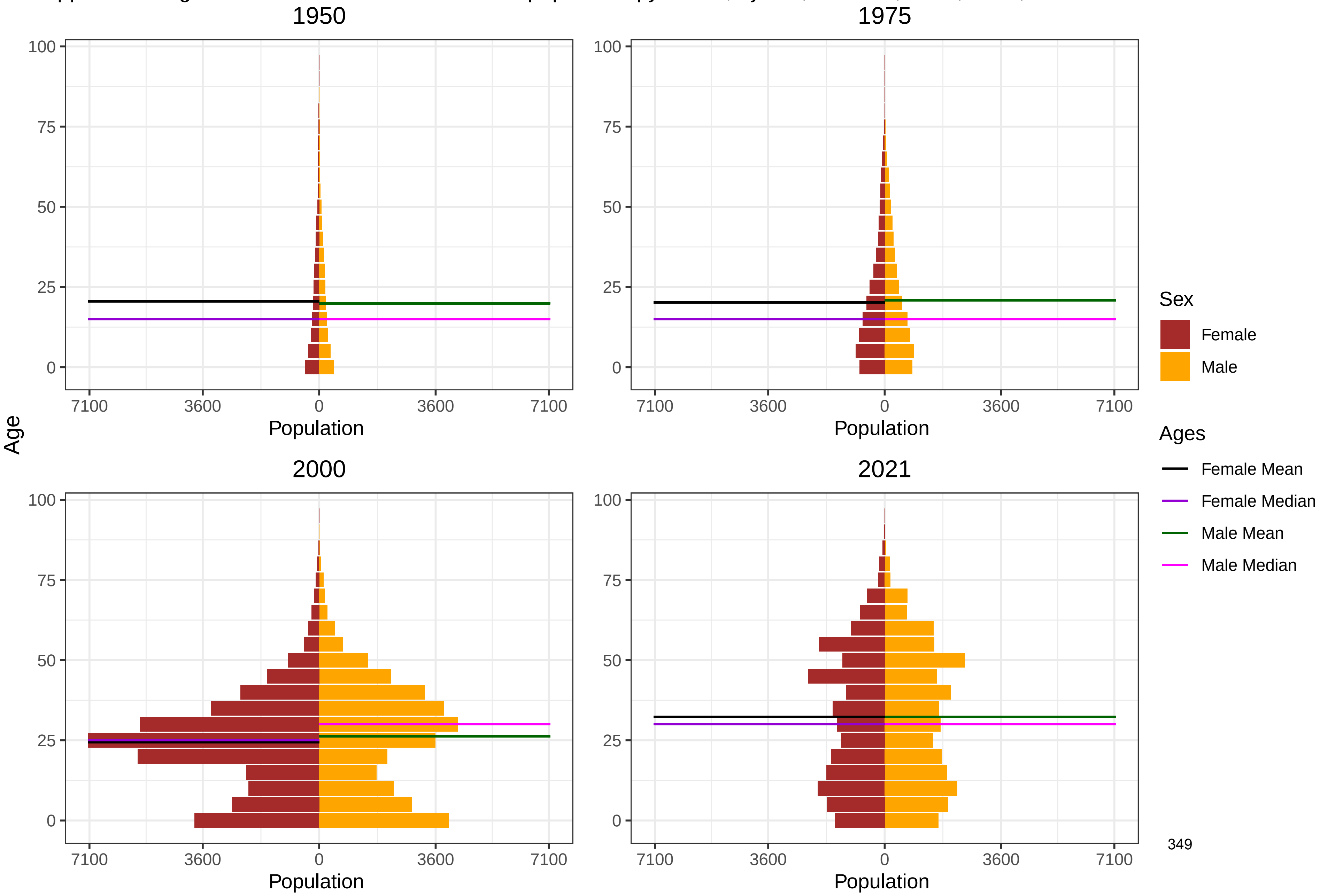

Appendix 2 Figure S9: Norway population pyramids, by sex, for 1950, 1975, 2000, and 2021

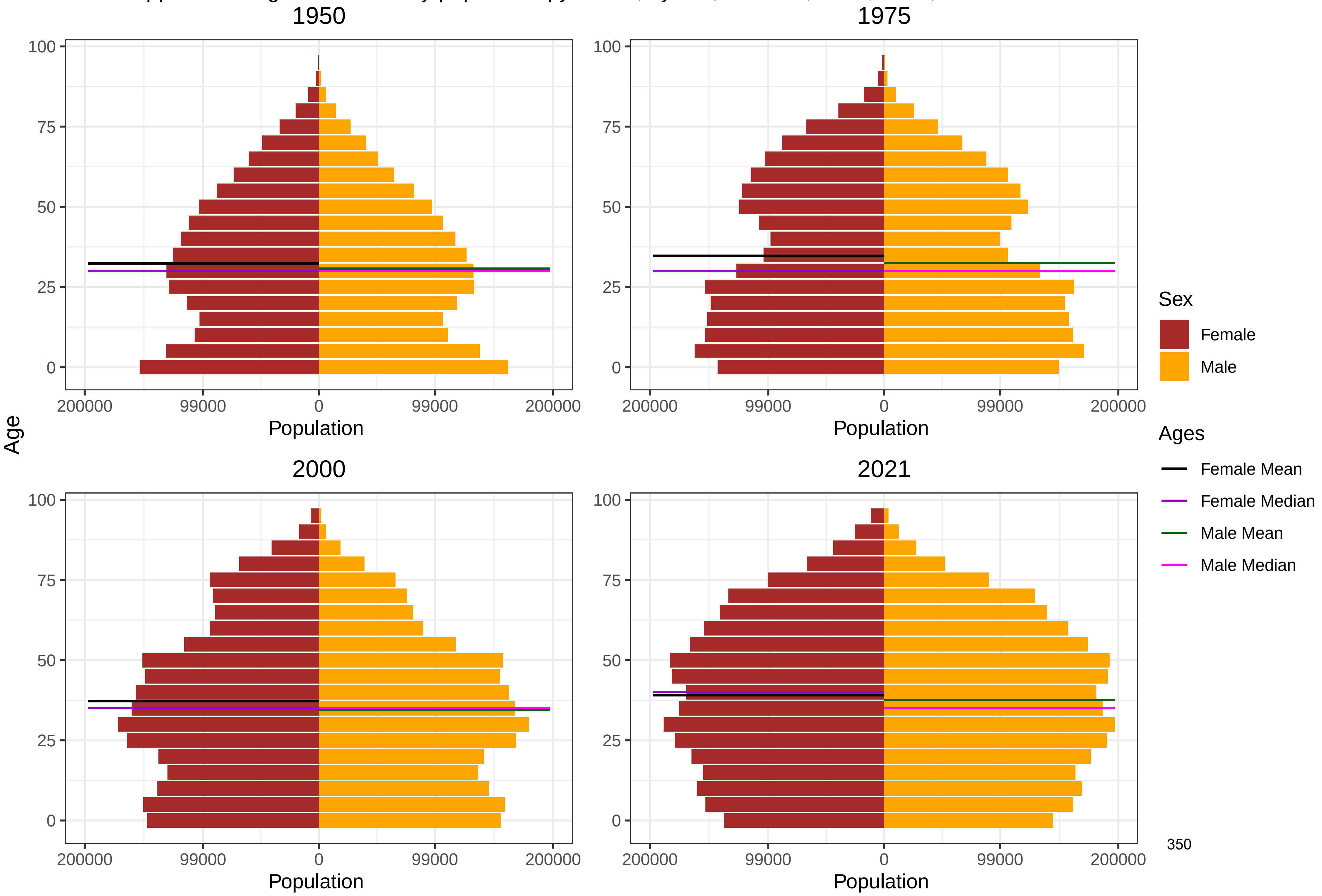

Appendix 2 Figure S9: Oman population pyramids, by sex, for 1950, 1975, 2000, and 2021

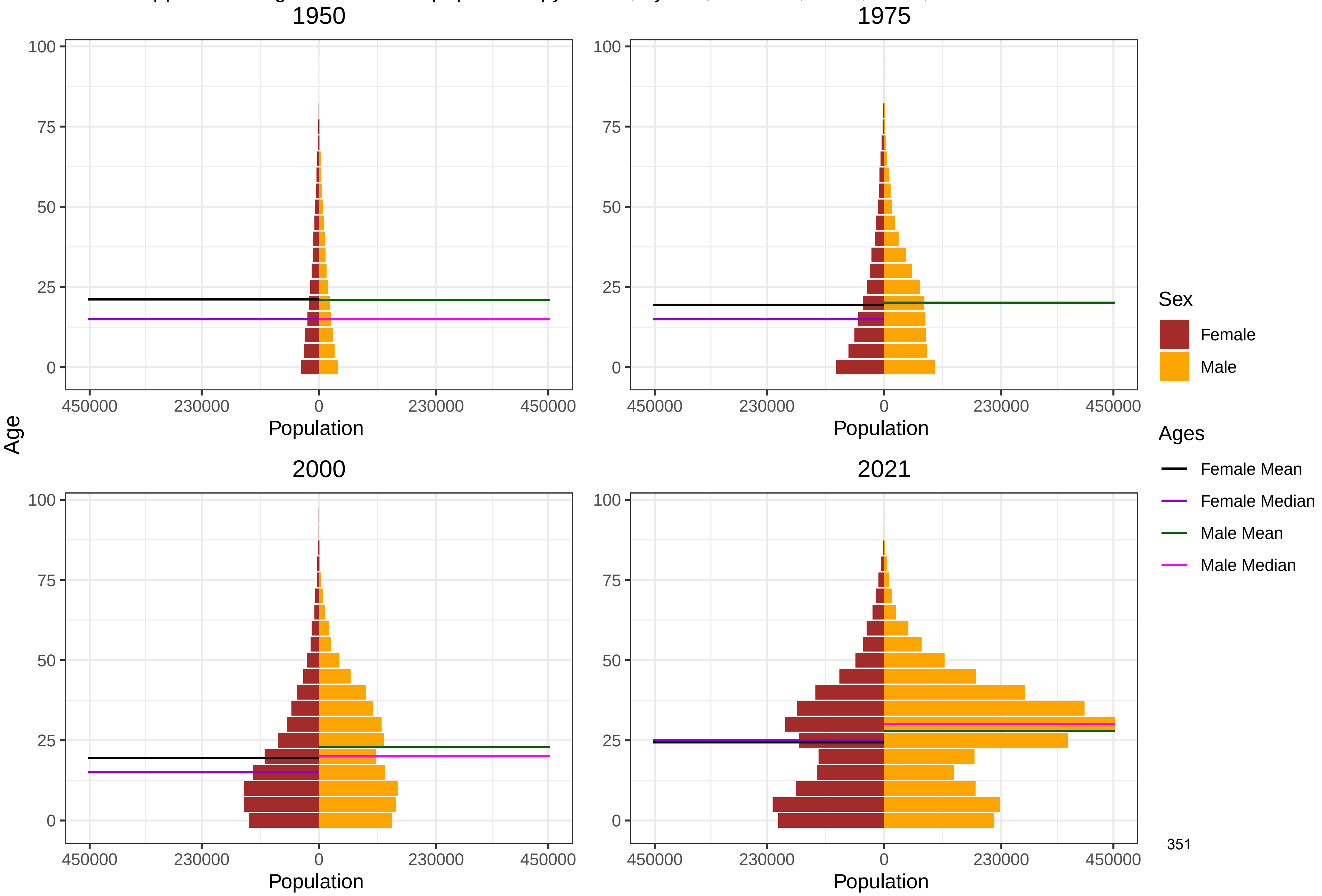

Appendix 2 Figure S9: Pakistan population pyramids, by sex, for 1950, 1975, 2000, and 2021

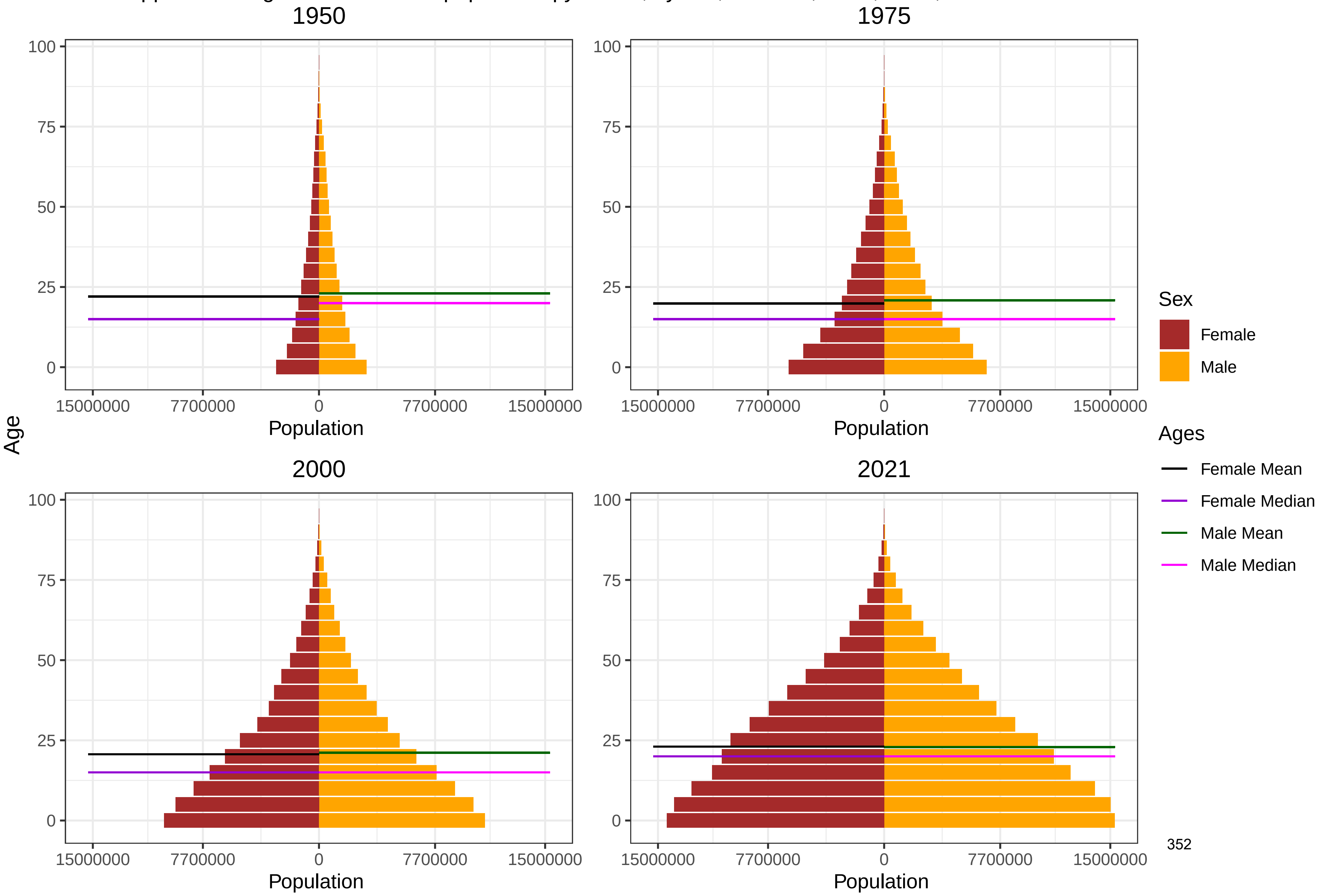

Appendix 2 Figure S9: Palau population pyramids, by sex, for 1950, 1975, 2000, and 2021

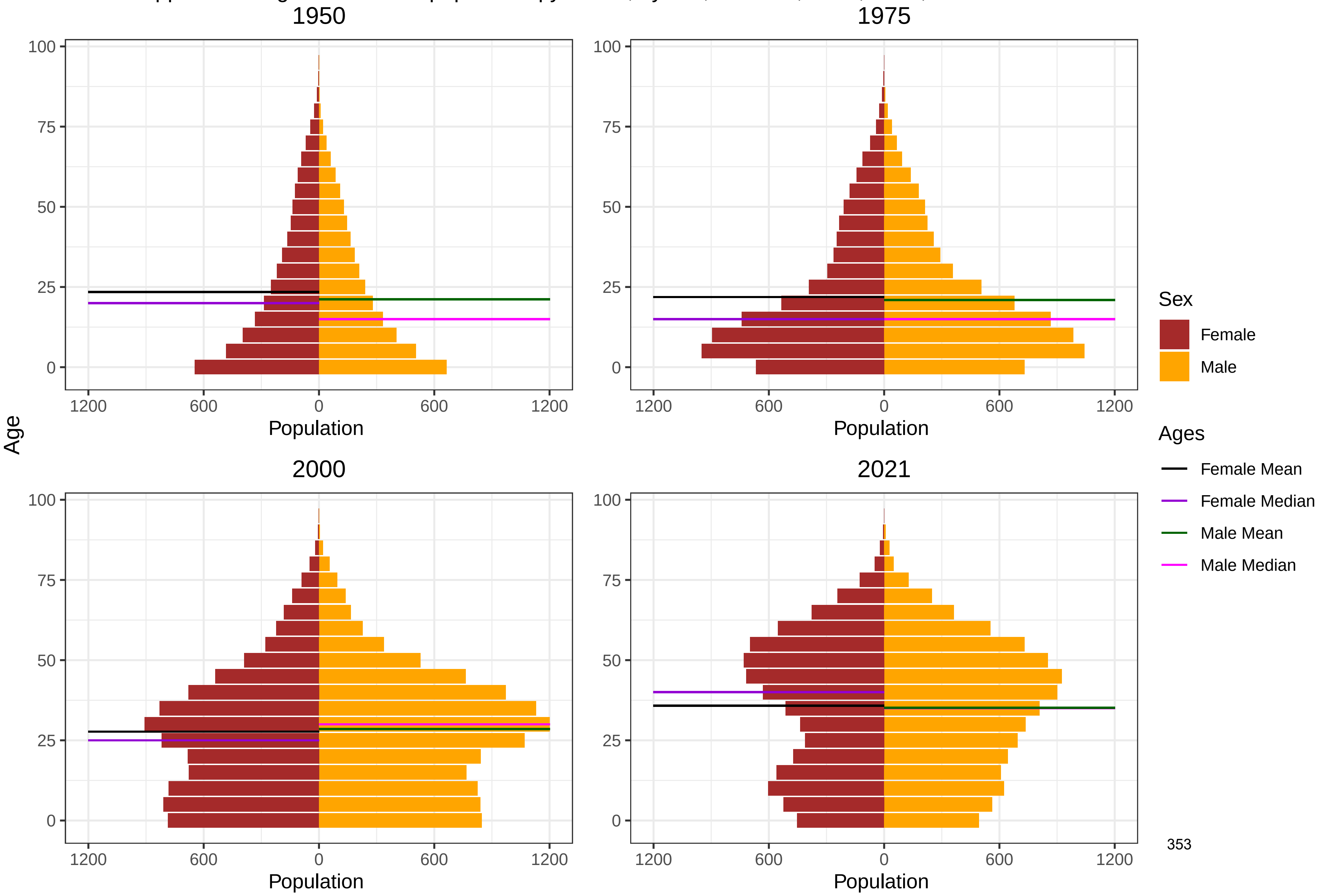

Appendix 2 Figure S9: Palestine population pyramids, by sex, for 1950, 1975, 2000, and 2021

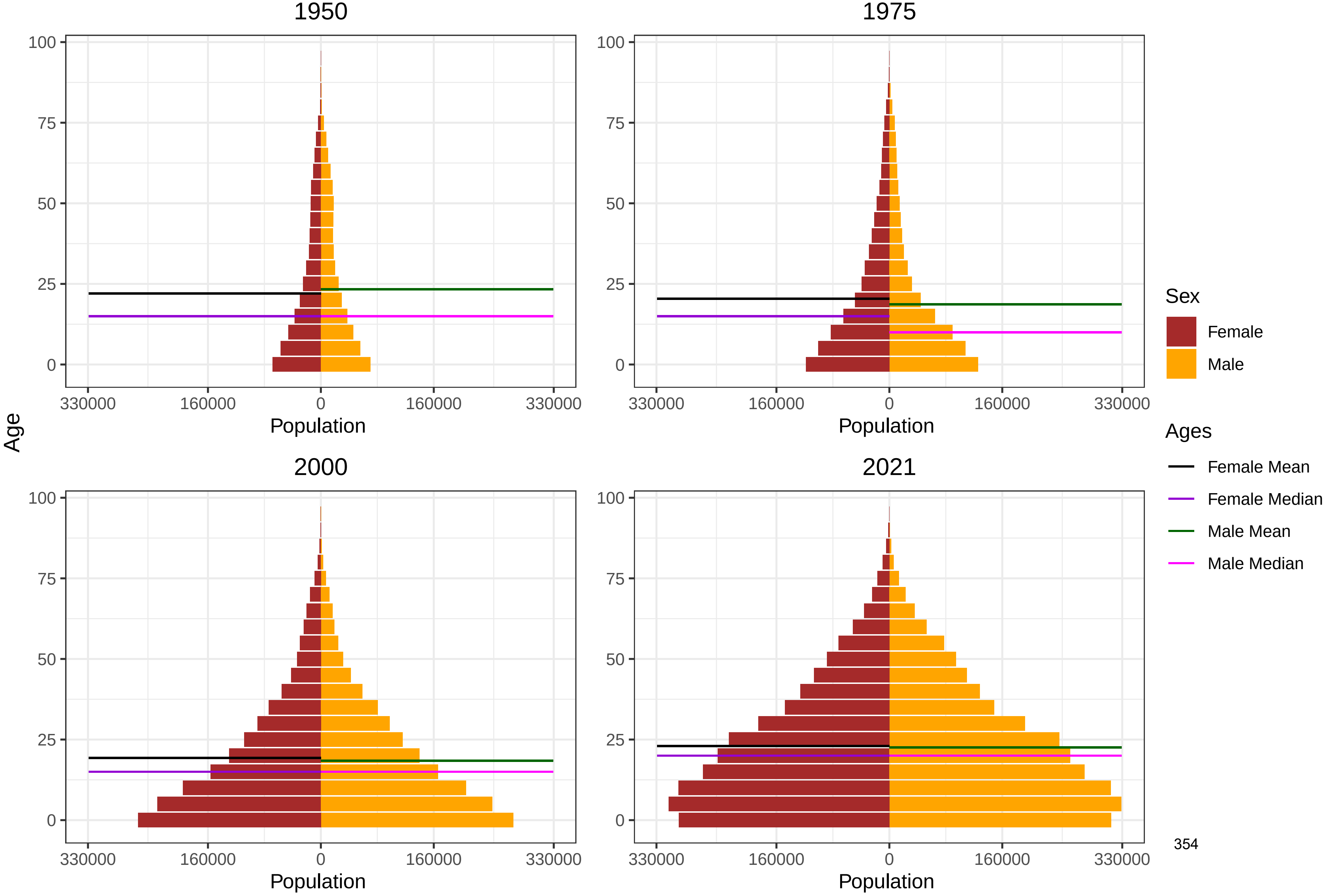

Appendix 2 Figure S9: Panama population pyramids, by sex, for 1950, 1975, 2000, and 2021

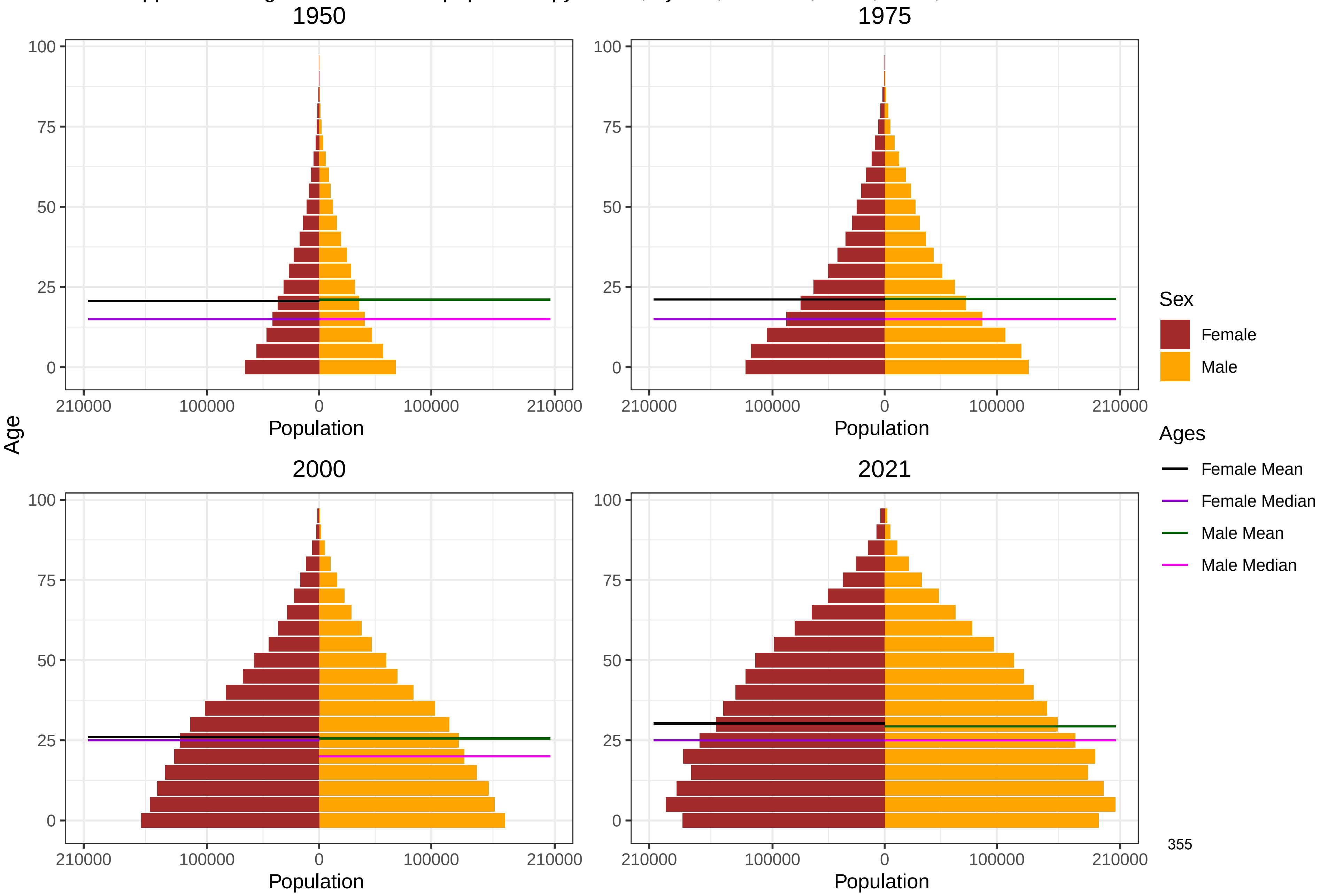

Appendix 2 Figure S9: Papua New Guinea population pyramids, by sex, for 1950, 1975, 2000, and 2021

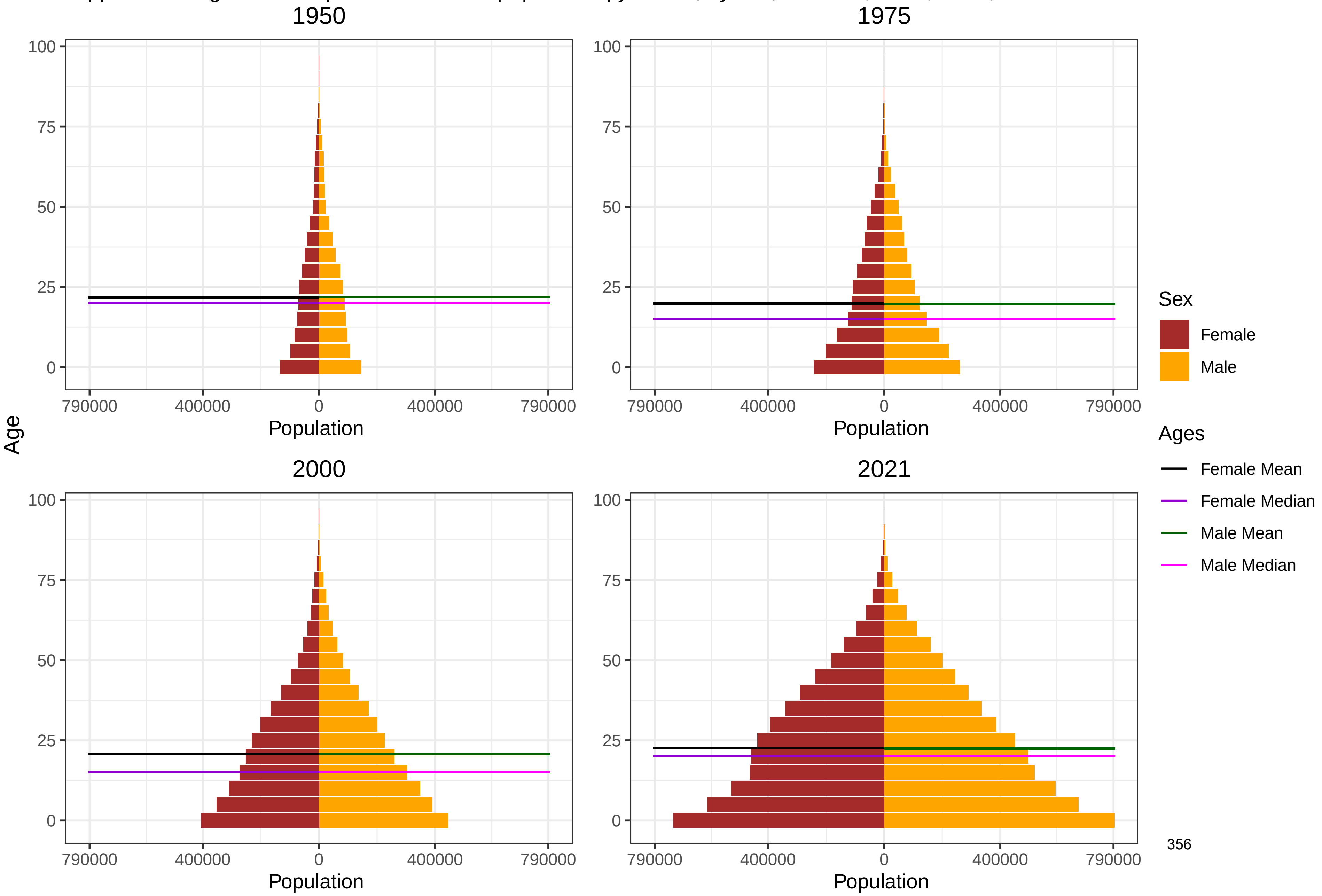

Appendix 2 Figure S9: Paraguay population pyramids, by sex, for 1950, 1975, 2000, and 2021

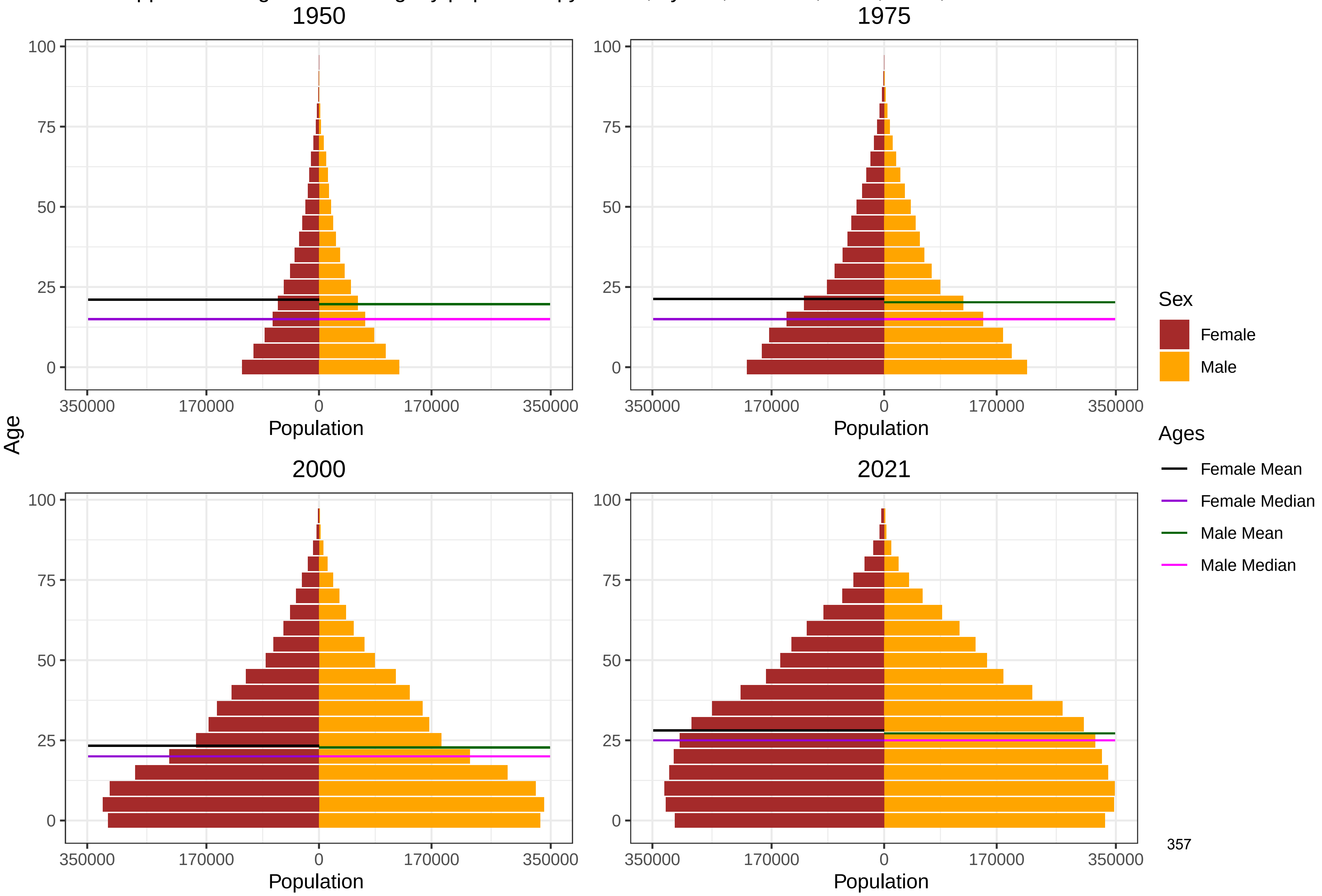

Appendix 2 Figure S9: Peru population pyramids, by sex, for 1950, 1975, 2000, and 2021

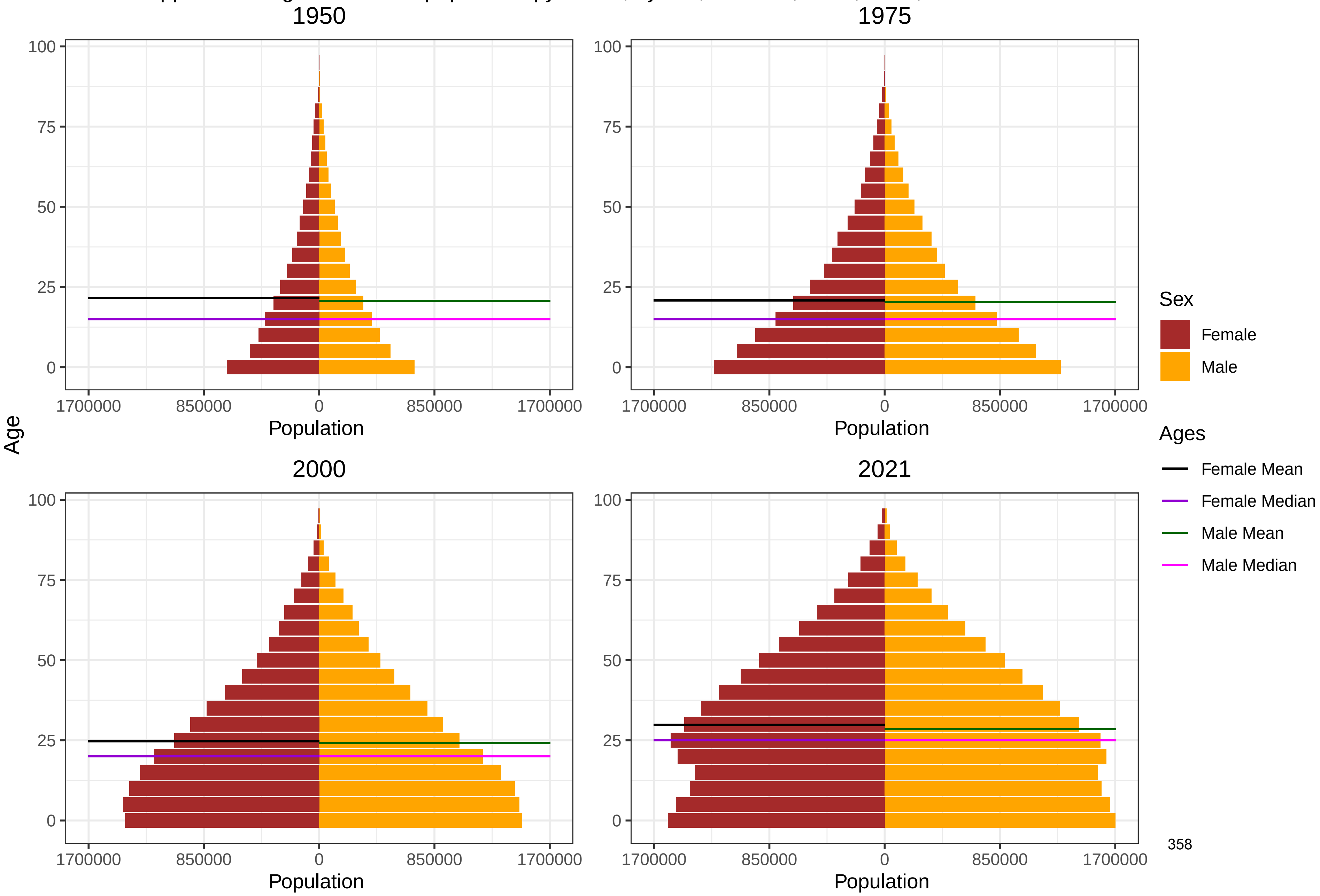

Appendix 2 Figure S9: Philippines population pyramids, by sex, for 1950, 1975, 2000, and 2021

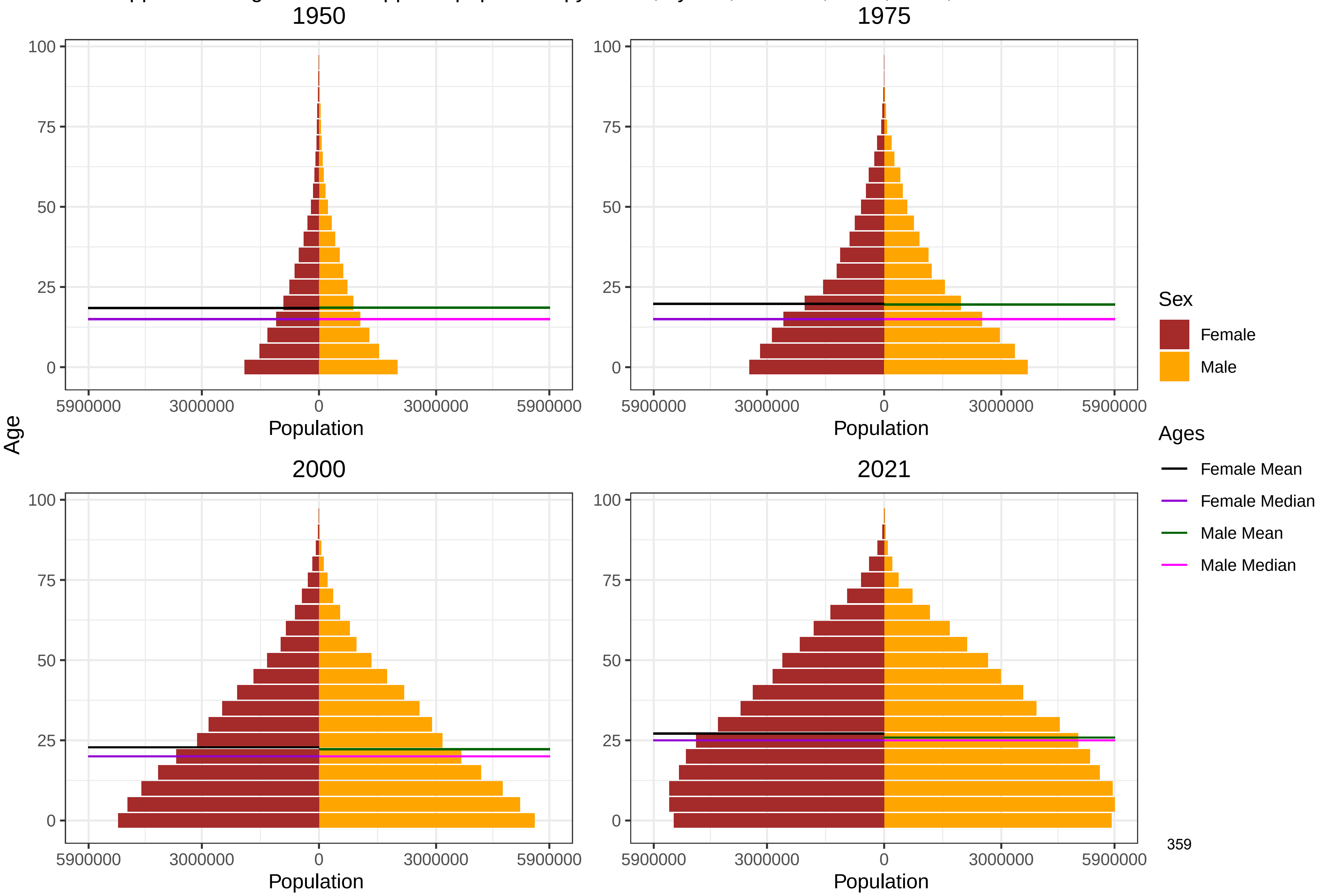

Appendix 2 Figure S9: Poland population pyramids, by sex, for 1950, 1975, 2000, and 2021

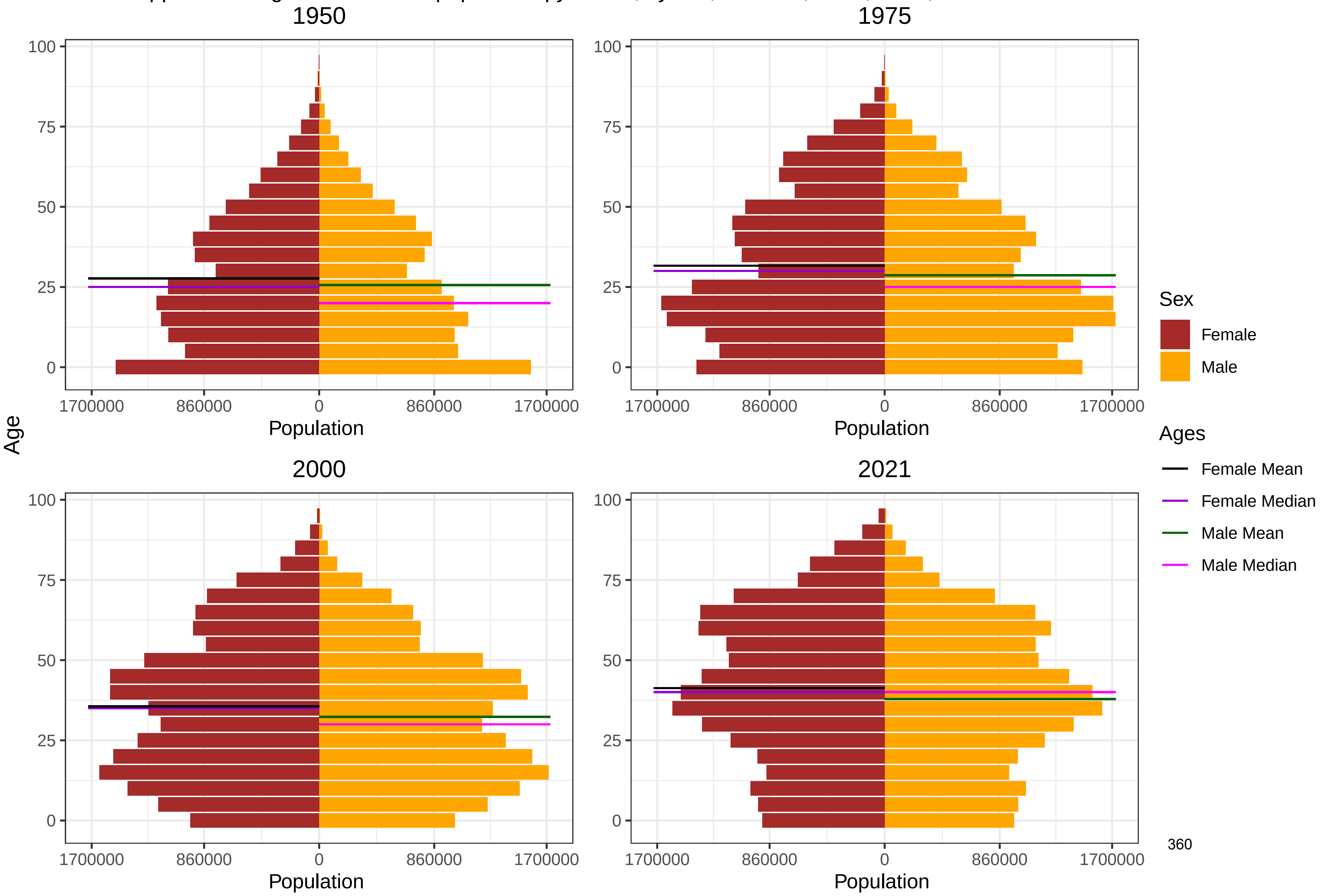

Appendix 2 Figure S9: Portugal population pyramids, by sex, for 1950, 1975, 2000, and 2021

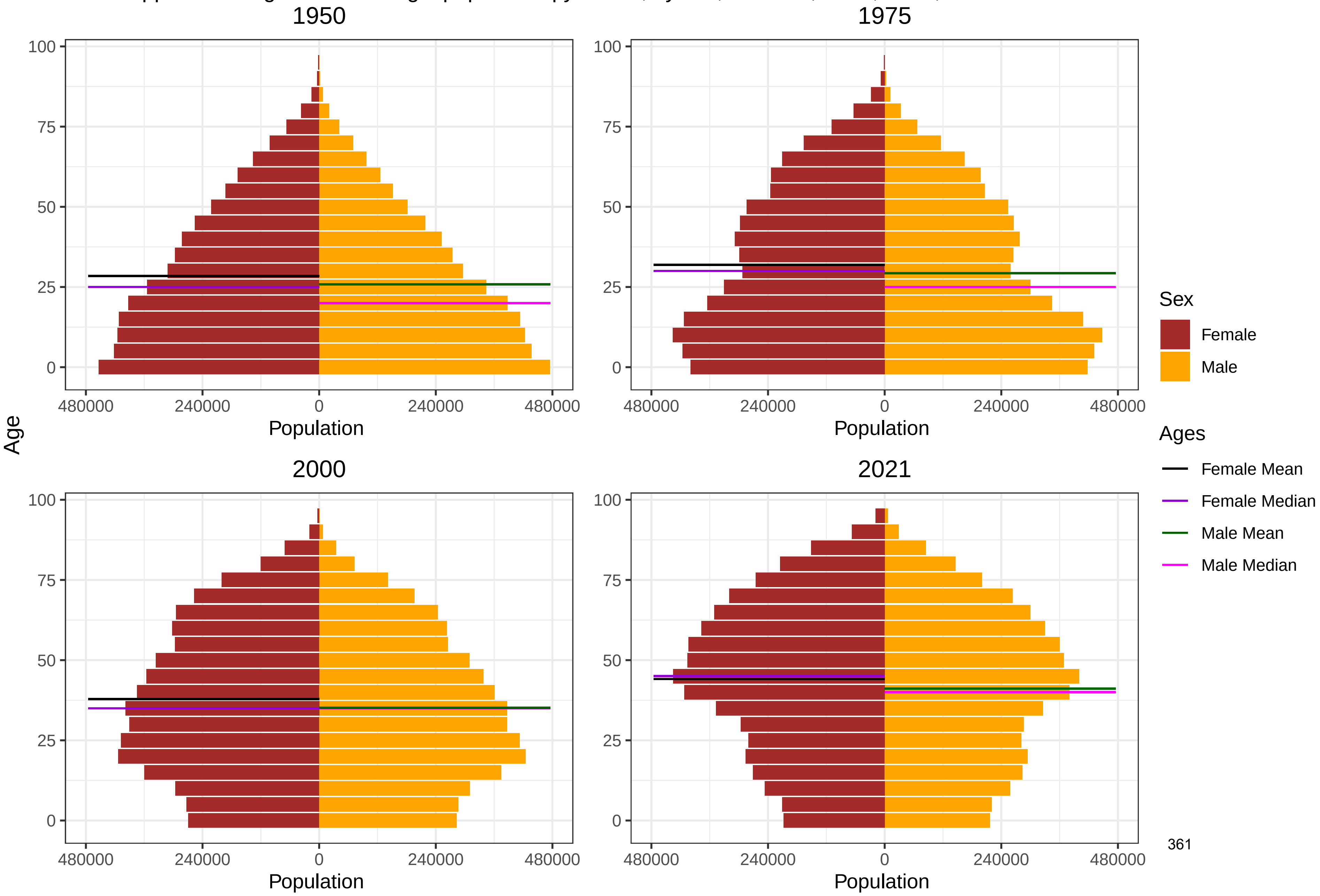

Appendix 2 Figure S9: Puerto Rico population pyramids, by sex, for 1950, 1975, 2000, and 2021

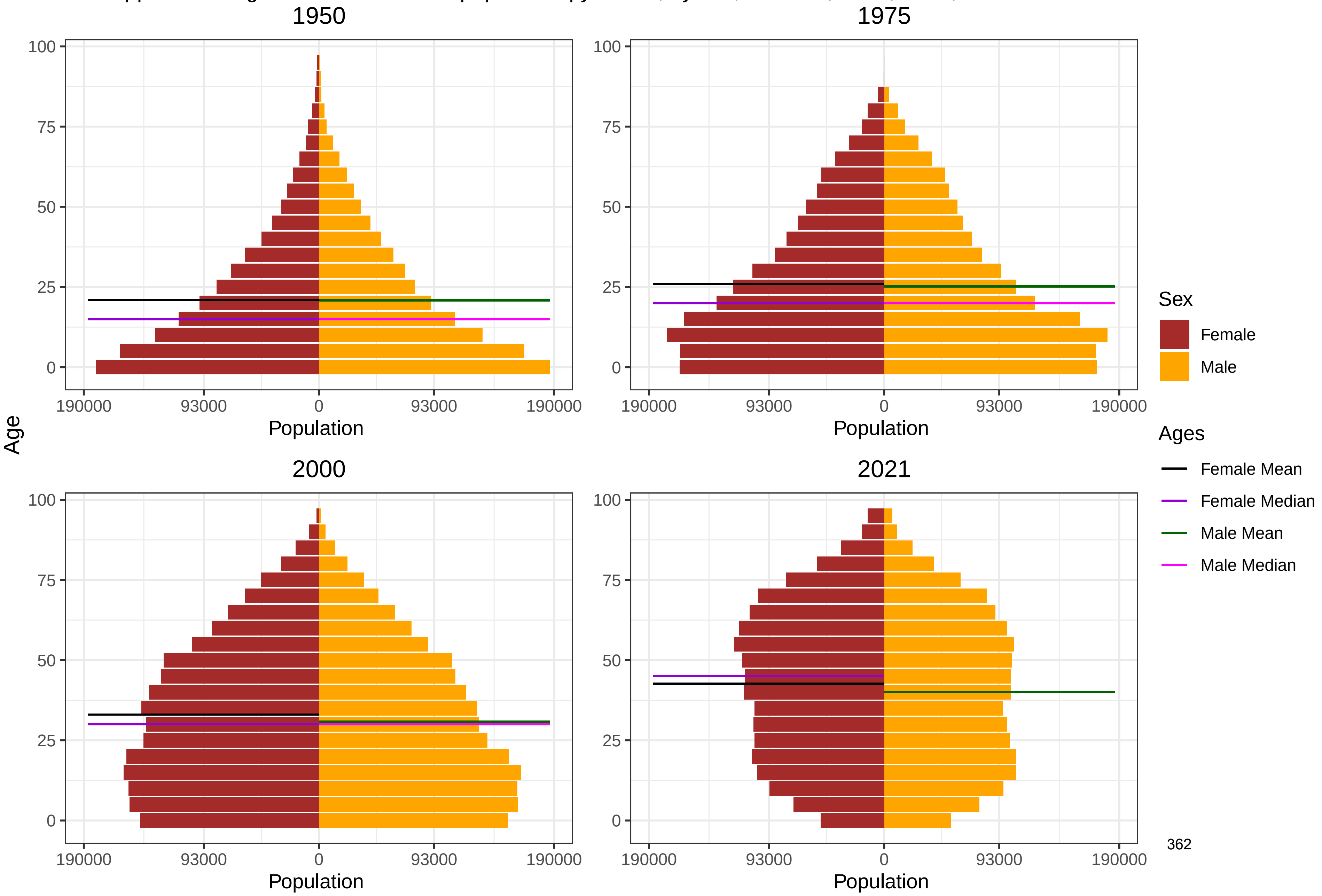

Appendix 2 Figure S9: Qatar population pyramids, by sex, for 1950, 1975, 2000, and 2021

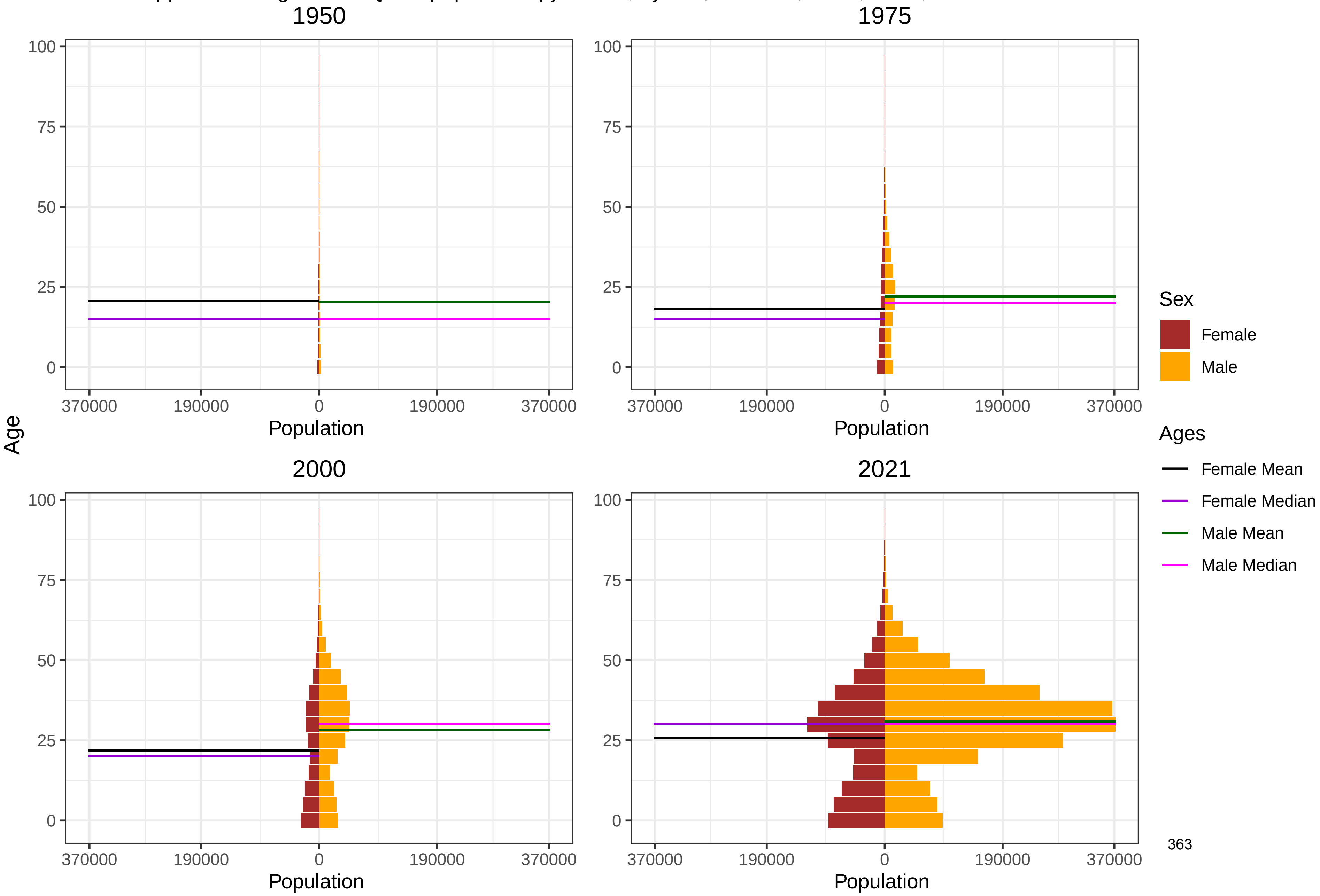

Appendix 2 Figure S9: Romania population pyramids, by sex, for 1950, 1975, 2000, and 2021

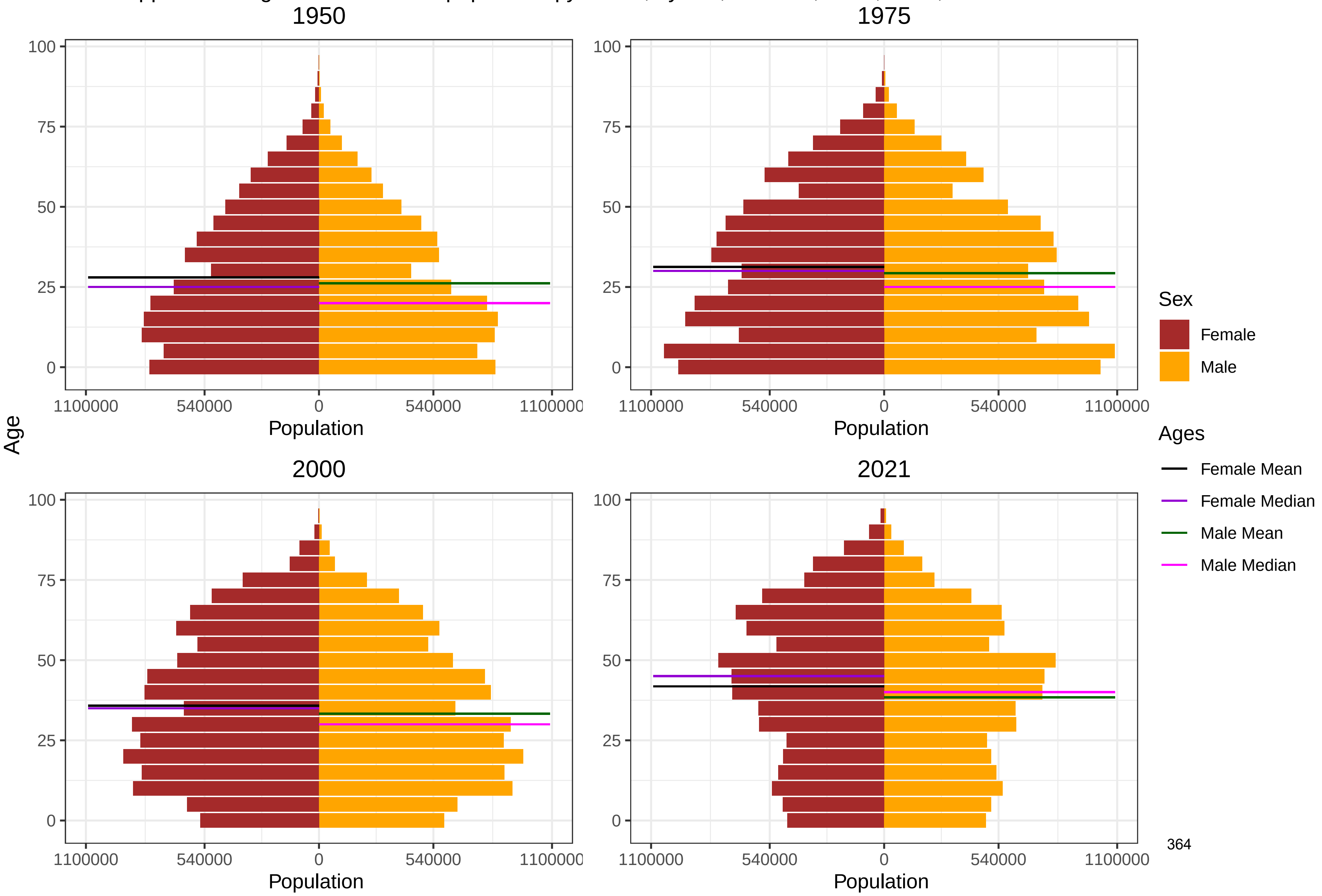

Appendix 2 Figure S9: Russia population pyramids, by sex, for 1950, 1975, 2000, and 2021

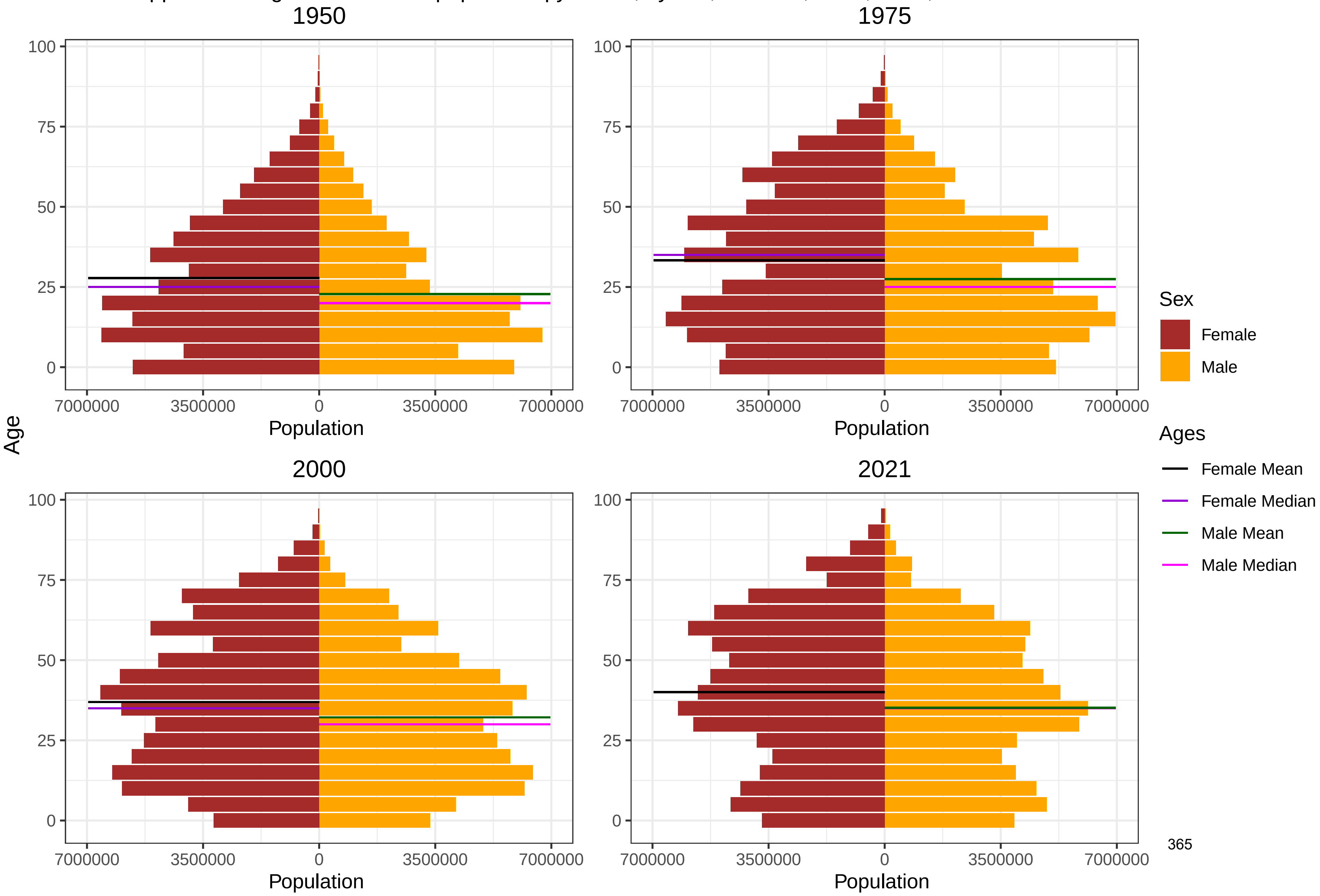

Appendix 2 Figure S9: Rwanda population pyramids, by sex, for 1950, 1975, 2000, and 2021

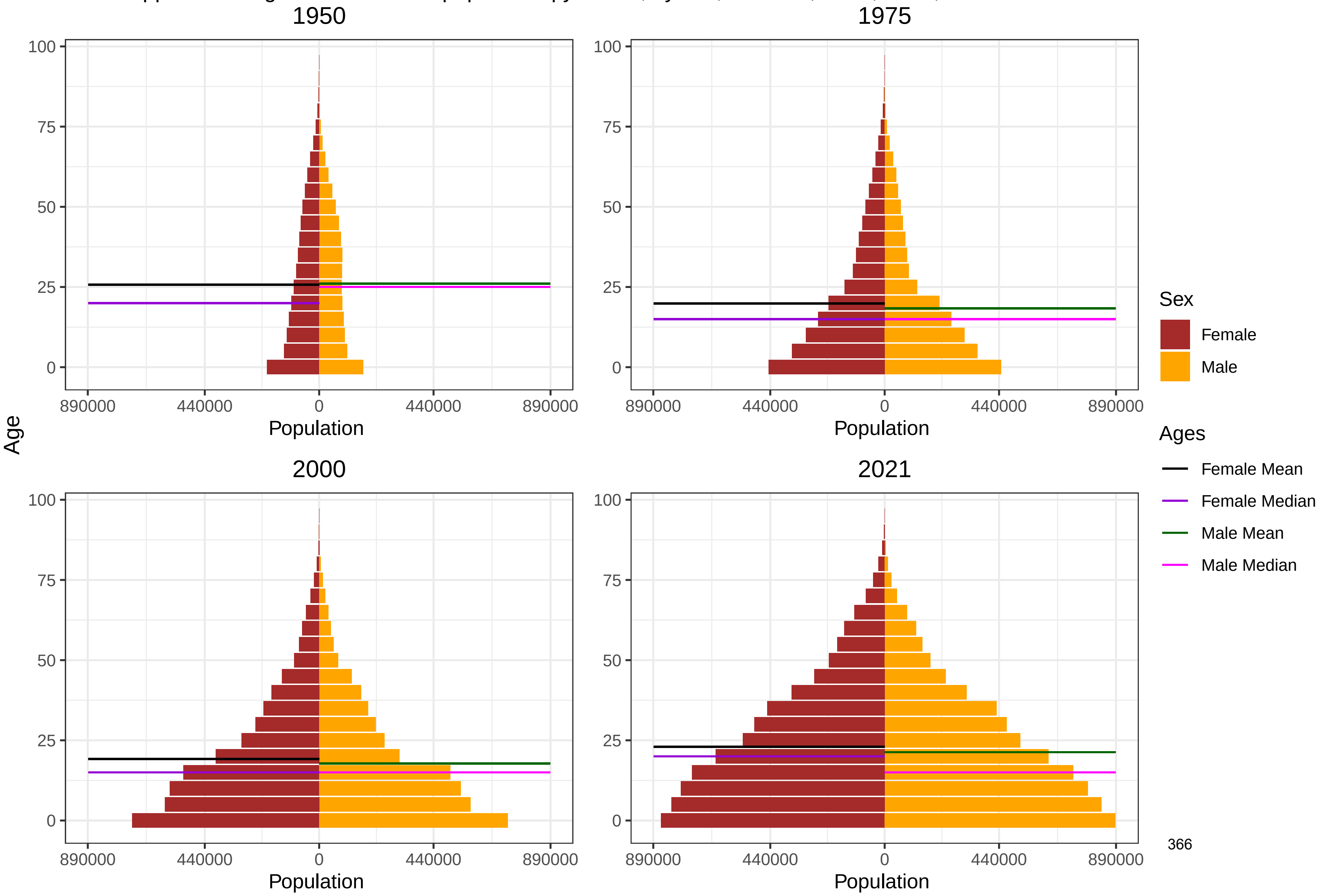

Appendix 2 Figure S9: Saint Kitts and Nevis population pyramids, by sex, for 1950, 1975, 2000, and 2021

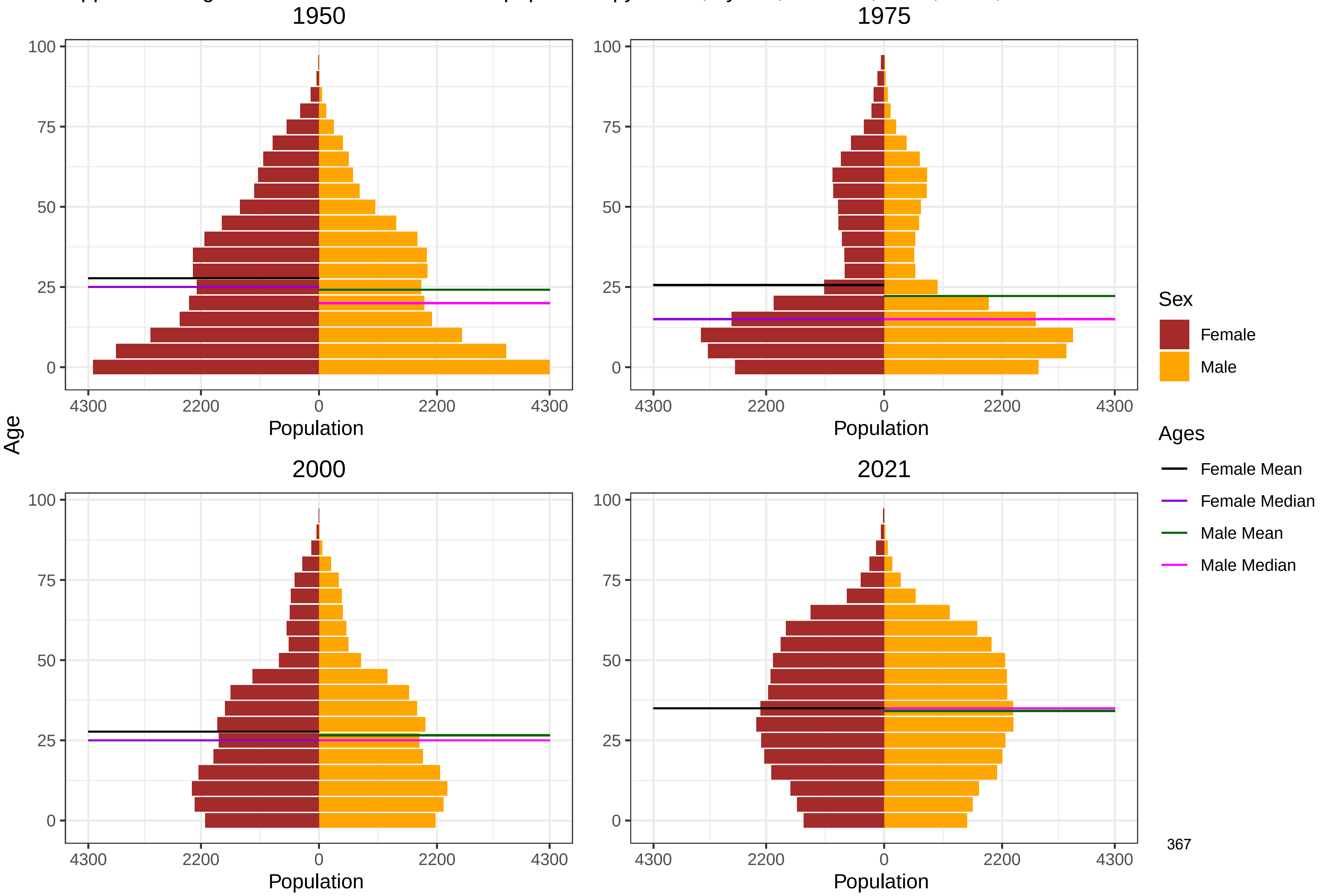

Appendix 2 Figure S9: Saint Lucia population pyramids, by sex, for 1950, 1975, 2000, and 2021

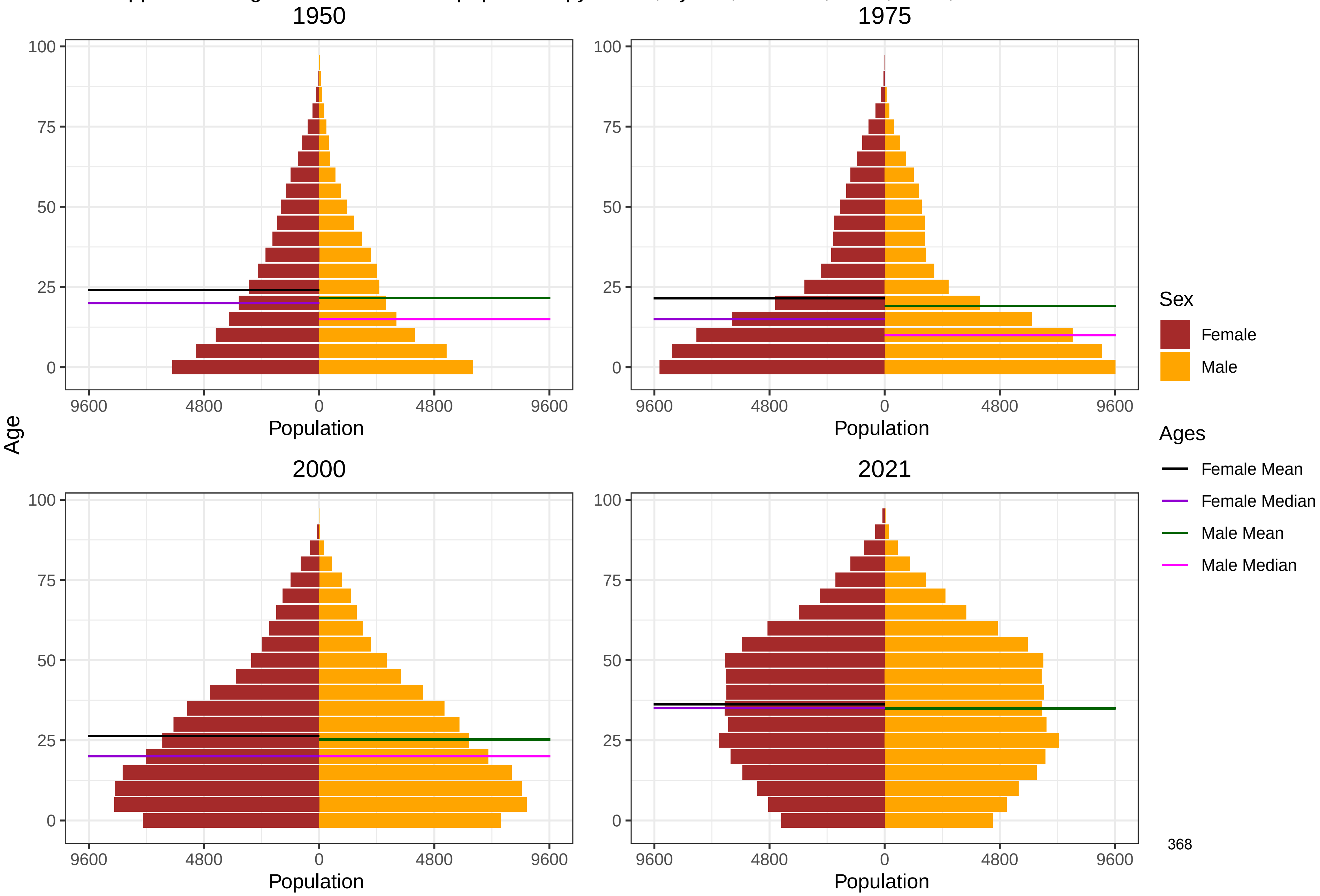

Appendix 2 Figure S9: Saint Vincent and the Grenadines population pyramids, by sex, for 1950, 1975, 2000, and 2021

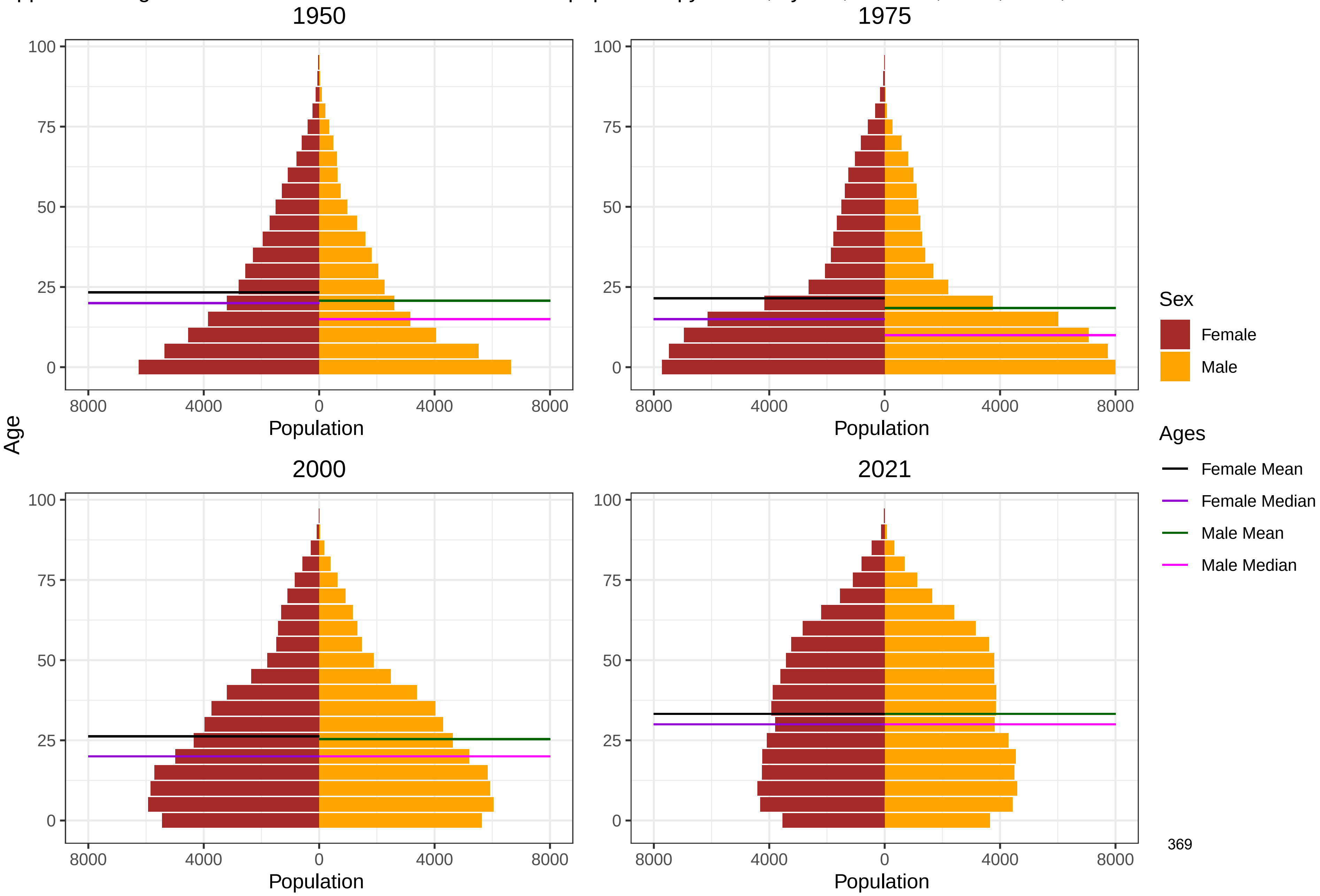

Appendix 2 Figure S9: Samoa population pyramids, by sex, for 1950, 1975, 2000, and 2021

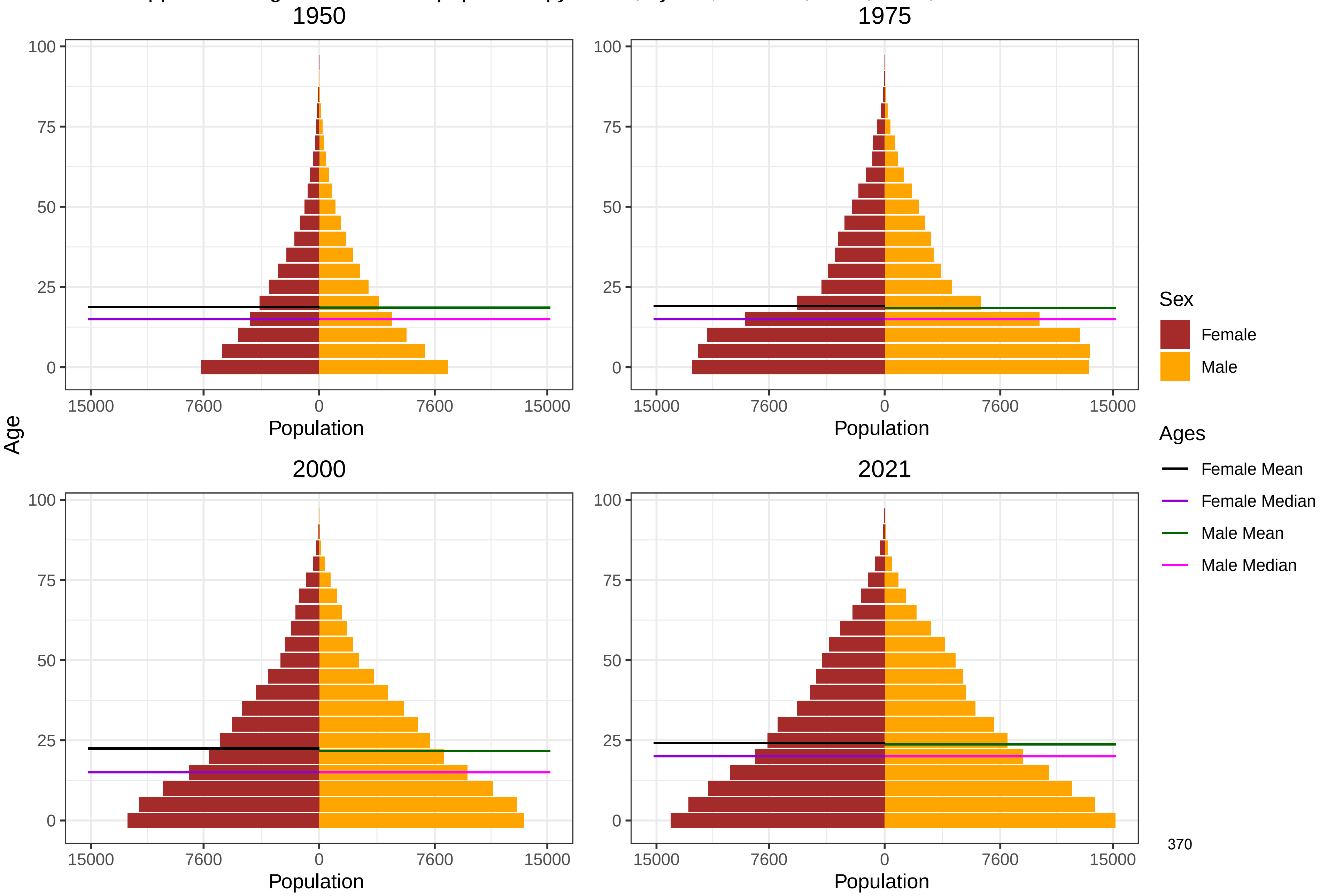

Appendix 2 Figure S9: San Marino population pyramids, by sex, for 1950, 1975, 2000, and 2021

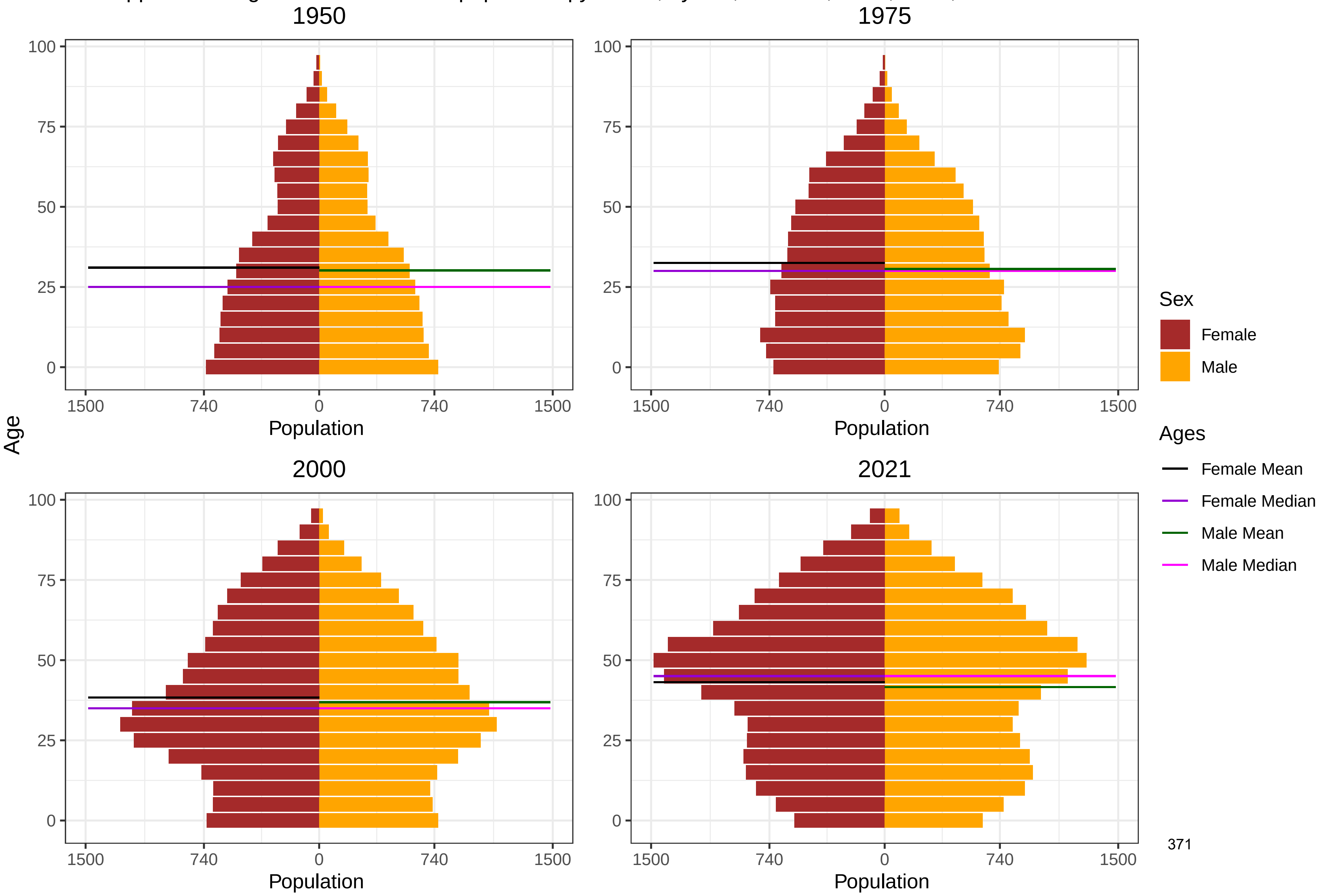

Appendix 2 Figure S9: Saudi Arabia population pyramids, by sex, for 1950, 1975, 2000, and 2021

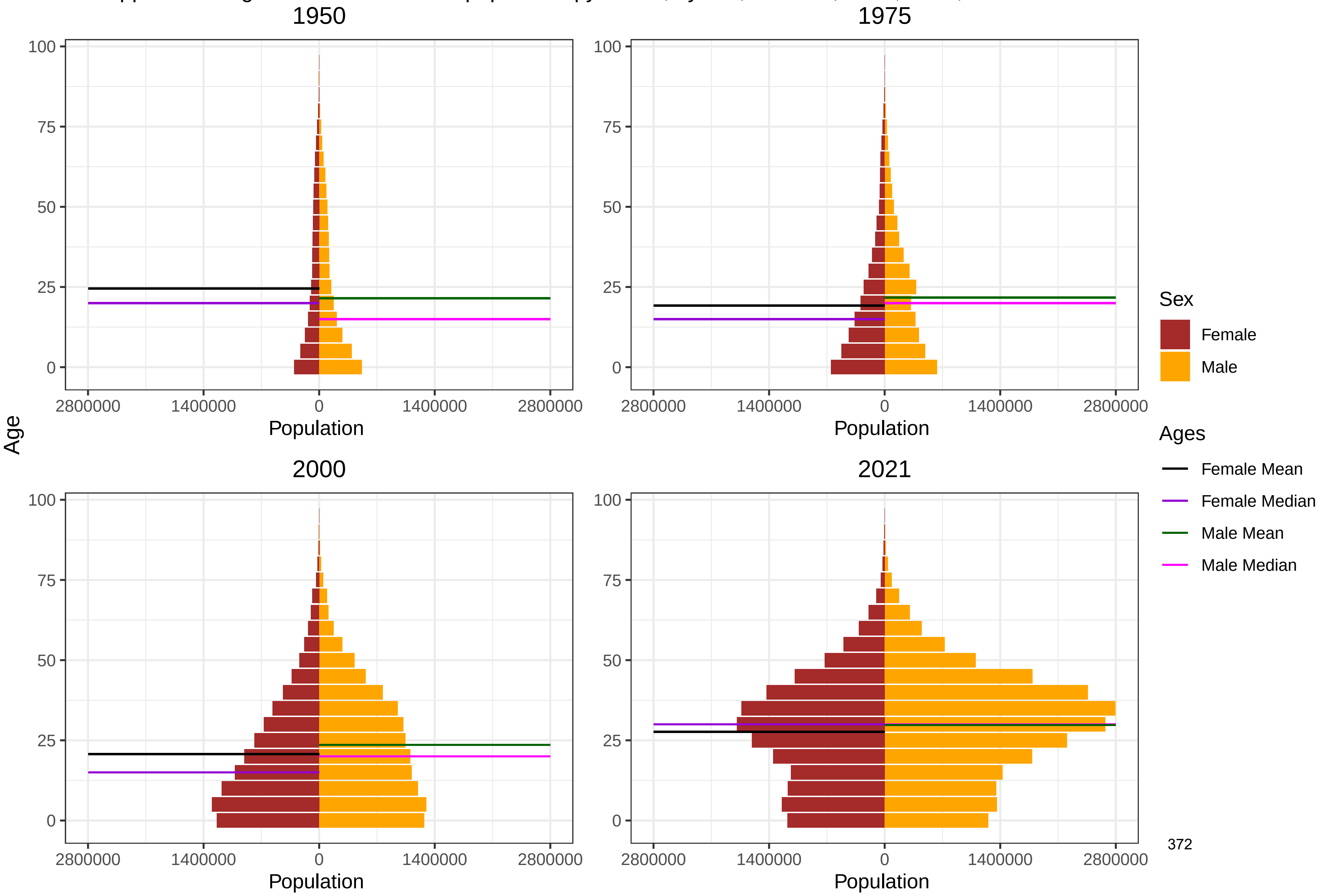

Appendix 2 Figure S9: Senegal population pyramids, by sex, for 1950, 1975, 2000, and 2021

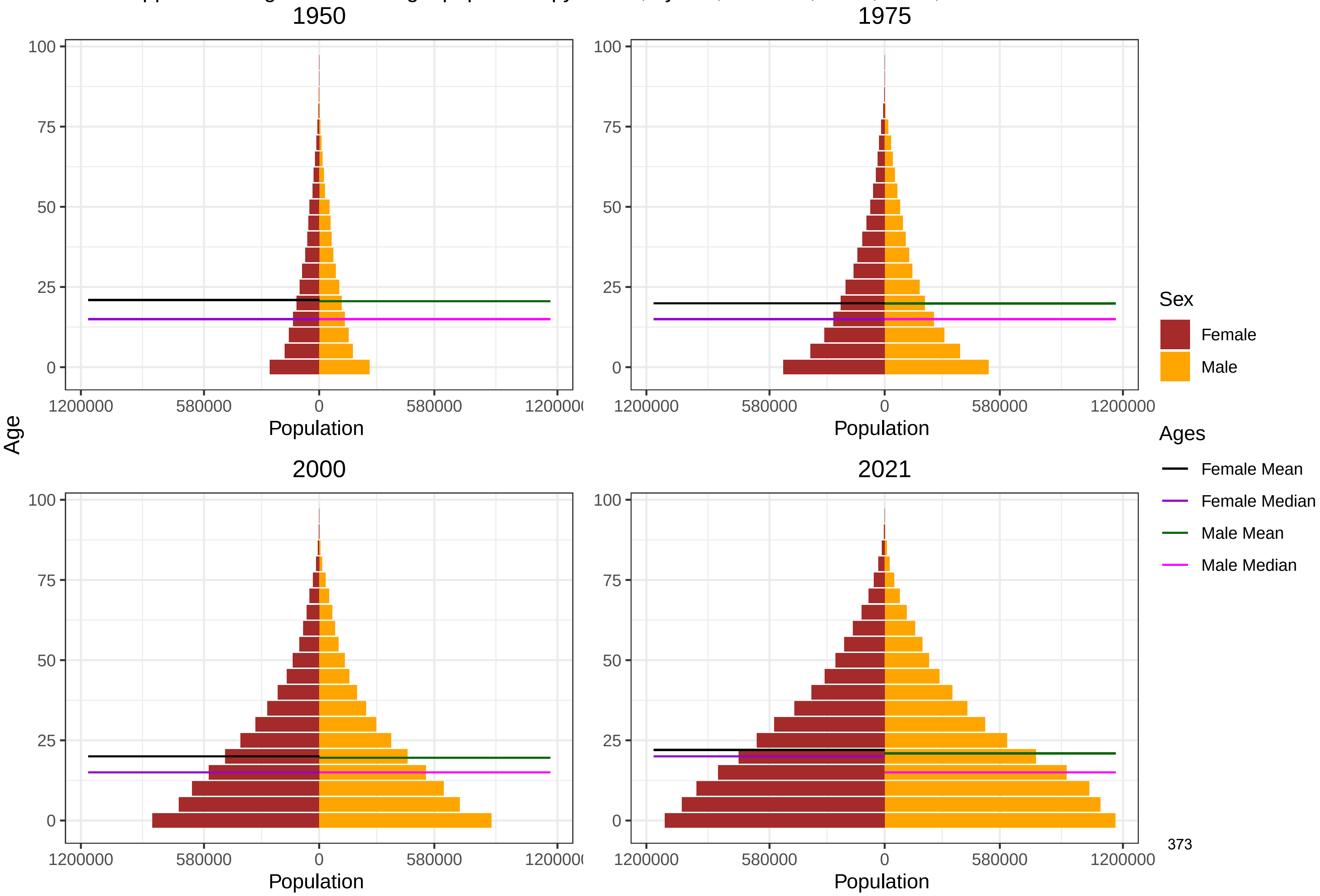

Appendix 2 Figure S9: Serbia population pyramids, by sex, for 1950, 1975, 2000, and 2021

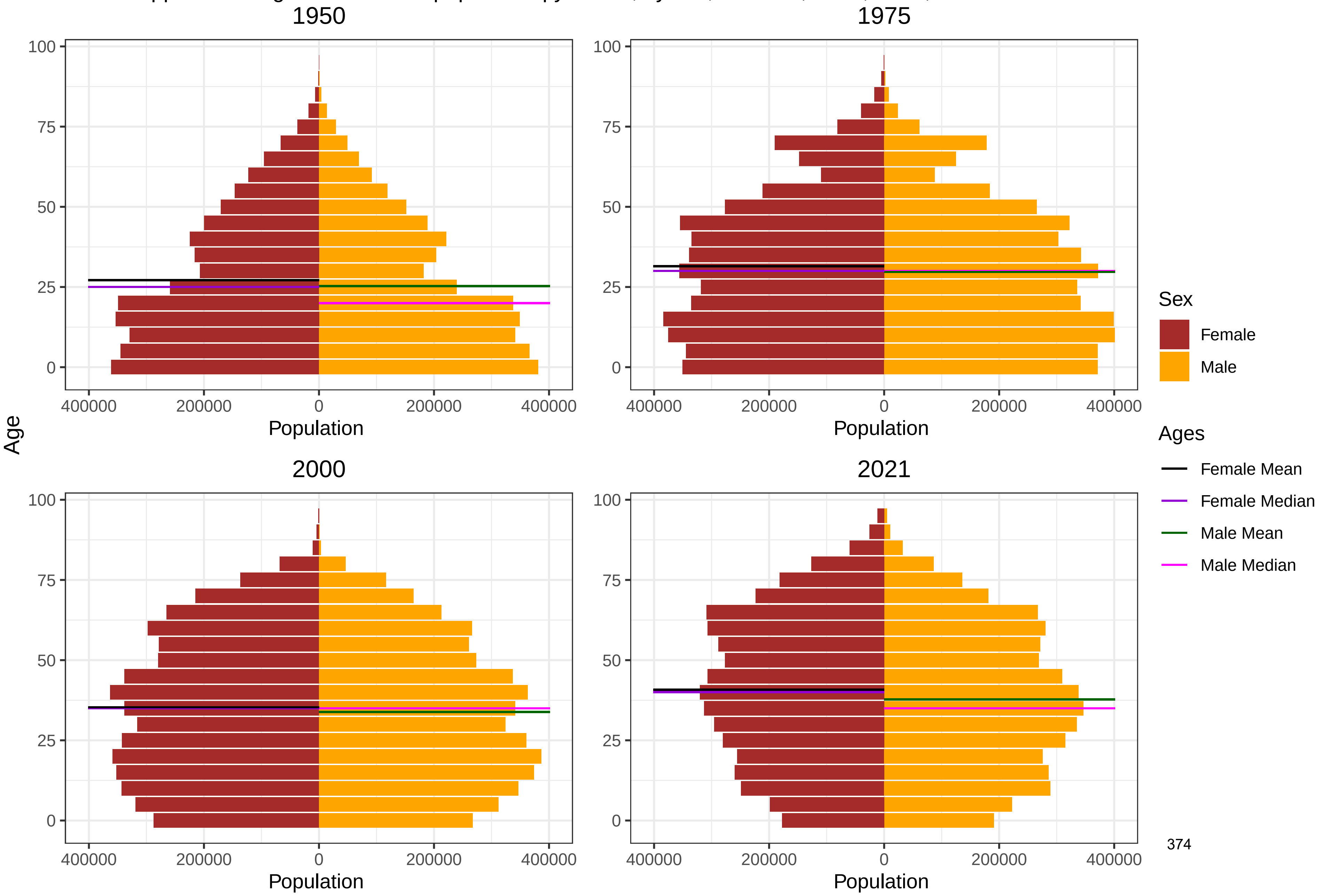

Appendix 2 Figure S9: Seychelles population pyramids, by sex, for 1950, 1975, 2000, and 2021

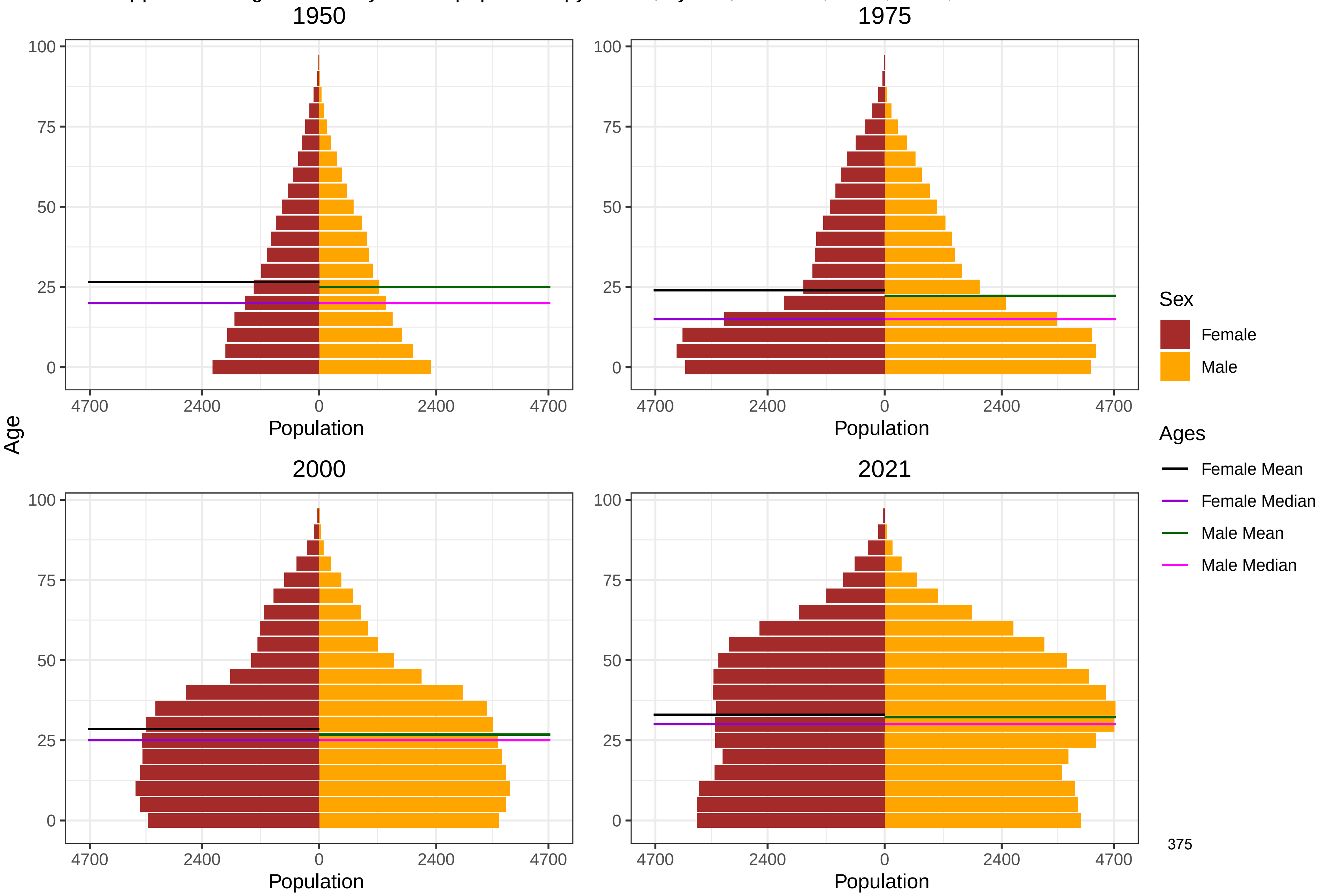

Appendix 2 Figure S9: Sierra Leone population pyramids, by sex, for 1950, 1975, 2000, and 2021

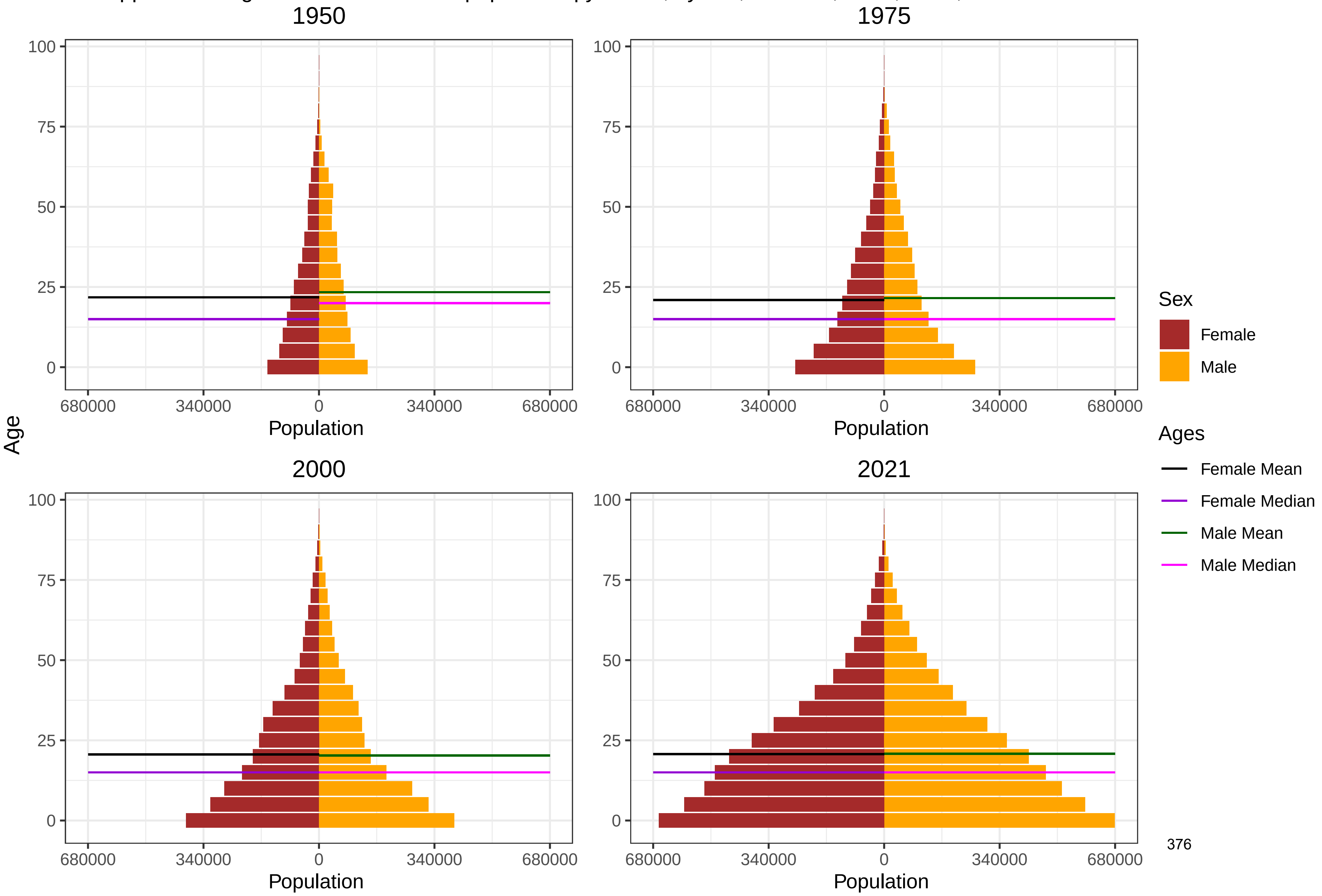

Appendix 2 Figure S9: Singapore population pyramids, by sex, for 1950, 1975, 2000, and 2021

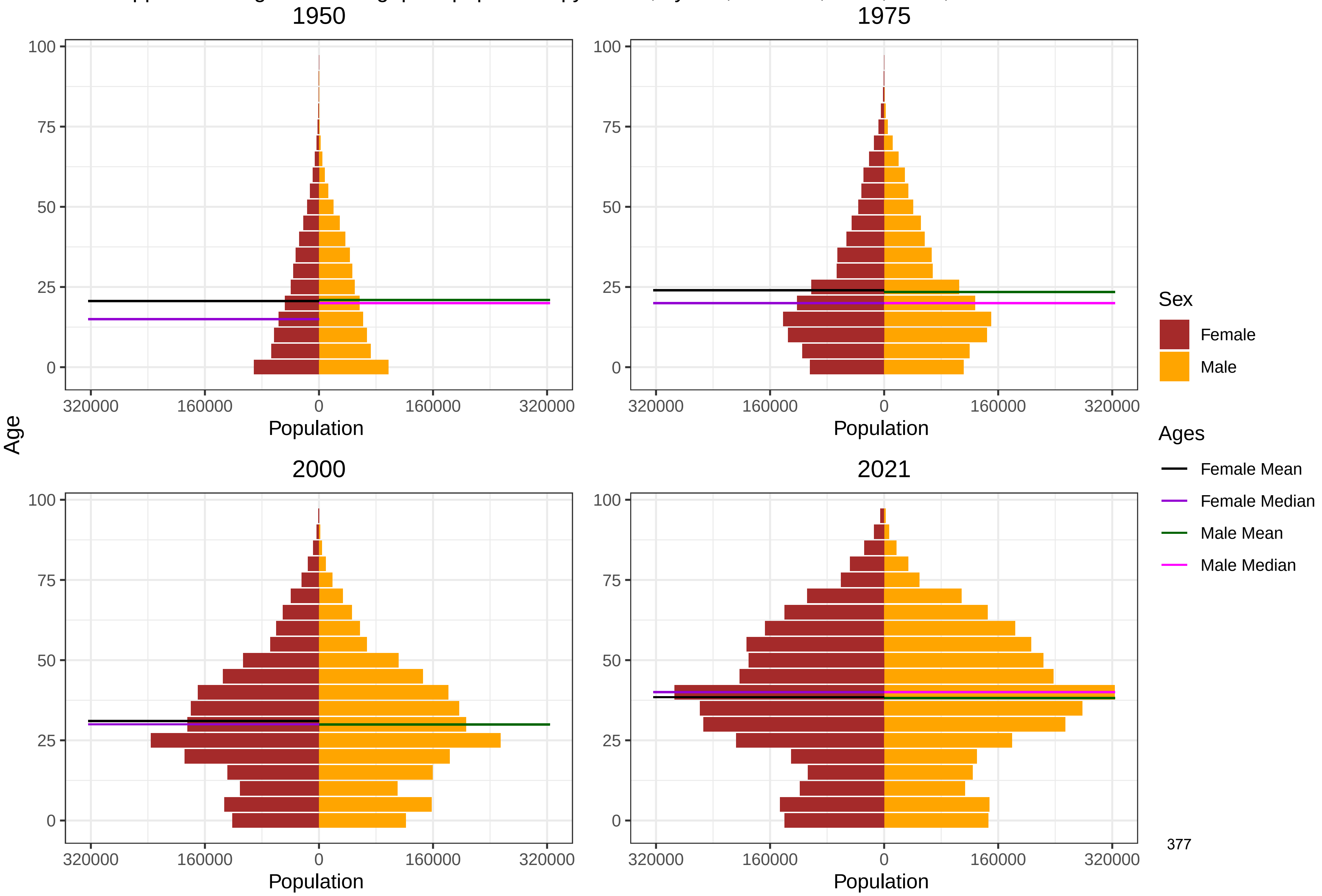

Appendix 2 Figure S9: Slovakia population pyramids, by sex, for 1950, 1975, 2000, and 2021

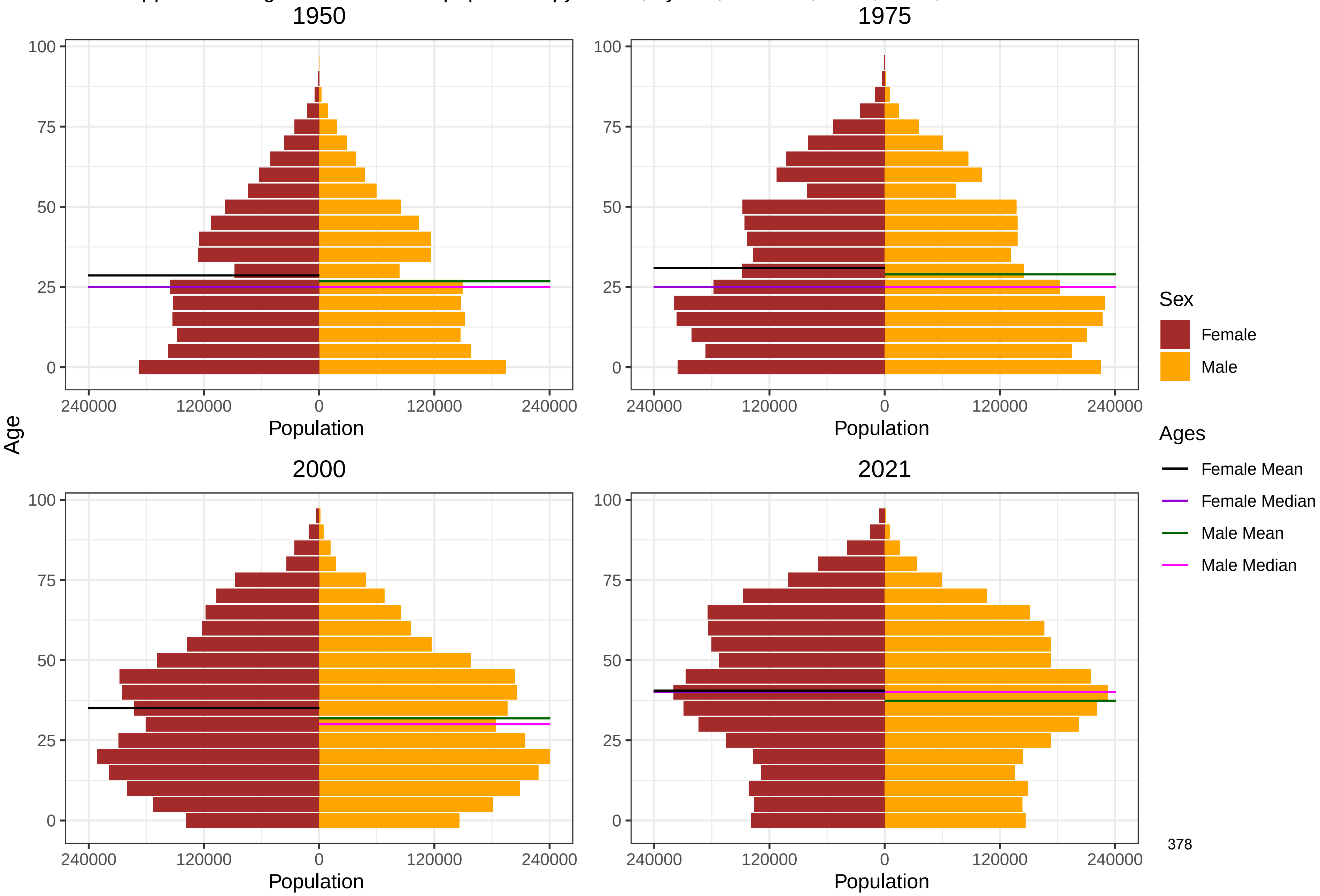

Appendix 2 Figure S9: Slovenia population pyramids, by sex, for 1950, 1975, 2000, and 2021

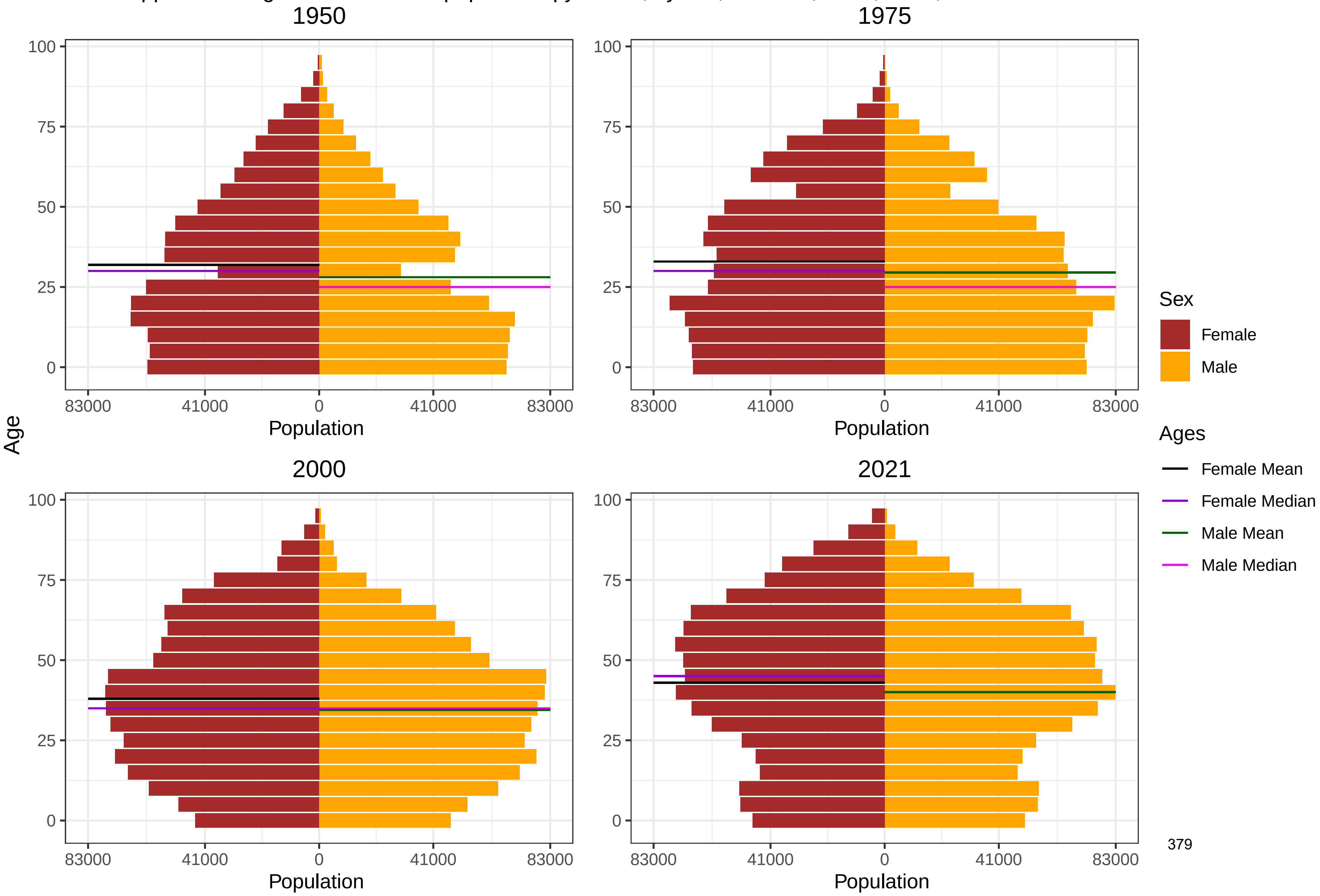

Appendix 2 Figure S9: Solomon Islands population pyramids, by sex, for 1950, 1975, 2000, and 2021

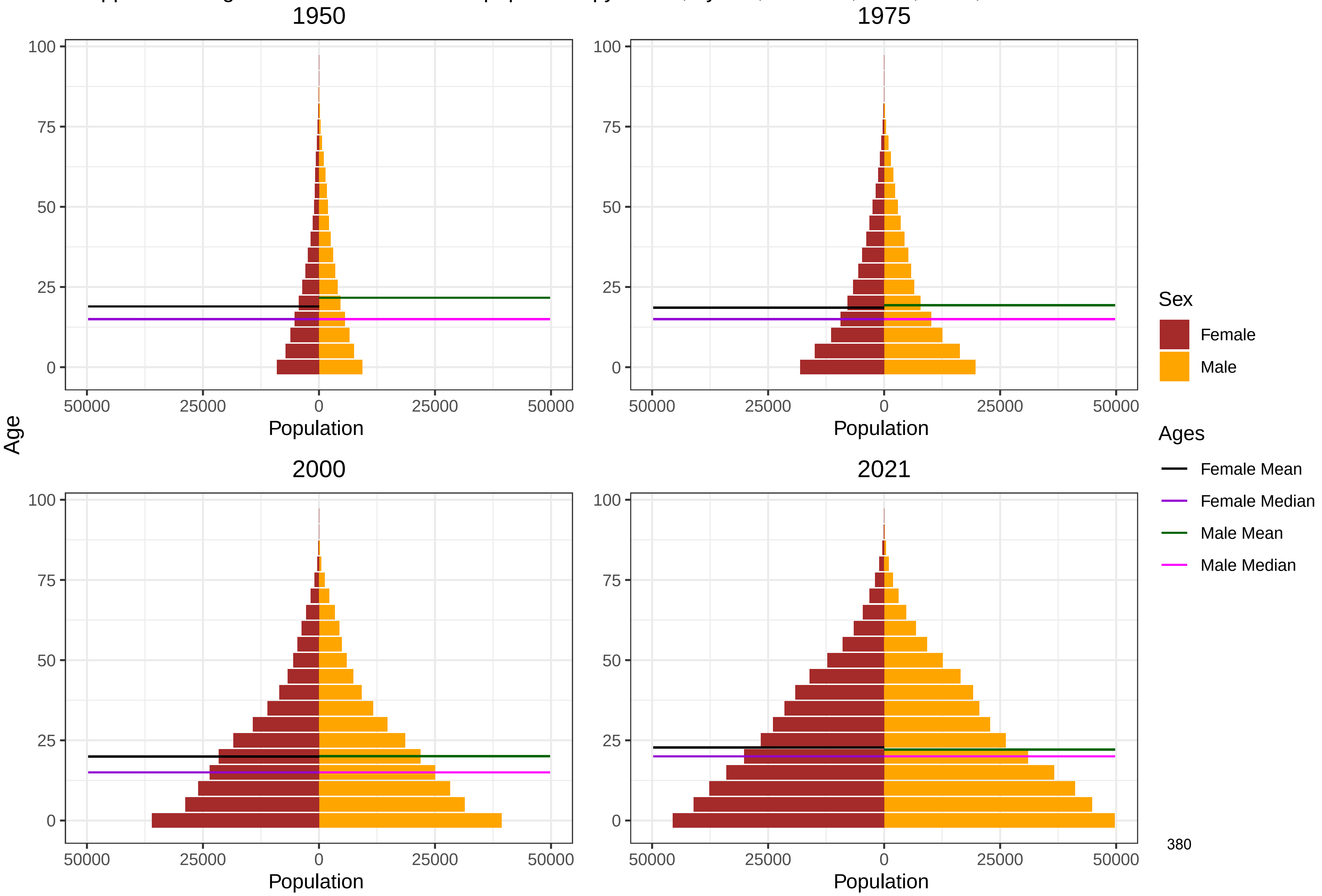

Appendix 2 Figure S9: Somalia population pyramids, by sex, for 1950, 1975, 2000, and 2021

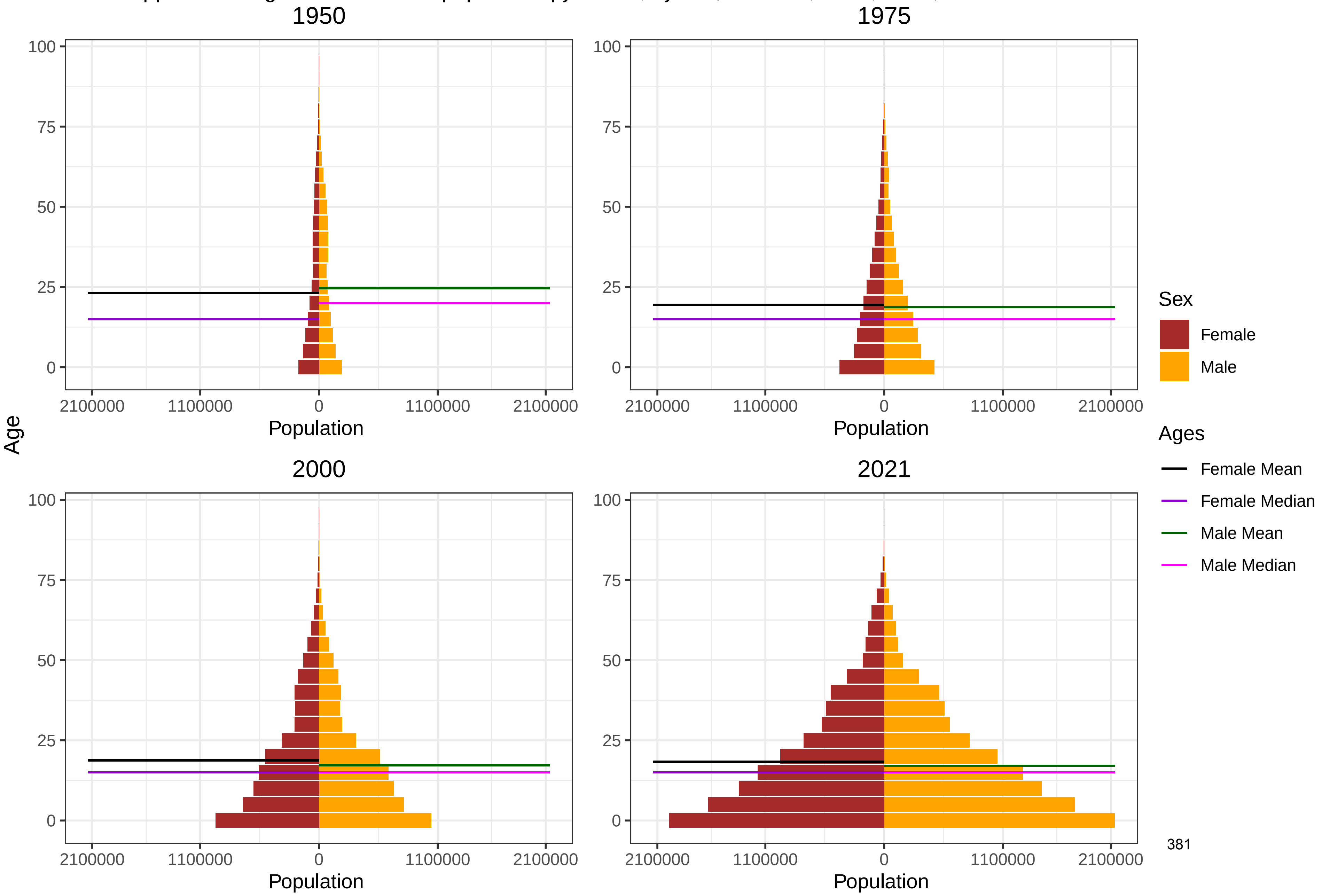

Appendix 2 Figure S9: South Africa population pyramids, by sex, for 1950, 1975, 2000, and 2021

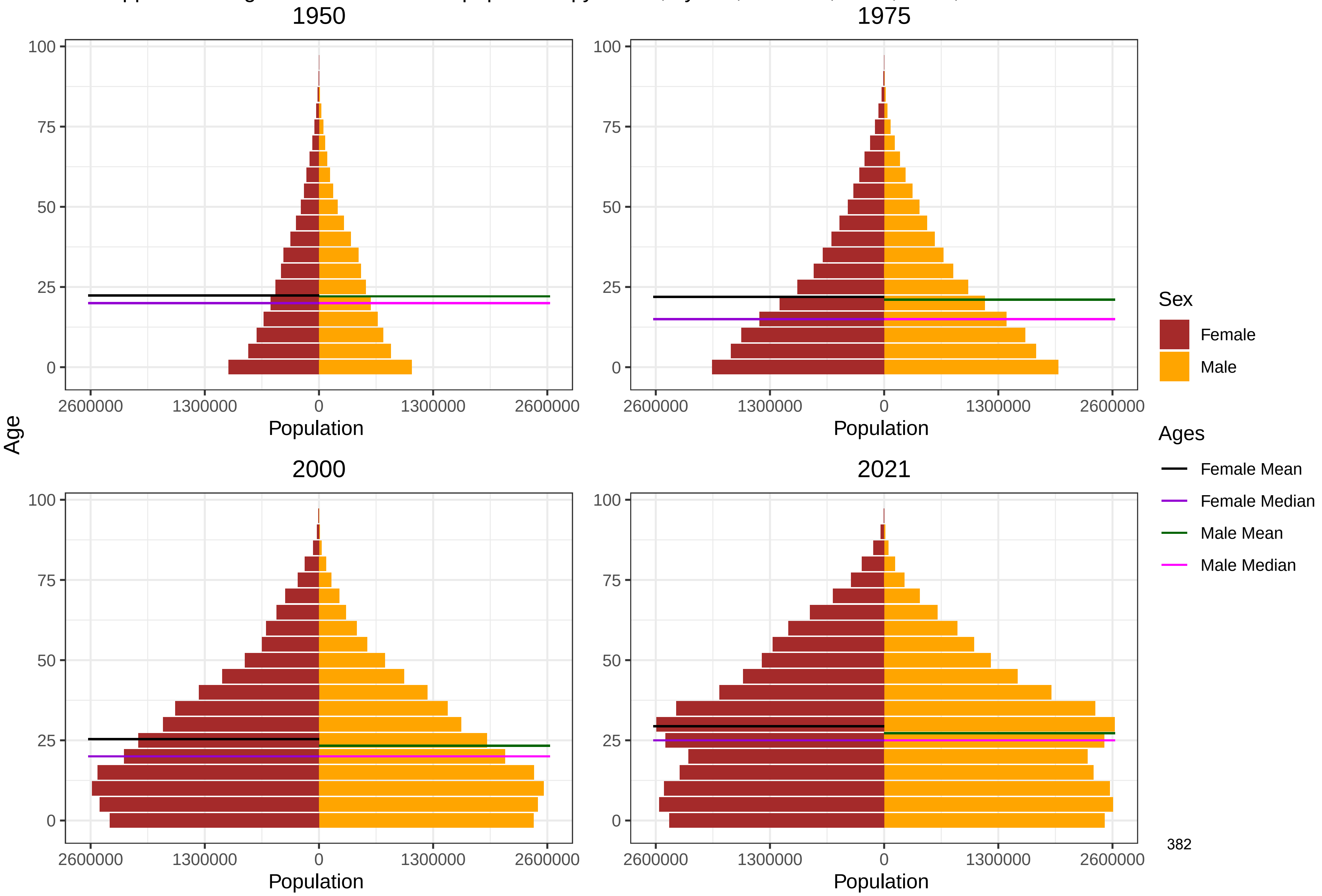

Appendix 2 Figure S9: South Korea population pyramids, by sex, for 1950, 1975, 2000, and 2021

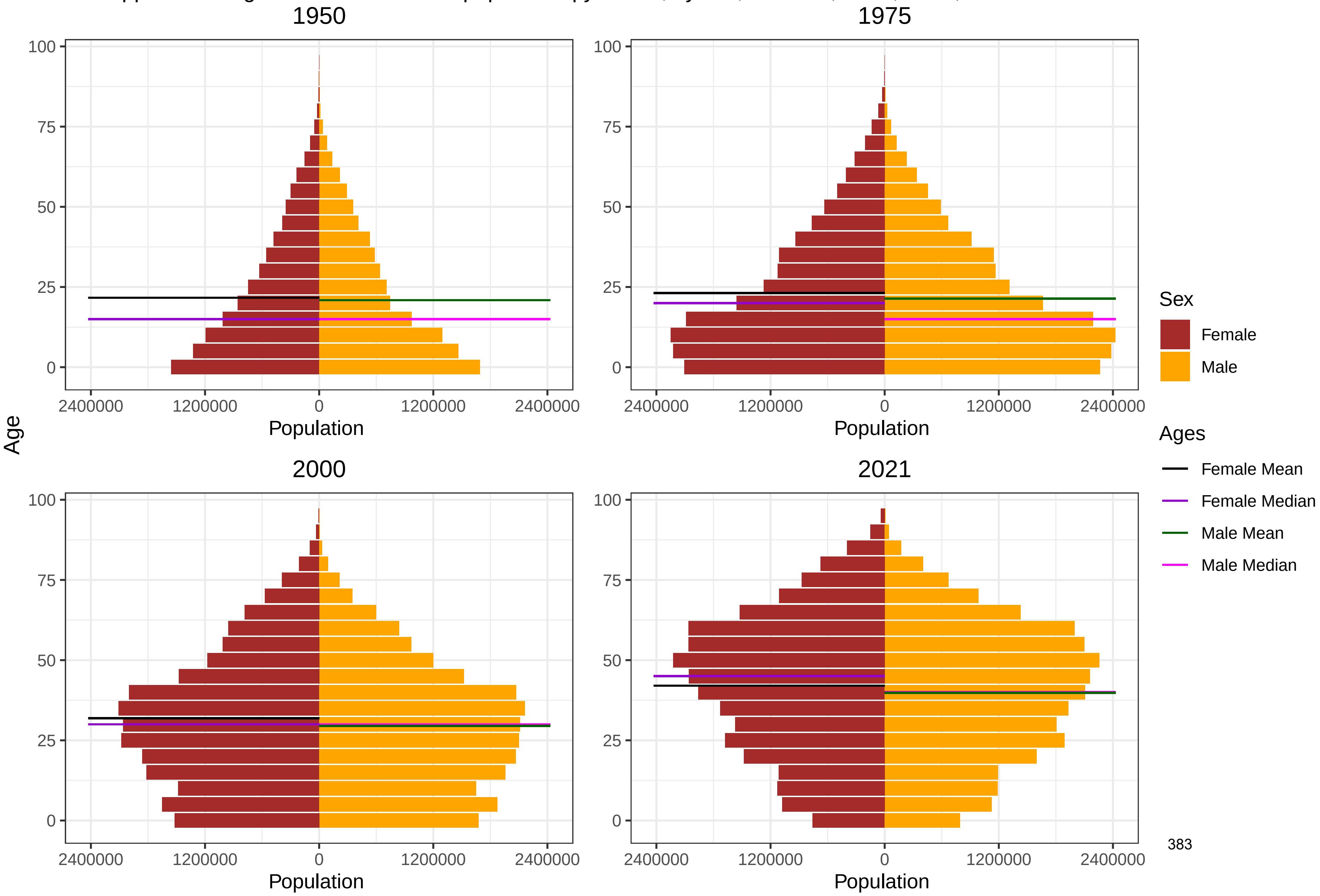

Appendix 2 Figure S9: South Sudan population pyramids, by sex, for 1950, 1975, 2000, and 2021

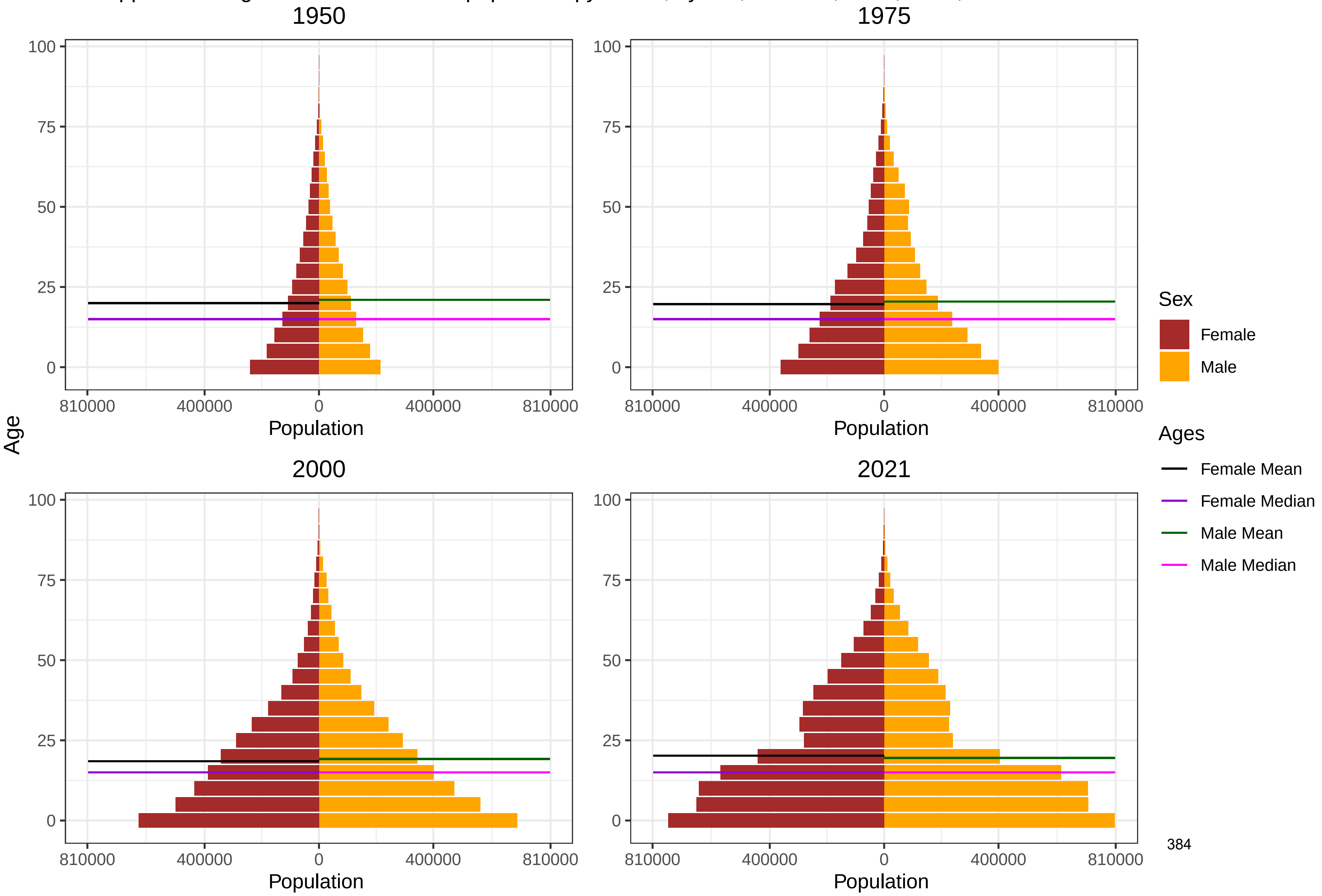

Appendix 2 Figure S9: Spain population pyramids, by sex, for 1950, 1975, 2000, and 2021

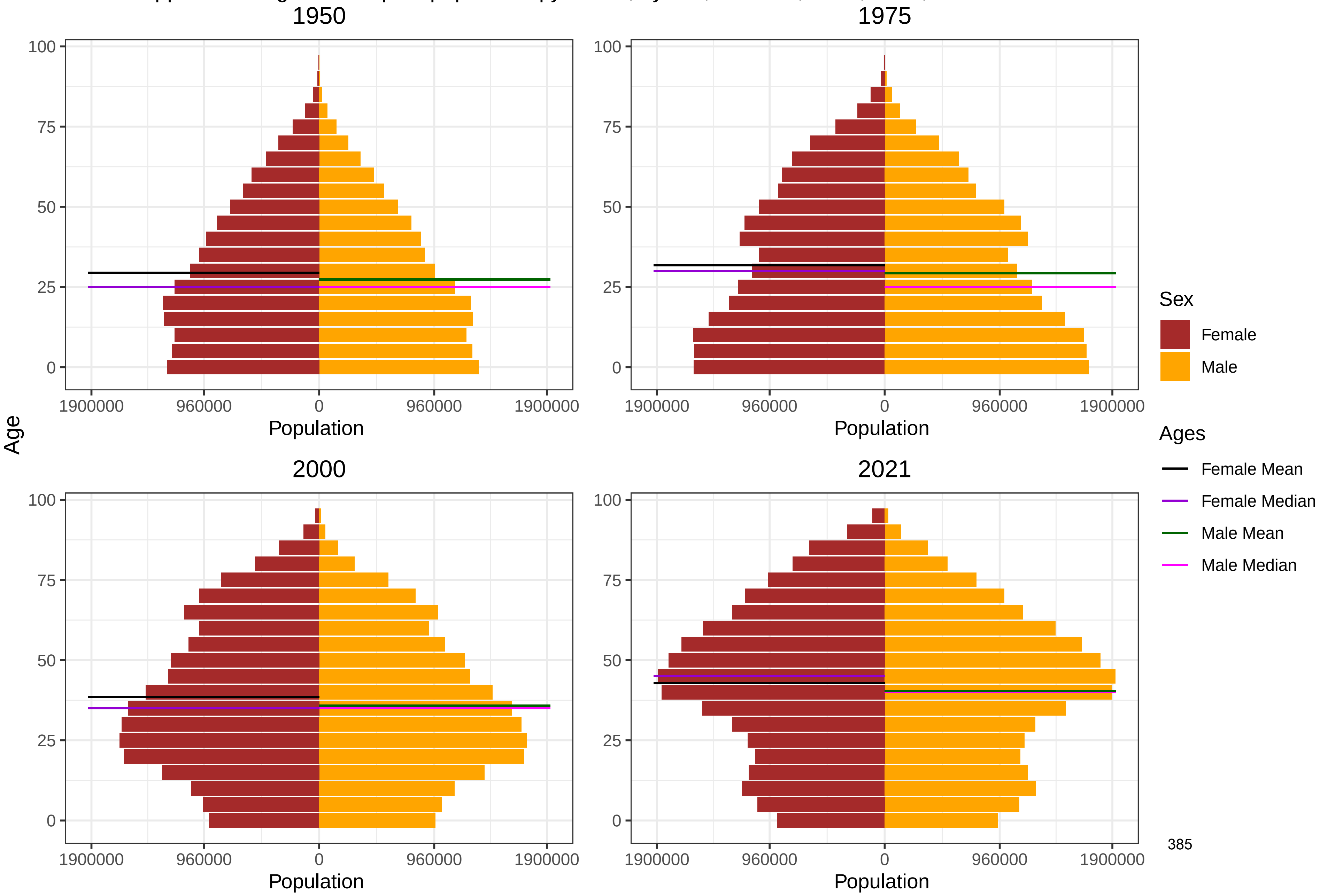

Appendix 2 Figure S9: Sri Lanka population pyramids, by sex, for 1950, 1975, 2000, and 2021

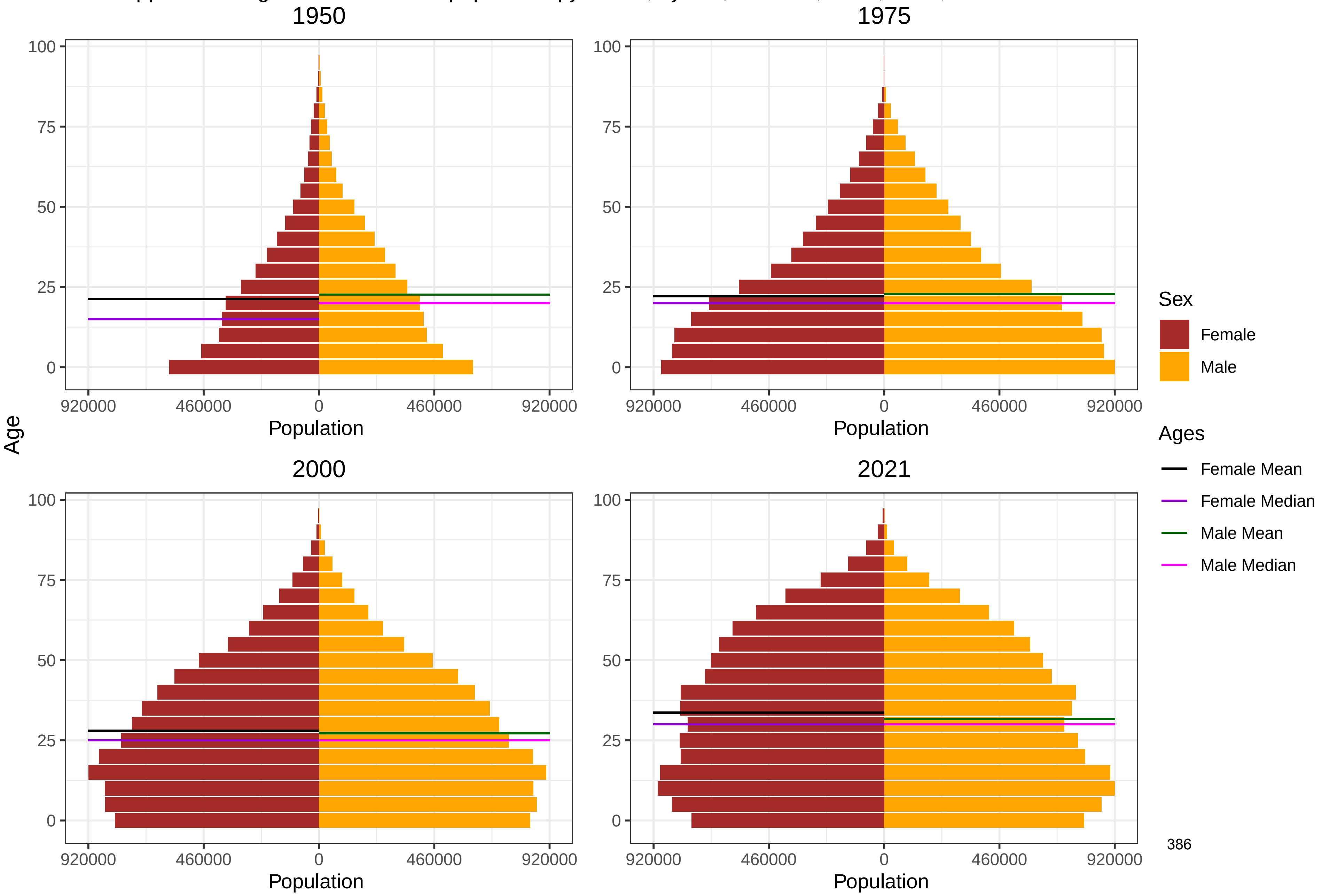

Appendix 2 Figure S9: Sudan population pyramids, by sex, for 1950, 1975, 2000, and 2021

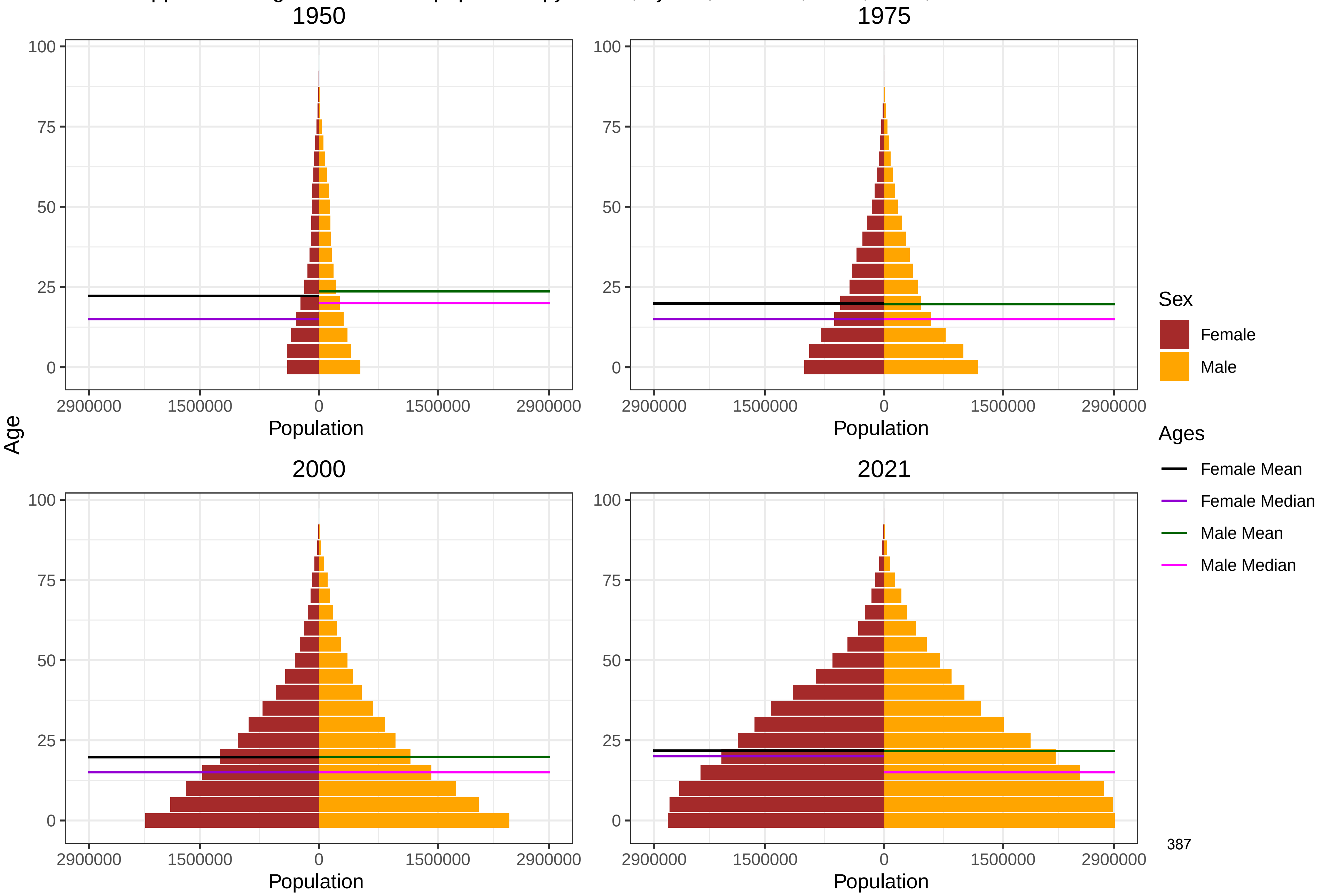

Appendix 2 Figure S9: Suriname population pyramids, by sex, for 1950, 1975, 2000, and 2021

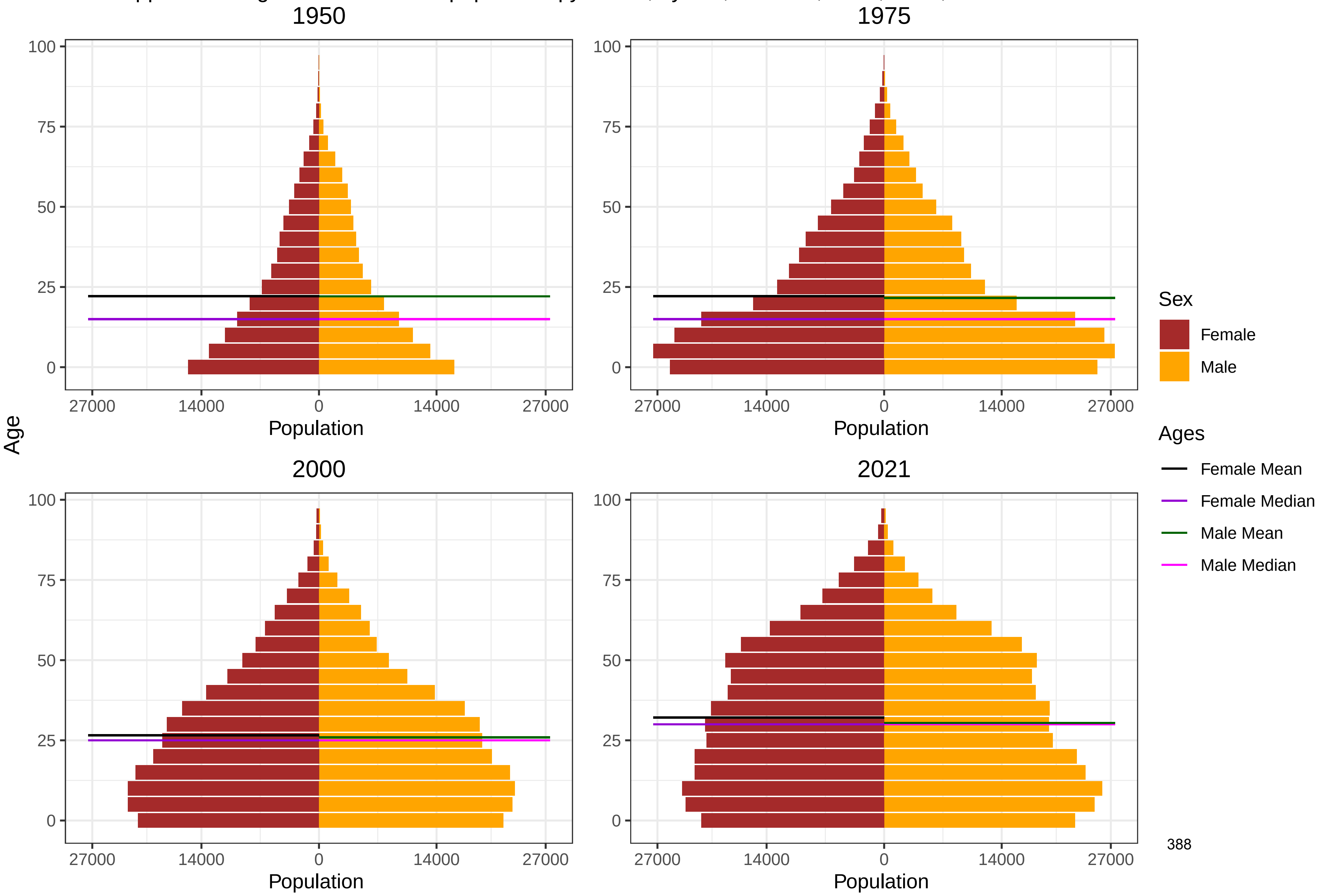

Appendix 2 Figure S9: Sweden population pyramids, by sex, for 1950, 1975, 2000, and 2021

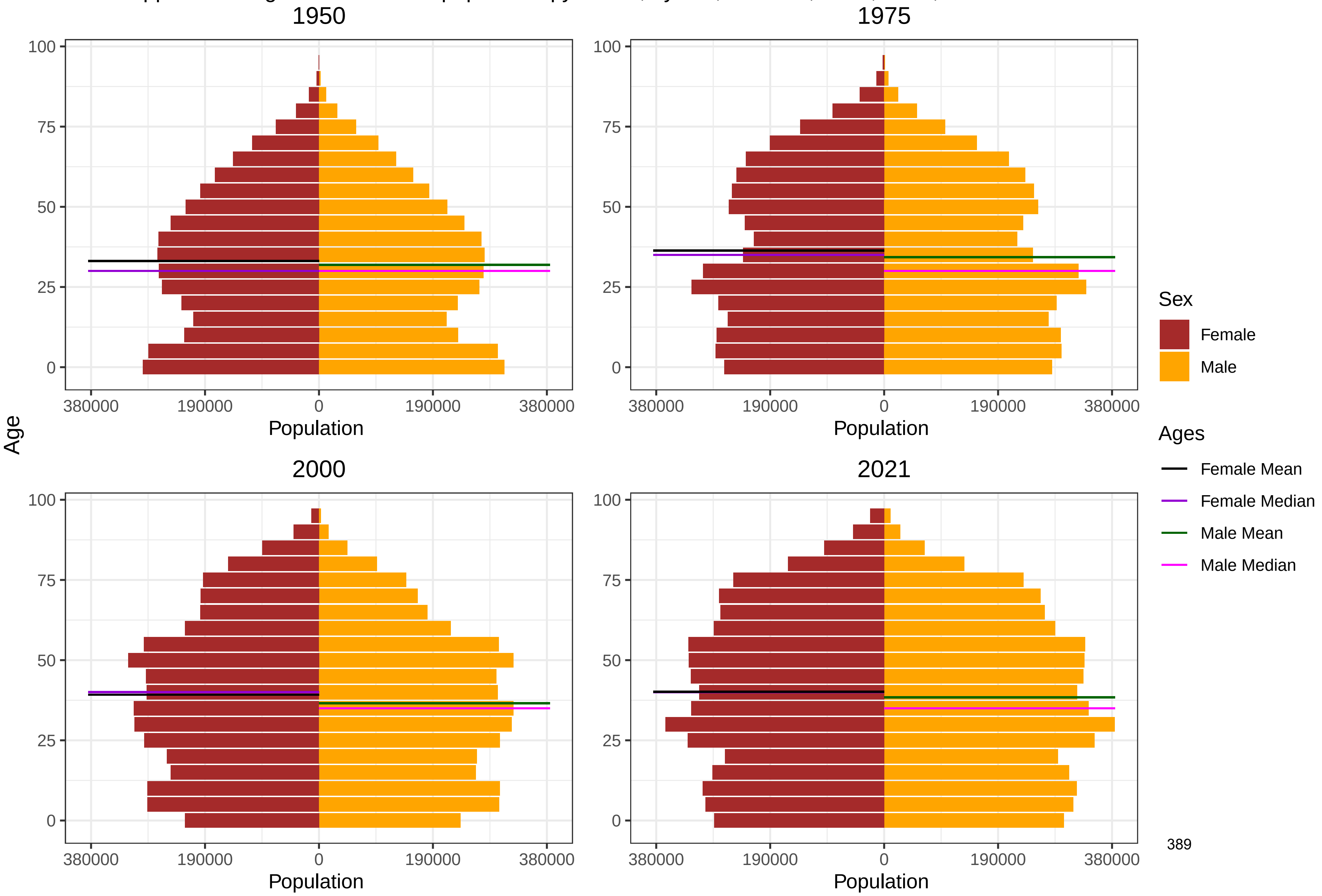

Appendix 2 Figure S9: Switzerland population pyramids, by sex, for 1950, 1975, 2000, and 2021

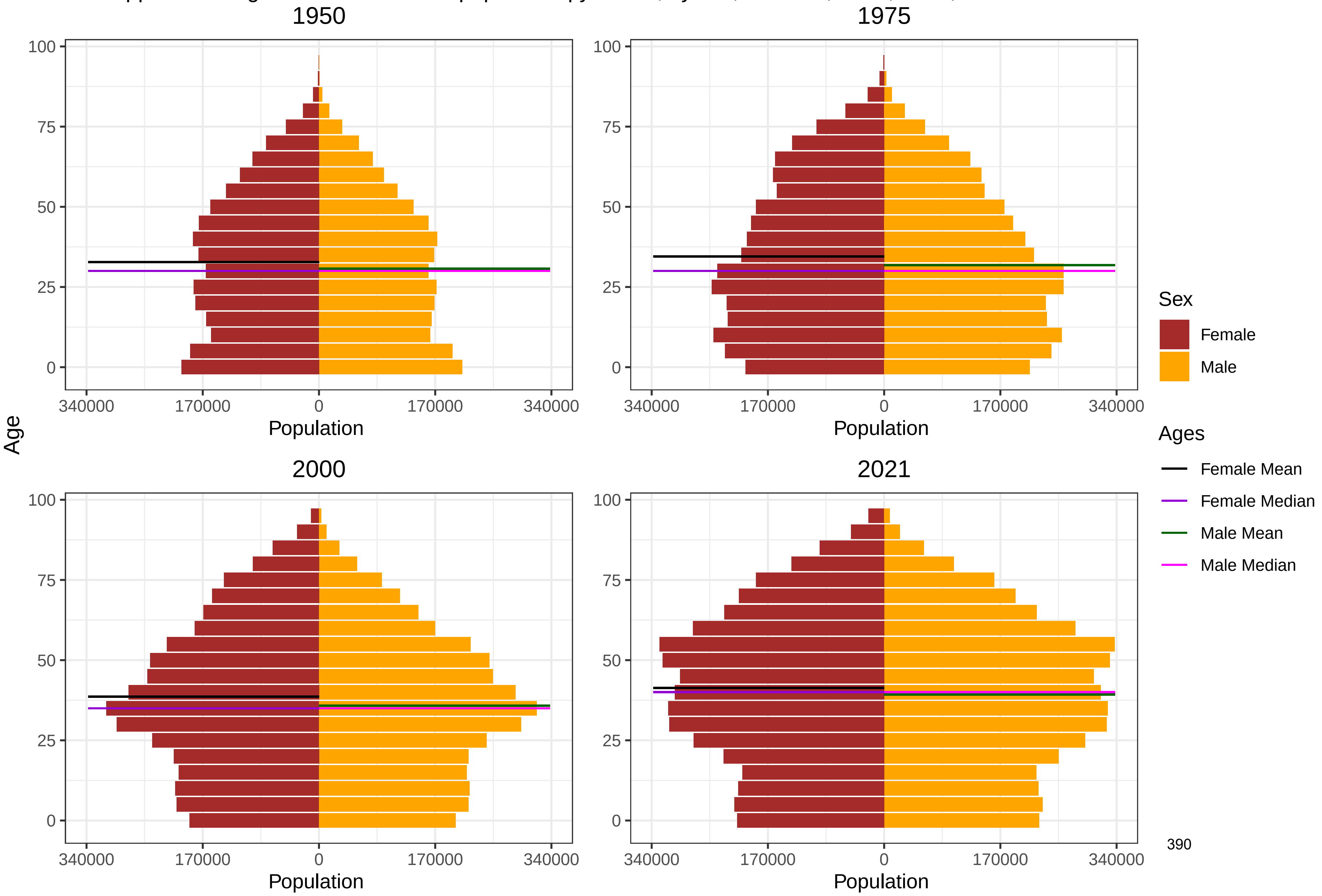

Appendix 2 Figure S9: Syria population pyramids, by sex, for 1950, 1975, 2000, and 2021

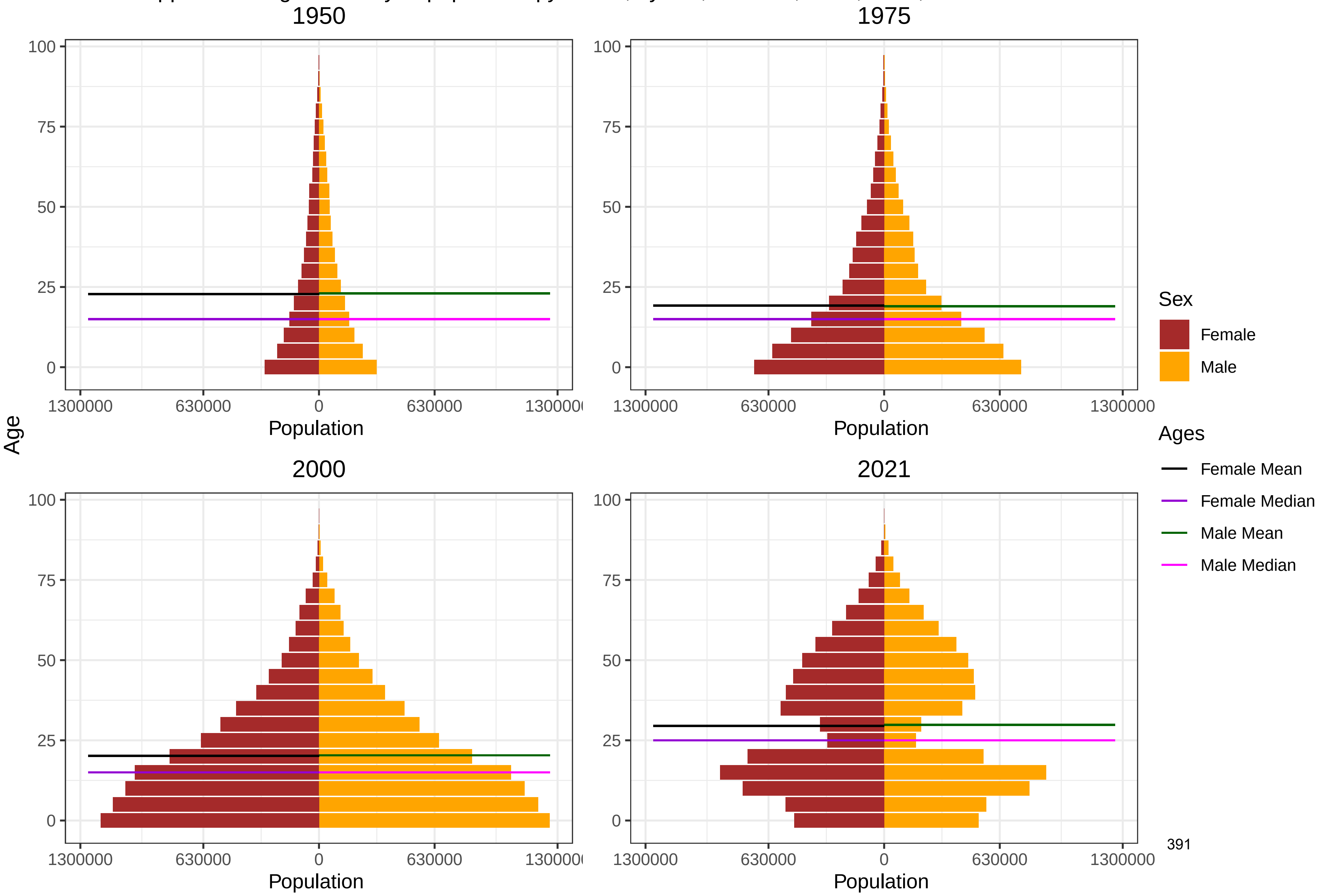

Appendix 2 Figure S9: São Tomé and Príncipe population pyramids, by sex, for 1950, 1975, 2000, and 2021

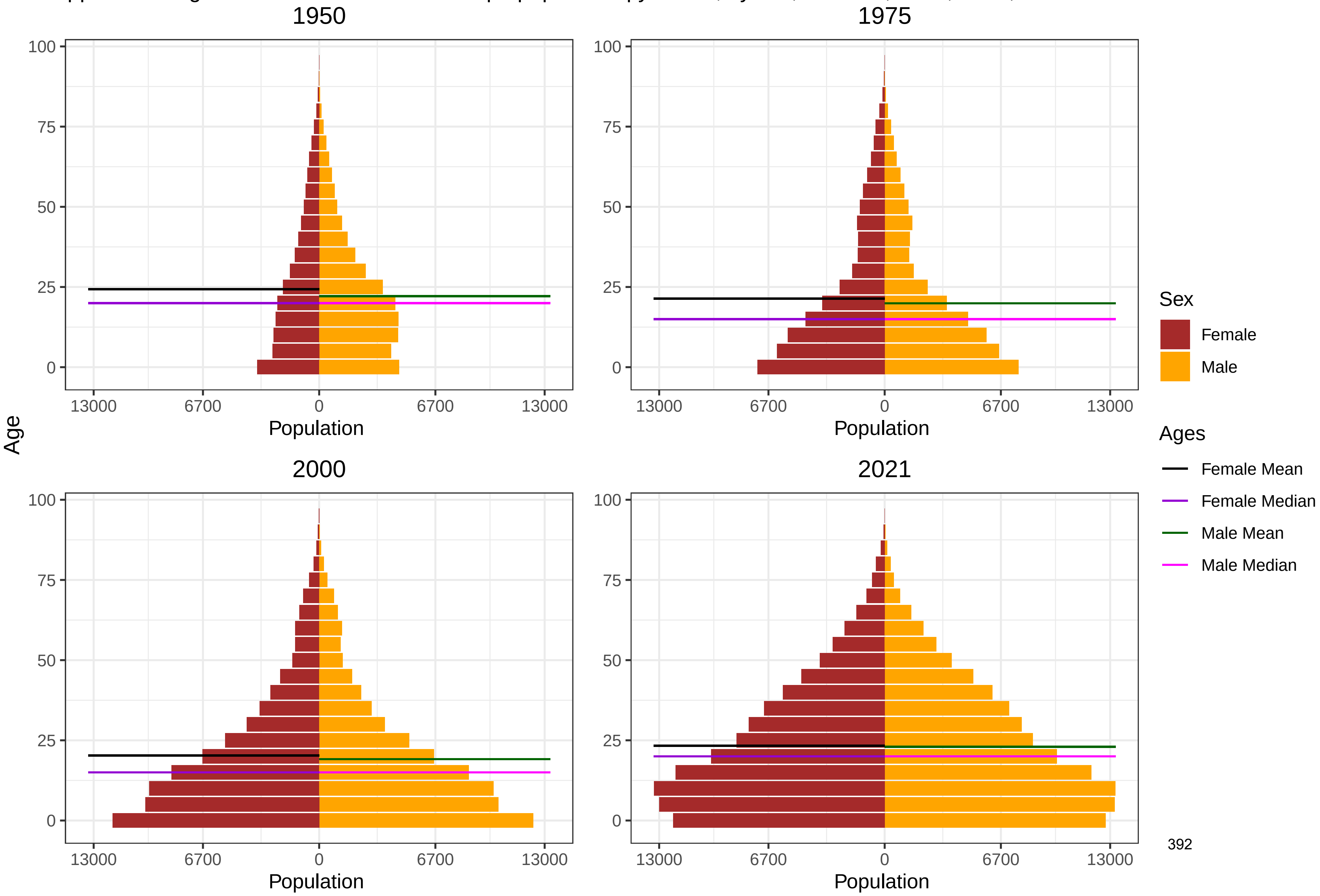

Appendix 2 Figure S9: Taiwan (province of China) population pyramids, by sex, for 1950, 1975, 2000, and 2021

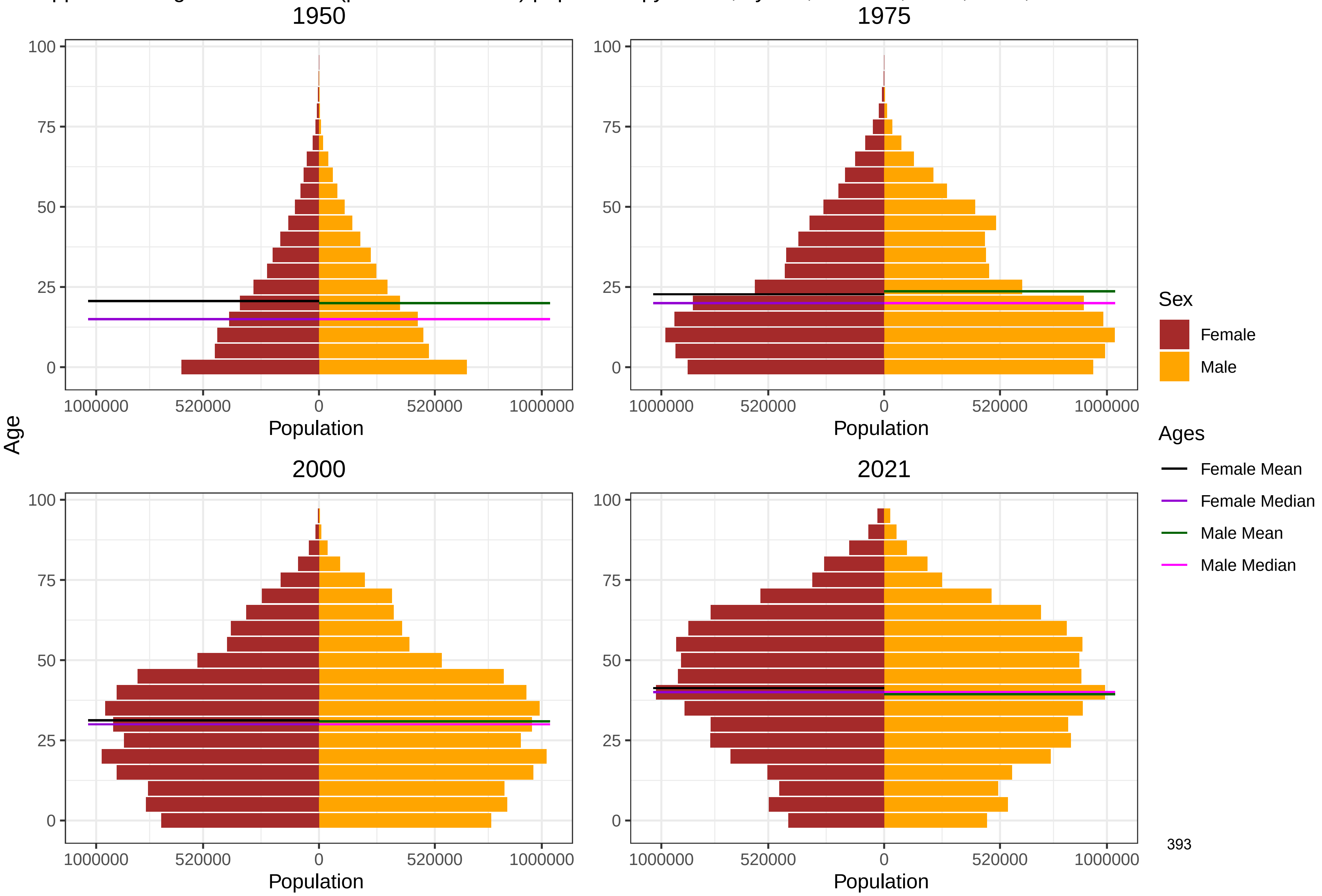

Appendix 2 Figure S9: Tajikistan population pyramids, by sex, for 1950, 1975, 2000, and 2021

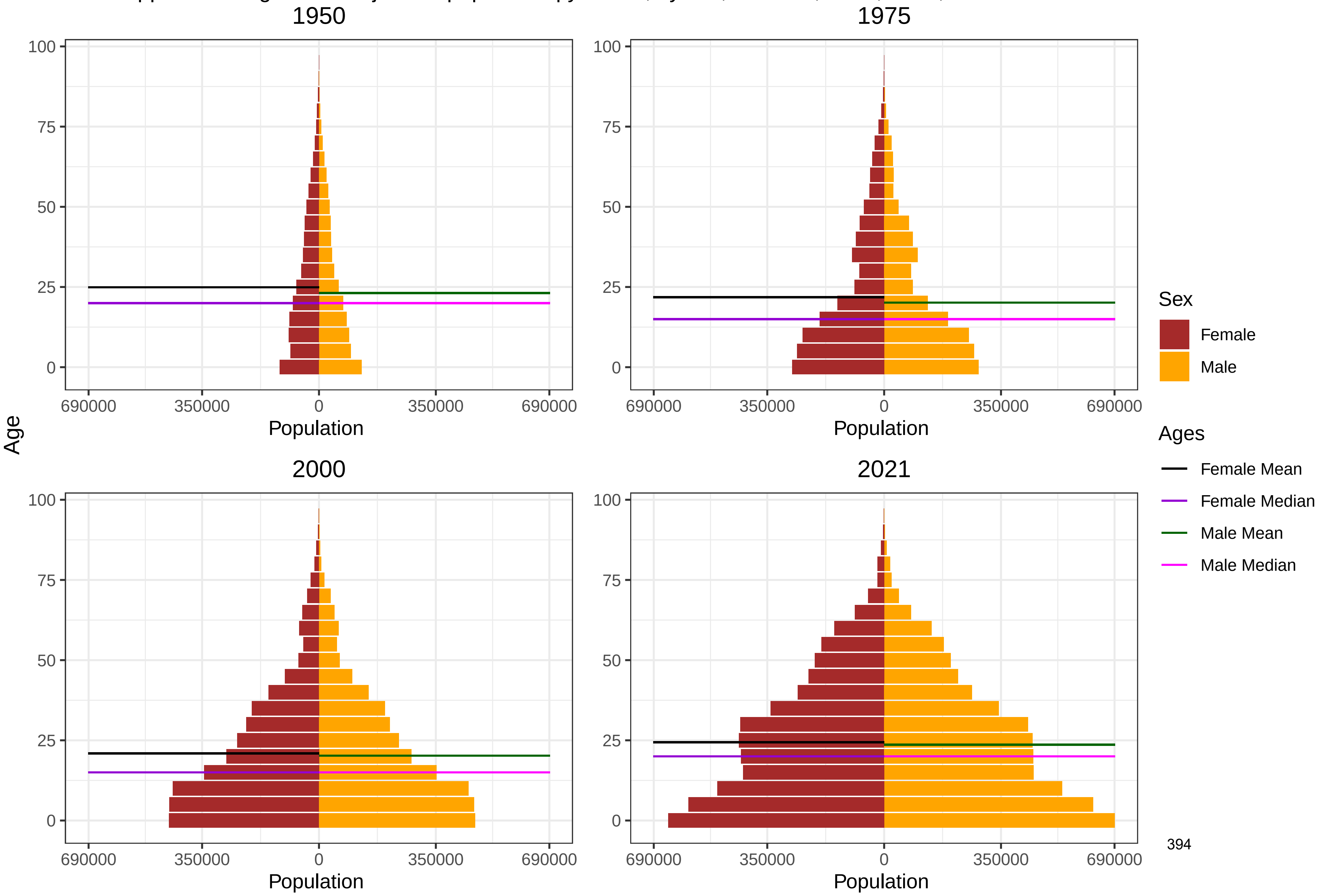

Appendix 2 Figure S9: Tanzania population pyramids, by sex, for 1950, 1975, 2000, and 2021

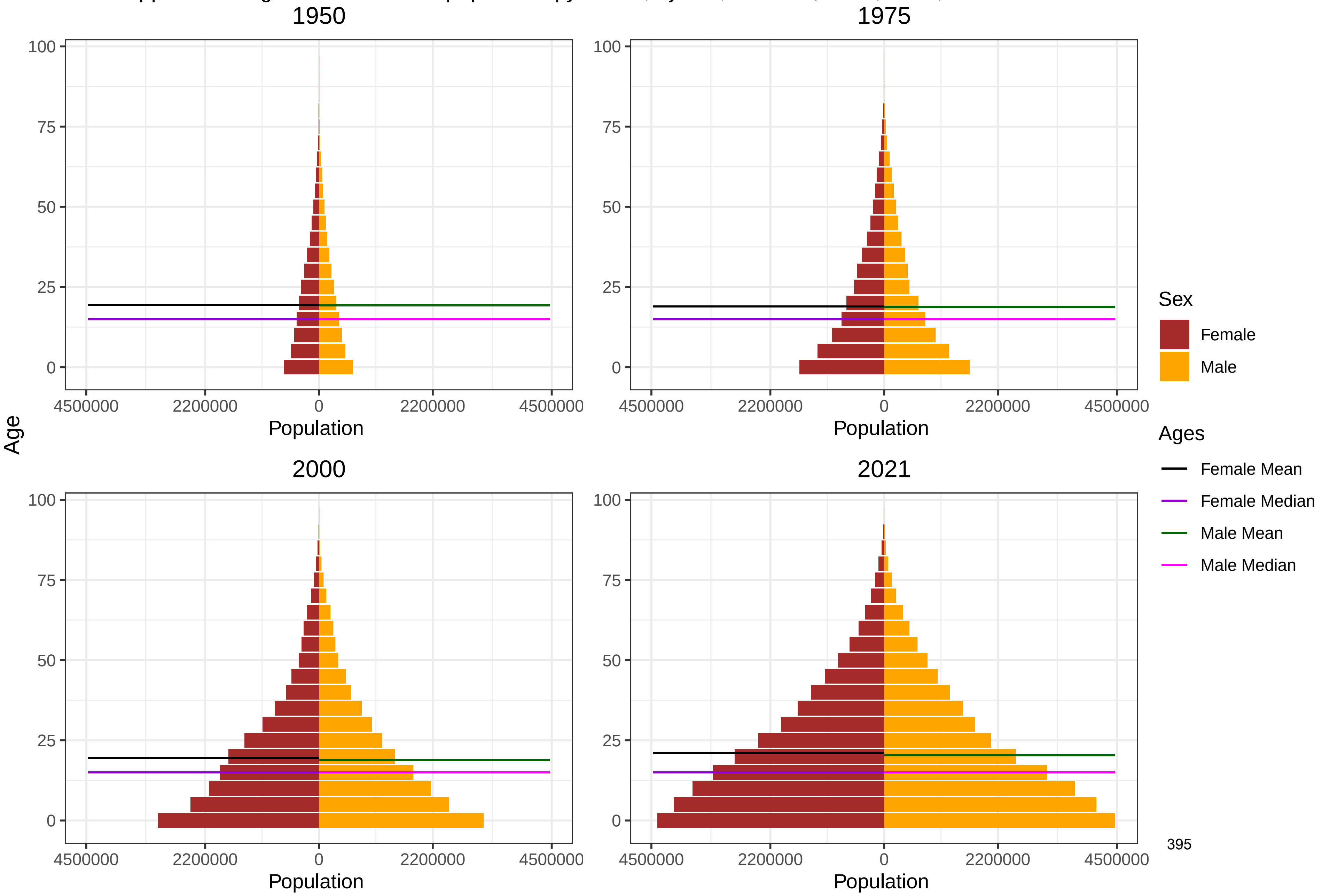

Appendix 2 Figure S9: Thailand population pyramids, by sex, for 1950, 1975, 2000, and 2021

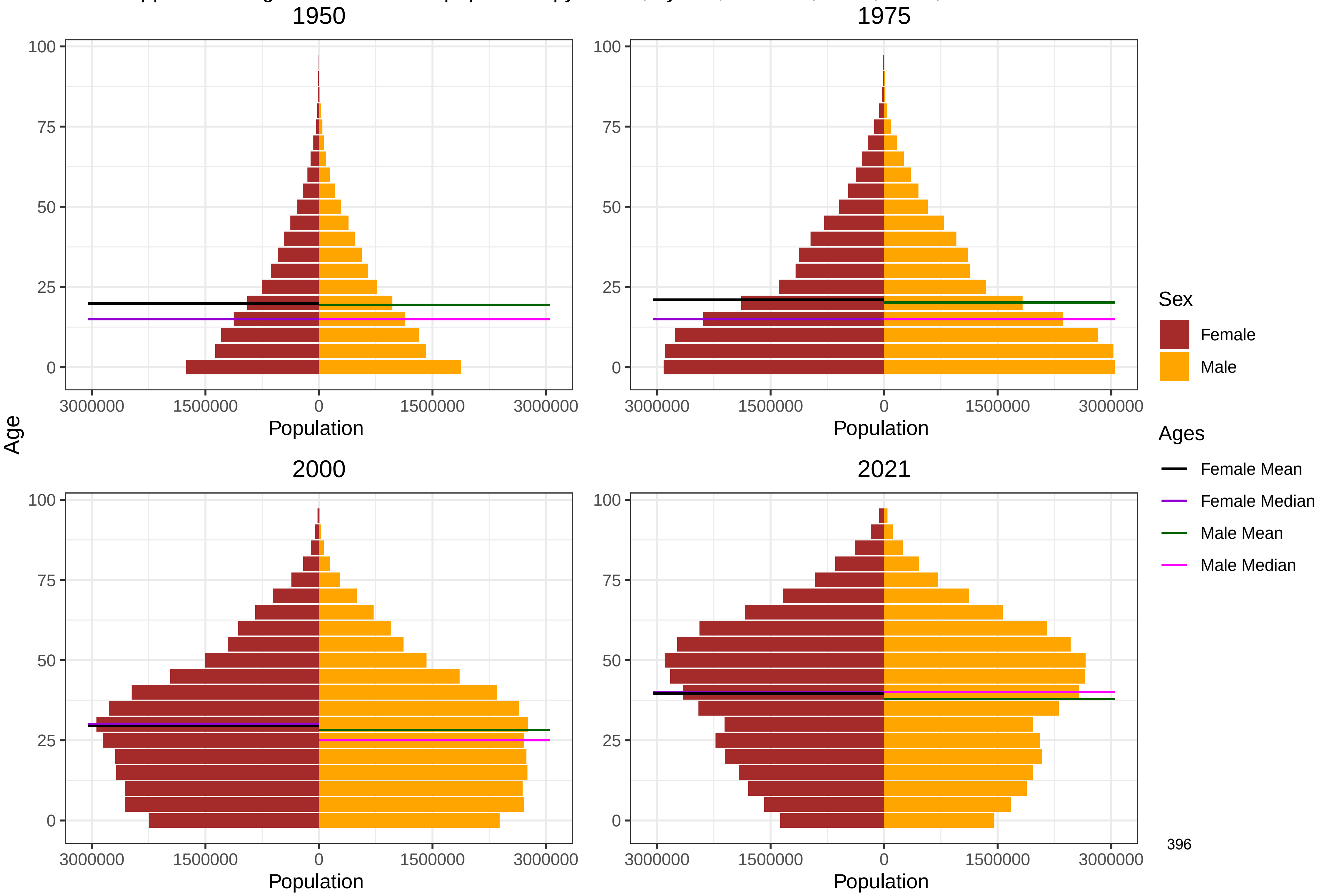

Appendix 2 Figure S9: The Bahamas population pyramids, by sex, for 1950, 1975, 2000, and 2021

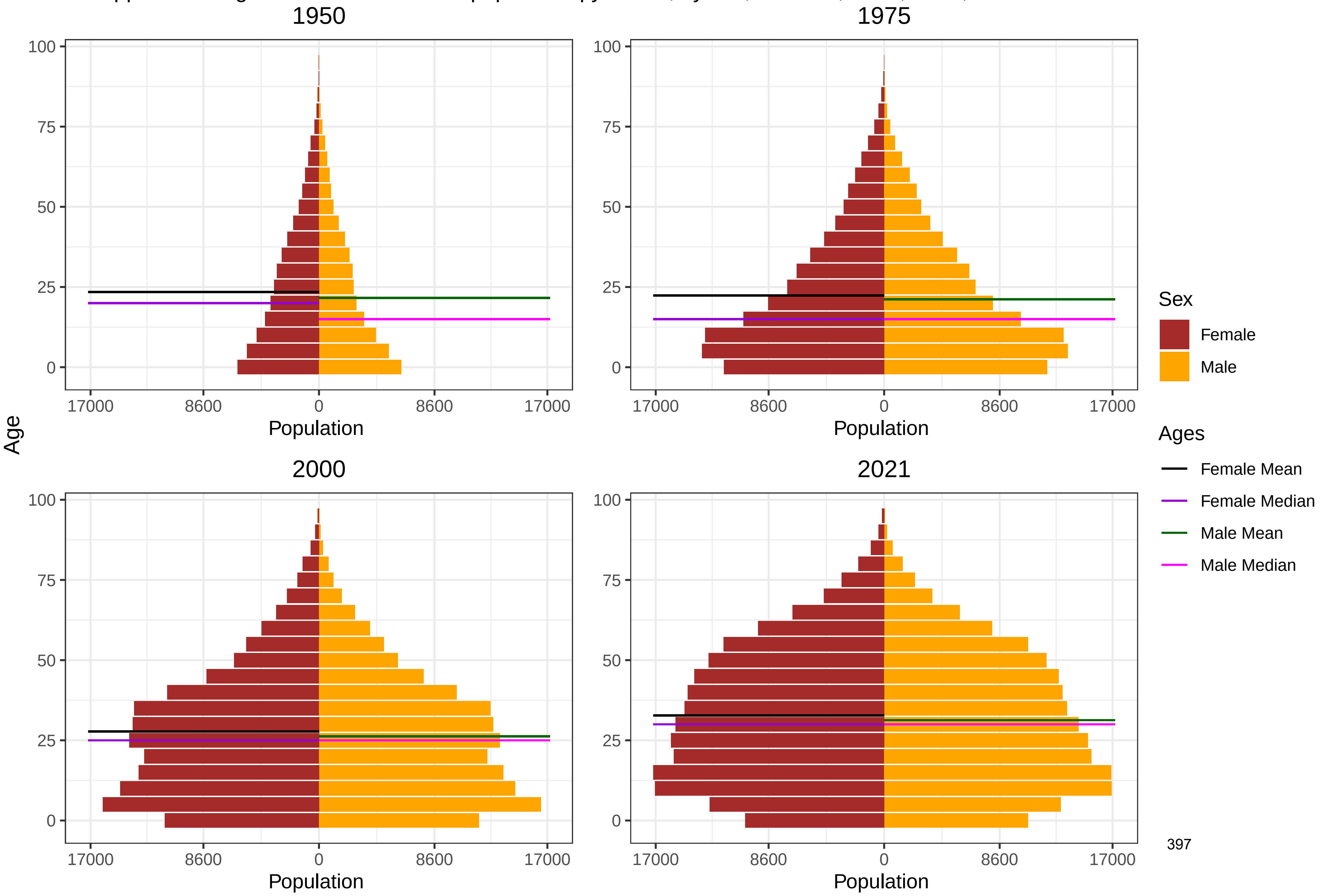

Appendix 2 Figure S9: The Gambia population pyramids, by sex, for 1950, 1975, 2000, and 2021

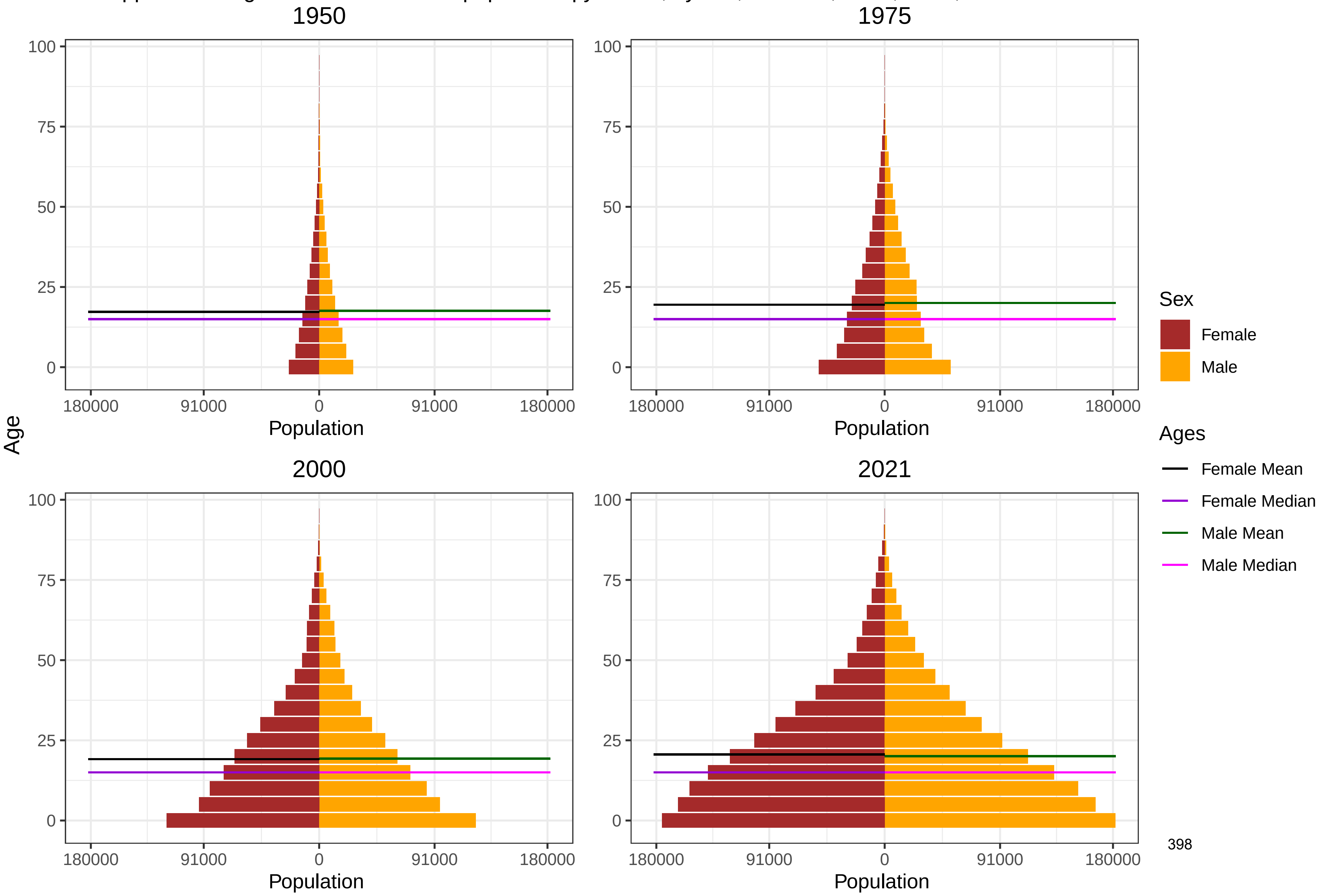

Appendix 2 Figure S9: Timor−Leste population pyramids, by sex, for 1950, 1975, 2000, and 2021

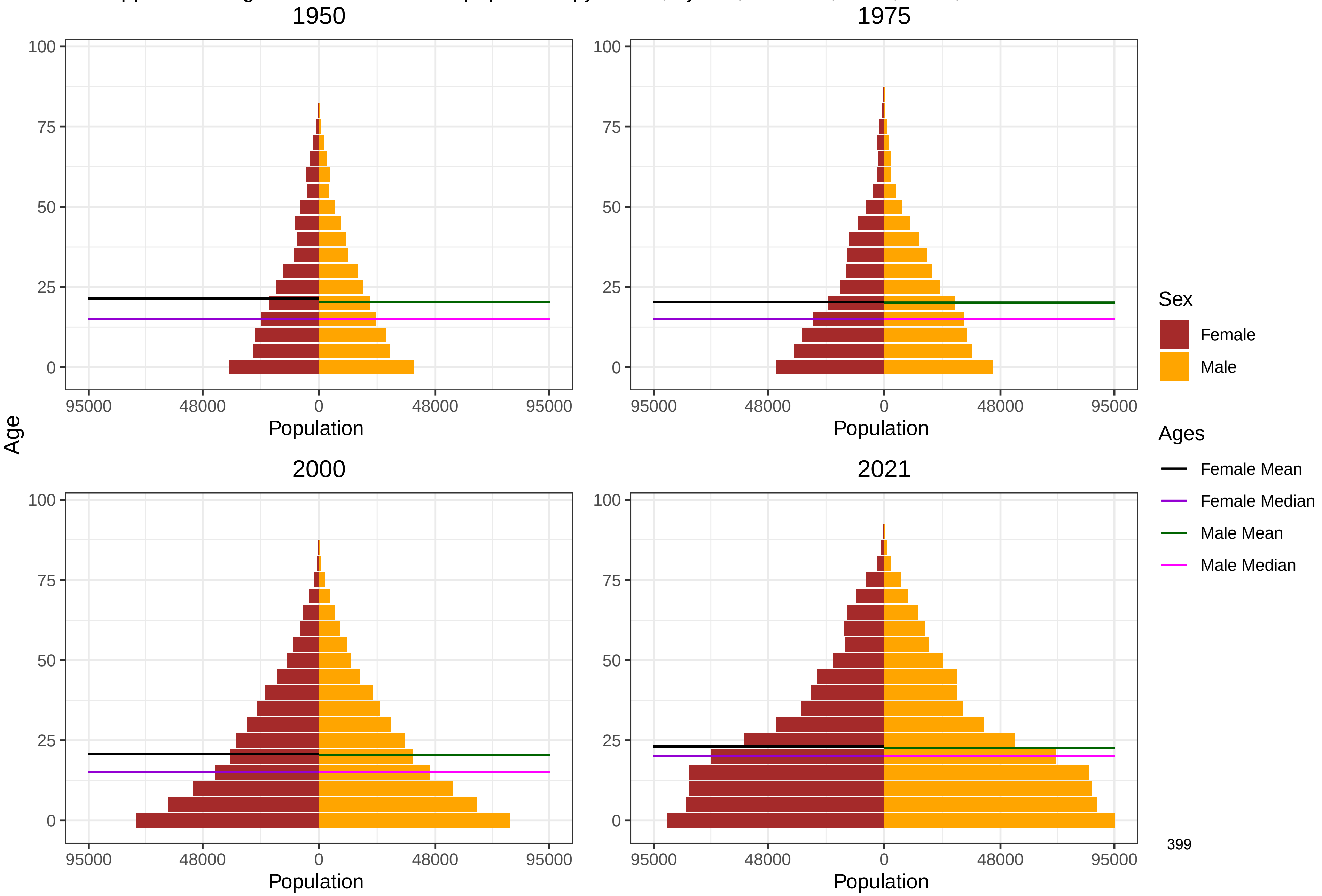

Appendix 2 Figure S9: Togo population pyramids, by sex, for 1950, 1975, 2000, and 2021

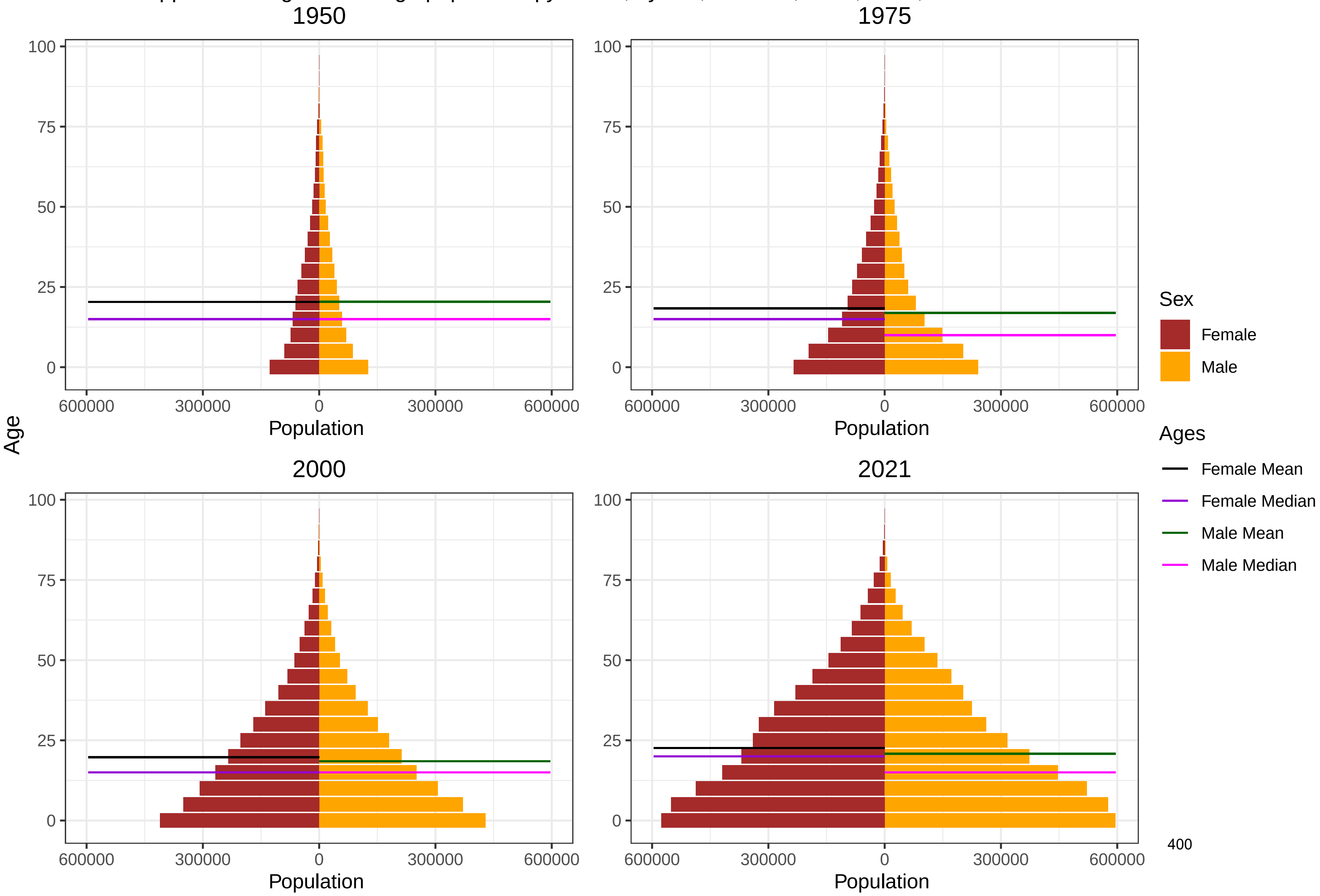

Appendix 2 Figure S9: Tokelau population pyramids, by sex, for 1950, 1975, 2000, and 2021

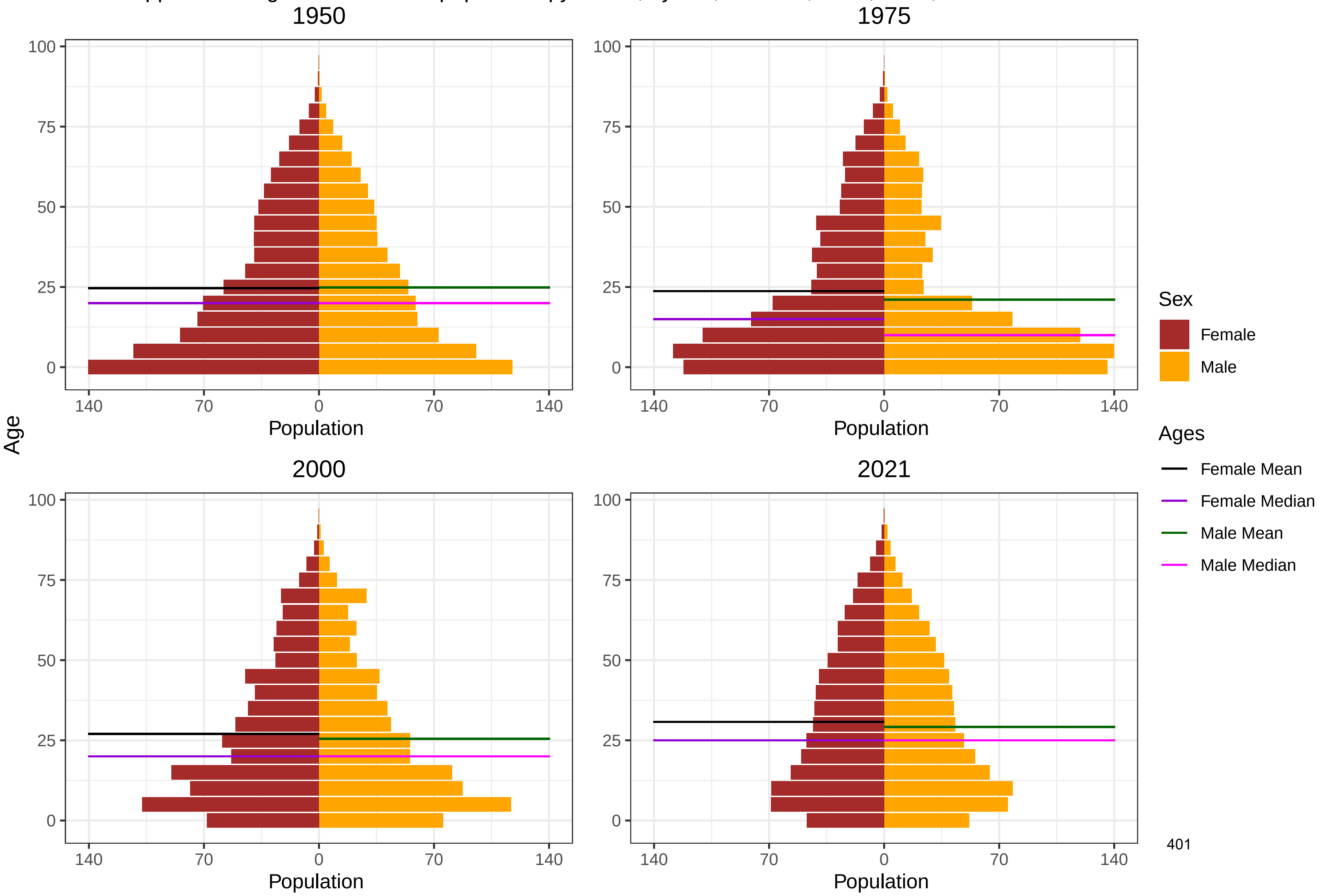

Appendix 2 Figure S9: Tonga population pyramids, by sex, for 1950, 1975, 2000, and 2021

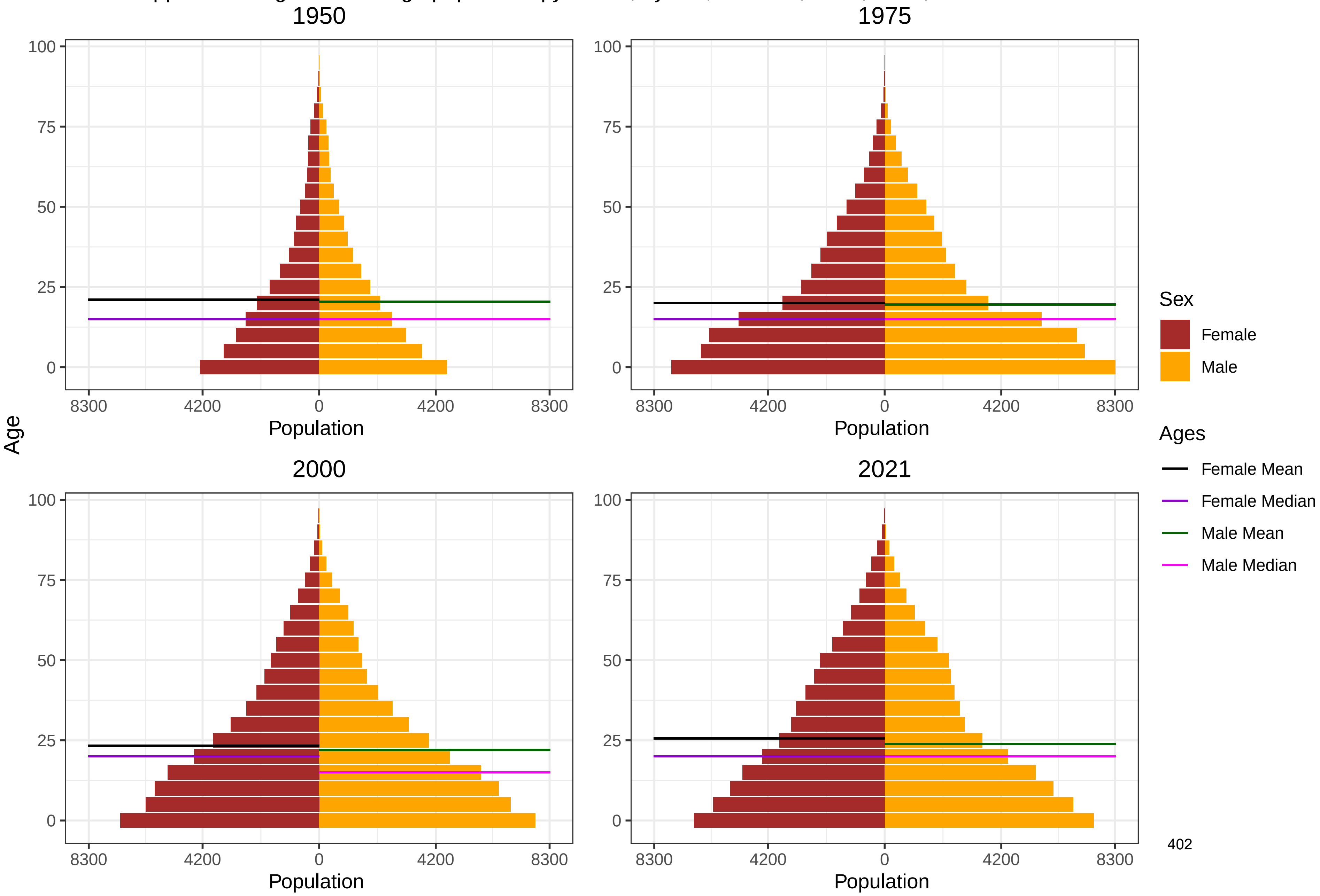

Appendix 2 Figure S9: Trinidad and Tobago population pyramids, by sex, for 1950, 1975, 2000, and 2021

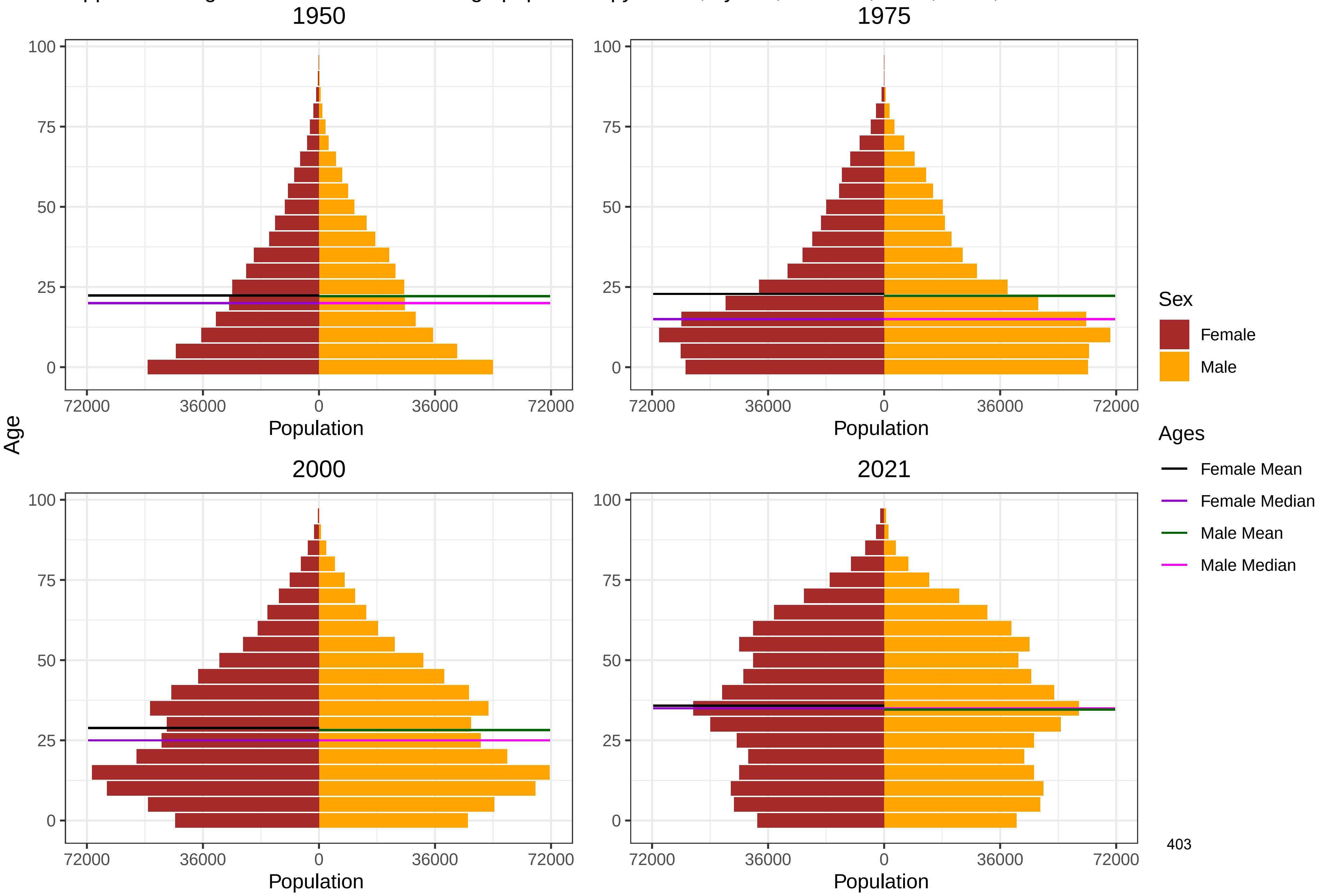

Appendix 2 Figure S9: Tunisia population pyramids, by sex, for 1950, 1975, 2000, and 2021

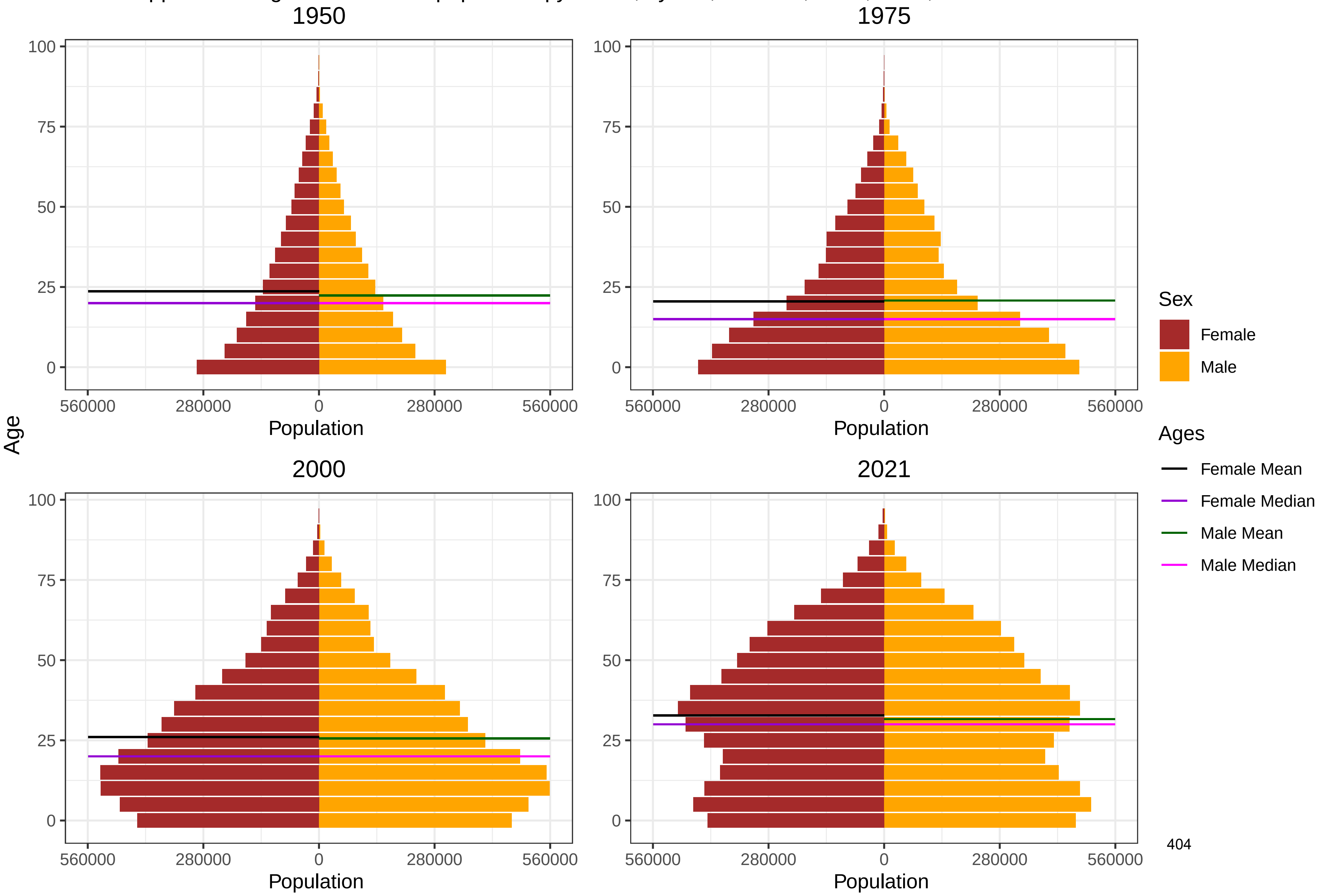

Appendix 2 Figure S9: Turkmenistan population pyramids, by sex, for 1950, 1975, 2000, and 2021

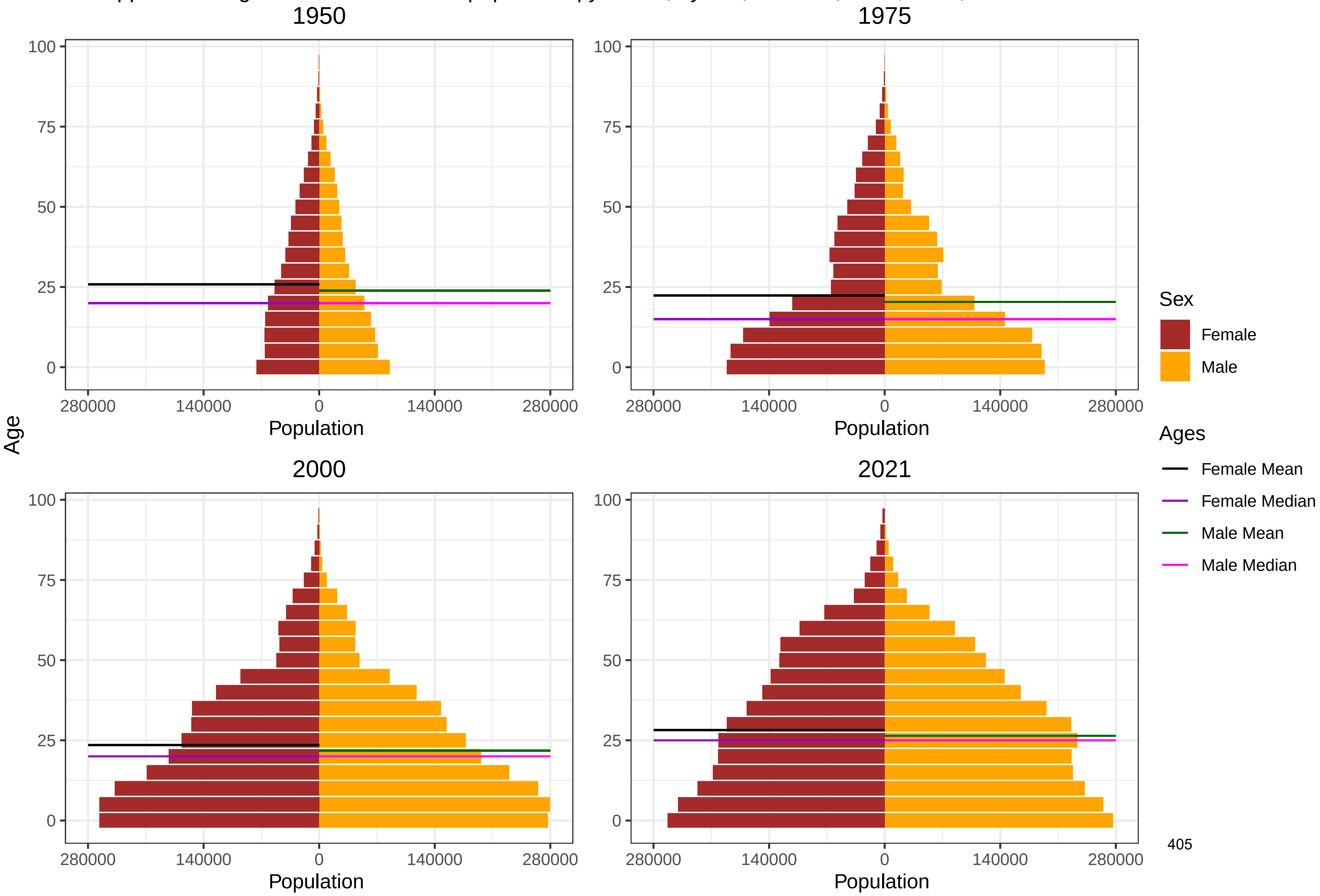

Appendix 2 Figure S9: Tuvalu population pyramids, by sex, for 1950, 1975, 2000, and 2021

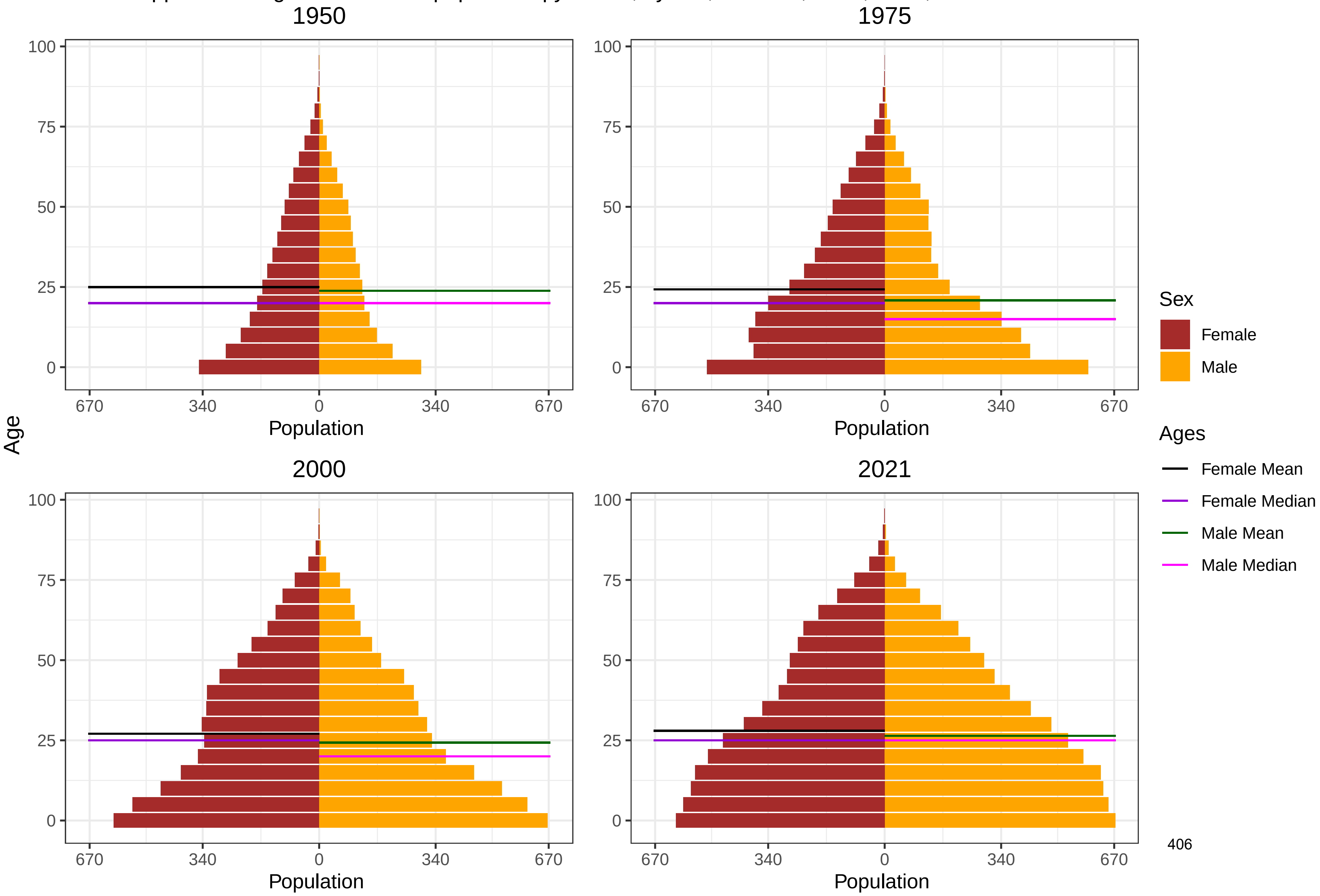

Appendix 2 Figure S9: Türkiye population pyramids, by sex, for 1950, 1975, 2000, and 2021

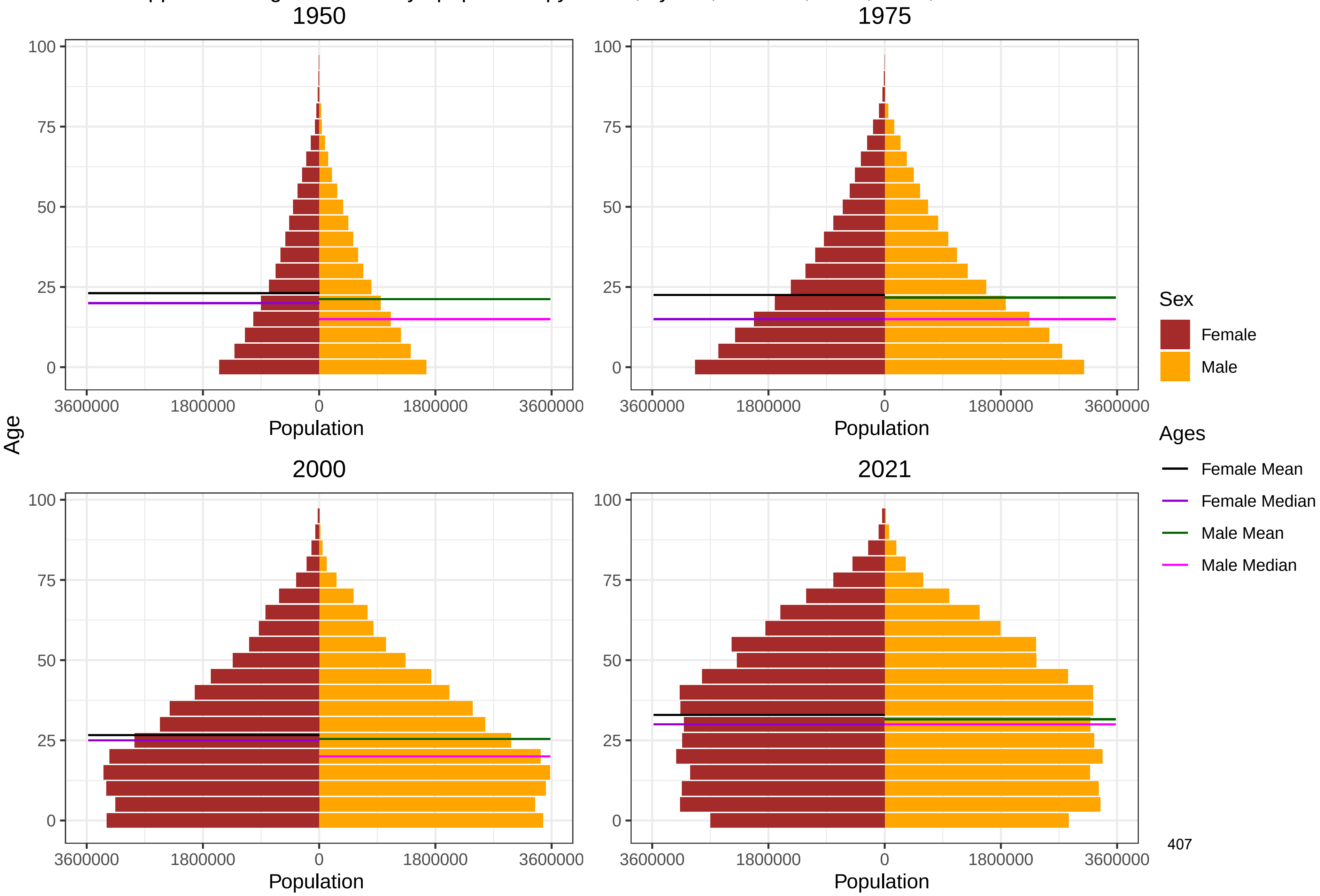

Appendix 2 Figure S9: UK population pyramids, by sex, for 1950, 1975, 2000, and 2021

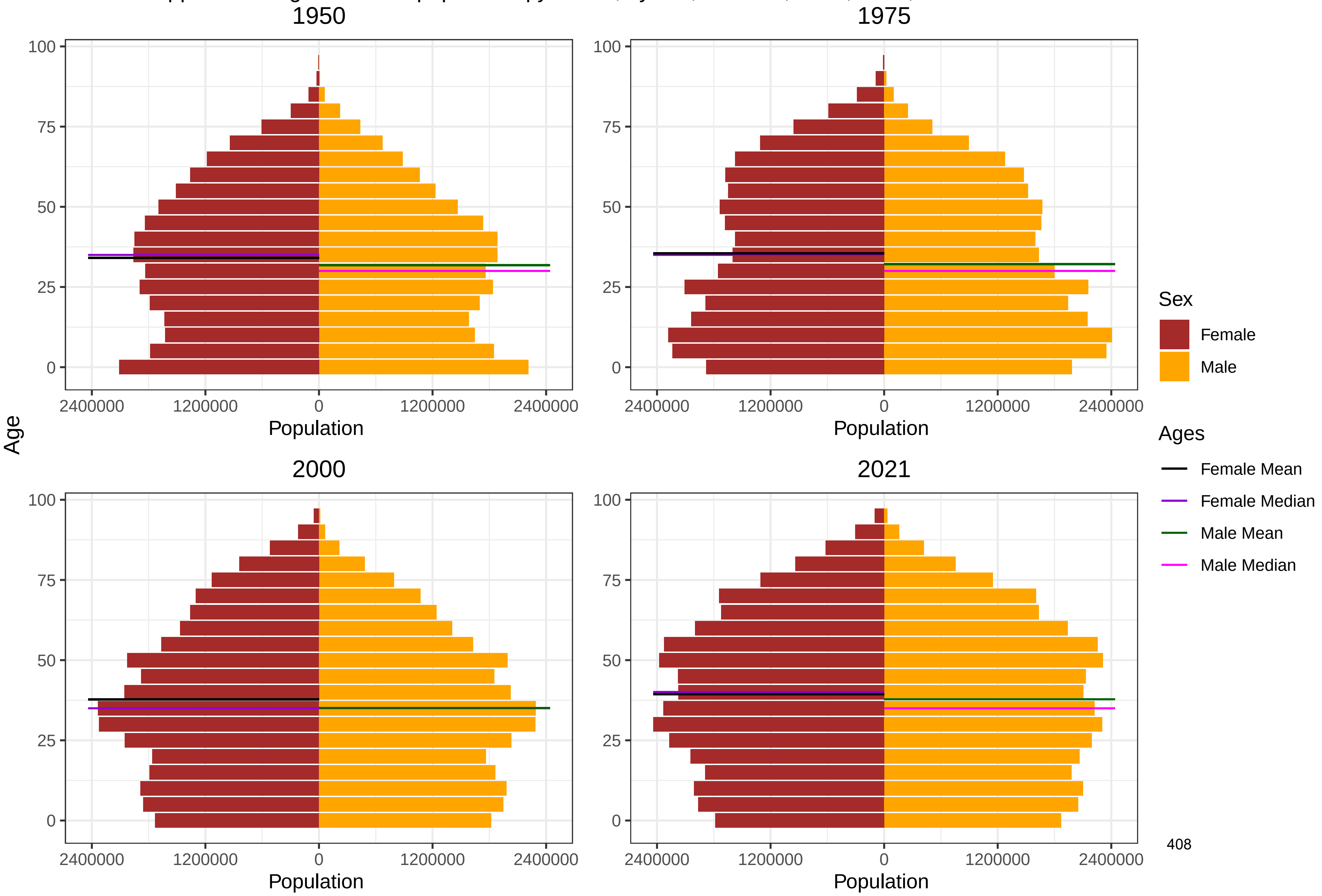

Appendix 2 Figure S9: USA population pyramids, by sex, for 1950, 1975, 2000, and 2021

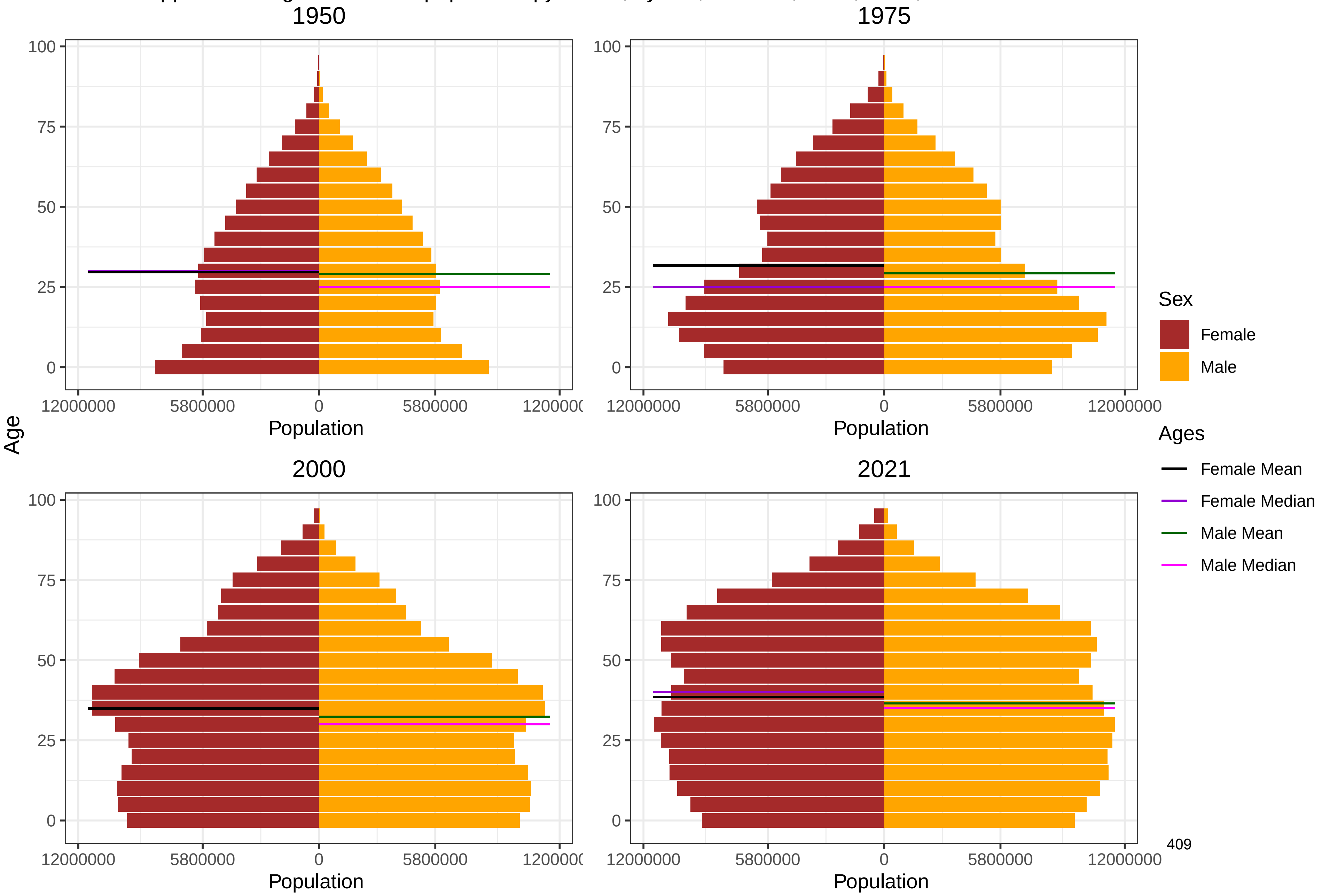

Appendix 2 Figure S9: Uganda population pyramids, by sex, for 1950, 1975, 2000, and 2021

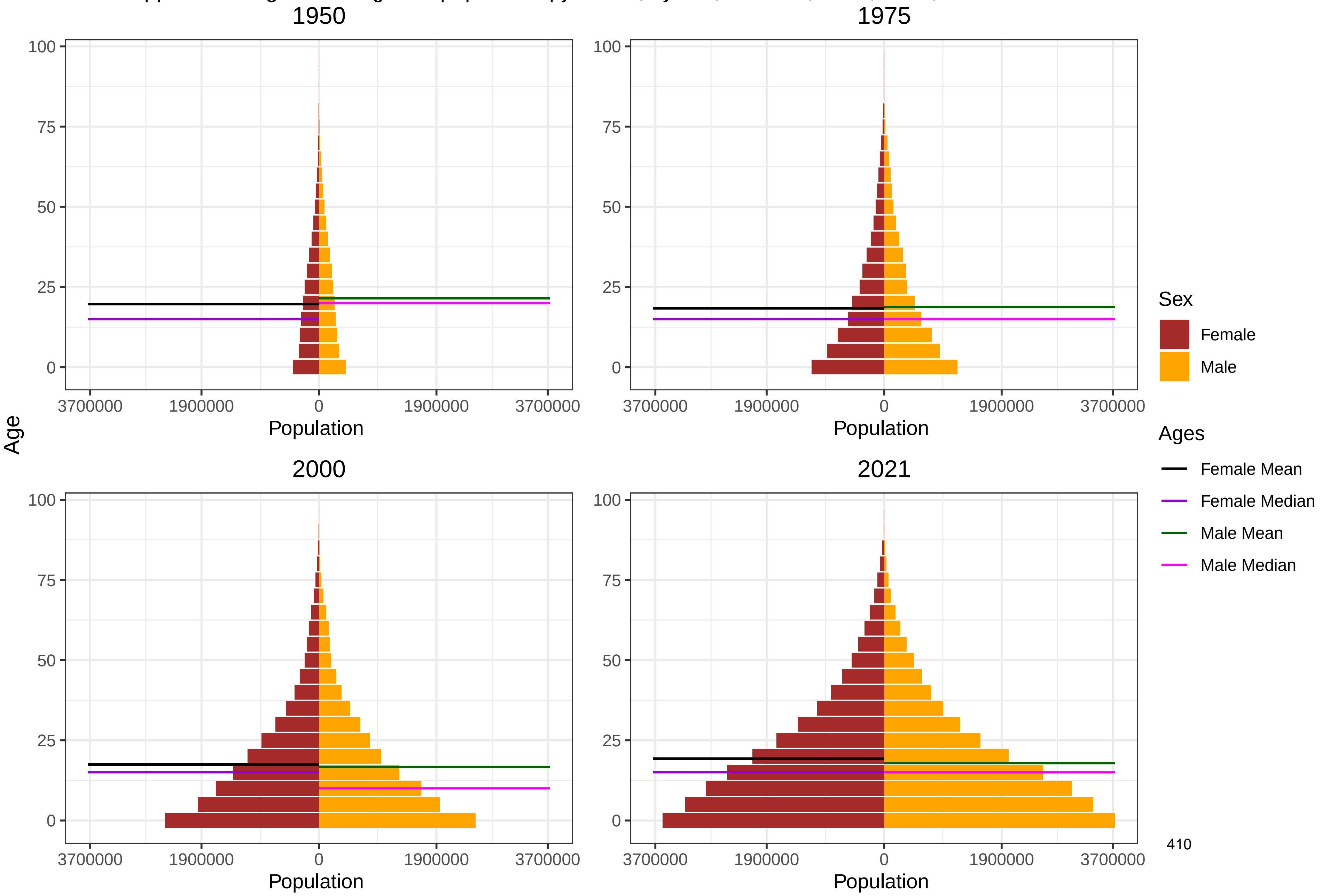

Appendix 2 Figure S9: Ukraine population pyramids, by sex, for 1950, 1975, 2000, and 2021

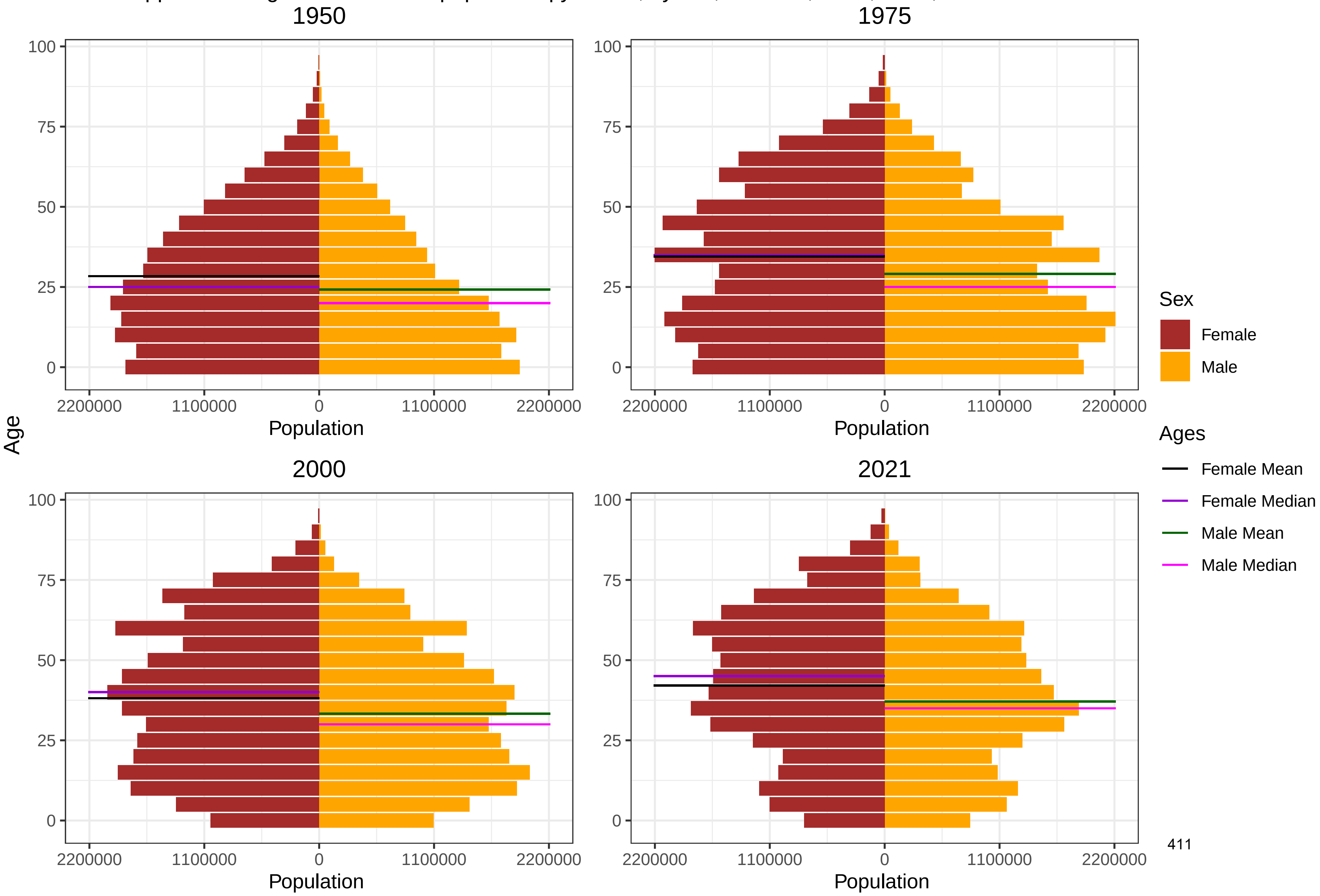

Appendix 2 Figure S9: United Arab Emirates population pyramids, by sex, for 1950, 1975, 2000, and 2021

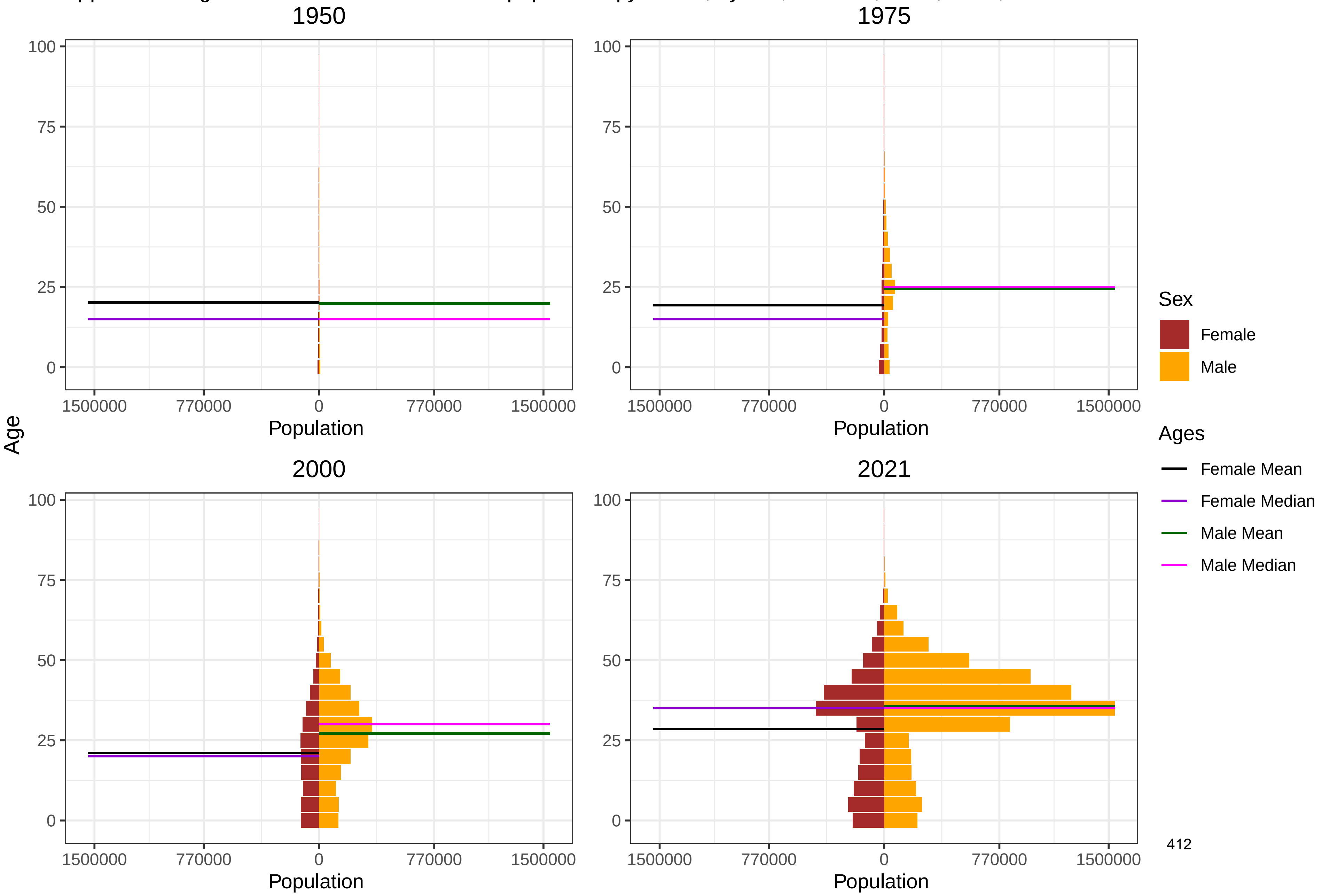

Appendix 2 Figure S9: Uruguay population pyramids, by sex, for 1950, 1975, 2000, and 2021

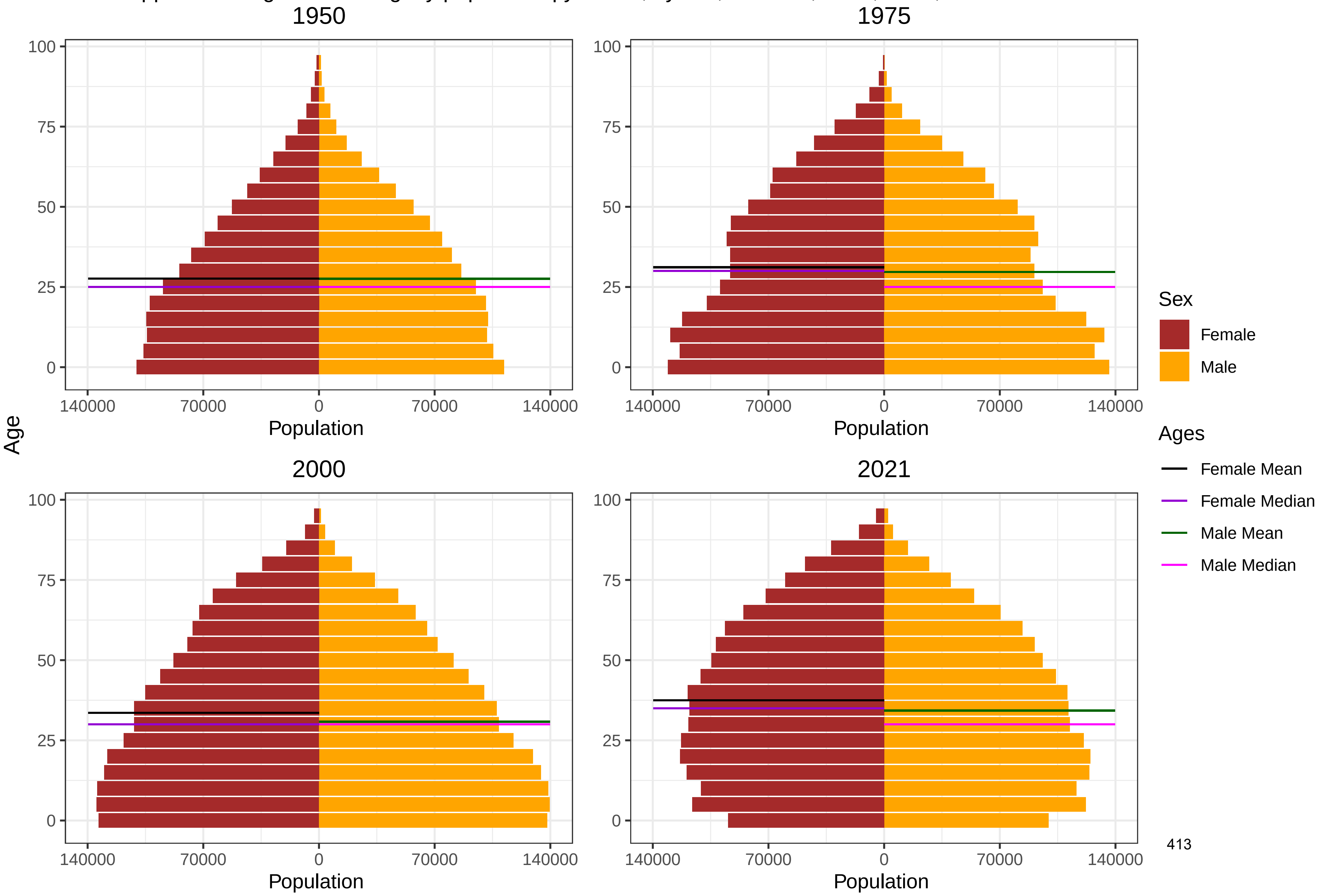

Appendix 2 Figure S9: Uzbekistan population pyramids, by sex, for 1950, 1975, 2000, and 2021

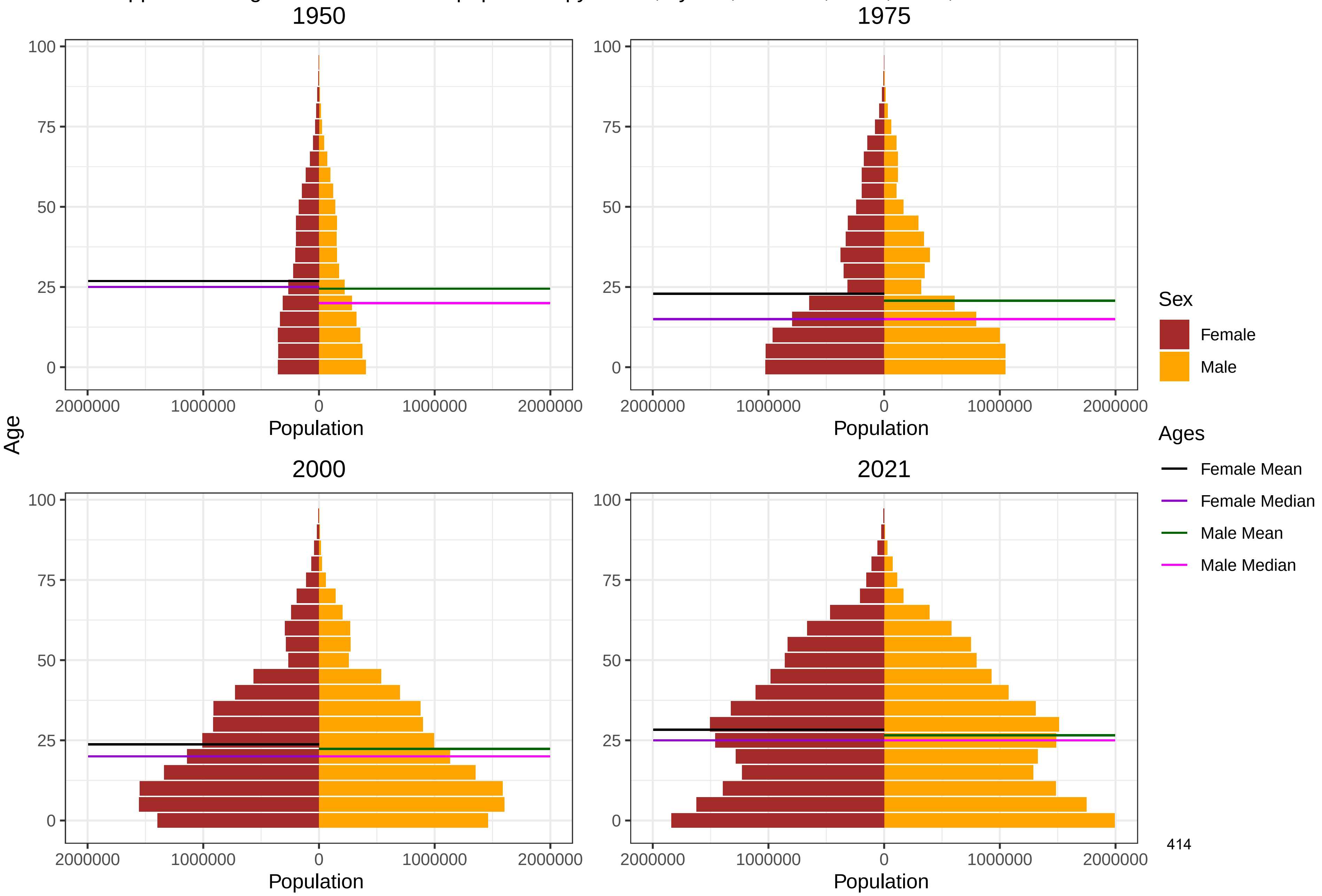

Appendix 2 Figure S9: Vanuatu population pyramids, by sex, for 1950, 1975, 2000, and 2021

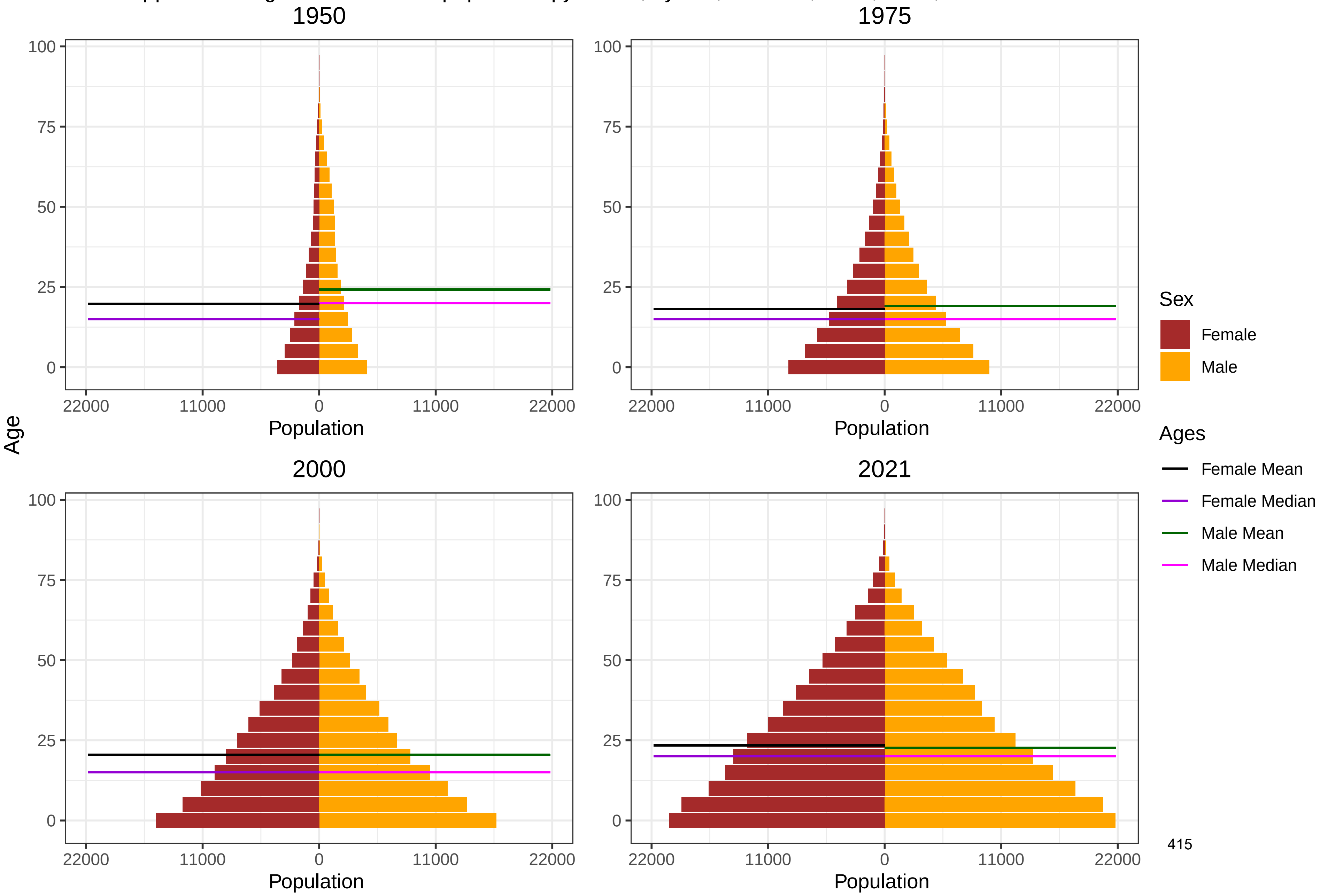

Appendix 2 Figure S9: Venezuela population pyramids, by sex, for 1950, 1975, 2000, and 2021

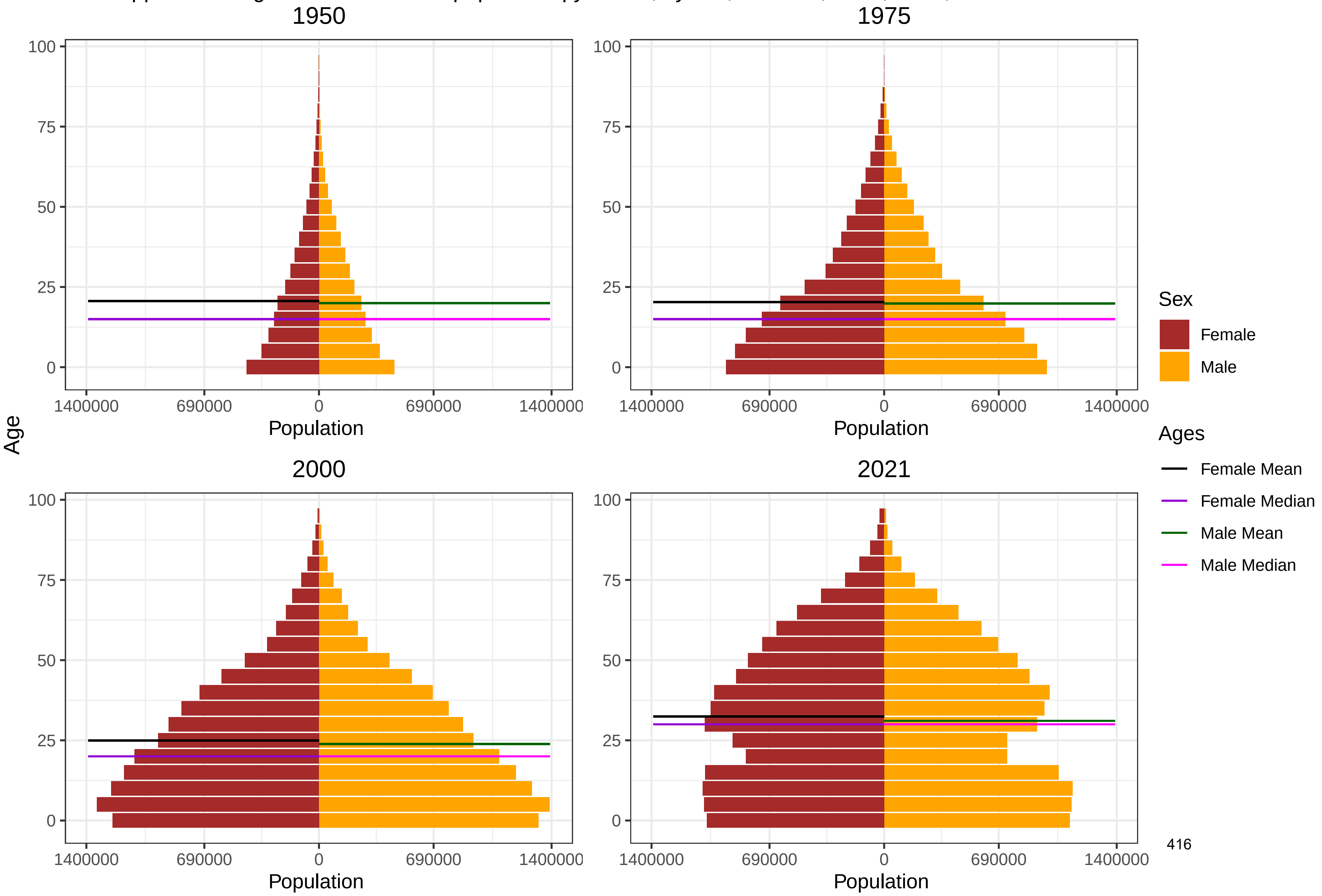

Appendix 2 Figure S9: Viet Nam population pyramids, by sex, for 1950, 1975, 2000, and 2021

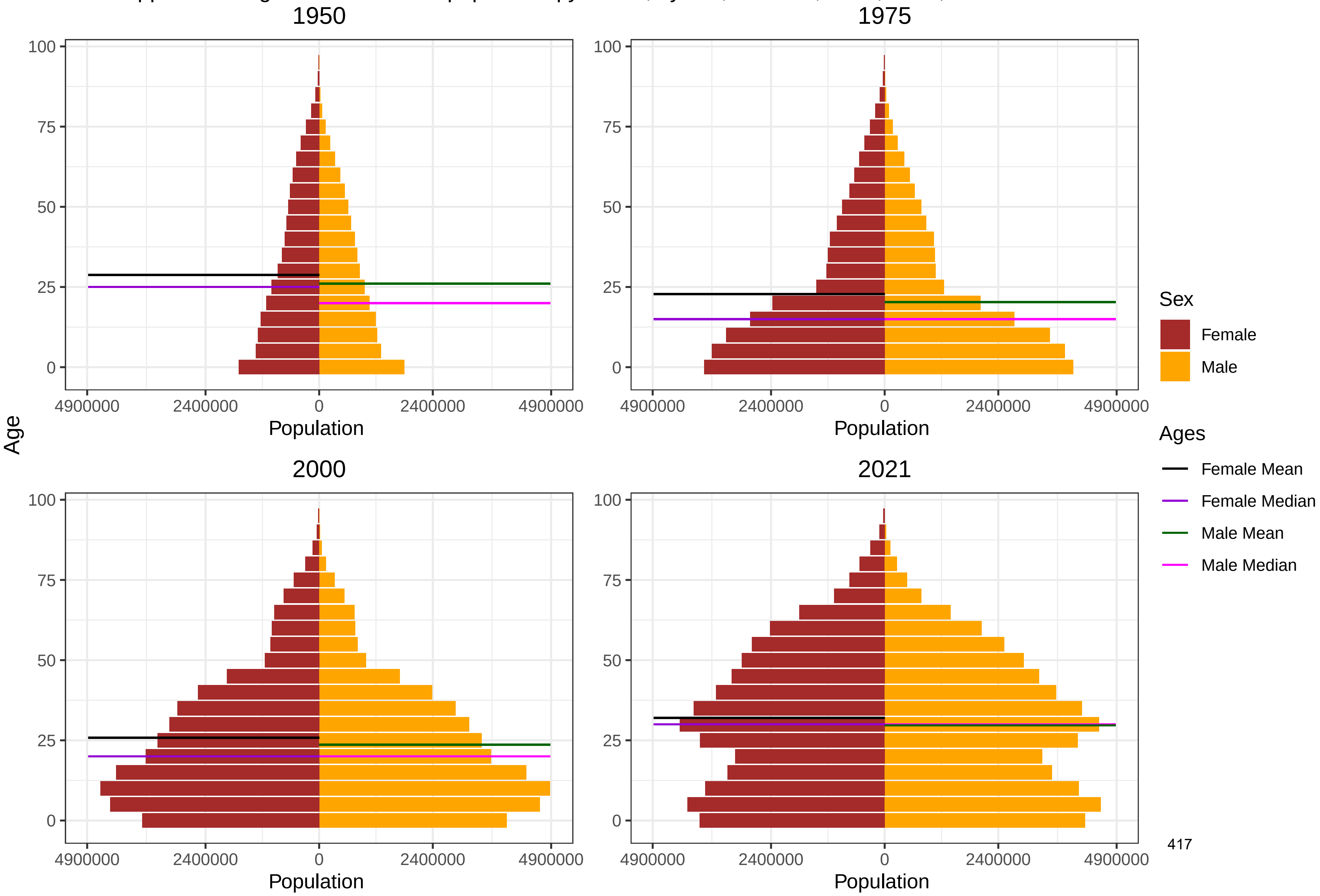

Appendix 2 Figure S9: Virgin Islands population pyramids, by sex, for 1950, 1975, 2000, and 2021

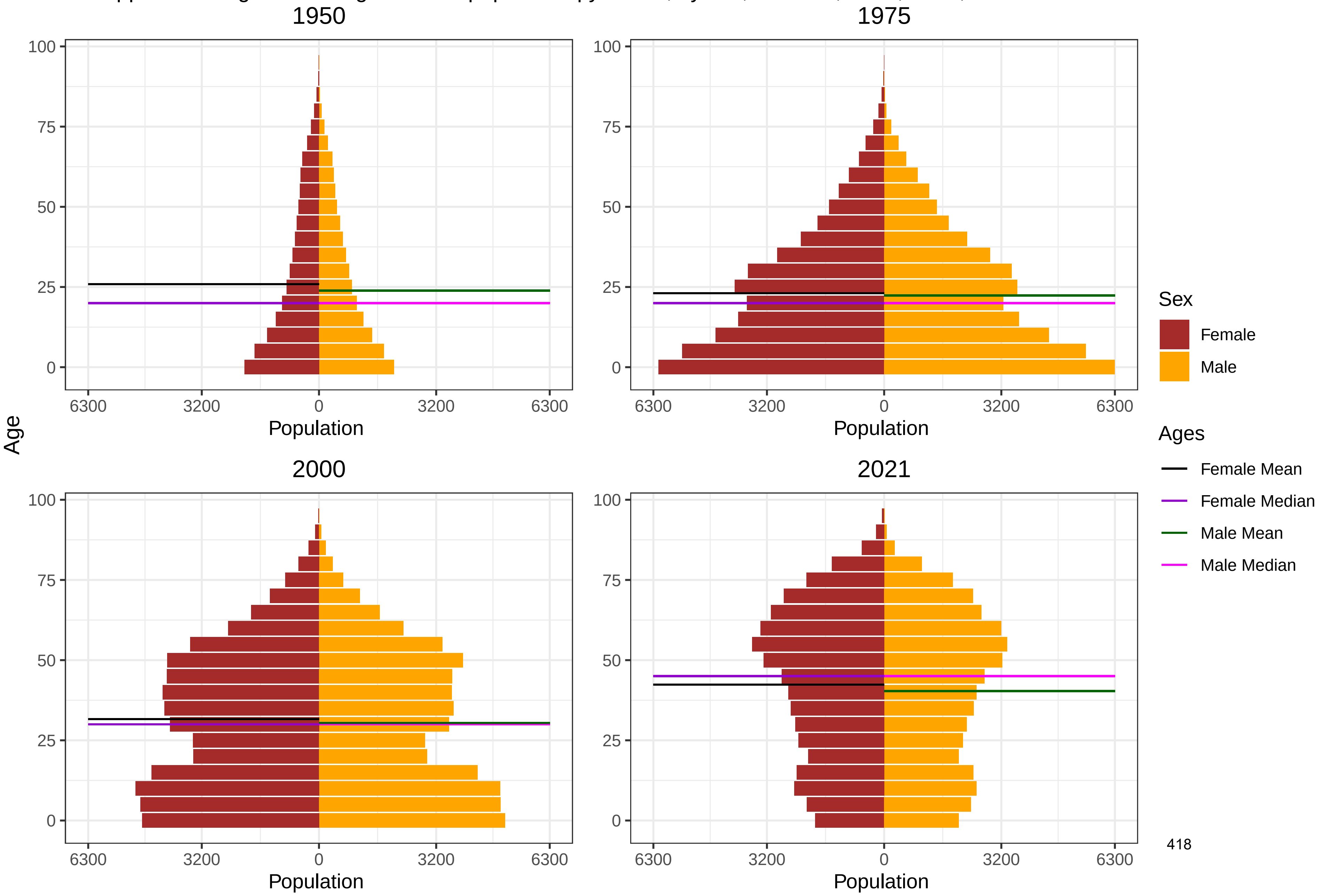

Appendix 2 Figure S9: Yemen population pyramids, by sex, for 1950, 1975, 2000, and 2021

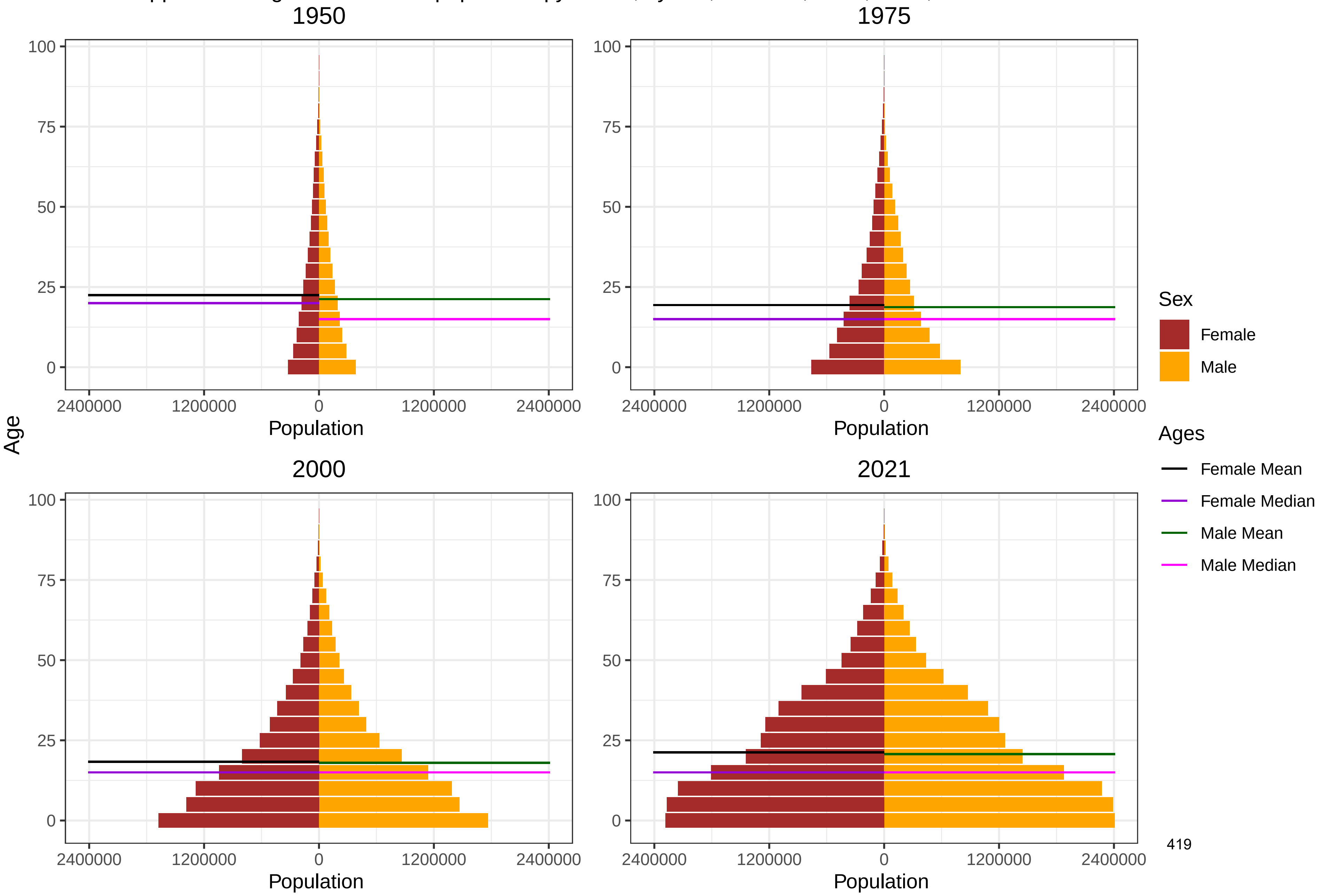

Appendix 2 Figure S9: Zambia population pyramids, by sex, for 1950, 1975, 2000, and 2021

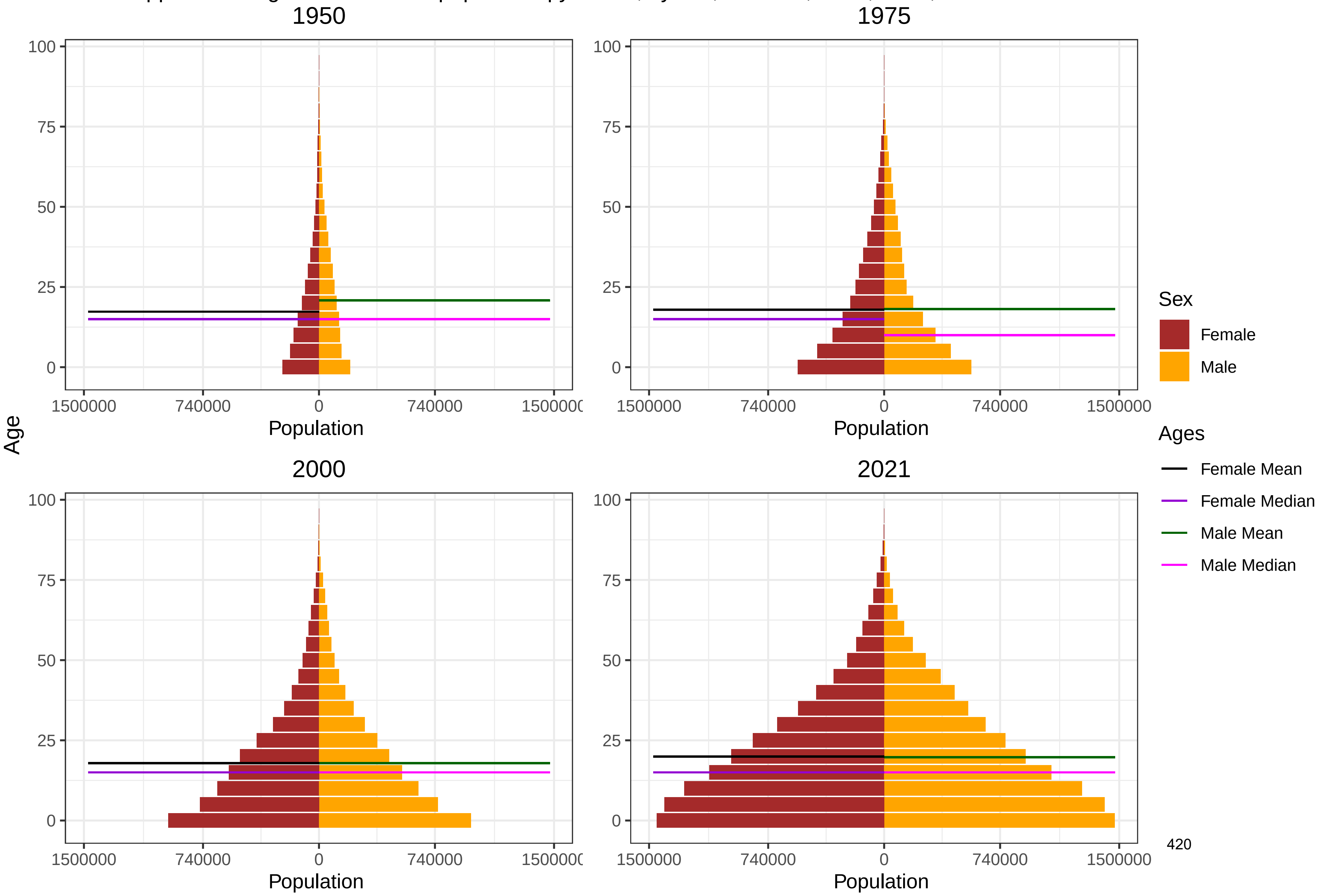

Appendix 2 Figure S9: Zimbabwe population pyramids, by sex, for 1950, 1975, 2000, and 2021

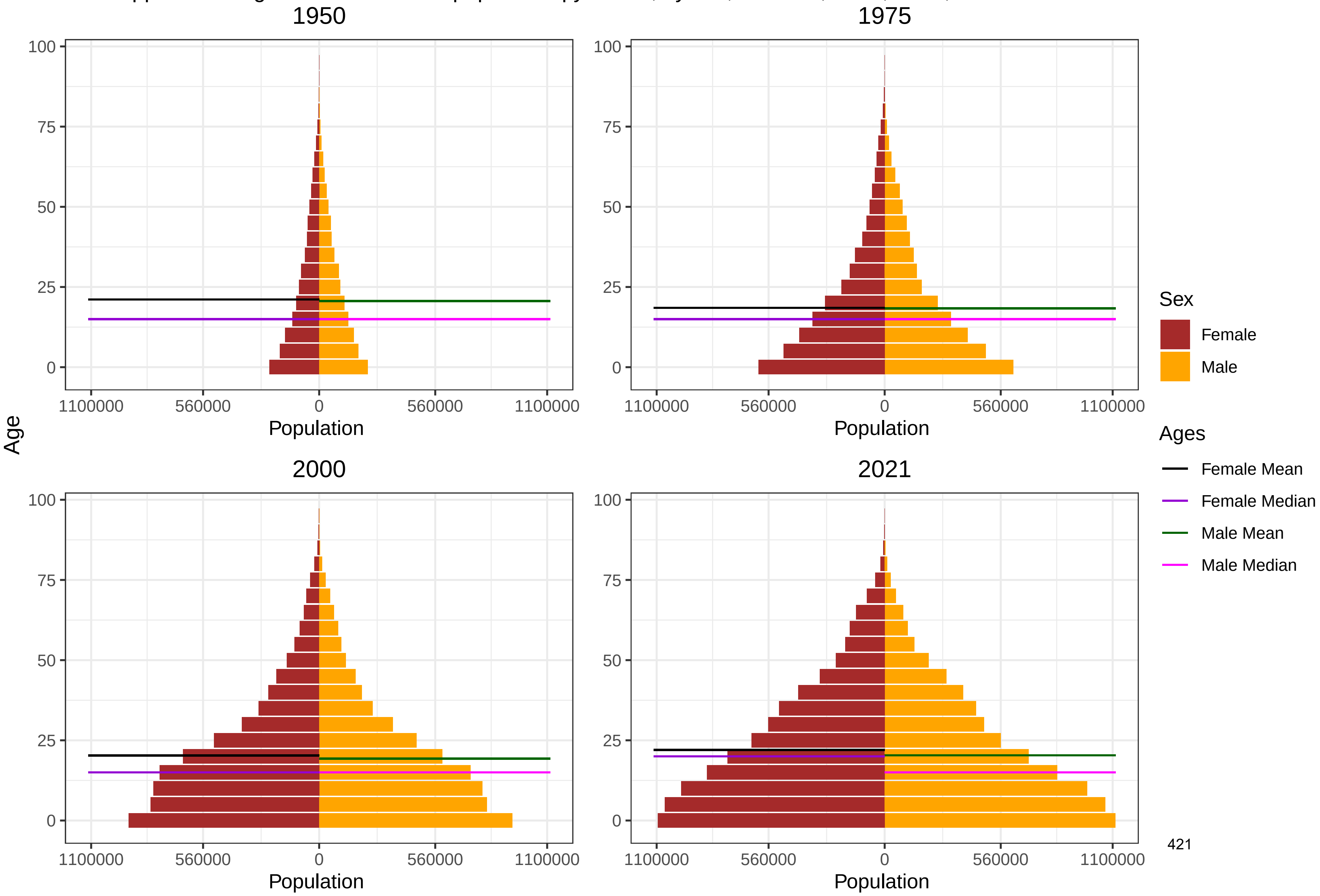

Appendix 2 Figure S10: Comparison of COVID excess mortality estimates (pseudo−log) between GBD 2021 and WHO, by location

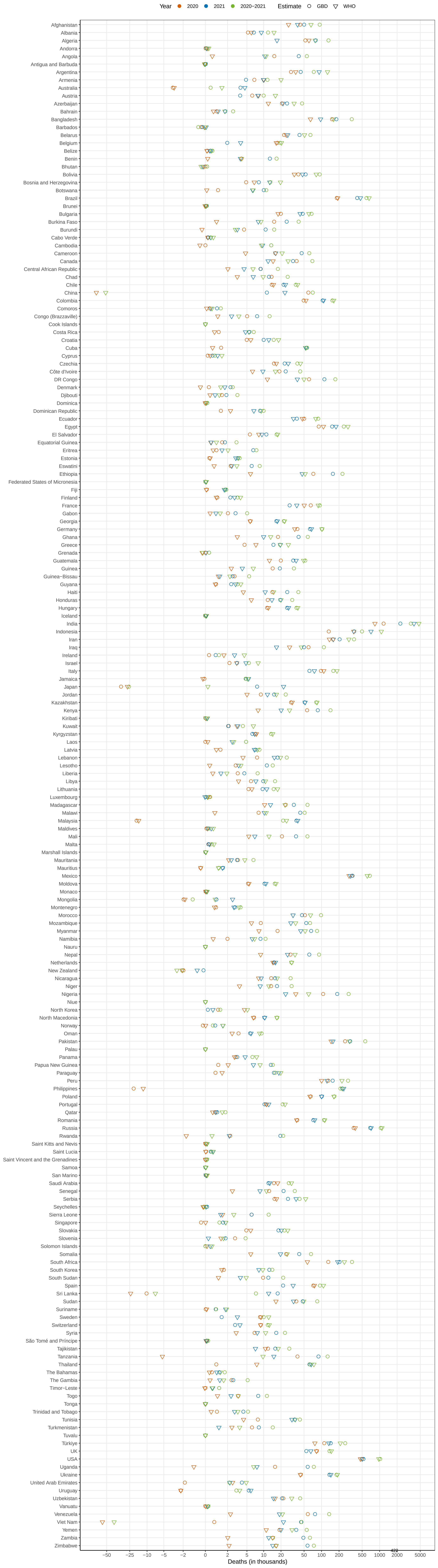

Appendix 2 Figure S11: Comparison of under 5 mortality estimates for 2021 between GBD 2021 and UNICEF 2022, by location

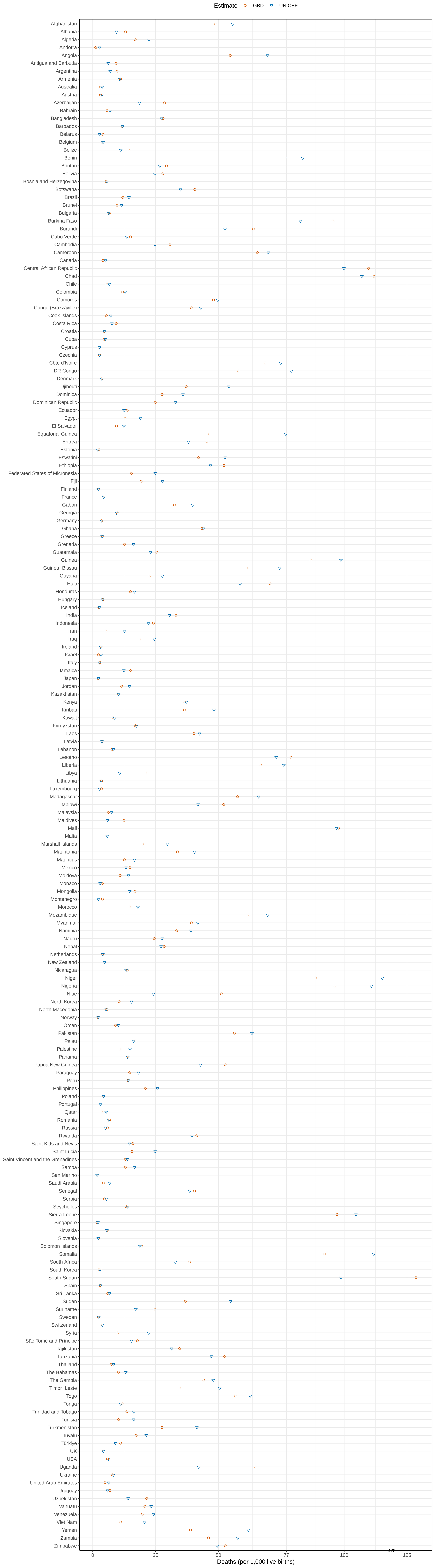

Appendix 2 Figure S12: Percent difference between GBD 2021 and WPP 2022
adult mortality estimates, by location and sex, for 1950, 1975, 2000, 2015, 2021

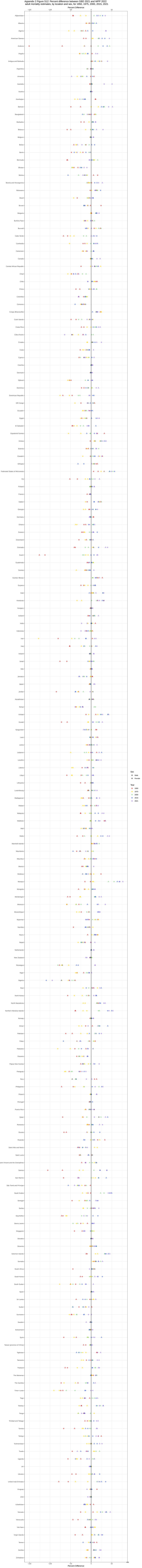

Appendix 2 Figure S13: Comparison of life expectancy at birth estimates for 2021 between GBD 2021 and WPP 2022, by sex and location

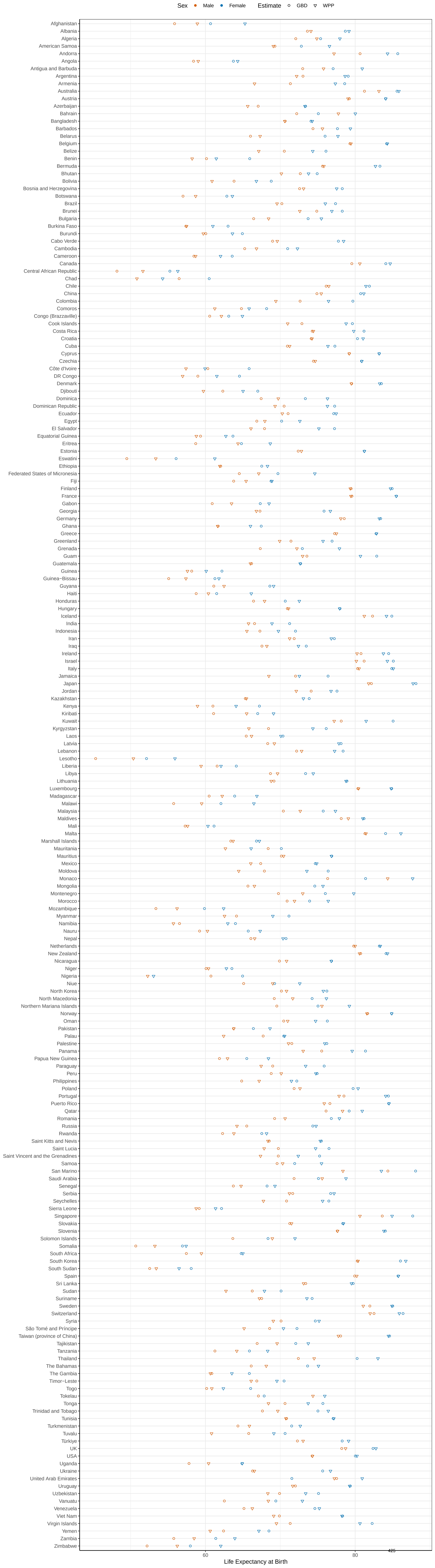

Appendix 2 Figure S14: Comparison of life expectancy at birth estimates for countries in GBD 2021 and WPP 2022 for 1950, both sexes combined

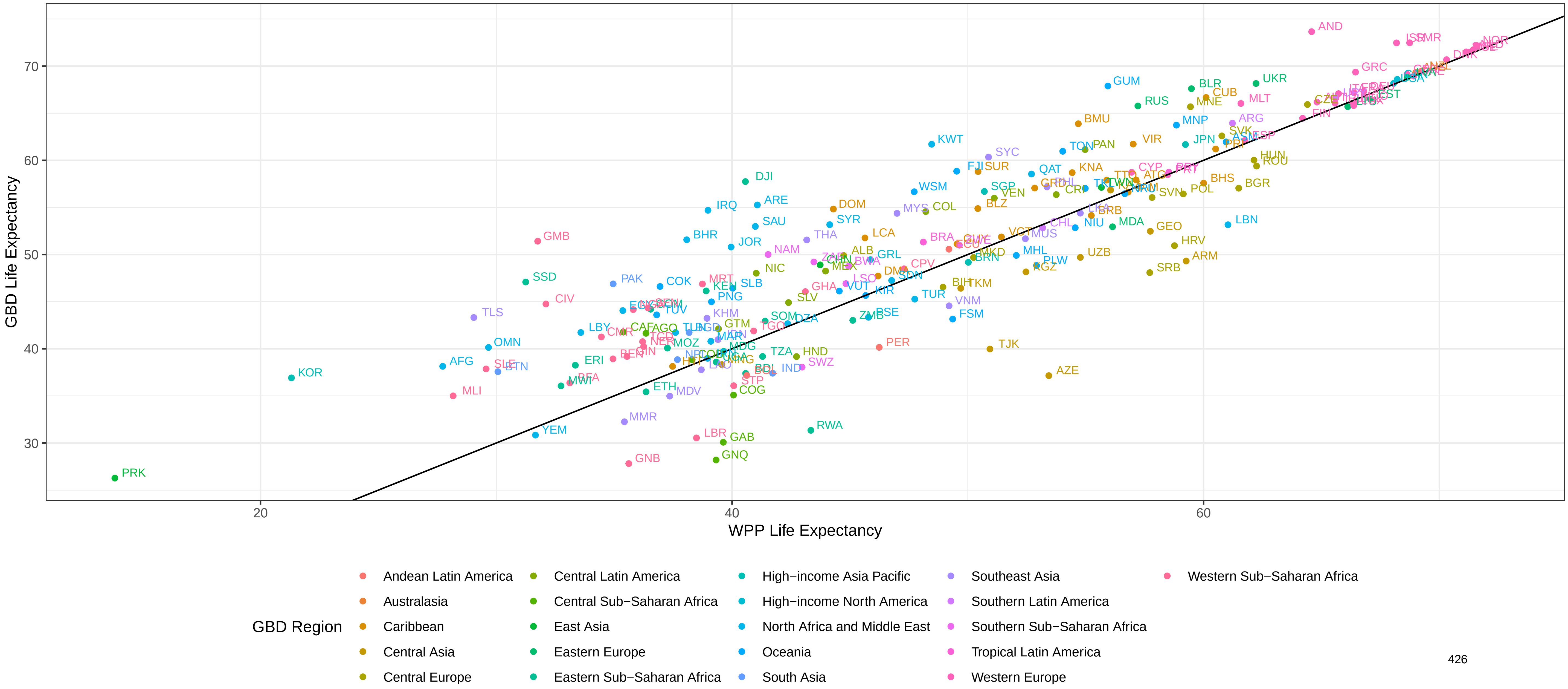

Appendix 2 Figure S14: Comparison of life expectancy at birth estimates for countries in GBD 2021 and WPP 2022 for 1955, both sexes combined

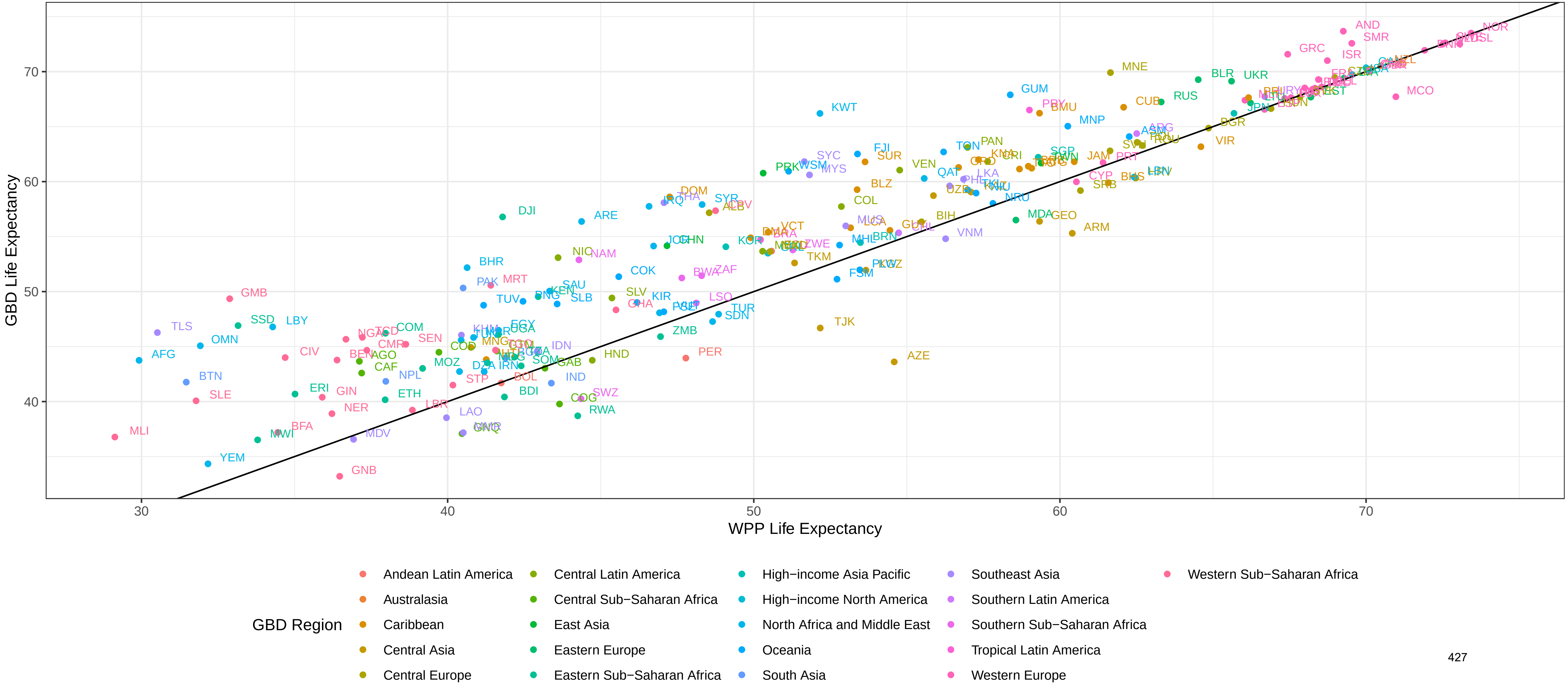

Appendix 2 Figure S14: Comparison of life expectancy at birth estimates for countries in GBD 2021 and WPP 2022 for 1960, both sexes combined

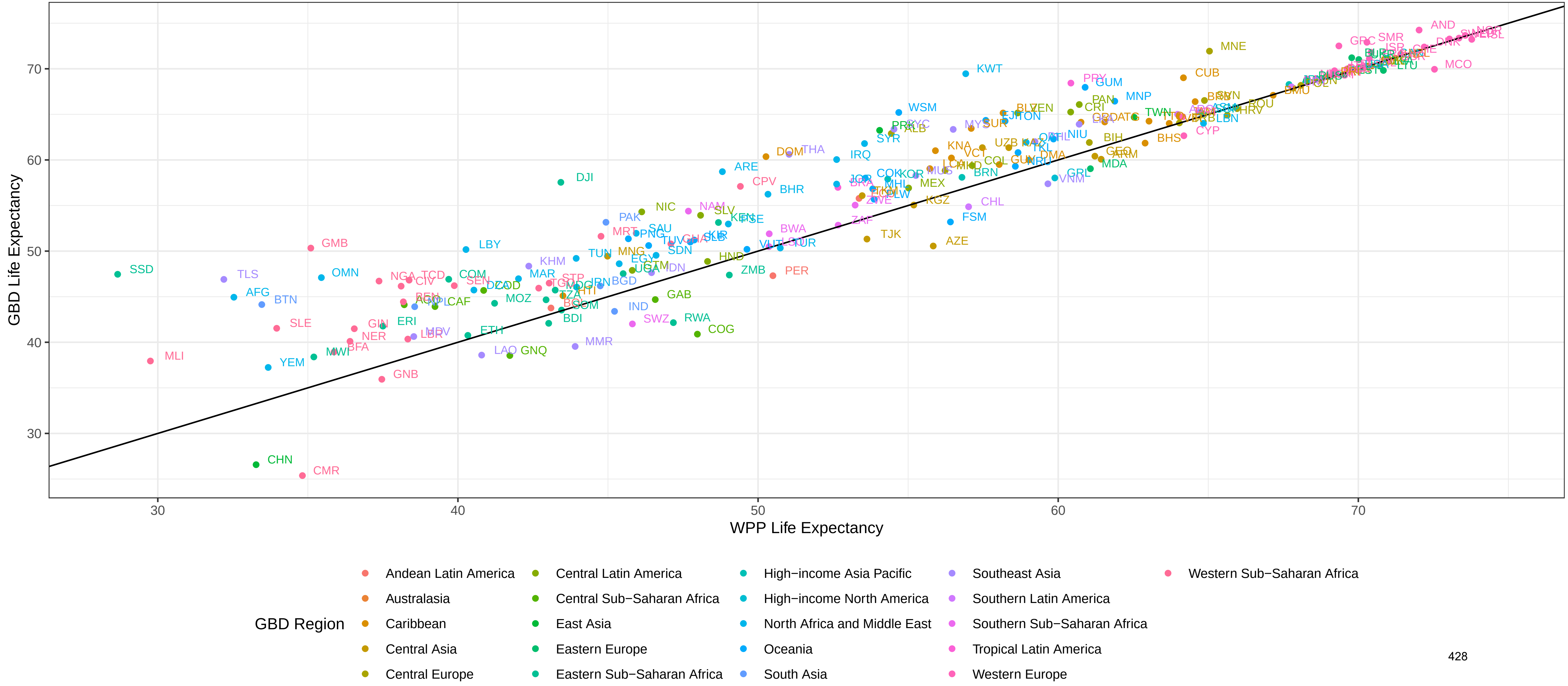

Appendix 2 Figure S14: Comparison of life expectancy at birth estimates for countries in GBD 2021 and WPP 2022 for 1965, both sexes combined

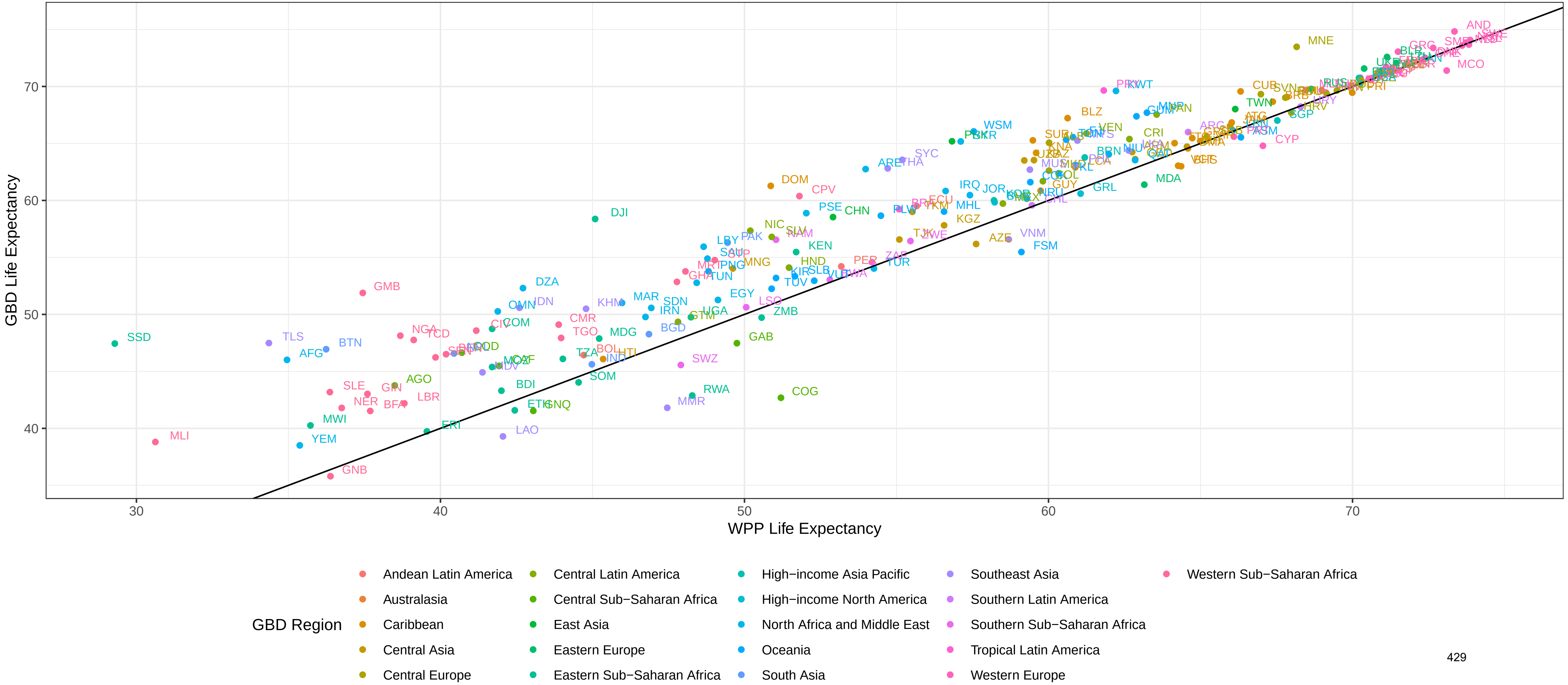

Appendix 2 Figure S14: Comparison of life expectancy at birth estimates for countries in GBD 2021 and WPP 2022 for 1970, both sexes combined

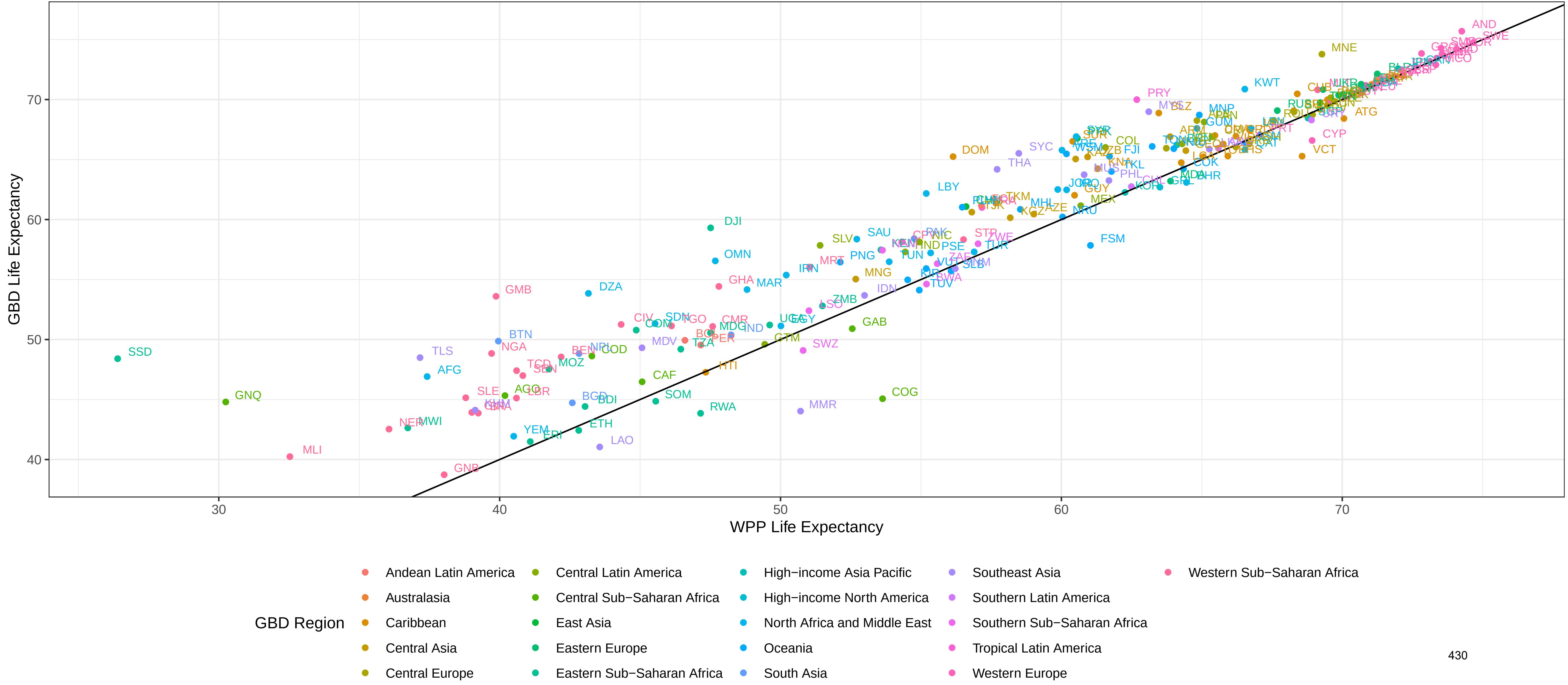

Appendix 2 Figure S14: Comparison of life expectancy at birth estimates for countries in GBD 2021 and WPP 2022 for 1975, both sexes combined

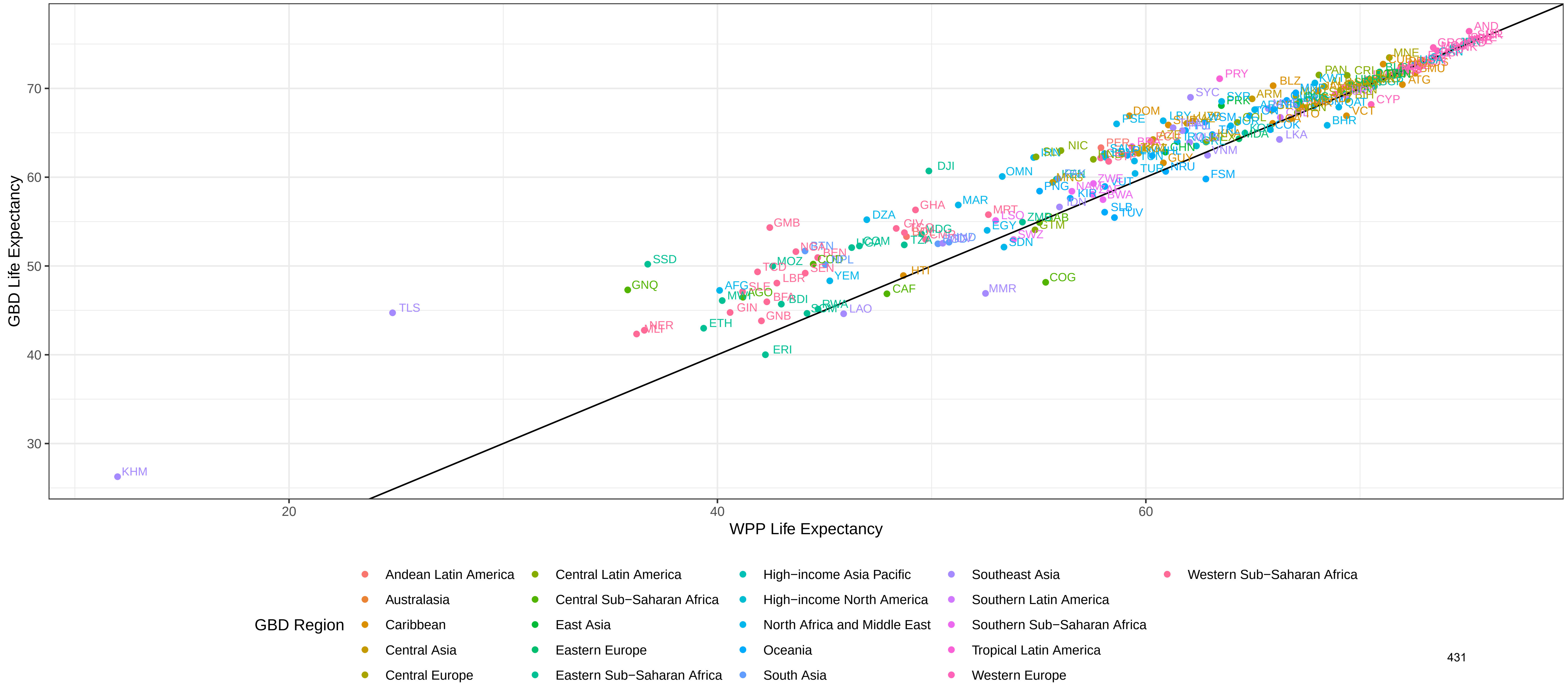

Appendix 2 Figure S14: Comparison of life expectancy at birth estimates for countries in GBD 2021 and WPP 2022 for 1980, both sexes combined

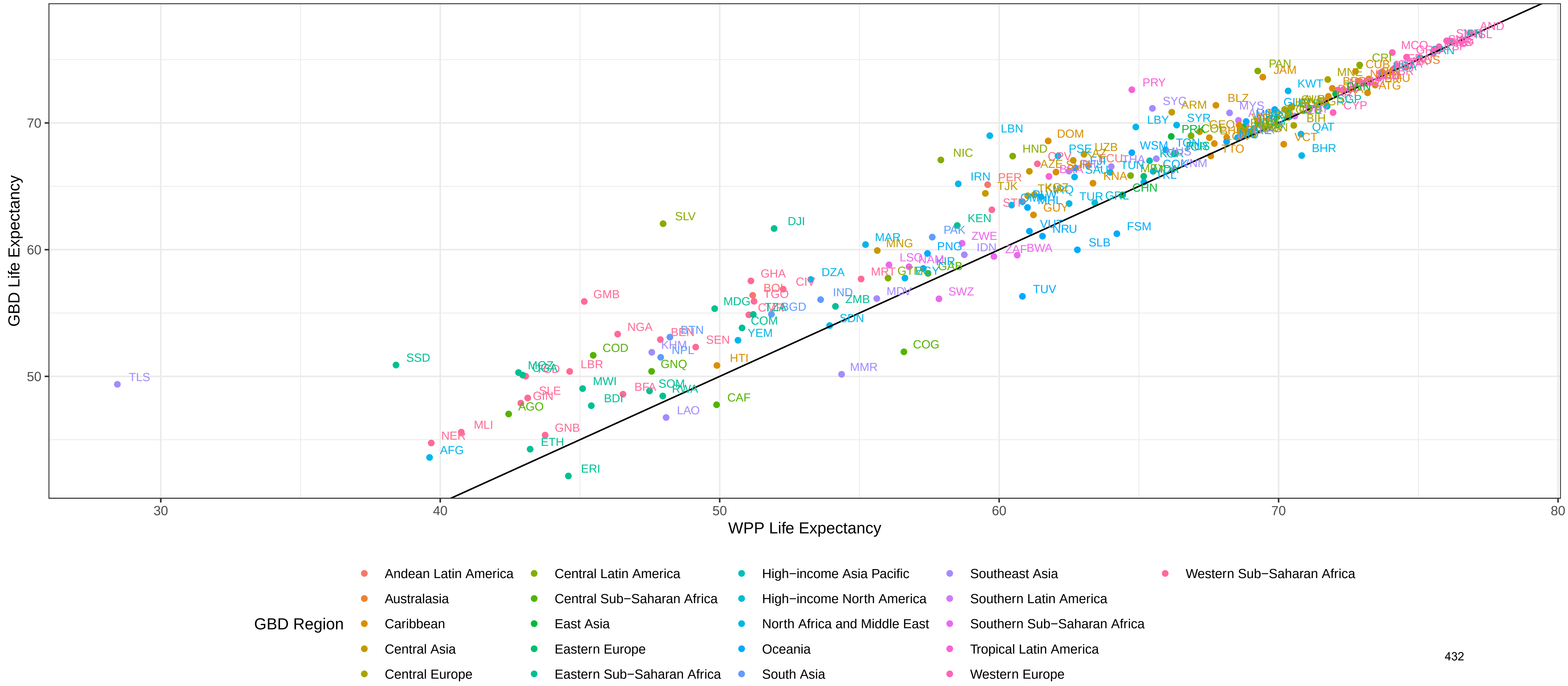

Appendix 2 Figure S14: Comparison of life expectancy at birth estimates for countries in GBD 2021 and WPP 2022 for 1985, both sexes combined

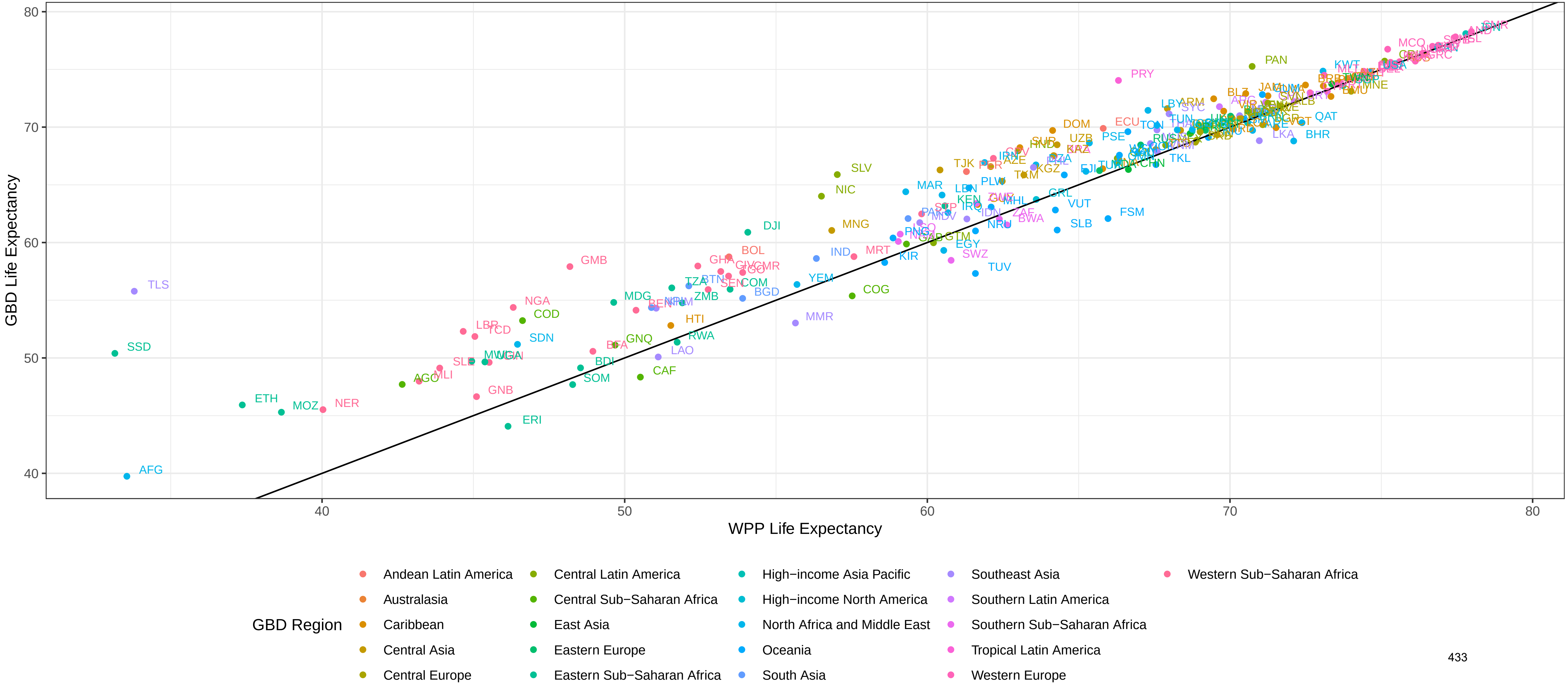

Appendix 2 Figure S14: Comparison of life expectancy at birth estimates for countries in GBD 2021 and WPP 2022 for 1990, both sexes combined

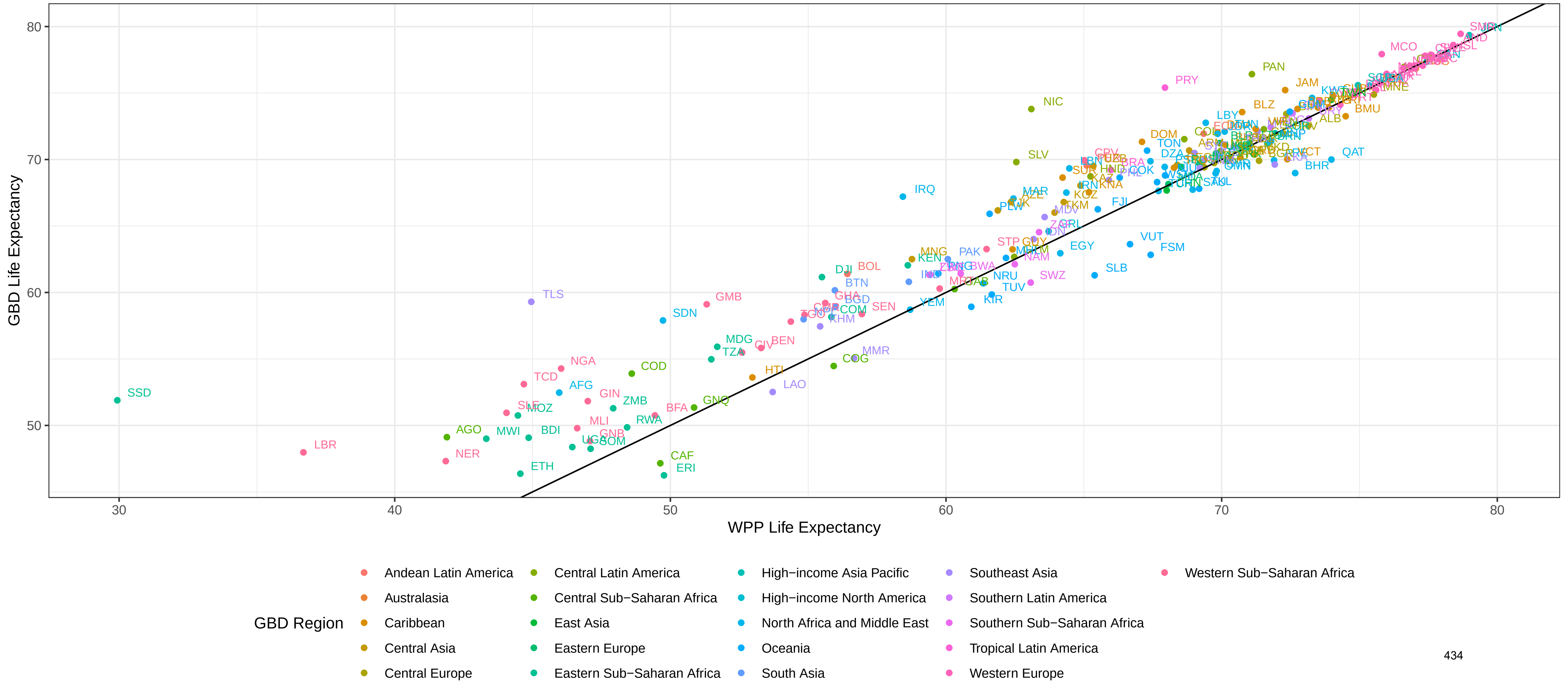

Appendix 2 Figure S14: Comparison of life expectancy at birth estimates for countries in GBD 2021 and WPP 2022 for 1995, both sexes combined

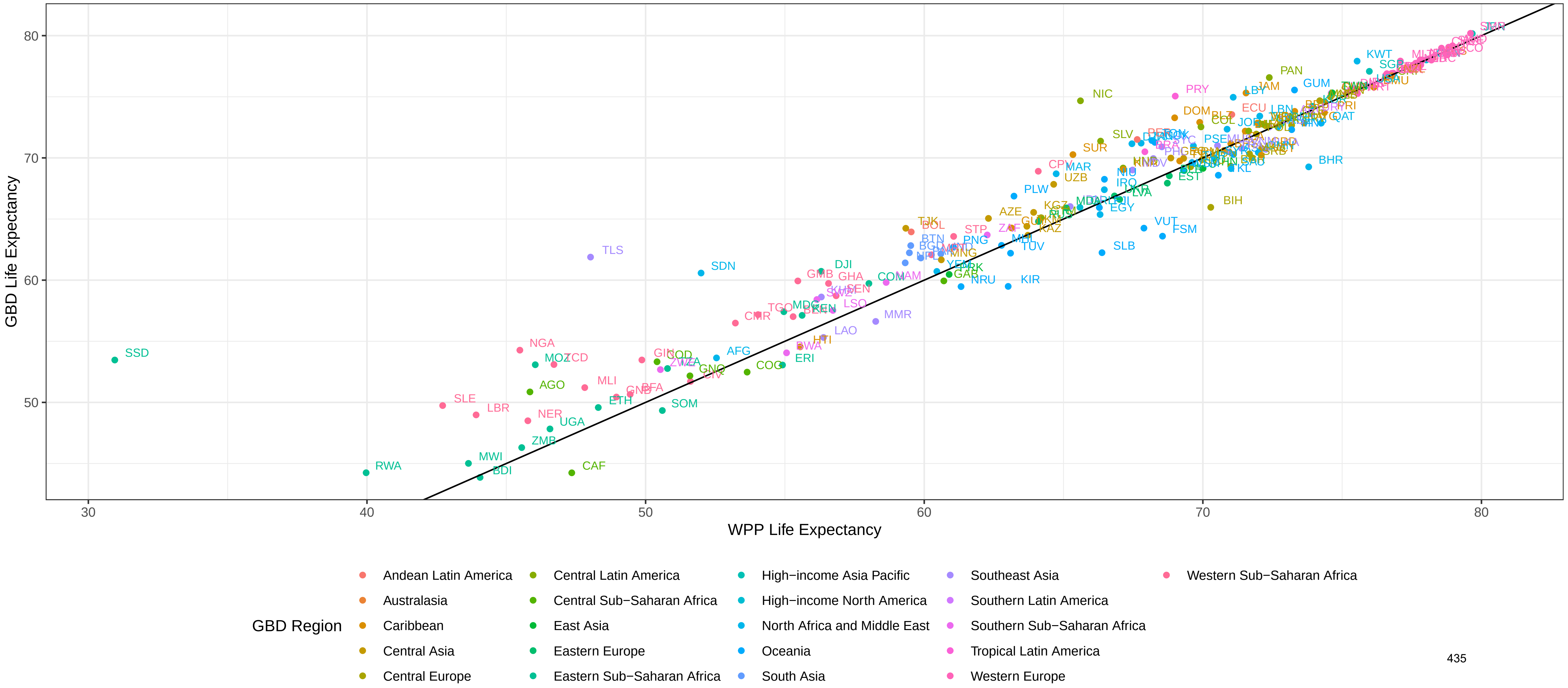

Appendix 2 Figure S14: Comparison of life expectancy at birth estimates for countries in GBD 2021 and WPP 2022 for 2000, both sexes combined

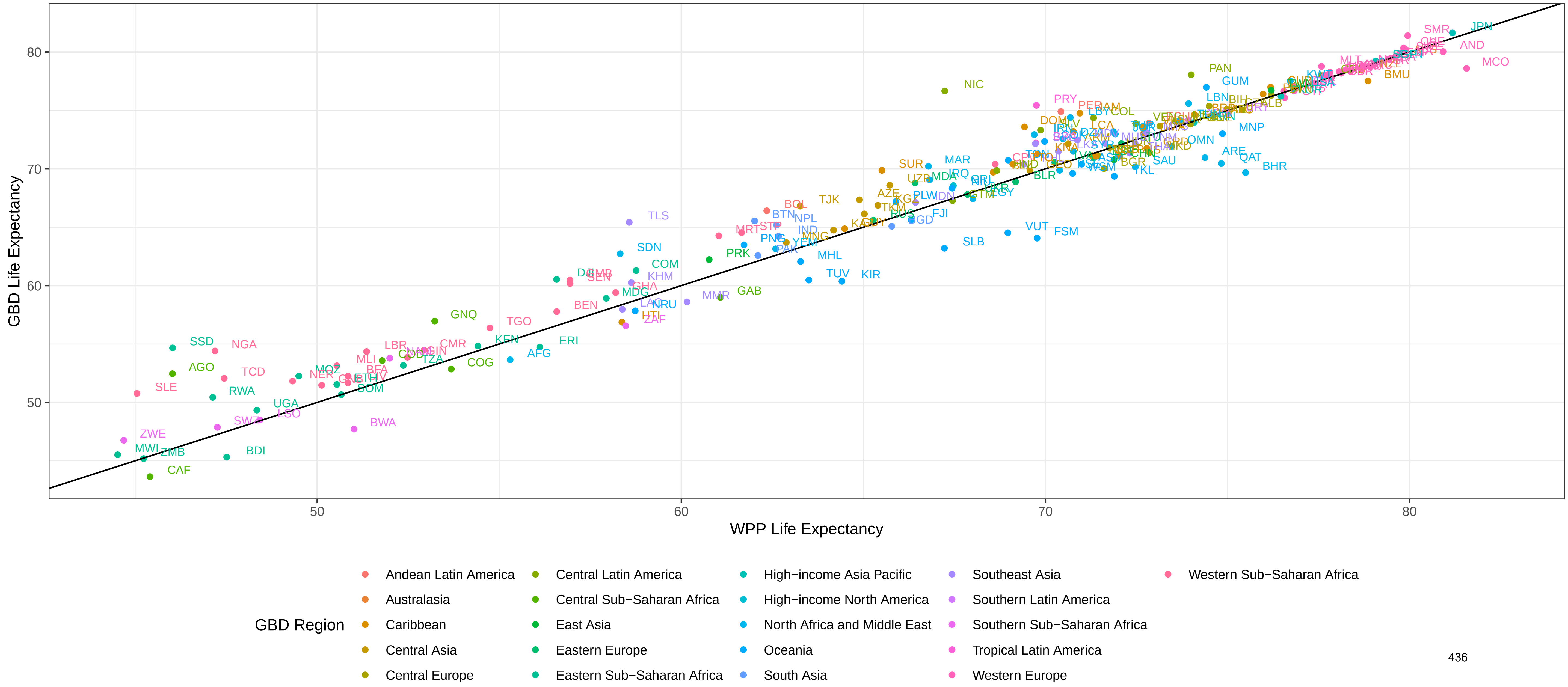

Appendix 2 Figure S14: Comparison of life expectancy at birth estimates for countries in GBD 2021 and WPP 2022 for 2005, both sexes combined

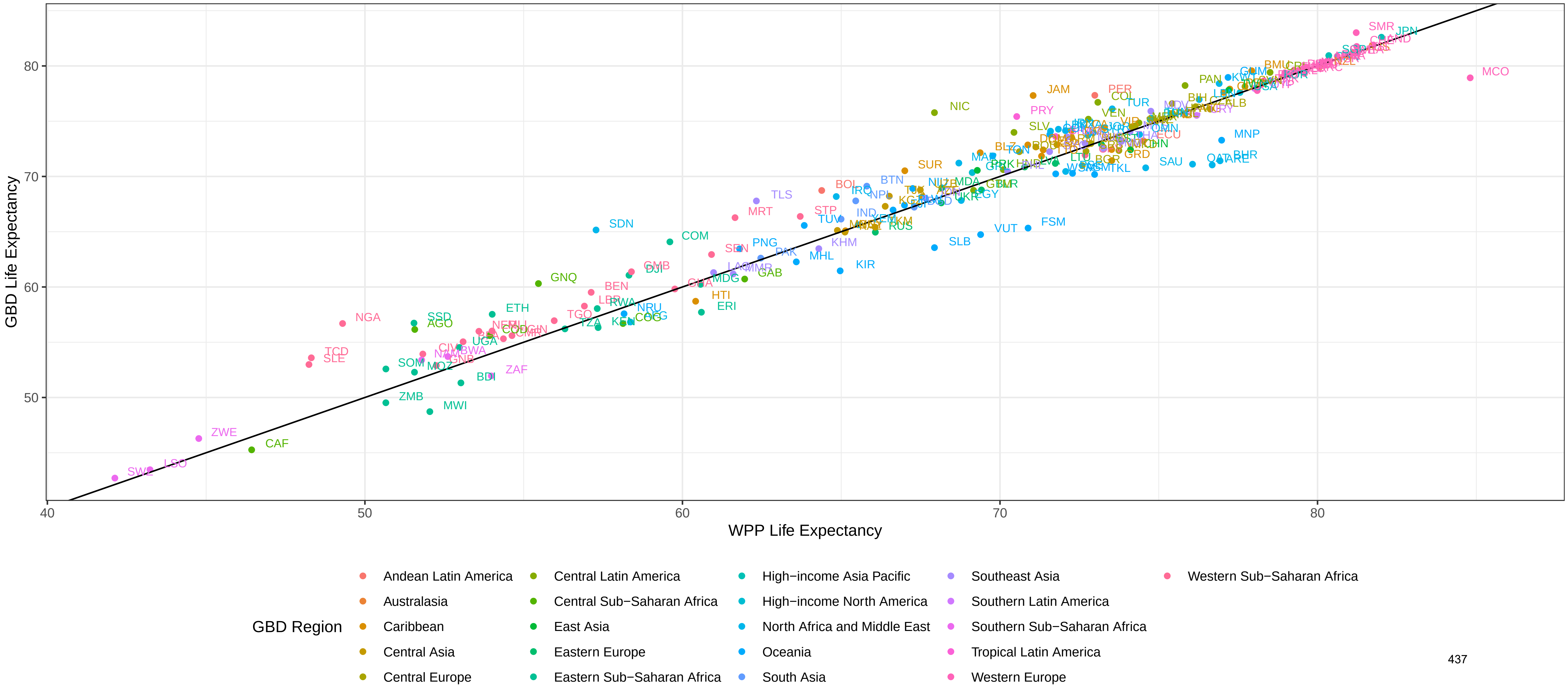

Appendix 2 Figure S14: Comparison of life expectancy at birth estimates for countries in GBD 2021 and WPP 2022 for 2010, both sexes combined

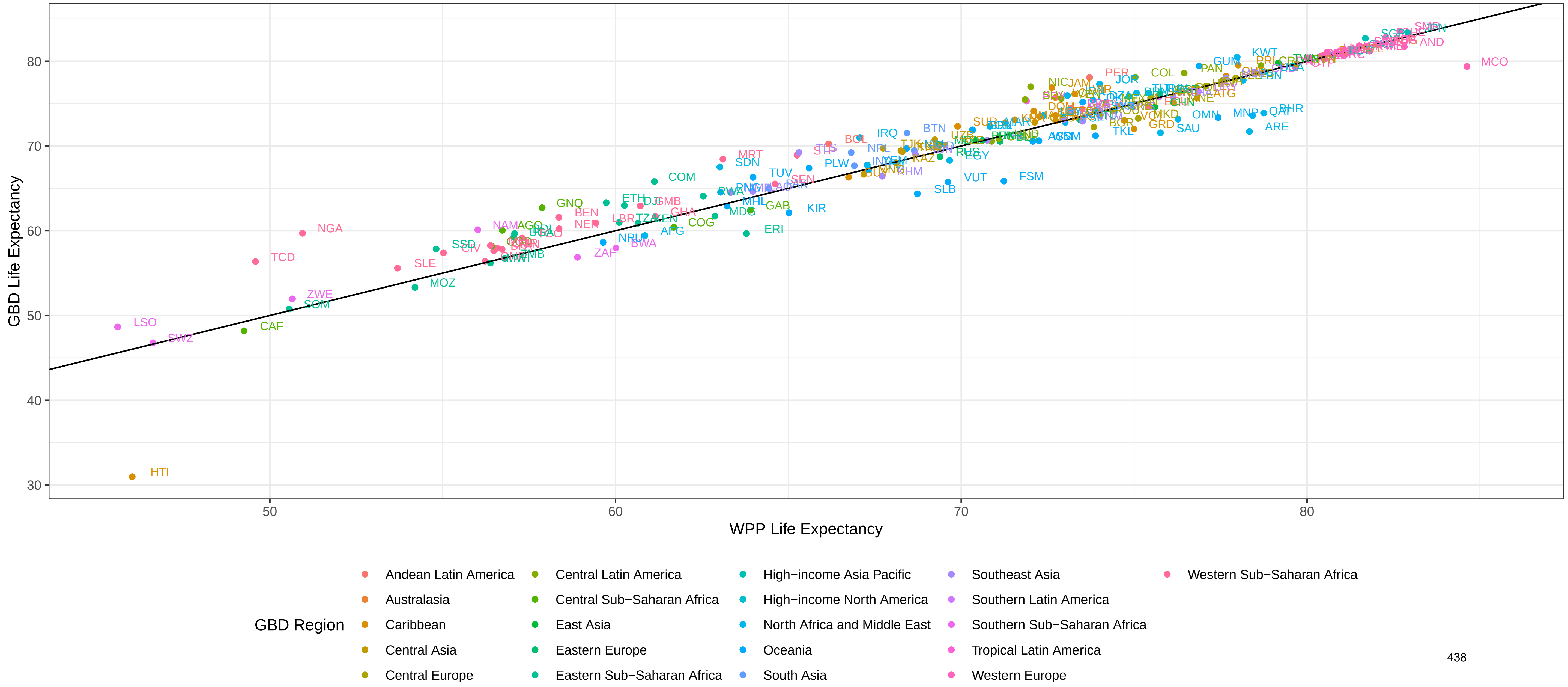

Appendix 2 Figure S14: Comparison of life expectancy at birth estimates for countries in GBD 2021 and WPP 2022 for 2015, both sexes combined

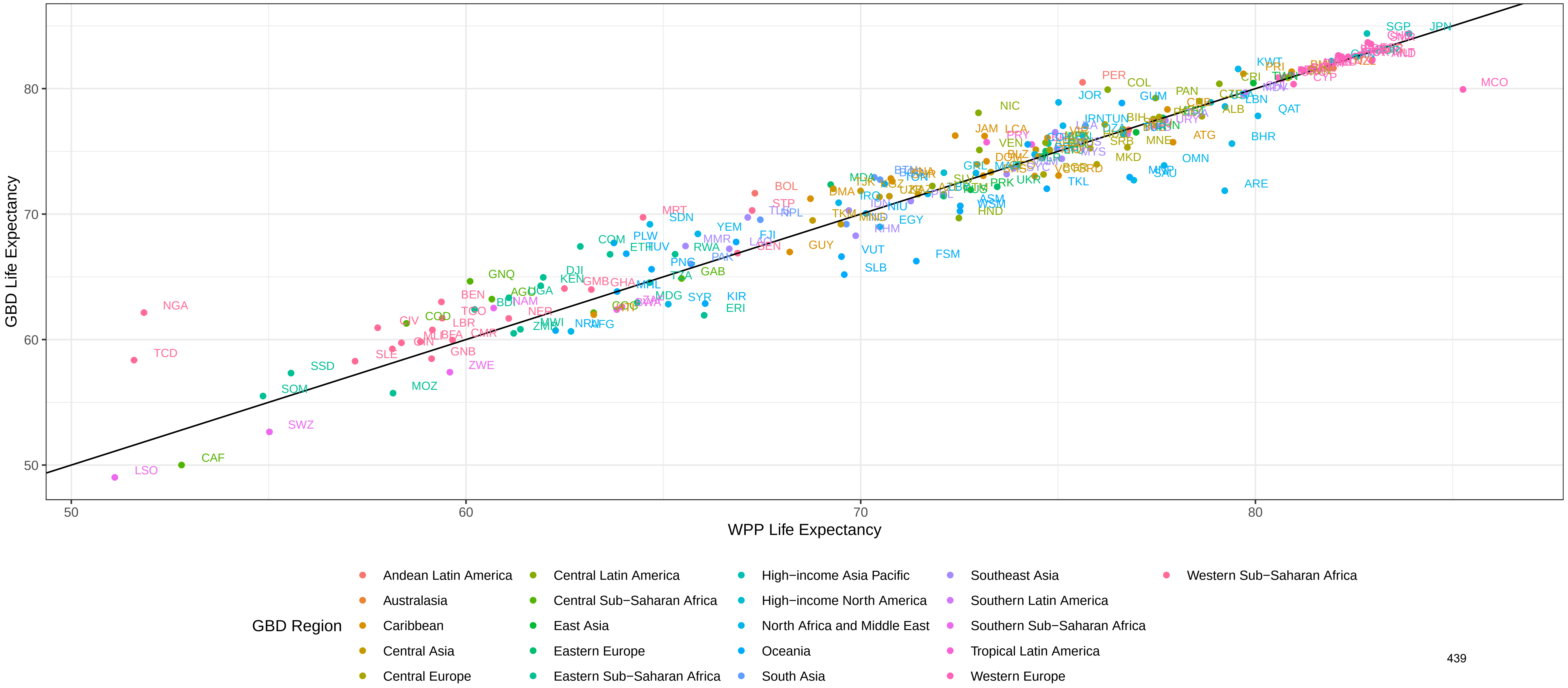

Appendix 2 Figure S14: Comparison of life expectancy at birth estimates for countries in GBD 2021 and WPP 2022 for 2020, both sexes combined

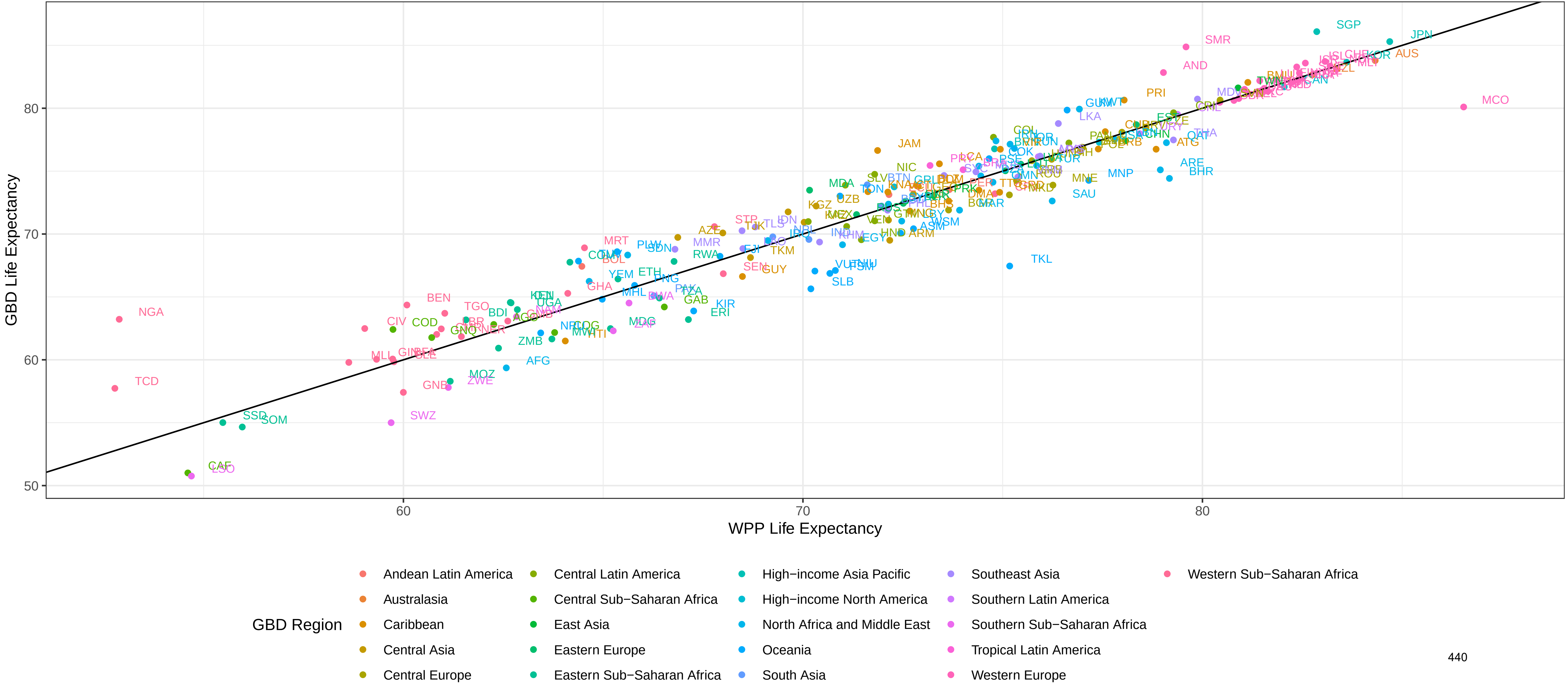

Appendix 2 Figure S14: Comparison of life expectancy at birth estimates for countries in GBD 2021 and WPP 2022 for 2021, both sexes combined

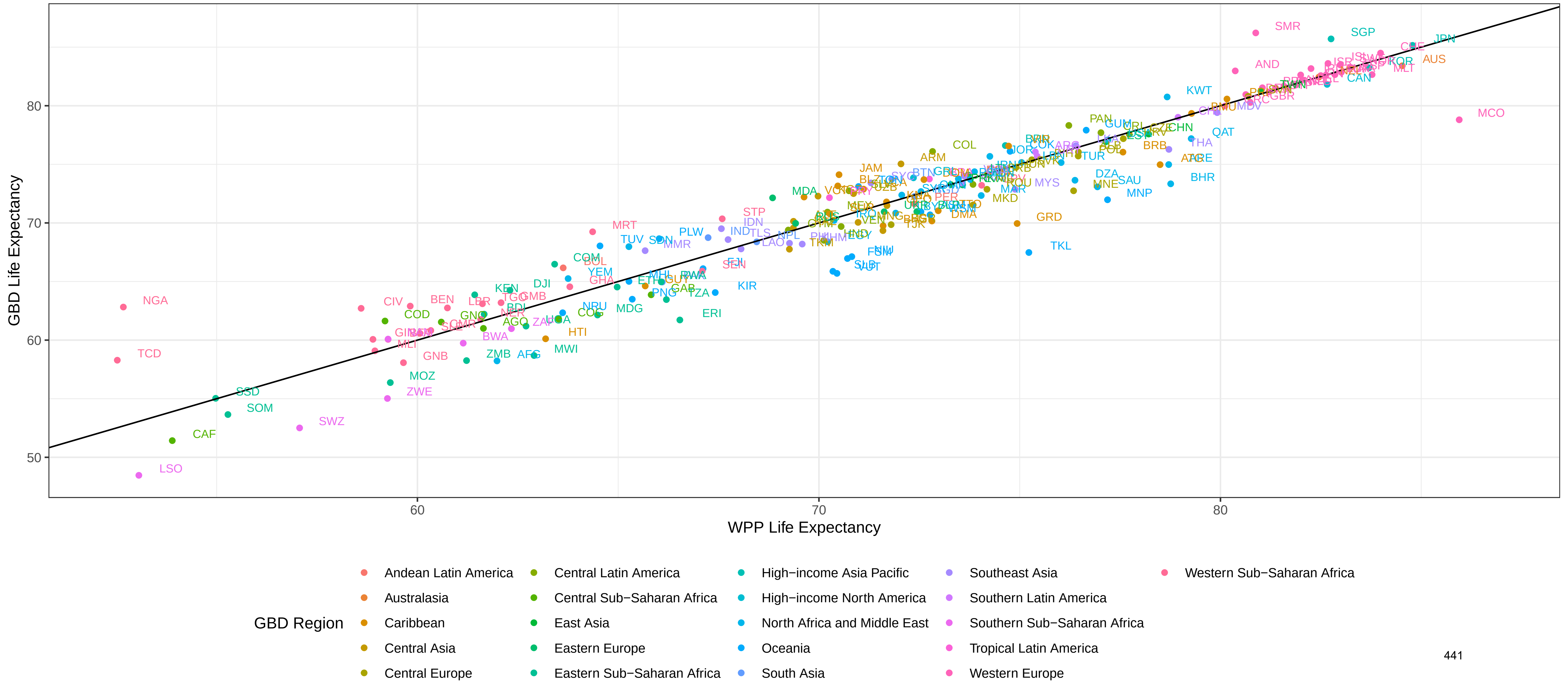

Appendix 2 Figure S15a: Percent difference between GBD 2021 and WPP 2022 population estimates, by location and sex, all ages, for 1950, 1975, 2000, 2015, 2021

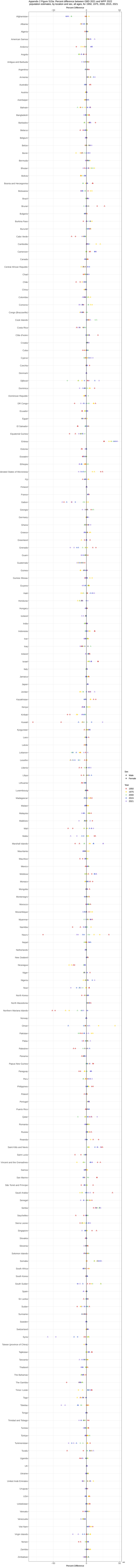

Appendix 2 Figure S15b: Percent difference between GBD 2021 and WPP 2022 population estimates, by location and sex, ages <15 and >65 for 2021

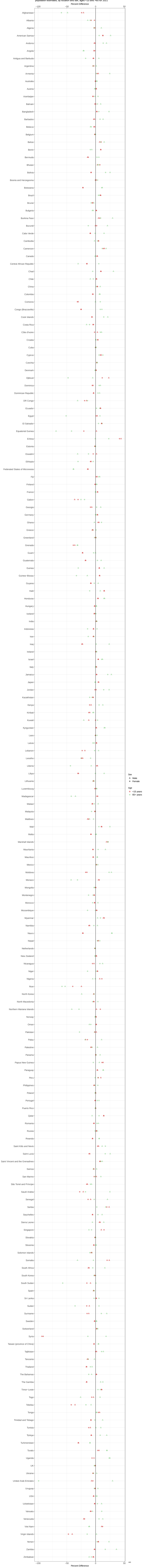

Appendix 2 Table S1: Under-5 death numbers (in thousands) with 95% UIs by location, both sexes combined, 1950, 1960, 1970, 1980, 1990, 2000, 2010, 2019, 2020, 2021

Location	1950	1960	1970	1980	1990	2000	2010	2019	2020	2021
Global	20031·2 (17201·9 to 23013·8)	31582·9 (24999·9 to 43648·6)	16069·3 (15098·7 to 17082·4)	13517·5 (12694·3 to 14302·7)	11283·2 (10716·3 to 11891·5)	9213·1 (8733·6 to 9728·9)	7257·2 (6723·6 to 7829·9)	5210·9 (4497·5 to 6012·4)	4894·9 (4189·4 to 5710·0)	4664·6 (3977·5 to 5504·0)
Central Europe, eastern Europe, and central Asia	899·3 (803·8 to 1009·1)	541·5 (506·8 to 578·0)	302·9 (281·4 to 327·0)	267·1 (255·4 to 279·9)	213·0 (203·7 to 223·4)	123·0 (115·8 to 130·5)	91·9 (85·2 to 99·0)	66·5 (58·6 to 75·1)	61·7 (53·9 to 70·5)	59·0 (51·7 to 67·6)
Central Asia	219·4 (184·6 to 259·6)	198·2 (171·5 to 226·8)	124·0 (107·5 to 142·7)	114·4 (106·3 to 123·6)	111·1 (103·7 to 119·1)	72·0 (65·8 to 78·8)	58·2 (52·0 to 64·9)	47·6 (40·6 to 55·6)	44·7 (38·0 to 52·5)	42·6 (36·0 to 50·4)
Armenia	14·3 (11·7 to 17·5)	11·7 (9·8 to 13·8)	5·5 (4·6 to 6·5)	4·6 (4·0 to 5·3)	3·2 (2·8 to 3·7)	1·3 (1·1 to 1·4)	0·8 (0·7 to 0·9)	0·5 (0·4 to 0·6)	0·5 (0·4 to 0·6)	0·4 (0·3 to 0·5)
Azerbaijan	49·3 (40·9 to 58·1)	45·7 (39·4 to 52·0)	22·8 (19·8 to 26·8)	14·1 (12·3 to 16·0)	14·6 (12·9 to 16·3)	8·8 (7·8 to 10·0)	6·9 (5·9 to 8·1)	4·7 (3·8 to 5·6)	4·3 (3·5 to 5·2)	3·9 (3·2 to 4·7)
Georgia	20·3 (16·3 to 24·8)	14·6 (12·3 to 17·1)	7·8 (6·3 to 9·6)	5·1 (4·5 to 5·9)	3·7 (3·2 to 4·2)	1·8 (1·6 to 2·0)	0·9 (0·8 to 1·1)	0·5 (0·4 to 0·6)	0·5 (0·4 to 0·6)	0·4 (0·3 to 0·6)
Kazakhstan	30·3 (24·3 to 37·9)	30·5 (25·8 to 35·8)	17·5 (14·6 to 20·8)	16·6 (14·7 to 18·4)	13·7 (12·3 to 15·2)	8·0 (7·0 to 9·1)	8·3 (7·0 to 9·6)	4·7 (3·9 to 5·6)	4·5 (3·7 to 5·3)	4·1 (3·4 to 5·0)
Kyrgyzstan	12·8 (9·6 to 16·3)	12·2 (10·3 to 14·2)	8·4 (6·9 to 10·1)	8·5 (7·5 to 9·6)	7·6 (6·9 to 8·4)	4·6 (4·1 to 5·1)	4·0 (3·9 to 4·2)	3·0 (2·8 to 3·2)	2·8 (2·6 to 3·0)	2·7 (2·3 to 3·0)
Mongolia	9·0 (7·0 to 11·3)	8·3 (7·0 to 9·5)	7·0 (6·1 to 8·0)	6·9 (6·1 to 7·7)	6·1 (5·6 to 6·7)	2·9 (2·6 to 3·2)	2·1 (1·8 to 2·4)	1·5 (1·2 to 1·8)	1·4 (1·1 to 1·6)	1·3 (1·1 to 1·6)
Tajikistan	25·7 (20·4 to 31·8)	23·5 (19·9 to 27·4)	15·0 (12·7 to 17·6)	15·0 (13·3 to 16·8)	16·5 (15·0 to 17·9)	12·8 (11·4 to 14·3)	10·4 (8·9 to 12·0)	10·9 (9·0 to 13·1)	10·1 (8·3 to 12·2)	9·7 (8·0 to 11·9)
Turkmenistan	11·4 (9·2 to 13·9)	10·2 (8·8 to 11·7)	7·2 (5·9 to 8·9)	8·4 (7·3 to 9·7)	8·8 (7·8 to 9·9)	6·6 (5·8 to 7·4)	4·5 (3·9 to 5·1)	3·4 (2·7 to 4·1)	3·2 (2·6 to 3·8)	3·0 (2·4 to 3·7)
Uzbekistan	46·5 (35·3 to 59·9)	41·6 (34·0 to 49·3)	32·6 (27·7 to 38·4)	35·3 (31·7 to 38·8)	37·0 (33·7 to 40·6)	25·1 (22·1 to 28·4)	20·4 (17·2 to 23·8)	18·5 (15·4 to 22·3)	17·6 (14·5 to 21·3)	17·0 (14·0 to 20·7)
Central Europe	397·6 (380·9 to 415·0)	153·5 (149·8 to 157·2)	92·6 (90·5 to 94·6)	61·7 (60·3 to 63·0)	38·8 (37·8 to 39·7)	16·6 (16·0 to 17·2)	9·1 (8·9 to 9·4)	6·0 (5·6 to 6·4)	5·6 (5·2 to 6·0)	5·3 (4·8 to 5·9)
Albania	12·1 (9·8 to 15·0)	9·1 (7·7 to 10·6)	5·8 (4·9 to 6·7)	3·4 (2·9 to 4·0)	3·2 (2·8 to 3·6)	1·5 (1·3 to 1·8)	0·6 (0·5 to 0·7)	0·4 (0·4 to 0·5)	0·4 (0·3 to 0·5)	0·4 (0·3 to 0·4)
Bosnia and Herzegovina	27·6 (24·1 to 31·4)	14·1 (12·7 to 15·6)	6·4 (5·8 to 6·9)	3·1 (2·7 to 3·4)	1·1 (1·1 to 1·2)	0·4 (0·4 to 0·5)	0·2 (0·2 to 0·3)	0·2 (0·2 to 0·2)	0·2 (0·2 to 0·2)	0·1 (0·1 to 0·2)
Bulgaria	33·2 (31·3 to 35·2)	7·8 (7·6 to 8·0)	4·4 (4·3 to 4·5)	3·2 (3·1 to 3·3)	2·0 (1·9 to 2·1)	1·2 (1·1 to 1·3)	0·8 (0·8 to 0·9)	0·4 (0·4 to 0·5)	0·4 (0·3 to 0·4)	0·4 (0·3 to 0·4)
Croatia	19·5 (18·3 to 20·9)	6·3 (6·1 to 6·4)	2·8 (2·7 to 2·9)	1·5 (1·5 to 1·6)	0·7 (0·6 to 0·7)	0·4 (0·3 to 0·4)	0·2 (0·2 to 0·2)	0·2 (0·1 to 0·2)	0·2 (0·1 to 0·2)	0·2 (0·1 to 0·2)
Czechia	9·8 (9·1 to 10·4)	3·1 (3·0 to 3·2)	3·5 (3·4 to 3·6)	2·9 (2·8 to 3·0)	1·5 (1·5 to 1·6)	0·5 (0·4 to 0·5)	0·4 (0·4 to 0·4)	0·3 (0·3 to 0·4)	0·3 (0·3 to 0·3)	0·3 (0·2 to 0·3)
Hungary	25·0 (23·1 to 26·8)	7·8 (7·5 to 8·0)	6·1 (5·9 to 6·3)	3·7 (3·6 to 3·9)	2·1 (2·0 to 2·2)	1·0 (1·0 to 1·1)	0·5 (0·5 to 0·6)	0·4 (0·4 to 0·4)	0·4 (0·3 to 0·4)	0·4 (0·3 to 0·4)
Montenegro	1·3 (1·1 to 1·5)	0·8 (0·8 to 0·9)	0·5 (0·4 to 0·5)	0·3 (0·3 to 0·3)	0·2 (0·1 to 0·2)	0·1 (0·1 to 0·1)	0·1 (0·1 to 0·1)	0·0 (0·0 to 0·0)	0·0 (0·0 to 0·0)	0·0 (0·0 to 0·0)
North Macedonia	13·8 (11·9 to 16·0)	7·3 (6·5 to 8·1)	4·0 (3·7 to 4·3)	2·5 (2·2 to 2·7)	1·3 (1·3 to 1·4)	0·4 (0·4 to 0·4)	0·2 (0·2 to 0·2)	0·1 (0·1 to 0·2)	0·1 (0·1 to 0·1)	0·1 (0·1 to 0·1)
Poland	131·0 (126·9 to 135·2)	42·1 (41·4 to 42·8)	20·4 (20·0 to 20·9)	16·9 (16·5 to 17·3)	10·1 (9·8 to 10·3)	3·5 (3·4 to 3·7)	2·4 (2·3 to 2·5)	1·6 (1·5 to 1·7)	1·5 (1·4 to 1·6)	1·5 (1·3 to 1·7)
Romania	60·3 (51·4 to 70·5)	31·9 (30·9 to 32·8)	24·2 (23·6 to 24·8)	14·3 (13·8 to 14·7)	10·7 (10·3 to 11·1)	5·1 (4·9 to 5·3)	2·5 (2·3 to 2·6)	1·4 (1·3 to 1·5)	1·3 (1·2 to 1·4)	1·2 (1·1 to 1·3)
Serbia	46·5 (44·1 to 49·2)	16·7 (16·3 to 17·0)	10·2 (9·9 to 10·5)	6·3 (6·1 to 6·5)	3·9 (3·8 to 4·1)	1·5 (1·3 to 1·8)	0·6 (0·5 to 0·6)	0·4 (0·4 to 0·4)	0·4 (0·3 to 0·4)	0·3 (0·3 to 0·4)
Slovakia	9·1 (8·5 to 9·6)	3·1 (3·0 to 3·2)	2·5 (2·4 to 2·5)	2·2 (2·2 to 2·3)	1·1 (1·1 to 1·2)	0·5 (0·5 to 0·6)	0·4 (0·4 to 0·4)	0·4 (0·3 to 0·4)	0·3 (0·3 to 0·4)	0·3 (0·3 to 0·4)
Slovenia	5·0 (4·7 to 5·4)	2·0 (1·9 to 2·0)	1·0 (1·0 to 1·1)	0·5 (0·5 to 0·6)	0·2 (0·2 to 0·2)	0·1 (0·1 to 0·1)	0·1 (0·1 to 0·1)	0·0 (0·0 to 0·0)	0·0 (0·0 to 0·0)	0·0 (0·0 to 0·0)
Eastern Europe	282·3 (219·8 to 361·2)	189·8 (182·6 to 197·5)	86·3 (81·5 to 91·6)	91·1 (87·4 to 95·2)	63·1 (60·6 to 65·7)	34·4 (33·4 to 35·5)	24·6 (23·9 to 25·2)	12·8 (12·2 to 13·5)	11·4 (10·8 to 12·0)	11·1 (10·3 to 11·9)
Belarus	16·3 (12·2 to 21·0)	11·5 (9·6 to 13·5)	5·1 (4·3 to 6·0)	3·9 (3·3 to 4·5)	2·7 (2·3 to 3·1)	1·6 (1·3 to 1·8)	0·9 (0·7 to 1·0)	0·4 (0·4 to 0·6)	0·4 (0·3 to 0·5)	0·3 (0·3 to 0·4)
Estonia	1·1 (0·8 to 1·5)	0·7 (0·7 to 0·8)	0·5 (0·4 to 0·5)	0·5 (0·5 to 0·5)	0·4 (0·4 to 0·4)	0·1 (0·1 to 0·2)	0·1 (0·1 to 0·1)	0·0 (0·0 to 0·0)	0·0 (0·0 to 0·0)	0·0 (0·0 to 0·0)
Latvia	1·4 (1·0 to 1·9)	1·1 (1·1 to 1·2)	0·7 (0·7 to 0·8)	0·8 (0·7 to 0·8)	0·7 (0·6 to 0·7)	0·3 (0·3 to 0·3)	0·2 (0·1 to 0·2)	0·1 (0·1 to 0·1)	0·1 (0·1 to 0·1)	0·1 (0·1 to 0·1)
Lithuania	4·8 (3·6 to 6·2)	2·9 (2·8 to 3·0)	1·3 (1·2 to 1·3)	1·0 (1·0 to 1·1)	0·8 (0·8 to 0·9)	0·4 (0·4 to 0·4)	0·2 (0·2 to 0·2)	0·1 (0·1 to 0·1)	0·1 (0·1 to 0·1)	0·1 (0·1 to 0·1)
Moldova	14·5 (11·6 to 18·1)	9·8 (8·2 to 11·5)	4·9 (3·9 to 6·0)	4·3 (3·8 to 5·0)	2·6 (2·3 to 3·0)	1·2 (1·0 to 1·3)	0·7 (0·6 to 0·8)	0·4 (0·3 to 0·5)	0·4 (0·3 to 0·5)	0·3 (0·2 to 0·4)
Russia	198·6 (142·8 to 270·5)	129·0 (126·4 to 131·5)	53·9 (52·4 to 55·5)	61·7 (60·3 to 63·2)	43·2 (42·3 to 44·2)	24·9 (24·1 to 25·7)	17·3 (16·8 to 17·8)	9·1 (8·7 to 9·6)	8·0 (7·6 to 8·4)	8·1 (7·6 to 8·6)
Ukraine	45·7 (34·8 to 59·1)	34·7 (29·6 to 40·8)	20·0 (16·6 to 23·8)	18·9 (15·9 to 22·0)	12·7 (11·2 to 14·5)	6·0 (5·8 to 6·2)	5·3 (5·1 to 5·5)	2·7 (2·4 to 3·0)	2·5 (2·1 to 2·8)	2·2 (1·7 to 2·6)
High income	843·0 (806·5 to 882·8)	640·7 (628·2 to 653·6)	408·7 (399·7 to 418·0)	209·8 (205·6 to 214·4)	137·6 (135·4 to 140·0)	89·0 (87·7 to 90·3)	71·2 (70·1 to 72·4)	55·3 (54·1 to 56·5)	51·3 (49·1 to 53·6)	47·9 (44·0 to 52·2)
Australasia	7·6 (7·3 to 8·0)	7·4 (7·3 to 7·6)	6·8 (6·7 to 7·0)	3·7 (3·6 to 3·8)	3·0 (3·0 to 3·2)	2·0 (1·9 to 2·1)	1·9 (1·8 to 2·0)	1·5 (1·4 to 1·6)	1·4 (1·3 to 1·5)	1·2 (1·0 to 1·4)
Australia	6·0 (5·6 to 6·3)	5·7 (5·6 to 5·9)	5·6 (5·4 to 5·7)	2·9 (2·8 to 3·0)	2·4 (2·3 to 2·5)	1·6 (1·5 to 1·6)	1·5 (1·4 to 1·5)	1·2 (1·1 to 1·3)	1·1 (1·0 to 1·2)	0·9 (0·7 to 1·0)
New Zealand	1·7 (1·5 to 1·8)	1·7 (1·7 to 1·8)	1·3 (1·2 to 1·3)	0·8 (0·7 to 0·8)	0·6 (0·6 to 0·7)	0·4 (0·4 to 0·5)	0·4 (0·4 to 0·5)	0·3 (0·3 to 0·3)	0·3 (0·2 to 0·3)	0·3 (0·3 to 0·3)
High-income Asia Pacific	289·4 (262·6 to 321·5)	162·4 (151·0 to 174·2)	89·5 (81·7 to 97·0)	37·8 (34·5 to 41·4)	16·5 (15·2 to 17·9)	9·6 (9·4 to 9·9)	5·5 (5·3 to 5·7)	3·5 (3·3 to 3·6)	3·0 (2·8 to 3·2)	2·7 (2·5 to 2·9)
Brunei	0·6 (0·5 to 0·6)	0·5 (0·4 to 0·5)	0·2 (0·2 to 0·3)	0·1 (0·1 to 0·1)	0·1 (0·1 to 0·1)	0·1 (0·1 to 0·1)	0·1 (0·1 to 0·1)	0·1 (0·1 to 0·1)	0·1 (0·1 to 0·1)	0·1 (0·0 to 0·1)
Japan	141·6 (133·9 to 149·7)	62·3 (60·6 to 64·2)	32·7 (31·7 to 33·6)	15·9 (15·4 to 16·4)	7·9 (7·6 to 8·1)	5·3 (5·1 to 5·5)	3·4 (3·3 to 3·6)	2·3 (2·2 to 2·4)	2·0 (1·8 to 2·2)	1·8 (1·6 to 2·1)
Aichi	5·1 (4·3 to 5·8)	2·7 (2·4 to 3·0)	1·7 (1·6 to 1·9)	0·8 (0·8 to 0·9)	0·4 (0·4 to 0·4)	0·3 (0·3 to 0·3)	0·2 (0·2 to 0·2)	0·1 (0·1 to 0·2)	0·1 (0·1 to 0·1)	0·1 (0·1 to 0·1)
Akita	2·1 (1·8 to 2·4)	0·9 (0·8 to 1·0)	0·4 (0·3 to 0·4)	0·2 (0·2 to 0·2)	0·1 (0·1 to 0·1)	0·0 (0·0 to 0·0)	0·0 (0·0 to 0·0)	0·0 (0·0 to 0·0)	0·0 (0·0 to 0·0)	0·0 (0·0 to 0·0)
Aomori	2·6 (2·2 to 2·9)	1·2 (1·1 to 1·3)	0·5 (0·5 to 0·6)	0·3 (0·2 to 0·3)	0·1 (0·1 to 0·1)	0·1 (0·1 to 0·1)	0·0 (0·0 to 0·0)	0·0 (0·0 to 0·0)	0·0 (0·0 to 0·0)	0·0 (0·0 to 0·0)
Chiba	3·4 (2·9 to 4·0)	1·4 (1·3 to 1·6)	1·0 (0·9 to 1·2)	0·6 (0·6 to 0·7)	0·3 (0·3 to 0·3)	0·2 (0·2 to 0·3)	0·2 (0·2 to 0·2)	0·1 (0·1 to 0·1)	0·1 (0·1 to 0·1)	0·1 (0·1 to 0·1)
Ehime	2·6 (2·3 to 2·9)	1·0 (0·9 to 1·1)	0·4 (0·4 to 0·5)	0·2 (0·2 to 0·2)	0·1 (0·1 to 0·1)	0·1 (0·1 to 0·1)	0·0 (0·0 to 0·0)	0·0 (0·0 to 0·0)	0·0 (0·0 to 0·0)	0·0 (0·0 to 0·0)
Fukui	1·1 (1·0 to 1·4)	0·5 (0·4 to 0·5)	0·2 (0·2 to 0·2)	0·1 (0·1 to 0·1)	0·1 (0·1 to 0·1)	0·0 (0·0 to 0·0)	0·0 (0·0 to 0·0)	0·0 (0·0 to 0·0)	0·0 (0·0 to 0·0)	0·0 (0·0 to 0·0)
Fukuoka	5·6 (4·7 to 6·5)	2·4 (2·2 to 2·7)	1·2 (1·0 to 1·3)	0·6 (0·6 to 0·6)	0·3 (0·3 to 0·3)	0·2 (0·2 to 0·2)	0·2 (0·1 to 0·2)	0·1 (0·1 to 0·1)	0·1 (0·1 to 0·1)	0·1 (0·1 to 0·1)
Fukushima	4·6 (4·0 to 5·2)	1·7 (1·6 to 1·9)	0·7 (0·7 to 0·8)	0·4 (0·3 to 0·4)	0·2 (0·2 to 0·2)	0·1 (0·1 to 0·1)	0·1 (0·1 to 0·1)	0·0 (0·0 to 0·0)	0·0 (0·0 to 0·0)	0·0 (0·0 to 0·0)
Gifu	2·3 (2·0 to 2·7)	1·1 (1·0 to 1·2)	0·6 (0·5 to 0·6)	0·3 (0·3 to 0·3)	0·1 (0·1 to 0·1)	0·1 (0·1 to 0·1)	0·1 (0·1 to 0·1)	0·0 (0·0 to 0·0)	0·0 (0·0 to 0·0)	0·0 (0·0 to 0·0)
Gunma	2·9 (2·5 to 3·4)	1·1 (1·0 to 1·2)	0·6 (0·5 to 0·6)	0·3 (0·2 to 0·3)	0·1 (0·1 to 0·1)	0·1 (0·1 to 0·1)	0·1 (0·0 to 0·1)	0·0 (0·0 to 0·0)	0·0 (0·0 to 0·0)	0·0 (0·0 to 0·0)
Hiroshima	3·6 (3·2 to 4·1)	1·5 (1·3 to 1·6)	0·8 (0·7 to 0·9)	0·4 (0·4 to 0·4)	0·2 (0·2 to 0·2)	0·1 (0·1 to 0·1)	0·1 (0·1 to 0·1)	0·1 (0·0 to 0·1)	0·0 (0·0 to 0·1)	0·0 (0·0 to 0·0)
Hokkaidō	6·9 (5·8 to 8·2)	3·3 (2·9 to 3·6)	1·7 (1·5 to 1·9)	0·8 (0·8 to 0·8)	0·3 (0·3 to 0·4)	0·2 (0·2 to 0·2)	0·1 (0·1 to 0·1)	0·1 (0·1 to 0·1)	0·1 (0·1 to 0·1)	0·1 (0·1 to 0·1)
Hyōgo	4·9 (4·1 to 5·7)	2·3 (2·1 to 2·6)	1·4 (1·2 to 1·6)	0·7 (0·6 to 0·7)	0·3 (0·3 to 0·4)	0·2 (0·2 to 0·2)	0·1 (0·1 to 0·1)	0·1 (0·1 to 0·1)	0·1 (0·1 to 0·1)	0·1 (0·1 to 0·1)
Ibaraki	4·5 (3·7 to 5·3)	1·6 (1·4 to 1·8)	0·8 (0·7 to 0·9)	0·4 (0·4 to 0·5)	0·2 (0·2 to 0·2)	0·1 (0·1 to 0·1)	0·1 (0·1 to 0·1)	0·1 (0·1 to 0·1)	0·1 (0·0 to 0·1)	0·0 (0·0 to 0·1)
Ishikawa	1·5 (1·3 to 1·8)	0·6 (0·6 to 0·7)	0·3 (0·3 to 0·4)	0·2 (0·1 to 0·2)	0·1 (0·1 to 0·1)	0·1 (0·0 to 0·1)	0·0 (0·0 to 0·0)	0·0 (0·0 to 0·0)	0·0 (0·0 to 0·0)	0·0 (0·0 to 0·0)
Iwate	3·0 (2·6 to 3·4)	1·3 (1·2 to 1·4)	0·6 (0·5 to 0·6)	0·2 (0·2 to 0·3)	0·1 (0·1 to 0·1)	0·1 (0·0 to 0·1)	0·0 (0·0 to 0·0)	0·0 (0·0 to 0·0)	0·0 (0·0 to 0·0)	0·0 (0·0 to 0·0)
Kagawa	1·4 (1·2 to 1·7)	0·6 (0·5 to 0·6)	0·3 (0·2 to 0·3)	0·1 (0·1 to 0·1)	0·1 (0·1 to 0·1)	0·0 (0·0 to 0·0)	0·0 (0·0 to 0·0)	0·0 (0·0 to 0·0)	0·0 (0·0 to 0·0)	0·0 (0·0 to 0·0)
Kagoshima	4·5 (4·0 to 5·2)	1·7 (1·5 to 1·8)	0·6 (0·6 to 0·7)	0·3 (0·3 to 0·3)	0·1 (0·1 to 0·1)	0·1 (0·1 to 0·1)	0·1 (0·0 to 0·1)	0·0 (0·0 to 0·0)	0·0 (0·0 to 0·0)	0·0 (0·0 to 0·0)
Kanagawa	3·6 (2·8 to 4·5)	2·0 (1·7 to 2·3)	1·6 (1·4 to 1·7)	0·9 (0·8 to 0·9)	0·5 (0·4 to 0·5)	0·4 (0·3 to 0·4)	0·3 (0·2 to 0·3)	0·2 (0·2 to 0·2)	0·1 (0·1 to 0·1)	0·1 (0·1 to 0·1)
Kōchi	1·8 (1·5 to 2·0)	0·6 (0·6 to 0·7)	0·3 (0·2 to 0·3)	0·1 (0·1 to 0·1)	0·1 (0·1 to 0·1)	0·0 (0·0 to 0·0)	0·0 (0·0 to 0·0)	0·0 (0·0 to 0·0)	0·0 (0·0 to 0·0)	0·0 (0·0 to 0·0)
Kumamoto	3·5 (3·0 to 3·9)	1·4 (1·3 to 1·5)	0·6 (0·5 to 0·6)	0·3 (0·3 to 0·3)	0·1 (0·1 to 0·1)	0·1 (0·1 to 0·1)	0·1 (0·0 to 0·1)	0·0 (0·0 to 0·0)	0·0 (0·0 to 0·0)	0·0 (0·0 to 0·0)

Kyōto	2·6 (2·2 to 2·9)	1·1 (1·0 to 1·2)	0·6 (0·5 to 0·6)	0·3 (0·3 to 0·3)	0·2 (0·1 to 0·2)	0·1 (0·1 to 0·1)	0·1 (0·1 to 0·1)	0·0 (0·0 to 0·1)	0·0 (0·0 to 0·0)	0·0 (0·0 to 0·0)
Mie	2·3 (2·0 to 2·6)	1·0 (0·9 to 1·1)	0·5 (0·4 to 0·5)	0·2 (0·2 to 0·2)	0·1 (0·1 to 0·1)	0·1 (0·1 to 0·1)	0·1 (0·1 to 0·1)	0·0 (0·0 to 0·0)	0·0 (0·0 to 0·0)	0·0 (0·0 to 0·0)
Miyagi	2·9 (2·5 to 3·3)	1·2 (1·2 to 1·3)	0·6 (0·5 to 0·6)	0·3 (0·3 to 0·3)	0·1 (0·1 to 0·2)	0·1 (0·1 to 0·1)	0·1 (0·1 to 0·1)	0·0 (0·0 to 0·0)	0·0 (0·0 to 0·0)	0·0 (0·0 to 0·0)
Miyazaki	2·2 (1·9 to 2·4)	0·9 (0·8 to 1·0)	0·4 (0·3 to 0·4)	0·2 (0·2 to 0·2)	0·1 (0·1 to 0·1)	0·1 (0·0 to 0·1)	0·0 (0·0 to 0·0)	0·0 (0·0 to 0·0)	0·0 (0·0 to 0·0)	0·0 (0·0 to 0·0)
Nagano	3·8 (3·3 to 4·4)	1·4 (1·3 to 1·5)	0·6 (0·6 to 0·6)	0·3 (0·3 to 0·3)	0·1 (0·1 to 0·1)	0·1 (0·1 to 0·1)	0·0 (0·0 to 0·1)	0·0 (0·0 to 0·0)	0·0 (0·0 to 0·0)	0·0 (0·0 to 0·0)
Nagasaki	3·1 (2·7 to 3·5)	1·2 (1·1 to 1·3)	0·5 (0·5 to 0·5)	0·2 (0·2 to 0·2)	0·1 (0·1 to 0·1)	0·1 (0·1 to 0·1)	0·0 (0·0 to 0·0)	0·0 (0·0 to 0·0)	0·0 (0·0 to 0·0)	0·0 (0·0 to 0·0)
Nara	1·1 (0·9 to 1·3)	0·5 (0·4 to 0·5)	0·3 (0·2 to 0·3)	0·2 (0·2 to 0·2)	0·1 (0·1 to 0·1)	0·1 (0·0 to 0·1)	0·0 (0·0 to 0·0)	0·0 (0·0 to 0·0)	0·0 (0·0 to 0·0)	0·0 (0·0 to 0·0)
Niigata	4·5 (3·9 to 5·1)	1·7 (1·6 to 1·9)	0·7 (0·7 to 0·8)	0·3 (0·3 to 0·4)	0·1 (0·1 to 0·2)	0·1 (0·1 to 0·1)	0·1 (0·0 to 0·1)	0·0 (0·0 to 0·0)	0·0 (0·0 to 0·0)	0·0 (0·0 to 0·0)
Ōita	2·2 (1·9 to 2·5)	0·9 (0·8 to 0·9)	0·4 (0·3 to 0·4)	0·2 (0·2 to 0·2)	0·1 (0·1 to 0·1)	0·0 (0·0 to 0·1)	0·0 (0·0 to 0·0)	0·0 (0·0 to 0·0)	0·0 (0·0 to 0·0)	0·0 (0·0 to 0·0)
Okayama	2·5 (2·2 to 2·9)	1·0 (1·0 to 1·1)	0·5 (0·4 to 0·5)	0·2 (0·2 to 0·2)	0·1 (0·1 to 0·1)	0·1 (0·1 to 0·1)	0·1 (0·0 to 0·1)	0·0 (0·0 to 0·0)	0·0 (0·0 to 0·0)	0·0 (0·0 to 0·0)
Okinawa	2·1 (1·8 to 2·4)	0·9 (0·8 to 0·9)	0·5 (0·4 to 0·5)	0·2 (0·2 to 0·2)	0·1 (0·1 to 0·1)	0·1 (0·1 to 0·1)	0·1 (0·1 to 0·1)	0·0 (0·0 to 0·0)	0·0 (0·0 to 0·0)	0·0 (0·0 to 0·0)
Ōsaka	5·8 (4·7 to 7·2)	3·4 (2·9 to 3·8)	2·3 (2·1 to 2·5)	1·1 (1·0 to 1·1)	0·6 (0·5 to 0·6)	0·4 (0·4 to 0·4)	0·2 (0·2 to 0·2)	0·2 (0·1 to 0·2)	0·1 (0·1 to 0·1)	0·1 (0·1 to 0·1)
Saga	1·8 (1·6 to 2·1)	0·7 (0·6 to 0·7)	0·3 (0·2 to 0·3)	0·1 (0·1 to 0·1)	0·1 (0·1 to 0·1)	0·0 (0·0 to 0·0)	0·0 (0·0 to 0·0)	0·0 (0·0 to 0·0)	0·0 (0·0 to 0·0)	0·0 (0·0 to 0·0)
Saitama	3·6 (3·0 to 4·2)	1·6 (1·4 to 1·8)	1·3 (1·2 to 1·5)	0·7 (0·7 to 0·8)	0·4 (0·4 to 0·4)	0·3 (0·3 to 0·3)	0·2 (0·2 to 0·2)	0·1 (0·1 to 0·1)	0·1 (0·1 to 0·1)	0·1 (0·1 to 0·1)
Shiga	1·5 (1·2 to 1·7)	0·6 (0·5 to 0·6)	0·3 (0·2 to 0·3)	0·2 (0·1 to 0·2)	0·1 (0·1 to 0·1)	0·1 (0·1 to 0·1)	0·0 (0·0 to 0·0)	0·0 (0·0 to 0·0)	0·0 (0·0 to 0·0)	0·0 (0·0 to 0·0)
Shimane	1·6 (1·3 to 1·9)	0·6 (0·5 to 0·7)	0·2 (0·2 to 0·3)	0·1 (0·1 to 0·1)	0·1 (0·0 to 0·1)	0·0 (0·0 to 0·0)	0·0 (0·0 to 0·0)	0·0 (0·0 to 0·0)	0·0 (0·0 to 0·0)	0·0 (0·0 to 0·0)
Shizuoka	3·8 (3·3 to 4·3)	1·7 (1·5 to 1·8)	0·9 (0·8 to 1·0)	0·4 (0·4 to 0·5)	0·2 (0·2 to 0·2)	0·1 (0·1 to 0·2)	0·1 (0·1 to 0·1)	0·1 (0·1 to 0·1)	0·1 (0·1 to 0·1)	0·1 (0·0 to 0·1)
Tochigi	3·0 (2·6 to 3·4)	1·1 (1·0 to 1·2)	0·5 (0·5 to 0·6)	0·3 (0·3 to 0·3)	0·1 (0·1 to 0·2)	0·1 (0·1 to 0·1)	0·1 (0·1 to 0·1)	0·0 (0·0 to 0·0)	0·0 (0·0 to 0·0)	0·0 (0·0 to 0·0)
Tokushima	1·4 (1·2 to 1·7)	0·5 (0·5 to 0·6)	0·2 (0·2 to 0·3)	0·1 (0·1 to 0·1)	0·1 (0·0 to 0·1)	0·0 (0·0 to 0·0)	0·0 (0·0 to 0·0)	0·0 (0·0 to 0·0)	0·0 (0·0 to 0·0)	0·0 (0·0 to 0·0)
Tōkyō	7·9 (6·1 to 10·1)	5·1 (4·3 to 5·9)	3·0 (2·7 to 3·4)	1·3 (1·2 to 1·3)	0·6 (0·6 to 0·7)	0·4 (0·4 to 0·5)	0·3 (0·3 to 0·3)	0·2 (0·2 to 0·2)	0·2 (0·2 to 0·2)	0·2 (0·2 to 0·2)
Tottori	1·1 (0·9 to 1·3)	0·4 (0·4 to 0·5)	0·2 (0·2 to 0·2)	0·1 (0·1 to 0·1)	0·0 (0·0 to 0·0)	0·0 (0·0 to 0·0)	0·0 (0·0 to 0·0)	0·0 (0·0 to 0·0)	0·0 (0·0 to 0·0)	0·0 (0·0 to 0·0)
Toyama	1·7 (1·5 to 1·9)	0·7 (0·7 to 0·8)	0·4 (0·3 to 0·4)	0·2 (0·1 to 0·2)	0·1 (0·1 to 0·1)	0·0 (0·0 to 0·1)	0·0 (0·0 to 0·0)	0·0 (0·0 to 0·0)	0·0 (0·0 to 0·0)	0·0 (0·0 to 0·0)
Wakayama	1·8 (1·5 to 2·0)	0·7 (0·6 to 0·8)	0·3 (0·3 to 0·4)	0·2 (0·1 to 0·2)	0·1 (0·1 to 0·1)	0·0 (0·0 to 0·0)	0·0 (0·0 to 0·0)	0·0 (0·0 to 0·0)	0·0 (0·0 to 0·0)	0·0 (0·0 to 0·0)
Yamagata	2·4 (2·1 to 2·7)	1·0 (0·9 to 1·0)	0·4 (0·3 to 0·4)	0·2 (0·2 to 0·2)	0·1 (0·1 to 0·1)	0·0 (0·0 to 0·1)	0·0 (0·0 to 0·0)	0·0 (0·0 to 0·0)	0·0 (0·0 to 0·0)	0·0 (0·0 to 0·0)
Yamaguchi	2·5 (2·2 to 2·9)	1·1 (1·0 to 1·1)	0·5 (0·4 to 0·5)	0·2 (0·2 to 0·2)	0·1 (0·1 to 0·1)	0·1 (0·1 to 0·1)	0·0 (0·0 to 0·0)	0·0 (0·0 to 0·0)	0·0 (0·0 to 0·0)	0·0 (0·0 to 0·0)
Yamanashi	1·4 (1·1 to 1·6)	0·5 (0·4 to 0·6)	0·2 (0·2 to 0·2)	0·1 (0·1 to 0·1)	0·1 (0·1 to 0·1)	0·0 (0·0 to 0·0)	0·0 (0·0 to 0·0)	0·0 (0·0 to 0·0)	0·0 (0·0 to 0·0)	0·0 (0·0 to 0·0)
South Korea	141·8 (116·4 to 173·0)	96·7 (85·6 to 108·5)	55·2 (47·8 to 62·7)	21·1 (17·8 to 24·7)	8·1 (6·9 to 9·5)	4·1 (3·9 to 4·2)	1·9 (1·8 to 2·0)	1·0 (1·0 to 1·1)	0·9 (0·8 to 0·9)	0·7 (0·5 to 0·8)
Singapore	5·4 (5·1 to 5·8)	3·0 (2·9 to 3·1)	1·3 (1·2 to 1·4)	0·7 (0·6 to 0·7)	0·4 (0·4 to 0·5)	0·2 (0·2 to 0·2)	0·1 (0·1 to 0·2)	0·1 (0·1 to 0·1)	0·1 (0·1 to 0·1)	0·1 (0·1 to 0·1)
High-income North America	145·5 (141·5 to 149·5)	145·8 (144·1 to 147·5)	93·3 (91·6 to 94·9)	58·6 (57·4 to 59·7)	48·8 (47·9 to 49·7)	35·3 (34·6 to 35·9)	31·0 (30·4 to 31·5)	26·1 (25·6 to 26·6)	24·6 (24·0 to 25·3)	23·1 (21·1 to 25·2)
Canada	17·1 (16·4 to 17·7)	14·7 (14·4 to 14·9)	7·8 (7·6 to 8·0)	4·6 (4·4 to 4·7)	3·3 (3·2 to 3·4)	2·0 (1·9 to 2·1)	2·1 (2·0 to 2·2)	1·8 (1·7 to 1·9)	1·8 (1·7 to 1·9)	1·5 (1·2 to 1·8)
Greenland	0·2 (0·1 to 0·2)	0·1 (0·1 to 0·1)	0·1 (0·1 to 0·1)	0·0 (0·0 to 0·0)	0·0 (0·0 to 0·0)	0·0 (0·0 to 0·0)	0·0 (0·0 to 0·0)	0·0 (0·0 to 0·0)	0·0 (0·0 to 0·0)	0·0 (0·0 to 0·0)
USA	128·2 (124·4 to 132·3)	131·0 (129·4 to 132·6)	85·4 (83·9 to 86·9)	54·0 (52·9 to 55·0)	45·5 (44·6 to 46·4)	33·3 (32·6 to 33·9)	28·9 (28·3 to 29·4)	24·3 (23·8 to 24·8)	22·8 (22·3 to 23·4)	21·6 (19·7 to 23·6)
Alabama	3·6 (3·1 to 4·1)	3·1 (3·0 to 3·2)	1·9 (1·8 to 2·0)	1·1 (1·1 to 1·2)	0·8 (0·8 to 0·9)	0·7 (0·7 to 0·7)	0·6 (0·6 to 0·6)	0·5 (0·5 to 0·6)	0·5 (0·5 to 0·5)	0·5 (0·4 to 0·5)
Alaska	0·2 (0·1 to 0·2)	0·3 (0·3 to 0·3)	0·2 (0·2 to 0·2)	0·2 (0·2 to 0·2)	0·1 (0·1 to 0·2)	0·1 (0·1 to 0·1)	0·1 (0·1 to 0·1)	0·1 (0·1 to 0·1)	0·1 (0·1 to 0·1)	0·1 (0·1 to 0·1)
Arizona	1·0 (0·7 to 1·3)	1·4 (1·3 to 1·5)	0·9 (0·8 to 0·9)	0·8 (0·7 to 0·8)	0·8 (0·7 to 0·8)	0·7 (0·7 to 0·8)	0·7 (0·6 to 0·7)	0·5 (0·5 to 0·5)	0·5 (0·5 to 0·5)	0·5 (0·4 to 0·5)
Arkansas	1·8 (1·2 to 2·4)	1·4 (1·3 to 1·4)	0·9 (0·9 to 0·9)	0·6 (0·5 to 0·6)	0·4 (0·4 to 0·5)	0·4 (0·4 to 0·4)	0·3 (0·3 to 0·4)	0·3 (0·3 to 0·3)	0·3 (0·3 to 0·3)	0·3 (0·2 to 0·3)
California	8·3 (7·0 to 9·9)	10·1 (9·6 to 10·5)	7·2 (6·9 to 7·5)	5·4 (5·0 to 5·7)	6·0 (5·8 to 6·2)	3·6 (3·4 to 3·8)	2·9 (2·8 to 3·0)	2·2 (2·1 to 2·3)	2·0 (1·9 to 2·1)	1·8 (1·7 to 2·0)
Colorado	1·2 (1·0 to 1·3)	1·3 (1·3 to 1·4)	0·9 (0·9 to 1·0)	0·6 (0·6 to 0·7)	0·5 (0·5 to 0·6)	0·5 (0·5 to 0·5)	0·5 (0·4 to 0·5)	0·3 (0·3 to 0·4)	0·3 (0·3 to 0·3)	0·3 (0·3 to 0·3)
Connecticut	1·4 (1·0 to 1·8)	1·4 (1·4 to 1·5)	0·9 (0·9 to 1·0)	0·5 (0·5 to 0·5)	0·5 (0·4 to 0·5)	0·3 (0·3 to 0·3)	0·2 (0·2 to 0·2)	0·2 (0·2 to 0·2)	0·2 (0·1 to 0·2)	0·2 (0·1 to 0·2)
Delaware	0·3 (0·2 to 0·4)	0·3 (0·3 to 0·3)	0·2 (0·2 to 0·2)	0·1 (0·1 to 0·2)	0·1 (0·1 to 0·1)	0·1 (0·1 to 0·1)	0·1 (0·1 to 0·1)	0·1 (0·1 to 0·1)	0·1 (0·1 to 0·1)	0·1 (0·1 to 0·1)
Washington, DC	1·0 (0·7 to 1·4)	0·8 (0·8 to 0·9)	0·5 (0·5 to 0·6)	0·3 (0·3 to 0·3)	0·3 (0·3 to 0·4)	0·1 (0·1 to 0·1)	0·1 (0·1 to 0·1)	0·1 (0·1 to 0·1)	0·1 (0·1 to 0·1)	0·1 (0·0 to 0·1)
Florida	2·9 (2·4 to 3·4)	4·0 (3·9 to 4·2)	3·0 (2·9 to 3·1)	2·4 (2·3 to 2·5)	2·4 (2·3 to 2·5)	1·8 (1·7 to 1·9)	1·7 (1·7 to 1·8)	1·6 (1·5 to 1·6)	1·5 (1·4 to 1·5)	1·4 (1·2 to 1·5)
Georgia	3·9 (3·3 to 4·7)	4·0 (3·9 to 4·2)	2·5 (2·4 to 2·6)	1·6 (1·5 to 1·7)	1·6 (1·5 to 1·7)	1·3 (1·3 to 1·4)	1·1 (1·0 to 1·1)	1·0 (1·0 to 1·1)	0·9 (0·9 to 1·0)	0·9 (0·8 to 1·0)
Hawaii	0·5 (0·4 to 0·6)	0·5 (0·5 to 0·5)	0·3 (0·3 to 0·3)	0·2 (0·2 to 0·2)	0·2 (0·2 to 0·2)	0·1 (0·1 to 0·2)	0·1 (0·1 to 0·1)	0·1 (0·1 to 0·1)	0·1 (0·1 to 0·1)	0·1 (0·1 to 0·1)
Idaho	0·5 (0·4 to 0·6)	0·5 (0·4 to 0·5)	0·3 (0·3 to 0·3)	0·3 (0·3 to 0·3)	0·2 (0·2 to 0·2)	0·2 (0·2 to 0·2)	0·1 (0·1 to 0·2)	0·1 (0·1 to 0·1)	0·1 (0·1 to 0·1)	0·1 (0·1 to 0·1)
Illinois	6·7 (5·8 to 7·7)	7·0 (6·8 to 7·2)	5·0 (4·9 to 5·2)	3·3 (3·1 to 3·4)	2·5 (2·4 to 2·6)	1·8 (1·7 to 1·9)	1·3 (1·2 to 1·3)	0·9 (0·9 to 1·0)	0·8 (0·8 to 0·9)	0·8 (0·7 to 0·9)
Indiana	2·8 (2·4 to 3·2)	3·1 (3·0 to 3·2)	2·2 (2·1 to 2·3)	1·2 (1·2 to 1·3)	1·0 (0·9 to 1·0)	0·8 (0·8 to 0·9)	0·7 (0·7 to 0·8)	0·6 (0·6 to 0·7)	0·6 (0·6 to 0·6)	0·6 (0·5 to 0·6)
Iowa	1·6 (1·2 to 2·0)	1·6 (1·5 to 1·7)	1·0 (0·9 to 1·0)	0·6 (0·6 to 0·7)	0·4 (0·3 to 0·4)	0·3 (0·3 to 0·3)	0·2 (0·2 to 0·2)	0·2 (0·2 to 0·2)	0·2 (0·2 to 0·2)	0·2 (0·2 to 0·2)
Kansas	1·3 (0·9 to 1·6)	1·3 (1·2 to 1·3)	0·8 (0·7 to 0·8)	0·5 (0·5 to 0·6)	0·4 (0·4 to 0·4)	0·3 (0·3 to 0·4)	0·3 (0·3 to 0·3)	0·2 (0·2 to 0·3)	0·3 (0·2 to 0·3)	0·2 (0·2 to 0·3)
Kentucky	2·7 (1·9 to 3·6)	2·4 (2·3 to 2·5)	1·4 (1·3 to 1·5)	0·9 (0·8 to 0·9)	0·6 (0·5 to 0·6)	0·5 (0·4 to 0·5)	0·4 (0·4 to 0·5)	0·3 (0·3 to 0·3)	0·4 (0·3 to 0·4)	0·3 (0·3 to 0·4)
Louisiana	3·4 (2·8 to 4·1)	3·5 (3·3 to 3·6)	2·1 (2·0 to 2·2)	1·4 (1·4 to 1·5)	1·0 (0·9 to 1·0)	0·8 (0·7 to 0·8)	0·6 (0·5 to 0·6)	0·5 (0·5 to 0·6)	0·5 (0·5 to 0·5)	0·5 (0·4 to 0·5)
Maine	0·8 (0·6 to 1·0)	0·7 (0·7 to 0·7)	0·4 (0·4 to 0·4)	0·2 (0·2 to 0·2)	0·1 (0·1 to 0·1)	0·1 (0·1 to 0·1)	0·1 (0·1 to 0·1)	0·1 (0·1 to 0·1)	0·1 (0·1 to 0·1)	0·1 (0·1 to 0·1)
Maryland	2·1 (1·8 to 2·4)	2·4 (2·3 to 2·5)	1·5 (1·4 to 1·6)	0·9 (0·9 to 1·0)	0·9 (0·8 to 0·9)	0·7 (0·6 to 0·7)	0·6 (0·5 to 0·6)	0·5 (0·4 to 0·5)	0·4 (0·4 to 0·5)	0·4 (0·4 to 0·5)
Massachusetts	3·3 (2·3 to 4·5)	2·9 (2·8 to 3·0)	1·8 (1·7 to 1·9)	0·9 (0·8 to 0·9)	0·7 (0·7 to 0·8)	0·5 (0·4 to 0·5)	0·4 (0·4 to 0·4)	0·3 (0·3 to 0·3)	0·3 (0·3 to 0·3)	0·3 (0·2 to 0·3)
Michigan	4·8 (4·0 to 5·5)	5·4 (5·2 to 5·6)	3·9 (3·8 to 4·0)	2·1 (2·0 to 2·2)	1·8 (1·7 to 1·9)	1·3 (1·2 to 1·3)	0·9 (0·9 to 0·9)	0·8 (0·7 to 0·8)	0·8 (0·7 to 0·8)	0·7 (0·6 to 0·8)
Minnesota	2·0 (1·4 to 2·8)	2·1 (2·0 to 2·2)	1·3 (1·3 to 1·4)	0·8 (0·7 to 0·9)	0·6 (0·5 to 0·6)	0·4 (0·4 to 0·5)	0·4 (0·4 to 0·4)	0·4 (0·3 to 0·4)	0·3 (0·3 to 0·4)	0·3 (0·3 to 0·4)
Mississippi	3·6 (2·5 to 4·8)	2·9 (2·8 to 3·0)	1·7 (1·6 to 1·8)	1·0 (0·9 to 1·0)	0·6 (0·6 to 0·7)	0·6 (0·5 to 0·6)	0·5 (0·4 to 0·5)	0·4 (0·4 to 0·4)	0·4 (0·3 to 0·4)	0·3 (0·3 to 0·4)
Missouri	3·0 (2·6 to 3·4)	2·8 (2·7 to 2·9)	1·8 (1·8 to 1·9)	1·2 (1·1 to 1·2)	0·9 (0·8 to 0·9)	0·7 (0·6 to 0·7)	0·6 (0·6 to 0·6)	0·5 (0·5 to 0·5)	0·5 (0·5 to 0·5)	0·5 (0·4 to 0·5)
Montana	0·5 (0·4 to 0·6)	0·5 (0·5 to 0·5)	0·3 (0·3 to 0·3)	0·2 (0·2 to 0·2)	0·1 (0·1 to 0·1)	0·1 (0·1 to 0·1)	0·1 (0·1 to 0·1)	0·1 (0·1 to 0·1)	0·1 (0·1 to 0·1)	0·1 (0·1 to 0·1)
Nebraska	0·9 (0·7 to 1·2)	0·9 (0·8 to 0·9)	0·5 (0·5 to 0·6)	0·4 (0·3 to 0·4)	0·2 (0·2 to 0·2)	0·2 (0·2 to 0·2)	0·2 (0·2 to 0·2)	0·2 (0·1 to 0·2)	0·2 (0·1 to 0·2)	0·1 (0·1 to 0·2)
Nevada	0·2 (0·1 to 0·2)	0·3 (0·3 to 0·3)	0·2 (0·2 to 0·3)	0·2 (0·2 to 0·2)	0·2 (0·2 to 0·2)	0·2 (0·2 to 0·2)	0·2 (0·2 to 0·3)	0·2 (0·2 to 0·3)	0·2 (0·2 to 0·2)	0·2 (0·2 to 0·2)
New Hampshire	0·4 (0·3 to 0·5)	0·4 (0·4 to 0·4)	0·3 (0·3 to 0·3)	0·2 (0·1 to 0·2)	0·1 (0·1 to 0·1)	0·1 (0·1 to 0·1)	0·1 (0·1 to 0·1)	0·1 (0·0 to 0·1)	0·1 (0·0 to 0·1)	0·1 (0·0 to 0·1)
New Jersey	3·7 (3·1 to 4·5)	3·9 (3·7 to 4·0)	2·7 (2·6 to 2·8)	1·4 (1·3 to 1·5)	1·3 (1·2 to 1·3)	0·8 (0·8 to 0·9)	0·6 (0·6 to 0·6)	0·5 (0·4 to 0·5)	0·4 (0·4 to 0·5)	0·4 (0·4 to 0·5)
New Mexico	1·1 (0·7 to 1·5)	1·2 (1·1 to 1·2)	0·6 (0·5 to 0·6)	0·4 (0·4 to 0·4)	0·3 (0·3 to 0·3)	0·2 (0·2 to 0·2)	0·2 (0·2 to 0·2)	0·2 (0·1 to 0·2)	0·1 (0·1 to 0·2)	0·1 (0·1 to 0·2)

New York	11·2 (9·5 to 12·9)	10·7 (10·5 to 11·0)	7·0 (6·8 to 7·2)	3·5 (3·4 to 3·7)	3·4 (3·3 to 3·6)	1·9 (1·9 to 2·0)	1·5 (1·4 to 1·5)	1·1 (1·0 to 1·2)	1·0 (0·9 to 1·0)	0·9 (0·8 to 1·0)
North Carolina	4·5 (3·9 to 5·3)	4·1 (4·0 to 4·3)	2·7 (2·6 to 2·8)	1·4 (1·4 to 1·5)	1·3 (1·2 to 1·3)	1·2 (1·1 to 1·3)	1·0 (1·0 to 1·1)	0·9 (0·9 to 1·0)	0·9 (0·9 to 1·0)	0·9 (0·8 to 1·0)
North Dakota	0·5 (0·4 to 0·8)	0·5 (0·4 to 0·5)	0·2 (0·2 to 0·2)	0·2 (0·2 to 0·2)	0·1 (0·1 to 0·1)	0·1 (0·1 to 0·1)	0·1 (0·1 to 0·1)	0·1 (0·1 to 0·1)	0·1 (0·1 to 0·1)	0·1 (0·1 to 0·1)
Ohio	5·6 (4·7 to 6·4)	6·4 (6·2 to 6·6)	4·2 (4·1 to 4·3)	2·5 (2·4 to 2·6)	1·8 (1·8 to 1·9)	1·4 (1·3 to 1·4)	1·2 (1·2 to 1·3)	1·0 (1·0 to 1·1)	1·0 (0·9 to 1·0)	0·9 (0·9 to 1·0)
Oklahoma	1·6 (1·3 to 1·8)	1·6 (1·5 to 1·6)	1·1 (1·0 to 1·1)	0·8 (0·7 to 0·8)	0·5 (0·5 to 0·6)	0·5 (0·5 to 0·5)	0·5 (0·4 to 0·5)	0·4 (0·4 to 0·4)	0·3 (0·3 to 0·4)	0·3 (0·3 to 0·4)
Oregon	1·0 (0·7 to 1·2)	1·0 (1·0 to 1·1)	0·7 (0·6 to 0·7)	0·6 (0·5 to 0·6)	0·4 (0·4 to 0·4)	0·3 (0·3 to 0·3)	0·3 (0·2 to 0·3)	0·2 (0·2 to 0·2)	0·2 (0·2 to 0·2)	0·2 (0·2 to 0·2)
Pennsylvania	7·7 (6·5 to 8·9)	6·9 (6·7 to 7·1)	4·4 (4·2 to 4·5)	2·4 (2·3 to 2·5)	1·9 (1·8 to 2·0)	1·2 (1·1 to 1·2)	1·1 (1·1 to 1·2)	0·9 (0·8 to 0·9)	0·9 (0·8 to 0·9)	0·8 (0·7 to 0·9)
Rhode Island	0·6 (0·4 to 0·8)	0·5 (0·5 to 0·6)	0·3 (0·3 to 0·4)	0·2 (0·2 to 0·2)	0·1 (0·1 to 0·2)	0·1 (0·1 to 0·1)	0·1 (0·1 to 0·1)	0·1 (0·1 to 0·1)	0·1 (0·1 to 0·1)	0·1 (0·0 to 0·1)
South Carolina	2·5 (2·2 to 2·9)	2·5 (2·4 to 2·6)	1·4 (1·3 to 1·5)	1·0 (0·9 to 1·0)	0·8 (0·8 to 0·9)	0·6 (0·5 to 0·6)	0·5 (0·4 to 0·5)	0·5 (0·4 to 0·5)	0·4 (0·4 to 0·4)	0·4 (0·4 to 0·4)
South Dakota	0·6 (0·4 to 0·7)	0·5 (0·5 to 0·6)	0·3 (0·3 to 0·3)	0·2 (0·2 to 0·2)	0·1 (0·1 to 0·1)	0·1 (0·1 to 0·1)	0·1 (0·1 to 0·1)	0·1 (0·1 to 0·1)	0·1 (0·1 to 0·1)	0·1 (0·1 to 0·1)
Tennessee	3·2 (2·8 to 3·7)	2·9 (2·8 to 3·0)	1·8 (1·7 to 1·9)	1·1 (1·0 to 1·1)	0·9 (0·9 to 1·0)	0·8 (0·8 to 0·9)	0·7 (0·7 to 0·8)	0·7 (0·6 to 0·7)	0·6 (0·6 to 0·6)	0·6 (0·5 to 0·6)
Texas	7·9 (6·8 to 9·0)	8·8 (8·6 to 9·1)	5·8 (5·6 to 6·0)	4·2 (4·0 to 4·3)	3·2 (3·1 to 3·3)	2·6 (2·5 to 2·7)	2·8 (2·7 to 2·9)	2·5 (2·4 to 2·6)	2·4 (2·3 to 2·4)	2·3 (2·0 to 2·5)
Utah	0·5 (0·4 to 0·7)	0·6 (0·6 to 0·7)	0·5 (0·5 to 0·5)	0·5 (0·5 to 0·6)	0·3 (0·3 to 0·3)	0·3 (0·3 to 0·3)	0·3 (0·3 to 0·3)	0·3 (0·3 to 0·3)	0·3 (0·3 to 0·3)	0·3 (0·2 to 0·3)
Vermont	0·3 (0·2 to 0·4)	0·3 (0·3 to 0·3)	0·2 (0·2 to 0·2)	0·1 (0·1 to 0·1)	0·1 (0·1 to 0·1)	0·0 (0·0 to 0·0)	0·0 (0·0 to 0·0)	0·0 (0·0 to 0·0)	0·0 (0·0 to 0·0)	0·0 (0·0 to 0·0)
Virginia	3·3 (2·8 to 4·0)	3·3 (3·2 to 3·4)	2·0 (1·9 to 2·1)	1·2 (1·2 to 1·3)	1·1 (1·1 to 1·2)	0·8 (0·8 to 0·9)	0·8 (0·7 to 0·8)	0·6 (0·6 to 0·7)	0·6 (0·6 to 0·7)	0·6 (0·5 to 0·6)
Washington	1·6 (1·4 to 1·8)	1·8 (1·7 to 1·8)	1·2 (1·2 to 1·3)	0·9 (0·9 to 1·0)	0·7 (0·7 to 0·8)	0·5 (0·5 to 0·5)	0·5 (0·4 to 0·5)	0·4 (0·4 to 0·4)	0·4 (0·4 to 0·4)	0·4 (0·3 to 0·4)
West Virginia	1·8 (1·3 to 2·3)	1·3 (1·2 to 1·3)	0·8 (0·8 to 0·8)	0·4 (0·4 to 0·5)	0·3 (0·2 to 0·3)	0·2 (0·2 to 0·2)	0·2 (0·2 to 0·2)	0·1 (0·1 to 0·2)	0·1 (0·1 to 0·2)	0·1 (0·1 to 0·2)
Wisconsin	2·5 (2·2 to 2·9)	2·5 (2·4 to 2·6)	1·5 (1·4 to 1·5)	0·9 (0·9 to 1·0)	0·7 (0·6 to 0·7)	0·5 (0·5 to 0·6)	0·5 (0·4 to 0·5)	0·4 (0·4 to 0·5)	0·4 (0·4 to 0·4)	0·4 (0·3 to 0·4)
Wyoming	0·3 (0·2 to 0·3)	0·3 (0·3 to 0·3)	0·2 (0·1 to 0·2)	0·1 (0·1 to 0·2)	0·1 (0·1 to 0·1)	0·1 (0·0 to 0·1)	0·1 (0·0 to 0·1)	0·0 (0·0 to 0·0)	0·0 (0·0 to 0·0)	0·0 (0·0 to 0·0)
Southern Latin America	66·7 (64·4 to 69·2)	84·4 (83·2 to 85·7)	65·3 (64·3 to 66·3)	38·2 (37·5 to 38·8)	26·7 (26·2 to 27·2)	17·1 (16·6 to 17·7)	13·3 (13·0 to 13·7)	8·6 (8·3 to 8·9)	7·6 (6·6 to 8·6)	6·6 (5·4 to 8·1)
Argentina	34·2 (32·7 to 35·8)	37·3 (36·5 to 38·0)	38·5 (37·7 to 39·2)	26·7 (26·1 to 27·3)	19·7 (19·3 to 20·1)	13·4 (12·9 to 13·9)	10·6 (10·3 to 10·9)	6·7 (6·5 to 7·0)	6·1 (5·2 to 7·0)	5·2 (4·1 to 6·5)
Chile	29·8 (28·6 to 31·0)	43·7 (43·0 to 44·3)	23·7 (23·2 to 24·2)	9·2 (8·9 to 9·4)	5·7 (5·5 to 5·9)	2·9 (2·8 to 3·0)	2·2 (2·2 to 2·3)	1·5 (1·5 to 1·6)	1·3 (1·2 to 1·4)	1·2 (1·0 to 1·3)
Uruguay	2·7 (2·5 to 2·9)	3·5 (3·3 to 3·6)	3·1 (3·0 to 3·3)	2·2 (2·1 to 2·3)	1·3 (1·2 to 1·4)	0·9 (0·8 to 0·9)	0·5 (0·4 to 0·6)	0·3 (0·3 to 0·3)	0·3 (0·2 to 0·3)	0·2 (0·2 to 0·3)
Western Europe	333·8 (322·7 to 346·2)	240·7 (238·2 to 243·3)	153·8 (151·8 to 155·8)	71·5 (70·5 to 72·6)	42·7 (42·0 to 43·4)	24·9 (24·4 to 25·5)	19·5 (19·1 to 19·9)	15·7 (15·1 to 16·2)	14·7 (14·1 to 15·4)	14·3 (13·3 to 15·5)
Andorra	0·0 (0·0 to 0·0)	0·0 (0·0 to 0·0)	0·0 (0·0 to 0·0)	0·0 (0·0 to 0·0)	0·0 (0·0 to 0·0)	0·0 (0·0 to 0·0)	0·0 (0·0 to 0·0)	0·0 (0·0 to 0·0)	0·0 (0·0 to 0·0)	0·0 (0·0 to 0·0)
Austria	6·0 (5·7 to 6·4)	5·5 (5·4 to 5·7)	3·3 (3·2 to 3·4)	1·5 (1·5 to 1·6)	0·9 (0·8 to 0·9)	0·4 (0·4 to 0·5)	0·4 (0·3 to 0·4)	0·3 (0·3 to 0·3)	0·3 (0·3 to 0·3)	0·3 (0·2 to 0·3)
Belgium	8·9 (8·4 to 9·4)	5·4 (5·3 to 5·6)	3·5 (3·4 to 3·6)	1·8 (1·7 to 1·9)	1·2 (1·1 to 1·3)	0·7 (0·7 to 0·7)	0·6 (0·5 to 0·6)	0·5 (0·5 to 0·5)	0·4 (0·4 to 0·5)	0·4 (0·3 to 0·5)
Cyprus	2·1 (1·7 to 2·6)	1·6 (1·3 to 1·8)	0·7 (0·7 to 0·8)	0·4 (0·3 to 0·4)	0·2 (0·2 to 0·2)	0·1 (0·1 to 0·1)	0·0 (0·0 to 0·1)	0·0 (0·0 to 0·0)	0·0 (0·0 to 0·0)	0·0 (0·0 to 0·0)
Denmark	2·6 (2·4 to 2·9)	1·9 (1·9 to 2·0)	1·2 (1·2 to 1·3)	0·6 (0·5 to 0·6)	0·6 (0·5 to 0·6)	0·4 (0·4 to 0·4)	0·2 (0·2 to 0·3)	0·2 (0·2 to 0·2)	0·2 (0·2 to 0·2)	0·2 (0·2 to 0·3)
Finland	5·0 (4·6 to 5·3)	2·1 (2·0 to 2·2)	1·0 (1·0 to 1·1)	0·6 (0·5 to 0·6)	0·4 (0·4 to 0·5)	0·2 (0·2 to 0·3)	0·2 (0·2 to 0·2)	0·1 (0·1 to 0·1)	0·1 (0·1 to 0·1)	0·1 (0·1 to 0·1)
France	47·4 (45·3 to 49·5)	22·4 (22·0 to 22·8)	15·3 (15·0 to 15·6)	9·7 (9·4 to 10·0)	6·7 (6·5 to 6·9)	4·1 (4·0 to 4·3)	3·4 (3·3 to 3·5)	3·0 (2·9 to 3·1)	3·1 (2·9 to 3·2)	2·8 (2·5 to 3·1)
Germany	61·3 (52·4 to 71·2)	50·8 (49·8 to 51·7)	27·6 (27·0 to 28·2)	12·8 (12·5 to 13·2)	7·7 (7·4 to 7·9)	4·1 (3·9 to 4·2)	2·8 (2·7 to 2·9)	2·9 (2·7 to 3·0)	2·8 (2·6 to 2·9)	2·8 (2·6 to 3·0)
Greece	9·6 (9·0 to 10·2)	7·0 (6·8 to 7·3)	4·6 (4·5 to 4·8)	2·9 (2·8 to 3·0)	1·1 (1·0 to 1·1)	0·6 (0·6 to 0·7)	0·5 (0·5 to 0·5)	0·4 (0·3 to 0·4)	0·3 (0·3 to 0·4)	0·3 (0·3 to 0·4)
Iceland	0·1 (0·1 to 0·1)	0·1 (0·1 to 0·1)	0·1 (0·1 to 0·1)	0·0 (0·0 to 0·0)	0·0 (0·0 to 0·0)	0·0 (0·0 to 0·0)	0·0 (0·0 to 0·0)	0·0 (0·0 to 0·0)	0·0 (0·0 to 0·0)	0·0 (0·0 to 0·0)
Ireland	3·3 (3·1 to 3·6)	2·1 (2·0 to 2·1)	1·4 (1·4 to 1·5)	1·0 (1·0 to 1·0)	0·5 (0·5 to 0·5)	0·4 (0·4 to 0·4)	0·3 (0·3 to 0·3)	0·2 (0·2 to 0·2)	0·2 (0·2 to 0·2)	0·2 (0·2 to 0·2)
Israel	3·0 (2·7 to 3·2)	2·1 (2·0 to 2·2)	2·1 (2·0 to 2·1)	1·7 (1·6 to 1·8)	1·2 (1·2 to 1·3)	0·9 (0·9 to 1·0)	0·7 (0·7 to 0·8)	0·7 (0·6 to 0·7)	0·5 (0·5 to 0·6)	0·4 (0·4 to 0·5)
Italy	58·8 (56·9 to 60·7)	47·0 (46·3 to 47·7)	30·8 (30·2 to 31·3)	10·8 (10·6 to 11·1)	5·4 (5·2 to 5·5)	2·9 (2·8 to 3·0)	2·2 (2·1 to 2·3)	1·2 (1·2 to 1·3)	1·2 (1·1 to 1·3)	1·2 (1·0 to 1·3)
Luxembourg	0·3 (0·2 to 0·3)	0·2 (0·2 to 0·2)	0·1 (0·1 to 0·1)	0·1 (0·0 to 0·1)	0·0 (0·0 to 0·0)	0·0 (0·0 to 0·0)	0·0 (0·0 to 0·0)	0·0 (0·0 to 0·0)	0·0 (0·0 to 0·0)	0·0 (0·0 to 0·0)
Malta	0·8 (0·7 to 0·9)	0·3 (0·3 to 0·4)	0·1 (0·1 to 0·2)	0·1 (0·1 to 0·1)	0·1 (0·1 to 0·1)	0·0 (0·0 to 0·0)	0·0 (0·0 to 0·0)	0·0 (0·0 to 0·0)	0·0 (0·0 to 0·0)	0·0 (0·0 to 0·0)
Monaco	0·0 (0·0 to 0·0)	0·0 (0·0 to 0·0)	0·0 (0·0 to 0·0)	0·0 (0·0 to 0·0)	0·0 (0·0 to 0·0)	0·0 (0·0 to 0·0)	0·0 (0·0 to 0·0)	0·0 (0·0 to 0·0)	0·0 (0·0 to 0·0)	0·0 (0·0 to 0·0)
Netherlands	6·6 (6·2 to 7·0)	5·1 (4·9 to 5·2)	3·7 (3·6 to 3·9)	1·9 (1·8 to 2·0)	1·7 (1·6 to 1·8)	1·3 (1·2 to 1·3)	0·8 (0·8 to 0·9)	0·7 (0·7 to 0·8)	0·7 (0·7 to 0·8)	0·7 (0·6 to 0·7)
Norway	1·9 (1·7 to 2·1)	1·4 (1·4 to 1·5)	1·0 (1·0 to 1·1)	0·5 (0·5 to 0·5)	0·5 (0·5 to 0·5)	0·3 (0·3 to 0·3)	0·2 (0·2 to 0·2)	0·1 (0·1 to 0·1)	0·1 (0·1 to 0·1)	0·1 (0·1 to 0·1)
Agder	0·1 (0·1 to 0·1)	0·1 (0·1 to 0·1)	0·1 (0·1 to 0·1)	0·0 (0·0 to 0·0)	0·0 (0·0 to 0·0)	0·0 (0·0 to 0·0)	0·0 (0·0 to 0·0)	0·0 (0·0 to 0·0)	0·0 (0·0 to 0·0)	0·0 (0·0 to 0·0)
Innlandet	0·2 (0·2 to 0·2)	0·1 (0·1 to 0·1)	0·1 (0·1 to 0·1)	0·0 (0·0 to 0·0)	0·0 (0·0 to 0·0)	0·0 (0·0 to 0·0)	0·0 (0·0 to 0·0)	0·0 (0·0 to 0·0)	0·0 (0·0 to 0·0)	0·0 (0·0 to 0·0)
Møre og Romsdal	0·1 (0·1 to 0·2)	0·1 (0·1 to 0·1)	0·1 (0·1 to 0·1)	0·0 (0·0 to 0·0)	0·0 (0·0 to 0·0)	0·0 (0·0 to 0·0)	0·0 (0·0 to 0·0)	0·0 (0·0 to 0·0)	0·0 (0·0 to 0·0)	0·0 (0·0 to 0·0)
Nordland	0·2 (0·2 to 0·2)	0·1 (0·1 to 0·1)	0·1 (0·1 to 0·1)	0·0 (0·0 to 0·0)	0·0 (0·0 to 0·0)	0·0 (0·0 to 0·0)	0·0 (0·0 to 0·0)	0·0 (0·0 to 0·0)	0·0 (0·0 to 0·0)	0·0 (0·0 to 0·0)
Oslo	0·1 (0·1 to 0·2)	0·1 (0·1 to 0·1)	0·1 (0·1 to 0·1)	0·0 (0·0 to 0·1)	0·1 (0·1 to 0·1)	0·0 (0·0 to 0·0)	0·0 (0·0 to 0·0)	0·0 (0·0 to 0·0)	0·0 (0·0 to 0·0)	0·0 (0·0 to 0·0)
Rogaland	0·1 (0·1 to 0·2)	0·1 (0·1 to 0·1)	0·1 (0·1 to 0·1)	0·0 (0·0 to 0·1)	0·0 (0·0 to 0·1)	0·0 (0·0 to 0·0)	0·0 (0·0 to 0·0)	0·0 (0·0 to 0·0)	0·0 (0·0 to 0·0)	0·0 (0·0 to 0·0)
Troms og Finnmark	0·2 (0·2 to 0·2)	0·1 (0·1 to 0·1)	0·1 (0·1 to 0·1)	0·0 (0·0 to 0·0)	0·0 (0·0 to 0·0)	0·0 (0·0 to 0·0)	0·0 (0·0 to 0·0)	0·0 (0·0 to 0·0)	0·0 (0·0 to 0·0)	0·0 (0·0 to 0·0)
Trøndelag	0·2 (0·1 to 0·2)	0·1 (0·1 to 0·1)	0·1 (0·1 to 0·1)	0·0 (0·0 to 0·0)	0·0 (0·0 to 0·0)	0·0 (0·0 to 0·0)	0·0 (0·0 to 0·0)	0·0 (0·0 to 0·0)	0·0 (0·0 to 0·0)	0·0 (0·0 to 0·0)
Vestfold og Telemark	0·1 (0·1 to 0·2)	0·1 (0·1 to 0·1)	0·1 (0·1 to 0·1)	0·0 (0·0 to 0·0)	0·0 (0·0 to 0·0)	0·0 (0·0 to 0·0)	0·0 (0·0 to 0·0)	0·0 (0·0 to 0·0)	0·0 (0·0 to 0·0)	0·0 (0·0 to 0·0)
Vestland	0·2 (0·2 to 0·3)	0·2 (0·2 to 0·2)	0·1 (0·1 to 0·1)	0·1 (0·1 to 0·1)	0·1 (0·1 to 0·1)	0·0 (0·0 to 0·0)	0·0 (0·0 to 0·0)	0·0 (0·0 to 0·0)	0·0 (0·0 to 0·0)	0·0 (0·0 to 0·0)
Viken	0·3 (0·2 to 0·3)	0·2 (0·2 to 0·2)	0·2 (0·2 to 0·2)	0·1 (0·1 to 0·1)	0·1 (0·1 to 0·1)	0·1 (0·0 to 0·1)	0·0 (0·0 to 0·0)	0·0 (0·0 to 0·0)	0·0 (0·0 to 0·0)	0·0 (0·0 to 0·0)
Portugal	31·6 (30·3 to 33·0)	22·2 (21·8 to 22·7)	12·9 (12·5 to 13·2)	4·5 (4·3 to 4·6)	1·7 (1·6 to 1·7)	0·8 (0·8 to 0·9)	0·4 (0·4 to 0·4)	0·3 (0·3 to 0·3)	0·3 (0·2 to 0·3)	0·2 (0·2 to 0·3)
San Marino	0·0 (0·0 to 0·0)	0·0 (0·0 to 0·0)	0·0 (0·0 to 0·0)	0·0 (0·0 to 0·0)	0·0 (0·0 to 0·0)	0·0 (0·0 to 0·0)	0·0 (0·0 to 0·0)	0·0 (0·0 to 0·0)	0·0 (0·0 to 0·0)	0·0 (0·0 to 0·0)
Spain	54·2 (52·7 to 55·8)	34·2 (33·6 to 34·9)	21·1 (20·6 to 21·6)	8·3 (8·1 to 8·5)	3·8 (3·6 to 3·9)	2·1 (2·0 to 2·2)	1·9 (1·8 to 2·0)	1·2 (1·1 to 1·3)	1·1 (1·0 to 1·2)	1·0 (0·9 to 1·1)
Sweden	2·6 (2·4 to 2·9)	2·1 (2·0 to 2·2)	1·5 (1·4 to 1·5)	0·8 (0·7 to 0·8)	0·8 (0·8 to 0·9)	0·4 (0·3 to 0·4)	0·3 (0·3 to 0·4)	0·3 (0·3 to 0·3)	0·3 (0·3 to 0·3)	0·3 (0·2 to 0·3)
Stockholm	0·3 (0·3 to 0·4)	0·3 (0·2 to 0·3)	0·3 (0·2 to 0·3)	0·2 (0·1 to 0·2)	0·2 (0·1 to 0·2)	0·1 (0·1 to 0·1)	0·1 (0·1 to 0·1)	0·1 (0·1 to 0·1)	0·1 (0·1 to 0·1)	0·1 (0·0 to 0·1)
Sweden except Stockholm	2·3 (2·1 to 2·5)	1·8 (1·7 to 1·9)	1·2 (1·1 to 1·3)	0·6 (0·6 to 0·6)	0·7 (0·6 to 0·7)	0·3 (0·3 to 0·3)	0·3 (0·2 to 0·3)	0·2 (0·2 to 0·3)	0·2 (0·2 to 0·3)	0·2 (0·2 to 0·2)
Switzerland	3·2 (3·0 to 3·5)	2·5 (2·4 to 2·6)	1·9 (1·8 to 2·0)	0·8 (0·8 to 0·9)	0·7 (0·7 to 0·8)	0·5 (0·4 to 0·5)	0·4 (0·3 to 0·4)	0·3 (0·3 to 0·3)	0·3 (0·3 to 0·4)	0·3 (0·3 to 0·4)
UK	24·2 (23·3 to 25·2)	24·5 (24·0 to 24·9)	19·6 (19·2 to 20·0)	10·8 (10·5 to 11·0)	7·5 (7·3 to 7·8)	4·6 (4·3 to 4·9)	4·1 (4·0 to 4·3)	3·1 (3·0 to 3·3)	2·6 (2·4 to 2·8)	2·9 (2·6 to 3·2)
England	18·4 (17·3 to 19·4)	19·1 (18·7 to 19·6)	15·7 (15·4 to 16·1)	8·7 (8·4 to 9·0)	6·3 (6·1 to 6·5)	3·9 (3·7 to 4·1)	3·6 (3·4 to 3·7)	2·7 (2·6 to 2·8)	2·2 (2·1 to 2·4)	2·5 (2·3 to 2·8)
East Midlands	1·5 (1·2 to 1·8)	1·5 (1·4 to 1·7)	1·3 (1·2 to 1·4)	0·7 (0·7 to 0·8)	0·5 (0·5 to 0·5)	0·3 (0·3 to 0·3)	0·3 (0·3 to 0·3)	0·2 (0·2 to 0·2)	0·2 (0·2 to 0·2)	0·2 (0·2 to 0·2)
Derby	0·1 (0·1 to 0·2)	0·1 (0·1 to 0·1)	0·1 (0·1 to 0·1)	0·1 (0·0 to 0·1)	0·0 (0·0 to 0·0)	0·0 (0·0 to 0·0)	0·0 (0·0 to 0·0)	0·0 (0·0 to 0·0)	0·0 (0·0 to 0·0)	0·0 (0·0 to 0·0)

Derbyshire	0·3 (0·2 to 0·3)	0·3 (0·2 to 0·3)	0·2 (0·2 to 0·2)	0·1 (0·1 to 0·1)	0·1 (0·1 to 0·1)	0·0 (0·0 to 0·0)	0·0 (0·0 to 0·0)	0·0 (0·0 to 0·0)	0·0 (0·0 to 0·0)	0·0 (0·0 to 0·0)
Leicester	0·2 (0·1 to 0·2)	0·2 (0·1 to 0·2)	0·2 (0·1 to 0·2)	0·1 (0·1 to 0·1)	0·1 (0·1 to 0·1)	0·0 (0·0 to 0·0)	0·0 (0·0 to 0·0)	0·0 (0·0 to 0·0)	0·0 (0·0 to 0·0)	0·0 (0·0 to 0·0)
Leicestershire	0·2 (0·1 to 0·2)	0·2 (0·2 to 0·2)	0·1 (0·1 to 0·2)	0·1 (0·1 to 0·1)	0·1 (0·1 to 0·1)	0·0 (0·0 to 0·0)	0·0 (0·0 to 0·0)	0·0 (0·0 to 0·0)	0·0 (0·0 to 0·0)	0·0 (0·0 to 0·0)
Lincolnshire	0·2 (0·1 to 0·2)	0·2 (0·2 to 0·2)	0·2 (0·1 to 0·2)	0·1 (0·1 to 0·1)	0·1 (0·1 to 0·1)	0·0 (0·0 to 0·0)	0·0 (0·0 to 0·0)	0·0 (0·0 to 0·0)	0·0 (0·0 to 0·0)	0·0 (0·0 to 0·0)
Northamptonshire	0·2 (0·2 to 0·2)	0·2 (0·2 to 0·2)	0·2 (0·2 to 0·2)	0·1 (0·1 to 0·1)	0·1 (0·1 to 0·1)	0·0 (0·0 to 0·1)	0·0 (0·0 to 0·1)	0·0 (0·0 to 0·0)	0·0 (0·0 to 0·0)	0·0 (0·0 to 0·0)
Nottingham	0·1 (0·1 to 0·2)	0·2 (0·1 to 0·2)	0·1 (0·1 to 0·1)	0·1 (0·1 to 0·1)	0·1 (0·0 to 0·1)	0·0 (0·0 to 0·0)	0·0 (0·0 to 0·0)	0·0 (0·0 to 0·0)	0·0 (0·0 to 0·0)	0·0 (0·0 to 0·0)
Nottinghamshire	0·2 (0·2 to 0·3)	0·3 (0·2 to 0·3)	0·2 (0·2 to 0·2)	0·1 (0·1 to 0·1)	0·1 (0·1 to 0·1)	0·0 (0·0 to 0·1)	0·0 (0·0 to 0·0)	0·0 (0·0 to 0·0)	0·0 (0·0 to 0·0)	0·0 (0·0 to 0·0)
Rutland	0·0 (0·0 to 0·0)	0·0 (0·0 to 0·0)	0·0 (0·0 to 0·0)	0·0 (0·0 to 0·0)	0·0 (0·0 to 0·0)	0·0 (0·0 to 0·0)	0·0 (0·0 to 0·0)	0·0 (0·0 to 0·0)	0·0 (0·0 to 0·0)	0·0 (0·0 to 0·0)
East of England	1·5 (1·3 to 1·7)	1·6 (1·5 to 1·7)	1·3 (1·2 to 1·5)	0·8 (0·7 to 0·8)	0·5 (0·5 to 0·5)	0·3 (0·3 to 0·4)	0·3 (0·3 to 0·4)	0·3 (0·2 to 0·3)	0·2 (0·2 to 0·2)	0·2 (0·2 to 0·3)
Bedford	0·1 (0·0 to 0·1)	0·1 (0·1 to 0·1)	0·0 (0·0 to 0·1)	0·0 (0·0 to 0·0)	0·0 (0·0 to 0·0)	0·0 (0·0 to 0·0)	0·0 (0·0 to 0·0)	0·0 (0·0 to 0·0)	0·0 (0·0 to 0·0)	0·0 (0·0 to 0·0)
Cambridgeshire	0·1 (0·1 to 0·1)	0·1 (0·1 to 0·1)	0·1 (0·1 to 0·1)	0·1 (0·1 to 0·1)	0·0 (0·0 to 0·0)	0·0 (0·0 to 0·0)	0·0 (0·0 to 0·0)	0·0 (0·0 to 0·0)	0·0 (0·0 to 0·0)	0·0 (0·0 to 0·0)
Central Bedfordshire	0·1 (0·0 to 0·1)	0·1 (0·1 to 0·1)	0·1 (0·1 to 0·1)	0·0 (0·0 to 0·0)	0·0 (0·0 to 0·0)	0·0 (0·0 to 0·0)	0·0 (0·0 to 0·0)	0·0 (0·0 to 0·0)	0·0 (0·0 to 0·0)	0·0 (0·0 to 0·0)
Essex	0·4 (0·3 to 0·4)	0·4 (0·3 to 0·4)	0·3 (0·3 to 0·4)	0·2 (0·2 to 0·2)	0·1 (0·1 to 0·1)	0·1 (0·1 to 0·1)	0·1 (0·1 to 0·1)	0·1 (0·1 to 0·1)	0·0 (0·0 to 0·1)	0·0 (0·0 to 0·1)
Hertfordshire	0·3 (0·2 to 0·4)	0·3 (0·3 to 0·4)	0·2 (0·2 to 0·3)	0·1 (0·1 to 0·2)	0·1 (0·1 to 0·1)	0·1 (0·1 to 0·1)	0·1 (0·1 to 0·1)	0·0 (0·0 to 0·0)	0·0 (0·0 to 0·0)	0·0 (0·0 to 0·1)
Luton	0·1 (0·1 to 0·1)	0·1 (0·1 to 0·1)	0·1 (0·1 to 0·1)	0·0 (0·0 to 0·1)	0·0 (0·0 to 0·0)	0·0 (0·0 to 0·0)	0·0 (0·0 to 0·0)	0·0 (0·0 to 0·0)	0·0 (0·0 to 0·0)	0·0 (0·0 to 0·0)
Norfolk	0·2 (0·2 to 0·3)	0·2 (0·2 to 0·3)	0·2 (0·2 to 0·2)	0·1 (0·1 to 0·1)	0·1 (0·1 to 0·1)	0·0 (0·0 to 0·0)	0·0 (0·0 to 0·0)	0·0 (0·0 to 0·0)	0·0 (0·0 to 0·0)	0·0 (0·0 to 0·0)
Peterborough	0·0 (0·0 to 0·1)	0·0 (0·0 to 0·1)	0·0 (0·0 to 0·0)	0·0 (0·0 to 0·0)	0·0 (0·0 to 0·0)	0·0 (0·0 to 0·0)	0·0 (0·0 to 0·0)	0·0 (0·0 to 0·0)	0·0 (0·0 to 0·0)	0·0 (0·0 to 0·0)
Southend-on-Sea	0·1 (0·1 to 0·1)	0·1 (0·1 to 0·1)	0·0 (0·0 to 0·1)	0·0 (0·0 to 0·0)	0·0 (0·0 to 0·0)	0·0 (0·0 to 0·0)	0·0 (0·0 to 0·0)	0·0 (0·0 to 0·0)	0·0 (0·0 to 0·0)	0·0 (0·0 to 0·0)
Suffolk	0·2 (0·2 to 0·2)	0·2 (0·2 to 0·2)	0·2 (0·1 to 0·2)	0·1 (0·1 to 0·1)	0·1 (0·1 to 0·1)	0·0 (0·0 to 0·0)	0·0 (0·0 to 0·0)	0·0 (0·0 to 0·0)	0·0 (0·0 to 0·0)	0·0 (0·0 to 0·0)
Thurrock	0·0 (0·0 to 0·1)	0·1 (0·0 to 0·1)	0·0 (0·0 to 0·1)	0·0 (0·0 to 0·0)	0·0 (0·0 to 0·0)	0·0 (0·0 to 0·0)	0·0 (0·0 to 0·0)	0·0 (0·0 to 0·0)	0·0 (0·0 to 0·0)	0·0 (0·0 to 0·0)
Greater London	3·6 (3·3 to 3·9)	3·5 (3·4 to 3·7)	2·7 (2·6 to 2·8)	1·4 (1·4 to 1·5)	1·1 (1·0 to 1·1)	0·8 (0·7 to 0·8)	0·7 (0·7 to 0·7)	0·5 (0·5 to 0·5)	0·4 (0·4 to 0·4)	0·4 (0·4 to 0·5)
Barking and Dagenham	0·1 (0·1 to 0·2)	0·1 (0·1 to 0·1)	0·1 (0·1 to 0·1)	0·0 (0·0 to 0·0)	0·0 (0·0 to 0·0)	0·0 (0·0 to 0·0)	0·0 (0·0 to 0·0)	0·0 (0·0 to 0·0)	0·0 (0·0 to 0·0)	0·0 (0·0 to 0·0)
Barnet	0·1 (0·1 to 0·2)	0·1 (0·1 to 0·1)	0·1 (0·1 to 0·1)	0·0 (0·0 to 0·1)	0·0 (0·0 to 0·0)	0·0 (0·0 to 0·0)	0·0 (0·0 to 0·0)	0·0 (0·0 to 0·0)	0·0 (0·0 to 0·0)	0·0 (0·0 to 0·0)
Bexley	0·1 (0·1 to 0·1)	0·1 (0·1 to 0·1)	0·1 (0·1 to 0·1)	0·0 (0·0 to 0·0)	0·0 (0·0 to 0·0)	0·0 (0·0 to 0·0)	0·0 (0·0 to 0·0)	0·0 (0·0 to 0·0)	0·0 (0·0 to 0·0)	0·0 (0·0 to 0·0)
Brent	0·1 (0·1 to 0·2)	0·2 (0·1 to 0·2)	0·1 (0·1 to 0·1)	0·1 (0·1 to 0·1)	0·0 (0·0 to 0·0)	0·0 (0·0 to 0·0)	0·0 (0·0 to 0·0)	0·0 (0·0 to 0·0)	0·0 (0·0 to 0·0)	0·0 (0·0 to 0·0)
Bromley	0·1 (0·1 to 0·1)	0·1 (0·1 to 0·1)	0·1 (0·1 to 0·1)	0·0 (0·0 to 0·0)	0·0 (0·0 to 0·0)	0·0 (0·0 to 0·0)	0·0 (0·0 to 0·0)	0·0 (0·0 to 0·0)	0·0 (0·0 to 0·0)	0·0 (0·0 to 0·0)
Camden	0·1 (0·1 to 0·1)	0·1 (0·1 to 0·1)	0·1 (0·1 to 0·1)	0·0 (0·0 to 0·0)	0·0 (0·0 to 0·0)	0·0 (0·0 to 0·0)	0·0 (0·0 to 0·0)	0·0 (0·0 to 0·0)	0·0 (0·0 to 0·0)	0·0 (0·0 to 0·0)
Croydon	0·2 (0·1 to 0·2)	0·2 (0·1 to 0·2)	0·1 (0·1 to 0·1)	0·1 (0·1 to 0·1)	0·1 (0·0 to 0·1)	0·0 (0·0 to 0·0)	0·0 (0·0 to 0·0)	0·0 (0·0 to 0·0)	0·0 (0·0 to 0·0)	0·0 (0·0 to 0·0)
Ealing	0·1 (0·1 to 0·2)	0·1 (0·1 to 0·1)	0·1 (0·1 to 0·1)	0·1 (0·1 to 0·1)	0·0 (0·0 to 0·0)	0·0 (0·0 to 0·0)	0·0 (0·0 to 0·0)	0·0 (0·0 to 0·0)	0·0 (0·0 to 0·0)	0·0 (0·0 to 0·0)
Enfield	0·1 (0·1 to 0·1)	0·1 (0·1 to 0·1)	0·1 (0·1 to 0·1)	0·0 (0·0 to 0·1)	0·0 (0·0 to 0·0)	0·0 (0·0 to 0·0)	0·0 (0·0 to 0·0)	0·0 (0·0 to 0·0)	0·0 (0·0 to 0·0)	0·0 (0·0 to 0·0)
Greenwich	0·1 (0·1 to 0·2)	0·1 (0·1 to 0·1)	0·1 (0·1 to 0·1)	0·1 (0·1 to 0·1)	0·0 (0·0 to 0·0)	0·0 (0·0 to 0·0)	0·0 (0·0 to 0·0)	0·0 (0·0 to 0·0)	0·0 (0·0 to 0·0)	0·0 (0·0 to 0·0)
Hackney	0·1 (0·1 to 0·2)	0·1 (0·1 to 0·2)	0·1 (0·1 to 0·1)	0·1 (0·1 to 0·1)	0·0 (0·0 to 0·1)	0·0 (0·0 to 0·0)	0·0 (0·0 to 0·0)	0·0 (0·0 to 0·0)	0·0 (0·0 to 0·0)	0·0 (0·0 to 0·0)
Hammersmith and Fulham	0·1 (0·1 to 0·1)	0·1 (0·1 to 0·1)	0·1 (0·1 to 0·1)	0·0 (0·0 to 0·0)	0·0 (0·0 to 0·0)	0·0 (0·0 to 0·0)	0·0 (0·0 to 0·0)	0·0 (0·0 to 0·0)	0·0 (0·0 to 0·0)	0·0 (0·0 to 0·0)
Haringey	0·1 (0·1 to 0·1)	0·1 (0·1 to 0·1)	0·1 (0·1 to 0·1)	0·0 (0·0 to 0·1)	0·0 (0·0 to 0·0)	0·0 (0·0 to 0·0)	0·0 (0·0 to 0·0)	0·0 (0·0 to 0·0)	0·0 (0·0 to 0·0)	0·0 (0·0 to 0·0)
Harrow	0·1 (0·1 to 0·1)	0·1 (0·1 to 0·1)	0·1 (0·0 to 0·1)	0·0 (0·0 to 0·0)	0·0 (0·0 to 0·0)	0·0 (0·0 to 0·0)	0·0 (0·0 to 0·0)	0·0 (0·0 to 0·0)	0·0 (0·0 to 0·0)	0·0 (0·0 to 0·0)
Havering	0·1 (0·1 to 0·1)	0·1 (0·1 to 0·1)	0·1 (0·1 to 0·1)	0·0 (0·0 to 0·0)	0·0 (0·0 to 0·0)	0·0 (0·0 to 0·0)	0·0 (0·0 to 0·0)	0·0 (0·0 to 0·0)	0·0 (0·0 to 0·0)	0·0 (0·0 to 0·0)
Hillingdon	0·1 (0·1 to 0·1)	0·1 (0·1 to 0·1)	0·1 (0·1 to 0·1)	0·0 (0·0 to 0·0)	0·0 (0·0 to 0·0)	0·0 (0·0 to 0·0)	0·0 (0·0 to 0·0)	0·0 (0·0 to 0·0)	0·0 (0·0 to 0·0)	0·0 (0·0 to 0·0)
Hounslow	0·1 (0·1 to 0·1)	0·1 (0·1 to 0·1)	0·1 (0·1 to 0·1)	0·0 (0·0 to 0·0)	0·0 (0·0 to 0·0)	0·0 (0·0 to 0·0)	0·0 (0·0 to 0·0)	0·0 (0·0 to 0·0)	0·0 (0·0 to 0·0)	0·0 (0·0 to 0·0)
Islington	0·1 (0·1 to 0·1)	0·1 (0·1 to 0·1)	0·1 (0·1 to 0·1)	0·0 (0·0 to 0·0)	0·0 (0·0 to 0·0)	0·0 (0·0 to 0·0)	0·0 (0·0 to 0·0)	0·0 (0·0 to 0·0)	0·0 (0·0 to 0·0)	0·0 (0·0 to 0·0)
Kensington and Chelsea	0·1 (0·0 to 0·1)	0·1 (0·1 to 0·1)	0·1 (0·1 to 0·1)	0·0 (0·0 to 0·0)	0·0 (0·0 to 0·0)	0·0 (0·0 to 0·0)	0·0 (0·0 to 0·0)	0·0 (0·0 to 0·0)	0·0 (0·0 to 0·0)	0·0 (0·0 to 0·0)
Kingston upon Thames	0·0 (0·0 to 0·1)	0·0 (0·0 to 0·0)	0·0 (0·0 to 0·0)	0·0 (0·0 to 0·0)	0·0 (0·0 to 0·0)	0·0 (0·0 to 0·0)	0·0 (0·0 to 0·0)	0·0 (0·0 to 0·0)	0·0 (0·0 to 0·0)	0·0 (0·0 to 0·0)
Lambeth	0·2 (0·2 to 0·2)	0·2 (0·2 to 0·2)	0·2 (0·1 to 0·2)	0·1 (0·1 to 0·1)	0·1 (0·0 to 0·1)	0·0 (0·0 to 0·0)	0·0 (0·0 to 0·0)	0·0 (0·0 to 0·0)	0·0 (0·0 to 0·0)	0·0 (0·0 to 0·0)
Lewisham	0·2 (0·1 to 0·2)	0·1 (0·1 to 0·2)	0·1 (0·1 to 0·1)	0·1 (0·1 to 0·1)	0·0 (0·0 to 0·0)	0·0 (0·0 to 0·0)	0·0 (0·0 to 0·0)	0·0 (0·0 to 0·0)	0·0 (0·0 to 0·0)	0·0 (0·0 to 0·0)
Merton	0·1 (0·1 to 0·1)	0·1 (0·1 to 0·1)	0·1 (0·1 to 0·1)	0·0 (0·0 to 0·0)	0·0 (0·0 to 0·0)	0·0 (0·0 to 0·0)	0·0 (0·0 to 0·0)	0·0 (0·0 to 0·0)	0·0 (0·0 to 0·0)	0·0 (0·0 to 0·0)
Newham	0·2 (0·1 to 0·2)	0·2 (0·1 to 0·2)	0·1 (0·1 to 0·1)	0·1 (0·1 to 0·1)	0·1 (0·0 to 0·1)	0·0 (0·0 to 0·1)	0·0 (0·0 to 0·0)	0·0 (0·0 to 0·0)	0·0 (0·0 to 0·0)	0·0 (0·0 to 0·0)
Redbridge	0·1 (0·1 to 0·1)	0·1 (0·1 to 0·1)	0·1 (0·1 to 0·1)	0·0 (0·0 to 0·0)	0·0 (0·0 to 0·0)	0·0 (0·0 to 0·0)	0·0 (0·0 to 0·0)	0·0 (0·0 to 0·0)	0·0 (0·0 to 0·0)	0·0 (0·0 to 0·0)
Richmond upon Thames	0·1 (0·1 to 0·1)	0·0 (0·0 to 0·1)	0·0 (0·0 to 0·0)	0·0 (0·0 to 0·0)	0·0 (0·0 to 0·0)	0·0 (0·0 to 0·0)	0·0 (0·0 to 0·0)	0·0 (0·0 to 0·0)	0·0 (0·0 to 0·0)	0·0 (0·0 to 0·0)
Southwark	0·1 (0·1 to 0·2)	0·1 (0·1 to 0·2)	0·1 (0·1 to 0·1)	0·1 (0·0 to 0·1)	0·1 (0·0 to 0·1)	0·0 (0·0 to 0·0)	0·0 (0·0 to 0·0)	0·0 (0·0 to 0·0)	0·0 (0·0 to 0·0)	0·0 (0·0 to 0·0)
Sutton	0·1 (0·1 to 0·1)	0·1 (0·1 to 0·1)	0·0 (0·0 to 0·1)	0·0 (0·0 to 0·0)	0·0 (0·0 to 0·0)	0·0 (0·0 to 0·0)	0·0 (0·0 to 0·0)	0·0 (0·0 to 0·0)	0·0 (0·0 to 0·0)	0·0 (0·0 to 0·0)
Tower Hamlets	0·1 (0·1 to 0·2)	0·1 (0·1 to 0·2)	0·1 (0·1 to 0·1)	0·1 (0·1 to 0·1)	0·0 (0·0 to 0·0)	0·0 (0·0 to 0·0)	0·0 (0·0 to 0·0)	0·0 (0·0 to 0·0)	0·0 (0·0 to 0·0)	0·0 (0·0 to 0·0)
Waltham Forest	0·1 (0·1 to 0·2)	0·1 (0·1 to 0·2)	0·1 (0·1 to 0·1)	0·1 (0·0 to 0·1)	0·0 (0·0 to 0·0)	0·0 (0·0 to 0·0)	0·0 (0·0 to 0·0)	0·0 (0·0 to 0·0)	0·0 (0·0 to 0·0)	0·0 (0·0 to 0·0)
Wandsworth	0·1 (0·1 to 0·2)	0·1 (0·1 to 0·1)	0·1 (0·1 to 0·1)	0·1 (0·1 to 0·1)	0·0 (0·0 to 0·0)	0·0 (0·0 to 0·0)	0·0 (0·0 to 0·0)	0·0 (0·0 to 0·0)	0·0 (0·0 to 0·0)	0·0 (0·0 to 0·0)
Westminster	0·1 (0·1 to 0·1)	0·1 (0·1 to 0·1)	0·1 (0·1 to 0·1)	0·0 (0·0 to 0·1)	0·0 (0·0 to 0·0)	0·0 (0·0 to 0·0)	0·0 (0·0 to 0·0)	0·0 (0·0 to 0·0)	0·0 (0·0 to 0·0)	0·0 (0·0 to 0·0)
North East England	1·0 (0·8 to 1·3)	1·1 (1·0 to 1·3)	1·0 (0·9 to 1·0)	0·5 (0·5 to 0·5)	0·3 (0·3 to 0·3)	0·2 (0·2 to 0·2)	0·1 (0·1 to 0·1)	0·1 (0·1 to 0·1)	0·1 (0·1 to 0·1)	0·1 (0·1 to 0·1)
County Durham	0·2 (0·1 to 0·2)	0·2 (0·2 to 0·2)	0·2 (0·2 to 0·2)	0·1 (0·1 to 0·1)	0·1 (0·1 to 0·1)	0·0 (0·0 to 0·0)	0·0 (0·0 to 0·0)	0·0 (0·0 to 0·0)	0·0 (0·0 to 0·0)	0·0 (0·0 to 0·0)
Darlington	0·0 (0·0 to 0·1)	0·0 (0·0 to 0·1)	0·0 (0·0 to 0·0)	0·0 (0·0 to 0·0)	0·0 (0·0 to 0·0)	0·0 (0·0 to 0·0)	0·0 (0·0 to 0·0)	0·0 (0·0 to 0·0)	0·0 (0·0 to 0·0)	0·0 (0·0 to 0·0)
Gateshead	0·1 (0·1 to 0·1)	0·1 (0·1 to 0·1)	0·1 (0·1 to 0·1)	0·0 (0·0 to 0·0)	0·0 (0·0 to 0·0)	0·0 (0·0 to 0·0)	0·0 (0·0 to 0·0)	0·0 (0·0 to 0·0)	0·0 (0·0 to 0·0)	0·0 (0·0 to 0·0)
Hartlepool	0·0 (0·0 to 0·1)	0·1 (0·0 to 0·1)	0·0 (0·0 to 0·1)	0·0 (0·0 to 0·0)	0·0 (0·0 to 0·0)	0·0 (0·0 to 0·0)	0·0 (0·0 to 0·0)	0·0 (0·0 to 0·0)	0·0 (0·0 to 0·0)	0·0 (0·0 to 0·0)
Middlesbrough	0·1 (0·1 to 0·1)	0·1 (0·1 to 0·1)	0·1 (0·1 to 0·1)	0·0 (0·0 to 0·0)	0·0 (0·0 to 0·0)	0·0 (0·0 to 0·0)	0·0 (0·0 to 0·0)	0·0 (0·0 to 0·0)	0·0 (0·0 to 0·0)	0·0 (0·0 to 0·0)
Newcastle upon Tyne	0·1 (0·1 to 0·1)	0·1 (0·1 to 0·1)	0·1 (0·1 to 0·1)	0·0 (0·0 to 0·0)	0·0 (0·0 to 0·0)	0·0 (0·0 to 0·0)	0·0 (0·0 to 0·0)	0·0 (0·0 to 0·0)	0·0 (0·0 to 0·0)	0·0 (0·0 to 0·0)
North Tyneside	0·1 (0·1 to 0·1)	0·1 (0·1 to 0·1)	0·1 (0·1 to 0·1)	0·0 (0·0 to 0·0)	0·0 (0·0 to 0·0)	0·0 (0·0 to 0·0)	0·0 (0·0 to 0·0)	0·0 (0·0 to 0·0)	0·0 (0·0 to 0·0)	0·0 (0·0 to 0·0)
Northumberland	0·1 (0·1 to 0·1)	0·1 (0·1 to 0·1)	0·1 (0·1 to 0·1)	0·0 (0·0 to 0·1)	0·0 (0·0 to 0·0)	0·0 (0·0 to 0·0)	0·0 (0·0 to 0·0)	0·0 (0·0 to 0·0)	0·0 (0·0 to 0·0)	0·0 (0·0 to 0·0)
Redcar and Cleveland	0·1 (0·0 to 0·1)	0·1 (0·1 to 0·1)	0·1 (0·1 to 0·1)	0·0 (0·0 to 0·0)	0·0 (0·0 to 0·0)	0·0 (0·0 to 0·0)	0·0 (0·0 to 0·0)	0·0 (0·0 to 0·0)	0·0 (0·0 to 0·0)	0·0 (0·0 to 0·0)
South Tyneside	0·1 (0·0 to 0·1)	0·1 (0·1 to 0·1)	0·1 (0·0 to 0·1)	0·0 (0·0 to 0·0)	0·0 (0·0 to 0·0)	0·0 (0·0 to 0·0)	0·0 (0·0 to 0·0)	0·0 (0·0 to 0·0)	0·0 (0·0 to 0·0)	0·0 (0·0 to 0·0)

Stockton-on-Tees	0·1 (0·0 to 0·1)	0·1 (0·1 to 0·1)	0·1 (0·1 to 0·1)	0·0 (0·0 to 0·0)	0·0 (0·0 to 0·0)	0·0 (0·0 to 0·0)	0·0 (0·0 to 0·0)	0·0 (0·0 to 0·0)	0·0 (0·0 to 0·0)	0·0 (0·0 to 0·0)
Sunderland	0·1 (0·1 to 0·2)	0·1 (0·1 to 0·2)	0·1 (0·1 to 0·1)	0·1 (0·1 to 0·1)	0·0 (0·0 to 0·0)	0·0 (0·0 to 0·0)	0·0 (0·0 to 0·0)	0·0 (0·0 to 0·0)	0·0 (0·0 to 0·0)	0·0 (0·0 to 0·0)
North West England	3·0 (2·7 to 3·3)	3·2 (3·1 to 3·3)	2·6 (2·5 to 2·7)	1·4 (1·3 to 1·5)	1·0 (0·9 to 1·0)	0·6 (0·5 to 0·6)	0·5 (0·5 to 0·5)	0·4 (0·4 to 0·4)	0·3 (0·3 to 0·4)	0·4 (0·3 to 0·4)
Blackburn with Darwen	0·1 (0·1 to 0·1)	0·1 (0·1 to 0·1)	0·1 (0·1 to 0·1)	0·0 (0·0 to 0·0)	0·0 (0·0 to 0·0)	0·0 (0·0 to 0·0)	0·0 (0·0 to 0·0)	0·0 (0·0 to 0·0)	0·0 (0·0 to 0·0)	0·0 (0·0 to 0·0)
Blackpool	0·1 (0·1 to 0·1)	0·1 (0·1 to 0·1)	0·0 (0·0 to 0·0)	0·0 (0·0 to 0·0)	0·0 (0·0 to 0·0)	0·0 (0·0 to 0·0)	0·0 (0·0 to 0·0)	0·0 (0·0 to 0·0)	0·0 (0·0 to 0·0)	0·0 (0·0 to 0·0)
Bolton	0·1 (0·1 to 0·2)	0·1 (0·1 to 0·1)	0·1 (0·1 to 0·1)	0·1 (0·1 to 0·1)	0·0 (0·0 to 0·0)	0·0 (0·0 to 0·0)	0·0 (0·0 to 0·0)	0·0 (0·0 to 0·0)	0·0 (0·0 to 0·0)	0·0 (0·0 to 0·0)
Bury	0·1 (0·1 to 0·1)	0·1 (0·1 to 0·1)	0·1 (0·1 to 0·1)	0·0 (0·0 to 0·0)	0·0 (0·0 to 0·0)	0·0 (0·0 to 0·0)	0·0 (0·0 to 0·0)	0·0 (0·0 to 0·0)	0·0 (0·0 to 0·0)	0·0 (0·0 to 0·0)
Cheshire East	0·1 (0·1 to 0·1)	0·1 (0·1 to 0·1)	0·1 (0·1 to 0·1)	0·1 (0·0 to 0·1)	0·0 (0·0 to 0·0)	0·0 (0·0 to 0·0)	0·0 (0·0 to 0·0)	0·0 (0·0 to 0·0)	0·0 (0·0 to 0·0)	0·0 (0·0 to 0·0)
Cheshire West and Chester	0·1 (0·1 to 0·1)	0·1 (0·1 to 0·1)	0·1 (0·1 to 0·1)	0·0 (0·0 to 0·1)	0·0 (0·0 to 0·0)	0·0 (0·0 to 0·0)	0·0 (0·0 to 0·0)	0·0 (0·0 to 0·0)	0·0 (0·0 to 0·0)	0·0 (0·0 to 0·0)
Cumbria	0·2 (0·2 to 0·2)	0·2 (0·2 to 0·2)	0·2 (0·1 to 0·2)	0·1 (0·1 to 0·1)	0·1 (0·1 to 0·1)	0·0 (0·0 to 0·0)	0·0 (0·0 to 0·0)	0·0 (0·0 to 0·0)	0·0 (0·0 to 0·0)	0·0 (0·0 to 0·0)
Halton	0·0 (0·0 to 0·0)	0·0 (0·0 to 0·0)	0·0 (0·0 to 0·0)	0·0 (0·0 to 0·0)	0·0 (0·0 to 0·0)	0·0 (0·0 to 0·0)	0·0 (0·0 to 0·0)	0·0 (0·0 to 0·0)	0·0 (0·0 to 0·0)	0·0 (0·0 to 0·0)
Knowsley	0·1 (0·1 to 0·1)	0·1 (0·1 to 0·1)	0·1 (0·1 to 0·1)	0·0 (0·0 to 0·1)	0·0 (0·0 to 0·0)	0·0 (0·0 to 0·0)	0·0 (0·0 to 0·0)	0·0 (0·0 to 0·0)	0·0 (0·0 to 0·0)	0·0 (0·0 to 0·0)
Lancashire	0·4 (0·4 to 0·5)	0·5 (0·4 to 0·5)	0·4 (0·3 to 0·4)	0·2 (0·2 to 0·2)	0·2 (0·1 to 0·2)	0·1 (0·1 to 0·1)	0·1 (0·1 to 0·1)	0·1 (0·1 to 0·1)	0·1 (0·0 to 0·1)	0·1 (0·0 to 0·1)
Liverpool	0·2 (0·2 to 0·3)	0·3 (0·2 to 0·3)	0·2 (0·2 to 0·2)	0·1 (0·1 to 0·1)	0·1 (0·1 to 0·1)	0·0 (0·0 to 0·0)	0·0 (0·0 to 0·0)	0·0 (0·0 to 0·0)	0·0 (0·0 to 0·0)	0·0 (0·0 to 0·0)
Manchester	0·3 (0·2 to 0·3)	0·3 (0·3 to 0·3)	0·3 (0·2 to 0·3)	0·1 (0·1 to 0·1)	0·1 (0·1 to 0·1)	0·1 (0·1 to 0·1)	0·1 (0·1 to 0·1)	0·0 (0·0 to 0·1)	0·0 (0·0 to 0·0)	0·0 (0·0 to 0·1)
Oldham	0·1 (0·1 to 0·1)	0·1 (0·1 to 0·1)	0·1 (0·1 to 0·1)	0·1 (0·1 to 0·1)	0·0 (0·0 to 0·0)	0·0 (0·0 to 0·0)	0·0 (0·0 to 0·0)	0·0 (0·0 to 0·0)	0·0 (0·0 to 0·0)	0·0 (0·0 to 0·0)
Rochdale	0·1 (0·1 to 0·1)	0·1 (0·1 to 0·1)	0·1 (0·1 to 0·1)	0·1 (0·1 to 0·1)	0·0 (0·0 to 0·0)	0·0 (0·0 to 0·0)	0·0 (0·0 to 0·0)	0·0 (0·0 to 0·0)	0·0 (0·0 to 0·0)	0·0 (0·0 to 0·0)
Salford	0·1 (0·1 to 0·2)	0·1 (0·1 to 0·2)	0·1 (0·1 to 0·1)	0·1 (0·1 to 0·1)	0·0 (0·0 to 0·0)	0·0 (0·0 to 0·0)	0·0 (0·0 to 0·0)	0·0 (0·0 to 0·0)	0·0 (0·0 to 0·0)	0·0 (0·0 to 0·0)
Sefton	0·1 (0·1 to 0·1)	0·1 (0·1 to 0·1)	0·1 (0·1 to 0·1)	0·0 (0·0 to 0·0)	0·0 (0·0 to 0·0)	0·0 (0·0 to 0·0)	0·0 (0·0 to 0·0)	0·0 (0·0 to 0·0)	0·0 (0·0 to 0·0)	0·0 (0·0 to 0·0)
St Helens	0·1 (0·1 to 0·1)	0·1 (0·1 to 0·1)	0·1 (0·1 to 0·1)	0·0 (0·0 to 0·0)	0·0 (0·0 to 0·0)	0·0 (0·0 to 0·0)	0·0 (0·0 to 0·0)	0·0 (0·0 to 0·0)	0·0 (0·0 to 0·0)	0·0 (0·0 to 0·0)
Stockport	0·1 (0·1 to 0·1)	0·1 (0·1 to 0·1)	0·1 (0·1 to 0·1)	0·0 (0·0 to 0·1)	0·0 (0·0 to 0·0)	0·0 (0·0 to 0·0)	0·0 (0·0 to 0·0)	0·0 (0·0 to 0·0)	0·0 (0·0 to 0·0)	0·0 (0·0 to 0·0)
Tameside	0·1 (0·1 to 0·1)	0·1 (0·1 to 0·1)	0·1 (0·1 to 0·1)	0·0 (0·0 to 0·1)	0·0 (0·0 to 0·0)	0·0 (0·0 to 0·0)	0·0 (0·0 to 0·0)	0·0 (0·0 to 0·0)	0·0 (0·0 to 0·0)	0·0 (0·0 to 0·0)
Trafford	0·1 (0·1 to 0·1)	0·1 (0·1 to 0·1)	0·1 (0·1 to 0·1)	0·0 (0·0 to 0·1)	0·0 (0·0 to 0·0)	0·0 (0·0 to 0·0)	0·0 (0·0 to 0·0)	0·0 (0·0 to 0·0)	0·0 (0·0 to 0·0)	0·0 (0·0 to 0·0)
Warrington	0·1 (0·0 to 0·1)	0·1 (0·1 to 0·1)	0·1 (0·0 to 0·1)	0·0 (0·0 to 0·0)	0·0 (0·0 to 0·0)	0·0 (0·0 to 0·0)	0·0 (0·0 to 0·0)	0·0 (0·0 to 0·0)	0·0 (0·0 to 0·0)	0·0 (0·0 to 0·0)
Wigan	0·1 (0·1 to 0·1)	0·1 (0·1 to 0·1)	0·1 (0·1 to 0·1)	0·1 (0·1 to 0·1)	0·0 (0·0 to 0·0)	0·0 (0·0 to 0·0)	0·0 (0·0 to 0·0)	0·0 (0·0 to 0·0)	0·0 (0·0 to 0·0)	0·0 (0·0 to 0·0)
Wirral	0·2 (0·1 to 0·2)	0·2 (0·2 to 0·2)	0·1 (0·1 to 0·1)	0·1 (0·1 to 0·1)	0·0 (0·0 to 0·0)	0·0 (0·0 to 0·0)	0·0 (0·0 to 0·0)	0·0 (0·0 to 0·0)	0·0 (0·0 to 0·0)	0·0 (0·0 to 0·0)
South East England	2·1 (1·9 to 2·3)	2·1 (2·0 to 2·2)	1·7 (1·6 to 1·8)	1·1 (1·1 to 1·2)	0·9 (0·8 to 0·9)	0·5 (0·4 to 0·5)	0·5 (0·4 to 0·5)	0·4 (0·4 to 0·4)	0·3 (0·3 to 0·3)	0·3 (0·3 to 0·4)
Bracknell Forest	0·0 (0·0 to 0·0)	0·0 (0·0 to 0·0)	0·0 (0·0 to 0·0)	0·0 (0·0 to 0·0)	0·0 (0·0 to 0·0)	0·0 (0·0 to 0·0)	0·0 (0·0 to 0·0)	0·0 (0·0 to 0·0)	0·0 (0·0 to 0·0)	0·0 (0·0 to 0·0)
Brighton and Hove	0·1 (0·1 to 0·1)	0·1 (0·1 to 0·1)	0·1 (0·1 to 0·1)	0·0 (0·0 to 0·0)	0·0 (0·0 to 0·0)	0·0 (0·0 to 0·0)	0·0 (0·0 to 0·0)	0·0 (0·0 to 0·0)	0·0 (0·0 to 0·0)	0·0 (0·0 to 0·0)
Buckinghamshire	0·1 (0·1 to 0·1)	0·1 (0·1 to 0·1)	0·1 (0·1 to 0·1)	0·1 (0·1 to 0·1)	0·1 (0·0 to 0·1)	0·0 (0·0 to 0·0)	0·0 (0·0 to 0·0)	0·0 (0·0 to 0·0)	0·0 (0·0 to 0·0)	0·0 (0·0 to 0·0)
East Sussex	0·1 (0·1 to 0·1)	0·1 (0·1 to 0·1)	0·1 (0·1 to 0·1)	0·1 (0·0 to 0·1)	0·0 (0·0 to 0·0)	0·0 (0·0 to 0·0)	0·0 (0·0 to 0·0)	0·0 (0·0 to 0·0)	0·0 (0·0 to 0·0)	0·0 (0·0 to 0·0)
Hampshire	0·3 (0·2 to 0·3)	0·3 (0·3 to 0·4)	0·3 (0·3 to 0·3)	0·2 (0·2 to 0·2)	0·1 (0·1 to 0·1)	0·1 (0·1 to 0·1)	0·1 (0·1 to 0·1)	0·1 (0·0 to 0·1)	0·0 (0·0 to 0·1)	0·1 (0·0 to 0·1)
Isle of Wight	0·1 (0·0 to 0·1)	0·0 (0·0 to 0·0)	0·0 (0·0 to 0·0)	0·0 (0·0 to 0·0)	0·0 (0·0 to 0·0)	0·0 (0·0 to 0·0)	0·0 (0·0 to 0·0)	0·0 (0·0 to 0·0)	0·0 (0·0 to 0·0)	0·0 (0·0 to 0·0)
Kent	0·4 (0·3 to 0·5)	0·4 (0·4 to 0·4)	0·3 (0·3 to 0·4)	0·2 (0·2 to 0·2)	0·2 (0·1 to 0·2)	0·1 (0·1 to 0·1)	0·1 (0·1 to 0·1)	0·1 (0·1 to 0·1)	0·1 (0·1 to 0·1)	0·1 (0·1 to 0·1)
Medway	0·1 (0·1 to 0·1)	0·1 (0·1 to 0·1)	0·1 (0·1 to 0·1)	0·1 (0·1 to 0·1)	0·0 (0·0 to 0·0)	0·0 (0·0 to 0·0)	0·0 (0·0 to 0·0)	0·0 (0·0 to 0·0)	0·0 (0·0 to 0·0)	0·0 (0·0 to 0·0)
Milton Keynes	0·0 (0·0 to 0·0)	0·0 (0·0 to 0·0)	0·0 (0·0 to 0·0)	0·0 (0·0 to 0·0)	0·0 (0·0 to 0·0)	0·0 (0·0 to 0·0)	0·0 (0·0 to 0·0)	0·0 (0·0 to 0·0)	0·0 (0·0 to 0·0)	0·0 (0·0 to 0·0)
Oxfordshire	0·1 (0·1 to 0·1)	0·1 (0·1 to 0·1)	0·1 (0·1 to 0·1)	0·1 (0·1 to 0·1)	0·1 (0·1 to 0·1)	0·0 (0·0 to 0·0)	0·0 (0·0 to 0·0)	0·0 (0·0 to 0·0)	0·0 (0·0 to 0·0)	0·0 (0·0 to 0·0)
Portsmouth	0·1 (0·1 to 0·1)	0·1 (0·1 to 0·1)	0·1 (0·1 to 0·1)	0·0 (0·0 to 0·0)	0·0 (0·0 to 0·0)	0·0 (0·0 to 0·0)	0·0 (0·0 to 0·0)	0·0 (0·0 to 0·0)	0·0 (0·0 to 0·0)	0·0 (0·0 to 0·0)
Reading	0·0 (0·0 to 0·0)	0·0 (0·0 to 0·0)	0·0 (0·0 to 0·0)	0·0 (0·0 to 0·0)	0·0 (0·0 to 0·0)	0·0 (0·0 to 0·0)	0·0 (0·0 to 0·0)	0·0 (0·0 to 0·0)	0·0 (0·0 to 0·0)	0·0 (0·0 to 0·0)
Slough	0·0 (0·0 to 0·1)	0·0 (0·0 to 0·1)	0·0 (0·0 to 0·0)	0·0 (0·0 to 0·0)	0·0 (0·0 to 0·0)	0·0 (0·0 to 0·0)	0·0 (0·0 to 0·0)	0·0 (0·0 to 0·0)	0·0 (0·0 to 0·0)	0·0 (0·0 to 0·0)
Southampton	0·1 (0·1 to 0·1)	0·1 (0·1 to 0·1)	0·1 (0·1 to 0·1)	0·0 (0·0 to 0·0)	0·0 (0·0 to 0·0)	0·0 (0·0 to 0·0)	0·0 (0·0 to 0·0)	0·0 (0·0 to 0·0)	0·0 (0·0 to 0·0)	0·0 (0·0 to 0·0)
Surrey	0·3 (0·3 to 0·4)	0·3 (0·3 to 0·3)	0·2 (0·2 to 0·2)	0·1 (0·1 to 0·2)	0·1 (0·1 to 0·1)	0·1 (0·0 to 0·1)	0·0 (0·0 to 0·1)	0·0 (0·0 to 0·0)	0·0 (0·0 to 0·0)	0·0 (0·0 to 0·0)
West Berkshire	0·0 (0·0 to 0·0)	0·0 (0·0 to 0·0)	0·0 (0·0 to 0·0)	0·0 (0·0 to 0·0)	0·0 (0·0 to 0·0)	0·0 (0·0 to 0·0)	0·0 (0·0 to 0·0)	0·0 (0·0 to 0·0)	0·0 (0·0 to 0·0)	0·0 (0·0 to 0·0)
West Sussex	0·2 (0·2 to 0·2)	0·2 (0·1 to 0·2)	0·1 (0·1 to 0·1)	0·1 (0·1 to 0·1)	0·1 (0·1 to 0·1)	0·0 (0·0 to 0·0)	0·0 (0·0 to 0·0)	0·0 (0·0 to 0·0)	0·0 (0·0 to 0·0)	0·0 (0·0 to 0·0)
Windsor and Maidenhead	0·0 (0·0 to 0·0)	0·0 (0·0 to 0·0)	0·0 (0·0 to 0·0)	0·0 (0·0 to 0·0)	0·0 (0·0 to 0·0)	0·0 (0·0 to 0·0)	0·0 (0·0 to 0·0)	0·0 (0·0 to 0·0)	0·0 (0·0 to 0·0)	0·0 (0·0 to 0·0)
Wokingham	0·0 (0·0 to 0·0)	0·0 (0·0 to 0·0)	0·0 (0·0 to 0·0)	0·0 (0·0 to 0·0)	0·0 (0·0 to 0·0)	0·0 (0·0 to 0·0)	0·0 (0·0 to 0·0)	0·0 (0·0 to 0·0)	0·0 (0·0 to 0·0)	0·0 (0·0 to 0·0)
South West England	1·3 (1·1 to 1·5)	1·3 (1·2 to 1·4)	1·1 (1·0 to 1·2)	0·7 (0·6 to 0·7)	0·5 (0·5 to 0·5)	0·3 (0·3 to 0·3)	0·3 (0·2 to 0·3)	0·2 (0·2 to 0·2)	0·2 (0·1 to 0·2)	0·2 (0·1 to 0·2)
Bath and North East Somerset	0·0 (0·0 to 0·1)	0·0 (0·0 to 0·0)	0·0 (0·0 to 0·0)	0·0 (0·0 to 0·0)	0·0 (0·0 to 0·0)	0·0 (0·0 to 0·0)	0·0 (0·0 to 0·0)	0·0 (0·0 to 0·0)	0·0 (0·0 to 0·0)	0·0 (0·0 to 0·0)
Bournemouth	0·0 (0·0 to 0·1)	0·0 (0·0 to 0·0)	0·0 (0·0 to 0·0)	0·0 (0·0 to 0·0)	0·0 (0·0 to 0·0)	0·0 (0·0 to 0·0)	0·0 (0·0 to 0·0)	0·0 (0·0 to 0·0)	0·0 (0·0 to 0·0)	0·0 (0·0 to 0·0)
Bristol, City of	0·2 (0·1 to 0·2)	0·2 (0·1 to 0·2)	0·1 (0·1 to 0·2)	0·1 (0·1 to 0·1)	0·1 (0·0 to 0·1)	0·0 (0·0 to 0·0)	0·0 (0·0 to 0·0)	0·0 (0·0 to 0·0)	0·0 (0·0 to 0·0)	0·0 (0·0 to 0·0)
Cornwall	0·1 (0·1 to 0·2)	0·1 (0·1 to 0·1)	0·1 (0·1 to 0·1)	0·1 (0·1 to 0·1)	0·0 (0·0 to 0·1)	0·0 (0·0 to 0·0)	0·0 (0·0 to 0·0)	0·0 (0·0 to 0·0)	0·0 (0·0 to 0·0)	0·0 (0·0 to 0·0)
Devon	0·2 (0·1 to 0·2)	0·2 (0·1 to 0·2)	0·1 (0·1 to 0·1)	0·1 (0·1 to 0·1)	0·1 (0·1 to 0·1)	0·0 (0·0 to 0·0)	0·0 (0·0 to 0·0)	0·0 (0·0 to 0·0)	0·0 (0·0 to 0·0)	0·0 (0·0 to 0·0)
Dorset	0·1 (0·1 to 0·1)	0·1 (0·1 to 0·1)	0·1 (0·1 to 0·1)	0·0 (0·0 to 0·0)	0·0 (0·0 to 0·0)	0·0 (0·0 to 0·0)	0·0 (0·0 to 0·0)	0·0 (0·0 to 0·0)	0·0 (0·0 to 0·0)	0·0 (0·0 to 0·0)
Gloucestershire	0·1 (0·1 to 0·2)	0·1 (0·1 to 0·2)	0·1 (0·1 to 0·1)	0·1 (0·1 to 0·1)	0·1 (0·1 to 0·1)	0·0 (0·0 to 0·0)	0·0 (0·0 to 0·0)	0·0 (0·0 to 0·0)	0·0 (0·0 to 0·0)	0·0 (0·0 to 0·0)
North Somerset	0·0 (0·0 to 0·1)	0·0 (0·0 to 0·0)	0·0 (0·0 to 0·0)	0·0 (0·0 to 0·0)	0·0 (0·0 to 0·0)	0·0 (0·0 to 0·0)	0·0 (0·0 to 0·0)	0·0 (0·0 to 0·0)	0·0 (0·0 to 0·0)	0·0 (0·0 to 0·0)
Plymouth	0·1 (0·1 to 0·1)	0·1 (0·1 to 0·1)	0·1 (0·1 to 0·1)	0·1 (0·0 to 0·1)	0·0 (0·0 to 0·0)	0·0 (0·0 to 0·0)	0·0 (0·0 to 0·0)	0·0 (0·0 to 0·0)	0·0 (0·0 to 0·0)	0·0 (0·0 to 0·0)
Poole	0·0 (0·0 to 0·0)	0·0 (0·0 to 0·0)	0·0 (0·0 to 0·0)	0·0 (0·0 to 0·0)	0·0 (0·0 to 0·0)	0·0 (0·0 to 0·0)	0·0 (0·0 to 0·0)	0·0 (0·0 to 0·0)	0·0 (0·0 to 0·0)	0·0 (0·0 to 0·0)
Somerset	0·1 (0·1 to 0·1)	0·1 (0·1 to 0·1)	0·1 (0·1 to 0·1)	0·1 (0·1 to 0·1)	0·0 (0·0 to 0·0)	0·0 (0·0 to 0·0)	0·0 (0·0 to 0·0)	0·0 (0·0 to 0·0)	0·0 (0·0 to 0·0)	0·0 (0·0 to 0·0)
South Gloucestershire	0·0 (0·0 to 0·1)	0·1 (0·1 to 0·1)	0·0 (0·0 to 0·1)	0·0 (0·0 to 0·0)	0·0 (0·0 to 0·0)	0·0 (0·0 to 0·0)	0·0 (0·0 to 0·0)	0·0 (0·0 to 0·0)	0·0 (0·0 to 0·0)	0·0 (0·0 to 0·0)
Swindon	0·1 (0·0 to 0·1)	0·1 (0·1 to 0·1)	0·1 (0·1 to 0·1)	0·0 (0·0 to 0·0)	0·0 (0·0 to 0·0)	0·0 (0·0 to 0·0)	0·0 (0·0 to 0·0)	0·0 (0·0 to 0·0)	0·0 (0·0 to 0·0)	0·0 (0·0 to 0·0)
Torbay	0·0 (0·0 to 0·0)	0·0 (0·0 to 0·0)	0·0 (0·0 to 0·0)	0·0 (0·0 to 0·0)	0·0 (0·0 to 0·0)	0·0 (0·0 to 0·0)	0·0 (0·0 to 0·0)	0·0 (0·0 to 0·0)	0·0 (0·0 to 0·0)	0·0 (0·0 to 0·0)
Wiltshire	0·1 (0·1 to 0·1)	0·1 (0·1 to 0·1)	0·1 (0·1 to 0·1)	0·1 (0·1 to 0·1)	0·0 (0·0 to 0·0)	0·0 (0·0 to 0·0)	0·0 (0·0 to 0·0)	0·0 (0·0 to 0·0)	0·0 (0·0 to 0·0)	0·0 (0·0 to 0·0)
West Midlands	2·2 (2·0 to 2·4)	2·5 (2·4 to 2·6)	2·1 (2·0 to 2·2)	1·1 (1·0 to 1·2)	0·9 (0·8 to 0·9)	0·5 (0·5 to 0·5)	0·5 (0·5 to 0·5)	0·4 (0·4 to 0·4)	0·3 (0·3 to 0·4)	0·4 (0·3 to 0·4)
Birmingham	0·6 (0·5 to 0·6)	0·6 (0·6 to 0·7)	0·5 (0·5 to 0·6)	0·3 (0·3 to 0·3)	0·2 (0·2 to 0·3)	0·2 (0·1 to 0·2)	0·1 (0·1 to 0·2)	0·1 (0·1 to 0·1)	0·1 (0·1 to 0·1)	0·1 (0·1 to 0·1)

Coventry	0·2 (0·1 to 0·2)	0·2 (0·2 to 0·2)	0·2 (0·2 to 0·2)	0·1 (0·1 to 0·1)	0·1 (0·1 to 0·1)	0·0 (0·0 to 0·0)	0·0 (0·0 to 0·0)	0·0 (0·0 to 0·0)	0·0 (0·0 to 0·0)	0·0 (0·0 to 0·0)
Dudley	0·1 (0·1 to 0·1)	0·1 (0·1 to 0·1)	0·1 (0·1 to 0·1)	0·0 (0·0 to 0·1)	0·0 (0·0 to 0·1)	0·0 (0·0 to 0·0)	0·0 (0·0 to 0·0)	0·0 (0·0 to 0·0)	0·0 (0·0 to 0·0)	0·0 (0·0 to 0·0)
Herefordshire, County of	0·1 (0·0 to 0·1)	0·1 (0·0 to 0·1)	0·0 (0·0 to 0·1)	0·0 (0·0 to 0·0)	0·0 (0·0 to 0·0)	0·0 (0·0 to 0·0)	0·0 (0·0 to 0·0)	0·0 (0·0 to 0·0)	0·0 (0·0 to 0·0)	0·0 (0·0 to 0·0)
Sandwell	0·2 (0·2 to 0·2)	0·2 (0·2 to 0·2)	0·2 (0·2 to 0·2)	0·1 (0·1 to 0·1)	0·1 (0·1 to 0·1)	0·0 (0·0 to 0·0)	0·0 (0·0 to 0·0)	0·0 (0·0 to 0·0)	0·0 (0·0 to 0·0)	0·0 (0·0 to 0·0)
Shropshire	0·1 (0·1 to 0·1)	0·1 (0·1 to 0·1)	0·1 (0·1 to 0·1)	0·0 (0·0 to 0·0)	0·0 (0·0 to 0·0)	0·0 (0·0 to 0·0)	0·0 (0·0 to 0·0)	0·0 (0·0 to 0·0)	0·0 (0·0 to 0·0)	0·0 (0·0 to 0·0)
Solihull	0·1 (0·1 to 0·1)	0·1 (0·1 to 0·1)	0·1 (0·1 to 0·1)	0·0 (0·0 to 0·0)	0·0 (0·0 to 0·0)	0·0 (0·0 to 0·0)	0·0 (0·0 to 0·0)	0·0 (0·0 to 0·0)	0·0 (0·0 to 0·0)	0·0 (0·0 to 0·0)
Staffordshire	0·2 (0·2 to 0·3)	0·3 (0·3 to 0·3)	0·3 (0·2 to 0·3)	0·1 (0·1 to 0·2)	0·1 (0·1 to 0·1)	0·1 (0·0 to 0·1)	0·1 (0·0 to 0·1)	0·0 (0·0 to 0·0)	0·0 (0·0 to 0·0)	0·0 (0·0 to 0·1)
Stoke-on-Trent	0·1 (0·1 to 0·2)	0·2 (0·1 to 0·2)	0·1 (0·1 to 0·1)	0·1 (0·1 to 0·1)	0·0 (0·0 to 0·1)	0·0 (0·0 to 0·0)	0·0 (0·0 to 0·0)	0·0 (0·0 to 0·0)	0·0 (0·0 to 0·0)	0·0 (0·0 to 0·0)
Telford and Wrekin	0·0 (0·0 to 0·0)	0·0 (0·0 to 0·0)	0·0 (0·0 to 0·0)	0·0 (0·0 to 0·0)	0·0 (0·0 to 0·0)	0·0 (0·0 to 0·0)	0·0 (0·0 to 0·0)	0·0 (0·0 to 0·0)	0·0 (0·0 to 0·0)	0·0 (0·0 to 0·0)
Walsall	0·1 (0·1 to 0·1)	0·2 (0·1 to 0·2)	0·1 (0·1 to 0·1)	0·1 (0·1 to 0·1)	0·0 (0·0 to 0·0)	0·0 (0·0 to 0·0)	0·0 (0·0 to 0·0)	0·0 (0·0 to 0·0)	0·0 (0·0 to 0·0)	0·0 (0·0 to 0·0)
Warwickshire	0·1 (0·1 to 0·2)	0·2 (0·1 to 0·2)	0·1 (0·1 to 0·1)	0·1 (0·1 to 0·1)	0·1 (0·1 to 0·1)	0·0 (0·0 to 0·0)	0·0 (0·0 to 0·0)	0·0 (0·0 to 0·0)	0·0 (0·0 to 0·0)	0·0 (0·0 to 0·0)
Wolverhampton	0·1 (0·1 to 0·1)	0·1 (0·1 to 0·1)	0·1 (0·1 to 0·1)	0·1 (0·1 to 0·1)	0·0 (0·0 to 0·1)	0·0 (0·0 to 0·0)	0·0 (0·0 to 0·0)	0·0 (0·0 to 0·0)	0·0 (0·0 to 0·0)	0·0 (0·0 to 0·0)
Worcestershire	0·1 (0·1 to 0·2)	0·1 (0·1 to 0·2)	0·1 (0·1 to 0·1)	0·1 (0·1 to 0·1)	0·1 (0·1 to 0·1)	0·0 (0·0 to 0·0)	0·0 (0·0 to 0·0)	0·0 (0·0 to 0·0)	0·0 (0·0 to 0·0)	0·0 (0·0 to 0·0)
Yorkshire and the Humber	2·2 (1·9 to 2·5)	2·3 (2·1 to 2·5)	1·9 (1·8 to 2·0)	1·0 (0·9 to 1·1)	0·7 (0·7 to 0·7)	0·5 (0·4 to 0·5)	0·4 (0·4 to 0·4)	0·3 (0·3 to 0·3)	0·2 (0·2 to 0·2)	0·3 (0·2 to 0·3)
Barnsley	0·1 (0·1 to 0·1)	0·1 (0·1 to 0·1)	0·1 (0·1 to 0·1)	0·0 (0·0 to 0·1)	0·0 (0·0 to 0·0)	0·0 (0·0 to 0·0)	0·0 (0·0 to 0·0)	0·0 (0·0 to 0·0)	0·0 (0·0 to 0·0)	0·0 (0·0 to 0·0)
Bradford	0·3 (0·2 to 0·4)	0·3 (0·3 to 0·3)	0·2 (0·2 to 0·3)	0·1 (0·1 to 0·2)	0·1 (0·1 to 0·1)	0·1 (0·1 to 0·1)	0·1 (0·1 to 0·1)	0·0 (0·0 to 0·1)	0·0 (0·0 to 0·0)	0·0 (0·0 to 0·1)
Calderdale	0·1 (0·1 to 0·1)	0·1 (0·1 to 0·1)	0·1 (0·1 to 0·1)	0·0 (0·0 to 0·0)	0·0 (0·0 to 0·0)	0·0 (0·0 to 0·0)	0·0 (0·0 to 0·0)	0·0 (0·0 to 0·0)	0·0 (0·0 to 0·0)	0·0 (0·0 to 0·0)
Doncaster	0·1 (0·1 to 0·1)	0·1 (0·1 to 0·1)	0·1 (0·1 to 0·1)	0·1 (0·1 to 0·1)	0·0 (0·0 to 0·0)	0·0 (0·0 to 0·0)	0·0 (0·0 to 0·0)	0·0 (0·0 to 0·0)	0·0 (0·0 to 0·0)	0·0 (0·0 to 0·0)
East Riding of Yorkshire	0·1 (0·1 to 0·1)	0·1 (0·1 to 0·1)	0·1 (0·1 to 0·1)	0·0 (0·0 to 0·0)	0·0 (0·0 to 0·0)	0·0 (0·0 to 0·0)	0·0 (0·0 to 0·0)	0·0 (0·0 to 0·0)	0·0 (0·0 to 0·0)	0·0 (0·0 to 0·0)
Kingston upon Hull, City of	0·1 (0·1 to 0·2)	0·2 (0·1 to 0·2)	0·1 (0·1 to 0·1)	0·1 (0·1 to 0·1)	0·0 (0·0 to 0·1)	0·0 (0·0 to 0·0)	0·0 (0·0 to 0·0)	0·0 (0·0 to 0·0)	0·0 (0·0 to 0·0)	0·0 (0·0 to 0·0)
Kirklees	0·2 (0·2 to 0·2)	0·2 (0·2 to 0·2)	0·2 (0·2 to 0·2)	0·1 (0·1 to 0·1)	0·1 (0·1 to 0·1)	0·0 (0·0 to 0·0)	0·0 (0·0 to 0·0)	0·0 (0·0 to 0·0)	0·0 (0·0 to 0·0)	0·0 (0·0 to 0·0)
Leeds	0·3 (0·3 to 0·4)	0·3 (0·3 to 0·4)	0·3 (0·2 to 0·3)	0·1 (0·1 to 0·2)	0·1 (0·1 to 0·1)	0·1 (0·1 to 0·1)	0·1 (0·0 to 0·1)	0·0 (0·0 to 0·0)	0·0 (0·0 to 0·0)	0·0 (0·0 to 0·0)
North East Lincolnshire	0·1 (0·1 to 0·1)	0·1 (0·1 to 0·1)	0·1 (0·1 to 0·1)	0·0 (0·0 to 0·0)	0·0 (0·0 to 0·0)	0·0 (0·0 to 0·0)	0·0 (0·0 to 0·0)	0·0 (0·0 to 0·0)	0·0 (0·0 to 0·0)	0·0 (0·0 to 0·0)
North Lincolnshire	0·1 (0·0 to 0·1)	0·1 (0·1 to 0·1)	0·1 (0·0 to 0·1)	0·0 (0·0 to 0·0)	0·0 (0·0 to 0·0)	0·0 (0·0 to 0·0)	0·0 (0·0 to 0·0)	0·0 (0·0 to 0·0)	0·0 (0·0 to 0·0)	0·0 (0·0 to 0·0)
North Yorkshire	0·2 (0·1 to 0·2)	0·2 (0·2 to 0·2)	0·1 (0·1 to 0·1)	0·1 (0·1 to 0·1)	0·1 (0·0 to 0·1)	0·0 (0·0 to 0·0)	0·0 (0·0 to 0·0)	0·0 (0·0 to 0·0)	0·0 (0·0 to 0·0)	0·0 (0·0 to 0·0)
Rotherham	0·1 (0·1 to 0·1)	0·1 (0·1 to 0·1)	0·1 (0·1 to 0·1)	0·1 (0·0 to 0·1)	0·0 (0·0 to 0·0)	0·0 (0·0 to 0·0)	0·0 (0·0 to 0·0)	0·0 (0·0 to 0·0)	0·0 (0·0 to 0·0)	0·0 (0·0 to 0·0)
Sheffield	0·2 (0·2 to 0·3)	0·2 (0·2 to 0·2)	0·2 (0·1 to 0·2)	0·1 (0·1 to 0·1)	0·1 (0·1 to 0·1)	0·0 (0·0 to 0·1)	0·0 (0·0 to 0·0)	0·0 (0·0 to 0·0)	0·0 (0·0 to 0·0)	0·0 (0·0 to 0·0)
Wakefield	0·1 (0·1 to 0·2)	0·2 (0·1 to 0·2)	0·1 (0·1 to 0·1)	0·1 (0·1 to 0·1)	0·0 (0·0 to 0·0)	0·0 (0·0 to 0·0)	0·0 (0·0 to 0·0)	0·0 (0·0 to 0·0)	0·0 (0·0 to 0·0)	0·0 (0·0 to 0·0)
York	0·1 (0·0 to 0·1)	0·1 (0·0 to 0·1)	0·0 (0·0 to 0·0)	0·0 (0·0 to 0·0)	0·0 (0·0 to 0·0)	0·0 (0·0 to 0·0)	0·0 (0·0 to 0·0)	0·0 (0·0 to 0·0)	0·0 (0·0 to 0·0)	0·0 (0·0 to 0·0)
Northern Ireland	1·1 (1·0 to 1·3)	1·0 (0·9 to 1·0)	0·8 (0·8 to 0·9)	0·5 (0·4 to 0·5)	0·2 (0·2 to 0·3)	0·1 (0·1 to 0·2)	0·1 (0·1 to 0·2)	0·1 (0·1 to 0·1)	0·1 (0·1 to 0·1)	0·1 (0·1 to 0·1)
Scotland	3·5 (3·3 to 3·8)	3·1 (3·0 to 3·2)	2·0 (1·9 to 2·1)	1·0 (0·9 to 1·0)	0·6 (0·6 to 0·7)	0·3 (0·3 to 0·4)	0·3 (0·2 to 0·3)	0·2 (0·2 to 0·2)	0·2 (0·1 to 0·2)	0·2 (0·2 to 0·2)
Wales	1·2 (0·9 to 1·6)	1·2 (1·0 to 1·4)	1·0 (0·9 to 1·2)	0·6 (0·5 to 0·6)	0·3 (0·3 to 0·4)	0·2 (0·2 to 0·2)	0·2 (0·2 to 0·2)	0·1 (0·1 to 0·1)	0·1 (0·1 to 0·1)	0·1 (0·1 to 0·1)
Latin America and Caribbean	1318·6 (1165·1 to 1496·2)	1229·1 (1138·7 to 1325·9)	1098·5 (1023·2 to 1173·1)	823·2 (777·4 to 871·2)	538·2 (509·0 to 568·4)	362·4 (337·1 to 388·8)	317·2 (289·1 to 346·8)	181·2 (150·3 to 215·3)	164·1 (136·5 to 196·9)	154·8 (125·2 to 189·9)
Andean Latin America	228·2 (200·1 to 261·5)	225·3 (207·4 to 244·8)	200·5 (189·4 to 212·9)	130·7 (122·6 to 139·2)	84·9 (79·4 to 91·1)	53·8 (49·7 to 58·0)	33·2 (29·1 to 37·6)	25·4 (21·1 to 30·4)	21·9 (18·0 to 26·5)	20·6 (16·2 to 25·7)
Bolivia	51·8 (43·5 to 61·3)	50·3 (44·7 to 56·8)	42·9 (39·3 to 46·5)	33·5 (31·1 to 35·9)	24·8 (23·2 to 26·6)	17·0 (15·6 to 18·5)	11·2 (9·9 to 12·5)	7·6 (6·4 to 9·0)	7·1 (6·0 to 8·3)	6·8 (5·7 to 8·0)
Ecuador	29·8 (24·4 to 36·1)	33·0 (30·4 to 35·8)	29·8 (27·5 to 32·2)	20·6 (18·9 to 22·6)	12·3 (11·2 to 13·5)	11·3 (9·8 to 13·0)	7·8 (6·4 to 9·4)	5·6 (4·4 to 6·9)	4·9 (3·9 to 6·2)	4·4 (3·4 to 5·8)
Peru	146·6 (120·5 to 179·6)	142·0 (127·1 to 156·2)	127·8 (118·6 to 137·4)	76·6 (69·7 to 83·9)	47·9 (43·5 to 52·9)	25·5 (22·7 to 28·1)	14·3 (12·3 to 16·6)	12·2 (9·6 to 15·2)	9·9 (7·8 to 12·5)	9·4 (6·4 to 12·8)
Caribbean	113·4 (98·9 to 130·2)	110·5 (103·8 to 117·2)	95·4 (89·8 to 101·0)	75·0 (69·6 to 80·9)	62·6 (57·8 to 67·9)	43·8 (40·3 to 47·7)	102·6 (95·1 to 110·7)	33·3 (27·9 to 39·4)	32·5 (27·0 to 38·9)	32·5 (26·9 to 39·0)
Antigua and Barbuda	0·2 (0·2 to 0·3)	0·1 (0·1 to 0·2)	0·1 (0·1 to 0·1)	0·0 (0·0 to 0·0)	0·0 (0·0 to 0·0)	0·0 (0·0 to 0·0)	0·0 (0·0 to 0·0)	0·0 (0·0 to 0·0)	0·0 (0·0 to 0·0)	0·0 (0·0 to 0·0)
The Bahamas	0·3 (0·3 to 0·4)	0·3 (0·2 to 0·3)	0·2 (0·2 to 0·2)	0·2 (0·1 to 0·2)	0·1 (0·1 to 0·1)	0·1 (0·1 to 0·1)	0·1 (0·1 to 0·1)	0·1 (0·1 to 0·1)	0·0 (0·0 to 0·1)	0·0 (0·0 to 0·1)
Barbados	1·7 (1·3 to 2·1)	0·6 (0·5 to 0·7)	0·2 (0·2 to 0·3)	0·1 (0·1 to 0·1)	0·1 (0·1 to 0·1)	0·1 (0·0 to 0·1)	0·0 (0·0 to 0·1)	0·0 (0·0 to 0·0)	0·0 (0·0 to 0·0)	0·0 (0·0 to 0·0)
Belize	0·5 (0·4 to 0·6)	0·5 (0·4 to 0·6)	0·5 (0·4 to 0·5)	0·4 (0·3 to 0·4)	0·3 (0·3 to 0·3)	0·2 (0·2 to 0·2)	0·1 (0·1 to 0·2)	0·1 (0·1 to 0·1)	0·1 (0·1 to 0·1)	0·1 (0·1 to 0·1)
Bermuda	0·1 (0·1 to 0·1)	0·0 (0·0 to 0·1)	0·0 (0·0 to 0·0)	0·0 (0·0 to 0·0)	0·0 (0·0 to 0·0)	0·0 (0·0 to 0·0)	0·0 (0·0 to 0·0)	0·0 (0·0 to 0·0)	0·0 (0·0 to 0·0)	0·0 (0·0 to 0·0)
Cuba	9·4 (6·2 to 13·9)	10·0 (9·7 to 10·3)	10·6 (10·3 to 10·9)	3·3 (3·2 to 3·4)	2·5 (2·4 to 2·6)	1·3 (1·2 to 1·3)	0·8 (0·7 to 0·8)	0·7 (0·6 to 0·7)	0·6 (0·6 to 0·7)	0·5 (0·4 to 0·5)
Dominica	0·5 (0·4 to 0·6)	0·4 (0·3 to 0·4)	0·2 (0·1 to 0·2)	0·0 (0·0 to 0·1)	0·0 (0·0 to 0·0)	0·0 (0·0 to 0·0)	0·0 (0·0 to 0·0)	0·0 (0·0 to 0·0)	0·0 (0·0 to 0·0)	0·0 (0·0 to 0·0)
Dominican Republic	23·7 (19·0 to 29·2)	28·4 (25·9 to 31·3)	24·0 (21·7 to 26·5)	18·0 (16·1 to 20·1)	14·6 (13·1 to 16·2)	9·1 (8·0 to 10·1)	6·6 (5·5 to 7·7)	5·6 (4·4 to 6·9)	5·4 (4·4 to 6·7)	5·3 (4·3 to 6·4)
Grenada	0·5 (0·4 to 0·7)	0·4 (0·3 to 0·4)	0·2 (0·1 to 0·2)	0·1 (0·1 to 0·1)	0·1 (0·0 to 0·1)	0·0 (0·0 to 0·0)	0·0 (0·0 to 0·0)	0·0 (0·0 to 0·0)	0·0 (0·0 to 0·0)	0·0 (0·0 to 0·0)
Guyana	3·4 (2·8 to 4·2)	2·8 (2·5 to 3·1)	2·2 (2·0 to 2·5)	1·6 (1·4 to 1·8)	1·4 (1·2 to 1·6)	0·8 (0·7 to 0·9)	0·5 (0·4 to 0·6)	0·4 (0·3 to 0·5)	0·4 (0·3 to 0·5)	0·3 (0·3 to 0·4)
Haiti	50·0 (41·5 to 59·8)	48·3 (43·7 to 52·8)	44·9 (41·3 to 48·7)	42·8 (39·0 to 47·0)	37·4 (34·1 to 41·0)	28·0 (25·4 to 31·0)	91·2 (84·5 to 98·7)	24·0 (20·0 to 28·4)	23·5 (19·5 to 28·0)	24·0 (19·9 to 28·8)
Jamaica	6·2 (5·0 to 7·5)	5·6 (5·1 to 6·2)	3·7 (3·3 to 4·1)	2·2 (2·0 to 2·5)	1·7 (1·4 to 1·9)	1·1 (0·9 to 1·4)	0·8 (0·7 to 0·9)	0·6 (0·4 to 0·7)	0·5 (0·4 to 0·7)	0·5 (0·4 to 0·7)
Puerto Rico	8·2 (7·6 to 8·7)	4·2 (4·1 to 4·3)	2·2 (2·1 to 2·3)	1·5 (1·5 to 1·6)	1·0 (1·0 to 1·1)	0·6 (0·6 to 0·7)	0·4 (0·3 to 0·4)	0·1 (0·1 to 0·2)	0·2 (0·1 to 0·2)	0·1 (0·1 to 0·1)
Saint Kitts and Nevis	0·3 (0·2 to 0·3)	0·3 (0·3 to 0·3)	0·1 (0·1 to 0·1)	0·1 (0·1 to 0·1)	0·0 (0·0 to 0·0)	0·0 (0·0 to 0·0)	0·0 (0·0 to 0·0)	0·0 (0·0 to 0·0)	0·0 (0·0 to 0·0)	0·0 (0·0 to 0·0)
Saint Lucia	0·5 (0·4 to 0·7)	0·6 (0·5 to 0·7)	0·3 (0·3 to 0·4)	0·1 (0·1 to 0·2)	0·1 (0·1 to 0·1)	0·1 (0·0 to 0·1)	0·0 (0·0 to 0·1)	0·0 (0·0 to 0·0)	0·0 (0·0 to 0·0)	0·0 (0·0 to 0·0)
Saint Vincent and the Grenadines	0·6 (0·5 to 0·7)	0·6 (0·5 to 0·7)	0·3 (0·3 to 0·4)	0·2 (0·1 to 0·2)	0·1 (0·1 to 0·1)	0·1 (0·0 to 0·1)	0·0 (0·0 to 0·0)	0·0 (0·0 to 0·0)	0·0 (0·0 to 0·0)	0·0 (0·0 to 0·0)
Suriname	0·7 (0·5 to 0·9)	0·9 (0·8 to 1·1)	0·9 (0·7 to 1·1)	0·6 (0·6 to 0·7)	0·5 (0·4 to 0·5)	0·4 (0·3 to 0·4)	0·3 (0·2 to 0·3)	0·2 (0·2 to 0·3)	0·2 (0·2 to 0·3)	0·2 (0·2 to 0·3)
Trinidad and Tobago	2·4 (1·9 to 3·0)	2·2 (2·0 to 2·5)	1·3 (1·2 to 1·5)	1·1 (1·0 to 1·3)	0·7 (0·6 to 0·8)	0·5 (0·4 to 0·6)	0·3 (0·3 to 0·4)	0·2 (0·2 to 0·3)	0·2 (0·2 to 0·3)	0·2 (0·2 to 0·3)
Virgin Islands	0·1 (0·0 to 0·1)	0·1 (0·0 to 0·1)	0·1 (0·1 to 0·1)	0·1 (0·1 to 0·1)	0·0 (0·0 to 0·0)	0·0 (0·0 to 0·0)	0·0 (0·0 to 0·0)	0·0 (0·0 to 0·0)	0·0 (0·0 to 0·0)	0·0 (0·0 to 0·0)
Central Latin America	462·8 (400·8 to 534·5)	439·8 (411·4 to 467·6)	415·3 (390·5 to 439·2)	311·8 (291·3 to 332·7)	220·4 (205·9 to 236·4)	144·7 (132·7 to 157·6)	102·0 (89·7 to 115·6)	72·4 (58·9 to 86·8)	65·2 (52·6 to 80·6)	60·4 (46·7 to 77·3)
Colombia	73·6 (57·0 to 92·7)	79·3 (69·8 to 88·6)	65·2 (58·2 to 72·8)	43·1 (37·6 to 49·0)	30·5 (26·8 to 34·1)	23·4 (20·0 to 27·1)	15·1 (11·6 to 19·3)	10·3 (7·8 to 13·4)	9·4 (7·0 to 12·3)	8·1 (5·8 to 11·0)
Costa Rica	5·7 (5·4 to 6·0)	5·4 (5·3 to 5·5)	4·6 (4·5 to 4·8)	1·7 (1·6 to 1·7)	1·4 (1·3 to 1·5)	1·0 (0·9 to 1·0)	0·8 (0·7 to 0·8)	0·6 (0·6 to 0·7)	0·5 (0·5 to 0·6)	0·5 (0·5 to 0·6)
El Salvador	25·8 (20·4 to 33·1)	25·2 (22·3 to 28·7)	23·0 (20·7 to 25·4)	16·6 (14·9 to 18·3)	8·9 (7·9 to 10·0)	4·5 (3·9 to 5·1)	2·3 (2·0 to 2·6)	1·4 (1·1 to 1·7)	1·2 (0·9 to 1·5)	1·1 (0·8 to 1·5)
Guatemala	34·0 (27·3 to 42·2)	35·6 (31·4 to 40·0)	35·5 (32·1 to 39·4)	36·0 (32·9 to 39·5)	26·3 (24·0 to 28·8)	19·0 (16·9 to 21·3)	12·1 (10·4 to 14·4)	9·7 (7·9 to 11·5)	8·2 (6·6 to 10·0)	7·6 (6·0 to 9·8)
Honduras	17·7 (14·4 to 22·1)	17·0 (15·0 to 19·1)	15·4 (13·9 to 17·2)	13·4 (12·1 to 14·7)	9·6 (8·7 to 10·7)	6·9 (6·1 to 7·9)	5·2 (4·3 to 6·4)	3·8 (3·1 to 4·6)	3·5 (2·9 to 4·2)	3·3 (2·7 to 4·0)
Mexico	254·4 (206·2 to 311·6)	234·6 (214·4 to 256·4)	227·2 (207·1 to 247·5)	166·6 (149·0 to 184·5)	115·5 (105·2 to 128·2)	71·7 (64·4 to 79·7)	51·4 (45·0 to 58·5)	31·8 (25·3 to 39·1)	29·5 (23·6 to 37·3)	28·1 (22·0 to 36·0)

Aguascalientes	1·6 (1·3 to 2·0)	1·4 (1·3 to 1·6)	1·4 (1·2 to 1·5)	1·1 (0·9 to 1·3)	0·9 (0·8 to 1·0)	0·6 (0·5 to 0·7)	0·4 (0·4 to 0·5)	0·3 (0·3 to 0·4)	0·3 (0·2 to 0·4)	0·3 (0·2 to 0·4)
Baja California	2·0 (1·5 to 2·7)	2·8 (2·6 to 3·1)	3·3 (3·0 to 3·6)	1·6 (1·4 to 1·8)	1·6 (1·5 to 1·8)	1·4 (1·2 to 1·5)	1·3 (1·1 to 1·5)	0·7 (0·6 to 0·9)	0·7 (0·6 to 0·9)	0·7 (0·5 to 0·9)
Baja California Sur	0·5 (0·4 to 0·7)	0·5 (0·4 to 0·5)	0·5 (0·4 to 0·6)	0·4 (0·3 to 0·4)	0·2 (0·2 to 0·2)	0·2 (0·2 to 0·2)	0·2 (0·2 to 0·3)	0·2 (0·1 to 0·2)	0·2 (0·1 to 0·2)	0·2 (0·1 to 0·2)
Campeche	1·0 (0·8 to 1·3)	1·0 (0·9 to 1·1)	1·2 (1·0 to 1·3)	1·1 (1·0 to 1·3)	0·8 (0·7 to 0·9)	0·5 (0·4 to 0·5)	0·4 (0·3 to 0·4)	0·2 (0·2 to 0·3)	0·2 (0·2 to 0·3)	0·2 (0·2 to 0·3)
Chiapas	8·4 (6·4 to 10·9)	8·9 (8·0 to 9·8)	9·4 (8·5 to 10·4)	8·5 (7·4 to 9·7)	7·6 (6·7 to 8·6)	4·3 (3·7 to 5·0)	2·6 (2·2 to 3·0)	2·3 (1·9 to 2·8)	2·1 (1·7 to 2·7)	2·2 (1·7 to 2·8)
Chihuahua	8·7 (6·6 to 11·1)	8·5 (7·6 to 9·4)	7·8 (7·1 to 8·7)	4·9 (4·3 to 5·5)	3·3 (2·9 to 3·8)	2·7 (2·3 to 3·1)	1·6 (1·3 to 1·8)	1·1 (0·9 to 1·3)	1·1 (0·8 to 1·4)	1·0 (0·8 to 1·4)
Coahuila	7·6 (5·9 to 9·7)	6·4 (5·7 to 7·0)	5·6 (4·9 to 6·2)	3·7 (3·2 to 4·5)	2·4 (2·0 to 2·8)	1·5 (1·3 to 1·7)	1·0 (0·8 to 1·1)	0·8 (0·7 to 1·0)	0·8 (0·6 to 1·0)	0·8 (0·6 to 1·0)
Colima	1·6 (1·2 to 1·9)	1·4 (1·3 to 1·5)	1·3 (1·1 to 1·5)	0·9 (0·7 to 1·1)	0·5 (0·4 to 0·6)	0·3 (0·3 to 0·3)	0·2 (0·2 to 0·3)	0·1 (0·1 to 0·2)	0·1 (0·1 to 0·2)	0·1 (0·1 to 0·1)
Durango	7·2 (5·8 to 8·9)	6·0 (5·4 to 6·6)	5·0 (4·5 to 5·5)	3·3 (2·7 to 4·0)	2·1 (1·8 to 2·4)	1·2 (1·1 to 1·5)	0·8 (0·7 to 0·9)	0·5 (0·4 to 0·6)	0·4 (0·4 to 0·6)	0·4 (0·3 to 0·6)
Guanajuato	15·4 (11·1 to 20·4)	13·7 (12·5 to 15·0)	12·8 (11·1 to 14·6)	9·6 (8·7 to 10·5)	6·5 (6·0 to 7·1)	3·0 (2·7 to 3·4)	2·3 (2·0 to 2·6)	1·5 (1·2 to 1·9)	1·4 (1·1 to 1·8)	1·3 (1·0 to 1·6)
Guerrero	11·8 (9·5 to 14·6)	11·9 (10·0 to 13·9)	12·2 (10·5 to 13·9)	9·4 (8·3 to 10·6)	7·2 (6·3 to 8·1)	5·0 (4·3 to 5·7)	3·4 (2·7 to 4·1)	1·6 (1·2 to 2·1)	1·5 (1·1 to 1·9)	1·4 (1·0 to 1·8)
Hidalgo	8·3 (6·5 to 10·5)	6·7 (6·0 to 7·4)	5·9 (5·3 to 6·5)	3·7 (3·2 to 4·2)	2·6 (2·2 to 3·0)	1·6 (1·4 to 1·9)	1·0 (0·9 to 1·2)	0·6 (0·5 to 0·8)	0·5 (0·4 to 0·7)	0·5 (0·4 to 0·7)
Jalisco	15·8 (12·0 to 20·3)	14·5 (13·1 to 16·0)	13·3 (11·7 to 14·8)	8·2 (7·1 to 9·4)	5·8 (5·0 to 6·8)	4·1 (3·6 to 4·7)	2·9 (2·6 to 3·3)	1·9 (1·5 to 2·3)	1·9 (1·5 to 2·4)	1·9 (1·4 to 2·4)
México	11·1 (8·3 to 14·6)	11·5 (10·4 to 12·7)	16·2 (14·6 to 17·6)	18·1 (16·0 to 20·2)	14·5 (13·1 to 16·0)	9·0 (8·0 to 10·1)	7·3 (6·5 to 8·3)	4·3 (3·4 to 5·3)	3·7 (2·9 to 4·6)	3·0 (2·3 to 3·8)
Mexico City	20·8 (14·6 to 28·5)	19·5 (17·4 to 21·7)	18·1 (16·5 to 19·8)	13·2 (12·0 to 14·5)	6·7 (6·1 to 7·3)	3·8 (3·4 to 4·2)	3·0 (2·6 to 3·4)	1·5 (1·2 to 1·8)	1·5 (1·2 to 1·9)	1·8 (1·4 to 2·3)
Michoacán de Ocampo	17·6 (14·2 to 21·7)	15·6 (13·1 to 18·3)	13·3 (11·6 to 15·0)	9·1 (8·0 to 10·2)	6·1 (5·4 to 6·9)	3·5 (3·1 to 4·0)	2·7 (2·2 to 3·3)	1·4 (1·1 to 1·8)	1·4 (1·0 to 1·8)	1·3 (1·0 to 1·8)
Morelos	2·9 (2·3 to 3·6)	2·8 (2·6 to 3·1)	3·0 (2·7 to 3·4)	2·3 (2·0 to 2·6)	1·4 (1·3 to 1·6)	0·9 (0·7 to 1·0)	0·7 (0·6 to 0·8)	0·4 (0·3 to 0·5)	0·4 (0·3 to 0·4)	0·3 (0·3 to 0·4)
Nayarit	3·4 (2·7 to 4·2)	3·0 (2·7 to 3·3)	2·6 (2·4 to 2·9)	1·6 (1·3 to 1·9)	1·0 (0·9 to 1·1)	0·6 (0·5 to 0·7)	0·4 (0·3 to 0·4)	0·3 (0·2 to 0·3)	0·2 (0·2 to 0·3)	0·2 (0·2 to 0·3)
Nuevo León	6·0 (4·7 to 7·6)	5·8 (5·2 to 6·4)	5·8 (5·2 to 6·3)	3·5 (3·0 to 4·0)	2·3 (1·9 to 2·7)	1·9 (1·6 to 2·2)	1·3 (1·1 to 1·5)	1·1 (0·9 to 1·4)	1·0 (0·8 to 1·2)	1·0 (0·8 to 1·3)
Oaxaca	15·7 (12·2 to 19·4)	14·0 (11·7 to 16·4)	13·2 (11·5 to 14·8)	9·0 (7·9 to 10·2)	5·6 (5·0 to 6·4)	3·5 (3·1 to 4·0)	2·5 (2·0 to 3·2)	1·2 (0·9 to 1·6)	1·2 (0·9 to 1·5)	1·1 (0·8 to 1·5)
Puebla	20·5 (14·9 to 27·2)	17·7 (16·1 to 19·4)	16·6 (15·0 to 18·4)	11·8 (10·4 to 13·4)	8·1 (7·0 to 9·2)	5·1 (4·5 to 5·9)	3·6 (3·2 to 4·1)	2·2 (1·8 to 2·7)	2·1 (1·6 to 2·6)	1·9 (1·5 to 2·4)
Querétaro	3·4 (2·5 to 4·6)	2·9 (2·6 to 3·1)	2·6 (2·3 to 2·9)	2·2 (1·9 to 2·4)	1·7 (1·5 to 1·9)	0·9 (0·8 to 1·0)	0·7 (0·6 to 0·8)	0·5 (0·4 to 0·6)	0·5 (0·4 to 0·6)	0·4 (0·3 to 0·6)
Quintana Roo	0·2 (0·2 to 0·3)	0·3 (0·3 to 0·3)	0·4 (0·4 to 0·4)	0·6 (0·5 to 0·7)	0·6 (0·5 to 0·7)	0·5 (0·4 to 0·6)	0·5 (0·5 to 0·6)	0·4 (0·3 to 0·5)	0·3 (0·3 to 0·4)	0·3 (0·3 to 0·4)
San Luis Potosí	9·9 (7·8 to 12·3)	8·3 (6·9 to 9·7)	7·4 (6·4 to 8·4)	4·5 (3·9 to 5·1)	3·1 (2·8 to 3·5)	1·9 (1·7 to 2·2)	1·4 (1·1 to 1·7)	0·7 (0·5 to 0·9)	0·6 (0·5 to 0·8)	0·6 (0·5 to 0·8)
Sinaloa	5·9 (4·6 to 7·5)	5·5 (5·0 to 6·1)	5·5 (5·0 to 6·0)	3·6 (3·1 to 4·2)	2·2 (1·9 to 2·7)	1·4 (1·2 to 1·7)	0·8 (0·7 to 0·9)	0·6 (0·5 to 0·7)	0·5 (0·4 to 0·6)	0·5 (0·4 to 0·7)
Sonora	4·2 (3·3 to 5·4)	4·1 (3·7 to 4·5)	4·1 (3·7 to 4·4)	2·6 (2·3 to 3·0)	1·7 (1·4 to 2·0)	1·4 (1·2 to 1·7)	1·0 (0·9 to 1·2)	0·7 (0·5 to 0·9)	0·6 (0·4 to 0·7)	0·5 (0·4 to 0·7)
Tabasco	3·2 (2·5 to 4·3)	3·4 (3·1 to 3·8)	4·0 (3·7 to 4·4)	3·4 (3·0 to 3·9)	2·5 (2·2 to 2·8)	1·4 (1·1 to 1·6)	1·1 (0·9 to 1·2)	0·8 (0·6 to 1·0)	0·7 (0·6 to 0·9)	0·6 (0·5 to 0·8)
Tamaulipas	6·2 (4·8 to 7·9)	6·0 (5·4 to 6·6)	5·7 (5·2 to 6·3)	3·5 (3·1 to 4·0)	2·2 (1·9 to 2·5)	1·3 (1·2 to 1·5)	1·2 (1·0 to 1·4)	0·8 (0·7 to 1·0)	0·8 (0·6 to 1·0)	0·8 (0·6 to 1·0)
Tlaxcala	3·4 (2·4 to 4·5)	2·7 (2·5 to 3·0)	2·3 (2·0 to 2·5)	1·8 (1·6 to 2·0)	1·3 (1·2 to 1·4)	0·8 (0·7 to 0·9)	0·6 (0·5 to 0·7)	0·4 (0·3 to 0·4)	0·3 (0·3 to 0·4)	0·3 (0·2 to 0·4)
Veracruz de Ignacio de la Llave	20·1 (15·4 to 26·2)	19·1 (17·3 to 21·1)	19·6 (17·3 to 21·8)	14·0 (12·2 to 15·9)	9·9 (8·7 to 11·3)	5·6 (4·9 to 6·3)	3·2 (2·8 to 3·7)	1·9 (1·5 to 2·4)	1·8 (1·5 to 2·3)	1·6 (1·2 to 2·1)
Yucatán	3·8 (2·9 to 4·9)	3·4 (3·1 to 3·8)	3·1 (2·8 to 3·5)	2·5 (2·1 to 2·9)	1·7 (1·5 to 2·0)	1·0 (0·9 to 1·1)	0·7 (0·6 to 0·8)	0·4 (0·3 to 0·5)	0·5 (0·4 to 0·6)	0·4 (0·3 to 0·6)
Zacatecas	6·3 (5·0 to 7·8)	5·3 (4·8 to 5·8)	4·1 (3·6 to 4·5)	2·8 (2·3 to 3·4)	1·6 (1·4 to 1·8)	0·9 (0·7 to 1·0)	0·6 (0·5 to 0·7)	0·4 (0·3 to 0·5)	0·4 (0·3 to 0·4)	0·3 (0·3 to 0·4)
Nicaragua	14·3 (11·6 to 17·8)	14·8 (13·1 to 16·6)	14·4 (13·1 to 15·9)	12·6 (11·7 to 13·7)	8·8 (8·0 to 9·7)	4·8 (4·2 to 5·4)	3·0 (2·5 to 3·5)	2·1 (1·6 to 2·7)	2·0 (1·5 to 2·5)	1·8 (1·3 to 2·3)
Panama	3·3 (2·6 to 4·0)	3·6 (3·2 to 4·0)	3·0 (2·6 to 3·3)	2·0 (1·7 to 2·2)	1·6 (1·4 to 1·8)	1·5 (1·3 to 1·7)	1·3 (1·1 to 1·5)	1·2 (1·0 to 1·4)	1·1 (0·9 to 1·3)	1·0 (0·8 to 1·3)
Venezuela	34·1 (32·8 to 35·5)	24·4 (24·0 to 24·9)	26·9 (26·3 to 27·4)	19·8 (19·2 to 20·4)	17·9 (17·3 to 18·5)	12·0 (11·2 to 12·7)	10·7 (10·3 to 11·2)	11·5 (9·0 to 14·5)	9·8 (7·4 to 12·6)	8·9 (6·6 to 11·6)
Tropical Latin America	514·2 (384·0 to 668·4)	453·5 (393·0 to 516·8)	387·2 (340·9 to 434·6)	305·8 (274·9 to 341·1)	170·2 (153·8 to 186·6)	120·0 (104·7 to 136·1)	79·4 (66·9 to 93·3)	50·1 (40·6 to 61·2)	44·6 (36·3 to 53·8)	41·4 (33·8 to 50·3)
Brazil	507·9 (377·3 to 661·8)	447·6 (387·0 to 510·8)	381·4 (335·4 to 428·7)	300·6 (270·1 to 335·8)	165·9 (149·3 to 182·1)	116·4 (101·2 to 132·5)	76·7 (64·5 to 90·1)	48·0 (38·7 to 58·5)	42·5 (34·6 to 51·5)	39·5 (32·4 to 47·8)
Acre	1·4 (1·0 to 2·1)	1·3 (1·1 to 1·5)	1·3 (1·1 to 1·5)	1·3 (1·1 to 1·5)	0·8 (0·7 to 0·9)	0·8 (0·7 to 1·0)	0·6 (0·5 to 0·8)	0·4 (0·3 to 0·5)	0·3 (0·3 to 0·4)	0·3 (0·2 to 0·4)
Alagoas	18·3 (12·5 to 25·2)	16·0 (13·4 to 18·9)	14·8 (12·5 to 17·1)	13·7 (12·0 to 15·6)	8·2 (6·9 to 9·6)	5·1 (4·4 to 6·0)	2·5 (1·9 to 3·1)	1·2 (0·9 to 1·5)	1·0 (0·7 to 1·2)	0·9 (0·7 to 1·1)
Amapá	0·5 (0·4 to 0·8)	0·6 (0·5 to 0·7)	0·6 (0·5 to 0·7)	0·3 (0·3 to 0·3)	0·2 (0·2 to 0·2)	0·3 (0·3 to 0·4)	0·3 (0·3 to 0·4)	0·3 (0·3 to 0·4)	0·3 (0·3 to 0·4)	0·3 (0·2 to 0·4)
Amazonas	4·6 (2·8 to 7·1)	3·7 (3·0 to 4·5)	3·4 (2·8 to 4·0)	3·4 (2·8 to 4·1)	2·0 (1·7 to 2·4)	2·1 (1·8 to 2·4)	1·7 (1·5 to 1·9)	1·5 (1·3 to 1·8)	1·4 (1·2 to 1·6)	1·4 (1·2 to 1·6)
Bahia	56·1 (37·0 to 81·8)	51·3 (41·4 to 62·2)	48·1 (41·3 to 55·2)	35·9 (31·5 to 40·8)	21·4 (18·8 to 24·4)	14·4 (12·2 to 16·9)	9·1 (7·2 to 11·3)	4·7 (3·7 to 5·9)	4·0 (3·1 to 5·1)	3·6 (2·8 to 4·6)
Ceará	47·9 (32·8 to 67·1)	43·1 (36·1 to 51·2)	36·0 (30·7 to 42·1)	26·1 (21·9 to 31·1)	12·3 (10·8 to 14·1)	8·3 (7·1 to 9·7)	4·4 (3·4 to 5·4)	2·5 (1·9 to 3·2)	2·0 (1·5 to 2·6)	1·7 (1·3 to 2·3)
Distrito Federal	0·3 (0·2 to 0·5)	0·6 (0·5 to 0·7)	1·8 (1·5 to 2·1)	1·9 (1·7 to 2·1)	1·1 (1·0 to 1·2)	0·9 (0·7 to 1·0)	0·7 (0·6 to 0·7)	0·5 (0·4 to 0·6)	0·5 (0·4 to 0·5)	0·4 (0·4 to 0·5)
Espírito Santo	5·8 (4·3 to 7·6)	5·4 (4·5 to 6·3)	4·7 (4·0 to 5·6)	3·3 (3·0 to 3·7)	2·0 (1·8 to 2·2)	1·4 (1·2 to 1·7)	1·3 (1·0 to 1·6)	0·8 (0·6 to 1·0)	0·7 (0·6 to 1·0)	0·7 (0·6 to 0·9)
Goiás	12·9 (8·4 to 18·6)	13·4 (11·2 to 15·8)	10·9 (9·0 to 12·9)	6·9 (5·9 to 8·0)	3·7 (3·2 to 4·3)	3·1 (2·4 to 3·8)	2·0 (1·6 to 2·5)	1·5 (1·1 to 1·9)	1·4 (1·1 to 1·7)	1·3 (1·0 to 1·6)
Maranhão	27·2 (21·4 to 34·4)	30·6 (25·9 to 36·2)	29·1 (25·2 to 33·4)	24·4 (21·4 to 27·5)	13·7 (12·1 to 15·5)	9·5 (8·2 to 11·1)	6·6 (5·2 to 8·2)	3·2 (2·4 to 4·1)	2·6 (2·0 to 3·3)	2·4 (1·8 to 3·0)
Mato Grosso	1·6 (1·1 to 2·2)	2·2 (1·8 to 2·6)	2·7 (2·3 to 3·2)	2·3 (2·0 to 2·7)	1·8 (1·5 to 2·0)	1·4 (1·1 to 1·8)	1·1 (0·9 to 1·4)	0·9 (0·7 to 1·2)	0·9 (0·7 to 1·2)	0·9 (0·7 to 1·1)
Mato Grosso do Sul	2·1 (1·3 to 3·1)	2·4 (1·9 to 2·9)	2·5 (2·0 to 3·0)	2·0 (1·8 to 2·2)	1·3 (1·1 to 1·4)	1·1 (1·0 to 1·3)	0·8 (0·7 to 0·9)	0·6 (0·5 to 0·7)	0·6 (0·5 to 0·7)	0·5 (0·5 to 0·6)
Minas Gerais	63·6 (44·4 to 89·0)	51·4 (42·5 to 60·8)	35·0 (29·5 to 41·2)	23·1 (19·9 to 26·5)	13·0 (11·1 to 14·9)	9·5 (7·7 to 11·5)	5·9 (4·8 to 7·3)	3·9 (3·0 to 4·9)	3·5 (2·7 to 4·4)	3·2 (2·5 to 4·1)
Pará	13·6 (9·4 to 18·7)	11·9 (10·0 to 14·0)	11·4 (9·4 to 13·6)	11·1 (9·5 to 13·0)	6·8 (5·9 to 7·7)	5·7 (4·5 to 7·2)	4·7 (3·9 to 5·5)	2·9 (2·3 to 3·7)	2·7 (2·0 to 3·5)	2·5 (1·9 to 3·2)
Paraíba	34·1 (22·7 to 47·8)	26·7 (23·4 to 30·6)	19·2 (16·4 to 22·1)	11·6 (9·6 to 13·7)	5·7 (5·0 to 6·4)	3·5 (3·0 to 4·1)	2·2 (1·8 to 2·6)	1·2 (0·9 to 1·5)	1·0 (0·7 to 1·2)	0·9 (0·6 to 1·2)
Paraná	13·6 (9·5 to 19·0)	17·4 (14·5 to 20·5)	17·4 (14·4 to 20·5)	11·6 (10·4 to 12·7)	6·0 (5·4 to 6·6)	4·1 (3·5 to 4·7)	3·1 (2·4 to 3·8)	2·0 (1·6 to 2·5)	1·8 (1·4 to 2·3)	1·7 (1·3 to 2·1)
Pernambuco	64·6 (44·4 to 87·7)	56·1 (48·6 to 64·7)	47·1 (41·2 to 53·3)	30·8 (25·8 to 36·6)	15·4 (13·6 to 17·2)	9·8 (8·2 to 11·5)	5·6 (4·7 to 6·5)	2·6 (2·0 to 3·3)	2·3 (1·7 to 2·9)	2·1 (1·6 to 2·7)
Piauí	11·9 (7·7 to 16·8)	10·4 (8·9 to 12·1)	10·7 (8·9 to 12·7)	8·0 (6·9 to 9·3)	4·4 (3·8 to 5·0)	3·0 (2·6 to 3·5)	2·1 (1·7 to 2·5)	1·2 (0·9 to 1·5)	1·0 (0·7 to 1·2)	0·9 (0·7 to 1·1)
Rio de Janeiro	25·4 (13·8 to 41·6)	18·9 (15·5 to 22·9)	16·1 (13·2 to 19·3)	16·9 (15·4 to 18·6)	8·8 (8·0 to 9·6)	5·8 (5·1 to 6·5)	3·9 (3·5 to 4·3)	3·3 (2·7 to 3·9)	3·0 (2·6 to 3·5)	2·8 (2·5 to 3·2)
Rio Grande do Norte	20·4 (14·1 to 28·2)	16·4 (13·7 to 19·4)	12·5 (10·9 to 14·2)	8·3 (7·0 to 10·1)	4·8 (4·0 to 5·6)	3·0 (2·5 to 3·6)	1·8 (1·4 to 2·2)	0·9 (0·7 to 1·2)	0·8 (0·6 to 1·0)	0·7 (0·5 to 0·9)
Rio Grande do Sul	21·1 (13·9 to 29·6)	15·4 (12·8 to 18·7)	10·3 (8·5 to 12·7)	7·5 (6·8 to 8·3)	3·6 (3·3 to 4·0)	2·9 (2·6 to 3·3)	1·7 (1·5 to 1·9)	1·7 (1·4 to 2·0)	1·4 (1·2 to 1·7)	1·4 (1·2 to 1·6)
Rondônia	0·4 (0·2 to 0·6)	0·4 (0·4 to 0·5)	0·5 (0·4 to 0·6)	1·3 (1·1 to 1·6)	1·2 (1·0 to 1·4)	1·0 (0·9 to 1·2)	0·8 (0·6 to 0·9)	0·5 (0·3 to 0·6)	0·4 (0·3 to 0·5)	0·3 (0·2 to 0·4)
Roraima	0·2 (0·2 to 0·3)	0·2 (0·2 to 0·2)	0·2 (0·2 to 0·3)	0·2 (0·2 to 0·2)	0·2 (0·2 to 0·2)	0·2 (0·2 to 0·3)	0·3 (0·2 to 0·3)	0·3 (0·3 to 0·4)	0·3 (0·2 to 0·4)	0·2 (0·2 to 0·3)
Santa Catarina	7·1 (4·6 to 10·3)	5·8 (4·8 to 7·0)	4·9 (4·1 to 5·8)	4·0 (3·4 to 4·6)	2·6 (2·3 to 3·1)	2·1 (1·7 to 2·5)	1·4 (1·1 to 1·7)	1·1 (0·8 to 1·5)	1·1 (0·8 to 1·4)	1·0 (0·8 to 1·3)
São Paulo	43·1 (28·8 to 60·5)	37·5 (30·7 to 45·1)	33·2 (27·8 to 39·2)	38·9 (35·4 to 42·8)	21·2 (19·3 to 23·1)	14·1 (12·2 to 16·1)	10·2 (8·2 to 12·4)	7·2 (5·6 to 9·0)	6·6 (5·5 to 7·7)	6·3 (5·5 to 7·3)
Sergipe	7·8 (5·2 to 11·4)	6·2 (5·2 to 7·4)	4·8 (4·1 to 5·4)	4·0 (3·4 to 4·6)	2·4 (2·1 to 2·7)	1·8 (1·4 to 2·3)	1·1 (0·9 to 1·4)	0·7 (0·6 to 0·9)	0·7 (0·5 to 0·8)	0·6 (0·5 to 0·8)
Tocantins	2·0 (1·1 to 3·4)	2·2 (1·9 to 2·7)	2·4 (1·9 to 2·9)	1·9 (1·6 to 2·2)	1·4 (1·2 to 1·7)	1·3 (1·1 to 1·6)	0·9 (0·7 to 1·1)	0·5 (0·4 to 0·6)	0·4 (0·3 to 0·6)	0·4 (0·3 to 0·5)

Paraguay	6·3 (4·6 to 8·2)	5·9 (5·2 to 6·7)	5·8 (5·1 to 6·6)	5·1 (4·4 to 5·9)	4·2 (3·6 to 5·0)	3·6 (3·1 to 4·2)	2·7 (2·2 to 3·3)	2·2 (1·6 to 2·8)	2·1 (1·5 to 2·7)	1·9 (1·4 to 2·5)
North Africa and Middle East	1518·0 (1316·6 to 1738·6)	1509·7 (1388·1 to 1634·0)	1538·8 (1445·6 to 1641·2)	1352·9 (1280·9 to 1426·8)	880·0 (826·5 to 934·1)	620·7 (575·4 to 669·3)	466·2 (424·0 to 511·1)	298·2 (258·1 to 342·4)	263·4 (227·7 to 302·4)	242·9 (208·4 to 280·3)
North Africa and Middle East	1518·0 (1316·6 to 1738·6)	1509·7 (1388·1 to 1634·0)	1538·8 (1445·6 to 1641·2)	1352·9 (1280·9 to 1426·8)	880·0 (826·5 to 934·1)	620·7 (575·4 to 669·3)	466·2 (424·0 to 511·1)	298·2 (258·1 to 342·4)	263·4 (227·7 to 302·4)	242·9 (208·4 to 280·3)
Afghanistan	96·4 (75·2 to 119·2)	103·5 (88·8 to 120·0)	114·8 (98·9 to 134·5)	130·4 (113·1 to 148·5)	67·5 (60·7 to 75·3)	100·1 (90·5 to 110·4)	71·1 (61·1 to 81·8)	61·6 (51·2 to 73·5)	59·3 (49·2 to 70·9)	58·0 (48·1 to 69·8)
Algeria	134·3 (107·5 to 169·2)	126·0 (109·0 to 144·8)	115·6 (99·7 to 132·7)	102·1 (94·8 to 109·6)	44·2 (40·5 to 48·0)	26·1 (23·0 to 29·6)	24·6 (22·3 to 27·2)	19·1 (16·0 to 22·9)	17·0 (13·8 to 20·6)	15·5 (12·2 to 19·3)
Bahrain	1·0 (0·8 to 1·3)	1·0 (0·8 to 1·1)	0·5 (0·5 to 0·6)	0·3 (0·3 to 0·3)	0·3 (0·3 to 0·3)	0·2 (0·1 to 0·2)	0·2 (0·1 to 0·2)	0·1 (0·1 to 0·2)	0·1 (0·1 to 0·1)	0·1 (0·1 to 0·1)
Egypt	218·9 (168·1 to 273·5)	228·1 (203·7 to 252·8)	340·0 (311·5 to 372·0)	280·2 (253·5 to 308·9)	158·7 (143·1 to 175·2)	84·8 (74·4 to 96·3)	76·9 (66·2 to 88·8)	45·1 (36·7 to 55·0)	36·9 (29·8 to 45·1)	33·1 (27·1 to 40·7)
Iran	309·3 (249·6 to 373·1)	279·3 (239·8 to 323·0)	233·3 (203·9 to 266·1)	187·7 (165·8 to 211·9)	117·5 (105·3 to 130·1)	45·4 (39·7 to 51·4)	36·3 (32·1 to 41·1)	13·7 (12·3 to 15·2)	8·8 (8·1 to 9·6)	5·6 (4·7 to 6·7)
Alborz	3·1 (2·4 to 3·9)	4·4 (3·8 to 5·2)	4·3 (3·7 to 5·1)	3·1 (2·7 to 3·5)	1·6 (1·4 to 1·8)	0·8 (0·7 to 1·0)	0·7 (0·6 to 0·8)	0·3 (0·2 to 0·3)	0·2 (0·1 to 0·2)	0·1 (0·1 to 0·1)
Ardebil	9·2 (7·4 to 11·4)	8·1 (7·0 to 9·4)	7·1 (6·3 to 8·1)	6·2 (5·4 to 7·1)	3·7 (3·3 to 4·2)	1·3 (1·1 to 1·5)	0·7 (0·6 to 0·8)	0·2 (0·2 to 0·3)	0·2 (0·1 to 0·2)	0·1 (0·1 to 0·1)
Bushehr	2·0 (1·5 to 2·4)	2·3 (1·9 to 2·6)	2·3 (2·0 to 2·7)	2·0 (1·7 to 2·3)	1·2 (1·1 to 1·4)	0·5 (0·4 to 0·6)	0·5 (0·4 to 0·5)	0·2 (0·2 to 0·3)	0·2 (0·1 to 0·2)	0·1 (0·1 to 0·1)
Chahar Mahaal and Bakhtiari	3·0 (2·3 to 3·7)	2·6 (2·2 to 3·0)	2·3 (1·9 to 2·6)	2·1 (1·8 to 2·4)	1·5 (1·3 to 1·6)	0·5 (0·4 to 0·6)	0·4 (0·3 to 0·4)	0·1 (0·1 to 0·2)	0·1 (0·1 to 0·1)	0·1 (0·0 to 0·1)
East Azarbayejan	30·8 (24·9 to 37·2)	26·6 (22·9 to 30·7)	21·4 (18·7 to 24·3)	17·0 (14·9 to 19·5)	7·7 (6·8 to 8·6)	2·7 (2·3 to 3·2)	1·8 (1·6 to 2·0)	0·5 (0·5 to 0·6)	0·3 (0·3 to 0·4)	0·2 (0·2 to 0·2)
Fars	6·7 (5·1 to 8·5)	8·4 (7·0 to 9·8)	8·5 (7·3 to 9·9)	7·7 (6·7 to 8·8)	5·1 (4·4 to 5·8)	2·5 (2·1 to 3·0)	2·2 (1·9 to 2·5)	0·8 (0·7 to 1·0)	0·6 (0·5 to 0·7)	0·4 (0·3 to 0·4)
Gilan	10·5 (8·1 to 13·3)	10·2 (8·1 to 12·9)	8·2 (7·0 to 9·6)	5·2 (4·4 to 6·1)	11·2 (10·3 to 12·3)	0·8 (0·7 to 0·9)	0·4 (0·4 to 0·5)	0·1 (0·1 to 0·2)	0·1 (0·0 to 0·1)	0·0 (0·0 to 0·1)
Golestan	7·5 (6·0 to 9·3)	6·3 (5·3 to 7·4)	5·3 (4·6 to 6·1)	4·8 (4·1 to 5·5)	3·3 (2·8 to 3·7)	1·3 (1·2 to 1·5)	1·0 (0·9 to 1·1)	0·4 (0·3 to 0·5)	0·3 (0·2 to 0·3)	0·2 (0·1 to 0·2)
Hamadan	14·8 (11·7 to 18·0)	13·5 (11·6 to 15·6)	11·0 (9·6 to 12·5)	7·8 (6·8 to 8·9)	4·1 (3·6 to 4·6)	1·5 (1·3 to 1·7)	0·9 (0·8 to 1·0)	0·3 (0·3 to 0·4)	0·2 (0·2 to 0·2)	0·1 (0·1 to 0·1)
Hormozgan	2·9 (2·3 to 3·6)	3·4 (2·9 to 4·0)	3·1 (2·7 to 3·6)	2·9 (2·5 to 3·3)	2·4 (2·1 to 2·8)	1·2 (1·0 to 1·3)	1·0 (0·8 to 1·1)	0·4 (0·4 to 0·5)	0·3 (0·3 to 0·3)	0·2 (0·2 to 0·2)
Ilam	1·3 (1·0 to 1·6)	1·3 (1·1 to 1·5)	1·4 (1·2 to 1·6)	1·5 (1·3 to 1·7)	0·9 (0·8 to 1·1)	0·4 (0·3 to 0·5)	0·2 (0·2 to 0·3)	0·1 (0·1 to 0·1)	0·1 (0·1 to 0·1)	0·0 (0·0 to 0·1)
Isfahan	13·3 (10·3 to 16·7)	13·1 (11·2 to 15·3)	10·6 (9·1 to 12·3)	8·5 (7·4 to 9·8)	4·4 (3·8 to 5·0)	1·9 (1·5 to 2·3)	1·7 (1·5 to 2·0)	0·7 (0·6 to 0·8)	0·4 (0·3 to 0·5)	0·3 (0·2 to 0·3)
Kerman	16·5 (12·9 to 20·6)	10·6 (9·0 to 12·4)	7·6 (6·5 to 8·8)	6·3 (5·5 to 7·1)	3·3 (2·9 to 3·8)	1·5 (1·3 to 1·7)	1·8 (1·5 to 2·1)	0·8 (0·7 to 0·9)	0·6 (0·5 to 0·7)	0·4 (0·3 to 0·4)
Kermanshah	8·9 (7·1 to 11·2)	8·6 (7·3 to 10·0)	7·7 (6·7 to 8·9)	7·1 (6·3 to 8·1)	4·0 (3·5 to 4·5)	1·6 (1·4 to 1·8)	1·1 (0·9 to 1·2)	0·4 (0·3 to 0·4)	0·2 (0·2 to 0·3)	0·1 (0·1 to 0·2)
Khorasan-e-Razavi	37·9 (31·0 to 45·4)	33·5 (28·5 to 38·8)	26·8 (23·6 to 30·3)	22·3 (19·5 to 25·5)	12·5 (10·9 to 14·1)	5·2 (4·5 to 6·0)	4·6 (3·9 to 5·4)	1·4 (1·2 to 1·5)	0·9 (0·7 to 1·0)	0·6 (0·4 to 0·7)
Khuzestan	21·8 (16·9 to 28·0)	16·5 (13·8 to 19·6)	13·5 (11·7 to 15·8)	10·6 (9·1 to 12·2)	5·4 (4·8 to 6·1)	3·1 (2·6 to 3·7)	2·7 (2·4 to 3·1)	1·1 (1·0 to 1·2)	0·7 (0·6 to 0·8)	0·5 (0·4 to 0·5)
Kohgiluyeh and Boyer-Ahmad	1·2 (0·9 to 1·5)	1·3 (1·1 to 1·5)	1·4 (1·2 to 1·6)	1·6 (1·4 to 1·8)	1·2 (1·1 to 1·4)	0·6 (0·5 to 0·7)	0·3 (0·3 to 0·4)	0·1 (0·1 to 0·2)	0·1 (0·1 to 0·1)	0·1 (0·0 to 0·1)
Kurdistan	13·6 (11·1 to 16·5)	11·3 (9·8 to 13·0)	8·9 (7·8 to 10·1)	7·8 (6·8 to 8·8)	4·4 (3·8 to 4·9)	1·6 (1·4 to 1·8)	0·9 (0·8 to 1·0)	0·3 (0·3 to 0·4)	0·2 (0·2 to 0·2)	0·1 (0·1 to 0·2)
Lorestan	12·5 (9·7 to 15·9)	8·6 (7·3 to 10·1)	6·5 (5·6 to 7·5)	5·7 (5·0 to 6·7)	3·5 (3·1 to 4·1)	1·2 (1·0 to 1·4)	0·6 (0·5 to 0·7)	0·2 (0·2 to 0·2)	0·1 (0·1 to 0·1)	0·1 (0·1 to 0·1)
Markazi	3·1 (2·5 to 4·0)	5·2 (4·4 to 6·0)	6·1 (5·2 to 7·1)	4·2 (3·7 to 4·9)	1·9 (1·6 to 2·2)	0·7 (0·6 to 0·8)	0·5 (0·4 to 0·6)	0·2 (0·2 to 0·2)	0·1 (0·1 to 0·1)	0·1 (0·1 to 0·1)
Mazandaran	13·8 (10·5 to 17·8)	10·1 (8·5 to 12·0)	7·3 (6·2 to 8·6)	5·5 (4·8 to 6·4)	2·5 (2·2 to 3·0)	1·0 (0·9 to 1·2)	0·7 (0·6 to 0·8)	0·3 (0·2 to 0·3)	0·2 (0·1 to 0·2)	0·1 (0·1 to 0·1)
North Khorasan	3·8 (2·9 to 4·9)	3·6 (3·1 to 4·2)	3·3 (2·9 to 3·7)	2·9 (2·6 to 3·3)	2·1 (1·8 to 2·4)	0·8 (0·7 to 0·9)	0·5 (0·4 to 0·6)	0·2 (0·2 to 0·2)	0·1 (0·1 to 0·1)	0·1 (0·1 to 0·1)
Qazvin	9·0 (7·2 to 11·2)	7·7 (6·6 to 8·9)	5·7 (5·0 to 6·6)	3·7 (3·2 to 4·2)	1·8 (1·6 to 2·0)	0·8 (0·7 to 0·9)	0·5 (0·4 to 0·5)	0·2 (0·2 to 0·2)	0·1 (0·1 to 0·1)	0·1 (0·1 to 0·1)
Qom	1·2 (0·9 to 1·5)	2·0 (1·7 to 2·3)	2·4 (2·1 to 2·8)	1·9 (1·7 to 2·2)	1·0 (0·9 to 1·2)	0·5 (0·4 to 0·6)	0·4 (0·4 to 0·5)	0·2 (0·2 to 0·2)	0·1 (0·1 to 0·1)	0·1 (0·1 to 0·1)
Semnan	1·6 (1·3 to 2·0)	2·0 (1·7 to 2·3)	1·8 (1·6 to 2·1)	1·3 (1·1 to 1·5)	0·6 (0·5 to 0·7)	0·3 (0·2 to 0·3)	0·2 (0·2 to 0·3)	0·1 (0·1 to 0·1)	0·1 (0·0 to 0·1)	0·0 (0·0 to 0·1)
Sistan and Baluchistan	9·7 (7·4 to 12·4)	7·3 (6·3 to 8·5)	6·1 (5·3 to 6·9)	6·5 (5·7 to 7·5)	5·7 (5·0 to 6·4)	3·4 (3·0 to 3·9)	3·3 (2·9 to 3·7)	1·4 (1·2 to 1·6)	1·0 (0·9 to 1·2)	0·7 (0·6 to 0·8)
South Khorasan	4·9 (3·9 to 6·0)	4·7 (4·1 to 5·5)	4·3 (3·8 to 4·8)	3·7 (3·2 to 4·2)	2·2 (1·9 to 2·5)	0·7 (0·6 to 0·8)	0·4 (0·4 to 0·5)	0·2 (0·2 to 0·2)	0·1 (0·1 to 0·1)	0·1 (0·1 to 0·1)
Tehran	14·0 (10·8 to 18·0)	17·5 (14·3 to 21·0)	14·9 (12·5 to 17·8)	10·0 (8·2 to 11·8)	5·7 (4·6 to 6·9)	3·1 (2·6 to 3·7)	3·0 (2·4 to 3·5)	1·3 (1·1 to 1·4)	0·7 (0·5 to 0·9)	0·4 (0·3 to 0·6)
West Azarbayejan	11·9 (9·6 to 14·3)	13·1 (11·2 to 15·2)	12·5 (10·9 to 14·2)	10·8 (9·4 to 12·3)	5·8 (5·1 to 6·6)	2·6 (2·2 to 3·0)	2·2 (1·9 to 2·4)	0·7 (0·6 to 0·8)	0·5 (0·4 to 0·5)	0·3 (0·3 to 0·4)
Yazd	3·7 (2·9 to 4·5)	3·0 (2·6 to 3·6)	2·2 (1·9 to 2·6)	1·6 (1·4 to 1·8)	0·9 (0·8 to 1·0)	0·5 (0·4 to 0·6)	0·5 (0·5 to 0·6)	0·2 (0·2 to 0·3)	0·2 (0·1 to 0·2)	0·1 (0·1 to 0·1)
Zanjan	15·1 (11·7 to 18·8)	12·3 (10·7 to 14·1)	8·7 (7·7 to 9·9)	5·3 (4·6 to 5·9)	5·8 (5·3 to 6·2)	0·9 (0·8 to 1·1)	0·5 (0·5 to 0·6)	0·2 (0·2 to 0·2)	0·1 (0·1 to 0·1)	0·1 (0·1 to 0·1)
Iraq	39·2 (29·6 to 50·3)	44·8 (37·0 to 53·0)	49·8 (42·2 to 57·7)	43·6 (39·3 to 47·9)	39·4 (36·1 to 42·8)	38·0 (34·3 to 42·1)	29·9 (25·0 to 35·4)	20·0 (16·1 to 24·9)	17·2 (13·8 to 21·5)	15·7 (12·4 to 19·9)
Jordan	4·5 (3·7 to 5·7)	5·2 (4·4 to 6·0)	5·2 (4·7 to 5·8)	4·7 (4·3 to 5·2)	4·4 (3·9 to 4·9)	3·8 (3·4 to 4·3)	3·6 (3·1 to 4·1)	2·8 (2·4 to 3·4)	2·6 (2·1 to 3·2)	2·5 (2·0 to 3·0)
Kuwait	0·3 (0·2 to 0·4)	0·7 (0·6 to 0·8)	1·7 (1·7 to 1·8)	1·7 (1·6 to 1·8)	0·6 (0·6 to 0·7)	0·5 (0·4 to 0·5)	0·6 (0·6 to 0·7)	0·5 (0·4 to 0·5)	0·4 (0·4 to 0·5)	0·4 (0·3 to 0·5)
Lebanon	9·7 (7·2 to 12·5)	6·6 (5·4 to 8·0)	4·8 (4·0 to 5·6)	3·6 (3·1 to 4·2)	2·7 (2·2 to 3·2)	1·8 (1·5 to 2·2)	1·2 (0·9 to 1·4)	0·8 (0·6 to 1·1)	0·7 (0·5 to 0·9)	0·6 (0·4 to 0·9)
Libya	14·5 (12·1 to 17·5)	14·7 (12·8 to 16·7)	10·3 (8·9 to 11·6)	7·6 (6·6 to 8·8)	5·4 (4·5 to 6·4)	3·1 (2·6 to 3·7)	2·6 (2·1 to 3·1)	2·1 (1·7 to 2·7)	1·9 (1·5 to 2·4)	1·8 (1·4 to 2·2)
Morocco	140·0 (119·1 to 164·4)	145·4 (133·3 to 157·4)	129·9 (120·2 to 138·8)	99·8 (91·3 to 109·9)	60·2 (53·3 to 68·0)	40·8 (33·3 to 48·7)	25·4 (20·7 to 30·9)	12·1 (9·8 to 14·7)	10·5 (8·6 to 12·7)	9·5 (7·7 to 11·4)
Oman	6·6 (5·3 to 8·1)	7·4 (6·4 to 8·5)	6·0 (5·2 to 6·9)	4·8 (4·1 to 5·6)	2·4 (2·0 to 2·9)	0·8 (0·7 to 1·0)	0·7 (0·7 to 0·8)	0·9 (0·8 to 1·0)	0·8 (0·8 to 0·9)	0·7 (0·6 to 0·8)
Palestine	11·1 (8·9 to 13·7)	8·6 (7·4 to 9·9)	5·0 (4·3 to 5·9)	4·0 (3·5 to 4·5)	3·6 (3·2 to 3·9)	3·2 (2·9 to 3·7)	2·8 (2·4 to 3·2)	1·6 (1·3 to 2·0)	1·4 (1·1 to 1·8)	1·3 (1·0 to 1·7)
Qatar	0·2 (0·1 to 0·2)	0·3 (0·2 to 0·3)	0·3 (0·3 to 0·4)	0·2 (0·2 to 0·3)	0·2 (0·2 to 0·2)	0·1 (0·1 to 0·2)	0·2 (0·1 to 0·2)	0·2 (0·1 to 0·2)	0·2 (0·1 to 0·2)	0·1 (0·1 to 0·2)
Saudi Arabia	29·4 (22·6 to 37·3)	32·3 (27·4 to 37·7)	33·3 (28·5 to 38·7)	29·2 (24·7 to 34·0)	23·1 (18·8 to 28·1)	11·7 (9·5 to 14·3)	5·4 (4·8 to 6·1)	2·7 (2·2 to 3·2)	2·2 (1·7 to 2·7)	2·0 (1·5 to 2·5)
Sudan	57·0 (46·2 to 70·6)	63·2 (55·7 to 71·8)	97·0 (88·0 to 106·1)	122·3 (109·5 to 135·6)	124·5 (113·9 to 136·7)	110·6 (100·1 to 122·2)	82·4 (69·9 to 95·6)	49·9 (40·4 to 60·5)	45·8 (36·8 to 55·7)	42·5 (33·9 to 52·1)
Syria	34·1 (27·7 to 41·8)	29·1 (26·4 to 31·9)	25·1 (22·7 to 27·7)	23·1 (20·3 to 26·3)	20·8 (18·0 to 24·2)	9·1 (8·4 to 9·7)	7·5 (6·7 to 8·3)	2·8 (2·3 to 3·4)	2·3 (1·8 to 2·8)	2·0 (1·6 to 2·5)
Tunisia	54·8 (46·6 to 64·6)	44·8 (40·8 to 48·8)	30·7 (28·3 to 33·2)	19·3 (17·3 to 21·2)	11·4 (10·0 to 13·0)	5·6 (4·8 to 6·4)	3·8 (3·2 to 4·4)	2·2 (1·8 to 2·7)	1·9 (1·6 to 2·3)	1·7 (1·4 to 2·1)
Türkiye	256·0 (209·1 to 307·3)	267·6 (244·1 to 294·1)	233·8 (215·4 to 253·7)	192·9 (173·5 to 215·5)	107·0 (95·6 to 119·2)	59·0 (51·7 to 67·3)	29·8 (24·7 to 35·5)	15·7 (12·5 to 19·1)	12·9 (10·5 to 15·6)	11·4 (9·3 to 13·7)
United Arab Emirates	0·7 (0·6 to 0·9)	0·6 (0·5 to 0·7)	0·7 (0·6 to 0·8)	1·3 (1·1 to 1·5)	1·0 (0·9 to 1·1)	0·6 (0·5 to 0·6)	0·7 (0·7 to 0·8)	0·5 (0·5 to 0·6)	0·5 (0·4 to 0·5)	0·4 (0·3 to 0·4)
Yemen	99·8 (85·7 to 115·8)	100·2 (92·7 to 108·0)	100·4 (93·4 to 107·7)	93·5 (85·8 to 100·8)	84·8 (78·0 to 91·9)	75·0 (67·3 to 83·5)	60·2 (51·7 to 69·1)	43·4 (35·9 to 51·7)	39·8 (32·7 to 47·4)	37·8 (30·9 to 45·3)
South Asia	6446·6 (5070·3 to 8080·4)	6329·3 (5632·9 to 7142·8)	5402·6 (4906·5 to 5904·3)	4836·0 (4410·3 to 5271·8)	3671·3 (3382·6 to 3982·4)	2845·1 (2580·3 to 3118·4)	2110·1 (1877·6 to 2359·1)	1378·9 (1181·2 to 1604·2)	1256·0 (1066·5 to 1486·5)	1180·1 (994·9 to 1410·9)
South Asia	6446·6 (5070·3 to 8080·4)	6329·3 (5632·9 to 7142·8)	5402·6 (4906·5 to 5904·3)	4836·0 (4410·3 to 5271·8)	3671·3 (3382·6 to 3982·4)	2845·1 (2580·3 to 3118·4)	2110·1 (1877·6 to 2359·1)	1378·9 (1181·2 to 1604·2)	1256·0 (1066·5 to 1486·5)	1180·1 (994·9 to 1410·9)
Bangladesh	682·8 (575·9 to 798·8)	624·5 (564·5 to 682·8)	795·5 (738·8 to 855·0)	672·2 (617·6 to 728·1)	550·4 (509·2 to 589·4)	349·0 (319·1 to 381·6)	202·7 (179·2 to 228·8)	97·6 (79·8 to 118·9)	86·8 (70·1 to 106·2)	79·2 (63·4 to 98·0)
Bhutan	2·7 (2·2 to 3·4)	3·0 (2·5 to 3·5)	3·3 (2·7 to 3·9)	3·7 (3·1 to 4·3)	2·8 (2·5 to 3·1)	1·5 (1·3 to 1·7)	0·7 (0·6 to 0·8)	0·4 (0·3 to 0·5)	0·4 (0·3 to 0·5)	0·4 (0·3 to 0·5)
India	5187·4 (3860·1 to 6746·4)	5200·5 (4520·3 to 5986·0)	4069·1 (3592·5 to 4557·5)	3517·9 (3108·8 to 3929·8)	2555·4 (2290·8 to 2851·6)	2018·9 (1771·9 to 2277·3)	1461·0 (1256·3 to 1679·5)	874·8 (722·2 to 1044·6)	782·8 (638·8 to 958·6)	729·7 (589·8 to 902·4)
Nepal	135·5 (116·6 to 157·9)	129·2 (117·6 to 140·9)	124·1 (113·2 to 134·9)	140·8 (128·5 to 153·2)	104·8 (96·5 to 112·5)	64·0 (58·0 to 70·3)	32·9 (28·3 to 37·8)	20·3 (15·9 to 25·6)	19·1 (15·0 to 24·4)	18·2 (14·0 to 23·4)
Pakistan	438·1 (361·5 to 521·9)	372·1 (340·8 to 405·7)	410·6 (377·5 to 445·5)	501·4 (457·2 to 548·8)	457·9 (415·3 to 501·2)	411·6 (364·6 to 466·1)	412·8 (357·6 to 477·4)	385·8 (314·9 to 470·0)	366·9 (302·3 to 443·2)	352·6 (287·7 to 428·0)
Azad Jammu & Kashmir	11·7 (8·8 to 15·1)	9·6 (8·2 to 11·2)	10·0 (8·8 to 11·2)	11·9 (9·7 to 14·2)	9·8 (8·2 to 11·7)	7·0 (5·4 to 8·7)	5·4 (3·9 to 7·3)	4·4 (3·2 to 6·1)	4·1 (3·0 to 5·5)	3·9 (2·9 to 5·2)
Balochistan	10·3 (7·5 to 14·0)	6·8 (5·8 to 7·9)	9·1 (7·8 to 10·5)	16·3 (13·9 to 19·3)	18·1 (15·9 to 20·4)	19·4 (15·7 to 23·7)	26·0 (20·8 to 32·2)	30·5 (23·8 to 38·0)	28·9 (22·9 to 36·1)	28·7 (22·9 to 35·9)

Gilgit-Baltistan	3·5 (2·6 to 4·5)	2·7 (2·4 to 3·1)	3·1 (2·7 to 3·5)	4·0 (3·4 to 4·8)	3·6 (3·0 to 4·2)	3·3 (2·7 to 3·9)	3·0 (2·3 to 3·8)	2·9 (2·3 to 3·7)	2·7 (2·2 to 3·4)	2·7 (2·2 to 3·3)
Islamabad Capital Territory	1·9 (1·3 to 2·7)	1·6 (1·3 to 1·9)	1·4 (1·1 to 1·7)	1·3 (1·0 to 1·6)	0·9 (0·7 to 1·2)	0·8 (0·6 to 1·0)	0·8 (0·6 to 1·1)	0·7 (0·5 to 1·0)	0·7 (0·5 to 0·9)	0·7 (0·5 to 0·9)
Khyber Pakhtunkhwa	78·7 (59·7 to 103·4)	61·8 (54·8 to 69·1)	62·8 (55·2 to 70·6)	63·9 (55·3 to 74·0)	48·6 (42·6 to 55·6)	53·1 (45·0 to 61·4)	58·3 (45·9 to 72·9)	59·3 (45·9 to 74·9)	55·4 (43·6 to 69·8)	53·1 (42·4 to 66·2)
Punjab	254·7 (209·1 to 307·4)	216·7 (196·3 to 237·6)	237·2 (214·7 to 258·9)	297·6 (268·7 to 327·5)	270·6 (246·9 to 294·5)	229·7 (205·0 to 258·2)	222·9 (194·7 to 255·1)	205·6 (162·9 to 257·2)	196·9 (156·1 to 247·2)	189·8 (149·8 to 238·4)
Sindh	77·4 (58·8 to 98·0)	72·9 (63·9 to 82·4)	87·1 (78·5 to 96·6)	106·4 (95·1 to 120·8)	106·2 (90·9 to 121·3)	98·4 (82·6 to 116·2)	96·3 (81·1 to 113·9)	82·3 (65·3 to 104·3)	78·1 (61·8 to 99·0)	73·9 (58·5 to 93·7)
Southeast Asia, east Asia, and Oceania	6542·7 (5562·3 to 7715·2)	18555·9 (12186·9 to 30154·1)	4260·0 (3905·4 to 4653·9)	2667·3 (2455·8 to 2897·1)	2154·4 (1979·0 to 2353·4)	1213·3 (1121·9 to 1321·2)	648·6 (594·2 to 704·7)	423·1 (360·5 to 492·5)	380·9 (324·4 to 442·2)	352·4 (301·5 to 410·7)
East Asia	4312·8 (3488·7 to 5313·4)	16455·3 (10150·9 to 27985·5)	2542·3 (2268·3 to 2862·6)	1331·4 (1182·9 to 1494·8)	1257·8 (1114·7 to 1422·9)	617·7 (548·8 to 714·0)	229·1 (203·3 to 255·6)	127·1 (110·2 to 146·0)	105·3 (89·7 to 123·4)	90·0 (76·2 to 106·6)
China	4199·6 (3387·0 to 5189·2)	16383·4 (10082·4 to 27914·7)	2501·6 (2230·2 to 2821·7)	1306·3 (1159·1 to 1468·1)	1238·5 (1093·5 to 1403·9)	557·7 (503·0 to 613·4)	219·8 (194·0 to 246·5)	122·6 (105·8 to 141·1)	101·2 (85·6 to 118·7)	86·1 (72·3 to 102·2)
North Korea	66·4 (51·1 to 84·7)	46·9 (38·2 to 57·3)	30·9 (24·8 to 37·8)	18·9 (14·6 to 24·6)	16·5 (13·2 to 20·8)	57·7 (24·4 to 136·2)	8·4 (6·6 to 10·3)	3·8 (2·8 to 4·9)	3·4 (2·5 to 4·5)	3·1 (2·3 to 4·1)
Taiwan (province of China)	46·8 (45·2 to 48·7)	25·0 (24·5 to 25·4)	9·9 (9·6 to 10·2)	6·1 (5·9 to 6·4)	2·7 (2·6 to 2·9)	2·3 (2·1 to 2·4)	1·0 (0·9 to 1·0)	0·8 (0·7 to 0·9)	0·7 (0·7 to 0·8)	0·7 (0·7 to 0·8)
Oceania	22·6 (19·0 to 26·6)	20·9 (18·2 to 23·7)	17·4 (15·3 to 19·6)	15·0 (13·3 to 16·8)	15·0 (13·3 to 17·0)	16·3 (14·1 to 18·7)	18·7 (15·8 to 22·0)	19·8 (16·4 to 23·9)	19·7 (16·2 to 23·7)	19·8 (16·3 to 23·7)
American Samoa	0·1 (0·1 to 0·1)	0·0 (0·0 to 0·0)	0·0 (0·0 to 0·0)	0·0 (0·0 to 0·0)	0·0 (0·0 to 0·0)	0·0 (0·0 to 0·0)	0·0 (0·0 to 0·0)	0·0 (0·0 to 0·0)	0·0 (0·0 to 0·0)	0·0 (0·0 to 0·0)
Cook Islands	0·1 (0·1 to 0·2)	0·1 (0·1 to 0·1)	0·0 (0·0 to 0·0)	0·0 (0·0 to 0·0)	0·0 (0·0 to 0·0)	0·0 (0·0 to 0·0)	0·0 (0·0 to 0·0)	0·0 (0·0 to 0·0)	0·0 (0·0 to 0·0)	0·0 (0·0 to 0·0)
Fiji	1·0 (0·8 to 1·2)	0·9 (0·8 to 1·0)	0·7 (0·6 to 0·8)	0·6 (0·5 to 0·7)	0·5 (0·4 to 0·6)	0·5 (0·4 to 0·6)	0·5 (0·4 to 0·6)	0·4 (0·3 to 0·5)	0·4 (0·3 to 0·5)	0·3 (0·3 to 0·5)
Guam	0·1 (0·1 to 0·1)	0·1 (0·1 to 0·1)	0·1 (0·1 to 0·1)	0·0 (0·0 to 0·1)	0·0 (0·0 to 0·0)	0·0 (0·0 to 0·0)	0·0 (0·0 to 0·1)	0·0 (0·0 to 0·0)	0·0 (0·0 to 0·0)	0·0 (0·0 to 0·0)
Kiribati	0·3 (0·2 to 0·3)	0·2 (0·2 to 0·3)	0·2 (0·2 to 0·2)	0·2 (0·2 to 0·3)	0·2 (0·2 to 0·3)	0·2 (0·1 to 0·2)	0·1 (0·1 to 0·2)	0·1 (0·1 to 0·1)	0·1 (0·1 to 0·1)	0·1 (0·1 to 0·1)
Marshall Islands	0·1 (0·1 to 0·1)	0·1 (0·1 to 0·1)	0·1 (0·0 to 0·1)	0·1 (0·0 to 0·1)	0·0 (0·0 to 0·1)	0·1 (0·1 to 0·1)	0·0 (0·0 to 0·0)	0·0 (0·0 to 0·0)	0·0 (0·0 to 0·0)	0·0 (0·0 to 0·0)
Federated States of Micronesia	0·3 (0·3 to 0·4)	0·3 (0·2 to 0·3)	0·2 (0·2 to 0·2)	0·2 (0·1 to 0·2)	0·1 (0·1 to 0·2)	0·1 (0·1 to 0·1)	0·0 (0·0 to 0·1)	0·0 (0·0 to 0·0)	0·0 (0·0 to 0·0)	0·0 (0·0 to 0·0)
Nauru	0·0 (0·0 to 0·0)	0·0 (0·0 to 0·0)	0·0 (0·0 to 0·0)	0·0 (0·0 to 0·0)	0·0 (0·0 to 0·0)	0·0 (0·0 to 0·0)	0·0 (0·0 to 0·0)	0·0 (0·0 to 0·0)	0·0 (0·0 to 0·0)	0·0 (0·0 to 0·0)
Niue	0·0 (0·0 to 0·0)	0·0 (0·0 to 0·0)	0·0 (0·0 to 0·0)	0·0 (0·0 to 0·0)	0·0 (0·0 to 0·0)	0·0 (0·0 to 0·0)	0·0 (0·0 to 0·0)	0·0 (0·0 to 0·0)	0·0 (0·0 to 0·0)	0·0 (0·0 to 0·0)
Northern Mariana Islands	0·0 (0·0 to 0·0)	0·0 (0·0 to 0·0)	0·0 (0·0 to 0·0)	0·0 (0·0 to 0·0)	0·0 (0·0 to 0·0)	0·0 (0·0 to 0·0)	0·0 (0·0 to 0·0)	0·0 (0·0 to 0·0)	0·0 (0·0 to 0·0)	0·0 (0·0 to 0·0)
Palau	0·1 (0·1 to 0·1)	0·0 (0·0 to 0·0)	0·0 (0·0 to 0·0)	0·0 (0·0 to 0·0)	0·0 (0·0 to 0·0)	0·0 (0·0 to 0·0)	0·0 (0·0 to 0·0)	0·0 (0·0 to 0·0)	0·0 (0·0 to 0·0)	0·0 (0·0 to 0·0)
Papua New Guinea	17·0 (13·8 to 20·8)	15·9 (13·6 to 18·6)	13·4 (11·5 to 15·3)	11·6 (10·2 to 13·2)	12·0 (10·5 to 13·7)	13·4 (11·5 to 15·5)	16·1 (13·5 to 19·0)	17·5 (14·4 to 21·1)	17·5 (14·4 to 21·1)	17·6 (14·5 to 21·1)
Samoa	0·6 (0·5 to 0·8)	0·5 (0·4 to 0·6)	0·4 (0·3 to 0·4)	0·2 (0·2 to 0·3)	0·2 (0·1 to 0·2)	0·1 (0·1 to 0·1)	0·1 (0·1 to 0·1)	0·2 (0·1 to 0·2)	0·1 (0·1 to 0·1)	0·1 (0·1 to 0·1)
Solomon Islands	0·9 (0·8 to 1·1)	0·9 (0·8 to 1·1)	0·8 (0·6 to 0·9)	0·6 (0·5 to 0·7)	0·6 (0·5 to 0·7)	0·6 (0·5 to 0·7)	0·5 (0·4 to 0·6)	0·4 (0·3 to 0·5)	0·4 (0·3 to 0·5)	0·4 (0·3 to 0·5)
Tokelau	0·0 (0·0 to 0·0)	0·0 (0·0 to 0·0)	0·0 (0·0 to 0·0)	0·0 (0·0 to 0·0)	0·0 (0·0 to 0·0)	0·0 (0·0 to 0·0)	0·0 (0·0 to 0·0)	0·0 (0·0 to 0·0)	0·0 (0·0 to 0·0)	0·0 (0·0 to 0·0)
Tonga	0·1 (0·1 to 0·2)	0·1 (0·1 to 0·2)	0·1 (0·1 to 0·2)	0·1 (0·1 to 0·1)	0·1 (0·1 to 0·1)	0·1 (0·1 to 0·1)	0·1 (0·0 to 0·1)	0·0 (0·0 to 0·0)	0·0 (0·0 to 0·0)	0·0 (0·0 to 0·0)
Tuvalu	0·0 (0·0 to 0·1)	0·0 (0·0 to 0·0)	0·0 (0·0 to 0·0)	0·0 (0·0 to 0·0)	0·0 (0·0 to 0·0)	0·0 (0·0 to 0·0)	0·0 (0·0 to 0·0)	0·0 (0·0 to 0·0)	0·0 (0·0 to 0·0)	0·0 (0·0 to 0·0)
Vanuatu	0·4 (0·3 to 0·5)	0·5 (0·4 to 0·5)	0·4 (0·3 to 0·5)	0·3 (0·2 to 0·3)	0·2 (0·2 to 0·3)	0·2 (0·2 to 0·3)	0·2 (0·2 to 0·3)	0·2 (0·1 to 0·2)	0·2 (0·1 to 0·2)	0·2 (0·1 to 0·2)
Southeast Asia	2207·4 (1939·1 to 2503·7)	2079·7 (1899·4 to 2278·1)	1700·2 (1594·0 to 1808·8)	1321·0 (1242·3 to 1405·9)	881·6 (833·2 to 931·2)	579·3 (539·2 to 622·6)	400·8 (358·7 to 444·6)	276·1 (230·9 to 328·5)	255·9 (215·0 to 301·3)	242·6 (205·1 to 286·9)
Cambodia	55·9 (43·9 to 70·9)	55·9 (47·0 to 65·2)	54·2 (46·9 to 62·2)	55·6 (49·8 to 61·9)	51·0 (47·3 to 55·1)	33·7 (30·9 to 36·6)	17·7 (15·5 to 20·2)	12·0 (9·9 to 14·4)	11·6 (9·6 to 14·1)	11·0 (9·1 to 13·4)
Indonesia	1105·1 (937·7 to 1288·5)	1089·6 (988·2 to 1189·1)	886·0 (825·7 to 951·1)	632·3 (590·1 to 674·3)	392·4 (362·0 to 424·0)	254·2 (231·2 to 276·9)	179·2 (154·8 to 206·5)	119·1 (94·5 to 145·0)	111·2 (89·6 to 135·9)	106·6 (86·1 to 130·5)
Aceh	13·9 (11·0 to 17·3)	14·0 (12·8 to 15·3)	13·0 (11·7 to 14·4)	11·0 (10·0 to 12·1)	8·0 (7·2 to 8·8)	5·9 (5·4 to 6·4)	4·0 (3·5 to 4·6)	2·7 (2·2 to 3·3)	2·5 (2·0 to 3·1)	2·5 (2·0 to 3·0)
Bali	16·2 (12·7 to 20·7)	14·3 (13·1 to 15·8)	11·3 (10·2 to 12·5)	6·8 (6·1 to 7·5)	3·4 (3·0 to 3·8)	2·4 (2·2 to 2·7)	1·7 (1·5 to 2·0)	1·1 (0·9 to 1·5)	1·1 (0·8 to 1·3)	1·0 (0·8 to 1·3)
Bangka-Belitung Islands	4·6 (3·5 to 6·1)	4·3 (3·6 to 5·1)	3·7 (3·3 to 4·1)	3·0 (2·7 to 3·3)	2·0 (1·8 to 2·2)	1·2 (1·0 to 1·3)	0·9 (0·7 to 1·1)	0·8 (0·6 to 1·0)	0·7 (0·5 to 0·9)	0·7 (0·5 to 0·9)
Banten	38·4 (29·7 to 48·9)	36·3 (30·4 to 42·5)	30·8 (28·2 to 33·4)	27·7 (25·4 to 29·8)	26·9 (24·7 to 29·1)	15·9 (13·9 to 17·9)	9·9 (8·0 to 12·3)	6·3 (4·9 to 8·2)	5·8 (4·6 to 7·5)	5·5 (4·3 to 7·0)
Bengkulu	7·5 (6·1 to 9·3)	7·0 (6·4 to 7·7)	5·8 (5·3 to 6·4)	4·8 (4·4 to 5·2)	3·6 (3·3 to 3·9)	2·3 (2·1 to 2·6)	1·6 (1·4 to 1·8)	0·9 (0·7 to 1·2)	0·9 (0·7 to 1·1)	0·8 (0·7 to 1·0)
Gorontalo	4·2 (3·1 to 5·4)	4·9 (4·1 to 5·8)	4·7 (4·3 to 5·2)	3·2 (2·8 to 3·6)	2·0 (1·8 to 2·2)	1·7 (1·5 to 2·0)	1·5 (1·2 to 1·8)	1·0 (0·8 to 1·3)	0·9 (0·7 to 1·2)	0·9 (0·7 to 1·2)
Jakarta	27·3 (21·6 to 34·0)	30·8 (28·0 to 33·8)	26·1 (23·7 to 29·1)	19·4 (17·5 to 21·5)	11·9 (10·7 to 13·1)	7·0 (6·3 to 7·9)	4·8 (4·2 to 5·5)	2·7 (2·1 to 3·5)	2·5 (1·9 to 3·1)	2·3 (1·8 to 2·9)
Jambi	9·9 (7·9 to 12·2)	9·7 (8·9 to 10·6)	8·4 (7·7 to 9·3)	6·8 (6·3 to 7·4)	4·6 (4·1 to 5·1)	3·2 (2·9 to 3·5)	2·3 (2·0 to 2·6)	1·5 (1·2 to 1·8)	1·4 (1·1 to 1·7)	1·3 (1·1 to 1·6)
West Java	217·6 (183·7 to 256·1)	226·8 (206·6 to 247·8)	187·8 (172·9 to 203·3)	129·0 (119·4 to 138·7)	70·8 (65·0 to 76·5)	46·1 (40·4 to 52·0)	33·2 (28·4 to 38·0)	21·4 (16·9 to 26·2)	19·8 (15·5 to 24·5)	18·9 (15·0 to 23·2)
Central Java	165·3 (132·0 to 204·4)	162·1 (145·5 to 179·6)	124·1 (112·4 to 137·7)	77·4 (70·0 to 84·6)	42·5 (38·7 to 46·7)	23·0 (20·6 to 25·4)	14·9 (12·5 to 17·6)	9·4 (7·3 to 11·7)	8·7 (6·9 to 10·8)	8·3 (6·6 to 10·2)
East Java	207·6 (173·0 to 248·6)	197·7 (179·1 to 216·8)	147·9 (134·6 to 163·8)	95·9 (87·6 to 104·6)	52·3 (47·9 to 56·7)	30·1 (26·3 to 33·9)	19·0 (16·3 to 21·8)	11·0 (8·6 to 13·6)	10·1 (7·8 to 12·4)	9·6 (7·5 to 11·8)
West Kalimantan	15·5 (12·4 to 19·5)	18·0 (16·3 to 19·9)	17·9 (16·1 to 19·9)	15·6 (14·3 to 16·9)	9·7 (8·7 to 10·7)	5·8 (5·2 to 6·6)	3·8 (3·4 to 4·3)	2·4 (1·8 to 3·0)	2·2 (1·7 to 2·8)	2·1 (1·7 to 2·7)
South Kalimantan	23·2 (19·6 to 27·2)	25·8 (23·5 to 28·0)	21·0 (19·5 to 22·5)	15·0 (13·9 to 16·1)	9·3 (8·2 to 10·3)	5·9 (5·3 to 6·5)	3·9 (3·3 to 4·5)	2·4 (1·9 to 3·0)	2·2 (1·7 to 2·7)	2·1 (1·7 to 2·6)
Central Kalimantan	4·3 (3·5 to 5·3)	3·8 (3·5 to 4·2)	3·3 (3·0 to 3·6)	2·9 (2·6 to 3·1)	2·6 (2·3 to 2·8)	2·2 (2·0 to 2·4)	1·7 (1·5 to 1·9)	1·3 (1·1 to 1·6)	1·3 (1·0 to 1·5)	1·2 (1·0 to 1·5)
East Kalimantan	4·4 (3·5 to 5·6)	5·2 (4·8 to 5·7)	5·0 (4·4 to 5·5)	4·2 (3·8 to 4·6)	2·9 (2·6 to 3·3)	2·4 (2·2 to 2·7)	1·8 (1·6 to 2·0)	1·3 (1·0 to 1·5)	1·2 (0·9 to 1·4)	1·1 (0·9 to 1·4)
North Kalimantan	0·7 (0·5 to 0·8)	0·9 (0·8 to 1·0)	1·1 (1·0 to 1·2)	0·8 (0·7 to 0·9)	0·6 (0·5 to 0·7)	0·6 (0·5 to 0·7)	0·5 (0·4 to 0·6)	0·4 (0·3 to 0·5)	0·4 (0·3 to 0·5)	0·4 (0·3 to 0·5)
Riau Islands	1·8 (1·4 to 2·2)	1·5 (1·2 to 1·8)	1·2 (1·1 to 1·3)	1·3 (1·2 to 1·5)	1·6 (1·5 to 1·8)	1·4 (1·3 to 1·6)	1·2 (1·0 to 1·5)	0·8 (0·6 to 1·0)	0·7 (0·6 to 0·9)	0·7 (0·6 to 0·9)
Lampung	24·2 (19·5 to 29·9)	29·1 (26·3 to 32·2)	27·4 (25·2 to 30·1)	21·7 (20·0 to 23·5)	13·6 (12·4 to 14·8)	8·0 (7·3 to 8·7)	5·7 (4·9 to 6·6)	4·4 (3·4 to 5·5)	4·2 (3·3 to 5·1)	4·0 (3·2 to 5·0)
Maluku	10·2 (8·3 to 12·4)	8·6 (7·3 to 10·1)	6·8 (6·3 to 7·4)	5·8 (5·2 to 6·4)	4·7 (4·2 to 5·1)	3·5 (3·2 to 3·9)	2·6 (2·1 to 3·1)	1·6 (1·2 to 1·9)	1·5 (1·2 to 1·8)	1·4 (1·1 to 1·7)
North Maluku	4·5 (3·5 to 6·0)	3·9 (3·2 to 4·6)	3·2 (2·9 to 3·6)	3·1 (2·7 to 3·5)	2·6 (2·3 to 3·0)	2·1 (1·9 to 2·4)	1·6 (1·3 to 2·0)	1·1 (0·9 to 1·5)	1·1 (0·8 to 1·4)	1·0 (0·8 to 1·4)
West Nusa Tenggara	32·8 (26·9 to 39·2)	32·6 (29·8 to 35·3)	30·4 (28·2 to 32·8)	24·7 (23·1 to 26·4)	14·4 (13·0 to 16·0)	8·3 (7·5 to 9·1)	4·8 (4·0 to 5·6)	3·0 (2·3 to 3·7)	2·8 (2·2 to 3·4)	2·6 (2·1 to 3·2)
East Nusa Tenggara	27·8 (22·7 to 34·0)	25·4 (21·3 to 29·9)	21·4 (19·6 to 23·4)	15·5 (14·2 to 16·8)	10·7 (9·9 to 11·6)	8·9 (8·0 to 9·8)	6·4 (5·2 to 7·8)	4·1 (3·3 to 5·0)	3·9 (3·1 to 4·7)	3·7 (3·0 to 4·5)
Papua	2·0 (1·6 to 2·4)	2·7 (2·3 to 3·2)	3·1 (2·8 to 3·4)	3·2 (2·8 to 3·5)	3·3 (2·9 to 3·6)	3·9 (3·5 to 4·3)	5·1 (4·2 to 6·1)	4·9 (3·9 to 6·1)	4·7 (3·7 to 5·8)	4·5 (3·6 to 5·6)
West Papua	1·6 (1·3 to 2·0)	1·9 (1·6 to 2·2)	2·0 (1·8 to 2·2)	1·8 (1·6 to 2·0)	1·5 (1·4 to 1·7)	1·1 (1·0 to 1·2)	1·0 (0·8 to 1·2)	0·7 (0·6 to 0·9)	0·7 (0·5 to 0·8)	0·7 (0·5 to 0·8)
Riau	8·8 (7·2 to 10·8)	11·2 (10·2 to 12·4)	10·8 (9·9 to 11·7)	8·2 (7·5 to 8·9)	5·5 (5·1 to 6·0)	5·0 (4·4 to 5·6)	4·9 (4·3 to 5·5)	2·7 (2·2 to 3·3)	2·5 (2·0 to 3·1)	2·4 (1·9 to 3·0)
West Sulawesi	8·3 (6·2 to 10·7)	7·5 (6·3 to 8·8)	6·5 (5·9 to 7·1)	4·9 (4·5 to 5·4)	3·3 (2·9 to 3·6)	2·5 (2·3 to 2·7)	2·0 (1·7 to 2·4)	1·5 (1·1 to 1·9)	1·4 (1·1 to 1·8)	1·4 (1·0 to 1·7)
South Sulawesi	48·3 (39·5 to 57·4)	48·8 (44·7 to 53·0)	40·8 (37·4 to 44·6)	28·6 (26·5 to 30·9)	16·1 (14·3 to 17·9)	10·1 (9·3 to 11·0)	6·4 (5·4 to 7·4)	3·8 (3·0 to 4·8)	3·6 (2·9 to 4·4)	3·4 (2·8 to 4·2)
Central Sulawesi	10·6 (8·9 to 12·7)	9·9 (9·1 to 10·7)	8·8 (8·1 to 9·7)	7·3 (6·8 to 7·8)	5·2 (4·7 to 5·7)	4·3 (3·9 to 4·7)	3·3 (2·9 to 3·8)	2·2 (1·8 to 2·7)	2·1 (1·7 to 2·6)	2·0 (1·6 to 2·5)
Southeast Sulawesi	8·5 (7·0 to 10·2)	9·0 (7·7 to 10·5)	8·5 (7·8 to 9·3)	6·6 (6·1 to 7·1)	4·5 (4·1 to 4·9)	3·4 (3·1 to 3·8)	2·7 (2·2 to 3·4)	2·2 (1·7 to 2·7)	2·1 (1·7 to 2·6)	2·1 (1·6 to 2·5)
North Sulawesi	8·5 (6·8 to 10·5)	7·1 (6·4 to 7·8)	4·9 (4·4 to 5·4)	3·0 (2·7 to 3·4)	2·2 (2·0 to 2·5)	1·7 (1·6 to 1·9)	1·5 (1·3 to 1·7)	1·2 (1·0 to 1·5)	1·1 (0·9 to 1·4)	1·1 (0·9 to 1·4)
West Sumatra	49·6 (42·0 to 58·8)	37·3 (33·6 to 41·0)	25·4 (23·2 to 27·8)	17·1 (15·8 to 18·5)	9·7 (8·9 to 10·6)	6·5 (5·8 to 7·2)	3·9 (3·4 to 4·5)	2·1 (1·6 to 2·6)	1·9 (1·5 to 2·4)	1·8 (1·5 to 2·2)
South Sumatra	26·5 (22·4 to 31·6)	27·2 (24·7 to 29·8)	23·6 (21·8 to 25·9)	18·7 (17·1 to 20·2)	13·4 (12·3 to 14·7)	9·3 (8·1 to 10·3)	6·5 (5·6 to 7·5)	4·5 (3·5 to 5·5)	4·2 (3·3 to 5·1)	4·0 (3·2 to 4·9)

North Sumatra	47·0 (38·6 to 57·4)	48·1 (43·8 to 52·6)	40·8 (37·6 to 44·4)	33·5 (30·9 to 36·1)	24·9 (22·6 to 27·5)	17·5 (16·0 to 19·3)	13·6 (11·5 to 15·9)	11·3 (9·0 to 13·8)	10·7 (8·7 to 13·2)	10·4 (8·4 to 12·8)
Yogyakarta	23·5 (17·7 to 30·4)	16·2 (13·7 to 18·7)	8·4 (7·5 to 9·5)	3·8 (3·4 to 4·2)	1·9 (1·6 to 2·1)	1·0 (0·9 to 1·1)	0·7 (0·6 to 0·9)	0·6 (0·4 to 0·7)	0·5 (0·4 to 0·7)	0·5 (0·4 to 0·6)
Laos	26·9 (21·3 to 33·3)	31·3 (26·6 to 36·3)	34·0 (28·3 to 40·5)	33·1 (28·1 to 38·3)	28·7 (25·6 to 31·7)	22·7 (20·3 to 25·2)	11·9 (10·5 to 13·3)	7·7 (6·2 to 9·4)	7·4 (5·8 to 9·1)	7·0 (5·4 to 8·8)
Malaysia	38·2 (35·3 to 41·0)	31·6 (31·1 to 32·1)	19·7 (19·3 to 20·2)	12·5 (11·1 to 14·2)	8·5 (8·2 to 8·8)	4·8 (4·6 to 5·0)	3·5 (3·4 to 3·7)	3·7 (3·5 to 3·9)	3·2 (3·0 to 3·5)	3·0 (2·7 to 3·4)
Maldives	1·1 (0·9 to 1·4)	1·2 (1·0 to 1·4)	1·1 (1·0 to 1·2)	0·9 (0·8 to 1·0)	0·6 (0·5 to 0·6)	0·2 (0·2 to 0·2)	0·1 (0·1 to 0·2)	0·1 (0·1 to 0·1)	0·1 (0·1 to 0·1)	0·1 (0·1 to 0·1)
Mauritius	3·1 (2·8 to 3·3)	2·6 (2·5 to 2·7)	1·7 (1·6 to 1·8)	1·0 (0·9 to 1·0)	0·5 (0·5 to 0·5)	0·3 (0·3 to 0·4)	0·2 (0·2 to 0·2)	0·2 (0·2 to 0·2)	0·2 (0·2 to 0·2)	0·2 (0·1 to 0·2)
Myanmar	308·8 (245·6 to 383·3)	309·2 (262·0 to 361·0)	284·8 (245·1 to 328·2)	249·7 (209·6 to 292·5)	162·0 (140·8 to 184·8)	114·7 (99·8 to 131·3)	70·5 (60·9 to 80·3)	46·5 (38·0 to 56·9)	43·8 (35·6 to 54·1)	42·1 (33·9 to 53·2)
Philippines	189·7 (148·0 to 241·7)	167·2 (146·2 to 189·0)	143·9 (130·3 to 158·0)	136·8 (124·3 to 150·2)	107·1 (95·1 to 119·8)	80·3 (70·2 to 91·2)	73·8 (64·0 to 84·7)	57·2 (47·4 to 68·9)	50·8 (42·6 to 60·3)	47·6 (39·3 to 57·6)
Seychelles	0·1 (0·1 to 0·1)	0·2 (0·1 to 0·2)	0·1 (0·1 to 0·1)	0·1 (0·0 to 0·1)	0·0 (0·0 to 0·0)	0·0 (0·0 to 0·0)	0·0 (0·0 to 0·0)	0·0 (0·0 to 0·0)	0·0 (0·0 to 0·0)	0·0 (0·0 to 0·0)
Sri Lanka	53·2 (49·5 to 57·2)	32·9 (31·9 to 33·9)	25·3 (24·2 to 26·4)	18·9 (18·1 to 19·7)	7·4 (6·9 to 8·0)	5·7 (5·4 to 6·1)	4·3 (4·1 to 4·6)	2·2 (1·8 to 2·8)	2·0 (1·6 to 2·6)	1·8 (1·4 to 2·3)
Thailand	173·4 (143·1 to 207·6)	144·0 (132·0 to 156·4)	93·5 (85·4 to 102·0)	57·9 (51·7 to 64·3)	29·8 (25·7 to 34·2)	15·7 (13·4 to 18·3)	7·8 (7·4 to 8·2)	4·8 (4·5 to 5·2)	4·3 (3·9 to 4·7)	4·0 (3·5 to 4·5)
Timor-Leste	6·1 (5·0 to 7·5)	6·5 (5·6 to 7·5)	5·6 (5·0 to 6·3)	5·3 (4·9 to 5·8)	4·6 (4·2 to 5·0)	2·9 (2·6 to 3·3)	1·6 (1·3 to 1·9)	1·4 (1·1 to 1·7)	1·4 (1·1 to 1·7)	1·4 (1·2 to 1·7)
Viet Nam	242·7 (187·1 to 308·4)	204·0 (169·4 to 242·7)	147·5 (130·4 to 167·3)	114·9 (105·2 to 124·8)	87·8 (79·8 to 95·6)	43·1 (38·4 to 48·5)	29·4 (25·5 to 33·7)	20·7 (16·3 to 25·9)	19·5 (15·4 to 24·9)	17·5 (13·7 to 22·5)
Sub-Saharan Africa	2462·9 (2073·9 to 2902·3)	2776·7 (2454·5 to 3119·5)	3057·8 (2817·8 to 3305·9)	3361·2 (3181·6 to 3539·1)	3688·6 (3529·8 to 3856·3)	3959·6 (3760·5 to 4151·0)	3552·0 (3295·6 to 3813·3)	2807·7 (2407·3 to 3264·8)	2717·3 (2300·2 to 3225·7)	2627·6 (2208·5 to 3144·0)
Central sub-Saharan Africa	260·3 (213·8 to 311·7)	314·4 (273·2 to 358·3)	347·3 (307·4 to 390·4)	379·3 (342·2 to 417·0)	426·1 (395·4 to 456·9)	489·4 (453·4 to 528·8)	435·1 (390·4 to 479·6)	291·3 (248·8 to 339·7)	271·4 (231·8 to 320·8)	258·6 (219·7 to 307·1)
Angola	62·2 (50·1 to 76·6)	66·1 (56·6 to 76·5)	66·0 (56·7 to 76·2)	78·5 (68·6 to 88·9)	100·6 (91·8 to 110·1)	112·8 (103·6 to 122·6)	93·8 (81·3 to 107·1)	68·2 (56·3 to 81·1)	65·6 (54·7 to 77·9)	65·3 (54·3 to 78·0)
Central African Republic	15·1 (12·0 to 19·1)	17·9 (15·0 to 20·9)	20·0 (17·4 to 22·9)	21·0 (18·6 to 23·7)	23·5 (21·5 to 25·7)	28·0 (25·3 to 30·9)	28·7 (24·1 to 34·0)	22·4 (18·0 to 27·7)	21·6 (17·4 to 26·8)	20·6 (16·6 to 25·8)
Congo (Brazzaville)	11·6 (8·9 to 15·4)	12·4 (10·8 to 14·2)	12·5 (10·8 to 14·5)	10·3 (8·9 to 12·0)	9·2 (8·1 to 10·4)	11·7 (10·4 to 13·2)	9·5 (8·1 to 10·9)	6·0 (5·0 to 7·2)	5·4 (4·4 to 6·5)	5·0 (4·2 to 6·1)
DR Congo	163·2 (129·0 to 202·1)	210·1 (177·4 to 245·1)	242·3 (210·1 to 277·3)	263·9 (235·0 to 293·8)	286·8 (262·2 to 311·1)	330·7 (299·5 to 366·1)	297·9 (260·3 to 335·4)	191·2 (159·4 to 228·6)	175·5 (146·1 to 213·5)	164·6 (136·9 to 204·4)
Equatorial Guinea	3·0 (2·4 to 3·7)	2·8 (2·4 to 3·2)	2·4 (2·0 to 2·8)	2·4 (2·0 to 2·7)	3·2 (2·8 to 3·7)	3·4 (2·8 to 4·0)	2·5 (2·0 to 3·1)	1·9 (1·4 to 2·5)	1·8 (1·3 to 2·5)	1·8 (1·3 to 2·4)
Gabon	5·1 (4·0 to 6·5)	5·1 (4·3 to 5·9)	4·2 (3·5 to 5·0)	3·1 (2·7 to 3·6)	2·8 (2·4 to 3·2)	2·9 (2·4 to 3·4)	2·7 (2·2 to 3·3)	1·7 (1·2 to 2·2)	1·5 (1·1 to 2·1)	1·4 (1·0 to 1·9)
Eastern sub-Saharan Africa	935·8 (778·7 to 1102·3)	1058·3 (927·2 to 1192·3)	1161·8 (1059·9 to 1269·8)	1338·5 (1264·1 to 1415·5)	1485·7 (1411·1 to 1557·3)	1453·6 (1369·1 to 1544·0)	1130·8 (1015·0 to 1245·1)	826·8 (687·8 to 992·9)	803·9 (658·4 to 983·3)	786·9 (639·7 to 978·3)
Burundi	39·8 (31·3 to 49·4)	39·9 (34·3 to 45·7)	42·6 (38·0 to 47·7)	43·5 (39·8 to 47·6)	46·2 (42·7 to 50·0)	41·5 (37·7 to 45·6)	37·4 (33·1 to 42·2)	29·5 (23·7 to 36·4)	29·9 (23·6 to 37·9)	29·6 (23·0 to 38·3)
Comoros	1·5 (1·2 to 1·8)	2·0 (1·7 to 2·3)	2·5 (2·2 to 2·8)	2·7 (2·5 to 3·0)	2·5 (2·2 to 2·7)	2·0 (1·8 to 2·4)	1·4 (1·1 to 1·7)	0·9 (0·7 to 1·1)	0·8 (0·7 to 1·0)	0·8 (0·7 to 1·0)
Djibouti	0·4 (0·3 to 0·5)	0·5 (0·5 to 0·6)	0·8 (0·7 to 0·9)	1·1 (0·9 to 1·2)	1·5 (1·3 to 1·7)	1·8 (1·5 to 2·1)	1·7 (1·4 to 2·1)	1·3 (1·0 to 1·6)	1·2 (1·0 to 1·4)	1·1 (0·9 to 1·4)
Eritrea	14·9 (11·7 to 18·5)	18·4 (15·7 to 21·3)	20·8 (18·0 to 23·9)	21·7 (19·4 to 24·1)	21·6 (19·0 to 24·4)	13·9 (11·7 to 16·3)	12·3 (9·6 to 15·4)	9·4 (7·1 to 12·2)	9·0 (6·8 to 11·8)	8·8 (6·6 to 11·7)
Ethiopia	253·8 (200·6 to 314·3)	293·1 (251·0 to 336·1)	328·4 (292·7 to 366·7)	403·7 (368·9 to 439·9)	451·3 (418·8 to 485·1)	423·6 (388·5 to 461·4)	268·4 (237·0 to 301·8)	189·1 (152·8 to 232·4)	183·7 (147·3 to 227·8)	179·5 (143·1 to 225·2)
Addis Ababa	4·1 (3·1 to 5·1)	5·5 (4·5 to 6·5)	6·4 (5·5 to 7·3)	6·6 (5·8 to 7·4)	5·2 (4·6 to 5·9)	3·3 (2·8 to 3·9)	1·6 (1·3 to 1·9)	0·9 (0·7 to 1·1)	0·8 (0·7 to 1·0)	0·8 (0·6 to 1·0)
Afar	7·2 (5·6 to 9·0)	4·2 (3·6 to 4·8)	2·7 (2·4 to 3·1)	3·7 (3·4 to 4·1)	7·3 (6·7 to 7·9)	11·2 (10·0 to 12·5)	7·2 (6·1 to 8·5)	4·5 (3·7 to 5·6)	4·3 (3·4 to 5·4)	4·1 (3·3 to 5·1)
Amhara	75·3 (59·0 to 95·2)	88·2 (75·2 to 101·7)	97·4 (86·3 to 109·9)	116·2 (105·9 to 127·1)	122·4 (112·9 to 131·9)	104·9 (95·0 to 115·1)	53·7 (47·0 to 60·5)	33·7 (27·2 to 41·7)	32·5 (25·9 to 40·9)	31·5 (24·8 to 39·4)
Benishangul-Gumuz	5·8 (4·6 to 7·1)	6·3 (5·3 to 7·2)	6·1 (5·4 to 6·9)	6·5 (5·8 to 7·2)	5·7 (5·1 to 6·3)	5·0 (4·3 to 5·8)	3·6 (3·1 to 4·1)	2·6 (2·1 to 3·2)	2·6 (2·0 to 3·2)	2·5 (2·0 to 3·1)
Dire Dawa	1·5 (1·1 to 1·8)	2·3 (1·9 to 2·6)	3·1 (2·7 to 3·5)	3·2 (2·8 to 3·5)	2·4 (2·2 to 2·6)	1·5 (1·3 to 1·6)	0·8 (0·7 to 0·9)	0·5 (0·4 to 0·6)	0·5 (0·4 to 0·6)	0·4 (0·3 to 0·6)
Gambella	1·2 (1·0 to 1·6)	1·7 (1·4 to 1·9)	1·9 (1·7 to 2·2)	2·1 (1·9 to 2·4)	1·8 (1·7 to 2·0)	1·5 (1·3 to 1·7)	1·0 (0·9 to 1·1)	0·6 (0·5 to 0·7)	0·6 (0·4 to 0·7)	0·5 (0·4 to 0·7)
Harari	0·9 (0·7 to 1·1)	1·2 (1·0 to 1·3)	1·4 (1·3 to 1·6)	1·4 (1·3 to 1·6)	1·1 (1·0 to 1·2)	0·7 (0·6 to 0·8)	0·4 (0·4 to 0·5)	0·3 (0·2 to 0·3)	0·3 (0·2 to 0·3)	0·2 (0·2 to 0·3)
Oromia	77·2 (60·1 to 97·3)	84·5 (72·2 to 96·6)	91·9 (81·7 to 103·0)	117·5 (107·1 to 128·4)	151·2 (138·7 to 163·4)	155·8 (141·5 to 170·4)	103·8 (92·2 to 116·5)	77·2 (62·5 to 93·8)	75·2 (60·8 to 92·9)	74·1 (59·2 to 93·5)
Somali	10·5 (8·1 to 13·3)	10·4 (8·9 to 12·1)	10·7 (9·4 to 12·2)	14·0 (12·6 to 15·4)	20·2 (18·3 to 22·3)	26·6 (24·0 to 29·4)	23·6 (20·2 to 27·1)	19·6 (16·0 to 23·6)	19·0 (15·4 to 23·1)	18·6 (15·1 to 22·8)
Southern Nations, Nationalities, and Peoples	51·9 (41·1 to 64·5)	68·3 (58·4 to 78·4)	83·7 (74·0 to 94·3)	105·4 (95·8 to 115·2)	109·7 (100·2 to 120·3)	94·3 (85·6 to 103·2)	61·8 (54·3 to 69·6)	42·7 (34·1 to 53·0)	41·7 (32·8 to 53·1)	40·6 (32·0 to 51·5)
Tigray	18·4 (14·4 to 23·1)	20·6 (17·5 to 23·6)	23·0 (20·4 to 25·9)	27·1 (24·6 to 29·8)	24·2 (21·8 to 26·7)	18·8 (17·0 to 20·7)	10·8 (9·6 to 12·1)	6·5 (5·4 to 8·0)	6·3 (5·1 to 7·7)	6·0 (4·9 to 7·4)
Kenya	74·1 (63·2 to 86·1)	80·5 (74·5 to 86·5)	85·8 (80·1 to 91·9)	86·6 (80·6 to 92·9)	92·7 (85·5 to 99·9)	100·6 (90·4 to 111·3)	78·4 (66·5 to 91·6)	49·3 (40·1 to 59·7)	45·5 (37·0 to 55·2)	43·7 (35·3 to 53·5)
Baringo	1·0 (0·8 to 1·2)	1·3 (1·2 to 1·4)	1·6 (1·5 to 1·8)	1·7 (1·5 to 1·8)	1·9 (1·6 to 2·1)	1·8 (1·6 to 2·1)	1·4 (1·1 to 1·7)	1·0 (0·8 to 1·2)	0·9 (0·7 to 1·1)	0·8 (0·7 to 1·0)
Bomet	0·4 (0·3 to 0·5)	0·5 (0·4 to 0·5)	0·6 (0·5 to 0·7)	0·7 (0·6 to 0·8)	0·8 (0·7 to 0·9)	1·0 (0·8 to 1·1)	1·4 (1·1 to 1·6)	0·7 (0·6 to 0·9)	0·6 (0·5 to 0·8)	0·6 (0·5 to 0·7)
Bungoma	2·9 (2·3 to 3·6)	3·1 (2·8 to 3·3)	3·3 (3·0 to 3·6)	3·7 (3·4 to 3·9)	4·4 (3·9 to 4·8)	6·9 (6·1 to 7·8)	3·6 (3·1 to 4·2)	2·3 (1·9 to 2·7)	2·2 (1·8 to 2·6)	2·3 (1·8 to 2·8)
Busia	3·2 (2·6 to 4·0)	3·3 (3·0 to 3·6)	3·0 (2·7 to 3·2)	2·7 (2·5 to 2·9)	3·1 (2·8 to 3·4)	3·3 (2·8 to 3·8)	2·4 (2·0 to 2·9)	1·4 (1·1 to 1·7)	1·3 (1·1 to 1·7)	1·4 (1·1 to 1·8)
Elgeyo Marakwet	0·7 (0·6 to 0·9)	1·0 (0·9 to 1·1)	1·2 (1·1 to 1·3)	0·9 (0·8 to 1·0)	0·9 (0·8 to 1·0)	1·1 (0·9 to 1·3)	0·4 (0·3 to 0·5)	0·2 (0·2 to 0·3)	0·2 (0·2 to 0·3)	0·2 (0·2 to 0·3)
Embu	1·5 (1·2 to 1·9)	1·5 (1·3 to 1·6)	1·2 (1·1 to 1·3)	1·0 (1·0 to 1·1)	1·0 (0·8 to 1·1)	0·9 (0·8 to 1·0)	0·7 (0·6 to 0·8)	0·4 (0·3 to 0·5)	0·3 (0·3 to 0·4)	0·3 (0·2 to 0·4)
Garissa	0·4 (0·3 to 0·4)	0·4 (0·4 to 0·5)	0·6 (0·5 to 0·6)	0·8 (0·7 to 0·8)	0·6 (0·5 to 0·7)	1·1 (0·9 to 1·3)	1·3 (1·1 to 1·5)	1·1 (0·9 to 1·3)	1·0 (0·9 to 1·2)	1·0 (0·8 to 1·2)
Homa Bay	8·5 (7·2 to 10·0)	8·2 (7·6 to 8·9)	8·1 (7·3 to 9·0)	7·2 (6·5 to 7·8)	6·9 (6·4 to 7·5)	7·4 (6·7 to 8·2)	4·2 (3·7 to 4·9)	2·2 (1·8 to 2·7)	1·9 (1·5 to 2·3)	1·7 (1·4 to 2·1)
Isiolo	0·4 (0·3 to 0·5)	0·5 (0·5 to 0·6)	0·6 (0·6 to 0·7)	0·7 (0·6 to 0·8)	0·8 (0·7 to 0·9)	0·8 (0·7 to 0·8)	0·7 (0·6 to 0·8)	0·6 (0·5 to 0·8)	0·6 (0·5 to 0·7)	0·6 (0·5 to 0·7)
Kajiado	0·3 (0·2 to 0·3)	0·3 (0·3 to 0·4)	0·5 (0·4 to 0·5)	0·7 (0·6 to 0·8)	0·8 (0·7 to 0·9)	1·1 (0·9 to 1·2)	1·2 (1·0 to 1·4)	1·0 (0·8 to 1·2)	0·9 (0·7 to 1·1)	0·9 (0·7 to 1·1)
Kakamega	4·4 (3·6 to 5·3)	5·0 (4·6 to 5·4)	5·5 (5·1 to 6·0)	5·8 (5·4 to 6·3)	7·3 (6·5 to 8·0)	5·4 (4·8 to 6·0)	4·9 (4·2 to 5·7)	2·5 (2·1 to 3·1)	2·3 (1·8 to 2·8)	2·3 (1·8 to 2·8)
Kericho	0·4 (0·3 to 0·5)	0·7 (0·6 to 0·8)	1·1 (1·0 to 1·2)	1·6 (1·4 to 1·7)	1·5 (1·3 to 1·6)	1·7 (1·5 to 1·9)	1·0 (0·8 to 1·1)	0·9 (0·7 to 1·1)	0·9 (0·7 to 1·1)	0·9 (0·7 to 1·1)
Kiambu	1·4 (1·0 to 1·8)	1·3 (1·1 to 1·4)	1·3 (1·2 to 1·5)	1·5 (1·3 to 1·7)	1·6 (1·4 to 1·9)	2·5 (2·2 to 2·9)	2·2 (1·8 to 2·6)	1·6 (1·2 to 2·0)	1·5 (1·2 to 1·8)	1·4 (1·1 to 1·8)
Kilifi	3·5 (2·9 to 4·2)	3·8 (3·5 to 4·2)	4·1 (3·8 to 4·4)	4·4 (3·9 to 4·9)	4·0 (3·6 to 4·4)	4·0 (3·5 to 4·5)	3·1 (2·6 to 3·6)	2·0 (1·6 to 2·4)	1·8 (1·4 to 2·2)	1·7 (1·3 to 2·0)
Kirinyaga	1·6 (1·3 to 1·9)	1·8 (1·6 to 2·0)	1·8 (1·6 to 2·0)	1·0 (0·9 to 1·1)	0·7 (0·6 to 0·9)	0·8 (0·7 to 1·0)	0·7 (0·6 to 0·8)	0·4 (0·3 to 0·5)	0·4 (0·3 to 0·5)	0·4 (0·3 to 0·5)
Kisii	2·3 (1·7 to 3·0)	3·3 (3·0 to 3·7)	4·0 (3·7 to 4·4)	4·5 (3·8 to 5·2)	4·8 (4·2 to 5·4)	4·5 (3·9 to 5·1)	2·1 (1·7 to 2·6)	1·0 (0·8 to 1·3)	1·0 (0·8 to 1·2)	0·9 (0·7 to 1·1)
Kisumu	3·0 (2·2 to 3·9)	3·4 (3·1 to 3·8)	3·9 (3·6 to 4·2)	3·9 (3·3 to 4·5)	4·6 (4·1 to 5·1)	4·4 (3·8 to 5·0)	2·4 (1·9 to 3·0)	1·3 (1·0 to 1·7)	1·2 (1·0 to 1·5)	1·2 (0·9 to 1·5)
Kitui	3·1 (2·5 to 3·9)	3·4 (3·1 to 3·7)	3·2 (3·0 to 3·5)	2·5 (2·3 to 2·7)	2·5 (2·1 to 2·8)	2·5 (2·2 to 2·9)	1·6 (1·4 to 1·9)	0·9 (0·7 to 1·1)	0·8 (0·6 to 1·0)	0·7 (0·6 to 0·9)
Kwale	2·2 (1·9 to 2·7)	2·8 (2·6 to 3·0)	2·8 (2·6 to 3·0)	2·5 (2·2 to 2·9)	2·2 (1·9 to 2·5)	2·0 (1·7 to 2·3)	1·8 (1·5 to 2·1)	1·2 (0·9 to 1·5)	1·1 (0·9 to 1·4)	1·0 (0·8 to 1·3)
Laikipia	0·2 (0·1 to 0·2)	0·2 (0·1 to 0·2)	0·2 (0·2 to 0·3)	0·3 (0·3 to 0·4)	0·4 (0·3 to 0·5)	0·6 (0·5 to 0·7)	0·4 (0·3 to 0·5)	0·2 (0·1 to 0·3)	0·2 (0·1 to 0·2)	0·2 (0·1 to 0·2)
Lamu	0·3 (0·2 to 0·4)	0·3 (0·3 to 0·3)	0·3 (0·3 to 0·3)	0·3 (0·2 to 0·3)	0·3 (0·3 to 0·4)	0·3 (0·3 to 0·4)	0·3 (0·2 to 0·3)	0·2 (0·1 to 0·2)	0·2 (0·1 to 0·2)	0·1 (0·1 to 0·2)
Machakos	1·7 (1·4 to 2·2)	2·1 (1·9 to 2·3)	2·5 (2·3 to 2·7)	2·6 (2·3 to 2·9)	2·3 (2·0 to 2·7)	2·2 (1·9 to 2·6)	1·2 (1·0 to 1·5)	0·7 (0·6 to 0·9)	0·7 (0·5 to 0·9)	0·6 (0·5 to 0·8)
Makueni	2·2 (1·7 to 2·8)	1·9 (1·7 to 2·2)	1·8 (1·6 to 2·0)	1·6 (1·4 to 1·8)	1·5 (1·3 to 1·8)	1·3 (1·1 to 1·5)	1·0 (0·8 to 1·2)	0·4 (0·3 to 0·5)	0·4 (0·3 to 0·5)	0·3 (0·3 to 0·4)
Mandera	0·4 (0·4 to 0·6)	0·5 (0·5 to 0·6)	0·6 (0·5 to 0·7)	0·6 (0·6 to 0·7)	0·8 (0·7 to 0·9)	1·3 (1·0 to 1·6)	3·5 (3·1 to 4·0)	2·6 (2·1 to 3·1)	2·3 (2·0 to 2·8)	2·2 (1·9 to 2·6)
Marsabit	0·3 (0·2 to 0·3)	0·3 (0·3 to 0·3)	0·4 (0·4 to 0·5)	0·6 (0·6 to 0·7)	0·6 (0·6 to 0·7)	0·6 (0·6 to 0·7)	0·8 (0·7 to 1·0)	0·8 (0·7 to 1·0)	0·8 (0·6 to 0·9)	0·7 (0·6 to 0·9)

Meru	2·3 (1·8 to 3·0)	2·7 (2·4 to 3·0)	2·8 (2·5 to 3·0)	2·4 (2·2 to 2·6)	2·5 (2·1 to 2·9)	3·0 (2·6 to 3·4)	2·9 (2·5 to 3·4)	1·7 (1·4 to 2·1)	1·5 (1·2 to 1·9)	1·4 (1·1 to 1·7)
Migori	4·2 (3·4 to 5·1)	4·5 (4·1 to 5·0)	4·8 (4·4 to 5·2)	5·4 (4·9 to 5·9)	6·2 (5·7 to 6·7)	7·1 (6·4 to 7·8)	4·6 (3·9 to 5·4)	2·9 (2·3 to 3·5)	2·6 (2·1 to 3·2)	2·4 (2·0 to 3·0)
Mombasa	1·4 (1·0 to 1·7)	1·6 (1·5 to 1·8)	1·7 (1·5 to 1·8)	1·3 (1·1 to 1·4)	1·3 (1·1 to 1·5)	1·5 (1·3 to 1·7)	1·3 (1·1 to 1·6)	0·7 (0·5 to 0·8)	0·6 (0·4 to 0·7)	0·5 (0·4 to 0·7)
Murang'a	2·8 (2·3 to 3·5)	2·6 (2·3 to 2·8)	2·3 (2·0 to 2·6)	1·6 (1·5 to 1·9)	1·5 (1·2 to 1·7)	1·2 (1·0 to 1·3)	1·0 (0·8 to 1·2)	0·6 (0·4 to 0·7)	0·5 (0·4 to 0·7)	0·5 (0·4 to 0·6)
Nairobi	2·8 (2·0 to 3·7)	2·5 (2·0 to 3·0)	2·5 (2·2 to 2·9)	3·3 (2·9 to 3·7)	3·0 (2·6 to 3·5)	4·2 (3·5 to 4·8)	3·0 (2·2 to 3·9)	1·6 (1·1 to 2·1)	1·4 (1·0 to 1·9)	1·2 (0·9 to 1·7)
Nakuru	0·4 (0·3 to 0·5)	0·5 (0·4 to 0·6)	0·8 (0·7 to 0·9)	1·3 (1·2 to 1·5)	1·7 (1·4 to 1·9)	2·2 (1·9 to 2·5)	1·9 (1·5 to 2·3)	1·3 (1·0 to 1·6)	1·2 (0·9 to 1·5)	1·1 (0·9 to 1·4)
Nandi	0·6 (0·4 to 0·8)	0·7 (0·6 to 0·8)	0·9 (0·8 to 1·0)	1·0 (0·9 to 1·1)	1·1 (1·0 to 1·3)	1·1 (1·0 to 1·3)	0·9 (0·7 to 1·1)	0·6 (0·4 to 0·7)	0·5 (0·4 to 0·6)	0·5 (0·4 to 0·6)
Narok	0·5 (0·4 to 0·6)	0·6 (0·5 to 0·7)	0·8 (0·7 to 0·9)	0·9 (0·7 to 1·0)	1·5 (1·2 to 1·7)	1·0 (0·8 to 1·1)	1·4 (1·2 to 1·6)	1·1 (0·8 to 1·4)	1·0 (0·8 to 1·3)	1·0 (0·8 to 1·2)
Nyamira	0·7 (0·6 to 0·9)	0·7 (0·6 to 0·8)	0·7 (0·6 to 0·7)	0·7 (0·6 to 0·7)	0·6 (0·5 to 0·6)	0·7 (0·6 to 0·8)	0·6 (0·5 to 0·7)	0·3 (0·3 to 0·4)	0·3 (0·2 to 0·4)	0·3 (0·2 to 0·4)
Nyandarua	0·7 (0·6 to 0·8)	0·7 (0·7 to 0·8)	0·7 (0·6 to 0·8)	0·5 (0·4 to 0·6)	0·6 (0·4 to 0·7)	0·7 (0·6 to 0·9)	0·7 (0·6 to 0·9)	0·4 (0·3 to 0·5)	0·3 (0·3 to 0·4)	0·3 (0·3 to 0·4)
Nyeri	1·8 (1·4 to 2·3)	1·4 (1·2 to 1·6)	1·2 (1·0 to 1·3)	0·9 (0·8 to 1·0)	0·8 (0·7 to 1·0)	0·8 (0·7 to 1·0)	0·6 (0·4 to 0·7)	0·3 (0·3 to 0·4)	0·3 (0·2 to 0·4)	0·3 (0·2 to 0·3)
Samburu	0·1 (0·1 to 0·2)	0·2 (0·2 to 0·2)	0·3 (0·3 to 0·3)	0·3 (0·3 to 0·4)	0·5 (0·4 to 0·6)	0·6 (0·5 to 0·7)	0·7 (0·5 to 0·8)	0·6 (0·5 to 0·8)	0·6 (0·5 to 0·7)	0·6 (0·5 to 0·7)
Siaya	3·6 (3·0 to 4·4)	4·5 (4·2 to 4·9)	4·7 (4·4 to 5·1)	5·0 (4·3 to 5·8)	6·1 (5·5 to 6·7)	6·1 (5·4 to 6·8)	3·4 (2·9 to 3·9)	1·8 (1·5 to 2·2)	1·7 (1·4 to 2·1)	1·8 (1·4 to 2·2)
Taita Taveta	0·7 (0·6 to 0·9)	0·8 (0·7 to 0·9)	0·9 (0·9 to 1·0)	0·7 (0·6 to 0·8)	0·6 (0·6 to 0·7)	0·6 (0·5 to 0·7)	0·4 (0·3 to 0·5)	0·2 (0·2 to 0·3)	0·2 (0·1 to 0·2)	0·2 (0·1 to 0·2)
Tana River	0·3 (0·2 to 0·4)	0·3 (0·3 to 0·4)	0·4 (0·3 to 0·4)	0·6 (0·5 to 0·7)	0·6 (0·5 to 0·7)	0·8 (0·7 to 1·0)	0·8 (0·7 to 0·9)	0·6 (0·5 to 0·7)	0·5 (0·4 to 0·7)	0·5 (0·4 to 0·6)
Tharaka Nithi	0·6 (0·4 to 0·7)	0·4 (0·4 to 0·5)	0·4 (0·3 to 0·4)	0·3 (0·2 to 0·3)	0·3 (0·3 to 0·3)	0·3 (0·3 to 0·3)	0·2 (0·2 to 0·2)	0·2 (0·2 to 0·3)	0·2 (0·2 to 0·3)	0·2 (0·2 to 0·3)
Trans Nzoia	0·2 (0·2 to 0·3)	0·3 (0·3 to 0·3)	0·6 (0·5 to 0·6)	1·2 (1·1 to 1·3)	1·2 (1·1 to 1·4)	1·4 (1·2 to 1·6)	1·1 (0·9 to 1·4)	0·6 (0·5 to 0·8)	0·6 (0·5 to 0·7)	0·6 (0·4 to 0·7)
Turkana	0·5 (0·4 to 0·6)	0·8 (0·7 to 0·9)	1·2 (1·1 to 1·4)	1·2 (1·1 to 1·4)	1·3 (1·1 to 1·5)	1·8 (1·6 to 2·1)	2·3 (2·0 to 2·7)	1·5 (1·2 to 1·9)	1·5 (1·2 to 1·8)	1·4 (1·1 to 1·7)
Uasin Gishu	0·2 (0·2 to 0·3)	0·4 (0·3 to 0·4)	0·6 (0·5 to 0·6)	0·9 (0·8 to 1·0)	1·1 (1·0 to 1·2)	1·3 (1·1 to 1·5)	1·3 (1·1 to 1·6)	1·0 (0·8 to 1·2)	0·9 (0·7 to 1·1)	0·9 (0·7 to 1·1)
Vihiga	2·3 (1·9 to 2·9)	1·9 (1·7 to 2·1)	1·5 (1·3 to 1·8)	1·2 (1·1 to 1·4)	1·5 (1·3 to 1·7)	1·3 (1·1 to 1·5)	1·1 (0·9 to 1·3)	0·5 (0·4 to 0·6)	0·4 (0·3 to 0·5)	0·4 (0·3 to 0·5)
Wajir	0·7 (0·6 to 0·9)	0·7 (0·6 to 0·9)	0·9 (0·8 to 1·1)	1·1 (1·0 to 1·3)	0·9 (0·8 to 1·0)	2·1 (1·7 to 2·5)	2·6 (2·3 to 3·0)	2·3 (1·9 to 2·8)	2·3 (1·9 to 2·7)	2·2 (1·9 to 2·6)
West Pokot	0·3 (0·3 to 0·4)	0·4 (0·4 to 0·5)	0·7 (0·6 to 0·7)	1·2 (1·1 to 1·3)	1·4 (1·3 to 1·6)	1·4 (1·2 to 1·5)	1·4 (1·1 to 1·7)	1·0 (0·8 to 1·2)	0·9 (0·7 to 1·1)	0·8 (0·7 to 1·0)
Madagascar	61·1 (48·3 to 74·6)	63·6 (54·3 to 73·6)	61·6 (54·9 to 68·6)	66·9 (61·3 to 72·8)	72·8 (66·7 to 79·3)	73·5 (66·5 to 81·2)	72·5 (63·6 to 82·4)	56·2 (45·8 to 68·9)	52·2 (41·6 to 64·9)	48·9 (39·0 to 62·0)
Malawi	51·8 (41·4 to 63·6)	63·0 (55·3 to 70·9)	71·2 (65·1 to 77·5)	84·0 (78·0 to 90·5)	101·8 (95·7 to 108·0)	84·1 (77·6 to 90·4)	56·2 (50·6 to 62·1)	33·5 (27·9 to 40·1)	31·7 (26·5 to 38·3)	29·6 (24·3 to 35·8)
Mozambique	97·9 (79·6 to 121·3)	102·5 (85·7 to 119·2)	110·2 (96·2 to 126·4)	131·1 (120·0 to 142·7)	130·7 (120·6 to 140·5)	128·6 (118·6 to 140·2)	102·3 (90·5 to 115·7)	73·9 (59·4 to 91·7)	72·3 (57·4 to 91·6)	68·5 (54·0 to 88·1)
Rwanda	48·7 (37·9 to 62·0)	43·9 (39·2 to 48·7)	50·6 (45·7 to 55·6)	53·7 (49·0 to 58·5)	51·4 (46·5 to 56·6)	47·6 (43·0 to 52·8)	25·8 (22·0 to 30·0)	16·5 (13·3 to 20·0)	15·6 (12·7 to 19·0)	15·1 (12·3 to 18·3)
Somalia	26·1 (20·6 to 32·5)	33·3 (28·5 to 38·5)	43·0 (36·7 to 49·7)	60·7 (53·0 to 68·6)	71·4 (64·5 to 78·9)	76·2 (67·3 to 86·0)	101·7 (88·1 to 116·8)	86·4 (70·5 to 105·4)	86·0 (70·3 to 105·5)	86·0 (70·2 to 105·5)
South Sudan	27·8 (21·2 to 35·4)	33·3 (27·6 to 39·9)	39·6 (33·2 to 46·6)	44·6 (37·5 to 52·1)	50·1 (43·5 to 57·0)	47·5 (42·1 to 53·2)	50·1 (41·9 to 58·9)	47·0 (37·7 to 58·7)	47·0 (37·4 to 58·8)	47·5 (37·6 to 59·9)
Uganda	82·8 (65·7 to 101·3)	95·5 (82·3 to 109·8)	105·6 (96·3 to 115·5)	130·3 (120·5 to 140·1)	150·3 (141·0 to 159·5)	163·5 (153·5 to 174·1)	128·7 (116·8 to 141·9)	92·7 (76·8 to 112·5)	95·2 (76·1 to 120·2)	101·1 (78·6 to 131·0)
Tanzania	120·8 (95·7 to 149·7)	143·6 (123·0 to 165·0)	152·4 (138·0 to 167·3)	160·5 (149·8 to 172·5)	180·4 (169·1 to 192·4)	184·4 (170·0 to 198·8)	143·8 (127·3 to 162·1)	108·8 (89·0 to 132·5)	103·5 (83·6 to 128·4)	98·2 (79·1 to 123·4)
Zambia	33·9 (27·3 to 42·5)	44·6 (38·2 to 51·6)	46·3 (42·1 to 50·5)	46·7 (43·2 to 50·1)	60·2 (56·1 to 64·6)	63·8 (58·0 to 70·0)	49·3 (43·1 to 56·2)	31·5 (24·9 to 39·2)	29·7 (23·6 to 37·8)	27·9 (21·9 to 35·4)
Southern sub-Saharan Africa	140·4 (110·3 to 175·3)	146·7 (123·2 to 171·6)	149·6 (132·6 to 167·8)	153·3 (140·6 to 166·7)	122·6 (113·7 to 132·5)	126·9 (117·3 to 136·7)	107·0 (97·3 to 117·0)	76·2 (64·1 to 90·2)	73·1 (60·5 to 88·4)	71·4 (59·0 to 87·7)
Botswana	2·6 (2·0 to 3·4)	3·1 (2·5 to 3·6)	3·3 (2·9 to 3·7)	3·1 (2·7 to 3·4)	2·9 (2·6 to 3·3)	3·5 (3·0 to 4·1)	2·5 (2·0 to 3·1)	2·0 (1·6 to 2·6)	2·0 (1·5 to 2·6)	2·0 (1·5 to 2·6)
Eswatini	3·7 (3·0 to 4·7)	4·3 (3·7 to 5·0)	4·0 (3·4 to 4·7)	3·3 (2·9 to 3·8)	2·5 (2·3 to 2·8)	3·3 (3·0 to 3·6)	2·7 (2·4 to 3·0)	1·3 (1·1 to 1·7)	1·3 (1·0 to 1·6)	1·2 (1·0 to 1·6)
Lesotho	6·2 (5·1 to 7·5)	6·5 (5·9 to 7·1)	7·5 (6·9 to 8·2)	6·3 (5·6 to 7·1)	4·8 (4·3 to 5·4)	5·0 (4·5 to 5·6)	4·3 (3·7 to 4·9)	3·4 (2·8 to 4·1)	3·4 (2·8 to 4·1)	3·4 (2·7 to 4·1)
Namibia	3·5 (2·7 to 4·5)	3·7 (3·1 to 4·5)	3·5 (3·0 to 4·0)	3·7 (3·3 to 4·0)	3·5 (3·2 to 3·8)	3·7 (3·3 to 4·1)	3·2 (2·8 to 3·8)	2·1 (1·6 to 2·6)	2·0 (1·5 to 2·5)	1·9 (1·5 to 2·5)
South Africa	104·2 (79·8 to 132·8)	106·7 (88·5 to 125·6)	106·8 (93·0 to 122·6)	107·2 (96·4 to 118·9)	81·9 (75·1 to 89·5)	79·2 (71·6 to 86·9)	59·3 (52·0 to 67·1)	41·6 (34·9 to 49·6)	39·6 (32·9 to 48·0)	38·4 (31·6 to 47·1)
Eastern Cape	37·1 (28·2 to 48·1)	31·9 (26·4 to 37·5)	25·2 (21·9 to 28·9)	24·1 (21·4 to 26·9)	18·7 (16·8 to 20·9)	14·5 (12·8 to 16·1)	9·7 (8·5 to 11·0)	6·3 (5·2 to 7·5)	6·0 (5·0 to 7·4)	5·8 (4·8 to 7·2)
Free State	9·6 (7·2 to 12·7)	10·5 (8·5 to 12·5)	9·9 (8·5 to 11·4)	9·7 (8·5 to 10·9)	6·1 (5·5 to 6·9)	5·7 (5·1 to 6·4)	4·0 (3·6 to 4·6)	2·5 (2·1 to 3·1)	2·3 (1·9 to 2·8)	2·2 (1·8 to 2·7)
Gauteng	7·8 (5·6 to 10·7)	9·2 (7·4 to 11·2)	10·4 (8·8 to 12·2)	11·6 (10·1 to 13·3)	9·5 (8·2 to 10·7)	13·5 (11·9 to 15·2)	11·6 (10·1 to 13·2)	8·8 (7·2 to 10·5)	8·2 (6·7 to 10·1)	7·9 (6·5 to 9·8)
KwaZulu-Natal	21·8 (16·7 to 28·5)	24·0 (19·9 to 28·4)	26·4 (23·1 to 30·2)	25·6 (22·9 to 28·2)	19·4 (17·3 to 21·8)	19·8 (17·7 to 21·8)	12·9 (11·2 to 14·6)	8·4 (6·9 to 10·1)	8·3 (6·8 to 10·1)	8·0 (6·6 to 9·8)
Limpopo	7·2 (5·4 to 9·4)	8·7 (7·2 to 10·4)	12·1 (10·4 to 14·0)	13·0 (11·5 to 14·6)	9·5 (8·5 to 10·5)	7·3 (6·5 to 8·2)	6·6 (5·7 to 7·5)	5·5 (4·6 to 6·5)	5·3 (4·4 to 6·3)	5·2 (4·4 to 6·4)
Mpumalanga	8·2 (6·2 to 10·5)	8·5 (7·0 to 10·0)	7·6 (6·6 to 8·8)	7·6 (6·8 to 8·5)	5·9 (5·2 to 6·6)	5·8 (5·1 to 6·5)	4·8 (4·2 to 5·4)	3·9 (3·2 to 4·7)	3·8 (3·1 to 4·6)	3·7 (3·1 to 4·6)
North West	7·0 (5·2 to 9·1)	8·2 (6·8 to 9·6)	9·1 (7·9 to 10·5)	9·2 (8·2 to 10·5)	7·4 (6·6 to 8·3)	7·7 (6·7 to 8·7)	5·3 (4·6 to 6·0)	3·0 (2·5 to 3·6)	2·8 (2·3 to 3·3)	2·6 (2·2 to 3·2)
Northern Cape	2·7 (2·0 to 3·6)	2·7 (2·2 to 3·3)	2·6 (2·3 to 3·1)	2·4 (2·1 to 2·7)	1·7 (1·5 to 1·9)	1·3 (1·1 to 1·5)	1·4 (1·2 to 1·6)	1·2 (1·0 to 1·5)	1·1 (0·9 to 1·4)	1·1 (0·9 to 1·4)
Western Cape	2·7 (1·9 to 3·8)	3·0 (2·4 to 3·7)	3·5 (2·9 to 4·1)	4·1 (3·5 to 4·8)	3·7 (3·2 to 4·2)	3·8 (3·3 to 4·3)	3·0 (2·5 to 3·4)	2·0 (1·6 to 2·4)	1·8 (1·5 to 2·2)	1·7 (1·4 to 2·1)
Zimbabwe	20·1 (15·4 to 25·4)	22·5 (18·8 to 26·4)	24·5 (21·7 to 27·6)	29·7 (27·1 to 32·5)	26·9 (24·9 to 29·0)	32·2 (29·7 to 34·6)	35·0 (31·9 to 38·6)	25·7 (21·7 to 30·7)	24·8 (20·6 to 29·9)	24·6 (20·2 to 30·2)
Western sub-Saharan Africa	1126·5 (945·4 to 1319·6)	1257·3 (1119·6 to 1400·2)	1399·0 (1298·2 to 1501·6)	1490·1 (1413·8 to 1567·6)	1654·2 (1583·9 to 1726·0)	1889·8 (1797·8 to 1986·6)	1879·1 (1751·8 to 2007·6)	1613·4 (1393·1 to 1854·1)	1568·9 (1342·1 to 1830·2)	1510·6 (1279·0 to 1779·9)
Benin	27·6 (21·8 to 34·4)	30·6 (27·2 to 34·2)	32·1 (29·6 to 34·9)	33·6 (31·0 to 36·2)	40·2 (37·3 to 43·3)	42·4 (39·1 to 45·8)	45·6 (41·3 to 50·1)	41·4 (34·2 to 49·4)	40·3 (32·7 to 49·1)	39·6 (32·0 to 49·1)
Burkina Faso	70·4 (56·9 to 85·8)	80·1 (69·1 to 91·2)	87·6 (78·7 to 96·9)	92·4 (86·0 to 99·5)	93·7 (87·4 to 100·3)	102·9 (94·1 to 111·9)	103·0 (89·0 to 117·8)	91·2 (74·9 to 110·8)	92·8 (75·6 to 113·9)	87·8 (71·1 to 108·8)
Cabo Verde	1·2 (1·1 to 1·3)	1·5 (1·4 to 1·6)	1·6 (1·5 to 1·6)	0·9 (0·8 to 1·0)	0·8 (0·8 to 0·9)	0·6 (0·5 to 0·7)	0·3 (0·3 to 0·4)	0·2 (0·1 to 0·2)	0·1 (0·1 to 0·2)	0·1 (0·1 to 0·2)
Cameroon	62·0 (52·6 to 72·3)	66·1 (60·0 to 72·1)	64·5 (59·2 to 69·7)	63·7 (59·3 to 68·3)	63·0 (57·7 to 68·1)	78·4 (72·1 to 84·9)	88·6 (79·0 to 99·7)	72·0 (59·8 to 84·8)	71·5 (59·0 to 85·6)	67·6 (55·6 to 80·4)
Chad	35·5 (28·2 to 43·9)	38·5 (32·6 to 44·5)	45·7 (40·2 to 51·6)	54·0 (49·7 to 58·7)	61·0 (56·3 to 65·7)	76·0 (69·8 to 83·4)	84·6 (75·6 to 94·4)	91·4 (76·5 to 108·5)	92·3 (77·8 to 110·1)	92·9 (77·9 to 112·4)
Côte d'Ivoire	40·6 (34·4 to 47·3)	53·9 (49·9 to 58·3)	61·8 (57·7 to 66·2)	63·1 (58·4 to 67·7)	79·6 (73·5 to 85·2)	92·3 (84·8 to 100·5)	89·3 (78·4 to 100·1)	70·0 (59·4 to 82·8)	67·7 (57·4 to 80·4)	64·4 (54·3 to 76·1)
The Gambia	2·3 (1·8 to 3·0)	2·7 (2·2 to 3·2)	3·7 (3·2 to 4·2)	5·1 (4·6 to 5·7)	5·6 (5·0 to 6·2)	5·7 (5·1 to 6·5)	4·8 (4·2 to 5·5)	3·7 (3·0 to 4·5)	3·5 (2·8 to 4·3)	3·4 (2·7 to 4·2)
Ghana	57·2 (45·3 to 72·3)	69·4 (62·6 to 76·2)	77·5 (71·1 to 84·0)	78·7 (73·0 to 84·8)	72·7 (67·2 to 78·6)	65·5 (60·4 to 71·2)	63·2 (56·0 to 70·9)	48·5 (38·6 to 60·2)	45·4 (35·6 to 57·4)	42·0 (32·3 to 53·9)
Guinea	43·0 (35·2 to 52·3)	50·3 (42·9 to 57·7)	59·3 (52·2 to 67·4)	62·4 (56·1 to 68·8)	64·0 (59·0 to 69·3)	62·2 (56·6 to 67·7)	54·8 (48·6 to 61·4)	45·8 (38·4 to 54·3)	44·8 (37·4 to 53·7)	42·6 (35·3 to 51·5)
Guinea-Bissau	12·2 (10·2 to 14·4)	9·8 (8·3 to 11·3)	7·9 (6·9 to 9·1)	8·9 (7·8 to 10·0)	9·1 (8·1 to 10·3)	8·4 (7·6 to 9·3)	6·5 (5·6 to 7·5)	4·7 (3·9 to 5·7)	4·6 (3·8 to 5·6)	4·4 (3·6 to 5·4)
Liberia	15·8 (12·0 to 20·2)	18·9 (16·3 to 21·6)	20·9 (18·7 to 23·2)	24·0 (21·5 to 26·9)	28·4 (25·6 to 31·1)	21·6 (19·6 to 23·8)	15·3 (13·2 to 17·6)	12·0 (9·6 to 15·1)	11·9 (9·2 to 15·3)	10·9 (8·4 to 14·4)
Mali	67·6 (55·5 to 82·4)	84·5 (74·1 to 95·5)	102·9 (94·5 to 111·4)	104·0 (96·4 to 112·0)	102·0 (95·3 to 108·7)	103·8 (95·2 to 112·3)	109·1 (98·3 to 120·4)	104·1 (87·1 to 123·9)	104·9 (87·1 to 127·2)	101·2 (83·9 to 123·5)
Mauritania	6·6 (5·4 to 7·9)	7·5 (6·8 to 8·2)	7·5 (6·9 to 8·2)	8·6 (7·9 to 9·4)	8·4 (7·7 to 9·2)	8·4 (7·6 to 9·2)	7·4 (6·6 to 8·4)	5·2 (4·4 to 6·2)	4·9 (4·1 to 5·8)	4·6 (3·8 to 5·5)
Niger	52·5 (43·0 to 62·8)	66·4 (57·8 to 75·3)	83·3 (75·3 to 91·5)	102·7 (95·8 to 110·4)	120·4 (111·9 to 130·1)	132·5 (119·8 to 146·3)	99·9 (84·7 to 116·5)	100·2 (82·9 to 120·8)	100·1 (82·0 to 123·5)	100·0 (80·9 to 124·5)
Nigeria	539·8 (430·1 to 663·8)	571·7 (498·5 to 649·1)	624·4 (558·7 to 692·5)	668·2 (624·6 to 715·6)	789·6 (746·2 to 833·6)	975·9 (921·8 to 1035·8)	1010·7 (928·2 to 1094·6)	854·4 (733·0 to 991·4)	818·2 (693·9 to 966·2)	787·0 (662·3 to 938·3)
São Tomé and Príncipe	0·9 (0·9 to 1·0)	0·8 (0·7 to 0·8)	0·5 (0·4 to 0·5)	0·4 (0·4 to 0·4)	0·5 (0·4 to 0·5)	0·4 (0·4 to 0·5)	0·2 (0·2 to 0·3)	0·1 (0·1 to 0·1)	0·1 (0·1 to 0·1)	0·1 (0·1 to 0·1)

Senegal	38·1 (31·7 to 45·5)	46·1 (42·3 to 50·3)	59·3 (54·8 to 63·9)	57·8 (53·8 to 62·5)	46·4 (43·2 to 49·7)	46·7 (43·5 to 50·3)	34·0 (30·9 to 37·3)	22·4 (18·8 to 26·5)	20·7 (17·3 to 24·5)	19·3 (16·1 to 23·0)
Sierra Leone	31·7 (25·0 to 39·0)	35·7 (30·4 to 41·5)	37·0 (31·6 to 43·0)	40·2 (35·7 to 45·1)	46·6 (43·1 to 50·3)	42·4 (39·3 to 45·8)	39·3 (35·2 to 43·8)	30·1 (24·4 to 36·7)	30·4 (24·0 to 38·2)	28·9 (22·8 to 36·4)
Togo	21·3 (17·0 to 26·5)	22·9 (19·7 to 26·3)	21·6 (19·4 to 23·9)	21·1 (19·3 to 23·2)	22·3 (20·2 to 24·3)	23·4 (20·9 to 26·0)	22·4 (19·7 to 25·1)	15·8 (12·9 to 19·2)	14·8 (12·1 to 18·4)	13·8 (11·1 to 17·4)

Appendix 2 Table S2a: Under-5 mortality rates with 95% UIs by location, both sexes combined, 1950, 1960, 1970, 1980, 1990, 2000, 2010, 2019, 2020, 2021

Location	1950	1960	1970	1980	1990	2000	2010	2019	2020	2021
Global	234 (204 to 265)	289 (235 to 381)	138 (130 to 146)	110 (104 to 117)	84·5 (80·4 to 88·9)	70·9 (67·3 to 74·7)	52·6 (48·8 to 56·6)	38·3 (33·2 to 44·1)	36·8 (31·6 to 42·8)	35·7 (30·5 to 42)
Central Europe, eastern Europe, and central Asia	124 (111 to 138)	72 (67·5 to 76·7)	48 (44·6 to 51·7)	37·5 (35·9 to 39·2)	31 (29·7 to 32·5)	26·6 (25 to 28·2)	16·5 (15·3 to 17·8)	12·8 (11·3 to 14·5)	12·2 (10·7 to 13·9)	12 (10·5 to 13·7)
Central Asia	252 (216 to 292)	148 (129 to 168)	92·3 (80·5 to 105)	69·4 (64·6 to 74·8)	56·1 (52·4 to 60)	48·8 (44·7 to 53·3)	32·3 (28·9 to 36)	23·2 (19·8 to 27·1)	21·8 (18·5 to 25·5)	20·9 (17·6 to 24·6)
Armenia	276 (233 to 329)	160 (135 to 187)	91·7 (76·3 to 107)	60·8 (53·5 to 69·1)	41·8 (37·2 to 47·5)	30·4 (26·9 to 34·3)	17·5 (15 to 20·2)	12·7 (10·4 to 15·5)	13·6 (11·2 to 16·4)	11·1 (8·97 to 13·8)
Azerbaijan	405 (349 to 459)	261 (229 to 292)	145 (127 to 168)	89·4 (78·8 to 101)	77·6 (69·1 to 86·4)	66·5 (59 to 75·1)	42 (35·7 to 48·9)	31·8 (26·1 to 38·1)	30·4 (25·2 to 36·4)	28·6 (23·4 to 34·7)
Georgia	218 (178 to 260)	138 (118 to 160)	86·4 (70·1 to 105)	55·8 (48·6 to 64)	41·3 (36·2 to 47)	35·2 (30·9 to 40·3)	16·8 (14·1 to 19·8)	10·3 (8·42 to 12·7)	9·98 (8·08 to 12·3)	9·75 (7·73 to 12·2)
Kazakhstan	129 (104 to 159)	89·1 (75·7 to 104)	53·1 (44·4 to 62·9)	46·1 (40·9 to 51·1)	36·4 (32·8 to 40·3)	36·3 (32·1 to 41·1)	23·7 (20·2 to 27·4)	11·8 (9·82 to 14·1)	11 (9·21 to 13·2)	10·2 (8·38 to 12·3)
Kyrgyzstan	227 (176 to 280)	152 (130 to 176)	102 (84·2 to 121)	77·7 (68·5 to 87·6)	56·9 (51·6 to 62·6)	43·1 (38·6 to 47·8)	26·6 (25·5 to 27·6)	18·4 (17·3 to 19·5)	17·4 (16 to 18·9)	17 (14·9 to 19)
Mongolia	378 (308 to 454)	221 (191 to 250)	146 (128 to 165)	110 (99 to 122)	84·3 (77·3 to 91·6)	54·7 (49 to 60·4)	30·2 (26·6 to 34·4)	18·9 (15·8 to 22·5)	17·5 (14·7 to 20·9)	16·9 (14 to 20·5)
Tajikistan	381 (316 to 450)	233 (201 to 268)	135 (115 to 156)	100 (89·3 to 111)	79·7 (72·9 to 86·4)	66·2 (59·1 to 73·4)	43·9 (37·9 to 50·5)	38·9 (32·3 to 46·5)	35·8 (29·6 to 43·2)	34·5 (28·5 to 42·2)
Turkmenistan	273 (227 to 326)	155 (135 to 177)	99·3 (81·3 to 121)	85·9 (74·8 to 98·2)	70 (62·4 to 78·5)	59·2 (52·2 to 66·4)	41·3 (35·9 to 47·6)	30·2 (24·7 to 36·3)	28·5 (23·3 to 34·4)	27·5 (22·2 to 33·5)
Uzbekistan	264 (208 to 327)	116 (95·7 to 136)	83·7 (71·6 to 97·8)	64·8 (58·5 to 71·3)	52·1 (47·6 to 57·1)	44·7 (39·4 to 50·4)	32·7 (27·6 to 38·1)	23·6 (19·6 to 28·3)	22·2 (18·4 to 26·8)	21·5 (17·7 to 26)
Central Europe	169 (163 to 176)	74·2 (72·6 to 75·9)	47·6 (46·5 to 48·6)	29·3 (28·7 to 30)	22·2 (21·7 to 22·7)	13·5 (13·1 to 14)	7·41 (7·18 to 7·64)	5·37 (5·05 to 5·7)	5·16 (4·8 to 5·54)	5·04 (4·54 to 5·58)
Albania	264 (220 to 317)	138 (118 to 159)	83·3 (71·1 to 96·8)	48·3 (41·5 to 55·7)	38·9 (34·6 to 43·8)	28·2 (24·3 to 32·7)	17·5 (14·7 to 20·5)	14·8 (12·2 to 17·9)	14·7 (12 to 17·9)	13·1 (10·7 to 16)
Bosnia and Herzegovina	280 (250 to 312)	120 (109 to 132)	73·1 (67 to 79)	38·6 (34·8 to 42·7)	16·9 (15·8 to 17·9)	11·1 (10·3 to 11·9)	6·73 (6·13 to 7·41)	6·73 (6·03 to 7·47)	6·4 (5·54 to 7·35)	5·25 (4·41 to 6·25)
Bulgaria	187 (177 to 197)	56·8 (55·3 to 58·4)	32·4 (31·3 to 33·4)	25·4 (24·5 to 26·2)	19·5 (18·7 to 20·3)	17·5 (16·6 to 18·4)	11·4 (10·8 to 12·1)	6·85 (6·26 to 7·56)	6·3 (5·79 to 6·86)	6·61 (5·85 to 7·4)
Croatia	222 (209 to 236)	76·1 (74·1 to 78·2)	42·1 (40·3 to 44)	21·5 (20·7 to 22·5)	11·8 (11 to 12·6)	7·99 (7·47 to 8·55)	5·15 (4·75 to 5·56)	4·39 (3·92 to 4·93)	4·56 (4·03 to 5·12)	4·58 (3·84 to 5·42)
Czechia	54·1 (50·7 to 57·7)	23·7 (23 to 24·5)	23·6 (22·8 to 24·4)	18·5 (17·8 to 19·1)	12 (11·5 to 12·5)	5·31 (4·99 to 5·67)	3·44 (3·22 to 3·66)	3·07 (2·79 to 3·36)	2·81 (2·55 to 3·09)	2·7 (2·32 to 3·13)
Hungary	129 (120 to 138)	53 (51·4 to 54·5)	40·2 (38·8 to 41·5)	24·8 (23·9 to 25·7)	16·9 (16·2 to 17·6)	10·5 (9·91 to 11·1)	5·94 (5·56 to 6·34)	4·32 (3·91 to 4·78)	4·15 (3·71 to 4·63)	4·04 (3·44 to 4·68)
Montenegro	97·2 (85·5 to 111)	62·3 (58·9 to 66·1)	41·1 (38·4 to 43·9)	24·8 (22·8 to 26·8)	15·9 (14·3 to 17·6)	12·3 (11 to 13·7)	7·22 (6·57 to 7·92)	3·26 (2·84 to 3·73)	4·13 (3·46 to 4·96)	3·85 (3·19 to 4·66)
North Macedonia	241 (212 to 273)	173 (156 to 190)	100 (92·5 to 107)	60·8 (54·7 to 67)	38·6 (36·9 to 40·4)	15·5 (14·5 to 16·5)	9·82 (9·05 to 10·6)	6·89 (6·21 to 7·69)	6·38 (5·73 to 7·07)	5·58 (4·92 to 6·29)
Poland	170 (165 to 175)	63·2 (62·1 to 64·2)	38 (37·2 to 38·8)	24·6 (24 to 25·1)	18·2 (17·8 to 18·7)	9·47 (9·11 to 9·84)	5·82 (5·59 to 6·04)	4·31 (4·06 to 4·58)	4·12 (3·8 to 4·49)	4·38 (3·87 to 4·96)
Romania	145 (125 to 167)	90·6 (87·9 to 93·1)	56·7 (55·2 to 58·1)	36·1 (35·1 to 37·2)	33·2 (32 to 34·3)	22·5 (21·5 to 23·5)	11·5 (10·9 to 12·1)	7·42 (6·93 to 7·94)	7·07 (6·63 to 7·52)	6·74 (6·13 to 7·37)
Serbia	278 (267 to 291)	95·4 (93·3 to 97·4)	70 (68·2 to 72)	39·4 (38·2 to 40·7)	28·4 (27·4 to 29·4)	14·7 (12·5 to 17·1)	6·57 (5·69 to 7·6)	5·45 (5·01 to 5·88)	5·31 (4·94 to 5·71)	4·68 (4·21 to 5·17)
Slovakia	98·2 (92·7 to 104)	35·2 (34·1 to 36·4)	30·6 (29·5 to 31·8)	23·5 (22·7 to 24·4)	14·4 (13·7 to 15·1)	9·85 (9·28 to 10·5)	6·79 (6·34 to 7·25)	6·09 (5·61 to 6·59)	6·04 (5·6 to 6·53)	5·76 (5·11 to 6·42)
Slovenia	161 (150 to 172)	70·3 (67·9 to 72·8)	36·7 (35·3 to 38·2)	18·1 (17·3 to 19)	9·94 (9·26 to 10·7)	5·41 (4·99 to 5·85)	3·28 (2·99 to 3·6)	2·27 (2·01 to 2·55)	2·31 (2·07 to 2·59)	2·24 (1·97 to 2·52)
Eastern Europe	69·2 (54·4 to 87·9)	46·5 (44·8 to 48·4)	28·4 (26·8 to 30·1)	27 (26 to 28·3)	20·4 (19·6 to 21·2)	17·9 (17·4 to 18·4)	9·68 (9·42 to 9·94)	6·37 (6·05 to 6·69)	5·92 (5·62 to 6·25)	6·05 (5·6 to 6·52)
Belarus	83·5 (63·5 to 107)	57·2 (47·9 to 67)	33·6 (28·4 to 39·6)	24·7 (20·9 to 28·6)	18·2 (15·7 to 21·2)	16·9 (14·4 to 19·7)	8·01 (6·68 to 9·47)	4·81 (3·82 to 5·99)	4·15 (3·12 to 5·35)	4·01 (3·11 to 5·26)
Estonia	57·1 (42·2 to 75)	36·7 (35 to 38·5)	22·3 (21·1 to 23·4)	21·8 (20·7 to 23)	16·9 (16 to 17·8)	11·1 (10·3 to 12)	4·6 (4·19 to 4·99)	2·36 (2·06 to 2·71)	2·32 (2·04 to 2·66)	2·52 (2·2 to 2·89)
Latvia	41·8 (30·6 to 55·1)	32·3 (30·8 to 33·8)	21·4 (20·5 to 22·4)	21·1 (20·2 to 22)	17·2 (16·4 to 17·9)	13·5 (12·7 to 14·3)	7·65 (7·14 to 8·24)	4·04 (3·62 to 4·51)	4·03 (3·58 to 4·49)	3·73 (3·22 to 4·28)
Lithuania	85·4 (64·5 to 111)	48·1 (46·3 to 49·8)	22·6 (21·7 to 23·6)	19·8 (18·9 to 20·6)	14·6 (13·9 to 15·3)	10·8 (10·2 to 11·5)	6·07 (5·65 to 6·51)	3·86 (3·48 to 4·27)	3·57 (3·23 to 3·94)	3·53 (3·15 to 3·94)
Moldova	182 (149 to 223)	112 (94·6 to 131)	71·2 (57·1 to 87)	51·3 (44·7 to 59·3)	32·2 (28·4 to 36·6)	27·4 (23·9 to 31·3)	16·5 (14·1 to 19·1)	12·5 (9·8 to 15·8)	12·5 (9·72 to 16·1)	10·9 (8·2 to 14·4)
Russia	69·6 (50·5 to 93·9)	46·4 (45·5 to 47·3)	27·7 (26·9 to 28·5)	27·4 (26·8 to 28·1)	20·9 (20·4 to 21·4)	18·8 (18·2 to 19·4)	9·53 (9·25 to 9·82)	6·01 (5·7 to 6·31)	5·51 (5·26 to 5·78)	5·83 (5·47 to 6·22)
Ukraine	54·5 (41·8 to 70·1)	38·8 (33·1 to 45·5)	26·3 (21·8 to 31·2)	24·7 (20·9 to 28·7)	18·6 (16·3 to 21·1)	15·3 (14·8 to 15·9)	10·5 (10·1 to 10·9)	8·49 (7·6 to 9·53)	8·28 (7·19 to 9·51)	7·77 (6·17 to 9·23)
High income	60·6 (58 to 63·4)	44·4 (43·5 to 45·3)	29·3 (28·7 to 30)	16·8 (16·4 to 17·1)	11 (10·9 to 11·2)	7·55 (7·44 to 7·67)	5·98 (5·88 to 6·08)	5·14 (5·03 to 5·24)	4·86 (4·65 to 5·08)	4·57 (4·2 to 4·98)
Australasia	30·9 (29·4 to 32·3)	24·9 (24·4 to 25·5)	20·9 (20·4 to 21·4)	13·3 (12·8 to 13·7)	9·7 (9·4 to 10)	6·58 (6·34 to 6·82)	5·34 (5·11 to 5·59)	4·2 (3·95 to 4·44)	3·91 (3·6 to 4·22)	3·31 (2·82 to 3·83)
Australia	30·3 (28·5 to 32·1)	24·2 (23·6 to 24·9)	21·1 (20·6 to 21·7)	12·9 (12·4 to 13·4)	9·45 (9·12 to 9·82)	6·31 (6·05 to 6·59)	5·04 (4·82 to 5·28)	4 (3·77 to 4·24)	3·82 (3·49 to 4·16)	2·99 (2·5 to 3·55)
New Zealand	33·2 (30·6 to 36·2)	27·4 (26·3 to 28·5)	20 (19·1 to 21)	14·9 (14·1 to 15·7)	10·8 (10·1 to 11·5)	7·73 (7·19 to 8·27)	6·75 (6·29 to 7·27)	5·17 (4·75 to 5·61)	4·38 (4·06 to 4·71)	4·82 (4·26 to 5·35)
High-income Asia Pacific	89·9 (81·9 to 99·4)	59·8 (55·7 to 64)	30·5 (27·9 to 33)	15·1 (13·8 to 16·6)	8·31 (7·68 to 9·02)	5·21 (5·07 to 5·35)	3·47 (3·35 to 3·59)	2·72 (2·61 to 2·84)	2·45 (2·3 to 2·61)	2·21 (2·05 to 2·38)
Brunei	217 (199 to 236)	120 (113 to 126)	52·5 (49·7 to 55·3)	20·8 (18·9 to 22·8)	11·5 (10·4 to 12·7)	10·2 (8·88 to 11·7)	10·2 (9·14 to 11·4)	10·7 (9·16 to 12·5)	10·5 (8·72 to 12·6)	9·66 (7·68 to 12·1)
Japan	59·7 (56·6 to 63)	38 (37 to 39·2)	16·6 (16·1 to 17)	9·7 (9·42 to 10)	6·23 (6·02 to 6·45)	4·41 (4·24 to 4·58)	3·17 (3·04 to 3·3)	2·5 (2·38 to 2·62)	2·24 (2·06 to 2·44)	2·11 (1·88 to 2·38)
Aichi	52 (44·8 to 59·8)	35·8 (32 to 39·3)	15 (13·5 to 16·7)	8·98 (8·57 to 9·4)	5·84 (5·51 to 6·18)	4·19 (3·95 to 4·46)	3·23 (3 to 3·45)	2·41 (2·24 to 2·58)	2·22 (2·06 to 2·39)	2·13 (1·87 to 2·41)
Akita	52·5 (45·2 to 60·6)	38·1 (35·3 to 41·3)	19·2 (17·8 to 20·9)	9·99 (9·32 to 10·8)	6·7 (6·2 to 7·21)	4·33 (3·89 to 4·82)	3·19 (2·86 to 3·52)	2·69 (2·43 to 2·97)	2·47 (2·08 to 2·89)	2·31 (1·97 to 2·7)
Aomori	61·4 (53·8 to 69·6)	41·3 (38·3 to 44·7)	20·4 (18·8 to 22)	11·3 (10·7 to 12)	7·26 (6·75 to 7·83)	5·16 (4·78 to 5·56)	3·6 (3·31 to 3·92)	3·04 (2·78 to 3·31)	2·72 (2·32 to 3·17)	2·55 (2·17 to 2·98)
Chiba	57 (48·3 to 67·1)	36 (32·4 to 40·2)	14·1 (12·5 to 15·9)	8·96 (8·48 to 9·46)	5·47 (5·17 to 5·83)	4·33 (4·09 to 4·59)	3·3 (3·05 to 3·55)	2·61 (2·41 to 2·82)	2·35 (2·18 to 2·55)	2·21 (1·94 to 2·52)
Ehime	59 (52·2 to 66·5)	39·5 (36·5 to 42·6)	18·3 (16·9 to 19·9)	10·2 (9·56 to 10·9)	6·57 (6·1 to 7·07)	4·3 (3·99 to 4·65)	2·83 (2·56 to 3·08)	2·36 (2·15 to 2·57)	2·06 (1·76 to 2·39)	1·96 (1·66 to 2·29)
Fukui	55 (46·1 to 64·9)	36·5 (32·7 to 40·4)	16·6 (14·7 to 18·8)	8·75 (8 to 9·55)	6·45 (5·83 to 7·1)	4·77 (4·34 to 5·26)	3·06 (2·79 to 3·35)	2·44 (2·2 to 2·71)	2·66 (2·24 to 3·13)	2·49 (2·11 to 2·92)
Fukuoka	54·3 (46 to 62·9)	35·3 (31·9 to 39·1)	16·5 (14·6 to 18·5)	9·2 (8·74 to 9·69)	6·47 (6·13 to 6·82)	4·63 (4·38 to 4·89)	3·31 (3·07 to 3·57)	2·81 (2·6 to 3·01)	2·48 (2·28 to 2·69)	2·38 (2·09 to 2·73)
Fukushima	73·2 (63·7 to 83·3)	45·2 (41·9 to 49·1)	22·7 (20·9 to 24·6)	11·6 (11·1 to 12·2)	7·39 (7 to 7·82)	4·98 (4·64 to 5·32)	3·38 (3·15 to 3·61)	3 (2·78 to 3·24)	2·82 (2·47 to 3·23)	2·7 (2·34 to 3·13)
Gifu	55·6 (48·1 to 64·8)	38·7 (35·9 to 41·5)	17·4 (16·2 to 18·6)	9·8 (9·32 to 10·3)	5·77 (5·36 to 6·19)	4·27 (3·99 to 4·57)	3·38 (3·16 to 3·62)	2·66 (2·45 to 2·87)	2·31 (2 to 2·66)	2·18 (1·88 to 2·52)
Gunma	64·8 (56·1 to 73·8)	42·8 (39·5 to 46·5)	18·3 (16·8 to 19·8)	10 (9·49 to 10·6)	6·23 (5·77 to 6·69)	4·52 (4·23 to 4·84)	3·15 (2·94 to 3·37)	2·42 (2·21 to 2·63)	2·27 (1·96 to 2·6)	2·17 (1·86 to 2·52)
Hiroshima	64·2 (56·8 to 72·6)	40·9 (38 to 44·2)	17·2 (15·8 to 18·7)	10·2 (9·65 to 10·7)	6·29 (5·92 to 6·66)	4·03 (3·72 to 4·36)	3·2 (2·9 to 3·54)	2·49 (2·3 to 2·69)	2·16 (1·89 to 2·46)	2·02 (1·75 to 2·35)
Hokkaidō	52·3 (44·2 to 61·4)	36·2 (32·2 to 40)	18 (16 to 20·2)	10·3 (9·82 to 10·7)	6·05 (5·7 to 6·41)	4·11 (3·81 to 4·4)	2·98 (2·79 to 3·2)	2·64 (2·4 to 2·89)	2·48 (2·28 to 2·69)	2·41 (2·11 to 2·75)
Hyōgo	54·3 (45·8 to 63·3)	34·8 (31·1 to 38·8)	15·3 (13·5 to 17·2)	9·22 (8·79 to 9·7)	6·08 (5·74 to 6·45)	4·28 (4 to 4·55)	2·88 (2·69 to 3·1)	2·22 (2·07 to 2·39)	2 (1·85 to 2·16)	1·87 (1·65 to 2·14)
Ibaraki	76 (63·9 to 89·6)	45·7 (40·8 to 51)	20·4 (18·1 to 23)	11·3 (10·7 to 12)	6·32 (5·87 to 6·77)	4·53 (4·24 to 4·86)	3·42 (3·2 to 3·66)	3·07 (2·83 to 3·31)	2·91 (2·66 to 3·16)	2·79 (2·44 to 3·2)
Ishikawa	55·6 (47·9 to 65)	40·3 (37·2 to 43·5)	17·9 (16·5 to 19·4)	9·94 (9·32 to 10·6)	6·22 (5·66 to 6·83)	4·63 (4·26 to 5·04)	3·17 (2·9 to 3·46)	2·36 (2·14 to 2·61)	2·07 (1·76 to 2·45)	1·93 (1·65 to 2·27)
Iwate	70·7 (61·1 to 80·8)	47·7 (43·9 to 51·7)	24·2 (22·3 to 26·2)	12·4 (11·7 to 13·3)	6·13 (5·68 to 6·61)	4·29 (3·94 to 4·68)	3·76 (3·4 to 4·12)	3·14 (2·87 to 3·44)	2·62 (2·25 to 3·06)	2·47 (2·12 to 2·89)
Kagawa	56·7 (48·9 to 66)	40 (37 to 43)	17·4 (16·2 to 18·9)	9·49 (8·77 to 10·3)	6·32 (5·76 to 6·97)	4·09 (3·71 to 4·49)	3·33 (3·05 to 3·63)	2·5 (2·28 to 2·74)	2·35 (1·99 to 2·8)	2·23 (1·89 to 2·62)
Kagoshima	81·5 (71·3 to 92·5)	44·3 (40·9 to 48·1)	23·4 (21·6 to 25·3)	11·9 (11·2 to 12·5)	6·92 (6·51 to 7·37)	5·03 (4·73 to 5·37)	3·52 (3·27 to 3·76)	2·9 (2·65 to 3·14)	2·66 (2·3 to 3·06)	2·52 (2·15 to 2·92)
Kanagawa	50 (39·5 to 62·9)	31·4 (26·9 to 35·9)	13 (11·7 to 14·4)	8·8 (8·34 to 9·27)	5·88 (5·52 to 6·23)	4·42 (4·13 to 4·72)	3·27 (3·04 to 3·53)	2·52 (2·28 to 2·77)	2·01 (1·87 to 2·16)	1·8 (1·58 to 2·06)
Kōchi	74·5 (64·2 to 84·6)	46·3 (43 to 50·1)	21·3 (19·5 to 23·1)	10·9 (10 to 11·9)	7·52 (6·94 to 8·16)	4·88 (4·46 to 5·39)	3·84 (3·5 to 4·2)	2·98 (2·69 to 3·3)	2·88 (2·44 to 3·38)	2·73 (2·31 to 3·2)
Kumamoto	65·5 (57·7 to 73·8)	43·7 (40·6 to 47)	21·7 (20·1 to 23·3)	11·3 (10·7 to 11·9)	6·64 (6·18 to 7·16)	4·59 (4·29 to 4·94)	3·16 (2·83 to 3·5)	2·56 (2·34 to 2·77)	2·55 (2·23 to 2·9)	2·32 (2·01 to 2·7)

Kyōto	55·4 (47·8 to 63·3)	35·3 (32·6 to 38·4)	14·3 (13·1 to 15·6)	8·6 (8·07 to 9·1)	6·29 (5·83 to 6·77)	4·27 (3·94 to 4·62)	3·11 (2·89 to 3·34)	2·65 (2·45 to 2·86)	2·13 (1·85 to 2·45)	2·01 (1·74 to 2·32)
Mie	59·4 (52·1 to 68·1)	40·8 (38 to 43·8)	17·8 (16·5 to 19·1)	9·99 (9·45 to 10·5)	6·39 (5·93 to 6·9)	4·74 (4·44 to 5·09)	3·47 (3·22 to 3·75)	2·35 (2·15 to 2·56)	2·19 (1·88 to 2·53)	2·06 (1·76 to 2·39)
Miyagi	60·6 (52·8 to 69)	39·4 (36·6 to 42·4)	18·6 (17·2 to 19·9)	10·3 (9·76 to 10·9)	6·17 (5·82 to 6·49)	4·03 (3·75 to 4·31)	3·16 (2·92 to 3·4)	2·61 (2·35 to 2·88)	2·3 (1·98 to 2·65)	2·15 (1·85 to 2·48)
Miyazaki	64·9 (57·1 to 72·9)	42·7 (39·4 to 46·2)	20·7 (19 to 22·5)	10·8 (10 to 11·7)	7·28 (6·8 to 7·82)	4·82 (4·44 to 5·22)	3·36 (3·05 to 3·69)	2·75 (2·52 to 2·99)	2·61 (2·25 to 3·05)	2·48 (2·12 to 2·9)
Nagano	73·1 (63·9 to 83·6)	44·2 (41·1 to 48)	18·2 (16·8 to 19·5)	9·6 (9·11 to 10·1)	6·04 (5·71 to 6·42)	3·59 (3·36 to 3·85)	2·66 (2·48 to 2·85)	2·12 (1·94 to 2·29)	2·01 (1·74 to 2·3)	1·91 (1·65 to 2·22)
Nagasaki	59·5 (52·5 to 67·3)	34·3 (31·7 to 36·9)	17·5 (16·3 to 18·7)	9·43 (8·82 to 10·1)	6·18 (5·73 to 6·67)	4·73 (4·39 to 5·07)	3·56 (3·3 to 3·81)	2·86 (2·61 to 3·11)	2·82 (2·43 to 3·22)	2·73 (2·34 to 3·18)
Nara	58·9 (51·5 to 67·5)	36·8 (34 to 39·8)	15·4 (14·2 to 16·7)	9·74 (9·05 to 10·4)	6·04 (5·62 to 6·49)	4·05 (3·72 to 4·39)	3·32 (3 to 3·65)	2·73 (2·52 to 2·95)	2·62 (2·24 to 3·04)	2·51 (2·15 to 2·91)
Niigata	64 (55·6 to 72·9)	40·9 (37·8 to 44·2)	18·6 (17·2 to 20)	10·1 (9·6 to 10·7)	5·58 (5·26 to 5·95)	4·43 (4·08 to 4·73)	2·75 (2·52 to 3·01)	2·34 (2·13 to 2·55)	2·19 (1·88 to 2·54)	2·11 (1·82 to 2·45)
Ōita	62·7 (55·2 to 72·2)	41·4 (38·1 to 44·8)	20·2 (18·5 to 22)	10·7 (10·2 to 11·3)	6·33 (5·83 to 6·86)	4·2 (3·85 to 4·58)	3·28 (2·98 to 3·6)	2·51 (2·28 to 2·76)	2·3 (1·97 to 2·7)	2·18 (1·85 to 2·55)
Okayama	59·5 (52·2 to 67·3)	39·2 (36·6 to 42)	16·1 (14·8 to 17·4)	9·11 (8·56 to 9·65)	6·54 (6·14 to 6·98)	4·61 (4·27 to 4·95)	3·08 (2·87 to 3·29)	2·56 (2·35 to 2·76)	2·28 (1·97 to 2·62)	2·14 (1·85 to 2·49)
Okinawa	113 (99·4 to 128)	45·5 (42·1 to 49·2)	21·3 (19·7 to 23)	10·7 (10 to 11·3)	7·27 (6·79 to 7·78)	5·16 (4·78 to 5·6)	3·29 (2·95 to 3·64)	2·44 (2·23 to 2·65)	2·41 (2·07 to 2·8)	2·27 (1·95 to 2·65)
Ōsaka	55 (44·5 to 67·6)	34·2 (30 to 38·5)	13·9 (12·6 to 15·1)	9·18 (8·78 to 9·61)	6·43 (6·06 to 6·78)	4·25 (4·01 to 4·5)	3·01 (2·81 to 3·24)	2·44 (2·2 to 2·69)	2·13 (1·97 to 2·31)	1·9 (1·67 to 2·17)
Saga	65·6 (57·1 to 75·8)	39·9 (36·9 to 43)	19·2 (17·7 to 20·8)	10·2 (9·47 to 11)	6·83 (6·3 to 7·44)	4·74 (4·34 to 5·18)	2·93 (2·66 to 3·22)	2·23 (2·02 to 2·45)	2·08 (1·77 to 2·44)	1·99 (1·68 to 2·33)
Saitama	57·4 (49 to 66·9)	35·5 (31·8 to 39·2)	14·3 (12·8 to 15·8)	9·18 (8·68 to 9·71)	5·9 (5·59 to 6·21)	4·31 (4·06 to 4·56)	3·06 (2·86 to 3·26)	2·29 (2·13 to 2·44)	1·92 (1·79 to 2·08)	1·77 (1·55 to 2·01)
Shiga	65·1 (55·7 to 75·6)	42·1 (38·7 to 45·5)	17·1 (15·7 to 18·6)	9·21 (8·54 to 9·93)	6·23 (5·74 to 6·78)	4·62 (4·29 to 4·96)	3·17 (2·93 to 3·45)	2·36 (2·16 to 2·58)	2·13 (1·82 to 2·47)	2·01 (1·71 to 2·36)
Shimane	60·4 (49·9 to 72·6)	41·7 (36·3 to 47·6)	20·1 (17·3 to 23·6)	10 (9·24 to 10·9)	6·66 (6·1 to 7·27)	4·63 (4·17 to 5·14)	3·1 (2·81 to 3·4)	2·44 (2·21 to 2·71)	2·23 (1·87 to 2·65)	2·06 (1·75 to 2·43)
Shizuoka	53·3 (46·5 to 61)	33·4 (30·8 to 36·4)	14·8 (13·6 to 16·2)	8·54 (8·1 to 9·05)	5·96 (5·63 to 6·3)	4·01 (3·72 to 4·29)	2·97 (2·75 to 3·19)	2·66 (2·44 to 2·87)	2·49 (2·27 to 2·71)	2·42 (2·13 to 2·77)
Tochigi	65·7 (57·9 to 74·7)	42·2 (39·2 to 45·7)	19·2 (17·7 to 20·7)	10·8 (10·2 to 11·4)	6·94 (6·45 to 7·44)	5·2 (4·83 to 5·62)	3·56 (3·3 to 3·83)	2·93 (2·69 to 3·17)	2·64 (2·28 to 3·05)	2·49 (2·15 to 2·9)
Tokushima	59·5 (49·5 to 72)	39·9 (35·3 to 45)	19 (16·9 to 21·3)	10·3 (9·47 to 11·1)	6·7 (6·06 to 7·34)	5·17 (4·76 to 5·6)	4·16 (3·82 to 4·53)	3·22 (2·86 to 3·6)	3·02 (2·53 to 3·57)	2·83 (2·4 to 3·35)
Tōkyō	46·5 (36·2 to 59·1)	31·4 (26·6 to 36·6)	14·1 (12·6 to 15·6)	8·92 (8·48 to 9·37)	5·99 (5·65 to 6·34)	4·52 (4·25 to 4·81)	2·97 (2·75 to 3·19)	2·12 (1·92 to 2·32)	1·85 (1·74 to 1·99)	1·78 (1·58 to 2·02)
Tottori	65·9 (55·2 to 79)	42·8 (38 to 48)	20·4 (18·1 to 23·3)	10·3 (9·46 to 11·2)	6·34 (5·7 to 7·02)	4·16 (3·69 to 4·68)	3·53 (3·2 to 3·88)	2·96 (2·66 to 3·27)	2·61 (2·19 to 3·09)	2·48 (2·1 to 2·93)
Toyama	59·9 (52 to 67·8)	45·1 (41·5 to 48·9)	20·1 (18·5 to 21·7)	10·8 (9·97 to 11·6)	7·2 (6·58 to 7·86)	4·73 (4·32 to 5·19)	3·26 (3 to 3·54)	2·33 (2·1 to 2·58)	2·12 (1·79 to 2·48)	2 (1·71 to 2·36)
Wakayama	69·2 (59·4 to 79·7)	42·8 (39·1 to 47)	18·7 (17·2 to 20·3)	11·3 (10·5 to 12·1)	6·07 (5·56 to 6·61)	4·22 (3·84 to 4·65)	3·11 (2·82 to 3·39)	2·35 (2·12 to 2·62)	2·4 (2·01 to 2·85)	2·32 (1·97 to 2·75)
Yamagata	62·4 (54·6 to 71·1)	42·1 (39 to 45·5)	20·4 (18·9 to 22·1)	10·2 (9·51 to 10·8)	5·77 (5·32 to 6·28)	4·39 (3·96 to 4·86)	3·45 (3·16 to 3·75)	3·03 (2·77 to 3·3)	2·75 (2·35 to 3·22)	2·58 (2·2 to 3·01)
Yamaguchi	57·9 (50·9 to 66·9)	40·4 (37·2 to 43·9)	18·3 (16·8 to 20)	10·7 (10·1 to 11·5)	6·68 (6·19 to 7·21)	4·59 (4·21 to 5·06)	3·28 (2·95 to 3·61)	2·97 (2·72 to 3·21)	2·71 (2·34 to 3·14)	2·59 (2·23 to 3·02)
Yamanashi	63·2 (53·4 to 75·3)	38 (34·2 to 42·2)	16·9 (15·1 to 18·8)	9·46 (8·75 to 10·2)	6·48 (5·89 to 7·12)	4·7 (4·27 to 5·18)	2·88 (2·61 to 3·13)	2·28 (2·06 to 2·51)	2·12 (1·81 to 2·5)	1·98 (1·66 to 2·33)
South Korea	185 (155 to 221)	95·7 (85·2 to 107)	59·5 (51·7 to 67·2)	26 (22 to 30·3)	12·2 (10·3 to 14·2)	6·86 (6·6 to 7·12)	4·16 (3·97 to 4·36)	3·39 (3·16 to 3·64)	3·09 (2·88 to 3·3)	2·48 (2 to 2·95)
Singapore	116 (109 to 124)	49·8 (48 to 51·5)	30·4 (28·5 to 32·5)	17 (15·8 to 18·3)	9·05 (8·33 to 9·76)	4 (3·58 to 4·47)	2·8 (2·49 to 3·12)	1·83 (1·62 to 2·08)	1·58 (1·39 to 1·79)	1·66 (1·4 to 1·98)
High-income North America	37·2 (36·2 to 38·2)	31·4 (31·1 to 31·8)	23·4 (23 to 23·8)	14·9 (14·6 to 15·2)	10·8 (10·6 to 11)	8·14 (8 to 8·3)	7·09 (6·96 to 7·22)	6·37 (6·25 to 6·51)	6·1 (5·94 to 6·27)	5·71 (5·23 to 6·24)
Canada	45·4 (43·8 to 47·2)	30·9 (30·4 to 31·5)	21·4 (20·8 to 21·9)	12·6 (12·2 to 13)	8·24 (7·96 to 8·53)	5·83 (5·61 to 6·07)	5·53 (5·32 to 5·76)	4·77 (4·54 to 5)	4·91 (4·55 to 5·29)	4·03 (3·36 to 4·82)
Greenland	166 (149 to 186)	90·3 (85·3 to 95·7)	55·3 (51·1 to 59·8)	44·6 (39·2 to 51·2)	32 (28·1 to 36·1)	20·4 (18·1 to 22·8)	12·4 (10·8 to 14·1)	9·98 (8·42 to 11·6)	10·7 (9·12 to 12·3)	10·6 (8·98 to 12·3)
USA	36·3 (35·2 to 37·4)	31·5 (31·1 to 31·9)	23·6 (23·2 to 24)	15·1 (14·8 to 15·4)	11 (10·8 to 11·3)	8·33 (8·17 to 8·5)	7·24 (7·1 to 7·38)	6·53 (6·4 to 6·67)	6·22 (6·06 to 6·37)	5·88 (5·37 to 6·43)
Alabama	43·5 (38·1 to 50·2)	39·1 (37·8 to 40·3)	28·9 (27·9 to 29·9)	18·1 (17·2 to 19·2)	13·2 (12·5 to 13·9)	11·3 (10·6 to 12·1)	10 (9·4 to 10·6)	9·09 (8·6 to 9·6)	8·31 (7·86 to 8·83)	8·06 (7·28 to 9·03)
Alaska	46·4 (35·4 to 58·3)	46 (43·6 to 48·4)	25·5 (23·8 to 27·2)	17·7 (16·5 to 19)	12·1 (11·3 to 12·9)	9·13 (8·48 to 9·81)	7·26 (6·71 to 7·82)	7·61 (6·93 to 8·33)	7·34 (6·55 to 8·16)	7·25 (6·01 to 8·69)
Arizona	46·2 (35 to 60·1)	40·6 (38·5 to 42·8)	24·4 (23·2 to 25·7)	16·3 (15·1 to 17·5)	11·4 (10·7 to 12·2)	8·78 (8·29 to 9·32)	7·44 (6·99 to 7·92)	6·67 (6·28 to 7·07)	6·59 (6·2 to 7·02)	6·15 (5·56 to 6·81)
Arkansas	38·2 (26·7 to 51·6)	34·4 (32·9 to 35·9)	25·7 (24·5 to 26·9)	15·3 (14·4 to 16·2)	12 (11·2 to 12·8)	10·1 (9·46 to 10·7)	8·79 (8·25 to 9·4)	8·38 (7·82 to 8·96)	8·62 (7·96 to 9·3)	8·2 (6·84 to 9·8)
California	34·2 (28·7 to 40·5)	27·5 (26·3 to 28·7)	21·2 (20·4 to 22·2)	14 (13·2 to 14·9)	10·6 (10·2 to 11)	7·07 (6·77 to 7·41)	5·82 (5·57 to 6·05)	5·14 (4·91 to 5·37)	4·74 (4·53 to 4·96)	4·42 (4·01 to 4·87)
Colorado	38·2 (33·4 to 43·9)	32·1 (30·8 to 33·3)	22·6 (21·4 to 23·9)	12·4 (11·6 to 13·2)	9·87 (9·27 to 10·5)	7·46 (7·02 to 7·94)	6·81 (6·39 to 7·24)	5·44 (5·08 to 5·83)	4·91 (4·51 to 5·31)	4·51 (4·06 to 5·03)
Connecticut	31·8 (23·4 to 40·9)	25·1 (24·1 to 26·1)	18·7 (17·9 to 19·6)	12·6 (11·9 to 13·3)	8·89 (8·42 to 9·38)	6·88 (6·49 to 7·26)	5·88 (5·47 to 6·3)	4·83 (4·4 to 5·31)	4·69 (4·22 to 5·16)	4·45 (3·65 to 5·43)
Delaware	37·6 (25·4 to 51·5)	28·4 (26·6 to 30·3)	22·2 (20·8 to 23·7)	16 (14·5 to 17·5)	12·2 (11·3 to 13·2)	10·4 (9·51 to 11·3)	9·02 (8·38 to 9·71)	7·99 (7·16 to 8·87)	7·3 (6·48 to 8·24)	7·13 (5·9 to 8·66)
Washington, DC	64 (44·5 to 88·4)	51·5 (48·7 to 54·4)	41·3 (38·4 to 44·5)	37·9 (34·9 to 41·1)	40·4 (37·6 to 43·3)	19·2 (17·3 to 21·1)	13·3 (11·2 to 15·5)	7·93 (7·02 to 8·88)	7·2 (6·3 to 8·2)	6·96 (5·63 to 8·56)
Florida	45 (38·3 to 52·9)	37·3 (36·2 to 38·4)	27·1 (26·2 to 28·1)	18·4 (17·6 to 19·2)	12·6 (12·1 to 13·1)	9·32 (8·92 to 9·77)	8·19 (7·87 to 8·52)	7·23 (6·9 to 7·55)	6·82 (6·53 to 7·12)	6·43 (5·82 to 7·13)
Georgia	42·2 (35·9 to 49·9)	39·5 (37·8 to 41·4)	27·5 (26·4 to 28·8)	17·7 (16·7 to 18·7)	14·1 (13·4 to 14·7)	10·2 (9·69 to 10·6)	7·88 (7·4 to 8·4)	8·37 (7·97 to 8·82)	7·59 (7·2 to 8·01)	7·36 (6·66 to 8·14)
Hawaii	32·4 (24·8 to 40·5)	29·2 (27·9 to 30·7)	20·8 (19·7 to 22)	12·9 (12·1 to 13·8)	9·83 (9·17 to 10·5)	8·88 (8·26 to 9·49)	7·21 (6·68 to 7·71)	6·85 (6·27 to 7·5)	6·35 (5·76 to 7·02)	6·19 (5·17 to 7·47)
Idaho	30·5 (23·2 to 38·3)	28·7 (27·2 to 30·3)	20·7 (19·6 to 21·9)	13·1 (12·3 to 13·8)	10 (9·45 to 10·7)	7·83 (7·35 to 8·31)	6·14 (5·75 to 6·55)	5·04 (4·58 to 5·56)	4·97 (4·52 to 5·45)	4·5 (3·74 to 5·47)
Illinois	35·2 (30·1 to 40·4)	30·5 (29·6 to 31·4)	25·4 (24·7 to 26·1)	17·6 (17 to 18·2)	12·6 (12 to 13·1)	9·78 (9·4 to 10·2)	7·89 (7·52 to 8·29)	6·53 (6·2 to 6·83)	6·28 (5·97 to 6·59)	6·04 (5·43 to 6·68)
Indiana	30·6 (26·9 to 35)	28·6 (27·8 to 29·4)	22·4 (21·6 to 23·2)	14·1 (13·4 to 14·8)	10·9 (10·4 to 11·4)	9·26 (8·76 to 9·76)	8·51 (8·1 to 8·94)	7·55 (7·12 to 8·03)	7·49 (7·07 to 7·95)	7·04 (6·33 to 7·79)
Iowa	27·3 (20·3 to 34·8)	25·8 (24·8 to 26·8)	20·6 (19·8 to 21·6)	12·8 (12·1 to 13·6)	8·76 (8·29 to 9·26)	6·98 (6·55 to 7·42)	5·64 (5·29 to 6)	5·61 (5·18 to 6·05)	5·29 (4·85 to 5·76)	4·98 (4·12 to 5·98)
Kansas	29·8 (22·4 to 38·3)	26·8 (25·6 to 27·9)	20·8 (19·9 to 21·8)	13·2 (12·4 to 13·9)	9·62 (9·09 to 10·2)	8·44 (8·01 to 8·92)	7·63 (7·2 to 8·08)	6·72 (6·22 to 7·24)	7·25 (6·66 to 7·83)	6·49 (5·43 to 7·64)
Kentucky	37 (25·9 to 49·8)	34·3 (33·1 to 35·5)	23·5 (22·5 to 24·6)	15·1 (14·3 to 15·9)	10·3 (9·69 to 10·9)	8·33 (7·84 to 8·9)	8·01 (7·49 to 8·55)	6·12 (5·75 to 6·52)	6·82 (6·39 to 7·28)	6·38 (5·31 to 7·69)
Louisiana	46·2 (38·4 to 55·3)	39 (37·6 to 40·6)	28·2 (27·1 to 29·3)	17·9 (17·1 to 18·8)	13·4 (12·7 to 14·1)	11·4 (10·8 to 12)	9·26 (8·74 to 9·85)	9·12 (8·6 to 9·68)	8·63 (8·13 to 9·12)	8·24 (7·36 to 9·22)
Maine	36·3 (27·7 to 45·8)	31·7 (30·3 to 33)	22·9 (21·7 to 24)	11·3 (10·6 to 12)	7·71 (7·19 to 8·23)	6·13 (5·71 to 6·59)	6·62 (6·11 to 7·11)	6·04 (5·49 to 6·64)	6·34 (5·73 to 7·06)	5·88 (4·87 to 7·19)
Maryland	37·2 (32·7 to 42·5)	32·4 (31·5 to 33·5)	21·5 (20·5 to 22·6)	15·9 (14·9 to 16·9)	10·9 (10·4 to 11·5)	8·84 (8·34 to 9·35)	7·6 (7·17 to 8·07)	6·61 (6·23 to 7)	6·32 (5·93 to 6·73)	6·16 (5·55 to 6·88)
Massachusetts	33·4 (23·5 to 44·9)	25·7 (24·8 to 26·6)	19·2 (18·4 to 20·1)	12 (11·3 to 12·6)	7·89 (7·47 to 8·33)	5·64 (5·31 to 6·02)	5·13 (4·79 to 5·48)	4·2 (3·91 to 4·52)	3·89 (3·58 to 4·21)	3·56 (2·96 to 4·17)
Michigan	31·1 (26·4 to 36·1)	27·9 (27 to 29)	23 (22·3 to 23·7)	14·5 (13·9 to 15·2)	11·6 (11 to 12·2)	9·17 (8·8 to 9·57)	7·83 (7·44 to 8·23)	7·11 (6·78 to 7·46)	6·99 (6·62 to 7·36)	6·34 (5·76 to 7)
Minnesota	29·2 (20·4 to 39·6)	26·3 (25·4 to 27·2)	19·4 (18·5 to 20·4)	11·6 (10·9 to 12·4)	7·72 (7·28 to 8·19)	6·33 (5·94 to 6·73)	5·4 (5·08 to 5·75)	5·4 (5·05 to 5·75)	4·98 (4·62 to 5·36)	4·72 (3·96 to 5·59)
Mississippi	58·6 (41·5 to 78)	50·3 (48·8 to 52)	35·7 (34·1 to 37·4)	20·8 (19·8 to 22·1)	15 (14·3 to 15·9)	13·3 (12·5 to 14·1)	11·4 (10·7 to 12·1)	10·4 (9·75 to 11·1)	10 (9·28 to 10·8)	9·77 (8·3 to 11·4)
Missouri	35·2 (30·9 to 40·4)	29·8 (28·8 to 31)	23·2 (22·3 to 24·1)	14·9 (14·2 to 15·6)	10·9 (10·3 to 11·5)	8·53 (8·06 to 9·02)	7·67 (7·24 to 8·12)	7·18 (6·79 to 7·65)	6·96 (6·52 to 7·4)	6·77 (6·05 to 7·53)
Montana	33·8 (25·8 to 42·5)	31·2 (29·8 to 32·7)	24·5 (23·2 to 25·8)	14 (13·1 to 14·8)	9·74 (9·04 to 10·4)	7·82 (7·29 to 8·4)	7·01 (6·48 to 7·55)	5·78 (5·19 to 6·4)	5·75 (5·18 to 6·43)	5·26 (4·33 to 6·41)
Nebraska	31·8 (23·5 to 41·1)	27·6 (26·4 to 28·9)	20·8 (19·7 to 22)	13·2 (12·4 to 14)	8·9 (8·36 to 9·5)	8·03 (7·51 to 8·59)	6·22 (5·78 to 6·67)	5·99 (5·42 to 6·59)	6·02 (5·48 to 6·63)	5·51 (4·51 to 6·73)
Nevada	45·2 (34·4 to 56·6)	39·7 (37·2 to 42·1)	25·7 (24·3 to 27·1)	14·3 (13·4 to 15·3)	9·68 (9·12 to 10·2)	7·63 (7·23 to 8·07)	6·66 (6·24 to 7·1)	6·51 (6·06 to 7·02)	5·88 (5·43 to 6·34)	5·66 (4·75 to 6·76)
New Hampshire	32·4 (23·8 to 42·1)	28·2 (26·8 to 29·7)	20 (18·8 to 21·2)	11·6 (10·8 to 12·5)	7·82 (7·3 to 8·36)	5·64 (5·25 to 6·06)	5·17 (4·74 to 5·61)	4·26 (3·82 to 4·76)	4·46 (4 to 5·03)	4·05 (3·3 to 4·99)
New Jersey	35·9 (30·3 to 42·6)	29·2 (28·2 to 30·3)	22·5 (21·7 to 23·4)	14·3 (13·6 to 15)	10·6 (10·1 to 11·1)	7·28 (6·9 to 7·68)	5·63 (5·31 to 5·96)	4·5 (4·23 to 4·79)	4·25 (4 to 4·52)	3·88 (3·48 to 4·3)
New Mexico	51·5 (36 to 69·5)	41·2 (39·2 to 43·4)	26·8 (25·5 to 28·2)	15·2 (14·3 to 16)	10·8 (10·1 to 11·4)	7·89 (7·41 to 8·43)	6·96 (6·47 to 7·53)	6·67 (6·04 to 7·36)	6·44 (5·85 to 7·07)	6·17 (5·07 to 7·44)

New York	36·4 (31 to 41·8)	29·9 (29·1 to 30·6)	23·3 (22·6 to 24)	14·8 (14·3 to 15·3)	12 (11·6 to 12·4)	7·7 (7·4 to 8·01)	6·14 (5·87 to 6·41)	4·85 (4·62 to 5·11)	4·33 (4·13 to 4·53)	4·04 (3·62 to 4·46)
North Carolina	42·6 (36·5 to 49·5)	37·8 (36·8 to 38·9)	28·3 (27·3 to 29·4)	16·7 (16 to 17·5)	12·2 (11·7 to 12·8)	10·1 (9·56 to 10·7)	8·17 (7·81 to 8·57)	7·97 (7·58 to 8·37)	7·93 (7·51 to 8·35)	7·75 (7 to 8·59)
North Dakota	33·7 (23·5 to 45·8)	29 (27·2 to 30·9)	18·6 (17·1 to 20)	13·6 (12·5 to 15)	9·41 (8·56 to 10·4)	8·33 (7·61 to 9·08)	7·37 (6·62 to 8·14)	6·88 (6·24 to 7·59)	6·51 (5·82 to 7·23)	6·23 (5·21 to 7·45)
Ohio	30·1 (25·6 to 34·6)	27·9 (27·2 to 28·7)	21·5 (20·8 to 22·3)	14·7 (14·2 to 15·4)	10·7 (10·3 to 11·1)	8·82 (8·47 to 9·18)	8·75 (8·36 to 9·15)	7·84 (7·46 to 8·2)	7·37 (7·07 to 7·71)	7·04 (6·39 to 7·76)
Oklahoma	31·6 (27·5 to 36·5)	31·8 (30·6 to 33)	24·4 (23·3 to 25·7)	14·9 (14 to 15·7)	10·9 (10·3 to 11·5)	9·78 (9·15 to 10·4)	9·14 (8·57 to 9·69)	8·17 (7·66 to 8·7)	7·09 (6·56 to 7·63)	6·96 (6·17 to 7·82)
Oregon	29·7 (21·9 to 37·8)	28·2 (27 to 29·5)	19·4 (18·5 to 20·4)	13·2 (12·6 to 14)	9·04 (8·51 to 9·58)	6·6 (6·23 to 7)	5·62 (5·3 to 5·97)	5·34 (4·94 to 5·74)	4·9 (4·5 to 5·36)	4·53 (3·75 to 5·47)
Pennsylvania	33·9 (28·8 to 39·1)	28·7 (27·9 to 29·4)	23·1 (22·5 to 23·9)	15 (14·4 to 15·6)	10·7 (10·3 to 11·1)	8·04 (7·67 to 8·41)	7·84 (7·46 to 8·24)	6·55 (6·23 to 6·84)	6·37 (6·06 to 6·7)	6·11 (5·51 to 6·76)
Rhode Island	35·1 (26·4 to 44·5)	28·9 (27·3 to 30·5)	22 (20·6 to 23·3)	13·7 (12·8 to 14·7)	9·42 (8·7 to 10·2)	7·39 (6·85 to 8·01)	7·2 (6·59 to 7·77)	6·11 (5·46 to 6·81)	5·35 (4·74 to 6·02)	4·94 (4·02 to 6·08)
South Carolina	42·5 (37 to 48·7)	41·3 (39·9 to 42·8)	27·5 (26 to 29)	19·3 (18·3 to 20·2)	14·1 (13·4 to 14·9)	10·3 (9·75 to 10·9)	8·32 (7·81 to 8·83)	7·92 (7·47 to 8·39)	7·26 (6·79 to 7·72)	6·96 (6·24 to 7·67)
South Dakota	33·9 (25·7 to 43·1)	32·5 (30·9 to 34·1)	23·8 (22·6 to 25·1)	14·5 (13·5 to 15·4)	11·3 (10·6 to 12·1)	8·74 (8·14 to 9·4)	7·95 (7·38 to 8·58)	7·84 (7·22 to 8·52)	7·84 (7·09 to 8·65)	7·45 (6·18 to 9)
Tennessee	39·9 (35 to 45·7)	35·5 (34·5 to 36·6)	25·5 (24·5 to 26·5)	15·8 (15 to 16·6)	12·1 (11·5 to 12·7)	10·4 (9·81 to 11)	9·01 (8·47 to 9·63)	8·29 (7·81 to 8·82)	7·48 (7·04 to 7·94)	7·14 (6·49 to 7·89)
Texas	39·7 (34·1 to 45·2)	36 (35·2 to 37)	26·2 (25·4 to 27·1)	15·6 (15 to 16·2)	10·2 (9·78 to 10·6)	7·45 (7·17 to 7·74)	7·14 (6·9 to 7·4)	6·78 (6·5 to 7·05)	6·5 (6·27 to 6·74)	6·16 (5·58 to 6·78)
Utah	27·6 (20·5 to 35·7)	24·1 (22·9 to 25·3)	18·2 (17·2 to 19·3)	12·6 (11·9 to 13·3)	8·58 (8·1 to 9·12)	6·5 (6·11 to 6·91)	5·86 (5·54 to 6·18)	6·27 (5·92 to 6·65)	6 (5·52 to 6·5)	5·51 (4·62 to 6·6)
Vermont	34·3 (25·1 to 45·6)	30·6 (28·8 to 32·4)	19·4 (18·2 to 20·7)	11·7 (10·8 to 12·7)	7·76 (7·11 to 8·52)	6·38 (5·86 to 6·97)	5·3 (4·82 to 5·82)	4·7 (4·16 to 5·31)	4·27 (3·75 to 4·84)	4·09 (3·34 to 5)
Virginia	40·4 (33·7 to 47·9)	35·4 (34·3 to 36·6)	24·2 (23·3 to 25·2)	15·6 (14·9 to 16·4)	11·2 (10·6 to 11·7)	8·1 (7·65 to 8·56)	7·71 (7·36 to 8·06)	6·48 (6·14 to 6·82)	6·5 (6·08 to 6·95)	6·2 (5·66 to 6·84)
Washington	29·2 (25·4 to 33·2)	28 (27 to 28·9)	21 (20 to 22)	13·1 (12·4 to 13·8)	8·7 (8·21 to 9·17)	6·15 (5·79 to 6·52)	5·21 (4·9 to 5·52)	4·98 (4·66 to 5·3)	4·85 (4·54 to 5·19)	4·31 (3·89 to 4·73)
West Virginia	37·2 (27·6 to 47·4)	31·9 (30·7 to 33·3)	26·3 (25·2 to 27·5)	14·9 (14·1 to 15·7)	11·4 (10·7 to 12·2)	9·19 (8·58 to 9·87)	8·96 (8·38 to 9·59)	7·79 (7·05 to 8·55)	8·36 (7·57 to 9·23)	7·77 (6·37 to 9·31)
Wisconsin	32 (28 to 36·9)	27·4 (26·5 to 28·3)	18·9 (18·1 to 19·6)	11·9 (11·3 to 12·5)	8·88 (8·43 to 9·34)	7·53 (7·12 to 7·96)	6·55 (6·15 to 6·95)	6·64 (6·23 to 7·05)	6·39 (6·02 to 6·8)	6·05 (5·45 to 6·69)
Wyoming	36·9 (27 to 48·1)	33·8 (31·7 to 36)	24·5 (23 to 26·2)	13·7 (12·7 to 14·8)	10·4 (9·56 to 11·4)	7·92 (7·22 to 8·68)	6·84 (6·25 to 7·45)	6·31 (5·62 to 7·03)	6·09 (5·4 to 6·81)	6·02 (4·95 to 7·28)
Southern Latin America	96·3 (93·1 to 99·6)	105 (104 to 107)	76·3 (75·1 to 77·4)	38·1 (37·4 to 38·7)	25·3 (24·9 to 25·8)	17·3 (16·8 to 17·8)	13·1 (12·7 to 13·4)	10 (9·69 to 10·4)	9·38 (8·2 to 10·6)	8·51 (6·87 to 10·4)
Argentina	77·2 (73·9 to 80·5)	78·7 (77·1 to 80·2)	69·7 (68·4 to 71)	38·4 (37·6 to 39·2)	28·4 (27·8 to 29)	19·3 (18·6 to 20)	14·5 (14·1 to 14·9)	11·1 (10·7 to 11·5)	10·7 (9·18 to 12·3)	9·7 (7·68 to 12·1)
Chile	148 (142 to 153)	162 (160 to 165)	94·7 (92·8 to 96·6)	36·5 (35·5 to 37·5)	18·8 (18·2 to 19·4)	11·7 (11·3 to 12·1)	9·07 (8·7 to 9·44)	7·34 (6·92 to 7·78)	6·1 (5·58 to 6·7)	5·66 (4·93 to 6·44)
Uruguay	56·8 (51·9 to 61·9)	59·1 (56·6 to 61·8)	57·8 (55·4 to 60·2)	40·8 (39·1 to 42·3)	23·2 (22·1 to 24·2)	16·5 (15·4 to 17·7)	11·7 (10·1 to 13·6)	7·95 (7·24 to 8·62)	7·49 (6·28 to 8·75)	6·84 (5·46 to 8·51)
Western Europe	56·8 (54·9 to 58·8)	40·2 (39·8 to 40·6)	26·5 (26·2 to 26·8)	14·9 (14·6 to 15·1)	9·25 (9·11 to 9·4)	5·78 (5·66 to 5·9)	4·27 (4·18 to 4·35)	3·74 (3·6 to 3·87)	3·56 (3·41 to 3·72)	3·49 (3·23 to 3·77)
Andorra	21·1 (14·2 to 30·5)	22·1 (17·2 to 27·8)	15·8 (12·7 to 19·7)	12·5 (10·4 to 15·1)	7·6 (6·3 to 9·14)	3·82 (3·1 to 4·65)	2·86 (2·41 to 3·38)	1·9 (1·57 to 2·28)	1·81 (1·48 to 2·17)	1·16 (0·766 to 1·54)
Austria	55·6 (52·4 to 58·8)	43·8 (42·7 to 45·1)	29·1 (28·2 to 30)	17·1 (16·4 to 17·8)	9·5 (9·03 to 10)	5·68 (5·31 to 6·06)	4·53 (4·21 to 4·87)	3·58 (3·32 to 3·87)	3·55 (3·28 to 3·82)	3·1 (2·74 to 3·51)
Belgium	60·9 (57·6 to 64·4)	34·3 (33·4 to 35·2)	24·5 (23·8 to 25·2)	14·7 (14·1 to 15·3)	9·71 (9·25 to 10·1)	5·97 (5·65 to 6·3)	4·37 (4·1 to 4·64)	4·14 (3·86 to 4·45)	3·76 (3·4 to 4·18)	3·69 (3·05 to 4·44)
Cyprus	134 (109 to 164)	97·5 (84·5 to 112)	59·1 (52·9 to 65·2)	28·1 (26·5 to 29·7)	13·8 (12·8 to 14·8)	6·95 (6·38 to 7·63)	3·55 (3·22 to 3·89)	2·53 (2·23 to 2·9)	2·46 (2·12 to 2·86)	2·45 (2·04 to 2·91)
Denmark	33·6 (30·8 to 36·4)	25·5 (24·5 to 26·5)	16·8 (16 to 17·6)	10·1 (9·49 to 10·7)	9·15 (8·55 to 9·79)	5·57 (5·25 to 5·93)	3·93 (3·66 to 4·25)	3·52 (3·19 to 3·87)	3·52 (3·22 to 3·85)	3·63 (3·2 to 4·09)
Finland	49·6 (46 to 53·2)	26 (25·1 to 27·1)	16·4 (15·6 to 17·2)	8·81 (8·28 to 9·38)	6·77 (6·34 to 7·24)	4·26 (3·95 to 4·61)	2·95 (2·69 to 3·23)	2·47 (2·2 to 2·74)	2·27 (2·04 to 2·51)	2·22 (1·92 to 2·56)
France	55·3 (53 to 57·7)	26·6 (26·1 to 27·1)	17·6 (17·3 to 18)	12·3 (12 to 12·6)	8·76 (8·52 to 9·01)	5·45 (5·28 to 5·62)	4·26 (4·12 to 4·41)	4·24 (4·08 to 4·41)	4·39 (4·15 to 4·64)	4·04 (3·64 to 4·46)
Germany	52·5 (45 to 60·7)	41·1 (40·3 to 41·9)	26·2 (25·6 to 26·7)	15·2 (14·9 to 15·6)	8·65 (8·41 to 8·89)	5·35 (5·16 to 5·55)	4·07 (3·92 to 4·23)	3·62 (3·46 to 3·78)	3·52 (3·36 to 3·66)	3·53 (3·3 to 3·77)
Greece	58·1 (54·7 to 62)	42·9 (41·7 to 44·1)	30·4 (29·5 to 31·4)	19·3 (18·6 to 20)	10·3 (9·74 to 10·8)	6·17 (5·82 to 6·54)	4·32 (4·05 to 4·58)	4·4 (4·01 to 4·79)	4 (3·62 to 4·42)	3·93 (3·4 to 4·5)
Iceland	30·6 (26·3 to 35·5)	21·3 (19·8 to 22·9)	15·8 (14·6 to 17·1)	9·7 (8·81 to 10·7)	6·43 (5·76 to 7·13)	3·9 (3·44 to 4·43)	2·63 (2·3 to 3·03)	2·06 (1·76 to 2·42)	2·45 (2·06 to 2·87)	2·43 (1·98 to 2·94)
Ireland	49·2 (45·5 to 52·9)	32·2 (31·1 to 33·4)	21·4 (20·6 to 22·3)	13·5 (12·8 to 14·1)	9·23 (8·66 to 9·79)	7·03 (6·52 to 7·49)	4·37 (4·04 to 4·72)	3·6 (3·24 to 4)	3·27 (2·97 to 3·58)	3·36 (2·93 to 3·83)
Israel	61·4 (56·6 to 66·6)	37·7 (36·3 to 39·2)	26·1 (25·1 to 27·1)	18·3 (17·7 to 19·1)	11·5 (11 to 12·1)	6·66 (6·32 to 7·01)	4·45 (4·21 to 4·72)	3·6 (3·37 to 3·83)	2·9 (2·68 to 3·13)	2·3 (1·97 to 2·65)
Italy	63·2 (61·3 to 65·1)	52 (51·2 to 52·7)	33·8 (33·3 to 34·4)	16·7 (16·3 to 17·1)	9·62 (9·36 to 9·89)	5·48 (5·29 to 5·67)	3·93 (3·77 to 4·09)	2·91 (2·75 to 3·05)	2·98 (2·75 to 3·25)	2·92 (2·57 to 3·28)
Luxembourg	61·5 (54·2 to 69·2)	37·2 (34·8 to 39·7)	23·7 (22 to 25·6)	12·8 (11·7 to 13·9)	8·82 (8·04 to 9·7)	4·25 (3·83 to 4·73)	3·03 (2·67 to 3·44)	2·83 (2·44 to 3·26)	3·67 (3·15 to 4·25)	3·47 (2·87 to 4·16)
Malta	73 (65·5 to 81·1)	41·2 (39 to 43·7)	27·1 (25·1 to 29·2)	15·8 (14·6 to 17·2)	10·7 (9·74 to 11·8)	7·44 (6·68 to 8·26)	6·54 (5·88 to 7·28)	6·45 (5·52 to 7·52)	5·39 (4·45 to 6·43)	5·28 (4·21 to 6·56)
Monaco	78·7 (60·1 to 102)	45·5 (37 to 56·2)	21·3 (16·4 to 26·8)	12·8 (9·73 to 16·8)	7·29 (5·68 to 9·45)	4·73 (3·7 to 6·02)	3·16 (2·44 to 3·96)	2·34 (1·82 to 2·96)	3·82 (3·73 to 3·96)	3·82 (3·75 to 3·94)
Netherlands	27·6 (26 to 29·4)	21 (20·4 to 21·6)	15·8 (15·3 to 16·2)	10·7 (10·2 to 11·1)	8·73 (8·37 to 9·11)	6·28 (5·97 to 6·59)	4·43 (4·2 to 4·66)	4·15 (3·9 to 4·42)	4·18 (3·94 to 4·42)	3·83 (3·49 to 4·21)
Norway	30 (27·2 to 33·1)	22·7 (21·7 to 23·8)	15·7 (15 to 16·5)	9·94 (9·36 to 10·5)	8·68 (8·18 to 9·2)	4·82 (4·48 to 5·17)	3·22 (2·96 to 3·52)	2·45 (2·2 to 2·71)	2·11 (1·91 to 2·34)	2·12 (1·84 to 2·44)
Agder	31·8 (26·1 to 37·8)	26 (24·1 to 28)	17·4 (15·9 to 18·9)	11·3 (10·2 to 12·3)	9·85 (8·85 to 11)	5·49 (5·01 to 5·95)	3·7 (3·39 to 4·04)	2·83 (2·5 to 3·23)	2·3 (2·03 to 2·62)	2·48 (2·05 to 2·95)
Innlandet	29·4 (25·5 to 33·8)	22·3 (20·8 to 23·9)	15·8 (14·8 to 16·9)	10·1 (9·22 to 11·1)	8·78 (7·94 to 9·67)	5·09 (4·62 to 5·55)	3·47 (3·15 to 3·82)	2·7 (2·38 to 3·04)	2·28 (2·02 to 2·58)	2·26 (1·93 to 2·64)
Møre og Romsdal	34·4 (28·9 to 40·7)	23·9 (22·2 to 25·9)	17·6 (16·1 to 19·1)	10·7 (9·66 to 11·9)	8·46 (7·57 to 9·5)	4·62 (4·23 to 5·03)	3·13 (2·8 to 3·48)	2·6 (2·29 to 2·96)	2·21 (1·95 to 2·49)	2·38 (1·98 to 2·84)
Nordland	36·4 (31·6 to 42)	27·9 (26 to 29·7)	17·4 (16·1 to 18·8)	10·5 (9·51 to 11·5)	8·4 (7·49 to 9·32)	4·83 (4·4 to 5·29)	3·92 (3·53 to 4·38)	3·12 (2·74 to 3·54)	2·63 (2·33 to 2·95)	2·85 (2·38 to 3·4)
Oslo	18·6 (14 to 23·9)	16·8 (15·4 to 18·2)	13·7 (12·4 to 15·3)	10·2 (9·14 to 11·4)	10·4 (9·39 to 11·5)	5·53 (4·89 to 6·2)	2·98 (2·66 to 3·34)	2·03 (1·8 to 2·32)	1·75 (1·56 to 1·97)	1·67 (1·44 to 1·96)
Rogaland	31·1 (25·5 to 37·6)	23·8 (21·8 to 25·8)	16·5 (15 to 18)	10·5 (9·48 to 11·6)	8·27 (7·52 to 9·11)	4·7 (4·35 to 5·08)	3·19 (2·88 to 3·54)	2·41 (2·12 to 2·72)	2·11 (1·88 to 2·4)	2·1 (1·78 to 2·45)
Troms og Finnmark	47·1 (41·3 to 54·2)	31·7 (29·6 to 33·8)	19·6 (18 to 21·2)	11·5 (10·4 to 12·7)	9·92 (9 to 10·9)	5·39 (4·97 to 5·78)	3·67 (3·36 to 4·04)	2·84 (2·47 to 3·25)	2·47 (2·18 to 2·81)	2·65 (2·17 to 3·19)
Trøndelag	29·4 (24·9 to 34·4)	20·8 (19·2 to 22·6)	15 (13·8 to 16·2)	9·62 (8·71 to 10·6)	7·72 (6·96 to 8·47)	4·15 (3·83 to 4·47)	2·88 (2·62 to 3·18)	2·24 (1·97 to 2·54)	1·93 (1·71 to 2·17)	1·87 (1·61 to 2·17)
Vestfold og Telemark	28 (24·2 to 31·9)	22·6 (21·1 to 24·1)	15·7 (14·5 to 16·9)	9·73 (8·9 to 10·6)	8·08 (7·33 to 8·77)	5·14 (4·67 to 5·63)	3·5 (3·2 to 3·85)	2·55 (2·23 to 2·88)	2·25 (2·01 to 2·51)	2·22 (1·92 to 2·56)
Vestland	29·7 (25·1 to 34·8)	21·1 (19·7 to 22·5)	14·5 (13·5 to 15·6)	9·01 (8·22 to 9·77)	7·83 (7·1 to 8·51)	4·25 (3·9 to 4·59)	3·09 (2·84 to 3·38)	2·49 (2·2 to 2·82)	2·14 (1·92 to 2·39)	2·31 (1·93 to 2·71)
Viken	26·1 (22·4 to 30)	20·8 (19·4 to 22·4)	14·5 (13·4 to 15·6)	9·02 (8·31 to 9·75)	8·55 (7·84 to 9·32)	4·59 (4·06 to 5·19)	3·14 (2·82 to 3·46)	2·41 (2·15 to 2·7)	2·12 (1·91 to 2·36)	2·04 (1·79 to 2·32)
Portugal	149 (144 to 156)	105 (103 to 107)	73·4 (71·5 to 75·2)	28·2 (27·3 to 29·1)	14·4 (13·7 to 15)	7·38 (6·96 to 7·8)	3·79 (3·53 to 4·06)	3·64 (3·37 to 3·95)	3·11 (2·86 to 3·36)	2·93 (2·56 to 3·32)
San Marino	24·1 (16·6 to 34·5)	20·2 (16·3 to 24·8)	16·2 (13·6 to 19·2)	12 (9·92 to 14·6)	8·59 (7·1 to 10·3)	5·01 (4·01 to 6·16)	3·01 (2·49 to 3·63)	2·52 (1·93 to 3·26)	2·25 (1·66 to 3·05)	1·66 (1·12 to 2·33)
Spain	93·8 (91·1 to 96·4)	53·4 (52·4 to 54·4)	32 (31·2 to 32·8)	14·5 (14·2 to 14·9)	9·36 (9·03 to 9·71)	5·41 (5·18 to 5·63)	3·94 (3·78 to 4·11)	3·32 (3·16 to 3·5)	3·09 (2·91 to 3·3)	2·97 (2·71 to 3·25)
Sweden	22·7 (20·9 to 24·5)	20·3 (19·5 to 21·2)	13·2 (12·7 to 13·8)	8·04 (7·62 to 8·48)	6·77 (6·39 to 7·13)	3·87 (3·62 to 4·13)	2·99 (2·8 to 3·2)	2·54 (2·32 to 2·79)	2·62 (2·42 to 2·83)	2·26 (2·01 to 2·51)
Stockholm	18·9 (15·3 to 23·6)	18·3 (15·8 to 21)	13 (11·4 to 14·7)	8·53 (7·7 to 9·39)	6·68 (6·08 to 7·32)	3·83 (3·46 to 4·23)	2·7 (2·4 to 3·01)	2·24 (1·97 to 2·57)	2·31 (2 to 2·64)	1·93 (1·6 to 2·31)
Sweden except Stockholm	23·3 (21·3 to 25·5)	20·7 (19·7 to 21·7)	13·3 (12·7 to 14)	7·92 (7·51 to 8·34)	6·8 (6·42 to 7·19)	3·88 (3·63 to 4·14)	3·09 (2·9 to 3·3)	2·63 (2·4 to 2·89)	2·72 (2·51 to 2·93)	2·37 (2·12 to 2·64)
Switzerland	38·5 (35·7 to 41·5)	27·4 (26·4 to 28·4)	19·6 (18·8 to 20·4)	11·2 (10·6 to 11·8)	8·74 (8·26 to 9·21)	6·12 (5·75 to 6·51)	4·4 (4·11 to 4·71)	3·7 (3·4 to 4·04)	4 (3·7 to 4·33)	3·74 (3·31 to 4·21)
UK	27·5 (26·4 to 28·6)	27·3 (26·9 to 27·8)	21·7 (21·3 to 22·1)	15 (14·6 to 15·4)	9·54 (9·23 to 9·86)	6·74 (6·33 to 7·16)	5·2 (5·03 to 5·38)	4·35 (4·18 to 4·53)	3·72 (3·46 to 3·99)	4·15 (3·76 to 4·58)
England	26·2 (24·7 to 27·6)	26·7 (26 to 27·3)	21·3 (20·9 to 21·8)	14·8 (14·4 to 15·3)	9·6 (9·3 to 9·93)	6·82 (6·41 to 7·24)	5·27 (5·1 to 5·47)	4·37 (4·2 to 4·54)	3·73 (3·47 to 3·99)	4·17 (3·78 to 4·6)
East Midlands	26·4 (21·9 to 32)	26·5 (24·4 to 28·5)	21·3 (19·5 to 23·5)	14·4 (13·4 to 15·4)	9·56 (9·13 to 10)	6·7 (6·16 to 7·27)	5·28 (4·95 to 5·65)	4·58 (4·32 to 4·84)	3·92 (3·54 to 4·33)	4·33 (3·88 to 4·82)
Derby	36·9 (29·1 to 46·6)	32·8 (28·5 to 37·7)	26·8 (23·8 to 30·1)	18·4 (16·2 to 20·8)	11·3 (10·2 to 12·3)	7·94 (7·05 to 8·92)	6·54 (5·78 to 7·42)	5·69 (5·15 to 6·3)	5·12 (4·49 to 5·82)	6·09 (5·4 to 6·92)

Derbyshire	26 (20·5 to 32·6)	24·7 (21·8 to 27·7)	19·8 (17·4 to 22·3)	13·3 (12·3 to 14·2)	8·2 (7·56 to 8·91)	5·72 (5·18 to 6·29)	4·37 (4·09 to 4·69)	3·95 (3·66 to 4·24)	3·56 (3·19 to 3·95)	3·98 (3·56 to 4·44)
Leicester	32·7 (25·7 to 41)	34·6 (30 to 39·6)	29·8 (26·6 to 33·3)	19·4 (17·2 to 21·6)	12·1 (11·2 to 13)	9·18 (8·1 to 10·4)	7·35 (6·58 to 8·2)	6·36 (5·82 to 6·93)	5·64 (4·88 to 6·48)	6·33 (5·55 to 7·19)
Leicestershire	20·6 (16·8 to 24·8)	22·6 (20·6 to 24·7)	17·8 (16 to 19·8)	12·1 (10·8 to 13·6)	8·59 (8 to 9·21)	5·54 (5·02 to 6·1)	4·49 (4·18 to 4·82)	4·02 (3·7 to 4·34)	3·48 (3·14 to 3·81)	3·96 (3·56 to 4·37)
Lincolnshire	25·7 (20·3 to 32·4)	25·2 (22·2 to 28·3)	20 (17·9 to 22·4)	13·3 (12·2 to 14·5)	9·34 (8·6 to 10·2)	6·58 (5·93 to 7·3)	5·07 (4·64 to 5·51)	3·6 (3·32 to 3·89)	3·3 (2·94 to 3·66)	3·67 (3·25 to 4·11)
Northamptonshire	26·6 (21·1 to 33·4)	26·4 (23·7 to 29·4)	20·8 (18·4 to 23·6)	13·9 (12·3 to 15·7)	10·3 (9·48 to 11·3)	6·45 (5·85 to 7·09)	5·22 (4·88 to 5·57)	4·66 (4·3 to 5·08)	3·15 (2·85 to 3·49)	3·17 (2·79 to 3·56)
Nottingham	29·7 (22·6 to 38·4)	30·7 (26·5 to 35·3)	25·7 (22·8 to 28·7)	18·4 (16 to 21·3)	12 (11·2 to 13)	10·1 (9·08 to 11·2)	6·94 (6·4 to 7·52)	6·15 (5·59 to 6·75)	5·44 (4·76 to 6·23)	5·87 (5·09 to 6·73)
Nottinghamshire	24·2 (19·1 to 30·2)	24·2 (21·7 to 26·8)	19·4 (17·2 to 21·8)	13·1 (12·2 to 14)	8·26 (7·63 to 8·95)	6 (5·48 to 6·56)	4·72 (4·38 to 5·09)	4·27 (3·95 to 4·62)	3·91 (3·53 to 4·34)	4·33 (3·9 to 4·83)
Rutland	14·2 (11 to 18·5)	15·1 (11·9 to 18·8)	12 (10·7 to 13·5)	9·85 (8·55 to 11·3)	6·72 (5·66 to 7·85)	4·39 (3·59 to 5·3)	3·21 (2·57 to 3·98)	2·21 (1·93 to 2·51)	1·53 (1·33 to 1·75)	1·55 (1·37 to 1·74)
East of England	22·4 (19·7 to 25·3)	22·8 (21 to 24·7)	18·3 (16·7 to 20)	12·5 (11·6 to 13·5)	7·52 (7·18 to 7·87)	5·51 (5·1 to 5·96)	4·57 (4·3 to 4·85)	3·69 (3·5 to 3·91)	3·15 (2·86 to 3·47)	3·49 (3·13 to 3·9)
Bedford	26·8 (21·9 to 32·8)	26·6 (23·8 to 29·9)	21·7 (19·3 to 24·3)	15·2 (13·2 to 17·2)	9·76 (8·72 to 11)	6·98 (6·18 to 7·84)	5·9 (5·28 to 6·55)	5·02 (4·58 to 5·53)	4·3 (3·81 to 4·8)	4·32 (3·82 to 4·85)
Cambridgeshire	14 (11·3 to 17·2)	19·2 (16·8 to 21·8)	15·2 (13·5 to 16·9)	10·7 (9·49 to 11·9)	6·62 (6·09 to 7·16)	5·34 (4·84 to 5·92)	4·35 (4·05 to 4·66)	3·45 (3·2 to 3·72)	2·92 (2·62 to 3·25)	3·25 (2·92 to 3·6)
Central Bedfordshire	21·3 (17·6 to 25·6)	21·8 (18·7 to 25·2)	17·3 (15·2 to 19·5)	12 (10·6 to 13·5)	7·38 (6·71 to 8·07)	5·18 (4·63 to 5·84)	4·03 (3·64 to 4·43)	3·36 (3·08 to 3·67)	2·98 (2·63 to 3·34)	3·37 (3·02 to 3·74)
Essex	21·8 (18 to 26)	21·9 (19·5 to 24·6)	17·3 (15·1 to 19·7)	11·9 (10·6 to 13·2)	7·2 (6·71 to 7·69)	5 (4·52 to 5·52)	4·32 (3·96 to 4·69)	3·43 (3·18 to 3·7)	2·84 (2·47 to 3·23)	3·13 (2·61 to 3·72)
Hertfordshire	21 (16·9 to 25·5)	20·6 (17·6 to 23·5)	16·8 (14·9 to 18·8)	11·9 (10·6 to 13·5)	6·93 (6·43 to 7·47)	4·99 (4·52 to 5·47)	3·98 (3·63 to 4·32)	3·3 (3·04 to 3·57)	2·94 (2·53 to 3·38)	3·27 (2·73 to 3·87)
Luton	30·8 (25·9 to 36·4)	30·1 (26·3 to 34·3)	24·8 (22·2 to 27·4)	16·8 (14·7 to 19)	10·3 (9·3 to 11·5)	9·48 (8·37 to 10·7)	7·49 (6·71 to 8·29)	6 (5·39 to 6·64)	5·2 (4·5 to 5·95)	5·56 (4·95 to 6·22)
Norfolk	24·8 (20·1 to 30·3)	24·9 (21·6 to 28·2)	19·7 (17·6 to 21·9)	13·4 (12·4 to 14·5)	7·85 (7·3 to 8·47)	5·61 (4·99 to 6·27)	4·64 (4·25 to 5·04)	3·58 (3·32 to 3·87)	3·13 (2·79 to 3·47)	3·52 (3·13 to 3·89)
Peterborough	27·2 (20·6 to 34·3)	25·5 (21·1 to 30)	21·4 (19 to 23·9)	14·2 (12·7 to 16)	9·06 (8·18 to 10)	8·01 (6·96 to 9·09)	6·41 (5·75 to 7·12)	5·22 (4·71 to 5·71)	4·15 (3·67 to 4·67)	4·49 (3·99 to 5·02)
Southend-on-Sea	34·4 (28 to 41·6)	28·4 (25 to 32·1)	23 (20·5 to 25·6)	14·6 (12·8 to 16·5)	8·26 (7·39 to 9·15)	5·56 (4·9 to 6·26)	4·41 (3·9 to 4·87)	3·66 (3·23 to 4·13)	3·09 (2·67 to 3·58)	3·45 (3·01 to 3·93)
Suffolk	23·4 (19·8 to 27·4)	23·1 (20·1 to 26·3)	18·1 (16·1 to 20·5)	12·1 (10·7 to 13·6)	7·18 (6·63 to 7·69)	4·94 (4·42 to 5·48)	4·26 (3·94 to 4·59)	3·38 (3·1 to 3·69)	2·89 (2·6 to 3·22)	3·29 (2·95 to 3·64)
Thurrock	21·2 (17·3 to 25·8)	25·1 (22·1 to 28·7)	20·7 (18·5 to 23·1)	13·6 (11·8 to 15·5)	8·06 (7·28 to 8·87)	5·93 (5·23 to 6·67)	4·72 (4·29 to 5·17)	4·24 (3·93 to 4·58)	3·42 (2·99 to 3·89)	3·83 (3·37 to 4·33)
Greater London	28·7 (26·1 to 31·5)	30·1 (28·9 to 31·4)	23·9 (22·9 to 24·9)	16·8 (15·9 to 17·8)	10·6 (10·2 to 11)	7·81 (7·26 to 8·35)	5·65 (5·38 to 5·94)	4·16 (3·99 to 4·35)	3·51 (3·26 to 3·77)	3·9 (3·49 to 4·34)
Barking and Dagenham	48 (40 to 56·5)	38·5 (33·3 to 43·6)	31 (28·9 to 33·3)	19·6 (17·3 to 22·2)	10·7 (10·1 to 11·4)	6·83 (5·98 to 7·69)	5·57 (5·02 to 6·18)	4·47 (4·16 to 4·81)	3·66 (3·39 to 3·95)	3·98 (3·53 to 4·44)
Barnet	25 (20·9 to 30)	25·8 (24 to 27·9)	20·1 (18·6 to 21·7)	13·7 (12·7 to 14·9)	7·78 (7·3 to 8·33)	5·67 (5·13 to 6·28)	4·33 (4 to 4·68)	3·23 (2·98 to 3·51)	2·77 (2·49 to 3·06)	3·23 (2·89 to 3·58)
Bexley	24·3 (20·8 to 28·7)	21·1 (19·8 to 22·5)	16 (14·9 to 17·1)	11·1 (10·1 to 12·1)	6·97 (6·44 to 7·63)	5·37 (4·83 to 5·95)	4·33 (3·96 to 4·71)	3·68 (3·36 to 4·02)	3·59 (3·26 to 3·93)	4·1 (3·65 to 4·56)
Brent	31·6 (25·9 to 38·3)	33·4 (30·3 to 36·8)	26·5 (24·4 to 28·9)	18·4 (16·8 to 20·1)	12·6 (11·5 to 13·8)	10·2 (9·25 to 11·4)	6·93 (6·43 to 7·44)	4·76 (4·34 to 5·17)	3·92 (3·56 to 4·29)	4·41 (3·86 to 5)
Bromley	23·8 (20·2 to 28·4)	21·2 (18·1 to 24·4)	16·2 (15 to 17·5)	11·6 (10·1 to 13·3)	7·75 (7·22 to 8·29)	5·14 (4·64 to 5·7)	3·55 (3·23 to 3·86)	2·96 (2·7 to 3·24)	2·63 (2·42 to 2·85)	2·92 (2·62 to 3·25)
Camden	15·6 (13·5 to 18·2)	31 (29 to 33)	26·1 (24·2 to 28·2)	19·1 (17·5 to 20·8)	11·2 (10·2 to 12·2)	7·26 (6·52 to 8·12)	4·9 (4·49 to 5·34)	3·3 (3 to 3·62)	2·88 (2·57 to 3·2)	3·14 (2·72 to 3·59)
Croydon	30·2 (25·5 to 35·5)	26·8 (24·9 to 28·7)	20·7 (19·3 to 22·1)	14·6 (13·5 to 15·8)	10·5 (9·76 to 11·3)	7·92 (7·15 to 8·78)	5·64 (5·15 to 6·19)	4·56 (4·2 to 4·94)	3·77 (3·43 to 4·13)	4·08 (3·62 to 4·55)
Ealing	28 (23·6 to 33·1)	27·7 (25·8 to 29·7)	21·5 (19·9 to 23)	14·8 (13·6 to 16·2)	9·76 (9·16 to 10·4)	7·11 (6·47 to 7·84)	5·17 (4·76 to 5·65)	3·65 (3·34 to 3·97)	3·25 (3 to 3·5)	3·61 (3·22 to 4·03)
Enfield	30·8 (26 to 36·6)	26·9 (25·2 to 28·7)	20·4 (18·9 to 21·9)	14·3 (13 to 15·7)	8·95 (8·35 to 9·6)	7·47 (6·63 to 8·37)	6·01 (5·38 to 6·72)	4·3 (3·97 to 4·63)	3·55 (3·2 to 3·91)	4·21 (3·73 to 4·7)
Greenwich	41·6 (34·9 to 49·1)	37·8 (35·1 to 40·5)	29·9 (27·6 to 32·2)	20·2 (18·4 to 22·1)	11·1 (10·1 to 12·2)	7·54 (6·84 to 8·28)	5·99 (5·51 to 6·47)	5·05 (4·63 to 5·53)	4·46 (4·12 to 4·81)	5·07 (4·51 to 5·65)
Hackney	36·1 (30·9 to 42·4)	34·2 (31·8 to 36·5)	28·3 (26·3 to 30·5)	20·3 (18·6 to 22)	13·6 (12·8 to 14·5)	10·8 (9·8 to 11·9)	7·4 (6·85 to 7·97)	5·38 (5·02 to 5·76)	4·36 (4·01 to 4·7)	4·31 (3·73 to 4·93)
Hammersmith and Fulham	17·8 (14·9 to 21)	28·3 (26·6 to 30·2)	22·2 (20·5 to 24·1)	17·1 (15·6 to 18·8)	12·5 (11·1 to 14)	7·81 (7·06 to 8·67)	5·13 (4·66 to 5·65)	3·78 (3·44 to 4·16)	3·05 (2·78 to 3·32)	3·43 (2·99 to 3·86)
Haringey	25·8 (21·9 to 30·5)	31·3 (29 to 33·7)	24·4 (22·6 to 26·4)	18·2 (16·4 to 20·1)	12·3 (11·2 to 13·4)	10·3 (9·21 to 11·3)	6·64 (6·14 to 7·18)	4·26 (3·9 to 4·62)	3·55 (3·28 to 3·84)	3·94 (3·51 to 4·41)
Harrow	26·9 (22·4 to 32·2)	23·1 (21·5 to 24·9)	17·4 (16·3 to 18·5)	12 (10·9 to 13·2)	8·87 (8·24 to 9·55)	7·99 (6·98 to 9·03)	6·6 (5·91 to 7·33)	5·06 (4·61 to 5·5)	4·15 (3·77 to 4·52)	4·69 (4·19 to 5·23)
Havering	26·5 (21·7 to 32·2)	23·2 (19·8 to 26·7)	18·4 (16·9 to 20)	12·6 (11 to 14·4)	7·62 (7·01 to 8·33)	5·37 (4·74 to 6·15)	4·25 (3·88 to 4·63)	3·08 (2·79 to 3·39)	2·64 (2·32 to 2·97)	3·11 (2·75 to 3·49)
Hillingdon	27·7 (23 to 33·2)	25·2 (23·3 to 27)	19·9 (18·5 to 21·4)	13·8 (12·8 to 15)	8·59 (7·93 to 9·35)	6·08 (5·39 to 6·81)	5·13 (4·64 to 5·65)	3·78 (3·48 to 4·08)	3·33 (3·02 to 3·67)	3·73 (3·33 to 4·17)
Hounslow	27·7 (22·7 to 33·2)	28·4 (24·1 to 32·7)	22 (20·4 to 23·9)	14·9 (13·1 to 16·8)	10·1 (9·16 to 11·2)	7·51 (6·75 to 8·33)	6·13 (5·47 to 6·84)	5 (4·57 to 5·43)	4·46 (4·08 to 4·87)	4·86 (4·33 to 5·42)
Islington	26·6 (22 to 31·8)	32·1 (27 to 37·4)	25·9 (23·9 to 28·3)	19·7 (17·1 to 22·6)	11·2 (10·3 to 12·2)	8·12 (7·26 to 9·04)	5·11 (4·68 to 5·56)	3·46 (3·22 to 3·73)	2·8 (2·59 to 3·02)	3·1 (2·77 to 3·45)
Kensington and Chelsea	10·3 (8·44 to 12·4)	34 (29 to 39·4)	30·2 (27·7 to 32·7)	21·5 (19·4 to 23·9)	11·3 (9·87 to 12·8)	6·22 (5·46 to 7·09)	4·62 (3·95 to 5·39)	3·88 (3·54 to 4·25)	3·13 (2·85 to 3·43)	3·49 (3·08 to 3·94)
Kingston upon Thames	21 (16·3 to 26·1)	21·2 (18 to 24·3)	15·6 (14·3 to 16·9)	11·4 (10·2 to 12·6)	7·7 (6·98 to 8·46)	4·77 (4·04 to 5·57)	4·24 (3·71 to 4·83)	4·19 (3·83 to 4·59)	3·3 (3·01 to 3·59)	3·7 (3·27 to 4·13)
Lambeth	35·4 (30·1 to 41·7)	38·3 (35·7 to 40·9)	31·3 (28·7 to 33·8)	24·2 (22·4 to 26·2)	13·1 (12·1 to 14·2)	10·1 (9·06 to 11·2)	7·2 (6·55 to 7·86)	5·09 (4·67 to 5·5)	4·15 (3·78 to 4·54)	4·82 (4·25 to 5·46)
Lewisham	35·1 (30·3 to 41)	32·3 (30·4 to 34·4)	25·6 (24 to 27·5)	18·7 (17·1 to 20·7)	11·6 (10·8 to 12·5)	9·23 (8·24 to 10·3)	6·19 (5·71 to 6·71)	4·46 (4·03 to 4·93)	4·18 (3·75 to 4·64)	4·67 (4·09 to 5·27)
Merton	32·4 (27·6 to 38)	29 (27·1 to 31)	22·2 (20·7 to 23·6)	15·5 (14·1 to 17)	9·56 (8·83 to 10·4)	5·88 (5·1 to 6·71)	4·99 (4·51 to 5·54)	3·51 (3·24 to 3·8)	3·05 (2·82 to 3·3)	3·44 (3·08 to 3·81)
Newham	40·9 (31·7 to 51·2)	36·7 (32·4 to 41·4)	29·7 (26·6 to 33·1)	20 (17·8 to 22·5)	12·6 (11·7 to 13·6)	10·5 (9·51 to 11·6)	7·4 (6·74 to 8·07)	5·06 (4·64 to 5·49)	4·31 (3·89 to 4·74)	4·47 (3·92 to 5·07)
Redbridge	26·2 (21·7 to 30·9)	26·1 (24·5 to 27·7)	20·1 (18·8 to 21·4)	13·5 (12·2 to 14·8)	9·26 (8·5 to 10)	6·86 (6 to 7·72)	5·09 (4·53 to 5·72)	3·77 (3·51 to 4·07)	3·23 (2·98 to 3·48)	3·49 (3·12 to 3·89)
Richmond upon Thames	21 (17·8 to 24·7)	22·1 (20·8 to 23·5)	16·6 (15·4 to 17·8)	12·5 (11·6 to 13·6)	7·63 (6·79 to 8·51)	4·72 (4·2 to 5·3)	3·58 (3·23 to 3·98)	2·83 (2·58 to 3·1)	2·63 (2·41 to 2·85)	2·96 (2·63 to 3·31)
Southwark	35·9 (29·4 to 43·7)	30·5 (26·1 to 35·1)	25 (23·1 to 27·2)	17·4 (15·3 to 19·7)	13·5 (12·5 to 14·5)	11 (9·86 to 12·2)	6·85 (6·33 to 7·43)	4·66 (4·28 to 5·05)	3·7 (3·35 to 4·06)	4·12 (3·64 to 4·64)
Sutton	29·8 (25·5 to 35·1)	25·7 (24 to 27·6)	19·4 (18 to 20·8)	13·4 (12·3 to 14·7)	8·21 (7·46 to 8·98)	5·57 (5·03 to 6·19)	4·61 (4·21 to 5·02)	3·98 (3·66 to 4·34)	3·27 (2·98 to 3·58)	3·85 (3·44 to 4·3)
Tower Hamlets	52·8 (44·3 to 62·4)	41·5 (38·1 to 45·2)	37·5 (34·4 to 40·8)	24·4 (22·1 to 26·9)	11·3 (10·2 to 12·5)	8·58 (7·76 to 9·58)	6·28 (5·69 to 6·88)	4·77 (4·35 to 5·21)	3·83 (3·46 to 4·21)	4 (3·56 to 4·48)
Waltham Forest	38·9 (31·8 to 46·9)	35·6 (32 to 39·5)	26·6 (24 to 29·3)	18·3 (16·3 to 20·6)	12·5 (11·4 to 13·7)	9·51 (8·46 to 10·6)	6·59 (6·08 to 7·16)	4·14 (3·81 to 4·48)	3·45 (3·12 to 3·81)	3·98 (3·54 to 4·46)
Wandsworth	26·7 (22·7 to 31·2)	31 (29 to 33·1)	24 (22·3 to 25·7)	18·3 (16·5 to 20·2)	11·6 (10·6 to 12·8)	7·11 (6·24 to 8·04)	4·79 (4·39 to 5·21)	3·45 (3·17 to 3·75)	2·7 (2·44 to 2·96)	3·01 (2·67 to 3·38)
Westminster	19·4 (16·1 to 23)	40·4 (33·7 to 47·5)	35·9 (32·6 to 39·4)	26·7 (23·4 to 30·7)	13·8 (12·6 to 15·1)	8·38 (7·6 to 9·29)	4·69 (4·29 to 5·1)	3·22 (2·93 to 3·53)	2·42 (2·16 to 2·69)	2·84 (2·49 to 3·22)
North East England	25·5 (20·4 to 30·9)	25·6 (21·9 to 29·8)	21·6 (20·1 to 23·3)	14·3 (13·3 to 15·4)	9·48 (8·97 to 9·98)	6·65 (6·12 to 7·26)	4·58 (4·3 to 4·85)	3·8 (3·4 to 4·25)	3·22 (2·78 to 3·68)	3·67 (3·19 to 4·17)
County Durham	24·6 (18·7 to 31·4)	24·8 (21·7 to 28)	20·7 (18·6 to 22·9)	13·7 (12·4 to 15·2)	8·64 (7·91 to 9·38)	6·8 (6·15 to 7·54)	4·65 (4·31 to 5·02)	3·87 (3·37 to 4·43)	3·44 (3 to 3·9)	3·92 (3·43 to 4·43)
Darlington	28·2 (21·6 to 36·3)	28·2 (22·5 to 34·3)	23·4 (21·1 to 25·7)	15·7 (13·6 to 17·9)	9·94 (8·85 to 11·1)	7·01 (5·81 to 8·52)	4·93 (4·16 to 5·86)	3·9 (3·41 to 4·43)	3·39 (2·88 to 3·99)	4·03 (3·43 to 4·65)
Gateshead	28·5 (21·6 to 36·5)	28·5 (24·7 to 33·1)	24·2 (22 to 26·7)	16·3 (14·6 to 18·2)	10·8 (9·7 to 12·1)	6·65 (5·72 to 7·68)	5·09 (4·49 to 5·72)	4·24 (3·74 to 4·84)	3·56 (3·05 to 4·12)	3·94 (3·39 to 4·51)
Hartlepool	31·1 (24·5 to 38·9)	31·6 (25·8 to 38·1)	27·2 (24·7 to 29·9)	18·7 (16·7 to 21)	10·8 (9·77 to 12)	7·29 (6·32 to 8·39)	4·95 (4·22 to 5·76)	3·43 (2·98 to 3·94)	2·6 (2·23 to 3)	3·04 (2·63 to 3·5)
Middlesbrough	31·2 (24·5 to 39·5)	29·5 (25·9 to 33·4)	25·7 (23·7 to 28)	17·1 (15·4 to 19·1)	11·4 (10·5 to 12·3)	8·28 (7·45 to 9·22)	5·65 (5·19 to 6·11)	4·41 (3·84 to 5·07)	3·79 (3·22 to 4·35)	4·29 (3·74 to 4·9)
Newcastle upon Tyne	20·3 (15·4 to 25·8)	23·2 (19·8 to 27·2)	19·9 (18 to 22)	13·3 (12 to 14·5)	9·87 (8·91 to 10·9)	6·59 (5·95 to 7·26)	4·57 (4·22 to 4·92)	4·16 (3·7 to 4·7)	3·4 (2·91 to 3·87)	3·89 (3·39 to 4·4)
North Tyneside	25 (19 to 32·1)	23·1 (18·3 to 29·4)	19·5 (17·7 to 21·5)	12·4 (10·9 to 14·2)	8·91 (8·01 to 9·91)	5·91 (5·26 to 6·58)	3·98 (3·6 to 4·37)	3·45 (3 to 3·96)	2·84 (2·43 to 3·29)	3·18 (2·72 to 3·66)
Northumberland	25·6 (19·8 to 33·2)	24·4 (19·4 to 30·6)	19·9 (17·7 to 22·3)	12·7 (11·2 to 14·2)	8·7 (7·84 to 9·6)	5·95 (5·15 to 6·79)	4·01 (3·62 to 4·41)	3·34 (2·93 to 3·79)	2·86 (2·41 to 3·35)	3·17 (2·74 to 3·63)
Redcar and Cleveland	25·7 (19·4 to 33·7)	26·6 (20·9 to 33·7)	21·7 (19·7 to 23·9)	15 (12·5 to 17·5)	9·36 (8·62 to 10·2)	6·09 (5·34 to 6·91)	4·26 (3·63 to 4·93)	3·57 (3·1 to 4·1)	2·64 (2·18 to 3·13)	3·15 (2·66 to 3·68)
South Tyneside	23·2 (17·8 to 29·5)	21·7 (17·6 to 26·3)	18·8 (17·1 to 20·7)	12·5 (11·2 to 14)	7·77 (7·04 to 8·58)	5·87 (5·12 to 6·72)	3·93 (3·34 to 4·56)	3·07 (2·66 to 3·54)	2·83 (2·37 to 3·32)	3·35 (2·83 to 3·9)

Stockton-on-Tees	25·4 (19·3 to 32·6)	25·2 (21·7 to 29·6)	21·3 (19·5 to 23·3)	14·5 (13 to 16·1)	9·49 (8·76 to 10·3)	6·58 (5·9 to 7·29)	4·8 (4·39 to 5·2)	4·15 (3·61 to 4·72)	3·63 (3·07 to 4·21)	4·28 (3·64 to 4·93)
Sunderland	26·1 (19·9 to 33·4)	26·4 (21·1 to 32·7)	22·2 (20 to 24·8)	14·5 (12·8 to 16·3)	9·88 (8·96 to 10·8)	7·1 (6·46 to 7·86)	4·5 (4·16 to 4·84)	3·57 (3·14 to 4·05)	2·96 (2·54 to 3·4)	3·29 (2·85 to 3·77)
North West England	27·8 (24·8 to 31)	27·8 (26·7 to 29·1)	22·4 (21·4 to 23·3)	15·6 (14·8 to 16·5)	9·9 (9·45 to 10·4)	7·36 (6·86 to 7·91)	5·72 (5·44 to 6·03)	5·2 (4·98 to 5·43)	4·36 (4·06 to 4·66)	4·93 (4·43 to 5·47)
Blackburn with Darwen	37·4 (29·5 to 46·1)	32·1 (27·9 to 36·5)	25·5 (23·5 to 27·6)	17 (15 to 19·2)	13·5 (12·4 to 14·7)	10·6 (9·49 to 11·8)	8·26 (7·62 to 8·92)	6·5 (5·89 to 7·17)	5·13 (4·69 to 5·59)	5·86 (5·26 to 6·44)
Blackpool	36·7 (29·9 to 45)	30·6 (28·3 to 33)	24·5 (22·7 to 26·4)	16·6 (15 to 18·2)	10·8 (9·7 to 12)	8·7 (7·68 to 9·84)	7·35 (6·65 to 8·1)	6·67 (6·11 to 7·31)	5·25 (4·8 to 5·76)	6·11 (5·53 to 6·74)
Bolton	31·9 (26·6 to 37·7)	31·3 (28·7 to 34·2)	24·7 (22·9 to 26·6)	16·9 (15·4 to 18·4)	11·1 (10·3 to 12·1)	7·78 (7·09 to 8·5)	6·26 (5·71 to 6·86)	5·69 (5·22 to 6·21)	4·63 (4·23 to 5·05)	5·35 (4·86 to 5·89)
Bury	27·3 (22·2 to 33·2)	29·3 (25·6 to 33·3)	22·8 (21·2 to 24·6)	16 (14·1 to 18·1)	9·97 (9·08 to 11)	6·29 (5·66 to 6·95)	5·01 (4·55 to 5·52)	4·9 (4·44 to 5·36)	4·29 (3·83 to 4·72)	4·86 (4·32 to 5·44)
Cheshire East	24·1 (20·5 to 28·2)	24·2 (22·6 to 25·9)	18·8 (17·6 to 20)	13·6 (12·6 to 14·8)	8·5 (7·78 to 9·34)	5·61 (5·12 to 6·11)	4·41 (4·07 to 4·79)	4·02 (3·7 to 4·36)	3·39 (3·1 to 3·67)	3·74 (3·38 to 4·12)
Cheshire West and Chester	21·2 (17·9 to 24·6)	21·4 (19·9 to 22·9)	16·8 (15·7 to 18)	12·4 (11·3 to 13·5)	8·1 (7·47 to 8·82)	5·57 (5·06 to 6·07)	5 (4·6 to 5·43)	4·16 (3·76 to 4·56)	3·43 (3·12 to 3·74)	3·8 (3·44 to 4·17)
Cumbria	28·1 (23·9 to 32·6)	27·4 (24 to 31)	21·8 (20·1 to 23·7)	15·4 (13·8 to 17·1)	9·15 (8·45 to 9·85)	5·8 (5·27 to 6·34)	4·47 (4·13 to 4·81)	3·96 (3·66 to 4·28)	3·43 (3·11 to 3·76)	3·84 (3·49 to 4·22)
Halton	23·7 (19·3 to 28·9)	25·9 (24 to 28)	20·6 (19 to 22·2)	13·7 (12·5 to 14·9)	9·7 (8·79 to 10·7)	7·7 (6·91 to 8·45)	5·44 (4·97 to 5·94)	4·26 (3·94 to 4·59)	3·64 (3·27 to 3·99)	4·1 (3·66 to 4·58)
Knowsley	27·5 (22·6 to 32·7)	27·2 (23·6 to 31·2)	24·1 (22·3 to 25·7)	17·5 (16 to 19·2)	8·9 (8·13 to 9·76)	7·12 (6·36 to 7·98)	4·97 (4·28 to 5·71)	4·57 (4·18 to 4·95)	3·79 (3·37 to 4·19)	4·24 (3·78 to 4·74)
Lancashire	29·2 (24·3 to 35·6)	28·1 (25·7 to 30·7)	21·9 (19·4 to 24·3)	15 (13·6 to 16·5)	10·3 (9·54 to 11·1)	7·38 (6·6 to 8·23)	6·04 (5·43 to 6·68)	5·21 (4·86 to 5·6)	4·2 (3·68 to 4·74)	4·65 (3·88 to 5·54)
Liverpool	24·2 (19·9 to 29·5)	26·7 (23·7 to 29·9)	22·7 (20·7 to 24·7)	15·8 (14·3 to 17·6)	8·89 (8·12 to 9·63)	7·84 (7·14 to 8·55)	6·09 (5·69 to 6·52)	5·87 (5·43 to 6·36)	5·02 (4·61 to 5·47)	5·89 (5·31 to 6·47)
Manchester	29·1 (24 to 35·2)	32·9 (28·4 to 37·4)	28·1 (25·5 to 31)	20·5 (18·1 to 23)	12·5 (11·4 to 13·8)	10·6 (9·59 to 11·7)	7·28 (6·81 to 7·8)	6·86 (6·38 to 7·36)	5·81 (5·05 to 6·61)	6·66 (5·45 to 7·97)
Oldham	34·3 (28·6 to 39·9)	33·4 (30·4 to 36·8)	26·6 (24·3 to 28·9)	18·1 (16·3 to 20·1)	11·6 (10·5 to 12·6)	8·89 (7·95 to 9·82)	7·57 (6·99 to 8·21)	7·17 (6·67 to 7·78)	6·4 (5·76 to 7·04)	7·07 (6·38 to 7·8)
Rochdale	35·6 (30·1 to 41)	33·9 (31·4 to 36·4)	27·5 (25·6 to 29·2)	18·5 (17 to 20·1)	11·5 (10·6 to 12·4)	8·41 (7·63 to 9·19)	6·14 (5·69 to 6·6)	5·98 (5·49 to 6·51)	4·86 (4·38 to 5·36)	5·65 (5·14 to 6·22)
Salford	29·9 (25 to 35·8)	30·9 (28 to 33·8)	25·3 (23 to 27·5)	18 (16·5 to 19·6)	10·3 (9·48 to 11·2)	7·88 (6·85 to 8·97)	6·02 (5·4 to 6·65)	5·33 (4·93 to 5·76)	4·53 (4·13 to 4·95)	5·27 (4·8 to 5·81)
Sefton	24·5 (20·8 to 28·4)	21·9 (20·4 to 23·6)	17·6 (16·1 to 19)	12·5 (11·4 to 13·6)	8·35 (7·63 to 9·19)	6·2 (5·66 to 6·82)	5·01 (4·61 to 5·44)	4·84 (4·43 to 5·27)	4·07 (3·69 to 4·44)	4·61 (4·16 to 5·12)
St Helens	23·8 (19·2 to 29)	24·7 (21·3 to 28·1)	19·7 (18·3 to 21·4)	14·1 (12·3 to 16·1)	9·05 (8·11 to 10·1)	7·47 (6·64 to 8·34)	4·73 (4·33 to 5·17)	4·05 (3·71 to 4·46)	3·55 (3·21 to 3·91)	3·83 (3·47 to 4·23)
Stockport	24 (20·5 to 27·7)	23 (21·5 to 24·6)	18·2 (17 to 19·3)	13 (11·9 to 14·1)	8·67 (7·94 to 9·49)	6·1 (5·57 to 6·65)	4·81 (4·44 to 5·24)	4·97 (4·59 to 5·4)	4·01 (3·65 to 4·36)	4·51 (4·09 to 4·97)
Tameside	31·1 (26·1 to 36·6)	31·4 (28·7 to 34·2)	24·9 (22·9 to 27)	16·9 (15·3 to 18·6)	9·44 (8·56 to 10·4)	6·66 (5·98 to 7·46)	5·18 (4·73 to 5·68)	4·39 (4·02 to 4·78)	3·74 (3·34 to 4·15)	4·21 (3·79 to 4·68)
Trafford	29 (24·9 to 33·6)	28·6 (26·6 to 30·7)	23·1 (21·6 to 24·6)	16·9 (15·6 to 18·4)	9·79 (8·9 to 10·7)	6·31 (5·68 to 6·97)	4·17 (3·78 to 4·6)	3·86 (3·5 to 4·23)	3·08 (2·75 to 3·41)	3·43 (3·06 to 3·8)
Warrington	21·6 (17·4 to 26·3)	22·6 (20·9 to 24·5)	17·6 (16·4 to 18·8)	12·4 (11·2 to 13·8)	9·53 (8·66 to 10·6)	6·73 (6·06 to 7·46)	5·18 (4·73 to 5·68)	4·58 (4·13 to 5·04)	3·8 (3·4 to 4·2)	4·16 (3·69 to 4·64)
Wigan	22·9 (19·4 to 26·6)	25·1 (23·3 to 27)	19·3 (18·1 to 20·6)	13·5 (12·4 to 14·7)	8·54 (7·87 to 9·25)	6·88 (6·21 to 7·54)	4·82 (4·42 to 5·24)	4·37 (4·04 to 4·7)	3·97 (3·62 to 4·33)	4·43 (3·98 to 4·89)
Wirral	30·4 (25·5 to 35·9)	27·8 (25·8 to 30)	22·2 (20·9 to 23·6)	15·2 (13·9 to 16·6)	9·38 (8·57 to 10·3)	6·73 (5·93 to 7·62)	5·49 (4·99 to 6·05)	4·85 (4·48 to 5·27)	4·06 (3·67 to 4·49)	4·75 (4·27 to 5·25)
South East England	21·9 (19·8 to 24·3)	22·4 (21·4 to 23·6)	17·4 (16·4 to 18·2)	12·8 (12·1 to 13·5)	8·55 (8·22 to 8·91)	5·33 (4·91 to 5·75)	4·25 (4 to 4·52)	3·81 (3·64 to 3·99)	3·28 (3 to 3·56)	3·62 (3·24 to 4·06)
Bracknell Forest	15·5 (12·1 to 19·4)	18 (15·3 to 20·9)	14·4 (13·2 to 15·7)	10·4 (9·01 to 11·8)	7·13 (6·25 to 8·07)	4·73 (4·03 to 5·51)	3·6 (3·08 to 4·2)	2·44 (2·17 to 2·73)	1·62 (1·41 to 1·86)	1·75 (1·53 to 2·01)
Brighton and Hove	25 (20·7 to 29·5)	25·4 (21·7 to 29·4)	19·9 (18 to 21·9)	15·9 (14·3 to 17·9)	10·3 (9·48 to 11·3)	7·15 (6·39 to 7·93)	5·34 (4·87 to 5·82)	3·88 (3·53 to 4·27)	3·08 (2·7 to 3·46)	3·22 (2·79 to 3·71)
Buckinghamshire	18·2 (15·1 to 21·5)	17·8 (16·5 to 19·1)	13·7 (12·8 to 14·7)	10·2 (9·35 to 11·1)	8·54 (7·91 to 9·19)	5·46 (4·93 to 6·05)	4·71 (4·23 to 5·23)	3·96 (3·67 to 4·25)	3·48 (3·05 to 3·93)	3·99 (3·53 to 4·5)
East Sussex	27·5 (23·1 to 32·1)	23·5 (21·7 to 25·3)	17·3 (16 to 18·7)	12·2 (11·1 to 13·3)	8·36 (7·72 to 9·02)	5·57 (5·06 to 6·12)	4·69 (4·18 to 5·23)	4·17 (3·87 to 4·48)	3·33 (3·01 to 3·65)	3·54 (3·15 to 3·96)
Hampshire	20·6 (17·4 to 24)	22·8 (21 to 24·8)	17·7 (16·3 to 19·2)	13·7 (12·6 to 14·9)	7·88 (7·29 to 8·5)	4·7 (4·21 to 5·25)	3·82 (3·5 to 4·13)	3·83 (3·53 to 4·17)	3·62 (3·17 to 4·09)	4·01 (3·35 to 4·73)
Isle of Wight	38·7 (31·5 to 47)	31 (26·6 to 35·6)	22·8 (20·9 to 25)	16·2 (14·1 to 18·5)	10·3 (9·1 to 11·7)	4·77 (4·08 to 5·51)	3·37 (2·88 to 3·92)	3·25 (2·9 to 3·6)	2·73 (2·33 to 3·14)	2·82 (2·46 to 3·24)
Kent	24·3 (20·8 to 28)	23·8 (22·3 to 25·3)	18 (16·2 to 20)	12·9 (11·8 to 14)	9·07 (8·5 to 9·72)	5·09 (4·56 to 5·66)	4·04 (3·78 to 4·3)	4 (3·76 to 4·27)	3·52 (3·09 to 3·96)	3·96 (3·3 to 4·71)
Medway	22·1 (17·9 to 27·4)	26·9 (24·6 to 29·5)	21 (19·3 to 22·7)	14·8 (13·3 to 16·2)	10·2 (9·33 to 11·2)	6·62 (5·92 to 7·3)	4·61 (4·24 to 5·03)	4·06 (3·67 to 4·48)	3·53 (3·16 to 3·93)	3·56 (3·13 to 4·02)
Milton Keynes	23·8 (19·1 to 29·2)	24·8 (20·9 to 28·7)	18·3 (16·7 to 20·2)	12·8 (11·2 to 14·4)	10·2 (9·21 to 11·3)	7·55 (6·78 to 8·48)	5·7 (5·12 to 6·36)	4·74 (4·28 to 5·24)	3·99 (3·51 to 4·5)	4·21 (3·67 to 4·79)
Oxfordshire	13 (10·5 to 15·4)	17·3 (15·9 to 18·7)	13·6 (12·7 to 14·5)	10·7 (9·72 to 11·6)	7·52 (6·94 to 8·13)	5·12 (4·66 to 5·66)	4·42 (4·04 to 4·86)	3·27 (3·05 to 3·51)	2·6 (2·31 to 2·9)	2·92 (2·6 to 3·25)
Portsmouth	23·5 (19 to 28·9)	28·4 (24·8 to 32)	22·7 (20·8 to 24·9)	16·5 (14·7 to 18·5)	10·9 (9·94 to 12)	6·89 (6·11 to 7·66)	4·06 (3·7 to 4·45)	3·44 (3·13 to 3·75)	2·59 (2·29 to 2·9)	2·72 (2·38 to 3·1)
Reading	16·5 (13·7 to 19·7)	22·3 (20·7 to 23·9)	17·8 (16·2 to 19·5)	14·2 (12·8 to 15·7)	10·1 (9·21 to 11)	8·3 (7·37 to 9·28)	5·77 (5·3 to 6·28)	4·48 (4·11 to 4·91)	3·9 (3·5 to 4·31)	4·21 (3·72 to 4·73)
Slough	28·8 (23·1 to 35·6)	28·2 (25·9 to 30·6)	23·7 (21·9 to 25·7)	15·9 (14·4 to 17·5)	10·9 (9·79 to 12)	7·37 (6·42 to 8·49)	6·23 (5·45 to 6·99)	5·27 (4·85 to 5·7)	4·77 (4·24 to 5·38)	5·13 (4·48 to 5·82)
Southampton	23 (18·7 to 28·2)	28·6 (26·2 to 31·1)	23·2 (21 to 25·4)	16·4 (14·7 to 18·2)	10·4 (9·57 to 11·3)	6·56 (5·74 to 7·46)	4·88 (4·46 to 5·36)	4·4 (4·05 to 4·78)	3·65 (3·17 to 4·17)	3·93 (3·47 to 4·46)
Surrey	22·8 (19·5 to 26·5)	21·1 (19·8 to 22·3)	16·5 (15·2 to 18)	12·8 (11·7 to 14)	7·64 (7·07 to 8·26)	4·44 (4·04 to 4·87)	3·48 (3·22 to 3·75)	3·32 (3·1 to 3·56)	2·84 (2·44 to 3·26)	3·2 (2·76 to 3·66)
West Berkshire	15·5 (12·2 to 19·3)	16·2 (13·5 to 19)	12·3 (11 to 13·7)	9·27 (8·03 to 10·7)	7·68 (6·85 to 8·61)	5·26 (4·59 to 5·96)	3·92 (3·53 to 4·35)	3·25 (2·94 to 3·58)	2·68 (2·34 to 3·06)	3·15 (2·77 to 3·57)
West Sussex	26·9 (21·3 to 33·4)	22·7 (18·7 to 26·7)	17·2 (15·4 to 19·2)	12·2 (10·9 to 13·7)	7·85 (7·28 to 8·5)	4·77 (4·28 to 5·24)	4·07 (3·78 to 4·39)	3·68 (3·42 to 3·95)	3·16 (2·83 to 3·49)	3·56 (3·18 to 3·96)
Windsor and Maidenhead	15·4 (12·6 to 18·5)	16·7 (14·3 to 19·2)	12·9 (11·7 to 14·2)	9·8 (8·92 to 10·8)	7·31 (6·56 to 8·07)	5·36 (4·69 to 6·12)	3·88 (3·37 to 4·5)	3·29 (2·97 to 3·64)	2·74 (2·37 to 3·15)	3·05 (2·64 to 3·49)
Wokingham	12·2 (9·57 to 15·1)	14·4 (12·1 to 16·8)	11 (9·97 to 12·1)	9·1 (7·9 to 10·5)	6·69 (5·97 to 7·52)	4·21 (3·61 to 4·87)	3·69 (3·19 to 4·28)	3·59 (3·25 to 3·98)	2·7 (2·33 to 3·08)	2·9 (2·53 to 3·31)
South West England	22·6 (18·8 to 27)	23·4 (21·8 to 25·1)	18·7 (17·2 to 20·5)	13·4 (12·5 to 14·4)	8·32 (7·88 to 8·73)	5·71 (5·26 to 6·22)	4·4 (4·16 to 4·64)	3·27 (3·08 to 3·45)	2·86 (2·5 to 3·22)	3·19 (2·8 to 3·6)
Bath and North East Somerset	21·2 (16·2 to 27·7)	21·4 (17·7 to 25·4)	17·2 (15·2 to 19·5)	13·5 (11·4 to 15·8)	7·66 (6·89 to 8·53)	4·78 (4·2 to 5·42)	3·17 (2·67 to 3·73)	2·46 (2·22 to 2·69)	2·22 (1·89 to 2·55)	2·47 (2·12 to 2·8)
Bournemouth	22·5 (17·1 to 29·8)	21·9 (19·9 to 24·2)	17·8 (16 to 19·8)	12·6 (11·2 to 14·2)	7·58 (6·69 to 8·6)	6·01 (5·31 to 6·79)	4·13 (3·74 to 4·52)	3·25 (2·84 to 3·69)	2·06 (1·69 to 2·44)	2·13 (1·7 to 2·54)
Bristol, City of	21·5 (17·1 to 26·7)	27·3 (25·2 to 29·6)	22·9 (20·8 to 25·4)	16·7 (15·2 to 18·2)	9·95 (9·11 to 10·8)	6·78 (6·13 to 7·5)	4·48 (4·18 to 4·78)	3·26 (2·98 to 3·57)	2·84 (2·5 to 3·22)	3·18 (2·8 to 3·56)
Cornwall	25·4 (20·2 to 31·6)	23·9 (22·1 to 26)	18·7 (16·9 to 20·5)	12·6 (11·5 to 13·8)	8·13 (7·45 to 8·85)	5·67 (5·1 to 6·23)	5·03 (4·64 to 5·46)	3·68 (3·41 to 3·99)	3·33 (2·91 to 3·75)	3·71 (3·26 to 4·17)
Devon	23·6 (19 to 29·1)	23·3 (21·3 to 25·5)	18·1 (16·4 to 20·2)	13 (11·9 to 14·2)	8·73 (8·01 to 9·49)	5·41 (4·78 to 6·08)	4·44 (3·92 to 5·01)	3·42 (3·17 to 3·68)	3·01 (2·57 to 3·46)	3·34 (2·92 to 3·77)
Dorset	23·6 (18·8 to 29·2)	22·1 (20 to 24·4)	17·2 (15·4 to 19·1)	12·4 (11·1 to 13·9)	7·28 (6·7 to 7·94)	5·22 (4·63 to 5·85)	4·28 (3·98 to 4·58)	3·06 (2·81 to 3·32)	2·75 (2·37 to 3·14)	3·03 (2·61 to 3·43)
Gloucestershire	21·6 (17·2 to 26·5)	22·2 (20·5 to 23·9)	17·8 (16·2 to 19·6)	12·9 (11·7 to 14·1)	8·22 (7·61 to 8·85)	5·62 (5·07 to 6·25)	4·25 (3·97 to 4·54)	3·19 (2·95 to 3·46)	2·89 (2·49 to 3·29)	3·32 (2·9 to 3·77)
North Somerset	24·3 (18 to 31·3)	23·3 (21·7 to 25·1)	18·4 (16·5 to 20·4)	13·2 (12 to 14·7)	7·43 (6·58 to 8·39)	5·78 (5·15 to 6·49)	4·28 (3·89 to 4·7)	3·08 (2·75 to 3·43)	2·42 (2·07 to 2·8)	2·74 (2·37 to 3·12)
Plymouth	24·4 (19·5 to 29·6)	26·5 (24·4 to 28·8)	21·9 (19·4 to 24·6)	15·5 (13·9 to 17·1)	9·06 (8·23 to 9·95)	7·03 (6·13 to 8·01)	5·35 (4·88 to 5·88)	3·72 (3·36 to 4·1)	3·24 (2·79 to 3·7)	3·54 (3·1 to 4)
Poole	23·5 (17·9 to 29·8)	23 (19·2 to 27)	18·1 (16·3 to 20)	12·8 (11·6 to 14·2)	8·05 (7·12 to 9·17)	5·9 (5·14 to 6·82)	4·13 (3·57 to 4·8)	2·99 (2·57 to 3·42)	1·81 (1·55 to 2·1)	1·86 (1·55 to 2·17)
Somerset	21·6 (16·5 to 27·1)	21 (19 to 22·9)	16·6 (14·8 to 18·6)	11·8 (10·8 to 12·9)	7·11 (6·51 to 7·76)	5·28 (4·76 to 5·82)	4·44 (4·15 to 4·73)	3·46 (3·25 to 3·69)	3·25 (2·79 to 3·71)	3·61 (3·15 to 4·08)
South Gloucestershire	18 (13·9 to 23·1)	19·8 (18·2 to 21·4)	15·6 (14·1 to 17·2)	11·4 (10·2 to 12·7)	7·77 (7·11 to 8·53)	5·13 (4·59 to 5·72)	3·43 (3·17 to 3·7)	2·78 (2·58 to 3·02)	2·56 (2·17 to 2·91)	2·86 (2·48 to 3·25)
Swindon	28·8 (21·5 to 37·6)	29 (24·1 to 34·6)	24·5 (21·8 to 27·5)	17·7 (15·1 to 20·6)	9·52 (8·65 to 10·4)	6·45 (5·77 to 7·22)	4·33 (4 to 4·68)	3·1 (2·86 to 3·37)	2·6 (2·23 to 2·96)	2·83 (2·43 to 3·22)
Torbay	25·9 (21·2 to 31·1)	23·1 (19·1 to 27·3)	17·8 (15·9 to 19·8)	11·9 (10·7 to 13·3)	8·44 (7·57 to 9·36)	6·06 (5·09 to 7·17)	5·4 (4·62 to 6·2)	3·67 (3·29 to 4·11)	2·85 (2·43 to 3·3)	3·19 (2·77 to 3·65)
Wiltshire	19·3 (15·7 to 23·7)	22·6 (20·6 to 24·8)	17·9 (16·2 to 19·7)	13·1 (11·8 to 14·3)	8·59 (7·84 to 9·42)	5·38 (4·68 to 6·11)	4·3 (3·92 to 4·68)	3·16 (2·88 to 3·49)	3·1 (2·65 to 3·57)	3·59 (3·11 to 4·1)
West Midlands	27·5 (24·9 to 30·4)	28·6 (27·4 to 29·9)	23·6 (22·5 to 24·6)	16·2 (15·3 to 17·2)	11·7 (10·9 to 12·4)	8·07 (7·48 to 8·69)	6·62 (6·3 to 6·99)	6 (5·7 to 6·33)	5·14 (4·77 to 5·51)	5·82 (5·2 to 6·46)
Birmingham	33·4 (30 to 37)	33·5 (31·9 to 35·3)	28·7 (26·5 to 31)	19·4 (17·8 to 21·1)	14·4 (13·3 to 15·7)	10·9 (9·95 to 12)	8·61 (7·91 to 9·31)	7·99 (7·21 to 8·87)	6·58 (5·91 to 7·23)	7·46 (6·34 to 8·61)

Coventry	29·6 (24·1 to 35·5)	32·3 (30 to 34·7)	27·4 (24·9 to 30·1)	19·9 (18 to 21·8)	13·7 (12·5 to 14·8)	8·61 (7·78 to 9·49)	6·07 (5·49 to 6·76)	5·09 (4·65 to 5·58)	4·54 (4·09 to 5)	5·19 (4·69 to 5·73)
Dudley	23·7 (19·8 to 28·2)	24·9 (23 to 26·8)	20 (18·5 to 21·4)	13·3 (12·1 to 14·5)	11·7 (10·6 to 12·8)	6·97 (6·16 to 7·88)	5·46 (4·95 to 6·04)	4·84 (4·37 to 5·37)	4·17 (3·76 to 4·61)	4·69 (4·22 to 5·18)
Herefordshire, County of	28·8 (23·4 to 34·7)	26·8 (22·8 to 31)	21·6 (20 to 23·3)	15·2 (13·4 to 17·3)	8·58 (7·57 to 9·65)	4·9 (4·25 to 5·68)	4·46 (3·86 to 5·09)	4·66 (4·14 to 5·18)	3·9 (3·47 to 4·36)	4·46 (3·97 to 4·95)
Sandwell	39·2 (32·9 to 46·8)	37·2 (34·6 to 39·9)	31·5 (29·3 to 33·8)	21·7 (19·9 to 23·5)	13·2 (11·9 to 14·7)	9·35 (8·39 to 10·4)	8·01 (7·31 to 8·79)	6·92 (6·37 to 7·48)	5·76 (5·21 to 6·34)	6·84 (6·15 to 7·58)
Shropshire	21·4 (17·5 to 25·5)	22·6 (19·1 to 26·5)	18 (16·7 to 19·5)	12·8 (11·1 to 14·6)	8·98 (8·1 to 9·95)	5·62 (5·03 to 6·28)	4·31 (3·9 to 4·75)	3·82 (3·42 to 4·27)	3·44 (3·05 to 3·85)	3·88 (3·41 to 4·38)
Solihull	21·1 (17·3 to 25·4)	23·6 (21·9 to 25·4)	19 (17·4 to 20·7)	14·8 (13·4 to 16·4)	9·38 (8·39 to 10·4)	5·62 (4·91 to 6·37)	4·73 (4·21 to 5·27)	4·49 (4·05 to 4·97)	4·12 (3·71 to 4·56)	4·6 (4·14 to 5·09)
Staffordshire	21·9 (18·2 to 25·9)	24·8 (21·6 to 28)	20 (18·2 to 22)	14·1 (12·8 to 15·6)	9·74 (8·78 to 10·8)	6·54 (5·9 to 7·24)	5·72 (5·27 to 6·2)	5·23 (4·78 to 5·71)	4·74 (4·2 to 5·33)	5·3 (4·45 to 6·24)
Stoke-on-Trent	35·4 (29 to 42·2)	35·4 (32·8 to 38·1)	30·6 (28·5 to 32·7)	20·2 (18·4 to 22·1)	12·9 (11·7 to 14·4)	10·1 (9·08 to 11·2)	8·38 (7·62 to 9·16)	8·05 (7·23 to 8·91)	7·05 (6·36 to 7·73)	7·83 (6·99 to 8·7)
Telford and Wrekin	24·5 (19·8 to 30)	28·1 (26·1 to 30·2)	22·5 (21 to 24·1)	15·3 (14 to 16·8)	11·9 (10·6 to 13·3)	7·48 (6·65 to 8·39)	5·96 (5·33 to 6·61)	4·91 (4·37 to 5·46)	4·1 (3·7 to 4·53)	4·6 (4·12 to 5·08)
Walsall	28·8 (24·1 to 34·1)	29·4 (27·5 to 31·7)	24·7 (22·9 to 26·6)	17·1 (15·5 to 18·8)	11·5 (10·3 to 12·8)	8·63 (7·68 to 9·66)	8·39 (7·6 to 9·19)	7·23 (6·6 to 7·92)	5·83 (5·14 to 6·52)	6·71 (6·01 to 7·41)
Warwickshire	19·7 (16·3 to 23·4)	21·3 (19·8 to 22·9)	17 (15·9 to 18·2)	12·1 (11 to 13·2)	9·42 (8·56 to 10·3)	5·74 (5·16 to 6·35)	4·76 (4·31 to 5·29)	4·29 (3·92 to 4·71)	3·9 (3·5 to 4·34)	4·43 (3·99 to 4·9)
Wolverhampton	29·5 (24·5 to 35·4)	28·6 (26·3 to 31·1)	23·9 (22·1 to 25·8)	16·4 (14·8 to 18)	12·8 (11·5 to 14·3)	9·37 (8·46 to 10·4)	7·23 (6·56 to 7·95)	6·05 (5·42 to 6·77)	5·16 (4·64 to 5·69)	5·78 (5·11 to 6·46)
Worcestershire	20·2 (16·9 to 24·1)	21·9 (20·2 to 23·4)	17·3 (16 to 18·6)	11·7 (10·7 to 12·7)	9·43 (8·62 to 10·2)	6·35 (5·77 to 6·93)	4·85 (4·45 to 5·29)	4·65 (4·26 to 5·07)	4·13 (3·75 to 4·5)	4·58 (4·11 to 5·04)
Yorkshire and the Humber	29·9 (26 to 33·9)	29·3 (27·3 to 31·3)	23·2 (22 to 24·4)	16·1 (15·1 to 17·2)	10·4 (9·88 to 10·9)	8·01 (7·42 to 8·62)	6·02 (5·66 to 6·38)	4·63 (4·36 to 4·93)	3·93 (3·6 to 4·26)	4·47 (3·99 to 4·97)
Barnsley	31 (26 to 36·7)	29·5 (26·8 to 32·8)	23·3 (21·5 to 25·2)	15·9 (14·1 to 17·7)	10·2 (9·23 to 11·2)	7·37 (6·64 to 8·15)	5·22 (4·76 to 5·72)	3·83 (3·43 to 4·26)	3·45 (3·04 to 3·88)	4·1 (3·62 to 4·62)
Bradford	40·6 (32·6 to 50·3)	36·6 (32·4 to 41·1)	29·7 (27·2 to 32·4)	19·4 (17·4 to 21·8)	13·5 (12·2 to 14·7)	11·7 (10·5 to 13)	8·87 (8·11 to 9·62)	6·72 (6·21 to 7·28)	5·81 (5·05 to 6·57)	6·57 (5·46 to 7·79)
Calderdale	37·2 (31·2 to 44·5)	32·3 (29 to 35·8)	25 (22·9 to 26·8)	17·2 (15·4 to 19·1)	12·4 (11·3 to 13·6)	8·95 (8·15 to 9·77)	7·1 (6·51 to 7·72)	5·14 (4·67 to 5·67)	4·18 (3·74 to 4·66)	4·66 (4·14 to 5·17)
Doncaster	27·9 (23·2 to 33·1)	27 (24·1 to 30·2)	22·1 (20·3 to 23·7)	14·6 (13·2 to 16·2)	9·78 (8·94 to 10·6)	7·96 (7·22 to 8·72)	6·21 (5·76 to 6·72)	4·7 (4·27 to 5·14)	4·18 (3·77 to 4·6)	4·87 (4·37 to 5·38)
East Riding of Yorkshire	25·3 (20·1 to 31·2)	23·1 (20·2 to 26·4)	17·8 (16·4 to 19·2)	12·2 (10·8 to 13·8)	8·07 (7·41 to 8·77)	5·97 (5·46 to 6·51)	4·24 (3·92 to 4·61)	3·14 (2·82 to 3·44)	2·8 (2·48 to 3·17)	3·19 (2·82 to 3·57)
Kingston upon Hull, City of	30·3 (25·7 to 36·2)	32·6 (29·1 to 36)	26·7 (24·5 to 28·7)	18 (16·2 to 19·9)	11·1 (10·3 to 11·9)	8·04 (7·3 to 8·81)	5·75 (5·35 to 6·21)	4·67 (4·21 to 5·16)	3·97 (3·53 to 4·45)	4·53 (4·02 to 5·04)
Kirklees	35·2 (29·9 to 40·8)	32·5 (29·2 to 36·4)	25·4 (23·2 to 27·6)	17·7 (16 to 19·7)	10·9 (10 to 11·7)	8·78 (8·07 to 9·59)	7·07 (6·44 to 7·74)	5·91 (5·45 to 6·4)	4·97 (4·53 to 5·44)	5·55 (5·02 to 6·11)
Leeds	28·1 (22·4 to 35)	29·3 (25·7 to 33·1)	23·3 (21·3 to 25·5)	16·5 (14·9 to 18·3)	10·4 (9·57 to 11·3)	7·64 (6·99 to 8·31)	5·47 (4·96 to 6)	4·48 (4·11 to 4·87)	3·92 (3·4 to 4·49)	4·46 (3·67 to 5·32)
North East Lincolnshire	27·5 (21·9 to 34·2)	26·6 (23·3 to 30·6)	21·7 (20·2 to 23·4)	15·4 (13·6 to 17·4)	8·58 (7·83 to 9·44)	7 (6·31 to 7·72)	5·78 (5·33 to 6·28)	4·75 (4·29 to 5·2)	3·86 (3·42 to 4·35)	4·36 (3·84 to 4·88)
North Lincolnshire	26·7 (20·4 to 34·5)	25·9 (22·8 to 29·3)	20·6 (19 to 22·2)	14·1 (12·7 to 15·6)	10 (9·05 to 11·1)	7·3 (6·53 to 8·22)	4·95 (4·47 to 5·44)	3·78 (3·42 to 4·19)	3·02 (2·66 to 3·41)	3·38 (2·96 to 3·8)
North Yorkshire	26·8 (22·5 to 31·6)	25·5 (23·3 to 27·8)	19·6 (18 to 21·3)	14 (12·7 to 15·6)	8·02 (7·38 to 8·67)	5·23 (4·77 to 5·73)	4·11 (3·85 to 4·37)	3·08 (2·83 to 3·32)	2·69 (2·43 to 2·98)	3·01 (2·7 to 3·32)
Rotherham	28·3 (23·7 to 33·7)	28·7 (25·9 to 31·6)	22·7 (20·9 to 24·3)	15·5 (13·9 to 17·1)	10·5 (9·75 to 11·4)	7·8 (7·1 to 8·52)	6·04 (5·6 to 6·5)	4·59 (4·17 to 5·05)	3·55 (3·16 to 3·95)	4·08 (3·65 to 4·52)
Sheffield	25 (21 to 29·9)	26·2 (23·3 to 29·2)	20·2 (18 to 22·5)	14·7 (13·2 to 16·3)	10·3 (9·4 to 11·3)	8·31 (7·5 to 9·16)	5·84 (5·32 to 6·39)	4·5 (4·16 to 4·86)	3·47 (3·15 to 3·82)	3·99 (3·57 to 4·39)
Wakefield	31·5 (26·3 to 37·5)	31 (27·7 to 34·3)	24·9 (22·9 to 27)	17·3 (15·7 to 19)	9·89 (9·13 to 10·7)	7·72 (7·07 to 8·43)	5·72 (5·21 to 6·22)	3·96 (3·63 to 4·31)	3·46 (3·14 to 3·82)	4·07 (3·7 to 4·49)
York	22·6 (17·4 to 28·4)	24·6 (21·9 to 27·7)	19·1 (17·6 to 20·7)	13·9 (12·4 to 15·5)	8·91 (8·09 to 9·8)	6·81 (6·13 to 7·6)	4·81 (4·41 to 5·24)	3·57 (3·18 to 3·96)	2·88 (2·52 to 3·27)	3·21 (2·77 to 3·64)
Northern Ireland	38·4 (34·8 to 42·6)	31·2 (30 to 32·5)	25·8 (24·7 to 26·8)	17·4 (16·3 to 18·5)	9·23 (8·6 to 9·91)	6·39 (5·82 to 7·04)	5·6 (5·12 to 6·11)	4·92 (4·36 to 5·51)	4·17 (3·69 to 4·7)	3·57 (2·84 to 4·3)
Scotland	34·1 (31·7 to 36·6)	30·5 (29·6 to 31·6)	22·5 (21·7 to 23·4)	14·9 (14·1 to 15·7)	9·46 (8·86 to 10·1)	6·39 (5·86 to 6·94)	4·46 (4·11 to 4·81)	3·94 (3·57 to 4·36)	3·44 (3·12 to 3·78)	4·27 (3·81 to 4·72)
Wales	26·5 (19 to 35·5)	28 (23·2 to 32·7)	22·9 (19·5 to 27·2)	15·8 (14·5 to 17·3)	8·79 (8·25 to 9·35)	6·3 (5·78 to 6·85)	4·75 (4·44 to 5·09)	4·05 (3·68 to 4·41)	3·84 (3·4 to 4·31)	4·04 (3·42 to 4·69)
Latin America and Caribbean	220 (198 to 246)	156 (146 to 168)	118 (111 to 126)	80·5 (76·2 to 85)	51·8 (49 to 54·6)	34·2 (31·8 to 36·6)	31 (28·3 to 33·8)	18·6 (15·4 to 22)	17·2 (14·3 to 20·6)	16·5 (13·4 to 20·2)
Andean Latin America	338 (302 to 376)	266 (247 to 286)	201 (191 to 212)	120 (113 to 127)	73·5 (68·8 to 78·7)	45·2 (41·9 to 48·6)	27·9 (24·5 to 31·5)	20·3 (16·9 to 24·3)	17·6 (14·4 to 21·2)	16·7 (13·1 to 20·8)
Bolivia	375 (326 to 427)	303 (273 to 335)	231 (213 to 248)	159 (149 to 169)	109 (103 to 117)	71·2 (65·5 to 77)	44·8 (39·9 to 50)	30·8 (26 to 36·1)	29 (24·3 to 33·9)	27·9 (23·5 to 32·7)
Ecuador	217 (181 to 256)	167 (155 to 180)	126 (117 to 136)	81·6 (74·9 to 89·1)	42 (38·6 to 46)	33·6 (29·2 to 38·4)	22·4 (18·4 to 26·9)	16·7 (13·3 to 20·5)	14·9 (11·8 to 18·7)	13·7 (10·5 to 17·9)
Peru	367 (314 to 428)	293 (267 to 318)	222 (207 to 236)	122 (112 to 133)	75 (68·4 to 82·6)	41·5 (37 to 45·6)	24·1 (20·7 to 28)	18·3 (14·4 to 22·7)	14·7 (11·6 to 18·6)	14 (9·55 to 19·1)
Caribbean	178 (157 to 201)	134 (126 to 142)	108 (102 to 114)	91·1 (84·8 to 98·1)	70 (64·8 to 75·7)	51·4 (47·3 to 55·8)	123 (115 to 132)	41·3 (34·6 to 48·7)	40·6 (33·8 to 48·3)	40·8 (33·9 to 48·8)
Antigua and Barbuda	122 (99 to 151)	73·7 (62·8 to 86·7)	38·5 (32·3 to 45·6)	22 (18·6 to 26)	13·4 (11·1 to 16)	13·8 (11 to 17·1)	11·9 (9·9 to 14·1)	10·7 (8·8 to 12·8)	10·2 (8·28 to 12·4)	9·31 (7·98 to 10·7)
The Bahamas	117 (94·4 to 146)	75·3 (64·6 to 88)	44·5 (38 to 52·5)	31·7 (26·8 to 37·1)	22 (18·3 to 26·2)	16 (13·4 to 19·2)	13·3 (11·1 to 15·8)	20·5 (17·5 to 24·6)	10·5 (7·8 to 14)	10·2 (7·81 to 13·5)
Barbados	228 (186 to 276)	82 (69·8 to 96·1)	46·2 (38·9 to 54)	24·9 (21·1 to 29·8)	18·1 (15·2 to 21·2)	14·5 (12·2 to 17·3)	14 (11·8 to 16·7)	12·1 (8·97 to 15·9)	11·9 (8·51 to 16·2)	11·7 (8·25 to 16·3)
Belize	175 (144 to 212)	120 (105 to 138)	93·6 (82·6 to 106)	65·5 (59 to 72·4)	44·6 (40 to 49·5)	29·9 (26·2 to 33·6)	19·7 (17·2 to 22·6)	15·9 (13·2 to 19·1)	15·8 (13·1 to 19)	14·4 (11·9 to 17·5)
Bermuda	71 (58·2 to 86·1)	41·5 (37·8 to 45·5)	27·4 (24·6 to 30·8)	17·1 (15·1 to 19·3)	10·3 (8·83 to 11·8)	5·59 (4·72 to 6·63)	4·96 (4·2 to 5·84)	3·85 (3·1 to 4·73)	3·76 (2·94 to 4·7)	3·78 (3·23 to 4·51)
Cuba	57·8 (38·2 to 84·3)	47·6 (46·3 to 48·9)	43·4 (42·2 to 44·7)	22·9 (22 to 23·8)	13·7 (13·1 to 14·4)	8·47 (8·02 to 8·91)	5·9 (5·57 to 6·31)	6·26 (5·85 to 6·69)	6·1 (5·64 to 6·57)	4·56 (3·9 to 5·28)
Dominica	254 (210 to 302)	139 (121 to 160)	64·4 (54·8 to 75·3)	23·9 (20·1 to 28·3)	18·8 (15·8 to 22)	18·8 (15·6 to 22·3)	23·2 (19·4 to 27·5)	27·9 (21·2 to 35·9)	28 (21·1 to 36·8)	27·6 (20·2 to 37·1)
Dominican Republic	211 (173 to 253)	176 (162 to 191)	131 (119 to 143)	89·2 (80·1 to 99)	65·9 (59·1 to 72·8)	41·2 (36·5 to 45·9)	33·2 (28 to 38·8)	26 (20·9 to 32·3)	25·4 (20·5 to 31·3)	24·9 (20·2 to 30·1)
Grenada	156 (125 to 190)	97·7 (83·6 to 113)	60·3 (50·6 to 70·9)	37·5 (31·2 to 45·6)	23·3 (19·1 to 28·4)	16·8 (13·4 to 20·6)	14·8 (11·8 to 18·7)	13·8 (10·9 to 17·1)	13·5 (10·6 to 16·8)	12·6 (10·1 to 15·6)
Guyana	176 (147 to 210)	109 (98·4 to 121)	85·5 (76·5 to 95·9)	63·6 (55·6 to 72)	54·6 (47·7 to 62·3)	39·5 (35 to 44·1)	32 (27·5 to 37·2)	25·5 (19·6 to 32·4)	23·8 (17·9 to 30·7)	22·7 (17 to 29·7)
Haiti	368 (316 to 423)	263 (240 to 285)	236 (219 to 254)	197 (181 to 214)	151 (139 to 164)	105 (95·6 to 116)	278 (261 to 298)	71·6 (60 to 84·3)	69·8 (58 to 82·7)	70·6 (59·2 to 84·1)
Jamaica	140 (116 to 168)	86·8 (78·5 to 95·1)	56·5 (50·5 to 63·1)	37·8 (33·6 to 42·7)	28·9 (25·2 to 32·8)	21·8 (17·5 to 26·8)	18·6 (15·7 to 22·2)	16·2 (12·4 to 20·6)	15·7 (11·7 to 20·6)	15 (11 to 20·1)
Puerto Rico	104 (97·1 to 111)	56·2 (54·6 to 58)	32·8 (31·5 to 34)	21·4 (20·4 to 22·4)	15·5 (14·7 to 16·3)	11·2 (10·5 to 12)	8·96 (7·99 to 9·99)	7·18 (6·46 to 7·96)	7·89 (7·11 to 8·77)	6·43 (5·36 to 7·72)
Saint Kitts and Nevis	153 (137 to 170)	138 (132 to 144)	70·1 (64·9 to 75·7)	56·2 (50·3 to 62·7)	31·9 (28·7 to 35·5)	22·4 (19·5 to 25·5)	18·9 (16 to 22·5)	17·3 (14·1 to 21·2)	16·3 (13 to 20·4)	15·9 (12·5 to 20·4)
Saint Lucia	191 (157 to 231)	143 (123 to 164)	76·7 (65·9 to 89·2)	38·4 (32·4 to 45·2)	23·8 (19·8 to 28·3)	18·9 (15·6 to 22·7)	19·3 (15·5 to 23·7)	16·6 (12·3 to 22)	15·8 (11·6 to 21·3)	15·6 (11·2 to 21·2)
Saint Vincent and the Grenadines	208 (171 to 249)	148 (128 to 169)	92 (78 to 108)	55·9 (46·9 to 66·4)	31·5 (25·6 to 38)	24·9 (20·7 to 30)	19·6 (16·5 to 23·3)	14·2 (11·2 to 17·8)	13·3 (10·3 to 17·2)	13 (9·64 to 17·2)
Suriname	96·3 (77 to 119)	81 (68·5 to 94·4)	67·6 (56·3 to 80·4)	66·4 (58·4 to 74·8)	51·4 (45·2 to 58·4)	40 (34·1 to 46·8)	30·3 (25·6 to 35·4)	27·1 (21·4 to 34·1)	25·4 (19·8 to 32·5)	24·8 (18·9 to 32)
Trinidad and Tobago	95·4 (76·7 to 117)	69·8 (62·8 to 77·4)	51·1 (45·5 to 57·8)	36·7 (32·4 to 41·2)	27·1 (23 to 31·5)	26·5 (22·2 to 31·4)	17·8 (14·5 to 22·2)	14·4 (11·4 to 18)	13·7 (10·6 to 17·5)	13·6 (10·2 to 18)
Virgin Islands	67 (56·5 to 78·6)	47·4 (42·7 to 52·5)	31·4 (28·2 to 34·8)	26·9 (24·1 to 29·9)	17·6 (15·2 to 20·1)	11·2 (9·51 to 13·1)	8·43 (6·93 to 10·2)	6·87 (5·4 to 8·61)	7·17 (5·77 to 8·84)	5·93 (4·78 to 7·27)
Central Latin America	206 (181 to 235)	139 (131 to 147)	103 (97·1 to 109)	70·3 (65·9 to 74·9)	44·7 (41·8 to 47·9)	29·6 (27·1 to 32·1)	21·4 (18·8 to 24·2)	17·4 (14·2 to 20·8)	16·2 (13·1 to 20)	15·4 (11·9 to 19·7)
Colombia	152 (119 to 188)	118 (105 to 131)	83·4 (74·7 to 92·6)	53·5 (46·8 to 60·5)	33·5 (29·5 to 37·5)	26 (22·2 to 30)	19·6 (15 to 25)	14·7 (11·2 to 19·1)	13·6 (10·1 to 17·8)	11·9 (8·65 to 16·3)
Costa Rica	154 (145 to 163)	93·9 (91·8 to 96)	81·8 (79·4 to 84·3)	24 (23 to 25·1)	17·3 (16·4 to 18·2)	12·6 (11·9 to 13·3)	10·8 (10·2 to 11·5)	9·86 (9·08 to 10·7)	9·07 (8·19 to 10)	9·36 (8·22 to 10·7)
El Salvador	289 (236 to 353)	201 (179 to 226)	146 (132 to 160)	93·2 (84·2 to 102)	52·3 (46·5 to 58·7)	28·9 (25·3 to 32·6)	17·3 (14·9 to 19·8)	11·1 (8·68 to 13·8)	10 (7·71 to 12·8)	9·46 (7·08 to 12·5)
Guatemala	258 (213 to 311)	202 (180 to 224)	169 (154 to 186)	122 (112 to 133)	77·3 (70·9 to 84·4)	49·4 (44·1 to 55·2)	35·2 (30·1 to 41·5)	30·6 (25·1 to 36·3)	26·7 (21·7 to 32·5)	25·5 (20 to 32·6)
Honduras	286 (240 to 345)	190 (169 to 212)	136 (123 to 150)	89·1 (80·9 to 97·1)	54·5 (49·1 to 60·4)	35·8 (31·3 to 40·5)	25·2 (20·7 to 30·5)	17·3 (14·2 to 21)	15·8 (13·1 to 19·1)	15 (12·2 to 18·2)
Mexico	222 (184 to 265)	146 (134 to 159)	104 (95·5 to 113)	73·2 (65·8 to 80·8)	46 (42 to 50·9)	29 (26·1 to 32·2)	20·9 (18·3 to 23·8)	15·7 (12·5 to 19·2)	15·1 (12·1 to 19)	14·8 (11·6 to 18·9)

Aguascalientes	184 (151 to 223)	116 (106 to 127)	83·1 (74·3 to 91·5)	58·1 (47·6 to 68·6)	37·4 (32·4 to 43·2)	23·2 (19·7 to 27·5)	16·5 (14 to 19·4)	12·9 (10·3 to 16·3)	12·9 (10·3 to 16·3)	13 (10·1 to 16·6)
Baja California	194 (143 to 253)	122 (111 to 134)	87·4 (80 to 95)	43·8 (39·9 to 48·1)	33·8 (30·6 to 37·3)	23·5 (21·1 to 26·1)	20 (17·5 to 22·9)	13·3 (10·8 to 16·5)	13·8 (10·9 to 17·2)	13·5 (10·6 to 17·5)
Baja California Sur	217 (168 to 272)	133 (121 to 147)	90·5 (77·8 to 104)	54·9 (48·6 to 61·4)	24·8 (22·5 to 27·5)	19·5 (17·1 to 21·9)	17·3 (15 to 19·7)	13·7 (10·6 to 17·4)	14·3 (11·1 to 18)	13·9 (10·6 to 17·8)
Campeche	211 (172 to 256)	140 (127 to 153)	103 (92·1 to 113)	76·7 (66·6 to 87)	45·8 (40·9 to 51·6)	27 (23·6 to 30·8)	20·8 (18·1 to 23·8)	16·5 (13·1 to 20·5)	15·3 (12·1 to 19·2)	14·7 (11·5 to 18·7)
Chiapas	208 (165 to 264)	160 (146 to 176)	126 (114 to 139)	95·3 (83·6 to 108)	59·4 (52·4 to 67·4)	34·2 (29·3 to 39·8)	18·6 (16·2 to 21·3)	18·7 (15·1 to 22·8)	17·6 (13·9 to 22·2)	18·4 (14·3 to 23·7)
Chihuahua	213 (166 to 266)	148 (134 to 162)	108 (97·2 to 118)	78·1 (69·2 to 87·8)	48 (41·8 to 55)	34·4 (29·8 to 39·4)	22 (18·8 to 25·4)	18·7 (14·7 to 23·1)	19 (15 to 24)	19·2 (15 to 24·6)
Coahuila	233 (186 to 287)	151 (137 to 166)	107 (94·9 to 119)	74·1 (62·9 to 87·6)	41·7 (35·9 to 48·7)	26·8 (22·7 to 31·2)	16·1 (14 to 18·6)	16·1 (12·9 to 19·9)	15·3 (12·1 to 19·2)	15·5 (12·1 to 19·8)
Colima	282 (232 to 338)	177 (163 to 194)	119 (103 to 137)	75·2 (61·7 to 89·5)	40·8 (35·2 to 47·1)	25·4 (22·1 to 29·2)	18·4 (15·8 to 21·3)	11·9 (9·2 to 15)	12 (9·38 to 15·2)	11·7 (9·07 to 15)
Durango	247 (203 to 298)	159 (146 to 174)	111 (99·4 to 122)	76·9 (62·8 to 91·6)	46·9 (41·6 to 53·3)	30·5 (25·9 to 35·9)	21·1 (18·2 to 24·3)	14·7 (11·8 to 18·3)	13·9 (11·1 to 17·6)	14·2 (11·1 to 18)
Guanajuato	236 (176 to 301)	156 (144 to 170)	114 (99·5 to 129)	81·7 (74·2 to 89·2)	49·9 (45·7 to 54·5)	23·9 (21·3 to 26·7)	18·2 (16 to 20·5)	14·3 (11·5 to 17·7)	13·7 (10·9 to 17·1)	12·9 (10·2 to 16·6)
Guerrero	275 (228 to 330)	204 (174 to 234)	156 (136 to 176)	111 (97·9 to 123)	73·1 (64·1 to 82·8)	49·6 (43·1 to 56·5)	36·4 (29·6 to 43·9)	22·1 (16·8 to 28·2)	21 (15·8 to 27·4)	20·6 (15·1 to 27·3)
Hidalgo	230 (185 to 284)	152 (138 to 166)	110 (99·1 to 120)	67·5 (59 to 76·5)	41 (35·3 to 47·3)	27·4 (23·2 to 32·3)	17·5 (15·1 to 20·1)	12·7 (10·2 to 15·8)	11·6 (9·28 to 14·6)	11·2 (8·4 to 14·8)
Jalisco	188 (146 to 236)	126 (115 to 138)	87·3 (76·9 to 96·9)	55·3 (48 to 63·3)	35·9 (30·9 to 41·9)	26·2 (22·9 to 30)	18·2 (16 to 20·5)	13·4 (10·7 to 16·6)	13·8 (11 to 17·4)	14·1 (11 to 18·1)
México	201 (153 to 255)	128 (117 to 141)	89·8 (81·7 to 97·6)	70·6 (62·7 to 78·2)	48·6 (44·3 to 53·6)	28·2 (25·1 to 31·6)	23 (20·2 to 26)	17·1 (13·8 to 21·1)	15·3 (12·2 to 19·4)	12·9 (10 to 16·6)
Mexico City	169 (122 to 225)	95·4 (85·8 to 105)	69·2 (63·2 to 75·3)	57·2 (52·3 to 62·4)	33·1 (30·3 to 36·2)	23 (20·6 to 25·5)	20·9 (18·3 to 23·7)	14 (11·3 to 17·3)	15·3 (12·2 to 19)	19·1 (15 to 24)
Michoacán de Ocampo	254 (209 to 303)	171 (146 to 198)	120 (106 to 135)	80·9 (71·1 to 90·6)	50·5 (44·7 to 57·1)	33·1 (28·9 to 37·4)	26 (21·2 to 31·6)	16·4 (12·3 to 20·8)	16 (11·9 to 21)	15·9 (11·6 to 21·3)
Morelos	249 (202 to 300)	156 (143 to 171)	108 (97·4 to 120)	71·5 (62·3 to 81·3)	40·4 (35·8 to 45·4)	25·1 (21·5 to 29·2)	18·2 (15·7 to 20·9)	13 (10·3 to 16·3)	12·4 (9·85 to 15·6)	12·4 (9·66 to 15·8)
Nayarit	247 (203 to 297)	158 (144 to 172)	103 (93 to 114)	61·6 (50·6 to 73·3)	39·4 (34·6 to 44·9)	24·1 (20·6 to 28·4)	16·1 (13·8 to 18·5)	13·8 (11 to 17·2)	12·8 (9·85 to 16·4)	12·6 (9·47 to 16·6)
Nuevo León	185 (149 to 230)	116 (105 to 128)	78·6 (71·5 to 85·7)	48·3 (41·3 to 55·7)	28·7 (24·5 to 33·8)	21·4 (18·2 to 25·2)	13·8 (12 to 15·8)	12·1 (9·68 to 15)	11 (8·82 to 13·8)	11·3 (8·86 to 14·4)
Oaxaca	255 (206 to 308)	186 (158 to 215)	146 (129 to 162)	100 (88·3 to 112)	55·9 (49·6 to 63·4)	36·1 (31·5 to 41·2)	27 (21·5 to 33·6)	16·8 (12·4 to 21·4)	16·2 (12 to 21·2)	15·9 (11·6 to 21·2)
Puebla	278 (212 to 354)	195 (179 to 212)	140 (127 to 155)	94·5 (83·1 to 106)	58·2 (51 to 66)	36·3 (31·7 to 41·2)	25·4 (22·3 to 28·7)	17·8 (14·5 to 22)	17·5 (14 to 21·8)	16·6 (13·1 to 21·1)
Querétaro	260 (197 to 332)	163 (150 to 177)	111 (100 to 122)	78·9 (70·8 to 86·7)	46·5 (41·9 to 51·7)	23·3 (21 to 25·8)	18·2 (15·8 to 20·7)	12·1 (9·61 to 15)	11·5 (9·15 to 14·3)	11·6 (9·05 to 14·7)
Quintana Roo	183 (150 to 222)	127 (117 to 138)	92·5 (83·9 to 101)	64 (52·9 to 76·6)	35·7 (31 to 41·1)	22·9 (19·7 to 26·8)	18·1 (15·7 to 20·9)	12·6 (10·1 to 15·7)	11·4 (9·01 to 14·5)	10·9 (8·5 to 14·1)
San Luis Potosí	257 (210 to 311)	171 (145 to 196)	122 (107 to 138)	74·7 (65·2 to 83·9)	47·2 (42·1 to 52·9)	31·5 (27·3 to 36·1)	24 (19·6 to 29·2)	15 (11·2 to 19·1)	14·5 (10·8 to 18·9)	14·4 (10·5 to 19·1)
Sinaloa	208 (166 to 257)	136 (124 to 149)	92·1 (83·6 to 100)	57 (49·6 to 65)	33·7 (28·2 to 40·3)	21·7 (18·1 to 26)	13·4 (11·7 to 15·3)	12·3 (9·86 to 15·2)	10·8 (8·64 to 13·6)	11·2 (8·7 to 14·5)
Sonora	172 (137 to 216)	115 (104 to 127)	84·5 (76·7 to 92·2)	58·9 (52·3 to 66)	33·7 (28·6 to 39·8)	26·1 (22·4 to 30·2)	19·1 (16·7 to 21·8)	16·6 (13·1 to 20·8)	14·4 (11·3 to 18·5)	14·3 (11·1 to 18·6)
Tabasco	202 (160 to 257)	140 (128 to 154)	109 (99·5 to 119)	79 (69·6 to 89·2)	48·4 (43·1 to 54·7)	27·7 (22·7 to 33·1)	21 (18·4 to 23·8)	19·6 (15·7 to 24)	19·6 (15·7 to 24·6)	17·2 (13·3 to 22·2)
Tamaulipas	192 (150 to 239)	127 (115 to 139)	88·6 (80·6 to 96·8)	60·6 (53·4 to 68·3)	35·5 (31·4 to 40·2)	21 (18·3 to 23·9)	17·9 (15·7 to 20·5)	16 (12·8 to 19·9)	15·3 (12·1 to 19·1)	15·8 (12·3 to 20)
Tlaxcala	261 (197 to 331)	170 (156 to 185)	117 (105 to 129)	88·3 (79·6 to 97·1)	52·2 (46·8 to 58·2)	29·8 (26·5 to 33·5)	22·4 (19·6 to 25·5)	15·5 (12·4 to 19·3)	15·4 (12·1 to 19·7)	14·5 (11·1 to 19)
Veracruz de Ignacio de la Llave	228 (181 to 287)	157 (143 to 172)	114 (101 to 126)	76·7 (67·2 to 86·8)	50·4 (44·3 to 57·5)	31·8 (28 to 36·1)	19·9 (17·5 to 22·8)	16·6 (13·4 to 20·6)	16·5 (13·3 to 20·5)	15·2 (11·8 to 19·7)
Yucatán	187 (147 to 234)	136 (123 to 149)	93·6 (84·5 to 103)	69·1 (58·9 to 80·4)	43·1 (37·5 to 49·3)	25·8 (22·4 to 29·3)	17·5 (15·2 to 19·8)	12·5 (9·98 to 15·4)	13·4 (10·5 to 17·1)	13·5 (10·5 to 17·5)
Zacatecas	202 (166 to 245)	132 (120 to 144)	89·4 (79·7 to 99)	64·6 (53·7 to 76·9)	38·1 (33·4 to 43·5)	24·2 (20·4 to 28·8)	17·6 (15·2 to 20·3)	13 (10·5 to 16·1)	12·1 (9·57 to 15·3)	11·6 (8·94 to 14·8)
Nicaragua	278 (232 to 333)	210 (188 to 233)	158 (144 to 173)	101 (93·8 to 109)	62·1 (56·5 to 68·1)	36 (31·9 to 40·9)	22·5 (18·8 to 26·4)	15·9 (12·3 to 20·2)	15·2 (11·6 to 19·6)	13·8 (10·3 to 18)
Panama	106 (87 to 128)	82·7 (74·7 to 91)	56·1 (50·1 to 62·6)	36·1 (31·6 to 41)	26·9 (23·1 to 30·8)	22·6 (19·4 to 26·2)	18 (15·2 to 21·5)	16·3 (13·4 to 19·5)	15·2 (12·4 to 18·6)	14·1 (11 to 17·8)
Venezuela	160 (155 to 167)	75·3 (74·2 to 76·6)	68·3 (66·9 to 69·7)	41·3 (40·2 to 42·5)	32·9 (31·8 to 34)	23·2 (21·8 to 24·6)	18·6 (17·9 to 19·3)	22·7 (17·8 to 28·5)	21·1 (16·1 to 27·3)	19·7 (14·8 to 25·8)
Tropical Latin America	212 (164 to 267)	150 (131 to 169)	115 (102 to 129)	78·7 (71·1 to 87·4)	50·2 (45·5 to 54·9)	32·8 (28·7 to 37·1)	22·7 (19·2 to 26·7)	14·2 (11·5 to 17·3)	12·8 (10·4 to 15·4)	12 (9·85 to 14·6)
Brazil	214 (165 to 270)	152 (133 to 171)	117 (103 to 130)	79·6 (71·8 to 88·5)	50·9 (45·9 to 55·7)	33 (28·8 to 37·5)	22·8 (19·2 to 26·8)	14·1 (11·4 to 17·2)	12·7 (10·3 to 15·3)	11·9 (9·81 to 14·4)
Acre	249 (177 to 339)	173 (149 to 199)	136 (115 to 160)	102 (87·1 to 117)	65·7 (57 to 75·2)	49·2 (41·2 to 57·7)	34·1 (28·8 to 39·9)	18·7 (14 to 24·1)	17·3 (12·9 to 22·4)	16·3 (12·5 to 20·6)
Alagoas	324 (237 to 419)	252 (216 to 290)	201 (174 to 229)	165 (146 to 186)	108 (92 to 126)	59·9 (51·4 to 69·5)	30·2 (23·5 to 37·7)	14 (10·9 to 17·7)	11·6 (8·8 to 14·7)	10·8 (8·26 to 13·8)
Amapá	275 (196 to 374)	176 (153 to 203)	114 (94·4 to 137)	42·4 (37·8 to 47·5)	26 (23·1 to 29)	26·5 (22·9 to 30·5)	22·3 (19·6 to 25·5)	22·8 (18·4 to 28)	20·7 (16·9 to 25·4)	19·7 (16·4 to 23·4)
Amazonas	187 (119 to 275)	116 (95·9 to 140)	88·4 (74·1 to 103)	64·7 (54 to 77·1)	40 (33·5 to 47·5)	30·4 (26·7 to 34·5)	21·8 (19·4 to 24·3)	18·6 (15·6 to 22·4)	16·9 (14·3 to 19·9)	17 (14·8 to 19·5)
Bahia	232 (161 to 321)	179 (147 to 213)	145 (126 to 164)	95·6 (84·3 to 108)	73 (64·3 to 82·5)	45·4 (38·7 to 53·1)	29·8 (23·7 to 37)	16 (12·5 to 20·1)	13·8 (10·7 to 17·6)	12·6 (9·75 to 16·1)
Ceará	336 (246 to 439)	261 (224 to 302)	189 (163 to 217)	130 (110 to 153)	73·3 (64·5 to 83·5)	43·3 (36·9 to 50·4)	22·4 (17·5 to 27·4)	12·3 (9·33 to 15·5)	9·9 (7·56 to 12·6)	8·55 (6·46 to 11·1)
Distrito Federal	173 (117 to 238)	117 (99·5 to 136)	83 (69·4 to 99·3)	46·4 (42·2 to 51·3)	28 (25·4 to 31·1)	18·7 (16·4 to 21·2)	15·6 (13·8 to 17·6)	11 (9·01 to 13·5)	11 (9·27 to 12·9)	10·8 (9·32 to 12·6)
Espírito Santo	155 (117 to 200)	107 (90·1 to 124)	86·9 (74·5 to 103)	51·5 (46·8 to 56·7)	34·4 (31·2 to 38·2)	22·8 (19·6 to 26·4)	22·6 (18 to 27·9)	13·5 (10·4 to 17·3)	12·6 (9·61 to 16·2)	12·4 (9·45 to 15·9)
Goiás	256 (176 to 348)	184 (156 to 214)	122 (102 to 144)	70·5 (60·4 to 80·9)	43·2 (37·5 to 49·6)	31·9 (25·3 to 39·6)	23·4 (18·6 to 28·9)	15·7 (12·2 to 19·9)	14·7 (11·4 to 18·6)	14·2 (11 to 17·9)
Maranhão	314 (257 to 380)	248 (214 to 286)	200 (175 to 226)	136 (120 to 152)	86 (76·2 to 96·6)	52·4 (45·3 to 60·9)	32·4 (25·8 to 40)	14·6 (11 to 18·8)	11·9 (9·11 to 15)	11 (8·53 to 13·8)
Mato Grosso	170 (123 to 227)	141 (119 to 165)	107 (90·6 to 125)	58 (49·8 to 66·7)	40·5 (35·1 to 46·7)	29·1 (23 to 36·4)	22·5 (17·9 to 27·9)	16·7 (13·2 to 21·1)	16·3 (12·6 to 20·7)	15·9 (12·2 to 20·3)
Mato Grosso do Sul	145 (94·4 to 211)	99·9 (80·6 to 122)	72·6 (60·3 to 86·8)	45·9 (41·5 to 50·7)	31·1 (28·1 to 34·6)	26·8 (23·3 to 30·6)	20·1 (17·9 to 22·6)	13·4 (10·9 to 16·3)	13 (11 to 15·4)	12·2 (10·4 to 14·2)
Minas Gerais	180 (130 to 243)	125 (104 to 146)	91·6 (77·9 to 107)	58·6 (50·8 to 66·8)	38·5 (32·9 to 44·1)	28·2 (22·9 to 33·9)	19·9 (16 to 24·4)	13·2 (10·3 to 16·5)	12 (9·36 to 15·1)	11·2 (8·74 to 14·3)
Pará	256 (184 to 334)	173 (148 to 200)	129 (108 to 152)	87·8 (75·5 to 102)	57·8 (50·3 to 65·9)	38·5 (30·5 to 48·3)	29·3 (24·8 to 34·5)	17·7 (13·8 to 22·4)	16·7 (12·5 to 21·2)	15·6 (12 to 20)
Paraíba	364 (261 to 475)	272 (242 to 305)	193 (167 to 219)	128 (107 to 149)	77·3 (68·2 to 87·3)	43·6 (36·8 to 51·2)	26·1 (21·5 to 30·9)	13·7 (10·7 to 17·1)	11·2 (8·37 to 14·6)	10·4 (7·69 to 13·8)
Paraná	137 (96·7 to 185)	99·2 (83·1 to 116)	78·5 (65·7 to 92·2)	54·9 (49·8 to 60·4)	33·1 (30 to 36·4)	22·8 (19·7 to 26·5)	19·9 (15·7 to 24·5)	12·6 (9·98 to 15·8)	11·6 (8·85 to 14·7)	10·9 (8·24 to 13·9)
Pernambuco	351 (259 to 445)	267 (236 to 302)	205 (181 to 229)	132 (112 to 155)	84·6 (75·1 to 94·4)	48·5 (41 to 56·8)	28·6 (24·1 to 33·6)	13·8 (10·8 to 17·3)	12·2 (9·41 to 15·5)	11·5 (8·98 to 14·6)
Piauí	234 (160 to 316)	174 (152 to 199)	148 (125 to 173)	97·4 (84·4 to 112)	64·4 (55·5 to 73·7)	40·9 (35·1 to 47·3)	26·9 (22·3 to 32·1)	15·2 (11·7 to 19·2)	13·1 (9·84 to 16·5)	11·6 (8·78 to 14·9)
Rio de Janeiro	137 (77·7 to 217)	83·2 (68·7 to 100)	66·9 (55·2 to 80)	61 (55·6 to 66·9)	37·8 (34·5 to 41·4)	23·6 (20·8 to 26·6)	17·7 (15·8 to 19·9)	15·1 (12·6 to 18)	14·2 (12 to 16·5)	13·5 (11·9 to 15·4)
Rio Grande do Norte	393 (292 to 500)	284 (243 to 327)	188 (166 to 211)	118 (99·8 to 141)	75·5 (63·8 to 87·9)	41·7 (35·1 to 48·9)	24·9 (19·6 to 31)	12·8 (9·92 to 16·2)	10·6 (7·95 to 13·6)	9·83 (7·44 to 12·7)
Rio Grande do Sul	131 (89 to 179)	83·5 (69·9 to 100)	58·5 (48·6 to 71·4)	39·1 (35·6 to 43·1)	22·6 (20·6 to 25)	18·6 (16·3 to 21)	13·3 (11·7 to 14·9)	13·1 (10·9 to 15·5)	11·4 (9·74 to 13·3)	11·5 (10·1 to 13·1)
Rondônia	252 (146 to 389)	150 (123 to 180)	102 (85·3 to 121)	75·1 (63·3 to 88·9)	44·9 (38·4 to 52·3)	31·7 (26·8 to 37·2)	24·8 (19·8 to 30·6)	14·4 (11·1 to 18·3)	13·6 (10·3 to 17·4)	9·56 (6·47 to 13)
Roraima	267 (190 to 357)	180 (155 to 206)	128 (107 to 153)	62·4 (55·5 to 70·3)	38·3 (33·6 to 43·8)	25·8 (22·3 to 29·5)	24·1 (20·9 to 27·8)	24·6 (20·1 to 29·7)	22·1 (18·1 to 26·4)	17·8 (14·4 to 21·9)
Santa Catarina	117 (77 to 166)	73·1 (60·4 to 87·7)	60·1 (50 to 71·1)	42·5 (36·4 to 48·6)	31·1 (26·8 to 36)	23·3 (19·3 to 27·9)	17·3 (13·9 to 21·3)	11·3 (8·14 to 15·7)	11·3 (8·69 to 14·5)	10·6 (8·17 to 13·6)
São Paulo	116 (78·9 to 159)	78·2 (64·5 to 93·3)	62·4 (52·6 to 73·4)	53·2 (48·6 to 58·5)	33·7 (30·7 to 36·7)	21·7 (18·8 to 24·7)	17·2 (13·9 to 20·9)	12·4 (9·65 to 15·5)	11·6 (9·73 to 13·7)	11·5 (9·89 to 13·2)
Sergipe	253 (177 to 348)	186 (158 to 217)	132 (114 to 149)	101 (88 to 116)	64·4 (56·4 to 73)	41·6 (32·8 to 52·3)	25·6 (20·7 to 31·5)	16·4 (12·6 to 20·6)	14·8 (11·4 to 18·7)	13·6 (10·5 to 17·3)
Tocantins	253 (147 to 396)	195 (164 to 229)	145 (121 to 174)	89·2 (76·1 to 104)	57·6 (48·2 to 68·2)	39·2 (33·6 to 45·8)	24·3 (19·7 to 29·7)	13·6 (10·5 to 17·4)	11·9 (8·88 to 15·5)	9·97 (6·95 to 13·5)

Paraguay	116 (87·5 to 149)	79·8 (70·4 to 89·5)	67·9 (60·1 to 76·4)	48·7 (42·1 to 55·5)	33 (28·3 to 38·8)	27·3 (23·2 to 32)	20·2 (16·6 to 24·4)	16·6 (12·3 to 21·4)	15·8 (11·4 to 20·7)	14·7 (10·5 to 19·6)
North Africa and Middle East	315 (280 to 353)	250 (232 to 268)	192 (181 to 203)	128 (122 to 135)	80·1 (75·4 to 84·8)	55·3 (51·3 to 59·5)	35·4 (32·3 to 38·8)	23·6 (20·5 to 27)	21·4 (18·5 to 24·5)	20·2 (17·4 to 23·3)
North Africa and Middle East	315 (280 to 353)	250 (232 to 268)	192 (181 to 203)	128 (122 to 135)	80·1 (75·4 to 84·8)	55·3 (51·3 to 59·5)	35·4 (32·3 to 38·8)	23·6 (20·5 to 27)	21·4 (18·5 to 24·5)	20·2 (17·4 to 23·3)
Afghanistan	354 (290 to 418)	251 (220 to 286)	226 (198 to 260)	217 (191 to 243)	159 (145 to 176)	130 (118 to 142)	79·6 (68·8 to 91·1)	54·3 (45·2 to 64·5)	51 (42·5 to 60·7)	48·7 (40·5 to 58·4)
Algeria	320 (265 to 388)	256 (225 to 288)	190 (167 to 215)	130 (122 to 139)	57·1 (52·4 to 61·9)	40·1 (35·4 to 45·3)	28 (25·4 to 31)	19·8 (16·5 to 23·6)	18 (14·7 to 21·8)	16·9 (13·4 to 21)
Bahrain	195 (156 to 243)	150 (129 to 172)	76·1 (65 to 89·5)	30·2 (28·2 to 32·4)	23·6 (22·3 to 25)	11·8 (11 to 12·8)	8·15 (7·56 to 8·74)	7·09 (6·39 to 7·91)	6·07 (5·22 to 6·94)	5·69 (4·77 to 6·67)
Egypt	315 (253 to 377)	276 (251 to 301)	236 (218 to 255)	154 (141 to 168)	84·4 (76·4 to 92·9)	44·6 (39·2 to 50·3)	28·1 (24·2 to 32·4)	16·4 (13·4 to 19·9)	13·9 (11·2 to 16·9)	12·8 (10·5 to 15·7)
Iran	378 (316 to 440)	288 (253 to 326)	199 (176 to 224)	102 (90·9 to 115)	71·3 (64·1 to 78·9)	39·9 (34·9 to 45)	26·6 (23·5 to 30)	11·1 (9·99 to 12·4)	7·7 (7·06 to 8·41)	5·25 (4·36 to 6·21)
Alborz	322 (262 to 387)	272 (236 to 311)	177 (153 to 204)	76·9 (67 to 87·9)	44·6 (38·7 to 50·6)	29·1 (24·2 to 34·5)	19 (16·5 to 21·8)	7·96 (6·75 to 9·31)	4·82 (4·17 to 5·5)	3·35 (2·65 to 4·13)
Ardebil	387 (321 to 459)	315 (276 to 355)	241 (215 to 270)	140 (123 to 157)	98 (86·7 to 110)	53·8 (47·8 to 60·7)	30·6 (26·9 to 34·5)	12·6 (11·2 to 14·4)	8·99 (8·07 to 10·1)	6·05 (5·19 to 7·01)
Bushehr	332 (269 to 396)	259 (223 to 295)	190 (165 to 217)	93·4 (80·9 to 107)	58·6 (50·8 to 66·3)	36·2 (31·6 to 41·1)	26·4 (22·3 to 30·9)	12 (10·3 to 13·8)	8·33 (7·24 to 9·55)	5·63 (4·83 to 6·55)
Chahar Mahaal and Bakhtiari	292 (233 to 355)	224 (193 to 256)	163 (141 to 186)	88·5 (76·5 to 101)	63·5 (54·8 to 71·5)	33·9 (29·3 to 39·1)	20·3 (17·2 to 23·7)	8·35 (7·22 to 9·52)	5·06 (4·43 to 5·7)	3·41 (2·77 to 4·14)
East Azarbayejan	411 (347 to 475)	324 (286 to 364)	239 (213 to 268)	133 (117 to 151)	77·9 (69 to 87·1)	43·6 (36·9 to 51·4)	26·6 (23·5 to 30·3)	9·73 (8·51 to 11·1)	6·36 (5·51 to 7·28)	4·33 (3·43 to 5·33)
Fars	255 (201 to 312)	191 (163 to 220)	132 (114 to 152)	68·7 (59·8 to 78·6)	52·1 (45·6 to 58·9)	37·5 (31·7 to 44·3)	28·1 (24·6 to 32·2)	12·9 (11·2 to 14·7)	9·21 (7·86 to 10·9)	6·24 (4·87 to 7·81)
Gilan	313 (250 to 383)	231 (188 to 282)	149 (129 to 171)	70 (59·4 to 80·7)	180 (166 to 196)	25·9 (22 to 30)	13·8 (12·1 to 15·4)	5·67 (4·93 to 6·66)	2·7 (2·03 to 3·34)	1·88 (1·31 to 2·5)
Golestan	330 (274 to 397)	259 (224 to 298)	190 (167 to 215)	101 (87·6 to 116)	74·5 (65·2 to 84·2)	44·7 (38·9 to 51·1)	27·1 (23·6 to 30·8)	12·1 (10·5 to 13·8)	8·54 (7·58 to 9·68)	5·79 (5 to 6·69)
Hamadan	467 (392 to 541)	371 (328 to 414)	274 (242 to 305)	134 (118 to 152)	81·2 (71·2 to 90·7)	48 (42·3 to 54·5)	28·6 (25·2 to 32·3)	12·9 (11·2 to 14·7)	9·25 (8·36 to 10·2)	6·25 (5·49 to 7·16)
Hormozgan	375 (312 to 446)	265 (230 to 301)	187 (163 to 212)	100 (87·6 to 113)	78 (68·9 to 88·4)	46·8 (40·7 to 53·5)	28·7 (25·1 to 32·5)	12·3 (10·8 to 14)	8·76 (7·98 to 9·69)	5·93 (5·2 to 6·71)
Ilam	305 (244 to 366)	242 (209 to 276)	180 (158 to 203)	103 (90·1 to 117)	66·7 (58·4 to 75·3)	42·2 (35·4 to 49·7)	26 (22·4 to 29·8)	11·9 (10·3 to 13·7)	8·35 (6·65 to 10·2)	5·7 (4·11 to 7·53)
Isfahan	318 (255 to 385)	267 (232 to 303)	171 (148 to 195)	81·7 (71·2 to 93·4)	47·9 (41·9 to 54·1)	30 (24·2 to 36·3)	23 (20 to 26·6)	10·5 (8·81 to 12·3)	7·09 (5·87 to 8·49)	4·89 (3·7 to 6·3)
Kerman	417 (343 to 493)	296 (257 to 337)	200 (175 to 227)	97·5 (85·5 to 110)	61·1 (53·6 to 68·9)	37·2 (32·4 to 42·4)	31·5 (27·1 to 36·3)	15·2 (13·3 to 17·1)	11·6 (10·4 to 13·2)	7·95 (6·87 to 9·23)
Kermanshah	369 (305 to 442)	296 (258 to 336)	216 (191 to 244)	121 (107 to 137)	80·6 (70·5 to 90·6)	50·4 (44·2 to 57·4)	31·7 (27·2 to 36·6)	13·1 (11·5 to 15)	9·23 (8·12 to 10·6)	6·21 (5·31 to 7·26)
Khorasan-e-Razavi	489 (419 to 557)	382 (334 to 429)	274 (246 to 305)	139 (122 to 157)	86·1 (75·7 to 96·5)	51·6 (44·3 to 59·4)	36·3 (30·9 to 42·2)	12·1 (10·9 to 13·3)	8·3 (6·91 to 9·69)	5·55 (4·08 to 7·13)
Khuzestan	336 (272 to 410)	245 (209 to 285)	172 (150 to 198)	89·2 (77 to 102)	53·2 (47 to 59·8)	38·1 (32·1 to 44·8)	26·9 (23·8 to 30·5)	11·8 (10·7 to 13·1)	8·48 (7·59 to 9·48)	5·85 (4·8 to 7·02)
Kohgiluyeh and Boyer-Ahmad	325 (265 to 389)	233 (202 to 268)	176 (153 to 200)	105 (91·2 to 120)	73·3 (64·5 to 82·4)	46·5 (40·4 to 53·1)	26 (22·6 to 29·8)	10·6 (9·1 to 12·2)	7·1 (6·22 to 8·08)	4·77 (3·92 to 5·72)
Kurdistan	467 (398 to 539)	404 (360 to 450)	305 (274 to 338)	179 (159 to 200)	111 (98·5 to 124)	61·2 (54·2 to 68·7)	33·2 (29 to 37·7)	12·9 (11·4 to 14·8)	9·29 (8·28 to 10·4)	6·33 (5·47 to 7·26)
Lorestan	387 (314 to 469)	276 (238 to 317)	195 (171 to 221)	104 (90·3 to 119)	67·5 (59·1 to 77·7)	36·4 (31·5 to 41·7)	18 (15·2 to 21·2)	7·34 (6·31 to 8·54)	4·14 (3·56 to 4·73)	2·79 (2·28 to 3·34)
Markazi	335 (272 to 404)	284 (246 to 324)	213 (186 to 243)	103 (89·7 to 117)	63·8 (55·4 to 72·5)	37·9 (32·4 to 43·5)	24·9 (21·2 to 28·9)	11 (9·61 to 12·3)	7·4 (6·65 to 8·21)	4·98 (4·22 to 5·84)
Mazandaran	266 (209 to 332)	204 (174 to 237)	137 (118 to 159)	66 (56·8 to 76·2)	39 (33·2 to 45·3)	25·5 (21·9 to 29·9)	16·5 (14·3 to 18·9)	7·72 (6·57 to 8·92)	4·34 (3·77 to 4·92)	2·91 (2·28 to 3·59)
North Khorasan	432 (350 to 519)	348 (306 to 393)	265 (238 to 296)	141 (125 to 158)	91·1 (80·7 to 102)	50·4 (43·4 to 57·7)	27·9 (24·2 to 31·8)	11·5 (10·1 to 13·1)	8 (7·12 to 8·95)	5·37 (4·59 to 6·22)
Qazvin	437 (365 to 515)	364 (320 to 410)	255 (226 to 286)	123 (109 to 140)	68·6 (60·2 to 76·7)	42·5 (37·1 to 48·4)	23·3 (19·8 to 27·1)	9·8 (8·49 to 11·1)	6·35 (5·52 to 7·24)	4·25 (3·39 to 5·17)
Qom	294 (236 to 359)	241 (208 to 277)	178 (154 to 202)	87·5 (76·6 to 99·5)	50·1 (43·1 to 57·6)	29·8 (25·7 to 34·7)	20·3 (17·5 to 23·2)	8·42 (7·38 to 9·63)	5·21 (4·46 to 6·07)	3·51 (2·75 to 4·35)
Semnan	356 (291 to 423)	301 (263 to 343)	206 (180 to 236)	96·1 (83·6 to 110)	55·8 (47·9 to 64·7)	36·1 (29·8 to 43·3)	24·4 (21·2 to 28)	10·6 (8·99 to 12·3)	7·16 (5·45 to 8·93)	4·9 (3·39 to 6·55)
Sistan and Baluchistan	461 (373 to 554)	342 (300 to 385)	254 (226 to 282)	144 (126 to 162)	99·5 (88·1 to 111)	60·5 (53·3 to 68·8)	39·7 (35·5 to 44·4)	15·7 (13·7 to 18)	12·4 (11·1 to 13·8)	8·61 (7·57 to 9·72)
South Khorasan	428 (362 to 501)	349 (308 to 392)	267 (239 to 297)	149 (131 to 168)	97·9 (86·4 to 110)	56·3 (49 to 63·8)	30·7 (26·1 to 35·7)	12·2 (10·7 to 13·8)	8·45 (7·54 to 9·55)	5·61 (4·83 to 6·49)
Tehran	253 (201 to 314)	179 (148 to 212)	106 (89·4 to 125)	46·6 (38·5 to 55)	29·7 (24·2 to 36)	21·5 (17·9 to 25·4)	16·2 (13·3 to 19·3)	7·76 (6·67 to 8·97)	4·54 (3·19 to 5·93)	3·07 (1·84 to 4·48)
West Azarbayejan	397 (335 to 460)	332 (292 to 375)	251 (224 to 281)	137 (120 to 155)	82 (72·1 to 92·7)	50·8 (44·1 to 58·5)	34·1 (30·4 to 38·2)	12·4 (11 to 14·1)	8·95 (8·02 to 9·99)	6·19 (5·3 to 7·16)
Yazd	415 (347 to 487)	269 (232 to 309)	176 (154 to 201)	81·7 (71 to 93)	47·9 (41·5 to 55)	32 (27·2 to 36·8)	25·7 (22·7 to 28·7)	11·9 (10·3 to 13·5)	8·48 (7·49 to 9·56)	5·88 (4·87 to 6·99)
Zanjan	511 (426 to 594)	446 (399 to 494)	321 (289 to 355)	161 (143 to 180)	184 (171 to 197)	50·1 (43 to 58·4)	27·4 (24·2 to 31)	12 (10·4 to 13·6)	8·52 (7·41 to 9·73)	5·8 (4·78 to 6·92)
Iraq	189 (146 to 237)	148 (124 to 173)	118 (101 to 136)	76·2 (68·7 to 83·5)	57·4 (52·7 to 62·3)	45·8 (41·4 to 50·7)	32·4 (27·1 to 38·3)	22·9 (18·5 to 28·4)	20·1 (16·2 to 25·1)	18·8 (14·8 to 23·7)
Jordan	206 (171 to 251)	133 (115 to 153)	83·1 (74·9 to 92·3)	51·2 (46·8 to 56·1)	33·5 (30·2 to 37)	25·9 (23 to 28·8)	17·8 (15·3 to 20·5)	13·1 (10·9 to 15·7)	12 (9·98 to 14·7)	11·5 (9·43 to 14·1)
Kuwait	134 (94·8 to 185)	62·9 (54·9 to 72·2)	50·5 (48·7 to 52·4)	34·8 (33·4 to 36·4)	18·1 (16·5 to 20)	11·4 (10·5 to 12·3)	11·1 (10·2 to 12)	9·28 (8·31 to 10·3)	8·3 (7·33 to 9·31)	8·07 (6·65 to 9·69)
Lebanon	207 (159 to 262)	95 (78·1 to 113)	64·2 (53·5 to 75·1)	49·3 (42·2 to 56·8)	31·6 (26·7 to 37·4)	21·2 (17·6 to 25·3)	13·5 (10·9 to 16·8)	8·9 (6·47 to 12·1)	8·06 (5·79 to 11·1)	7·71 (5·44 to 10·9)
Libya	335 (287 to 389)	225 (200 to 253)	121 (106 to 135)	63·3 (55·2 to 73·1)	41 (34·4 to 48·5)	25·1 (20·9 to 29·9)	22·1 (17·8 to 26·8)	24·4 (19·1 to 30·5)	22·7 (18 to 28·4)	21·6 (16·9 to 27)
Morocco	348 (306 to 394)	252 (234 to 270)	187 (174 to 198)	126 (116 to 139)	76·6 (68·1 to 86·2)	50·8 (41·8 to 60·4)	31·4 (25·7 to 38·1)	17·8 (14·4 to 21·6)	16 (13·1 to 19·2)	14·8 (12·1 to 17·8)
Oman	335 (277 to 397)	241 (211 to 272)	155 (135 to 174)	85·4 (73 to 98·8)	33·9 (28·7 to 40·3)	15·2 (13·3 to 17·4)	11·6 (10·9 to 12·2)	11·4 (10·5 to 12·4)	10·3 (9·39 to 11·3)	9·07 (8·01 to 10·2)
Palestine	308 (256 to 366)	202 (176 to 231)	100 (86·3 to 116)	65·7 (58·5 to 73·2)	41·4 (37 to 45·7)	28·4 (25·3 to 31·9)	21 (18·2 to 24·3)	12·8 (10·2 to 16·1)	11·5 (9·15 to 14·7)	10·8 (8·57 to 13·9)
Qatar	149 (118 to 188)	110 (92·5 to 129)	68·7 (58·3 to 80·5)	28·8 (23·7 to 34·2)	17·2 (14·3 to 20·4)	10·9 (9·08 to 13)	6·56 (5·33 to 7·91)	4·61 (3·69 to 5·75)	4·45 (3·58 to 5·56)	3·64 (2·89 to 4·62)
Saudi Arabia	152 (119 to 190)	193 (166 to 222)	138 (119 to 159)	76·2 (64·7 to 88·4)	45·5 (37·1 to 55·1)	23·5 (19·2 to 28·8)	10·6 (9·31 to 11·9)	5·41 (4·41 to 6·53)	4·5 (3·48 to 5·59)	4·22 (3·22 to 5·27)
Sudan	251 (209 to 302)	231 (206 to 260)	213 (195 to 231)	193 (175 to 212)	149 (137 to 162)	104 (94·8 to 115)	67·6 (57·7 to 78·1)	42 (34·1 to 50·6)	39·2 (31·6 to 47·4)	36·8 (29·5 to 45)
Syria	219 (182 to 262)	134 (122 to 146)	88·8 (80·6 to 97·3)	62·8 (55·3 to 71·1)	45·6 (39·7 to 52·7)	18·2 (16·8 to 19·4)	14·1 (12·7 to 15·5)	13·6 (11 to 16·7)	11·2 (8·98 to 13·7)	10 (7·96 to 12·4)
Tunisia	334 (292 to 380)	235 (216 to 253)	154 (143 to 167)	87·2 (78·8 to 95·6)	51·7 (45·4 to 58·6)	30·3 (26 to 34·7)	19·2 (16·1 to 22·4)	12·3 (10 to 14·8)	11 (8·99 to 13·3)	10·3 (8·35 to 12·5)
Türkiye	298 (251 to 346)	250 (230 to 271)	182 (169 to 196)	123 (112 to 137)	71·9 (64·5 to 79·8)	41·7 (36·7 to 47·5)	23·2 (19·3 to 27·6)	13·9 (11·2 to 17)	12·1 (9·79 to 14·6)	11·1 (9·12 to 13·4)
United Arab Emirates	187 (152 to 230)	156 (133 to 179)	79·4 (68·1 to 92)	38·4 (32·9 to 45·4)	20·8 (18·1 to 23·8)	11·8 (10·9 to 12·7)	8·45 (7·62 to 9·39)	5·96 (5·31 to 6·67)	5·74 (5·07 to 6·53)	4·85 (4·08 to 5·66)
Yemen	466 (416 to 520)	371 (349 to 394)	293 (274 to 312)	189 (175 to 202)	131 (121 to 141)	92 (82·9 to 102)	58·1 (50·1 to 66·4)	43·9 (36·4 to 52·1)	40·8 (33·6 to 48·4)	38·9 (32 to 46·5)
South Asia	346 (284 to 414)	268 (242 to 298)	199 (182 to 216)	150 (138 to 163)	106 (98·4 to 115)	79 (72 to 86·3)	58 (51·8 to 64·6)	42 (36·1 to 48·7)	38·9 (33·1 to 45·9)	37·1 (31·4 to 44·2)
South Asia	346 (284 to 414)	268 (242 to 298)	199 (182 to 216)	150 (138 to 163)	106 (98·4 to 115)	79 (72 to 86·3)	58 (51·8 to 64·6)	42 (36·1 to 48·7)	38·9 (33·1 to 45·9)	37·1 (31·4 to 44·2)
Bangladesh	314 (270 to 360)	260 (238 to 280)	249 (233 to 265)	181 (168 to 195)	129 (120 to 137)	85·5 (78·5 to 93)	53·6 (47·5 to 60·3)	32·7 (26·8 to 39·6)	29·9 (24·3 to 36·5)	28 (22·5 to 34·6)
Bhutan	366 (303 to 435)	280 (243 to 320)	223 (190 to 261)	192 (165 to 220)	128 (113 to 141)	86·1 (76·7 to 97·1)	47·4 (39·6 to 55·5)	31·9 (25·2 to 39·5)	30·9 (24·2 to 38·2)	29·3 (22·8 to 36·6)
India	358 (281 to 440)	275 (244 to 310)	195 (174 to 216)	146 (130 to 162)	101 (91·2 to 112)	76·2 (67·2 to 85·6)	55·6 (48 to 63·6)	38·2 (31·6 to 45·4)	34·9 (28·6 to 42·5)	33·1 (26·9 to 40·8)
Nepal	350 (309 to 397)	286 (262 to 309)	229 (211 to 247)	200 (185 to 216)	135 (125 to 144)	82·3 (74·8 to 90)	50·6 (43·7 to 57·8)	31·6 (24·8 to 39·7)	29·8 (23·4 to 37·8)	28·4 (22 to 36·4)
Pakistan	275 (233 to 320)	204 (188 to 220)	158 (147 to 171)	137 (126 to 149)	109 (99·5 to 119)	88·8 (79 to 99·9)	73·2 (63·8 to 84·3)	61·7 (50·7 to 74·7)	58·6 (48·5 to 70·4)	56·3 (46·2 to 68)
Azad Jammu & Kashmir	296 (232 to 365)	220 (191 to 253)	173 (154 to 193)	148 (123 to 175)	112 (94·5 to 132)	86·6 (67·3 to 107)	62·7 (46 to 84·5)	53·2 (38·9 to 71·7)	49·9 (37·1 to 66·8)	48·4 (36·6 to 63·7)
Balochistan	215 (161 to 281)	163 (140 to 189)	111 (95·8 to 126)	99·1 (84·6 to 116)	88·8 (78·5 to 99·5)	79·3 (64·9 to 96·3)	76·1 (61·4 to 93·6)	67·6 (53·3 to 83·6)	62·9 (50·1 to 77·9)	61·1 (49 to 76)

Gilgit-Baltistan	192 (148 to 245)	219 (197 to 243)	178 (155 to 201)	154 (129 to 179)	115 (97·2 to 134)	88·3 (72·7 to 106)	63·2 (49·2 to 78·4)	50·2 (39·3 to 63·4)	46·9 (37·3 to 58·5)	45·3 (36·6 to 56·1)
Islamabad Capital Territory	271 (197 to 360)	242 (207 to 284)	162 (133 to 191)	102 (82·2 to 127)	56·7 (45·1 to 71·9)	34·3 (26·7 to 42·5)	22·5 (16·5 to 29·4)	16·5 (12·3 to 21·8)	15·3 (11·2 to 20·6)	14·6 (10·6 to 19·8)
Khyber Pakhtunkhwa	285 (224 to 357)	191 (171 to 211)	145 (128 to 162)	112 (98 to 129)	77 (67·9 to 87·6)	68·8 (58·6 to 79·1)	56·8 (44·9 to 70·6)	50·6 (39·4 to 63·6)	47·2 (37·3 to 59·1)	45 (36·1 to 55·9)
Punjab	272 (229 to 318)	208 (190 to 225)	166 (151 to 180)	150 (137 to 164)	119 (109 to 129)	95·3 (85·5 to 106)	78·8 (69·1 to 89·6)	66·4 (52·9 to 82·3)	63·6 (50·8 to 79·2)	61·4 (48·8 to 76·5)
Sindh	296 (235 to 361)	205 (181 to 228)	155 (141 to 170)	129 (116 to 146)	111 (96 to 126)	91·9 (77·8 to 108)	76 (64·3 to 89·4)	61·3 (48·9 to 77·1)	58·3 (46·4 to 73·4)	55·3 (44 to 69·7)
Southeast Asia, east Asia, and Oceania	239 (207 to 277)	454 (331 to 637)	111 (102 to 120)	82 (75·7 to 88·7)	59 (54·3 to 64·2)	42·6 (39·4 to 46·3)	23·8 (21·8 to 25·8)	15·4 (13·2 to 17·9)	15 (12·8 to 17·3)	14·6 (12·6 to 17)
East Asia	221 (182 to 267)	537 (385 to 744)	96·1 (86·2 to 108)	67·7 (60·4 to 75·7)	52·5 (46·7 to 59·2)	38 (33·8 to 43·8)	15·5 (13·7 to 17·3)	8·1 (7·02 to 9·29)	7·71 (6·6 to 8·99)	7·27 (6·18 to 8·56)
China	223 (183 to 270)	549 (396 to 757)	97·7 (87·6 to 110)	69·4 (61·8 to 77·6)	53·6 (47·5 to 60·5)	36 (32·5 to 39·5)	15·4 (13·6 to 17·3)	8·06 (6·94 to 9·26)	7·67 (6·53 to 8·95)	7·23 (6·12 to 8·56)
North Korea	181 (143 to 225)	97·6 (80 to 118)	64·2 (52 to 78·2)	43·5 (33·7 to 56·2)	31·8 (25·5 to 39·8)	113 (50·2 to 249)	21·9 (17·3 to 27)	12 (8·92 to 15·6)	11·2 (8·27 to 14·7)	10·5 (7·81 to 13·9)
Taiwan (province of China)	144 (139 to 151)	59·9 (58·9 to 60·9)	25·9 (25·2 to 26·7)	15·1 (14·6 to 15·6)	8·48 (8·1 to 8·9)	8·15 (7·64 to 8·67)	4·94 (4·6 to 5·31)	4·55 (4·19 to 4·96)	4·3 (3·93 to 4·7)	4·62 (4·09 to 5·15)
Oceania	220 (189 to 254)	146 (128 to 165)	105 (92·5 to 118)	79·4 (70·9 to 88·6)	68·2 (60·7 to 76·7)	60·4 (52·5 to 69·1)	56 (47·3 to 65·5)	48·9 (40·5 to 58·9)	47·7 (39·5 to 57·1)	47·1 (38·9 to 56·1)
American Samoa	90 (77·2 to 105)	51·9 (47·3 to 57)	36·8 (33·4 to 40·8)	21·9 (18·9 to 25·3)	17 (15·2 to 18·9)	14·7 (12·9 to 16·8)	12·3 (10·8 to 14)	12·6 (10·2 to 15·6)	13·4 (10·7 to 16·8)	12·1 (9·4 to 15·5)
Cook Islands	187 (164 to 212)	88·8 (80·8 to 96·6)	55·2 (49·4 to 61·6)	37·7 (33·1 to 42·6)	25·5 (22·1 to 29·4)	13·8 (11·1 to 17)	5·81 (4·42 to 7·52)	2·79 (2·25 to 3·43)	5·38 (5·37 to 5·45)	5·43 (5·43 to 5·46)
Fiji	84·2 (70·3 to 100)	56·2 (50·6 to 61·9)	43·2 (38·5 to 48·2)	33·1 (28 to 38·8)	25·4 (20·8 to 30·7)	26 (20·6 to 32·7)	24·1 (20 to 28·7)	20·4 (16 to 25·7)	19·5 (15·2 to 24·9)	19·3 (14·6 to 25·2)
Guam	58·3 (50·1 to 67·2)	36·6 (33·7 to 39·5)	27·9 (25·6 to 30·5)	17 (15·5 to 18·7)	12·3 (11·2 to 13·6)	11·6 (10·5 to 12·9)	14·2 (12·8 to 15·8)	14 (12·1 to 16·2)	12·7 (10·4 to 15)	12 (9·65 to 14·9)
Kiribati	234 (194 to 278)	162 (142 to 183)	124 (106 to 143)	94·7 (79·7 to 113)	82·8 (69·8 to 97)	63·1 (52·3 to 74·7)	48·7 (39·9 to 58·6)	38·7 (31·9 to 46·4)	37·5 (30·8 to 45·7)	36·4 (29·6 to 44·7)
Marshall Islands	171 (134 to 213)	92 (76·3 to 108)	57·8 (47·8 to 69·8)	39·7 (33·6 to 46·8)	32·8 (29·1 to 36·8)	37·9 (33·2 to 43·4)	28·8 (24·2 to 34·2)	21·5 (16·9 to 27·5)	20·7 (16·1 to 27·1)	19·9 (15·3 to 26·2)
Federated States of Micronesia	273 (224 to 329)	125 (107 to 144)	81·5 (69·2 to 94·7)	53·4 (44·5 to 63·7)	41·3 (34·1 to 49·5)	36·2 (31 to 42·2)	21·7 (17·6 to 26·2)	16·5 (13 to 20·7)	15·9 (12·5 to 19·9)	15·4 (12·2 to 19·1)
Nauru	103 (82·1 to 129)	69·5 (58·2 to 81·4)	53·9 (45·6 to 63·9)	38·9 (32·3 to 46·2)	34·4 (28·6 to 40·4)	46·5 (40·3 to 52·8)	41·6 (34·8 to 49)	26·5 (20 to 34·6)	25·4 (19 to 33·9)	24·5 (18·2 to 33)
Niue	166 (138 to 196)	70·7 (62·3 to 79·8)	46 (39·5 to 53·3)	30·6 (25·3 to 36·3)	24·7 (20·2 to 29·6)	28·8 (23·6 to 34·7)	23·2 (18·5 to 28·9)	20·7 (15·9 to 27·1)	50·8 (50·6 to 52·1)	51·1 (51 to 52·5)
Northern Mariana Islands	61·7 (46 to 81·9)	35·2 (28·6 to 43·1)	19·8 (15·3 to 25·9)	12·5 (9·67 to 16·3)	8·94 (7·14 to 11)	7·23 (5·97 to 8·61)	7·53 (6·11 to 9·09)	7·19 (5·83 to 8·77)	7·22 (5·49 to 9·24)	6·21 (5·02 to 7·36)
Palau	199 (154 to 251)	125 (102 to 148)	72·8 (60 to 88)	48·6 (39·1 to 58·6)	32·4 (25·1 to 40·4)	23·3 (18·8 to 28·6)	21·3 (17·4 to 25·9)	17·4 (14·2 to 21·3)	17·1 (14·1 to 21·1)	16·9 (13·9 to 20·8)
Papua New Guinea	258 (215 to 306)	171 (148 to 197)	122 (106 to 139)	95·2 (83·9 to 108)	82·9 (73 to 94·2)	71·3 (61·4 to 81·9)	64·5 (54·4 to 75·9)	54·8 (45·2 to 65·8)	53·5 (44·3 to 64·1)	52·7 (43·5 to 62·8)
Samoa	166 (137 to 199)	97·9 (85·3 to 112)	66·7 (56·8 to 77)	43·1 (36 to 51·8)	29·9 (23·7 to 37·7)	21·7 (17·1 to 27·1)	17·4 (13·6 to 21·7)	25·5 (22·4 to 29·2)	15·5 (12·6 to 19)	13 (10·1 to 16·6)
Solomon Islands	205 (170 to 246)	153 (134 to 174)	101 (86 to 118)	61 (51 to 72·6)	45·5 (37·4 to 54·7)	34·5 (28 to 41·3)	27·7 (22·4 to 33·8)	20·6 (16·6 to 25·5)	20·8 (16·8 to 25·6)	19·5 (15·6 to 24·2)
Tokelau	108 (84·9 to 139)	76·6 (62·3 to 92·2)	54 (42·6 to 66)	43·6 (35·3 to 52·5)	29 (23·5 to 35·2)	21·2 (17 to 26·9)	13·4 (10·6 to 17)	9·99 (7·4 to 13·5)	62·9 (62·9 to 62·9)	64 (64 to 64)
Tonga	72·2 (55·8 to 93·1)	48·3 (40·4 to 57·3)	41·2 (34·9 to 48·1)	34·7 (29·1 to 40·9)	25·5 (21·3 to 30·2)	20·9 (17·3 to 25)	17·4 (14·1 to 21·3)	12·5 (9·87 to 15·7)	12·1 (9·44 to 15·3)	11·7 (9·05 to 14·9)
Tuvalu	262 (207 to 322)	167 (139 to 198)	135 (111 to 164)	110 (90·8 to 133)	74·8 (63·4 to 87·1)	53·5 (46·8 to 61·4)	25·7 (21 to 30·8)	18·9 (14·6 to 24)	18·1 (13·8 to 23·2)	17·3 (13·2 to 22·5)
Vanuatu	201 (166 to 236)	158 (139 to 178)	102 (87 to 118)	53·5 (44·6 to 63·3)	37·3 (31·1 to 44)	34·9 (29·6 to 40·6)	28·8 (24·5 to 33·4)	21·9 (17·4 to 27·5)	21·7 (17·1 to 27·6)	20·7 (16·3 to 26·6)
Southeast Asia	280 (250 to 313)	194 (178 to 211)	144 (135 to 152)	104 (98·4 to 111)	71·3 (67·5 to 75·2)	48·4 (45·1 to 52)	32·8 (29·4 to 36·3)	24 (20·1 to 28·5)	22·5 (19 to 26·4)	21·5 (18·2 to 25·4)
Cambodia	274 (222 to 335)	213 (182 to 245)	182 (160 to 206)	162 (146 to 179)	121 (112 to 130)	92·6 (85·3 to 100)	48·9 (42·8 to 55·6)	33·1 (27·5 to 39·6)	32·2 (26·8 to 39)	30·7 (25·5 to 37·4)
Indonesia	311 (271 to 355)	232 (212 to 251)	171 (160 to 183)	121 (113 to 128)	83 (76·8 to 89·4)	53·7 (48·9 to 58·3)	36·9 (32 to 42·4)	26·6 (21·1 to 32·3)	25 (20·2 to 30·5)	24·1 (19·5 to 29·5)
Aceh	250 (205 to 302)	179 (164 to 195)	140 (127 to 155)	103 (94·1 to 113)	77·7 (70·1 to 85·5)	55·9 (51·3 to 60·4)	40·5 (35 to 46·3)	30·8 (24·7 to 37·5)	29 (23·2 to 35·5)	27·9 (22·6 to 34·4)
Bali	263 (213 to 323)	179 (164 to 196)	131 (118 to 144)	90·2 (81·9 to 99·5)	55·2 (49·2 to 61·4)	37·5 (33·7 to 41·6)	27·3 (23·3 to 31·7)	20 (15 to 25·5)	18·8 (14·4 to 23·6)	18·3 (14·3 to 22·8)
Bangka-Belitung Islands	297 (237 to 373)	221 (187 to 258)	167 (151 to 184)	117 (106 to 130)	87·1 (78·4 to 96·4)	50 (43·9 to 56·4)	34·1 (27·4 to 42)	28·5 (21·7 to 37·2)	26·8 (20·5 to 34·7)	25·8 (20 to 33)
Banten	358 (288 to 435)	339 (293 to 387)	249 (231 to 268)	167 (154 to 179)	111 (103 to 120)	62·6 (54·8 to 70·4)	41·5 (33·5 to 51·5)	30·3 (23·4 to 39·2)	28·3 (22·2 to 36·4)	27 (21·2 to 34·2)
Bengkulu	403 (339 to 477)	305 (280 to 331)	218 (200 to 239)	146 (135 to 157)	96·4 (87·9 to 105)	61·5 (55·9 to 67·1)	40·7 (35·8 to 45·6)	26·7 (21·3 to 33·1)	25 (19·7 to 31)	24 (18·8 to 29·7)
Gorontalo	291 (223 to 364)	258 (220 to 298)	196 (179 to 215)	158 (140 to 178)	118 (106 to 130)	86 (75·8 to 96·7)	64·9 (52·6 to 79·3)	47 (35·9 to 61·7)	44·5 (33·6 to 58·2)	42·7 (32·8 to 55·1)
Jakarta	206 (166 to 251)	190 (174 to 207)	129 (118 to 143)	86·9 (78·8 to 95·6)	56·7 (51·1 to 62·6)	37 (33 to 41·6)	25·7 (22·2 to 29·5)	18·2 (13·9 to 23·2)	17 (13·2 to 21·3)	16·5 (13 to 20·6)
Jambi	304 (252 to 362)	237 (218 to 256)	176 (161 to 191)	119 (109 to 129)	81 (72·4 to 89)	51·3 (47·1 to 56·1)	34·7 (30·7 to 38·8)	25·9 (20·9 to 31·5)	24·3 (19·7 to 29·7)	23·5 (18·9 to 28·7)
West Java	364 (319 to 412)	294 (271 to 318)	214 (199 to 229)	146 (136 to 156)	94·3 (86·8 to 102)	56·8 (49·9 to 63·8)	37·7 (32·4 to 43·1)	25·9 (20·6 to 31·7)	24·3 (19·1 to 29·9)	23·3 (18·6 to 28·6)
Central Java	284 (232 to 342)	192 (174 to 211)	139 (127 to 153)	94·1 (85·6 to 103)	62·8 (57·4 to 68·7)	39·1 (35·1 to 43·1)	26·1 (22 to 30·8)	17·4 (13·6 to 21·7)	16·2 (12·9 to 20·1)	15·6 (12·5 to 19·3)
East Java	276 (237 to 321)	201 (184 to 219)	151 (139 to 166)	112 (103 to 122)	77·5 (71·2 to 83·7)	48·7 (42·7 to 54·7)	31·5 (27·1 to 36·2)	21·3 (16·7 to 26·3)	20 (15·5 to 24·6)	19·4 (15·3 to 23·9)
West Kalimantan	270 (222 to 329)	236 (214 to 257)	196 (178 to 215)	145 (133 to 156)	97·2 (88·3 to 107)	59·5 (52·6 to 66·7)	36·2 (32·3 to 40·5)	22·6 (17·3 to 28·6)	21·1 (16·5 to 26·3)	20·2 (16 to 25·1)
South Kalimantan	396 (347 to 448)	347 (321 to 372)	259 (243 to 275)	189 (177 to 202)	129 (116 to 142)	76·2 (68·6 to 84)	48·5 (41·3 to 56)	35·5 (28·1 to 44·3)	33·4 (26·5 to 41·1)	32·1 (25·6 to 39·7)
Central Kalimantan	219 (180 to 261)	158 (145 to 173)	114 (104 to 124)	80·5 (73·8 to 87·4)	65·6 (59·1 to 72·2)	48·8 (44·4 to 53·3)	34·5 (30·7 to 38·6)	27·6 (22·3 to 33·6)	26·1 (21·1 to 31·8)	25·3 (20·4 to 31)
East Kalimantan	198 (160 to 242)	203 (188 to 219)	155 (139 to 170)	102 (92·7 to 112)	65·3 (58·9 to 71·9)	42 (38·2 to 46)	26·1 (23 to 29·4)	19·6 (15·8 to 24)	18·3 (14·8 to 22·3)	17·6 (14·2 to 21·6)
North Kalimantan	170 (136 to 207)	219 (200 to 239)	183 (166 to 201)	127 (113 to 143)	84·3 (73·1 to 95·7)	59·5 (51·8 to 67·9)	40·4 (33·8 to 48·2)	31·7 (24·6 to 39·7)	29·8 (23·2 to 37·5)	28·7 (22·9 to 35·2)
Riau Islands	197 (161 to 241)	172 (144 to 202)	118 (107 to 130)	81·6 (72·8 to 90·9)	60·4 (54·5 to 66·2)	43·8 (39·4 to 48·1)	28·3 (22·9 to 34·9)	20·2 (16·2 to 24·7)	18·9 (15·3 to 23·1)	18·3 (14·7 to 22·4)
Lampung	254 (208 to 307)	234 (213 to 256)	171 (158 to 186)	117 (109 to 127)	79·6 (72·9 to 86·5)	49·4 (45·3 to 53·7)	35·5 (30·3 to 41)	28·5 (22·3 to 35·3)	26·9 (21·2 to 33·2)	26 (20·7 to 32·3)
Maluku	420 (360 to 486)	295 (255 to 336)	219 (203 to 236)	161 (147 to 176)	121 (110 to 132)	87·2 (79·4 to 95·8)	64·7 (53·9 to 77·7)	39 (30·9 to 48)	36·1 (28·8 to 44·4)	34·1 (27·7 to 41·7)
North Maluku	346 (279 to 428)	251 (211 to 293)	190 (172 to 207)	149 (132 to 167)	112 (100 to 125)	83 (73·3 to 93·1)	62·6 (51·2 to 76·8)	44·9 (34·3 to 59·2)	42·1 (32·1 to 55·3)	40·1 (31·1 to 52·2)
West Nusa Tenggara	400 (343 to 460)	331 (306 to 355)	270 (252 to 287)	204 (192 to 216)	137 (124 to 151)	83·7 (76·2 to 91·6)	46·9 (39·9 to 54·4)	31·9 (25·5 to 39·4)	29·9 (24 to 36·7)	28·7 (23 to 35·2)
East Nusa Tenggara	392 (331 to 456)	270 (232 to 310)	209 (192 to 227)	149 (137 to 161)	104 (95·7 to 111)	75 (68·1 to 82·5)	53·7 (44·3 to 65)	35·9 (29 to 43·5)	33·5 (27·1 to 40·5)	31·8 (25·6 to 38·6)
Papua	138 (114 to 167)	174 (146 to 205)	150 (137 to 164)	122 (110 to 135)	98·1 (88·3 to 107)	73·9 (66·9 to 81·3)	62·4 (51·9 to 74·3)	49·7 (39·5 to 60·9)	47·3 (37·9 to 57·7)	45·8 (36·7 to 56·1)
West Papua	142 (114 to 175)	139 (117 to 164)	121 (110 to 133)	89·3 (80·1 to 98·8)	69·3 (62·4 to 76·4)	58 (51·9 to 64·4)	48·3 (40 to 58·1)	31·3 (24·8 to 38·3)	29·5 (23·6 to 36·1)	28·3 (22·6 to 34·6)
Riau	234 (196 to 282)	223 (205 to 244)	172 (159 to 185)	111 (103 to 120)	76·3 (70·2 to 82·5)	49·5 (43·8 to 55·4)	36·8 (32·3 to 41·4)	23·2 (18·4 to 28·5)	21·7 (17·4 to 26·7)	20·8 (16·6 to 25·8)
West Sulawesi	470 (378 to 565)	347 (301 to 396)	271 (249 to 294)	199 (183 to 216)	136 (123 to 148)	88·5 (80·6 to 97·2)	65·6 (54·3 to 78·9)	48·6 (37·7 to 62·9)	45·7 (35 to 58·8)	43·5 (33·9 to 55)
South Sulawesi	336 (286 to 386)	249 (230 to 269)	183 (169 to 197)	132 (123 to 142)	87 (78 to 96·5)	55 (50·6 to 59·6)	36·7 (31·2 to 42·5)	23·3 (18·5 to 29)	21·9 (17·5 to 26·8)	21·1 (17 to 25·8)
Central Sulawesi	329 (286 to 381)	266 (247 to 287)	196 (181 to 213)	142 (132 to 151)	103 (93·6 to 112)	70·7 (64·2 to 77·9)	52·2 (45·5 to 59)	39·6 (32·1 to 47·8)	37·4 (30·2 to 45·5)	35·9 (29·2 to 43·7)
Southeast Sulawesi	352 (302 to 408)	292 (254 to 330)	232 (215 to 251)	153 (142 to 165)	102 (93·9 to 110)	66·9 (60·5 to 73·5)	50·5 (41·2 to 61·5)	42·9 (34 to 52·5)	41 (33 to 50·6)	39·9 (31·8 to 48·9)
North Sulawesi	248 (204 to 299)	155 (141 to 171)	107 (97·6 to 117)	65·2 (59 to 72·6)	50·2 (45·6 to 55·1)	38·6 (34·9 to 42·7)	33·5 (29·1 to 38·4)	28·2 (22·3 to 34·8)	26·8 (21·1 to 33)	26 (20·5 to 32·3)
West Sumatra	534 (473 to 598)	297 (272 to 323)	203 (187 to 219)	130 (120 to 140)	85·4 (78·5 to 92·5)	57·7 (51·7 to 63·8)	37 (32·3 to 42·2)	20·2 (15·9 to 24·8)	18·6 (14·9 to 22·8)	17·7 (14·3 to 21·6)
South Sumatra	309 (267 to 356)	241 (221 to 262)	177 (165 to 193)	114 (105 to 123)	83 (76·1 to 90·4)	57·1 (50·3 to 63·7)	38·7 (33·4 to 44·5)	30·7 (24·2 to 37·6)	28·9 (23·1 to 35·5)	27·8 (22·4 to 34·1)

North Sumatra	279 (235 to 330)	189 (173 to 205)	137 (127 to 148)	98·6 (91·6 to 106)	75·6 (68·8 to 82·9)	53·4 (48·8 to 58·6)	42 (35·7 to 48·8)	36·8 (29·6 to 44·9)	35 (28·3 to 42·9)	33·8 (27·2 to 41·6)
Yogyakarta	325 (256 to 400)	163 (141 to 188)	91·8 (81·9 to 102)	51·5 (47·2 to 56·8)	32·5 (28·9 to 36·8)	20·8 (18·7 to 23·2)	14·8 (12·3 to 17·5)	13·9 (10·5 to 17·8)	12·9 (9·84 to 16·4)	12·5 (9·86 to 15·5)
Laos	322 (262 to 387)	308 (268 to 349)	276 (236 to 320)	228 (197 to 259)	168 (151 to 184)	119 (107 to 131)	69·9 (62 to 78·1)	43·8 (35·2 to 53·2)	42·2 (33·4 to 51·9)	40·2 (31·3 to 50·3)
Malaysia	148 (137 to 158)	95·2 (93·7 to 96·7)	56·1 (54·8 to 57·4)	30·4 (26·8 to 34·3)	17·3 (16·6 to 17·9)	9·05 (8·69 to 9·41)	7·09 (6·78 to 7·41)	7·62 (7·22 to 8·04)	6·71 (6·2 to 7·26)	6·24 (5·55 to 7·01)
Maldives	362 (302 to 423)	298 (256 to 342)	209 (185 to 234)	138 (123 to 154)	63·7 (56·7 to 71·4)	31·6 (27·1 to 36·4)	19·3 (16·6 to 22·1)	13·9 (11·5 to 17·2)	12·4 (10·1 to 15·3)	12·5 (10·1 to 15·6)
Mauritius	155 (144 to 167)	103 (100 to 106)	78·2 (74·7 to 81·7)	39·6 (37·4 to 41·8)	21·4 (19·9 to 23·2)	17·1 (15·7 to 18·5)	14·2 (13·1 to 15·3)	15·6 (13·9 to 17·3)	16·1 (13·3 to 19·1)	12·6 (10·5 to 14·3)
Myanmar	412 (344 to 486)	307 (267 to 349)	249 (218 to 283)	193 (165 to 223)	144 (126 to 162)	108 (94·4 to 122)	66·9 (58 to 75·8)	43·1 (35·3 to 52·5)	40·7 (33·2 to 50)	39·2 (31·7 to 49·3)
Philippines	198 (157 to 247)	135 (119 to 152)	97·7 (88·9 to 107)	78·9 (71·9 to 86·3)	54 (48·2 to 60·3)	36·1 (31·6 to 40·9)	30·2 (26·2 to 34·5)	24·8 (20·5 to 29·7)	22·2 (18·7 to 26·3)	21 (17·3 to 25·3)
Seychelles	101 (87·7 to 117)	95·7 (89·3 to 102)	73·2 (68·7 to 78·2)	32·7 (29·7 to 35·7)	18·6 (16·9 to 20·6)	13·3 (11·7 to 14·9)	14 (12·4 to 15·7)	14·2 (12·2 to 16·5)	14·1 (11·9 to 16·6)	13·3 (10·8 to 16·4)
Sri Lanka	186 (174 to 200)	94·5 (91·8 to 97)	68·3 (65·4 to 71·2)	45·6 (43·7 to 47·5)	20·7 (19·3 to 22·3)	16·6 (15·7 to 17·7)	11·7 (11 to 12·4)	7·11 (5·79 to 8·75)	6·63 (5·16 to 8·28)	5·97 (4·61 to 7·71)
Thailand	194 (164 to 228)	119 (109 to 128)	75·4 (69·1 to 82)	48·5 (43·4 to 53·6)	28·5 (24·7 to 32·7)	17·7 (15·2 to 20·6)	10 (9·54 to 10·6)	8·43 (7·85 to 9·06)	7·64 (6·95 to 8·38)	7·39 (6·54 to 8·3)
Timor-Leste	308 (254 to 369)	256 (223 to 288)	249 (224 to 275)	215 (198 to 234)	137 (126 to 149)	83·7 (74·7 to 93·3)	47·1 (39·4 to 55·3)	36·7 (30 to 44·1)	36 (29·4 to 43·6)	35·2 (29 to 42·7)
Viet Nam	287 (230 to 350)	138 (116 to 162)	93·1 (82·7 to 105)	64 (58·7 to 69·3)	45·2 (41·2 to 49·1)	28·2 (25·1 to 31·6)	18·2 (15·8 to 20·8)	12·7 (10 to 15·8)	12·2 (9·63 to 15·5)	11·1 (8·73 to 14·3)
Sub-Saharan Africa	314 (271 to 361)	261 (234 to 289)	230 (213 to 247)	197 (188 to 207)	173 (166 to 180)	148 (141 to 154)	107 (99·4 to 114)	76·4 (65·9 to 88·3)	73·6 (62·7 to 86·9)	70·7 (59·7 to 84)
Central sub-Saharan Africa	328 (277 to 380)	248 (219 to 279)	221 (198 to 245)	195 (177 to 213)	172 (161 to 184)	154 (143 to 165)	106 (95·9 to 116)	65·6 (56·3 to 76·1)	61·2 (52·5 to 72)	58·3 (49·7 to 68·9)
Angola	310 (257 to 369)	270 (235 to 307)	243 (212 to 277)	225 (199 to 251)	210 (194 to 228)	167 (155 to 180)	96·8 (84·4 to 110)	58·5 (48·5 to 69·3)	55·7 (46·6 to 65·9)	54·7 (45·7 to 65·1)
Central African Republic	262 (214 to 321)	238 (203 to 274)	211 (186 to 239)	201 (180 to 224)	191 (176 to 206)	180 (164 to 197)	155 (131 to 181)	120 (97·4 to 147)	115 (93·4 to 141)	110 (89·2 to 136)
Congo (Brazzaville)	365 (295 to 452)	266 (234 to 299)	218 (191 to 247)	141 (123 to 161)	104 (92 to 116)	104 (92·7 to 116)	65·2 (56·2 to 75·1)	45·1 (37·5 to 53·9)	41·2 (33·9 to 49·4)	39·2 (32·4 to 47·3)
DR Congo	338 (279 to 402)	241 (208 to 276)	217 (191 to 245)	192 (172 to 211)	166 (153 to 179)	153 (139 to 168)	110 (96·7 to 123)	66·5 (55·8 to 79·1)	61·4 (51·4 to 74·3)	57·8 (48·3 to 71·4)
Equatorial Guinea	485 (412 to 564)	303 (263 to 345)	234 (201 to 268)	178 (155 to 201)	164 (144 to 185)	121 (102 to 144)	68·3 (54·7 to 83·9)	48·5 (36·6 to 63·2)	47·7 (35·5 to 64)	46·3 (34·6 to 62·3)
Gabon	488 (406 to 572)	242 (207 to 278)	176 (148 to 206)	109 (95·1 to 125)	80·1 (70 to 90·7)	69·8 (59 to 82)	58 (47·6 to 69·9)	37·7 (28·2 to 49·5)	34·8 (25·7 to 47·3)	32·5 (23·6 to 44·5)
Eastern sub-Saharan Africa	336 (288 to 384)	260 (231 to 288)	226 (208 to 245)	197 (187 to 207)	172 (164 to 180)	134 (127 to 142)	89·1 (80·3 to 97·6)	61·5 (51·4 to 73·4)	59·5 (49 to 72·4)	57·9 (47·4 to 71·6)
Burundi	346 (281 to 413)	279 (244 to 314)	252 (227 to 278)	216 (199 to 233)	182 (169 to 196)	158 (144 to 172)	96 (85·3 to 108)	65·6 (53 to 80·4)	65·4 (51·9 to 82·5)	63·9 (50 to 82)
Comoros	270 (225 to 319)	234 (203 to 266)	196 (174 to 219)	165 (150 to 180)	126 (114 to 140)	104 (90·6 to 119)	74·5 (60·4 to 89·5)	52·1 (42·2 to 63)	49·7 (40·4 to 60·4)	48 (39 to 58·9)
Djibouti	128 (102 to 156)	139 (118 to 162)	126 (108 to 148)	104 (91·4 to 119)	99 (87·4 to 112)	88·1 (75·3 to 101)	64·4 (53·1 to 76·7)	42 (34·1 to 51)	39·2 (31·8 to 47·7)	37·2 (30·1 to 45·6)
Eritrea	314 (256 to 379)	262 (227 to 297)	230 (202 to 260)	190 (171 to 208)	151 (134 to 168)	94·5 (80·5 to 110)	69·9 (54·9 to 87·3)	49·4 (37·7 to 63·9)	47·1 (35·5 to 61·2)	45·5 (34·4 to 60·3)
Ethiopia	365 (303 to 431)	277 (242 to 311)	259 (234 to 285)	234 (216 to 253)	192 (179 to 205)	143 (132 to 155)	86 (76·3 to 96·2)	56·2 (45·7 to 68·6)	54·1 (43·6 to 66·7)	52·2 (41·8 to 65·1)
Addis Ababa	161 (127 to 198)	152 (127 to 179)	134 (116 to 152)	103 (92·4 to 115)	88·4 (77·8 to 99·4)	62·7 (52·8 to 73·5)	29·6 (24·9 to 34·3)	18·1 (14·3 to 22·6)	17·1 (13·5 to 21·2)	16·5 (13·2 to 20·7)
Afar	600 (514 to 688)	341 (301 to 380)	296 (264 to 330)	264 (243 to 287)	199 (183 to 214)	177 (160 to 196)	116 (99·1 to 135)	73·1 (59·7 to 89·6)	69·9 (56 to 87·1)	66·9 (53·5 to 83·2)
Amhara	380 (313 to 454)	280 (244 to 315)	262 (236 to 292)	241 (221 to 261)	196 (182 to 210)	151 (137 to 164)	87·1 (76·7 to 97·6)	55·2 (44·9 to 67·8)	53·1 (42·5 to 66·5)	50·9 (40·3 to 63·3)
Benishangul-Gumuz	531 (454 to 607)	319 (278 to 357)	300 (269 to 332)	285 (260 to 312)	241 (218 to 263)	185 (163 to 211)	108 (94·6 to 123)	69·7 (56·5 to 85·3)	68·1 (54·3 to 85)	65·3 (52·2 to 80·7)
Dire Dawa	272 (219 to 331)	295 (256 to 332)	283 (254 to 315)	248 (226 to 272)	211 (193 to 231)	133 (119 to 148)	68·8 (59 to 79·3)	43·3 (34·5 to 54·6)	41·7 (32·2 to 55)	39·3 (30·4 to 50·4)
Gambella	474 (390 to 561)	338 (295 to 381)	320 (286 to 356)	296 (269 to 324)	241 (221 to 262)	149 (134 to 165)	84·9 (73·6 to 97)	49·4 (39·2 to 62·1)	47·7 (37·5 to 61·4)	45·2 (35 to 58)
Harari	337 (277 to 403)	349 (309 to 388)	320 (289 to 352)	265 (242 to 289)	206 (187 to 226)	121 (107 to 135)	69·4 (60·1 to 79·6)	43·7 (34·6 to 54·2)	41·6 (32·6 to 52·9)	39·2 (30·6 to 50·1)
Oromia	377 (309 to 450)	280 (244 to 313)	259 (234 to 287)	230 (211 to 248)	191 (176 to 205)	140 (128 to 152)	83·1 (74·2 to 92·9)	55·2 (45 to 66·6)	53·1 (43·1 to 65·2)	51·4 (41·2 to 64·3)
Somali	318 (258 to 384)	226 (195 to 257)	209 (186 to 234)	174 (158 to 189)	145 (132 to 158)	139 (126 to 152)	102 (88·4 to 117)	76·2 (62·8 to 91·3)	73·7 (60 to 89)	71·3 (58·2 to 86·7)
Southern Nations, Nationalities, and Peoples	339 (280 to 403)	289 (253 to 324)	274 (247 to 302)	256 (236 to 276)	214 (197 to 232)	151 (138 to 164)	90·3 (79·7 to 101)	56·7 (45·6 to 70·1)	54·8 (43·4 to 69·2)	52·6 (41·7 to 66·4)
Tigray	391 (323 to 464)	281 (245 to 316)	260 (233 to 288)	244 (224 to 266)	179 (163 to 196)	117 (106 to 128)	67·4 (60·2 to 75·1)	39·3 (32·4 to 47·8)	37·4 (30·6 to 45·7)	35·5 (29 to 43·4)
Kenya	269 (235 to 306)	187 (174 to 200)	147 (138 to 157)	111 (104 to 119)	96·2 (89·1 to 103)	85·2 (76·9 to 93·9)	58·2 (49·6 to 67·6)	39·9 (32·5 to 48)	37·5 (30·6 to 45·3)	36·6 (29·7 to 44·7)
Baringo	245 (203 to 293)	196 (179 to 213)	185 (170 to 201)	155 (141 to 168)	130 (112 to 147)	103 (90 to 116)	77·7 (63·8 to 93·1)	58·5 (46·8 to 71·4)	55·5 (44·8 to 67·5)	53·5 (43·3 to 65·1)
Bomet	111 (85·4 to 141)	75·6 (66·1 to 85·8)	72·6 (63·4 to 82·5)	66·2 (58·4 to 74·4)	52·6 (46·8 to 58·5)	44 (37·3 to 51·9)	40·5 (33·3 to 48·5)	28·5 (22·6 to 35·5)	27 (21·7 to 33·2)	25·8 (20·6 to 31·7)
Bungoma	326 (273 to 385)	221 (203 to 238)	168 (155 to 180)	134 (126 to 144)	104 (94·1 to 115)	100 (89·1 to 113)	62·7 (54·2 to 72·1)	38·4 (31·8 to 46)	36·3 (29·9 to 43·9)	37·9 (30·9 to 46·2)
Busia	530 (458 to 612)	330 (302 to 358)	245 (229 to 262)	156 (145 to 169)	135 (122 to 148)	128 (112 to 147)	83·5 (69·9 to 98·4)	54·2 (43·7 to 66·9)	53·5 (42·7 to 66·7)	58·2 (46·1 to 72·2)
Elgeyo Marakwet	180 (147 to 220)	121 (110 to 134)	134 (122 to 145)	117 (107 to 128)	76·8 (68·6 to 84·8)	61·7 (53·2 to 70·4)	34·8 (28·2 to 42·1)	23·7 (18·6 to 29·5)	24 (19·3 to 29·4)	22 (17·6 to 27·3)
Embu	344 (286 to 412)	225 (206 to 244)	141 (131 to 152)	85·6 (79·2 to 92·7)	66·9 (57·4 to 76·6)	56·3 (48·6 to 64·8)	47 (38·7 to 55·6)	32·7 (25·6 to 40·9)	31·1 (24·2 to 38·9)	29·8 (23·2 to 37·2)
Garissa	193 (157 to 232)	172 (149 to 196)	135 (118 to 154)	128 (114 to 143)	98·1 (85·2 to 111)	69·8 (56·2 to 84·9)	46·7 (40·1 to 54·1)	29·6 (24·3 to 35·5)	27·4 (22·7 to 32·8)	25·9 (21·8 to 30·7)
Homa Bay	550 (492 to 612)	368 (344 to 393)	332 (303 to 360)	261 (241 to 281)	213 (199 to 229)	195 (178 to 212)	108 (94·4 to 125)	64·7 (52·9 to 78·6)	59 (48·2 to 71·7)	55·7 (44·8 to 68·3)
Isiolo	375 (313 to 444)	386 (355 to 419)	365 (337 to 394)	290 (261 to 319)	221 (197 to 246)	165 (146 to 185)	121 (105 to 140)	83·1 (68 to 99·9)	79·2 (63·5 to 95·9)	75·7 (60·9 to 91·8)
Kajiado	152 (122 to 186)	108 (97·1 to 119)	104 (94·9 to 114)	85·8 (77·3 to 94·7)	60·8 (54·1 to 67·9)	52·8 (45·7 to 60·2)	36·9 (30·6 to 43·7)	22·7 (18·4 to 27·8)	20·7 (17 to 25·2)	19·5 (16 to 23·6)
Kakamega	328 (278 to 385)	224 (207 to 240)	181 (169 to 195)	140 (131 to 151)	143 (130 to 157)	126 (113 to 139)	81·1 (69·3 to 93·5)	53·2 (43·5 to 64)	49·5 (40·1 to 60)	51·4 (41·6 to 62·9)
Kericho	55·8 (44·2 to 69·3)	63·1 (56·4 to 70)	68·4 (62 to 75·1)	71·8 (65·1 to 79·2)	52·3 (46·2 to 58·3)	54·3 (47·7 to 61·4)	39·9 (33·8 to 47·1)	30·8 (24·7 to 37·7)	29·5 (23·8 to 35·9)	28·6 (23·2 to 34·9)
Kiambu	146 (112 to 184)	87·5 (76·7 to 98·9)	59·6 (52·8 to 67·4)	48·6 (43·3 to 54·5)	44·9 (38·5 to 51·3)	52·1 (45 to 60)	42·5 (35·7 to 50·3)	29·8 (23·1 to 37·4)	28 (21·9 to 34·9)	26·9 (21·3 to 33·3)
Kilifi	383 (328 to 444)	285 (265 to 306)	231 (216 to 246)	174 (157 to 194)	130 (119 to 141)	101 (90 to 113)	65·4 (55·8 to 75·7)	42·4 (34·1 to 51·5)	39·4 (31·6 to 48·3)	36·8 (29·3 to 45·1)
Kirinyaga	317 (273 to 368)	210 (192 to 230)	161 (145 to 178)	78 (69 to 87·7)	58·3 (49 to 68·7)	60·8 (51·5 to 71·7)	49·3 (41·1 to 58·3)	43·7 (34·6 to 53·9)	42·7 (33·8 to 52·7)	41·7 (32·8 to 51·7)
Kisii	198 (153 to 252)	176 (161 to 192)	151 (140 to 163)	126 (108 to 147)	114 (101 to 128)	90·9 (79·3 to 102)	50·1 (40·6 to 61)	33·9 (26·9 to 42)	33·4 (26·4 to 41·2)	32·6 (26 to 40·4)
Kisumu	268 (207 to 336)	186 (169 to 204)	173 (160 to 186)	151 (129 to 175)	142 (127 to 157)	130 (113 to 148)	69·6 (55·4 to 86·4)	44·2 (34·7 to 54·9)	41·7 (32·9 to 51·6)	42·6 (33·1 to 53·4)
Kitui	371 (308 to 444)	229 (213 to 245)	166 (156 to 178)	104 (95·7 to 113)	81·9 (71·5 to 92·4)	73 (62·8 to 83·6)	46·6 (39 to 54·4)	30·7 (24·6 to 37·8)	28·2 (22·6 to 34·7)	26·7 (21·4 to 32·9)
Kwale	364 (312 to 421)	295 (275 to 315)	224 (208 to 239)	162 (143 to 183)	122 (107 to 137)	90·2 (77·9 to 103)	66·6 (55·4 to 78·8)	43·6 (34·1 to 54)	40·6 (32·2 to 49·7)	38·3 (30·5 to 47)
Laikipia	131 (91·3 to 180)	90·8 (75 to 107)	70·5 (62·9 to 79)	52·4 (46·4 to 58·2)	46·9 (37·2 to 57·7)	49·4 (42·1 to 57·1)	29·7 (22·4 to 38·7)	18·8 (12·9 to 26·1)	16·8 (11·6 to 23·2)	15·8 (11 to 22·1)
Lamu	468 (385 to 554)	318 (286 to 355)	222 (200 to 244)	126 (110 to 143)	125 (110 to 142)	107 (90·9 to 124)	80 (66·6 to 93·4)	57 (43·4 to 72·4)	53·8 (41·5 to 68·2)	51 (39·4 to 63·3)
Machakos	184 (149 to 228)	143 (131 to 155)	113 (105 to 122)	79·1 (70·8 to 88·9)	61·5 (53·3 to 70·7)	58·8 (50·7 to 67·9)	38·6 (31·5 to 46·9)	26·4 (20·9 to 32·7)	24·5 (19·4 to 30·7)	23·1 (18·3 to 28·9)
Makueni	340 (276 to 412)	172 (153 to 192)	132 (119 to 145)	93·9 (81·8 to 106)	79·1 (67·2 to 91·3)	59·3 (51·2 to 67·6)	39 (32·5 to 46·3)	23·5 (18·7 to 28·9)	21·2 (16·7 to 26·4)	19·6 (15·5 to 24·5)
Mandera	168 (137 to 202)	140 (121 to 161)	128 (112 to 147)	121 (107 to 136)	117 (102 to 133)	76·5 (62·2 to 92·4)	77·2 (68·5 to 86·8)	58·1 (47·6 to 69·4)	54 (45·1 to 64·4)	51·6 (43·6 to 61·2)
Marsabit	187 (147 to 235)	205 (182 to 231)	166 (148 to 184)	136 (119 to 153)	110 (97·1 to 125)	81·5 (73 to 90·5)	72·3 (61·8 to 84·1)	55·2 (45·8 to 65·7)	51·8 (42·3 to 62·3)	50 (41 to 60·5)

Meru	192 (155 to 239)	129 (116 to 142)	96·3 (88·3 to 105)	61·7 (56·4 to 67)	54·2 (46·4 to 62·8)	54·6 (47·7 to 62)	54·6 (45·9 to 63·8)	45·8 (37·5 to 55·9)	43·6 (34·9 to 53·5)	42 (33·8 to 51·5)
Migori	444 (381 to 516)	314 (289 to 340)	277 (258 to 297)	253 (231 to 274)	224 (209 to 240)	196 (179 to 214)	111 (94·3 to 128)	70·4 (57·3 to 85·6)	65·2 (52·7 to 78·8)	62·2 (50·9 to 75·8)
Mombasa	164 (126 to 206)	191 (173 to 212)	142 (132 to 153)	96·1 (84·4 to 109)	82·8 (72·5 to 93·9)	74·4 (64·4 to 84·7)	52·1 (42·7 to 62·3)	34·3 (26·9 to 42·9)	31·7 (24·6 to 40·3)	29·4 (22·8 to 37·4)
Murang'a	302 (254 to 360)	147 (134 to 162)	101 (89·1 to 113)	56·6 (50·2 to 63·6)	46·1 (39·1 to 53·6)	46·2 (39·7 to 53·4)	43·2 (35·5 to 51·8)	33·3 (26·1 to 42)	32·2 (25·2 to 40·4)	31·1 (24·4 to 39)
Nairobi	173 (128 to 224)	177 (146 to 209)	110 (95·6 to 126)	75·7 (67·4 to 84·8)	69·4 (59·5 to 80·3)	66·3 (56·7 to 76·5)	35·8 (26·6 to 46·3)	22·6 (16 to 30·7)	20·5 (14·6 to 28·5)	19·3 (13·8 to 26·7)
Nakuru	62·5 (49·1 to 78)	55·6 (49·8 to 62)	54·2 (48·7 to 60)	52·6 (47·3 to 58·5)	46·8 (40·7 to 53·1)	49·6 (43·4 to 56·2)	43·5 (35·1 to 52·9)	33·7 (25·9 to 43)	32·4 (25·3 to 40·8)	31·1 (24·3 to 39·2)
Nandi	141 (105 to 186)	91·8 (81·8 to 103)	78·4 (71·2 to 85·6)	68·4 (60·8 to 76·6)	57·3 (50·1 to 64·4)	45·5 (39·8 to 51·4)	32·1 (26·5 to 38·3)	22·5 (17·8 to 28·1)	21·2 (17 to 26·2)	20·9 (16·5 to 26)
Narok	188 (148 to 234)	123 (102 to 147)	114 (97·5 to 133)	70·7 (60·7 to 81·8)	76·5 (64·3 to 89·7)	48·3 (40·9 to 56·4)	36·1 (31·1 to 41·8)	26·5 (20·3 to 33·5)	24·7 (19·5 to 30·7)	23·6 (19·2 to 28·9)
Nyamira	185 (148 to 230)	96·4 (86·9 to 108)	77 (68·7 to 85·6)	64·7 (56·7 to 72·7)	51·2 (46·1 to 57·2)	50 (43·1 to 57·5)	36·3 (29·3 to 43·8)	25·6 (20·4 to 31·6)	25·1 (20 to 31·3)	24·4 (19·3 to 30·3)
Nyandarua	186 (156 to 224)	125 (114 to 137)	82·1 (73·8 to 91·5)	45·9 (39·7 to 52·9)	38·3 (30·3 to 46·7)	43·4 (37·8 to 49·8)	50·9 (41·8 to 60·1)	42·6 (33·1 to 53·3)	41·3 (32·6 to 51·7)	40·2 (31·9 to 50)
Nyeri	263 (206 to 326)	123 (109 to 138)	76·3 (67·1 to 86·2)	44·8 (39·2 to 51·4)	41·3 (35·5 to 48·2)	45·9 (38·4 to 54·1)	39·3 (31·5 to 47·4)	33·6 (26·6 to 41·5)	32 (25·3 to 40)	31·2 (24·3 to 39·2)
Samburu	78·5 (59 to 104)	69·7 (60·3 to 80·1)	78·1 (69·1 to 88·4)	84·7 (74·6 to 95·8)	88 (75·3 to 102)	77·9 (68·5 to 88·9)	69·1 (57·2 to 82·2)	54·2 (42·2 to 67·6)	52·7 (42 to 65·1)	50·8 (41·4 to 62·3)
Siaya	382 (325 to 440)	287 (267 to 308)	238 (223 to 254)	204 (177 to 234)	201 (184 to 220)	186 (167 to 206)	103 (87·7 to 118)	61·2 (49·6 to 74·5)	57·8 (46·7 to 71·3)	61·2 (49·2 to 76·3)
Taita Taveta	261 (218 to 311)	188 (171 to 205)	169 (155 to 183)	107 (93·8 to 122)	76·1 (67 to 85·3)	74·1 (63·1 to 85·5)	48·5 (39·8 to 57·4)	29·1 (22·5 to 36·6)	27·1 (21·1 to 34·3)	25·5 (19·8 to 32·2)
Tana River	279 (216 to 349)	165 (145 to 185)	136 (120 to 152)	118 (104 to 134)	96·8 (84·8 to 110)	98 (83·6 to 113)	74·9 (64·1 to 86·9)	51·3 (41·2 to 62·2)	48·2 (38·4 to 58·5)	46 (37·4 to 55·4)
Tharaka Nithi	343 (280 to 417)	137 (123 to 155)	110 (97·6 to 123)	74·2 (65 to 84·3)	76·2 (65·2 to 87·8)	75·9 (66 to 87·4)	41 (33·1 to 48·8)	28·1 (22·5 to 34·6)	26·8 (21 to 33·2)	25·3 (19·9 to 31·7)
Trans Nzoia	89·6 (72·2 to 109)	94·3 (86·4 to 103)	88 (80·3 to 95·9)	86·1 (78·1 to 93·9)	66 (58·2 to 73·6)	55·7 (48·4 to 63·4)	39·5 (32·4 to 47·5)	28 (22 to 34·8)	25·6 (20·4 to 31·9)	25·5 (20·1 to 32·1)
Turkana	107 (81·5 to 136)	118 (104 to 134)	139 (121 to 159)	153 (138 to 169)	118 (103 to 132)	83·9 (73·3 to 94·9)	65·2 (56·3 to 75·6)	46·1 (36·4 to 56·8)	44·9 (36·5 to 54·7)	43·5 (35·4 to 52·4)
Uasin Gishu	63·8 (50·8 to 79·3)	60 (53·5 to 66·5)	55·4 (50·1 to 61·1)	56·8 (50·9 to 63)	53·1 (46·7 to 59·3)	45·7 (39·7 to 52·3)	37·5 (30·8 to 45·2)	28·4 (22·4 to 35·2)	26·8 (21·3 to 33)	26·3 (21 to 32·3)
Vihiga	383 (320 to 450)	159 (143 to 177)	120 (103 to 137)	94·6 (82·1 to 108)	101 (88·7 to 114)	91 (77·1 to 105)	67·9 (55·9 to 81·5)	47·6 (37·8 to 58·2)	45·7 (36·1 to 56·5)	47·4 (37·6 to 59·1)
Wajir	254 (208 to 309)	239 (211 to 272)	192 (172 to 214)	170 (152 to 189)	131 (115 to 148)	110 (90·5 to 131)	88·2 (78·3 to 98·7)	59·3 (49 to 71·1)	56·6 (47 to 67·3)	53·9 (45·5 to 63·7)
West Pokot	189 (154 to 227)	136 (124 to 149)	135 (122 to 148)	143 (131 to 155)	125 (112 to 140)	87·7 (76·7 to 99·6)	70·3 (57·9 to 84·5)	54·5 (43·4 to 66·2)	52·3 (42·7 to 63)	50·5 (41·1 to 61·3)
Madagascar	314 (257 to 371)	240 (209 to 274)	190 (171 to 210)	164 (151 to 178)	145 (133 to 157)	110 (100 to 121)	87·4 (77 to 99)	64·9 (53·2 to 79)	60·9 (48·9 to 75·2)	57·6 (46·2 to 72·4)
Malawi	369 (307 to 436)	351 (316 to 388)	310 (287 to 334)	250 (234 to 266)	214 (202 to 225)	162 (151 to 174)	95·3 (86·1 to 105)	58·4 (48·9 to 69·6)	55·6 (46·6 to 66·9)	52·1 (43 to 62·7)
Mozambique	348 (294 to 413)	290 (249 to 328)	248 (220 to 279)	226 (209 to 245)	220 (205 to 235)	160 (149 to 174)	105 (93·9 to 118)	68·2 (55·3 to 83·9)	66·2 (53 to 83·3)	62·2 (49·4 to 79·3)
Rwanda	449 (361 to 546)	280 (255 to 306)	259 (237 to 281)	210 (193 to 226)	164 (150 to 179)	143 (130 to 157)	73·1 (62·8 to 84·6)	45·5 (36·9 to 54·9)	42·9 (35·1 to 52)	41·4 (33·7 to 49·8)
Somalia	256 (207 to 312)	248 (215 to 281)	238 (206 to 271)	188 (166 to 210)	192 (175 to 211)	158 (141 to 177)	146 (128 to 166)	98·8 (81·3 to 119)	95·3 (78·3 to 116)	92·3 (75·9 to 112)
South Sudan	248 (194 to 305)	248 (210 to 290)	233 (199 to 269)	219 (188 to 250)	202 (178 to 227)	150 (135 to 167)	125 (105 to 146)	139 (113 to 170)	132 (107 to 162)	129 (103 to 159)
Uganda	378 (315 to 441)	242 (211 to 275)	205 (188 to 223)	200 (186 to 213)	172 (162 to 182)	138 (130 to 146)	89·7 (81·7 to 98·5)	59·6 (49·6 to 72)	61·1 (49 to 76·6)	64·6 (50·6 to 83)
Tanzania	345 (284 to 409)	257 (224 to 290)	215 (197 to 233)	177 (167 to 190)	156 (147 to 166)	125 (116 to 134)	82 (73 to 92)	58 (47·7 to 70·2)	55·3 (44·9 to 68·1)	52·4 (42·4 to 65·6)
Zambia	304 (253 to 368)	250 (219 to 282)	194 (178 to 210)	164 (152 to 175)	166 (155 to 177)	142 (130 to 154)	86 (75·6 to 97·4)	51·1 (40·7 to 63·3)	48·8 (38·9 to 61·6)	46·1 (36·5 to 58·1)
Southern sub-Saharan Africa	202 (162 to 247)	157 (133 to 181)	126 (113 to 141)	104 (95·5 to 112)	76 (70·7 to 81·9)	78 (72·3 to 83·8)	60·2 (54·9 to 65·6)	45·2 (38·2 to 53·3)	44 (36·6 to 52·9)	43·6 (36·2 to 53·2)
Botswana	173 (134 to 219)	130 (109 to 152)	109 (95·8 to 123)	77·6 (69·5 to 86·3)	63·8 (56·4 to 71·8)	72·7 (62·3 to 84·9)	51·5 (42·4 to 63)	41·9 (32·1 to 53·6)	40·9 (30·9 to 53·2)	40·6 (30·3 to 53·9)
Eswatini	313 (259 to 380)	261 (227 to 297)	191 (165 to 217)	124 (109 to 140)	79·4 (72·5 to 86·6)	94·5 (85·9 to 103)	80 (70·4 to 90·1)	44·9 (36 to 55·6)	43 (34·4 to 53·5)	42·1 (33·4 to 53·8)
Lesotho	223 (189 to 263)	184 (168 to 200)	172 (159 to 186)	114 (103 to 127)	89·3 (79·5 to 100)	97 (87·7 to 107)	87·6 (76 to 99·8)	78·5 (65 to 93·2)	78·6 (65·2 to 93·4)	78·8 (64·6 to 94·5)
Namibia	201 (159 to 252)	144 (120 to 170)	107 (93·4 to 122)	92·2 (83·4 to 101)	69·3 (63·2 to 75·8)	66·1 (58·7 to 73·8)	52·6 (45·6 to 60·9)	35·4 (28 to 44·3)	34·1 (26·9 to 43·1)	33·4 (26·1 to 43)
South Africa	203 (159 to 252)	160 (135 to 186)	130 (114 to 148)	109 (98·4 to 120)	78·2 (71·8 to 85·1)	77·3 (70·1 to 84·5)	52·8 (46·5 to 59·6)	40·2 (33·8 to 47·7)	39 (32·5 to 47·1)	38·6 (31·9 to 47·1)
Eastern Cape	337 (268 to 416)	214 (181 to 247)	180 (158 to 204)	141 (126 to 157)	104 (93·8 to 115)	94·3 (84 to 104)	68 (59·9 to 76·2)	49·9 (41·6 to 59·5)	49·2 (41 to 59·9)	48·5 (39·9 to 59·7)
Free State	207 (158 to 265)	183 (151 to 215)	158 (138 to 181)	140 (124 to 155)	97·6 (87·3 to 109)	101 (89·2 to 112)	71·3 (63·1 to 80·3)	55·4 (46·7 to 66·3)	51·8 (43·2 to 62·9)	51·1 (42·6 to 62·4)
Gauteng	91·7 (66·5 to 123)	96·2 (77·6 to 116)	83·4 (71·4 to 97·3)	83·4 (72·9 to 95·1)	61·4 (53·6 to 69·1)	72·1 (63·9 to 80·9)	44·5 (38·7 to 50·5)	33·7 (27·9 to 40·3)	32·4 (26·6 to 39·7)	31·8 (26·1 to 39·2)
KwaZulu-Natal	198 (155 to 252)	174 (147 to 202)	140 (124 to 159)	116 (105 to 128)	83·6 (74·8 to 93·2)	87·1 (78·3 to 95·7)	55·6 (48·7 to 63)	40·4 (33·2 to 48·3)	40·6 (33·5 to 49)	40·1 (33 to 48·9)
Limpopo	203 (157 to 259)	140 (117 to 163)	111 (96·1 to 127)	86·1 (76·8 to 96·4)	60·7 (54·6 to 67·2)	55·5 (49·5 to 62·2)	51·5 (44·7 to 58·5)	46·5 (38·9 to 55·2)	45·2 (37·8 to 53·7)	45·4 (38·1 to 55)
Mpumalanga	213 (165 to 266)	168 (141 to 196)	149 (131 to 171)	117 (105 to 130)	76·6 (68·7 to 85·6)	74·1 (65·6 to 82·8)	51·5 (45 to 58·1)	41 (34·1 to 49)	39·7 (32·9 to 47·9)	39·6 (32·8 to 48·4)
North West	198 (152 to 250)	180 (152 to 209)	146 (127 to 167)	122 (109 to 137)	88·4 (79·2 to 98·6)	92·2 (81·5 to 104)	67·9 (59·6 to 76·6)	52·5 (44·2 to 62)	49·9 (41·5 to 59·9)	49·3 (41 to 59·8)
Northern Cape	156 (119 to 202)	137 (112 to 164)	121 (104 to 141)	98·8 (86·8 to 112)	78·7 (69·6 to 88·2)	72·6 (63·5 to 82·7)	61·4 (53·5 to 69·4)	48·9 (41·1 to 58·4)	45·7 (38·5 to 55·4)	44·9 (37·4 to 55)
Western Cape	77·7 (54·7 to 107)	63·3 (51 to 78·2)	57·6 (48·6 to 67·5)	57·8 (49·7 to 66·3)	45·9 (39·9 to 52·4)	41·1 (35·9 to 46·6)	27 (23·1 to 31·2)	19·7 (16·3 to 23·8)	18·4 (15·2 to 22·4)	17·9 (14·8 to 22·1)
Zimbabwe	182 (143 to 226)	132 (111 to 154)	105 (92·8 to 117)	89·8 (82·1 to 98·1)	70·4 (65·3 to 75·7)	78·5 (72·7 to 84·2)	75·6 (69·2 to 83·3)	55 (46·4 to 65·2)	53·2 (44·3 to 63·8)	52·7 (43·6 to 64·5)
Western sub-Saharan Africa	321 (275 to 367)	288 (260 to 316)	259 (242 to 275)	218 (208 to 229)	192 (185 to 200)	169 (161 to 177)	128 (120 to 136)	94·1 (81·8 to 107)	90·7 (78·1 to 105)	86·3 (73·5 to 101)
Benin	381 (317 to 452)	292 (263 to 323)	245 (228 to 263)	198 (184 to 212)	174 (162 to 186)	141 (131 to 152)	113 (103 to 124)	83·7 (69·4 to 99·2)	80·1 (65·5 to 96·9)	77·3 (62·8 to 95·2)
Burkina Faso	373 (314 to 436)	351 (310 to 391)	297 (270 to 324)	248 (232 to 264)	208 (195 to 221)	180 (165 to 194)	140 (122 to 159)	103 (85·2 to 124)	103 (84·5 to 125)	95·5 (77·9 to 117)
Cabo Verde	292 (268 to 321)	162 (156 to 168)	160 (155 to 165)	85·4 (80·2 to 90·6)	65·1 (61·3 to 69)	50·3 (42·9 to 59)	27·4 (23 to 32·6)	17·2 (13·6 to 21·6)	15·8 (12·3 to 20·3)	15 (11·3 to 19·7)
Cameroon	323 (281 to 366)	259 (238 to 280)	207 (192 to 222)	171 (160 to 182)	136 (126 to 146)	129 (119 to 139)	101 (90·4 to 113)	70·4 (58·8 to 82·4)	69·6 (57·9 to 83)	65·5 (54·3 to 77·6)
Chad	360 (298 to 425)	275 (238 to 311)	262 (235 to 291)	236 (219 to 254)	200 (187 to 214)	181 (168 to 197)	145 (131 to 161)	117 (99·2 to 138)	115 (97·7 to 136)	112 (94·6 to 134)
Côte d'Ivoire	275 (237 to 315)	267 (249 to 286)	215 (201 to 229)	159 (148 to 170)	147 (137 to 157)	139 (128 to 150)	108 (95·8 to 121)	75·3 (64·2 to 88·5)	72·5 (61·7 to 85·5)	68·5 (58·2 to 80·6)
The Gambia	193 (149 to 242)	224 (188 to 260)	190 (165 to 216)	165 (148 to 183)	129 (116 to 143)	102 (91·5 to 114)	68·2 (59·4 to 77·5)	49·2 (40·2 to 60)	46·4 (37·4 to 57)	44·2 (35·3 to 55·4)
Ghana	266 (217 to 323)	213 (194 to 232)	179 (166 to 193)	149 (138 to 160)	123 (114 to 132)	98·8 (91·3 to 107)	75·9 (67·6 to 84·9)	50·3 (40·2 to 62·1)	47·1 (37·1 to 59·2)	43·4 (33·6 to 55·5)
Guinea	357 (303 to 416)	332 (292 to 373)	310 (278 to 345)	267 (243 to 290)	225 (209 to 241)	178 (163 to 193)	133 (119 to 148)	95·6 (80·9 to 112)	92·6 (77·9 to 110)	86·8 (72·7 to 104)
Guinea-Bissau	490 (431 to 551)	362 (317 to 406)	287 (255 to 323)	244 (217 to 272)	203 (183 to 225)	163 (148 to 178)	102 (88·4 to 116)	66·9 (55·3 to 80·6)	64·8 (53·5 to 78·6)	61·8 (50·9 to 75·1)
Liberia	500 (412 to 590)	350 (310 to 389)	296 (269 to 324)	247 (223 to 273)	247 (226 to 268)	170 (155 to 186)	96·1 (83·6 to 110)	73·2 (58·8 to 90·9)	72·7 (56·6 to 93·1)	66·9 (51·7 to 87·8)
Mali	402 (344 to 470)	362 (324 to 400)	335 (312 to 359)	281 (263 to 299)	237 (223 to 251)	193 (178 to 207)	146 (132 to 160)	106 (89·4 to 125)	104 (87·1 to 125)	97·7 (81·4 to 118)
Mauritania	249 (209 to 291)	188 (171 to 205)	146 (135 to 158)	128 (117 to 139)	100 (91·4 to 109)	82·6 (74·9 to 90·9)	57·9 (51·4 to 65·3)	38·5 (32·3 to 45·4)	35·9 (30·1 to 42·3)	33·7 (28·3 to 40·2)
Niger	345 (291 to 403)	359 (321 to 398)	334 (306 to 361)	304 (287 to 323)	283 (266 to 302)	223 (204 to 243)	125 (107 to 145)	95·1 (79·2 to 114)	92 (75·7 to 113)	88·7 (72·1 to 110)
Nigeria	309 (254 to 368)	281 (250 to 313)	260 (236 to 285)	223 (209 to 237)	203 (193 to 213)	183 (174 to 193)	142 (131 to 153)	106 (91·7 to 122)	101 (86·4 to 118)	96·3 (81·8 to 114)
São Tomé and Príncipe	411 (383 to 442)	267 (254 to 280)	146 (140 to 153)	112 (104 to 119)	105 (96·8 to 115)	77·9 (69·5 to 86·8)	39·6 (33·9 to 45·7)	20·3 (15·8 to 25·8)	18·5 (14·2 to 24·1)	17·8 (13·5 to 23·2)

Senegal	297<br>(250 to 347)	273<br>(253 to 294)	263<br>(246 to 281)	201<br>(188 to 215)	138<br>(129 to 148)	119<br>(111 to 128)	71·7<br>(65·4 to 78·3)	46·7<br>(39·2 to 55)	43·3<br>(36·4 to 51·1)	40·5<br>(33·9 to 47·9)
Sierra Leone	376<br>(308 to 447)	331<br>(288 to 374)	293<br>(256 to 333)	263<br>(237 to 290)	237<br>(221 to 253)	217<br>(203 to 232)	155<br>(140 to 171)	103<br>(84·5 to 124)	103<br>(82·3 to 128)	97·2<br>(77·3 to 121)
Togo	321<br>(262 to 387)	267<br>(233 to 301)	215<br>(194 to 235)	167<br>(153 to 182)	142<br>(129 to 154)	122<br>(110 to 135)	94<br>(83·1 to 105)	63·4<br>(52·1 to 76·7)	60·1<br>(49·3 to 74·1)	56·7<br>(45·7 to 70·8)

Appendix 2 Table S2b: Under-5 mortality rates with 95% UIs by location, females, 1950, 1960, 1970, 1980, 1990, 2000, 2010, 2019, 2020, 2021

Location	1950	1960	1970	1980	1990	2000	2010	2019	2020	2021
Global	226 (200 to 253)	279 (227 to 365)	133 (126 to 139)	107 (101 to 112)	81·6 (78·2 to 85·3)	68·5 (65·3 to 71·7)	50·5 (47·3 to 53·8)	36·4 (32 to 41·2)	34·5 (30·1 to 39·9)	33·4 (29 to 38·8)
Central Europe, eastern Europe, and central Asia	116 (105 to 127)	66·2 (62·7 to 69·8)	43·7 (40·9 to 46·8)	33·5 (32·2 to 34·8)	27·5 (26·5 to 28·7)	23·5 (22·3 to 24·8)	14·5 (13·5 to 15·6)	11·3 (10 to 12·6)	10·7 (9·44 to 12·1)	10·5 (9·24 to 11·9)
Central Asia	248 (217 to 280)	141 (125 to 157)	86·1 (75·9 to 97·7)	63·6 (59·6 to 67·8)	50·5 (47·5 to 53·7)	43·6 (40·2 to 47·3)	28·6 (25·7 to 31·7)	20·3 (17·4 to 23·6)	19·1 (16·3 to 22·4)	18·2 (15·5 to 21·6)
Armenia	270 (230 to 315)	151 (127 to 175)	83·6 (70·6 to 97·2)	54·7 (48·6 to 61·4)	38·1 (33·8 to 43·5)	26·5 (23·5 to 29·9)	15·4 (13·4 to 17·7)	11·3 (9·33 to 13·9)	12·1 (9·95 to 14·7)	9·86 (7·94 to 12·3)
Azerbaijan	409 (360 to 459)	262 (232 to 291)	143 (126 to 165)	86·9 (77·4 to 96·7)	72·7 (65 to 80·4)	61·4 (54·5 to 69·7)	38·6 (33·4 to 44·4)	28·5 (23·4 to 34)	27·1 (22·4 to 32·5)	25·2 (20·5 to 30·8)
Georgia	203 (168 to 239)	126 (107 to 145)	77·4 (63·2 to 94·3)	49·1 (43 to 56·2)	35·7 (31·4 to 40·5)	30·6 (26·7 to 35)	15 (12·5 to 17·6)	9·31 (7·63 to 11·4)	9·06 (7·36 to 11·2)	8·87 (7·07 to 11·1)
Kazakhstan	121 (99·4 to 147)	82·4 (70·1 to 96·2)	48·8 (41 to 57·5)	40·7 (36·2 to 44·9)	31·7 (28·7 to 35·2)	31·3 (27·8 to 35·2)	20·8 (17·7 to 24)	10·3 (8·59 to 12·3)	9·64 (7·98 to 11·6)	8·87 (7·32 to 10·8)
Kyrgyzstan	218 (173 to 266)	141 (122 to 160)	94·4 (77·8 to 112)	70·9 (62·3 to 80·2)	51·3 (46·4 to 56·2)	38·4 (34·5 to 42·6)	23·9 (23 to 24·9)	16·6 (15·6 to 17·7)	15·7 (14·3 to 17·2)	15·3 (13·5 to 17·2)
Mongolia	362 (297 to 434)	211 (182 to 238)	138 (122 to 154)	103 (92·8 to 114)	77·9 (71·6 to 85)	49·6 (44·6 to 54·8)	27 (23·9 to 30·6)	16·8 (14·1 to 19·9)	15·8 (13·3 to 18·9)	15·2 (12·6 to 18·5)
Tajikistan	392 (332 to 459)	232 (201 to 265)	131 (113 to 151)	95·4 (86·5 to 105)	74·1 (68 to 81)	60·4 (54·3 to 67)	38·6 (33·6 to 43·9)	33·7 (28·1 to 40)	31 (25·6 to 37·1)	29·9 (24·7 to 36·1)
Turkmenistan	274 (231 to 321)	145 (127 to 163)	91·4 (74·6 to 111)	78·7 (69·1 to 89·6)	62·8 (56·5 to 69·7)	51·5 (45·8 to 57·3)	35·9 (31·4 to 41·1)	26·3 (21·7 to 31·6)	24·8 (20·3 to 29·9)	23·9 (19·4 to 29·2)
Uzbekistan	259 (210 to 314)	106 (89·1 to 123)	75·2 (64·1 to 87·7)	58·3 (52·4 to 63·9)	46·1 (42·1 to 50·7)	40 (35·5 to 45)	28·9 (24·4 to 33·9)	20·7 (17·1 to 24·8)	19·5 (16·2 to 23·4)	18·8 (15·6 to 22·7)
Central Europe	160 (154 to 166)	69·3 (68 to 70·6)	43·9 (43 to 44·8)	26·3 (25·8 to 26·9)	19·6 (19·2 to 20)	12 (11·6 to 12·3)	6·63 (6·46 to 6·8)	4·86 (4·61 to 5·13)	4·69 (4·4 to 4·99)	4·58 (4·19 to 4·99)
Albania	279 (234 to 335)	145 (126 to 165)	85·3 (73·4 to 99·3)	47 (40·7 to 53·5)	37·9 (34·1 to 42)	26·1 (23 to 29·7)	15·9 (13·7 to 18·2)	13·6 (11·5 to 16·2)	13·6 (11 to 16·5)	12 (10 to 14·4)
Bosnia and Herzegovina	280 (251 to 311)	120 (109 to 132)	72·9 (67 to 78·8)	36·3 (32·5 to 40·2)	15·1 (14 to 16)	9·57 (8·87 to 10·3)	5·95 (5·42 to 6·55)	6·27 (5·68 to 6·89)	5·94 (5·16 to 6·81)	4·93 (4·22 to 5·72)
Bulgaria	177 (164 to 189)	52·4 (50·8 to 54·2)	28·6 (27·6 to 29·6)	22·3 (21·5 to 23·1)	17 (16·3 to 17·8)	15·5 (14·6 to 16·4)	10 (9·63 to 10·5)	6·26 (5·76 to 6·86)	5·83 (5·38 to 6·34)	6·09 (5·51 to 6·71)
Croatia	214 (195 to 233)	72·4 (69·9 to 75)	39·7 (37·8 to 41·6)	20·2 (19·3 to 21·3)	10·3 (9·61 to 11)	7·28 (6·91 to 7·66)	4·75 (4·39 to 5·1)	3·93 (3·51 to 4·39)	4·08 (3·64 to 4·55)	4·1 (3·52 to 4·75)
Czechia	48·4 (44·8 to 52·5)	20·3 (19·6 to 21·1)	20·1 (19·3 to 20·8)	15·6 (15 to 16·2)	10·3 (9·85 to 10·7)	4·68 (4·43 to 4·97)	2·98 (2·79 to 3·18)	2·66 (2·45 to 2·89)	2·38 (2·18 to 2·61)	2·3 (2 to 2·63)
Hungary	117 (107 to 127)	47·4 (45·9 to 49)	35·9 (34·6 to 37·3)	21·7 (20·9 to 22·6)	15 (14·4 to 15·6)	9·41 (8·88 to 9·97)	5·58 (5·25 to 5·96)	3·92 (3·56 to 4·32)	3·75 (3·4 to 4·15)	3·67 (3·2 to 4·17)
Montenegro	105 (92 to 120)	66·3 (62·6 to 70·5)	42·9 (40·1 to 45·9)	25 (22·9 to 27·1)	15·1 (13·6 to 16·8)	11·2 (9·98 to 12·4)	6·48 (5·88 to 7·12)	2·81 (2·46 to 3·19)	3·74 (3·1 to 4·49)	3·49 (2·87 to 4·22)
North Macedonia	255 (221 to 291)	183 (166 to 200)	105 (98·4 to 112)	62·8 (56·3 to 69·2)	38 (36·1 to 39·9)	14·1 (13·4 to 15)	9·01 (8·27 to 9·77)	6·09 (5·53 to 6·72)	5·92 (5·32 to 6·52)	5·14 (4·58 to 5·72)
Poland	153 (148 to 159)	55·9 (54·5 to 57·3)	33·2 (32·2 to 34·3)	21·3 (20·8 to 21·9)	16 (15·5 to 16·4)	8·49 (8·16 to 8·83)	5·27 (5·06 to 5·48)	3·96 (3·74 to 4·22)	3·83 (3·54 to 4·18)	4·06 (3·64 to 4·51)
Romania	141 (123 to 162)	83·9 (81·5 to 86·3)	52·4 (50·9 to 54)	33·2 (32·1 to 34·4)	29·6 (28·5 to 30·7)	20 (19·1 to 21)	10·2 (9·73 to 10·7)	6·68 (6·24 to 7·2)	6·38 (6·01 to 6·77)	6·07 (5·6 to 6·57)
Serbia	262 (250 to 276)	87 (84·5 to 89·7)	64 (61·8 to 66·3)	34·5 (33·3 to 35·8)	22·3 (21·5 to 23·1)	11·8 (10·1 to 13·7)	5·74 (4·99 to 6·64)	4·9 (4·51 to 5·25)	4·72 (4·38 to 5·08)	4·17 (3·75 to 4·6)
Slovakia	89·7 (83·5 to 96·7)	31·4 (30·3 to 32·6)	26·7 (25·6 to 27·8)	20·1 (19·3 to 20·9)	12·5 (11·9 to 13·2)	8·73 (8·31 to 9·17)	6·03 (5·62 to 6·43)	5·39 (5·03 to 5·75)	5·39 (4·98 to 5·85)	5·14 (4·67 to 5·67)
Slovenia	161 (148 to 173)	67·5 (64·7 to 70·5)	34·5 (33 to 36)	16·6 (15·7 to 17·4)	8·86 (8·23 to 9·56)	4·77 (4·38 to 5·16)	3·02 (2·74 to 3·32)	2·02 (1·81 to 2·25)	2·02 (1·83 to 2·23)	1·96 (1·76 to 2·17)
Eastern Europe	61·4 (48·7 to 77·4)	40·7 (39·6 to 41·9)	24·7 (23·5 to 26)	23·3 (22·4 to 24·3)	17·5 (16·8 to 18·2)	15·5 (15·1 to 16)	8·52 (8·33 to 8·71)	5·69 (5·44 to 5·93)	5·28 (5·03 to 5·55)	5·35 (4·95 to 5·75)
Belarus	74·1 (57 to 93·5)	49·8 (42 to 58·5)	28·7 (24·3 to 33·9)	20·9 (17·7 to 24·2)	15·4 (13·2 to 17·8)	14·2 (12·1 to 16·5)	6·84 (5·72 to 8·07)	4·13 (3·3 to 5·12)	3·55 (2·72 to 4·57)	3·42 (2·71 to 4·42)
Estonia	49·5 (37·1 to 64·5)	31·3 (29·7 to 32·9)	18·9 (17·9 to 19·9)	18·4 (17·4 to 19·3)	14·2 (13·5 to 15)	9·77 (9·1 to 10·5)	4·26 (3·87 to 4·64)	2·24 (2 to 2·52)	2·2 (1·97 to 2·46)	2·4 (2·14 to 2·69)
Latvia	35·5 (26·2 to 46·4)	27·7 (26·3 to 29·1)	18·1 (17·3 to 19)	17·8 (17 to 18·6)	14·7 (14 to 15·4)	12·2 (11·5 to 13)	7·11 (6·62 to 7·67)	3·77 (3·41 to 4·16)	3·74 (3·35 to 4·11)	3·46 (3·04 to 3·91)
Lithuania	76·9 (59·7 to 96·8)	42·5 (40·7 to 44·3)	19·6 (18·8 to 20·5)	16·9 (16·2 to 17·7)	12·8 (12·2 to 13·5)	9·68 (9·08 to 10·3)	5·45 (5·12 to 5·77)	3·47 (3·15 to 3·81)	3·21 (2·93 to 3·5)	3·17 (2·89 to 3·49)
Moldova	163 (136 to 198)	99·1 (85·7 to 113)	61·8 (50·5 to 74·6)	44·1 (38·5 to 50·8)	27·9 (24·7 to 31·7)	24·4 (21·3 to 27·6)	14·4 (12·3 to 16·6)	10·8 (8·73 to 13·4)	10·9 (8·46 to 14·2)	9·43 (7·24 to 12·2)
Russia	61·5 (44·7 to 83·1)	40·4 (39·3 to 41·5)	23·9 (23·1 to 24·7)	23·5 (22·8 to 24·2)	17·9 (17·4 to 18·3)	16·2 (15·7 to 16·7)	8·36 (8·11 to 8·61)	5·35 (5·17 to 5·54)	4·88 (4·66 to 5·13)	5·11 (4·78 to 5·45)
Ukraine	49·1 (38·3 to 62·3)	34·8 (29·7 to 40·5)	23·3 (19·5 to 27·7)	21·9 (18·7 to 25·3)	16·4 (14·3 to 18·7)	13·7 (13·1 to 14·3)	9·39 (9·03 to 9·78)	7·69 (6·9 to 8·64)	7·59 (6·65 to 8·68)	7·14 (5·68 to 8·54)
High income	55·7 (53·5 to 58·2)	40·3 (39·6 to 41·1)	26·3 (25·8 to 26·8)	15 (14·7 to 15·3)	9·85 (9·7 to 10)	6·8 (6·71 to 6·89)	5·42 (5·35 to 5·48)	4·65 (4·59 to 4·72)	4·42 (4·25 to 4·59)	4·16 (3·88 to 4·47)
Australasia	27·4 (25·8 to 29·1)	22 (21·5 to 22·6)	18·3 (17·8 to 18·8)	11·6 (11·2 to 12)	8·51 (8·24 to 8·82)	5·86 (5·65 to 6·08)	4·75 (4·54 to 4·96)	3·79 (3·56 to 4·02)	3·56 (3·31 to 3·83)	2·99 (2·61 to 3·42)
Australia	26·9 (25 to 28·8)	21·4 (20·8 to 22·2)	18·4 (17·8 to 18·9)	11·3 (10·8 to 11·7)	8·28 (7·98 to 8·61)	5·61 (5·39 to 5·86)	4·45 (4·25 to 4·66)	3·59 (3·37 to 3·81)	3·47 (3·2 to 3·78)	2·7 (2·29 to 3·15)
New Zealand	29·4 (26·9 to 32·2)	24·3 (23·2 to 25·4)	17·7 (16·8 to 18·8)	13·2 (12·4 to 14)	9·52 (8·94 to 10·1)	6·92 (6·42 to 7·41)	6·14 (5·72 to 6·61)	4·78 (4·4 to 5·2)	4 (3·71 to 4·31)	4·38 (3·98 to 4·77)
High-income Asia Pacific	85·8 (78·3 to 95·1)	56·2 (52·6 to 60)	28·1 (25·8 to 30·3)	14·1 (12·8 to 15·4)	7·71 (7·12 to 8·35)	4·81 (4·67 to 4·96)	3·22 (3·1 to 3·34)	2·55 (2·46 to 2·65)	2·31 (2·18 to 2·46)	2·1 (1·96 to 2·25)
Brunei	196 (178 to 214)	107 (101 to 113)	47·4 (44·7 to 50·3)	19 (17·2 to 20·8)	10·6 (9·7 to 11·6)	9·37 (8·22 to 10·6)	9·27 (8·31 to 10·4)	9·63 (8·4 to 11)	9·6 (8·14 to 11·3)	8·72 (7·12 to 10·7)
Japan	54·5 (51·5 to 57·8)	34·1 (33·1 to 35)	14·6 (14·1 to 15·1)	8·63 (8·37 to 8·92)	5·63 (5·43 to 5·85)	4·06 (3·89 to 4·23)	2·97 (2·84 to 3·09)	2·38 (2·27 to 2·49)	2·14 (1·97 to 2·33)	2·02 (1·81 to 2·27)
Aichi	47·8 (40·8 to 55·4)	32·2 (29·2 to 35)	13·4 (12 to 14·9)	8·04 (7·65 to 8·49)	5·23 (4·92 to 5·55)	3·86 (3·62 to 4·15)	3·06 (2·83 to 3·3)	2·36 (2·19 to 2·54)	2·18 (2·02 to 2·35)	2·09 (1·83 to 2·39)
Akita	48·2 (41·1 to 56·4)	34 (31·3 to 37·1)	16·9 (15·8 to 18·3)	8·88 (8·27 to 9·63)	6·03 (5·56 to 6·53)	3·87 (3·47 to 4·3)	2·85 (2·59 to 3·12)	2·42 (2·19 to 2·67)	2·23 (1·87 to 2·64)	2·08 (1·78 to 2·43)
Aomori	55·3 (48·2 to 63·6)	36·4 (33·4 to 39·6)	17·8 (16·4 to 19·2)	9·96 (9·34 to 10·6)	6·66 (6·19 to 7·2)	4·94 (4·56 to 5·36)	3·57 (3·28 to 3·88)	3·08 (2·81 to 3·35)	2·74 (2·3 to 3·24)	2·57 (2·19 to 2·98)
Chiba	49·8 (42·4 to 58·8)	31·5 (28·4 to 34·9)	12·2 (10·8 to 13·8)	7·84 (7·44 to 8·27)	4·97 (4·68 to 5·32)	4·01 (3·75 to 4·29)	3·12 (2·87 to 3·36)	2·56 (2·36 to 2·78)	2·33 (2·15 to 2·53)	2·19 (1·93 to 2·49)
Ehime	55·2 (48·4 to 61·9)	36·1 (33·2 to 39·1)	16·5 (15·2 to 18)	9·22 (8·6 to 9·86)	5·98 (5·55 to 6·47)	3·96 (3·66 to 4·29)	2·62 (2·39 to 2·84)	2·2 (2·02 to 2·39)	1·95 (1·64 to 2·3)	1·86 (1·59 to 2·16)
Fukui	50·7 (42·1 to 60)	33·5 (30 to 37·4)	15·1 (13·4 to 17)	7·96 (7·27 to 8·66)	5·79 (5·25 to 6·37)	4·31 (3·89 to 4·76)	2·83 (2·61 to 3·07)	2·28 (2·06 to 2·52)	2·49 (2·07 to 2·98)	2·33 (1·98 to 2·73)
Fukuoka	50·7 (42·4 to 59·3)	32·2 (29·1 to 35·4)	14·9 (13·1 to 16·8)	8·33 (7·96 to 8·77)	5·84 (5·52 to 6·18)	4·24 (3·97 to 4·51)	3·09 (2·87 to 3·34)	2·72 (2·51 to 2·93)	2·43 (2·24 to 2·64)	2·34 (2·05 to 2·69)
Fukushima	67·8 (59·2 to 77·4)	40·3 (37·1 to 43·7)	20 (18·4 to 21·7)	10·3 (9·74 to 10·9)	6·67 (6·27 to 7·1)	4·54 (4·21 to 4·89)	3·11 (2·9 to 3·33)	2·79 (2·6 to 2·98)	2·63 (2·32 to 2·99)	2·52 (2·19 to 2·92)
Gifu	52·1 (44·5 to 61·6)	35·7 (32·8 to 38·8)	15·8 (14·8 to 16·8)	8·94 (8·43 to 9·45)	5·23 (4·83 to 5·68)	3·89 (3·64 to 4·17)	3·1 (2·91 to 3·29)	2·45 (2·27 to 2·64)	2·15 (1·82 to 2·5)	2·02 (1·75 to 2·33)
Gunma	56·7 (49 to 65)	37 (33·7 to 40·6)	15·6 (14·4 to 16·8)	8·62 (8·14 to 9·15)	5·47 (5·05 to 5·89)	4·1 (3·86 to 4·36)	2·96 (2·74 to 3·18)	2·3 (2·11 to 2·49)	2·17 (1·84 to 2·52)	2·08 (1·79 to 2·39)
Hiroshima	58·5 (51·6 to 65·6)	36·4 (33·4 to 39·4)	15·2 (14 to 16·4)	9 (8·47 to 9·54)	5·7 (5·33 to 6·08)	3·77 (3·47 to 4·12)	3·07 (2·76 to 3·4)	2·43 (2·26 to 2·62)	2·11 (1·86 to 2·4)	1·98 (1·73 to 2·29)
Hokkaidō	47·7 (40·3 to 56·1)	32·5 (29·2 to 35·6)	16 (14·1 to 18)	9·17 (8·68 to 9·68)	5·49 (5·16 to 5·84)	3·78 (3·48 to 4·09)	2·73 (2·54 to 2·92)	2·41 (2·18 to 2·65)	2·28 (2·09 to 2·48)	2·22 (1·94 to 2·55)
Hyōgo	47·2 (39·5 to 55·4)	29·8 (26·8 to 32·9)	13 (11·4 to 14·6)	7·9 (7·54 to 8·32)	5·36 (5·04 to 5·71)	3·87 (3·61 to 4·13)	2·63 (2·44 to 2·84)	2·08 (1·94 to 2·26)	1·87 (1·73 to 2·03)	1·76 (1·55 to 2·02)
Ibaraki	70·2 (59·1 to 82·8)	40·7 (36·1 to 45·5)	17·9 (15·8 to 20·2)	9·92 (9·36 to 10·6)	5·61 (5·19 to 6·03)	4·15 (3·87 to 4·45)	3·26 (3·04 to 3·5)	2·97 (2·76 to 3·18)	2·82 (2·58 to 3·06)	2·71 (2·36 to 3·13)
Ishikawa	51·5 (44·4 to 60·4)	37 (33·8 to 40·6)	16·2 (15 to 17·5)	9·05 (8·47 to 9·69)	5·8 (5·26 to 6·39)	4·3 (3·94 to 4·69)	2·87 (2·61 to 3·14)	2·13 (1·93 to 2·34)	1·87 (1·58 to 2·24)	1·75 (1·5 to 2·05)
Iwate	65·3 (55·6 to 76)	43·3 (39·5 to 47·2)	21·7 (19·8 to 23·7)	11·2 (10·4 to 12·1)	5·63 (5·2 to 6·13)	4·06 (3·72 to 4·42)	3·58 (3·23 to 3·93)	2·99 (2·75 to 3·24)	2·53 (2·14 to 2·96)	2·38 (2·06 to 2·76)
Kagawa	51·2 (43·9 to 59·7)	35·3 (32·3 to 38·5)	15·3 (14·2 to 16·5)	8·37 (7·7 to 9·1)	5·69 (5·15 to 6·3)	3·78 (3·43 to 4·15)	3·1 (2·85 to 3·37)	2·36 (2·17 to 2·56)	2·22 (1·85 to 2·67)	2·1 (1·8 to 2·47)
Kagoshima	75·5 (65·5 to 86·3)	40·5 (36·6 to 44·6)	21·1 (19·6 to 22·6)	10·6 (10 to 11·3)	6·29 (5·89 to 6·75)	4·6 (4·3 to 4·94)	3·26 (3·04 to 3·48)	2·68 (2·47 to 2·9)	2·45 (2·08 to 2·86)	2·32 (2 to 2·67)
Kanagawa	45·4 (35·7 to 57)	28 (24·1 to 32·2)	11·4 (10·2 to 12·6)	7·88 (7·39 to 8·4)	5·39 (5·03 to 5·72)	4·11 (3·83 to 4·4)	3·09 (2·85 to 3·34)	2·4 (2·12 to 2·68)	1·92 (1·78 to 2·08)	1·73 (1·51 to 1·97)
Kōchi	72·3 (62·8 to 82·3)	43·1 (39·7 to 46·9)	19·5 (18·1 to 21·1)	10·1 (9·27 to 11)	7·03 (6·44 to 7·68)	4·58 (4·17 to 5·1)	3·66 (3·32 to 4·03)	2·82 (2·56 to 3·11)	2·72 (2·27 to 3·23)	2·57 (2·19 to 3)
Kumamoto	58·9 (51·6 to 67·1)	38·9 (35·9 to 42·3)	19·1 (17·6 to 20·6)	9·9 (9·44 to 10·4)	5·9 (5·47 to 6·38)	4·14 (3·83 to 4·47)	2·93 (2·62 to 3·25)	2·4 (2·2 to 2·6)	2·42 (2·09 to 2·77)	2·2 (1·91 to 2·53)

Kyōto	49·6 (43 to 56·5)	31 (28·5 to 33·7)	12·4 (11·4 to 13·6)	7·54 (7·1 to 7·97)	5·64 (5·21 to 6·12)	3·96 (3·63 to 4·32)	3 (2·78 to 3·25)	2·64 (2·44 to 2·84)	2·12 (1·85 to 2·43)	2 (1·74 to 2·32)
Mie	54·9 (47·6 to 63·5)	36·9 (34·2 to 40)	15·9 (14·6 to 17·2)	8·99 (8·44 to 9·55)	5·81 (5·36 to 6·3)	4·33 (4·03 to 4·65)	3·18 (2·95 to 3·42)	2·17 (1·98 to 2·34)	2·03 (1·72 to 2·37)	1·91 (1·63 to 2·21)
Miyagi	55·3 (47·8 to 64)	35 (32·4 to 37·8)	16·3 (15 to 17·5)	9·13 (8·58 to 9·69)	5·59 (5·25 to 5·94)	3·77 (3·48 to 4·06)	3·03 (2·81 to 3·27)	2·52 (2·26 to 2·78)	2·23 (1·93 to 2·56)	2·09 (1·81 to 2·4)
Miyazaki	58·8 (51 to 66·8)	38 (34·8 to 41·6)	18·2 (16·7 to 19·9)	9·57 (8·86 to 10·4)	6·49 (6·03 to 6·99)	4·39 (4·03 to 4·75)	3·06 (2·78 to 3·36)	2·51 (2·32 to 2·73)	2·4 (2·04 to 2·81)	2·28 (1·96 to 2·63)
Nagano	65·2 (56·7 to 74·7)	38·7 (35·7 to 42·2)	15·8 (14·6 to 17·1)	8·45 (7·97 to 8·94)	5·54 (5·2 to 5·94)	3·38 (3·14 to 3·63)	2·52 (2·35 to 2·69)	2·04 (1·89 to 2·19)	1·94 (1·66 to 2·25)	1·85 (1·6 to 2·12)
Nagasaki	53·8 (47·4 to 60·8)	30·2 (27·7 to 32·9)	15·2 (14·2 to 16·2)	8·31 (7·72 to 8·92)	5·6 (5·15 to 6·07)	4·42 (4·09 to 4·77)	3·41 (3·19 to 3·65)	2·84 (2·61 to 3·04)	2·6 (2·2 to 3·03)	2·51 (2·18 to 2·9)
Nara	53·4 (46·4 to 61·1)	33·2 (30·4 to 36·2)	13·8 (12·7 to 14·9)	8·71 (8·03 to 9·39)	5·51 (5·11 to 5·95)	3·79 (3·48 to 4·14)	3·12 (2·82 to 3·44)	2·61 (2·4 to 2·8)	2·5 (2·11 to 2·95)	2·39 (2·06 to 2·78)
Niigata	58 (50·3 to 66·3)	36·1 (33·2 to 39·3)	16·4 (15·1 to 17·6)	8·88 (8·38 to 9·45)	5·06 (4·76 to 5·4)	4·05 (3·71 to 4·37)	2·6 (2·39 to 2·83)	2·21 (2·03 to 2·4)	2·09 (1·8 to 2·4)	2·02 (1·74 to 2·34)
Ōita	59·2 (52·1 to 68·4)	38·4 (35·3 to 41·9)	18·5 (16·9 to 20·2)	9·88 (9·29 to 10·5)	5·88 (5·41 to 6·38)	3·93 (3·6 to 4·32)	3·1 (2·81 to 3·39)	2·37 (2·17 to 2·6)	2·19 (1·85 to 2·59)	2·07 (1·78 to 2·4)
Okayama	55·2 (47·6 to 63·4)	36 (33·3 to 39·1)	14·6 (13·4 to 15·9)	8·34 (7·85 to 8·82)	5·99 (5·58 to 6·45)	4·18 (3·89 to 4·5)	2·8 (2·64 to 2·98)	2·32 (2·15 to 2·51)	2·1 (1·81 to 2·4)	1·97 (1·71 to 2·29)
Okinawa	102 (89·7 to 116)	41 (37·6 to 44·9)	19 (17·3 to 20·7)	9·6 (8·99 to 10·2)	6·71 (6·27 to 7·18)	4·8 (4·44 to 5·24)	3·09 (2·76 to 3·42)	2·32 (2·13 to 2·52)	2·29 (1·99 to 2·65)	2·16 (1·86 to 2·52)
Ōsaka	50 (40 to 62·9)	30·4 (26·5 to 34·6)	12·3 (11·2 to 13·4)	8·19 (7·77 to 8·63)	5·89 (5·53 to 6·24)	3·94 (3·7 to 4·19)	2·81 (2·6 to 3·05)	2·34 (2·08 to 2·62)	2·04 (1·87 to 2·23)	1·82 (1·6 to 2·09)
Saga	59 (51·2 to 69)	36·1 (33·1 to 39·2)	17·1 (15·7 to 18·7)	9·15 (8·49 to 9·92)	6·14 (5·64 to 6·71)	4·39 (4·02 to 4·79)	2·76 (2·48 to 3·06)	2·12 (1·93 to 2·32)	1·97 (1·65 to 2·34)	1·88 (1·61 to 2·19)
Saitama	49·5 (42 to 57·5)	30·4 (27·3 to 33·5)	12·1 (10·8 to 13·4)	7·88 (7·41 to 8·36)	5·19 (4·91 to 5·49)	3·92 (3·67 to 4·18)	2·79 (2·58 to 3·01)	2·07 (1·92 to 2·23)	1·79 (1·66 to 1·94)	1·64 (1·43 to 1·89)
Shiga	57·6 (48·3 to 67·8)	36·7 (33·6 to 40)	14·8 (13·5 to 16·2)	8·09 (7·49 to 8·75)	5·59 (5·11 to 6·11)	4·3 (4·01 to 4·63)	3·02 (2·79 to 3·27)	2·26 (2·08 to 2·44)	2·06 (1·75 to 2·41)	1·95 (1·67 to 2·26)
Shimane	56 (46·9 to 66·6)	38·2 (33·2 to 44)	18·2 (15·9 to 21·2)	9·15 (8·42 to 9·95)	6·13 (5·63 to 6·66)	4·23 (3·81 to 4·71)	2·82 (2·54 to 3·1)	2·21 (2 to 2·45)	2·02 (1·68 to 2·44)	1·87 (1·59 to 2·19)
Shizuoka	47·2 (41·1 to 54)	29 (26·7 to 31·5)	12·7 (11·7 to 13·9)	7·43 (7·03 to 7·9)	5·33 (5·03 to 5·65)	3·66 (3·37 to 3·96)	2·82 (2·62 to 3·02)	2·59 (2·38 to 2·8)	2·42 (2·2 to 2·64)	2·36 (2·06 to 2·7)
Tochigi	60·4 (53·4 to 68)	38·4 (35·2 to 42·3)	17·1 (15·8 to 18·6)	9·69 (9·09 to 10·3)	6·19 (5·73 to 6·7)	4·81 (4·42 to 5·21)	3·41 (3·19 to 3·62)	2·9 (2·67 to 3·12)	2·62 (2·28 to 3·02)	2·48 (2·15 to 2·88)
Tokushima	54·2 (45 to 64·9)	35·9 (31·6 to 40·8)	16·8 (15 to 19)	9·15 (8·44 to 9·87)	6·01 (5·45 to 6·63)	4·63 (4·24 to 5·04)	3·74 (3·41 to 4·1)	2·93 (2·61 to 3·26)	2·76 (2·29 to 3·32)	2·59 (2·21 to 3·02)
Tōkyō	43·2 (33·3 to 55·2)	28·6 (24·3 to 33·6)	12·7 (11·4 to 14·1)	8·08 (7·62 to 8·59)	5·45 (5·11 to 5·78)	4·08 (3·82 to 4·37)	2·77 (2·56 to 3)	2·02 (1·8 to 2·25)	1·78 (1·67 to 1·9)	1·71 (1·51 to 1·95)
Tottori	58·4 (49 to 69·9)	37·9 (33·5 to 42·8)	17·9 (15·9 to 20·4)	9·07 (8·36 to 9·92)	5·77 (5·19 to 6·42)	3·86 (3·4 to 4·34)	3·31 (2·98 to 3·67)	2·78 (2·52 to 3·06)	2·46 (2·04 to 2·96)	2·34 (1·99 to 2·76)
Toyama	56 (48·8 to 63·6)	41·2 (37·4 to 45)	18·1 (16·8 to 19·6)	9·78 (9·02 to 10·6)	6·67 (6·11 to 7·23)	4·48 (4·1 to 4·91)	3·15 (2·88 to 3·42)	2·29 (2·07 to 2·52)	2·07 (1·74 to 2·44)	1·96 (1·68 to 2·3)
Wakayama	68 (58·4 to 78·3)	41·1 (37 to 45·5)	17·7 (16·4 to 19·2)	10·7 (9·97 to 11·6)	5·92 (5·43 to 6·45)	4·16 (3·76 to 4·6)	3·09 (2·81 to 3·4)	2·35 (2·13 to 2·61)	2·41 (1·99 to 2·89)	2·33 (1·97 to 2·73)
Yamagata	57·1 (49·7 to 65)	37·8 (34·7 to 41·5)	18·1 (16·8 to 19·6)	9·08 (8·47 to 9·74)	5·31 (4·88 to 5·77)	4·02 (3·63 to 4·44)	3·07 (2·86 to 3·31)	2·64 (2·43 to 2·87)	2·64 (2·25 to 3·09)	2·48 (2·15 to 2·87)
Yamaguchi	52·6 (45·8 to 60·7)	35·9 (32·9 to 39·1)	16·1 (14·7 to 17·7)	9·45 (8·83 to 10·2)	6 (5·53 to 6·51)	4·24 (3·88 to 4·66)	3·1 (2·77 to 3·42)	2·83 (2·61 to 3·04)	2·57 (2·2 to 3)	2·46 (2·13 to 2·82)
Yamanashi	58·4 (49·2 to 70·1)	34·9 (31·5 to 38·9)	15·3 (13·7 to 17·1)	8·56 (7·89 to 9·28)	5·99 (5·44 to 6·58)	4·38 (3·93 to 4·83)	2·69 (2·44 to 2·96)	2·14 (1·95 to 2·35)	2·01 (1·69 to 2·41)	1·88 (1·59 to 2·19)
South Korea	187 (157 to 224)	93 (83·2 to 103)	56·3 (49·1 to 63·6)	25·2 (21·3 to 29·2)	11·7 (9·85 to 13·6)	6·41 (6·15 to 6·7)	3·82 (3·62 to 4·03)	3·08 (2·86 to 3·32)	2·81 (2·61 to 3·02)	2·27 (1·82 to 2·72)
Singapore	112 (103 to 123)	45·4 (43·4 to 47·3)	27·3 (25·5 to 29·2)	15·3 (14·2 to 16·5)	8·4 (7·71 to 9·08)	3·5 (3·14 to 3·9)	2·44 (2·17 to 2·74)	1·84 (1·63 to 2·08)	1·63 (1·44 to 1·84)	1·74 (1·49 to 2·04)
High-income North America	32·4 (31·2 to 33·7)	27·5 (27·1 to 27·8)	20·5 (20·1 to 20·8)	13·1 (12·9 to 13·4)	9·58 (9·4 to 9·74)	7·29 (7·17 to 7·43)	6·38 (6·28 to 6·49)	5·74 (5·62 to 5·86)	5·52 (5·39 to 5·66)	5·18 (4·77 to 5·63)
Canada	40·4 (38·6 to 42·4)	27·2 (26·6 to 27·8)	18·7 (18·2 to 19·2)	11 (10·7 to 11·4)	7·3 (7·03 to 7·56)	5·27 (5·06 to 5·48)	5·09 (4·87 to 5·31)	4·34 (4·17 to 4·5)	4·46 (4·15 to 4·79)	3·69 (3·13 to 4·34)
Greenland	160 (143 to 179)	85·5 (80 to 91·1)	51·7 (47·3 to 56·1)	39·5 (34·7 to 45·3)	28·8 (25·6 to 32·5)	18·6 (16·5 to 20·8)	11·9 (10·4 to 13·5)	9·64 (8·21 to 11·2)	10·4 (8·91 to 11·9)	10·2 (8·8 to 11·8)
USA	31·6 (30·3 to 33)	27·5 (27·1 to 27·9)	20·6 (20·3 to 21)	13·3 (13·1 to 13·6)	9·79 (9·61 to 9·97)	7·46 (7·33 to 7·6)	6·5 (6·38 to 6·63)	5·87 (5·75 to 6·01)	5·63 (5·5 to 5·76)	5·33 (4·89 to 5·79)
Alabama	38·9 (33·2 to 46)	35·1 (33·8 to 36·5)	25·5 (24·4 to 26·5)	16·1 (15·3 to 17)	11·3 (10·7 to 12)	9·84 (9·23 to 10·5)	8·93 (8·4 to 9·49)	8·06 (7·59 to 8·57)	7·29 (6·83 to 7·83)	7·09 (6·46 to 7·86)
Alaska	41·7 (31·8 to 52·2)	40·4 (38 to 42·8)	22·4 (20·9 to 24)	15·4 (14·4 to 16·6)	10·6 (9·91 to 11·4)	8·06 (7·49 to 8·6)	6·38 (5·87 to 6·87)	6·71 (6·2 to 7·26)	6·42 (5·85 to 7·02)	6·38 (5·4 to 7·51)
Arizona	40·4 (31 to 52·6)	35·5 (33·6 to 37·4)	21·5 (20·4 to 22·8)	13·9 (12·9 to 15)	10·1 (9·52 to 10·7)	7·81 (7·34 to 8·37)	6·72 (6·28 to 7·17)	5·99 (5·6 to 6·39)	5·95 (5·56 to 6·36)	5·56 (5·07 to 6·1)
Arkansas	33 (23·5 to 44)	30·1 (28·7 to 31·5)	22·2 (21·1 to 23·4)	13·5 (12·7 to 14·4)	10·7 (10·1 to 11·6)	8·85 (8·24 to 9·49)	7·71 (7·21 to 8·27)	7·64 (7·11 to 8·19)	7·83 (7·17 to 8·48)	7·47 (6·36 to 8·77)
California	29·2 (25 to 33·9)	23·7 (22·6 to 24·8)	18·3 (17·6 to 19·1)	12·3 (11·5 to 13·1)	9·48 (9·13 to 9·83)	6·47 (6·18 to 6·8)	5·29 (5·07 to 5·5)	4·64 (4·41 to 4·88)	4·31 (4·09 to 4·53)	4·05 (3·63 to 4·49)
Colorado	32·1 (27·6 to 37·5)	27 (25·9 to 28·2)	19·1 (17·9 to 20·2)	10·9 (10·2 to 11·6)	8·44 (7·89 to 9·07)	6·69 (6·24 to 7·16)	6·02 (5·63 to 6·42)	5·09 (4·7 to 5·48)	4·64 (4·23 to 5·05)	4·25 (3·8 to 4·71)
Connecticut	26·7 (19·6 to 34·1)	21·1 (20·2 to 22·1)	16 (15·2 to 16·8)	11·1 (10·4 to 11·7)	7·9 (7·49 to 8·35)	6·16 (5·79 to 6·56)	5·31 (4·92 to 5·68)	4·44 (4·06 to 4·85)	4·2 (3·84 to 4·56)	4·03 (3·31 to 4·84)
Delaware	33 (22·5 to 45·4)	25·1 (23·5 to 26·9)	19·7 (18·4 to 21·2)	14·2 (12·9 to 15·4)	11 (10·2 to 11·9)	9·47 (8·67 to 10·3)	8·31 (7·69 to 8·95)	7·45 (6·78 to 8·17)	6·83 (6·21 to 7·59)	6·69 (5·63 to 7·94)
Washington, DC	56·7 (39·2 to 79·7)	45·7 (43 to 48·6)	36·2 (33·6 to 39)	32·8 (30 to 35·6)	35·6 (32·9 to 38·4)	17 (15·4 to 18·7)	12·2 (10·4 to 14)	7·39 (6·52 to 8·32)	6·75 (5·87 to 7·72)	6·56 (5·31 to 8)
Florida	39·2 (33·2 to 46)	32·3 (31·2 to 33·4)	23·7 (22·8 to 24·7)	16·3 (15·6 to 17·1)	11·3 (10·8 to 11·7)	8·45 (8·06 to 8·92)	7·34 (7·03 to 7·69)	6·35 (6·03 to 6·67)	6·09 (5·81 to 6·39)	5·73 (5·18 to 6·36)
Georgia	37·2 (31·6 to 43·6)	34·3 (32·7 to 36·1)	24·1 (23·1 to 25·3)	15·8 (14·8 to 16·8)	12·5 (11·9 to 13·1)	8·93 (8·54 to 9·33)	7·15 (6·69 to 7·66)	7·85 (7·45 to 8·27)	7·23 (6·83 to 7·64)	7 (6·37 to 7·71)
Hawaii	28·4 (21·7 to 35·6)	25·2 (24 to 26·5)	18 (17 to 19·1)	11·2 (10·5 to 12)	8·56 (7·97 to 9·17)	7·76 (7·17 to 8·3)	6·18 (5·76 to 6·59)	5·92 (5·45 to 6·42)	5·48 (5·07 to 5·96)	5·34 (4·52 to 6·34)
Idaho	25·4 (19·4 to 32·1)	24·3 (23 to 25·7)	17·7 (16·7 to 18·8)	11·4 (10·7 to 12·1)	8·74 (8·18 to 9·35)	6·91 (6·47 to 7·36)	5·52 (5·25 to 5·82)	4·64 (4·25 to 5·03)	4·55 (4·19 to 4·91)	4·13 (3·46 to 4·91)
Illinois	30·4 (25·2 to 36·1)	26·6 (25·7 to 27·5)	22·3 (21·6 to 23·1)	15·6 (15 to 16·2)	11·3 (10·8 to 11·8)	8·84 (8·46 to 9·22)	7·1 (6·75 to 7·47)	5·82 (5·5 to 6·13)	5·67 (5·45 to 5·91)	5·44 (4·96 to 5·95)
Indiana	26·4 (22·8 to 31·1)	24·9 (24·1 to 25·7)	19·6 (18·8 to 20·4)	12·3 (11·7 to 13)	9·44 (8·97 to 9·96)	8·21 (7·75 to 8·72)	7·69 (7·26 to 8·15)	6·79 (6·36 to 7·24)	6·85 (6·43 to 7·29)	6·46 (5·82 to 7·08)
Iowa	23·4 (17·4 to 30)	22·2 (21·3 to 23·1)	17·8 (17·1 to 18·6)	11 (10·4 to 11·7)	7·48 (7·05 to 7·91)	6·08 (5·77 to 6·43)	4·96 (4·66 to 5·27)	4·98 (4·64 to 5·35)	4·72 (4·37 to 5·08)	4·45 (3·7 to 5·29)
Kansas	25·6 (19·3 to 32·9)	22·9 (21·9 to 23·9)	18·2 (17·3 to 19·1)	11·7 (11 to 12·4)	8·43 (7·95 to 8·96)	7·4 (6·98 to 7·86)	6·8 (6·42 to 7·23)	6 (5·6 to 6·45)	6·47 (5·88 to 7·05)	5·83 (4·96 to 6·75)
Kentucky	32·6 (23·6 to 43·3)	30·7 (29·4 to 32·1)	20·5 (19·7 to 21·3)	13·2 (12·5 to 14)	8·8 (8·29 to 9·38)	7·24 (6·8 to 7·73)	7·15 (6·78 to 7·54)	5·48 (5·13 to 5·83)	6·15 (5·71 to 6·64)	5·75 (4·84 to 6·79)
Louisiana	41·1 (33·3 to 50·8)	34·9 (33·5 to 36·5)	25·1 (24 to 26·2)	15·9 (15·2 to 16·7)	12 (11·4 to 12·7)	10·2 (9·63 to 10·8)	8·2 (7·69 to 8·77)	8·22 (7·69 to 8·76)	7·88 (7·43 to 8·33)	7·48 (6·77 to 8·23)
Maine	31·1 (23·6 to 39·2)	27·1 (25·8 to 28·3)	19·7 (18·7 to 20·7)	9·76 (9·12 to 10·4)	6·92 (6·46 to 7·43)	5·65 (5·25 to 6·1)	6·41 (5·89 to 6·94)	6·09 (5·58 to 6·62)	6·02 (5·54 to 6·54)	5·86 (4·92 to 6·97)
Maryland	32·7 (28·1 to 38·5)	28·6 (27·7 to 29·6)	19·1 (18·1 to 20·1)	14·4 (13·6 to 15·4)	10·1 (9·49 to 10·7)	8·16 (7·67 to 8·68)	6·9 (6·47 to 7·37)	5·9 (5·54 to 6·27)	5·68 (5·34 to 6·06)	5·57 (5·01 to 6·19)
Massachusetts	28·2 (19·9 to 37·4)	21·7 (20·8 to 22·5)	16·5 (15·8 to 17·3)	10·4 (9·82 to 11)	7·06 (6·66 to 7·49)	5·06 (4·74 to 5·44)	4·58 (4·27 to 4·9)	3·79 (3·51 to 4·07)	3·54 (3·24 to 3·86)	3·24 (2·71 to 3·79)
Michigan	26·8 (22·5 to 31·7)	24·3 (23·4 to 25·2)	20·2 (19·5 to 21)	12·8 (12·2 to 13·4)	10·3 (9·81 to 10·8)	8·04 (7·68 to 8·4)	7·09 (6·69 to 7·48)	6·42 (6·07 to 6·79)	6·45 (6·09 to 6·81)	5·78 (5·31 to 6·32)
Minnesota	24·8 (17·3 to 33·3)	22·6 (21·7 to 23·4)	16·5 (15·7 to 17·4)	9·92 (9·26 to 10·6)	6·66 (6·25 to 7·08)	5·44 (5·12 to 5·8)	4·68 (4·38 to 5·01)	4·77 (4·47 to 5·08)	4·29 (3·94 to 4·63)	4·08 (3·44 to 4·75)
Mississippi	52·3 (37·9 to 68·9)	45·2 (43·4 to 47·1)	31·7 (30·1 to 33·2)	18·4 (17·4 to 19·6)	13·5 (12·8 to 14·4)	11·8 (11·1 to 12·6)	10·3 (9·57 to 10·9)	9·04 (8·44 to 9·66)	8·76 (8·02 to 9·51)	8·55 (7·35 to 9·91)
Missouri	30·9 (26·3 to 36·2)	26·3 (25·2 to 27·4)	20·4 (19·5 to 21·3)	13·3 (12·5 to 14)	9·5 (9·02 to 10)	7·58 (7·12 to 8·09)	6·8 (6·39 to 7·26)	6·43 (6·05 to 6·87)	6·29 (5·9 to 6·69)	6·09 (5·49 to 6·72)
Montana	28·5 (22·1 to 35·3)	26·9 (25·7 to 28·2)	21·5 (20·3 to 22·8)	12·5 (11·7 to 13·3)	8·56 (7·94 to 9·23)	6·92 (6·45 to 7·43)	6·3 (5·8 to 6·83)	5·18 (4·76 to 5·63)	5·19 (4·77 to 5·65)	4·76 (3·95 to 5·73)
Nebraska	27·2 (20 to 35·2)	23·7 (22·6 to 24·9)	17·9 (16·9 to 19)	11·6 (10·9 to 12·3)	7·95 (7·41 to 8·49)	7·25 (6·75 to 7·81)	5·73 (5·27 to 6·19)	5·46 (4·98 to 5·95)	5·5 (5·07 to 5·98)	5·03 (4·16 to 6·02)
Nevada	40 (30 to 50·3)	34·6 (32·6 to 36·7)	22·2 (20·9 to 23·5)	12·6 (11·8 to 13·5)	8·55 (8·04 to 9·05)	6·84 (6·47 to 7·26)	5·96 (5·56 to 6·41)	5·85 (5·49 to 6·28)	5·28 (4·96 to 5·62)	5·1 (4·34 to 5·95)
New Hampshire	27·8 (20·2 to 36·6)	24·1 (22·7 to 25·5)	17·3 (16·4 to 18·3)	10·1 (9·44 to 10·8)	6·69 (6·23 to 7·15)	5·01 (4·66 to 5·38)	4·63 (4·24 to 5·04)	3·84 (3·42 to 4·28)	4·03 (3·58 to 4·54)	3·65 (2·95 to 4·45)
New Jersey	31·3 (26·1 to 37·6)	25·6 (24·5 to 26·7)	19·8 (18·9 to 20·6)	12·8 (12·2 to 13·5)	9·6 (9·08 to 10·1)	6·58 (6·19 to 6·98)	5·09 (4·77 to 5·41)	4·03 (3·77 to 4·32)	3·85 (3·6 to 4·13)	3·54 (3·17 to 3·88)
New Mexico	46 (32·4 to 62·1)	36·3 (34·4 to 38·3)	23·5 (22·3 to 24·8)	13 (12·3 to 13·6)	9·26 (8·7 to 9·85)	6·96 (6·53 to 7·47)	6·13 (5·69 to 6·65)	5·91 (5·4 to 6·46)	5·78 (5·36 to 6·23)	5·53 (4·6 to 6·57)

New York	31·7 (26·9 to 36·4)	26·1 (25·3 to 26·9)	20·5 (19·8 to 21·2)	13·3 (12·8 to 13·8)	10·8 (10·5 to 11·3)	6·92 (6·62 to 7·23)	5·64 (5·35 to 5·92)	4·33 (4·1 to 4·59)	3·9 (3·71 to 4·09)	3·66 (3·25 to 4·08)
North Carolina	37·8 (31·8 to 45·2)	33·4 (32·2 to 34·7)	25·1 (24 to 26·1)	14·8 (14·1 to 15·6)	10·8 (10·2 to 11·3)	9·02 (8·5 to 9·57)	7·29 (6·94 to 7·7)	7·16 (6·79 to 7·57)	7·14 (6·73 to 7·53)	6·99 (6·38 to 7·71)
North Dakota	29·1 (20·2 to 39·2)	24·7 (23 to 26·4)	15·8 (14·6 to 17)	11·6 (10·6 to 12·6)	8·17 (7·47 to 9)	7·44 (6·79 to 8·09)	6·73 (6·04 to 7·42)	6·24 (5·75 to 6·77)	6·03 (5·51 to 6·6)	5·75 (4·86 to 6·77)
Ohio	25·9 (21·4 to 30·8)	24·5 (23·7 to 25·3)	18·8 (18·2 to 19·5)	13 (12·4 to 13·6)	9·42 (9 to 9·85)	7·86 (7·53 to 8·22)	7·8 (7·42 to 8·2)	6·97 (6·6 to 7·36)	6·66 (6·36 to 6·99)	6·35 (5·81 to 6·94)
Oklahoma	27·2 (23·5 to 32)	27·3 (26·2 to 28·4)	20·6 (19·6 to 21·8)	12·8 (12 to 13·6)	9·53 (8·95 to 10·1)	8·59 (8·02 to 9·17)	8·11 (7·61 to 8·65)	7·18 (6·69 to 7·69)	6·25 (5·74 to 6·8)	6·13 (5·51 to 6·87)
Oregon	25·1 (18·5 to 31·6)	24 (22·9 to 25·1)	16·9 (16·1 to 17·7)	11·6 (11 to 12·2)	7·78 (7·29 to 8·27)	5·91 (5·56 to 6·32)	5·12 (4·8 to 5·45)	4·83 (4·48 to 5·19)	4·42 (4·07 to 4·77)	4·11 (3·41 to 4·88)
Pennsylvania	29·3 (24·1 to 34·9)	25·1 (24·3 to 26)	20·4 (19·8 to 21·1)	13·4 (12·8 to 14)	9·43 (9·04 to 9·86)	7·18 (6·83 to 7·54)	7·03 (6·65 to 7·44)	5·79 (5·47 to 6·13)	5·74 (5·44 to 6·05)	5·51 (5·01 to 6·07)
Rhode Island	31·3 (23 to 40·6)	25·3 (23·9 to 26·8)	19·3 (18·1 to 20·5)	12·4 (11·5 to 13·4)	8·35 (7·76 to 8·96)	6·64 (6·12 to 7·24)	6·59 (6·01 to 7·14)	5·63 (5 to 6·31)	4·95 (4·49 to 5·44)	4·52 (3·67 to 5·46)
South Carolina	37·3 (31·8 to 43·8)	36·6 (35·4 to 38·1)	24·4 (23·1 to 25·7)	17·3 (16·4 to 18·2)	12·6 (11·9 to 13·4)	9·1 (8·53 to 9·65)	7·22 (6·76 to 7·65)	6·78 (6·36 to 7·2)	6·3 (5·81 to 6·74)	6·02 (5·46 to 6·59)
South Dakota	29·1 (21·9 to 36·9)	28·1 (26·6 to 29·6)	20·9 (19·8 to 22·2)	12·8 (11·9 to 13·6)	9·7 (9·11 to 10·4)	7·58 (7·05 to 8·14)	7·05 (6·52 to 7·62)	7·04 (6·6 to 7·53)	6·96 (6·42 to 7·56)	6·71 (5·62 to 7·95)
Tennessee	34·9 (29·7 to 40·8)	31·5 (30·4 to 32·6)	22·5 (21·5 to 23·5)	13·9 (13·1 to 14·6)	10·6 (10 to 11·2)	9·36 (8·77 to 10)	8·04 (7·55 to 8·59)	7·43 (6·95 to 7·92)	6·54 (6·16 to 6·95)	6·3 (5·76 to 6·95)
Texas	34·8 (29·7 to 39·7)	31·8 (30·9 to 32·8)	22·8 (22 to 23·6)	13·8 (13·2 to 14·3)	8·95 (8·59 to 9·33)	6·73 (6·46 to 7·03)	6·47 (6·21 to 6·74)	6·2 (5·9 to 6·52)	5·95 (5·72 to 6·18)	5·66 (5·08 to 6·25)
Utah	23·2 (17·2 to 29·9)	20·7 (19·6 to 21·8)	15·4 (14·5 to 16·4)	10·7 (10·1 to 11·4)	7·47 (7·05 to 7·98)	5·64 (5·29 to 5·98)	4·97 (4·68 to 5·24)	5·27 (4·99 to 5·55)	5·12 (4·75 to 5·52)	4·67 (3·94 to 5·55)
Vermont	30 (21·7 to 40·1)	26·5 (25·1 to 28)	16·7 (15·6 to 17·9)	10·3 (9·47 to 11·2)	7·11 (6·62 to 7·66)	6·02 (5·51 to 6·57)	5·08 (4·64 to 5·54)	4·48 (3·91 to 5·09)	4·16 (3·63 to 4·73)	3·97 (3·23 to 4·86)
Virginia	35·9 (29·6 to 43·9)	31·1 (30 to 32·2)	21·2 (20·3 to 22·2)	13·8 (13·1 to 14·5)	9·91 (9·46 to 10·3)	7·14 (6·73 to 7·57)	6·98 (6·65 to 7·34)	5·84 (5·49 to 6·19)	5·96 (5·56 to 6·38)	5·68 (5·19 to 6·19)
Washington	24·7 (21·2 to 28·7)	23·5 (22·7 to 24·4)	17·9 (17 to 18·8)	11·2 (10·7 to 11·9)	7·67 (7·21 to 8·15)	5·57 (5·22 to 5·94)	4·69 (4·4 to 4·98)	4·55 (4·26 to 4·86)	4·44 (4·18 to 4·73)	3·96 (3·58 to 4·35)
West Virginia	32·1 (23·9 to 40·7)	27·8 (26·6 to 29·2)	22·8 (21·7 to 23·9)	13·1 (12·3 to 13·9)	10 (9·41 to 10·7)	8·16 (7·62 to 8·78)	7·93 (7·46 to 8·41)	6·84 (6·21 to 7·48)	7·34 (6·71 to 8·04)	6·84 (5·68 to 8·07)
Wisconsin	27·6 (23·5 to 32·4)	24 (23 to 24·9)	16·4 (15·8 to 17)	10·5 (10 to 11·1)	7·77 (7·34 to 8·21)	6·82 (6·41 to 7·23)	5·87 (5·5 to 6·24)	6·19 (5·81 to 6·57)	6 (5·63 to 6·4)	5·66 (5·1 to 6·24)
Wyoming	31 (22·6 to 40·3)	28·6 (26·8 to 30·5)	21·4 (20·1 to 23)	12·1 (11·2 to 13·1)	9·33 (8·65 to 10)	7·15 (6·52 to 7·83)	6·24 (5·74 to 6·74)	5·86 (5·33 to 6·4)	5·69 (5·16 to 6·23)	5·67 (4·78 to 6·7)
Southern Latin America	90·3 (87 to 93·5)	99·3 (97·9 to 101)	70·8 (69·6 to 72)	34·3 (33·7 to 35)	22·6 (22·2 to 23)	15·4 (15 to 15·9)	11·7 (11·4 to 12)	9·01 (8·71 to 9·29)	8·43 (7·41 to 9·5)	7·7 (6·26 to 9·38)
Argentina	71·3 (67·7 to 75)	74·1 (72·3 to 75·8)	64·9 (63·5 to 66·4)	34·7 (33·9 to 35·5)	25·3 (24·7 to 25·8)	17·3 (16·6 to 17·9)	13 (12·6 to 13·4)	9·91 (9·56 to 10·3)	9·63 (8·28 to 11·1)	8·79 (6·98 to 11)
Chile	142 (136 to 148)	153 (150 to 156)	87·9 (86 to 89·6)	33·1 (32·2 to 33·9)	16·9 (16·3 to 17·5)	10·6 (10·2 to 11)	8·29 (7·94 to 8·67)	6·77 (6·45 to 7·09)	5·46 (5·02 to 5·97)	5·09 (4·5 to 5·7)
Uruguay	50·8 (45·7 to 56·2)	54 (51·5 to 56·8)	51·8 (49·5 to 54·3)	36·4 (34·8 to 38)	20·4 (19·4 to 21·4)	14·4 (13·4 to 15·4)	10·3 (8·85 to 11·9)	7·07 (6·47 to 7·66)	6·68 (5·72 to 7·72)	6·07 (4·95 to 7·4)
Western Europe	51·6 (50·1 to 53·3)	36 (35·6 to 36·4)	23·4 (23·1 to 23·8)	13·1 (12·9 to 13·3)	8·17 (8·05 to 8·3)	5·2 (5·11 to 5·29)	3·89 (3·82 to 3·95)	3·39 (3·3 to 3·49)	3·24 (3·11 to 3·36)	3·18 (2·99 to 3·38)
Andorra	17·2 (11·6 to 24·6)	18·9 (14·9 to 23·6)	14·5 (11·9 to 17·8)	13 (10·9 to 15·5)	8·04 (6·63 to 9·62)	3·9 (3·19 to 4·69)	2·82 (2·37 to 3·34)	1·93 (1·58 to 2·31)	1·84 (1·51 to 2·19)	1·27 (0·857 to 1·66)
Austria	48·7 (45·5 to 52·1)	38·5 (37·2 to 39·8)	25·1 (24·3 to 25·9)	14·7 (14·1 to 15·4)	8·46 (8·01 to 8·95)	5·13 (4·81 to 5·49)	4·09 (3·8 to 4·38)	3·21 (2·97 to 3·46)	3·23 (2·98 to 3·47)	2·82 (2·54 to 3·13)
Belgium	53·4 (49·7 to 58)	29·9 (29 to 30·8)	21·1 (20·3 to 21·7)	12·8 (12·3 to 13·4)	8·31 (7·89 to 8·71)	5·22 (4·97 to 5·5)	3·77 (3·55 to 4·03)	3·64 (3·39 to 3·89)	3·38 (3·09 to 3·73)	3·3 (2·76 to 3·89)
Cyprus	124 (103 to 149)	89·3 (78·2 to 102)	53·3 (47·7 to 58·9)	24·8 (23·4 to 26·2)	12·4 (11·5 to 13·5)	6·6 (6·03 to 7·26)	3·48 (3·14 to 3·81)	2·5 (2·21 to 2·83)	2·44 (2·13 to 2·82)	2·43 (2·04 to 2·88)
Denmark	28·2 (25·8 to 30·8)	21·7 (20·8 to 22·6)	14 (13·4 to 14·7)	8·54 (8·05 to 9·05)	7·9 (7·35 to 8·46)	4·96 (4·63 to 5·3)	3·62 (3·34 to 3·91)	3·12 (2·84 to 3·42)	3·08 (2·83 to 3·36)	3·19 (2·86 to 3·55)
Finland	43·7 (40·2 to 47·5)	22·6 (21·6 to 23·7)	14·1 (13·4 to 14·8)	7·67 (7·19 to 8·16)	6·12 (5·72 to 6·55)	3·82 (3·53 to 4·15)	2·7 (2·46 to 2·97)	2·25 (2 to 2·5)	1·99 (1·8 to 2·19)	1·96 (1·73 to 2·22)
France	49·3 (47 to 51·8)	23·2 (22·7 to 23·7)	15·3 (15 to 15·7)	10·5 (10·2 to 10·9)	7·44 (7·23 to 7·66)	4·77 (4·61 to 4·94)	3·82 (3·68 to 3·97)	3·75 (3·61 to 3·92)	3·93 (3·74 to 4·14)	3·62 (3·31 to 3·95)
Germany	46·2 (39·7 to 53·3)	36·2 (35·4 to 36·9)	22·7 (22·3 to 23·2)	13·3 (12·9 to 13·7)	7·5 (7·3 to 7·72)	4·77 (4·59 to 4·96)	3·66 (3·56 to 3·76)	3·29 (3·15 to 3·43)	3·25 (3·11 to 3·39)	3·26 (3·08 to 3·46)
Greece	57·4 (52·9 to 61·9)	41·1 (39·6 to 42·6)	27·9 (26·9 to 28·9)	17·2 (16·5 to 17·9)	9·43 (8·91 to 9·92)	5·55 (5·24 to 5·89)	3·95 (3·71 to 4·2)	4·1 (3·74 to 4·47)	3·68 (3·35 to 4·05)	3·58 (3·11 to 4·08)
Iceland	27·5 (23·4 to 32)	19·3 (18 to 20·8)	14·4 (13·2 to 15·5)	8·97 (8·14 to 9·84)	6·02 (5·4 to 6·69)	3·64 (3·28 to 4·03)	2·46 (2·16 to 2·83)	1·92 (1·67 to 2·2)	2·3 (1·97 to 2·63)	2·28 (1·89 to 2·69)
Ireland	43·3 (39·6 to 47·5)	28·2 (27·1 to 29·4)	19 (18·1 to 19·9)	12 (11·4 to 12·5)	8·02 (7·5 to 8·57)	6·16 (5·76 to 6·54)	3·95 (3·66 to 4·26)	3·34 (3·03 to 3·7)	3·1 (2·83 to 3·38)	3·21 (2·85 to 3·59)
Israel	64·7 (59 to 71·1)	36·8 (35·2 to 38·3)	24·1 (23·1 to 25)	17·1 (16·4 to 17·9)	10·9 (10·3 to 11·4)	6·13 (5·8 to 6·46)	4·14 (3·9 to 4·41)	3·27 (3·09 to 3·45)	2·63 (2·43 to 2·82)	2·11 (1·79 to 2·47)
Italy	57·6 (55 to 60·1)	46·9 (45·8 to 48·2)	30·3 (29·6 to 31·1)	14·8 (14·4 to 15·3)	8·74 (8·42 to 9·02)	5·05 (4·86 to 5·24)	3·62 (3·46 to 3·78)	2·68 (2·56 to 2·8)	2·73 (2·56 to 2·93)	2·68 (2·4 to 2·96)
Luxembourg	51 (44·7 to 57·8)	32·1 (29·9 to 34·4)	20·8 (19·2 to 22·4)	11·2 (10·3 to 12·2)	7·7 (7·01 to 8·47)	3·86 (3·47 to 4·28)	2·81 (2·48 to 3·19)	2·6 (2·3 to 2·94)	3·38 (2·97 to 3·83)	3·2 (2·72 to 3·75)
Malta	67·7 (60·3 to 76·1)	37·3 (35·2 to 39·7)	24·3 (22·5 to 26·3)	14 (12·8 to 15·2)	9·33 (8·46 to 10·3)	6·48 (5·8 to 7·22)	5·96 (5·35 to 6·65)	5·94 (5·2 to 6·75)	4·99 (4·21 to 5·77)	4·88 (4·02 to 5·89)
Monaco	77·7 (59·2 to 100)	46·8 (38·3 to 56·9)	20·8 (16·2 to 26·1)	12·8 (9·73 to 16·5)	7·45 (5·82 to 9·55)	4·85 (3·83 to 6·12)	3·17 (2·46 to 3·96)	2·3 (1·81 to 2·89)	3·76 (3·74 to 3·85)	3·78 (3·75 to 3·84)
Netherlands	24·4 (22·8 to 26·1)	18·1 (17·6 to 18·7)	13·5 (13·1 to 14)	9·27 (8·84 to 9·66)	7·53 (7·19 to 7·87)	5·59 (5·29 to 5·9)	3·98 (3·79 to 4·17)	3·75 (3·53 to 4·01)	3·79 (3·57 to 4·01)	3·48 (3·21 to 3·77)
Norway	27·1 (24·5 to 29·9)	20·3 (19·3 to 21·3)	14 (13·3 to 14·7)	8·89 (8·36 to 9·45)	7·86 (7·39 to 8·39)	4·44 (4·12 to 4·75)	3·01 (2·77 to 3·26)	2·29 (2·08 to 2·51)	1·98 (1·8 to 2·17)	1·99 (1·76 to 2·23)
Agder	26·8 (22 to 31·9)	21·6 (19·9 to 23·4)	14·5 (13·2 to 15·7)	9·45 (8·54 to 10·4)	8·56 (7·62 to 9·63)	5·12 (4·66 to 5·61)	3·63 (3·33 to 4)	2·83 (2·52 to 3·19)	2·27 (2·01 to 2·54)	2·44 (2·06 to 2·83)
Innlandet	26·6 (23·1 to 30·8)	19·8 (18·3 to 21·4)	14·1 (13·1 to 15·1)	9·04 (8·21 to 9·92)	7·88 (7·17 to 8·63)	4·6 (4·16 to 5·02)	3·11 (2·84 to 3·42)	2·4 (2·14 to 2·69)	2·03 (1·81 to 2·29)	2 (1·73 to 2·31)
Møre og Romsdal	31·2 (25·8 to 37·1)	21·9 (20·3 to 23·7)	15·9 (14·4 to 17·5)	9·54 (8·66 to 10·5)	7·61 (6·82 to 8·54)	4·13 (3·77 to 4·5)	2·78 (2·49 to 3·09)	2·32 (2·05 to 2·62)	1·95 (1·74 to 2·18)	2·1 (1·79 to 2·46)
Nordland	34 (29·2 to 39·5)	25·4 (23·6 to 27·2)	15·8 (14·5 to 17·3)	9·54 (8·66 to 10·5)	7·81 (6·91 to 8·78)	4·65 (4·25 to 5·1)	3·84 (3·48 to 4·26)	3·06 (2·71 to 3·45)	2·58 (2·31 to 2·85)	2·8 (2·39 to 3·28)
Oslo	16·8 (12·6 to 21·8)	15·3 (14·1 to 16·6)	12·5 (11·1 to 14·1)	9·34 (8·32 to 10·6)	9·79 (8·77 to 10·9)	5·15 (4·59 to 5·71)	2·78 (2·49 to 3·13)	1·92 (1·71 to 2·18)	1·66 (1·48 to 1·88)	1·58 (1·37 to 1·84)
Rogaland	26·9 (21·9 to 32·5)	20·5 (18·7 to 22·2)	14·2 (12·8 to 15·6)	9·08 (8·2 to 10)	7·35 (6·62 to 8·16)	4·36 (4 to 4·74)	3·02 (2·74 to 3·34)	2·3 (2·03 to 2·61)	2·04 (1·82 to 2·3)	2·03 (1·75 to 2·37)
Troms og Finnmark	43·4 (37·9 to 50)	28·8 (26·7 to 30·9)	17·7 (16·2 to 19·3)	10·4 (9·36 to 11·4)	9·06 (8·25 to 9·94)	5·01 (4·6 to 5·4)	3·46 (3·17 to 3·82)	2·69 (2·37 to 3·05)	2·33 (2·06 to 2·61)	2·51 (2·1 to 2·95)
Trøndelag	27·3 (23·1 to 32·2)	19·3 (17·6 to 21)	13·8 (12·6 to 15)	8·88 (7·93 to 9·84)	7·12 (6·37 to 7·87)	3·81 (3·5 to 4·11)	2·65 (2·41 to 2·94)	2·05 (1·82 to 2·28)	1·76 (1·57 to 1·96)	1·71 (1·48 to 1·96)
Vestfold og Telemark	26·5 (22·7 to 30·6)	21·2 (19·7 to 22·7)	14·7 (13·6 to 15·9)	9·18 (8·34 to 10·1)	7·68 (6·98 to 8·36)	4·98 (4·52 to 5·44)	3·47 (3·18 to 3·8)	2·52 (2·22 to 2·83)	2·21 (1·99 to 2·46)	2·2 (1·93 to 2·5)
Vestland	27 (22·8 to 31·7)	19·1 (17·7 to 20·5)	13 (12·1 to 13·9)	8·1 (7·35 to 8·84)	6·93 (6·28 to 7·61)	3·75 (3·43 to 4·07)	2·73 (2·51 to 3·01)	2·2 (1·96 to 2·46)	1·91 (1·71 to 2·12)	2·05 (1·76 to 2·36)
Viken	22·5 (19·3 to 26)	17·9 (16·8 to 19·3)	12·4 (11·5 to 13·5)	7·85 (7·24 to 8·49)	7·56 (6·87 to 8·35)	4·18 (3·7 to 4·72)	2·9 (2·63 to 3·2)	2·23 (1·99 to 2·5)	1·98 (1·76 to 2·22)	1·9 (1·68 to 2·15)
Portugal	141 (133 to 150)	98 (94·9 to 101)	65·5 (63·3 to 67·8)	24·6 (23·7 to 25·6)	12·5 (11·9 to 13)	6·48 (6·12 to 6·82)	3·32 (3·08 to 3·58)	3·19 (2·96 to 3·47)	2·78 (2·57 to 2·99)	2·61 (2·33 to 2·89)
San Marino	23·4 (16·1 to 33·3)	19·6 (16·2 to 23·8)	15·6 (13·2 to 18·2)	11·5 (9·52 to 13·9)	8·37 (7·02 to 9·93)	4·96 (4·05 to 6·01)	3·01 (2·51 to 3·6)	2·52 (1·95 to 3·29)	2·25 (1·67 to 3·06)	1·75 (1·17 to 2·47)
Spain	88·1 (85 to 91·6)	47·8 (47 to 48·6)	28·5 (27·7 to 29·2)	12·7 (12·3 to 13)	8·31 (8 to 8·63)	4·92 (4·71 to 5·14)	3·66 (3·5 to 3·83)	2·98 (2·83 to 3·15)	2·77 (2·6 to 2·96)	2·67 (2·47 to 2·9)
Sweden	20·8 (19·1 to 22·6)	18·5 (17·6 to 19·4)	12 (11·4 to 12·6)	7·29 (6·87 to 7·72)	6·08 (5·7 to 6·44)	3·47 (3·25 to 3·71)	2·74 (2·56 to 2·93)	2·34 (2·14 to 2·58)	2·38 (2·2 to 2·56)	2·08 (1·89 to 2·28)
Stockholm	17·9 (14·5 to 22·2)	16·9 (14·5 to 19·5)	11·9 (10·4 to 13·5)	7·88 (7·14 to 8·67)	6·11 (5·55 to 6·73)	3·56 (3·21 to 3·93)	2·52 (2·3 to 2·76)	2·09 (1·87 to 2·37)	2·13 (1·85 to 2·43)	1·82 (1·52 to 2·16)
Sweden except Stockholm	21·3 (19·4 to 23·4)	18·8 (17·7 to 19·8)	12 (11·3 to 12·7)	7·16 (6·71 to 7·62)	6·07 (5·7 to 6·45)	3·45 (3·2 to 3·69)	2·82 (2·63 to 3·01)	2·42 (2·19 to 2·68)	2·46 (2·27 to 2·66)	2·17 (1·98 to 2·37)
Switzerland	34·1 (31·1 to 37·1)	23·6 (22·7 to 24·6)	16·8 (16·1 to 17·5)	9·62 (9·08 to 10·2)	7·66 (7·22 to 8·11)	5·41 (5·09 to 5·74)	4·1 (3·84 to 4·39)	3·47 (3·19 to 3·76)	3·71 (3·43 to 4·02)	3·48 (3·14 to 3·85)
UK	24·9 (23·8 to 26·1)	24·7 (24·1 to 25·3)	19·5 (19·1 to 20)	13·5 (13·1 to 13·9)	8·62 (8·33 to 8·93)	6·17 (5·8 to 6·55)	4·78 (4·6 to 4·97)	4·01 (3·87 to 4·17)	3·36 (3·12 to 3·6)	3·75 (3·39 to 4·14)
England	23·8 (22·4 to 25·3)	24·3 (23·5 to 25·1)	19·3 (18·9 to 19·9)	13·4 (13 to 13·9)	8·75 (8·45 to 9·1)	6·28 (5·89 to 6·68)	4·89 (4·7 to 5·09)	4·07 (3·91 to 4·22)	3·37 (3·12 to 3·61)	3·76 (3·4 to 4·15)
East Midlands	24·4 (20·2 to 29·5)	24·2 (22·4 to 26·1)	19·4 (17·8 to 21·3)	13·1 (12·1 to 14)	8·73 (8·32 to 9·15)	6·22 (5·7 to 6·74)	4·91 (4·59 to 5·25)	4·28 (4·02 to 4·55)	3·61 (3·26 to 3·98)	3·97 (3·54 to 4·44)
Derby	35·4 (27·7 to 45·2)	30·9 (27·3 to 35·3)	25·2 (22·5 to 28·2)	16·9 (14·9 to 19·3)	10·4 (9·4 to 11·5)	7·12 (6·29 to 8·03)	5·69 (5·03 to 6·52)	4·92 (4·45 to 5·45)	4·34 (3·81 to 4·94)	5·16 (4·58 to 5·86)

Derbyshire	24·2 (19·3 to 30·3)	23·2 (20 to 26·3)	18·5 (16·4 to 21)	12·5 (11·5 to 13·5)	7·7 (7·1 to 8·34)	5·43 (4·89 to 5·99)	4·08 (3·8 to 4·38)	3·68 (3·4 to 3·98)	3·29 (2·94 to 3·64)	3·67 (3·26 to 4·1)
Leicester	29·3 (22·9 to 36·7)	30·7 (26·5 to 35·1)	26·4 (23·5 to 29·8)	17·1 (15 to 19·2)	10·8 (9·94 to 11·6)	8·31 (7·31 to 9·42)	6·64 (5·96 to 7·39)	5·79 (5·29 to 6·3)	5·02 (4·35 to 5·75)	5·64 (4·91 to 6·41)
Leicestershire	19·3 (15·6 to 23·2)	21 (19·1 to 22·8)	16·4 (14·7 to 18·4)	11·3 (9·92 to 12·8)	7·95 (7·39 to 8·58)	5·2 (4·72 to 5·75)	4·2 (3·88 to 4·57)	3·76 (3·44 to 4·11)	3·24 (2·93 to 3·54)	3·67 (3·28 to 4·1)
Lincolnshire	23·2 (18·3 to 29·1)	22·4 (19·6 to 25·1)	17·7 (15·7 to 19·9)	11·8 (10·8 to 12·9)	8·46 (7·76 to 9·22)	6·12 (5·52 to 6·78)	4·76 (4·34 to 5·19)	3·4 (3·12 to 3·67)	2·99 (2·66 to 3·32)	3·31 (2·93 to 3·73)
Northamptonshire	23·2 (18·3 to 29·1)	23·3 (20·6 to 26·1)	18·2 (16·1 to 20·7)	12·2 (10·8 to 13·7)	9·04 (8·28 to 9·86)	5·73 (5·2 to 6·32)	4·81 (4·44 to 5·21)	4·44 (4·06 to 4·87)	2·98 (2·7 to 3·29)	3 (2·64 to 3·37)
Nottingham	29·3 (22·2 to 38·1)	28·9 (25·1 to 33)	24·2 (21·4 to 27·2)	17·1 (14·8 to 19·8)	11·1 (10·3 to 12·1)	9·52 (8·53 to 10·6)	6·5 (5·97 to 7·08)	5·69 (5·14 to 6·3)	5·13 (4·47 to 5·86)	5·52 (4·76 to 6·4)
Nottinghamshire	22·2 (17·7 to 27·8)	22·2 (19·7 to 24·7)	17·7 (15·9 to 19·7)	12 (11·1 to 12·9)	7·7 (7·07 to 8·36)	5·77 (5·26 to 6·34)	4·58 (4·26 to 4·94)	4·16 (3·81 to 4·54)	3·68 (3·32 to 4·05)	4·07 (3·64 to 4·54)
Rutland	14·8 (11·5 to 19·2)	13·6 (10·8 to 17)	10·8 (9·54 to 12·2)	8·8 (7·62 to 10·2)	6·18 (5·22 to 7·23)	4·14 (3·41 to 4·97)	2·92 (2·35 to 3·56)	2·02 (1·79 to 2·28)	1·41 (1·2 to 1·62)	1·42 (1·24 to 1·6)
East of England	20 (17·6 to 22·5)	20·3 (18·6 to 21·9)	16·2 (14·8 to 17·7)	11·1 (10·3 to 12)	6·78 (6·45 to 7·11)	5·05 (4·66 to 5·45)	4·23 (3·96 to 4·49)	3·43 (3·24 to 3·63)	2·88 (2·63 to 3·16)	3·18 (2·85 to 3·55)
Bedford	23·1 (19·1 to 28·2)	24 (21·4 to 26·9)	19·6 (17·4 to 22)	13·7 (11·9 to 15·5)	8·81 (7·95 to 9·8)	6·39 (5·66 to 7·17)	5·41 (4·87 to 5·99)	4·63 (4·21 to 5·11)	3·95 (3·48 to 4·44)	3·96 (3·48 to 4·47)
Cambridgeshire	13·2 (10·6 to 16·2)	16·6 (14·6 to 18·7)	13·1 (11·6 to 14·7)	9·31 (8·24 to 10·4)	5·85 (5·37 to 6·33)	4·75 (4·3 to 5·27)	3·85 (3·56 to 4·18)	3·06 (2·83 to 3·3)	2·56 (2·28 to 2·86)	2·85 (2·54 to 3·17)
Central Bedfordshire	18·4 (15·3 to 22)	19 (16·1 to 22)	15·1 (13·3 to 16·9)	10·3 (9·11 to 11·7)	6·47 (5·89 to 7·11)	4·53 (4·04 to 5·1)	3·52 (3·16 to 3·92)	2·95 (2·7 to 3·23)	2·57 (2·27 to 2·89)	2·91 (2·58 to 3·24)
Essex	19·6 (16 to 23·6)	20 (17·9 to 22·3)	15·7 (13·6 to 18)	10·8 (9·73 to 12)	6·6 (6·09 to 7·13)	4·64 (4·19 to 5·13)	4·02 (3·67 to 4·4)	3·2 (2·93 to 3·5)	2·62 (2·28 to 2·99)	2·89 (2·41 to 3·45)
Hertfordshire	18·5 (14·9 to 22·6)	18·4 (15·6 to 21·1)	15 (13·3 to 16·8)	10·7 (9·52 to 12·2)	6·38 (5·86 to 6·92)	4·73 (4·25 to 5·21)	3·83 (3·48 to 4·19)	3·17 (2·88 to 3·45)	2·7 (2·3 to 3·1)	2·99 (2·48 to 3·59)
Luton	25·8 (21·7 to 30·6)	25·6 (22·5 to 29·1)	21·1 (18·8 to 23·4)	14·3 (12·5 to 16·1)	8·95 (8·04 to 9·93)	8·5 (7·47 to 9·58)	6·86 (6·15 to 7·54)	5·53 (4·99 to 6·12)	4·73 (4·09 to 5·42)	5·05 (4·46 to 5·67)
Norfolk	21·5 (17·5 to 26·3)	21·8 (18·8 to 25)	17·2 (15·4 to 19·1)	11·7 (10·8 to 12·8)	7·01 (6·49 to 7·56)	5·11 (4·53 to 5·74)	4·31 (3·94 to 4·68)	3·34 (3·08 to 3·61)	2·87 (2·58 to 3·17)	3·23 (2·9 to 3·56)
Peterborough	24·5 (18·5 to 30·9)	22·7 (18·8 to 26·6)	19 (16·8 to 21·2)	12·8 (11·3 to 14·4)	8·11 (7·29 to 9·04)	7·18 (6·22 to 8·17)	5·84 (5·19 to 6·56)	4·76 (4·32 to 5·23)	3·78 (3·39 to 4·25)	4·08 (3·66 to 4·55)
Southend-on-Sea	31·6 (25·8 to 38·6)	25·4 (22·6 to 28·4)	20·4 (18·2 to 22·9)	13·1 (11·6 to 14·9)	7·53 (6·73 to 8·39)	5·15 (4·57 to 5·78)	4·09 (3·59 to 4·56)	3·4 (3 to 3·86)	2·87 (2·46 to 3·33)	3·2 (2·79 to 3·65)
Suffolk	21·2 (17·9 to 25·1)	20·1 (17·5 to 22·9)	15·7 (13·8 to 17·7)	10·5 (9·32 to 11·8)	6·3 (5·81 to 6·79)	4·33 (3·89 to 4·84)	3·75 (3·44 to 4·07)	2·98 (2·73 to 3·24)	2·51 (2·27 to 2·79)	2·85 (2·56 to 3·15)
Thurrock	18·8 (15·2 to 22·9)	22·5 (19·8 to 25·7)	18·7 (16·6 to 21)	12·1 (10·7 to 13·6)	7·6 (6·9 to 8·37)	6·04 (5·39 to 6·7)	4·87 (4·42 to 5·33)	4·43 (4·1 to 4·76)	3·56 (3·07 to 4·09)	3·99 (3·48 to 4·54)
Greater London	25·7 (23·2 to 28·3)	27·6 (26·2 to 29·1)	21·8 (20·8 to 22·8)	15·3 (14·5 to 16·2)	9·66 (9·27 to 10)	7·18 (6·67 to 7·72)	5·24 (4·96 to 5·54)	3·88 (3·71 to 4·07)	3·12 (2·89 to 3·35)	3·45 (3·08 to 3·86)
Barking and Dagenham	40·1 (33·2 to 47·2)	31·3 (27 to 35·5)	25·3 (23·6 to 27·2)	15·8 (14 to 17·9)	8·73 (8·18 to 9·3)	5·48 (4·79 to 6·17)	4·52 (4·08 to 5·01)	3·63 (3·37 to 3·9)	2·86 (2·63 to 3·11)	3·09 (2·74 to 3·46)
Barnet	24·2 (20 to 29·3)	25·7 (23·8 to 27·8)	19·9 (18·3 to 21·6)	13·7 (12·6 to 14·8)	7·76 (7·23 to 8·32)	5·74 (5·17 to 6·37)	4·41 (4·03 to 4·81)	3·31 (3·03 to 3·61)	2·73 (2·45 to 3·03)	3·18 (2·83 to 3·55)
Bexley	21·3 (18·1 to 25·2)	18·9 (17·8 to 20·1)	14·3 (13·3 to 15·4)	10 (9·04 to 11·1)	6·33 (5·84 to 6·88)	4·92 (4·43 to 5·48)	4 (3·69 to 4·34)	3·41 (3·1 to 3·74)	3·21 (2·92 to 3·51)	3·66 (3·26 to 4·06)
Brent	27·3 (22·1 to 33·6)	29·7 (27 to 32·5)	23·7 (21·6 to 25·9)	16·5 (14·9 to 18·2)	11·3 (10·3 to 12·5)	9·14 (8·25 to 10·2)	6·32 (5·8 to 6·84)	4·37 (3·98 to 4·78)	3·43 (3·1 to 3·77)	3·85 (3·36 to 4·37)
Bromley	21·8 (18·4 to 26·1)	19·9 (16·8 to 23·2)	15·1 (14 to 16·4)	10·9 (9·48 to 12·6)	7·37 (6·79 to 7·94)	4·93 (4·45 to 5·44)	3·4 (3·07 to 3·75)	2·84 (2·57 to 3·1)	2·42 (2·22 to 2·63)	2·68 (2·4 to 2·99)
Camden	12·5 (10·7 to 14·6)	27·9 (26·2 to 29·8)	23·8 (22·1 to 25·5)	17·1 (15·6 to 18·6)	10·2 (9·26 to 11·2)	6·71 (6·02 to 7·51)	4·65 (4·22 to 5·12)	3·14 (2·84 to 3·46)	2·63 (2·34 to 2·96)	2·86 (2·47 to 3·28)
Croydon	26·9 (22·7 to 31·7)	24·3 (22·5 to 26·1)	18·7 (17·1 to 20·1)	13·3 (12·1 to 14·5)	9·57 (8·78 to 10·3)	7·18 (6·47 to 7·96)	5·08 (4·59 to 5·64)	4·08 (3·72 to 4·46)	3·26 (2·95 to 3·61)	3·52 (3·1 to 3·95)
Ealing	25·3 (21·2 to 30·3)	24·4 (22·7 to 26·2)	18·8 (17·3 to 20·3)	13·1 (11·8 to 14·4)	8·49 (7·93 to 9·09)	6·32 (5·73 to 6·99)	4·77 (4·38 to 5·21)	3·47 (3·16 to 3·81)	2·84 (2·6 to 3·08)	3·15 (2·8 to 3·51)
Enfield	24·6 (20·7 to 29·2)	21·7 (20·3 to 23·1)	16·4 (15·2 to 17·6)	11·5 (10·5 to 12·6)	7·48 (6·96 to 8·05)	6·46 (5·75 to 7·26)	5·34 (4·74 to 6·02)	3·91 (3·58 to 4·23)	2·74 (2·45 to 3·03)	3·23 (2·85 to 3·61)
Greenwich	35 (29·3 to 41·5)	31·2 (29 to 33·3)	24·6 (22·6 to 26·6)	16·6 (15·1 to 18·3)	9·14 (8·32 to 10·1)	6·21 (5·63 to 6·82)	4·95 (4·56 to 5·34)	4·17 (3·81 to 4·58)	3·53 (3·23 to 3·82)	4 (3·54 to 4·45)
Hackney	35·4 (30·3 to 41·9)	33·5 (31 to 36)	27·4 (25·4 to 29·8)	19·7 (18 to 21·5)	12·8 (12 to 13·8)	10·1 (9·16 to 11·2)	6·86 (6·28 to 7·48)	5·01 (4·66 to 5·4)	3·9 (3·58 to 4·25)	3·86 (3·28 to 4·45)
Hammersmith and Fulham	15·6 (13 to 18·6)	25·2 (23·8 to 26·8)	19·7 (18·1 to 21·5)	15·3 (13·9 to 16·7)	11·2 (10 to 12·6)	7·11 (6·42 to 7·89)	4·63 (4·24 to 5·09)	3·43 (3·13 to 3·76)	2·65 (2·42 to 2·89)	2·98 (2·59 to 3·36)
Haringey	21 (17·7 to 25)	26 (24·1 to 28)	20·2 (18·7 to 21·9)	15·1 (13·5 to 16·6)	10·4 (9·48 to 11·3)	8·73 (7·86 to 9·66)	5·9 (5·44 to 6·37)	3·91 (3·55 to 4·27)	2·85 (2·62 to 3·09)	3·15 (2·79 to 3·54)
Harrow	24·3 (20·3 to 29·1)	21·1 (19·7 to 22·8)	16 (14·9 to 17·1)	10·9 (9·86 to 12)	8·19 (7·61 to 8·8)	7·55 (6·58 to 8·51)	6·23 (5·63 to 6·87)	4·81 (4·39 to 5·23)	3·78 (3·44 to 4·14)	4·26 (3·81 to 4·79)
Havering	23·2 (18·8 to 28·2)	20·5 (17·4 to 23·8)	16·3 (15 to 17·7)	11 (9·64 to 12·5)	6·83 (6·29 to 7·47)	4·84 (4·33 to 5·49)	3·87 (3·53 to 4·2)	2·8 (2·54 to 3·09)	2·29 (2·02 to 2·59)	2·68 (2·35 to 3·04)
Hillingdon	24·5 (20·3 to 29·3)	22·3 (20·7 to 24)	17·6 (16·3 to 19)	12·3 (11·3 to 13·4)	7·67 (7·07 to 8·35)	5·54 (4·89 to 6·23)	4·66 (4·28 to 5·09)	3·45 (3·16 to 3·72)	2·86 (2·59 to 3·15)	3·2 (2·84 to 3·57)
Hounslow	24·6 (20 to 29·5)	25·4 (21·4 to 29·5)	19·7 (18·2 to 21·3)	13·2 (11·6 to 15)	9·26 (8·46 to 10·2)	7·09 (6·35 to 7·87)	5·85 (5·17 to 6·55)	4·8 (4·35 to 5·24)	4·16 (3·75 to 4·58)	4·53 (4·01 to 5·05)
Islington	26·5 (21·8 to 31·7)	32·7 (27·2 to 38·4)	26·4 (24·3 to 28·7)	19·9 (17·2 to 22·8)	11·4 (10·5 to 12·3)	8·32 (7·43 to 9·33)	5·27 (4·86 to 5·68)	3·58 (3·34 to 3·85)	2·78 (2·55 to 3·02)	3·07 (2·73 to 3·42)
Kensington and Chelsea	8·01 (6·58 to 9·65)	30·5 (25·7 to 35·5)	27·1 (24·9 to 29·5)	19 (17·1 to 21)	10 (8·76 to 11·5)	5·58 (4·89 to 6·38)	4·22 (3·6 to 4·94)	3·55 (3·21 to 3·9)	2·68 (2·43 to 2·94)	2·98 (2·61 to 3·37)
Kingston upon Thames	18·5 (14·4 to 23)	19 (15·9 to 22·1)	14 (12·7 to 15·4)	10·3 (9·14 to 11·5)	7 (6·35 to 7·73)	4·4 (3·72 to 5·16)	3·92 (3·47 to 4·43)	3·89 (3·53 to 4·28)	3·01 (2·73 to 3·3)	3·36 (2·95 to 3·77)
Lambeth	34·5 (29·2 to 41)	37·2 (34·7 to 39·9)	30·3 (27·8 to 32·9)	23·1 (21·4 to 25·1)	12·6 (11·6 to 13·7)	9·77 (8·76 to 10·9)	6·93 (6·3 to 7·61)	4·9 (4·5 to 5·3)	3·87 (3·49 to 4·27)	4·47 (3·91 to 5·06)
Lewisham	32·5 (27·9 to 38·2)	30·1 (28·1 to 32·3)	23·7 (22·1 to 25·7)	17·3 (15·7 to 19·2)	10·7 (9·94 to 11·6)	8·6 (7·67 to 9·67)	5·81 (5·34 to 6·3)	4·19 (3·79 to 4·61)	3·73 (3·32 to 4·15)	4·15 (3·61 to 4·69)
Merton	27·7 (23·5 to 32·6)	25 (23·4 to 26·6)	19·2 (17·9 to 20·5)	13·2 (12 to 14·5)	8·18 (7·51 to 8·93)	5·08 (4·41 to 5·8)	4·33 (3·87 to 4·82)	3·04 (2·82 to 3·28)	2·51 (2·31 to 2·73)	2·82 (2·52 to 3·14)
Newham	37·7 (29·4 to 47·6)	33·6 (29·5 to 37·9)	27·2 (24·2 to 30·4)	18·3 (16·2 to 20·5)	11·5 (10·6 to 12·4)	9·74 (8·79 to 10·7)	7·08 (6·41 to 7·82)	4·87 (4·43 to 5·33)	3·95 (3·55 to 4·39)	4·09 (3·56 to 4·65)
Redbridge	24·9 (20·7 to 29·1)	24·5 (23·1 to 26·1)	18·7 (17·4 to 20)	12·6 (11·4 to 13·9)	8·68 (8·01 to 9·32)	6·58 (5·74 to 7·36)	5 (4·51 to 5·54)	3·79 (3·51 to 4·08)	3·1 (2·88 to 3·35)	3·36 (3 to 3·74)
Richmond upon Thames	19·6 (16·6 to 23·1)	20·9 (19·4 to 22·5)	15·6 (14·5 to 16·7)	12 (11 to 13)	7·28 (6·48 to 8·16)	4·6 (4·13 to 5·17)	3·54 (3·19 to 3·94)	2·82 (2·56 to 3·09)	2·47 (2·26 to 2·7)	2·77 (2·5 to 3·07)
Southwark	31·9 (26·1 to 39·2)	27·5 (23·3 to 31·9)	22·5 (20·7 to 24·3)	15·7 (13·8 to 17·8)	12·2 (11·2 to 13·3)	9·96 (8·9 to 11·1)	6·35 (5·85 to 6·94)	4·33 (3·95 to 4·71)	3·3 (2·98 to 3·63)	3·66 (3·23 to 4·14)
Sutton	27·6 (23·7 to 32·7)	24·5 (23 to 26·2)	18·5 (17·1 to 19·9)	12·9 (11·7 to 14·1)	7·89 (7·14 to 8·69)	5·39 (4·86 to 5·98)	4·48 (4·04 to 4·96)	3·88 (3·56 to 4·23)	3·04 (2·77 to 3·33)	3·58 (3·18 to 4)
Tower Hamlets	52·7 (44·4 to 62·8)	41 (37·8 to 44·7)	37 (33·4 to 40·6)	24·2 (21·6 to 26·8)	11 (10·1 to 12·1)	8·18 (7·46 to 9·06)	6·01 (5·42 to 6·6)	4·58 (4·17 to 5·02)	3·52 (3·15 to 3·91)	3·68 (3·24 to 4·13)
Waltham Forest	38·6 (31·5 to 46·7)	35·3 (31·8 to 39·2)	26·2 (23·2 to 29·5)	18 (15·8 to 20·4)	12·1 (11 to 13·3)	9·07 (8·11 to 10·2)	6·24 (5·7 to 6·86)	3·92 (3·59 to 4·26)	3·12 (2·8 to 3·45)	3·58 (3·16 to 4·02)
Wandsworth	24·7 (20·8 to 28·9)	29·1 (27·2 to 31·2)	22·5 (20·7 to 24·3)	17 (15·2 to 18·9)	10·8 (9·82 to 12)	6·56 (5·73 to 7·47)	4·39 (4·04 to 4·75)	3·14 (2·89 to 3·42)	2·36 (2·11 to 2·63)	2·62 (2·31 to 2·95)
Westminster	15·8 (13·1 to 18·8)	36·3 (30 to 43·1)	32·4 (29·3 to 35·6)	24 (21 to 27·6)	12·5 (11·2 to 13·6)	7·59 (6·84 to 8·42)	4·27 (3·86 to 4·68)	2·94 (2·67 to 3·22)	2·14 (1·89 to 2·4)	2·5 (2·17 to 2·85)
North East England	23·4 (18·8 to 28·6)	23·7 (20·3 to 27·3)	19·9 (18·6 to 21·4)	13·2 (12·3 to 14·2)	8·8 (8·3 to 9·31)	6·22 (5·71 to 6·75)	4·3 (4·02 to 4·56)	3·57 (3·18 to 4)	3·1 (2·7 to 3·52)	3·53 (3·08 to 4·02)
County Durham	20·6 (15·6 to 26·2)	20·6 (18·1 to 23·2)	17·2 (15·5 to 19)	11·5 (10·3 to 12·7)	7·3 (6·65 to 8)	5·97 (5·35 to 6·63)	4·16 (3·81 to 4·52)	3·49 (3·03 to 4·02)	3·15 (2·76 to 3·53)	3·58 (3·14 to 4·03)
Darlington	21·8 (16·6 to 28·1)	23·1 (18·5 to 28·2)	19·2 (17·4 to 21·2)	12·9 (11·2 to 14·7)	8·06 (7·17 to 9·06)	5·63 (4·68 to 6·78)	4·02 (3·39 to 4·77)	3·19 (2·81 to 3·6)	2·86 (2·46 to 3·34)	3·39 (2·92 to 3·9)
Gateshead	26 (19·6 to 33·5)	26·2 (22·5 to 30·4)	22·1 (20·1 to 24·5)	14·9 (13·3 to 16·7)	9·93 (8·84 to 11·1)	6·16 (5·31 to 7·12)	4·72 (4·18 to 5·26)	3·95 (3·49 to 4·47)	3·4 (2·94 to 3·89)	3·75 (3·26 to 4·27)
Hartlepool	27·9 (21·9 to 35)	28·3 (22·9 to 34·1)	24·2 (22 to 26·5)	16·5 (14·8 to 18·6)	9·66 (8·71 to 10·8)	6·5 (5·71 to 7·41)	4·47 (3·83 to 5·18)	3·12 (2·7 to 3·57)	2·46 (2·12 to 2·82)	2·86 (2·49 to 3·28)
Middlesbrough	30 (23·4 to 37·9)	27·6 (24·3 to 31·1)	23·9 (22·1 to 25·9)	15·9 (14·1 to 17·9)	10·7 (9·79 to 11·6)	7·87 (7·05 to 8·77)	5·42 (4·89 to 5·94)	4·27 (3·7 to 4·94)	3·82 (3·27 to 4·38)	4·32 (3·77 to 4·93)
Newcastle upon Tyne	20·8 (15·9 to 26·5)	23·4 (20 to 27·5)	20·1 (18·3 to 22·1)	13·4 (12·2 to 14·6)	9·98 (9 to 11·1)	6·54 (5·91 to 7·23)	4·44 (4·06 to 4·85)	3·99 (3·54 to 4·5)	3·3 (2·84 to 3·75)	3·77 (3·3 to 4·27)
North Tyneside	24·5 (18·5 to 31·6)	23 (18·1 to 29·1)	19·4 (17·5 to 21·4)	12·5 (10·8 to 14·2)	8·94 (7·99 to 10)	5·96 (5·29 to 6·63)	4·08 (3·66 to 4·5)	3·52 (3·05 to 4·04)	3·02 (2·6 to 3·5)	3·38 (2·9 to 3·87)
Northumberland	24·6 (19 to 31·9)	24·3 (19·6 to 30·2)	19·8 (17·7 to 22·1)	12·6 (11·2 to 14·2)	8·71 (7·84 to 9·58)	5·91 (5·08 to 6·77)	3·98 (3·6 to 4·37)	3·3 (2·91 to 3·75)	2·94 (2·47 to 3·41)	3·24 (2·8 to 3·74)
Redcar and Cleveland	22·8 (17·2 to 29·8)	24 (19 to 30·2)	19·5 (17·6 to 21·6)	13·5 (11·2 to 15·7)	8·5 (7·79 to 9·25)	5·62 (4·98 to 6·32)	4 (3·42 to 4·62)	3·34 (2·9 to 3·86)	2·51 (2·09 to 2·97)	2·99 (2·52 to 3·47)
South Tyneside	21·5 (16·5 to 27·7)	20·4 (16·7 to 24·7)	17·7 (16·1 to 19·4)	11·9 (10·7 to 13·4)	7·43 (6·74 to 8·17)	5·67 (4·97 to 6·43)	3·77 (3·25 to 4·33)	2·95 (2·55 to 3·4)	2·78 (2·33 to 3·26)	3·28 (2·77 to 3·82)

Stockton-on-Tees	21·3 (16·3 to 27·7)	21·3 (18·3 to 25)	18 (16·5 to 19·6)	12·2 (10·9 to 13·6)	8·11 (7·42 to 8·83)	5·8 (5·19 to 6·44)	4·21 (3·81 to 4·62)	3·66 (3·19 to 4·18)	3·27 (2·8 to 3·78)	3·85 (3·29 to 4·44)
Sunderland	25·2 (19 to 32·5)	25·9 (20·6 to 32·3)	21·7 (19·4 to 24·4)	14·1 (12·3 to 16)	9·57 (8·67 to 10·5)	6·95 (6·29 to 7·72)	4·42 (4·04 to 4·79)	3·5 (3·09 to 3·96)	2·97 (2·55 to 3·39)	3·3 (2·84 to 3·8)
North West England	25·6 (22·6 to 28·6)	25·4 (24·2 to 26·8)	20·4 (19·4 to 21·4)	14·2 (13·4 to 15)	9·07 (8·64 to 9·54)	6·8 (6·3 to 7·29)	5·32 (5·02 to 5·62)	4·84 (4·62 to 5·07)	4·11 (3·82 to 4·42)	4·64 (4·13 to 5·18)
Blackburn with Darwen	36 (28·3 to 45·4)	30 (25·7 to 34·4)	23·7 (21·8 to 25·7)	15·8 (13·9 to 18)	12·5 (11·5 to 13·7)	9·83 (8·84 to 10·9)	7·59 (7 to 8·21)	5·99 (5·4 to 6·66)	4·78 (4·31 to 5·26)	5·44 (4·87 to 6·04)
Blackpool	36·3 (29·5 to 44·9)	30·6 (28·5 to 32·8)	24·4 (22·4 to 26·5)	16·5 (14·9 to 18·1)	10·8 (9·72 to 12·1)	8·68 (7·7 to 9·79)	7·31 (6·58 to 8·1)	6·63 (6·05 to 7·27)	5·2 (4·7 to 5·74)	6·03 (5·44 to 6·67)
Bolton	27·1 (22·4 to 32·4)	26·6 (24·6 to 29)	21 (19·4 to 22·8)	14·4 (13·1 to 15·6)	9·64 (8·88 to 10·5)	6·97 (6·34 to 7·61)	5·69 (5·19 to 6·25)	5·23 (4·82 to 5·69)	4·34 (3·92 to 4·77)	5 (4·49 to 5·54)
Bury	26·2 (21·1 to 31·9)	29 (24·9 to 33·4)	22·5 (20·9 to 24·3)	15·7 (13·8 to 17·7)	9·91 (8·97 to 11)	6·23 (5·59 to 6·9)	5 (4·51 to 5·54)	4·91 (4·43 to 5·39)	4·31 (3·82 to 4·8)	4·88 (4·31 to 5·49)
Cheshire East	20·9 (17·8 to 24·3)	21·7 (20·3 to 23·3)	16·8 (15·6 to 18)	12·2 (11·2 to 13·3)	7·67 (6·98 to 8·47)	5·08 (4·62 to 5·55)	4·05 (3·7 to 4·45)	3·68 (3·37 to 4·02)	3·13 (2·82 to 3·42)	3·44 (3·08 to 3·83)
Cheshire West and Chester	17·6 (15 to 20·3)	18 (16·7 to 19·2)	14·2 (13·2 to 15·2)	10·5 (9·52 to 11·4)	7·02 (6·45 to 7·68)	5·11 (4·66 to 5·59)	4·68 (4·27 to 5·11)	3·93 (3·55 to 4·35)	3·25 (2·94 to 3·56)	3·59 (3·24 to 3·97)
Cumbria	25·6 (21·6 to 30·1)	24·6 (21·1 to 28)	19·5 (17·9 to 21·3)	13·8 (12·3 to 15·3)	8·29 (7·62 to 8·94)	5·48 (4·99 to 6·01)	4·28 (3·91 to 4·66)	3·8 (3·49 to 4·13)	3·38 (3·06 to 3·7)	3·78 (3·4 to 4·18)
Halton	23·1 (18·7 to 28·3)	25·1 (23 to 27·4)	19·9 (18·1 to 21·6)	13·3 (12·1 to 14·5)	9·5 (8·62 to 10·5)	7·51 (6·78 to 8·24)	5·31 (4·84 to 5·77)	4·18 (3·89 to 4·49)	3·42 (3·06 to 3·79)	3·85 (3·44 to 4·29)
Knowsley	24·9 (20·4 to 29·7)	24·5 (21·1 to 28·4)	21·7 (20 to 23·3)	15·8 (14·4 to 17·4)	8·12 (7·38 to 8·9)	6·63 (5·93 to 7·45)	4·64 (3·97 to 5·35)	4·25 (3·87 to 4·64)	3·48 (3·06 to 3·9)	3·89 (3·45 to 4·39)
Lancashire	27·9 (22·9 to 34·3)	26·5 (24·2 to 28·9)	20·4 (18 to 22·9)	13·9 (12·5 to 15·5)	9·4 (8·63 to 10·2)	6·64 (5·92 to 7·44)	5·42 (4·79 to 6·1)	4·71 (4·37 to 5·14)	3·89 (3·39 to 4·42)	4·29 (3·54 to 5·21)
Liverpool	21·5 (17·8 to 26·4)	23·3 (20·5 to 26·4)	19·8 (18 to 21·8)	13·8 (12·3 to 15·4)	7·88 (7·19 to 8·59)	7·07 (6·47 to 7·74)	5·57 (5·19 to 5·96)	5·39 (4·96 to 5·81)	4·69 (4·29 to 5·16)	5·49 (4·91 to 6·08)
Manchester	26 (21·4 to 31·5)	28·6 (24·3 to 33)	24·4 (22·1 to 27)	17·8 (15·7 to 20)	11 (9·96 to 12)	9·46 (8·54 to 10·4)	6·61 (6·13 to 7·09)	6·26 (5·77 to 6·78)	5·41 (4·66 to 6·19)	6·19 (5·01 to 7·5)
Oldham	31·4 (25·9 to 37·1)	30·8 (28·1 to 34)	24·5 (22·3 to 26·8)	16·4 (14·8 to 18·2)	10·6 (9·67 to 11·6)	8·18 (7·37 to 9·06)	6·91 (6·33 to 7·52)	6·58 (6·1 to 7·13)	6·06 (5·46 to 6·72)	6·68 (5·98 to 7·4)
Rochdale	34·7 (29·3 to 40·5)	32·3 (29·7 to 35·1)	26 (24·3 to 27·8)	17·5 (16 to 19·1)	11 (10·1 to 12)	8·21 (7·46 to 8·97)	6·05 (5·57 to 6·54)	5·9 (5·39 to 6·44)	4·82 (4·31 to 5·35)	5·6 (5·04 to 6·21)
Salford	29·3 (24·1 to 35·2)	29·4 (26·8 to 32·1)	24 (21·7 to 26·4)	16·8 (15·3 to 18·4)	9·88 (9·11 to 10·7)	7·64 (6·61 to 8·73)	5·71 (5·2 to 6·26)	4·98 (4·58 to 5·37)	4·23 (3·84 to 4·63)	4·91 (4·43 to 5·44)
Sefton	22·3 (19 to 26)	20·1 (18·6 to 21·7)	16 (14·7 to 17·4)	11·4 (10·4 to 12·5)	7·59 (6·92 to 8·39)	5·48 (4·99 to 6·01)	4·4 (4·08 to 4·73)	4·24 (3·87 to 4·62)	3·63 (3·23 to 4·01)	4·09 (3·64 to 4·58)
St Helens	20·4 (16·4 to 25·1)	21·5 (18·4 to 24·7)	17·1 (15·9 to 18·6)	12·3 (10·7 to 14·1)	8·01 (7·16 to 8·9)	6·83 (6·1 to 7·63)	4·36 (3·96 to 4·79)	3·77 (3·43 to 4·16)	3·31 (2·97 to 3·67)	3·56 (3·22 to 3·95)
Stockport	21·1 (17·8 to 24·7)	20·7 (19·4 to 22)	16·3 (15·2 to 17·4)	11·6 (10·7 to 12·6)	7·86 (7·17 to 8·66)	5·54 (5·05 to 6·08)	4·42 (4·03 to 4·86)	4·56 (4·18 to 4·94)	3·66 (3·31 to 4·01)	4·11 (3·71 to 4·56)
Tameside	26·3 (22 to 30·9)	27 (24·7 to 29·6)	21·4 (19·6 to 23·3)	14·4 (13 to 15·8)	8·18 (7·4 to 9·07)	5·97 (5·36 to 6·7)	4·76 (4·32 to 5·25)	4·06 (3·71 to 4·42)	3·54 (3·13 to 3·94)	3·97 (3·53 to 4·43)
Trafford	27·5 (23·5 to 32·1)	27·3 (25·1 to 29·7)	22 (20·5 to 23·5)	16 (14·8 to 17·4)	9·37 (8·46 to 10·4)	6·13 (5·52 to 6·8)	4·05 (3·63 to 4·5)	3·74 (3·37 to 4·12)	2·98 (2·62 to 3·33)	3·3 (2·94 to 3·71)
Warrington	19·6 (15·5 to 24)	20·3 (18·9 to 21·9)	15·8 (14·7 to 16·9)	11·3 (10·1 to 12·6)	8·63 (7·83 to 9·56)	6·11 (5·47 to 6·76)	4·72 (4·27 to 5·22)	4·2 (3·78 to 4·63)	3·5 (3·09 to 3·9)	3·82 (3·36 to 4·34)
Wigan	21 (17·6 to 24·6)	23·3 (21·7 to 25·1)	17·9 (16·5 to 19·3)	12·5 (11·4 to 13·7)	7·95 (7·26 to 8·66)	6·34 (5·72 to 6·98)	4·44 (4·09 to 4·8)	3·98 (3·69 to 4·29)	3·69 (3·38 to 4·01)	4·1 (3·65 to 4·54)
Wirral	30·3 (25·2 to 35·7)	27·7 (25·6 to 30)	22·1 (20·7 to 23·6)	15·1 (13·7 to 16·6)	9·4 (8·61 to 10·3)	6·82 (6·05 to 7·72)	5·62 (5·13 to 6·17)	5 (4·62 to 5·42)	4·2 (3·76 to 4·68)	4·91 (4·4 to 5·45)
South East England	20 (17·9 to 22·3)	20·2 (19·1 to 21·4)	15·6 (14·7 to 16·4)	11·6 (10·9 to 12·2)	7·78 (7·45 to 8·12)	4·94 (4·55 to 5·35)	3·98 (3·73 to 4·25)	3·58 (3·42 to 3·75)	2·9 (2·65 to 3·16)	3·2 (2·83 to 3·6)
Bracknell Forest	14·3 (11·1 to 17·9)	16·3 (14 to 18·9)	13 (11·7 to 14·4)	9·36 (8·05 to 10·8)	6·51 (5·69 to 7·39)	4·36 (3·72 to 5·07)	3·32 (2·84 to 3·9)	2·26 (2·02 to 2·54)	1·46 (1·24 to 1·71)	1·58 (1·36 to 1·84)
Brighton and Hove	22·7 (18·9 to 26·7)	22·8 (19·2 to 26·6)	17·8 (16 to 19·6)	14·4 (12·9 to 16·3)	9·42 (8·58 to 10·3)	6·6 (5·88 to 7·32)	4·94 (4·47 to 5·47)	3·62 (3·29 to 3·97)	2·73 (2·35 to 3·13)	2·85 (2·43 to 3·33)
Buckinghamshire	16·7 (13·7 to 20)	16·3 (15·2 to 17·6)	12·6 (11·6 to 13·6)	9·55 (8·68 to 10·5)	8·16 (7·49 to 8·85)	5·39 (4·84 to 5·99)	4·64 (4·17 to 5·16)	3·88 (3·59 to 4·17)	3·31 (2·92 to 3·72)	3·79 (3·35 to 4·26)
East Sussex	23·4 (19·7 to 27·3)	21·1 (19·6 to 22·6)	15·6 (14·2 to 17)	11 (9·96 to 12·1)	7·72 (7·07 to 8·38)	5·3 (4·82 to 5·85)	4·6 (4·09 to 5·16)	4·1 (3·77 to 4·43)	2·98 (2·7 to 3·26)	3·17 (2·84 to 3·53)
Hampshire	19·3 (16·1 to 22·5)	21 (19·3 to 22·9)	16·3 (14·8 to 17·8)	12·5 (11·4 to 13·8)	7·17 (6·55 to 7·79)	4·27 (3·78 to 4·78)	3·5 (3·17 to 3·85)	3·53 (3·2 to 3·87)	3·07 (2·67 to 3·5)	3·38 (2·81 to 4·03)
Isle of Wight	33·9 (27·8 to 40·6)	25·8 (22 to 29·7)	18·8 (17·2 to 20·5)	13·5 (11·7 to 15·4)	8·6 (7·67 to 9·68)	4·01 (3·46 to 4·61)	2·84 (2·43 to 3·31)	2·74 (2·45 to 3·02)	2·34 (2·02 to 2·66)	2·43 (2·14 to 2·76)
Kent	21·5 (18·4 to 24·9)	21·1 (19·7 to 22·4)	16 (14·3 to 17·7)	11·4 (10·4 to 12·5)	8·17 (7·59 to 8·81)	4·63 (4·13 to 5·16)	3·69 (3·42 to 3·97)	3·69 (3·42 to 3·96)	3·07 (2·65 to 3·49)	3·44 (2·82 to 4·12)
Medway	17·7 (14·3 to 21·8)	22·1 (20·2 to 24·1)	17·2 (15·9 to 18·7)	12·1 (10·9 to 13·3)	8·59 (7·81 to 9·43)	5·72 (5·11 to 6·31)	3·96 (3·66 to 4·29)	3·5 (3·17 to 3·85)	2·79 (2·45 to 3·14)	2·8 (2·41 to 3·22)
Milton Keynes	21·6 (17·2 to 26·5)	23·5 (19·7 to 27·5)	17·5 (15·8 to 19·2)	12 (10·5 to 13·5)	9·68 (8·79 to 10·7)	7·18 (6·41 to 8·04)	5·47 (4·89 to 6·09)	4·57 (4·11 to 5·09)	3·7 (3·26 to 4·18)	3·91 (3·43 to 4·44)
Oxfordshire	12·4 (10·1 to 14·9)	15 (13·9 to 16·2)	11·8 (11 to 12·7)	9·34 (8·47 to 10·2)	6·63 (6·1 to 7·2)	4·64 (4·19 to 5·12)	4·09 (3·72 to 4·54)	3·07 (2·85 to 3·31)	2·32 (2·07 to 2·59)	2·61 (2·32 to 2·9)
Portsmouth	22·2 (17·9 to 27·5)	25·2 (22 to 28·5)	20·2 (18·4 to 22·3)	14·2 (12·7 to 15·9)	9·54 (8·7 to 10·5)	6·14 (5·45 to 6·86)	3·64 (3·29 to 4·03)	3·09 (2·82 to 3·39)	2·23 (1·93 to 2·56)	2·34 (2 to 2·71)
Reading	16 (13·2 to 19·1)	22 (20·4 to 23·7)	17·6 (15·8 to 19·4)	14 (12·6 to 15·6)	10·1 (9·23 to 11·1)	8·43 (7·53 to 9·47)	5·89 (5·37 to 6·47)	4·6 (4·21 to 5·05)	3·86 (3·39 to 4·35)	4·16 (3·63 to 4·72)
Slough	29·3 (23·4 to 36·3)	27·5 (25·1 to 30)	23 (21·3 to 24·8)	15·5 (13·9 to 17·3)	10·7 (9·74 to 11·8)	7·2 (6·24 to 8·31)	6·1 (5·45 to 6·78)	5·19 (4·81 to 5·61)	4·52 (3·97 to 5·18)	4·85 (4·2 to 5·58)
Southampton	23·3 (18·9 to 28·6)	28·5 (26·1 to 31·1)	23 (20·8 to 25·1)	16 (14·4 to 17·7)	10·1 (9·23 to 10·9)	6·3 (5·51 to 7·14)	4·69 (4·34 to 5·08)	4·22 (3·91 to 4·54)	3·33 (2·87 to 3·81)	3·58 (3·1 to 4·08)
Surrey	21·3 (18 to 25)	19·1 (18 to 20·3)	14·9 (13·7 to 16·3)	11·6 (10·5 to 12·9)	6·97 (6·41 to 7·57)	4·13 (3·74 to 4·53)	3·28 (3·03 to 3·57)	3·13 (2·9 to 3·4)	2·46 (2·11 to 2·83)	2·77 (2·38 to 3·19)
West Berkshire	14·5 (11·4 to 18·2)	15·2 (12·6 to 17·9)	11·5 (10·4 to 12·8)	8·72 (7·58 to 10)	7·28 (6·5 to 8·11)	5·12 (4·49 to 5·81)	3·86 (3·45 to 4·3)	3·22 (2·89 to 3·58)	2·53 (2·17 to 2·91)	2·97 (2·56 to 3·43)
West Sussex	22·7 (17·9 to 28·4)	19·3 (15·8 to 22·8)	14·7 (13·1 to 16·3)	10·3 (9·18 to 11·7)	6·78 (6·25 to 7·34)	4·2 (3·78 to 4·62)	3·63 (3·35 to 3·97)	3·29 (3·04 to 3·57)	2·74 (2·47 to 3·02)	3·08 (2·75 to 3·43)
Windsor and Maidenhead	15 (12·2 to 18)	15·9 (13·4 to 18·5)	12·4 (11·2 to 13·6)	9·54 (8·66 to 10·5)	7·35 (6·67 to 8·03)	5·58 (4·86 to 6·37)	4·1 (3·53 to 4·78)	3·49 (3·14 to 3·88)	2·84 (2·42 to 3·31)	3·16 (2·71 to 3·64)
Wokingham	11·4 (8·96 to 14·2)	13·3 (11·1 to 15·8)	10·1 (9·22 to 11·1)	8·51 (7·39 to 9·85)	6·28 (5·58 to 7·07)	4·03 (3·52 to 4·59)	3·55 (3·08 to 4·14)	3·45 (3·09 to 3·84)	2·44 (2·07 to 2·83)	2·62 (2·25 to 3·02)
South West England	21·2 (17·6 to 25·3)	21·6 (20·1 to 23·2)	17·2 (15·9 to 18·8)	12·4 (11·5 to 13·3)	7·74 (7·33 to 8·15)	5·37 (4·93 to 5·86)	4·16 (3·93 to 4·4)	3·11 (2·93 to 3·29)	2·55 (2·23 to 2·87)	2·84 (2·49 to 3·2)
Bath and North East Somerset	21·1 (16·1 to 27·4)	21 (17·3 to 24·9)	16·8 (14·9 to 18·9)	13·3 (11·2 to 15·6)	7·6 (6·83 to 8·48)	4·79 (4·25 to 5·4)	3·19 (2·7 to 3·71)	2·47 (2·24 to 2·71)	2·06 (1·74 to 2·38)	2·29 (1·95 to 2·61)
Bournemouth	20·3 (15·2 to 26·8)	19·6 (17·9 to 21·3)	15·8 (14·2 to 17·7)	11·4 (10·1 to 12·9)	6·96 (6·13 to 7·9)	5·63 (4·99 to 6·39)	3·81 (3·4 to 4·23)	3 (2·62 to 3·42)	1·76 (1·43 to 2·08)	1·82 (1·43 to 2·2)
Bristol, City of	20·4 (15·9 to 25·4)	25·1 (23 to 27·3)	20·9 (18·8 to 23·2)	15·2 (13·9 to 16·6)	8·88 (8·14 to 9·68)	5·91 (5·34 to 6·52)	3·93 (3·63 to 4·23)	2·86 (2·61 to 3·14)	2·36 (2·08 to 2·66)	2·62 (2·31 to 2·95)
Cornwall	23·4 (18·4 to 29·1)	22·1 (20·3 to 24)	17·2 (15·6 to 18·9)	11·7 (10·7 to 12·8)	7·73 (7·04 to 8·45)	5·67 (5·08 to 6·27)	5·1 (4·64 to 5·59)	3·8 (3·49 to 4·15)	3·21 (2·81 to 3·63)	3·58 (3·13 to 4·05)
Devon	20·9 (16·8 to 26)	19·8 (18·2 to 21·6)	15·4 (14 to 17·1)	11·1 (10·2 to 12·1)	7·65 (7·01 to 8·33)	4·93 (4·33 to 5·56)	4·13 (3·67 to 4·61)	3·23 (2·95 to 3·51)	2·68 (2·31 to 3·09)	2·97 (2·59 to 3·39)
Dorset	22·5 (17·9 to 28·3)	20·6 (18·7 to 22·6)	16 (14·3 to 17·8)	11·7 (10·4 to 13·2)	7·01 (6·41 to 7·64)	5·01 (4·44 to 5·65)	4·09 (3·75 to 4·43)	2·93 (2·69 to 3·21)	2·4 (2·08 to 2·73)	2·64 (2·28 to 2·98)
Gloucestershire	20·4 (16·2 to 25·2)	20·8 (19·1 to 22·4)	16·7 (15·1 to 18·4)	12·2 (11 to 13·3)	7·9 (7·32 to 8·52)	5·41 (4·86 to 6·04)	4·05 (3·74 to 4·36)	3·03 (2·8 to 3·3)	2·57 (2·19 to 2·93)	2·94 (2·54 to 3·37)
North Somerset	23·9 (17·6 to 30·9)	22·5 (21 to 24)	17·6 (15·9 to 19·4)	13 (11·7 to 14·4)	7·31 (6·52 to 8·16)	5·67 (5·06 to 6·36)	4·28 (3·91 to 4·69)	3·09 (2·76 to 3·45)	2·24 (1·9 to 2·58)	2·53 (2·16 to 2·89)
Plymouth	22·6 (18 to 27·7)	24·3 (22·3 to 26·3)	19·9 (17·6 to 22·3)	14 (12·6 to 15·4)	8·01 (7·28 to 8·79)	6·13 (5·35 to 6·98)	4·65 (4·27 to 5·05)	3·24 (2·92 to 3·56)	2·64 (2·27 to 3·01)	2·87 (2·5 to 3·24)
Poole	22·3 (17 to 28·3)	22·9 (19·5 to 26·3)	18 (16·3 to 20)	12·7 (11·4 to 14·1)	7·93 (6·97 to 9)	5·81 (5·08 to 6·66)	4·18 (3·59 to 4·89)	3·04 (2·62 to 3·47)	1·72 (1·48 to 1·96)	1·76 (1·49 to 2·03)
Somerset	20·1 (15·4 to 25·1)	19·3 (17·6 to 21·2)	15·4 (13·7 to 17·2)	10·9 (9·8 to 12·1)	6·71 (6·12 to 7·33)	5·05 (4·51 to 5·58)	4·28 (3·98 to 4·6)	3·36 (3·13 to 3·58)	2·93 (2·52 to 3·34)	3·24 (2·83 to 3·68)
South Gloucestershire	17 (12·9 to 21·7)	19·3 (17·7 to 20·9)	15·2 (13·7 to 16·8)	11·1 (9·82 to 12·5)	7·53 (6·87 to 8·26)	4·88 (4·38 to 5·42)	3·24 (2·96 to 3·54)	2·63 (2·42 to 2·86)	2·25 (1·93 to 2·56)	2·5 (2·16 to 2·84)
Swindon	28·1 (20·7 to 36·5)	28·4 (23·3 to 34·1)	23·9 (21·3 to 26·7)	17·1 (14·5 to 19·9)	9·38 (8·56 to 10·3)	6·33 (5·65 to 7·03)	4·31 (3·94 to 4·7)	3·1 (2·86 to 3·37)	2·45 (2·11 to 2·8)	2·66 (2·27 to 3·04)
Torbay	22·3 (18·1 to 26·9)	20·7 (17·4 to 24·2)	16 (14·3 to 17·9)	10·6 (9·51 to 11·8)	7·7 (6·9 to 8·56)	5·62 (4·82 to 6·53)	4·97 (4·32 to 5·67)	3·4 (3·07 to 3·8)	2·46 (2·1 to 2·85)	2·75 (2·37 to 3·15)
Wiltshire	18·4 (14·7 to 22·6)	20·4 (18·5 to 22·4)	16·1 (14·5 to 17·7)	11·8 (10·7 to 13)	7·78 (7·09 to 8·53)	4·95 (4·3 to 5·66)	3·97 (3·62 to 4·32)	2·94 (2·69 to 3·24)	2·74 (2·34 to 3·16)	3·17 (2·74 to 3·62)
West Midlands	24·9 (22·4 to 27·7)	25·8 (24·5 to 27·2)	21·2 (20·2 to 22·3)	14·5 (13·7 to 15·4)	10·5 (9·83 to 11·3)	7·34 (6·81 to 7·91)	6·02 (5·69 to 6·39)	5·44 (5·16 to 5·73)	4·46 (4·14 to 4·8)	5·04 (4·5 to 5·62)
Birmingham	30·4 (27·1 to 34·1)	29·9 (28 to 31·8)	25·5 (23·1 to 27·8)	17·1 (15·6 to 18·8)	13 (12 to 14·2)	9·93 (8·97 to 11)	7·79 (7·09 to 8·54)	7·14 (6·37 to 8·03)	5·68 (5·09 to 6·24)	6·43 (5·46 to 7·48)

Coventry	27·2 (21·9 to 33·3)	29·1 (27 to 31·2)	24·6 (22·2 to 27·2)	17·9 (16·1 to 19·8)	12·7 (11·7 to 13·8)	8·16 (7·33 to 9·05)	5·91 (5·38 to 6·53)	4·98 (4·57 to 5·42)	4·18 (3·79 to 4·6)	4·77 (4·33 to 5·25)
Dudley	20·5 (17·1 to 24·5)	22·4 (20·7 to 24·3)	18 (16·5 to 19·4)	12 (10·9 to 13·1)	10·7 (9·66 to 11·8)	6·35 (5·57 to 7·18)	4·87 (4·42 to 5·37)	4·29 (3·89 to 4·74)	3·52 (3·17 to 3·89)	3·95 (3·57 to 4·37)
Herefordshire, County of	26·8 (21·9 to 32·2)	24·1 (20·7 to 27·6)	19·4 (17·6 to 21·1)	13·6 (11·9 to 15·6)	7·77 (6·85 to 8·72)	4·48 (3·86 to 5·15)	4·14 (3·62 to 4·68)	4·32 (3·86 to 4·76)	3·45 (3·02 to 3·9)	3·93 (3·47 to 4·42)
Sandwell	37·4 (31·5 to 44·6)	34·2 (31·7 to 37)	28·9 (26·4 to 31·3)	19·8 (18 to 21·6)	12·1 (10·9 to 13·4)	8·56 (7·63 to 9·52)	7·41 (6·67 to 8·22)	6·47 (5·95 to 7·01)	5·15 (4·65 to 5·69)	6·09 (5·46 to 6·74)
Shropshire	19·8 (16·3 to 23·5)	21 (17·4 to 24·8)	16·7 (15·3 to 18·1)	11·8 (10·2 to 13·7)	8·1 (7·24 to 9·02)	5·1 (4·55 to 5·72)	3·91 (3·5 to 4·33)	3·46 (3·09 to 3·87)	2·98 (2·6 to 3·4)	3·35 (2·92 to 3·8)
Solihull	17·1 (14 to 20·7)	20·1 (18·7 to 21·7)	16·2 (14·8 to 17·5)	12·6 (11·4 to 14)	7·95 (7·15 to 8·83)	4·9 (4·27 to 5·58)	4·09 (3·68 to 4·51)	3·88 (3·49 to 4·29)	3·41 (2·99 to 3·85)	3·8 (3·36 to 4·25)
Staffordshire	18·8 (15·5 to 22·1)	21·4 (18·5 to 24·4)	17·2 (15·5 to 19)	12·1 (10·9 to 13·5)	8·39 (7·58 to 9·27)	5·66 (5·1 to 6·29)	5·06 (4·65 to 5·49)	4·71 (4·28 to 5·15)	4·08 (3·61 to 4·59)	4·55 (3·81 to 5·4)
Stoke-on-Trent	32·7 (26·8 to 39·1)	32·5 (30 to 35·2)	28 (25·6 to 30·2)	18·4 (16·8 to 19·9)	11·8 (10·7 to 13·1)	9·2 (8·27 to 10·2)	7·39 (6·66 to 8·16)	7·01 (6·32 to 7·8)	5·85 (5·26 to 6·45)	6·47 (5·78 to 7·19)
Telford and Wrekin	21·2 (17·1 to 26)	24·6 (22·8 to 26·5)	19·7 (18·2 to 21·2)	13·4 (12·2 to 14·7)	10·5 (9·48 to 11·7)	6·57 (5·86 to 7·38)	5·29 (4·76 to 5·84)	4·36 (3·89 to 4·83)	3·46 (3·06 to 3·89)	3·87 (3·43 to 4·33)
Walsall	26·3 (22 to 31·1)	27·1 (25·1 to 29·4)	22·6 (20·7 to 24·7)	15·6 (14 to 17·3)	10·5 (9·39 to 11·6)	7·9 (7·01 to 8·8)	7·8 (7·17 to 8·47)	6·7 (6·11 to 7·38)	5·13 (4·48 to 5·82)	5·89 (5·21 to 6·57)
Warwickshire	19·4 (16 to 23·2)	20·9 (19·4 to 22·5)	16·6 (15·3 to 17·9)	11·8 (10·7 to 13)	9·16 (8·26 to 9·99)	5·54 (5·01 to 6·12)	4·52 (4·05 to 5·08)	4·03 (3·68 to 4·42)	3·59 (3·24 to 4)	4·08 (3·67 to 4·53)
Wolverhampton	26·6 (22·1 to 31·8)	24·9 (22·8 to 27·2)	20·7 (19 to 22·7)	14·2 (12·8 to 15·7)	11·1 (10 to 12·4)	8·41 (7·61 to 9·28)	6·5 (5·83 to 7·22)	5·47 (4·88 to 6·13)	4·46 (4·01 to 4·94)	4·98 (4·43 to 5·59)
Worcestershire	18·3 (15·4 to 21·7)	19·9 (18·2 to 21·6)	15·7 (14·5 to 17)	10·6 (9·67 to 11·6)	8·51 (7·73 to 9·32)	5·79 (5·27 to 6·33)	4·37 (3·99 to 4·8)	4·18 (3·83 to 4·56)	3·51 (3·17 to 3·85)	3·88 (3·45 to 4·32)
Yorkshire and the Humber	27·4 (23·8 to 31·2)	26·6 (24·7 to 28·4)	20·9 (19·8 to 22·1)	14·5 (13·6 to 15·5)	9·33 (8·87 to 9·8)	7·27 (6·71 to 7·83)	5·51 (5·16 to 5·87)	4·25 (4 to 4·53)	3·6 (3·3 to 3·91)	4·08 (3·63 to 4·56)
Barnsley	27·3 (22·6 to 32·6)	25·8 (23·2 to 28·8)	20·3 (18·6 to 22·1)	13·8 (12·2 to 15·4)	8·96 (8·03 to 9·95)	6·64 (5·97 to 7·37)	4·77 (4·29 to 5·25)	3·51 (3·17 to 3·9)	3·14 (2·78 to 3·53)	3·71 (3·3 to 4·18)
Bradford	39·3 (31·4 to 48·9)	35·4 (30·6 to 40·2)	28·4 (25·8 to 31·1)	18·5 (16·3 to 20·9)	12·7 (11·5 to 14)	11·1 (9·95 to 12·2)	8·54 (7·69 to 9·41)	6·55 (5·99 to 7·18)	5·69 (4·87 to 6·49)	6·41 (5·3 to 7·67)
Calderdale	31·6 (26 to 38·1)	27·5 (24·8 to 30·3)	21·3 (19·5 to 23)	14·7 (13·1 to 16·4)	10·5 (9·61 to 11·5)	7·69 (6·98 to 8·4)	6·02 (5·53 to 6·54)	4·39 (3·96 to 4·87)	3·53 (3·12 to 3·99)	3·92 (3·46 to 4·39)
Doncaster	24·8 (20·6 to 29·4)	24 (21·4 to 26·9)	19·6 (18 to 21·1)	13·1 (11·8 to 14·5)	8·7 (8 to 9·44)	7·12 (6·5 to 7·8)	5·57 (5·14 to 6·04)	4·21 (3·82 to 4·63)	3·71 (3·33 to 4·12)	4·3 (3·84 to 4·8)
East Riding of Yorkshire	23·7 (18·8 to 29·4)	21·9 (19 to 25·1)	16·8 (15·5 to 18·3)	11·5 (10·2 to 13)	7·6 (6·93 to 8·26)	5·55 (5·05 to 6·05)	3·99 (3·66 to 4·35)	2·95 (2·66 to 3·24)	2·61 (2·29 to 2·98)	2·96 (2·61 to 3·33)
Kingston upon Hull, City of	25·7 (21·7 to 30·7)	27·6 (24·8 to 30·4)	22·7 (20·8 to 24·4)	15·4 (13·9 to 17·1)	9·79 (9·07 to 10·6)	7·66 (6·95 to 8·43)	5·6 (5·14 to 6·09)	4·59 (4·11 to 5·08)	3·85 (3·37 to 4·35)	4·38 (3·86 to 4·92)
Kirklees	30·8 (26·1 to 36)	28·5 (25·5 to 32)	22·1 (20·1 to 24·3)	15·3 (13·8 to 17·1)	9·49 (8·69 to 10·3)	7·63 (7·03 to 8·33)	6·18 (5·61 to 6·76)	5·14 (4·73 to 5·55)	4·47 (4·09 to 4·92)	4·98 (4·5 to 5·51)
Leeds	27·2 (21·7 to 34)	27·6 (24 to 31·5)	21·8 (20 to 23·8)	15·4 (13·8 to 17·1)	9·62 (8·85 to 10·5)	6·91 (6·32 to 7·55)	4·87 (4·4 to 5·41)	3·98 (3·63 to 4·35)	3·45 (2·96 to 3·95)	3·91 (3·19 to 4·71)
North East Lincolnshire	24·6 (19·4 to 30·9)	24 (21 to 27·8)	19·5 (18·1 to 21)	13·7 (12·1 to 15·6)	7·8 (7·09 to 8·61)	6·38 (5·75 to 7·04)	5·35 (4·86 to 5·87)	4·4 (3·98 to 4·82)	3·47 (3·05 to 3·93)	3·9 (3·44 to 4·39)
North Lincolnshire	23·5 (17·7 to 30·6)	22·1 (19·5 to 24·9)	17·5 (16·1 to 19·1)	12 (10·8 to 13·3)	8·57 (7·71 to 9·47)	6·14 (5·52 to 6·91)	4·21 (3·76 to 4·64)	3·22 (2·9 to 3·57)	2·58 (2·24 to 2·93)	2·87 (2·5 to 3·26)
North Yorkshire	23·8 (20 to 28·2)	21·6 (19·8 to 23·5)	16·5 (15 to 17·9)	11·8 (10·6 to 13·1)	6·74 (6·2 to 7·31)	4·41 (4·02 to 4·84)	3·48 (3·25 to 3·71)	2·6 (2·39 to 2·81)	2·26 (2·03 to 2·53)	2·51 (2·26 to 2·81)
Rotherham	25·9 (21·6 to 30·6)	26·5 (24·2 to 29)	21 (19·3 to 22·7)	14·2 (12·7 to 15·7)	9·53 (8·73 to 10·4)	7·2 (6·56 to 7·9)	5·64 (5·25 to 6·08)	4·28 (3·88 to 4·71)	3·31 (2·91 to 3·72)	3·79 (3·35 to 4·24)
Sheffield	23·1 (19·2 to 27·6)	23·5 (20·8 to 26·3)	18 (16 to 20·1)	13 (11·6 to 14·7)	9·29 (8·44 to 10·3)	7·65 (6·88 to 8·41)	5·47 (4·99 to 6)	4·26 (3·94 to 4·59)	3·28 (2·98 to 3·61)	3·76 (3·39 to 4·17)
Wakefield	30 (25·2 to 35·8)	29·3 (26·2 to 32·1)	23·4 (21·3 to 25·6)	16·3 (14·6 to 18)	9·14 (8·38 to 9·9)	7·13 (6·47 to 7·82)	5·3 (4·84 to 5·83)	3·67 (3·35 to 4)	3·22 (2·9 to 3·56)	3·77 (3·42 to 4·16)
York	20·5 (15·7 to 26·2)	22·1 (19·6 to 24·7)	17 (15·4 to 18·7)	12·4 (11·1 to 13·9)	8·07 (7·32 to 8·88)	6·23 (5·59 to 6·97)	4·41 (4 to 4·83)	3·3 (2·92 to 3·66)	2·69 (2·3 to 3·09)	2·98 (2·56 to 3·43)
Northern Ireland	34·4 (30·9 to 38·5)	28·1 (26·9 to 29·4)	23·2 (22·1 to 24·4)	15·7 (14·6 to 16·7)	8·4 (7·82 to 9·06)	5·84 (5·3 to 6·41)	5·09 (4·67 to 5·52)	4·66 (4·17 to 5·19)	3·9 (3·5 to 4·34)	3·39 (2·7 to 4·1)
Scotland	30·1 (27·7 to 32·9)	26·5 (25·5 to 27·5)	19·5 (18·7 to 20·3)	12·9 (12·2 to 13·7)	8·01 (7·47 to 8·55)	5·58 (5·18 to 6)	3·91 (3·61 to 4·24)	3·43 (3·12 to 3·77)	3·1 (2·81 to 3·42)	3·91 (3·49 to 4·35)
Wales	23·9 (17·2 to 31·6)	24·9 (20·8 to 29·1)	20 (17 to 23·7)	13·8 (12·6 to 15·1)	7·54 (7·07 to 8·02)	5·43 (4·98 to 5·9)	4·08 (3·79 to 4·4)	3·47 (3·14 to 3·77)	3·28 (2·92 to 3·65)	3·44 (2·92 to 4·01)
Latin America and Caribbean	213 (192 to 236)	149 (140 to 159)	112 (105 to 118)	74·4 (70·7 to 78·1)	47·5 (45·1 to 50)	31 (29 to 33)	28·8 (26·4 to 31·2)	16·9 (14·2 to 19·9)	15·6 (13·3 to 18·5)	15 (12·4 to 18·1)
Andean Latin America	336 (301 to 377)	260 (242 to 278)	193 (184 to 203)	115 (108 to 122)	70·1 (65·5 to 74·9)	42 (39·1 to 45)	25·8 (22·9 to 29·1)	18·8 (15·8 to 22·1)	16·2 (13·6 to 19·2)	15·4 (12·3 to 19)
Bolivia	373 (328 to 421)	297 (267 to 329)	219 (204 to 234)	152 (142 to 161)	104 (97·4 to 112)	67 (61·6 to 73·1)	41·8 (37·3 to 46·6)	28·5 (24·5 to 32·8)	26·8 (23·1 to 30·9)	25·8 (22·3 to 29·8)
Ecuador	216 (181 to 255)	164 (151 to 176)	120 (111 to 129)	77·7 (71·1 to 84·9)	38·8 (35·4 to 42·6)	30·5 (26·5 to 35)	20·3 (16·8 to 24·3)	15·1 (12·2 to 18·5)	13·4 (10·7 to 16·7)	12·4 (9·54 to 16·2)
Peru	366 (311 to 430)	287 (262 to 312)	215 (202 to 228)	118 (108 to 130)	72·3 (65·8 to 79·6)	38·7 (34·8 to 42·6)	22·3 (19·2 to 25·9)	17 (13·5 to 21)	13·7 (10·9 to 17·1)	13·1 (9·01 to 17·6)
Caribbean	165 (147 to 184)	125 (119 to 131)	101 (95·4 to 106)	84·3 (78·5 to 90·5)	66·2 (61·7 to 71·3)	48 (44·7 to 51·5)	120 (112 to 129)	38·3 (32·5 to 44·3)	37·5 (32 to 44)	37·8 (32·1 to 44·4)
Antigua and Barbuda	118 (96 to 146)	69·8 (60·6 to 80·5)	36·5 (31·2 to 42·4)	21 (17·6 to 24·7)	12·9 (10·8 to 15·3)	13·6 (11 to 16·5)	11·9 (10·1 to 13·9)	10·9 (9·13 to 12·9)	10 (8·32 to 11·8)	9·6 (8·33 to 10·9)
The Bahamas	110 (90·1 to 135)	69·9 (60·3 to 81·2)	41·1 (35·8 to 47·6)	29·4 (24·9 to 34·3)	20·3 (16·9 to 24·3)	14·7 (12·6 to 17·3)	12·3 (10·5 to 14·2)	19·6 (16·9 to 23·1)	9·65 (7·38 to 12·6)	9·44 (7·41 to 12·1)
Barbados	214 (178 to 258)	75 (64·1 to 88·3)	40·8 (34·7 to 47·3)	22·1 (18·9 to 26)	16·1 (13·6 to 18·7)	14·5 (12·5 to 16·9)	13·4 (11·6 to 15·6)	11·8 (9 to 15·2)	11·6 (8·55 to 15·3)	11·5 (8·28 to 15·5)
Belize	159 (131 to 193)	112 (97 to 128)	86·8 (77·7 to 96·8)	59·6 (53·8 to 65·7)	40·6 (36·3 to 45·2)	27·4 (24·2 to 30·9)	17·9 (15·8 to 20·3)	14·3 (12 to 17·1)	14 (11·7 to 16·9)	12·9 (10·8 to 15·7)
Bermuda	64·2 (52·6 to 78·4)	37·4 (34·3 to 40·9)	25·2 (22·5 to 28·3)	16·3 (14·3 to 18·3)	10·1 (8·79 to 11·5)	5·56 (4·75 to 6·55)	4·97 (4·22 to 5·83)	3·88 (3·19 to 4·67)	3·78 (3·03 to 4·63)	3·92 (3·38 to 4·58)
Cuba	53·8 (35·8 to 78·7)	44·3 (42·7 to 45·8)	39·2 (37·9 to 40·6)	20·2 (19·4 to 21)	11·9 (11·3 to 12·5)	7·38 (6·99 to 7·79)	5·15 (4·83 to 5·51)	5·42 (5·03 to 5·77)	5·24 (4·84 to 5·65)	3·94 (3·33 to 4·59)
Dominica	243 (202 to 284)	133 (116 to 151)	59·7 (50·7 to 70)	21·6 (18·1 to 25·5)	17 (14·5 to 19·8)	17·2 (14·4 to 20·4)	21·4 (18 to 25·2)	25·6 (19·9 to 32·3)	25·7 (19·8 to 33·1)	25·3 (19·1 to 33·3)
Dominican Republic	201 (165 to 245)	163 (149 to 178)	123 (112 to 135)	80·8 (72·2 to 89·5)	62·3 (56·4 to 68·5)	37·5 (33·4 to 41·7)	30·3 (25·6 to 35·4)	23·7 (19·1 to 29·4)	23 (18·7 to 28·2)	22·5 (18·6 to 26·9)
Grenada	154 (128 to 183)	94·7 (82·2 to 108)	56·9 (48·3 to 66·4)	35·1 (28·8 to 42·3)	21·6 (18 to 25·9)	15·4 (12·6 to 18·5)	13·8 (11·2 to 17)	12·8 (10·3 to 15·7)	12·5 (10 to 15·3)	11·7 (9·49 to 14·3)
Guyana	165 (140 to 193)	101 (91 to 112)	77·4 (70·5 to 86·2)	57·8 (50·6 to 65·8)	49·1 (42·9 to 55·7)	35·2 (31·2 to 39·3)	28·7 (24·6 to 33·1)	22·3 (17·3 to 27·8)	20·8 (16 to 26·4)	19·9 (15·2 to 25·4)
Haiti	334 (289 to 381)	244 (224 to 264)	222 (206 to 237)	185 (169 to 202)	144 (132 to 156)	99·4 (90·6 to 109)	273 (255 to 292)	66·7 (56·7 to 77·4)	65 (55·4 to 75·9)	65·8 (56·3 to 76·7)
Jamaica	127 (104 to 153)	81·2 (73·3 to 89)	52 (46·9 to 57·8)	34·7 (30·7 to 39·1)	26·7 (23·5 to 30)	20·1 (16·1 to 24·9)	17·1 (14·4 to 20·5)	14·9 (11·6 to 18·6)	14·5 (11 to 18·5)	13·8 (10·4 to 18)
Puerto Rico	101 (93·1 to 109)	51·2 (49·5 to 53·2)	29 (27·8 to 30·3)	18·7 (17·9 to 19·6)	13·9 (13·2 to 14·6)	10·1 (9·5 to 10·8)	7·94 (7·08 to 8·86)	6·45 (5·86 to 7·12)	7·38 (6·71 to 8·14)	5·84 (4·94 to 6·89)
Saint Kitts and Nevis	154 (138 to 173)	133 (127 to 140)	66·5 (61·2 to 72·1)	53 (47·9 to 58·8)	31·8 (28·4 to 35·2)	22·9 (20·1 to 26·1)	18·5 (15·8 to 21·9)	16·6 (13·8 to 19·8)	15·7 (12·9 to 19·1)	15·3 (12·3 to 19·1)
Saint Lucia	178 (150 to 212)	136 (119 to 153)	70·6 (60·4 to 82·2)	34·4 (29·3 to 40·2)	21·8 (18·4 to 25·5)	17·7 (15 to 20·7)	18·3 (15·1 to 22)	15·3 (11·6 to 19·7)	14·5 (11 to 19)	14·3 (10·6 to 19)
Saint Vincent and the Grenadines	206 (172 to 243)	147 (128 to 168)	88·8 (75 to 104)	52·3 (44·1 to 62·1)	28·9 (23·5 to 34·6)	22·8 (19 to 27·3)	17·7 (15·1 to 20·6)	13·1 (10·5 to 16)	12·3 (9·73 to 15·4)	12 (9·19 to 15·4)
Suriname	89·1 (71·3 to 110)	74·1 (64 to 85)	60·7 (51·3 to 71·1)	58·5 (51·6 to 66)	45·4 (40·2 to 51·5)	36·2 (30·9 to 42·3)	26·9 (22·7 to 31·4)	24 (19·2 to 29·7)	22·4 (17·9 to 28·3)	21·9 (17·1 to 27·8)
Trinidad and Tobago	88 (69·8 to 109)	63·6 (57·2 to 70·6)	46·1 (41 to 51·8)	32·6 (28·8 to 36·6)	23·6 (20·2 to 27·4)	23·9 (20·1 to 28·4)	16·3 (13·3 to 20·3)	13·4 (10·8 to 16·4)	12·7 (10·1 to 16)	12·6 (9·76 to 16·3)
Virgin Islands	62·4 (52·5 to 73·6)	43·4 (38·9 to 47·9)	28·1 (25·3 to 31·1)	23·9 (21·5 to 26·5)	15·3 (13·2 to 17·5)	9·86 (8·32 to 11·5)	7·47 (6·18 to 8·95)	6·15 (4·93 to 7·59)	6·4 (5·25 to 7·77)	5·31 (4·37 to 6·39)
Central Latin America	202 (178 to 229)	134 (127 to 142)	98·4 (93·3 to 103)	65 (61·1 to 69)	40·6 (38·2 to 43·5)	26·6 (24·4 to 28·8)	19·2 (17 to 21·6)	15·6 (12·9 to 18·5)	14·5 (12 to 17·6)	13·8 (11 to 17·3)
Colombia	149 (117 to 187)	114 (102 to 125)	81·2 (72·9 to 90·1)	48·6 (42·8 to 54·7)	30 (26·6 to 33·6)	23·4 (20 to 27)	17·7 (13·8 to 22·2)	13·3 (10·2 to 16·9)	12·1 (9·19 to 15·7)	10·6 (7·82 to 14·2)
Costa Rica	145 (135 to 156)	88·5 (85·8 to 91·1)	75·6 (73·3 to 78·3)	21 (20·1 to 22)	15·3 (14·5 to 16·1)	11·1 (10·5 to 11·8)	9·71 (9·15 to 10·3)	9·33 (8·76 to 9·99)	8·54 (7·78 to 9·36)	8·78 (7·85 to 9·8)
El Salvador	281 (231 to 343)	190 (172 to 211)	137 (124 to 150)	86·2 (77·9 to 94·5)	48·2 (43 to 53·9)	26·5 (23·2 to 30)	15·8 (13·8 to 17·9)	10·3 (8·23 to 12·7)	9·39 (7·41 to 11·8)	8·85 (6·78 to 11·4)
Guatemala	258 (213 to 307)	198 (180 to 217)	165 (151 to 180)	116 (106 to 127)	73·6 (67·4 to 80·4)	45·3 (40·4 to 50·5)	32·2 (27·8 to 37·5)	27·6 (23·1 to 32·3)	23·9 (19·9 to 28·8)	22·9 (18·2 to 28·7)
Honduras	274 (230 to 329)	183 (164 to 204)	133 (120 to 146)	84·9 (77·2 to 92·3)	50·3 (45·2 to 55·9)	33·3 (29·4 to 37·6)	22·8 (18·7 to 27·6)	15·5 (13 to 18·7)	14·3 (12 to 16·9)	13·5 (11·2 to 16·1)
Mexico	218 (181 to 259)	141 (130 to 154)	99·5 (91·4 to 107)	67·5 (60·9 to 74)	41·6 (37·9 to 46)	25·8 (23·2 to 28·7)	18·7 (16·4 to 21·3)	14 (11·3 to 17·1)	13·5 (10·9 to 16·7)	13·2 (10·6 to 16·6)

Aguascalientes	178 (144 to 215)	109 (99 to 120)	77·1 (69 to 85·3)	52·3 (42·5 to 62·1)	32·9 (28·7 to 37·8)	20·3 (17·5 to 23·7)	14·7 (12·5 to 17·3)	11·6 (9·27 to 14·6)	11·5 (9·39 to 14·2)	11·6 (9·25 to 14·5)
Baja California	186 (136 to 244)	116 (105 to 127)	81·9 (74·8 to 89)	38·5 (34·8 to 42·5)	29·8 (27 to 33·2)	20·8 (18·4 to 23·3)	17·8 (15·7 to 20·3)	12 (9·8 to 14·9)	12·4 (10 to 15·2)	12·2 (9·74 to 15·4)
Baja California Sur	208 (161 to 261)	125 (114 to 138)	84 (72·5 to 96·9)	50·2 (45·1 to 55·4)	22·4 (20·2 to 25)	17·7 (15·5 to 20)	15·7 (13·6 to 17·8)	12·3 (9·62 to 15·4)	12·8 (10·1 to 15·9)	12·5 (9·79 to 15·6)
Campeche	204 (167 to 247)	131 (119 to 144)	95·6 (86 to 105)	71·7 (62·1 to 81·6)	41·9 (37·2 to 47·3)	23·8 (20·7 to 27·4)	18·3 (15·9 to 20·9)	14·6 (11·6 to 18·1)	13·2 (10·7 to 16·2)	12·7 (10·2 to 15·9)
Chiapas	194 (153 to 244)	148 (137 to 161)	116 (106 to 127)	87·2 (76·9 to 97·9)	54·8 (48·4 to 62)	31·2 (26·6 to 36·4)	17·1 (14·8 to 19·6)	17·3 (14 to 21·1)	16·3 (13 to 20·3)	17 (13·4 to 21·5)
Chihuahua	199 (154 to 250)	137 (125 to 150)	98·5 (89·1 to 108)	70·9 (63·2 to 79·3)	42·5 (37·3 to 48·5)	30·1 (26 to 34·7)	19·6 (16·7 to 22·6)	16·7 (13·4 to 20·7)	17 (13·7 to 21·1)	17·1 (13·6 to 21·7)
Coahuila	225 (177 to 279)	145 (133 to 158)	102 (90·2 to 114)	66·7 (56·3 to 79·4)	37 (31·7 to 43·2)	23·8 (20 to 27·8)	14·5 (12·6 to 16·7)	14·5 (11·8 to 17·9)	13·7 (11 to 17·1)	13·8 (11 to 17·5)
Colima	274 (226 to 331)	169 (155 to 185)	112 (95·6 to 131)	69·4 (58 to 81·2)	37·2 (32·1 to 42·9)	23·2 (20·1 to 26·6)	16·6 (14·4 to 19)	10·7 (8·25 to 13·6)	10·7 (8·49 to 13·3)	10·4 (8·13 to 13·2)
Durango	251 (205 to 303)	159 (146 to 174)	109 (97·6 to 119)	70·9 (57·7 to 84·4)	42·9 (37·5 to 48·8)	27·4 (23·5 to 32)	18·9 (16·5 to 21·6)	13 (10·5 to 16)	12 (9·74 to 14·8)	12·2 (9·74 to 15·2)
Guanajuato	225 (169 to 286)	147 (136 to 159)	106 (93·2 to 118)	75·2 (68·1 to 82·6)	44·9 (41 to 49·3)	21 (18·6 to 23·4)	16·1 (14·2 to 18·1)	12·7 (10·3 to 15·6)	12·1 (9·78 to 14·9)	11·4 (9·13 to 14·3)
Guerrero	270 (223 to 325)	199 (167 to 230)	151 (131 to 170)	105 (93·6 to 117)	67·1 (58·8 to 75·9)	44·6 (38·8 to 50·8)	32·8 (26·7 to 39·4)	19·8 (15·2 to 25·2)	19·1 (14·6 to 24·6)	18·8 (14·1 to 24·5)
Hidalgo	211 (168 to 263)	138 (126 to 150)	98·6 (89·2 to 108)	62·5 (54·7 to 71)	37·8 (32·9 to 43·2)	24·6 (20·8 to 28·8)	15·4 (13·3 to 17·6)	11·3 (9·09 to 14·1)	10·4 (8·38 to 13)	9·97 (7·64 to 12·9)
Jalisco	180 (139 to 228)	117 (108 to 128)	80·6 (70·7 to 89·6)	49·4 (43 to 56·5)	32 (27·7 to 37·2)	22·9 (20·1 to 26·4)	16 (14·1 to 17·9)	11·6 (9·34 to 14·4)	12·2 (9·83 to 15·1)	12·5 (9·97 to 15·7)
México	185 (142 to 234)	115 (106 to 126)	79·6 (72 to 86·7)	63·6 (56·6 to 70·3)	43·5 (39·5 to 47·8)	24·7 (21·9 to 27·6)	20·3 (17·8 to 23)	15·2 (12·3 to 18·7)	13·6 (10·9 to 16·8)	11·4 (9·05 to 14·3)
Mexico City	176 (129 to 232)	97·5 (88·7 to 107)	69·6 (63·6 to 76)	53·4 (48·4 to 58·8)	29·4 (26·9 to 32·3)	20·5 (18·3 to 22·8)	18·7 (16·3 to 21·2)	12·5 (10·2 to 15·3)	13·7 (11·1 to 16·7)	17 (13·7 to 21)
Michoacán de Ocampo	243 (198 to 293)	162 (136 to 190)	112 (98·6 to 126)	73·7 (64·7 to 82·9)	44·8 (39·7 to 50·4)	28·9 (25·2 to 32·8)	23 (18·8 to 27·8)	14·7 (11 to 18·6)	14·6 (11·1 to 18·8)	14·4 (10·7 to 19)
Morelos	231 (187 to 279)	141 (129 to 154)	96·7 (87·2 to 107)	63·1 (54·7 to 72·1)	35·4 (31·4 to 40·1)	21·9 (19 to 25·3)	16·2 (14·1 to 18·5)	11·7 (9·43 to 14·5)	11 (8·93 to 13·6)	11 (8·77 to 13·8)
Nayarit	254 (207 to 307)	157 (144 to 172)	100 (90·7 to 111)	56·9 (47·9 to 66·7)	35·2 (30·9 to 40·1)	21·5 (18·5 to 25·4)	14·6 (12·6 to 16·7)	12·5 (10 to 15·5)	11·5 (9 to 14·6)	11·3 (8·68 to 14·7)
Nuevo León	172 (137 to 213)	106 (97·5 to 116)	71 (64·6 to 77·1)	43·7 (37·7 to 50·3)	25·7 (21·9 to 30·3)	19 (16·3 to 22)	12·4 (10·8 to 14·1)	11 (8·91 to 13·5)	9·85 (8·06 to 12·1)	10·1 (8·18 to 12·7)
Oaxaca	252 (203 to 305)	182 (154 to 210)	142 (124 to 159)	96·7 (85·1 to 108)	52·4 (46·7 to 59·3)	32·7 (28·7 to 37·2)	24·6 (19·5 to 30·6)	15·4 (11·4 to 19·4)	15 (11·3 to 19·3)	14·6 (10·8 to 19·2)
Puebla	292 (225 to 366)	205 (190 to 221)	146 (133 to 161)	89·1 (79 to 99·6)	53·4 (46·8 to 60·6)	32·6 (28·6 to 36·8)	22·7 (19·9 to 25·6)	15·9 (13·1 to 19·4)	15·5 (12·6 to 19·1)	14·8 (11·9 to 18·4)
Querétaro	254 (191 to 325)	155 (142 to 169)	105 (94 to 116)	73·2 (65·6 to 81)	43·5 (39·1 to 48·4)	21 (18·8 to 23·3)	16 (13·9 to 18)	10·5 (8·46 to 13)	10·1 (8·16 to 12·3)	10·2 (8·14 to 12·7)
Quintana Roo	184 (150 to 223)	125 (115 to 136)	89·8 (81·3 to 97·8)	61·3 (50·4 to 73·3)	32·7 (28·3 to 37·8)	20·6 (17·9 to 23·8)	15·9 (13·9 to 18·3)	11·1 (9·02 to 13·8)	10·1 (8·17 to 12·6)	9·69 (7·75 to 12·2)
San Luis Potosí	262 (214 to 318)	173 (146 to 201)	121 (107 to 138)	69·5 (60·6 to 78·5)	42·3 (37·8 to 47·7)	28·1 (24·3 to 32·1)	21·6 (17·6 to 26)	13·6 (10·2 to 17·2)	13 (9·95 to 16·9)	12·9 (9·69 to 16·9)
Sinaloa	205 (161 to 255)	131 (120 to 143)	87·4 (78·8 to 95·4)	52·5 (45·8 to 59·7)	30·5 (25·6 to 36·2)	19·2 (15·9 to 23·1)	11·9 (10·3 to 13·6)	11 (8·81 to 13·5)	9·55 (7·73 to 11·9)	9·9 (7·79 to 12·5)
Sonora	174 (140 to 217)	116 (105 to 126)	84·3 (77·3 to 91·2)	53·5 (47·5 to 60)	29·9 (25·2 to 35·4)	22·9 (19·7 to 26·4)	17·1 (15·1 to 19·4)	15 (11·9 to 18·9)	12·8 (10·3 to 16·1)	12·6 (10 to 16)
Tabasco	191 (150 to 244)	129 (119 to 140)	102 (92·8 to 111)	73·1 (64·5 to 82·1)	43·3 (38·6 to 48·9)	24·3 (20 to 29·3)	18·4 (16·1 to 20·8)	17·1 (13·9 to 21·1)	17·1 (13·9 to 21·1)	15 (11·8 to 18·9)
Tamaulipas	202 (158 to 253)	131 (120 to 143)	89·9 (82·5 to 97·4)	56·1 (50·1 to 63)	31·4 (27·7 to 35·5)	18·7 (16·2 to 21·4)	15·9 (14 to 18)	14·2 (11·4 to 17·6)	13·5 (10·9 to 16·5)	13·9 (11·2 to 17·2)
Tlaxcala	247 (187 to 312)	162 (150 to 175)	109 (98·8 to 120)	82 (73·2 to 90·7)	48·1 (42·9 to 53·4)	26·3 (23·3 to 29·6)	19·9 (17·4 to 22·6)	13·8 (11·1 to 17·1)	13·4 (10·7 to 16·7)	12·5 (9·88 to 15·9)
Veracruz de Ignacio de la Llave	230 (183 to 289)	153 (141 to 167)	109 (97·4 to 121)	71·6 (63·3 to 80·6)	46·1 (40·2 to 52·7)	28·7 (25·5 to 32·4)	18·2 (16 to 20·9)	15·2 (12·4 to 18·9)	15·1 (12·3 to 18·4)	14·1 (11·2 to 17·8)
Yucatán	188 (147 to 233)	134 (123 to 146)	91·4 (81·9 to 100)	65·2 (55·5 to 76)	40 (34·8 to 46·3)	23·6 (20·4 to 27·1)	15·9 (13·8 to 18·1)	11·3 (9·08 to 13·8)	12·2 (9·71 to 15·3)	12·2 (9·69 to 15·5)
Zacatecas	188 (152 to 231)	122 (111 to 133)	81·2 (72·4 to 89·4)	57·8 (47·9 to 69)	33·8 (29·6 to 38·6)	21 (17·9 to 24·8)	15·5 (13·5 to 17·7)	11·4 (9·16 to 14)	10·7 (8·57 to 13·2)	10·2 (8·04 to 12·8)
Nicaragua	264 (225 to 315)	199 (179 to 221)	145 (132 to 159)	92 (85·3 to 99·5)	55 (50·3 to 59·8)	32 (28·3 to 36·3)	19·9 (16·7 to 23·2)	14 (11 to 17·5)	13·5 (10·4 to 16·9)	12·1 (9·35 to 15·5)
Panama	100 (81·7 to 122)	77·8 (70·2 to 85·7)	53·1 (47·8 to 58·7)	33·8 (29·7 to 38·3)	24·7 (21·4 to 28)	20·7 (17·9 to 24·1)	16·5 (14 to 19·7)	14·9 (12·5 to 17·7)	14·1 (11·6 to 16·9)	13 (10·5 to 16·1)
Venezuela	158 (151 to 165)	72·1 (70·8 to 73·3)	64·3 (62·8 to 66)	37·5 (36·6 to 38·5)	29·3 (28·3 to 30·4)	20·7 (19·4 to 22)	16·6 (16 to 17·3)	20·3 (16·3 to 25)	18·9 (14·7 to 23·9)	17·6 (13·6 to 22·5)
Tropical Latin America	201 (154 to 253)	139 (123 to 156)	106 (94·4 to 118)	71·6 (64·5 to 79·5)	45·2 (40·9 to 49·4)	29·4 (25·9 to 33·3)	20·5 (17·4 to 24)	12·9 (10·4 to 15·6)	11·7 (9·66 to 14·1)	11 (9·1 to 13·3)
Brazil	203 (155 to 256)	141 (124 to 158)	107 (95·4 to 120)	72·3 (65·1 to 80·4)	45·8 (41·3 to 50·2)	29·6 (25·9 to 33·6)	20·6 (17·4 to 24)	12·8 (10·3 to 15·5)	11·6 (9·58 to 14)	10·9 (9·04 to 13·1)
Acre	228 (160 to 306)	158 (136 to 180)	122 (104 to 144)	90·7 (77·5 to 105)	58·2 (50·7 to 66·4)	43·7 (36·7 to 51·4)	31 (26·1 to 36·4)	16·6 (12·5 to 21·4)	15·4 (11·5 to 19·8)	14·6 (11·1 to 18·6)
Alagoas	327 (235 to 424)	253 (218 to 292)	201 (173 to 229)	152 (135 to 170)	97·7 (83·1 to 114)	53·4 (45·7 to 62)	27·2 (21·1 to 33·8)	12·5 (9·88 to 15·7)	10·3 (7·88 to 13·1)	9·66 (7·53 to 12·4)
Amapá	267 (190 to 359)	167 (145 to 192)	106 (88·3 to 127)	38·4 (34·1 to 43·1)	23 (20·5 to 25·4)	23·4 (20·1 to 26·8)	20·4 (17·8 to 23·4)	20·8 (17 to 25·5)	19 (15·6 to 23·4)	18·1 (15·1 to 21·5)
Amazonas	181 (116 to 264)	111 (92·2 to 134)	83·8 (69·8 to 98·9)	58·4 (48·6 to 69·9)	35·9 (30·2 to 42·4)	26·7 (23·5 to 30·5)	19·9 (17·6 to 22·2)	16·6 (14 to 19·9)	15·2 (12·8 to 17·8)	15·3 (13·5 to 17·3)
Bahia	206 (141 to 285)	160 (131 to 189)	129 (112 to 145)	85·7 (75·4 to 97·2)	65·5 (57·6 to 74·2)	41 (35 to 47·7)	27 (21·4 to 33·6)	14·4 (11·4 to 18)	12·7 (9·8 to 16·1)	11·6 (8·93 to 14·8)
Ceará	304 (218 to 403)	233 (200 to 270)	167 (144 to 193)	119 (99·9 to 141)	65·5 (57·8 to 74·7)	39·1 (33·6 to 45·1)	20·2 (15·7 to 24·7)	11·1 (8·51 to 14·1)	8·87 (6·81 to 11·3)	7·68 (5·86 to 9·9)
Distrito Federal	196 (132 to 271)	112 (95·7 to 130)	78 (65·4 to 93·6)	42·7 (38·7 to 47·5)	25·5 (23·1 to 28·4)	16·8 (14·6 to 19·1)	13·9 (12·3 to 15·8)	9·8 (8·02 to 12·1)	9·98 (8·48 to 11·7)	9·76 (8·46 to 11·3)
Espírito Santo	146 (110 to 188)	99·9 (83·9 to 117)	80·6 (69·3 to 94·8)	46·9 (42·7 to 51·4)	31·1 (28·1 to 34·5)	20·6 (17·7 to 23·7)	20·2 (16·1 to 24·9)	12·4 (9·47 to 15·8)	11·6 (9 to 14·9)	11·4 (8·79 to 14·7)
Goiás	241 (165 to 326)	167 (142 to 195)	109 (90·2 to 128)	64·1 (55 to 73·9)	38·3 (33·2 to 44·3)	28·3 (22·5 to 35·3)	21·2 (16·9 to 26·3)	14·2 (11 to 17·9)	13·5 (10·4 to 17·1)	13 (10·1 to 16·4)
Maranhão	301 (244 to 364)	234 (201 to 273)	186 (162 to 211)	125 (110 to 139)	76·5 (67·1 to 86·1)	46·8 (40·4 to 54·5)	29 (22·8 to 36·1)	12·9 (9·73 to 16·8)	10·6 (8·21 to 13·2)	9·82 (7·69 to 12·3)
Mato Grosso	172 (123 to 230)	142 (119 to 166)	107 (90 to 124)	53·9 (46·3 to 61·9)	36·2 (31·1 to 41·9)	26 (20·6 to 32·6)	20·3 (16·2 to 25·1)	15·3 (12 to 19·2)	14·9 (11·6 to 18·9)	14·6 (11·3 to 18·5)
Mato Grosso do Sul	134 (87·5 to 195)	91·5 (73·7 to 112)	65·9 (54·4 to 79)	41·5 (37·4 to 45·9)	27·9 (25·1 to 31)	24·6 (21·2 to 28)	18·4 (16·4 to 20·8)	12·3 (10·1 to 15)	12·1 (10·2 to 14·2)	11·3 (9·83 to 13·1)
Minas Gerais	171 (123 to 232)	117 (99·4 to 137)	85·7 (72·6 to 101)	53·4 (46·2 to 61·3)	34·1 (29·3 to 39)	25·3 (20·7 to 30·4)	18 (14·5 to 22·2)	12 (9·42 to 15·1)	11 (8·6 to 13·8)	10·3 (8·06 to 13)
Pará	246 (184 to 318)	162 (138 to 189)	120 (100 to 143)	81 (69·8 to 93·8)	52·3 (45·5 to 59·8)	34·7 (27·4 to 43·2)	26·3 (22 to 30·9)	15·9 (12·4 to 20·1)	15 (11·5 to 19)	14 (10·8 to 17·8)
Paraíba	350 (248 to 461)	262 (232 to 294)	185 (160 to 211)	117 (98·7 to 136)	70·4 (61·8 to 80·1)	38·6 (32·5 to 45·5)	23·5 (19·4 to 28)	12·5 (9·72 to 15·8)	9·95 (7·43 to 13)	9·16 (6·86 to 12·1)
Paraná	126 (89·2 to 170)	90·9 (76 to 106)	71·4 (60·5 to 82·9)	50 (45·2 to 55·2)	29·6 (26·8 to 32·6)	20·3 (17·4 to 23·7)	17·9 (14·2 to 22·2)	11·4 (8·95 to 14·4)	10·6 (8·26 to 13·1)	9·91 (7·62 to 12·6)
Pernambuco	337 (246 to 436)	253 (224 to 287)	192 (169 to 214)	122 (103 to 143)	76·6 (67·6 to 85·8)	43·4 (36·9 to 50·8)	25·8 (21·7 to 30·2)	12·6 (9·79 to 15·8)	11·3 (8·8 to 14·3)	10·7 (8·36 to 13·6)
Piauí	216 (145 to 293)	158 (138 to 182)	133 (112 to 157)	86·6 (75·2 to 99·6)	56·9 (48·8 to 65·3)	36·5 (31·5 to 42·6)	24·5 (20·3 to 29·2)	13·8 (10·7 to 17·4)	11·9 (9·08 to 15)	10·6 (8·05 to 13·5)
Rio de Janeiro	128 (72 to 204)	74·8 (61·9 to 90·2)	60 (49·6 to 71·7)	54·8 (49·6 to 60·4)	34·6 (31·4 to 37·9)	21·3 (18·6 to 24)	16·1 (14·5 to 17·9)	13·8 (11·6 to 16·5)	13·2 (11·2 to 15·4)	12·6 (11·1 to 14·4)
Rio Grande do Norte	375 (285 to 478)	270 (231 to 309)	176 (155 to 197)	110 (92·2 to 131)	67·6 (57·2 to 78·8)	37·4 (31·5 to 44·2)	22·4 (17·6 to 28)	11·8 (9·13 to 14·9)	10·2 (7·75 to 13·1)	9·46 (7·18 to 12·2)
Rio Grande do Sul	146 (100 to 200)	85·5 (71·8 to 103)	59·5 (49·5 to 72·4)	35·4 (32 to 38·9)	20·3 (18·5 to 22·5)	16·8 (14·8 to 18·9)	12·1 (10·8 to 13·5)	11·8 (9·83 to 14·1)	10·5 (8·95 to 12·2)	10·5 (9·23 to 12)
Rondônia	237 (136 to 371)	140 (115 to 168)	93·9 (80·3 to 110)	68·7 (57·9 to 81·2)	39·5 (34 to 45·9)	27·9 (23·6 to 32·9)	22·6 (18·1 to 27·8)	13·2 (10·3 to 16·7)	12·4 (9·43 to 15·9)	8·72 (5·96 to 11·8)
Roraima	253 (178 to 342)	171 (147 to 197)	120 (100 to 142)	56·8 (50·6 to 63·6)	34·5 (30·2 to 39·5)	23·5 (20·2 to 27)	21·6 (18·8 to 24·8)	22·3 (18·3 to 27)	20·4 (16·6 to 24·6)	16·3 (13·3 to 20·1)
Santa Catarina	112 (73·6 to 162)	64·3 (52·8 to 77·2)	52·5 (43·5 to 62·2)	37·3 (32·1 to 42·6)	27·3 (23·7 to 31·6)	20·9 (17·4 to 24·8)	15·6 (12·5 to 19·3)	10·3 (7·32 to 14·2)	10·3 (7·97 to 13·2)	9·69 (7·55 to 12·3)
São Paulo	107 (72·5 to 147)	70·3 (57·9 to 83·3)	55·9 (47·3 to 66·2)	47·6 (43 to 52·4)	30·8 (28·1 to 33·9)	19·8 (17·2 to 22·4)	15·7 (12·6 to 19·1)	11·3 (8·79 to 14·3)	10·8 (9·13 to 12·7)	10·7 (9·35 to 12·2)
Sergipe	234 (167 to 319)	173 (147 to 202)	121 (105 to 137)	92·6 (80·5 to 106)	58·9 (51·4 to 67)	37·2 (29·2 to 46·6)	23·1 (18·7 to 28·3)	14·7 (11·2 to 18·5)	13·4 (10·3 to 17·1)	12·3 (9·56 to 15·7)
Tocantins	237 (135 to 370)	181 (153 to 212)	133 (111 to 159)	80·4 (68·5 to 93·7)	51·5 (42·8 to 61·1)	35·1 (30·4 to 40·7)	22 (17·9 to 27·1)	12·5 (9·74 to 16·1)	11·1 (8·33 to 14·3)	9·16 (6·55 to 12·2)

Paraguay	112 (84·6 to 146)	75·5 (66·8 to 84·6)	63·7 (56·1 to 72·1)	45·7 (39·9 to 52·2)	30·1 (25·7 to 35·3)	24·7 (21·1 to 29)	18·1 (15 to 21·6)	14·8 (11·3 to 19)	14·4 (10·6 to 18·6)	13·2 (9·7 to 17·4)
North Africa and Middle East	310 (278 to 343)	245 (230 to 261)	190 (181 to 200)	125 (119 to 130)	76·5 (72·8 to 80·4)	51·6 (48·3 to 55)	32·8 (30·2 to 35·5)	21·7 (19·1 to 24·6)	19·8 (17·4 to 22·4)	18·7 (16·4 to 21·2)
North Africa and Middle East	310 (278 to 343)	245 (230 to 261)	190 (181 to 200)	125 (119 to 130)	76·5 (72·8 to 80·4)	51·6 (48·3 to 55)	32·8 (30·2 to 35·5)	21·7 (19·1 to 24·6)	19·8 (17·4 to 22·4)	18·7 (16·4 to 21·2)
Afghanistan	365 (301 to 430)	269 (237 to 302)	240 (211 to 274)	228 (201 to 255)	162 (148 to 177)	128 (117 to 141)	77·1 (66·5 to 88·2)	51·8 (43·6 to 61·1)	48·7 (40·9 to 57·5)	46·4 (38·8 to 55·1)
Algeria	311 (256 to 375)	248 (219 to 278)	187 (165 to 207)	126 (117 to 134)	51·7 (47·2 to 56·2)	36·6 (32·6 to 41)	25·9 (23·6 to 28·5)	18·3 (15·3 to 21·9)	16·7 (13·5 to 20·3)	15·7 (12·3 to 19·5)
Bahrain	215 (175 to 262)	150 (130 to 170)	74·5 (65·1 to 85)	29·1 (27·1 to 31·4)	22·7 (21·4 to 24·1)	11·1 (10·4 to 11·9)	7·8 (7·21 to 8·4)	6·86 (6·26 to 7·56)	5·93 (5·15 to 6·72)	5·57 (4·76 to 6·45)
Egypt	315 (256 to 373)	281 (257 to 307)	245 (226 to 264)	156 (143 to 171)	85·9 (77·9 to 94·5)	42·4 (37·5 to 47·6)	26·4 (23 to 30·2)	15·3 (12·5 to 18·4)	13 (10·6 to 15·6)	12 (9·95 to 14·3)
Iran	355 (302 to 407)	272 (241 to 307)	187 (166 to 208)	94 (83·6 to 105)	65·6 (59·1 to 72·2)	35·8 (31·8 to 40·2)	23·8 (21·1 to 26·8)	9·99 (9·04 to 11)	7·06 (6·46 to 7·73)	4·87 (4·02 to 5·79)
Alborz	317 (259 to 377)	237 (206 to 270)	151 (131 to 174)	64·5 (56·1 to 73·6)	37·9 (32·8 to 43·2)	24·9 (20·7 to 29·5)	16·4 (14·3 to 18·7)	7·01 (5·96 to 8·14)	4·33 (3·72 to 4·97)	3·05 (2·39 to 3·76)
Ardebil	376 (316 to 441)	297 (261 to 335)	227 (204 to 251)	128 (113 to 143)	90·4 (79·8 to 102)	49·1 (44·1 to 54·5)	27·9 (24·4 to 31·4)	11·6 (10·3 to 13·2)	8·49 (7·63 to 9·54)	5·78 (4·96 to 6·69)
Bushehr	329 (269 to 390)	259 (224 to 295)	188 (165 to 214)	90·4 (78 to 103)	55·7 (48·1 to 63·1)	34 (29·8 to 38·1)	24·5 (20·6 to 28·8)	11 (9·47 to 12·7)	7·91 (6·87 to 9·07)	5·4 (4·65 to 6·32)
Chahar Mahaal and Bakhtiari	285 (229 to 342)	216 (187 to 248)	155 (137 to 176)	82·4 (70·9 to 93·7)	58·5 (50·8 to 66·3)	30·8 (27·2 to 34·8)	18·3 (15·4 to 21·4)	7·58 (6·59 to 8·6)	4·76 (4·16 to 5·38)	3·24 (2·63 to 3·92)
East Azarbayejan	399 (341 to 458)	313 (276 to 351)	230 (205 to 256)	124 (109 to 140)	71·9 (63·3 to 80·5)	39·8 (33·6 to 46·7)	24·5 (21·5 to 27·8)	9·01 (7·99 to 10·2)	6·13 (5·33 to 7)	4·21 (3·34 to 5·13)
Fars	238 (190 to 289)	181 (157 to 207)	123 (108 to 140)	63 (55·1 to 71·9)	47·5 (42 to 52·9)	33·7 (28·9 to 39·6)	25·2 (22·1 to 28·8)	11·5 (10·1 to 13·1)	8·44 (7·17 to 9·88)	5·77 (4·5 to 7·23)
Gilan	274 (223 to 331)	211 (174 to 256)	135 (119 to 154)	62·6 (53·3 to 72·3)	176 (162 to 192)	23·1 (19·6 to 26·8)	12·3 (10·8 to 13·9)	5·2 (4·53 to 6·01)	2·62 (2·03 to 3·24)	1·85 (1·28 to 2·44)
Golestan	311 (262 to 371)	240 (207 to 276)	174 (153 to 196)	90·7 (77·8 to 103)	66·5 (58·1 to 75·8)	39·6 (34·7 to 44·8)	23·9 (20·8 to 27·3)	10·7 (9·37 to 12·2)	7·7 (6·8 to 8·74)	5·28 (4·54 to 6·1)
Hamadan	450 (386 to 515)	367 (325 to 412)	270 (241 to 300)	129 (113 to 144)	76·8 (67·3 to 86·5)	44·8 (40 to 50·3)	26·5 (23·4 to 30·1)	12 (10·5 to 13·6)	8·87 (8·02 to 9·82)	6·06 (5·29 to 6·87)
Hormozgan	334 (280 to 393)	247 (215 to 281)	172 (152 to 195)	90·5 (79·2 to 102)	70·8 (62 to 80·6)	42·1 (36·7 to 48)	25·7 (22·5 to 29·2)	11 (9·72 to 12·5)	8·02 (7·28 to 8·91)	5·49 (4·79 to 6·26)
Ilam	286 (231 to 341)	239 (206 to 272)	176 (155 to 197)	98·2 (85·9 to 111)	62·6 (54·7 to 71)	38·9 (32·6 to 46·5)	23·5 (20·1 to 27·1)	10·6 (9·23 to 12·2)	7·63 (6·06 to 9·33)	5·26 (3·77 to 6·89)
Isfahan	298 (242 to 358)	247 (215 to 280)	155 (136 to 177)	72·3 (63 to 82·4)	42 (36·9 to 47·2)	26·2 (21·2 to 31·6)	20·2 (17·7 to 23·1)	9·3 (7·98 to 10·7)	6·43 (5·34 to 7·65)	4·49 (3·34 to 5·72)
Kerman	391 (327 to 458)	281 (246 to 320)	188 (166 to 213)	89·5 (78 to 101)	55·5 (48·7 to 62·8)	33·3 (29·4 to 37·8)	28·4 (24·8 to 32·4)	13·7 (12·1 to 15·5)	10·7 (9·51 to 12·1)	7·37 (6·39 to 8·56)
Kermanshah	348 (291 to 411)	290 (253 to 329)	210 (187 to 236)	115 (102 to 129)	75·5 (65·9 to 85)	46·7 (41·4 to 52·3)	29·3 (24·9 to 34)	12·1 (10·6 to 13·8)	8·74 (7·64 to 10)	5·93 (5·07 to 6·95)
Khorasan-e-Razavi	472 (408 to 535)	374 (328 to 421)	268 (242 to 295)	133 (116 to 151)	81·2 (71·1 to 91·6)	48 (41·5 to 54·9)	33·4 (28·4 to 38·6)	11 (9·91 to 12·2)	7·79 (6·33 to 9·27)	5·27 (3·78 to 6·94)
Khuzestan	285 (232 to 345)	212 (181 to 246)	148 (129 to 169)	75·6 (65 to 86·2)	45·2 (40·2 to 50·7)	32·6 (27·7 to 37·9)	23·4 (20·8 to 26·4)	10·5 (9·46 to 11·5)	7·64 (6·83 to 8·54)	5·33 (4·37 to 6·38)
Kohgiluyeh and Boyer-Ahmad	309 (255 to 367)	226 (196 to 259)	170 (149 to 191)	99·2 (86·6 to 112)	68·4 (59·8 to 77·2)	42·6 (37·6 to 48·1)	23·4 (20·3 to 26·9)	9·47 (8·19 to 10·8)	6·47 (5·67 to 7·37)	4·39 (3·61 to 5·3)
Kurdistan	439 (377 to 504)	395 (355 to 438)	301 (271 to 330)	173 (154 to 192)	105 (93·1 to 119)	56·9 (51 to 63·4)	30·4 (26·5 to 34·5)	11·8 (10·5 to 13·3)	8·7 (7·79 to 9·75)	5·98 (5·15 to 6·89)
Lorestan	359 (295 to 432)	265 (230 to 304)	184 (163 to 209)	95·7 (83 to 110)	61·2 (53·1 to 70·6)	32·4 (28·3 to 37)	15·8 (13·2 to 18·7)	6·46 (5·6 to 7·48)	3·71 (3·19 to 4·23)	2·53 (2·04 to 3·08)
Markazi	314 (258 to 379)	271 (235 to 309)	203 (179 to 229)	94·9 (83·1 to 108)	58·9 (51 to 67·4)	34·8 (30·3 to 39·4)	23·1 (19·6 to 27)	10·3 (8·99 to 11·6)	7·19 (6·45 to 7·96)	4·9 (4·15 to 5·69)
Mazandaran	231 (182 to 287)	173 (147 to 201)	115 (99·3 to 133)	54·8 (47·3 to 63·1)	33 (28·3 to 38·4)	21·8 (19 to 25·3)	14·3 (12·4 to 16·3)	6·84 (5·9 to 7·85)	3·92 (3·41 to 4·48)	2·66 (2·05 to 3·31)
North Khorasan	420 (343 to 501)	337 (297 to 379)	256 (229 to 285)	133 (118 to 149)	85·4 (75·4 to 96·4)	46·9 (40·9 to 53·2)	25·8 (22·2 to 29·6)	10·8 (9·48 to 12·2)	7·75 (6·87 to 8·68)	5·27 (4·51 to 6·1)
Qazvin	411 (346 to 480)	355 (313 to 399)	248 (221 to 277)	117 (103 to 132)	63·7 (56 to 71·4)	38·8 (34·4 to 43·7)	20·9 (17·9 to 24·2)	8·82 (7·69 to 9·95)	5·89 (5·09 to 6·7)	3·99 (3·18 to 4·86)
Qom	283 (231 to 339)	232 (201 to 265)	170 (149 to 192)	81 (71·3 to 91·2)	45·8 (39·7 to 52·5)	26·9 (23·4 to 31·1)	18·3 (16 to 20·9)	7·76 (6·83 to 8·87)	5 (4·28 to 5·84)	3·41 (2·68 to 4·25)
Semnan	354 (297 to 415)	284 (248 to 323)	192 (168 to 218)	87·1 (76 to 99·3)	50 (42·9 to 58·2)	32 (26·4 to 38·6)	21·8 (18·8 to 25·1)	9·54 (8·08 to 11·2)	6·63 (5·07 to 8·25)	4·6 (3·17 to 6·09)
Sistan and Baluchistan	411 (337 to 492)	305 (267 to 345)	227 (202 to 251)	126 (110 to 142)	88·1 (77·8 to 98·9)	53·5 (47·6 to 60·4)	35·2 (31·4 to 39·2)	13·9 (12·1 to 15·9)	11·1 (9·91 to 12·4)	7·81 (6·84 to 8·82)
South Khorasan	411 (353 to 478)	321 (283 to 361)	244 (221 to 269)	134 (117 to 150)	88·2 (77·7 to 99·1)	50·1 (44·3 to 56·1)	27·3 (23·1 to 31·9)	10·9 (9·51 to 12·3)	7·69 (6·82 to 8·71)	5·17 (4·44 to 6·01)
Tehran	241 (195 to 294)	163 (136 to 191)	93·9 (80 to 110)	40·3 (33·6 to 47·1)	25·4 (20·8 to 30·4)	18·2 (15·1 to 21·4)	13·6 (11·2 to 16·2)	6·57 (5·71 to 7·52)	3·82 (2·62 to 5·06)	2·61 (1·48 to 3·89)
West Azarbayejan	378 (322 to 434)	320 (282 to 360)	242 (217 to 268)	128 (113 to 145)	75·5 (66·4 to 85·4)	46·2 (40·4 to 52·7)	31 (27·7 to 34·6)	11·3 (10·1 to 12·7)	8·36 (7·58 to 9·3)	5·84 (5·02 to 6·71)
Yazd	388 (328 to 453)	261 (229 to 296)	170 (151 to 191)	77·1 (66·8 to 87·2)	44·5 (38·7 to 50·2)	29·4 (25·1 to 33·9)	23·5 (20·8 to 26·4)	10·8 (9·54 to 12·1)	7·93 (6·89 to 9·04)	5·56 (4·49 to 6·79)
Zanjan	481 (404 to 558)	436 (390 to 483)	315 (284 to 347)	155 (137 to 172)	178 (167 to 191)	45·8 (39·5 to 52·8)	24·4 (21·7 to 27·4)	10·7 (9·43 to 12·1)	7·74 (6·75 to 8·79)	5·32 (4·35 to 6·34)
Iraq	173 (136 to 214)	138 (117 to 159)	108 (94·1 to 123)	68·1 (61·5 to 74·7)	52·2 (47·7 to 56·6)	41·9 (38·5 to 45·7)	29·9 (25·1 to 35·2)	21 (17·2 to 25·5)	18·4 (15 to 22·5)	17·2 (14 to 21·2)
Jordan	216 (183 to 260)	135 (119 to 152)	83·1 (75·5 to 91·6)	50·2 (46·3 to 54·5)	31·8 (28·7 to 34·9)	24 (21·4 to 26·8)	16·3 (14·4 to 18·5)	12·1 (10·3 to 14·3)	11·2 (9·49 to 13·2)	10·7 (9·02 to 12·8)
Kuwait	92·4 (67·5 to 126)	61·1 (54·3 to 68·3)	49·7 (47·7 to 51·7)	32·3 (30·8 to 33·9)	16·6 (15·1 to 18·4)	10·5 (9·74 to 11·4)	10·2 (9·36 to 11)	8·46 (7·57 to 9·45)	7·67 (6·84 to 8·59)	7·37 (6·12 to 8·73)
Lebanon	209 (161 to 259)	95·7 (79·7 to 114)	64·1 (53·9 to 74·8)	48·8 (42·1 to 56·2)	31·6 (26·7 to 37·3)	20·7 (17·2 to 24·6)	12·9 (10·6 to 15·8)	8·43 (6·21 to 11·4)	7·67 (5·64 to 10·4)	7·33 (5·27 to 10·2)
Libya	335 (290 to 388)	230 (207 to 255)	119 (106 to 133)	60·4 (53·5 to 68·5)	37·8 (31·7 to 44·7)	23·3 (19·6 to 27·4)	21·1 (17·1 to 25·3)	22·2 (17·7 to 27·2)	20·7 (16·7 to 25·4)	19·7 (15·8 to 24·2)
Morocco	333 (294 to 376)	241 (225 to 257)	177 (166 to 188)	119 (110 to 129)	71·4 (63·8 to 79·4)	45·2 (37·6 to 53·1)	27 (22·3 to 32·5)	15·2 (12·4 to 18·4)	13·6 (11·2 to 16·3)	12·6 (10·3 to 15·2)
Oman	334 (280 to 393)	239 (214 to 265)	151 (134 to 169)	80·7 (70 to 92·2)	31·4 (26·9 to 36·8)	14 (12·2 to 15·9)	10·6 (10·1 to 11·3)	10·5 (9·79 to 11·3)	9·61 (8·92 to 10·4)	8·5 (7·62 to 9·38)
Palestine	307 (258 to 359)	203 (177 to 231)	99·3 (84·9 to 115)	66·7 (59·8 to 73·9)	41·3 (37·1 to 45·4)	27·1 (24·4 to 30·3)	19·3 (16·8 to 22·2)	11·9 (9·51 to 14·8)	10·6 (8·45 to 13·4)	10 (7·93 to 12·8)
Qatar	138 (110 to 172)	113 (95·6 to 133)	68·8 (58·5 to 80·1)	28 (23·3 to 33·3)	16·2 (13·5 to 19·2)	10·1 (8·37 to 11·9)	6·08 (4·94 to 7·34)	4·33 (3·5 to 5·37)	4·16 (3·37 to 5·19)	3·45 (2·74 to 4·32)
Saudi Arabia	179 (144 to 218)	172 (149 to 195)	121 (106 to 139)	66·8 (57·7 to 76·4)	40 (32·9 to 47·7)	20·6 (16·9 to 25·1)	9·51 (8·55 to 10·5)	4·93 (4·05 to 5·91)	4·12 (3·24 to 5·08)	3·87 (3·01 to 4·79)
Sudan	280 (239 to 328)	250 (223 to 280)	214 (196 to 233)	195 (180 to 209)	144 (133 to 157)	97 (88·8 to 106)	61·1 (52·7 to 70)	37·2 (30·7 to 44·1)	34·8 (28·6 to 41·4)	32·8 (26·8 to 39·3)
Syria	232 (192 to 276)	137 (125 to 150)	87·2 (80·4 to 94·1)	60·3 (53·1 to 68·2)	41·5 (36·4 to 47·1)	16·3 (15·3 to 17·4)	12·5 (11·3 to 13·7)	12 (9·95 to 14·6)	9·89 (8·06 to 11·9)	8·87 (7·21 to 10·8)
Tunisia	319 (283 to 361)	227 (209 to 244)	151 (140 to 163)	85·1 (77·6 to 92·4)	48·1 (43·1 to 53·7)	27·4 (23·7 to 31·3)	17·1 (14·6 to 19·8)	11 (9·18 to 13)	9·85 (8·22 to 11·7)	9·23 (7·65 to 11)
Türkiye	280 (240 to 323)	244 (226 to 263)	182 (170 to 196)	118 (108 to 130)	67·5 (60·6 to 75)	38 (33·4 to 43·1)	21·4 (17·8 to 25·4)	13 (10·5 to 15·7)	11·3 (9·22 to 13·6)	10·4 (8·52 to 12·4)
United Arab Emirates	175 (144 to 211)	146 (128 to 165)	72·8 (63·1 to 83·2)	34·9 (30·7 to 40·2)	18·7 (16·6 to 20·9)	10·5 (9·83 to 11·2)	7·6 (6·92 to 8·37)	5·57 (4·94 to 6·24)	5·21 (4·59 to 5·91)	4·49 (3·8 to 5·22)
Yemen	479 (430 to 529)	352 (332 to 370)	282 (263 to 301)	183 (172 to 193)	128 (118 to 138)	89·5 (81·1 to 98·7)	55·7 (49·1 to 62·7)	41·7 (35·3 to 48·6)	38·8 (32·7 to 45·2)	37 (31 to 43·4)
South Asia	337 (279 to 400)	263 (238 to 289)	197 (181 to 213)	151 (139 to 163)	107 (99·5 to 116)	80·5 (73·5 to 87·7)	59·2 (53·2 to 65·5)	42·4 (36·7 to 48·7)	37·9 (32·6 to 44·3)	36·1 (30·6 to 42·8)
South Asia	337 (279 to 400)	263 (238 to 289)	197 (181 to 213)	151 (139 to 163)	107 (99·5 to 116)	80·5 (73·5 to 87·7)	59·2 (53·2 to 65·5)	42·4 (36·7 to 48·7)	37·9 (32·6 to 44·3)	36·1 (30·6 to 42·8)
Bangladesh	304 (262 to 347)	250 (230 to 272)	239 (227 to 252)	183 (170 to 196)	127 (118 to 136)	83·4 (76·6 to 91·1)	52·9 (47·2 to 59·3)	32·1 (26·7 to 38·5)	29·4 (24·4 to 35·3)	27·5 (22·7 to 33·2)
Bhutan	374 (313 to 435)	281 (245 to 318)	225 (193 to 264)	194 (168 to 222)	126 (113 to 140)	84·3 (74·8 to 95·3)	45·7 (38·2 to 53·4)	30·5 (24 to 37·8)	29·5 (23·1 to 36·7)	28·1 (21·8 to 34·9)
India	348 (275 to 423)	269 (241 to 301)	194 (174 to 215)	146 (131 to 162)	103 (93·1 to 114)	78·9 (69·9 to 88·1)	57·8 (50·1 to 66·2)	39·4 (32·7 to 46·7)	33·5 (27·6 to 40·8)	31·8 (25·5 to 39·3)
Nepal	340 (301 to 382)	280 (258 to 304)	226 (208 to 246)	201 (188 to 214)	136 (126 to 145)	81·7 (74·6 to 89·6)	49·6 (43·3 to 56·5)	30·7 (24·7 to 38·1)	29 (23·3 to 36·1)	27·6 (22 to 34·4)
Pakistan	284 (241 to 327)	208 (193 to 224)	161 (149 to 174)	140 (130 to 151)	107 (98·2 to 117)	86·9 (77·5 to 97·6)	70·9 (62·2 to 80·7)	59·2 (49·3 to 70)	58·1 (48·8 to 69)	55·7 (46·6 to 66·3)
Azad Jammu & Kashmir	255 (201 to 314)	203 (181 to 228)	164 (145 to 183)	142 (119 to 166)	107 (90·2 to 125)	81·9 (63·8 to 101)	58·7 (43·2 to 79·6)	49·4 (37·1 to 65·2)	48 (35·9 to 64·2)	46·6 (35·6 to 61·4)
Balochistan	203 (156 to 261)	154 (133 to 177)	104 (92 to 117)	94·8 (81·1 to 111)	85·1 (74·9 to 94·8)	76·6 (62·5 to 92·5)	73 (59·6 to 88·4)	64·1 (51·7 to 77·3)	61·5 (49·1 to 75·8)	59·6 (48·2 to 73·6)

Gilgit-Baltistan	222 (171 to 279)	208 (189 to 229)	170 (148 to 194)	150 (126 to 175)	110 (93·1 to 128)	83·8 (68·8 to 102)	59·2 (46·4 to 73·3)	46·7 (37·4 to 57·1)	45·2 (36·3 to 55·9)	43·7 (35·5 to 53·8)
Islamabad Capital Territory	289 (214 to 382)	235 (203 to 274)	156 (127 to 186)	98·3 (79·5 to 122)	54·1 (43 to 69·9)	32·3 (25·1 to 40·3)	20·9 (15·2 to 27·2)	15·1 (11·2 to 20)	14·7 (10·7 to 19·7)	14·1 (10·2 to 18·8)
Khyber Pakhtunkhwa	320 (257 to 395)	201 (181 to 221)	148 (132 to 164)	112 (97 to 129)	73·5 (65·1 to 83·4)	64·7 (55·5 to 73·8)	52·3 (42 to 63·9)	46·3 (36·9 to 57·2)	44·1 (35·2 to 55·7)	42·1 (33·8 to 52·7)
Punjab	276 (232 to 324)	211 (192 to 229)	169 (155 to 183)	154 (141 to 168)	117 (107 to 126)	93 (83·4 to 104)	76 (67·5 to 86·2)	63·6 (51 to 78·9)	62·8 (51 to 76·6)	60·6 (49·5 to 74)
Sindh	308 (247 to 370)	212 (189 to 238)	159 (145 to 174)	134 (122 to 150)	114 (98·7 to 129)	93·3 (78·9 to 109)	76·5 (65·4 to 89)	61·2 (49·8 to 75·2)	60·7 (48·8 to 75·8)	57·6 (46·1 to 71·5)
Southeast Asia, east Asia, and Oceania	234 (205 to 267)	440 (322 to 616)	105 (97·1 to 114)	77·1 (71·4 to 83·1)	55 (51·2 to 59)	39·3 (36·5 to 43)	21·4 (19·8 to 23·1)	13·8 (11·8 to 15·8)	13·3 (11·5 to 15·4)	13 (11·3 to 15)
East Asia	223 (187 to 265)	525 (380 to 725)	93 (83·6 to 104)	64·8 (57·7 to 72·4)	49·7 (44·7 to 55)	35·3 (31·3 to 41·1)	13·7 (12·3 to 15·3)	7·1 (6·21 to 8·08)	6·77 (5·82 to 7·84)	6·39 (5·46 to 7·48)
China	225 (188 to 269)	538 (391 to 738)	94·7 (84·9 to 106)	66·5 (59 to 74·4)	50·7 (45·6 to 56·3)	33·3 (30 to 36·6)	13·7 (12·2 to 15·3)	7·05 (6·13 to 8·06)	6·73 (5·75 to 7·83)	6·35 (5·39 to 7·46)
North Korea	172 (138 to 210)	91·1 (75·7 to 109)	58·8 (48·7 to 70·2)	39·9 (30·9 to 51·6)	29·1 (23·2 to 36·2)	107 (46·4 to 238)	19·5 (15·7 to 23·8)	10·7 (7·92 to 13·8)	9·98 (7·34 to 13)	9·37 (6·92 to 12·3)
Taiwan (province of China)	154 (146 to 162)	59·7 (58·3 to 61·1)	24·6 (23·8 to 25·4)	13·7 (13·2 to 14·2)	7·8 (7·47 to 8·18)	7·52 (7·09 to 7·96)	4·59 (4·26 to 4·96)	4·2 (3·88 to 4·58)	3·93 (3·57 to 4·32)	4·23 (3·72 to 4·73)
Oceania	224 (196 to 256)	142 (128 to 156)	101 (90·9 to 112)	75·7 (68·9 to 82·8)	64·9 (57·9 to 73·1)	57·3 (50·6 to 64·7)	52·9 (45·8 to 60·9)	46 (39·1 to 54·1)	44·9 (38 to 52·8)	44·3 (37·4 to 51·9)
American Samoa	92·1 (78·1 to 109)	50·7 (46·4 to 55·3)	36·1 (33·2 to 39·5)	21·6 (18·7 to 24·9)	16·8 (15 to 18·7)	14·6 (12·7 to 16·6)	12·2 (10·7 to 13·9)	12·5 (10·4 to 15·2)	13·3 (10·9 to 16·3)	12·1 (9·61 to 15·1)
Cook Islands	206 (182 to 232)	92 (83·8 to 100)	55·6 (50·2 to 62·1)	37·1 (32·8 to 41·8)	23·7 (20·7 to 27)	13·2 (10·8 to 16·1)	5·67 (4·35 to 7·34)	2·75 (2·25 to 3·32)	5·59 (5·59 to 5·61)	5·65 (5·65 to 5·65)
Fiji	77·2 (64·9 to 92·1)	52·7 (47·5 to 57·8)	40·8 (36·3 to 45·7)	30·9 (26·2 to 36·1)	23·6 (19·9 to 27·9)	24·4 (19·5 to 30·3)	22·3 (18·6 to 26·4)	18·9 (15·2 to 23·3)	18·1 (14·5 to 22·6)	17·9 (13·9 to 22·8)
Guam	51·7 (44·5 to 59·3)	35 (32·3 to 37·8)	26·4 (24·2 to 29·1)	16·3 (15·1 to 17·7)	12·5 (11·6 to 13·7)	12·1 (10·9 to 13·4)	14·7 (13·3 to 16·2)	14·5 (12·5 to 16·6)	13·6 (11·2 to 16·2)	13 (10·4 to 15·9)
Kiribati	239 (199 to 285)	172 (150 to 192)	130 (112 to 149)	98·9 (83·7 to 118)	85·9 (73·6 to 99·4)	64·8 (54·4 to 75·8)	49·8 (41·2 to 59·2)	39·4 (32·6 to 47·5)	38·2 (31·7 to 45·9)	37·1 (30·4 to 45·1)
Marshall Islands	167 (133 to 206)	84·4 (71·3 to 99·1)	52·8 (44·1 to 63)	36·2 (31·1 to 42·3)	30·2 (26·9 to 33·9)	35·1 (31 to 40)	26·6 (22·6 to 31·1)	19·8 (16 to 24·9)	19·1 (15·2 to 24·2)	18·4 (14·5 to 23·5)
Federated States of Micronesia	278 (231 to 330)	123 (107 to 139)	79 (68·6 to 90·3)	51·4 (43·6 to 60·5)	39·7 (33·4 to 46·8)	34·5 (29·6 to 40)	20·6 (16·9 to 24·3)	15·7 (12·7 to 19·3)	15·2 (12·2 to 18·8)	14·7 (11·9 to 17·9)
Nauru	121 (98·3 to 147)	72·3 (61·1 to 84·4)	55·4 (47 to 65·3)	39·2 (33·2 to 45·5)	33·8 (28·3 to 39·9)	45·7 (39·8 to 51·8)	40·5 (34·1 to 47·7)	25·5 (19·7 to 32·3)	24·5 (18·7 to 31·7)	23·5 (18 to 30·7)
Niue	157 (131 to 189)	67·9 (59·9 to 76·9)	44·2 (38 to 50·9)	29 (24·3 to 34)	23·7 (19·8 to 27·9)	26·9 (22·5 to 32·3)	22·2 (18 to 27·1)	19·7 (15·4 to 25·4)	52·9 (52·8 to 53·7)	53·3 (53·2 to 54)
Northern Mariana Islands	59·2 (45·4 to 76·8)	33·6 (27·6 to 40·5)	19 (14·8 to 24·7)	12 (9·3 to 15·5)	8·47 (6·83 to 10·3)	6·87 (5·71 to 8·14)	7·18 (5·88 to 8·6)	6·93 (5·58 to 8·44)	6·94 (5·3 to 8·92)	6·05 (4·85 to 7·18)
Palau	208 (163 to 261)	131 (108 to 156)	75·7 (62·5 to 91·4)	50·4 (41 to 60·5)	33·1 (25·9 to 41·3)	23·8 (19·6 to 28·7)	21·8 (18 to 26·1)	17·8 (14·7 to 21·5)	17·6 (14·6 to 21)	17·3 (14·4 to 20·9)
Papua New Guinea	271 (229 to 316)	166 (147 to 187)	118 (104 to 131)	90·7 (81·6 to 101)	78·8 (69·6 to 89·9)	67·5 (59·1 to 77)	60·9 (52·2 to 70·6)	51·6 (43·6 to 60·7)	50·3 (42·6 to 59·2)	49·6 (41·9 to 58)
Samoa	162 (133 to 196)	93·3 (81·7 to 106)	62·8 (53·5 to 72·2)	40·1 (33·4 to 47·9)	27·8 (22·2 to 35)	20·8 (16·4 to 26)	17·1 (13·5 to 21·1)	25·5 (22·5 to 29·2)	15·5 (12·7 to 19)	13·1 (10·2 to 16·6)
Solomon Islands	199 (165 to 239)	155 (136 to 176)	101 (85·9 to 117)	59·9 (50·6 to 71)	44·4 (37·2 to 52·7)	33·5 (27·3 to 40·2)	26·8 (21·7 to 32·7)	19·9 (16·1 to 24·4)	20·1 (16·3 to 24·5)	18·8 (15 to 23·3)
Tokelau	102 (80·7 to 131)	77·3 (63 to 92·9)	56·5 (44·7 to 69·1)	45·9 (37·2 to 55)	29·7 (24·1 to 36·1)	21·5 (17·3 to 26·9)	13·3 (10·6 to 16·6)	9·91 (7·46 to 13)	65·9 (65·9 to 65·9)	67·1 (67·1 to 67·1)
Tonga	72·6 (57·1 to 93·3)	48·7 (41·3 to 56·8)	41·4 (35·7 to 47·6)	34·7 (29·5 to 40·6)	25·5 (21·2 to 30·2)	20·8 (17·4 to 24·4)	17·3 (14·2 to 20·6)	12·5 (10 to 15·3)	12 (9·65 to 15)	11·7 (9·24 to 14·5)
Tuvalu	197 (157 to 241)	140 (116 to 164)	114 (94·5 to 140)	93·7 (77·2 to 113)	63·1 (53·7 to 73·5)	45·7 (40 to 52·3)	21·3 (17·5 to 25·5)	15·7 (12·1 to 19·7)	15 (11·4 to 19·1)	14·3 (10·9 to 18·6)
Vanuatu	205 (172 to 242)	156 (139 to 174)	99·8 (85·4 to 116)	51·6 (43·4 to 61)	35·8 (29·9 to 42·5)	33·6 (29 to 38·5)	27·6 (23·9 to 31·5)	20·9 (17·1 to 25·6)	20·7 (16·8 to 25·5)	19·8 (16·1 to 24·7)
Southeast Asia	258 (233 to 288)	179 (166 to 193)	132 (125 to 139)	96·2 (91·2 to 102)	64·7 (61·4 to 67·9)	44·1 (41·3 to 47)	29·3 (26·5 to 32·3)	21·1 (17·9 to 24·9)	19·8 (16·9 to 23)	18·9 (16·3 to 22·1)
Cambodia	258 (210 to 312)	200 (175 to 228)	168 (148 to 188)	150 (137 to 163)	111 (104 to 120)	85·2 (79 to 91·6)	44·2 (39·2 to 49·3)	29·7 (25·2 to 35·1)	28·8 (24·5 to 34·3)	27·5 (23·4 to 32·5)
Indonesia	281 (245 to 321)	216 (199 to 232)	158 (148 to 168)	113 (106 to 121)	76·8 (70·9 to 82·9)	49·8 (45·6 to 54)	33·2 (28·7 to 38·1)	23 (18·5 to 27·7)	21·7 (17·7 to 26·1)	20·9 (17·2 to 25·1)
Aceh	198 (160 to 242)	147 (134 to 159)	114 (103 to 127)	86·7 (78·7 to 95)	65·7 (58·8 to 73·6)	48·1 (44 to 52·1)	33·8 (29·3 to 38·5)	24·8 (20·1 to 30·3)	23·3 (18·9 to 28·2)	22·5 (18·3 to 27·2)
Bali	257 (210 to 316)	188 (172 to 205)	137 (124 to 150)	97·3 (88·4 to 107)	60·2 (53·8 to 66·7)	40·8 (36·8 to 45)	28·1 (24 to 32·3)	19·6 (14·9 to 25·1)	18·5 (14·3 to 23·3)	18 (14·3 to 22·4)
Bangka-Belitung Islands	284 (225 to 356)	208 (174 to 244)	153 (140 to 168)	109 (98 to 120)	78·7 (70·5 to 86·7)	44·5 (38·7 to 50·1)	30·1 (23·9 to 37·5)	24·9 (19·2 to 32·3)	23·4 (17·9 to 30)	22·4 (17·5 to 28·7)
Banten	325 (257 to 397)	323 (276 to 372)	235 (216 to 255)	159 (145 to 171)	102 (92·9 to 112)	55·7 (48·6 to 63·2)	35·3 (28·5 to 43·7)	24·9 (19·3 to 32·1)	23·2 (18·2 to 29·7)	22·1 (17·6 to 27·9)
Bengkulu	336 (283 to 400)	258 (235 to 281)	183 (169 to 200)	126 (117 to 135)	80·7 (73·2 to 88)	50·6 (46·2 to 55·1)	33·4 (29·4 to 38·1)	21·6 (17·2 to 26·5)	20·1 (16·1 to 24·8)	19·2 (15·5 to 23·6)
Gorontalo	277 (209 to 354)	268 (227 to 312)	204 (186 to 223)	169 (149 to 191)	122 (110 to 135)	85·9 (75·6 to 96·5)	62·6 (50·4 to 76·9)	43·9 (33·1 to 58·5)	41·4 (31·3 to 54·7)	39·8 (30·1 to 52·4)
Jakarta	179 (144 to 218)	165 (153 to 177)	110 (102 to 120)	76·3 (68·5 to 84·4)	49·8 (45·1 to 54·9)	33·3 (30 to 37·3)	23 (20 to 26·3)	16 (12·4 to 20·2)	14·9 (11·7 to 18·5)	14·4 (11·7 to 17·6)
Jambi	290 (242 to 341)	228 (212 to 245)	167 (154 to 182)	117 (108 to 126)	76·9 (68·8 to 85·2)	48 (43·9 to 52·3)	31·2 (27·2 to 35·5)	22·5 (18·3 to 27·2)	21·1 (17·2 to 25·5)	20·3 (16·6 to 24·6)
West Java	305 (266 to 350)	251 (231 to 269)	181 (168 to 196)	128 (118 to 137)	83·3 (76·6 to 90·2)	53·1 (46·5 to 59·9)	34·6 (30 to 39·4)	23·1 (18·6 to 28)	21·6 (17·3 to 26·1)	20·7 (16·8 to 25)
Central Java	250 (203 to 303)	176 (161 to 190)	126 (116 to 138)	87·1 (78·7 to 95·8)	57·2 (52 to 62·9)	35·6 (32·2 to 39·4)	22·9 (19·4 to 27)	14·8 (11·7 to 18)	13·7 (11 to 16·7)	13·2 (10·7 to 16)
East Java	281 (243 to 325)	213 (195 to 232)	158 (144 to 173)	118 (109 to 129)	80 (73·4 to 87)	48·9 (43·1 to 54·9)	29·6 (25·5 to 34)	19 (15 to 23·5)	17·8 (14·1 to 21·8)	17·3 (13·8 to 21·3)
West Kalimantan	243 (194 to 301)	221 (199 to 243)	184 (162 to 206)	139 (126 to 151)	91·2 (82 to 100)	55·6 (48·9 to 63·1)	33·5 (30·3 to 37·5)	20·4 (15·9 to 25·7)	19 (15·2 to 23·4)	18·2 (14·7 to 22·2)
South Kalimantan	324 (280 to 370)	286 (263 to 307)	213 (198 to 228)	159 (148 to 171)	109 (96·9 to 120)	64·6 (58·4 to 71·2)	41·6 (35·5 to 47·9)	29·7 (23·8 to 36·5)	28 (22·6 to 33·9)	26·9 (21·9 to 32·4)
Central Kalimantan	206 (171 to 245)	149 (137 to 160)	105 (96·8 to 114)	76·5 (69·7 to 83·4)	60·5 (54·4 to 66·7)	44·8 (41 to 48·7)	30·5 (27·3 to 34·2)	23·9 (19·5 to 28·6)	22·6 (18·5 to 27)	21·8 (17·9 to 26·1)
East Kalimantan	173 (139 to 212)	175 (161 to 189)	132 (118 to 145)	88·4 (80·9 to 96·8)	59·5 (53·5 to 65·8)	40·7 (37·1 to 44·6)	26·4 (23·4 to 29·5)	20 (16·3 to 24·3)	18·7 (15·3 to 22·7)	18·1 (14·7 to 21·9)
North Kalimantan	163 (131 to 201)	208 (190 to 227)	170 (155 to 187)	120 (106 to 134)	78·4 (68·3 to 89·4)	55·2 (48·5 to 62·5)	37·7 (31·3 to 44·9)	29·4 (23·2 to 36·6)	27·7 (21·9 to 34·1)	26·5 (21·2 to 32·4)
Riau Islands	181 (150 to 217)	156 (129 to 187)	105 (95·4 to 117)	74·1 (66·8 to 82·1)	54·4 (49·3 to 59·4)	39·3 (35·8 to 42·9)	25·4 (20·3 to 31·8)	17·9 (14·5 to 21·5)	16·7 (13·7 to 20·2)	16·2 (13·3 to 19·5)
Lampung	231 (189 to 278)	215 (196 to 233)	154 (143 to 168)	108 (99·1 to 117)	71·6 (65·3 to 77·9)	43·9 (40·4 to 47·8)	30·6 (26·5 to 35·3)	24 (19·1 to 29·2)	22·6 (18·2 to 27·6)	21·8 (17·8 to 26·4)
Maluku	368 (312 to 426)	265 (231 to 298)	194 (180 to 208)	147 (134 to 161)	109 (99·4 to 121)	80·7 (73 to 88·9)	60·6 (50·1 to 73·5)	35·4 (28·4 to 43·4)	32·9 (26·4 to 40)	31 (25·7 to 37·5)
North Maluku	306 (245 to 378)	223 (190 to 259)	165 (151 to 180)	133 (118 to 148)	99·2 (87·5 to 112)	74·8 (65·7 to 84·4)	56·9 (46·3 to 70·9)	39·7 (30·2 to 52·9)	37·1 (28·2 to 48·6)	35·4 (27·3 to 46·5)
West Nusa Tenggara	354 (302 to 406)	306 (283 to 326)	246 (229 to 263)	188 (176 to 200)	127 (115 to 140)	79·2 (72 to 87·2)	43·6 (37·6 to 50)	28·7 (23·3 to 34·9)	27 (22 to 32·6)	25·8 (21·2 to 31·1)
East Nusa Tenggara	334 (282 to 389)	246 (211 to 284)	189 (174 to 205)	139 (128 to 150)	97·2 (88·5 to 106)	70·7 (64·1 to 77·9)	50·2 (40·9 to 61·4)	32·3 (26·4 to 38·9)	30·1 (24·8 to 36·3)	28·6 (23·6 to 34·3)
Papua	128 (107 to 152)	165 (139 to 193)	140 (128 to 154)	116 (104 to 129)	90·5 (81·6 to 98·7)	67·2 (60·7 to 74·2)	56·1 (46·2 to 67·4)	43·5 (35·1 to 53)	41·4 (33·7 to 49·5)	40 (33·1 to 47·8)
West Papua	129 (106 to 156)	129 (107 to 154)	110 (101 to 121)	85·3 (77 to 94·2)	66·9 (60·3 to 73·6)	55·6 (50·3 to 61·3)	45·6 (37·2 to 55·6)	28·9 (23·2 to 35·2)	27·3 (22·1 to 33)	26·2 (21·4 to 31·6)
Riau	207 (174 to 245)	193 (175 to 212)	145 (133 to 158)	96·5 (87·9 to 106)	64·8 (59·5 to 70·1)	42·4 (37·9 to 47·1)	30·4 (26·7 to 34·2)	18·1 (14·6 to 21·9)	16·9 (13·7 to 20·5)	16·2 (13·2 to 19·7)
West Sulawesi	398 (316 to 489)	325 (278 to 371)	255 (235 to 276)	192 (177 to 208)	129 (117 to 141)	84·2 (76·4 to 92·9)	61·2 (50·1 to 75·2)	44·7 (33·9 to 58·2)	42 (31·6 to 54·6)	40 (30·6 to 51·4)
South Sulawesi	317 (269 to 365)	260 (240 to 281)	191 (175 to 207)	140 (131 to 152)	89·8 (80 to 99·8)	55·1 (50·9 to 59·5)	33·6 (28·5 to 39·3)	19·7 (15·8 to 24·2)	18·5 (15·1 to 22·5)	17·8 (14·6 to 21·5)
Central Sulawesi	300 (258 to 348)	244 (225 to 263)	179 (165 to 195)	133 (123 to 142)	93·5 (85·5 to 102)	64·1 (58·4 to 70·3)	45·8 (40 to 51·7)	34·5 (28·2 to 41·6)	32·5 (26·7 to 39·1)	31·2 (25·6 to 37·7)
Southeast Sulawesi	279 (239 to 324)	245 (210 to 285)	195 (181 to 211)	133 (123 to 143)	87·7 (80·5 to 94·7)	56·6 (50·9 to 62·5)	41·4 (32·9 to 51·3)	34·5 (27·4 to 41·9)	32·9 (26·4 to 40)	32·1 (26·1 to 39)
North Sulawesi	224 (184 to 271)	142 (130 to 155)	95·5 (87·2 to 105)	58·7 (53·1 to 65)	44·5 (40·4 to 48·7)	34·1 (31 to 37·5)	29·5 (25·6 to 33·9)	24 (19·3 to 29·3)	22·8 (18·5 to 27·6)	22·1 (17·9 to 26·8)
West Sumatra	475 (415 to 539)	274 (248 to 297)	186 (170 to 202)	122 (113 to 131)	80·5 (73·7 to 87·3)	55·6 (49·5 to 61·8)	37 (32·2 to 42·1)	19·6 (15·6 to 24)	18·2 (14·6 to 22·3)	17·3 (14·2 to 20·9)
South Sumatra	287 (246 to 329)	229 (210 to 248)	166 (153 to 182)	109 (100 to 117)	76·8 (69·9 to 83·6)	52·2 (46·2 to 58·4)	33·6 (28·8 to 38·7)	25·4 (20·2 to 31)	23·9 (19·2 to 29·2)	23 (18·6 to 28)

North Sumatra	251 (213 to 296)	174 (160 to 189)	124 (114 to 135)	91 (84 to 97·8)	67·2 (60·5 to 74·4)	46·2 (42·3 to 50·4)	35·1 (29·8 to 40·9)	30·2 (24·6 to 36·6)	28·6 (23·4 to 34·9)	27·7 (22·9 to 33·5)
Yogyakarta	281 (220 to 347)	146 (128 to 167)	80·3 (72·1 to 89·2)	46·3 (41·9 to 51·8)	29·3 (26·2 to 33·1)	19·4 (17·6 to 21·4)	13·3 (11 to 15·8)	12·2 (9·52 to 15·6)	11·3 (8·85 to 14·2)	11 (8·77 to 13·6)
Laos	302 (253 to 353)	298 (261 to 335)	266 (232 to 301)	219 (191 to 249)	159 (143 to 175)	110 (99·4 to 121)	63·8 (56·6 to 71·4)	39·7 (32·7 to 47·3)	38·3 (31 to 46·1)	36·4 (29·1 to 44·5)
Malaysia	133 (122 to 145)	86·7 (84·6 to 88·8)	50·5 (49·1 to 52)	27·3 (24·1 to 30·9)	15·6 (14·9 to 16·2)	8·22 (7·87 to 8·58)	6·4 (6·16 to 6·65)	7·01 (6·64 to 7·41)	6·19 (5·71 to 6·68)	5·77 (5·1 to 6·46)
Maldives	349 (295 to 404)	290 (252 to 330)	199 (178 to 222)	131 (117 to 145)	60·3 (53·7 to 67·6)	30 (26·1 to 34·6)	17·9 (15·5 to 20·6)	12·7 (10·5 to 15·6)	11·6 (9·55 to 14·2)	11·4 (9·38 to 14·2)
Mauritius	150 (138 to 163)	99 (95·3 to 103)	73·3 (69·7 to 77·2)	35·9 (33·9 to 38)	18·5 (17·1 to 20·1)	15 (13·8 to 16·4)	12·8 (11·7 to 13·8)	14·1 (12·6 to 15·8)	14·6 (12·3 to 17·1)	11·5 (9·71 to 12·8)
Myanmar	388 (334 to 447)	283 (246 to 319)	229 (201 to 259)	176 (151 to 201)	130 (114 to 148)	97·3 (84·7 to 110)	59·6 (51·8 to 67·2)	38·1 (31·5 to 46·3)	36 (29·7 to 44·3)	34·7 (28·3 to 43·1)
Philippines	187 (148 to 233)	125 (111 to 140)	88·3 (80·4 to 96·7)	72·4 (65·7 to 79·2)	47·8 (42·7 to 53·3)	32·1 (28·5 to 35·9)	27·3 (23·7 to 31·2)	22·4 (18·6 to 26·9)	20 (17 to 23·4)	18·9 (16 to 22·4)
Seychelles	96·1 (82·9 to 112)	91·1 (84·8 to 97)	69 (64·5 to 73·9)	30·4 (27·6 to 33·3)	17·5 (15·8 to 19·4)	12·5 (11 to 14)	13 (11·8 to 14·3)	13·3 (11·6 to 15·1)	13·7 (11·9 to 15·8)	12·9 (10·7 to 15·5)
Sri Lanka	188 (174 to 204)	91·4 (88·2 to 94·3)	64·6 (61·6 to 67·9)	41·2 (39·3 to 43)	18·8 (17·4 to 20·3)	15·2 (14·3 to 16·2)	10·5 (9·91 to 11·2)	6·4 (5·23 to 7·82)	5·99 (4·73 to 7·37)	5·38 (4·24 to 6·8)
Thailand	186 (157 to 219)	109 (101 to 119)	70·3 (64·3 to 76·5)	43 (38·6 to 47·2)	25·1 (21·7 to 28·7)	16·1 (13·9 to 18·8)	9·01 (8·6 to 9·44)	7·56 (7·05 to 8·09)	6·86 (6·2 to 7·6)	6·65 (5·8 to 7·56)
Timor-Leste	333 (279 to 389)	255 (227 to 283)	245 (223 to 268)	213 (197 to 230)	133 (122 to 144)	79·7 (71·3 to 88·5)	43·9 (37·5 to 51)	34 (28·7 to 40·1)	33·4 (28 to 39·5)	32·6 (27·6 to 38·6)
Viet Nam	257 (209 to 311)	119 (101 to 139)	79·6 (70·6 to 89·4)	54·7 (50·6 to 58·8)	38 (34·6 to 41·2)	23·5 (21 to 26·3)	15·2 (13·3 to 17·4)	10·6 (8·53 to 13·1)	10·2 (8·18 to 12·8)	9·31 (7·39 to 11·8)
Sub-Saharan Africa	300 (263 to 339)	249 (226 to 273)	218 (205 to 231)	187 (179 to 195)	164 (159 to 170)	140 (134 to 146)	100 (94·5 to 106)	71·2 (62·5 to 80·8)	68·2 (59 to 78·9)	65·4 (56·3 to 76·3)
Central sub-Saharan Africa	300 (258 to 343)	235 (209 to 261)	209 (189 to 228)	184 (170 to 199)	164 (154 to 173)	146 (136 to 156)	99 (90·4 to 107)	60·3 (52·5 to 68·8)	56·2 (49 to 64·7)	53·5 (46·6 to 62·1)
Angola	291 (244 to 342)	254 (226 to 282)	229 (205 to 255)	211 (190 to 234)	200 (186 to 215)	159 (149 to 171)	88·5 (78·8 to 99)	52·7 (44·9 to 61·4)	50·3 (43 to 58·5)	49·4 (42·1 to 58)
Central African Republic	258 (210 to 318)	235 (200 to 269)	207 (183 to 233)	196 (177 to 216)	182 (169 to 195)	170 (156 to 186)	145 (123 to 169)	111 (91·2 to 135)	107 (87·5 to 131)	101 (83·3 to 126)
Congo (Brazzaville)	336 (269 to 419)	245 (219 to 270)	202 (177 to 231)	129 (112 to 149)	97 (86·1 to 108)	97 (86·8 to 108)	60 (52 to 69·2)	41·2 (34·4 to 48·9)	37·6 (31·2 to 44·8)	35·7 (29·8 to 42·9)
DR Congo	303 (253 to 352)	229 (200 to 259)	205 (184 to 227)	182 (165 to 198)	158 (147 to 170)	145 (131 to 159)	103 (91·4 to 115)	61·7 (53 to 71·6)	56·9 (48·7 to 67·1)	53·4 (45·8 to 63·9)
Equatorial Guinea	440 (379 to 506)	288 (251 to 327)	221 (191 to 251)	167 (148 to 187)	156 (138 to 175)	116 (96·4 to 137)	63·9 (51·4 to 78·3)	44·6 (34·5 to 56·6)	44 (33·6 to 57·6)	42·7 (32·6 to 56·2)
Gabon	427 (358 to 501)	218 (189 to 246)	157 (135 to 182)	97·2 (85·7 to 111)	70·7 (62·3 to 79·5)	60·5 (52 to 70·2)	49·1 (40·8 to 58·6)	31·6 (23·9 to 41)	29·1 (21·9 to 38·9)	27·1 (20·1 to 36·8)
Eastern sub-Saharan Africa	325 (284 to 366)	250 (225 to 274)	214 (199 to 229)	187 (179 to 195)	162 (156 to 169)	126 (120 to 133)	82·7 (75·9 to 89·4)	56·6 (48·3 to 66·6)	54·4 (45·7 to 65)	52·9 (43·9 to 64·2)
Burundi	343 (281 to 408)	277 (243 to 313)	249 (225 to 273)	209 (193 to 225)	173 (161 to 186)	148 (135 to 162)	88·9 (78·9 to 99·3)	60·2 (48·3 to 74·2)	60·1 (46·7 to 76·3)	58·6 (45·6 to 76)
Comoros	255 (217 to 297)	222 (197 to 248)	187 (169 to 206)	156 (142 to 170)	119 (108 to 130)	98·9 (87·3 to 111)	71·5 (58·6 to 85·4)	49·9 (41·2 to 59·1)	47·5 (39·4 to 56·5)	45·9 (38·1 to 55)
Djibouti	106 (85·8 to 128)	127 (111 to 145)	116 (101 to 133)	95·5 (85 to 107)	91·3 (81 to 103)	83·1 (72·3 to 93·9)	60·4 (50·7 to 71)	39 (32·2 to 46·5)	36·3 (30 to 43·2)	34·4 (28·5 to 41·3)
Eritrea	289 (235 to 350)	241 (209 to 275)	210 (184 to 238)	173 (156 to 190)	139 (124 to 154)	86 (73·8 to 99·3)	63·1 (49·7 to 78·5)	44·3 (33·8 to 57·4)	42·2 (31·6 to 55·5)	40·8 (30·6 to 54·2)
Ethiopia	376 (320 to 437)	262 (233 to 290)	245 (225 to 266)	222 (207 to 238)	180 (169 to 192)	133 (123 to 143)	78·4 (70·7 to 86·5)	50·8 (42·3 to 60·6)	47·2 (38·9 to 57·5)	45·4 (37·3 to 55·6)
Addis Ababa	184 (148 to 224)	146 (122 to 171)	127 (112 to 143)	96·5 (86·4 to 107)	81·1 (71·8 to 90·3)	57·3 (48·7 to 67·2)	26·6 (22·8 to 30·6)	16·2 (13·1 to 19·8)	14·8 (12 to 18·1)	14·4 (11·6 to 17·8)
Afar	639 (561 to 717)	341 (307 to 375)	298 (269 to 327)	273 (251 to 294)	202 (187 to 217)	178 (160 to 197)	114 (99·7 to 130)	70·2 (58·4 to 84·2)	65·6 (54 to 80)	62·6 (51·4 to 76·2)
Amhara	398 (335 to 467)	269 (239 to 300)	254 (232 to 280)	233 (214 to 254)	185 (172 to 198)	139 (127 to 152)	78 (69·7 to 86·4)	48·9 (40·5 to 58·9)	44·8 (36·5 to 55·5)	42·9 (34·5 to 52·7)
Benishangul-Gumuz	534 (463 to 603)	302 (266 to 339)	285 (260 to 313)	272 (248 to 298)	231 (208 to 253)	179 (157 to 205)	102 (90 to 114)	64·2 (53·3 to 76·8)	61 (49·5 to 74·9)	58·3 (47·9 to 70·7)
Dire Dawa	304 (251 to 363)	278 (244 to 311)	267 (241 to 294)	233 (212 to 257)	195 (178 to 214)	120 (107 to 132)	61·1 (52·9 to 69·8)	38·1 (30·9 to 46·6)	35·3 (27·9 to 46)	33·1 (26·2 to 42·2)
Gambella	380 (313 to 452)	290 (256 to 324)	274 (248 to 301)	260 (238 to 282)	233 (215 to 251)	143 (129 to 158)	79·4 (70 to 89·2)	45·4 (36·9 to 55·7)	42·6 (34·2 to 54)	40·3 (32·1 to 50·7)
Harari	375 (315 to 438)	338 (303 to 373)	310 (283 to 337)	255 (233 to 277)	195 (178 to 213)	115 (102 to 128)	65 (56·6 to 73·9)	40·7 (33·1 to 49·4)	37·5 (30 to 46·9)	35·3 (28·1 to 44)
Oromia	375 (315 to 441)	261 (232 to 288)	243 (221 to 266)	213 (200 to 227)	176 (163 to 189)	131 (120 to 141)	77 (69·6 to 84·8)	50·6 (42·4 to 60·2)	47·2 (39·2 to 57·1)	45·6 (37·7 to 55·6)
Somali	349 (286 to 415)	234 (203 to 265)	216 (194 to 239)	181 (165 to 198)	147 (134 to 161)	138 (125 to 151)	99·6 (86·3 to 113)	73·6 (61·7 to 86·2)	68·8 (57 to 81·9)	66·4 (55·5 to 79·4)
Southern Nations, Nationalities, and Peoples	345 (290 to 403)	263 (233 to 292)	251 (229 to 276)	237 (218 to 255)	200 (185 to 217)	137 (126 to 150)	79·4 (70·6 to 87·9)	49·1 (40·3 to 59·5)	45·8 (36·7 to 56·8)	43·8 (35·3 to 54·5)
Tigray	402 (340 to 468)	267 (236 to 297)	248 (226 to 272)	233 (215 to 254)	166 (150 to 183)	106 (95·7 to 116)	59·2 (53·4 to 65·4)	33·8 (28·3 to 40·7)	30·6 (25·4 to 37·1)	29·1 (24·1 to 35·2)
Kenya	263 (232 to 298)	178 (167 to 189)	140 (131 to 148)	105 (98·8 to 112)	91·4 (84·9 to 98)	81·5 (74·3 to 88·5)	55·1 (47·5 to 63·5)	37·5 (31 to 44·7)	34·7 (28·8 to 41·2)	33·9 (28·2 to 40·4)
Baringo	231 (190 to 278)	175 (159 to 191)	168 (154 to 183)	140 (128 to 152)	118 (102 to 134)	93·6 (82·2 to 105)	71·3 (58·4 to 85·5)	53·5 (43 to 65)	49·3 (40·2 to 59·7)	47·5 (38·5 to 58)
Bomet	118 (92·2 to 149)	73 (64·7 to 81·6)	69·4 (60·6 to 79·1)	63·3 (55·8 to 71)	50·2 (44·9 to 55·9)	42 (35·9 to 49)	38·8 (32·5 to 45·7)	27·1 (22 to 33·1)	25 (20·3 to 30·4)	23·9 (19·6 to 28·9)
Bungoma	289 (243 to 343)	193 (180 to 206)	145 (133 to 156)	120 (112 to 128)	96·7 (87·1 to 107)	95·8 (85·8 to 107)	59 (51·1 to 67·9)	36·1 (30·1 to 42·8)	33·5 (28·2 to 40)	35·4 (29·3 to 42·3)
Busia	477 (408 to 556)	290 (268 to 313)	214 (200 to 230)	135 (125 to 146)	119 (108 to 130)	116 (102 to 133)	75·1 (63·2 to 88·6)	48·3 (39·1 to 59·2)	45·6 (36·9 to 56·1)	50·8 (41·1 to 62·2)
Elgeyo Marakwet	165 (135 to 199)	117 (107 to 126)	128 (117 to 140)	111 (101 to 122)	70·8 (63·6 to 78·6)	56·8 (49·5 to 64·2)	31·7 (25·9 to 38·1)	21·3 (16·9 to 26·4)	20·9 (17·2 to 25·2)	19·1 (15·6 to 23·1)
Embu	349 (289 to 418)	225 (206 to 244)	139 (130 to 149)	83·2 (76·9 to 90·6)	61·5 (53·2 to 70·4)	50·4 (43·7 to 57·5)	42·5 (35·2 to 50·5)	29·4 (23·2 to 36·4)	27·9 (22·2 to 34·4)	26·7 (21·3 to 32·8)
Garissa	194 (157 to 233)	175 (152 to 198)	136 (119 to 155)	129 (116 to 143)	98·6 (85·7 to 112)	69·5 (56·2 to 84·7)	46·4 (40·3 to 53·2)	29·1 (24 to 34·8)	26·9 (22·7 to 31·7)	25·4 (21·7 to 29·5)
Homa Bay	533 (476 to 593)	342 (324 to 360)	308 (281 to 335)	241 (223 to 260)	201 (187 to 216)	187 (172 to 202)	103 (90·9 to 118)	61·3 (50·6 to 73·9)	55·7 (46·5 to 66·2)	52·7 (43·3 to 63)
Isiolo	389 (324 to 461)	380 (353 to 408)	362 (334 to 391)	286 (259 to 315)	212 (189 to 236)	155 (139 to 173)	111 (97·6 to 126)	75·2 (62 to 90·1)	72·1 (58·2 to 87·4)	68·9 (55·9 to 83·7)
Kajiado	163 (132 to 199)	113 (102 to 123)	108 (99·1 to 119)	89 (80·4 to 98)	61·2 (54·5 to 67·9)	50·6 (44·6 to 56·6)	32·6 (27·5 to 38·1)	19·8 (16·1 to 23·9)	17·4 (14·5 to 20·9)	16·5 (13·8 to 19·6)
Kakamega	281 (241 to 329)	204 (189 to 220)	166 (153 to 178)	133 (125 to 142)	142 (129 to 156)	126 (114 to 138)	80·8 (69·9 to 92·6)	52·6 (43·5 to 63·1)	47·9 (39·6 to 57·2)	50·4 (41·5 to 60·8)
Kericho	58·4 (45·8 to 72·4)	59 (52·4 to 65·8)	64·1 (58·7 to 70)	67·9 (62 to 74·3)	51·4 (45·4 to 57·3)	54·6 (48·3 to 60·9)	39·6 (33·6 to 46·2)	30·3 (24·4 to 36·8)	28·1 (23·2 to 33·8)	27·3 (22·4 to 32·7)
Kiambu	134 (105 to 167)	78·1 (69·2 to 87·2)	52·7 (47 to 59·3)	43 (38·4 to 48)	43·1 (36·8 to 49·6)	52·5 (45·7 to 60·2)	43·1 (36·1 to 50·7)	30·1 (23·8 to 37·2)	27·2 (21·7 to 33·5)	26·1 (21 to 31·8)
Kilifi	411 (353 to 473)	303 (283 to 324)	245 (229 to 259)	179 (162 to 199)	132 (121 to 142)	97·1 (87·2 to 109)	60·3 (51·7 to 69·5)	38·6 (31·3 to 46·2)	35·6 (29·1 to 42·6)	33·1 (27·1 to 39·7)
Kirinyaga	299 (258 to 349)	210 (191 to 229)	162 (146 to 179)	77 (68·2 to 86·8)	56·8 (48 to 66·8)	57·3 (48·4 to 67·4)	45·8 (38·1 to 53·7)	40·4 (32·4 to 49·8)	38·1 (30·2 to 47·3)	37·2 (29·5 to 46·2)
Kisii	195 (149 to 249)	167 (151 to 184)	143 (131 to 156)	119 (103 to 137)	106 (94·7 to 119)	84·3 (74·4 to 94)	46·5 (38·1 to 56·1)	31·3 (25 to 38·4)	29·6 (23·8 to 35·9)	29 (23·6 to 35·2)
Kisumu	264 (204 to 334)	174 (157 to 192)	161 (149 to 173)	140 (122 to 160)	132 (118 to 147)	119 (104 to 135)	62·1 (49·7 to 76·4)	39·1 (30·8 to 48·7)	37·5 (29·9 to 45·6)	38·6 (30·4 to 47·3)
Kitui	311 (256 to 377)	198 (186 to 211)	143 (134 to 153)	89·5 (82·6 to 97)	74·7 (65·6 to 84·5)	66·1 (57·3 to 75·3)	41·3 (35 to 48·1)	27 (22 to 32·9)	24·8 (20·2 to 30)	23·5 (19·3 to 28·5)
Kwale	318 (271 to 371)	283 (268 to 299)	214 (199 to 230)	154 (136 to 174)	117 (102 to 131)	84·9 (74·2 to 96)	62·2 (52·3 to 73·4)	40·4 (32 to 49·4)	36·8 (29·8 to 44·4)	34·7 (28·3 to 41·8)
Laikipia	115 (80·1 to 160)	77·3 (64·2 to 91·3)	61·5 (54·7 to 69)	45·2 (40·1 to 50·2)	41 (32·5 to 50·3)	43·9 (37·9 to 50·1)	27·2 (20·8 to 35)	17·2 (11·9 to 23·8)	15 (10·7 to 20·1)	14·1 (10·2 to 19·2)
Lamu	467 (386 to 553)	340 (308 to 377)	236 (212 to 259)	133 (118 to 151)	133 (117 to 150)	112 (96·6 to 129)	83·9 (70·3 to 97·7)	59·4 (45·3 to 75·1)	55·2 (43 to 69·5)	52·3 (40·9 to 64·2)
Machakos	173 (140 to 214)	134 (125 to 144)	105 (97·9 to 114)	71·3 (63·5 to 80)	58·2 (50 to 67)	61 (53·2 to 69·5)	40·8 (33·7 to 49·3)	27·9 (22·2 to 34·3)	26·1 (20·8 to 32·3)	24·6 (19·6 to 30·5)
Makueni	354 (288 to 431)	183 (164 to 203)	140 (127 to 154)	98·8 (86·8 to 112)	81·5 (69 to 94·2)	58·1 (51·2 to 65·2)	36·6 (31·1 to 42·9)	21·6 (17·5 to 26·3)	20 (16·1 to 24·6)	18·5 (15 to 22·7)
Mandera	184 (150 to 221)	138 (121 to 159)	124 (109 to 141)	114 (103 to 127)	111 (96·7 to 126)	72·5 (59·3 to 87·1)	72·3 (63·8 to 81·2)	54 (44·4 to 64·7)	47 (39·8 to 55·4)	44·9 (38·8 to 52·1)
Marsabit	182 (145 to 225)	196 (176 to 218)	155 (139 to 170)	125 (111 to 141)	99·9 (89 to 112)	72·5 (65·7 to 79·3)	64·4 (55·4 to 74·3)	48·8 (41·2 to 57·3)	46·3 (38·6 to 54·5)	44·7 (37·3 to 52·9)

Meru	191 (154 to 236)	127 (115 to 140)	94·3 (86·2 to 104)	58·7 (53·7 to 63·6)	50·1 (42·6 to 58·5)	49·6 (43·9 to 55·9)	49·7 (42·3 to 57·4)	41·6 (34·3 to 50·4)	39·6 (32·2 to 47·7)	38·1 (31·3 to 45·9)
Migori	425 (362 to 496)	294 (270 to 316)	258 (242 to 276)	234 (214 to 254)	210 (195 to 224)	187 (172 to 201)	106 (90·6 to 121)	67 (54·6 to 81)	60·7 (50·2 to 72·1)	58 (48·4 to 69·4)
Mombasa	188 (149 to 231)	189 (172 to 206)	138 (128 to 148)	91·8 (80·4 to 105)	78·4 (68 to 89·6)	71 (61·7 to 80·2)	49·5 (41·4 to 59)	32·5 (25·9 to 39·9)	29·9 (23·5 to 37·2)	27·7 (21·8 to 34·7)
Murang'a	280 (238 to 330)	143 (132 to 155)	97·3 (85·9 to 110)	52·7 (47·1 to 58·8)	43·5 (36·8 to 50·4)	43·3 (37·6 to 49·6)	38·9 (32·2 to 46·3)	29·5 (23·3 to 36·9)	27·5 (21·9 to 33·9)	26·6 (21·3 to 32·8)
Nairobi	197 (146 to 254)	170 (139 to 202)	99·3 (86 to 114)	68·2 (61·7 to 75·4)	62·2 (53·8 to 71·5)	62·5 (54·4 to 71·3)	34·5 (25·5 to 44·6)	21·7 (15·4 to 29·9)	18·7 (13·6 to 25·4)	17·6 (13 to 23·9)
Nakuru	64·5 (51 to 80)	56 (50·2 to 62·1)	55·3 (50 to 61·3)	52·1 (46·5 to 58·3)	46 (40·2 to 52·2)	49·1 (43·1 to 55·4)	42·9 (34·5 to 51·9)	32·9 (25·9 to 41·3)	30·9 (24·4 to 38·5)	29·8 (23·7 to 37)
Nandi	125 (93·4 to 164)	87·8 (77·6 to 98·3)	74·9 (68·2 to 81·9)	65·4 (58 to 73·4)	52·8 (45·7 to 59·5)	40·7 (35·8 to 45·4)	28·5 (23·8 to 33·7)	19·8 (15·9 to 24·4)	18 (14·6 to 21·9)	18 (14·4 to 22·1)
Narok	178 (141 to 222)	114 (96 to 134)	104 (89·6 to 121)	62·8 (54·4 to 72·1)	69·6 (59·2 to 80·7)	43·1 (36·6 to 49·9)	31·9 (27·7 to 36·6)	23·3 (17·8 to 29·4)	21·2 (17·1 to 25·9)	20·2 (16·8 to 24·3)
Nyamira	225 (181 to 278)	105 (95·1 to 118)	83·3 (74·5 to 92·4)	69·6 (61·1 to 77·9)	54·6 (49·4 to 60·5)	52·9 (45·9 to 60·5)	37·6 (30·7 to 44·9)	26·4 (21·2 to 32·4)	25·2 (20·2 to 31·2)	24·5 (19·6 to 30·3)
Nyandarua	179 (150 to 215)	115 (105 to 125)	73·8 (65·9 to 82·3)	40·4 (34·8 to 46·6)	33·5 (27 to 40·8)	38·3 (33·9 to 43·2)	45·2 (37·9 to 53·2)	37·9 (29·7 to 47·1)	35 (27·8 to 43·5)	34 (27 to 42·3)
Nyeri	288 (228 to 353)	128 (113 to 143)	78·5 (69·7 to 88·1)	45·5 (40 to 51·7)	41 (35·5 to 47·4)	43·7 (37·5 to 50·5)	36·7 (29·7 to 44)	31·3 (25·3 to 38)	28·2 (22·7 to 34·8)	27·5 (22 to 33·8)
Samburu	78·7 (60·1 to 102)	64·3 (56 to 73·2)	72·3 (64·1 to 81·5)	79·2 (70·1 to 89·4)	82·4 (70·2 to 95·8)	73·5 (64·1 to 83·9)	65·2 (54·1 to 78·1)	50·8 (39·7 to 63·2)	49·4 (39·9 to 60)	47·5 (39·6 to 57)
Siaya	364 (309 to 422)	285 (264 to 306)	234 (219 to 251)	198 (173 to 226)	197 (179 to 216)	185 (168 to 203)	99·5 (84·2 to 115)	58·3 (47·8 to 70·5)	54·5 (44·9 to 66·1)	58·5 (47·6 to 71·6)
Taita Taveta	263 (221 to 314)	180 (166 to 195)	162 (149 to 175)	101 (88·2 to 115)	70·8 (62·1 to 79·6)	68·9 (59·8 to 78·4)	45 (37·3 to 53·1)	26·6 (20·9 to 32·8)	25·4 (20·3 to 31·3)	23·9 (18·9 to 29·7)
Tana River	253 (199 to 317)	156 (139 to 175)	127 (112 to 143)	111 (97·2 to 127)	91·7 (80·2 to 104)	93·5 (80·7 to 106)	72·3 (62·6 to 83·2)	49·5 (40·2 to 59·2)	46·7 (38·1 to 56·3)	44·6 (36·7 to 53·4)
Tharaka Nithi	342 (282 to 413)	132 (119 to 146)	106 (94·1 to 118)	70·4 (61·8 to 79·4)	72·3 (62 to 83·2)	72·6 (64·1 to 82)	38·3 (31·6 to 45·1)	26 (21·2 to 31·6)	24·6 (19·7 to 30·1)	23·2 (18·6 to 28·8)
Trans Nzoia	91·7 (73·9 to 111)	90·3 (83·8 to 97·2)	85·1 (77·4 to 93·3)	83·2 (74·9 to 91·7)	63·5 (56·2 to 70·5)	53·6 (47 to 60·8)	37·8 (30·9 to 45·4)	26·5 (21·1 to 32·8)	24·2 (19·6 to 29·8)	24·2 (19·6 to 29·8)
Turkana	103 (80·3 to 131)	117 (104 to 131)	137 (119 to 157)	151 (137 to 165)	112 (98·5 to 125)	79·4 (69·9 to 89)	61·6 (53·2 to 71·4)	42·9 (34·2 to 52·8)	41·4 (34·3 to 49·6)	40 (33·4 to 47·3)
Uasin Gishu	65·9 (52·5 to 82·4)	60·9 (54·7 to 67·3)	56·4 (51·1 to 62·2)	57·1 (51·5 to 63·2)	52·6 (46·6 to 59)	43·9 (38·1 to 50·2)	36·1 (29·5 to 43·2)	27·2 (21·7 to 33·1)	24·9 (20·1 to 30·3)	24·7 (20·1 to 29·9)
Vihiga	371 (312 to 436)	155 (141 to 171)	116 (100 to 132)	91·1 (79·6 to 104)	97·5 (86·1 to 110)	88·5 (76·5 to 101)	66·2 (55·2 to 78·4)	46 (37·1 to 56)	44·6 (35·7 to 54·5)	46·3 (37·4 to 57)
Wajir	297 (244 to 359)	240 (214 to 269)	190 (171 to 210)	168 (151 to 185)	128 (113 to 144)	107 (88·5 to 127)	84·5 (74·9 to 94·4)	56·1 (46·1 to 67·3)	54·2 (46 to 63·3)	51·5 (44·3 to 59·4)
West Pokot	190 (155 to 227)	147 (136 to 161)	144 (131 to 157)	152 (140 to 166)	133 (119 to 148)	93·7 (82·2 to 106)	74·8 (61·8 to 89·2)	57·9 (46·5 to 70·1)	55·2 (45·6 to 65·7)	53·3 (43·8 to 64·3)
Madagascar	293 (241 to 349)	231 (201 to 263)	182 (166 to 198)	158 (145 to 171)	137 (127 to 148)	104 (94·1 to 114)	82·3 (72·1 to 93·1)	60·8 (49·3 to 74·4)	56·9 (45·8 to 70·9)	53·8 (42·9 to 68·1)
Malawi	342 (287 to 400)	334 (306 to 363)	294 (276 to 313)	235 (220 to 249)	204 (193 to 215)	153 (142 to 165)	88·4 (80·8 to 96·3)	53·7 (45·9 to 62·9)	51·2 (43·7 to 60·2)	47·8 (40·5 to 56·5)
Mozambique	335 (288 to 395)	279 (244 to 312)	239 (214 to 264)	217 (202 to 232)	211 (197 to 225)	152 (142 to 164)	98·9 (89·7 to 109)	63·1 (52·5 to 75·8)	61·4 (50·4 to 75·5)	57·5 (46·9 to 71·4)
Rwanda	456 (365 to 556)	288 (262 to 315)	233 (212 to 254)	202 (185 to 218)	157 (143 to 171)	134 (122 to 148)	68 (58·3 to 78·4)	42·1 (34·5 to 50·6)	39·7 (32·8 to 47·6)	38·3 (31·8 to 45·2)
Somalia	245 (203 to 292)	238 (212 to 264)	229 (204 to 256)	179 (162 to 197)	183 (168 to 199)	150 (135 to 165)	138 (123 to 153)	91·8 (77·4 to 108)	88·6 (75 to 105)	85·7 (72·3 to 102)
South Sudan	216 (173 to 264)	233 (202 to 267)	220 (192 to 249)	206 (181 to 233)	188 (167 to 210)	143 (130 to 158)	117 (99·2 to 136)	131 (109 to 156)	124 (103 to 148)	121 (99·6 to 146)
Uganda	362 (301 to 425)	232 (203 to 261)	184 (170 to 199)	187 (173 to 199)	159 (150 to 168)	124 (117 to 132)	81·5 (74·7 to 88·7)	53·8 (44·5 to 64·5)	55·5 (44·5 to 69·8)	59·1 (46 to 75·8)
Tanzania	329 (275 to 389)	251 (223 to 278)	208 (192 to 224)	169 (159 to 180)	149 (140 to 159)	121 (112 to 130)	77·7 (70·2 to 85·4)	54·6 (46·1 to 64·7)	52 (43·2 to 62·8)	49·3 (40·9 to 60·2)
Zambia	280 (237 to 331)	243 (216 to 269)	185 (172 to 199)	157 (146 to 169)	158 (148 to 167)	134 (123 to 145)	80 (71·6 to 89·1)	47·9 (39·1 to 58)	45·8 (37·3 to 56·1)	43·2 (35·2 to 53)
Southern sub-Saharan Africa	193 (159 to 230)	148 (128 to 169)	119 (107 to 131)	95·5 (88·6 to 102)	69·9 (65·3 to 75·2)	72·9 (68·3 to 77·7)	56·1 (51·8 to 60·7)	42·1 (36 to 48·9)	40·6 (34·7 to 47·5)	40·2 (34·2 to 48)
Botswana	168 (132 to 208)	123 (106 to 141)	104 (91·9 to 115)	72 (65·3 to 79·1)	59 (52·1 to 66·2)	67·9 (58·6 to 78·5)	48·4 (40·2 to 58·4)	39·1 (29·9 to 50·1)	38·2 (29 to 50·2)	37·9 (28·5 to 50·1)
Eswatini	285 (240 to 341)	244 (214 to 277)	176 (154 to 198)	113 (99·4 to 127)	70·4 (64·6 to 76·6)	84·6 (77·3 to 92·3)	72·5 (63·6 to 81·8)	40·4 (33·2 to 48·9)	38·7 (31·7 to 47·3)	37·8 (30·8 to 47)
Lesotho	188 (158 to 221)	172 (157 to 187)	163 (152 to 174)	108 (96·9 to 120)	78·8 (70·3 to 88·4)	85·4 (77·3 to 93·5)	77 (67·3 to 87·3)	69·1 (58·4 to 80·7)	69·2 (58·6 to 80·9)	69·4 (58·3 to 81·9)
Namibia	196 (158 to 238)	140 (119 to 164)	103 (91·7 to 116)	87·1 (80·1 to 93·7)	63·5 (58·1 to 69·3)	59·9 (53·7 to 66·9)	47·5 (41·6 to 54)	31·8 (25·9 to 39)	30·6 (24·7 to 37·8)	30 (24·1 to 37·5)
South Africa	197 (159 to 239)	152 (129 to 174)	122 (108 to 138)	101 (91·9 to 110)	72·8 (67·4 to 79·2)	73·6 (67·3 to 79·9)	50 (44·1 to 56·3)	37·9 (32·3 to 44·4)	36·3 (30·9 to 42·7)	35·9 (30·5 to 42·6)
Eastern Cape	286 (229 to 350)	190 (161 to 219)	161 (142 to 182)	127 (115 to 139)	95 (86·7 to 105)	88·3 (80·3 to 95·9)	64·4 (56·7 to 72·4)	47·4 (39·7 to 56·2)	44·7 (38·1 to 53·3)	44 (37·2 to 52·8)
Free State	218 (170 to 271)	177 (148 to 207)	151 (132 to 172)	133 (119 to 147)	92·2 (82·4 to 103)	96·8 (87·3 to 106)	68·1 (60·8 to 76·4)	52·7 (44·6 to 62·3)	48·5 (41·2 to 57·8)	47·9 (40·9 to 56·9)
Gauteng	98·7 (73·1 to 130)	89·3 (73·2 to 107)	76·8 (66·3 to 88·7)	77·4 (68·3 to 87·6)	59·4 (52 to 66·8)	69·1 (61·2 to 77·7)	41·4 (36·2 to 47·1)	31·1 (25·9 to 37·1)	29·5 (24·8 to 35·5)	29 (24·4 to 35·1)
KwaZulu-Natal	205 (163 to 254)	175 (149 to 201)	136 (122 to 152)	107 (96·6 to 117)	75·7 (67·7 to 84·8)	82·7 (74·7 to 90·3)	53 (46·7 to 59·7)	38·4 (32·5 to 45)	39·1 (33·4 to 45·8)	38·7 (33·2 to 45·3)
Limpopo	174 (137 to 214)	132 (113 to 151)	104 (91·1 to 116)	81·3 (72·8 to 90·5)	57·6 (52 to 63·6)	53·4 (47·7 to 59·5)	49·1 (42·5 to 55·9)	44 (37·5 to 50·9)	41·4 (35·6 to 48·1)	41·6 (35·9 to 48·7)
Mpumalanga	212 (168 to 259)	163 (139 to 187)	146 (129 to 167)	113 (102 to 124)	73·9 (66·3 to 82)	71·2 (63·4 to 78·8)	49 (43 to 55·4)	38·8 (32·4 to 46·1)	37·8 (31·8 to 44·8)	37·7 (31·7 to 45·1)
North West	193 (151 to 239)	167 (143 to 193)	134 (118 to 154)	111 (100 to 124)	84·7 (75·8 to 94·2)	90·6 (81 to 100)	65·6 (58 to 74)	50·5 (42·6 to 59·1)	47·1 (39·9 to 55·4)	46·5 (39·4 to 55·6)
Northern Cape	173 (135 to 220)	137 (114 to 162)	120 (104 to 140)	96·8 (85·2 to 110)	73·6 (65 to 82·3)	68·5 (59·9 to 77·9)	58·4 (50·9 to 65·8)	46·5 (39·3 to 55·3)	42·7 (36·7 to 50·7)	42 (35·8 to 50·3)
Western Cape	77·2 (56·3 to 104)	60·3 (49·1 to 73·4)	54·7 (46·8 to 63·2)	53·8 (46·9 to 61·1)	42·2 (36·7 to 48·1)	38 (33·5 to 42·8)	25·3 (21·7 to 29·1)	18·5 (15·4 to 22·1)	17·2 (14·6 to 20·6)	16·8 (14·2 to 20·2)
Zimbabwe	169 (135 to 205)	126 (109 to 144)	97·4 (86·6 to 108)	80·6 (73·5 to 87·7)	62·8 (58·3 to 67·2)	71 (66·6 to 75·5)	69·5 (63·8 to 76·2)	50·6 (43·7 to 58·5)	49 (41·8 to 57·5)	48·6 (41 to 57·8)
Western sub-Saharan Africa	307 (267 to 347)	275 (251 to 299)	247 (233 to 260)	208 (199 to 217)	184 (178 to 190)	162 (156 to 169)	121 (114 to 127)	88·4 (78·1 to 99·2)	84·8 (74·5 to 97)	80·5 (70·2 to 92·3)
Benin	360 (298 to 426)	289 (263 to 317)	238 (222 to 254)	189 (176 to 204)	167 (156 to 178)	135 (126 to 146)	107 (97·6 to 116)	77·9 (64·8 to 93·1)	74·6 (61 to 91·3)	72·1 (58·4 to 89·6)
Burkina Faso	374 (318 to 429)	345 (311 to 377)	289 (267 to 312)	238 (224 to 253)	201 (189 to 212)	174 (161 to 188)	134 (120 to 150)	98·1 (83·2 to 116)	98·1 (82·2 to 117)	90·7 (75·8 to 108)
Cabo Verde	274 (248 to 303)	154 (147 to 161)	152 (147 to 158)	80·2 (75·3 to 85·4)	60·9 (57·5 to 64·4)	46·3 (39·4 to 54·1)	24·9 (21 to 29·4)	15·5 (12·4 to 19·4)	14·3 (11·2 to 18·2)	13·6 (10·3 to 17·9)
Cameroon	291 (255 to 329)	242 (225 to 260)	195 (183 to 208)	160 (150 to 170)	129 (119 to 139)	122 (114 to 130)	94·8 (85·9 to 105)	65·6 (56·3 to 75·3)	65·2 (55·2 to 76·4)	61·1 (51·8 to 70·6)
Chad	338 (285 to 395)	257 (228 to 285)	246 (225 to 270)	226 (210 to 242)	193 (182 to 205)	174 (162 to 190)	139 (126 to 152)	111 (96·5 to 127)	109 (94·5 to 126)	106 (91·6 to 123)
Côte d'Ivoire	262 (229 to 296)	251 (234 to 270)	199 (187 to 210)	144 (135 to 155)	137 (126 to 147)	130 (120 to 141)	100 (89·9 to 110)	68·5 (59·4 to 78·9)	66·1 (57·4 to 76·4)	62·4 (54 to 72·1)
The Gambia	181 (143 to 223)	196 (165 to 225)	167 (148 to 188)	146 (131 to 162)	117 (105 to 129)	94·2 (84·6 to 105)	63·2 (55·5 to 71·4)	45·4 (38·1 to 54)	42·8 (35·7 to 51·4)	40·8 (33·6 to 49·6)
Ghana	249 (208 to 300)	199 (183 to 215)	165 (153 to 177)	140 (130 to 151)	117 (109 to 125)	92·6 (86 to 99·8)	70·5 (63·2 to 79·2)	46·2 (37·8 to 56·1)	43·2 (35 to 53·2)	39·8 (31·6 to 49·6)
Guinea	329 (280 to 383)	327 (290 to 362)	307 (279 to 338)	262 (238 to 285)	220 (204 to 238)	170 (156 to 185)	125 (112 to 138)	88·7 (76·7 to 102)	85·9 (74·2 to 100)	80·3 (69·3 to 93·9)
Guinea-Bissau	447 (396 to 499)	324 (284 to 365)	256 (229 to 284)	218 (196 to 240)	183 (167 to 200)	148 (136 to 161)	91·6 (79·9 to 104)	59·8 (50·5 to 70·9)	58 (48·8 to 68·9)	55·3 (46·3 to 65·7)
Liberia	452 (368 to 544)	322 (285 to 359)	279 (254 to 307)	236 (214 to 260)	234 (213 to 254)	161 (147 to 177)	91·2 (79·9 to 103)	68·9 (55·6 to 85·6)	68·7 (53·3 to 88·5)	63 (49 to 83)
Mali	375 (323 to 436)	352 (314 to 391)	325 (302 to 349)	273 (256 to 290)	228 (214 to 241)	184 (171 to 198)	134 (121 to 147)	96·8 (81·7 to 115)	95·3 (79·4 to 115)	88·9 (73·8 to 108)
Mauritania	220 (185 to 254)	177 (162 to 192)	140 (130 to 151)	121 (112 to 130)	94·2 (86·2 to 103)	75·7 (69 to 83·5)	52·4 (47·1 to 58·7)	34·4 (29·3 to 40)	32 (27·4 to 37·2)	30 (25·7 to 35·2)
Niger	333 (284 to 382)	335 (304 to 364)	321 (298 to 343)	302 (285 to 321)	283 (267 to 302)	222 (205 to 238)	120 (105 to 136)	90·3 (76·9 to 105)	87·4 (73·9 to 104)	84·2 (70·6 to 101)
Nigeria	303 (252 to 354)	268 (241 to 297)	247 (225 to 269)	210 (197 to 222)	193 (184 to 202)	176 (167 to 186)	135 (126 to 145)	100 (87·9 to 113)	94·5 (82·5 to 108)	90 (78·2 to 104)
São Tomé and Príncipe	442 (412 to 474)	271 (258 to 285)	147 (141 to 155)	109 (102 to 116)	102 (93·1 to 111)	75·5 (67·6 to 83·9)	37·1 (32·2 to 42·3)	18·8 (15 to 23·5)	17·2 (13·5 to 21·8)	16·6 (13 to 21)

Senegal	288 (245 to 335)	267 (249 to 287)	260 (243 to 277)	193 (180 to 207)	130 (121 to 140)	112 (104 to 121)	67·3 (61·2 to 73·4)	42·9 (36·7 to 49·8)	39·8 (34·1 to 45·8)	37·2 (31·9 to 43·1)
Sierra Leone	364 (303 to 426)	324 (286 to 363)	287 (255 to 319)	257 (235 to 281)	230 (215 to 245)	207 (192 to 222)	146 (132 to 161)	96 (80·1 to 114)	96·2 (78·1 to 118)	90·3 (73·7 to 110)
Togo	296 (248 to 349)	245 (216 to 276)	201 (183 to 220)	162 (148 to 176)	134 (122 to 145)	114 (102 to 126)	86·3 (77 to 96·4)	57·6 (48·5 to 68·2)	54·4 (45·5 to 65·6)	51·3 (42·3 to 62·3)

Appendix 2 Table S2c: Under-5 mortality rates with 95% UIs by location, males, 1950, 1960, 1970, 1980, 1990, 2000, 2010, 2019, 2020, 2021

Location	1950	1960	1970	1980	1990	2000	2010	2019	2020	2021
Global	241 (207 to 276)	299 (242 to 396)	142 (133 to 151)	114 (107 to 121)	87·3 (82·5 to 92·4)	73·2 (69 to 77·5)	54·5 (50 to 59·1)	40·1 (34·4 to 46·7)	38·9 (33 to 45·7)	37·8 (31·8 to 45)
Central Europe, eastern Europe, and central Asia	132 (117 to 149)	77·4 (72·3 to 83·1)	52 (48·1 to 56·3)	41·2 (39·3 to 43·3)	34·4 (32·8 to 36·2)	29·5 (27·6 to 31·4)	18·3 (16·9 to 19·8)	14·3 (12·5 to 16·2)	13·6 (11·8 to 15·5)	13·3 (11·6 to 15·3)
Central Asia	257 (215 to 302)	155 (133 to 177)	98·2 (84·6 to 113)	75 (69·1 to 81·3)	61·4 (57·2 to 66)	53·6 (48·8 to 58·8)	35·8 (31·8 to 40·1)	25·8 (21·9 to 30·3)	24·3 (20·5 to 28·5)	23·3 (19·6 to 27·6)
Armenia	282 (234 to 341)	168 (140 to 197)	99·2 (82 to 117)	66·6 (57·6 to 76·3)	45·3 (40·3 to 51·4)	33·8 (29·9 to 38·2)	19·4 (16·5 to 22·5)	13·9 (11·3 to 17)	14·9 (12·2 to 18·1)	12·1 (9·84 to 15·2)
Azerbaijan	401 (339 to 463)	259 (226 to 293)	147 (127 to 171)	91·8 (79·7 to 105)	82·2 (72·6 to 91·7)	71 (62·7 to 80·2)	44·9 (37·7 to 52·9)	34·7 (28·3 to 41·7)	33·4 (27·6 to 40)	31·5 (25·8 to 38·4)
Georgia	231 (189 to 279)	150 (126 to 175)	95 (76·9 to 115)	62·1 (53·9 to 71·8)	46·4 (40·6 to 52·8)	39·2 (34·6 to 44·8)	18·5 (15·5 to 21·7)	11·3 (9·14 to 13·9)	10·8 (8·77 to 13·5)	10·6 (8·38 to 13·4)
Kazakhstan	136 (108 to 169)	95·4 (79·8 to 111)	57·2 (47·5 to 68·4)	51·3 (45·4 to 57·1)	40·8 (36·8 to 45·3)	40·9 (35·9 to 46·4)	26·4 (22·5 to 30·7)	13·2 (11 to 15·8)	12·3 (10·3 to 14·7)	11·4 (9·41 to 13·9)
Kyrgyzstan	235 (180 to 294)	162 (137 to 191)	109 (90·1 to 130)	84·2 (73·9 to 95·1)	62·4 (56·4 to 68·8)	47·5 (42·6 to 53)	29·1 (27·8 to 30·3)	20·1 (18·8 to 21·4)	19 (17·4 to 20·6)	18·5 (16·3 to 20·8)
Mongolia	394 (317 to 476)	231 (200 to 264)	154 (133 to 177)	118 (105 to 131)	90·4 (82·9 to 98·1)	59·5 (53·2 to 65·9)	33·1 (29·2 to 38)	20·9 (17·4 to 25)	19·1 (16 to 23)	18·4 (15·2 to 22·5)
Tajikistan	370 (301 to 448)	235 (200 to 272)	137 (116 to 161)	104 (91·7 to 118)	84·9 (77·2 to 93)	71·7 (64 to 79·5)	49 (41·8 to 56·8)	43·7 (36·1 to 52·5)	40·3 (33·2 to 49)	38·9 (32·1 to 47·8)
Turkmenistan	272 (221 to 331)	165 (141 to 190)	107 (87·2 to 131)	92·8 (80 to 107)	76·8 (67·8 to 86·8)	66·5 (58·2 to 75)	46·4 (40·1 to 53·7)	33·9 (27·6 to 40·9)	32·1 (26·2 to 38·5)	30·9 (25·1 to 37·6)
Uzbekistan	268 (207 to 339)	126 (103 to 150)	92 (78·3 to 108)	71·2 (64·3 to 78·5)	57·8 (52·7 to 63·4)	49·1 (43 to 55·6)	36·2 (30·5 to 42·4)	26·2 (21·7 to 31·5)	24·7 (20·4 to 29·8)	23·9 (19·7 to 29)
Central Europe	178 (170 to 186)	78·9 (76·7 to 81)	51·1 (49·8 to 52·3)	32·2 (31·4 to 32·9)	24·7 (24 to 25·4)	15 (14·4 to 15·6)	8·14 (7·85 to 8·44)	5·85 (5·47 to 6·26)	5·61 (5·19 to 6·06)	5·48 (4·86 to 6·16)
Albania	249 (207 to 299)	131 (111 to 152)	81·4 (69 to 95)	49·4 (42 to 57·7)	39·9 (35·1 to 45·6)	30·1 (25·5 to 35·4)	18·9 (15·6 to 22·4)	16 (13 to 19·6)	15·8 (12·9 to 19·3)	14·1 (11·3 to 17·6)
Bosnia and Herzegovina	281 (246 to 318)	120 (108 to 131)	73·2 (66·5 to 79·5)	40·8 (36·4 to 45·3)	18·6 (17·4 to 19·7)	12·6 (11·7 to 13·5)	7·47 (6·74 to 8·24)	7·16 (6·35 to 8·03)	6·84 (5·85 to 7·95)	5·55 (4·55 to 6·71)
Bulgaria	196 (186 to 208)	61 (58·8 to 63·1)	35·9 (34·6 to 37·2)	28·3 (27·3 to 29·4)	21·8 (20·8 to 22·8)	19·4 (18·4 to 20·5)	12·7 (11·9 to 13·7)	7·4 (6·68 to 8·2)	6·74 (6·15 to 7·37)	7·11 (6·15 to 8·11)
Croatia	229 (216 to 243)	79·7 (76·9 to 82·4)	44·3 (42·1 to 46·4)	22·7 (21·6 to 23·8)	13·1 (12·3 to 14·1)	8·67 (7·98 to 9·41)	5·53 (5·07 to 6)	4·83 (4·26 to 5·45)	5·01 (4·37 to 5·68)	5·03 (4·16 to 6·03)
Czechia	59·4 (55·3 to 63·5)	26·9 (26 to 27·9)	26·9 (26 to 27·9)	21·2 (20·4 to 22)	13·6 (13 to 14·3)	5·9 (5·48 to 6·36)	3·87 (3·61 to 4·13)	3·45 (3·11 to 3·82)	3·22 (2·9 to 3·55)	3·09 (2·63 to 3·61)
Hungary	141 (131 to 150)	58·2 (56·3 to 60·1)	44·2 (42·6 to 45·8)	27·7 (26·6 to 28·9)	18·8 (17·9 to 19·6)	11·5 (10·8 to 12·2)	6·27 (5·84 to 6·73)	4·71 (4·21 to 5·27)	4·52 (4·01 to 5·1)	4·39 (3·67 to 5·17)
Montenegro	89·6 (78·7 to 103)	58·5 (55·3 to 62·1)	39·3 (36·7 to 42·4)	24·6 (22·6 to 26·6)	16·6 (14·9 to 18·5)	13·4 (11·9 to 14·9)	7·91 (7·15 to 8·71)	3·69 (3·18 to 4·25)	4·5 (3·75 to 5·41)	4·19 (3·48 to 5·08)
North Macedonia	228 (200 to 257)	163 (147 to 180)	94·9 (86·7 to 103)	59 (53·1 to 65·3)	39·3 (37·3 to 41·4)	16·8 (15·5 to 18·1)	10·6 (9·72 to 11·5)	7·65 (6·8 to 8·61)	6·82 (6·06 to 7·66)	5·99 (5·21 to 6·88)
Poland	186 (179 to 193)	69·9 (68·4 to 71·4)	42·5 (41·4 to 43·6)	27·7 (27 to 28·4)	20·4 (19·8 to 21)	10·4 (9·99 to 10·8)	6·34 (6·09 to 6·58)	4·64 (4·34 to 4·96)	4·4 (4·04 to 4·8)	4·69 (4·09 to 5·36)
Romania	148 (124 to 174)	96·9 (93·3 to 101)	60·7 (58·8 to 62·6)	38·9 (37·5 to 40·4)	36·6 (35·2 to 37·9)	24·8 (23·7 to 26)	12·7 (12 to 13·4)	8·11 (7·56 to 8·71)	7·72 (7·19 to 8·3)	7·37 (6·61 to 8·18)
Serbia	294 (275 to 312)	103 (100 to 106)	75·6 (73·1 to 78)	44·1 (42·7 to 45·7)	34·9 (33·6 to 36·2)	17·6 (15 to 20·5)	7·35 (6·33 to 8·49)	5·96 (5·44 to 6·47)	5·86 (5·45 to 6·32)	5·15 (4·63 to 5·71)
Slovakia	106 (99·4 to 113)	38·8 (37·4 to 40·2)	34·4 (33·1 to 35·8)	26·8 (25·8 to 27·9)	16·1 (15·4 to 17)	10·9 (10·1 to 11·7)	7·52 (6·99 to 8·07)	6·75 (6·12 to 7·39)	6·66 (6·13 to 7·25)	6·35 (5·54 to 7·18)
Slovenia	161 (149 to 173)	72·8 (70 to 75·7)	38·7 (36·8 to 40·6)	19·6 (18·6 to 20·6)	11 (10·2 to 11·7)	6·01 (5·54 to 6·53)	3·52 (3·19 to 3·87)	2·51 (2·19 to 2·84)	2·59 (2·28 to 2·92)	2·5 (2·17 to 2·85)
Eastern Europe	76·6 (59·5 to 97·9)	52 (49·6 to 54·6)	32 (30 to 34·2)	30·6 (29·2 to 32·1)	23 (22·1 to 24)	20·1 (19·4 to 20·8)	10·8 (10·4 to 11·1)	7·02 (6·62 to 7·44)	6·53 (6·16 to 6·92)	6·72 (6·19 to 7·25)
Belarus	92·2 (69·2 to 119)	64·1 (53·9 to 75·4)	38·3 (32·3 to 45·4)	28·3 (23·9 to 32·9)	20·9 (17·9 to 24·4)	19·4 (16·5 to 22·6)	9·1 (7·62 to 10·8)	5·45 (4·29 to 6·81)	4·72 (3·51 to 6·12)	4·55 (3·49 to 6·04)
Estonia	64·1 (46·8 to 84·7)	41·8 (39·7 to 44)	25·5 (24·1 to 26·9)	25·1 (23·7 to 26·4)	19·4 (18·4 to 20·6)	12·4 (11·4 to 13·5)	4·92 (4·48 to 5·37)	2·47 (2·11 to 2·89)	2·44 (2·1 to 2·83)	2·64 (2·24 to 3·11)
Latvia	48·1 (35·1 to 63·8)	36·7 (34·9 to 38·6)	24·6 (23·4 to 25·8)	24·2 (23·2 to 25·3)	19·5 (18·6 to 20·5)	14·7 (13·7 to 15·7)	8·16 (7·59 to 8·81)	4·29 (3·77 to 4·85)	4·31 (3·77 to 4·87)	3·99 (3·39 to 4·64)
Lithuania	93·5 (69 to 123)	53·3 (51·3 to 55·4)	25·5 (24·4 to 26·7)	22·5 (21·4 to 23·5)	16·3 (15·5 to 17·1)	11·9 (11·2 to 12·6)	6·66 (6·14 to 7·23)	4·23 (3·77 to 4·74)	3·91 (3·5 to 4·36)	3·86 (3·4 to 4·39)
Moldova	199 (160 to 248)	125 (103 to 147)	80·1 (63·6 to 99·1)	58·2 (50·1 to 67·2)	36·3 (31·9 to 41·2)	30·3 (26·2 to 34·9)	18·5 (15·8 to 21·4)	14 (10·9 to 18)	13·9 (10·9 to 18)	12·3 (9·15 to 16·6)
Russia	77·2 (55·5 to 105)	52·1 (50·8 to 53·3)	31·4 (30·4 to 32·3)	31·1 (30·3 to 32)	23·7 (23·2 to 24·4)	21·2 (20·5 to 21·9)	10·6 (10·3 to 11)	6·62 (6·21 to 7·05)	6·11 (5·82 to 6·43)	6·52 (6·11 to 6·96)
Ukraine	59·7 (44·9 to 77·1)	42·7 (36·2 to 50·2)	29·2 (23·9 to 35)	27·5 (23 to 32·2)	20·6 (18·1 to 23·4)	16·9 (16·2 to 17·6)	11·5 (11·1 to 12)	9·25 (8·25 to 10·4)	8·92 (7·67 to 10·3)	8·36 (6·68 to 9·99)
High income	65·2 (61·9 to 68·3)	48·2 (47·1 to 49·4)	32·2 (31·4 to 33)	18·4 (18 to 18·9)	12·2 (11·9 to 12·4)	8·27 (8·11 to 8·42)	6·52 (6·4 to 6·65)	5·61 (5·45 to 5·76)	5·29 (5·03 to 5·55)	4·96 (4·51 to 5·47)
Australasia	34·1 (32·4 to 35·9)	27·6 (26·9 to 28·4)	23·5 (22·9 to 24)	14·8 (14·3 to 15·4)	10·8 (10·5 to 11·2)	7·26 (6·98 to 7·54)	5·9 (5·64 to 6·2)	4·58 (4·29 to 4·88)	4·25 (3·87 to 4·62)	3·61 (3·03 to 4·26)
Australia	33·4 (31·4 to 35·5)	26·9 (26·1 to 27·7)	23·7 (23 to 24·4)	14·4 (13·9 to 15)	10·6 (10·1 to 11)	6·98 (6·67 to 7·29)	5·6 (5·34 to 5·89)	4·39 (4·11 to 4·67)	4·14 (3·75 to 4·55)	3·27 (2·68 to 3·94)
New Zealand	36·8 (33·8 to 40·3)	30·4 (29·1 to 31·6)	22·2 (21·2 to 23·3)	16·5 (15·7 to 17·5)	12 (11·1 to 12·8)	8·49 (7·89 to 9·1)	7·33 (6·83 to 7·94)	5·53 (5·08 to 6·06)	4·74 (4·35 to 5·12)	5·23 (4·52 to 5·92)
High-income Asia Pacific	93·8 (85 to 104)	63·1 (58·5 to 67·9)	32·7 (29·7 to 35·7)	16·1 (14·7 to 17·7)	8·87 (8·15 to 9·64)	5·58 (5·41 to 5·76)	3·7 (3·57 to 3·86)	2·88 (2·74 to 3·02)	2·58 (2·41 to 2·76)	2·32 (2·13 to 2·51)
Brunei	236 (217 to 257)	132 (124 to 140)	57·4 (54·1 to 60·6)	22·4 (20·3 to 24·6)	12·2 (10·9 to 13·7)	11 (9·49 to 12·8)	11·1 (9·92 to 12·4)	11·7 (9·87 to 13·9)	11·3 (9·18 to 13·9)	10·5 (8·22 to 13·4)
Japan	64·7 (61 to 68·6)	41·8 (40·3 to 43·3)	18·4 (17·8 to 18·9)	10·7 (10·4 to 11·1)	6·79 (6·54 to 7·04)	4·75 (4·56 to 4·94)	3·36 (3·22 to 3·51)	2·62 (2·47 to 2·76)	2·33 (2·14 to 2·55)	2·19 (1·94 to 2·5)
Aichi	56 (47·9 to 64·9)	39·2 (34·6 to 43·5)	16·6 (14·9 to 18·4)	9·86 (9·4 to 10·3)	6·42 (6·05 to 6·79)	4·5 (4·22 to 4·78)	3·39 (3·15 to 3·63)	2·45 (2·27 to 2·63)	2·26 (2·1 to 2·44)	2·17 (1·9 to 2·47)
Akita	56·5 (49 to 64·6)	42 (38·7 to 45·4)	21·4 (19·6 to 23·4)	11 (10·2 to 11·9)	7·34 (6·77 to 7·93)	4·78 (4·28 to 5·33)	3·51 (3·1 to 3·92)	2·95 (2·65 to 3·28)	2·7 (2·29 to 3·15)	2·52 (2·12 to 2·96)
Aomori	67·2 (59 to 75·7)	46·1 (42·7 to 49·8)	22·8 (20·9 to 24·6)	12·6 (11·8 to 13·4)	7·84 (7·26 to 8·49)	5·37 (4·95 to 5·8)	3·63 (3·33 to 3·97)	3·01 (2·72 to 3·3)	2·71 (2·34 to 3·13)	2·53 (2·12 to 3)
Chiba	64·3 (54·2 to 75·8)	40·3 (35·7 to 45·4)	15·9 (14 to 17·9)	10 (9·41 to 10·6)	5·95 (5·58 to 6·33)	4·64 (4·37 to 4·91)	3·47 (3·21 to 3·74)	2·65 (2·45 to 2·87)	2·38 (2·2 to 2·57)	2·23 (1·96 to 2·55)
Ehime	62·6 (54·9 to 71)	42·7 (39·4 to 46·5)	20 (18·3 to 22)	11·1 (10·4 to 12)	7·13 (6·59 to 7·72)	4·63 (4·26 to 5·04)	3·02 (2·71 to 3·33)	2·5 (2·27 to 2·76)	2·16 (1·86 to 2·49)	2·05 (1·72 to 2·42)
Fukui	59·2 (49·6 to 70)	39·2 (35·1 to 43·8)	18·1 (15·8 to 20·5)	9·49 (8·66 to 10·5)	7·06 (6·34 to 7·8)	5·2 (4·72 to 5·72)	3·27 (2·93 to 3·65)	2·6 (2·32 to 2·91)	2·82 (2·4 to 3·3)	2·64 (2·21 to 3·15)
Fukuoka	57·7 (48·8 to 66·8)	38·1 (33·9 to 42·7)	18 (15·9 to 20·2)	10 (9·43 to 10·6)	7·06 (6·68 to 7·48)	4·99 (4·74 to 5·28)	3·51 (3·24 to 3·82)	2·9 (2·68 to 3·12)	2·52 (2·31 to 2·74)	2·42 (2·12 to 2·76)
Fukushima	78·2 (67·7 to 89·5)	49·9 (46·1 to 54·1)	25·3 (23·3 to 27·5)	12·9 (12·2 to 13·6)	8·06 (7·62 to 8·54)	5·39 (4·98 to 5·78)	3·63 (3·34 to 3·93)	3·21 (2·94 to 3·5)	3·01 (2·6 to 3·47)	2·88 (2·47 to 3·35)
Gifu	59 (51·3 to 68)	41·4 (38·6 to 44·4)	18·9 (17·5 to 20·3)	10·6 (10·1 to 11·2)	6·28 (5·84 to 6·73)	4·63 (4·3 to 4·98)	3·64 (3·36 to 3·95)	2·86 (2·62 to 3·12)	2·47 (2·15 to 2·82)	2·33 (1·98 to 2·72)
Gunma	72·4 (62·4 to 82·8)	48·3 (44·8 to 52·1)	20·8 (18·9 to 22·7)	11·4 (10·7 to 12·1)	6·95 (6·42 to 7·47)	4·93 (4·57 to 5·31)	3·33 (3·1 to 3·57)	2·53 (2·3 to 2·77)	2·36 (2·04 to 2·71)	2·25 (1·91 to 2·64)
Hiroshima	69·5 (60·7 to 79·1)	45·2 (41·9 to 49)	19·2 (17·6 to 20·8)	11·3 (10·7 to 11·9)	6·84 (6·43 to 7·28)	4·27 (3·94 to 4·62)	3·32 (3 to 3·67)	2·54 (2·34 to 2·77)	2·2 (1·92 to 2·53)	2·06 (1·77 to 2·41)
Hokkaidō	56·6 (48·2 to 66·4)	39·6 (35·1 to 44·3)	20 (17·7 to 22·5)	11·3 (10·8 to 11·9)	6·58 (6·17 to 7·01)	4·42 (4·11 to 4·75)	3·22 (2·99 to 3·48)	2·87 (2·59 to 3·15)	2·67 (2·44 to 2·92)	2·59 (2·26 to 2·96)
Hyōgo	61 (51·3 to 71·4)	39·6 (35 to 44·6)	17·5 (15·4 to 19·6)	10·5 (9·88 to 11·1)	6·76 (6·38 to 7·19)	4·67 (4·36 to 4·97)	3·12 (2·9 to 3·37)	2·35 (2·19 to 2·54)	2·11 (1·96 to 2·29)	1·98 (1·74 to 2·26)
Ibaraki	81·4 (68·1 to 96·4)	50·6 (45·2 to 56·2)	22·8 (20·2 to 25·8)	12·7 (12 to 13·5)	7 (6·52 to 7·5)	4·89 (4·54 to 5·26)	3·58 (3·33 to 3·85)	3·16 (2·88 to 3·44)	3 (2·74 to 3·28)	2·86 (2·5 to 3·29)
Ishikawa	59·5 (50·6 to 69·7)	43·3 (40·2 to 46·8)	19·5 (17·8 to 21·3)	10·8 (10 to 11·6)	6·62 (6·01 to 7·29)	4·96 (4·54 to 5·4)	3·46 (3·16 to 3·79)	2·58 (2·31 to 2·86)	2·25 (1·92 to 2·65)	2·1 (1·77 to 2·5)
Iwate	75·8 (65·8 to 86·4)	51·9 (47·9 to 56·3)	26·5 (24·5 to 28·8)	13·6 (12·8 to 14·6)	6·6 (6·08 to 7·13)	4·51 (4·14 to 4·93)	3·94 (3·54 to 4·33)	3·28 (2·95 to 3·63)	2·72 (2·34 to 3·16)	2·56 (2·16 to 3·04)
Kagawa	61·8 (53·4 to 71·8)	44·5 (41·2 to 47·9)	19·4 (17·8 to 21·2)	10·5 (9·74 to 11·5)	6·93 (6·29 to 7·65)	4·38 (3·96 to 4·82)	3·55 (3·23 to 3·9)	2·63 (2·37 to 2·93)	2·48 (2·1 to 2·93)	2·34 (1·97 to 2·79)
Kagoshima	87·2 (76 to 99·3)	48 (44·7 to 51·5)	25·6 (23·4 to 27·9)	13 (12·3 to 13·7)	7·53 (7·06 to 8·02)	5·45 (5·1 to 5·84)	3·76 (3·47 to 4·1)	3·1 (2·79 to 3·4)	2·86 (2·49 to 3·26)	2·71 (2·28 to 3·17)
Kanagawa	54·3 (42·5 to 68·5)	34·5 (29·8 to 39·4)	14·6 (13·1 to 16·2)	9·67 (9·17 to 10·2)	6·34 (5·95 to 6·74)	4·72 (4·39 to 5·06)	3·45 (3·19 to 3·73)	2·63 (2·41 to 2·87)	2·09 (1·94 to 2·25)	1·88 (1·64 to 2·15)
Kōchi	76·5 (65·5 to 87·3)	49·4 (45·7 to 53·5)	22·9 (20·8 to 25·2)	11·7 (10·7 to 12·8)	7·99 (7·33 to 8·71)	5·16 (4·67 to 5·74)	4 (3·64 to 4·38)	3·13 (2·78 to 3·48)	3·03 (2·59 to 3·55)	2·87 (2·41 to 3·4)
Kumamoto	71·7 (62·7 to 81·4)	48·2 (44·7 to 52·1)	24·2 (22·4 to 26)	12·6 (11·9 to 13·4)	7·34 (6·8 to 7·92)	5·02 (4·66 to 5·39)	3·38 (3·01 to 3·76)	2·7 (2·46 to 2·96)	2·68 (2·35 to 3·05)	2·45 (2·08 to 2·86)

Kyōto	60·8 (52·3 to 70·1)	39·4 (36·4 to 42·6)	16·1 (14·7 to 17·5)	9·59 (8·96 to 10·2)	6·89 (6·38 to 7·4)	4·57 (4·21 to 4·9)	3·2 (2·97 to 3·45)	2·66 (2·45 to 2·9)	2·14 (1·84 to 2·48)	2·01 (1·73 to 2·34)
Mie	63·6 (55·9 to 72·9)	44·4 (41·2 to 47·9)	19·6 (18·1 to 21)	10·9 (10·3 to 11·6)	6·94 (6·44 to 7·48)	5·14 (4·78 to 5·53)	3·76 (3·46 to 4·08)	2·53 (2·29 to 2·79)	2·35 (2·04 to 2·7)	2·2 (1·86 to 2·59)
Miyagi	65·6 (57·4 to 74·6)	43·5 (40 to 46·9)	20·8 (19·1 to 22·2)	11·4 (10·8 to 12·1)	6·71 (6·31 to 7·1)	4·28 (3·97 to 4·59)	3·29 (3·02 to 3·57)	2·7 (2·4 to 3·02)	2·36 (2·02 to 2·74)	2·21 (1·88 to 2·57)
Miyazaki	70·8 (62·4 to 79·7)	47·2 (43·6 to 51·2)	23·1 (21·2 to 25·1)	12 (11·1 to 13)	8·04 (7·49 to 8·63)	5·23 (4·8 to 5·7)	3·65 (3·31 to 4·01)	2·97 (2·7 to 3·27)	2·82 (2·44 to 3·27)	2·68 (2·27 to 3·17)
Nagano	80·5 (69·5 to 92·6)	49·5 (45·9 to 53·5)	20·4 (18·9 to 21·9)	10·7 (10·1 to 11·3)	6·51 (6·13 to 6·94)	3·79 (3·53 to 4·07)	2·79 (2·57 to 3·04)	2·19 (1·97 to 2·41)	2·08 (1·8 to 2·38)	1·97 (1·69 to 2·31)
Nagasaki	64·9 (57·2 to 73·6)	38·1 (35·5 to 40·9)	19·6 (18·3 to 21·1)	10·5 (9·79 to 11·3)	6·74 (6·23 to 7·28)	5·03 (4·66 to 5·39)	3·69 (3·38 to 4·01)	2·88 (2·6 to 3·18)	3·03 (2·62 to 3·48)	2·93 (2·48 to 3·47)
Nara	64·2 (55·6 to 73·8)	40·1 (37 to 43·3)	17 (15·5 to 18·6)	10·7 (9·96 to 11·5)	6·54 (6·08 to 7·04)	4·29 (3·93 to 4·67)	3·5 (3·16 to 3·87)	2·85 (2·61 to 3·1)	2·73 (2·36 to 3·15)	2·61 (2·22 to 3·09)
Niigata	69·7 (60·4 to 79·5)	45·5 (42 to 49·2)	20·7 (19·2 to 22·3)	11·3 (10·7 to 11·9)	6·09 (5·71 to 6·54)	4·79 (4·43 to 5·12)	2·89 (2·62 to 3·18)	2·47 (2·23 to 2·72)	2·28 (1·95 to 2·66)	2·2 (1·87 to 2·58)
Ōita	66 (57·6 to 76·2)	44·2 (40·6 to 48)	21·8 (19·9 to 23·8)	11·5 (10·9 to 12·2)	6·75 (6·2 to 7·32)	4·44 (4·06 to 4·86)	3·45 (3·12 to 3·81)	2·64 (2·36 to 2·93)	2·41 (2·07 to 2·82)	2·28 (1·93 to 2·69)
Okayama	63·6 (55·7 to 71·8)	42·3 (39·4 to 45·4)	17·6 (16·1 to 19)	9·84 (9·16 to 10·5)	7·07 (6·63 to 7·54)	5·01 (4·64 to 5·41)	3·34 (3·07 to 3·61)	2·78 (2·52 to 3·03)	2·45 (2·1 to 2·82)	2·31 (1·97 to 2·69)
Okinawa	124 (108 to 142)	49·8 (46·1 to 53·5)	23·5 (21·7 to 25·3)	11·7 (11 to 12·4)	7·81 (7·23 to 8·45)	5·5 (5·05 to 5·95)	3·49 (3·11 to 3·89)	2·56 (2·32 to 2·82)	2·52 (2·15 to 2·94)	2·38 (2·02 to 2·78)
Ōsaka	59·6 (48·5 to 72·6)	37·7 (33·4 to 42·4)	15·4 (14 to 16·8)	10·1 (9·66 to 10·6)	6·93 (6·51 to 7·34)	4·55 (4·28 to 4·81)	3·21 (2·99 to 3·45)	2·54 (2·29 to 2·78)	2·21 (2·04 to 2·42)	1·98 (1·74 to 2·25)
Saga	71·9 (62·9 to 82·8)	43·5 (40·1 to 46·9)	21·2 (19·5 to 23·1)	11·2 (10·3 to 12·1)	7·48 (6·87 to 8·14)	5·08 (4·62 to 5·57)	3·1 (2·81 to 3·41)	2·34 (2·1 to 2·6)	2·19 (1·87 to 2·55)	2·08 (1·75 to 2·46)
Saitama	64·8 (55 to 75·5)	40·3 (36 to 44·6)	16·3 (14·6 to 18)	10·4 (9·85 to 11)	6·58 (6·23 to 6·94)	4·68 (4·4 to 4·97)	3·31 (3·12 to 3·52)	2·49 (2·31 to 2·67)	2·05 (1·91 to 2·23)	1·88 (1·65 to 2·14)
Shiga	72·2 (62·1 to 83)	47·1 (43·3 to 51)	19·3 (17·7 to 21·2)	10·3 (9·49 to 11)	6·84 (6·27 to 7·47)	4·91 (4·54 to 5·31)	3·32 (3·03 to 3·64)	2·46 (2·21 to 2·72)	2·2 (1·88 to 2·55)	2·07 (1·74 to 2·46)
Shimane	64·7 (52·7 to 79·2)	45 (39 to 51·5)	21·8 (18·5 to 25·7)	10·8 (9·91 to 11·8)	7·17 (6·55 to 7·87)	5·01 (4·47 to 5·61)	3·37 (3·04 to 3·7)	2·66 (2·39 to 2·97)	2·42 (2·05 to 2·86)	2·25 (1·88 to 2·67)
Shizuoka	59·2 (51·7 to 68)	37·6 (34·6 to 41)	16·7 (15·4 to 18·3)	9·6 (9·04 to 10·2)	6·55 (6·16 to 6·95)	4·34 (4·04 to 4·65)	3·11 (2·87 to 3·37)	2·72 (2·49 to 2·97)	2·55 (2·33 to 2·78)	2·48 (2·18 to 2·83)
Tochigi	70·8 (61·1 to 81·7)	45·8 (42·6 to 49·3)	21·1 (19·4 to 22·9)	11·9 (11·2 to 12·6)	7·64 (7·12 to 8·2)	5·58 (5·16 to 6·04)	3·7 (3·4 to 4·03)	2·96 (2·7 to 3·24)	2·65 (2·28 to 3·1)	2·51 (2·14 to 2·92)
Tokushima	64·4 (53·6 to 78·5)	43·7 (38·6 to 49·3)	21·1 (18·6 to 23·8)	11·3 (10·4 to 12·3)	7·34 (6·62 to 8·07)	5·69 (5·22 to 6·16)	4·55 (4·17 to 4·96)	3·49 (3·05 to 3·93)	3·26 (2·75 to 3·84)	3·06 (2·57 to 3·63)
Tōkyō	49·7 (38·4 to 63)	34 (29 to 39·5)	15·4 (13·7 to 17·1)	9·72 (9·25 to 10·2)	6·51 (6·11 to 6·9)	4·94 (4·63 to 5·27)	3·16 (2·94 to 3·4)	2·21 (2·03 to 2·4)	1·93 (1·79 to 2·08)	1·85 (1·63 to 2·09)
Tottori	73·3 (60·6 to 87·4)	47·5 (42·2 to 53·2)	22·7 (20 to 26)	11·4 (10·4 to 12·5)	6·89 (6·18 to 7·66)	4·44 (3·94 to 5)	3·74 (3·37 to 4·1)	3·12 (2·78 to 3·49)	2·75 (2·34 to 3·24)	2·61 (2·18 to 3·12)
Toyama	63·7 (55·1 to 72·7)	48·8 (45·3 to 52·7)	21·8 (19·9 to 23·9)	11·7 (10·8 to 12·7)	7·71 (6·98 to 8·52)	4·96 (4·5 to 5·45)	3·37 (3·07 to 3·69)	2·38 (2·12 to 2·64)	2·15 (1·83 to 2·53)	2·04 (1·73 to 2·45)
Wakayama	70·3 (59·8 to 81·6)	44·4 (40·7 to 48·5)	19·6 (17·9 to 21·6)	11·8 (11 to 12·7)	6·21 (5·64 to 6·79)	4·28 (3·87 to 4·73)	3·12 (2·82 to 3·41)	2·35 (2·1 to 2·63)	2·4 (2·03 to 2·81)	2·32 (1·94 to 2·76)
Yamagata	67·3 (58·4 to 77·4)	46 (42·7 to 49·6)	22·5 (20·8 to 24·5)	11·2 (10·4 to 11·9)	6·21 (5·66 to 6·8)	4·74 (4·26 to 5·25)	3·8 (3·44 to 4·18)	3·39 (3·07 to 3·72)	2·84 (2·44 to 3·32)	2·67 (2·26 to 3·16)
Yamaguchi	63 (54·8 to 73)	44·7 (41·1 to 48·8)	20·5 (18·7 to 22·4)	11·9 (11·2 to 12·8)	7·32 (6·77 to 7·89)	4·93 (4·48 to 5·43)	3·45 (3·1 to 3·82)	3·12 (2·82 to 3·41)	2·84 (2·46 to 3·29)	2·72 (2·31 to 3·22)
Yamanashi	67·7 (56·6 to 81·1)	40·8 (36·5 to 45·3)	18·3 (16·4 to 20·4)	10·3 (9·46 to 11·2)	6·95 (6·29 to 7·67)	5·01 (4·55 to 5·53)	3·06 (2·76 to 3·34)	2·41 (2·15 to 2·69)	2·22 (1·9 to 2·62)	2·07 (1·74 to 2·47)
South Korea	183 (153 to 218)	98·1 (87·1 to 110)	62·5 (54 to 70·9)	26·7 (22·5 to 31·3)	12·6 (10·7 to 14·7)	7·26 (6·96 to 7·57)	4·47 (4·26 to 4·71)	3·68 (3·42 to 3·96)	3·36 (3·11 to 3·61)	2·68 (2·17 to 3·18)
Singapore	120 (110 to 130)	53·9 (51·5 to 56·1)	33·2 (31 to 35·6)	18·5 (17·1 to 20)	9·65 (8·85 to 10·5)	4·49 (3·99 to 5·03)	3·15 (2·81 to 3·51)	1·83 (1·6 to 2·1)	1·53 (1·33 to 1·75)	1·59 (1·32 to 1·91)
High-income North America	41·8 (40·4 to 43·2)	35·2 (34·7 to 35·7)	26·2 (25·7 to 26·7)	16·5 (16·2 to 16·9)	12 (11·7 to 12·2)	8·95 (8·76 to 9·14)	7·76 (7·61 to 7·92)	6·98 (6·82 to 7·15)	6·65 (6·44 to 6·86)	6·22 (5·66 to 6·88)
Canada	50·2 (48 to 52·5)	34·5 (33·7 to 35·2)	23·8 (23·2 to 24·5)	14 (13·5 to 14·5)	9·13 (8·82 to 9·46)	6·37 (6·11 to 6·64)	5·95 (5·7 to 6·2)	5·18 (4·87 to 5·48)	5·33 (4·91 to 5·78)	4·35 (3·57 to 5·26)
Greenland	172 (153 to 192)	94·7 (88·9 to 101)	58·7 (53·8 to 63·7)	49·5 (43·3 to 57)	35·2 (30·7 to 39·8)	22·1 (19·6 to 24·7)	12·9 (11·1 to 14·8)	10·3 (8·56 to 12·2)	11 (9·24 to 12·9)	10·9 (9·09 to 12·8)
USA	40·9 (39·5 to 42·4)	35·3 (34·8 to 35·8)	26·4 (25·9 to 26·9)	16·8 (16·4 to 17·1)	12·2 (12 to 12·5)	9·16 (8·97 to 9·36)	7·93 (7·77 to 8·1)	7·17 (6·99 to 7·33)	6·78 (6·58 to 6·98)	6·4 (5·82 to 7·05)
Alabama	47·9 (42·4 to 54·7)	42·8 (41·2 to 44·4)	32·2 (30·9 to 33·6)	20·1 (19·1 to 21·3)	15 (14·2 to 15·9)	12·8 (11·9 to 13·7)	11 (10·3 to 11·8)	10·1 (9·52 to 10·7)	9·28 (8·78 to 9·86)	8·99 (8 to 10·2)
Alaska	50·7 (38·6 to 64·2)	51·4 (48·6 to 54·3)	28·5 (26·6 to 30·6)	19·8 (18·5 to 21·3)	13·5 (12·5 to 14·5)	10·1 (9·34 to 11)	8·09 (7·47 to 8·72)	8·46 (7·63 to 9·39)	8·2 (7·25 to 9·27)	8·07 (6·59 to 9·88)
Arizona	51·7 (38·6 to 67·6)	45·4 (42·9 to 48)	27·1 (25·6 to 28·6)	18·5 (17·2 to 19·9)	12·7 (11·8 to 13·6)	9·71 (9·15 to 10·3)	8·14 (7·58 to 8·73)	7·33 (6·89 to 7·76)	7·21 (6·77 to 7·68)	6·72 (5·98 to 7·53)
Arkansas	43·3 (29·9 to 59·1)	38·4 (36·7 to 40·4)	29 (27·6 to 30·5)	16·9 (15·9 to 18)	13·2 (12·4 to 14·2)	11·3 (10·6 to 12·1)	9·82 (9·17 to 10·5)	9·08 (8·42 to 9·73)	9·38 (8·67 to 10·2)	8·89 (7·3 to 10·7)
California	38·9 (32·2 to 46·5)	31·1 (29·8 to 32·5)	24 (23 to 25·1)	15·6 (14·7 to 16·6)	11·7 (11·2 to 12·1)	7·63 (7·28 to 8·01)	6·33 (6·06 to 6·62)	5·61 (5·36 to 5·85)	5·15 (4·92 to 5·43)	4·78 (4·33 to 5·26)
Colorado	44 (38·8 to 50·4)	36·9 (35·3 to 38·4)	26 (24·5 to 27·5)	13·9 (13 to 14·8)	11·2 (10·5 to 12)	8·2 (7·7 to 8·72)	7·56 (7·06 to 8·07)	5·77 (5·4 to 6·21)	5·17 (4·74 to 5·61)	4·76 (4·23 to 5·38)
Connecticut	36·6 (27·2 to 47·6)	28·9 (27·7 to 30·1)	21·3 (20·4 to 22·4)	14·1 (13·4 to 14·9)	9·83 (9·27 to 10·4)	7·55 (7·08 to 7·98)	6·42 (5·98 to 6·9)	5·21 (4·69 to 5·79)	5·15 (4·56 to 5·77)	4·86 (3·96 to 6·02)
Delaware	41·9 (28·3 to 57·8)	31·5 (29·4 to 33·7)	24·6 (23 to 26·2)	17·7 (16·1 to 19·4)	13·3 (12·3 to 14·4)	11·2 (10·2 to 12·2)	9·7 (8·91 to 10·5)	8·5 (7·48 to 9·6)	7·75 (6·77 to 8·97)	7·54 (6·11 to 9·38)
Washington, DC	71·1 (50·1 to 96·9)	57·2 (54 to 60·4)	46·2 (42·7 to 50·3)	42·9 (39·4 to 46·4)	45 (41·8 to 48·3)	21·3 (19·2 to 23·6)	14·3 (11·9 to 17)	8·45 (7·44 to 9·46)	7·63 (6·67 to 8·68)	7·34 (5·86 to 9·14)
Florida	50·6 (42·5 to 59·6)	42·1 (40·8 to 43·4)	30·4 (29·3 to 31·5)	20·3 (19·4 to 21·2)	13·8 (13·3 to 14·4)	10·1 (9·69 to 10·6)	9·01 (8·66 to 9·36)	8·07 (7·7 to 8·44)	7·52 (7·18 to 7·86)	7·1 (6·4 to 7·88)
Georgia	47 (39·6 to 56·1)	44·5 (42·4 to 46·8)	30·7 (29·4 to 32·3)	19·6 (18·4 to 20·7)	15·6 (14·8 to 16·4)	11·3 (10·8 to 11·9)	8·58 (8·05 to 9·15)	8·87 (8·42 to 9·36)	7·94 (7·5 to 8·42)	7·7 (6·89 to 8·59)
Hawaii	36·1 (27·7 to 45·2)	33 (31·4 to 34·9)	23·5 (22·2 to 24·9)	14·5 (13·6 to 15·4)	11 (10·3 to 11·8)	9·94 (9·25 to 10·7)	8·18 (7·53 to 8·75)	7·74 (7·02 to 8·54)	7·17 (6·42 to 8·06)	7·01 (5·77 to 8·54)
Idaho	35·4 (27 to 44·5)	32·8 (31·1 to 34·7)	23·5 (22·2 to 24·9)	14·7 (13·8 to 15·5)	11·2 (10·5 to 11·9)	8·7 (8·12 to 9·26)	6·73 (6·21 to 7·28)	5·42 (4·87 to 6·05)	5·37 (4·82 to 5·98)	4·84 (3·98 to 5·94)
Illinois	39·8 (34·7 to 44·9)	34·2 (33·1 to 35·2)	28·2 (27·4 to 29·2)	19·5 (18·8 to 20·2)	13·8 (13·2 to 14·4)	10·7 (10·2 to 11·2)	8·65 (8·23 to 9·08)	7·2 (6·85 to 7·55)	6·86 (6·46 to 7·28)	6·61 (5·89 to 7·37)
Indiana	34·6 (30·7 to 39·4)	32·1 (31·2 to 33·2)	25·2 (24·2 to 26·1)	15·8 (15 to 16·6)	12·3 (11·7 to 12·9)	10·2 (9·68 to 10·8)	9·29 (8·85 to 9·75)	8·27 (7·77 to 8·81)	8·1 (7·61 to 8·62)	7·6 (6·79 to 8·55)
Iowa	30·9 (22·9 to 39·9)	29·2 (28 to 30·4)	23·3 (22·2 to 24·5)	14·6 (13·7 to 15·5)	9·99 (9·41 to 10·6)	7·83 (7·26 to 8·39)	6·29 (5·88 to 6·72)	6·21 (5·68 to 6·74)	5·84 (5·26 to 6·45)	5·49 (4·48 to 6·67)
Kansas	33·6 (24·8 to 43·4)	30·5 (29·1 to 31·8)	23·3 (22·2 to 24·5)	14·6 (13·7 to 15·4)	10·8 (10·1 to 11·4)	9·43 (8·93 to 10)	8·42 (7·9 to 8·94)	7·39 (6·76 to 8·03)	7·98 (7·33 to 8·64)	7·11 (5·87 to 8·53)
Kentucky	41·3 (28·4 to 56·7)	37·7 (36·1 to 39·3)	26·3 (25 to 27·7)	16·8 (15·9 to 17·8)	11·6 (11 to 12·4)	9·36 (8·78 to 10)	8·84 (8·14 to 9·53)	6·74 (6·29 to 7·22)	7·45 (7·01 to 7·95)	6·98 (5·74 to 8·59)
Louisiana	51 (43·3 to 60·1)	43 (41·2 to 44·9)	31·1 (29·8 to 32·5)	19·8 (18·8 to 20·8)	14·7 (13·9 to 15·5)	12·4 (11·8 to 13·1)	10·3 (9·67 to 11)	9·99 (9·42 to 10·6)	9·36 (8·74 to 9·91)	8·97 (7·93 to 10·1)
Maine	41·2 (31·3 to 52·1)	36·1 (34·4 to 37·7)	25·9 (24·5 to 27·3)	12·8 (11·9 to 13·6)	8·45 (7·82 to 9·05)	6·59 (6·09 to 7·1)	6·82 (6·25 to 7·35)	5·99 (5·33 to 6·68)	6·63 (5·9 to 7·55)	5·89 (4·77 to 7·41)
Maryland	41·4 (36·9 to 47·1)	36·1 (34·7 to 37·5)	23·8 (22·6 to 25)	17·2 (16·2 to 18·4)	11·7 (11·1 to 12·4)	9·49 (8·94 to 10·1)	8·28 (7·79 to 8·81)	7·29 (6·86 to 7·74)	6·93 (6·46 to 7·42)	6·72 (6·02 to 7·59)
Massachusetts	38·3 (26·6 to 51·8)	29·5 (28·5 to 30·6)	21·7 (20·8 to 22·8)	13·4 (12·6 to 14·2)	8·69 (8·21 to 9·2)	6·19 (5·78 to 6·62)	5·66 (5·24 to 6·08)	4·59 (4·25 to 4·96)	4·21 (3·87 to 4·57)	3·86 (3·21 to 4·58)
Michigan	35·3 (30·1 to 41)	31·4 (30·3 to 32·7)	25·7 (24·9 to 26·5)	16·2 (15·5 to 16·9)	12·8 (12·1 to 13·5)	10·2 (9·79 to 10·7)	8·54 (8·09 to 8·98)	7·77 (7·41 to 8·16)	7·5 (7·07 to 7·91)	6·88 (6·21 to 7·68)
Minnesota	33·6 (23·3 to 45·7)	29·9 (28·8 to 30·9)	22·1 (21·1 to 23·3)	13·2 (12·3 to 14·1)	8·74 (8·19 to 9·29)	7·17 (6·71 to 7·65)	6·08 (5·7 to 6·49)	6 (5·58 to 6·43)	5·65 (5·23 to 6·08)	5·34 (4·42 to 6·38)
Mississippi	64·7 (44·4 to 87·9)	55·2 (53·2 to 57·2)	39·6 (37·6 to 41·7)	23·2 (21·9 to 24·6)	16·5 (15·6 to 17·4)	14·7 (13·8 to 15·6)	12·5 (11·7 to 13·3)	11·7 (10·9 to 12·6)	11·3 (10·4 to 12·1)	10·9 (9·15 to 13)
Missouri	39·3 (34·8 to 44·8)	33·2 (31·9 to 34·5)	25·8 (24·7 to 26·9)	16·5 (15·7 to 17·3)	12·2 (11·5 to 12·9)	9·43 (8·88 to 9·98)	8·5 (7·98 to 9·02)	7·9 (7·43 to 8·42)	7·6 (7·08 to 8·12)	7·41 (6·53 to 8·3)
Montana	38·9 (29·7 to 49·4)	35·4 (33·6 to 37·2)	27·3 (25·8 to 28·9)	15·3 (14·3 to 16·4)	10·9 (10 to 11·7)	8·68 (8·02 to 9·35)	7·7 (7·09 to 8·32)	6·35 (5·64 to 7·14)	6·29 (5·55 to 7·17)	5·74 (4·65 to 7·1)
Nebraska	36·1 (27·1 to 46·3)	31·2 (29·7 to 32·8)	23·5 (22·3 to 24·9)	14·7 (13·8 to 15·6)	9·81 (9·22 to 10·5)	8·78 (8·18 to 9·41)	6·69 (6·21 to 7·22)	6·5 (5·84 to 7·24)	6·52 (5·85 to 7·29)	5·98 (4·81 to 7·36)
Nevada	50·2 (38·3 to 63·2)	44·6 (41·6 to 47·4)	29 (27·5 to 30·6)	15·9 (14·8 to 17·1)	10·7 (10·1 to 11·4)	8·38 (7·9 to 8·88)	7·32 (6·83 to 7·82)	7·14 (6·56 to 7·83)	6·44 (5·86 to 7·1)	6·2 (5·13 to 7·52)
New Hampshire	36·8 (27·4 to 47·5)	32·1 (30·4 to 33·9)	22·5 (21·1 to 24)	13·1 (12·1 to 14·1)	8·89 (8·28 to 9·49)	6·23 (5·78 to 6·73)	5·69 (5·22 to 6·18)	4·67 (4·2 to 5·24)	4·88 (4·37 to 5·51)	4·43 (3·61 to 5·48)
New Jersey	40·2 (34·1 to 47·5)	32·6 (31·3 to 34)	25·1 (24·2 to 26·1)	15·8 (14·9 to 16·6)	11·5 (10·9 to 12·1)	7·94 (7·5 to 8·39)	6·15 (5·79 to 6·54)	4·96 (4·64 to 5·29)	4·63 (4·33 to 4·94)	4·22 (3·75 to 4·7)
New Mexico	56·7 (39·2 to 76·4)	45·9 (43·6 to 48·6)	30 (28·4 to 31·7)	17·3 (16·3 to 18·4)	12·2 (11·5 to 12·9)	8·79 (8·21 to 9·39)	7·76 (7·21 to 8·39)	7·4 (6·63 to 8·23)	7·08 (6·29 to 7·93)	6·78 (5·5 to 8·29)

New York	40·9 (34·5 to 47·5)	33·5 (32·5 to 34·4)	26 (25·2 to 26·8)	16·2 (15·6 to 16·9)	13 (12·5 to 13·5)	8·44 (8·09 to 8·8)	6·62 (6·33 to 6·94)	5·35 (5·08 to 5·63)	4·73 (4·51 to 4·98)	4·4 (3·94 to 4·85)
North Carolina	47·2 (40·8 to 54·7)	42 (40·7 to 43·4)	31·4 (30·2 to 32·6)	18·5 (17·6 to 19·4)	13·7 (13·1 to 14·3)	11·2 (10·6 to 11·8)	9·02 (8·57 to 9·49)	8·74 (8·29 to 9·22)	8·69 (8·19 to 9·18)	8·47 (7·6 to 9·48)
North Dakota	38·1 (26·2 to 52·2)	33·2 (31·1 to 35·3)	21·3 (19·5 to 23)	15·6 (14·2 to 17·1)	10·6 (9·54 to 11·7)	9·18 (8·36 to 10)	7·98 (7·15 to 8·85)	7·49 (6·66 to 8·43)	6·96 (6·1 to 7·92)	6·69 (5·52 to 8·11)
Ohio	34·3 (29·7 to 38·9)	31·2 (30·3 to 32·1)	24·1 (23·3 to 25)	16·4 (15·8 to 17·2)	11·9 (11·4 to 12·4)	9·73 (9·33 to 10·1)	9·65 (9·22 to 10·1)	8·67 (8·26 to 9·07)	8·05 (7·7 to 8·44)	7·7 (6·89 to 8·54)
Oklahoma	35·8 (31·5 to 40·8)	36·1 (34·6 to 37·6)	28 (26·6 to 29·5)	16·9 (15·8 to 17·9)	12·1 (11·5 to 12·9)	10·9 (10·2 to 11·6)	10·1 (9·46 to 10·7)	9·11 (8·55 to 9·73)	7·89 (7·32 to 8·52)	7·76 (6·79 to 8·78)
Oregon	34·1 (25·2 to 44)	32·2 (30·8 to 33·6)	21·8 (20·7 to 23·1)	14·8 (14 to 15·6)	10·2 (9·61 to 10·9)	7·26 (6·83 to 7·69)	6·1 (5·73 to 6·48)	5·82 (5·35 to 6·31)	5·36 (4·82 to 5·97)	4·93 (4·03 to 5·95)
Pennsylvania	38·4 (33·1 to 43·6)	32·1 (31·2 to 33)	25·7 (24·9 to 26·6)	16·5 (15·8 to 17·2)	11·9 (11·4 to 12·4)	8·86 (8·43 to 9·3)	8·61 (8·19 to 9·04)	7·26 (6·92 to 7·61)	6·98 (6·62 to 7·36)	6·68 (5·96 to 7·46)
Rhode Island	38·7 (29·6 to 48·4)	32·3 (30·4 to 34·3)	24·5 (22·9 to 26)	15 (13·9 to 16·1)	10·4 (9·54 to 11·4)	8·12 (7·5 to 8·83)	7·77 (7·07 to 8·47)	6·57 (5·87 to 7·32)	5·72 (4·97 to 6·58)	5·35 (4·34 to 6·64)
South Carolina	47·4 (41·4 to 54·6)	45·7 (43·8 to 47·7)	30·5 (28·6 to 32·4)	21·2 (20 to 22·3)	15·6 (14·7 to 16·5)	11·5 (10·9 to 12·2)	9·39 (8·76 to 10)	9·02 (8·51 to 9·58)	8·18 (7·68 to 8·71)	7·87 (6·98 to 8·73)
South Dakota	38·6 (29·3 to 48·9)	36·6 (34·8 to 38·6)	26·5 (25 to 28)	16·2 (15·1 to 17·3)	12·9 (12 to 13·9)	9·84 (9·12 to 10·6)	8·8 (8·13 to 9·53)	8·6 (7·79 to 9·49)	8·67 (7·7 to 9·77)	8·16 (6·66 to 10)
Tennessee	44·9 (39·8 to 50·9)	39·3 (38 to 40·7)	28·4 (27·1 to 29·5)	17·6 (16·8 to 18·5)	13·6 (12·8 to 14·3)	11·4 (10·7 to 12·1)	9·93 (9·33 to 10·6)	9·12 (8·57 to 9·74)	8·37 (7·86 to 8·9)	7·94 (7·14 to 8·84)
Texas	44·4 (37·7 to 51)	40·1 (39 to 41·3)	29·5 (28·6 to 30·5)	17·3 (16·6 to 18)	11·3 (10·9 to 11·7)	8·13 (7·82 to 8·47)	7·79 (7·51 to 8·08)	7·33 (7·05 to 7·62)	7·02 (6·76 to 7·32)	6·63 (5·96 to 7·31)
Utah	32 (23·7 to 41·4)	27·3 (26 to 28·7)	20·9 (19·7 to 22·1)	14·4 (13·6 to 15·2)	9·62 (9·06 to 10·3)	7·31 (6·87 to 7·82)	6·71 (6·33 to 7·09)	7·23 (6·76 to 7·69)	6·83 (6·22 to 7·46)	6·31 (5·25 to 7·61)
Vermont	38·3 (28·4 to 50)	34·4 (32·1 to 36·7)	21·9 (20·5 to 23·4)	13 (12 to 14·2)	8·37 (7·54 to 9·33)	6·72 (6·13 to 7·33)	5·5 (4·93 to 6·13)	4·9 (4·34 to 5·53)	4·39 (3·84 to 4·99)	4·21 (3·41 to 5·18)
Virginia	44·5 (37·4 to 52·3)	39·6 (38·2 to 41)	27 (26 to 28·1)	17·3 (16·5 to 18·2)	12·3 (11·7 to 13)	9·01 (8·47 to 9·53)	8·4 (8·03 to 8·84)	7·09 (6·72 to 7·45)	7·01 (6·54 to 7·51)	6·7 (6·04 to 7·46)
Washington	33·5 (29·7 to 38)	32·2 (31 to 33·4)	23·9 (22·8 to 25·2)	14·8 (14·1 to 15·7)	9·68 (9·11 to 10·2)	6·7 (6·31 to 7·12)	5·71 (5·34 to 6·07)	5·39 (5·03 to 5·75)	5·25 (4·87 to 5·64)	4·63 (4·16 to 5·13)
West Virginia	42·1 (31·1 to 54·4)	35·9 (34·3 to 37·5)	29·7 (28·3 to 31)	16·6 (15·7 to 17·5)	12·8 (12 to 13·7)	10·2 (9·48 to 11)	9·96 (9·21 to 10·8)	8·71 (7·81 to 9·68)	9·33 (8·33 to 10·4)	8·66 (7 to 10·5)
Wisconsin	36·4 (32·1 to 41·7)	30·6 (29·5 to 31·7)	21·2 (20·2 to 22·2)	13·2 (12·4 to 13·9)	9·93 (9·4 to 10·5)	8·2 (7·73 to 8·68)	7·2 (6·76 to 7·66)	7·07 (6·57 to 7·57)	6·77 (6·32 to 7·22)	6·41 (5·75 to 7·13)
Wyoming	42·5 (31·1 to 55·7)	38·8 (36·4 to 41·3)	27·5 (25·7 to 29·4)	15·3 (14·2 to 16·5)	11·5 (10·4 to 12·6)	8·63 (7·85 to 9·49)	7·41 (6·71 to 8·16)	6·72 (5·86 to 7·65)	6·46 (5·58 to 7·38)	6·35 (5·08 to 7·83)
Southern Latin America	102 (98·2 to 106)	111 (109 to 113)	81·5 (80·1 to 83)	41·7 (40·9 to 42·5)	28 (27·4 to 28·6)	19·1 (18·5 to 19·7)	14·3 (13·9 to 14·7)	11 (10·6 to 11·4)	10·3 (8·97 to 11·6)	9·29 (7·45 to 11·5)
Argentina	82·9 (79·3 to 87)	83·1 (81·3 to 84·9)	74·4 (72·8 to 76·1)	42·1 (41·1 to 43)	31·4 (30·6 to 32·2)	21·3 (20·5 to 22·1)	15·9 (15·5 to 16·4)	12·2 (11·7 to 12·6)	11·7 (10·1 to 13·4)	10·6 (8·34 to 13·2)
Chile	153 (146 to 161)	171 (168 to 174)	101 (98·6 to 104)	39·9 (38·6 to 41·2)	20·6 (19·9 to 21·3)	12·7 (12·2 to 13·3)	9·82 (9·41 to 10·2)	7·89 (7·34 to 8·47)	6·73 (6·1 to 7·42)	6·22 (5·32 to 7·19)
Uruguay	62·5 (56·8 to 68·4)	64 (61·2 to 67·1)	63·5 (60·6 to 66·3)	45 (43 to 46·8)	25·8 (24·6 to 27)	18·6 (17·3 to 20)	13·1 (11·2 to 15·2)	8·77 (7·96 to 9·58)	8·25 (6·86 to 9·7)	7·57 (5·94 to 9·53)
Western Europe	61·6 (59·2 to 64·1)	44·2 (43·5 to 44·7)	29·4 (28·9 to 29·8)	16·6 (16·3 to 16·8)	10·3 (10·1 to 10·5)	6·33 (6·18 to 6·48)	4·63 (4·51 to 4·74)	4·07 (3·89 to 4·23)	3·88 (3·68 to 4·07)	3·8 (3·47 to 4·15)
Andorra	25 (16·6 to 36)	25 (19·4 to 31·6)	17 (13·3 to 21·4)	12·1 (9·92 to 14·7)	7·21 (5·96 to 8·71)	3·75 (2·99 to 4·6)	2·9 (2·43 to 3·44)	1·87 (1·53 to 2·26)	1·78 (1·45 to 2·15)	1·06 (0·672 to 1·44)
Austria	62 (57·9 to 65·9)	48·9 (47·4 to 50·4)	32·8 (31·7 to 34)	19·3 (18·5 to 20·3)	10·5 (9·92 to 11)	6·2 (5·75 to 6·67)	4·95 (4·57 to 5·35)	3·92 (3·62 to 4·27)	3·85 (3·53 to 4·18)	3·37 (2·93 to 3·85)
Belgium	68 (64·5 to 72)	38·5 (37·3 to 39·7)	27·7 (26·9 to 28·6)	16·4 (15·7 to 17·1)	11 (10·5 to 11·6)	6·68 (6·28 to 7·1)	4·94 (4·64 to 5·26)	4·62 (4·29 to 4·97)	4·12 (3·7 to 4·61)	4·06 (3·31 to 4·95)
Cyprus	144 (114 to 178)	105 (90·6 to 121)	64·5 (57·5 to 71·9)	31·1 (29·2 to 33)	15·1 (14 to 16·3)	7·29 (6·67 to 8·03)	3·61 (3·27 to 3·98)	2·56 (2·22 to 2·97)	2·48 (2·1 to 2·93)	2·46 (2·04 to 2·94)
Denmark	38·6 (35·3 to 42·3)	29·1 (27·9 to 30·3)	19·5 (18·5 to 20·4)	11·5 (10·8 to 12·2)	10·3 (9·67 to 11·1)	6·16 (5·78 to 6·58)	4·23 (3·92 to 4·58)	3·89 (3·52 to 4·31)	3·94 (3·59 to 4·33)	4·04 (3·5 to 4·62)
Finland	55·1 (50·6 to 59·4)	29·3 (28·1 to 30·4)	18·7 (17·8 to 19·6)	9·89 (9·29 to 10·5)	7·39 (6·9 to 7·92)	4·69 (4·32 to 5·05)	3·19 (2·91 to 3·49)	2·67 (2·37 to 2·98)	2·54 (2·26 to 2·84)	2·46 (2·11 to 2·88)
France	61·1 (58·1 to 64)	29·9 (29·2 to 30·5)	19·8 (19·3 to 20·2)	13·9 (13·6 to 14·4)	10 (9·73 to 10·3)	6·09 (5·89 to 6·3)	4·68 (4·52 to 4·85)	4·7 (4·51 to 4·9)	4·83 (4·54 to 5·13)	4·44 (3·95 to 4·96)
Germany	58·4 (49·5 to 68·1)	45·8 (44·8 to 46·7)	29·4 (28·7 to 30·1)	17·1 (16·6 to 17·5)	9·74 (9·45 to 10)	5·9 (5·69 to 6·13)	4·47 (4·26 to 4·68)	3·92 (3·73 to 4·12)	3·77 (3·59 to 3·95)	3·79 (3·49 to 4·1)
Greece	58·7 (54·8 to 63·2)	44·6 (43·1 to 46)	32·8 (31·6 to 33·9)	21·3 (20·5 to 22·2)	11 (10·4 to 11·7)	6·76 (6·34 to 7·17)	4·66 (4·37 to 4·96)	4·68 (4·23 to 5·13)	4·31 (3·85 to 4·79)	4·26 (3·64 to 4·96)
Iceland	33·5 (28·8 to 39·2)	23·2 (21·3 to 25)	17·2 (15·7 to 18·8)	10·4 (9·4 to 11·5)	6·81 (6·06 to 7·59)	4·15 (3·58 to 4·79)	2·78 (2·43 to 3·22)	2·2 (1·83 to 2·63)	2·59 (2·13 to 3·1)	2·57 (2·05 to 3·18)
Ireland	54·7 (50·1 to 59)	36 (34·5 to 37·6)	23·7 (22·7 to 24·8)	14·9 (14·1 to 15·6)	10·4 (9·72 to 11)	7·84 (7·25 to 8·41)	4·77 (4·39 to 5·19)	3·84 (3·41 to 4·3)	3·43 (3·09 to 3·79)	3·5 (2·99 to 4·06)
Israel	58·4 (53·6 to 63·4)	38·5 (36·9 to 40·3)	27·9 (26·8 to 29·1)	19·5 (18·7 to 20·4)	12·2 (11·6 to 12·7)	7·17 (6·8 to 7·55)	4·74 (4·46 to 5·03)	3·91 (3·64 to 4·22)	3·16 (2·88 to 3·43)	2·48 (2·14 to 2·86)
Italy	68·5 (65·8 to 71·3)	56·7 (55·6 to 57·9)	37·2 (36·4 to 37·9)	18·4 (17·9 to 18·9)	10·5 (10·1 to 10·8)	5·89 (5·66 to 6·13)	4·22 (4·04 to 4·42)	3·12 (2·93 to 3·3)	3·21 (2·91 to 3·55)	3·15 (2·73 to 3·61)
Luxembourg	71·6 (62·9 to 80·6)	42 (39·3 to 45·1)	26·6 (24·6 to 28·7)	14·3 (13 to 15·6)	9·9 (9·02 to 10·9)	4·63 (4·15 to 5·16)	3·25 (2·85 to 3·71)	3·04 (2·58 to 3·58)	3·94 (3·31 to 4·66)	3·72 (3 to 4·55)
Malta	77·9 (69·5 to 86·8)	44·9 (42·3 to 47·7)	29·8 (27·5 to 32·2)	17·5 (16·1 to 19·1)	12·1 (10·9 to 13·3)	8·36 (7·5 to 9·29)	7·09 (6·34 to 7·92)	6·93 (5·8 to 8·25)	5·78 (4·64 to 7·01)	5·65 (4·4 to 7·18)
Monaco	79·7 (60·5 to 104)	44·3 (35·3 to 55·5)	21·9 (16·7 to 27·7)	12·9 (9·67 to 17·1)	7·17 (5·51 to 9·43)	4·65 (3·59 to 6)	3·19 (2·43 to 4·02)	2·39 (1·83 to 3·06)	3·87 (3·71 to 4·08)	3·86 (3·72 to 4·05)
Netherlands	30·7 (28·5 to 33)	23·7 (23 to 24·5)	17·8 (17·3 to 18·4)	12 (11·5 to 12·5)	9·88 (9·46 to 10·3)	6·93 (6·57 to 7·26)	4·85 (4·57 to 5·15)	4·54 (4·24 to 4·87)	4·55 (4·26 to 4·86)	4·17 (3·73 to 4·65)
Norway	32·7 (29·7 to 36·1)	24·9 (23·8 to 26·2)	17·3 (16·5 to 18·2)	10·9 (10·3 to 11·6)	9·46 (8·89 to 10)	5·18 (4·79 to 5·58)	3·43 (3·14 to 3·76)	2·6 (2·32 to 2·9)	2·24 (2 to 2·5)	2·25 (1·92 to 2·63)
Agder	36·6 (30·1 to 43·5)	30·2 (28·1 to 32·5)	20·2 (18·4 to 21·9)	13 (11·7 to 14·2)	11·1 (9·98 to 12·3)	5·83 (5·31 to 6·33)	3·76 (3·41 to 4·15)	2·83 (2·47 to 3·26)	2·34 (2·03 to 2·71)	2·52 (2·03 to 3·05)
Innlandet	32·1 (27·8 to 36·8)	24·7 (22·9 to 26·5)	17·4 (16·2 to 18·6)	11·2 (10·2 to 12·2)	9·63 (8·61 to 10·7)	5·55 (5·01 to 6·08)	3·82 (3·45 to 4·21)	2·97 (2·59 to 3·38)	2·52 (2·19 to 2·89)	2·5 (2·09 to 2·97)
Møre og Romsdal	37·5 (31·2 to 44·4)	25·9 (23·9 to 28)	19·2 (17·6 to 20·8)	11·9 (10·6 to 13·2)	9·27 (8·21 to 10·5)	5·09 (4·62 to 5·55)	3·47 (3·07 to 3·89)	2·88 (2·52 to 3·28)	2·45 (2·13 to 2·82)	2·64 (2·16 to 3·2)
Nordland	38·6 (33·4 to 44·7)	30·3 (28·1 to 32·4)	18·8 (17·5 to 20·3)	11·4 (10·3 to 12·5)	8·95 (8·03 to 9·95)	5·01 (4·53 to 5·49)	4 (3·56 to 4·48)	3·18 (2·77 to 3·65)	2·67 (2·33 to 3·05)	2·9 (2·35 to 3·53)
Oslo	20·3 (15·4 to 26·2)	18·2 (16·6 to 19·9)	14·9 (13·4 to 16·6)	11 (9·79 to 12·3)	11 (9·96 to 12·1)	5·89 (5·17 to 6·64)	3·17 (2·8 to 3·58)	2·14 (1·87 to 2·44)	1·84 (1·63 to 2·07)	1·75 (1·48 to 2·08)
Rogaland	35·2 (29 to 42·5)	26·9 (24·6 to 29·3)	18·7 (17·1 to 20·3)	11·9 (10·7 to 13·1)	9·15 (8·29 to 10·1)	5·02 (4·62 to 5·47)	3·35 (3 to 3·74)	2·5 (2·2 to 2·86)	2·18 (1·91 to 2·5)	2·16 (1·81 to 2·56)
Troms og Finnmark	50·7 (44·2 to 58·3)	34·4 (32·2 to 36·9)	21·4 (19·6 to 23·2)	12·5 (11·3 to 13·8)	10·7 (9·65 to 11·8)	5·74 (5·3 to 6·18)	3·87 (3·5 to 4·28)	2·98 (2·56 to 3·45)	2·6 (2·26 to 3·01)	2·79 (2·24 to 3·43)
Trøndelag	31·4 (26·5 to 36·7)	22·3 (20·6 to 24·2)	16·1 (14·8 to 17·5)	10·3 (9·35 to 11·3)	8·3 (7·45 to 9·16)	4·48 (4·12 to 4·86)	3·1 (2·8 to 3·42)	2·41 (2·1 to 2·77)	2·08 (1·82 to 2·38)	2·03 (1·7 to 2·39)
Vestfold og Telemark	29·4 (25·6 to 33·3)	24 (22·3 to 25·6)	16·6 (15·3 to 18)	10·3 (9·36 to 11·2)	8·46 (7·62 to 9·29)	5·3 (4·79 to 5·82)	3·53 (3·19 to 3·93)	2·58 (2·24 to 2·95)	2·28 (2 to 2·59)	2·24 (1·89 to 2·64)
Vestland	32·1 (27 to 37·8)	23 (21·5 to 24·5)	15·9 (14·7 to 17·2)	9·87 (9·03 to 10·7)	8·68 (7·86 to 9·46)	4·73 (4·35 to 5·11)	3·42 (3·11 to 3·75)	2·76 (2·4 to 3·16)	2·36 (2·1 to 2·66)	2·55 (2·09 to 3·04)
Viken	29·5 (25·2 to 34·1)	23·6 (22 to 25·4)	16·4 (15·1 to 17·6)	10·1 (9·26 to 11)	9·47 (8·72 to 10·3)	4·98 (4·38 to 5·65)	3·36 (2·98 to 3·76)	2·58 (2·28 to 2·9)	2·26 (2·03 to 2·51)	2·17 (1·86 to 2·51)
Portugal	157 (148 to 167)	111 (108 to 114)	80·8 (78·2 to 83·4)	31·5 (30·4 to 32·7)	16·1 (15·3 to 17)	8·24 (7·75 to 8·74)	4·23 (3·93 to 4·55)	4·07 (3·74 to 4·44)	3·42 (3·11 to 3·74)	3·24 (2·78 to 3·73)
San Marino	24·8 (16·9 to 35·4)	20·7 (16·5 to 25·8)	16·7 (13·9 to 20·2)	12·4 (10·3 to 15·2)	8·8 (7·22 to 10·6)	5·06 (3·97 to 6·31)	3·02 (2·48 to 3·65)	2·51 (1·92 to 3·25)	2·24 (1·64 to 3·03)	1·58 (1·07 to 2·23)
Spain	99·2 (94 to 104)	58·6 (57·2 to 60·2)	35·4 (34·4 to 36·4)	16·2 (15·8 to 16·7)	10·3 (9·96 to 10·8)	5·86 (5·59 to 6·13)	4·2 (4·02 to 4·39)	3·64 (3·46 to 3·85)	3·39 (3·19 to 3·62)	3·26 (2·93 to 3·61)
Sweden	24·4 (22·5 to 26·5)	22·1 (21·1 to 23·2)	14·5 (13·8 to 15·2)	8·75 (8·26 to 9·25)	7·43 (7 to 7·84)	4·25 (3·97 to 4·54)	3·23 (3·01 to 3·47)	2·72 (2·48 to 2·98)	2·84 (2·61 to 3·09)	2·43 (2·13 to 2·75)
Stockholm	19·9 (16·1 to 24·9)	19·6 (16·9 to 22·6)	14·1 (12·3 to 16)	9·14 (8·19 to 10·1)	7·21 (6·57 to 7·93)	4·09 (3·67 to 4·52)	2·86 (2·51 to 3·24)	2·38 (2·06 to 2·76)	2·48 (2·14 to 2·85)	2·03 (1·67 to 2·46)
Sweden except Stockholm	25·2 (22·8 to 27·7)	22·6 (21·4 to 23·7)	14·5 (13·9 to 15·3)	8·65 (8·18 to 9·13)	7·49 (7·05 to 7·93)	4·29 (4·01 to 4·6)	3·36 (3·13 to 3·59)	2·83 (2·58 to 3·11)	2·96 (2·72 to 3·21)	2·56 (2·25 to 2·9)
Switzerland	42·7 (39·4 to 46·2)	31 (29·8 to 32·1)	22·2 (21·3 to 23·2)	12·8 (12 to 13·5)	9·77 (9·21 to 10·3)	6·79 (6·34 to 7·25)	4·68 (4·34 to 5·07)	3·92 (3·59 to 4·3)	4·27 (3·92 to 4·65)	3·99 (3·48 to 4·56)
UK	30 (28·7 to 31·4)	29·9 (29·2 to 30·5)	23·7 (23·2 to 24·3)	16·4 (15·9 to 16·9)	10·4 (10 to 10·8)	7·29 (6·79 to 7·77)	5·59 (5·39 to 5·81)	4·66 (4·45 to 4·88)	4·07 (3·77 to 4·37)	4·54 (4·11 to 5·03)
England	28·4 (26·7 to 30·1)	29 (28·1 to 29·8)	23·2 (22·6 to 23·9)	16·1 (15·6 to 16·7)	10·4 (10 to 10·8)	7·33 (6·85 to 7·81)	5·64 (5·44 to 5·86)	4·67 (4·46 to 4·87)	4·07 (3·78 to 4·37)	4·56 (4·13 to 5·03)
East Midlands	28·4 (23·4 to 34·3)	28·6 (26·3 to 30·9)	23·1 (21 to 25·6)	15·6 (14·5 to 16·8)	10·4 (9·85 to 10·8)	7·15 (6·57 to 7·8)	5·64 (5·27 to 6·07)	4·86 (4·58 to 5·18)	4·22 (3·8 to 4·69)	4·67 (4·18 to 5·22)
Derby	38·3 (30·5 to 47·9)	34·5 (29·5 to 40·3)	28·3 (25 to 32·1)	19·8 (17·3 to 22·6)	12·1 (10·9 to 13·3)	8·71 (7·71 to 9·82)	7·35 (6·47 to 8·29)	6·44 (5·8 to 7·15)	5·86 (5·12 to 6·69)	6·99 (6·16 to 7·97)

Derbyshire	27·7 (21·8 to 35·1)	26·2 (23·3 to 29·1)	21 (18·4 to 23·7)	14 (12·9 to 15·1)	8·68 (7·96 to 9·49)	6 (5·43 to 6·59)	4·64 (4·33 to 5·01)	4·21 (3·88 to 4·55)	3·81 (3·41 to 4·26)	4·28 (3·79 to 4·8)
Leicester	35·8 (28·1 to 45·2)	38·3 (33·1 to 44·1)	33 (29·4 to 36·9)	21·7 (19·2 to 24·1)	13·3 (12·3 to 14·4)	10 (8·82 to 11·3)	8·03 (7·11 to 9·06)	6·91 (6·26 to 7·59)	6·23 (5·36 to 7·23)	7·01 (6·09 to 8·03)
Leicestershire	21·8 (17·7 to 26·5)	24·1 (21·8 to 26·5)	19 (17·2 to 21·3)	13 (11·5 to 14·4)	9·19 (8·51 to 9·95)	5·85 (5·29 to 6·47)	4·76 (4·43 to 5·13)	4·25 (3·91 to 4·62)	3·71 (3·32 to 4·08)	4·22 (3·79 to 4·67)
Lincolnshire	28·1 (22 to 35·6)	28 (24·6 to 31·5)	22·2 (19·9 to 24·8)	14·8 (13·5 to 16·1)	10·2 (9·33 to 11·2)	7·02 (6·29 to 7·81)	5·36 (4·89 to 5·88)	3·79 (3·47 to 4·11)	3·59 (3·19 to 4·02)	4 (3·55 to 4·49)
Northamptonshire	30 (23·7 to 37·8)	29·4 (26·4 to 32·6)	23·3 (20·5 to 26·4)	15·6 (13·6 to 17·6)	11·6 (10·6 to 12·7)	7·12 (6·44 to 7·86)	5·61 (5·24 to 5·97)	4·88 (4·49 to 5·31)	3·31 (2·98 to 3·71)	3·34 (2·92 to 3·78)
Nottingham	30 (22·8 to 38·8)	32·4 (27·6 to 37·8)	27·2 (24·2 to 30·5)	19·7 (16·9 to 22·8)	12·9 (11·9 to 14·1)	10·6 (9·51 to 11·9)	7·35 (6·77 to 7·99)	6·57 (5·93 to 7·23)	5·74 (4·99 to 6·59)	6·19 (5·36 to 7·11)
Nottinghamshire	26·1 (20·5 to 32·8)	26·1 (23·5 to 28·9)	21·1 (18·5 to 23·8)	14·2 (13·2 to 15·2)	8·8 (8·14 to 9·55)	6·21 (5·65 to 6·81)	4·85 (4·48 to 5·27)	4·38 (4·04 to 4·75)	4·13 (3·69 to 4·62)	4·59 (4·12 to 5·14)
Rutland	13·8 (10·7 to 17·9)	16·5 (13 to 20·6)	13·2 (11·7 to 14·7)	10·9 (9·41 to 12·5)	7·22 (6·04 to 8·48)	4·61 (3·75 to 5·63)	3·49 (2·77 to 4·35)	2·39 (2·08 to 2·73)	1·66 (1·43 to 1·9)	1·68 (1·5 to 1·86)
East of England	24·7 (21·6 to 28·1)	25·3 (23·2 to 27·5)	20·3 (18·4 to 22·2)	13·9 (12·8 to 15)	8·22 (7·86 to 8·63)	5·94 (5·47 to 6·45)	4·9 (4·6 to 5·19)	3·94 (3·72 to 4·18)	3·41 (3·07 to 3·79)	3·78 (3·37 to 4·24)
Bedford	30·6 (24·9 to 37·6)	29 (25·8 to 32·8)	23·6 (20·9 to 26·4)	16·5 (14·3 to 18·7)	10·7 (9·4 to 12·1)	7·55 (6·65 to 8·53)	6·38 (5·68 to 7·11)	5·4 (4·89 to 5·98)	4·64 (4·1 to 5·2)	4·67 (4·1 to 5·26)
Cambridgeshire	14·7 (11·9 to 17·9)	21·6 (18·9 to 24·6)	17·1 (15·2 to 19·2)	12 (10·6 to 13·3)	7·35 (6·74 to 7·98)	5·91 (5·34 to 6·56)	4·83 (4·5 to 5·15)	3·83 (3·52 to 4·14)	3·26 (2·92 to 3·62)	3·63 (3·28 to 4·04)
Central Bedfordshire	24·1 (19·7 to 29·1)	24·4 (20·9 to 28·2)	19·4 (17 to 21·9)	13·7 (12 to 15·4)	8·23 (7·48 to 9·03)	5·8 (5·14 to 6·53)	4·51 (4·11 to 4·93)	3·75 (3·41 to 4·11)	3·37 (2·97 to 3·78)	3·81 (3·41 to 4·23)
Essex	24 (20 to 28·3)	23·7 (20·9 to 26·9)	18·8 (16·4 to 21·5)	12·9 (11·5 to 14·4)	7·77 (7·3 to 8·28)	5·34 (4·82 to 5·94)	4·6 (4·21 to 5·01)	3·64 (3·39 to 3·92)	3·04 (2·64 to 3·48)	3·35 (2·8 to 3·98)
Hertfordshire	23·3 (18·8 to 28·3)	22·6 (19·6 to 25·8)	18·5 (16·4 to 20·9)	13·1 (11·5 to 14·9)	7·46 (6·9 to 8·06)	5·23 (4·73 to 5·76)	4·12 (3·77 to 4·47)	3·42 (3·17 to 3·69)	3·16 (2·72 to 3·67)	3·53 (2·96 to 4·15)
Luton	35·7 (30·2 to 42·2)	34·3 (29·8 to 39·3)	28·2 (25·3 to 31·2)	19·1 (16·7 to 21·6)	11·7 (10·5 to 12·9)	10·4 (9·19 to 11·8)	8·09 (7·22 to 9·05)	6·44 (5·76 to 7·18)	5·64 (4·86 to 6·48)	6·04 (5·35 to 6·76)
Norfolk	27·9 (22·5 to 34·5)	27·9 (24·3 to 31·4)	22 (19·5 to 24·6)	15 (13·8 to 16·3)	8·65 (8·04 to 9·38)	6·08 (5·41 to 6·81)	4·95 (4·52 to 5·41)	3·8 (3·5 to 4·15)	3·37 (2·97 to 3·77)	3·8 (3·36 to 4·22)
Peterborough	29·7 (22·6 to 37·7)	28·2 (23·6 to 33)	23·7 (20·8 to 26·6)	15·6 (13·9 to 17·7)	10 (9·02 to 11·1)	8·83 (7·67 to 10·1)	6·95 (6·2 to 7·72)	5·65 (5·06 to 6·23)	4·5 (3·95 to 5·1)	4·88 (4·3 to 5·47)
Southend-on-Sea	36·9 (30·1 to 45)	31·3 (27·3 to 35·8)	25·4 (22·7 to 28·3)	16 (14 to 18·2)	8·97 (7·97 to 9·97)	5·95 (5·16 to 6·73)	4·71 (4·19 to 5·2)	3·9 (3·45 to 4·44)	3·31 (2·86 to 3·85)	3·69 (3·22 to 4·21)
Suffolk	25·4 (21·6 to 29·8)	26·1 (22·6 to 29·8)	20·5 (18·1 to 23·1)	13·6 (12 to 15·4)	8·01 (7·41 to 8·63)	5·51 (4·91 to 6·13)	4·74 (4·39 to 5·12)	3·76 (3·44 to 4·11)	3·25 (2·9 to 3·63)	3·71 (3·32 to 4·12)
Thurrock	23·6 (19 to 28·9)	27·5 (24·2 to 31·6)	22·6 (20·1 to 25·2)	15 (12·8 to 17·3)	8·49 (7·63 to 9·46)	5·82 (5·06 to 6·66)	4·56 (4·13 to 5)	4·06 (3·74 to 4·42)	3·28 (2·87 to 3·74)	3·67 (3·25 to 4·12)
Greater London	31·8 (28·7 to 34·9)	32·6 (31·3 to 34)	25·9 (24·7 to 27)	18·2 (17·2 to 19·2)	11·4 (11 to 11·9)	8·4 (7·77 to 9·05)	6·03 (5·73 to 6·36)	4·43 (4·22 to 4·66)	3·89 (3·59 to 4·18)	4·34 (3·89 to 4·85)
Barking and Dagenham	55 (45·9 to 64·8)	45·1 (39·1 to 51·1)	36·2 (33·8 to 38·9)	23·1 (20·4 to 26·2)	12·7 (11·8 to 13·5)	8·14 (7·13 to 9·17)	6·58 (5·92 to 7·31)	5·28 (4·9 to 5·68)	4·44 (4·09 to 4·8)	4·83 (4·29 to 5·39)
Barnet	25·9 (21·7 to 31)	25·9 (23·9 to 28·2)	20·2 (18·7 to 21·8)	13·8 (12·6 to 15)	7·8 (7·29 to 8·37)	5·59 (5·06 to 6·21)	4·25 (3·93 to 4·59)	3·15 (2·9 to 3·44)	2·81 (2·52 to 3·12)	3·29 (2·93 to 3·65)
Bexley	27·4 (23·3 to 32·2)	23·2 (21·6 to 25)	17·6 (16·3 to 18·9)	12 (10·9 to 13·2)	7·6 (6·95 to 8·34)	5·81 (5·18 to 6·47)	4·64 (4·19 to 5·12)	3·94 (3·57 to 4·33)	3·94 (3·57 to 4·35)	4·51 (4·02 to 5·03)
Brent	35·8 (29·7 to 43)	36·8 (33·2 to 40·8)	29·2 (26·8 to 31·7)	20·2 (18·5 to 21·9)	13·8 (12·6 to 15·1)	11·3 (10·2 to 12·6)	7·51 (6·99 to 8·09)	5·12 (4·62 to 5·59)	4·37 (3·94 to 4·83)	4·94 (4·31 to 5·61)
Bromley	25·7 (21·6 to 30·6)	22·4 (19·3 to 25·5)	17·1 (15·7 to 18·7)	12·2 (10·6 to 14)	8·1 (7·52 to 8·67)	5·34 (4·79 to 5·97)	3·69 (3·38 to 4)	3·07 (2·78 to 3·37)	2·83 (2·6 to 3·08)	3·15 (2·82 to 3·53)
Camden	19·3 (16·6 to 22·5)	33·8 (31·4 to 36·5)	28·2 (26 to 30·7)	21 (19·1 to 23·1)	12·1 (11·1 to 13·2)	7·78 (6·95 to 8·7)	5·13 (4·73 to 5·58)	3·46 (3·13 to 3·79)	3·11 (2·76 to 3·46)	3·4 (2·96 to 3·89)
Croydon	33·5 (28·2 to 39·9)	29·2 (27·1 to 31·4)	22·7 (21·1 to 24·2)	15·9 (14·6 to 17·2)	11·4 (10·7 to 12·3)	8·63 (7·76 to 9·62)	6·17 (5·66 to 6·74)	5·02 (4·62 to 5·42)	4·25 (3·88 to 4·66)	4·62 (4·11 to 5·15)
Ealing	30·5 (25·6 to 35·7)	30·9 (28·5 to 33·2)	24·1 (22·4 to 25·8)	16·5 (15 to 18)	11 (10·3 to 11·7)	7·87 (7·15 to 8·68)	5·55 (5·09 to 6·09)	3·82 (3·48 to 4·15)	3·64 (3·36 to 3·94)	4·05 (3·61 to 4·51)
Enfield	36·8 (31 to 43·7)	31·9 (29·8 to 34)	24·2 (22·5 to 25·9)	16·9 (15·4 to 18·5)	10·4 (9·67 to 11·1)	8·45 (7·53 to 9·43)	6·65 (5·99 to 7·38)	4·68 (4·33 to 5·04)	4·33 (3·89 to 4·76)	5·14 (4·55 to 5·74)
Greenwich	47·8 (40 to 56·5)	44·2 (41 to 47·4)	34·9 (32·3 to 37·7)	23·5 (21·4 to 25·8)	13 (11·9 to 14·4)	8·82 (7·98 to 9·69)	6·98 (6·41 to 7·56)	5·9 (5·41 to 6·46)	5·36 (4·96 to 5·8)	6·1 (5·4 to 6·79)
Hackney	36·7 (31·4 to 42·8)	34·8 (32·3 to 37·5)	29·1 (27 to 31·3)	20·8 (19·1 to 22·7)	14·4 (13·4 to 15·4)	11·4 (10·3 to 12·7)	7·92 (7·34 to 8·52)	5·75 (5·33 to 6·21)	4·8 (4·42 to 5·17)	4·75 (4·15 to 5·38)
Hammersmith and Fulham	20·1 (16·8 to 23·7)	31·3 (29·2 to 33·6)	24·7 (22·6 to 26·9)	18·9 (17·2 to 21)	13·7 (12·1 to 15·5)	8·47 (7·63 to 9·37)	5·6 (5·03 to 6·24)	4·12 (3·71 to 4·58)	3·42 (3·11 to 3·77)	3·86 (3·36 to 4·39)
Haringey	30·5 (26 to 35·9)	36·4 (33·7 to 39·2)	28·4 (26·2 to 30·6)	21·1 (19·1 to 23·4)	14·2 (12·9 to 15·5)	11·8 (10·5 to 13·1)	7·36 (6·73 to 8·03)	4·6 (4·21 to 4·98)	4·23 (3·92 to 4·56)	4·7 (4·19 to 5·27)
Harrow	29·3 (24·2 to 35·1)	24·8 (22·9 to 27·1)	18·8 (17·6 to 20·1)	13·1 (11·9 to 14·5)	9·53 (8·77 to 10·3)	8·41 (7·36 to 9·51)	6·96 (6·18 to 7·83)	5·31 (4·82 to 5·82)	4·5 (4·08 to 4·93)	5·1 (4·54 to 5·68)
Havering	29·7 (24·4 to 35·9)	25·6 (21·7 to 29·6)	20·3 (18·6 to 22·1)	14 (12·2 to 16·2)	8·35 (7·67 to 9·15)	5·85 (5·08 to 6·77)	4·61 (4·17 to 5·08)	3·34 (3·01 to 3·69)	2·97 (2·61 to 3·36)	3·51 (3·1 to 3·94)
Hillingdon	30·8 (25·6 to 36·8)	27·9 (25·7 to 30·1)	22·2 (20·5 to 23·9)	15·3 (14 to 16·7)	9·48 (8·68 to 10·3)	6·59 (5·81 to 7·4)	5·56 (4·97 to 6·23)	4·09 (3·76 to 4·45)	3·78 (3·43 to 4·18)	4·23 (3·76 to 4·73)
Hounslow	30·6 (25·2 to 36·7)	31·2 (26·8 to 35·9)	24·3 (22·3 to 26·6)	16·5 (14·5 to 18·7)	11 (9·75 to 12·2)	7·9 (7·04 to 8·78)	6·39 (5·71 to 7·14)	5·18 (4·71 to 5·68)	4·74 (4·33 to 5·16)	5·18 (4·62 to 5·81)
Islington	26·7 (22·2 to 31·9)	31·6 (26·8 to 36·5)	25·5 (23·3 to 28)	19·5 (16·9 to 22·4)	11·1 (10·1 to 12·1)	7·92 (7 to 8·87)	4·96 (4·48 to 5·46)	3·35 (3·09 to 3·63)	2·83 (2·62 to 3·05)	3·13 (2·79 to 3·5)
Kensington and Chelsea	13·2 (10·8 to 16)	37·4 (32 to 43·2)	33·1 (30·3 to 36)	24·1 (21·7 to 26·9)	12·5 (10·9 to 14·2)	6·84 (5·97 to 7·78)	4·99 (4·28 to 5·87)	4·19 (3·81 to 4·6)	3·55 (3·2 to 3·91)	3·96 (3·48 to 4·5)
Kingston upon Thames	23·4 (18 to 29·4)	23·3 (20 to 26·7)	17·1 (15·7 to 18·5)	12·4 (11·1 to 13·8)	8·36 (7·56 to 9·25)	5·12 (4·31 to 6·01)	4·54 (3·9 to 5·23)	4·49 (4·09 to 4·93)	3·58 (3·27 to 3·9)	4·03 (3·57 to 4·49)
Lambeth	36·3 (30·8 to 42·6)	39·2 (36·5 to 42·2)	32·2 (29·5 to 35)	25·2 (23·2 to 27·4)	13·6 (12·5 to 14·7)	10·4 (9·31 to 11·6)	7·45 (6·81 to 8·12)	5·26 (4·78 to 5·75)	4·42 (4·03 to 4·82)	5·15 (4·54 to 5·84)
Lewisham	37·7 (32·5 to 44)	34·4 (32·1 to 36·8)	27·4 (25·4 to 29·5)	20 (18·2 to 22·2)	12·4 (11·6 to 13·4)	9·83 (8·72 to 11·1)	6·55 (6·02 to 7·17)	4·72 (4·23 to 5·26)	4·61 (4·12 to 5·13)	5·16 (4·49 to 5·82)
Merton	36·8 (31·3 to 43·1)	32·7 (30·4 to 35·3)	25 (23·3 to 26·7)	17·6 (16·1 to 19·4)	10·9 (10 to 11·8)	6·65 (5·76 to 7·56)	5·63 (5·09 to 6·26)	3·96 (3·63 to 4·32)	3·57 (3·29 to 3·86)	4·03 (3·6 to 4·48)
Newham	43·9 (34 to 55·3)	39·7 (34·9 to 44·9)	32·1 (28·7 to 35·6)	21·7 (19·2 to 24·4)	13·6 (12·6 to 14·8)	11·3 (10·1 to 12·5)	7·72 (7 to 8·39)	5·25 (4·82 to 5·68)	4·66 (4·2 to 5·11)	4·85 (4·26 to 5·47)
Redbridge	27·6 (22·6 to 32·8)	27·5 (25·7 to 29·6)	21·3 (20 to 22·9)	14·3 (12·9 to 15·8)	9·82 (8·94 to 10·7)	7·12 (6·23 to 8·04)	5·17 (4·54 to 5·92)	3·76 (3·45 to 4·09)	3·34 (3·07 to 3·62)	3·62 (3·23 to 4·04)
Richmond upon Thames	22·4 (18·7 to 26·5)	23·3 (21·8 to 24·8)	17·5 (16·1 to 18·9)	13 (12 to 14·2)	7·97 (7·07 to 8·9)	4·83 (4·29 to 5·44)	3·61 (3·25 to 4·04)	2·85 (2·59 to 3·13)	2·78 (2·53 to 3·04)	3·14 (2·76 to 3·58)
Southwark	39·8 (32·9 to 48·2)	33·5 (28·8 to 38·3)	27·5 (25·2 to 30·1)	19 (16·7 to 21·4)	14·7 (13·6 to 15·8)	12·1 (10·8 to 13·4)	7·35 (6·77 to 7·98)	4·97 (4·53 to 5·44)	4·09 (3·7 to 4·51)	4·56 (4·01 to 5·17)
Sutton	32 (27·1 to 37·9)	26·8 (24·8 to 29)	20·3 (18·8 to 21·8)	13·9 (12·6 to 15·2)	8·51 (7·73 to 9·29)	5·75 (5·18 to 6·47)	4·73 (4·35 to 5·13)	4·08 (3·74 to 4·44)	3·48 (3·19 to 3·8)	4·11 (3·67 to 4·6)
Tower Hamlets	52·9 (44·4 to 62·6)	41·9 (38·5 to 45·8)	38 (34·9 to 41·3)	24·7 (22·4 to 27·1)	11·6 (10·3 to 13)	8·97 (8·04 to 10·1)	6·53 (5·91 to 7·18)	4·94 (4·49 to 5·45)	4·11 (3·7 to 4·53)	4·31 (3·82 to 4·84)
Waltham Forest	39·2 (31·7 to 47·7)	35·8 (32·1 to 39·7)	26·9 (24·6 to 29·5)	18·6 (16·6 to 20·9)	12·8 (11·7 to 14·1)	9·93 (8·84 to 11·1)	6·92 (6·35 to 7·53)	4·34 (3·99 to 4·71)	3·77 (3·4 to 4·17)	4·35 (3·86 to 4·89)
Wandsworth	28·7 (24·3 to 33·7)	32·8 (30·4 to 35·1)	25·4 (23·6 to 27·3)	19·6 (17·7 to 21·7)	12·4 (11·2 to 13·7)	7·64 (6·7 to 8·68)	5·17 (4·71 to 5·67)	3·74 (3·41 to 4·08)	3·01 (2·71 to 3·32)	3·37 (2·98 to 3·77)
Westminster	23·6 (19·4 to 28·3)	44·4 (37·3 to 52·1)	39·2 (35·3 to 43·2)	29·3 (25·5 to 33·9)	15·2 (13·8 to 16·6)	9·14 (8·27 to 10·2)	5·09 (4·65 to 5·53)	3·48 (3·15 to 3·84)	2·68 (2·4 to 2·97)	3·15 (2·76 to 3·59)
North East England	27·5 (22·1 to 33·2)	27·5 (23·5 to 32·2)	23·3 (21·5 to 25·2)	15·4 (14·3 to 16·6)	10·1 (9·54 to 10·7)	7·06 (6·47 to 7·73)	4·85 (4·54 to 5·18)	4·01 (3·57 to 4·51)	3·34 (2·85 to 3·83)	3·81 (3·28 to 4·35)
County Durham	28·4 (21·7 to 36)	28·7 (25 to 32·5)	24 (21·5 to 26·7)	15·9 (14·2 to 17·6)	9·93 (9·1 to 10·8)	7·6 (6·84 to 8·44)	5·11 (4·76 to 5·51)	4·24 (3·68 to 4·84)	3·73 (3·2 to 4·27)	4·24 (3·7 to 4·79)
Darlington	34·9 (26·8 to 45)	32·9 (26·2 to 39·9)	27·2 (24·5 to 29·9)	18·3 (15·9 to 20·9)	11·7 (10·4 to 13·2)	8·39 (6·93 to 10·2)	5·83 (4·9 to 6·93)	4·6 (4 to 5·24)	3·91 (3·25 to 4·66)	4·66 (3·93 to 5·42)
Gateshead	31 (23·4 to 39·8)	30·8 (26·5 to 35·8)	26·1 (23·6 to 28·9)	17·6 (15·7 to 19·8)	11·7 (10·4 to 13·1)	7·12 (6·12 to 8·24)	5·43 (4·74 to 6·15)	4·51 (3·96 to 5·15)	3·72 (3·13 to 4·33)	4·11 (3·52 to 4·75)
Hartlepool	34·3 (26·7 to 42·9)	34·9 (28·3 to 42·1)	30·2 (27·3 to 33·4)	20·7 (18·4 to 23·4)	11·9 (10·7 to 13·2)	8·06 (6·88 to 9·34)	5·4 (4·56 to 6·33)	3·73 (3·24 to 4·3)	2·74 (2·33 to 3·19)	3·2 (2·75 to 3·7)
Middlesbrough	32·3 (25·2 to 40·5)	31·5 (27·5 to 35·6)	27·5 (25·1 to 30·3)	18·3 (16·3 to 20·4)	12·1 (11·1 to 13·1)	8·68 (7·76 to 9·71)	5·88 (5·43 to 6·34)	4·55 (3·93 to 5·22)	3·76 (3·16 to 4·36)	4·27 (3·68 to 4·89)
Newcastle upon Tyne	19·8 (15 to 25·3)	22·9 (19·5 to 27)	19·7 (17·7 to 21·9)	13·2 (11·9 to 14·5)	9·76 (8·84 to 10·8)	6·64 (5·96 to 7·35)	4·71 (4·35 to 5·07)	4·33 (3·82 to 4·92)	3·49 (2·96 to 4)	4·01 (3·49 to 4·56)
North Tyneside	25·5 (19·4 to 32·8)	23·3 (18·5 to 29·3)	19·7 (17·6 to 21·8)	12·4 (10·9 to 14·2)	8·88 (7·98 to 9·81)	5·86 (5·22 to 6·56)	3·89 (3·53 to 4·26)	3·38 (2·93 to 3·91)	2·67 (2·26 to 3·13)	3 (2·54 to 3·46)
Northumberland	26·7 (20·7 to 34·7)	24·4 (19·2 to 30·9)	19·9 (17·7 to 22·6)	12·7 (11·2 to 14·4)	8·68 (7·79 to 9·63)	5·99 (5·18 to 6·81)	4·03 (3·61 to 4·47)	3·36 (2·93 to 3·84)	2·8 (2·34 to 3·26)	3·1 (2·67 to 3·56)
Redcar and Cleveland	28·5 (21·3 to 37·2)	29·1 (22·6 to 37·3)	23·8 (21·5 to 26·2)	16·4 (13·6 to 19·2)	10·2 (9·33 to 11·1)	6·53 (5·69 to 7·49)	4·52 (3·8 to 5·29)	3·79 (3·27 to 4·38)	2·77 (2·27 to 3·29)	3·3 (2·78 to 3·86)
South Tyneside	24·9 (19·1 to 31·8)	23 (18·5 to 28)	19·9 (18 to 22)	13·1 (11·6 to 14·8)	8·1 (7·29 to 8·96)	6·06 (5·24 to 7)	4·07 (3·43 to 4·79)	3·18 (2·73 to 3·69)	2·88 (2·39 to 3·4)	3·41 (2·88 to 3·98)

Stockton-on-Tees	29·4 (22·3 to 37·5)	28·9 (24·7 to 33·9)	24·5 (22·2 to 26·9)	16·7 (14·9 to 18·5)	10·8 (9·96 to 11·7)	7·31 (6·55 to 8·1)	5·37 (4·91 to 5·8)	4·62 (3·99 to 5·28)	3·97 (3·32 to 4·65)	4·69 (3·98 to 5·44)
Sunderland	27 (20·5 to 34·2)	26·8 (21·6 to 33·2)	22·7 (20·5 to 25·2)	14·9 (13·1 to 16·6)	10·2 (9·21 to 11·1)	7·24 (6·58 to 8·01)	4·58 (4·24 to 4·92)	3·63 (3·2 to 4·14)	2·96 (2·53 to 3·39)	3·29 (2·84 to 3·79)
North West England	29·9 (26·7 to 33·3)	30·1 (28·9 to 31·3)	24·3 (23·2 to 25·2)	17 (16·1 to 17·9)	10·7 (10·2 to 11·2)	7·89 (7·32 to 8·54)	6·11 (5·79 to 6·46)	5·54 (5·28 to 5·8)	4·59 (4·26 to 4·92)	5·2 (4·67 to 5·78)
Blackburn with Darwen	38·6 (30·6 to 47·8)	34·2 (30·1 to 38·8)	27·3 (24·8 to 29·7)	18·2 (15·9 to 20·5)	14·5 (13·3 to 15·8)	11·3 (10·1 to 12·6)	8·91 (8·16 to 9·69)	7 (6·34 to 7·72)	5·47 (5·01 to 5·97)	6·27 (5·6 to 6·9)
Blackpool	37 (29·9 to 45·4)	30·6 (27·9 to 33·3)	24·6 (22·8 to 26·6)	16·7 (14·9 to 18·5)	10·7 (9·58 to 12)	8·71 (7·63 to 9·96)	7·39 (6·67 to 8·19)	6·71 (6·11 to 7·38)	5·3 (4·84 to 5·78)	6·18 (5·58 to 6·8)
Bolton	36·3 (30·5 to 42·6)	35·7 (32·7 to 39·2)	28·1 (26·2 to 30·4)	19·3 (17·5 to 21·1)	12·5 (11·5 to 13·7)	8·55 (7·73 to 9·37)	6·8 (6·18 to 7·45)	6·13 (5·57 to 6·71)	4·91 (4·46 to 5·37)	5·68 (5·15 to 6·23)
Bury	28·5 (23 to 34·9)	29·6 (25·8 to 33·5)	23·1 (21·2 to 25·2)	16·3 (14·4 to 18·4)	10 (9·03 to 11·1)	6·35 (5·68 to 7·04)	5·01 (4·55 to 5·53)	4·9 (4·41 to 5·41)	4·27 (3·83 to 4·7)	4·85 (4·32 to 5·4)
Cheshire East	27·2 (22·8 to 32)	26·6 (24·6 to 28·6)	20·7 (19·4 to 22·1)	14·9 (13·7 to 16·3)	9·3 (8·49 to 10·2)	6·11 (5·57 to 6·67)	4·75 (4·39 to 5·17)	4·33 (3·97 to 4·74)	3·63 (3·34 to 3·93)	4·01 (3·64 to 4·41)
Cheshire West and Chester	24·6 (20·7 to 28·6)	24·6 (22·9 to 26·4)	19·4 (18 to 20·6)	14·3 (13 to 15·6)	9·13 (8·42 to 9·91)	6 (5·42 to 6·58)	5·3 (4·87 to 5·77)	4·37 (3·95 to 4·8)	3·59 (3·26 to 3·92)	3·99 (3·61 to 4·37)
Cumbria	30·4 (25·7 to 35·7)	30·1 (26·5 to 33·7)	24 (22·1 to 26·2)	17 (15·2 to 18·8)	9·97 (9·18 to 10·8)	6·1 (5·51 to 6·7)	4·65 (4·29 to 5·04)	4·12 (3·8 to 4·45)	3·48 (3·14 to 3·83)	3·91 (3·52 to 4·32)
Halton	24·2 (19·6 to 29·5)	26·6 (24·6 to 28·8)	21·2 (19·5 to 22·9)	14·1 (12·8 to 15·4)	9·89 (8·9 to 10·9)	7·89 (7·04 to 8·72)	5·57 (5·06 to 6·15)	4·33 (3·96 to 4·71)	3·85 (3·46 to 4·23)	4·34 (3·85 to 4·9)
Knowsley	29·9 (24·6 to 35·5)	29·8 (26 to 33·9)	26·3 (24·3 to 28·2)	19·1 (17·3 to 21)	9·65 (8·77 to 10·6)	7·57 (6·75 to 8·51)	5·29 (4·57 to 6·07)	4·87 (4·42 to 5·33)	4·08 (3·64 to 4·53)	4·58 (4·06 to 5·11)
Lancashire	30·5 (25 to 37·1)	29·5 (26·9 to 32·3)	23·3 (20·9 to 25·6)	16·1 (14·6 to 17·6)	11·1 (10·3 to 12)	8·08 (7·23 to 9·03)	6·62 (6·03 to 7·26)	5·69 (5·34 to 6·12)	4·5 (3·95 to 5·07)	4·98 (4·18 to 5·93)
Liverpool	26·6 (21·7 to 32·3)	29·8 (26·7 to 33·2)	25·4 (23·2 to 27·6)	17·8 (16 to 19·9)	9·84 (9·01 to 10·7)	8·55 (7·72 to 9·37)	6·59 (6·07 to 7·09)	6·33 (5·79 to 6·89)	5·33 (4·87 to 5·82)	6·26 (5·64 to 6·89)
Manchester	31·8 (26 to 38·5)	36·9 (32 to 41·8)	31·5 (28·6 to 34·8)	23 (20·3 to 26)	14 (12·7 to 15·4)	11·7 (10·5 to 12·9)	7·91 (7·38 to 8·5)	7·43 (6·88 to 7·98)	6·19 (5·43 to 7·02)	7·1 (5·89 to 8·5)
Oldham	37 (30·9 to 42·9)	35·8 (32·5 to 39·8)	28·5 (26·1 to 31)	19·6 (17·7 to 21·8)	12·5 (11·3 to 13·8)	9·55 (8·55 to 10·6)	8·21 (7·56 to 8·96)	7·75 (7·17 to 8·45)	6·73 (6·04 to 7·45)	7·45 (6·7 to 8·2)
Rochdale	36·4 (30·9 to 42)	35·3 (32·8 to 38)	28·8 (26·7 to 30·8)	19·5 (17·8 to 21·3)	11·9 (11 to 12·9)	8·6 (7·79 to 9·43)	6·22 (5·79 to 6·72)	6·06 (5·54 to 6·63)	4·89 (4·42 to 5·4)	5·7 (5·17 to 6·27)
Salford	30·5 (25·5 to 36·4)	32·2 (29·1 to 35·5)	26·4 (24·1 to 28·9)	19·1 (17·4 to 20·9)	10·7 (9·76 to 11·6)	8·1 (7·02 to 9·3)	6·3 (5·59 to 7·03)	5·67 (5·2 to 6·15)	4·82 (4·35 to 5·32)	5·61 (5·05 to 6·23)
Sefton	26·5 (22·6 to 31)	23·7 (21·8 to 25·7)	19 (17·3 to 20·8)	13·5 (12·3 to 14·7)	9·07 (8·27 to 10)	6·89 (6·27 to 7·57)	5·58 (5·11 to 6·1)	5·4 (4·93 to 5·92)	4·48 (4·1 to 4·86)	5·09 (4·59 to 5·62)
St Helens	27·2 (21·9 to 32·8)	27·7 (24·2 to 31·4)	22·2 (20·4 to 24·2)	15·8 (13·7 to 18)	10 (8·99 to 11·2)	8·07 (7·12 to 9·08)	5·09 (4·67 to 5·58)	4·33 (3·94 to 4·78)	3·78 (3·43 to 4·16)	4·09 (3·69 to 4·53)
Stockport	26·9 (23 to 31)	25·2 (23·4 to 27·3)	19·9 (18·5 to 21·2)	14·3 (13·1 to 15·6)	9·44 (8·66 to 10·3)	6·62 (6·03 to 7·26)	5·18 (4·78 to 5·66)	5·36 (4·91 to 5·87)	4·34 (3·96 to 4·72)	4·89 (4·43 to 5·38)
Tameside	35·6 (29·5 to 42·2)	35·5 (32·4 to 38·7)	28·2 (25·9 to 30·6)	19·3 (17·4 to 21·2)	10·6 (9·62 to 11·7)	7·33 (6·56 to 8·27)	5·59 (5·1 to 6·13)	4·71 (4·29 to 5·16)	3·94 (3·54 to 4·36)	4·45 (3·99 to 4·93)
Trafford	30·5 (26·2 to 35·4)	29·8 (27·7 to 32)	24·1 (22·3 to 26)	17·8 (16·3 to 19·4)	10·2 (9·28 to 11·2)	6·47 (5·84 to 7·14)	4·3 (3·89 to 4·75)	3·98 (3·58 to 4·38)	3·19 (2·85 to 3·53)	3·54 (3·18 to 3·92)
Warrington	23·5 (19·1 to 28·7)	24·8 (22·6 to 27·1)	19·3 (18 to 20·6)	13·5 (12·1 to 15·1)	10·4 (9·36 to 11·5)	7·34 (6·62 to 8·18)	5·62 (5·12 to 6·16)	4·94 (4·43 to 5·46)	4·08 (3·66 to 4·53)	4·47 (3·96 to 4·99)
Wigan	24·7 (20·9 to 28·7)	26·8 (24·6 to 29·1)	20·7 (19·4 to 22)	14·5 (13·2 to 15·9)	9·11 (8·36 to 9·86)	7·39 (6·65 to 8·12)	5·19 (4·7 to 5·66)	4·73 (4·35 to 5·13)	4·24 (3·84 to 4·66)	4·74 (4·24 to 5·24)
Wirral	30·5 (25·6 to 36)	27·9 (25·8 to 30·2)	22·3 (20·8 to 23·7)	15·3 (14 to 16·7)	9·35 (8·53 to 10·3)	6·64 (5·8 to 7·54)	5·36 (4·83 to 5·97)	4·71 (4·34 to 5·16)	3·92 (3·57 to 4·33)	4·59 (4·15 to 5·07)
South East England	23·7 (21·4 to 26·4)	24·5 (23·3 to 25·8)	19 (17·9 to 20)	14 (13·2 to 14·8)	9·28 (8·89 to 9·69)	5·7 (5·23 to 6·18)	4·51 (4·24 to 4·79)	4·04 (3·84 to 4·24)	3·64 (3·33 to 3·96)	4·02 (3·58 to 4·51)
Bracknell Forest	16·5 (13 to 20·8)	19·6 (16·5 to 22·9)	15·7 (14·5 to 16·8)	11·3 (9·83 to 12·9)	7·72 (6·79 to 8·77)	5·1 (4·33 to 5·98)	3·88 (3·31 to 4·51)	2·6 (2·31 to 2·92)	1·77 (1·55 to 2·02)	1·92 (1·69 to 2·19)
Brighton and Hove	27·1 (22·3 to 32·2)	27·9 (23·8 to 32·1)	21·8 (19·7 to 24)	17·3 (15·5 to 19·5)	11·2 (10·3 to 12·3)	7·66 (6·83 to 8·52)	5·71 (5·21 to 6·25)	4·14 (3·72 to 4·61)	3·41 (3·01 to 3·81)	3·58 (3·11 to 4·08)
Buckinghamshire	19·6 (16·4 to 23·2)	19·2 (17·6 to 20·7)	14·7 (13·7 to 15·8)	10·9 (9·88 to 11·9)	8·91 (8·23 to 9·59)	5·53 (4·99 to 6·13)	4·78 (4·27 to 5·32)	4·02 (3·72 to 4·34)	3·65 (3·19 to 4·13)	4·17 (3·68 to 4·72)
East Sussex	31·7 (26·4 to 37·5)	25·7 (23·6 to 27·8)	19 (17·5 to 20·5)	13·4 (12·1 to 14·6)	8·97 (8·25 to 9·75)	5·82 (5·28 to 6·4)	4·76 (4·28 to 5·29)	4·24 (3·92 to 4·56)	3·64 (3·27 to 4·02)	3·88 (3·43 to 4·39)
Hampshire	21·8 (18·3 to 25·5)	24·5 (22·5 to 26·7)	19·1 (17·7 to 20·5)	14·7 (13·6 to 16)	8·55 (7·96 to 9·24)	5·11 (4·54 to 5·73)	4·12 (3·8 to 4·46)	4·12 (3·78 to 4·46)	4·15 (3·63 to 4·68)	4·59 (3·85 to 5·46)
Isle of Wight	42·8 (34·8 to 52·2)	36 (30·9 to 41·3)	26·6 (24·3 to 29·2)	18·7 (16·2 to 21·5)	11·9 (10·4 to 13·5)	5·49 (4·67 to 6·39)	3·88 (3·29 to 4·52)	3·74 (3·31 to 4·15)	3·09 (2·63 to 3·59)	3·2 (2·75 to 3·71)
Kent	27 (23·1 to 31·1)	26·3 (24·6 to 28·1)	20 (17·9 to 22·1)	14·2 (13 to 15·5)	9·94 (9·26 to 10·6)	5·53 (4·96 to 6·13)	4·36 (4·11 to 4·64)	4·3 (4·05 to 4·57)	3·95 (3·51 to 4·43)	4·45 (3·76 to 5·25)
Medway	26·4 (21·3 to 32·7)	31·4 (28·7 to 34·5)	24·4 (22·5 to 26·4)	17·2 (15·5 to 18·9)	11·8 (10·7 to 12·9)	7·44 (6·65 to 8·21)	5·23 (4·76 to 5·74)	4·58 (4·16 to 5·07)	4·23 (3·81 to 4·71)	4·26 (3·76 to 4·81)
Milton Keynes	26·2 (20·9 to 32·3)	25·9 (22 to 30)	19·1 (17·3 to 21·1)	13·5 (11·9 to 15·4)	10·7 (9·56 to 11·9)	7·91 (7·06 to 8·87)	5·93 (5·3 to 6·58)	4·9 (4·39 to 5·42)	4·27 (3·75 to 4·83)	4·51 (3·93 to 5·15)
Oxfordshire	13·4 (10·8 to 16)	19·4 (17·7 to 21)	15·3 (14·3 to 16·4)	11·9 (10·9 to 12·9)	8·38 (7·72 to 9·07)	5·59 (5·09 to 6·17)	4·73 (4·33 to 5·18)	3·46 (3·21 to 3·74)	2·86 (2·54 to 3·21)	3·22 (2·85 to 3·6)
Portsmouth	24·6 (20 to 30·2)	31·4 (27·1 to 35·4)	25·1 (23·1 to 27·6)	18·7 (16·6 to 21)	12·3 (11·1 to 13·5)	7·59 (6·74 to 8·44)	4·45 (4·05 to 4·89)	3·76 (3·41 to 4·12)	2·93 (2·61 to 3·24)	3·08 (2·7 to 3·48)
Reading	17·1 (14·1 to 20·4)	22·5 (20·8 to 24·4)	18 (16·5 to 19·7)	14·4 (12·9 to 16)	10·1 (9·16 to 11·1)	8·17 (7·25 to 9·14)	5·65 (5·2 to 6·15)	4·36 (4 to 4·81)	3·94 (3·57 to 4·35)	4·25 (3·77 to 4·8)
Slough	28·4 (22·8 to 35·3)	28·8 (26·6 to 31·2)	24·4 (22·3 to 26·6)	16·2 (14·6 to 17·9)	11 (9·83 to 12·3)	7·53 (6·51 to 8·67)	6·35 (5·46 to 7·28)	5·34 (4·91 to 5·83)	5·01 (4·45 to 5·61)	5·39 (4·73 to 6·12)
Southampton	22·6 (18·3 to 27·9)	28·7 (26·2 to 31·4)	23·4 (21·3 to 25·7)	16·8 (15 to 18·7)	10·7 (9·82 to 11·7)	6·81 (5·92 to 7·75)	5·06 (4·55 to 5·63)	4·57 (4·14 to 5·01)	3·96 (3·46 to 4·5)	4·27 (3·77 to 4·84)
Surrey	24·2 (20·6 to 28·2)	22·9 (21·4 to 24·5)	18 (16·6 to 19·5)	13·9 (12·7 to 15·1)	8·28 (7·64 to 8·98)	4·73 (4·26 to 5·22)	3·68 (3·4 to 3·95)	3·5 (3·28 to 3·74)	3·2 (2·74 to 3·68)	3·62 (3·12 to 4·12)
West Berkshire	16·5 (13·1 to 20·5)	17·1 (14·3 to 20)	13 (11·5 to 14·5)	9·8 (8·39 to 11·4)	8·06 (7·16 to 9·12)	5·4 (4·68 to 6·18)	3·98 (3·57 to 4·42)	3·28 (2·94 to 3·62)	2·82 (2·48 to 3·2)	3·32 (2·95 to 3·75)
West Sussex	31 (24·6 to 38·4)	25·9 (21·5 to 30·3)	19·7 (17·6 to 21·9)	13·9 (12·4 to 15·6)	8·88 (8·25 to 9·6)	5·31 (4·76 to 5·84)	4·47 (4·17 to 4·82)	4·03 (3·76 to 4·32)	3·56 (3·17 to 3·97)	4·01 (3·56 to 4·49)
Windsor and Maidenhead	15·8 (12·8 to 19·1)	17·5 (15·1 to 20)	13·5 (12·2 to 14·8)	10·1 (9·05 to 11·2)	7·26 (6·42 to 8·17)	5·16 (4·5 to 5·88)	3·67 (3·19 to 4·24)	3·1 (2·78 to 3·44)	2·64 (2·32 to 3·01)	2·94 (2·58 to 3·33)
Wokingham	12·9 (10·1 to 16)	15·4 (13·1 to 17·9)	11·8 (10·6 to 13)	9·65 (8·35 to 11·2)	7·08 (6·29 to 8·03)	4·39 (3·69 to 5·17)	3·81 (3·28 to 4·44)	3·72 (3·36 to 4·16)	2·94 (2·56 to 3·34)	3·17 (2·77 to 3·6)
South West England	24 (19·8 to 28·8)	25·1 (23·4 to 27·1)	20·1 (18·5 to 22·1)	14·4 (13·4 to 15·5)	8·86 (8·37 to 9·37)	6·03 (5·53 to 6·56)	4·63 (4·35 to 4·9)	3·42 (3·22 to 3·62)	3·16 (2·75 to 3·58)	3·53 (3·08 to 3·99)
Bath and North East Somerset	21·4 (16·3 to 27·9)	21·8 (18·1 to 26)	17·7 (15·5 to 20·2)	13·7 (11·6 to 16·1)	7·71 (6·9 to 8·62)	4·76 (4·11 to 5·44)	3·16 (2·63 to 3·77)	2·44 (2·21 to 2·69)	2·37 (2 to 2·74)	2·64 (2·26 to 3·01)
Bournemouth	24·6 (18·7 to 32·7)	24·2 (21·7 to 26·9)	19·7 (17·6 to 22)	13·6 (12·2 to 15·3)	8·16 (7·17 to 9·24)	6·35 (5·56 to 7·22)	4·44 (4·04 to 4·86)	3·48 (3·02 to 4)	2·35 (1·93 to 2·78)	2·42 (1·96 to 2·88)
Bristol, City of	22·5 (17·7 to 28·1)	29·4 (27·1 to 32)	24·9 (22·4 to 27·6)	18·1 (16·3 to 19·8)	11 (10 to 11·9)	7·61 (6·86 to 8·46)	5 (4·7 to 5·31)	3·64 (3·33 to 4)	3·3 (2·89 to 3·75)	3·7 (3·25 to 4·16)
Cornwall	27·4 (21·7 to 34·2)	25·7 (23·6 to 28)	20·1 (18·2 to 22·3)	13·5 (12·3 to 14·8)	8·52 (7·75 to 9·3)	5·67 (5·08 to 6·28)	4·96 (4·57 to 5·37)	3·58 (3·31 to 3·88)	3·43 (2·99 to 3·86)	3·83 (3·37 to 4·32)
Devon	26 (20·8 to 31·9)	26·6 (24·1 to 29·2)	20·6 (18·6 to 23)	14·8 (13·5 to 16·1)	9·75 (8·92 to 10·7)	5·87 (5·2 to 6·59)	4·74 (4·13 to 5·41)	3·6 (3·32 to 3·89)	3·31 (2·82 to 3·82)	3·68 (3·2 to 4·18)
Dorset	24·6 (19·5 to 30·4)	23·5 (21 to 26·1)	18·4 (16·5 to 20·6)	13·1 (11·7 to 14·6)	7·54 (6·89 to 8·22)	5·43 (4·78 to 6·1)	4·46 (4·17 to 4·76)	3·18 (2·91 to 3·48)	3·09 (2·63 to 3·55)	3·41 (2·92 to 3·87)
Gloucestershire	22·8 (18·2 to 28)	23·5 (21·7 to 25·4)	18·8 (17·1 to 20·9)	13·7 (12·3 to 15·1)	8·53 (7·85 to 9·22)	5·82 (5·23 to 6·51)	4·45 (4·16 to 4·76)	3·35 (3·08 to 3·64)	3·2 (2·74 to 3·65)	3·68 (3·21 to 4·19)
North Somerset	24·7 (18·4 to 31·9)	24·1 (22·1 to 26·3)	19·1 (17 to 21·4)	13·5 (12 to 15·1)	7·54 (6·58 to 8·62)	5·89 (5·21 to 6·64)	4·29 (3·85 to 4·72)	3·08 (2·74 to 3·45)	2·59 (2·2 to 2·99)	2·94 (2·54 to 3·35)
Plymouth	26·2 (21 to 31·7)	28·6 (26 to 31·5)	23·7 (20·8 to 26·9)	17 (15·1 to 19·1)	10·1 (9·08 to 11·1)	7·87 (6·85 to 8·98)	6·01 (5·44 to 6·66)	4·16 (3·75 to 4·62)	3·8 (3·27 to 4·35)	4·17 (3·63 to 4·72)
Poole	24·7 (18·9 to 31·3)	23·1 (19 to 27·5)	18·1 (16·2 to 20·2)	12·9 (11·6 to 14·4)	8·16 (7·17 to 9·34)	5·99 (5·16 to 6·95)	4·09 (3·53 to 4·75)	2·94 (2·54 to 3·37)	1·89 (1·6 to 2·22)	1·96 (1·6 to 2·32)
Somerset	22·8 (17·5 to 28·8)	22·5 (20·2 to 24·7)	17·8 (15·8 to 19·9)	12·7 (11·6 to 13·8)	7·5 (6·85 to 8·23)	5·5 (4·98 to 6·1)	4·6 (4·31 to 4·91)	3·57 (3·33 to 3·82)	3·57 (3·05 to 4·1)	3·96 (3·44 to 4·52)
South Gloucestershire	19·1 (14·7 to 24·7)	20·2 (18·5 to 22)	15·9 (14·3 to 17·8)	11·7 (10·5 to 13)	8·01 (7·23 to 8·88)	5·36 (4·79 to 5·98)	3·61 (3·34 to 3·9)	2·93 (2·69 to 3·19)	2·85 (2·42 to 3·26)	3·19 (2·76 to 3·65)
Swindon	29·5 (22·1 to 38·4)	29·6 (24·5 to 34·9)	25·2 (22·2 to 28·4)	18·3 (15·5 to 21·3)	9·65 (8·75 to 10·6)	6·57 (5·84 to 7·39)	4·35 (4·03 to 4·71)	3·1 (2·84 to 3·39)	2·74 (2·33 to 3·14)	2·99 (2·56 to 3·41)
Torbay	29·8 (24·1 to 35·9)	25·3 (20·9 to 30·2)	19·4 (17·5 to 21·6)	13·2 (11·7 to 14·7)	9·14 (8·15 to 10·2)	6·48 (5·34 to 7·79)	5·81 (4·94 to 6·76)	3·94 (3·51 to 4·44)	3·23 (2·75 to 3·75)	3·63 (3·15 to 4·15)
Wiltshire	20·1 (16·4 to 24·8)	24·7 (22·4 to 27·1)	19·6 (17·8 to 21·8)	14·3 (12·9 to 15·6)	9·37 (8·53 to 10·3)	5·79 (5·06 to 6·58)	4·61 (4·17 to 5·09)	3·37 (3·04 to 3·77)	3·45 (2·94 to 3·98)	4 (3·46 to 4·59)
West Midlands	29·9 (27 to 33·1)	31·3 (30·1 to 32·5)	25·9 (24·7 to 27)	17·8 (16·8 to 18·9)	12·7 (11·8 to 13·6)	8·77 (8·09 to 9·48)	7·19 (6·84 to 7·6)	6·53 (6·18 to 6·9)	5·77 (5·35 to 6·21)	6·55 (5·86 to 7·3)
Birmingham	36·2 (32·3 to 40·3)	37 (35·2 to 38·9)	31·8 (29·3 to 34·2)	21·5 (19·8 to 23·3)	15·7 (14·5 to 17)	11·9 (10·8 to 13·1)	9·39 (8·63 to 10·1)	8·79 (8·01 to 9·66)	7·43 (6·68 to 8·2)	8·43 (7·16 to 9·77)

Coventry	31·7 (25·9 to 38·2)	35·3 (32·5 to 38·3)	30·1 (27·6 to 32·9)	21·8 (19·8 to 24)	14·5 (13·2 to 15·8)	9·04 (8·11 to 9·97)	6·23 (5·55 to 6·97)	5·2 (4·71 to 5·75)	4·87 (4·37 to 5·4)	5·59 (5·01 to 6·18)
Dudley	26·8 (22·6 to 31·9)	27·2 (25·1 to 29·3)	21·8 (20·3 to 23·3)	14·5 (13·1 to 15·9)	12·6 (11·4 to 13·8)	7·55 (6·67 to 8·54)	6·03 (5·39 to 6·74)	5·36 (4·82 to 5·99)	4·78 (4·29 to 5·31)	5·39 (4·83 to 5·96)
Herefordshire, County of	30·6 (24·9 to 36·7)	29·4 (24·7 to 34·2)	23·8 (22·2 to 25·5)	16·8 (14·8 to 19·1)	9·35 (8·2 to 10·6)	5·3 (4·56 to 6·19)	4·77 (4·11 to 5·5)	4·97 (4·4 to 5·59)	4·33 (3·87 to 4·82)	4·95 (4·43 to 5·5)
Sandwell	40·8 (33·8 to 49·3)	39·9 (37·1 to 42·7)	34·1 (31·9 to 36·3)	23·5 (21·6 to 25·5)	14·3 (12·8 to 15·9)	10·1 (8·98 to 11·3)	8·58 (7·88 to 9·41)	7·35 (6·75 to 8·01)	6·35 (5·7 to 7·01)	7·55 (6·78 to 8·36)
Shropshire	22·9 (18·6 to 27·5)	24·2 (20·5 to 28·3)	19·3 (17·8 to 20·9)	13·7 (11·8 to 15·6)	9·8 (8·85 to 10·8)	6·11 (5·46 to 6·85)	4·68 (4·26 to 5·14)	4·16 (3·71 to 4·66)	3·87 (3·43 to 4·31)	4·37 (3·84 to 4·93)
Solihull	25·2 (20·6 to 30·3)	26·9 (25 to 28·9)	21·7 (19·6 to 23·7)	16·8 (15·3 to 18·7)	10·7 (9·58 to 12)	6·28 (5·47 to 7·13)	5·33 (4·69 to 6·01)	5·07 (4·56 to 5·63)	4·78 (4·34 to 5·26)	5·35 (4·83 to 5·91)
Staffordshire	25 (20·5 to 29·5)	28 (24·6 to 31·4)	22·7 (20·7 to 24·9)	16 (14·5 to 17·8)	11 (9·92 to 12·2)	7·39 (6·63 to 8·19)	6·35 (5·81 to 6·93)	5·73 (5·22 to 6·26)	5·36 (4·73 to 6·03)	6 (5·04 to 7·05)
Stoke-on-Trent	37·8 (30·8 to 45·5)	38·1 (35·1 to 41·3)	33 (30·9 to 35·3)	21·8 (19·7 to 24)	14 (12·6 to 15·6)	11 (9·81 to 12·2)	9·35 (8·49 to 10·2)	9·07 (8·13 to 10)	8·23 (7·44 to 9·04)	9·16 (8·14 to 10·2)
Telford and Wrekin	27·6 (22·4 to 33·6)	31·4 (29 to 33·9)	25·2 (23·5 to 27)	17·2 (15·6 to 19)	13·2 (11·7 to 14·8)	8·34 (7·41 to 9·41)	6·61 (5·86 to 7·36)	5·43 (4·84 to 6·09)	4·71 (4·27 to 5·2)	5·29 (4·74 to 5·84)
Walsall	31·2 (25·9 to 37·3)	31·6 (29·3 to 34·2)	26·6 (24·9 to 28·5)	18·6 (16·9 to 20·4)	12·4 (11 to 13·9)	9·34 (8·3 to 10·5)	8·95 (8·05 to 9·94)	7·73 (7·04 to 8·5)	6·49 (5·75 to 7·24)	7·49 (6·74 to 8·24)
Warwickshire	19·9 (16·5 to 23·9)	21·7 (20 to 23·4)	17·4 (16·2 to 18·6)	12·4 (11·2 to 13·6)	9·67 (8·75 to 10·6)	5·93 (5·3 to 6·57)	5 (4·53 to 5·55)	4·55 (4·13 to 5·02)	4·19 (3·75 to 4·65)	4·77 (4·27 to 5·28)
Wolverhampton	32·1 (26·6 to 38·6)	32·1 (29·4 to 35)	26·8 (25 to 28·8)	18·4 (16·7 to 20·2)	14·4 (12·9 to 16·1)	10·3 (9·23 to 11·4)	7·93 (7·17 to 8·71)	6·6 (5·94 to 7·41)	5·83 (5·24 to 6·47)	6·54 (5·76 to 7·32)
Worcestershire	22·1 (18·3 to 26·3)	23·7 (21·9 to 25·4)	18·8 (17·4 to 20·4)	12·7 (11·5 to 13·8)	10·3 (9·39 to 11·2)	6·87 (6·22 to 7·53)	5·31 (4·86 to 5·78)	5·1 (4·65 to 5·6)	4·71 (4·29 to 5·16)	5·23 (4·7 to 5·77)
Yorkshire and the Humber	32·2 (28·1 to 36·7)	31·9 (29·7 to 34)	25·4 (24 to 26·7)	17·6 (16·6 to 18·8)	11·3 (10·8 to 11·9)	8·71 (8·04 to 9·4)	6·51 (6·12 to 6·92)	4·99 (4·69 to 5·34)	4·23 (3·88 to 4·59)	4·83 (4·31 to 5·39)
Barnsley	34·5 (28·9 to 40·7)	33·1 (30 to 36·5)	26·2 (24 to 28·4)	17·9 (15·9 to 20)	11·4 (10·4 to 12·5)	8·06 (7·21 to 8·89)	5·65 (5·14 to 6·18)	4·12 (3·67 to 4·62)	3·74 (3·29 to 4·21)	4·46 (3·93 to 5·03)
Bradford	41·8 (33·7 to 51·5)	37·9 (33·7 to 42·4)	30·9 (28·3 to 33·9)	20·2 (18·2 to 22·7)	14·3 (12·9 to 15·6)	12·3 (11 to 13·7)	9·18 (8·5 to 9·91)	6·88 (6·37 to 7·43)	5·92 (5·2 to 6·64)	6·71 (5·61 to 7·91)
Calderdale	42·6 (35·7 to 50·5)	36·7 (32·7 to 40·8)	28·4 (26·2 to 30·6)	19·5 (17·5 to 21·7)	14·1 (12·9 to 15·5)	10·1 (9·25 to 11·1)	8·15 (7·46 to 8·91)	5·87 (5·32 to 6·51)	4·81 (4·33 to 5·36)	5·37 (4·77 to 5·98)
Doncaster	30·9 (25·7 to 36·8)	29·8 (26·6 to 33·4)	24·4 (22·3 to 26·3)	16·1 (14·4 to 18)	10·8 (9·86 to 11·9)	8·76 (7·95 to 9·65)	6·83 (6·3 to 7·39)	5·17 (4·66 to 5·68)	4·63 (4·18 to 5·08)	5·41 (4·86 to 5·98)
East Riding of Yorkshire	26·8 (21·2 to 33·3)	24·2 (21·2 to 27·6)	18·7 (17 to 20·4)	12·9 (11·4 to 14·5)	8·51 (7·77 to 9·28)	6·37 (5·78 to 6·98)	4·48 (4·13 to 4·89)	3·31 (2·97 to 3·65)	2·98 (2·65 to 3·34)	3·41 (3·01 to 3·82)
Kingston upon Hull, City of	34·6 (29·3 to 41·2)	37·2 (33·3 to 41·3)	30·4 (27·9 to 32·8)	20·4 (18·4 to 22·5)	12·3 (11·3 to 13·2)	8·38 (7·6 to 9·21)	5·9 (5·48 to 6·39)	4·74 (4·25 to 5·29)	4·09 (3·63 to 4·56)	4·67 (4·15 to 5·19)
Kirklees	39·4 (33·3 to 45·8)	36·3 (32·5 to 40·6)	28·4 (26 to 30·8)	19·9 (18 to 22·1)	12·2 (11·3 to 13·1)	9·9 (9·05 to 10·8)	7·95 (7·25 to 8·7)	6·65 (6·1 to 7·22)	5·46 (4·95 to 5·99)	6·11 (5·5 to 6·72)
Leeds	28·9 (22·8 to 36·3)	30·8 (27·1 to 35)	24·8 (22·3 to 27·4)	17·5 (15·7 to 19·5)	11·2 (10·2 to 12·1)	8·33 (7·59 to 9·1)	6·05 (5·5 to 6·63)	4·96 (4·53 to 5·43)	4·37 (3·8 to 4·99)	4·99 (4·13 to 5·93)
North East Lincolnshire	30·3 (24·2 to 37·7)	29·1 (25·6 to 33·2)	23·8 (21·9 to 25·7)	17·1 (15 to 19·2)	9·32 (8·45 to 10·3)	7·59 (6·81 to 8·42)	6·19 (5·72 to 6·73)	5·07 (4·55 to 5·58)	4·23 (3·74 to 4·76)	4·79 (4·23 to 5·38)
North Lincolnshire	29·5 (22·7 to 38)	29·5 (25·8 to 33·5)	23·5 (21·8 to 25·3)	16 (14·4 to 17·8)	11·4 (10·3 to 12·6)	8·43 (7·51 to 9·48)	5·65 (5·11 to 6·23)	4·32 (3·9 to 4·79)	3·45 (3·05 to 3·86)	3·86 (3·39 to 4·35)
North Yorkshire	29·4 (24·6 to 34·8)	29·2 (26·7 to 31·8)	22·5 (20·8 to 24·4)	16·2 (14·6 to 18)	9·24 (8·5 to 9·97)	6·01 (5·48 to 6·61)	4·71 (4·41 to 5·02)	3·53 (3·24 to 3·83)	3·1 (2·81 to 3·41)	3·47 (3·12 to 3·84)
Rotherham	30·7 (25·6 to 36·9)	30·7 (27·4 to 34·2)	24·3 (22·3 to 26·1)	16·7 (15 to 18·5)	11·5 (10·5 to 12·5)	8·38 (7·64 to 9·23)	6·41 (5·89 to 6·96)	4·87 (4·38 to 5·37)	3·77 (3·39 to 4·16)	4·35 (3·9 to 4·81)
Sheffield	26·7 (22·3 to 31·8)	28·8 (25·7 to 32·1)	22·3 (19·9 to 25)	16·2 (14·7 to 18)	11·2 (10·2 to 12·2)	8·94 (8 to 9·96)	6·19 (5·6 to 6·79)	4·73 (4·32 to 5·15)	3·66 (3·29 to 4·06)	4·21 (3·74 to 4·66)
Wakefield	32·9 (27·4 to 39·6)	32·7 (29·1 to 36·4)	26·4 (24·3 to 28·5)	18·3 (16·5 to 20·1)	10·6 (9·75 to 11·4)	8·29 (7·54 to 9·1)	6·12 (5·51 to 6·72)	4·23 (3·86 to 4·62)	3·69 (3·33 to 4·06)	4·36 (3·93 to 4·82)
York	24·6 (19 to 31)	27 (23·8 to 30·6)	21·1 (19·5 to 22·8)	15·4 (13·6 to 17·2)	9·72 (8·79 to 10·7)	7·36 (6·58 to 8·25)	5·19 (4·75 to 5·67)	3·83 (3·39 to 4·26)	3·07 (2·69 to 3·48)	3·43 (2·97 to 3·87)
Northern Ireland	42·2 (38·1 to 46·8)	34·1 (32·6 to 35·7)	28·2 (26·9 to 29·4)	19·1 (17·9 to 20·3)	10 (9·3 to 10·8)	6·92 (6·2 to 7·68)	6·08 (5·49 to 6·71)	5·18 (4·52 to 5·87)	4·42 (3·84 to 5·07)	3·74 (2·92 to 4·56)
Scotland	37·8 (34·9 to 40·8)	34·4 (33·1 to 35·6)	25·4 (24·4 to 26·5)	16·7 (15·9 to 17·7)	10·8 (10·1 to 11·6)	7·15 (6·49 to 7·85)	4·98 (4·59 to 5·4)	4·42 (3·97 to 4·91)	3·76 (3·38 to 4·15)	4·6 (4·04 to 5·18)
Wales	29 (20·8 to 39)	31 (25·3 to 36·5)	25·5 (21·7 to 30·3)	17·7 (16·2 to 19·4)	9·99 (9·38 to 10·6)	7·13 (6·51 to 7·78)	5·39 (5·02 to 5·77)	4·63 (4·22 to 5·07)	4·4 (3·87 to 4·96)	4·63 (3·94 to 5·42)
Latin America and Caribbean	228 (203 to 256)	164 (152 to 176)	125 (116 to 134)	86·3 (81·4 to 91·7)	55·9 (52·8 to 59·2)	37·2 (34·5 to 40)	33·1 (30·1 to 36·3)	20·2 (16·6 to 24·1)	18·6 (15·3 to 22·6)	17·9 (14·3 to 22·2)
Andean Latin America	340 (302 to 380)	271 (251 to 293)	208 (196 to 220)	124 (117 to 132)	76·7 (71·8 to 82)	48·2 (44·4 to 52·4)	29·9 (26·1 to 34)	21·8 (17·9 to 26·4)	18·8 (15·2 to 23·1)	17·8 (13·7 to 22·5)
Bolivia	377 (325 to 435)	309 (277 to 342)	241 (220 to 262)	166 (154 to 177)	115 (107 to 123)	75·1 (68·8 to 82)	47·6 (42·1 to 53·4)	32·9 (27·3 to 39·3)	31 (25·6 to 36·9)	29·8 (24·6 to 35·6)
Ecuador	217 (181 to 256)	171 (157 to 184)	132 (122 to 143)	85·3 (78·2 to 93·3)	45·2 (41·3 to 49·5)	36·5 (31·8 to 42)	24·4 (19·8 to 29·5)	18·2 (14·3 to 22·6)	16·3 (12·8 to 20·6)	15 (11·5 to 19·6)
Peru	369 (313 to 429)	298 (270 to 325)	228 (212 to 245)	126 (115 to 138)	77·6 (70·6 to 85·5)	44·1 (39·3 to 48·7)	25·7 (22 to 30·1)	19·5 (15·2 to 24·6)	15·6 (12·1 to 19·9)	14·8 (9·95 to 20·4)
Caribbean	190 (166 to 218)	143 (133 to 153)	115 (108 to 122)	97·6 (90·6 to 105)	73·6 (68 to 79·8)	54·6 (49·7 to 59·9)	126 (117 to 136)	44·2 (36·5 to 52·9)	43·5 (35·6 to 52·5)	43·6 (35·5 to 53·1)
Antigua and Barbuda	126 (101 to 156)	77·6 (64·9 to 92·6)	40·5 (33·4 to 48·7)	22·9 (19·4 to 27·2)	13·8 (11·4 to 16·7)	14·1 (11 to 17·7)	11·8 (9·65 to 14·4)	10·5 (8·46 to 12·8)	10·4 (8·3 to 12·8)	9·04 (7·61 to 10·5)
The Bahamas	124 (97·9 to 158)	80·4 (68·4 to 95·2)	47·8 (40·1 to 57·1)	33·9 (28·3 to 39·9)	23·7 (19·7 to 28·2)	17·2 (14·1 to 21)	14·3 (11·7 to 17·3)	21·4 (17·9 to 26)	11·3 (8·24 to 15·3)	11 (8·19 to 14·8)
Barbados	240 (195 to 294)	88·6 (74·7 to 104)	51·4 (42·9 to 60·4)	27·6 (23·2 to 33·2)	20 (16·8 to 23·7)	14·6 (12 to 17·5)	14·5 (12 to 17·6)	12·4 (9·02 to 16·5)	12·1 (8·47 to 16·9)	12 (8·26 to 17)
Belize	190 (156 to 230)	129 (112 to 147)	100 (87·6 to 115)	71·2 (63·7 to 79)	48·5 (43·4 to 54·2)	32·3 (28 to 36·5)	21·3 (18·5 to 24·7)	17·4 (14·3 to 21)	17·6 (14·4 to 21·3)	15·8 (12·9 to 19·4)
Bermuda	77·3 (63·5 to 94·2)	45·3 (40·9 to 50·2)	29·5 (26·2 to 33)	18 (15·9 to 20·3)	10·4 (8·92 to 12·2)	5·61 (4·72 to 6·7)	4·96 (4·15 to 5·86)	3·82 (3·01 to 4·78)	3·73 (2·86 to 4·76)	3·64 (3·08 to 4·42)
Cuba	61·7 (40 to 90·9)	50·7 (48·9 to 52·3)	47·4 (45·7 to 48·9)	25·4 (24·4 to 26·6)	15·4 (14·7 to 16·2)	9·48 (8·96 to 9·99)	6·61 (6·22 to 7·06)	7·05 (6·55 to 7·56)	6·89 (6·36 to 7·46)	5·13 (4·38 to 5·96)
Dominica	265 (218 to 320)	146 (126 to 167)	68·8 (58·7 to 80·4)	26·1 (22 to 31·1)	20·5 (17 to 24·3)	20·3 (16·7 to 24·2)	25 (20·5 to 29·8)	30·1 (22·6 to 39·4)	30·3 (22·4 to 40·3)	29·8 (21·2 to 40·7)
Dominican Republic	219 (179 to 263)	188 (171 to 205)	138 (125 to 152)	97·1 (86·8 to 108)	69·4 (61·3 to 77·5)	44·7 (39·7 to 50)	35·9 (30·3 to 42·2)	28·2 (22·5 to 35·3)	27·8 (22·2 to 34·3)	27·1 (21·8 to 33·4)
Grenada	157 (123 to 196)	101 (84·9 to 118)	63·5 (52·8 to 75·9)	39·9 (33·2 to 48·5)	25 (20 to 31)	18·1 (14·2 to 22·5)	15·8 (12·4 to 20·2)	14·8 (11·5 to 18·5)	14·4 (11·2 to 18·1)	13·5 (10·5 to 16·9)
Guyana	186 (152 to 226)	117 (105 to 131)	93 (82·2 to 105)	69 (60·4 to 78·2)	59·7 (52 to 68·1)	43·4 (38·4 to 48·6)	35·2 (30 to 41)	28·4 (21·5 to 36·7)	26·6 (19·6 to 34·9)	25·4 (18·6 to 33·7)
Haiti	401 (342 to 465)	280 (253 to 307)	250 (230 to 271)	209 (191 to 228)	158 (144 to 172)	111 (99·9 to 124)	284 (265 to 304)	76·4 (62·9 to 91·5)	74·5 (61·2 to 89·9)	75·2 (61·4 to 91·2)
Jamaica	154 (128 to 186)	92·1 (83 to 101)	60·9 (53·8 to 68·1)	40·9 (36·2 to 46·2)	31·1 (26·8 to 35·5)	23·4 (18·8 to 29)	20·1 (16·9 to 23·9)	17·4 (13·1 to 22·6)	16·9 (12·4 to 22·6)	16·2 (11·6 to 22·1)
Puerto Rico	107 (98 to 116)	60·9 (58·8 to 63·1)	36·4 (34·9 to 37·9)	23·9 (22·8 to 25·1)	17·1 (16 to 18·1)	12·2 (11·3 to 13)	9·93 (8·83 to 11·1)	7·86 (7·04 to 8·74)	8·37 (7·44 to 9·39)	7 (5·73 to 8·5)
Saint Kitts and Nevis	152 (135 to 170)	142 (135 to 150)	73·4 (67·8 to 79·7)	59·3 (52·6 to 66·6)	32 (28·7 to 35·9)	21·9 (19·1 to 25·1)	19·4 (16·1 to 23·1)	17·9 (14·3 to 22·4)	16·9 (13·2 to 21·6)	16·5 (12·6 to 21·5)
Saint Lucia	205 (167 to 254)	149 (126 to 175)	82·6 (70·7 to 96·6)	42·2 (35·3 to 50·2)	25·8 (20·9 to 31·1)	20·1 (16·2 to 24·4)	20·3 (15·9 to 25·4)	17·9 (13 to 24·2)	17 (12·1 to 23·4)	16·8 (11·7 to 23·3)
Saint Vincent and the Grenadines	211 (169 to 256)	148 (128 to 171)	95 (80·2 to 112)	59·3 (49·6 to 71·2)	34·1 (27·5 to 41·2)	27 (22·2 to 32·7)	21·5 (17·8 to 25·9)	15·3 (11·8 to 19·5)	14·3 (10·8 to 18·9)	13·9 (10·1 to 18·9)
Suriname	103 (81·6 to 129)	87·6 (73·1 to 103)	74·3 (60·8 to 89·7)	74·1 (65·1 to 83·6)	57·2 (50·2 to 65·2)	43·7 (37·1 to 51·2)	33·6 (28·3 to 39·4)	30·1 (23·5 to 38·3)	28·2 (21·7 to 36·6)	27·5 (20·6 to 36)
Trinidad and Tobago	103 (82·8 to 126)	75·7 (68 to 84·1)	55·9 (49·7 to 63·6)	40·7 (35·8 to 45·6)	30·4 (25·8 to 35·5)	28·9 (24·2 to 34·4)	19·3 (15·6 to 24·2)	15·5 (12 to 19·6)	14·7 (11·1 to 19)	14·5 (10·6 to 19·7)
Virgin Islands	71·4 (60 to 84·2)	51·3 (46·1 to 56·9)	34·6 (30·7 to 38·5)	29·9 (26·5 to 33·4)	19·7 (17 to 22·5)	12·6 (10·5 to 14·7)	9·35 (7·63 to 11·4)	7·56 (5·84 to 9·6)	7·9 (6·27 to 9·86)	6·52 (5·15 to 8·1)
Central Latin America	210 (184 to 240)	144 (134 to 153)	107 (101 to 114)	75·5 (70·4 to 80·8)	48·7 (45·4 to 52·3)	32·4 (29·7 to 35·4)	23·5 (20·6 to 26·7)	19·1 (15·4 to 23·1)	17·8 (14·2 to 22·2)	16·9 (12·9 to 22)
Colombia	155 (121 to 194)	122 (107 to 137)	85·5 (76·3 to 95·3)	58·2 (50·5 to 66·3)	36·9 (32·5 to 41·5)	28·5 (24·3 to 33)	21·4 (16·2 to 27·5)	16·2 (12·2 to 21)	14·9 (10·9 to 19·8)	13·1 (9·35 to 18·2)
Costa Rica	162 (152 to 173)	99·1 (96 to 102)	87·7 (84·6 to 91·1)	26·9 (25·7 to 28·2)	19·2 (18·2 to 20·2)	14 (13·2 to 14·8)	11·9 (11·1 to 12·7)	10·4 (9·35 to 11·4)	9·58 (8·58 to 10·6)	9·92 (8·52 to 11·5)
El Salvador	294 (238 to 362)	211 (187 to 239)	155 (140 to 170)	99·9 (90·1 to 110)	56·3 (50 to 63·3)	31·1 (27·3 to 35·4)	18·6 (15·9 to 21·7)	11·8 (9·09 to 14·9)	10·6 (8·05 to 13·8)	10 (7·35 to 13·5)
Guatemala	258 (212 to 312)	205 (181 to 231)	173 (156 to 191)	128 (117 to 139)	80·8 (74·3 to 88·3)	53·3 (47·5 to 59·6)	38·2 (32·3 to 45·3)	33·6 (27·1 to 40·5)	29·3 (23·5 to 36·2)	28 (21·6 to 36·4)
Honduras	296 (246 to 358)	197 (174 to 220)	140 (126 to 154)	93 (83·8 to 102)	58·6 (52·7 to 65)	38 (33 to 43·2)	27·5 (22·4 to 33·5)	19 (15·4 to 23·3)	17·4 (14·1 to 21·2)	16·4 (13·2 to 20·3)
Mexico	226 (186 to 271)	151 (138 to 164)	109 (99·2 to 119)	78·7 (70·5 to 87·3)	50·3 (45·8 to 55·8)	32·1 (28·9 to 35·6)	23·1 (20·1 to 26·4)	17·3 (13·7 to 21·3)	16·6 (13·1 to 21·1)	16·3 (12·6 to 21·1)

Aguascalientes	189 (155 to 231)	122 (111 to 134)	88·7 (79 to 98)	63·8 (52·5 to 75·8)	41·9 (36·1 to 48·6)	25·9 (21·9 to 31·1)	18·3 (15·4 to 21·6)	14·2 (11·3 to 17·8)	14·2 (11·2 to 18·3)	14·3 (10·9 to 18·6)
Baja California	203 (151 to 262)	128 (115 to 141)	92·7 (84·7 to 101)	48·9 (44·3 to 53·7)	37·8 (34·2 to 41·7)	26·2 (23·6 to 29·1)	22·1 (19·2 to 25·4)	14·6 (11·6 to 18·1)	15·1 (11·7 to 19·1)	14·9 (11·4 to 19·4)
Baja California Sur	225 (173 to 284)	141 (127 to 156)	96·6 (82·7 to 112)	59·4 (51·6 to 67)	27·1 (24·7 to 30·2)	21·3 (18·8 to 24·2)	18·8 (16·2 to 21·7)	15·1 (11·6 to 19·3)	15·7 (12 to 20·1)	15·3 (11·5 to 20)
Campeche	218 (177 to 265)	148 (134 to 162)	110 (98·1 to 122)	81·6 (70·8 to 92·7)	49·6 (44·4 to 55·6)	30·2 (26·3 to 34·4)	23·2 (20·1 to 26·8)	18·4 (14·6 to 22·9)	17·3 (13·5 to 21·9)	16·7 (12·7 to 21·6)
Chiapas	221 (174 to 279)	171 (154 to 190)	136 (122 to 151)	103 (90·4 to 117)	63·8 (56·3 to 72·9)	37·1 (31·6 to 43)	20·1 (17·4 to 23)	20·1 (16 to 24·6)	18·9 (14·6 to 24)	19·8 (15 to 25·7)
Chihuahua	227 (177 to 285)	158 (142 to 174)	116 (105 to 128)	85 (74·7 to 96)	53·3 (46·3 to 61·2)	38·6 (33·4 to 43·9)	24·4 (20·9 to 28·2)	20·5 (16·2 to 25·5)	20·9 (16·2 to 26·6)	21·2 (16·3 to 27·7)
Coahuila	242 (191 to 300)	156 (139 to 172)	112 (99 to 125)	81 (68·9 to 94·9)	46·3 (40 to 53·9)	29·6 (25·3 to 34·5)	17·7 (15·3 to 20·3)	17·6 (13·9 to 21·8)	16·8 (13 to 21·3)	17·1 (13·2 to 21·9)
Colima	289 (236 to 348)	184 (168 to 201)	125 (110 to 143)	80·7 (65·2 to 97·5)	44·3 (37·9 to 51·4)	27·6 (23·9 to 31·8)	20·2 (17·3 to 23·5)	13 (10·1 to 16·4)	13·3 (10·3 to 17·1)	13 (9·89 to 16·9)
Durango	243 (198 to 295)	159 (144 to 174)	113 (101 to 125)	82·7 (67·8 to 98·4)	50·7 (44·9 to 57·1)	33·6 (28·3 to 39·9)	23·3 (19·9 to 27)	16·4 (13·1 to 20·5)	15·8 (12·4 to 20·1)	16·1 (12·3 to 20·7)
Guanajuato	246 (184 to 318)	165 (150 to 181)	121 (104 to 139)	87·2 (80·1 to 95·8)	54·8 (50·4 to 59·9)	26·7 (23·7 to 29·9)	20·2 (17·7 to 22·8)	15·9 (12·7 to 19·7)	15·3 (11·9 to 19·2)	14·4 (11 to 18·6)
Guerrero	279 (230 to 335)	209 (180 to 239)	160 (141 to 182)	117 (102 to 131)	78·9 (69·2 to 89·8)	54·5 (47·3 to 62·2)	39·9 (32·5 to 48·6)	24·4 (18·4 to 31·4)	22·8 (16·9 to 30·3)	22·3 (16·2 to 30·2)
Hidalgo	248 (199 to 305)	165 (148 to 181)	120 (109 to 132)	72·3 (63·2 to 82·4)	44·1 (37·7 to 51·1)	30·2 (25·4 to 35·8)	19·5 (16·9 to 22·5)	14·1 (11·2 to 17·5)	12·8 (10·1 to 16·2)	12·4 (9·15 to 16·6)
Jalisco	195 (152 to 247)	134 (120 to 149)	93·7 (83 to 104)	60·9 (52·8 to 70)	39·6 (33·9 to 46·5)	29·3 (25·6 to 33·7)	20·3 (17·8 to 23)	15·1 (12 to 18·9)	15·4 (12 to 19·6)	15·7 (12·1 to 20·4)
México	215 (164 to 274)	141 (126 to 155)	99·6 (90·5 to 108)	77·5 (68·5 to 86·3)	53·7 (48·7 to 59·1)	31·6 (28·1 to 35·4)	25·7 (22·6 to 29·1)	19 (15·2 to 23·6)	17·1 (13·4 to 21·8)	14·4 (11 to 18·7)
Mexico City	162 (116 to 218)	93·4 (83 to 104)	68·8 (62·5 to 75·7)	60·8 (55·6 to 66·3)	36·7 (33·7 to 40·3)	25·5 (22·9 to 28·3)	23·1 (20·1 to 26·2)	15·5 (12·4 to 19·2)	16·9 (13·2 to 21·3)	21·1 (16·4 to 27·1)
Michoacán de Ocampo	264 (220 to 313)	180 (155 to 206)	128 (113 to 144)	87·9 (77·2 to 98·5)	56·1 (49·6 to 63·8)	37·1 (32·5 to 42)	29 (23·6 to 35·2)	18·1 (13·5 to 23·1)	17·5 (12·8 to 23·1)	17·3 (12·4 to 23·4)
Morelos	264 (215 to 318)	170 (154 to 186)	119 (107 to 133)	79·6 (69 to 90·8)	45·2 (40·1 to 50·7)	28·1 (23·8 to 32·8)	20·2 (17·3 to 23·3)	14·3 (11·3 to 18)	13·8 (10·7 to 17·6)	13·8 (10·5 to 17·8)
Nayarit	242 (198 to 289)	158 (144 to 174)	105 (94 to 118)	66·2 (53·6 to 79·5)	43·5 (38 to 49·8)	26·7 (22·5 to 31·5)	17·6 (15 to 20·3)	15·1 (11·9 to 19)	14 (10·6 to 18·3)	13·8 (10·3 to 18·5)
Nuevo León	197 (156 to 246)	125 (111 to 138)	85·7 (77·8 to 93·9)	52·6 (44·7 to 61·3)	31·6 (26·9 to 37·2)	23·8 (20·1 to 28·2)	15·2 (13·2 to 17·4)	13·1 (10·4 to 16·4)	12·1 (9·53 to 15·3)	12·4 (9·54 to 16·1)
Oaxaca	259 (207 to 312)	190 (161 to 220)	150 (133 to 167)	104 (91·2 to 116)	59·3 (52·3 to 67·4)	39·5 (34·1 to 45·2)	29·3 (23·5 to 36·3)	18·2 (13·4 to 23·2)	17·5 (12·7 to 23·2)	17·1 (12·3 to 23)
Puebla	265 (200 to 341)	185 (169 to 202)	135 (122 to 149)	99·7 (87·4 to 113)	62·8 (54·9 to 71·4)	39·9 (34·6 to 45·7)	28·1 (24·8 to 31·8)	19·7 (15·8 to 24·5)	19·5 (15·4 to 24·6)	18·5 (14·2 to 23·8)
Querétaro	266 (201 to 340)	170 (155 to 185)	118 (105 to 131)	84·4 (75·7 to 92·5)	49·4 (44·3 to 55·1)	25·6 (23 to 28·3)	20·3 (17·6 to 23·3)	13·6 (10·7 to 17)	12·9 (10·1 to 16·3)	13 (9·98 to 16·7)
Quintana Roo	182 (148 to 221)	129 (118 to 141)	95·1 (85·7 to 105)	66·6 (55·2 to 79·4)	38·5 (33·6 to 44·4)	25·1 (21·3 to 29·8)	20·2 (17·5 to 23·5)	14 (11·2 to 17·5)	12·6 (9·85 to 16·2)	12·1 (9·24 to 15·8)
San Luis Potosí	252 (205 to 304)	169 (144 to 194)	122 (107 to 139)	79·8 (69·6 to 90·1)	51·9 (46·2 to 58·4)	34·9 (30·2 to 39·8)	26·4 (21·4 to 32·1)	16·3 (12 to 20·9)	15·9 (11·6 to 21·1)	15·9 (11·4 to 21·5)
Sinaloa	211 (167 to 264)	141 (127 to 155)	96·5 (87·1 to 105)	61·4 (53·6 to 70·3)	36·9 (30·7 to 44·3)	24·1 (20·2 to 28·8)	14·9 (12·9 to 17)	13·6 (10·8 to 16·9)	12 (9·5 to 15·3)	12·5 (9·56 to 16·4)
Sonora	170 (132 to 214)	115 (102 to 128)	84·8 (75·8 to 93·7)	64·1 (56·5 to 72·4)	37·5 (31·9 to 44)	29·2 (25·2 to 33·7)	21·1 (18·3 to 24·1)	18·2 (14·3 to 22·9)	16 (12·2 to 20·8)	15·9 (12·2 to 21)
Tabasco	212 (167 to 269)	151 (135 to 167)	117 (105 to 128)	84·7 (74·6 to 96)	53·4 (47·2 to 60·5)	31 (25·4 to 37)	23·5 (20·6 to 26·8)	22 (17·5 to 26·9)	22 (17·4 to 27·9)	19·5 (14·9 to 25·2)
Tamaulipas	182 (142 to 227)	123 (110 to 135)	87·4 (78·3 to 96·7)	64·9 (56·7 to 73·7)	39·4 (34·9 to 44·7)	23·2 (20·2 to 26·5)	19·9 (17·2 to 22·8)	17·8 (14·1 to 22·2)	17·1 (13·3 to 21·6)	17·6 (13·5 to 22·6)
Tlaxcala	276 (207 to 357)	178 (161 to 195)	124 (111 to 139)	94·3 (85·1 to 103)	56·3 (50·4 to 62·8)	33·1 (29·4 to 37·2)	24·9 (21·7 to 28·4)	17·2 (13·6 to 21·5)	17·4 (13·3 to 22·6)	16·5 (12·4 to 21·9)
Veracruz de Ignacio de la Llave	228 (179 to 288)	161 (145 to 177)	118 (105 to 131)	81·7 (71 to 93·4)	54·7 (48·2 to 62·1)	34·8 (30·2 to 39·7)	21·6 (19 to 24·7)	17·9 (14·3 to 22·2)	17·9 (14·3 to 22·6)	16·4 (12·5 to 21·7)
Yucatán	187 (144 to 235)	137 (122 to 152)	95·6 (86·1 to 107)	72·7 (61·6 to 85·2)	46·2 (40·2 to 53·1)	27·9 (24·3 to 31·7)	19 (16·5 to 21·7)	13·6 (10·8 to 16·8)	14·6 (11·2 to 18·9)	14·7 (11·2 to 19·3)
Zacatecas	213 (174 to 258)	141 (128 to 154)	97·3 (86·5 to 109)	71·1 (58·9 to 84·5)	42·4 (36·9 to 48·3)	27·2 (22·7 to 32·7)	19·8 (16·9 to 22·9)	14·6 (11·7 to 18·2)	13·6 (10·5 to 17·4)	12·9 (9·81 to 16·9)
Nicaragua	290 (238 to 351)	221 (197 to 247)	170 (154 to 186)	110 (101 to 119)	68·9 (62·2 to 75·8)	39·8 (35 to 45·1)	24·9 (20·6 to 29·5)	17·7 (13·5 to 22·6)	16·9 (12·7 to 21·9)	15·3 (11·2 to 20·2)
Panama	112 (91·7 to 135)	87·5 (78·9 to 96)	59 (52·3 to 66·5)	38·3 (33·5 to 43·6)	28·9 (24·7 to 33·3)	24·3 (20·8 to 28·2)	19·4 (16·4 to 23·1)	17·6 (14·3 to 21·3)	16·2 (13 to 20·1)	15·1 (11·6 to 19·4)
Venezuela	163 (155 to 172)	78·5 (76·8 to 80·4)	72·1 (70·4 to 73·7)	45 (43·5 to 46·7)	36·2 (34·9 to 37·6)	25·6 (24 to 27·2)	20·4 (19·6 to 21·3)	25 (19·3 to 31·8)	23·3 (17·3 to 30·5)	21·7 (15·9 to 28·7)
Tropical Latin America	222 (170 to 280)	160 (139 to 181)	124 (110 to 139)	85·5 (77 to 95·1)	55 (49·7 to 60·3)	36 (31·4 to 40·7)	24·8 (20·9 to 29·1)	15·5 (12·5 to 18·8)	13·8 (11·2 to 16·7)	13 (10·5 to 15·9)
Brazil	224 (171 to 284)	162 (140 to 184)	126 (111 to 141)	86·5 (77·7 to 96·2)	55·8 (50·3 to 61·2)	36·2 (31·5 to 41·2)	25 (21 to 29·3)	15·4 (12·4 to 18·7)	13·7 (11 to 16·6)	12·9 (10·4 to 15·8)
Acre	268 (188 to 364)	188 (161 to 217)	148 (126 to 175)	112 (95·4 to 129)	72·8 (62·8 to 84)	54·3 (45·6 to 63·9)	37 (31·4 to 43·3)	20·6 (15·5 to 26·7)	19 (14·1 to 24·9)	18 (13·7 to 22·9)
Alagoas	321 (232 to 420)	250 (213 to 291)	201 (173 to 232)	178 (156 to 201)	117 (99·9 to 136)	66·1 (56·3 to 76·7)	33 (25·7 to 41·5)	15·4 (11·9 to 19·6)	12·8 (9·68 to 16·3)	11·8 (8·95 to 15·3)
Amapá	283 (199 to 387)	185 (160 to 214)	121 (101 to 145)	46·3 (41·2 to 52·2)	29 (25·7 to 32·5)	29·5 (25·4 to 33·9)	24·2 (21·2 to 27·6)	24·7 (19·9 to 30·5)	22·2 (18·1 to 27·4)	21·2 (17·4 to 25·4)
Amazonas	193 (121 to 291)	120 (98·7 to 145)	92·7 (77·3 to 109)	70·8 (59·3 to 84·4)	44 (36·9 to 52)	34 (29·8 to 38·7)	23·7 (20·9 to 26·6)	20·5 (17·1 to 24·6)	18·5 (15·5 to 22)	18·7 (16 to 21·7)
Bahia	257 (175 to 358)	197 (161 to 235)	160 (138 to 183)	105 (92·2 to 119)	80·1 (70·3 to 90·6)	49·6 (42·2 to 58·2)	32·5 (25·7 to 40·4)	17·5 (13·6 to 22·2)	14·9 (11·5 to 19)	13·6 (10·5 to 17·5)
Ceará	363 (266 to 470)	286 (244 to 331)	209 (181 to 243)	140 (119 to 164)	80·6 (70·7 to 91·7)	47·2 (39·9 to 55·3)	24·6 (19 to 30·3)	13·4 (10·1 to 16·9)	10·9 (8·23 to 13·9)	9·38 (7·01 to 12·4)
Distrito Federal	156 (106 to 216)	121 (102 to 143)	87·7 (73·3 to 105)	49·9 (45·1 to 55)	30·4 (27·3 to 34)	20·4 (18 to 23·1)	17·2 (15·1 to 19·5)	12·1 (9·93 to 14·9)	11·9 (10 to 14·1)	11·8 (10·1 to 14)
Espírito Santo	164 (124 to 215)	113 (95·1 to 132)	92·9 (78·8 to 110)	55·8 (50·6 to 61·7)	37·6 (34·1 to 41·7)	25 (21·2 to 29·1)	24·9 (19·8 to 30·7)	14·6 (11·2 to 18·6)	13·5 (10·3 to 17·3)	13·3 (10 to 17·4)
Goiás	270 (187 to 367)	200 (169 to 233)	136 (112 to 160)	76·6 (65·8 to 87·9)	47·8 (41·4 to 55·1)	35·3 (27·9 to 44·1)	25·5 (20·2 to 31·6)	17·1 (13·2 to 21·6)	15·9 (12·2 to 20·2)	15·4 (11·8 to 19·5)
Maranhão	325 (265 to 395)	261 (225 to 302)	213 (186 to 241)	146 (129 to 164)	95·1 (84·3 to 107)	57·7 (49·8 to 67·4)	35·7 (28·3 to 44·1)	16·1 (12·2 to 20·8)	13·1 (10·1 to 16·7)	12·1 (9·36 to 15·3)
Mato Grosso	168 (121 to 225)	141 (119 to 165)	107 (90·8 to 125)	61·9 (53·1 to 70·9)	44·6 (38·5 to 51·4)	31·9 (25·1 to 40)	24·6 (19·6 to 30·5)	18·1 (14·1 to 22·9)	17·6 (13·6 to 22·5)	17·2 (13·2 to 22·1)
Mato Grosso do Sul	154 (98·7 to 228)	108 (86·7 to 131)	78·8 (65·9 to 94·6)	50 (45·3 to 55·5)	34·1 (30·8 to 37·9)	28·9 (25·1 to 33)	21·7 (19·2 to 24·4)	14·4 (11·8 to 17·6)	14 (11·7 to 16·8)	13 (11 to 15·4)
Minas Gerais	188 (135 to 255)	131 (109 to 155)	97·2 (81·9 to 114)	63·5 (55 to 72·3)	42·7 (36·3 to 49·2)	30·9 (25 to 37·5)	21·7 (17·5 to 26·7)	14·3 (11·2 to 17·8)	12·9 (9·99 to 16·3)	12·1 (9·33 to 15·4)
Pará	265 (186 to 354)	183 (156 to 212)	138 (115 to 162)	94·3 (80·8 to 110)	63 (54·3 to 72·4)	42·2 (33·3 to 53·1)	32·3 (27·1 to 38·1)	19·4 (15·1 to 24·6)	18·2 (13·6 to 23·3)	17 (13 to 22·2)
Paraíba	378 (273 to 494)	281 (249 to 317)	201 (173 to 229)	138 (116 to 162)	83·9 (74 to 94·7)	48·5 (40·9 to 56·9)	28·5 (23·5 to 33·9)	14·8 (11·6 to 18·4)	12·5 (9·28 to 16·3)	11·5 (8·47 to 15·4)
Paraná	147 (104 to 202)	107 (89·4 to 125)	85·2 (70·5 to 101)	59·6 (54 to 65·4)	36·4 (33 to 40)	25·2 (21·8 to 29·1)	21·7 (17·2 to 26·8)	13·8 (10·8 to 17·2)	12·6 (9·42 to 16·1)	11·8 (8·89 to 15·2)
Pernambuco	363 (270 to 462)	281 (247 to 318)	217 (193 to 243)	142 (120 to 168)	92·3 (81·7 to 103)	53·4 (44·9 to 62·5)	31·3 (26·4 to 36·9)	15 (11·7 to 18·9)	13·1 (10 to 16·6)	12·3 (9·42 to 15·8)
Piauí	249 (171 to 337)	189 (164 to 217)	162 (136 to 189)	108 (93·2 to 125)	71·6 (61·6 to 81·8)	45·1 (38·7 to 52·3)	29·2 (24 to 34·9)	16·5 (12·7 to 21·1)	14·2 (10·7 to 18)	12·6 (9·37 to 16·4)
Rio de Janeiro	146 (82·9 to 232)	91 (75 to 109)	73·5 (59·9 to 88·3)	66·9 (60·8 to 73·5)	41 (37·4 to 45·1)	25·8 (22·7 to 29·2)	19·3 (17 to 21·8)	16·4 (13·7 to 19·6)	15·1 (12·7 to 17·7)	14·4 (12·5 to 16·5)
Rio Grande do Norte	409 (302 to 524)	298 (254 to 345)	200 (176 to 226)	125 (106 to 150)	83 (69·8 to 96·5)	45·8 (38·4 to 54·1)	27·4 (21·5 to 34·1)	13·8 (10·6 to 17·5)	10·9 (8·11 to 14·1)	10·2 (7·61 to 13·2)
Rio Grande do Sul	119 (78·4 to 166)	81·5 (67·9 to 98·1)	57·6 (47·8 to 70·3)	42·7 (38·6 to 47)	24·9 (22·6 to 27·5)	20·2 (17·7 to 23)	14·4 (12·6 to 16·3)	14·3 (11·9 to 17)	12·3 (10·4 to 14·4)	12·5 (10·8 to 14·2)
Rondônia	267 (154 to 410)	160 (132 to 191)	109 (90·3 to 131)	81·1 (68·2 to 96·4)	49·9 (42·6 to 58·3)	35·4 (29·9 to 41·4)	26·8 (21·3 to 33)	15·5 (11·9 to 19·8)	14·7 (11·1 to 18·8)	10·3 (6·97 to 14·2)
Roraima	281 (199 to 373)	189 (162 to 218)	137 (114 to 163)	67·8 (60·2 to 76·3)	41·9 (36·6 to 48)	28 (24·2 to 32·2)	26·5 (22·9 to 30·7)	26·8 (21·8 to 32·7)	23·7 (19·5 to 28·4)	19·1 (15·4 to 23·9)
Santa Catarina	120 (79·5 to 173)	81·4 (67·4 to 97·7)	67·5 (55·9 to 79·4)	47·4 (40·5 to 54·7)	34·6 (29·7 to 40·2)	25·7 (21·1 to 30·8)	18·8 (15·1 to 23·2)	12·3 (8·89 to 17)	12·2 (9·36 to 15·7)	11·5 (8·7 to 14·8)
São Paulo	123 (85·1 to 169)	85·7 (70·3 to 103)	68·6 (57·6 to 80·4)	58·6 (53·6 to 64·4)	36·4 (33·1 to 39·9)	23·5 (20·4 to 26·7)	18·7 (15 to 22·8)	13·3 (10·4 to 16·8)	12·3 (10·3 to 14·6)	12·2 (10·4 to 14·2)
Sergipe	271 (186 to 377)	198 (167 to 232)	142 (122 to 163)	110 (95·3 to 126)	69·7 (60·9 to 79·2)	45·8 (36·1 to 57·5)	28 (22·5 to 34·5)	18 (13·9 to 22·7)	16·2 (12·4 to 20·4)	14·8 (11·4 to 19)
Tocantins	268 (158 to 415)	208 (176 to 245)	157 (130 to 186)	97·5 (83·5 to 114)	63·5 (53·1 to 75)	43·1 (36·7 to 50·8)	26·5 (21·4 to 32·4)	14·7 (11·4 to 18·8)	12·8 (9·38 to 16·7)	10·7 (7·43 to 14·8)

Paraguay	120 (90·6 to 154)	83·9 (73·5 to 94·7)	71·9 (63·3 to 80·8)	51·5 (44·3 to 59·1)	35·8 (30·8 to 42·1)	29·8 (25·4 to 34·9)	22·2 (18 to 27·1)	18·2 (13·3 to 23·8)	17·1 (12·2 to 22·8)	16 (11·3 to 21·7)
North Africa and Middle East	320 (281 to 362)	254 (233 to 275)	194 (182 to 207)	132 (124 to 139)	83·4 (78 to 89)	58·7 (53·9 to 63·8)	38 (34·2 to 41·9)	25·3 (21·6 to 29·4)	22·9 (19·6 to 26·7)	21·7 (18·4 to 25·3)
North Africa and Middle East	320 (281 to 362)	254 (233 to 275)	194 (182 to 207)	132 (124 to 139)	83·4 (78 to 89)	58·7 (53·9 to 63·8)	38 (34·2 to 41·9)	25·3 (21·6 to 29·4)	22·9 (19·6 to 26·7)	21·7 (18·4 to 25·3)
Afghanistan	343 (279 to 410)	235 (203 to 270)	213 (186 to 246)	206 (180 to 232)	157 (141 to 174)	131 (118 to 144)	81·9 (70·3 to 94·2)	56·5 (47·1 to 67·8)	53·1 (44 to 63·9)	50·8 (41·9 to 61·7)
Algeria	328 (271 to 398)	262 (230 to 297)	194 (167 to 223)	135 (125 to 144)	62·1 (57·1 to 67·8)	43·5 (37·9 to 49·1)	30 (27·1 to 33·1)	21·2 (17·6 to 25·2)	19·3 (15·7 to 23·4)	18·1 (14·3 to 22·5)
Bahrain	179 (139 to 227)	150 (128 to 175)	77·7 (64·8 to 93·6)	31·3 (29 to 33·7)	24·4 (22·9 to 26·1)	12·6 (11·5 to 13·7)	8·48 (7·83 to 9·16)	7·3 (6·51 to 8·25)	6·2 (5·23 to 7·15)	5·81 (4·77 to 6·89)
Egypt	315 (249 to 384)	271 (245 to 297)	228 (210 to 246)	152 (138 to 167)	83 (75 to 91·3)	46·6 (40·8 to 52·9)	29·8 (25·2 to 34·7)	17·4 (14·1 to 21·3)	14·7 (11·7 to 18·1)	13·6 (11 to 17)
Iran	399 (330 to 470)	303 (264 to 345)	210 (184 to 239)	110 (97·4 to 125)	76·8 (68·6 to 85·1)	43·7 (38·2 to 49·9)	29·2 (25·8 to 33·1)	12·2 (10·9 to 13·7)	8·3 (7·56 to 9·09)	5·62 (4·67 to 6·7)
Alborz	326 (262 to 394)	303 (262 to 347)	200 (173 to 231)	88·5 (76·9 to 101)	50·9 (44·2 to 57·8)	33·1 (27·5 to 39·3)	21·5 (18·6 to 24·6)	8·85 (7·47 to 10·4)	5·27 (4·58 to 6·02)	3·63 (2·87 to 4·47)
Ardebil	396 (325 to 476)	330 (289 to 373)	254 (225 to 287)	150 (132 to 170)	105 (92·8 to 117)	58·2 (50·9 to 66·5)	33·1 (29 to 37·5)	13·6 (11·9 to 15·5)	9·45 (8·44 to 10·6)	6·31 (5·35 to 7·37)
Bushehr	336 (268 to 406)	258 (222 to 297)	191 (165 to 220)	96·3 (83·1 to 111)	61·5 (52·8 to 69·3)	38·4 (33 to 44·1)	28·3 (23·9 to 33·2)	12·8 (10·9 to 14·9)	8·72 (7·58 to 10)	5·84 (4·98 to 6·85)
Chahar Mahaal and Bakhtiari	299 (236 to 366)	230 (197 to 266)	170 (145 to 197)	94·3 (81·4 to 108)	68·2 (59 to 77)	36·9 (31·4 to 43)	22·3 (19 to 26)	9·07 (7·81 to 10·4)	5·35 (4·65 to 6·07)	3·57 (2·86 to 4·35)
East Azarbayejan	421 (350 to 497)	334 (293 to 378)	248 (218 to 280)	141 (124 to 162)	83·5 (73·8 to 93·9)	47·2 (39·6 to 55·4)	28·6 (25·2 to 32·5)	10·4 (8·95 to 12·1)	6·58 (5·67 to 7·58)	4·43 (3·51 to 5·51)
Fars	271 (210 to 338)	199 (168 to 232)	140 (118 to 164)	74·1 (64·1 to 85·4)	56·6 (49·3 to 64·1)	41 (34·3 to 48·7)	30·8 (26·8 to 35·3)	14·1 (12·1 to 16·1)	9·94 (8·44 to 11·8)	6·68 (5·2 to 8·37)
Gilan	354 (277 to 439)	249 (202 to 308)	162 (138 to 188)	77 (65·3 to 89·1)	184 (169 to 200)	28·6 (24·4 to 33·1)	15·1 (13·2 to 17·1)	6·13 (5·28 to 7·21)	2·77 (2·04 to 3·48)	1·92 (1·31 to 2·61)
Golestan	348 (283 to 421)	278 (239 to 321)	206 (179 to 234)	112 (96·2 to 128)	82·1 (71·9 to 93·1)	49·6 (42·5 to 57·2)	30·1 (26·3 to 34·3)	13·4 (11·6 to 15·4)	9·34 (8·27 to 10·6)	6·28 (5·41 to 7·26)
Hamadan	482 (398 to 566)	374 (329 to 421)	277 (244 to 312)	138 (121 to 157)	85·3 (74·7 to 95·7)	51 (44·3 to 58·9)	30·5 (26·8 to 34·7)	13·7 (11·9 to 15·7)	9·6 (8·67 to 10·6)	6·44 (5·55 to 7·43)
Hormozgan	418 (346 to 500)	281 (244 to 320)	200 (174 to 229)	109 (95·5 to 124)	84·9 (74·8 to 96·2)	51·3 (44·2 to 59·2)	31·4 (27·6 to 35·9)	13·6 (11·8 to 15·4)	9·46 (8·57 to 10·5)	6·34 (5·54 to 7·26)
Ilam	324 (255 to 394)	246 (210 to 281)	184 (159 to 210)	107 (93·4 to 122)	70·7 (61·7 to 79·9)	45·4 (38·2 to 53·3)	28·4 (24·6 to 32·7)	13·1 (11·2 to 15·2)	9·03 (7·17 to 11·1)	6·11 (4·38 to 8·16)
Isfahan	337 (267 to 411)	286 (247 to 327)	185 (159 to 214)	90·6 (78·4 to 104)	53·6 (46·6 to 60·5)	33·7 (27·3 to 40·9)	25·7 (22·1 to 29·7)	11·6 (9·61 to 13·7)	7·71 (6·39 to 9·32)	5·27 (3·99 to 6·77)
Kerman	442 (358 to 526)	309 (267 to 354)	211 (183 to 242)	105 (92·1 to 119)	66·5 (57·9 to 75·5)	40·9 (35·2 to 47)	34·5 (29·3 to 40·2)	16·6 (14·6 to 18·8)	12·5 (11·1 to 14·2)	8·5 (7·3 to 9·82)
Kermanshah	389 (317 to 471)	301 (261 to 344)	222 (194 to 253)	127 (111 to 145)	85·3 (74·5 to 96·1)	53·9 (46·5 to 62·2)	34 (29·2 to 39·1)	14·1 (12·2 to 16·2)	9·68 (8·49 to 11·1)	6·45 (5·49 to 7·6)
Khorasan-e-Razavi	504 (429 to 580)	388 (339 to 440)	279 (248 to 314)	144 (126 to 164)	90·8 (79·5 to 102)	55 (47·1 to 63·8)	39·1 (33·3 to 45·5)	13·1 (11·8 to 14·5)	8·79 (7·38 to 10·2)	5·82 (4·35 to 7·37)
Khuzestan	384 (310 to 470)	276 (236 to 321)	195 (169 to 225)	102 (88·3 to 117)	60·9 (53·4 to 68·4)	43·3 (36·2 to 51·3)	30·3 (26·4 to 34·5)	13·2 (11·7 to 14·6)	9·28 (8·24 to 10·4)	6·35 (5·2 to 7·65)
Kohgiluyeh and Boyer-Ahmad	341 (271 to 414)	240 (207 to 275)	182 (158 to 208)	110 (95·3 to 127)	78·1 (68·3 to 87·4)	50·2 (43·1 to 57·9)	28·5 (24·8 to 32·7)	11·7 (9·97 to 13·6)	7·7 (6·73 to 8·81)	5·12 (4·18 to 6·12)
Kurdistan	493 (416 to 574)	412 (365 to 461)	309 (276 to 345)	185 (163 to 208)	116 (103 to 130)	65·3 (57 to 74·2)	35·8 (31·2 to 41)	14 (12·2 to 16·1)	9·85 (8·72 to 11·2)	6·66 (5·71 to 7·72)
Lorestan	414 (331 to 507)	286 (245 to 331)	204 (177 to 233)	111 (96·3 to 129)	73·5 (64·3 to 84·3)	40·2 (34·5 to 46·3)	20·2 (16·9 to 23·7)	8·16 (6·99 to 9·53)	4·54 (3·88 to 5·2)	3·03 (2·46 to 3·63)
Markazi	355 (284 to 433)	297 (256 to 341)	223 (193 to 258)	110 (95·4 to 127)	68·5 (59·2 to 78)	40·8 (34·6 to 47·5)	26·5 (22·6 to 30·7)	11·6 (10·1 to 13·1)	7·59 (6·79 to 8·52)	5·06 (4·2 to 5·99)
Mazandaran	298 (233 to 372)	233 (198 to 270)	158 (135 to 183)	76·6 (65·9 to 88·6)	44·7 (38 to 52)	29·1 (24·7 to 34·3)	18·5 (16·1 to 21·3)	8·55 (7·24 to 9·95)	4·73 (4·12 to 5·39)	3·14 (2·45 to 3·89)
North Khorasan	443 (354 to 538)	359 (315 to 405)	274 (244 to 308)	148 (130 to 167)	96·5 (85·2 to 109)	53·7 (45·5 to 62·5)	29·9 (26 to 34)	12·3 (10·7 to 14)	8·24 (7·3 to 9·28)	5·47 (4·62 to 6·38)
Qazvin	464 (383 to 554)	372 (325 to 421)	262 (229 to 297)	129 (113 to 148)	73·3 (64 to 82·7)	45·9 (39·5 to 53)	25·6 (21·7 to 29·8)	10·7 (9·19 to 12·3)	6·78 (5·86 to 7·78)	4·5 (3·57 to 5·52)
Qom	304 (239 to 377)	250 (214 to 288)	185 (159 to 213)	93·6 (81·4 to 107)	54·2 (46·2 to 62·9)	32·6 (27·8 to 38·2)	22·1 (19 to 25·6)	9·05 (7·89 to 10·5)	5·4 (4·58 to 6·3)	3·61 (2·79 to 4·49)
Semnan	358 (290 to 431)	317 (275 to 362)	220 (189 to 254)	104 (90·4 to 121)	61·3 (52·6 to 71·3)	40 (33·1 to 47·6)	26·9 (23·5 to 30·9)	11·6 (9·82 to 13·6)	7·66 (5·77 to 9·64)	5·2 (3·58 to 6·98)
Sistan and Baluchistan	503 (403 to 603)	374 (327 to 422)	278 (248 to 311)	161 (141 to 182)	110 (97·5 to 123)	67·3 (58·8 to 77·1)	44 (39·2 to 49·3)	17·5 (15·1 to 19·9)	13·6 (12·1 to 15·1)	9·37 (8·22 to 10·6)
South Khorasan	443 (370 to 519)	374 (330 to 419)	289 (256 to 324)	163 (144 to 185)	107 (94·4 to 120)	62·2 (53·4 to 71·2)	33·9 (28·8 to 39·3)	13·4 (11·7 to 15·3)	9·17 (8·15 to 10·4)	6·03 (5·19 to 7)
Tehran	264 (205 to 331)	194 (160 to 232)	117 (98 to 139)	52·5 (43 to 62·4)	33·9 (27·3 to 41·5)	24·7 (20·5 to 29·2)	18·7 (15·3 to 22·3)	8·89 (7·54 to 10·3)	5·22 (3·68 to 6·79)	3·5 (2·15 to 5·08)
West Azarbayejan	416 (345 to 489)	343 (301 to 388)	260 (229 to 293)	145 (127 to 166)	88·2 (77·3 to 100)	55·2 (47·2 to 64·1)	37 (32·6 to 41·9)	13·4 (11·8 to 15·4)	9·5 (8·41 to 10·7)	6·52 (5·53 to 7·59)
Yazd	445 (368 to 529)	276 (236 to 320)	182 (156 to 212)	86·1 (74·5 to 98·8)	51·3 (44·1 to 59·3)	34·5 (29·4 to 39·8)	27·7 (24·4 to 31·2)	12·9 (11·1 to 14·7)	9 (7·96 to 10·3)	6·19 (5·23 to 7·26)
Zanjan	540 (446 to 633)	455 (405 to 506)	326 (292 to 363)	167 (148 to 188)	189 (176 to 203)	54·4 (46·2 to 64)	30·2 (26·4 to 34·4)	13·2 (11·4 to 15·2)	9·25 (8·01 to 10·6)	6·25 (5·12 to 7·51)
Iraq	204 (155 to 259)	157 (129 to 186)	127 (107 to 147)	83·6 (75·4 to 92·2)	62·2 (57 to 67·9)	49·4 (44·1 to 55·4)	34·8 (29 to 41·1)	24·8 (19·7 to 31·2)	21·7 (17·1 to 27·6)	20·3 (15·7 to 26·1)
Jordan	197 (160 to 244)	132 (112 to 154)	83 (73·9 to 93·3)	52·2 (47·3 to 57·9)	35 (31·4 to 38·8)	27·6 (24·4 to 31)	19·1 (16·2 to 22·3)	14 (11·4 to 17·1)	12·8 (10·5 to 15·9)	12·3 (9·91 to 15·4)
Kuwait	271 (191 to 369)	64·6 (55·3 to 75·8)	51·4 (49·1 to 53·4)	37·3 (35·6 to 39·1)	19·5 (17·6 to 21·5)	12·2 (11·2 to 13·3)	12 (10·9 to 13)	10·1 (8·96 to 11·3)	8·91 (7·81 to 10)	8·73 (7·08 to 10·6)
Lebanon	206 (155 to 266)	94·3 (76·9 to 113)	64·3 (53·3 to 75·9)	49·7 (42 to 57·8)	31·6 (26·7 to 37·6)	21·6 (17·8 to 26)	14·1 (11·2 to 17·7)	9·33 (6·67 to 12·8)	8·43 (5·96 to 11·9)	8·05 (5·59 to 11·5)
Libya	335 (284 to 392)	221 (193 to 252)	122 (106 to 138)	66·1 (56·6 to 77·4)	44 (36·4 to 52·3)	26·8 (22 to 32·3)	23·1 (18·5 to 28·4)	26·5 (20·4 to 33·4)	24·7 (19·2 to 31·2)	23·5 (18·2 to 29·7)
Morocco	362 (315 to 413)	262 (240 to 284)	195 (181 to 208)	134 (122 to 148)	81·5 (71·8 to 92·4)	56·2 (46 to 67·2)	35·6 (28·9 to 43·3)	20·2 (16·4 to 24·7)	18·2 (14·8 to 22)	16·9 (13·8 to 20·4)
Oman	338 (274 to 406)	243 (209 to 279)	158 (137 to 179)	89·9 (75·7 to 106)	36·3 (30·4 to 43·6)	16·4 (14·2 to 19)	12·5 (11·7 to 13·2)	12·2 (11·1 to 13·5)	11 (9·84 to 12·2)	9·62 (8·37 to 10·9)
Palestine	310 (252 to 375)	201 (174 to 232)	101 (86·5 to 117)	64·9 (57·1 to 72·9)	41·6 (36·7 to 46·2)	29·6 (26 to 33·6)	22·5 (19·5 to 26·1)	13·8 (10·9 to 17·3)	12·3 (9·73 to 15·7)	11·6 (9·11 to 15)
Qatar	169 (133 to 215)	108 (90 to 126)	68·6 (58·1 to 80·4)	29·5 (24·2 to 35·3)	18·2 (15 to 21·6)	11·8 (9·73 to 14)	7·02 (5·69 to 8·51)	4·89 (3·87 to 6·14)	4·73 (3·79 to 5·96)	3·84 (3·01 to 4·83)
Saudi Arabia	136 (104 to 173)	214 (183 to 248)	155 (133 to 181)	85·3 (71·6 to 100)	50·7 (40·7 to 62·1)	26·2 (21·3 to 32·2)	11·6 (9·99 to 13·2)	5·86 (4·66 to 7·15)	4·86 (3·71 to 6·06)	4·55 (3·45 to 5·73)
Sudan	231 (187 to 282)	219 (194 to 247)	211 (193 to 230)	192 (171 to 215)	153 (140 to 167)	111 (99·9 to 122)	73·6 (62 to 85·9)	46·4 (37·1 to 56·7)	43·3 (34·3 to 53·2)	40·6 (32 to 50·5)
Syria	207 (170 to 249)	131 (118 to 144)	90·3 (80·8 to 100)	65·2 (57·1 to 74·2)	49·5 (42·4 to 57·4)	19·9 (18·3 to 21·5)	15·6 (14 to 17·2)	15·1 (12 to 18·6)	12·4 (9·82 to 15·3)	11·1 (8·72 to 13·9)
Tunisia	347 (299 to 402)	242 (221 to 263)	157 (146 to 170)	89·2 (79·5 to 98·5)	55·1 (47·5 to 63·3)	33 (28 to 38)	21·1 (17·4 to 24·9)	13·4 (10·9 to 16·3)	12 (9·7 to 14·7)	11·2 (8·98 to 13·9)
Türkiye	314 (261 to 372)	254 (232 to 279)	182 (167 to 198)	128 (115 to 143)	76 (68 to 84·7)	45·2 (39·5 to 51·8)	24·9 (20·7 to 29·8)	14·9 (11·8 to 18·1)	12·8 (10·3 to 15·6)	11·8 (9·58 to 14·3)
United Arab Emirates	198 (158 to 247)	165 (139 to 193)	85·8 (72·7 to 100)	41·8 (35 to 50·3)	22·8 (19·6 to 26·6)	13 (11·9 to 14·1)	9·26 (8·3 to 10·3)	6·33 (5·65 to 7·09)	6·24 (5·46 to 7·09)	5·19 (4·35 to 6·1)
Yemen	459 (402 to 518)	389 (361 to 420)	304 (283 to 325)	194 (176 to 211)	134 (123 to 144)	94·5 (84·7 to 106)	60·4 (51·4 to 70·2)	45·9 (37·3 to 55·4)	42·6 (34·5 to 51·5)	40·7 (32·9 to 49·4)
South Asia	354 (288 to 422)	274 (246 to 306)	200 (183 to 218)	149 (136 to 162)	105 (97·2 to 114)	77·7 (70·7 to 85·3)	56·9 (50·5 to 63·7)	41·7 (35·5 to 48·9)	39·9 (33·5 to 47·2)	38 (31·9 to 45·7)
South Asia	354 (288 to 422)	274 (246 to 306)	200 (183 to 218)	149 (136 to 162)	105 (97·2 to 114)	77·7 (70·7 to 85·3)	56·9 (50·5 to 63·7)	41·7 (35·5 to 48·9)	39·9 (33·5 to 47·2)	38 (31·9 to 45·7)
Bangladesh	325 (278 to 373)	269 (245 to 292)	257 (238 to 278)	180 (165 to 194)	131 (121 to 139)	87·4 (80·3 to 95·1)	54·2 (47·9 to 61·4)	33·1 (26·6 to 40·7)	30·4 (24·1 to 37·7)	28·5 (22·4 to 35·6)
Bhutan	358 (290 to 434)	279 (242 to 321)	222 (187 to 262)	190 (162 to 219)	129 (114 to 143)	87·8 (78·2 to 99·2)	49 (40·5 to 58·3)	33·2 (26·1 to 41·3)	32·1 (25·3 to 40·1)	30·5 (23·6 to 38·5)
India	368 (286 to 454)	281 (248 to 320)	196 (174 to 218)	145 (129 to 161)	99·5 (89·4 to 111)	73·8 (64·9 to 83)	53·5 (46·1 to 61·3)	37 (30·4 to 44·3)	36·1 (29·3 to 44·1)	34·3 (27·8 to 42·4)
Nepal	362 (314 to 417)	291 (265 to 317)	232 (212 to 252)	200 (181 to 218)	135 (125 to 145)	82·7 (74·9 to 91)	51·4 (43·9 to 59·2)	32·4 (24·9 to 41·8)	30·6 (23·5 to 39·5)	29·2 (21·9 to 38·3)
Pakistan	268 (225 to 315)	200 (183 to 218)	156 (144 to 169)	135 (122 to 148)	110 (100 to 121)	90·5 (80·2 to 102)	75·5 (65·1 to 87·7)	64·1 (51·5 to 77·8)	59·2 (48 to 72·3)	56·9 (45·9 to 70·1)
Azad Jammu & Kashmir	345 (269 to 425)	236 (201 to 276)	182 (162 to 203)	154 (127 to 184)	117 (96·8 to 140)	91·1 (71·2 to 112)	66·5 (48·7 to 89·9)	56·7 (40·6 to 77·8)	51·6 (38 to 69·2)	50·1 (37·7 to 66·5)
Balochistan	225 (168 to 298)	170 (145 to 198)	116 (99·1 to 133)	103 (87·3 to 121)	92·2 (81·3 to 104)	81·8 (66·9 to 100)	79 (63 to 98·3)	70·9 (55·2 to 89·4)	64·2 (50·9 to 80)	62·4 (49·5 to 78·9)

Gilgit-Baltistan	173 (131 to 225)	229 (204 to 257)	184 (161 to 207)	157 (131 to 184)	119 (100 to 139)	92·2 (76·1 to 111)	66·7 (51·9 to 83·1)	53·4 (41 to 68)	48·4 (38·1 to 60·9)	46·8 (37·5 to 58·4)
Islamabad Capital Territory	256 (185 to 346)	247 (209 to 292)	167 (138 to 196)	105 (84·9 to 131)	59 (47 to 74·4)	36·1 (28·2 to 44·6)	24 (17·4 to 31·3)	17·7 (13·1 to 23·5)	15·8 (11·4 to 21·5)	15·1 (10·8 to 20·5)
Khyber Pakhtunkhwa	255 (198 to 322)	182 (162 to 202)	142 (125 to 159)	113 (98·5 to 131)	80·5 (70·3 to 92·4)	72·8 (61·4 to 84·6)	61 (47·6 to 76·7)	54·7 (41·9 to 70·1)	50 (39·3 to 62·9)	47·8 (38·1 to 59·2)
Punjab	269 (227 to 316)	205 (186 to 223)	163 (147 to 178)	147 (132 to 162)	121 (110 to 131)	97·4 (87·1 to 109)	81·3 (70·4 to 93·4)	69 (54·7 to 85·3)	64·2 (50·4 to 81·7)	62·1 (48·2 to 78·8)
Sindh	285 (223 to 354)	198 (174 to 223)	151 (136 to 168)	124 (111 to 141)	108 (92·9 to 124)	90·6 (76·3 to 106)	75·5 (63·6 to 88·9)	61·4 (48·1 to 79)	56·1 (44 to 71·1)	53·2 (42·2 to 67·4)
Southeast Asia, east Asia, and Oceania	243 (207 to 286)	466 (338 to 657)	116 (106 to 127)	86·4 (79·4 to 93·8)	62·5 (56·9 to 69)	45·5 (42 to 49·5)	25·9 (23·6 to 28·3)	16·9 (14·4 to 19·9)	16·4 (13·9 to 19·1)	16·1 (13·6 to 18·8)
East Asia	219 (178 to 269)	547 (390 to 760)	98·9 (87·7 to 112)	70·3 (62 to 78·9)	55 (48·1 to 63)	40·2 (35·8 to 46·2)	17 (15·1 to 19)	8·96 (7·77 to 10·3)	8·51 (7·25 to 9·95)	8·03 (6·8 to 9·55)
China	221 (179 to 271)	559 (400 to 773)	100 (89·1 to 113)	72 (63·5 to 80·8)	56 (49·1 to 64·2)	38·3 (34·6 to 42·1)	16·9 (15 to 19)	8·92 (7·69 to 10·3)	8·48 (7·21 to 9·95)	7·99 (6·73 to 9·53)
North Korea	190 (147 to 241)	104 (84 to 127)	69·4 (55·1 to 85·9)	47 (36·4 to 61·1)	34·3 (27·6 to 42·9)	118 (53·3 to 260)	24·2 (18·9 to 30)	13·3 (9·78 to 17·2)	12·4 (9·13 to 16·2)	11·6 (8·59 to 15·3)
Taiwan (province of China)	136 (128 to 146)	60 (58·7 to 61·4)	27·2 (26·3 to 28·1)	16·3 (15·7 to 17)	9·11 (8·65 to 9·6)	8·73 (8·12 to 9·36)	5·27 (4·88 to 5·7)	4·87 (4·45 to 5·36)	4·64 (4·24 to 5·08)	4·98 (4·41 to 5·59)
Oceania	216 (182 to 256)	150 (129 to 172)	109 (94·1 to 123)	82·8 (72·5 to 94·1)	71·2 (63·1 to 80·6)	63·3 (54·1 to 73·3)	58·8 (49·4 to 69·6)	51·5 (41·9 to 62·7)	50·3 (40·7 to 61·2)	49·6 (40·3 to 60·4)
American Samoa	88 (75·7 to 102)	53·1 (48 to 59·1)	37·5 (33·4 to 42·1)	22·1 (19 to 25·8)	17·1 (15·2 to 19·2)	14·8 (12·9 to 17)	12·3 (10·7 to 14·2)	12·6 (10 to 15·9)	13·5 (10·5 to 17·2)	12·2 (9·18 to 16)
Cook Islands	171 (148 to 195)	86·1 (78·3 to 93·9)	54·8 (48·8 to 61·6)	38·2 (33·3 to 43·5)	27·1 (23·4 to 31·4)	14·3 (11·4 to 17·9)	5·93 (4·5 to 7·7)	2·83 (2·24 to 3·53)	5·18 (5·17 to 5·3)	5·23 (5·22 to 5·29)
Fiji	91·6 (75·6 to 109)	59·5 (53·4 to 66)	45·4 (40·4 to 50·9)	35·2 (29·6 to 41·3)	27 (21·7 to 33·4)	27·5 (21·4 to 34·9)	25·8 (21·2 to 30·9)	21·8 (16·8 to 27·9)	20·8 (15·8 to 27·2)	20·6 (15·2 to 27·4)
Guam	65·9 (56·3 to 77·1)	38 (34·8 to 41·4)	29·3 (26·8 to 32·2)	17·7 (15·9 to 19·7)	12 (10·8 to 13·5)	11·2 (10 to 12·4)	13·8 (12·3 to 15·4)	13·6 (11·6 to 15·9)	11·8 (9·7 to 14·2)	11·2 (8·89 to 13·9)
Kiribati	229 (189 to 274)	153 (133 to 174)	118 (101 to 137)	90·7 (75·8 to 108)	79·8 (66·2 to 94·6)	61·6 (50·3 to 74)	47·7 (38·5 to 58·2)	38 (31 to 46·2)	36·9 (30·1 to 45·5)	35·8 (28·7 to 44·4)
Marshall Islands	174 (134 to 221)	98·9 (81·2 to 118)	62·3 (50·9 to 76·4)	42·9 (35·9 to 51·1)	35·2 (31·1 to 39·8)	40·4 (35·2 to 46·7)	30·8 (25·3 to 37)	23 (17·7 to 29·9)	22·2 (16·7 to 29·4)	21·3 (16 to 28·6)
Federated States of Micronesia	269 (218 to 330)	127 (107 to 148)	83·8 (69·7 to 99·5)	55·3 (45·2 to 66·8)	42·8 (34·8 to 51·9)	37·9 (32 to 44·5)	22·7 (18 to 28)	17·3 (13·4 to 21·9)	16·7 (12·9 to 21·3)	16·1 (12·5 to 20·4)
Nauru	89·8 (70·1 to 115)	66·9 (56·2 to 78·7)	52·5 (44 to 62·4)	38·7 (31·5 to 46·7)	34·8 (28·5 to 41·2)	47·2 (40·7 to 54·2)	42·5 (35·1 to 50·7)	27·4 (20·3 to 36·3)	26·3 (19·1 to 35·9)	25·4 (18·4 to 35)
Niue	174 (145 to 206)	73·2 (64·4 to 82·9)	47·7 (40·8 to 55·3)	32 (26·1 to 38·7)	25·6 (20·6 to 31·1)	30·8 (25 to 37·4)	24·2 (19·1 to 30·7)	21·6 (16·2 to 28·7)	48·9 (48·7 to 50·7)	49·2 (49 to 51)
Northern Mariana Islands	63·9 (46·4 to 86·5)	36·6 (29·4 to 45·5)	20·5 (15·7 to 26·8)	13 (9·96 to 17)	9·39 (7·43 to 11·7)	7·57 (6·19 to 9·09)	7·86 (6·32 to 9·61)	7·43 (6·03 to 9·07)	7·48 (5·64 to 9·62)	6·36 (5·13 to 7·51)
Palau	191 (146 to 243)	119 (97 to 142)	70·2 (57·6 to 84·5)	47 (37·4 to 57·2)	31·7 (24·5 to 39·6)	22·8 (18·1 to 28·3)	20·9 (16·8 to 25·6)	17 (13·7 to 21·1)	16·7 (13·6 to 20·9)	16·4 (13·4 to 20·6)
Papua New Guinea	248 (203 to 301)	176 (148 to 207)	127 (108 to 147)	99·3 (85·9 to 114)	86·6 (75·8 to 98·8)	74·8 (63·4 to 87·1)	67·8 (56·4 to 80·7)	57·8 (46·9 to 70·6)	56·3 (45·7 to 68·7)	55·5 (45·1 to 67·6)
Samoa	170 (141 to 201)	102 (88 to 117)	70·4 (59·7 to 82·2)	46 (38·3 to 55·4)	31·9 (25 to 40·6)	22·6 (17·6 to 28·3)	17·6 (13·6 to 22·1)	25·5 (22·3 to 29·3)	15·4 (12·5 to 18·9)	12·9 (9·97 to 16·5)
Solomon Islands	211 (174 to 255)	151 (131 to 172)	102 (85·8 to 119)	62·1 (51·1 to 74·5)	46·5 (37·6 to 56·8)	35·4 (28·6 to 42·5)	28·5 (23 to 34·6)	21·3 (16·8 to 26·6)	21·5 (17·3 to 26·5)	20·2 (16·1 to 25·1)
Tokelau	114 (88·5 to 148)	74·7 (60·6 to 90·5)	51·5 (40·7 to 63)	41·9 (33·8 to 51·2)	28·3 (22·8 to 34·5)	21 (16·5 to 26·8)	13·5 (10·6 to 17·4)	10·1 (7·31 to 14)	60·1 (60·1 to 60·1)	61·1 (61·1 to 61·1)
Tonga	71·7 (54·3 to 93·9)	48 (39·4 to 57·7)	41 (34·2 to 48·5)	34·7 (28·6 to 41·5)	25·6 (21·3 to 30·5)	21 (17·1 to 25·4)	17·6 (13·9 to 21·9)	12·6 (9·72 to 16·1)	12·2 (9·31 to 15·6)	11·8 (8·87 to 15·2)
Tuvalu	332 (262 to 407)	191 (159 to 226)	152 (125 to 185)	125 (103 to 149)	84·8 (71·9 to 98·9)	60·4 (52·6 to 69·4)	29·6 (24·2 to 35·7)	21·9 (16·9 to 27·8)	20·9 (15·9 to 26·8)	20 (15·2 to 25·9)
Vanuatu	197 (162 to 233)	159 (138 to 183)	103 (87·5 to 121)	55·2 (45·6 to 66)	38·7 (32·3 to 45·6)	36·1 (30·4 to 42·6)	30 (24·9 to 35·4)	22·9 (17·7 to 29·5)	22·6 (17·3 to 29·5)	21·6 (16·6 to 28·3)
Southeast Asia	301 (266 to 339)	207 (189 to 227)	155 (144 to 165)	112 (105 to 119)	77·5 (73·1 to 82·1)	52·5 (48·6 to 56·6)	36 (32·2 to 40)	26·7 (22·2 to 32)	25·1 (20·9 to 29·7)	24 (20·1 to 28·5)
Cambodia	290 (231 to 361)	224 (190 to 260)	195 (169 to 224)	174 (156 to 194)	129 (120 to 140)	99·7 (90·9 to 109)	53·5 (45·9 to 61·5)	36·4 (29·6 to 44·2)	35·4 (29·1 to 43·5)	33·8 (27·5 to 41·9)
Indonesia	342 (296 to 393)	246 (224 to 269)	184 (171 to 197)	127 (119 to 136)	88·8 (81·8 to 96)	57·3 (51·9 to 62·7)	40·5 (35 to 46·5)	29·9 (23·6 to 36·7)	28·2 (22·5 to 34·7)	27·2 (21·7 to 33·5)
Aceh	300 (244 to 363)	209 (192 to 228)	164 (148 to 180)	118 (108 to 129)	88·8 (80 to 97·8)	63·2 (57·8 to 68·7)	46·7 (40 to 54)	36·3 (28·9 to 44·4)	34·2 (27·3 to 42·2)	33 (26·4 to 41)
Bali	270 (213 to 335)	171 (156 to 188)	125 (113 to 138)	83·6 (75·3 to 92·2)	50·5 (44·9 to 56·3)	34·5 (30·7 to 38·9)	26·6 (22·5 to 31·1)	20·2 (14·9 to 25·9)	19 (14·3 to 24)	18·5 (14·4 to 23·3)
Bangka-Belitung Islands	308 (243 to 393)	234 (198 to 273)	179 (162 to 197)	125 (113 to 140)	95·1 (85·1 to 106)	55·3 (48·6 to 62·5)	37·8 (30·6 to 46·4)	32 (24·1 to 41·7)	30·1 (22·8 to 39·1)	28·9 (22·2 to 37·3)
Banten	391 (314 to 476)	353 (305 to 406)	262 (241 to 285)	174 (160 to 188)	119 (110 to 129)	68·9 (60·1 to 77·9)	47·2 (37·9 to 58·1)	35·2 (27 to 45·7)	33 (25·6 to 42·7)	31·5 (24·7 to 40·1)
Bengkulu	466 (390 to 553)	348 (320 to 376)	250 (227 to 275)	165 (152 to 179)	111 (101 to 121)	71·6 (64·6 to 78·6)	47·4 (41·6 to 53·5)	31·4 (24·9 to 39)	29·5 (23·1 to 36·7)	28·3 (22 to 35·4)
Gorontalo	306 (240 to 381)	247 (212 to 284)	188 (172 to 206)	148 (131 to 166)	113 (102 to 124)	86·1 (75·5 to 97·6)	67·1 (53·9 to 82·5)	50 (38·3 to 64·9)	47·3 (36·2 to 61·1)	45·4 (35·2 to 57·7)
Jakarta	230 (184 to 284)	213 (192 to 234)	147 (132 to 165)	96·8 (87·8 to 107)	63·2 (56·7 to 70·3)	40·5 (35·8 to 46·3)	28·2 (24·2 to 32·5)	20·4 (15·4 to 26·1)	19 (14·6 to 24)	18·4 (14·3 to 23·2)
Jambi	318 (261 to 385)	244 (223 to 268)	183 (165 to 202)	121 (110 to 133)	84·8 (75·3 to 93·8)	54·3 (49·1 to 60·5)	38 (33·6 to 42·5)	29 (23·1 to 35·8)	27·3 (21·6 to 33·7)	26·3 (20·9 to 32·4)
West Java	419 (364 to 478)	335 (305 to 364)	244 (226 to 261)	163 (151 to 175)	105 (96·5 to 113)	60·2 (52·5 to 68·1)	40·6 (34·9 to 46·6)	28·6 (22·4 to 35·8)	26·9 (20·8 to 33·4)	25·8 (20·2 to 32)
Central Java	319 (261 to 383)	207 (186 to 230)	152 (137 to 168)	101 (91·1 to 111)	68·1 (62 to 75·1)	42·4 (37·9 to 47·1)	29·1 (24·5 to 34·4)	20 (15·4 to 25·1)	18·6 (14·5 to 23·3)	17·9 (14·1 to 22·4)
East Java	270 (229 to 315)	190 (172 to 208)	145 (132 to 159)	106 (96·4 to 116)	75·1 (68·9 to 81·2)	48·5 (42·1 to 54·7)	33·3 (28·1 to 38·7)	23·5 (18·1 to 29·2)	22 (16·9 to 27·4)	21·4 (16·6 to 26·7)
West Kalimantan	297 (245 to 362)	249 (227 to 273)	207 (188 to 227)	150 (137 to 162)	103 (93·3 to 113)	63·1 (55·2 to 71·1)	38·6 (33·8 to 43·8)	24·7 (18·8 to 31·2)	23 (17·8 to 28·9)	22 (17·3 to 27·8)
South Kalimantan	461 (400 to 520)	402 (371 to 431)	301 (282 to 320)	217 (202 to 232)	148 (132 to 162)	86·8 (78·2 to 96·3)	54·8 (46 to 63·7)	40·8 (31·9 to 51·4)	38·5 (30·2 to 48·1)	37 (28·9 to 46·2)
Central Kalimantan	231 (187 to 278)	167 (151 to 186)	122 (110 to 134)	84·3 (76·7 to 92·3)	70·3 (62·7 to 78·1)	52·5 (47·5 to 58·1)	38·2 (33·8 to 43)	31 (24·9 to 38·2)	29·4 (23·5 to 36·3)	28·5 (22·7 to 35·4)
East Kalimantan	219 (177 to 268)	229 (211 to 249)	175 (158 to 194)	114 (103 to 126)	70·6 (63·4 to 78·3)	43·3 (39·1 to 47·9)	25·8 (22·4 to 29·7)	19·2 (15·2 to 23·8)	17·8 (14·2 to 22·1)	17·2 (13·6 to 21·4)
North Kalimantan	175 (138 to 216)	230 (209 to 251)	194 (175 to 215)	133 (119 to 151)	89·9 (77·4 to 103)	63·5 (54·8 to 73·7)	42·9 (35·4 to 51·4)	33·8 (25·8 to 42·7)	31·8 (24·5 to 40·2)	30·6 (24·1 to 38·2)
Riau Islands	213 (169 to 264)	186 (156 to 218)	130 (117 to 144)	88·7 (78·9 to 99·7)	66·2 (59·2 to 73·1)	48·1 (42·7 to 53·6)	31 (25·5 to 37·8)	22·3 (17·7 to 27·6)	20·9 (16·5 to 26)	20·3 (16·1 to 25·1)
Lampung	277 (226 to 333)	252 (229 to 277)	185 (170 to 203)	126 (116 to 137)	87·1 (78·9 to 95·4)	54·4 (49·4 to 59·8)	40 (33·8 to 46·6)	32·7 (25·2 to 41)	30·9 (24·1 to 38·7)	29·9 (23·4 to 37·7)
Maluku	472 (404 to 549)	322 (275 to 370)	242 (222 to 264)	175 (158 to 192)	131 (120 to 144)	93·3 (84·6 to 103)	68·6 (57·2 to 81·6)	42·3 (33·3 to 52)	39·3 (31·1 to 48·5)	37 (29·7 to 45·7)
North Maluku	383 (306 to 476)	276 (230 to 326)	212 (191 to 233)	164 (145 to 184)	123 (110 to 138)	90·6 (79·5 to 102)	67·9 (55·5 to 83·2)	49·8 (38·3 to 65·1)	46·7 (35·8 to 60·8)	44·5 (34·6 to 57·7)
West Nusa Tenggara	448 (378 to 520)	355 (327 to 384)	291 (270 to 311)	218 (204 to 232)	147 (132 to 161)	88 (79·1 to 96·8)	50 (42 to 58·7)	34·8 (27·5 to 43·5)	32·7 (25·7 to 40·5)	31·3 (24·7 to 38·8)
East Nusa Tenggara	451 (380 to 530)	292 (250 to 337)	227 (208 to 248)	159 (144 to 171)	110 (102 to 118)	78·9 (71·1 to 87·5)	57 (47·5 to 68·1)	39·3 (31·5 to 48·3)	36·7 (29·4 to 45)	34·8 (27·7 to 42·9)
Papua	149 (121 to 184)	183 (152 to 216)	159 (142 to 175)	127 (113 to 142)	105 (93·6 to 116)	80 (71·9 to 89)	68·2 (56·9 to 80·9)	55·4 (43·6 to 68·8)	52·8 (41·6 to 65·7)	51·2 (40 to 63·6)
West Papua	155 (122 to 194)	149 (126 to 174)	130 (118 to 144)	93 (83·1 to 104)	71·7 (64·2 to 79·6)	60·2 (53·4 to 67·8)	50·7 (42·5 to 60·4)	33·4 (26·2 to 41·5)	31·5 (24·9 to 38·8)	30·3 (23·7 to 37·7)
Riau	260 (213 to 319)	252 (230 to 276)	196 (181 to 211)	125 (114 to 136)	87 (79·6 to 94·4)	56·1 (49·1 to 63·3)	42·8 (37·4 to 48·1)	27·8 (22·1 to 34·3)	26·1 (20·7 to 32·4)	25 (19·8 to 31·3)
West Sulawesi	550 (449 to 652)	368 (317 to 420)	285 (261 to 310)	206 (187 to 225)	143 (128 to 157)	92·5 (83·4 to 102)	69·7 (57 to 83·7)	52·3 (40·5 to 66·4)	49·2 (38·2 to 62·9)	46·9 (36·7 to 58·8)
South Sulawesi	358 (299 to 412)	238 (220 to 258)	175 (162 to 189)	124 (115 to 134)	84·3 (75·2 to 93·3)	54·9 (50·2 to 59·8)	39·7 (33·5 to 45·8)	26·7 (21·1 to 33·4)	25·1 (19·7 to 31)	24·2 (19·2 to 29·9)
Central Sulawesi	356 (306 to 418)	286 (263 to 311)	212 (195 to 231)	150 (139 to 161)	111 (100 to 121)	76·8 (69·2 to 85·3)	58·2 (50·4 to 65·7)	44·3 (35·5 to 54·1)	41·9 (33·5 to 51·4)	40·3 (32·1 to 49·4)
Southeast Sulawesi	426 (365 to 493)	335 (295 to 375)	266 (245 to 288)	172 (159 to 185)	115 (106 to 125)	76·6 (69·1 to 84·5)	59 (48·9 to 70·8)	50·7 (40·2 to 62·3)	48·6 (38·6 to 60·2)	47·2 (37·3 to 58·1)
North Sulawesi	271 (219 to 328)	167 (151 to 186)	117 (106 to 129)	71·3 (63·9 to 79·9)	55·5 (50·2 to 61·5)	42·8 (38·6 to 47·5)	37·2 (32·1 to 42·8)	32·1 (25 to 39·8)	30·5 (23·6 to 38·1)	29·6 (22·9 to 37·2)
West Sumatra	592 (525 to 661)	319 (292 to 348)	219 (201 to 239)	138 (127 to 150)	90 (82·8 to 98)	59·8 (53 to 66·3)	36·9 (32·1 to 42·3)	20·7 (16·2 to 25·6)	19 (15 to 23·6)	18·1 (14·3 to 22·2)
South Sumatra	332 (283 to 387)	253 (231 to 274)	188 (172 to 205)	119 (109 to 129)	88·8 (81·1 to 97)	61·8 (54 to 69·4)	43·5 (37·4 to 50·2)	35·6 (28 to 44)	33·6 (26·5 to 41·6)	32·4 (25·6 to 40·2)

North Sumatra	307 (255 to 370)	202 (184 to 221)	149 (137 to 162)	106 (97·8 to 115)	83·4 (75·8 to 91·6)	60·1 (54·2 to 66·6)	48·5 (41 to 56·2)	43·2 (34·4 to 53·3)	41 (32·9 to 50·6)	39·6 (31·7 to 49·2)
Yogyakarta	370 (289 to 456)	179 (154 to 207)	103 (90·7 to 115)	56·4 (51·3 to 62·3)	35·5 (31·3 to 40·6)	22·1 (19·5 to 25·1)	16·2 (13·6 to 19·4)	15·5 (11·5 to 20·1)	14·3 (10·9 to 18·5)	14 (10·8 to 17·8)
Laos	348 (278 to 426)	318 (274 to 364)	286 (239 to 339)	236 (203 to 269)	176 (157 to 194)	127 (113 to 140)	75·7 (66·9 to 84·7)	47·7 (37·6 to 58·7)	46 (35·6 to 57·4)	43·9 (33·4 to 55·7)
Malaysia	166 (153 to 179)	103 (101 to 105)	61·4 (59·7 to 63)	33·3 (29·3 to 37·7)	18·9 (18·1 to 19·6)	9·83 (9·4 to 10·2)	7·74 (7·34 to 8·14)	8·18 (7·7 to 8·7)	7·2 (6·64 to 7·8)	6·67 (5·9 to 7·5)
Maldives	374 (306 to 447)	306 (259 to 354)	219 (192 to 249)	145 (128 to 162)	66·9 (59·5 to 75·4)	33 (28·3 to 38·2)	20·5 (17·6 to 23·5)	15 (12·3 to 18·6)	13·2 (10·6 to 16·4)	13·4 (10·8 to 17)
Mauritius	160 (148 to 174)	107 (103 to 111)	82·8 (78·8 to 86·7)	43·1 (40·5 to 45·8)	24·1 (22·3 to 26·2)	19 (17·5 to 20·7)	15·5 (14·3 to 16·7)	16·9 (15 to 19·1)	17·6 (14·4 to 20·9)	13·7 (11·2 to 15·7)
Myanmar	433 (353 to 520)	329 (285 to 376)	268 (234 to 305)	210 (177 to 243)	156 (136 to 177)	118 (103 to 134)	73·7 (63·7 to 83·8)	47·7 (39·2 to 58)	45·2 (36·7 to 56)	43·6 (35 to 55)
Philippines	208 (163 to 263)	145 (128 to 163)	106 (96·6 to 116)	84·9 (77·3 to 93·2)	59·8 (53·2 to 66·8)	39·8 (34·4 to 45·4)	32·8 (28·3 to 37·7)	27 (22·2 to 32·5)	24·3 (20·2 to 29)	22·9 (18·6 to 27·8)
Seychelles	106 (91·5 to 123)	99·9 (92·8 to 107)	77·1 (71·9 to 82·6)	34·8 (31·5 to 38·3)	19·6 (17·7 to 21·7)	14·1 (12·4 to 15·9)	14·8 (12·9 to 16·9)	15 (12·6 to 17·9)	14·5 (12 to 17·5)	13·7 (10·8 to 17·3)
Sri Lanka	185 (170 to 200)	97·5 (94·2 to 101)	71·9 (68·6 to 75·5)	49·9 (47·6 to 52·3)	22·6 (21 to 24·3)	18 (16·9 to 19·2)	12·8 (12 to 13·7)	7·78 (6·29 to 9·59)	7·25 (5·57 to 9·22)	6·53 (4·92 to 8·61)
Thailand	201 (169 to 236)	127 (117 to 138)	80·1 (73·1 to 87·6)	53·7 (47·6 to 59·8)	31·8 (27·5 to 36·6)	19·2 (16·3 to 22·3)	11 (10·4 to 11·7)	9·25 (8·57 to 10)	8·37 (7·63 to 9·18)	8·09 (7·18 to 9·08)
Timor-Leste	287 (232 to 350)	256 (220 to 294)	253 (223 to 284)	217 (198 to 239)	141 (128 to 154)	87·4 (77 to 98·4)	50 (41·1 to 59·5)	39·1 (31·3 to 48·1)	38·5 (30·7 to 47·7)	37·5 (30·4 to 46·4)
Viet Nam	314 (248 to 387)	156 (130 to 183)	106 (93 to 120)	72·8 (66·1 to 79·5)	52 (47·4 to 56·6)	32·6 (29 to 36·5)	21 (18·1 to 24·1)	14·6 (11·5 to 18·4)	14 (10·9 to 17·9)	12·8 (9·92 to 16·7)
Sub-Saharan Africa	329 (278 to 383)	272 (242 to 304)	241 (221 to 262)	207 (195 to 219)	181 (172 to 190)	155 (146 to 163)	113 (104 to 122)	81·4 (69·2 to 95·3)	78·8 (65·9 to 94·2)	75·7 (62·9 to 91·5)
Central sub-Saharan Africa	357 (299 to 420)	261 (228 to 297)	232 (205 to 261)	205 (184 to 225)	180 (166 to 194)	162 (149 to 175)	113 (101 to 126)	70·6 (59·8 to 83·4)	66 (55·6 to 79)	62·9 (52·9 to 75·5)
Angola	328 (268 to 395)	285 (244 to 329)	255 (219 to 296)	238 (208 to 269)	220 (201 to 242)	175 (161 to 191)	105 (89·9 to 120)	64·1 (52·1 to 77·1)	61 (50·2 to 73·1)	59·8 (49·2 to 72·2)
Central African Republic	266 (216 to 325)	241 (205 to 277)	214 (188 to 244)	206 (182 to 230)	199 (181 to 218)	190 (170 to 210)	164 (138 to 191)	128 (104 to 157)	123 (100 to 151)	117 (94·7 to 146)
Congo (Brazzaville)	393 (315 to 489)	286 (248 to 326)	232 (203 to 266)	151 (132 to 173)	110 (97·7 to 124)	110 (98 to 124)	70·2 (60·1 to 81·2)	48·8 (40·4 to 58·7)	44·7 (36·3 to 53·9)	42·6 (35 to 51·8)
DR Congo	379 (309 to 455)	254 (216 to 294)	229 (198 to 261)	201 (178 to 224)	173 (157 to 189)	160 (146 to 177)	116 (102 to 131)	71·2 (58·5 to 86)	65·8 (54·1 to 81·7)	62 (50·7 to 78)
Equatorial Guinea	531 (446 to 623)	318 (273 to 364)	246 (209 to 285)	188 (161 to 217)	171 (149 to 194)	127 (106 to 150)	72·3 (57·4 to 89·4)	52·1 (38·4 to 69·1)	51·1 (37·3 to 69·7)	49·6 (36·2 to 68·3)
Gabon	549 (453 to 642)	266 (225 to 309)	194 (161 to 230)	120 (104 to 139)	89·3 (77·4 to 102)	78·9 (65·8 to 93·6)	66·7 (54 to 80·8)	43·6 (32·3 to 57·8)	40·3 (29·5 to 55·4)	37·6 (27·1 to 51·9)
Eastern sub-Saharan Africa	346 (292 to 401)	270 (237 to 303)	238 (216 to 261)	207 (194 to 220)	181 (172 to 190)	142 (133 to 151)	95·1 (84·5 to 105)	66·1 (54·3 to 80·4)	64·4 (52·2 to 79·7)	62·7 (50·5 to 78·5)
Burundi	349 (283 to 417)	280 (244 to 316)	255 (228 to 283)	223 (204 to 242)	191 (176 to 206)	167 (152 to 183)	103 (91·3 to 116)	70·9 (57·4 to 86·9)	70·6 (55·7 to 88·9)	69 (54·3 to 88·6)
Comoros	284 (234 to 342)	245 (210 to 282)	204 (178 to 232)	173 (156 to 190)	133 (119 to 148)	109 (93·6 to 125)	77·2 (62 to 94)	54·3 (43·2 to 66·8)	51·7 (41·2 to 64·4)	50 (39·9 to 63)
Djibouti	155 (122 to 190)	150 (125 to 177)	136 (115 to 161)	113 (97 to 129)	106 (92·4 to 120)	92·5 (78·4 to 108)	68·1 (55·4 to 82·1)	44·8 (35·7 to 55·3)	41·8 (33·4 to 51·8)	39·7 (31·5 to 49·8)
Eritrea	337 (275 to 403)	280 (243 to 318)	248 (217 to 281)	205 (184 to 226)	161 (143 to 181)	102 (86·6 to 120)	76·2 (59·5 to 94·7)	54·1 (41·1 to 70)	51·6 (38·8 to 67)	49·9 (37·5 to 66·3)
Ethiopia	358 (291 to 430)	292 (251 to 332)	272 (243 to 303)	246 (226 to 267)	203 (188 to 218)	152 (140 to 166)	93 (81·6 to 105)	61·4 (49 to 76·1)	60·7 (47·9 to 75·7)	58·6 (46·1 to 73·9)
Addis Ababa	146 (112 to 185)	158 (131 to 186)	140 (120 to 162)	110 (97·3 to 123)	95·4 (83 to 109)	67·9 (56·6 to 80·4)	32·5 (26·9 to 38)	19·9 (15·4 to 25·3)	19·2 (15 to 24)	18·6 (14·6 to 23·5)
Afar	572 (479 to 668)	340 (295 to 386)	294 (258 to 333)	257 (234 to 281)	196 (179 to 213)	176 (159 to 196)	118 (99·4 to 139)	75·8 (60·5 to 94·5)	73·9 (57·9 to 93·2)	70·8 (55·5 to 89·3)
Amhara	367 (294 to 448)	289 (248 to 330)	270 (239 to 303)	247 (227 to 269)	205 (191 to 221)	161 (147 to 176)	95·6 (82·7 to 108)	61·3 (49 to 76·4)	60·9 (48·2 to 76·4)	58·5 (45·9 to 73·7)
Benishangul-Gumuz	530 (445 to 615)	334 (288 to 377)	314 (279 to 352)	298 (269 to 325)	251 (226 to 276)	191 (169 to 217)	115 (98·8 to 133)	74·8 (59·4 to 93·2)	74·9 (58·8 to 94·2)	71·8 (56·4 to 90·3)
Dire Dawa	252 (196 to 314)	310 (267 to 352)	298 (264 to 334)	261 (236 to 288)	227 (205 to 250)	146 (130 to 163)	76·1 (65 to 88·3)	48·3 (37·7 to 61·2)	47·7 (36·3 to 63·4)	45 (34·1 to 58·2)
Gambella	613 (510 to 717)	405 (351 to 459)	387 (342 to 434)	345 (313 to 380)	247 (224 to 273)	155 (139 to 173)	90·1 (77·2 to 104)	53·2 (41·3 to 68·4)	52·5 (40·3 to 68·4)	49·9 (38·1 to 65)
Harari	313 (252 to 382)	359 (313 to 404)	329 (294 to 367)	276 (250 to 302)	216 (195 to 238)	126 (112 to 143)	73·5 (63 to 85)	46·6 (36·1 to 58·8)	45·5 (34·8 to 58·6)	42·9 (33 to 55·3)
Oromia	379 (303 to 461)	297 (255 to 338)	275 (244 to 307)	245 (222 to 269)	205 (187 to 222)	148 (134 to 163)	88·9 (78·1 to 100)	59·6 (47·5 to 73·5)	58·6 (46·7 to 73·2)	56·7 (44·6 to 71·9)
Somali	287 (228 to 353)	218 (187 to 250)	203 (178 to 230)	167 (151 to 184)	143 (130 to 158)	140 (126 to 154)	105 (89·9 to 121)	78·6 (63·5 to 96·2)	78·2 (62·8 to 96)	75·8 (61 to 93·6)
Southern Nations, Nationalities, and Peoples	338 (273 to 409)	313 (269 to 354)	296 (265 to 329)	274 (251 to 298)	227 (208 to 247)	164 (149 to 179)	100 (87·9 to 113)	63·9 (50·7 to 80)	63·3 (49·7 to 80·7)	61 (47·5 to 77·7)
Tigray	382 (309 to 462)	294 (251 to 334)	271 (240 to 304)	254 (231 to 279)	191 (173 to 210)	127 (115 to 140)	75 (66·1 to 84·9)	44·5 (36·1 to 54·7)	43·7 (35·4 to 53·9)	41·7 (33·6 to 51·3)
Kenya	275 (239 to 316)	195 (179 to 211)	155 (144 to 166)	117 (109 to 126)	101 (92·6 to 109)	88·8 (79 to 99·3)	61·1 (51·4 to 71·5)	42·1 (34 to 51·2)	40·2 (32·3 to 49·3)	39·1 (31·2 to 48·4)
Baringo	262 (216 to 312)	217 (197 to 237)	201 (184 to 219)	169 (155 to 184)	141 (121 to 160)	112 (97·1 to 126)	83·6 (68·7 to 100)	63·2 (50·3 to 77·8)	61·3 (48·9 to 75·2)	59·1 (47·4 to 73)
Bomet	105 (79·4 to 136)	78·3 (67·4 to 90)	75·6 (65·3 to 86·1)	69·1 (60·7 to 78·5)	54·9 (48·5 to 61·5)	45·9 (38·2 to 54·9)	42·2 (34 to 51·3)	29·8 (23·4 to 37·7)	28·9 (22·8 to 36)	27·6 (21·8 to 34·3)
Bungoma	359 (302 to 424)	247 (225 to 269)	190 (176 to 204)	148 (137 to 159)	112 (101 to 123)	104 (91·7 to 118)	66·3 (56·7 to 76·7)	40·8 (33·4 to 49·5)	39 (31·6 to 47·6)	40·3 (32·2 to 49·9)
Busia	582 (506 to 668)	366 (333 to 400)	273 (255 to 292)	177 (163 to 191)	150 (135 to 166)	140 (121 to 161)	91·6 (75·9 to 108)	60 (48·1 to 74·5)	61·2 (48·4 to 77·2)	65·3 (51 to 82)
Elgeyo Marakwet	195 (156 to 240)	126 (111 to 142)	139 (127 to 152)	124 (113 to 135)	82·6 (73 to 91·6)	66·4 (56·3 to 76·8)	37·8 (30·1 to 46·2)	26 (20·3 to 32·7)	26·9 (21·2 to 33·6)	24·9 (19·5 to 31·4)
Embu	339 (282 to 408)	224 (205 to 244)	143 (132 to 154)	87·8 (80·5 to 95·9)	72·1 (61·5 to 82·8)	62 (53 to 71·7)	51·3 (42 to 61·4)	36 (27·7 to 45·4)	34·2 (26 to 43·2)	32·7 (25·1 to 41·6)
Garissa	192 (157 to 232)	169 (145 to 195)	134 (117 to 153)	127 (112 to 144)	97·7 (84 to 112)	70·2 (56·5 to 86·7)	47·1 (39·8 to 55·4)	30·2 (24·4 to 36·6)	27·9 (22·8 to 33·8)	26·4 (21·8 to 31·8)
Homa Bay	563 (504 to 627)	393 (364 to 424)	355 (323 to 385)	281 (257 to 304)	225 (209 to 243)	202 (181 to 223)	113 (97·3 to 131)	68 (55 to 83·3)	62·1 (49·5 to 77·3)	58·6 (46·5 to 73·8)
Isiolo	362 (300 to 434)	392 (356 to 431)	368 (339 to 398)	293 (263 to 323)	229 (203 to 256)	174 (152 to 196)	130 (111 to 152)	90·6 (73·2 to 110)	85·8 (68 to 106)	82·1 (65·4 to 101)
Kajiado	139 (112 to 171)	104 (91·9 to 116)	99·6 (90·7 to 109)	82·5 (74·1 to 91·3)	60·4 (53·2 to 67·8)	54·9 (46·8 to 63·6)	41 (33·6 to 49·1)	25·6 (20·5 to 31·5)	23·7 (19·2 to 29·3)	22·4 (18·1 to 27·5)
Kakamega	379 (319 to 446)	242 (224 to 261)	196 (182 to 211)	146 (135 to 159)	144 (130 to 159)	126 (112 to 141)	81·5 (68·7 to 94·6)	53·7 (43·4 to 65·3)	51 (40·6 to 62·8)	52·4 (41·6 to 65·2)
Kericho	53·8 (42·2 to 66·8)	67·2 (60·1 to 74·8)	72·6 (64·8 to 80·7)	75·7 (67·5 to 84)	53·3 (47 to 59·6)	54 (46·9 to 61·8)	40·3 (33·6 to 47·8)	31·2 (24·8 to 38·6)	30·8 (24·5 to 38·1)	29·9 (23·7 to 37·2)
Kiambu	156 (118 to 201)	96·5 (83·9 to 110)	66·1 (58·5 to 75·2)	53·9 (48·1 to 60·7)	46·6 (39·6 to 53·7)	51·8 (44·6 to 60)	42 (34·8 to 49·8)	29·5 (22·7 to 37·5)	28·8 (22·1 to 36·5)	27·7 (21·5 to 34·7)
Kilifi	357 (304 to 418)	267 (247 to 289)	218 (204 to 233)	170 (152 to 189)	129 (117 to 140)	104 (93 to 118)	70·3 (59·6 to 81·7)	46·1 (36·7 to 56·6)	43·1 (33·9 to 53·5)	40·3 (31·4 to 50·4)
Kirinyaga	340 (293 to 396)	211 (191 to 232)	160 (143 to 178)	79 (69·9 to 88·7)	59·8 (50·1 to 70·4)	64·2 (54·2 to 75·8)	52·7 (43·5 to 63)	46·8 (36·9 to 58·4)	47·1 (37 to 58·7)	46 (36 to 57·7)
Kisii	202 (154 to 256)	184 (169 to 202)	159 (147 to 172)	133 (113 to 156)	121 (107 to 137)	97·1 (83·3 to 111)	53·5 (42·9 to 66)	36·4 (28·5 to 45·3)	37 (28·9 to 46·3)	36 (28·3 to 45·3)
Kisumu	272 (209 to 341)	198 (180 to 217)	184 (171 to 198)	162 (136 to 191)	152 (136 to 168)	141 (121 to 161)	76·8 (61 to 95·3)	49·1 (38·4 to 61)	45·9 (35·5 to 57·6)	46·5 (35·7 to 59·2)
Kitui	431 (358 to 513)	258 (238 to 277)	188 (175 to 202)	118 (108 to 127)	88·6 (77 to 101)	79·4 (67·9 to 91·9)	51·6 (42·7 to 60·6)	34·3 (27·1 to 42·6)	31·4 (24·8 to 39·3)	29·8 (23·5 to 37·2)
Kwale	416 (358 to 480)	305 (281 to 331)	233 (215 to 251)	170 (150 to 192)	127 (111 to 144)	95·1 (81·2 to 110)	70·6 (58 to 84·5)	46·6 (35·9 to 58·6)	44·1 (34·3 to 54·9)	41·7 (32·8 to 51·8)
Laikipia	144 (101 to 198)	104 (86 to 123)	79 (70·2 to 88·6)	59·4 (52·5 to 66·1)	52·5 (41·4 to 64·6)	54·7 (46·1 to 63·7)	32·1 (23·9 to 41·9)	20·3 (13·9 to 28·2)	18·5 (12·5 to 26·2)	17·4 (11·7 to 25)
Lamu	469 (387 to 558)	296 (265 to 334)	209 (189 to 231)	118 (102 to 136)	118 (103 to 135)	101 (85·3 to 119)	76·3 (63·3 to 89·7)	54·7 (41·6 to 70·4)	52·4 (39·7 to 67)	49·7 (38·1 to 62·8)
Machakos	196 (156 to 245)	151 (136 to 167)	121 (111 to 131)	86·8 (77·2 to 97·3)	64·7 (55·9 to 74·6)	56·8 (48·6 to 65·8)	36·5 (29·5 to 44·5)	24·9 (19·6 to 31·2)	22·9 (17·9 to 29·1)	21·7 (16·8 to 27·7)
Makueni	326 (263 to 395)	160 (143 to 181)	124 (112 to 137)	89·1 (77·2 to 101)	76·7 (65·1 to 88·6)	60·4 (51·2 to 70·1)	41·3 (34 to 49·6)	25·3 (19·8 to 31·7)	22·3 (17·3 to 28·3)	20·7 (16 to 26·3)
Mandera	156 (126 to 190)	141 (121 to 164)	132 (115 to 152)	127 (112 to 144)	122 (107 to 140)	80·3 (64·7 to 98)	82 (72·3 to 92·6)	62·1 (50·4 to 74·5)	60·8 (50 to 73·8)	58·2 (48·2 to 70·1)
Marsabit	191 (147 to 243)	213 (188 to 243)	176 (157 to 197)	145 (127 to 165)	120 (105 to 137)	89·8 (79·2 to 101)	79·7 (67·4 to 93·3)	61·2 (50·2 to 73·4)	57 (45·8 to 69·9)	55·1 (44·1 to 68)

Meru	194 (155 to 243)	130 (117 to 145)	98·3 (89·2 to 108)	64·6 (58·7 to 70·6)	58·2 (49·5 to 67·2)	59·3 (51·3 to 68·2)	59·3 (49·3 to 70)	49·8 (40·3 to 61·2)	47·5 (37·4 to 59·2)	45·7 (36 to 57·1)
Migori	463 (398 to 536)	332 (304 to 363)	295 (270 to 320)	270 (247 to 294)	238 (220 to 257)	206 (185 to 227)	116 (97·4 to 135)	73·8 (59·5 to 90·1)	69·4 (55·4 to 85·1)	66·2 (53·1 to 81·9)
Mombasa	149 (112 to 192)	193 (172 to 218)	146 (134 to 159)	100 (87·9 to 114)	87 (76·2 to 98·9)	77·5 (66·7 to 89·3)	54·5 (44·1 to 65·3)	36·1 (27·9 to 45·9)	33·4 (25·4 to 43·3)	31·1 (23·6 to 39·9)
Murang'a	329 (274 to 395)	151 (135 to 170)	104 (91·9 to 117)	60·4 (53 to 68·8)	48·6 (40·9 to 57)	49 (41·7 to 57·4)	47·4 (38·5 to 57·2)	37 (28·6 to 47)	36·6 (28·4 to 46·8)	35·4 (27·3 to 45·1)
Nairobi	155 (113 to 202)	183 (153 to 215)	121 (105 to 138)	83·2 (72·7 to 94·4)	76·6 (65 to 89·3)	70·1 (58·9 to 81·8)	37·1 (27·6 to 47·7)	23·4 (16·6 to 32)	22·2 (15·5 to 31·5)	21 (14·6 to 29·5)
Nakuru	60·5 (47·2 to 75·9)	55·3 (49·1 to 61·9)	53 (47·3 to 58·7)	53·2 (47·6 to 58·9)	47·6 (41·2 to 53·7)	50·1 (43·5 to 57·1)	44·1 (35·6 to 54)	34·4 (26·2 to 44·4)	33·7 (25·8 to 43)	32·4 (24·9 to 41·3)
Nandi	161 (118 to 215)	95·7 (84·9 to 108)	81·8 (73·8 to 90·2)	71·2 (63·3 to 79·3)	61·5 (53·8 to 69·2)	50 (43·5 to 57·2)	35·6 (29·2 to 42·6)	25 (19·6 to 31·4)	24·2 (19·1 to 30·2)	23·8 (18·6 to 29·9)
Narok	195 (153 to 246)	132 (108 to 160)	124 (106 to 145)	78·4 (66·5 to 91·5)	83·2 (69·2 to 98·4)	53·2 (44·7 to 62·6)	40·1 (34·1 to 46·8)	29·6 (22·6 to 37·5)	28·1 (22 to 35·2)	26·8 (21·5 to 33·5)
Nyamira	154 (123 to 192)	87·8 (78·9 to 98·6)	70·9 (63·4 to 79·1)	60 (52·5 to 67·6)	48 (42·9 to 54)	47·3 (40·4 to 54·6)	35 (27·9 to 42·8)	24·9 (19·6 to 31)	25 (19·6 to 31·5)	24·3 (19 to 30·5)
Nyandarua	192 (160 to 231)	135 (121 to 150)	90·2 (80·8 to 101)	51·2 (44·3 to 59)	42·8 (33·6 to 52·6)	48·3 (41·6 to 55·9)	56·2 (45·8 to 67·3)	47·1 (36·3 to 59·2)	47·2 (37·1 to 59·4)	45·9 (35·9 to 57·4)
Nyeri	241 (185 to 302)	117 (104 to 132)	74·1 (64·3 to 84·4)	44·2 (38·3 to 51·7)	41·7 (35·4 to 49)	47·9 (39·3 to 57·8)	41·7 (33·1 to 50·7)	35·9 (27·9 to 45·1)	35·6 (27·6 to 44·9)	34·7 (26·6 to 44)
Samburu	79 (58·4 to 106)	75·1 (64 to 87·4)	83·8 (73·6 to 95·3)	90·2 (78·7 to 103)	93·5 (80 to 109)	82·2 (72 to 93·3)	72·9 (60·3 to 86·7)	57·5 (44·5 to 72·1)	56 (43·9 to 70·2)	54 (43 to 67·3)
Siaya	402 (342 to 467)	289 (269 to 312)	241 (225 to 258)	210 (180 to 243)	206 (187 to 226)	187 (166 to 209)	106 (91·1 to 122)	64·1 (51·6 to 78·8)	61·1 (48·1 to 76·6)	63·9 (50·1 to 80·8)
Taita Taveta	261 (216 to 315)	196 (176 to 217)	176 (161 to 191)	113 (98·4 to 129)	81 (71·2 to 91·3)	79 (65·9 to 92)	51·9 (42·2 to 62·1)	31·4 (24 to 40·1)	28·7 (22 to 37·2)	27 (20·7 to 34·5)
Tana River	306 (237 to 385)	172 (150 to 196)	144 (127 to 162)	125 (110 to 142)	101 (88·8 to 116)	102 (85·8 to 118)	77·4 (65·4 to 90·5)	53·1 (42·3 to 64·8)	49·4 (38·8 to 60·5)	47·3 (37·8 to 58·1)
Tharaka Nithi	345 (278 to 424)	142 (125 to 163)	114 (101 to 128)	77·8 (67·7 to 89·1)	79·9 (68·1 to 92·9)	79·1 (67·4 to 92·7)	43·5 (34·6 to 52·5)	30·1 (23·7 to 37·6)	28·9 (22·3 to 36·5)	27·3 (21·2 to 34·6)
Trans Nzoia	87·7 (69·9 to 108)	98·3 (88·4 to 110)	90·9 (82·9 to 99)	88·8 (80·6 to 97)	68·6 (60 to 77·1)	57·7 (49·5 to 66·4)	41·3 (33·8 to 49·8)	29·4 (22·9 to 37·2)	26·9 (21·1 to 33·9)	26·8 (20·8 to 34·3)
Turkana	108 (81·3 to 140)	121 (104 to 139)	141 (123 to 161)	156 (139 to 174)	123 (107 to 140)	88·2 (75·8 to 100)	68·7 (58·9 to 80·1)	49·1 (38·3 to 61·1)	48·3 (38·4 to 59·5)	46·7 (37·2 to 57·4)
Uasin Gishu	61·6 (49 to 77·3)	59·1 (52·8 to 65·7)	54·5 (48·9 to 60·2)	56·5 (50·2 to 63)	53·5 (46·8 to 60·1)	47·4 (40·9 to 54·2)	38·7 (31·6 to 46·8)	29·6 (22·9 to 37·1)	28·5 (22·5 to 35·8)	27·9 (21·9 to 34·9)
Vihiga	394 (327 to 464)	163 (144 to 183)	124 (106 to 142)	98·1 (83·8 to 113)	104 (91·2 to 118)	93·4 (78·1 to 109)	69·5 (56 to 84·3)	49·2 (38·8 to 60·9)	46·8 (36·6 to 58·9)	48·6 (37·8 to 61·2)
Wajir	222 (179 to 273)	239 (208 to 275)	194 (172 to 218)	172 (152 to 192)	134 (118 to 152)	113 (92 to 136)	91·8 (80·7 to 103)	62·4 (51 to 74·9)	58·9 (48 to 71·2)	56·2 (46·5 to 67·8)
West Pokot	188 (152 to 226)	125 (113 to 139)	126 (113 to 138)	134 (122 to 145)	117 (104 to 131)	81·9 (71 to 93·8)	65·8 (53·9 to 79·9)	51·2 (40·6 to 62·8)	49·4 (39·7 to 60·7)	47·8 (38·5 to 59·2)
Madagascar	335 (274 to 398)	249 (215 to 284)	198 (175 to 221)	170 (156 to 185)	152 (138 to 165)	116 (105 to 128)	92·3 (81 to 104)	68·8 (56·4 to 83·7)	64·6 (52 to 80·2)	61·2 (49·2 to 76·4)
Malawi	397 (326 to 475)	368 (326 to 413)	325 (297 to 355)	264 (246 to 282)	223 (210 to 236)	171 (159 to 184)	102 (90·8 to 114)	63 (51·9 to 75·9)	59·9 (49·1 to 72·9)	56·2 (45·5 to 69)
Mozambique	361 (299 to 433)	300 (255 to 346)	258 (225 to 293)	236 (216 to 256)	228 (212 to 245)	168 (154 to 183)	112 (97·9 to 128)	73·1 (58·1 to 91·8)	70·9 (55·4 to 91·4)	66·7 (52 to 87·1)
Rwanda	440 (357 to 532)	273 (248 to 299)	284 (259 to 307)	217 (199 to 235)	172 (157 to 188)	151 (138 to 168)	78 (66·8 to 90·3)	48·8 (38·9 to 59·5)	46 (37 to 56·1)	44·3 (35·3 to 54)
Somalia	266 (213 to 329)	256 (219 to 296)	245 (209 to 285)	195 (170 to 222)	201 (181 to 222)	166 (145 to 188)	154 (132 to 177)	105 (84·7 to 130)	102 (81·6 to 126)	98·4 (79·3 to 122)
South Sudan	282 (219 to 351)	261 (217 to 309)	246 (206 to 287)	230 (194 to 266)	215 (187 to 242)	157 (138 to 176)	132 (110 to 155)	147 (117 to 183)	139 (110 to 175)	136 (107 to 172)
Uganda	392 (327 to 458)	252 (218 to 288)	226 (205 to 247)	213 (198 to 229)	185 (174 to 195)	150 (141 to 160)	97·4 (87·7 to 108)	65·1 (54·1 to 79)	66·4 (53·4 to 83·2)	69·8 (54·8 to 89·5)
Tanzania	360 (293 to 432)	262 (225 to 299)	222 (201 to 244)	186 (173 to 200)	163 (152 to 173)	130 (120 to 139)	86·2 (75·5 to 98·5)	61·3 (49·3 to 76·1)	58·4 (46·6 to 73·9)	55·5 (43·8 to 70·5)
Zambia	330 (271 to 406)	256 (219 to 295)	203 (184 to 222)	171 (158 to 183)	174 (161 to 186)	150 (136 to 164)	92 (79·5 to 106)	54·2 (42·4 to 68·7)	51·7 (40·3 to 66·7)	48·9 (37·7 to 63·1)
Southern sub-Saharan Africa	209 (164 to 261)	164 (138 to 193)	134 (118 to 151)	112 (102 to 122)	81·8 (75·6 to 88·7)	82·9 (76·3 to 90·1)	64·1 (57·8 to 70·7)	48·3 (40·1 to 57·7)	47·2 (38·6 to 57·9)	46·8 (38 to 58·1)
Botswana	178 (135 to 229)	136 (111 to 163)	114 (99·2 to 131)	83 (73·3 to 93·6)	68·5 (60·5 to 77·5)	77·4 (65·2 to 91·3)	54·5 (44·4 to 67·3)	44·5 (34 to 57·3)	43·5 (32·7 to 56·9)	43·2 (32·4 to 57·2)
Eswatini	341 (279 to 419)	277 (239 to 319)	206 (175 to 238)	136 (119 to 153)	88·1 (79·7 to 96·7)	104 (94·4 to 114)	87·1 (76·1 to 98·4)	49·2 (38·9 to 61·8)	47·1 (37 to 59·8)	46·1 (35·9 to 60)
Lesotho	257 (216 to 304)	195 (178 to 214)	181 (165 to 198)	120 (107 to 135)	99·2 (88·2 to 112)	108 (96·6 to 119)	97·8 (84·1 to 113)	87·5 (71·2 to 105)	87·6 (71·6 to 105)	87·9 (71 to 107)
Namibia	206 (160 to 262)	148 (122 to 176)	110 (94·8 to 128)	97·1 (86·4 to 108)	74·9 (67·7 to 82·5)	72 (63·8 to 80·8)	57·6 (49·2 to 67·2)	38·9 (30·3 to 49·6)	37·5 (28·9 to 48·5)	36·7 (28·1 to 48·3)
South Africa	208 (160 to 263)	169 (141 to 197)	137 (120 to 158)	117 (104 to 130)	83·2 (75·8 to 91·2)	80·8 (72·7 to 89·3)	55·6 (48·5 to 63·1)	42·4 (35·2 to 50·8)	41·7 (33·8 to 51·4)	41·2 (33·3 to 51·3)
Eastern Cape	391 (310 to 485)	236 (198 to 276)	198 (172 to 225)	155 (136 to 173)	112 (101 to 125)	100 (88 to 112)	71·5 (63 to 80·6)	52·3 (43·6 to 62·9)	53·6 (43·5 to 66·2)	52·7 (42·4 to 66·1)
Free State	195 (146 to 254)	189 (155 to 224)	165 (142 to 191)	147 (129 to 164)	103 (91·2 to 115)	104 (90·7 to 118)	74·4 (65·1 to 84·3)	58 (48·4 to 70·5)	54·9 (45 to 67·5)	54·1 (44·3 to 67·5)
Gauteng	86·9 (61·5 to 119)	103 (82·6 to 125)	89·7 (75·9 to 106)	89·3 (77·3 to 103)	63·4 (55·1 to 72·1)	75·1 (66·2 to 84·6)	47·5 (41·2 to 54)	36·2 (29·9 to 43·7)	35·3 (28·3 to 43·8)	34·6 (27·9 to 43·4)
KwaZulu-Natal	189 (144 to 247)	173 (144 to 204)	144 (124 to 165)	126 (113 to 139)	91·2 (81 to 102)	91·2 (81·6 to 101)	58·2 (50·4 to 66·6)	42·3 (33·9 to 51·8)	42·1 (33·2 to 52·1)	41·5 (32·9 to 52·4)
Limpopo	237 (178 to 308)	147 (120 to 175)	117 (101 to 137)	90·7 (80·1 to 103)	63·7 (56·9 to 70·9)	57·5 (51·1 to 64·8)	53·9 (46·7 to 61·5)	48·9 (40·1 to 59·2)	48·8 (39·9 to 59·4)	49·1 (40 to 60·6)
Mpumalanga	215 (162 to 276)	174 (144 to 205)	152 (132 to 175)	121 (108 to 136)	79·2 (70·2 to 88·9)	76·9 (67·9 to 86·8)	53·9 (47 to 61·3)	43·2 (35·4 to 52·3)	41·6 (33·8 to 50·9)	41·4 (33·8 to 51·4)
North West	201 (152 to 258)	192 (160 to 225)	157 (137 to 182)	132 (116 to 149)	92 (82·2 to 103)	93·8 (81·6 to 107)	70 (61·1 to 79·2)	54·5 (45·3 to 65·1)	52·6 (42·9 to 64)	52 (42·4 to 64·1)
Northern Cape	140 (103 to 186)	137 (110 to 167)	121 (103 to 143)	101 (87·7 to 115)	83·6 (73·5 to 94·3)	76·4 (66·6 to 86·6)	64·2 (56·1 to 72·9)	51·1 (42·8 to 61·7)	48·7 (39·8 to 59·9)	47·8 (39·1 to 59·6)
Western Cape	78·1 (53·6 to 110)	66·1 (52·2 to 82·6)	60·2 (50·1 to 71·5)	61·5 (52 to 71·6)	49·5 (42·6 to 56·9)	44 (38·1 to 50·6)	28·7 (24·5 to 33·3)	20·9 (17·2 to 25·5)	19·6 (15·9 to 24·3)	19·1 (15·5 to 23·9)
Zimbabwe	196 (151 to 248)	137 (114 to 163)	111 (97·7 to 126)	98·6 (89·6 to 108)	77·7 (71·5 to 84)	85·7 (78·5 to 92·9)	81·6 (73·8 to 90·7)	59·2 (49 to 71·5)	57·3 (46·7 to 70·3)	56·8 (45·9 to 71)
Western sub-Saharan Africa	334 (283 to 387)	300 (269 to 332)	270 (250 to 290)	228 (217 to 241)	200 (191 to 210)	175 (166 to 185)	134 (124 to 144)	99·6 (85·4 to 115)	96·4 (81·6 to 113)	91·8 (77·1 to 109)
Benin	404 (335 to 478)	295 (263 to 328)	251 (232 to 272)	206 (190 to 221)	180 (166 to 194)	147 (136 to 159)	119 (108 to 132)	89·2 (73·5 to 107)	85·3 (69·6 to 103)	82·2 (66·6 to 101)
Burkina Faso	373 (307 to 444)	356 (308 to 403)	305 (274 to 335)	256 (240 to 275)	214 (200 to 230)	185 (169 to 202)	145 (124 to 168)	108 (87 to 133)	108 (86·7 to 134)	100 (79·8 to 126)
Cabo Verde	310 (283 to 342)	170 (163 to 178)	168 (162 to 174)	90·3 (84·9 to 96·1)	69·2 (64·7 to 73·7)	54·2 (45·9 to 63·5)	29·8 (24·7 to 35·8)	18·8 (14·7 to 23·5)	17·3 (13·3 to 22·2)	16·4 (12·2 to 21·5)
Cameroon	355 (306 to 406)	275 (250 to 299)	218 (200 to 237)	181 (168 to 195)	143 (131 to 154)	135 (123 to 147)	106 (93·8 to 121)	74·9 (61·4 to 89·2)	73·9 (60·1 to 89·1)	69·6 (56·5 to 84)
Chad	380 (309 to 454)	292 (247 to 335)	277 (245 to 312)	245 (225 to 267)	207 (190 to 224)	187 (171 to 205)	152 (135 to 170)	124 (103 to 148)	121 (101 to 146)	118 (97·5 to 145)
Côte d'Ivoire	287 (244 to 333)	282 (261 to 303)	230 (213 to 248)	174 (161 to 186)	157 (146 to 168)	147 (135 to 159)	116 (101 to 131)	81·6 (68·7 to 97·2)	78·4 (65·8 to 93·7)	74·4 (62 to 88·6)
The Gambia	204 (155 to 259)	252 (210 to 295)	212 (183 to 243)	182 (163 to 203)	141 (126 to 157)	110 (97·5 to 123)	73 (62·8 to 83·7)	52·7 (42·2 to 65·1)	49·7 (39·5 to 62·7)	47·4 (37·1 to 60·9)
Ghana	282 (226 to 346)	227 (205 to 249)	193 (176 to 209)	157 (145 to 169)	128 (118 to 138)	105 (96 to 114)	81·1 (71·9 to 90·8)	54·2 (42·6 to 67·8)	50·8 (39·3 to 64·9)	46·9 (35·5 to 61)
Guinea	389 (325 to 455)	338 (292 to 382)	312 (276 to 350)	271 (247 to 295)	230 (213 to 248)	185 (169 to 201)	140 (124 to 157)	102 (84·8 to 122)	98·9 (80·9 to 120)	92·9 (75·9 to 114)
Guinea-Bissau	527 (460 to 596)	396 (347 to 447)	317 (276 to 360)	270 (237 to 303)	222 (198 to 248)	177 (159 to 196)	111 (96·5 to 127)	73·6 (60 to 89·8)	71·2 (57·6 to 87·8)	68 (55 to 84·2)
Liberia	546 (453 to 640)	377 (334 to 419)	311 (282 to 341)	257 (231 to 285)	260 (237 to 282)	178 (163 to 194)	101 (87·2 to 116)	77·3 (62 to 95·8)	76·4 (59·9 to 97·7)	70·5 (54·3 to 92·8)
Mali	431 (365 to 506)	371 (330 to 410)	345 (320 to 370)	288 (269 to 308)	245 (230 to 260)	201 (184 to 216)	156 (142 to 172)	115 (96·8 to 137)	113 (94·5 to 136)	106 (88·4 to 128)
Mauritania	278 (231 to 326)	199 (179 to 219)	152 (140 to 165)	134 (122 to 146)	106 (95·8 to 116)	89 (80·5 to 98·5)	63·1 (55 to 71·7)	42·4 (34·9 to 50·7)	39·5 (32·6 to 47·3)	37·2 (30·6 to 45)
Niger	368 (304 to 434)	383 (337 to 430)	346 (312 to 379)	306 (287 to 325)	283 (266 to 303)	224 (202 to 247)	131 (110 to 153)	99·6 (81·3 to 122)	96·4 (77·5 to 121)	93 (73·8 to 118)
Nigeria	316 (256 to 382)	293 (258 to 329)	274 (247 to 300)	235 (220 to 252)	213 (200 to 226)	189 (179 to 201)	149 (136 to 162)	112 (94·7 to 131)	107 (90 to 128)	102 (84·7 to 124)
São Tomé and Príncipe	385 (355 to 419)	262 (249 to 276)	145 (137 to 154)	114 (106 to 122)	109 (99·6 to 119)	80·2 (71·4 to 89·8)	42 (35·4 to 49·3)	21·7 (16·5 to 28)	19·8 (14·9 to 26·2)	18·9 (14 to 25·2)

Senegal	307 (257 to 361)	280 (256 to 303)	266 (248 to 285)	209 (196 to 224)	147 (136 to 157)	126 (117 to 136)	75·9 (68·9 to 83·4)	50·3 (41·6 to 59·6)	46·6 (38·4 to 55·9)	43·7 (35·8 to 52·6)
Sierra Leone	389 (314 to 468)	337 (291 to 385)	300 (257 to 346)	267 (237 to 299)	243 (225 to 260)	227 (211 to 244)	164 (147 to 182)	110 (88·7 to 134)	110 (86·4 to 139)	104 (81 to 131)
Togo	359 (287 to 438)	287 (249 to 325)	228 (204 to 251)	172 (157 to 188)	150 (136 to 164)	130 (117 to 144)	101 (88·9 to 114)	69·1 (55·8 to 85·4)	65·5 (52·5 to 82·2)	61·8 (48·7 to 78·6)

Appendix 2 Table S3a: Age-standardised mortality rates (per 100 000) with 95% UIs by location, both sexes combined, 1950, 1960, 1970, 1980, 1990, 2000, 2010, 2019, 2020, 2021

Location	1950	1960	1970	1980	1990	2000	2010	2019	2020	2021
Global	1980 (1860 to 2120)	2080 (1860 to 2460)	1440 (1390 to 1510)	1270 (1230 to 1320)	1110 (1080 to 1140)	1010 (986 to 1040)	855 (829 to 881)	736 (700 to 773)	795 (757 to 838)	835 (796 to 879)
Central Europe, eastern Europe, and central Asia	1360 (1260 to 1470)	1070 (1030 to 1110)	1050 (1020 to 1080)	1080 (1050 to 1110)	1030 (993 to 1060)	1120 (1090 to 1160)	930 (902 to 960)	756 (714 to 801)	854 (805 to 907)	948 (888 to 1010)
Central Asia	1980 (1670 to 2340)	1440 (1320 to 1570)	1210 (1140 to 1280)	1060 (1020 to 1100)	1020 (983 to 1050)	1090 (1040 to 1150)	966 (909 to 1030)	803 (735 to 877)	954 (868 to 1050)	982 (876 to 1100)
Armenia	1840 (1520 to 2220)	1270 (1110 to 1450)	995 (877 to 1130)	838 (761 to 930)	908 (841 to 982)	898 (837 to 970)	814 (753 to 877)	649 (576 to 736)	958 (845 to 1090)	752 (648 to 876)
Azerbaijan	2630 (2190 to 3150)	1640 (1460 to 1850)	1270 (1160 to 1380)	1090 (1000 to 1190)	1050 (968 to 1140)	1020 (941 to 1110)	965 (885 to 1050)	829 (717 to 959)	1060 (926 to 1220)	1050 (892 to 1230)
Georgia	1780 (1470 to 2130)	1320 (1170 to 1490)	1100 (998 to 1210)	993 (908 to 1080)	974 (899 to 1050)	956 (847 to 1070)	805 (734 to 888)	737 (664 to 817)	854 (771 to 945)	981 (877 to 1110)
Kazakhstan	1650 (1360 to 1990)	1360 (1200 to 1530)	1170 (1080 to 1280)	1050 (969 to 1140)	1050 (970 to 1140)	1300 (1200 to 1400)	1120 (1090 to 1150)	882 (805 to 969)	1050 (956 to 1160)	1170 (1040 to 1320)
Kyrgyzstan	2200 (1830 to 2610)	1700 (1470 to 1970)	1450 (1280 to 1650)	1160 (1050 to 1280)	1080 (988 to 1180)	1100 (1010 to 1200)	1000 (920 to 1090)	753 (658 to 877)	914 (767 to 1080)	871 (712 to 1050)
Mongolia	2720 (2220 to 3320)	2030 (1710 to 2410)	1760 (1530 to 1980)	1460 (1290 to 1630)	1360 (1230 to 1490)	1390 (1270 to 1520)	1190 (1090 to 1300)	938 (825 to 1060)	900 (822 to 984)	1010 (920 to 1120)
Tajikistan	2520 (2060 to 3030)	1840 (1600 to 2120)	1340 (1150 to 1540)	1160 (1030 to 1300)	1090 (995 to 1200)	1090 (1000 to 1200)	1020 (918 to 1130)	771 (657 to 910)	984 (830 to 1160)	1040 (867 to 1230)
Turkmenistan	2200 (1830 to 2620)	1640 (1480 to 1830)	1340 (1220 to 1470)	1180 (1080 to 1280)	1120 (1040 to 1210)	1130 (992 to 1290)	939 (846 to 1040)	943 (776 to 1130)	1110 (915 to 1330)	1150 (938 to 1390)
Uzbekistan	1870 (1510 to 2290)	1300 (1140 to 1470)	1140 (1050 to 1240)	1030 (955 to 1100)	911 (845 to 980)	1010 (875 to 1180)	948 (782 to 1190)	796 (622 to 1040)	886 (677 to 1200)	869 (647 to 1200)
Central Europe	1560 (1500 to 1630)	1120 (1090 to 1160)	1070 (1040 to 1100)	1040 (1010 to 1080)	1000 (972 to 1040)	883 (856 to 913)	730 (717 to 742)	640 (616 to 666)	719 (690 to 749)	787 (752 to 825)
Albania	1660 (1460 to 1890)	1110 (994 to 1230)	940 (776 to 1120)	895 (752 to 1050)	854 (789 to 922)	713 (657 to 780)	591 (524 to 666)	618 (535 to 722)	708 (595 to 831)	757 (631 to 901)
Bosnia and Herzegovina	2050 (1880 to 2240)	1400 (1300 to 1530)	1120 (1040 to 1210)	1000 (930 to 1080)	797 (734 to 867)	713 (616 to 818)	629 (576 to 690)	637 (571 to 715)	723 (644 to 813)	759 (623 to 916)
Bulgaria	1390 (1290 to 1490)	967 (879 to 1070)	938 (849 to 1040)	1020 (933 to 1140)	1230 (1130 to 1340)	1200 (1100 to 1290)	983 (960 to 1010)	902 (820 to 984)	1020 (928 to 1130)	1220 (1100 to 1360)
Croatia	2230 (2070 to 2410)	1480 (1380 to 1600)	1200 (1110 to 1300)	970 (902 to 1050)	1000 (930 to 1090)	816 (746 to 894)	668 (621 to 717)	576 (511 to 651)	620 (551 to 708)	673 (596 to 768)
Czechia	1270 (1190 to 1370)	1070 (998 to 1160)	1130 (1050 to 1200)	1070 (999 to 1160)	986 (915 to 1070)	764 (754 to 774)	611 (600 to 622)	530 (490 to 576)	596 (549 to 653)	629 (573 to 692)
Hungary	1450 (1350 to 1560)	1170 (1090 to 1250)	1120 (1040 to 1200)	1140 (1070 to 1220)	1090 (1020 to 1170)	928 (863 to 1000)	763 (709 to 828)	661 (590 to 750)	712 (629 to 814)	781 (690 to 886)
Montenegro	1100 (1010 to 1200)	776 (712 to 848)	729 (668 to 797)	770 (707 to 840)	725 (668 to 788)	787 (718 to 862)	740 (683 to 802)	851 (751 to 972)	994 (877 to 1140)	1150 (1010 to 1330)
North Macedonia	1850 (1690 to 2020)	1390 (1280 to 1510)	1120 (1040 to 1200)	903 (836 to 976)	994 (905 to 1090)	1280 (1180 to 1390)	1150 (1020 to 1310)	987 (830 to 1180)	1110 (917 to 1340)	1250 (1020 to 1540)
Poland	1560 (1460 to 1680)	1050 (986 to 1130)	990 (924 to 1060)	983 (906 to 1070)	957 (887 to 1040)	801 (740 to 871)	661 (654 to 667)	583 (539 to 632)	664 (608 to 723)	715 (651 to 790)
Romania	1510 (1260 to 1810)	1160 (1060 to 1280)	1110 (1010 to 1220)	1120 (1030 to 1230)	1070 (990 to 1160)	970 (894 to 1050)	823 (763 to 887)	709 (631 to 796)	792 (704 to 907)	890 (779 to 1020)
Serbia	2110 (1960 to 2250)	1380 (1280 to 1470)	1280 (1180 to 1380)	1170 (1090 to 1260)	1200 (1110 to 1300)	1150 (1060 to 1250)	853 (792 to 919)	745 (666 to 830)	855 (753 to 969)	897 (794 to 1030)
Slovakia	1320 (1300 to 1350)	987 (972 to 1000)	1040 (1030 to 1060)	1000 (986 to 1010)	968 (955 to 982)	806 (794 to 820)	702 (692 to 714)	608 (558 to 666)	660 (601 to 726)	788 (714 to 872)
Slovenia	1520 (1420 to 1640)	1060 (982 to 1150)	1020 (943 to 1110)	962 (886 to 1050)	762 (686 to 854)	663 (646 to 678)	512 (498 to 527)	434 (398 to 474)	489 (446 to 535)	472 (428 to 522)
Eastern Europe	1120 (1010 to 1260)	955 (905 to 1010)	988 (943 to 1040)	1080 (1030 to 1140)	1020 (964 to 1080)	1240 (1170 to 1320)	1030 (977 to 1090)	803 (729 to 888)	901 (812 to 1010)	1030 (917 to 1150)
Belarus	967 (762 to 1200)	799 (732 to 871)	804 (750 to 862)	912 (850 to 985)	914 (848 to 988)	1050 (975 to 1140)	965 (893 to 1050)	779 (687 to 886)	918 (779 to 1090)	1040 (849 to 1270)
Estonia	1190 (967 to 1460)	1030 (1010 to 1060)	1010 (982 to 1030)	1040 (1020 to 1070)	1020 (996 to 1040)	942 (922 to 965)	670 (652 to 688)	541 (494 to 592)	553 (505 to 605)	647 (583 to 718)
Latvia	1030 (804 to 1270)	956 (888 to 1040)	953 (885 to 1030)	1040 (970 to 1120)	1020 (949 to 1100)	950 (889 to 1020)	806 (789 to 823)	674 (619 to 733)	705 (646 to 776)	842 (766 to 925)
Lithuania	1090 (862 to 1360)	903 (838 to 968)	882 (826 to 944)	942 (876 to 1010)	915 (848 to 988)	853 (793 to 921)	816 (759 to 882)	658 (606 to 716)	734 (671 to 801)	796 (720 to 878)
Moldova	1960 (1630 to 2340)	1720 (1530 to 1920)	1410 (1290 to 1530)	1230 (1140 to 1320)	1130 (1050 to 1220)	1060 (965 to 1160)	955 (857 to 1070)	719 (596 to 872)	786 (668 to 942)	877 (732 to 1080)
Russia	1140 (972 to 1330)	986 (915 to 1060)	1020 (953 to 1090)	1120 (1040 to 1210)	1030 (959 to 1120)	1280 (1180 to 1390)	1060 (989 to 1130)	795 (703 to 902)	928 (815 to 1060)	1050 (929 to 1210)
Ukraine	1040 (886 to 1220)	873 (808 to 942)	938 (881 to 1000)	1020 (951 to 1110)	996 (928 to 1080)	1260 (1170 to 1360)	1000 (891 to 1140)	879 (698 to 1080)	877 (688 to 1130)	1010 (758 to 1310)
High income	1250 (1210 to 1280)	1040 (1020 to 1070)	960 (939 to 983)	795 (776 to 815)	670 (655 to 685)	569 (556 to 583)	469 (459 to 481)	428 (417 to 440)	463 (452 to 475)	464 (452 to 478)
Australasia	1080 (1020 to 1140)	981 (923 to 1050)	977 (922 to 1040)	780 (732 to 830)	653 (614 to 697)	519 (488 to 552)	424 (399 to 453)	379 (354 to 406)	356 (335 to 380)	370 (349 to 392)
Australia	1080 (1020 to 1160)	983 (915 to 1060)	982 (915 to 1060)	768 (711 to 829)	643 (597 to 694)	510 (474 to 551)	418 (391 to 451)	372 (343 to 403)	350 (326 to 377)	364 (341 to 391)
New Zealand	1070 (998 to 1150)	970 (901 to 1050)	958 (891 to 1030)	838 (783 to 902)	702 (650 to 757)	564 (524 to 609)	458 (416 to 504)	419 (390 to 453)	391 (360 to 422)	400 (366 to 437)
High-income Asia Pacific	1800 (1700 to 1910)	1270 (1200 to 1350)	1030 (974 to 1100)	779 (735 to 827)	593 (560 to 630)	466 (440 to 494)	381 (360 to 405)	323 (304 to 344)	314 (298 to 332)	321 (303 to 343)
Brunei	2100 (1910 to 2300)	1550 (1400 to 1710)	1130 (1000 to 1250)	976 (858 to 1100)	1010 (900 to 1120)	822 (747 to 909)	728 (656 to 803)	713 (619 to 817)	654 (558 to 766)	673 (572 to 790)
Japan	1500 (1410 to 1620)	1150 (1080 to 1230)	923 (862 to 994)	686 (641 to 735)	530 (496 to 569)	427 (400 to 459)	365 (340 to 394)	316 (294 to 342)	307 (288 to 329)	312 (290 to 336)
Aichi	1470 (1260 to 1710)	1140 (956 to 1340)	919 (772 to 1080)	695 (634 to 766)	541 (495 to 595)	436 (398 to 480)	366 (333 to 404)	315 (284 to 350)	305 (279 to 337)	308 (278 to 342)
Akita	1570 (1350 to 1830)	1230 (1020 to 1450)	997 (850 to 1170)	738 (670 to 808)	556 (507 to 614)	452 (410 to 502)	394 (357 to 438)	341 (304 to 382)	332 (299 to 370)	347 (308 to 391)
Aomori	1660 (1420 to 1930)	1240 (1050 to 1470)	996 (830 to 1190)	734 (664 to 808)	572 (518 to 631)	476 (432 to 523)	416 (374 to 463)	376 (333 to 420)	362 (327 to 403)	370 (328 to 414)
Chiba	1520 (1280 to 1750)	1170 (982 to 1380)	914 (766 to 1090)	685 (622 to 756)	518 (472 to 575)	428 (389 to 475)	366 (332 to 407)	317 (285 to 356)	310 (281 to 346)	312 (279 to 350)
Ehime	1510 (1270 to 1760)	1140 (960 to 1360)	903 (758 to 1070)	664 (602 to 728)	522 (473 to 576)	435 (393 to 477)	365 (331 to 404)	324 (289 to 365)	317 (287 to 351)	317 (282 to 355)
Fukui	1370 (1160 to 1610)	1050 (889 to 1230)	852 (727 to 1010)	625 (569 to 693)	497 (449 to 551)	396 (359 to 439)	351 (317 to 390)	291 (258 to 327)	294 (265 to 327)	302 (268 to 341)
Fukuoka	1490 (1260 to 1750)	1140 (955 to 1340)	934 (782 to 1130)	695 (632 to 773)	538 (488 to 600)	429 (389 to 477)	363 (330 to 403)	314 (281 to 354)	305 (277 to 338)	309 (277 to 346)
Fukushima	1650 (1430 to 1910)	1240 (1050 to 1450)	999 (843 to 1190)	710 (640 to 785)	532 (480 to 587)	442 (400 to 486)	382 (346 to 423)	346 (308 to 387)	340 (307 to 376)	342 (306 to 383)
Gifu	1500 (1280 to 1770)	1170 (986 to 1380)	956 (815 to 1130)	720 (653 to 795)	529 (480 to 586)	430 (388 to 477)	361 (327 to 400)	316 (283 to 355)	301 (272 to 334)	309 (276 to 347)
Gunma	1570 (1340 to 1830)	1220 (1020 to 1430)	963 (813 to 1150)	712 (644 to 787)	531 (481 to 587)	429 (388 to 474)	376 (340 to 415)	330 (294 to 368)	322 (290 to 357)	330 (295 to 369)
Hiroshima	1530 (1310 to 1770)	1150 (966 to 1360)	906 (762 to 1090)	679 (617 to 747)	524 (477 to 581)	416 (378 to 462)	352 (320 to 390)	308 (276 to 344)	290 (264 to 323)	295 (262 to 332)
Hokkaidō	1530 (1290 to 1790)	1170 (971 to 1370)	960 (819 to 1130)	708 (645 to 778)	537 (490 to 589)	425 (385 to 468)	368 (335 to 406)	328 (296 to 363)	323 (294 to 355)	330 (296 to 366)
Hyōgo	1470 (1250 to 1720)	1130 (957 to 1330)	932 (790 to 1110)	705 (642 to 776)	555 (504 to 615)	440 (401 to 485)	368 (333 to 407)	306 (275 to 342)	300 (270 to 332)	303 (271 to 338)
Ibaraki	1690 (1420 to 1960)	1260 (1070 to 1470)	993 (834 to 1160)	718 (655 to 785)	558 (506 to 614)	451 (411 to 494)	388 (354 to 428)	341 (307 to 379)	327 (297 to 360)	332 (300 to 367)
Ishikawa	1390 (1170 to 1620)	1100 (923 to 1290)	883 (746 to 1040)	683 (620 to 757)	511 (463 to 563)	418 (379 to 460)	352 (318 to 392)	298 (266 to 334)	292 (264 to 325)	302 (269 to 340)
Iwate	1650 (1410 to 1910)	1240 (1060 to 1460)	982 (827 to 1160)	707 (638 to 781)	543 (492 to 599)	432 (392 to 478)	387 (350 to 430)	357 (319 to 400)	332 (299 to 368)	338 (299 to 376)
Kagawa	1450 (1220 to 1690)	1110 (928 to 1320)	864 (730 to 1020)	634 (575 to 704)	512 (464 to 567)	422 (384 to 470)	363 (327 to 405)	310 (276 to 350)	306 (276 to 344)	307 (272 to 348)
Kagoshima	1700 (1470 to 1970)	1260 (1070 to 1480)	1020 (865 to 1200)	718 (652 to 789)	537 (486 to 593)	432 (390 to 476)	372 (334 to 411)	332 (294 to 371)	316 (285 to 349)	321 (286 to 359)
Kanagawa	1370 (1170 to 1580)	1040 (866 to 1230)	836 (709 to 989)	653 (595 to 721)	516 (469 to 566)	422 (386 to 465)	354 (322 to 390)	300 (270 to 333)	294 (267 to 326)	300 (269 to 334)
Kōchi	1650 (1400 to 1910)	1230 (1040 to 1450)	988 (847 to 1150)	700 (634 to 770)	528 (479 to 584)	430 (388 to 475)	375 (341 to 414)	322 (286 to 360)	314 (283 to 350)	319 (283 to 360)
Kumamoto	1570 (1340 to 1810)	1200 (1000 to 1420)	962 (813 to 1120)	677 (618 to 747)	499 (452 to 551)	393 (356 to 436)	339 (306 to 378)	293 (262 to 331)	285 (258 to 316)	297 (266 to 334)

Kyōto	1420 (1210 to 1650)	1070 (901 to 1280)	858 (718 to 1020)	650 (586 to 715)	520 (469 to 575)	421 (382 to 465)	352 (316 to 388)	288 (256 to 322)	280 (254 to 312)	290 (255 to 323)
Mie	1480 (1260 to 1720)	1140 (957 to 1350)	902 (752 to 1060)	680 (611 to 756)	540 (489 to 602)	427 (385 to 474)	366 (329 to 410)	314 (278 to 353)	298 (267 to 330)	310 (275 to 348)
Miyagi	1560 (1330 to 1800)	1190 (995 to 1400)	970 (812 to 1150)	705 (642 to 782)	534 (486 to 591)	427 (386 to 473)	362 (328 to 402)	312 (279 to 349)	304 (276 to 337)	306 (274 to 346)
Miyazaki	1600 (1370 to 1860)	1210 (1020 to 1420)	963 (820 to 1150)	702 (636 to 774)	525 (477 to 582)	418 (380 to 462)	360 (328 to 400)	326 (292 to 367)	311 (281 to 345)	327 (292 to 372)
Nagano	1520 (1300 to 1770)	1150 (964 to 1370)	904 (769 to 1060)	653 (594 to 718)	489 (440 to 540)	395 (358 to 438)	334 (300 to 369)	289 (256 to 325)	283 (255 to 313)	282 (251 to 316)
Nagasaki	1560 (1330 to 1840)	1190 (1000 to 1390)	985 (830 to 1150)	713 (648 to 788)	538 (489 to 593)	426 (387 to 471)	370 (336 to 412)	324 (290 to 364)	308 (278 to 340)	328 (292 to 369)
Nara	1480 (1270 to 1720)	1150 (958 to 1360)	934 (791 to 1100)	704 (643 to 777)	544 (496 to 600)	417 (376 to 463)	353 (319 to 392)	307 (274 to 346)	283 (256 to 313)	297 (264 to 335)
Niigata	1550 (1330 to 1800)	1190 (1020 to 1400)	970 (820 to 1140)	709 (646 to 789)	514 (467 to 573)	413 (375 to 458)	355 (322 to 398)	327 (294 to 370)	315 (285 to 350)	320 (284 to 361)
Ōita	1560 (1330 to 1830)	1180 (1000 to 1420)	942 (794 to 1120)	694 (629 to 770)	528 (479 to 588)	423 (381 to 470)	348 (314 to 390)	310 (275 to 351)	306 (274 to 340)	289 (256 to 324)
Okayama	1470 (1260 to 1710)	1130 (951 to 1340)	881 (738 to 1040)	656 (594 to 726)	508 (463 to 561)	409 (371 to 451)	353 (318 to 392)	310 (277 to 347)	296 (267 to 326)	293 (262 to 327)
Okinawa	1670 (1450 to 1950)	1170 (943 to 1420)	977 (814 to 1150)	624 (563 to 692)	459 (415 to 509)	397 (359 to 440)	359 (323 to 398)	321 (286 to 360)	314 (284 to 348)	325 (287 to 364)
Ōsaka	1510 (1290 to 1760)	1160 (979 to 1370)	947 (797 to 1100)	737 (669 to 812)	586 (532 to 644)	457 (414 to 505)	387 (350 to 428)	331 (297 to 368)	325 (294 to 358)	327 (294 to 365)
Saga	1530 (1290 to 1800)	1150 (971 to 1360)	918 (786 to 1070)	669 (606 to 738)	522 (473 to 581)	427 (387 to 475)	363 (330 to 402)	315 (283 to 355)	312 (281 to 348)	309 (272 to 347)
Saitama	1510 (1290 to 1770)	1150 (967 to 1370)	902 (758 to 1070)	696 (635 to 766)	537 (489 to 592)	427 (388 to 469)	372 (338 to 410)	322 (290 to 356)	314 (285 to 345)	319 (287 to 354)
Shiga	1530 (1320 to 1770)	1210 (1030 to 1420)	991 (850 to 1150)	713 (646 to 784)	540 (491 to 595)	411 (372 to 454)	343 (310 to 380)	295 (264 to 330)	289 (262 to 319)	276 (245 to 308)
Shimane	1500 (1270 to 1780)	1110 (941 to 1320)	908 (772 to 1060)	643 (580 to 716)	485 (439 to 539)	398 (359 to 444)	355 (321 to 397)	299 (264 to 341)	282 (253 to 315)	303 (268 to 342)
Shizuoka	1420 (1210 to 1660)	1080 (898 to 1290)	871 (727 to 1030)	656 (594 to 729)	506 (459 to 562)	413 (376 to 458)	360 (326 to 399)	317 (285 to 354)	309 (281 to 343)	310 (277 to 346)
Tochigi	1600 (1370 to 1860)	1230 (1020 to 1440)	977 (830 to 1160)	735 (667 to 808)	567 (513 to 626)	449 (406 to 496)	393 (354 to 433)	343 (307 to 383)	329 (296 to 364)	331 (296 to 370)
Tokushima	1590 (1380 to 1850)	1220 (1030 to 1440)	980 (830 to 1140)	723 (657 to 795)	541 (493 to 595)	430 (389 to 472)	382 (345 to 424)	331 (296 to 372)	297 (266 to 330)	341 (303 to 382)
Tōkyō	1340 (1150 to 1560)	1040 (892 to 1230)	864 (731 to 1020)	651 (595 to 716)	536 (489 to 590)	434 (396 to 478)	362 (330 to 399)	306 (277 to 339)	298 (272 to 329)	302 (272 to 335)
Tottori	1510 (1280 to 1780)	1150 (974 to 1350)	929 (800 to 1080)	679 (610 to 750)	531 (478 to 587)	427 (386 to 471)	371 (333 to 411)	333 (296 to 375)	317 (284 to 352)	319 (279 to 360)
Toyama	1490 (1260 to 1750)	1150 (966 to 1370)	915 (781 to 1080)	670 (611 to 746)	507 (462 to 560)	405 (367 to 447)	352 (320 to 392)	305 (272 to 342)	296 (265 to 331)	307 (273 to 345)
Wakayama	1550 (1330 to 1820)	1200 (1010 to 1420)	963 (821 to 1130)	725 (652 to 797)	561 (508 to 618)	448 (403 to 498)	392 (354 to 433)	343 (305 to 386)	332 (300 to 369)	329 (290 to 369)
Yamagata	1580 (1340 to 1840)	1200 (1010 to 1410)	981 (826 to 1160)	699 (628 to 776)	516 (464 to 573)	424 (384 to 473)	354 (318 to 394)	328 (292 to 369)	306 (277 to 340)	322 (284 to 363)
Yamaguchi	1500 (1270 to 1760)	1150 (969 to 1370)	918 (772 to 1090)	704 (642 to 779)	530 (480 to 585)	436 (394 to 484)	377 (340 to 422)	328 (293 to 369)	320 (289 to 358)	320 (285 to 361)
Yamanashi	1530 (1300 to 1790)	1150 (969 to 1360)	916 (774 to 1070)	676 (614 to 743)	520 (472 to 573)	421 (382 to 465)	354 (321 to 390)	321 (285 to 359)	299 (270 to 329)	308 (276 to 346)
South Korea	3140 (2900 to 3420)	1810 (1670 to 1960)	1560 (1440 to 1680)	1290 (1210 to 1390)	945 (886 to 1020)	703 (658 to 754)	467 (435 to 502)	364 (338 to 393)	357 (333 to 383)	375 (348 to 405)
Singapore	1750 (1640 to 1880)	1310 (1200 to 1440)	1120 (1050 to 1200)	936 (877 to 998)	696 (650 to 745)	528 (494 to 569)	389 (362 to 418)	294 (274 to 320)	284 (262 to 308)	293 (268 to 318)
High-income North America	1070 (1010 to 1140)	983 (926 to 1050)	921 (866 to 980)	752 (707 to 803)	669 (629 to 712)	617 (580 to 657)	523 (492 to 556)	502 (470 to 538)	577 (543 to 615)	586 (550 to 627)
Canada	1070 (997 to 1140)	937 (872 to 1010)	834 (777 to 898)	705 (658 to 756)	609 (564 to 658)	530 (495 to 569)	434 (405 to 470)	399 (369 to 434)	427 (397 to 459)	418 (390 to 449)
Greenland	2250 (1990 to 2540)	1730 (1580 to 1890)	1360 (1250 to 1480)	1390 (1260 to 1510)	1360 (1290 to 1440)	1060 (983 to 1170)	860 (807 to 917)	750 (673 to 848)	766 (680 to 872)	758 (669 to 874)
USA	1070 (1010 to 1150)	988 (926 to 1060)	929 (869 to 997)	757 (706 to 815)	675 (631 to 725)	626 (585 to 674)	533 (498 to 572)	515 (478 to 558)	595 (555 to 640)	607 (564 to 655)
Alabama	1180 (986 to 1390)	1040 (945 to 1150)	990 (898 to 1090)	813 (739 to 900)	754 (686 to 832)	736 (670 to 814)	685 (624 to 755)	658 (591 to 737)	769 (698 to 851)	816 (703 to 945)
Alaska	1240 (1040 to 1460)	1080 (976 to 1190)	933 (843 to 1030)	822 (746 to 911)	695 (629 to 769)	622 (563 to 687)	567 (514 to 626)	524 (470 to 587)	542 (490 to 601)	651 (568 to 740)
Arizona	1090 (911 to 1310)	960 (866 to 1060)	892 (804 to 978)	717 (649 to 794)	626 (568 to 690)	593 (539 to 652)	504 (456 to 555)	508 (454 to 567)	620 (564 to 685)	654 (563 to 756)
Arkansas	1030 (855 to 1230)	921 (838 to 1020)	896 (814 to 991)	762 (695 to 843)	725 (661 to 800)	711 (648 to 786)	651 (592 to 720)	650 (588 to 722)	747 (677 to 829)	782 (671 to 907)
California	1000 (855 to 1180)	928 (848 to 1020)	872 (796 to 951)	723 (659 to 793)	653 (595 to 714)	562 (511 to 616)	455 (414 to 497)	429 (388 to 473)	494 (451 to 541)	513 (447 to 588)
Colorado	1020 (855 to 1220)	908 (828 to 1000)	853 (778 to 940)	684 (621 to 754)	602 (548 to 662)	562 (509 to 622)	482 (438 to 530)	461 (415 to 513)	526 (478 to 581)	538 (455 to 625)
Connecticut	1040 (863 to 1220)	953 (866 to 1050)	845 (766 to 932)	712 (646 to 789)	599 (544 to 660)	566 (513 to 623)	463 (420 to 510)	449 (404 to 504)	526 (476 to 582)	478 (401 to 568)
Delaware	1180 (988 to 1380)	1050 (947 to 1160)	988 (898 to 1090)	785 (712 to 868)	713 (649 to 784)	646 (585 to 710)	555 (504 to 612)	538 (484 to 600)	601 (547 to 663)	619 (542 to 708)
Washington, DC	1240 (1040 to 1480)	1150 (1040 to 1270)	1180 (1070 to 1300)	972 (876 to 1080)	997 (900 to 1100)	789 (711 to 876)	574 (518 to 639)	525 (469 to 591)	626 (559 to 697)	591 (504 to 687)
Florida	1010 (847 to 1210)	921 (836 to 1020)	880 (798 to 976)	721 (655 to 803)	638 (580 to 707)	595 (542 to 659)	503 (459 to 556)	495 (446 to 555)	560 (509 to 619)	602 (513 to 700)
Georgia	1160 (968 to 1370)	1050 (950 to 1160)	1010 (918 to 1110)	819 (742 to 909)	752 (681 to 829)	705 (638 to 781)	601 (545 to 664)	560 (502 to 624)	650 (588 to 717)	699 (603 to 813)
Hawaii	990 (830 to 1180)	880 (797 to 972)	744 (673 to 822)	589 (532 to 653)	541 (488 to 598)	483 (437 to 537)	431 (388 to 480)	413 (371 to 463)	416 (375 to 463)	434 (362 to 513)
Idaho	991 (829 to 1180)	904 (822 to 1000)	861 (779 to 960)	682 (618 to 759)	613 (555 to 678)	576 (522 to 637)	520 (472 to 577)	501 (449 to 559)	549 (498 to 606)	610 (523 to 706)
Illinois	1100 (920 to 1290)	1030 (932 to 1130)	977 (885 to 1080)	781 (708 to 863)	700 (634 to 770)	643 (581 to 713)	529 (479 to 583)	505 (451 to 565)	600 (542 to 662)	572 (487 to 672)
Indiana	1050 (884 to 1230)	988 (890 to 1090)	940 (848 to 1040)	769 (692 to 852)	685 (618 to 759)	678 (611 to 754)	595 (539 to 658)	601 (535 to 671)	699 (628 to 770)	699 (592 to 806)
Iowa	944 (796 to 1110)	882 (799 to 975)	844 (762 to 926)	666 (599 to 735)	581 (525 to 639)	570 (514 to 630)	514 (464 to 561)	516 (460 to 574)	587 (531 to 648)	565 (478 to 673)
Kansas	942 (790 to 1120)	879 (801 to 974)	826 (751 to 909)	690 (625 to 764)	607 (551 to 668)	604 (548 to 671)	550 (499 to 609)	555 (500 to 619)	634 (575 to 700)	636 (540 to 752)
Kentucky	1090 (913 to 1270)	998 (907 to 1100)	992 (901 to 1090)	810 (737 to 895)	733 (667 to 808)	721 (655 to 793)	668 (608 to 736)	665 (598 to 739)	753 (684 to 829)	804 (688 to 945)
Louisiana	1170 (981 to 1400)	1060 (958 to 1170)	1020 (920 to 1120)	847 (766 to 939)	776 (702 to 857)	749 (679 to 828)	662 (603 to 732)	654 (587 to 734)	791 (716 to 873)	800 (683 to 916)
Maine	1110 (930 to 1320)	1000 (905 to 1110)	949 (857 to 1050)	737 (661 to 821)	639 (575 to 707)	617 (557 to 685)	530 (480 to 585)	534 (477 to 599)	547 (494 to 612)	596 (506 to 703)
Maryland	1160 (976 to 1370)	1070 (971 to 1180)	971 (875 to 1070)	783 (708 to 869)	708 (639 to 781)	653 (590 to 720)	515 (467 to 565)	503 (448 to 559)	577 (520 to 635)	558 (472 to 653)
Massachusetts	1050 (876 to 1250)	985 (899 to 1080)	888 (806 to 977)	721 (656 to 796)	624 (566 to 687)	578 (526 to 638)	473 (430 to 520)	457 (411 to 509)	517 (469 to 569)	471 (396 to 550)
Michigan	1070 (901 to 1280)	996 (908 to 1090)	947 (862 to 1040)	770 (700 to 846)	692 (630 to 759)	654 (596 to 720)	571 (519 to 627)	543 (488 to 601)	632 (576 to 692)	634 (542 to 738)
Minnesota	958 (798 to 1150)	893 (812 to 986)	816 (738 to 904)	652 (591 to 722)	580 (529 to 639)	548 (498 to 606)	462 (420 to 512)	458 (412 to 512)	514 (466 to 569)	509 (433 to 597)
Mississippi	1200 (1010 to 1420)	1070 (966 to 1170)	1040 (941 to 1150)	838 (757 to 929)	770 (697 to 850)	774 (702 to 857)	700 (630 to 774)	711 (637 to 794)	854 (768 to 948)	869 (735 to 1010)
Missouri	1050 (880 to 1240)	969 (880 to 1060)	945 (853 to 1040)	769 (698 to 845)	686 (620 to 752)	673 (608 to 740)	594 (537 to 651)	587 (525 to 646)	679 (616 to 745)	681 (582 to 796)
Montana	1080 (908 to 1280)	995 (905 to 1100)	895 (812 to 987)	744 (671 to 826)	624 (567 to 688)	605 (544 to 670)	543 (490 to 602)	519 (466 to 579)	579 (522 to 641)	610 (526 to 710)
Nebraska	928 (766 to 1110)	879 (793 to 968)	832 (750 to 917)	682 (613 to 756)	614 (555 to 677)	569 (516 to 629)	509 (458 to 562)	504 (450 to 564)	566 (509 to 628)	561 (475 to 654)
Nevada	1350 (1150 to 1600)	1120 (1010 to 1250)	1020 (922 to 1130)	813 (732 to 901)	735 (664 to 808)	671 (604 to 743)	576 (521 to 634)	544 (488 to 604)	628 (568 to 693)	668 (578 to 772)
New Hampshire	1100 (924 to 1310)	974 (881 to 1070)	916 (826 to 1010)	706 (639 to 782)	630 (568 to 694)	578 (521 to 646)	486 (438 to 538)	496 (444 to 555)	516 (465 to 573)	534 (456 to 624)
New Jersey	1110 (920 to 1320)	1020 (923 to 1120)	942 (853 to 1030)	772 (700 to 849)	680 (618 to 745)	610 (552 to 669)	489 (443 to 538)	452 (405 to 501)	564 (511 to 618)	498 (422 to 581)
New Mexico	1100 (910 to 1320)	961 (868 to 1060)	912 (826 to 1010)	728 (660 to 806)	640 (584 to 709)	596 (540 to 659)	541 (491 to 598)	557 (499 to 621)	656 (591 to 722)	686 (587 to 797)

New York	1120 (952 to 1300)	1040 (948 to 1150)	966 (879 to 1070)	781 (712 to 861)	702 (641 to 773)	582 (530 to 644)	474 (432 to 524)	427 (386 to 476)	543 (494 to 601)	487 (417 to 563)
North Carolina	1130 (929 to 1340)	1040 (953 to 1150)	981 (889 to 1080)	783 (708 to 863)	711 (645 to 781)	686 (621 to 756)	572 (519 to 627)	561 (505 to 621)	626 (567 to 691)	665 (568 to 772)
North Dakota	973 (828 to 1150)	893 (808 to 994)	828 (748 to 923)	678 (612 to 755)	580 (527 to 643)	563 (510 to 627)	505 (455 to 559)	485 (433 to 544)	547 (492 to 607)	490 (425 to 571)
Ohio	1080 (907 to 1270)	999 (910 to 1100)	957 (870 to 1060)	795 (723 to 883)	692 (632 to 768)	668 (608 to 738)	590 (538 to 650)	597 (538 to 665)	679 (620 to 749)	698 (600 to 822)
Oklahoma	998 (833 to 1190)	918 (834 to 1020)	905 (822 to 996)	758 (688 to 841)	693 (629 to 766)	715 (650 to 790)	670 (608 to 741)	651 (586 to 726)	741 (670 to 818)	790 (681 to 915)
Oregon	997 (835 to 1180)	927 (839 to 1020)	843 (763 to 928)	702 (636 to 778)	636 (576 to 701)	590 (534 to 654)	510 (461 to 561)	480 (429 to 535)	506 (456 to 557)	559 (471 to 662)
Pennsylvania	1140 (952 to 1340)	1050 (953 to 1150)	978 (892 to 1080)	794 (725 to 877)	688 (627 to 756)	643 (587 to 711)	548 (498 to 605)	534 (480 to 595)	609 (555 to 670)	616 (524 to 713)
Rhode Island	1100 (928 to 1300)	987 (895 to 1090)	902 (815 to 995)	723 (652 to 796)	637 (579 to 702)	588 (532 to 649)	511 (461 to 563)	487 (436 to 541)	543 (488 to 600)	511 (434 to 593)
South Carolina	1260 (1050 to 1500)	1110 (1010 to 1230)	1030 (936 to 1130)	833 (755 to 922)	748 (679 to 821)	715 (646 to 792)	620 (561 to 684)	607 (544 to 676)	707 (644 to 778)	745 (638 to 871)
South Dakota	996 (846 to 1170)	926 (846 to 1020)	852 (769 to 940)	697 (630 to 775)	604 (548 to 670)	585 (531 to 649)	512 (465 to 565)	518 (462 to 578)	589 (533 to 651)	554 (482 to 637)
Tennessee	1090 (919 to 1300)	996 (904 to 1100)	969 (881 to 1070)	787 (714 to 873)	740 (670 to 818)	723 (658 to 799)	646 (586 to 712)	646 (579 to 718)	739 (671 to 818)	785 (679 to 909)
Texas	1010 (842 to 1200)	920 (841 to 1010)	897 (815 to 990)	755 (687 to 837)	685 (622 to 755)	641 (584 to 706)	547 (499 to 600)	518 (467 to 574)	616 (561 to 676)	653 (561 to 752)
Utah	982 (828 to 1160)	894 (815 to 987)	830 (753 to 915)	668 (608 to 738)	580 (527 to 641)	558 (507 to 616)	500 (456 to 552)	488 (440 to 544)	542 (495 to 601)	564 (487 to 656)
Vermont	1110 (943 to 1300)	995 (900 to 1100)	902 (812 to 999)	730 (659 to 809)	641 (578 to 706)	583 (525 to 645)	494 (445 to 549)	478 (425 to 537)	489 (440 to 544)	525 (451 to 610)
Virginia	1150 (968 to 1360)	1040 (939 to 1150)	953 (864 to 1050)	782 (704 to 864)	690 (624 to 760)	638 (576 to 703)	525 (475 to 582)	503 (448 to 559)	565 (510 to 624)	593 (504 to 697)
Washington	1020 (848 to 1210)	931 (842 to 1020)	878 (793 to 968)	695 (628 to 769)	621 (562 to 683)	574 (519 to 631)	489 (443 to 539)	463 (412 to 515)	488 (442 to 537)	516 (439 to 608)
West Virginia	1110 (937 to 1330)	1010 (917 to 1100)	1030 (927 to 1140)	822 (745 to 909)	728 (663 to 802)	736 (667 to 813)	686 (621 to 753)	694 (621 to 771)	766 (694 to 848)	860 (742 to 1010)
Wisconsin	1010 (838 to 1200)	944 (859 to 1040)	858 (778 to 948)	694 (631 to 768)	628 (572 to 693)	593 (538 to 656)	507 (462 to 561)	507 (454 to 566)	576 (524 to 637)	561 (473 to 665)
Wyoming	1070 (902 to 1280)	970 (875 to 1080)	917 (820 to 1020)	756 (681 to 842)	646 (580 to 717)	621 (559 to 694)	556 (502 to 617)	542 (479 to 609)	586 (526 to 651)	654 (570 to 749)
Southern Latin America	1340 (1270 to 1400)	1270 (1220 to 1340)	1140 (1080 to 1200)	942 (898 to 992)	818 (778 to 863)	708 (670 to 745)	634 (601 to 668)	581 (534 to 631)	617 (565 to 676)	636 (578 to 696)
Argentina	1230 (1150 to 1310)	1180 (1100 to 1270)	1130 (1060 to 1210)	944 (885 to 1010)	832 (776 to 896)	740 (686 to 795)	670 (628 to 713)	631 (556 to 717)	672 (591 to 774)	679 (591 to 780)
Chile	1810 (1690 to 1940)	1620 (1530 to 1750)	1210 (1120 to 1290)	955 (879 to 1040)	787 (727 to 854)	618 (571 to 673)	544 (489 to 612)	463 (429 to 503)	508 (465 to 556)	531 (484 to 583)
Uruguay	1050 (976 to 1120)	1010 (928 to 1100)	1020 (922 to 1130)	918 (832 to 1010)	805 (726 to 892)	717 (653 to 793)	636 (591 to 685)	611 (539 to 693)	571 (506 to 647)	694 (612 to 787)
Western Europe	1180 (1130 to 1220)	1000 (976 to 1030)	946 (923 to 972)	813 (793 to 834)	681 (669 to 695)	565 (556 to 577)	456 (450 to 463)	408 (396 to 421)	440 (426 to 454)	429 (415 to 445)
Andorra	824 (632 to 1000)	794 (610 to 969)	715 (543 to 898)	631 (474 to 832)	553 (434 to 705)	496 (402 to 604)	429 (362 to 499)	395 (317 to 488)	390 (310 to 483)	386 (294 to 493)
Austria	1260 (1180 to 1350)	1100 (1020 to 1190)	1060 (981 to 1140)	901 (836 to 969)	712 (663 to 766)	572 (533 to 616)	473 (467 to 479)	420 (386 to 459)	451 (412 to 494)	437 (399 to 484)
Belgium	1280 (1200 to 1370)	1060 (982 to 1140)	1010 (944 to 1080)	867 (802 to 938)	699 (649 to 760)	597 (553 to 643)	490 (484 to 495)	428 (395 to 464)	484 (444 to 528)	426 (388 to 472)
Cyprus	1960 (1760 to 2150)	1800 (1640 to 1980)	1660 (1440 to 1920)	1370 (1250 to 1490)	1200 (1100 to 1300)	898 (824 to 976)	652 (592 to 716)	557 (484 to 635)	530 (457 to 613)	532 (453 to 626)
Denmark	1030 (968 to 1110)	911 (852 to 978)	833 (780 to 892)	786 (776 to 795)	736 (727 to 745)	640 (632 to 649)	529 (521 to 536)	445 (409 to 483)	442 (405 to 488)	447 (406 to 493)
Finland	1430 (1340 to 1540)	1130 (1060 to 1210)	1040 (1020 to 1050)	795 (786 to 804)	716 (707 to 724)	588 (580 to 595)	477 (469 to 485)	409 (376 to 446)	408 (374 to 445)	410 (372 to 457)
France	1140 (1070 to 1230)	1030 (947 to 1110)	924 (854 to 999)	784 (726 to 850)	634 (596 to 682)	541 (503 to 582)	438 (410 to 469)	379 (344 to 416)	412 (374 to 455)	395 (356 to 439)
Germany	1160 (1010 to 1330)	1020 (946 to 1090)	1030 (970 to 1110)	872 (815 to 930)	728 (722 to 734)	575 (570 to 581)	484 (480 to 489)	449 (412 to 491)	459 (419 to 504)	467 (423 to 522)
Greece	920 (861 to 987)	828 (772 to 894)	775 (720 to 838)	753 (699 to 812)	671 (623 to 725)	609 (566 to 655)	501 (468 to 538)	459 (410 to 521)	476 (423 to 547)	513 (448 to 595)
Iceland	874 (827 to 927)	836 (812 to 859)	801 (775 to 827)	650 (632 to 669)	587 (567 to 608)	494 (477 to 512)	438 (418 to 458)	375 (339 to 415)	363 (327 to 404)	362 (322 to 404)
Ireland	1280 (1200 to 1370)	1090 (1010 to 1170)	1030 (962 to 1100)	950 (881 to 1030)	808 (752 to 872)	688 (636 to 749)	472 (435 to 512)	411 (375 to 448)	408 (372 to 449)	398 (359 to 444)
Israel	752 (699 to 811)	849 (790 to 918)	918 (850 to 995)	825 (758 to 898)	644 (584 to 712)	566 (510 to 626)	430 (396 to 468)	369 (323 to 419)	375 (328 to 436)	379 (327 to 441)
Italy	1130 (1060 to 1210)	989 (918 to 1070)	901 (838 to 970)	812 (756 to 877)	645 (602 to 692)	526 (490 to 567)	427 (423 to 430)	374 (347 to 406)	425 (390 to 466)	402 (368 to 441)
Luxembourg	1240 (1150 to 1330)	1120 (1050 to 1200)	1080 (1010 to 1160)	917 (848 to 996)	748 (693 to 810)	585 (541 to 636)	462 (444 to 479)	398 (363 to 436)	419 (381 to 464)	399 (358 to 444)
Malta	1150 (1070 to 1230)	1140 (1040 to 1240)	1050 (959 to 1160)	980 (886 to 1090)	692 (622 to 775)	567 (511 to 628)	454 (419 to 493)	378 (332 to 431)	376 (331 to 435)	395 (342 to 461)
Monaco	1130 (984 to 1290)	975 (822 to 1130)	869 (740 to 1000)	714 (623 to 801)	596 (470 to 694)	564 (448 to 668)	529 (453 to 609)	483 (408 to 563)	497 (415 to 588)	558 (470 to 655)
Netherlands	980 (968 to 991)	870 (863 to 878)	840 (833 to 847)	706 (699 to 712)	649 (642 to 656)	596 (590 to 602)	469 (463 to 475)	427 (395 to 466)	460 (422 to 501)	455 (413 to 501)
Norway	896 (837 to 954)	825 (770 to 882)	796 (743 to 854)	699 (652 to 745)	648 (606 to 694)	560 (552 to 567)	454 (448 to 461)	382 (354 to 415)	372 (340 to 406)	377 (341 to 412)
Agder	964 (866 to 1070)	823 (745 to 910)	794 (717 to 880)	716 (648 to 788)	671 (621 to 726)	572 (550 to 594)	477 (456 to 496)	412 (360 to 467)	400 (346 to 459)	405 (338 to 487)
Innlandet	875 (786 to 972)	801 (726 to 880)	753 (682 to 829)	681 (619 to 747)	657 (608 to 708)	608 (587 to 629)	489 (471 to 507)	410 (363 to 464)	372 (325 to 422)	375 (317 to 441)
Møre og Romsdal	871 (780 to 975)	784 (709 to 869)	750 (676 to 830)	664 (601 to 735)	583 (539 to 627)	518 (497 to 540)	400 (382 to 419)	364 (322 to 416)	367 (318 to 422)	364 (301 to 437)
Nordland	978 (879 to 1090)	821 (742 to 907)	806 (730 to 893)	679 (615 to 750)	671 (620 to 727)	564 (540 to 588)	469 (448 to 490)	404 (354 to 460)	382 (330 to 436)	386 (319 to 461)
Oslo	709 (634 to 791)	845 (772 to 931)	814 (740 to 899)	738 (670 to 811)	694 (643 to 748)	569 (551 to 588)	442 (426 to 460)	360 (318 to 408)	364 (320 to 414)	355 (301 to 418)
Rogaland	876 (783 to 975)	814 (739 to 896)	774 (693 to 857)	689 (623 to 762)	608 (563 to 655)	540 (520 to 560)	450 (433 to 467)	370 (328 to 415)	376 (329 to 426)	373 (316 to 437)
Troms og Finnmark	1000 (897 to 1120)	938 (848 to 1030)	878 (790 to 972)	756 (682 to 836)	717 (662 to 773)	624 (600 to 648)	479 (458 to 500)	408 (355 to 466)	414 (362 to 475)	439 (369 to 526)
Trøndelag	917 (822 to 1020)	844 (768 to 934)	830 (751 to 922)	715 (648 to 792)	668 (619 to 723)	546 (525 to 566)	452 (434 to 468)	395 (348 to 447)	373 (324 to 428)	373 (313 to 444)
Vestfold og Telemark	866 (781 to 959)	814 (738 to 898)	793 (714 to 878)	715 (647 to 787)	653 (608 to 703)	576 (556 to 596)	474 (456 to 492)	396 (349 to 446)	375 (327 to 426)	383 (320 to 455)
Vestland	883 (794 to 975)	804 (736 to 884)	770 (700 to 849)	643 (587 to 706)	588 (544 to 633)	514 (497 to 532)	424 (409 to 439)	357 (317 to 402)	352 (312 to 401)	350 (295 to 413)
Viken	1130 (1020 to 1250)	842 (764 to 927)	816 (746 to 901)	713 (651 to 781)	652 (606 to 702)	563 (547 to 579)	461 (447 to 475)	381 (343 to 424)	371 (330 to 416)	387 (330 to 449)
Portugal	1460 (1370 to 1570)	1190 (1110 to 1270)	1140 (1060 to 1230)	934 (870 to 1010)	829 (770 to 896)	686 (638 to 738)	499 (466 to 536)	422 (375 to 473)	446 (394 to 503)	443 (389 to 509)
San Marino	858 (722 to 1070)	867 (709 to 1040)	790 (689 to 904)	670 (578 to 772)	532 (436 to 641)	446 (381 to 526)	364 (328 to 403)	362 (302 to 440)	321 (258 to 399)	285 (210 to 376)
Spain	1350 (1270 to 1450)	997 (930 to 1070)	925 (864 to 987)	718 (667 to 772)	647 (601 to 693)	526 (522 to 530)	420 (417 to 424)	374 (344 to 408)	423 (387 to 463)	388 (353 to 432)
Sweden	1040 (967 to 1110)	885 (827 to 947)	768 (761 to 775)	701 (694 to 708)	603 (597 to 609)	514 (508 to 520)	432 (426 to 436)	376 (345 to 411)	399 (362 to 440)	370 (330 to 418)
Stockholm	1050 (840 to 1280)	903 (721 to 1100)	796 (658 to 944)	739 (720 to 760)	644 (627 to 662)	524 (510 to 539)	441 (428 to 454)	378 (331 to 431)	427 (367 to 495)	338 (277 to 408)
Sweden except Stockholm	1030 (954 to 1120)	882 (814 to 953)	763 (734 to 789)	693 (686 to 701)	594 (587 to 601)	512 (506 to 518)	430 (424 to 435)	377 (344 to 414)	395 (356 to 437)	380 (339 to 426)
Switzerland	1110 (1040 to 1190)	958 (895 to 1030)	862 (809 to 925)	703 (656 to 749)	592 (555 to 632)	483 (454 to 517)	387 (382 to 393)	327 (299 to 359)	362 (330 to 400)	330 (298 to 367)
UK	1160 (1100 to 1230)	1050 (992 to 1120)	949 (892 to 1010)	847 (801 to 904)	716 (673 to 764)	612 (576 to 651)	475 (447 to 507)	443 (415 to 475)	488 (459 to 521)	493 (466 to 522)
England	1140 (1070 to 1220)	1020 (954 to 1100)	928 (863 to 994)	831 (775 to 898)	703 (653 to 758)	600 (558 to 647)	463 (431 to 501)	433 (401 to 472)	480 (445 to 518)	485 (454 to 519)
East Midlands	1160 (975 to 1400)	1050 (850 to 1280)	947 (788 to 1140)	840 (754 to 936)	700 (629 to 774)	606 (547 to 674)	473 (425 to 523)	450 (390 to 518)	495 (430 to 576)	506 (436 to 587)
Derby	1230 (971 to 1550)	1120 (825 to 1450)	992 (779 to 1230)	897 (772 to 1030)	742 (646 to 846)	608 (527 to 697)	523 (455 to 594)	467 (383 to 556)	525 (431 to 639)	570 (461 to 693)

Derbyshire	1210 (954 to 1520)	1060 (808 to 1370)	973 (779 to 1210)	848 (743 to 972)	721 (636 to 824)	607 (536 to 690)	464 (409 to 530)	457 (384 to 548)	489 (408 to 592)	498 (411 to 613)
Leicester	1250 (982 to 1550)	1150 (863 to 1490)	1030 (813 to 1290)	907 (787 to 1040)	745 (652 to 845)	686 (601 to 781)	542 (471 to 617)	506 (419 to 610)	554 (457 to 665)	556 (452 to 664)
Leicestershire	1050 (820 to 1320)	963 (718 to 1250)	864 (694 to 1070)	778 (680 to 891)	640 (560 to 724)	556 (488 to 627)	430 (375 to 487)	388 (323 to 461)	437 (362 to 528)	442 (363 to 529)
Lincolnshire	1140 (895 to 1410)	1030 (777 to 1330)	933 (740 to 1170)	838 (732 to 960)	687 (604 to 775)	598 (526 to 674)	461 (406 to 523)	463 (389 to 552)	499 (416 to 598)	483 (400 to 587)
Northamptonshire	1110 (888 to 1390)	997 (759 to 1290)	902 (715 to 1120)	804 (699 to 921)	686 (602 to 776)	591 (522 to 670)	475 (415 to 536)	454 (378 to 542)	502 (420 to 603)	585 (462 to 735)
Nottingham	1260 (979 to 1590)	1170 (895 to 1540)	1050 (832 to 1290)	934 (819 to 1070)	786 (688 to 893)	743 (654 to 840)	570 (498 to 650)	496 (410 to 602)	601 (496 to 730)	569 (463 to 692)
Nottinghamshire	1150 (909 to 1440)	1040 (782 to 1370)	935 (756 to 1170)	828 (724 to 952)	693 (613 to 784)	608 (538 to 685)	474 (420 to 540)	462 (387 to 551)	514 (427 to 622)	507 (419 to 612)
Rutland	1010 (765 to 1270)	926 (674 to 1220)	802 (616 to 1020)	725 (620 to 850)	630 (546 to 730)	504 (433 to 579)	400 (344 to 468)	352 (284 to 433)	347 (280 to 436)	414 (332 to 515)
East of England	1080 (912 to 1270)	970 (779 to 1180)	876 (732 to 1040)	783 (701 to 874)	657 (592 to 725)	562 (508 to 622)	438 (396 to 487)	411 (358 to 473)	445 (387 to 511)	442 (383 to 512)
Bedford	1110 (853 to 1430)	1020 (757 to 1370)	885 (704 to 1110)	778 (670 to 900)	668 (585 to 763)	597 (523 to 681)	480 (418 to 548)	462 (381 to 563)	529 (435 to 651)	475 (384 to 588)
Cambridgeshire	1000 (795 to 1260)	931 (702 to 1250)	835 (656 to 1040)	766 (664 to 880)	638 (560 to 722)	550 (485 to 621)	404 (354 to 461)	387 (322 to 466)	418 (347 to 502)	396 (325 to 487)
Central Bedfordshire	1090 (856 to 1370)	1010 (738 to 1350)	867 (684 to 1090)	762 (658 to 877)	653 (573 to 744)	546 (477 to 629)	437 (376 to 502)	388 (318 to 473)	424 (342 to 514)	430 (350 to 528)
Essex	1110 (895 to 1360)	976 (741 to 1240)	902 (717 to 1100)	800 (702 to 916)	654 (586 to 735)	554 (494 to 623)	439 (389 to 496)	424 (358 to 503)	451 (378 to 539)	466 (390 to 565)
Hertfordshire	1080 (859 to 1330)	949 (707 to 1240)	879 (704 to 1090)	791 (689 to 913)	657 (578 to 742)	568 (502 to 643)	428 (379 to 486)	396 (331 to 471)	446 (370 to 534)	413 (338 to 497)
Luton	1180 (922 to 1490)	1090 (817 to 1430)	955 (759 to 1200)	842 (722 to 971)	726 (628 to 824)	642 (555 to 732)	497 (434 to 563)	466 (380 to 556)	563 (454 to 675)	521 (417 to 637)
Norfolk	1050 (845 to 1300)	950 (727 to 1220)	841 (667 to 1040)	756 (661 to 868)	657 (578 to 740)	551 (492 to 626)	449 (399 to 510)	419 (351 to 501)	444 (372 to 532)	451 (376 to 556)
Peterborough	1170 (912 to 1500)	1080 (821 to 1430)	950 (748 to 1180)	813 (703 to 941)	723 (632 to 819)	625 (548 to 710)	508 (443 to 583)	458 (379 to 552)	510 (419 to 623)	487 (396 to 594)
Southend-on-Sea	1160 (913 to 1440)	1060 (769 to 1400)	906 (718 to 1130)	786 (681 to 912)	688 (595 to 786)	605 (530 to 688)	475 (412 to 542)	442 (364 to 536)	492 (404 to 601)	526 (426 to 644)
Suffolk	1040 (817 to 1310)	955 (718 to 1260)	852 (676 to 1060)	770 (672 to 890)	635 (562 to 721)	549 (483 to 624)	421 (370 to 482)	404 (338 to 486)	414 (345 to 499)	422 (346 to 515)
Thurrock	1170 (926 to 1470)	1100 (823 to 1450)	970 (769 to 1210)	854 (739 to 991)	711 (617 to 810)	578 (504 to 660)	477 (414 to 547)	380 (313 to 463)	468 (384 to 568)	495 (400 to 605)
Greater London	1090 (927 to 1280)	984 (814 to 1180)	894 (748 to 1060)	808 (727 to 905)	696 (627 to 770)	604 (546 to 668)	427 (385 to 473)	383 (332 to 438)	454 (396 to 525)	460 (398 to 533)
Barking and Dagenham	1250 (1030 to 1520)	1120 (861 to 1440)	1000 (807 to 1220)	882 (769 to 1020)	752 (658 to 853)	678 (592 to 772)	523 (457 to 601)	473 (390 to 574)	593 (489 to 713)	578 (470 to 703)
Barnet	985 (769 to 1210)	886 (667 to 1140)	784 (625 to 968)	699 (607 to 805)	616 (542 to 701)	518 (456 to 588)	394 (344 to 451)	334 (276 to 404)	403 (331 to 484)	419 (345 to 512)
Bexley	1010 (806 to 1250)	906 (698 to 1160)	806 (634 to 978)	716 (622 to 825)	610 (529 to 689)	529 (463 to 598)	413 (358 to 471)	380 (314 to 459)	465 (380 to 560)	447 (364 to 540)
Brent	1100 (869 to 1370)	986 (756 to 1280)	887 (711 to 1090)	804 (697 to 928)	682 (601 to 776)	593 (524 to 678)	421 (370 to 482)	380 (314 to 463)	454 (380 to 551)	447 (366 to 551)
Bromley	971 (771 to 1220)	870 (673 to 1130)	778 (618 to 967)	708 (614 to 812)	606 (530 to 687)	529 (463 to 599)	373 (326 to 425)	336 (280 to 401)	386 (317 to 463)	377 (305 to 464)
Camden	1100 (883 to 1360)	1040 (786 to 1340)	966 (783 to 1190)	918 (799 to 1050)	760 (668 to 858)	733 (645 to 828)	467 (409 to 533)	395 (326 to 478)	369 (305 to 444)	371 (305 to 448)
Croydon	1040 (837 to 1270)	914 (695 to 1170)	841 (662 to 1020)	782 (684 to 893)	677 (599 to 764)	577 (509 to 654)	417 (366 to 476)	406 (342 to 488)	491 (412 to 595)	448 (374 to 548)
Ealing	1110 (868 to 1360)	989 (756 to 1270)	908 (721 to 1110)	829 (725 to 946)	697 (608 to 791)	582 (510 to 658)	418 (365 to 474)	391 (322 to 471)	493 (409 to 584)	471 (386 to 577)
Enfield	1040 (835 to 1300)	932 (720 to 1210)	829 (661 to 1030)	748 (656 to 849)	639 (559 to 726)	575 (504 to 651)	398 (345 to 454)	399 (329 to 481)	437 (360 to 530)	435 (356 to 531)
Greenwich	1180 (934 to 1440)	1030 (783 to 1340)	928 (742 to 1140)	828 (720 to 949)	725 (638 to 823)	664 (584 to 753)	470 (412 to 536)	431 (358 to 523)	496 (410 to 607)	506 (415 to 617)
Hackney	1160 (933 to 1430)	1070 (821 to 1370)	1000 (804 to 1240)	948 (830 to 1080)	820 (721 to 931)	682 (598 to 771)	481 (423 to 550)	436 (362 to 525)	518 (431 to 627)	858 (654 to 1080)
Hammersmith and Fulham	1160 (915 to 1440)	1070 (816 to 1410)	990 (783 to 1230)	936 (810 to 1080)	828 (721 to 955)	688 (603 to 784)	462 (403 to 533)	405 (332 to 488)	516 (425 to 631)	477 (390 to 582)
Haringey	1130 (895 to 1400)	1020 (781 to 1300)	921 (745 to 1130)	844 (734 to 972)	696 (612 to 789)	660 (581 to 747)	481 (424 to 549)	397 (332 to 478)	471 (392 to 572)	448 (365 to 545)
Harrow	993 (780 to 1240)	893 (680 to 1180)	800 (635 to 986)	713 (619 to 830)	592 (517 to 672)	510 (450 to 579)	362 (315 to 414)	326 (270 to 395)	377 (311 to 454)	371 (299 to 456)
Havering	1020 (813 to 1260)	905 (680 to 1150)	816 (650 to 1010)	742 (642 to 849)	614 (535 to 695)	536 (466 to 605)	417 (365 to 476)	376 (309 to 450)	450 (370 to 539)	453 (368 to 548)
Hillingdon	1020 (825 to 1280)	925 (715 to 1190)	836 (664 to 1030)	756 (660 to 871)	653 (576 to 741)	594 (524 to 673)	429 (376 to 490)	411 (344 to 493)	492 (410 to 596)	504 (416 to 608)
Hounslow	1090 (871 to 1350)	982 (750 to 1260)	885 (706 to 1100)	796 (696 to 919)	700 (617 to 793)	624 (550 to 706)	438 (386 to 498)	382 (317 to 456)	462 (389 to 559)	445 (364 to 542)
Islington	1150 (915 to 1430)	1060 (814 to 1350)	976 (774 to 1200)	904 (789 to 1030)	794 (697 to 898)	756 (664 to 855)	520 (455 to 593)	444 (370 to 533)	482 (397 to 580)	538 (442 to 648)
Kensington and Chelsea	1050 (832 to 1300)	1000 (763 to 1280)	930 (744 to 1130)	888 (774 to 1010)	759 (665 to 865)	528 (465 to 599)	368 (322 to 419)	338 (278 to 410)	358 (295 to 432)	420 (341 to 513)
Kingston upon Thames	975 (767 to 1220)	876 (678 to 1140)	784 (620 to 974)	710 (614 to 821)	614 (541 to 698)	524 (459 to 599)	349 (305 to 402)	326 (269 to 394)	396 (329 to 485)	390 (314 to 474)
Lambeth	1240 (990 to 1510)	1130 (837 to 1460)	1040 (827 to 1300)	915 (800 to 1050)	774 (679 to 890)	695 (614 to 792)	489 (426 to 559)	411 (337 to 499)	492 (409 to 601)	537 (435 to 660)
Lewisham	1160 (929 to 1420)	1030 (797 to 1340)	920 (733 to 1120)	826 (719 to 942)	747 (652 to 848)	701 (614 to 795)	490 (430 to 562)	434 (359 to 523)	502 (414 to 603)	513 (419 to 629)
Merton	1050 (837 to 1300)	942 (721 to 1210)	842 (668 to 1040)	752 (655 to 860)	658 (578 to 743)	549 (481 to 624)	394 (344 to 453)	360 (297 to 436)	470 (390 to 571)	459 (376 to 561)
Newham	1210 (967 to 1480)	1060 (808 to 1380)	976 (776 to 1180)	877 (762 to 1010)	766 (669 to 871)	707 (619 to 802)	509 (445 to 578)	451 (373 to 541)	600 (492 to 734)	571 (466 to 698)
Redbridge	1020 (823 to 1260)	907 (689 to 1180)	822 (662 to 1020)	714 (620 to 815)	624 (543 to 709)	534 (468 to 605)	390 (340 to 442)	348 (288 to 417)	473 (391 to 570)	437 (356 to 527)
Richmond upon Thames	992 (781 to 1250)	905 (698 to 1180)	821 (656 to 1020)	755 (655 to 870)	607 (527 to 693)	503 (440 to 572)	339 (295 to 389)	310 (258 to 377)	360 (298 to 434)	372 (300 to 454)
Southwark	1170 (938 to 1440)	1040 (786 to 1320)	953 (766 to 1190)	871 (758 to 1000)	878 (771 to 1000)	683 (599 to 778)	490 (430 to 562)	406 (337 to 493)	522 (434 to 632)	537 (441 to 656)
Sutton	1020 (808 to 1260)	903 (686 to 1160)	794 (633 to 984)	702 (602 to 812)	629 (550 to 715)	555 (487 to 632)	393 (344 to 450)	368 (306 to 452)	400 (333 to 488)	456 (371 to 559)
Tower Hamlets	1290 (1030 to 1600)	1160 (893 to 1470)	1090 (889 to 1330)	1020 (893 to 1170)	850 (745 to 963)	778 (684 to 886)	517 (455 to 591)	429 (356 to 512)	613 (513 to 740)	611 (505 to 746)
Waltham Forest	1130 (907 to 1400)	1000 (771 to 1280)	899 (725 to 1100)	806 (701 to 927)	684 (602 to 778)	645 (572 to 730)	474 (417 to 541)	401 (334 to 481)	445 (374 to 535)	455 (375 to 560)
Wandsworth	1130 (902 to 1390)	1030 (799 to 1350)	944 (746 to 1160)	849 (744 to 980)	776 (684 to 880)	644 (572 to 732)	425 (374 to 480)	410 (341 to 494)	471 (389 to 568)	455 (373 to 555)
Westminster	1070 (839 to 1340)	1020 (778 to 1310)	949 (748 to 1160)	905 (788 to 1040)	736 (641 to 833)	602 (528 to 684)	378 (331 to 433)	344 (287 to 419)	374 (310 to 449)	390 (320 to 478)
North East England	1270 (1060 to 1500)	1140 (914 to 1430)	1040 (863 to 1230)	920 (822 to 1040)	798 (715 to 888)	673 (606 to 750)	525 (470 to 588)	507 (438 to 591)	561 (486 to 659)	567 (484 to 670)
County Durham	1280 (1020 to 1600)	1130 (846 to 1460)	1040 (824 to 1270)	920 (802 to 1060)	803 (707 to 910)	678 (597 to 774)	537 (472 to 612)	513 (423 to 615)	574 (473 to 696)	567 (462 to 685)
Darlington	1250 (972 to 1580)	1140 (839 to 1500)	1030 (812 to 1290)	903 (776 to 1050)	786 (686 to 896)	674 (590 to 771)	517 (452 to 598)	500 (408 to 612)	501 (413 to 625)	594 (475 to 734)
Gateshead	1300 (1030 to 1620)	1190 (899 to 1590)	1080 (849 to 1340)	947 (818 to 1090)	794 (694 to 907)	699 (610 to 801)	517 (449 to 593)	504 (414 to 612)	578 (470 to 703)	551 (447 to 688)
Hartlepool	1360 (1080 to 1680)	1230 (924 to 1600)	1120 (895 to 1380)	981 (841 to 1130)	836 (730 to 954)	728 (632 to 833)	561 (482 to 643)	579 (472 to 709)	576 (476 to 704)	608 (497 to 747)
Middlesbrough	1360 (1080 to 1690)	1240 (919 to 1640)	1130 (894 to 1390)	1000 (863 to 1170)	889 (772 to 1030)	744 (648 to 862)	589 (512 to 677)	615 (500 to 757)	675 (550 to 832)	708 (571 to 880)
Newcastle upon Tyne	1260 (984 to 1540)	1150 (865 to 1520)	1050 (830 to 1280)	920 (792 to 1070)	832 (728 to 957)	684 (597 to 784)	533 (464 to 612)	503 (412 to 605)	578 (475 to 705)	608 (493 to 756)
North Tyneside	1220 (974 to 1520)	1110 (823 to 1480)	998 (799 to 1240)	881 (764 to 1020)	775 (679 to 886)	665 (582 to 765)	524 (456 to 598)	471 (385 to 569)	554 (454 to 678)	531 (432 to 651)
Northumberland	1210 (952 to 1520)	1090 (817 to 1420)	986 (784 to 1210)	908 (789 to 1030)	735 (645 to 841)	599 (526 to 685)	459 (402 to 530)	454 (376 to 545)	499 (415 to 606)	491 (403 to 600)
Redcar and Cleveland	1270 (996 to 1630)	1160 (874 to 1540)	1040 (832 to 1300)	917 (785 to 1070)	781 (675 to 900)	662 (578 to 762)	512 (442 to 589)	528 (429 to 642)	532 (431 to 657)	619 (501 to 774)
South Tyneside	1290 (1010 to 1620)	1170 (871 to 1540)	1050 (829 to 1300)	923 (795 to 1070)	811 (702 to 931)	677 (590 to 773)	540 (467 to 623)	522 (426 to 644)	585 (476 to 724)	568 (462 to 707)

Stockton-on-Tees	1220 (962 to 1520)	1110 (814 to 1460)	999 (783 to 1260)	871 (753 to 1000)	780 (680 to 892)	629 (552 to 715)	494 (429 to 567)	498 (406 to 608)	538 (442 to 656)	564 (461 to 695)
Sunderland	1260 (1020 to 1570)	1150 (857 to 1490)	1030 (834 to 1270)	915 (802 to 1060)	821 (720 to 930)	721 (635 to 821)	579 (505 to 668)	536 (441 to 654)	620 (513 to 753)	594 (483 to 729)
North West England	1240 (1040 to 1430)	1110 (918 to 1340)	1010 (853 to 1180)	901 (812 to 1000)	776 (701 to 860)	664 (601 to 736)	524 (475 to 584)	488 (428 to 558)	552 (479 to 632)	557 (482 to 645)
Blackburn with Darwen	1310 (1040 to 1620)	1200 (903 to 1560)	1060 (844 to 1310)	943 (816 to 1080)	839 (734 to 952)	733 (642 to 832)	560 (492 to 637)	520 (426 to 626)	583 (482 to 702)	627 (510 to 760)
Blackpool	1320 (1060 to 1620)	1210 (931 to 1550)	1060 (845 to 1310)	932 (804 to 1070)	841 (737 to 954)	825 (728 to 932)	726 (635 to 826)	690 (570 to 835)	658 (537 to 791)	744 (609 to 904)
Bolton	1280 (1020 to 1580)	1170 (908 to 1510)	1040 (847 to 1270)	927 (804 to 1060)	783 (693 to 885)	690 (609 to 784)	539 (473 to 612)	512 (429 to 611)	553 (458 to 663)	574 (472 to 697)
Bury	1190 (945 to 1480)	1100 (840 to 1420)	959 (756 to 1200)	847 (731 to 974)	755 (656 to 861)	632 (555 to 720)	501 (435 to 576)	459 (379 to 559)	556 (457 to 675)	542 (444 to 664)
Cheshire East	1080 (840 to 1330)	987 (753 to 1260)	883 (708 to 1090)	820 (713 to 942)	698 (618 to 797)	575 (504 to 654)	447 (391 to 508)	401 (334 to 482)	460 (383 to 551)	435 (357 to 533)
Cheshire West and Chester	1120 (878 to 1380)	1030 (776 to 1350)	934 (754 to 1140)	839 (718 to 961)	716 (626 to 809)	583 (510 to 662)	457 (399 to 520)	433 (360 to 524)	506 (417 to 610)	490 (402 to 600)
Cumbria	1180 (937 to 1450)	1080 (830 to 1410)	970 (782 to 1180)	869 (762 to 995)	747 (661 to 844)	606 (537 to 688)	474 (418 to 541)	454 (383 to 541)	494 (416 to 596)	508 (420 to 613)
Halton	1270 (1010 to 1570)	1200 (913 to 1540)	1070 (861 to 1330)	952 (829 to 1100)	800 (699 to 915)	717 (634 to 820)	552 (482 to 632)	520 (429 to 629)	571 (474 to 691)	564 (456 to 688)
Knowsley	1300 (1030 to 1640)	1210 (919 to 1560)	1090 (870 to 1340)	973 (846 to 1120)	832 (726 to 950)	737 (652 to 837)	576 (500 to 665)	541 (449 to 654)	612 (505 to 737)	643 (524 to 786)
Lancashire	1240 (1000 to 1500)	1070 (829 to 1360)	976 (785 to 1220)	894 (781 to 1020)	764 (677 to 861)	640 (567 to 722)	496 (441 to 563)	494 (416 to 585)	547 (458 to 649)	574 (478 to 685)
Liverpool	1290 (1020 to 1570)	1150 (872 to 1490)	1080 (874 to 1310)	946 (822 to 1080)	828 (731 to 939)	766 (676 to 869)	613 (537 to 702)	565 (470 to 678)	641 (533 to 770)	676 (561 to 828)
Manchester	1320 (1070 to 1620)	1170 (871 to 1490)	1080 (867 to 1320)	972 (840 to 1110)	881 (769 to 996)	776 (678 to 876)	605 (525 to 691)	549 (454 to 661)	687 (560 to 822)	703 (569 to 858)
Oldham	1300 (1030 to 1620)	1200 (928 to 1560)	1050 (840 to 1310)	938 (818 to 1070)	810 (707 to 921)	694 (611 to 790)	560 (488 to 638)	528 (436 to 638)	623 (511 to 753)	589 (482 to 716)
Rochdale	1290 (1040 to 1570)	1160 (883 to 1490)	1040 (825 to 1280)	913 (797 to 1050)	823 (720 to 935)	693 (605 to 790)	576 (503 to 657)	523 (431 to 629)	615 (509 to 743)	613 (496 to 758)
Salford	1320 (1070 to 1600)	1210 (935 to 1570)	1100 (878 to 1330)	991 (870 to 1130)	851 (751 to 964)	732 (648 to 830)	592 (520 to 676)	505 (427 to 605)	619 (518 to 744)	597 (495 to 725)
Sefton	1180 (914 to 1460)	1060 (810 to 1390)	957 (773 to 1170)	856 (746 to 975)	710 (627 to 808)	631 (556 to 718)	536 (472 to 612)	502 (416 to 601)	555 (462 to 668)	532 (439 to 650)
St Helens	1280 (1010 to 1570)	1190 (919 to 1520)	1060 (861 to 1300)	983 (856 to 1120)	797 (704 to 904)	670 (587 to 764)	553 (483 to 631)	536 (442 to 648)	586 (483 to 701)	605 (497 to 738)
Stockport	1110 (882 to 1370)	1020 (779 to 1300)	914 (733 to 1140)	845 (733 to 971)	727 (643 to 820)	605 (537 to 682)	486 (429 to 560)	432 (361 to 512)	477 (396 to 569)	476 (389 to 577)
Tameside	1280 (1010 to 1570)	1180 (893 to 1530)	1040 (832 to 1290)	924 (800 to 1060)	780 (689 to 886)	702 (617 to 796)	564 (492 to 645)	487 (401 to 586)	618 (506 to 748)	577 (471 to 707)
Trafford	1130 (893 to 1400)	1050 (804 to 1350)	931 (749 to 1150)	833 (721 to 962)	720 (628 to 819)	603 (527 to 685)	461 (400 to 528)	381 (314 to 456)	476 (389 to 574)	431 (351 to 527)
Warrington	1180 (911 to 1460)	1100 (830 to 1440)	986 (791 to 1220)	885 (769 to 1010)	775 (679 to 882)	632 (551 to 723)	508 (440 to 580)	492 (405 to 590)	553 (453 to 668)	550 (445 to 669)
Wigan	1260 (1010 to 1550)	1170 (892 to 1540)	1030 (832 to 1280)	918 (802 to 1050)	794 (700 to 898)	705 (625 to 799)	554 (487 to 632)	503 (419 to 605)	588 (484 to 712)	541 (441 to 659)
Wirral	1220 (972 to 1520)	1090 (824 to 1430)	980 (776 to 1200)	871 (759 to 994)	757 (671 to 857)	647 (570 to 736)	510 (449 to 583)	490 (404 to 590)	517 (428 to 619)	569 (466 to 692)
South East England	1050 (890 to 1240)	942 (769 to 1140)	855 (723 to 1000)	773 (692 to 863)	646 (582 to 714)	556 (504 to 614)	429 (388 to 476)	404 (352 to 463)	438 (384 to 503)	433 (374 to 499)
Bracknell Forest	998 (790 to 1250)	930 (701 to 1220)	821 (643 to 1010)	740 (632 to 864)	636 (554 to 730)	523 (454 to 599)	400 (350 to 459)	350 (285 to 425)	384 (312 to 470)	346 (278 to 424)
Brighton and Hove	1120 (886 to 1410)	1040 (791 to 1360)	906 (732 to 1130)	819 (714 to 952)	740 (651 to 841)	643 (563 to 731)	510 (447 to 584)	442 (366 to 535)	529 (435 to 642)	461 (376 to 559)
Buckinghamshire	966 (768 to 1200)	893 (662 to 1140)	810 (636 to 1000)	758 (656 to 879)	640 (560 to 730)	530 (465 to 606)	411 (362 to 470)	363 (300 to 438)	384 (319 to 466)	410 (336 to 503)
East Sussex	1070 (846 to 1330)	963 (739 to 1240)	845 (674 to 1030)	748 (653 to 854)	613 (540 to 694)	531 (468 to 601)	433 (383 to 492)	436 (362 to 520)	450 (379 to 539)	466 (384 to 565)
Hampshire	1040 (833 to 1280)	916 (686 to 1180)	853 (688 to 1060)	757 (662 to 870)	631 (560 to 712)	540 (481 to 610)	410 (363 to 464)	381 (322 to 455)	408 (344 to 489)	396 (331 to 478)
Isle of Wight	1140 (910 to 1430)	1030 (775 to 1350)	880 (705 to 1100)	772 (660 to 897)	680 (594 to 772)	566 (491 to 648)	465 (404 to 532)	445 (364 to 539)	459 (374 to 560)	476 (380 to 587)
Kent	1110 (896 to 1380)	970 (756 to 1230)	901 (715 to 1100)	827 (725 to 942)	676 (596 to 759)	586 (518 to 659)	446 (392 to 504)	439 (370 to 526)	479 (400 to 571)	473 (394 to 573)
Medway	1110 (863 to 1380)	1030 (775 to 1350)	889 (705 to 1100)	781 (674 to 906)	696 (607 to 791)	621 (545 to 703)	514 (450 to 587)	467 (386 to 556)	533 (440 to 646)	484 (394 to 598)
Milton Keynes	1070 (841 to 1330)	1000 (755 to 1300)	880 (697 to 1100)	788 (675 to 906)	672 (587 to 767)	566 (491 to 639)	458 (400 to 525)	408 (336 to 487)	482 (393 to 576)	451 (368 to 548)
Oxfordshire	957 (741 to 1220)	886 (670 to 1150)	808 (645 to 997)	743 (642 to 857)	621 (540 to 702)	548 (480 to 622)	418 (366 to 475)	373 (306 to 446)	406 (334 to 486)	403 (327 to 495)
Portsmouth	1150 (909 to 1420)	1080 (805 to 1390)	942 (748 to 1170)	851 (746 to 973)	752 (660 to 853)	680 (598 to 772)	518 (458 to 588)	463 (383 to 557)	547 (455 to 668)	511 (418 to 624)
Reading	1040 (820 to 1310)	982 (729 to 1290)	868 (698 to 1080)	791 (681 to 909)	698 (604 to 795)	610 (534 to 692)	491 (429 to 560)	460 (377 to 550)	585 (474 to 705)	488 (396 to 593)
Slough	1130 (888 to 1420)	1060 (797 to 1350)	945 (756 to 1170)	859 (742 to 997)	761 (668 to 870)	647 (561 to 742)	498 (434 to 568)	460 (377 to 554)	528 (438 to 643)	571 (467 to 708)
Southampton	1140 (911 to 1410)	1070 (802 to 1390)	942 (755 to 1170)	826 (713 to 945)	731 (637 to 832)	637 (562 to 724)	494 (436 to 561)	458 (374 to 558)	552 (456 to 673)	503 (405 to 615)
Surrey	1010 (792 to 1250)	884 (666 to 1150)	824 (656 to 1020)	758 (658 to 868)	618 (543 to 699)	530 (465 to 597)	392 (343 to 442)	379 (311 to 456)	411 (339 to 488)	397 (325 to 475)
West Berkshire	962 (741 to 1210)	898 (676 to 1160)	786 (623 to 981)	712 (609 to 820)	606 (526 to 694)	523 (458 to 595)	402 (350 to 459)	357 (292 to 437)	384 (315 to 473)	408 (322 to 501)
West Sussex	1020 (808 to 1260)	915 (690 to 1160)	830 (675 to 1020)	753 (652 to 871)	620 (545 to 709)	537 (471 to 614)	429 (377 to 490)	412 (345 to 497)	421 (350 to 510)	439 (359 to 543)
Windsor and Maidenhead	934 (730 to 1170)	866 (639 to 1110)	754 (601 to 932)	680 (585 to 786)	611 (528 to 701)	496 (430 to 569)	379 (327 to 437)	344 (280 to 420)	408 (331 to 501)	396 (320 to 489)
Wokingham	894 (681 to 1150)	833 (622 to 1090)	723 (557 to 905)	650 (557 to 763)	541 (474 to 623)	440 (384 to 505)	341 (298 to 393)	332 (273 to 410)	374 (307 to 461)	432 (351 to 529)
South West England	1050 (886 to 1240)	949 (766 to 1160)	860 (716 to 1010)	775 (692 to 868)	647 (585 to 721)	549 (496 to 611)	432 (388 to 482)	415 (364 to 477)	434 (378 to 502)	462 (399 to 536)
Bath and North East Somerset	973 (761 to 1220)	885 (671 to 1160)	777 (610 to 978)	685 (585 to 790)	607 (530 to 694)	516 (453 to 588)	394 (343 to 451)	378 (312 to 459)	432 (359 to 524)	431 (351 to 523)
Bournemouth	1080 (849 to 1330)	983 (729 to 1270)	870 (679 to 1080)	779 (674 to 898)	702 (612 to 803)	622 (546 to 709)	495 (430 to 564)	470 (390 to 564)	574 (470 to 690)	912 (711 to 1160)
Bristol, City of	1100 (873 to 1370)	1000 (762 to 1300)	923 (721 to 1130)	831 (719 to 962)	694 (610 to 790)	605 (535 to 691)	492 (429 to 563)	462 (384 to 552)	513 (425 to 618)	511 (419 to 626)
Cornwall	1100 (882 to 1350)	986 (750 to 1260)	896 (710 to 1110)	802 (702 to 916)	660 (584 to 747)	560 (495 to 633)	445 (393 to 505)	443 (374 to 520)	457 (381 to 549)	478 (394 to 574)
Devon	1030 (812 to 1260)	915 (687 to 1210)	848 (680 to 1060)	768 (667 to 875)	622 (550 to 707)	535 (474 to 608)	423 (371 to 482)	410 (343 to 489)	405 (334 to 492)	417 (342 to 509)
Dorset	1030 (817 to 1280)	926 (698 to 1210)	825 (661 to 1030)	727 (630 to 843)	594 (526 to 678)	490 (428 to 557)	375 (328 to 425)	366 (307 to 437)	379 (314 to 460)	359 (293 to 438)
Gloucestershire	1020 (795 to 1250)	913 (675 to 1190)	835 (656 to 1030)	790 (688 to 913)	652 (571 to 740)	545 (478 to 622)	431 (378 to 491)	435 (366 to 513)	452 (377 to 542)	466 (384 to 570)
North Somerset	1020 (800 to 1280)	921 (699 to 1220)	805 (647 to 995)	708 (614 to 821)	612 (539 to 698)	523 (460 to 604)	447 (392 to 514)	397 (330 to 482)	429 (357 to 523)	434 (355 to 531)
Plymouth	1150 (926 to 1440)	1060 (801 to 1390)	953 (752 to 1180)	855 (737 to 982)	713 (626 to 811)	625 (548 to 711)	494 (433 to 566)	458 (380 to 548)	473 (389 to 570)	476 (388 to 584)
Poole	1060 (826 to 1310)	964 (720 to 1260)	854 (664 to 1060)	765 (662 to 895)	646 (567 to 741)	516 (454 to 590)	412 (356 to 473)	407 (335 to 493)	456 (374 to 555)	747 (571 to 953)
Somerset	1040 (814 to 1300)	936 (714 to 1220)	854 (676 to 1050)	780 (679 to 899)	646 (571 to 740)	546 (483 to 622)	428 (377 to 486)	422 (353 to 508)	438 (366 to 533)	457 (378 to 561)
South Gloucestershire	981 (768 to 1270)	904 (681 to 1190)	817 (642 to 1030)	707 (607 to 822)	603 (528 to 687)	550 (483 to 627)	398 (348 to 456)	364 (302 to 439)	407 (336 to 497)	422 (346 to 514)
Swindon	1090 (863 to 1360)	1010 (763 to 1310)	908 (721 to 1120)	815 (708 to 941)	679 (600 to 765)	579 (510 to 656)	439 (383 to 498)	387 (321 to 458)	477 (398 to 573)	424 (347 to 524)
Torbay	1120 (883 to 1390)	1010 (753 to 1310)	884 (709 to 1110)	784 (671 to 911)	672 (587 to 765)	629 (548 to 723)	519 (452 to 594)	526 (432 to 636)	467 (381 to 566)	511 (413 to 630)
Wiltshire	1020 (797 to 1280)	930 (693 to 1210)	832 (648 to 1030)	763 (659 to 874)	666 (586 to 759)	534 (469 to 604)	420 (366 to 478)	382 (318 to 451)	404 (333 to 485)	439 (358 to 532)
West Midlands	1200 (1020 to 1390)	1080 (886 to 1330)	979 (830 to 1150)	876 (785 to 981)	736 (660 to 823)	624 (560 to 693)	480 (432 to 535)	455 (396 to 528)	518 (448 to 595)	519 (444 to 611)
Birmingham	1230 (987 to 1510)	1080 (839 to 1370)	1000 (808 to 1220)	916 (800 to 1050)	776 (679 to 883)	661 (582 to 748)	483 (426 to 553)	472 (392 to 566)	574 (476 to 688)	583 (478 to 716)

Coventry	1220 (973 to 1510)	1110 (818 to 1440)	995 (803 to 1230)	870 (756 to 1000)	759 (662 to 873)	637 (559 to 728)	512 (447 to 589)	486 (404 to 594)	544 (451 to 667)	547 (445 to 677)
Dudley	1140 (904 to 1430)	1050 (792 to 1370)	923 (734 to 1140)	833 (723 to 961)	715 (622 to 819)	622 (545 to 710)	480 (420 to 547)	454 (372 to 556)	506 (416 to 610)	525 (425 to 644)
Herefordshire, County of	1140 (901 to 1430)	1060 (791 to 1390)	920 (739 to 1140)	806 (700 to 930)	636 (554 to 722)	552 (483 to 630)	441 (385 to 505)	418 (345 to 508)	428 (353 to 522)	482 (393 to 589)
Sandwell	1300 (1040 to 1600)	1200 (921 to 1560)	1050 (839 to 1290)	911 (798 to 1040)	779 (678 to 894)	701 (614 to 800)	567 (499 to 651)	507 (421 to 618)	583 (486 to 704)	605 (493 to 752)
Shropshire	1130 (876 to 1410)	1050 (780 to 1370)	920 (737 to 1140)	806 (697 to 932)	674 (588 to 771)	565 (492 to 640)	436 (382 to 497)	391 (322 to 477)	426 (352 to 517)	493 (400 to 601)
Solihull	1040 (819 to 1310)	969 (730 to 1270)	838 (656 to 1060)	735 (640 to 844)	615 (536 to 711)	551 (484 to 624)	404 (352 to 465)	399 (325 to 486)	488 (408 to 584)	445 (363 to 542)
Staffordshire	1220 (975 to 1490)	1080 (825 to 1370)	993 (792 to 1250)	884 (773 to 1020)	748 (657 to 853)	624 (550 to 706)	464 (408 to 524)	454 (384 to 544)	514 (432 to 614)	478 (394 to 584)
Stoke-on-Trent	1350 (1070 to 1670)	1240 (940 to 1620)	1120 (894 to 1380)	1010 (875 to 1160)	826 (722 to 941)	697 (612 to 794)	584 (507 to 670)	553 (454 to 668)	671 (552 to 809)	630 (515 to 767)
Telford and Wrekin	1220 (940 to 1530)	1140 (870 to 1490)	1000 (799 to 1240)	882 (770 to 1030)	727 (637 to 838)	630 (555 to 722)	491 (430 to 568)	491 (410 to 599)	559 (464 to 678)	633 (520 to 785)
Walsall	1250 (989 to 1550)	1160 (869 to 1470)	1020 (813 to 1280)	914 (792 to 1060)	785 (684 to 896)	649 (569 to 740)	525 (458 to 599)	490 (408 to 588)	540 (442 to 651)	556 (454 to 677)
Warwickshire	1120 (877 to 1400)	1030 (764 to 1340)	924 (730 to 1150)	844 (740 to 968)	703 (618 to 810)	584 (512 to 666)	446 (388 to 510)	429 (358 to 523)	477 (392 to 578)	460 (378 to 567)
Wolverhampton	1250 (977 to 1570)	1150 (871 to 1500)	1020 (816 to 1260)	900 (781 to 1040)	782 (682 to 900)	668 (585 to 768)	554 (482 to 637)	509 (419 to 618)	594 (483 to 714)	609 (495 to 752)
Worcestershire	1090 (848 to 1350)	1000 (765 to 1300)	906 (716 to 1120)	824 (719 to 947)	686 (600 to 784)	592 (518 to 674)	465 (409 to 532)	433 (362 to 522)	478 (395 to 578)	462 (372 to 570)
Yorkshire and the Humber	1210 (1030 to 1410)	1090 (893 to 1310)	980 (814 to 1140)	871 (783 to 971)	736 (662 to 813)	619 (556 to 687)	496 (448 to 550)	474 (414 to 543)	525 (454 to 601)	526 (455 to 608)
Barnsley	1290 (1030 to 1600)	1180 (895 to 1530)	1030 (810 to 1280)	897 (768 to 1030)	755 (657 to 854)	649 (567 to 739)	508 (441 to 576)	498 (412 to 598)	560 (460 to 684)	594 (485 to 719)
Bradford	1270 (998 to 1570)	1120 (847 to 1450)	1020 (811 to 1260)	898 (781 to 1030)	768 (676 to 868)	669 (589 to 759)	519 (455 to 589)	510 (424 to 609)	597 (490 to 721)	556 (451 to 672)
Calderdale	1260 (1020 to 1560)	1140 (874 to 1470)	1010 (797 to 1240)	900 (781 to 1040)	774 (677 to 880)	627 (544 to 713)	526 (457 to 599)	460 (375 to 551)	526 (430 to 637)	545 (445 to 664)
Doncaster	1250 (987 to 1540)	1120 (841 to 1450)	989 (780 to 1220)	867 (749 to 998)	771 (672 to 870)	668 (583 to 757)	540 (473 to 610)	513 (420 to 611)	581 (478 to 703)	569 (464 to 695)
East Riding of Yorkshire	1120 (888 to 1380)	1010 (759 to 1310)	897 (697 to 1110)	794 (685 to 914)	677 (595 to 765)	586 (509 to 664)	458 (397 to 519)	406 (331 to 490)	433 (354 to 524)	456 (372 to 556)
Kingston upon Hull, City of	1260 (1000 to 1540)	1130 (854 to 1430)	994 (808 to 1210)	867 (751 to 980)	760 (668 to 863)	700 (613 to 799)	586 (514 to 672)	582 (482 to 702)	653 (539 to 793)	652 (529 to 803)
Kirklees	1270 (1010 to 1570)	1150 (870 to 1480)	1040 (824 to 1280)	950 (827 to 1100)	767 (673 to 870)	641 (555 to 731)	508 (445 to 577)	483 (399 to 574)	559 (458 to 673)	509 (414 to 617)
Leeds	1190 (958 to 1460)	1040 (793 to 1330)	968 (780 to 1190)	867 (764 to 983)	722 (638 to 810)	605 (534 to 680)	495 (439 to 557)	493 (414 to 585)	548 (449 to 654)	567 (465 to 680)
North East Lincolnshire	1220 (967 to 1520)	1110 (842 to 1460)	957 (761 to 1190)	837 (728 to 960)	765 (670 to 864)	632 (552 to 717)	511 (448 to 584)	500 (417 to 596)	524 (432 to 631)	542 (444 to 655)
North Lincolnshire	1220 (983 to 1500)	1130 (852 to 1480)	984 (775 to 1220)	870 (753 to 1010)	764 (669 to 872)	620 (540 to 710)	483 (422 to 556)	444 (365 to 541)	480 (393 to 588)	481 (393 to 597)
North Yorkshire	1120 (903 to 1400)	1010 (755 to 1310)	913 (724 to 1120)	813 (714 to 927)	673 (593 to 762)	554 (486 to 633)	444 (393 to 506)	414 (347 to 496)	444 (369 to 538)	432 (356 to 526)
Rotherham	1260 (1000 to 1560)	1160 (869 to 1530)	1010 (806 to 1260)	904 (791 to 1030)	758 (667 to 858)	637 (556 to 727)	503 (442 to 572)	500 (415 to 597)	577 (476 to 694)	549 (446 to 668)
Sheffield	1190 (958 to 1460)	1070 (829 to 1370)	972 (776 to 1190)	863 (755 to 998)	744 (653 to 845)	617 (542 to 705)	503 (443 to 573)	480 (402 to 576)	519 (432 to 627)	551 (452 to 675)
Wakefield	1300 (1050 to 1610)	1200 (923 to 1540)	1070 (858 to 1320)	962 (831 to 1110)	774 (682 to 876)	637 (557 to 725)	521 (457 to 594)	519 (436 to 621)	558 (464 to 670)	579 (471 to 700)
York	1110 (863 to 1380)	1030 (761 to 1360)	898 (713 to 1120)	801 (700 to 926)	672 (592 to 768)	538 (471 to 615)	419 (368 to 478)	417 (347 to 507)	453 (376 to 549)	486 (400 to 599)
Northern Ireland	1260 (1180 to 1350)	1220 (1140 to 1320)	1060 (976 to 1140)	990 (915 to 1060)	835 (776 to 898)	664 (612 to 719)	512 (474 to 554)	458 (423 to 497)	490 (452 to 531)	494 (456 to 534)
Scotland	1310 (1220 to 1400)	1400 (1300 to 1510)	1210 (1120 to 1310)	968 (901 to 1040)	823 (766 to 890)	703 (654 to 759)	560 (518 to 605)	519 (459 to 581)	556 (495 to 630)	564 (493 to 648)
Wales	1220 (1130 to 1330)	1090 (1010 to 1180)	973 (900 to 1050)	859 (796 to 929)	718 (666 to 775)	635 (586 to 684)	505 (468 to 545)	478 (442 to 521)	520 (486 to 560)	510 (478 to 548)
Latin America and Caribbean	1910 (1800 to 2020)	1540 (1480 to 1610)	1350 (1300 to 1400)	1070 (1030 to 1110)	900 (869 to 931)	755 (730 to 783)	713 (686 to 740)	622 (583 to 666)	759 (711 to 816)	828 (766 to 899)
Andean Latin America	2360 (2130 to 2610)	1890 (1740 to 2050)	1750 (1640 to 1900)	1100 (1030 to 1190)	906 (836 to 981)	731 (669 to 799)	634 (577 to 696)	596 (529 to 677)	868 (776 to 975)	972 (869 to 1100)
Bolivia	2870 (2530 to 3240)	2330 (1970 to 2770)	1950 (1690 to 2250)	1600 (1400 to 1830)	1340 (1190 to 1510)	1090 (973 to 1220)	925 (784 to 1100)	828 (677 to 1050)	1300 (1090 to 1600)	1450 (1220 to 1780)
Ecuador	1770 (1510 to 2060)	1510 (1390 to 1640)	1390 (1270 to 1530)	1110 (1020 to 1200)	807 (757 to 868)	709 (639 to 782)	693 (632 to 756)	604 (516 to 711)	848 (722 to 1000)	796 (652 to 958)
Peru	2400 (2100 to 2730)	1910 (1710 to 2120)	1860 (1690 to 2080)	999 (885 to 1120)	839 (741 to 950)	645 (558 to 748)	530 (457 to 612)	541 (463 to 629)	784 (670 to 913)	946 (826 to 1090)
Caribbean	1660 (1510 to 1830)	1370 (1290 to 1460)	1220 (1160 to 1280)	1060 (1000 to 1110)	968 (906 to 1030)	849 (804 to 891)	1300 (1220 to 1390)	745 (669 to 820)	787 (705 to 872)	933 (822 to 1050)
Antigua and Barbuda	1540 (1390 to 1690)	1190 (1090 to 1290)	1000 (928 to 1090)	807 (696 to 943)	776 (708 to 852)	755 (695 to 830)	722 (660 to 793)	719 (627 to 832)	672 (579 to 782)	766 (680 to 871)
The Bahamas	1560 (1270 to 1900)	1330 (1120 to 1560)	1180 (1080 to 1290)	958 (870 to 1050)	846 (770 to 924)	853 (782 to 929)	770 (704 to 842)	846 (704 to 1010)	867 (703 to 1060)	1030 (842 to 1260)
Barbados	1490 (1340 to 1660)	1070 (986 to 1150)	1050 (974 to 1130)	908 (839 to 977)	785 (729 to 844)	739 (688 to 799)	673 (628 to 726)	679 (547 to 817)	641 (507 to 790)	681 (532 to 840)
Belize	1640 (1480 to 1830)	987 (897 to 1090)	853 (784 to 932)	776 (716 to 846)	699 (643 to 762)	949 (882 to 1020)	733 (683 to 790)	685 (593 to 790)	738 (637 to 852)	787 (653 to 947)
Bermuda	1260 (1130 to 1410)	1240 (1150 to 1340)	1010 (934 to 1090)	832 (768 to 903)	852 (787 to 929)	604 (554 to 652)	460 (411 to 515)	424 (365 to 497)	414 (357 to 485)	526 (444 to 624)
Cuba	1290 (1060 to 1560)	1170 (1050 to 1310)	1140 (1060 to 1220)	751 (699 to 810)	729 (650 to 827)	615 (571 to 667)	565 (521 to 612)	571 (508 to 640)	572 (507 to 657)	830 (716 to 965)
Dominica	2060 (1830 to 2320)	1360 (1240 to 1480)	1070 (991 to 1160)	910 (824 to 996)	924 (848 to 1000)	859 (778 to 942)	821 (748 to 904)	835 (716 to 996)	833 (717 to 1000)	1030 (820 to 1320)
Dominican Republic	1540 (1350 to 1750)	1200 (1070 to 1340)	967 (878 to 1060)	901 (816 to 1000)	794 (700 to 901)	692 (618 to 777)	696 (583 to 832)	658 (550 to 784)	724 (613 to 856)	735 (609 to 895)
Grenada	1450 (1240 to 1690)	1110 (963 to 1280)	1030 (868 to 1200)	960 (815 to 1140)	945 (850 to 1050)	902 (826 to 984)	918 (845 to 999)	832 (725 to 954)	829 (709 to 959)	1110 (876 to 1420)
Guyana	2070 (1840 to 2300)	1500 (1350 to 1660)	1420 (1280 to 1600)	1450 (1260 to 1650)	1360 (1220 to 1510)	1210 (1080 to 1350)	1150 (1040 to 1270)	1070 (863 to 1320)	1200 (948 to 1520)	1450 (1040 to 1990)
Haiti	2780 (2360 to 3260)	2390 (1930 to 2920)	2250 (1910 to 2620)	2020 (1750 to 2320)	1870 (1580 to 2180)	1670 (1440 to 1930)	3730 (3240 to 4310)	1330 (1020 to 1730)	1560 (1180 to 2030)	1750 (1280 to 2420)
Jamaica	1660 (1530 to 1790)	1160 (1080 to 1240)	981 (908 to 1060)	734 (678 to 800)	669 (618 to 725)	680 (629 to 734)	598 (554 to 649)	626 (498 to 778)	620 (474 to 791)	757 (593 to 959)
Puerto Rico	1220 (1130 to 1310)	1100 (1020 to 1180)	1050 (974 to 1130)	876 (817 to 936)	760 (704 to 820)	619 (580 to 664)	495 (462 to 531)	438 (405 to 473)	459 (428 to 494)	463 (386 to 543)
Saint Kitts and Nevis	1320 (1090 to 1600)	1180 (1040 to 1360)	1110 (1030 to 1200)	1210 (1120 to 1310)	1220 (1110 to 1320)	969 (884 to 1070)	871 (764 to 987)	837 (680 to 1040)	839 (671 to 1060)	959 (769 to 1190)
Saint Lucia	1770 (1610 to 1950)	1390 (1260 to 1530)	1200 (1090 to 1320)	1040 (959 to 1120)	1100 (1020 to 1190)	846 (783 to 918)	647 (601 to 700)	655 (543 to 781)	690 (564 to 830)	875 (670 to 1140)
Saint Vincent and the Grenadines	1760 (1590 to 1950)	1320 (1200 to 1450)	1120 (1020 to 1230)	1020 (934 to 1110)	994 (914 to 1080)	920 (847 to 994)	832 (757 to 909)	830 (728 to 945)	830 (712 to 968)	906 (761 to 1070)
Suriname	1500 (1380 to 1640)	1240 (1110 to 1390)	1070 (957 to 1190)	1110 (992 to 1270)	945 (835 to 1080)	884 (821 to 956)	774 (707 to 848)	778 (684 to 887)	777 (666 to 907)	910 (699 to 1260)
Trinidad and Tobago	1700 (1580 to 1830)	1310 (1220 to 1410)	1440 (1350 to 1560)	1330 (1240 to 1430)	1070 (995 to 1150)	915 (853 to 986)	741 (684 to 801)	702 (598 to 827)	756 (613 to 930)	931 (692 to 1230)
Virgin Islands	1480 (1260 to 1710)	1520 (1360 to 1670)	1330 (1180 to 1490)	1000 (892 to 1130)	849 (752 to 951)	786 (694 to 875)	636 (559 to 722)	619 (509 to 742)	585 (486 to 698)	617 (484 to 805)
Central Latin America	1950 (1850 to 2060)	1570 (1500 to 1640)	1330 (1280 to 1390)	1060 (1010 to 1120)	861 (825 to 896)	715 (683 to 746)	640 (613 to 668)	616 (581 to 652)	809 (768 to 852)	854 (794 to 917)
Colombia	1700 (1570 to 1850)	1580 (1440 to 1750)	1070 (985 to 1180)	930 (787 to 1090)	803 (735 to 879)	674 (609 to 748)	534 (484 to 589)	464 (430 to 503)	565 (522 to 613)	642 (548 to 741)
Costa Rica	1580 (1460 to 1700)	1130 (1040 to 1240)	1070 (985 to 1170)	700 (638 to 776)	597 (538 to 660)	564 (527 to 605)	493 (457 to 529)	475 (423 to 539)	491 (433 to 562)	566 (498 to 644)
El Salvador	2160 (1970 to 2400)	1590 (1460 to 1730)	1480 (1370 to 1600)	1170 (1030 to 1340)	830 (766 to 899)	715 (638 to 801)	636 (581 to 698)	642 (561 to 741)	729 (614 to 868)	798 (644 to 982)
Guatemala	2510 (2310 to 2750)	2450 (2240 to 2710)	2170 (2000 to 2380)	1530 (1420 to 1650)	1390 (1260 to 1540)	1150 (1060 to 1240)	925 (827 to 1040)	829 (766 to 896)	881 (811 to 956)	1040 (909 to 1190)
Honduras	2470 (2170 to 2790)	1990 (1790 to 2200)	1590 (1420 to 1770)	940 (835 to 1060)	907 (802 to 1020)	947 (783 to 1130)	943 (765 to 1150)	933 (802 to 1100)	1130 (969 to 1340)	1240 (1070 to 1460)
Mexico	2220 (2030 to 2420)	1640 (1530 to 1760)	1470 (1370 to 1580)	1140 (1070 to 1220)	906 (843 to 970)	727 (679 to 780)	677 (633 to 724)	656 (608 to 707)	914 (855 to 977)	923 (857 to 997)

Aguascalientes	1940 (1690 to 2230)	1520 (1320 to 1740)	1420 (1230 to 1630)	1110 (1010 to 1240)	950 (853 to 1050)	754 (683 to 830)	653 (591 to 722)	624 (560 to 696)	828 (748 to 912)	860 (771 to 959)
Baja California	2180 (1880 to 2540)	1680 (1450 to 1930)	1550 (1330 to 1810)	1170 (1050 to 1280)	1100 (995 to 1200)	945 (858 to 1040)	783 (709 to 861)	730 (658 to 810)	1070 (965 to 1180)	979 (880 to 1090)
Baja California Sur	2100 (1780 to 2410)	1560 (1350 to 1780)	1410 (1230 to 1610)	1100 (996 to 1220)	837 (754 to 924)	738 (669 to 814)	663 (600 to 731)	675 (602 to 755)	900 (812 to 993)	908 (814 to 1010)
Campeche	2000 (1710 to 2330)	1500 (1300 to 1720)	1340 (1180 to 1530)	1020 (921 to 1130)	852 (770 to 939)	660 (596 to 729)	639 (582 to 707)	649 (584 to 725)	874 (793 to 963)	880 (788 to 983)
Chiapas	2170 (1820 to 2530)	1620 (1420 to 1870)	1460 (1270 to 1670)	1170 (1060 to 1300)	888 (804 to 986)	748 (680 to 828)	687 (621 to 756)	713 (643 to 790)	974 (879 to 1070)	959 (857 to 1070)
Chihuahua	2220 (1910 to 2560)	1740 (1510 to 2000)	1570 (1360 to 1800)	1250 (1130 to 1390)	1170 (1060 to 1290)	933 (844 to 1030)	981 (890 to 1080)	803 (723 to 899)	1210 (1100 to 1340)	965 (868 to 1080)
Coahuila	2320 (1990 to 2720)	1790 (1570 to 2060)	1630 (1410 to 1880)	1300 (1170 to 1440)	1070 (969 to 1190)	807 (728 to 895)	720 (652 to 792)	687 (611 to 770)	997 (902 to 1100)	865 (773 to 971)
Colima	2600 (2260 to 3000)	1890 (1630 to 2180)	1680 (1470 to 1920)	1290 (1160 to 1420)	960 (867 to 1060)	734 (660 to 807)	684 (619 to 754)	694 (620 to 777)	918 (831 to 1020)	1050 (940 to 1180)
Durango	2220 (1900 to 2570)	1620 (1400 to 1850)	1430 (1220 to 1640)	1070 (970 to 1200)	829 (751 to 913)	656 (591 to 721)	761 (690 to 835)	612 (546 to 683)	784 (711 to 864)	734 (659 to 815)
Guanajuato	2250 (1930 to 2600)	1730 (1500 to 1970)	1560 (1370 to 1790)	1140 (1030 to 1260)	1040 (942 to 1150)	759 (687 to 835)	644 (585 to 710)	719 (644 to 805)	970 (880 to 1070)	1040 (931 to 1150)
Guerrero	2510 (2170 to 2890)	1930 (1680 to 2230)	1720 (1490 to 1980)	1420 (1280 to 1580)	1010 (902 to 1120)	796 (718 to 881)	724 (646 to 808)	642 (564 to 727)	778 (695 to 870)	783 (691 to 886)
Hidalgo	2450 (2110 to 2850)	1790 (1560 to 2040)	1610 (1410 to 1840)	1260 (1140 to 1380)	833 (751 to 914)	610 (554 to 668)	578 (525 to 632)	579 (520 to 637)	738 (670 to 807)	779 (663 to 903)
Jalisco	2060 (1770 to 2370)	1560 (1350 to 1780)	1390 (1220 to 1610)	1100 (1000 to 1210)	961 (871 to 1050)	774 (704 to 846)	685 (626 to 750)	679 (612 to 752)	870 (792 to 953)	970 (872 to 1080)
México	2150 (1840 to 2490)	1670 (1440 to 1920)	1490 (1290 to 1710)	1280 (1170 to 1410)	1030 (936 to 1120)	781 (709 to 856)	675 (617 to 743)	656 (593 to 726)	1040 (954 to 1140)	867 (782 to 964)
Mexico City	1810 (1540 to 2100)	1350 (1170 to 1560)	1240 (1060 to 1450)	1010 (924 to 1120)	857 (774 to 956)	716 (652 to 793)	671 (611 to 743)	629 (567 to 704)	1010 (918 to 1120)	1160 (1050 to 1300)
Michoacán de Ocampo	2420 (2050 to 2810)	1740 (1510 to 2010)	1530 (1340 to 1750)	1190 (1070 to 1320)	826 (746 to 915)	688 (624 to 766)	638 (579 to 703)	625 (561 to 697)	778 (704 to 858)	920 (830 to 1030)
Morelos	2340 (2040 to 2680)	1690 (1440 to 1960)	1500 (1310 to 1750)	1170 (1050 to 1300)	898 (808 to 994)	692 (623 to 767)	637 (574 to 702)	697 (622 to 778)	931 (842 to 1030)	1090 (979 to 1220)
Nayarit	2190 (1890 to 2510)	1590 (1370 to 1840)	1360 (1190 to 1570)	1030 (925 to 1140)	801 (724 to 888)	634 (573 to 704)	644 (584 to 710)	565 (509 to 628)	708 (640 to 782)	758 (680 to 847)
Nuevo León	1920 (1640 to 2220)	1430 (1230 to 1630)	1300 (1120 to 1510)	1020 (918 to 1130)	875 (789 to 964)	730 (657 to 805)	658 (595 to 724)	622 (560 to 694)	835 (757 to 920)	872 (780 to 972)
Oaxaca	2660 (2300 to 3040)	2050 (1790 to 2350)	1860 (1630 to 2160)	1510 (1370 to 1670)	1000 (909 to 1100)	727 (659 to 802)	652 (594 to 722)	620 (557 to 694)	774 (704 to 855)	883 (796 to 984)
Puebla	2750 (2360 to 3170)	1980 (1710 to 2280)	1730 (1510 to 1990)	1320 (1190 to 1460)	988 (891 to 1090)	778 (705 to 857)	676 (614 to 742)	688 (616 to 765)	978 (887 to 1070)	1120 (1010 to 1250)
Querétaro	2330 (1990 to 2730)	1740 (1500 to 1980)	1520 (1320 to 1760)	1250 (1100 to 1410)	1020 (920 to 1120)	799 (724 to 880)	665 (604 to 734)	656 (590 to 731)	882 (800 to 969)	979 (879 to 1090)
Quintana Roo	2200 (1920 to 2510)	1810 (1580 to 2070)	1730 (1500 to 1980)	1450 (1290 to 1620)	1170 (1040 to 1310)	879 (789 to 976)	868 (780 to 968)	847 (745 to 959)	1040 (930 to 1170)	1010 (886 to 1150)
San Luis Potosí	2350 (2000 to 2710)	1660 (1440 to 1920)	1460 (1270 to 1710)	947 (860 to 1040)	800 (730 to 875)	648 (590 to 714)	605 (552 to 664)	600 (540 to 667)	836 (763 to 918)	848 (762 to 943)
Sinaloa	2030 (1730 to 2390)	1500 (1310 to 1750)	1330 (1140 to 1540)	1030 (931 to 1130)	775 (698 to 854)	652 (589 to 715)	668 (606 to 733)	559 (503 to 621)	763 (692 to 848)	723 (649 to 807)
Sonora	1970 (1710 to 2310)	1580 (1360 to 1820)	1470 (1270 to 1700)	1200 (1050 to 1370)	1040 (932 to 1140)	830 (752 to 911)	770 (695 to 846)	735 (658 to 818)	1030 (934 to 1130)	950 (852 to 1050)
Tabasco	2180 (1890 to 2530)	1660 (1430 to 1900)	1530 (1330 to 1760)	1270 (1150 to 1400)	898 (814 to 993)	714 (647 to 788)	710 (648 to 780)	744 (668 to 828)	1060 (960 to 1160)	923 (783 to 1080)
Tamaulipas	2050 (1730 to 2440)	1500 (1310 to 1750)	1360 (1180 to 1570)	1090 (992 to 1200)	848 (768 to 932)	671 (605 to 738)	667 (610 to 730)	625 (562 to 694)	796 (726 to 872)	754 (678 to 838)
Tlaxcala	2300 (1940 to 2660)	1670 (1440 to 1950)	1430 (1240 to 1660)	1140 (995 to 1290)	880 (798 to 975)	707 (638 to 782)	621 (561 to 685)	612 (547 to 686)	930 (842 to 1030)	994 (894 to 1110)
Veracruz de Ignacio de la Llave	2210 (1890 to 2580)	1640 (1430 to 1870)	1460 (1260 to 1670)	1050 (954 to 1160)	834 (759 to 920)	678 (616 to 750)	648 (590 to 711)	673 (607 to 748)	856 (784 to 941)	862 (743 to 993)
Yucatán	1980 (1690 to 2290)	1650 (1440 to 1890)	1520 (1320 to 1750)	1270 (1150 to 1400)	966 (869 to 1070)	750 (680 to 831)	702 (638 to 776)	664 (593 to 740)	868 (787 to 966)	880 (787 to 990)
Zacatecas	1880 (1610 to 2200)	1420 (1240 to 1630)	1270 (1090 to 1460)	906 (821 to 1000)	743 (669 to 824)	649 (585 to 720)	624 (566 to 690)	622 (556 to 693)	862 (782 to 952)	879 (792 to 977)
Nicaragua	1910 (1730 to 2130)	1580 (1430 to 1740)	1430 (1300 to 1570)	953 (796 to 1120)	662 (600 to 736)	577 (525 to 636)	578 (522 to 641)	518 (443 to 606)	715 (613 to 845)	815 (678 to 992)
Panama	1230 (1120 to 1350)	1020 (944 to 1100)	982 (915 to 1060)	686 (637 to 746)	597 (554 to 650)	537 (500 to 578)	515 (479 to 553)	472 (415 to 536)	579 (507 to 665)	538 (425 to 668)
Venezuela	1480 (1380 to 1580)	1160 (1080 to 1260)	1190 (1110 to 1280)	982 (913 to 1060)	787 (737 to 845)	715 (663 to 767)	631 (584 to 680)	703 (565 to 876)	912 (736 to 1120)	987 (782 to 1240)
Tropical Latin America	1850 (1670 to 2060)	1530 (1400 to 1650)	1320 (1210 to 1420)	1080 (1010 to 1150)	921 (860 to 988)	777 (726 to 834)	685 (639 to 739)	609 (541 to 692)	681 (600 to 781)	750 (656 to 871)
Brazil	1850 (1660 to 2060)	1550 (1420 to 1680)	1330 (1220 to 1440)	1090 (1010 to 1160)	929 (866 to 999)	781 (728 to 839)	686 (639 to 741)	608 (538 to 690)	681 (600 to 784)	747 (652 to 868)
Acre	1980 (1630 to 2340)	1560 (1330 to 1820)	1270 (1080 to 1470)	1010 (905 to 1120)	896 (799 to 997)	805 (722 to 892)	681 (612 to 750)	658 (546 to 780)	784 (642 to 945)	773 (624 to 936)
Alagoas	2230 (1820 to 2670)	1860 (1600 to 2150)	1550 (1330 to 1810)	1220 (1100 to 1370)	981 (879 to 1090)	755 (683 to 837)	716 (642 to 805)	640 (531 to 771)	719 (602 to 882)	723 (598 to 888)
Amapá	2080 (1720 to 2470)	1600 (1380 to 1830)	1220 (1050 to 1410)	894 (788 to 1000)	982 (866 to 1110)	830 (737 to 938)	662 (584 to 754)	750 (612 to 922)	869 (700 to 1070)	786 (630 to 990)
Amazonas	1620 (1330 to 1960)	1310 (1100 to 1560)	1150 (961 to 1350)	882 (785 to 990)	802 (706 to 895)	663 (595 to 737)	609 (540 to 682)	611 (508 to 741)	826 (684 to 1010)	894 (728 to 1110)
Bahia	1740 (1460 to 2080)	1490 (1280 to 1730)	1290 (1120 to 1470)	925 (838 to 1030)	818 (735 to 910)	721 (657 to 799)	729 (657 to 814)	585 (491 to 706)	642 (534 to 780)	653 (532 to 796)
Ceará	2240 (1870 to 2700)	1830 (1580 to 2110)	1460 (1250 to 1680)	1030 (890 to 1190)	677 (603 to 763)	649 (578 to 728)	602 (536 to 674)	596 (493 to 730)	671 (551 to 819)	652 (521 to 810)
Distrito Federal	2060 (1760 to 2410)	1770 (1510 to 2040)	1600 (1380 to 1860)	1420 (1280 to 1570)	1170 (1050 to 1300)	909 (822 to 1010)	740 (669 to 818)	623 (521 to 740)	751 (624 to 906)	770 (632 to 931)
Espírito Santo	1710 (1450 to 2010)	1490 (1260 to 1740)	1330 (1130 to 1560)	1110 (1010 to 1230)	916 (822 to 1020)	779 (704 to 861)	669 (603 to 746)	583 (484 to 699)	682 (568 to 825)	733 (601 to 892)
Goiás	2510 (2110 to 2930)	2100 (1790 to 2430)	1610 (1390 to 1910)	1180 (1030 to 1350)	915 (824 to 1030)	732 (662 to 814)	686 (618 to 768)	630 (527 to 771)	736 (615 to 902)	844 (693 to 1040)
Maranhão	2300 (1970 to 2700)	1940 (1680 to 2230)	1660 (1420 to 1970)	1190 (1040 to 1370)	940 (842 to 1050)	788 (709 to 875)	944 (835 to 1060)	756 (625 to 930)	845 (700 to 1030)	806 (652 to 1010)
Mato Grosso	1470 (1240 to 1740)	1290 (1100 to 1510)	1100 (925 to 1280)	819 (729 to 919)	858 (765 to 964)	772 (693 to 863)	671 (605 to 743)	589 (494 to 703)	738 (617 to 891)	820 (673 to 1000)
Mato Grosso do Sul	1610 (1350 to 1910)	1410 (1180 to 1670)	1250 (1050 to 1460)	993 (898 to 1100)	898 (804 to 1000)	799 (718 to 887)	711 (641 to 792)	604 (501 to 725)	698 (574 to 854)	809 (654 to 991)
Minas Gerais	1860 (1590 to 2180)	1590 (1350 to 1850)	1380 (1170 to 1610)	1130 (1030 to 1240)	923 (835 to 1020)	708 (642 to 778)	627 (567 to 690)	562 (475 to 666)	604 (504 to 721)	707 (581 to 853)
Pará	1850 (1550 to 2170)	1480 (1260 to 1730)	1260 (1060 to 1480)	1010 (890 to 1140)	839 (746 to 946)	698 (616 to 784)	679 (603 to 766)	643 (529 to 783)	767 (624 to 942)	739 (595 to 920)
Paraíba	2330 (1910 to 2820)	1810 (1560 to 2100)	1410 (1210 to 1630)	1030 (925 to 1160)	744 (668 to 831)	658 (596 to 725)	679 (615 to 752)	695 (581 to 834)	640 (529 to 781)	683 (558 to 840)
Paraná	1700 (1420 to 1990)	1490 (1260 to 1750)	1370 (1150 to 1620)	1190 (1070 to 1320)	998 (895 to 1110)	836 (758 to 923)	718 (646 to 793)	601 (501 to 719)	666 (552 to 803)	828 (671 to 1020)
Pernambuco	2470 (2060 to 2940)	2010 (1720 to 2310)	1610 (1390 to 1860)	1180 (1060 to 1310)	1050 (945 to 1160)	873 (788 to 963)	693 (624 to 762)	649 (539 to 771)	750 (623 to 904)	740 (606 to 894)
Piauí	1700 (1410 to 2020)	1420 (1220 to 1640)	1250 (1060 to 1450)	933 (813 to 1080)	722 (633 to 815)	630 (558 to 704)	638 (560 to 728)	576 (470 to 709)	646 (525 to 787)	632 (502 to 797)
Rio de Janeiro	1660 (1380 to 1990)	1410 (1190 to 1670)	1280 (1090 to 1480)	1190 (1090 to 1320)	1140 (1030 to 1260)	895 (818 to 985)	749 (682 to 825)	677 (574 to 804)	788 (661 to 950)	839 (696 to 1020)
Rio Grande do Norte	2370 (1950 to 2840)	1760 (1480 to 2060)	1280 (1100 to 1490)	890 (790 to 1010)	686 (609 to 770)	613 (550 to 683)	601 (537 to 671)	575 (480 to 682)	642 (537 to 768)	658 (539 to 800)
Rio Grande do Sul	1590 (1330 to 1860)	1360 (1160 to 1590)	1180 (992 to 1370)	1030 (936 to 1140)	915 (827 to 1010)	806 (735 to 883)	679 (616 to 748)	616 (516 to 746)	640 (534 to 764)	760 (630 to 918)
Rondônia	2550 (2090 to 3100)	2160 (1850 to 2470)	1900 (1650 to 2190)	1900 (1690 to 2110)	1290 (1140 to 1450)	916 (822 to 1020)	680 (603 to 763)	596 (490 to 718)	692 (566 to 849)	709 (570 to 878)
Roraima	2650 (2260 to 3100)	2220 (1910 to 2530)	1920 (1650 to 2250)	1750 (1510 to 2000)	1620 (1420 to 1860)	1250 (1090 to 1430)	1020 (882 to 1160)	888 (710 to 1090)	1020 (822 to 1270)	924 (729 to 1160)
Santa Catarina	1460 (1220 to 1720)	1260 (1070 to 1490)	1140 (953 to 1340)	982 (889 to 1080)	897 (803 to 991)	810 (729 to 890)	652 (590 to 716)	570 (478 to 673)	624 (516 to 749)	730 (599 to 883)
São Paulo	1520 (1290 to 1800)	1330 (1130 to 1570)	1190 (1030 to 1410)	1110 (1010 to 1220)	1020 (933 to 1130)	857 (784 to 935)	679 (620 to 746)	592 (504 to 698)	669 (568 to 796)	769 (643 to 923)
Sergipe	1970 (1640 to 2350)	1650 (1410 to 1930)	1340 (1150 to 1560)	1090 (986 to 1220)	1000 (904 to 1120)	805 (731 to 892)	676 (608 to 753)	618 (517 to 748)	706 (586 to 860)	713 (587 to 879)
Tocantins	2050 (1640 to 2510)	1720 (1470 to 1990)	1340 (1140 to 1580)	938 (792 to 1110)	839 (738 to 946)	753 (669 to 841)	744 (662 to 834)	682 (562 to 824)	744 (612 to 905)	724 (588 to 891)

Paraguay	1840 (1550 to 2160)	894 (786 to 1020)	884 (783 to 994)	755 (672 to 849)	642 (566 to 731)	643 (559 to 731)	665 (575 to 778)	684 (548 to 858)	678 (535 to 862)	896 (706 to 1130)
North Africa and Middle East	2360 (2150 to 2620)	1990 (1830 to 2150)	1650 (1540 to 1770)	1350 (1280 to 1430)	1170 (1110 to 1230)	966 (914 to 1020)	842 (798 to 890)	784 (734 to 842)	921 (857 to 994)	990 (915 to 1080)
North Africa and Middle East	2360 (2150 to 2620)	1990 (1830 to 2150)	1650 (1540 to 1770)	1350 (1280 to 1430)	1170 (1110 to 1230)	966 (914 to 1020)	842 (798 to 890)	784 (734 to 842)	921 (857 to 994)	990 (915 to 1080)
Afghanistan	2940 (2250 to 4050)	2500 (1990 to 3160)	2360 (1960 to 2870)	2580 (2120 to 3270)	2010 (1630 to 2440)	2020 (1580 to 2570)	1650 (1220 to 2230)	1510 (1060 to 2170)	1980 (1410 to 2920)	2090 (1500 to 2980)
Algeria	2440 (2200 to 2720)	2190 (1920 to 2490)	1820 (1580 to 2090)	1740 (1570 to 1910)	1050 (930 to 1180)	873 (779 to 974)	794 (723 to 871)	725 (614 to 859)	889 (751 to 1060)	962 (795 to 1180)
Bahrain	2150 (1750 to 2580)	1880 (1570 to 2200)	1570 (1300 to 1870)	1460 (1320 to 1620)	1350 (1240 to 1470)	1330 (1220 to 1440)	1110 (1030 to 1200)	800 (719 to 898)	925 (829 to 1030)	1020 (906 to 1150)
Egypt	2470 (2230 to 2750)	2110 (1960 to 2280)	2000 (1810 to 2200)	1720 (1570 to 1890)	1620 (1480 to 1780)	1300 (1190 to 1420)	1340 (1210 to 1480)	1160 (1060 to 1260)	1320 (1160 to 1490)	1420 (1210 to 1660)
Iran	2630 (2360 to 2920)	2140 (1930 to 2400)	1550 (1410 to 1720)	1080 (969 to 1190)	1000 (918 to 1100)	774 (706 to 850)	637 (592 to 686)	572 (523 to 623)	633 (578 to 699)	811 (738 to 900)
Alborz	2350 (1990 to 2740)	1960 (1650 to 2340)	1350 (1140 to 1620)	900 (747 to 1100)	865 (729 to 1020)	787 (674 to 934)	629 (556 to 720)	596 (507 to 715)	680 (579 to 816)	891 (756 to 1060)
Ardebil	2660 (2290 to 3100)	2250 (1880 to 2640)	1710 (1450 to 2030)	1190 (1010 to 1440)	984 (836 to 1160)	724 (633 to 833)	615 (553 to 689)	655 (565 to 757)	718 (618 to 827)	911 (781 to 1060)
Bushehr	2310 (1960 to 2690)	1950 (1640 to 2310)	1490 (1240 to 1760)	1030 (864 to 1230)	873 (744 to 1020)	741 (640 to 843)	662 (592 to 737)	583 (501 to 670)	637 (547 to 736)	801 (688 to 927)
Chahar Mahaal and Bakhtiari	2070 (1760 to 2430)	1770 (1490 to 2090)	1380 (1160 to 1630)	1030 (864 to 1230)	897 (763 to 1040)	714 (612 to 834)	591 (522 to 666)	505 (433 to 583)	543 (465 to 634)	670 (576 to 789)
East Azarbayejan	2860 (2460 to 3280)	2360 (1980 to 2810)	1750 (1490 to 2050)	1200 (1010 to 1420)	1090 (932 to 1280)	918 (796 to 1060)	786 (701 to 875)	702 (605 to 811)	748 (637 to 880)	914 (778 to 1070)
Fars	2050 (1750 to 2420)	1750 (1470 to 2070)	1360 (1150 to 1620)	1070 (894 to 1280)	982 (825 to 1160)	843 (725 to 981)	732 (646 to 828)	637 (535 to 748)	693 (586 to 818)	872 (740 to 1030)
Gilan	2140 (1830 to 2490)	1800 (1490 to 2190)	1330 (1110 to 1590)	971 (803 to 1150)	2140 (1860 to 2460)	820 (708 to 952)	688 (615 to 773)	611 (523 to 712)	676 (571 to 786)	868 (744 to 1010)
Golestan	2470 (2110 to 2890)	2120 (1790 to 2500)	1630 (1380 to 1940)	1160 (959 to 1380)	1030 (875 to 1230)	878 (766 to 1000)	716 (642 to 802)	738 (638 to 845)	798 (690 to 923)	1000 (860 to 1170)
Hamadan	3330 (2870 to 3840)	2720 (2310 to 3200)	1980 (1700 to 2330)	1280 (1080 to 1510)	981 (829 to 1160)	737 (640 to 857)	658 (586 to 739)	566 (489 to 659)	609 (517 to 712)	746 (639 to 879)
Hormozgan	2510 (2150 to 2940)	2210 (1890 to 2650)	1600 (1350 to 1910)	1180 (978 to 1420)	1070 (904 to 1270)	879 (768 to 1020)	696 (616 to 783)	600 (515 to 703)	662 (567 to 780)	844 (724 to 1000)
Ilam	2330 (1990 to 2720)	2020 (1690 to 2410)	1590 (1330 to 1890)	1140 (954 to 1350)	960 (816 to 1120)	793 (690 to 912)	686 (609 to 767)	654 (569 to 760)	718 (615 to 831)	890 (767 to 1040)
Isfahan	2190 (1870 to 2550)	1880 (1570 to 2210)	1330 (1110 to 1590)	904 (750 to 1090)	807 (679 to 954)	670 (573 to 780)	556 (496 to 620)	527 (448 to 618)	593 (506 to 696)	778 (659 to 909)
Kerman	2910 (2500 to 3400)	2330 (1970 to 2740)	1720 (1450 to 2020)	1210 (1020 to 1420)	1070 (903 to 1260)	944 (820 to 1090)	756 (675 to 854)	669 (576 to 766)	720 (619 to 833)	879 (748 to 1030)
Kermanshah	2850 (2450 to 3300)	2420 (2050 to 2860)	1850 (1550 to 2190)	1300 (1090 to 1550)	1130 (955 to 1340)	937 (812 to 1080)	739 (658 to 831)	622 (526 to 732)	691 (591 to 812)	883 (752 to 1030)
Khorasan-e-Razavi	3460 (2960 to 3980)	2810 (2400 to 3270)	2020 (1720 to 2330)	1290 (1080 to 1520)	1130 (959 to 1330)	943 (818 to 1090)	733 (656 to 821)	637 (547 to 743)	702 (596 to 814)	885 (753 to 1030)
Khuzestan	2370 (2010 to 2790)	1970 (1650 to 2320)	1500 (1260 to 1760)	1120 (940 to 1330)	935 (802 to 1100)	822 (724 to 939)	672 (607 to 747)	626 (542 to 720)	697 (598 to 810)	912 (783 to 1060)
Kohgiluyeh and Boyer-Ahmad	2240 (1900 to 2620)	1870 (1570 to 2180)	1480 (1240 to 1730)	1130 (947 to 1350)	889 (746 to 1050)	676 (582 to 784)	546 (483 to 621)	510 (436 to 597)	558 (471 to 658)	705 (600 to 834)
Kurdistan	3430 (2960 to 3970)	2930 (2500 to 3450)	2160 (1840 to 2530)	1440 (1220 to 1710)	1100 (942 to 1300)	814 (711 to 938)	674 (600 to 754)	545 (461 to 628)	592 (511 to 700)	749 (640 to 881)
Lorestan	2700 (2310 to 3170)	2190 (1840 to 2580)	1650 (1390 to 1940)	1200 (1010 to 1430)	989 (844 to 1160)	808 (704 to 933)	705 (629 to 794)	608 (520 to 707)	681 (581 to 798)	896 (770 to 1060)
Markazi	2600 (2220 to 3020)	2240 (1890 to 2650)	1700 (1420 to 1990)	1170 (982 to 1410)	971 (819 to 1150)	783 (672 to 915)	592 (528 to 664)	501 (430 to 591)	568 (482 to 665)	748 (632 to 865)
Mazandaran	1890 (1600 to 2230)	1620 (1360 to 1920)	1230 (1040 to 1450)	910 (762 to 1080)	776 (655 to 916)	670 (571 to 777)	592 (527 to 663)	531 (453 to 615)	595 (508 to 699)	771 (658 to 910)
North Khorasan	3030 (2600 to 3560)	2560 (2180 to 3000)	1970 (1660 to 2340)	1300 (1090 to 1540)	1100 (925 to 1300)	868 (753 to 1010)	659 (588 to 745)	609 (518 to 705)	679 (573 to 792)	884 (746 to 1040)
Qazvin	2930 (2510 to 3390)	2470 (2050 to 2990)	1790 (1420 to 2210)	1160 (962 to 1370)	928 (789 to 1080)	795 (687 to 927)	659 (590 to 732)	587 (503 to 681)	657 (559 to 766)	846 (719 to 992)
Qom	2180 (1860 to 2550)	1870 (1560 to 2200)	1430 (1200 to 1690)	1060 (876 to 1260)	946 (795 to 1130)	836 (721 to 961)	690 (614 to 767)	528 (451 to 613)	606 (517 to 711)	824 (707 to 962)
Semnan	2600 (2210 to 3040)	2230 (1900 to 2630)	1570 (1320 to 1840)	1070 (894 to 1290)	894 (762 to 1050)	788 (677 to 910)	647 (577 to 732)	565 (482 to 664)	644 (543 to 766)	856 (734 to 1000)
Sistan and Baluchistan	3220 (2710 to 3780)	2590 (2210 to 3070)	1970 (1660 to 2310)	1350 (1130 to 1610)	1110 (938 to 1310)	827 (708 to 964)	680 (602 to 770)	626 (529 to 742)	692 (584 to 819)	906 (772 to 1070)
South Khorasan	2810 (2400 to 3260)	2400 (2010 to 2810)	1800 (1550 to 2110)	1160 (970 to 1380)	907 (771 to 1070)	663 (578 to 759)	574 (514 to 645)	486 (419 to 564)	549 (474 to 644)	704 (596 to 818)
Tehran	1650 (1400 to 1950)	1320 (1100 to 1550)	940 (785 to 1110)	693 (571 to 835)	655 (543 to 790)	578 (493 to 671)	488 (428 to 552)	458 (384 to 538)	514 (438 to 603)	667 (568 to 782)
West Azarbayejan	2890 (2510 to 3340)	2450 (2090 to 2860)	1840 (1560 to 2180)	1240 (1040 to 1480)	1150 (972 to 1350)	972 (847 to 1110)	763 (685 to 853)	726 (630 to 837)	777 (668 to 904)	970 (825 to 1130)
Yazd	2500 (2150 to 2920)	1910 (1620 to 2260)	1400 (1180 to 1660)	1030 (852 to 1220)	888 (750 to 1040)	792 (675 to 925)	697 (618 to 780)	570 (487 to 666)	680 (578 to 798)	932 (798 to 1090)
Zanjan	3350 (2860 to 3940)	2820 (2370 to 3380)	1990 (1630 to 2450)	1230 (1030 to 1470)	2150 (1860 to 2490)	800 (686 to 928)	638 (571 to 709)	542 (467 to 627)	609 (524 to 710)	780 (670 to 907)
Iraq	1590 (1360 to 1830)	1270 (1120 to 1430)	1150 (991 to 1340)	1060 (933 to 1210)	1020 (882 to 1180)	947 (741 to 1200)	872 (632 to 1200)	826 (591 to 1150)	1180 (879 to 1610)	1110 (818 to 1570)
Jordan	2020 (1480 to 2700)	1620 (1270 to 2060)	1310 (1110 to 1540)	1040 (919 to 1170)	857 (744 to 991)	814 (681 to 965)	597 (524 to 681)	475 (399 to 557)	629 (523 to 744)	719 (596 to 852)
Kuwait	1210 (953 to 1510)	861 (732 to 1000)	824 (767 to 889)	788 (737 to 844)	675 (608 to 751)	593 (538 to 660)	470 (436 to 510)	388 (347 to 440)	506 (448 to 576)	465 (382 to 563)
Lebanon	1740 (1380 to 2130)	1210 (957 to 1510)	1050 (876 to 1270)	1010 (822 to 1270)	1000 (815 to 1260)	699 (595 to 810)	601 (534 to 676)	508 (424 to 608)	631 (527 to 764)	780 (652 to 938)
Libya	2460 (2110 to 2850)	1950 (1630 to 2290)	1240 (1090 to 1400)	882 (781 to 1010)	759 (641 to 900)	699 (619 to 788)	771 (685 to 868)	809 (632 to 1010)	923 (732 to 1150)	971 (767 to 1230)
Morocco	2540 (2160 to 2990)	2190 (1860 to 2560)	1710 (1480 to 2010)	1370 (1210 to 1560)	1040 (923 to 1180)	910 (800 to 1050)	855 (708 to 1030)	822 (649 to 962)	986 (807 to 1140)	953 (782 to 1100)
Oman	2780 (1910 to 4270)	2270 (1640 to 3160)	1610 (1230 to 2120)	1250 (979 to 1600)	1020 (820 to 1270)	876 (720 to 1050)	824 (729 to 926)	717 (611 to 828)	926 (809 to 1050)	940 (822 to 1070)
Palestine	2400 (1860 to 3050)	1760 (1460 to 2130)	1470 (1260 to 1730)	1140 (919 to 1460)	1120 (942 to 1360)	1080 (962 to 1220)	977 (890 to 1080)	727 (653 to 818)	774 (680 to 887)	897 (786 to 1030)
Qatar	1570 (1110 to 2290)	1400 (1070 to 1870)	1240 (970 to 1570)	1140 (956 to 1350)	1250 (1070 to 1450)	1210 (1030 to 1400)	960 (823 to 1120)	544 (450 to 660)	682 (571 to 820)	676 (561 to 819)
Saudi Arabia	1960 (1600 to 2370)	1910 (1540 to 2330)	1510 (1240 to 1830)	1130 (924 to 1390)	1100 (899 to 1320)	1020 (913 to 1150)	931 (854 to 1020)	810 (686 to 966)	892 (754 to 1070)	862 (717 to 1050)
Sudan	2180 (1810 to 2650)	2010 (1690 to 2400)	1880 (1630 to 2150)	1700 (1490 to 1930)	1480 (1290 to 1720)	1250 (1030 to 1530)	1020 (850 to 1250)	906 (721 to 1170)	1120 (900 to 1400)	1170 (900 to 1500)
Syria	1600 (1330 to 1930)	1170 (973 to 1380)	940 (845 to 1050)	837 (711 to 979)	1040 (856 to 1240)	1020 (898 to 1170)	875 (737 to 1040)	981 (776 to 1230)	964 (761 to 1240)	1010 (802 to 1300)
Tunisia	2600 (2220 to 3040)	2160 (1910 to 2470)	1780 (1610 to 1960)	1270 (1130 to 1430)	820 (727 to 927)	763 (644 to 895)	676 (522 to 845)	604 (462 to 772)	663 (500 to 849)	869 (701 to 1070)
Türkiye	2160 (1820 to 2510)	1800 (1540 to 2080)	1430 (1240 to 1650)	1140 (1000 to 1280)	1040 (951 to 1150)	752 (637 to 883)	644 (580 to 718)	627 (567 to 693)	757 (661 to 866)	774 (663 to 897)
United Arab Emirates	1630 (1230 to 2140)	1420 (1140 to 1730)	1100 (887 to 1340)	1060 (846 to 1310)	1040 (844 to 1240)	1010 (853 to 1190)	1110 (934 to 1310)	1050 (851 to 1300)	800 (635 to 991)	826 (652 to 1020)
Yemen	3530 (2490 to 5350)	2940 (2200 to 4150)	2560 (1990 to 3380)	1820 (1480 to 2250)	1490 (1240 to 1800)	1270 (1040 to 1540)	1040 (849 to 1300)	1080 (832 to 1430)	1220 (947 to 1600)	1360 (1040 to 1780)
South Asia	2810 (2500 to 3170)	2400 (2170 to 2670)	2000 (1850 to 2150)	1610 (1510 to 1710)	1390 (1300 to 1480)	1180 (1110 to 1270)	1030 (964 to 1110)	920 (827 to 1020)	1020 (920 to 1150)	1090 (973 to 1220)
South Asia	2810 (2500 to 3170)	2400 (2170 to 2670)	2000 (1850 to 2150)	1610 (1510 to 1710)	1390 (1300 to 1480)	1180 (1110 to 1270)	1030 (964 to 1110)	920 (827 to 1020)	1020 (920 to 1150)	1090 (973 to 1220)
Bangladesh	2500 (2140 to 2920)	2180 (1870 to 2530)	2320 (2020 to 2690)	1590 (1400 to 1800)	1440 (1310 to 1600)	1210 (1090 to 1340)	992 (895 to 1100)	762 (618 to 924)	876 (711 to 1060)	883 (715 to 1080)
Bhutan	2890 (2380 to 3450)	2420 (1960 to 2930)	2010 (1530 to 2590)	1810 (1370 to 2410)	1390 (1120 to 1720)	1090 (936 to 1270)	834 (721 to 976)	761 (628 to 890)	755 (625 to 891)	771 (638 to 918)
India	2940 (2560 to 3380)	2510 (2230 to 2840)	2030 (1850 to 2230)	1650 (1520 to 1790)	1390 (1290 to 1510)	1160 (1080 to 1270)	1020 (939 to 1120)	918 (809 to 1040)	1010 (888 to 1150)	1090 (948 to 1240)
Nepal	2780 (2380 to 3210)	2420 (2080 to 2780)	2090 (1780 to 2440)	1920 (1640 to 2250)	1540 (1330 to 1770)	1130 (1010 to 1280)	959 (835 to 1100)	911 (749 to 1130)	1050 (876 to 1290)	1200 (1000 to 1450)
Pakistan	2090 (1810 to 2380)	1720 (1520 to 1950)	1410 (1270 to 1580)	1290 (1170 to 1420)	1250 (1110 to 1420)	1310 (1190 to 1450)	1180 (997 to 1380)	1050 (905 to 1240)	1290 (1110 to 1480)	1320 (1130 to 1540)
Azad Jammu & Kashmir	2100 (1660 to 2560)	1760 (1450 to 2130)	1450 (1200 to 1730)	1300 (1080 to 1560)	1230 (979 to 1540)	1260 (1010 to 1560)	1080 (822 to 1410)	984 (734 to 1300)	1180 (900 to 1570)	1320 (988 to 1840)
Balochistan	1730 (1380 to 2120)	1490 (1200 to 1820)	1230 (1000 to 1500)	1170 (963 to 1410)	1250 (1000 to 1570)	1380 (1110 to 1680)	1300 (976 to 1660)	1180 (919 to 1520)	1500 (1210 to 1840)	1430 (1130 to 1790)

Gilgit-Baltistan	2090 (1530 to 2780)	1970 (1560 to 2460)	1650 (1360 to 1990)	1490 (1240 to 1780)	1420 (1100 to 1810)	1490 (1130 to 1920)	1290 (903 to 1870)	1160 (767 to 1760)	1590 (1090 to 2260)	1490 (1010 to 2180)
Islamabad Capital Territory	2070 (1650 to 2550)	1740 (1380 to 2140)	1250 (1030 to 1530)	985 (815 to 1190)	880 (705 to 1090)	932 (742 to 1170)	860 (648 to 1100)	807 (626 to 1000)	1030 (831 to 1260)	1070 (842 to 1350)
Khyber Pakhtunkhwa	2160 (1770 to 2590)	1740 (1430 to 2060)	1420 (1190 to 1670)	1240 (1040 to 1460)	1190 (953 to 1500)	1330 (1070 to 1600)	1220 (945 to 1550)	1070 (842 to 1350)	1360 (1120 to 1690)	1350 (1030 to 1760)
Punjab	2090 (1800 to 2430)	1740 (1510 to 2000)	1450 (1280 to 1630)	1340 (1200 to 1490)	1290 (1120 to 1480)	1330 (1180 to 1500)	1170 (983 to 1400)	1050 (892 to 1260)	1240 (1050 to 1460)	1320 (1110 to 1560)
Sindh	2070 (1670 to 2500)	1660 (1380 to 2000)	1340 (1120 to 1610)	1210 (1020 to 1430)	1210 (979 to 1480)	1280 (1050 to 1530)	1160 (913 to 1470)	1040 (832 to 1300)	1330 (1100 to 1600)	1310 (1050 to 1630)
Southeast Asia, east Asia, and Oceania	2150 (1940 to 2390)	3150 (2440 to 4370)	1440 (1280 to 1630)	1390 (1260 to 1530)	1170 (1080 to 1270)	1030 (963 to 1100)	856 (801 to 915)	706 (631 to 792)	706 (632 to 791)	727 (650 to 815)
East Asia	2100 (1840 to 2400)	3680 (2680 to 5410)	1380 (1170 to 1620)	1400 (1240 to 1580)	1190 (1070 to 1320)	1030 (945 to 1120)	820 (750 to 894)	656 (560 to 761)	652 (558 to 755)	644 (556 to 752)
China	2070 (1800 to 2370)	3770 (2730 to 5540)	1400 (1170 to 1640)	1420 (1250 to 1610)	1200 (1080 to 1340)	1030 (944 to 1130)	827 (754 to 903)	658 (560 to 767)	655 (558 to 761)	646 (555 to 758)
North Korea	4060 (2870 to 5910)	1250 (923 to 1710)	1100 (838 to 1410)	1040 (796 to 1330)	1020 (810 to 1240)	1450 (1060 to 2270)	985 (834 to 1150)	848 (684 to 1050)	842 (669 to 1060)	827 (648 to 1050)
Taiwan (province of China)	1660 (1420 to 1930)	1280 (1260 to 1290)	1050 (1040 to 1060)	879 (872 to 886)	746 (739 to 753)	627 (621 to 634)	495 (489 to 501)	449 (415 to 489)	428 (394 to 469)	442 (402 to 486)
Oceania	2270 (1920 to 2700)	1900 (1650 to 2180)	1640 (1430 to 1860)	1460 (1270 to 1680)	1400 (1190 to 1650)	1310 (1150 to 1470)	1220 (1080 to 1370)	1150 (985 to 1340)	1170 (994 to 1360)	1390 (1100 to 1750)
American Samoa	1360 (1170 to 1600)	1280 (1160 to 1420)	1250 (1120 to 1380)	1080 (977 to 1180)	1020 (934 to 1100)	1000 (921 to 1090)	998 (906 to 1100)	977 (856 to 1120)	1030 (906 to 1190)	971 (834 to 1140)
Cook Islands	2540 (2280 to 2830)	1740 (1570 to 1940)	1350 (1180 to 1520)	1270 (1150 to 1410)	1110 (992 to 1230)	906 (809 to 1010)	730 (651 to 819)	682 (581 to 792)	681 (574 to 804)	675 (562 to 807)
Fiji	1690 (1520 to 1870)	1330 (1200 to 1460)	1250 (1120 to 1410)	1180 (1070 to 1310)	1280 (1120 to 1480)	1450 (1320 to 1600)	1260 (1140 to 1390)	1200 (985 to 1450)	1210 (971 to 1490)	1460 (1110 to 1850)
Guam	974 (863 to 1100)	1140 (1050 to 1240)	1140 (1050 to 1240)	1070 (994 to 1160)	832 (774 to 898)	589 (548 to 637)	518 (479 to 560)	556 (506 to 615)	523 (480 to 571)	562 (496 to 636)
Kiribati	2470 (2110 to 2840)	2120 (1770 to 2480)	1890 (1550 to 2230)	1670 (1370 to 1980)	1700 (1470 to 1920)	1640 (1450 to 1850)	1550 (1320 to 1790)	1450 (1200 to 1730)	1440 (1190 to 1720)	1420 (1180 to 1720)
Marshall Islands	2330 (1660 to 3340)	1890 (1440 to 2500)	1630 (1310 to 2090)	1470 (1270 to 1700)	1580 (1420 to 1750)	1590 (1420 to 1790)	1550 (1370 to 1750)	1420 (1170 to 1760)	1410 (1140 to 1750)	1400 (1120 to 1740)
Federated States of Micronesia	2600 (2190 to 3030)	2090 (1710 to 2550)	1830 (1420 to 2330)	1620 (1310 to 2000)	1520 (1280 to 1790)	1420 (1230 to 1640)	1320 (1120 to 1560)	1260 (1020 to 1570)	1260 (1010 to 1580)	1250 (1000 to 1590)
Nauru	1840 (1530 to 2220)	1720 (1460 to 2020)	1700 (1460 to 1990)	1690 (1370 to 2090)	1740 (1480 to 2040)	1980 (1740 to 2260)	1920 (1650 to 2220)	1650 (1330 to 2020)	1630 (1300 to 2020)	1620 (1290 to 2010)
Niue	1930 (1650 to 2240)	1430 (1190 to 1700)	1250 (1040 to 1450)	1100 (909 to 1310)	1110 (936 to 1300)	1130 (957 to 1290)	1060 (900 to 1230)	1020 (843 to 1240)	1130 (953 to 1330)	1120 (942 to 1330)
Northern Mariana Islands	1350 (1110 to 1680)	1270 (1050 to 1560)	1130 (959 to 1320)	1030 (870 to 1260)	951 (807 to 1150)	851 (736 to 974)	834 (716 to 957)	828 (695 to 971)	740 (608 to 894)	940 (772 to 1080)
Palau	2190 (1780 to 2650)	1770 (1430 to 2140)	1520 (1230 to 1830)	1400 (1140 to 1670)	1290 (1070 to 1530)	1190 (1010 to 1390)	1200 (1020 to 1390)	1140 (945 to 1350)	1130 (930 to 1360)	1130 (926 to 1370)
Papua New Guinea	2450 (1950 to 3100)	2070 (1710 to 2470)	1750 (1440 to 2060)	1560 (1280 to 1890)	1460 (1150 to 1840)	1310 (1100 to 1550)	1260 (1070 to 1440)	1180 (956 to 1440)	1200 (967 to 1480)	1500 (1120 to 2000)
Samoa	1540 (1340 to 1750)	1050 (943 to 1160)	1180 (1070 to 1290)	1070 (956 to 1200)	1130 (980 to 1290)	1060 (929 to 1220)	991 (864 to 1140)	1030 (884 to 1210)	1000 (855 to 1180)	990 (843 to 1180)
Solomon Islands	2530 (2160 to 2920)	2220 (1850 to 2580)	1960 (1620 to 2270)	1710 (1430 to 2040)	1640 (1380 to 1960)	1500 (1270 to 1810)	1430 (1180 to 1730)	1340 (1080 to 1700)	1340 (1080 to 1720)	1330 (1060 to 1700)
Tokelau	1770 (1460 to 2120)	1550 (1240 to 1880)	1390 (1130 to 1660)	1320 (1110 to 1550)	1180 (999 to 1380)	1080 (929 to 1270)	964 (811 to 1160)	879 (718 to 1080)	1030 (880 to 1210)	1030 (871 to 1220)
Tonga	1570 (1310 to 1830)	1380 (1210 to 1550)	1250 (1080 to 1430)	1120 (985 to 1260)	930 (801 to 1080)	951 (832 to 1070)	885 (754 to 1030)	840 (692 to 1010)	835 (683 to 1010)	832 (677 to 1010)
Tuvalu	2580 (2110 to 3090)	2160 (1780 to 2610)	1950 (1620 to 2300)	1850 (1590 to 2140)	1660 (1450 to 1880)	1630 (1440 to 1860)	1270 (1100 to 1470)	1180 (995 to 1420)	1170 (979 to 1400)	1160 (966 to 1380)
Vanuatu	2590 (2190 to 3000)	2290 (1890 to 2750)	1930 (1570 to 2280)	1600 (1350 to 1900)	1460 (1230 to 1740)	1400 (1200 to 1620)	1310 (1170 to 1470)	1220 (1070 to 1400)	1220 (1080 to 1400)	1380 (1170 to 1650)
Southeast Asia	2320 (2110 to 2560)	1820 (1670 to 1990)	1610 (1490 to 1740)	1340 (1250 to 1440)	1150 (1080 to 1210)	1030 (961 to 1100)	927 (876 to 979)	817 (753 to 880)	827 (753 to 904)	942 (828 to 1060)
Cambodia	2540 (2070 to 3090)	2160 (1790 to 2580)	2380 (1990 to 2850)	1970 (1560 to 2470)	1660 (1430 to 1940)	1490 (1320 to 1670)	1170 (1020 to 1340)	1060 (856 to 1310)	1060 (844 to 1310)	1180 (927 to 1480)
Indonesia	2660 (2350 to 3000)	2150 (1890 to 2440)	1780 (1580 to 2010)	1440 (1310 to 1580)	1220 (1120 to 1340)	1100 (946 to 1280)	1060 (937 to 1180)	954 (806 to 1090)	1020 (857 to 1160)	1120 (899 to 1350)
Aceh	2200 (1820 to 2610)	1830 (1470 to 2270)	1620 (1300 to 1990)	1360 (1160 to 1590)	1200 (1040 to 1390)	1080 (857 to 1340)	1140 (956 to 1340)	1030 (814 to 1240)	1100 (862 to 1310)	1210 (923 to 1500)
Bali	2470 (2060 to 2910)	2040 (1630 to 2530)	1780 (1410 to 2210)	1490 (1260 to 1760)	1220 (1060 to 1410)	1090 (882 to 1350)	1000 (822 to 1170)	907 (715 to 1090)	960 (763 to 1150)	1060 (813 to 1310)
Bangka-Belitung Islands	2860 (2380 to 3390)	2400 (1910 to 3010)	2090 (1660 to 2540)	1760 (1480 to 2060)	1570 (1350 to 1810)	1410 (1130 to 1720)	1320 (1130 to 1510)	1180 (977 to 1390)	1240 (1020 to 1460)	1440 (1120 to 1790)
Banten	3290 (2770 to 3870)	2820 (2320 to 3480)	2240 (1830 to 2750)	1700 (1450 to 2000)	1310 (1150 to 1510)	1090 (882 to 1350)	1120 (941 to 1310)	1010 (794 to 1220)	1070 (849 to 1310)	1160 (900 to 1460)
Bengkulu	3370 (2870 to 3920)	2790 (2290 to 3390)	2290 (1870 to 2770)	1830 (1560 to 2130)	1500 (1300 to 1730)	1340 (1070 to 1630)	1240 (1050 to 1430)	1080 (890 to 1280)	1150 (949 to 1360)	1240 (991 to 1540)
Gorontalo	2790 (2360 to 3310)	2360 (1910 to 2930)	1980 (1620 to 2410)	1630 (1390 to 1900)	1380 (1200 to 1590)	1200 (984 to 1500)	1210 (1010 to 1410)	1060 (844 to 1310)	1130 (912 to 1390)	1240 (946 to 1560)
Jakarta	1960 (1630 to 2310)	1670 (1360 to 2080)	1360 (1100 to 1680)	1110 (961 to 1290)	1010 (873 to 1150)	995 (789 to 1240)	963 (787 to 1130)	862 (676 to 1030)	926 (725 to 1110)	1020 (779 to 1280)
Jambi	2990 (2540 to 3460)	2520 (2060 to 3090)	2190 (1770 to 2640)	1840 (1560 to 2110)	1500 (1290 to 1730)	1240 (988 to 1540)	1140 (955 to 1320)	1010 (806 to 1200)	1070 (850 to 1260)	1190 (902 to 1450)
West Java	2980 (2550 to 3460)	2420 (2010 to 2890)	1920 (1580 to 2280)	1470 (1280 to 1670)	1200 (1060 to 1350)	1030 (841 to 1240)	983 (837 to 1150)	900 (715 to 1100)	959 (768 to 1150)	1070 (821 to 1330)
Central Java	2320 (1960 to 2750)	1860 (1520 to 2280)	1550 (1270 to 1880)	1230 (1080 to 1410)	1020 (907 to 1150)	1010 (829 to 1240)	982 (820 to 1140)	890 (712 to 1050)	948 (770 to 1120)	1060 (811 to 1310)
East Java	2390 (2060 to 2770)	1960 (1620 to 2330)	1650 (1350 to 2000)	1360 (1200 to 1560)	1190 (1060 to 1350)	1070 (868 to 1320)	1000 (845 to 1170)	904 (734 to 1080)	968 (785 to 1160)	1060 (824 to 1320)
West Kalimantan	2600 (2190 to 3060)	2370 (1930 to 2860)	2140 (1760 to 2580)	1790 (1540 to 2060)	1480 (1280 to 1710)	1310 (1050 to 1620)	1180 (1010 to 1370)	1030 (859 to 1220)	1060 (905 to 1250)	1190 (962 to 1460)
South Kalimantan	3270 (2770 to 3800)	2830 (2330 to 3510)	2260 (1830 to 2750)	1770 (1530 to 2060)	1580 (1370 to 1810)	1500 (1200 to 1890)	1380 (1170 to 1600)	1210 (985 to 1440)	1300 (1060 to 1550)	1430 (1120 to 1780)
Central Kalimantan	2410 (2010 to 2850)	2010 (1600 to 2470)	1780 (1440 to 2180)	1550 (1290 to 1810)	1370 (1180 to 1600)	1140 (916 to 1410)	1160 (979 to 1340)	1050 (836 to 1250)	1130 (903 to 1340)	1250 (973 to 1560)
East Kalimantan	2080 (1720 to 2470)	1940 (1550 to 2410)	1680 (1350 to 2060)	1370 (1160 to 1610)	1290 (1100 to 1490)	1310 (1070 to 1610)	1230 (1070 to 1410)	1070 (897 to 1260)	1130 (955 to 1340)	1260 (998 to 1550)
North Kalimantan	1850 (1530 to 2240)	1750 (1430 to 2210)	1520 (1220 to 1870)	1190 (1020 to 1390)	954 (807 to 1130)	775 (602 to 995)	742 (607 to 898)	667 (510 to 845)	726 (570 to 910)	859 (626 to 1120)
Riau Islands	2190 (1840 to 2620)	1910 (1490 to 2380)	1660 (1330 to 2080)	1450 (1200 to 1720)	1270 (1080 to 1480)	949 (771 to 1160)	940 (780 to 1090)	872 (679 to 1050)	927 (724 to 1120)	1030 (768 to 1290)
Lampung	2560 (2140 to 3060)	2190 (1770 to 2720)	1780 (1440 to 2160)	1400 (1200 to 1630)	1180 (1030 to 1370)	983 (797 to 1230)	952 (793 to 1140)	872 (694 to 1090)	925 (734 to 1160)	1040 (799 to 1330)
Maluku	3570 (3050 to 4130)	2770 (2260 to 3410)	2320 (1870 to 2840)	1940 (1650 to 2290)	1650 (1420 to 1910)	1400 (1120 to 1740)	1420 (1180 to 1660)	1210 (978 to 1460)	1270 (1020 to 1510)	1350 (1070 to 1650)
North Maluku	3410 (2890 to 4000)	2840 (2310 to 3460)	2480 (2070 to 3050)	2180 (1890 to 2510)	1730 (1490 to 2000)	1450 (1160 to 1800)	1400 (1190 to 1650)	1210 (960 to 1450)	1290 (1030 to 1550)	1400 (1080 to 1740)
West Nusa Tenggara	3540 (3040 to 4070)	3030 (2490 to 3630)	2610 (2150 to 3210)	2100 (1810 to 2410)	1690 (1470 to 1930)	1430 (1150 to 1780)	1240 (1040 to 1450)	1110 (872 to 1320)	1170 (917 to 1400)	1240 (965 to 1520)
East Nusa Tenggara	3260 (2780 to 3740)	2590 (2090 to 3150)	2200 (1780 to 2660)	1790 (1540 to 2070)	1510 (1310 to 1730)	1290 (1040 to 1610)	1150 (954 to 1360)	1030 (794 to 1260)	1080 (852 to 1300)	1210 (907 to 1500)
Papua	1980 (1630 to 2350)	1920 (1560 to 2430)	1800 (1450 to 2220)	1610 (1380 to 1870)	1490 (1290 to 1720)	1430 (1150 to 1770)	1460 (1220 to 1710)	1250 (997 to 1530)	1320 (1030 to 1640)	1410 (1060 to 1760)
West Papua	2090 (1730 to 2460)	1950 (1550 to 2420)	1830 (1470 to 2250)	1590 (1350 to 1860)	1390 (1190 to 1600)	1250 (1000 to 1560)	1400 (1200 to 1610)	1090 (891 to 1310)	1160 (954 to 1390)	1250 (994 to 1550)
Riau	2310 (1920 to 2720)	2090 (1700 to 2610)	1790 (1440 to 2250)	1430 (1220 to 1650)	1240 (1070 to 1440)	1110 (892 to 1390)	1050 (873 to 1230)	944 (741 to 1130)	1020 (805 to 1230)	1140 (854 to 1420)
West Sulawesi	3900 (3290 to 4600)	3150 (2570 to 3830)	2700 (2200 to 3280)	2220 (1900 to 2570)	1850 (1600 to 2130)	1550 (1230 to 1920)	1360 (1140 to 1570)	1170 (908 to 1430)	1240 (976 to 1530)	1390 (1060 to 1750)
South Sulawesi	2900 (2450 to 3360)	2390 (1970 to 2930)	2000 (1610 to 2470)	1630 (1400 to 1900)	1340 (1170 to 1530)	1150 (910 to 1440)	1100 (910 to 1280)	978 (769 to 1180)	1040 (817 to 1240)	1110 (859 to 1360)
Central Sulawesi	3260 (2790 to 3810)	2630 (2130 to 3220)	2180 (1760 to 2670)	1780 (1520 to 2070)	1650 (1430 to 1900)	1500 (1210 to 1860)	1340 (1130 to 1550)	1170 (930 to 1390)	1230 (982 to 1480)	1390 (1060 to 1730)
Southeast Sulawesi	3120 (2640 to 3610)	2750 (2210 to 3350)	2390 (1950 to 2890)	1870 (1590 to 2160)	1550 (1340 to 1790)	1450 (1180 to 1800)	1350 (1150 to 1560)	1190 (932 to 1450)	1270 (993 to 1540)	1370 (1060 to 1710)
North Sulawesi	2450 (2040 to 2870)	2040 (1650 to 2510)	1800 (1440 to 2250)	1550 (1320 to 1790)	1420 (1250 to 1620)	1280 (1030 to 1590)	1200 (1020 to 1370)	1060 (847 to 1260)	1140 (913 to 1380)	1240 (955 to 1530)
West Sumatra	3690 (3160 to 4320)	2450 (2030 to 3010)	1930 (1550 to 2350)	1490 (1270 to 1720)	1270 (1110 to 1460)	1190 (950 to 1460)	1170 (990 to 1340)	997 (813 to 1180)	1060 (876 to 1250)	1150 (910 to 1420)
South Sumatra	2520 (2130 to 2990)	2030 (1670 to 2490)	1660 (1360 to 2010)	1280 (1100 to 1490)	1150 (1010 to 1320)	1030 (820 to 1250)	995 (822 to 1180)	906 (710 to 1130)	970 (772 to 1220)	1070 (823 to 1360)

North Sumatra	2540 (2140 to 2970)	2060 (1670 to 2530)	1750 (1420 to 2190)	1470 (1250 to 1700)	1320 (1150 to 1530)	1290 (1040 to 1580)	1230 (1040 to 1420)	1100 (868 to 1340)	1160 (911 to 1390)	1250 (961 to 1550)
Yogyakarta	2530 (2110 to 2960)	1830 (1480 to 2280)	1470 (1160 to 1830)	1210 (1020 to 1450)	1110 (971 to 1270)	1040 (843 to 1270)	992 (854 to 1140)	926 (779 to 1080)	975 (820 to 1130)	1110 (883 to 1360)
Laos	3170 (2310 to 4430)	2970 (2260 to 3880)	2750 (2220 to 3450)	2340 (1950 to 2800)	1960 (1640 to 2330)	1620 (1390 to 1890)	1230 (1040 to 1440)	1050 (844 to 1270)	1040 (825 to 1260)	1150 (911 to 1420)
Malaysia	1510 (1250 to 1820)	1080 (1010 to 1150)	874 (818 to 937)	870 (726 to 1030)	843 (784 to 911)	832 (777 to 888)	808 (724 to 893)	740 (654 to 842)	656 (575 to 747)	883 (767 to 1020)
Maldives	3330 (2780 to 3940)	2750 (2310 to 3200)	2090 (1840 to 2390)	1650 (1510 to 1820)	1180 (1090 to 1280)	835 (770 to 910)	573 (518 to 642)	466 (406 to 530)	462 (400 to 530)	522 (430 to 622)
Mauritius	1890 (1750 to 2030)	1630 (1500 to 1780)	1300 (1190 to 1410)	1170 (1070 to 1290)	1000 (934 to 1080)	857 (792 to 930)	734 (677 to 796)	715 (627 to 822)	686 (606 to 783)	791 (676 to 926)
Myanmar	3440 (2870 to 4090)	2840 (2340 to 3400)	2500 (2080 to 2980)	2100 (1760 to 2480)	1800 (1480 to 2170)	1600 (1300 to 1950)	1230 (1050 to 1440)	1020 (842 to 1230)	1050 (862 to 1280)	1160 (922 to 1440)
Philippines	1300 (1160 to 1480)	1190 (1090 to 1290)	1240 (1140 to 1340)	1100 (1020 to 1180)	1020 (937 to 1100)	907 (832 to 988)	911 (839 to 988)	874 (789 to 979)	851 (758 to 951)	1160 (1020 to 1340)
Seychelles	1410 (1270 to 1570)	1190 (1090 to 1290)	1130 (1030 to 1230)	840 (767 to 925)	950 (863 to 1050)	862 (789 to 945)	828 (762 to 898)	822 (722 to 949)	715 (617 to 830)	835 (716 to 972)
Sri Lanka	1580 (1470 to 1700)	1290 (1180 to 1410)	1300 (1200 to 1400)	1170 (1090 to 1260)	1010 (892 to 1160)	870 (762 to 1000)	754 (697 to 816)	608 (461 to 785)	554 (326 to 784)	663 (454 to 886)
Thailand	1730 (1510 to 1980)	1220 (1100 to 1350)	1120 (1020 to 1230)	1040 (943 to 1150)	859 (775 to 950)	817 (726 to 922)	636 (573 to 702)	567 (482 to 665)	567 (463 to 690)	624 (480 to 792)
Timor-Leste	2360 (1860 to 2940)	2120 (1810 to 2490)	2010 (1660 to 2410)	1920 (1670 to 2240)	1400 (1230 to 1600)	1120 (975 to 1260)	981 (866 to 1100)	977 (808 to 1170)	967 (791 to 1170)	1120 (892 to 1400)
Viet Nam	2180 (1780 to 2660)	1510 (1220 to 1870)	1570 (1310 to 1880)	1130 (945 to 1370)	980 (846 to 1170)	863 (772 to 977)	843 (757 to 937)	769 (655 to 879)	764 (645 to 875)	802 (667 to 939)
Sub-Saharan Africa	2590 (2260 to 2970)	2360 (2090 to 2610)	2080 (1900 to 2270)	1840 (1720 to 1970)	1750 (1660 to 1840)	1840 (1740 to 1950)	1500 (1410 to 1590)	1220 (1120 to 1340)	1410 (1290 to 1530)	1570 (1440 to 1710)
Central sub-Saharan Africa	2890 (2420 to 3410)	2520 (2110 to 2950)	2280 (1960 to 2620)	2050 (1780 to 2350)	1900 (1680 to 2150)	1920 (1670 to 2210)	1610 (1330 to 1950)	1360 (1050 to 1780)	1550 (1200 to 2000)	1710 (1320 to 2200)
Angola	2660 (2190 to 3150)	2510 (2150 to 2940)	2380 (1990 to 2810)	2280 (1850 to 2760)	2150 (1690 to 2710)	1930 (1610 to 2310)	1480 (1260 to 1740)	1280 (1040 to 1590)	1450 (1190 to 1780)	1710 (1400 to 2090)
Central African Republic	2910 (2230 to 3840)	2700 (2160 to 3370)	2490 (2090 to 2920)	2380 (2060 to 2720)	2470 (2180 to 2770)	2880 (2460 to 3360)	2450 (1990 to 2920)	2160 (1650 to 2740)	2620 (2030 to 3300)	2570 (1990 to 3270)
Congo (Brazzaville)	3410 (2610 to 4430)	3000 (2480 to 3680)	2660 (2230 to 3110)	2180 (1840 to 2550)	2030 (1730 to 2360)	2120 (1850 to 2430)	1570 (1360 to 1810)	1400 (1130 to 1700)	1610 (1320 to 1950)	1700 (1370 to 2060)
DR Congo	2930 (2420 to 3480)	2470 (2000 to 3010)	2210 (1860 to 2600)	1970 (1660 to 2300)	1800 (1540 to 2090)	1850 (1540 to 2240)	1610 (1260 to 2090)	1350 (946 to 1950)	1530 (1090 to 2170)	1680 (1180 to 2400)
Equatorial Guinea	3870 (3280 to 4560)	3140 (2590 to 3760)	2610 (2100 to 3160)	2170 (1800 to 2580)	2130 (1740 to 2550)	1650 (1290 to 2100)	1290 (1010 to 1680)	1250 (964 to 1630)	1580 (1250 to 1990)	1620 (1250 to 2100)
Gabon	3430 (2750 to 4100)	2530 (2120 to 2990)	2100 (1670 to 2560)	1640 (1320 to 2020)	1570 (1350 to 1820)	1670 (1420 to 1960)	1420 (1180 to 1680)	1250 (991 to 1530)	1460 (1200 to 1750)	1540 (1260 to 1840)
Eastern sub-Saharan Africa	2850 (2480 to 3260)	2500 (2240 to 2790)	2260 (2070 to 2480)	2030 (1900 to 2180)	2010 (1900 to 2120)	2000 (1880 to 2140)	1470 (1380 to 1560)	1200 (1090 to 1320)	1380 (1260 to 1520)	1610 (1470 to 1770)
Burundi	3140 (2320 to 4230)	2750 (2180 to 3450)	2560 (2120 to 3080)	2320 (1990 to 2690)	2270 (1960 to 2590)	2540 (2150 to 2970)	1480 (1300 to 1700)	1270 (1030 to 1540)	1390 (1110 to 1700)	1530 (1240 to 1850)
Comoros	2490 (1990 to 3150)	2310 (1940 to 2750)	2030 (1690 to 2410)	1830 (1550 to 2160)	1570 (1320 to 1840)	1380 (1180 to 1600)	1130 (949 to 1340)	1030 (815 to 1340)	1100 (866 to 1400)	1230 (976 to 1580)
Djibouti	1620 (1190 to 2260)	1580 (1200 to 2080)	1470 (1170 to 1820)	1340 (1100 to 1610)	1390 (1120 to 1680)	1450 (1150 to 1810)	1310 (1040 to 1670)	1140 (871 to 1510)	1400 (1100 to 1790)	1460 (1140 to 1850)
Eritrea	3200 (2340 to 4560)	2910 (2240 to 3810)	2960 (2240 to 4550)	2780 (2450 to 3160)	2500 (2180 to 2860)	1980 (1570 to 2430)	1680 (1250 to 2260)	1470 (1050 to 2090)	1500 (1070 to 2120)	1730 (1210 to 2470)
Ethiopia	3340 (2470 to 4700)	2960 (2330 to 3770)	2810 (2370 to 3360)	2680 (2390 to 2970)	2510 (2240 to 2770)	2070 (1840 to 2340)	1250 (1140 to 1390)	992 (835 to 1170)	1180 (992 to 1400)	1380 (1170 to 1660)
Addis Ababa	2320 (1680 to 3340)	2260 (1680 to 3020)	2120 (1640 to 2750)	1980 (1660 to 2380)	2080 (1770 to 2430)	2160 (1760 to 2620)	1220 (1060 to 1420)	1000 (791 to 1280)	1340 (1050 to 1700)	1690 (1330 to 2160)
Afar	5460 (3890 to 7710)	4280 (3170 to 5780)	4060 (3120 to 5210)	4090 (3460 to 4840)	3710 (3160 to 4310)	3140 (2630 to 3740)	1930 (1640 to 2250)	1510 (1150 to 1960)	1880 (1440 to 2410)	2290 (1790 to 2900)
Amhara	3260 (2380 to 4690)	2840 (2170 to 3680)	2680 (2140 to 3330)	2530 (2160 to 2940)	2480 (2130 to 2820)	2160 (1850 to 2530)	1250 (1090 to 1420)	960 (772 to 1200)	1090 (870 to 1340)	1220 (989 to 1510)
Benishangul-Gumuz	4910 (3630 to 6960)	4010 (3020 to 5240)	3870 (3010 to 4930)	3780 (3150 to 4460)	3410 (2930 to 3990)	2960 (2460 to 3530)	1790 (1520 to 2100)	1270 (968 to 1640)	1260 (937 to 1660)	1260 (941 to 1700)
Dire Dawa	2820 (2030 to 3950)	2790 (2040 to 3760)	2660 (2030 to 3470)	2440 (2020 to 2960)	2290 (1910 to 2720)	1990 (1590 to 2450)	1110 (932 to 1300)	899 (696 to 1150)	1230 (959 to 1580)	1580 (1220 to 2040)
Gambella	3390 (2420 to 4920)	2810 (2130 to 3770)	2690 (2060 to 3530)	2630 (2180 to 3150)	2560 (2150 to 3020)	2310 (1880 to 2850)	1510 (1260 to 1790)	1100 (852 to 1400)	1510 (1180 to 1910)	1950 (1560 to 2480)
Harari	3660 (2630 to 5180)	3510 (2620 to 4760)	3190 (2450 to 4170)	2860 (2330 to 3450)	2620 (2170 to 3130)	2110 (1710 to 2600)	1170 (981 to 1420)	946 (736 to 1250)	1350 (1060 to 1750)	1790 (1400 to 2320)
Oromia	3410 (2510 to 4780)	3000 (2300 to 4000)	2850 (2270 to 3600)	2710 (2330 to 3130)	2490 (2160 to 2860)	1920 (1640 to 2250)	1140 (987 to 1310)	912 (738 to 1120)	1110 (902 to 1380)	1330 (1070 to 1640)
Somali	3010 (2170 to 4350)	2680 (1980 to 3660)	2570 (1940 to 3370)	2420 (2010 to 2910)	2230 (1880 to 2610)	2030 (1670 to 2440)	1430 (1210 to 1670)	1210 (929 to 1560)	1560 (1210 to 2030)	1930 (1510 to 2500)
Southern Nations, Nationalities, and Peoples	3310 (2420 to 4750)	3060 (2330 to 4060)	2930 (2330 to 3660)	2820 (2430 to 3250)	2570 (2220 to 2970)	2020 (1690 to 2480)	1380 (1170 to 1600)	1100 (853 to 1390)	1290 (1010 to 1660)	1500 (1160 to 1920)
Tigray	3750 (2700 to 5400)	3300 (2500 to 4380)	3170 (2420 to 4040)	3090 (2600 to 3610)	2670 (2260 to 3080)	2160 (1790 to 2600)	1180 (1010 to 1390)	956 (743 to 1260)	1130 (881 to 1470)	1330 (1020 to 1710)
Kenya	2230 (1980 to 2520)	1820 (1600 to 2060)	1550 (1370 to 1790)	1300 (1160 to 1450)	1280 (1150 to 1430)	1730 (1520 to 1960)	1430 (1310 to 1570)	1130 (986 to 1290)	1390 (1220 to 1570)	1500 (1310 to 1690)
Baringo	2380 (1960 to 2860)	2140 (1670 to 2750)	2010 (1630 to 2470)	1770 (1430 to 2160)	1610 (1300 to 1930)	1660 (1380 to 1990)	1470 (1210 to 1760)	1180 (941 to 1530)	1400 (1120 to 1760)	1500 (1190 to 1850)
Bomet	1180 (957 to 1440)	1050 (819 to 1340)	974 (788 to 1200)	882 (711 to 1080)	916 (750 to 1120)	1440 (1100 to 1850)	1220 (994 to 1480)	948 (776 to 1190)	1170 (967 to 1400)	1270 (1060 to 1510)
Bungoma	2470 (2050 to 2990)	1930 (1500 to 2450)	1590 (1310 to 1970)	1350 (1110 to 1640)	1220 (1000 to 1470)	1400 (1160 to 1690)	1110 (919 to 1310)	951 (772 to 1200)	1120 (923 to 1380)	1210 (991 to 1510)
Busia	3740 (3080 to 4450)	2640 (2060 to 3290)	2030 (1640 to 2480)	1480 (1200 to 1800)	1480 (1190 to 1820)	2100 (1630 to 2630)	1480 (1190 to 1820)	1110 (884 to 1410)	1380 (1120 to 1710)	1530 (1250 to 1880)
Elgeyo Marakwet	1880 (1540 to 2310)	1680 (1300 to 2160)	1640 (1320 to 2020)	1490 (1200 to 1810)	1330 (1060 to 1670)	1510 (1220 to 1840)	1350 (1140 to 1610)	1180 (967 to 1460)	1340 (1090 to 1630)	1390 (1140 to 1720)
Embu	2470 (2050 to 3010)	1870 (1490 to 2370)	1380 (1120 to 1720)	1040 (843 to 1280)	959 (794 to 1180)	1190 (920 to 1520)	1110 (886 to 1370)	953 (756 to 1230)	1130 (932 to 1400)	1200 (993 to 1490)
Garissa	2120 (1710 to 2600)	1930 (1470 to 2530)	1720 (1360 to 2190)	1590 (1260 to 1950)	1500 (1190 to 1870)	1600 (1260 to 2030)	1480 (1190 to 1800)	1250 (1020 to 1520)	1670 (1400 to 1980)	1850 (1530 to 2200)
Homa Bay	3860 (3270 to 4570)	2780 (2230 to 3430)	2420 (2020 to 2890)	1930 (1600 to 2290)	1880 (1520 to 2320)	3550 (2780 to 4400)	2550 (2080 to 3140)	1710 (1290 to 2190)	2010 (1570 to 2540)	2080 (1660 to 2620)
Isiolo	3190 (2610 to 3840)	2920 (2300 to 3680)	2620 (2170 to 3180)	2060 (1690 to 2470)	1700 (1400 to 2060)	1770 (1360 to 2240)	1560 (1240 to 1920)	1240 (977 to 1590)	1560 (1270 to 1910)	1700 (1390 to 2060)
Kajiado	1670 (1350 to 2050)	1500 (1140 to 1940)	1410 (1130 to 1790)	1240 (990 to 1590)	1190 (936 to 1500)	1520 (1210 to 1850)	1330 (1100 to 1590)	1110 (893 to 1350)	1440 (1200 to 1730)	1570 (1310 to 1870)
Kakamega	2620 (2150 to 3130)	2050 (1640 to 2570)	1710 (1400 to 2090)	1400 (1160 to 1680)	1450 (1210 to 1750)	1950 (1560 to 2400)	1440 (1200 to 1730)	1070 (870 to 1350)	1320 (1100 to 1620)	1450 (1210 to 1750)
Kericho	1260 (976 to 1570)	1220 (910 to 1630)	1130 (879 to 1410)	1030 (818 to 1270)	1020 (811 to 1270)	1370 (1060 to 1740)	1170 (923 to 1480)	983 (774 to 1260)	1170 (939 to 1460)	1240 (995 to 1540)
Kiambu	1900 (1520 to 2320)	1620 (1230 to 2090)	1400 (1100 to 1740)	1280 (1020 to 1570)	1320 (1060 to 1600)	1720 (1390 to 2090)	1460 (1230 to 1730)	1200 (945 to 1470)	1500 (1230 to 1780)	1620 (1330 to 1930)
Kilifi	2760 (2300 to 3280)	2190 (1760 to 2730)	1840 (1520 to 2240)	1480 (1220 to 1790)	1310 (1090 to 1580)	1520 (1220 to 1880)	1280 (1050 to 1540)	1010 (805 to 1290)	1260 (1030 to 1580)	1380 (1130 to 1690)
Kirinyaga	2500 (2060 to 3000)	1980 (1540 to 2590)	1610 (1280 to 2000)	1120 (899 to 1420)	1090 (875 to 1360)	1390 (1080 to 1730)	1250 (1010 to 1580)	1080 (838 to 1370)	1270 (1020 to 1580)	1350 (1100 to 1650)
Kisii	2250 (1810 to 2730)	2070 (1620 to 2650)	1830 (1470 to 2270)	1590 (1290 to 1970)	1580 (1300 to 1940)	1950 (1610 to 2350)	1590 (1340 to 1870)	1310 (1030 to 1600)	1560 (1260 to 1880)	1660 (1330 to 2020)
Kisumu	2350 (1930 to 2870)	1950 (1520 to 2450)	1740 (1420 to 2140)	1540 (1270 to 1870)	1650 (1350 to 2000)	2830 (2280 to 3580)	2190 (1770 to 2670)	1630 (1220 to 2070)	2030 (1620 to 2490)	2160 (1750 to 2630)
Kitui	2590 (2150 to 3110)	1980 (1560 to 2490)	1620 (1320 to 1990)	1270 (1030 to 1550)	1160 (960 to 1430)	1520 (1200 to 1900)	1260 (1030 to 1540)	1010 (812 to 1260)	1180 (978 to 1470)	1260 (1020 to 1520)
Kwale	2860 (2370 to 3460)	2400 (1870 to 3030)	1950 (1590 to 2420)	1570 (1270 to 1900)	1410 (1140 to 1750)	1590 (1250 to 2020)	1480 (1200 to 1800)	1180 (922 to 1490)	1410 (1100 to 1760)	1510 (1200 to 1860)
Laikipia	1450 (1140 to 1790)	1250 (945 to 1620)	1090 (861 to 1380)	920 (732 to 1160)	993 (792 to 1250)	1470 (1160 to 1920)	1290 (1030 to 1600)	1030 (810 to 1280)	1270 (1030 to 1570)	1370 (1120 to 1670)
Lamu	3220 (2660 to 3940)	2400 (1860 to 3100)	1860 (1490 to 2310)	1280 (1010 to 1590)	1270 (994 to 1600)	1450 (1110 to 1880)	1280 (1020 to 1600)	1060 (827 to 1380)	1330 (1070 to 1650)	1450 (1160 to 1810)
Machakos	1740 (1400 to 2120)	1470 (1130 to 1920)	1250 (1010 to 1540)	1010 (820 to 1230)	937 (783 to 1140)	1330 (1050 to 1690)	1180 (972 to 1420)	978 (787 to 1220)	1170 (971 to 1430)	1260 (1040 to 1520)
Makueni	2160 (1760 to 2620)	1530 (1200 to 1910)	1280 (1050 to 1570)	1050 (844 to 1280)	1020 (846 to 1250)	1840 (1360 to 2460)	1470 (1190 to 1790)	1020 (836 to 1260)	1200 (1010 to 1450)	1260 (1060 to 1500)
Mandera	1930 (1570 to 2360)	1780 (1360 to 2260)	1670 (1320 to 2080)	1580 (1260 to 1960)	1610 (1290 to 2030)	1740 (1370 to 2170)	1690 (1360 to 2030)	1400 (1080 to 1730)	2040 (1660 to 2450)	2360 (1930 to 2820)
Marsabit	1950 (1560 to 2440)	1840 (1420 to 2330)	1610 (1290 to 2020)	1360 (1090 to 1700)	1240 (1010 to 1530)	1380 (1070 to 1760)	1370 (1090 to 1700)	1150 (896 to 1460)	1400 (1140 to 1750)	1510 (1230 to 1890)

Meru	1710 (1400 to 2080)	1430 (1100 to 1810)	1210 (993 to 1490)	997 (817 to 1240)	974 (811 to 1170)	1280 (1020 to 1640)	1230 (1030 to 1510)	1040 (842 to 1320)	1260 (1050 to 1570)	1370 (1140 to 1670)
Migori	3130 (2620 to 3740)	2420 (1920 to 3020)	2080 (1730 to 2510)	1840 (1540 to 2200)	1770 (1450 to 2140)	2660 (2070 to 3380)	2250 (1780 to 2800)	1560 (1180 to 2050)	1930 (1510 to 2440)	2060 (1650 to 2580)
Mombasa	1890 (1510 to 2350)	1710 (1330 to 2220)	1380 (1100 to 1750)	1080 (865 to 1350)	1130 (899 to 1420)	1650 (1280 to 2100)	1330 (1080 to 1640)	1010 (798 to 1300)	1410 (1160 to 1730)	1580 (1310 to 1920)
Murang'a	2160 (1800 to 2570)	1560 (1220 to 1960)	1280 (1040 to 1580)	1050 (849 to 1300)	1060 (849 to 1290)	1390 (1110 to 1720)	1310 (1080 to 1560)	1040 (820 to 1300)	1230 (987 to 1520)	1310 (1070 to 1590)
Nairobi	2080 (1700 to 2500)	1700 (1340 to 2100)	1310 (1080 to 1600)	1080 (898 to 1310)	1200 (973 to 1450)	1800 (1480 to 2170)	1470 (1260 to 1690)	1180 (989 to 1400)	1610 (1370 to 1870)	1780 (1530 to 2060)
Nakuru	1300 (1020 to 1630)	1230 (944 to 1560)	1140 (910 to 1450)	1040 (839 to 1290)	1070 (857 to 1330)	1490 (1210 to 1830)	1180 (978 to 1420)	937 (766 to 1140)	1200 (1000 to 1430)	1310 (1090 to 1530)
Nandi	1720 (1380 to 2130)	1510 (1160 to 1950)	1360 (1090 to 1740)	1230 (991 to 1550)	1240 (1000 to 1520)	1620 (1330 to 1960)	1330 (1120 to 1580)	1070 (860 to 1320)	1270 (1040 to 1560)	1350 (1100 to 1610)
Narok	1970 (1580 to 2390)	1750 (1340 to 2240)	1650 (1310 to 2060)	1410 (1110 to 1740)	1480 (1180 to 1840)	1820 (1450 to 2220)	1490 (1240 to 1800)	1200 (969 to 1480)	1540 (1270 to 1840)	1690 (1400 to 2000)
Nyamira	2060 (1640 to 2540)	1750 (1350 to 2230)	1570 (1230 to 1970)	1420 (1120 to 1740)	1440 (1180 to 1750)	2070 (1690 to 2520)	2020 (1700 to 2410)	1620 (1250 to 2040)	1900 (1490 to 2340)	1990 (1560 to 2470)
Nyandarua	2180 (1750 to 2680)	1830 (1390 to 2390)	1530 (1190 to 1920)	1320 (1020 to 1670)	1330 (1060 to 1590)	1720 (1380 to 2100)	1720 (1430 to 2060)	1440 (1130 to 1760)	1710 (1350 to 2070)	1810 (1470 to 2170)
Nyeri	2210 (1790 to 2710)	1660 (1280 to 2100)	1360 (1070 to 1730)	1180 (926 to 1480)	1230 (975 to 1490)	1680 (1330 to 2060)	1430 (1150 to 1720)	1190 (904 to 1480)	1430 (1140 to 1760)	1530 (1230 to 1850)
Samburu	1390 (1090 to 1740)	1330 (1000 to 1730)	1270 (1010 to 1610)	1200 (962 to 1490)	1250 (979 to 1560)	1620 (1280 to 2000)	1470 (1180 to 1850)	1200 (948 to 1540)	1500 (1220 to 1880)	1640 (1340 to 2030)
Siaya	2710 (2190 to 3260)	2180 (1690 to 2810)	1790 (1470 to 2190)	1520 (1240 to 1840)	1630 (1330 to 1980)	2610 (2070 to 3340)	1750 (1430 to 2140)	1200 (973 to 1480)	1420 (1160 to 1740)	1530 (1260 to 1830)
Taita Taveta	2200 (1760 to 2690)	1820 (1400 to 2370)	1650 (1330 to 2080)	1260 (1010 to 1590)	1180 (926 to 1500)	1620 (1250 to 2060)	1580 (1250 to 1940)	1200 (914 to 1510)	1460 (1180 to 1800)	1580 (1260 to 1910)
Tana River	2630 (2120 to 3230)	2170 (1640 to 2850)	1960 (1540 to 2450)	1770 (1400 to 2230)	1730 (1350 to 2150)	2180 (1760 to 2650)	1990 (1660 to 2390)	1580 (1260 to 1940)	1890 (1510 to 2290)	2020 (1620 to 2460)
Tharaka Nithi	2500 (2050 to 3040)	1820 (1370 to 2340)	1640 (1290 to 2060)	1450 (1140 to 1800)	1440 (1160 to 1780)	1740 (1400 to 2130)	1430 (1200 to 1700)	1260 (997 to 1540)	1370 (1100 to 1700)	1380 (1120 to 1720)
Trans Nzoia	1380 (1070 to 1750)	1290 (985 to 1660)	1180 (926 to 1460)	1050 (851 to 1300)	1050 (841 to 1310)	1500 (1160 to 1920)	1310 (1050 to 1600)	1040 (822 to 1310)	1260 (1020 to 1560)	1350 (1100 to 1660)
Turkana	1610 (1270 to 2000)	1580 (1200 to 2060)	1590 (1260 to 1980)	1560 (1270 to 1940)	1530 (1220 to 1930)	2210 (1640 to 2980)	1750 (1380 to 2170)	1270 (960 to 1630)	1680 (1350 to 2090)	1870 (1520 to 2300)
Uasin Gishu	1210 (938 to 1550)	1150 (863 to 1520)	1060 (829 to 1360)	970 (781 to 1210)	1010 (816 to 1270)	1400 (1120 to 1710)	1200 (971 to 1450)	952 (770 to 1240)	1190 (985 to 1470)	1300 (1070 to 1580)
Vihiga	2540 (2120 to 3050)	1710 (1320 to 2190)	1460 (1190 to 1840)	1290 (1060 to 1590)	1340 (1080 to 1670)	1780 (1420 to 2220)	1480 (1200 to 1790)	1180 (902 to 1510)	1360 (1090 to 1690)	1460 (1160 to 1830)
Wajir	2640 (2150 to 3200)	2360 (1830 to 3010)	2050 (1650 to 2540)	1810 (1480 to 2210)	1690 (1360 to 2100)	1770 (1440 to 2160)	1680 (1360 to 2060)	1430 (1080 to 1780)	1770 (1420 to 2180)	1930 (1550 to 2350)
West Pokot	1850 (1480 to 2290)	1660 (1270 to 2140)	1620 (1300 to 2020)	1540 (1240 to 1900)	1470 (1190 to 1820)	1500 (1210 to 1850)	1380 (1120 to 1700)	1150 (899 to 1470)	1410 (1130 to 1770)	1510 (1190 to 1890)
Madagascar	2650 (2300 to 3030)	2250 (1930 to 2650)	1960 (1710 to 2250)	1690 (1480 to 1930)	1720 (1530 to 1920)	1570 (1370 to 1800)	1410 (1140 to 1720)	1270 (1010 to 1560)	1530 (1250 to 1830)	1610 (1320 to 1930)
Malawi	3150 (2600 to 3750)	2860 (2390 to 3420)	2460 (2130 to 2840)	1970 (1740 to 2200)	2040 (1830 to 2270)	2520 (2210 to 2880)	1730 (1540 to 1960)	1380 (1190 to 1600)	1510 (1300 to 1740)	2010 (1760 to 2290)
Mozambique	2610 (2140 to 3150)	2350 (1990 to 2750)	2120 (1820 to 2440)	1930 (1700 to 2200)	1900 (1670 to 2190)	1890 (1680 to 2130)	1960 (1700 to 2250)	1630 (1340 to 1930)	1710 (1400 to 2040)	2120 (1770 to 2480)
Rwanda	3500 (2980 to 4060)	2710 (2250 to 3230)	2590 (2180 to 3040)	2250 (1950 to 2560)	2250 (2030 to 2500)	2230 (1980 to 2490)	1220 (1070 to 1400)	1050 (854 to 1290)	1090 (880 to 1350)	1400 (1150 to 1700)
Somalia	2750 (2280 to 3250)	2720 (2220 to 3310)	2590 (2160 to 3050)	2320 (1930 to 2760)	2340 (1970 to 2730)	2240 (1820 to 2730)	2460 (1910 to 3210)	1850 (1350 to 2590)	2330 (1710 to 3300)	2600 (1900 to 3620)
South Sudan	2230 (1790 to 2770)	2180 (1800 to 2640)	2090 (1670 to 2600)	1920 (1570 to 2310)	1890 (1550 to 2290)	1760 (1430 to 2180)	1550 (1270 to 1920)	1640 (1280 to 2100)	1930 (1550 to 2440)	1990 (1570 to 2560)
Uganda	2720 (2240 to 3250)	2210 (1900 to 2570)	1940 (1680 to 2290)	1970 (1660 to 2360)	2140 (1840 to 2460)	2200 (1930 to 2470)	1470 (1280 to 1670)	1150 (970 to 1390)	1290 (1080 to 1540)	1610 (1320 to 1940)
Tanzania	2810 (2330 to 3370)	2500 (2100 to 2920)	2120 (1820 to 2490)	1670 (1460 to 1920)	1680 (1480 to 1880)	1840 (1630 to 2050)	1360 (1210 to 1520)	1100 (922 to 1340)	1260 (1060 to 1520)	1450 (1230 to 1730)
Zambia	2470 (2020 to 2990)	2190 (1780 to 2710)	1820 (1540 to 2150)	1670 (1450 to 1890)	2000 (1780 to 2250)	2680 (2360 to 3000)	1730 (1520 to 1970)	1420 (1130 to 1780)	1650 (1340 to 2050)	2150 (1730 to 2680)
Southern sub-Saharan Africa	2030 (1820 to 2270)	1810 (1650 to 1980)	1600 (1480 to 1740)	1390 (1250 to 1560)	1150 (1040 to 1250)	1820 (1700 to 1960)	1770 (1660 to 1880)	1250 (1120 to 1400)	1560 (1400 to 1750)	1800 (1620 to 2030)
Botswana	2420 (2010 to 2890)	2250 (1870 to 2680)	2040 (1700 to 2460)	1660 (1380 to 1950)	1480 (1190 to 1780)	2450 (1960 to 3010)	1650 (1430 to 1880)	1180 (994 to 1380)	1250 (1040 to 1490)	1910 (1590 to 2290)
Eswatini	3110 (2610 to 3650)	2810 (2330 to 3330)	2230 (1830 to 2650)	1760 (1460 to 2100)	1520 (1290 to 1780)	2560 (2100 to 3080)	2760 (2320 to 3250)	1870 (1490 to 2300)	2280 (1890 to 2750)	2940 (2370 to 3520)
Lesotho	2340 (1960 to 2750)	2090 (1750 to 2490)	1920 (1610 to 2300)	1540 (1300 to 1830)	1390 (1190 to 1640)	2400 (2000 to 2840)	2560 (2210 to 2910)	2290 (1900 to 2680)	2580 (2170 to 2990)	3340 (2790 to 3930)
Namibia	2110 (1710 to 2560)	1870 (1520 to 2240)	1670 (1410 to 1970)	1610 (1350 to 1900)	1430 (1260 to 1610)	2050 (1800 to 2350)	1550 (1340 to 1790)	1310 (1050 to 1620)	1450 (1180 to 1770)	1970 (1620 to 2350)
South Africa	1970 (1780 to 2170)	1760 (1620 to 1930)	1560 (1440 to 1710)	1360 (1190 to 1580)	1080 (966 to 1200)	1620 (1500 to 1760)	1660 (1540 to 1800)	1150 (1010 to 1310)	1470 (1300 to 1680)	1640 (1450 to 1880)
Eastern Cape	2110 (1790 to 2470)	1670 (1440 to 1940)	1450 (1230 to 1680)	1190 (951 to 1460)	1030 (877 to 1200)	1870 (1680 to 2090)	1970 (1780 to 2190)	1480 (1240 to 1780)	2190 (1830 to 2620)	1960 (1630 to 2380)
Free State	1910 (1630 to 2230)	1740 (1490 to 2000)	1540 (1310 to 1790)	1360 (1090 to 1690)	1190 (1020 to 1390)	1950 (1740 to 2180)	2240 (1990 to 2480)	1360 (1130 to 1620)	1740 (1440 to 2080)	1930 (1580 to 2340)
Gauteng	2140 (1840 to 2470)	1990 (1720 to 2290)	1840 (1580 to 2140)	1740 (1420 to 2140)	1270 (1070 to 1500)	1550 (1400 to 1730)	1480 (1340 to 1640)	1030 (860 to 1240)	1270 (1070 to 1520)	1440 (1200 to 1720)
KwaZulu-Natal	1910 (1660 to 2210)	1770 (1520 to 2030)	1550 (1350 to 1790)	1340 (1090 to 1630)	1160 (987 to 1350)	1950 (1740 to 2160)	1770 (1590 to 1950)	984 (827 to 1170)	1380 (1140 to 1650)	1560 (1300 to 1860)
Limpopo	1540 (1300 to 1830)	1350 (1150 to 1580)	1180 (1010 to 1380)	978 (775 to 1220)	716 (605 to 842)	1130 (1000 to 1280)	1630 (1460 to 1800)	1240 (1030 to 1460)	1460 (1210 to 1770)	1880 (1580 to 2260)
Mpumalanga	1820 (1550 to 2140)	1650 (1410 to 1910)	1480 (1280 to 1730)	1250 (1000 to 1530)	920 (775 to 1090)	1530 (1370 to 1710)	1670 (1500 to 1860)	1050 (879 to 1270)	1250 (1030 to 1490)	1610 (1320 to 1930)
North West	1770 (1510 to 2080)	1620 (1380 to 1870)	1390 (1190 to 1610)	1200 (968 to 1500)	826 (696 to 988)	1700 (1500 to 1930)	2210 (1970 to 2490)	1830 (1510 to 2220)	1940 (1600 to 2420)	2250 (1860 to 2790)
Northern Cape	1570 (1310 to 1830)	1430 (1230 to 1670)	1290 (1100 to 1510)	1110 (897 to 1400)	1120 (952 to 1320)	1950 (1750 to 2210)	1920 (1710 to 2190)	1340 (1110 to 1630)	1610 (1330 to 1980)	2010 (1640 to 2470)
Western Cape	1700 (1450 to 1980)	1570 (1340 to 1840)	1480 (1270 to 1730)	1460 (1180 to 1820)	1230 (1040 to 1460)	1270 (1150 to 1430)	1080 (976 to 1200)	928 (781 to 1120)	1220 (1010 to 1470)	1340 (1110 to 1640)
Zimbabwe	2110 (1530 to 2940)	1860 (1380 to 2410)	1670 (1330 to 2050)	1490 (1280 to 1710)	1390 (1180 to 1620)	2620 (2190 to 3070)	2260 (2030 to 2530)	1730 (1430 to 2060)	1980 (1660 to 2360)	2600 (2160 to 3060)
Western sub-Saharan Africa	2460 (2040 to 2990)	2360 (2010 to 2770)	2030 (1800 to 2320)	1750 (1590 to 1920)	1660 (1540 to 1790)	1680 (1550 to 1840)	1410 (1300 to 1540)	1190 (1080 to 1330)	1340 (1200 to 1490)	1420 (1290 to 1590)
Benin	2660 (1930 to 3610)	2350 (1880 to 2970)	2060 (1730 to 2470)	1790 (1570 to 2050)	1610 (1440 to 1800)	1520 (1350 to 1700)	1310 (1160 to 1480)	1150 (976 to 1360)	1220 (1040 to 1460)	1400 (1190 to 1670)
Burkina Faso	3150 (2240 to 4500)	2820 (2160 to 3660)	2390 (1980 to 2900)	2040 (1780 to 2350)	1900 (1660 to 2170)	1840 (1610 to 2110)	1540 (1360 to 1740)	1330 (1160 to 1540)	1520 (1340 to 1770)	1600 (1390 to 1850)
Cabo Verde	1820 (1590 to 2070)	1480 (1340 to 1630)	1390 (1250 to 1550)	969 (868 to 1080)	849 (756 to 949)	847 (713 to 1020)	689 (598 to 784)	809 (671 to 963)	843 (688 to 1010)	842 (681 to 1030)
Cameroon	2630 (1990 to 3440)	4210 (3480 to 5080)	1950 (1610 to 2310)	1710 (1460 to 1980)	1510 (1330 to 1700)	1810 (1580 to 2080)	1590 (1340 to 1900)	1290 (1010 to 1670)	1430 (1130 to 1850)	1650 (1320 to 2090)
Chad	2540 (1740 to 3690)	2160 (1580 to 3030)	2080 (1610 to 2710)	1910 (1590 to 2280)	1740 (1550 to 1950)	1900 (1640 to 2180)	1640 (1440 to 1890)	1460 (1160 to 1820)	1740 (1410 to 2130)	1690 (1350 to 2080)
Côte d'Ivoire	2350 (1990 to 2710)	2230 (1860 to 2690)	1880 (1600 to 2220)	1550 (1350 to 1800)	1660 (1450 to 1900)	1970 (1730 to 2220)	1600 (1410 to 1810)	1230 (1030 to 1470)	1410 (1190 to 1680)	1420 (1190 to 1720)
The Gambia	1970 (1590 to 2400)	1960 (1620 to 2340)	1760 (1470 to 2080)	1630 (1360 to 1940)	1470 (1240 to 1750)	1420 (1230 to 1650)	1340 (1130 to 1550)	1250 (1010 to 1510)	1520 (1250 to 1810)	1530 (1240 to 1840)
Ghana	2250 (1920 to 2620)	1940 (1670 to 2270)	1710 (1470 to 2020)	1540 (1300 to 1830)	1480 (1260 to 1750)	1490 (1310 to 1690)	1400 (1230 to 1580)	1170 (949 to 1460)	1280 (1040 to 1580)	1400 (1140 to 1710)
Guinea	2710 (2230 to 3230)	2510 (2060 to 3070)	2310 (1930 to 2760)	2030 (1740 to 2370)	1780 (1540 to 2050)	1720 (1530 to 1960)	1560 (1320 to 1810)	1330 (1080 to 1650)	1600 (1310 to 1960)	1650 (1350 to 2030)
Guinea-Bissau	3970 (3400 to 4590)	3260 (2770 to 3750)	3000 (2500 to 3560)	2480 (2120 to 2910)	2250 (1940 to 2600)	2100 (1760 to 2470)	1830 (1580 to 2080)	1630 (1320 to 1990)	2090 (1740 to 2510)	2020 (1630 to 2440)
Liberia	3230 (2570 to 4020)	2550 (2160 to 2960)	2210 (1880 to 2620)	1840 (1590 to 2150)	1950 (1690 to 2230)	1710 (1480 to 1980)	1400 (1220 to 1630)	1220 (967 to 1550)	1420 (1140 to 1780)	1440 (1150 to 1840)
Mali	3110 (2580 to 3670)	2880 (2370 to 3470)	2670 (2290 to 3160)	2230 (1950 to 2560)	1930 (1720 to 2150)	1720 (1550 to 1910)	1460 (1300 to 1630)	1290 (1080 to 1540)	1560 (1310 to 1880)	1730 (1450 to 2070)
Mauritania	2280 (1760 to 3110)	1990 (1600 to 2480)	1700 (1400 to 2060)	1620 (1380 to 1890)	1490 (1300 to 1680)	1210 (1070 to 1380)	995 (851 to 1170)	916 (741 to 1150)	1090 (900 to 1340)	1080 (870 to 1340)
Niger	2630 (1980 to 3550)	2560 (2010 to 3230)	2350 (1990 to 2780)	2220 (1970 to 2510)	2020 (1830 to 2250)	1780 (1590 to 1990)	1380 (1170 to 1620)	1230 (998 to 1520)	1420 (1170 to 1740)	1460 (1190 to 1800)
Nigeria	2330 (1700 to 3420)	2130 (1600 to 2870)	1960 (1580 to 2470)	1660 (1410 to 1970)	1630 (1430 to 1860)	1650 (1410 to 1940)	1350 (1160 to 1570)	1130 (971 to 1330)	1250 (1070 to 1450)	1320 (1150 to 1530)
São Tomé and Príncipe	2860 (2560 to 3220)	2190 (1990 to 2410)	1480 (1350 to 1610)	1210 (1110 to 1320)	1230 (1100 to 1360)	1210 (1070 to 1370)	1060 (914 to 1200)	958 (823 to 1110)	1040 (903 to 1200)	1060 (913 to 1220)

Senegal	2340<br>(1750 to 3130)	2210<br>(1770 to 2710)	2160<br>(1830 to 2520)	1840<br>(1610 to 2100)	1500<br>(1350 to 1680)	1400<br>(1250 to 1550)	1140<br>(1010 to 1290)	1040<br>(843 to 1290)	1210<br>(992 to 1490)	1340<br>(1090 to 1620)
Sierra Leone	2800<br>(2170 to 3550)	2510<br>(2060 to 3030)	2240<br>(1870 to 2680)	2010<br>(1700 to 2370)	1840<br>(1590 to 2120)	1880<br>(1680 to 2150)	1670<br>(1440 to 1930)	1340<br>(1090 to 1640)	1530<br>(1260 to 1890)	1490<br>(1230 to 1840)
Togo	2540<br>(2050 to 3130)	2280<br>(1880 to 2710)	1900<br>(1620 to 2240)	1610<br>(1410 to 1850)	1530<br>(1310 to 1780)	1670<br>(1400 to 1960)	1540<br>(1330 to 1770)	1260<br>(1000 to 1600)	1350<br>(1070 to 1710)	1460<br>(1170 to 1860)

Appendix 2 Table S3b: Age-standardised mortality rates (per 100 000) with 95% UIs by location, females, 1950, 1960, 1970, 1980, 1990, 2000, 2010, 2019, 2020, 2021

Location	1950	1960	1970	1980	1990	2000	2010	2019	2020	2021
Global	1760 (1640 to 1870)	1870 (1660 to 2220)	1270 (1210 to 1330)	1110 (1070 to 1160)	953 (925 to 985)	866 (841 to 891)	717 (695 to 740)	615 (582 to 650)	652 (619 to 688)	681 (646 to 720)
Central Europe, eastern Europe, and central Asia	1190 (1100 to 1290)	909 (877 to 942)	856 (830 to 884)	854 (825 to 882)	801 (774 to 830)	849 (820 to 881)	700 (678 to 723)	578 (544 to 616)	649 (609 to 694)	737 (688 to 792)
Central Asia	1700 (1450 to 2000)	1220 (1120 to 1330)	996 (941 to 1060)	872 (838 to 906)	824 (796 to 852)	882 (840 to 929)	774 (729 to 825)	651 (596 to 710)	760 (693 to 834)	785 (701 to 873)
Armenia	1630 (1370 to 1940)	1130 (984 to 1280)	864 (762 to 974)	716 (648 to 791)	751 (696 to 814)	773 (720 to 840)	670 (617 to 725)	527 (467 to 600)	692 (611 to 793)	592 (510 to 689)
Azerbaijan	2320 (1950 to 2740)	1450 (1280 to 1630)	1090 (989 to 1190)	911 (835 to 1000)	848 (776 to 921)	854 (781 to 934)	808 (739 to 883)	696 (605 to 808)	854 (740 to 986)	850 (722 to 994)
Georgia	1440 (1190 to 1710)	1050 (939 to 1180)	844 (764 to 934)	770 (704 to 844)	768 (708 to 831)	744 (654 to 835)	576 (525 to 635)	523 (471 to 580)	626 (564 to 695)	729 (649 to 822)
Kazakhstan	1320 (1090 to 1590)	1080 (956 to 1220)	923 (840 to 1020)	805 (741 to 878)	815 (754 to 884)	973 (898 to 1060)	860 (830 to 891)	686 (628 to 751)	816 (737 to 905)	899 (798 to 1010)
Kyrgyzstan	1830 (1520 to 2170)	1450 (1250 to 1680)	1170 (1020 to 1340)	950 (859 to 1060)	856 (780 to 941)	865 (785 to 946)	771 (702 to 844)	586 (505 to 687)	703 (589 to 831)	674 (552 to 814)
Mongolia	2530 (2080 to 3060)	1890 (1590 to 2230)	1600 (1400 to 1810)	1260 (1110 to 1420)	1130 (1020 to 1240)	1140 (1030 to 1250)	933 (842 to 1030)	709 (630 to 797)	673 (606 to 747)	767 (690 to 862)
Tajikistan	2350 (1950 to 2820)	1670 (1470 to 1920)	1200 (1030 to 1370)	1030 (918 to 1150)	948 (853 to 1050)	980 (889 to 1080)	922 (829 to 1030)	701 (595 to 836)	851 (711 to 1030)	890 (724 to 1080)
Turkmenistan	1930 (1620 to 2290)	1410 (1260 to 1570)	1150 (1040 to 1260)	1010 (921 to 1100)	940 (868 to 1010)	914 (802 to 1050)	756 (683 to 841)	764 (631 to 919)	882 (731 to 1060)	912 (746 to 1100)
Uzbekistan	1660 (1370 to 2020)	1160 (1020 to 1310)	980 (898 to 1070)	885 (816 to 960)	758 (702 to 817)	856 (738 to 990)	797 (656 to 1000)	677 (531 to 879)	741 (566 to 999)	726 (542 to 1010)
Central Europe	1430 (1380 to 1490)	995 (966 to 1030)	908 (880 to 936)	852 (825 to 881)	791 (766 to 817)	698 (676 to 723)	565 (555 to 575)	494 (475 to 514)	547 (525 to 570)	604 (578 to 633)
Albania	1590 (1390 to 1820)	996 (896 to 1100)	810 (677 to 952)	722 (609 to 845)	665 (614 to 719)	552 (504 to 606)	488 (426 to 558)	530 (456 to 617)	587 (496 to 690)	619 (522 to 738)
Bosnia and Herzegovina	1940 (1780 to 2120)	1350 (1240 to 1460)	954 (885 to 1030)	834 (775 to 900)	670 (619 to 728)	588 (502 to 683)	513 (469 to 563)	530 (476 to 596)	590 (525 to 665)	614 (510 to 742)
Bulgaria	1280 (1180 to 1380)	866 (787 to 955)	827 (747 to 915)	881 (802 to 982)	1010 (926 to 1110)	986 (904 to 1070)	784 (758 to 810)	700 (633 to 768)	778 (703 to 860)	951 (860 to 1060)
Croatia	2100 (1950 to 2280)	1380 (1280 to 1490)	1030 (950 to 1120)	781 (725 to 843)	762 (706 to 824)	635 (576 to 702)	530 (491 to 571)	456 (403 to 516)	490 (435 to 559)	530 (469 to 606)
Czechia	1180 (1100 to 1270)	894 (831 to 966)	911 (851 to 975)	853 (793 to 919)	753 (698 to 817)	604 (592 to 616)	478 (468 to 489)	418 (382 to 456)	462 (423 to 507)	481 (438 to 530)
Hungary	1290 (1210 to 1400)	1040 (972 to 1120)	944 (878 to 1020)	918 (856 to 979)	841 (787 to 906)	709 (658 to 766)	584 (543 to 635)	518 (462 to 587)	556 (491 to 637)	605 (534 to 689)
Montenegro	1010 (928 to 1110)	672 (620 to 732)	596 (548 to 651)	647 (594 to 703)	564 (517 to 615)	648 (592 to 715)	623 (570 to 680)	708 (621 to 809)	789 (696 to 912)	924 (807 to 1070)
North Macedonia	1730 (1580 to 1910)	1390 (1280 to 1510)	1090 (1010 to 1180)	804 (744 to 876)	857 (779 to 943)	1130 (1040 to 1230)	1060 (943 to 1210)	865 (728 to 1040)	1000 (822 to 1220)	1080 (883 to 1330)
Poland	1360 (1270 to 1450)	897 (840 to 960)	808 (755 to 867)	756 (697 to 821)	721 (667 to 780)	605 (559 to 658)	485 (479 to 491)	431 (399 to 468)	485 (443 to 528)	530 (482 to 586)
Romania	1410 (1190 to 1680)	1070 (973 to 1170)	989 (898 to 1090)	985 (903 to 1080)	910 (841 to 984)	798 (735 to 866)	643 (596 to 692)	538 (478 to 605)	590 (523 to 673)	680 (595 to 777)
Serbia	1980 (1850 to 2120)	1280 (1190 to 1380)	1110 (1020 to 1200)	1000 (934 to 1080)	1030 (953 to 1120)	1030 (952 to 1120)	727 (674 to 784)	626 (560 to 700)	697 (614 to 792)	740 (654 to 851)
Slovakia	1200 (1170 to 1220)	842 (823 to 862)	866 (848 to 885)	790 (772 to 807)	726 (710 to 742)	604 (589 to 619)	532 (519 to 546)	471 (430 to 517)	508 (461 to 558)	607 (548 to 675)
Slovenia	1220 (1130 to 1310)	915 (842 to 993)	834 (768 to 900)	782 (718 to 851)	593 (531 to 664)	504 (485 to 522)	388 (371 to 405)	327 (295 to 361)	370 (335 to 408)	351 (316 to 391)
Eastern Europe	952 (826 to 1090)	793 (751 to 837)	784 (747 to 824)	831 (787 to 877)	781 (741 to 828)	902 (852 to 956)	748 (710 to 793)	597 (539 to 667)	671 (600 to 757)	790 (703 to 895)
Belarus	833 (663 to 1030)	661 (608 to 719)	645 (602 to 692)	708 (658 to 764)	707 (655 to 766)	764 (708 to 826)	666 (615 to 722)	557 (492 to 636)	661 (561 to 781)	752 (615 to 915)
Estonia	1010 (822 to 1230)	850 (820 to 879)	804 (776 to 832)	802 (775 to 829)	770 (744 to 796)	693 (668 to 718)	476 (455 to 498)	396 (358 to 439)	397 (360 to 438)	464 (414 to 518)
Latvia	876 (690 to 1080)	797 (738 to 869)	770 (714 to 830)	796 (738 to 861)	775 (717 to 837)	694 (646 to 747)	585 (564 to 604)	499 (454 to 546)	514 (470 to 567)	607 (550 to 673)
Lithuania	948 (756 to 1170)	769 (711 to 828)	698 (651 to 749)	718 (664 to 773)	694 (642 to 752)	618 (574 to 668)	568 (528 to 618)	470 (432 to 512)	514 (469 to 560)	571 (515 to 631)
Moldova	1720 (1440 to 2040)	1510 (1350 to 1680)	1230 (1130 to 1340)	1050 (974 to 1130)	952 (886 to 1030)	856 (778 to 942)	727 (653 to 813)	546 (454 to 669)	600 (511 to 717)	664 (556 to 816)
Russia	955 (778 to 1160)	811 (752 to 872)	801 (747 to 857)	849 (785 to 915)	785 (729 to 851)	914 (844 to 992)	762 (711 to 817)	590 (522 to 669)	690 (606 to 790)	819 (722 to 944)
Ukraine	913 (743 to 1110)	742 (687 to 801)	764 (718 to 816)	809 (751 to 878)	782 (728 to 844)	952 (886 to 1030)	764 (678 to 868)	665 (509 to 851)	664 (505 to 881)	769 (538 to 1040)
High income	1070 (1040 to 1100)	876 (856 to 898)	777 (759 to 795)	629 (614 to 645)	525 (513 to 537)	450 (440 to 461)	374 (365 to 382)	342 (333 to 352)	366 (357 to 376)	366 (356 to 377)
Australasia	918 (868 to 973)	804 (757 to 858)	777 (733 to 828)	605 (567 to 644)	514 (483 to 549)	416 (391 to 443)	348 (327 to 371)	312 (291 to 334)	291 (274 to 311)	301 (284 to 319)
Australia	914 (856 to 982)	800 (743 to 863)	778 (726 to 839)	591 (548 to 637)	504 (469 to 545)	407 (378 to 439)	340 (318 to 367)	304 (280 to 330)	285 (265 to 307)	294 (275 to 315)
New Zealand	943 (880 to 1010)	818 (758 to 884)	775 (721 to 833)	670 (624 to 723)	567 (525 to 612)	464 (430 to 501)	390 (355 to 430)	355 (329 to 385)	326 (299 to 352)	341 (312 to 373)
High-income Asia Pacific	1510 (1430 to 1610)	1090 (1030 to 1160)	864 (814 to 918)	637 (601 to 677)	459 (433 to 487)	342 (324 to 364)	275 (260 to 293)	237 (223 to 252)	228 (217 to 242)	234 (221 to 250)
Brunei	2110 (1910 to 2340)	1550 (1410 to 1710)	1090 (975 to 1210)	896 (794 to 1000)	894 (802 to 993)	769 (694 to 853)	668 (603 to 738)	640 (549 to 745)	575 (491 to 674)	591 (502 to 698)
Japan	1340 (1260 to 1440)	984 (923 to 1050)	770 (719 to 829)	561 (524 to 601)	411 (385 to 442)	312 (292 to 335)	263 (245 to 284)	232 (215 to 250)	223 (209 to 239)	227 (211 to 245)
Aichi	1350 (1110 to 1650)	1000 (801 to 1240)	789 (633 to 973)	583 (531 to 643)	430 (393 to 474)	325 (296 to 357)	266 (241 to 295)	232 (209 to 259)	223 (204 to 247)	228 (206 to 254)
Akita	1380 (1120 to 1680)	1040 (838 to 1280)	833 (674 to 1000)	599 (540 to 662)	428 (389 to 474)	324 (293 to 360)	274 (247 to 307)	254 (224 to 288)	228 (204 to 255)	261 (229 to 293)
Aomori	1420 (1170 to 1720)	1030 (805 to 1280)	792 (635 to 968)	574 (520 to 636)	428 (386 to 474)	336 (304 to 371)	287 (256 to 319)	265 (233 to 300)	258 (233 to 290)	263 (232 to 297)
Chiba	1370 (1110 to 1650)	1020 (814 to 1250)	774 (621 to 967)	566 (514 to 627)	402 (363 to 448)	317 (288 to 352)	268 (242 to 298)	234 (210 to 262)	226 (204 to 252)	226 (201 to 254)
Ehime	1310 (1060 to 1610)	949 (751 to 1180)	725 (587 to 882)	528 (475 to 581)	399 (361 to 439)	315 (284 to 348)	258 (232 to 288)	237 (210 to 268)	233 (209 to 264)	228 (201 to 259)
Fukui	1220 (963 to 1490)	894 (720 to 1100)	708 (578 to 856)	531 (479 to 591)	395 (354 to 441)	288 (258 to 322)	258 (230 to 288)	216 (190 to 243)	216 (193 to 241)	222 (195 to 252)
Fukuoka	1310 (1060 to 1610)	956 (764 to 1190)	761 (614 to 967)	552 (501 to 611)	404 (364 to 451)	311 (283 to 346)	259 (234 to 290)	229 (204 to 258)	221 (200 to 246)	225 (202 to 252)
Fukushima	1480 (1220 to 1790)	1060 (867 to 1300)	835 (683 to 1020)	579 (522 to 645)	409 (369 to 454)	320 (288 to 354)	275 (248 to 307)	248 (220 to 279)	248 (223 to 277)	249 (219 to 281)
Gifu	1390 (1140 to 1680)	1050 (851 to 1290)	847 (689 to 1030)	618 (560 to 682)	428 (387 to 473)	324 (291 to 359)	266 (240 to 295)	235 (208 to 264)	223 (201 to 248)	229 (203 to 259)
Gunma	1430 (1160 to 1740)	1060 (858 to 1300)	826 (677 to 1010)	610 (551 to 672)	425 (384 to 471)	317 (286 to 352)	274 (248 to 306)	243 (215 to 273)	231 (206 to 258)	241 (213 to 271)
Hiroshima	1360 (1110 to 1660)	975 (776 to 1210)	748 (598 to 935)	551 (500 to 609)	406 (368 to 450)	295 (267 to 328)	250 (225 to 279)	221 (195 to 249)	208 (188 to 230)	213 (189 to 243)
Hokkaidō	1360 (1100 to 1660)	989 (787 to 1220)	787 (638 to 981)	578 (526 to 636)	409 (373 to 449)	303 (275 to 334)	263 (238 to 292)	242 (218 to 268)	239 (217 to 263)	244 (218 to 273)
Hyōgo	1320 (1070 to 1600)	976 (777 to 1200)	778 (623 to 975)	577 (522 to 637)	430 (390 to 478)	324 (294 to 358)	267 (241 to 297)	223 (199 to 248)	216 (194 to 240)	219 (196 to 246)
Ibaraki	1520 (1240 to 1840)	1090 (872 to 1340)	836 (670 to 1040)	585 (532 to 641)	437 (396 to 483)	331 (300 to 364)	281 (254 to 311)	248 (223 to 276)	236 (213 to 260)	244 (218 to 272)
Ishikawa	1240 (979 to 1530)	946 (761 to 1170)	740 (608 to 898)	589 (532 to 657)	400 (362 to 442)	310 (281 to 343)	254 (228 to 283)	226 (200 to 255)	217 (195 to 242)	223 (197 to 252)
Iwate	1460 (1190 to 1780)	1050 (851 to 1280)	812 (663 to 978)	571 (514 to 633)	417 (378 to 461)	310 (278 to 343)	270 (243 to 301)	254 (226 to 286)	232 (208 to 258)	247 (219 to 278)
Kagawa	1280 (1050 to 1570)	939 (753 to 1170)	715 (593 to 867)	517 (468 to 577)	390 (352 to 436)	315 (283 to 350)	266 (239 to 298)	236 (208 to 269)	211 (189 to 237)	227 (201 to 261)
Kagoshima	1510 (1240 to 1800)	1080 (874 to 1320)	852 (700 to 1030)	585 (529 to 643)	417 (376 to 459)	312 (281 to 346)	267 (239 to 297)	237 (208 to 267)	228 (205 to 253)	228 (200 to 257)
Kanagawa	1250 (1010 to 1520)	902 (719 to 1130)	701 (557 to 876)	533 (485 to 588)	403 (366 to 443)	310 (283 to 343)	257 (233 to 284)	224 (200 to 249)	216 (197 to 239)	220 (197 to 246)
Kōchi	1430 (1170 to 1730)	1030 (833 to 1280)	800 (668 to 960)	556 (503 to 615)	401 (361 to 443)	308 (276 to 341)	258 (230 to 286)	237 (207 to 268)	211 (187 to 237)	228 (200 to 259)
Kumamoto	1390 (1130 to 1680)	1020 (817 to 1230)	796 (648 to 970)	552 (501 to 611)	383 (348 to 423)	287 (261 to 319)	246 (222 to 275)	209 (185 to 237)	207 (185 to 231)	211 (186 to 239)

Kyōto	1290 (1040 to 1570)	940 (757 to 1160)	728 (573 to 912)	537 (484 to 594)	410 (368 to 454)	305 (276 to 339)	254 (228 to 283)	216 (190 to 243)	206 (185 to 230)	210 (186 to 237)
Mie	1340 (1100 to 1620)	992 (799 to 1230)	764 (614 to 934)	557 (502 to 621)	419 (378 to 466)	314 (284 to 349)	264 (237 to 296)	230 (200 to 259)	222 (198 to 248)	227 (199 to 257)
Miyagi	1380 (1140 to 1660)	1020 (816 to 1240)	797 (644 to 984)	586 (532 to 652)	414 (375 to 460)	309 (278 to 342)	259 (233 to 288)	226 (200 to 255)	225 (204 to 251)	226 (202 to 256)
Miyazaki	1400 (1150 to 1710)	1020 (820 to 1250)	794 (655 to 964)	564 (511 to 621)	394 (356 to 437)	297 (269 to 330)	259 (235 to 287)	231 (204 to 261)	219 (197 to 243)	240 (212 to 274)
Nagano	1370 (1130 to 1680)	991 (790 to 1220)	757 (611 to 927)	540 (488 to 594)	387 (346 to 430)	291 (263 to 322)	243 (218 to 270)	218 (192 to 246)	210 (189 to 232)	210 (185 to 238)
Nagasaki	1360 (1110 to 1650)	987 (797 to 1220)	791 (648 to 960)	570 (517 to 631)	405 (367 to 449)	306 (276 to 338)	262 (237 to 293)	236 (210 to 269)	227 (204 to 255)	234 (207 to 265)
Nara	1330 (1090 to 1610)	997 (798 to 1230)	794 (649 to 959)	590 (535 to 654)	437 (396 to 485)	314 (283 to 350)	264 (238 to 293)	236 (208 to 266)	212 (190 to 237)	220 (195 to 249)
Niigata	1380 (1140 to 1660)	1020 (814 to 1240)	807 (650 to 1000)	585 (533 to 648)	400 (359 to 444)	292 (264 to 326)	245 (221 to 274)	234 (208 to 266)	228 (205 to 255)	229 (202 to 259)
Ōita	1400 (1150 to 1690)	1030 (831 to 1270)	805 (669 to 972)	567 (512 to 627)	409 (368 to 458)	312 (280 to 347)	249 (224 to 279)	226 (199 to 258)	229 (206 to 255)	203 (179 to 228)
Okayama	1300 (1070 to 1560)	959 (768 to 1190)	731 (590 to 895)	531 (480 to 588)	386 (350 to 429)	293 (266 to 325)	248 (222 to 275)	220 (195 to 249)	214 (193 to 238)	205 (182 to 232)
Okinawa	1420 (1160 to 1720)	956 (735 to 1200)	761 (619 to 922)	492 (444 to 548)	334 (302 to 371)	273 (246 to 303)	249 (223 to 278)	232 (206 to 262)	217 (196 to 243)	229 (202 to 256)
Ōsaka	1380 (1130 to 1680)	1010 (819 to 1260)	802 (643 to 987)	605 (549 to 668)	456 (415 to 502)	334 (303 to 368)	279 (252 to 308)	237 (213 to 263)	231 (210 to 254)	232 (208 to 260)
Saga	1350 (1080 to 1650)	967 (776 to 1200)	746 (615 to 903)	537 (485 to 594)	395 (358 to 439)	304 (273 to 339)	259 (234 to 288)	236 (207 to 268)	229 (204 to 259)	221 (193 to 250)
Saitama	1380 (1100 to 1660)	1010 (809 to 1240)	778 (625 to 955)	577 (524 to 636)	425 (385 to 469)	321 (291 to 354)	274 (248 to 303)	238 (213 to 264)	233 (210 to 258)	233 (209 to 260)
Shiga	1380 (1130 to 1640)	1040 (846 to 1270)	832 (682 to 1000)	590 (533 to 653)	431 (391 to 475)	301 (270 to 332)	253 (228 to 281)	219 (194 to 248)	216 (194 to 240)	205 (181 to 230)
Shimane	1280 (1030 to 1610)	921 (745 to 1130)	733 (605 to 882)	506 (454 to 566)	358 (321 to 398)	270 (244 to 301)	248 (223 to 278)	220 (193 to 253)	196 (174 to 221)	207 (180 to 238)
Shizuoka	1270 (1010 to 1550)	923 (733 to 1140)	725 (582 to 897)	532 (481 to 593)	394 (355 to 437)	302 (274 to 336)	261 (236 to 291)	232 (208 to 260)	226 (203 to 250)	227 (200 to 255)
Tochigi	1450 (1180 to 1760)	1070 (851 to 1310)	822 (678 to 1010)	607 (550 to 669)	447 (404 to 492)	332 (300 to 367)	286 (258 to 315)	255 (225 to 288)	240 (217 to 266)	245 (217 to 275)
Tokushima	1400 (1140 to 1690)	1030 (824 to 1260)	811 (667 to 972)	592 (538 to 653)	416 (377 to 461)	311 (280 to 343)	288 (259 to 320)	251 (220 to 285)	212 (187 to 237)	256 (224 to 289)
Tōkyō	1230 (1000 to 1470)	909 (741 to 1120)	726 (591 to 896)	536 (490 to 590)	415 (378 to 458)	324 (296 to 356)	262 (239 to 289)	222 (199 to 246)	216 (196 to 239)	218 (196 to 242)
Tottori	1280 (1030 to 1570)	962 (782 to 1160)	756 (632 to 906)	536 (481 to 593)	399 (356 to 441)	300 (271 to 331)	260 (232 to 289)	262 (229 to 297)	236 (210 to 264)	229 (199 to 262)
Toyama	1330 (1090 to 1620)	978 (773 to 1210)	756 (617 to 914)	546 (494 to 610)	388 (350 to 431)	292 (263 to 323)	248 (224 to 276)	212 (187 to 240)	210 (187 to 236)	218 (193 to 249)
Wakayama	1400 (1150 to 1690)	1040 (847 to 1270)	819 (678 to 983)	598 (538 to 660)	434 (390 to 479)	327 (292 to 365)	282 (253 to 313)	248 (219 to 281)	246 (218 to 275)	235 (205 to 266)
Yamagata	1420 (1150 to 1730)	1060 (852 to 1290)	840 (684 to 1010)	571 (513 to 633)	405 (364 to 450)	315 (284 to 351)	257 (231 to 287)	245 (215 to 278)	216 (194 to 240)	241 (211 to 273)
Yamaguchi	1310 (1060 to 1600)	956 (770 to 1180)	739 (598 to 904)	554 (502 to 613)	398 (360 to 442)	314 (284 to 350)	272 (244 to 303)	239 (210 to 270)	228 (204 to 256)	231 (207 to 262)
Yamanashi	1360 (1110 to 1640)	974 (768 to 1190)	748 (618 to 906)	544 (491 to 601)	399 (361 to 443)	308 (279 to 343)	254 (228 to 281)	247 (218 to 278)	226 (201 to 253)	214 (190 to 243)
South Korea	2260 (2090 to 2460)	1530 (1410 to 1660)	1300 (1200 to 1410)	1050 (981 to 1120)	738 (692 to 794)	544 (509 to 585)	349 (326 to 374)	274 (253 to 296)	266 (248 to 285)	282 (262 to 305)
Singapore	1360 (1270 to 1470)	1060 (976 to 1170)	900 (841 to 965)	769 (718 to 822)	574 (536 to 616)	429 (401 to 463)	314 (292 to 338)	242 (224 to 262)	224 (206 to 243)	237 (217 to 258)
High-income North America	907 (854 to 964)	799 (753 to 853)	719 (676 to 765)	578 (544 to 618)	523 (491 to 557)	507 (477 to 541)	435 (409 to 462)	412 (385 to 442)	467 (439 to 498)	473 (443 to 505)
Canada	940 (879 to 1000)	778 (722 to 838)	652 (606 to 701)	536 (500 to 574)	471 (436 to 507)	426 (398 to 458)	358 (334 to 388)	329 (304 to 358)	351 (327 to 378)	340 (317 to 365)
Greenland	1930 (1700 to 2180)	1400 (1250 to 1530)	1170 (1070 to 1290)	1230 (1110 to 1340)	1190 (1110 to 1280)	926 (848 to 1040)	711 (656 to 772)	598 (536 to 680)	620 (554 to 705)	613 (544 to 702)
USA	904 (847 to 967)	801 (751 to 861)	725 (678 to 778)	582 (544 to 626)	529 (494 to 567)	516 (482 to 555)	444 (414 to 476)	422 (391 to 457)	480 (448 to 516)	489 (455 to 528)
Alabama	1010 (806 to 1250)	843 (764 to 928)	763 (692 to 847)	611 (555 to 676)	578 (526 to 638)	588 (533 to 654)	569 (518 to 627)	535 (479 to 598)	626 (567 to 691)	653 (546 to 776)
Alaska	998 (827 to 1190)	859 (775 to 954)	717 (647 to 798)	591 (533 to 658)	538 (484 to 595)	513 (462 to 570)	474 (429 to 525)	443 (395 to 499)	433 (390 to 480)	515 (449 to 592)
Arizona	863 (685 to 1070)	719 (649 to 797)	676 (609 to 747)	547 (495 to 608)	485 (440 to 536)	484 (437 to 532)	414 (374 to 456)	405 (362 to 451)	489 (443 to 541)	512 (429 to 605)
Arkansas	865 (679 to 1080)	736 (665 to 820)	684 (621 to 755)	566 (516 to 628)	560 (510 to 620)	576 (523 to 637)	536 (486 to 593)	535 (482 to 594)	620 (562 to 686)	637 (533 to 760)
California	832 (671 to 1030)	738 (674 to 810)	688 (628 to 753)	567 (517 to 620)	516 (469 to 566)	469 (427 to 515)	380 (346 to 416)	346 (313 to 382)	389 (355 to 427)	407 (340 to 481)
Colorado	841 (664 to 1050)	736 (668 to 816)	662 (600 to 726)	530 (481 to 586)	480 (434 to 532)	476 (432 to 526)	408 (371 to 450)	381 (341 to 423)	426 (386 to 469)	437 (357 to 523)
Connecticut	876 (695 to 1070)	780 (707 to 863)	668 (603 to 738)	553 (498 to 613)	481 (436 to 532)	464 (418 to 512)	381 (346 to 421)	363 (325 to 408)	423 (381 to 471)	386 (315 to 470)
Delaware	974 (800 to 1180)	854 (771 to 951)	764 (694 to 847)	614 (555 to 681)	580 (525 to 641)	539 (488 to 596)	465 (421 to 514)	431 (385 to 484)	491 (443 to 545)	495 (429 to 572)
Washington, DC	1040 (831 to 1280)	936 (845 to 1030)	931 (841 to 1030)	742 (665 to 826)	722 (651 to 802)	617 (555 to 688)	482 (432 to 536)	431 (381 to 489)	496 (444 to 554)	474 (401 to 560)
Florida	833 (657 to 1040)	730 (663 to 809)	669 (606 to 743)	545 (495 to 610)	487 (442 to 541)	479 (436 to 532)	408 (371 to 452)	397 (357 to 444)	444 (405 to 491)	475 (399 to 565)
Georgia	976 (778 to 1210)	844 (763 to 932)	776 (702 to 858)	626 (567 to 695)	582 (527 to 644)	579 (523 to 640)	504 (456 to 557)	467 (418 to 519)	536 (483 to 593)	570 (472 to 679)
Hawaii	860 (700 to 1050)	732 (660 to 813)	597 (537 to 663)	450 (402 to 501)	418 (377 to 464)	382 (344 to 425)	334 (300 to 372)	318 (284 to 358)	316 (283 to 356)	335 (283 to 395)
Idaho	803 (641 to 999)	692 (624 to 772)	657 (591 to 736)	518 (468 to 577)	478 (431 to 527)	488 (441 to 541)	441 (398 to 489)	427 (380 to 479)	453 (410 to 503)	500 (429 to 585)
Illinois	924 (746 to 1140)	835 (760 to 920)	766 (693 to 844)	603 (546 to 665)	550 (499 to 605)	530 (478 to 587)	439 (397 to 483)	414 (368 to 460)	480 (434 to 531)	463 (383 to 557)
Indiana	884 (698 to 1100)	804 (727 to 888)	739 (667 to 819)	592 (531 to 658)	540 (487 to 600)	560 (500 to 620)	496 (448 to 551)	500 (443 to 558)	572 (515 to 632)	572 (474 to 682)
Iowa	766 (605 to 950)	701 (636 to 778)	650 (587 to 715)	495 (444 to 547)	449 (402 to 495)	467 (419 to 519)	424 (384 to 465)	418 (372 to 466)	477 (430 to 530)	454 (374 to 555)
Kansas	773 (610 to 961)	702 (636 to 778)	635 (576 to 704)	526 (474 to 586)	467 (422 to 518)	496 (449 to 553)	450 (406 to 501)	465 (417 to 522)	516 (466 to 572)	521 (423 to 639)
Kentucky	916 (727 to 1130)	806 (731 to 891)	763 (694 to 842)	611 (553 to 678)	567 (513 to 627)	587 (532 to 649)	560 (508 to 619)	558 (501 to 625)	619 (562 to 682)	661 (546 to 794)
Louisiana	994 (786 to 1240)	866 (783 to 955)	794 (717 to 881)	646 (584 to 719)	603 (545 to 667)	612 (554 to 676)	544 (493 to 602)	539 (481 to 601)	643 (581 to 712)	645 (541 to 758)
Maine	919 (742 to 1130)	804 (723 to 894)	736 (664 to 816)	557 (499 to 623)	508 (456 to 560)	510 (461 to 568)	443 (398 to 489)	438 (388 to 490)	438 (391 to 490)	482 (401 to 572)
Maryland	979 (790 to 1200)	881 (795 to 971)	758 (682 to 838)	611 (549 to 678)	563 (508 to 622)	548 (495 to 607)	433 (391 to 479)	410 (365 to 456)	463 (416 to 515)	451 (372 to 546)
Massachusetts	890 (706 to 1120)	798 (728 to 876)	687 (621 to 756)	564 (512 to 626)	495 (449 to 546)	481 (437 to 532)	388 (353 to 428)	372 (334 to 415)	420 (380 to 464)	382 (311 to 464)
Michigan	903 (722 to 1130)	806 (735 to 887)	740 (672 to 815)	593 (536 to 654)	549 (501 to 603)	544 (494 to 600)	479 (435 to 526)	449 (404 to 496)	516 (467 to 566)	515 (424 to 612)
Minnesota	786 (621 to 982)	724 (657 to 800)	625 (564 to 695)	492 (445 to 546)	453 (412 to 498)	452 (410 to 502)	382 (347 to 424)	380 (340 to 424)	426 (386 to 473)	419 (347 to 506)
Mississippi	1030 (825 to 1270)	889 (805 to 980)	803 (727 to 892)	634 (570 to 701)	589 (532 to 650)	628 (565 to 694)	576 (520 to 638)	571 (510 to 636)	689 (617 to 766)	690 (575 to 821)
Missouri	870 (683 to 1080)	785 (712 to 860)	735 (662 to 810)	593 (537 to 654)	536 (484 to 589)	557 (502 to 614)	494 (446 to 543)	476 (426 to 526)	548 (495 to 603)	546 (453 to 652)
Montana	849 (695 to 1040)	771 (697 to 858)	681 (616 to 755)	563 (506 to 627)	495 (447 to 551)	492 (442 to 546)	457 (412 to 505)	433 (384 to 490)	461 (414 to 514)	492 (420 to 568)
Nebraska	749 (602 to 924)	701 (630 to 775)	632 (567 to 700)	513 (461 to 572)	473 (427 to 525)	470 (425 to 521)	424 (380 to 470)	424 (378 to 475)	464 (416 to 515)	465 (394 to 548)
Nevada	1020 (826 to 1250)	833 (746 to 932)	788 (708 to 870)	622 (557 to 693)	573 (518 to 633)	552 (494 to 614)	485 (439 to 539)	440 (393 to 490)	496 (447 to 547)	531 (460 to 615)
New Hampshire	893 (720 to 1100)	771 (699 to 855)	705 (636 to 783)	557 (503 to 620)	498 (449 to 551)	480 (431 to 537)	409 (368 to 455)	409 (365 to 459)	417 (375 to 465)	435 (362 to 515)
New Jersey	956 (762 to 1180)	846 (766 to 932)	751 (681 to 825)	610 (552 to 674)	545 (494 to 598)	505 (457 to 556)	405 (368 to 447)	371 (333 to 412)	448 (405 to 491)	404 (330 to 487)
New Mexico	903 (720 to 1110)	786 (706 to 869)	708 (636 to 784)	535 (483 to 596)	499 (452 to 555)	486 (436 to 542)	442 (400 to 491)	439 (392 to 494)	521 (468 to 574)	536 (446 to 644)

New York	963 (782 to 1160)	862 (786 to 953)	770 (701 to 850)	615 (560 to 679)	549 (501 to 605)	481 (438 to 533)	392 (358 to 434)	348 (315 to 388)	428 (390 to 472)	391 (328 to 472)
North Carolina	936 (738 to 1160)	836 (762 to 919)	743 (673 to 816)	579 (524 to 640)	545 (494 to 598)	559 (506 to 616)	474 (430 to 521)	459 (412 to 510)	510 (461 to 563)	536 (444 to 639)
North Dakota	809 (674 to 971)	715 (645 to 797)	619 (559 to 692)	510 (458 to 568)	433 (390 to 480)	447 (404 to 499)	410 (368 to 455)	392 (346 to 443)	426 (379 to 475)	385 (332 to 448)
Ohio	915 (741 to 1130)	823 (749 to 909)	754 (684 to 831)	620 (565 to 690)	552 (503 to 611)	556 (505 to 613)	497 (452 to 548)	494 (447 to 551)	557 (509 to 612)	569 (474 to 687)
Oklahoma	811 (643 to 1010)	715 (650 to 792)	683 (618 to 752)	569 (514 to 635)	550 (496 to 611)	592 (536 to 658)	560 (506 to 618)	541 (487 to 605)	613 (555 to 677)	643 (540 to 774)
Oregon	793 (627 to 1000)	723 (656 to 799)	642 (576 to 709)	528 (474 to 586)	500 (453 to 553)	497 (449 to 551)	431 (389 to 475)	395 (352 to 440)	412 (372 to 456)	450 (369 to 544)
Pennsylvania	974 (784 to 1180)	862 (786 to 952)	776 (706 to 854)	619 (564 to 682)	548 (497 to 603)	526 (478 to 581)	452 (410 to 500)	438 (395 to 486)	494 (450 to 546)	498 (414 to 593)
Rhode Island	931 (749 to 1140)	814 (731 to 900)	707 (636 to 777)	549 (494 to 608)	500 (454 to 554)	488 (438 to 540)	419 (378 to 466)	398 (354 to 446)	434 (390 to 482)	409 (346 to 483)
South Carolina	1060 (849 to 1330)	892 (808 to 985)	794 (717 to 872)	628 (567 to 696)	584 (527 to 641)	574 (518 to 637)	510 (461 to 563)	496 (442 to 552)	573 (517 to 633)	596 (497 to 710)
South Dakota	794 (644 to 972)	727 (659 to 803)	649 (586 to 718)	512 (462 to 570)	455 (411 to 505)	468 (423 to 521)	417 (376 to 463)	422 (374 to 474)	458 (413 to 514)	436 (374 to 507)
Tennessee	935 (738 to 1160)	813 (736 to 900)	749 (681 to 828)	592 (538 to 656)	565 (510 to 625)	583 (529 to 648)	535 (485 to 590)	526 (473 to 587)	597 (540 to 662)	627 (519 to 743)
Texas	834 (663 to 1050)	732 (669 to 808)	691 (627 to 764)	576 (524 to 639)	524 (476 to 579)	531 (484 to 585)	460 (420 to 506)	426 (384 to 473)	498 (453 to 546)	524 (442 to 613)
Utah	825 (653 to 1020)	709 (644 to 782)	644 (586 to 716)	526 (476 to 582)	466 (422 to 515)	477 (434 to 529)	428 (389 to 473)	422 (380 to 470)	462 (421 to 512)	480 (410 to 560)
Vermont	894 (739 to 1080)	797 (716 to 886)	691 (620 to 768)	554 (502 to 615)	503 (452 to 554)	487 (438 to 543)	414 (373 to 462)	395 (351 to 448)	402 (357 to 452)	426 (363 to 494)
Virginia	962 (770 to 1210)	842 (760 to 932)	735 (667 to 812)	594 (532 to 656)	540 (488 to 598)	532 (480 to 588)	441 (400 to 488)	421 (375 to 471)	468 (422 to 516)	488 (404 to 583)
Washington	813 (642 to 1020)	732 (659 to 807)	673 (607 to 743)	538 (487 to 596)	489 (441 to 538)	477 (431 to 526)	411 (371 to 453)	388 (346 to 433)	403 (364 to 443)	426 (351 to 519)
West Virginia	920 (732 to 1140)	794 (722 to 878)	800 (723 to 889)	620 (561 to 689)	562 (509 to 618)	610 (549 to 675)	568 (514 to 626)	582 (518 to 648)	622 (561 to 690)	699 (583 to 833)
Wisconsin	849 (683 to 1060)	772 (699 to 855)	670 (607 to 740)	531 (480 to 590)	493 (448 to 545)	485 (440 to 536)	421 (381 to 466)	422 (377 to 472)	470 (427 to 519)	462 (377 to 562)
Wyoming	842 (693 to 1020)	769 (689 to 859)	675 (604 to 752)	573 (514 to 644)	510 (455 to 566)	517 (464 to 578)	474 (428 to 526)	450 (396 to 509)	475 (425 to 530)	527 (458 to 611)
Southern Latin America	1140 (1090 to 1200)	1080 (1030 to 1130)	922 (878 to 969)	752 (716 to 790)	647 (614 to 682)	552 (523 to 582)	507 (480 to 533)	470 (431 to 511)	489 (448 to 537)	505 (458 to 554)
Argentina	1030 (960 to 1100)	960 (900 to 1030)	895 (836 to 959)	744 (697 to 799)	654 (611 to 703)	572 (531 to 615)	534 (500 to 569)	510 (450 to 580)	534 (469 to 614)	540 (470 to 621)
Chile	1630 (1520 to 1750)	1460 (1370 to 1570)	1050 (980 to 1130)	812 (747 to 882)	652 (601 to 707)	504 (467 to 549)	443 (399 to 498)	378 (350 to 410)	404 (369 to 443)	424 (386 to 464)
Uruguay	833 (773 to 897)	840 (771 to 920)	826 (748 to 916)	726 (658 to 804)	620 (557 to 687)	546 (497 to 602)	488 (453 to 527)	472 (417 to 534)	444 (393 to 504)	528 (464 to 599)
Western Europe	1020 (988 to 1060)	848 (825 to 872)	772 (752 to 794)	648 (631 to 665)	535 (526 to 546)	446 (438 to 455)	364 (359 to 370)	329 (320 to 340)	351 (340 to 363)	342 (331 to 355)
Andorra	596 (437 to 732)	600 (451 to 739)	548 (416 to 676)	475 (358 to 624)	402 (310 to 519)	363 (294 to 442)	322 (269 to 379)	312 (247 to 387)	295 (233 to 366)	296 (227 to 376)
Austria	1120 (1050 to 1200)	926 (858 to 997)	869 (806 to 936)	728 (675 to 784)	571 (532 to 614)	457 (426 to 492)	378 (373 to 383)	338 (310 to 369)	359 (329 to 394)	349 (319 to 386)
Belgium	1060 (986 to 1130)	884 (819 to 950)	821 (769 to 879)	681 (630 to 738)	544 (504 to 591)	464 (429 to 500)	390 (385 to 396)	347 (321 to 378)	390 (357 to 426)	340 (309 to 377)
Cyprus	1750 (1590 to 1910)	1630 (1470 to 1800)	1400 (1220 to 1620)	1150 (1050 to 1260)	1040 (963 to 1130)	794 (726 to 861)	542 (489 to 594)	471 (410 to 543)	462 (398 to 534)	452 (386 to 529)
Denmark	975 (914 to 1050)	816 (762 to 877)	697 (652 to 748)	624 (616 to 633)	594 (585 to 604)	533 (524 to 541)	442 (434 to 449)	369 (339 to 400)	367 (336 to 404)	369 (336 to 406)
Finland	1210 (1130 to 1300)	951 (891 to 1020)	815 (803 to 827)	591 (582 to 600)	538 (529 to 547)	449 (441 to 457)	361 (354 to 368)	320 (294 to 348)	319 (292 to 349)	316 (287 to 353)
France	974 (909 to 1040)	838 (774 to 909)	729 (673 to 788)	590 (546 to 640)	464 (434 to 499)	399 (371 to 429)	328 (307 to 351)	288 (262 to 316)	312 (282 to 344)	298 (269 to 332)
Germany	961 (842 to 1110)	846 (788 to 910)	830 (780 to 894)	706 (660 to 753)	583 (578 to 588)	460 (455 to 464)	394 (390 to 398)	365 (334 to 398)	369 (337 to 406)	373 (338 to 418)
Greece	852 (796 to 913)	747 (697 to 806)	670 (623 to 725)	637 (592 to 686)	572 (531 to 618)	521 (484 to 562)	428 (399 to 460)	378 (338 to 431)	387 (343 to 442)	414 (361 to 480)
Iceland	755 (717 to 798)	735 (707 to 762)	672 (646 to 700)	516 (494 to 538)	496 (476 to 518)	418 (400 to 437)	374 (356 to 393)	313 (283 to 348)	300 (270 to 335)	323 (288 to 360)
Ireland	1200 (1120 to 1280)	971 (898 to 1050)	871 (810 to 934)	773 (715 to 838)	651 (606 to 702)	555 (513 to 604)	390 (360 to 423)	347 (316 to 379)	341 (310 to 375)	332 (300 to 371)
Israel	740 (688 to 798)	800 (744 to 865)	836 (772 to 904)	743 (684 to 809)	574 (519 to 637)	495 (446 to 549)	374 (345 to 407)	315 (276 to 358)	313 (273 to 362)	315 (271 to 367)
Italy	1020 (957 to 1090)	853 (791 to 925)	749 (698 to 808)	644 (601 to 696)	504 (470 to 542)	411 (383 to 444)	339 (336 to 342)	302 (280 to 328)	338 (311 to 371)	322 (295 to 354)
Luxembourg	1160 (1080 to 1250)	964 (896 to 1040)	855 (796 to 919)	734 (677 to 798)	579 (535 to 630)	452 (417 to 491)	371 (357 to 387)	326 (297 to 359)	340 (308 to 377)	319 (287 to 355)
Malta	1070 (990 to 1150)	1010 (919 to 1100)	923 (841 to 1020)	855 (770 to 945)	576 (517 to 642)	464 (418 to 517)	378 (347 to 412)	310 (272 to 354)	310 (272 to 358)	348 (301 to 407)
Monaco	1000 (853 to 1170)	837 (692 to 989)	727 (617 to 856)	565 (489 to 640)	462 (335 to 558)	448 (339 to 539)	436 (366 to 520)	396 (324 to 472)	407 (330 to 488)	448 (368 to 534)
Netherlands	924 (913 to 934)	770 (762 to 778)	691 (684 to 698)	543 (536 to 549)	503 (496 to 509)	482 (476 to 488)	392 (387 to 397)	366 (338 to 400)	389 (357 to 425)	383 (348 to 422)
Norway	823 (766 to 878)	719 (670 to 772)	640 (597 to 688)	539 (502 to 575)	500 (466 to 536)	444 (436 to 452)	373 (366 to 380)	323 (297 to 352)	315 (286 to 344)	322 (292 to 352)
Agder	901 (798 to 1010)	707 (636 to 784)	619 (555 to 687)	548 (489 to 608)	500 (458 to 548)	449 (424 to 475)	391 (369 to 414)	363 (316 to 416)	342 (291 to 396)	332 (269 to 408)
Innlandet	878 (780 to 985)	754 (678 to 831)	651 (586 to 719)	549 (494 to 608)	505 (466 to 550)	506 (481 to 531)	396 (374 to 418)	349 (301 to 398)	316 (275 to 364)	324 (265 to 392)
Møre og Romsdal	778 (690 to 876)	709 (639 to 788)	594 (531 to 664)	507 (452 to 562)	457 (418 to 501)	403 (379 to 426)	314 (294 to 334)	302 (262 to 349)	310 (266 to 361)	311 (244 to 389)
Nordland	906 (811 to 1020)	712 (641 to 789)	652 (587 to 724)	510 (460 to 572)	500 (459 to 546)	430 (405 to 454)	366 (342 to 388)	346 (302 to 401)	315 (267 to 366)	312 (248 to 383)
Oslo	642 (572 to 718)	691 (628 to 760)	629 (571 to 696)	568 (513 to 621)	547 (506 to 591)	454 (435 to 474)	367 (349 to 385)	304 (267 to 346)	320 (278 to 369)	316 (266 to 374)
Rogaland	771 (678 to 869)	702 (635 to 777)	632 (562 to 701)	542 (487 to 601)	480 (443 to 523)	434 (413 to 455)	371 (352 to 390)	302 (264 to 345)	321 (276 to 367)	322 (271 to 384)
Troms og Finnmark	943 (832 to 1060)	783 (704 to 867)	704 (629 to 784)	539 (484 to 599)	522 (476 to 568)	481 (454 to 508)	388 (363 to 413)	345 (295 to 399)	326 (282 to 382)	334 (270 to 409)
Trøndelag	880 (778 to 985)	772 (699 to 858)	680 (610 to 756)	563 (505 to 626)	514 (472 to 560)	436 (413 to 460)	374 (355 to 394)	337 (294 to 385)	322 (277 to 374)	324 (266 to 395)
Vestfold og Telemark	766 (680 to 859)	697 (629 to 772)	631 (566 to 705)	544 (490 to 603)	497 (457 to 540)	455 (434 to 479)	399 (379 to 420)	336 (290 to 385)	324 (277 to 375)	336 (274 to 407)
Vestland	815 (735 to 909)	714 (649 to 789)	629 (570 to 695)	488 (440 to 539)	457 (419 to 496)	402 (384 to 423)	340 (324 to 358)	296 (261 to 337)	287 (250 to 329)	289 (238 to 350)
Viken	1060 (953 to 1180)	744 (676 to 823)	660 (599 to 730)	559 (509 to 617)	510 (470 to 551)	450 (433 to 468)	387 (371 to 404)	321 (287 to 360)	313 (277 to 354)	336 (283 to 395)
Portugal	1320 (1230 to 1420)	1040 (969 to 1110)	971 (904 to 1040)	762 (710 to 825)	658 (612 to 713)	537 (499 to 577)	387 (361 to 416)	326 (290 to 366)	345 (306 to 390)	338 (298 to 389)
San Marino	663 (552 to 858)	682 (553 to 823)	613 (520 to 712)	512 (422 to 605)	413 (328 to 512)	333 (276 to 407)	262 (222 to 306)	268 (210 to 340)	273 (217 to 346)	233 (167 to 311)
Spain	1200 (1120 to 1290)	871 (812 to 930)	784 (732 to 837)	584 (543 to 629)	497 (462 to 531)	393 (389 to 397)	316 (312 to 319)	284 (261 to 310)	321 (295 to 352)	292 (266 to 324)
Sweden	964 (899 to 1030)	791 (740 to 847)	639 (631 to 646)	546 (540 to 553)	474 (468 to 480)	418 (413 to 424)	359 (354 to 364)	320 (292 to 350)	334 (303 to 369)	318 (277 to 362)
Stockholm	958 (717 to 1240)	795 (592 to 1020)	659 (513 to 842)	568 (547 to 591)	501 (484 to 518)	421 (405 to 438)	364 (349 to 379)	318 (279 to 361)	355 (304 to 413)	295 (239 to 366)
Sweden except Stockholm	966 (884 to 1050)	790 (722 to 858)	635 (598 to 664)	542 (534 to 550)	468 (461 to 476)	418 (411 to 424)	358 (352 to 364)	321 (292 to 354)	331 (298 to 366)	324 (283 to 369)
Switzerland	986 (922 to 1060)	818 (764 to 878)	707 (663 to 759)	551 (514 to 589)	452 (423 to 482)	379 (356 to 407)	315 (309 to 320)	275 (252 to 303)	295 (269 to 327)	270 (244 to 302)
UK	1020 (967 to 1080)	877 (826 to 935)	769 (722 to 816)	681 (643 to 729)	581 (546 to 619)	510 (480 to 543)	401 (377 to 428)	378 (354 to 405)	408 (384 to 436)	409 (386 to 434)
England	982 (920 to 1050)	847 (789 to 913)	749 (696 to 803)	668 (623 to 722)	569 (530 to 614)	500 (465 to 541)	391 (364 to 423)	368 (340 to 401)	399 (371 to 432)	400 (375 to 428)
East Midlands	998 (791 to 1240)	863 (666 to 1090)	764 (612 to 941)	677 (605 to 762)	564 (506 to 625)	503 (453 to 558)	398 (357 to 442)	386 (335 to 445)	414 (358 to 482)	418 (359 to 489)
Derby	1080 (784 to 1450)	949 (662 to 1250)	813 (604 to 1070)	727 (613 to 850)	601 (519 to 686)	501 (430 to 573)	434 (374 to 494)	406 (332 to 485)	440 (356 to 539)	469 (373 to 576)

Derbyshire	986 (741 to 1290)	844 (604 to 1160)	772 (579 to 1010)	682 (591 to 788)	577 (510 to 659)	506 (446 to 573)	394 (346 to 456)	388 (324 to 471)	410 (340 to 499)	414 (342 to 506)
Leicester	1100 (805 to 1450)	972 (691 to 1300)	848 (632 to 1110)	750 (641 to 865)	590 (518 to 667)	560 (488 to 638)	442 (384 to 508)	422 (346 to 510)	485 (393 to 589)	457 (370 to 553)
Leicestershire	911 (648 to 1240)	798 (563 to 1100)	699 (518 to 915)	618 (531 to 714)	517 (455 to 588)	464 (408 to 528)	354 (309 to 400)	330 (276 to 393)	354 (292 to 431)	367 (299 to 441)
Lincolnshire	975 (714 to 1290)	841 (588 to 1160)	750 (555 to 978)	682 (588 to 789)	554 (484 to 626)	495 (435 to 564)	389 (341 to 445)	394 (329 to 472)	404 (335 to 491)	405 (329 to 490)
Northamptonshire	960 (706 to 1290)	822 (578 to 1120)	732 (545 to 960)	646 (554 to 747)	555 (486 to 631)	487 (427 to 553)	397 (348 to 452)	394 (327 to 473)	420 (348 to 505)	480 (370 to 602)
Nottingham	1100 (805 to 1470)	976 (696 to 1370)	835 (644 to 1070)	747 (642 to 870)	614 (535 to 701)	607 (531 to 689)	473 (411 to 543)	419 (345 to 511)	501 (408 to 614)	457 (363 to 568)
Nottinghamshire	994 (726 to 1310)	858 (598 to 1160)	754 (571 to 1000)	663 (571 to 771)	567 (501 to 642)	508 (449 to 579)	407 (356 to 466)	412 (345 to 499)	452 (374 to 547)	424 (344 to 521)
Rutland	906 (652 to 1240)	799 (543 to 1110)	669 (488 to 894)	608 (501 to 737)	525 (445 to 615)	423 (358 to 494)	340 (287 to 405)	323 (257 to 408)	258 (206 to 329)	335 (261 to 426)
East of England	931 (741 to 1150)	804 (622 to 1030)	708 (563 to 880)	634 (562 to 717)	537 (484 to 595)	468 (423 to 520)	372 (334 to 413)	352 (305 to 405)	371 (321 to 427)	360 (310 to 422)
Bedford	971 (694 to 1330)	850 (598 to 1180)	698 (514 to 927)	603 (509 to 713)	551 (476 to 633)	480 (418 to 554)	403 (349 to 472)	389 (316 to 478)	446 (365 to 549)	379 (304 to 484)
Cambridgeshire	872 (628 to 1180)	763 (529 to 1080)	656 (491 to 877)	613 (524 to 716)	512 (446 to 585)	467 (410 to 531)	338 (296 to 390)	325 (268 to 394)	349 (287 to 422)	323 (262 to 400)
Central Bedfordshire	996 (728 to 1340)	879 (613 to 1240)	727 (526 to 973)	639 (540 to 748)	566 (496 to 652)	462 (400 to 535)	370 (318 to 425)	333 (270 to 410)	328 (262 to 402)	384 (311 to 477)
Essex	924 (680 to 1220)	796 (575 to 1070)	728 (550 to 936)	644 (557 to 741)	538 (477 to 606)	462 (410 to 523)	374 (330 to 424)	373 (314 to 442)	378 (317 to 452)	379 (315 to 466)
Hertfordshire	902 (664 to 1210)	778 (556 to 1070)	709 (530 to 936)	644 (554 to 751)	539 (474 to 613)	479 (423 to 542)	368 (324 to 420)	343 (285 to 411)	383 (320 to 462)	343 (280 to 420)
Luton	1070 (767 to 1450)	941 (655 to 1280)	801 (588 to 1050)	712 (602 to 841)	577 (498 to 660)	522 (450 to 597)	431 (374 to 496)	385 (311 to 466)	454 (363 to 549)	398 (312 to 502)
Norfolk	910 (670 to 1210)	779 (550 to 1060)	670 (490 to 871)	599 (516 to 696)	528 (466 to 599)	454 (399 to 519)	370 (324 to 421)	336 (280 to 404)	360 (302 to 432)	364 (295 to 448)
Peterborough	1030 (740 to 1390)	900 (632 to 1240)	749 (554 to 1010)	659 (558 to 782)	591 (511 to 673)	518 (449 to 594)	428 (374 to 499)	388 (318 to 471)	419 (339 to 520)	402 (321 to 493)
Southend-on-Sea	1060 (776 to 1390)	926 (628 to 1270)	767 (576 to 1010)	666 (564 to 788)	576 (502 to 668)	502 (436 to 577)	415 (356 to 475)	387 (313 to 478)	413 (334 to 512)	423 (337 to 522)
Suffolk	921 (672 to 1230)	799 (560 to 1090)	698 (519 to 936)	631 (541 to 734)	517 (456 to 588)	457 (400 to 522)	358 (315 to 411)	351 (294 to 427)	346 (289 to 418)	341 (277 to 420)
Thurrock	1030 (746 to 1410)	924 (638 to 1280)	775 (575 to 1030)	684 (574 to 806)	582 (501 to 664)	496 (430 to 574)	404 (349 to 468)	345 (280 to 423)	438 (354 to 538)	411 (329 to 522)
Greater London	942 (761 to 1150)	815 (656 to 1010)	721 (574 to 875)	645 (575 to 722)	558 (502 to 618)	502 (454 to 556)	352 (317 to 392)	320 (277 to 365)	365 (318 to 424)	362 (310 to 422)
Barking and Dagenham	1040 (784 to 1350)	886 (643 to 1190)	772 (569 to 992)	680 (578 to 789)	596 (518 to 682)	548 (476 to 630)	416 (362 to 483)	376 (306 to 456)	457 (368 to 556)	444 (359 to 549)
Barnet	872 (648 to 1130)	755 (545 to 1030)	650 (477 to 839)	571 (485 to 664)	510 (447 to 584)	442 (387 to 507)	340 (295 to 390)	281 (232 to 342)	329 (272 to 400)	323 (261 to 397)
Bexley	877 (651 to 1140)	749 (540 to 989)	647 (473 to 835)	566 (486 to 662)	502 (431 to 573)	445 (387 to 507)	321 (276 to 368)	349 (286 to 421)	411 (336 to 495)	359 (291 to 446)
Brent	948 (701 to 1250)	818 (589 to 1110)	724 (533 to 948)	647 (555 to 756)	553 (487 to 630)	499 (438 to 574)	351 (305 to 404)	298 (242 to 366)	362 (299 to 446)	315 (256 to 391)
Bromley	861 (645 to 1130)	743 (523 to 1000)	646 (477 to 844)	571 (488 to 661)	487 (424 to 558)	447 (391 to 509)	323 (278 to 370)	293 (237 to 356)	318 (259 to 386)	292 (232 to 362)
Camden	926 (676 to 1220)	842 (603 to 1120)	761 (577 to 975)	711 (606 to 827)	615 (540 to 698)	543 (475 to 619)	382 (330 to 442)	348 (286 to 422)	306 (250 to 371)	319 (258 to 392)
Croydon	914 (678 to 1190)	780 (553 to 1060)	711 (529 to 910)	659 (566 to 765)	570 (503 to 642)	494 (435 to 562)	340 (296 to 392)	341 (285 to 413)	384 (320 to 468)	354 (288 to 434)
Ealing	937 (698 to 1230)	803 (577 to 1080)	714 (536 to 931)	645 (555 to 745)	551 (481 to 628)	479 (416 to 547)	321 (279 to 366)	320 (260 to 387)	413 (341 to 497)	359 (291 to 438)
Enfield	902 (666 to 1200)	771 (544 to 1040)	664 (497 to 874)	583 (493 to 676)	511 (445 to 580)	491 (427 to 559)	344 (298 to 395)	314 (257 to 381)	348 (286 to 426)	330 (267 to 410)
Greenwich	996 (743 to 1330)	836 (611 to 1120)	733 (558 to 939)	647 (547 to 764)	579 (508 to 660)	554 (483 to 631)	388 (338 to 449)	366 (303 to 444)	434 (359 to 537)	409 (332 to 500)
Hackney	1000 (739 to 1300)	884 (633 to 1180)	803 (614 to 1030)	750 (647 to 867)	668 (582 to 763)	580 (509 to 658)	414 (361 to 474)	344 (284 to 416)	379 (313 to 458)	624 (474 to 789)
Hammersmith and Fulham	979 (725 to 1280)	875 (624 to 1190)	781 (592 to 1010)	725 (623 to 843)	638 (555 to 739)	556 (485 to 639)	366 (317 to 421)	324 (262 to 398)	412 (335 to 507)	355 (287 to 437)
Haringey	971 (717 to 1270)	846 (606 to 1120)	739 (560 to 965)	669 (571 to 780)	566 (495 to 646)	531 (464 to 604)	376 (329 to 433)	320 (264 to 387)	380 (312 to 468)	346 (277 to 430)
Harrow	889 (653 to 1170)	772 (552 to 1070)	680 (506 to 880)	602 (516 to 715)	479 (416 to 546)	433 (378 to 496)	300 (260 to 346)	281 (230 to 345)	287 (234 to 348)	345 (273 to 431)
Havering	884 (647 to 1160)	752 (530 to 1000)	666 (498 to 859)	601 (514 to 700)	490 (424 to 557)	454 (394 to 517)	349 (304 to 399)	328 (265 to 397)	369 (301 to 447)	371 (295 to 453)
Hillingdon	887 (664 to 1160)	766 (546 to 1040)	671 (492 to 865)	596 (508 to 701)	540 (474 to 616)	497 (438 to 568)	365 (316 to 421)	326 (267 to 394)	410 (338 to 499)	423 (348 to 519)
Hounslow	945 (706 to 1250)	823 (587 to 1100)	723 (538 to 947)	650 (558 to 765)	567 (498 to 642)	512 (450 to 590)	360 (314 to 412)	312 (256 to 378)	354 (294 to 434)	348 (280 to 428)
Islington	990 (733 to 1320)	879 (631 to 1200)	790 (596 to 1020)	734 (629 to 857)	625 (546 to 711)	596 (516 to 678)	400 (345 to 457)	374 (307 to 455)	415 (340 to 505)	463 (374 to 563)
Kensington and Chelsea	906 (671 to 1190)	838 (598 to 1130)	754 (576 to 977)	705 (607 to 814)	557 (485 to 639)	417 (363 to 482)	302 (261 to 345)	283 (230 to 350)	296 (239 to 362)	335 (269 to 416)
Kingston upon Thames	841 (618 to 1120)	722 (526 to 982)	623 (467 to 822)	554 (468 to 651)	497 (431 to 567)	450 (389 to 520)	298 (260 to 348)	286 (231 to 353)	299 (245 to 370)	338 (270 to 421)
Lambeth	1040 (769 to 1360)	910 (641 to 1220)	805 (596 to 1050)	728 (624 to 842)	603 (526 to 694)	593 (522 to 683)	402 (349 to 466)	334 (274 to 405)	373 (305 to 458)	421 (339 to 531)
Lewisham	996 (741 to 1310)	848 (600 to 1150)	739 (553 to 954)	658 (564 to 767)	602 (523 to 689)	575 (504 to 656)	418 (365 to 483)	346 (285 to 426)	424 (348 to 514)	350 (280 to 435)
Merton	931 (685 to 1210)	804 (578 to 1080)	703 (516 to 919)	624 (536 to 721)	539 (471 to 615)	475 (416 to 543)	327 (283 to 378)	306 (249 to 376)	377 (309 to 462)	363 (294 to 446)
Newham	1040 (773 to 1320)	882 (629 to 1220)	794 (594 to 1040)	714 (607 to 835)	598 (523 to 682)	575 (502 to 658)	405 (352 to 461)	369 (302 to 446)	472 (391 to 577)	449 (363 to 555)
Redbridge	892 (665 to 1170)	759 (542 to 1020)	670 (495 to 880)	560 (478 to 653)	494 (426 to 564)	448 (390 to 512)	324 (281 to 371)	280 (230 to 337)	332 (273 to 405)	351 (282 to 434)
Richmond upon Thames	871 (639 to 1160)	760 (544 to 1030)	666 (495 to 866)	602 (514 to 703)	500 (433 to 575)	447 (387 to 514)	297 (257 to 344)	278 (229 to 343)	266 (217 to 325)	301 (240 to 375)
Southwark	994 (742 to 1300)	843 (604 to 1140)	754 (555 to 987)	686 (584 to 800)	664 (581 to 762)	571 (500 to 652)	406 (355 to 467)	342 (281 to 417)	446 (364 to 544)	409 (332 to 512)
Sutton	913 (669 to 1200)	777 (555 to 1070)	667 (503 to 881)	588 (495 to 683)	534 (461 to 612)	484 (418 to 555)	325 (283 to 377)	318 (262 to 390)	336 (278 to 413)	354 (285 to 441)
Tower Hamlets	1060 (804 to 1380)	911 (666 to 1230)	826 (626 to 1060)	751 (644 to 873)	638 (559 to 726)	598 (525 to 683)	400 (350 to 464)	391 (327 to 470)	488 (407 to 596)	527 (429 to 647)
Waltham Forest	992 (737 to 1330)	839 (613 to 1120)	731 (549 to 956)	648 (558 to 756)	563 (493 to 644)	548 (483 to 624)	379 (328 to 435)	325 (267 to 394)	349 (292 to 425)	345 (280 to 428)
Wandsworth	990 (728 to 1310)	873 (632 to 1170)	777 (578 to 1010)	678 (582 to 792)	609 (534 to 690)	544 (481 to 623)	353 (309 to 400)	324 (268 to 395)	381 (314 to 462)	358 (290 to 435)
Westminster	888 (650 to 1190)	820 (588 to 1080)	742 (553 to 949)	699 (599 to 807)	590 (514 to 677)	480 (418 to 552)	326 (284 to 375)	291 (236 to 358)	306 (250 to 374)	310 (248 to 379)
North East England	1090 (877 to 1360)	945 (724 to 1230)	840 (660 to 1050)	741 (654 to 846)	650 (581 to 731)	566 (506 to 633)	451 (403 to 505)	434 (375 to 510)	474 (409 to 558)	479 (407 to 566)
County Durham	1070 (780 to 1420)	902 (644 to 1260)	821 (606 to 1080)	740 (639 to 863)	674 (588 to 767)	585 (509 to 668)	458 (400 to 525)	443 (362 to 542)	495 (407 to 606)	479 (383 to 587)
Darlington	1080 (784 to 1450)	949 (669 to 1320)	842 (613 to 1120)	740 (629 to 876)	648 (558 to 750)	566 (491 to 652)	441 (381 to 511)	440 (355 to 539)	411 (333 to 510)	539 (425 to 662)
Gateshead	1100 (816 to 1470)	969 (683 to 1350)	854 (626 to 1120)	751 (643 to 880)	627 (544 to 722)	562 (489 to 644)	440 (380 to 508)	425 (344 to 521)	494 (399 to 606)	484 (387 to 607)
Hartlepool	1170 (859 to 1530)	1020 (722 to 1400)	906 (679 to 1190)	798 (678 to 936)	682 (592 to 780)	602 (520 to 692)	486 (414 to 560)	497 (401 to 612)	474 (383 to 584)	546 (434 to 678)
Middlesbrough	1200 (884 to 1600)	1060 (736 to 1460)	942 (690 to 1230)	837 (711 to 989)	724 (627 to 838)	633 (549 to 741)	500 (432 to 582)	524 (424 to 647)	558 (451 to 686)	606 (487 to 765)
Newcastle upon Tyne	1080 (804 to 1440)	951 (664 to 1310)	839 (622 to 1080)	733 (626 to 857)	664 (575 to 763)	574 (498 to 660)	464 (403 to 537)	433 (354 to 530)	477 (388 to 582)	496 (395 to 617)
North Tyneside	1080 (790 to 1410)	944 (665 to 1300)	828 (617 to 1090)	721 (615 to 847)	642 (560 to 739)	550 (478 to 634)	432 (373 to 496)	395 (321 to 482)	435 (352 to 531)	452 (366 to 568)
Northumberland	1060 (783 to 1410)	926 (654 to 1290)	821 (609 to 1080)	739 (632 to 859)	612 (533 to 702)	510 (446 to 582)	396 (346 to 455)	380 (313 to 461)	418 (347 to 510)	412 (334 to 510)
Redcar and Cleveland	1090 (805 to 1480)	958 (682 to 1310)	842 (624 to 1100)	733 (621 to 869)	615 (528 to 713)	557 (485 to 646)	455 (390 to 529)	470 (379 to 580)	443 (358 to 554)	579 (465 to 731)
South Tyneside	1110 (814 to 1460)	960 (676 to 1330)	841 (616 to 1090)	736 (624 to 866)	657 (569 to 760)	547 (473 to 632)	465 (400 to 544)	444 (358 to 553)	500 (402 to 617)	458 (369 to 572)

Stockton-on-Tees	1050 (760 to 1410)	918 (642 to 1280)	805 (595 to 1070)	703 (599 to 824)	625 (543 to 719)	528 (459 to 604)	428 (368 to 497)	437 (355 to 534)	460 (371 to 562)	473 (383 to 590)
Sunderland	1100 (815 to 1440)	956 (675 to 1290)	841 (632 to 1080)	734 (634 to 852)	659 (576 to 749)	606 (530 to 696)	499 (436 to 578)	456 (376 to 560)	545 (450 to 661)	462 (372 to 569)
North West England	1070 (868 to 1310)	928 (734 to 1160)	819 (663 to 1010)	729 (652 to 817)	628 (567 to 697)	551 (498 to 609)	445 (401 to 496)	419 (366 to 480)	464 (402 to 531)	468 (408 to 545)
Blackburn with Darwen	1160 (860 to 1500)	1020 (731 to 1380)	873 (662 to 1130)	769 (658 to 892)	671 (586 to 767)	598 (521 to 683)	464 (400 to 531)	438 (357 to 529)	485 (391 to 589)	513 (412 to 624)
Blackpool	1170 (873 to 1530)	1030 (760 to 1400)	880 (670 to 1120)	769 (654 to 890)	653 (571 to 750)	645 (563 to 736)	564 (491 to 648)	550 (448 to 670)	522 (426 to 636)	592 (478 to 736)
Bolton	1110 (828 to 1440)	969 (704 to 1320)	837 (628 to 1080)	737 (627 to 856)	636 (559 to 723)	573 (503 to 653)	454 (395 to 521)	423 (348 to 509)	464 (384 to 562)	498 (408 to 609)
Bury	1060 (786 to 1380)	935 (658 to 1260)	781 (593 to 1040)	682 (579 to 800)	611 (532 to 701)	533 (466 to 610)	433 (374 to 501)	407 (331 to 499)	468 (382 to 572)	495 (401 to 612)
Cheshire East	935 (683 to 1240)	812 (586 to 1110)	700 (531 to 910)	644 (548 to 750)	570 (500 to 650)	474 (417 to 541)	391 (342 to 445)	329 (272 to 398)	381 (317 to 462)	352 (285 to 436)
Cheshire West and Chester	991 (734 to 1300)	866 (614 to 1190)	768 (580 to 998)	679 (580 to 787)	592 (516 to 671)	493 (430 to 559)	383 (332 to 440)	370 (304 to 449)	446 (363 to 542)	404 (329 to 500)
Cumbria	1030 (756 to 1340)	904 (656 to 1240)	794 (602 to 1030)	707 (611 to 808)	607 (538 to 688)	509 (447 to 580)	419 (369 to 480)	377 (315 to 454)	422 (352 to 511)	410 (338 to 500)
Halton	1140 (834 to 1470)	1020 (724 to 1380)	882 (656 to 1160)	785 (671 to 921)	663 (579 to 760)	605 (528 to 692)	488 (422 to 563)	443 (366 to 547)	515 (423 to 628)	454 (366 to 555)
Knowsley	1170 (865 to 1530)	1040 (744 to 1400)	903 (673 to 1190)	807 (691 to 940)	678 (588 to 776)	598 (526 to 686)	482 (414 to 559)	476 (392 to 580)	533 (439 to 642)	600 (489 to 739)
Lancashire	1020 (774 to 1330)	865 (634 to 1160)	784 (592 to 1020)	725 (629 to 829)	624 (551 to 705)	533 (473 to 604)	425 (375 to 481)	422 (355 to 500)	452 (377 to 539)	482 (399 to 578)
Liverpool	1130 (829 to 1480)	969 (700 to 1330)	900 (682 to 1160)	779 (674 to 902)	671 (591 to 762)	632 (556 to 722)	508 (443 to 583)	490 (408 to 590)	548 (452 to 667)	589 (482 to 717)
Manchester	1120 (832 to 1430)	949 (669 to 1290)	856 (652 to 1110)	760 (653 to 872)	688 (597 to 778)	613 (534 to 698)	494 (426 to 565)	482 (394 to 584)	564 (461 to 676)	575 (463 to 702)
Oldham	1140 (839 to 1490)	1010 (739 to 1370)	860 (641 to 1120)	753 (648 to 870)	648 (565 to 737)	573 (501 to 656)	476 (413 to 545)	452 (371 to 549)	500 (410 to 604)	514 (417 to 629)
Rochdale	1140 (848 to 1470)	983 (704 to 1310)	843 (636 to 1090)	734 (634 to 851)	682 (594 to 776)	587 (512 to 673)	494 (429 to 567)	468 (385 to 563)	538 (441 to 654)	510 (412 to 634)
Salford	1130 (839 to 1490)	992 (710 to 1350)	862 (656 to 1120)	763 (654 to 888)	686 (600 to 780)	616 (542 to 700)	489 (428 to 561)	434 (358 to 523)	502 (416 to 605)	517 (421 to 632)
Sefton	1050 (754 to 1390)	906 (636 to 1230)	795 (602 to 1030)	707 (606 to 817)	580 (508 to 662)	532 (463 to 610)	444 (389 to 509)	425 (354 to 517)	457 (380 to 553)	427 (348 to 524)
St Helens	1110 (828 to 1460)	984 (704 to 1330)	841 (632 to 1100)	804 (686 to 933)	646 (565 to 737)	554 (479 to 633)	471 (410 to 541)	462 (378 to 556)	467 (384 to 567)	547 (444 to 669)
Stockport	978 (711 to 1270)	853 (619 to 1150)	745 (553 to 965)	686 (592 to 795)	588 (518 to 668)	516 (454 to 589)	419 (365 to 480)	370 (307 to 449)	395 (328 to 476)	403 (325 to 499)
Tameside	1130 (840 to 1490)	1000 (711 to 1350)	865 (646 to 1130)	763 (653 to 885)	632 (554 to 726)	558 (485 to 640)	470 (405 to 538)	430 (350 to 524)	580 (473 to 707)	477 (385 to 593)
Trafford	1040 (756 to 1350)	923 (641 to 1270)	793 (594 to 1050)	704 (599 to 816)	581 (508 to 665)	521 (454 to 598)	378 (329 to 435)	333 (272 to 402)	418 (339 to 508)	361 (291 to 460)
Warrington	1050 (770 to 1390)	938 (661 to 1290)	810 (614 to 1060)	726 (618 to 844)	641 (559 to 730)	529 (458 to 605)	452 (390 to 520)	420 (338 to 507)	449 (364 to 542)	480 (390 to 593)
Wigan	1100 (816 to 1430)	978 (702 to 1340)	832 (635 to 1090)	731 (628 to 843)	633 (557 to 718)	591 (524 to 672)	456 (400 to 522)	457 (375 to 551)	499 (406 to 607)	459 (372 to 564)
Wirral	1070 (793 to 1410)	926 (672 to 1280)	809 (597 to 1060)	713 (609 to 824)	613 (538 to 697)	541 (472 to 612)	457 (401 to 526)	438 (357 to 530)	437 (360 to 524)	479 (394 to 592)
South East England	908 (729 to 1120)	784 (614 to 985)	692 (558 to 856)	620 (551 to 698)	526 (473 to 582)	468 (424 to 518)	365 (331 to 406)	343 (298 to 395)	368 (320 to 422)	358 (307 to 415)
Bracknell Forest	890 (643 to 1200)	787 (556 to 1070)	661 (491 to 862)	594 (494 to 708)	508 (438 to 589)	440 (380 to 506)	362 (313 to 418)	296 (238 to 367)	292 (234 to 362)	288 (228 to 359)
Brighton and Hove	1010 (749 to 1340)	891 (635 to 1230)	748 (573 to 995)	678 (579 to 797)	563 (490 to 643)	526 (459 to 605)	418 (363 to 481)	376 (310 to 461)	482 (396 to 592)	381 (310 to 468)
Buckinghamshire	868 (632 to 1160)	759 (532 to 1040)	664 (494 to 857)	614 (518 to 723)	526 (458 to 605)	460 (402 to 524)	360 (314 to 410)	299 (246 to 365)	318 (262 to 385)	336 (271 to 414)
East Sussex	952 (698 to 1250)	816 (588 to 1100)	688 (531 to 884)	605 (518 to 702)	508 (445 to 575)	441 (390 to 500)	362 (318 to 410)	363 (298 to 437)	366 (305 to 441)	383 (311 to 468)
Hampshire	875 (649 to 1150)	757 (526 to 1020)	699 (536 to 896)	604 (519 to 696)	516 (454 to 584)	457 (406 to 516)	348 (307 to 394)	324 (272 to 388)	344 (290 to 411)	333 (275 to 402)
Isle of Wight	993 (729 to 1310)	853 (605 to 1150)	694 (521 to 914)	606 (504 to 713)	528 (455 to 605)	460 (395 to 531)	384 (333 to 445)	366 (294 to 444)	358 (288 to 440)	391 (307 to 491)
Kent	921 (692 to 1190)	787 (563 to 1050)	722 (537 to 940)	655 (565 to 752)	547 (482 to 617)	498 (439 to 563)	378 (333 to 426)	377 (316 to 451)	397 (332 to 473)	391 (322 to 473)
Medway	992 (719 to 1320)	874 (620 to 1180)	722 (541 to 937)	641 (543 to 749)	558 (484 to 638)	515 (451 to 588)	436 (379 to 502)	394 (322 to 475)	433 (352 to 527)	398 (316 to 492)
Milton Keynes	935 (692 to 1270)	822 (571 to 1130)	682 (512 to 899)	608 (512 to 716)	560 (486 to 644)	477 (411 to 543)	406 (350 to 462)	365 (298 to 440)	412 (335 to 493)	400 (322 to 493)
Oxfordshire	860 (615 to 1160)	752 (520 to 1030)	668 (501 to 876)	600 (513 to 697)	510 (442 to 581)	459 (400 to 522)	356 (310 to 406)	305 (249 to 369)	352 (288 to 422)	327 (264 to 400)
Portsmouth	1010 (745 to 1340)	894 (639 to 1210)	747 (554 to 971)	670 (573 to 780)	586 (512 to 673)	564 (490 to 647)	428 (374 to 491)	405 (334 to 498)	431 (355 to 530)	422 (340 to 520)
Reading	895 (656 to 1200)	793 (560 to 1100)	660 (497 to 869)	595 (503 to 698)	539 (463 to 620)	502 (437 to 574)	404 (348 to 467)	381 (308 to 457)	465 (374 to 570)	408 (327 to 503)
Slough	1030 (738 to 1390)	911 (646 to 1240)	782 (596 to 1020)	715 (607 to 852)	617 (533 to 709)	545 (471 to 629)	397 (343 to 460)	381 (308 to 467)	444 (364 to 551)	448 (359 to 564)
Southampton	988 (726 to 1310)	880 (626 to 1190)	734 (552 to 959)	652 (550 to 757)	580 (499 to 663)	534 (468 to 609)	427 (373 to 489)	394 (321 to 479)	433 (355 to 531)	396 (316 to 491)
Surrey	854 (626 to 1140)	730 (525 to 986)	669 (506 to 859)	616 (529 to 716)	514 (449 to 586)	442 (389 to 499)	332 (290 to 376)	335 (274 to 404)	365 (300 to 438)	331 (269 to 402)
West Berkshire	844 (599 to 1130)	742 (530 to 1010)	615 (463 to 826)	551 (465 to 646)	490 (420 to 569)	452 (391 to 517)	356 (305 to 410)	309 (247 to 380)	339 (274 to 417)	311 (245 to 390)
West Sussex	893 (647 to 1200)	758 (538 to 1030)	666 (500 to 874)	594 (504 to 692)	509 (445 to 583)	455 (397 to 522)	370 (323 to 423)	338 (282 to 410)	344 (285 to 417)	360 (293 to 447)
Windsor and Maidenhead	886 (633 to 1170)	785 (549 to 1080)	664 (501 to 879)	605 (506 to 708)	525 (452 to 608)	406 (350 to 467)	336 (287 to 390)	313 (254 to 386)	360 (285 to 441)	342 (271 to 427)
Wokingham	820 (584 to 1120)	720 (508 to 989)	597 (438 to 792)	540 (453 to 644)	473 (409 to 552)	397 (344 to 460)	307 (265 to 356)	292 (237 to 361)	350 (285 to 432)	348 (280 to 429)
South West England	908 (720 to 1110)	786 (616 to 999)	694 (549 to 860)	627 (557 to 708)	523 (474 to 586)	457 (411 to 510)	364 (326 to 408)	350 (306 to 404)	362 (315 to 418)	379 (327 to 446)
Bath and North East Somerset	866 (623 to 1160)	757 (531 to 1030)	645 (485 to 856)	572 (481 to 672)	489 (424 to 561)	433 (376 to 501)	325 (281 to 377)	306 (249 to 375)	368 (301 to 452)	341 (274 to 420)
Bournemouth	961 (687 to 1260)	846 (606 to 1140)	733 (546 to 958)	662 (562 to 779)	560 (486 to 646)	506 (440 to 581)	397 (342 to 458)	405 (330 to 488)	472 (383 to 573)	703 (544 to 888)
Bristol, City of	942 (688 to 1240)	825 (608 to 1100)	742 (555 to 970)	664 (569 to 781)	542 (474 to 626)	492 (430 to 565)	421 (365 to 485)	384 (315 to 463)	460 (377 to 565)	409 (329 to 505)
Cornwall	933 (689 to 1210)	807 (575 to 1110)	711 (532 to 940)	641 (553 to 741)	533 (469 to 605)	475 (417 to 540)	384 (336 to 437)	370 (312 to 439)	366 (302 to 433)	394 (322 to 478)
Devon	865 (640 to 1130)	733 (519 to 1010)	671 (491 to 882)	623 (535 to 722)	504 (443 to 573)	447 (395 to 506)	341 (297 to 391)	350 (293 to 421)	332 (273 to 403)	353 (289 to 437)
Dorset	900 (650 to 1200)	771 (553 to 1050)	670 (498 to 891)	590 (504 to 691)	489 (429 to 558)	400 (348 to 457)	307 (266 to 352)	311 (255 to 374)	318 (261 to 389)	295 (238 to 368)
Gloucestershire	876 (640 to 1160)	750 (534 to 1020)	668 (493 to 871)	632 (544 to 734)	526 (461 to 595)	455 (398 to 522)	372 (326 to 426)	367 (305 to 440)	365 (304 to 440)	385 (315 to 474)
North Somerset	900 (649 to 1200)	781 (557 to 1100)	663 (494 to 878)	586 (499 to 691)	506 (443 to 583)	435 (379 to 504)	375 (324 to 436)	330 (272 to 403)	352 (289 to 435)	368 (295 to 460)
Plymouth	974 (706 to 1260)	863 (622 to 1170)	745 (556 to 976)	669 (568 to 776)	567 (498 to 646)	499 (436 to 572)	413 (362 to 477)	374 (303 to 454)	407 (331 to 493)	392 (314 to 490)
Poole	912 (661 to 1200)	800 (562 to 1120)	683 (501 to 907)	613 (518 to 732)	540 (467 to 628)	430 (370 to 497)	356 (307 to 411)	356 (291 to 436)	375 (304 to 458)	581 (448 to 744)
Somerset	904 (654 to 1190)	780 (563 to 1070)	693 (522 to 890)	635 (544 to 747)	533 (469 to 612)	464 (408 to 529)	367 (322 to 419)	348 (289 to 424)	372 (310 to 457)	369 (303 to 458)
South Gloucestershire	854 (624 to 1150)	756 (527 to 1030)	659 (481 to 862)	557 (469 to 658)	513 (447 to 591)	470 (409 to 541)	334 (289 to 386)	339 (277 to 408)	347 (285 to 425)	362 (291 to 449)
Swindon	955 (691 to 1280)	843 (583 to 1170)	738 (542 to 976)	671 (569 to 792)	536 (469 to 613)	492 (427 to 561)	379 (332 to 434)	340 (279 to 407)	408 (335 to 499)	347 (279 to 434)
Torbay	958 (694 to 1290)	841 (597 to 1150)	717 (534 to 948)	637 (534 to 754)	532 (460 to 614)	504 (433 to 584)	428 (370 to 492)	438 (354 to 537)	394 (319 to 480)	413 (329 to 515)
Wiltshire	895 (646 to 1200)	785 (556 to 1080)	683 (509 to 878)	613 (525 to 716)	534 (468 to 612)	447 (388 to 506)	370 (322 to 422)	314 (258 to 375)	343 (282 to 416)	362 (293 to 443)
West Midlands	1020 (820 to 1270)	887 (702 to 1120)	781 (625 to 968)	696 (615 to 779)	592 (527 to 664)	514 (463 to 572)	404 (363 to 450)	382 (330 to 442)	426 (368 to 487)	433 (370 to 509)
Birmingham	1020 (761 to 1340)	871 (629 to 1170)	799 (607 to 1030)	724 (628 to 829)	616 (537 to 700)	530 (468 to 598)	396 (346 to 455)	385 (318 to 462)	457 (376 to 548)	465 (377 to 571)

Coventry	1050 (780 to 1410)	914 (635 to 1250)	797 (602 to 1040)	689 (592 to 803)	602 (523 to 697)	526 (461 to 603)	410 (357 to 474)	375 (308 to 467)	444 (367 to 544)	437 (352 to 553)
Dudley	984 (716 to 1330)	860 (628 to 1210)	730 (549 to 950)	651 (552 to 760)	565 (491 to 652)	504 (442 to 577)	398 (344 to 456)	397 (321 to 485)	430 (351 to 518)	474 (380 to 583)
Herefordshire, County of	1040 (742 to 1370)	913 (640 to 1270)	771 (570 to 1020)	678 (576 to 790)	532 (461 to 612)	447 (387 to 518)	376 (326 to 434)	348 (285 to 421)	354 (290 to 432)	403 (327 to 501)
Sandwell	1130 (842 to 1500)	996 (716 to 1360)	844 (642 to 1130)	727 (622 to 840)	601 (521 to 695)	575 (500 to 662)	475 (416 to 551)	413 (338 to 509)	474 (391 to 571)	502 (406 to 623)
Shropshire	986 (714 to 1330)	865 (613 to 1180)	726 (537 to 946)	637 (540 to 741)	542 (468 to 624)	456 (397 to 521)	369 (319 to 422)	340 (279 to 415)	358 (293 to 436)	451 (362 to 560)
Solihull	916 (667 to 1240)	812 (580 to 1100)	670 (495 to 895)	580 (494 to 677)	512 (446 to 594)	469 (409 to 538)	352 (304 to 407)	348 (283 to 423)	433 (358 to 526)	387 (311 to 477)
Staffordshire	992 (739 to 1330)	853 (616 to 1190)	776 (579 to 1030)	695 (602 to 809)	606 (531 to 694)	525 (461 to 595)	399 (351 to 455)	389 (328 to 465)	427 (357 to 508)	387 (317 to 475)
Stoke-on-Trent	1170 (859 to 1540)	1030 (728 to 1430)	899 (681 to 1170)	818 (699 to 948)	660 (572 to 758)	566 (495 to 647)	496 (431 to 572)	466 (383 to 570)	592 (489 to 709)	526 (424 to 648)
Telford and Wrekin	1040 (756 to 1380)	925 (656 to 1290)	776 (571 to 1020)	685 (581 to 806)	563 (488 to 648)	533 (466 to 618)	422 (370 to 488)	408 (335 to 500)	468 (385 to 574)	528 (427 to 660)
Walsall	1100 (819 to 1470)	966 (678 to 1330)	822 (612 to 1070)	728 (621 to 842)	636 (551 to 728)	539 (468 to 621)	424 (370 to 487)	395 (323 to 482)	429 (350 to 522)	455 (368 to 562)
Warwickshire	973 (711 to 1310)	850 (606 to 1150)	739 (551 to 982)	672 (582 to 779)	579 (505 to 666)	483 (424 to 552)	374 (327 to 430)	364 (300 to 444)	397 (328 to 481)	387 (316 to 476)
Wolverhampton	1090 (800 to 1440)	952 (676 to 1300)	808 (614 to 1060)	716 (608 to 838)	631 (548 to 729)	538 (467 to 621)	457 (392 to 527)	402 (328 to 491)	486 (392 to 585)	504 (407 to 630)
Worcestershire	950 (692 to 1280)	834 (589 to 1140)	736 (548 to 972)	662 (569 to 770)	559 (488 to 639)	504 (444 to 574)	402 (350 to 461)	372 (308 to 448)	382 (314 to 461)	397 (320 to 493)
Yorkshire and the Humber	1040 (839 to 1280)	897 (698 to 1140)	787 (618 to 962)	696 (619 to 784)	597 (538 to 659)	519 (464 to 578)	420 (377 to 466)	402 (350 to 459)	436 (376 to 500)	439 (378 to 510)
Barnsley	1120 (826 to 1470)	982 (711 to 1330)	823 (598 to 1090)	720 (609 to 841)	615 (535 to 700)	542 (472 to 620)	439 (379 to 497)	408 (335 to 493)	448 (363 to 544)	494 (396 to 607)
Bradford	1100 (818 to 1440)	943 (671 to 1270)	840 (620 to 1090)	735 (635 to 848)	621 (547 to 703)	556 (484 to 632)	438 (381 to 496)	440 (362 to 528)	502 (413 to 612)	466 (374 to 566)
Calderdale	1090 (795 to 1440)	944 (676 to 1290)	803 (604 to 1060)	710 (602 to 832)	622 (541 to 712)	528 (456 to 604)	444 (385 to 512)	404 (326 to 488)	442 (359 to 537)	463 (370 to 569)
Doncaster	1070 (807 to 1430)	919 (658 to 1260)	785 (572 to 1010)	690 (587 to 802)	651 (568 to 739)	564 (488 to 641)	449 (390 to 514)	456 (373 to 547)	513 (419 to 627)	496 (401 to 608)
East Riding of Yorkshire	985 (726 to 1300)	853 (618 to 1170)	734 (541 to 963)	649 (551 to 753)	548 (478 to 621)	492 (426 to 560)	394 (339 to 447)	351 (285 to 429)	353 (288 to 428)	374 (302 to 457)
Kingston upon Hull, City of	1080 (809 to 1430)	930 (667 to 1260)	799 (601 to 1030)	687 (586 to 795)	608 (530 to 694)	568 (492 to 655)	470 (408 to 544)	455 (373 to 550)	520 (424 to 630)	525 (427 to 653)
Kirklees	1080 (806 to 1420)	945 (685 to 1270)	829 (628 to 1070)	762 (649 to 885)	627 (549 to 713)	551 (478 to 632)	422 (368 to 480)	408 (334 to 492)	468 (382 to 566)	426 (343 to 521)
Leeds	989 (741 to 1340)	844 (601 to 1160)	775 (579 to 1010)	688 (595 to 790)	582 (514 to 656)	502 (442 to 566)	411 (363 to 466)	406 (337 to 483)	457 (376 to 546)	457 (369 to 554)
North East Lincolnshire	1090 (815 to 1460)	951 (668 to 1310)	795 (598 to 1040)	695 (592 to 813)	637 (558 to 731)	524 (457 to 601)	423 (368 to 485)	417 (344 to 501)	429 (351 to 519)	477 (386 to 592)
North Lincolnshire	1040 (758 to 1380)	912 (636 to 1250)	760 (550 to 1010)	668 (564 to 788)	612 (533 to 699)	528 (456 to 608)	408 (354 to 475)	370 (302 to 450)	366 (299 to 452)	399 (320 to 500)
North Yorkshire	968 (725 to 1300)	838 (604 to 1160)	740 (561 to 951)	660 (568 to 770)	545 (476 to 621)	477 (416 to 546)	391 (343 to 446)	354 (293 to 429)	369 (306 to 449)	352 (287 to 428)
Rotherham	1070 (792 to 1440)	943 (664 to 1330)	783 (576 to 1040)	678 (579 to 789)	600 (523 to 683)	522 (456 to 599)	434 (379 to 500)	415 (344 to 498)	472 (386 to 573)	470 (378 to 580)
Sheffield	1020 (764 to 1350)	872 (638 to 1150)	778 (568 to 1010)	690 (595 to 806)	603 (528 to 691)	511 (449 to 587)	425 (374 to 485)	418 (349 to 501)	445 (371 to 542)	474 (388 to 589)
Wakefield	1110 (814 to 1470)	974 (702 to 1320)	830 (618 to 1080)	754 (643 to 875)	627 (551 to 715)	540 (468 to 617)	434 (379 to 495)	434 (362 to 528)	446 (371 to 532)	498 (406 to 609)
York	988 (731 to 1290)	876 (626 to 1230)	737 (549 to 967)	651 (555 to 767)	535 (467 to 613)	436 (380 to 503)	355 (308 to 407)	356 (292 to 435)	404 (329 to 491)	395 (320 to 487)
Northern Ireland	1210 (1130 to 1300)	1140 (1060 to 1230)	880 (815 to 949)	787 (728 to 844)	679 (630 to 730)	554 (510 to 599)	430 (398 to 465)	390 (358 to 423)	414 (382 to 450)	415 (382 to 451)
Scotland	1610 (1500 to 1720)	1240 (1150 to 1340)	1040 (967 to 1130)	793 (737 to 856)	674 (626 to 729)	582 (540 to 629)	470 (435 to 508)	442 (392 to 496)	468 (416 to 530)	469 (412 to 538)
Wales	1040 (960 to 1140)	893 (814 to 978)	783 (717 to 853)	687 (635 to 744)	580 (538 to 628)	529 (489 to 571)	427 (396 to 463)	430 (397 to 469)	470 (438 to 508)	470 (438 to 504)
Latin America and Caribbean	1720 (1630 to 1820)	1380 (1320 to 1440)	1190 (1150 to 1240)	928 (896 to 964)	766 (740 to 793)	627 (606 to 650)	602 (579 to 626)	509 (477 to 546)	597 (559 to 642)	653 (605 to 709)
Andean Latin America	2230 (2030 to 2450)	1790 (1660 to 1930)	1710 (1600 to 1850)	1050 (971 to 1120)	816 (757 to 883)	658 (604 to 718)	568 (516 to 624)	533 (473 to 607)	699 (625 to 789)	778 (692 to 880)
Bolivia	2660 (2340 to 2990)	2160 (1830 to 2550)	1790 (1540 to 2100)	1490 (1310 to 1710)	1280 (1130 to 1450)	1030 (927 to 1160)	886 (753 to 1050)	789 (653 to 992)	1110 (945 to 1360)	1220 (1020 to 1490)
Ecuador	1710 (1480 to 1970)	1450 (1340 to 1580)	1370 (1250 to 1510)	1040 (956 to 1130)	728 (684 to 784)	609 (551 to 669)	582 (523 to 639)	513 (440 to 604)	663 (568 to 783)	637 (528 to 764)
Peru	2280 (2000 to 2580)	1810 (1630 to 2010)	1860 (1680 to 2050)	953 (848 to 1070)	733 (650 to 827)	575 (495 to 660)	474 (411 to 544)	482 (410 to 562)	621 (532 to 730)	743 (644 to 866)
Caribbean	1490 (1360 to 1640)	1240 (1170 to 1320)	1100 (1040 to 1150)	947 (898 to 993)	870 (816 to 928)	753 (711 to 793)	1320 (1240 to 1420)	642 (577 to 708)	664 (595 to 738)	769 (679 to 866)
Antigua and Barbuda	1330 (1210 to 1470)	1060 (974 to 1160)	871 (798 to 949)	694 (596 to 810)	600 (539 to 666)	657 (596 to 730)	593 (541 to 662)	642 (554 to 744)	589 (506 to 690)	649 (576 to 738)
The Bahamas	1380 (1130 to 1670)	1180 (994 to 1380)	1020 (936 to 1120)	785 (713 to 863)	688 (626 to 753)	701 (639 to 765)	622 (568 to 680)	720 (608 to 854)	686 (558 to 834)	804 (657 to 977)
Barbados	1340 (1210 to 1500)	949 (877 to 1030)	913 (847 to 993)	803 (743 to 870)	681 (627 to 740)	654 (600 to 708)	625 (578 to 679)	609 (488 to 731)	582 (459 to 715)	607 (476 to 750)
Belize	1540 (1400 to 1710)	889 (809 to 974)	801 (728 to 880)	710 (652 to 775)	623 (568 to 686)	756 (698 to 818)	628 (584 to 680)	583 (508 to 679)	612 (530 to 707)	651 (547 to 779)
Bermuda	1110 (991 to 1250)	981 (894 to 1070)	784 (714 to 862)	594 (536 to 655)	628 (573 to 691)	468 (422 to 510)	315 (275 to 366)	298 (252 to 360)	292 (250 to 349)	370 (311 to 446)
Cuba	1110 (921 to 1340)	1050 (939 to 1170)	973 (908 to 1040)	629 (584 to 677)	640 (571 to 725)	523 (485 to 568)	476 (440 to 517)	468 (416 to 526)	464 (409 to 532)	642 (549 to 752)
Dominica	1870 (1670 to 2100)	1280 (1170 to 1400)	972 (898 to 1060)	809 (731 to 892)	771 (697 to 850)	726 (658 to 797)	692 (632 to 762)	704 (608 to 836)	702 (606 to 840)	838 (677 to 1060)
Dominican Republic	1460 (1290 to 1660)	1150 (1020 to 1280)	899 (807 to 994)	828 (739 to 925)	720 (639 to 813)	557 (492 to 633)	586 (498 to 692)	533 (451 to 632)	562 (476 to 669)	580 (480 to 706)
Grenada	1340 (1150 to 1540)	1000 (872 to 1150)	901 (768 to 1050)	849 (722 to 999)	807 (724 to 897)	621 (563 to 684)	659 (604 to 722)	700 (608 to 799)	695 (594 to 805)	895 (718 to 1130)
Guyana	1850 (1650 to 2070)	1330 (1190 to 1500)	1240 (1110 to 1380)	1320 (1150 to 1500)	1170 (1040 to 1320)	1040 (924 to 1170)	990 (880 to 1100)	902 (724 to 1110)	992 (784 to 1250)	1160 (866 to 1540)
Haiti	2450 (2070 to 2870)	2140 (1730 to 2620)	2050 (1750 to 2370)	1880 (1620 to 2160)	1830 (1560 to 2130)	1700 (1460 to 1970)	4130 (3600 to 4770)	1310 (1010 to 1700)	1460 (1110 to 1900)	1600 (1200 to 2140)
Jamaica	1500 (1390 to 1630)	1040 (968 to 1120)	880 (813 to 950)	656 (605 to 718)	611 (562 to 661)	594 (549 to 643)	498 (458 to 542)	550 (440 to 679)	544 (424 to 686)	639 (505 to 795)
Puerto Rico	1100 (1020 to 1190)	984 (914 to 1050)	887 (823 to 955)	710 (662 to 760)	580 (535 to 626)	475 (444 to 510)	375 (349 to 403)	322 (297 to 350)	335 (310 to 362)	340 (287 to 397)
Saint Kitts and Nevis	1210 (996 to 1460)	1070 (942 to 1230)	986 (902 to 1080)	1100 (1010 to 1200)	1070 (970 to 1180)	829 (752 to 913)	708 (620 to 804)	658 (531 to 816)	659 (526 to 837)	743 (597 to 926)
Saint Lucia	1810 (1660 to 1980)	1350 (1220 to 1490)	1130 (1020 to 1230)	924 (852 to 1000)	941 (867 to 1030)	694 (637 to 754)	491 (450 to 536)	481 (399 to 573)	518 (424 to 624)	673 (520 to 865)
Saint Vincent and the Grenadines	1570 (1420 to 1740)	1260 (1140 to 1380)	1010 (916 to 1110)	905 (827 to 985)	904 (826 to 999)	787 (720 to 861)	727 (661 to 796)	688 (600 to 794)	686 (582 to 804)	728 (610 to 864)
Suriname	1310 (1190 to 1440)	1100 (982 to 1240)	921 (819 to 1030)	948 (844 to 1080)	837 (738 to 955)	738 (682 to 803)	633 (576 to 694)	636 (556 to 727)	635 (546 to 736)	726 (565 to 982)
Trinidad and Tobago	1540 (1430 to 1670)	1120 (1040 to 1210)	1310 (1220 to 1410)	1190 (1110 to 1290)	918 (850 to 995)	772 (714 to 835)	601 (553 to 654)	552 (471 to 650)	593 (484 to 729)	715 (538 to 923)
Virgin Islands	1240 (1060 to 1430)	1290 (1150 to 1420)	1050 (933 to 1160)	881 (786 to 988)	714 (636 to 795)	643 (570 to 717)	473 (410 to 533)	421 (349 to 500)	364 (304 to 429)	404 (316 to 529)
Central Latin America	1850 (1760 to 1950)	1480 (1420 to 1540)	1230 (1180 to 1280)	954 (907 to 1000)	753 (722 to 783)	596 (570 to 621)	530 (507 to 552)	506 (478 to 535)	630 (598 to 664)	671 (624 to 721)
Colombia	1690 (1560 to 1840)	1590 (1450 to 1750)	1040 (951 to 1140)	845 (720 to 986)	672 (615 to 736)	534 (483 to 593)	431 (390 to 474)	384 (355 to 416)	433 (400 to 470)	495 (425 to 570)
Costa Rica	1530 (1410 to 1660)	1140 (1040 to 1250)	1020 (935 to 1120)	595 (539 to 659)	508 (458 to 562)	466 (434 to 502)	395 (365 to 424)	378 (336 to 428)	376 (332 to 430)	436 (384 to 497)
El Salvador	2100 (1910 to 2310)	1540 (1420 to 1680)	1380 (1280 to 1500)	924 (820 to 1050)	653 (602 to 709)	529 (469 to 589)	492 (448 to 541)	490 (428 to 568)	554 (465 to 663)	603 (489 to 739)
Guatemala	2590 (2370 to 2820)	2610 (2390 to 2890)	2110 (1940 to 2310)	1330 (1230 to 1430)	1240 (1120 to 1370)	1000 (925 to 1080)	793 (710 to 892)	725 (670 to 783)	723 (666 to 786)	848 (745 to 965)
Honduras	2370 (2100 to 2670)	1850 (1660 to 2040)	1480 (1320 to 1660)	902 (800 to 1020)	792 (695 to 902)	929 (753 to 1140)	925 (747 to 1130)	853 (714 to 1040)	992 (838 to 1190)	1070 (902 to 1290)
Mexico	2080 (1900 to 2260)	1500 (1400 to 1620)	1300 (1220 to 1400)	1070 (1010 to 1150)	849 (790 to 909)	649 (606 to 696)	580 (543 to 622)	549 (509 to 593)	718 (673 to 768)	738 (684 to 797)

Aguascalientes	1860 (1580 to 2240)	1410 (1200 to 1670)	1290 (1110 to 1500)	1080 (973 to 1200)	913 (820 to 1010)	696 (629 to 769)	605 (546 to 668)	546 (487 to 611)	662 (599 to 731)	691 (618 to 774)
Baja California	1990 (1660 to 2400)	1490 (1230 to 1790)	1300 (1080 to 1560)	1030 (926 to 1130)	934 (847 to 1030)	792 (718 to 872)	638 (578 to 705)	574 (516 to 638)	820 (741 to 903)	721 (647 to 805)
Baja California Sur	1880 (1580 to 2200)	1330 (1130 to 1560)	1140 (966 to 1330)	915 (820 to 1020)	646 (579 to 718)	627 (563 to 692)	577 (521 to 634)	587 (521 to 655)	763 (689 to 842)	681 (610 to 759)
Campeche	1910 (1610 to 2280)	1440 (1210 to 1700)	1260 (1070 to 1480)	1100 (986 to 1220)	895 (806 to 993)	649 (586 to 720)	600 (545 to 666)	571 (513 to 640)	726 (659 to 802)	733 (656 to 820)
Chiapas	2040 (1690 to 2430)	1540 (1300 to 1840)	1320 (1100 to 1600)	1160 (1050 to 1280)	870 (784 to 967)	712 (646 to 787)	630 (570 to 693)	663 (595 to 734)	838 (760 to 925)	845 (756 to 941)
Chihuahua	2180 (1820 to 2590)	1660 (1390 to 1960)	1470 (1230 to 1750)	1190 (1070 to 1360)	1050 (957 to 1160)	803 (723 to 889)	727 (659 to 802)	651 (584 to 728)	947 (862 to 1050)	781 (698 to 873)
Coahuila	2270 (1900 to 2740)	1690 (1440 to 1990)	1500 (1260 to 1780)	1220 (1110 to 1360)	999 (900 to 1110)	729 (656 to 811)	632 (569 to 697)	609 (543 to 686)	834 (752 to 924)	732 (653 to 821)
Colima	2340 (1950 to 2800)	1650 (1380 to 1940)	1370 (1160 to 1620)	1060 (949 to 1180)	918 (827 to 1020)	674 (605 to 750)	600 (542 to 666)	535 (476 to 604)	690 (622 to 769)	799 (718 to 898)
Durango	2110 (1780 to 2520)	1470 (1220 to 1770)	1240 (1050 to 1470)	1030 (930 to 1150)	758 (686 to 834)	579 (523 to 636)	574 (521 to 635)	524 (468 to 589)	625 (566 to 687)	614 (549 to 681)
Guanajuato	2160 (1800 to 2550)	1580 (1350 to 1870)	1410 (1190 to 1660)	1100 (992 to 1220)	1010 (915 to 1120)	673 (611 to 743)	567 (515 to 627)	587 (526 to 655)	749 (679 to 826)	827 (742 to 923)
Guerrero	2300 (1920 to 2730)	1700 (1420 to 2050)	1450 (1220 to 1720)	1180 (1050 to 1300)	816 (725 to 907)	672 (602 to 747)	547 (485 to 618)	492 (433 to 559)	569 (504 to 636)	575 (501 to 662)
Hidalgo	2240 (1890 to 2640)	1650 (1360 to 1960)	1420 (1190 to 1700)	1140 (1030 to 1260)	776 (697 to 855)	537 (486 to 590)	486 (438 to 532)	478 (428 to 530)	589 (532 to 645)	612 (510 to 728)
Jalisco	1930 (1620 to 2270)	1480 (1240 to 1750)	1290 (1080 to 1540)	1100 (1010 to 1210)	933 (847 to 1030)	724 (659 to 793)	601 (549 to 661)	556 (502 to 618)	692 (631 to 759)	773 (693 to 859)
México	2000 (1650 to 2380)	1510 (1250 to 1790)	1310 (1090 to 1540)	1130 (1030 to 1240)	935 (852 to 1020)	731 (663 to 801)	606 (554 to 666)	573 (518 to 636)	806 (737 to 884)	706 (638 to 785)
Mexico City	1720 (1450 to 2030)	1260 (1060 to 1490)	1110 (927 to 1330)	980 (893 to 1080)	824 (740 to 919)	645 (586 to 715)	596 (543 to 660)	535 (484 to 599)	757 (692 to 843)	904 (815 to 1010)
Michoacán de Ocampo	2160 (1770 to 2510)	1580 (1310 to 1900)	1330 (1120 to 1580)	1070 (970 to 1190)	771 (695 to 854)	618 (558 to 688)	544 (492 to 601)	497 (445 to 556)	608 (549 to 672)	720 (645 to 801)
Morelos	2100 (1780 to 2520)	1520 (1260 to 1830)	1300 (1090 to 1550)	1060 (956 to 1180)	848 (761 to 941)	643 (577 to 711)	562 (504 to 619)	565 (505 to 630)	709 (641 to 788)	824 (735 to 920)
Nayarit	2070 (1720 to 2450)	1460 (1230 to 1730)	1200 (1020 to 1410)	932 (835 to 1040)	713 (641 to 795)	560 (502 to 621)	506 (456 to 561)	469 (421 to 526)	566 (512 to 629)	578 (517 to 648)
Nuevo León	1800 (1510 to 2130)	1370 (1130 to 1630)	1220 (1010 to 1460)	1020 (918 to 1120)	860 (779 to 949)	668 (602 to 738)	556 (505 to 613)	529 (475 to 589)	671 (609 to 745)	708 (632 to 787)
Oaxaca	2540 (2120 to 2990)	1910 (1610 to 2280)	1700 (1430 to 2050)	1450 (1310 to 1610)	990 (897 to 1090)	675 (612 to 746)	559 (506 to 618)	533 (478 to 597)	621 (563 to 687)	718 (647 to 800)
Puebla	2610 (2190 to 3050)	1910 (1610 to 2260)	1620 (1350 to 1900)	1230 (1110 to 1350)	943 (845 to 1040)	700 (632 to 769)	602 (546 to 662)	596 (532 to 664)	786 (712 to 865)	920 (825 to 1030)
Querétaro	2190 (1820 to 2650)	1580 (1330 to 1870)	1350 (1120 to 1610)	1180 (1020 to 1370)	928 (835 to 1020)	700 (633 to 772)	582 (528 to 645)	566 (510 to 631)	719 (654 to 793)	790 (709 to 877)
Quintana Roo	2030 (1720 to 2410)	1600 (1360 to 1890)	1490 (1260 to 1760)	1200 (1070 to 1350)	1040 (912 to 1170)	802 (714 to 902)	810 (724 to 910)	774 (676 to 884)	901 (801 to 1020)	861 (751 to 979)
San Luis Potosí	2180 (1830 to 2570)	1580 (1320 to 1880)	1330 (1110 to 1590)	914 (831 to 1010)	782 (710 to 858)	587 (532 to 647)	523 (476 to 577)	509 (457 to 567)	672 (613 to 739)	681 (611 to 761)
Sinaloa	1820 (1520 to 2160)	1350 (1130 to 1610)	1140 (940 to 1380)	947 (856 to 1040)	683 (611 to 754)	554 (499 to 608)	491 (444 to 541)	434 (389 to 483)	576 (522 to 638)	563 (504 to 627)
Sonora	1920 (1590 to 2290)	1480 (1230 to 1760)	1340 (1120 to 1610)	1150 (1010 to 1310)	944 (847 to 1040)	747 (671 to 822)	663 (600 to 732)	587 (526 to 655)	788 (717 to 868)	741 (662 to 822)
Tabasco	1990 (1660 to 2380)	1530 (1290 to 1820)	1380 (1160 to 1660)	1200 (1090 to 1330)	921 (834 to 1020)	695 (628 to 770)	627 (570 to 689)	650 (582 to 724)	873 (790 to 965)	764 (631 to 916)
Tamaulipas	1920 (1600 to 2300)	1420 (1170 to 1710)	1240 (1040 to 1510)	998 (905 to 1090)	808 (729 to 890)	598 (538 to 658)	535 (486 to 587)	488 (437 to 543)	620 (562 to 680)	597 (535 to 665)
Tlaxcala	2170 (1790 to 2600)	1580 (1310 to 1880)	1320 (1090 to 1560)	1160 (984 to 1340)	900 (813 to 998)	661 (597 to 730)	569 (512 to 630)	530 (471 to 595)	728 (655 to 809)	795 (708 to 890)
Veracruz de Ignacio de la Llave	2060 (1740 to 2450)	1460 (1220 to 1730)	1240 (1040 to 1470)	958 (871 to 1060)	765 (694 to 843)	596 (541 to 660)	573 (521 to 630)	575 (517 to 639)	692 (630 to 762)	693 (577 to 816)
Yucatán	2010 (1700 to 2360)	1650 (1410 to 1930)	1520 (1260 to 1810)	1290 (1170 to 1420)	968 (871 to 1070)	726 (656 to 808)	660 (599 to 729)	580 (520 to 651)	721 (650 to 799)	743 (664 to 837)
Zacatecas	1710 (1430 to 2040)	1280 (1080 to 1520)	1080 (913 to 1310)	862 (781 to 954)	717 (646 to 793)	597 (536 to 663)	542 (491 to 599)	513 (457 to 578)	662 (600 to 736)	684 (612 to 767)
Nicaragua	1800 (1640 to 2000)	1490 (1340 to 1630)	1280 (1160 to 1420)	800 (673 to 948)	548 (494 to 610)	468 (422 to 520)	479 (430 to 532)	434 (371 to 508)	552 (473 to 653)	633 (520 to 770)
Panama	1080 (984 to 1190)	939 (867 to 1020)	932 (867 to 1010)	594 (550 to 644)	504 (466 to 551)	440 (408 to 476)	403 (374 to 434)	380 (334 to 433)	451 (394 to 518)	427 (343 to 524)
Venezuela	1390 (1290 to 1480)	1070 (991 to 1160)	1100 (1020 to 1180)	865 (804 to 930)	667 (624 to 716)	528 (490 to 566)	473 (438 to 510)	536 (434 to 666)	681 (554 to 834)	732 (584 to 912)
Tropical Latin America	1540 (1380 to 1730)	1260 (1140 to 1370)	1080 (992 to 1180)	888 (827 to 951)	744 (693 to 798)	622 (582 to 666)	540 (504 to 582)	481 (428 to 547)	531 (469 to 609)	589 (514 to 681)
Brazil	1530 (1370 to 1730)	1270 (1160 to 1390)	1090 (996 to 1190)	893 (832 to 958)	749 (698 to 806)	624 (583 to 670)	540 (503 to 583)	480 (426 to 547)	531 (467 to 613)	587 (511 to 680)
Acre	1490 (1200 to 1830)	1170 (968 to 1400)	955 (804 to 1130)	786 (696 to 873)	654 (584 to 733)	668 (593 to 740)	533 (478 to 590)	495 (409 to 591)	598 (485 to 722)	587 (474 to 712)
Alagoas	1940 (1550 to 2370)	1600 (1340 to 1900)	1330 (1120 to 1570)	1030 (931 to 1150)	826 (736 to 925)	625 (564 to 694)	537 (478 to 603)	506 (419 to 613)	561 (469 to 692)	572 (470 to 706)
Amapá	1810 (1470 to 2210)	1410 (1190 to 1660)	1100 (941 to 1290)	827 (729 to 932)	811 (705 to 917)	667 (583 to 754)	487 (425 to 562)	583 (466 to 721)	621 (497 to 775)	579 (455 to 725)
Amazonas	1380 (1090 to 1700)	1120 (910 to 1370)	980 (800 to 1190)	746 (660 to 836)	698 (614 to 778)	557 (497 to 623)	495 (438 to 556)	474 (393 to 576)	634 (527 to 770)	720 (586 to 897)
Bahia	1450 (1180 to 1780)	1240 (1050 to 1470)	1080 (917 to 1280)	801 (722 to 895)	627 (560 to 701)	542 (488 to 602)	573 (518 to 640)	437 (366 to 527)	476 (397 to 570)	490 (401 to 599)
Ceará	1800 (1440 to 2210)	1470 (1240 to 1730)	1160 (959 to 1380)	846 (701 to 1000)	562 (493 to 638)	536 (472 to 608)	467 (406 to 530)	458 (373 to 562)	516 (423 to 639)	490 (390 to 621)
Distrito Federal	1760 (1460 to 2120)	1450 (1200 to 1700)	1340 (1120 to 1590)	1210 (1080 to 1340)	964 (865 to 1080)	763 (689 to 844)	602 (542 to 667)	521 (434 to 622)	601 (498 to 727)	617 (505 to 745)
Espírito Santo	1410 (1180 to 1700)	1230 (996 to 1490)	1110 (916 to 1340)	916 (824 to 1010)	757 (680 to 846)	607 (548 to 674)	526 (472 to 587)	458 (379 to 550)	528 (438 to 640)	580 (474 to 709)
Goiás	2090 (1700 to 2510)	1720 (1420 to 2070)	1310 (1080 to 1620)	976 (849 to 1130)	763 (688 to 862)	604 (543 to 671)	578 (520 to 646)	509 (425 to 624)	583 (488 to 716)	670 (552 to 830)
Maranhão	1680 (1410 to 2000)	1400 (1190 to 1630)	1170 (988 to 1400)	839 (704 to 984)	739 (661 to 836)	773 (690 to 866)	747 (656 to 851)	665 (541 to 817)	680 (558 to 836)	651 (531 to 824)
Mato Grosso	1290 (1040 to 1560)	1130 (934 to 1350)	965 (787 to 1170)	709 (627 to 803)	703 (624 to 792)	623 (552 to 702)	533 (480 to 593)	466 (392 to 559)	584 (486 to 709)	652 (532 to 795)
Mato Grosso do Sul	1330 (1060 to 1640)	1150 (940 to 1410)	1020 (840 to 1230)	817 (735 to 907)	755 (676 to 844)	643 (580 to 713)	571 (516 to 635)	490 (407 to 592)	559 (458 to 687)	643 (520 to 790)
Minas Gerais	1560 (1260 to 1900)	1330 (1090 to 1590)	1150 (937 to 1400)	947 (860 to 1040)	767 (691 to 848)	580 (527 to 638)	498 (451 to 551)	450 (382 to 533)	478 (398 to 570)	565 (463 to 684)
Pará	1590 (1310 to 1910)	1260 (1050 to 1510)	1070 (876 to 1290)	855 (746 to 975)	709 (628 to 809)	582 (509 to 659)	539 (471 to 612)	497 (405 to 612)	582 (472 to 716)	566 (453 to 704)
Paraíba	2020 (1620 to 2540)	1580 (1340 to 1880)	1230 (1020 to 1460)	905 (814 to 1020)	658 (592 to 738)	519 (470 to 573)	469 (425 to 522)	444 (371 to 533)	491 (404 to 600)	526 (430 to 648)
Paraná	1400 (1150 to 1720)	1240 (1010 to 1510)	1140 (936 to 1400)	991 (892 to 1100)	851 (764 to 943)	714 (645 to 788)	578 (518 to 639)	480 (400 to 574)	522 (432 to 628)	646 (525 to 793)
Pernambuco	2150 (1730 to 2610)	1740 (1470 to 2030)	1410 (1190 to 1660)	1010 (913 to 1120)	881 (792 to 977)	706 (636 to 780)	552 (497 to 607)	511 (426 to 608)	586 (487 to 705)	589 (480 to 712)
Piauí	1400 (1130 to 1710)	1160 (960 to 1380)	1020 (834 to 1230)	763 (642 to 908)	587 (510 to 675)	531 (467 to 605)	487 (420 to 561)	451 (360 to 562)	500 (400 to 621)	482 (384 to 617)
Rio de Janeiro	1340 (1070 to 1650)	1120 (910 to 1390)	1010 (828 to 1220)	946 (863 to 1040)	877 (795 to 975)	693 (634 to 763)	596 (542 to 658)	546 (461 to 651)	628 (526 to 753)	682 (566 to 823)
Rio Grande do Norte	2010 (1630 to 2480)	1490 (1250 to 1770)	1080 (900 to 1280)	760 (663 to 872)	575 (509 to 649)	504 (450 to 566)	457 (406 to 514)	442 (369 to 528)	492 (410 to 589)	510 (420 to 623)
Rio Grande do Sul	1330 (1090 to 1600)	1110 (897 to 1330)	954 (778 to 1160)	825 (750 to 910)	728 (656 to 807)	638 (581 to 702)	537 (488 to 591)	492 (413 to 595)	504 (421 to 604)	602 (497 to 726)
Rondônia	2020 (1600 to 2510)	1730 (1440 to 2050)	1580 (1340 to 1860)	2000 (1800 to 2230)	1110 (982 to 1260)	740 (663 to 826)	585 (520 to 662)	476 (392 to 579)	552 (447 to 679)	534 (429 to 670)
Roraima	2100 (1720 to 2510)	1760 (1480 to 2100)	1510 (1260 to 1820)	1970 (1650 to 2300)	1190 (1020 to 1390)	1070 (916 to 1230)	802 (672 to 939)	690 (548 to 855)	817 (643 to 1050)	716 (561 to 927)
Santa Catarina	1190 (968 to 1450)	1020 (822 to 1240)	925 (754 to 1140)	804 (725 to 886)	732 (657 to 811)	649 (586 to 714)	525 (475 to 579)	462 (387 to 549)	493 (407 to 590)	581 (474 to 704)
São Paulo	1230 (1010 to 1500)	1070 (877 to 1300)	967 (792 to 1170)	894 (815 to 980)	816 (742 to 897)	662 (605 to 721)	541 (494 to 594)	477 (408 to 564)	528 (448 to 628)	609 (510 to 731)
Sergipe	1670 (1380 to 2010)	1410 (1190 to 1670)	1140 (967 to 1370)	967 (869 to 1080)	833 (750 to 938)	685 (619 to 761)	507 (453 to 568)	473 (396 to 570)	531 (441 to 651)	538 (439 to 662)
Tocantins	1670 (1300 to 2120)	1400 (1170 to 1670)	1090 (908 to 1300)	766 (635 to 922)	694 (603 to 797)	659 (582 to 742)	585 (520 to 663)	538 (443 to 660)	575 (467 to 711)	529 (427 to 656)

Paraguay	1740 (1490 to 2050)	808 (709 to 923)	822 (718 to 944)	698 (611 to 796)	572 (497 to 663)	547 (472 to 633)	549 (459 to 646)	543 (440 to 687)	532 (423 to 672)	687 (544 to 871)
North Africa and Middle East	2080 (1900 to 2300)	1760 (1630 to 1890)	1470 (1380 to 1570)	1200 (1140 to 1260)	1070 (1020 to 1130)	862 (814 to 912)	755 (714 to 797)	713 (668 to 765)	808 (750 to 872)	846 (781 to 918)
North Africa and Middle East	2080 (1900 to 2300)	1760 (1630 to 1890)	1470 (1380 to 1570)	1200 (1140 to 1260)	1070 (1020 to 1130)	862 (814 to 912)	755 (714 to 797)	713 (668 to 765)	808 (750 to 872)	846 (781 to 918)
Afghanistan	2830 (2160 to 3870)	2440 (1960 to 3080)	2340 (1940 to 2830)	2430 (2060 to 2910)	2000 (1630 to 2410)	2000 (1570 to 2550)	1660 (1230 to 2240)	1490 (1060 to 2150)	1820 (1300 to 2690)	1880 (1350 to 2700)
Algeria	2230 (2000 to 2510)	1880 (1650 to 2130)	1700 (1480 to 1940)	1720 (1560 to 1890)	1090 (973 to 1220)	827 (732 to 918)	863 (791 to 944)	809 (695 to 953)	904 (773 to 1070)	944 (793 to 1140)
Bahrain	1900 (1550 to 2280)	1620 (1350 to 1910)	1330 (1120 to 1560)	1220 (1090 to 1370)	1180 (1070 to 1290)	1180 (1080 to 1300)	1010 (930 to 1100)	740 (667 to 831)	826 (741 to 921)	889 (789 to 997)
Egypt	2530 (2300 to 2800)	2200 (2050 to 2370)	2250 (2040 to 2470)	1960 (1790 to 2140)	1870 (1710 to 2050)	1460 (1340 to 1600)	1640 (1480 to 1810)	1530 (1410 to 1680)	1600 (1430 to 1780)	1620 (1410 to 1840)
Iran	2100 (1890 to 2350)	1740 (1560 to 1940)	1270 (1150 to 1410)	869 (779 to 964)	918 (836 to 1010)	706 (644 to 777)	567 (527 to 611)	525 (477 to 575)	580 (526 to 641)	678 (611 to 755)
Alborz	1970 (1640 to 2360)	1610 (1330 to 1960)	1130 (930 to 1370)	763 (616 to 947)	833 (681 to 1010)	832 (694 to 1000)	721 (626 to 830)	674 (560 to 816)	733 (609 to 900)	830 (690 to 994)
Ardebil	2180 (1840 to 2570)	1840 (1540 to 2210)	1420 (1190 to 1710)	987 (805 to 1190)	897 (741 to 1070)	677 (594 to 775)	496 (444 to 558)	535 (459 to 621)	593 (502 to 689)	706 (595 to 828)
Bushehr	1940 (1600 to 2280)	1650 (1350 to 1970)	1270 (1040 to 1520)	858 (704 to 1040)	774 (647 to 930)	653 (567 to 750)	567 (509 to 630)	511 (438 to 591)	558 (472 to 647)	649 (549 to 759)
Chahar Mahaal and Bakhtiari	1660 (1390 to 1980)	1430 (1180 to 1710)	1120 (920 to 1340)	810 (659 to 990)	761 (632 to 906)	603 (512 to 711)	466 (407 to 527)	392 (327 to 470)	429 (359 to 512)	503 (423 to 605)
East Azarbayejan	2330 (1960 to 2710)	1930 (1610 to 2300)	1440 (1190 to 1720)	976 (800 to 1180)	1020 (842 to 1240)	883 (753 to 1030)	713 (633 to 803)	661 (551 to 783)	701 (584 to 836)	796 (671 to 947)
Fars	1590 (1330 to 1900)	1380 (1130 to 1650)	1070 (884 to 1290)	812 (652 to 997)	844 (689 to 1030)	738 (623 to 871)	646 (565 to 738)	571 (470 to 679)	621 (515 to 749)	724 (600 to 863)
Gilan	1700 (1430 to 2000)	1470 (1180 to 1820)	1090 (885 to 1330)	784 (637 to 959)	2270 (1960 to 2640)	747 (636 to 869)	631 (559 to 711)	584 (487 to 687)	641 (533 to 761)	740 (618 to 882)
Golestan	1970 (1640 to 2340)	1690 (1380 to 2050)	1320 (1080 to 1620)	952 (776 to 1180)	912 (747 to 1100)	779 (679 to 899)	624 (557 to 703)	648 (551 to 753)	694 (595 to 807)	810 (692 to 944)
Hamadan	2670 (2260 to 3140)	2240 (1860 to 2710)	1630 (1370 to 1940)	1010 (822 to 1220)	877 (727 to 1040)	698 (601 to 813)	577 (512 to 646)	525 (442 to 620)	570 (476 to 679)	642 (540 to 764)
Hormozgan	1960 (1640 to 2300)	1830 (1510 to 2200)	1290 (1060 to 1550)	938 (760 to 1160)	918 (754 to 1100)	754 (647 to 884)	589 (520 to 668)	520 (435 to 614)	572 (480 to 684)	670 (557 to 802)
Ilam	1830 (1520 to 2190)	1630 (1330 to 1970)	1280 (1060 to 1550)	926 (742 to 1140)	837 (686 to 996)	691 (594 to 802)	581 (515 to 650)	552 (475 to 646)	605 (510 to 712)	706 (604 to 841)
Isfahan	1750 (1460 to 2090)	1510 (1240 to 1830)	1070 (870 to 1290)	728 (597 to 903)	750 (610 to 907)	637 (533 to 738)	525 (465 to 597)	513 (428 to 605)	571 (479 to 674)	671 (564 to 803)
Kerman	2290 (1920 to 2700)	1850 (1550 to 2230)	1360 (1120 to 1630)	922 (758 to 1130)	898 (740 to 1080)	809 (697 to 942)	668 (593 to 760)	611 (509 to 716)	655 (547 to 767)	742 (617 to 884)
Kermanshah	2180 (1850 to 2580)	1910 (1570 to 2310)	1450 (1200 to 1770)	1010 (844 to 1210)	964 (789 to 1150)	830 (714 to 965)	671 (591 to 760)	584 (484 to 702)	641 (535 to 766)	748 (626 to 886)
Khorasan-e-Razavi	2840 (2400 to 3310)	2340 (1960 to 2790)	1680 (1420 to 2010)	1070 (868 to 1280)	1020 (838 to 1220)	858 (733 to 1000)	643 (569 to 723)	578 (482 to 686)	633 (526 to 751)	739 (620 to 871)
Khuzestan	1820 (1530 to 2170)	1550 (1280 to 1870)	1200 (995 to 1450)	889 (734 to 1090)	843 (705 to 1020)	774 (681 to 885)	577 (519 to 643)	582 (492 to 680)	644 (546 to 755)	767 (652 to 908)
Kohgiluyeh and Boyer-Ahmad	1820 (1520 to 2170)	1530 (1260 to 1850)	1220 (1010 to 1490)	906 (737 to 1090)	840 (687 to 1020)	669 (572 to 789)	518 (457 to 587)	500 (418 to 597)	544 (449 to 658)	622 (515 to 748)
Kurdistan	2710 (2290 to 3170)	2390 (2000 to 2870)	1770 (1490 to 2090)	1160 (951 to 1400)	943 (786 to 1120)	708 (612 to 813)	563 (497 to 632)	494 (415 to 577)	538 (453 to 643)	624 (524 to 740)
Lorestan	2130 (1790 to 2540)	1760 (1440 to 2130)	1330 (1100 to 1600)	937 (768 to 1150)	848 (708 to 1020)	700 (606 to 813)	549 (485 to 622)	463 (376 to 550)	533 (437 to 636)	665 (557 to 787)
Markazi	1980 (1640 to 2340)	1760 (1450 to 2100)	1350 (1120 to 1620)	905 (740 to 1100)	872 (720 to 1040)	719 (614 to 840)	550 (488 to 620)	477 (404 to 564)	538 (453 to 634)	638 (539 to 748)
Mazandaran	1500 (1230 to 1790)	1290 (1060 to 1570)	1010 (820 to 1210)	735 (594 to 895)	724 (586 to 890)	645 (545 to 764)	533 (474 to 602)	502 (418 to 600)	558 (461 to 665)	656 (549 to 786)
North Khorasan	2520 (2090 to 2970)	2130 (1780 to 2590)	1640 (1360 to 1960)	1080 (881 to 1300)	994 (818 to 1200)	815 (702 to 952)	601 (530 to 676)	589 (495 to 688)	647 (544 to 773)	761 (636 to 905)
Qazvin	2400 (2030 to 2810)	2100 (1710 to 2560)	1510 (1210 to 1900)	955 (778 to 1170)	813 (684 to 971)	699 (601 to 818)	524 (465 to 587)	462 (386 to 546)	526 (437 to 627)	634 (529 to 756)
Qom	1760 (1450 to 2080)	1540 (1260 to 1840)	1190 (984 to 1420)	840 (681 to 1010)	931 (773 to 1120)	880 (755 to 1010)	788 (700 to 877)	600 (509 to 701)	658 (556 to 779)	782 (664 to 918)
Semnan	2110 (1770 to 2510)	1810 (1490 to 2190)	1280 (1060 to 1550)	835 (669 to 1020)	744 (618 to 895)	648 (550 to 764)	529 (465 to 599)	463 (380 to 554)	534 (442 to 645)	655 (546 to 777)
Sistan and Baluchistan	2490 (2060 to 2930)	2030 (1700 to 2440)	1560 (1280 to 1890)	1070 (876 to 1310)	956 (794 to 1150)	738 (618 to 864)	587 (506 to 672)	565 (462 to 681)	623 (510 to 759)	743 (621 to 893)
South Khorasan	2330 (1950 to 2710)	1930 (1600 to 2310)	1460 (1220 to 1720)	948 (786 to 1160)	801 (669 to 972)	602 (523 to 696)	499 (446 to 561)	432 (368 to 506)	486 (408 to 578)	578 (490 to 683)
Tehran	1420 (1180 to 1680)	1130 (932 to 1360)	813 (664 to 984)	603 (488 to 755)	600 (488 to 743)	531 (447 to 620)	442 (384 to 505)	425 (342 to 512)	474 (391 to 562)	554 (462 to 663)
West Azarbayejan	2300 (1960 to 2700)	1990 (1660 to 2370)	1500 (1260 to 1820)	998 (818 to 1220)	1040 (845 to 1240)	909 (782 to 1050)	682 (609 to 762)	691 (594 to 801)	729 (614 to 862)	838 (706 to 988)
Yazd	2070 (1730 to 2460)	1610 (1350 to 1920)	1180 (961 to 1430)	811 (659 to 987)	781 (643 to 940)	703 (592 to 834)	599 (527 to 681)	516 (430 to 614)	611 (505 to 725)	752 (633 to 896)
Zanjan	2700 (2250 to 3220)	2360 (1930 to 2860)	1670 (1350 to 2090)	987 (805 to 1190)	2270 (1970 to 2640)	660 (563 to 768)	491 (438 to 548)	430 (364 to 503)	493 (418 to 588)	592 (504 to 699)
Iraq	1330 (1140 to 1520)	1090 (965 to 1230)	990 (870 to 1140)	819 (716 to 933)	863 (746 to 998)	807 (632 to 1040)	738 (537 to 1010)	691 (501 to 946)	931 (704 to 1270)	885 (658 to 1240)
Jordan	1720 (1260 to 2290)	1380 (1090 to 1750)	1100 (940 to 1290)	837 (730 to 951)	906 (784 to 1040)	970 (817 to 1130)	664 (576 to 761)	490 (411 to 574)	601 (503 to 711)	657 (548 to 776)
Kuwait	949 (757 to 1170)	736 (642 to 838)	697 (648 to 754)	682 (634 to 732)	584 (529 to 646)	479 (433 to 533)	387 (357 to 421)	260 (230 to 296)	302 (266 to 347)	310 (262 to 367)
Lebanon	1480 (1190 to 1810)	1020 (817 to 1260)	886 (740 to 1070)	836 (688 to 1060)	808 (659 to 1010)	637 (545 to 732)	499 (441 to 567)	412 (346 to 490)	495 (412 to 599)	599 (500 to 721)
Libya	2180 (1870 to 2510)	1750 (1470 to 2040)	1120 (986 to 1270)	808 (717 to 920)	679 (573 to 802)	622 (550 to 700)	709 (628 to 798)	706 (556 to 875)	782 (620 to 972)	816 (646 to 1030)
Morocco	2190 (1890 to 2540)	1940 (1670 to 2250)	1520 (1320 to 1770)	1230 (1090 to 1390)	970 (866 to 1090)	864 (763 to 985)	820 (688 to 982)	769 (623 to 903)	876 (723 to 1010)	848 (694 to 985)
Oman	2350 (1610 to 3610)	1930 (1400 to 2670)	1380 (1060 to 1810)	1030 (817 to 1310)	823 (658 to 1020)	673 (557 to 804)	613 (546 to 684)	535 (462 to 613)	688 (597 to 783)	694 (608 to 789)
Palestine	2030 (1580 to 2560)	1520 (1260 to 1820)	1150 (982 to 1360)	973 (782 to 1230)	952 (795 to 1150)	880 (780 to 990)	819 (741 to 908)	628 (563 to 708)	660 (580 to 753)	743 (650 to 851)
Qatar	1260 (891 to 1840)	1170 (883 to 1580)	1030 (813 to 1290)	935 (766 to 1110)	1010 (866 to 1170)	924 (800 to 1070)	834 (720 to 957)	512 (428 to 615)	572 (483 to 683)	570 (480 to 680)
Saudi Arabia	1800 (1480 to 2170)	1680 (1370 to 2040)	1350 (1130 to 1640)	1040 (859 to 1270)	1010 (835 to 1210)	943 (837 to 1070)	832 (747 to 934)	736 (602 to 910)	786 (638 to 963)	763 (615 to 952)
Sudan	2030 (1710 to 2440)	1880 (1610 to 2210)	1740 (1530 to 1990)	1610 (1420 to 1820)	1400 (1230 to 1620)	1180 (982 to 1420)	963 (805 to 1170)	849 (679 to 1090)	984 (796 to 1250)	1010 (804 to 1300)
Syria	1450 (1210 to 1730)	1040 (885 to 1210)	834 (741 to 942)	740 (634 to 863)	996 (830 to 1180)	1070 (944 to 1220)	900 (768 to 1050)	908 (721 to 1140)	913 (724 to 1150)	947 (757 to 1210)
Tunisia	2310 (1990 to 2680)	1940 (1720 to 2200)	1570 (1420 to 1740)	1050 (942 to 1180)	697 (619 to 785)	614 (522 to 715)	542 (422 to 676)	485 (373 to 615)	524 (401 to 664)	664 (534 to 812)
Türkiye	1740 (1470 to 2020)	1500 (1290 to 1730)	1190 (1030 to 1370)	921 (810 to 1040)	836 (756 to 921)	554 (470 to 650)	505 (444 to 570)	517 (461 to 578)	611 (533 to 697)	618 (531 to 718)
United Arab Emirates	1470 (1120 to 1910)	1290 (1040 to 1570)	1000 (812 to 1210)	1040 (832 to 1280)	988 (801 to 1180)	904 (762 to 1060)	1330 (1130 to 1550)	2280 (1900 to 2750)	1870 (1520 to 2270)	1790 (1450 to 2190)
Yemen	3150 (2210 to 4770)	2540 (1900 to 3580)	2210 (1730 to 2880)	1610 (1320 to 2000)	1360 (1140 to 1620)	1160 (959 to 1400)	948 (777 to 1170)	935 (718 to 1240)	1030 (801 to 1360)	1130 (867 to 1470)
South Asia	2650 (2330 to 3010)	2300 (2040 to 2580)	1960 (1800 to 2140)	1550 (1450 to 1660)	1310 (1220 to 1410)	1080 (1010 to 1170)	913 (848 to 985)	834 (748 to 930)	888 (790 to 1000)	931 (815 to 1050)
South Asia	2650 (2330 to 3010)	2300 (2040 to 2580)	1960 (1800 to 2140)	1550 (1450 to 1660)	1310 (1220 to 1410)	1080 (1010 to 1170)	913 (848 to 985)	834 (748 to 930)	888 (790 to 1000)	931 (815 to 1050)
Bangladesh	2300 (1980 to 2700)	2040 (1770 to 2360)	2290 (2010 to 2640)	1550 (1370 to 1740)	1350 (1230 to 1490)	1070 (969 to 1200)	878 (792 to 968)	697 (568 to 842)	769 (625 to 931)	771 (624 to 944)
Bhutan	2740 (2270 to 3250)	2280 (1860 to 2760)	1930 (1470 to 2480)	1760 (1340 to 2350)	1390 (1120 to 1720)	1090 (938 to 1270)	785 (675 to 920)	709 (588 to 829)	703 (582 to 830)	712 (590 to 845)
India	2760 (2350 to 3190)	2390 (2060 to 2730)	1970 (1780 to 2180)	1580 (1460 to 1720)	1310 (1200 to 1430)	1060 (977 to 1170)	898 (825 to 980)	825 (727 to 934)	866 (756 to 1000)	916 (781 to 1060)
Nepal	2510 (2140 to 2890)	2210 (1900 to 2550)	1940 (1660 to 2250)	1820 (1560 to 2120)	1500 (1300 to 1710)	1060 (952 to 1190)	876 (766 to 1000)	815 (671 to 1020)	910 (756 to 1120)	1010 (845 to 1240)
Pakistan	2120 (1820 to 2470)	1810 (1550 to 2080)	1540 (1350 to 1740)	1280 (1150 to 1420)	1230 (1090 to 1390)	1290 (1140 to 1460)	1140 (957 to 1350)	1020 (837 to 1250)	1180 (980 to 1420)	1200 (987 to 1430)
Azad Jammu & Kashmir	2000 (1560 to 2500)	1790 (1420 to 2250)	1550 (1230 to 1910)	1280 (1020 to 1600)	1220 (919 to 1600)	1240 (945 to 1620)	1070 (760 to 1460)	976 (681 to 1390)	1110 (794 to 1540)	1200 (858 to 1740)
Balochistan	1720 (1330 to 2180)	1540 (1190 to 1930)	1310 (1030 to 1700)	1140 (910 to 1430)	1220 (934 to 1590)	1360 (1050 to 1730)	1270 (920 to 1740)	1170 (845 to 1580)	1380 (1020 to 1810)	1330 (975 to 1800)

Gilgit-Baltistan	2130 (1520 to 2860)	1970 (1530 to 2540)	1710 (1330 to 2140)	1430 (1150 to 1820)	1380 (1040 to 1820)	1470 (1070 to 1970)	1280 (841 to 1870)	1150 (713 to 1800)	1440 (921 to 2150)	1380 (881 to 2110)
Islamabad Capital Territory	2180 (1710 to 2720)	1850 (1390 to 2350)	1380 (1090 to 1740)	995 (788 to 1230)	875 (670 to 1130)	904 (696 to 1190)	799 (587 to 1100)	729 (531 to 961)	878 (672 to 1120)	901 (686 to 1170)
Khyber Pakhtunkhwa	2260 (1810 to 2770)	1830 (1450 to 2270)	1530 (1250 to 1890)	1220 (996 to 1480)	1170 (909 to 1530)	1320 (1020 to 1670)	1180 (881 to 1540)	1060 (782 to 1420)	1250 (958 to 1620)	1240 (925 to 1630)
Punjab	2120 (1780 to 2520)	1830 (1540 to 2140)	1570 (1360 to 1810)	1330 (1170 to 1520)	1260 (1080 to 1490)	1300 (1120 to 1500)	1130 (923 to 1400)	1010 (811 to 1280)	1140 (928 to 1440)	1190 (964 to 1450)
Sindh	2140 (1690 to 2620)	1770 (1410 to 2190)	1470 (1180 to 1830)	1210 (994 to 1480)	1200 (943 to 1520)	1260 (999 to 1580)	1130 (844 to 1510)	1020 (762 to 1350)	1210 (941 to 1540)	1190 (904 to 1550)
Southeast Asia, east Asia, and Oceania	1880 (1660 to 2130)	2890 (2240 to 4040)	1280 (1110 to 1490)	1230 (1090 to 1390)	1010 (916 to 1110)	862 (797 to 933)	677 (628 to 730)	549 (479 to 629)	548 (478 to 628)	562 (484 to 646)
East Asia	1860 (1570 to 2170)	3410 (2500 to 5050)	1240 (1030 to 1520)	1240 (1060 to 1460)	1020 (894 to 1160)	848 (767 to 934)	629 (568 to 694)	494 (411 to 592)	491 (407 to 590)	485 (398 to 588)
China	1850 (1560 to 2170)	3500 (2560 to 5190)	1250 (1030 to 1540)	1260 (1070 to 1490)	1030 (901 to 1180)	846 (760 to 935)	631 (568 to 698)	494 (408 to 596)	492 (404 to 595)	485 (395 to 593)
North Korea	2440 (1730 to 3540)	1070 (798 to 1470)	950 (729 to 1210)	895 (690 to 1150)	876 (698 to 1070)	1270 (922 to 2030)	838 (701 to 989)	706 (564 to 891)	699 (548 to 889)	686 (531 to 878)
Taiwan (province of China)	1510 (1310 to 1730)	1120 (1100 to 1130)	905 (894 to 916)	747 (738 to 756)	623 (614 to 631)	505 (498 to 512)	380 (374 to 386)	334 (309 to 364)	317 (292 to 348)	328 (298 to 362)
Oceania	2040 (1730 to 2420)	1680 (1450 to 1950)	1440 (1250 to 1640)	1280 (1120 to 1490)	1260 (1070 to 1480)	1200 (1050 to 1350)	1130 (1010 to 1260)	1050 (904 to 1210)	1050 (904 to 1220)	1200 (983 to 1460)
American Samoa	1250 (1070 to 1460)	1110 (1010 to 1230)	1050 (940 to 1170)	899 (801 to 994)	821 (749 to 898)	858 (776 to 946)	891 (803 to 988)	886 (762 to 1030)	929 (804 to 1080)	878 (736 to 1040)
Cook Islands	2440 (2190 to 2720)	1750 (1570 to 1970)	1280 (1100 to 1440)	1110 (992 to 1240)	929 (818 to 1050)	716 (622 to 816)	557 (494 to 633)	516 (426 to 625)	519 (421 to 637)	517 (418 to 637)
Fiji	1620 (1450 to 1780)	1090 (970 to 1210)	951 (850 to 1070)	930 (836 to 1040)	1040 (881 to 1230)	1190 (1080 to 1320)	1110 (995 to 1230)	1020 (832 to 1240)	1020 (827 to 1260)	1200 (917 to 1500)
Guam	860 (759 to 970)	957 (874 to 1050)	903 (828 to 992)	952 (873 to 1030)	821 (758 to 894)	533 (491 to 582)	401 (369 to 437)	439 (393 to 490)	379 (342 to 418)	413 (364 to 463)
Kiribati	2100 (1790 to 2450)	1830 (1510 to 2150)	1640 (1330 to 1960)	1450 (1180 to 1730)	1470 (1260 to 1680)	1400 (1230 to 1590)	1340 (1140 to 1550)	1250 (1040 to 1500)	1240 (1030 to 1490)	1230 (1020 to 1480)
Marshall Islands	1900 (1330 to 2730)	1550 (1150 to 2100)	1350 (1070 to 1740)	1210 (1020 to 1440)	1310 (1180 to 1450)	1490 (1330 to 1670)	1450 (1270 to 1650)	1320 (1070 to 1620)	1310 (1060 to 1610)	1290 (1040 to 1600)
Federated States of Micronesia	2320 (1950 to 2700)	1830 (1480 to 2250)	1600 (1220 to 2030)	1420 (1130 to 1770)	1340 (1110 to 1580)	1250 (1080 to 1450)	1150 (979 to 1360)	1100 (892 to 1370)	1100 (885 to 1380)	1090 (872 to 1390)
Nauru	1950 (1620 to 2330)	1830 (1540 to 2150)	1700 (1460 to 2000)	1500 (1220 to 1850)	1450 (1240 to 1690)	1670 (1470 to 1900)	1630 (1400 to 1880)	1400 (1140 to 1720)	1390 (1110 to 1710)	1370 (1090 to 1710)
Niue	1780 (1520 to 2060)	1290 (1060 to 1550)	1020 (846 to 1220)	894 (732 to 1080)	927 (768 to 1100)	937 (780 to 1100)	883 (734 to 1050)	849 (685 to 1040)	973 (816 to 1160)	971 (806 to 1150)
Northern Mariana Islands	1230 (1010 to 1530)	1160 (947 to 1430)	1040 (853 to 1250)	948 (771 to 1180)	863 (697 to 1070)	737 (620 to 874)	683 (576 to 808)	687 (564 to 830)	560 (457 to 678)	765 (628 to 867)
Palau	1920 (1550 to 2340)	1520 (1240 to 1850)	1280 (1030 to 1590)	1180 (954 to 1440)	1140 (938 to 1350)	1110 (945 to 1280)	1190 (1010 to 1370)	1200 (1000 to 1420)	1190 (986 to 1430)	1180 (974 to 1420)
Papua New Guinea	2220 (1780 to 2830)	1840 (1520 to 2220)	1570 (1300 to 1880)	1430 (1170 to 1750)	1360 (1070 to 1710)	1250 (1050 to 1470)	1200 (1020 to 1370)	1100 (902 to 1350)	1120 (902 to 1380)	1320 (1020 to 1700)
Samoa	1420 (1250 to 1610)	1070 (967 to 1180)	1000 (916 to 1100)	795 (687 to 909)	959 (813 to 1110)	936 (806 to 1100)	911 (784 to 1070)	972 (815 to 1150)	941 (781 to 1130)	931 (773 to 1120)
Solomon Islands	2230 (1890 to 2600)	1960 (1620 to 2320)	1710 (1380 to 2010)	1470 (1210 to 1770)	1430 (1190 to 1710)	1330 (1110 to 1590)	1260 (1030 to 1530)	1180 (944 to 1500)	1190 (946 to 1520)	1170 (933 to 1500)
Tokelau	1670 (1370 to 2010)	1490 (1190 to 1830)	1350 (1100 to 1630)	1250 (1040 to 1490)	1120 (944 to 1350)	1030 (865 to 1240)	911 (747 to 1130)	827 (658 to 1040)	1000 (842 to 1190)	995 (834 to 1200)
Tonga	1400 (1160 to 1650)	1260 (1100 to 1420)	1150 (988 to 1320)	966 (841 to 1100)	774 (662 to 901)	736 (636 to 843)	726 (610 to 856)	698 (567 to 865)	694 (558 to 860)	692 (553 to 865)
Tuvalu	2170 (1770 to 2600)	1890 (1540 to 2300)	1720 (1410 to 2080)	1650 (1380 to 1940)	1490 (1280 to 1730)	1440 (1260 to 1640)	1120 (944 to 1310)	1040 (848 to 1290)	1030 (837 to 1290)	1020 (824 to 1280)
Vanuatu	2060 (1710 to 2430)	1820 (1470 to 2220)	1560 (1230 to 1880)	1320 (1080 to 1590)	1220 (1000 to 1450)	1160 (987 to 1350)	1080 (957 to 1220)	1010 (888 to 1160)	1010 (889 to 1160)	1120 (958 to 1330)
Southeast Asia	1990 (1780 to 2210)	1590 (1440 to 1740)	1370 (1260 to 1490)	1180 (1090 to 1280)	1000 (940 to 1060)	895 (832 to 962)	784 (734 to 835)	680 (622 to 749)	684 (613 to 758)	762 (674 to 861)
Cambodia	2290 (1880 to 2750)	1960 (1630 to 2330)	1900 (1580 to 2290)	1700 (1350 to 2140)	1490 (1280 to 1750)	1340 (1190 to 1500)	1000 (878 to 1150)	913 (733 to 1120)	907 (722 to 1120)	996 (783 to 1260)
Indonesia	2280 (1970 to 2640)	1900 (1600 to 2240)	1610 (1380 to 1880)	1340 (1210 to 1500)	1140 (1040 to 1260)	1040 (886 to 1210)	951 (819 to 1070)	842 (691 to 998)	880 (728 to 1050)	949 (766 to 1160)
Aceh	1940 (1520 to 2370)	1660 (1270 to 2150)	1510 (1150 to 1970)	1330 (1080 to 1630)	1160 (969 to 1370)	1030 (810 to 1310)	1070 (870 to 1290)	965 (705 to 1220)	1010 (758 to 1270)	1080 (811 to 1370)
Bali	2260 (1830 to 2800)	1940 (1480 to 2530)	1740 (1300 to 2240)	1520 (1230 to 1840)	1190 (1010 to 1410)	1040 (825 to 1310)	944 (758 to 1130)	832 (642 to 1050)	863 (676 to 1100)	926 (703 to 1200)
Bangka-Belitung Islands	2610 (2080 to 3240)	2230 (1690 to 2870)	1990 (1540 to 2510)	1750 (1420 to 2110)	1590 (1290 to 1910)	1420 (1130 to 1790)	1280 (1060 to 1520)	1120 (884 to 1380)	1150 (908 to 1450)	1280 (984 to 1620)
Banten	2720 (2180 to 3320)	2440 (1930 to 3080)	1980 (1530 to 2540)	1560 (1290 to 1900)	1200 (1010 to 1410)	1040 (822 to 1290)	1000 (809 to 1220)	893 (682 to 1120)	934 (715 to 1170)	992 (736 to 1270)
Bengkulu	3070 (2540 to 3670)	2650 (2050 to 3390)	2280 (1820 to 2910)	1960 (1620 to 2320)	1660 (1390 to 1960)	1420 (1120 to 1770)	1260 (1020 to 1520)	1080 (839 to 1350)	1120 (887 to 1390)	1170 (901 to 1480)
Gorontalo	2750 (2200 to 3330)	2440 (1880 to 3070)	2100 (1620 to 2630)	1830 (1500 to 2190)	1640 (1400 to 1940)	1550 (1240 to 1970)	1550 (1270 to 1870)	1320 (988 to 1680)	1350 (1030 to 1740)	1410 (1060 to 1810)
Jakarta	1860 (1480 to 2310)	1610 (1240 to 2080)	1360 (1060 to 1750)	1170 (966 to 1430)	1020 (852 to 1200)	917 (721 to 1160)	856 (684 to 1030)	755 (584 to 974)	795 (620 to 1010)	854 (640 to 1110)
Jambi	2770 (2270 to 3370)	2400 (1850 to 3090)	2150 (1630 to 2720)	1890 (1540 to 2240)	1500 (1260 to 1780)	1210 (951 to 1540)	1080 (900 to 1290)	941 (713 to 1150)	972 (737 to 1210)	1050 (784 to 1320)
West Java	2380 (1960 to 2860)	1990 (1600 to 2480)	1630 (1300 to 2030)	1300 (1100 to 1540)	1090 (942 to 1250)	983 (782 to 1200)	898 (744 to 1080)	800 (618 to 1010)	835 (652 to 1050)	908 (689 to 1150)
Central Java	1950 (1570 to 2350)	1610 (1270 to 2060)	1380 (1080 to 1750)	1140 (966 to 1340)	949 (820 to 1100)	928 (741 to 1150)	873 (712 to 1040)	772 (594 to 964)	807 (628 to 998)	880 (685 to 1120)
East Java	2040 (1690 to 2440)	1700 (1360 to 2140)	1450 (1170 to 1810)	1220 (1050 to 1420)	1070 (935 to 1220)	952 (763 to 1190)	836 (686 to 997)	738 (571 to 928)	780 (615 to 977)	846 (642 to 1070)
West Kalimantan	2480 (2020 to 3040)	2330 (1820 to 2960)	2180 (1710 to 2770)	1940 (1610 to 2270)	1550 (1300 to 1830)	1300 (1030 to 1630)	1130 (929 to 1350)	973 (773 to 1200)	990 (778 to 1230)	1070 (833 to 1360)
South Kalimantan	2740 (2240 to 3250)	2430 (1920 to 3030)	2020 (1570 to 2610)	1670 (1390 to 2000)	1470 (1250 to 1730)	1390 (1080 to 1780)	1220 (990 to 1460)	1050 (800 to 1300)	1110 (843 to 1380)	1200 (907 to 1530)
Central Kalimantan	2250 (1800 to 2820)	1920 (1450 to 2470)	1760 (1340 to 2260)	1600 (1300 to 1930)	1410 (1190 to 1680)	1100 (870 to 1390)	1080 (887 to 1270)	958 (723 to 1200)	1000 (760 to 1250)	1080 (819 to 1380)
East Kalimantan	1940 (1540 to 2390)	1830 (1380 to 2370)	1660 (1280 to 2140)	1440 (1160 to 1760)	1360 (1130 to 1620)	1280 (1020 to 1590)	1160 (964 to 1370)	976 (768 to 1220)	1010 (803 to 1290)	1090 (840 to 1400)
North Kalimantan	1400 (1130 to 1730)	1380 (1120 to 1730)	1220 (978 to 1510)	984 (812 to 1190)	766 (627 to 922)	634 (490 to 826)	565 (444 to 698)	505 (373 to 672)	543 (401 to 715)	632 (453 to 845)
Riau Islands	2020 (1600 to 2540)	1790 (1350 to 2320)	1600 (1190 to 2080)	1440 (1130 to 1800)	1260 (1020 to 1520)	1030 (821 to 1280)	1030 (831 to 1220)	914 (691 to 1150)	942 (704 to 1200)	1000 (744 to 1280)
Lampung	2070 (1660 to 2550)	1810 (1390 to 2300)	1510 (1160 to 1900)	1220 (1010 to 1460)	994 (837 to 1160)	899 (723 to 1150)	873 (717 to 1060)	784 (602 to 1020)	814 (633 to 1060)	889 (688 to 1140)
Maluku	3260 (2670 to 3890)	2650 (2070 to 3340)	2300 (1760 to 2910)	2030 (1640 to 2450)	1750 (1450 to 2070)	1530 (1190 to 1920)	1500 (1210 to 1810)	1260 (965 to 1580)	1290 (1000 to 1640)	1340 (1030 to 1680)
North Maluku	3190 (2600 to 3840)	2740 (2150 to 3410)	2480 (1970 to 3150)	2260 (1880 to 2680)	1970 (1660 to 2330)	1760 (1400 to 2240)	1730 (1420 to 2060)	1470 (1110 to 1830)	1510 (1140 to 1860)	1560 (1180 to 1960)
West Nusa Tenggara	3130 (2600 to 3700)	2800 (2200 to 3520)	2490 (1990 to 3130)	2120 (1760 to 2530)	1700 (1430 to 2010)	1440 (1120 to 1850)	1190 (969 to 1440)	1050 (793 to 1320)	1090 (817 to 1360)	1130 (862 to 1430)
East Nusa Tenggara	2820 (2340 to 3370)	2340 (1830 to 2950)	2060 (1590 to 2630)	1760 (1440 to 2120)	1530 (1280 to 1800)	1290 (1030 to 1630)	1090 (883 to 1320)	963 (721 to 1230)	988 (745 to 1250)	1070 (793 to 1390)
Papua	2020 (1610 to 2510)	2010 (1530 to 2620)	1930 (1480 to 2490)	1830 (1500 to 2200)	1680 (1400 to 1990)	1520 (1190 to 1910)	1450 (1180 to 1740)	1210 (912 to 1560)	1250 (937 to 1620)	1300 (953 to 1680)
West Papua	2220 (1780 to 2730)	2120 (1610 to 2720)	2060 (1590 to 2610)	1910 (1570 to 2320)	1730 (1450 to 2060)	1600 (1260 to 2000)	1740 (1460 to 2050)	1330 (1030 to 1700)	1360 (1070 to 1710)	1400 (1090 to 1750)
Riau	1960 (1550 to 2390)	1790 (1380 to 2280)	1570 (1210 to 2040)	1310 (1060 to 1560)	1200 (1010 to 1430)	1080 (844 to 1370)	941 (758 to 1130)	821 (625 to 1040)	865 (672 to 1090)	943 (702 to 1210)
West Sulawesi	3590 (2960 to 4330)	3120 (2410 to 3920)	2770 (2190 to 3490)	2410 (2000 to 2850)	2010 (1710 to 2370)	1650 (1300 to 2090)	1450 (1160 to 1740)	1230 (924 to 1570)	1260 (963 to 1620)	1350 (1010 to 1720)
South Sulawesi	2580 (2140 to 3120)	2240 (1760 to 2810)	1920 (1450 to 2440)	1620 (1330 to 1970)	1300 (1100 to 1530)	1100 (855 to 1400)	1000 (808 to 1200)	865 (654 to 1090)	902 (692 to 1130)	951 (715 to 1210)
Central Sulawesi	2770 (2260 to 3320)	2280 (1740 to 2930)	1930 (1510 to 2470)	1640 (1350 to 1970)	1650 (1390 to 1950)	1540 (1220 to 1940)	1360 (1100 to 1630)	1160 (893 to 1470)	1190 (917 to 1490)	1290 (955 to 1650)
Southeast Sulawesi	2870 (2320 to 3430)	2640 (2050 to 3320)	2400 (1870 to 3040)	2030 (1660 to 2410)	1740 (1480 to 2040)	1540 (1220 to 1950)	1330 (1090 to 1600)	1150 (872 to 1470)	1190 (905 to 1520)	1260 (931 to 1600)
North Sulawesi	2290 (1820 to 2790)	1940 (1480 to 2510)	1760 (1330 to 2290)	1580 (1290 to 1920)	1410 (1200 to 1670)	1240 (989 to 1550)	1130 (934 to 1350)	990 (758 to 1230)	1040 (779 to 1300)	1100 (835 to 1400)
West Sumatra	3240 (2700 to 3840)	2240 (1790 to 2850)	1810 (1410 to 2270)	1460 (1190 to 1750)	1220 (1020 to 1420)	1090 (872 to 1370)	1040 (842 to 1250)	869 (665 to 1080)	910 (720 to 1120)	969 (728 to 1220)
South Sumatra	2180 (1770 to 2650)	1800 (1440 to 2250)	1500 (1170 to 1900)	1200 (988 to 1410)	1060 (907 to 1240)	952 (752 to 1180)	893 (730 to 1070)	801 (619 to 1030)	841 (664 to 1070)	904 (681 to 1180)

North Sumatra	2210 (1820 to 2680)	1830 (1400 to 2360)	1610 (1220 to 2120)	1400 (1150 to 1670)	1220 (1020 to 1460)	1160 (916 to 1430)	1050 (858 to 1250)	917 (682 to 1160)	959 (728 to 1200)	1020 (767 to 1330)
Yogyakarta	2280 (1850 to 2780)	1720 (1300 to 2220)	1440 (1080 to 1870)	1230 (1000 to 1520)	1150 (973 to 1350)	1090 (863 to 1360)	957 (790 to 1160)	851 (668 to 1070)	879 (707 to 1110)	967 (751 to 1230)
Laos	2670 (1920 to 3740)	2570 (1950 to 3390)	2410 (1940 to 3030)	2110 (1750 to 2530)	1770 (1480 to 2110)	1470 (1260 to 1710)	1070 (914 to 1260)	912 (740 to 1100)	900 (721 to 1100)	976 (782 to 1200)
Malaysia	1300 (1080 to 1560)	965 (901 to 1030)	677 (633 to 728)	726 (607 to 857)	730 (676 to 791)	712 (666 to 762)	720 (648 to 794)	664 (587 to 756)	614 (538 to 699)	758 (661 to 878)
Maldives	3040 (2550 to 3550)	2500 (2120 to 2900)	1920 (1690 to 2180)	1830 (1670 to 2020)	1210 (1120 to 1310)	818 (742 to 903)	517 (462 to 583)	414 (360 to 473)	414 (360 to 475)	449 (376 to 533)
Mauritius	1770 (1640 to 1900)	1490 (1370 to 1620)	1130 (1040 to 1230)	932 (847 to 1020)	777 (716 to 841)	691 (635 to 753)	578 (530 to 632)	576 (504 to 661)	542 (476 to 625)	628 (538 to 733)
Myanmar	2950 (2460 to 3490)	2410 (1980 to 2890)	2100 (1750 to 2500)	1800 (1510 to 2120)	1560 (1280 to 1880)	1390 (1140 to 1700)	1030 (881 to 1210)	834 (692 to 1020)	857 (706 to 1050)	934 (750 to 1160)
Philippines	1200 (1070 to 1370)	1170 (1070 to 1270)	1160 (1060 to 1260)	1070 (985 to 1160)	920 (844 to 1000)	775 (709 to 848)	783 (715 to 856)	736 (660 to 827)	702 (620 to 798)	934 (794 to 1090)
Seychelles	1230 (1080 to 1400)	1030 (948 to 1120)	888 (810 to 969)	680 (612 to 755)	678 (612 to 756)	647 (585 to 715)	639 (583 to 698)	644 (554 to 750)	564 (484 to 655)	671 (574 to 785)
Sri Lanka	1560 (1450 to 1690)	1360 (1250 to 1490)	1220 (1120 to 1310)	1070 (999 to 1160)	788 (693 to 906)	638 (558 to 736)	570 (527 to 616)	485 (369 to 617)	446 (278 to 617)	522 (362 to 687)
Thailand	1530 (1340 to 1750)	1070 (953 to 1190)	930 (832 to 1040)	833 (736 to 937)	695 (617 to 781)	665 (577 to 757)	515 (454 to 578)	436 (367 to 520)	435 (348 to 532)	474 (364 to 602)
Timor-Leste	2280 (1810 to 2860)	2000 (1720 to 2350)	1920 (1580 to 2310)	1810 (1560 to 2110)	1410 (1220 to 1610)	1110 (968 to 1270)	921 (812 to 1040)	891 (749 to 1060)	879 (729 to 1060)	985 (799 to 1220)
Viet Nam	1750 (1440 to 2140)	1220 (992 to 1510)	1160 (976 to 1430)	923 (771 to 1120)	778 (669 to 938)	651 (574 to 747)	620 (535 to 706)	565 (474 to 662)	561 (468 to 659)	588 (484 to 699)
Sub-Saharan Africa	2290 (2000 to 2620)	2080 (1860 to 2320)	1880 (1730 to 2050)	1670 (1560 to 1790)	1580 (1490 to 1660)	1680 (1580 to 1800)	1360 (1270 to 1440)	1090 (1000 to 1190)	1220 (1110 to 1340)	1330 (1220 to 1450)
Central sub-Saharan Africa	2320 (1930 to 2750)	2070 (1740 to 2440)	1900 (1640 to 2190)	1740 (1520 to 2010)	1670 (1480 to 1890)	1730 (1500 to 1990)	1470 (1210 to 1780)	1210 (926 to 1580)	1340 (1030 to 1740)	1450 (1110 to 1890)
Angola	2210 (1820 to 2630)	2090 (1780 to 2460)	1960 (1630 to 2310)	1900 (1540 to 2300)	1850 (1450 to 2330)	1700 (1410 to 2030)	1300 (1110 to 1540)	1130 (903 to 1400)	1240 (1010 to 1540)	1430 (1170 to 1760)
Central African Republic	2410 (1820 to 3220)	2260 (1770 to 2880)	2090 (1720 to 2480)	2000 (1710 to 2310)	2100 (1830 to 2390)	2630 (2170 to 3120)	2180 (1730 to 2640)	1840 (1370 to 2370)	2170 (1640 to 2790)	2120 (1600 to 2720)
Congo (Brazzaville)	2850 (2150 to 3710)	2530 (2070 to 3140)	2270 (1880 to 2680)	1870 (1540 to 2200)	1810 (1520 to 2120)	2070 (1780 to 2380)	1590 (1370 to 1840)	1380 (1100 to 1690)	1510 (1220 to 1850)	1560 (1250 to 1900)
DR Congo	2290 (1880 to 2740)	1990 (1610 to 2430)	1830 (1540 to 2160)	1680 (1430 to 1970)	1600 (1370 to 1850)	1670 (1380 to 2030)	1480 (1160 to 1930)	1200 (839 to 1740)	1320 (932 to 1890)	1430 (987 to 2040)
Equatorial Guinea	3250 (2770 to 3820)	2670 (2190 to 3200)	2210 (1780 to 2680)	1840 (1520 to 2200)	1810 (1470 to 2190)	1500 (1160 to 1920)	1310 (981 to 1730)	1140 (845 to 1520)	1370 (1060 to 1780)	1390 (1060 to 1840)
Gabon	2770 (2220 to 3330)	2050 (1710 to 2440)	1690 (1350 to 2070)	1310 (1050 to 1620)	1270 (1080 to 1500)	1490 (1250 to 1780)	1240 (1000 to 1500)	1060 (811 to 1320)	1200 (956 to 1470)	1250 (998 to 1530)
Eastern sub-Saharan Africa	2570 (2240 to 2960)	2260 (2020 to 2530)	2070 (1910 to 2270)	1860 (1730 to 1990)	1800 (1700 to 1910)	1810 (1690 to 1950)	1310 (1230 to 1400)	1060 (962 to 1170)	1180 (1070 to 1300)	1340 (1230 to 1480)
Burundi	2760 (2030 to 3760)	2430 (1910 to 3060)	2260 (1870 to 2720)	2040 (1740 to 2380)	2030 (1730 to 2350)	2300 (1940 to 2720)	1370 (1200 to 1570)	1130 (915 to 1370)	1200 (968 to 1480)	1300 (1050 to 1590)
Comoros	2330 (1850 to 2930)	2180 (1830 to 2590)	1900 (1590 to 2250)	1690 (1440 to 1990)	1460 (1230 to 1710)	1320 (1130 to 1520)	1070 (907 to 1270)	969 (770 to 1250)	1010 (803 to 1300)	1110 (877 to 1420)
Djibouti	1450 (1080 to 2020)	1450 (1110 to 1900)	1340 (1070 to 1650)	1220 (1010 to 1460)	1220 (994 to 1480)	1290 (1020 to 1620)	1170 (915 to 1500)	1010 (758 to 1360)	1170 (915 to 1520)	1210 (939 to 1560)
Eritrea	2800 (2050 to 3980)	2580 (1970 to 3400)	2490 (1950 to 3250)	2290 (2010 to 2620)	2040 (1750 to 2350)	1650 (1280 to 2060)	1450 (1070 to 1960)	1290 (915 to 1850)	1310 (927 to 1860)	1480 (1030 to 2130)
Ethiopia	3100 (2270 to 4450)	2660 (2080 to 3430)	2550 (2100 to 3090)	2450 (2130 to 2790)	2260 (1990 to 2540)	1970 (1740 to 2260)	1150 (1030 to 1290)	898 (743 to 1080)	1020 (845 to 1210)	1150 (956 to 1400)
Addis Ababa	2150 (1490 to 3150)	2060 (1450 to 2850)	1970 (1450 to 2640)	1860 (1460 to 2330)	1990 (1630 to 2390)	2420 (1910 to 3020)	1150 (949 to 1400)	938 (695 to 1240)	1170 (887 to 1530)	1420 (1080 to 1840)
Afar	5640 (4010 to 8240)	4300 (3160 to 5870)	4150 (3140 to 5400)	4270 (3510 to 5230)	3940 (3300 to 4630)	4170 (3440 to 5010)	2250 (1900 to 2640)	1780 (1340 to 2330)	1980 (1490 to 2590)	2210 (1670 to 2880)
Amhara	3080 (2200 to 4450)	2590 (1900 to 3520)	2470 (1920 to 3160)	2360 (1940 to 2840)	2240 (1910 to 2570)	1960 (1610 to 2380)	1050 (901 to 1230)	783 (618 to 1010)	865 (684 to 1100)	958 (767 to 1210)
Benishangul-Gumuz	4980 (3630 to 7110)	4060 (2950 to 5390)	3990 (2990 to 5230)	4010 (3240 to 4840)	3780 (3150 to 4570)	3550 (2940 to 4250)	2050 (1700 to 2440)	1480 (1070 to 1990)	1460 (1060 to 1980)	1450 (1020 to 2020)
Dire Dawa	2560 (1780 to 3710)	2440 (1750 to 3340)	2360 (1720 to 3200)	2170 (1720 to 2730)	1970 (1560 to 2440)	1830 (1380 to 2390)	984 (793 to 1220)	790 (601 to 1040)	1020 (791 to 1340)	1260 (957 to 1680)
Gambella	2690 (1910 to 3960)	2340 (1740 to 3220)	2250 (1680 to 3020)	2210 (1750 to 2710)	1990 (1600 to 2460)	1790 (1390 to 2300)	1070 (846 to 1360)	776 (615 to 999)	1060 (836 to 1340)	1370 (1070 to 1740)
Harari	3490 (2470 to 5060)	3290 (2400 to 4530)	3020 (2270 to 4010)	2650 (2100 to 3270)	2230 (1790 to 2740)	1940 (1480 to 2540)	1050 (819 to 1380)	845 (613 to 1140)	1120 (854 to 1530)	1440 (1090 to 1850)
Oromia	3120 (2250 to 4430)	2690 (2050 to 3640)	2590 (1990 to 3360)	2470 (2030 to 2920)	2210 (1880 to 2560)	1820 (1510 to 2210)	1130 (963 to 1330)	889 (704 to 1120)	1010 (812 to 1270)	1150 (922 to 1470)
Somali	3190 (2210 to 4700)	2800 (2030 to 3840)	2730 (1990 to 3570)	2630 (2110 to 3230)	2580 (2120 to 3070)	2480 (2020 to 2980)	1440 (1170 to 1720)	1220 (920 to 1630)	1430 (1070 to 1910)	1650 (1260 to 2180)
Southern Nations, Nationalities, and Peoples	2920 (2070 to 4230)	2600 (1920 to 3510)	2490 (1900 to 3170)	2390 (1970 to 2860)	2160 (1820 to 2550)	1820 (1480 to 2230)	1230 (1030 to 1480)	964 (726 to 1250)	1080 (817 to 1430)	1220 (943 to 1610)
Tigray	3460 (2430 to 5040)	2960 (2170 to 4070)	2860 (2120 to 3810)	2810 (2290 to 3400)	2420 (2010 to 2860)	2000 (1580 to 2450)	1090 (899 to 1310)	888 (655 to 1190)	1000 (759 to 1330)	1130 (855 to 1520)
Kenya	1970 (1700 to 2300)	1620 (1390 to 1900)	1420 (1220 to 1690)	1220 (1060 to 1400)	1180 (1040 to 1330)	1550 (1330 to 1830)	1260 (1120 to 1400)	974 (804 to 1160)	1140 (969 to 1340)	1220 (1040 to 1410)
Baringo	2030 (1580 to 2610)	1830 (1350 to 2480)	1770 (1370 to 2290)	1600 (1250 to 2080)	1420 (1100 to 1840)	1330 (1040 to 1680)	1200 (951 to 1520)	1000 (743 to 1370)	1160 (885 to 1540)	1220 (942 to 1590)
Bomet	1040 (801 to 1330)	911 (669 to 1230)	869 (670 to 1140)	799 (617 to 1040)	820 (650 to 1060)	1300 (939 to 1750)	1070 (828 to 1360)	808 (650 to 1060)	950 (777 to 1190)	1000 (811 to 1220)
Bungoma	2110 (1670 to 2730)	1680 (1260 to 2220)	1420 (1090 to 1870)	1240 (970 to 1590)	1100 (848 to 1390)	1210 (934 to 1560)	1010 (797 to 1230)	873 (660 to 1160)	988 (770 to 1280)	1060 (832 to 1390)
Busia	3140 (2480 to 3890)	2260 (1690 to 2980)	1790 (1380 to 2330)	1320 (1020 to 1710)	1290 (962 to 1690)	1750 (1310 to 2330)	1300 (959 to 1710)	956 (691 to 1290)	1150 (890 to 1490)	1260 (983 to 1660)
Elgeyo Marakwet	1700 (1300 to 2240)	1560 (1130 to 2120)	1570 (1200 to 2100)	1450 (1100 to 1870)	1260 (948 to 1680)	1300 (977 to 1680)	1220 (963 to 1540)	1120 (851 to 1470)	1230 (944 to 1630)	1260 (964 to 1640)
Embu	2380 (1900 to 2970)	1820 (1400 to 2370)	1360 (1050 to 1780)	1040 (808 to 1350)	912 (699 to 1190)	1080 (813 to 1490)	967 (739 to 1280)	798 (627 to 1100)	920 (736 to 1200)	967 (785 to 1250)
Garissa	1980 (1480 to 2580)	1820 (1310 to 2510)	1630 (1200 to 2190)	1530 (1140 to 2030)	1420 (1050 to 1870)	1430 (1010 to 1910)	1380 (1030 to 1770)	1200 (890 to 1550)	1470 (1150 to 1840)	1580 (1240 to 1950)
Homa Bay	3250 (2660 to 3940)	2300 (1810 to 2950)	2060 (1670 to 2570)	1670 (1340 to 2070)	1630 (1230 to 2110)	3080 (2280 to 4050)	2230 (1680 to 2880)	1370 (955 to 1930)	1580 (1160 to 2120)	1620 (1240 to 2150)
Isiolo	3010 (2360 to 3760)	2740 (2090 to 3530)	2500 (1980 to 3140)	2010 (1610 to 2530)	1600 (1240 to 2000)	1540 (1130 to 2060)	1320 (962 to 1760)	1030 (753 to 1440)	1250 (958 to 1660)	1330 (1040 to 1710)
Kajiado	1580 (1220 to 2080)	1420 (1040 to 1970)	1380 (1040 to 1810)	1240 (952 to 1670)	1170 (844 to 1620)	1390 (995 to 1860)	1190 (879 to 1510)	1010 (737 to 1350)	1230 (944 to 1610)	1300 (1020 to 1650)
Kakamega	2190 (1730 to 2760)	1800 (1390 to 2360)	1530 (1210 to 1990)	1280 (993 to 1640)	1290 (1020 to 1610)	1650 (1230 to 2170)	1280 (986 to 1620)	928 (698 to 1230)	1110 (877 to 1440)	1200 (962 to 1520)
Kericho	1170 (890 to 1580)	1140 (813 to 1600)	1070 (804 to 1460)	989 (757 to 1290)	924 (699 to 1230)	1150 (841 to 1560)	938 (685 to 1280)	801 (628 to 1120)	924 (731 to 1240)	973 (766 to 1280)
Kiambu	1550 (1180 to 2020)	1340 (992 to 1810)	1200 (876 to 1630)	1110 (830 to 1470)	1120 (848 to 1460)	1420 (1010 to 1820)	1130 (875 to 1440)	923 (656 to 1220)	1140 (872 to 1440)	1230 (946 to 1550)
Kilifi	2700 (2180 to 3300)	2160 (1710 to 2760)	1870 (1510 to 2350)	1550 (1200 to 1950)	1390 (1080 to 1760)	1550 (1160 to 2050)	1190 (926 to 1510)	915 (696 to 1240)	1090 (862 to 1430)	1170 (905 to 1500)
Kirinyaga	2230 (1760 to 2800)	1830 (1380 to 2410)	1520 (1130 to 1990)	1070 (811 to 1420)	964 (733 to 1270)	1140 (839 to 1550)	1000 (745 to 1350)	835 (623 to 1130)	972 (754 to 1290)	1030 (798 to 1390)
Kisii	2220 (1680 to 2790)	2060 (1510 to 2760)	1870 (1390 to 2470)	1670 (1260 to 2150)	1620 (1230 to 2110)	1940 (1490 to 2440)	1650 (1330 to 2020)	1340 (988 to 1750)	1500 (1100 to 1900)	1560 (1160 to 2040)
Kisumu	1770 (1400 to 2220)	1440 (1090 to 1910)	1330 (1040 to 1710)	1180 (940 to 1520)	1220 (935 to 1590)	2160 (1560 to 2900)	1770 (1310 to 2290)	1190 (820 to 1660)	1490 (1100 to 1940)	1580 (1230 to 2020)
Kitui	2120 (1700 to 2640)	1690 (1280 to 2250)	1430 (1100 to 1860)	1150 (898 to 1490)	1060 (836 to 1350)	1390 (1030 to 1850)	1000 (773 to 1280)	788 (622 to 1040)	911 (731 to 1190)	963 (781 to 1230)
Kwale	2570 (2010 to 3260)	2280 (1720 to 2990)	1920 (1490 to 2500)	1620 (1250 to 2090)	1510 (1150 to 1940)	1720 (1260 to 2320)	1330 (994 to 1710)	1030 (730 to 1380)	1180 (862 to 1570)	1250 (915 to 1650)
Laikipia	1310 (988 to 1760)	1150 (799 to 1640)	1020 (766 to 1360)	882 (682 to 1160)	922 (684 to 1250)	1320 (961 to 1780)	1130 (837 to 1540)	882 (657 to 1200)	1040 (809 to 1370)	1090 (864 to 1400)
Lamu	3140 (2500 to 3970)	2460 (1830 to 3210)	1930 (1470 to 2530)	1380 (1040 to 1810)	1370 (1010 to 1790)	1500 (1050 to 2080)	1140 (816 to 1520)	916 (677 to 1250)	1090 (850 to 1460)	1180 (910 to 1580)
Machakos	1520 (1160 to 1960)	1320 (983 to 1760)	1160 (892 to 1520)	967 (746 to 1260)	896 (704 to 1140)	1290 (959 to 1680)	1050 (813 to 1340)	823 (647 to 1090)	956 (771 to 1230)	1010 (825 to 1300)
Makueni	1950 (1540 to 2430)	1360 (1030 to 1750)	1170 (910 to 1490)	961 (745 to 1210)	922 (735 to 1170)	1710 (1200 to 2350)	1310 (1020 to 1680)	859 (702 to 1100)	975 (819 to 1220)	1010 (842 to 1250)
Mandera	1870 (1440 to 2400)	1680 (1230 to 2260)	1580 (1180 to 2090)	1500 (1140 to 1980)	1500 (1120 to 1980)	1530 (1080 to 2100)	1530 (1140 to 1940)	1320 (948 to 1750)	1720 (1320 to 2190)	1920 (1500 to 2370)
Marsabit	1800 (1370 to 2330)	1720 (1280 to 2310)	1510 (1170 to 1970)	1310 (1010 to 1700)	1160 (879 to 1530)	1220 (891 to 1680)	1170 (859 to 1570)	972 (716 to 1360)	1140 (861 to 1560)	1210 (944 to 1670)

Meru	1610 (1300 to 2030)	1370 (1030 to 1820)	1200 (938 to 1580)	1020 (791 to 1340)	951 (740 to 1240)	1190 (903 to 1610)	1090 (862 to 1400)	898 (697 to 1220)	1050 (840 to 1390)	1110 (905 to 1440)
Migori	2710 (2180 to 3360)	2100 (1620 to 2720)	1860 (1480 to 2340)	1670 (1350 to 2080)	1590 (1240 to 2020)	2360 (1730 to 3210)	2020 (1480 to 2630)	1330 (905 to 1890)	1570 (1140 to 2140)	1660 (1250 to 2190)
Mombasa	1930 (1460 to 2520)	1750 (1300 to 2350)	1430 (1090 to 1880)	1160 (863 to 1560)	1210 (848 to 1630)	1750 (1260 to 2400)	1190 (911 to 1600)	863 (664 to 1190)	1130 (908 to 1470)	1250 (1010 to 1620)
Murang'a	1860 (1510 to 2280)	1380 (1040 to 1810)	1160 (896 to 1500)	951 (718 to 1260)	911 (696 to 1170)	1160 (852 to 1530)	1100 (824 to 1370)	872 (655 to 1160)	1010 (767 to 1310)	1060 (832 to 1360)
Nairobi	1970 (1560 to 2470)	1590 (1230 to 2070)	1230 (959 to 1580)	1040 (827 to 1350)	1170 (902 to 1490)	1770 (1340 to 2230)	1370 (1140 to 1610)	1090 (858 to 1370)	1370 (1100 to 1670)	1480 (1220 to 1810)
Nakuru	1040 (772 to 1370)	997 (712 to 1380)	941 (690 to 1250)	870 (663 to 1140)	864 (653 to 1130)	1160 (846 to 1550)	986 (762 to 1260)	794 (635 to 1020)	967 (795 to 1210)	1040 (855 to 1280)
Nandi	1190 (915 to 1580)	1080 (769 to 1480)	996 (746 to 1320)	912 (683 to 1200)	877 (672 to 1150)	1080 (815 to 1410)	848 (658 to 1090)	739 (586 to 966)	878 (706 to 1150)	936 (735 to 1190)
Narok	1720 (1300 to 2260)	1530 (1080 to 2170)	1480 (1100 to 1940)	1290 (951 to 1700)	1320 (958 to 1740)	1530 (1130 to 2060)	1240 (956 to 1620)	999 (751 to 1330)	1240 (938 to 1570)	1340 (1070 to 1680)
Nyamira	1980 (1520 to 2530)	1630 (1210 to 2170)	1480 (1100 to 1980)	1350 (1010 to 1770)	1340 (1010 to 1690)	1830 (1390 to 2330)	1890 (1520 to 2330)	1520 (1090 to 2030)	1700 (1250 to 2220)	1750 (1290 to 2290)
Nyandarua	1800 (1350 to 2370)	1520 (1090 to 2110)	1310 (944 to 1800)	1150 (833 to 1530)	1120 (797 to 1470)	1390 (997 to 1830)	1410 (1110 to 1790)	1110 (783 to 1470)	1300 (945 to 1690)	1380 (1040 to 1770)
Nyeri	2240 (1730 to 2800)	1680 (1260 to 2270)	1420 (1070 to 1900)	1250 (917 to 1650)	1300 (954 to 1640)	1770 (1300 to 2270)	1470 (1110 to 1850)	1180 (799 to 1610)	1330 (966 to 1770)	1380 (1020 to 1810)
Samburu	1280 (946 to 1700)	1240 (874 to 1730)	1190 (875 to 1590)	1140 (863 to 1490)	1130 (835 to 1540)	1320 (971 to 1770)	1200 (884 to 1620)	997 (747 to 1380)	1210 (933 to 1620)	1310 (1000 to 1710)
Siaya	2380 (1900 to 2930)	2000 (1540 to 2590)	1710 (1360 to 2160)	1480 (1160 to 1890)	1550 (1180 to 1990)	2420 (1820 to 3210)	1690 (1320 to 2170)	1100 (842 to 1460)	1250 (977 to 1630)	1320 (1060 to 1690)
Taita Taveta	1960 (1530 to 2520)	1620 (1200 to 2170)	1500 (1140 to 2000)	1190 (894 to 1590)	1130 (805 to 1520)	1560 (1110 to 2120)	1480 (1070 to 1900)	1090 (752 to 1490)	1250 (921 to 1630)	1320 (987 to 1710)
Tana River	2250 (1690 to 2950)	1930 (1350 to 2650)	1800 (1300 to 2410)	1690 (1210 to 2280)	1680 (1190 to 2170)	2100 (1510 to 2710)	1790 (1400 to 2230)	1400 (1050 to 1830)	1610 (1210 to 2080)	1700 (1260 to 2230)
Tharaka Nithi	2280 (1770 to 2890)	1630 (1160 to 2190)	1510 (1120 to 2000)	1340 (1010 to 1770)	1310 (976 to 1700)	1550 (1160 to 2020)	1150 (916 to 1420)	1010 (716 to 1310)	1090 (804 to 1410)	1100 (803 to 1440)
Trans Nzoia	1280 (952 to 1720)	1210 (870 to 1680)	1110 (844 to 1450)	1020 (774 to 1320)	976 (729 to 1280)	1310 (941 to 1740)	1110 (822 to 1480)	871 (668 to 1190)	1010 (804 to 1330)	1070 (851 to 1390)
Turkana	1370 (1040 to 1830)	1370 (1020 to 1850)	1370 (1060 to 1790)	1360 (1060 to 1740)	1340 (960 to 1790)	1840 (1290 to 2500)	1480 (1050 to 2000)	1060 (753 to 1510)	1340 (1020 to 1800)	1460 (1140 to 1880)
Uasin Gishu	1150 (871 to 1550)	1120 (797 to 1570)	1070 (783 to 1440)	1010 (770 to 1340)	1030 (763 to 1380)	1300 (968 to 1700)	1060 (796 to 1380)	838 (655 to 1160)	990 (797 to 1310)	1060 (862 to 1380)
Vihiga	2330 (1890 to 2890)	1580 (1180 to 2060)	1380 (1060 to 1810)	1240 (961 to 1610)	1290 (980 to 1700)	1670 (1260 to 2200)	1440 (1090 to 1830)	1110 (777 to 1490)	1240 (893 to 1610)	1300 (968 to 1710)
Wajir	2520 (1950 to 3210)	2180 (1630 to 2900)	1920 (1440 to 2520)	1730 (1330 to 2280)	1560 (1200 to 2020)	1500 (1110 to 1950)	1500 (1150 to 1900)	1340 (944 to 1770)	1560 (1160 to 2020)	1650 (1250 to 2100)
West Pokot	1870 (1410 to 2460)	1730 (1260 to 2390)	1710 (1290 to 2240)	1710 (1330 to 2220)	1550 (1190 to 2010)	1390 (1070 to 1800)	1270 (983 to 1650)	1070 (795 to 1450)	1260 (961 to 1690)	1320 (1020 to 1760)
Madagascar	2370 (2050 to 2700)	1990 (1700 to 2350)	1980 (1740 to 2260)	1590 (1390 to 1810)	1590 (1420 to 1780)	1490 (1300 to 1700)	1330 (1080 to 1630)	1220 (972 to 1480)	1390 (1140 to 1670)	1440 (1170 to 1730)
Malawi	2780 (2320 to 3290)	2610 (2150 to 3130)	2300 (1990 to 2660)	1880 (1650 to 2110)	1900 (1690 to 2140)	2340 (2010 to 2710)	1510 (1330 to 1740)	1170 (976 to 1420)	1270 (1060 to 1540)	1630 (1400 to 1920)
Mozambique	2380 (1970 to 2850)	2150 (1830 to 2510)	1940 (1680 to 2240)	1800 (1590 to 2040)	1690 (1480 to 1940)	1630 (1430 to 1850)	1660 (1420 to 1920)	1340 (1070 to 1620)	1410 (1110 to 1710)	1710 (1390 to 2050)
Rwanda	3260 (2750 to 3840)	2510 (2090 to 3000)	2320 (1940 to 2730)	2080 (1800 to 2380)	2060 (1850 to 2300)	2040 (1800 to 2300)	1100 (965 to 1270)	936 (761 to 1150)	966 (779 to 1200)	1190 (977 to 1450)
Somalia	2470 (2050 to 2920)	2460 (2010 to 3000)	2340 (1960 to 2760)	2100 (1740 to 2500)	2070 (1730 to 2440)	1990 (1600 to 2450)	2170 (1680 to 2870)	1640 (1190 to 2300)	1990 (1440 to 2830)	2190 (1600 to 3060)
South Sudan	1960 (1590 to 2430)	1970 (1640 to 2390)	1860 (1500 to 2320)	1710 (1420 to 2050)	1640 (1360 to 1980)	1520 (1240 to 1910)	1370 (1110 to 1700)	1430 (1120 to 1830)	1620 (1300 to 2050)	1660 (1310 to 2160)
Uganda	2340 (1920 to 2820)	1920 (1650 to 2230)	1680 (1470 to 1990)	1690 (1420 to 2020)	1850 (1560 to 2150)	1920 (1660 to 2200)	1250 (1080 to 1430)	945 (790 to 1150)	1050 (869 to 1280)	1290 (1060 to 1570)
Tanzania	2510 (2090 to 3020)	2280 (1920 to 2680)	1940 (1670 to 2280)	1520 (1340 to 1740)	1520 (1350 to 1710)	1690 (1490 to 1910)	1260 (1110 to 1410)	1000 (836 to 1210)	1110 (932 to 1340)	1240 (1040 to 1490)
Zambia	2150 (1770 to 2570)	1970 (1610 to 2440)	1660 (1410 to 1940)	1540 (1350 to 1740)	1870 (1650 to 2110)	2450 (2140 to 2790)	1490 (1280 to 1730)	1230 (956 to 1570)	1390 (1100 to 1770)	1750 (1410 to 2190)
Southern sub-Saharan Africa	1720 (1530 to 1930)	1540 (1390 to 1680)	1370 (1260 to 1490)	1190 (1060 to 1340)	945 (858 to 1040)	1600 (1470 to 1730)	1560 (1470 to 1660)	1070 (962 to 1210)	1300 (1170 to 1470)	1480 (1330 to 1660)
Botswana	2010 (1660 to 2420)	1860 (1520 to 2240)	1710 (1380 to 2070)	1390 (1130 to 1660)	1220 (942 to 1500)	2030 (1520 to 2660)	1470 (1230 to 1730)	1040 (867 to 1230)	1080 (897 to 1310)	1550 (1290 to 1850)
Eswatini	2510 (2080 to 2960)	2270 (1870 to 2710)	1790 (1460 to 2140)	1400 (1160 to 1690)	1220 (1020 to 1440)	2200 (1750 to 2740)	2320 (1880 to 2810)	1540 (1160 to 1940)	1860 (1490 to 2270)	2380 (1890 to 2870)
Lesotho	1820 (1520 to 2200)	1680 (1390 to 2030)	1540 (1280 to 1860)	1230 (1030 to 1480)	1100 (931 to 1300)	2060 (1680 to 2510)	2180 (1840 to 2510)	1950 (1580 to 2340)	2170 (1790 to 2560)	2740 (2260 to 3220)
Namibia	1750 (1410 to 2140)	1580 (1280 to 1900)	1420 (1190 to 1690)	1370 (1140 to 1620)	1190 (1040 to 1350)	1800 (1550 to 2110)	1280 (1070 to 1510)	1070 (819 to 1350)	1180 (922 to 1480)	1560 (1260 to 1890)
South Africa	1690 (1510 to 1870)	1500 (1360 to 1640)	1340 (1220 to 1480)	1170 (1010 to 1370)	882 (784 to 990)	1410 (1300 to 1530)	1460 (1360 to 1590)	974 (858 to 1110)	1220 (1080 to 1400)	1340 (1180 to 1530)
Eastern Cape	1780 (1480 to 2110)	1450 (1210 to 1700)	1270 (1050 to 1540)	1040 (818 to 1300)	874 (731 to 1030)	1570 (1390 to 1760)	1720 (1540 to 1920)	1200 (999 to 1430)	1730 (1440 to 2080)	1540 (1270 to 1880)
Free State	1730 (1430 to 2060)	1540 (1260 to 1820)	1370 (1130 to 1670)	1240 (965 to 1590)	970 (810 to 1150)	1730 (1530 to 1940)	1960 (1750 to 2180)	1150 (937 to 1370)	1440 (1180 to 1740)	1580 (1300 to 1930)
Gauteng	1680 (1390 to 1990)	1550 (1280 to 1850)	1440 (1200 to 1730)	1350 (1070 to 1690)	1010 (829 to 1210)	1390 (1250 to 1560)	1350 (1210 to 1510)	903 (746 to 1110)	1080 (891 to 1310)	1190 (979 to 1440)
KwaZulu-Natal	1840 (1530 to 2170)	1690 (1420 to 1970)	1490 (1240 to 1780)	1270 (1010 to 1590)	911 (768 to 1090)	1690 (1520 to 1890)	1540 (1390 to 1700)	844 (713 to 1020)	1150 (955 to 1360)	1280 (1060 to 1540)
Limpopo	1350 (1100 to 1640)	1220 (1020 to 1450)	1080 (877 to 1330)	912 (711 to 1150)	632 (521 to 758)	919 (811 to 1050)	1360 (1210 to 1500)	1010 (824 to 1200)	1190 (971 to 1440)	1500 (1250 to 1820)
Mpumalanga	1570 (1300 to 1890)	1400 (1170 to 1690)	1280 (1070 to 1550)	1070 (853 to 1340)	780 (642 to 934)	1310 (1170 to 1460)	1460 (1310 to 1620)	904 (747 to 1100)	1050 (862 to 1280)	1320 (1070 to 1610)
North West	1610 (1310 to 1930)	1460 (1220 to 1740)	1270 (1060 to 1530)	1120 (878 to 1420)	711 (587 to 857)	1610 (1410 to 1840)	2130 (1860 to 2420)	1630 (1340 to 2030)	1680 (1370 to 2120)	1880 (1520 to 2350)
Northern Cape	1480 (1210 to 1770)	1320 (1090 to 1570)	1180 (970 to 1450)	1010 (790 to 1290)	1010 (841 to 1210)	1690 (1510 to 1900)	1710 (1500 to 1950)	1130 (930 to 1400)	1350 (1090 to 1680)	1640 (1340 to 2040)
Western Cape	1390 (1130 to 1680)	1270 (1050 to 1550)	1200 (984 to 1480)	1170 (921 to 1490)	996 (821 to 1210)	1050 (948 to 1190)	914 (824 to 1020)	785 (663 to 953)	1010 (841 to 1230)	1090 (895 to 1340)
Zimbabwe	1750 (1270 to 2470)	1570 (1170 to 2060)	1420 (1130 to 1760)	1280 (1090 to 1480)	1200 (1010 to 1410)	2380 (1910 to 2870)	2080 (1860 to 2360)	1560 (1260 to 1860)	1730 (1410 to 2080)	2170 (1800 to 2570)
Western sub-Saharan Africa	2230 (1840 to 2710)	2100 (1800 to 2470)	1880 (1660 to 2150)	1640 (1480 to 1830)	1560 (1420 to 1680)	1570 (1420 to 1730)	1300 (1190 to 1440)	1090 (970 to 1220)	1180 (1050 to 1340)	1240 (1100 to 1380)
Benin	2390 (1730 to 3250)	2150 (1720 to 2720)	1890 (1590 to 2250)	1650 (1450 to 1880)	1450 (1300 to 1620)	1350 (1200 to 1510)	1160 (1030 to 1310)	998 (850 to 1190)	1050 (896 to 1260)	1170 (997 to 1400)
Burkina Faso	2760 (1970 to 3940)	2500 (1920 to 3230)	2140 (1780 to 2610)	1850 (1620 to 2130)	1740 (1530 to 1990)	1680 (1470 to 1930)	1360 (1200 to 1540)	1160 (1010 to 1340)	1290 (1130 to 1500)	1340 (1170 to 1550)
Cabo Verde	1730 (1520 to 1950)	1430 (1300 to 1570)	1350 (1220 to 1500)	888 (795 to 989)	741 (658 to 828)	684 (580 to 818)	562 (499 to 630)	585 (475 to 704)	609 (493 to 739)	609 (486 to 748)
Cameroon	2360 (1780 to 3100)	2900 (2370 to 3510)	1840 (1530 to 2180)	1640 (1410 to 1890)	1400 (1240 to 1580)	1660 (1440 to 1930)	1450 (1210 to 1760)	1150 (902 to 1490)	1240 (978 to 1620)	1390 (1100 to 1770)
Chad	2270 (1570 to 3280)	1950 (1430 to 2680)	1880 (1450 to 2440)	1750 (1460 to 2090)	1630 (1460 to 1820)	1730 (1510 to 1980)	1510 (1320 to 1730)	1340 (1080 to 1660)	1520 (1240 to 1870)	1480 (1190 to 1830)
Côte d'Ivoire	2060 (1750 to 2380)	1970 (1650 to 2390)	1690 (1450 to 1980)	1420 (1230 to 1640)	1410 (1210 to 1610)	1700 (1450 to 1960)	1410 (1190 to 1650)	1050 (877 to 1290)	1170 (985 to 1420)	1180 (982 to 1430)
The Gambia	1660 (1350 to 2030)	1650 (1380 to 1970)	1500 (1270 to 1780)	1410 (1180 to 1680)	1280 (1090 to 1520)	1260 (1080 to 1470)	1200 (1020 to 1410)	1110 (897 to 1360)	1300 (1050 to 1570)	1300 (1040 to 1580)
Ghana	1990 (1710 to 2310)	1740 (1500 to 2030)	1560 (1340 to 1830)	1430 (1210 to 1690)	1380 (1170 to 1630)	1380 (1200 to 1560)	1220 (1070 to 1380)	1010 (808 to 1280)	1080 (875 to 1350)	1160 (939 to 1430)
Guinea	2490 (2080 to 2940)	2400 (2000 to 2910)	2250 (1910 to 2670)	2030 (1750 to 2360)	1800 (1570 to 2070)	1650 (1460 to 1870)	1460 (1250 to 1690)	1250 (1020 to 1540)	1420 (1160 to 1740)	1450 (1190 to 1790)
Guinea-Bissau	3300 (2810 to 3820)	2700 (2280 to 3140)	2400 (1990 to 2870)	2090 (1760 to 2470)	1920 (1660 to 2240)	1840 (1540 to 2170)	1630 (1390 to 1870)	1440 (1150 to 1760)	1760 (1450 to 2140)	1700 (1370 to 2070)
Liberia	2780 (2200 to 3490)	2240 (1920 to 2580)	2000 (1700 to 2360)	1770 (1530 to 2060)	1820 (1570 to 2080)	1670 (1450 to 1930)	1390 (1210 to 1610)	1190 (948 to 1510)	1330 (1060 to 1670)	1330 (1050 to 1700)
Mali	2830 (2360 to 3350)	2700 (2240 to 3250)	2530 (2180 to 2990)	2170 (1910 to 2490)	1940 (1730 to 2140)	1760 (1600 to 1960)	1440 (1280 to 1610)	1250 (1050 to 1510)	1430 (1200 to 1740)	1530 (1280 to 1850)
Mauritania	2060 (1580 to 2820)	1860 (1500 to 2330)	1630 (1360 to 1970)	1580 (1350 to 1840)	1490 (1300 to 1680)	1250 (1100 to 1410)	1020 (881 to 1200)	924 (756 to 1160)	1040 (859 to 1280)	1020 (829 to 1270)
Niger	2360 (1790 to 3160)	2320 (1840 to 2900)	2160 (1840 to 2540)	2090 (1860 to 2350)	1940 (1760 to 2150)	1740 (1560 to 1940)	1310 (1120 to 1520)	1170 (953 to 1430)	1290 (1060 to 1580)	1310 (1070 to 1620)
Nigeria	2170 (1530 to 3170)	1970 (1460 to 2680)	1820 (1430 to 2310)	1560 (1290 to 1890)	1530 (1300 to 1770)	1550 (1280 to 1850)	1260 (1030 to 1520)	1050 (854 to 1280)	1120 (928 to 1360)	1170 (970 to 1410)
São Tomé and Príncipe	2810 (2520 to 3140)	2060 (1870 to 2260)	1340 (1230 to 1450)	1110 (1000 to 1220)	1130 (1020 to 1260)	1190 (1060 to 1350)	991 (861 to 1130)	868 (733 to 1020)	920 (787 to 1080)	930 (796 to 1090)

Senegal	2100 (1570 to 2790)	2000 (1610 to 2460)	1960 (1670 to 2280)	1680 (1480 to 1910)	1360 (1220 to 1520)	1280 (1150 to 1420)	1040 (914 to 1180)	950 (771 to 1180)	1060 (868 to 1310)	1150 (940 to 1400)
Sierra Leone	2470 (1930 to 3110)	2220 (1830 to 2670)	1990 (1680 to 2360)	1800 (1530 to 2110)	1650 (1440 to 1890)	1720 (1540 to 1970)	1600 (1380 to 1840)	1280 (1050 to 1570)	1410 (1170 to 1740)	1370 (1130 to 1690)
Togo	2240 (1820 to 2730)	2030 (1680 to 2410)	1740 (1500 to 2040)	1520 (1330 to 1730)	1420 (1210 to 1640)	1480 (1240 to 1750)	1350 (1160 to 1580)	1090 (865 to 1390)	1150 (914 to 1470)	1230 (986 to 1580)

Appendix 2 Table S3c: Age-standardised mortality rates (per 100 000) with 95% UIs by location, males, 1950, 1960, 1970, 1980, 1990, 2000, 2010, 2019, 2020, 2021										
**Location**	**1950**	**1960**	**1970**	**1980**	**1990**	**2000**	**2010**	**2019**	**2020**	**2021**
Global	2230 (2080 to 2390)	2330 (2090 to 2720)	1670 (1590 to 1750)	1480 (1420 to 1540)	1300 (1250 to 1350)	1190 (1160 to 1230)	1020 (985 to 1060)	879 (831 to 930)	964 (910 to 1020)	1020 (962 to 1080)
Central Europe, eastern Europe, and central Asia	1620 (1490 to 1760)	1320 (1280 to 1370)	1360 (1320 to 1400)	1430 (1390 to 1470)	1360 (1330 to 1410)	1500 (1450 to 1550)	1240 (1210 to 1280)	997 (940 to 1060)	1140 (1070 to 1210)	1240 (1160 to 1320)
Central Asia	2330 (1950 to 2750)	1740 (1580 to 1900)	1510 (1430 to 1600)	1340 (1290 to 1390)	1290 (1250 to 1340)	1380 (1300 to 1460)	1230 (1150 to 1310)	1010 (919 to 1100)	1220 (1110 to 1340)	1250 (1110 to 1400)
Armenia	2090 (1710 to 2550)	1450 (1260 to 1650)	1160 (1010 to 1320)	1000 (904 to 1120)	1110 (1030 to 1210)	1050 (974 to 1140)	990 (916 to 1070)	801 (710 to 904)	1270 (1120 to 1450)	963 (830 to 1130)
Azerbaijan	3040 (2520 to 3640)	1930 (1700 to 2190)	1580 (1440 to 1730)	1400 (1280 to 1530)	1410 (1290 to 1540)	1250 (1150 to 1370)	1170 (1070 to 1280)	1000 (865 to 1150)	1340 (1160 to 1540)	1330 (1130 to 1560)
Georgia	2220 (1840 to 2630)	1710 (1520 to 1940)	1530 (1390 to 1690)	1430 (1310 to 1550)	1320 (1210 to 1430)	1280 (1120 to 1440)	1140 (1040 to 1260)	1050 (946 to 1160)	1180 (1070 to 1310)	1360 (1210 to 1530)
Kazakhstan	2070 (1710 to 2490)	1780 (1570 to 2000)	1550 (1420 to 1710)	1430 (1300 to 1550)	1410 (1300 to 1530)	1770 (1620 to 1920)	1480 (1420 to 1540)	1160 (1060 to 1280)	1430 (1290 to 1580)	1580 (1390 to 1790)
Kyrgyzstan	2670 (2240 to 3170)	2050 (1740 to 2410)	1840 (1590 to 2130)	1480 (1310 to 1660)	1440 (1310 to 1580)	1430 (1300 to 1590)	1310 (1190 to 1440)	1000 (865 to 1170)	1230 (1030 to 1450)	1160 (943 to 1400)
Mongolia	2960 (2390 to 3640)	2190 (1820 to 2640)	1930 (1670 to 2200)	1700 (1480 to 1910)	1660 (1480 to 1830)	1720 (1560 to 1900)	1510 (1360 to 1690)	1230 (1070 to 1390)	1190 (1070 to 1330)	1340 (1190 to 1520)
Tajikistan	2720 (2200 to 3300)	2040 (1760 to 2370)	1520 (1290 to 1800)	1320 (1170 to 1500)	1270 (1150 to 1420)	1230 (1100 to 1360)	1120 (999 to 1260)	844 (710 to 1000)	1120 (934 to 1350)	1200 (976 to 1440)
Turkmenistan	2500 (2070 to 2990)	1940 (1730 to 2180)	1590 (1440 to 1740)	1410 (1290 to 1530)	1360 (1260 to 1480)	1440 (1260 to 1640)	1200 (1080 to 1330)	1180 (974 to 1420)	1420 (1170 to 1690)	1480 (1200 to 1790)
Uzbekistan	2120 (1700 to 2600)	1470 (1280 to 1670)	1350 (1230 to 1470)	1220 (1130 to 1320)	1120 (1040 to 1210)	1210 (1050 to 1420)	1160 (949 to 1440)	956 (744 to 1250)	1080 (827 to 1460)	1060 (790 to 1460)
Central Europe	1710 (1640 to 1780)	1290 (1260 to 1330)	1290 (1250 to 1330)	1290 (1250 to 1340)	1280 (1240 to 1330)	1120 (1090 to 1160)	944 (928 to 961)	826 (795 to 859)	943 (905 to 982)	1020 (979 to 1070)
Albania	1760 (1530 to 2010)	1260 (1130 to 1400)	1130 (915 to 1350)	1160 (968 to 1360)	1160 (1070 to 1250)	960 (878 to 1050)	720 (626 to 821)	710 (602 to 839)	844 (707 to 985)	913 (754 to 1100)
Bosnia and Herzegovina	2190 (2010 to 2400)	1480 (1360 to 1610)	1330 (1240 to 1430)	1240 (1160 to 1340)	952 (878 to 1040)	877 (744 to 1030)	778 (714 to 854)	764 (685 to 857)	884 (785 to 996)	936 (751 to 1140)
Bulgaria	1500 (1390 to 1620)	1080 (978 to 1200)	1060 (960 to 1170)	1180 (1080 to 1320)	1470 (1360 to 1610)	1450 (1330 to 1570)	1230 (1190 to 1270)	1160 (1050 to 1270)	1340 (1220 to 1490)	1580 (1430 to 1770)
Croatia	2390 (2220 to 2590)	1620 (1500 to 1750)	1450 (1350 to 1570)	1240 (1160 to 1340)	1400 (1290 to 1510)	1100 (996 to 1210)	859 (800 to 923)	729 (646 to 824)	785 (698 to 898)	856 (758 to 977)
Czechia	1280 (1200 to 1380)	1320 (1230 to 1430)	1420 (1330 to 1520)	1390 (1290 to 1490)	1310 (1220 to 1430)	985 (971 to 998)	785 (773 to 798)	672 (619 to 732)	770 (708 to 844)	818 (746 to 900)
Hungary	1640 (1540 to 1770)	1320 (1230 to 1420)	1340 (1240 to 1440)	1440 (1350 to 1540)	1430 (1330 to 1540)	1230 (1150 to 1330)	1020 (944 to 1100)	856 (765 to 970)	925 (818 to 1060)	1020 (901 to 1160)
Montenegro	1220 (1120 to 1340)	928 (844 to 1020)	941 (858 to 1030)	938 (855 to 1030)	933 (854 to 1020)	967 (877 to 1070)	875 (802 to 956)	1030 (902 to 1170)	1260 (1110 to 1440)	1460 (1270 to 1680)
North Macedonia	1980 (1820 to 2160)	1390 (1280 to 1520)	1160 (1070 to 1250)	1010 (934 to 1090)	1150 (1050 to 1270)	1450 (1340 to 1590)	1220 (1080 to 1390)	1130 (945 to 1350)	1200 (994 to 1450)	1430 (1170 to 1760)
Poland	1830 (1710 to 1970)	1280 (1190 to 1370)	1250 (1170 to 1340)	1300 (1190 to 1410)	1290 (1190 to 1390)	1070 (990 to 1160)	900 (891 to 908)	781 (724 to 847)	907 (831 to 989)	962 (874 to 1060)
Romania	1630 (1350 to 1970)	1280 (1170 to 1410)	1250 (1140 to 1380)	1280 (1170 to 1390)	1260 (1160 to 1360)	1170 (1080 to 1270)	1040 (966 to 1120)	919 (818 to 1030)	1050 (931 to 1200)	1160 (1010 to 1320)
Serbia	2250 (2090 to 2420)	1490 (1380 to 1590)	1500 (1380 to 1620)	1360 (1270 to 1480)	1400 (1300 to 1520)	1270 (1170 to 1380)	998 (926 to 1070)	886 (793 to 987)	1050 (926 to 1190)	1100 (968 to 1260)
Slovakia	1530 (1510 to 1560)	1240 (1220 to 1260)	1260 (1240 to 1280)	1260 (1240 to 1280)	1280 (1260 to 1300)	1080 (1060 to 1100)	937 (920 to 955)	793 (727 to 867)	870 (790 to 958)	1030 (931 to 1140)
Slovenia	2030 (1890 to 2190)	1260 (1160 to 1360)	1290 (1190 to 1400)	1200 (1100 to 1300)	997 (893 to 1120)	887 (861 to 912)	682 (659 to 706)	576 (524 to 628)	650 (590 to 712)	631 (575 to 697)
Eastern Europe	1410 (1220 to 1630)	1250 (1180 to 1310)	1360 (1290 to 1420)	1510 (1440 to 1590)	1400 (1330 to 1480)	1750 (1650 to 1850)	1430 (1360 to 1520)	1100 (994 to 1210)	1240 (1110 to 1390)	1370 (1210 to 1540)
Belarus	1180 (921 to 1480)	1030 (943 to 1120)	1060 (994 to 1140)	1240 (1150 to 1330)	1220 (1140 to 1320)	1460 (1360 to 1580)	1410 (1310 to 1530)	1130 (996 to 1290)	1340 (1140 to 1580)	1500 (1230 to 1840)
Estonia	1510 (1210 to 1850)	1360 (1320 to 1400)	1350 (1310 to 1390)	1450 (1420 to 1490)	1410 (1370 to 1440)	1320 (1290 to 1350)	982 (953 to 1010)	764 (698 to 836)	791 (720 to 867)	931 (842 to 1030)
Latvia	1280 (996 to 1590)	1230 (1140 to 1330)	1240 (1150 to 1340)	1420 (1330 to 1530)	1400 (1300 to 1500)	1330 (1250 to 1440)	1150 (1120 to 1180)	942 (861 to 1020)	999 (917 to 1100)	1200 (1090 to 1310)
Lithuania	1290 (1010 to 1620)	1100 (1030 to 1190)	1150 (1070 to 1230)	1250 (1160 to 1350)	1230 (1130 to 1330)	1190 (1110 to 1290)	1190 (1100 to 1280)	942 (868 to 1020)	1070 (970 to 1170)	1140 (1030 to 1260)
Moldova	2300 (1910 to 2750)	2020 (1800 to 2260)	1650 (1520 to 1800)	1480 (1360 to 1590)	1370 (1280 to 1480)	1350 (1230 to 1490)	1260 (1130 to 1400)	944 (778 to 1140)	1030 (886 to 1230)	1170 (979 to 1440)
Russia	1470 (1190 to 1790)	1310 (1220 to 1410)	1430 (1330 to 1530)	1610 (1490 to 1740)	1460 (1350 to 1580)	1830 (1690 to 1980)	1480 (1380 to 1580)	1080 (959 to 1230)	1280 (1120 to 1460)	1390 (1220 to 1610)
Ukraine	1270 (1010 to 1550)	1100 (1020 to 1190)	1260 (1180 to 1340)	1390 (1290 to 1500)	1340 (1250 to 1450)	1720 (1600 to 1850)	1370 (1210 to 1550)	1190 (923 to 1500)	1180 (891 to 1590)	1380 (956 to 1880)
High income	1450 (1420 to 1490)	1250 (1220 to 1280)	1210 (1180 to 1240)	1020 (1000 to 1050)	871 (851 to 890)	728 (712 to 746)	591 (578 to 605)	533 (519 to 547)	580 (566 to 595)	582 (566 to 598)
Australasia	1260 (1190 to 1330)	1190 (1120 to 1270)	1230 (1160 to 1310)	1010 (952 to 1080)	837 (786 to 893)	649 (611 to 691)	516 (486 to 550)	457 (426 to 489)	430 (404 to 459)	448 (423 to 476)
Australia	1280 (1200 to 1370)	1200 (1120 to 1300)	1240 (1160 to 1340)	1000 (928 to 1080)	828 (768 to 895)	640 (595 to 691)	511 (479 to 552)	450 (415 to 488)	424 (395 to 458)	445 (416 to 477)
New Zealand	1200 (1120 to 1290)	1150 (1060 to 1240)	1190 (1110 to 1280)	1070 (998 to 1150)	882 (817 to 957)	698 (647 to 751)	542 (492 to 596)	493 (458 to 534)	467 (430 to 506)	468 (428 to 514)
High-income Asia Pacific	2150 (2030 to 2280)	1510 (1420 to 1600)	1250 (1180 to 1330)	965 (911 to 1030)	778 (734 to 826)	638 (602 to 678)	523 (494 to 556)	431 (405 to 459)	420 (398 to 445)	429 (404 to 457)
Brunei	2090 (1870 to 2330)	1540 (1380 to 1730)	1160 (1030 to 1290)	1080 (944 to 1210)	1130 (1010 to 1260)	881 (793 to 989)	796 (712 to 881)	882 (764 to 1030)	796 (679 to 933)	822 (698 to 961)
Japan	1730 (1630 to 1860)	1360 (1280 to 1460)	1120 (1050 to 1210)	852 (796 to 914)	698 (653 to 749)	590 (552 to 634)	501 (468 to 542)	422 (392 to 457)	411 (385 to 441)	417 (388 to 450)
Aichi	1650 (1340 to 1980)	1310 (1040 to 1610)	1090 (868 to 1350)	843 (768 to 930)	696 (635 to 763)	590 (537 to 650)	496 (451 to 546)	414 (372 to 461)	405 (368 to 446)	404 (364 to 447)
Akita	1830 (1500 to 2200)	1480 (1180 to 1840)	1240 (1000 to 1530)	927 (837 to 1020)	748 (679 to 830)	644 (581 to 720)	569 (513 to 633)	451 (398 to 508)	473 (419 to 528)	457 (401 to 519)
Aomori	1970 (1600 to 2350)	1540 (1240 to 1890)	1270 (1020 to 1610)	953 (862 to 1050)	777 (701 to 861)	680 (617 to 750)	601 (542 to 669)	527 (463 to 593)	501 (451 to 556)	513 (454 to 577)
Chiba	1730 (1420 to 2090)	1380 (1090 to 1690)	1100 (864 to 1360)	841 (762 to 931)	680 (617 to 755)	578 (525 to 645)	492 (446 to 549)	414 (371 to 465)	408 (369 to 456)	413 (369 to 462)
Ehime	1760 (1450 to 2130)	1380 (1100 to 1710)	1130 (894 to 1400)	845 (767 to 930)	698 (633 to 773)	607 (546 to 672)	515 (465 to 573)	435 (388 to 492)	422 (381 to 470)	430 (380 to 485)
Fukui	1620 (1290 to 1980)	1270 (1000 to 1560)	1040 (841 to 1270)	752 (683 to 834)	637 (576 to 707)	556 (499 to 617)	481 (434 to 535)	386 (339 to 433)	390 (350 to 439)	403 (353 to 459)
Fukuoka	1730 (1420 to 2100)	1380 (1100 to 1690)	1170 (941 to 1440)	896 (814 to 993)	736 (669 to 819)	607 (551 to 674)	513 (466 to 574)	430 (385 to 483)	418 (380 to 463)	422 (378 to 474)
Fukushima	1890 (1570 to 2270)	1470 (1170 to 1810)	1210 (977 to 1520)	886 (802 to 978)	703 (635 to 778)	613 (552 to 675)	523 (472 to 583)	466 (412 to 525)	452 (404 to 503)	457 (407 to 515)
Gifu	1650 (1340 to 2010)	1320 (1030 to 1640)	1080 (868 to 1370)	845 (766 to 933)	665 (605 to 740)	579 (521 to 643)	487 (441 to 542)	417 (371 to 470)	396 (356 to 443)	407 (360 to 463)
Gunma	1780 (1460 to 2140)	1410 (1100 to 1730)	1130 (885 to 1420)	839 (757 to 924)	673 (609 to 743)	582 (524 to 645)	507 (458 to 562)	432 (384 to 486)	431 (385 to 477)	432 (384 to 484)
Hiroshima	1750 (1420 to 2110)	1370 (1090 to 1680)	1110 (884 to 1390)	848 (769 to 936)	687 (624 to 764)	585 (531 to 647)	492 (447 to 547)	420 (376 to 469)	394 (357 to 441)	396 (352 to 448)
Hokkaidō	1750 (1440 to 2130)	1370 (1070 to 1670)	1160 (932 to 1440)	867 (792 to 954)	709 (646 to 778)	593 (538 to 653)	513 (465 to 564)	442 (396 to 489)	432 (393 to 476)	443 (398 to 494)
Hyōgo	1680 (1370 to 2030)	1340 (1060 to 1650)	1130 (905 to 1400)	874 (792 to 962)	731 (664 to 807)	606 (552 to 668)	503 (455 to 556)	412 (370 to 463)	408 (367 to 452)	408 (365 to 456)
Ibaraki	1920 (1570 to 2280)	1480 (1180 to 1820)	1200 (960 to 1490)	892 (816 to 977)	720 (653 to 794)	613 (559 to 671)	524 (475 to 578)	448 (400 to 501)	432 (390 to 478)	434 (388 to 481)
Ishikawa	1660 (1330 to 2030)	1320 (1050 to 1630)	1080 (869 to 1330)	804 (728 to 894)	670 (605 to 741)	573 (521 to 631)	488 (441 to 546)	390 (345 to 439)	387 (349 to 433)	402 (352 to 452)
Iwate	1900 (1570 to 2270)	1480 (1180 to 1840)	1210 (966 to 1500)	898 (811 to 988)	723 (653 to 796)	608 (552 to 673)	550 (495 to 613)	488 (435 to 550)	460 (412 to 514)	452 (397 to 508)
Kagawa	1670 (1370 to 2040)	1320 (1040 to 1660)	1060 (854 to 1320)	796 (719 to 883)	684 (617 to 761)	576 (521 to 642)	495 (444 to 554)	402 (354 to 456)	426 (380 to 480)	405 (356 to 461)
Kagoshima	1980 (1640 to 2390)	1530 (1200 to 1870)	1270 (1020 to 1590)	914 (828 to 1010)	716 (649 to 794)	612 (552 to 677)	521 (467 to 577)	452 (399 to 508)	426 (382 to 474)	438 (386 to 492)
Kanagawa	1550 (1260 to 1860)	1220 (955 to 1520)	1010 (807 to 1280)	814 (742 to 896)	666 (605 to 733)	573 (520 to 631)	476 (434 to 526)	391 (352 to 433)	386 (351 to 426)	394 (354 to 439)
Kōchi	1920 (1570 to 2300)	1490 (1190 to 1840)	1230 (987 to 1480)	891 (802 to 983)	708 (639 to 785)	610 (549 to 672)	542 (491 to 603)	430 (378 to 483)	446 (399 to 501)	433 (383 to 492)
Kumamoto	1810 (1480 to 2180)	1430 (1140 to 1760)	1180 (944 to 1460)	848 (773 to 938)	668 (602 to 740)	551 (498 to 610)	472 (426 to 526)	403 (358 to 454)	384 (346 to 428)	408 (362 to 460)

Kyōto	1600 (1300 to 1940)	1260 (1000 to 1590)	1030 (803 to 1280)	803 (723 to 883)	678 (613 to 751)	589 (532 to 654)	486 (436 to 537)	380 (337 to 423)	374 (338 to 415)	390 (343 to 439)
Mie	1680 (1370 to 2030)	1340 (1060 to 1660)	1080 (857 to 1360)	849 (762 to 946)	712 (644 to 793)	593 (531 to 660)	503 (450 to 563)	418 (370 to 469)	390 (348 to 433)	412 (363 to 464)
Miyagi	1790 (1470 to 2140)	1430 (1140 to 1770)	1190 (945 to 1490)	866 (789 to 962)	703 (640 to 778)	593 (538 to 660)	500 (453 to 558)	420 (376 to 472)	400 (361 to 443)	406 (361 to 458)
Miyazaki	1860 (1510 to 2210)	1450 (1160 to 1800)	1170 (937 to 1480)	882 (797 to 981)	717 (648 to 794)	604 (548 to 671)	504 (454 to 563)	453 (402 to 512)	431 (390 to 483)	440 (387 to 505)
Nagano	1760 (1450 to 2090)	1370 (1080 to 1700)	1100 (876 to 1380)	807 (732 to 892)	633 (566 to 700)	540 (488 to 602)	454 (408 to 503)	377 (331 to 425)	372 (334 to 412)	368 (328 to 415)
Nagasaki	1840 (1530 to 2230)	1460 (1160 to 1810)	1250 (1000 to 1580)	920 (833 to 1020)	739 (670 to 818)	610 (552 to 677)	528 (475 to 588)	440 (393 to 499)	412 (372 to 458)	450 (396 to 510)
Nara	1690 (1390 to 2050)	1340 (1050 to 1650)	1110 (869 to 1390)	857 (782 to 946)	694 (630 to 769)	563 (507 to 628)	476 (429 to 531)	398 (354 to 451)	375 (338 to 415)	396 (351 to 447)
Niigata	1810 (1480 to 2200)	1430 (1160 to 1770)	1190 (958 to 1480)	873 (792 to 978)	680 (618 to 758)	586 (530 to 648)	505 (457 to 565)	442 (395 to 500)	424 (381 to 470)	433 (384 to 489)
Ōita	1780 (1460 to 2190)	1380 (1100 to 1730)	1120 (893 to 1400)	865 (781 to 958)	700 (629 to 780)	586 (529 to 654)	488 (439 to 548)	421 (372 to 480)	403 (360 to 451)	403 (356 to 457)
Okayama	1690 (1380 to 2050)	1340 (1060 to 1670)	1070 (851 to 1340)	819 (743 to 906)	680 (617 to 747)	576 (520 to 636)	498 (447 to 552)	422 (374 to 474)	398 (357 to 440)	402 (359 to 451)
Okinawa	2030 (1680 to 2480)	1500 (1170 to 1870)	1310 (1030 to 1650)	829 (748 to 924)	654 (588 to 727)	572 (516 to 636)	499 (449 to 556)	425 (378 to 479)	427 (383 to 474)	436 (384 to 491)
Ōsaka	1720 (1420 to 2060)	1360 (1080 to 1680)	1130 (912 to 1400)	912 (829 to 1000)	769 (696 to 844)	632 (572 to 698)	532 (480 to 588)	449 (402 to 501)	443 (401 to 488)	446 (401 to 499)
Saga	1800 (1470 to 2180)	1400 (1120 to 1750)	1150 (925 to 1410)	854 (771 to 950)	710 (646 to 788)	613 (553 to 678)	510 (461 to 566)	416 (371 to 471)	418 (375 to 466)	423 (372 to 484)
Saitama	1710 (1420 to 2030)	1340 (1060 to 1640)	1070 (842 to 1320)	853 (776 to 936)	692 (630 to 760)	572 (521 to 628)	494 (449 to 547)	422 (379 to 469)	409 (370 to 449)	418 (377 to 466)
Shiga	1780 (1470 to 2130)	1440 (1170 to 1780)	1210 (977 to 1470)	882 (799 to 974)	698 (628 to 772)	572 (518 to 633)	467 (423 to 518)	386 (343 to 435)	375 (337 to 417)	362 (320 to 410)
Shimane	1840 (1470 to 2240)	1360 (1080 to 1690)	1130 (900 to 1390)	827 (745 to 923)	670 (605 to 748)	581 (521 to 649)	502 (455 to 564)	401 (350 to 455)	387 (346 to 439)	424 (373 to 483)
Shizuoka	1630 (1340 to 1960)	1290 (1020 to 1600)	1060 (835 to 1340)	823 (747 to 910)	665 (604 to 737)	568 (517 to 628)	490 (443 to 545)	419 (374 to 469)	410 (371 to 456)	409 (364 to 458)
Tochigi	1830 (1510 to 2210)	1450 (1150 to 1760)	1180 (949 to 1500)	903 (818 to 996)	730 (664 to 807)	609 (551 to 672)	533 (481 to 588)	445 (395 to 498)	434 (390 to 485)	432 (384 to 483)
Tokushima	1840 (1510 to 2210)	1440 (1150 to 1790)	1190 (948 to 1460)	890 (807 to 978)	720 (653 to 794)	605 (545 to 666)	506 (455 to 564)	436 (387 to 489)	406 (362 to 453)	447 (393 to 508)
Tōkyō	1520 (1250 to 1840)	1230 (988 to 1500)	1040 (834 to 1290)	800 (730 to 882)	701 (639 to 770)	582 (530 to 641)	489 (446 to 538)	412 (373 to 458)	402 (366 to 442)	407 (368 to 454)
Tottori	1880 (1510 to 2290)	1410 (1130 to 1750)	1160 (951 to 1410)	882 (793 to 978)	724 (651 to 801)	613 (552 to 681)	525 (471 to 586)	423 (374 to 482)	416 (371 to 467)	432 (376 to 490)
Toyama	1740 (1390 to 2130)	1390 (1110 to 1730)	1140 (918 to 1400)	842 (763 to 939)	678 (613 to 748)	569 (514 to 631)	498 (451 to 557)	428 (381 to 485)	412 (367 to 464)	426 (377 to 480)
Wakayama	1770 (1450 to 2140)	1400 (1120 to 1750)	1150 (920 to 1450)	895 (805 to 990)	745 (672 to 823)	623 (556 to 693)	546 (491 to 606)	467 (411 to 528)	446 (399 to 497)	456 (399 to 518)
Yamagata	1810 (1470 to 2180)	1420 (1120 to 1770)	1170 (932 to 1480)	876 (787 to 974)	672 (606 to 754)	576 (520 to 642)	485 (434 to 541)	433 (383 to 492)	423 (378 to 472)	422 (371 to 479)
Yamaguchi	1770 (1450 to 2130)	1410 (1130 to 1730)	1160 (930 to 1440)	926 (844 to 1030)	723 (654 to 803)	616 (559 to 688)	529 (477 to 590)	446 (396 to 507)	439 (396 to 494)	436 (385 to 496)
Yamanashi	1770 (1460 to 2160)	1390 (1100 to 1710)	1140 (916 to 1410)	851 (766 to 934)	686 (624 to 757)	578 (521 to 636)	482 (434 to 536)	410 (359 to 464)	384 (343 to 424)	419 (370 to 475)
South Korea	4100 (3790 to 4470)	2150 (1990 to 2330)	1890 (1740 to 2040)	1630 (1530 to 1750)	1260 (1190 to 1360)	943 (883 to 1010)	638 (594 to 687)	488 (452 to 525)	481 (449 to 517)	499 (464 to 541)
Singapore	2330 (2170 to 2510)	1640 (1500 to 1800)	1390 (1300 to 1490)	1130 (1060 to 1200)	844 (792 to 903)	650 (606 to 700)	485 (450 to 521)	358 (333 to 388)	357 (328 to 387)	359 (329 to 391)
High-income North America	1250 (1180 to 1330)	1190 (1120 to 1270)	1170 (1100 to 1250)	985 (925 to 1050)	867 (815 to 923)	761 (715 to 811)	632 (595 to 672)	608 (569 to 652)	705 (663 to 751)	718 (674 to 769)
Canada	1190 (1110 to 1280)	1100 (1020 to 1190)	1040 (971 to 1120)	921 (858 to 988)	797 (740 to 860)	670 (626 to 722)	530 (494 to 574)	482 (445 to 523)	516 (480 to 555)	511 (476 to 547)
Greenland	2960 (2650 to 3290)	2330 (2110 to 2560)	1580 (1400 to 1760)	1570 (1410 to 1720)	1540 (1440 to 1650)	1190 (1110 to 1290)	1010 (909 to 1100)	898 (795 to 1030)	907 (801 to 1040)	900 (791 to 1040)
USA	1260 (1180 to 1340)	1200 (1120 to 1290)	1190 (1110 to 1270)	992 (926 to 1070)	875 (817 to 939)	771 (721 to 829)	644 (601 to 691)	624 (578 to 675)	728 (679 to 784)	744 (692 to 805)
Alabama	1360 (1080 to 1690)	1270 (1150 to 1400)	1280 (1160 to 1410)	1090 (990 to 1210)	998 (909 to 1100)	935 (850 to 1030)	829 (754 to 917)	804 (722 to 900)	938 (853 to 1040)	1010 (844 to 1220)
Alaska	1360 (1110 to 1650)	1220 (1100 to 1350)	1120 (1000 to 1240)	1060 (956 to 1180)	870 (784 to 966)	743 (670 to 821)	667 (600 to 741)	605 (538 to 680)	655 (588 to 731)	790 (670 to 920)
Arizona	1310 (1030 to 1620)	1210 (1090 to 1330)	1140 (1020 to 1250)	923 (836 to 1030)	797 (721 to 881)	720 (655 to 793)	606 (548 to 668)	624 (557 to 695)	766 (696 to 845)	812 (681 to 972)
Arkansas	1190 (938 to 1510)	1120 (1020 to 1240)	1140 (1040 to 1260)	1010 (916 to 1120)	945 (857 to 1040)	886 (806 to 979)	790 (716 to 876)	784 (706 to 875)	891 (806 to 989)	948 (789 to 1130)
California	1190 (959 to 1470)	1150 (1050 to 1260)	1110 (1010 to 1210)	933 (848 to 1020)	826 (754 to 906)	680 (620 to 746)	544 (496 to 593)	524 (474 to 578)	614 (559 to 672)	635 (532 to 751)
Colorado	1210 (961 to 1500)	1100 (999 to 1220)	1090 (993 to 1200)	894 (813 to 984)	769 (700 to 847)	676 (610 to 745)	572 (521 to 628)	552 (495 to 614)	639 (580 to 707)	654 (530 to 779)
Connecticut	1210 (968 to 1500)	1160 (1050 to 1280)	1080 (979 to 1200)	940 (853 to 1040)	768 (699 to 845)	706 (638 to 782)	569 (515 to 629)	553 (495 to 618)	648 (588 to 717)	588 (471 to 728)
Delaware	1390 (1120 to 1690)	1260 (1140 to 1400)	1280 (1160 to 1410)	1030 (936 to 1150)	900 (811 to 995)	785 (708 to 868)	665 (603 to 733)	664 (592 to 748)	727 (657 to 807)	765 (644 to 896)
Washington, DC	1500 (1190 to 1870)	1430 (1290 to 1590)	1520 (1370 to 1680)	1300 (1170 to 1450)	1370 (1230 to 1510)	1020 (911 to 1130)	695 (625 to 780)	637 (565 to 718)	780 (693 to 874)	730 (596 to 874)
Florida	1200 (945 to 1480)	1130 (1020 to 1250)	1130 (1020 to 1260)	937 (851 to 1040)	826 (752 to 914)	732 (667 to 812)	614 (559 to 679)	608 (546 to 683)	692 (629 to 766)	748 (615 to 899)
Georgia	1380 (1090 to 1690)	1300 (1180 to 1430)	1320 (1200 to 1460)	1090 (989 to 1210)	994 (897 to 1100)	880 (798 to 976)	726 (658 to 803)	671 (601 to 746)	787 (711 to 868)	854 (717 to 1010)
Hawaii	1100 (866 to 1380)	1000 (902 to 1110)	886 (799 to 985)	740 (664 to 827)	679 (610 to 751)	600 (540 to 668)	545 (489 to 609)	520 (465 to 586)	527 (474 to 588)	545 (428 to 672)
Idaho	1160 (913 to 1450)	1110 (1000 to 1230)	1080 (978 to 1210)	881 (794 to 983)	787 (707 to 876)	684 (616 to 758)	612 (555 to 678)	582 (519 to 650)	654 (589 to 727)	730 (600 to 876)
Illinois	1280 (1030 to 1570)	1240 (1130 to 1370)	1250 (1130 to 1380)	1030 (933 to 1140)	911 (824 to 1000)	798 (722 to 883)	645 (583 to 711)	615 (549 to 686)	742 (670 to 819)	703 (576 to 853)
Indiana	1240 (977 to 1540)	1200 (1080 to 1320)	1200 (1080 to 1320)	1020 (914 to 1120)	892 (803 to 986)	843 (757 to 935)	720 (652 to 799)	722 (643 to 806)	849 (763 to 937)	850 (694 to 1020)
Iowa	1130 (904 to 1420)	1090 (979 to 1200)	1090 (984 to 1200)	904 (813 to 998)	772 (694 to 853)	715 (644 to 788)	627 (563 to 689)	632 (563 to 703)	719 (648 to 793)	698 (567 to 850)
Kansas	1120 (882 to 1410)	1080 (980 to 1190)	1070 (965 to 1180)	917 (830 to 1020)	802 (728 to 885)	752 (680 to 840)	672 (609 to 744)	660 (590 to 735)	774 (703 to 858)	770 (621 to 930)
Kentucky	1260 (999 to 1540)	1210 (1100 to 1330)	1270 (1150 to 1400)	1070 (971 to 1180)	965 (877 to 1060)	905 (821 to 996)	801 (727 to 885)	790 (712 to 881)	907 (823 to 998)	970 (804 to 1160)
Louisiana	1370 (1090 to 1700)	1280 (1160 to 1410)	1300 (1180 to 1440)	1110 (1010 to 1240)	1010 (915 to 1120)	931 (844 to 1040)	807 (733 to 892)	788 (706 to 880)	960 (871 to 1060)	980 (811 to 1160)
Maine	1320 (1050 to 1630)	1220 (1100 to 1360)	1220 (1100 to 1350)	990 (888 to 1100)	828 (741 to 920)	760 (684 to 846)	639 (575 to 711)	644 (576 to 724)	670 (602 to 748)	723 (575 to 886)
Maryland	1360 (1060 to 1700)	1310 (1180 to 1440)	1250 (1130 to 1390)	1030 (931 to 1140)	911 (825 to 1010)	792 (715 to 875)	618 (562 to 679)	615 (549 to 682)	713 (643 to 787)	687 (552 to 845)
Massachusetts	1240 (975 to 1530)	1220 (1110 to 1340)	1170 (1070 to 1290)	957 (868 to 1050)	815 (738 to 897)	717 (648 to 789)	584 (529 to 644)	566 (507 to 629)	639 (580 to 703)	582 (475 to 711)
Michigan	1240 (997 to 1530)	1200 (1100 to 1320)	1200 (1100 to 1330)	1010 (918 to 1110)	890 (811 to 978)	801 (727 to 883)	685 (624 to 754)	654 (588 to 728)	769 (700 to 844)	774 (636 to 927)
Minnesota	1130 (896 to 1400)	1070 (976 to 1190)	1050 (952 to 1160)	866 (784 to 960)	760 (690 to 840)	679 (616 to 750)	561 (511 to 625)	551 (495 to 616)	619 (558 to 684)	615 (497 to 759)
Mississippi	1380 (1110 to 1700)	1260 (1140 to 1390)	1330 (1200 to 1470)	1110 (996 to 1230)	1020 (922 to 1130)	971 (874 to 1070)	855 (768 to 945)	882 (789 to 986)	1060 (948 to 1170)	1090 (901 to 1290)
Missouri	1240 (982 to 1530)	1180 (1070 to 1290)	1210 (1100 to 1340)	1010 (916 to 1110)	899 (813 to 987)	830 (749 to 912)	718 (648 to 788)	713 (639 to 788)	828 (750 to 910)	836 (692 to 1010)
Montana	1280 (1020 to 1590)	1210 (1090 to 1330)	1130 (1020 to 1250)	966 (872 to 1080)	793 (717 to 876)	748 (674 to 834)	640 (574 to 711)	611 (543 to 686)	703 (630 to 782)	735 (604 to 891)
Nebraska	1110 (866 to 1420)	1080 (972 to 1180)	1080 (971 to 1200)	906 (815 to 1010)	813 (731 to 898)	703 (634 to 781)	616 (552 to 684)	600 (534 to 671)	685 (617 to 761)	672 (544 to 813)
Nevada	1610 (1320 to 1980)	1370 (1220 to 1540)	1260 (1140 to 1410)	1040 (935 to 1150)	930 (840 to 1030)	799 (718 to 885)	675 (608 to 746)	659 (590 to 735)	774 (698 to 853)	818 (677 to 985)
New Hampshire	1340 (1060 to 1660)	1220 (1090 to 1350)	1190 (1070 to 1320)	920 (832 to 1030)	820 (734 to 913)	710 (636 to 792)	583 (522 to 648)	597 (530 to 672)	631 (566 to 707)	649 (525 to 799)
New Jersey	1290 (1030 to 1590)	1220 (1110 to 1340)	1190 (1080 to 1310)	999 (908 to 1100)	868 (788 to 952)	753 (682 to 828)	598 (541 to 656)	552 (495 to 611)	705 (639 to 774)	613 (496 to 737)
New Mexico	1280 (1010 to 1590)	1140 (1020 to 1250)	1150 (1040 to 1270)	963 (871 to 1070)	815 (737 to 900)	727 (658 to 809)	653 (590 to 722)	686 (611 to 766)	802 (726 to 885)	850 (697 to 1030)

New York	1290 (1040 to 1570)	1240 (1140 to 1380)	1230 (1120 to 1350)	1020 (926 to 1120)	914 (835 to 1010)	721 (656 to 796)	579 (528 to 640)	525 (473 to 586)	683 (621 to 756)	605 (500 to 721)
North Carolina	1340 (1070 to 1650)	1290 (1170 to 1410)	1280 (1160 to 1410)	1060 (961 to 1170)	946 (856 to 1040)	859 (778 to 949)	697 (632 to 766)	682 (613 to 756)	764 (692 to 843)	819 (675 to 982)
North Dakota	1120 (895 to 1380)	1060 (958 to 1180)	1060 (952 to 1180)	878 (790 to 983)	769 (692 to 858)	712 (638 to 796)	619 (558 to 688)	591 (525 to 666)	686 (617 to 765)	609 (508 to 729)
Ohio	1250 (1000 to 1550)	1200 (1090 to 1330)	1220 (1110 to 1350)	1040 (942 to 1160)	896 (816 to 993)	823 (748 to 910)	708 (644 to 781)	718 (645 to 802)	822 (750 to 908)	850 (705 to 1020)
Oklahoma	1190 (944 to 1490)	1140 (1040 to 1260)	1180 (1070 to 1300)	1010 (917 to 1120)	889 (806 to 982)	876 (792 to 965)	802 (729 to 882)	776 (698 to 865)	888 (804 to 980)	958 (802 to 1140)
Oregon	1200 (950 to 1490)	1140 (1030 to 1260)	1080 (980 to 1200)	927 (842 to 1030)	814 (735 to 898)	705 (636 to 781)	605 (547 to 665)	578 (518 to 644)	612 (552 to 673)	683 (552 to 841)
Pennsylvania	1320 (1050 to 1620)	1260 (1150 to 1390)	1240 (1130 to 1370)	1040 (947 to 1150)	889 (811 to 977)	805 (735 to 889)	670 (608 to 740)	649 (584 to 725)	745 (677 to 821)	754 (622 to 894)
Rhode Island	1290 (1020 to 1600)	1200 (1090 to 1320)	1170 (1060 to 1300)	978 (882 to 1080)	840 (757 to 929)	732 (660 to 814)	633 (569 to 697)	599 (535 to 669)	683 (610 to 762)	640 (514 to 788)
South Carolina	1470 (1160 to 1820)	1380 (1250 to 1530)	1350 (1220 to 1480)	1120 (1010 to 1240)	980 (891 to 1070)	902 (814 to 999)	758 (687 to 837)	736 (659 to 821)	862 (781 to 948)	917 (756 to 1100)
South Dakota	1180 (949 to 1460)	1120 (1020 to 1240)	1080 (975 to 1200)	928 (838 to 1040)	802 (725 to 892)	736 (665 to 819)	626 (566 to 696)	625 (555 to 702)	740 (664 to 821)	690 (581 to 810)
Tennessee	1260 (995 to 1560)	1210 (1100 to 1340)	1240 (1140 to 1370)	1050 (945 to 1160)	989 (898 to 1090)	912 (828 to 1000)	784 (712 to 862)	788 (705 to 877)	904 (822 to 1000)	971 (801 to 1160)
Texas	1200 (963 to 1490)	1130 (1040 to 1250)	1150 (1050 to 1270)	988 (900 to 1090)	900 (817 to 990)	782 (712 to 860)	651 (593 to 715)	625 (563 to 691)	752 (687 to 824)	801 (670 to 938)
Utah	1140 (909 to 1430)	1100 (993 to 1220)	1050 (950 to 1160)	848 (768 to 937)	729 (661 to 810)	655 (593 to 726)	582 (531 to 645)	561 (506 to 626)	631 (575 to 700)	656 (534 to 796)
Vermont	1350 (1090 to 1660)	1230 (1100 to 1360)	1180 (1060 to 1310)	987 (890 to 1100)	832 (741 to 924)	712 (639 to 790)	592 (533 to 659)	571 (507 to 644)	588 (528 to 659)	637 (531 to 772)
Virginia	1350 (1080 to 1650)	1260 (1140 to 1400)	1230 (1120 to 1360)	1040 (940 to 1160)	904 (817 to 995)	783 (708 to 866)	632 (570 to 702)	598 (534 to 663)	678 (613 to 750)	715 (587 to 864)
Washington	1210 (961 to 1500)	1140 (1030 to 1260)	1130 (1020 to 1240)	901 (816 to 997)	796 (721 to 875)	697 (629 to 769)	584 (529 to 642)	549 (491 to 611)	586 (530 to 644)	619 (510 to 750)
West Virginia	1300 (1040 to 1640)	1230 (1120 to 1360)	1290 (1160 to 1430)	1090 (984 to 1200)	963 (876 to 1060)	904 (818 to 997)	827 (745 to 909)	817 (727 to 910)	922 (834 to 1020)	1030 (856 to 1240)
Wisconsin	1170 (919 to 1450)	1130 (1030 to 1250)	1090 (983 to 1200)	913 (828 to 1010)	816 (741 to 899)	738 (670 to 818)	616 (561 to 683)	607 (545 to 679)	700 (636 to 771)	676 (548 to 823)
Wyoming	1270 (1020 to 1560)	1160 (1040 to 1290)	1180 (1060 to 1320)	973 (872 to 1090)	830 (744 to 927)	754 (670 to 842)	646 (578 to 721)	635 (552 to 718)	698 (622 to 778)	784 (664 to 919)
Southern Latin America	1540 (1470 to 1610)	1500 (1430 to 1570)	1390 (1320 to 1460)	1180 (1120 to 1240)	1030 (978 to 1090)	903 (856 to 952)	794 (753 to 836)	722 (664 to 784)	778 (713 to 852)	801 (730 to 877)
Argentina	1440 (1340 to 1540)	1420 (1330 to 1530)	1410 (1320 to 1510)	1190 (1120 to 1280)	1060 (987 to 1140)	953 (886 to 1030)	843 (790 to 898)	788 (694 to 894)	851 (747 to 979)	858 (748 to 987)
Chile	2000 (1870 to 2150)	1810 (1700 to 1950)	1390 (1290 to 1490)	1130 (1040 to 1230)	947 (875 to 1030)	758 (700 to 827)	666 (599 to 748)	565 (523 to 614)	636 (584 to 697)	661 (602 to 727)
Uruguay	1280 (1190 to 1380)	1200 (1100 to 1310)	1240 (1120 to 1380)	1160 (1050 to 1280)	1040 (938 to 1160)	937 (851 to 1030)	830 (770 to 892)	799 (704 to 903)	740 (653 to 838)	919 (812 to 1040)
Western Europe	1370 (1320 to 1420)	1210 (1180 to 1250)	1200 (1170 to 1230)	1050 (1030 to 1080)	889 (872 to 907)	728 (715 to 742)	573 (565 to 582)	505 (491 to 521)	548 (532 to 566)	536 (519 to 556)
Andorra	976 (767 to 1180)	942 (716 to 1160)	864 (644 to 1100)	784 (589 to 1030)	701 (554 to 887)	629 (507 to 760)	536 (449 to 630)	477 (379 to 597)	487 (388 to 607)	477 (356 to 617)
Austria	1450 (1360 to 1550)	1350 (1250 to 1450)	1340 (1240 to 1440)	1170 (1090 to 1250)	926 (863 to 996)	737 (685 to 794)	597 (589 to 605)	521 (480 to 570)	566 (517 to 620)	544 (497 to 603)
Belgium	1630 (1530 to 1740)	1280 (1180 to 1370)	1250 (1170 to 1340)	1130 (1040 to 1220)	929 (863 to 1010)	785 (730 to 848)	619 (611 to 626)	528 (487 to 572)	599 (550 to 655)	533 (486 to 592)
Cyprus	2180 (1950 to 2420)	1980 (1780 to 2200)	1950 (1680 to 2270)	1710 (1570 to 1870)	1430 (1320 to 1550)	1020 (928 to 1120)	808 (728 to 896)	650 (560 to 748)	589 (504 to 682)	618 (522 to 728)
Denmark	1090 (1020 to 1180)	1020 (951 to 1090)	996 (932 to 1070)	996 (984 to 1010)	929 (916 to 942)	789 (777 to 801)	642 (632 to 651)	538 (496 to 584)	534 (489 to 590)	540 (491 to 598)
Finland	1750 (1630 to 1890)	1370 (1280 to 1460)	1350 (1330 to 1360)	1100 (1090 to 1120)	977 (965 to 991)	785 (773 to 797)	625 (615 to 635)	517 (475 to 563)	513 (469 to 558)	520 (473 to 580)
France	1400 (1310 to 1500)	1310 (1210 to 1430)	1220 (1120 to 1320)	1070 (992 to 1160)	878 (824 to 943)	740 (688 to 796)	583 (546 to 626)	496 (451 to 546)	540 (489 to 595)	518 (466 to 574)
Germany	1410 (1230 to 1630)	1260 (1170 to 1360)	1410 (1320 to 1510)	1210 (1130 to 1290)	960 (952 to 968)	739 (732 to 746)	593 (587 to 599)	550 (504 to 600)	568 (518 to 624)	581 (526 to 650)
Greece	998 (934 to 1070)	925 (859 to 999)	905 (840 to 978)	893 (829 to 963)	783 (727 to 845)	705 (655 to 759)	590 (551 to 634)	554 (495 to 629)	583 (519 to 668)	630 (551 to 732)
Iceland	1020 (962 to 1080)	949 (914 to 986)	942 (904 to 976)	807 (776 to 839)	692 (666 to 721)	588 (565 to 616)	517 (492 to 542)	446 (400 to 494)	435 (392 to 483)	405 (359 to 454)
Ireland	1360 (1270 to 1460)	1220 (1130 to 1310)	1220 (1130 to 1310)	1170 (1080 to 1260)	1010 (944 to 1090)	864 (800 to 942)	571 (527 to 621)	487 (444 to 531)	487 (443 to 535)	474 (426 to 530)
Israel	762 (707 to 820)	897 (834 to 968)	1000 (927 to 1080)	913 (838 to 995)	725 (657 to 803)	649 (587 to 718)	495 (455 to 538)	430 (376 to 488)	447 (391 to 519)	451 (389 to 526)
Italy	1260 (1180 to 1350)	1150 (1070 to 1250)	1090 (1010 to 1180)	1040 (967 to 1120)	834 (779 to 896)	685 (638 to 740)	549 (544 to 554)	468 (434 to 510)	537 (494 to 589)	505 (463 to 554)
Luxembourg	1330 (1230 to 1430)	1310 (1220 to 1410)	1370 (1270 to 1470)	1170 (1090 to 1280)	998 (922 to 1090)	781 (713 to 850)	594 (568 to 620)	493 (449 to 543)	521 (472 to 579)	499 (449 to 554)
Malta	1230 (1140 to 1330)	1290 (1180 to 1410)	1210 (1100 to 1330)	1130 (1030 to 1260)	846 (761 to 948)	707 (635 to 784)	551 (507 to 600)	462 (404 to 526)	452 (398 to 522)	443 (385 to 516)
Monaco	1320 (1160 to 1510)	1180 (998 to 1370)	1080 (916 to 1230)	929 (804 to 1050)	784 (643 to 902)	713 (585 to 829)	642 (549 to 744)	586 (508 to 675)	604 (511 to 703)	688 (589 to 798)
Netherlands	1040 (1030 to 1050)	980 (970 to 990)	1010 (1000 to 1020)	922 (912 to 933)	864 (854 to 874)	764 (755 to 773)	577 (569 to 584)	505 (466 to 550)	550 (505 to 599)	549 (500 to 603)
Norway	979 (914 to 1040)	943 (880 to 1010)	982 (915 to 1060)	899 (838 to 960)	845 (790 to 906)	720 (708 to 732)	562 (552 to 571)	454 (420 to 492)	441 (404 to 482)	442 (400 to 484)
Agder	1030 (914 to 1160)	949 (858 to 1060)	1020 (914 to 1130)	925 (832 to 1020)	908 (836 to 991)	755 (720 to 792)	600 (566 to 633)	466 (404 to 536)	476 (411 to 553)	502 (405 to 625)
Innlandet	869 (770 to 979)	847 (763 to 935)	857 (773 to 948)	824 (746 to 911)	841 (778 to 907)	734 (701 to 767)	612 (583 to 640)	486 (427 to 553)	439 (383 to 508)	435 (360 to 532)
Møre og Romsdal	1010 (893 to 1150)	863 (773 to 963)	939 (847 to 1050)	858 (774 to 958)	746 (683 to 811)	676 (639 to 714)	508 (479 to 540)	434 (374 to 503)	428 (362 to 498)	418 (332 to 527)
Nordland	1060 (941 to 1190)	935 (845 to 1040)	971 (875 to 1080)	875 (789 to 968)	888 (816 to 966)	754 (716 to 797)	613 (578 to 647)	470 (408 to 542)	460 (395 to 533)	472 (374 to 582)
Oslo	803 (702 to 914)	1070 (975 to 1180)	1100 (997 to 1220)	1010 (918 to 1120)	927 (852 to 1000)	751 (718 to 787)	550 (522 to 579)	435 (381 to 495)	417 (363 to 480)	402 (324 to 490)
Rogaland	1020 (904 to 1150)	940 (848 to 1040)	944 (844 to 1050)	866 (780 to 961)	773 (711 to 836)	676 (644 to 712)	550 (524 to 578)	454 (398 to 515)	442 (381 to 504)	432 (360 to 518)
Troms og Finnmark	1070 (949 to 1200)	1100 (987 to 1210)	1070 (955 to 1190)	1010 (912 to 1120)	969 (885 to 1050)	831 (790 to 873)	592 (558 to 628)	477 (411 to 548)	520 (448 to 603)	565 (454 to 704)
Trøndelag	963 (855 to 1080)	928 (839 to 1020)	1000 (908 to 1110)	897 (811 to 995)	868 (799 to 942)	693 (659 to 725)	552 (524 to 580)	464 (404 to 527)	432 (374 to 500)	428 (351 to 516)
Vestfold og Telemark	992 (885 to 1110)	944 (850 to 1040)	990 (885 to 1100)	933 (846 to 1040)	865 (798 to 939)	748 (715 to 784)	571 (540 to 601)	470 (411 to 536)	435 (375 to 502)	436 (352 to 538)
Vestland	964 (858 to 1080)	911 (828 to 1010)	943 (855 to 1050)	839 (764 to 925)	760 (701 to 823)	666 (637 to 696)	533 (508 to 558)	433 (381 to 491)	434 (379 to 496)	423 (343 to 513)
Viken	1200 (1070 to 1340)	944 (856 to 1040)	993 (906 to 1100)	898 (813 to 987)	836 (775 to 902)	716 (689 to 744)	555 (533 to 575)	453 (403 to 507)	443 (388 to 500)	448 (370 to 535)
Portugal	1640 (1530 to 1760)	1380 (1280 to 1470)	1360 (1270 to 1460)	1160 (1080 to 1260)	1060 (988 to 1150)	883 (819 to 952)	642 (599 to 691)	544 (484 to 610)	575 (508 to 650)	578 (507 to 664)
San Marino	1070 (910 to 1290)	1070 (888 to 1280)	997 (872 to 1140)	863 (755 to 988)	680 (554 to 825)	587 (496 to 687)	488 (443 to 536)	470 (401 to 566)	371 (301 to 456)	341 (256 to 445)
Spain	1560 (1460 to 1670)	1160 (1080 to 1240)	1110 (1040 to 1180)	888 (826 to 956)	840 (780 to 901)	697 (691 to 703)	555 (550 to 560)	489 (449 to 535)	555 (507 to 608)	511 (465 to 569)
Sweden	1120 (1050 to 1200)	990 (923 to 1060)	919 (910 to 930)	900 (890 to 910)	775 (766 to 784)	642 (633 to 650)	522 (514 to 529)	441 (404 to 482)	474 (431 to 522)	431 (376 to 497)
Stockholm	1190 (886 to 1520)	1060 (780 to 1410)	999 (768 to 1270)	1020 (980 to 1050)	869 (835 to 900)	678 (653 to 702)	545 (523 to 565)	455 (397 to 519)	519 (445 to 604)	392 (308 to 494)
Sweden except Stockholm	1110 (1020 to 1210)	981 (905 to 1060)	907 (864 to 947)	882 (871 to 893)	758 (749 to 768)	635 (626 to 644)	518 (510 to 526)	440 (403 to 484)	468 (422 to 519)	442 (387 to 506)
Switzerland	1270 (1180 to 1360)	1130 (1060 to 1220)	1070 (1000 to 1140)	914 (852 to 975)	791 (742 to 846)	626 (587 to 670)	482 (475 to 489)	389 (355 to 426)	444 (406 to 491)	402 (362 to 447)
UK	1360 (1290 to 1440)	1310 (1240 to 1400)	1230 (1150 to 1300)	1100 (1040 to 1180)	915 (858 to 976)	751 (708 to 801)	566 (532 to 604)	520 (487 to 558)	582 (547 to 622)	590 (558 to 626)
England	1370 (1290 to 1470)	1290 (1200 to 1390)	1210 (1120 to 1290)	1080 (1010 to 1170)	898 (832 to 969)	736 (686 to 795)	551 (512 to 596)	511 (472 to 556)	576 (535 to 622)	585 (547 to 628)
East Midlands	1390 (1100 to 1740)	1310 (1010 to 1680)	1220 (950 to 1520)	1080 (964 to 1220)	892 (802 to 992)	746 (673 to 829)	565 (507 to 626)	525 (458 to 605)	593 (515 to 691)	610 (522 to 712)
Derby	1450 (1060 to 1920)	1380 (977 to 1900)	1270 (935 to 1650)	1160 (989 to 1350)	947 (820 to 1080)	751 (652 to 863)	633 (548 to 719)	540 (438 to 658)	633 (514 to 776)	696 (554 to 875)

Derbyshire	1490 (1100 to 1930)	1340 (956 to 1830)	1260 (910 to 1650)	1090 (947 to 1260)	921 (812 to 1060)	742 (653 to 847)	550 (482 to 630)	547 (456 to 654)	588 (492 to 715)	605 (490 to 751)
Leicester	1500 (1090 to 1970)	1420 (1000 to 1930)	1320 (975 to 1760)	1150 (987 to 1340)	964 (846 to 1100)	848 (739 to 968)	661 (575 to 755)	606 (494 to 731)	623 (512 to 757)	663 (533 to 796)
Leicestershire	1250 (920 to 1670)	1200 (841 to 1660)	1110 (826 to 1470)	1020 (891 to 1180)	821 (721 to 933)	682 (600 to 775)	527 (462 to 602)	462 (383 to 549)	543 (450 to 655)	534 (428 to 649)
Lincolnshire	1350 (982 to 1800)	1280 (902 to 1780)	1190 (880 to 1570)	1060 (915 to 1210)	874 (768 to 993)	735 (643 to 830)	547 (479 to 619)	546 (452 to 655)	618 (512 to 742)	577 (469 to 715)
Northamptonshire	1330 (978 to 1780)	1250 (854 to 1680)	1150 (870 to 1540)	1040 (897 to 1220)	871 (765 to 989)	733 (645 to 830)	571 (497 to 648)	524 (432 to 627)	593 (493 to 711)	704 (541 to 893)
Nottingham	1510 (1110 to 2020)	1470 (1060 to 2040)	1380 (1050 to 1790)	1230 (1070 to 1430)	1040 (914 to 1190)	923 (813 to 1040)	686 (597 to 785)	587 (483 to 709)	721 (594 to 889)	714 (578 to 875)
Nottinghamshire	1360 (997 to 1800)	1290 (922 to 1760)	1190 (888 to 1580)	1080 (925 to 1250)	866 (763 to 983)	747 (656 to 845)	555 (487 to 636)	516 (436 to 617)	581 (484 to 708)	603 (496 to 728)
Rutland	1170 (826 to 1580)	1120 (792 to 1590)	1000 (712 to 1360)	894 (753 to 1080)	765 (655 to 887)	613 (518 to 715)	469 (398 to 552)	385 (298 to 492)	456 (362 to 580)	501 (392 to 649)
East of England	1290 (1040 to 1570)	1210 (937 to 1550)	1130 (906 to 1400)	1010 (900 to 1140)	827 (746 to 916)	688 (622 to 762)	520 (469 to 578)	483 (420 to 553)	533 (465 to 611)	539 (461 to 631)
Bedford	1330 (952 to 1790)	1280 (905 to 1780)	1170 (862 to 1560)	1040 (885 to 1230)	834 (722 to 955)	758 (666 to 870)	573 (494 to 658)	549 (453 to 670)	624 (512 to 769)	594 (478 to 736)
Cambridgeshire	1190 (859 to 1570)	1170 (833 to 1630)	1100 (808 to 1440)	986 (847 to 1150)	815 (714 to 926)	659 (576 to 747)	486 (426 to 555)	465 (383 to 561)	503 (415 to 607)	486 (396 to 604)
Central Bedfordshire	1240 (881 to 1650)	1190 (845 to 1650)	1080 (802 to 1410)	944 (800 to 1100)	772 (674 to 878)	663 (576 to 761)	520 (448 to 600)	450 (364 to 553)	531 (426 to 656)	479 (385 to 593)
Essex	1360 (1030 to 1730)	1240 (880 to 1670)	1170 (887 to 1520)	1050 (912 to 1210)	821 (731 to 925)	684 (606 to 769)	519 (460 to 586)	483 (406 to 576)	538 (449 to 644)	566 (470 to 683)
Hertfordshire	1340 (968 to 1760)	1220 (842 to 1690)	1160 (858 to 1520)	1020 (882 to 1180)	829 (729 to 940)	692 (607 to 787)	502 (442 to 571)	463 (383 to 556)	524 (434 to 633)	502 (406 to 608)
Luton	1370 (982 to 1810)	1300 (934 to 1770)	1180 (856 to 1550)	1030 (870 to 1200)	940 (814 to 1070)	788 (676 to 906)	567 (494 to 650)	567 (459 to 684)	697 (561 to 841)	670 (529 to 824)
Norfolk	1250 (919 to 1650)	1190 (856 to 1630)	1090 (819 to 1410)	990 (853 to 1150)	835 (734 to 942)	680 (600 to 773)	545 (481 to 621)	521 (436 to 622)	550 (458 to 664)	559 (461 to 692)
Peterborough	1380 (993 to 1860)	1340 (947 to 1880)	1240 (932 to 1620)	1020 (868 to 1190)	908 (790 to 1030)	765 (668 to 871)	604 (525 to 687)	544 (445 to 662)	633 (516 to 770)	597 (480 to 732)
Southend-on-Sea	1340 (947 to 1780)	1280 (912 to 1750)	1140 (855 to 1540)	990 (850 to 1160)	856 (743 to 986)	755 (656 to 863)	551 (478 to 634)	506 (406 to 624)	580 (473 to 716)	646 (519 to 793)
Suffolk	1220 (900 to 1610)	1170 (818 to 1630)	1070 (796 to 1420)	970 (837 to 1130)	803 (706 to 914)	673 (594 to 767)	493 (430 to 568)	464 (388 to 560)	493 (407 to 593)	518 (425 to 638)
Thurrock	1370 (990 to 1810)	1350 (934 to 1880)	1250 (933 to 1640)	1100 (948 to 1310)	893 (772 to 1020)	695 (600 to 795)	571 (492 to 656)	420 (338 to 509)	492 (403 to 598)	580 (464 to 711)
Greater London	1340 (1090 to 1620)	1260 (1010 to 1580)	1180 (945 to 1450)	1080 (966 to 1210)	899 (809 to 995)	743 (670 to 823)	517 (468 to 574)	459 (397 to 526)	559 (489 to 645)	576 (496 to 675)
Barking and Dagenham	1510 (1150 to 1970)	1420 (1040 to 1910)	1320 (1010 to 1730)	1200 (1040 to 1400)	988 (867 to 1120)	870 (752 to 990)	661 (574 to 754)	595 (484 to 726)	772 (632 to 934)	751 (602 to 912)
Barnet	1180 (861 to 1580)	1110 (796 to 1510)	1010 (769 to 1340)	915 (791 to 1070)	773 (676 to 880)	628 (548 to 716)	464 (402 to 533)	401 (330 to 490)	495 (408 to 600)	537 (433 to 661)
Bexley	1210 (900 to 1610)	1140 (826 to 1540)	1050 (786 to 1360)	957 (817 to 1100)	772 (671 to 879)	647 (564 to 740)	532 (463 to 611)	418 (339 to 508)	528 (431 to 638)	554 (447 to 680)
Brent	1340 (994 to 1760)	1250 (890 to 1690)	1150 (864 to 1510)	1040 (897 to 1220)	848 (746 to 970)	703 (618 to 803)	497 (437 to 571)	477 (393 to 579)	556 (461 to 676)	602 (492 to 741)
Bromley	1160 (846 to 1550)	1090 (798 to 1490)	1000 (744 to 1310)	936 (804 to 1080)	807 (706 to 922)	660 (575 to 749)	438 (380 to 503)	390 (319 to 472)	468 (382 to 568)	482 (390 to 604)
Camden	1380 (1030 to 1800)	1360 (995 to 1850)	1320 (996 to 1720)	1270 (1090 to 1460)	950 (833 to 1080)	985 (862 to 1110)	574 (500 to 656)	447 (368 to 542)	437 (360 to 530)	428 (348 to 522)
Croydon	1250 (918 to 1670)	1140 (832 to 1570)	1060 (789 to 1390)	987 (852 to 1140)	847 (747 to 961)	690 (609 to 785)	509 (447 to 583)	486 (403 to 587)	623 (517 to 757)	565 (466 to 694)
Ealing	1370 (991 to 1790)	1280 (918 to 1740)	1220 (905 to 1570)	1100 (954 to 1280)	901 (782 to 1020)	720 (632 to 820)	531 (465 to 604)	477 (393 to 575)	584 (483 to 697)	605 (490 to 742)
Enfield	1280 (929 to 1680)	1200 (868 to 1630)	1100 (839 to 1400)	1020 (877 to 1180)	836 (728 to 949)	698 (610 to 792)	460 (398 to 526)	514 (426 to 619)	557 (458 to 675)	573 (468 to 701)
Greenwich	1430 (1050 to 1850)	1330 (935 to 1780)	1230 (924 to 1620)	1120 (965 to 1300)	942 (826 to 1080)	824 (724 to 940)	572 (496 to 652)	502 (416 to 612)	561 (458 to 687)	616 (502 to 762)
Hackney	1420 (1070 to 1840)	1360 (978 to 1840)	1330 (1000 to 1760)	1270 (1090 to 1460)	1010 (895 to 1150)	807 (708 to 914)	549 (484 to 627)	545 (446 to 667)	687 (566 to 834)	1140 (857 to 1440)
Hammersmith and Fulham	1440 (1060 to 1890)	1400 (1010 to 1900)	1340 (1010 to 1750)	1280 (1100 to 1500)	1090 (943 to 1250)	874 (766 to 996)	587 (510 to 680)	508 (411 to 617)	643 (527 to 787)	633 (512 to 788)
Haringey	1380 (1020 to 1820)	1310 (968 to 1740)	1220 (925 to 1600)	1140 (973 to 1310)	897 (787 to 1020)	843 (743 to 956)	615 (540 to 706)	489 (405 to 595)	577 (477 to 708)	565 (456 to 695)
Harrow	1170 (864 to 1540)	1100 (784 to 1520)	996 (743 to 1300)	901 (767 to 1050)	767 (673 to 874)	620 (545 to 706)	439 (381 to 508)	379 (310 to 461)	482 (399 to 585)	397 (320 to 496)
Havering	1230 (903 to 1630)	1140 (802 to 1560)	1050 (785 to 1370)	959 (822 to 1110)	804 (700 to 908)	654 (568 to 745)	508 (442 to 590)	438 (360 to 523)	556 (450 to 673)	559 (448 to 687)
Hillingdon	1230 (926 to 1650)	1170 (857 to 1620)	1090 (815 to 1450)	1010 (867 to 1160)	815 (715 to 929)	731 (643 to 835)	504 (441 to 577)	504 (421 to 612)	580 (482 to 701)	590 (482 to 721)
Hounslow	1310 (978 to 1740)	1230 (901 to 1670)	1140 (864 to 1460)	1030 (885 to 1200)	893 (783 to 1010)	777 (688 to 874)	534 (469 to 612)	464 (384 to 561)	588 (490 to 709)	553 (449 to 679)
Islington	1420 (1050 to 1830)	1350 (1000 to 1800)	1280 (961 to 1660)	1180 (1020 to 1370)	1020 (892 to 1160)	972 (851 to 1100)	666 (580 to 764)	523 (434 to 632)	554 (456 to 669)	618 (502 to 756)
Kensington and Chelsea	1320 (974 to 1720)	1320 (965 to 1770)	1260 (944 to 1630)	1210 (1040 to 1400)	1060 (932 to 1210)	681 (597 to 775)	449 (393 to 514)	399 (329 to 489)	429 (352 to 518)	516 (411 to 640)
Kingston upon Thames	1200 (888 to 1580)	1140 (817 to 1590)	1060 (782 to 1390)	978 (833 to 1140)	799 (700 to 914)	632 (546 to 729)	417 (360 to 484)	374 (304 to 454)	509 (418 to 627)	446 (356 to 551)
Lambeth	1550 (1180 to 2000)	1480 (1050 to 1990)	1410 (1080 to 1830)	1210 (1050 to 1410)	1020 (886 to 1180)	796 (700 to 909)	582 (506 to 668)	501 (408 to 613)	639 (532 to 781)	685 (550 to 842)
Lewisham	1420 (1060 to 1840)	1320 (939 to 1800)	1220 (928 to 1580)	1120 (964 to 1300)	953 (832 to 1090)	874 (761 to 991)	575 (499 to 660)	544 (450 to 662)	602 (494 to 724)	722 (587 to 890)
Merton	1250 (933 to 1640)	1180 (834 to 1610)	1070 (805 to 1420)	974 (842 to 1130)	844 (732 to 963)	656 (572 to 751)	475 (413 to 548)	426 (350 to 522)	583 (483 to 718)	575 (466 to 713)
Newham	1470 (1090 to 1900)	1350 (969 to 1820)	1260 (940 to 1640)	1140 (977 to 1320)	1010 (883 to 1150)	868 (756 to 990)	633 (551 to 721)	544 (448 to 658)	752 (617 to 917)	712 (583 to 869)
Redbridge	1240 (926 to 1630)	1160 (844 to 1580)	1080 (808 to 1410)	978 (842 to 1130)	819 (713 to 929)	651 (567 to 739)	468 (406 to 534)	430 (352 to 521)	650 (533 to 790)	538 (436 to 652)
Richmond upon Thames	1200 (866 to 1570)	1160 (822 to 1570)	1090 (811 to 1460)	1020 (877 to 1180)	777 (675 to 886)	583 (508 to 668)	394 (342 to 458)	350 (285 to 427)	475 (391 to 581)	463 (369 to 576)
Southwark	1460 (1080 to 1890)	1350 (970 to 1830)	1280 (983 to 1680)	1160 (1000 to 1340)	1190 (1040 to 1360)	820 (718 to 933)	586 (511 to 675)	482 (398 to 590)	613 (506 to 742)	691 (562 to 848)
Sutton	1210 (893 to 1600)	1120 (809 to 1550)	1010 (753 to 1340)	906 (773 to 1050)	782 (681 to 895)	660 (579 to 756)	477 (413 to 546)	429 (350 to 531)	476 (394 to 586)	582 (472 to 718)
Tower Hamlets	1590 (1200 to 2050)	1510 (1100 to 2030)	1490 (1150 to 1930)	1420 (1240 to 1650)	1120 (987 to 1280)	1000 (882 to 1140)	641 (562 to 734)	468 (389 to 563)	751 (624 to 905)	705 (580 to 864)
Waltham Forest	1360 (990 to 1730)	1270 (913 to 1720)	1170 (865 to 1540)	1060 (917 to 1230)	869 (756 to 992)	785 (693 to 892)	596 (522 to 682)	490 (408 to 589)	558 (465 to 678)	589 (479 to 730)
Wandsworth	1380 (1020 to 1810)	1310 (966 to 1790)	1230 (922 to 1590)	1150 (1000 to 1340)	1040 (919 to 1170)	791 (695 to 900)	520 (455 to 594)	520 (428 to 628)	589 (485 to 712)	586 (474 to 713)
Westminster	1340 (990 to 1740)	1330 (976 to 1840)	1290 (982 to 1660)	1230 (1070 to 1450)	926 (807 to 1050)	764 (669 to 870)	431 (373 to 498)	403 (334 to 490)	445 (370 to 535)	474 (384 to 588)
North East England	1500 (1210 to 1840)	1410 (1080 to 1830)	1330 (1050 to 1650)	1190 (1060 to 1350)	1020 (912 to 1130)	822 (736 to 919)	617 (552 to 692)	593 (512 to 688)	664 (574 to 783)	669 (568 to 794)
County Durham	1510 (1160 to 1960)	1410 (1000 to 1960)	1330 (1000 to 1720)	1190 (1030 to 1380)	990 (868 to 1120)	811 (708 to 923)	635 (557 to 723)	594 (483 to 715)	662 (542 to 796)	666 (534 to 815)
Darlington	1470 (1090 to 1960)	1400 (966 to 1950)	1310 (977 to 1730)	1150 (976 to 1350)	991 (862 to 1130)	827 (717 to 954)	611 (529 to 714)	568 (458 to 703)	611 (502 to 769)	650 (511 to 827)
Gateshead	1550 (1130 to 2060)	1490 (1060 to 2070)	1410 (1060 to 1830)	1250 (1080 to 1460)	1050 (912 to 1200)	895 (778 to 1030)	610 (529 to 706)	595 (485 to 724)	667 (545 to 809)	624 (501 to 784)
Hartlepool	1590 (1190 to 2090)	1510 (1080 to 2050)	1420 (1060 to 1860)	1260 (1060 to 1470)	1060 (920 to 1230)	903 (781 to 1030)	653 (561 to 754)	674 (545 to 836)	698 (570 to 871)	676 (538 to 842)
Middlesbrough	1600 (1200 to 2100)	1510 (1060 to 2060)	1420 (1060 to 1860)	1250 (1060 to 1470)	1130 (973 to 1300)	887 (765 to 1030)	699 (597 to 809)	724 (588 to 894)	825 (670 to 1020)	830 (660 to 1030)
Newcastle upon Tyne	1500 (1100 to 1980)	1450 (1020 to 2000)	1380 (1030 to 1780)	1220 (1050 to 1430)	1100 (958 to 1260)	827 (724 to 949)	614 (530 to 709)	588 (480 to 710)	702 (579 to 865)	746 (591 to 933)
North Tyneside	1430 (1060 to 1900)	1360 (947 to 1940)	1260 (943 to 1670)	1130 (964 to 1330)	983 (857 to 1130)	831 (721 to 954)	643 (554 to 740)	566 (460 to 696)	710 (574 to 875)	628 (501 to 778)
Northumberland	1400 (1030 to 1830)	1320 (933 to 1820)	1220 (922 to 1610)	1160 (999 to 1320)	912 (793 to 1050)	722 (633 to 826)	536 (467 to 622)	542 (446 to 659)	595 (491 to 730)	586 (472 to 727)
Redcar and Cleveland	1520 (1120 to 2040)	1450 (1030 to 2020)	1340 (1000 to 1790)	1200 (1010 to 1410)	1030 (894 to 1190)	808 (701 to 930)	581 (498 to 676)	596 (478 to 737)	633 (511 to 786)	661 (525 to 840)
South Tyneside	1530 (1140 to 2000)	1450 (1010 to 1990)	1360 (1010 to 1760)	1210 (1020 to 1410)	1040 (896 to 1200)	860 (745 to 991)	638 (549 to 740)	619 (501 to 768)	690 (562 to 874)	712 (567 to 890)

Stockton-on-Tees	1440 (1060 to 1940)	1370 (961 to 1920)	1280 (952 to 1700)	1130 (959 to 1310)	1000 (870 to 1150)	761 (663 to 873)	576 (500 to 665)	568 (466 to 694)	636 (522 to 777)	673 (542 to 841)
Sunderland	1490 (1100 to 1990)	1410 (1010 to 1960)	1320 (999 to 1720)	1200 (1030 to 1400)	1070 (928 to 1210)	891 (783 to 1020)	681 (590 to 790)	636 (521 to 776)	712 (586 to 872)	767 (625 to 952)
North West England	1480 (1200 to 1800)	1400 (1120 to 1770)	1310 (1070 to 1600)	1180 (1050 to 1320)	999 (902 to 1110)	821 (747 to 909)	622 (560 to 693)	568 (496 to 651)	653 (567 to 746)	658 (564 to 770)
Blackburn with Darwen	1550 (1160 to 2020)	1490 (1070 to 2000)	1370 (1020 to 1770)	1230 (1050 to 1430)	1090 (955 to 1250)	920 (806 to 1050)	675 (591 to 772)	612 (499 to 744)	702 (579 to 846)	752 (611 to 934)
Blackpool	1550 (1170 to 2030)	1490 (1070 to 2010)	1360 (1020 to 1770)	1200 (1020 to 1400)	1130 (982 to 1280)	1070 (946 to 1220)	926 (806 to 1060)	853 (701 to 1030)	824 (674 to 995)	919 (746 to 1130)
Bolton	1520 (1120 to 2010)	1460 (1060 to 1980)	1370 (1040 to 1710)	1210 (1040 to 1400)	1010 (887 to 1150)	852 (748 to 969)	641 (561 to 727)	616 (513 to 741)	647 (533 to 780)	653 (533 to 802)
Bury	1410 (1050 to 1850)	1370 (977 to 1840)	1260 (927 to 1630)	1120 (961 to 1310)	975 (848 to 1110)	774 (675 to 885)	586 (508 to 675)	522 (429 to 645)	660 (538 to 799)	590 (476 to 731)
Cheshire East	1280 (948 to 1690)	1250 (906 to 1670)	1170 (879 to 1530)	1090 (946 to 1260)	885 (778 to 1010)	713 (619 to 816)	517 (450 to 590)	489 (407 to 588)	554 (458 to 668)	535 (435 to 659)
Cheshire West and Chester	1320 (954 to 1760)	1270 (917 to 1740)	1190 (896 to 1560)	1090 (929 to 1270)	898 (781 to 1020)	708 (615 to 806)	548 (476 to 626)	507 (415 to 618)	580 (478 to 701)	592 (478 to 725)
Cumbria	1400 (1020 to 1810)	1320 (943 to 1820)	1230 (924 to 1590)	1110 (962 to 1290)	949 (834 to 1080)	740 (652 to 842)	539 (472 to 618)	542 (453 to 650)	574 (480 to 690)	623 (506 to 759)
Halton	1470 (1090 to 1930)	1450 (1050 to 1980)	1350 (1030 to 1750)	1210 (1050 to 1410)	1000 (876 to 1150)	872 (765 to 1000)	631 (551 to 727)	612 (503 to 746)	638 (526 to 778)	696 (567 to 859)
Knowsley	1530 (1140 to 2000)	1480 (1090 to 2030)	1380 (1050 to 1810)	1240 (1070 to 1450)	1070 (929 to 1230)	936 (822 to 1070)	695 (601 to 806)	622 (513 to 752)	705 (579 to 849)	689 (561 to 851)
Lancashire	1530 (1170 to 1950)	1370 (998 to 1850)	1280 (971 to 1670)	1160 (1010 to 1340)	974 (863 to 1100)	789 (697 to 890)	584 (516 to 665)	576 (484 to 684)	660 (550 to 786)	685 (568 to 826)
Liverpool	1560 (1160 to 2010)	1450 (1040 to 1930)	1380 (1050 to 1820)	1220 (1050 to 1420)	1070 (948 to 1220)	954 (839 to 1080)	743 (650 to 850)	649 (539 to 780)	749 (620 to 900)	773 (637 to 956)
Manchester	1630 (1230 to 2100)	1520 (1100 to 2040)	1450 (1090 to 1870)	1320 (1140 to 1520)	1170 (1010 to 1320)	995 (871 to 1130)	736 (635 to 845)	628 (516 to 752)	832 (678 to 993)	854 (690 to 1050)
Oldham	1540 (1130 to 2020)	1490 (1080 to 1990)	1360 (1040 to 1750)	1240 (1070 to 1440)	1070 (932 to 1220)	854 (750 to 973)	663 (580 to 762)	617 (508 to 756)	769 (630 to 926)	675 (546 to 821)
Rochdale	1520 (1120 to 1970)	1460 (1050 to 1940)	1350 (1010 to 1720)	1200 (1040 to 1400)	1040 (909 to 1180)	830 (727 to 949)	675 (590 to 772)	581 (476 to 700)	698 (575 to 846)	727 (578 to 905)
Salford	1600 (1190 to 2050)	1560 (1140 to 2100)	1480 (1140 to 1880)	1360 (1180 to 1580)	1100 (972 to 1250)	898 (788 to 1020)	718 (629 to 818)	584 (488 to 707)	751 (622 to 898)	682 (554 to 836)
Sefton	1400 (1040 to 1830)	1330 (950 to 1860)	1240 (940 to 1610)	1110 (960 to 1280)	916 (803 to 1040)	776 (681 to 883)	658 (575 to 758)	596 (490 to 719)	682 (567 to 832)	661 (541 to 808)
St Helens	1510 (1120 to 1940)	1480 (1090 to 2010)	1380 (1030 to 1790)	1250 (1080 to 1440)	1030 (905 to 1180)	836 (732 to 958)	655 (568 to 748)	621 (508 to 753)	718 (588 to 864)	670 (539 to 827)
Stockport	1340 (992 to 1760)	1290 (930 to 1730)	1200 (896 to 1560)	1090 (938 to 1270)	945 (830 to 1070)	739 (648 to 838)	572 (500 to 659)	504 (415 to 604)	574 (474 to 690)	560 (457 to 691)
Tameside	1510 (1090 to 1980)	1450 (1040 to 1950)	1330 (998 to 1730)	1190 (1020 to 1380)	1000 (876 to 1130)	907 (796 to 1030)	681 (588 to 785)	551 (456 to 668)	660 (534 to 798)	693 (559 to 844)
Trafford	1310 (966 to 1740)	1270 (915 to 1700)	1160 (865 to 1500)	1050 (896 to 1210)	933 (815 to 1070)	719 (626 to 819)	569 (492 to 657)	437 (357 to 528)	541 (443 to 660)	509 (406 to 631)
Warrington	1370 (978 to 1800)	1340 (981 to 1790)	1260 (934 to 1630)	1140 (978 to 1310)	975 (849 to 1110)	774 (674 to 887)	573 (495 to 660)	583 (479 to 714)	670 (552 to 820)	631 (503 to 764)
Wigan	1500 (1120 to 1940)	1450 (1040 to 2010)	1340 (1010 to 1760)	1220 (1050 to 1420)	1050 (922 to 1190)	870 (766 to 982)	680 (592 to 778)	552 (458 to 667)	677 (555 to 824)	626 (506 to 771)
Wirral	1460 (1060 to 1910)	1370 (954 to 1880)	1270 (944 to 1650)	1140 (983 to 1330)	990 (874 to 1120)	804 (709 to 917)	573 (500 to 654)	552 (452 to 663)	607 (504 to 733)	678 (550 to 839)
South East England	1260 (1030 to 1530)	1190 (930 to 1470)	1110 (884 to 1360)	1010 (899 to 1140)	821 (738 to 905)	677 (612 to 746)	509 (458 to 563)	478 (416 to 546)	523 (457 to 599)	523 (449 to 605)
Bracknell Forest	1180 (874 to 1600)	1160 (818 to 1590)	1080 (784 to 1440)	972 (827 to 1140)	828 (720 to 952)	640 (551 to 738)	448 (382 to 516)	416 (336 to 512)	496 (401 to 604)	423 (331 to 534)
Brighton and Hove	1330 (986 to 1780)	1300 (948 to 1750)	1190 (885 to 1580)	1060 (913 to 1240)	1010 (889 to 1150)	799 (702 to 916)	624 (544 to 718)	517 (424 to 627)	576 (476 to 705)	549 (445 to 682)
Buckinghamshire	1120 (814 to 1490)	1100 (776 to 1490)	1030 (737 to 1370)	981 (842 to 1150)	806 (707 to 926)	623 (543 to 720)	476 (416 to 548)	446 (363 to 542)	476 (391 to 576)	509 (410 to 631)
East Sussex	1270 (938 to 1700)	1200 (856 to 1630)	1110 (822 to 1460)	978 (838 to 1130)	771 (677 to 879)	657 (577 to 745)	523 (462 to 600)	525 (434 to 629)	557 (464 to 668)	569 (465 to 694)
Hampshire	1260 (947 to 1640)	1150 (806 to 1560)	1080 (791 to 1460)	987 (858 to 1140)	797 (706 to 898)	656 (580 to 738)	487 (428 to 554)	451 (381 to 536)	486 (411 to 585)	471 (388 to 569)
Isle of Wight	1370 (1010 to 1780)	1300 (910 to 1780)	1170 (866 to 1540)	1030 (873 to 1210)	896 (775 to 1020)	715 (619 to 823)	566 (486 to 658)	539 (439 to 663)	575 (466 to 709)	572 (456 to 705)
Kent	1370 (1040 to 1760)	1240 (901 to 1650)	1180 (884 to 1550)	1100 (956 to 1260)	868 (763 to 979)	707 (622 to 798)	531 (464 to 602)	513 (433 to 611)	578 (482 to 689)	568 (468 to 695)
Medway	1290 (942 to 1710)	1260 (890 to 1740)	1130 (831 to 1500)	993 (847 to 1170)	898 (779 to 1030)	773 (677 to 881)	612 (531 to 702)	560 (459 to 674)	651 (538 to 787)	585 (471 to 730)
Milton Keynes	1270 (908 to 1650)	1250 (885 to 1690)	1170 (847 to 1580)	1050 (879 to 1230)	832 (724 to 950)	691 (595 to 790)	522 (456 to 602)	460 (377 to 553)	563 (456 to 683)	505 (410 to 624)
Oxfordshire	1120 (824 to 1480)	1090 (751 to 1510)	1020 (736 to 1330)	955 (823 to 1110)	777 (673 to 882)	672 (583 to 765)	495 (426 to 566)	457 (375 to 550)	471 (386 to 564)	497 (400 to 615)
Portsmouth	1370 (1010 to 1800)	1350 (961 to 1830)	1250 (930 to 1670)	1130 (980 to 1310)	996 (873 to 1130)	839 (733 to 953)	632 (552 to 725)	530 (430 to 643)	688 (569 to 840)	615 (500 to 757)
Reading	1270 (941 to 1720)	1270 (911 to 1730)	1200 (892 to 1570)	1100 (943 to 1290)	928 (801 to 1060)	759 (660 to 866)	596 (515 to 682)	554 (453 to 669)	725 (583 to 880)	574 (463 to 701)
Slough	1300 (965 to 1750)	1280 (896 to 1760)	1180 (884 to 1570)	1070 (906 to 1260)	961 (836 to 1110)	772 (669 to 887)	619 (537 to 708)	556 (456 to 674)	629 (515 to 766)	719 (581 to 892)
Southampton	1370 (1030 to 1790)	1350 (972 to 1850)	1260 (927 to 1640)	1090 (925 to 1270)	943 (816 to 1070)	769 (678 to 876)	571 (499 to 654)	536 (441 to 654)	698 (575 to 851)	642 (516 to 788)
Surrey	1250 (940 to 1620)	1140 (804 to 1600)	1080 (782 to 1450)	984 (851 to 1140)	771 (675 to 872)	656 (574 to 741)	465 (407 to 524)	429 (350 to 513)	461 (379 to 552)	473 (384 to 572)
West Berkshire	1140 (813 to 1530)	1120 (793 to 1520)	1040 (749 to 1370)	948 (805 to 1110)	771 (666 to 888)	619 (534 to 708)	459 (398 to 531)	414 (338 to 510)	436 (352 to 535)	525 (414 to 650)
West Sussex	1220 (909 to 1600)	1160 (807 to 1600)	1100 (822 to 1450)	1010 (866 to 1170)	784 (688 to 900)	652 (575 to 747)	503 (440 to 581)	508 (422 to 611)	522 (430 to 630)	538 (438 to 672)
Windsor and Maidenhead	1040 (745 to 1390)	1020 (724 to 1440)	913 (674 to 1210)	806 (677 to 964)	738 (635 to 850)	625 (543 to 724)	435 (371 to 504)	380 (302 to 470)	465 (378 to 579)	458 (366 to 569)
Wokingham	1020 (734 to 1410)	1010 (710 to 1410)	922 (667 to 1230)	826 (700 to 975)	639 (555 to 736)	498 (432 to 574)	382 (331 to 440)	381 (307 to 475)	402 (325 to 494)	529 (425 to 658)
South West England	1270 (1010 to 1570)	1190 (920 to 1500)	1120 (878 to 1390)	1000 (884 to 1120)	826 (746 to 921)	674 (610 to 751)	515 (462 to 576)	493 (431 to 567)	522 (454 to 604)	561 (482 to 655)
Bath and North East Somerset	1160 (832 to 1540)	1100 (792 to 1510)	996 (729 to 1300)	873 (738 to 1020)	784 (679 to 899)	632 (556 to 724)	480 (415 to 554)	468 (383 to 571)	512 (423 to 622)	545 (441 to 670)
Bournemouth	1290 (930 to 1740)	1230 (857 to 1680)	1120 (815 to 1460)	986 (836 to 1150)	922 (798 to 1060)	785 (688 to 896)	614 (532 to 706)	541 (448 to 656)	690 (568 to 842)	1140 (875 to 1470)
Bristol, City of	1360 (1010 to 1770)	1280 (903 to 1760)	1210 (908 to 1560)	1090 (930 to 1270)	920 (806 to 1050)	762 (673 to 876)	576 (499 to 658)	555 (458 to 674)	572 (473 to 692)	632 (513 to 785)
Cornwall	1320 (954 to 1740)	1240 (872 to 1700)	1170 (874 to 1540)	1040 (899 to 1210)	844 (741 to 959)	674 (595 to 765)	521 (457 to 591)	540 (450 to 642)	575 (476 to 694)	586 (482 to 712)
Devon	1260 (917 to 1670)	1170 (812 to 1580)	1110 (825 to 1450)	974 (841 to 1120)	793 (698 to 901)	654 (573 to 747)	525 (459 to 600)	480 (399 to 572)	494 (409 to 599)	492 (399 to 602)
Dorset	1220 (889 to 1600)	1150 (801 to 1600)	1060 (770 to 1390)	934 (807 to 1100)	743 (649 to 850)	608 (533 to 690)	458 (399 to 524)	431 (361 to 516)	450 (373 to 544)	432 (348 to 528)
Gloucestershire	1230 (866 to 1610)	1160 (811 to 1580)	1100 (799 to 1460)	1030 (891 to 1200)	838 (732 to 950)	670 (586 to 768)	499 (435 to 573)	517 (432 to 616)	561 (465 to 675)	570 (464 to 702)
North Somerset	1210 (883 to 1650)	1150 (815 to 1590)	1040 (772 to 1380)	913 (776 to 1070)	771 (676 to 887)	642 (559 to 742)	536 (468 to 620)	480 (396 to 591)	525 (434 to 647)	515 (418 to 639)
Plymouth	1400 (1030 to 1860)	1350 (949 to 1860)	1270 (959 to 1660)	1130 (966 to 1320)	922 (801 to 1050)	801 (700 to 913)	594 (515 to 681)	558 (458 to 674)	550 (448 to 670)	572 (457 to 708)
Poole	1270 (897 to 1700)	1210 (861 to 1660)	1130 (830 to 1480)	1010 (860 to 1190)	803 (701 to 931)	637 (556 to 732)	481 (419 to 559)	467 (384 to 572)	553 (452 to 678)	941 (705 to 1220)
Somerset	1240 (904 to 1650)	1170 (818 to 1620)	1100 (804 to 1460)	994 (853 to 1160)	804 (707 to 921)	657 (581 to 750)	502 (440 to 573)	510 (424 to 615)	521 (432 to 640)	565 (463 to 695)
South Gloucestershire	1170 (839 to 1580)	1120 (797 to 1570)	1050 (772 to 1390)	942 (809 to 1110)	730 (638 to 839)	654 (569 to 754)	477 (414 to 550)	392 (325 to 470)	480 (393 to 591)	491 (396 to 609)
Swindon	1280 (941 to 1690)	1230 (863 to 1670)	1150 (848 to 1530)	1020 (875 to 1200)	891 (782 to 1010)	697 (613 to 796)	508 (441 to 583)	441 (363 to 524)	556 (462 to 675)	512 (413 to 636)
Torbay	1350 (980 to 1780)	1270 (906 to 1740)	1160 (868 to 1550)	1030 (870 to 1220)	890 (769 to 1020)	802 (699 to 924)	630 (544 to 730)	630 (514 to 766)	546 (439 to 658)	622 (494 to 773)
Wiltshire	1200 (859 to 1610)	1140 (783 to 1550)	1050 (777 to 1400)	990 (843 to 1150)	858 (751 to 978)	654 (574 to 745)	481 (417 to 550)	468 (390 to 558)	478 (394 to 579)	532 (430 to 648)
West Midlands	1450 (1170 to 1760)	1370 (1070 to 1730)	1280 (1020 to 1570)	1150 (1030 to 1300)	942 (844 to 1050)	774 (695 to 859)	575 (517 to 644)	544 (470 to 636)	629 (545 to 725)	620 (525 to 732)
Birmingham	1550 (1160 to 1970)	1410 (1020 to 1870)	1330 (1010 to 1710)	1220 (1050 to 1420)	1000 (881 to 1150)	837 (736 to 958)	589 (516 to 675)	576 (479 to 696)	716 (593 to 859)	726 (594 to 895)

Coventry	1440 (1070 to 1900)	1370 (952 to 1880)	1270 (932 to 1660)	1120 (966 to 1300)	972 (846 to 1120)	789 (689 to 902)	638 (553 to 739)	628 (520 to 769)	671 (555 to 821)	682 (551 to 843)
Dudley	1380 (992 to 1800)	1330 (942 to 1820)	1220 (887 to 1600)	1120 (966 to 1300)	937 (818 to 1070)	796 (691 to 912)	585 (507 to 673)	522 (424 to 642)	596 (487 to 725)	580 (468 to 717)
Herefordshire, County of	1300 (942 to 1780)	1260 (882 to 1690)	1130 (846 to 1470)	989 (845 to 1150)	776 (672 to 885)	697 (607 to 795)	521 (450 to 603)	500 (410 to 615)	511 (422 to 627)	573 (462 to 708)
Sandwell	1550 (1160 to 2050)	1490 (1050 to 2010)	1370 (1040 to 1760)	1190 (1030 to 1380)	1050 (910 to 1200)	873 (763 to 1000)	682 (594 to 789)	620 (519 to 754)	718 (592 to 872)	725 (582 to 905)
Shropshire	1330 (973 to 1800)	1300 (916 to 1800)	1200 (889 to 1580)	1070 (914 to 1230)	863 (750 to 988)	717 (620 to 816)	522 (456 to 602)	451 (368 to 553)	507 (415 to 617)	531 (427 to 651)
Solihull	1240 (882 to 1650)	1220 (860 to 1670)	1110 (794 to 1450)	980 (847 to 1160)	765 (664 to 885)	663 (578 to 759)	469 (407 to 544)	460 (377 to 561)	556 (461 to 672)	512 (410 to 636)
Staffordshire	1500 (1130 to 1930)	1370 (991 to 1880)	1300 (978 to 1690)	1150 (996 to 1320)	949 (833 to 1080)	758 (662 to 862)	545 (476 to 620)	535 (450 to 646)	624 (522 to 743)	597 (489 to 738)
Stoke-on-Trent	1600 (1190 to 2080)	1550 (1110 to 2090)	1460 (1110 to 1850)	1330 (1140 to 1540)	1080 (941 to 1230)	875 (765 to 997)	690 (597 to 789)	668 (547 to 809)	770 (632 to 936)	758 (613 to 932)
Telford and Wrekin	1430 (1040 to 1920)	1410 (994 to 1880)	1310 (971 to 1700)	1170 (1010 to 1380)	970 (850 to 1130)	764 (667 to 875)	575 (498 to 668)	589 (490 to 729)	668 (552 to 814)	763 (618 to 958)
Walsall	1490 (1060 to 2000)	1430 (1020 to 1900)	1330 (982 to 1710)	1200 (1040 to 1400)	1010 (878 to 1160)	803 (701 to 920)	652 (566 to 748)	606 (501 to 730)	678 (554 to 822)	674 (541 to 832)
Warwickshire	1320 (969 to 1740)	1280 (891 to 1760)	1200 (886 to 1580)	1100 (947 to 1270)	874 (764 to 1010)	726 (634 to 831)	537 (468 to 619)	508 (424 to 624)	575 (473 to 701)	549 (445 to 683)
Wolverhampton	1490 (1090 to 1960)	1430 (1020 to 1970)	1330 (998 to 1720)	1180 (1000 to 1370)	983 (860 to 1120)	832 (722 to 958)	673 (580 to 777)	637 (519 to 776)	724 (584 to 875)	739 (596 to 922)
Worcestershire	1310 (953 to 1750)	1270 (898 to 1740)	1170 (861 to 1520)	1080 (931 to 1260)	875 (766 to 997)	709 (618 to 808)	543 (471 to 619)	508 (423 to 615)	594 (487 to 715)	536 (429 to 664)
Yorkshire and the Humber	1450 (1160 to 1750)	1370 (1060 to 1700)	1270 (1020 to 1580)	1140 (1020 to 1280)	943 (848 to 1040)	758 (681 to 840)	592 (534 to 655)	562 (492 to 644)	634 (549 to 728)	630 (539 to 730)
Barnsley	1500 (1100 to 1950)	1430 (1020 to 1960)	1310 (988 to 1710)	1130 (962 to 1320)	954 (831 to 1090)	802 (695 to 916)	593 (516 to 676)	612 (504 to 740)	705 (577 to 860)	714 (570 to 868)
Bradford	1550 (1140 to 2010)	1420 (1020 to 1960)	1310 (970 to 1720)	1160 (995 to 1340)	993 (871 to 1130)	825 (727 to 935)	622 (545 to 711)	592 (488 to 709)	708 (575 to 850)	659 (532 to 806)
Calderdale	1540 (1120 to 1970)	1460 (1030 to 1990)	1360 (1020 to 1770)	1220 (1040 to 1410)	1010 (884 to 1160)	759 (657 to 864)	631 (547 to 721)	527 (428 to 644)	628 (512 to 763)	644 (514 to 791)
Doncaster	1460 (1070 to 1910)	1370 (985 to 1850)	1260 (964 to 1670)	1120 (955 to 1300)	941 (819 to 1070)	808 (700 to 923)	649 (566 to 736)	574 (466 to 682)	657 (541 to 798)	645 (518 to 798)
East Riding of Yorkshire	1320 (974 to 1730)	1250 (880 to 1700)	1150 (824 to 1520)	1010 (861 to 1170)	867 (757 to 985)	721 (628 to 819)	538 (468 to 612)	471 (387 to 569)	532 (431 to 643)	551 (444 to 679)
Kingston upon Hull, City of	1520 (1120 to 1970)	1430 (1020 to 1940)	1310 (990 to 1710)	1160 (990 to 1350)	1000 (876 to 1140)	883 (772 to 1010)	729 (635 to 833)	734 (609 to 895)	816 (674 to 994)	801 (640 to 990)
Kirklees	1550 (1150 to 2040)	1450 (1030 to 1950)	1370 (1020 to 1770)	1250 (1080 to 1450)	981 (861 to 1130)	770 (669 to 880)	616 (535 to 704)	582 (474 to 696)	678 (554 to 820)	617 (499 to 750)
Leeds	1490 (1120 to 1920)	1350 (964 to 1840)	1280 (952 to 1700)	1160 (1010 to 1340)	924 (813 to 1040)	744 (654 to 838)	599 (527 to 673)	606 (507 to 726)	671 (552 to 807)	717 (586 to 870)
North East Lincolnshire	1430 (1050 to 1850)	1350 (978 to 1850)	1210 (887 to 1620)	1060 (918 to 1230)	949 (827 to 1080)	775 (677 to 883)	618 (536 to 708)	596 (493 to 719)	633 (518 to 765)	613 (496 to 743)
North Lincolnshire	1440 (1060 to 1860)	1390 (991 to 1890)	1280 (946 to 1690)	1150 (984 to 1350)	975 (846 to 1120)	741 (642 to 857)	576 (501 to 659)	531 (436 to 645)	626 (509 to 767)	579 (466 to 724)
North Yorkshire	1340 (985 to 1750)	1260 (888 to 1720)	1170 (864 to 1540)	1040 (902 to 1200)	860 (756 to 972)	660 (580 to 760)	509 (448 to 582)	492 (411 to 587)	538 (450 to 654)	532 (436 to 648)
Rotherham	1480 (1100 to 1970)	1430 (1040 to 1910)	1320 (978 to 1720)	1240 (1070 to 1430)	1000 (878 to 1130)	809 (705 to 926)	597 (521 to 679)	604 (496 to 730)	707 (580 to 858)	644 (522 to 792)
Sheffield	1430 (1070 to 1870)	1360 (977 to 1820)	1270 (960 to 1670)	1140 (982 to 1320)	958 (836 to 1090)	768 (672 to 878)	598 (525 to 683)	553 (463 to 672)	605 (502 to 736)	638 (524 to 788)
Wakefield	1530 (1150 to 2010)	1490 (1070 to 1970)	1410 (1090 to 1820)	1270 (1090 to 1470)	1000 (873 to 1130)	768 (673 to 876)	631 (552 to 722)	620 (516 to 746)	707 (589 to 849)	678 (546 to 826)
York	1300 (944 to 1740)	1260 (873 to 1720)	1150 (859 to 1510)	1030 (893 to 1210)	879 (774 to 1010)	689 (602 to 795)	503 (441 to 577)	492 (408 to 598)	510 (420 to 618)	600 (491 to 738)
Northern Ireland	1310 (1230 to 1410)	1310 (1220 to 1420)	1290 (1190 to 1400)	1300 (1200 to 1400)	1070 (998 to 1160)	826 (762 to 895)	612 (566 to 662)	542 (500 to 590)	585 (537 to 633)	589 (541 to 640)
Scotland	1270 (1190 to 1370)	1610 (1500 to 1740)	1430 (1320 to 1550)	1240 (1150 to 1330)	1050 (972 to 1130)	868 (806 to 939)	674 (625 to 731)	610 (541 to 685)	661 (587 to 749)	677 (591 to 776)
Wales	1450 (1340 to 1590)	1360 (1250 to 1480)	1260 (1160 to 1380)	1130 (1050 to 1220)	917 (848 to 991)	781 (723 to 844)	601 (555 to 650)	531 (491 to 579)	574 (535 to 622)	555 (518 to 595)
Latin America and Caribbean	2110 (1990 to 2240)	1720 (1640 to 1790)	1520 (1450 to 1580)	1220 (1170 to 1260)	1040 (1010 to 1080)	897 (868 to 930)	838 (808 to 869)	749 (704 to 800)	947 (889 to 1020)	1030 (958 to 1120)
Andean Latin America	2500 (2220 to 2810)	2000 (1830 to 2180)	1800 (1670 to 1960)	1160 (1070 to 1260)	1000 (920 to 1080)	808 (736 to 885)	703 (640 to 772)	662 (580 to 760)	1050 (933 to 1190)	1190 (1060 to 1340)
Bolivia	3100 (2660 to 3600)	2540 (2130 to 3040)	2130 (1820 to 2480)	1730 (1490 to 1980)	1420 (1240 to 1620)	1150 (1010 to 1300)	968 (812 to 1160)	870 (699 to 1110)	1510 (1250 to 1860)	1720 (1450 to 2110)
Ecuador	1820 (1540 to 2160)	1560 (1430 to 1700)	1410 (1290 to 1560)	1180 (1080 to 1280)	886 (832 to 952)	813 (730 to 902)	812 (722 to 908)	703 (592 to 833)	1060 (898 to 1260)	977 (796 to 1190)
Peru	2540 (2200 to 2910)	2010 (1780 to 2260)	1880 (1690 to 2110)	1040 (919 to 1180)	949 (838 to 1080)	719 (619 to 838)	588 (505 to 683)	603 (506 to 718)	959 (812 to 1120)	1170 (1020 to 1340)
Caribbean	1850 (1670 to 2050)	1510 (1420 to 1600)	1360 (1290 to 1430)	1170 (1100 to 1230)	1070 (1000 to 1150)	957 (906 to 1010)	1290 (1200 to 1380)	858 (772 to 944)	925 (828 to 1030)	1120 (984 to 1270)
Antigua and Barbuda	1950 (1760 to 2150)	1430 (1320 to 1570)	1230 (1130 to 1350)	951 (817 to 1110)	1010 (925 to 1110)	877 (799 to 967)	908 (822 to 998)	842 (728 to 975)	770 (664 to 898)	908 (804 to 1030)
The Bahamas	1790 (1450 to 2180)	1530 (1280 to 1800)	1360 (1240 to 1500)	1190 (1070 to 1300)	1050 (947 to 1150)	1040 (945 to 1140)	962 (879 to 1060)	1000 (829 to 1210)	1090 (880 to 1340)	1320 (1070 to 1610)
Barbados	1800 (1620 to 2010)	1320 (1220 to 1440)	1340 (1240 to 1450)	1090 (1000 to 1170)	916 (843 to 994)	842 (778 to 914)	738 (681 to 799)	762 (616 to 922)	711 (554 to 882)	768 (598 to 954)
Belize	1730 (1550 to 1950)	1100 (987 to 1220)	909 (829 to 1000)	842 (770 to 929)	776 (710 to 851)	1150 (1060 to 1240)	834 (774 to 904)	785 (676 to 913)	862 (745 to 999)	922 (760 to 1120)
Bermuda	1420 (1270 to 1590)	1620 (1480 to 1760)	1330 (1230 to 1450)	1170 (1070 to 1270)	1170 (1070 to 1280)	789 (729 to 854)	661 (594 to 729)	590 (510 to 687)	572 (495 to 664)	729 (620 to 856)
Cuba	1450 (1190 to 1760)	1280 (1150 to 1440)	1310 (1230 to 1410)	870 (810 to 939)	820 (730 to 928)	716 (663 to 776)	663 (613 to 718)	687 (611 to 776)	693 (615 to 793)	1050 (898 to 1210)
Dominica	2340 (2060 to 2640)	1470 (1340 to 1610)	1210 (1110 to 1330)	1060 (949 to 1160)	1160 (1070 to 1260)	1080 (967 to 1190)	1010 (911 to 1120)	999 (854 to 1190)	995 (851 to 1190)	1260 (989 to 1630)
Dominican Republic	1610 (1400 to 1840)	1250 (1090 to 1410)	1030 (915 to 1150)	970 (861 to 1090)	869 (762 to 993)	840 (724 to 965)	809 (667 to 975)	788 (659 to 947)	897 (747 to 1090)	900 (738 to 1100)
Grenada	1640 (1380 to 1920)	1290 (1110 to 1500)	1230 (1020 to 1430)	1110 (935 to 1320)	1130 (1010 to 1260)	1380 (1260 to 1520)	1810 (1660 to 1970)	1180 (1040 to 1360)	1170 (1010 to 1360)	1450 (1160 to 1860)
Guyana	2350 (2090 to 2630)	1720 (1530 to 1940)	1640 (1450 to 1840)	1580 (1340 to 1830)	1560 (1380 to 1760)	1380 (1220 to 1580)	1320 (1170 to 1500)	1250 (980 to 1560)	1440 (1130 to 1840)	1780 (1260 to 2490)
Haiti	3150 (2680 to 3700)	2680 (2160 to 3260)	2490 (2100 to 2890)	2180 (1880 to 2510)	1900 (1610 to 2220)	1630 (1390 to 1890)	3300 (2860 to 3840)	1340 (1020 to 1750)	1660 (1240 to 2180)	1910 (1360 to 2700)
Jamaica	1860 (1710 to 2010)	1320 (1230 to 1420)	1100 (1020 to 1190)	828 (761 to 907)	734 (677 to 797)	774 (712 to 839)	715 (660 to 778)	712 (541 to 913)	703 (515 to 928)	889 (688 to 1130)
Puerto Rico	1350 (1250 to 1450)	1220 (1140 to 1310)	1220 (1130 to 1310)	1080 (1000 to 1160)	968 (899 to 1050)	790 (740 to 849)	638 (593 to 686)	573 (529 to 621)	607 (563 to 656)	609 (504 to 718)
Saint Kitts and Nevis	1550 (1280 to 1870)	1400 (1220 to 1620)	1330 (1230 to 1460)	1350 (1230 to 1470)	1470 (1350 to 1610)	1200 (1090 to 1330)	1080 (944 to 1220)	1070 (866 to 1320)	1070 (852 to 1350)	1240 (986 to 1530)
Saint Lucia	1750 (1590 to 1950)	1430 (1300 to 1580)	1300 (1170 to 1430)	1180 (1090 to 1280)	1330 (1220 to 1440)	1050 (966 to 1140)	862 (797 to 936)	876 (727 to 1040)	906 (742 to 1090)	1120 (852 to 1480)
Saint Vincent and the Grenadines	2010 (1810 to 2240)	1430 (1300 to 1590)	1300 (1170 to 1440)	1400 (1290 to 1530)	1140 (1040 to 1250)	1080 (990 to 1170)	967 (883 to 1050)	988 (866 to 1130)	986 (847 to 1160)	1100 (928 to 1300)
Suriname	1720 (1560 to 1880)	1390 (1240 to 1560)	1230 (1100 to 1360)	1310 (1150 to 1510)	1060 (929 to 1210)	1040 (958 to 1130)	930 (849 to 1020)	942 (821 to 1080)	942 (796 to 1100)	1130 (844 to 1600)
Trinidad and Tobago	1900 (1770 to 2050)	1550 (1440 to 1670)	1600 (1500 to 1730)	1480 (1380 to 1590)	1230 (1140 to 1340)	1090 (1010 to 1170)	894 (822 to 970)	866 (737 to 1020)	937 (756 to 1150)	1180 (866 to 1570)
Virgin Islands	1870 (1610 to 2140)	1780 (1590 to 1980)	1730 (1530 to 1940)	1150 (1020 to 1310)	1020 (900 to 1160)	953 (840 to 1070)	838 (730 to 959)	868 (704 to 1040)	879 (720 to 1050)	900 (704 to 1160)
Central Latin America	2050 (1940 to 2170)	1660 (1590 to 1740)	1440 (1380 to 1500)	1180 (1120 to 1240)	975 (933 to 1020)	845 (807 to 881)	762 (728 to 794)	739 (696 to 782)	1010 (964 to 1070)	1070 (992 to 1150)
Colombia	1710 (1570 to 1870)	1580 (1440 to 1750)	1120 (1030 to 1220)	1020 (857 to 1210)	940 (860 to 1030)	826 (747 to 916)	649 (589 to 716)	554 (513 to 599)	722 (666 to 784)	820 (698 to 950)
Costa Rica	1620 (1500 to 1750)	1150 (1050 to 1270)	1120 (1030 to 1230)	808 (734 to 898)	690 (622 to 763)	669 (624 to 720)	603 (557 to 648)	588 (524 to 667)	627 (553 to 716)	716 (631 to 814)
El Salvador	2220 (2000 to 2470)	1640 (1500 to 1790)	1590 (1470 to 1720)	1430 (1250 to 1640)	1030 (950 to 1110)	942 (838 to 1060)	820 (748 to 898)	839 (735 to 972)	959 (795 to 1150)	1060 (851 to 1300)
Guatemala	2440 (2230 to 2680)	2310 (2100 to 2560)	2230 (2050 to 2450)	1730 (1600 to 1870)	1560 (1420 to 1730)	1310 (1210 to 1420)	1080 (964 to 1210)	953 (881 to 1030)	1070 (981 to 1160)	1270 (1100 to 1450)
Honduras	2570 (2250 to 2920)	2130 (1920 to 2380)	1710 (1510 to 1930)	981 (853 to 1140)	1050 (905 to 1210)	973 (767 to 1210)	964 (771 to 1200)	1020 (855 to 1210)	1290 (1100 to 1510)	1450 (1240 to 1710)
Mexico	2350 (2150 to 2560)	1790 (1680 to 1940)	1630 (1520 to 1760)	1240 (1160 to 1330)	986 (918 to 1060)	817 (762 to 878)	783 (733 to 837)	774 (718 to 835)	1130 (1060 to 1210)	1130 (1050 to 1220)

Aguascalientes	2020 (1720 to 2370)	1630 (1360 to 1940)	1550 (1280 to 1830)	1190 (1080 to 1330)	990 (892 to 1100)	821 (741 to 909)	712 (643 to 789)	719 (641 to 806)	1010 (917 to 1120)	1050 (944 to 1170)
Baja California	2350 (1960 to 2750)	1870 (1550 to 2200)	1760 (1480 to 2120)	1320 (1200 to 1450)	1250 (1130 to 1370)	1110 (1000 to 1220)	936 (849 to 1030)	889 (796 to 989)	1330 (1200 to 1460)	1250 (1120 to 1390)
Baja California Sur	2330 (1940 to 2740)	1850 (1560 to 2160)	1700 (1440 to 1990)	1280 (1150 to 1420)	1030 (923 to 1140)	850 (769 to 942)	747 (674 to 827)	769 (687 to 864)	1040 (936 to 1150)	1140 (1020 to 1280)
Campeche	2080 (1700 to 2500)	1560 (1350 to 1850)	1410 (1220 to 1640)	1020 (918 to 1130)	888 (802 to 978)	704 (634 to 777)	695 (632 to 770)	738 (660 to 826)	1030 (927 to 1130)	1030 (924 to 1160)
Chiapas	2270 (1840 to 2740)	1750 (1500 to 2070)	1590 (1370 to 1850)	1210 (1090 to 1340)	928 (840 to 1030)	813 (737 to 898)	746 (675 to 822)	771 (693 to 856)	1110 (1000 to 1230)	1080 (963 to 1200)
Chihuahua	2260 (1900 to 2670)	1820 (1520 to 2160)	1660 (1380 to 1980)	1350 (1230 to 1500)	1280 (1170 to 1420)	1070 (965 to 1180)	1250 (1130 to 1370)	967 (872 to 1090)	1500 (1370 to 1650)	1160 (1050 to 1300)
Coahuila	2370 (2000 to 2820)	1890 (1590 to 2230)	1740 (1440 to 2080)	1400 (1260 to 1550)	1150 (1040 to 1280)	889 (800 to 986)	812 (731 to 899)	769 (687 to 869)	1170 (1060 to 1290)	1010 (899 to 1130)
Colima	2880 (2440 to 3380)	2220 (1860 to 2620)	1990 (1680 to 2320)	1520 (1360 to 1680)	1010 (917 to 1120)	822 (735 to 908)	779 (703 to 860)	866 (771 to 970)	1160 (1050 to 1290)	1330 (1190 to 1500)
Durango	2340 (1930 to 2790)	1780 (1510 to 2060)	1600 (1300 to 1880)	1150 (1040 to 1270)	922 (829 to 1010)	738 (662 to 815)	958 (870 to 1050)	704 (629 to 788)	957 (866 to 1050)	863 (771 to 960)
Guanajuato	2360 (1990 to 2770)	1860 (1570 to 2200)	1700 (1440 to 2000)	1250 (1120 to 1380)	1080 (981 to 1200)	854 (769 to 942)	733 (664 to 808)	869 (779 to 974)	1210 (1100 to 1340)	1270 (1140 to 1420)
Guerrero	2720 (2330 to 3180)	2230 (1890 to 2600)	2030 (1720 to 2420)	1680 (1500 to 1870)	1220 (1090 to 1380)	945 (842 to 1050)	929 (823 to 1050)	836 (724 to 965)	1050 (927 to 1180)	1060 (923 to 1210)
Hidalgo	2650 (2200 to 3160)	1980 (1700 to 2290)	1820 (1570 to 2100)	1400 (1260 to 1530)	929 (835 to 1020)	693 (627 to 762)	680 (615 to 747)	694 (622 to 766)	906 (822 to 995)	971 (806 to 1150)
Jalisco	2180 (1820 to 2560)	1650 (1400 to 1950)	1500 (1270 to 1800)	1140 (1040 to 1250)	1020 (918 to 1110)	852 (775 to 932)	781 (712 to 856)	815 (735 to 902)	1070 (970 to 1170)	1190 (1070 to 1320)
México	2300 (1920 to 2700)	1840 (1540 to 2200)	1660 (1390 to 1970)	1470 (1340 to 1610)	1120 (1020 to 1230)	859 (780 to 943)	758 (691 to 835)	755 (683 to 836)	1310 (1200 to 1430)	1050 (949 to 1170)
Mexico City	1910 (1570 to 2240)	1530 (1240 to 1860)	1400 (1140 to 1730)	1080 (978 to 1200)	938 (832 to 1060)	809 (736 to 898)	766 (698 to 845)	742 (670 to 832)	1320 (1200 to 1470)	1480 (1330 to 1660)
Michoacán de Ocampo	2640 (2190 to 3170)	1930 (1670 to 2290)	1720 (1470 to 2040)	1330 (1200 to 1480)	921 (832 to 1020)	782 (709 to 869)	743 (675 to 818)	764 (688 to 851)	966 (878 to 1060)	1140 (1030 to 1280)
Morelos	2580 (2210 to 3000)	1910 (1570 to 2270)	1700 (1430 to 2020)	1310 (1180 to 1460)	977 (877 to 1080)	766 (688 to 850)	725 (654 to 802)	846 (756 to 948)	1180 (1070 to 1310)	1410 (1250 to 1570)
Nayarit	2300 (1970 to 2680)	1760 (1470 to 2120)	1530 (1290 to 1840)	1120 (1010 to 1250)	891 (802 to 987)	712 (644 to 793)	784 (709 to 867)	665 (595 to 739)	857 (771 to 947)	949 (847 to 1060)
Nuevo León	2030 (1680 to 2400)	1500 (1260 to 1810)	1390 (1160 to 1680)	1060 (950 to 1170)	927 (836 to 1030)	807 (727 to 895)	769 (696 to 848)	725 (653 to 808)	1010 (919 to 1120)	1060 (945 to 1180)
Oaxaca	2780 (2390 to 3230)	2200 (1870 to 2570)	2020 (1710 to 2380)	1620 (1470 to 1790)	1060 (966 to 1180)	818 (740 to 901)	758 (687 to 838)	721 (648 to 806)	949 (862 to 1050)	1080 (964 to 1200)
Puebla	2890 (2410 to 3390)	2070 (1740 to 2440)	1840 (1550 to 2180)	1450 (1310 to 1600)	1070 (968 to 1180)	887 (804 to 976)	764 (695 to 839)	798 (715 to 886)	1200 (1090 to 1320)	1350 (1210 to 1510)
Querétaro	2470 (2060 to 2920)	1900 (1610 to 2230)	1700 (1400 to 2040)	1340 (1150 to 1550)	1110 (1010 to 1230)	909 (823 to 1000)	762 (690 to 844)	757 (683 to 844)	1060 (967 to 1170)	1200 (1070 to 1330)
Quintana Roo	2310 (1960 to 2690)	1960 (1650 to 2280)	1890 (1590 to 2240)	1640 (1430 to 1850)	1270 (1130 to 1460)	958 (849 to 1080)	939 (828 to 1050)	927 (792 to 1080)	1180 (1040 to 1340)	1160 (995 to 1340)
San Luis Potosí	2460 (2040 to 2910)	1780 (1520 to 2110)	1610 (1370 to 1920)	1000 (903 to 1110)	861 (785 to 946)	722 (655 to 796)	693 (631 to 761)	700 (628 to 778)	1020 (923 to 1110)	1030 (923 to 1150)
Sinaloa	2220 (1820 to 2720)	1700 (1450 to 2010)	1530 (1300 to 1810)	1130 (1020 to 1250)	871 (783 to 961)	754 (680 to 830)	853 (774 to 940)	693 (622 to 768)	965 (873 to 1070)	896 (804 to 1000)
Sonora	2030 (1710 to 2440)	1680 (1390 to 2010)	1580 (1300 to 1910)	1280 (1080 to 1530)	1120 (1010 to 1240)	934 (844 to 1030)	890 (803 to 977)	890 (796 to 994)	1280 (1160 to 1410)	1170 (1050 to 1300)
Tabasco	2340 (1980 to 2730)	1790 (1490 to 2120)	1660 (1380 to 1990)	1380 (1250 to 1520)	928 (839 to 1030)	778 (700 to 857)	804 (731 to 884)	850 (766 to 945)	1250 (1130 to 1370)	1090 (900 to 1320)
Tamaulipas	2150 (1720 to 2630)	1620 (1380 to 1920)	1480 (1250 to 1760)	1210 (1100 to 1330)	933 (843 to 1030)	753 (678 to 830)	812 (738 to 890)	779 (699 to 864)	994 (905 to 1090)	930 (834 to 1030)
Tlaxcala	2420 (2040 to 2840)	1770 (1490 to 2130)	1540 (1280 to 1840)	1170 (995 to 1360)	916 (826 to 1020)	775 (695 to 857)	683 (617 to 756)	709 (630 to 794)	1160 (1050 to 1290)	1220 (1100 to 1370)
Veracruz de Ignacio de la Llave	2380 (1990 to 2810)	1840 (1600 to 2140)	1700 (1410 to 2010)	1160 (1060 to 1290)	915 (831 to 1010)	768 (699 to 847)	730 (664 to 804)	784 (707 to 871)	1040 (951 to 1150)	1050 (877 to 1270)
Yucatán	1950 (1610 to 2310)	1640 (1390 to 1950)	1520 (1290 to 1810)	1300 (1180 to 1440)	1010 (908 to 1120)	794 (718 to 884)	761 (687 to 845)	756 (675 to 848)	1030 (934 to 1140)	1030 (922 to 1160)
Zacatecas	2010 (1680 to 2410)	1580 (1340 to 1850)	1440 (1190 to 1680)	966 (872 to 1070)	795 (714 to 884)	720 (650 to 800)	709 (639 to 785)	740 (660 to 827)	1080 (974 to 1190)	1090 (977 to 1220)
Nicaragua	2040 (1830 to 2290)	1690 (1530 to 1870)	1600 (1430 to 1790)	1140 (949 to 1340)	817 (739 to 910)	734 (668 to 808)	698 (629 to 774)	619 (530 to 726)	919 (788 to 1090)	1040 (858 to 1280)
Panama	1380 (1260 to 1510)	1090 (1010 to 1180)	1030 (956 to 1120)	777 (721 to 845)	691 (642 to 754)	638 (594 to 689)	632 (589 to 681)	569 (502 to 646)	718 (629 to 823)	658 (513 to 821)
Venezuela	1600 (1480 to 1710)	1270 (1180 to 1380)	1290 (1200 to 1390)	1100 (1020 to 1190)	916 (859 to 983)	934 (866 to 1000)	808 (750 to 872)	895 (717 to 1120)	1190 (954 to 1470)	1300 (1020 to 1630)
Tropical Latin America	2160 (1930 to 2400)	1810 (1650 to 1970)	1570 (1440 to 1710)	1290 (1210 to 1380)	1120 (1050 to 1200)	953 (889 to 1020)	854 (798 to 921)	760 (674 to 864)	862 (762 to 989)	944 (826 to 1100)
Brazil	2160 (1930 to 2420)	1830 (1670 to 1990)	1590 (1450 to 1730)	1310 (1220 to 1400)	1130 (1050 to 1210)	959 (893 to 1030)	856 (798 to 925)	759 (672 to 866)	862 (761 to 996)	940 (821 to 1090)
Acre	2270 (1850 to 2730)	1840 (1520 to 2190)	1550 (1270 to 1850)	1250 (1110 to 1390)	1210 (1080 to 1360)	922 (831 to 1030)	836 (753 to 922)	886 (733 to 1050)	1050 (866 to 1270)	1040 (836 to 1260)
Alagoas	2550 (2050 to 3070)	2130 (1770 to 2560)	1790 (1500 to 2200)	1430 (1290 to 1600)	1150 (1030 to 1280)	900 (813 to 1000)	926 (816 to 1040)	811 (670 to 979)	914 (763 to 1120)	908 (747 to 1110)
Amapá	2310 (1870 to 2790)	1770 (1460 to 2110)	1320 (1090 to 1580)	956 (828 to 1100)	1160 (991 to 1340)	1000 (867 to 1160)	864 (739 to 1000)	942 (741 to 1190)	1180 (934 to 1470)	1030 (812 to 1340)
Amazonas	1810 (1440 to 2250)	1470 (1160 to 1810)	1300 (1040 to 1600)	1020 (891 to 1150)	902 (790 to 1020)	770 (685 to 866)	725 (634 to 818)	758 (631 to 918)	1040 (856 to 1270)	1080 (878 to 1340)
Bahia	2060 (1670 to 2470)	1760 (1450 to 2100)	1510 (1260 to 1810)	1060 (962 to 1190)	1040 (929 to 1160)	928 (838 to 1030)	907 (813 to 1020)	760 (627 to 926)	843 (701 to 1030)	851 (690 to 1050)
Ceará	2730 (2220 to 3350)	2250 (1890 to 2680)	1790 (1480 to 2150)	1230 (1010 to 1500)	805 (706 to 914)	776 (675 to 884)	756 (653 to 862)	761 (616 to 940)	857 (685 to 1060)	851 (668 to 1090)
Distrito Federal	2240 (1870 to 2670)	2000 (1640 to 2380)	1900 (1580 to 2260)	1730 (1550 to 1900)	1660 (1490 to 1850)	1070 (963 to 1180)	921 (830 to 1020)	758 (628 to 904)	959 (797 to 1160)	985 (813 to 1190)
Espírito Santo	1970 (1610 to 2360)	1720 (1390 to 2080)	1540 (1250 to 1890)	1320 (1190 to 1460)	1090 (970 to 1210)	963 (869 to 1070)	828 (747 to 921)	724 (602 to 870)	862 (716 to 1040)	915 (750 to 1120)
Goiás	2920 (2410 to 3500)	2440 (2030 to 2910)	1890 (1550 to 2280)	1380 (1190 to 1630)	1090 (971 to 1220)	864 (781 to 964)	806 (727 to 900)	760 (637 to 930)	906 (757 to 1110)	1040 (854 to 1290)
Maranhão	2990 (2510 to 3520)	2600 (2180 to 3040)	2290 (1880 to 2750)	1740 (1460 to 2070)	1320 (1170 to 1490)	797 (706 to 900)	1140 (1000 to 1300)	854 (688 to 1060)	1020 (828 to 1260)	976 (770 to 1230)
Mato Grosso	1620 (1310 to 1960)	1420 (1140 to 1730)	1200 (966 to 1460)	910 (804 to 1030)	995 (873 to 1140)	906 (801 to 1020)	801 (722 to 886)	710 (594 to 845)	889 (745 to 1070)	988 (810 to 1200)
Mato Grosso do Sul	1850 (1500 to 2240)	1610 (1290 to 1980)	1440 (1160 to 1770)	1170 (1060 to 1300)	1030 (920 to 1150)	955 (858 to 1060)	855 (774 to 950)	727 (607 to 874)	851 (703 to 1040)	992 (801 to 1220)
Minas Gerais	2150 (1770 to 2580)	1840 (1510 to 2240)	1620 (1320 to 1970)	1340 (1220 to 1470)	1100 (992 to 1210)	851 (774 to 937)	772 (700 to 851)	690 (583 to 818)	753 (628 to 900)	875 (721 to 1050)
Pará	2130 (1730 to 2570)	1710 (1400 to 2100)	1470 (1180 to 1810)	1170 (1010 to 1350)	970 (842 to 1100)	812 (709 to 931)	820 (714 to 936)	799 (647 to 1000)	968 (780 to 1200)	929 (734 to 1170)
Paraíba	2650 (2140 to 3210)	2040 (1680 to 2430)	1590 (1310 to 1900)	1170 (1050 to 1310)	838 (754 to 935)	822 (740 to 903)	942 (852 to 1040)	1040 (869 to 1240)	827 (683 to 1010)	878 (718 to 1080)
Paraná	1930 (1570 to 2340)	1700 (1390 to 2070)	1570 (1270 to 1910)	1390 (1250 to 1540)	1150 (1030 to 1270)	976 (883 to 1080)	877 (788 to 968)	742 (618 to 887)	835 (693 to 1010)	1040 (845 to 1280)
Pernambuco	2810 (2310 to 3380)	2290 (1910 to 2720)	1840 (1530 to 2200)	1380 (1230 to 1530)	1240 (1120 to 1370)	1070 (963 to 1180)	862 (778 to 949)	820 (679 to 974)	961 (796 to 1160)	933 (763 to 1120)
Piauí	2020 (1630 to 2450)	1700 (1410 to 2030)	1520 (1230 to 1820)	1150 (969 to 1350)	893 (774 to 1030)	737 (637 to 842)	817 (696 to 954)	720 (571 to 896)	816 (651 to 1010)	811 (629 to 1030)
Rio de Janeiro	2020 (1630 to 2500)	1730 (1400 to 2120)	1610 (1300 to 1940)	1530 (1400 to 1690)	1460 (1320 to 1620)	1150 (1050 to 1260)	942 (858 to 1040)	838 (705 to 996)	994 (835 to 1200)	1040 (865 to 1260)
Rio Grande do Norte	2730 (2210 to 3370)	2040 (1700 to 2420)	1490 (1240 to 1810)	1030 (905 to 1170)	809 (716 to 911)	735 (652 to 823)	771 (684 to 866)	732 (614 to 869)	824 (690 to 989)	837 (682 to 1020)
Rio Grande do Sul	1850 (1510 to 2240)	1620 (1320 to 1960)	1430 (1150 to 1740)	1280 (1160 to 1410)	1140 (1030 to 1260)	1010 (922 to 1110)	854 (779 to 941)	768 (644 to 930)	812 (678 to 969)	964 (799 to 1160)
Rondônia	2820 (2300 to 3460)	2430 (2030 to 2860)	2180 (1840 to 2590)	1850 (1620 to 2090)	1450 (1260 to 1630)	1090 (976 to 1210)	788 (687 to 897)	715 (579 to 880)	830 (679 to 1030)	885 (702 to 1100)
Roraima	3030 (2550 to 3590)	2580 (2180 to 3000)	2280 (1900 to 2740)	1710 (1410 to 2040)	1960 (1670 to 2280)	1420 (1200 to 1680)	1240 (1060 to 1450)	1080 (830 to 1390)	1200 (949 to 1540)	1130 (858 to 1450)
Santa Catarina	1700 (1360 to 2070)	1490 (1200 to 1840)	1340 (1080 to 1650)	1170 (1060 to 1290)	1080 (961 to 1190)	996 (898 to 1100)	797 (719 to 874)	695 (579 to 821)	781 (646 to 939)	906 (744 to 1090)
São Paulo	1790 (1460 to 2160)	1580 (1280 to 1930)	1430 (1180 to 1750)	1360 (1240 to 1490)	1260 (1150 to 1390)	1090 (994 to 1190)	846 (772 to 930)	730 (621 to 862)	847 (719 to 1010)	970 (813 to 1160)
Sergipe	2300 (1860 to 2790)	1910 (1560 to 2320)	1570 (1300 to 1900)	1230 (1100 to 1370)	1220 (1100 to 1370)	932 (844 to 1030)	894 (806 to 997)	797 (668 to 970)	928 (776 to 1130)	934 (767 to 1150)
Tocantins	2380 (1900 to 2960)	2020 (1660 to 2410)	1620 (1340 to 1970)	1140 (920 to 1370)	1000 (868 to 1150)	832 (729 to 944)	911 (791 to 1030)	833 (677 to 1030)	929 (759 to 1150)	942 (748 to 1170)

Paraguay	1950 (1630 to 2330)	1020 (885 to 1180)	955 (823 to 1100)	817 (704 to 952)	718 (612 to 836)	746 (633 to 860)	791 (667 to 948)	846 (671 to 1070)	846 (654 to 1080)	1140 (889 to 1450)
North Africa and Middle East	2630 (2380 to 2920)	2220 (2040 to 2410)	1830 (1700 to 1970)	1510 (1410 to 1600)	1260 (1190 to 1320)	1070 (1010 to 1130)	931 (880 to 986)	852 (796 to 916)	1030 (955 to 1120)	1130 (1040 to 1230)
North Africa and Middle East	2630 (2380 to 2920)	2220 (2040 to 2410)	1830 (1700 to 1970)	1510 (1410 to 1600)	1260 (1190 to 1320)	1070 (1010 to 1130)	931 (880 to 986)	852 (796 to 916)	1030 (955 to 1120)	1130 (1040 to 1230)
Afghanistan	3010 (2300 to 4180)	2530 (2020 to 3220)	2370 (1950 to 2890)	2720 (2150 to 3770)	2020 (1620 to 2440)	2030 (1600 to 2590)	1640 (1210 to 2220)	1530 (1080 to 2200)	2150 (1530 to 3140)	2320 (1670 to 3330)
Algeria	2640 (2360 to 2940)	2510 (2200 to 2860)	1930 (1660 to 2250)	1770 (1580 to 1960)	1060 (936 to 1210)	922 (819 to 1040)	761 (690 to 837)	693 (579 to 828)	914 (770 to 1090)	1010 (827 to 1240)
Bahrain	2440 (2000 to 2920)	2190 (1820 to 2580)	1850 (1530 to 2210)	1720 (1550 to 1920)	1560 (1430 to 1720)	1470 (1340 to 1610)	1230 (1130 to 1340)	870 (780 to 978)	1030 (917 to 1150)	1160 (1020 to 1310)
Egypt	2470 (2220 to 2780)	2070 (1920 to 2240)	1990 (1800 to 2190)	1660 (1510 to 1820)	1470 (1340 to 1610)	1220 (1120 to 1340)	1230 (1120 to 1360)	1070 (986 to 1170)	1290 (1120 to 1480)	1430 (1200 to 1700)
Iran	3070 (2750 to 3420)	2530 (2280 to 2840)	1830 (1660 to 2020)	1290 (1150 to 1420)	1080 (982 to 1190)	834 (757 to 918)	708 (659 to 765)	623 (567 to 680)	691 (630 to 765)	947 (856 to 1050)
Alborz	2630 (2180 to 3100)	2300 (1890 to 2810)	1580 (1260 to 1940)	1040 (811 to 1340)	881 (699 to 1090)	758 (633 to 920)	611 (525 to 714)	594 (480 to 747)	695 (574 to 858)	996 (835 to 1200)
Ardebil	3030 (2560 to 3560)	2620 (2160 to 3140)	1980 (1610 to 2420)	1380 (1120 to 1720)	1060 (864 to 1270)	769 (662 to 891)	735 (661 to 825)	785 (662 to 914)	854 (726 to 997)	1140 (975 to 1340)
Bushehr	2610 (2150 to 3110)	2240 (1830 to 2720)	1710 (1390 to 2090)	1190 (962 to 1480)	969 (782 to 1170)	821 (706 to 945)	746 (665 to 836)	651 (546 to 766)	713 (594 to 844)	955 (812 to 1130)
Chahar Mahaal and Bakhtiari	2410 (1980 to 2870)	2100 (1720 to 2550)	1640 (1340 to 2000)	1250 (999 to 1540)	1020 (818 to 1240)	817 (685 to 971)	718 (627 to 815)	629 (524 to 744)	667 (553 to 803)	858 (724 to 1030)
East Azarbayejan	3280 (2780 to 3860)	2750 (2270 to 3320)	2040 (1680 to 2440)	1410 (1140 to 1720)	1160 (938 to 1400)	951 (809 to 1120)	860 (761 to 969)	744 (622 to 875)	796 (655 to 961)	1030 (868 to 1230)
Fars	2460 (2040 to 2950)	2130 (1740 to 2560)	1660 (1340 to 2030)	1340 (1070 to 1650)	1110 (896 to 1390)	933 (776 to 1110)	818 (713 to 945)	706 (572 to 852)	768 (631 to 930)	1020 (858 to 1240)
Gilan	2630 (2200 to 3110)	2190 (1750 to 2700)	1610 (1280 to 2000)	1190 (954 to 1480)	2000 (1710 to 2330)	878 (739 to 1040)	741 (654 to 842)	642 (527 to 760)	717 (588 to 854)	1000 (846 to 1180)
Golestan	2870 (2400 to 3400)	2510 (2030 to 3000)	1940 (1560 to 2360)	1370 (1080 to 1700)	1150 (930 to 1410)	970 (838 to 1110)	811 (723 to 908)	838 (708 to 986)	915 (775 to 1080)	1220 (1030 to 1450)
Hamadan	3870 (3290 to 4510)	3190 (2650 to 3810)	2330 (1950 to 2800)	1560 (1260 to 1920)	1080 (884 to 1330)	786 (676 to 918)	755 (668 to 859)	621 (515 to 752)	665 (544 to 798)	860 (715 to 1020)
Hormozgan	3000 (2510 to 3590)	2570 (2120 to 3110)	1900 (1550 to 2320)	1400 (1120 to 1780)	1210 (981 to 1490)	987 (836 to 1170)	802 (696 to 919)	681 (557 to 823)	749 (615 to 911)	1020 (859 to 1220)
Ilam	2690 (2270 to 3180)	2330 (1880 to 2840)	1820 (1470 to 2230)	1320 (1060 to 1630)	1060 (865 to 1290)	878 (752 to 1030)	788 (697 to 891)	753 (628 to 896)	830 (695 to 990)	1090 (921 to 1290)
Isfahan	2590 (2150 to 3060)	2250 (1830 to 2710)	1590 (1280 to 1980)	1090 (859 to 1390)	849 (673 to 1050)	693 (585 to 825)	590 (522 to 674)	550 (455 to 670)	625 (514 to 748)	887 (745 to 1050)
Kerman	3420 (2870 to 4060)	2790 (2310 to 3310)	2070 (1700 to 2540)	1510 (1220 to 1850)	1230 (982 to 1500)	1060 (900 to 1250)	840 (737 to 960)	724 (604 to 859)	783 (652 to 941)	1020 (852 to 1200)
Kermanshah	3330 (2840 to 3880)	2850 (2360 to 3430)	2180 (1780 to 2650)	1550 (1260 to 1910)	1260 (1030 to 1560)	1030 (872 to 1220)	817 (722 to 930)	674 (546 to 818)	754 (624 to 911)	1030 (862 to 1220)
Khorasan-e-Razavi	3990 (3350 to 4640)	3270 (2730 to 3860)	2340 (1950 to 2800)	1520 (1200 to 1850)	1240 (1010 to 1530)	1020 (863 to 1200)	826 (733 to 935)	697 (577 to 836)	771 (641 to 918)	1030 (870 to 1220)
Khuzestan	2860 (2340 to 3420)	2370 (1930 to 2860)	1780 (1470 to 2170)	1360 (1080 to 1650)	1020 (827 to 1240)	858 (748 to 997)	766 (690 to 856)	675 (570 to 792)	753 (635 to 893)	1060 (897 to 1240)
Kohgiluyeh and Boyer-Ahmad	2620 (2160 to 3110)	2200 (1800 to 2640)	1730 (1400 to 2100)	1360 (1100 to 1680)	925 (741 to 1140)	689 (582 to 813)	582 (508 to 667)	532 (439 to 643)	585 (480 to 713)	792 (665 to 948)
Kurdistan	4000 (3410 to 4640)	3390 (2840 to 4060)	2490 (2070 to 2980)	1690 (1400 to 2040)	1240 (1020 to 1510)	907 (783 to 1060)	784 (694 to 886)	600 (497 to 718)	651 (540 to 778)	878 (742 to 1050)
Lorestan	3150 (2640 to 3770)	2560 (2120 to 3070)	1930 (1550 to 2340)	1440 (1170 to 1790)	1110 (900 to 1350)	900 (770 to 1060)	859 (759 to 975)	773 (660 to 907)	850 (724 to 1010)	1170 (997 to 1380)
Markazi	2990 (2520 to 3530)	2630 (2160 to 3190)	2010 (1640 to 2420)	1430 (1150 to 1790)	1070 (846 to 1310)	851 (714 to 1020)	645 (568 to 734)	532 (438 to 641)	606 (500 to 731)	865 (719 to 1020)
Mazandaran	2250 (1860 to 2710)	1950 (1580 to 2360)	1460 (1180 to 1810)	1100 (881 to 1370)	818 (654 to 1010)	690 (576 to 814)	652 (574 to 738)	562 (461 to 669)	634 (526 to 762)	888 (750 to 1040)
North Khorasan	3520 (2950 to 4190)	3030 (2520 to 3650)	2320 (1920 to 2820)	1530 (1220 to 1870)	1190 (968 to 1430)	923 (785 to 1090)	730 (643 to 828)	639 (524 to 760)	720 (604 to 846)	1010 (853 to 1190)
Qazvin	3440 (2890 to 4080)	2890 (2340 to 3530)	2080 (1630 to 2650)	1390 (1120 to 1720)	1040 (849 to 1270)	881 (745 to 1040)	794 (704 to 891)	716 (605 to 839)	794 (662 to 936)	1070 (911 to 1260)
Qom	2490 (2070 to 2980)	2180 (1770 to 2630)	1660 (1340 to 2030)	1270 (1020 to 1550)	971 (773 to 1200)	828 (704 to 965)	658 (580 to 739)	522 (432 to 620)	612 (510 to 728)	905 (766 to 1060)
Semnan	2970 (2510 to 3540)	2650 (2200 to 3190)	1880 (1540 to 2280)	1340 (1070 to 1660)	1050 (840 to 1270)	925 (781 to 1080)	768 (673 to 873)	669 (559 to 796)	756 (624 to 906)	1070 (900 to 1260)
Sistan and Baluchistan	3790 (3180 to 4500)	3090 (2590 to 3720)	2340 (1930 to 2790)	1620 (1300 to 1980)	1240 (999 to 1540)	904 (754 to 1070)	773 (669 to 892)	691 (568 to 864)	761 (620 to 931)	1070 (897 to 1280)
South Khorasan	3300 (2760 to 3850)	2840 (2360 to 3410)	2110 (1740 to 2520)	1350 (1090 to 1670)	1010 (832 to 1220)	722 (621 to 828)	652 (580 to 736)	546 (462 to 643)	614 (520 to 725)	838 (708 to 985)
Tehran	1850 (1500 to 2240)	1500 (1210 to 1830)	1070 (851 to 1320)	784 (608 to 1000)	707 (562 to 874)	620 (512 to 743)	531 (456 to 614)	493 (402 to 597)	557 (462 to 663)	776 (654 to 915)
West Azarbayejan	3370 (2860 to 3940)	2870 (2390 to 3430)	2140 (1750 to 2590)	1470 (1190 to 1830)	1250 (1020 to 1520)	1020 (886 to 1170)	844 (751 to 948)	765 (641 to 900)	833 (689 to 993)	1110 (934 to 1310)
Yazd	2980 (2480 to 3550)	2280 (1860 to 2790)	1670 (1340 to 2050)	1270 (1020 to 1590)	991 (790 to 1220)	867 (724 to 1030)	826 (722 to 936)	623 (514 to 747)	743 (609 to 894)	1110 (939 to 1310)
Zanjan	3970 (3320 to 4710)	3320 (2730 to 4020)	2350 (1870 to 2980)	1500 (1220 to 1860)	2030 (1740 to 2370)	933 (790 to 1090)	788 (705 to 884)	660 (559 to 774)	729 (616 to 865)	982 (831 to 1150)
Iraq	1840 (1570 to 2120)	1450 (1280 to 1640)	1300 (1110 to 1560)	1280 (1130 to 1470)	1190 (1020 to 1370)	1090 (840 to 1390)	1010 (732 to 1380)	970 (688 to 1350)	1440 (1070 to 1980)	1370 (999 to 1930)
Jordan	2280 (1670 to 3050)	1860 (1450 to 2360)	1530 (1280 to 1790)	1250 (1100 to 1420)	810 (689 to 945)	703 (578 to 849)	570 (491 to 660)	473 (393 to 562)	665 (545 to 791)	782 (643 to 926)
Kuwait	1690 (1300 to 2140)	972 (801 to 1160)	953 (884 to 1030)	892 (827 to 959)	758 (675 to 852)	667 (603 to 742)	526 (486 to 571)	478 (428 to 542)	653 (578 to 742)	580 (471 to 712)
Lebanon	1960 (1560 to 2420)	1390 (1100 to 1740)	1220 (1010 to 1480)	1190 (966 to 1510)	1220 (991 to 1530)	766 (646 to 897)	711 (626 to 799)	615 (511 to 738)	787 (656 to 946)	992 (823 to 1200)
Libya	2740 (2320 to 3180)	2180 (1820 to 2550)	1390 (1210 to 1570)	960 (846 to 1100)	834 (697 to 993)	770 (679 to 874)	831 (737 to 938)	908 (706 to 1130)	1060 (840 to 1320)	1120 (884 to 1420)
Morocco	2820 (2380 to 3340)	2430 (2040 to 2850)	1900 (1630 to 2240)	1500 (1320 to 1710)	1110 (981 to 1260)	958 (837 to 1110)	890 (727 to 1080)	877 (683 to 1020)	1100 (899 to 1270)	1060 (866 to 1230)
Oman	3220 (2220 to 4940)	2670 (1940 to 3710)	1890 (1440 to 2500)	1510 (1180 to 1940)	1250 (1000 to 1530)	1090 (883 to 1310)	1060 (937 to 1200)	939 (800 to 1090)	1230 (1090 to 1380)	1220 (1070 to 1390)
Palestine	2720 (2110 to 3480)	2010 (1660 to 2440)	1830 (1560 to 2150)	1350 (1080 to 1720)	1360 (1150 to 1650)	1370 (1220 to 1540)	1250 (1140 to 1380)	890 (799 to 994)	955 (840 to 1090)	1120 (984 to 1280)
Qatar	1920 (1350 to 2800)	1650 (1230 to 2220)	1440 (1120 to 1830)	1260 (1030 to 1530)	1420 (1200 to 1670)	1420 (1210 to 1650)	1090 (928 to 1280)	566 (459 to 693)	776 (643 to 938)	766 (629 to 932)
Saudi Arabia	2130 (1740 to 2560)	2130 (1710 to 2590)	1650 (1350 to 2010)	1200 (967 to 1500)	1170 (941 to 1400)	1090 (971 to 1230)	1000 (912 to 1090)	859 (732 to 1010)	962 (818 to 1140)	928 (782 to 1120)
Sudan	2310 (1910 to 2820)	2100 (1770 to 2530)	1990 (1710 to 2290)	1780 (1550 to 2030)	1560 (1350 to 1810)	1320 (1090 to 1620)	1080 (894 to 1320)	955 (758 to 1240)	1230 (991 to 1550)	1300 (993 to 1670)
Syria	1750 (1440 to 2110)	1290 (1060 to 1540)	1040 (906 to 1200)	931 (782 to 1100)	1080 (881 to 1290)	1020 (888 to 1190)	918 (769 to 1090)	1140 (904 to 1430)	1070 (845 to 1390)	1120 (882 to 1440)
Tunisia	2980 (2530 to 3490)	2490 (2200 to 2850)	2070 (1860 to 2300)	1610 (1440 to 1810)	946 (837 to 1070)	922 (775 to 1090)	830 (640 to 1040)	741 (565 to 961)	825 (620 to 1060)	1110 (893 to 1360)
Türkiye	2690 (2260 to 3150)	2230 (1890 to 2590)	1750 (1490 to 2040)	1410 (1230 to 1600)	1280 (1160 to 1420)	1030 (873 to 1220)	833 (736 to 940)	752 (664 to 853)	926 (797 to 1060)	954 (806 to 1120)
United Arab Emirates	1800 (1350 to 2360)	1540 (1230 to 1880)	1180 (940 to 1480)	1080 (863 to 1330)	1070 (867 to 1290)	1100 (921 to 1290)	990 (819 to 1180)	738 (577 to 930)	561 (433 to 705)	622 (482 to 774)
Yemen	3960 (2800 to 6040)	3450 (2580 to 4880)	3020 (2330 to 4020)	2090 (1690 to 2580)	1650 (1370 to 1990)	1380 (1120 to 1680)	1140 (927 to 1420)	1240 (947 to 1620)	1420 (1100 to 1850)	1600 (1210 to 2120)
South Asia	2970 (2580 to 3390)	2490 (2200 to 2810)	2030 (1860 to 2210)	1660 (1540 to 1770)	1450 (1360 to 1560)	1280 (1200 to 1370)	1150 (1070 to 1240)	1010 (902 to 1130)	1170 (1040 to 1320)	1270 (1130 to 1440)
South Asia	2970 (2580 to 3390)	2490 (2200 to 2810)	2030 (1860 to 2210)	1660 (1540 to 1770)	1450 (1360 to 1560)	1280 (1200 to 1370)	1150 (1070 to 1240)	1010 (902 to 1130)	1170 (1040 to 1320)	1270 (1130 to 1440)
Bangladesh	2660 (2260 to 3120)	2280 (1950 to 2670)	2340 (2020 to 2720)	1620 (1420 to 1840)	1520 (1370 to 1690)	1310 (1180 to 1460)	1090 (985 to 1210)	821 (658 to 999)	974 (789 to 1180)	988 (801 to 1210)
Bhutan	3140 (2580 to 3760)	2620 (2120 to 3180)	2130 (1610 to 2760)	1870 (1410 to 2500)	1390 (1110 to 1720)	1100 (936 to 1270)	879 (763 to 1030)	810 (668 to 949)	804 (665 to 950)	828 (684 to 988)
India	3130 (2680 to 3640)	2640 (2260 to 3030)	2090 (1870 to 2320)	1710 (1570 to 1860)	1470 (1350 to 1600)	1260 (1160 to 1380)	1150 (1050 to 1260)	1020 (888 to 1170)	1160 (1010 to 1350)	1280 (1110 to 1490)
Nepal	3090 (2640 to 3540)	2650 (2270 to 3060)	2250 (1910 to 2640)	2020 (1720 to 2360)	1590 (1370 to 1830)	1200 (1070 to 1360)	1040 (907 to 1200)	1020 (835 to 1260)	1210 (1010 to 1480)	1410 (1180 to 1700)
Pakistan	2050 (1730 to 2410)	1650 (1390 to 1940)	1320 (1160 to 1510)	1290 (1160 to 1450)	1270 (1130 to 1450)	1340 (1190 to 1500)	1210 (991 to 1450)	1080 (857 to 1340)	1380 (1160 to 1670)	1430 (1190 to 1720)
Azad Jammu & Kashmir	2230 (1700 to 2840)	1750 (1350 to 2250)	1390 (1100 to 1710)	1320 (1030 to 1660)	1250 (936 to 1630)	1270 (955 to 1660)	1100 (753 to 1530)	994 (669 to 1470)	1250 (892 to 1740)	1440 (1020 to 2010)
Balochistan	1740 (1290 to 2310)	1460 (1100 to 1880)	1170 (890 to 1490)	1200 (922 to 1530)	1280 (966 to 1710)	1400 (1050 to 1800)	1320 (924 to 1780)	1190 (855 to 1660)	1600 (1230 to 2060)	1510 (1130 to 1990)

Gilgit-Baltistan	2060 (1480 to 2900)	1970 (1460 to 2570)	1600 (1240 to 2020)	1540 (1210 to 1930)	1460 (1100 to 1930)	1510 (1080 to 2040)	1300 (824 to 2020)	1170 (705 to 1820)	1730 (1170 to 2530)	1610 (1040 to 2430)
Islamabad Capital Territory	1980 (1470 to 2550)	1660 (1260 to 2150)	1170 (894 to 1520)	980 (754 to 1260)	885 (661 to 1180)	955 (703 to 1280)	909 (634 to 1240)	873 (627 to 1170)	1160 (882 to 1490)	1220 (903 to 1600)
Khyber Pakhtunkhwa	2070 (1590 to 2620)	1660 (1290 to 2080)	1330 (1030 to 1690)	1260 (991 to 1560)	1210 (906 to 1590)	1340 (1030 to 1690)	1250 (916 to 1710)	1080 (771 to 1460)	1460 (1120 to 1890)	1450 (1030 to 1990)
Punjab	2080 (1710 to 2460)	1670 (1380 to 1980)	1350 (1160 to 1570)	1340 (1170 to 1540)	1310 (1110 to 1540)	1360 (1150 to 1580)	1220 (950 to 1500)	1090 (841 to 1390)	1340 (1080 to 1640)	1440 (1180 to 1770)
Sindh	2020 (1560 to 2570)	1580 (1240 to 2000)	1250 (970 to 1580)	1200 (950 to 1480)	1220 (939 to 1580)	1290 (1000 to 1640)	1190 (864 to 1590)	1060 (773 to 1420)	1440 (1120 to 1810)	1410 (1070 to 1820)
Southeast Asia, east Asia, and Oceania	2450 (2180 to 2770)	3450 (2700 to 4730)	1660 (1430 to 1930)	1580 (1390 to 1790)	1360 (1230 to 1510)	1240 (1150 to 1330)	1080 (997 to 1180)	905 (778 to 1040)	907 (781 to 1050)	937 (810 to 1080)
East Asia	2400 (2040 to 2800)	4010 (2990 to 5820)	1570 (1270 to 1920)	1620 (1360 to 1890)	1410 (1230 to 1600)	1280 (1150 to 1410)	1080 (969 to 1190)	870 (710 to 1040)	866 (712 to 1040)	856 (702 to 1030)
China	2350 (1980 to 2750)	4110 (3060 to 6010)	1580 (1270 to 1930)	1640 (1380 to 1920)	1430 (1240 to 1630)	1290 (1160 to 1420)	1100 (983 to 1220)	879 (712 to 1050)	876 (717 to 1060)	864 (706 to 1050)
North Korea	6070 (4300 to 8860)	1510 (1110 to 2090)	1340 (1010 to 1720)	1270 (970 to 1610)	1260 (1010 to 1490)	1750 (1310 to 2690)	1230 (1070 to 1400)	1060 (878 to 1290)	1060 (861 to 1290)	1040 (833 to 1270)
Taiwan (province of China)	1870 (1590 to 2200)	1490 (1470 to 1510)	1210 (1200 to 1220)	1010 (1000 to 1020)	861 (851 to 871)	742 (733 to 751)	616 (608 to 623)	580 (537 to 633)	555 (511 to 608)	572 (522 to 630)
Oceania	2470 (2090 to 2940)	2110 (1840 to 2410)	1840 (1610 to 2070)	1650 (1440 to 1880)	1540 (1310 to 1820)	1410 (1230 to 1590)	1310 (1160 to 1460)	1250 (1060 to 1470)	1270 (1080 to 1490)	1570 (1220 to 2030)
American Samoa	1480 (1260 to 1750)	1470 (1330 to 1620)	1450 (1310 to 1590)	1270 (1160 to 1380)	1220 (1120 to 1320)	1160 (1060 to 1250)	1110 (1010 to 1210)	1070 (937 to 1220)	1130 (1010 to 1300)	1060 (925 to 1230)
Cook Islands	2670 (2380 to 2970)	1740 (1560 to 1920)	1430 (1250 to 1610)	1430 (1300 to 1570)	1270 (1150 to 1400)	1090 (975 to 1200)	910 (813 to 1010)	865 (749 to 984)	858 (738 to 996)	850 (720 to 997)
Fiji	1780 (1600 to 1960)	1540 (1380 to 1710)	1540 (1370 to 1730)	1540 (1380 to 1720)	1630 (1390 to 1900)	1820 (1660 to 2010)	1500 (1350 to 1670)	1490 (1220 to 1790)	1510 (1240 to 1830)	1850 (1420 to 2330)
Guam	1090 (971 to 1230)	1340 (1240 to 1460)	1460 (1350 to 1600)	1190 (1090 to 1290)	841 (776 to 916)	640 (590 to 696)	637 (585 to 691)	680 (618 to 759)	685 (624 to 760)	732 (640 to 838)
Kiribati	2810 (2420 to 3200)	2460 (2070 to 2840)	2210 (1840 to 2550)	1950 (1630 to 2270)	1980 (1740 to 2220)	1950 (1730 to 2190)	1810 (1560 to 2090)	1700 (1430 to 2040)	1690 (1420 to 2020)	1670 (1390 to 2010)
Marshall Islands	2690 (1930 to 3870)	2240 (1720 to 2950)	1940 (1560 to 2460)	1740 (1490 to 2040)	1860 (1670 to 2060)	1690 (1500 to 1910)	1640 (1410 to 1900)	1520 (1240 to 1890)	1510 (1220 to 1870)	1500 (1200 to 1870)
Federated States of Micronesia	2940 (2510 to 3390)	2400 (2010 to 2890)	2080 (1630 to 2640)	1820 (1490 to 2230)	1700 (1440 to 2020)	1600 (1390 to 1860)	1490 (1280 to 1770)	1430 (1170 to 1780)	1430 (1150 to 1800)	1420 (1140 to 1810)
Nauru	1800 (1490 to 2160)	1670 (1420 to 1980)	1690 (1440 to 1990)	1810 (1460 to 2250)	1980 (1670 to 2330)	2290 (1990 to 2650)	2230 (1910 to 2590)	1940 (1560 to 2390)	1920 (1540 to 2370)	1910 (1520 to 2360)
Niue	2110 (1800 to 2440)	1610 (1320 to 1920)	1550 (1310 to 1790)	1390 (1170 to 1630)	1340 (1160 to 1540)	1360 (1180 to 1530)	1270 (1110 to 1450)	1230 (1040 to 1460)	1320 (1130 to 1550)	1320 (1120 to 1540)
Northern Mariana Islands	1500 (1230 to 1840)	1390 (1160 to 1680)	1230 (1070 to 1400)	1110 (957 to 1320)	1020 (887 to 1220)	952 (833 to 1070)	974 (843 to 1100)	960 (814 to 1110)	919 (749 to 1120)	1120 (915 to 1290)
Palau	2590 (2140 to 3100)	2110 (1720 to 2510)	1790 (1460 to 2110)	1640 (1350 to 1910)	1430 (1190 to 1710)	1250 (1040 to 1470)	1210 (1030 to 1400)	1090 (894 to 1300)	1090 (876 to 1310)	1090 (879 to 1320)
Papua New Guinea	2650 (2120 to 3360)	2280 (1890 to 2730)	1910 (1580 to 2250)	1680 (1380 to 2020)	1550 (1220 to 1950)	1370 (1140 to 1620)	1310 (1120 to 1500)	1240 (1010 to 1520)	1280 (1020 to 1570)	1670 (1210 to 2270)
Samoa	1650 (1420 to 1890)	1020 (915 to 1140)	1360 (1240 to 1500)	1410 (1270 to 1580)	1320 (1160 to 1480)	1190 (1060 to 1340)	1080 (909 to 1250)	1090 (956 to 1260)	1060 (924 to 1230)	1050 (914 to 1230)
Solomon Islands	2730 (2340 to 3130)	2400 (2010 to 2760)	2150 (1810 to 2480)	1900 (1610 to 2250)	1820 (1530 to 2170)	1660 (1400 to 2000)	1580 (1320 to 1930)	1490 (1210 to 1890)	1490 (1210 to 1920)	1480 (1200 to 1890)
Tokelau	1890 (1560 to 2250)	1620 (1300 to 1940)	1440 (1180 to 1700)	1410 (1200 to 1630)	1240 (1060 to 1430)	1140 (998 to 1310)	1020 (883 to 1200)	935 (788 to 1110)	1070 (926 to 1230)	1060 (914 to 1240)
Tonga	1750 (1480 to 2020)	1510 (1340 to 1690)	1360 (1180 to 1550)	1290 (1130 to 1450)	1110 (958 to 1280)	1210 (1050 to 1370)	1080 (932 to 1240)	1010 (846 to 1200)	1000 (833 to 1190)	1000 (825 to 1200)
Tuvalu	3150 (2610 to 3760)	2550 (2120 to 3020)	2260 (1870 to 2720)	2110 (1790 to 2470)	1870 (1650 to 2120)	1860 (1670 to 2100)	1440 (1250 to 1670)	1330 (1090 to 1610)	1320 (1080 to 1620)	1300 (1060 to 1600)
Vanuatu	2880 (2440 to 3310)	2600 (2170 to 3080)	2200 (1820 to 2560)	1820 (1560 to 2150)	1680 (1430 to 1990)	1620 (1400 to 1870)	1530 (1370 to 1710)	1440 (1270 to 1640)	1430 (1270 to 1640)	1650 (1400 to 2000)
Southeast Asia	2670 (2410 to 2950)	2080 (1890 to 2280)	1890 (1750 to 2040)	1520 (1410 to 1640)	1320 (1230 to 1400)	1190 (1100 to 1280)	1090 (1030 to 1170)	974 (884 to 1060)	993 (899 to 1090)	1150 (999 to 1330)
Cambodia	2790 (2270 to 3400)	2370 (1950 to 2830)	2880 (2400 to 3440)	2310 (1830 to 2930)	1870 (1610 to 2190)	1700 (1510 to 1910)	1410 (1230 to 1590)	1280 (1030 to 1550)	1270 (1020 to 1550)	1450 (1140 to 1830)
Indonesia	2980 (2590 to 3430)	2390 (2040 to 2780)	1960 (1680 to 2280)	1540 (1370 to 1730)	1310 (1180 to 1450)	1180 (993 to 1380)	1180 (1010 to 1360)	1080 (883 to 1270)	1170 (957 to 1390)	1320 (1010 to 1630)
Aceh	2470 (1990 to 3040)	2000 (1520 to 2600)	1720 (1310 to 2200)	1380 (1110 to 1690)	1240 (1030 to 1480)	1140 (870 to 1460)	1220 (961 to 1510)	1110 (840 to 1410)	1210 (923 to 1550)	1380 (976 to 1770)
Bali	2620 (2100 to 3190)	2130 (1620 to 2770)	1820 (1380 to 2350)	1460 (1170 to 1780)	1260 (1050 to 1500)	1140 (890 to 1430)	1070 (816 to 1300)	992 (721 to 1260)	1070 (790 to 1330)	1200 (888 to 1560)
Bangka-Belitung Islands	3040 (2460 to 3720)	2550 (1940 to 3300)	2190 (1680 to 2780)	1780 (1410 to 2160)	1550 (1290 to 1830)	1390 (1100 to 1750)	1360 (1100 to 1640)	1240 (932 to 1550)	1330 (1020 to 1650)	1600 (1160 to 2100)
Banten	3640 (3000 to 4410)	3090 (2430 to 3940)	2460 (1930 to 3100)	1840 (1490 to 2230)	1430 (1200 to 1710)	1140 (884 to 1450)	1240 (969 to 1520)	1130 (828 to 1440)	1230 (909 to 1560)	1350 (982 to 1750)
Bengkulu	3680 (3020 to 4390)	2930 (2330 to 3640)	2290 (1770 to 2880)	1710 (1390 to 2090)	1370 (1140 to 1630)	1270 (983 to 1590)	1220 (952 to 1480)	1080 (808 to 1380)	1180 (880 to 1530)	1310 (961 to 1750)
Gorontalo	2860 (2300 to 3490)	2290 (1760 to 2950)	1840 (1430 to 2370)	1430 (1160 to 1750)	1130 (940 to 1360)	892 (705 to 1130)	920 (722 to 1150)	845 (617 to 1120)	949 (697 to 1250)	1090 (766 to 1480)
Jakarta	2060 (1610 to 2570)	1730 (1330 to 2240)	1350 (1030 to 1750)	1040 (845 to 1250)	987 (814 to 1190)	1080 (828 to 1360)	1080 (847 to 1320)	984 (719 to 1230)	1080 (806 to 1350)	1200 (876 to 1560)
Jambi	3160 (2550 to 3810)	2630 (2080 to 3310)	2230 (1730 to 2810)	1790 (1450 to 2140)	1490 (1250 to 1780)	1260 (986 to 1590)	1190 (934 to 1470)	1080 (802 to 1370)	1160 (872 to 1440)	1320 (965 to 1720)
West Java	3410 (2860 to 4020)	2770 (2230 to 3400)	2180 (1740 to 2670)	1630 (1370 to 1930)	1310 (1120 to 1520)	1070 (865 to 1340)	1070 (856 to 1330)	1010 (750 to 1290)	1090 (823 to 1380)	1250 (897 to 1610)
Central Java	2650 (2180 to 3220)	2090 (1630 to 2670)	1730 (1330 to 2160)	1340 (1100 to 1620)	1100 (935 to 1300)	1110 (880 to 1380)	1110 (891 to 1340)	1030 (794 to 1250)	1110 (875 to 1390)	1270 (937 to 1610)
East Java	2680 (2240 to 3200)	2220 (1760 to 2740)	1900 (1490 to 2380)	1550 (1300 to 1830)	1360 (1150 to 1570)	1210 (967 to 1520)	1210 (970 to 1440)	1110 (847 to 1360)	1200 (928 to 1470)	1330 (993 to 1690)
West Kalimantan	2730 (2210 to 3300)	2420 (1870 to 3060)	2100 (1630 to 2650)	1650 (1340 to 2000)	1420 (1190 to 1690)	1320 (1020 to 1670)	1240 (994 to 1500)	1080 (813 to 1360)	1140 (879 to 1420)	1310 (982 to 1680)
South Kalimantan	3740 (3080 to 4450)	3220 (2570 to 4040)	2500 (1950 to 3130)	1880 (1570 to 2270)	1680 (1410 to 1980)	1620 (1260 to 2070)	1570 (1280 to 1880)	1390 (1050 to 1720)	1520 (1170 to 1900)	1700 (1260 to 2180)
Central Kalimantan	2550 (2040 to 3140)	2090 (1620 to 2660)	1800 (1390 to 2290)	1500 (1190 to 1860)	1340 (1100 to 1620)	1170 (905 to 1490)	1240 (966 to 1500)	1140 (868 to 1430)	1250 (963 to 1570)	1420 (1030 to 1840)
East Kalimantan	2200 (1780 to 2720)	2040 (1590 to 2610)	1710 (1300 to 2170)	1320 (1080 to 1610)	1240 (1020 to 1450)	1350 (1060 to 1690)	1290 (1080 to 1550)	1150 (898 to 1410)	1240 (961 to 1530)	1410 (1050 to 1820)
North Kalimantan	2090 (1650 to 2620)	2000 (1560 to 2580)	1760 (1360 to 2260)	1380 (1130 to 1710)	1140 (911 to 1430)	913 (674 to 1220)	915 (708 to 1170)	833 (595 to 1100)	915 (669 to 1210)	1090 (749 to 1470)
Riau Islands	2330 (1840 to 2850)	2030 (1530 to 2620)	1730 (1320 to 2240)	1470 (1160 to 1810)	1270 (1030 to 1540)	870 (679 to 1110)	860 (670 to 1090)	833 (592 to 1100)	913 (662 to 1210)	1050 (736 to 1410)
Lampung	2850 (2320 to 3490)	2460 (1920 to 3170)	2000 (1530 to 2530)	1550 (1250 to 1900)	1350 (1120 to 1610)	1060 (837 to 1350)	1030 (797 to 1300)	958 (699 to 1250)	1030 (753 to 1350)	1190 (849 to 1590)
Maluku	3840 (3200 to 4600)	2880 (2240 to 3700)	2340 (1800 to 2910)	1860 (1500 to 2270)	1540 (1250 to 1870)	1270 (964 to 1630)	1330 (1030 to 1670)	1160 (871 to 1520)	1260 (944 to 1600)	1370 (977 to 1780)
North Maluku	3600 (2910 to 4360)	2930 (2310 to 3680)	2490 (2000 to 3100)	2090 (1740 to 2520)	1510 (1250 to 1800)	1190 (915 to 1500)	1110 (873 to 1400)	979 (721 to 1280)	1100 (817 to 1430)	1250 (890 to 1660)
West Nusa Tenggara	3870 (3240 to 4610)	3230 (2560 to 3980)	2720 (2120 to 3420)	2090 (1700 to 2460)	1680 (1420 to 1970)	1420 (1110 to 1800)	1300 (1010 to 1600)	1160 (872 to 1480)	1260 (944 to 1600)	1360 (994 to 1770)
East Nusa Tenggara	3670 (3040 to 4390)	2810 (2180 to 3530)	2330 (1800 to 2970)	1810 (1490 to 2200)	1490 (1250 to 1760)	1300 (1020 to 1660)	1210 (941 to 1530)	1110 (803 to 1450)	1170 (876 to 1510)	1350 (963 to 1790)
Papua	1930 (1490 to 2410)	1850 (1400 to 2380)	1660 (1260 to 2180)	1410 (1130 to 1710)	1340 (1120 to 1610)	1370 (1070 to 1750)	1460 (1150 to 1800)	1280 (955 to 1650)	1370 (1020 to 1800)	1490 (1040 to 1960)
West Papua	1960 (1540 to 2440)	1780 (1340 to 2300)	1620 (1240 to 2070)	1320 (1040 to 1670)	1120 (915 to 1370)	995 (759 to 1280)	1170 (940 to 1450)	922 (691 to 1210)	1020 (778 to 1320)	1150 (827 to 1540)
Riau	2570 (2050 to 3180)	2350 (1840 to 2990)	2000 (1530 to 2580)	1550 (1260 to 1870)	1270 (1060 to 1510)	1140 (892 to 1470)	1160 (902 to 1440)	1070 (787 to 1330)	1170 (878 to 1460)	1330 (955 to 1730)
West Sulawesi	4260 (3520 to 5180)	3180 (2500 to 3920)	2610 (2060 to 3290)	2010 (1640 to 2430)	1700 (1400 to 2020)	1440 (1120 to 1830)	1260 (1010 to 1570)	1120 (814 to 1480)	1220 (890 to 1610)	1420 (1010 to 1920)
South Sulawesi	3180 (2610 to 3820)	2540 (2000 to 3250)	2090 (1610 to 2650)	1660 (1350 to 2000)	1390 (1160 to 1650)	1210 (912 to 1540)	1220 (934 to 1480)	1120 (844 to 1400)	1200 (909 to 1500)	1320 (978 to 1680)
Central Sulawesi	3640 (3020 to 4360)	2930 (2290 to 3710)	2400 (1860 to 3030)	1910 (1560 to 2330)	1660 (1370 to 1970)	1470 (1150 to 1860)	1330 (1030 to 1640)	1170 (863 to 1500)	1270 (944 to 1640)	1490 (1060 to 1960)
Southeast Sulawesi	3390 (2800 to 4090)	2840 (2230 to 3600)	2340 (1860 to 2970)	1670 (1370 to 2030)	1340 (1110 to 1600)	1350 (1050 to 1730)	1360 (1070 to 1690)	1240 (898 to 1620)	1350 (1010 to 1730)	1500 (1060 to 1970)
North Sulawesi	2620 (2080 to 3180)	2140 (1660 to 2740)	1840 (1430 to 2360)	1510 (1200 to 1830)	1430 (1210 to 1660)	1320 (1030 to 1670)	1270 (996 to 1520)	1140 (857 to 1440)	1250 (941 to 1590)	1390 (1000 to 1800)
West Sumatra	4250 (3510 to 5040)	2710 (2140 to 3460)	2070 (1590 to 2600)	1530 (1240 to 1830)	1340 (1130 to 1590)	1320 (1020 to 1690)	1340 (1090 to 1590)	1160 (903 to 1410)	1260 (1000 to 1540)	1390 (1060 to 1760)
South Sumatra	2810 (2300 to 3460)	2250 (1760 to 2870)	1820 (1430 to 2330)	1380 (1110 to 1690)	1250 (1050 to 1490)	1100 (862 to 1400)	1110 (865 to 1370)	1020 (719 to 1340)	1110 (816 to 1430)	1240 (859 to 1650)

North Sumatra	2790 (2260 to 3380)	2230 (1730 to 2830)	1880 (1450 to 2450)	1530 (1240 to 1850)	1430 (1200 to 1690)	1440 (1130 to 1800)	1450 (1160 to 1750)	1320 (994 to 1640)	1410 (1050 to 1740)	1540 (1140 to 1970)
Yogyakarta	2810 (2270 to 3410)	1960 (1490 to 2550)	1510 (1130 to 1950)	1180 (912 to 1490)	1060 (880 to 1280)	993 (770 to 1230)	1030 (828 to 1240)	1010 (778 to 1260)	1090 (838 to 1340)	1270 (930 to 1650)
Laos	3600 (2610 to 5030)	3410 (2610 to 4460)	3160 (2550 to 3930)	2620 (2180 to 3130)	2170 (1820 to 2570)	1790 (1540 to 2090)	1400 (1190 to 1640)	1200 (961 to 1460)	1190 (942 to 1450)	1350 (1050 to 1670)
Malaysia	1700 (1420 to 2050)	1180 (1110 to 1260)	1110 (1030 to 1180)	1020 (847 to 1210)	962 (894 to 1040)	956 (892 to 1020)	890 (795 to 986)	820 (726 to 933)	703 (619 to 802)	1010 (872 to 1180)
Maldives	3530 (2930 to 4190)	2900 (2430 to 3390)	2200 (1930 to 2520)	1550 (1410 to 1710)	1160 (1070 to 1260)	848 (772 to 930)	619 (550 to 702)	508 (440 to 581)	502 (433 to 581)	581 (474 to 700)
Mauritius	2060 (1920 to 2200)	1800 (1640 to 1960)	1510 (1390 to 1650)	1470 (1340 to 1600)	1300 (1200 to 1400)	1070 (987 to 1160)	924 (855 to 1000)	872 (764 to 1000)	850 (750 to 972)	981 (836 to 1150)
Myanmar	4040 (3390 to 4780)	3350 (2780 to 3990)	2940 (2470 to 3470)	2430 (2050 to 2860)	2080 (1720 to 2500)	1850 (1500 to 2240)	1490 (1270 to 1730)	1260 (1040 to 1510)	1310 (1080 to 1590)	1470 (1160 to 1830)
Philippines	1400 (1230 to 1590)	1200 (1090 to 1320)	1330 (1210 to 1470)	1160 (1070 to 1270)	1140 (1040 to 1260)	1060 (957 to 1170)	1080 (976 to 1200)	1050 (927 to 1190)	1040 (912 to 1180)	1440 (1220 to 1720)
Seychelles	1650 (1480 to 1850)	1390 (1270 to 1510)	1530 (1390 to 1660)	1040 (943 to 1160)	1310 (1180 to 1450)	1130 (1030 to 1240)	1060 (982 to 1150)	1020 (893 to 1180)	865 (747 to 1010)	1020 (879 to 1190)
Sri Lanka	1600 (1490 to 1720)	1230 (1130 to 1360)	1370 (1270 to 1480)	1250 (1170 to 1350)	1230 (1080 to 1430)	1130 (988 to 1290)	981 (905 to 1060)	768 (586 to 1010)	691 (380 to 1000)	847 (563 to 1150)
Thailand	1930 (1680 to 2220)	1400 (1230 to 1590)	1360 (1200 to 1520)	1310 (1170 to 1500)	1060 (928 to 1190)	984 (839 to 1140)	769 (666 to 879)	715 (589 to 858)	715 (561 to 891)	796 (608 to 1020)
Timor-Leste	2490 (1960 to 3120)	2290 (1940 to 2700)	2120 (1740 to 2550)	2030 (1760 to 2380)	1400 (1230 to 1590)	1120 (966 to 1280)	1040 (900 to 1190)	1060 (866 to 1290)	1060 (854 to 1280)	1260 (986 to 1600)
Viet Nam	2740 (2250 to 3320)	1930 (1560 to 2390)	2090 (1740 to 2490)	1430 (1200 to 1720)	1270 (1090 to 1510)	1180 (1040 to 1320)	1170 (1060 to 1270)	1060 (916 to 1190)	1050 (905 to 1180)	1110 (937 to 1280)
Sub-Saharan Africa	2900 (2520 to 3330)	2650 (2350 to 2940)	2300 (2080 to 2530)	2010 (1860 to 2170)	1930 (1820 to 2050)	2010 (1890 to 2140)	1650 (1550 to 1770)	1370 (1250 to 1510)	1620 (1500 to 1770)	1840 (1690 to 2020)
Central sub-Saharan Africa	3310 (2770 to 3890)	2860 (2400 to 3350)	2600 (2230 to 2980)	2350 (2030 to 2680)	2160 (1900 to 2440)	2150 (1870 to 2480)	1780 (1480 to 2150)	1540 (1200 to 2000)	1820 (1430 to 2330)	2050 (1600 to 2620)
Angola	3100 (2560 to 3660)	2910 (2500 to 3380)	2770 (2330 to 3270)	2640 (2150 to 3200)	2460 (1940 to 3100)	2190 (1830 to 2620)	1680 (1430 to 1980)	1480 (1210 to 1820)	1720 (1420 to 2080)	2070 (1710 to 2530)
Central African Republic	3460 (2700 to 4540)	3240 (2640 to 3990)	2970 (2540 to 3450)	2830 (2480 to 3200)	2900 (2580 to 3220)	3190 (2790 to 3660)	2780 (2320 to 3280)	2530 (1980 to 3170)	3190 (2530 to 3980)	3160 (2480 to 3950)
Congo (Brazzaville)	4030 (3110 to 5230)	3560 (2960 to 4340)	3130 (2670 to 3620)	2560 (2190 to 2950)	2300 (1980 to 2640)	2200 (1940 to 2510)	1560 (1340 to 1770)	1430 (1170 to 1700)	1730 (1430 to 2060)	1860 (1520 to 2240)
DR Congo	3330 (2760 to 3970)	2760 (2250 to 3360)	2490 (2090 to 2910)	2230 (1880 to 2600)	2030 (1740 to 2350)	2080 (1740 to 2510)	1790 (1390 to 2300)	1530 (1080 to 2210)	1810 (1290 to 2540)	2020 (1420 to 2870)
Equatorial Guinea	4610 (3940 to 5430)	3670 (3070 to 4360)	3050 (2490 to 3660)	2550 (2140 to 3000)	2520 (2080 to 3000)	1870 (1470 to 2350)	1280 (1050 to 1630)	1410 (1130 to 1780)	1870 (1520 to 2300)	1940 (1510 to 2430)
Gabon	4300 (3500 to 5090)	3160 (2700 to 3630)	2610 (2110 to 3150)	2040 (1660 to 2490)	1920 (1670 to 2210)	1890 (1630 to 2190)	1630 (1400 to 1890)	1490 (1220 to 1770)	1790 (1500 to 2100)	1900 (1590 to 2230)
Eastern sub-Saharan Africa	3150 (2740 to 3590)	2760 (2450 to 3090)	2460 (2240 to 2710)	2210 (2060 to 2380)	2220 (2100 to 2350)	2190 (2060 to 2330)	1640 (1540 to 1750)	1350 (1220 to 1490)	1600 (1460 to 1770)	1910 (1740 to 2110)
Burundi	3600 (2680 to 4830)	3160 (2510 to 3930)	2930 (2440 to 3500)	2670 (2300 to 3060)	2550 (2230 to 2890)	2790 (2400 to 3270)	1600 (1400 to 1830)	1410 (1140 to 1710)	1560 (1260 to 1910)	1750 (1430 to 2120)
Comoros	2670 (2110 to 3390)	2460 (2050 to 2940)	2170 (1800 to 2570)	1980 (1670 to 2340)	1690 (1420 to 1980)	1450 (1230 to 1690)	1190 (996 to 1410)	1110 (870 to 1440)	1190 (937 to 1530)	1380 (1100 to 1770)
Djibouti	1830 (1340 to 2550)	1740 (1310 to 2280)	1610 (1270 to 2020)	1470 (1200 to 1780)	1550 (1250 to 1880)	1610 (1290 to 2000)	1440 (1160 to 1810)	1270 (981 to 1650)	1610 (1280 to 2040)	1690 (1330 to 2130)
Eritrea	3650 (2690 to 5200)	3330 (2570 to 4350)	3540 (2610 to 6010)	3350 (2980 to 3770)	3040 (2670 to 3460)	2420 (1990 to 2940)	2000 (1530 to 2690)	1710 (1240 to 2450)	1750 (1260 to 2460)	2090 (1490 to 2960)
Ethiopia	3670 (2710 to 5170)	3320 (2560 to 4280)	3110 (2600 to 3760)	2920 (2570 to 3290)	2730 (2420 to 3040)	2150 (1900 to 2450)	1340 (1200 to 1500)	1080 (886 to 1310)	1330 (1100 to 1610)	1610 (1340 to 1960)
Addis Ababa	2530 (1790 to 3700)	2500 (1830 to 3440)	2310 (1740 to 3050)	2120 (1680 to 2550)	2180 (1820 to 2600)	1930 (1530 to 2390)	1310 (1080 to 1550)	1090 (804 to 1420)	1550 (1190 to 1980)	2060 (1580 to 2610)
Afar	5330 (3820 to 7710)	4260 (3100 to 5800)	3990 (3020 to 5280)	3930 (3250 to 4780)	3520 (2950 to 4170)	2550 (2050 to 3090)	1780 (1440 to 2130)	1370 (1010 to 1810)	1820 (1380 to 2380)	2320 (1800 to 2980)
Amhara	3520 (2520 to 5070)	3120 (2330 to 4060)	2900 (2270 to 3620)	2690 (2260 to 3150)	2690 (2280 to 3100)	2320 (1970 to 2750)	1430 (1220 to 1660)	1140 (860 to 1470)	1310 (1010 to 1660)	1500 (1170 to 1930)
Benishangul-Gumuz	4800 (3480 to 6900)	3900 (2900 to 5270)	3700 (2800 to 4820)	3540 (2890 to 4320)	3110 (2610 to 3690)	2540 (2070 to 3090)	1620 (1310 to 1970)	1130 (820 to 1510)	1130 (781 to 1570)	1130 (813 to 1580)
Dire Dawa	3380 (2380 to 4870)	3440 (2480 to 4750)	3260 (2440 to 4270)	2960 (2440 to 3630)	2740 (2280 to 3260)	2160 (1730 to 2640)	1250 (1020 to 1530)	1020 (762 to 1360)	1480 (1120 to 1940)	1950 (1470 to 2510)
Gambella	5300 (3800 to 7620)	4280 (3200 to 5840)	4100 (3090 to 5340)	3900 (3250 to 4710)	3440 (2920 to 4010)	2730 (2210 to 3330)	1920 (1560 to 2300)	1450 (1070 to 1930)	2030 (1530 to 2670)	2640 (2080 to 3430)
Harari	4260 (3070 to 6090)	4250 (3120 to 5790)	3950 (3060 to 5260)	3620 (2960 to 4390)	3420 (2860 to 4050)	2330 (1900 to 2920)	1320 (1080 to 1590)	1070 (782 to 1440)	1620 (1230 to 2110)	2220 (1700 to 2940)
Oromia	3800 (2790 to 5380)	3410 (2550 to 4640)	3190 (2510 to 4030)	2980 (2530 to 3530)	2770 (2370 to 3230)	2000 (1700 to 2360)	1150 (964 to 1340)	934 (728 to 1220)	1200 (946 to 1520)	1490 (1160 to 1890)
Somali	2760 (1960 to 4010)	2460 (1830 to 3350)	2270 (1710 to 3040)	2020 (1640 to 2490)	1790 (1460 to 2170)	1720 (1370 to 2130)	1430 (1170 to 1730)	1200 (892 to 1590)	1630 (1220 to 2160)	2080 (1590 to 2690)
Southern Nations, Nationalities, and Peoples	3830 (2750 to 5450)	3610 (2660 to 4870)	3410 (2650 to 4340)	3220 (2720 to 3810)	2920 (2500 to 3400)	2190 (1780 to 2800)	1510 (1260 to 1780)	1240 (909 to 1630)	1490 (1140 to 1930)	1780 (1350 to 2300)
Tigray	4120 (2920 to 5880)	3700 (2750 to 5090)	3500 (2650 to 4560)	3360 (2790 to 4010)	2910 (2440 to 3430)	2320 (1890 to 2830)	1280 (1060 to 1530)	1030 (760 to 1360)	1270 (937 to 1700)	1540 (1160 to 2030)
Kenya	2450 (2100 to 2860)	1990 (1680 to 2320)	1680 (1410 to 1990)	1380 (1180 to 1600)	1390 (1220 to 1570)	1950 (1700 to 2210)	1660 (1510 to 1830)	1330 (1130 to 1540)	1700 (1460 to 1930)	1860 (1600 to 2100)
Baringo	2680 (2130 to 3330)	2420 (1810 to 3190)	2240 (1710 to 2910)	1940 (1480 to 2530)	1830 (1410 to 2280)	2130 (1700 to 2620)	1820 (1440 to 2220)	1400 (1030 to 1860)	1710 (1290 to 2210)	1850 (1410 to 2330)
Bomet	1340 (994 to 1750)	1200 (867 to 1640)	1100 (798 to 1480)	978 (732 to 1330)	1030 (819 to 1350)	1650 (1270 to 2190)	1440 (1130 to 1790)	1120 (890 to 1460)	1450 (1180 to 1780)	1600 (1310 to 1940)
Bungoma	2840 (2220 to 3560)	2200 (1640 to 2970)	1770 (1350 to 2300)	1460 (1110 to 1890)	1360 (1030 to 1740)	1660 (1280 to 2050)	1250 (994 to 1540)	1050 (799 to 1400)	1290 (1010 to 1630)	1430 (1100 to 1830)
Busia	4200 (3380 to 5160)	2930 (2210 to 3810)	2240 (1730 to 2870)	1610 (1220 to 2090)	1650 (1240 to 2150)	2470 (1780 to 3170)	1720 (1280 to 2240)	1320 (958 to 1730)	1710 (1310 to 2200)	1900 (1500 to 2390)
Elgeyo Marakwet	2050 (1570 to 2620)	1790 (1320 to 2410)	1710 (1280 to 2250)	1520 (1140 to 1960)	1390 (1040 to 1810)	1760 (1330 to 2200)	1520 (1250 to 1830)	1240 (917 to 1600)	1460 (1110 to 1820)	1550 (1170 to 1940)
Embu	2560 (1980 to 3240)	1920 (1420 to 2530)	1390 (1010 to 1850)	1030 (740 to 1400)	1010 (788 to 1340)	1310 (966 to 1820)	1310 (954 to 1690)	1150 (834 to 1550)	1400 (1060 to 1830)	1520 (1170 to 1940)
Garissa	2270 (1730 to 2960)	2040 (1450 to 2850)	1800 (1330 to 2440)	1630 (1180 to 2170)	1560 (1150 to 2050)	1750 (1340 to 2260)	1560 (1190 to 1920)	1290 (963 to 1640)	1840 (1490 to 2230)	2070 (1670 to 2530)
Homa Bay	4280 (3550 to 5170)	3180 (2470 to 4040)	2780 (2230 to 3450)	2200 (1750 to 2760)	2190 (1680 to 2790)	4240 (3270 to 5330)	3140 (2440 to 4000)	2270 (1550 to 3040)	2740 (2000 to 3590)	2850 (2130 to 3690)
Isiolo	3360 (2640 to 4130)	3070 (2350 to 4020)	2740 (2150 to 3470)	2120 (1640 to 2720)	1800 (1400 to 2320)	1990 (1450 to 2580)	1810 (1360 to 2300)	1470 (1020 to 2000)	1890 (1440 to 2400)	2080 (1590 to 2640)
Kajiado	1730 (1300 to 2230)	1550 (1100 to 2140)	1440 (1050 to 1960)	1230 (898 to 1660)	1210 (871 to 1580)	1700 (1260 to 2180)	1520 (1230 to 1840)	1230 (938 to 1550)	1700 (1370 to 2060)	1880 (1530 to 2260)
Kakamega	2930 (2290 to 3610)	2220 (1660 to 2850)	1850 (1410 to 2380)	1500 (1170 to 1910)	1610 (1240 to 2050)	2320 (1740 to 2930)	1680 (1330 to 2130)	1260 (919 to 1660)	1630 (1260 to 2040)	1800 (1440 to 2260)
Kericho	1310 (943 to 1730)	1270 (900 to 1750)	1170 (829 to 1560)	1060 (780 to 1420)	1080 (801 to 1430)	1580 (1140 to 2070)	1440 (1060 to 1840)	1200 (882 to 1600)	1460 (1110 to 1890)	1570 (1210 to 1960)
Kiambu	2230 (1710 to 2860)	1890 (1340 to 2520)	1620 (1200 to 2090)	1470 (1090 to 1890)	1570 (1230 to 1930)	2170 (1750 to 2630)	1930 (1610 to 2280)	1590 (1240 to 1970)	2010 (1640 to 2400)	2180 (1760 to 2580)
Kilifi	2780 (2220 to 3450)	2190 (1690 to 2830)	1810 (1390 to 2320)	1420 (1110 to 1840)	1240 (965 to 1580)	1470 (1130 to 1900)	1380 (1080 to 1710)	1130 (836 to 1470)	1500 (1170 to 1920)	1660 (1320 to 2080)
Kirinyaga	2760 (2190 to 3460)	2120 (1570 to 2880)	1710 (1270 to 2240)	1190 (846 to 1580)	1230 (895 to 1610)	1720 (1250 to 2230)	1600 (1240 to 2030)	1400 (986 to 1840)	1680 (1240 to 2120)	1780 (1400 to 2250)
Kisii	2290 (1740 to 2910)	2080 (1520 to 2780)	1790 (1350 to 2330)	1520 (1150 to 1990)	1530 (1170 to 1950)	1950 (1510 to 2420)	1510 (1230 to 1780)	1270 (910 to 1630)	1630 (1260 to 2030)	1790 (1400 to 2210)
Kisumu	2770 (2210 to 3470)	2380 (1780 to 3130)	2160 (1660 to 2770)	1930 (1450 to 2500)	2170 (1690 to 2740)	3870 (3040 to 4760)	2910 (2350 to 3620)	2340 (1650 to 3030)	2920 (2260 to 3680)	3110 (2420 to 3900)
Kitui	3070 (2440 to 3830)	2290 (1720 to 2950)	1830 (1390 to 2390)	1390 (1040 to 1820)	1280 (970 to 1650)	1670 (1230 to 2180)	1610 (1270 to 1990)	1320 (954 to 1730)	1570 (1190 to 1980)	1670 (1280 to 2100)
Kwale	3170 (2480 to 3970)	2490 (1870 to 3330)	1980 (1490 to 2600)	1530 (1150 to 1960)	1310 (988 to 1750)	1460 (1090 to 1930)	1630 (1260 to 2030)	1360 (983 to 1770)	1690 (1250 to 2120)	1830 (1420 to 2290)
Laikipia	1550 (1140 to 2030)	1320 (922 to 1830)	1140 (798 to 1560)	956 (673 to 1290)	1050 (816 to 1410)	1610 (1180 to 2200)	1470 (1110 to 1870)	1210 (919 to 1560)	1560 (1270 to 1960)	1720 (1390 to 2110)
Lamu	3290 (2570 to 4140)	2340 (1720 to 3100)	1790 (1350 to 2430)	1190 (858 to 1590)	1190 (888 to 1620)	1400 (1030 to 1940)	1430 (1050 to 1880)	1200 (842 to 1660)	1570 (1190 to 2060)	1730 (1340 to 2210)
Machakos	1880 (1440 to 2390)	1570 (1140 to 2150)	1310 (971 to 1730)	1050 (776 to 1390)	976 (758 to 1260)	1380 (1020 to 1810)	1350 (1070 to 1650)	1180 (873 to 1530)	1460 (1140 to 1820)	1580 (1250 to 1970)
Makueni	2390 (1840 to 2980)	1730 (1270 to 2320)	1440 (1060 to 1930)	1160 (849 to 1530)	1160 (882 to 1520)	2040 (1430 to 2700)	1710 (1320 to 2120)	1240 (948 to 1600)	1510 (1210 to 1860)	1610 (1300 to 1980)
Mandera	2060 (1580 to 2660)	1910 (1350 to 2590)	1780 (1330 to 2350)	1660 (1230 to 2170)	1710 (1290 to 2240)	1930 (1450 to 2470)	1790 (1410 to 2210)	1460 (1050 to 1880)	2280 (1810 to 2790)	2690 (2160 to 3280)
Marsabit	2050 (1550 to 2640)	1920 (1420 to 2500)	1680 (1250 to 2260)	1410 (1040 to 1870)	1320 (989 to 1750)	1550 (1110 to 2060)	1580 (1190 to 2030)	1340 (938 to 1810)	1670 (1250 to 2170)	1840 (1380 to 2360)

Meru	1800 (1390 to 2330)	1480 (1050 to 2000)	1220 (884 to 1620)	973 (718 to 1300)	996 (780 to 1270)	1380 (1030 to 1850)	1410 (1090 to 1770)	1210 (884 to 1610)	1530 (1180 to 1960)	1680 (1310 to 2120)
Migori	3500 (2820 to 4300)	2730 (2070 to 3580)	2330 (1810 to 2980)	2030 (1600 to 2560)	1990 (1530 to 2540)	3070 (2280 to 4040)	2600 (2010 to 3250)	1910 (1320 to 2550)	2450 (1890 to 3170)	2660 (2070 to 3310)
Mombasa	1860 (1380 to 2460)	1700 (1220 to 2280)	1350 (1000 to 1830)	1030 (747 to 1350)	1070 (843 to 1400)	1590 (1180 to 2150)	1480 (1130 to 1910)	1170 (865 to 1600)	1700 (1350 to 2110)	1930 (1560 to 2380)
Murang'a	2520 (2000 to 3120)	1780 (1320 to 2380)	1440 (1080 to 1890)	1170 (860 to 1550)	1240 (938 to 1540)	1730 (1360 to 2150)	1630 (1360 to 1930)	1280 (932 to 1640)	1560 (1210 to 1970)	1670 (1310 to 2060)
Nairobi	2140 (1670 to 2700)	1770 (1360 to 2260)	1360 (1040 to 1760)	1110 (869 to 1440)	1220 (925 to 1560)	1850 (1430 to 2290)	1550 (1290 to 1820)	1250 (1000 to 1560)	1820 (1530 to 2150)	2050 (1730 to 2410)
Nakuru	1510 (1120 to 1970)	1420 (1010 to 1900)	1300 (943 to 1750)	1200 (883 to 1570)	1290 (942 to 1640)	1910 (1500 to 2390)	1450 (1160 to 1760)	1120 (870 to 1430)	1490 (1220 to 1840)	1650 (1320 to 2020)
Nandi	2130 (1630 to 2710)	1880 (1380 to 2490)	1720 (1260 to 2260)	1580 (1180 to 2090)	1650 (1260 to 2090)	2320 (1830 to 2820)	2000 (1660 to 2410)	1520 (1160 to 1920)	1810 (1390 to 2250)	1920 (1500 to 2350)
Narok	2170 (1680 to 2740)	1930 (1410 to 2590)	1810 (1340 to 2380)	1520 (1120 to 1990)	1620 (1210 to 2090)	2150 (1680 to 2700)	1790 (1490 to 2180)	1440 (1110 to 1810)	1920 (1550 to 2320)	2110 (1690 to 2530)
Nyamira	2200 (1700 to 2850)	1920 (1400 to 2580)	1690 (1240 to 2250)	1510 (1110 to 1940)	1560 (1210 to 1960)	2370 (1850 to 2970)	2180 (1750 to 2670)	1740 (1260 to 2250)	2130 (1630 to 2710)	2270 (1770 to 2890)
Nyandarua	2480 (1890 to 3150)	2100 (1520 to 2820)	1740 (1280 to 2310)	1490 (1070 to 1940)	1560 (1210 to 1940)	2140 (1710 to 2650)	2140 (1760 to 2560)	1900 (1430 to 2370)	2270 (1790 to 2790)	2410 (1940 to 2930)
Nyeri	2190 (1690 to 2780)	1630 (1180 to 2210)	1290 (940 to 1740)	1090 (769 to 1490)	1150 (870 to 1490)	1580 (1180 to 2020)	1390 (1080 to 1730)	1200 (913 to 1600)	1560 (1230 to 1970)	1710 (1380 to 2110)
Samburu	1490 (1080 to 1970)	1430 (1020 to 1920)	1370 (976 to 1850)	1270 (949 to 1700)	1370 (973 to 1840)	2000 (1460 to 2610)	1840 (1400 to 2370)	1470 (1040 to 1990)	1900 (1440 to 2430)	2100 (1600 to 2650)
Siaya	2850 (2270 to 3490)	2240 (1690 to 2940)	1830 (1410 to 2350)	1550 (1210 to 1960)	1680 (1280 to 2130)	2870 (2170 to 3740)	1900 (1460 to 2470)	1360 (1080 to 1750)	1710 (1370 to 2170)	1860 (1510 to 2290)
Taita Taveta	2420 (1830 to 3120)	2010 (1450 to 2770)	1790 (1340 to 2360)	1350 (988 to 1820)	1240 (897 to 1680)	1700 (1230 to 2270)	1720 (1310 to 2180)	1320 (937 to 1720)	1710 (1320 to 2130)	1880 (1480 to 2330)
Tana River	2860 (2240 to 3630)	2320 (1670 to 3120)	2090 (1530 to 2760)	1840 (1380 to 2410)	1770 (1310 to 2300)	2260 (1770 to 2840)	2200 (1770 to 2700)	1760 (1310 to 2280)	2200 (1710 to 2800)	2380 (1860 to 2980)
Tharaka Nithi	2810 (2190 to 3490)	2080 (1520 to 2790)	1840 (1350 to 2490)	1580 (1150 to 2060)	1610 (1210 to 2050)	1990 (1590 to 2480)	1810 (1480 to 2190)	1570 (1210 to 2060)	1730 (1350 to 2210)	1760 (1360 to 2280)
Trans Nzoia	1430 (1040 to 1900)	1340 (948 to 1800)	1210 (872 to 1640)	1080 (801 to 1420)	1110 (846 to 1460)	1660 (1210 to 2210)	1540 (1150 to 1930)	1240 (911 to 1610)	1560 (1220 to 1960)	1700 (1350 to 2130)
Turkana	1790 (1330 to 2320)	1740 (1250 to 2380)	1750 (1290 to 2310)	1730 (1300 to 2250)	1730 (1280 to 2240)	2660 (1820 to 3860)	2070 (1610 to 2560)	1520 (1050 to 1980)	2100 (1620 to 2600)	2360 (1870 to 2890)
Uasin Gishu	1230 (882 to 1660)	1160 (818 to 1600)	1050 (740 to 1440)	936 (681 to 1290)	996 (766 to 1300)	1510 (1160 to 1880)	1370 (1100 to 1670)	1080 (834 to 1420)	1420 (1130 to 1780)	1570 (1260 to 1950)
Vihiga	2770 (2210 to 3480)	1870 (1360 to 2550)	1570 (1160 to 2080)	1350 (1010 to 1780)	1400 (1030 to 1820)	1920 (1420 to 2430)	1530 (1190 to 1920)	1250 (903 to 1710)	1520 (1140 to 1950)	1650 (1260 to 2140)
Wajir	2690 (2100 to 3400)	2500 (1850 to 3280)	2180 (1640 to 2790)	1890 (1440 to 2420)	1810 (1350 to 2360)	2000 (1580 to 2520)	1810 (1410 to 2260)	1490 (1070 to 1940)	1920 (1480 to 2390)	2120 (1670 to 2600)
West Pokot	1840 (1380 to 2330)	1600 (1130 to 2190)	1530 (1110 to 2060)	1410 (1040 to 1860)	1390 (1030 to 1800)	1610 (1180 to 2020)	1530 (1160 to 1950)	1250 (881 to 1670)	1610 (1200 to 2090)	1750 (1320 to 2230)
Madagascar	2880 (2490 to 3300)	2430 (2080 to 2870)	1960 (1690 to 2250)	1780 (1560 to 2040)	1840 (1640 to 2050)	1650 (1440 to 1890)	1480 (1200 to 1810)	1330 (1050 to 1640)	1680 (1380 to 2010)	1790 (1470 to 2150)
Malawi	3480 (2870 to 4180)	3110 (2570 to 3720)	2630 (2260 to 3070)	2070 (1820 to 2340)	2210 (1960 to 2480)	2750 (2430 to 3110)	2020 (1790 to 2280)	1650 (1390 to 1920)	1820 (1550 to 2110)	2510 (2190 to 2870)
Mozambique	2950 (2410 to 3560)	2620 (2200 to 3060)	2320 (1990 to 2690)	2070 (1810 to 2360)	2140 (1870 to 2460)	2210 (1960 to 2480)	2350 (2080 to 2650)	2000 (1710 to 2300)	2110 (1790 to 2470)	2680 (2310 to 3100)
Rwanda	3840 (3310 to 4370)	2990 (2490 to 3530)	2910 (2460 to 3410)	2460 (2130 to 2800)	2490 (2250 to 2740)	2480 (2230 to 2750)	1390 (1230 to 1600)	1210 (982 to 1480)	1270 (1030 to 1570)	1710 (1410 to 2060)
Somalia	3000 (2500 to 3530)	2950 (2420 to 3580)	2820 (2340 to 3300)	2540 (2120 to 3000)	2620 (2220 to 3030)	2540 (2090 to 3080)	2840 (2210 to 3660)	2120 (1550 to 2940)	2790 (2060 to 3950)	3180 (2320 to 4450)
South Sudan	2490 (2000 to 3120)	2380 (1950 to 2890)	2310 (1840 to 2880)	2090 (1700 to 2540)	2080 (1700 to 2520)	1940 (1590 to 2390)	1700 (1400 to 2080)	1820 (1420 to 2330)	2220 (1780 to 2800)	2290 (1810 to 2940)
Uganda	3000 (2470 to 3580)	2460 (2110 to 2870)	2190 (1890 to 2570)	2240 (1900 to 2680)	2450 (2140 to 2790)	2530 (2250 to 2830)	1770 (1550 to 1990)	1420 (1210 to 1700)	1610 (1360 to 1900)	2060 (1690 to 2460)
Tanzania	3100 (2570 to 3720)	2740 (2300 to 3200)	2320 (1990 to 2710)	1830 (1580 to 2110)	1840 (1630 to 2060)	2000 (1790 to 2240)	1480 (1310 to 1650)	1200 (1010 to 1460)	1430 (1200 to 1710)	1700 (1440 to 2020)
Zambia	2750 (2230 to 3330)	2370 (1920 to 2940)	1960 (1640 to 2320)	1790 (1550 to 2030)	2120 (1890 to 2380)	2890 (2570 to 3260)	1980 (1760 to 2250)	1620 (1300 to 2020)	1940 (1600 to 2390)	2620 (2130 to 3240)
Southern sub-Saharan Africa	2350 (2090 to 2640)	2110 (1920 to 2330)	1870 (1710 to 2040)	1640 (1470 to 1840)	1400 (1280 to 1530)	2140 (2000 to 2300)	2050 (1930 to 2190)	1490 (1330 to 1660)	1900 (1700 to 2140)	2250 (2030 to 2540)
Botswana	2800 (2360 to 3320)	2640 (2240 to 3120)	2410 (2040 to 2890)	2000 (1690 to 2310)	1820 (1500 to 2150)	3040 (2570 to 3570)	1920 (1680 to 2170)	1380 (1170 to 1640)	1500 (1250 to 1790)	2420 (2030 to 2920)
Eswatini	3860 (3310 to 4450)	3470 (2940 to 4060)	2810 (2340 to 3290)	2240 (1880 to 2620)	1930 (1660 to 2220)	3030 (2560 to 3570)	3360 (2890 to 3920)	2340 (1950 to 2800)	2940 (2530 to 3450)	3830 (3100 to 4540)
Lesotho	3110 (2680 to 3560)	2790 (2370 to 3250)	2570 (2180 to 3030)	2090 (1790 to 2460)	1920 (1660 to 2220)	2990 (2580 to 3440)	3140 (2730 to 3560)	2780 (2360 to 3220)	3220 (2770 to 3690)	4310 (3670 to 5050)
Namibia	2450 (2010 to 2960)	2200 (1800 to 2610)	1970 (1660 to 2300)	1910 (1610 to 2230)	1750 (1550 to 1950)	2370 (2120 to 2670)	1920 (1690 to 2180)	1650 (1380 to 1990)	1840 (1550 to 2210)	2570 (2170 to 3040)
South Africa	2250 (2020 to 2520)	2040 (1840 to 2260)	1810 (1650 to 1980)	1590 (1380 to 1860)	1320 (1180 to 1480)	1920 (1780 to 2090)	1940 (1800 to 2100)	1370 (1200 to 1560)	1810 (1590 to 2070)	2050 (1800 to 2350)
Eastern Cape	2540 (2110 to 3050)	1970 (1640 to 2360)	1680 (1390 to 2020)	1400 (1100 to 1760)	1240 (1050 to 1480)	2320 (2100 to 2600)	2350 (2120 to 2610)	1880 (1560 to 2270)	2870 (2380 to 3460)	2580 (2110 to 3160)
Free State	2130 (1720 to 2580)	1980 (1610 to 2360)	1750 (1420 to 2110)	1510 (1170 to 1890)	1460 (1220 to 1750)	2260 (2020 to 2520)	2630 (2350 to 2910)	1660 (1370 to 1980)	2170 (1790 to 2600)	2430 (1960 to 2970)
Gauteng	2460 (2070 to 2880)	2340 (1950 to 2760)	2190 (1820 to 2610)	2130 (1690 to 2660)	1560 (1290 to 1860)	1740 (1570 to 1960)	1640 (1460 to 1830)	1180 (976 to 1440)	1500 (1250 to 1810)	1740 (1440 to 2110)
KwaZulu-Natal	2010 (1660 to 2440)	1860 (1520 to 2240)	1620 (1320 to 1980)	1420 (1110 to 1780)	1480 (1240 to 1740)	2320 (2080 to 2570)	2100 (1890 to 2310)	1170 (978 to 1420)	1720 (1420 to 2070)	1980 (1660 to 2390)
Limpopo	1750 (1410 to 2130)	1480 (1210 to 1820)	1290 (1030 to 1590)	1070 (806 to 1410)	849 (699 to 1010)	1490 (1330 to 1660)	2120 (1900 to 2380)	1620 (1320 to 1960)	1920 (1580 to 2360)	2520 (2090 to 3050)
Mpumalanga	2060 (1690 to 2510)	1890 (1540 to 2290)	1690 (1360 to 2050)	1440 (1120 to 1810)	1090 (901 to 1320)	1820 (1630 to 2040)	1960 (1760 to 2180)	1250 (1020 to 1530)	1510 (1230 to 1820)	2020 (1650 to 2460)
North West	1910 (1570 to 2320)	1760 (1440 to 2130)	1510 (1240 to 1830)	1290 (997 to 1650)	961 (785 to 1170)	1850 (1630 to 2100)	2370 (2100 to 2680)	2090 (1680 to 2540)	2260 (1840 to 2820)	2740 (2250 to 3390)
Northern Cape	1700 (1350 to 2060)	1580 (1290 to 1920)	1420 (1150 to 1740)	1230 (964 to 1580)	1250 (1050 to 1500)	2290 (2060 to 2580)	2210 (1950 to 2530)	1590 (1280 to 1960)	1940 (1560 to 2430)	2490 (2030 to 3090)
Western Cape	2050 (1670 to 2480)	1910 (1550 to 2310)	1820 (1450 to 2190)	1810 (1420 to 2320)	1520 (1240 to 1840)	1540 (1390 to 1730)	1280 (1160 to 1430)	1090 (904 to 1330)	1470 (1210 to 1770)	1650 (1350 to 2030)
Zimbabwe	2530 (1850 to 3490)	2220 (1680 to 2880)	1980 (1590 to 2410)	1740 (1510 to 1990)	1610 (1370 to 1860)	2920 (2470 to 3380)	2470 (2210 to 2780)	1950 (1650 to 2310)	2320 (1970 to 2730)	3200 (2690 to 3730)
Western sub-Saharan Africa	2720 (2260 to 3310)	2640 (2220 to 3120)	2210 (1920 to 2550)	1860 (1670 to 2090)	1770 (1600 to 1960)	1790 (1610 to 1990)	1520 (1370 to 1700)	1310 (1160 to 1490)	1520 (1340 to 1710)	1640 (1450 to 1850)
Benin	2990 (2180 to 4040)	2580 (2070 to 3270)	2260 (1880 to 2710)	1950 (1700 to 2230)	1780 (1590 to 1990)	1700 (1510 to 1910)	1490 (1320 to 1690)	1320 (1120 to 1570)	1430 (1220 to 1710)	1680 (1430 to 2000)
Burkina Faso	3410 (2430 to 4900)	3100 (2360 to 4030)	2630 (2170 to 3210)	2240 (1950 to 2590)	2070 (1820 to 2380)	2020 (1760 to 2310)	1740 (1530 to 1960)	1520 (1330 to 1770)	1780 (1570 to 2070)	1890 (1640 to 2200)
Cabo Verde	1940 (1690 to 2230)	1540 (1380 to 1710)	1420 (1280 to 1590)	1080 (960 to 1210)	998 (881 to 1130)	1070 (890 to 1300)	854 (726 to 985)	1120 (932 to 1330)	1170 (963 to 1390)	1170 (960 to 1400)
Cameroon	2940 (2210 to 3840)	5600 (4650 to 6750)	2060 (1700 to 2450)	1770 (1500 to 2050)	1620 (1430 to 1830)	1970 (1720 to 2250)	1740 (1480 to 2050)	1450 (1140 to 1840)	1640 (1300 to 2100)	1950 (1570 to 2450)
Chad	2830 (1920 to 4140)	2400 (1740 to 3390)	2320 (1780 to 3030)	2090 (1730 to 2490)	1860 (1640 to 2090)	2060 (1780 to 2370)	1770 (1540 to 2030)	1570 (1240 to 1950)	1930 (1560 to 2370)	1870 (1490 to 2310)
Côte d'Ivoire	2620 (2210 to 3030)	2470 (2050 to 3010)	2070 (1750 to 2430)	1680 (1450 to 1950)	1900 (1670 to 2150)	2230 (1970 to 2500)	1790 (1580 to 2030)	1400 (1160 to 1670)	1640 (1390 to 1950)	1670 (1400 to 2000)
The Gambia	2240 (1810 to 2710)	2270 (1870 to 2710)	2030 (1670 to 2430)	1860 (1540 to 2230)	1650 (1380 to 1960)	1590 (1360 to 1860)	1480 (1260 to 1710)	1400 (1130 to 1680)	1770 (1460 to 2090)	1790 (1470 to 2140)
Ghana	2500 (2110 to 2910)	2140 (1820 to 2500)	1870 (1590 to 2210)	1670 (1400 to 1980)	1590 (1350 to 1880)	1620 (1420 to 1820)	1610 (1440 to 1810)	1390 (1130 to 1710)	1540 (1270 to 1880)	1720 (1410 to 2070)
Guinea	2960 (2410 to 3550)	2630 (2140 to 3240)	2350 (1950 to 2840)	2020 (1720 to 2370)	1760 (1520 to 2030)	1800 (1590 to 2050)	1650 (1410 to 1930)	1420 (1140 to 1750)	1780 (1450 to 2170)	1860 (1500 to 2280)
Guinea-Bissau	4570 (3920 to 5280)	3770 (3240 to 4310)	3570 (3010 to 4200)	2860 (2460 to 3320)	2590 (2240 to 2980)	2390 (2000 to 2810)	2070 (1790 to 2330)	1860 (1520 to 2230)	2500 (2100 to 2970)	2400 (1950 to 2920)
Liberia	3690 (2930 to 4570)	2860 (2410 to 3320)	2420 (2050 to 2850)	1900 (1630 to 2230)	2080 (1810 to 2380)	1750 (1510 to 2030)	1410 (1220 to 1640)	1250 (984 to 1590)	1510 (1210 to 1890)	1550 (1240 to 1970)
Mali	3410 (2820 to 4050)	3070 (2520 to 3720)	2820 (2410 to 3360)	2290 (1990 to 2640)	1930 (1700 to 2160)	1680 (1510 to 1870)	1480 (1320 to 1660)	1320 (1090 to 1580)	1690 (1410 to 2020)	1910 (1600 to 2300)
Mauritania	2490 (1920 to 3370)	2120 (1700 to 2640)	1780 (1470 to 2150)	1660 (1410 to 1950)	1480 (1290 to 1680)	1180 (1030 to 1340)	966 (824 to 1140)	909 (726 to 1140)	1150 (936 to 1400)	1130 (903 to 1400)
Niger	3000 (2240 to 4080)	2880 (2250 to 3650)	2580 (2180 to 3060)	2360 (2090 to 2660)	2100 (1890 to 2340)	1810 (1620 to 2040)	1450 (1230 to 1710)	1300 (1050 to 1610)	1560 (1280 to 1910)	1610 (1310 to 1990)
Nigeria	2550 (1830 to 3720)	2330 (1710 to 3200)	2130 (1660 to 2720)	1770 (1430 to 2150)	1740 (1440 to 2050)	1750 (1430 to 2110)	1440 (1170 to 1760)	1220 (988 to 1510)	1390 (1140 to 1700)	1500 (1250 to 1810)
São Tomé and Príncipe	2950 (2610 to 3330)	2350 (2130 to 2580)	1650 (1500 to 1800)	1340 (1210 to 1480)	1450 (1290 to 1630)	1240 (1090 to 1410)	1130 (967 to 1280)	1060 (927 to 1210)	1180 (1030 to 1360)	1210 (1060 to 1390)

Senegal	2620<br>(1960 to 3510)	2450<br>(1940 to 3030)	2360<br>(1990 to 2760)	2010<br>(1750 to 2290)	1650<br>(1480 to 1840)	1520<br>(1360 to 1690)	1250<br>(1080 to 1420)	1140<br>(920 to 1400)	1380<br>(1130 to 1670)	1550<br>(1270 to 1870)
Sierra Leone	3150<br>(2450 to 3980)	2790<br>(2280 to 3360)	2470<br>(2050 to 2980)	2210<br>(1850 to 2620)	2020<br>(1750 to 2340)	2030<br>(1820 to 2340)	1750<br>(1500 to 2020)	1390<br>(1140 to 1730)	1650<br>(1360 to 2030)	1610<br>(1330 to 1990)
Togo	2860<br>(2300 to 3560)	2540<br>(2080 to 3010)	2080<br>(1760 to 2460)	1720<br>(1490 to 1980)	1660<br>(1420 to 1940)	1890<br>(1600 to 2210)	1780<br>(1570 to 2030)	1490<br>(1190 to 1870)	1620<br>(1290 to 2030)	1780<br>(1430 to 2250)

Appendix 2 Table S4a: Life expectancy at birth with 95% UIs by location, both sexes combined, 1950, 1960, 1970, 1980, 1990, 2000, 2010, 2019, 2020, 2021

Location	1950	1960	1970	1980	1990	2000	2010	2019	2020	2021
Global	49 (46·7 to 51·3)	46 (39·7 to 50)	58·7 (57·8 to 59·5)	62·2 (61·5 to 62·9)	65·5 (65 to 66)	67·2 (66·8 to 67·7)	70·5 (70·1 to 71)	73·3 (72·6 to 74)	72·4 (71·6 to 73·1)	71·7 (70·9 to 72·5)
Central Europe, eastern Europe, and central Asia	60·8 (59·2 to 62·3)	66·7 (66·3 to 67·1)	68·2 (67·9 to 68·5)	68·3 (68·1 to 68·4)	69·4 (69·3 to 69·5)	67·9 (67·8 to 68·1)	70·9 (70·8 to 71·1)	74·1 (73·8 to 74·4)	72·7 (72·4 to 73)	71·5 (71 to 71·8)
Central Asia	48·8 (44·4 to 53)	58 (56 to 60)	63·4 (62·2 to 64·7)	66·6 (66·1 to 67·1)	67·9 (67·5 to 68·3)	67·1 (66·6 to 67·5)	69·9 (69·6 to 70·3)	73·1 (72·5 to 73·6)	71·1 (70·4 to 71·7)	70·8 (69·8 to 71·8)
Armenia	49·3 (44·2 to 53·9)	60·1 (57·4 to 62·9)	66·9 (64·9 to 68·9)	70·8 (69·7 to 72)	70·7 (70·2 to 71·1)	72·2 (71·8 to 72·5)	73·9 (73·6 to 74·1)	76·7 (76·4 to 77)	69·5 (69·1 to 69·9)	75 (74·1 to 76)
Azerbaijan	37·1 (32·5 to 42)	50·6 (47·6 to 53·6)	60·4 (58·7 to 62)	66·2 (65·2 to 67·1)	66·8 (66 to 67·6)	67·3 (66·5 to 68·1)	70·1 (69·4 to 70·8)	72·9 (72 to 73·7)	69·7 (69·1 to 70·4)	70·1 (69·2 to 71·2)
Georgia	52·5 (48 to 56·9)	60·4 (58·3 to 62·6)	65·7 (64·2 to 67·3)	69·3 (68·5 to 70·1)	69·6 (69·1 to 70)	69·8 (68·7 to 71)	72·8 (72·5 to 73·1)	74·6 (74·3 to 74·9)	73·2 (72·9 to 73·4)	71·5 (71·2 to 71·7)
Kazakhstan	56·8 (53 to 60·5)	61·3 (59·8 to 62·9)	65 (64 to 66)	67 (66·4 to 67·6)	68 (67·4 to 68·7)	64·8 (64·2 to 65·3)	68 (67·5 to 68·5)	72·7 (72·1 to 73·3)	70·9 (70·4 to 71·4)	69·6 (68·7 to 70·4)
Kyrgyzstan	48·2 (43·7 to 52·9)	55·1 (52 to 57·9)	60·2 (57·6 to 62·5)	64·3 (63·1 to 65·5)	66·8 (65·8 to 67·8)	66·9 (66·1 to 67·6)	69·4 (68·7 to 70·1)	74 (73·2 to 74·9)	71·8 (70·6 to 72·9)	72·3 (70·7 to 73·9)
Mongolia	38·3 (32·4 to 44·1)	49·4 (46·2 to 52·6)	55 (52·5 to 57·6)	59·9 (58·1 to 61·9)	62·5 (61·6 to 63·5)	63·7 (62·7 to 64·6)	66·7 (65·8 to 67·5)	70·5 (69 to 72)	71·1 (70·2 to 72)	70 (69·1 to 71)
Tajikistan	40 (34·1 to 45·8)	51·3 (48·3 to 54·2)	60·6 (57·8 to 63·2)	64·4 (62·8 to 66·1)	66·2 (65·2 to 67·2)	66·8 (65·8 to 67·9)	69·7 (68·4 to 70·9)	73·2 (71·7 to 74·7)	70·1 (68·8 to 71·5)	69·3 (67·8 to 71)
Turkmenistan	46·4 (41·5 to 50·8)	56·1 (54·2 to 57·9)	61·4 (59·6 to 63)	64·3 (63·2 to 65·3)	66 (65·4 to 66·6)	66·1 (65·6 to 66·7)	69·3 (68·8 to 69·8)	70 (67·7 to 72·5)	68·1 (65·9 to 70·4)	67·8 (65·5 to 70·1)
Uzbekistan	49·7 (43·7 to 55·3)	61·3 (59·1 to 63·6)	65·2 (64 to 66·4)	67·5 (66·9 to 68·2)	69·5 (69·1 to 69·9)	68·6 (68 to 69·2)	70·7 (70·1 to 71·3)	73·4 (72·7 to 74·2)	72·2 (70·9 to 73·5)	72·5 (70·8 to 74·2)
Central Europe	56·8 (56 to 57·5)	66·7 (66·6 to 66·9)	68·9 (68·8 to 69)	70 (70 to 70·1)	70·7 (70·6 to 70·7)	72·7 (72·6 to 72·8)	75·3 (75·3 to 75·4)	77 (76·9 to 77·1)	75·8 (75·7 to 75·9)	74·7 (74·5 to 74·8)
Albania	49·9 (45·9 to 53·4)	62·9 (61·1 to 64·7)	68·3 (65·7 to 71)	71·3 (69·3 to 73·4)	72·5 (72·1 to 73)	75 (74·5 to 75·6)	78 (76·9 to 79·1)	78 (76·9 to 79)	76·9 (75·9 to 78)	76 (74·7 to 77·5)
Bosnia and Herzegovina	46·5 (44·5 to 48·5)	61·9 (61 to 62·7)	66·3 (65·8 to 66·8)	69·8 (69·5 to 70·2)	73·4 (73·2 to 73·6)	75·4 (74·5 to 76·2)	77·1 (76·7 to 77·4)	77·2 (76·9 to 77·6)	75·9 (75·7 to 76·2)	75·4 (73·8 to 77·1)
Bulgaria	57 (56·4 to 57·7)	69·3 (69·1 to 69·5)	71·4 (71·2 to 71·5)	71·1 (71 to 71·3)	69·9 (69·6 to 70·2)	70 (69·7 to 70·4)	72·2 (71·9 to 72·5)	73·5 (73·1 to 73·9)	71·9 (71·6 to 72·3)	69·9 (69·4 to 70·3)
Croatia	50·9 (50·1 to 51·7)	64·9 (64·7 to 65·1)	68·7 (68·5 to 69)	71·1 (70·9 to 71·3)	71·9 (71·8 to 72·1)	74·5 (73·9 to 75·2)	76·9 (76·7 to 77·1)	78·8 (78·5 to 79)	78·1 (77·8 to 78·4)	77·2 (76·9 to 77·5)
Czechia	65·9 (65·6 to 66·2)	70·3 (70·2 to 70·5)	69·6 (69·5 to 69·7)	70·4 (70·3 to 70·6)	71·5 (71·4 to 71·6)	75·1 (75 to 75·2)	77·8 (77·6 to 78)	79·5 (79·3 to 79·7)	78·4 (78·3 to 78·6)	77·6 (77·3 to 77·8)
Hungary	60 (59·4 to 60·7)	68·2 (68 to 68·3)	69·2 (69 to 69·3)	69·1 (68·9 to 69·2)	69·4 (69·3 to 69·5)	71·8 (71·7 to 71·9)	74·7 (74·6 to 74·8)	76·7 (76·4 to 76·9)	75·8 (75·6 to 76)	74·5 (74·3 to 74·6)
Montenegro	65·7 (64·5 to 66·7)	71·9 (71·2 to 72·7)	73·8 (73 to 74·6)	73·4 (72·7 to 74·2)	74·9 (74·4 to 75·4)	73·8 (73·4 to 74·2)	75·1 (74·7 to 75·5)	75·2 (74·6 to 75·8)	73·9 (73·4 to 74·3)	72·7 (72·1 to 73·3)
North Macedonia	49·7 (47·5 to 51·7)	58·8 (57·7 to 60)	65·9 (65·4 to 66·5)	70·2 (69·7 to 70·7)	70·4 (70 to 70·7)	71·4 (71·1 to 71·7)	73·3 (73 to 73·5)	75·1 (74·7 to 75·5)	73·1 (72·7 to 73·5)	71·5 (70·4 to 72·7)
Poland	56·4 (56·1 to 56·7)	67·9 (67·8 to 68)	70·2 (70·1 to 70·2)	70·5 (70·4 to 70·5)	71 (71 to 71·1)	73·9 (73·8 to 73·9)	76·4 (76·3 to 76·4)	78 (77·9 to 78·1)	76·6 (76·5 to 76·7)	75·7 (75·6 to 75·8)
Romania	59·4 (56·2 to 62·4)	65·6 (65·4 to 65·8)	68·3 (68·1 to 68·4)	69·4 (69·3 to 69·6)	69·7 (69·6 to 69·8)	71·1 (71 to 71·2)	73·7 (73·6 to 73·8)	75·5 (75·4 to 75·6)	74·2 (74·1 to 74·4)	72·9 (72·8 to 73)
Serbia	48·1 (47·2 to 48·8)	64·1 (63·9 to 64·2)	66 (65·9 to 66·2)	69 (68·9 to 69·1)	70·1 (70 to 70·2)	71·1 (70·9 to 71·3)	74·2 (74 to 74·3)	76 (75·8 to 76·1)	74·6 (74·5 to 74·7)	74·1 (74 to 74·3)
Slovakia	62·6 (62·2 to 63)	70·3 (70·1 to 70·5)	69·9 (69·7 to 70)	70·6 (70·4 to 70·8)	71 (70·9 to 71·2)	73·7 (73·4 to 73·8)	75·8 (75·6 to 76)	77·7 (77·5 to 77·9)	76·9 (76·7 to 77·1)	74·7 (74·6 to 74·9)
Slovenia	56·1 (55·3 to 56·7)	66·5 (66·2 to 66·8)	69 (68·7 to 69·2)	71·3 (71 to 71·5)	74·2 (73·9 to 74·5)	76·3 (76 to 76·6)	79·7 (79·4 to 80)	81·6 (81·2 to 82·1)	80·7 (80·3 to 81)	80·8 (80·4 to 81·3)
Eastern Europe	66·1 (64·2 to 67·9)	69·2 (69 to 69·3)	69·6 (69·5 to 69·7)	68·3 (68·2 to 68·4)	69·8 (69·7 to 69·8)	66·5 (66·4 to 66·5)	69·4 (69·4 to 69·4)	73·3 (72·9 to 73·7)	72 (71·5 to 72·4)	70·4 (69·8 to 70·9)
Belarus	67·6 (63·5 to 71·4)	71·2 (70·4 to 72)	72·1 (71·6 to 72·6)	71 (70·6 to 71·3)	71·2 (71 to 71·5)	68·9 (68·6 to 69·1)	70·5 (70·4 to 70·7)	74·4 (74·2 to 74·5)	72·4 (71·3 to 73·5)	71 (69·2 to 72·7)
Estonia	66·5 (62·8 to 69·8)	69·3 (69 to 69·6)	70·4 (70·1 to 70·7)	69·6 (69·3 to 69·8)	70 (69·7 to 70·3)	70·9 (70·6 to 71·3)	76·1 (75·8 to 76·5)	79 (78·5 to 79·4)	78·7 (78·3 to 79·1)	76·9 (76·5 to 77·3)
Latvia	68·8 (65·2 to 72·1)	70·3 (70 to 70·6)	70·4 (70·2 to 70·7)	69·2 (68·9 to 69·4)	69·7 (69·4 to 69·9)	70·6 (70·3 to 70·8)	73·2 (73 to 73·5)	76 (75·6 to 76·3)	75·6 (75·1 to 76)	73·2 (73 to 73·5)
Lithuania	65·7 (61·6 to 69·6)	69·8 (69·5 to 70·1)	71·3 (71 to 71·5)	70·5 (70·3 to 70·7)	71·3 (71·1 to 71·5)	72·2 (72 to 72·4)	73·1 (72·9 to 73·3)	76·4 (76·1 to 76·6)	75 (74·8 to 75·3)	74·1 (73·8 to 74·4)
Moldova	52·9 (48·4 to 57)	59 (56·8 to 61·3)	63·2 (62 to 64·5)	65·8 (65·2 to 66·3)	68·1 (67·8 to 68·5)	68·8 (68·5 to 69·1)	70·2 (69·9 to 70·4)	74·7 (73·5 to 75·7)	73·5 (73·1 to 73·8)	72·1 (71 to 73·2)
Russia	65·8 (63·2 to 68·2)	68·7 (68·6 to 68·8)	69·1 (69 to 69·1)	67·6 (67·6 to 67·7)	69·4 (69·4 to 69·5)	65·6 (65·6 to 65·7)	68·7 (68·7 to 68·8)	73·3 (73·3 to 73·4)	71·5 (71·5 to 71·6)	70 (69·9 to 70)
Ukraine	68·2 (66 to 70·4)	71 (70·5 to 71·5)	70·8 (70·4 to 71·2)	69·7 (69·4 to 70)	70·5 (70·3 to 70·7)	67·8 (67·7 to 67·9)	70·7 (70·6 to 70·7)	72·6 (70·7 to 74·3)	72·6 (70·5 to 74·6)	71 (68·5 to 73·6)
High income	64·7 (64·5 to 65)	69 (68·9 to 69)	70·8 (70·7 to 70·9)	73·8 (73·7 to 73·8)	76·1 (76·1 to 76·1)	78·3 (78·2 to 78·3)	80·5 (80·5 to 80·5)	81·5 (81·5 to 81·6)	80·6 (80·6 to 80·7)	80·6 (80·5 to 80·7)
Australasia	69·4 (69·2 to 69·5)	71·1 (71 to 71·1)	71·2 (71·1 to 71·2)	74·2 (74·1 to 74·3)	76·6 (76·5 to 76·7)	79·5 (79·4 to 79·5)	81·8 (81·7 to 81·9)	83 (82·9 to 83·1)	83·6 (83·5 to 83·7)	83·2 (83·2 to 83·3)
Australia	69·3 (69·1 to 69·5)	71 (71 to 71·1)	71·1 (71 to 71·2)	74·4 (74·3 to 74·5)	76·8 (76·8 to 76·9)	79·7 (79·6 to 79·7)	82 (81·9 to 82)	83·2 (83·1 to 83·3)	83·8 (83·7 to 83·9)	83·4 (83·3 to 83·5)
New Zealand	69·5 (69·2 to 69·7)	71·2 (71 to 71·3)	71·6 (71·4 to 71·7)	73·3 (73·2 to 73·4)	75·5 (75·4 to 75·6)	78·5 (78·3 to 78·6)	80·9 (80·8 to 81·1)	81·9 (81·8 to 82·1)	82·6 (82·5 to 82·8)	82·4 (82·3 to 82·6)
High-income Asia Pacific	55·5 (54·7 to 56·1)	65·5 (65·1 to 65·8)	70 (69·8 to 70·3)	74·4 (74·2 to 74·5)	77·8 (77·7 to 77·8)	80·6 (80·5 to 80·6)	82·9 (82·8 to 82·9)	84·7 (84·7 to 84·7)	85 (85 to 85·1)	84·8 (84·8 to 84·9)
Brunei	49·2 (47·8 to 50·6)	58·1 (56·6 to 59·6)	66·2 (65·1 to 67·4)	70 (68·8 to 71·2)	71·2 (70·2 to 72·2)	74 (73·2 to 74·7)	75·8 (75·1 to 76·5)	76·4 (75·5 to 77·3)	76·8 (75·5 to 77·9)	76·6 (75·4 to 77·7)
Japan	61·7 (61·5 to 61·9)	68·3 (68·2 to 68·4)	72·6 (72·5 to 72·6)	76·5 (76·4 to 76·5)	79·4 (79·3 to 79·4)	81·6 (81·6 to 81·7)	83·4 (83·3 to 83·4)	85 (84·9 to 85)	85·3 (85·2 to 85·3)	85·2 (85·1 to 85·2)
Aichi	63·2 (61·2 to 65·2)	69 (67·2 to 70·9)	73 (71·3 to 74·7)	76·5 (76·4 to 76·7)	79·3 (79·1 to 79·5)	81·5 (81·3 to 81·6)	83·4 (83·2 to 83·5)	85 (84·8 to 85·2)	85·3 (85·1 to 85·5)	85·2 (85 to 85·5)
Akita	61·7 (59·4 to 63·8)	67·6 (65·8 to 69·7)	71·7 (70 to 73·4)	75·8 (75·6 to 76·1)	78·9 (78·6 to 79·2)	81 (80·7 to 81·3)	82·5 (82·2 to 82·9)	84·1 (83·7 to 84·6)	84·4 (84 to 84·9)	84 (83·5 to 84·5)
Aomori	59·6 (57·4 to 61·8)	66·5 (64·5 to 68·6)	71 (69·1 to 72·7)	75·3 (75 to 75·5)	78·3 (78 to 78·6)	80·4 (80·1 to 80·7)	82 (81·7 to 82·3)	83·2 (82·7 to 83·6)	83·7 (83·3 to 84)	83·4 (82·9 to 83·9)
Chiba	62·2 (60 to 64·3)	68·4 (66·5 to 70·5)	73·1 (71·2 to 74·7)	76·7 (76·5 to 76·9)	79·7 (79·5 to 79·9)	81·6 (81·5 to 81·8)	83·4 (83·1 to 83·5)	84·9 (84·7 to 85·1)	85·2 (85 to 85·4)	85·1 (84·9 to 85·4)
Ehime	61·4 (59·2 to 63·8)	68 (65·9 to 70)	72·4 (70·5 to 74·2)	76·6 (76·3 to 76·9)	79·5 (79·2 to 79·7)	81·4 (81·1 to 81·7)	83·4 (83·1 to 83·7)	84·7 (84·3 to 85·2)	85 (84·6 to 85·4)	85 (84·5 to 85·5)
Fukui	61·1 (58·4 to 63·8)	69·3 (67·4 to 71·2)	73·5 (71·9 to 75)	77·5 (77·2 to 77·8)	80·1 (79·8 to 80·4)	82·6 (82·3 to 82·9)	83·9 (83·6 to 84·3)	85·9 (85·4 to 86·4)	85·8 (85·3 to 86·3)	85·6 (85 to 86·1)
Fukuoka	62 (59·8 to 64·2)	68·3 (66·3 to 70·2)	72·2 (70·1 to 73·9)	76·2 (76 to 76·3)	79·1 (78·9 to 79·2)	81·6 (81·4 to 81·8)	83·5 (83·2 to 83·7)	85 (84·8 to 85·3)	85·4 (85·1 to 85·6)	85·2 (85 to 85·5)
Fukushima	59·6 (57·5 to 61·8)	66·9 (64·9 to 68·8)	71·3 (69·4 to 73)	76 (75·7 to 76·2)	79·3 (79 to 79·5)	81·3 (81 to 81·6)	82·9 (82·6 to 83·2)	84 (83·7 to 84·4)	84·2 (83·9 to 84·6)	84·2 (83·8 to 84·6)
Gifu	62·5 (60·2 to 64·7)	68·5 (66·5 to 70·3)	72·6 (70·9 to 74·2)	76·4 (76·2 to 76·6)	79·6 (79·3 to 79·8)	81·6 (81·3 to 81·9)	83·5 (83·2 to 83·8)	85 (84·6 to 85·3)	85·5 (85·1 to 85·8)	85·2 (84·9 to 85·6)
Gunma	61 (58·7 to 63·1)	67·5 (65·7 to 69·3)	72·3 (70·5 to 73·9)	76·3 (76·1 to 76·6)	79·5 (79·2 to 79·7)	81·6 (81·3 to 81·9)	83·1 (82·8 to 83·3)	84·5 (84·2 to 84·9)	84·8 (84·4 to 85·1)	84·6 (84·2 to 84·9)
Hiroshima	61·2 (59 to 63·4)	68·1 (66 to 70·2)	72·8 (70·9 to 74·5)	76·6 (76·4 to 76·8)	79·5 (79·3 to 79·7)	81·9 (81·7 to 82·2)	83·8 (83·5 to 84)	85·2 (84·9 to 85·6)	85·9 (85·6 to 86·2)	85·7 (85·3 to 86·1)
Hokkaidō	61·6 (59·4 to 63·7)	67·9 (65·9 to 69·8)	71·8 (69·9 to 73·5)	76 (75·8 to 76·1)	79·1 (78·9 to 79·3)	81·7 (81·5 to 81·9)	83·3 (83·1 to 83·5)	84·6 (84·3 to 84·8)	84·8 (84·6 to 85)	84·6 (84·3 to 84·9)
Hyōgo	62·7 (60·4 to 64·8)	68·7 (66·9 to 70·5)	72·6 (70·8 to 74·2)	76·2 (76 to 76·4)	78·9 (78·7 to 79·1)	81·4 (81·2 to 81·5)	83·3 (83·1 to 83·5)	85·3 (85 to 85·6)	85·5 (85·3 to 85·8)	85·4 (85·2 to 85·7)
Ibaraki	59·1 (56·6 to 61·4)	66·7 (64·7 to 68·6)	71·6 (69·8 to 73·3)	75·9 (75·7 to 76·1)	78·8 (78·6 to 79·1)	81 (80·8 to 81·3)	82·7 (82·5 to 82·9)	84·2 (83·8 to 84·5)	84·6 (84·3 to 84·9)	84·5 (84·1 to 84·8)
Ishikawa	60·9 (58·2 to 63·7)	68·7 (66·8 to 70·6)	73 (71·4 to 74·6)	76·8 (76·5 to 77)	79·8 (79·6 to 80·1)	82 (81·7 to 82·3)	83·9 (83·5 to 84·2)	85·7 (85·2 to 86·1)	85·9 (85·5 to 86·3)	85·6 (85·1 to 86)
Iwate	59·5 (57·1 to 61·8)	66·6 (64·7 to 68·4)	71·3 (69·5 to 73)	75·9 (75·6 to 76·2)	79 (78·8 to 79·3)	81·5 (81·2 to 81·8)	82·7 (82·4 to 83)	83·6 (83·2 to 84·1)	84·5 (84 to 84·8)	84·3 (83·8 to 84·8)
Kagawa	62·3 (60 to 64·7)	68·5 (66·4 to 70·5)	73·2 (71·5 to 74·7)	77·2 (77 to 77·5)	79·7 (79·4 to 80)	81·8 (81·5 to 82·1)	83·5 (83·2 to 83·8)	85·2 (84·8 to 85·7)	85·4 (85 to 85·9)	85·4 (85 to 85·9)
Kagoshima	58·1 (55·7 to 60·5)	66·3 (64·3 to 68·2)	70·7 (68·8 to 72·5)	75·7 (75·5 to 76)	79·1 (78·8 to 79·4)	81·5 (81·2 to 81·7)	83·1 (82·9 to 83·4)	84·4 (84 to 84·8)	85 (84·6 to 85·3)	84·8 (84·4 to 85·3)
Kanagawa	64·2 (62 to 66·2)	70·1 (68·2 to 72)	74 (72·3 to 75·6)	77·1 (77 to 77·3)	79·7 (79·5 to 79·8)	81·8 (81·6 to 81·9)	83·7 (83·5 to 83·9)	85·5 (85·3 to 85·7)	85·7 (85·6 to 85·9)	85·5 (85·3 to 85·8)
Kōchi	58·8 (56·5 to 61·1)	66·3 (64 to 68·4)	71 (69·3 to 72·7)	75·8 (75·5 to 76·1)	79·2 (78·9 to 79·5)	81·4 (81·1 to 81·8)	83 (82·6 to 83·4)	84·8 (84·3 to 85·3)	85·1 (84·6 to 85·5)	84·9 (84·4 to 85·4)
Kumamoto	60·5 (58·2 to 62·6)	67·3 (65·3 to 69·1)	71·7 (69·9 to 73·4)	76·6 (76·3 to 76·8)	80 (79·7 to 80·2)	82·6 (82·3 to 82·8)	84·2 (83·8 to 84·5)	85·7 (85·3 to 86·2)	86·1 (85·8 to 86·4)	85·7 (85·2 to 86·1)

Kyōto	63·3 (61·1 to 65·4)	69·3 (67·4 to 71·3)	73·5 (71·6 to 75·2)	77·1 (76·8 to 77·3)	79·6 (79·4 to 79·9)	82 (81·7 to 82·2)	83·9 (83·7 to 84·2)	86 (85·6 to 86·4)	86·4 (86 to 86·7)	86 (85·6 to 86·4)
Mie	62·4 (60·1 to 64·5)	68·5 (66·5 to 70·3)	73 (71·2 to 74·6)	76·7 (76·4 to 76·9)	79·3 (79·1 to 79·6)	81·7 (81·4 to 82)	83·4 (83·1 to 83·7)	85·1 (84·7 to 85·5)	85·6 (85·2 to 85·9)	85·2 (84·8 to 85·7)
Miyagi	61·6 (59·4 to 63·8)	68·1 (66·2 to 70·1)	72·2 (70·3 to 73·9)	76·4 (76·2 to 76·6)	79·4 (79·2 to 79·6)	81·7 (81·4 to 81·9)	83·5 (83·2 to 83·8)	85·1 (84·7 to 85·4)	85·4 (85·1 to 85·7)	85·4 (85 to 85·8)
Miyazaki	60 (57·7 to 62·3)	66·9 (64·8 to 69)	71·6 (69·8 to 73·3)	76 (75·7 to 76·3)	79·3 (79 to 79·6)	81·8 (81·5 to 82·1)	83·5 (83·2 to 83·8)	84·6 (84·1 to 85·1)	85·1 (84·7 to 85·5)	84·6 (84·1 to 85·1)
Nagano	61·2 (59 to 63·3)	68·2 (66·3 to 70·2)	73 (71·3 to 74·7)	77·2 (76·9 to 77·4)	80·4 (80·1 to 80·6)	82·5 (82·2 to 82·8)	84·3 (84 to 84·6)	85·8 (85·4 to 86·2)	86·1 (85·7 to 86·4)	86·1 (85·7 to 86·5)
Nagasaki	60·9 (58·6 to 63·3)	67·7 (65·8 to 69·7)	71·5 (69·7 to 73·3)	75·9 (75·6 to 76·1)	79·1 (78·8 to 79·4)	81·6 (81·3 to 81·9)	83·2 (82·9 to 83·5)	84·7 (84·3 to 85·2)	85·3 (84·9 to 85·7)	84·6 (84·1 to 85·1)
Nara	62·6 (60·3 to 64·7)	68·7 (66·7 to 70·5)	72·8 (71 to 74·3)	76·3 (76·1 to 76·6)	79·3 (79 to 79·5)	82 (81·7 to 82·3)	83·8 (83·4 to 84·1)	85·2 (84·8 to 85·6)	86·1 (85·7 to 86·4)	85·6 (85·1 to 86·1)
Niigata	61·5 (59·3 to 63·4)	68·1 (66·1 to 69·9)	72·3 (70·6 to 73·9)	76·5 (76·3 to 76·7)	79·9 (79·6 to 80·1)	82 (81·8 to 82·3)	83·7 (83·4 to 83·9)	84·6 (84·2 to 85)	85 (84·7 to 85·3)	84·9 (84·5 to 85·3)
Ōita	60·8 (58·6 to 63·1)	67·6 (65·5 to 69·5)	72·1 (70·2 to 73·7)	76·2 (75·9 to 76·4)	79·4 (79·1 to 79·7)	81·8 (81·5 to 82·1)	83·9 (83·6 to 84·2)	85·2 (84·7 to 85·7)	85·3 (84·9 to 85·7)	86 (85·5 to 86·4)
Okayama	62·2 (59·9 to 64·5)	68·5 (66·5 to 70·6)	73·3 (71·4 to 75)	77·2 (76·9 to 77·4)	79·8 (79·6 to 80·1)	82·1 (81·8 to 82·4)	83·7 (83·4 to 84)	85·2 (84·8 to 85·6)	85·7 (85·3 to 86)	85·8 (85·4 to 86·2)
Okinawa	56·5 (54 to 58·8)	66·7 (64·5 to 68·9)	70·7 (68·8 to 72·7)	77·1 (76·8 to 77·4)	80·6 (80·3 to 80·8)	82·4 (82·1 to 82·7)	83·6 (83·3 to 83·9)	85 (84·5 to 85·4)	85·2 (84·9 to 85·6)	84·8 (84·3 to 85·3)
Ōsaka	62·4 (60·3 to 64·5)	68·5 (66·7 to 70·3)	72·5 (70·9 to 74·1)	75·8 (75·6 to 75·9)	78·2 (78·1 to 78·4)	80·9 (80·8 to 81·1)	82·8 (82·6 to 82·9)	84·5 (84·3 to 84·7)	84·8 (84·6 to 85)	84·7 (84·5 to 84·9)
Saga	60·9 (58·5 to 63)	67·8 (65·8 to 69·8)	72·2 (70·6 to 73·7)	76·5 (76·2 to 76·8)	79·4 (79·1 to 79·7)	81·7 (81·3 to 82)	83·5 (83·1 to 83·9)	85 (84·5 to 85·5)	85·2 (84·8 to 85·6)	85·3 (84·8 to 85·8)
Saitama	62·5 (60·3 to 64·7)	68·8 (66·9 to 70·8)	73·2 (71·5 to 74·9)	76·5 (76·3 to 76·6)	79·3 (79·1 to 79·5)	81·7 (81·5 to 81·9)	83·2 (83 to 83·4)	84·8 (84·5 to 85)	85 (84·8 to 85·2)	84·9 (84·7 to 85·2)
Shiga	61·9 (59·7 to 64)	68 (66 to 69·9)	72·2 (70·7 to 73·7)	76·4 (76·2 to 76·7)	79·4 (79·2 to 79·7)	82·1 (81·9 to 82·4)	84·1 (83·7 to 84·3)	85·6 (85·2 to 86)	85·8 (85·5 to 86·2)	86·3 (85·9 to 86·7)
Shimane	59 (56·4 to 61·6)	67·9 (65·7 to 69·9)	72·2 (70·4 to 73·8)	76·9 (76·5 to 77·2)	80·3 (79·9 to 80·6)	82·6 (82·2 to 83)	83·8 (83·5 to 84·2)	86 (85·4 to 86·6)	86·5 (86 to 86·9)	85·8 (85·2 to 86·3)
Shizuoka	63·2 (60·9 to 65·3)	69·5 (67·5 to 71·4)	73·5 (71·6 to 75·1)	77·1 (76·9 to 77·3)	79·9 (79·7 to 80·1)	82 (81·8 to 82·3)	83·5 (83·3 to 83·8)	84·9 (84·6 to 85·2)	85·2 (84·9 to 85·5)	85·2 (84·9 to 85·5)
Tochigi	60·5 (58·3 to 62·8)	67·3 (65·3 to 69·2)	71·8 (70·1 to 73·6)	75·7 (75·5 to 75·9)	78·6 (78·4 to 78·8)	81·1 (80·8 to 81·3)	82·6 (82·3 to 82·8)	84·1 (83·8 to 84·5)	84·6 (84·3 to 84·9)	84·5 (84·1 to 84·9)
Tokushima	60·5 (58 to 62·7)	67·1 (65 to 69·2)	71·6 (69·9 to 73·2)	75·8 (75·5 to 76)	79 (78·7 to 79·3)	81·6 (81·3 to 81·9)	83 (82·6 to 83·3)	84·6 (84·1 to 85·2)	85·7 (85·3 to 86·2)	84·3 (83·8 to 84·8)
Tōkyō	64·6 (62·4 to 66·7)	70 (68·1 to 71·9)	73·5 (71·8 to 75·2)	77·1 (77 to 77·2)	79·3 (79·2 to 79·4)	81·4 (81·3 to 81·6)	83·5 (83·3 to 83·6)	85·3 (85·2 to 85·5)	85·6 (85·5 to 85·8)	85·5 (85·4 to 85·7)
Tottori	58·9 (56·1 to 61·6)	67·8 (65·9 to 69·8)	72·3 (70·8 to 73·7)	76·6 (76·2 to 76·8)	79·3 (78·9 to 79·6)	81·6 (81·2 to 82)	83·2 (82·8 to 83·5)	84·4 (83·9 to 85)	84·9 (84·5 to 85·4)	84·9 (84·3 to 85·4)
Toyama	61·8 (59·6 to 64)	67·9 (65·8 to 69·7)	72·4 (70·7 to 74)	76·6 (76·3 to 76·9)	79·8 (79·5 to 80·1)	82·4 (82·1 to 82·7)	83·9 (83·5 to 84·2)	85·5 (85 to 86)	85·8 (85·4 to 86·3)	85·5 (85 to 86)
Wakayama	60·9 (58·6 to 63·2)	67·6 (65·7 to 69·4)	72·1 (70·2 to 73·7)	75·9 (75·7 to 76·2)	78·8 (78·5 to 79·1)	81·1 (80·8 to 81·4)	82·6 (82·3 to 83)	84·1 (83·6 to 84·6)	84·4 (84 to 84·8)	84·6 (84·1 to 85·1)
Yamagata	61·1 (58·9 to 63·4)	67·6 (65·7 to 69·4)	71·7 (70 to 73·2)	76·2 (75·9 to 76·5)	79·8 (79·5 to 80·1)	81·9 (81·5 to 82·2)	83·8 (83·5 to 84·2)	84·8 (84·3 to 85·4)	85·5 (85·1 to 85·9)	85 (84·5 to 85·4)
Yamaguchi	61·6 (59·4 to 63·9)	68 (65·9 to 69·9)	72·5 (70·6 to 74·2)	76·2 (75·9 to 76·4)	79·3 (79·1 to 79·6)	81·4 (81·1 to 81·7)	83 (82·7 to 83·4)	84·6 (84·1 to 85)	84·8 (84·5 to 85·2)	84·8 (84·4 to 85·3)
Yamanashi	61 (58·6 to 63·4)	68 (65·9 to 70)	72·5 (70·8 to 74·1)	76·5 (76·2 to 76·8)	79·5 (79·2 to 79·8)	81·8 (81·5 to 82·1)	83·7 (83·3 to 84)	84·8 (84·3 to 85·4)	85·5 (85·1 to 85·9)	85·3 (84·8 to 85·7)
South Korea	36·9 (34·8 to 38·8)	57·9 (56·8 to 59)	62·3 (61·3 to 63·2)	67 (66·7 to 67·3)	72 (71·8 to 72·1)	76·2 (76·2 to 76·3)	80·7 (80·7 to 80·8)	83·4 (83·4 to 83·5)	83·7 (83·6 to 83·7)	83·2 (83·1 to 83·4)
Singapore	56·7 (56·1 to 57·2)	65·1 (64·9 to 65·3)	68·5 (68·3 to 68·7)	71·3 (71·2 to 71·5)	75·6 (75·4 to 75·7)	79·2 (79·1 to 79·4)	82·7 (82·6 to 82·8)	85·7 (85·5 to 85·9)	86·1 (85·9 to 86·3)	85·7 (85·5 to 85·9)
High-income North America	68·2 (68·1 to 68·2)	70 (70 to 70·1)	71 (71 to 71·1)	74 (74 to 74)	75·7 (75·7 to 75·8)	77·1 (77·1 to 77·1)	79 (79 to 79·1)	79·4 (79·4 to 79·5)	77·7 (77·7 to 77·8)	77·6 (77·4 to 77·7)
Canada	68·6 (68·4 to 68·7)	71·2 (71·1 to 71·2)	72·7 (72·7 to 72·8)	75·2 (75·1 to 75·2)	77·3 (77·3 to 77·4)	79·3 (79·2 to 79·3)	81·4 (81·4 to 81·5)	82·3 (82·2 to 82·4)	81·7 (81·6 to 81·8)	81·8 (81·7 to 82)
Greenland	49·5 (47·4 to 51·5)	58 (57 to 59·1)	62·7 (61·6 to 63·8)	63·7 (62·2 to 65·2)	64·6 (63·6 to 65·6)	68·6 (67·1 to 69·9)	71·9 (70·9 to 72·9)	74 (72·6 to 75·1)	73·7 (72·4 to 74·8)	73·8 (72·4 to 75)
USA	68·2 (68·1 to 68·2)	69·9 (69·9 to 70)	70·9 (70·8 to 70·9)	73·9 (73·9 to 73·9)	75·6 (75·5 to 75·6)	76·9 (76·8 to 76·9)	78·8 (78·7 to 78·8)	79·1 (79·1 to 79·1)	77·3 (77·3 to 77·3)	77·1 (77 to 77·2)
Alabama	65·9 (63·8 to 68·2)	68·2 (67·9 to 68·4)	69·3 (69·1 to 69·5)	72·6 (72·4 to 72·8)	73·8 (73·6 to 74·1)	74·4 (74·2 to 74·7)	75·4 (75·2 to 75·6)	75·7 (75·5 to 75·9)	73·7 (73·5 to 73·9)	73 (71·5 to 74·4)
Alaska	63·8 (61·5 to 66·1)	67·7 (67·2 to 68·3)	69·8 (69·3 to 70·2)	72·2 (71·8 to 72·7)	75 (74·6 to 75·4)	76·7 (76·3 to 77)	77·8 (77·5 to 78·2)	78·5 (78 to 79)	78 (77·5 to 78·4)	75·7 (74·5 to 76·8)
Arizona	66·7 (64·4 to 69·1)	69·2 (68·9 to 69·6)	71 (70·7 to 71·2)	74·3 (74·1 to 74·6)	76·4 (76·2 to 76·7)	77·3 (77·1 to 77·5)	79·3 (79·1 to 79·5)	79·1 (78·9 to 79·3)	76·5 (76·3 to 76·7)	76 (74·6 to 77·3)
Arkansas	68·2 (65·8 to 70·6)	70·2 (69·9 to 70·5)	71 (70·7 to 71·3)	73·6 (73·4 to 73·9)	74·5 (74·2 to 74·7)	75 (74·7 to 75·2)	76 (75·7 to 76·2)	76 (75·7 to 76·2)	74 (73·8 to 74·3)	73·5 (71·9 to 75·1)
California	69·3 (67·2 to 71·4)	70·9 (70·8 to 71·1)	71·8 (71·7 to 71·9)	74·5 (74·4 to 74·6)	76 (75·9 to 76·1)	78·3 (78·2 to 78·4)	80·8 (80·7 to 80·8)	81·4 (81·3 to 81·5)	79·6 (79·6 to 79·7)	79·3 (78 to 80·5)
Colorado	68·7 (66·6 to 70·9)	71·1 (70·8 to 71·4)	72·3 (72 to 72·5)	75·5 (75·2 to 75·7)	77·3 (77 to 77·5)	78·4 (78·2 to 78·6)	80·1 (79·9 to 80·3)	80·5 (80·3 to 80·7)	78·9 (78·8 to 79·1)	78·8 (77·2 to 80·3)
Connecticut	69·4 (67·2 to 71·5)	71·1 (70·9 to 71·4)	72·7 (72·5 to 73)	75 (74·8 to 75·3)	77·3 (77·1 to 77·6)	78·3 (78 to 78·5)	80·6 (80·4 to 80·9)	80·9 (80·6 to 81·2)	79·1 (78·8 to 79·3)	80·3 (78·7 to 81·8)
Delaware	66·4 (64·3 to 68·4)	69·4 (69 to 69·8)	70·2 (69·9 to 70·5)	73·4 (73·1 to 73·8)	75 (74·7 to 75·4)	76·4 (76·1 to 76·8)	78·2 (77·9 to 78·5)	78·4 (78 to 78·8)	77·1 (76·7 to 77·5)	76·7 (75·6 to 77·9)
Washington, DC	63·7 (60·8 to 66·3)	66·1 (65·8 to 66·5)	65·4 (65·1 to 65·8)	68·7 (68·3 to 69·1)	67·3 (66·8 to 67·7)	72·4 (72 to 72·8)	77·3 (76·8 to 77·7)	78·6 (78·1 to 79·1)	76·2 (75·7 to 76·7)	77 (75·5 to 78·7)
Florida	67·9 (65·6 to 70·3)	69·9 (69·7 to 70·1)	70·8 (70·6 to 71)	74·1 (73·9 to 74·2)	76 (75·8 to 76·1)	77·2 (77·1 to 77·3)	79·3 (79·2 to 79·4)	79·5 (79·3 to 79·6)	77·9 (77·8 to 78)	77 (75·6 to 78·5)
Georgia	65·9 (63·5 to 68·2)	67·8 (67·6 to 68)	68·6 (68·4 to 68·8)	72·4 (72·2 to 72·6)	73·9 (73·7 to 74·1)	75·2 (75·1 to 75·4)	77·3 (77·2 to 77·5)	78 (77·9 to 78·2)	76·1 (75·9 to 76·2)	75·3 (73·9 to 76·6)
Hawaii	69·2 (67 to 71·3)	71·5 (71·2 to 71·9)	74 (73·7 to 74·4)	77·2 (76·8 to 77·6)	78·4 (78·1 to 78·8)	79·9 (79·5 to 80·2)	81·2 (80·9 to 81·5)	81·7 (81·4 to 82·1)	81·6 (81·2 to 82)	81·2 (79·6 to 82·8)
Idaho	69·3 (67·1 to 71·4)	71·3 (70·9 to 71·8)	72·1 (71·7 to 72·5)	75·4 (75 to 75·8)	77 (76·6 to 77·4)	78·1 (77·7 to 78·4)	79·3 (79 to 79·6)	79·8 (79·4 to 80·1)	78·7 (78·4 to 79)	77·5 (76·1 to 78·9)
Illinois	68·1 (66 to 70·2)	69·6 (69·5 to 69·7)	70·2 (70·1 to 70·3)	73·5 (73·3 to 73·6)	75·1 (75 to 75·2)	76·5 (76·4 to 76·7)	78·9 (78·8 to 79)	79·4 (79·3 to 79·5)	77·3 (77·1 to 77·4)	77·9 (76·5 to 79·4)
Indiana	69 (67 to 71·1)	70·3 (70·2 to 70·5)	71·1 (70·9 to 71·2)	73·9 (73·7 to 74·1)	75·6 (75·4 to 75·8)	75·9 (75·8 to 76·1)	77·4 (77·2 to 77·6)	77·2 (77 to 77·4)	75·3 (75·2 to 75·5)	75·3 (73·9 to 76·8)
Iowa	70·8 (68·7 to 72·8)	72·2 (72 to 72·4)	72·7 (72·5 to 72·9)	75·9 (75·6 to 76·2)	77·8 (77·6 to 78·1)	78·3 (78 to 78·5)	79·4 (79·2 to 79·7)	79·3 (79 to 79·6)	77·8 (77·6 to 78·1)	78·3 (76·8 to 79·7)
Kansas	70·5 (68·4 to 72·8)	72 (71·7 to 72·2)	72·9 (72·6 to 73·2)	75·3 (75·1 to 75·6)	77·1 (76·9 to 77·4)	77·4 (77·1 to 77·7)	78·4 (78·1 to 78·7)	78·3 (78 to 78·6)	76·7 (76·4 to 77)	76·7 (75·2 to 78·3)
Kentucky	68 (65·7 to 70·2)	69·6 (69·4 to 69·8)	70 (69·8 to 70·2)	73 (72·8 to 73·2)	74·6 (74·4 to 74·9)	75·2 (75 to 75·4)	75·8 (75·6 to 76)	75·8 (75·6 to 76)	73·9 (73·7 to 74·1)	73·2 (71·7 to 74·7)
Louisiana	65·9 (63·6 to 68·2)	68·1 (67·8 to 68·3)	68·9 (68·7 to 69·1)	71·9 (71·7 to 72·1)	73·4 (73·2 to 73·6)	74·1 (73·9 to 74·3)	75·7 (75·5 to 75·9)	75·8 (75·6 to 76)	73·3 (73 to 73·5)	73·2 (71·7 to 74·7)
Maine	67·9 (65·7 to 69·9)	70·1 (69·8 to 70·5)	71 (70·6 to 71·3)	74·6 (74·2 to 74·9)	76·7 (76·4 to 77·1)	77·4 (77·1 to 77·8)	79 (78·7 to 79·4)	78·8 (78·4 to 79·2)	78·4 (78 to 78·8)	77·5 (75·9 to 79)
Maryland	67·1 (65 to 69·1)	68·7 (68·5 to 68·9)	70·4 (70·2 to 70·6)	73·5 (73·3 to 73·7)	75 (74·8 to 75·2)	76·3 (76·1 to 76·5)	79·2 (79 to 79·4)	79·3 (79·1 to 79·5)	77·6 (77·4 to 77·8)	78·1 (76·5 to 79·8)
Massachusetts	69·1 (67 to 71·2)	70·7 (70·5 to 70·8)	72 (71·9 to 72·2)	75 (74·8 to 75·2)	76·9 (76·8 to 77·1)	78·2 (78 to 78·4)	80·5 (80·3 to 80·6)	80·8 (80·6 to 81)	79·6 (79·4 to 79·7)	80·6 (79·1 to 82·1)
Michigan	68·8 (66·8 to 70·8)	70·2 (70·1 to 70·4)	70·8 (70·7 to 71)	73·8 (73·7 to 74)	75·3 (75·2 to 75·5)	76·4 (76·2 to 76·5)	77·9 (77·8 to 78·1)	78·5 (78·3 to 78·6)	76·6 (76·4 to 76·7)	76·6 (75·2 to 78·1)
Minnesota	70·5 (68·1 to 72·7)	71·9 (71·7 to 72·2)	73·2 (73 to 73·4)	76·2 (76 to 76·4)	77·9 (77·7 to 78·1)	78·8 (78·6 to 79·1)	80·7 (80·5 to 81)	80·8 (80·6 to 81)	79·5 (79·3 to 79·7)	79·7 (78·3 to 81·1)
Mississippi	65 (62·5 to 67·4)	67·5 (67·3 to 67·8)	68·4 (68·1 to 68·6)	71·9 (71·7 to 72·2)	73·4 (73·1 to 73·7)	73·5 (73·3 to 73·8)	74·9 (74·6 to 75·1)	74·6 (74·3 to 74·8)	72 (71·8 to 72·3)	71·9 (70·3 to 73·5)
Missouri	68·7 (66·6 to 70·6)	70·4 (70·2 to 70·6)	70·8 (70·6 to 70·9)	73·8 (73·6 to 74)	75·4 (75·2 to 75·6)	76 (75·8 to 76·2)	77·4 (77·2 to 77·5)	77·3 (77·1 to 77·5)	75·4 (75·3 to 75·6)	75·5 (73·9 to 77)
Montana	67·6 (65·4 to 69·8)	69·7 (69·3 to 70·1)	71·1 (70·7 to 71·5)	74 (73·6 to 74·4)	76·7 (76·3 to 77·1)	77·3 (76·9 to 77·7)	78·5 (78·1 to 78·9)	79 (78·6 to 79·4)	77·6 (77·2 to 78)	77 (75·5 to 78·5)
Nebraska	70·6 (68·4 to 72·8)	72·1 (71·7 to 72·4)	72·7 (72·4 to 73)	75·5 (75·2 to 75·8)	77·1 (76·7 to 77·4)	78·2 (77·9 to 78·5)	79·5 (79·2 to 79·8)	79·6 (79·2 to 79·9)	78·2 (77·9 to 78·5)	78·4 (77 to 79·8)
Nevada	62·6 (60·2 to 64·9)	67·3 (66·8 to 67·9)	69·3 (68·9 to 69·7)	72·6 (72·2 to 73)	74·5 (74·2 to 74·8)	75·9 (75·7 to 76·2)	77·8 (77·6 to 78·1)	78·5 (78·2 to 78·8)	76·6 (76·3 to 76·8)	75·9 (74·5 to 77·3)
New Hampshire	68 (66 to 70·1)	70·6 (70·2 to 71)	71·6 (71·2 to 72)	75·2 (74·9 to 75·6)	76·9 (76·6 to 77·3)	78·3 (78 to 78·6)	80·2 (79·8 to 80·5)	79·8 (79·4 to 80·2)	79·5 (79·1 to 79·9)	79·1 (77·6 to 80·5)
New Jersey	68·1 (66 to 70·2)	70 (69·8 to 70·1)	71·1 (70·9 to 71·2)	74 (73·8 to 74·1)	75·6 (75·5 to 75·8)	77·4 (77·2 to 77·6)	80 (79·8 to 80·2)	80·8 (80·7 to 81)	78·3 (78·1 to 78·5)	79·8 (78·3 to 81·2)
New Mexico	66·6 (63·9 to 69·1)	69·5 (69·1 to 69·9)	70·6 (70·2 to 70·9)	74 (73·7 to 74·4)	76 (75·7 to 76·4)	77·2 (76·9 to 77·5)	78·3 (78 to 78·7)	77·6 (77·3 to 77·9)	75·2 (74·9 to 75·6)	74·9 (73·2 to 76·5)

New York	68 (66·1 to 69·9)	69·7 (69·6 to 69·8)	70·6 (70·5 to 70·7)	73·6 (73·5 to 73·7)	74·8 (74·7 to 75)	77·8 (77·7 to 77·9)	80·3 (80·2 to 80·4)	81·5 (81·4 to 81·6)	78·6 (78·5 to 78·7)	80 (78·6 to 81·4)
North Carolina	66·7 (64·5 to 69·1)	68·3 (68·1 to 68·5)	69·4 (69·3 to 69·6)	73·1 (72·9 to 73·3)	74·8 (74·6 to 74·9)	75·6 (75·5 to 75·8)	77·9 (77·7 to 78·1)	78 (77·8 to 78·1)	76·5 (76·3 to 76·6)	75·8 (74·3 to 77·4)
North Dakota	69·7 (67·7 to 71·9)	71·7 (71·4 to 72·1)	72·9 (72·5 to 73·3)	75·7 (75·3 to 76)	77·8 (77·4 to 78·1)	78·3 (78 to 78·7)	79·4 (79·1 to 79·8)	79·8 (79·3 to 80·3)	78·4 (78 to 78·8)	79·7 (78·4 to 81)
Ohio	68·8 (66·8 to 70·8)	70·3 (70·2 to 70·4)	70·9 (70·8 to 71)	73·4 (73·3 to 73·6)	75·5 (75·3 to 75·6)	76·3 (76·1 to 76·4)	77·5 (77·4 to 77·7)	77·2 (77·1 to 77·3)	75·6 (75·5 to 75·8)	75·3 (73·8 to 76·7)
Oklahoma	69·3 (67·1 to 71·5)	70·8 (70·5 to 71·1)	71·3 (71 to 71·5)	73·7 (73·5 to 74)	75·3 (75·1 to 75·5)	75·1 (74·9 to 75·3)	75·7 (75·4 to 75·9)	76 (75·8 to 76·2)	74·4 (74·2 to 74·6)	73·5 (72 to 75)
Oregon	69·5 (67·4 to 71·7)	71 (70·7 to 71·3)	72·5 (72·2 to 72·8)	75 (74·8 to 75·3)	76·6 (76·3 to 76·9)	77·9 (77·6 to 78·1)	79·5 (79·3 to 79·7)	80·1 (79·8 to 80·3)	79·4 (79·2 to 79·6)	78·3 (76·7 to 79·8)
Pennsylvania	67·9 (65·9 to 69·9)	69·6 (69·5 to 69·7)	70·5 (70·4 to 70·6)	73·5 (73·4 to 73·7)	75·6 (75·4 to 75·7)	76·7 (76·5 to 76·8)	78·5 (78·3 to 78·6)	78·7 (78·6 to 78·8)	77·2 (77 to 77·3)	77 (75·6 to 78·5)
Rhode Island	68·3 (66·2 to 70·3)	70·6 (70·3 to 70·9)	71·9 (71·5 to 72·2)	74·8 (74·4 to 75·1)	76·6 (76·3 to 77)	77·9 (77·5 to 78·2)	79·5 (79·1 to 79·8)	80 (79·6 to 80·5)	78·9 (78·5 to 79·3)	79·6 (78·2 to 81)
South Carolina	64·4 (62·2 to 66·7)	66·5 (66·2 to 66·7)	68·3 (68·1 to 68·5)	72 (71·8 to 72·2)	73·8 (73·6 to 74·1)	74·8 (74·6 to 75)	76·7 (76·5 to 76·9)	76·9 (76·7 to 77·1)	74·8 (74·6 to 75)	74·3 (72·7 to 75·9)
South Dakota	69·1 (67·1 to 71·2)	70·9 (70·5 to 71·2)	72·2 (71·8 to 72·5)	74·9 (74·5 to 75·3)	77 (76·7 to 77·4)	77·6 (77·3 to 78)	79·2 (78·8 to 79·5)	78·9 (78·4 to 79·3)	77·4 (76·9 to 77·8)	78·1 (76·8 to 79·4)
Tennessee	67·7 (65·4 to 69·7)	69·5 (69·3 to 69·7)	70 (69·8 to 70·3)	73·2 (73 to 73·4)	74·3 (74·1 to 74·5)	74·8 (74·7 to 75)	76·2 (76 to 76·4)	76 (75·8 to 76·2)	74·2 (74 to 74·3)	73·5 (71·9 to 75·1)
Texas	68·6 (66·5 to 70·8)	70·4 (70·3 to 70·6)	71 (70·9 to 71·1)	73·7 (73·6 to 73·9)	75·3 (75·2 to 75·4)	76·6 (76·5 to 76·7)	78·5 (78·4 to 78·6)	79·1 (79 to 79·2)	77 (76·9 to 77·1)	76·3 (75 to 77·6)
Utah	69·9 (67·6 to 71·9)	71·7 (71·4 to 72·1)	72·9 (72·5 to 73·2)	75·9 (75·6 to 76·2)	77·9 (77·6 to 78·2)	78·6 (78·4 to 78·9)	79·8 (79·5 to 80)	80·1 (79·8 to 80·3)	79 (78·7 to 79·2)	78·5 (77·2 to 79·7)
Vermont	67·6 (65·6 to 69·6)	70·4 (70·1 to 70·8)	71·9 (71·5 to 72·3)	74·7 (74·4 to 75·1)	76·7 (76·3 to 77·1)	78·1 (77·7 to 78·4)	80 (79·6 to 80·4)	80·2 (79·8 to 80·7)	79·9 (79·4 to 80·4)	79·1 (77·9 to 80·2)
Virginia	66·9 (64·8 to 68·9)	68·9 (68·7 to 69·1)	70·3 (70·2 to 70·5)	73·5 (73·3 to 73·7)	75·4 (75·3 to 75·6)	76·8 (76·7 to 77)	79·1 (78·9 to 79·3)	79·5 (79·4 to 79·7)	78·1 (77·9 to 78·2)	77·5 (76·1 to 79)
Washington	69·5 (67·4 to 71·5)	71·2 (70·9 to 71·4)	71·9 (71·7 to 72·1)	75·2 (75 to 75·4)	76·9 (76·7 to 77·1)	78·2 (78 to 78·4)	80 (79·8 to 80·2)	80·6 (80·4 to 80·8)	79·9 (79·7 to 80)	79·3 (77·9 to 80·9)
West Virginia	67·6 (65·3 to 69·7)	69·6 (69·4 to 69·9)	69·3 (69·1 to 69·6)	72·7 (72·5 to 73)	74·7 (74·3 to 75)	74·9 (74·6 to 75·2)	75·4 (75·1 to 75·7)	74·8 (74·5 to 75·2)	73·3 (73 to 73·6)	72 (70·4 to 73·5)
Wisconsin	69·7 (67·6 to 71·7)	71·2 (71 to 71·4)	72·6 (72·4 to 72·8)	75·5 (75·2 to 75·7)	76·9 (76·7 to 77·1)	77·8 (77·6 to 78)	79·6 (79·4 to 79·8)	79·5 (79·3 to 79·7)	78 (77·8 to 78·2)	78·4 (76·8 to 79·9)
Wyoming	67·4 (65·1 to 69·5)	69·8 (69·3 to 70·3)	70·8 (70·4 to 71·2)	73·9 (73·5 to 74·3)	76·3 (75·9 to 76·7)	77 (76·6 to 77·3)	78·2 (77·8 to 78·5)	78·4 (77·9 to 78·9)	77·3 (76·8 to 77·8)	76 (74·9 to 77·2)
Southern Latin America	61·2 (60·9 to 61·4)	62·2 (62·1 to 62·3)	65·3 (65·2 to 65·4)	70·1 (70·1 to 70·2)	72·7 (72·7 to 72·8)	74·9 (74·8 to 75)	76·6 (76·5 to 76·6)	77·9 (77·8 to 77·9)	77·2 (77 to 77·3)	76·8 (76·6 to 77·1)
Argentina	63·9 (63·7 to 64·2)	65 (64·9 to 65·1)	66 (65·8 to 66·1)	70·2 (70·1 to 70·3)	72·4 (72·4 to 72·5)	74·2 (74·1 to 74·3)	75·8 (75·7 to 75·9)	76·9 (76·8 to 77)	76·1 (76 to 76·3)	76·1 (75·7 to 76·3)
Chile	52·8 (52·5 to 53·2)	54·9 (54·6 to 55·1)	62·7 (62·6 to 62·9)	69·8 (69·7 to 69·9)	73·5 (73·4 to 73·6)	76·9 (76·9 to 77)	78·6 (78·6 to 78·7)	80·6 (80·4 to 80·7)	79·5 (79·4 to 79·6)	79 (78·9 to 79·2)
Uruguay	66·6 (66·3 to 67)	68 (67·8 to 68·3)	68·3 (68·1 to 68·5)	70·5 (70·4 to 70·7)	73·1 (72·9 to 73·2)	74·7 (74·6 to 74·9)	76·4 (76·1 to 76·7)	77·1 (77 to 77·4)	78 (77·7 to 78·3)	75·7 (75·3 to 76)
Western Europe	66·9 (66·7 to 67·1)	70·1 (70·1 to 70·2)	71·6 (71·6 to 71·6)	74 (74 to 74)	76·3 (76·3 to 76·3)	78·6 (78·6 to 78·6)	81·1 (81 to 81·1)	82·3 (82·3 to 82·4)	81·6 (81·6 to 81·7)	81·8 (81·7 to 81·9)
Andorra	73·7 (70·7 to 76·9)	74·2 (71·7 to 77·3)	75·7 (72·7 to 78·7)	77·1 (73·8 to 80)	78·6 (75·6 to 81·3)	80 (77·7 to 82·4)	81·7 (80 to 83·5)	82·7 (80·6 to 84·9)	82·8 (80·7 to 85)	83 (80·5 to 85·6)
Austria	66·2 (65·9 to 66·4)	68·8 (68·7 to 68·9)	70·1 (70 to 70·3)	72·7 (72·6 to 72·8)	75·8 (75·8 to 75·9)	78·4 (78·4 to 78·5)	80·7 (80·6 to 80·8)	82·1 (81·9 to 82·2)	81·4 (81·3 to 81·5)	81·7 (81·5 to 81·8)
Belgium	66 (65·8 to 66·3)	70 (69·9 to 70·1)	71 (70·9 to 71·1)	73·2 (73·1 to 73·3)	76 (75·9 to 76·1)	77·9 (77·8 to 78)	80·2 (80·1 to 80·3)	81·8 (81·6 to 81·9)	80·6 (80·5 to 80·7)	81·8 (81·6 to 81·9)
Cyprus	58·7 (55·7 to 61·5)	62·7 (60·9 to 64·3)	66·6 (65·7 to 67·4)	70·8 (70·6 to 71)	74·4 (74·1 to 74·7)	76·1 (75·5 to 76·7)	79·3 (78·6 to 79·8)	80·8 (80·1 to 81·4)	81·3 (80·6 to 82·1)	81·2 (80·4 to 82)
Denmark	70·7 (70·4 to 70·9)	72·4 (72·2 to 72·5)	73·5 (73·3 to 73·6)	74·3 (74·2 to 74·4)	75·1 (75 to 75·3)	77 (76·9 to 77·2)	79·5 (79·4 to 79·6)	81·5 (81·3 to 81·7)	81·6 (81·4 to 81·7)	81·5 (81·3 to 81·7)
Finland	64·5 (64·2 to 64·7)	69·1 (69 to 69·3)	70·5 (70·3 to 70·6)	74 (73·9 to 74·1)	75·4 (75·2 to 75·5)	78 (77·9 to 78·2)	80·4 (80·3 to 80·6)	82·2 (82 to 82·4)	82·3 (82·1 to 82·4)	82·2 (82 to 82·4)
France	67·2 (67 to 67·3)	71·1 (71·1 to 71·2)	72·5 (72·4 to 72·5)	74·5 (74·5 to 74·6)	77 (77 to 77·1)	79 (79 to 79·1)	81·4 (81·3 to 81·4)	83 (82·9 to 83·1)	82·1 (82 to 82·1)	82·6 (82·5 to 82·7)
Germany	67·3 (66·7 to 67·9)	69·5 (69·4 to 69·6)	70·5 (70·5 to 70·6)	73·2 (73·1 to 73·2)	75·5 (75·5 to 75·6)	78·3 (78·3 to 78·4)	80·4 (80·4 to 80·4)	81·3 (81·3 to 81·4)	81·2 (81·1 to 81·2)	81 (80·9 to 81)
Greece	69·4 (69 to 69·7)	72·5 (72·4 to 72·7)	73·8 (73·7 to 74)	75·2 (75·1 to 75·3)	77·1 (77 to 77·1)	78·3 (78·3 to 78·4)	80·3 (80·2 to 80·4)	81·2 (81 to 81·3)	80·8 (80·6 to 80·9)	80 (79·8 to 80·2)
Iceland	71·5 (70·6 to 72·3)	73·2 (72·9 to 73·6)	73·8 (73·4 to 74·3)	76·4 (76·1 to 76·8)	78 (77·6 to 78·4)	80·2 (79·8 to 80·6)	81·7 (81·3 to 82·2)	83·3 (82·7 to 83·9)	83·6 (83 to 84·2)	83·6 (82·9 to 84·3)
Ireland	66·1 (65·8 to 66·3)	70·1 (70 to 70·3)	71·2 (71 to 71·3)	72·6 (72·5 to 72·8)	74·9 (74·7 to 75)	76·7 (76·5 to 76·8)	80·7 (80·6 to 80·9)	82·3 (82·1 to 82·6)	82·4 (82·2 to 82·6)	82·6 (82·4 to 82·8)
Israel	72·5 (72 to 72·9)	71·8 (71·6 to 72)	71·7 (71·5 to 71·8)	74 (73·9 to 74·2)	77·2 (77·1 to 77·3)	78·8 (78·7 to 78·9)	81·8 (81·7 to 81·9)	83·4 (83·3 to 83·5)	83·3 (83·2 to 83·4)	83·2 (82·9 to 83·4)
Italy	67·1 (66·9 to 67·2)	69·8 (69·7 to 69·8)	71·7 (71·6 to 71·8)	74·1 (74·1 to 74·1)	77 (77 to 77·1)	79·6 (79·5 to 79·6)	82 (81·9 to 82)	83·4 (83·3 to 83·4)	82·2 (82·2 to 82·3)	82·7 (82·6 to 82·7)
Luxembourg	65·8 (65·1 to 66·5)	68·9 (68·6 to 69·3)	69·9 (69·6 to 70·3)	72·6 (72·3 to 72·9)	75·2 (74·9 to 75·5)	78·3 (77·9 to 78·6)	81 (80·7 to 81·4)	82·7 (82·2 to 83·2)	82·2 (81·7 to 82·7)	82·6 (82 to 83·2)
Malta	66 (65·3 to 66·7)	68·9 (68·6 to 69·2)	70·8 (70·5 to 71·1)	72·6 (72·3 to 72·9)	76·4 (76·1 to 76·8)	78·8 (78·4 to 79·2)	81·2 (80·8 to 81·5)	83 (82·4 to 83·6)	83·1 (82·5 to 83·6)	82·7 (81·9 to 83·3)
Monaco	66·3 (64·4 to 68·1)	69·9 (68·3 to 71·8)	72·9 (71·2 to 74·6)	75·6 (74·2 to 76·9)	77·9 (76·1 to 80·4)	78·6 (76·4 to 81·1)	79·4 (78·2 to 80·5)	80·5 (79 to 81·9)	80·1 (78·4 to 81·8)	78·8 (77·2 to 80·4)
Netherlands	71·7 (71·6 to 71·9)	73·4 (73·3 to 73·5)	73·7 (73·6 to 73·7)	75·8 (75·7 to 75·9)	77 (76·9 to 77·1)	78·2 (78·1 to 78·3)	80·9 (80·8 to 81)	82 (81·9 to 82·1)	81·3 (81·2 to 81·5)	81·5 (81·4 to 81·7)
Norway	72·2 (71·9 to 72·4)	73·7 (73·5 to 73·8)	74·2 (74·1 to 74·3)	75·9 (75·7 to 76)	76·9 (76·7 to 77)	78·8 (78·7 to 78·9)	81·2 (81·1 to 81·3)	83·1 (82·9 to 83·2)	83·3 (83·2 to 83·5)	83·3 (83·1 to 83·4)
Agder	72·1 (71·2 to 72·8)	73·4 (73 to 73·8)	74·2 (73·7 to 74·6)	75·5 (75 to 75·9)	76·3 (75·9 to 76·7)	78·5 (78·1 to 78·9)	80·7 (80·3 to 81·1)	82·3 (81·8 to 82·9)	82·7 (82·1 to 83·2)	82·7 (81·4 to 83·8)
Innlandet	72·9 (72·3 to 73·6)	74·2 (73·9 to 74·6)	75 (74·6 to 75·4)	76·2 (75·9 to 76·6)	76·7 (76·4 to 77·1)	78 (77·6 to 78·3)	80·4 (80·1 to 80·8)	82·4 (81·8 to 83)	83·3 (82·8 to 83·9)	83·3 (82·3 to 84·4)
Møre og Romsdal	72·6 (71·7 to 73·3)	74·8 (74·4 to 75·2)	75·1 (74·7 to 75·5)	76·6 (76·1 to 77)	78·2 (77·8 to 78·7)	79·8 (79·4 to 80·3)	82·6 (82·1 to 83)	83·6 (83 to 84·2)	83·5 (82·8 to 84·2)	83·7 (82·3 to 85)
Nordland	71·4 (70·6 to 72·2)	73·5 (73 to 73·9)	73·9 (73·5 to 74·3)	76 (75·5 to 76·4)	76·5 (76 to 76·9)	78·8 (78·3 to 79·3)	80·8 (80·3 to 81·3)	82·5 (81·8 to 83·1)	83 (82·4 to 83·7)	83 (81·7 to 84·4)
Oslo	75·1 (74·3 to 75·9)	73·5 (73·1 to 73·8)	73·8 (73·5 to 74·2)	74·9 (74·6 to 75·3)	75·5 (75·1 to 75·9)	78·3 (77·9 to 78·7)	81·5 (81·1 to 81·8)	83·7 (83·2 to 84·2)	83·6 (83·1 to 84·1)	83·9 (82·8 to 85)
Rogaland	72·3 (71·4 to 73·2)	73·6 (73·2 to 74)	74·3 (74 to 74·8)	76 (75·6 to 76·4)	77·7 (77·3 to 78·1)	79·3 (78·9 to 79·7)	81·4 (81 to 81·8)	83·4 (82·9 to 83·9)	83·3 (82·8 to 83·7)	83·4 (82·4 to 84·3)
Troms og Finnmark	69·3 (68·4 to 70·1)	71·5 (71·1 to 71·9)	72·5 (72 to 72·9)	74·6 (74·2 to 75)	75·5 (75·1 to 76)	77·7 (77·2 to 78·1)	80·6 (80·2 to 81·1)	82·4 (81·8 to 83)	82·2 (81·5 to 82·8)	81·7 (80·3 to 83)
Trøndelag	72·2 (71·5 to 72·9)	73·8 (73·4 to 74·1)	74·2 (73·9 to 74·6)	75·8 (75·5 to 76·2)	76·8 (76·5 to 77·2)	79·3 (78·9 to 79·7)	81·4 (81 to 81·8)	82·9 (82·3 to 83·4)	83·4 (82·8 to 83·9)	83·5 (82·4 to 84·4)
Vestfold og Telemark	72·6 (71·9 to 73·4)	73·7 (73·3 to 74·1)	74·2 (73·8 to 74·5)	75·7 (75·3 to 76·1)	76·8 (76·4 to 77·2)	78·4 (78 to 78·8)	80·8 (80·4 to 81·1)	82·8 (82·3 to 83·4)	83·2 (82·7 to 83·8)	83·1 (82 to 84·3)
Vestland	72·8 (72·2 to 73·5)	74·2 (73·8 to 74·6)	74·8 (74·5 to 75·2)	77 (76·6 to 77·3)	78·1 (77·8 to 78·4)	79·9 (79·5 to 80·2)	81·9 (81·6 to 82·3)	83·7 (83·2 to 84·3)	83·9 (83·4 to 84·4)	84 (83 to 85·1)
Viken	70·4 (69·7 to 71)	73·8 (73·5 to 74·1)	74·2 (73·9 to 74·5)	75·8 (75·5 to 76·2)	76·8 (76·5 to 77·1)	78·8 (78·5 to 79·1)	81·1 (80·8 to 81·4)	83·2 (82·8 to 83·6)	83·4 (83 to 83·8)	83·1 (82·2 to 84)
Portugal	58·5 (58·1 to 58·9)	64·7 (64·6 to 64·9)	67·1 (66·9 to 67·2)	71·7 (71·5 to 71·8)	74·1 (74 to 74·2)	76·7 (76·6 to 76·8)	80·1 (80 to 80·2)	82 (81·9 to 82·1)	81·5 (81·4 to 81·6)	81·5 (81·4 to 81·7)
San Marino	72·5 (68·9 to 75·2)	72·9 (71 to 75)	74·3 (73 to 75·4)	76·5 (75·1 to 78)	79·5 (78·4 to 80·6)	81·4 (80·5 to 82·3)	83·6 (82·7 to 84·4)	83·6 (81·9 to 85)	84·9 (82·9 to 86·7)	86·2 (83·3 to 89)
Spain	62·1 (61·9 to 62·3)	69·4 (69·4 to 69·5)	72 (71·9 to 72·1)	75·5 (75·4 to 75·5)	76·9 (76·8 to 76·9)	79·4 (79·3 to 79·4)	82 (82 to 82·1)	83·2 (83·2 to 83·3)	82·1 (82·1 to 82·2)	82·9 (82·8 to 82·9)
Sweden	71·5 (71·3 to 71·6)	73·3 (73·2 to 73·4)	74·7 (74·7 to 74·8)	76 (75·9 to 76·1)	77·9 (77·8 to 78)	79·9 (79·8 to 80)	81·8 (81·7 to 81·9)	83·3 (83·1 to 83·4)	82·8 (82·7 to 82·9)	83·5 (82·8 to 84·2)
Stockholm	71·6 (69·3 to 73·8)	73·2 (71 to 75·3)	74·3 (72·2 to 76·5)	75·3 (75 to 75·6)	77 (76·7 to 77·3)	79·6 (79·3 to 79·9)	81·6 (81·3 to 81·9)	83·3 (83 to 83·6)	82·5 (82·2 to 82·8)	84·5 (83·4 to 85·6)
Sweden except Stockholm	71·5 (71·1 to 72)	73·3 (72·9 to 73·7)	74·9 (74·4 to 75·3)	76·2 (76·1 to 76·3)	78·1 (78 to 78·2)	80 (79·9 to 80·1)	81·8 (81·7 to 81·9)	83·2 (83·1 to 83·4)	82·9 (82·8 to 83)	83·2 (82·6 to 83·9)
Switzerland	68·9 (68·7 to 69·2)	71·6 (71·5 to 71·7)	73·2 (73·1 to 73·3)	75·8 (75·7 to 75·9)	77·8 (77·7 to 77·9)	80·3 (80·2 to 80·4)	82·9 (82·7 to 83)	84·7 (84·5 to 84·8)	83·7 (83·6 to 83·9)	84·5 (84·3 to 84·7)
UK	69·1 (69 to 69·2)	70·8 (70·8 to 70·9)	71·9 (71·9 to 72)	73·5 (73·5 to 73·6)	75·7 (75·7 to 75·8)	77·8 (77·8 to 77·9)	80·6 (80·5 to 80·6)	81·4 (81·4 to 81·5)	80·5 (80·4 to 80·5)	80·3 (80·2 to 80·3)
England	69·5 (69·4 to 69·6)	71·1 (71·1 to 71·2)	72·2 (72·1 to 72·2)	73·8 (73·7 to 73·8)	76 (75·9 to 76)	78·1 (78 to 78·1)	80·8 (80·8 to 80·9)	81·7 (81·6 to 81·7)	80·7 (80·6 to 80·8)	80·5 (80·4 to 80·6)
East Midlands	69·4 (67·4 to 71·3)	71 (69·1 to 72·9)	72 (70·1 to 73·9)	73·7 (73·2 to 74·2)	76·1 (75·9 to 76·2)	78 (77·8 to 78·2)	80·7 (80·5 to 80·9)	81·4 (81·2 to 81·6)	80·5 (80·3 to 80·7)	80·1 (79·6 to 80·7)
Derby	68·3 (65·5 to 71)	70·1 (67·7 to 72·7)	71·2 (68·8 to 73·5)	72·8 (71·9 to 73·6)	75·2 (74·8 to 75·7)	77·8 (77·4 to 78·3)	79·5 (79·1 to 80)	80·9 (80·3 to 81·4)	79·8 (79·3 to 80·3)	78·7 (78 to 79·5)

Derbyshire	69 (66·6 to 71·7)	70·9 (68·6 to 73·4)	71·8 (69·4 to 74·1)	73·6 (72·9 to 74·3)	75·8 (75·5 to 76·2)	78·1 (77·8 to 78·4)	81 (80·7 to 81·3)	81·3 (80·9 to 81·7)	80·7 (80·3 to 81·1)	80·4 (79·7 to 81)
Leicester	68·1 (65·5 to 70·8)	69·6 (66·9 to 72·2)	70·5 (68 to 73)	72·5 (71·7 to 73·3)	75 (74·6 to 75·4)	76·3 (75·9 to 76·7)	78·9 (78·5 to 79·4)	80·2 (79·6 to 80·7)	79 (78·6 to 79·5)	78·9 (78·2 to 79·6)
Leicestershire	71·1 (68·4 to 73·6)	72·4 (69·9 to 75)	73·4 (70·9 to 75·7)	74·9 (74·1 to 75·6)	77·2 (76·9 to 77·5)	79·2 (78·8 to 79·5)	81·8 (81·4 to 82·1)	82·9 (82·5 to 83·3)	81·7 (81·3 to 82·1)	81·6 (80·9 to 82·2)
Lincolnshire	69·7 (66·9 to 72·4)	71·3 (68·7 to 73·9)	72·3 (69·8 to 74·9)	73·9 (73·2 to 74·7)	76·3 (75·9 to 76·6)	78·1 (77·8 to 78·5)	80·9 (80·6 to 81·3)	81·1 (80·7 to 81·5)	80·5 (80·1 to 80·9)	80·6 (80 to 81·3)
Northamptonshire	69·9 (67·3 to 72·4)	71·6 (69 to 74·1)	72·7 (70·2 to 75·1)	74·3 (73·5 to 75)	76·2 (75·9 to 76·6)	78·3 (77·9 to 78·6)	80·6 (80·3 to 81)	81·4 (80·9 to 81·8)	80·6 (80·2 to 81)	78·9 (77·5 to 80·6)
Nottingham	68·2 (65·6 to 71)	69·6 (67·1 to 72)	70·5 (68 to 72·7)	72·2 (71·4 to 72·9)	74·3 (73·9 to 74·7)	75·1 (74·7 to 75·5)	78·4 (78 to 78·8)	80 (79·4 to 80·6)	78·2 (77·7 to 78·6)	78·6 (77·8 to 79·4)
Nottinghamshire	69·7 (67·1 to 72·2)	71·3 (68·8 to 73·7)	72·3 (69·9 to 74·6)	73·9 (73·2 to 74·6)	76·3 (76 to 76·6)	78·1 (77·8 to 78·4)	80·8 (80·5 to 81·1)	81·4 (81 to 81·8)	80·5 (80·1 to 80·8)	80·4 (79·8 to 81)
Rutland	72 (69·3 to 74·7)	73·6 (70·8 to 76·1)	74·7 (72 to 77·2)	75·9 (74·7 to 77)	77·5 (76·7 to 78·2)	80·1 (79·4 to 80·9)	82·5 (81·7 to 83·4)	83·9 (82·8 to 84·9)	84·1 (83·1 to 85·1)	82·4 (81·2 to 83·6)
East of England	70·8 (69·1 to 72·5)	72·4 (70·5 to 74·2)	73·3 (71·4 to 75·2)	74·8 (74·3 to 75·4)	77 (76·8 to 77·2)	79 (78·8 to 79·2)	81·6 (81·4 to 81·7)	82·4 (82·2 to 82·6)	81·6 (81·5 to 81·8)	81·6 (81·1 to 82·1)
Bedford	70·3 (67·4 to 72·9)	71·8 (69·2 to 74·4)	73 (70·5 to 75·4)	74·7 (73·8 to 75·6)	76·6 (76·1 to 77·1)	78·1 (77·7 to 78·6)	80·5 (80 to 81)	81 (80·3 to 81·7)	79·8 (79·2 to 80·4)	80·7 (79·9 to 81·4)
Cambridgeshire	72·1 (69·5 to 74·5)	73·1 (70·6 to 75·5)	74 (71·6 to 76·4)	75·3 (74·5 to 76)	77·4 (77·1 to 77·8)	79·3 (78·9 to 79·6)	82·4 (82·1 to 82·8)	83 (82·6 to 83·4)	82·3 (81·9 to 82·6)	82·8 (82·1 to 83·4)
Central Bedfordshire	70·9 (68·4 to 73·4)	72·4 (69·7 to 74·9)	73·6 (70·9 to 76·1)	75·2 (74·4 to 76)	77 (76·6 to 77·4)	79·3 (78·9 to 79·7)	81·6 (81·1 to 82·1)	82·9 (82·3 to 83·5)	82·1 (81·5 to 82·6)	82 (81·2 to 82·7)
Essex	70·6 (68·4 to 72·9)	72·3 (70·1 to 74·6)	73·1 (70·8 to 75·6)	74·7 (74 to 75·3)	77·1 (76·8 to 77·3)	79·2 (78·9 to 79·5)	81·6 (81·3 to 81·9)	82·2 (81·9 to 82·6)	81·6 (81·3 to 81·9)	81·2 (80·6 to 81·7)
Hertfordshire	71·1 (68·7 to 73·5)	72·8 (70·3 to 75·4)	73·5 (71·1 to 75·8)	74·8 (74·1 to 75·6)	77·2 (76·9 to 77·5)	79·1 (78·8 to 79·4)	81·9 (81·6 to 82·2)	82·9 (82·5 to 83·2)	81·9 (81·6 to 82·2)	82·5 (81·9 to 83)
Luton	69·2 (66·7 to 71·8)	70·8 (68·1 to 73·2)	71·9 (69·4 to 74·3)	73·7 (72·8 to 74·6)	75·6 (75·1 to 76·1)	77·2 (76·7 to 77·6)	79·9 (79·5 to 80·4)	80·9 (80·3 to 81·5)	78·9 (78·4 to 79·4)	79·5 (78·7 to 80·3)
Norfolk	70·9 (68·2 to 73·3)	72·5 (70 to 75)	73·7 (71·5 to 76)	75·2 (74·5 to 75·9)	77·1 (76·8 to 77·4)	79·1 (78·8 to 79·4)	81·3 (81 to 81·6)	82·1 (81·7 to 82·5)	81·6 (81·2 to 81·9)	81·5 (80·8 to 82)
Peterborough	69·5 (66·7 to 72·1)	71·2 (68·4 to 73·7)	72·1 (69·6 to 74·7)	74·1 (73·2 to 75)	75·7 (75·3 to 76·2)	77·6 (77·1 to 78)	79·8 (79·3 to 80·3)	81·1 (80·4 to 81·7)	80 (79·4 to 80·5)	80·4 (79·7 to 81·2)
Southend-on-Sea	69·4 (66·8 to 71·9)	71·5 (68·9 to 74)	72·8 (70·3 to 75)	74·7 (73·8 to 75·6)	76·4 (75·9 to 76·9)	78·1 (77·6 to 78·5)	80·7 (80·2 to 81·1)	81·6 (80·9 to 82·2)	80·7 (80·1 to 81·2)	79·9 (79·2 to 80·7)
Suffolk	71·1 (68·5 to 73·6)	72·6 (70·1 to 75)	73·7 (71·2 to 76)	75·1 (74·4 to 75·9)	77·4 (77·1 to 77·8)	79·3 (78·9 to 79·6)	82 (81·7 to 82·4)	82·6 (82·2 to 83)	82·4 (82 to 82·7)	82·1 (81·5 to 82·7)
Thurrock	69·9 (67·1 to 72·2)	71 (68·4 to 73·7)	71·9 (69·3 to 74·3)	73·7 (72·7 to 74·5)	75·9 (75·5 to 76·4)	78·6 (78·1 to 79·1)	80·6 (80·1 to 81·1)	83·1 (82·4 to 83·8)	80·8 (80·2 to 81·5)	80·2 (79·3 to 81)
Greater London	69·7 (68 to 71·5)	71·3 (69·6 to 73)	72·3 (70·6 to 74·1)	73·9 (73·4 to 74·3)	75·9 (75·7 to 76)	77·8 (77·7 to 78)	81·7 (81·5 to 81·8)	83·1 (82·9 to 83·3)	81·3 (81·1 to 81·4)	81 (80·5 to 81·5)
Barking and Dagenham	67 (64·5 to 69·4)	69·3 (66·9 to 71·8)	70·6 (68·3 to 72·9)	72·7 (71·8 to 73·5)	75 (74·6 to 75·5)	76·7 (76·2 to 77·2)	79·4 (78·9 to 79·9)	80·8 (80·1 to 81·5)	78·3 (77·7 to 78·8)	78·4 (77·6 to 79·2)
Barnet	71·4 (68·9 to 73·9)	73 (70·7 to 75·4)	74·3 (72 to 76·7)	75·9 (75·2 to 76·6)	77·7 (77·3 to 78·1)	79·8 (79·4 to 80·2)	82·6 (82·2 to 83)	84·7 (84·1 to 85·3)	82·6 (82·1 to 83)	82·2 (81·5 to 82·9)
Bexley	71·1 (68·4 to 73·6)	72·9 (70·5 to 75·2)	74·1 (71·9 to 76·4)	75·7 (74·9 to 76·5)	77·7 (77·3 to 78·2)	79·5 (79·1 to 80)	82·2 (81·7 to 82·7)	83·2 (82·5 to 83·9)	81·2 (80·6 to 81·7)	81·5 (80·7 to 82·3)
Brent	69·4 (66·8 to 72)	71·1 (68·7 to 73·7)	72·4 (70·1 to 74·6)	73·8 (73·1 to 74·7)	75·9 (75·5 to 76·3)	77·9 (77·5 to 78·3)	81·8 (81·4 to 82·2)	83·2 (82·5 to 83·8)	81·2 (80·7 to 81·7)	81·4 (80·6 to 82·1)
Bromley	71·7 (69·1 to 74·1)	73·5 (71·2 to 75·8)	74·6 (72·3 to 76·8)	75·8 (75·1 to 76·6)	77·8 (77·5 to 78·3)	79·6 (79·2 to 80)	83·3 (82·8 to 83·7)	84·6 (84 to 85·2)	83·2 (82·7 to 83·7)	83·4 (82·6 to 84·2)
Camden	70·2 (67·6 to 72·7)	70·6 (68·2 to 73·1)	71·3 (68·9 to 73·6)	72·2 (71·4 to 72·9)	74·5 (74 to 74·9)	75·6 (75·2 to 76)	80·7 (80·3 to 81·1)	82·8 (82·1 to 83·4)	83·6 (83 to 84·1)	83·4 (82·5 to 84·2)
Croydon	70·4 (68 to 72·7)	72·4 (69·9 to 74·7)	73·4 (71·2 to 75·7)	74·6 (73·9 to 75·4)	76·5 (76·1 to 76·9)	78·4 (78 to 78·8)	82 (81·6 to 82·4)	82·6 (82·1 to 83·2)	80·5 (80·1 to 80·8)	81·3 (80·5 to 82)
Ealing	69·5 (66·9 to 72·1)	71·3 (68·8 to 73·6)	72·2 (69·9 to 74·6)	73·5 (72·8 to 74·3)	75·8 (75·4 to 76·3)	78·1 (77·8 to 78·5)	82 (81·6 to 82·5)	83·1 (82·5 to 83·6)	80·5 (80·1 to 80·9)	80·9 (80·2 to 81·7)
Enfield	70·3 (67·7 to 72·8)	72·3 (69·8 to 74·6)	73·5 (71·2 to 75·8)	75 (74·2 to 75·9)	77·1 (76·7 to 77·5)	78·6 (78·2 to 79)	82·5 (82·1 to 83)	82·7 (82·1 to 83·3)	81·7 (81·2 to 82·2)	81·7 (81 to 82·5)
Greenwich	68·1 (65·7 to 70·7)	70·2 (67·9 to 72·6)	71·6 (69·2 to 73·8)	73·4 (72·5 to 74·2)	75·4 (75 to 75·8)	76·7 (76·3 to 77·1)	80·6 (80·2 to 81·1)	81·8 (81·2 to 82·4)	80·2 (79·7 to 80·7)	79·8 (79·1 to 80·6)
Hackney	68·5 (66·1 to 71)	70·1 (67·7 to 72·6)	70·7 (68·3 to 72·9)	71·7 (70·9 to 72·5)	73·5 (73·2 to 73·9)	76·1 (75·7 to 76·5)	80·2 (79·7 to 80·6)	81·6 (81 to 82·2)	79·8 (79·3 to 80·3)	74 (72·2 to 76·1)
Hammersmith and Fulham	69·4 (66·8 to 72·2)	70·3 (67·9 to 72·6)	71·1 (68·8 to 73·6)	72 (71·2 to 72·8)	73·3 (72·9 to 73·8)	76·4 (75·9 to 76·8)	80·8 (80·4 to 81·3)	82·5 (81·8 to 83·2)	79·7 (79·1 to 80·2)	80·7 (79·9 to 81·5)
Haringey	69·5 (66·8 to 72)	70·8 (68·5 to 73·3)	72 (69·7 to 74·3)	73·3 (72·5 to 74·1)	75·7 (75·3 to 76·2)	76·7 (76·3 to 77·1)	80·3 (79·8 to 80·7)	82·7 (82·1 to 83·3)	80·8 (80·4 to 81·3)	81·2 (80·5 to 82)
Harrow	71·2 (68·6 to 73·7)	73·1 (70·7 to 75·4)	74·2 (71·9 to 76·6)	75·8 (75 to 76·6)	78 (77·5 to 78·4)	79·8 (79·4 to 80·3)	83·5 (83 to 84)	84·9 (84·2 to 85·5)	83·4 (82·8 to 84)	83·4 (82·7 to 84·2)
Havering	70·8 (68·3 to 73·3)	72·8 (70·4 to 75·2)	73·8 (71·8 to 76·2)	75·3 (74·6 to 76·1)	77·6 (77·1 to 78)	79·4 (79 to 79·8)	82 (81·5 to 82·5)	83·4 (82·7 to 84)	81·4 (80·9 to 82)	81·3 (80·5 to 82·1)
Hillingdon	70·7 (68·1 to 73·2)	72·5 (70 to 74·8)	73·5 (71·3 to 75·7)	74·9 (74·1 to 75·7)	76·9 (76·4 to 77·3)	78·2 (77·8 to 78·6)	81·6 (81·2 to 82·1)	82·4 (81·8 to 83)	80·5 (80·1 to 81)	80·2 (79·5 to 80·9)
Hounslow	69·9 (67·4 to 72·4)	71·5 (69·1 to 74·1)	72·7 (70·3 to 75)	74·2 (73·5 to 75·1)	75·9 (75·5 to 76·3)	77·6 (77·2 to 78)	81·4 (81 to 81·9)	83·1 (82·5 to 83·7)	81·1 (80·5 to 81·6)	81·3 (80·5 to 82·2)
Islington	69·1 (66·4 to 71·7)	70·3 (67·8 to 72·7)	71·1 (68·7 to 73·4)	72·2 (71·4 to 73·1)	74 (73·5 to 74·4)	75·2 (74·8 to 75·7)	79·5 (79 to 79·9)	81·5 (80·9 to 82·1)	80·6 (80·1 to 81·1)	79·4 (78·6 to 80·2)
Kensington and Chelsea	71·1 (68·6 to 73·6)	71 (68·6 to 73·5)	71·6 (69·2 to 73·8)	72·5 (71·8 to 73·3)	74·8 (74·4 to 75·2)	79·5 (79 to 79·9)	83·4 (82·8 to 83·9)	84·5 (83·7 to 85·3)	84 (83·4 to 84·6)	82·3 (81·4 to 83·2)
Kingston upon Thames	71·7 (69·1 to 74·1)	73·3 (71 to 75·7)	74·4 (72·1 to 76·6)	75·8 (74·9 to 76·7)	77·6 (77·1 to 78·1)	79·7 (79·2 to 80·1)	84 (83·4 to 84·5)	84·9 (84·1 to 85·6)	82·9 (82·3 to 83·5)	82·9 (82·1 to 83·8)
Lambeth	67·6 (65 to 70·1)	69·1 (66·7 to 71·5)	70 (67·6 to 72·3)	71·7 (71 to 72·6)	73·9 (73·5 to 74·3)	75·8 (75·4 to 76·2)	80 (79·6 to 80·4)	82·3 (81·7 to 82·9)	80·4 (79·9 to 80·9)	79·5 (78·8 to 80·2)
Lewisham	68·6 (66·1 to 71)	70·7 (68·3 to 73)	71·9 (69·8 to 74·1)	73·6 (72·8 to 74·4)	74·9 (74·5 to 75·3)	76 (75·7 to 76·4)	80·1 (79·7 to 80·5)	81·7 (81·1 to 82·3)	80·3 (79·8 to 80·7)	79·7 (78·9 to 80·5)
Merton	70·2 (67·7 to 72·6)	72·1 (69·8 to 74·3)	73·3 (71 to 75·5)	75 (74·1 to 75·8)	76·7 (76·3 to 77·2)	79·1 (78·7 to 79·6)	82·7 (82·1 to 83·2)	83·8 (83·1 to 84·5)	81 (80·5 to 81·6)	81·3 (80·5 to 82·1)
Newham	67·7 (65·1 to 70·3)	69·7 (67·4 to 72·2)	70·8 (68·6 to 73·1)	72·6 (71·8 to 73·4)	74·4 (74 to 74·8)	75·5 (75·1 to 75·9)	79·6 (79·1 to 80)	81·3 (80·8 to 81·9)	78·2 (77·7 to 78·6)	78·5 (77·7 to 79·2)
Redbridge	70·8 (68·4 to 73·3)	72·5 (69·8 to 74·7)	73·6 (71·3 to 75·8)	75·5 (74·6 to 76·3)	77·3 (76·9 to 77·8)	79·4 (78·9 to 79·8)	82·8 (82·3 to 83·2)	84·2 (83·6 to 84·8)	80·9 (80·4 to 81·4)	81·7 (81 to 82·6)
Richmond upon Thames	71·5 (69·1 to 73·9)	72·9 (70·5 to 75·5)	73·9 (71·4 to 76·1)	75 (74·2 to 75·8)	77·8 (77·3 to 78·2)	80·2 (79·7 to 80·7)	84·3 (83·8 to 84·9)	85·4 (84·7 to 86·2)	83·9 (83·2 to 84·6)	83·6 (82·7 to 84·4)
Southwark	68·4 (65·7 to 70·9)	70·5 (68 to 73·1)	71·4 (69 to 73·8)	72·6 (71·8 to 73·5)	72·8 (72·4 to 73·2)	76·1 (75·7 to 76·5)	80 (79·6 to 80·5)	82·4 (81·9 to 83)	79·8 (79·4 to 80·3)	79·5 (78·8 to 80·2)
Sutton	70·7 (68·2 to 73·2)	72·8 (70·5 to 75·2)	74·2 (71·8 to 76·4)	75·9 (75 to 76·8)	77·4 (76·9 to 77·8)	79·1 (78·6 to 79·5)	82·7 (82·2 to 83·2)	83·6 (82·8 to 84·3)	82·7 (82·2 to 83·3)	81·4 (80·6 to 82·1)
Tower Hamlets	66·3 (63·5 to 68·8)	68·6 (66·1 to 71)	69 (66·6 to 71·3)	70·4 (69·6 to 71·3)	73·2 (72·8 to 73·7)	74·8 (74·4 to 75·2)	79·4 (79 to 79·8)	81·8 (81·3 to 82·4)	78·1 (77·7 to 78·6)	78·1 (77·5 to 78·8)
Waltham Forest	68·7 (66·2 to 71·3)	70·7 (68·4 to 73)	72·1 (69·9 to 74·4)	73·9 (73·1 to 74·7)	76·1 (75·7 to 76·5)	77·1 (76·7 to 77·5)	80·5 (80·1 to 80·9)	82·7 (82·1 to 83·3)	81·5 (80·9 to 82)	81·1 (80·3 to 81·9)
Wandsworth	69·4 (67 to 71·8)	70·7 (68·4 to 72·9)	71·7 (69·4 to 73·9)	73·1 (72·4 to 73·9)	74·6 (74·2 to 74·9)	77·2 (76·9 to 77·6)	81·8 (81·4 to 82·2)	82·5 (81·9 to 83·1)	81·1 (80·7 to 81·6)	81·4 (80·8 to 82·2)
Westminster	70·4 (67·9 to 73)	70·5 (67·8 to 72·8)	71 (68·7 to 73·6)	71·9 (71 to 72·7)	74·8 (74·4 to 75·2)	77·9 (77·5 to 78·3)	83·1 (82·6 to 83·6)	84·3 (83·6 to 85)	83·4 (82·8 to 84)	83 (82·2 to 83·8)
North East England	68·2 (66·3 to 70·1)	69·8 (68 to 71·7)	70·8 (68·9 to 72·6)	72·5 (71·9 to 73·1)	74·5 (74·2 to 74·7)	76·7 (76·5 to 77)	79·5 (79·2 to 79·7)	80 (79·8 to 80·3)	79·1 (78·8 to 79·3)	78·8 (78·2 to 79·4)
County Durham	68·1 (65·5 to 70·6)	69·9 (67·6 to 72·3)	70·8 (68·5 to 73·1)	72·5 (71·8 to 73·2)	74·5 (74·1 to 74·8)	76·7 (76·4 to 77·1)	79·3 (78·9 to 79·7)	80 (79·5 to 80·4)	78·8 (78·4 to 79·2)	78·7 (78 to 79·4)
Darlington	68·4 (65·7 to 71)	70 (67·4 to 72·5)	70·9 (68·3 to 73·3)	72·7 (71·8 to 73·6)	74·7 (74·1 to 75·2)	76·8 (76·3 to 77·3)	79·7 (79·1 to 80·2)	80·3 (79·6 to 81)	80·2 (79·6 to 80·9)	78·5 (77·6 to 79·4)
Gateshead	67·7 (65·1 to 70·3)	69·3 (66·6 to 71·7)	70·2 (67·8 to 72·5)	72 (71·2 to 72·8)	74·4 (74 to 74·8)	76·3 (75·8 to 76·7)	79·7 (79·2 to 80·1)	80·1 (79·5 to 80·8)	78·9 (78·4 to 79·4)	79·2 (78·4 to 80)
Hartlepool	67 (64·3 to 69·7)	68·7 (66·2 to 71·4)	69·6 (67·2 to 72)	71·5 (70·5 to 72·5)	73·8 (73·3 to 74·4)	75·8 (75·2 to 76·3)	78·7 (78·1 to 79·3)	78·6 (77·9 to 79·4)	78·7 (78 to 79·3)	77·7 (76·6 to 78·7)
Middlesbrough	67 (64·2 to 69·5)	68·7 (66·2 to 71·3)	69·5 (67 to 72)	71·3 (70·5 to 72·2)	73·1 (72·6 to 73·5)	75·5 (75 to 75·9)	78·1 (77·6 to 78·7)	77·9 (77·2 to 78·6)	77·2 (76·5 to 77·8)	76·5 (75·7 to 77·4)
Newcastle upon Tyne	68·7 (66·2 to 71·3)	70 (67·3 to 72·4)	70·8 (68·5 to 73·1)	72·5 (71·7 to 73·2)	73·8 (73·4 to 74·1)	76·4 (75·9 to 76·8)	79·3 (78·8 to 79·7)	80·1 (79·6 to 80·7)	78·9 (78·4 to 79·4)	78·3 (77·6 to 79·1)
North Tyneside	68·8 (66·2 to 71·5)	70·5 (67·8 to 73·1)	71·5 (69 to 73·7)	73·1 (72·3 to 74)	74·9 (74·5 to 75·3)	77 (76·5 to 77·4)	79·6 (79·1 to 80)	80·9 (80·3 to 81·5)	79·3 (78·8 to 79·9)	79·8 (79 to 80·6)
Northumberland	69 (66·3 to 71·6)	70·6 (68·2 to 73)	71·7 (69·4 to 74)	73 (72·2 to 73·8)	75·6 (75·2 to 75·9)	78·2 (77·8 to 78·6)	80·9 (80·5 to 81·4)	81·3 (80·8 to 81·8)	80·5 (80 to 80·9)	80·6 (79·8 to 81·3)
Redcar and Cleveland	68·2 (65·1 to 70·9)	69·7 (67 to 72·3)	70·7 (68·2 to 73·1)	72·5 (71·6 to 73·4)	74·7 (74·3 to 75·2)	77 (76·5 to 77·5)	79·8 (79·3 to 80·3)	79·7 (78·9 to 80·4)	79·4 (78·7 to 80)	77·7 (76·8 to 78·6)
South Tyneside	68·2 (65·6 to 70·8)	69·9 (67·4 to 72·5)	70·8 (68·4 to 73·3)	72·5 (71·6 to 73·5)	74·3 (73·9 to 74·8)	76·7 (76·2 to 77·2)	79·2 (78·7 to 79·7)	79·8 (79·1 to 80·5)	78·6 (78 to 79·2)	78·7 (77·8 to 79·6)

Stockton-on-Tees	68·8 (66 to 71·4)	70·4 (67·8 to 72·8)	71·3 (68·8 to 73·6)	73·2 (72·4 to 74)	74·7 (74·3 to 75·2)	77·5 (77·1 to 78)	80·2 (79·7 to 80·6)	80·3 (79·6 to 80·9)	79·5 (79 to 80·1)	79 (78·2 to 79·8)
Sunderland	68·3 (65·7 to 70·7)	69·9 (67·3 to 72·5)	70·9 (68·5 to 73·2)	72·6 (71·8 to 73·3)	74·1 (73·7 to 74·5)	76 (75·6 to 76·4)	78·4 (78 to 78·9)	79·5 (78·9 to 80·1)	78 (77·5 to 78·5)	78·2 (77·4 to 79)
North West England	68·3 (66·8 to 70·1)	70 (68·4 to 71·6)	71·1 (69·4 to 72·7)	72·7 (72·2 to 73·2)	74·7 (74·6 to 74·9)	76·8 (76·6 to 76·9)	79·4 (79·2 to 79·5)	80·4 (80·2 to 80·5)	79·2 (79 to 79·3)	78·9 (78·4 to 79·4)
Blackburn with Darwen	67·3 (64·7 to 69·9)	69·2 (66·7 to 71·6)	70·4 (68·2 to 72·7)	72·1 (71·3 to 72·9)	73·6 (73·2 to 74·1)	75·4 (74·9 to 75·9)	78·5 (78 to 79)	79·7 (79 to 80·3)	78·4 (77·8 to 79)	77·4 (76·6 to 78·2)
Blackpool	67·2 (64·6 to 69·7)	69·2 (66·8 to 71·5)	70·5 (68·3 to 72·8)	72·4 (71·6 to 73·2)	73·7 (73·2 to 74·1)	74·1 (73·7 to 74·6)	75·5 (75·1 to 76)	76·3 (75·6 to 77)	76·9 (76·4 to 77·4)	75·4 (74·6 to 76·2)
Bolton	67·8 (65·2 to 70·5)	69·4 (67 to 71·9)	70·5 (68·3 to 72·8)	72·2 (71·5 to 73·1)	74·6 (74·2 to 74·9)	76·3 (75·9 to 76·7)	79·1 (78·7 to 79·5)	79·8 (79·3 to 80·4)	79 (78·6 to 79·5)	78·7 (78 to 79·4)
Bury	69·1 (66·7 to 71·6)	70·5 (68·2 to 72·7)	71·8 (69·4 to 74·1)	73·5 (72·7 to 74·3)	75·2 (74·7 to 75·6)	77·5 (77·1 to 77·9)	80 (79·5 to 80·5)	81·1 (80·5 to 81·7)	79·3 (78·8 to 79·8)	79·3 (78·7 to 80·1)
Cheshire East	70·5 (68·1 to 73)	71·9 (69·6 to 74·2)	73 (70·8 to 75·2)	74·1 (73·4 to 74·9)	76·3 (76 to 76·7)	78·7 (78·4 to 79·1)	81·3 (80·9 to 81·7)	82·5 (82 to 83)	81·3 (80·8 to 81·7)	81·8 (81·1 to 82·4)
Cheshire West and Chester	70·1 (67·5 to 72·6)	71·6 (69·2 to 73·7)	72·4 (70·1 to 74·6)	73·8 (73 to 74·5)	75·9 (75·5 to 76·3)	78·5 (78·1 to 78·8)	81 (80·6 to 81·4)	81·8 (81·2 to 82·3)	80·4 (80 to 80·9)	80·5 (79·9 to 81·2)
Cumbria	69 (66·5 to 71·5)	70·6 (68·2 to 72·9)	71·6 (69·4 to 73·9)	73·2 (72·5 to 73·9)	75·3 (74·9 to 75·6)	77·9 (77·5 to 78·3)	80·6 (80·3 to 81)	81·2 (80·8 to 81·7)	80·4 (80 to 80·8)	80 (79·4 to 80·7)
Halton	68·3 (65·8 to 70·9)	69·6 (67·2 to 72·1)	70·5 (68·2 to 73·1)	72·2 (71·3 to 73)	74·4 (74 to 74·9)	75·9 (75·5 to 76·4)	78·9 (78·4 to 79·4)	79·7 (79 to 80·4)	78·8 (78·3 to 79·4)	78·6 (77·7 to 79·4)
Knowsley	67·8 (65 to 70·5)	69·3 (67 to 71·6)	70·1 (67·9 to 72·4)	71·7 (70·9 to 72·5)	74 (73·6 to 74·4)	75·6 (75·1 to 76)	78·4 (77·9 to 78·8)	79·3 (78·6 to 80)	77·8 (77·2 to 78·4)	77·3 (76·5 to 78)
Lancashire	68·4 (66·1 to 70·7)	70·4 (68·1 to 72·7)	71·5 (69·1 to 73·8)	72·9 (72·2 to 73·6)	75 (74·7 to 75·3)	77·3 (77 to 77·6)	80 (79·7 to 80·3)	80·4 (80 to 80·8)	79·3 (79 to 79·6)	78·7 (78·1 to 79·3)
Liverpool	67·7 (65·2 to 70·1)	69·3 (66·8 to 71·8)	69·9 (67·7 to 72·2)	71·7 (71 to 72·5)	73·6 (73·3 to 74)	74·8 (74·4 to 75·1)	77·6 (77·2 to 77·9)	78·7 (78·3 to 79·2)	77·5 (77·2 to 77·9)	76·8 (76·2 to 77·5)
Manchester	67·2 (64·7 to 69·5)	68·8 (66·4 to 71·3)	69·6 (67·2 to 71·9)	71·1 (70·3 to 71·9)	72·7 (72·3 to 73)	74·1 (73·7 to 74·5)	77·3 (77 to 77·7)	79 (78·5 to 79·4)	76·7 (76·3 to 77)	76·3 (75·7 to 76·9)
Oldham	67·5 (64·9 to 70)	69·1 (66·8 to 71·3)	70·4 (68 to 72·7)	72·2 (71·4 to 72·9)	74·2 (73·8 to 74·6)	76·1 (75·7 to 76·5)	78·6 (78·2 to 79·1)	79·5 (78·9 to 80)	77·7 (77·2 to 78·1)	78·1 (77·4 to 78·8)
Rochdale	67·5 (65 to 69·8)	69·3 (67 to 71·6)	70·5 (68·2 to 72·9)	72·4 (71·6 to 73·2)	74·1 (73·6 to 74·5)	76·2 (75·8 to 76·6)	78·3 (77·9 to 78·7)	79·5 (78·9 to 80·1)	78 (77·5 to 78·4)	77·6 (76·8 to 78·4)
Salford	67·3 (64·9 to 69·8)	68·8 (66·5 to 71·3)	69·7 (67·5 to 72)	71·2 (70·5 to 72)	73·4 (73 to 73·7)	75·4 (75 to 75·8)	78 (77·6 to 78·4)	80 (79·4 to 80·5)	77·9 (77·4 to 78·3)	78·2 (77·5 to 78·9)
Sefton	69·3 (66·8 to 71·8)	71 (68·6 to 73·4)	72 (69·9 to 74·3)	73·5 (72·7 to 74·2)	75·8 (75·4 to 76·2)	77·4 (77 to 77·8)	79·3 (78·9 to 79·7)	80·1 (79·6 to 80·7)	79·1 (78·6 to 79·6)	79·4 (78·6 to 80·1)
St Helens	68·3 (65·7 to 70·8)	69·6 (67·3 to 72)	70·6 (68·4 to 72·9)	71·9 (71·1 to 72·7)	74·6 (74·2 to 74·9)	76·7 (76·3 to 77·1)	78·9 (78·4 to 79·3)	79·4 (78·8 to 80)	78·5 (77·9 to 79)	78·1 (77·3 to 78·9)
Stockport	70·1 (67·5 to 72·6)	71·6 (69·4 to 73·9)	72·6 (70·2 to 75)	73·7 (73 to 74·5)	75·9 (75·5 to 76·2)	78·1 (77·7 to 78·4)	80·4 (80 to 80·8)	81·7 (81·2 to 82·3)	80·8 (80·3 to 81·2)	80·8 (80·1 to 81·5)
Tameside	67·9 (65·4 to 70·5)	69·4 (66·9 to 71·7)	70·6 (68·4 to 72·9)	72·4 (71·6 to 73·1)	74·5 (74·1 to 75)	76·2 (75·7 to 76·6)	78·6 (78·2 to 79)	80·5 (79·9 to 81·1)	78·2 (77·7 to 78·7)	78·5 (77·7 to 79·2)
Trafford	69·7 (67·3 to 72·1)	71·1 (68·9 to 73·3)	72·2 (69·9 to 74·4)	73·8 (73 to 74·5)	75·7 (75·3 to 76·1)	78·1 (77·7 to 78·5)	81 (80·5 to 81·5)	83·1 (82·5 to 83·7)	81·1 (80·6 to 81·6)	81·9 (81·1 to 82·6)
Warrington	69·5 (66·9 to 72·1)	70·8 (68·3 to 73)	71·7 (69·4 to 74)	73·2 (72·4 to 73·9)	74·9 (74·5 to 75·3)	77·6 (77·2 to 78·1)	80 (79·6 to 80·4)	80·7 (80 to 81·2)	79·7 (79·2 to 80·1)	79·8 (79·1 to 80·4)
Wigan	68·5 (66 to 71)	69·8 (67·4 to 72·2)	71 (68·8 to 73·5)	72·6 (71·9 to 73·4)	74·7 (74·3 to 75)	76·3 (75·9 to 76·7)	78·9 (78·5 to 79·3)	80 (79·5 to 80·6)	78·7 (78·3 to 79·1)	79·3 (78·6 to 80)
Wirral	68·5 (65·9 to 70·9)	70·4 (68·1 to 72·8)	71·5 (69·2 to 73·9)	73·1 (72·4 to 73·8)	75·1 (74·7 to 75·5)	77 (76·6 to 77·4)	79·7 (79·3 to 80·1)	80·4 (79·9 to 81)	79·7 (79·3 to 80·2)	78·8 (78·1 to 79·5)
South East England	71·1 (69·3 to 72·9)	72·6 (70·9 to 74·3)	73·6 (72 to 75·2)	74·9 (74·4 to 75·3)	77·1 (77 to 77·3)	79·1 (78·9 to 79·2)	81·8 (81·6 to 81·9)	82·6 (82·4 to 82·7)	81·8 (81·7 to 82)	81·8 (81·4 to 82·3)
Bracknell Forest	72·1 (69·5 to 74·5)	73·4 (70·8 to 75·6)	74·2 (71·9 to 76·5)	75·4 (74·5 to 76·4)	77·2 (76·7 to 77·8)	79·7 (79·2 to 80·2)	82·5 (82 to 83·1)	83·9 (83·2 to 84·8)	82·8 (82·1 to 83·6)	84·1 (83·1 to 84·9)
Brighton and Hove	70·1 (67·5 to 72·6)	71·7 (69·4 to 73·9)	72·8 (70·4 to 74·8)	74·1 (73·2 to 74·8)	75·2 (74·8 to 75·6)	77·1 (76·7 to 77·5)	79·8 (79·4 to 80·2)	81·5 (81 to 82·1)	80 (79·5 to 80·4)	81·2 (80·5 to 81·9)
Buckinghamshire	72·4 (70 to 75)	73·7 (71·4 to 76·1)	74·5 (72·2 to 76·7)	75·3 (74·6 to 76·1)	77·4 (77·1 to 77·8)	79·7 (79·3 to 80)	82·2 (81·9 to 82·6)	83·6 (83·2 to 84·1)	83·1 (82·7 to 83·5)	82·5 (81·8 to 83·1)
East Sussex	70·5 (68 to 72·9)	72·5 (70·1 to 74·7)	73·7 (71·5 to 76)	75·2 (74·5 to 75·9)	77·7 (77·3 to 78)	79·4 (79 to 79·8)	81·6 (81·2 to 82)	81·7 (81·2 to 82·1)	81·5 (81·1 to 81·9)	81·1 (80·5 to 81·7)
Hampshire	71·4 (68·9 to 73·7)	73 (70·6 to 75·4)	73·7 (71·2 to 75·9)	75·1 (74·4 to 75·8)	77·5 (77·3 to 77·8)	79·5 (79·3 to 79·8)	82·3 (82 to 82·6)	83·2 (82·8 to 83·6)	82·6 (82·3 to 82·9)	82·8 (82·2 to 83·4)
Isle of Wight	69·1 (66·4 to 71·8)	71·4 (68·9 to 73·7)	73 (70·6 to 75·2)	74·7 (73·8 to 75·7)	76·4 (75·8 to 76·9)	78·9 (78·3 to 79·4)	80·9 (80·4 to 81·4)	81·5 (80·7 to 82·2)	81·2 (80·6 to 81·8)	80·7 (79·9 to 81·5)
Kent	70·3 (68 to 72·7)	72·1 (69·8 to 74·3)	72·9 (70·7 to 75)	74·1 (73·4 to 74·8)	76·5 (76·3 to 76·8)	78·6 (78·3 to 78·9)	81·4 (81·1 to 81·7)	81·8 (81·5 to 82·1)	80·9 (80·6 to 81·2)	80·9 (80·3 to 81·4)
Medway	70·4 (67·8 to 73·1)	71·6 (69·1 to 73·8)	73 (70·6 to 75·1)	74·7 (73·9 to 75·4)	76·1 (75·7 to 76·5)	77·7 (77·3 to 78·1)	79·8 (79·4 to 80·2)	81 (80·4 to 81·6)	79·7 (79·2 to 80·2)	80·5 (79·8 to 81·2)
Milton Keynes	70·7 (68·1 to 73·3)	72·1 (69·6 to 74·4)	73·2 (70·6 to 75·5)	74·6 (73·6 to 75·5)	76·5 (76 to 77)	78·8 (78·3 to 79·2)	81 (80·6 to 81·4)	82·3 (81·8 to 82·9)	80·9 (80·4 to 81·3)	81·4 (80·7 to 82·1)
Oxfordshire	72·6 (70 to 75·3)	73·7 (71·3 to 76)	74·5 (72·2 to 76·5)	75·4 (74·7 to 76·1)	77·7 (77·4 to 78)	79·3 (79 to 79·6)	82·1 (81·8 to 82·4)	83·5 (83 to 83·9)	82·7 (82·4 to 83·1)	82·7 (82·1 to 83·3)
Portsmouth	69·8 (67·2 to 72·4)	71 (68·5 to 73·3)	72·1 (69·6 to 74·5)	73·5 (72·7 to 74·3)	75·1 (74·6 to 75·5)	76·7 (76·3 to 77·1)	79·7 (79·3 to 80·1)	81 (80·5 to 81·6)	79·6 (79·1 to 80·1)	80·1 (79·4 to 80·8)
Reading	71·4 (68·8 to 73·9)	72·4 (69·9 to 74·7)	73·3 (70·8 to 75·4)	74·4 (73·5 to 75·2)	76 (75·6 to 76·5)	77·8 (77·3 to 78·3)	80·2 (79·7 to 80·7)	81 (80·3 to 81·7)	78·8 (78·3 to 79·4)	80·5 (79·8 to 81·3)
Slough	69·8 (67·1 to 72·4)	71·3 (68·7 to 73·6)	72 (69·6 to 74·4)	73·5 (72·6 to 74·3)	75 (74·5 to 75·5)	77·2 (76·7 to 77·6)	80 (79·5 to 80·5)	81 (80·4 to 81·7)	79·7 (79·1 to 80·2)	78·8 (78 to 79·6)
Southampton	69·9 (67·2 to 72·4)	71 (68·3 to 73·4)	72 (69·7 to 74·3)	73·7 (72·9 to 74·6)	75·4 (74·9 to 75·8)	77·3 (76·9 to 77·8)	80·1 (79·7 to 80·6)	81·1 (80·5 to 81·7)	79·3 (78·8 to 79·8)	80·3 (79·6 to 81)
Surrey	71·7 (69·3 to 74)	73·5 (71 to 75·7)	74·1 (71·7 to 76·4)	75·2 (74·5 to 75·8)	77·8 (77·5 to 78·1)	79·8 (79·5 to 80·1)	82·8 (82·5 to 83·1)	83·4 (83 to 83·8)	82·7 (82·4 to 83)	82·9 (82·4 to 83·5)
West Berkshire	72·6 (69·9 to 75·2)	73·9 (71·5 to 76·1)	74·9 (72·4 to 77)	76 (75·1 to 76·9)	77·8 (77·3 to 78·3)	79·7 (79·2 to 80·2)	82·4 (81·9 to 83)	83·7 (83 to 84·4)	83·1 (82·6 to 83·7)	82·5 (81·8 to 83·4)
West Sussex	71·1 (68·7 to 73·5)	73 (70·4 to 75·3)	73·9 (71·7 to 76·1)	75·2 (74·5 to 75·9)	77·6 (77·3 to 77·9)	79·5 (79·2 to 79·8)	81·8 (81·5 to 82·2)	82·4 (82 to 82·8)	82·3 (81·9 to 82·6)	81·8 (81·2 to 82·3)
Windsor and Maidenhead	73·1 (70·6 to 75·5)	74·4 (72·1 to 76·8)	75·4 (73·2 to 77·6)	76·6 (75·7 to 77·5)	77·8 (77·3 to 78·3)	80·2 (79·7 to 80·7)	83·1 (82·5 to 83·6)	84·1 (83·4 to 84·8)	82·7 (82·1 to 83·2)	82·8 (82 to 83·6)
Wokingham	73·7 (71·3 to 76·4)	74·9 (72·5 to 77·2)	76 (73·8 to 78·4)	77·1 (76·1 to 78)	79·2 (78·7 to 79·6)	81·6 (81·1 to 82·1)	84·2 (83·6 to 84·7)	84·4 (83·7 to 85·2)	83·4 (82·8 to 84)	81·9 (81·1 to 82·7)
South West England	70·8 (69 to 72·7)	72·4 (70·6 to 74·2)	73·4 (71·6 to 75·2)	74·8 (74·3 to 75·3)	77·1 (76·9 to 77·2)	79·2 (79 to 79·4)	81·7 (81·5 to 81·9)	82·3 (82·1 to 82·5)	81·9 (81·7 to 82·1)	81·2 (80·7 to 81·7)
Bath and North East Somerset	72·1 (69·5 to 74·7)	73·7 (71·2 to 76)	74·8 (72·5 to 77)	76·3 (75·4 to 77·2)	77·8 (77·3 to 78·3)	79·9 (79·4 to 80·4)	82·7 (82·2 to 83·2)	83·2 (82·6 to 83·9)	82·2 (81·6 to 82·8)	82·2 (81·4 to 82·9)
Bournemouth	70·7 (68·2 to 73·3)	72·4 (70 to 74·8)	73·5 (71 to 75·8)	74·9 (74 to 75·8)	76·1 (75·6 to 76·6)	77·8 (77·3 to 78·2)	80·2 (79·7 to 80·6)	81·1 (80·5 to 81·7)	79·5 (78·9 to 80)	73·8 (71·9 to 75·7)
Bristol, City of	70·3 (67·6 to 72·8)	71·5 (69 to 74)	72·2 (69·8 to 74·6)	73·7 (72·9 to 74·5)	76 (75·6 to 76·4)	77·9 (77·5 to 78·3)	80·2 (79·8 to 80·6)	81·2 (80·7 to 81·7)	80·4 (80 to 80·8)	80·1 (79·5 to 80·8)
Cornwall	70·2 (67·7 to 72·6)	72 (69·6 to 74·4)	72·9 (70·6 to 75·3)	74·4 (73·6 to 75·2)	76·9 (76·5 to 77·2)	79 (78·6 to 79·4)	81·4 (81 to 81·7)	81·6 (81·2 to 82·1)	81·3 (80·9 to 81·7)	80·9 (80·2 to 81·5)
Devon	70·9 (68·4 to 73·4)	72·7 (70 to 75·2)	73·5 (71·2 to 76)	74·9 (74·2 to 75·7)	77·5 (77·2 to 77·9)	79·5 (79·2 to 79·9)	82 (81·6 to 82·3)	82·5 (82·1 to 83)	82·6 (82·2 to 82·9)	82·2 (81·6 to 82·9)
Dorset	71·1 (68·5 to 73·7)	72·9 (70·3 to 75·3)	74 (71·7 to 76·3)	75·6 (74·8 to 76·3)	78 (77·7 to 78·4)	80·4 (80 to 80·8)	83·2 (82·8 to 83·6)	83·6 (83 to 84)	83·3 (82·8 to 83·7)	83·7 (83 to 84·4)
Gloucestershire	71·3 (68·9 to 74·1)	72·9 (70·3 to 75·4)	73·8 (71·4 to 76·1)	74·8 (74 to 75·5)	77 (76·6 to 77·3)	79·3 (78·9 to 79·6)	81·8 (81·4 to 82·1)	82·1 (81·6 to 82·5)	81·7 (81·4 to 82·1)	81·3 (80·7 to 82)
North Somerset	71·4 (68·8 to 73·9)	73·1 (70·6 to 75·5)	74·3 (72 to 76·5)	75·9 (75 to 76·7)	77·7 (77·3 to 78·2)	79·7 (79·2 to 80·1)	81·3 (80·9 to 81·8)	82·7 (82·1 to 83·4)	82·1 (81·6 to 82·6)	81·9 (81·1 to 82·7)
Plymouth	69·7 (66·9 to 72·3)	71 (68·5 to 73·5)	72 (69·5 to 74·3)	73·4 (72·6 to 74·2)	75·8 (75·4 to 76·2)	77·6 (77·2 to 78)	80·2 (79·7 to 80·6)	81·2 (80·6 to 81·8)	80·9 (80·5 to 81·4)	80·8 (80·1 to 81·5)
Poole	70·9 (68·4 to 73·5)	72·5 (70 to 75)	73·6 (71·2 to 76)	75 (74·1 to 75·9)	77·1 (76·6 to 77·6)	79·8 (79·3 to 80·4)	82·2 (81·7 to 82·8)	82·5 (81·8 to 83·2)	81·6 (80·9 to 82·2)	76 (74·1 to 78·1)
Somerset	71·1 (68·5 to 73·7)	72·8 (70·2 to 75·3)	73·6 (71·3 to 76)	74·9 (74·1 to 75·6)	77·2 (76·9 to 77·6)	79·4 (79·1 to 79·8)	81·9 (81·5 to 82·3)	82·1 (81·7 to 82·6)	81·9 (81·4 to 82·2)	81·4 (80·7 to 82·1)
South Gloucestershire	72·1 (69·4 to 74·6)	73·4 (70·9 to 75·7)	74·2 (71·8 to 76·6)	75·8 (74·9 to 76·7)	77·9 (77·5 to 78·4)	79·6 (79·2 to 80)	82·6 (82·2 to 83·1)	83·7 (83·1 to 84·3)	82·7 (82·1 to 83·2)	82·3 (81·6 to 83)
Swindon	70·2 (67·5 to 72·7)	71·7 (69 to 74·1)	72·5 (70·1 to 74·9)	74 (73·1 to 74·9)	76·4 (76 to 76·9)	78·5 (78·1 to 79)	81·5 (81 to 82)	83 (82·4 to 83·6)	81·1 (80·6 to 81·6)	82 (81·2 to 82·8)
Torbay	70·1 (67·3 to 72·6)	72 (69·4 to 74·6)	73·2 (70·7 to 75·5)	74·8 (73·9 to 75·7)	76·6 (76 to 77·1)	77·6 (77·1 to 78·1)	79·6 (79·1 to 80·1)	79·8 (79·1 to 80·5)	80·8 (80·1 to 81·4)	79·9 (79 to 80·8)
Wiltshire	71·6 (68·9 to 74·1)	72·9 (70·5 to 75·5)	73·9 (71·6 to 76·3)	75·1 (74·3 to 75·8)	76·9 (76·6 to 77·3)	79·6 (79·2 to 80)	82 (81·7 to 82·4)	83·2 (82·7 to 83·6)	82·7 (82·3 to 83·1)	82 (81·3 to 82·6)
West Midlands	69 (67·2 to 70·9)	70·5 (68·8 to 72·2)	71·5 (69·7 to 73·3)	73·2 (72·7 to 73·7)	75·4 (75·2 to 75·6)	77·6 (77·4 to 77·7)	80·4 (80·2 to 80·6)	81·2 (81 to 81·3)	79·9 (79·7 to 80·1)	79·8 (79·3 to 80·3)
Birmingham	68·2 (66 to 70·7)	70 (67·8 to 72·3)	70·9 (68·6 to 73·1)	72·6 (71·9 to 73·3)	74·5 (74·2 to 74·8)	76·5 (76·1 to 76·8)	80·1 (79·8 to 80·5)	80·5 (80·2 to 80·9)	78·5 (78·2 to 78·9)	78·2 (77·6 to 78·9)

Coventry	68·7 (65·9 to 71·1)	70·1 (67·4 to 72·5)	71 (68·6 to 73·4)	72·8 (72 to 73·6)	74·7 (74·3 to 75·1)	77·1 (76·7 to 77·5)	79·6 (79·2 to 80)	80·5 (80 to 81)	79·3 (78·8 to 79·7)	79·2 (78·4 to 79·9)
Dudley	69·9 (67·3 to 72·6)	71·3 (68·8 to 73·6)	72·5 (70 to 75)	74 (73·3 to 74·7)	75·8 (75·4 to 76·2)	77·7 (77·4 to 78·1)	80·5 (80·1 to 80·9)	81·3 (80·8 to 81·8)	80·3 (79·9 to 80·7)	79·9 (79·3 to 80·6)
Herefordshire, County of	69·8 (67·1 to 72·5)	71·4 (69 to 73·8)	72·6 (70·2 to 75)	74·4 (73·5 to 75·3)	77·3 (76·8 to 77·8)	79·1 (78·6 to 79·6)	81·4 (80·9 to 81·9)	82·1 (81·4 to 82·7)	82 (81·4 to 82·6)	80·8 (80·1 to 81·6)
Sandwell	67·4 (64·8 to 69·9)	69·1 (66·6 to 71·6)	70·2 (67·7 to 72·5)	72·3 (71·5 to 73)	74·4 (74 to 74·8)	76·1 (75·6 to 76·5)	78·5 (78·1 to 78·9)	79·9 (79·4 to 80·4)	78·5 (78 to 78·9)	77·9 (77·2 to 78·6)
Shropshire	70·2 (67·6 to 73·1)	71·6 (69 to 74)	72·7 (70·2 to 75)	74·4 (73·6 to 75·2)	76·6 (76·2 to 77)	78·9 (78·5 to 79·3)	81·6 (81·2 to 82)	82·8 (82·3 to 83·4)	82·1 (81·6 to 82·6)	80·8 (80·2 to 81·5)
Solihull	71·3 (68·6 to 73·8)	72·6 (70·2 to 74·9)	73·7 (71·4 to 76·1)	75·3 (74·5 to 76·2)	77·5 (77·1 to 78)	79·1 (78·6 to 79·6)	82·4 (81·9 to 82·9)	82·6 (81·9 to 83·2)	80·8 (80·3 to 81·3)	81·5 (80·7 to 82·3)
Staffordshire	69·3 (67·1 to 71·8)	70·9 (68·6 to 73·2)	71·7 (69·3 to 74·1)	73·3 (72·6 to 74)	75·5 (75·2 to 75·8)	77·9 (77·6 to 78·2)	81 (80·6 to 81·3)	81·5 (81·1 to 81·9)	80·3 (79·9 to 80·6)	80·8 (80·2 to 81·4)
Stoke-on-Trent	67·1 (64·5 to 69·8)	68·6 (66·2 to 71)	69·4 (67·1 to 71·8)	71·2 (70·4 to 72)	73·9 (73·5 to 74·2)	76·1 (75·7 to 76·5)	78·1 (77·7 to 78·6)	79 (78·5 to 79·6)	77·3 (76·8 to 77·7)	77·5 (76·8 to 78·3)
Telford and Wrekin	69·1 (66·3 to 71·8)	70·2 (67·8 to 72·7)	71·4 (68·9 to 73·7)	73·1 (72·2 to 74)	75·6 (75·1 to 76·1)	77·6 (77·1 to 78·1)	80·2 (79·7 to 80·7)	80·4 (79·7 to 81)	79·3 (78·8 to 79·8)	78·2 (77·4 to 79)
Walsall	68·4 (65·8 to 71·1)	69·9 (67·3 to 72·4)	70·9 (68·4 to 73·3)	72·6 (71·8 to 73·3)	74·6 (74·2 to 75)	77 (76·6 to 77·4)	79·3 (78·9 to 79·8)	80·3 (79·8 to 80·9)	79·4 (79 to 79·9)	79 (78·3 to 79·8)
Warwickshire	70·4 (67·8 to 73)	71·7 (69·3 to 74·1)	72·6 (70·1 to 75·1)	73·9 (73·1 to 74·7)	76·2 (75·8 to 76·5)	78·6 (78·3 to 79)	81·4 (81 to 81·7)	82·1 (81·7 to 82·5)	81 (80·6 to 81·4)	81·2 (80·5 to 81·8)
Wolverhampton	68·4 (65·7 to 71·1)	70 (67·6 to 72·4)	71 (68·7 to 73·3)	72·8 (72 to 73·5)	74·5 (74·1 to 74·9)	76·5 (76·1 to 76·9)	78·8 (78·3 to 79·2)	79·9 (79·3 to 80·5)	78·5 (78·1 to 79)	78·2 (77·5 to 78·9)
Worcestershire	70·7 (68·1 to 73·4)	72·1 (69·7 to 74·5)	73 (70·8 to 75·4)	74·4 (73·7 to 75·1)	76·6 (76·3 to 76·9)	78·6 (78·2 to 78·9)	81 (80·6 to 81·3)	81·9 (81·4 to 82·3)	80·9 (80·5 to 81·2)	81·1 (80·4 to 81·8)
Yorkshire and the Humber	68·6 (66·8 to 70·4)	70·3 (68·4 to 72·1)	71·4 (69·7 to 73·1)	73·1 (72·7 to 73·7)	75·4 (75·2 to 75·6)	77·6 (77·4 to 77·8)	80·1 (79·9 to 80·2)	80·8 (80·6 to 81)	79·8 (79·6 to 80)	79·7 (79·2 to 80·2)
Barnsley	68 (65·4 to 70·8)	69·6 (67·1 to 72·1)	71 (68·5 to 73·2)	72·8 (72·1 to 73·7)	75·1 (74·7 to 75·5)	77·2 (76·8 to 77·6)	79·9 (79·4 to 80·3)	80·3 (79·7 to 80·9)	79·1 (78·6 to 79·6)	78·1 (77·4 to 78·9)
Bradford	67·4 (64·7 to 70)	69·5 (66·9 to 71·9)	70·6 (68·1 to 72·9)	72·5 (71·8 to 73·3)	74·6 (74·3 to 75)	76·5 (76·1 to 76·9)	79·4 (79·1 to 79·8)	80·1 (79·6 to 80·6)	78·5 (78·1 to 78·8)	78·9 (78·3 to 79·6)
Calderdale	67·8 (65·1 to 70·2)	69·7 (67·2 to 72·1)	70·9 (68·7 to 73·3)	72·6 (71·9 to 73·4)	74·6 (74·2 to 75·1)	77·4 (77 to 77·9)	79·4 (78·9 to 79·9)	81·1 (80·5 to 81·7)	80 (79·5 to 80·5)	79·6 (78·8 to 80·3)
Doncaster	68·5 (65·8 to 71)	70·2 (67·6 to 72·7)	71·5 (69·2 to 73·8)	73·3 (72·5 to 74)	74·9 (74·5 to 75·3)	76·8 (76·4 to 77·2)	79·2 (78·8 to 79·6)	80·1 (79·5 to 80·6)	78·8 (78·3 to 79·2)	78·9 (78·2 to 79·6)
East Riding of Yorkshire	70·1 (67·5 to 72·5)	71·8 (69·3 to 74·2)	73 (70·6 to 75·4)	74·5 (73·8 to 75·4)	76·7 (76·3 to 77)	78·6 (78·2 to 79)	81·1 (80·7 to 81·5)	82·5 (82 to 83·1)	81·9 (81·4 to 82·3)	81·3 (80·7 to 82)
Kingston upon Hull, City of	68·2 (65·4 to 70·7)	69·8 (67·2 to 72·3)	71·1 (68·9 to 73·2)	73 (72·2 to 73·8)	74·8 (74·4 to 75·2)	76 (75·6 to 76·4)	78·1 (77·6 to 78·5)	78·5 (78 to 79·1)	77·4 (76·9 to 77·8)	77·2 (76·5 to 77·9)
Kirklees	67·8 (65·3 to 70·5)	69·6 (67·2 to 72·1)	70·7 (68·3 to 73)	72·2 (71·5 to 73)	75 (74·6 to 75·3)	77·3 (76·9 to 77·7)	79·8 (79·4 to 80·2)	80·6 (80·1 to 81·1)	79·3 (79 to 79·7)	80·1 (79·4 to 80·7)
Leeds	68·7 (66·2 to 71·2)	70·5 (68·1 to 72·9)	71·3 (69 to 73·8)	73 (72·3 to 73·7)	75·4 (75·1 to 75·7)	77·8 (77·4 to 78·1)	80·1 (79·8 to 80·4)	80·5 (80·1 to 80·9)	79·4 (79·1 to 79·8)	79 (78·4 to 79·7)
North East Lincolnshire	68·9 (65·9 to 71·5)	70·7 (68 to 73·1)	72 (69·6 to 74·3)	73·8 (72·9 to 74·7)	75 (74·5 to 75·4)	77·3 (76·8 to 77·7)	79·7 (79·2 to 80·2)	80·2 (79·5 to 80·8)	79·9 (79·3 to 80·4)	79·3 (78·5 to 80·2)
North Lincolnshire	68·9 (66·3 to 71·3)	70·5 (68 to 72·9)	71·6 (69·2 to 74·1)	73·3 (72·5 to 74·2)	74·9 (74·4 to 75·4)	77·6 (77·1 to 78·1)	80·4 (79·9 to 80·9)	81·5 (80·9 to 82·2)	80·7 (80·1 to 81·2)	80·8 (80 to 81·6)
North Yorkshire	69·9 (67·3 to 72·3)	71·5 (69 to 74)	72·6 (70·2 to 75)	74·2 (73·5 to 74·9)	76·7 (76·4 to 77·1)	79·1 (78·8 to 79·5)	81·4 (81·1 to 81·8)	82·3 (81·9 to 82·8)	81·7 (81·3 to 82·1)	82 (81·3 to 82·6)
Rotherham	68·4 (65·8 to 70·9)	69·9 (67·4 to 72·3)	71·2 (68·8 to 73·6)	72·8 (72 to 73·6)	75·1 (74·7 to 75·5)	77·4 (77 to 77·8)	80 (79·6 to 80·5)	80·2 (79·7 to 80·8)	78·9 (78·4 to 79·3)	79·1 (78·4 to 79·8)
Sheffield	69·1 (66·7 to 71·6)	70·7 (68·1 to 73·2)	71·7 (69·6 to 74)	73·4 (72·7 to 74·1)	75·4 (75 to 75·7)	77·7 (77·4 to 78·1)	80 (79·7 to 80·4)	80·8 (80·4 to 81·3)	80·2 (79·8 to 80·6)	79·4 (78·8 to 80)
Wakefield	67·8 (65·2 to 70·3)	69·3 (66·9 to 71·8)	70·3 (68·1 to 72·5)	72 (71·2 to 72·7)	75 (74·6 to 75·3)	77·3 (76·9 to 77·7)	79·5 (79·2 to 79·9)	79·8 (79·3 to 80·4)	78·9 (78·5 to 79·4)	78·6 (77·9 to 79·3)
York	70·4 (67·7 to 73)	71·7 (69·1 to 74·2)	72·9 (70·4 to 75·1)	74·4 (73·6 to 75·2)	76·6 (76·1 to 77·1)	79·3 (78·8 to 79·8)	82 (81·5 to 82·5)	82·2 (81·5 to 82·8)	81·6 (81 to 82·1)	80·8 (80 to 81·5)
Northern Ireland	67·5 (67·2 to 67·8)	69·9 (69·7 to 70·1)	70·8 (70·7 to 71)	71·7 (71·5 to 71·8)	74·4 (74·2 to 74·5)	77·3 (77·1 to 77·5)	79·7 (79·5 to 79·8)	81 (80·8 to 81·2)	80·3 (80·1 to 80·5)	80·3 (79·9 to 80·6)
Scotland	67·1 (66·9 to 67·3)	68·9 (68·8 to 69)	70·3 (70·2 to 70·4)	71·9 (71·8 to 72)	74 (73·9 to 74·1)	76 (75·9 to 76·1)	78·6 (78·5 to 78·7)	79·5 (79·3 to 79·6)	78·7 (78·6 to 78·9)	78·5 (78·4 to 78·7)
Wales	68·6 (67·8 to 69·3)	70·3 (69·7 to 70·8)	71·5 (70·9 to 72)	73·3 (73·1 to 73·6)	75·7 (75·6 to 75·9)	77·4 (77·3 to 77·6)	79·9 (79·8 to 80)	80·6 (80·4 to 80·8)	79·8 (79·6 to 79·9)	79·9 (79·7 to 80·1)
Latin America and Caribbean	50 (48·2 to 51·7)	56·8 (55·9 to 57·7)	60·7 (60·1 to 61·4)	65·9 (65·5 to 66·4)	69·7 (69·3 to 69·9)	72·7 (72·4 to 73)	73·4 (73·1 to 73·8)	76 (75·4 to 76·6)	73·4 (72·8 to 74)	72·3 (71·5 to 73)
Andean Latin America	41·4 (38·5 to 44·3)	48·4 (46·6 to 50·1)	52·2 (51 to 53·4)	63·7 (62·6 to 64·7)	68·5 (67·6 to 69·4)	73 (72 to 73·9)	75·6 (74·5 to 76·7)	76·9 (75·3 to 78·3)	72·2 (70·7 to 73·6)	71·1 (69·8 to 72·4)
Bolivia	37·2 (33·6 to 40·7)	43·8 (40·6 to 46·7)	49·9 (47·7 to 52)	56·4 (54·7 to 58·1)	61·4 (59·8 to 62·9)	66·4 (64·8 to 67·9)	70·2 (68 to 72·4)	72·7 (69·6 to 75·1)	67·4 (65·2 to 69·4)	66·2 (64·1 to 67·9)
Ecuador	50·6 (47·1 to 53·6)	55·8 (54·9 to 56·7)	61·2 (60·4 to 61·9)	66·6 (66·1 to 67·2)	71·9 (71·6 to 72·3)	73·9 (72·8 to 75)	74·7 (73·7 to 75·6)	77·2 (76 to 78·3)	73·1 (71·8 to 74·5)	74 (72·1 to 75·7)
Peru	40·1 (36·2 to 43·9)	47·3 (45·1 to 49·6)	49·5 (48 to 51·1)	65·1 (63·5 to 66·8)	69·6 (68·2 to 71·1)	74·9 (73·4 to 76·5)	78·1 (76·5 to 79·7)	78·2 (76·4 to 79·9)	73·5 (71·6 to 75·5)	71·6 (70·2 to 73)
Caribbean	54·8 (52·5 to 57·2)	60·3 (59·2 to 61·4)	63·2 (62·3 to 64·2)	66·1 (65·2 to 66·9)	67·9 (67·1 to 68·6)	70·2 (69·4 to 70·9)	57·8 (56·2 to 59·2)	72·5 (70·9 to 74)	71·8 (70·1 to 73·4)	69·6 (67·7 to 71·3)
Antigua and Barbuda	57·9 (55·7 to 59·9)	64·2 (63·1 to 65·2)	68·4 (67·6 to 69·2)	72·4 (71·1 to 73·6)	73·9 (73·2 to 74·7)	74·5 (73·9 to 75·1)	75·7 (75 to 76·3)	75·7 (74·6 to 76·8)	76·7 (75·6 to 77·8)	75 (74·8 to 75·1)
The Bahamas	57·6 (53·2 to 61·6)	61·8 (59·1 to 64·7)	65·3 (64·2 to 66·4)	68·8 (67·8 to 69·8)	71·1 (70·2 to 72)	71·1 (70·3 to 71·9)	72·9 (72·1 to 73·8)	71·2 (68·9 to 73·3)	71·8 (69·4 to 74·1)	69·8 (67·5 to 71·8)
Barbados	54·1 (50·8 to 57·1)	66·4 (65·2 to 67·4)	69·1 (68·4 to 69·7)	72·7 (72·2 to 73·2)	73·8 (73·3 to 74·3)	74·6 (74·1 to 75·2)	76 (75·5 to 76·5)	76·1 (74·1 to 78·1)	76·8 (74·4 to 79)	76 (73·7 to 78·3)
Belize	54·9 (52·2 to 57·3)	65·2 (63·4 to 66·9)	68·9 (67·6 to 70·1)	71·4 (70·4 to 72·4)	73·6 (72·8 to 74·3)	69·7 (69·2 to 70·2)	73·5 (73 to 73·9)	74·7 (73·6 to 75·7)	73·8 (73 to 74·7)	73·2 (71·8 to 74·7)
Bermuda	63·9 (62·2 to 65·5)	67·1 (66·4 to 67·8)	70 (69·3 to 70·7)	73 (72·4 to 73·6)	73·3 (72·7 to 73·7)	77·5 (76·9 to 78·1)	80·8 (79·8 to 81·6)	81·8 (80·1 to 83)	82·1 (80·5 to 83·2)	79·3 (77·5 to 80·8)
Cuba	66·7 (63·1 to 69·9)	69 (68·8 to 69·2)	70·5 (70·3 to 70·6)	74·1 (73·9 to 74·2)	74·8 (74·6 to 74·9)	77 (76·8 to 77·1)	78·3 (78·1 to 78·4)	78·1 (77·9 to 78·3)	78·1 (78 to 78·3)	73·9 (73 to 74·9)
Dominica	47·7 (43·9 to 51·1)	60 (58·4 to 61·5)	67 (66 to 67·9)	72·1 (71·5 to 72·7)	72 (71·5 to 72·7)	73 (72·3 to 73·7)	73·1 (72·3 to 73·8)	72·6 (70·5 to 74·2)	72·6 (70·3 to 74·4)	70·2 (67·4 to 72·7)
Dominican Republic	54·8 (51·7 to 57·9)	60·4 (58·3 to 62·5)	65·2 (63·8 to 66·6)	68·6 (67·4 to 69·8)	71·3 (69·9 to 72·7)	73·6 (72·4 to 74·8)	74·1 (71·7 to 76·4)	74·8 (73 to 76·6)	73·8 (72·2 to 75·3)	73·7 (71·8 to 75·5)
Grenada	57·1 (53·8 to 60·4)	64·1 (61·6 to 66·8)	66·9 (64·3 to 69·5)	68·9 (66·2 to 71·3)	70·1 (68·9 to 71·3)	71·8 (71·2 to 72·4)	72 (71·4 to 72·6)	73·2 (72·3 to 74·2)	73·3 (72·2 to 74·4)	69·9 (66·9 to 72·2)
Guyana	51·1 (48·6 to 53·4)	59·5 (58 to 60·9)	62 (60·3 to 63·7)	62·7 (60·8 to 64·8)	63·2 (61·9 to 64·5)	64·9 (63·6 to 66·1)	66·3 (65·2 to 67·4)	67·9 (64·9 to 70·9)	66·6 (63·5 to 69·8)	64·6 (60·6 to 68·6)
Haiti	38·1 (34 to 42·4)	45·1 (42·2 to 48·3)	47·3 (44·6 to 50·1)	50·9 (48·6 to 53·2)	53·6 (51·6 to 55·8)	56·9 (54·9 to 59)	31 (29 to 33)	63·1 (59·6 to 66·5)	61·5 (58·3 to 64·8)	60·1 (56·5 to 63·6)
Jamaica	56·6 (54·7 to 58·3)	64·8 (64·1 to 65·4)	67·5 (66·9 to 68)	73·6 (73·2 to 74·1)	75·2 (74·8 to 75·6)	74·8 (74·3 to 75·2)	76·9 (76·5 to 77·2)	76·4 (73·9 to 79)	76·6 (73·6 to 79·9)	74·1 (71·3 to 76·9)
Puerto Rico	61·2 (60·7 to 61·7)	69·1 (68·9 to 69·4)	71·3 (71 to 71·5)	73·5 (73·3 to 73·7)	73·9 (73·7 to 74·1)	76·4 (76·2 to 76·6)	79·5 (79·3 to 79·8)	81·3 (80·9 to 81·6)	80·7 (80·3 to 81)	80·6 (78·5 to 82·8)
Saint Kitts and Nevis	58·7 (57·1 to 60·2)	61 (60·2 to 61·8)	64·2 (63·3 to 65·1)	65·2 (64·4 to 66·1)	67·5 (66·9 to 68·2)	71·3 (70·4 to 72·2)	72·8 (72·1 to 73·4)	73·3 (71·9 to 74·9)	73·4 (71·6 to 75·1)	71·8 (70·1 to 73·5)
Saint Lucia	51·8 (48·7 to 54·1)	59 (57·1 to 60·9)	64·7 (63·3 to 66)	68·4 (67·6 to 69·2)	70·1 (69·5 to 70·7)	73·2 (72·7 to 73·7)	75·7 (75·1 to 76·3)	76·1 (74·3 to 77·9)	75·6 (73·5 to 77·5)	72·9 (69·7 to 75·6)
Saint Vincent and the Grenadines	51·9 (49·1 to 54·4)	60·2 (58·4 to 62)	65·3 (63·9 to 66·6)	68·3 (67 to 69·5)	70 (69·2 to 70·9)	71·1 (70·4 to 71·7)	73 (72·4 to 73·7)	73·3 (72·1 to 74·3)	73·3 (71·9 to 74·7)	72·2 (70·5 to 73·7)
Suriname	58·8 (57·1 to 60·4)	63·5 (61·9 to 65)	66·5 (64·9 to 68)	66·1 (65·3 to 66·9)	68·6 (67·9 to 69·4)	69·9 (69·2 to 70·5)	72·3 (71·7 to 72·9)	72·5 (71·3 to 73·5)	72·6 (70·9 to 74·1)	70·8 (66·9 to 73·6)
Trinidad and Tobago	57·9 (56·4 to 59·2)	64·3 (63·6 to 64·9)	65·2 (64·6 to 65·8)	67·4 (67 to 67·8)	69·4 (68·9 to 69·8)	70·4 (69·9 to 70·9)	73·6 (73·1 to 74)	74·3 (72·4 to 76·1)	73·5 (70·8 to 76)	71 (67·7 to 74·4)
Virgin Islands	61·7 (59·7 to 63·7)	64 (62·6 to 65·4)	66·3 (64·8 to 67·7)	69·8 (68·3 to 71·3)	72·3 (70·8 to 73·8)	73·6 (72·2 to 74·9)	76·1 (74·6 to 77·5)	76·2 (73·7 to 78·4)	76·7 (74·2 to 78·9)	76·6 (73·1 to 79·5)
Central Latin America	49·7 (47·9 to 51·4)	57·5 (56·9 to 58·1)	61·9 (61·4 to 62·3)	66·5 (65·9 to 67)	70·7 (70·4 to 70·9)	73·7 (73·5 to 73·9)	75·5 (75·2 to 75·8)	76·1 (75·5 to 76·7)	72·5 (71·9 to 73·1)	71·9 (70·9 to 72·8)
Colombia	54·6 (52·3 to 56·7)	59·4 (58·5 to 60·4)	66 (65·3 to 66·7)	69 (67 to 71·1)	71·5 (71·2 to 71·9)	74·4 (74 to 74·7)	78·1 (77·7 to 78·5)	80·2 (79·9 to 80·6)	77·7 (77·3 to 78)	76·1 (74·5 to 77·8)
Costa Rica	56·4 (55·7 to 57)	65·3 (64·9 to 65·6)	66·9 (66·6 to 67·2)	74·6 (74·2 to 74·9)	77 (76·6 to 77·4)	78 (77·7 to 78·2)	79·5 (79·2 to 79·7)	80·1 (79·7 to 80·4)	79·6 (79·3 to 80)	77·7 (77·3 to 78·1)
El Salvador	44·9 (40·8 to 48·3)	53·9 (52·2 to 55·5)	57·8 (56·8 to 58·9)	62 (61·1 to 63)	69·8 (69·3 to 70·4)	73·3 (72·9 to 73·7)	75·5 (75·2 to 75·8)	75·5 (75·2 to 75·8)	73·9 (72·4 to 75·5)	72·7 (70·6 to 74·9)
Guatemala	42·1 (39·1 to 44·8)	47·9 (46·4 to 49·4)	49·6 (48·4 to 50·7)	57·8 (56·7 to 58·7)	62·7 (62·1 to 63·2)	67·3 (66·8 to 67·7)	70·6 (70·1 to 71)	72·2 (71·7 to 72·7)	71 (70·6 to 71·5)	69·4 (67·8 to 71)
Honduras	39·2 (35·6 to 42·3)	48·9 (47 to 50·7)	57·3 (55·8 to 58·7)	67·4 (66·2 to 68·4)	68·7 (67·3 to 70·3)	69·9 (67·3 to 72·3)	70·9 (68·1 to 73·5)	71·6 (69·3 to 73·3)	69·5 (67·4 to 71·2)	68·5 (66·3 to 70·3)
Mexico	48·2 (45·6 to 50·6)	56·9 (56 to 57·8)	61·2 (60·5 to 61·8)	65·8 (65·3 to 66·4)	70·6 (70·3 to 71)	74 (73·8 to 74·3)	75·1 (74·8 to 75·3)	75·6 (75·3 to 75·8)	71 (70·7 to 71·2)	70·9 (70·6 to 71·2)

Aguascalientes	53·2 (50 to 56·3)	60·8 (58·7 to 63)	63·9 (62·1 to 65·7)	68·1 (67·2 to 69)	72·5 (72 to 73)	75 (74·5 to 75·4)	76·2 (75·9 to 76·6)	76·7 (76·3 to 77·1)	73 (72·6 to 73·3)	72·6 (72·2 to 73)
Baja California	49·8 (45·8 to 53·8)	58·3 (56·1 to 60·4)	61·6 (59·3 to 63·6)	67·6 (67·1 to 68)	69·6 (69·2 to 69·9)	71·2 (70·9 to 71·5)	73·3 (73 to 73·5)	74·1 (73·7 to 74·4)	68·8 (68·5 to 69·1)	69·6 (69·2 to 69·9)
Baja California Sur	49·5 (45·9 to 53·3)	58·7 (56·5 to 60·8)	62·8 (60·8 to 64·8)	68·1 (67·5 to 68·8)	72·9 (72·4 to 73·3)	74·6 (74·2 to 75)	75·9 (75·6 to 76·3)	76 (75·5 to 76·4)	72·3 (71·9 to 72·7)	71·6 (71·2 to 72·1)
Campeche	51·2 (47·7 to 54·4)	59·5 (57·4 to 61·7)	63·4 (61·5 to 65·2)	67·8 (66·9 to 68·7)	71·8 (71·2 to 72·3)	75·4 (75 to 75·9)	76 (75·7 to 76·4)	75·8 (75·3 to 76·3)	72 (71·6 to 72·4)	72 (71·5 to 72·4)
Chiapas	49·3 (45·5 to 52·8)	56·1 (53·8 to 58·2)	60 (57·9 to 62)	64·3 (63·4 to 65·2)	69·9 (69·2 to 70·5)	73 (72·5 to 73·4)	74·9 (74·6 to 75·2)	74·6 (74·2 to 74·9)	70·7 (70·3 to 71)	71 (70·5 to 71·4)
Chihuahua	49 (45·2 to 52·7)	56·7 (54·7 to 58·9)	60·8 (58·7 to 62·7)	65·2 (64·4 to 65·9)	68·4 (67·9 to 68·9)	71·3 (70·9 to 71·7)	68·3 (68 to 68·6)	72·5 (72·1 to 72·9)	66·8 (66·3 to 67·1)	69·9 (69·3 to 70·3)
Coahuila	47·9 (44 to 51·4)	56·8 (54·6 to 58·9)	60·8 (58·7 to 62·6)	65·3 (64·3 to 66·2)	70·5 (69·9 to 70·9)	73·5 (73·1 to 73·9)	74·5 (74·2 to 74·8)	75·2 (74·8 to 75·5)	70·3 (69·9 to 70·6)	72 (71·6 to 72·4)
Colima	42·5 (38·7 to 46)	53 (50·8 to 55·2)	58·3 (56·2 to 60·4)	64·2 (63·1 to 65·3)	70·2 (69·6 to 70·7)	74·4 (73·9 to 74·8)	75·6 (75·2 to 76)	74·5 (74·1 to 75)	71·1 (70·7 to 71·4)	69·5 (69 to 69·9)
Durango	47·1 (43·7 to 50·6)	56·6 (54·2 to 58·8)	61·4 (59·3 to 63·5)	66·2 (65·1 to 67·3)	71·3 (70·7 to 71·8)	75·1 (74·6 to 75·6)	73·3 (72·9 to 73·7)	76·6 (76·2 to 77)	73·3 (72·9 to 73·6)	74 (73·6 to 74·5)
Guanajuato	47·8 (43·1 to 52)	56·5 (54·5 to 58·7)	60·6 (58·5 to 62·7)	66·1 (65·5 to 66·7)	70·9 (70·5 to 71·3)	74·9 (74·6 to 75·1)	76·1 (75·9 to 76·4)	74·4 (74 to 74·6)	70·2 (69·9 to 70·4)	69·7 (69·4 to 70)
Guerrero	43·2 (39·7 to 46·6)	51·3 (48·6 to 54·2)	55·9 (53·7 to 58·2)	61·4 (60·3 to 62·5)	67·8 (66·8 to 68·7)	71·9 (71·1 to 72·5)	73·3 (72·5 to 74·2)	75·2 (74·3 to 76·1)	72·6 (71·6 to 73·5)	72·6 (71·6 to 73·6)
Hidalgo	44·9 (41 to 48·3)	53·7 (51·5 to 56)	58 (55·5 to 60·1)	63 (62·3 to 63·7)	71·4 (70·9 to 71·9)	76 (75·6 to 76·5)	77·1 (76·8 to 77·5)	77·3 (76·9 to 77·6)	73·9 (73·6 to 74·2)	73·6 (72·2 to 75)
Jalisco	51·7 (48·1 to 55·1)	59·2 (57·2 to 61·2)	63·1 (61·1 to 65·1)	68 (67·4 to 68·6)	71·2 (70·7 to 71·6)	74 (73·6 to 74·3)	75·5 (75·2 to 75·7)	75·4 (75·1 to 75·6)	72·1 (71·9 to 72·4)	70·7 (70·3 to 71)
México	49·7 (46 to 53·3)	57·9 (55·9 to 60·1)	62·2 (60·2 to 64)	64·9 (64·3 to 65·5)	69·6 (69·2 to 69·9)	73·5 (73·2 to 73·8)	75·2 (75 to 75·5)	75·7 (75·3 to 76)	69·3 (69 to 69·6)	72 (71·6 to 72·3)
Mexico City	54·6 (50·9 to 58·2)	62·9 (61 to 64·8)	65·7 (64 to 67·4)	68·6 (68 to 69·1)	71·7 (71·1 to 72·5)	74·4 (74·2 to 74·7)	75·5 (75·2 to 75·7)	76·5 (76·2 to 76·8)	69·9 (69·6 to 70·1)	67·4 (67 to 67·9)
Michoacán de Ocampo	44·8 (41·4 to 47·9)	54 (51·1 to 56·8)	59·1 (56·8 to 61·1)	64·2 (63·4 to 65)	71·5 (70·9 to 72)	74·5 (74·1 to 74·9)	75·5 (75 to 75·9)	75·8 (75·3 to 76·2)	72·7 (72·2 to 73)	70·4 (69·9 to 70·8)
Morelos	45·9 (42·3 to 49·3)	55·8 (53·5 to 58)	60·6 (58·3 to 62·6)	65·5 (64·7 to 66·3)	71·4 (70·9 to 71·8)	75·2 (74·8 to 75·5)	76 (75·7 to 76·3)	74·8 (74·5 to 75·2)	70·8 (70·5 to 71·1)	68·7 (68·3 to 69·1)
Nayarit	47·3 (43·8 to 50·6)	56·8 (54·6 to 58·9)	62·2 (60·3 to 64·2)	68·2 (67·3 to 69·1)	72 (71·4 to 72·5)	75·9 (75·4 to 76·4)	75·8 (75·4 to 76·2)	77·5 (77 to 77·9)	74·6 (74·2 to 75)	73·6 (73·1 to 74)
Nuevo León	54·2 (50·8 to 57·4)	62·1 (60·2 to 64·1)	65·6 (63·8 to 67·4)	70·5 (69·9 to 71·1)	72·8 (72·4 to 73·2)	75·1 (74·8 to 75·4)	75·9 (75·6 to 76·1)	76·7 (76·4 to 77)	72·7 (72·5 to 73)	72 (71·6 to 72·3)
Oaxaca	42·4 (39 to 45·8)	50·6 (48·2 to 53·3)	54·5 (52·1 to 56·6)	60 (59·1 to 60·8)	68·4 (67·8 to 69)	73·5 (73 to 73·9)	75·2 (74·7 to 75·7)	76·2 (75·7 to 76·6)	73·1 (72·6 to 73·5)	71·6 (71·1 to 72)
Puebla	41·5 (36·9 to 45·9)	50·7 (48·5 to 53)	55·8 (53·7 to 57·8)	61·8 (61 to 62·7)	68·9 (68·3 to 69·5)	72·9 (72·4 to 73·3)	75 (74·7 to 75·3)	75·1 (74·7 to 75·4)	70·1 (69·8 to 70·4)	68·6 (68·2 to 69)
Querétaro	45·8 (41·1 to 50·5)	55·9 (53·7 to 58·2)	60·9 (58·8 to 63·1)	65·4 (64 to 66·8)	70·3 (69·8 to 70·8)	73·9 (73·6 to 74·2)	76 (75·7 to 76·3)	76·8 (76·5 to 77·1)	73 (72·7 to 73·2)	71·4 (71 to 71·8)
Quintana Roo	51·1 (48 to 53·9)	57·8 (55·8 to 60)	60·9 (58·9 to 62·8)	65·1 (63·7 to 66·4)	70·7 (69·8 to 71·5)	73·3 (72·6 to 74·1)	73·7 (72·9 to 74·4)	74 (72·9 to 75)	71·7 (70·8 to 72·5)	71·9 (70·9 to 72·8)
San Luis Potosí	45·8 (42·3 to 49·3)	55·3 (52·6 to 58·1)	59·9 (57·6 to 62·1)	68·1 (67·3 to 68·9)	72·1 (71·5 to 72·6)	75·6 (75·1 to 76)	76·4 (75·9 to 76·8)	76·7 (76·3 to 77·2)	72·1 (71·7 to 72·5)	71·8 (71·4 to 72·2)
Sinaloa	50·5 (46·8 to 53·9)	58·6 (56·3 to 60·7)	63 (61 to 65·1)	68·1 (67·4 to 68·8)	73 (72·4 to 73·5)	75·7 (75·2 to 76·1)	75 (74·7 to 75·3)	77·7 (77·3 to 78)	73·5 (73·2 to 73·8)	73·9 (73·6 to 74·3)
Sonora	53·3 (49·8 to 56·2)	60·3 (58·1 to 62·4)	63·3 (61·3 to 65·4)	67·1 (65·6 to 68·6)	70·9 (70·4 to 71·4)	73·1 (72·7 to 73·5)	73·9 (73·6 to 74·2)	74·1 (73·7 to 74·5)	69·6 (69·2 to 69·9)	70·2 (69·8 to 70·6)
Tabasco	49·5 (45·7 to 52·8)	57 (54·7 to 59·2)	60·2 (58 to 62·4)	63·9 (63·1 to 64·6)	71 (70·4 to 71·5)	74·4 (73·9 to 74·9)	74·4 (74·1 to 74·7)	73·7 (73·2 to 74·1)	68·9 (68·5 to 69·3)	71·1 (69·4 to 72·9)
Tamaulipas	51·4 (47·7 to 54·8)	59·2 (56·9 to 61·3)	63·1 (61·1 to 64·9)	67·1 (66·4 to 67·7)	72·1 (71·7 to 72·5)	75·3 (74·9 to 75·6)	75·2 (74·9 to 75·5)	76·1 (75·7 to 76·5)	72·5 (72·2 to 72·9)	73·3 (72·8 to 73·6)
Tlaxcala	46 (41·4 to 50·6)	55·9 (53·7 to 58·2)	61·3 (59·2 to 63·4)	66·1 (64·6 to 67·6)	71·2 (70·7 to 71·7)	74·8 (74·4 to 75·2)	76·4 (76 to 76·7)	76·6 (76·2 to 77·1)	71 (70·7 to 71·4)	70·3 (69·8 to 70·7)
Veracruz de Ignacio de la Llave	47·4 (43·4 to 51)	55·9 (53·8 to 58)	60·2 (58·1 to 62·4)	65·9 (65·1 to 66·7)	70·9 (70·3 to 71·4)	74·4 (74·1 to 74·8)	75·5 (75·2 to 75·8)	75·1 (74·8 to 75·4)	71·7 (71·4 to 72)	72 (70·4 to 73·5)
Yucatán	52·9 (49·4 to 56·2)	59·3 (57·3 to 61·3)	63·1 (61·3 to 65)	67·1 (66·3 to 68)	71·7 (71·1 to 72·2)	74·8 (74·3 to 75·1)	75·8 (75·5 to 76·1)	76·2 (75·9 to 76·6)	72·8 (72·5 to 73·2)	72·4 (72 to 72·8)
Zacatecas	52·4 (49 to 55·5)	60·4 (58·1 to 62·6)	64·6 (62·7 to 66·5)	69·9 (68·9 to 70·8)	73·8 (73·2 to 74·2)	76 (75·5 to 76·4)	76·3 (76 to 76·6)	76·1 (75·7 to 76·5)	71·6 (71·2 to 71·9)	71·1 (70·6 to 71·5)
Nicaragua	48 (44·3 to 51·2)	54·3 (52·6 to 55·9)	58·1 (56·7 to 59·5)	67·1 (65·1 to 69·1)	73·8 (73·2 to 74·4)	76·7 (76·2 to 77·1)	77 (76·6 to 77·4)	78·7 (78·3 to 79·1)	74·7 (74·3 to 75·2)	73·3 (72 to 74·4)
Panama	61·1 (59·4 to 62·8)	66·1 (65·2 to 66·9)	68·1 (67·5 to 68·7)	74·1 (73·6 to 74·6)	76·4 (76 to 76·9)	78·1 (77·7 to 78·5)	78·6 (78·2 to 79)	79·9 (79·4 to 80·3)	77·2 (76·8 to 77·7)	78·3 (76·2 to 80·8)
Venezuela	56 (55·5 to 56·4)	65·2 (65 to 65·3)	66·3 (66·1 to 66·5)	69·4 (69·3 to 69·5)	72·3 (72·1 to 72·4)	73·9 (73·7 to 74)	75·6 (75·5 to 75·7)	74 (71 to 76·8)	70·6 (67·8 to 73·2)	69·7 (67 to 72·3)
Tropical Latin America	51·5 (47·9 to 54·7)	57·2 (55·7 to 58·6)	61·2 (60·1 to 62·2)	65·9 (65·3 to 66·5)	69·4 (69 to 69·7)	72·3 (71·9 to 72·6)	74·5 (74·2 to 74·8)	76·5 (76·2 to 76·8)	75·1 (74·9 to 75·4)	73·7 (73·4 to 73·9)
Brazil	51·3 (47·6 to 54·6)	57 (55·5 to 58·4)	61 (59·9 to 62)	65·8 (65·1 to 66·4)	69·2 (68·9 to 69·6)	72·2 (71·9 to 72·5)	74·5 (74·1 to 74·8)	76·5 (76·3 to 76·8)	75·1 (74·9 to 75·3)	73·7 (73·5 to 73·9)
Acre	48·9 (43·1 to 54·2)	56·1 (53·5 to 58·8)	61 (58·6 to 63·5)	65·8 (64·5 to 67)	69·2 (68·4 to 70)	71·7 (70·9 to 72·4)	74·2 (73·6 to 74·7)	75·4 (74·8 to 75·9)	73·3 (72·7 to 73·7)	73·4 (72·9 to 73·9)
Alagoas	43·2 (37 to 49·1)	49·3 (46·2 to 52·3)	54·4 (51·6 to 57·1)	59·8 (58·3 to 61·2)	66 (64·6 to 67·3)	71·6 (70·9 to 72·3)	73·3 (72·3 to 74·2)	76 (75·1 to 76·8)	74·3 (73·9 to 74·8)	74·2 (73·9 to 74·6)
Amapá	47·1 (40·8 to 52·7)	55·9 (53·5 to 58·4)	63·2 (61 to 65·4)	70·9 (69·9 to 71·8)	71·3 (70·3 to 72·4)	72·5 (71·3 to 73·7)	74·9 (73·7 to 76·1)	73·7 (72·3 to 75·1)	72·3 (71 to 73·6)	73·1 (71·5 to 74·5)
Amazonas	54·7 (48·7 to 59·8)	61·2 (58·5 to 64)	64·4 (62 to 66·8)	69·2 (68 to 70·3)	71·9 (71·1 to 72·8)	74·8 (74·1 to 75·4)	76 (75·4 to 76·7)	76·4 (76 to 76·7)	72·5 (72·1 to 72·8)	71 (70·6 to 71·5)
Bahia	51·7 (45·9 to 56·9)	56·6 (53·6 to 59·6)	60·6 (58·4 to 62·8)	67·8 (66·8 to 68·8)	70·4 (69·5 to 71·2)	73 (72·2 to 73·6)	73·2 (72·5 to 74)	76·8 (76 to 77·6)	75·6 (74·9 to 76·3)	75·3 (74·6 to 76·1)
Ceará	43 (36·4 to 49)	49·6 (46·5 to 52·7)	56·6 (53·8 to 59·3)	64·7 (62 to 67·1)	73·2 (72 to 74·4)	74·8 (73·5 to 75·9)	76·4 (75·4 to 77·4)	76·8 (75·6 to 78·1)	75·4 (74·3 to 76·4)	75·8 (74·5 to 77)
Distrito Federal	53·1 (48·5 to 56·9)	58·9 (56·5 to 61·3)	62·8 (60·6 to 64·8)	66·8 (66·4 to 67·2)	69·7 (69·4 to 70)	72·5 (72·2 to 72·7)	75·2 (74·9 to 75·5)	78·2 (77·9 to 78·5)	76 (75·8 to 76·3)	75·2 (75 to 75·5)
Espírito Santo	55·3 (51·8 to 58·6)	59·9 (57·4 to 62·5)	62·7 (60·4 to 64·9)	67·7 (67·2 to 68·1)	70·7 (70·3 to 71)	72·4 (72·1 to 72·8)	74·5 (74 to 74·9)	77·1 (76·7 to 77·4)	75·1 (74·7 to 75·4)	74·1 (73·8 to 74·4)
Goiás	44 (38·7 to 49·3)	50·2 (47·6 to 52·9)	57·4 (54·9 to 59·7)	64·9 (63·5 to 66·4)	69·8 (69·2 to 70·5)	73·2 (72·5 to 73·7)	74·4 (73·9 to 74·8)	76·1 (75·7 to 76·4)	74·1 (73·7 to 74·4)	71·9 (71·6 to 72·3)
Maranhão	43·4 (39·1 to 47·5)	48·9 (46·2 to 51·8)	53·6 (50·9 to 56·1)	61·8 (59·7 to 64)	67·7 (66·6 to 68·8)	71·5 (70·4 to 72·4)	70·7 (69·8 to 71·6)	74 (72·8 to 75·2)	73 (72 to 73·9)	73·4 (72·3 to 74·5)
Mato Grosso	56·8 (52·3 to 60·9)	60·1 (57·4 to 62·8)	64·1 (61·6 to 66·8)	70·6 (69·5 to 71·7)	70·5 (69·6 to 71·4)	72·4 (71·4 to 73·4)	74·7 (74·1 to 75·1)	76·8 (76·4 to 77·2)	73·8 (73·4 to 74·2)	72·1 (71·7 to 72·4)
Mato Grosso do Sul	56·4 (52 to 60·4)	60·7 (58·1 to 63·3)	63·9 (61·4 to 66·3)	68·9 (68·5 to 69·4)	71 (70·6 to 71·3)	72·3 (72 to 72·7)	74·2 (73·9 to 74·5)	76·7 (76·4 to 77)	74·9 (74·6 to 75·2)	72·6 (72·3 to 72·9)
Minas Gerais	52·7 (48·2 to 56·6)	57·8 (55·2 to 60·2)	61·5 (59·1 to 63·8)	66·1 (65·5 to 66·7)	70·2 (69·8 to 70·6)	73·9 (73·4 to 74·3)	75·9 (75·5 to 76·2)	77·7 (77·5 to 78)	76·9 (76·6 to 77·1)	74·7 (74·4 to 75)
Pará	49·7 (44·6 to 54·7)	56·9 (54·3 to 59·5)	61·5 (58·8 to 64)	66·9 (65·3 to 68·3)	70·8 (69·8 to 71·8)	73·6 (72·5 to 74·8)	74·2 (73·1 to 75·2)	75·6 (74·3 to 76·8)	73·5 (72·3 to 74·6)	73·9 (72·6 to 75·1)
Paraíba	41·1 (34·2 to 47·9)	49·3 (46·3 to 52·2)	57 (54·4 to 59·7)	64·7 (63·1 to 66·3)	71·6 (70·8 to 72·3)	74·6 (73·9 to 75·2)	74·7 (74·2 to 75·1)	75·3 (74·9 to 75·6)	76 (75·6 to 76·3)	75 (74·6 to 75·3)
Paraná	56·7 (53 to 59·8)	60·4 (58 to 62·8)	62·8 (60·6 to 65)	66·4 (66 to 66·8)	69·8 (69·5 to 70·1)	72·3 (72 to 72·5)	74 (73·6 to 74·4)	76·9 (76·6 to 77·2)	75·6 (75·3 to 75·9)	72·4 (72·1 to 72·8)
Pernambuco	40·4 (34·4 to 46)	47·5 (44·6 to 50·3)	53·8 (51·4 to 56·2)	62 (60·3 to 63·5)	66 (65·3 to 66·8)	69·8 (69·2 to 70·4)	73·9 (73·5 to 74·3)	75·7 (75·3 to 75·9)	73·9 (73·5 to 74·3)	74 (73·5 to 74·4)
Piauí	52·3 (46·4 to 57·8)	57·8 (55·1 to 60·3)	61 (58·2 to 63·6)	67·7 (65·8 to 69·5)	72·7 (71·6 to 74)	75·2 (74 to 76·4)	75·5 (74·3 to 76·6)	77·3 (76 to 78·7)	75·8 (74·5 to 77)	76·1 (74·8 to 77·5)
Rio de Janeiro	56·1 (50·6 to 60·4)	61·3 (58·9 to 63·8)	63·6 (61·4 to 65·8)	65·2 (64·7 to 65·6)	66·7 (66·4 to 66·9)	70·5 (70·3 to 70·7)	73·4 (73·2 to 73·5)	74·9 (74·7 to 75·2)	73·1 (72·8 to 73·3)	72·1 (71·9 to 72·3)
Rio Grande do Norte	39·7 (32·9 to 46·1)	49·2 (45·6 to 52·7)	58·7 (56·1 to 60·9)	67·6 (65·7 to 69·2)	73·1 (71·9 to 74·3)	75·6 (74·7 to 76·5)	76·4 (75·5 to 77·1)	77·3 (76·9 to 77·7)	76 (75·6 to 76·3)	75·6 (75·2 to 75·9)
Rio Grande do Sul	57·8 (54·1 to 61·2)	62·5 (60·2 to 64·7)	65·8 (63·8 to 68)	68·6 (68·4 to 68·9)	71·1 (70·9 to 71·3)	72·9 (72·7 to 73·1)	75·2 (75 to 75·3)	76·6 (76·4 to 76·8)	76·2 (76 to 76·4)	73·8 (73·6 to 73·9)
Rondônia	45·1 (36·6 to 51·8)	52·4 (49·9 to 55·1)	57·3 (54·9 to 59·4)	59·6 (58·4 to 60·8)	67 (66 to 67·8)	71·9 (71·3 to 72·6)	74·4 (73·4 to 75·3)	76·9 (75·8 to 77·9)	74·9 (74 to 75·8)	74·7 (73·6 to 75·8)
Roraima	43·2 (37·9 to 48)	50 (47·4 to 52·5)	55 (52·6 to 57·6)	62·3 (60·3 to 64·2)	64·4 (62·6 to 66·1)	68·8 (67·1 to 70·4)	71·6 (70 to 73)	72·3 (70·3 to 74)	70·7 (69 to 72·4)	71·7 (69·8 to 73·5)
Santa Catarina	59·9 (55·9 to 63·1)	64·1 (61·9 to 66·5)	66·3 (64·1 to 68·4)	69·3 (68·8 to 69·8)	71 (70·6 to 71·4)	73 (72·6 to 73·4)	75·7 (75·3 to 76·1)	77·8 (77·4 to 78·1)	76·7 (76·4 to 76·9)	74·6 (74·3 to 74·9)
São Paulo	59 (55·9 to 62·3)	62·9 (60·6 to 65·2)	65·4 (63·1 to 67·3)	67 (66·6 to 67·4)	69 (68·7 to 69·2)	71·6 (71·3 to 71·8)	75·1 (74·8 to 75·4)	77·2 (77 to 77·5)	75·7 (75·5 to 75·9)	73·6 (73·3 to 73·9)
Sergipe	49·2 (42·8 to 54·3)	55·1 (52·2 to 57·8)	61 (58·7 to 63·2)	65 (63·9 to 66·1)	69·4 (68·7 to 70·1)	72 (71·2 to 72·8)	74·6 (74 to 75·1)	76·2 (75·7 to 76·6)	74·5 (74·1 to 74·9)	74·3 (73·9 to 74·7)
Tocantins	48·3 (39·3 to 55·1)	53·7 (50·8 to 56·7)	60·1 (57·4 to 62·8)	68 (65·8 to 70·2)	71·8 (70·5 to 73)	73·2 (72·2 to 74·2)	73·8 (72·9 to 74·8)	75·1 (74 to 76·3)	74·2 (73·1 to 75·1)	74·5 (73·4 to 75·6)

Paraguay	58·7 (55·4 to 62)	68·4 (66·6 to 70·2)	70 (68·7 to 71·3)	72·6 (71·3 to 74)	75·4 (74 to 76·7)	75·4 (73·9 to 77·2)	75·3 (73·9 to 76·6)	75·4 (73·4 to 77·4)	75·4 (73·4 to 77·5)	72·2 (69·9 to 74·2)
North Africa and Middle East	43·2 (40 to 46·3)	48·8 (46·7 to 50·9)	54·4 (52·8 to 56)	60·3 (59 to 61·3)	65·4 (64·6 to 66·3)	69·2 (68·4 to 69·9)	72 (71·1 to 72·8)	73·6 (72·6 to 74·5)	72 (70·9 to 73)	71·1 (70 to 72·2)
North Africa and Middle East	43·2 (40 to 46·3)	48·8 (46·7 to 50·9)	54·4 (52·8 to 56)	60·3 (59 to 61·3)	65·4 (64·6 to 66·3)	69·2 (68·4 to 69·9)	72 (71·1 to 72·8)	73·6 (72·6 to 74·5)	72 (70·9 to 73)	71·1 (70 to 72·2)
Afghanistan	38·1 (34·2 to 42·2)	44·9 (41·5 to 48·6)	46·9 (43·3 to 50·5)	43·6 (34·2 to 48·6)	52·5 (49·9 to 55·4)	53·7 (51·3 to 56·2)	59·4 (56·5 to 62·2)	61·6 (59 to 64·3)	59·4 (57 to 61·7)	58·2 (56·3 to 60·3)
Algeria	42·7 (38·4 to 46·2)	45·7 (43·3 to 48·1)	53·8 (50·9 to 56·6)	57·6 (56·7 to 58·6)	69·9 (68·7 to 71)	72·6 (71·4 to 73·7)	75·4 (74·7 to 76·1)	77·1 (75·8 to 78·2)	74·6 (73·5 to 75·7)	73·6 (72·3 to 74·9)
Bahrain	51·6 (46·8 to 56·1)	56·2 (53 to 59·3)	63·1 (60·2 to 65·8)	67·4 (66·5 to 68·3)	69 (68·5 to 69·5)	69·7 (69·1 to 70·2)	73·9 (73·4 to 74·3)	76 (75 to 76·8)	74·4 (73·5 to 75·4)	73·3 (72·3 to 74·4)
Egypt	44 (40·1 to 48)	48·6 (46·9 to 50·3)	51·1 (49·8 to 52·3)	57·8 (56·7 to 58·7)	63 (62·4 to 63·5)	67·4 (67 to 67·8)	68·3 (68 to 68·6)	70·5 (70·2 to 70·7)	69·1 (68 to 70·4)	68·4 (66·7 to 70)
Iran	39 (35·1 to 42·9)	46 (43·4 to 48·5)	55·4 (53·5 to 57·1)	65·2 (64·2 to 66·2)	67·5 (66·8 to 68·1)	72·9 (72·4 to 73·3)	75·9 (75·7 to 76·2)	78·1 (77·7 to 78·3)	77·4 (77·1 to 77·7)	74·4 (74·1 to 74·6)
Alborz	43·3 (38·8 to 47·7)	48·4 (45·3 to 51·4)	58·8 (56·2 to 61·4)	69·4 (67·2 to 71·4)	71·7 (69·9 to 73·3)	73·8 (72·6 to 75)	76·8 (75·9 to 77·7)	78·1 (76·7 to 79·5)	77·2 (76 to 78·3)	73·8 (72·9 to 74·7)
Ardebil	38·6 (34·1 to 42·9)	44·3 (41·3 to 47·2)	52 (49·3 to 54·7)	62·1 (60 to 64·1)	67·1 (65·4 to 68·8)	73·3 (72·6 to 74·1)	76·4 (75·9 to 76·9)	76·6 (75·7 to 77·4)	76·1 (75·2 to 77)	73·1 (72·3 to 73·8)
Bushehr	43·2 (38·9 to 47·6)	49·1 (46·1 to 52)	56·6 (54 to 59·1)	66·6 (64·5 to 68·4)	70·5 (68·9 to 72·1)	73·8 (73·1 to 74·5)	75·6 (75·2 to 76·1)	78 (77·2 to 78·9)	77·5 (76·6 to 78·4)	74·7 (73·9 to 75·5)
Chahar Mahaal and Bakhtiari	46·9 (42·5 to 51·2)	52·3 (49·5 to 54·9)	59 (56·7 to 61·6)	66·4 (64·4 to 68·3)	69·9 (68·3 to 71·5)	74·4 (73·3 to 75·5)	77·4 (76·7 to 78·1)	79·8 (78·7 to 80·8)	79·4 (78·3 to 80·3)	76·8 (75·9 to 77·6)
East Azarbayejan	36·3 (31·9 to 40·6)	43 (39·9 to 45·9)	51·7 (49·1 to 54·4)	62·6 (60·3 to 64·8)	67·3 (65·5 to 69)	71·4 (70·3 to 72·4)	74·4 (73·8 to 75)	76·6 (75·5 to 77·6)	76·4 (75·3 to 77·4)	73·8 (73 to 74·7)
Fars	48·6 (44·3 to 52·7)	53·8 (51·3 to 56·5)	60·5 (58·2 to 62·8)	66·4 (64·3 to 68·4)	69·1 (67·2 to 70·7)	72 (70·7 to 73·2)	74·2 (73·3 to 75·1)	76·9 (75·5 to 78·1)	76·5 (75·4 to 77·7)	73·6 (72·5 to 74·6)
Gilan	45·3 (40·5 to 49·7)	51·6 (47·7 to 55·2)	60·1 (57·7 to 62·5)	68·1 (66·1 to 69·9)	45·5 (43·4 to 47·4)	73·4 (72·4 to 74·3)	75·9 (75·3 to 76·6)	77·9 (76·7 to 79)	77·3 (76·3 to 78·4)	74·2 (73·4 to 75)
Golestan	42·2 (37·9 to 46·4)	47·6 (44·6 to 50·5)	54·9 (52·6 to 57·3)	64·3 (62·3 to 66·3)	67·1 (65·3 to 68·8)	70·8 (70·1 to 71·5)	74·1 (73·6 to 74·6)	74·6 (73·8 to 75·6)	74·3 (73·4 to 75·2)	71·5 (70·7 to 72·2)
Hamadan	31·6 (27·1 to 35·9)	38·5 (35·4 to 41·7)	48 (45·4 to 50·8)	60·8 (58·6 to 62·9)	67·5 (65·6 to 69·3)	72·9 (71·9 to 73·9)	75 (74·3 to 75·7)	77·8 (76·6 to 79)	77·2 (76·1 to 78·3)	74·8 (73·8 to 75·7)
Hormozgan	40·2 (35·7 to 44·6)	46·2 (43·4 to 48·8)	55·4 (52·9 to 58)	64 (61·7 to 66·2)	66·4 (64·5 to 68·2)	70·8 (69·7 to 71·8)	74·6 (73·8 to 75·4)	77·3 (76·1 to 78·6)	76·8 (75·6 to 78)	73·8 (72·8 to 74·8)
Ilam	44·4 (40·3 to 48·4)	49·2 (46·1 to 52·1)	56 (53·5 to 58·5)	64·6 (62·4 to 66·6)	68·7 (67·1 to 70·4)	72·6 (71·7 to 73·6)	75·2 (74·5 to 75·8)	76·8 (75·8 to 77·8)	76·4 (75·5 to 77·4)	73·6 (72·8 to 74·5)
Isfahan	44·7 (40·2 to 49)	49·3 (46·1 to 52·5)	59·3 (56·8 to 61·8)	69 (67 to 70·9)	72·3 (70·6 to 73·9)	75·4 (74·3 to 76·4)	77·9 (77·2 to 78·5)	79·1 (78 to 80·3)	78·3 (77·2 to 79·3)	75 (74·1 to 75·9)
Kerman	35·9 (31·2 to 40·8)	44·4 (41·5 to 47·6)	53·7 (51 to 56·1)	63·4 (61·2 to 65·4)	67·5 (65·7 to 69·3)	70·7 (69·6 to 71·7)	74·2 (73·4 to 74·9)	76·6 (75·4 to 77·8)	76·3 (75·2 to 77·4)	73·7 (72·7 to 74·7)
Kermanshah	38·1 (33·6 to 42·5)	43·6 (40·5 to 46·4)	51·7 (49·1 to 54·3)	61·7 (59·4 to 63·9)	65·6 (63·7 to 67·6)	69·6 (68·5 to 70·7)	73·6 (72·8 to 74·4)	76·8 (75·6 to 78)	76·1 (74·9 to 77·3)	73·1 (72·2 to 74·1)
Khorasan-e-Razavi	29·9 (25·8 to 34·3)	37·4 (34·1 to 40·7)	47·7 (45 to 50·4)	61 (58·7 to 63·3)	65·6 (63·7 to 67·4)	69·8 (68·6 to 70·9)	73·7 (73 to 74·5)	76·8 (75·7 to 78)	76·3 (75·4 to 77·3)	73·4 (72·5 to 74·3)
Khuzestan	42·8 (37·8 to 47·5)	49·5 (46·3 to 52·3)	57·3 (54·6 to 59·6)	64·7 (62·5 to 66·8)	69·6 (68 to 71·1)	72·1 (71·4 to 72·9)	75 (74·6 to 75·4)	76·6 (75·6 to 77·6)	75·9 (75 to 76·9)	72·7 (71·9 to 73·5)
Kohgiluyeh and Boyer-Ahmad	44·2 (39·8 to 48·6)	50·9 (48·2 to 53·5)	57·3 (54·9 to 59·7)	63·9 (61·7 to 65·9)	69·2 (67·3 to 71)	74 (72·9 to 75·2)	77·6 (76·8 to 78·3)	79·1 (77·8 to 80·4)	78·5 (77·3 to 79·6)	75·7 (74·6 to 76·6)
Kurdistan	30·9 (26·8 to 34·9)	35·9 (32·8 to 38·8)	45·2 (42·6 to 47·8)	57·3 (54·9 to 59·6)	64·7 (62·8 to 66·6)	71·4 (70·4 to 72·3)	74·8 (74·2 to 75·4)	78·4 (77·5 to 79·5)	77·9 (76·9 to 78·9)	75·1 (74·2 to 76)
Lorestan	38·4 (33·1 to 43·4)	46·4 (43·2 to 49·4)	54·6 (52 to 57·2)	63·2 (61·1 to 65·2)	68·2 (66·4 to 69·8)	72·6 (71·7 to 73·6)	75·4 (74·7 to 76·1)	77·6 (76·5 to 78·8)	76·9 (75·9 to 78)	73·3 (72·6 to 74·2)
Markazi	41·1 (36·4 to 45·5)	45·5 (42·6 to 48·4)	53·3 (50·5 to 56)	63·6 (61·4 to 65·6)	68·5 (66·7 to 70·4)	72·6 (71·5 to 73·6)	76·7 (76 to 77·5)	79·5 (78·4 to 80·7)	78·5 (77·4 to 79·7)	75·2 (74·3 to 76·1)
Mazandaran	49·5 (45 to 53·7)	54·7 (51·9 to 57·5)	62 (59·5 to 64·3)	69·1 (67·3 to 70·8)	73 (71·4 to 74·7)	75·4 (74·3 to 76·4)	77·1 (76·4 to 77·8)	79 (77·8 to 80·2)	78·1 (77·1 to 79·2)	75 (74·1 to 75·8)
North Khorasan	34·4 (29 to 39·5)	40·4 (37·4 to 43·5)	48·5 (45·8 to 51)	60·9 (58·5 to 63)	65·4 (63·5 to 67·1)	70·5 (69·5 to 71·6)	75·1 (74·3 to 75·8)	77 (75·8 to 78·1)	76·2 (75·2 to 77·2)	73 (72·1 to 73·8)
Qazvin	34·8 (29·9 to 39·2)	40·5 (37·3 to 43·7)	50·7 (47·9 to 53·3)	63 (60·9 to 65·1)	69·1 (67·4 to 70·7)	72·6 (71·6 to 73·5)	75·9 (75·3 to 76·5)	78 (76·9 to 79)	77·3 (76·2 to 78·4)	74·1 (73·3 to 74·9)
Qom	45·9 (41·4 to 50·2)	50·6 (47·7 to 53·4)	57·8 (55·1 to 60·1)	65·6 (63·6 to 67·6)	70·3 (68·7 to 71·8)	73 (72·1 to 73·8)	76·1 (75·6 to 76·6)	79·4 (78·5 to 80·4)	78·4 (77·4 to 79·4)	74·8 (74 to 75·5)
Semnan	40·1 (35·6 to 44·4)	44·8 (41·8 to 47·9)	54·9 (52·2 to 57·7)	65·1 (63·1 to 67)	70·2 (68·6 to 71·8)	73 (71·9 to 73·9)	76·1 (75·4 to 76·7)	78·4 (77·3 to 79·6)	77·6 (76·5 to 78·6)	74 (73·1 to 74·7)
Sistan and Baluchistan	32·4 (27·1 to 37·9)	40·6 (37·7 to 43·5)	49 (46·4 to 51·6)	60 (57·6 to 62·1)	64·6 (62·6 to 66·8)	70·5 (69·1 to 71·8)	73·9 (72·8 to 75)	75·9 (74·2 to 77·7)	75 (73·5 to 76·4)	71·8 (70·5 to 73)
South Khorasan	35·8 (31·3 to 40·1)	41·6 (38·6 to 44·5)	50 (47·4 to 52·8)	62·6 (60·2 to 64·7)	68·3 (66·4 to 70)	74·3 (73·4 to 75·2)	77 (76·4 to 77·6)	79·9 (79 to 80·9)	78·9 (78 to 79·8)	75·9 (75·1 to 76·7)
Tehran	52·4 (47·7 to 56·6)	59·3 (56·4 to 62·2)	67·6 (65·4 to 69·7)	74·4 (72·3 to 76·2)	75·8 (74 to 77·6)	77·6 (76·5 to 78·8)	79·8 (79 to 80·7)	81 (79·7 to 82·2)	80·1 (79 to 81·1)	77·1 (76·1 to 77·9)
West Azarbayejan	36·6 (32·5 to 40·6)	41·9 (38·8 to 44·9)	50·3 (47·5 to 53)	61·6 (59·5 to 63·7)	66·4 (64·7 to 68·2)	70·4 (69·5 to 71·3)	74·4 (73·9 to 75)	76·3 (75·3 to 77·2)	75·9 (75 to 76·8)	73·1 (72·2 to 73·9)
Yazd	38·5 (33·6 to 43·2)	48·9 (45·9 to 51·9)	58·1 (55·6 to 60·4)	66·2 (64·2 to 68·2)	70·9 (69·3 to 72·5)	73·3 (72·3 to 74·4)	75·5 (74·7 to 76·2)	78·4 (77·2 to 79·6)	77·2 (76·1 to 78·2)	73·1 (72·3 to 73·9)
Zanjan	29·6 (24·2 to 34·8)	34·9 (31·7 to 38·1)	46 (43·3 to 48·8)	60·4 (58·2 to 62·6)	44·9 (43 to 46·7)	72·1 (71·2 to 73)	76·1 (75·7 to 76·5)	78·8 (78 to 79·7)	78·1 (77·2 to 78·9)	75 (74·3 to 75·7)
Iraq	54·7 (51 to 58·4)	60 (57·7 to 62·4)	62·5 (58·9 to 64·8)	64·2 (62·3 to 66)	67·2 (65·3 to 69·1)	69·1 (66·1 to 71·9)	71 (68 to 74)	72·9 (70·3 to 75·6)	69·5 (67·6 to 71·5)	70·2 (68·3 to 72·5)
Jordan	50·8 (45·6 to 55·3)	57·4 (54·4 to 60·4)	62·5 (60·9 to 64·1)	67·6 (66·1 to 69·2)	71·9 (70·4 to 73·4)	73 (71·1 to 74·8)	77·3 (76·4 to 78·3)	80·2 (78·7 to 81·8)	77·1 (75·6 to 78·7)	75·7 (74·1 to 77·3)
Kuwait	61·7 (56·5 to 66·5)	69·5 (66·8 to 71·9)	70·9 (70·5 to 71·3)	72·5 (72·2 to 72·8)	74·6 (74 to 75·3)	77·5 (77·2 to 77·8)	80·5 (80·2 to 80·8)	82·8 (82·5 to 83·1)	79·9 (79·6 to 80·2)	80·7 (79·2 to 82·3)
Lebanon	53·2 (47·5 to 58·7)	64 (60·6 to 67·5)	67·6 (64·6 to 70·3)	69 (66·4 to 71·2)	69·3 (67·3 to 70·9)	75·6 (74·2 to 77)	77·8 (76·9 to 78·7)	79·9 (78·7 to 81)	77·5 (76·3 to 78·7)	75·2 (74 to 76·2)
Libya	41·7 (37·9 to 45·6)	50·2 (47·3 to 53·4)	62·2 (60·4 to 64·1)	69·7 (68·3 to 71·1)	72·8 (70·9 to 74·7)	74·4 (73·2 to 75·6)	73·6 (72·5 to 74·6)	72·4 (69·7 to 75)	71 (68·4 to 73·4)	70·8 (68·2 to 73·1)
Morocco	40·8 (36·6 to 44·6)	47 (44·1 to 50)	54·2 (51·6 to 56·7)	60·4 (58·4 to 62·4)	67·1 (65·3 to 68·7)	70·2 (68·4 to 71·9)	72·3 (69·9 to 74·6)	73·9 (72 to 76·4)	71·9 (70·3 to 73·9)	72·3 (70·7 to 74·3)
Oman	40·1 (34·7 to 45·4)	47·1 (43·1 to 51·4)	56·6 (53 to 59·8)	63·5 (60 to 66·8)	69 (65·9 to 72·1)	71·9 (69·7 to 74·4)	73·2 (71·8 to 74·5)	75·4 (73·9 to 77)	74·1 (72·8 to 75·5)	72·7 (71·4 to 73·9)
Palestine	43·3 (38 to 48·4)	53 (49·4 to 56·5)	57·2 (54·4 to 59·8)	67·4 (64·3 to 69·9)	69·4 (67·3 to 71·3)	69·9 (68·8 to 70·9)	72·8 (72·2 to 73·4)	76·1 (75·2 to 76·9)	75·4 (74·3 to 76·5)	73·8 (72·6 to 74·9)
Qatar	58·5 (53·6 to 62·8)	61·9 (58·9 to 65·2)	65·8 (63 to 68·5)	69·1 (67 to 71·3)	70 (68·4 to 71·6)	70·5 (68·9 to 72)	73·6 (72 to 75·1)	78·9 (77 to 80·8)	77·3 (75·5 to 79)	77·2 (75·4 to 78·9)
Saudi Arabia	53 (48·3 to 57·5)	52 (48·2 to 55·5)	58·4 (54·8 to 61·8)	65·7 (62·1 to 68·8)	67·7 (64·9 to 70·8)	70·2 (68·7 to 71·5)	71·6 (70·7 to 72·3)	73·7 (71·9 to 75·4)	72·6 (70·7 to 74·3)	73·1 (71·1 to 75)
Sudan	47·2 (42·2 to 52)	49·5 (46·1 to 52·8)	51·3 (48·8 to 53·6)	54 (51·8 to 56·3)	57·9 (55·6 to 60·3)	62·7 (60·2 to 65·3)	67·5 (64·6 to 70·2)	70·7 (67·2 to 73·5)	68·3 (65·3 to 70·9)	68 (64·9 to 70·8)
Syria	53·2 (49 to 57·2)	61·8 (59 to 64·9)	66·9 (65·3 to 68·6)	69·8 (67·4 to 72·2)	69·1 (66·9 to 71·4)	71·5 (70·1 to 72·8)	74·2 (72·6 to 75·8)	70·7 (68·3 to 73·1)	72·4 (69·9 to 74·6)	72·4 (69·9 to 74·6)
Tunisia	41·7 (37·4 to 45·8)	49·2 (47·2 to 51·4)	56·5 (55·4 to 57·6)	66·1 (64·9 to 67·2)	72·1 (70·9 to 73·3)	74·2 (72·4 to 75·8)	76·2 (73·4 to 79)	77·8 (75 to 80·5)	76·8 (74 to 79·7)	73·7 (71·5 to 75·9)
Türkiye	45·3 (40·7 to 49·7)	50·3 (47·4 to 53·4)	57·3 (54·7 to 59·8)	63·6 (61·5 to 65·6)	67·6 (66·4 to 68·9)	73·2 (71·9 to 74·4)	76·2 (75·2 to 77·3)	77·4 (76·2 to 78·5)	75·4 (74·2 to 76·8)	75·2 (73·7 to 76·7)
United Arab Emirates	55·3 (50·1 to 59·9)	58·7 (55·2 to 62·2)	65·8 (62·4 to 69)	68·8 (65·8 to 71·7)	69·9 (67·6 to 72·3)	71 (69·3 to 72·7)	71·7 (70·3 to 73·2)	73·1 (71·7 to 74·6)	75·1 (73·4 to 76·8)	75 (73·6 to 76·6)
Yemen	30·8 (27·1 to 34·6)	37·2 (34·4 to 40·3)	41·9 (38·8 to 45)	52·8 (50·2 to 55·7)	58·7 (55·8 to 61·7)	63·2 (60 to 66·2)	67·8 (64·6 to 70·7)	67·1 (63·9 to 69·9)	66·2 (63·3 to 68·8)	65·3 (62·2 to 67·9)
South Asia	38·5 (34·6 to 42·3)	44·3 (42 to 46·6)	50·3 (49 to 51·7)	56·2 (55·2 to 57·2)	60·6 (59·9 to 61·4)	64·1 (63·4 to 64·8)	67·5 (66·8 to 68·1)	70·3 (69·5 to 71·1)	69·2 (68·3 to 69·9)	68·5 (67·6 to 69·3)
South Asia	38·5 (34·6 to 42·3)	44·3 (42 to 46·6)	50·3 (49 to 51·7)	56·2 (55·2 to 57·2)	60·6 (59·9 to 61·4)	64·1 (63·4 to 64·8)	67·5 (66·8 to 68·1)	70·3 (69·5 to 71·1)	69·2 (68·3 to 69·9)	68·5 (67·6 to 69·3)
Bangladesh	41·7 (38 to 45·1)	46·2 (43·5 to 48·9)	44·7 (42·6 to 46·7)	54·9 (53·1 to 57)	58·9 (57·6 to 60·1)	65·1 (63·8 to 66·3)	69·5 (68·4 to 70·5)	73·7 (71·5 to 76)	72·2 (70·1 to 74·3)	72·3 (70 to 74·3)
Bhutan	37·6 (32 to 43·3)	44·1 (39·9 to 48·5)	49·9 (45·2 to 54·3)	53·1 (49·2 to 57·1)	60·2 (57·4 to 62·9)	65·5 (63·3 to 67·8)	71·5 (69·3 to 73·7)	73·7 (71·2 to 76·3)	73·9 (71·5 to 76·5)	73·7 (71·3 to 76·2)
India	37·4 (32·6 to 41·9)	43·4 (40·8 to 45·9)	50·4 (48·8 to 52)	56·1 (54·9 to 57·2)	60·8 (59·9 to 61·7)	64·2 (63·4 to 65)	67·7 (66·9 to 68·4)	70·5 (69·7 to 71·3)	69·6 (68·7 to 70·4)	68·7 (67·8 to 69·6)
Nepal	38·8 (35·3 to 42·3)	43·9 (41 to 46·7)	48·8 (46·2 to 51·7)	51·5 (48·9 to 54·2)	58 (55·8 to 60·1)	65·2 (63·5 to 66·8)	69·2 (67·4 to 71·1)	71·3 (68·9 to 73·4)	69·8 (67·7 to 71·7)	68·4 (66·4 to 70·1)
Pakistan	46·9 (43·7 to 50·2)	53·2 (51·1 to 55·1)	58·4 (57 to 59·8)	61 (59·8 to 62·2)	62·5 (61·6 to 63·4)	62·6 (61·4 to 63·8)	65 (63·2 to 66·7)	67·3 (65·1 to 69·4)	65·1 (63·2 to 66·9)	65 (63·1 to 66·9)
Azad Jammu & Kashmir	46·1 (41·2 to 51·3)	52·2 (48·6 to 55·7)	57·5 (54·9 to 60·1)	60·4 (57·4 to 63·5)	62·8 (60·1 to 65·4)	63·5 (60·4 to 66·5)	67 (63·8 to 70·2)	68·9 (65·7 to 71·9)	66·9 (64·1 to 69·4)	65·8 (63·3 to 68·1)
Balochistan	52·9 (47·9 to 57·6)	57·5 (54·2 to 60·6)	62·8 (60·1 to 65·6)	64·1 (61·5 to 66·9)	63·5 (61 to 65·8)	62·4 (59·6 to 65·2)	63·5 (60·4 to 66·7)	65·4 (62 to 68·6)	62·9 (60·3 to 65·5)	63·6 (60·8 to 66·2)

Gilgit-Baltistan	50·4 (46·4 to 54·5)	50·2 (47·1 to 53·4)	55 (52·2 to 58)	57·9 (54·8 to 60·9)	60·3 (57·7 to 63·3)	60·8 (58 to 63·4)	64·5 (61·3 to 67·7)	66·9 (63·6 to 70·3)	63·5 (60·8 to 65·8)	64·3 (61·5 to 67·4)
Islamabad Capital Territory	47·2 (41·2 to 52·8)	51·6 (47·9 to 55)	60·5 (57·4 to 63·6)	66·9 (64·1 to 69·5)	70·4 (68 to 72·6)	70·7 (68·1 to 73·4)	72·8 (70 to 75·7)	74 (71·5 to 76·8)	71·3 (69·2 to 73·6)	70·9 (68·6 to 73·5)
Khyber Pakhtunkhwa	45·9 (41·2 to 50·5)	53·5 (50·7 to 56·4)	58·9 (56·5 to 61·5)	62·6 (60·2 to 65·2)	64·8 (62·4 to 67)	63·5 (60·9 to 66)	65·1 (62·1 to 67·9)	67·7 (64·4 to 70·7)	65·1 (62·4 to 67·4)	65·4 (62·2 to 68·4)
Punjab	46·9 (43·6 to 50·2)	52·8 (50·5 to 55)	57·7 (56·1 to 59·4)	59·8 (58·2 to 61·5)	61·6 (60·3 to 63)	62·1 (60·6 to 63·8)	64·8 (62·7 to 67)	67·1 (64·8 to 69·6)	65·3 (63 to 67·4)	64·7 (62·6 to 66·8)
Sindh	46·3 (41·4 to 51·2)	53·8 (50·9 to 56·8)	59·5 (56·9 to 61·9)	62·5 (60·2 to 64·7)	63 (60·7 to 65·3)	62·9 (60·5 to 65·5)	65·2 (62·4 to 68)	67·6 (64·4 to 70·4)	64·8 (62·5 to 67)	65·2 (62·5 to 67·9)
Southeast Asia, east Asia, and Oceania	47 (44·2 to 49·5)	32·7 (21·4 to 40·6)	59·6 (57·8 to 61·2)	63·3 (62 to 64·6)	67 (66 to 68)	69·7 (69·1 to 70·3)	73·1 (72·5 to 73·7)	75·7 (74·6 to 76·8)	75·7 (74·6 to 76·9)	75·4 (74·1 to 76·6)
East Asia	48·3 (45 to 51·4)	27·4 (15·2 to 36·9)	61·3 (59·2 to 63·4)	64·5 (63 to 66·2)	67·8 (66·6 to 69·1)	70·6 (69·8 to 71·5)	74·6 (73·8 to 75·3)	77·3 (75·8 to 78·7)	77·4 (75·9 to 78·8)	77·6 (76 to 79·1)
China	48·9 (45·6 to 52·1)	26·6 (14·4 to 36·2)	61·1 (59 to 63·3)	64·3 (62·7 to 66)	67·7 (66·4 to 68·9)	70·8 (70 to 71·6)	74·6 (73·8 to 75·3)	77·3 (75·8 to 78·8)	77·4 (75·9 to 78·9)	77·6 (76 to 79·2)
North Korea	26·3 (23·9 to 28·6)	63·2 (59·5 to 66·6)	66·8 (63·4 to 70·3)	68·9 (65·3 to 72·1)	69·9 (66·6 to 73)	62·2 (49·9 to 68·5)	70·7 (68·4 to 72·9)	72·9 (70·2 to 75·2)	73 (70·4 to 75·3)	73·3 (70·7 to 75·7)
Taiwan (province of China)	57·1 (54·5 to 59·6)	64·7 (64·5 to 64·8)	69·7 (69·6 to 69·9)	72·3 (72·2 to 72·4)	74·5 (74·4 to 74·6)	76·7 (76·6 to 76·8)	79·8 (79·7 to 79·9)	81 (80·9 to 81·2)	81·6 (81·5 to 81·7)	81·3 (81·1 to 81·4)
Oceania	47·8 (44·7 to 51·1)	53·9 (51·3 to 56·5)	58·2 (55·9 to 60·5)	61·3 (58·9 to 63·5)	62·7 (60·4 to 65)	64·1 (62·3 to 65·9)	65·2 (63·2 to 67)	66·5 (64·3 to 68·6)	66·4 (64·1 to 68·4)	64·4 (61·6 to 67·1)
American Samoa	62 (59·5 to 64·2)	65·2 (63·9 to 66·5)	66·4 (65·2 to 67·7)	69·3 (68·1 to 70·6)	70·3 (69·2 to 71·2)	70·4 (69·5 to 71·3)	70·6 (69·4 to 71·5)	70·8 (68·9 to 72·5)	70·1 (68·2 to 71·6)	71 (68·7 to 72·9)
Cook Islands	46·6 (44·5 to 48·8)	58 (56·6 to 59·5)	64·2 (62·7 to 65·8)	66·2 (64·8 to 67·5)	68·6 (67·1 to 70)	72·3 (71 to 73·6)	75·2 (73·8 to 76·4)	76·1 (74·3 to 77·8)	76 (74·1 to 77·8)	76·1 (74·2 to 78)
Fiji	58·8 (57·3 to 60·3)	64·3 (63·6 to 65·1)	65·3 (64·5 to 66·1)	66·4 (65·5 to 67·2)	66·3 (64·5 to 67·9)	65·6 (65·1 to 66·1)	67·2 (66·5 to 68)	68·3 (65·5 to 70·9)	68·2 (65·2 to 71·1)	66·1 (62·9 to 69·6)
Guam	67·9 (65·9 to 69·6)	68 (67·3 to 68·6)	67·6 (66·9 to 68·2)	71·1 (70·6 to 71·6)	73·6 (73·1 to 74·1)	77 (76·5 to 77·5)	79·4 (78·8 to 80·1)	78·3 (77·1 to 79·4)	79·9 (78·8 to 80·9)	77·9 (76·2 to 79·8)
Kiribati	45·7 (42·1 to 48·9)	51·2 (48·3 to 54·3)	55 (52·2 to 57·9)	58·5 (55·8 to 61·2)	58·9 (56·8 to 61)	60·4 (58·1 to 62·4)	62·1 (59·5 to 64·7)	63·7 (60·6 to 66·4)	63·9 (60·7 to 66·6)	64·1 (60·9 to 66·8)
Marshall Islands	49·9 (45·5 to 54·3)	56·8 (53·2 to 60·2)	60·8 (57·4 to 63·9)	63·3 (61·4 to 65·3)	62·6 (61·5 to 63·8)	62·1 (60·3 to 63·7)	62·9 (61 to 64·8)	64·6 (61·5 to 67·6)	64·8 (61·6 to 67·9)	65 (61·5 to 68·1)
Federated States of Micronesia	43·2 (39·2 to 47·2)	53·2 (50·3 to 55·9)	57·8 (55·1 to 60·5)	61·3 (58·4 to 64·1)	62·8 (60·2 to 65·5)	64·1 (61·8 to 66·3)	65·9 (63·4 to 68·2)	66·8 (63·6 to 69·6)	66·9 (63·7 to 69·8)	67 (63·6 to 69·9)
Nauru	56·5 (52·6 to 60·2)	59·3 (56·5 to 61·9)	60·2 (57·5 to 62·8)	61·1 (58 to 64)	60·7 (58·3 to 63·2)	57·8 (55·9 to 59·9)	58·6 (56·1 to 61·1)	61·9 (58·6 to 64·8)	62·1 (58·7 to 65·1)	62·3 (58·8 to 65·4)
Niue	52·8 (49·7 to 55·7)	62·3 (59·8 to 64·8)	65·9 (63·5 to 68·5)	68·5 (65·6 to 71·2)	68·8 (66·2 to 71·2)	68·3 (66·1 to 70·6)	69·7 (67·1 to 72·1)	70·3 (67·6 to 72·8)	67·1 (65·1 to 68·9)	67·1 (65·1 to 69)
Northern Mariana Islands	63·7 (59·7 to 67·3)	66·4 (63·7 to 69·2)	68·7 (66 to 71·4)	70·1 (67·1 to 72·6)	71·4 (68·7 to 73·6)	73 (71·2 to 74·9)	73·4 (71·6 to 75·4)	73·5 (71·3 to 75·7)	74·3 (71·6 to 76·9)	72 (70·7 to 74·2)
Palau	48·8 (43·6 to 53·8)	55·7 (52 to 59·3)	61 (57·8 to 64·3)	63·8 (60·6 to 66·8)	65·9 (62·9 to 68·7)	67·2 (65 to 69·5)	67·4 (65·2 to 69·7)	68·5 (66 to 70·9)	68·6 (66·1 to 71)	68·7 (66·1 to 71·1)
Papua New Guinea	45 (41·2 to 49)	51·4 (48·2 to 54·6)	56·5 (53·5 to 59·5)	59·7 (56·7 to 62·7)	61·4 (58·4 to 64·3)	63·5 (61·1 to 65·9)	64·5 (62·3 to 66·9)	66·1 (63·6 to 68·5)	65·9 (63·4 to 68·4)	63·5 (60·3 to 66·7)
Samoa	56·7 (53·7 to 59·5)	65·2 (63·4 to 66·9)	65·5 (64·1 to 66·8)	67·7 (66·1 to 69·1)	68·3 (66·4 to 70·1)	69·6 (67·5 to 71·5)	70·6 (68·7 to 72·4)	69·5 (67·3 to 71·4)	70·4 (68·1 to 72·4)	70·7 (68·3 to 72·8)
Solomon Islands	46·4 (43 to 49·7)	51 (48·2 to 54)	55·7 (52·9 to 58·6)	60 (57·1 to 62·8)	61·3 (58·4 to 64)	63·2 (60·3 to 65·9)	64·4 (61·3 to 67·3)	65·7 (62·5 to 68·7)	65·6 (62·4 to 68·5)	65·9 (62·6 to 68·7)
Tokelau	57 (53·4 to 60·1)	60·8 (57·3 to 64·5)	64 (60·8 to 67·3)	65·3 (62·4 to 68·4)	67·8 (65·2 to 70·4)	69·4 (66·8 to 71·7)	71·2 (68·3 to 73·6)	72·6 (69·7 to 75·2)	67·5 (65·4 to 69·4)	67·5 (65·3 to 69·5)
Tonga	61 (57·5 to 63·9)	64·3 (62·5 to 66)	66·1 (64·2 to 68)	67·9 (66·5 to 69·2)	70·7 (69·1 to 72·3)	70·7 (69·5 to 71·9)	71·9 (69·6 to 74)	72·9 (70·4 to 75·3)	73 (70·4 to 75·5)	73·1 (70·4 to 75·6)
Tuvalu	43·6 (38·4 to 49·1)	50·6 (46·6 to 54·8)	54·1 (50·7 to 57·3)	56·3 (53·4 to 58·9)	59·8 (57·6 to 62·1)	60·5 (58·5 to 62·3)	66·3 (64 to 68·4)	67·6 (65·3 to 69·7)	67·8 (65·5 to 69·9)	68 (65·7 to 70·1)
Vanuatu	46·1 (42·8 to 49·7)	50·2 (47·1 to 53·4)	55·9 (53 to 59)	61·5 (58·4 to 64·3)	63·6 (60·6 to 66·2)	64·5 (62 to 66·8)	65·8 (63·9 to 67·6)	67 (65·1 to 68·8)	67·1 (65 to 68·9)	65·7 (63·3 to 67·8)
Southeast Asia	43·7 (41 to 46·3)	51·6 (49·6 to 53·3)	55·4 (54·1 to 56·8)	61·2 (60 to 62·4)	65·3 (64·5 to 66·1)	67·9 (67·2 to 68·6)	70·5 (69·7 to 71·2)	72·6 (71·6 to 73·6)	72·5 (71·3 to 73·6)	71 (69·4 to 72·5)
Cambodia	43·2 (37·8 to 48·3)	48·4 (44·3 to 52·2)	44·1 (40·8 to 47·2)	51·9 (48·6 to 54·8)	57·5 (55·8 to 59)	60·2 (58·7 to 61·7)	66·4 (64·8 to 68·2)	69·2 (66·5 to 71·9)	69·4 (66·5 to 72·1)	68·2 (65·3 to 71)
Indonesia	41 (38·1 to 43·9)	47·6 (45·4 to 49·8)	53·7 (51·9 to 55·5)	59·6 (58·3 to 60·9)	64 (63·1 to 64·9)	67·1 (66·1 to 68)	69 (67·8 to 70·2)	71·2 (69·6 to 73)	70·6 (68·9 to 72·4)	69·5 (67·3 to 71·9)
Aceh	46·9 (43·2 to 50·6)	52·8 (50 to 55·8)	56·8 (54 to 59·6)	61·4 (59·1 to 63·5)	64·4 (62·8 to 66·2)	67·2 (65·5 to 68·9)	67·8 (65·8 to 69·6)	69·9 (67·5 to 72·5)	69·3 (66·8 to 72)	68·3 (65·7 to 71·3)
Bali	44·4 (40·2 to 48·3)	50·9 (48·1 to 53·8)	55·6 (53 to 58·3)	60·5 (58·4 to 62·7)	65·6 (64·1 to 67·1)	68·6 (67·2 to 70·2)	70·6 (68·8 to 72·6)	72·5 (70·3 to 74·9)	71·9 (69·7 to 74·5)	70·8 (68·3 to 73·7)
Bangka-Belitung Islands	40·3 (35·6 to 44·7)	46·2 (42·9 to 49·3)	51·3 (48·8 to 53·7)	56·5 (54·2 to 58·8)	60 (58·2 to 61·9)	63·7 (62·3 to 65·2)	65·7 (63·8 to 67·5)	67·9 (65·6 to 70·3)	67·4 (65·2 to 69·8)	65·8 (63 to 68·7)
Banten	35·4 (30·9 to 39·6)	38·7 (35·1 to 42·5)	46·5 (44 to 49·1)	54·8 (52·4 to 57)	61·6 (59·9 to 63·3)	66·8 (65·1 to 68·4)	68 (66·2 to 70)	70·3 (67·9 to 72·7)	69·7 (67·3 to 72·3)	68·9 (66·3 to 71·5)
Bengkulu	33·3 (29 to 37·3)	40·1 (37·4 to 42·8)	47·4 (45·1 to 49·9)	54·4 (52·4 to 56·4)	60·1 (58·4 to 61·8)	63·8 (62·2 to 65·4)	66·4 (64·5 to 68·4)	69·4 (67·2 to 71·7)	68·8 (66·5 to 71·2)	68 (65·3 to 70·7)
Gorontalo	40·5 (36 to 45)	44·7 (41·3 to 48·2)	50·7 (48·1 to 53·3)	55·8 (53·2 to 58·2)	60·4 (58·5 to 62·2)	63·8 (62 to 65·6)	64·9 (62·6 to 67·2)	68 (65·4 to 70·7)	67·4 (64·7 to 70·1)	66·6 (63·7 to 69·6)
Jakarta	50·5 (46·8 to 54·2)	53·9 (50·9 to 56·7)	60·1 (57·5 to 62·7)	65·2 (63·1 to 67·1)	68·2 (66·7 to 69·8)	69·9 (68·4 to 71·5)	71·2 (69·5 to 73·2)	73·2 (71 to 75·6)	72·5 (70·4 to 75)	71·5 (68·9 to 74·2)
Jambi	39·2 (35·8 to 42·9)	44·8 (42·1 to 47·6)	50·1 (47·6 to 52·6)	55·7 (53·8 to 57·9)	61 (59·3 to 62·8)	65·6 (64·1 to 67·1)	68·2 (66·4 to 70·1)	70·5 (68·3 to 72·9)	70·1 (67·8 to 72·6)	68·9 (66·4 to 71·7)
West Java	37 (33·6 to 40·3)	43·2 (40·7 to 45·7)	50·8 (48·5 to 53·3)	58·2 (56·3 to 60·1)	63·9 (62·4 to 65·3)	68 (66·6 to 69·4)	70 (68·2 to 71·8)	72·1 (69·8 to 74·6)	71·5 (69·2 to 73·9)	70·3 (67·7 to 73)
Central Java	44·7 (40·5 to 48·6)	52·1 (49·3 to 54·9)	57·6 (55·1 to 60)	63·3 (61·3 to 65·1)	67·8 (66·4 to 69·2)	69·4 (68·1 to 70·9)	71 (69·3 to 72·8)	72·9 (71 to 75·2)	72·2 (70·2 to 74·4)	71 (68·5 to 73·8)
East Java	44·3 (41·1 to 47·3)	50·6 (48·1 to 53·1)	55·9 (53·4 to 58·3)	60·8 (59 to 62·7)	64·7 (63·3 to 66)	68 (66·5 to 69·3)	70·2 (68·4 to 71·9)	72·3 (70·1 to 74·5)	71·6 (69·4 to 73·8)	70·5 (68 to 73·2)
West Kalimantan	43·1 (39·3 to 46·8)	45·9 (43·1 to 48·7)	49·7 (47·1 to 52·2)	55 (52·9 to 57·1)	60·4 (58·7 to 61·9)	64·3 (62·7 to 65·8)	67·5 (65·8 to 69·2)	70·3 (68·2 to 72·4)	70·1 (67·9 to 72·1)	68·9 (66·3 to 71·3)
South Kalimantan	34 (30·7 to 37·4)	38·1 (35·7 to 40·5)	45·9 (43·4 to 48·2)	53·1 (51·1 to 55·1)	57·9 (56·2 to 59·6)	61·3 (59·7 to 63)	64·2 (62·3 to 66·2)	67·2 (64·8 to 69·7)	66·5 (64·1 to 69)	65·5 (62·7 to 68·4)
Central Kalimantan	46·5 (43·4 to 49·9)	52·1 (49·2 to 55·1)	56·4 (54·1 to 59)	60·4 (58·3 to 62·5)	63·1 (61·5 to 64·7)	67 (65·3 to 68·4)	67·9 (66·2 to 69·9)	69·8 (67·4 to 72·5)	69·2 (66·9 to 71·6)	68·1 (65·2 to 71·1)
East Kalimantan	49·9 (46·6 to 53·3)	50·8 (48·1 to 53·5)	55·5 (52·9 to 58·3)	61·2 (59·2 to 63·4)	64 (62·4 to 65·7)	65·2 (63·9 to 66·5)	67·1 (65·4 to 68·8)	69·7 (67·6 to 71·9)	69·1 (66·9 to 71·3)	68·1 (65·3 to 70·6)
North Kalimantan	53·2 (49·7 to 56·7)	52·3 (49·3 to 55)	56·4 (53·8 to 59·1)	62·7 (60·4 to 65)	68 (65·7 to 70·1)	72·3 (70·3 to 74·3)	73·8 (71·5 to 76)	75·6 (72·8 to 78·3)	74·6 (71·8 to 77·5)	72·8 (69·5 to 76·1)
Riau Islands	49·1 (45·7 to 52·5)	52·4 (49 to 55·7)	57·4 (54·7 to 60)	61·2 (58·9 to 63·6)	64·5 (62·7 to 66·4)	69·8 (68·2 to 71·2)	71·2 (69·3 to 73·1)	72·9 (70·9 to 75·5)	72·3 (70·2 to 74·9)	71·1 (68·5 to 74·1)
Lampung	44 (40·1 to 47·5)	47·4 (44·6 to 50·3)	53·9 (51·3 to 56·6)	60·3 (58·1 to 62·2)	64·8 (63 to 66·4)	69·1 (67·4 to 70·5)	70·6 (68·5 to 72·5)	72·3 (69·9 to 74·8)	71·7 (69·3 to 74·2)	70·5 (67·7 to 73·3)
Maluku	31·6 (27·9 to 35·6)	40·6 (37·2 to 43·9)	47·1 (44·6 to 49·5)	52·7 (50·3 to 55)	57·5 (55·5 to 59·3)	61·5 (59·7 to 63·3)	63 (60·8 to 65·2)	67 (64·5 to 69·6)	66·6 (64·1 to 69·2)	66·1 (63·5 to 68·8)
North Maluku	35·1 (30·3 to 39·4)	41·8 (38·6 to 45·1)	47·1 (44·6 to 49·5)	51·3 (49·1 to 53·5)	57 (55 to 58·9)	60·8 (59 to 62·6)	63 (60·6 to 65·2)	66·4 (63·8 to 69·2)	65·8 (63·3 to 68·6)	65·1 (62·2 to 68·1)
West Nusa Tenggara	32·4 (28·9 to 36)	37·5 (35·2 to 40)	42·9 (40·5 to 45·3)	49·5 (47·5 to 51·7)	56·3 (54·5 to 58·2)	61·7 (60 to 63·2)	66 (64·1 to 68·1)	68·8 (66·6 to 71·7)	68·4 (66·1 to 71)	67·8 (65·2 to 70·5)
East Nusa Tenggara	34·5 (30·8 to 38·3)	43 (39·7 to 46·2)	48·7 (46·2 to 51·1)	54·8 (52·8 to 56·9)	59·7 (58 to 61·3)	63·6 (62 to 65·2)	66·8 (64·7 to 68·9)	69·6 (67·1 to 72·4)	69·3 (66·9 to 71·9)	68·2 (65·4 to 71·1)
Papua	53·1 (49·8 to 56·2)	52 (48·7 to 55)	54·5 (51·7 to 57·2)	57·6 (55·3 to 59·9)	60 (58·3 to 61·8)	62 (60·4 to 63·7)	62·6 (60·6 to 64·9)	65·6 (62·9 to 68·3)	65·2 (62·4 to 68)	64·7 (61·8 to 67·8)
West Papua	51·9 (49 to 55·4)	53·2 (50·2 to 56·4)	55·4 (52·7 to 58)	59·2 (56·9 to 61·5)	62·4 (60·6 to 64·4)	64·7 (63·1 to 66·5)	64 (62 to 65·8)	68·5 (65·9 to 71)	67·9 (65·4 to 70·4)	67·2 (64·6 to 69·9)
Riau	46·6 (43·1 to 50·1)	48·6 (45·8 to 51·5)	53·7 (51·2 to 56·5)	60·1 (58·1 to 62·2)	64·1 (62·5 to 65·7)	67·2 (65·7 to 68·8)	69·2 (67·3 to 71·2)	71·7 (69·7 to 74·2)	71 (68·8 to 73·5)	69·8 (67 to 72·8)
West Sulawesi	28·2 (23·1 to 33·3)	36·2 (32·9 to 39·8)	42·1 (39·7 to 44·7)	48·7 (46·5 to 50·8)	54·8 (53 to 56·6)	60·2 (58·5 to 61·8)	63·6 (61·5 to 65·7)	66·8 (64 to 69·9)	66·4 (63·7 to 69·2)	65·4 (62·4 to 68·4)
South Sulawesi	38·5 (35·1 to 42)	45·1 (42·4 to 47·8)	51·3 (48·8 to 53·9)	57 (55 to 59·1)	62·4 (60·7 to 64)	66·6 (65 to 68·1)	68·6 (66·8 to 70·7)	71·2 (68·9 to 73·9)	70·6 (68·5 to 73·2)	69·9 (67·5 to 72·6)
Central Sulawesi	36·5 (33·1 to 39·7)	42·7 (39·9 to 45·2)	49·2 (46·7 to 51·8)	55·1 (53 to 57·3)	58·3 (56·6 to 60·1)	61·6 (60·1 to 63·1)	64·6 (62·7 to 66·6)	67·5 (65 to 70)	67 (64·7 to 69·8)	65·8 (63·1 to 68·9)
Southeast Sulawesi	36·4 (32·8 to 40·1)	40·8 (37·4 to 44)	46 (43·5 to 48·6)	53·7 (51·6 to 56)	59·3 (57·7 to 61)	62·3 (60·8 to 64)	64·6 (62·7 to 66·6)	67 (64·3 to 69·8)	66·4 (63·9 to 69·2)	65·7 (63 to 68·5)
North Sulawesi	45·1 (41·5 to 48·9)	52 (49·5 to 54·8)	56·7 (54·4 to 59·3)	61·3 (59·3 to 63·3)	63·5 (62 to 65)	65·9 (64·5 to 67·3)	67·4 (65·8 to 69·3)	69·6 (67·2 to 72·2)	68·9 (66·5 to 71·5)	68·1 (65·4 to 71)
West Sumatra	27 (23·3 to 30·6)	42·7 (39·8 to 45·4)	51·1 (48·5 to 53·7)	58·6 (56·5 to 60·9)	63·3 (61·6 to 64·8)	65·8 (64 to 67·4)	67·5 (65·8 to 69·5)	70·9 (68·8 to 73·2)	70·3 (68·1 to 72·6)	69·4 (67 to 72·1)
South Sumatra	42 (38·6 to 45·3)	48·4 (45·6 to 51·2)	54·8 (52·1 to 57·2)	61·8 (59·9 to 63·9)	65 (63·5 to 66·4)	68·1 (66·5 to 69·5)	69·8 (67·8 to 71·7)	71·7 (69·1 to 74·2)	71 (68·4 to 73·3)	70 (67 to 72·9)

North Sumatra	43·2 (39·6 to 46·7)	50·4 (47·6 to 53·2)	55·6 (53·1 to 58·3)	60·3 (58·4 to 62·4)	63·1 (61·5 to 64·7)	64·9 (63·3 to 66·4)	66·5 (64·6 to 68·5)	68·6 (66·1 to 71·3)	68·1 (65·7 to 70·7)	67·3 (64·6 to 70·4)
Yogyakarta	41·5 (36·7 to 46·1)	53·4 (50·2 to 56·5)	60·6 (58 to 63·2)	65·8 (63·6 to 67·9)	68·3 (66·9 to 69·8)	69·8 (68·5 to 71·1)	70·9 (69·2 to 72·5)	72·2 (70·3 to 74·1)	71·7 (69·6 to 73·4)	70·2 (67·6 to 72·7)
Laos	37·8 (32·5 to 42·9)	38·6 (34·5 to 42·7)	41 (36·6 to 45·3)	46·8 (43 to 50·6)	52·5 (49·6 to 55·4)	58 (55·8 to 60·2)	64·6 (62·5 to 67)	68·6 (65·6 to 71·6)	68·8 (65·8 to 71·9)	67·8 (64·6 to 70·9)
Malaysia	54·4 (51·4 to 57·4)	63·4 (63·1 to 63·6)	69 (68·8 to 69·2)	70·8 (68·3 to 73·2)	72·1 (72 to 72·2)	73·1 (73 to 73·2)	74·2 (73·1 to 75·2)	74·8 (74·7 to 74·9)	76·2 (76·1 to 76·3)	72·9 (72·1 to 73·4)
Maldives	35 (30·2 to 39·8)	40·6 (36·9 to 44·4)	49·3 (46·7 to 52)	56·1 (54·9 to 57·2)	65·7 (64·8 to 66·5)	72·5 (71·7 to 73·4)	78 (77·3 to 78·7)	80·6 (79·5 to 81·8)	80·7 (79·6 to 81·8)	79·4 (77·6 to 81·1)
Mauritius	51·7 (50·8 to 52·5)	58·3 (57·9 to 58·7)	63·7 (63·3 to 64·2)	67·2 (66·8 to 67·5)	70 (69·7 to 70·3)	72·2 (71·8 to 72·5)	74·2 (73·9 to 74·6)	74·4 (73·9 to 74·9)	74·9 (74·4 to 75·4)	73·4 (72·5 to 74·8)
Myanmar	32·3 (27 to 37·7)	39·5 (35·8 to 43·2)	44 (40·5 to 47·8)	50·2 (46·9 to 53·5)	55·1 (52 to 57·9)	58·6 (56·4 to 60·9)	64·5 (62·3 to 66·8)	69 (66·5 to 71·2)	68·8 (66·2 to 71)	67·6 (64·9 to 70·2)
Philippines	57·2 (53·7 to 60·1)	62 (60·6 to 63·3)	63·3 (62·3 to 64·2)	66·2 (65·3 to 67·1)	68·5 (67·6 to 69·2)	70·4 (69·6 to 71·1)	70·7 (69·9 to 71·4)	71·4 (70·5 to 72·3)	71·9 (71 to 72·8)	68·3 (66·9 to 69·5)
Seychelles	60·3 (58·6 to 62)	63·4 (62·3 to 64·4)	65·5 (64·7 to 66·4)	71·2 (70·5 to 71·9)	70·5 (69·9 to 71)	72·2 (71·6 to 72·7)	73·1 (72·5 to 73·7)	73·2 (72·4 to 74)	74·7 (73·8 to 75·5)	73·4 (72·5 to 74·3)
Sri Lanka	54·4 (53·5 to 55·2)	63·9 (63·7 to 64·2)	65·9 (65·6 to 66·1)	68·9 (68·7 to 69·1)	69·6 (68·9 to 70·3)	71·5 (71·4 to 71·6)	74·1 (73·9 to 74·2)	77·5 (74·6 to 80·3)	78·8 (74·6 to 84·5)	76·6 (73·2 to 80·5)
Thailand	51·5 (48·5 to 54·5)	60·6 (58·9 to 62·3)	64·2 (62·8 to 65·5)	66·6 (65·3 to 67·8)	71 (69·8 to 72·2)	71·3 (69·9 to 72·9)	75·8 (74·6 to 76·9)	77·4 (75·8 to 78·9)	77·5 (75·3 to 79·4)	76·3 (73·5 to 79·1)
Timor-Leste	43·3 (38 to 48·5)	46·9 (43·3 to 50·5)	48·5 (45·3 to 51·7)	49·4 (46·6 to 52)	59·3 (57·2 to 61·4)	65·4 (63·6 to 67·2)	69·2 (67·8 to 70·8)	70·2 (67·9 to 72·3)	70·3 (67·9 to 72·5)	68·6 (66·1 to 71)
Viet Nam	44·6 (38·9 to 49·9)	57·4 (53·7 to 60·8)	55·9 (53·2 to 58·4)	66·3 (63·5 to 68·8)	69·4 (67 to 71·4)	72·1 (70·5 to 73·7)	72·9 (71·7 to 74·3)	74·4 (72·7 to 76·3)	74·5 (72·8 to 76·5)	74 (72·1 to 76·1)
Sub-Saharan Africa	41·4 (37·4 to 45·1)	44·7 (42 to 47·2)	48·5 (46·6 to 50·5)	52 (50·7 to 53·4)	53·5 (52·4 to 54·6)	52·9 (51·6 to 54·1)	58·7 (57·6 to 59·9)	63·9 (62·1 to 65·4)	62·4 (60·5 to 64)	61·3 (59·5 to 62·7)
Central sub-Saharan Africa	39·4 (34·7 to 44)	44·9 (41·6 to 48·3)	47·7 (44·6 to 50·9)	50·6 (48 to 53·2)	52·6 (50·7 to 54·6)	52·7 (51 to 54·6)	58·2 (56·1 to 60·3)	63·1 (60·5 to 65·7)	61·9 (59·5 to 64·2)	61 (58·7 to 63·1)
Angola	41·6 (36·6 to 46·6)	44·1 (40·4 to 47·7)	45·3 (41·5 to 49·2)	47 (43·6 to 50·4)	49·1 (46·4 to 51·9)	52·5 (50·5 to 54·4)	60·1 (57·6 to 62·6)	64·1 (60·8 to 67·3)	62·8 (59·7 to 65·8)	61 (58·2 to 63·7)
Central African Republic	41·8 (36·9 to 46·4)	43·9 (40·1 to 48·3)	46·5 (43·4 to 49·8)	47·8 (45·3 to 50·3)	47·2 (45·1 to 49·3)	43·6 (41 to 46·4)	48·2 (44·9 to 51·6)	52·5 (48·3 to 56·1)	51 (47·2 to 54·2)	51·4 (47·6 to 54·9)
Congo (Brazzaville)	35·1 (29·7 to 39·7)	40·9 (37·8 to 43·9)	45·1 (41·7 to 48·5)	51·9 (49·5 to 54·6)	54·5 (52·5 to 56·6)	52·9 (50·9 to 54·6)	60·4 (58·3 to 62·5)	63·4 (60·6 to 66·1)	62·2 (59·6 to 64·6)	61·8 (59·2 to 64·2)
DR Congo	38·8 (33·6 to 43·9)	45·7 (42·2 to 49·4)	48·6 (45·3 to 52·2)	51·7 (48·8 to 54·4)	53·9 (51·9 to 55·9)	53·6 (51·4 to 55·7)	58·2 (56·1 to 60·4)	63·5 (60·6 to 66·3)	62·4 (59·9 to 64·9)	61·6 (59·3 to 64·1)
Equatorial Guinea	28·2 (23 to 33·3)	38·5 (34·9 to 42·2)	44·8 (41·3 to 48·7)	50·4 (46·8 to 53·9)	51·3 (48·4 to 54·6)	57 (53·4 to 60·5)	62·7 (58·6 to 66·7)	64·4 (59·5 to 68·7)	61·8 (57·4 to 65·6)	61·5 (57·2 to 65·3)
Gabon	30·1 (24·1 to 36·3)	44·7 (40·5 to 48·7)	50·9 (47 to 54·9)	58·1 (55·3 to 61)	60·3 (58·2 to 62·5)	59 (56·5 to 61·4)	62·4 (59·8 to 65)	65·8 (62·3 to 69·2)	64·2 (61 to 67·1)	63·9 (60·6 to 66·7)
Eastern sub-Saharan Africa	38·9 (35 to 42·6)	44·1 (41·5 to 46·7)	47·2 (45·3 to 49·1)	50·2 (48·9 to 51·5)	50·9 (49·8 to 52)	51·7 (50·2 to 53·1)	59·7 (58·5 to 61)	64·8 (63 to 66·4)	63·2 (61·4 to 64·7)	61·5 (59·8 to 63)
Burundi	37·4 (32 to 42·7)	42·1 (38·7 to 45·5)	44·4 (41·5 to 47·4)	47·7 (45·1 to 50·2)	49·1 (46·6 to 51·7)	45·3 (43·3 to 47·5)	59·7 (57·7 to 61·7)	64·2 (61·9 to 66·6)	63·2 (60·7 to 65·6)	62·2 (59·9 to 64·4)
Comoros	44·2 (40·1 to 48·3)	46·9 (43·5 to 50·4)	50·8 (47·8 to 53·7)	53·8 (50·9 to 56·7)	58·2 (55·6 to 60·8)	61·3 (58·8 to 63·9)	65·8 (63·6 to 68·2)	68·1 (65·5 to 70·5)	67·8 (65·2 to 70)	66·5 (64·2 to 68·5)
Djibouti	57·7 (53·5 to 61·8)	57·5 (53·4 to 61·5)	59·3 (55·7 to 63·1)	61·7 (58·5 to 64·9)	61·2 (58 to 64·1)	60·5 (57 to 63·9)	63 (59·3 to 66·6)	66·6 (62·5 to 70·1)	64·5 (61 to 67·5)	64·3 (60·9 to 67·2)
Eritrea	38·3 (33·4 to 43·1)	41·8 (38·1 to 45·8)	41·5 (26·1 to 47)	42·1 (39·9 to 44·3)	46·2 (44·3 to 48·2)	54·7 (52 to 57·6)	59·7 (56·3 to 63)	63·2 (59·4 to 66·7)	63·2 (59·5 to 66·5)	61·7 (58·3 to 64·7)
Ethiopia	35·4 (31·4 to 39·2)	40·8 (38·3 to 43·3)	42·4 (40·2 to 44·6)	44·3 (42·7 to 45·9)	46·4 (45·3 to 47·6)	51·5 (49·9 to 52·9)	63·3 (62·3 to 64·4)	68·4 (66·8 to 69·9)	66·4 (64·7 to 67·8)	64·5 (63·1 to 65·8)
Addis Ababa	50·4 (46·7 to 53·8)	51·2 (48·2 to 53·9)	53·1 (50·5 to 55·4)	55·5 (53·1 to 57·8)	53·6 (51·9 to 55·3)	53·3 (50·3 to 56·1)	66·2 (64·5 to 67·8)	70·1 (67·9 to 72)	66·8 (65 to 68·4)	64 (62·4 to 65·4)
Afar	19·5 (15·3 to 23·7)	31·8 (29 to 34·6)	34·2 (31·6 to 37)	34·8 (32·6 to 37·1)	38·6 (36·6 to 40·5)	41·4 (39·3 to 43·5)	54·4 (52·2 to 56·5)	60·8 (58·4 to 63·4)	58·8 (56·5 to 61)	56·9 (55 to 58·6)
Amhara	35·4 (30·9 to 39·8)	41·6 (38·8 to 44·3)	43·3 (40·8 to 45·6)	45·2 (43·2 to 47·1)	45·7 (44·4 to 47·1)	50·5 (48·5 to 52·3)	63·4 (61·8 to 64·9)	69·2 (67·1 to 70·9)	67·8 (65·9 to 69·6)	66·4 (64·6 to 67·9)
Benishangul-Gumuz	23·1 (19·1 to 27)	33·5 (30·5 to 36·4)	34·8 (32·2 to 37·5)	35·5 (32·9 to 37·9)	38·5 (36·3 to 40·7)	43 (40·8 to 45·1)	56·5 (54·5 to 58·5)	63·7 (61·2 to 66·4)	64 (61·2 to 66·7)	64·1 (61·4 to 66·7)
Dire Dawa	41·8 (37·9 to 45·7)	41·1 (38·2 to 44·3)	42·4 (39·5 to 45·2)	45·2 (42·6 to 47·7)	47·2 (45 to 49·4)	51·8 (48·8 to 54·6)	65·7 (63·7 to 67·7)	70·5 (68·4 to 72·5)	66·8 (64·9 to 68·4)	64 (62·2 to 65·4)
Gambella	30·8 (25·4 to 35·8)	39·3 (35·9 to 42·7)	40·9 (37·8 to 44)	42·2 (39·7 to 44·8)	44·1 (42·1 to 46·2)	48·8 (45·6 to 51·4)	59·3 (56·6 to 61·7)	66·9 (64·6 to 69·1)	63·4 (61·3 to 65·2)	60·5 (58·8 to 62·1)
Harari	34·4 (30·4 to 38·3)	34·7 (31·6 to 37·9)	37·4 (34·5 to 40·4)	41·3 (38·7 to 44)	44·9 (42·9 to 46·9)	52·1 (49·6 to 54·8)	64·9 (62·8 to 67·1)	69·8 (67·3 to 72)	65·7 (63·7 to 67·4)	62·4 (60·7 to 63·8)
Oromia	34·7 (30·4 to 38·7)	40·6 (37·9 to 43·2)	42·3 (39·9 to 44·9)	44·3 (42·4 to 46·3)	46·9 (45·4 to 48·4)	53·2 (51·6 to 54·8)	65 (63·6 to 66·5)	69·8 (68 to 71·6)	67·5 (65·6 to 69·1)	65·4 (63·7 to 66·8)
Somali	39·3 (34·8 to 43·3)	44·8 (41·8 to 47·9)	46·4 (43·6 to 49·5)	48·8 (46·2 to 51·4)	50·8 (49 to 52·8)	52·3 (50·4 to 54·3)	60·4 (58·4 to 62·4)	64·5 (61·9 to 67)	61·7 (59·5 to 63·7)	59·3 (57·4 to 61·1)
Southern Nations, Nationalities, and Peoples	36·5 (32·6 to 40·2)	39·7 (37 to 42·6)	41·2 (38·6 to 43·5)	42·6 (40·6 to 44·7)	45·6 (44·1 to 47·4)	52·3 (46·6 to 54·8)	61·8 (60·2 to 63·4)	66·9 (64·7 to 69·1)	65·2 (63·1 to 67·2)	63·5 (61·7 to 65·4)
Tigray	32·6 (28·3 to 36·6)	38·8 (36·1 to 41·7)	40·4 (37·9 to 42·9)	41·4 (39·2 to 43·5)	46·2 (44·5 to 47·8)	52·1 (50·2 to 53·9)	65·1 (63·3 to 66·8)	70 (67·7 to 72)	68·1 (66·1 to 70)	66·2 (64·2 to 68)
Kenya	46·1 (43·3 to 48·8)	53·1 (51·2 to 55)	57·5 (55·6 to 59·3)	61·9 (60·4 to 63·4)	62 (60·3 to 63·5)	54·8 (52·2 to 57·4)	60·9 (59·8 to 61·9)	66·8 (65·3 to 68·4)	64·6 (63·1 to 66)	63·9 (62·5 to 65·2)
Baringo	46·3 (42·2 to 50)	50·2 (47·1 to 53·2)	51·6 (48·7 to 54·7)	54·9 (51·8 to 57·8)	57 (54·2 to 60)	56·3 (53·6 to 58·8)	60·6 (58·1 to 62·9)	65·7 (62·7 to 68·6)	64 (61·3 to 66·6)	63·4 (60·9 to 65·6)
Bomet	64·1 (60·9 to 67·4)	67·4 (64·6 to 70)	68·5 (65·5 to 71)	70·1 (67·4 to 72·6)	69·2 (65·4 to 71·8)	59 (53·5 to 64)	63·6 (60 to 66·8)	70 (66·9 to 72·5)	67·8 (65·4 to 69·8)	67·2 (65 to 68·9)
Bungoma	42·4 (38 to 46·3)	50·9 (47·9 to 54)	56·4 (53·1 to 59·1)	60·4 (57·8 to 63)	62·7 (59·9 to 65·4)	59 (55·7 to 62·3)	66 (63·6 to 68·4)	70·2 (67·4 to 72·5)	68·4 (66·1 to 70·5)	67·4 (65 to 69·4)
Busia	27·4 (22·5 to 31·9)	41·1 (38·4 to 44)	48·9 (45·9 to 51·9)	58 (54·9 to 60·9)	57·9 (54·4 to 61·2)	49·5 (45·3 to 53·8)	59·5 (56·3 to 62·9)	66·6 (63·4 to 69·6)	64·1 (61·3 to 66·6)	62·7 (60·2 to 65)
Elgeyo Marakwet	53·3 (49·7 to 56·8)	57·6 (54·5 to 60·8)	57·3 (54·2 to 60·4)	59·5 (56·4 to 62·4)	62·4 (59·3 to 65·6)	59·5 (56·3 to 62·6)	63·9 (61·7 to 66)	67·7 (65 to 70·1)	66·3 (64 to 68·6)	66·1 (63·7 to 68·3)
Embu	41·5 (36·7 to 45·8)	51·2 (48·3 to 54·2)	59·8 (56·7 to 62·8)	66·6 (63·9 to 69·2)	68·3 (65·5 to 70·7)	63·4 (58·5 to 67·5)	65·9 (62·5 to 69·1)	70·2 (66·8 to 73)	68·3 (65·7 to 70·5)	67·8 (65·2 to 69·7)
Garissa	50·3 (46·6 to 54·1)	53 (49·5 to 56·6)	56·4 (52·7 to 59·7)	57·9 (54·9 to 61)	59·7 (56·6 to 63)	58·9 (54·8 to 62·6)	62·5 (59·6 to 65·3)	66·7 (64·2 to 69·1)	63·5 (61·4 to 65·5)	62·5 (60·5 to 64·4)
Homa Bay	25·8 (22·2 to 29·3)	38·3 (36 to 40·9)	42·1 (39·2 to 45)	48·9 (46·3 to 51·8)	49·8 (45·7 to 53·2)	36·3 (32·4 to 40·4)	46·4 (43·2 to 49·3)	57 (53·1 to 60·9)	56 (52·8 to 59)	56·4 (53·4 to 59)
Isiolo	35·4 (31·1 to 39·8)	36·7 (33·7 to 39·6)	39·3 (36·2 to 42·1)	46·4 (43·3 to 49·7)	52·3 (49·2 to 55·7)	51·3 (46·5 to 55·8)	56·9 (53·2 to 60·5)	63·2 (59·7 to 66·8)	60·8 (57·9 to 63·6)	60·1 (57·5 to 62·6)
Kajiado	56·4 (52·8 to 59·9)	60 (56·8 to 63·1)	61 (57·7 to 64·2)	63·8 (60·5 to 66·7)	64·7 (61·1 to 68)	58·7 (54·7 to 62·3)	63·3 (60·7 to 65·7)	68 (65·2 to 70·6)	65·2 (63 to 67·3)	64·4 (62·2 to 66·3)
Kakamega	41·4 (37·3 to 45·2)	49·8 (46·9 to 52·6)	54·6 (51·7 to 57·6)	59·6 (56·8 to 62·3)	58 (55·1 to 60·7)	51·2 (47·2 to 55·1)	59·9 (57·2 to 62·6)	67·2 (64·4 to 69·8)	65 (62·4 to 67·2)	63·9 (61·8 to 66)
Kericho	65·3 (61·8 to 68·6)	65·5 (62·1 to 68·7)	66·2 (63·1 to 69·3)	67·4 (64·4 to 69·9)	67·9 (64·5 to 70·5)	61 (56·7 to 65)	65·7 (62·3 to 68·9)	69·9 (66·6 to 72·7)	68 (65·3 to 70·4)	67·4 (64·8 to 69·5)
Kiambu	54·3 (50·4 to 57·8)	59·7 (56·5 to 62·8)	63·3 (60·3 to 66·3)	65·5 (62·9 to 68·3)	64·8 (62·1 to 67·6)	57·8 (54·7 to 61)	62·4 (60·3 to 64·5)	66·9 (64·4 to 69·5)	64·5 (62·3 to 66·7)	63·8 (61·9 to 65·7)
Kilifi	37·9 (34·1 to 41·9)	46 (43·2 to 48·8)	51·1 (48·3 to 53·8)	57·1 (53·8 to 59·8)	60 (56·8 to 63)	56·7 (52·3 to 60·8)	62·7 (59·8 to 65·5)	68·8 (65·8 to 71·4)	66·4 (64 to 68·5)	65·7 (63·4 to 67·6)
Kirinyaga	42·5 (38·7 to 46·2)	50·9 (48 to 54)	56·4 (53·2 to 59·5)	65·8 (62·8 to 68·7)	67 (63·8 to 70)	61·1 (57·4 to 64·7)	64·7 (61·8 to 67·4)	67·9 (64·7 to 70·9)	66·1 (63·5 to 68·6)	65·5 (63·1 to 67·8)
Kisii	49·1 (45 to 53·1)	51·4 (48·4 to 54·3)	54·5 (51·5 to 57·4)	57·8 (54·8 to 60·8)	58·1 (55·3 to 60·9)	53·8 (50·8 to 56·9)	61·3 (59·3 to 63·3)	66·1 (63·5 to 68·6)	64·2 (62 to 66·4)	63·5 (61·4 to 65·7)
Kisumu	45·5 (40·7 to 49·9)	52·1 (49·1 to 55·2)	54·5 (51·5 to 57·2)	57·4 (54·2 to 60·4)	55·4 (52·1 to 58·6)	43·2 (39·2 to 47)	52 (49 to 55·2)	59·6 (56·1 to 63·4)	57·6 (54·8 to 60·3)	57·2 (54·6 to 59·7)
Kitui	39·7 (34·8 to 44·2)	50·1 (47·1 to 52·9)	56 (53 to 58·9)	62·6 (59·7 to 65·4)	64·5 (61·6 to 67·4)	58 (53·1 to 62·2)	63·8 (60·8 to 66·8)	69·3 (66·2 to 71·9)	67·7 (65·2 to 70·1)	67·3 (64·9 to 69·5)
Kwale	38 (34 to 41·7)	44 (41·2 to 47)	50·3 (47·2 to 53·3)	56·5 (53·3 to 59·6)	59·2 (55·8 to 62·3)	56·4 (52·3 to 60·3)	60·5 (57·8 to 63·2)	66·4 (63·6 to 69·5)	64·7 (62·1 to 67·3)	64·1 (61·7 to 66·5)
Laikipia	59·7 (55 to 63·8)	63·8 (60·5 to 67·1)	66·7 (63·4 to 69·8)	70 (67·1 to 72·7)	68·3 (64·8 to 71·4)	59·5 (54·5 to 63·8)	64·1 (60·4 to 67·3)	69·4 (66·1 to 72·4)	67·1 (64·4 to 69·6)	66·5 (64 to 68·5)
Lamu	32 (26·8 to 37·1)	43 (39·6 to 46·3)	51·2 (47·7 to 54·6)	61·6 (58·1 to 64·7)	61 (57·3 to 64·4)	58 (53·1 to 62·6)	62·5 (58·9 to 65·9)	67·4 (63·7 to 70·5)	64·8 (62 to 67·4)	64 (61·3 to 66·3)
Machakos	54·4 (50·1 to 58·3)	58·9 (55·7 to 61·9)	62·7 (59·5 to 65·7)	67·3 (64·5 to 70·1)	68·8 (66·2 to 71·1)	61 (56·2 to 65·3)	65·4 (62·7 to 68)	70·1 (67·5 to 72·7)	68·1 (65·9 to 70·2)	67·6 (65·3 to 69·5)
Makueni	44·3 (39·1 to 49·1)	57·1 (53·8 to 59·9)	61·5 (58·6 to 64·3)	66·3 (63·4 to 69·1)	66·5 (63·3 to 69·4)	53·5 (47·7 to 59·6)	60·2 (57·3 to 63·3)	68·9 (66·2 to 71·2)	67·4 (65·3 to 69·3)	67·2 (65·2 to 68·8)
Mandera	53 (49·4 to 56·5)	55·6 (52·4 to 59)	57·1 (54 to 60·5)	58·3 (55·1 to 61·3)	57·7 (54·4 to 60·7)	56·8 (53·2 to 60·4)	58·7 (56·2 to 61·2)	63·5 (60·5 to 66·4)	59·5 (57·3 to 61·7)	58·1 (56·2 to 60)
Marsabit	52·1 (47·7 to 56·2)	52·5 (49·3 to 55·9)	56·2 (52·8 to 59·4)	60·1 (56·9 to 63·4)	62·3 (59 to 65·4)	59·5 (54·3 to 64)	61·1 (57·6 to 64·6)	65·6 (62·4 to 68·9)	63·7 (60·9 to 66·3)	62·9 (60·2 to 65·3)

Meru	54·2 (50·1 to 57·9)	59·9 (56·9 to 63)	63·8 (60·8 to 66·7)	68·2 (65·3 to 70·9)	68·5 (65·8 to 71·1)	61·8 (56·6 to 65·8)	63·5 (60·7 to 66·1)	67·9 (64·9 to 70·6)	65·8 (63·3 to 68)	65·1 (62·8 to 67·2)
Migori	33·4 (28·8 to 37·6)	43 (40·1 to 45·7)	47 (44·1 to 49·5)	50·1 (47·3 to 52·8)	51 (47·9 to 54·2)	41·5 (36·8 to 46)	49·9 (46·4 to 53·3)	59·5 (55·3 to 63·5)	57·7 (54·7 to 60·8)	57·5 (54·7 to 60·1)
Mombasa	53·3 (49·5 to 57·2)	54·1 (50·9 to 57·1)	59·7 (56·4 to 62·9)	65·5 (62·3 to 68·4)	64·2 (60·3 to 67·6)	55·5 (51·1 to 60)	62 (59 to 65)	68·9 (65·7 to 71·8)	65·3 (63·1 to 67·2)	64·1 (62 to 66)
Murang'a	45·7 (41·7 to 49·8)	57·8 (54·8 to 60·8)	62·8 (59·8 to 65·9)	67·9 (65·1 to 70·5)	68·3 (65·5 to 70·7)	61·8 (57·5 to 65·5)	64·3 (61·7 to 67)	69 (66·1 to 71·7)	67·1 (64·5 to 69·5)	66·6 (64·2 to 68·7)
Nairobi	51·3 (47·3 to 55·4)	54·9 (51·3 to 58·3)	62 (58·9 to 64·7)	66·4 (63·8 to 69·1)	64·1 (61·2 to 67·2)	55·4 (52·2 to 58·4)	62·6 (60·8 to 64·4)	67·5 (65·4 to 69·7)	64·1 (62·4 to 65·9)	63·1 (61·4 to 64·6)
Nakuru	64·5 (61 to 67·6)	65·7 (62·4 to 68·7)	66·8 (63·5 to 69·6)	68·1 (65·4 to 70·8)	67·1 (63·8 to 70)	59·2 (55·2 to 62·9)	64·8 (61·9 to 67·2)	70·2 (67·3 to 72·6)	67·4 (65·1 to 69·3)	66·6 (64·6 to 68·3)
Nandi	56·4 (52·1 to 60·3)	60·7 (57·3 to 63·9)	62·8 (59·6 to 65·9)	64·5 (61·4 to 67·4)	64·3 (61·2 to 67·2)	58·3 (54·7 to 61·7)	63·5 (61 to 65·8)	68·7 (66 to 71·3)	66·9 (64·4 to 69·1)	66·3 (64·1 to 68·5)
Narok	52·1 (48 to 56)	56·9 (53·4 to 60·2)	58 (54·6 to 61·2)	62·5 (59·4 to 65·5)	60·7 (57·4 to 63·9)	55·9 (52 to 59·6)	61·6 (58·6 to 64·1)	67 (64 to 69·6)	64·3 (61·9 to 66·4)	63·5 (61·1 to 65·5)
Nyamira	51·4 (47·6 to 55·1)	58 (54·8 to 61·1)	60·6 (57·3 to 63·8)	62·8 (60 to 65·8)	62·8 (60·1 to 65·5)	54·2 (50·5 to 57·6)	57·7 (55·4 to 59·8)	62·8 (59·9 to 65·7)	61·2 (58·7 to 63·7)	60·8 (58·4 to 63·3)
Nyandarua	50·2 (46·4 to 53·9)	55·9 (52·4 to 59·4)	60·7 (57·4 to 64·1)	65·2 (62·2 to 68·4)	65·2 (62·3 to 67·9)	58·5 (54·7 to 61·8)	59·6 (57·4 to 61·8)	63·5 (60·5 to 66·6)	61·8 (59·2 to 64·4)	61·3 (58·9 to 63·7)
Nyeri	46·8 (42·3 to 51·1)	57·6 (54·3 to 60·7)	62·8 (59·4 to 65·8)	66·9 (63·9 to 69·9)	66 (63·3 to 69)	57·8 (53·8 to 61·6)	62·7 (59·9 to 65·5)	66·7 (63·5 to 70·1)	64·9 (62·1 to 67·5)	64·4 (61·9 to 66·6)
Samburu	62·8 (59·3 to 66·2)	63·9 (60·6 to 67)	63·9 (60·6 to 66·9)	64·3 (61·2 to 67·2)	62·6 (59·1 to 66·1)	55·7 (51·8 to 59·6)	59·6 (56·5 to 62·8)	64·9 (61·6 to 68·1)	62·5 (59·7 to 65·1)	61·7 (59·2 to 64·1)
Siaya	38·6 (34·5 to 42·6)	46·2 (43·3 to 49·1)	51·4 (48·5 to 54)	55·5 (52·3 to 58·7)	53·4 (49·8 to 56·9)	42·2 (37·9 to 46·7)	53·9 (50·6 to 57·2)	64 (60·8 to 66·9)	62·4 (59·6 to 65)	61·7 (59·2 to 63·9)
Taita Taveta	46·9 (42·8 to 50·8)	53·2 (49·9 to 56·5)	55·5 (52·3 to 58·8)	62·5 (58·9 to 65·6)	63·9 (59·9 to 67·3)	56 (51·3 to 60·8)	59·4 (56·5 to 62·7)	66·6 (63·4 to 69·8)	64·6 (62 to 67·2)	64 (61·7 to 66·3)
Tana River	43·4 (38·6 to 47·8)	51·4 (48 to 54·9)	54·1 (50·8 to 57·4)	56·5 (53·3 to 59·7)	57 (53·9 to 60·4)	51·1 (48 to 54·4)	55·3 (53 to 57·7)	61·6 (58·5 to 64·3)	59·9 (57·4 to 62·6)	59·4 (56·8 to 61·7)
Tharaka Nithi	41·5 (36·3 to 46·5)	55·5 (52·4 to 58·8)	58·3 (55·1 to 61·4)	61·9 (58·9 to 65)	61·5 (58·6 to 64·4)	56·7 (53·2 to 59·9)	63·1 (61·1 to 65·2)	66·2 (63·4 to 69)	65·4 (62·9 to 68·1)	65·4 (62·5 to 68)
Trans Nzoia	62·2 (58·6 to 65·8)	63·1 (60 to 66·2)	64·7 (61·5 to 67·8)	66·4 (63·4 to 69·2)	66·4 (63·2 to 69·4)	58·9 (54·4 to 63·2)	63·3 (60·1 to 66·7)	68·9 (65·9 to 71·7)	66·9 (64·1 to 69·2)	66·3 (63·9 to 68·4)
Turkana	58·8 (55·1 to 62·3)	58·6 (55·3 to 61·8)	57·6 (54·4 to 60·9)	57·2 (53·9 to 60·3)	57·5 (53·8 to 61·4)	49·4 (42·1 to 54·3)	56 (52·3 to 59·6)	64·3 (60·4 to 68)	61·4 (58·5 to 64)	60·5 (57·9 to 62·9)
Uasin Gishu	65·6 (62·2 to 69)	66·6 (63·1 to 69·8)	67·9 (64·6 to 71)	68·9 (66 to 71·4)	67·7 (64·6 to 70·5)	60·8 (57 to 64·4)	65·2 (62·6 to 67·7)	70·4 (67·5 to 72·7)	67·9 (65·7 to 69·9)	67·1 (65 to 68·8)
Vihiga	39·7 (35·1 to 44·1)	55·6 (52·5 to 58·6)	59·8 (56·3 to 63)	62·8 (59·5 to 65·7)	61 (57·7 to 64·1)	54·2 (50·2 to 57·9)	60·3 (57·5 to 63·2)	66·3 (63·2 to 69·3)	64·8 (61·9 to 67·5)	64 (61·4 to 66·4)
Wajir	44 (40·1 to 47·8)	46·7 (43·4 to 49·9)	50·9 (47·4 to 54·2)	53·9 (50·9 to 56·7)	56·4 (53·3 to 59·3)	55·7 (52·7 to 58·9)	58·6 (56·1 to 61·2)	63·2 (60·1 to 66·3)	61 (58·4 to 63·8)	60·2 (58 to 62·6)
West Pokot	53·1 (49·3 to 56·9)	57 (53·8 to 60·1)	57·3 (54·2 to 60·7)	57·6 (54·6 to 60·6)	58·7 (55·6 to 61·9)	58·7 (55·9 to 61·8)	62·1 (59·5 to 64·7)	66·6 (63·6 to 69·5)	64·3 (61·7 to 66·8)	63·6 (61·2 to 65·9)
Madagascar	39·7 (35·9 to 43·6)	45·7 (43·1 to 48·4)	50·6 (48·1 to 52·9)	55·3 (53·5 to 57·1)	55·9 (54·4 to 57·4)	58·9 (57·1 to 60·9)	61·7 (59·2 to 64·4)	64·4 (61·5 to 67·4)	62·5 (60·1 to 64·9)	62·1 (59·9 to 64·5)
Malawi	36·1 (30·9 to 41·2)	38·4 (34·9 to 42)	42·6 (40·2 to 45·1)	49 (47·1 to 51·1)	49 (47·3 to 50·6)	45·5 (42·9 to 48)	56·2 (54·4 to 57·7)	62·5 (60 to 64·7)	61·7 (59·2 to 63·8)	58·7 (56·7 to 60·6)
Mozambique	40·1 (34·6 to 45·2)	44·3 (40·3 to 48·1)	47·5 (44·6 to 50·5)	50·3 (48 to 52·6)	50·8 (49 to 52·4)	52·3 (50·4 to 54·1)	53·3 (51·5 to 55·3)	58·7 (56·1 to 61·4)	58·3 (55·4 to 61·1)	56·4 (54 to 58·6)
Rwanda	31·3 (25·9 to 36·7)	42·2 (39·4 to 44·8)	43·9 (41·2 to 46·5)	48·5 (46·2 to 50·8)	49·9 (48 to 51·6)	50·4 (48·7 to 52·2)	64·1 (62·1 to 65·9)	68·1 (65·1 to 70·7)	67·8 (65 to 70·4)	65 (62·7 to 67·1)
Somalia	42·9 (38·1 to 47·8)	43·5 (39·7 to 47·5)	44·9 (41·4 to 48·9)	48·8 (45·7 to 52·1)	48·2 (45·7 to 50·9)	50·7 (48·1 to 53·4)	50·8 (47·9 to 53·7)	56·7 (52·8 to 60·6)	54·7 (51·2 to 57·8)	53·6 (50·1 to 56·9)
South Sudan	47·1 (41·6 to 52·6)	47·5 (43·1 to 51·5)	48·4 (44·4 to 52·9)	50·9 (46·6 to 55·4)	51·9 (48·6 to 55·4)	54·7 (51·4 to 57·8)	57·8 (54 to 61·5)	56·6 (51·8 to 61·1)	55 (50·5 to 58·8)	55 (50·5 to 59·1)
Uganda	38·6 (33·2 to 44)	47·5 (44 to 51·1)	51·2 (48·6 to 53·8)	50·1 (48·2 to 52·1)	48·4 (45·4 to 51·5)	49·3 (47·4 to 51·2)	59·3 (57·3 to 61·3)	65·4 (62·8 to 67·8)	64 (61·3 to 66·3)	61·2 (58·7 to 63·7)
Tanzania	39·2 (33·8 to 44·3)	44·7 (41·2 to 48·2)	49·2 (46·7 to 51·9)	54·9 (52·7 to 57)	55 (53·2 to 56·7)	53·2 (50·9 to 55·4)	61 (59·4 to 62·7)	66·3 (63·8 to 68·5)	64·9 (62·6 to 67)	63·5 (61·4 to 65·3)
Zambia	43 (37·4 to 47·9)	47·4 (43·4 to 51·4)	52·8 (49·7 to 55·7)	55·5 (53·5 to 57·6)	51·3 (49·1 to 53·3)	45·2 (42·8 to 47·7)	56·7 (54·6 to 58·6)	62·4 (58·9 to 65·7)	60·9 (57·7 to 64)	58·3 (55·4 to 61)
Southern sub-Saharan Africa	49·1 (45·6 to 52·2)	52·9 (50·7 to 55)	56·3 (54·8 to 57·7)	59·6 (58·5 to 60·7)	63·7 (62·7 to 64·6)	54 (52·6 to 55·3)	55·7 (55 to 56·3)	63·7 (62·5 to 64·7)	61·2 (60 to 62·1)	59·3 (58·2 to 60·3)
Botswana	48·8 (44 to 53·1)	51·9 (48·9 to 55·1)	54·6 (51·8 to 57·3)	59·6 (56·9 to 62·1)	61·4 (58·4 to 64·5)	47·7 (43·5 to 51·9)	58 (55·6 to 60)	65 (63 to 66·9)	64·5 (62·5 to 66·4)	59·7 (58 to 61·6)
Eswatini	38 (32·9 to 42·8)	42 (38·3 to 45·8)	49·1 (45·5 to 52·7)	56·1 (53·3 to 59·1)	60·7 (58·6 to 62·9)	47·9 (43·5 to 52)	46·8 (43·8 to 49·5)	56·6 (52·9 to 60·5)	55 (51·8 to 58·1)	52·5 (49·6 to 55·5)
Lesotho	46·9 (43·2 to 50·5)	50·5 (47·8 to 53·2)	52·4 (49·6 to 55·2)	58·8 (56·3 to 61·2)	61·3 (59 to 63·5)	48·5 (45 to 51·7)	48·6 (46·3 to 51)	51·7 (48·9 to 54·4)	50·8 (48·4 to 53·2)	48·5 (46·5 to 50·5)
Namibia	50 (44·8 to 54·8)	54·4 (50·4 to 58·5)	57·4 (54·5 to 60·5)	58·7 (56·8 to 60·7)	62·1 (60·5 to 63·7)	53·8 (50·9 to 56·2)	60·1 (57·8 to 62·4)	64·3 (60·8 to 67·3)	63·4 (60·1 to 66·3)	60·1 (57·4 to 62·5)
South Africa	49·2 (46 to 52·1)	52·8 (51 to 54·7)	56·3 (54·9 to 57·7)	59·5 (58·3 to 60·7)	64·5 (63·8 to 65·3)	56·6 (56 to 57·1)	56·9 (56·4 to 57·4)	65·4 (64·7 to 66)	62·3 (61·7 to 62·9)	61 (60·3 to 61·6)
Eastern Cape	42·8 (37·5 to 47·5)	51·6 (48·6 to 54·6)	55·7 (52·9 to 58·3)	60·7 (58·3 to 62·9)	64·3 (62·8 to 66)	52·7 (51·6 to 53·8)	52 (51 to 52·9)	59·7 (58·4 to 60·8)	55 (54 to 56)	56·1 (55 to 57·3)
Free State	49·2 (45·2 to 52·9)	51·9 (49 to 55)	55·2 (52·7 to 57·8)	58·1 (55·7 to 60·4)	61·7 (60·2 to 63·3)	51·5 (50·4 to 52·7)	50·2 (49·4 to 50·9)	62·2 (61 to 63·2)	59·3 (58·2 to 60·4)	57·9 (56·7 to 59·1)
Gauteng	52·6 (49·7 to 55·6)	53·8 (51·4 to 56·3)	55·9 (53·4 to 58·4)	56·7 (54·8 to 58·8)	62·9 (61·4 to 64·5)	59·1 (58 to 60·1)	60·8 (59·9 to 61·7)	68 (66·6 to 69·4)	65·6 (64·3 to 66·9)	64·1 (62·9 to 65·5)
KwaZulu-Natal	49·4 (45·7 to 52·9)	51·8 (48·9 to 54·9)	55·6 (53 to 58·1)	59·2 (57 to 61·3)	62·7 (61·1 to 64·4)	51·9 (50·9 to 52·9)	54·6 (54 to 55·3)	67·8 (66·8 to 68·8)	63·5 (62·4 to 64·4)	61·8 (60·7 to 62·9)
Limpopo	53·9 (49·7 to 57·7)	58·5 (55·8 to 61·3)	62·1 (59·4 to 64·6)	66·2 (63·7 to 68·7)	72 (70·4 to 73·6)	63·7 (62·3 to 64·8)	56·8 (55·8 to 57·9)	63·3 (62 to 64·6)	61 (59·6 to 62·3)	57·8 (56·5 to 59·1)
Mpumalanga	50·3 (46·4 to 54)	53·7 (51·1 to 56·6)	56·4 (53·6 to 59)	60·8 (58·6 to 62·8)	67·3 (65·8 to 68·8)	58 (57 to 58·8)	56·3 (55·4 to 57·1)	66·5 (65·2 to 67·8)	64·4 (63·1 to 65·7)	61·2 (60 to 62·4)
North West	51·2 (47·1 to 54·7)	53·5 (50·6 to 56·5)	57·7 (54·9 to 60·3)	61·2 (58·8 to 63·7)	68·8 (67·3 to 70·5)	54·4 (52·6 to 55·9)	50·5 (49 to 51·9)	56·7 (54·8 to 58·5)	56·6 (54·7 to 58·4)	55·2 (53·4 to 56·8)
Northern Cape	55·5 (51·8 to 58·9)	57·8 (55·2 to 60·6)	60·4 (57·8 to 63)	63·8 (61·7 to 65·9)	64·2 (62·6 to 65·8)	54 (53·2 to 54·8)	54·4 (53·2 to 55·5)	62·5 (60·9 to 64·2)	60·3 (58·6 to 61·9)	57·6 (56·1 to 59·1)
Western Cape	57·7 (55 to 60·4)	59·8 (57·3 to 62·4)	61 (58·5 to 63·3)	61·4 (59·4 to 63·5)	64·6 (63·2 to 66)	64·4 (63·8 to 64·8)	67·5 (66·9 to 68)	70·2 (69·3 to 71·1)	66·8 (66 to 67·7)	65·7 (64·7 to 66·6)
Zimbabwe	51 (46·1 to 55·6)	55 (51·3 to 58·9)	58 (55·2 to 61·1)	60·5 (58·6 to 62·6)	61·3 (58·5 to 63·9)	46·8 (43·2 to 50·6)	52 (50·2 to 53·7)	58·9 (56 to 61·7)	57·8 (55 to 60·4)	55 (52·5 to 57·3)
Western sub-Saharan Africa	42·3 (38·4 to 46·2)	43·3 (40·7 to 45·9)	48·1 (46·2 to 50·2)	52·4 (51 to 53·9)	54·2 (53 to 55·5)	54·4 (53·1 to 55·8)	59·4 (57·9 to 60·8)	63·8 (61·7 to 65·6)	62·5 (60·4 to 64·5)	62·1 (59·9 to 63·8)
Benin	38·9 (33·1 to 44·6)	44·4 (41·1 to 47·9)	48·6 (45·8 to 51·1)	52·9 (50·6 to 55·1)	55·8 (54·2 to 57·4)	57·8 (56 to 59·6)	61·6 (59·9 to 63·3)	64·9 (62·5 to 67·2)	64·4 (61·9 to 66·5)	62·9 (60·5 to 65)
Burkina Faso	36·4 (31·3 to 41·3)	38·9 (35·4 to 42·5)	43·9 (41·2 to 46·6)	48·6 (46·3 to 50·7)	50·8 (48·8 to 52·6)	52·2 (50 to 54·4)	57·6 (55·7 to 59·6)	61·7 (59·1 to 64)	60·1 (57·3 to 62·4)	60·1 (57·6 to 62·3)
Cabo Verde	48·5 (45·3 to 51·5)	57·1 (55·7 to 58·6)	58·1 (56·7 to 59·5)	66·8 (65·4 to 68·2)	69·9 (68·4 to 71·5)	70·4 (67·2 to 73·1)	74·3 (72·6 to 76·2)	73·6 (71·6 to 75·7)	73·2 (71·1 to 75·4)	73·2 (71·1 to 75·4)
Cameroon	41·3 (37·6 to 45·2)	25·4 (23·7 to 27·1)	51·1 (48·5 to 53·9)	54·9 (52·8 to 57·2)	58·3 (56·6 to 60·1)	54·5 (52·4 to 56·5)	57·9 (55·4 to 60·5)	63·1 (59·8 to 66·2)	62 (58·9 to 64·9)	60·8 (58 to 63·2)
Chad	40·8 (35·1 to 46·4)	46·8 (42·6 to 51·6)	47·4 (43·8 to 50·8)	50 (47·7 to 52·4)	53·1 (51·3 to 54·9)	52·1 (49·8 to 54·2)	56·4 (54·2 to 58·5)	59·5 (55·8 to 63·1)	57·7 (54·3 to 61)	58·3 (54·5 to 61·5)
Côte d'Ivoire	44·8 (41·1 to 48·5)	46·2 (43·1 to 49·1)	51·3 (48·8 to 53·8)	56·9 (54·9 to 58·9)	55·5 (53·2 to 57·7)	51·7 (49·4 to 54·1)	57·4 (55·4 to 59·2)	63·9 (60·9 to 66·5)	62·5 (59·8 to 64·9)	62·7 (59·9 to 65·1)
The Gambia	51·4 (46·3 to 56·5)	50·3 (46·1 to 54·2)	53·6 (50·3 to 56·9)	55·9 (52·9 to 58·7)	59·1 (56·5 to 61·8)	60·5 (58·5 to 62·7)	62·9 (60·9 to 65·2)	65·2 (62·5 to 67·7)	63·1 (60·8 to 65·3)	63·2 (60·9 to 65·5)
Ghana	46·1 (42 to 50)	50·8 (48 to 53·6)	54·4 (51·5 to 57·2)	57·5 (54·9 to 60·2)	59·2 (56·8 to 61·4)	59·4 (57·4 to 61·5)	61·7 (59·9 to 63·5)	66 (63·3 to 68·7)	65·3 (62·7 to 67·6)	64·6 (62·3 to 66·7)
Guinea	39·2 (34·2 to 44·3)	41·5 (37·1 to 45·8)	43·9 (40·2 to 48)	47·9 (45·1 to 50·8)	51·8 (49·8 to 54)	53·9 (51·8 to 55·9)	57·8 (55·6 to 60·1)	62 (58·5 to 65·4)	60 (56·5 to 62·9)	60·1 (56·6 to 63)
Guinea-Bissau	27·8 (23·5 to 32·2)	35·9 (32 to 39·8)	38·7 (35·6 to 41·8)	45·4 (42·1 to 48·7)	48·8 (45·8 to 51·8)	51·5 (49·1 to 53·9)	56·4 (53·9 to 59)	59·9 (57·1 to 62·8)	57·4 (55·1 to 59·6)	58·1 (55·6 to 60·7)
Liberia	30·5 (24·7 to 37)	40·3 (36·7 to 44·3)	45·1 (41·9 to 48·3)	50·4 (47·7 to 53·1)	48 (45·9 to 50·1)	54·4 (52·5 to 56·3)	60·9 (58·7 to 63·1)	64·3 (60·6 to 67·8)	62·5 (58·5 to 65·8)	62·7 (58·9 to 66)
Mali	35 (29·8 to 39·9)	37·9 (34·7 to 41·4)	40·2 (38 to 42·7)	45·6 (43·4 to 47·7)	49·8 (48·3 to 51·3)	53·1 (51·7 to 54·6)	58·3 (56·4 to 60·1)	62 (59·6 to 64·2)	59·8 (57·3 to 62)	59·1 (56·8 to 61)
Mauritania	46·9 (43 to 50·8)	51·6 (48·8 to 54·8)	56 (53·2 to 58·9)	57·7 (55·4 to 60·1)	60·3 (58·6 to 62·1)	64·3 (62·4 to 66·1)	68·4 (66·2 to 70·6)	70·8 (67·9 to 73·5)	68·9 (66·3 to 71·2)	69·2 (66·5 to 71·7)
Niger	40·2 (35·2 to 45·3)	40·1 (35·9 to 44·5)	42·5 (39·3 to 45·8)	44·7 (42·6 to 46·8)	47·3 (45·6 to 49·1)	51·8 (50 to 53·8)	60·2 (57·3 to 63·3)	63·4 (59·5 to 66·8)	61·8 (58·1 to 65·1)	61·8 (58·1 to 65)
Nigeria	44·2 (40·1 to 47·9)	46·7 (44 to 49·5)	48·8 (46·6 to 51·3)	53·3 (51·5 to 55·3)	54·3 (52·4 to 56·2)	54·4 (52·3 to 56·6)	59·7 (57·5 to 61·8)	64·3 (62 to 66·3)	63·2 (61·2 to 65·3)	62·8 (60·8 to 64·6)
São Tomé and Príncipe	36·1 (33·4 to 38·5)	46·5 (45 to 47·9)	58·3 (57·2 to 59·5)	63·1 (62·1 to 64·2)	63·3 (61·9 to 64·7)	64·5 (62·9 to 66·2)	68·9 (67·2 to 70·7)	71·5 (69·3 to 73·4)	70·6 (68·5 to 72·4)	70·4 (68·3 to 72·1)

Senegal	44·3 (40·4 to 48·6)	46·2 (43·3 to 49·1)	47 (44·7 to 49·6)	52·3 (50·4 to 54·1)	58·4 (56·7 to 59·9)	60·2 (58·9 to 61·5)	65·5 (64·2 to 66·9)	68·4 (65·8 to 70·9)	66·8 (64·4 to 69)	65·9 (63·5 to 67·9)
Sierra Leone	37·9 (32·2 to 43·7)	41·5 (37·1 to 45·9)	45·1 (40·7 to 49·4)	48·3 (44·8 to 51·7)	51 (48·7 to 53·3)	50·8 (48·9 to 52·6)	55·6 (53·1 to 58·1)	61·6 (58·1 to 65·3)	59·8 (55·7 to 63·6)	60·6 (56·5 to 64·1)
Togo	41·9 (36·3 to 47·5)	45·9 (41·9 to 50·3)	51·1 (47·8 to 54·4)	55·9 (53·7 to 58·4)	57·8 (55·9 to 59·8)	56·4 (54·2 to 58·6)	59·1 (56·8 to 61·6)	64·2 (60·4 to 67·5)	63·7 (60·1 to 66·8)	63·1 (59·6 to 66·2)

Appendix 2 Table S4b: Life expectancy at birth with 95% UIs by location, females, 1950, 1960, 1970, 1980, 1990, 2000, 2010, 2019, 2020, 2021

Location	1950	1960	1970	1980	1990	2000	2010	2019	2020	2021
Global	51·6 (49·4 to 53·8)	48·3 (42 to 52·3)	61·1 (60·2 to 61·9)	64·7 (64 to 65·4)	68·1 (67·6 to 68·6)	69·8 (69·4 to 70·3)	73·3 (72·8 to 73·7)	76 (75·2 to 76·7)	75·3 (74·5 to 76)	74·8 (74 to 75·5)
Central Europe, eastern Europe, and central Asia	63·8 (62·3 to 65·3)	69·8 (69·4 to 70·1)	71·9 (71·6 to 72·1)	72·6 (72·5 to 72·8)	73·8 (73·7 to 73·9)	73·2 (73·1 to 73·3)	75·7 (75·6 to 75·8)	78·2 (77·9 to 78·5)	76·9 (76·6 to 77·3)	75·5 (75 to 75·9)
Central Asia	51·9 (47·8 to 55·8)	61·4 (59·5 to 63·2)	67·1 (65·9 to 68·2)	70·3 (69·8 to 70·8)	71·6 (71·3 to 72)	71 (70·6 to 71·4)	73·6 (73·2 to 73·9)	76·3 (75·7 to 76·7)	74·6 (74 to 75·2)	74·3 (73·3 to 75·2)
Armenia	52·2 (47·5 to 56·5)	63·1 (60·5 to 65·6)	70 (68·3 to 71·7)	73·8 (72·7 to 74·8)	73·9 (73·5 to 74·3)	74·9 (74·6 to 75·2)	77·1 (76·8 to 77·4)	79·8 (79·3 to 80·1)	76 (75·6 to 76·4)	78·6 (77·8 to 79·4)
Azerbaijan	39·2 (34·9 to 43·8)	53·3 (50·4 to 56·5)	63·5 (61·8 to 65·1)	69·3 (68·4 to 70·2)	70·6 (69·8 to 71·4)	70·6 (69·7 to 71·4)	73 (72·4 to 73·7)	75·6 (74·8 to 76·3)	73·3 (72·6 to 74)	73·4 (72·5 to 74·3)
Georgia	57 (53 to 61·1)	64·9 (63 to 66·8)	70·3 (68·9 to 71·7)	73·3 (72·5 to 74)	73·7 (73·2 to 74·1)	74 (72·8 to 75·1)	77·9 (77·5 to 78·2)	79·5 (79·1 to 80)	77·7 (77·4 to 78)	75·8 (75·5 to 76·2)
Kazakhstan	61·3 (57·7 to 64·9)	65·8 (64·3 to 67·3)	69·5 (68·5 to 70·6)	72 (71·3 to 72·6)	72·6 (72·1 to 73·2)	70·5 (69·9 to 71)	73 (72·5 to 73·5)	76·8 (76·3 to 77·3)	75 (74·5 to 75·5)	73·9 (73·1 to 74·7)
Kyrgyzstan	51·9 (47·5 to 56·5)	58·9 (56·1 to 61·6)	64·3 (61·8 to 66·6)	68·7 (67·3 to 69·9)	70·9 (70·1 to 71·8)	71·4 (70·6 to 72·2)	73·8 (73 to 74·6)	77·7 (76·8 to 78·5)	75·7 (74·6 to 76·8)	76·1 (74·7 to 77·6)
Mongolia	39·9 (34·1 to 45·5)	50·8 (47·8 to 53·8)	56·7 (54·2 to 59·2)	62·5 (60·6 to 64·4)	65·5 (64·5 to 66·6)	67 (66 to 68·1)	71·1 (70·1 to 72·1)	75·3 (74 to 76·4)	75·8 (74·8 to 76·8)	74·6 (73·5 to 75·7)
Tajikistan	40·6 (35·1 to 46·3)	53 (50·1 to 55·7)	62·7 (60 to 65·2)	66·6 (65 to 68)	68·6 (67·5 to 69·8)	69 (67·7 to 70·1)	71·7 (70·5 to 72·8)	74·9 (73·5 to 76·5)	72·6 (71·1 to 74·1)	72·1 (70·4 to 73·7)
Turkmenistan	48·8 (44·2 to 53)	59·4 (57·5 to 61·1)	64·7 (63 to 66·2)	67·5 (66·6 to 68·5)	69·3 (68·7 to 69·8)	70 (69·5 to 70·5)	73·1 (72·6 to 73·5)	73·4 (71·3 to 75·7)	71·8 (69·7 to 73·9)	71·5 (69·4 to 73·7)
Uzbekistan	52·1 (46·6 to 57·3)	64·3 (62·3 to 66·3)	68·2 (67 to 69·3)	70·4 (69·6 to 71·1)	72·7 (72·3 to 73·1)	71·5 (70·8 to 72·1)	73·4 (72·7 to 74)	75·8 (75·1 to 76·5)	74·9 (73·7 to 75·9)	75·1 (73·6 to 76·6)
Central Europe	58·9 (58·2 to 59·5)	68·9 (68·8 to 69)	71·7 (71·6 to 71·8)	73·4 (73·4 to 73·5)	74·6 (74·5 to 74·7)	76·4 (76·3 to 76·5)	79 (78·9 to 79)	80·4 (80·3 to 80·6)	79·4 (79·4 to 79·5)	78·3 (78·2 to 78·5)
Albania	50·2 (46 to 53·9)	64·4 (62·5 to 66·4)	70·4 (67·9 to 73·1)	74·1 (72·2 to 76·1)	75·7 (75·2 to 76·1)	78·4 (77·7 to 79)	80·4 (79·4 to 81·5)	80·1 (79·1 to 81·1)	79·3 (78·4 to 80·2)	78·7 (77·6 to 79·9)
Bosnia and Herzegovina	47·5 (45·4 to 49·4)	63·1 (62·2 to 63·9)	68·8 (68·2 to 69·4)	72·6 (72·2 to 73)	76·2 (75·9 to 76·5)	78 (76·9 to 79·2)	79·8 (79·5 to 80·2)	79·7 (79·3 to 80·1)	78·7 (78·4 to 79·1)	78·3 (76·9 to 79·8)
Bulgaria	58·9 (58 to 59·8)	71·2 (71 to 71·5)	73·5 (73·3 to 73·7)	73·8 (73·6 to 74)	73·5 (73·1 to 73·8)	73·7 (73·3 to 74)	75·9 (75·6 to 76·3)	77·4 (77 to 77·8)	75·9 (75·5 to 76·3)	73·7 (73·3 to 74·1)
Croatia	52·9 (51·5 to 54·1)	66·7 (66·4 to 66·9)	71·6 (71·2 to 71·9)	74·5 (74·3 to 74·8)	75·7 (75·5 to 76)	78·1 (77·4 to 78·8)	80 (79·7 to 80·3)	81·7 (81·4 to 82·1)	81·1 (80·8 to 81·4)	80·3 (80 to 80·6)
Czechia	68·1 (67·8 to 68·4)	73·2 (73·1 to 73·4)	73·2 (73 to 73·4)	74·1 (74 to 74·3)	75·6 (75·4 to 75·8)	78·4 (78·2 to 78·6)	80·9 (80·7 to 81·1)	82·3 (82·1 to 82·6)	81·5 (81·3 to 81·7)	80·9 (80·6 to 81·1)
Hungary	62·4 (61·7 to 63·1)	70·3 (70·1 to 70·5)	72 (71·8 to 72·2)	72·7 (72·6 to 72·9)	73·8 (73·7 to 74)	76·1 (75·9 to 76·3)	78·5 (78·3 to 78·7)	79·9 (79·7 to 80·1)	79·1 (78·9 to 79·4)	78 (77·8 to 78·2)
Montenegro	66·4 (65·2 to 67·6)	73·9 (73·2 to 74·5)	76·3 (75·6 to 77)	75·8 (75·2 to 76·4)	78·3 (77·7 to 78·8)	76·7 (76·2 to 77·2)	77·7 (77·2 to 78·2)	78·1 (77·5 to 78·6)	77·2 (76·7 to 77·7)	76 (75·4 to 76·6)
North Macedonia	49·3 (46·8 to 51·6)	58·5 (57·2 to 59·7)	66·7 (66·1 to 67·4)	71·8 (71·2 to 72·4)	72·6 (72·1 to 73·1)	73·7 (73·3 to 74)	75·4 (75 to 75·7)	77 (76·5 to 77·4)	75·3 (74·8 to 75·7)	74·2 (73·2 to 75·3)
Poland	59·6 (59·3 to 60)	70·8 (70·6 to 70·9)	73·5 (73·4 to 73·6)	74·8 (74·7 to 74·9)	75·6 (75·5 to 75·7)	78 (77·9 to 78·1)	80·5 (80·4 to 80·6)	81·8 (81·7 to 81·9)	80·7 (80·6 to 80·8)	79·7 (79·6 to 79·8)
Romania	60·9 (57·9 to 63·7)	67·5 (67·3 to 67·7)	70·4 (70·2 to 70·6)	72 (71·8 to 72·1)	73 (72·9 to 73·2)	74·7 (74·6 to 74·8)	77·5 (77·4 to 77·6)	79·4 (79·3 to 79·6)	78·4 (78·2 to 78·5)	76·8 (76·7 to 77)
Serbia	49·9 (49 to 50·7)	65·5 (65·3 to 65·8)	68·6 (68·3 to 68·8)	71·7 (71·5 to 71·9)	73 (72·8 to 73·1)	73·8 (73·6 to 74)	76·7 (76·5 to 76·9)	78·4 (78·2 to 78·6)	77·4 (77·2 to 77·6)	76·7 (76·5 to 76·9)
Slovakia	64·4 (63·9 to 64·9)	72·8 (72·5 to 73·1)	73·2 (72·9 to 73·4)	74·6 (74·3 to 74·8)	75·6 (75·3 to 75·9)	77·9 (77·6 to 78·2)	79·6 (79·4 to 79·9)	81 (80·7 to 81·3)	80·3 (80 to 80·6)	78·3 (78·1 to 78·6)
Slovenia	59·5 (58·5 to 60·5)	69·1 (68·6 to 69·5)	72·3 (71·9 to 72·7)	75 (74·6 to 75·4)	78 (77·7 to 78·4)	80 (79·6 to 80·4)	83 (82·5 to 83·4)	84·7 (84·1 to 85·3)	83·7 (83·2 to 84·1)	84 (83·4 to 84·6)
Eastern Europe	69·5 (67·3 to 71·6)	72·6 (72·5 to 72·7)	73·8 (73·7 to 73·9)	73·3 (73·3 to 73·4)	74·6 (74·5 to 74·7)	72·9 (72·9 to 72·9)	75·1 (75·1 to 75·1)	78 (77·5 to 78·5)	76·7 (76·2 to 77·2)	74·9 (74·2 to 75·5)
Belarus	70·6 (66·9 to 74)	74·5 (73·8 to 75·2)	75·9 (75·4 to 76·3)	75·4 (75·1 to 75·7)	75·8 (75·5 to 76)	74·7 (74·4 to 74·9)	76·6 (76·4 to 76·8)	79·2 (78·9 to 79·4)	77·4 (76·4 to 78·4)	76 (74·4 to 77·5)
Estonia	70 (66·8 to 72·9)	73 (72·5 to 73·4)	74·5 (74·1 to 74·9)	74·4 (74 to 74·8)	75 (74·6 to 75·4)	76·2 (75·7 to 76·6)	80·8 (80·3 to 81·3)	82·7 (82·1 to 83·3)	82·7 (82·2 to 83·2)	81·2 (80·6 to 81·8)
Latvia	72 (68·8 to 74·9)	73·5 (73·2 to 73·9)	74·3 (73·9 to 74·6)	74·2 (73·9 to 74·5)	74·7 (74·4 to 75)	76 (75·6 to 76·3)	78·1 (77·8 to 78·5)	80·1 (79·7 to 80·6)	80 (79·5 to 80·4)	78·1 (77·7 to 78·5)
Lithuania	68·7 (65·1 to 72·1)	72·7 (72·3 to 73·1)	75·3 (75 to 75·7)	75·4 (75·1 to 75·7)	76·1 (75·8 to 76·4)	77·5 (77·2 to 77·8)	78·7 (78·4 to 79)	81 (80·6 to 81·4)	80·1 (79·7 to 80·4)	78·9 (78·5 to 79·3)
Moldova	56·5 (52·3 to 60·1)	62·2 (60·3 to 64·2)	66·4 (65·3 to 67·5)	69 (68·4 to 69·4)	71·5 (71·2 to 71·9)	72·5 (72·2 to 72·8)	74·7 (74·4 to 75)	78·9 (77·9 to 79·9)	77·6 (77·2 to 78)	76·4 (75·4 to 77·3)
Russia	69·5 (66·4 to 72·3)	72·3 (72·2 to 72·4)	73·6 (73·5 to 73·7)	73 (73 to 73·1)	74·5 (74·4 to 74·6)	72·5 (72·4 to 72·5)	74·8 (74·7 to 74·8)	78·1 (78 to 78·1)	76·4 (76·3 to 76·4)	74·3 (74·3 to 74·4)
Ukraine	70·8 (67·9 to 73·4)	73·7 (73·3 to 74·2)	74·2 (73·9 to 74·6)	74 (73·7 to 74·2)	74·8 (74·6 to 75)	73·5 (73·4 to 73·5)	75·4 (75·3 to 75·5)	77·1 (75·1 to 79·2)	77·2 (74·8 to 79·3)	75·7 (72·7 to 78·6)
High income	67·7 (67·5 to 67·8)	71·8 (71·8 to 71·9)	74·1 (74 to 74·1)	77·1 (77·1 to 77·2)	79·4 (79·4 to 79·4)	81·2 (81·2 to 81·2)	83·1 (83·1 to 83·1)	84 (84 to 84·1)	83·3 (83·3 to 83·4)	83·3 (83·3 to 83·4)
Australasia	71·9 (71·8 to 72·1)	74·1 (74 to 74·2)	74·6 (74·5 to 74·7)	77·7 (77·6 to 77·8)	79·7 (79·6 to 79·8)	82·1 (82 to 82·2)	84 (83·9 to 84)	85 (84·9 to 85·1)	85·7 (85·6 to 85·8)	85·3 (85·3 to 85·4)
Australia	72 (71·8 to 72·2)	74·2 (74·1 to 74·3)	74·6 (74·5 to 74·7)	78 (77·9 to 78·1)	80 (79·9 to 80·1)	82·3 (82·2 to 82·4)	84·2 (84·1 to 84·3)	85·3 (85·2 to 85·4)	85·9 (85·8 to 86)	85·6 (85·5 to 85·7)
New Zealand	71·5 (71·2 to 71·8)	73·8 (73·6 to 74)	74·7 (74·5 to 74·9)	76·3 (76·2 to 76·5)	78·4 (78·2 to 78·5)	80·8 (80·7 to 81)	82·8 (82·6 to 82·9)	83·8 (83·6 to 83·9)	84·6 (84·4 to 84·7)	84·1 (83·9 to 84·3)
High-income Asia Pacific	59·6 (59 to 60·2)	68·1 (67·7 to 68·4)	72·9 (72·7 to 73·2)	77·2 (77·1 to 77·4)	80·9 (80·9 to 81)	84·1 (84 to 84·1)	86·2 (86·2 to 86·3)	87·6 (87·6 to 87·7)	88 (88 to 88·1)	87·8 (87·7 to 87·8)
Brunei	49·5 (47·8 to 51·3)	58·3 (56·8 to 59·7)	67 (66 to 68·1)	71·2 (70 to 72·3)	73·1 (72·2 to 74)	75·2 (74·5 to 76)	77·1 (76·4 to 77·9)	77·8 (76·8 to 78·8)	78·3 (77·2 to 79·3)	78·3 (77·1 to 79·3)
Japan	63·5 (63·3 to 63·7)	70·7 (70·6 to 70·8)	75·2 (75·2 to 75·3)	79·1 (79 to 79·1)	82·3 (82·2 to 82·3)	85·1 (85 to 85·1)	86·7 (86·6 to 86·7)	87·9 (87·8 to 87·9)	88·3 (88·2 to 88·3)	88·1 (88 to 88·2)
Aichi	64·4 (61·3 to 67·2)	70·9 (68·5 to 73·4)	75·2 (73 to 77·3)	78·8 (78·5 to 79)	81·9 (81·7 to 82·1)	84·6 (84·4 to 84·9)	86·6 (86·3 to 86·8)	87·8 (87·5 to 88·1)	88·2 (87·9 to 88·5)	88 (87·7 to 88·3)
Akita	64·1 (60·8 to 66·9)	70·5 (68 to 72·8)	74·7 (72·8 to 76·6)	78·7 (78·4 to 79)	82 (81·7 to 82·3)	84·7 (84·3 to 85)	86·3 (85·9 to 86·7)	87 (86·4 to 87·6)	88·1 (87·6 to 88·6)	86·8 (86·2 to 87·4)
Aomori	62·6 (59·4 to 65·6)	70 (67·2 to 72·6)	74·6 (72·5 to 76·7)	78·7 (78·3 to 79)	81·9 (81·5 to 82·2)	84·4 (84 to 84·8)	86 (85·5 to 86·5)	86·8 (86·2 to 87·4)	87·1 (86·6 to 87·6)	86·9 (86·3 to 87·5)
Chiba	63·9 (60·6 to 66·9)	70·8 (68·2 to 73·4)	75·6 (73·3 to 77·7)	79·2 (79 to 79·5)	82·6 (82·3 to 82·8)	84·9 (84·7 to 85·2)	86·5 (86·2 to 86·8)	87·8 (87·4 to 88·1)	88·1 (87·8 to 88·4)	88·1 (87·8 to 88·5)
Ehime	63·7 (60·2 to 66·9)	70·9 (68·2 to 73·7)	75·7 (73·5 to 77·8)	79·6 (79·2 to 79·9)	82·5 (82·2 to 82·9)	85 (84·6 to 85·3)	86·9 (86·5 to 87·4)	87·7 (87·1 to 88·3)	87·9 (87·4 to 88·4)	88·1 (87·4 to 88·7)
Fukui	62·8 (58·9 to 66·5)	71·8 (69·2 to 74·4)	76 (74·1 to 78)	79·6 (79·2 to 80)	82·7 (82·3 to 83·2)	86 (85·5 to 86·5)	87·1 (86·6 to 87·7)	88·6 (87·9 to 89·3)	88·7 (88·1 to 89·3)	88·4 (87·7 to 89·1)
Fukuoka	64·1 (60·9 to 67·1)	71·1 (68·4 to 73·6)	75·2 (72·8 to 77·4)	79·2 (78·9 to 79·4)	82·4 (82·2 to 82·7)	85·2 (84·9 to 85·5)	86·9 (86·6 to 87·1)	88 (87·6 to 88·3)	88·4 (88·1 to 88·7)	88·2 (87·8 to 88·6)
Fukushima	61·5 (58·2 to 64·6)	69·5 (67 to 72·2)	74·1 (72 to 76·1)	78·7 (78·4 to 79)	82·3 (82 to 82·7)	84·9 (84·5 to 85·3)	86·3 (86 to 86·7)	87·3 (86·8 to 87·8)	87·3 (86·9 to 87·8)	87·4 (86·8 to 87·9)
Gifu	63·5 (60·2 to 66·6)	70·2 (67·6 to 72·7)	74·5 (72·4 to 76·5)	78·5 (78·2 to 78·8)	82 (81·7 to 82·3)	84·7 (84·3 to 85)	86·5 (86·2 to 86·9)	87·7 (87·3 to 88·2)	88·2 (87·8 to 88·7)	88 (87·5 to 88·4)
Gunma	62·6 (59·3 to 65·7)	69·8 (67·1 to 72·3)	74·7 (72·6 to 76·7)	78·6 (78·3 to 78·9)	82·1 (81·7 to 82·4)	84·9 (84·5 to 85·2)	86·3 (85·9 to 86·6)	87·4 (86·9 to 87·9)	87·9 (87·5 to 88·3)	87·5 (87 to 88)
Hiroshima	63·2 (60 to 66·3)	70·8 (68·1 to 73·5)	75·7 (73·2 to 77·8)	79·4 (79·1 to 79·6)	82·5 (82·2 to 82·8)	85·6 (85·2 to 85·9)	87·2 (86·8 to 87·6)	88·3 (87·9 to 88·8)	88·9 (88·5 to 89·4)	88·7 (88·2 to 89·2)
Hokkaidō	63·6 (60·4 to 66·6)	70·6 (68 to 73·2)	74·8 (72·4 to 77)	78·7 (78·5 to 78·9)	82·2 (82 to 82·5)	85·5 (85·2 to 85·8)	86·7 (86·4 to 87)	87·5 (87·2 to 87·9)	87·7 (87·4 to 88)	87·5 (87·1 to 87·9)
Hyōgo	64·5 (61·3 to 67·5)	71·2 (68·6 to 73·8)	75·3 (73 to 77·4)	78·9 (78·6 to 79·1)	81·9 (81·6 to 82·1)	84·7 (84·4 to 84·9)	86·5 (86·2 to 86·8)	88·2 (87·9 to 88·6)	88·5 (88·2 to 88·9)	88·4 (88 to 88·8)
Ibaraki	61 (57·7 to 64·1)	69·3 (66·6 to 71·9)	74·3 (72 to 76·5)	78·7 (78·4 to 79)	81·8 (81·4 to 82)	84·4 (84·1 to 84·8)	86 (85·7 to 86·4)	87·2 (86·8 to 87·7)	87·8 (87·4 to 88·1)	87·4 (86·9 to 87·9)
Ishikawa	62·6 (58·6 to 66·4)	71·1 (68·4 to 73·5)	75·6 (73·6 to 77·4)	78·9 (78·5 to 79·3)	82·7 (82·3 to 83)	85·3 (84·8 to 85·7)	87·1 (86·7 to 87·6)	88·2 (87·6 to 88·9)	88·6 (88·1 to 89·1)	88·3 (87·7 to 88·9)
Iwate	61·5 (58·1 to 64·7)	69·3 (66·7 to 71·9)	74·2 (72·3 to 76·1)	78·7 (78·4 to 79·1)	82·1 (81·8 to 82·5)	85·1 (84·8 to 85·5)	86·4 (86 to 86·9)	87 (86·5 to 87·6)	87·9 (87·4 to 88·4)	87·3 (86·8 to 87·9)
Kagawa	64·3 (60·8 to 67·5)	71·1 (68·3 to 73·7)	75·9 (73·9 to 77·8)	79·8 (79·4 to 80·2)	82·8 (82·4 to 83·2)	85·1 (84·7 to 85·5)	86·7 (86·3 to 87·2)	87·9 (87·2 to 88·6)	89 (88·4 to 89·5)	88·3 (87·6 to 88·9)
Kagoshima	60·2 (56·7 to 63·3)	68·9 (66·3 to 71·5)	73·5 (71·3 to 75·5)	78·5 (78·2 to 78·8)	82·1 (81·8 to 82·4)	85·1 (84·7 to 85·4)	86·5 (86·1 to 86·9)	87·7 (87·1 to 88·2)	88·1 (87·6 to 88·6)	88·1 (87·6 to 88·7)
Kanagawa	65·7 (62·6 to 68·5)	72·3 (69·6 to 74·9)	76·5 (74·2 to 78·6)	79·6 (79·4 to 79·9)	82·5 (82·3 to 82·7)	85·1 (84·8 to 85·3)	86·9 (86·6 to 87·1)	88·2 (87·9 to 88·4)	88·5 (88·3 to 88·8)	88·3 (88·1 to 88·6)
Kōchi	61·4 (57·8 to 64·7)	69·5 (66·6 to 72·1)	74·4 (72·4 to 76·2)	79 (78·6 to 79·3)	82·5 (82 to 82·8)	85·1 (84·7 to 85·5)	86·8 (86·4 to 87·3)	87·7 (87 to 88·5)	88·8 (88·2 to 89·4)	88·1 (87·4 to 88·8)
Kumamoto	62·6 (59·2 to 65·8)	70 (67·3 to 72·5)	74·6 (72·4 to 76·6)	79·3 (79 to 79·6)	83 (82·6 to 83·3)	85·9 (85·5 to 86·3)	87·3 (86·9 to 87·7)	88·8 (88·2 to 89·4)	89 (88·6 to 89·5)	88·8 (88·2 to 89·3)

Kyōto	64·7 (61·5 to 67·7)	71·5 (68·8 to 74)	75·9 (73·5 to 78·1)	79·5 (79·2 to 79·8)	82·4 (82 to 82·7)	85·5 (85·2 to 86)	87·2 (86·8 to 87·7)	88·7 (88·1 to 89·2)	89·2 (88·7 to 89·6)	88·9 (88·4 to 89·4)
Mie	64 (60·9 to 67)	70·7 (68 to 73·3)	75·4 (73·3 to 77·5)	79·2 (78·9 to 79·5)	82·2 (81·9 to 82·5)	85·1 (84·7 to 85·5)	86·7 (86·3 to 87·1)	88 (87·4 to 88·5)	88·3 (87·9 to 88·8)	88·1 (87·6 to 88·6)
Miyagi	63·6 (60·4 to 66·6)	70·7 (68·1 to 73·3)	75·1 (72·9 to 77·1)	78·8 (78·5 to 79·1)	82·3 (82 to 82·6)	85·2 (84·8 to 85·5)	86·8 (86·4 to 87·2)	88·1 (87·6 to 88·6)	88·2 (87·8 to 88·6)	88·2 (87·7 to 88·7)
Miyazaki	62·4 (58·9 to 65·5)	69·8 (67·2 to 72·4)	74·6 (72·5 to 76·6)	79 (78·7 to 79·4)	82·6 (82·2 to 82·9)	85·6 (85·2 to 86)	86·8 (86·4 to 87·2)	87·9 (87·3 to 88·5)	88·4 (87·9 to 88·9)	87·6 (87 to 88·2)
Nagano	62·9 (59·7 to 65·9)	70·6 (68 to 73·1)	75·5 (73·5 to 77·4)	79·5 (79·2 to 79·8)	82·9 (82·6 to 83·2)	85·7 (85·4 to 86·1)	87·4 (87 to 87·7)	88·4 (87·9 to 88·9)	88·8 (88·4 to 89·2)	88·8 (88·3 to 89·2)
Nagasaki	63·3 (60 to 66·5)	70·7 (67·8 to 73·3)	74·7 (72·5 to 76·8)	78·8 (78·5 to 79·2)	82·4 (82·1 to 82·7)	85·2 (84·9 to 85·6)	86·7 (86·3 to 87·1)	87·7 (87·2 to 88·3)	88·1 (87·6 to 88·6)	87·8 (87·3 to 88·4)
Nara	64·3 (61·1 to 67·3)	71 (68·3 to 73·4)	75·3 (73·3 to 77·2)	78·8 (78·5 to 79·2)	81·9 (81·6 to 82·2)	85 (84·6 to 85·3)	86·6 (86·2 to 87)	87·7 (87·1 to 88·2)	88·7 (88·2 to 89·2)	88·3 (87·8 to 88·9)
Niigata	63·6 (60·4 to 66·5)	70·8 (68·2 to 73·3)	75·2 (72·8 to 77·3)	79·1 (78·9 to 79·4)	82·7 (82·4 to 83)	85·8 (85·4 to 86·1)	87·3 (86·9 to 87·7)	87·7 (87·2 to 88·2)	88 (87·6 to 88·4)	88 (87·4 to 88·5)
Ōita	62·7 (59·5 to 65·7)	69·9 (67·2 to 72·4)	74·5 (72·5 to 76·3)	78·8 (78·5 to 79·2)	82·3 (82 to 82·7)	85·1 (84·7 to 85·5)	87·2 (86·8 to 87·6)	88·2 (87·6 to 88·8)	88 (87·5 to 88·5)	89·2 (88·6 to 89·8)
Okayama	64·2 (61 to 67·4)	71·1 (68·4 to 73·7)	76 (73·8 to 78)	79·9 (79·6 to 80·2)	82·9 (82·6 to 83·3)	85·6 (85·3 to 86)	87·2 (86·8 to 87·6)	88·3 (87·8 to 88·8)	88·6 (88·2 to 89·1)	89 (88·4 to 89·5)
Okinawa	59·7 (56·2 to 63·1)	70·2 (67·3 to 72·9)	74·7 (72·5 to 76·7)	80·2 (79·9 to 80·6)	84·2 (83·9 to 84·5)	86·5 (86·1 to 86·9)	87·4 (87 to 87·8)	88·2 (87·6 to 88·8)	88·8 (88·4 to 89·3)	88·3 (87·7 to 88·9)
Ōsaka	63·9 (60·8 to 66·9)	70·8 (68·2 to 73·2)	75 (72·8 to 77·1)	78·4 (78·2 to 78·6)	81·2 (81·1 to 81·4)	84·4 (84·2 to 84·7)	86·1 (85·9 to 86·4)	87·7 (87·4 to 88)	88 (87·7 to 88·2)	88 (87·7 to 88·2)
Saga	63·2 (59·7 to 66·3)	70·6 (67·8 to 73·2)	75·2 (73·3 to 77·1)	79·3 (78·9 to 79·7)	82·7 (82·2 to 83·1)	85·5 (85 to 85·9)	86·9 (86·4 to 87·4)	87·8 (87·1 to 88·4)	88·1 (87·5 to 88·6)	88·5 (87·8 to 89·1)
Saitama	64 (60·8 to 67)	70·9 (68·2 to 73·4)	75·4 (73·1 to 77·5)	78·9 (78·7 to 79·1)	82 (81·7 to 82·2)	84·9 (84·6 to 85·1)	86·3 (86 to 86·6)	87·6 (87·3 to 88)	87·8 (87·6 to 88·1)	87·8 (87·5 to 88·2)
Shiga	63·6 (60·5 to 66·7)	70·3 (67·7 to 72·7)	74·8 (72·8 to 76·7)	78·9 (78·5 to 79·2)	82 (81·7 to 82·3)	85·4 (85 to 85·7)	87 (86·7 to 87·4)	88·3 (87·8 to 88·8)	88·4 (88 to 88·9)	88·9 (88·4 to 89·4)
Shimane	61·5 (57·4 to 65·3)	70·8 (68·2 to 73·4)	75·3 (73·3 to 77·1)	79·9 (79·5 to 80·3)	83·8 (83·4 to 84·3)	86·9 (86·4 to 87·4)	87·6 (87·1 to 88·1)	89·1 (88·3 to 90)	89·9 (89·2 to 90·5)	89·4 (88·7 to 90·1)
Shizuoka	65·1 (61·9 to 68·1)	71·9 (69·3 to 74·4)	76·1 (73·8 to 78·2)	79·7 (79·5 to 80)	82·7 (82·4 to 83)	85·4 (85 to 85·7)	86·7 (86·4 to 87)	87·8 (87·4 to 88·3)	88·2 (87·8 to 88·5)	88·1 (87·6 to 88·6)
Tochigi	62·3 (58·9 to 65·4)	69·7 (67 to 72·2)	74·5 (72·5 to 76·6)	78·3 (78 to 78·7)	81·4 (81·1 to 81·7)	84·4 (84 to 84·7)	85·9 (85·5 to 86·2)	87 (86·5 to 87·5)	87·6 (87·2 to 88)	87·4 (86·9 to 87·9)
Tokushima	62·7 (59·3 to 65·8)	69·9 (67·2 to 72·6)	74·5 (72·5 to 76·4)	78·6 (78·2 to 79)	82·1 (81·7 to 82·5)	85·2 (84·7 to 85·6)	85·9 (85·5 to 86·4)	87·4 (86·6 to 88·2)	88·9 (88·3 to 89·6)	87·1 (86·4 to 87·8)
Tōkyō	65·9 (62·9 to 68·9)	72·1 (69·7 to 74·6)	76 (73·8 to 78)	79·5 (79·3 to 79·7)	82·2 (82 to 82·4)	84·6 (84·4 to 84·8)	86·7 (86·5 to 86·9)	88·3 (88·1 to 88·5)	88·6 (88·4 to 88·8)	88·5 (88·3 to 88·8)
Tottori	61·7 (57·7 to 65·4)	70·8 (68·4 to 73·4)	75·4 (73·5 to 77·1)	79·6 (79·2 to 80)	82·6 (82·2 to 83)	85·3 (84·9 to 85·8)	86·7 (86·3 to 87·2)	86·8 (86·1 to 87·5)	87·7 (87·1 to 88·3)	87·9 (87·3 to 88·6)
Toyama	63·5 (60·1 to 66·5)	70·3 (67·7 to 72·9)	75·1 (73·2 to 77)	79·2 (78·9 to 79·6)	82·9 (82·5 to 83·2)	86 (85·5 to 86·4)	87·4 (87 to 87·8)	89 (88·3 to 89·7)	89·1 (88·6 to 89·7)	88·8 (88·1 to 89·4)
Wakayama	62·5 (59·2 to 65·6)	69·8 (67·1 to 72·3)	74·6 (72·6 to 76·4)	78·5 (78·2 to 78·9)	81·8 (81·4 to 82·2)	84·6 (84·2 to 85)	86 (85·6 to 86·4)	87·2 (86·6 to 87·8)	87·3 (86·8 to 87·8)	87·8 (87·2 to 88·4)
Yamagata	62·9 (59·7 to 65·8)	69·8 (67·2 to 72·2)	74·1 (72·3 to 76)	78·9 (78·5 to 79·2)	82·6 (82·2 to 82·9)	85·1 (84·7 to 85·5)	87·1 (86·6 to 87·5)	87·8 (87·1 to 88·5)	89 (88·4 to 89·5)	87·8 (87·2 to 88·4)
Yamaguchi	63·9 (60·5 to 67)	70·9 (68·1 to 73·5)	75·6 (73·5 to 77·7)	79·2 (78·9 to 79·6)	82·6 (82·2 to 82·9)	85 (84·6 to 85·4)	86·4 (86 to 86·8)	87·6 (87 to 88·1)	88 (87·5 to 88·5)	87·9 (87·3 to 88·4)
Yamanashi	63 (59·5 to 66·2)	70·7 (67·9 to 73·4)	75·5 (73·6 to 77·3)	79·4 (79 to 79·8)	82·6 (82·2 to 82·9)	85·2 (84·7 to 85·6)	87 (86·6 to 87·4)	87·4 (86·7 to 88·1)	88·1 (87·6 to 88·6)	88·6 (88 to 89·2)
South Korea	46·5 (44·2 to 48·6)	61·2 (60·1 to 62·3)	65·8 (65 to 66·7)	70·8 (70·5 to 71·1)	75·9 (75·7 to 76·1)	79·7 (79·7 to 79·8)	84 (83·9 to 84)	86·2 (86·1 to 86·3)	86·5 (86·4 to 86·6)	86 (85·9 to 86·2)
Singapore	60·5 (59·9 to 61·1)	68·4 (68·2 to 68·7)	71·9 (71·7 to 72·1)	74·3 (74·1 to 74·5)	78·2 (78 to 78·3)	81·7 (81·5 to 81·9)	85 (84·8 to 85·1)	87·6 (87·4 to 87·8)	88·3 (88·1 to 88·6)	87·7 (87·5 to 87·9)
High-income North America	71·1 (71 to 71·2)	73·4 (73·4 to 73·5)	74·9 (74·8 to 74·9)	77·7 (77·7 to 77·8)	79·1 (79·1 to 79·1)	79·7 (79·6 to 79·7)	81·4 (81·3 to 81·4)	81·9 (81·9 to 82)	80·5 (80·5 to 80·6)	80·4 (80·3 to 80·6)
Canada	70·9 (70·7 to 71)	74·2 (74·1 to 74·3)	76·3 (76·3 to 76·4)	78·9 (78·8 to 78·9)	80·6 (80·5 to 80·6)	81·8 (81·7 to 81·9)	83·6 (83·5 to 83·6)	84·4 (84·3 to 84·5)	83·9 (83·8 to 84)	84·1 (83·9 to 84·2)
Greenland	52·2 (50·2 to 54·3)	61·3 (60·1 to 62·6)	65·9 (64·7 to 67·1)	66·3 (64·8 to 67·6)	67·5 (66·3 to 68·6)	71·1 (69·5 to 72·4)	74·9 (73·7 to 76)	77·3 (76 to 78·2)	76·8 (75·7 to 77·8)	76·9 (75·7 to 77·9)
USA	71·2 (71 to 71·3)	73·4 (73·3 to 73·4)	74·7 (74·7 to 74·8)	77·6 (77·6 to 77·7)	79 (78·9 to 79)	79·5 (79·4 to 79·5)	81·1 (81·1 to 81·2)	81·6 (81·6 to 81·7)	80·2 (80·1 to 80·2)	80 (79·9 to 80·2)
Alabama	68·7 (65·7 to 71·7)	71·7 (71·4 to 72)	73·6 (73·3 to 73·9)	76·9 (76·6 to 77·2)	77·7 (77·4 to 78)	77·7 (77·4 to 78)	78 (77·8 to 78·3)	78·7 (78·4 to 79)	76·8 (76·5 to 77·1)	76·4 (74·6 to 78·3)
Alaska	68·2 (65·8 to 70·5)	71·7 (71 to 72·4)	74·3 (73·7 to 74·8)	77 (76·5 to 77·6)	78·6 (78·1 to 79)	79·3 (78·9 to 79·8)	80·2 (79·8 to 80·7)	80·8 (80·2 to 81·4)	81 (80·4 to 81·6)	79 (77·7 to 80·3)
Arizona	71 (68 to 73·9)	74 (73·4 to 74·4)	75·3 (74·9 to 75·8)	78·2 (77·9 to 78·6)	79·9 (79·6 to 80·2)	80·1 (79·8 to 80·4)	81·8 (81·6 to 82·1)	82·1 (81·8 to 82·3)	79·8 (79·6 to 80·1)	79·4 (77·8 to 81·1)
Arkansas	71·4 (68·3 to 74·4)	73·9 (73·5 to 74·4)	75·3 (74·8 to 75·7)	77·9 (77·5 to 78·3)	78·1 (77·7 to 78·6)	78 (77·6 to 78·3)	78·8 (78·4 to 79·1)	78·7 (78·4 to 79·1)	76·8 (76·5 to 77·2)	76·6 (74·7 to 78·5)
California	72·4 (69·6 to 75)	74·6 (74·4 to 74·7)	75·4 (75·3 to 75·6)	78 (77·9 to 78·1)	79·3 (79·2 to 79·4)	80·6 (80·5 to 80·7)	82·9 (82·8 to 83)	83·9 (83·8 to 84)	82·6 (82·5 to 82·7)	82·2 (80·7 to 83·7)
Colorado	72·2 (69·3 to 74·9)	74·5 (74·1 to 74·9)	76·1 (75·8 to 76·5)	78·9 (78·6 to 79·3)	80·2 (79·9 to 80·6)	80·5 (80·2 to 80·8)	82·1 (81·8 to 82·4)	82·8 (82·5 to 83·1)	81·7 (81·4 to 81·9)	81·5 (79·8 to 83·1)
Connecticut	72·2 (69·5 to 74·8)	74·3 (74 to 74·7)	76·2 (75·9 to 76·5)	78·5 (78·2 to 78·8)	80·2 (79·9 to 80·5)	80·8 (80·5 to 81·1)	82·9 (82·6 to 83·2)	83·4 (83 to 83·8)	81·8 (81·5 to 82·2)	82·8 (81·1 to 84·6)
Delaware	70 (67·3 to 72·4)	72·8 (72·2 to 73·3)	74·2 (73·7 to 74·6)	76·9 (76·4 to 77·4)	77·9 (77·5 to 78·4)	78·9 (78·5 to 79·3)	80·6 (80·1 to 80·9)	81·3 (80·7 to 81·8)	80 (79·4 to 80·5)	79·9 (78·7 to 81·1)
Washington, DC	67·3 (64 to 70·4)	69·8 (69·3 to 70·2)	70 (69·5 to 70·4)	73·3 (72·8 to 73·8)	73·4 (72·8 to 73·9)	76·4 (75·9 to 76·9)	79·7 (79·2 to 80·3)	81·3 (80·6 to 82)	79·6 (79 to 80·2)	80·2 (78·4 to 81·8)
Florida	71·4 (68·3 to 74·4)	73·8 (73·6 to 74·1)	75·2 (74·9 to 75·4)	78·2 (77·9 to 78·4)	79·7 (79·6 to 79·9)	80·1 (80 to 80·3)	81·9 (81·8 to 82·1)	82·2 (82·1 to 82·4)	81 (80·8 to 81·1)	80·3 (78·7 to 81·9)
Georgia	69·1 (65·9 to 72)	71·5 (71·2 to 71·8)	73·1 (72·8 to 73·3)	76·4 (76·2 to 76·7)	77·6 (77·3 to 77·8)	78 (77·8 to 78·2)	79·7 (79·5 to 79·9)	80·4 (80·2 to 80·6)	78·9 (78·7 to 79·1)	78·2 (76·6 to 79·9)
Hawaii	71·7 (69·1 to 74·2)	74·1 (73·7 to 74·6)	77·2 (76·7 to 77·6)	80·8 (80·3 to 81·2)	81·7 (81·3 to 82·1)	82·7 (82·2 to 83·1)	84·2 (83·8 to 84·6)	84·8 (84·2 to 85·2)	84·8 (84·2 to 85·3)	84·2 (82·7 to 85·6)
Idaho	73 (70·2 to 75·7)	75·4 (74·8 to 75·9)	76·3 (75·8 to 76·8)	79·2 (78·7 to 79·7)	80·2 (79·7 to 80·7)	80·2 (79·8 to 80·6)	81·3 (80·9 to 81·7)	81·8 (81·3 to 82·2)	81·1 (80·7 to 81·6)	80·1 (78·8 to 81·3)
Illinois	71·1 (68·3 to 73·6)	72·9 (72·7 to 73·1)	74 (73·9 to 74·2)	77·2 (77 to 77·4)	78·5 (78·3 to 78·7)	79·1 (79 to 79·3)	81·3 (81·1 to 81·5)	81·9 (81·7 to 82·1)	80·3 (80·1 to 80·5)	80·7 (79 to 82·6)
Indiana	72 (69·2 to 74·6)	73·6 (73·4 to 73·9)	74·8 (74·5 to 75)	77·6 (77·4 to 77·9)	78·8 (78·5 to 79·1)	78·5 (78·3 to 78·8)	79·8 (79·6 to 80·1)	79·7 (79·5 to 79·9)	78·2 (78 to 78·4)	78·2 (76·4 to 80)
Iowa	74·1 (71·4 to 76·7)	75·7 (75·4 to 76)	76·5 (76·2 to 76·9)	79·8 (79·5 to 80·2)	81·1 (80·6 to 81·4)	80·7 (80·4 to 81·1)	81·7 (81·4 to 82·1)	81·9 (81·5 to 82·3)	80·5 (80·1 to 80·9)	81·1 (79·4 to 82·8)
Kansas	73·8 (71·1 to 76·3)	75·5 (75·1 to 75·9)	76·8 (76·4 to 77·2)	79 (78·6 to 79·4)	80·5 (80·1 to 80·9)	80 (79·6 to 80·3)	81 (80·6 to 81·3)	80·6 (80·2 to 81)	79·5 (79·1 to 79·8)	79·4 (77·6 to 81·2)
Kentucky	71·1 (68·2 to 73·8)	73·2 (72·9 to 73·5)	74·3 (74 to 74·6)	77·1 (76·8 to 77·5)	78·2 (77·9 to 78·5)	78 (77·7 to 78·3)	78·3 (78 to 78·6)	78·3 (78 to 78·6)	76·9 (76·6 to 77·2)	76·3 (74·3 to 78·1)
Louisiana	69 (66 to 72·2)	71·5 (71·2 to 71·8)	73 (72·7 to 73·3)	76·1 (75·8 to 76·4)	77·2 (76·9 to 77·5)	77·1 (76·8 to 77·4)	78·6 (78·3 to 78·9)	78·6 (78·3 to 78·9)	76·5 (76·2 to 76·8)	76·5 (74·8 to 78·3)
Maine	71·2 (68·6 to 73·7)	73·7 (73·3 to 74·1)	74·9 (74·5 to 75·3)	78·4 (78 to 78·8)	79·7 (79·2 to 80·1)	79·8 (79·4 to 80·3)	81·3 (80·8 to 81·7)	81·4 (80·8 to 81·9)	81·4 (80·8 to 81·8)	80·4 (78·8 to 82)
Maryland	70 (67·3 to 72·8)	72 (71·7 to 72·3)	74·2 (74 to 74·5)	77 (76·7 to 77·3)	78·3 (78 to 78·6)	78·8 (78·5 to 79)	81·4 (81·1 to 81·6)	82 (81·7 to 82·3)	80·6 (80·3 to 80·8)	80·9 (79 to 82·8)
Massachusetts	72 (69·1 to 74·6)	74 (73·8 to 74·3)	75·8 (75·6 to 76·1)	78·3 (78·1 to 78·6)	80 (79·7 to 80·2)	80·5 (80·3 to 80·8)	82·8 (82·5 to 83)	83·2 (83 to 83·5)	82·1 (81·8 to 82·3)	83 (81·4 to 84·6)
Michigan	71·6 (68·9 to 74·2)	73·6 (73·4 to 73·8)	74·6 (74·4 to 74·8)	77·5 (77·2 to 77·7)	78·5 (78·3 to 78·7)	78·8 (78·6 to 79)	80·2 (80 to 80·4)	80·9 (80·7 to 81·2)	79·4 (79·1 to 79·6)	79·5 (77·7 to 81·2)
Minnesota	73·7 (70·9 to 76·5)	75·2 (74·9 to 75·5)	77 (76·7 to 77·3)	79·9 (79·6 to 80·2)	81 (80·6 to 81·3)	81·2 (80·9 to 81·4)	82·9 (82·7 to 83·2)	83 (82·7 to 83·3)	81·8 (81·5 to 82·1)	82 (80·4 to 83·6)
Mississippi	67·9 (64·6 to 71)	70·7 (70·4 to 71·1)	72·8 (72·5 to 73·2)	76·3 (75·9 to 76·6)	77·3 (77 to 77·7)	76·6 (76·3 to 77)	77·7 (77·4 to 78·1)	77·8 (77·4 to 78·2)	75·4 (75·1 to 75·7)	75·5 (73·6 to 77·4)
Missouri	71·9 (69·1 to 74·6)	73·8 (73·5 to 74·1)	74·7 (74·5 to 75)	77·5 (77·3 to 77·8)	78·8 (78·5 to 79·1)	78·6 (78·3 to 78·8)	79·9 (79·6 to 80·1)	80·2 (79·9 to 80·4)	78·5 (78·3 to 78·8)	78·6 (76·8 to 80·6)
Montana	72 (69·4 to 74·3)	73·9 (73·4 to 74·3)	75·3 (74·9 to 75·8)	78 (77·6 to 78·4)	79·7 (79·3 to 80·2)	80 (79·6 to 80·4)	80·8 (80·3 to 81·2)	81·3 (80·8 to 81·8)	80·6 (80 to 81·1)	79·9 (78·6 to 81·2)
Nebraska	74·1 (71·5 to 76·8)	75·5 (75·1 to 75·9)	76·8 (76·4 to 77·1)	79·3 (78·9 to 79·7)	80·4 (80 to 80·8)	80·6 (80·2 to 81)	81·8 (81·4 to 82·1)	81·7 (81·3 to 82·1)	80·7 (80·3 to 81·2)	80·8 (79·3 to 82·2)
Nevada	68·2 (65·3 to 71)	72·1 (71·4 to 72·7)	73·6 (73·1 to 74·2)	76·6 (76·1 to 77·1)	78 (77·6 to 78·4)	78·7 (78·3 to 79)	80·1 (79·8 to 80·4)	81·2 (80·8 to 81·6)	79·7 (79·4 to 80·1)	79 (77·7 to 80·3)
New Hampshire	71·7 (69·1 to 74·2)	74·3 (73·8 to 74·8)	75·6 (75·1 to 76)	78·5 (78 to 78·9)	80 (79·6 to 80·4)	80·7 (80·3 to 81·1)	82·2 (81·8 to 82·6)	82·2 (81·7 to 82·7)	82·1 (81·6 to 82·6)	81·7 (80·2 to 83·1)
New Jersey	70·7 (68 to 73·3)	72·9 (72·7 to 73·1)	74·6 (74·3 to 74·8)	77·3 (77·1 to 77·5)	78·7 (78·4 to 78·9)	79·8 (79·6 to 80·1)	82·3 (82 to 82·5)	83·2 (82·9 to 83·4)	81·2 (81 to 81·5)	82·3 (80·6 to 84·1)
New Mexico	70·3 (67·1 to 73·2)	72·9 (72·4 to 73·4)	74·6 (74·1 to 75·1)	78·6 (78·1 to 79)	79·6 (79·1 to 80·1)	80·1 (79·6 to 80·5)	81·1 (80·6 to 81·5)	80·9 (80·5 to 81·4)	78·7 (78·3 to 79·2)	78·5 (76·5 to 80·4)

New York	70·6 (68·2 to 73)	72·8 (72·6 to 72·9)	74·1 (74 to 74·3)	77·1 (76·9 to 77·2)	78·4 (78·3 to 78·6)	80·3 (80·1 to 80·4)	82·5 (82·4 to 82·7)	83·9 (83·7 to 84·1)	81·6 (81·5 to 81·8)	82·7 (81 to 84·3)
North Carolina	70·2 (67·1 to 73·1)	72·2 (71·9 to 72·5)	74 (73·8 to 74·3)	77·5 (77·3 to 77·8)	78·5 (78·3 to 78·7)	78·5 (78·3 to 78·7)	80·4 (80·1 to 80·6)	80·6 (80·4 to 80·8)	79·4 (79·2 to 79·6)	78·9 (77·2 to 80·7)
North Dakota	73 (70·8 to 75·2)	75·3 (74·8 to 75·8)	77·1 (76·7 to 77·5)	79·5 (79 to 79·9)	81·4 (81 to 81·8)	81·2 (80·7 to 81·6)	82 (81·6 to 82·5)	82·5 (81·9 to 83·1)	81·6 (81 to 82·2)	82·7 (81·3 to 83·9)
Ohio	71·6 (69 to 74·2)	73·5 (73·3 to 73·7)	74·5 (74·4 to 74·7)	76·9 (76·7 to 77·1)	78·5 (78·4 to 78·7)	78·7 (78·5 to 78·9)	79·8 (79·7 to 80)	79·8 (79·6 to 80)	78·4 (78·2 to 78·7)	78·2 (76·5 to 79·9)
Oklahoma	72·8 (70 to 75·6)	74·7 (74·3 to 75·1)	75·6 (75·3 to 76)	77·9 (77·5 to 78·2)	78·5 (78·1 to 78·8)	77·7 (77·4 to 78·1)	78·2 (77·9 to 78·6)	78·6 (78·3 to 79)	77·1 (76·8 to 77·4)	76·6 (74·7 to 78·4)
Oregon	73·3 (70·4 to 76)	74·9 (74·4 to 75·3)	76·5 (76·1 to 76·9)	78·9 (78·5 to 79·3)	79·8 (79·4 to 80·1)	80 (79·7 to 80·4)	81·6 (81·2 to 81·9)	82·5 (82·1 to 82·8)	82 (81·6 to 82·3)	81·1 (79·2 to 82·9)
Pennsylvania	70·6 (68 to 73·3)	72·7 (72·6 to 72·9)	74·1 (73·9 to 74·3)	77·1 (76·9 to 77·3)	78·7 (78·5 to 78·8)	79·4 (79·2 to 79·5)	81 (80·8 to 81·2)	81·3 (81·1 to 81·5)	80 (79·8 to 80·2)	79·9 (78·2 to 81·7)
Rhode Island	71·3 (68·6 to 73·8)	73·7 (73·3 to 74·1)	75·5 (75 to 75·9)	78·6 (78·1 to 79)	79·8 (79·3 to 80·2)	80·3 (79·8 to 80·7)	81·9 (81·4 to 82·4)	82·5 (81·9 to 83)	81·7 (81·1 to 82·2)	82·3 (80·8 to 83·6)
South Carolina	67·5 (64·3 to 70·6)	70·3 (70 to 70·7)	72·7 (72·4 to 73·1)	76·3 (76 to 76·7)	77·5 (77·1 to 77·8)	78 (77·7 to 78·3)	79·4 (79·2 to 79·7)	79·7 (79·4 to 80)	78 (77·7 to 78·3)	77·6 (75·8 to 79·4)
South Dakota	73·1 (70·6 to 75·4)	74·8 (74·3 to 75·3)	76·2 (75·8 to 76·6)	79·2 (78·7 to 79·6)	80·7 (80·2 to 81·2)	80·5 (80·1 to 81)	81·8 (81·3 to 82·2)	81·5 (80·9 to 82·1)	80·6 (80 to 81·2)	81·2 (79·8 to 82·5)
Tennessee	70·5 (67·6 to 73·2)	72·9 (72·6 to 73·2)	74·2 (74 to 74·5)	77·4 (77·1 to 77·7)	78·1 (77·9 to 78·4)	77·9 (77·6 to 78·2)	78·8 (78·6 to 79·1)	78·9 (78·7 to 79·2)	77·4 (77·1 to 77·6)	76·9 (75 to 78·9)
Texas	72 (69 to 74·6)	74·1 (73·9 to 74·3)	75·1 (74·9 to 75·3)	77·7 (77·6 to 77·9)	79·1 (78·9 to 79·2)	79·2 (79·1 to 79·3)	80·7 (80·6 to 80·9)	81·6 (81·4 to 81·7)	79·8 (79·7 to 80)	79·3 (77·9 to 80·9)
Utah	73 (70·4 to 75·6)	75·4 (74·9 to 75·8)	76·7 (76·3 to 77·1)	79·2 (78·8 to 79·6)	80·7 (80·3 to 81·1)	80·6 (80·3 to 81)	81·7 (81·4 to 82)	81·9 (81·6 to 82·2)	81 (80·7 to 81·4)	80·6 (79·4 to 81·8)
Vermont	71·7 (69·3 to 73·8)	74·1 (73·5 to 74·6)	75·9 (75·5 to 76·3)	78·5 (78 to 78·9)	79·8 (79·4 to 80·2)	80·3 (79·9 to 80·8)	82·1 (81·7 to 82·6)	82·5 (82 to 83·2)	82·3 (81·7 to 82·9)	81·7 (80·5 to 82·9)
Virginia	70·1 (67·1 to 72·9)	72·4 (72·1 to 72·7)	74·5 (74·2 to 74·7)	77·4 (77·1 to 77·7)	78·8 (78·5 to 79)	79·2 (79 to 79·5)	81·3 (81 to 81·5)	81·8 (81·5 to 82)	80·6 (80·3 to 80·8)	80·2 (78·4 to 81·9)
Washington	73·1 (70·4 to 75·8)	74·9 (74·6 to 75·3)	75·8 (75·5 to 76·1)	78·7 (78·4 to 79)	80 (79·7 to 80·4)	80·5 (80·2 to 80·8)	82·1 (81·8 to 82·4)	82·7 (82·4 to 82·9)	82·3 (82 to 82·5)	81·7 (79·9 to 83·4)
West Virginia	71·1 (68·1 to 73·9)	73·6 (73·2 to 74)	73·5 (73·1 to 73·9)	76·9 (76·4 to 77·3)	78·3 (77·8 to 78·7)	77·6 (77·1 to 78)	78·1 (77·7 to 78·5)	77·5 (77 to 78)	76·6 (76·1 to 77)	75·3 (73·3 to 77·3)
Wisconsin	72·7 (70·1 to 75·3)	74·4 (74·1 to 74·7)	76·2 (76 to 76·5)	79 (78·7 to 79·3)	80 (79·7 to 80·3)	80·3 (80 to 80·6)	81·8 (81·6 to 82·1)	81·8 (81·5 to 82)	80·6 (80·3 to 80·9)	80·8 (79·2 to 82·6)
Wyoming	71·9 (69·6 to 74·1)	74 (73·4 to 74·6)	75·7 (75·2 to 76·2)	77·9 (77·5 to 78·4)	79·4 (78·9 to 79·9)	79·4 (78·9 to 79·8)	80·3 (79·9 to 80·8)	80·9 (80·3 to 81·5)	80·2 (79·6 to 80·8)	79·1 (77·9 to 80·3)
Southern Latin America	64 (63·7 to 64·2)	65·2 (65·1 to 65·4)	69 (68·9 to 69·1)	73·7 (73·6 to 73·8)	76·3 (76·2 to 76·4)	78·4 (78·4 to 78·5)	79·6 (79·6 to 79·7)	80·6 (80·5 to 80·7)	80·2 (80 to 80·3)	79·9 (79·6 to 80·1)
Argentina	66·9 (66·7 to 67·2)	68·4 (68·2 to 68·5)	69·9 (69·7 to 70)	73·8 (73·7 to 73·9)	76 (75·9 to 76·1)	77·9 (77·8 to 78)	79 (78·9 to 79)	79·7 (79·6 to 79·8)	79·2 (79 to 79·3)	79·1 (78·8 to 79·3)
Chile	55·2 (54·8 to 55·6)	57·2 (56·9 to 57·5)	65·7 (65·6 to 65·9)	72·9 (72·7 to 73)	76·7 (76·6 to 76·8)	79·8 (79·7 to 79·9)	81·3 (81·2 to 81·4)	83 (82·9 to 83·2)	82·3 (82·2 to 82·5)	81·9 (81·7 to 82·1)
Uruguay	70·2 (69·8 to 70·7)	71·2 (70·9 to 71·5)	71·9 (71·7 to 72·1)	74·2 (74 to 74·4)	76·9 (76·7 to 77·1)	78·6 (78·4 to 78·8)	80 (79·7 to 80·3)	80·5 (80·3 to 80·8)	81·2 (80·9 to 81·5)	79·4 (79 to 79·7)
Western Europe	69·2 (69·1 to 69·3)	72·8 (72·8 to 72·8)	74·6 (74·6 to 74·7)	77·2 (77·2 to 77·3)	79·5 (79·5 to 79·5)	81·5 (81·5 to 81·6)	83·6 (83·5 to 83·6)	84·6 (84·5 to 84·6)	84 (84 to 84·1)	84·2 (84·1 to 84·3)
Andorra	77·9 (75·2 to 81·3)	77·9 (75·6 to 80·8)	79 (76·7 to 81·5)	80·4 (77·4 to 83)	82·3 (79·5 to 84·8)	83·5 (81·5 to 85·6)	84·8 (83·1 to 86·5)	85·2 (83·2 to 87·2)	85·7 (83·7 to 87·7)	85·7 (83·5 to 87·9)
Austria	68·6 (68·3 to 68·8)	71·9 (71·8 to 72)	73·5 (73·4 to 73·6)	76·1 (76 to 76·2)	79 (78·8 to 79·1)	81·3 (81·2 to 81·4)	83·2 (83·1 to 83·4)	84·4 (84·2 to 84·5)	83·8 (83·7 to 84)	84·1 (83·9 to 84·2)
Belgium	68·9 (68·5 to 69·1)	73 (72·8 to 73·1)	74·2 (74·1 to 74·4)	76·7 (76·6 to 76·8)	79·3 (79·2 to 79·4)	81 (80·9 to 81·2)	82·8 (82·7 to 82·9)	84 (83·9 to 84·2)	83 (82·9 to 83·1)	84·2 (84 to 84·4)
Cyprus	61·7 (59·1 to 64·1)	65·4 (64 to 66·9)	69·7 (68·9 to 70·4)	73·4 (73·1 to 73·7)	76·3 (76 to 76·6)	78·1 (77·5 to 78·6)	81·3 (80·8 to 81·9)	82·9 (82·2 to 83·5)	83·3 (82·6 to 83·9)	83·2 (82·5 to 83·9)
Denmark	71·9 (71·6 to 72·2)	74·2 (74·1 to 74·4)	76 (75·8 to 76·1)	77·4 (77·2 to 77·5)	77·9 (77·8 to 78·1)	79·3 (79·2 to 79·5)	81·6 (81·4 to 81·7)	83·5 (83·3 to 83·7)	83·5 (83·4 to 83·7)	83·5 (83·3 to 83·7)
Finland	68·1 (67·7 to 68·4)	72·5 (72·3 to 72·6)	74·6 (74·4 to 74·7)	78·3 (78·1 to 78·4)	79·4 (79·2 to 79·6)	81·5 (81·3 to 81·6)	83·7 (83·5 to 83·8)	84·9 (84·6 to 85·1)	84·9 (84·7 to 85·1)	84·9 (84·7 to 85·2)
France	69·8 (69·6 to 70)	74·3 (74·3 to 74·4)	76·1 (76·1 to 76·2)	78·6 (78·5 to 78·6)	81·1 (81·1 to 81·2)	82·7 (82·6 to 82·7)	84·6 (84·5 to 84·6)	85·8 (85·7 to 85·9)	85·1 (85 to 85·1)	85·5 (85·4 to 85·6)
Germany	70·2 (69·6 to 70·8)	72·4 (72·3 to 72·4)	73·6 (73·5 to 73·6)	76·2 (76·2 to 76·3)	78·6 (78·5 to 78·7)	81·2 (81·1 to 81·2)	82·8 (82·8 to 82·9)	83·6 (83·6 to 83·7)	83·5 (83·5 to 83·6)	83·4 (83·3 to 83·5)
Greece	70·9 (70·5 to 71·2)	74·2 (74 to 74·4)	76 (75·8 to 76·1)	77·6 (77·4 to 77·7)	79·4 (79·3 to 79·5)	80·8 (80·7 to 80·9)	82·7 (82·6 to 82·8)	83·7 (83·6 to 83·9)	83·4 (83·3 to 83·6)	82·8 (82·6 to 83)
Iceland	74 (73·2 to 74·7)	75·4 (74·9 to 75·9)	76·6 (76·1 to 77·1)	79·5 (79 to 80)	80·2 (79·7 to 80·6)	82·1 (81·7 to 82·6)	83·4 (82·9 to 83·9)	85·2 (84·5 to 85·8)	85·6 (85 to 86·2)	84·9 (84·2 to 85·5)
Ireland	67·2 (66·9 to 67·5)	71·9 (71·7 to 72·1)	73·6 (73·5 to 73·8)	75·5 (75·3 to 75·7)	77·6 (77·4 to 77·8)	79·3 (79·2 to 79·5)	82·9 (82·7 to 83)	84·1 (83·8 to 84·3)	84·3 (84·1 to 84·5)	84·5 (84·2 to 84·7)
Israel	72·7 (72·2 to 73·3)	72·8 (72·7 to 73)	73·5 (73·4 to 73·7)	75·7 (75·6 to 75·9)	78·8 (78·6 to 78·9)	80·6 (80·5 to 80·8)	83·4 (83·3 to 83·5)	85·1 (84·9 to 85·2)	85·2 (85 to 85·3)	85·1 (84·9 to 85·3)
Italy	68·9 (68·7 to 69·1)	72·3 (72·2 to 72·4)	74·6 (74·5 to 74·7)	77·4 (77·3 to 77·5)	80·3 (80·2 to 80·3)	82·4 (82·4 to 82·5)	84·4 (84·3 to 84·4)	85·5 (85·4 to 85·5)	84·5 (84·5 to 84·6)	84·9 (84·8 to 85)
Luxembourg	68·2 (67·6 to 68·8)	71·8 (71·4 to 72·1)	73·6 (73·3 to 74)	75·9 (75·6 to 76·3)	78·7 (78·4 to 79·1)	81·4 (81 to 81·7)	83·4 (83 to 83·8)	84·8 (84·2 to 85·3)	84·3 (83·8 to 84·8)	84·9 (84·4 to 85·4)
Malta	67·4 (66·7 to 68·1)	70·8 (70·4 to 71·2)	73 (72·6 to 73·4)	74·7 (74·3 to 75)	78·7 (78·4 to 79·1)	81·1 (80·7 to 81·6)	83·3 (82·8 to 83·8)	85·1 (84·5 to 85·7)	85·1 (84·5 to 85·7)	84·1 (83·4 to 84·7)
Monaco	68·1 (65·9 to 70·3)	72·3 (70·4 to 74·3)	75·3 (73·5 to 77)	78·6 (77·2 to 80)	81 (79·1 to 84·2)	81·4 (79·2 to 84·2)	81·7 (80·2 to 83·2)	82·7 (81·1 to 84·6)	82·4 (80·7 to 84·3)	81·4 (79·8 to 83·2)
Netherlands	72·9 (72·7 to 73)	75·4 (75·2 to 75·5)	76·5 (76·4 to 76·6)	79·2 (79·1 to 79·3)	80·1 (80 to 80·3)	80·7 (80·6 to 80·8)	82·8 (82·7 to 82·9)	83·6 (83·4 to 83·7)	83 (82·9 to 83·2)	83·2 (83·1 to 83·4)
Norway	73·7 (73·4 to 74)	75·9 (75·7 to 76·1)	77·3 (77·2 to 77·5)	79·3 (79·2 to 79·5)	80·1 (80 to 80·3)	81·6 (81·4 to 81·7)	83·4 (83·2 to 83·6)	84·8 (84·6 to 85)	85 (84·8 to 85·2)	84·9 (84·7 to 85·1)
Agder	73·7 (72·7 to 74·7)	76 (75·5 to 76·5)	77·7 (77·1 to 78·2)	79·2 (78·7 to 79·8)	80·1 (79·5 to 80·6)	81·4 (80·8 to 82)	82·9 (82·3 to 83·4)	83·7 (83 to 84·5)	84·3 (83·5 to 85·1)	84·6 (83·1 to 86·1)
Innlandet	73·5 (72·6 to 74·3)	75·6 (75·1 to 76·1)	77·2 (76·7 to 77·7)	79·1 (78·6 to 79·6)	80 (79·5 to 80·6)	80·2 (79·7 to 80·7)	82·8 (82·2 to 83·3)	84 (83·3 to 84·9)	85 (84·2 to 85·8)	84·9 (83·5 to 86·2)
Møre og Romsdal	74·3 (73·2 to 75·2)	76·7 (76·1 to 77·2)	78·2 (77·7 to 78·8)	80 (79·5 to 80·6)	81·2 (80·6 to 81·7)	82·6 (82·1 to 83·2)	85·1 (84·5 to 85·7)	85·4 (84·5 to 86·3)	85·2 (84·4 to 86·1)	85·3 (83·5 to 86·9)
Nordland	72·7 (71·7 to 73·7)	75·8 (75·2 to 76·3)	77·2 (76·6 to 77·7)	79·8 (79·2 to 80·4)	80·2 (79·6 to 80·7)	81·9 (81·3 to 82·5)	83·5 (82·9 to 84·2)	84·1 (83·3 to 85)	85 (84·1 to 85·9)	85·2 (83·5 to 86·9)
Oslo	76·6 (75·7 to 77·4)	76·4 (76 to 76·8)	77·4 (77 to 77·9)	78·5 (78 to 79)	78·7 (78·3 to 79·2)	81·1 (80·7 to 81·6)	83·5 (83 to 84)	85·4 (84·7 to 86·1)	84·9 (84·3 to 85·6)	85·1 (84 to 86·2)
Rogaland	74·3 (73·3 to 75·3)	76·1 (75·6 to 76·6)	77·3 (76·8 to 77·8)	79·3 (78·8 to 79·8)	80·7 (80·2 to 81·2)	81·9 (81·5 to 82·4)	83·5 (83·1 to 84)	85·4 (84·7 to 86·1)	84·9 (84·1 to 85·6)	84·9 (83·8 to 86)
Troms og Finnmark	70·8 (69·7 to 71·9)	74·4 (73·8 to 74·9)	75·9 (75·3 to 76·5)	79·1 (78·5 to 79·7)	79·6 (79 to 80·2)	80·8 (80·2 to 81·4)	83 (82·4 to 83·6)	84·2 (83·3 to 85)	84·7 (83·8 to 85·5)	84·5 (82·8 to 86·1)
Trøndelag	73·7 (72·8 to 74·6)	75·6 (75·1 to 76·1)	77·1 (76·7 to 77·6)	79·2 (78·7 to 79·7)	80·1 (79·6 to 80·6)	81·9 (81·4 to 82·4)	83·4 (82·9 to 83·9)	84·5 (83·8 to 85·2)	84·9 (84·1 to 85·6)	84·9 (83·6 to 86·3)
Vestfold og Telemark	74·6 (73·6 to 75·5)	76·1 (75·6 to 76·6)	77·4 (76·8 to 77·9)	79·2 (78·7 to 79·7)	80·2 (79·7 to 80·7)	81·2 (80·7 to 81·7)	82·7 (82·2 to 83·2)	84·5 (83·8 to 85·2)	84·7 (84 to 85·4)	84·5 (83·1 to 85·9)
Vestland	74·3 (73·5 to 75·1)	76·2 (75·7 to 76·6)	77·7 (77·3 to 78·2)	80·5 (80·1 to 81)	81·2 (80·7 to 81·6)	82·7 (82·2 to 83·2)	84·3 (83·8 to 84·8)	85·6 (85 to 86·3)	85·9 (85·2 to 86·6)	85·9 (84·5 to 87·2)
Viken	72 (71·2 to 72·7)	75·9 (75·5 to 76·3)	77·3 (76·9 to 77·7)	79·1 (78·7 to 79·5)	80 (79·6 to 80·3)	81·5 (81·1 to 81·8)	83·1 (82·7 to 83·5)	84·8 (84·4 to 85·4)	85·1 (84·6 to 85·6)	84·5 (83·5 to 85·6)
Portugal	60·9 (60·3 to 61·5)	67·4 (67·1 to 67·6)	70·2 (70 to 70·4)	75·1 (75 to 75·3)	77·6 (77·5 to 77·7)	80·1 (80 to 80·2)	83·1 (83 to 83·2)	84·8 (84·6 to 84·9)	84·3 (84·2 to 84·4)	84·4 (84·3 to 84·6)
San Marino	76·2 (72·7 to 78·8)	76·4 (74·5 to 78·5)	77·8 (76·5 to 79·2)	79·9 (78·4 to 81·7)	82·4 (80·7 to 83·9)	84·5 (83·1 to 85·8)	87·6 (85·9 to 89·3)	86·6 (84·6 to 88·5)	86·4 (84·4 to 88·2)	88·1 (85·3 to 91)
Spain	64·5 (64·2 to 64·7)	71·8 (71·7 to 71·9)	74·6 (74·6 to 74·7)	78·5 (78·4 to 78·5)	80·4 (80·4 to 80·5)	82·9 (82·8 to 82·9)	85 (84·9 to 85·1)	86 (85·9 to 86·1)	85 (84·9 to 85)	85·7 (85·6 to 85·8)
Sweden	72·7 (72·5 to 72·9)	75 (74·9 to 75·2)	77·3 (77·2 to 77·4)	79·2 (79·1 to 79·3)	80·8 (80·6 to 80·9)	82·2 (82·1 to 82·3)	83·8 (83·6 to 83·9)	84·9 (84·8 to 85·1)	84·6 (84·5 to 84·7)	85 (84·1 to 85·9)
Stockholm	72·9 (69·9 to 75·8)	75·1 (72·2 to 78·2)	77 (74·2 to 79·6)	78·7 (78·3 to 79·1)	80·1 (79·8 to 80·5)	82·1 (81·7 to 82·4)	83·7 (83·3 to 84)	85 (84·6 to 85·4)	84·3 (84 to 84·7)	85·8 (84·4 to 87·1)
Sweden except Stockholm	72·7 (72·1 to 73·3)	75·1 (74·5 to 75·7)	77·4 (76·9 to 78·1)	79·3 (79·1 to 79·4)	80·9 (80·7 to 81)	82·3 (82·1 to 82·4)	83·8 (83·6 to 83·9)	84·9 (84·7 to 85)	84·6 (84·5 to 84·8)	84·8 (84 to 85·7)
Switzerland	71·1 (70·8 to 71·4)	74·3 (74·1 to 74·4)	76·2 (76·1 to 76·3)	79·1 (79 to 79·2)	81·2 (81·1 to 81·3)	83·1 (83 to 83·3)	85 (84·9 to 85·2)	86·3 (86·2 to 86·5)	85·7 (85·5 to 85·8)	86·4 (86·2 to 86·6)
UK	71·3 (71·2 to 71·4)	73·6 (73·5 to 73·7)	74·9 (74·9 to 75)	76·5 (76·4 to 76·5)	78·4 (78·4 to 78·5)	80·1 (80 to 80·1)	82·5 (82·4 to 82·6)	83·2 (83·1 to 83·3)	82·5 (82·4 to 82·6)	82·4 (82·3 to 82·5)
England	71·8 (71·7 to 71·9)	74 (73·9 to 74)	75·2 (75·1 to 75·3)	76·7 (76·6 to 76·8)	78·6 (78·6 to 78·7)	80·3 (80·2 to 80·3)	82·8 (82·7 to 82·8)	83·5 (83·4 to 83·5)	82·7 (82·7 to 82·8)	82·6 (82·5 to 82·7)
East Midlands	71·7 (69·3 to 74·3)	73·9 (71·5 to 76·2)	75·1 (72·7 to 77·4)	76·6 (75·9 to 77·3)	78·8 (78·5 to 79·1)	80·3 (80 to 80·5)	82·6 (82·3 to 82·9)	83 (82·7 to 83·3)	82·5 (82·2 to 82·8)	82·3 (81·6 to 82·9)
Derby	70·4 (66·8 to 73·9)	72·8 (69·5 to 75·8)	74·2 (71·2 to 77·1)	75·7 (74·5 to 76·8)	78 (77·4 to 78·6)	80·3 (79·7 to 80·9)	81·7 (81·1 to 82·3)	82·5 (81·7 to 83·3)	81·8 (81·2 to 82·5)	81 (80 to 82)

Derbyshire	72 (68·9 to 75·2)	74·2 (71 to 77·3)	75·1 (72·2 to 77·9)	76·5 (75·5 to 77·5)	78·7 (78·2 to 79·1)	80·3 (79·9 to 80·8)	82·8 (82·4 to 83·2)	83·1 (82·5 to 83·6)	82·6 (82·1 to 83·1)	82·4 (81·6 to 83·3)
Leicester	70·4 (66·8 to 73·7)	72·4 (69·2 to 75·4)	73·5 (70·6 to 76·6)	75·2 (74·2 to 76·3)	78·2 (77·6 to 78·7)	79 (78·4 to 79·6)	81·4 (80·8 to 82)	82·2 (81·6 to 83)	80·8 (80·2 to 81·5)	81·4 (80·4 to 82·2)
Leicestershire	73·2 (69·5 to 76·6)	75·1 (71·9 to 78·1)	76·4 (73·3 to 79·2)	77·8 (76·8 to 78·8)	79·8 (79·4 to 80·3)	81·2 (80·8 to 81·7)	83·8 (83·3 to 84·3)	84·5 (83·9 to 85·1)	83·9 (83·4 to 84·4)	83·5 (82·7 to 84·4)
Lincolnshire	72·1 (68·6 to 75·5)	74·3 (71·1 to 77·6)	75·4 (72·3 to 78·5)	76·7 (75·7 to 77·6)	79 (78·5 to 79·4)	80·4 (79·9 to 80·9)	82·9 (82·4 to 83·3)	82·9 (82·3 to 83·5)	82·8 (82·3 to 83·2)	82·6 (81·7 to 83·4)
Northamptonshire	72·2 (68·5 to 75·5)	74·5 (71 to 77·6)	75·6 (72·4 to 78·7)	77·2 (76·2 to 78·2)	78·9 (78·4 to 79·4)	80·6 (80·2 to 81·1)	82·6 (82·1 to 83·1)	83 (82·4 to 83·5)	82·6 (82 to 83·1)	81·2 (79·7 to 82·8)
Nottingham	70·5 (67 to 73·9)	72·6 (69·4 to 75·7)	73·9 (70·7 to 76·6)	75·3 (74·3 to 76·4)	77·5 (77 to 78)	77·8 (77·3 to 78·4)	80·7 (80·1 to 81·3)	82 (81·1 to 82·7)	80·4 (79·7 to 81)	81·1 (80 to 82·1)
Nottinghamshire	71·9 (68·3 to 75·1)	74·1 (70·9 to 77·2)	75·3 (72·3 to 78·2)	76·8 (75·8 to 77·7)	78·9 (78·4 to 79·3)	80·2 (79·8 to 80·7)	82·5 (82 to 82·9)	82·7 (82·1 to 83·2)	82 (81·5 to 82·4)	82·4 (81·6 to 83·2)
Rutland	73·7 (70·1 to 77)	75·8 (72·3 to 78·8)	77·2 (73·9 to 80·2)	78·1 (76·5 to 79·7)	79·7 (78·6 to 80·7)	82·1 (81·1 to 83·2)	84·3 (83·1 to 85·5)	84·8 (83·2 to 86·2)	86·8 (85·4 to 88·2)	84·8 (83·3 to 86·1)
East of England	73 (70·6 to 75·3)	75·1 (72·7 to 77·4)	76·3 (73·8 to 78·6)	77·6 (76·9 to 78·3)	79·5 (79·2 to 79·7)	81·1 (80·9 to 81·4)	83·4 (83·1 to 83·6)	84 (83·8 to 84·3)	83·6 (83·3 to 83·8)	83·8 (83·1 to 84·4)
Bedford	72·6 (68·9 to 76)	74·7 (71·5 to 77·8)	76·4 (73·1 to 79·3)	78 (76·8 to 79·2)	79·1 (78·3 to 79·7)	80·7 (80 to 81·4)	82·5 (81·8 to 83·2)	83 (82 to 83·9)	81·9 (81·1 to 82·6)	83·2 (82·1 to 84·3)
Cambridgeshire	74·1 (70·6 to 77·4)	76 (72·6 to 79·1)	77·3 (74·2 to 80·1)	78·2 (77·2 to 79·1)	80 (79·6 to 80·5)	81·2 (80·7 to 81·7)	84·3 (83·8 to 84·8)	84·7 (84·1 to 85·4)	84·1 (83·6 to 84·7)	84·8 (84 to 85·6)
Central Bedfordshire	72·5 (68·7 to 75·7)	74·6 (71·3 to 77·8)	76·1 (72·8 to 79·1)	77·5 (76·4 to 78·6)	78·8 (78·3 to 79·3)	81·2 (80·6 to 81·8)	83·4 (82·8 to 84)	84·5 (83·6 to 85·3)	84·7 (84 to 85·4)	83·2 (82·2 to 84·2)
Essex	73·1 (69·8 to 76·4)	75·1 (72·2 to 78)	76 (73·1 to 78·9)	77·4 (76·6 to 78·2)	79·5 (79·1 to 79·8)	81·3 (80·9 to 81·7)	83·3 (82·9 to 83·7)	83·6 (83·2 to 84·1)	83·5 (83·1 to 83·9)	83·4 (82·7 to 84·2)
Hertfordshire	73·6 (70·3 to 76·6)	75·6 (72·7 to 78·7)	76·4 (73·4 to 79·1)	77·5 (76·7 to 78·4)	79·6 (79·2 to 80)	81 (80·6 to 81·4)	83·5 (83·1 to 83·9)	84·3 (83·8 to 84·8)	83·5 (83·1 to 83·9)	84·4 (83·6 to 85·1)
Luton	71·2 (67·5 to 74·7)	73·3 (69·8 to 76·6)	74·6 (71·3 to 77·7)	76·1 (74·9 to 77·3)	78·5 (77·9 to 79·1)	79·7 (79·1 to 80·4)	81·7 (81·1 to 82·4)	83 (82·1 to 83·8)	81·4 (80·7 to 82·2)	82·6 (81·5 to 83·7)
Norfolk	73·1 (69·9 to 76·3)	75·4 (72·2 to 78·6)	76·8 (73·9 to 79·7)	78·2 (77·2 to 79·1)	79·7 (79·3 to 80·1)	81·4 (80·9 to 81·8)	83·4 (83 to 83·9)	84·4 (83·9 to 85)	83·8 (83·3 to 84·3)	83·7 (82·9 to 84·4)
Peterborough	71·8 (67·9 to 75·3)	74·1 (70·7 to 77·2)	75·6 (72·3 to 78·7)	77 (75·8 to 78·2)	78·3 (77·6 to 79)	79·9 (79·2 to 80·5)	81·8 (81·1 to 82·5)	83 (82·1 to 83·8)	82·1 (81·3 to 83)	82·6 (81·5 to 83·7)
Southend-on-Sea	71 (67·6 to 74·3)	73·6 (70·5 to 77)	75·2 (72 to 78·2)	77 (75·8 to 78·1)	78·7 (78 to 79·3)	80·3 (79·7 to 80·9)	82·3 (81·6 to 82·9)	83 (82·2 to 83·9)	82·7 (81·9 to 83·4)	82·3 (81·3 to 83·4)
Suffolk	73 (69·5 to 76·2)	75·3 (71·8 to 78·4)	76·5 (73·3 to 79·5)	77·7 (76·8 to 78·7)	79·9 (79·5 to 80·4)	81·4 (81 to 81·9)	83·8 (83·3 to 84·2)	84·1 (83·6 to 84·7)	84·2 (83·7 to 84·7)	84·3 (83·6 to 85·2)
Thurrock	72 (68·3 to 75·3)	73·8 (70·5 to 77)	75·2 (71·9 to 78·2)	76·7 (75·5 to 77·8)	78·5 (77·8 to 79·1)	80·4 (79·7 to 81·1)	82·5 (81·7 to 83·2)	84·1 (83·2 to 85)	82 (81·2 to 82·8)	82·3 (81·1 to 83·5)
Greater London	72·1 (69·8 to 74·3)	74·1 (71·9 to 76·2)	75·4 (73·2 to 77·7)	76·9 (76·3 to 77·6)	78·7 (78·5 to 79)	80·1 (79·9 to 80·3)	83·8 (83·6 to 84·1)	85·1 (84·8 to 85·3)	83·7 (83·4 to 83·9)	83·7 (83·1 to 84·4)
Barking and Dagenham	70·2 (66·8 to 73·4)	73·1 (69·8 to 76·3)	74·5 (71·4 to 77·7)	76·3 (75·2 to 77·4)	78·1 (77·5 to 78·7)	79·3 (78·7 to 79·9)	82·1 (81·4 to 82·7)	83·4 (82·5 to 84·4)	81·3 (80·5 to 82·1)	81·6 (80·4 to 82·7)
Barnet	73·2 (70·1 to 76·5)	75·3 (72·1 to 78·2)	76·9 (73·9 to 79·8)	78·4 (77·4 to 79·4)	80 (79·5 to 80·5)	81·6 (81 to 82·1)	84·2 (83·7 to 84·8)	86·5 (85·6 to 87·3)	84·7 (84·1 to 85·3)	84·9 (84 to 85·7)
Bexley	73·3 (69·9 to 76·7)	75·7 (72·7 to 78·6)	77·1 (74·2 to 80·1)	78·6 (77·6 to 79·7)	80·1 (79·5 to 80·7)	81·5 (80·9 to 82·2)	84·8 (84·2 to 85·5)	84·2 (83·3 to 85)	82·6 (81·8 to 83·3)	83·9 (82·9 to 85)
Brent	71·9 (68·5 to 75·2)	74 (70·9 to 77)	75·3 (72·2 to 78·3)	76·8 (75·7 to 77·8)	78·7 (78·2 to 79·2)	80·1 (79·6 to 80·7)	83·8 (83·2 to 84·4)	85·8 (84·9 to 86·7)	83·7 (83 to 84·5)	85·2 (84·2 to 86·2)
Bromley	73·5 (70·1 to 76·7)	75·7 (72·7 to 78·8)	77·1 (74·2 to 80)	78·5 (77·5 to 79·6)	80·4 (79·8 to 80·9)	81·5 (80·9 to 82)	84·8 (84·2 to 85·4)	86 (85·1 to 86·9)	85·2 (84·5 to 85·9)	86·1 (85·1 to 87·1)
Camden	73 (69·5 to 76·5)	73·9 (70·8 to 76·9)	74·8 (71·8 to 77·6)	75·7 (74·6 to 76·8)	77·6 (77·1 to 78·2)	79·3 (78·8 to 79·9)	83 (82·3 to 83·6)	84·2 (83·3 to 85)	85·5 (84·7 to 86·4)	84·9 (83·8 to 86·1)
Croydon	72·4 (68·9 to 75·6)	74·7 (71·6 to 78)	75·8 (72·9 to 78·7)	76·9 (75·9 to 77·9)	78·7 (78·1 to 79·2)	80·3 (79·8 to 80·8)	84·3 (83·7 to 84·8)	84·7 (83·9 to 85·6)	83·2 (82·6 to 83·8)	84 (83 to 85)
Ealing	72·2 (68·9 to 75·5)	74·5 (71·3 to 77·5)	75·7 (72·7 to 78·5)	77 (75·9 to 78)	78·9 (78·4 to 79·5)	80·5 (79·9 to 81)	85 (84·4 to 85·7)	85·4 (84·5 to 86·3)	82·6 (82 to 83·2)	84·1 (83 to 85·1)
Enfield	72·8 (69·3 to 76·1)	75·2 (72·3 to 78·2)	76·7 (73·8 to 79·6)	78·3 (77·1 to 79·4)	79·9 (79·3 to 80·4)	80·4 (79·9 to 81)	84·1 (83·5 to 84·7)	85·3 (84·5 to 86·1)	84·2 (83·6 to 84·9)	84·7 (83·8 to 85·8)
Greenwich	70·9 (67·4 to 74·2)	73·6 (70·3 to 76·7)	75·1 (72·1 to 78)	76·7 (75·6 to 77·9)	78·3 (77·8 to 78·9)	79 (78·4 to 79·5)	82·8 (82·2 to 83·4)	83·6 (82·8 to 84·5)	81·9 (81·2 to 82·6)	82·5 (81·4 to 83·5)
Hackney	70·9 (67·3 to 74·1)	73 (70 to 76·2)	74 (71·1 to 76·9)	74·9 (73·8 to 76)	76·5 (76 to 77·1)	78·3 (77·8 to 78·9)	82 (81·4 to 82·7)	84·2 (83·4 to 85·1)	83·3 (82·5 to 84·1)	78 (76·2 to 79·8)
Hammersmith and Fulham	72·1 (68·7 to 75·5)	73·5 (70·3 to 76·6)	74·7 (71·6 to 77·7)	75·6 (74·4 to 76·7)	77·2 (76·5 to 77·7)	79·1 (78·5 to 79·6)	83·4 (82·8 to 84·1)	84·9 (84 to 85·9)	82·4 (81·6 to 83·2)	84·1 (83·1 to 85·1)
Haringey	72 (68·5 to 75·3)	73·9 (70·9 to 76·9)	75·3 (72·5 to 78·1)	76·5 (75·4 to 77·7)	78·5 (77·9 to 79)	79·5 (78·9 to 80)	83·1 (82·5 to 83·7)	85 (84·2 to 85·9)	83·2 (82·6 to 83·9)	84·2 (83·1 to 85·1)
Harrow	73 (69·6 to 76·4)	75·2 (72·2 to 78·2)	76·5 (73·3 to 79·2)	77·9 (76·8 to 79)	80·5 (79·9 to 81·1)	81·7 (81·1 to 82·3)	85·5 (84·9 to 86·2)	86·5 (85·4 to 87·4)	86·4 (85·5 to 87·3)	84·3 (83·2 to 85·4)
Havering	73·1 (69·7 to 76·3)	75·5 (72·5 to 78·5)	76·7 (73·9 to 79·6)	78 (77 to 79·1)	80·3 (79·7 to 80·9)	81·3 (80·8 to 81·9)	84 (83·4 to 84·6)	84·8 (83·9 to 85·7)	83·6 (82·9 to 84·3)	83·5 (82·5 to 84·7)
Hillingdon	73 (69·7 to 76)	75·3 (72·1 to 78·2)	76·6 (73·6 to 79·6)	78 (76·9 to 79)	79·2 (78·7 to 79·7)	80·3 (79·8 to 80·9)	83·5 (82·9 to 84)	84·9 (84 to 85·8)	82·7 (82 to 83·3)	82·3 (81·4 to 83·2)
Hounslow	72·2 (68·5 to 75·6)	74·3 (71·1 to 77·4)	75·6 (72·3 to 78·6)	77 (75·8 to 78)	78·6 (78 to 79·2)	80 (79·4 to 80·5)	83·6 (82·9 to 84·2)	85·3 (84·4 to 86·2)	84·1 (83·3 to 84·8)	84·1 (83·1 to 85·2)
Islington	71·4 (67·6 to 74·8)	73·1 (69·8 to 76·1)	74·2 (71·2 to 77·2)	75·2 (74 to 76·4)	77·4 (76·8 to 78)	78·2 (77·6 to 78·8)	82·5 (81·8 to 83·1)	83·4 (82·5 to 84·2)	82·4 (81·7 to 83·2)	81·2 (80·2 to 82·2)
Kensington and Chelsea	73·4 (69·8 to 76·5)	73·8 (70·7 to 77)	74·8 (71·8 to 77·5)	75·8 (74·7 to 76·9)	78·8 (78·1 to 79·5)	82·1 (81·5 to 82·8)	85·5 (84·7 to 86·2)	86·4 (85·3 to 87·5)	86·1 (85·1 to 87·1)	84·7 (83·4 to 85·9)
Kingston upon Thames	74 (70·5 to 77·2)	76·1 (73 to 79)	77·6 (74·5 to 80·5)	78·9 (77·7 to 80·1)	80·2 (79·5 to 80·8)	81·4 (80·8 to 82·1)	85·6 (84·9 to 86·4)	86·3 (85·3 to 87·4)	85·9 (85 to 86·8)	84·5 (83·4 to 85·7)
Lambeth	70·4 (67 to 73·6)	72·5 (69·3 to 75·4)	73·8 (70·7 to 76·9)	75·2 (74·1 to 76·2)	77·4 (76·8 to 77·9)	78·2 (77·6 to 78·7)	82·4 (81·8 to 82·9)	84·6 (83·8 to 85·4)	83·5 (82·8 to 84·2)	82·2 (81·1 to 83·2)
Lewisham	71·1 (67·5 to 74·3)	73·6 (70·6 to 76·8)	75·1 (72 to 78)	76·6 (75·4 to 77·6)	77·8 (77·3 to 78·3)	78·6 (78 to 79·1)	82 (81·4 to 82·6)	84·2 (83·4 to 85)	82·2 (81·6 to 82·9)	84·1 (83 to 85·3)
Merton	72·2 (69 to 75·5)	74·6 (71·4 to 77·5)	76 (73 to 79)	77·4 (76·3 to 78·5)	79·3 (78·6 to 79·9)	80·9 (80·3 to 81·5)	84·7 (83·9 to 85·3)	85·6 (84·6 to 86·5)	83·4 (82·7 to 84·2)	83·9 (82·8 to 85)
Newham	70·1 (66·8 to 73·5)	72·7 (69·6 to 75·9)	73·9 (70·8 to 76·8)	75·5 (74·3 to 76·6)	77·7 (77 to 78·2)	78·2 (77·6 to 78·8)	82·3 (81·7 to 82·9)	83·5 (82·7 to 84·3)	81 (80·4 to 81·7)	81·5 (80·5 to 82·5)
Redbridge	72·8 (69·4 to 76)	75·1 (72 to 78·1)	76·4 (73·4 to 79·2)	78·5 (77·3 to 79·6)	80·2 (79·6 to 80·7)	81·4 (80·8 to 82)	84·7 (84·1 to 85·4)	86·5 (85·6 to 87·4)	84·8 (84 to 85·6)	84·3 (83·1 to 85·4)
Richmond upon Thames	73·5 (70 to 76·7)	75·4 (72·3 to 78·6)	76·8 (73·8 to 79·7)	77·9 (76·7 to 79·1)	80·1 (79·5 to 80·7)	81·5 (80·9 to 82·2)	85·7 (85 to 86·4)	86·6 (85·5 to 87·6)	87 (86·1 to 88)	85·7 (84·5 to 86·9)
Southwark	71·1 (67·6 to 74·5)	73·7 (70·6 to 76·9)	74·9 (71·9 to 78)	76·1 (75 to 77·2)	76·6 (76·1 to 77·1)	78·6 (78 to 79·1)	82·3 (81·7 to 82·8)	84·3 (83·5 to 85·1)	81·7 (81 to 82·3)	82·6 (81·6 to 83·5)
Sutton	72·5 (68·9 to 75·7)	75 (72 to 78)	76·6 (73·8 to 79·4)	78·1 (77 to 79·2)	79·4 (78·7 to 80)	80·7 (80 to 81·3)	84·7 (84 to 85·4)	85·1 (84·1 to 86·1)	84·6 (83·8 to 85·4)	84·1 (83 to 85·2)
Tower Hamlets	69·3 (65·6 to 72·7)	72·3 (69·1 to 75·3)	73·2 (70·1 to 76·2)	74·7 (73·6 to 75·9)	77·2 (76·6 to 77·8)	78·2 (77·7 to 78·8)	82·4 (81·8 to 83)	82·9 (82·1 to 83·7)	80·7 (80·1 to 81·4)	79·9 (78·9 to 80·9)
Waltham Forest	70·8 (67·1 to 74·2)	73·4 (70·3 to 76·3)	75·1 (72 to 78·1)	76·7 (75·6 to 77·8)	78·5 (78 to 79·1)	79·1 (78·6 to 79·7)	83 (82·4 to 83·6)	84·9 (84 to 85·7)	84·1 (83·3 to 84·8)	84·1 (83·1 to 85·2)
Wandsworth	71·5 (68 to 74·9)	73·4 (70·2 to 76·3)	74·6 (71·5 to 77·5)	76·2 (75·1 to 77·2)	77·7 (77·2 to 78·2)	79·3 (78·8 to 79·9)	83·8 (83·3 to 84·4)	84·9 (84·1 to 85·7)	83·3 (82·7 to 84)	84 (82·9 to 85)
Westminster	73·3 (69·8 to 76·6)	73·8 (70·7 to 76·8)	74·7 (71·9 to 77·8)	75·6 (74·5 to 76·8)	78 (77·4 to 78·6)	80·6 (80 to 81·2)	84·6 (84 to 85·3)	86·1 (85·1 to 87)	85·6 (84·8 to 86·4)	85·4 (84·4 to 86·5)
North East England	70·6 (67·9 to 73·1)	72·7 (70·3 to 75·2)	73·8 (71·4 to 76·2)	75·4 (74·7 to 76·2)	77·1 (76·8 to 77·4)	78·9 (78·6 to 79·2)	81·3 (81 to 81·6)	81·9 (81·5 to 82·3)	81·1 (80·7 to 81·4)	80·9 (80·1 to 81·6)
County Durham	70·9 (67·4 to 74·1)	73·2 (70 to 76·3)	74·1 (71 to 77·1)	75·5 (74·4 to 76·5)	76·8 (76·3 to 77·3)	78·5 (78·1 to 79)	81·2 (80·7 to 81·7)	81·7 (81·1 to 82·4)	80·7 (80·1 to 81·2)	80·8 (79·8 to 81·7)
Darlington	70·9 (67·3 to 74·3)	72·9 (69·6 to 76·2)	73·9 (70·6 to 77·2)	75·5 (74·2 to 76·8)	77·2 (76·5 to 78)	79 (78·2 to 79·8)	81·6 (80·8 to 82·3)	81·8 (80·8 to 82·8)	82·4 (81·5 to 83·3)	79·9 (78·8 to 81·1)
Gateshead	70·4 (66·8 to 73·7)	72·5 (69·1 to 75·5)	73·6 (70·5 to 76·7)	75·2 (74·2 to 76·4)	77·5 (76·9 to 78·1)	79 (78·4 to 79·7)	81·6 (80·9 to 82·2)	82·1 (81·3 to 83)	81 (80·3 to 81·7)	80·9 (79·9 to 82)
Hartlepool	69·5 (65·9 to 73·2)	71·7 (68·4 to 75·1)	72·8 (69·4 to 75·9)	74·4 (73·1 to 75·7)	76·6 (75·9 to 77·3)	78·3 (77·4 to 79)	80·5 (79·8 to 81·3)	80·5 (79·5 to 81·6)	81·1 (80·2 to 82)	79·3 (78 to 80·6)
Middlesbrough	69 (65·4 to 72·7)	71·3 (68 to 74·5)	72·3 (69·1 to 75·5)	73·9 (72·7 to 75·1)	75·8 (75·1 to 76·5)	77·7 (77 to 78·3)	80·2 (79·5 to 80·9)	80 (79·1 to 80·9)	79·5 (78·7 to 80·3)	78·6 (77·6 to 79·8)
Newcastle upon Tyne	70·9 (67·4 to 74·1)	72·8 (69·6 to 76·2)	73·9 (71·1 to 77)	75·6 (74·5 to 76·6)	76·7 (76·1 to 77·3)	78·3 (78·1 to 79·3)	81 (80·4 to 81·6)	81·9 (81·1 to 82·8)	81·1 (80·5 to 81·8)	80·7 (79·7 to 81·7)
North Tyneside	70·8 (67·2 to 74·3)	72·9 (70 to 76·1)	74·1 (71·1 to 77)	75·9 (74·6 to 76·9)	77·3 (76·7 to 77·8)	79·3 (78·7 to 79·9)	81·8 (81·1 to 82·4)	82·9 (82 to 83·7)	82 (81·2 to 82·7)	81·7 (80·6 to 82·8)
Northumberland	70·9 (67·4 to 74·4)	73 (69·7 to 76·4)	74·2 (71·1 to 77)	75·7 (74·5 to 76·7)	77·9 (77·3 to 78·4)	80·1 (79·6 to 80·7)	82·7 (82·1 to 83·2)	83·2 (82·5 to 84)	82·5 (81·8 to 83·1)	82·6 (81·7 to 83·5)
Redcar and Cleveland	70·7 (67 to 74·2)	72·7 (69·5 to 76)	73·9 (70·8 to 76·9)	75·6 (74·4 to 76·8)	77·8 (77·1 to 78·5)	79·2 (78·5 to 79·9)	81·2 (80·5 to 82)	81·1 (80·2 to 82·1)	81·6 (80·7 to 82·5)	79 (77·8 to 80·1)
South Tyneside	70·6 (67·1 to 74·1)	72·9 (69·3 to 76·1)	74 (70·8 to 77·3)	75·6 (74·3 to 76·8)	77·1 (76·5 to 77·7)	79·4 (78·7 to 80)	81 (80·4 to 81·8)	81·7 (80·8 to 82·7)	80·6 (79·8 to 81·4)	81·2 (80 to 82·4)

Stockton-on-Tees	71·2 (67·5 to 74·8)	73·4 (70 to 76·7)	74·5 (71·3 to 77·6)	76·2 (75 to 77·4)	77·6 (77 to 78·2)	79·7 (79·1 to 80·4)	81·9 (81·2 to 82·5)	81·8 (80·9 to 82·7)	81·3 (80·5 to 82·1)	81·1 (80 to 82·1)
Sunderland	70·5 (67·1 to 73·9)	72·6 (69·2 to 75·8)	73·8 (70·7 to 76·7)	75·6 (74·5 to 76·5)	77 (76·4 to 77·5)	78·2 (77·6 to 78·8)	80·3 (79·7 to 80·9)	81·4 (80·6 to 82·2)	79·7 (79 to 80·3)	81·1 (80·1 to 82·2)
North West England	70·6 (68·4 to 72·8)	72·8 (70·6 to 74·9)	74·1 (71·9 to 76·3)	75·6 (75 to 76·2)	77·5 (77·3 to 77·7)	79·1 (78·9 to 79·4)	81·4 (81·2 to 81·6)	82·2 (81·9 to 82·4)	81·2 (81 to 81·4)	81 (80·4 to 81·6)
Blackburn with Darwen	69·3 (65·6 to 72·6)	71·8 (68·5 to 75)	73·3 (70·5 to 76·3)	75 (73·9 to 76)	76·7 (76 to 77·3)	78·2 (77·5 to 78·9)	80·9 (80·2 to 81·6)	81·7 (80·8 to 82·6)	80·6 (79·8 to 81·4)	79·9 (78·8 to 81)
Blackpool	69·2 (65·9 to 72·5)	71·7 (68·6 to 74·6)	73·2 (70·5 to 76)	75 (73·8 to 76)	77 (76·3 to 77·6)	77·4 (76·8 to 78·1)	78·8 (78·2 to 79·4)	79·3 (78·4 to 80·1)	79·7 (79 to 80·5)	78·5 (77·5 to 79·6)
Bolton	70·3 (67 to 73·7)	72·5 (69·4 to 75·5)	73·9 (71·1 to 76·9)	75·5 (74·5 to 76·6)	77·4 (76·8 to 77·9)	78·8 (78·2 to 79·3)	81·1 (80·6 to 81·7)	82·1 (81·3 to 82·8)	81·2 (80·6 to 81·8)	80·7 (79·8 to 81·6)
Bury	71 (67·8 to 74·3)	73 (69·9 to 76·2)	74·7 (71·5 to 77·5)	76·4 (75·3 to 77·6)	77·9 (77·3 to 78·5)	79·6 (79 to 80·2)	81·7 (81·1 to 82·4)	82·5 (81·6 to 83·4)	81·4 (80·6 to 82·1)	80·6 (79·6 to 81·5)
Cheshire East	72·7 (69·5 to 76·1)	74·8 (71·6 to 77·7)	76·3 (73·4 to 79)	77·3 (76·4 to 78·2)	78·9 (78·4 to 79·3)	81 (80·5 to 81·5)	82·8 (82·3 to 83·4)	84·6 (83·9 to 85·3)	83·3 (82·7 to 83·9)	84 (83·1 to 84·9)
Cheshire West and Chester	72·2 (68·7 to 75·4)	74·2 (70·8 to 77·4)	75·2 (72·2 to 78)	76·6 (75·6 to 77·6)	78·3 (77·8 to 78·9)	80·5 (80 to 81)	83 (82·4 to 83·5)	83·5 (82·7 to 84·2)	81·9 (81·3 to 82·5)	82·7 (81·8 to 83·6)
Cumbria	71·2 (67·8 to 74·5)	73·3 (70·2 to 76·3)	74·6 (71·7 to 77·4)	76·1 (75·2 to 77)	78 (77·5 to 78·4)	80·1 (79·6 to 80·5)	82·1 (81·6 to 82·5)	83·3 (82·6 to 84)	82·3 (81·8 to 82·8)	82·4 (81·6 to 83·3)
Halton	70·2 (66·9 to 73·6)	72·1 (68·9 to 75·1)	73·4 (70·3 to 76·5)	74·8 (73·5 to 76·1)	76·9 (76·3 to 77·5)	78·1 (77·5 to 78·9)	80·4 (79·7 to 81·1)	81·6 (80·7 to 82·5)	80 (79·2 to 80·9)	81·1 (80 to 82·3)
Knowsley	69·8 (66·3 to 73·2)	71·9 (68·7 to 75)	73 (69·8 to 76)	74·4 (73·4 to 75·4)	76·7 (76·1 to 77·3)	78·3 (77·7 to 78·9)	80·6 (79·9 to 81·2)	80·9 (80 to 81·8)	79·6 (78·8 to 80·4)	78·4 (77·3 to 79·4)
Lancashire	71·1 (68·1 to 74·1)	73·5 (70·5 to 76·5)	74·7 (71·7 to 77·7)	75·8 (75 to 76·7)	77·7 (77·3 to 78·1)	79·6 (79·3 to 80)	81·9 (81·5 to 82·3)	82·2 (81·7 to 82·7)	81·5 (81·1 to 81·9)	80·8 (80 to 81·5)
Liverpool	69·9 (66·4 to 73·4)	72·1 (68·7 to 75·1)	72·7 (69·8 to 75·7)	74·5 (73·4 to 75·5)	76·5 (76 to 77)	77·4 (76·9 to 77·8)	79·9 (79·4 to 80·4)	80·5 (79·9 to 81·1)	79·4 (78·9 to 80)	78·6 (77·7 to 79·4)
Manchester	69·9 (66·6 to 73·2)	72·2 (69 to 75·2)	73·2 (70·2 to 76·1)	74·7 (73·8 to 75·7)	76·2 (75·7 to 76·6)	77·4 (76·9 to 77·9)	80 (79·5 to 80·4)	80·5 (79·9 to 81·2)	79 (78·6 to 79·5)	78·8 (78·1 to 79·6)
Oldham	69·7 (66·4 to 73)	71·9 (68·8 to 74·8)	73·4 (70·5 to 76·5)	75·2 (74·2 to 76·3)	77·1 (76·6 to 77·7)	78·7 (78·1 to 79·3)	80·6 (80 to 81·3)	81·3 (80·5 to 82·1)	80·3 (79·6 to 80·9)	79·8 (78·8 to 80·8)
Rochdale	69·5 (66·3 to 72·8)	72 (69·1 to 74·8)	73·5 (70·5 to 76·5)	75·4 (74·4 to 76·5)	76·5 (75·9 to 77)	78·4 (77·8 to 79)	80·3 (79·7 to 80·9)	81 (80·1 to 81·8)	79·8 (79·2 to 80·5)	80 (78·9 to 81)
Salford	69·8 (66·5 to 73·2)	72 (68·8 to 75·1)	73·4 (70·5 to 76·2)	75 (73·8 to 76)	76·3 (75·7 to 76·9)	77·7 (77·1 to 78·3)	80·3 (79·8 to 80·9)	81·8 (81 to 82·6)	80·4 (79·8 to 81·1)	80·1 (79·2 to 81)
Sefton	71·2 (67·8 to 74·5)	73·5 (70·4 to 76·7)	74·7 (71·7 to 77·7)	76·1 (75·1 to 77·1)	78·4 (77·9 to 79)	79·6 (79·1 to 80·1)	81·5 (80·9 to 82·1)	82·1 (81·3 to 82·8)	81·3 (80·7 to 81·9)	81·9 (81 to 82·9)
St Helens	70·7 (67·3 to 73·9)	72·7 (69·6 to 75·9)	74·1 (71 to 77)	74·8 (73·7 to 75·8)	77·3 (76·7 to 77·9)	79·2 (78·5 to 79·7)	80·8 (80·2 to 81·5)	81·2 (80·4 to 82·1)	81·2 (80·5 to 81·9)	79·5 (78·5 to 80·5)
Stockport	72·2 (68·9 to 75·5)	74·3 (71·2 to 77·2)	75·6 (72·6 to 78·6)	76·7 (75·6 to 77·6)	78·5 (78 to 79)	80 (79·5 to 80·5)	82·1 (81·6 to 82·7)	83·4 (82·7 to 84·2)	82·8 (82·2 to 83·4)	82·7 (81·7 to 83·6)
Tameside	70·1 (66·5 to 73·4)	72·1 (68·9 to 75·1)	73·5 (70·6 to 76·4)	75·1 (74 to 76·2)	77·3 (76·8 to 77·9)	79·1 (78·5 to 79·7)	80·9 (80·3 to 81·4)	82 (81·1 to 82·8)	79·1 (78·4 to 79·8)	80·7 (79·7 to 81·7)
Trafford	71·2 (67·9 to 74·4)	73·2 (70 to 76·1)	74·6 (71·4 to 77·3)	76·1 (74·9 to 77·1)	78·4 (77·9 to 79)	79·9 (79·3 to 80·4)	83·2 (82·5 to 83·8)	84·5 (83·6 to 85·3)	82·6 (81·9 to 83·3)	83·8 (82·8 to 84·7)
Warrington	71·4 (67·8 to 74·8)	73·4 (69·8 to 76·4)	74·6 (71·5 to 77·5)	75·9 (74·8 to 77)	77·4 (76·9 to 78)	79·8 (79·3 to 80·4)	81·5 (80·9 to 82)	82·4 (81·5 to 83·2)	82·1 (81·5 to 82·7)	81·3 (80·4 to 82·3)
Wigan	70·8 (67·3 to 74·1)	72·7 (69·7 to 75·7)	74·2 (71·2 to 76·9)	75·7 (74·8 to 76·8)	77·5 (77·1 to 78)	78·6 (78·1 to 79·1)	81·2 (80·6 to 81·7)	81·3 (80·5 to 82)	80·8 (80·2 to 81·4)	81·3 (80·4 to 82·2)
Wirral	70·4 (67·1 to 73·8)	72·8 (69·6 to 75·8)	74·2 (71·2 to 77·3)	75·8 (74·8 to 76·9)	77·8 (77·3 to 78·3)	79·3 (78·8 to 79·8)	81·1 (80·6 to 81·7)	81·7 (81 to 82·5)	81·8 (81·2 to 82·4)	80·8 (80 to 81·7)
South East England	73·2 (70·9 to 75·4)	75·3 (73·2 to 77·5)	76·5 (74·3 to 78·6)	77·8 (77·1 to 78·4)	79·7 (79·5 to 79·9)	81·1 (80·9 to 81·3)	83·5 (83·3 to 83·7)	84·2 (84 to 84·5)	83·7 (83·5 to 83·9)	83·9 (83·3 to 84·4)
Bracknell Forest	73·9 (70·6 to 77·1)	75·8 (72·7 to 78·8)	77·2 (74·2 to 80)	78·3 (77·1 to 79·6)	80 (79·2 to 80·7)	81·7 (80·9 to 82·4)	83·6 (82·9 to 84·3)	85·6 (84·6 to 86·6)	85·9 (84·8 to 87)	86·1 (84·7 to 87·4)
Brighton and Hove	72 (68·6 to 75)	74·1 (71·1 to 77)	75·6 (72·6 to 78·3)	76·6 (75·5 to 77·7)	78·8 (78·2 to 79·4)	79·8 (79·2 to 80·3)	82·1 (81·6 to 82·7)	83·3 (82·6 to 84)	81·2 (80·6 to 81·8)	83·3 (82·4 to 84·2)
Buckinghamshire	74 (70·6 to 77·3)	76 (73 to 79)	77·2 (74·4 to 79·8)	78 (77 to 79)	79·8 (79·3 to 80·2)	81·3 (80·9 to 81·8)	83·7 (83·1 to 84·2)	85·5 (84·9 to 86·1)	84·9 (84·3 to 85·5)	84·5 (83·6 to 85·2)
East Sussex	72·6 (69·3 to 76)	75 (72 to 78)	76·6 (73·9 to 79·3)	78·1 (77·1 to 79)	80 (79·5 to 80·5)	81·6 (81·1 to 82·1)	83·6 (83·1 to 84)	83·7 (83·1 to 84·3)	83·7 (83·2 to 84·2)	83·2 (82·4 to 84)
Hampshire	73·7 (70·6 to 76·9)	75·7 (72·6 to 78·7)	76·4 (73·4 to 79·1)	78 (77·1 to 78·9)	80 (79·6 to 80·4)	81·5 (81·1 to 81·8)	84·1 (83·7 to 84·4)	84·8 (84·4 to 85·3)	84·3 (83·9 to 84·7)	84·5 (83·8 to 85·2)
Isle of Wight	71·6 (68·2 to 74·8)	74·5 (71·5 to 77·3)	76·4 (73·3 to 79·3)	77·9 (76·7 to 79·4)	79·6 (78·8 to 80·3)	81·3 (80·6 to 82)	83·1 (82·3 to 83·7)	83·6 (82·6 to 84·6)	83·9 (83·1 to 84·7)	83 (82 to 84·1)
Kent	72·9 (70·1 to 75·9)	75·1 (72 to 78·2)	76 (73 to 78·9)	77·2 (76·3 to 78)	79·2 (78·9 to 79·6)	80·5 (80·2 to 80·9)	83·2 (82·8 to 83·6)	83·5 (83 to 83·9)	82·9 (82·5 to 83·3)	83 (82·3 to 83·7)
Medway	72·4 (68·9 to 75·9)	74·4 (71·2 to 77·3)	76 (73 to 79)	77·3 (76·2 to 78·5)	78·9 (78·3 to 79·4)	80 (79·5 to 80·6)	81·7 (81·1 to 82·3)	82·8 (82 to 83·6)	82·1 (81·4 to 82·7)	82·8 (81·8 to 83·7)
Milton Keynes	73 (69·5 to 76·1)	75 (71·8 to 78·1)	76·7 (73·6 to 79·4)	77·9 (76·7 to 79·1)	78·8 (78·2 to 79·4)	80·7 (80·2 to 81·3)	82·4 (81·9 to 83)	83·5 (82·7 to 84·3)	82·6 (82 to 83·2)	82·9 (82 to 83·8)
Oxfordshire	74·2 (71 to 77·7)	76·1 (72·9 to 79·1)	77·1 (74·2 to 79·8)	78·2 (77·2 to 79·1)	80·1 (79·7 to 80·6)	81·3 (80·9 to 81·8)	83·8 (83·4 to 84·2)	85·4 (84·9 to 86)	84·2 (83·8 to 84·8)	84·8 (84 to 85·5)
Portsmouth	72 (68·5 to 75·1)	73·9 (70·8 to 76·9)	75·5 (72·5 to 78·5)	76·8 (75·7 to 77·9)	78·3 (77·7 to 79)	79 (78·5 to 79·7)	81·9 (81·3 to 82·5)	82·6 (81·7 to 83·4)	82·1 (81·4 to 82·8)	82·3 (81·3 to 83·2)
Reading	73·8 (70·2 to 77)	75·5 (72·3 to 78·5)	77 (74 to 79·7)	78·1 (77 to 79·3)	79·2 (78·6 to 79·9)	80·2 (79·5 to 80·8)	82·4 (81·7 to 83·1)	83·1 (82·3 to 84)	81·5 (80·8 to 82·2)	82·7 (81·7 to 83·6)
Slough	71·4 (67·9 to 74·8)	73·6 (70·6 to 76·6)	74·8 (71·8 to 77·6)	75·9 (74·7 to 77·1)	77·7 (76·9 to 78·4)	79·3 (78·7 to 80)	82·6 (81·9 to 83·3)	83·1 (82·2 to 84)	81·7 (80·9 to 82·5)	81·6 (80·6 to 82·7)
Southampton	72·2 (68·5 to 75·5)	73·9 (70·8 to 76·8)	75·5 (72·6 to 78·4)	77 (75·9 to 78·1)	78·4 (77·9 to 79)	79·6 (79·1 to 80·2)	81·9 (81·3 to 82·4)	82·8 (82 to 83·6)	81·9 (81·3 to 82·6)	82·8 (81·8 to 83·8)
Surrey	74 (70·6 to 77)	76·1 (73·2 to 79)	77 (74·1 to 79·7)	77·9 (77 to 78·8)	80 (79·7 to 80·4)	81·9 (81·5 to 82·2)	84·5 (84·2 to 84·9)	84·7 (84·2 to 85·2)	84·1 (83·7 to 84·5)	84·8 (84·2 to 85·4)
West Berkshire	74·6 (71·3 to 77·8)	76·6 (73·4 to 79·4)	78·1 (75·1 to 80·8)	79·2 (77·9 to 80·4)	80·4 (79·6 to 81·1)	81·4 (80·7 to 82·1)	83·8 (83·1 to 84·5)	85·2 (84·3 to 86·2)	84·5 (83·7 to 85·2)	85·2 (84·2 to 86·2)
West Sussex	73·3 (69·9 to 76·5)	75·8 (72·6 to 78·9)	77 (74·1 to 79·7)	78·3 (77·4 to 79·3)	80·1 (79·7 to 80·5)	81·5 (81·1 to 81·9)	83·5 (83 to 83·9)	84·4 (83·9 to 85)	84·3 (83·9 to 84·8)	83·8 (83·1 to 84·5)
Windsor and Maidenhead	74 (70·7 to 77·2)	75·9 (72·8 to 78·9)	77·2 (74·2 to 79·8)	78·1 (77 to 79·3)	79·6 (79 to 80·3)	82·5 (81·8 to 83·2)	84·4 (83·6 to 85·1)	85·1 (84·1 to 86)	83·9 (83·1 to 84·8)	84·3 (83·3 to 85·3)
Wokingham	75·1 (71·7 to 78·3)	77 (73·9 to 79·8)	78·5 (75·7 to 81·4)	79·4 (78·2 to 80·6)	80·8 (80 to 81·5)	82·7 (82 to 83·4)	85·3 (84·5 to 86)	85·7 (84·8 to 86·7)	84·1 (83·2 to 85)	84·2 (83·1 to 85·3)
South West England	73·1 (70·6 to 75·5)	75·2 (73 to 77·5)	76·4 (74 to 78·7)	77·6 (76·9 to 78·3)	79·7 (79·4 to 79·9)	81·4 (81·1 to 81·6)	83·6 (83·3 to 83·8)	84·1 (83·8 to 84·4)	83·8 (83·5 to 84·1)	83·4 (82·8 to 83·9)
Bath and North East Somerset	73·9 (70·2 to 77·1)	75·9 (72·8 to 78·9)	77·3 (74·3 to 80·2)	78·6 (77·4 to 79·8)	80·4 (79·7 to 81·1)	81·9 (81·2 to 82·6)	84·7 (84·1 to 85·4)	85·4 (84·4 to 86·2)	83·9 (83·1 to 84·6)	84·5 (83·4 to 85·5)
Bournemouth	72·6 (69·1 to 76)	74·7 (71·5 to 77·7)	75·9 (72·9 to 79)	77·1 (75·9 to 78·3)	79 (78·3 to 79·6)	80·3 (79·7 to 80·9)	82·7 (82·1 to 83·4)	82·8 (81·9 to 83·6)	81·6 (80·7 to 82·3)	77·1 (75·5 to 78·8)
Bristol, City of	72·7 (69·2 to 76)	74·4 (71·3 to 77·3)	75·4 (72·6 to 78·3)	76·8 (75·8 to 77·8)	79·2 (78·7 to 79·7)	80·4 (79·9 to 81)	82·1 (81·6 to 82·6)	83·3 (82·6 to 83·9)	81·7 (81·1 to 82·3)	82·6 (81·8 to 83·5)
Cornwall	72·7 (69·5 to 75·9)	74·9 (72 to 77·7)	76·1 (73 to 79)	77·4 (76·3 to 78·4)	79·5 (79·1 to 80)	80·9 (80·5 to 81·4)	83 (82·5 to 83·5)	83·6 (82·9 to 84·2)	83·7 (83·1 to 84·2)	83 (82·1 to 83·7)
Devon	73·6 (70·1 to 76·8)	75·9 (72·8 to 79·1)	76·8 (73·7 to 79·8)	77·8 (76·9 to 78·7)	80·1 (79·7 to 80·6)	81·7 (81·2 to 82·1)	84·2 (83·8 to 84·7)	84·2 (83·7 to 84·8)	84·6 (84·1 to 85·1)	84·1 (83·3 to 84·9)
Dorset	73·2 (69·7 to 76·5)	75·5 (72·1 to 78·5)	76·9 (73·8 to 79·7)	78·2 (77·2 to 79·2)	80·4 (79·9 to 80·9)	82·7 (82·1 to 83·2)	85·2 (84·7 to 85·8)	85·2 (84·4 to 85·9)	85 (84·4 to 85·7)	85·7 (84·7 to 86·6)
Gloucestershire	73·5 (70 to 76·8)	75·7 (72·7 to 78·7)	76·9 (73·9 to 79·8)	77·7 (76·6 to 78·6)	79·6 (79·1 to 80)	81·4 (81 to 81·9)	83·4 (83 to 83·9)	83·9 (83·4 to 84·4)	84 (83·5 to 84·5)	83·3 (82·5 to 84·1)
North Somerset	73·2 (69·9 to 76·6)	75·5 (72·5 to 78·5)	77 (73·9 to 79·9)	78·3 (77·2 to 79·4)	80 (79·4 to 80·6)	81·8 (81·2 to 82·5)	83·3 (82·6 to 83·9)	84·6 (83·7 to 85·5)	84·2 (83·5 to 84·9)	83·7 (82·6 to 84·7)
Plymouth	72·3 (68·8 to 75·7)	74·2 (71·3 to 77·1)	75·5 (72·4 to 78·5)	76·8 (75·7 to 77·9)	78·7 (78·2 to 79·3)	80·4 (79·8 to 80·9)	82·3 (81·6 to 82·8)	83·5 (82·7 to 84·3)	82·7 (82 to 83·3)	83 (82 to 83·9)
Poole	73·2 (69·6 to 76·4)	75·2 (71·9 to 78·1)	76·6 (73·4 to 79·7)	77·8 (76·6 to 79·1)	79·3 (78·6 to 80)	81·9 (81·2 to 82·6)	83·8 (83·1 to 84·5)	84 (83·1 to 84·9)	83·6 (82·7 to 84·5)	79·1 (77·3 to 81)
Somerset	73·2 (69·9 to 76·6)	75·4 (72·3 to 78·4)	76·5 (73·6 to 79·3)	77·6 (76·6 to 78·6)	79·6 (79·2 to 80·1)	81·3 (80·8 to 81·8)	83·5 (83·1 to 84)	84·1 (83·5 to 84·8)	83·5 (83 to 84·1)	83·7 (82·8 to 84·5)
South Gloucestershire	74·2 (70·5 to 77·4)	75·9 (72·6 to 78·9)	77·1 (74·1 to 80·1)	78·7 (77·5 to 79·9)	79·9 (79·3 to 80·5)	81·4 (80·8 to 81·9)	84·5 (83·8 to 85·1)	84·4 (83·6 to 85·2)	84·2 (83·5 to 84·9)	83·9 (83 to 84·8)
Swindon	72·3 (68·6 to 75·6)	74·3 (71·1 to 77·6)	75·4 (72·3 to 78·4)	76·6 (75·4 to 77·7)	79·3 (78·6 to 79·9)	80·5 (79·9 to 81·2)	83·1 (82·5 to 83·8)	84·4 (83·5 to 85·2)	82·7 (82·1 to 83·4)	84·1 (83·1 to 85·2)
Torbay	72·6 (68·8 to 76)	74·7 (71·3 to 77·7)	76·2 (73·1 to 79·2)	77·5 (76·2 to 78·8)	79·5 (78·7 to 80·3)	80·3 (79·7 to 81)	81·9 (81·2 to 82·6)	82 (81 to 82·9)	82·9 (82 to 83·8)	82·5 (81·3 to 83·6)
Wiltshire	73·5 (70 to 77)	75·4 (72·3 to 78·4)	76·7 (73·7 to 79·4)	77·9 (76·9 to 78·9)	79·6 (79·1 to 80·1)	81·6 (81·1 to 82·2)	83·4 (82·9 to 84)	85·1 (84·4 to 85·8)	84·4 (83·8 to 84·9)	83·9 (83·1 to 84·7)
West Midlands	71·5 (69 to 73·8)	73·6 (71·3 to 75·8)	74·8 (72·4 to 77·1)	76·3 (75·7 to 77)	78·2 (77·9 to 78·4)	79·9 (79·7 to 80·2)	82·4 (82·2 to 82·7)	83·1 (82·8 to 83·4)	82·1 (81·8 to 82·4)	81·9 (81·2 to 82·5)
Birmingham	71·1 (68 to 74·3)	73·3 (70·2 to 76·3)	74·3 (71·4 to 77·2)	75·8 (75 to 76·7)	77·5 (77·1 to 77·9)	79·3 (78·9 to 79·7)	82·5 (82·1 to 82·9)	82·9 (82·4 to 83·4)	81·2 (80·8 to 81·7)	80·9 (80·1 to 81·8)

Coventry	71 (67·3 to 74·5)	73·1 (69·9 to 76·2)	74·3 (71·2 to 77·2)	76·1 (75 to 77·1)	77·7 (77·2 to 78·2)	79·5 (78·9 to 80)	82·2 (81·6 to 82·7)	83·3 (82·5 to 84)	81·7 (81 to 82·3)	81·8 (80·8 to 82·7)
Dudley	72·4 (68·7 to 75·5)	74·3 (71·2 to 77·3)	75·8 (72·8 to 78·6)	77·2 (76·2 to 78·2)	78·8 (78·2 to 79·3)	80·2 (79·7 to 80·8)	82·6 (82·1 to 83·2)	82·8 (82 to 83·5)	82·1 (81·5 to 82·7)	81·3 (80·4 to 82·2)
Herefordshire, County of	71·5 (68·1 to 75)	73·8 (70·6 to 76·8)	75·2 (72 to 78·2)	76·7 (75·5 to 77·9)	79·5 (78·8 to 80·1)	81·5 (80·9 to 82·2)	83·2 (82·5 to 83·9)	84·1 (83·2 to 85)	84·1 (83·3 to 84·8)	82·7 (81·7 to 83·7)
Sandwell	69·8 (66·1 to 73·1)	72·1 (69 to 75·2)	73·6 (70·6 to 76·6)	75·5 (74·5 to 76·5)	77·8 (77·3 to 78·3)	78·7 (78·1 to 79·2)	80·7 (80·1 to 81·2)	82·3 (81·5 to 83)	80·9 (80·3 to 81·6)	80·2 (79·2 to 81·2)
Shropshire	72·4 (68·8 to 75·9)	74·4 (71·3 to 77·3)	76 (72·8 to 78·9)	77·5 (76·4 to 78·5)	79·3 (78·7 to 79·8)	81·3 (80·8 to 81·9)	83·4 (82·9 to 84)	84·3 (83·5 to 85·1)	83·9 (83·3 to 84·6)	82·1 (81·2 to 82·9)
Solihull	73·5 (70·2 to 76·7)	75·3 (72·2 to 78·3)	76·9 (73·8 to 79·9)	78·5 (77·4 to 79·6)	79·8 (79·2 to 80·5)	81 (80·3 to 81·6)	83·9 (83·2 to 84·6)	84·1 (83·2 to 84·9)	82·1 (81·4 to 82·9)	83·1 (82·1 to 84·1)
Staffordshire	72·4 (69·2 to 75·6)	74·3 (70·9 to 77·4)	75·2 (72·1 to 78)	76·6 (75·8 to 77·5)	78·2 (77·7 to 78·6)	80 (79·6 to 80·4)	82·7 (82·3 to 83·1)	83·2 (82·7 to 83·7)	82·3 (81·8 to 82·7)	83 (82·3 to 83·9)
Stoke-on-Trent	69·5 (66·1 to 72·8)	71·7 (68·4 to 74·8)	72·8 (69·8 to 75·9)	74·3 (73·2 to 75·4)	76·8 (76·3 to 77·4)	78·8 (78·2 to 79·4)	80·2 (79·6 to 80·7)	81 (80·2 to 81·8)	79 (78·3 to 79·6)	79·8 (78·8 to 80·7)
Telford and Wrekin	71·7 (68·2 to 75·1)	73·6 (70·4 to 76·4)	75·1 (72·1 to 78)	76·6 (75·5 to 77·8)	78·7 (78·1 to 79·4)	79·6 (79 to 80·3)	82 (81·3 to 82·6)	82·5 (81·6 to 83·4)	81·4 (80·7 to 82·2)	80·3 (79·3 to 81·3)
Walsall	70·7 (67·2 to 74)	72·8 (69·5 to 76·1)	74·2 (71 to 77·3)	75·7 (74·7 to 76·8)	77·3 (76·8 to 77·8)	79·3 (78·8 to 79·9)	81·8 (81·2 to 82·5)	82·7 (81·9 to 83·4)	82 (81·3 to 82·7)	81·4 (80·4 to 82·3)
Warwickshire	72·5 (68·9 to 75·8)	74·5 (71·4 to 77·3)	75·7 (72·6 to 78·6)	76·9 (76 to 77·9)	78·6 (78·2 to 79)	80·9 (80·4 to 81·3)	83·3 (82·9 to 83·8)	83·8 (83·3 to 84·4)	83 (82·4 to 83·5)	83·1 (82·2 to 83·9)
Wolverhampton	70·9 (67·3 to 74)	73·1 (69·9 to 76·3)	74·5 (71·3 to 77·3)	76 (75 to 77·1)	77·4 (76·9 to 78)	79·3 (78·8 to 79·9)	81·1 (80·5 to 81·7)	82·6 (81·7 to 83·4)	80·9 (80·3 to 81·6)	80·4 (79·4 to 81·3)
Worcestershire	72·8 (69·4 to 76·1)	74·8 (71·6 to 78)	75·9 (72·7 to 78·7)	77·2 (76·2 to 78·1)	79·1 (78·7 to 79·5)	80·5 (80·1 to 80·9)	82·7 (82·2 to 83·1)	83·5 (82·9 to 84·1)	83·3 (82·8 to 83·8)	82·8 (82 to 83·6)
Yorkshire and the Humber	71 (68·5 to 73·4)	73·2 (70·9 to 75·5)	74·6 (72·3 to 77)	76·2 (75·5 to 76·9)	78·1 (77·8 to 78·3)	79·8 (79·6 to 80·1)	82 (81·7 to 82·3)	82·7 (82·4 to 83)	81·9 (81·6 to 82·2)	81·8 (81·1 to 82·4)
Barnsley	70·4 (66·7 to 73·8)	72·7 (69·4 to 75·6)	74·3 (71 to 77·3)	75·9 (74·9 to 77·1)	77·8 (77·2 to 78·4)	79·4 (78·8 to 80)	81·6 (81 to 82·1)	82·6 (81·7 to 83·4)	81·6 (80·9 to 82·2)	80·4 (79·3 to 81·4)
Bradford	69·6 (66·3 to 72·9)	72·2 (69·1 to 75·4)	73·5 (70·5 to 76·4)	75·4 (74·4 to 76·3)	77·4 (76·9 to 77·9)	78·9 (78·4 to 79·4)	81·4 (80·9 to 81·9)	81·8 (81·1 to 82·4)	80·5 (80·1 to 81)	81·1 (80·3 to 81·9)
Calderdale	70·5 (67 to 74)	72·9 (69·7 to 75·9)	74·5 (71·5 to 77·2)	76 (74·8 to 77·2)	77·6 (77 to 78·2)	79·7 (79·1 to 80·3)	81·4 (80·8 to 82·1)	82·6 (81·8 to 83·4)	82 (81·3 to 82·7)	81·5 (80·5 to 82·5)
Doncaster	70·9 (67·3 to 74·1)	73·3 (70·1 to 76·5)	74·8 (71·9 to 78)	76·4 (75·4 to 77·4)	77·1 (76·5 to 77·6)	79 (78·4 to 79·5)	81·4 (80·8 to 82)	81·5 (80·8 to 82·2)	80·4 (79·8 to 80·9)	80·7 (79·8 to 81·6)
East Riding of Yorkshire	72·1 (68·7 to 75·3)	74·3 (71 to 77·2)	75·8 (72·6 to 78·6)	77·2 (76·1 to 78·2)	79·2 (78·7 to 79·7)	80·5 (80 to 81·1)	82·8 (82·2 to 83·3)	84 (83·3 to 84·8)	84 (83·3 to 84·6)	83·5 (82·6 to 84·4)
Kingston upon Hull, City of	70·7 (67 to 73·9)	72·9 (69·6 to 75·9)	74·3 (71·4 to 77·2)	76·2 (75·1 to 77·2)	77·6 (77 to 78·2)	78·7 (78·1 to 79·3)	80·7 (80·1 to 81·3)	81·4 (80·7 to 82·2)	80·2 (79·5 to 80·8)	80 (79 to 80·9)
Kirklees	70·5 (67·2 to 73·8)	72·7 (69·6 to 75·8)	74·1 (71·1 to 77)	75·4 (74·4 to 76·4)	77·6 (77·1 to 78·1)	79·2 (78·7 to 79·7)	81·9 (81·4 to 82·5)	82·5 (81·9 to 83·1)	81·4 (80·8 to 81·9)	82·1 (81·2 to 83)
Leeds	71·3 (67·7 to 74·5)	73·6 (70·1 to 76·9)	74·5 (71·6 to 77·5)	76·2 (75·3 to 77·1)	78·2 (77·8 to 78·7)	80·1 (79·7 to 80·6)	82·3 (81·8 to 82·7)	82·7 (82·1 to 83·2)	81·5 (81 to 82)	81·4 (80·6 to 82·2)
North East Lincolnshire	70·9 (67·2 to 74·1)	73·2 (70·1 to 76·3)	74·7 (71·7 to 77·7)	76·4 (75·2 to 77·5)	77·4 (76·8 to 78)	79·7 (79·1 to 80·4)	82 (81·2 to 82·6)	82·3 (81·4 to 83·2)	82·2 (81·4 to 82·9)	81 (80 to 82·1)
North Lincolnshire	71·5 (67·9 to 74·9)	73·8 (70·7 to 76·9)	75·4 (72·3 to 78·4)	76·9 (75·8 to 78)	77·8 (77·2 to 78·5)	79·7 (79 to 80·3)	82·4 (81·7 to 83)	83·5 (82·6 to 84·4)	83·5 (82·7 to 84·3)	82·9 (81·8 to 83·8)
North Yorkshire	72·2 (68·6 to 75·4)	74·4 (71·3 to 77·4)	75·7 (72·7 to 78·4)	77·1 (76·1 to 78)	79·4 (78·9 to 79·8)	80·9 (80·4 to 81·3)	82·9 (82·4 to 83·4)	84 (83·4 to 84·6)	83·6 (83·1 to 84·2)	84·1 (83·3 to 84·9)
Rotherham	71 (67·4 to 74·4)	73·2 (69·9 to 76·3)	74·8 (71·6 to 78·1)	76·6 (75·6 to 77·7)	78 (77·4 to 78·6)	79·8 (79·2 to 80·3)	81·7 (81 to 82·2)	82·3 (81·6 to 83·1)	81·1 (80·5 to 81·8)	81 (79·9 to 81·9)
Sheffield	71·5 (68·1 to 74·7)	73·7 (70·5 to 76·6)	74·9 (72 to 78·1)	76·4 (75·5 to 77·4)	78 (77·6 to 78·5)	80 (79·5 to 80·4)	82 (81·5 to 82·5)	82·4 (81·8 to 82·9)	82 (81·6 to 82·5)	81·2 (80·4 to 82)
Wakefield	70·3 (66·8 to 73·8)	72·5 (69·3 to 75·5)	74 (70·9 to 76·8)	75·3 (74·3 to 76·3)	77·6 (77 to 78·1)	79·4 (78·8 to 79·9)	81·7 (81·2 to 82·2)	81·9 (81·3 to 82·6)	81·4 (80·8 to 82·1)	80·5 (79·6 to 81·4)
York	72·3 (69·2 to 75·6)	74·2 (71 to 77·3)	75·7 (72·7 to 78·6)	77·2 (76·1 to 78·2)	79·4 (78·8 to 80·1)	81·7 (81 to 82·3)	83·8 (83·1 to 84·4)	83·9 (83 to 84·8)	82·9 (82·1 to 83·6)	83 (82 to 83·9)
Northern Ireland	68·7 (68·3 to 69)	71·7 (71·4 to 71·9)	73·6 (73·4 to 73·8)	75 (74·7 to 75·2)	77·2 (77 to 77·5)	79·6 (79·3 to 79·8)	81·8 (81·5 to 82)	82·8 (82·5 to 83·1)	82·3 (82 to 82·6)	82·3 (81·9 to 82·7)
Scotland	68 (67·8 to 68·2)	71·3 (71·1 to 71·4)	73·1 (73 to 73·3)	74·8 (74·7 to 75)	76·7 (76·6 to 76·9)	78·6 (78·4 to 78·7)	80·8 (80·6 to 81)	81·4 (81·2 to 81·6)	80·9 (80·7 to 81·1)	80·8 (80·6 to 81)
Wales	71·1 (70·3 to 71·8)	73·2 (72·6 to 73·9)	74·6 (73·9 to 75·2)	76·3 (76 to 76·6)	78·5 (78·3 to 78·7)	79·7 (79·5 to 79·9)	81·9 (81·7 to 82·1)	81·9 (81·7 to 82·1)	81·1 (80·9 to 81·3)	81·1 (80·8 to 81·3)
Latin America and Caribbean	52·4 (50·6 to 54)	59·3 (58·4 to 60)	63·2 (62·6 to 63·8)	68·9 (68·5 to 69·3)	72·7 (72·5 to 73)	75·8 (75·6 to 76·1)	76·1 (75·7 to 76·5)	79 (78·4 to 79·5)	77·1 (76·5 to 77·6)	75·9 (75·2 to 76·6)
Andean Latin America	42·5 (39·5 to 45·3)	49·7 (48·1 to 51·3)	52·4 (51·3 to 53·6)	65·1 (64 to 66)	70·6 (69·7 to 71·4)	74·8 (74 to 75·7)	77·3 (76·2 to 78·5)	78·5 (77 to 79·9)	75·3 (73·8 to 76·6)	74·3 (72·9 to 75·5)
Bolivia	38·2 (34·9 to 41·4)	45·2 (42·2 to 48)	51·8 (49·4 to 54·2)	57·9 (56·3 to 59·5)	62·4 (60·8 to 64)	67·4 (65·8 to 68·8)	71 (68·8 to 73·1)	73·5 (70·6 to 75·6)	69·7 (67·5 to 71·6)	68·8 (66·7 to 70·5)
Ecuador	51·2 (47·9 to 54·2)	56·8 (55·9 to 57·7)	62·3 (61·6 to 63)	68·6 (68 to 69·1)	74·2 (73·8 to 74·5)	76·6 (75·7 to 77·5)	77·5 (76·6 to 78·6)	79·5 (78·4 to 80·6)	76·6 (75·4 to 77·7)	77·1 (75·5 to 78·7)
Peru	41·2 (36·9 to 45·1)	48·7 (46·6 to 50·7)	49 (47·4 to 50·5)	66·3 (64·6 to 67·9)	72 (70·7 to 73·3)	76·8 (75·4 to 78·2)	79·7 (78·2 to 81·1)	79·8 (78 to 81·6)	76·7 (74·9 to 78·4)	74·9 (73·4 to 76·3)
Caribbean	57 (54·9 to 59·2)	62·3 (61·2 to 63·3)	65·2 (64·4 to 66·1)	68·1 (67·4 to 68·9)	69·8 (69·1 to 70·5)	72·1 (71·4 to 72·9)	56·4 (54·7 to 58)	74·9 (73·4 to 76·3)	74·5 (72·8 to 76)	72·5 (70·7 to 74·1)
Antigua and Barbuda	60·3 (58·1 to 62·1)	65·9 (64·9 to 67)	70·6 (69·6 to 71·5)	74·8 (73·6 to 76)	77·4 (76·5 to 78·2)	76·8 (76 to 77·5)	78 (77·1 to 78·8)	77·1 (76 to 78·4)	78·5 (77·5 to 79·6)	77·1 (76·7 to 77·3)
The Bahamas	60·1 (56·1 to 63·9)	64·3 (61·7 to 67)	68·3 (67·2 to 69·3)	72·3 (71·3 to 73·3)	74·5 (73·6 to 75·4)	74·4 (73·6 to 75·3)	76·3 (75·5 to 77·1)	73·7 (71·6 to 75·6)	75·4 (73·3 to 77·5)	73·6 (71·7 to 75·4)
Barbados	56·5 (53·5 to 59·3)	68·4 (67·2 to 69·4)	71·4 (70·7 to 72·1)	74·8 (74·2 to 75·4)	76·1 (75·5 to 76·6)	76·7 (76 to 77·4)	77·2 (76·6 to 77·8)	77·6 (75·7 to 79·6)	78·1 (76 to 80·2)	77·6 (75·5 to 79·7)
Belize	56·6 (54·1 to 58·7)	67·1 (65·5 to 68·9)	70·2 (68·9 to 71·5)	73 (72·1 to 74)	75·6 (74·6 to 76·5)	73·3 (72·7 to 73·8)	76·2 (75·6 to 76·7)	77·2 (76·3 to 78·1)	76·8 (76 to 77·6)	76·1 (74·9 to 77·3)
Bermuda	66·5 (64·9 to 68·1)	70·3 (69·4 to 71·2)	73·8 (72·9 to 74·7)	77·6 (76·7 to 78·5)	77·4 (76·7 to 78·1)	80·8 (80·1 to 81·7)	84·9 (83·5 to 86·1)	85·5 (83·7 to 86·8)	85·7 (84 to 86·9)	83·3 (81·5 to 84·7)
Cuba	68·9 (65·5 to 71·8)	70·8 (70·5 to 71)	72·3 (72·1 to 72·5)	76·2 (76 to 76·4)	76·7 (76·5 to 76·9)	79·2 (79 to 79·3)	80·4 (80·2 to 80·6)	80·6 (80·4 to 80·9)	80·8 (80·5 to 81)	77·3 (76·3 to 78·3)
Dominica	49·7 (46·4 to 52·9)	61·3 (59·7 to 62·9)	68·7 (67·6 to 69·7)	73·9 (73 to 74·7)	74·7 (73·8 to 75·5)	75·4 (74·6 to 76·2)	75·7 (74·9 to 76·4)	75·2 (73·3 to 76·8)	75·3 (73·2 to 76·9)	73·3 (70·8 to 75·5)
Dominican Republic	56·3 (53·2 to 59·5)	61·8 (59·7 to 63·9)	66·9 (65·3 to 68·5)	70·4 (69 to 71·7)	73·4 (72·2 to 74·6)	76·9 (75·6 to 78·2)	76·9 (75 to 78·8)	78 (76·5 to 79·6)	77·5 (76 to 79)	77·3 (75·5 to 78·9)
Grenada	58·9 (56 to 61·9)	66 (63·6 to 68·5)	69·3 (66·7 to 71·7)	71·1 (68·6 to 73·3)	72·6 (71·4 to 73·7)	76·5 (75·7 to 77·3)	75·9 (75·3 to 76·6)	75·5 (74·5 to 76·5)	75·7 (74·5 to 76·7)	72·9 (70·5 to 74·9)
Guyana	52·9 (50·5 to 55)	61·6 (59·9 to 63·3)	64·8 (63·3 to 66·3)	65·5 (63·8 to 67·5)	66·5 (65·1 to 67·9)	67·8 (66·5 to 69·2)	69·6 (68·3 to 70·8)	71·2 (68·4 to 74·1)	70·3 (67·3 to 73·3)	68·6 (65 to 72·1)
Haiti	41·4 (37·6 to 45·7)	47·6 (44·6 to 50·7)	49·4 (46·9 to 52)	52·5 (50·3 to 54·8)	54·1 (52·1 to 56·1)	56·6 (54·7 to 58·8)	27·6 (25·6 to 29·4)	63·6 (60·3 to 66·9)	62·6 (59·4 to 65·7)	61·5 (58·2 to 64·6)
Jamaica	58·6 (56·9 to 60·2)	66·7 (66 to 67·3)	69·5 (68·9 to 70·1)	75·2 (74·7 to 75·7)	76·5 (76·1 to 77)	76·8 (76·3 to 77·3)	79·4 (79 to 79·8)	78·2 (75·6 to 81)	78·4 (75·5 to 81·3)	76·4 (73·7 to 78·9)
Puerto Rico	62·9 (62·3 to 63·6)	71·3 (70·9 to 71·6)	74·2 (73·9 to 74·5)	76·9 (76·6 to 77·1)	78·2 (77·9 to 78·5)	80·2 (79·9 to 80·5)	83·2 (82·8 to 83·6)	85·1 (84·6 to 85·6)	84·7 (84·2 to 85·2)	84·5 (82·8 to 86·4)
Saint Kitts and Nevis	60·2 (58·5 to 61·7)	62·9 (61·9 to 63·9)	66·6 (65·4 to 67·8)	67·5 (66·5 to 68·3)	69·2 (68·4 to 70·1)	73·5 (72·4 to 74·4)	75·7 (75 to 76·4)	76·8 (75·4 to 78·3)	76·8 (75·2 to 78·4)	75·5 (73·9 to 77·1)
Saint Lucia	53·6 (51 to 55·5)	60·6 (58·7 to 62·3)	66·5 (65·2 to 67·7)	70·5 (69·6 to 71·3)	72·5 (71·9 to 73·2)	76·2 (75·6 to 76·9)	79·4 (78·7 to 80·1)	80 (78·3 to 81·7)	79·2 (77·4 to 80·9)	76·5 (73·8 to 78·9)
Saint Vincent and the Grenadines	53·3 (50·7 to 55·7)	61·1 (59·3 to 62·7)	67·3 (65·9 to 68·7)	70·2 (69 to 71·3)	71·8 (70·9 to 72·7)	73·5 (72·7 to 74·4)	75·1 (74·4 to 75·9)	76 (74·8 to 77·1)	76 (74·6 to 77·4)	75·2 (73·7 to 76·6)
Suriname	61·2 (59·5 to 62·8)	65·5 (64 to 66·9)	69·1 (67·4 to 70·8)	69 (68·2 to 69·8)	71·1 (70·2 to 71·8)	73 (72·2 to 73·7)	75·6 (74·9 to 76·3)	75·6 (74·6 to 76·7)	75·8 (74·2 to 77·1)	74·2 (70·9 to 76·7)
Trinidad and Tobago	59·2 (57·8 to 60·5)	66·7 (66 to 67·5)	67·3 (66·7 to 67·9)	69·8 (69·3 to 70·3)	71·9 (71·4 to 72·4)	73 (72·5 to 73·5)	76·8 (76·3 to 77·4)	77·9 (76·3 to 79·6)	77·1 (74·8 to 79·3)	75 (72 to 78)
Virgin Islands	64·8 (62·8 to 66·7)	67·3 (65·8 to 68·8)	69·8 (68·4 to 71·2)	72·3 (71 to 73·7)	75·4 (74·2 to 76·6)	77 (75·8 to 78)	80·6 (79·4 to 81·8)	81·7 (79·8 to 83·4)	83·1 (81·3 to 84·8)	82·3 (79·4 to 84·6)
Central Latin America	51 (49·3 to 52·6)	58·9 (58·4 to 59·5)	63·7 (63·3 to 64·1)	69·4 (68·9 to 69·9)	73·5 (73·2 to 73·7)	76·7 (76·5 to 77)	78·5 (78·2 to 78·7)	79·1 (78·6 to 79·6)	76·4 (75·9 to 76·9)	75·7 (74·9 to 76·5)
Colombia	56 (53·5 to 58)	60·6 (59·8 to 61·5)	67·4 (66·6 to 68)	71·2 (69·4 to 73)	75 (74·7 to 75·3)	78·4 (78·1 to 78·7)	81·2 (80·9 to 81·6)	82·8 (82·5 to 83·1)	81·4 (81 to 81·7)	79·7 (78·2 to 81·2)
Costa Rica	57·4 (56·6 to 58·3)	66·4 (66 to 66·9)	68·3 (67·9 to 68·7)	77·2 (76·8 to 77·6)	79·3 (78·9 to 79·6)	80·5 (80·2 to 80·9)	82·3 (82 to 82·7)	83·1 (82·7 to 83·4)	83 (82·7 to 83·4)	81·2 (80·8 to 81·5)
El Salvador	46·2 (42·2 to 49·4)	55·5 (54 to 56·9)	60·3 (59·2 to 61·3)	67·6 (67 to 68·3)	74·4 (73·8 to 75)	78·5 (78 to 78·9)	79·7 (79·3 to 80)	79·7 (79·4 to 80·1)	78·2 (76·8 to 79·6)	77·2 (75·4 to 79·1)
Guatemala	41·8 (39 to 44·5)	48 (46·6 to 49·3)	51 (49·9 to 52)	61·7 (60·8 to 62·5)	65·4 (64·9 to 65·9)	70·3 (69·9 to 70·7)	73·6 (73·1 to 73·9)	74·6 (74·2 to 75)	74·3 (73·9 to 74·6)	72·7 (71·3 to 74·1)
Honduras	40·5 (37·1 to 43·5)	50·6 (48·8 to 52·5)	58·8 (57·3 to 60·3)	68·8 (67·7 to 70)	71 (69·4 to 72·5)	70·7 (67·9 to 73·3)	71·8 (69 to 74·3)	73 (70·4 to 75·1)	71·4 (69·1 to 73·3)	70·7 (68·4 to 72·6)
Mexico	49·7 (47·1 to 52·1)	58·5 (57·6 to 59·3)	63·2 (62·6 to 63·8)	68·9 (68·4 to 69·4)	73·2 (72·9 to 73·5)	76·4 (76·1 to 76·6)	77·7 (77·5 to 77·9)	78·5 (78·3 to 78·8)	75 (74·8 to 75·2)	74·7 (74·4 to 74·9)

Aguascalientes	54·9 (50·9 to 58·5)	62·6 (59·8 to 65·4)	66·2 (63·9 to 68·3)	70·8 (69·9 to 71·7)	74·4 (73·9 to 74·9)	76·8 (76·3 to 77·2)	77·9 (77·6 to 78·4)	79·1 (78·6 to 79·5)	76·4 (76 to 76·7)	75·9 (75·4 to 76·4)
Baja California	52·6 (47·9 to 56·9)	60·9 (57·9 to 63·7)	64·7 (62 to 67·4)	71·5 (71 to 72)	73·1 (72·6 to 73·5)	75·1 (74·7 to 75·4)	77 (76·7 to 77·4)	78·2 (77·8 to 78·6)	73·6 (73·2 to 73·9)	75 (74·6 to 75·3)
Baja California Sur	52·1 (47·7 to 56·2)	61·4 (58·7 to 64·1)	66·2 (63·7 to 68·5)	72·3 (71·5 to 73)	76·6 (76·1 to 77·1)	77·6 (77·2 to 78·1)	78·3 (77·8 to 78·7)	78·7 (78·1 to 79·1)	75·2 (74·8 to 75·6)	76 (75·4 to 76·5)
Campeche	52·7 (48·9 to 56·2)	60·9 (58·1 to 63·6)	65 (62·7 to 67·2)	69·6 (68·7 to 70·5)	73·7 (73·1 to 74·2)	77 (76·6 to 77·5)	77·8 (77·4 to 78·2)	78·2 (77·6 to 78·7)	74·9 (74·5 to 75·3)	74·8 (74·2 to 75·3)
Chiapas	50·6 (46·6 to 54·6)	57·3 (54·3 to 60·3)	61·6 (58·7 to 64·3)	66·4 (65·5 to 67·2)	71·5 (70·8 to 72·1)	74·7 (74·2 to 75·2)	76·5 (76·2 to 76·8)	76·2 (75·8 to 76·5)	73·2 (72·9 to 73·6)	73·1 (72·7 to 73·5)
Chihuahua	50·6 (46·3 to 54·7)	58·1 (55·3 to 61·1)	62·4 (59·6 to 64·9)	68 (67·3 to 68·6)	71·6 (71 to 72·1)	74·4 (74 to 74·7)	74·7 (74·3 to 75)	76·4 (76 to 76·8)	71·6 (71·2 to 71·9)	73·9 (73·4 to 74·3)
Coahuila	49·2 (45·1 to 53·2)	57·9 (55 to 60·8)	62·4 (59·7 to 64·8)	67·9 (66·9 to 68·8)	72·7 (72·2 to 73·2)	75·5 (75·1 to 76)	76·8 (76·5 to 77·2)	77·3 (76·8 to 77·7)	73·2 (72·8 to 73·5)	74·6 (74·1 to 75)
Colima	45 (40·7 to 48·9)	55·8 (53 to 58·7)	62 (59·5 to 64·6)	68·9 (67·8 to 69·9)	72·8 (72·2 to 73·4)	76·9 (76·4 to 77·3)	78·2 (77·8 to 78·7)	79 (78·5 to 79·6)	75·9 (75·4 to 76·3)	74·2 (73·6 to 74·7)
Durango	48·4 (44·4 to 52·8)	58·1 (55·1 to 60·9)	63·5 (60·9 to 65·9)	69·3 (68·3 to 70·4)	74 (73·5 to 74·6)	77·3 (76·8 to 77·8)	77·6 (77·2 to 78)	79 (78·5 to 79·5)	76·7 (76·3 to 77·1)	76·8 (76·3 to 77·3)
Guanajuato	50 (45 to 54·3)	58·6 (55·7 to 61·3)	63 (60·3 to 65·6)	69 (68·4 to 69·7)	73·3 (72·9 to 73·7)	77·1 (76·8 to 77·3)	78·3 (78 to 78·6)	78·2 (77·8 to 78·5)	74·8 (74·5 to 75·1)	73·8 (73·4 to 74·1)
Guerrero	44·7 (40·8 to 48·7)	53 (49·5 to 56·6)	58·2 (55·2 to 61)	65·1 (64 to 66·1)	71·6 (70·6 to 72·5)	74·7 (74 to 75·4)	77·7 (76·7 to 78·5)	79·3 (78·3 to 80·4)	77·4 (76·4 to 78·3)	77·2 (76·1 to 78·4)
Hidalgo	47·3 (42·9 to 51·2)	56 (52·8 to 59·2)	60·6 (57·3 to 63·4)	66·9 (66·2 to 67·6)	74·2 (73·6 to 74·7)	78·3 (77·8 to 78·7)	79·7 (79·3 to 80·1)	80·1 (79·7 to 80·5)	77·5 (77·1 to 77·8)	77·2 (75·3 to 79)
Jalisco	53·4 (49·4 to 57)	60·9 (58 to 63·7)	65 (62·4 to 67·5)	70·3 (69·7 to 70·9)	73·5 (73 to 73·9)	76·1 (75·8 to 76·4)	77·9 (77·7 to 78·2)	78·7 (78·4 to 79)	75·9 (75·7 to 76·2)	74·5 (74·1 to 74·9)
México	52·2 (48 to 56·1)	60·4 (57·4 to 63·3)	64·8 (62 to 67·2)	68·7 (68·2 to 69·3)	72·5 (72·1 to 72·8)	75·7 (75·4 to 75·9)	77·4 (77·1 to 77·6)	78·3 (77·9 to 78·6)	73·7 (73·5 to 74)	75·5 (75·1 to 75·8)
Mexico City	55·1 (51 to 59·1)	63·9 (61·4 to 66·3)	67·4 (64·9 to 69·5)	71·2 (70·5 to 71·8)	74·2 (73·5 to 74·9)	76·6 (76·3 to 76·9)	77·7 (77·5 to 78)	79·2 (79 to 79·5)	74·6 (74·3 to 74·8)	71·8 (71·3 to 72·2)
Michoacán de Ocampo	47·7 (43·9 to 51·9)	56·7 (53·1 to 60)	62·1 (59·2 to 64·9)	68·1 (67·3 to 68·9)	74·4 (73·8 to 74·9)	77 (76·6 to 77·4)	78·2 (77·8 to 78·7)	79·5 (79·1 to 79·9)	76·8 (76·4 to 77·2)	74·7 (74·2 to 75·2)
Morelos	49·2 (45·1 to 53)	58·9 (56 to 61·9)	63·9 (61·4 to 66·4)	69·7 (68·9 to 70·5)	74·1 (73·6 to 74·6)	77·3 (76·9 to 77·6)	78·2 (77·8 to 78·6)	78·6 (78·2 to 79)	75·6 (75·2 to 75·9)	73·8 (73·3 to 74·2)
Nayarit	47·6 (43·7 to 51·8)	57·7 (54·6 to 60·5)	63·7 (61·4 to 66)	70·8 (70 to 71·7)	74·8 (74·2 to 75·3)	78·1 (77·6 to 78·6)	79·3 (78·8 to 79·7)	80·2 (79·7 to 80·7)	77·9 (77·4 to 78·3)	77·5 (77 to 78)
Nuevo León	55·8 (51·8 to 59·2)	63·4 (60·8 to 65·9)	67 (64·5 to 69·4)	72·4 (71·8 to 72·9)	74·8 (74·4 to 75·3)	77·1 (76·8 to 77·4)	78·6 (78·4 to 78·9)	79·4 (79·1 to 79·7)	76·1 (75·9 to 76·4)	75·3 (75 to 75·7)
Oaxaca	43·7 (39·7 to 47·7)	52 (48·9 to 55·4)	56·3 (53·4 to 59·1)	63·2 (62·3 to 64)	70·9 (70·3 to 71·5)	75·8 (75·4 to 76·3)	77·8 (77·3 to 78·3)	78·7 (78·3 to 79·1)	76·7 (76·3 to 77·1)	75 (74·5 to 75·4)
Puebla	41·9 (37·2 to 46·6)	51·2 (48·3 to 54·1)	57 (54·1 to 59·7)	65·5 (64·6 to 66·2)	71·5 (70·9 to 72·1)	75·5 (75·1 to 75·9)	77·2 (76·8 to 77·5)	77·8 (77·4 to 78·1)	74 (73·7 to 74·3)	72·3 (71·8 to 72·7)
Querétaro	47·7 (42·7 to 52·7)	58 (54·9 to 60·8)	63·3 (60·5 to 66·1)	68·8 (67·4 to 70·1)	73·1 (72·6 to 73·6)	76·6 (76·2 to 76·9)	78·3 (78 to 78·7)	79·4 (79 to 79·8)	76·4 (76·1 to 76·7)	75 (74·6 to 75·4)
Quintana Roo	52·5 (48·7 to 56)	59·5 (56·8 to 62·1)	63·2 (60·6 to 65·5)	68·8 (67·4 to 70·2)	73·6 (72·7 to 74·4)	75·4 (74·6 to 76·2)	75·9 (75·2 to 76·6)	76·4 (75·3 to 77·4)	74·7 (73·8 to 75·5)	75 (74 to 75·9)
San Luis Potosí	46·4 (42·4 to 50·3)	56·1 (52·7 to 59·5)	61·6 (58·6 to 64·3)	70·3 (69·5 to 71)	74·3 (73·8 to 74·8)	77·6 (77·2 to 78·1)	78·7 (78·3 to 79·2)	79·4 (78·9 to 79·9)	75·7 (75·3 to 76·1)	75·4 (75 to 75·9)
Sinaloa	52·6 (48·2 to 56·8)	60·9 (58 to 63·7)	65·7 (62·9 to 68·4)	71·5 (70·8 to 72·1)	75·8 (75·3 to 76·4)	78·5 (78·1 to 79)	79·8 (79·5 to 80·2)	81·3 (80·8 to 81·7)	77·8 (77·4 to 78·1)	77·8 (77·3 to 78·2)
Sonora	54·1 (50 to 58)	61·3 (58·4 to 63·9)	64·8 (62 to 67·4)	69·8 (68·1 to 71·4)	73·7 (73·2 to 74·2)	75·8 (75·4 to 76·2)	76·9 (76·5 to 77·2)	77·9 (77·5 to 78·3)	74·1 (73·7 to 74·4)	74·7 (74·2 to 75·1)
Tabasco	51·8 (47·7 to 55·7)	59·3 (56·2 to 62·2)	62·7 (59·8 to 65·2)	67·5 (66·7 to 68·2)	73·1 (72·5 to 73·6)	76·4 (75·9 to 76·9)	76·9 (76·5 to 77·3)	76·4 (76 to 76·9)	72·2 (71·8 to 72·6)	74·4 (72·2 to 76·4)
Tamaulipas	52 (47·7 to 55·7)	60·2 (57·3 to 62·9)	64·5 (61·7 to 67·1)	70·2 (69·6 to 70·8)	74·6 (74·1 to 75)	77·4 (77 to 77·8)	78·5 (78·2 to 78·9)	79·6 (79·2 to 80·1)	76·5 (76·1 to 76·9)	76·9 (76·4 to 77·3)
Tlaxcala	47·8 (43 to 52·4)	57·3 (54·4 to 59·9)	63·1 (60·1 to 65·7)	68·5 (67 to 70·1)	73 (72·5 to 73·6)	76·8 (76·3 to 77·2)	78 (77·6 to 78·4)	79 (78·5 to 79·5)	75·1 (74·7 to 75·4)	74 (73·5 to 74·5)
Veracruz de Ignacio de la Llave	49 (44·5 to 52·9)	57·9 (54·9 to 60·7)	62·8 (60·1 to 65·4)	69·5 (68·8 to 70·1)	73·3 (72·7 to 73·9)	76·8 (76·4 to 77·2)	77·7 (77·4 to 78)	77·9 (77·5 to 78·2)	75·2 (74·9 to 75·5)	75·5 (73·5 to 77·3)
Yucatán	52·5 (48·6 to 56·2)	59 (56·2 to 61·6)	63·2 (60·6 to 65·7)	68·4 (67·5 to 69·3)	73·3 (72·7 to 73·8)	76·2 (75·7 to 76·6)	77·6 (77·2 to 77·9)	78·4 (78 to 78·8)	75·6 (75·2 to 76)	75·1 (74·6 to 75·5)
Zacatecas	54·8 (50·8 to 58·4)	62·5 (59·6 to 65·2)	67·1 (64·6 to 69·5)	72·5 (71·5 to 73·4)	75·7 (75·3 to 76·2)	78 (77·5 to 78·4)	78·6 (78·2 to 79)	79·4 (78·9 to 79·8)	76·1 (75·7 to 76·5)	75·8 (75·3 to 76·3)
Nicaragua	49·5 (46·1 to 52·3)	55·8 (54·2 to 57·4)	60·5 (59·1 to 61·9)	70·3 (68·3 to 72·3)	77 (76·3 to 77·6)	80·1 (79·5 to 80·6)	79·6 (79·2 to 80)	81·1 (80·6 to 81·5)	78·3 (77·8 to 78·6)	76·8 (75·6 to 77·9)
Panama	63·2 (61·5 to 64·9)	67·4 (66·5 to 68·5)	69·2 (68·6 to 69·8)	76·4 (75·8 to 76·9)	78·9 (78·4 to 79·4)	80·9 (80·4 to 81·4)	82 (81·5 to 82·4)	82·7 (82·2 to 83·2)	80·6 (80·1 to 81·1)	81·4 (79·5 to 83·5)
Venezuela	57·1 (56·6 to 57·6)	66·8 (66·5 to 67)	68·4 (68·2 to 68·6)	72·3 (72·1 to 72·4)	75·2 (75 to 75·3)	78·7 (78·5 to 78·9)	80·1 (79·9 to 80·2)	78·4 (75·8 to 80·7)	75·4 (73 to 77·6)	74·6 (72·3 to 76·9)
Tropical Latin America	55·4 (51·8 to 58·8)	61·3 (59·7 to 62·7)	65·1 (63·9 to 66·2)	69·5 (68·9 to 70·1)	73·2 (72·9 to 73·6)	76 (75·7 to 76·3)	78·2 (77·8 to 78·4)	79·9 (79·6 to 80·2)	78·7 (78·5 to 79)	77·3 (77·1 to 77·6)
Brazil	55·4 (51·6 to 58·8)	61·1 (59·4 to 62·5)	64·9 (63·7 to 66·1)	69·4 (68·8 to 70)	73·1 (72·8 to 73·5)	76 (75·7 to 76·3)	78·2 (77·8 to 78·4)	79·9 (79·7 to 80·2)	78·7 (78·5 to 79)	77·4 (77·2 to 77·6)
Acre	54·6 (48·6 to 60)	61·4 (58·4 to 64·5)	66·1 (63·3 to 68·8)	70·4 (69·1 to 71·6)	74·1 (73·3 to 74·9)	75·1 (74·3 to 75·7)	77·8 (77·2 to 78·4)	79·4 (78·7 to 80)	77·1 (76·5 to 77·6)	77·2 (76·6 to 77·8)
Alagoas	46 (39·2 to 52·5)	52·3 (48·8 to 55·5)	57·6 (54·3 to 60·5)	63·6 (62·2 to 64·9)	69·6 (68·3 to 70·9)	75 (74·2 to 75·7)	78·3 (77·3 to 79·3)	79·6 (78·5 to 80·6)	78·3 (77·8 to 78·9)	78 (77·6 to 78·4)
Amapá	50·2 (43·4 to 56·2)	58·9 (56·1 to 61·6)	65·6 (63·1 to 67·9)	72·7 (71·6 to 73·7)	74·8 (73·7 to 75·9)	76·5 (75·4 to 77·6)	79·4 (78·1 to 80·5)	77·7 (76·2 to 79·2)	77 (75·5 to 78·3)	77·6 (76 to 79·2)
Amazonas	58·1 (51·8 to 63·5)	64·5 (61·3 to 67·6)	67·5 (64·9 to 70·3)	72·2 (71 to 73·4)	74·8 (73·9 to 75·6)	77·6 (76·8 to 78·3)	79·2 (78·5 to 80)	80 (79·6 to 80·4)	76·4 (76 to 76·8)	74·6 (74·1 to 75)
Bahia	56 (50·2 to 61·3)	60·5 (57·1 to 63·6)	64·2 (61·3 to 66·8)	70·6 (69·6 to 71·5)	74·7 (73·8 to 75·6)	77·6 (76·6 to 78·4)	77·3 (76·5 to 78)	81·3 (80·4 to 82·3)	80·2 (79·5 to 81)	79·9 (78·9 to 80·7)
Ceará	48·2 (41·2 to 54·7)	54·8 (51·5 to 58·1)	61·5 (58·2 to 64·5)	68·6 (65·8 to 71·3)	76·6 (75·3 to 78)	78 (76·6 to 79·3)	80·3 (79·1 to 81·6)	80·7 (79·3 to 81·9)	79·3 (78·2 to 80·4)	79·8 (78·5 to 81·1)
Distrito Federal	55 (49·3 to 59·7)	62·6 (59·9 to 65·2)	65·8 (63·3 to 68·2)	69·6 (69·2 to 70)	73 (72·6 to 73·3)	75·8 (75·4 to 76·1)	78·5 (78·2 to 78·8)	80·8 (80·4 to 81·1)	79·2 (78·9 to 79·5)	78·4 (78·1 to 78·7)
Espírito Santo	59·6 (55·9 to 63·3)	63·9 (60·9 to 66·9)	66·5 (63·7 to 69)	71·2 (70·7 to 71·6)	74·3 (74 to 74·7)	76·7 (76·3 to 77)	78·5 (78 to 78·9)	80·6 (80·2 to 80·9)	78·8 (78·5 to 79·2)	77·6 (77·2 to 78)
Goiás	48·3 (42·3 to 53·7)	54·8 (51·5 to 57·8)	61·8 (58·7 to 64·8)	68·4 (66·6 to 70·1)	73·1 (72·4 to 73·8)	76·4 (75·8 to 76·9)	77·5 (77 to 77·9)	79·4 (79 to 79·7)	77·7 (77·3 to 78)	75·6 (75·3 to 76)
Maranhão	49·8 (44·9 to 54·3)	55·7 (52·5 to 59)	60·7 (57·6 to 63·4)	68·6 (66 to 71·2)	72·3 (71·1 to 73·3)	72·7 (71·6 to 73·7)	74·3 (73·2 to 75·3)	76·6 (75·4 to 77·8)	76·6 (75·5 to 77·6)	76·8 (75·5 to 78)
Mato Grosso	59·6 (54·9 to 64·1)	62·8 (59·7 to 66)	66·7 (63·7 to 69·7)	73·3 (72 to 74·5)	74·3 (73·4 to 75·2)	76·1 (75·1 to 77·1)	78·3 (77·8 to 78·8)	80·2 (79·7 to 80·6)	77·5 (77·1 to 77·9)	75·8 (75·3 to 76·2)
Mato Grosso do Sul	60·6 (55·7 to 65·2)	64·7 (61·6 to 67·7)	67·6 (64·7 to 70·3)	72 (71·5 to 72·6)	74·3 (73·8 to 74·7)	76 (75·6 to 76·4)	77·8 (77·4 to 78·2)	79·7 (79·3 to 80·1)	78·1 (77·8 to 78·5)	76·1 (75·7 to 76·4)
Minas Gerais	56·4 (51·7 to 60·6)	61·3 (58·2 to 64·5)	65 (62·1 to 67·8)	69·2 (68·6 to 69·8)	73·6 (73·1 to 74)	77 (76·6 to 77·4)	79·3 (78·9 to 79·7)	80·8 (80·5 to 81·1)	80·1 (79·9 to 80·4)	78 (77·7 to 78·3)
Pará	53·2 (47·9 to 58·2)	60·6 (57·6 to 63·6)	65 (61·6 to 67·9)	70·2 (68·5 to 71·7)	73·9 (72·8 to 75)	76·6 (75·4 to 77·9)	78 (76·7 to 79·1)	79·4 (77·9 to 80·8)	77·5 (76·2 to 78·9)	77·9 (76·4 to 79·4)
Paraíba	44·4 (36·7 to 51·6)	52·4 (49·1 to 55·6)	59·9 (56·7 to 62·8)	67·5 (66 to 69)	73·9 (73·1 to 74·7)	78·3 (77·6 to 78·9)	80·3 (79·8 to 80·8)	81·2 (80·7 to 81·6)	79·9 (79·5 to 80·3)	78·9 (78·6 to 79·3)
Paraná	60·7 (56·8 to 64·5)	64·2 (61·3 to 67·1)	66·4 (63·7 to 69)	69·5 (69·1 to 69·9)	72·9 (72·6 to 73·2)	75·4 (75·1 to 75·7)	77·7 (77·3 to 78)	80·1 (79·8 to 80·4)	79·1 (78·9 to 79·4)	76·3 (75·9 to 76·7)
Pernambuco	43·9 (37·4 to 50·5)	51·1 (47·7 to 54·1)	57·1 (54·2 to 59·8)	65·2 (63·6 to 66·6)	69·8 (69 to 70·5)	74 (73·4 to 74·5)	77·7 (77·3 to 78·1)	79·3 (78·9 to 79·6)	77·7 (77·3 to 78)	77·6 (77·1 to 78)
Piauí	56·5 (50·5 to 62·2)	62 (58·8 to 64·9)	65·1 (62 to 68·2)	71·3 (69 to 73·6)	76 (74·7 to 77·3)	77·9 (76·5 to 79·3)	79·8 (78·2 to 81·2)	80·8 (79·2 to 82·5)	79·6 (78·2 to 81)	80·1 (78·4 to 81·8)
Rio de Janeiro	60·9 (55·3 to 65·5)	66 (63 to 69)	68·2 (65·3 to 70·8)	69·4 (69 to 69·9)	71·7 (71·4 to 72)	75 (74·7 to 75·2)	77·1 (76·9 to 77·3)	78·3 (78·1 to 78·6)	76·6 (76·3 to 76·8)	75·4 (75·2 to 75·6)
Rio Grande do Norte	43·6 (36·5 to 50)	53·1 (49·1 to 56·7)	62·4 (59·4 to 65·3)	70·7 (68·8 to 72·5)	76·2 (75 to 77·5)	78·7 (77·8 to 79·6)	80·3 (79·4 to 81·2)	81·1 (80·7 to 81·5)	79·9 (79·5 to 80·3)	79·3 (78·9 to 79·8)
Rio Grande do Sul	60·5 (56·1 to 64·5)	66 (63·1 to 68·9)	69·2 (66·4 to 71·9)	72·3 (72 to 72·6)	74·9 (74·7 to 75·2)	76·6 (76·4 to 76·8)	78·7 (78·5 to 78·9)	79·8 (79·5 to 80)	79·5 (79·3 to 79·7)	77·2 (76·9 to 77·4)
Rondônia	50 (41·3 to 56·8)	57·1 (53·7 to 60·4)	61·1 (58·3 to 63·5)	62·2 (60·9 to 63·3)	70·9 (70·1 to 71·7)	75·3 (74·7 to 76)	77·6 (76·8 to 78·4)	80·1 (79·1 to 81·2)	78·3 (77·5 to 79·2)	78·7 (77·6 to 79·9)
Roraima	47·5 (41·5 to 53)	54·3 (51·3 to 57·3)	59·3 (56·2 to 62)	65·5 (63·3 to 67·5)	69·5 (67·8 to 71·4)	72·4 (70·8 to 74)	75·1 (73·5 to 76·7)	76·5 (74·5 to 78·5)	74·4 (72·5 to 76·3)	75·6 (73·4 to 77·5)
Santa Catarina	63·9 (59·5 to 67·5)	68·2 (65·5 to 71)	70·1 (67·4 to 72·7)	72·7 (72·2 to 73·2)	74·6 (74·1 to 74·9)	76·5 (76·1 to 76·9)	78·9 (78·6 to 79·3)	80·6 (80·2 to 81)	79·9 (79·6 to 80·2)	77·8 (77·5 to 78·1)
São Paulo	63·4 (59·5 to 67)	67·2 (64·3 to 70)	69·3 (66·7 to 71·8)	70·8 (70·4 to 71·2)	73·3 (73 to 73·5)	76 (75·8 to 76·2)	78·6 (78·3 to 78·8)	80·2 (79·9 to 80·4)	79 (78·8 to 79·2)	77 (76·8 to 77·3)
Sergipe	53·1 (47·4 to 58·2)	58·7 (55·6 to 61·7)	64·3 (61·5 to 66·9)	67·9 (66·8 to 68·9)	72·5 (71·7 to 73·2)	75·1 (74·2 to 75·8)	78·8 (78·3 to 79·4)	80·1 (79·6 to 80·6)	78·8 (78·3 to 79·2)	78·5 (78·1 to 79)
Tocantins	52·6 (43·3 to 59·7)	57·9 (54·6 to 61·2)	64·1 (61 to 67·2)	71·6 (69·1 to 73·8)	74·8 (73·5 to 76·1)	75·6 (74·7 to 76·6)	77·3 (76·4 to 78·3)	78·7 (77·4 to 79·9)	78 (76·9 to 79·1)	78·8 (77·5 to 80·1)

Paraguay	59·8 (56·5 to 63·1)	70·1 (68·1 to 71·9)	71·3 (69·6 to 72·9)	74 (72·4 to 75·5)	77·2 (75·5 to 78·8)	77·9 (76·2 to 79·6)	78·2 (76·6 to 79·7)	78·6 (76·7 to 80·4)	78·7 (76·6 to 80·7)	75·9 (73·8 to 77·6)
North Africa and Middle East	45·8 (42·8 to 48·6)	51·4 (49·5 to 53·3)	56·7 (55·2 to 58·2)	62·8 (61·7 to 63·7)	67·2 (66·5 to 67·9)	71·1 (70·4 to 71·9)	73·9 (73·1 to 74·6)	75·4 (74·5 to 76·2)	74·3 (73·3 to 75·2)	73·7 (72·6 to 74·7)
North Africa and Middle East	45·8 (42·8 to 48·6)	51·4 (49·5 to 53·3)	56·7 (55·2 to 58·2)	62·8 (61·7 to 63·7)	67·2 (66·5 to 67·9)	71·1 (70·4 to 71·9)	73·9 (73·1 to 74·6)	75·4 (74·5 to 76·2)	74·3 (73·3 to 75·2)	73·7 (72·6 to 74·7)
Afghanistan	38 (34·1 to 42)	44·3 (40·8 to 47·9)	46·2 (42·6 to 49·9)	45·3 (40·6 to 49)	52·5 (50 to 55·2)	54·1 (51·7 to 56·5)	59·8 (57 to 62·6)	62·8 (60·2 to 65·5)	61·1 (58·7 to 63·5)	60·7 (58·5 to 62·8)
Algeria	44·5 (40·3 to 48·2)	49·9 (47·6 to 52·3)	55·4 (52·7 to 58)	59·1 (58·1 to 60·1)	71·2 (70·1 to 72·2)	74 (72·9 to 75·1)	76 (75·5 to 76·6)	77·5 (76·5 to 78·5)	76 (75·1 to 77)	75·4 (74·3 to 76·4)
Bahrain	52·7 (47·9 to 57·3)	58·6 (55·4 to 61·8)	65·5 (62·9 to 68·1)	69·9 (68·8 to 70·9)	70·5 (69·9 to 71·2)	71·3 (70·6 to 71·9)	75 (74·4 to 75·6)	76·8 (75·9 to 77·6)	75·8 (74·9 to 76·7)	75·1 (74·1 to 76)
Egypt	45·5 (41·6 to 49·3)	49·8 (48 to 51·5)	52·5 (51·1 to 53·8)	59·1 (58·1 to 60·1)	63·7 (63·1 to 64·3)	68·7 (68·2 to 69)	69·3 (69 to 69·6)	71·3 (71·1 to 71·6)	70·6 (69·6 to 71·7)	70·2 (68·7 to 71·6)
Iran	43·7 (40·2 to 47·3)	50·3 (47·9 to 52·6)	59·4 (57·7 to 61)	69·2 (68·2 to 70·1)	69·5 (68·8 to 70·2)	75 (74·5 to 75·4)	78·1 (77·8 to 78·3)	79·6 (79·3 to 80)	79·1 (78·8 to 79·5)	77·2 (76·8 to 77·6)
Alborz	46·5 (42 to 51·1)	53·3 (50·1 to 56·4)	62·9 (60·1 to 65·7)	72·4 (69·7 to 74·8)	73·5 (71·4 to 75·5)	75 (73·7 to 76·3)	77·6 (76·8 to 78·5)	79 (77·6 to 80·3)	78·4 (77·1 to 79·7)	76·5 (75·3 to 77·6)
Ardebil	42·3 (37·8 to 47)	48·4 (45·1 to 51·7)	55·9 (52·9 to 58·6)	66 (63·5 to 68·3)	69·2 (67 to 71)	74·7 (73·9 to 75·6)	79·4 (78·8 to 79·9)	79·3 (78·5 to 80·3)	78·8 (77·8 to 79·8)	76·7 (75·8 to 77·6)
Bushehr	46·3 (41·8 to 50·9)	52 (48·6 to 55·5)	59·5 (56·6 to 62·3)	69·6 (67 to 72)	72·7 (70·8 to 74·6)	75·9 (75·1 to 76·7)	77·9 (77·4 to 78·4)	79·8 (78·9 to 80·9)	79·4 (78·5 to 80·4)	77·6 (76·8 to 78·5)
Chahar Mahaal and Bakhtiari	50·9 (46·5 to 55·4)	56·3 (53·2 to 59·2)	62·9 (60·1 to 65·6)	70·8 (68·3 to 73·2)	72·8 (70·9 to 74·7)	77 (75·9 to 78·2)	80·5 (79·6 to 81·3)	82·7 (81·3 to 84)	82·1 (80·8 to 83·3)	80·3 (79·2 to 81·4)
East Azarbayejan	40·2 (35·8 to 44·6)	46·9 (43·4 to 50·2)	55·5 (52·7 to 58·7)	66·4 (63·3 to 69)	69·7 (67·7 to 71·5)	73·3 (72·1 to 74·4)	76·6 (75·9 to 77·2)	78·2 (77 to 79·3)	78 (76·9 to 79·2)	76·4 (75·3 to 77·5)
Fars	53·6 (49·4 to 57·6)	58·2 (55·3 to 61·3)	64·9 (62 to 67·5)	71·5 (68·7 to 73·8)	72·4 (70·2 to 74·4)	74·9 (73·6 to 76·2)	76·8 (75·9 to 77·7)	79·2 (77·8 to 80·6)	78·8 (77·5 to 80·1)	76·9 (75·6 to 78)
Gilan	50·9 (46·5 to 55·3)	56 (52 to 59·5)	64 (61·1 to 66·8)	72 (69·6 to 74·3)	42·9 (40·5 to 44·9)	75·5 (74·5 to 76·6)	78 (77·3 to 78·7)	79·3 (78·1 to 80·6)	78·9 (77·7 to 80·2)	76·9 (75·8 to 78)
Golestan	46·8 (42·4 to 50·9)	52·2 (48·9 to 55·6)	59·2 (56·1 to 62)	68 (65 to 70·4)	69·8 (67·6 to 71·9)	73·1 (72·3 to 73·8)	76·9 (76·3 to 77·3)	77 (75·9 to 78·1)	76·8 (75·7 to 77·8)	74·8 (73·9 to 75·8)
Hamadan	35·6 (31 to 39·9)	42 (38·5 to 45·6)	51·7 (48·5 to 54·6)	65·6 (63 to 68·1)	70·2 (68 to 72·1)	74·6 (73·7 to 75·6)	77·6 (77 to 78·2)	79·5 (78·3 to 80·7)	79·1 (78 to 80·2)	77·7 (76·6 to 78·8)
Hormozgan	46 (41·7 to 50·4)	49·8 (46·8 to 52·8)	59·8 (56·8 to 62·5)	68·2 (65·6 to 70·6)	69·5 (67·4 to 71·6)	73·5 (72·3 to 74·6)	77·5 (76·6 to 78·3)	79·6 (78·4 to 81)	79·1 (78 to 80·5)	77·2 (76·2 to 78·5)
Ilam	49 (44·9 to 53·4)	52·9 (49·6 to 56·2)	59·7 (56·9 to 62·5)	68·2 (65·8 to 70·5)	71·3 (69·3 to 73·2)	75 (73·8 to 76)	77·7 (77 to 78·4)	78·9 (77·7 to 80·1)	78·5 (77·2 to 79·6)	76·6 (75·5 to 77·8)
Isfahan	49·4 (44·8 to 54·2)	54 (50·8 to 57·3)	63·6 (60·6 to 66·4)	72·7 (70·5 to 74·9)	74·5 (72·7 to 76·4)	77·2 (76 to 78·3)	79·4 (78·6 to 80)	80·3 (79·1 to 81·5)	79·7 (78·5 to 80·9)	77·7 (76·6 to 78·7)
Kerman	40·8 (36·2 to 45·5)	48·9 (45·3 to 52·4)	58·2 (55·2 to 61·1)	68·5 (65·9 to 70·8)	71·1 (68·8 to 73·1)	73·9 (72·8 to 75)	76·8 (75·9 to 77·5)	78·6 (77·3 to 79·9)	78·3 (77 to 79·5)	76·7 (75·5 to 77·8)
Kermanshah	43·2 (38·9 to 47·4)	47·9 (44·6 to 51·2)	56·1 (53 to 58·9)	66·1 (63·4 to 68·5)	68·9 (66·8 to 71·2)	72·4 (71·2 to 73·6)	76 (75·1 to 76·8)	78·4 (77·1 to 79·8)	78 (76·8 to 79·3)	76·1 (75 to 77·3)
Khorasan-e-Razavi	33·6 (29·4 to 38)	40·8 (37·1 to 44·3)	51·1 (48·1 to 54·1)	64·5 (61·7 to 67·2)	68·2 (65·9 to 70·2)	72·1 (70·9 to 73·2)	76·1 (75·3 to 76·9)	78·6 (77·3 to 79·9)	78·2 (76·9 to 79·4)	76·3 (75·1 to 77·4)
Khuzestan	49·2 (44·5 to 53·7)	54·9 (51·6 to 58·3)	62 (59 to 64·9)	69·5 (66·9 to 71·9)	72·1 (70·1 to 74)	73·9 (73·2 to 74·6)	77·6 (77·1 to 78)	78·2 (77·1 to 79·4)	77·7 (76·6 to 78·8)	75·6 (74·5 to 76·6)
Kohgiluyeh and Boyer-Ahmad	48·3 (43·9 to 52·6)	54·6 (51·4 to 57·8)	60·9 (57·9 to 63·6)	68·4 (65·8 to 70·8)	71·2 (69·1 to 73·4)	75·2 (74 to 76·5)	79 (78·2 to 79·9)	80 (78·7 to 81·4)	79·7 (78·3 to 81)	78 (76·8 to 79·2)
Kurdistan	35·7 (31·4 to 40)	39·6 (36·5 to 43·1)	48·9 (45·9 to 52)	61·6 (58·9 to 64·4)	67·7 (65·5 to 69·8)	73·8 (72·8 to 74·8)	77·8 (77·1 to 78·3)	80·2 (79·1 to 81·3)	79·7 (78·7 to 80·9)	78 (77·1 to 79)
Lorestan	43·4 (38·3 to 48·2)	50·5 (46·9 to 53·9)	58·7 (55·7 to 61·5)	68 (65·4 to 70·4)	71·1 (69·1 to 73·1)	75·1 (74 to 76·1)	78·8 (78·1 to 79·5)	80·9 (79·7 to 82·7)	80 (78·8 to 81·5)	77·3 (76·3 to 78·6)
Markazi	46·4 (41·8 to 50·8)	50·2 (46·9 to 53·4)	57·7 (54·6 to 60·5)	68·6 (66·1 to 71)	71·5 (69·5 to 73·3)	75 (73·9 to 76)	78·5 (77·8 to 79·3)	80·7 (79·5 to 81·8)	80 (78·8 to 81·1)	77·9 (76·9 to 78·9)
Mazandaran	55 (50·5 to 59·3)	59·8 (56·5 to 62·8)	66·1 (63·3 to 68·7)	73·3 (70·8 to 75·5)	74·9 (72·9 to 76·9)	76·7 (75·5 to 77·8)	79 (78·2 to 79·8)	80·1 (78·7 to 81·5)	79·5 (78·3 to 80·8)	77·5 (76·3 to 78·7)
North Khorasan	37·9 (32·6 to 42·9)	44·1 (40·7 to 47·4)	52·1 (49·2 to 55·1)	64·3 (61·7 to 66·8)	67·9 (65·8 to 70)	72·3 (71·2 to 73·4)	76·9 (76·2 to 77·7)	77·9 (76·6 to 79·3)	77·5 (76·2 to 78·7)	75·5 (74·5 to 76·5)
Qazvin	39·2 (34·4 to 43·9)	43·8 (40·3 to 47·4)	54·1 (50·9 to 57)	66·9 (64·4 to 69·3)	71·7 (69·8 to 73·6)	74·8 (73·8 to 75·8)	79 (78·3 to 79·7)	81 (79·8 to 82·3)	80·2 (78·9 to 81·3)	77·9 (76·8 to 78·9)
Qom	50 (45·7 to 54·2)	54·3 (51 to 57·6)	61·2 (58·2 to 64)	70·2 (67·8 to 72·5)	72·3 (70·5 to 74)	74·5 (73·7 to 75·4)	76·9 (76·4 to 77·4)	79·9 (79 to 80·9)	79·3 (78·3 to 80·3)	77·1 (76·2 to 77·9)
Semnan	43·7 (39·2 to 48·1)	49·2 (45·9 to 52·5)	59 (56 to 61·9)	70·1 (67·3 to 72·4)	73·5 (71·6 to 75·4)	76·1 (74·8 to 77·2)	78·9 (78·1 to 79·7)	81 (79·6 to 82·3)	80 (78·7 to 81·3)	77·5 (76·5 to 78·6)
Sistan and Baluchistan	38·5 (33 to 43·7)	46·2 (43 to 49·5)	54·2 (51·3 to 57·1)	64·7 (61·9 to 67·3)	68 (65·5 to 70·2)	72·9 (71·4 to 74·5)	76·6 (75·4 to 77·8)	77·9 (75·8 to 80)	77 (75·2 to 78·6)	74·9 (73·3 to 76·4)
South Khorasan	39·7 (35·4 to 44·2)	46·7 (43·1 to 50·1)	54·8 (51·8 to 57·4)	66·5 (63·7 to 68·9)	70·9 (68·8 to 72·8)	76·1 (75·2 to 77·1)	79·1 (78·4 to 79·8)	81·5 (80·3 to 82·9)	80·5 (79·4 to 81·7)	78·6 (77·6 to 79·6)
Tehran	55·5 (51·2 to 59·6)	62·5 (59·3 to 65·5)	70·3 (67·7 to 72·8)	76·5 (74 to 78·8)	77·4 (75·3 to 79·4)	79 (77·7 to 80·5)	81·1 (80·1 to 82·2)	82 (80·4 to 83·6)	81·3 (80 to 82·5)	79·5 (78·2 to 80·7)
West Azarbayejan	41·1 (36·9 to 45·1)	45·9 (42·6 to 49·3)	54·3 (51·2 to 57)	65·8 (63·1 to 68·4)	69·2 (67 to 71·2)	72·5 (71·5 to 73·5)	76·6 (76·1 to 77·2)	78 (76·9 to 79·1)	77·7 (76·6 to 78·8)	75·8 (74·8 to 76·9)
Yazd	42·9 (38·1 to 47·3)	52·3 (49 to 55·5)	61·4 (58·7 to 64)	70·9 (68·4 to 73·1)	73·5 (71·6 to 75·4)	75·6 (74·4 to 76·8)	77·8 (77 to 78·6)	80 (78·7 to 81·3)	78·9 (77·7 to 80)	76·3 (75·3 to 77·2)
Zanjan	34·3 (29·1 to 39·6)	38·5 (35 to 41·9)	49·6 (46·5 to 52·6)	65 (62·4 to 67·5)	42·2 (40 to 44·2)	75·2 (74·2 to 76·2)	79·6 (79·1 to 80·1)	81·7 (80·6 to 82·8)	80·7 (79·7 to 81·8)	78·6 (77·7 to 79·5)
Iraq	58·6 (55·1 to 62)	63·2 (61·1 to 65·3)	65·6 (63·5 to 67·4)	70 (68·1 to 71·8)	70·3 (68·5 to 72·1)	71·8 (68·9 to 74·5)	73·8 (71 to 76·7)	75·6 (73 to 77·9)	72·8 (71 to 74·5)	73·5 (71·6 to 75·4)
Jordan	52·9 (48 to 57·2)	59·9 (56·8 to 62·7)	65·7 (64·1 to 67·1)	70·7 (69·1 to 72·5)	71·9 (70·5 to 73·3)	72·2 (70·6 to 73·8)	77·2 (76·2 to 78·1)	80·4 (79·1 to 81·8)	78·5 (77·1 to 79·9)	77·6 (76·1 to 78·9)
Kuwait	67·2 (63·1 to 71·1)	71·9 (70 to 73·9)	73·4 (72·9 to 74)	74·8 (74·4 to 75·2)	77·3 (76·8 to 77·8)	80·2 (79·8 to 80·5)	82·8 (82·5 to 83·2)	87·3 (86·8 to 87·7)	85·5 (85 to 85·9)	85·1 (84 to 86·2)
Lebanon	55·8 (50·4 to 61·1)	66·7 (63·5 to 69·8)	70·2 (67·4 to 72·6)	71·8 (69·4 to 73·8)	73·1 (71·2 to 74·5)	76·9 (75·7 to 78·2)	80 (79·1 to 80·9)	82·2 (81·1 to 83·3)	80·4 (79·3 to 81·3)	78·4 (77·4 to 79·3)
Libya	43·7 (40·1 to 47·5)	51·8 (49·1 to 54·9)	63·7 (62 to 65·6)	71·3 (70·1 to 72·6)	74·5 (72·7 to 76·4)	76·2 (75·2 to 77·3)	74·9 (74 to 75·9)	74·7 (72·2 to 77·1)	73·7 (71·2 to 75·9)	73·4 (70·9 to 75·4)
Morocco	43·7 (39·8 to 47·4)	49·1 (46·3 to 52)	56·5 (54·1 to 58·8)	62·4 (60·6 to 64·3)	68·3 (66·8 to 69·8)	71·3 (69·6 to 72·8)	73·1 (70·9 to 75·1)	74·8 (72·9 to 77·2)	73·5 (71·7 to 75·5)	73·9 (72·2 to 75·8)
Oman	42·9 (37·4 to 47·9)	50 (46·1 to 54)	59·5 (56·1 to 62·6)	66·9 (63·6 to 70)	72·3 (69·4 to 75·3)	75·7 (73·5 to 78)	77·3 (76 to 78·7)	79·1 (77·6 to 80·6)	77 (75·7 to 78·2)	76·3 (75·1 to 77·4)
Palestine	46·2 (41·2 to 51·2)	55·5 (52·1 to 59)	63·3 (60·5 to 65·7)	69·4 (66·5 to 71·8)	71·7 (69·7 to 73·4)	73·2 (72·2 to 74·1)	74·9 (74·3 to 75·5)	77·8 (76·9 to 78·7)	77·4 (76·3 to 78·4)	76·2 (75·2 to 77·2)
Qatar	62·5 (58 to 66·4)	64·8 (61·8 to 67·5)	68·9 (66·4 to 71·2)	72·6 (70·6 to 74·5)	72·7 (71·3 to 74·1)	73·7 (72·4 to 75·1)	75·6 (74·3 to 76·8)	79·9 (78·2 to 81·6)	79·2 (77·7 to 80·7)	79·2 (77·6 to 80·7)
Saudi Arabia	53·3 (48·6 to 58)	55 (51·3 to 58·2)	60·8 (57·5 to 64)	67·5 (64·1 to 70·4)	69·4 (66·7 to 72·1)	71·6 (70 to 73·1)	73·5 (72·4 to 74·5)	75·3 (73·3 to 77·3)	74·7 (72·7 to 76·6)	75·1 (72·9 to 77·2)
Sudan	47·1 (42·3 to 51·6)	49·9 (46·7 to 53)	52·5 (50·2 to 54·7)	54·8 (52·8 to 56·9)	59·2 (57 to 61·5)	64·1 (61·7 to 66·5)	68·8 (66 to 71·4)	71·8 (68·6 to 74·5)	70·3 (67·3 to 72·8)	70·1 (67·2 to 72·7)
Syria	54·6 (50·4 to 58·7)	63·7 (61·2 to 66·5)	69·1 (67·2 to 70·9)	72 (69·7 to 74·3)	70·7 (68·7 to 72·7)	72·8 (71·5 to 73·9)	75·6 (74·2 to 77)	74·5 (72·3 to 76·5)	74·9 (72·8 to 76·9)	74·7 (72·5 to 76·6)
Tunisia	44 (40·1 to 47·7)	50·9 (49·2 to 52·8)	57·8 (56·5 to 59)	68 (66·9 to 69)	74·4 (73·3 to 75·4)	76·9 (75·4 to 78·3)	78·9 (76·3 to 81·4)	80·3 (77·8 to 82·8)	79·6 (77·1 to 82·2)	77·1 (75·1 to 79)
Türkiye	50 (46 to 54·1)	54·1 (51·2 to 57·2)	60·7 (58·3 to 63·2)	67·4 (65·5 to 69·2)	71·3 (70 to 72·5)	77·6 (76·2 to 78·9)	79·6 (78·2 to 80·9)	80 (78·8 to 81·2)	78·5 (77·2 to 79·7)	78·3 (77 to 79·5)
United Arab Emirates	57·4 (52·7 to 61·7)	60·6 (57·2 to 64)	67·5 (64·4 to 70·7)	69·6 (66·7 to 72·3)	70·9 (68·7 to 73·2)	72·5 (70·9 to 74·2)	71·3 (70·1 to 72·5)	70·4 (69·5 to 71·2)	71·4 (70·6 to 72·3)	71·5 (70·8 to 72·3)
Yemen	32 (28·4 to 35·8)	40·5 (37·6 to 43·6)	45·5 (42·9 to 48·3)	55·3 (52·7 to 57·9)	60·5 (57·7 to 63·2)	64·7 (61·7 to 67·5)	69·4 (66·4 to 72·2)	70·1 (67 to 72·8)	69·3 (66·3 to 71·8)	68·5 (65·5 to 70·9)
South Asia	39·6 (35·6 to 43·5)	45·1 (42·5 to 47·5)	50·5 (49 to 52)	56·8 (55·7 to 57·8)	61·5 (60·8 to 62·3)	65·4 (64·6 to 66·1)	69·4 (68·6 to 70)	71·8 (70·8 to 72·6)	71·2 (70·3 to 72·1)	70·8 (69·8 to 71·8)
South Asia	39·6 (35·6 to 43·5)	45·1 (42·5 to 47·5)	50·5 (49 to 52)	56·8 (55·7 to 57·8)	61·5 (60·8 to 62·3)	65·4 (64·6 to 66·1)	69·4 (68·6 to 70)	71·8 (70·8 to 72·6)	71·2 (70·3 to 72·1)	70·8 (69·8 to 71·8)
Bangladesh	43·3 (39·6 to 46·9)	47·4 (44·6 to 50·1)	44·2 (42·1 to 46·1)	55·1 (53·1 to 57·1)	60·2 (59 to 61·4)	67·1 (65·8 to 68·2)	71·1 (70·2 to 72·1)	75 (72·9 to 77·1)	74 (71·9 to 76)	74·1 (72 to 76·1)
Bhutan	38 (32·8 to 43·4)	45 (40·9 to 49·3)	50·5 (45·7 to 54·9)	53·4 (49·6 to 57·2)	60·2 (57·5 to 62·9)	65·6 (63·3 to 67·8)	72·5 (70·3 to 74·7)	74·8 (72·4 to 77·3)	75 (72·6 to 77·5)	74·9 (72·6 to 77·3)
India	38·6 (33·8 to 43·2)	44·2 (41·2 to 47·1)	50·7 (48·9 to 52·6)	56·7 (55·4 to 57·9)	61·7 (60·7 to 62·7)	65·6 (64·6 to 66·5)	69·6 (68·8 to 70·4)	72·1 (71 to 73)	71·8 (70·7 to 72·7)	71·2 (70·2 to 72·4)
Nepal	40·8 (37·3 to 44·1)	45·5 (42·5 to 48·2)	50·1 (47·4 to 53)	52·3 (49·8 to 54·9)	58·4 (56·2 to 60·5)	66·3 (64·6 to 67·7)	70·6 (68·8 to 72·4)	73 (70·7 to 74·9)	71·8 (69·7 to 73·7)	70·8 (68·8 to 72·4)
Pakistan	46·1 (42·5 to 49·8)	52·1 (49·7 to 54·6)	57 (55·1 to 58·8)	61 (59·6 to 62·5)	62·9 (61·7 to 64)	62·9 (61·5 to 64·4)	65·7 (63·5 to 67·9)	68 (65 to 70·8)	66·4 (63·7 to 68·8)	66·4 (63·8 to 68·8)
Azad Jammu & Kashmir	48·6 (43·4 to 53·8)	52·6 (48·4 to 56·8)	56·9 (53·4 to 60·4)	61 (57·5 to 64·6)	63·2 (59·4 to 66·7)	63·9 (59·8 to 67·7)	67·5 (63·1 to 71·6)	69·4 (64·7 to 73·7)	68 (64 to 71·7)	67·2 (63·3 to 70·5)
Balochistan	53·4 (48·5 to 58·2)	57·4 (53·3 to 61·4)	62·1 (58·4 to 65·5)	64·8 (61·4 to 68·2)	64·1 (60·5 to 67·3)	62·8 (59·3 to 66·4)	64·1 (60·1 to 68·2)	65·9 (61·4 to 70·2)	64·2 (60·3 to 68·1)	64·7 (60·7 to 68·6)

Gilgit-Baltistan	48·8 (43·7 to 53·5)	50·6 (46·6 to 54·6)	54·8 (50·9 to 58·4)	58·7 (54·9 to 62·4)	61 (57·3 to 64·6)	61·3 (57·7 to 65·2)	65 (60·8 to 69·2)	67·4 (62·9 to 72·1)	65 (61·1 to 68·6)	65·6 (61·8 to 69·4)
Islamabad Capital Territory	45·6 (39·4 to 51·4)	50·9 (46·6 to 55·2)	59·2 (55·3 to 63·4)	67 (63·7 to 70·3)	70·7 (67·3 to 73·7)	71·4 (67·7 to 74·5)	73·9 (70·4 to 77·1)	75·4 (71·6 to 78·7)	73·4 (70·1 to 76·2)	73·2 (69·9 to 76·1)
Khyber Pakhtunkhwa	43·6 (38·4 to 48·6)	52·3 (48·5 to 56)	57·6 (54·4 to 60·9)	62·9 (59·8 to 66·1)	65·2 (61·9 to 68·4)	63·9 (60·6 to 67·1)	66·2 (62·4 to 69·9)	68·3 (64·2 to 72·1)	66·5 (62·8 to 69·8)	66·8 (63·2 to 70·3)
Punjab	46·5 (42·7 to 50·4)	51·8 (49·1 to 54·8)	56·3 (54 to 58·5)	59·7 (57·9 to 61·7)	62·1 (60·3 to 64)	62·6 (60·5 to 64·7)	65·6 (63·1 to 68·1)	67·9 (64·7 to 71·1)	66·6 (63·4 to 69·3)	66·2 (63·3 to 69·1)
Sindh	45·2 (40·3 to 50·3)	52·4 (48·6 to 56·1)	57·9 (54·7 to 61·2)	62·3 (59·2 to 65)	63·1 (60 to 66)	63·1 (60 to 66·3)	65·7 (61·8 to 69·2)	68·1 (64 to 71·7)	66·1 (62·6 to 69·5)	66·5 (63 to 69·8)
Southeast Asia, east Asia, and Oceania	49·6 (46·4 to 52·5)	34·5 (23·2 to 42·7)	62 (59·7 to 64)	65·4 (63·7 to 67)	69·4 (68·1 to 70·6)	72·3 (71·6 to 73·1)	76·2 (75·5 to 76·9)	78·8 (77·4 to 80·1)	78·8 (77·5 to 80·2)	78·6 (77·2 to 80)
East Asia	50·6 (46·8 to 54)	28·9 (16·6 to 38·7)	63·3 (60·2 to 66)	66·5 (64·3 to 68·6)	70·1 (68·5 to 71·7)	73·3 (72·3 to 74·2)	77·8 (76·9 to 78·6)	80·5 (78·7 to 82·1)	80·6 (78·8 to 82·3)	80·7 (78·9 to 82·5)
China	50·7 (46·9 to 54·3)	28 (15·8 to 37·9)	63 (59·9 to 65·8)	66·2 (64 to 68·5)	69·9 (68·3 to 71·6)	73·4 (72·5 to 74·4)	77·8 (76·9 to 78·7)	80·5 (78·7 to 82·2)	80·6 (78·7 to 82·3)	80·7 (78·9 to 82·6)
North Korea	41·2 (38 to 44·1)	66 (62·5 to 69)	69·4 (66·3 to 72·6)	71·4 (68 to 74·3)	72·4 (69·2 to 75·3)	64·8 (52·6 to 71·1)	73·4 (71·3 to 75·7)	75·8 (73·2 to 77·9)	76 (73·4 to 78·1)	76·2 (73·6 to 78·5)
Taiwan (province of China)	58·4 (56·1 to 60·6)	66·8 (66·6 to 67)	72·2 (72 to 72·3)	75 (74·8 to 75·1)	77·3 (77·1 to 77·4)	79·8 (79·6 to 79·9)	83 (82·8 to 83·2)	84·4 (84·3 to 84·6)	85 (84·8 to 85·2)	84·6 (84·4 to 84·8)
Oceania	49·2 (46·2 to 52·3)	56 (53·4 to 58·5)	60·5 (58·2 to 62·7)	63·5 (61·1 to 65·6)	64·5 (62·2 to 66·7)	65·7 (63·9 to 67·4)	66·6 (64·8 to 68·3)	68·1 (66 to 70)	68 (65·9 to 70)	66·6 (64·2 to 69)
American Samoa	63·2 (60·7 to 65·4)	67·6 (66·2 to 68·9)	69·4 (68·1 to 70·6)	72·3 (71·1 to 73·6)	73·8 (72·8 to 74·7)	73 (72 to 74)	72·6 (71·5 to 73·7)	72·6 (70·7 to 74·4)	72 (70·2 to 73·7)	72·8 (70·6 to 74·9)
Cook Islands	46·7 (44·7 to 48·7)	57·7 (56·3 to 59·2)	65·2 (63·7 to 66·7)	68·5 (67·1 to 69·9)	71·4 (69·8 to 73)	75·6 (74 to 77·1)	78·8 (77·4 to 80)	79·8 (77·7 to 81·7)	79·6 (77·6 to 81·5)	79·6 (77·6 to 81·6)
Fiji	59·2 (57·7 to 60·6)	66·6 (65·6 to 67·7)	69 (68 to 70)	69·7 (68·7 to 70·6)	69·1 (67·2 to 71)	68·2 (67·6 to 68·7)	69·2 (68·3 to 70·1)	70·4 (67·6 to 73·1)	70·4 (67·6 to 73·2)	68·8 (65·8 to 71·9)
Guam	70·1 (68·3 to 71·8)	70·6 (69·7 to 71·4)	71·3 (70·5 to 72·1)	73·8 (73·3 to 74·4)	75·8 (75·2 to 76·4)	78·6 (78 to 79·2)	82·9 (82·1 to 83·8)	82·9 (81·3 to 84·4)	85·7 (84·3 to 87·1)	82·9 (81·2 to 84·7)
Kiribati	48 (44·4 to 51·4)	53·3 (50·3 to 56·4)	57·3 (54·5 to 60·2)	60·9 (58·3 to 63·6)	61·5 (59·4 to 63·6)	63·5 (61·4 to 65·4)	65·1 (62·6 to 67·5)	66·6 (63·7 to 69·1)	66·8 (63·9 to 69·3)	67 (64·1 to 69·5)
Marshall Islands	53·6 (49·1 to 58)	60·9 (57·5 to 64·5)	64·8 (61·6 to 67·8)	67·2 (65·1 to 69·5)	66·3 (65 to 67·5)	63·9 (62·2 to 65·5)	64·6 (62·7 to 66·6)	66·4 (63·4 to 69·1)	66·6 (63·5 to 69·4)	66·8 (63·5 to 69·6)
Federated States of Micronesia	45·1 (41·3 to 48·8)	55·9 (53·1 to 58·5)	60·7 (58 to 63·5)	64 (61·3 to 66·8)	65·6 (62·9 to 68·2)	66·8 (64·7 to 68·9)	68·6 (66·3 to 70·8)	69·5 (66·6 to 72·2)	69·6 (66·6 to 72·3)	69·7 (66·6 to 72·4)
Nauru	54·5 (50·8 to 58·2)	58·2 (55·4 to 60·8)	60·5 (57·9 to 63·1)	63·6 (60·6 to 66·5)	64·3 (62·1 to 66·6)	61·5 (59·5 to 63·5)	62 (59·7 to 64·4)	65·3 (62 to 68·2)	65·5 (62·1 to 68·4)	65·7 (62·3 to 68·7)
Niue	54·5 (51·7 to 57·1)	64·1 (61·8 to 66·5)	69·4 (67 to 71·9)	72·1 (69·4 to 74·7)	71·9 (69·4 to 74·3)	71·6 (69·4 to 73·8)	72·7 (70·2 to 75)	73·3 (70·7 to 75·8)	69·2 (67·5 to 71)	69·2 (67·6 to 71·1)
Northern Mariana Islands	65·4 (61·6 to 68·7)	68·1 (65·3 to 70·8)	70·3 (67·5 to 73·2)	71·8 (68·7 to 74·4)	73·2 (70·4 to 75·8)	75·3 (73·2 to 77·2)	76·2 (74·3 to 78·1)	76·2 (73·9 to 78·3)	77·8 (75 to 80·5)	75 (73·8 to 77·1)
Palau	50·8 (45·3 to 55·8)	58·1 (54·3 to 61·8)	63·9 (60·5 to 67·2)	66·8 (63·7 to 69·8)	68·6 (65·8 to 71·4)	69·7 (67·7 to 71·9)	69·5 (67·4 to 71·5)	70·3 (68·1 to 72·4)	70·4 (68·2 to 72·5)	70·5 (68·2 to 72·6)
Papua New Guinea	45·9 (42 to 49·7)	53·4 (50·4 to 56·5)	58·4 (55·6 to 61·4)	61·3 (58·4 to 64·2)	62·8 (59·8 to 65·6)	64·4 (62·2 to 66·7)	65·5 (63·3 to 67·7)	67·3 (64·8 to 69·6)	67·2 (64·7 to 69·5)	65·5 (62·8 to 68·3)
Samoa	58 (55·3 to 60·6)	65·6 (64 to 67·1)	68·1 (66·9 to 69·3)	72·1 (70·3 to 73·7)	71·1 (69·1 to 73·1)	71·7 (69·4 to 73·6)	72 (70 to 73·9)	70·7 (68·4 to 72·8)	71·6 (69·3 to 73·8)	71·9 (69·5 to 74·2)
Solomon Islands	48·6 (45·3 to 51·9)	52·9 (49·9 to 55·8)	58·2 (55·5 to 61·1)	62·9 (60 to 65·7)	64·1 (61·3 to 66·7)	65·8 (63 to 68·4)	66·9 (64 to 69·7)	68·2 (65·2 to 71·1)	68·1 (65 to 70·8)	68·4 (65·2 to 71·1)
Tokelau	58·2 (54·7 to 61·2)	61·4 (58 to 65·1)	64·5 (61·3 to 67·7)	66·3 (63·3 to 69·4)	68·6 (65·9 to 71·3)	70·3 (67·6 to 72·7)	72·2 (69·2 to 74·8)	73·7 (70·6 to 76·4)	67·8 (65·6 to 69·9)	67·8 (65·6 to 70)
Tonga	62·9 (59·4 to 65·9)	65·8 (64·1 to 67·6)	67·5 (65·5 to 69·4)	70·1 (68·5 to 71·6)	73·1 (71·5 to 74·7)	73·9 (72·5 to 75·4)	74·6 (72·4 to 76·8)	75·5 (72·8 to 77·9)	75·6 (72·9 to 78·1)	75·7 (72·9 to 78·2)
Tuvalu	49·2 (44·5 to 54)	54·2 (50·3 to 58·1)	57·3 (54·3 to 60·3)	59·3 (56·7 to 61·9)	62·5 (60·2 to 64·7)	63·5 (61·5 to 65·3)	69 (66·7 to 71·3)	70·2 (67·4 to 72·6)	70·4 (67·6 to 72·9)	70·6 (67·8 to 73·2)
Vanuatu	49·9 (46·5 to 53·4)	54·2 (51·1 to 57·4)	59·8 (56·9 to 62·9)	65·2 (62·2 to 68)	67·2 (64·4 to 69·8)	68·1 (65·7 to 70·2)	69·3 (67·5 to 71)	70·5 (68·7 to 72·2)	70·5 (68·6 to 72·2)	69·4 (67·3 to 71·3)
Southeast Asia	47·2 (44·6 to 49·9)	54·5 (52·6 to 56·4)	59 (57·5 to 60·5)	63·8 (62·5 to 65)	67·9 (67·1 to 68·7)	70·5 (69·7 to 71·3)	73·3 (72·3 to 74·1)	75·5 (74·2 to 76·6)	75·5 (74·1 to 76·7)	74·3 (72·7 to 75·8)
Cambodia	45·4 (40·4 to 50·2)	50·2 (46·3 to 53·9)	51 (47·7 to 54)	55·4 (52·8 to 57·9)	59·6 (58 to 61·2)	62·4 (60·9 to 63·8)	69·2 (67·5 to 70·9)	71·7 (69·1 to 74·3)	71·9 (69·2 to 74·5)	71 (68·2 to 73·6)
Indonesia	44·4 (41·3 to 47·8)	50·2 (47·3 to 53·1)	55·8 (53·3 to 58·2)	61 (59·2 to 62·6)	65·4 (64·2 to 66·4)	68·3 (67·1 to 69·5)	70·8 (69·1 to 72·3)	73·2 (70·9 to 75·4)	72·8 (70·5 to 74·8)	72 (69·6 to 74·3)
Aceh	51·1 (46·9 to 55·5)	55·7 (51·7 to 59·6)	59 (54·9 to 62·7)	62·5 (59·4 to 65·5)	65·6 (63·4 to 68)	68·4 (66·3 to 70·6)	69·1 (66·6 to 71·5)	71·3 (67·9 to 74·9)	71 (67·6 to 74·2)	70·3 (67·2 to 73·6)
Bali	45·8 (41·1 to 50·3)	51·2 (47·3 to 55·1)	55·6 (51·8 to 59·6)	59·9 (57·1 to 63)	65·9 (63·8 to 67·8)	69·2 (67·2 to 71·3)	71·5 (69·2 to 73·9)	73·7 (70·6 to 76·7)	73·4 (70·3 to 76·2)	72·7 (69·6 to 75·6)
Bangka-Belitung Islands	42 (36·7 to 47)	47·7 (43·4 to 52)	52·5 (48·9 to 56)	57 (53·8 to 59·9)	60·2 (57·7 to 62·7)	63·9 (61·7 to 66)	66·6 (64 to 69)	69·1 (65·9 to 72·1)	68·9 (65·5 to 71·9)	67·9 (64·5 to 71)
Banten	39·3 (34·5 to 44·3)	41·4 (37·4 to 46)	49 (45·3 to 52·5)	56·5 (53·6 to 59·4)	63·6 (61·3 to 65·8)	67·9 (65·7 to 70)	69·9 (67·3 to 72·4)	72·4 (69·2 to 75·4)	72 (68·8 to 74·9)	71·5 (68·2 to 74·7)
Bengkulu	36·9 (33 to 41·2)	42·4 (38·6 to 46·3)	48·6 (45·2 to 51·9)	54·1 (51·1 to 57·1)	59·3 (57·2 to 61·5)	63·5 (61·3 to 65·7)	66·6 (64·1 to 69·3)	69·8 (66·5 to 72·9)	69·5 (66·2 to 72·7)	69·1 (65·8 to 72·3)
Gorontalo	40·9 (36·1 to 46·3)	43·6 (39·2 to 48)	49·3 (45·7 to 53)	53·4 (50·4 to 56·6)	57·5 (55·1 to 60)	60·2 (57·9 to 62·6)	61·7 (58·7 to 64·5)	65·4 (61·9 to 68·9)	65·4 (61·5 to 69)	65·1 (61·5 to 68·5)
Jakarta	52·3 (48·3 to 56·5)	55·4 (51·4 to 59·3)	60·9 (57 to 64·4)	64·9 (62 to 67·5)	68·4 (66·3 to 70·5)	71·3 (69·4 to 73·3)	73 (70·8 to 75·4)	75·1 (72·1 to 77·9)	74·6 (71·8 to 77·3)	73·9 (71 to 76·8)
Jambi	40·5 (36·3 to 44·7)	45·5 (41·7 to 49·3)	50·5 (46·8 to 54)	55·3 (52·5 to 58)	61·1 (58·9 to 63·3)	66·1 (64·1 to 68·1)	69·1 (66·7 to 71·5)	71·8 (68·8 to 75)	71·6 (68·5 to 74·7)	70·9 (67·9 to 74·1)
West Java	42·8 (39·1 to 46·7)	48 (44·4 to 51·5)	54·8 (51·4 to 58·1)	61 (58·5 to 63·4)	65·9 (64·1 to 67·8)	68·9 (66·9 to 70·7)	71·5 (69·2 to 73·7)	74 (71·1 to 76·7)	73·6 (70·7 to 76·3)	72·8 (69·7 to 75·4)
Central Java	48·9 (44·7 to 53·2)	55 (51·2 to 58·7)	60 (56·6 to 63·3)	64·9 (62·4 to 67·4)	69·2 (67·3 to 70·9)	70·9 (69·2 to 72·6)	72·8 (70·6 to 74·9)	75 (72·2 to 77·5)	74·5 (71·7 to 77·1)	73·6 (70·9 to 76·3)
East Java	46·6 (42·7 to 50·4)	52·4 (49·1 to 55·8)	57·7 (54·4 to 60·9)	62·5 (60·1 to 64·8)	66·4 (64·6 to 68·1)	69·8 (67·9 to 71·5)	73 (70·7 to 75·1)	75·3 (72·5 to 77·9)	74·7 (71·8 to 77·3)	73·9 (71·2 to 76·7)
West Kalimantan	44·7 (40·6 to 49·1)	46·5 (42·8 to 50·3)	49·6 (46 to 53·2)	53·9 (51·1 to 56·7)	60 (57·7 to 62·2)	64·7 (62·7 to 66·7)	68·4 (66·1 to 70·9)	71·4 (68·3 to 74·5)	71·3 (68·1 to 74·2)	70·6 (67·2 to 73·6)
South Kalimantan	39·4 (35·7 to 43·4)	42·9 (39·5 to 46·3)	49·6 (46·2 to 53·3)	55·4 (52·5 to 58·2)	59·9 (57·6 to 62·1)	63·2 (61·2 to 65·4)	66·8 (64·2 to 69·2)	69·9 (66·6 to 73)	69·4 (66·2 to 72·7)	68·7 (65·3 to 71·9)
Central Kalimantan	47·8 (43·9 to 52·4)	53 (49·3 to 57·1)	56·9 (53·4 to 60·6)	60 (57·2 to 63·1)	63 (60·7 to 65·2)	67·7 (65·7 to 69·6)	69·3 (67 to 71·8)	71·5 (68·2 to 74·6)	71·1 (68 to 74·1)	70·4 (67·1 to 73·5)
East Kalimantan	51·8 (47·7 to 56)	52·7 (48·9 to 56·5)	56·6 (53·1 to 60·2)	61 (58·3 to 63·9)	63·6 (61·4 to 65·8)	65·9 (63·8 to 67·8)	68·4 (65·8 to 70·6)	71·2 (68·1 to 74·3)	70·9 (67·7 to 73·8)	70·2 (67 to 73·3)
North Kalimantan	58·1 (54 to 62·3)	56·8 (53·1 to 59·9)	60·6 (57·4 to 63·9)	66·2 (63·3 to 69)	71·7 (69·2 to 74·1)	75·4 (73·2 to 77·8)	77·5 (75·1 to 80)	79·2 (76·1 to 82·5)	78·5 (75·4 to 81·4)	76·9 (73·7 to 80·2)
Riau Islands	50·9 (46·8 to 55)	53·9 (49·6 to 58·2)	58·5 (54·9 to 62·4)	61·8 (58·3 to 65·2)	65 (62·7 to 67·5)	68·9 (66·7 to 70·9)	70·2 (67·6 to 72·6)	72·4 (69·2 to 75·5)	72·2 (69 to 75·2)	71·6 (68·4 to 74·8)
Lampung	48·3 (44 to 52·6)	51·3 (47·3 to 55·1)	57·3 (53·6 to 60·7)	62·9 (60 to 65·7)	67·9 (65·8 to 69·9)	70·7 (68·8 to 72·4)	72·1 (69·5 to 74·2)	74·1 (70·8 to 76·8)	73·7 (70·5 to 76·6)	72·9 (69·7 to 75·8)
Maluku	34·4 (30·6 to 38·9)	42·1 (38·1 to 46·4)	48 (44·7 to 51·4)	52·5 (49·6 to 55·8)	57 (54·4 to 59·5)	60·7 (58·5 to 63·2)	62·4 (59·5 to 65·2)	66·7 (63·2 to 70·1)	66·7 (63·3 to 70)	66·5 (63·1 to 69·9)
North Maluku	37·2 (32·1 to 42)	43 (39·1 to 46·9)	47·8 (44·2 to 51·4)	51·2 (48 to 54·3)	55·4 (52·9 to 58·1)	58·6 (56·2 to 61·1)	60·2 (57·1 to 62·8)	64 (60·6 to 67·5)	64 (60·3 to 67·6)	63·8 (60·3 to 67·4)
West Nusa Tenggara	35·7 (32 to 39·6)	39·5 (36 to 42·8)	44·3 (41 to 47·6)	50 (47·2 to 52·8)	56·7 (54·4 to 59·1)	61·8 (59·6 to 64·1)	66·9 (64·3 to 69·5)	69·9 (66·6 to 73·2)	69·7 (66·5 to 72·9)	69·4 (66·4 to 72·5)
East Nusa Tenggara	38·7 (34·8 to 42·9)	45·3 (41·2 to 49·4)	50·4 (46·7 to 53·8)	55·4 (52·8 to 58·5)	59·9 (57·7 to 62·1)	63·9 (61·7 to 66)	67·8 (64·8 to 70·4)	70·9 (67·5 to 74·2)	70·8 (67·4 to 74)	70·1 (66·5 to 73·4)
Papua	52·8 (49 to 56·7)	51·4 (47·1 to 55·6)	53·5 (49·8 to 57)	55·7 (52·7 to 58·8)	58·4 (55·9 to 60·9)	61·5 (59·2 to 63·8)	63·1 (60·4 to 65·8)	66·5 (62·9 to 70·2)	66·4 (62·8 to 70)	66·2 (62·5 to 69·6)
West Papua	51·2 (47·2 to 55·3)	52 (48 to 56·2)	53·7 (49·9 to 57·4)	56·5 (53·1 to 59·5)	59·3 (56·8 to 61·9)	61·3 (59 to 63·7)	61·1 (58·4 to 63·6)	66·1 (62·6 to 69·7)	66 (62·6 to 69·1)	65·9 (62·3 to 69)
Riau	50·2 (46·2 to 54·4)	52·3 (48·4 to 56·4)	56·9 (53·2 to 60·3)	62·2 (59·5 to 65)	65·1 (62·8 to 67·1)	68 (65·9 to 69·9)	71·1 (68·6 to 73·7)	73·9 (70·9 to 76·8)	73·4 (70·4 to 76·1)	72·6 (69·4 to 75·7)
West Sulawesi	31·6 (26·5 to 36·9)	36·7 (32·8 to 41)	42 (38·8 to 45·2)	47·5 (44·6 to 50·4)	53·8 (51·4 to 56·2)	59·4 (57·2 to 61·6)	62·9 (60 to 65·7)	66·5 (63·1 to 70)	66·4 (62·9 to 70)	65·9 (62·5 to 69·5)
South Sulawesi	40·8 (36·9 to 45)	45·5 (41·8 to 49·2)	51·5 (48·2 to 55·4)	56·8 (53·9 to 59·6)	62·8 (60·6 to 65)	67·3 (65·3 to 69·4)	70·2 (67·5 to 72·5)	73·2 (70·2 to 76·2)	72·8 (69·6 to 75·6)	72·3 (69·4 to 75·1)
Central Sulawesi	40·1 (36·2 to 44·1)	45·8 (42·1 to 49·5)	51·9 (48·2 to 55·2)	56·9 (54·2 to 59·8)	58·9 (56·5 to 61·2)	61·6 (59·6 to 63·7)	64·8 (62·1 to 67·6)	68 (64·5 to 71·4)	67·9 (64·6 to 71·2)	67·2 (63·8 to 70·5)
Southeast Sulawesi	40·2 (36·4 to 44·5)	42·9 (39·2 to 47·1)	47·1 (43·6 to 50·9)	53·2 (50·3 to 56·3)	58·1 (55·9 to 60·2)	61·9 (59·8 to 64·1)	65·3 (62·5 to 67·8)	68·2 (64·7 to 71·6)	67·9 (64·5 to 71·2)	67·4 (64·2 to 70·9)
North Sulawesi	47·1 (42·9 to 51·5)	53·2 (49·5 to 56·9)	57·5 (54 to 61·3)	61·4 (58·5 to 64·4)	64·1 (62 to 66·1)	66·8 (64·8 to 68·7)	68·6 (66 to 71)	71 (67·8 to 74·4)	70·6 (67·5 to 73·9)	70·1 (66·9 to 73·2)
West Sumatra	30·9 (27 to 35·2)	44·9 (41·1 to 48·6)	52·8 (49·1 to 56·1)	59·4 (56·5 to 62·2)	64·3 (62·2 to 66·2)	67·3 (65·2 to 69·5)	69·5 (67 to 71·9)	73·2 (70 to 76·1)	72·8 (69·9 to 75·5)	72·2 (69·4 to 75·2)
South Sumatra	45·2 (40·8 to 49)	50·8 (47 to 54·3)	56·9 (53·5 to 60·3)	63·3 (60·7 to 66)	66·6 (64·5 to 68·5)	69·4 (67·4 to 71·4)	71·6 (69·1 to 73·9)	73·7 (70·3 to 76·7)	73·3 (70·1 to 76)	72·6 (69·4 to 75·5)

North Sumatra	46·5 (42·6 to 50·4)	52·7 (48·8 to 56·7)	57·5 (53·9 to 61·5)	61·5 (58·6 to 64·2)	64·8 (62·6 to 67)	67 (65 to 69·1)	69·4 (66·9 to 71·8)	71·8 (68·5 to 75)	71·4 (68·4 to 74·4)	70·8 (67·5 to 73·9)
Yogyakarta	44·8 (40·3 to 49·6)	55 (50·8 to 59)	61·5 (57·7 to 65·3)	65·9 (62·9 to 68·8)	68·1 (66 to 70·1)	69·4 (67·4 to 71·3)	71·6 (69·1 to 74)	73·5 (70·3 to 76·5)	73·3 (70·1 to 76)	72·3 (69·2 to 75·1)
Laos	41 (36·2 to 45·9)	41·4 (37·4 to 45·6)	44 (39·8 to 48·1)	48·8 (45·2 to 52·7)	54·6 (51·7 to 57·4)	60 (58 to 62·1)	67 (64·9 to 69·2)	70·9 (68·1 to 73·6)	71·1 (68·3 to 73·9)	70·4 (67·4 to 73·2)
Malaysia	57·5 (54·4 to 60·6)	65·4 (65·1 to 65·7)	73·5 (73·1 to 73·8)	73·7 (71·4 to 76)	74·5 (74·4 to 74·7)	75·6 (75·5 to 75·7)	76·4 (75·5 to 77·3)	77·1 (77 to 77·2)	78·1 (78 to 78·2)	75·7 (75·2 to 76·2)
Maldives	36·4 (32 to 40·9)	42 (38·4 to 45·8)	50·8 (48·5 to 53·3)	55 (53·9 to 56)	65·4 (64·6 to 66·2)	72·8 (71·9 to 73·7)	79·3 (78·6 to 80·1)	81·9 (80·9 to 83)	82 (81 to 83·1)	81·2 (79·7 to 82·6)
Mauritius	52·6 (51·6 to 53·5)	59·9 (59·3 to 60·5)	66·1 (65·6 to 66·7)	71 (70·5 to 71·5)	74·1 (73·6 to 74·5)	75·5 (75 to 75·9)	77·8 (77·3 to 78·2)	77·7 (77·1 to 78·3)	78·5 (77·9 to 79·1)	76·9 (76·1 to 78·1)
Myanmar	35·8 (30·9 to 40·7)	43·3 (39·6 to 46·9)	48 (44·7 to 51·6)	53·6 (50·5 to 56·6)	58·1 (55·2 to 60·8)	61·4 (59·4 to 63·6)	67·6 (65·5 to 69·8)	72·2 (69·8 to 74·2)	72 (69·6 to 74·1)	71·2 (68·7 to 73·5)
Philippines	58·8 (55·3 to 61·8)	63·4 (61·9 to 64·7)	65·7 (64·6 to 66·7)	69 (68·1 to 69·8)	71·8 (70·9 to 72·6)	73·8 (73 to 74·7)	74 (73·2 to 74·8)	74·7 (73·8 to 75·8)	75·2 (74·2 to 76·2)	72·2 (70·6 to 73·8)
Seychelles	62·9 (60·6 to 65)	66 (65 to 67)	69·4 (68·4 to 70·3)	74·6 (73·7 to 75·5)	75·5 (74·6 to 76·3)	76·6 (76 to 77·3)	77 (76·4 to 77·7)	76·9 (75·9 to 77·9)	78·3 (77·4 to 79·1)	76·5 (75·5 to 77·4)
Sri Lanka	54·1 (53 to 55)	64 (63·8 to 64·3)	67·4 (67·2 to 67·7)	71·2 (71 to 71·4)	74·1 (73·3 to 74·9)	76·5 (76·3 to 76·6)	78·2 (78·1 to 78·4)	80·4 (77·9 to 82·9)	81·5 (78 to 86)	79·7 (76·8 to 83·1)
Thailand	53·9 (50·8 to 56·9)	63·2 (61·6 to 64·9)	67·4 (65·8 to 69)	70·6 (69 to 72)	74·6 (73·2 to 75·8)	75·1 (73·7 to 76·7)	79·1 (77·8 to 80·3)	81·2 (79·7 to 82·6)	81·2 (79·2 to 83·3)	80·3 (77·8 to 82·6)
Timor-Leste	42·7 (37·5 to 47·9)	47·8 (44·2 to 51·2)	49·3 (46·1 to 52·5)	51·5 (48·5 to 54·2)	59·7 (57·5 to 61·9)	65·8 (63·8 to 67·7)	70·3 (68·8 to 71·9)	71·6 (69·5 to 73·7)	71·7 (69·6 to 73·8)	70·5 (68·2 to 72·8)
Viet Nam	50·3 (44·7 to 55·3)	62 (58·5 to 65·3)	63·6 (60·7 to 66·1)	69·9 (67·3 to 72·3)	73·2 (71 to 75)	76·4 (74·8 to 77·8)	77·4 (75·9 to 78·9)	78·7 (76·9 to 80·6)	78·8 (77 to 80·8)	78·3 (76·5 to 80·3)
Sub-Saharan Africa	43·9 (40·3 to 47·3)	47·4 (45 to 49·9)	50·8 (49·1 to 52·5)	54·1 (52·9 to 55·4)	55·6 (54·7 to 56·6)	54·5 (53·1 to 55·9)	60·5 (59·4 to 61·6)	66 (64·3 to 67·5)	64·9 (63·1 to 66·5)	64·1 (62·4 to 65·5)
Central sub-Saharan Africa	44 (39·4 to 48·6)	48·7 (45·5 to 52)	51·3 (48·3 to 54·4)	53·8 (51·2 to 56·3)	55 (53·2 to 56·9)	54·6 (52·7 to 56·5)	59·8 (57·9 to 61·9)	65·3 (62·7 to 67·8)	64·5 (62·1 to 66·8)	63·8 (61·5 to 66)
Angola	45·3 (40·7 to 50·1)	47·8 (44·3 to 51·2)	49·7 (45·9 to 53·4)	51·1 (47·7 to 54·3)	52·2 (49·4 to 54·9)	55 (53·1 to 57)	62·3 (59·8 to 64·7)	66·3 (63 to 69·5)	65·3 (62·1 to 68·3)	63·7 (60·8 to 66·6)
Central African Republic	45·3 (40·2 to 50·2)	47·3 (43·4 to 51·9)	49·9 (46·6 to 53·3)	51·1 (48·6 to 53·6)	50·3 (48·2 to 52·6)	45 (42 to 48·1)	50·4 (47 to 54)	55·8 (51·6 to 59·6)	54·6 (50·7 to 57·9)	55·2 (51·2 to 58·6)
Congo (Brazzaville)	39·3 (34·1 to 43·8)	44·8 (41·8 to 47·8)	48·6 (45·2 to 52·2)	55·4 (52·9 to 58·1)	56·9 (54·9 to 59·2)	53·4 (51·3 to 55·3)	60·3 (58·1 to 62·3)	63·8 (60·9 to 66·6)	63·2 (60·5 to 65·7)	63·1 (60·4 to 65·6)
DR Congo	44·2 (39·1 to 49·1)	49·6 (46·2 to 53·1)	52·1 (48·8 to 55·6)	54·5 (51·7 to 57·1)	56 (54·1 to 57·9)	55·3 (53·1 to 57·6)	59·7 (57·8 to 61·8)	65·7 (63 to 68·4)	65·1 (62·6 to 67·5)	64·5 (62·3 to 67)
Equatorial Guinea	32·8 (27·9 to 37·5)	42·1 (38·7 to 45·6)	48·3 (44·9 to 52)	53·7 (50·4 to 57)	54·5 (51·7 to 57·6)	58·6 (54·9 to 62·1)	62·1 (57·6 to 66·7)	65·8 (60·5 to 70·7)	63·7 (59 to 67·8)	63·7 (58·9 to 67·7)
Gabon	36·1 (30·4 to 42·2)	49·6 (45·6 to 53·5)	55·6 (51·9 to 59·4)	62·5 (59·8 to 65·2)	64·3 (62·3 to 66·5)	61 (58·2 to 63·7)	64·7 (61·7 to 67·6)	68·7 (65 to 72·2)	67·5 (64·2 to 70·6)	67·3 (64 to 70·2)
Eastern sub-Saharan Africa	40·8 (37·2 to 44·2)	46 (43·7 to 48·5)	49·3 (47·6 to 51·1)	52·3 (51 to 53·6)	53·1 (52 to 54·3)	53·3 (51·6 to 54·9)	61·7 (60·5 to 62·8)	67 (65·3 to 68·5)	65·9 (64·2 to 67·4)	64·5 (62·9 to 66)
Burundi	39·5 (33·8 to 45)	44·2 (40·6 to 47·8)	46·6 (43·7 to 49·8)	50·1 (47·6 to 52·8)	51·2 (48·5 to 53·9)	48·1 (45·7 to 50·6)	61·1 (59·1 to 63·1)	66·4 (64 to 68·7)	65·6 (63·2 to 68)	64·9 (62·6 to 67·2)
Comoros	45·7 (41·8 to 49·5)	48·2 (45·1 to 51·4)	52·2 (49·3 to 54·9)	55·5 (52·6 to 58·3)	59·6 (57·1 to 62·1)	62·2 (59·9 to 64·7)	66·7 (64·7 to 68·9)	69·3 (66·8 to 71·6)	69·1 (66·6 to 71·2)	68·2 (65·8 to 70·2)
Djibouti	60·4 (56·4 to 64)	59·4 (55·6 to 63)	61·3 (57·9 to 64·7)	63·6 (60·7 to 66·5)	63·7 (60·6 to 66·4)	62·6 (59 to 66·1)	64·7 (60·9 to 68·6)	68·6 (64·4 to 72·1)	67·1 (63·4 to 70·2)	67 (63·4 to 70)
Eritrea	41·4 (36·4 to 46·4)	44·7 (40·9 to 48·8)	46 (37·5 to 50·5)	48·4 (46·3 to 50·6)	52·3 (50·4 to 54·2)	58·8 (56 to 61·9)	62·8 (59·5 to 66·1)	66 (62·4 to 69·3)	66 (62·4 to 69·3)	64·8 (61·5 to 67·8)
Ethiopia	36·2 (32·1 to 40)	43·2 (40·4 to 45·9)	44·6 (42·1 to 47·1)	46·3 (44·3 to 48·2)	49 (47·7 to 50·5)	52·9 (51·3 to 54·5)	64·9 (63·6 to 66·1)	70·1 (68·1 to 72)	68·9 (66·9 to 70·7)	67·5 (65·7 to 69·2)
Addis Ababa	50·9 (46·7 to 55·2)	53·2 (49 to 57·1)	54·8 (51·4 to 58·3)	57·3 (53·8 to 60·4)	55·8 (53·3 to 58·3)	52 (48·3 to 55·5)	67·2 (64·7 to 69·5)	71·1 (67·6 to 74·3)	68·8 (65·7 to 71·5)	66·7 (64·1 to 69)
Afar	17·5 (13·6 to 21·6)	31·3 (28·1 to 34·8)	33·3 (30·1 to 37)	33·3 (29·9 to 36·5)	37·2 (34·4 to 40)	37·2 (34·8 to 39·6)	52·3 (49·9 to 54·5)	58·7 (55·4 to 61·8)	58·1 (55 to 61)	57·3 (54·5 to 60·1)
Amhara	35·5 (30·8 to 39·9)	43·5 (40·3 to 46·9)	44·9 (41·9 to 48)	46·6 (44·1 to 49·3)	48·8 (47 to 50·7)	52·7 (49·9 to 55·4)	66·4 (64·2 to 68·3)	72·4 (69·7 to 74·6)	71·5 (69 to 73·5)	70·3 (68 to 72·1)
Benishangul-Gumuz	22·5 (18·6 to 26·5)	33·6 (29·9 to 37·2)	34·5 (31 to 38)	34·7 (31·5 to 38)	37·3 (34·3 to 40·3)	40·8 (38 to 43·6)	54·8 (52·3 to 57·3)	62·1 (58·9 to 65·5)	62·5 (59·2 to 65·9)	62·7 (59·4 to 66·4)
Dire Dawa	42·4 (37·5 to 46·9)	44·3 (40·7 to 48)	45·3 (41·8 to 49)	48·1 (44·7 to 51·7)	51 (47·7 to 54·3)	53·7 (49·4 to 57·9)	67·7 (64·7 to 70·7)	72·6 (69·4 to 74·7)	69·8 (67·1 to 71·5)	67·5 (65·1 to 69·1)
Gambella	38·8 (33·5 to 43·6)	44·8 (41·1 to 48·6)	46·1 (42·7 to 49·6)	46·9 (43·8 to 50·2)	49·6 (46·7 to 52·7)	53·8 (49·6 to 58)	65 (61·3 to 68·4)	72 (69·5 to 74)	68·6 (66·5 to 70·3)	65·9 (64·1 to 67·5)
Harari	34·1 (29·9 to 38·5)	36·5 (32·9 to 40·1)	39·2 (35·8 to 42·8)	43·8 (40·4 to 47·1)	49·4 (46·5 to 52·4)	54·2 (50·3 to 58·3)	66·9 (63·3 to 70·2)	71·7 (67·9 to 74·7)	68·6 (65·5 to 70·9)	66 (63·5 to 68)
Oromia	36·3 (31·8 to 40·6)	43·2 (40·1 to 46·5)	44·7 (41·6 to 47·7)	46·7 (44·1 to 49·1)	49·9 (47·9 to 52·1)	54·8 (52·7 to 57)	65·4 (63·6 to 67·3)	70·5 (68·1 to 72·8)	69·1 (66·6 to 71·3)	67·7 (65·4 to 69·8)
Somali	37·1 (32·5 to 41·7)	43·6 (40·1 to 47·4)	44·9 (41·3 to 48·6)	46·8 (43·3 to 50)	48·3 (45·7 to 51)	49·5 (47·1 to 52·2)	60·5 (57·9 to 63)	64·5 (61·2 to 67·8)	63·1 (59·9 to 65·9)	61·6 (58·8 to 64·4)
Southern Nations, Nationalities, and Peoples	38·5 (34·3 to 42·8)	43·7 (40·3 to 47·1)	45 (41·8 to 48·1)	46·2 (43·5 to 49·1)	49·4 (47·3 to 51·6)	54·9 (51·9 to 57·5)	64·1 (61·9 to 66·1)	69·3 (66·4 to 72·1)	68·2 (65·3 to 70·7)	66·9 (64·4 to 69·2)
Tigray	33·5 (29 to 37·5)	41·4 (37·9 to 44·7)	42·8 (39·6 to 46·1)	43·7 (40·6 to 46·8)	49 (46·6 to 51·5)	54·5 (51·8 to 57·3)	66·7 (64·4 to 69·1)	71·4 (68·2 to 74·1)	70·2 (67·3 to 72·6)	68·9 (66·1 to 71·4)
Kenya	48·4 (45·3 to 51·5)	55·3 (52·9 to 57·9)	59·2 (56·5 to 61·6)	63·2 (61·3 to 65·3)	63·5 (61·3 to 65·3)	56 (52·7 to 59·5)	62·7 (61·3 to 64·3)	69·2 (67 to 71·6)	67·7 (65·8 to 69·6)	67·2 (65·2 to 68·9)
Baringo	49·5 (44·8 to 54·3)	53·7 (49·2 to 57·8)	54·4 (50·3 to 58·3)	57·1 (53·1 to 60·9)	59·5 (55·1 to 63·4)	59·8 (55·6 to 63·4)	63·9 (60·5 to 67·2)	68·6 (64·4 to 72·2)	67·3 (63·5 to 70·4)	66·8 (63·4 to 69·7)
Bomet	65·9 (61·5 to 69·9)	69·7 (65·4 to 73·1)	70·4 (66·6 to 73·9)	71·8 (68·2 to 74·9)	70·8 (66·3 to 74)	60·1 (53·8 to 66·6)	65·4 (60·7 to 69·5)	72·3 (68·4 to 75·2)	70·9 (67·7 to 73·2)	70·7 (68·1 to 72·9)
Bungoma	46·4 (41·5 to 51·2)	54·4 (50·4 to 58·5)	59·2 (54·8 to 63·3)	62·4 (58·4 to 66)	64·6 (60·6 to 68·6)	61·3 (56·5 to 65·6)	67·5 (64·2 to 70·7)	71·6 (67·9 to 75)	70·4 (67 to 73·3)	69·6 (66·3 to 72·4)
Busia	31·8 (26·5 to 36·7)	45·1 (41·3 to 49)	52·2 (47·7 to 56·2)	60·6 (56·3 to 64·5)	60·6 (55·9 to 65·2)	52·2 (46·8 to 57·7)	61·5 (57 to 66·2)	69·3 (64·9 to 73·4)	67·3 (63·5 to 70·7)	66 (62·4 to 69·1)
Elgeyo Marakwet	55·6 (51 to 60)	59·1 (54·6 to 63·3)	58·4 (53·8 to 62·6)	60·2 (55·9 to 64·2)	63·3 (58·8 to 67·7)	61·2 (56·7 to 65·9)	65·3 (62 to 68·4)	68·6 (64·8 to 72·3)	67·7 (64 to 71·1)	67·8 (64·2 to 71·1)
Embu	41·8 (36·3 to 46·8)	51·6 (47·7 to 55·3)	60·1 (55·7 to 64·3)	66·7 (62·8 to 70·4)	69·3 (65·2 to 73·1)	64·7 (58·6 to 69·9)	68·1 (63·5 to 72·1)	73 (68·4 to 75·8)	71·5 (67·9 to 74)	71·2 (67·8 to 73·3)
Garissa	51·4 (46·6 to 56·2)	53·9 (49·2 to 58·6)	57·3 (52·6 to 62)	58·5 (54 to 63)	60·5 (56 to 65)	60·4 (55·3 to 65·7)	63·7 (59·7 to 67·5)	67·6 (64 to 71·4)	65·6 (62·5 to 68·8)	64·9 (61·9 to 67·9)
Homa Bay	28·9 (24·5 to 33·1)	42·4 (39·1 to 45·6)	45·7 (42·1 to 49·6)	52·3 (48·8 to 55·7)	52·5 (47·3 to 57·6)	37·5 (32·7 to 42·9)	48 (43·5 to 52·6)	61·2 (55·6 to 66·4)	60·3 (55·8 to 64·2)	60·8 (57 to 64·2)
Isiolo	35·7 (30·7 to 41·1)	37·9 (34·3 to 41·7)	40·2 (36·2 to 44·2)	47·1 (43·2 to 51·1)	53·7 (49·7 to 58)	54 (47·8 to 60)	59·8 (54·6 to 65·1)	66·5 (61·1 to 71)	64·6 (60·1 to 68·3)	64·1 (60·2 to 67·2)
Kajiado	56·9 (52·1 to 61·4)	60·8 (56·3 to 64·8)	61·3 (56·9 to 65·2)	63·6 (59·1 to 67·4)	64·7 (59·7 to 69·6)	59·2 (53·8 to 64·9)	64·6 (60·7 to 68·8)	69·3 (65·1 to 73·5)	67·5 (63·7 to 70·8)	67·1 (63·9 to 70)
Kakamega	46 (41·5 to 50·4)	52·7 (48·5 to 56·9)	57·1 (52·9 to 60·8)	61·3 (57·3 to 65)	59·9 (56 to 63·7)	53·4 (48 to 58·8)	61·7 (57·8 to 65·7)	69·6 (65·8 to 73·2)	67·8 (64·6 to 70·7)	66·9 (63·8 to 69·7)
Kericho	66·4 (61·9 to 70·4)	66·8 (62·2 to 70·7)	67·3 (62·8 to 70·9)	68·2 (64·3 to 71·8)	69·2 (65 to 73)	63·2 (57·2 to 68·8)	68·9 (64 to 73·2)	72·8 (68·4 to 75·8)	71·4 (67·8 to 73·8)	71·1 (67·8 to 73·3)
Kiambu	58·3 (53·8 to 63)	63·3 (59 to 67·3)	66·2 (61·7 to 70·4)	68·1 (64·2 to 71·9)	67·6 (63·9 to 71·3)	60·6 (56·2 to 65·4)	66·7 (63·6 to 70)	71·3 (67·7 to 75·1)	69·2 (66·2 to 72·1)	68·5 (65·7 to 71)
Kilifi	37 (32·5 to 41·7)	45·4 (41·8 to 48·7)	50·3 (46·6 to 54)	56·2 (52·2 to 60)	58·9 (54·8 to 63·2)	55·9 (50·6 to 61·4)	63·5 (59·6 to 67·4)	70·4 (66·1 to 74·1)	68·7 (65·4 to 71·7)	68·2 (65·1 to 70·8)
Kirinyaga	44·9 (40·2 to 49·7)	52·1 (48·1 to 56·1)	57·3 (52·9 to 61·6)	66·6 (62·3 to 70·4)	68·8 (64·6 to 72·7)	63·8 (58·4 to 68·8)	68·1 (63·9 to 71·9)	71·9 (67·9 to 75·2)	70·4 (66·8 to 73·2)	69·8 (66·2 to 72·4)
Kisii	49·2 (44·2 to 54·1)	51·7 (47·4 to 56·2)	54·4 (49·9 to 58·5)	57·4 (53·4 to 61·5)	58 (54·1 to 61·9)	53·5 (49·7 to 57·3)	60·6 (58 to 63·4)	65·8 (62·4 to 69·6)	64·9 (61·6 to 68·6)	64·6 (61·5 to 68)
Kisumu	50·5 (45 to 55·7)	57·7 (53·7 to 61·6)	59·6 (55·7 to 63·3)	62·5 (58·5 to 66·3)	61 (56·3 to 65·4)	47·2 (41·5 to 53·3)	55·8 (51 to 60·4)	65·7 (60·2 to 70·8)	63·4 (59·4 to 67)	63·1 (59·7 to 65·9)
Kitui	45·5 (40·3 to 50·8)	54·1 (50·1 to 57·9)	59·2 (54·9 to 63·2)	64·8 (60·9 to 68·3)	66·2 (62·5 to 69·8)	59·4 (53·5 to 64·7)	67·6 (63·6 to 71·6)	73·3 (69·8 to 76)	71·9 (68·8 to 74·1)	71·5 (68·6 to 73·5)
Kwale	41·4 (36·9 to 46·2)	45·3 (41·4 to 49·1)	51 (46·7 to 55·1)	56·4 (51·9 to 60·5)	58·2 (54 to 62·7)	55 (50·4 to 60)	62 (58·4 to 65·9)	68·9 (64·6 to 73)	67·7 (64·1 to 71·1)	67·3 (63·9 to 70·6)
Laikipia	61·9 (56·6 to 66·9)	65·7 (60·6 to 70·3)	68·2 (63·7 to 72·1)	70·9 (67 to 74·3)	69·5 (65·1 to 73·6)	60·7 (54·6 to 66·6)	65·8 (60·5 to 70·3)	71·6 (67 to 75·3)	70·1 (66·4 to 73)	69·9 (66·6 to 72·4)
Lamu	32·2 (26·7 to 37·9)	41·6 (37·4 to 45·9)	50·1 (45·5 to 54·4)	60·1 (55·6 to 64·5)	59·5 (54·7 to 64·2)	56·8 (50·4 to 63)	64 (58·9 to 68·9)	69·6 (65·1 to 73·5)	67·7 (63·9 to 71)	67·1 (63·2 to 70)
Machakos	57 (52·2 to 62)	61 (57 to 64·8)	64·2 (60·1 to 68)	68·3 (64·5 to 71·8)	69·6 (66 to 73·1)	61 (55·5 to 66·5)	67 (63·1 to 70·7)	72·7 (69·2 to 75·5)	71·2 (67·9 to 73·5)	70·8 (67·9 to 72·9)
Makueni	45·6 (39·7 to 51·2)	58·7 (54·9 to 62·5)	62·8 (58·9 to 66·5)	67·5 (64 to 70·9)	68·1 (63·8 to 71·6)	54·4 (47·5 to 61·3)	61·8 (57·8 to 65·8)	71·5 (68·1 to 74·1)	70·6 (67·7 to 72·6)	70·7 (67·9 to 72·5)
Mandera	53 (48·3 to 57·4)	56·8 (52·1 to 60·9)	58·4 (54·2 to 62·6)	59·4 (55·2 to 63·6)	59 (54·6 to 63·4)	58·7 (53·7 to 63·9)	60·4 (57 to 64·1)	64·7 (60·6 to 68·7)	62·3 (58·9 to 65·5)	61·2 (58·6 to 64·1)
Marsabit	53·5 (48·6 to 58·3)	54 (49·8 to 58·1)	57·7 (53·4 to 61·9)	61·2 (57·1 to 65)	63·8 (59·1 to 68·1)	61·5 (55 to 67·2)	63·7 (58·6 to 68·6)	68·6 (63·7 to 72·7)	67·1 (62·7 to 70·6)	66·5 (62·5 to 69·9)

Meru	55·1 (50·5 to 59·5)	60·6 (56·7 to 64·4)	64 (59·7 to 67·8)	68 (64·2 to 71·7)	69 (65 to 72·7)	62·6 (56·9 to 67·6)	65·3 (61·4 to 68·8)	70·2 (66·1 to 73·6)	68·7 (64·8 to 71·4)	68·4 (65·1 to 70·9)
Migori	36·3 (31 to 41·3)	46·1 (42·2 to 49·8)	49·7 (45·7 to 53·6)	52·6 (48·9 to 56·1)	53·3 (48·9 to 57·6)	42·8 (37·4 to 48·7)	51·1 (46·6 to 56)	62·4 (56·9 to 67·9)	61·1 (56·8 to 65·3)	61 (57·2 to 64·6)
Mombasa	51·8 (47 to 56·8)	53·9 (49·4 to 58)	59·3 (54·9 to 63·6)	64·7 (59·8 to 68·9)	63·1 (57·7 to 68·4)	53·5 (48 to 59·7)	63·2 (58·5 to 67·4)	71·4 (66·9 to 74·5)	68·6 (65 to 71)	67·6 (64·7 to 69·6)
Murang'a	49·2 (44·8 to 53·6)	59·9 (55·7 to 63·8)	64·5 (60·4 to 68·3)	69·6 (65·6 to 73·1)	70·5 (67·1 to 73·7)	64·5 (59·2 to 69·5)	67·1 (63·7 to 70·9)	71·8 (67·8 to 75·3)	70·5 (67 to 73·3)	70·1 (66·9 to 72·6)
Nairobi	51 (45·7 to 55·9)	56·3 (52 to 60·5)	63·5 (59·6 to 67·3)	67·3 (63·6 to 70·5)	64·6 (60·6 to 68·8)	54·8 (50·6 to 59·3)	63·7 (61·2 to 66·2)	68·8 (65·5 to 71·9)	66·6 (63·9 to 69·1)	65·9 (63·4 to 68·3)
Nakuru	68·1 (63·9 to 72·1)	69·3 (65 to 73·1)	69·9 (66 to 73·7)	70·9 (67·2 to 74·2)	70·4 (66·4 to 74)	62·8 (57·2 to 68·3)	67·3 (63·3 to 71)	72·6 (69·1 to 75·3)	70·6 (67·5 to 72·9)	70·1 (67·6 to 72·1)
Nandi	63·3 (58·1 to 67·7)	66·6 (62·2 to 70·4)	68·1 (63·9 to 71·8)	69·7 (65·8 to 73·2)	70·2 (66·1 to 73·9)	65·2 (60·1 to 70·1)	71 (67 to 74·4)	74·5 (71 to 77·1)	72·9 (69·8 to 75)	72·3 (69·6 to 74·5)
Narok	54·9 (49·9 to 59·8)	59·6 (54·7 to 64·1)	60·5 (56 to 64·6)	64·3 (60·3 to 68·3)	62·7 (58 to 67·3)	58 (52·6 to 63·2)	64·5 (60·4 to 68·4)	70·1 (65·8 to 73·7)	68 (64·4 to 71)	67·3 (64·1 to 69·8)
Nyamira	50·5 (45·6 to 55·1)	59 (54·6 to 63·3)	61·5 (57·1 to 65·5)	63·6 (59·4 to 67·6)	64 (60·2 to 67·9)	55·7 (50·7 to 60·3)	58·8 (55·9 to 61·7)	64·1 (60·1 to 68·5)	63·2 (59·6 to 66·9)	63 (59·5 to 66·6)
Nyandarua	53·9 (48·9 to 58·5)	59·5 (55 to 63·9)	63·8 (58·7 to 68·1)	67·7 (63·5 to 71·8)	68·1 (64 to 72·3)	61·7 (56·6 to 66·6)	63·2 (59·9 to 66·5)	68·3 (64·3 to 72·6)	66·8 (63·2 to 70·5)	66·3 (62·9 to 69·5)
Nyeri	45·4 (40·1 to 50·5)	57 (52·6 to 61·3)	61·9 (57·4 to 66)	65·9 (61·9 to 69·8)	65·1 (61·2 to 69·3)	56·3 (51·7 to 61·2)	61·8 (58·2 to 65·7)	66·9 (62·3 to 72·2)	66·1 (61·9 to 70·1)	65·9 (62·2 to 69·6)
Samburu	64 (59·4 to 68·5)	65·4 (60·5 to 69·7)	65·3 (61·1 to 69·4)	65·5 (61·4 to 69·2)	64·2 (59·5 to 69·1)	59 (53·1 to 64·5)	62·7 (58 to 67·2)	67·9 (63·2 to 71·8)	65·9 (62 to 69·2)	65·4 (61·7 to 68·2)
Siaya	41·2 (36·7 to 45·9)	47·5 (43·8 to 51·2)	52·2 (48·2 to 55·6)	56·1 (52·2 to 60·1)	54·2 (49·9 to 58·8)	42·5 (37·4 to 48)	53·9 (49·6 to 58·5)	65·5 (60·5 to 69·4)	64·6 (60·5 to 67·9)	64 (60·7 to 66·8)
Taita Taveta	48·8 (43·6 to 53·6)	55·6 (51·2 to 59·8)	57·5 (53 to 61·8)	63·9 (59·2 to 67·8)	64·8 (59·7 to 69·9)	56·3 (50·5 to 62·1)	60·2 (56·2 to 65)	68·4 (63·7 to 73·1)	67·2 (63·5 to 71)	67 (63·3 to 70·3)
Tana River	46·8 (41·4 to 52·2)	53·7 (49·2 to 58·1)	56 (51 to 61)	57·7 (53·2 to 62·4)	57·7 (53·6 to 62·3)	51·5 (47·4 to 56·2)	57·3 (54·1 to 60·8)	64 (59·9 to 67·7)	62·8 (59·3 to 66·4)	62·4 (58·9 to 66)
Tharaka Nithi	43·1 (37·4 to 48·8)	57·6 (53·5 to 61·9)	60·1 (55·2 to 64·6)	63·5 (59·4 to 67·4)	63·4 (59·5 to 67·4)	58·6 (54 to 63·3)	67·2 (64·4 to 70·2)	70·3 (66·3 to 74·1)	69·6 (66·1 to 73·3)	69·6 (65·7 to 73·1)
Trans Nzoia	63·3 (58·4 to 67·6)	64·3 (59·9 to 68·2)	65·7 (61·6 to 69·6)	67·1 (63·3 to 70·6)	67·6 (63 to 71·9)	60·3 (54·3 to 66·2)	65·6 (61·1 to 70·2)	71·5 (67·3 to 74·7)	70·1 (66·7 to 72·9)	69·8 (66·6 to 72)
Turkana	61·6 (56·8 to 66)	61·2 (56·7 to 64·9)	60·3 (56 to 64·3)	59·7 (55·6 to 63·7)	59·7 (54·2 to 64·9)	51·6 (45·6 to 58·3)	58·3 (52·9 to 63·8)	67·3 (61·7 to 72)	65 (60·7 to 68·7)	64·5 (61·1 to 67·5)
Uasin Gishu	66·3 (61·6 to 70·3)	67·1 (62·4 to 71)	67·8 (63·5 to 71·9)	68·3 (64 to 72)	67·3 (62·6 to 71·4)	60·9 (55·7 to 66)	66·7 (62·6 to 70·6)	72·1 (67·9 to 75)	70·5 (67·3 to 72·8)	70 (67 to 72)
Vihiga	41·5 (36·2 to 46)	57 (53·2 to 61)	60·8 (56·2 to 65·2)	63·5 (59·3 to 67·4)	61·6 (57·1 to 66)	54·7 (49·5 to 59·7)	60·4 (56·6 to 64·7)	67·4 (63·2 to 71·8)	66·4 (62·6 to 70·3)	65·8 (62 to 69·5)
Wajir	42·9 (38·1 to 47·9)	47·9 (43·5 to 52·1)	52 (47·5 to 56·2)	54·8 (50·6 to 58·7)	57·7 (53·6 to 61·7)	58·5 (54·2 to 62·8)	60·6 (56·9 to 64·3)	64·5 (60·6 to 68·8)	63·1 (59·6 to 66·7)	62·7 (59·2 to 66·2)
West Pokot	52·8 (48 to 58)	55·9 (51·4 to 60·4)	56 (51·4 to 60·5)	55·5 (51·5 to 59·5)	57·2 (53·2 to 61·2)	58·8 (54·4 to 62·9)	62·9 (59·5 to 66·2)	67·5 (63·5 to 71·4)	65·8 (62·1 to 69·1)	65·4 (62·2 to 68·5)
Madagascar	40·4 (37 to 44·1)	46·3 (43·6 to 49)	51·7 (49·5 to 53·7)	56·5 (54·7 to 58·3)	57·4 (55·9 to 58·8)	60 (58·3 to 61·9)	62·8 (60·5 to 65·3)	65·3 (62·6 to 68·2)	64·1 (61·7 to 66·4)	63·9 (61·7 to 66·2)
Malawi	38·8 (33·8 to 43·7)	40·4 (36·9 to 44)	44·2 (41·8 to 46·6)	50·4 (48·4 to 52·4)	50·4 (48·5 to 52·3)	46·3 (43·2 to 49·2)	58·5 (56·3 to 60·4)	65·4 (62·2 to 68·4)	64·6 (61·6 to 67·5)	62·1 (59·5 to 64·5)
Mozambique	42·1 (37·1 to 46·9)	46·2 (42·5 to 49·8)	49·5 (46·6 to 52·3)	51·8 (49·6 to 54)	53·2 (51·4 to 54·8)	54·7 (52·8 to 56·7)	56 (54 to 58·2)	62·1 (59·3 to 65·1)	61·7 (58·6 to 64·8)	59·9 (57·4 to 62·4)
Rwanda	32·1 (26·1 to 37·7)	43 (40·2 to 45·8)	46·7 (44 to 49·4)	50 (47·7 to 52·4)	51·8 (50 to 53·6)	52 (50·1 to 54)	65·9 (63·9 to 67·7)	69·9 (67 to 72·5)	69·8 (67 to 72·3)	67·5 (65·2 to 69·7)
Somalia	45 (40·5 to 49·7)	45·5 (42 to 49·2)	46·8 (43·6 to 50·6)	50·8 (47·9 to 54)	50·9 (48·4 to 53·5)	53·2 (50·7 to 55·8)	53·6 (50·9 to 56·3)	59·5 (55·8 to 63·1)	57·7 (54·4 to 60·7)	56·9 (53·6 to 59·9)
South Sudan	50·3 (45·3 to 55·3)	49·6 (45·7 to 53·3)	51·1 (47·4 to 55·3)	53·3 (49·3 to 57·5)	54·8 (51·6 to 58)	57·3 (53·9 to 60·6)	60·1 (56 to 63·8)	59·2 (54·4 to 63·5)	58·1 (53·8 to 61·9)	58·1 (53·6 to 62)
Uganda	41·5 (35·9 to 47·1)	50·2 (46·7 to 53·6)	54·4 (51·7 to 56·9)	53·8 (51·9 to 55·9)	50·8 (47·4 to 54·4)	51·5 (49·3 to 53·9)	62 (59·7 to 64·2)	68·8 (66·1 to 71·3)	67·4 (64·5 to 70·1)	64·9 (62·2 to 67·3)
Tanzania	41·4 (36·3 to 46·4)	46·2 (43 to 49·6)	50·9 (48·4 to 53·5)	56·8 (54·8 to 58·7)	56·7 (54·8 to 58·4)	54·3 (51·6 to 56·8)	62·2 (60·6 to 64·1)	67·9 (65·5 to 70·1)	66·9 (64·6 to 69·1)	65·9 (63·8 to 67·8)
Zambia	46·1 (41·1 to 50·8)	49·3 (45·5 to 53·1)	54·7 (51·8 to 57·3)	57·1 (55·1 to 59·2)	52·5 (50·1 to 54·6)	46 (43·2 to 48·9)	59·1 (56·8 to 61·4)	64·7 (61·2 to 68·3)	63·6 (60·2 to 66·8)	61·4 (58·4 to 64·2)
Southern sub-Saharan Africa	52·5 (49·3 to 55·5)	56·3 (54·2 to 58·3)	59·6 (58·1 to 61)	62·9 (61·7 to 64·1)	67·4 (66·4 to 68·4)	56·3 (54·7 to 57·8)	57·8 (57·1 to 58·5)	66·6 (65·4 to 67·8)	64·5 (63·5 to 65·5)	63 (61·8 to 63·9)
Botswana	52·6 (47·9 to 57)	55·8 (52·7 to 59)	58·3 (55·5 to 61)	63·2 (60·6 to 65·7)	65 (61·8 to 68·4)	50·1 (44·4 to 55·6)	59·7 (56·6 to 62·5)	67·1 (64·8 to 69·3)	66·8 (64·5 to 69·2)	62·9 (60·9 to 65)
Eswatini	43·2 (38·2 to 47·7)	46·7 (43·1 to 50·3)	53·8 (50·4 to 57·3)	60·7 (58 to 63·6)	65·1 (63·1 to 67·2)	50·4 (44·7 to 55·4)	49·7 (45·8 to 53·2)	60·2 (56·2 to 64·7)	58·7 (55·2 to 62·2)	56·1 (53 to 59·2)
Lesotho	52·9 (49·1 to 56·4)	55 (52·3 to 57·7)	56·9 (54 to 59·6)	63·1 (60·7 to 65·4)	65·9 (63·5 to 68·2)	51·2 (46·8 to 55·1)	51·9 (49·2 to 54·5)	54·9 (51·7 to 58·3)	54·2 (51·2 to 57)	52·1 (49·7 to 54·6)
Namibia	53·4 (48·4 to 58)	57·5 (53·7 to 61·4)	60·7 (57·9 to 63·6)	62·1 (60·3 to 64)	65·6 (64 to 67·2)	56·1 (52·6 to 59)	63·6 (61 to 66·2)	67·8 (64·2 to 71·5)	67·1 (63·6 to 70·2)	64 (61·3 to 66·5)
South Africa	52·4 (49·5 to 55·2)	56·2 (54·3 to 58·1)	59·5 (58 to 61)	62·6 (61·3 to 63·9)	68·4 (67·6 to 69·3)	59·2 (58·6 to 59·9)	59 (58·5 to 59·5)	68·5 (67·8 to 69·2)	65·9 (65·2 to 66·5)	64·8 (64 to 65·5)
Eastern Cape	48·3 (43·5 to 52·9)	55·5 (52·1 to 59·1)	59·2 (55·7 to 62·4)	63·8 (60·9 to 66·5)	67·7 (65·8 to 69·6)	56·3 (54·9 to 57·6)	54·5 (53·3 to 55·7)	64·2 (62·8 to 65·4)	59·9 (58·7 to 61)	61·1 (59·5 to 62·5)
Free State	50·6 (46·4 to 54·9)	54·3 (50·8 to 58·1)	57·5 (54 to 61)	60·1 (56·9 to 63·1)	65·8 (63·7 to 68)	53·6 (52·2 to 55)	52·3 (51·3 to 53·2)	65·6 (64·2 to 66·9)	63·1 (61·6 to 64·4)	61·8 (60·2 to 63·4)
Gauteng	56·9 (53·3 to 60·6)	58·8 (55·6 to 62·1)	60·8 (57·5 to 64)	61·6 (58·8 to 64·3)	67·3 (65·3 to 69·3)	61·4 (60 to 62·7)	62·5 (61·4 to 63·7)	70·7 (68·9 to 72·3)	68·8 (67·1 to 70·4)	67·7 (66 to 69·3)
KwaZulu-Natal	50 (45·9 to 54)	52·8 (49·6 to 56·4)	56·7 (53·1 to 59·9)	60·6 (57·6 to 63·4)	67·6 (65·5 to 69·5)	54·7 (53·5 to 56·1)	57·4 (56·7 to 58·1)	70·8 (69·6 to 72)	67·1 (65·8 to 68·2)	65·6 (64·1 to 66·9)
Limpopo	57·4 (53·3 to 61·2)	60·5 (57·2 to 64)	63·8 (60·3 to 67·1)	67·6 (64·5 to 70·5)	74·1 (72 to 76·2)	67·4 (65·7 to 68·8)	59·8 (58·6 to 61)	67 (65·4 to 68·6)	64·8 (63 to 66·5)	61·7 (60 to 63·5)
Mpumalanga	53·1 (49·1 to 57·1)	56·9 (53·4 to 60·5)	59·4 (55·9 to 62·5)	63·7 (60·7 to 66·4)	70·3 (68·3 to 72·2)	61 (59·7 to 62)	58·8 (57·9 to 59·7)	69·4 (67·8 to 71)	67·6 (66 to 69·2)	64·8 (63·2 to 66·3)
North West	52·8 (48·7 to 57)	55·6 (52 to 59·2)	59·5 (56 to 62·9)	62·7 (59·7 to 65·7)	71·6 (69·5 to 73·6)	54·6 (52·4 to 56·6)	50·1 (48·4 to 51·9)	58·4 (55·9 to 61)	58·9 (56·3 to 61·4)	58 (55·2 to 60·5)
Northern Cape	56·1 (51·8 to 60)	59·6 (56·3 to 62·7)	62·2 (58·7 to 65·4)	65·6 (62·8 to 68·4)	66·3 (64·4 to 68·2)	56·6 (55·5 to 57·5)	56·4 (54·8 to 57·9)	65·8 (63·7 to 67·9)	63·8 (61·7 to 66)	61·4 (59·4 to 63·4)
Western Cape	62 (58·5 to 65·2)	64·1 (61 to 67·2)	65·3 (61·9 to 68·2)	65·8 (63·1 to 68·3)	68·8 (67 to 70·6)	68·1 (67·5 to 68·7)	70·5 (69·9 to 71·2)	73·4 (72·2 to 74·4)	70·5 (69·4 to 71·5)	69·5 (68·2 to 70·7)
Zimbabwe	54·8 (50·1 to 59·1)	58·3 (54·7 to 62·1)	61·2 (58·6 to 64·1)	63·6 (61·7 to 65·6)	63·8 (60·5 to 66·7)	47·8 (43·5 to 52·4)	53·5 (51·3 to 55·3)	61 (58·1 to 63·9)	60·2 (57·5 to 62·8)	58 (55·5 to 60·4)
Western sub-Saharan Africa	44·4 (40·7 to 48·1)	46·4 (43·8 to 49)	50 (48·1 to 51·9)	54 (52·4 to 55·5)	55·7 (54·4 to 57·1)	55·8 (54·1 to 57·3)	60·9 (59·3 to 62·5)	65·5 (63·5 to 67·4)	64·7 (62·5 to 66·6)	64·5 (62·5 to 66·3)
Benin	41·5 (35·8 to 47·3)	46 (42·6 to 49·2)	50·2 (47·5 to 52·8)	54·6 (52·4 to 56·8)	57·8 (56·3 to 59·3)	59·9 (58·1 to 61·6)	63·9 (62·3 to 65·6)	67·4 (65·1 to 69·6)	67 (64·7 to 69·2)	65·9 (63·5 to 68)
Burkina Faso	38·1 (33·1 to 43)	40·9 (37·5 to 44·2)	45·9 (43·2 to 48·5)	50·6 (48·3 to 52·8)	52·4 (50·5 to 54·2)	53·8 (51·6 to 56)	59·8 (57·9 to 61·7)	64·2 (61·8 to 66·5)	62·9 (60·2 to 65·1)	63 (60·7 to 65·1)
Cabo Verde	50·3 (47·1 to 53·2)	58·2 (56·9 to 59·5)	59·1 (57·8 to 60·5)	68·5 (67·1 to 69·9)	72·4 (70·9 to 73·8)	73·9 (71·1 to 76·3)	77·6 (76·1 to 79·1)	78·1 (76·2 to 80·1)	77·7 (75·9 to 79·7)	77·8 (75·8 to 79·8)
Cameroon	44·2 (40·6 to 48)	37·2 (35·1 to 39·3)	52·4 (49·8 to 55·1)	55·9 (53·9 to 58·1)	59·8 (58·1 to 61·6)	55·8 (53·6 to 58)	59·3 (56·6 to 62)	65·1 (61·8 to 68·2)	64·4 (61·1 to 67·3)	63·6 (60·6 to 66·1)
Chad	43·4 (38·1 to 48·5)	49·3 (45·4 to 53·6)	50·1 (46·8 to 53·1)	52·1 (49·9 to 54·4)	54·5 (53 to 56·1)	53·8 (51·5 to 55·9)	58 (55·8 to 60·1)	61·2 (57·8 to 64·5)	60 (56·7 to 63·1)	60·5 (56·9 to 63·5)
Côte d'Ivoire	47·5 (43·9 to 51·1)	48·9 (45·7 to 52)	53·8 (51·3 to 56·3)	59·1 (57·2 to 60·9)	58·4 (55·6 to 60·8)	53·7 (50·8 to 56·9)	59·4 (56·8 to 62)	66·6 (63·7 to 69·4)	65·5 (62·7 to 68·1)	65·8 (63·1 to 68·4)
The Gambia	54·8 (49·9 to 59·5)	54·4 (50·5 to 57·8)	57·2 (54·2 to 60·3)	59·2 (56·3 to 62)	61·9 (59·4 to 64·5)	62·7 (60·6 to 64·9)	64·8 (62·6 to 67·1)	67·2 (64·3 to 70)	65·7 (63·2 to 68)	65·9 (63·4 to 68·2)
Ghana	48·7 (45·1 to 52·4)	53·2 (50·4 to 55·9)	56·6 (53·7 to 59·1)	59·2 (56·5 to 61·7)	60·5 (58·1 to 62·7)	60·7 (58·6 to 62·9)	63·9 (62 to 65·8)	68·5 (65·8 to 71·2)	68 (65·4 to 70·4)	67·4 (65 to 69·6)
Guinea	41·5 (36·7 to 46·1)	42·3 (38·2 to 46·3)	44·3 (40·9 to 48)	48 (45·2 to 51)	51·9 (49·9 to 54)	54·8 (52·8 to 56·9)	59·1 (57 to 61·4)	63·3 (60·1 to 66·4)	62 (58·7 to 64·8)	62·2 (58·9 to 65·1)
Guinea-Bissau	32·1 (27·9 to 36·3)	40·4 (36·6 to 44·4)	44·5 (41·3 to 47·7)	49·3 (46 to 52·5)	52·2 (49·3 to 55)	54·2 (51·7 to 56·6)	58·8 (56·3 to 61·4)	62·6 (59·9 to 65·5)	60·7 (58·3 to 62·9)	61·3 (58·8 to 63·8)
Liberia	34·8 (28·6 to 41·2)	43·7 (40 to 47·4)	47·5 (44·2 to 50·7)	51·5 (48·9 to 54·1)	50·7 (48·5 to 52·9)	55 (53·1 to 57)	61·2 (59 to 63·4)	64·9 (61·3 to 68·3)	63·7 (59·8 to 67·1)	64·1 (60·1 to 67·4)
Mali	37·3 (32·2 to 42)	39·2 (35·8 to 42·6)	41·4 (39·1 to 43·9)	46·3 (44·2 to 48·2)	50·1 (48·8 to 51·5)	53·1 (51·7 to 54·7)	59 (57·2 to 60·8)	62·9 (60·5 to 65·2)	61·5 (58·9 to 63·6)	61·1 (58·8 to 63·2)
Mauritania	49·5 (45·9 to 53·3)	53 (50·2 to 56·2)	56·9 (54 to 59·7)	58·3 (56·2 to 60·6)	60·5 (58·9 to 62·3)	64·2 (62·4 to 65·9)	68·3 (66·3 to 70·4)	70·9 (68·1 to 73·4)	69·8 (67·2 to 72)	70·1 (67·4 to 72·5)
Niger	42·2 (37·4 to 47)	42·8 (38·9 to 46·8)	44·5 (41·5 to 47·6)	45·9 (43·8 to 47·9)	48·1 (46·4 to 49·7)	52·3 (50·6 to 54·1)	61·3 (58·6 to 64·1)	64·6 (60·9 to 67·7)	63·5 (60 to 66·5)	63·5 (60 to 66·6)
Nigeria	45·7 (41·5 to 49·5)	48·6 (45·6 to 51·6)	50·8 (48·3 to 53·8)	55 (52·4 to 57·5)	55·9 (53·5 to 58·4)	55·9 (53·1 to 58·7)	61·2 (58·3 to 64·2)	65·7 (62·8 to 68·5)	65·2 (62·3 to 67·8)	65 (62·2 to 67·4)
São Tomé and Príncipe	35 (32·7 to 37·3)	47·3 (45·8 to 48·7)	60 (58·9 to 61·2)	64·8 (63·6 to 65·9)	64·7 (63·3 to 66)	65 (63·4 to 66·6)	70 (68·3 to 71·6)	72·9 (70·7 to 75·1)	72·4 (70·1 to 74·3)	72·2 (70·1 to 74·1)

Senegal	46·5 (42·5 to 50·6)	48·1 (45·4 to 50·9)	48·7 (46·4 to 51·1)	54·2 (52·3 to 55·9)	60·4 (58·7 to 61·9)	61·9 (60·5 to 63·1)	67·1 (65·5 to 68·6)	70 (67·4 to 72·4)	68·9 (66·5 to 71·1)	68·2 (65·8 to 70·2)
Sierra Leone	40·3 (34·9 to 46)	43·7 (39·5 to 48)	47·3 (43·3 to 51·3)	50·4 (47·2 to 53·6)	53·1 (50·9 to 55·3)	52·7 (51 to 54·5)	56·6 (54·2 to 59·1)	62·7 (59·4 to 66·2)	61·4 (57·6 to 64·9)	62·1 (58·2 to 65·5)
Togo	44·8 (39·7 to 49·8)	48·6 (44·6 to 52·7)	53·1 (50 to 56·2)	57·2 (55 to 59·6)	59·4 (57·6 to 61·2)	58·4 (56 to 60·7)	61·4 (58·8 to 63·9)	66·8 (63·1 to 70)	66·5 (62·9 to 69·5)	66 (62·7 to 69)

Appendix 2 Table S4c: Life expectancy at birth with 95% UIs by location, males, 1950, 1960, 1970, 1980, 1990, 2000, 2010, 2019, 2020, 2021

Location	1950	1960	1970	1980	1990	2000	2010	2019	2020	2021
Global	46·7 (44·3 to 49·2)	43·8 (37·5 to 47·8)	56·4 (55·3 to 57·4)	59·8 (59 to 60·7)	63 (62·4 to 63·6)	64·8 (64·2 to 65·3)	68 (67·4 to 68·5)	70·8 (69·9 to 71·7)	69·7 (68·8 to 70·5)	69 (68 to 69·9)
Central Europe, eastern Europe, and central Asia	57·3 (55·4 to 59·2)	63·1 (62·6 to 63·5)	63·9 (63·6 to 64·3)	63·6 (63·4 to 63·7)	64·8 (64·7 to 64·9)	62·9 (62·7 to 63)	66·2 (66·1 to 66·3)	69·9 (69·5 to 70·3)	68·4 (68 to 68·8)	67·4 (66·9 to 67·9)
Central Asia	45·9 (41·3 to 50·3)	54·4 (52·2 to 56·5)	59·5 (58·1 to 60·8)	62·6 (62 to 63·2)	64 (63·5 to 64·4)	63·2 (62·7 to 63·8)	66·3 (65·9 to 66·7)	69·8 (69·2 to 70·4)	67·6 (66·9 to 68·3)	67·4 (66·4 to 68·5)
Armenia	46·5 (41·1 to 51·4)	56·9 (54 to 59·8)	63·6 (61·3 to 65·8)	67·5 (66·2 to 68·9)	67·3 (66·8 to 67·8)	69·3 (68·8 to 69·7)	70·5 (70·1 to 70·8)	73·4 (73 to 73·8)	63·7 (63·1 to 64·2)	71·3 (70·3 to 72·4)
Azerbaijan	35·1 (30·1 to 40·1)	47·4 (44·4 to 50·4)	56·8 (55 to 58·3)	62·5 (61·4 to 63·6)	62·7 (61·7 to 63·7)	64·1 (63 to 65·1)	67·2 (66·4 to 68)	70·1 (69·2 to 71)	66·4 (65·5 to 67·3)	67 (66 to 68·2)
Georgia	48·3 (43·6 to 52·9)	55·8 (53·5 to 58·3)	61 (59·2 to 62·6)	64·9 (64 to 65·7)	65·2 (64·8 to 65·7)	65·5 (64·2 to 66·8)	67·7 (67·3 to 68)	69·7 (69·4 to 70)	68·7 (68·4 to 69)	67·3 (67 to 67·5)
Kazakhstan	52·5 (48·6 to 56·3)	56·6 (54·9 to 58·4)	60·1 (58·9 to 61·2)	61·7 (60·9 to 62·5)	63·2 (62·5 to 63·9)	59·4 (58·6 to 60·1)	63·1 (62·4 to 63·8)	68·4 (67·7 to 69·1)	66·8 (66·2 to 67·5)	65·3 (64·4 to 66·2)
Kyrgyzstan	44·6 (40 to 49·2)	50·8 (47·3 to 54)	55·8 (53·1 to 58·3)	59·7 (58·2 to 61·3)	62·5 (61·4 to 63·6)	62·6 (61·4 to 63·6)	65·2 (64·2 to 66·1)	70·3 (69·2 to 71·4)	67·9 (66·6 to 69·2)	68·4 (66·6 to 70·2)
Mongolia	36·8 (30·6 to 42·9)	48·1 (44·6 to 51·6)	53·4 (50·7 to 56·2)	57·6 (55·4 to 59·7)	59·8 (58·6 to 61)	60·6 (59·4 to 61·7)	62·6 (61·4 to 63·8)	66 (64·3 to 67·6)	66·6 (65·3 to 67·8)	65·7 (64·3 to 67·1)
Tajikistan	39·3 (33·3 to 45·2)	49·6 (46·5 to 52·6)	58·4 (55·4 to 61·3)	62·2 (60·4 to 63·9)	63·7 (62·4 to 65)	64·7 (63·4 to 66)	67·9 (66·4 to 69·2)	71·7 (70 to 73·4)	67·8 (66·1 to 69·6)	66·9 (65·1 to 69·1)
Turkmenistan	44·3 (39·1 to 48·8)	52·8 (50·7 to 54·8)	57·9 (56·1 to 59·6)	60·8 (59·7 to 61·9)	62·6 (61·9 to 63·3)	62·3 (61·7 to 62·9)	65·6 (65 to 66·1)	66·7 (64·4 to 69·2)	64·7 (62·5 to 67·1)	64·3 (62 to 66·8)
Uzbekistan	47·3 (41·2 to 53·2)	58·3 (55·8 to 60·9)	62 (60·5 to 63·3)	64·4 (63·6 to 65·2)	66·1 (65·7 to 66·5)	65·7 (64·9 to 66·5)	68·1 (67·3 to 68·8)	71 (70·1 to 71·8)	69·6 (68·2 to 71)	69·9 (68·1 to 71·7)
Central Europe	54·6 (53·7 to 55·4)	64·4 (64·3 to 64·6)	66 (65·9 to 66·2)	66·7 (66·6 to 66·8)	66·9 (66·8 to 67)	69·1 (69 to 69·2)	71·7 (71·7 to 71·8)	73·7 (73·5 to 73·8)	72·3 (72·2 to 72·4)	71·3 (71·1 to 71·4)
Albania	49·5 (45·7 to 53)	61·3 (59·6 to 63·2)	66·1 (63·5 to 69)	68·7 (66·5 to 70·9)	69·8 (69·3 to 70·3)	71·9 (71·2 to 72·7)	75·7 (74·4 to 77)	76·1 (74·7 to 77·3)	74·7 (73·5 to 75·9)	73·6 (72·1 to 75·3)
Bosnia and Herzegovina	45·6 (43·2 to 47·7)	60·6 (59·7 to 61·5)	63·7 (63·2 to 64·2)	66·9 (66·5 to 67·3)	70·6 (70·3 to 70·8)	72·7 (71·4 to 74)	74·3 (74 to 74·6)	74·7 (74·4 to 75·1)	73·2 (72·9 to 73·5)	72·6 (70·8 to 74·6)
Bulgaria	55·3 (54·5 to 56)	67·5 (67·2 to 67·7)	69·3 (69·1 to 69·5)	68·6 (68·4 to 68·8)	66·6 (66·2 to 67·1)	66·6 (66·1 to 67·1)	68·7 (68·2 to 69·2)	69·9 (69·4 to 70·4)	68·3 (67·8 to 68·8)	66·4 (65·9 to 67)
Croatia	48·9 (48 to 49·8)	63 (62·7 to 63·2)	65·8 (65·5 to 66)	67·4 (67·2 to 67·6)	68·1 (67·9 to 68·3)	70·9 (70 to 71·7)	73·7 (73·4 to 73·9)	75·8 (75·5 to 76·1)	75·1 (74·8 to 75·4)	74·1 (73·8 to 74·4)
Czechia	63·9 (63·6 to 64·2)	67·3 (67·2 to 67·5)	66 (65·9 to 66·2)	66·8 (66·7 to 67)	67·6 (67·4 to 67·7)	71·7 (71·5 to 71·9)	74·6 (74·4 to 74·8)	76·6 (76·4 to 76·8)	75·4 (75·2 to 75·6)	74·4 (74·2 to 74·6)
Hungary	57·7 (57 to 58·3)	66 (65·8 to 66·2)	66·3 (66·1 to 66·5)	65·5 (65·3 to 65·6)	65·2 (65 to 65·3)	67·5 (67·4 to 67·7)	70·8 (70·6 to 70·9)	73·2 (73 to 73·4)	72·4 (72·2 to 72·6)	70·9 (70·7 to 71·1)
Montenegro	64·7 (63·6 to 65·8)	69·8 (69·1 to 70·6)	71·2 (70·3 to 72)	70·9 (70·1 to 71·7)	71·5 (70·9 to 72·2)	71 (70·3 to 71·6)	72·6 (71·9 to 73·2)	72·5 (71·7 to 73·2)	70·8 (70·2 to 71·4)	69·8 (69 to 70·5)
North Macedonia	50·4 (48·4 to 52·2)	59·1 (58 to 60·3)	65·2 (64·5 to 65·9)	68·8 (68·2 to 69·3)	68·3 (67·8 to 68·8)	69·3 (68·8 to 69·7)	71·3 (70·9 to 71·7)	73·4 (72·9 to 73·9)	71·2 (70·7 to 71·7)	69·2 (68 to 70·4)
Poland	53·1 (52·7 to 53·5)	64·7 (64·6 to 64·9)	66·6 (66·5 to 66·8)	66·2 (66·1 to 66·3)	66·6 (66·5 to 66·7)	69·7 (69·6 to 69·7)	72·1 (72·1 to 72·2)	74·1 (74 to 74·2)	72·6 (72·5 to 72·7)	71·8 (71·7 to 71·9)
Romania	57·8 (54·6 to 61·1)	63·7 (63·4 to 64)	66·1 (65·9 to 66·2)	66·9 (66·8 to 67·1)	66·6 (66·4 to 66·7)	67·7 (67·6 to 67·8)	70 (69·9 to 70·1)	71·8 (71·6 to 71·9)	70·4 (70·3 to 70·5)	69·2 (69·1 to 69·4)
Serbia	46·3 (45·1 to 47·5)	62·5 (62·3 to 62·8)	63·6 (63·4 to 63·8)	66·3 (66·1 to 66·5)	67·3 (67·1 to 67·5)	68·6 (68·3 to 68·9)	71·7 (71·5 to 71·9)	73·6 (73·4 to 73·8)	71·9 (71·8 to 72·1)	71·7 (71·5 to 71·8)
Slovakia	60·7 (60·2 to 61·2)	67·8 (67·5 to 68·1)	66·7 (66·4 to 66·9)	66·9 (66·7 to 67·2)	66·7 (66·5 to 66·9)	69·4 (69·2 to 69·6)	71·9 (71·7 to 72·1)	74·2 (74 to 74·5)	73·4 (73·2 to 73·7)	71·3 (71 to 71·5)
Slovenia	53 (52·2 to 53·8)	63·8 (63·4 to 64·3)	65·4 (65·1 to 65·8)	67·4 (67·1 to 67·8)	70·1 (69·8 to 70·5)	72·4 (72 to 72·7)	76·3 (75·8 to 76·6)	78·5 (78 to 79)	77·6 (77·2 to 78·1)	77·6 (77·2 to 78·1)
Eastern Europe	61·7 (59·1 to 64·2)	64·6 (64·4 to 64·8)	64·4 (64·2 to 64·5)	62·6 (62·5 to 62·7)	64·5 (64·5 to 64·6)	60·4 (60·4 to 60·5)	63·7 (63·7 to 63·8)	68·4 (67·7 to 69)	67·1 (66·4 to 67·7)	65·8 (65 to 66·6)
Belarus	63·8 (59·3 to 68)	66·9 (66 to 67·8)	67·5 (67 to 68)	65·9 (65·5 to 66·3)	66·3 (66 to 66·6)	63·3 (63 to 63·5)	64·6 (64·5 to 64·8)	69·3 (69·1 to 69·4)	67·4 (66·2 to 68·6)	66 (64·2 to 67·8)
Estonia	62·1 (58·1 to 65·8)	64·7 (64·2 to 65·2)	65·5 (65·1 to 65·9)	64·3 (63·9 to 64·7)	64·7 (64·4 to 65·1)	65·6 (65·2 to 66)	71 (70·6 to 71·4)	74·7 (74·2 to 75·2)	74·2 (73·7 to 74·7)	72·4 (71·9 to 72·9)
Latvia	64·6 (60·7 to 68·4)	66·2 (65·8 to 66·6)	66 (65·6 to 66·3)	63·8 (63·4 to 64)	64·4 (64·1 to 64·7)	65 (64·7 to 65·3)	68 (67·7 to 68·4)	71·4 (71 to 71·8)	70·8 (70·4 to 71·2)	68·3 (67·9 to 68·7)
Lithuania	62·2 (57·7 to 66·5)	66·4 (66·1 to 66·8)	66·8 (66·5 to 67·2)	65·4 (65·1 to 65·7)	66·2 (65·9 to 66·5)	66·7 (66·4 to 67)	67·5 (67·2 to 67·7)	71·4 (71·1 to 71·7)	69·8 (69·5 to 70·2)	69·2 (68·8 to 69·5)
Moldova	49·2 (44·5 to 53·6)	55·6 (53·1 to 58·1)	59·7 (58·3 to 61·1)	62·4 (61·7 to 63)	64·6 (64·2 to 64·9)	65 (64·5 to 65·3)	65·6 (65·3 to 65·9)	70·3 (69·1 to 71·5)	69·3 (68·8 to 69·7)	67·9 (66·7 to 69)
Russia	60·9 (57·4 to 64·4)	63·7 (63·6 to 63·8)	63·4 (63·3 to 63·5)	61·5 (61·5 to 61·6)	64 (63·9 to 64)	59·3 (59·3 to 59·4)	62·9 (62·8 to 62·9)	68·3 (68·3 to 68·4)	66·6 (66·6 to 66·7)	65·5 (65·5 to 65·6)
Ukraine	64·7 (61·5 to 67·9)	67·3 (66·7 to 67·9)	66·5 (66 to 66·9)	64·7 (64·3 to 65)	65·7 (65·4 to 65·9)	62·3 (62·2 to 62·4)	65·7 (65·7 to 65·8)	67·8 (65·2 to 70·5)	67·9 (64·8 to 70·8)	66·3 (62·7 to 70·1)
High income	61·9 (61·6 to 62·2)	66·1 (66 to 66·2)	67·5 (67·5 to 67·6)	70·4 (70·3 to 70·4)	72·7 (72·7 to 72·8)	75·2 (75·2 to 75·2)	77·7 (77·7 to 77·8)	79 (78·9 to 79)	78 (77·9 to 78)	77·9 (77·8 to 78)
Australasia	67 (66·8 to 67·2)	68·2 (68·1 to 68·3)	68 (67·9 to 68·1)	70·9 (70·8 to 71)	73·6 (73·5 to 73·6)	76·8 (76·7 to 76·9)	79·6 (79·5 to 79·7)	80·9 (80·8 to 81)	81·5 (81·4 to 81·7)	81·2 (81·1 to 81·2)
Australia	66·9 (66·7 to 67·1)	68·1 (68 to 68·3)	67·9 (67·8 to 68)	71 (70·9 to 71·1)	73·8 (73·7 to 73·9)	77 (76·9 to 77·1)	79·7 (79·6 to 79·8)	81·1 (80·9 to 81·2)	81·7 (81·6 to 81·8)	81·2 (81·1 to 81·3)
New Zealand	67·6 (67·3 to 67·9)	68·7 (68·5 to 68·9)	68·6 (68·5 to 68·8)	70·4 (70·2 to 70·5)	72·6 (72·4 to 72·8)	76 (75·8 to 76·1)	79 (78·8 to 79·2)	80·1 (79·9 to 80·3)	80·7 (80·6 to 80·9)	80·7 (80·5 to 80·9)
High-income Asia Pacific	51·8 (50·9 to 52·5)	62·9 (62·5 to 63·3)	67·2 (66·9 to 67·5)	71·4 (71·2 to 71·5)	74·4 (74·3 to 74·5)	76·9 (76·8 to 76·9)	79·4 (79·3 to 79·4)	81·7 (81·7 to 81·8)	82 (81·9 to 82·1)	81·8 (81·7 to 81·9)
Brunei	48·6 (46·9 to 50·4)	57·9 (56·3 to 59·5)	65·6 (64·4 to 66·9)	69 (67·7 to 70·2)	69·6 (68·4 to 70·7)	72·8 (71·7 to 73·8)	74·6 (73·6 to 75·6)	74·9 (73·7 to 76·1)	75·1 (73·8 to 76·3)	74·9 (73·6 to 76)
Japan	59·9 (59·6 to 60·1)	65·9 (65·8 to 66)	69·9 (69·9 to 70)	73·7 (73·7 to 73·8)	76·2 (76·2 to 76·3)	78 (78 to 78·1)	79·9 (79·9 to 80)	82 (81·9 to 82·1)	82·3 (82·2 to 82·3)	82·2 (82·1 to 82·2)
Aichi	62 (59·3 to 64·8)	67·2 (64·5 to 69·8)	70·9 (68·4 to 73·3)	74·2 (74 to 74·4)	76·6 (76·4 to 76·8)	78·1 (77·9 to 78·4)	80·1 (79·9 to 80·4)	82·3 (82 to 82·6)	82·5 (82·2 to 82·8)	82·6 (82·3 to 82·9)
Akita	59·4 (56·5 to 62·3)	64·9 (62 to 67·8)	68·7 (66 to 71·3)	72·7 (72·3 to 73·1)	75·5 (75·1 to 75·9)	77·1 (76·7 to 77·6)	78·5 (78 to 79)	81·1 (80·4 to 81·8)	80·7 (80·1 to 81·3)	81·1 (80·2 to 81·8)
Aomori	56·9 (54 to 59·9)	63·3 (60·5 to 66·2)	67·4 (64·6 to 70·1)	71·7 (71·4 to 72·1)	74·5 (74·1 to 74·8)	76·1 (75·8 to 76·5)	77·7 (77·3 to 78·2)	79·5 (78·9 to 80·1)	80 (79·5 to 80·5)	79·8 (79·1 to 80·4)
Chiba	60·5 (57·7 to 63·3)	66·2 (63·5 to 68·9)	70·6 (68 to 73)	74·1 (73·9 to 74·3)	76·7 (76·5 to 77)	78·3 (78·1 to 78·5)	80·2 (79·9 to 80·4)	82·2 (81·8 to 82·4)	82·4 (82·1 to 82·6)	82·3 (81·9 to 82·6)
Ehime	59·2 (56 to 62·2)	65·2 (62·4 to 68·1)	69·3 (66·5 to 71·9)	73·5 (73·1 to 73·9)	76·1 (75·7 to 76·5)	77·5 (77·1 to 78)	79·6 (79·1 to 80·1)	81·7 (81 to 82·3)	82 (81·4 to 82·5)	81·8 (81·1 to 82·5)
Fukui	59·3 (55·8 to 62·8)	67 (64·2 to 69·6)	70·9 (68·7 to 73)	75·2 (74·8 to 75·6)	77·2 (76·7 to 77·6)	78·9 (78·4 to 79·2)	80·5 (80·1 to 81)	83·1 (82·4 to 83·9)	82·9 (82·2 to 83·6)	82·7 (81·9 to 83·4)
Fukuoka	60 (57·1 to 62·8)	65·7 (62·9 to 68·4)	69·2 (66·3 to 71·7)	72·9 (72·7 to 73·2)	75·4 (75·2 to 75·6)	77·6 (77·3 to 77·9)	79·7 (79·4 to 80)	81·8 (81·4 to 82·1)	82·1 (81·8 to 82·4)	82 (81·7 to 82·4)
Fukushima	57·8 (54·9 to 60·5)	64·4 (61·7 to 67·2)	68·5 (65·7 to 71)	73·1 (72·8 to 73·4)	76 (75·6 to 76·3)	77·5 (77·2 to 77·9)	79·4 (79 to 79·8)	80·9 (80·4 to 81·4)	81·2 (80·7 to 81·7)	81·1 (80·6 to 81·7)
Gifu	61·5 (58·6 to 64·3)	66·9 (64·2 to 69·7)	70·8 (68·2 to 73·4)	74·3 (74 to 74·6)	77 (76·6 to 77·3)	78·3 (78 to 78·7)	80·3 (80 to 80·7)	82·2 (81·7 to 82·7)	82·7 (82·3 to 83·2)	82·5 (81·9 to 83·1)
Gunma	59·4 (56·4 to 62·2)	65·3 (62·7 to 68·1)	69·9 (67·1 to 72·4)	74 (73·6 to 74·3)	76·7 (76·4 to 77·1)	78·2 (77·8 to 78·6)	79·8 (79·4 to 80·2)	81·8 (81·2 to 82·3)	81·8 (81·3 to 82·3)	81·8 (81·2 to 82·3)
Hiroshima	59·4 (56·4 to 62·3)	65·6 (62·8 to 68·4)	70 (67·2 to 72·5)	73·8 (73·5 to 74·1)	76·3 (76 to 76·7)	78·1 (77·7 to 78·4)	80·1 (79·8 to 80·5)	82 (81·6 to 82·5)	82·7 (82·3 to 83·1)	82·7 (82·2 to 83·2)
Hokkaidō	59·8 (56·9 to 62·6)	65·6 (63 to 68·5)	69·1 (66·4 to 71·6)	73·3 (73 to 73·5)	75·9 (75·7 to 76·1)	77·7 (77·5 to 78)	79·6 (79·3 to 79·8)	81·5 (81·1 to 81·8)	81·7 (81·4 to 82)	81·5 (81·1 to 81·9)
Hyōgo	60·9 (58 to 63·8)	66·4 (63·7 to 69·1)	70 (67·4 to 72·5)	73·4 (73·2 to 73·6)	75·7 (75·5 to 75·9)	77·8 (77·5 to 78)	79·9 (79·7 to 80·2)	82·3 (81·9 to 82·6)	82·4 (82 to 82·8)	82·4 (82 to 82·8)
Ibaraki	57·3 (54·2 to 60·2)	64·2 (61·5 to 67)	68·8 (66·2 to 71·4)	73 (72·7 to 73·3)	75·8 (75·6 to 76·1)	77·6 (77·3 to 77·9)	79·4 (79·1 to 79·7)	81·3 (80·8 to 81·7)	81·7 (81·3 to 82·1)	81·7 (81·2 to 82·1)
Ishikawa	59 (55·7 to 62·2)	66·4 (63·8 to 69)	70·4 (67·9 to 72·7)	74·4 (74 to 74·8)	76·7 (76·4 to 77·1)	78·4 (78 to 78·8)	80·4 (79·9 to 80·8)	83 (82·4 to 83·7)	83·1 (82·6 to 83·7)	82·7 (82·1 to 83·3)
Iwate	57·7 (54·7 to 60·8)	64·1 (61·3 to 66·9)	68·5 (65·7 to 71·1)	72·9 (72·6 to 73·3)	75·7 (75·3 to 76·1)	77·6 (77·2 to 78)	78·8 (78·3 to 79·3)	80·3 (79·6 to 80·8)	80·9 (80·4 to 81·5)	81·3 (80·6 to 81·9)
Kagawa	60·3 (57·3 to 63·4)	66 (63 to 68·8)	70·5 (68 to 72·9)	74·5 (74·1 to 74·9)	76·3 (75·9 to 76·7)	78·3 (77·8 to 78·7)	80·1 (79·6 to 80·5)	82·4 (81·8 to 83·2)	81·9 (81·3 to 82·5)	82·5 (81·8 to 83·2)
Kagoshima	56·1 (53·1 to 59)	63·6 (61 to 66·5)	67·7 (64·9 to 70·3)	72·7 (72·3 to 73)	75·7 (75·4 to 76·1)	77·4 (77·1 to 77·8)	79·4 (79 to 79·9)	81·1 (80·5 to 81·7)	81·7 (81·2 to 82·2)	81·5 (80·9 to 82·2)
Kanagawa	62·8 (59·9 to 65·6)	68·1 (65·3 to 70·9)	71·7 (69·1 to 74·1)	74·6 (74·4 to 74·8)	76·8 (76·6 to 77)	78·5 (78·3 to 78·6)	80·6 (80·3 to 80·8)	82·8 (82·5 to 83·1)	83 (82·8 to 83·3)	82·8 (82·5 to 83·1)
Kōchi	56·5 (53·4 to 59·4)	63·4 (60·3 to 66·3)	67·7 (65·2 to 70·3)	72·4 (72 to 72·9)	75·6 (75·2 to 76·1)	77·4 (77 to 77·9)	79 (78·5 to 79·5)	81·7 (80·9 to 82·5)	81·3 (80·7 to 82)	81·7 (80·9 to 82·4)
Kumamoto	58·5 (55·6 to 61·3)	64·6 (61·9 to 67·5)	68·8 (66·1 to 71·4)	73·6 (73·2 to 74)	76·6 (76·2 to 76·9)	78·8 (78·3 to 79·2)	80·6 (80·2 to 81·1)	82·5 (81·9 to 83·1)	83 (82·5 to 83·5)	82·4 (81·8 to 83)

Kyōto	61·9 (59 to 64·6)	67·2 (64·5 to 70)	71·2 (68·6 to 73·7)	74·4 (74·1 to 74·7)	76·6 (76·2 to 76·9)	78·1 (77·8 to 78·4)	80·4 (80 to 80·7)	83·2 (82·7 to 83·7)	83·4 (82·9 to 83·8)	83 (82·4 to 83·5)
Mie	60·7 (57·7 to 63·6)	66·3 (63·4 to 69)	70·6 (67·9 to 73·1)	74 (73·6 to 74·3)	76·2 (75·8 to 76·5)	78·1 (77·7 to 78·5)	80 (79·6 to 80·4)	82·2 (81·6 to 82·8)	82·9 (82·4 to 83·4)	82·4 (81·8 to 82·9)
Miyagi	59·8 (56·9 to 62·5)	65·6 (62·9 to 68·2)	69·4 (66·7 to 72)	73·8 (73·5 to 74·1)	76·3 (76 to 76·6)	78 (77·7 to 78·4)	80 (79·6 to 80·4)	82 (81·5 to 82·4)	82·6 (82·1 to 83)	82·5 (81·9 to 83)
Miyazaki	57·9 (54·9 to 61)	64·3 (61·4 to 67·1)	68·7 (65·8 to 71·3)	72·8 (72·3 to 73·2)	75·6 (75·2 to 76·1)	77·7 (77·2 to 78·1)	79·9 (79·4 to 80·3)	81·2 (80·5 to 81·8)	81·7 (81·1 to 82·3)	81·5 (80·8 to 82·2)
Nagano	59·6 (56·8 to 62·4)	66 (63·3 to 68·7)	70·5 (67·8 to 73)	74·7 (74·3 to 75)	77·6 (77·3 to 77·9)	79·1 (78·7 to 79·5)	81·1 (80·7 to 81·5)	83·2 (82·6 to 83·7)	83·3 (82·8 to 83·8)	83·5 (82·9 to 84·1)
Nagasaki	58·7 (55·8 to 61·5)	64·9 (62·2 to 67·8)	68·3 (65·6 to 70·8)	72·7 (72·3 to 73·1)	75·5 (75·1 to 75·9)	77·6 (77·2 to 78)	79·3 (78·9 to 79·8)	81·5 (80·9 to 82·2)	82·2 (81·7 to 82·7)	81·3 (80·6 to 82)
Nara	60·9 (58·1 to 63·6)	66·5 (63·8 to 69·3)	70·2 (67·5 to 72·8)	73·7 (73·3 to 74·1)	76·4 (76 to 76·8)	78·7 (78·3 to 79·1)	80·7 (80·2 to 81·1)	82·6 (81·9 to 83·2)	83·3 (82·8 to 83·8)	82·7 (82·1 to 83·4)
Niigata	59·4 (56·5 to 62·2)	65·4 (62·6 to 68·1)	69·4 (66·7 to 72)	73·7 (73·4 to 74)	76·7 (76·4 to 77·1)	78·1 (77·8 to 78·4)	79·9 (79·5 to 80·3)	81·5 (81 to 82)	82 (81·5 to 82·4)	81·8 (81·2 to 82·3)
Ōita	59·1 (56·1 to 62·1)	65·3 (62·5 to 68·3)	69·6 (66·7 to 72·2)	73·3 (72·9 to 73·7)	76·2 (75·8 to 76·6)	78·2 (77·7 to 78·6)	80·3 (79·8 to 80·7)	82 (81·4 to 82·7)	82·5 (81·9 to 83·1)	82·5 (81·9 to 83·2)
Okayama	60·3 (57·5 to 63·2)	66·1 (63·3 to 68·9)	70·7 (67·9 to 73·2)	74·3 (74 to 74·6)	76·5 (76·1 to 76·8)	78·3 (78 to 78·7)	80·1 (79·7 to 80·5)	82·1 (81·5 to 82·6)	82·7 (82·2 to 83·1)	82·6 (82 to 83·2)
Okinawa	53·3 (50·1 to 56·3)	63·1 (60·1 to 66·2)	66·6 (63·6 to 69·4)	73·5 (73·1 to 73·9)	76·4 (76 to 76·9)	77·9 (77·5 to 78·3)	79·7 (79·2 to 80·1)	81·7 (81·1 to 82·4)	81·8 (81·3 to 82·3)	81·5 (80·9 to 82·1)
Ōsaka	60·8 (58 to 63·5)	66·3 (63·8 to 68·9)	70·1 (67·5 to 72·5)	73 (72·8 to 73·2)	75·1 (74·9 to 75·3)	77·2 (77 to 77·4)	79·3 (79·1 to 79·5)	81·3 (81·1 to 81·6)	81·5 (81·2 to 81·8)	81·4 (81·2 to 81·8)
Saga	58·7 (55·6 to 61·6)	65 (62·1 to 67·8)	69·1 (66·5 to 71·5)	73·3 (73 to 73·7)	75·8 (75·3 to 76·2)	77·5 (77 to 77·9)	79·7 (79·2 to 80·2)	82·2 (81·5 to 82·9)	82·2 (81·6 to 82·7)	82 (81·2 to 82·8)
Saitama	61·1 (58·4 to 63·8)	66·8 (64 to 69·5)	71·1 (68·5 to 73·5)	74 (73·8 to 74·2)	76·5 (76·3 to 76·8)	78·5 (78·3 to 78·7)	80·1 (79·9 to 80·4)	82 (81·7 to 82·3)	82·3 (82 to 82·6)	82·2 (81·8 to 82·5)
Shiga	60·2 (57·3 to 63·1)	65·7 (63 to 68·4)	69·7 (67·3 to 72·1)	73·8 (73·4 to 74·2)	76·6 (76·2 to 76·9)	78·6 (78·2 to 78·9)	80·9 (80·5 to 81·3)	83·1 (82·5 to 83·7)	83·3 (82·7 to 83·8)	83·7 (83 to 84·3)
Shimane	56·7 (53·3 to 60·1)	65·1 (62·1 to 68)	69·2 (66·5 to 71·7)	73·7 (73·2 to 74·1)	76·5 (76 to 76·9)	78·1 (77·5 to 78·5)	79·9 (79·4 to 80·4)	82·7 (81·9 to 83·5)	83 (82·3 to 83·6)	82·1 (81·4 to 83)
Shizuoka	61·4 (58·4 to 64·2)	67·1 (64·4 to 70)	70·9 (68·2 to 73·4)	74·3 (74 to 74·6)	76·9 (76·6 to 77·2)	78·5 (78·2 to 78·8)	80·3 (79·9 to 80·6)	82·1 (81·7 to 82·5)	82·3 (81·9 to 82·7)	82·4 (81·9 to 82·8)
Tochigi	58·9 (55·9 to 61·7)	64·9 (62·2 to 67·7)	69·2 (66·5 to 71·7)	73 (72·7 to 73·3)	75·6 (75·3 to 76)	77·6 (77·2 to 78)	79·3 (78·9 to 79·7)	81·4 (80·9 to 81·9)	81·6 (81·2 to 82·1)	81·8 (81·2 to 82·3)
Tokushima	58·5 (55·3 to 61·5)	64·6 (61·7 to 67·4)	68·7 (66·2 to 71·2)	72·8 (72·4 to 73·2)	75·7 (75·2 to 76·1)	77·7 (77·2 to 78·2)	79·7 (79·2 to 80·2)	81·6 (80·9 to 82·4)	82·4 (81·8 to 83)	81·4 (80·7 to 82·1)
Tōkyō	63·3 (60·5 to 66)	67·9 (65·4 to 70·6)	71·2 (68·6 to 73·6)	74·6 (74·4 to 74·7)	76·3 (76·1 to 76·4)	78·2 (78 to 78·3)	80·3 (80·1 to 80·4)	82·3 (82·1 to 82·6)	82·6 (82·4 to 82·8)	82·5 (82·2 to 82·7)
Tottori	56·2 (52·6 to 59·7)	64·9 (62·1 to 67·8)	69·1 (66·9 to 71·4)	73·3 (72·8 to 73·7)	75·6 (75·2 to 76)	77·5 (77 to 78)	79·4 (78·9 to 79·9)	81·9 (81·2 to 82·7)	82·1 (81·5 to 82·8)	81·8 (81 to 82·6)
Toyama	60·2 (57·3 to 63·1)	65·4 (62·7 to 68·2)	69·7 (67·2 to 72·1)	73·8 (73·4 to 74·1)	76·5 (76·1 to 76·9)	78·5 (78·1 to 78·9)	80·1 (79·6 to 80·5)	81·9 (81·2 to 82·5)	82·4 (81·8 to 82·9)	82·1 (81·4 to 82·8)
Wakayama	59·3 (56·3 to 62·2)	65·4 (62·4 to 68·2)	69·6 (66·9 to 72·1)	73·2 (72·8 to 73·5)	75·5 (75·1 to 75·9)	77·4 (76·9 to 77·8)	79·1 (78·5 to 79·6)	80·9 (80·2 to 81·6)	81·4 (80·8 to 82)	81·3 (80·6 to 82)
Yamagata	59·4 (56·6 to 62·3)	65·2 (62·3 to 68)	69·2 (66·6 to 71·8)	73·3 (73 to 73·7)	76·7 (76·3 to 77·1)	78·3 (77·9 to 78·8)	80·4 (79·9 to 80·9)	81·8 (81 to 82·4)	82 (81·4 to 82·5)	82 (81·3 to 82·7)
Yamaguchi	59·5 (56·4 to 62·4)	65·2 (62·4 to 68)	69·4 (66·7 to 71·9)	72·9 (72·6 to 73·3)	75·8 (75·4 to 76·2)	77·5 (77·1 to 77·9)	79·4 (79 to 79·9)	81·4 (80·8 to 82)	81·5 (81 to 82)	81·7 (81 to 82·3)
Yamanashi	59·1 (56 to 62·2)	65·4 (62·5 to 68·3)	69·5 (66·9 to 72)	73·6 (73·1 to 74)	76·3 (75·9 to 76·7)	78·2 (77·8 to 78·7)	80·3 (79·8 to 80·8)	82·3 (81·6 to 82·9)	83 (82·4 to 83·6)	82·1 (81·3 to 82·9)
South Korea	30·1 (28·2 to 31·9)	55 (53·8 to 56·1)	58·9 (58 to 59·9)	63·3 (63 to 63·7)	68 (67·8 to 68·2)	72·6 (72·5 to 72·6)	77·2 (77·1 to 77·3)	80·4 (80·3 to 80·5)	80·6 (80·5 to 80·7)	80·3 (80·1 to 80·5)
Singapore	53·8 (53·1 to 54·4)	62·2 (62 to 62·5)	65·5 (65·3 to 65·8)	68·7 (68·5 to 68·9)	73 (72·8 to 73·2)	76·8 (76·6 to 77)	80·3 (80·2 to 80·5)	83·6 (83·4 to 83·9)	83·7 (83·5 to 83·9)	83·6 (83·4 to 83·8)
High-income North America	65·5 (65·4 to 65·6)	67 (66·9 to 67)	67·4 (67·3 to 67·4)	70·3 (70·2 to 70·3)	72·3 (72·2 to 72·3)	74·4 (74·4 to 74·4)	76·6 (76·6 to 76·7)	76·9 (76·9 to 77)	75 (74·9 to 75)	74·8 (74·6 to 74·9)
Canada	66·6 (66·4 to 66·8)	68·5 (68·4 to 68·6)	69·5 (69·4 to 69·6)	71·6 (71·6 to 71·7)	74·1 (74 to 74·2)	76·6 (76·5 to 76·6)	79·2 (79·1 to 79·3)	80·2 (80·1 to 80·3)	79·5 (79·4 to 79·6)	79·5 (79·4 to 79·7)
Greenland	46·9 (44·9 to 49)	55·1 (53·6 to 56·6)	59·9 (58·3 to 61·5)	61·6 (59·8 to 63·3)	62·4 (61·1 to 63·8)	66·5 (65 to 67·9)	69·5 (68·1 to 71·1)	71·4 (69·7 to 72·8)	71·3 (69·7 to 72·6)	71·4 (69·7 to 72·7)
USA	65·5 (65·4 to 65·6)	66·8 (66·8 to 66·9)	67·2 (67·1 to 67·2)	70·1 (70·1 to 70·2)	72·1 (72 to 72·1)	74·2 (74·1 to 74·2)	76·3 (76·3 to 76·4)	76·6 (76·5 to 76·6)	74·5 (74·4 to 74·5)	74·3 (74·1 to 74·4)
Alabama	63·3 (60·1 to 66·3)	64·9 (64·6 to 65·1)	65·2 (64·9 to 65·5)	68·3 (68 to 68·6)	69·9 (69·6 to 70·1)	71·1 (70·9 to 71·4)	72·6 (72·4 to 72·9)	72·8 (72·5 to 73·1)	70·6 (70·4 to 70·9)	69·7 (67·6 to 71·8)
Alaska	61·7 (58·8 to 64·5)	65·3 (64·7 to 66)	66·7 (66 to 67·3)	68·7 (68·1 to 69·2)	72 (71·4 to 72·5)	74·2 (73·7 to 74·8)	75·7 (75·2 to 76·2)	76·5 (75·8 to 77·2)	75·4 (74·7 to 76)	72·9 (71·2 to 74·7)
Arizona	63·4 (60·3 to 66·9)	65·4 (65 to 65·8)	67·1 (66·7 to 67·5)	70·6 (70·3 to 71)	73 (72·7 to 73·3)	74·6 (74·3 to 74·9)	76·8 (76·6 to 77·1)	76·3 (76 to 76·6)	73·3 (73·1 to 73·6)	72·7 (70·7 to 74·7)
Arkansas	65·4 (62·1 to 68·7)	67 (66·5 to 67·4)	67 (66·6 to 67·4)	69·5 (69·1 to 69·8)	70·8 (70·4 to 71·2)	72 (71·6 to 72·3)	73·3 (72·9 to 73·6)	73·3 (72·9 to 73·7)	71·3 (71 to 71·7)	70·6 (68·5 to 72·8)
California	66·5 (63·7 to 69·4)	67·6 (67·5 to 67·8)	68·3 (68·2 to 68·5)	71·1 (70·9 to 71·2)	72·7 (72·6 to 72·8)	75·9 (75·8 to 76)	78·6 (78·4 to 78·7)	78·9 (78·8 to 79)	76·8 (76·6 to 76·9)	76·5 (74·6 to 78·4)
Colorado	65·8 (62·8 to 68·9)	68 (67·6 to 68·4)	68·7 (68·3 to 69)	72 (71·7 to 72·3)	74·2 (73·8 to 74·5)	76·2 (75·9 to 76·5)	78 (77·7 to 78·3)	78·2 (77·9 to 78·5)	76·3 (76 to 76·6)	76·2 (73·9 to 78·3)
Connecticut	66·8 (63·7 to 69·6)	68·1 (67·8 to 68·5)	69·3 (69 to 69·6)	71·4 (71·1 to 71·7)	74·2 (73·9 to 74·5)	75·6 (75·2 to 75·9)	78·1 (77·8 to 78·5)	78·3 (78 to 78·7)	76·3 (76 to 76·7)	77·7 (75·5 to 79·9)
Delaware	63·1 (60·3 to 66)	66·3 (65·8 to 66·8)	66·5 (66 to 67)	69·9 (69·4 to 70·4)	72 (71·5 to 72·5)	73·9 (73·4 to 74·4)	75·7 (75·2 to 76·2)	75·4 (74·8 to 76)	74·3 (73·7 to 74·8)	73·7 (71·9 to 75·5)
Washington, DC	60·2 (56·6 to 63·5)	62·4 (61·9 to 62·9)	60·8 (60·4 to 61·4)	64 (63·4 to 64·6)	61·4 (60·8 to 62)	68·3 (67·6 to 68·9)	74·6 (73·9 to 75·2)	75·7 (75 to 76·5)	72·8 (72·1 to 73·5)	73·9 (71·4 to 76·2)
Florida	64·8 (61·5 to 68)	66·4 (66·1 to 66·6)	66·7 (66·5 to 67)	70·1 (69·9 to 70·3)	72·2 (72 to 72·4)	74·3 (74·1 to 74·5)	76·6 (76·4 to 76·8)	76·7 (76·5 to 76·8)	74·9 (74·8 to 75·1)	74 (72 to 76)
Georgia	63 (59·7 to 66·1)	64·2 (64 to 64·5)	64·4 (64·1 to 64·6)	68·2 (68 to 68·4)	70 (69·8 to 70·2)	72·3 (72·1 to 72·5)	74·8 (74·6 to 75)	75·5 (75·3 to 75·7)	73·3 (73·1 to 73·5)	72·3 (70·4 to 74·4)
Hawaii	67·4 (64·3 to 70·2)	69·6 (69·1 to 70·2)	71·5 (71 to 72)	74·1 (73·6 to 74·7)	75·5 (75 to 76)	77·2 (76·7 to 77·8)	78·3 (77·8 to 78·8)	78·8 (78·2 to 79·4)	78·6 (78·1 to 79·2)	78·3 (75·9 to 80·8)
Idaho	66·4 (63·2 to 69·3)	68·1 (67·5 to 68·7)	68·5 (67·9 to 69·1)	71·9 (71·4 to 72·4)	73·9 (73·4 to 74·5)	76 (75·4 to 76·4)	77·3 (76·8 to 77·7)	77·8 (77·3 to 78·3)	76·5 (76 to 76·9)	75 (73 to 77)
Illinois	65·6 (62·8 to 68·4)	66·6 (66·5 to 66·8)	66·5 (66·4 to 66·7)	69·7 (69·5 to 69·8)	71·6 (71·4 to 71·8)	73·8 (73·6 to 73·9)	76·4 (76·2 to 76·6)	76·8 (76·6 to 77)	74·4 (74·2 to 74·5)	75·2 (73 to 77·3)
Indiana	66·4 (63·4 to 69·3)	67·3 (67·1 to 67·6)	67·5 (67·3 to 67·8)	70·2 (70 to 70·5)	72·2 (72 to 72·5)	73·2 (73 to 73·5)	74·9 (74·7 to 75·2)	74·7 (74·4 to 75)	72·6 (72·3 to 72·8)	72·6 (70·4 to 74·7)
Iowa	67·9 (64·9 to 70·9)	69 (68·7 to 69·3)	69·1 (68·8 to 69·4)	72 (71·6 to 72·3)	74·4 (74·1 to 74·8)	75·6 (75·3 to 76)	77 (76·7 to 77·4)	76·8 (76·4 to 77·1)	75·3 (75 to 75·6)	75·7 (73·7 to 77·8)
Kansas	67·7 (64·7 to 70·8)	68·8 (68·4 to 69·1)	69·2 (68·8 to 69·5)	71·6 (71·3 to 72)	73·7 (73·3 to 74)	74·8 (74·4 to 75·1)	75·9 (75·5 to 76·2)	76·1 (75·7 to 76·5)	74·1 (73·7 to 74·4)	74·1 (72 to 76·4)
Kentucky	65·2 (62·1 to 68·4)	66·4 (66·1 to 66·7)	66·1 (65·8 to 66·4)	69 (68·7 to 69·3)	71 (70·7 to 71·3)	72·2 (72 to 72·5)	73·3 (73·1 to 73·6)	73·3 (73 to 73·6)	71·1 (70·8 to 71·3)	70·4 (68·3 to 72·6)
Louisiana	63·2 (60·1 to 66·3)	64·8 (64·5 to 65·2)	65 (64·8 to 65·3)	67·7 (67·5 to 68)	69·5 (69·2 to 69·8)	71 (70·7 to 71·3)	72·9 (72·6 to 73·2)	73 (72·8 to 73·3)	70·2 (69·9 to 70·5)	70·1 (67·8 to 72·1)
Maine	65 (62·1 to 67·8)	66·9 (66·4 to 67·4)	67·3 (66·8 to 67·7)	70·8 (70·3 to 71·3)	73·6 (73·1 to 74·1)	74·9 (74·4 to 75·4)	76·7 (76·2 to 77·2)	76·3 (75·7 to 76·8)	75·5 (75 to 76·1)	74·7 (72·3 to 77·2)
Maryland	64·5 (61·5 to 67·3)	65·7 (65·5 to 66)	66·7 (66·4 to 66·9)	69·9 (69·6 to 70·1)	71·6 (71·3 to 71·9)	73·6 (73·4 to 73·9)	76·8 (76·5 to 77·1)	76·6 (76·3 to 76·9)	74·6 (74·3 to 74·8)	75·2 (72·9 to 77·6)
Massachusetts	66·5 (63·4 to 69·4)	67·4 (67·1 to 67·6)	68·1 (67·9 to 68·4)	71·3 (71·1 to 71·5)	73·6 (73·3 to 73·8)	75·7 (75·4 to 75·9)	78 (77·7 to 78·2)	78·3 (78 to 78·5)	77 (76·7 to 77·2)	78·1 (75·8 to 80·2)
Michigan	66·5 (63·7 to 69·2)	67·4 (67·2 to 67·5)	67·4 (67·2 to 67·5)	70·2 (70 to 70·4)	72 (71·8 to 72·2)	73·7 (73·6 to 73·9)	75·6 (75·3 to 75·8)	76 (75·8 to 76·2)	73·8 (73·6 to 74)	73·8 (71·7 to 76)
Minnesota	67·8 (64·7 to 70·8)	69 (68·7 to 69·3)	69·6 (69·3 to 69·9)	72·6 (72·3 to 72·8)	74·7 (74·4 to 75)	76·4 (76·1 to 76·7)	78·5 (78·2 to 78·7)	78·6 (78·3 to 78·9)	77·3 (77 to 77·5)	77·4 (75·3 to 79·5)
Mississippi	62·4 (59·1 to 65·7)	64·6 (64·2 to 64·9)	64·2 (63·9 to 64·6)	67·7 (67·4 to 68·1)	69·4 (69 to 69·7)	70·3 (70 to 70·6)	72 (71·7 to 72·3)	71·4 (71·1 to 71·8)	68·8 (68·4 to 69·1)	68·5 (66·4 to 70·7)
Missouri	65·9 (63 to 68·9)	67·2 (66·9 to 67·4)	67 (66·7 to 67·2)	70 (69·7 to 70·2)	71·9 (71·6 to 72·1)	73·3 (73 to 73·5)	74·8 (74·5 to 75)	74·5 (74·2 to 74·8)	72·5 (72·2 to 72·7)	72·5 (70·2 to 74·6)
Montana	64·5 (61·4 to 67·6)	66·4 (65·8 to 67)	67·5 (66·9 to 68·1)	70·4 (69·8 to 71)	73·7 (73·1 to 74·3)	74·7 (74·1 to 75·2)	76·3 (75·7 to 76·9)	76·8 (76·2 to 77·4)	74·9 (74·3 to 75·5)	74·4 (72·2 to 76·7)
Nebraska	67·7 (64·6 to 70·6)	68·9 (68·5 to 69·4)	69 (68·5 to 69·4)	71·7 (71·3 to 72·1)	73·7 (73·3 to 74·1)	75·7 (75·2 to 76·1)	77·3 (76·8 to 77·7)	77·4 (76·9 to 77·9)	75·8 (75·3 to 76·2)	76·1 (74 to 78·3)
Nevada	58·8 (55·8 to 61·6)	64·1 (63·4 to 64·8)	65·9 (65·3 to 66·4)	69·2 (68·7 to 69·7)	71·3 (70·9 to 71·7)	73·5 (73·1 to 73·9)	75·7 (75·3 to 76)	75·9 (75·6 to 76·3)	73·7 (73·4 to 74)	73 (71 to 75·2)
New Hampshire	64·7 (61·9 to 67·6)	67·3 (66·7 to 67·8)	67·9 (67·3 to 68·4)	71·9 (71·4 to 72·4)	73·7 (73·2 to 74·3)	75·9 (75·4 to 76·4)	78 (77·5 to 78·5)	77·5 (76·9 to 78)	77 (76·4 to 77·6)	76·6 (74·3 to 78·9)
New Jersey	65·7 (62·8 to 68·7)	67·2 (66·9 to 67·4)	67·7 (67·5 to 67·9)	70·5 (70·3 to 70·7)	72·4 (72·2 to 72·6)	74·8 (74·6 to 75)	77·6 (77·3 to 77·8)	78·4 (78·1 to 78·6)	75·4 (75·1 to 75·6)	77·1 (75 to 79·4)
New Mexico	63·7 (60·2 to 67)	66·5 (66 to 67·1)	66·9 (66·4 to 67·4)	69·8 (69·4 to 70·3)	72·6 (72·1 to 73)	74·4 (73·9 to 74·8)	75·7 (75·2 to 76·1)	74·5 (74 to 74·9)	72 (71·6 to 72·5)	71·5 (69·1 to 73·8)

New York	65·6 (63 to 68·2)	66·7 (66·6 to 66·9)	67 (66·9 to 67·1)	70 (69·8 to 70·1)	71·1 (70·9 to 71·2)	75·1 (75 to 75·3)	77·8 (77·7 to 78)	79 (78·8 to 79·1)	75·6 (75·5 to 75·8)	77·2 (75·2 to 79·3)
North Carolina	63·7 (60·7 to 66·8)	64·7 (64·5 to 65)	65·2 (64·9 to 65·4)	68·7 (68·5 to 69)	70·9 (70·7 to 71·1)	72·7 (72·5 to 72·9)	75·3 (75·1 to 75·5)	75·3 (75 to 75·5)	73·5 (73·3 to 73·7)	72·8 (70·6 to 75)
North Dakota	67·2 (64·2 to 70)	68·8 (68·3 to 69·3)	69·4 (68·9 to 69·9)	72·2 (71·7 to 72·7)	74·3 (73·8 to 74·9)	75·5 (75 to 76)	76·9 (76·4 to 77·4)	77·3 (76·7 to 77·9)	75·5 (74·9 to 76·1)	77 (75·2 to 78·9)
Ohio	66·4 (63·7 to 69·2)	67·4 (67·2 to 67·5)	67·4 (67·2 to 67·5)	69·8 (69·7 to 70)	72·2 (72 to 72·4)	73·6 (73·5 to 73·8)	75·2 (75 to 75·3)	74·6 (74·4 to 74·8)	72·9 (72·7 to 73·1)	72·4 (70·4 to 74·5)
Oklahoma	66·4 (63·3 to 69·4)	67·3 (67 to 67·6)	67·2 (66·9 to 67·5)	69·7 (69·4 to 70)	72 (71·6 to 72·3)	72·4 (72·1 to 72·7)	73·1 (72·8 to 73·4)	73·5 (73·2 to 73·8)	71·8 (71·5 to 72·1)	70·7 (68·6 to 72·8)
Oregon	66·5 (63·5 to 69·5)	67·7 (67·3 to 68·1)	68·8 (68·5 to 69·2)	71·4 (71 to 71·7)	73·4 (73 to 73·7)	75·6 (75·3 to 75·9)	77·4 (77·1 to 77·7)	77·7 (77·4 to 78·1)	76·9 (76·5 to 77·2)	75·6 (73·4 to 77·8)
Pennsylvania	65·5 (62·9 to 68·2)	66·7 (66·5 to 66·9)	67 (66·9 to 67·2)	69·9 (69·7 to 70)	72·2 (72 to 72·4)	73·8 (73·6 to 74)	75·9 (75·7 to 76·1)	76·1 (75·9 to 76·3)	74·4 (74·2 to 74·6)	74·2 (72·1 to 76·3)
Rhode Island	65·6 (62·7 to 68·4)	67·6 (67·1 to 68·1)	68·3 (67·8 to 68·8)	70·8 (70·3 to 71·3)	73·1 (72·6 to 73·7)	75·3 (74·7 to 75·8)	76·9 (76·3 to 77·4)	77·5 (76·9 to 78·1)	76·1 (75·6 to 76·7)	76·9 (74·7 to 79)
South Carolina	61·7 (58·6 to 64·9)	62·9 (62·6 to 63·2)	64·1 (63·8 to 64·4)	67·7 (67·4 to 68)	70·1 (69·8 to 70·4)	71·6 (71·3 to 71·8)	73·9 (73·7 to 74·2)	74 (73·7 to 74·3)	71·7 (71·5 to 72)	71·1 (68·8 to 73·3)
South Dakota	66 (63 to 69)	67·7 (67·2 to 68·2)	68·7 (68·2 to 69·1)	71 (70·4 to 71·5)	73·5 (72·9 to 74)	74·7 (74·2 to 75·3)	76·7 (76·1 to 77·2)	76·4 (75·7 to 77)	74·4 (73·8 to 75)	75·3 (73·4 to 77)
Tennessee	65·1 (62 to 68·2)	66·3 (66 to 66·6)	66·1 (65·8 to 66·3)	69·1 (68·8 to 69·3)	70·4 (70·2 to 70·7)	71·7 (71·5 to 71·9)	73·5 (73·3 to 73·8)	73·1 (72·9 to 73·4)	71 (70·8 to 71·3)	70·3 (68 to 72·4)
Texas	65·8 (62·8 to 68·8)	67·1 (66·9 to 67·3)	67·1 (66·9 to 67·3)	69·8 (69·7 to 70)	71·6 (71·4 to 71·7)	74 (73·9 to 74·1)	76·2 (76·1 to 76·3)	76·6 (76·5 to 76·7)	74·2 (74·1 to 74·3)	73·4 (71·6 to 75·3)
Utah	67·1 (63·9 to 70)	68·5 (67·9 to 69)	69·3 (68·9 to 69·8)	72·7 (72·2 to 73·1)	75·1 (74·7 to 75·6)	76·6 (76·2 to 77)	77·9 (77·4 to 78·2)	78·3 (77·9 to 78·7)	77 (76·6 to 77·3)	76·4 (74·4 to 78·4)
Vermont	64·1 (61·3 to 66·8)	67·1 (66·5 to 67·6)	68·2 (67·6 to 68·7)	71 (70·5 to 71·5)	73·5 (72·9 to 74)	75·7 (75·2 to 76·2)	77·8 (77·3 to 78·4)	77·9 (77·2 to 78·6)	77·5 (76·8 to 78·3)	76·6 (74·7 to 78·5)
Virginia	64·3 (61·3 to 67·2)	65·7 (65·4 to 65·9)	66·5 (66·2 to 66·7)	69·6 (69·4 to 69·8)	72 (71·8 to 72·2)	74·3 (74 to 74·5)	76·8 (76·6 to 77)	77·3 (77 to 77·5)	75·6 (75·3 to 75·8)	75 (73 to 77·2)
Washington	66·7 (63·8 to 69·6)	68 (67·7 to 68·3)	68·3 (68·1 to 68·6)	71·8 (71·5 to 72·1)	73·8 (73·5 to 74·1)	75·8 (75·5 to 76·1)	77·9 (77·6 to 78·1)	78·5 (78·2 to 78·8)	77·5 (77·3 to 77·8)	77 (74·9 to 79·1)
West Virginia	64·6 (61·4 to 67·7)	66·2 (65·8 to 66·6)	65·5 (65·1 to 65·9)	68·7 (68·4 to 69·1)	71 (70·6 to 71·4)	72·1 (71·7 to 72·5)	72·7 (72·3 to 73·1)	72·3 (71·8 to 72·8)	70·3 (69·9 to 70·7)	69 (66·9 to 71·3)
Wisconsin	67·2 (64·3 to 70·1)	68·3 (68 to 68·6)	69·3 (69 to 69·6)	72 (71·7 to 72·2)	73·7 (73·5 to 74)	75·2 (74·9 to 75·4)	77·3 (77 to 77·5)	77·3 (77 to 77·6)	75·5 (75·3 to 75·8)	76 (73·8 to 78·3)
Wyoming	64·1 (60·9 to 67)	66·5 (65·9 to 67·2)	66·7 (66·1 to 67·4)	70·3 (69·7 to 70·8)	73·2 (72·6 to 73·8)	74·6 (74·1 to 75·2)	76·2 (75·6 to 76·7)	76·2 (75·5 to 77)	74·8 (74·2 to 75·5)	73·5 (71·7 to 75·1)
Southern Latin America	58·8 (58·5 to 59·1)	59·5 (59·4 to 59·7)	62 (61·8 to 62·1)	66·8 (66·7 to 66·9)	69·3 (69·2 to 69·4)	71·4 (71·3 to 71·5)	73·5 (73·4 to 73·5)	75 (74·9 to 75·1)	74·1 (74 to 74·3)	73·8 (73·5 to 74·1)
Argentina	61·5 (61·2 to 61·7)	62·2 (62 to 62·3)	62·5 (62·4 to 62·7)	66·8 (66·7 to 66·9)	68·9 (68·8 to 69)	70·5 (70·4 to 70·6)	72·6 (72·5 to 72·7)	74 (73·9 to 74·1)	73·1 (72·9 to 73·2)	73 (72·7 to 73·3)
Chile	50·6 (50·2 to 51·1)	52·7 (52·4 to 52·9)	59·8 (59·6 to 60)	66·8 (66·6 to 66·9)	70·3 (70·2 to 70·5)	74·1 (73·9 to 74·2)	75·9 (75·8 to 76·1)	78·1 (77·9 to 78·2)	76·7 (76·5 to 76·8)	76·1 (76 to 76·3)
Uruguay	63·8 (63·3 to 64·2)	65·2 (64·9 to 65·5)	65 (64·7 to 65·3)	67·1 (66·9 to 67·3)	69·4 (69·2 to 69·6)	70·9 (70·7 to 71·1)	72·8 (72·5 to 73·1)	73·6 (73·4 to 73·9)	74·6 (74·2 to 75)	72 (71·6 to 72·4)
Western Europe	64·5 (64·3 to 64·7)	67·3 (67·3 to 67·4)	68·5 (68·4 to 68·5)	70·6 (70·6 to 70·7)	73 (72·9 to 73)	75·6 (75·5 to 75·6)	78·5 (78·4 to 78·5)	80 (79·9 to 80·1)	79·2 (79·2 to 79·2)	79·4 (79·3 to 79·4)
Andorra	71·2 (68 to 74·6)	71·8 (68·9 to 75·3)	73·2 (69·7 to 76·5)	74·5 (71 to 77·7)	75·8 (72·7 to 78·6)	77·2 (74·8 to 79·7)	79·2 (77·2 to 81·2)	80·6 (78·1 to 83)	80·4 (78·1 to 82·7)	80·7 (77·9 to 83·6)
Austria	63·6 (63·3 to 63·9)	65·5 (65·3 to 65·7)	66·5 (66·4 to 66·7)	69 (68·8 to 69·1)	72·4 (72·2 to 72·5)	75·3 (75·2 to 75·4)	77·9 (77·8 to 78·1)	79·7 (79·5 to 79·8)	78·9 (78·8 to 79)	79·2 (79·1 to 79·4)
Belgium	63·3 (63·1 to 63·6)	67·1 (66·9 to 67·2)	67·8 (67·7 to 68)	69·8 (69·7 to 69·9)	72·7 (72·6 to 72·8)	74·7 (74·6 to 74·9)	77·5 (77·4 to 77·6)	79·4 (79·2 to 79·6)	78·2 (78·1 to 78·4)	79·3 (79·1 to 79·5)
Cyprus	56·1 (52·8 to 59·3)	60·1 (57·9 to 62·2)	63·9 (62·7 to 65)	68·3 (68·1 to 68·6)	72·6 (72·3 to 73)	74·1 (73·3 to 75)	77·2 (76·3 to 78)	78·7 (77·8 to 79·7)	79·5 (78·6 to 80·4)	79·2 (78·2 to 80·1)
Denmark	69·5 (69·1 to 69·8)	70·6 (70·4 to 70·8)	71 (70·8 to 71·1)	71·3 (71·1 to 71·4)	72·3 (72·2 to 72·5)	74·7 (74·5 to 74·8)	77·4 (77·2 to 77·5)	79·5 (79·3 to 79·7)	79·6 (79·4 to 79·8)	79·5 (79·3 to 79·7)
Finland	60·7 (60·4 to 61)	65·6 (65·4 to 65·8)	66·3 (66·1 to 66·4)	69·5 (69·3 to 69·6)	71·2 (71 to 71·3)	74·4 (74·2 to 74·6)	77·1 (76·9 to 77·3)	79·5 (79·3 to 79·8)	79·6 (79·4 to 79·8)	79·5 (79·2 to 79·7)
France	64·5 (64·3 to 64·7)	67·8 (67·8 to 67·9)	68·8 (68·8 to 68·9)	70·5 (70·5 to 70·6)	73 (72·9 to 73)	75·3 (75·3 to 75·4)	78·1 (78 to 78·1)	80·1 (80 to 80·1)	79 (79 to 79·1)	79·6 (79·5 to 79·7)
Germany	64·4 (63·6 to 65·1)	66·5 (66·4 to 66·6)	67·2 (67·2 to 67·3)	69·7 (69·6 to 69·8)	72·1 (72·1 to 72·2)	75·3 (75·2 to 75·3)	77·9 (77·9 to 78)	79·1 (79 to 79·1)	78·8 (78·7 to 78·9)	78·5 (78·5 to 78·6)
Greece	67·8 (67·4 to 68·2)	70·7 (70·5 to 70·9)	71·7 (71·5 to 71·8)	72·8 (72·7 to 72·9)	74·7 (74·6 to 74·8)	75·9 (75·8 to 76)	77·8 (77·7 to 77·9)	78·7 (78·5 to 78·9)	78·2 (78 to 78·3)	77·2 (77 to 77·5)
Iceland	69 (68 to 69·9)	71·1 (70·6 to 71·6)	71·2 (70·7 to 71·8)	73·6 (73·1 to 74)	75·9 (75·4 to 76·4)	78·3 (77·8 to 78·7)	80 (79·5 to 80·5)	81·5 (80·8 to 82·2)	81·6 (81 to 82·2)	82·3 (81·6 to 83)
Ireland	65 (64·7 to 65·4)	68·5 (68·3 to 68·7)	69 (68·7 to 69·1)	70 (69·8 to 70·2)	72·2 (72 to 72·4)	74 (73·8 to 74·2)	78·6 (78·4 to 78·7)	80·5 (80·3 to 80·8)	80·6 (80·4 to 80·8)	80·8 (80·5 to 81)
Israel	72·2 (71·7 to 72·7)	70·9 (70·6 to 71·1)	69·9 (69·7 to 70·1)	72·4 (72·2 to 72·5)	75·5 (75·3 to 75·6)	76·8 (76·6 to 76·9)	80·1 (79·9 to 80·2)	81·7 (81·5 to 81·9)	81·3 (81·2 to 81·5)	81·2 (80·9 to 81·5)
Italy	65·2 (65 to 65·4)	67·3 (67·2 to 67·4)	68·8 (68·7 to 68·9)	70·8 (70·7 to 70·9)	73·7 (73·7 to 73·8)	76·5 (76·4 to 76·6)	79·3 (79·3 to 79·4)	81·1 (81·1 to 81·2)	79·9 (79·8 to 79·9)	80·3 (80·2 to 80·4)
Luxembourg	63·5 (62·6 to 64·3)	66·2 (65·8 to 66·7)	66·4 (65·9 to 66·9)	69·3 (68·8 to 69·7)	71·6 (71·2 to 72·1)	75 (74·5 to 75·5)	78·5 (78 to 79)	80·6 (80 to 81·1)	80·1 (79·5 to 80·6)	80·4 (79·8 to 81)
Malta	64·6 (63·7 to 65·5)	66·9 (66·5 to 67·3)	68·6 (68·3 to 69)	70·5 (70·1 to 70·9)	74·1 (73·6 to 74·5)	76·3 (75·9 to 76·7)	79 (78·5 to 79·4)	80·9 (80·2 to 81·6)	81 (80·4 to 81·6)	81·3 (80·6 to 82)
Monaco	64 (62·2 to 65·7)	67·3 (65·6 to 69·3)	70·2 (68·5 to 72)	72·3 (70·8 to 73·8)	74·7 (72·9 to 76·8)	75·9 (73·7 to 78)	77·1 (75·7 to 78·6)	78·2 (76·7 to 79·5)	77·8 (76·1 to 79·4)	76·3 (74·7 to 77·8)
Netherlands	70·6 (70·4 to 70·8)	71·4 (71·3 to 71·6)	70·9 (70·7 to 71)	72·5 (72·4 to 72·6)	73·8 (73·7 to 73·9)	75·5 (75·4 to 75·6)	78·8 (78·7 to 78·9)	80·3 (80·2 to 80·5)	79·6 (79·5 to 79·7)	79·8 (79·6 to 79·9)
Norway	70·6 (70·3 to 70·9)	71·5 (71·3 to 71·7)	71·3 (71·1 to 71·4)	72·5 (72·4 to 72·7)	73·7 (73·5 to 73·8)	76 (75·9 to 76·2)	79 (78·8 to 79·1)	81·3 (81·1 to 81·5)	81·6 (81·4 to 81·8)	81·7 (81·5 to 81·8)
Agder	70·5 (69·2 to 71·5)	70·9 (70·3 to 71·5)	71 (70·4 to 71·6)	72 (71·4 to 72·5)	72·8 (72·2 to 73·3)	75·6 (75·1 to 76·2)	78·4 (77·9 to 79)	80·9 (80 to 81·7)	81·1 (80·3 to 81·9)	80·7 (79·1 to 82·4)
Innlandet	72·5 (71·5 to 73·4)	73 (72·5 to 73·5)	73 (72·4 to 73·5)	73·6 (73·1 to 74·1)	73·7 (73·2 to 74·2)	75·7 (75·2 to 76·2)	78·1 (77·6 to 78·6)	80·7 (80 to 81·5)	81·6 (80·8 to 82·4)	81·8 (80·3 to 83·3)
Møre og Romsdal	70·9 (69·7 to 72)	73 (72·3 to 73·6)	72·1 (71·6 to 72·7)	73·3 (72·7 to 73·9)	75·4 (74·8 to 75·9)	77 (76·4 to 77·6)	80 (79·4 to 80·6)	81·8 (80·8 to 82·7)	81·9 (80·9 to 82·9)	82·2 (80·2 to 84·2)
Nordland	70·2 (69 to 71·2)	71·3 (70·7 to 71·9)	71 (70·4 to 71·5)	72·6 (72 to 73·2)	73·1 (72·5 to 73·7)	75·8 (75·2 to 76·4)	78·1 (77·5 to 78·8)	80·8 (80 to 81·8)	81·1 (80·2 to 82)	80·9 (79 to 82·9)
Oslo	73·3 (72 to 74·5)	70·2 (69·7 to 70·7)	69·9 (69·4 to 70·4)	71 (70·5 to 71·5)	72 (71·4 to 72·5)	75 (74·5 to 75·6)	79·1 (78·5 to 79·6)	81·8 (81·1 to 82·6)	82·2 (81·4 to 82·9)	82·6 (80·9 to 84·2)
Rogaland	70·3 (69·2 to 71·4)	71·2 (70·6 to 71·8)	71·5 (70·9 to 72)	72·7 (72·2 to 73·3)	74·8 (74·3 to 75·3)	76·6 (76·1 to 77·2)	79·3 (78·7 to 79·7)	81·3 (80·7 to 82·1)	81·6 (81 to 82·3)	81·9 (80·6 to 83·2)
Troms og Finnmark	67·9 (66·7 to 69)	69 (68·4 to 69·6)	69·5 (69 to 70·1)	70·9 (70·3 to 71·4)	71·9 (71·4 to 72·5)	74·7 (74·2 to 75·3)	78·3 (77·7 to 78·9)	80·7 (79·7 to 81·6)	79·9 (78·9 to 80·8)	79·2 (77·2 to 81)
Trøndelag	70·7 (69·6 to 71·6)	72 (71·4 to 72·5)	71·6 (71·1 to 72·1)	72·7 (72·2 to 73·2)	73·8 (73·2 to 74·2)	76·6 (76·1 to 77·2)	79·3 (78·8 to 79·8)	81·2 (80·5 to 81·9)	81·9 (81·2 to 82·6)	82·1 (80·7 to 83·4)
Vestfold og Telemark	70·8 (69·6 to 71·8)	71·4 (70·9 to 72)	71·2 (70·7 to 71·7)	72·3 (71·8 to 72·9)	73·5 (73 to 74)	75·5 (75 to 76)	78·8 (78·2 to 79·3)	81·1 (80·3 to 81·9)	81·7 (80·9 to 82·5)	81·7 (80·1 to 83·4)
Vestland	71·3 (70·2 to 72·2)	72·3 (71·7 to 72·8)	72 (71·5 to 72·5)	73·6 (73·1 to 74)	75 (74·5 to 75·5)	77 (76·5 to 77·5)	79·5 (79 to 80)	81·8 (81·1 to 82·6)	81·9 (81·2 to 82·6)	82·2 (80·6 to 83·7)
Viken	68·9 (67·9 to 69·8)	71·8 (71·3 to 72·2)	71·3 (70·9 to 71·7)	72·8 (72·3 to 73·2)	73·8 (73·4 to 74·2)	76·2 (75·7 to 76·6)	79·1 (78·7 to 79·5)	81·4 (80·8 to 82)	81·7 (81·1 to 82·2)	81·6 (80·3 to 83)
Portugal	55·9 (55·3 to 56·5)	62 (61·7 to 62·2)	63·8 (63·6 to 64)	68·1 (68 to 68·3)	70·6 (70·5 to 70·8)	73·3 (73·1 to 73·4)	77 (76·9 to 77·2)	79·1 (78·9 to 79·3)	78·6 (78·4 to 78·7)	78·5 (78·3 to 78·7)
San Marino	69·4 (65·8 to 72·3)	69·9 (68 to 72)	71·2 (69·8 to 72·2)	73·3 (72·1 to 74·7)	76·6 (75·3 to 78)	78·4 (77·4 to 79·6)	80·5 (79·6 to 81·3)	81 (79·5 to 82·2)	83·4 (81·3 to 85·4)	84·4 (81·4 to 87·1)
Spain	59·6 (59·3 to 60)	67 (66·8 to 67·1)	69·2 (69·1 to 69·3)	72·4 (72·3 to 72·5)	73·3 (73·2 to 73·4)	75·9 (75·8 to 76)	78·9 (78·9 to 79)	80·4 (80·3 to 80·5)	79·3 (79·2 to 79·4)	79·9 (79·8 to 80)
Sweden	70·3 (70·1 to 70·5)	71·5 (71·4 to 71·7)	72·3 (72·2 to 72·4)	73 (72·9 to 73·1)	75 (74·9 to 75·1)	77·5 (77·4 to 77·6)	79·8 (79·7 to 79·9)	81·6 (81·4 to 81·7)	81·1 (80·9 to 81·2)	82 (80·9 to 83)
Stockholm	70·1 (66·9 to 73·4)	71·1 (67·7 to 74·5)	71·5 (68·4 to 74·6)	71·7 (71·3 to 72·1)	73·7 (73·3 to 74·1)	76·9 (76·5 to 77·3)	79·4 (79·1 to 79·8)	81·5 (81·1 to 81·9)	80·7 (80·2 to 81)	83·2 (81·5 to 84·7)
Sweden except Stockholm	70·4 (69·8 to 71)	71·6 (71·1 to 72·3)	72·5 (71·9 to 73·2)	73·3 (73·1 to 73·4)	75·3 (75·2 to 75·4)	77·7 (77·5 to 77·8)	79·9 (79·7 to 80)	81·6 (81·4 to 81·8)	81·1 (81 to 81·3)	81·6 (80·7 to 82·7)
Switzerland	66·7 (66·4 to 67)	68·9 (68·8 to 69·1)	70·1 (70 to 70·3)	72·5 (72·3 to 72·6)	74·3 (74·2 to 74·4)	77·3 (77·2 to 77·5)	80·5 (80·4 to 80·7)	82·9 (82·7 to 83)	81·7 (81·5 to 81·8)	82·5 (82·3 to 82·7)
UK	66·9 (66·8 to 67)	67·9 (67·9 to 68)	68·8 (68·7 to 68·8)	70·5 (70·4 to 70·5)	72·9 (72·8 to 73)	75·4 (75·4 to 75·5)	78·5 (78·5 to 78·6)	79·6 (79·5 to 79·7)	78·5 (78·4 to 78·6)	78·2 (78·1 to 78·3)
England	67·1 (66·9 to 67·2)	68·2 (68·1 to 68·3)	69 (68·9 to 69·1)	70·7 (70·7 to 70·8)	73·1 (73·1 to 73·2)	75·7 (75·7 to 75·8)	78·9 (78·8 to 78·9)	79·9 (79·8 to 80)	78·7 (78·6 to 78·8)	78·4 (78·3 to 78·5)
East Midlands	67·1 (64·4 to 69·8)	68·2 (65·6 to 70·9)	69·1 (66·3 to 71·8)	70·8 (70·1 to 71·6)	73·3 (73 to 73·5)	75·7 (75·4 to 75·9)	78·7 (78·4 to 79)	79·7 (79·4 to 80)	78·6 (78·3 to 78·9)	78·1 (77·3 to 78·8)
Derby	66·1 (62 to 69·8)	67·5 (63·8 to 71·2)	68·3 (64·9 to 71·6)	69·9 (68·7 to 71)	72·4 (71·9 to 73)	75·3 (74·7 to 75·9)	77·4 (76·8 to 78)	79·2 (78·4 to 80)	77·8 (77·1 to 78·4)	76·5 (75·4 to 77·6)

Derbyshire	66·6 (63·2 to 70)	68·1 (64·8 to 71·5)	68·9 (65·4 to 72·2)	70·8 (69·8 to 71·7)	73 (72·6 to 73·5)	75·8 (75·4 to 76·3)	79·1 (78·6 to 79·6)	79·5 (79 to 80·1)	78·8 (78·3 to 79·3)	78·3 (77·4 to 79·2)
Leicester	65·7 (61·9 to 69·4)	66·7 (63·2 to 70·4)	67·5 (64 to 70·9)	69·6 (68·5 to 70·7)	71·9 (71·3 to 72·5)	73·6 (73 to 74·2)	76·5 (75·9 to 77·1)	78·1 (77·4 to 78·9)	77·3 (76·7 to 77·9)	76·6 (75·6 to 77·6)
Leicestershire	69 (65·2 to 72·6)	69·8 (66·2 to 73·4)	70·6 (66·9 to 73·7)	72 (71 to 73)	74·6 (74·1 to 75)	77 (76·6 to 77·5)	79·6 (79·1 to 80·1)	81·2 (80·6 to 81·8)	79·6 (79·1 to 80·1)	79·6 (78·7 to 80·5)
Lincolnshire	67·5 (63·6 to 71·2)	68·6 (64·8 to 72·3)	69·5 (66 to 72·8)	71·2 (70·2 to 72·2)	73·5 (73·1 to 74)	75·8 (75·3 to 76·3)	79 (78·5 to 79·4)	79·3 (78·7 to 79·8)	78·2 (77·7 to 78·8)	78·6 (77·6 to 79·5)
Northamptonshire	67·6 (63·9 to 71·3)	68·7 (65·3 to 72·5)	69·7 (66·2 to 73)	71·3 (70·3 to 72·4)	73·5 (73 to 73·9)	75·9 (75·3 to 76·3)	78·6 (78·1 to 79·1)	79·7 (79·1 to 80·3)	78·7 (78·2 to 79·3)	76·7 (75 to 78·8)
Nottingham	66 (62 to 69·7)	66·7 (63·1 to 70·3)	67·3 (63·9 to 70·4)	69 (67·9 to 70·1)	71·1 (70·6 to 71·6)	72·5 (71·9 to 73·1)	76·2 (75·6 to 76·8)	78·1 (77·3 to 78·9)	76·1 (75·4 to 76·7)	76·4 (75·4 to 77·4)
Nottinghamshire	67·6 (64·2 to 71)	68·6 (65·1 to 71·8)	69·4 (66 to 72·7)	71 (70 to 72)	73·7 (73·3 to 74·2)	75·8 (75·4 to 76·3)	79 (78·5 to 79·5)	80 (79·4 to 80·6)	78·9 (78·5 to 79·5)	78·5 (77·6 to 79·4)
Rutland	70·2 (66·3 to 73·9)	71·3 (67·3 to 74·9)	72·3 (68·5 to 75·8)	73·6 (72·1 to 75·2)	75·3 (74·3 to 76·3)	78 (77 to 79·1)	80·8 (79·7 to 82)	83 (81·5 to 84·6)	81·7 (80·4 to 83)	80·2 (78·3 to 82)
East of England	68·6 (65·9 to 71·2)	69·6 (67 to 72·1)	70·4 (67·7 to 73)	72·1 (71·3 to 72·8)	74·5 (74·2 to 74·7)	76·8 (76·5 to 77)	79·7 (79·4 to 79·9)	80·7 (80·4 to 80·9)	79·7 (79·4 to 80)	79·5 (78·8 to 80·2)
Bedford	67·9 (63·8 to 71·9)	69 (65·3 to 72·5)	69·7 (66·2 to 73)	71·5 (70·2 to 72·7)	74 (73·3 to 74·8)	75·5 (74·8 to 76·2)	78·4 (77·7 to 79·1)	79 (78·1 to 80)	77·8 (77 to 78·6)	78·2 (77 to 79·3)
Cambridgeshire	70·1 (66·3 to 73·8)	70·3 (66·9 to 73·9)	70·9 (67·4 to 74·3)	72·4 (71·4 to 73·5)	74·8 (74·3 to 75·3)	77·2 (76·7 to 77·7)	80·5 (80 to 81)	81·2 (80·6 to 81·8)	80·4 (79·9 to 81)	80·7 (79·8 to 81·6)
Central Bedfordshire	69·2 (65·4 to 73·3)	70·2 (66·6 to 73·7)	71·1 (67·7 to 74·4)	72·8 (71·7 to 74)	75·1 (74·4 to 75·7)	77·2 (76·6 to 77·8)	79·8 (79·1 to 80·4)	81·3 (80·4 to 82·2)	79·6 (78·9 to 80·3)	80·7 (79·7 to 81·8)
Essex	68·3 (65·2 to 71·5)	69·6 (66·3 to 72·8)	70·2 (67 to 73·3)	71·9 (71 to 72·8)	74·5 (74·2 to 74·9)	77 (76·5 to 77·3)	79·7 (79·3 to 80·1)	80·7 (80·3 to 81·2)	79·6 (79·2 to 80)	78·9 (78·1 to 79·8)
Hertfordshire	68·7 (65·1 to 72·3)	70 (66·4 to 73·6)	70·5 (67·2 to 73·6)	72·1 (71·1 to 73)	74·6 (74·2 to 75)	77 (76·5 to 77·4)	80·2 (79·7 to 80·6)	81·3 (80·8 to 81·8)	80·2 (79·8 to 80·7)	80·5 (79·6 to 81·3)
Luton	67·1 (63·3 to 71·1)	68·3 (64·7 to 71·6)	69·4 (66·1 to 72·9)	71·3 (70·1 to 72·4)	72·8 (72·2 to 73·3)	74·7 (74·1 to 75·4)	78·2 (77·5 to 78·8)	78·7 (77·9 to 79·6)	76·5 (75·8 to 77·2)	76·5 (75·3 to 77·6)
Norfolk	68·7 (65·2 to 72·3)	69·7 (66·3 to 73·1)	70·6 (67·3 to 73·8)	72·2 (71·2 to 73·2)	74·4 (74 to 74·8)	76·8 (76·3 to 77·3)	79·1 (78·7 to 79·6)	79·9 (79·3 to 80·5)	79·4 (78·9 to 79·9)	79·2 (78·3 to 80·1)
Peterborough	67·4 (63·4 to 71·3)	68·4 (64·7 to 72·1)	68·9 (65·6 to 72·3)	71·3 (70 to 72·6)	73·1 (72·5 to 73·8)	75·2 (74·6 to 75·8)	77·7 (77 to 78·4)	79·1 (78·2 to 80)	77·8 (77 to 78·5)	78·3 (77·3 to 79·3)
Southend-on-Sea	67·5 (63·6 to 71·4)	68·9 (65·4 to 72·3)	70 (66·5 to 73·5)	72·1 (70·9 to 73·4)	73·9 (73·2 to 74·5)	75·6 (75 to 76·3)	78·9 (78·3 to 79·6)	80 (79·1 to 81)	78·7 (78 to 79·5)	77·6 (76·6 to 78·7)
Suffolk	69·1 (65·5 to 72·6)	70 (66·3 to 73·5)	71 (67·5 to 74·2)	72·4 (71·4 to 73·5)	74·9 (74·4 to 75·3)	77 (76·6 to 77·5)	80·3 (79·8 to 80·8)	81·1 (80·5 to 81·7)	80·5 (80 to 81)	80 (79·1 to 80·9)
Thurrock	67·8 (63·8 to 71·7)	68·4 (64·8 to 72·2)	69 (65·4 to 72·4)	70·8 (69·5 to 72)	73·3 (72·7 to 73·9)	76·5 (75·8 to 77·2)	78·7 (78 to 79·4)	82 (81 to 83)	79·9 (79 to 80·8)	78·3 (77 to 79·5)
Greater London	67 (64·5 to 69·5)	68·1 (65·6 to 70·5)	69 (66·8 to 71·4)	70·7 (70 to 71·3)	72·8 (72·6 to 73)	75·4 (75·2 to 75·6)	79·4 (79·2 to 79·7)	81 (80·8 to 81·3)	78·9 (78·6 to 79·1)	78·4 (77·7 to 79·1)
Barking and Dagenham	64·2 (60·4 to 67·7)	66 (62·7 to 69·4)	67 (63·9 to 70·3)	69·1 (67·9 to 70·2)	71·8 (71·2 to 72·4)	73·9 (73·3 to 74·5)	76·6 (76 to 77·3)	78·1 (77·1 to 79)	75·4 (74·7 to 76·1)	75·4 (74·2 to 76·4)
Barnet	69·2 (65·6 to 72·8)	70·4 (66·9 to 73·8)	71·4 (68 to 74·6)	72·9 (71·8 to 74·1)	75·1 (74·6 to 75·7)	77·7 (77·2 to 78·2)	80·9 (80·3 to 81·4)	82·7 (81·8 to 83·5)	80·4 (79·8 to 81)	79·5 (78·4 to 80·5)
Bexley	68·8 (65 to 72·5)	70·2 (66·7 to 73·7)	71·1 (67·8 to 74·4)	72·6 (71·6 to 73·7)	75·1 (74·5 to 75·7)	77·3 (76·8 to 77·9)	79·4 (78·8 to 80·1)	82·1 (81·2 to 83)	79·7 (78·9 to 80·4)	79·1 (78 to 80·3)
Brent	66·8 (63 to 70·3)	68·1 (64·7 to 71·7)	69·3 (65·9 to 72·4)	70·8 (69·7 to 71·9)	73 (72·5 to 73·6)	75·6 (75·1 to 76·2)	79·9 (79·2 to 80·5)	80·6 (79·8 to 81·4)	78·7 (78·1 to 79·4)	78 (76·9 to 79)
Bromley	69·4 (65·6 to 73·2)	70·8 (67·3 to 74·3)	71·7 (68·4 to 75)	72·9 (71·8 to 73·9)	74·9 (74·4 to 75·4)	77·4 (76·9 to 77·9)	81·5 (80·9 to 82·1)	83 (82·1 to 83·8)	81·1 (80·4 to 81·8)	80·7 (79·7 to 81·8)
Camden	67·2 (63·6 to 70·7)	67·1 (63·7 to 70·7)	67·5 (64·2 to 70·7)	68·6 (67·5 to 69·7)	71·2 (70·6 to 71·8)	72·2 (71·7 to 72·7)	78·3 (77·7 to 78·9)	81·4 (80·4 to 82·2)	81·7 (80·8 to 82·5)	81·8 (80·6 to 83·1)
Croydon	68 (64·2 to 71·5)	69·6 (65·9 to 72·8)	70·7 (67·3 to 73·9)	72 (71 to 73·1)	74·1 (73·6 to 74·6)	76·4 (75·8 to 76·9)	79·7 (79·1 to 80·3)	80·4 (79·6 to 81·1)	77·7 (77·2 to 78·3)	78·5 (77·4 to 79·4)
Ealing	66·8 (63·2 to 70·5)	68 (64·3 to 71·4)	68·7 (65·5 to 71·9)	70·1 (69 to 71·1)	72·7 (72·2 to 73·3)	75·6 (75·2 to 76·1)	79·1 (78·5 to 79·7)	80·6 (79·8 to 81·4)	78·5 (77·9 to 79·1)	78 (76·9 to 78·9)
Enfield	67·5 (63·9 to 71·2)	69 (65·5 to 72·4)	70·1 (67·1 to 73·2)	71·6 (70·5 to 72·6)	74·1 (73·5 to 74·6)	76·5 (76 to 77)	80·9 (80·3 to 81·6)	80 (79·2 to 80·8)	79 (78·4 to 79·6)	78·6 (77·6 to 79·6)
Greenwich	65·3 (61·8 to 69)	66·9 (63·3 to 70·4)	68·1 (64·6 to 71·2)	69·9 (68·8 to 71·1)	72·3 (71·7 to 72·8)	74·2 (73·6 to 74·7)	78·3 (77·6 to 78·9)	80·1 (79·2 to 80·9)	78·6 (77·9 to 79·3)	77·4 (76·3 to 78·5)
Hackney	66·1 (62·5 to 69·7)	67·1 (63·4 to 70·7)	67·4 (64 to 70·5)	68·5 (67·5 to 69·6)	70·6 (70 to 71·1)	73·9 (73·2 to 74·4)	78·5 (77·8 to 79·2)	79 (78·2 to 79·8)	76·7 (76 to 77·4)	70·7 (68·7 to 72·9)
Hammersmith and Fulham	66·6 (63 to 70·3)	66·9 (63·5 to 70·5)	67·5 (64·6 to 70·7)	68·5 (67·4 to 69·7)	69·5 (69 to 70·1)	73·6 (73 to 74·2)	78·1 (77·4 to 78·7)	79·9 (79 to 80·8)	76·9 (76·1 to 77·6)	77·3 (76·1 to 78·4)
Haringey	66·7 (62·9 to 70·3)	67·5 (64·3 to 70·9)	68·5 (65·1 to 71·8)	69·9 (68·8 to 71·1)	72·8 (72·2 to 73·4)	73·9 (73·4 to 74·4)	77·4 (76·8 to 78)	80·3 (79·5 to 81·2)	78·5 (77·9 to 79·1)	78·4 (77·3 to 79·6)
Harrow	69·1 (65·3 to 72·7)	70·6 (67·3 to 74)	71·7 (68·3 to 74·8)	73·3 (72·3 to 74·5)	75·1 (74·5 to 75·7)	77·7 (77·1 to 78·3)	81·4 (80·7 to 82·1)	83·2 (82·2 to 84·2)	80·5 (79·8 to 81·2)	82·6 (81·5 to 83·8)
Havering	68·4 (64·7 to 71·9)	69·9 (66·5 to 73·6)	70·9 (67·7 to 74·1)	72·4 (71·4 to 73·5)	74·6 (74 to 75·1)	77·2 (76·7 to 77·8)	79·8 (79·1 to 80·5)	81·7 (80·7 to 82·6)	79·2 (78·4 to 79·8)	78·9 (77·8 to 79·9)
Hillingdon	68·3 (64·5 to 71·8)	69·5 (65·8 to 72·9)	70·3 (67·2 to 73·8)	71·8 (70·7 to 72·8)	74·3 (73·7 to 74·9)	75·9 (75·4 to 76·5)	79·8 (79·2 to 80·4)	80·2 (79·3 to 81)	78·5 (77·9 to 79·2)	78·2 (77·1 to 79·3)
Hounslow	67·5 (63·7 to 71·1)	68·6 (65·1 to 72·1)	69·6 (66·5 to 72·9)	71·4 (70·2 to 72·5)	73 (72·5 to 73·6)	75·1 (74·6 to 75·6)	79·2 (78·6 to 79·8)	80·9 (80·1 to 81·7)	78·3 (77·6 to 79)	78·7 (77·5 to 79·8)
Islington	66·5 (62·9 to 70·3)	67·3 (63·9 to 70·8)	68 (64·7 to 71·2)	69·1 (67·9 to 70·3)	70·6 (69·9 to 71·1)	72·3 (71·8 to 72·9)	76·6 (76 to 77·2)	79·6 (78·7 to 80·4)	78·8 (78·1 to 79·5)	77·6 (76·6 to 78·7)
Kensington and Chelsea	68·2 (64·5 to 71·9)	67·4 (64·1 to 70·8)	67·9 (64·7 to 71)	69 (68 to 70·2)	70·9 (70·4 to 71·5)	76·7 (76·1 to 77·3)	81·2 (80·4 to 82)	82·6 (81·6 to 83·7)	82 (81·1 to 82·8)	80 (78·8 to 81·1)
Kingston upon Thames	69·1 (65·5 to 72·6)	70·1 (66·7 to 73·3)	71 (67·6 to 74·2)	72·4 (71·2 to 73·5)	74·7 (74 to 75·4)	77·7 (77·1 to 78·3)	82·1 (81·3 to 82·8)	83·4 (82·3 to 84·4)	80·1 (79·3 to 81)	81·4 (80 to 82·7)
Lambeth	64·8 (61·2 to 68·1)	65·7 (62·4 to 69·1)	66·3 (63·3 to 69·4)	68·3 (67·1 to 69·4)	70·5 (69·9 to 71)	73·5 (73 to 74)	77·9 (77·3 to 78·5)	80 (79·2 to 80·9)	77·5 (77 to 78·1)	76·9 (75·9 to 77·9)
Lewisham	65·9 (62·4 to 69·6)	67·5 (64 to 71)	68·6 (65·6 to 71·6)	70·4 (69·3 to 71·5)	71·9 (71·3 to 72·4)	73·4 (72·9 to 73·9)	78·2 (77·6 to 78·8)	79·1 (78·3 to 79·9)	78·2 (77·5 to 78·8)	75·9 (74·9 to 77)
Merton	67·8 (64·3 to 71·4)	69·2 (65·9 to 72·5)	70·4 (67·2 to 73·7)	72·1 (70·9 to 73·3)	73·9 (73·3 to 74·5)	77·2 (76·6 to 77·7)	80·6 (79·9 to 81·2)	81·9 (81 to 82·9)	78·7 (77·9 to 79·4)	78·7 (77·5 to 79·9)
Newham	65·1 (61·3 to 68·8)	66·7 (63·4 to 70·3)	67·7 (64·4 to 71)	69·7 (68·6 to 70·8)	71·3 (70·7 to 71·8)	72·9 (72·3 to 73·5)	76·9 (76·4 to 77·5)	79·2 (78·4 to 80)	75·5 (75 to 76·1)	75·7 (74·6 to 76·6)
Redbridge	68·4 (64·6 to 71·9)	69·5 (66 to 72·8)	70·5 (67·2 to 73·7)	72·2 (71·1 to 73·3)	74·3 (73·8 to 74·9)	77·1 (76·6 to 77·7)	80·7 (80 to 81·4)	81·8 (81 to 82·7)	77·6 (77 to 78·2)	79·2 (78·2 to 80·3)
Richmond upon Thames	69·1 (65·5 to 72·8)	69·9 (66·4 to 73·4)	70·6 (67·1 to 73·8)	71·8 (70·6 to 73)	75 (74·4 to 75·6)	78·6 (78 to 79·3)	82·7 (81·9 to 83·4)	84·1 (83 to 85·2)	80·9 (80 to 81·9)	81·4 (80·2 to 82·5)
Southwark	65·5 (61·9 to 69·4)	67·1 (63·7 to 70·6)	67·8 (64·7 to 71·2)	69·1 (67·9 to 70·3)	69·3 (68·8 to 69·9)	73·6 (73·1 to 74·2)	77·8 (77·2 to 78·4)	80·5 (79·7 to 81·3)	78 (77·4 to 78·6)	76·6 (75·7 to 77·6)
Sutton	68·5 (64·9 to 72·3)	70·2 (66·7 to 73·7)	71·5 (68·1 to 74·6)	73·2 (72 to 74·4)	75 (74·4 to 75·6)	77·2 (76·5 to 77·8)	80·5 (79·9 to 81·3)	81·8 (80·8 to 82·9)	80·8 (80 to 81·6)	78·7 (77·7 to 79·8)
Tower Hamlets	63·7 (60 to 67·3)	65·3 (61·8 to 68·6)	65·4 (62·1 to 68·3)	66·8 (65·7 to 67·9)	69·8 (69·2 to 70·3)	71·9 (71·3 to 72·4)	76·8 (76·1 to 77·4)	80·8 (80 to 81·6)	75·9 (75·3 to 76·5)	76·6 (75·7 to 77·6)
Waltham Forest	66·5 (62·9 to 70·1)	67·9 (64·2 to 71·4)	69·1 (65·6 to 72·4)	70·9 (69·7 to 72)	73·5 (72·9 to 74)	74·9 (74·3 to 75·4)	77·9 (77·3 to 78·5)	80·5 (79·7 to 81·4)	78·9 (78·1 to 79·6)	78·3 (77·2 to 79·3)
Wandsworth	66·9 (63·2 to 70·5)	67·7 (64·3 to 71·4)	68·6 (65·5 to 71·7)	69·9 (68·9 to 70·8)	71·2 (70·8 to 71·7)	74·9 (74·4 to 75·4)	79·5 (79 to 80·1)	80 (79·2 to 80·8)	78·8 (78·2 to 79·5)	78·9 (78 to 79·8)
Westminster	67·5 (63·9 to 71·1)	66·9 (63·3 to 70·3)	67·3 (64·2 to 70·4)	68·2 (67·1 to 69·3)	71·6 (71 to 72·2)	75·3 (74·7 to 75·8)	81·7 (81 to 82·5)	82·5 (81·6 to 83·4)	81·4 (80·6 to 82·1)	80·8 (79·6 to 82)
North East England	66 (63·3 to 68·6)	67·1 (64·5 to 69·7)	67·8 (65·2 to 70·5)	69·6 (68·8 to 70·3)	71·7 (71·4 to 72)	74·4 (74·1 to 74·7)	77·6 (77·2 to 77·9)	78·2 (77·8 to 78·6)	77 (76·7 to 77·4)	76·8 (75·8 to 77·6)
County Durham	65·9 (62·5 to 69·4)	67·1 (63·7 to 70·5)	67·8 (64·7 to 71·1)	69·6 (68·7 to 70·6)	72 (71·6 to 72·5)	74·7 (74·2 to 75·2)	77·3 (76·8 to 77·8)	78·2 (77·6 to 78·9)	77 (76·4 to 77·6)	76·7 (75·6 to 77·7)
Darlington	65·9 (62·2 to 69·4)	67·2 (63·5 to 71)	67·9 (64·5 to 71·3)	69·9 (68·5 to 71·1)	72 (71·3 to 72·7)	74·4 (73·7 to 75·2)	77·7 (76·9 to 78·5)	78·7 (77·7 to 79·8)	78·1 (77·2 to 79)	77·1 (75·7 to 78·5)
Gateshead	65·4 (61·6 to 69·3)	66·4 (62·6 to 70)	67 (63·5 to 70·3)	68·8 (67·7 to 69·8)	71·2 (70·7 to 71·8)	73·5 (72·8 to 74·1)	77·7 (76·9 to 78·4)	78·2 (77·3 to 79)	76·9 (76·1 to 77·6)	77·5 (76·3 to 78·7)
Hartlepool	64·8 (61 to 68·5)	66 (62·4 to 69·6)	66·7 (63·2 to 69·8)	68·6 (67·3 to 69·9)	71·1 (70·4 to 71·8)	73·3 (72·5 to 74)	76·9 (76 to 77·7)	76·7 (75·6 to 77·8)	76·2 (75·2 to 77·1)	76 (74·3 to 77·5)
Middlesbrough	64·9 (61·1 to 68·6)	66·1 (62·5 to 70)	66·8 (63·5 to 70)	68·7 (67·5 to 69·9)	70·3 (69·7 to 70·9)	73·2 (72·5 to 73·8)	76 (75·3 to 76·8)	75·8 (74·8 to 76·8)	74·8 (73·9 to 75·6)	74·4 (73·1 to 75·6)
Newcastle upon Tyne	66·5 (62·7 to 70)	67·2 (63·8 to 70·6)	67·6 (64·5 to 70·8)	69·3 (68·2 to 70·3)	70·8 (70·2 to 71·3)	73·9 (73·3 to 74·5)	77·5 (76·9 to 78·1)	78·4 (77·6 to 79·2)	76·9 (76·2 to 77·5)	76·2 (75 to 77·2)
North Tyneside	66·8 (63·2 to 70·4)	68·1 (64·3 to 71·7)	68·7 (65·4 to 72)	70·3 (69·1 to 71·4)	72·3 (71·7 to 72·8)	74·5 (73·9 to 75·1)	77·2 (76·6 to 77·8)	78·8 (77·8 to 79·7)	76·8 (76 to 77·5)	77·7 (76·5 to 78·9)
Northumberland	67·1 (63·7 to 70·8)	68·4 (64·9 to 71·8)	69·2 (65·7 to 72·5)	70·4 (69·3 to 71·5)	73·1 (72·6 to 73·6)	76·1 (75·5 to 76·6)	79·1 (78·4 to 79·7)	79·3 (78·5 to 80)	78·4 (77·8 to 79·1)	78·5 (77·4 to 79·5)
Redcar and Cleveland	65·8 (61·9 to 69·5)	66·9 (63·2 to 70·5)	67·7 (64·3 to 71·1)	69·5 (68·3 to 70·8)	71·7 (71·1 to 72·3)	74·7 (74·1 to 75·4)	78·3 (77·5 to 79·1)	78·2 (77 to 79·2)	77·1 (76·2 to 78)	76·4 (75 to 77·8)
South Tyneside	65·9 (62·3 to 69·3)	67·2 (63·5 to 70·9)	67·8 (64·6 to 70·9)	69·5 (68·3 to 70·7)	71·5 (70·9 to 72·1)	74 (73·3 to 74·6)	77·2 (76·5 to 77·9)	77·8 (76·8 to 78·7)	76·5 (75·7 to 77·5)	76·2 (75·1 to 77·5)

Stockton-on-Tees	66·5 (62·7 to 70·1)	67·6 (64·1 to 71·1)	68·3 (64·9 to 71·6)	70·2 (69·1 to 71·3)	71·8 (71·2 to 72·4)	75·2 (74·5 to 75·8)	78·4 (77·7 to 79)	78·7 (77·7 to 79·6)	77·7 (77 to 78·5)	77 (75·7 to 78·1)
Sunderland	66·2 (62·4 to 69·8)	67·3 (63·4 to 71)	68 (64·6 to 71·2)	69·6 (68·5 to 70·6)	71·3 (70·8 to 71·8)	73·8 (73·2 to 74·3)	76·5 (75·9 to 77·1)	77·4 (76·6 to 78·3)	76·2 (75·4 to 76·9)	75·3 (74·2 to 76·5)
North West England	65·9 (63·6 to 68·2)	67·1 (64·7 to 69·4)	67·9 (65·6 to 70·4)	69·6 (68·9 to 70·3)	71·8 (71·6 to 72)	74·2 (74 to 74·5)	77·4 (77·1 to 77·6)	78·6 (78·4 to 78·8)	77·2 (76·9 to 77·4)	76·9 (76·2 to 77·6)
Blackburn with Darwen	65 (61·6 to 68·5)	66·4 (63 to 69·6)	67·3 (64·1 to 70·6)	69·1 (67·9 to 70·2)	70·6 (70 to 71·2)	72·6 (71·9 to 73·2)	76·2 (75·5 to 76·8)	77·7 (76·7 to 78·6)	76·3 (75·5 to 77·1)	75·2 (74·1 to 76·3)
Blackpool	65·1 (61·5 to 68·5)	66·6 (63 to 70·1)	67·6 (64·3 to 71)	69·5 (68·4 to 70·6)	70·4 (69·8 to 70·9)	71 (70·4 to 71·6)	72·5 (71·9 to 73·2)	73·6 (72·8 to 74·6)	74·3 (73·6 to 75)	72·5 (71·4 to 73·7)
Bolton	65·3 (61·6 to 69·1)	66·4 (63 to 69·7)	67·2 (64·4 to 70·4)	68·9 (67·9 to 69·9)	71·6 (71·1 to 72·1)	73·7 (73·2 to 74·2)	77 (76·4 to 77·5)	77·7 (77 to 78·4)	76·9 (76·3 to 77·6)	76·8 (75·8 to 77·9)
Bury	66·9 (63·2 to 70·4)	67·8 (64·5 to 70·9)	68·7 (65·6 to 71·9)	70·4 (69·3 to 71·5)	72·3 (71·8 to 72·9)	75·2 (74·7 to 75·8)	78·2 (77·5 to 78·9)	79·6 (78·7 to 80·5)	77·3 (76·6 to 77·9)	78·1 (77 to 79·1)
Cheshire East	68·2 (64·7 to 71·7)	69 (65·8 to 72·4)	69·7 (66·6 to 72·9)	70·9 (69·8 to 71·9)	73·6 (73·2 to 74·1)	76·3 (75·8 to 76·9)	79·7 (79·2 to 80·3)	80·4 (79·7 to 81·1)	79·3 (78·7 to 79·9)	79·6 (78·6 to 80·5)
Cheshire West and Chester	68 (64·2 to 71·7)	68·9 (65·6 to 72·3)	69·6 (66·5 to 72·6)	70·9 (69·8 to 71·9)	73·3 (72·9 to 73·8)	76·3 (75·8 to 76·8)	79 (78·4 to 79·6)	80 (79·2 to 80·7)	78·9 (78·3 to 79·5)	78·4 (77·4 to 79·4)
Cumbria	66·7 (63·3 to 70·4)	67·9 (64·4 to 71·4)	68·7 (65·6 to 72·1)	70·2 (69·2 to 71·2)	72·5 (72 to 72·9)	75·6 (75·1 to 76·1)	79·1 (78·6 to 79·6)	79·2 (78·6 to 79·9)	78·5 (77·9 to 79·1)	77·8 (76·8 to 78·7)
Halton	66·5 (62·8 to 70)	67·2 (63·9 to 70·7)	67·8 (64·6 to 71·2)	69·4 (68·3 to 70·5)	71·9 (71·2 to 72·5)	73·6 (73 to 74·3)	77·3 (76·5 to 78)	77·8 (76·8 to 78·8)	77·6 (76·8 to 78·4)	76·2 (75 to 77·4)
Knowsley	65·6 (61·8 to 69·1)	66·6 (63·3 to 69·9)	67·2 (64·1 to 70·4)	68·8 (67·7 to 69·9)	71·1 (70·5 to 71·6)	72·7 (72·1 to 73·3)	76 (75·4 to 76·7)	77·6 (76·7 to 78·5)	75·9 (75·1 to 76·7)	76·1 (74·9 to 77·3)
Lancashire	65·8 (62·7 to 68·9)	67·4 (64 to 70·7)	68·4 (65·1 to 71·6)	69·8 (68·9 to 70·8)	72·2 (71·8 to 72·6)	74·9 (74·5 to 75·3)	78·1 (77·6 to 78·6)	78·6 (78 to 79·1)	77·2 (76·8 to 77·6)	76·7 (75·8 to 77·6)
Liverpool	65·3 (62 to 68·8)	66·3 (62·9 to 69·8)	66·9 (63·6 to 70·2)	68·7 (67·8 to 69·7)	70·6 (70·2 to 71·1)	72·1 (71·7 to 72·6)	75·2 (74·7 to 75·7)	77 (76·3 to 77·6)	75·7 (75·2 to 76·2)	75·1 (74·2 to 76)
Manchester	64·4 (61·1 to 67·9)	65·4 (61·9 to 68·7)	66 (62·8 to 69·2)	67·5 (66·5 to 68·6)	69·3 (68·9 to 69·7)	71 (70·5 to 71·4)	74·8 (74·3 to 75·3)	77·4 (76·8 to 78)	74·5 (74 to 74·9)	74 (73·2 to 74·9)
Oldham	65·2 (61·4 to 68·7)	66·3 (63 to 69·6)	67·3 (64·2 to 70·6)	69·1 (68·1 to 70·1)	71·2 (70·7 to 71·7)	73·5 (72·9 to 74)	76·6 (75·9 to 77·2)	77·6 (76·7 to 78·3)	75·2 (74·6 to 75·9)	76·4 (75·4 to 77·5)
Rochdale	65·3 (61·7 to 68·9)	66·4 (63·2 to 69·9)	67·3 (64·2 to 70·7)	69·3 (68·2 to 70·4)	71·5 (71 to 72·1)	73·8 (73·2 to 74·5)	76·3 (75·7 to 76·9)	78 (77·1 to 78·9)	76·1 (75·5 to 76·8)	75·3 (74·1 to 76·4)
Salford	64·9 (61·3 to 68·5)	65·7 (62·4 to 69)	66·2 (63·3 to 69·5)	67·6 (66·6 to 68·6)	70·3 (69·8 to 70·9)	73 (72·4 to 73·5)	75·6 (75·1 to 76·2)	78·2 (77·4 to 79)	75·6 (74·9 to 76·2)	76·4 (75·3 to 77·5)
Sefton	67 (63·6 to 70·5)	68·2 (64·8 to 71·5)	69 (65·9 to 72·2)	70·5 (69·5 to 71·5)	72·9 (72·4 to 73·4)	74·9 (74·4 to 75·4)	76·9 (76·3 to 77·5)	78·1 (77·3 to 78·9)	76·9 (76·3 to 77·5)	76·8 (75·7 to 77·9)
St Helens	66 (62·5 to 69·6)	66·8 (63·3 to 70)	67·4 (64·3 to 70·7)	68·9 (67·9 to 70)	71·7 (71·1 to 72·3)	74·2 (73·6 to 74·7)	76·9 (76·2 to 77·5)	77·6 (76·7 to 78·5)	75·9 (75·2 to 76·7)	76·7 (75·5 to 77·8)
Stockport	67·7 (64·2 to 71·1)	68·7 (65·5 to 71·9)	69·4 (66·4 to 72·9)	70·6 (69·6 to 71·7)	73 (72·5 to 73·5)	75·9 (75·3 to 76·4)	78·6 (78 to 79·2)	80 (79·2 to 80·8)	78·7 (78·1 to 79·4)	78·9 (77·9 to 79·9)
Tameside	65·5 (62 to 69·3)	66·6 (63·3 to 69·9)	67·6 (64·6 to 70·9)	69·4 (68·4 to 70·4)	71·6 (71·1 to 72·1)	73·2 (72·6 to 73·7)	76·4 (75·8 to 77)	79 (78·1 to 79·8)	77·3 (76·7 to 78)	76·3 (75·3 to 77·4)
Trafford	67·8 (64 to 71·2)	68·8 (65·4 to 72·1)	69·7 (66·6 to 73)	71·2 (70·1 to 72·2)	72·8 (72·2 to 73·3)	76·1 (75·5 to 76·7)	78·7 (78·2 to 79·4)	81·6 (80·8 to 82·5)	79·6 (78·9 to 80·3)	80 (78·9 to 81·1)
Warrington	67·5 (64 to 71·4)	68·2 (64·8 to 71·5)	68·9 (65·5 to 72·3)	70·3 (69·3 to 71·4)	72·3 (71·7 to 72·8)	75·3 (74·7 to 75·9)	78·5 (77·9 to 79·1)	78·9 (78·1 to 79·8)	77·4 (76·8 to 78·1)	78·2 (77·3 to 79·1)
Wigan	66·2 (62·8 to 69·8)	67·1 (63·7 to 70·6)	67·9 (64·8 to 71·5)	69·4 (68·4 to 70·4)	71·7 (71·2 to 72·2)	73·9 (73·3 to 74·5)	76·7 (76·1 to 77·2)	78·8 (78·1 to 79·6)	76·8 (76·3 to 77·4)	77·3 (76·4 to 78·4)
Wirral	66·3 (62·7 to 69·9)	67·7 (64·3 to 71·2)	68·5 (65·1 to 71·9)	70·1 (69·1 to 71·2)	72·1 (71·6 to 72·6)	74·5 (73·9 to 75·1)	78·2 (77·6 to 78·8)	79 (78·2 to 79·8)	77·7 (77 to 78·3)	76·7 (75·6 to 77·7)
South East England	68·7 (66·2 to 71·2)	69·8 (67·3 to 72·2)	70·5 (68·1 to 73)	71·8 (71·1 to 72·5)	74·4 (74·2 to 74·6)	76·9 (76·7 to 77·1)	79·9 (79·7 to 80·1)	80·8 (80·6 to 81)	79·9 (79·7 to 80·1)	79·8 (79·2 to 80·5)
Bracknell Forest	70 (66·1 to 73·7)	70·7 (66·9 to 74·2)	71·2 (67·6 to 74·5)	72·4 (71·2 to 73·7)	74·3 (73·6 to 75)	77·5 (76·7 to 78·2)	81·3 (80·5 to 82·1)	82·2 (81·1 to 83·3)	79·8 (78·9 to 80·8)	81·9 (80·6 to 83·2)
Brighton and Hove	67·8 (64 to 71·5)	68·7 (65·3 to 72·1)	69·5 (66·2 to 72·8)	71·1 (69·9 to 72·2)	71·6 (71·1 to 72·1)	74·4 (73·8 to 75)	77·5 (77 to 78·1)	79·8 (79 to 80·6)	78·8 (78·2 to 79·5)	79·3 (78·3 to 80·2)
Buckinghamshire	70·6 (66·8 to 74·3)	71·2 (67·8 to 74·6)	71·8 (68·6 to 75·2)	72·6 (71·6 to 73·6)	74·9 (74·5 to 75·4)	77·8 (77·3 to 78·4)	80·7 (80·1 to 81·2)	81·6 (80·9 to 82·3)	81·1 (80·5 to 81·7)	80·4 (79·5 to 81·3)
East Sussex	68·2 (64·4 to 71·9)	69·6 (66·1 to 73·3)	70·5 (67·1 to 73·8)	72 (70·9 to 73·1)	74·9 (74·4 to 75·4)	77 (76·5 to 77·5)	79·5 (79 to 80)	79·6 (78·9 to 80·3)	79·3 (78·8 to 79·9)	79 (78·1 to 79·9)
Hampshire	69·1 (65·6 to 72·6)	70·2 (66·7 to 73·6)	70·9 (67·6 to 74·3)	72·1 (71·1 to 73)	74·9 (74·5 to 75·3)	77·4 (77·1 to 77·9)	80·5 (80·1 to 80·9)	81·5 (81 to 82)	80·8 (80·4 to 81·2)	81 (80·1 to 81·8)
Isle of Wight	66·7 (63 to 70·4)	68·2 (64·7 to 71·9)	69·5 (66 to 72·9)	71·4 (70·1 to 72·7)	73·1 (72·4 to 73·8)	76·3 (75·5 to 77)	78·7 (78 to 79·5)	79·4 (78·4 to 80·4)	78·6 (77·7 to 79·5)	78·5 (77·2 to 79·7)
Kent	67·7 (64·4 to 71)	69·1 (66·1 to 72·3)	69·8 (66·6 to 72·9)	70·9 (69·9 to 71·9)	73·7 (73·4 to 74·1)	76·4 (76 to 76·9)	79·4 (79 to 79·9)	80·1 (79·6 to 80·6)	78·9 (78·4 to 79·3)	78·8 (78 to 79·6)
Medway	68·3 (64·3 to 72·1)	69 (65·4 to 72·2)	70 (66·7 to 73·3)	71·9 (70·8 to 73)	73·2 (72·7 to 73·7)	75·3 (74·7 to 75·8)	77·8 (77·2 to 78·4)	79·1 (78·4 to 79·9)	77·5 (76·8 to 78·1)	78·3 (77·3 to 79·3)
Milton Keynes	68·6 (64·6 to 72·3)	69·4 (65·9 to 72·8)	70 (66·3 to 73·3)	71·3 (70·1 to 72·7)	74 (73·4 to 74·7)	76·6 (76 to 77·2)	79·5 (78·9 to 80·1)	81 (80·2 to 81·8)	79·1 (78·5 to 79·8)	79·9 (78·9 to 80·9)
Oxfordshire	70·9 (67·1 to 74·4)	71·2 (67·3 to 74·8)	71·8 (68·4 to 75·2)	72·6 (71·6 to 73·6)	75·2 (74·8 to 75·7)	77·2 (76·8 to 77·6)	80·3 (79·8 to 80·8)	81·5 (80·9 to 82·2)	81·2 (80·7 to 81·7)	80·7 (79·8 to 81·5)
Portsmouth	67·6 (63·8 to 71·1)	68 (64·4 to 71·4)	68·7 (65·4 to 71·9)	70·1 (69·1 to 71·2)	71·8 (71·2 to 72·3)	74·2 (73·7 to 74·8)	77·4 (76·8 to 78)	79·5 (78·7 to 80·4)	77·3 (76·7 to 77·9)	78 (77 to 79)
Reading	69 (65·2 to 72·5)	69·3 (65·6 to 72·7)	69·6 (66·2 to 73)	70·7 (69·5 to 71·9)	72·8 (72·2 to 73·4)	75·4 (74·8 to 76)	78 (77·3 to 78·7)	79 (78 to 80)	76·4 (75·7 to 77·1)	78·5 (77·4 to 79·6)
Slough	68·1 (63·8 to 71·8)	68·9 (65 to 72·4)	69·5 (65·9 to 72·7)	71 (69·8 to 72·3)	72·3 (71·6 to 72·9)	75 (74·3 to 75·6)	77·5 (76·9 to 78·2)	78·9 (78 to 79·9)	77·6 (76·9 to 78·4)	76·2 (75·1 to 77·3)
Southampton	67·7 (63·9 to 71·5)	68·1 (64·5 to 71·5)	68·7 (65·5 to 72·1)	70·5 (69·3 to 71·7)	72·3 (71·7 to 72·8)	75·1 (74·5 to 75·6)	78·4 (77·8 to 79)	79·4 (78·6 to 80·2)	76·8 (76·1 to 77·5)	77·9 (76·9 to 78·8)
Surrey	69·2 (66 to 72·6)	70·5 (67·1 to 73·8)	71·1 (67·6 to 74·5)	72·3 (71·3 to 73·2)	75·4 (74·9 to 75·7)	77·6 (77·2 to 78)	81 (80·5 to 81·4)	82 (81·5 to 82·6)	81·4 (80·9 to 81·8)	81·1 (80·3 to 81·9)
West Berkshire	70·6 (66·7 to 74·3)	71·2 (67·8 to 74·5)	71·7 (68·2 to 75·2)	72·9 (71·6 to 74·1)	75·1 (74·4 to 75·8)	77·9 (77·2 to 78·6)	81 (80·3 to 81·7)	82·2 (81·2 to 83·2)	81·7 (80·9 to 82·5)	80 (78·9 to 81·2)
West Sussex	68·6 (65 to 72)	69·8 (66·1 to 73·4)	70·5 (67·1 to 73·7)	71·8 (70·8 to 72·7)	74·9 (74·4 to 75·3)	77·3 (76·9 to 77·8)	80·1 (79·6 to 80·6)	80·3 (79·7 to 80·9)	80·1 (79·6 to 80·6)	79·6 (78·8 to 80·5)
Windsor and Maidenhead	71·8 (68 to 75·4)	72·5 (68·8 to 75·7)	73·4 (69·8 to 76·7)	74·8 (73·5 to 76·1)	75·7 (75 to 76·4)	77·8 (77 to 78·5)	81·6 (80·9 to 82·4)	83·1 (82·1 to 84·1)	81·4 (80·5 to 82·2)	81·3 (80·1 to 82·4)
Wokingham	72·1 (68 to 75·6)	72·6 (68·9 to 76·1)	73·3 (70 to 76·8)	74·5 (73·2 to 75·8)	77·3 (76·6 to 78)	80·3 (79·5 to 81)	83 (82·2 to 83·8)	83 (82 to 84·1)	82·6 (81·8 to 83·5)	79·7 (78·5 to 80·9)
South West England	68·5 (65·8 to 71·2)	69·5 (66·8 to 72·2)	70·3 (67·7 to 72·8)	71·8 (71·1 to 72·6)	74·3 (74·1 to 74·6)	76·9 (76·7 to 77·2)	79·7 (79·5 to 80)	80·5 (80·2 to 80·8)	80 (79·7 to 80·3)	79·1 (78·4 to 79·8)
Bath and North East Somerset	70 (66·3 to 73·6)	71·1 (67·3 to 74·6)	72 (68·6 to 75·3)	73·7 (72·4 to 75)	75 (74·3 to 75·6)	77·7 (77 to 78·3)	80·6 (79·8 to 81·2)	81 (80·1 to 82)	80·5 (79·7 to 81·2)	79·8 (78·8 to 80·9)
Bournemouth	68·4 (64·5 to 72·2)	69·6 (66 to 73)	70·5 (67·1 to 74)	72·2 (70·9 to 73·4)	73 (72·3 to 73·7)	75·1 (74·5 to 75·7)	77·7 (77 to 78·4)	79·5 (78·6 to 80·3)	77·6 (76·8 to 78·3)	71·1 (69·1 to 73·3)
Bristol, City of	67·7 (64 to 71·3)	68·4 (64·8 to 72·1)	69 (65·7 to 72)	70·5 (69·4 to 71·6)	72·7 (72·2 to 73·2)	75·3 (74·7 to 75·8)	78·3 (77·7 to 78·9)	79·1 (78·4 to 79·8)	79 (78·5 to 79·6)	77·8 (76·9 to 78·8)
Cornwall	67·9 (64·1 to 71·5)	69·1 (65·5 to 72·5)	69·7 (66·3 to 73·2)	71·3 (70·2 to 72·4)	74·2 (73·6 to 74·7)	76·9 (76·4 to 77·4)	79·6 (79·1 to 80·1)	79·6 (79 to 80·3)	79 (78·4 to 79·5)	78·8 (77·9 to 79·7)
Devon	68·4 (64·9 to 72)	69·5 (65·8 to 73·3)	70·2 (67 to 73·6)	71·9 (70·9 to 73)	74·8 (74·3 to 75·2)	77·3 (76·8 to 77·7)	79·6 (79·1 to 80·1)	80·8 (80·2 to 81·4)	80·5 (80·1 to 81)	80·4 (79·4 to 81·3)
Dorset	69·1 (65·4 to 72·7)	70·3 (66·6 to 73·9)	71·1 (67·7 to 74·5)	72·8 (71·7 to 73·9)	75·5 (75 to 76·1)	78 (77·5 to 78·5)	81·1 (80·5 to 81·6)	81·9 (81·1 to 82·6)	81·5 (80·9 to 82·1)	81·7 (80·6 to 82·8)
Gloucestershire	69 (65·3 to 72·9)	70 (66·4 to 73·6)	70·7 (67·3 to 74)	71·8 (70·8 to 72·8)	74·2 (73·7 to 74·7)	77 (76·5 to 77·5)	80·1 (79·6 to 80·6)	80·2 (79·6 to 80·9)	79·5 (79 to 80)	79·3 (78·4 to 80·2)
North Somerset	69·3 (65·4 to 72·9)	70·4 (66·9 to 74)	71·4 (68 to 74·7)	73·1 (71·9 to 74·4)	75·2 (74·5 to 75·8)	77·3 (76·6 to 78)	79·3 (78·6 to 80)	80·7 (79·8 to 81·6)	80 (79·2 to 80·7)	80 (78·9 to 81·1)
Plymouth	67·2 (63·4 to 71·2)	68 (64·3 to 71·3)	68·6 (65·3 to 71·9)	70·1 (69 to 71·1)	72·8 (72·2 to 73·3)	74·7 (74·2 to 75·3)	78 (77·4 to 78·6)	79·1 (78·2 to 79·8)	79·2 (78·6 to 79·8)	78·7 (77·7 to 79·7)
Poole	68·7 (64·7 to 72·3)	69·8 (66 to 73·1)	70·4 (67·1 to 73·9)	71·9 (70·6 to 73·2)	74·7 (73·9 to 75·4)	77·5 (76·8 to 78·3)	80·5 (79·7 to 81·2)	81 (80·1 to 82)	79·6 (78·7 to 80·5)	73·3 (71·1 to 75·6)
Somerset	69 (65·1 to 72·7)	70·1 (66·4 to 73·5)	70·7 (67·2 to 74·1)	72·1 (71 to 73·2)	74·7 (74·2 to 75·2)	77·4 (76·8 to 77·9)	80·1 (79·6 to 80·6)	80·1 (79·4 to 80·8)	80·1 (79·5 to 80·7)	79·2 (78·2 to 80·2)
South Gloucestershire	70 (66·2 to 73·7)	70·8 (67 to 74·2)	71·4 (67·8 to 74·9)	72·8 (71·6 to 73·9)	75·8 (75·2 to 76·4)	77·7 (77·1 to 78·2)	80·7 (80·1 to 81·3)	82·8 (82 to 83·7)	81·1 (80·3 to 81·8)	80·8 (79·7 to 81·8)
Swindon	68·2 (64·4 to 71·9)	69·2 (65·6 to 72·8)	69·8 (66·2 to 73)	71·4 (70·2 to 72·7)	73·6 (72·9 to 74·1)	76·4 (75·8 to 77·1)	79·8 (79·2 to 80·5)	81·6 (80·7 to 82·5)	79·4 (78·7 to 80·2)	80 (78·9 to 81·2)
Torbay	67·5 (63·6 to 71·3)	69 (65·5 to 72·9)	70 (66·4 to 73·1)	71·7 (70·4 to 72·9)	73·4 (72·7 to 74·1)	74·8 (74·1 to 75·5)	77·4 (76·6 to 78·1)	77·7 (76·7 to 78·8)	78·6 (77·7 to 79·6)	77·4 (76·2 to 78·7)
Wiltshire	69·6 (65·6 to 73·4)	70·4 (66·9 to 74)	71·2 (68 to 74·5)	72·2 (71·2 to 73·2)	74·2 (73·6 to 74·7)	77·5 (77 to 78)	80·6 (80 to 81·1)	81·2 (80·5 to 81·8)	81 (80·4 to 81·5)	80 (79 to 80·9)
West Midlands	66·6 (64 to 69·1)	67·6 (65·1 to 70·1)	68·3 (65·9 to 71)	70·1 (69·4 to 70·8)	72·5 (72·3 to 72·8)	75·1 (74·9 to 75·3)	78·3 (78·1 to 78·6)	79·2 (78·9 to 79·5)	77·8 (77·5 to 78)	77·7 (77 to 78·5)
Birmingham	65·4 (62·5 to 68·7)	66·7 (63·4 to 70)	67·6 (64·4 to 70·7)	69·4 (68·4 to 70·3)	71·4 (71 to 71·8)	73·6 (73·1 to 74)	77·7 (77·3 to 78·2)	78·2 (77·6 to 78·7)	76 (75·5 to 76·4)	75·6 (74·7 to 76·6)

Coventry	66·5 (62·8 to 70·1)	67·3 (63·7 to 70·8)	68·1 (64·8 to 71·5)	69·8 (68·7 to 70·9)	71·8 (71·2 to 72·3)	74·8 (74·2 to 75·3)	77·1 (76·6 to 77·6)	77·9 (77·2 to 78·6)	76·9 (76·3 to 77·5)	76·6 (75·6 to 77·6)
Dudley	67·5 (63·8 to 71·2)	68·4 (64·8 to 72)	69·3 (66·2 to 72·7)	70·8 (69·7 to 71·8)	72·7 (72·2 to 73·3)	75·1 (74·6 to 75·6)	78·3 (77·8 to 78·9)	79·7 (78·9 to 80·5)	78·6 (77·9 to 79·2)	78·6 (77·5 to 79·6)
Herefordshire, County of	68·1 (64 to 71·8)	69·1 (65·7 to 72·7)	70·1 (66·8 to 73·5)	72 (70·8 to 73·2)	75 (74·4 to 75·6)	76·6 (75·9 to 77·3)	79·6 (78·9 to 80·2)	80·2 (79·2 to 81·1)	79·9 (79·1 to 80·7)	78·9 (77·8 to 80·1)
Sandwell	65·1 (61·5 to 68·6)	66·2 (62·7 to 69·9)	67 (63·8 to 70·4)	69·1 (68 to 70·2)	71 (70·6 to 71·5)	73·4 (72·9 to 74)	76·3 (75·7 to 76·8)	77·6 (76·9 to 78·3)	76·1 (75·5 to 76·7)	75·7 (74·6 to 76·7)
Shropshire	68·2 (64·2 to 71·9)	68·9 (65·2 to 72·5)	69·6 (66·1 to 72·9)	71·4 (70·4 to 72·5)	73·9 (73·3 to 74·4)	76·3 (75·8 to 76·9)	79·7 (79·1 to 80·2)	81·3 (80·5 to 82·2)	80·2 (79·5 to 81)	79·6 (78·6 to 80·6)
Solihull	69 (65·2 to 72·8)	69·7 (66 to 73·2)	70·5 (67·1 to 74·3)	72·1 (70·9 to 73·3)	75 (74·5 to 75·6)	77 (76·4 to 77·6)	80·7 (80 to 81·4)	81·1 (80·1 to 81·9)	79·4 (78·7 to 80·1)	79·9 (78·8 to 81)
Staffordshire	66·9 (63·5 to 70·2)	67·9 (64·4 to 71·2)	68·5 (65·3 to 71·7)	70·1 (69·2 to 71·1)	72·8 (72·3 to 73·2)	75·7 (75·3 to 76·1)	79·2 (78·7 to 79·6)	79·8 (79·2 to 80·3)	78·3 (77·8 to 78·8)	78·6 (77·8 to 79·5)
Stoke-on-Trent	64·8 (61·2 to 68·5)	65·7 (62·2 to 69·3)	66·3 (63·2 to 69·8)	68·1 (67 to 69·1)	70·9 (70·4 to 71·4)	73·3 (72·7 to 73·8)	76·1 (75·5 to 76·7)	77·1 (76·4 to 77·8)	75·7 (75·1 to 76·3)	75·5 (74·4 to 76·5)
Telford and Wrekin	66·9 (63 to 70·7)	67·5 (64 to 71·2)	68·2 (64·8 to 71·7)	69·8 (68·5 to 71·1)	72·5 (71·8 to 73·1)	75·4 (74·8 to 76·1)	78·3 (77·6 to 79)	78·3 (77·4 to 79·2)	77·3 (76·5 to 78)	76·1 (75 to 77·2)
Walsall	66·1 (62·2 to 70·2)	67·1 (63·6 to 70·6)	67·7 (64·6 to 71·1)	69·4 (68·4 to 70·5)	71·8 (71·3 to 72·3)	74·5 (73·9 to 75·1)	76·8 (76·2 to 77·4)	78 (77·2 to 78·8)	77 (76·3 to 77·6)	76·8 (75·7 to 77·8)
Warwickshire	68·4 (64·7 to 72·1)	69·1 (65·4 to 72·6)	69·7 (66·1 to 73·1)	71 (69·9 to 72)	73·6 (73·2 to 74·1)	76·3 (75·8 to 76·8)	79·4 (78·9 to 79·9)	80·3 (79·7 to 81)	79 (78·5 to 79·6)	79·3 (78·3 to 80·2)
Wolverhampton	66·1 (62·4 to 69·8)	67·1 (63·6 to 70·6)	67·8 (64·4 to 71·1)	69·6 (68·6 to 70·7)	71·6 (71·1 to 72·1)	73·8 (73·2 to 74·3)	76·5 (75·9 to 77)	77·3 (76·5 to 78·1)	76·2 (75·6 to 76·9)	76·1 (75·1 to 77)
Worcestershire	68·6 (64·8 to 72·1)	69·3 (65·7 to 72·8)	70·1 (67 to 73·5)	71·5 (70·5 to 72·5)	74 (73·5 to 74·4)	76·5 (76 to 76·9)	79·3 (78·8 to 79·8)	80·2 (79·6 to 80·9)	78·6 (78·1 to 79·1)	79·3 (78·4 to 80·3)
Yorkshire and the Humber	66·2 (63·7 to 68·9)	67·4 (64·9 to 69·9)	68·2 (65·7 to 70·8)	70 (69·3 to 70·7)	72·6 (72·3 to 72·8)	75·3 (75·1 to 75·6)	78 (77·8 to 78·3)	78·9 (78·6 to 79·2)	77·8 (77·5 to 78)	77·6 (76·8 to 78·3)
Barnsley	65·9 (62·3 to 69·7)	67 (63·6 to 70·5)	68·1 (64·7 to 71·4)	69·9 (68·8 to 71)	72·4 (71·8 to 72·9)	74·9 (74·3 to 75·4)	78·1 (77·4 to 78·7)	78 (77·2 to 78·9)	76·9 (76·2 to 77·5)	75·9 (74·8 to 77)
Bradford	64·9 (61·4 to 68·6)	66·5 (62·9 to 70)	67·6 (64·2 to 70·9)	69·5 (68·5 to 70·6)	71·7 (71·2 to 72·2)	74 (73·5 to 74·5)	77·3 (76·8 to 77·8)	78·3 (77·7 to 79)	76·5 (75·9 to 77)	76·8 (75·8 to 77·7)
Calderdale	65 (61·4 to 68·8)	66·4 (63 to 70·1)	67·4 (64·1 to 70·5)	69·1 (68·1 to 70·3)	71·5 (71 to 72·2)	75·1 (74·5 to 75·7)	77·2 (76·6 to 77·9)	79·5 (78·6 to 80·4)	77·9 (77·2 to 78·7)	77·7 (76·6 to 78·8)
Doncaster	66·4 (62·6 to 70·3)	67·6 (64·1 to 71)	68·6 (65·2 to 71·5)	70·4 (69·4 to 71·3)	72·7 (72·2 to 73·2)	74·6 (74 to 75·1)	77 (76·4 to 77·5)	78·6 (77·8 to 79·4)	77·2 (76·6 to 77·8)	77·2 (76·2 to 78·2)
East Riding of Yorkshire	68·1 (64·5 to 71·7)	69·2 (65·7 to 72·7)	70·2 (66·9 to 73·7)	71·8 (70·8 to 72·9)	74 (73·5 to 74·5)	76·4 (75·9 to 76·9)	79·4 (78·8 to 79·9)	80·9 (80·1 to 81·7)	79·7 (79·1 to 80·4)	79·2 (78·2 to 80·2)
Kingston upon Hull, City of	65·6 (61·9 to 69·1)	66·7 (63·2 to 70·2)	67·8 (64·6 to 70·9)	69·7 (68·7 to 70·8)	71·8 (71·2 to 72·3)	73·3 (72·8 to 73·9)	75·6 (75 to 76·2)	75·8 (75 to 76·6)	74·7 (74·1 to 75·4)	74·7 (73·6 to 75·7)
Kirklees	65·2 (61·6 to 68·9)	66·5 (63·1 to 70)	67·4 (64·2 to 70·7)	68·9 (67·9 to 70)	72·2 (71·6 to 72·7)	75·2 (74·7 to 75·7)	77·5 (77 to 78·1)	78·7 (78 to 79·4)	77·4 (76·8 to 77·9)	78 (77 to 79·1)
Leeds	66·1 (62·7 to 69·6)	67·4 (64 to 70·8)	68·1 (64·7 to 71·4)	69·7 (68·8 to 70·8)	72·5 (72 to 72·9)	75·3 (74·8 to 75·8)	77·9 (77·4 to 78·4)	78·3 (77·7 to 78·8)	77·4 (76·9 to 77·8)	76·6 (75·7 to 77·5)
North East Lincolnshire	66·8 (63 to 70·6)	68·1 (64·5 to 71·6)	69·2 (65·9 to 72·6)	71·1 (69·9 to 72·2)	72·4 (71·7 to 73)	74·7 (74 to 75·4)	77·4 (76·8 to 78·1)	78·1 (77·1 to 79)	77·7 (77 to 78·5)	77·7 (76·5 to 78·8)
North Lincolnshire	66·7 (63·1 to 70·4)	67·7 (64·1 to 71·3)	68·5 (65·1 to 71·8)	70·1 (69 to 71·3)	72 (71·4 to 72·7)	75·4 (74·7 to 76·1)	78·4 (77·7 to 79·1)	79·5 (78·6 to 80·5)	78·1 (77·3 to 78·8)	78·9 (77·8 to 79·9)
North Yorkshire	67·6 (64 to 71·2)	68·7 (65·1 to 72·4)	69·6 (66·4 to 72·8)	71·2 (70·2 to 72·2)	73·9 (73·4 to 74·4)	77·2 (76·7 to 77·7)	79·9 (79·4 to 80·4)	80·6 (80 to 81·3)	79·8 (79·2 to 80·3)	79·9 (79 to 80·7)
Rotherham	66·2 (62·4 to 69·9)	67·2 (63·7 to 70·5)	68 (64·8 to 71·3)	69·2 (68·2 to 70·2)	72·2 (71·7 to 72·6)	74·9 (74·3 to 75·5)	78·3 (77·7 to 78·9)	78·1 (77·3 to 79)	76·7 (76·1 to 77·4)	77·3 (76·2 to 78·4)
Sheffield	66·8 (63·3 to 70·2)	67·8 (64·4 to 71·3)	68·6 (65·3 to 71·8)	70·3 (69·3 to 71·3)	72·6 (72·1 to 73)	75·3 (74·8 to 75·8)	78 (77·5 to 78·5)	79·2 (78·6 to 79·9)	78·4 (77·9 to 78·9)	77·6 (76·7 to 78·5)
Wakefield	65·7 (62·2 to 69·3)	66·5 (63·1 to 69·9)	67·1 (63·9 to 70·2)	68·8 (67·9 to 69·8)	72·3 (71·8 to 72·7)	75·1 (74·5 to 75·6)	77·3 (76·7 to 77·8)	77·7 (77 to 78·5)	76·5 (75·9 to 77·1)	76·7 (75·7 to 77·8)
York	68·3 (64·5 to 72·2)	69·1 (65·6 to 72·8)	70 (66·6 to 73·4)	71·5 (70·4 to 72·7)	73·6 (73 to 74·2)	76·7 (76·1 to 77·3)	80 (79·3 to 80·6)	80·4 (79·5 to 81·2)	80·2 (79·5 to 80·9)	78·6 (77·5 to 79·7)
Northern Ireland	66·3 (65·9 to 66·7)	68·1 (67·8 to 68·3)	68·1 (67·9 to 68·3)	68·4 (68·2 to 68·7)	71·4 (71·2 to 71·6)	74·8 (74·6 to 75·1)	77·5 (77·2 to 77·8)	79·1 (78·8 to 79·4)	78·4 (78·1 to 78·6)	78·3 (77·8 to 78·8)
Scotland	65·7 (65·5 to 66)	66·4 (66·3 to 66·6)	67·3 (67·2 to 67·5)	68·9 (68·7 to 69)	71·1 (70·9 to 71·2)	73·3 (73·1 to 73·5)	76·3 (76·1 to 76·5)	77·5 (77·2 to 77·7)	76·5 (76·3 to 76·7)	76·3 (76 to 76·5)
Wales	66·3 (65·4 to 67·1)	67·3 (66·6 to 68·1)	68·3 (67·7 to 69)	70·3 (70 to 70·6)	72·9 (72·7 to 73·1)	75 (74·8 to 75·2)	77·9 (77·7 to 78·1)	79·3 (79·1 to 79·6)	78·5 (78·3 to 78·7)	78·7 (78·5 to 79)
Latin America and Caribbean	47·9 (46 to 49·8)	54·5 (53·5 to 55·5)	58·5 (57·7 to 59·2)	63·2 (62·7 to 63·7)	66·7 (66·4 to 67)	69·7 (69·4 to 70)	70·7 (70·4 to 71·1)	73·1 (72·4 to 73·7)	70 (69·4 to 70·6)	68·9 (68·1 to 69·7)
Andean Latin America	40·4 (37·3 to 43·6)	47·1 (45·2 to 49)	51·9 (50·6 to 53·2)	62·3 (61·1 to 63·5)	66·6 (65·5 to 67·6)	71·1 (70·1 to 72·2)	73·9 (72·7 to 75)	75·3 (73·5 to 76·9)	69·5 (68 to 71·1)	68·3 (66·9 to 69·6)
Bolivia	36·3 (32·1 to 40·5)	42·4 (39·2 to 45·4)	48·2 (45·8 to 50·8)	55 (53 to 56·9)	60·4 (58·7 to 62)	65·4 (63·6 to 67·1)	69·4 (67 to 71·8)	71·8 (68·6 to 74·5)	65·3 (63 to 67·3)	63·8 (61·9 to 65·6)
Ecuador	49·9 (46 to 53·3)	54·8 (53·8 to 55·8)	60·1 (59·3 to 60·9)	64·8 (64·1 to 65·4)	69·8 (69·4 to 70·2)	71·4 (70·1 to 72·6)	71·9 (70·5 to 73·3)	74·9 (73·4 to 76·4)	70·1 (68·6 to 71·5)	71 (69 to 73·1)
Peru	39·1 (35·1 to 43)	46 (43·5 to 48·4)	50·1 (48·5 to 51·9)	64 (62·2 to 65·8)	67·3 (65·8 to 68·9)	73·1 (71·4 to 75)	76·7 (74·9 to 78·4)	76·6 (74·5 to 78·7)	70·8 (68·7 to 72·8)	68·8 (67·3 to 70·1)
Caribbean	52·8 (50·3 to 55·4)	58·5 (57·3 to 59·7)	61·4 (60·4 to 62·4)	64·1 (63·2 to 65)	66 (65·2 to 66·8)	68·2 (67·5 to 69)	59·1 (57·7 to 60·4)	70·2 (68·5 to 71·8)	69·3 (67·5 to 71)	66·9 (64·9 to 68·7)
Antigua and Barbuda	54·8 (52·5 to 56·8)	61·6 (60·3 to 62·9)	65·7 (64·6 to 66·8)	69·8 (68·4 to 71)	70·5 (69·7 to 71·6)	72·1 (71·3 to 72·9)	73·3 (72·5 to 74·1)	74·3 (73·1 to 75·3)	74·9 (73·7 to 76·1)	73 (72·7 to 73·3)
The Bahamas	54·8 (50·2 to 59·1)	59·2 (56·3 to 62·2)	62·4 (61·1 to 63·6)	65·4 (64·3 to 66·4)	67·7 (66·6 to 68·7)	67·7 (66·6 to 68·8)	69·5 (68·5 to 70·5)	68·6 (66·1 to 71)	68·3 (65·8 to 70·7)	66·1 (63·7 to 68·2)
Barbados	51 (47·3 to 54·2)	63·6 (62·3 to 64·7)	66·3 (65·4 to 67·1)	70·3 (69·7 to 71)	71·3 (70·7 to 71·9)	72·4 (71·7 to 73·1)	74·7 (74·1 to 75·3)	74·5 (72·3 to 76·7)	75·3 (72·7 to 77·8)	74·4 (71·8 to 76·8)
Belize	53·3 (50·2 to 56)	63·4 (61·3 to 65·3)	67·6 (66·1 to 69)	69·9 (68·6 to 71·1)	71·7 (70·8 to 72·5)	66·7 (66·1 to 67·4)	71 (70·4 to 71·6)	72·3 (71·1 to 73·6)	71·2 (70·3 to 72·1)	70·5 (69 to 72·3)
Bermuda	61·4 (59·7 to 63)	64·2 (63·3 to 65·1)	66·5 (65·6 to 67·3)	68·7 (68 to 69·4)	69·3 (68·7 to 69·9)	74·1 (73·5 to 74·8)	76·6 (75·8 to 77·5)	78 (76·5 to 79·4)	78·4 (76·9 to 79·6)	75·6 (73·9 to 77·1)
Cuba	65 (61·3 to 68·4)	67·6 (67·3 to 67·8)	69 (68·8 to 69·2)	72·3 (72 to 72·5)	73 (72·8 to 73·2)	74·9 (74·7 to 75·1)	76·2 (76 to 76·4)	75·7 (75·5 to 75·9)	75·6 (75·5 to 75·9)	70·9 (69·9 to 72·1)
Dominica	45·6 (41·4 to 49·2)	58·6 (56·8 to 60·2)	65 (63·9 to 66·1)	70·1 (69·3 to 70·8)	69·1 (68·4 to 69·7)	70·1 (69·4 to 71)	70·4 (69·6 to 71·2)	70·1 (68 to 71·9)	70·2 (67·8 to 72·2)	67·4 (64·4 to 70·3)
Dominican Republic	53·5 (50·2 to 56·8)	59·1 (56·8 to 61·5)	63·8 (61·9 to 65·6)	67 (65·2 to 68·7)	69·3 (67·7 to 71)	70·6 (68·9 to 72·4)	71·5 (68·8 to 74·4)	71·7 (69·6 to 73·9)	70·5 (68·5 to 72·5)	70·5 (68·3 to 72·5)
Grenada	54·6 (51·1 to 58·3)	61·6 (59 to 64·3)	64·1 (61·3 to 66·8)	66·3 (63·6 to 69)	67·6 (66·3 to 68·9)	67·7 (67 to 68·3)	68·2 (67·6 to 68·8)	70·7 (69·7 to 71·6)	70·8 (69·7 to 71·9)	67·3 (64·1 to 69·7)
Guyana	49·5 (46·8 to 52)	57·5 (55·8 to 59·2)	59·5 (57·6 to 61·3)	60·2 (58 to 62·5)	60·3 (58·7 to 62)	62·2 (60·5 to 63·8)	63·3 (61·7 to 65)	64·7 (61·4 to 68·1)	63·3 (59·9 to 66·9)	61·1 (57 to 65·4)
Haiti	35·2 (30·8 to 39·5)	42·9 (39·9 to 46·1)	45·4 (42·6 to 48·5)	49·3 (46·8 to 51·8)	53·2 (51 to 55·5)	57·2 (55·1 to 59·6)	35·4 (33·4 to 37·5)	62·7 (58·9 to 66·1)	60·4 (57 to 63·9)	58·8 (54·9 to 62·5)
Jamaica	54·5 (52·5 to 56·3)	62·8 (62·1 to 63·6)	65·4 (64·7 to 66·1)	72 (71·4 to 72·5)	73·9 (73·3 to 74·4)	72·7 (72·1 to 73·3)	74·6 (74·1 to 75)	74·7 (71·6 to 78)	75 (71·4 to 79·1)	72 (69·1 to 75·1)
Puerto Rico	59·7 (59 to 60·4)	67·2 (66·8 to 67·5)	68·5 (68·1 to 68·8)	70·2 (69·9 to 70·5)	69·8 (69·5 to 70·1)	72·6 (72·3 to 72·9)	75·8 (75·4 to 76·2)	77·3 (76·9 to 77·8)	76·6 (76·1 to 77)	76·6 (74·4 to 79·1)
Saint Kitts and Nevis	56·5 (54·9 to 57·9)	58·4 (57·2 to 59·6)	61·1 (60 to 62·1)	62·9 (61·8 to 64)	65·8 (64·9 to 66·6)	69·1 (68 to 70·1)	70 (69·3 to 70·7)	70·2 (68·6 to 71·7)	70·2 (68·4 to 72)	68·5 (66·7 to 70·2)
Saint Lucia	50·1 (46·6 to 53)	57·4 (55·4 to 59·4)	62·8 (61·1 to 64·3)	66·1 (65·2 to 67)	67·6 (66·9 to 68·3)	70·2 (69·5 to 70·8)	72·3 (71·6 to 73)	72·7 (70·7 to 74·6)	72·3 (70·1 to 74·4)	69·7 (66·4 to 72·7)
Saint Vincent and the Grenadines	50·4 (47·4 to 53·2)	59 (57 to 60·9)	62·7 (61·1 to 64·3)	65·9 (64·6 to 67·1)	68·2 (67·2 to 69·1)	68·8 (68 to 69·5)	71·1 (70·3 to 71·8)	71 (69·7 to 72·1)	71·1 (69·5 to 72·5)	69·7 (68 to 71·3)
Suriname	56·8 (54·9 to 58·5)	61·6 (59·9 to 63·3)	64·2 (62·6 to 65·7)	63·4 (62·4 to 64·4)	66·3 (65·4 to 67·2)	67 (66·2 to 67·9)	69·2 (68·5 to 70)	69·5 (68·1 to 70·7)	69·6 (67·6 to 71·4)	67·5 (63·4 to 70·7)
Trinidad and Tobago	56·6 (55 to 58·1)	61·9 (61·2 to 62·6)	63·2 (62·6 to 63·8)	65·2 (64·7 to 65·7)	67 (66·5 to 67·5)	68 (67·4 to 68·5)	70·6 (70 to 71·2)	70·9 (69 to 72·9)	70·2 (67·4 to 72·9)	67·6 (64·1 to 71·2)
Virgin Islands	58·8 (56·8 to 60·8)	61 (59·5 to 62·5)	63·2 (61·4 to 64·8)	67·2 (65·4 to 69·1)	69·2 (67·5 to 70·9)	70·1 (68·5 to 71·7)	71·5 (69·6 to 73·3)	70·9 (68 to 73·7)	70·8 (67·9 to 73·5)	71·3 (67·7 to 74·5)
Central Latin America	48·5 (46·5 to 50·2)	56·2 (55·5 to 56·8)	60·1 (59·6 to 60·5)	63·7 (63·1 to 64·3)	67·9 (67·6 to 68·2)	70·8 (70·5 to 71)	72·6 (72·2 to 72·8)	73·1 (72·4 to 73·8)	68·8 (68·1 to 69·4)	68·3 (67·3 to 69·3)
Colombia	53·3 (50·9 to 55·4)	58·2 (57·1 to 59·2)	64·7 (64 to 65·4)	66·8 (64·6 to 69·2)	68·2 (67·9 to 68·6)	70·5 (70·1 to 70·8)	75 (74·5 to 75·4)	77·6 (77·2 to 78)	74·1 (73·8 to 74·5)	72·6 (70·8 to 74·5)
Costa Rica	55·4 (54·5 to 56·2)	64·2 (63·6 to 64·8)	65·6 (65·1 to 66·1)	72·2 (71·7 to 72·6)	74·8 (74·4 to 75·2)	75·5 (75·2 to 75·9)	76·7 (76·4 to 77·1)	77·1 (76·7 to 77·4)	76·3 (76 to 76·6)	74·3 (73·9 to 74·6)
El Salvador	43·7 (39·4 to 47·2)	52·4 (50·4 to 54·2)	55·5 (54·4 to 56·6)	57·1 (55·8 to 58·3)	65·4 (64·7 to 65·9)	68·3 (67·9 to 68·7)	71 (70·6 to 71·3)	70·8 (70·5 to 71·2)	69·1 (67·4 to 71)	67·9 (65·4 to 70·4)
Guatemala	42·4 (39·2 to 45·2)	47·8 (46·1 to 49·5)	48·3 (47 to 49·5)	54·2 (52·8 to 55·3)	60·1 (59·5 to 60·6)	64·3 (63·8 to 64·7)	67·5 (67 to 68)	69·7 (69·1 to 70·2)	67·9 (67·3 to 68·3)	66·2 (64·4 to 67·9)
Honduras	38 (34·2 to 41·3)	47·2 (45·1 to 49·1)	55·9 (53·9 to 57·7)	66 (64·3 to 67·6)	66·5 (64·5 to 68·6)	69 (65·7 to 72·5)	70 (66·7 to 72·9)	70·1 (67·6 to 72·4)	67·7 (65·5 to 69·4)	66·4 (64·3 to 68·2)
Mexico	46·8 (44·1 to 49·2)	55·4 (54·5 to 56·3)	59·3 (58·6 to 60)	62·9 (62·3 to 63·5)	68·2 (67·7 to 68·5)	71·7 (71·4 to 72)	72·4 (72·2 to 72·7)	72·6 (72·3 to 72·9)	67·3 (66·9 to 67·5)	67·4 (67 to 67·7)

Aguascalientes	51·6 (48·2 to 55·2)	59·1 (56·1 to 62)	61·8 (59·1 to 64·4)	65·4 (64·4 to 66·4)	70·5 (69·9 to 71·1)	73·1 (72·5 to 73·6)	74·4 (73·9 to 74·9)	74·3 (73·7 to 74·8)	69·7 (69·2 to 70·1)	69·5 (68·9 to 70)
Baja California	47·6 (43·2 to 52·2)	56·1 (53·1 to 59)	58·9 (55·9 to 61·8)	63·9 (63·4 to 64·5)	66·4 (66 to 66·9)	67·7 (67·3 to 68·1)	69·9 (69·5 to 70·2)	70·4 (70 to 70·7)	64·8 (64·5 to 65·2)	65·1 (64·7 to 65·6)
Baja California Sur	47·4 (43·2 to 51·4)	56·4 (53·4 to 59·1)	60·2 (57·7 to 62·8)	64·7 (63·8 to 65·6)	69·8 (69·3 to 70·4)	72 (71·5 to 72·6)	73·8 (73·3 to 74·3)	73·6 (73 to 74·2)	69·8 (69·3 to 70·2)	68·1 (67·5 to 68·7)
Campeche	49·9 (45·9 to 53·9)	58·2 (55·2 to 61·1)	62 (59·2 to 64·4)	66·1 (65·1 to 67·2)	70 (69·2 to 70·6)	73·8 (73·2 to 74·3)	74·3 (73·8 to 74·8)	73·6 (72·9 to 74·2)	69·4 (68·9 to 69·9)	69·4 (68·8 to 70)
Chiapas	48·2 (43·9 to 52·2)	55 (52·1 to 57·8)	58·6 (55·8 to 61·2)	62·4 (61·3 to 63·5)	68·3 (67·5 to 69)	71·2 (70·6 to 71·7)	73·2 (72·9 to 73·6)	72·9 (72·5 to 73·3)	68·3 (67·8 to 68·6)	68·8 (68·3 to 69·3)
Chihuahua	47·5 (43·3 to 51·4)	55·5 (52·6 to 58·3)	59·4 (56·4 to 62)	62·5 (61·6 to 63·2)	65·5 (64·8 to 66·1)	68·4 (67·9 to 68·9)	62·8 (62·4 to 63·2)	68·9 (68·4 to 69·3)	62·5 (62·1 to 62·9)	66·1 (65·5 to 66·6)
Coahuila	46·7 (42·3 to 50·9)	55·7 (52·8 to 58·5)	59·5 (56·5 to 62·3)	62·9 (61·8 to 63·9)	68·3 (67·6 to 68·9)	71·6 (71·1 to 72·1)	72·4 (72 to 72·8)	73·3 (72·8 to 73·7)	67·6 (67·2 to 68)	69·6 (69·1 to 70·1)
Colima	40·3 (36·2 to 44·1)	50·5 (47·8 to 53·4)	55·2 (52·6 to 57·9)	60·2 (58·9 to 61·4)	67·8 (67 to 68·5)	72 (71·3 to 72·6)	73 (72·5 to 73·6)	70·4 (69·7 to 71)	66·8 (66·3 to 67·2)	65·3 (64·7 to 65·9)
Durango	46 (42 to 50)	55·2 (52·1 to 58·1)	59·7 (56·7 to 62·6)	63·3 (62 to 64·5)	68·6 (67·9 to 69·2)	73 (72·3 to 73·6)	69·4 (68·9 to 69·9)	74·4 (73·8 to 74·9)	70·1 (69·6 to 70·6)	71·4 (70·8 to 71·9)
Guanajuato	45·8 (41·1 to 50·5)	54·6 (51·8 to 57·5)	58·4 (55·4 to 61·3)	63·3 (62·6 to 63·9)	68·5 (68 to 69)	72·6 (72·2 to 72·9)	73·9 (73·6 to 74·2)	70·6 (70·2 to 71)	65·8 (65·5 to 66·1)	65·8 (65·4 to 66·3)
Guerrero	42 (38·3 to 45·7)	49·9 (46·9 to 53)	54·1 (51·5 to 56·9)	58·3 (56·9 to 59·7)	64·3 (63·1 to 65·4)	69 (68 to 70·1)	69·3 (68·1 to 70·4)	71 (69·6 to 72·4)	68·1 (66·8 to 69·4)	68·2 (66·8 to 69·7)
Hidalgo	42·6 (38·6 to 46·5)	51·7 (48·8 to 54·6)	55·7 (52·5 to 58·5)	59·6 (58·8 to 60·3)	68·8 (68·1 to 69·4)	73·7 (73·1 to 74·3)	74·6 (74·2 to 75)	74·5 (74 to 74·9)	70·5 (70·1 to 70·8)	70·2 (68·2 to 72·3)
Jalisco	50·1 (45·8 to 53·8)	57·7 (54·8 to 60·6)	61·3 (58·3 to 64)	65·7 (65 to 66·4)	68·9 (68·3 to 69·4)	71·7 (71·3 to 72·1)	73 (72·7 to 73·2)	72·1 (71·7 to 72·4)	68·6 (68·2 to 68·9)	67·1 (66·7 to 67·6)
México	47·5 (43·4 to 51·8)	55·8 (52·8 to 58·9)	59·8 (57·1 to 62·5)	61·3 (60·7 to 62)	66·8 (66·4 to 67·2)	71·3 (71 to 71·6)	73·1 (72·7 to 73·3)	73·1 (72·7 to 73·4)	65·3 (65 to 65·6)	68·5 (68·1 to 68·9)
Mexico City	53·9 (49·9 to 58)	61·8 (58·9 to 64·6)	64 (61·4 to 66·5)	65·9 (65·1 to 66·7)	69·2 (68·1 to 70·4)	72·1 (71·8 to 72·4)	73·1 (72·8 to 73·4)	73·7 (73·3 to 74)	65·5 (65·1 to 65·8)	63·3 (62·8 to 63·9)
Michoacán de Ocampo	42·3 (38·7 to 45·9)	51·6 (48·2 to 54·9)	56·4 (53·1 to 59·3)	60·5 (59·7 to 61·3)	68·5 (67·9 to 69·1)	71·8 (71·4 to 72·3)	72·7 (72·1 to 73·2)	72·2 (71·7 to 72·7)	68·7 (68·2 to 69·1)	66·2 (65·7 to 66·8)
Morelos	43 (39·2 to 47)	53 (50·1 to 56·1)	57·6 (54·5 to 60·7)	61·6 (60·7 to 62·4)	68·7 (68·1 to 69·3)	72·9 (72·4 to 73·5)	73·7 (73·2 to 74·2)	71·1 (70·6 to 71·6)	66·3 (65·9 to 66·7)	64·1 (63·6 to 64·6)
Nayarit	47 (43·1 to 50·6)	56 (53 to 58·9)	61·1 (58·3 to 63·9)	65·8 (64·8 to 66·9)	69·4 (68·6 to 70)	73·8 (73·2 to 74·5)	72·7 (72·2 to 73·2)	75 (74·3 to 75·6)	71·6 (71 to 72·1)	70·2 (69·5 to 70·7)
Nuevo León	52·9 (49·1 to 56·6)	61 (58·2 to 63·6)	64·3 (61·5 to 66·8)	68·6 (67·9 to 69·3)	70·8 (70·3 to 71·3)	73·2 (72·7 to 73·6)	73·2 (72·9 to 73·5)	74·3 (73·9 to 74·6)	69·6 (69·3 to 69·9)	68·9 (68·5 to 69·3)
Oaxaca	41·3 (37·5 to 45·1)	49·4 (46·2 to 52·8)	52·8 (50 to 55·7)	57·1 (56·1 to 58)	65·9 (65·2 to 66·6)	70·9 (70·4 to 71·4)	72·5 (71·9 to 73)	73·5 (73 to 74)	69·4 (68·9 to 69·9)	68·2 (67·6 to 68·7)
Puebla	41·2 (36·7 to 45·7)	50·2 (47·5 to 53·3)	54·8 (51·8 to 57·5)	58·4 (57·5 to 59·4)	66·3 (65·6 to 67)	70·1 (69·6 to 70·6)	72·7 (72·4 to 73·1)	72·3 (71·9 to 72·7)	66·3 (65·9 to 66·7)	65 (64·5 to 65·5)
Querétaro	44·2 (39·3 to 48·8)	54·1 (51·2 to 57·1)	58·8 (55·6 to 61·7)	62·3 (60·4 to 64·6)	67·6 (67 to 68·2)	71·2 (70·8 to 71·7)	73·6 (73·2 to 74)	74·2 (73·7 to 74·6)	69·7 (69·3 to 70·1)	68 (67·5 to 68·4)
Quintana Roo	50·1 (46·5 to 53·4)	56·7 (54·1 to 59·6)	59·4 (56·6 to 62·1)	62·5 (60·8 to 64·1)	68·4 (67·1 to 69·6)	71·5 (70·4 to 72·6)	71·7 (70·5 to 72·8)	71·7 (70·1 to 73·3)	69 (67·7 to 70·2)	69·1 (67·6 to 70·5)
San Luis Potosí	45·3 (41·4 to 49·2)	54·5 (51·3 to 57·9)	58·5 (55·4 to 61·1)	66 (65·1 to 66·9)	69·9 (69·3 to 70·5)	73·5 (73 to 74)	74·1 (73·6 to 74·7)	74·1 (73·6 to 74·6)	68·7 (68·2 to 69·1)	68·4 (67·8 to 68·9)
Sinaloa	48·6 (44·5 to 52·5)	56·6 (53·7 to 59·6)	60·8 (57·9 to 63·5)	65·2 (64·4 to 65·9)	70·5 (69·7 to 71·1)	73 (72·5 to 73·5)	70·7 (70·2 to 71)	74·3 (73·9 to 74·8)	69·7 (69·3 to 70·1)	70·4 (69·9 to 70·8)
Sonora	52·6 (48·6 to 56·2)	59·4 (56·3 to 62·3)	62·1 (59·1 to 64·8)	64·7 (62·4 to 66·8)	68·4 (67·8 to 68·9)	70·6 (70·2 to 71·1)	71·2 (70·8 to 71·6)	70·7 (70·2 to 71·1)	65·7 (65·3 to 66·1)	66·3 (65·8 to 66·8)
Tabasco	47·5 (43·1 to 51·4)	55 (51·9 to 58·2)	58·1 (55 to 61·1)	60·8 (59·9 to 61·6)	69 (68·3 to 69·6)	72·4 (71·8 to 72·9)	72 (71·5 to 72·4)	71 (70·4 to 71·5)	65·8 (65·3 to 66·2)	68 (65·5 to 70·7)
Tamaulipas	50·9 (47·1 to 54·8)	58·4 (55·4 to 61·2)	61·9 (59 to 64·7)	64·1 (63·4 to 64·9)	69·7 (69·1 to 70·2)	73·2 (72·7 to 73·6)	72·1 (71·7 to 72·5)	72·8 (72·4 to 73·3)	69 (68·5 to 69·4)	69·9 (69·4 to 70·4)
Tlaxcala	44·4 (39·1 to 49·5)	54·6 (51·7 to 57·8)	59·7 (56·6 to 62·6)	63·9 (61·8 to 66·1)	69·4 (68·7 to 70)	72·9 (72·4 to 73·4)	74·6 (74·1 to 75·1)	74·1 (73·5 to 74·7)	67·2 (66·7 to 67·6)	66·7 (66·1 to 67·3)
Veracruz de Ignacio de la Llave	46 (41·7 to 50)	54·2 (51·4 to 57·1)	58·1 (55·2 to 60·9)	62·7 (61·8 to 63·5)	68·5 (67·9 to 69·1)	72 (71·6 to 72·4)	73·3 (73 to 73·6)	72·3 (71·9 to 72·7)	68·3 (67·9 to 68·6)	68·6 (66·3 to 70·9)
Yucatán	53·2 (49·2 to 57·3)	59·5 (56·7 to 62·3)	63·1 (60·3 to 65·8)	65·9 (64·9 to 66·8)	70·1 (69·4 to 70·7)	73·3 (72·8 to 73·8)	74·1 (73·7 to 74·5)	74 (73·6 to 74·5)	70·2 (69·8 to 70·6)	69·8 (69·3 to 70·3)
Zacatecas	50·4 (46·5 to 54·2)	58·5 (55·6 to 61·6)	62·5 (59·3 to 65·2)	67·3 (66·2 to 68·4)	71·8 (71·1 to 72·4)	73·9 (73·3 to 74·5)	74 (73·5 to 74·5)	73 (72·4 to 73·5)	67·4 (66·9 to 67·8)	66·6 (66 to 67·2)
Nicaragua	46·6 (42·5 to 50·1)	52·8 (50·9 to 54·5)	55·8 (54 to 57·7)	64 (61·9 to 66·2)	70·5 (69·9 to 71·2)	73·2 (72·7 to 73·7)	74·3 (73·8 to 74·8)	76·2 (75·7 to 76·6)	71·4 (70·9 to 71·8)	69·9 (68·5 to 71·2)
Panama	59·4 (57·7 to 61)	64·9 (64·1 to 65·7)	67·1 (66·4 to 67·8)	72·1 (71·5 to 72·7)	74·2 (73·6 to 74·7)	75·5 (75 to 76)	75·5 (75·1 to 75·9)	77·3 (76·8 to 77·7)	74·3 (73·8 to 74·8)	75·5 (73·1 to 78·2)
Venezuela	54·8 (54·2 to 55·4)	63·5 (63·3 to 63·8)	64·3 (64·1 to 64·5)	66·7 (66·5 to 66·9)	69·5 (69·3 to 69·7)	69·7 (69·5 to 69·9)	71·4 (71·3 to 71·5)	69·7 (66·5 to 72·8)	66·1 (63 to 69)	65·1 (62·2 to 68·1)
Tropical Latin America	48·3 (44·6 to 51·7)	53·9 (52·2 to 55·4)	57·9 (56·6 to 59·1)	62·7 (62 to 63·3)	65·8 (65·4 to 66·1)	68·7 (68·3 to 69·1)	70·9 (70·5 to 71·2)	73·1 (72·8 to 73·4)	71·6 (71·3 to 71·8)	70·2 (69·9 to 70·4)
Brazil	48·1 (44·4 to 51·6)	53·6 (51·9 to 55·1)	57·6 (56·3 to 58·9)	62·5 (61·8 to 63·1)	65·6 (65·2 to 66)	68·6 (68·2 to 69)	70·8 (70·5 to 71·2)	73·1 (72·8 to 73·4)	71·5 (71·3 to 71·8)	70·2 (69·9 to 70·4)
Acre	45·7 (39·6 to 51·5)	52·8 (49·7 to 56·2)	57·6 (54·6 to 60·6)	62·2 (60·9 to 63·7)	65·4 (64·5 to 66·3)	68·7 (67·9 to 69·6)	71 (70·4 to 71·6)	71·8 (71·1 to 72·4)	69·9 (69·3 to 70·4)	70·1 (69·5 to 70·7)
Alagoas	40·9 (34·8 to 46·7)	46·7 (43·1 to 50·2)	51·6 (48·4 to 54·9)	56·3 (54·6 to 57·8)	62·6 (61·1 to 63·9)	68·4 (67·5 to 69·2)	68·5 (67·1 to 69·8)	72·2 (71 to 73·5)	70·3 (69·8 to 70·9)	70·4 (70 to 70·8)
Amapá	44·8 (38·4 to 50·8)	53·4 (50·3 to 56·6)	61·2 (58·3 to 64·1)	69·3 (68 to 70·6)	68·3 (66·7 to 69·8)	69 (67·2 to 70·7)	70·9 (69·2 to 72·7)	70 (67·7 to 72·1)	68·4 (66·5 to 70·3)	69·1 (66·8 to 71·4)
Amazonas	52·3 (45·9 to 58)	58·8 (55·3 to 62·3)	61·9 (58·8 to 65·1)	66·5 (65 to 67·9)	69·5 (68·3 to 70·6)	72·3 (71·2 to 73·3)	73·3 (72·2 to 74·4)	73·1 (72·6 to 73·5)	69·1 (68·7 to 69·5)	67·9 (67·4 to 68·4)
Bahia	48·1 (41·9 to 53·9)	53·3 (49·8 to 57)	57·5 (54·5 to 60·3)	65·2 (64·1 to 66·4)	66·5 (65·5 to 67·5)	69 (68 to 69·8)	69·4 (68·4 to 70·4)	72·4 (71·3 to 73·5)	71·1 (70·2 to 72)	71 (69·9 to 72·1)
Ceará	38·8 (32·5 to 44·9)	45·3 (41·8 to 48·9)	52·5 (49·3 to 55·9)	61·2 (57·7 to 64·3)	69·9 (68·3 to 71·4)	71·5 (69·7 to 73·3)	72·5 (70·9 to 74)	73 (71 to 74·8)	71·5 (69·9 to 73)	71·8 (69·8 to 73·6)
Distrito Federal	52·6 (48·1 to 56·5)	57 (54 to 60·1)	60·3 (57·4 to 62·9)	64·1 (63·6 to 64·6)	66·2 (65·8 to 66·6)	69 (68·7 to 69·4)	71·8 (71·4 to 72·1)	75·4 (75 to 75·8)	72·7 (72·4 to 73)	71·9 (71·6 to 72·2)
Espírito Santo	52·2 (48·3 to 56·1)	56·8 (53·6 to 60·1)	59·7 (56·7 to 62·7)	64·7 (64·2 to 65·2)	67·4 (66·9 to 67·8)	68·6 (68·2 to 69)	70·7 (70·2 to 71·3)	73·7 (73·3 to 74·1)	71·5 (71·1 to 71·8)	70·8 (70·3 to 71·2)
Goiás	40·8 (35·2 to 46·2)	46·8 (43·8 to 50)	54·1 (50·8 to 57·2)	62 (60·1 to 64)	66·9 (66 to 67·7)	70·2 (69·4 to 70·8)	71·5 (70·9 to 72)	73 (72·5 to 73·3)	70·7 (70·3 to 71·1)	68·5 (68·2 to 69)
Maranhão	39·2 (34·8 to 43·3)	44·2 (41·3 to 47·5)	48·6 (45·6 to 51·4)	56·4 (53·8 to 59)	63·1 (61·7 to 64·6)	70·4 (68·9 to 71·9)	67·6 (66·4 to 68·9)	71·4 (69·6 to 73·2)	69·6 (68·1 to 71·1)	70·1 (68·4 to 71·8)
Mato Grosso	54·9 (50·1 to 59·1)	58·2 (54·8 to 61·7)	62·3 (58·9 to 65·7)	68·5 (67·1 to 69·9)	67·5 (66·3 to 68·9)	69·5 (68·1 to 70·8)	71·6 (71 to 72·1)	74 (73·4 to 74·4)	70·7 (70·2 to 71·1)	68·9 (68·5 to 69·3)
Mato Grosso do Sul	53·5 (48·8 to 57·9)	57·9 (54·6 to 61·4)	61·3 (58·1 to 64·4)	66·5 (66 to 67)	68·2 (67·8 to 68·6)	69·2 (68·7 to 69·6)	70·9 (70·6 to 71·3)	73·8 (73·4 to 74·3)	71·9 (71·5 to 72·2)	69·4 (69 to 69·7)
Minas Gerais	49·9 (45·2 to 54·3)	54·9 (51·6 to 57·9)	58·7 (55·7 to 61·6)	63·3 (62·6 to 63·9)	67 (66·5 to 67·5)	70·8 (70·3 to 71·2)	72·4 (72 to 72·8)	74·7 (74·4 to 75)	73·7 (73·4 to 73·9)	71·5 (71·2 to 71·8)
Pará	46·8 (41·2 to 52·2)	53·9 (50·4 to 57·3)	58·4 (55·1 to 61·6)	64 (61·9 to 65·8)	68·1 (66·7 to 69·5)	71·1 (69·5 to 72·6)	70·8 (69·2 to 72·4)	72·1 (70·2 to 73·9)	69·9 (68·4 to 71·4)	70·4 (68·6 to 72·1)
Paraíba	38·3 (31·3 to 45·2)	46·7 (43·3 to 50·2)	54·3 (51·2 to 57·6)	62 (60·2 to 63·6)	69·1 (68·2 to 70)	71 (70·3 to 71·7)	69·8 (69·3 to 70·2)	70·5 (70·2 to 70·9)	72 (71·6 to 72·4)	71 (70·6 to 71·4)
Paraná	53·8 (49·7 to 57·5)	57·7 (54·6 to 60·7)	60·2 (57·3 to 63·2)	63·8 (63·3 to 64·2)	67 (66·7 to 67·3)	69·3 (68·9 to 69·6)	70·5 (70·1 to 70·9)	73·7 (73·4 to 74)	72·2 (71·8 to 72·4)	68·8 (68·4 to 69·3)
Pernambuco	37·5 (31·5 to 42·9)	44·2 (41 to 47·7)	50·8 (47·9 to 53·8)	58·9 (57·1 to 60·4)	62·3 (61·5 to 63·1)	65·7 (65 to 66·3)	70 (69·5 to 70·4)	71·9 (71·5 to 72·2)	70 (69·6 to 70·4)	70·3 (69·7 to 70·8)
Piauí	48·9 (42·9 to 54·5)	54·3 (51·2 to 57·5)	57·5 (54·1 to 60·9)	64·4 (62 to 66·7)	69·6 (68·1 to 71·1)	72·5 (70·9 to 74·3)	71·6 (69·9 to 73·2)	73·7 (71·7 to 75·9)	72·1 (70·3 to 74)	72·3 (70·3 to 74·4)
Rio de Janeiro	52·2 (46·6 to 57·2)	57·2 (54·1 to 60·5)	59·6 (56·5 to 62·6)	61·1 (60·6 to 61·6)	61·8 (61·5 to 62·1)	66 (65·8 to 66·3)	69·5 (69·2 to 69·7)	71·4 (71·1 to 71·7)	69·5 (69·2 to 69·8)	68·7 (68·5 to 68·9)
Rio Grande do Norte	36·5 (29·5 to 43·4)	46 (42·1 to 49·9)	55·5 (52·4 to 58·4)	64·7 (62·7 to 66·6)	70 (68·6 to 71·4)	72·6 (71·4 to 73·7)	72·5 (71·3 to 73·6)	73·5 (73 to 73·9)	72 (71·6 to 72·4)	71·8 (71·4 to 72·3)
Rio Grande do Sul	55·8 (52·1 to 59·5)	59·6 (56·7 to 62·6)	62·8 (59·9 to 65·6)	65·2 (64·9 to 65·6)	67·4 (67·2 to 67·7)	69·2 (69 to 69·5)	71·6 (71·4 to 71·9)	73·4 (73·1 to 73·7)	72·8 (72·6 to 73)	70·4 (70·2 to 70·6)
Rondônia	42·5 (33·8 to 49·5)	49·7 (46·7 to 52·8)	54·3 (51·4 to 57·3)	57·9 (56·3 to 59·5)	63·9 (62·7 to 65·1)	69·2 (68·3 to 70·1)	71·6 (70·2 to 72·8)	74 (72·5 to 75·6)	71·9 (70·5 to 73·3)	71·4 (69·8 to 72·9)
Roraima	40·5 (35·2 to 45·6)	47·3 (44·3 to 50·2)	52·2 (49·3 to 55·2)	60·1 (57·1 to 62·7)	61·2 (58·9 to 63·7)	65·9 (63·4 to 68·3)	68·7 (66·4 to 70·9)	68·8 (65·8 to 71·5)	67·7 (65·1 to 70·3)	68·7 (65·9 to 71·2)
Santa Catarina	57 (52·7 to 60·7)	60·9 (58 to 64·1)	63·2 (60·3 to 66·1)	66·2 (65·7 to 66·8)	67·8 (67·3 to 68·2)	69·7 (69·2 to 70·1)	72·5 (72·1 to 72·9)	75 (74·5 to 75·3)	73·6 (73·3 to 73·9)	71·4 (71·1 to 71·8)
São Paulo	55·6 (52 to 59·4)	59·4 (56·3 to 62·7)	62 (59 to 64·8)	63·6 (63·2 to 63·9)	65 (64·7 to 65·3)	67·4 (67·1 to 67·6)	71·7 (71·3 to 72)	74·2 (73·9 to 74·4)	72·3 (72·1 to 72·5)	70·2 (69·9 to 70·5)
Sergipe	45·6 (38·9 to 51·4)	51·9 (48·3 to 55·3)	57·9 (54·9 to 60·7)	62·3 (61 to 63·4)	66·4 (65·6 to 67·1)	69 (68·1 to 69·8)	70·5 (69·8 to 71)	72·1 (71·6 to 72·6)	70·3 (69·8 to 70·7)	70·1 (69·6 to 70·5)
Tocantins	45·1 (36 to 52·3)	50·5 (47 to 53·9)	57 (53·3 to 60·2)	65·1 (62·2 to 68·1)	69·3 (67·6 to 70·9)	71 (69·5 to 72·5)	70·9 (69·5 to 72·3)	71·9 (70·1 to 73·7)	70·8 (69·3 to 72·3)	71 (69·3 to 72·7)

Paraguay	57·7 (54·2 to 61)	66·6 (64·6 to 68·6)	68·6 (66·9 to 70·5)	71·3 (69·3 to 73·2)	73·7 (71·7 to 75·6)	73·1 (71·2 to 75·2)	72·7 (70·7 to 74·5)	72·5 (70·3 to 74·8)	72·5 (70·2 to 74·8)	69 (66·5 to 71·1)
North Africa and Middle East	41·3 (37·9 to 44·4)	46·6 (44·4 to 48·9)	52·5 (50·6 to 54·2)	58·1 (56·5 to 59·3)	63·8 (62·9 to 64·7)	67·4 (66·5 to 68·2)	70·3 (69·4 to 71·2)	72 (70·9 to 72·9)	70 (69 to 71·1)	68·9 (67·8 to 70·1)
North Africa and Middle East	41·3 (37·9 to 44·4)	46·6 (44·4 to 48·9)	52·5 (50·6 to 54·2)	58·1 (56·5 to 59·3)	63·8 (62·9 to 64·7)	67·4 (66·5 to 68·2)	70·3 (69·4 to 71·2)	72 (70·9 to 72·9)	70 (69 to 71·1)	68·9 (67·8 to 70·1)
Afghanistan	38·5 (34·4 to 42·6)	45·7 (42·2 to 49·3)	47·7 (44 to 51·2)	42·3 (29·8 to 48·7)	52·5 (49·8 to 55·5)	53·3 (50·8 to 55·9)	59·1 (56·1 to 62)	60·5 (57·9 to 63·2)	57·6 (55·3 to 60)	55·9 (54 to 57·9)
Algeria	41·1 (37 to 44·8)	42·1 (39·7 to 44·5)	52·5 (49·2 to 55·4)	56·3 (55·1 to 57·4)	68·6 (67·2 to 69·9)	71·2 (69·9 to 72·5)	74·9 (74·1 to 75·7)	76·6 (75·2 to 78)	73·3 (72·2 to 74·5)	72·1 (70·6 to 73·6)
Bahrain	50·9 (46 to 55·4)	54·6 (51·2 to 57·8)	61·3 (58·2 to 64·2)	65·8 (64·7 to 67)	67·9 (67·2 to 68·5)	68·6 (67·8 to 69·3)	73 (72·4 to 73·6)	75·3 (74·3 to 76·3)	73·5 (72·5 to 74·5)	72·2 (71·1 to 73·3)
Egypt	42·7 (38·5 to 46·8)	47·6 (45·8 to 49·3)	49·9 (48·7 to 51·1)	56·6 (55·6 to 57·6)	62·3 (61·7 to 62·9)	66·4 (66 to 66·9)	67·5 (67·1 to 67·8)	69·8 (69·5 to 70·1)	67·9 (66·4 to 69·4)	66·9 (65 to 68·7)
Iran	35·7 (31·5 to 40)	42·9 (40·2 to 45·4)	52·3 (50·3 to 54·2)	61·9 (60·7 to 63)	65·8 (64·9 to 66·6)	71·2 (70·6 to 71·7)	74 (73·6 to 74·4)	76·6 (76·1 to 77)	75·8 (75·3 to 76·2)	71·9 (71·5 to 72·3)
Alborz	41·3 (36·7 to 45·9)	44·5 (40·6 to 48·1)	55·4 (52·2 to 58·6)	66·9 (63·7 to 69·9)	70·3 (67·7 to 73·1)	73·1 (71·1 to 75·1)	76·3 (74·8 to 77·7)	77·4 (74·9 to 79·6)	76 (74·1 to 77·8)	71·5 (70·1 to 72·8)
Ardebil	36·2 (31·6 to 40·9)	41·2 (38 to 44·5)	49·2 (45·9 to 52·4)	59 (56·2 to 61·6)	65·3 (62·9 to 67·7)	72 (70·9 to 73·2)	73·8 (73·2 to 74·5)	74·1 (72·8 to 75·3)	73·7 (72·4 to 75)	70 (69 to 71)
Bushehr	41·3 (36·6 to 46)	47 (43·8 to 50·5)	54·4 (51·3 to 57·6)	64·1 (61·3 to 66·8)	68·6 (66·2 to 71·1)	72 (70·9 to 73·1)	73·8 (73·1 to 74·5)	76·5 (75·2 to 77·8)	75·9 (74·6 to 77·3)	72·3 (71·3 to 73·4)
Chahar Mahaal and Bakhtiari	44·3 (39·5 to 48·8)	49·4 (46 to 52·6)	56·1 (53·2 to 59·3)	62·9 (60 to 65·6)	67·5 (64·9 to 70)	72·2 (70·7 to 73·7)	74·7 (73·6 to 75·7)	77·1 (75·5 to 78·7)	76·8 (75·2 to 78·3)	73·7 (72·4 to 74·9)
East Azarbayejan	33·9 (29·2 to 38·3)	40·2 (36·9 to 43·4)	48·9 (46 to 52)	59·6 (56·7 to 62·6)	65·3 (62·8 to 67·8)	69·8 (68·2 to 71·3)	72·5 (71·5 to 73·4)	75·1 (73·4 to 76·8)	74·9 (73·3 to 76·5)	71·5 (70·2 to 72·8)
Fars	45·1 (40·4 to 49·8)	50·5 (47·5 to 53·7)	57·3 (54 to 60·2)	62·5 (59·7 to 65·2)	66·4 (63·4 to 69·1)	69·5 (67·6 to 71·5)	71·8 (70·5 to 73·2)	74·9 (72·8 to 76·9)	74·4 (72·6 to 76·3)	70·7 (69·2 to 72·2)
Gilan	40·4 (34·8 to 45·5)	47·8 (43·6 to 52·1)	56·8 (53·7 to 59·9)	64·5 (61·8 to 67·3)	48·4 (46·1 to 50·4)	71·4 (69·9 to 73)	74 (73 to 75)	76·6 (74·9 to 78·5)	75·8 (74·2 to 77·5)	71·7 (70·4 to 72·9)
Golestan	39·2 (34·6 to 43·9)	44·2 (40·8 to 47·5)	51·7 (48·8 to 54·8)	61·3 (58·3 to 64·1)	64·9 (62·3 to 67·4)	68·8 (67·7 to 69·8)	71·5 (70·8 to 72·2)	72·4 (70·9 to 73·9)	72 (70·4 to 73·6)	68·4 (67·2 to 69·6)
Hamadan	28·9 (24·2 to 33·9)	36·1 (33 to 39·4)	45·5 (42·3 to 48·8)	57·2 (54·4 to 60)	65·2 (62·4 to 67·9)	71·2 (69·7 to 72·7)	72·3 (71·3 to 73·4)	76·1 (74·1 to 78·1)	75·4 (73·7 to 77·2)	72·2 (70·6 to 73·7)
Hormozgan	35·7 (30·7 to 40·6)	43·7 (40·5 to 47)	52·3 (49·1 to 55·5)	60·8 (57·9 to 63·8)	64 (61·3 to 66·6)	68·6 (67 to 70·2)	72 (70·7 to 73·2)	75·2 (73·2 to 77·2)	74·7 (72·9 to 76·5)	70·8 (69·3 to 72·2)
Ilam	41·3 (36·7 to 45·9)	46·9 (43·5 to 50·2)	53·7 (50·6 to 56·7)	62·2 (59·2 to 65)	66·8 (64·4 to 69·3)	70·7 (69·3 to 72·1)	72·8 (71·9 to 73·8)	75 (73·4 to 76·6)	74·6 (73·1 to 76·1)	70·9 (69·7 to 72·2)
Isfahan	41·3 (36·4 to 45·8)	45·7 (42·2 to 49·3)	56 (52·9 to 59·1)	65·5 (63 to 68·4)	70·5 (67·8 to 73·1)	74 (72·3 to 75·7)	76·5 (75·4 to 77·6)	78·1 (76·3 to 80·1)	77 (75·3 to 78·6)	72·6 (71·3 to 73·9)
Kerman	32·6 (27·4 to 37·9)	41·2 (38 to 44·5)	50·3 (47·3 to 53·3)	59·3 (56·4 to 62·2)	64·6 (61·9 to 67·1)	68 (66·3 to 69·7)	71·8 (70·5 to 72·9)	74·9 (73 to 76·6)	74·5 (72·8 to 76·1)	71·1 (69·7 to 72·5)
Kermanshah	35 (30 to 39·8)	41 (37·7 to 44·1)	48·9 (45·7 to 52·2)	58·8 (55·9 to 61·6)	63 (60·4 to 65·7)	67·3 (65·6 to 69)	71·3 (70·1 to 72·5)	75·1 (73·1 to 77·2)	74·2 (72·4 to 76·1)	70·3 (68·9 to 71·7)
Khorasan-e-Razavi	27·5 (23·2 to 32)	35·1 (31·7 to 38·6)	45·2 (42·1 to 48·4)	58·4 (55·3 to 61·5)	63·3 (60·5 to 66)	67·7 (66·1 to 69·3)	71·5 (70·4 to 72·5)	75·2 (73·5 to 76·9)	74·6 (73 to 76·1)	70·9 (69·5 to 72·1)
Khuzestan	37·8 (32·2 to 43)	45·3 (41·8 to 48·8)	53·6 (50·5 to 56·6)	60·8 (57·9 to 63·6)	67·4 (65 to 69·9)	70·6 (69·6 to 71·7)	72·7 (72·1 to 73·3)	75·2 (73·6 to 76·7)	74·3 (72·9 to 75·8)	70·1 (69 to 71·3)
Kohgiluyeh and Boyer-Ahmad	41·1 (36·4 to 46·2)	48·1 (44·8 to 51·3)	54·6 (51·8 to 57·7)	60·4 (57·4 to 62·9)	67·6 (64·9 to 70·4)	73·1 (71·3 to 74·8)	76·3 (75·2 to 77·5)	78·2 (76·2 to 80·3)	77·4 (75·5 to 79·2)	73·5 (72·1 to 74·9)
Kurdistan	27·8 (23·5 to 32·3)	33·5 (30·4 to 36·5)	42·9 (39·8 to 45·9)	54·2 (51·2 to 57)	62·4 (59·6 to 65)	69·3 (68 to 70·7)	72·1 (71·1 to 73)	76·8 (75·1 to 78·6)	76·2 (74·6 to 77·8)	72·5 (71·2 to 73·8)
Lorestan	35 (29·4 to 40·4)	43·6 (40·3 to 47)	51·8 (48·6 to 54·9)	59·5 (56·8 to 62·2)	65·8 (63·3 to 68·4)	70·6 (69 to 72·1)	72·4 (71·4 to 73·4)	74·4 (73 to 75·9)	74 (72·6 to 75·3)	69·8 (68·8 to 70·8)
Markazi	38·1 (33·3 to 43)	42·6 (39·4 to 46)	50·3 (46·9 to 53·5)	59·9 (57 to 62·6)	65·9 (63·2 to 68·9)	70·4 (68·7 to 72·2)	75 (73·9 to 76·1)	78·5 (76·6 to 80·5)	77·2 (75·3 to 79)	72·7 (71·4 to 74)
Mazandaran	45·4 (40·4 to 50·3)	50·5 (47·2 to 53·8)	58·6 (55·3 to 61·7)	65·5 (62·6 to 68·2)	71·4 (68·8 to 74·1)	74·3 (72·6 to 75·9)	75·4 (74·2 to 76·5)	78 (76·1 to 80·1)	76·9 (75·2 to 78·5)	72·7 (71·4 to 73·9)
North Khorasan	32 (26·4 to 37·1)	37·6 (34·5 to 40·9)	45·6 (42·6 to 48·6)	58·1 (55·2 to 60·9)	63·3 (60·6 to 66)	68·8 (67·1 to 70·3)	73·1 (72·1 to 74·1)	75·9 (74·1 to 77·7)	74·9 (73·3 to 76·5)	70·7 (69·5 to 71·8)
Qazvin	31·4 (26·1 to 36·3)	38 (34·6 to 41·4)	48·1 (45 to 51·6)	59·8 (56·7 to 62·5)	67 (64·2 to 69·5)	70·6 (69·1 to 72·1)	73·1 (72·2 to 74)	75·3 (73·9 to 76·8)	74·7 (73·4 to 76·2)	70·9 (69·9 to 72)
Qom	43·4 (38·5 to 48·1)	47·8 (44·5 to 51·2)	55·2 (52·1 to 58·3)	62 (59·2 to 64·7)	68·6 (65·9 to 71)	71·8 (70·3 to 73·1)	75·4 (74·6 to 76·3)	79 (77·4 to 80·7)	77·5 (76·1 to 79·1)	72·7 (71·6 to 73·8)
Semnan	38 (33·2 to 42·8)	41·6 (38·2 to 44·9)	51·5 (48·3 to 54·8)	60·9 (58·2 to 63·5)	67·3 (65 to 69·9)	70·3 (68·7 to 71·8)	73·5 (72·5 to 74·5)	76·2 (74·6 to 77·8)	75·4 (73·8 to 77)	71 (69·9 to 72·1)
Sistan and Baluchistan	28·5 (22·9 to 34·4)	36·7 (33·5 to 39·9)	45·3 (42·2 to 48·3)	56·3 (53·4 to 59·1)	62 (59·2 to 65·1)	68·5 (66·4 to 70·7)	71·4 (69·8 to 73)	74·1 (71·4 to 76·6)	73·2 (71 to 75·3)	69 (67·3 to 70·7)
South Khorasan	33 (28·3 to 37·6)	38 (34·8 to 41·1)	46·6 (43·4 to 49·8)	59·5 (56·5 to 62·5)	66 (63·4 to 68·3)	72·6 (71·4 to 73·9)	75 (74·2 to 75·8)	78·2 (76·9 to 79·7)	77·4 (76 to 78·8)	73·5 (72·4 to 74·5)
Tehran	50·1 (44·9 to 54·7)	56·5 (53·1 to 60)	65·3 (62·3 to 68·1)	72·5 (69·5 to 75·2)	74·4 (71·8 to 77·1)	76·4 (74·6 to 78·2)	78·7 (77·4 to 79·9)	80 (78·1 to 81·9)	79 (77·4 to 80·4)	75 (73·7 to 76·2)
West Azarbayejan	33·6 (29 to 38·1)	39·1 (35·7 to 42·4)	47·6 (44·2 to 50·8)	58·4 (55·5 to 61·2)	64·2 (61·5 to 66·5)	68·6 (67·3 to 69·9)	72·4 (71·6 to 73·2)	74·7 (73·2 to 76·2)	74·3 (72·7 to 75·8)	70·6 (69·5 to 71·8)
Yazd	34·6 (29·3 to 39·9)	46 (42·7 to 49·5)	55·3 (52·1 to 58·2)	62·2 (59·3 to 64·8)	68·6 (66 to 71·2)	71·4 (69·7 to 73·1)	73·4 (72·2 to 74·5)	77 (75·2 to 78·8)	75·7 (74 to 77·3)	70·5 (69·4 to 71·7)
Zanjan	26·2 (20·7 to 31·7)	32·3 (29 to 35·7)	43·4 (40·3 to 46·5)	56·8 (54 to 59·5)	47·8 (45·9 to 49·6)	69·5 (68·2 to 70·6)	73·1 (72·4 to 73·7)	76·3 (75 to 77·6)	75·6 (74·4 to 77)	71·9 (70·9 to 72·9)
Iraq	51·5 (47·5 to 55·3)	57·3 (54·7 to 59·9)	59·8 (54·7 to 62·5)	59·4 (57·5 to 61·3)	64·5 (62·4 to 66·5)	66·6 (63·6 to 69·6)	68·5 (65·3 to 71·6)	70·6 (67·9 to 73·6)	66·8 (64·9 to 69)	67·5 (65·6 to 70)
Jordan	49·4 (44·3 to 53·9)	55·4 (52·4 to 58·5)	60 (58·1 to 61·7)	65·1 (63·5 to 66·8)	72·1 (70·3 to 73·9)	74·2 (71·9 to 76·5)	77·5 (76 to 78·9)	80·1 (78·1 to 82)	76 (74·2 to 78)	74·1 (72·4 to 75·9)
Kuwait	50·9 (42·8 to 57·7)	67·7 (64·7 to 70·8)	68·8 (68·3 to 69·4)	70·8 (70·3 to 71·3)	72·4 (71·5 to 73·4)	76 (75·6 to 76·4)	79·1 (78·7 to 79·4)	80·3 (79·9 to 80·6)	76·8 (76·5 to 77·1)	78·1 (76·3 to 80)
Lebanon	51·4 (45·7 to 57)	62 (58·4 to 65·5)	65·4 (62·3 to 68·2)	66·5 (63·8 to 68·8)	65·9 (63·8 to 67·6)	74·3 (72·7 to 75·9)	75·6 (74·6 to 76·7)	77·7 (76·3 to 78·9)	74·8 (73·4 to 76·1)	72·2 (70·9 to 73·3)
Libya	40·2 (36·1 to 44·3)	48·9 (45·9 to 52·2)	60·8 (58·9 to 62·9)	68·3 (66·7 to 69·8)	71·3 (69·3 to 73·3)	72·8 (71·6 to 74·1)	72·4 (71·2 to 73·5)	70·3 (67·4 to 73·1)	68·7 (66·1 to 71·3)	68·7 (66 to 71·1)
Morocco	38·6 (34·2 to 42·7)	45·2 (42·1 to 48·5)	52·2 (49·6 to 54·9)	58·6 (56·4 to 60·6)	65·8 (64 to 67·6)	69·2 (67·2 to 71·1)	71·5 (69 to 74)	72·9 (71·1 to 75·7)	70·4 (68·8 to 72·5)	70·9 (69·4 to 72·9)
Oman	38 (32·7 to 43·3)	44·7 (40·7 to 49·1)	54·1 (50·5 to 57·5)	61 (57·3 to 64·4)	66·6 (63·5 to 69·7)	69·4 (67·1 to 71·9)	70·6 (69·2 to 72)	73 (71·4 to 74·6)	72·3 (70·9 to 73·7)	70·5 (69·1 to 71·7)
Palestine	41·3 (35·7 to 46·5)	50·9 (47·1 to 54·4)	51·6 (48·6 to 54·3)	65·4 (62·2 to 68·1)	67·2 (65·1 to 69·3)	66·8 (65·7 to 67·9)	70·7 (70 to 71·4)	74·3 (73·3 to 75·3)	73·5 (72·3 to 74·8)	71·5 (70·3 to 72·8)
Qatar	54·7 (49·4 to 59·2)	59·8 (56·4 to 63·6)	64 (61 to 66·8)	67·5 (65 to 70)	68·7 (66·9 to 70·5)	69 (67·3 to 70·7)	72·6 (70·9 to 74·2)	78·4 (76·3 to 80·5)	76·2 (74·3 to 78)	76·1 (74·2 to 77·9)
Saudi Arabia	52·6 (48 to 57)	49·5 (45·5 to 53·2)	56·3 (52·5 to 59·9)	64·3 (60·5 to 67·6)	66·6 (63·6 to 69·7)	69·1 (67·6 to 70·4)	70·3 (69·2 to 71·1)	72·6 (70·8 to 74·3)	71·4 (69·5 to 73)	71·8 (69·9 to 73·6)
Sudan	47·4 (42·1 to 52·2)	49·4 (45·9 to 52·8)	50·4 (47·9 to 52·8)	53·3 (50·9 to 55·8)	56·7 (54·3 to 59·2)	61·5 (58·8 to 64·2)	66·4 (63·4 to 69·3)	69·7 (66·1 to 72·6)	66·7 (63·7 to 69·3)	66·3 (63·1 to 69·3)
Syria	52·1 (48·2 to 56)	60·1 (57·1 to 63·4)	65·1 (62·8 to 67·4)	67·9 (65·4 to 70·5)	67·8 (65·4 to 70·3)	70·4 (68·7 to 72)	72·9 (71 to 74·7)	66·3 (63·6 to 68·8)	69·6 (66·9 to 72·1)	70·1 (67·5 to 72·4)
Tunisia	39·7 (34·9 to 44·2)	47·7 (45·5 to 50)	55·5 (54·3 to 56·8)	64·6 (63·3 to 65·8)	70·1 (68·8 to 71·4)	71·8 (69·9 to 73·5)	73·9 (70·9 to 76·8)	75·4 (72·4 to 78·4)	74·3 (71·2 to 77·5)	70·8 (68·5 to 73·1)
Türkiye	41·3 (36·6 to 46·1)	47 (44 to 50·1)	54·4 (51·6 to 57·2)	60·3 (57·9 to 62·4)	64·4 (63 to 65·9)	69·3 (68 to 70·6)	73 (71·8 to 74·4)	74·9 (73·4 to 76·4)	72·6 (71·1 to 74·1)	72·3 (70·7 to 74)
United Arab Emirates	53·3 (47·8 to 58·2)	57·1 (53·4 to 60·6)	64·5 (60·9 to 67·9)	68·4 (65 to 71·5)	69·4 (66·8 to 71·9)	70·1 (68·3 to 71·5)	72·3 (70·6 to 74·1)	75·9 (73·9 to 78)	78·3 (76·1 to 80·4)	77·5 (75·7 to 79·6)
Yemen	29·7 (25·9 to 33·7)	34·4 (31·6 to 37·5)	38·8 (34·8 to 42·2)	50·6 (47·9 to 53·5)	57·1 (54·2 to 60·1)	61·7 (58·4 to 64·9)	66·3 (63 to 69·3)	64·3 (61·1 to 67·2)	63·5 (60·7 to 66·1)	62·4 (59·4 to 65·2)
South Asia	37·6 (33·3 to 41·7)	43·7 (41·1 to 46·2)	50·3 (48·6 to 51·9)	55·8 (54·7 to 56·9)	59·9 (59 to 60·7)	63 (62·2 to 63·8)	65·9 (65·1 to 66·6)	68·9 (67·9 to 69·9)	67·3 (66·3 to 68·2)	66·4 (65·4 to 67·4)
South Asia	37·6 (33·3 to 41·7)	43·7 (41·1 to 46·2)	50·3 (48·6 to 51·9)	55·8 (54·7 to 56·9)	59·9 (59 to 60·7)	63 (62·2 to 63·8)	65·9 (65·1 to 66·6)	68·9 (67·9 to 69·9)	67·3 (66·3 to 68·2)	66·4 (65·4 to 67·4)
Bangladesh	40·5 (36·9 to 44·1)	45·3 (42·6 to 48·2)	45·3 (43·1 to 47·5)	54·9 (53 to 56·9)	58 (56·7 to 59·2)	63·6 (62·2 to 64·8)	68·1 (67 to 69·1)	72·6 (70·2 to 75·1)	70·7 (68·4 to 72·8)	70·6 (68·3 to 72·8)
Bhutan	37 (31·2 to 42·9)	43·2 (38·9 to 47·6)	49·2 (44·6 to 53·7)	52·7 (48·8 to 56·9)	60·1 (57·3 to 62·9)	65·5 (63·2 to 67·8)	70·6 (68·4 to 72·9)	72·8 (70·2 to 75·4)	72·9 (70·4 to 75·6)	72·7 (70·2 to 75·2)
India	36·4 (31·3 to 41·3)	42·7 (39·7 to 45·6)	50·1 (48·3 to 51·9)	55·5 (54·3 to 56·8)	60 (59 to 61)	63·1 (62·1 to 64)	65·9 (65 to 66·8)	69 (68 to 70·1)	67·6 (66·5 to 68·7)	66·6 (65·4 to 67·7)
Nepal	37 (33·3 to 40·6)	42·4 (39·7 to 45·4)	47·7 (45 to 50·6)	50·8 (48·1 to 53·6)	57·6 (55·4 to 59·8)	64·2 (62·5 to 65·9)	67·9 (66 to 69·8)	69·7 (67·2 to 71·8)	67·8 (65·6 to 69·8)	66·1 (64·1 to 67·8)
Pakistan	47·6 (43·9 to 51·5)	54·1 (51·5 to 56·8)	59·6 (57·7 to 61·5)	61 (59·4 to 62·6)	62·2 (61 to 63·4)	62·3 (60·8 to 63·8)	64·4 (61·9 to 66·9)	66·8 (63·7 to 69·7)	64 (61·5 to 66·3)	63·8 (61·3 to 66·1)
Azad Jammu & Kashmir	43·2 (37·3 to 49·3)	51·8 (47·3 to 56·2)	57·9 (54·5 to 61·3)	60 (56·2 to 63·8)	62·4 (58·7 to 66)	63·2 (59·4 to 67·3)	66·6 (62 to 71·2)	68·7 (64 to 73·3)	66 (62·3 to 69·4)	64·5 (61·5 to 67·6)
Balochistan	52·4 (46·8 to 58·3)	57·6 (53·5 to 61·9)	63·5 (59·7 to 67·1)	63·6 (60 to 67·4)	63 (59·4 to 66·4)	62·1 (58·5 to 66·2)	63·2 (58·9 to 67·5)	65·1 (60·3 to 69·3)	61·9 (58·4 to 65·2)	62·7 (58·9 to 66·2)

Gilgit-Baltistan	51·6 (47·1 to 56·4)	50·1 (45·9 to 54·3)	55·4 (51·6 to 59·4)	57·2 (53·4 to 61·1)	59·7 (56·2 to 63·5)	60·5 (56·9 to 64·2)	64·2 (59·6 to 69)	66·7 (62·1 to 71·7)	62·2 (58·9 to 65·4)	63·3 (59·7 to 67·1)
Islamabad Capital Territory	48·6 (42·1 to 54·8)	52·2 (47·5 to 56·4)	61·5 (57·6 to 65·4)	66·9 (63·1 to 70·6)	70·2 (66·6 to 73·8)	70·3 (66·5 to 74)	72 (68 to 75·9)	73 (69·3 to 76·8)	69·7 (66·6 to 72·8)	69·3 (66·3 to 72·6)
Khyber Pakhtunkhwa	48·1 (43 to 53·3)	54·9 (51·2 to 58·4)	60·3 (56·7 to 64)	62·4 (59·1 to 66·1)	64·5 (61·2 to 68)	63·2 (59·9 to 66·7)	64·2 (60·1 to 68·2)	67·4 (62·9 to 71·7)	64 (60·4 to 67·4)	64·3 (60·4 to 68·5)
Punjab	47·3 (43·4 to 51·2)	53·7 (50·8 to 56·6)	59 (56·7 to 61·2)	59·9 (57·8 to 61·9)	61·2 (59·5 to 63·2)	61·7 (59·7 to 63·8)	64·2 (61·3 to 67·3)	66·5 (63·1 to 70)	64·2 (61·2 to 66·9)	63·4 (60·7 to 66·2)
Sindh	47·3 (41·8 to 53)	55 (51·2 to 58·8)	60·9 (57·6 to 64·4)	62·8 (59·7 to 65·9)	63 (59·8 to 66·2)	62·8 (59·5 to 66·4)	64·9 (60·9 to 69·1)	67·3 (62·8 to 71·5)	63·8 (60·9 to 66·7)	64·2 (60·8 to 67·7)
Southeast Asia, east Asia, and Oceania	44·8 (41·7 to 47·6)	31·1 (19·8 to 39·1)	57·3 (55·1 to 59·5)	61·3 (59·6 to 63·1)	64·9 (63·4 to 66·3)	67·4 (66·6 to 68·2)	70·4 (69·6 to 71·3)	72·9 (71·4 to 74·4)	72·9 (71·3 to 74·6)	72·5 (70·9 to 74·1)
East Asia	46·3 (42·7 to 49·8)	26·1 (14 to 35·5)	59·4 (56·5 to 62·3)	62·8 (60·6 to 65)	65·8 (64 to 67·6)	68·4 (67·4 to 69·5)	71·9 (70·7 to 73)	74·6 (72·5 to 76·5)	74·7 (72·6 to 76·8)	74·8 (72·7 to 77)
China	47·4 (43·6 to 51)	25·3 (13·3 to 34·8)	59·2 (56·2 to 62·2)	62·6 (60·4 to 64·9)	65·7 (63·9 to 67·5)	68·5 (67·5 to 69·6)	71·9 (70·7 to 73)	74·6 (72·5 to 76·6)	74·7 (72·5 to 76·8)	74·9 (72·7 to 77·1)
North Korea	18·5 (16·9 to 20·1)	60·2 (56·3 to 63·8)	63·7 (60 to 67·6)	65·9 (62·1 to 69·4)	66·8 (63·5 to 70·1)	59·3 (47·2 to 65·5)	67·6 (65·4 to 69·7)	69·8 (67·3 to 72·1)	69·9 (67·5 to 72·2)	70·1 (67·8 to 72·5)
Taiwan (province of China)	55·7 (52·9 to 58·3)	62·7 (62·5 to 62·9)	67·6 (67·5 to 67·8)	70·1 (70 to 70·2)	72·2 (72 to 72·3)	74·1 (74 to 74·3)	76·9 (76·7 to 77)	77·8 (77·7 to 78)	78·4 (78·2 to 78·5)	78·1 (77·9 to 78·2)
Oceania	46·8 (43·6 to 50·2)	52·3 (49·6 to 55)	56·5 (54·1 to 58·8)	59·6 (57·1 to 61·8)	61·1 (58·7 to 63·5)	62·8 (60·9 to 64·7)	63·9 (61·9 to 65·8)	65·1 (62·8 to 67·3)	64·9 (62·6 to 67·1)	62·5 (59·4 to 65·6)
American Samoa	60·8 (58·4 to 63)	63·1 (61·7 to 64·5)	63·9 (62·7 to 65·2)	66·7 (65·5 to 68)	67·4 (66·3 to 68·3)	68·1 (67·1 to 69)	68·6 (67·5 to 69·6)	69·2 (67·3 to 70·9)	68·3 (66·4 to 69·8)	69·3 (67 to 71·2)
Cook Islands	46·4 (44·2 to 48·7)	58·3 (56·9 to 59·9)	63·4 (61·8 to 64·9)	64·3 (62·9 to 65·5)	66·4 (64·9 to 67·8)	69·8 (68·4 to 71·2)	72·1 (70·7 to 73·4)	72·8 (71 to 74·5)	72·7 (70·9 to 74·5)	72·9 (70·9 to 74·7)
Fiji	58·5 (57 to 60)	62·9 (61·9 to 63·8)	62·7 (61·6 to 63·8)	63·9 (62·8 to 64·9)	63·9 (61·4 to 66·1)	63·4 (62·8 to 64)	65·4 (64·4 to 66·4)	66·3 (63·4 to 69·3)	66·2 (63·1 to 69·2)	63·8 (60·4 to 67·4)
Guam	65·9 (63·9 to 67·7)	65·9 (65·2 to 66·6)	64·8 (64·2 to 65·4)	68·7 (68·1 to 69·4)	72·1 (71·4 to 72·8)	75·8 (74·9 to 76·6)	76·3 (75·4 to 77·2)	74·5 (73·2 to 75·7)	74·7 (73·4 to 76)	73·5 (71·7 to 75·5)
Kiribati	44 (40·5 to 47·3)	49·4 (46·5 to 52·4)	52·8 (50·1 to 55·8)	56·2 (53·5 to 58·9)	56·4 (54·2 to 58·6)	57·3 (55·1 to 59·5)	59·2 (56·4 to 61·9)	60·7 (57·5 to 63·6)	60·9 (57·6 to 63·8)	61·1 (57·8 to 64)
Marshall Islands	47·4 (42·9 to 51·9)	53·6 (50·1 to 56·9)	57·6 (54·1 to 60·7)	60·1 (58 to 62·4)	59·6 (58·4 to 60·8)	60·5 (58·6 to 62·2)	61·4 (59·1 to 63·8)	63 (59·6 to 66·1)	63·2 (59·8 to 66·3)	63·4 (59·8 to 66·5)
Federated States of Micronesia	41·5 (37·3 to 45·6)	50·9 (48 to 53·6)	55·5 (52·6 to 58·4)	59 (56·1 to 61·8)	60·5 (57·6 to 63·4)	61·6 (59·2 to 63·9)	63·4 (60·8 to 65·9)	64·3 (61·1 to 67·3)	64·4 (61 to 67·4)	64·5 (61·1 to 67·5)
Nauru	57·5 (53·7 to 61·3)	59·9 (56·8 to 62·7)	60·1 (57·3 to 62·8)	59·5 (56·3 to 62·5)	58·1 (55·7 to 60·7)	55 (52·9 to 57·2)	55·6 (53·1 to 58·2)	58·8 (55·4 to 61·9)	59 (55·6 to 62·1)	59·2 (55·8 to 62·4)
Niue	51·3 (48·1 to 54·4)	60·4 (57·6 to 63·3)	62·4 (59·8 to 65·1)	64·9 (61·8 to 67·5)	65·8 (63·2 to 68·2)	65·4 (63·1 to 67·7)	66·9 (64·3 to 69·4)	67·5 (64·6 to 69·9)	65·1 (62·8 to 66·9)	65·1 (62·9 to 66·8)
Northern Mariana Islands	62·2 (58 to 66)	64·9 (62·2 to 67·7)	67·3 (64·6 to 69·7)	68·8 (65·8 to 71·2)	70·1 (67·4 to 72·1)	71·1 (69·3 to 73)	71 (69·3 to 73·1)	71·2 (69 to 73·5)	71·5 (68·9 to 74·1)	69·5 (68·1 to 71·9)
Palau	46·9 (41·9 to 51·7)	53·5 (50 to 57·1)	58·6 (55·3 to 62)	61·2 (58 to 64·3)	63·6 (60·5 to 66·5)	65·4 (62·9 to 68·1)	65·9 (63·6 to 68·4)	67·5 (64·7 to 70·2)	67·6 (64·8 to 70·4)	67·7 (64·9 to 70·5)
Papua New Guinea	44·4 (40·4 to 48·4)	49·8 (46·5 to 53·1)	55 (51·9 to 58)	58·4 (55·3 to 61·4)	60·3 (57·2 to 63·2)	62·7 (60·2 to 65·1)	63·7 (61·4 to 66·1)	65·1 (62·5 to 67·6)	64·8 (62·2 to 67·4)	61·9 (58·4 to 65·4)
Samoa	55·5 (52·3 to 58·4)	65 (63 to 66·9)	63·2 (61·7 to 64·7)	64·2 (62·6 to 65·7)	65·9 (63·9 to 67·9)	67·8 (65·6 to 69·7)	69·3 (67 to 71·7)	68·4 (66·3 to 70·2)	69·3 (67·1 to 71·2)	69·6 (67·2 to 71·5)
Solomon Islands	45·1 (41·5 to 48·6)	49·8 (46·9 to 52·9)	54 (51 to 57)	57·8 (54·9 to 60·7)	59·1 (56·1 to 61·9)	61·1 (58·1 to 63·9)	62·2 (59 to 65·2)	63·5 (60·2 to 66·6)	63·5 (60·2 to 66·4)	63·7 (60·3 to 66·5)
Tokelau	55·7 (51·8 to 59)	60·2 (56·6 to 63·9)	63·4 (60·3 to 66·7)	64·2 (61·4 to 67·2)	66·9 (64·4 to 69·5)	68·4 (65·9 to 70·6)	70·2 (67·5 to 72·4)	71·6 (68·9 to 74·1)	67·1 (65·1 to 69)	67·1 (65·1 to 69)
Tonga	59·2 (55·9 to 62·2)	62·8 (61·1 to 64·7)	64·9 (63 to 66·8)	65·9 (64·4 to 67·5)	68·5 (66·9 to 70·1)	68·1 (66·7 to 69·4)	69·4 (67·1 to 71·5)	70·4 (67·9 to 72·9)	70·5 (67·9 to 73)	70·6 (67·9 to 73·1)
Tuvalu	37·8 (32·2 to 43·7)	47·1 (43 to 51·3)	51 (46·7 to 54·9)	53·4 (49·9 to 56·6)	57·2 (54·9 to 59·5)	57·5 (55·4 to 59·3)	63·8 (61·3 to 66·1)	65·3 (62·4 to 68·2)	65·6 (62·5 to 68·4)	65·8 (62·7 to 68·7)
Vanuatu	44·7 (41·1 to 48·4)	48·2 (44·8 to 51·4)	53·5 (50·5 to 56·7)	58·9 (55·8 to 61·8)	60·9 (57·8 to 63·6)	61·7 (59 to 64)	62·8 (60·8 to 64·7)	64 (62 to 65·9)	64·1 (61·9 to 66)	62·5 (59·9 to 64·8)
Southeast Asia	40·8 (37·9 to 43·7)	49·1 (46·9 to 51·1)	52·2 (50·7 to 53·6)	58·8 (57·4 to 60·1)	62·8 (61·9 to 63·7)	65·4 (64·6 to 66·3)	67·8 (66·8 to 68·6)	69·8 (68·5 to 71·1)	69·7 (68·3 to 71)	67·9 (66·1 to 69·7)
Cambodia	41·4 (35·6 to 46·7)	46·7 (42·6 to 50·8)	38·8 (35·3 to 42·2)	48·2 (43·2 to 51·6)	55·2 (53·5 to 56·8)	57·8 (56·3 to 59·4)	63·5 (61·7 to 65·3)	66·4 (63·7 to 69·2)	66·6 (63·7 to 69·4)	65·2 (62·3 to 68·2)
Indonesia	38·2 (34·8 to 41·5)	45·6 (42·9 to 48·4)	51·8 (49·5 to 54·2)	58·3 (56·6 to 60·1)	62·7 (61·5 to 63·9)	66 (64·6 to 67·3)	67·4 (65·5 to 69·2)	69·3 (67 to 71·8)	68·6 (66·2 to 71)	67·3 (64·4 to 70·3)
Aceh	43·1 (38·5 to 47·7)	50·3 (46·7 to 54·2)	55 (51·2 to 58·7)	60·4 (57·4 to 63·5)	63·4 (61 to 65·8)	66·1 (63·7 to 68·6)	66·5 (63·6 to 69·3)	68·7 (65·2 to 72·1)	67·7 (64·4 to 71·2)	66·4 (62·8 to 70·2)
Bali	43·4 (38·8 to 48·1)	51 (47·2 to 54·6)	55·8 (52·3 to 59·6)	61·1 (58·3 to 64·1)	65·3 (63 to 67·4)	68 (66 to 70·2)	69·7 (67 to 72·6)	71·3 (68·1 to 74·7)	70·5 (67·4 to 73·7)	69·2 (65·9 to 72·6)
Bangka-Belitung Islands	39·1 (34·2 to 44)	45·1 (40·9 to 49·3)	50·3 (46·8 to 53·8)	56·1 (53 to 59·4)	59·8 (57·3 to 62·4)	63·6 (61·3 to 65·9)	64·9 (62·2 to 67·8)	66·8 (63·4 to 70·5)	66·2 (62·9 to 69·8)	64·1 (60·4 to 68)
Banten	32·8 (27·7 to 37·5)	36·9 (32·7 to 41)	44·7 (41·5 to 48)	53·3 (50·3 to 56·3)	59·9 (57·4 to 62·4)	65·8 (63·4 to 68·2)	66·3 (63·6 to 69·2)	68·5 (65·2 to 72)	67·7 (64·3 to 71·2)	66·7 (63·4 to 70·2)
Bengkulu	30 (25·1 to 34·9)	38·1 (34·8 to 41·3)	46·5 (43·3 to 50)	55 (52·1 to 58·1)	61 (58·7 to 63·4)	64·2 (61·8 to 66·5)	66·4 (63·6 to 69·3)	69·1 (65·7 to 72·6)	68·2 (64·7 to 71·7)	67 (63·3 to 70·6)
Gorontalo	40·1 (35 to 45)	46·3 (42 to 50·2)	52·6 (49·3 to 56·1)	58·5 (55·4 to 61·8)	63·8 (61·3 to 66·5)	68·7 (66·3 to 71·2)	69·2 (66·1 to 72·6)	71·3 (67·5 to 75·3)	70 (66·3 to 73·7)	68·4 (64·4 to 72·6)
Jakarta	48·9 (44·3 to 53·6)	52·6 (48·9 to 56·3)	59·7 (56·1 to 63·4)	65·8 (63 to 68·8)	68·2 (66 to 70·5)	68·6 (66·4 to 70·9)	69·6 (67·1 to 72·3)	71·5 (68·3 to 74·9)	70·5 (67·3 to 73·8)	69·3 (65·9 to 72·7)
Jambi	38·3 (34 to 42·8)	44·3 (40·8 to 47·9)	49·9 (46·3 to 53·4)	56·2 (53·3 to 59·3)	60·9 (58·4 to 63·1)	65·2 (63·1 to 67·5)	67·4 (64·7 to 70·3)	69·5 (66·3 to 73·2)	68·8 (65·5 to 72·1)	67·3 (63·8 to 70·9)
West Java	32·9 (29 to 36·5)	39·5 (36·5 to 42·7)	47·5 (44·6 to 50·7)	55·8 (53·4 to 58·5)	62 (60 to 63·8)	67·2 (65 to 69·2)	68·7 (66 to 71·3)	70·5 (67·3 to 74)	69·7 (66·6 to 72·9)	68·2 (64·9 to 71·8)
Central Java	41·3 (36·7 to 45·3)	49·7 (46·4 to 53·3)	55·4 (52·3 to 58·9)	61·7 (59 to 64·3)	66·4 (64·3 to 68·4)	68 (66·1 to 70)	69·2 (66·9 to 71·7)	70·9 (68 to 73·8)	70·1 (67·3 to 72·8)	68·6 (65·3 to 72)
East Java	43 (39·3 to 46·5)	49·3 (46 to 52·7)	54·2 (51·1 to 57·4)	59·2 (56·8 to 61·9)	62·9 (61·1 to 64·8)	66·1 (64·2 to 68)	67·5 (65·1 to 69·9)	69·5 (66·4 to 72·6)	68·7 (65·7 to 71·7)	67·5 (64·2 to 70·9)
West Kalimantan	41·6 (37·1 to 46·1)	45·5 (41·8 to 49·2)	49·9 (46·4 to 53·4)	56·3 (53·3 to 59·1)	60·8 (58·3 to 63)	64 (61·8 to 66·2)	66·7 (64·1 to 69·5)	69·5 (66·4 to 73)	69 (65·8 to 72·2)	67·5 (64 to 70·9)
South Kalimantan	29·5 (25·7 to 33·5)	34·2 (31·1 to 37·4)	42·7 (39·7 to 45·7)	51 (48·6 to 53·8)	56 (53·6 to 58·5)	59·6 (57·5 to 61·7)	62 (59·1 to 64·7)	64·8 (61·2 to 68·4)	64 (60·6 to 67·5)	62·8 (59 to 66·7)
Central Kalimantan	45·4 (41·1 to 49·4)	51·4 (47·7 to 55·2)	56·1 (52·6 to 59·7)	60·7 (57·7 to 63·9)	63·2 (60·7 to 65·7)	66·4 (64·3 to 68·7)	66·7 (64·1 to 69·8)	68·5 (65·4 to 71·9)	67·6 (64·3 to 70·9)	66·2 (62·5 to 70)
East Kalimantan	48·4 (44·1 to 52·7)	49·2 (45·7 to 52·6)	54·6 (50·9 to 58·4)	61·4 (58·6 to 64·3)	64·4 (62·1 to 66·6)	64·7 (62·9 to 66·8)	66·2 (63·6 to 68·7)	68·6 (65·1 to 71·7)	67·7 (64·4 to 70·9)	66·4 (62·9 to 69·9)
North Kalimantan	51 (46·9 to 55)	49·6 (46·2 to 53·1)	53·6 (50 to 57·1)	60·2 (57 to 63·1)	65·3 (62 to 68·4)	70 (67·1 to 72·7)	71·1 (68 to 74·1)	72·9 (69·2 to 76·5)	71·8 (68·4 to 75·4)	69·8 (66·1 to 73·9)
Riau Islands	47·7 (43·5 to 52·1)	51·1 (47·1 to 55·1)	56·4 (52·6 to 60·2)	60·8 (57·7 to 64·2)	64·2 (61·5 to 66·9)	70·8 (68·6 to 73)	72·3 (69·5 to 75)	73·4 (70·2 to 77·2)	72·4 (69·1 to 75·8)	70·8 (67·1 to 74·6)
Lampung	41·5 (37 to 45·6)	45 (41·4 to 48·4)	51·5 (48·3 to 55)	58·2 (55·6 to 61·2)	62·5 (60·2 to 64·7)	67·7 (65·5 to 69·8)	69·3 (66·5 to 72·2)	70·9 (67·4 to 74·4)	70·1 (66·7 to 73·8)	68·5 (64·9 to 72)
Maluku	29 (24·7 to 33·3)	39·4 (35·2 to 43·5)	46·5 (43·1 to 49·9)	53·2 (50 to 56·3)	58·2 (55·5 to 61)	62·6 (60 to 65·3)	63·8 (60·6 to 67·3)	67·4 (63·7 to 71·2)	66·6 (63 to 70·2)	65·8 (62·2 to 69·5)
North Maluku	33·3 (27·8 to 38·5)	40·9 (36·8 to 45)	46·6 (43·4 to 49·8)	51·5 (48·5 to 54·5)	58·9 (56·3 to 61·6)	63·7 (61 to 66·1)	66·5 (63·3 to 69·5)	69·3 (65·4 to 73·3)	68 (64·5 to 71·9)	66·7 (62·7 to 70·7)
West Nusa Tenggara	29·6 (25·4 to 33·9)	36 (33·1 to 39·3)	41·7 (38·7 to 44·8)	49·3 (46·5 to 52·1)	56·1 (53·8 to 58·4)	61·6 (59·5 to 63·8)	65·2 (62·5 to 68·1)	67·9 (64·5 to 71·5)	67·2 (63·9 to 70·7)	66·3 (62·7 to 69·9)
East Nusa Tenggara	30·8 (26·4 to 35·4)	41·3 (37·3 to 45·3)	47·3 (44 to 50·5)	54·3 (51·4 to 57·3)	59·7 (57·2 to 62)	63·4 (61·1 to 65·7)	65·9 (62·9 to 68·9)	68·5 (64·8 to 72·4)	68 (64·5 to 71·3)	66·5 (62·9 to 70·3)
Papua	53·5 (49·4 to 57·7)	52·8 (48·4 to 56·9)	55·7 (52 to 59·4)	59·7 (56·6 to 62·7)	61·5 (59·1 to 64·1)	62·4 (60 to 64·8)	62·3 (59·3 to 65·4)	64·9 (61·3 to 68·7)	64·3 (60·8 to 68·2)	63·6 (59·9 to 67·7)
West Papua	53·1 (49 to 57·3)	55 (51·1 to 59·2)	57·5 (53·7 to 61·1)	62·3 (58·9 to 65·6)	65·8 (63 to 68·5)	68·4 (65·9 to 70·8)	66·9 (64·1 to 69·5)	70·9 (67·3 to 74·4)	69·7 (66·3 to 73·2)	68·5 (64·6 to 72·5)
Riau	44·1 (39·9 to 48·2)	45·9 (42·5 to 49·5)	51·2 (47·8 to 54·8)	58·3 (55·4 to 61·3)	63·3 (60·9 to 65·5)	66·6 (64·3 to 68·7)	67·6 (64·8 to 70·6)	69·9 (66·9 to 73·3)	69 (66 to 72·4)	67·5 (64·1 to 71·2)
West Sulawesi	24·5 (19·2 to 30·2)	35·9 (32 to 40·1)	42·8 (39·7 to 45·9)	50·5 (47·6 to 53·6)	56·2 (53·7 to 58·7)	61·2 (58·7 to 63·4)	64·5 (61·5 to 67·5)	67·4 (63·3 to 71·3)	66·5 (62·7 to 70·2)	64·9 (61 to 68·8)
South Sulawesi	36·5 (32·5 to 40·6)	45 (41·8 to 48·4)	51·2 (48 to 54·6)	57·2 (54·6 to 60)	61·9 (59·7 to 64·3)	65·8 (63·6 to 68·1)	67·1 (64·5 to 70)	69·2 (65·9 to 72·9)	68·5 (65·6 to 71·8)	67·5 (64·4 to 70·9)
Central Sulawesi	33·9 (29·7 to 37·7)	40·5 (37·2 to 43·8)	47·2 (43·9 to 50·3)	53·7 (50·9 to 56·6)	57·9 (55·5 to 60·3)	61·6 (59·4 to 63·8)	64·4 (61·5 to 67·6)	67·2 (63·5 to 71·1)	66·4 (62·9 to 70)	64·7 (61 to 68·6)
Southeast Sulawesi	32·7 (28·4 to 37)	39·1 (35·3 to 43·3)	45·4 (42·1 to 48·6)	55 (52 to 57·9)	61·1 (58·8 to 63·6)	62·9 (60·6 to 65·3)	64·1 (61·3 to 67)	66 (62·1 to 70·1)	65·2 (61·5 to 68·8)	64·1 (60·2 to 68·1)
North Sulawesi	43·4 (39·1 to 48·1)	51 (47·3 to 54·9)	56·1 (52·5 to 59·4)	61·3 (58·6 to 64·3)	63 (61 to 65·3)	65·1 (63·1 to 67·3)	66·4 (63·8 to 69·2)	68·4 (64·9 to 72)	67·4 (64 to 70·8)	66·3 (62·7 to 70·3)
West Sumatra	23·2 (19·2 to 27·1)	40·6 (37·4 to 44·1)	49·5 (46·5 to 53·1)	58 (55·2 to 61)	62·2 (60·1 to 64·7)	64·1 (61·9 to 66·4)	65·6 (62·9 to 68·5)	68·8 (65·8 to 71·7)	68 (65 to 71·1)	66·9 (63·7 to 70·3)
South Sumatra	39·7 (35·5 to 43·7)	46·5 (43·1 to 50·2)	53 (49·7 to 56·4)	60·6 (57·7 to 63·6)	63·5 (61·2 to 65·7)	66·8 (64·5 to 68·9)	68·1 (65·3 to 71)	69·9 (66·3 to 73·7)	69 (65·4 to 72·4)	67·8 (64 to 71·9)

North Sumatra	40·8 (36·4 to 45·2)	48·7 (45·1 to 52·4)	54·1 (50·7 to 57·8)	59·3 (56·6 to 62·3)	61·6 (59·4 to 63·8)	62·9 (60·8 to 64·9)	63·7 (61·2 to 66·5)	65·7 (62·3 to 69·4)	65·1 (61·8 to 68·6)	64·2 (60·8 to 68)
Yogyakarta	38·3 (32·5 to 43·7)	52 (48·1 to 56)	59·9 (56·1 to 63·6)	66 (62·8 to 69·2)	68·7 (66·4 to 70·8)	70·3 (68·2 to 72·5)	70·2 (67·7 to 72·7)	70·9 (67·7 to 73·8)	70·1 (67·3 to 73·2)	68·3 (64·7 to 71·9)
Laos	34·8 (29·1 to 40·3)	36·2 (32·1 to 40·2)	38·4 (33·8 to 42·8)	44·9 (41 to 48·8)	50·6 (47·7 to 53·5)	56·1 (53·9 to 58·4)	62·4 (60·2 to 64·9)	66·4 (63·3 to 69·6)	66·7 (63·6 to 70)	65·4 (62·2 to 68·7)
Malaysia	51·8 (48·9 to 54·8)	61·8 (61·5 to 62·1)	65·7 (65·5 to 65·9)	68·2 (65·6 to 70·7)	69·9 (69·7 to 70)	70·8 (70·7 to 70·9)	72·2 (71 to 73·4)	72·8 (72·7 to 72·9)	74·7 (74·6 to 74·8)	70·4 (69·5 to 71·1)
Maldives	34 (28·8 to 39·2)	39·8 (35·9 to 43·8)	48·3 (45·4 to 51·2)	56·9 (55·6 to 58·2)	65·8 (65 to 66·7)	72·1 (71 to 73·2)	77 (76 to 77·9)	79·6 (78·3 to 80·8)	79·8 (78·6 to 81)	78·1 (76·1 to 80)
Mauritius	50·8 (49·8 to 51·6)	56·8 (56·3 to 57·3)	61·5 (61 to 62)	63·8 (63·3 to 64·2)	66·3 (65·9 to 66·7)	69 (68·6 to 69·5)	70·8 (70·3 to 71·2)	71·3 (70·7 to 71·8)	71·6 (71 to 72·2)	70·1 (69·1 to 71·6)
Myanmar	29·4 (24 to 35·1)	36·5 (32·7 to 40·2)	40·7 (36·9 to 44·5)	47·2 (43·8 to 50·7)	52·3 (49·1 to 55·3)	56 (53·7 to 58·3)	61·4 (59·1 to 63·7)	65·7 (63·1 to 68·1)	65·5 (62·9 to 67·9)	64·1 (61·3 to 66·9)
Philippines	55·8 (52 to 59·1)	60·8 (59·1 to 62·4)	61 (59·7 to 62·4)	63·6 (62·4 to 64·9)	65·4 (64·2 to 66·5)	67·3 (66·1 to 68·4)	67·6 (66·4 to 68·8)	68·4 (67·1 to 69·8)	68·8 (67·5 to 70·1)	64·8 (63 to 66·7)
Seychelles	57·8 (56·1 to 59·5)	60·8 (59·6 to 61·9)	61·9 (61·1 to 62·8)	67·8 (66·8 to 68·8)	66·1 (65·4 to 66·8)	68 (67·4 to 68·8)	69·6 (69 to 70·3)	70 (69·2 to 70·9)	71·6 (70·7 to 72·5)	70·8 (69·9 to 71·7)
Sri Lanka	54·8 (53·7 to 55·7)	63·9 (63·6 to 64·3)	64·6 (64·3 to 64·9)	67 (66·8 to 67·2)	65·8 (64·8 to 66·8)	67·1 (67 to 67·3)	70·1 (69·8 to 70·3)	74·4 (71·3 to 77·4)	76 (71·1 to 82·6)	73·4 (69·6 to 78·1)
Thailand	49·6 (46·5 to 52·6)	58·2 (55·8 to 60·3)	61·2 (59·4 to 63)	62·9 (61 to 64·7)	67·6 (66 to 69·2)	67·7 (65·6 to 69·9)	72·6 (70·6 to 74·4)	73·7 (71·4 to 75·9)	73·8 (70·9 to 76·6)	72·4 (69·1 to 75·8)
Timor-Leste	43·6 (38·1 to 48·9)	46 (42·3 to 49·7)	47·7 (44·4 to 51)	47·5 (44·7 to 50·2)	59 (56·8 to 61)	65·1 (63·3 to 67)	68·3 (66·5 to 70·1)	68·8 (66·4 to 71·2)	68·9 (66·4 to 71·3)	66·9 (64·2 to 69·6)
Viet Nam	39·6 (33·9 to 44·9)	53 (49·4 to 56·5)	49·1 (46·5 to 51·6)	62·5 (59·6 to 65·2)	65·4 (63 to 67·6)	67·9 (66·1 to 69·7)	68·6 (67·4 to 69·9)	70·2 (68·6 to 72·2)	70·4 (68·7 to 72·4)	69·9 (68 to 72)
Sub-Saharan Africa	39·3 (35 to 43·2)	42·3 (39·5 to 45)	46·4 (44·3 to 48·6)	50·1 (48·7 to 51·6)	51·5 (50·4 to 52·8)	51·5 (50·3 to 52·7)	57·1 (55·7 to 58·3)	61·8 (59·9 to 63·5)	60 (58 to 61·6)	58·7 (56·8 to 60·3)
Central sub-Saharan Africa	36·3 (31·3 to 41·2)	42·5 (39·1 to 46)	45 (41·8 to 48·3)	48 (45·3 to 50·7)	50·4 (48·4 to 52·4)	50·9 (49·1 to 52·7)	56·5 (54·4 to 58·6)	61 (58·3 to 63·6)	59·4 (57 to 61·7)	58·4 (56·1 to 60·5)
Angola	38·7 (33·4 to 43·7)	41·3 (37·6 to 45)	42·1 (38·1 to 46)	43·9 (40·4 to 47·3)	46·5 (43·8 to 49·3)	50·1 (48·2 to 52·1)	57·9 (55·3 to 60·5)	61·9 (58·7 to 65·2)	60·4 (57·4 to 63·4)	58·4 (55·6 to 61·1)
Central African Republic	39 (34·4 to 43·3)	41·1 (37·4 to 45·2)	43·6 (40·5 to 46·9)	44·9 (42·4 to 47·5)	44·4 (42·4 to 46·6)	42·4 (40 to 45)	46·2 (43 to 49·4)	49·5 (45·5 to 53·3)	48 (44·4 to 51·2)	48·2 (44·5 to 51·7)
Congo (Brazzaville)	31·6 (26·1 to 36·3)	37·5 (34·4 to 40·5)	41·8 (38·6 to 45·3)	48·7 (46·3 to 51·4)	52·1 (50·2 to 54·2)	52·2 (50·5 to 54)	60·6 (58·4 to 62·7)	63·1 (60·4 to 65·7)	61·2 (58·8 to 63·5)	60·6 (58·1 to 62·9)
DR Congo	35·4 (29·8 to 40·9)	43·5 (39·9 to 47·2)	46·3 (42·9 to 49·9)	49·3 (46·4 to 52·2)	51·9 (49·8 to 54)	51·7 (49·7 to 53·8)	56·5 (54·4 to 58·8)	61·4 (58·4 to 64·1)	59·9 (57·3 to 62·4)	59 (56·6 to 61·4)
Equatorial Guinea	24·3 (18·9 to 29·6)	35·7 (32 to 39·5)	42 (38·3 to 46)	47·5 (43·6 to 51·1)	48·4 (45·2 to 51·8)	55·2 (51·8 to 58·6)	63·4 (59·6 to 66·8)	62·7 (58·4 to 66·6)	59·7 (55·8 to 63·1)	59·3 (55·3 to 62·9)
Gabon	24·9 (19 to 31·2)	40·6 (36·2 to 44·8)	46·9 (42·9 to 50·9)	54·3 (51·4 to 57·2)	56·7 (54·6 to 58·9)	57 (54·9 to 59·4)	60·4 (57·9 to 62·8)	63·1 (59·7 to 66·4)	61·4 (58·2 to 64·2)	60·9 (57·8 to 63·6)
Eastern sub-Saharan Africa	37·3 (33·1 to 41·2)	42·4 (39·6 to 45·3)	45·3 (43 to 47·4)	48·4 (46·9 to 49·9)	48·9 (47·7 to 50)	50·3 (48·8 to 51·6)	58 (56·7 to 59·2)	62·7 (60·8 to 64·4)	60·8 (58·9 to 62·4)	58·9 (57·2 to 60·4)
Burundi	35·5 (30·4 to 40·5)	40·1 (36·8 to 43·4)	42·4 (39·4 to 45·3)	45·3 (42·8 to 47·7)	47·1 (44·8 to 49·5)	42·6 (40·8 to 44·5)	58·4 (56·4 to 60·5)	62·4 (60 to 64·9)	61·2 (58·6 to 63·5)	60 (57·7 to 62·3)
Comoros	42·7 (38·1 to 47·2)	45·7 (42·1 to 49·4)	49·5 (46·4 to 52·5)	52·4 (49·4 to 55·4)	56·8 (54·2 to 59·5)	60·3 (57·7 to 63·1)	64·9 (62·5 to 67·5)	66·9 (64·1 to 69·3)	66·4 (63·9 to 68·8)	64·8 (62·5 to 66·9)
Djibouti	54·8 (50·3 to 59·2)	55·7 (51·4 to 59·9)	57·5 (53·7 to 61·4)	59·9 (56·6 to 63·3)	59·1 (55·9 to 62·1)	58·9 (55·5 to 62·2)	61·7 (58 to 65·2)	65·1 (61 to 68·5)	62·7 (59·2 to 65·6)	62·3 (59 to 65·1)
Eritrea	35·5 (30·7 to 40·4)	39·1 (35·5 to 43·1)	37·8 (19·9 to 44)	36·9 (34·7 to 39)	41·2 (39·2 to 43·3)	50·9 (48·4 to 53·5)	56·5 (53 to 59·7)	60·4 (56·5 to 63·9)	60·4 (56·6 to 63·7)	58·7 (55·2 to 61·7)
Ethiopia	34·5 (30·2 to 38·7)	38·6 (35·8 to 41·6)	40·5 (37·9 to 43)	42·5 (40·5 to 44·6)	44·1 (42·7 to 45·4)	50·3 (48 to 51·9)	62 (60·6 to 63·4)	66·9 (64·7 to 69)	64·3 (62·3 to 66·3)	62 (60·3 to 63·7)
Addis Ababa	49·7 (45·5 to 53·6)	49·3 (45·2 to 53)	51·4 (47·9 to 55)	53·9 (50·7 to 57)	51·3 (49·1 to 53·5)	54·7 (51·6 to 57·9)	65·2 (63 to 67·5)	69·1 (66·5 to 72)	64·9 (62·7 to 67·1)	61·4 (59·7 to 63·2)
Afar	21·3 (16·6 to 26)	32·6 (29·5 to 36·1)	35·3 (31·8 to 38·5)	36·6 (33·8 to 39·3)	40·2 (38 to 42·4)	46·2 (43·6 to 48·7)	56·1 (53·4 to 58·9)	62·5 (59·1 to 66·1)	59·3 (56·3 to 62·2)	56·6 (54·5 to 58·7)
Amhara	35·1 (30·3 to 40·1)	40 (36·9 to 43·4)	42 (39 to 45·1)	44 (41·5 to 46·6)	43·1 (41·4 to 44·7)	48·6 (46·5 to 50·6)	61 (59 to 62·9)	66·5 (63·6 to 69·1)	64·7 (62 to 67·2)	63·1 (60·6 to 65·3)
Benishangul-Gumuz	23·9 (19·4 to 28·6)	34·1 (30·7 to 37·6)	35·7 (32·4 to 39)	36·9 (33·9 to 39·9)	40 (37·8 to 42·2)	45·7 (43 to 48·2)	58·1 (55·5 to 60·7)	65·4 (61·9 to 68·8)	65·6 (62·1 to 69·5)	65·5 (62 to 69·1)
Dire Dawa	40·2 (35·9 to 44·5)	37·6 (34·3 to 41·3)	39 (35·7 to 42·1)	41·9 (38·9 to 45)	43·8 (41·5 to 46·1)	50·2 (47·5 to 52·8)	63·9 (61·5 to 66·4)	68·7 (65·7 to 71·6)	64·2 (61·9 to 66·2)	60·9 (58·8 to 62·7)
Gambella	20 (14·7 to 25·4)	30·7 (26·9 to 34·4)	32 (28·8 to 35·5)	34 (31·2 to 37)	39·3 (37 to 41·4)	46·1 (43·4 to 48·7)	56·1 (53·5 to 58·8)	63·1 (59·8 to 66·3)	59·3 (56·4 to 61·8)	56·3 (54·1 to 58·2)
Harari	33·4 (29·1 to 37·7)	31·8 (28·5 to 35·2)	34·1 (30·9 to 37·2)	37·6 (34·7 to 40·6)	40·7 (38·4 to 43)	50·2 (47·6 to 52·8)	63 (60·5 to 65·6)	68·1 (64·7 to 71·3)	63·1 (60·8 to 65·4)	59·4 (57·5 to 61·2)
Oromia	33·3 (28·5 to 37·9)	38·1 (34·9 to 41·4)	40·1 (36·9 to 43)	42·2 (39·7 to 44·8)	44·4 (42·5 to 46·2)	51·8 (49·8 to 53·6)	64·7 (62·7 to 66·7)	69·3 (66·7 to 71·8)	66·1 (63·8 to 68·3)	63·5 (61·4 to 65·3)
Somali	42·4 (37·4 to 47)	47·1 (43·6 to 51)	49·1 (45·6 to 52·5)	52·2 (49·1 to 55·4)	54·2 (51·7 to 56·8)	54·7 (52 to 57·4)	60·4 (58 to 62·9)	64·4 (60·9 to 67·8)	60·7 (58 to 63·4)	57·9 (55·6 to 60)
Southern Nations, Nationalities, and Peoples	34·6 (30·4 to 38·9)	36·5 (33·2 to 40)	38·1 (35 to 41·1)	39·8 (37·3 to 42·4)	42·7 (40·8 to 44·7)	50·1 (41·8 to 53·4)	59·9 (57·9 to 62)	64·8 (61·8 to 67·9)	62·6 (60 to 65·3)	60·6 (58·3 to 63)
Tigray	31·7 (27·1 to 36·1)	36·6 (33·4 to 40·1)	38·4 (35·1 to 41·5)	39·6 (37 to 42·3)	43·8 (41·6 to 45·8)	50 (47·8 to 52·2)	63·6 (61·3 to 66)	68·7 (65·7 to 71·8)	66·2 (63·5 to 68·7)	63·9 (61·4 to 66·1)
Kenya	44·6 (41·4 to 47·4)	51·5 (49 to 54)	56 (53·3 to 58·6)	60·7 (58·6 to 63)	60·8 (58·9 to 62·6)	53·9 (51·3 to 56·3)	59·2 (57·8 to 60·5)	64·5 (62·5 to 66·7)	61·8 (60·1 to 63·7)	61 (59·4 to 62·6)
Baringo	44 (39·5 to 48)	47·5 (43·7 to 51·1)	49·3 (45·4 to 53·3)	53 (49 to 56·9)	54·9 (51·6 to 58·5)	53·6 (50·5 to 56·5)	57·9 (55·1 to 60·9)	63·3 (59·6 to 67·3)	61·2 (58 to 64·7)	60·5 (57·4 to 63·5)
Bomet	62·6 (58·2 to 66·9)	65·3 (61·4 to 69·2)	66·6 (62·4 to 70·9)	68·6 (64·4 to 72·5)	67·6 (63·6 to 70·5)	58·2 (52·7 to 62·9)	62·1 (58·5 to 65·5)	67·9 (64·3 to 70·6)	65·1 (62·2 to 67·3)	64·2 (61·5 to 66·2)
Bungoma	39·1 (34·5 to 43·5)	47·9 (44 to 51·9)	53·8 (49·6 to 57·7)	58·7 (55 to 62·5)	61 (57·3 to 64·6)	57·1 (53·6 to 60·8)	64·4 (61·5 to 67·4)	68·8 (65·1 to 72)	66·5 (63·4 to 69·2)	65·3 (62·4 to 67·9)
Busia	23·9 (19 to 28·9)	38 (34·5 to 41·9)	46·2 (42·6 to 49·8)	55·8 (51·7 to 59·7)	55·7 (51·7 to 60·1)	47·7 (43·6 to 52·3)	57·6 (53·4 to 61·9)	63·9 (60 to 68)	60·9 (57·5 to 64·2)	59·5 (56·3 to 62·5)
Elgeyo Marakwet	51·4 (46·6 to 56)	56·4 (51·9 to 60·5)	56·4 (52·3 to 60·5)	58·9 (54·6 to 63)	61·7 (57·9 to 66)	58·3 (54·9 to 62·1)	62·6 (60 to 65·2)	66·8 (63·2 to 70·5)	65·1 (62 to 68·5)	64·6 (61·7 to 67·9)
Embu	41·3 (36·2 to 46·3)	51 (47 to 55)	59·8 (55·4 to 64·4)	66·8 (62·5 to 71)	67·4 (63·3 to 70·7)	62·3 (56·7 to 66·6)	63·9 (59·8 to 68)	67·6 (63·4 to 71·5)	65·5 (62·1 to 68·5)	64·8 (61·7 to 67·3)
Garissa	49·6 (44·8 to 54·2)	52·3 (48·1 to 57·1)	55·8 (51·1 to 60·4)	57·6 (53·3 to 61·7)	59·3 (55·2 to 63·5)	58·1 (54 to 62·1)	61·9 (58·4 to 65·6)	66·1 (62·5 to 69·8)	62·2 (59·3 to 64·8)	60·9 (58·2 to 63·4)
Homa Bay	23·9 (20·2 to 27·5)	35·3 (32·4 to 38·3)	39 (35·6 to 42·7)	46 (42·7 to 49·4)	47·3 (43·4 to 51·7)	35·8 (32·1 to 39·4)	45 (41·1 to 48·5)	53·3 (48·7 to 58)	52·2 (48·1 to 56·1)	52·5 (48·9 to 56·2)
Isiolo	35·5 (30·8 to 40)	35·9 (32·3 to 39·6)	38·7 (35 to 42·4)	46 (41·7 to 50·2)	51·1 (47 to 55·2)	49·5 (44·9 to 54·4)	54·9 (51 to 59·1)	60·8 (56·3 to 65·6)	58·2 (54·7 to 61·6)	57·3 (54·3 to 60·4)
Kajiado	56·6 (52·1 to 61·1)	59·8 (55·5 to 64·3)	61 (56·3 to 65·5)	64·1 (59·6 to 68·3)	64·9 (60·8 to 69·2)	58·5 (54·3 to 62·6)	62·2 (59·3 to 65)	66·9 (63·6 to 70·2)	63·4 (60·6 to 65·8)	62·3 (59·9 to 64·3)
Kakamega	37·6 (32·9 to 42·4)	47·8 (44·2 to 51·8)	52·7 (48·9 to 56·5)	58·2 (54·5 to 62)	56·5 (52·9 to 60·3)	49·6 (45·7 to 53·8)	58·3 (55·1 to 61·6)	64·9 (61 to 68·8)	62·2 (59 to 65·2)	61·1 (58·2 to 63·7)
Kericho	64·9 (60·8 to 69·4)	64·8 (60·5 to 69·1)	65·5 (61·2 to 70)	66·9 (62·6 to 70·8)	67 (62·6 to 70·7)	59·9 (55·1 to 64·5)	63·3 (59·6 to 67·3)	67·4 (63·2 to 71·2)	65·1 (61·9 to 68·2)	64·4 (61·1 to 67·1)
Kiambu	51·5 (47 to 56)	57 (52·8 to 61·3)	61 (56·7 to 65·1)	63·3 (59·7 to 67·1)	62·4 (59·2 to 66·1)	55·6 (52·6 to 58·8)	59 (56·5 to 61·2)	63·2 (60·1 to 66·3)	60·8 (58·2 to 63·4)	60·1 (57·7 to 62·6)
Kilifi	39·1 (34·7 to 43·5)	46·8 (43·3 to 50·5)	52·1 (48·1 to 56·2)	58·1 (53·9 to 61·7)	61·3 (57·3 to 65·1)	57·9 (53·4 to 62·2)	62 (58·7 to 65·2)	67·2 (63·5 to 70·8)	64·1 (61·1 to 66·7)	63·2 (60·5 to 65·5)
Kirinyaga	40·2 (36 to 44·3)	50·1 (46·2 to 54·2)	55·8 (51·4 to 60)	65·2 (61 to 69·5)	65·4 (61·5 to 69·3)	58·9 (54·7 to 63·2)	61·7 (58·3 to 65·2)	64·5 (60·3 to 69·2)	62·5 (59·1 to 66·2)	62 (58·9 to 65·2)
Kisii	49 (44·2 to 53·8)	51·4 (47·3 to 55·4)	54·8 (50·8 to 58·9)	58·5 (54·3 to 62·7)	58·5 (55 to 62·3)	54·5 (51 to 58·2)	62·3 (59·8 to 64·9)	66·5 (63·3 to 70·3)	63·6 (60·6 to 66·4)	62·6 (59·9 to 65·2)
Kisumu	43 (37·8 to 47·7)	48·7 (45 to 52·4)	50·8 (46·8 to 54·4)	53·7 (49·6 to 57·7)	51·3 (47·9 to 54·9)	40·6 (37·5 to 43·7)	48·9 (45·6 to 51·8)	54·8 (51 to 59·4)	53·1 (50·1 to 56·5)	52·9 (49·7 to 56·1)
Kitui	34·7 (29·3 to 39·9)	46·7 (43·2 to 50·6)	53·2 (49·2 to 57·1)	60·6 (56·5 to 64·8)	62·8 (58·9 to 66·6)	56·8 (51·9 to 61·8)	60·4 (57·1 to 63·6)	65·5 (61·4 to 69·4)	63·9 (60·6 to 67·4)	63·4 (60·3 to 66·6)
Kwale	34·6 (30 to 38·9)	43·1 (39·3 to 46·9)	49·9 (45·6 to 54)	56·8 (52·6 to 61)	60·4 (56 to 64·7)	58·3 (53·6 to 62·9)	59·3 (56·2 to 62·6)	64·3 (60·4 to 68·5)	62·1 (59 to 65·5)	61·5 (58·5 to 64·5)
Laikipia	58·2 (52·8 to 63·4)	62·5 (57·7 to 66·8)	65·7 (61·2 to 70·2)	69·2 (65·2 to 73·3)	67·4 (63·2 to 70·6)	59·8 (53·5 to 63·7)	62·7 (58·6 to 66·8)	67·4 (63·5 to 70·6)	64·6 (61·5 to 67·1)	63·6 (60·9 to 65·8)
Lamu	31·9 (26·6 to 37·3)	44·6 (40·4 to 48·9)	52·6 (47·9 to 57·1)	63·2 (58·5 to 67·6)	62·6 (58 to 66·7)	59·5 (54·1 to 64·6)	61·5 (57·6 to 66·1)	65·8 (61 to 70·3)	62·8 (59·4 to 66·2)	61·8 (58·4 to 64·6)
Machakos	52·7 (47·8 to 57·5)	57·6 (53·4 to 61·7)	61·6 (57·5 to 65·8)	66·5 (62·1 to 70·5)	68·1 (64·6 to 71·3)	61·1 (55·8 to 65·9)	63·9 (60·9 to 67·2)	67·7 (64·1 to 71·3)	65·4 (62·5 to 68·3)	64·7 (62·1 to 67·4)
Makueni	43·4 (38·5 to 48·5)	55·8 (51·7 to 59·9)	60·2 (56 to 64·3)	65 (60·8 to 69·3)	64·9 (60·9 to 68·9)	53 (47·4 to 59·3)	58·7 (55·2 to 62·5)	66·4 (62·7 to 69·5)	64·5 (61·6 to 67)	64·1 (61·4 to 66·2)
Mandera	52·8 (48·6 to 57·1)	54·7 (50·3 to 59·3)	56·1 (51·9 to 60·3)	57·4 (53 to 61·9)	56·7 (52·6 to 60·5)	55·7 (51·8 to 59·8)	57·8 (54·9 to 61·1)	62·6 (58·7 to 66·7)	57·7 (54·9 to 60·3)	55·9 (53·3 to 58·1)
Marsabit	51·3 (46·3 to 56·2)	51·5 (47·3 to 55·8)	55·2 (50·5 to 59·5)	59·2 (54·7 to 63·6)	61·1 (56·9 to 65·5)	58·1 (53·1 to 63·1)	59·2 (55·2 to 63·1)	63·4 (59·1 to 68)	61·3 (57·9 to 64·7)	60·3 (57·2 to 63·5)

Meru	53·5 (48·7 to 58·3)	59·5 (55·2 to 63·8)	63·7 (59·3 to 68·4)	68·5 (64·3 to 72·6)	68·1 (64·6 to 71·3)	61·3 (56 to 65·7)	61·9 (58·5 to 65·4)	65·8 (62 to 69·7)	63·3 (60 to 66·3)	62·4 (59·4 to 65·1)
Migori	31·2 (26·5 to 35·6)	40·4 (36·9 to 43·9)	44·6 (40·7 to 48·3)	47·8 (44·3 to 51·5)	48·8 (45·2 to 52·7)	40·7 (36·2 to 45·1)	48·9 (45 to 52·5)	56·9 (52·5 to 61·5)	54·7 (51 to 58·2)	54·3 (51·1 to 57·4)
Mombasa	54·4 (49·6 to 59·5)	54·4 (50 to 58·6)	60 (55·4 to 64·7)	66·2 (61·9 to 70·6)	65·2 (60·6 to 68·8)	57·2 (52·4 to 61·7)	61·3 (57·7 to 65·1)	67·1 (63·1 to 70·7)	63 (60·3 to 65·4)	61·8 (59·2 to 63·9)
Murang'a	42·4 (37·6 to 47·2)	55·6 (51·3 to 59·8)	61 (56·6 to 65·2)	66·3 (62·5 to 70·2)	66·1 (62·7 to 69·5)	59·3 (55·3 to 63·1)	61·4 (59 to 63·8)	66·2 (62·8 to 70)	64·1 (60·9 to 67)	63·5 (60·6 to 66)
Nairobi	51·7 (47·2 to 56·4)	54·1 (50·1 to 58·2)	61 (56·9 to 64·8)	65·7 (61·9 to 69·2)	63·8 (60·2 to 67·7)	55·9 (52·5 to 59·7)	61·9 (59·6 to 64·2)	66·5 (63·5 to 69·6)	62·4 (60·1 to 64·6)	61·1 (59·1 to 62·9)
Nakuru	62·5 (58 to 66·7)	63·7 (59·4 to 67·9)	64·9 (60·7 to 69·1)	66·1 (62·5 to 70·2)	64·7 (60·9 to 68·8)	56·8 (53 to 60·8)	62·6 (59·5 to 65·5)	68 (64·7 to 70·8)	64·7 (61·9 to 66·9)	63·6 (61·2 to 65·6)
Nandi	52·4 (47·2 to 57)	57·1 (52·9 to 61·4)	59·1 (54·5 to 63·4)	60·9 (56·8 to 64·9)	60·1 (56·5 to 63·8)	54 (50·7 to 57·3)	58·3 (55·6 to 60·8)	63·9 (60·3 to 67·6)	62·2 (59 to 65·4)	61·6 (58·7 to 64·5)
Narok	50·3 (45·3 to 55)	54·9 (50·7 to 59·5)	56·2 (51·6 to 60·6)	61 (56·6 to 65)	59·3 (55·3 to 63·4)	54·6 (50·7 to 58·5)	59·3 (55·8 to 62)	64·4 (61 to 68·1)	61·3 (58·3 to 64·1)	60·4 (57·5 to 63)
Nyamira	51·8 (47·4 to 56·2)	56·9 (52·5 to 61·2)	59·8 (55·2 to 64·1)	62·1 (57·9 to 66·3)	61·6 (58 to 65·3)	53·2 (49·6 to 56·7)	56·7 (53·9 to 59·5)	61·5 (57·8 to 65·5)	59·4 (56·3 to 62·8)	58·7 (55·6 to 61·9)
Nyandarua	48·1 (43·7 to 52·5)	53·5 (49 to 58)	58·5 (54 to 63)	63·1 (59·1 to 67·6)	62·6 (58·8 to 66·1)	55·9 (52·5 to 59·5)	56·3 (53·5 to 59·1)	59·4 (55·7 to 63·2)	57·7 (54·3 to 60·8)	57·3 (54·3 to 60·2)
Nyeri	48·2 (42·9 to 53·1)	58·4 (53·9 to 62·7)	63·9 (59·4 to 68)	68·2 (64·1 to 72·5)	67·2 (63·6 to 70·9)	59·8 (55·3 to 64·3)	63·7 (60·2 to 67)	66·7 (62·6 to 69·9)	63·9 (60·7 to 66·4)	62·9 (60 to 65·2)
Samburu	61·8 (57·1 to 66)	62·6 (58 to 67)	62·6 (58 to 67·4)	63·3 (58·9 to 67·5)	61·3 (57·1 to 66·2)	53·6 (49·6 to 58·1)	57·2 (53·6 to 60·9)	62·3 (57·9 to 66·7)	59·6 (56·2 to 63)	58·7 (55·6 to 61·8)
Siaya	37 (32·4 to 41·7)	45·7 (42·1 to 49·3)	51 (47·2 to 54·7)	55 (50·7 to 58·9)	53 (48·7 to 57·3)	42·4 (37·8 to 46·8)	53·9 (49·5 to 57·9)	62·3 (57·8 to 65·6)	60 (56·1 to 63)	59·1 (56·1 to 61·7)
Taita Taveta	45·6 (40·8 to 50·3)	51·5 (47·1 to 55·7)	54·1 (49·9 to 58·5)	61·4 (56·6 to 65·8)	63·2 (58·3 to 67·7)	55·9 (50·8 to 61)	58·6 (55·2 to 62·4)	65·1 (61·1 to 69·6)	62·5 (59·4 to 65·7)	61·7 (58·9 to 64·6)
Tana River	40·9 (35·6 to 46·1)	49·9 (45·3 to 54·2)	52·8 (48·1 to 57·3)	55·6 (51·2 to 59·9)	56·6 (52·4 to 60·8)	51 (47·4 to 54·6)	53·9 (50·8 to 56·9)	59·6 (55·3 to 63·8)	57·6 (53·9 to 61)	57 (53·5 to 60)
Tharaka Nithi	39·9 (34·3 to 45·1)	53·3 (49·1 to 57·5)	56·4 (51·2 to 61·1)	60·5 (56·3 to 65·3)	59·7 (55·6 to 63·8)	54·9 (51·1 to 58·5)	59·3 (56·5 to 62·2)	62·7 (58·8 to 66·3)	61·9 (58·2 to 65·4)	61·9 (58 to 65·4)
Trans Nzoia	61·9 (57·3 to 66·1)	62·5 (58·3 to 66·5)	64·2 (59·6 to 68·6)	65·9 (61·7 to 69·7)	65·7 (61·6 to 69·3)	58·2 (53·4 to 63·1)	61·6 (58·1 to 65·7)	66·6 (62·8 to 70·1)	64·2 (60·9 to 67)	63·3 (60·4 to 65·8)
Turkana	57·3 (52·7 to 61·9)	57·1 (52·9 to 61·6)	56 (51·8 to 60·2)	55·5 (51·3 to 59·5)	55·8 (51·6 to 60·2)	48·1 (38·3 to 53·8)	54·4 (50·8 to 58)	61·9 (57·6 to 66·5)	58·4 (55·4 to 61·5)	57·3 (54·5 to 59·9)
Uasin Gishu	65·5 (61·1 to 70·1)	66·6 (61·9 to 70·9)	68·1 (63·6 to 72·6)	69·5 (65 to 73·3)	68·3 (64·5 to 71·3)	61·1 (57·2 to 65)	64·2 (61·3 to 67)	68·9 (65·4 to 71·8)	65·8 (63 to 68)	64·7 (62·3 to 66·8)
Vihiga	38·1 (32·8 to 43·1)	54·3 (50·2 to 58·5)	58·7 (53·9 to 63)	62·1 (57·7 to 66·4)	60·5 (56·6 to 64·8)	54·1 (49·8 to 58·7)	60·4 (57 to 63·8)	65·2 (61·2 to 69·5)	63·3 (59·4 to 66·8)	62·3 (58·9 to 65·4)
Wajir	45·1 (40·6 to 49·4)	45·8 (41·8 to 49·9)	49·9 (45·7 to 54)	53·2 (49·1 to 57·2)	55·3 (51·3 to 59·4)	54·1 (50·7 to 57·7)	57·5 (54·4 to 60·7)	62·4 (58·3 to 66·6)	59·7 (56·3 to 62·9)	58·8 (56·2 to 61·8)
West Pokot	53·5 (49·2 to 58)	58·4 (53·7 to 62·9)	58·9 (54·4 to 63·4)	59·7 (55·4 to 63·9)	60·3 (56·4 to 64·5)	59·1 (55·5 to 63·2)	61·4 (58·1 to 64·9)	65·7 (61·8 to 69·9)	62·9 (59·5 to 66·2)	62 (58·9 to 65)
Madagascar	39·4 (35·4 to 43·7)	45·8 (43·1 to 48·5)	49·4 (46·7 to 52·1)	54·3 (52·5 to 56·2)	54·6 (53 to 56·2)	57·9 (56 to 59·9)	60·7 (58 to 63·5)	63·4 (60·3 to 66·7)	61 (58·6 to 63·5)	60·5 (58·2 to 63)
Malawi	33·7 (28·1 to 39·2)	36·7 (33 to 40·6)	41·2 (38·5 to 44·2)	47·7 (45·6 to 49·9)	47·7 (45·9 to 49·4)	44·9 (42·6 to 47)	54 (52·2 to 55·7)	59·8 (57·1 to 62·4)	58·9 (56·4 to 61·4)	55·8 (53·7 to 57·7)
Mozambique	38 (32·4 to 43·3)	42·4 (38·4 to 46·6)	45·6 (42·6 to 48·6)	48·9 (46·5 to 51·3)	48·5 (46·8 to 50·3)	50·1 (48·3 to 51·8)	51 (49·3 to 52·9)	55·6 (53·1 to 58·1)	55·2 (52·4 to 57·8)	53·4 (51 to 55·5)
Rwanda	30·7 (25·8 to 35·6)	41·3 (38·6 to 43·9)	41·2 (38·6 to 43·9)	46·9 (44·7 to 49·2)	47·8 (46·1 to 49·6)	48·8 (47·2 to 50·3)	62 (60 to 63·9)	66 (63 to 68·7)	65·6 (62·9 to 68·3)	62·3 (60 to 64·3)
Somalia	41·4 (36·2 to 46·4)	42·1 (38 to 46·3)	43·4 (39·7 to 47·5)	47·2 (43·9 to 50·6)	45·9 (43·3 to 48·5)	48·3 (45·7 to 51)	48·1 (45·2 to 51·1)	54·1 (50·1 to 58·1)	51·8 (48·2 to 55·1)	50·7 (47·1 to 54)
South Sudan	44 (38 to 50)	45·7 (41 to 49·9)	46·2 (42 to 50·8)	49·1 (44·7 to 53·8)	49·9 (46·6 to 53·5)	52·8 (49·6 to 55·8)	56·2 (52·3 to 59·9)	54·4 (49·4 to 59)	52·5 (47·9 to 56·3)	52·6 (47·9 to 56·7)
Uganda	36·6 (31·4 to 41·9)	45·5 (42 to 49·1)	48·5 (45·8 to 51·1)	46·9 (45·1 to 48·9)	46·4 (43·6 to 49·1)	47·4 (45·6 to 49·1)	56·7 (54·9 to 58·5)	62·1 (59·5 to 64·4)	60·7 (58·1 to 63)	57·8 (55·3 to 60·3)
Tanzania	37·2 (31·5 to 42·6)	43·2 (39·7 to 47)	47·7 (45·1 to 50·4)	53·2 (50·8 to 55·5)	53·4 (51·6 to 55·2)	52·2 (50 to 54·2)	59·8 (58 to 61·6)	64·8 (62·1 to 67·1)	63 (60·7 to 65)	61·3 (59·2 to 63·1)
Zambia	40·5 (34·4 to 45·8)	46 (41·8 to 50·3)	51·3 (48 to 54·4)	54·2 (52·1 to 56·4)	50·3 (48·2 to 52·4)	44·6 (42·2 to 46·9)	54·6 (52·5 to 56·6)	60·3 (56·9 to 63·6)	58·7 (55·4 to 61·6)	55·8 (53 to 58·6)
Southern sub-Saharan Africa	46·2 (42·4 to 49·7)	49·9 (47·6 to 52·3)	53·3 (51·6 to 54·9)	56·5 (55·2 to 57·8)	60 (58·9 to 61)	51·6 (50·5 to 52·7)	53·4 (52·7 to 54·1)	60·6 (59·4 to 61·8)	57·9 (56·7 to 59)	55·9 (54·7 to 57)
Botswana	46 (41·2 to 50·4)	48·8 (45·6 to 52·1)	51·3 (48·5 to 54·2)	56·1 (53·2 to 58·8)	58 (55·1 to 60·9)	45·9 (42·3 to 49·3)	56·1 (53·7 to 58·4)	62·9 (60·3 to 65)	62·2 (59·7 to 64·2)	57 (55 to 58·9)
Eswatini	34 (28·7 to 38·9)	38·3 (34·5 to 42·2)	45·1 (41·4 to 48·8)	52 (49·1 to 55)	56·6 (54·5 to 58·9)	45·6 (42 to 48·8)	44·3 (41·7 to 46·7)	53·2 (49·7 to 56·6)	51·7 (48·7 to 54·5)	49·5 (46·9 to 52·2)
Lesotho	41·2 (37·4 to 44·7)	45·6 (43 to 48·2)	47·7 (45·1 to 50·4)	53·9 (51·5 to 56·3)	56·2 (53·9 to 58·3)	45·7 (42·8 to 48·2)	45·5 (43·2 to 47·6)	48·5 (45·9 to 51·2)	47·6 (45·4 to 49·9)	45·3 (43·5 to 47·2)
Namibia	47·6 (42·2 to 52·5)	51·8 (47·8 to 56)	54·6 (51·6 to 57·7)	55·5 (53·6 to 57·5)	58·9 (57·2 to 60·5)	51·6 (49·1 to 53·5)	56·8 (54·2 to 58·9)	60·7 (57·3 to 63·6)	59·9 (56·8 to 62·5)	56·5 (53·8 to 58·9)
South Africa	46·7 (43·2 to 49·8)	50 (47·8 to 52·1)	53·4 (51·7 to 55·1)	56·4 (55 to 57·9)	60·6 (59·6 to 61·6)	53·8 (53·2 to 54·4)	54·6 (54 to 55·1)	62·2 (61·3 to 63)	58·9 (58·1 to 59·6)	57·4 (56·6 to 58·3)
Eastern Cape	37·3 (31·4 to 42·9)	47·6 (43·9 to 51·4)	52 (48·3 to 55·5)	57·1 (53·9 to 60·2)	60·4 (58·3 to 62·6)	48·9 (47·9 to 50)	49·1 (48·2 to 50)	55·1 (53·4 to 56·5)	50·4 (49·2 to 51·8)	51·4 (49·8 to 53·1)
Free State	48·1 (43·6 to 52·2)	49·7 (46·1 to 53·4)	53 (49·5 to 56·2)	56·1 (53·1 to 59·2)	58·1 (56 to 60·1)	49·5 (48·5 to 50·5)	48·1 (47·4 to 48·7)	58·7 (57·4 to 60·1)	55·7 (54·4 to 57·1)	54·4 (52·9 to 55·8)
Gauteng	50·5 (47·4 to 53·8)	50·8 (47·8 to 53·9)	52·7 (49·6 to 55·7)	53·3 (50·8 to 55·9)	59·4 (57·5 to 61·5)	57 (55·8 to 58·2)	59·1 (57·9 to 60·3)	65·6 (63·6 to 67·6)	62·7 (60·9 to 64·5)	61·1 (59·3 to 62·8)
KwaZulu-Natal	48·8 (44·4 to 53)	50·8 (47·2 to 54·6)	54·6 (50·8 to 58·1)	57·8 (54·7 to 60·9)	57·7 (55·5 to 59·9)	48·9 (48 to 49·8)	51·6 (50·9 to 52·2)	64·5 (63 to 65·9)	59·7 (58·3 to 61)	57·9 (56·5 to 59·4)
Limpopo	50·4 (45·3 to 55·3)	56·7 (53·1 to 60·4)	60·3 (56·7 to 64)	64·5 (61 to 67·9)	69·2 (67 to 71·4)	59 (57·8 to 60)	53·4 (52·4 to 54·5)	59·2 (57·5 to 60·8)	57 (55·3 to 58·7)	53·9 (52·4 to 55·4)
Mpumalanga	48·1 (43·5 to 52·3)	51·1 (47·8 to 54·6)	54·1 (50·5 to 57·4)	58·1 (55·2 to 61)	64·2 (62·1 to 66·5)	55 (54·1 to 55·8)	53·8 (53 to 54·6)	63·6 (61·8 to 65·4)	61·2 (59·5 to 63)	57·8 (56·2 to 59·4)
North West	50·1 (45·4 to 54·4)	51·9 (48·2 to 55·8)	56·2 (52·6 to 59·6)	59·8 (56·7 to 63)	66·2 (64 to 68·5)	54 (52·5 to 55·5)	50·7 (49·4 to 52)	55·1 (53·1 to 57·1)	54·6 (52·7 to 56·6)	52·8 (51 to 54·6)
Northern Cape	54·8 (50·2 to 59)	56·1 (52·6 to 59·6)	58·7 (55·1 to 62·3)	62 (59·2 to 64·8)	62·2 (60 to 64·2)	51·6 (50·8 to 52·4)	52·4 (51 to 53·7)	59·4 (57·1 to 61·8)	57·1 (55 to 59·2)	54·3 (52·5 to 56·1)
Western Cape	54·4 (51 to 57·9)	56·2 (53·1 to 59·5)	57·3 (53·9 to 60·5)	57·7 (55 to 60·3)	60·8 (58·8 to 62·9)	60·7 (60·2 to 61·2)	64·4 (63·9 to 64·9)	67·1 (65·8 to 68·3)	63·4 (62·1 to 64·6)	62·1 (60·8 to 63·4)
Zimbabwe	47·7 (42·6 to 52·5)	52·2 (48·4 to 56·1)	55·2 (52·3 to 58·4)	57·8 (55·9 to 59·9)	59·1 (56·6 to 61·4)	45·9 (42·6 to 49·2)	50·4 (48·4 to 52·2)	56·7 (53·8 to 59·5)	55·3 (52·5 to 57·9)	52·2 (49·7 to 54·5)
Western sub-Saharan Africa	40·4 (36·1 to 44·5)	40·6 (37·8 to 43·4)	46·3 (44·1 to 48·7)	51 (49·3 to 52·9)	52·9 (51·4 to 54·5)	53·3 (51·7 to 55)	58 (56·2 to 59·6)	62·1 (59·7 to 64·2)	60·4 (58·1 to 62·6)	59·9 (57·6 to 61·9)
Benin	36·3 (30·6 to 42)	43 (39·6 to 46·5)	47 (44·3 to 49·6)	51·3 (49 to 53·5)	53·9 (52·3 to 55·5)	55·8 (54 to 57·6)	59·3 (57·6 to 61·2)	62·5 (60 to 64·8)	61·8 (59·3 to 63·9)	60·1 (57·8 to 62·1)
Burkina Faso	35·3 (30·1 to 40·5)	37·5 (33·7 to 41·2)	42·2 (39·3 to 45)	46·8 (44·5 to 48·9)	49·2 (47·1 to 51·2)	50·8 (48·6 to 52·9)	55·5 (53·5 to 57·6)	59·2 (56·6 to 61·7)	57·5 (54·7 to 60)	57·4 (54·9 to 59·6)
Cabo Verde	46·5 (43·2 to 49·7)	55·9 (54·3 to 57·7)	57·2 (55·6 to 58·8)	64·7 (63·1 to 66·3)	67 (65·2 to 68·8)	66·5 (63·2 to 69·5)	70·9 (68·6 to 73·2)	69·4 (67·2 to 71·6)	69 (66·8 to 71·2)	69 (66·8 to 71·2)
Cameroon	38·4 (34·6 to 42·5)	18·9 (17·7 to 20·2)	49·9 (47·3 to 52·7)	53·9 (51·6 to 56·3)	57 (55·3 to 58·8)	53·3 (51·4 to 55·2)	56·7 (54·3 to 59·2)	61·3 (58 to 64·4)	59·9 (56·9 to 62·8)	58·5 (55·7 to 60·8)
Chad	38·4 (32·3 to 44·6)	44·5 (39·9 to 49·6)	44·9 (41·1 to 48·5)	48 (45·7 to 50·4)	51·7 (49·8 to 53·7)	50·5 (48·3 to 52·7)	55 (52·7 to 57·3)	58·1 (54 to 61·8)	55·9 (52·3 to 59·3)	56·5 (52·5 to 59·8)
Côte d'Ivoire	42·7 (39 to 46·7)	44 (41 to 47)	49·2 (46·7 to 51·8)	55·1 (53·1 to 57·1)	53·3 (51·2 to 55·3)	50·3 (48·1 to 52·5)	55·8 (53·8 to 57·8)	61·7 (58·7 to 64·4)	60·1 (57·4 to 62·5)	60·3 (57·6 to 62·7)
The Gambia	49·1 (43·7 to 54·5)	46·9 (42·4 to 51)	50·5 (46·8 to 54·1)	53·1 (50·1 to 56·1)	56·8 (54·1 to 59·6)	58·6 (56·2 to 61)	61·3 (59·1 to 63·6)	63·3 (60·6 to 66·1)	60·8 (58·5 to 63)	60·9 (58·5 to 63·2)
Ghana	43·9 (39·6 to 48·1)	48·8 (46 to 51·7)	52·5 (49·6 to 55·4)	56 (53·3 to 58·7)	57·9 (55·5 to 60·2)	58·2 (56·2 to 60·3)	59·6 (57·8 to 61·3)	63·5 (60·8 to 66)	62·6 (60 to 64·9)	61·7 (59·5 to 63·9)
Guinea	36·8 (31·4 to 42·3)	40·8 (36·1 to 45·4)	43·7 (39·6 to 47·9)	47·8 (44·9 to 50·9)	51·9 (49·8 to 54)	53 (50·9 to 55)	56·6 (54·3 to 59)	60·7 (57·1 to 64·2)	58·3 (54·7 to 61·3)	58·2 (54·6 to 61·2)
Guinea-Bissau	24·6 (20·3 to 29·3)	32·6 (28·5 to 36·4)	34 (30·9 to 37·1)	42·2 (38·9 to 45·5)	45·9 (42·7 to 48·9)	48·9 (46·4 to 51·5)	54·1 (51·6 to 56·7)	57·3 (54·5 to 60·1)	54·4 (52 to 56·6)	55·1 (52·4 to 57·7)
Liberia	26·9 (21 to 33·2)	37·6 (34 to 41·7)	43·1 (40 to 46·3)	49·4 (46·5 to 52·2)	45·4 (43·4 to 47·5)	53·8 (51·8 to 55·7)	60·7 (58·4 to 62·9)	63·7 (60 to 67·2)	61·4 (57·4 to 64·7)	61·6 (57·7 to 64·8)
Mali	32·8 (27·4 to 37·9)	36·9 (33·6 to 40·4)	39·2 (36·9 to 41·7)	45 (42·8 to 47·2)	49·5 (47·8 to 51·3)	53·2 (51·6 to 54·8)	57·6 (55·7 to 59·6)	61·1 (58·6 to 63·5)	58·3 (55·8 to 60·4)	57·3 (55·1 to 59·2)
Mauritania	44·5 (40·3 to 48·7)	50·3 (47·4 to 53·5)	55·2 (52·2 to 58·1)	57·1 (54·6 to 59·7)	60·1 (58·3 to 62)	64·4 (62·5 to 66·4)	68·6 (66·3 to 70·8)	70·7 (67·8 to 73·5)	68·4 (65·4 to 70·4)	68·4 (65·6 to 71)
Niger	37·5 (32·2 to 43)	37·5 (33·1 to 42·2)	40·7 (37·3 to 44·2)	43·8 (41·8 to 46)	46·7 (44·9 to 48·5)	51·4 (49·4 to 53·6)	59·2 (56 to 62·4)	62·2 (58 to 65·8)	60·3 (56·4 to 63·7)	60·1 (56·3 to 63·4)
Nigeria	42·6 (38·2 to 46·9)	44·9 (41·5 to 48·2)	47 (44·2 to 49·9)	51·8 (49·2 to 54·5)	52·9 (50·3 to 55·5)	53·3 (50·6 to 56·2)	58·4 (55·3 to 61·3)	62·9 (59·6 to 65·9)	61·3 (58·5 to 64·1)	60·7 (58 to 63·1)
São Tomé and Príncipe	36·9 (34 to 39·6)	45·8 (44·3 to 47·3)	56·7 (55·5 to 58)	61·5 (60·4 to 62·6)	61·8 (60·2 to 63·3)	64·1 (62·2 to 66)	67·8 (66·1 to 69·8)	70 (67·9 to 71·8)	68·9 (66·9 to 70·6)	68·6 (66·5 to 70·3)

Senegal	42·3 (38·3 to 46·6)	44·5 (41·4 to 47·7)	45·6 (43·2 to 48·2)	50·7 (48·8 to 52·6)	56·6 (55 to 58·1)	58·6 (57·3 to 59·9)	64 (62·4 to 65·6)	66·9 (64·2 to 69·5)	64·9 (62·4 to 67·1)	63·7 (61·4 to 65·8)
Sierra Leone	35·8 (29·9 to 42)	39·9 (35·3 to 44·4)	43·4 (38·7 to 48)	46·6 (43 to 50·2)	49·2 (46·9 to 51·6)	48·9 (47 to 50·8)	54·6 (52·1 to 57·2)	60·6 (56·9 to 64·6)	58·4 (54·2 to 62·2)	59·2 (54·9 to 62·8)
Togo	38·6 (32·3 to 44·8)	43·5 (39·4 to 48)	49·3 (45·9 to 52·6)	54·7 (52·4 to 57·3)	56·2 (54·1 to 58·3)	54·4 (52·4 to 56·5)	56·8 (54·5 to 59·2)	61·5 (57·6 to 64·9)	60·9 (57·2 to 64·1)	60·2 (56·6 to 63·2)

Appendix 2 Table S5a: Population (in thousands) with 95% UIs by location, both sexes combined, 1950, 1960, 1970, 1980, 1990, 2000, 2010, 2019, 2020, 2021

Location	1950	1960	1970	1980	1990	2000	2010	2019	2020	2021
Global	2524671 (2476608 to 2576263)	3038848 (2967485 to 3114065)	3700586 (3606045 to 3798006)	4470486 (4375024 to 4569739)	5333623 (5231036 to 5444650)	6098778 (5976444 to 6222620)	6950756 (6814825 to 7089665)	7745328 (7542839 to 7959556)	7822364 (7614060 to 8040554)	7891353 (7666734 to 8131225)
Central Europe, eastern Europe, and central Asia	279593 (271048 to 287955)	320655 (310459 to 331135)	359786 (349402 to 370496)	392327 (380420 to 404423)	420895 (407281 to 433813)	417396 (403612 to 430773)	412557 (399008 to 425102)	418564 (395661 to 440080)	418563 (394748 to 441417)	417832 (393252 to 441078)
Central Asia	27907 (27124 to 28668)	35275 (34150 to 36348)	47362 (45941 to 48746)	58203 (56430 to 59943)	69312 (67115 to 71437)	74399 (70634 to 78129)	81917 (76182 to 87479)	93267 (84849 to 101897)	94580 (85750 to 103761)	95809 (85933 to 105623)
Armenia	1450 (1349 to 1551)	1882 (1745 to 2017)	2564 (2402 to 2736)	3172 (2933 to 3397)	3421 (3162 to 3683)	3318 (3069 to 3552)	3114 (2881 to 3350)	3025 (2656 to 3392)	3011 (2642 to 3385)	2995 (2605 to 3376)
Azerbaijan	3123 (2925 to 3331)	3944 (3650 to 4243)	5261 (4899 to 5591)	6271 (5779 to 6753)	7326 (6764 to 7851)	8284 (7704 to 8890)	9297 (8574 to 9976)	10356 (9021 to 11727)	10437 (9064 to 11820)	10500 (9082 to 11993)
Georgia	3681 (3428 to 3939)	4215 (3893 to 4511)	4795 (4456 to 5110)	5170 (4780 to 5555)	5523 (5116 to 5948)	4730 (4341 to 5117)	3995 (3621 to 4371)	3675 (3315 to 4054)	3645 (3292 to 4024)	3607 (3200 to 4005)
Kazakhstan	7612 (7109 to 8124)	9612 (8862 to 10330)	13001 (12050 to 13909)	14878 (13850 to 15990)	16393 (15117 to 17583)	14971 (13857 to 16126)	16205 (16109 to 16293)	18456 (16853 to 19991)	18717 (16987 to 20392)	18956 (17030 to 20835)
Kyrgyzstan	1747 (1625 to 1865)	2206 (2041 to 2369)	3020 (2826 to 3224)	3709 (3430 to 3978)	4464 (4140 to 4797)	5014 (4655 to 5379)	5653 (5264 to 6055)	6654 (5801 to 7448)	6762 (5806 to 7749)	6863 (5857 to 7900)
Mongolia	806 (756 to 857)	970 (885 to 1053)	1281 (1187 to 1375)	1698 (1576 to 1819)	2158 (2004 to 2321)	2445 (2274 to 2612)	2716 (2535 to 2906)	3223 (2989 to 3455)	3281 (3066 to 3485)	3336 (3085 to 3580)
Tajikistan	1663 (1555 to 1772)	2127 (1967 to 2281)	3005 (2800 to 3210)	4062 (3755 to 4346)	5369 (4968 to 5790)	6364 (5951 to 6796)	7790 (7313 to 8297)	9724 (8414 to 10935)	9947 (8516 to 11324)	10160 (8805 to 11593)
Turkmenistan	1261 (1179 to 1342)	1618 (1494 to 1745)	2228 (2080 to 2377)	2923 (2716 to 3140)	3699 (3424 to 3978)	4261 (3711 to 4832)	4634 (4163 to 5113)	5066 (4599 to 5526)	5114 (4611 to 5633)	5158 (4622 to 5702)
Uzbekistan	6564 (6108 to 6990)	8701 (8030 to 9364)	12207 (11396 to 13074)	16321 (15191 to 17416)	20959 (19365 to 22593)	25013 (21533 to 28655)	28513 (22712 to 34148)	33089 (24967 to 41580)	33667 (24640 to 42728)	34233 (24498 to 43631)
Central Europe	89204 (86772 to 91576)	101421 (98363 to 104498)	110914 (107555 to 114390)	120419 (116531 to 124397)	125092 (121089 to 129147)	122456 (118414 to 126469)	119169 (117163 to 121088)	116372 (111865 to 120811)	115911 (111198 to 120621)	115264 (109996 to 120347)
Albania	1265 (1183 to 1355)	1689 (1577 to 1808)	2192 (2032 to 2353)	2734 (2528 to 2937)	3305 (3066 to 3551)	3193 (2969 to 3433)	2888 (2672 to 3106)	2708 (2408 to 3009)	2689 (2358 to 3040)	2668 (2316 to 3021)
Bosnia and Herzegovina	2973 (2769 to 3177)	3495 (3233 to 3767)	3845 (3560 to 4115)	4220 (3916 to 4520)	4498 (4143 to 4860)	3976 (3489 to 4494)	3802 (3458 to 4138)	3391 (3031 to 3750)	3350 (2979 to 3723)	3303 (2901 to 3694)
Bulgaria	7140 (6641 to 7647)	7903 (7166 to 8669)	8485 (7662 to 9391)	8923 (8000 to 9754)	8680 (7968 to 9387)	7944 (7402 to 8576)	7436 (7388 to 7481)	6937 (6362 to 7556)	6873 (6263 to 7485)	6787 (6075 to 7429)
Croatia	3897 (3619 to 4185)	4187 (3868 to 4507)	4516 (4146 to 4856)	4832 (4475 to 5173)	4861 (4493 to 5241)	4574 (4248 to 4902)	4385 (4077 to 4698)	4253 (3753 to 4770)	4233 (3702 to 4724)	4209 (3676 to 4748)
Czechia	9114 (8430 to 9738)	9486 (8765 to 10184)	9793 (9160 to 10476)	10271 (9531 to 11008)	10294 (9472 to 11067)	10229 (10155 to 10304)	10474 (10398 to 10555)	10646 (9782 to 11503)	10647 (9744 to 11549)	10632 (9668 to 11611)
Hungary	9417 (8786 to 10042)	9990 (9325 to 10682)	10292 (9526 to 11022)	10617 (9953 to 11363)	10395 (9644 to 11131)	10203 (9440 to 10958)	9962 (9204 to 10689)	9689 (8528 to 10805)	9650 (8399 to 10847)	9597 (8426 to 10869)
Montenegro	412 (383 to 440)	480 (447 to 513)	539 (498 to 577)	593 (550 to 634)	626 (583 to 674)	637 (580 to 695)	633 (584 to 679)	623 (548 to 698)	621 (541 to 697)	618 (540 to 701)
North Macedonia	1320 (1231 to 1415)	1436 (1326 to 1543)	1667 (1548 to 1789)	1944 (1793 to 2084)	1992 (1820 to 2180)	2058 (1897 to 2226)	2143 (1881 to 2393)	2185 (1812 to 2566)	2183 (1786 to 2605)	2176 (1797 to 2588)
Poland	24548 (22821 to 26230)	29432 (27467 to 31388)	32570 (30352 to 34884)	35725 (32932 to 38709)	38173 (35142 to 41104)	38323 (35161 to 41335)	38382 (38119 to 38650)	38445 (35388 to 41377)	38373 (35211 to 41747)	38238 (34552 to 41891)
Romania	16586 (15400 to 17680)	18915 (17174 to 20710)	20813 (18836 to 22859)	22677 (20764 to 24665)	23380 (21556 to 25237)	22417 (20597 to 24302)	20687 (19157 to 22318)	19263 (17053 to 21572)	19118 (16668 to 21439)	18939 (16520 to 21513)
Serbia	6847 (6399 to 7328)	7751 (7226 to 8310)	8678 (7995 to 9365)	9408 (8714 to 10055)	9630 (8843 to 10366)	9669 (8884 to 10474)	9114 (8444 to 9775)	9015 (8070 to 10029)	8976 (7870 to 10145)	8920 (7752 to 10033)
Slovakia	3431 (3414 to 3447)	4071 (4053 to 4091)	4539 (4516 to 4561)	4979 (4954 to 5004)	5283 (5253 to 5311)	5390 (5357 to 5423)	5410 (5370 to 5451)	5439 (4972 to 5925)	5439 (4939 to 5965)	5429 (4896 to 5960)
Slovenia	1473 (1369 to 1570)	1579 (1455 to 1700)	1721 (1599 to 1858)	1877 (1733 to 2034)	1974 (1760 to 2184)	1993 (1979 to 2007)	2041 (2024 to 2058)	2074 (1914 to 2243)	2073 (1903 to 2255)	2070 (1886 to 2251)
Eastern Europe	162483 (154298 to 170850)	183959 (174240 to 194393)	201510 (191639 to 211876)	213705 (202314 to 225681)	226491 (213284 to 239110)	220541 (207623 to 233587)	211470 (199328 to 223117)	208924 (188559 to 228973)	208071 (186300 to 229377)	206759 (184806 to 227890)
Belarus	7398 (6902 to 7880)	8420 (7785 to 9051)	9277 (8691 to 9869)	9847 (9127 to 10552)	10444 (9646 to 11236)	10215 (9457 to 10978)	9655 (8896 to 10407)	9450 (8301 to 10621)	9399 (8123 to 10641)	9325 (8021 to 10622)
Estonia	1031 (1026 to 1035)	1205 (1199 to 1211)	1353 (1346 to 1359)	1473 (1465 to 1480)	1568 (1560 to 1577)	1394 (1385 to 1403)	1332 (1322 to 1343)	1315 (1206 to 1418)	1314 (1210 to 1424)	1311 (1188 to 1433)
Latvia	1898 (1767 to 2026)	2121 (1961 to 2271)	2365 (2201 to 2539)	2521 (2339 to 2701)	2658 (2462 to 2857)	2384 (2207 to 2542)	2116 (2100 to 2131)	1912 (1757 to 2068)	1893 (1729 to 2057)	1870 (1698 to 2049)
Lithuania	2406 (2237 to 2577)	2753 (2562 to 2956)	3131 (2923 to 3338)	3419 (3172 to 3671)	3674 (3405 to 3951)	3520 (3261 to 3776)	3092 (2841 to 3312)	2794 (2575 to 3026)	2763 (2526 to 3015)	2728 (2476 to 3006)
Moldova	2517 (2342 to 2687)	3041 (2811 to 3261)	3666 (3426 to 3909)	4101 (3815 to 4400)	4447 (4126 to 4774)	4197 (3806 to 4599)	3847 (3429 to 4264)	3649 (3063 to 4282)	3624 (3018 to 4200)	3593 (2973 to 4192)
Russia	109316 (102043 to 116949)	122736 (113951 to 132061)	133289 (124613 to 142784)	141262 (131018 to 152327)	150985 (138993 to 162537)	149212 (137387 to 161288)	145350 (135463 to 155215)	145939 (128166 to 164296)	145574 (126722 to 164875)	144854 (124993 to 163581)
Ukraine	37918 (35203 to 40496)	43683 (40579 to 46823)	48430 (45312 to 51431)	51082 (47022 to 54715)	52713 (48760 to 56465)	49618 (45980 to 53231)	46078 (40514 to 51749)	43865 (35601 to 52059)	43504 (35578 to 51406)	43078 (34633 to 51395)
High income	623299 (607003 to 638982)	701281 (684703 to 717170)	781533 (763324 to 799216)	848991 (828406 to 868398)	909017 (888816 to 929396)	968025 (944219 to 990224)	1036711 (1011130 to 1060798)	1086206 (1056493 to 1116741)	1089402 (1060822 to 1116988)	1091666 (1061851 to 1120812)
Australasia	10625 (10040 to 11210)	12979 (12176 to 13755)	15706 (14766 to 16637)	17892 (16820 to 19032)	20276 (19007 to 21564)	22749 (21311 to 24144)	25976 (24367 to 27539)	29931 (27982 to 31997)	30463 (28571 to 32355)	30961 (29169 to 32748)
Australia	8671 (8081 to 9245)	10545 (9730 to 11319)	12811 (11882 to 13737)	14647 (13585 to 15800)	16859 (15604 to 18092)	18885 (17447 to 20296)	21577 (19986 to 23074)	24944 (23003 to 27029)	25370 (23487 to 27221)	25792 (24001 to 27548)
New Zealand	1953 (1816 to 2084)	2434 (2253 to 2613)	2895 (2687 to 3099)	3246 (3010 to 3468)	3417 (3160 to 3674)	3864 (3576 to 4152)	4399 (3995 to 4832)	4987 (4603 to 5357)	5093 (4709 to 5504)	5170 (4717 to 5607)
High-income Asia Pacific	107073 (101028 to 112608)	123534 (116848 to 130279)	141806 (134011 to 149465)	160572 (152163 to 169030)	173380 (164261 to 182296)	180136 (170898 to 189541)	184776 (174807 to 194353)	186368 (176091 to 197244)	185944 (176720 to 195385)	185446 (175178 to 196125)
Brunei	62 (58 to 66)	87 (81 to 93)	135 (125 to 145)	192 (178 to 206)	259 (240 to 278)	333 (306 to 358)	395 (364 to 425)	442 (386 to 497)	447 (391 to 504)	451 (394 to 510)
Japan	85719 (79775 to 91136)	95900 (89643 to 102219)	107001 (99181 to 114496)	119467 (111296 to 127636)	125828 (117052 to 134168)	128988 (120101 to 137741)	129986 (120351 to 138961)	128408 (118748 to 138129)	128077 (119293 to 136180)	127697 (117992 to 137075)
Aichi	3468 (3141 to 3781)	4286 (3894 to 4677)	5500 (4988 to 6004)	6349 (5749 to 6918)	6809 (6179 to 7415)	7138 (6464 to 7785)	7511 (6769 to 8205)	7643 (6863 to 8431)	7645 (6905 to 8339)	7643 (6854 to 8416)
Akita	1338 (1210 to 1465)	1361 (1230 to 1494)	1272 (1153 to 1400)	1286 (1166 to 1408)	1255 (1133 to 1374)	1213 (1097 to 1331)	1111 (1002 to 1220)	990 (884 to 1098)	977 (883 to 1074)	963 (863 to 1067)
Aomori	1314 (1191 to 1446)	1454 (1318 to 1604)	1464 (1327 to 1612)	1561 (1418 to 1718)	1517 (1378 to 1672)	1507 (1368 to 1659)	1404 (1270 to 1548)	1274 (1143 to 1421)	1259 (1139 to 1385)	1243 (1114 to 1382)
Chiba	2185 (1966 to 2394)	2355 (2127 to 2577)	3439 (3105 to 3760)	4830 (4351 to 5282)	5651 (5093 to 6172)	6022 (5410 to 6580)	6268 (5633 to 6866)	6376 (5682 to 7017)	6382 (5744 to 6962)	6384 (5675 to 7064)
Ehime	1555 (1416 to 1707)	1529 (1396 to 1682)	1454 (1323 to 1600)	1540 (1403 to 1693)	1548 (1409 to 1698)	1522 (1386 to 1672)	1458 (1324 to 1601)	1371 (1236 to 1523)	1359 (1236 to 1494)	1347 (1215 to 1496)
Fukui	769 (696 to 845)	767 (692 to 845)	763 (691 to 841)	813 (735 to 894)	842 (761 to 928)	845 (762 to 931)	818 (739 to 899)	786 (704 to 873)	781 (707 to 859)	775 (695 to 864)
Fukuoka	3611 (3250 to 3952)	4067 (3666 to 4449)	4124 (3711 to 4516)	4644 (4188 to 5098)	4897 (4388 to 5382)	5098 (4587 to 5600)	5153 (4617 to 5661)	5211 (4625 to 5765)	5211 (4671 to 5705)	5208 (4642 to 5767)
Fukushima	2112 (1921 to 2316)	2089 (1889 to 2303)	1995 (1810 to 2197)	2083 (1886 to 2300)	2150 (1948 to 2367)	2170 (1967 to 2395)	2066 (1864 to 2274)	1881 (1689 to 2103)	1864 (1685 to 2062)	1847 (1652 to 2067)
Gifu	1575 (1427 to 1732)	1668 (1513 to 1831)	1802 (1628 to 1974)	2003 (1816 to 2203)	2110 (1900 to 2313)	2147 (1949 to 2363)	2121 (1914 to 2331)	2025 (1807 to 2249)	2011 (1811 to 2207)	1998 (1788 to 2222)
Gunma	1638 (1477 to 1798)	1612 (1454 to 1775)	1698 (1535 to 1869)	1889 (1712 to 2075)	2007 (1817 to 2204)	2058 (1866 to 2266)	2044 (1853 to 2244)	1977 (1778 to 2190)	1968 (1780 to 2165)	1958 (1754 to 2175)
Hiroshima	2125 (1921 to 2323)	2224 (2014 to 2437)	2487 (2254 to 2728)	2795 (2534 to 3066)	2902 (2624 to 3170)	2928 (2644 to 3202)	2894 (2613 to 3169)	2857 (2568 to 3156)	2843 (2572 to 3111)	2829 (2534 to 3133)
Hokkaidō	4389 (3998 to 4797)	5129 (4675 to 5609)	5309 (4828 to 5791)	5694 (5192 to 6233)	5758 (5247 to 6295)	5766 (5244 to 6322)	5630 (5129 to 6173)	5349 (4827 to 5912)	5311 (4838 to 5811)	5271 (4763 to 5829)
Hyōgo	3388 (3073 to 3705)	3980 (3606 to 4367)	4770 (4317 to 5216)	5245 (4761 to 5752)	5491 (4954 to 6023)	5639 (5112 to 6181)	5682 (5125 to 6246)	5568 (4997 to 6165)	5548 (5016 to 6091)	5525 (4960 to 6122)
Ibaraki	2085 (1898 to 2274)	2086 (1898 to 2279)	2195 (1996 to 2398)	2614 (2384 to 2856)	2903 (2631 to 3182)	3040 (2768 to 3320)	3024 (2744 to 3308)	2924 (2637 to 3230)	2912 (2649 to 3180)	2898 (2609 to 3195)
Ishikawa	977 (888 to 1071)	992 (899 to 1088)	1027 (932 to 1127)	1144 (1035 to 1259)	1188 (1080 to 1304)	1199 (1089 to 1318)	1188 (1072 to 1309)	1156 (1034 to 1281)	1151 (1040 to 1265)	1145 (1024 to 1269)
Iwate	1381 (1249 to 1509)	1476 (1336 to 1617)	1407 (1267 to 1547)	1455 (1315 to 1599)	1449 (1310 to 1591)	1444 (1302 to 1582)	1359 (1221 to 1492)	1248 (1117 to 1385)	1232 (1111 to 1352)	1215 (1088 to 1349)
Kagawa	965 (867 to 1056)	936 (841 to 1027)	930 (831 to 1022)	1022 (915 to 1123)	1045 (938 to 1146)	1042 (936 to 1146)	1005 (905 to 1106)	975 (867 to 1081)	968 (865 to 1063)	962 (854 to 1068)
Kagoshima	1860 (1693 to 2040)	2003 (1823 to 2199)	1775 (1618 to 1953)	1826 (1664 to 2010)	1837 (1673 to 2020)	1822 (1663 to 2006)	1741 (1578 to 1922)	1631 (1472 to 1815)	1617 (1473 to 1777)	1603 (1442 to 1783)
Kanagawa	2540 (2296 to 2777)	3506 (3180 to 3830)	5581 (5072 to 6098)	7054 (6409 to 7717)	8105 (7354 to 8857)	8614 (7822 to 9420)	9184 (8288 to 10033)	9353 (8368 to 10320)	9367 (8516 to 10226)	9376 (8416 to 10357)
Kōchi	893 (809 to 979)	871 (790 to 957)	807 (733 to 889)	850 (771 to 933)	840 (762 to 922)	829 (752 to 915)	777 (702 to 855)	713 (641 to 791)	704 (638 to 777)	696 (623 to 775)
Kumamoto	1872 (1697 to 2051)	1891 (1714 to 2074)	1743 (1580 to 1913)	1830 (1664 to 2002)	1879 (1704 to 2061)	1896 (1719 to 2080)	1852 (1672 to 2028)	1782 (1598 to 1976)	1771 (1604 to 1942)	1759 (1575 to 1956)

Kyōto	1872 (1693 to 2058)	2030 (1840 to 2235)	2300 (2084 to 2525)	2580 (2335 to 2842)	2647 (2392 to 2915)	2679 (2419 to 2951)	2656 (2402 to 2929)	2628 (2351 to 2926)	2618 (2366 to 2877)	2607 (2330 to 2909)
Mie	1496 (1349 to 1648)	1515 (1363 to 1667)	1581 (1424 to 1742)	1725 (1554 to 1909)	1831 (1647 to 2012)	1893 (1699 to 2092)	1887 (1687 to 2082)	1812 (1614 to 2024)	1801 (1620 to 1997)	1790 (1597 to 1990)
Miyagi	1699 (1534 to 1859)	1774 (1597 to 1939)	1862 (1674 to 2041)	2126 (1916 to 2326)	2289 (2068 to 2502)	2409 (2162 to 2635)	2386 (2152 to 2609)	2353 (2100 to 2606)	2343 (2110 to 2567)	2332 (2074 to 2579)
Miyazaki	1117 (1012 to 1220)	1156 (1046 to 1266)	1078 (975 to 1179)	1178 (1068 to 1291)	1195 (1083 to 1308)	1193 (1083 to 1308)	1158 (1048 to 1268)	1096 (982 to 1211)	1087 (985 to 1191)	1079 (963 to 1194)
Nagano	2103 (1910 to 2311)	2019 (1828 to 2223)	2004 (1816 to 2208)	2129 (1926 to 2346)	2202 (1991 to 2427)	2255 (2041 to 2480)	2197 (1990 to 2421)	2093 (1879 to 2332)	2081 (1889 to 2290)	2070 (1850 to 2292)
Nagasaki	1687 (1530 to 1846)	1787 (1620 to 1960)	1610 (1454 to 1765)	1627 (1474 to 1784)	1598 (1447 to 1749)	1547 (1400 to 1697)	1456 (1313 to 1600)	1352 (1210 to 1498)	1338 (1206 to 1472)	1323 (1181 to 1470)
Nara	780 (702 to 850)	795 (718 to 870)	950 (857 to 1038)	1234 (1116 to 1348)	1402 (1266 to 1532)	1467 (1324 to 1612)	1424 (1278 to 1561)	1355 (1207 to 1501)	1346 (1217 to 1477)	1337 (1197 to 1483)
Niigata	2514 (2267 to 2759)	2491 (2249 to 2730)	2410 (2172 to 2645)	2506 (2259 to 2742)	2527 (2280 to 2766)	2521 (2280 to 2763)	2421 (2178 to 2661)	2261 (2017 to 2500)	2237 (2020 to 2460)	2213 (1968 to 2453)
Ōita	1281 (1153 to 1407)	1261 (1133 to 1386)	1184 (1067 to 1305)	1256 (1130 to 1378)	1262 (1136 to 1389)	1243 (1116 to 1369)	1218 (1089 to 1339)	1153 (1024 to 1284)	1143 (1027 to 1257)	1133 (1005 to 1260)
Okayama	1698 (1547 to 1860)	1701 (1544 to 1871)	1746 (1588 to 1917)	1912 (1736 to 2102)	1966 (1792 to 2158)	1986 (1805 to 2178)	1973 (1790 to 2173)	1928 (1736 to 2144)	1918 (1743 to 2103)	1908 (1719 to 2116)
Okinawa	651 (587 to 717)	798 (685 to 914)	969 (874 to 1068)	1132 (1019 to 1245)	1242 (1120 to 1368)	1336 (1203 to 1468)	1421 (1276 to 1563)	1489 (1332 to 1653)	1494 (1349 to 1644)	1497 (1335 to 1667)
Ōsaka	3944 (3563 to 4341)	5596 (5062 to 6159)	7775 (7049 to 8571)	8633 (7830 to 9476)	8866 (8053 to 9737)	8940 (8103 to 9823)	8969 (8140 to 9849)	8978 (8062 to 9962)	8969 (8140 to 9833)	8956 (8034 to 9926)
Saga	968 (878 to 1058)	959 (869 to 1050)	859 (777 to 942)	885 (802 to 969)	898 (813 to 984)	895 (809 to 981)	868 (785 to 952)	831 (745 to 915)	826 (748 to 903)	821 (735 to 911)
Saitama	2192 (1992 to 2398)	2485 (2262 to 2725)	3953 (3600 to 4324)	5533 (5051 to 6039)	6519 (5933 to 7124)	7050 (6416 to 7726)	7331 (6650 to 8020)	7446 (6725 to 8224)	7453 (6775 to 8162)	7455 (6720 to 8250)
Shiga	880 (797 to 965)	858 (781 to 943)	885 (804 to 976)	1102 (998 to 1213)	1245 (1131 to 1370)	1365 (1240 to 1503)	1428 (1294 to 1575)	1437 (1296 to 1596)	1434 (1306 to 1574)	1432 (1288 to 1592)
Shimane	933 (837 to 1025)	907 (816 to 998)	793 (711 to 874)	804 (721 to 887)	799 (718 to 880)	778 (698 to 856)	733 (658 to 806)	691 (613 to 768)	685 (615 to 757)	680 (604 to 756)
Shizuoka	2533 (2291 to 2769)	2810 (2543 to 3086)	3163 (2847 to 3469)	3522 (3172 to 3867)	3746 (3375 to 4109)	3837 (3457 to 4217)	3831 (3441 to 4212)	3710 (3319 to 4119)	3692 (3320 to 4057)	3673 (3275 to 4083)
Tochigi	1583 (1437 to 1735)	1543 (1400 to 1688)	1618 (1467 to 1773)	1831 (1662 to 2010)	1975 (1788 to 2168)	2042 (1852 to 2240)	2036 (1843 to 2239)	1976 (1773 to 2190)	1965 (1780 to 2158)	1952 (1751 to 2164)
Tokushima	897 (814 to 981)	864 (786 to 947)	811 (737 to 887)	844 (766 to 926)	848 (773 to 928)	840 (763 to 920)	797 (723 to 873)	739 (664 to 818)	731 (660 to 802)	723 (648 to 801)
Tōkyō	6410 (5817 to 6973)	9811 (8909 to 10731)	11624 (10601 to 12669)	11799 (10740 to 12874)	11957 (10842 to 13056)	12182 (11072 to 13311)	13220 (11968 to 14469)	14109 (12709 to 15571)	14210 (12937 to 15503)	14303 (12847 to 15776)
Tottori	614 (554 to 679)	611 (552 to 674)	583 (528 to 643)	618 (558 to 685)	629 (568 to 695)	625 (566 to 692)	598 (539 to 662)	566 (507 to 633)	562 (508 to 620)	557 (498 to 624)
Toyama	1028 (932 to 1126)	1051 (950 to 1152)	1055 (953 to 1156)	1128 (1017 to 1237)	1143 (1034 to 1253)	1142 (1033 to 1252)	1115 (1005 to 1220)	1059 (948 to 1172)	1052 (950 to 1156)	1045 (933 to 1157)
Wakayama	1004 (908 to 1104)	1021 (922 to 1125)	1063 (963 to 1170)	1111 (1007 to 1222)	1097 (992 to 1208)	1091 (984 to 1203)	1017 (918 to 1118)	946 (846 to 1050)	936 (846 to 1029)	927 (827 to 1034)
Yamagata	1389 (1258 to 1523)	1348 (1220 to 1482)	1256 (1132 to 1381)	1281 (1154 to 1414)	1287 (1154 to 1420)	1271 (1147 to 1398)	1196 (1078 to 1319)	1100 (982 to 1223)	1087 (982 to 1199)	1075 (958 to 1194)
Yamaguchi	1575 (1419 to 1720)	1630 (1469 to 1788)	1547 (1397 to 1697)	1621 (1461 to 1776)	1605 (1445 to 1761)	1557 (1403 to 1709)	1480 (1331 to 1627)	1379 (1227 to 1531)	1364 (1226 to 1497)	1349 (1200 to 1499)
Yamanashi	829 (755 to 907)	796 (725 to 872)	780 (706 to 858)	822 (748 to 899)	870 (791 to 951)	905 (822 to 992)	878 (795 to 963)	830 (748 to 918)	824 (747 to 903)	817 (735 to 906)
South Korea	20146 (18762 to 21462)	25943 (24139 to 27846)	32595 (30312 to 34885)	38498 (35798 to 40978)	44245 (41092 to 47074)	46786 (43465 to 49893)	49318 (45867 to 52844)	51851 (47995 to 55747)	51723 (48248 to 55315)	51570 (47777 to 55127)
Singapore	1145 (1065 to 1221)	1604 (1461 to 1740)	2075 (1931 to 2210)	2415 (2264 to 2570)	3047 (2852 to 3254)	4029 (3745 to 4301)	5077 (4727 to 5428)	5668 (5234 to 6059)	5698 (5247 to 6148)	5727 (5262 to 6196)
High-income North America	168841 (158681 to 179005)	201998 (189365 to 213803)	231202 (217093 to 245383)	254382 (237935 to 270168)	281409 (264082 to 298852)	311426 (291744 to 330682)	342924 (322169 to 364096)	366859 (341618 to 392073)	368707 (345410 to 391587)	370175 (346061 to 393850)
Canada	14023 (13052 to 14971)	18298 (16941 to 19637)	21729 (20159 to 23310)	24471 (22760 to 26217)	27253 (25193 to 29412)	30302 (28135 to 32400)	33550 (30955 to 35903)	36801 (33816 to 39684)	37144 (34401 to 39899)	37469 (35051 to 40168)
Greenland	23 (22 to 25)	33 (31 to 35)	46 (43 to 49)	50 (50 to 50)	56 (55 to 56)	56 (56 to 57)	56 (56 to 57)	56 (51 to 61)	56 (51 to 61)	56 (51 to 61)
USA	154790 (144618 to 165123)	183661 (170728 to 195506)	209420 (195074 to 223358)	229854 (213131 to 245863)	254094 (236338 to 271660)	281061 (261390 to 300554)	309312 (288011 to 330613)	329996 (304603 to 354982)	331501 (307812 to 354588)	332643 (308381 to 356796)
Alabama	3118 (2821 to 3413)	3346 (3029 to 3660)	3553 (3200 to 3906)	3938 (3548 to 4324)	4127 (3733 to 4523)	4441 (4006 to 4864)	4790 (4336 to 5242)	5011 (4468 to 5549)	5025 (4530 to 5501)	5030 (4503 to 5556)
Alaska	130 (118 to 142)	232 (211 to 254)	314 (285 to 344)	410 (371 to 449)	563 (510 to 617)	631 (571 to 691)	708 (643 to 775)	733 (657 to 810)	732 (662 to 804)	731 (658 to 807)
Arizona	767 (696 to 844)	1334 (1209 to 1466)	1852 (1677 to 2038)	2763 (2493 to 3046)	3755 (3397 to 4137)	5144 (4660 to 5653)	6382 (5788 to 7029)	7124 (6364 to 7942)	7178 (6494 to 7886)	7220 (6480 to 8013)
Arkansas	1938 (1756 to 2123)	1830 (1655 to 2002)	1986 (1797 to 2173)	2311 (2088 to 2526)	2402 (2176 to 2625)	2676 (2428 to 2927)	2910 (2624 to 3187)	3009 (2701 to 3313)	3012 (2731 to 3294)	3011 (2706 to 3311)
California	10867 (9909 to 11895)	16099 (14662 to 17639)	20556 (18761 to 22498)	24100 (21905 to 26421)	30459 (27822 to 33343)	34085 (31071 to 37378)	37411 (34186 to 40969)	39387 (35650 to 43492)	39496 (36003 to 43301)	39561 (35813 to 43641)
Colorado	1355 (1228 to 1484)	1796 (1625 to 1966)	2290 (2078 to 2507)	2940 (2664 to 3218)	3372 (3072 to 3688)	4325 (3922 to 4742)	5037 (4582 to 5502)	5715 (5145 to 6305)	5784 (5240 to 6348)	5850 (5245 to 6448)
Connecticut	2058 (1870 to 2261)	2596 (2349 to 2848)	3113 (2819 to 3411)	3155 (2857 to 3459)	3346 (3044 to 3658)	3391 (3070 to 3722)	3562 (3233 to 3909)	3604 (3228 to 4001)	3600 (3256 to 3963)	3595 (3225 to 3979)
Delaware	326 (296 to 356)	457 (415 to 499)	566 (516 to 618)	602 (546 to 658)	681 (621 to 746)	784 (713 to 859)	905 (824 to 989)	986 (888 to 1088)	992 (901 to 1086)	996 (898 to 1099)
Washington, DC	819 (740 to 903)	783 (708 to 862)	775 (702 to 853)	645 (583 to 709)	616 (558 to 676)	582 (526 to 641)	619 (561 to 682)	685 (616 to 762)	690 (625 to 759)	695 (624 to 771)
Florida	2840 (2551 to 3119)	5069 (4566 to 5545)	7060 (6354 to 7745)	9950 (8938 to 10927)	13257 (11939 to 14544)	15956 (14335 to 17477)	18930 (17092 to 20741)	21355 (18999 to 23627)	21569 (19429 to 23625)	21750 (19400 to 23956)
Georgia	3511 (3180 to 3863)	4042 (3656 to 4441)	4736 (4283 to 5225)	5544 (4998 to 6122)	6635 (6000 to 7300)	8258 (7460 to 9073)	9802 (8860 to 10769)	10666 (9566 to 11860)	10726 (9702 to 11805)	10771 (9657 to 11936)
Hawaii	505 (457 to 553)	649 (586 to 709)	797 (719 to 876)	973 (875 to 1065)	1133 (1022 to 1243)	1218 (1097 to 1335)	1358 (1223 to 1493)	1447 (1293 to 1603)	1452 (1308 to 1594)	1456 (1300 to 1612)
Idaho	600 (543 to 658)	683 (617 to 747)	739 (664 to 811)	958 (862 to 1050)	1032 (932 to 1130)	1294 (1168 to 1419)	1571 (1420 to 1722)	1823 (1630 to 2019)	1848 (1665 to 2026)	1871 (1671 to 2068)
Illinois	8933 (8107 to 9816)	10322 (9350 to 11331)	11419 (10321 to 12570)	11570 (10459 to 12726)	11670 (10564 to 12846)	12267 (11064 to 13525)	12773 (11548 to 14098)	12811 (11445 to 14292)	12785 (11542 to 14116)	12747 (11467 to 14116)
Indiana	4034 (3644 to 4454)	4774 (4302 to 5273)	5346 (4824 to 5905)	5555 (5003 to 6142)	5661 (5114 to 6259)	5995 (5403 to 6639)	6448 (5823 to 7099)	6766 (6048 to 7550)	6786 (6129 to 7510)	6799 (6105 to 7565)
Iowa	2677 (2441 to 2932)	2824 (2558 to 3108)	2908 (2637 to 3206)	2946 (2659 to 3257)	2832 (2572 to 3123)	2888 (2613 to 3190)	3042 (2760 to 3355)	3180 (2861 to 3533)	3189 (2896 to 3511)	3196 (2866 to 3565)
Kansas	1954 (1771 to 2143)	2230 (2016 to 2444)	2312 (2092 to 2532)	2398 (2159 to 2628)	2525 (2288 to 2762)	2662 (2391 to 2917)	2839 (2562 to 3104)	2934 (2620 to 3243)	2936 (2650 to 3209)	2936 (2617 to 3239)
Kentucky	2997 (2721 to 3285)	3114 (2819 to 3411)	3328 (3011 to 3649)	3699 (3342 to 4055)	3766 (3413 to 4125)	4032 (3656 to 4421)	4341 (3937 to 4759)	4498 (4034 to 4979)	4504 (4079 to 4933)	4504 (4041 to 4968)
Louisiana	2744 (2482 to 3017)	3335 (3013 to 3670)	3763 (3399 to 4139)	4261 (3828 to 4687)	4308 (3896 to 4741)	4467 (4028 to 4917)	4531 (4105 to 4972)	4646 (4147 to 5173)	4652 (4197 to 5116)	4652 (4168 to 5143)
Maine	936 (847 to 1031)	993 (895 to 1093)	1026 (928 to 1131)	1141 (1023 to 1256)	1252 (1137 to 1374)	1264 (1142 to 1390)	1335 (1206 to 1470)	1363 (1222 to 1512)	1362 (1230 to 1501)	1360 (1221 to 1508)
Maryland	2398 (2174 to 2649)	3176 (2879 to 3507)	4041 (3659 to 4465)	4283 (3865 to 4734)	4881 (4429 to 5385)	5334 (4836 to 5884)	5841 (5306 to 6436)	6155 (5528 to 6862)	6173 (5583 to 6830)	6186 (5544 to 6860)
Massachusetts	4802 (4354 to 5239)	5275 (4808 to 5762)	5837 (5300 to 6388)	5824 (5283 to 6392)	6125 (5566 to 6695)	6300 (5700 to 6915)	6539 (5946 to 7157)	6979 (6260 to 7713)	7022 (6366 to 7701)	7064 (6374 to 7785)
Michigan	6528 (5947 to 7143)	8006 (7276 to 8729)	9132 (8291 to 10014)	9367 (8494 to 10255)	9484 (8634 to 10378)	9855 (8949 to 10799)	9822 (8918 to 10776)	10047 (9049 to 11122)	10066 (9176 to 11043)	10077 (9097 to 11113)
Minnesota	3048 (2761 to 3345)	3493 (3156 to 3830)	3914 (3530 to 4302)	4138 (3723 to 4547)	4466 (4045 to 4898)	4853 (4380 to 5326)	5283 (4764 to 5798)	5675 (5066 to 6283)	5707 (5150 to 6269)	5737 (5140 to 6337)
Mississippi	2219 (2009 to 2443)	2231 (2017 to 2455)	2289 (2064 to 2521)	2549 (2298 to 2809)	2629 (2381 to 2887)	2837 (2567 to 3127)	2977 (2692 to 3278)	2966 (2648 to 3301)	2957 (2667 to 3261)	2944 (2640 to 3263)
Missouri	4045 (3686 to 4447)	4424 (4032 to 4854)	4809 (4382 to 5292)	4983 (4526 to 5492)	5220 (4762 to 5738)	5538 (5021 to 6126)	5954 (5423 to 6572)	6151 (5567 to 6815)	6155 (5601 to 6775)	6151 (5529 to 6844)
Montana	604 (546 to 664)	691 (625 to 757)	718 (650 to 788)	797 (720 to 876)	815 (743 to 895)	908 (819 to 996)	985 (892 to 1079)	1078 (969 to 1195)	1086 (984 to 1195)	1093 (984 to 1206)
Nebraska	1353 (1223 to 1492)	1445 (1307 to 1596)	1529 (1382 to 1688)	1590 (1431 to 1761)	1611 (1459 to 1780)	1701 (1535 to 1876)	1821 (1647 to 2019)	1950 (1745 to 2177)	1962 (1777 to 2160)	1972 (1767 to 2193)
Nevada	164 (148 to 179)	292 (265 to 321)	508 (462 to 558)	819 (740 to 902)	1243 (1128 to 1366)	2029 (1834 to 2229)	2702 (2447 to 2973)	3083 (2768 to 3424)	3114 (2817 to 3424)	3140 (2819 to 3468)
New Hampshire	545 (494 to 599)	622 (567 to 682)	762 (693 to 837)	936 (849 to 1029)	1131 (1028 to 1241)	1227 (1116 to 1347)	1325 (1202 to 1459)	1375 (1241 to 1528)	1378 (1254 to 1513)	1380 (1246 to 1530)
New Jersey	4957 (4501 to 5464)	6214 (5649 to 6812)	7370 (6716 to 8108)	7470 (6778 to 8215)	7898 (7181 to 8667)	8393 (7637 to 9225)	8771 (7972 to 9637)	9236 (8324 to 10262)	9280 (8436 to 10197)	9318 (8404 to 10310)
New Mexico	697 (629 to 765)	974 (882 to 1065)	1054 (951 to 1158)	1322 (1193 to 1448)	1550 (1403 to 1696)	1823 (1651 to 2001)	2061 (1863 to 2254)	2120 (1901 to 2345)	2118 (1921 to 2323)	2111 (1899 to 2329)

New York	15182 (13746 to 16598)	17191 (15559 to 18794)	18720 (16966 to 20511)	17784 (16054 to 19455)	18332 (16569 to 20063)	18960 (17137 to 20746)	19264 (17382 to 21076)	20087 (17987 to 22157)	20155 (18201 to 22114)	20203 (18124 to 22279)
North Carolina	4132 (3747 to 4548)	4670 (4241 to 5114)	5247 (4760 to 5760)	5960 (5391 to 6561)	6786 (6176 to 7449)	8082 (7320 to 8890)	9616 (8728 to 10578)	10410 (9354 to 11565)	10457 (9461 to 11509)	10491 (9446 to 11580)
North Dakota	632 (571 to 695)	648 (582 to 711)	636 (573 to 701)	661 (595 to 730)	649 (586 to 714)	633 (571 to 695)	677 (612 to 745)	770 (688 to 858)	779 (704 to 858)	788 (705 to 874)
Ohio	8173 (7398 to 8967)	9935 (8957 to 10853)	10950 (9881 to 11965)	10942 (9822 to 12008)	11054 (9961 to 12111)	11228 (10110 to 12312)	11451 (10372 to 12540)	11772 (10494 to 13015)	11790 (10666 to 12907)	11797 (10550 to 13026)
Oklahoma	2271 (2052 to 2493)	2386 (2155 to 2613)	2644 (2393 to 2900)	3070 (2754 to 3375)	3209 (2902 to 3512)	3449 (3118 to 3788)	3721 (3372 to 4082)	3946 (3530 to 4351)	3962 (3572 to 4346)	3973 (3552 to 4377)
Oregon	1552 (1402 to 1707)	1811 (1635 to 1988)	2161 (1949 to 2376)	2669 (2403 to 2930)	2914 (2640 to 3201)	3418 (3073 to 3758)	3841 (3480 to 4223)	4209 (3769 to 4675)	4241 (3835 to 4659)	4270 (3834 to 4728)
Pennsylvania	10744 (9744 to 11774)	11594 (10479 to 12688)	12118 (10948 to 13284)	12020 (10855 to 13153)	12103 (10993 to 13243)	12193 (11020 to 13333)	12720 (11532 to 13919)	12988 (11635 to 14380)	12989 (11778 to 14181)	12976 (11679 to 14318)
Rhode Island	810 (736 to 887)	881 (801 to 964)	977 (885 to 1072)	961 (870 to 1053)	1023 (932 to 1123)	1038 (944 to 1138)	1046 (954 to 1147)	1092 (987 to 1204)	1096 (997 to 1203)	1101 (991 to 1216)
South Carolina	2152 (1947 to 2356)	2442 (2210 to 2682)	2679 (2433 to 2937)	3165 (2862 to 3469)	3566 (3254 to 3896)	4014 (3628 to 4402)	4651 (4219 to 5092)	5097 (4578 to 5647)	5131 (4662 to 5618)	5156 (4640 to 5711)
South Dakota	667 (604 to 730)	697 (632 to 762)	686 (622 to 752)	698 (631 to 767)	709 (643 to 777)	746 (676 to 817)	817 (742 to 895)	883 (794 to 977)	887 (806 to 972)	891 (799 to 981)
Tennessee	3352 (3044 to 3678)	3655 (3307 to 4015)	4054 (3666 to 4446)	4644 (4185 to 5109)	4991 (4527 to 5502)	5680 (5139 to 6245)	6369 (5781 to 6994)	6883 (6181 to 7642)	6920 (6270 to 7605)	6949 (6247 to 7695)
Texas	7891 (7137 to 8638)	9810 (8862 to 10705)	11598 (10481 to 12725)	14478 (13032 to 15869)	17383 (15742 to 19056)	20987 (19022 to 22954)	25490 (23166 to 27876)	28931 (26034 to 31941)	29218 (26541 to 31972)	29468 (26501 to 32430)
Utah	703 (634 to 768)	912 (822 to 993)	1098 (993 to 1198)	1486 (1339 to 1625)	1765 (1596 to 1926)	2243 (2020 to 2451)	2764 (2497 to 3018)	3235 (2895 to 3565)	3282 (2961 to 3584)	3328 (2976 to 3662)
Vermont	385 (349 to 425)	400 (361 to 440)	458 (414 to 507)	519 (467 to 574)	574 (521 to 633)	603 (544 to 667)	634 (574 to 699)	643 (577 to 714)	642 (580 to 710)	642 (575 to 714)
Virginia	3390 (3068 to 3745)	4066 (3670 to 4482)	4795 (4345 to 5288)	5436 (4908 to 6011)	6323 (5719 to 6974)	7139 (6459 to 7878)	8065 (7286 to 8859)	8600 (7709 to 9608)	8633 (7815 to 9517)	8655 (7782 to 9637)
Washington	2434 (2210 to 2678)	2922 (2649 to 3206)	3514 (3185 to 3861)	4203 (3809 to 4614)	4991 (4522 to 5497)	5900 (5325 to 6500)	6743 (6120 to 7403)	7631 (6860 to 8513)	7719 (7024 to 8497)	7803 (7028 to 8644)
West Virginia	2040 (1849 to 2245)	1906 (1732 to 2087)	1799 (1625 to 1982)	1971 (1780 to 2168)	1826 (1658 to 2004)	1794 (1626 to 1974)	1828 (1661 to 2013)	1798 (1610 to 2002)	1790 (1622 to 1972)	1778 (1594 to 1973)
Wisconsin	3513 (3172 to 3863)	4045 (3657 to 4437)	4540 (4087 to 4982)	4772 (4286 to 5241)	4990 (4512 to 5479)	5299 (4796 to 5814)	5678 (5130 to 6234)	5884 (5259 to 6538)	5892 (5325 to 6471)	5895 (5274 to 6523)
Wyoming	296 (268 to 328)	338 (305 to 371)	343 (309 to 379)	479 (430 to 528)	462 (417 to 510)	493 (442 to 544)	561 (506 to 617)	578 (515 to 644)	577 (520 to 637)	576 (513 to 641)
Southern Latin America	25712 (24530 to 26991)	30864 (29407 to 32308)	36116 (34360 to 37905)	42930 (40852 to 45090)	49538 (47015 to 52108)	55241 (52389 to 58209)	61231 (58115 to 64542)	66843 (61118 to 72802)	67359 (61028 to 73392)	67694 (61443 to 74178)
Argentina	17565 (16449 to 18825)	20651 (19186 to 21979)	24110 (22455 to 25716)	28761 (26837 to 30679)	33111 (30771 to 35419)	36797 (34190 to 39646)	41110 (38539 to 43898)	45006 (39411 to 50951)	45284 (39065 to 51180)	45487 (39215 to 51850)
Chile	5892 (5489 to 6279)	7628 (7104 to 8133)	9197 (8582 to 9827)	11212 (10283 to 12141)	13286 (12245 to 14359)	15143 (13878 to 16348)	16759 (14882 to 18563)	18420 (16957 to 19857)	18661 (17015 to 20371)	18798 (17096 to 20570)
Uruguay	2253 (2095 to 2406)	2583 (2359 to 2800)	2807 (2530 to 3095)	2954 (2662 to 3241)	3139 (2824 to 3483)	3298 (2989 to 3605)	3359 (3126 to 3601)	3412 (3010 to 3850)	3411 (3003 to 3838)	3406 (2987 to 3864)
Western Europe	311049 (299458 to 321971)	331906 (322917 to 340492)	356704 (347425 to 365169)	373214 (363827 to 382404)	384413 (376798 to 391436)	398473 (390838 to 405423)	421803 (415243 to 427581)	436205 (422886 to 448459)	436929 (423213 to 449960)	437389 (422281 to 450957)
Andorra	6 (5 to 6)	10 (9 to 11)	19 (17 to 21)	34 (31 to 38)	54 (54 to 55)	66 (65 to 66)	84 (83 to 85)	83 (76 to 90)	84 (77 to 92)	86 (78 to 94)
Austria	6910 (6455 to 7361)	7041 (6523 to 7562)	7426 (6887 to 7992)	7538 (7014 to 8105)	7768 (7226 to 8325)	8018 (7448 to 8595)	8371 (8300 to 8436)	8901 (8155 to 9650)	8944 (8168 to 9711)	8982 (8089 to 9783)
Belgium	8616 (8037 to 9163)	9125 (8482 to 9821)	9647 (9003 to 10302)	9832 (9081 to 10615)	9979 (9171 to 10741)	10257 (9508 to 11033)	10875 (10798 to 10956)	11415 (10533 to 12313)	11444 (10478 to 12456)	11469 (10320 to 12562)
Cyprus	489 (454 to 522)	592 (551 to 634)	650 (564 to 734)	670 (613 to 727)	778 (718 to 839)	918 (851 to 983)	1122 (1035 to 1207)	1319 (1167 to 1483)	1339 (1178 to 1504)	1358 (1173 to 1539)
Denmark	4273 (3975 to 4548)	4588 (4283 to 4910)	4931 (4583 to 5266)	5117 (5082 to 5150)	5144 (5106 to 5183)	5335 (5292 to 5378)	5535 (5487 to 5582)	5800 (5328 to 6260)	5827 (5266 to 6345)	5852 (5305 to 6409)
Finland	4027 (3742 to 4315)	4433 (4132 to 4727)	4632 (4609 to 4658)	4801 (4770 to 4831)	5010 (4978 to 5042)	5189 (5152 to 5227)	5383 (5342 to 5424)	5531 (5083 to 5988)	5534 (5063 to 6021)	5536 (4950 to 6058)
France	43144 (40166 to 46068)	46788 (43042 to 50538)	51902 (47905 to 56038)	54965 (50572 to 59182)	57771 (53647 to 61442)	59908 (55483 to 64350)	63753 (59530 to 67987)	66143 (60038 to 72367)	66282 (60015 to 72976)	66390 (59549 to 73473)
Germany	71368 (61506 to 80865)	73371 (68057 to 78632)	77452 (71972 to 82314)	78689 (73667 to 84101)	79941 (79371 to 80546)	82315 (81624 to 83040)	81640 (80936 to 82403)	84956 (77725 to 92266)	85202 (77264 to 93022)	85372 (76248 to 93979)
Greece	7774 (7258 to 8281)	8591 (7944 to 9213)	8935 (8263 to 9587)	9846 (9142 to 10584)	10389 (9615 to 11174)	11089 (10270 to 11918)	11073 (10301 to 11816)	10317 (9053 to 11467)	10250 (8910 to 11444)	10175 (8728 to 11509)
Iceland	141 (140 to 142)	174 (173 to 174)	204 (203 to 205)	227 (226 to 229)	254 (252 to 256)	279 (277 to 282)	318 (316 to 321)	344 (316 to 373)	347 (315 to 377)	350 (318 to 384)
Ireland	3050 (2854 to 3248)	2903 (2686 to 3123)	3034 (2833 to 3240)	3491 (3218 to 3758)	3602 (3335 to 3862)	3867 (3559 to 4169)	4599 (4233 to 4976)	4904 (4477 to 5349)	4923 (4457 to 5398)	4941 (4420 to 5452)
Israel	1557 (1451 to 1663)	2169 (2000 to 2326)	3041 (2805 to 3286)	3875 (3561 to 4213)	4961 (4472 to 5454)	6390 (5762 to 7074)	7846 (7196 to 8503)	9284 (8142 to 10522)	9439 (8105 to 10727)	9594 (8200 to 11048)
Italy	46705 (43482 to 49713)	50900 (46789 to 54813)	53864 (49829 to 57803)	56421 (52176 to 60403)	56799 (52809 to 60686)	56659 (52417 to 60668)	60325 (59855 to 60762)	60295 (55339 to 64965)	60073 (54656 to 65166)	59811 (54355 to 65105)
Luxembourg	302 (281 to 322)	315 (294 to 336)	340 (317 to 363)	362 (334 to 390)	381 (352 to 408)	434 (401 to 466)	503 (499 to 507)	618 (568 to 666)	631 (573 to 685)	644 (589 to 703)
Malta	334 (312 to 357)	330 (302 to 359)	323 (294 to 353)	340 (307 to 374)	371 (333 to 408)	402 (363 to 442)	424 (391 to 455)	440 (390 to 490)	441 (385 to 493)	442 (384 to 500)
Monaco	21 (20 to 23)	22 (20 to 23)	23 (22 to 26)	27 (25 to 29)	30 (28 to 32)	33 (31 to 35)	36 (32 to 39)	38 (34 to 41)	38 (34 to 41)	38 (34 to 41)
Netherlands	10038 (9981 to 10090)	11417 (11354 to 11480)	12979 (12906 to 13057)	14092 (13990 to 14186)	14922 (14813 to 15033)	15889 (15760 to 16021)	16593 (16444 to 16746)	17136 (15656 to 18591)	17176 (15721 to 18694)	17211 (15589 to 18893)
Norway	3278 (3069 to 3514)	3592 (3356 to 3839)	3885 (3621 to 4154)	4090 (3837 to 4378)	4246 (3965 to 4539)	4480 (4444 to 4517)	4864 (4824 to 4909)	5346 (4934 to 5752)	5383 (4927 to 5883)	5418 (4934 to 5963)
Agder	173 (156 to 191)	186 (169 to 204)	205 (186 to 226)	227 (207 to 250)	242 (225 to 258)	258 (255 to 261)	279 (276 to 282)	305 (272 to 341)	307 (271 to 346)	309 (270 to 350)
Innlandet	333 (303 to 366)	343 (312 to 378)	352 (319 to 387)	368 (334 to 405)	371 (346 to 397)	370 (366 to 375)	377 (372 to 381)	387 (346 to 433)	388 (342 to 438)	388 (339 to 441)
Møre og Romsdal	192 (174 to 210)	213 (193 to 234)	224 (203 to 246)	236 (215 to 258)	239 (223 to 256)	243 (241 to 246)	252 (249 to 255)	269 (241 to 300)	270 (239 to 304)	271 (236 to 307)
Nordland	221 (200 to 244)	237 (215 to 260)	241 (218 to 266)	245 (222 to 268)	240 (224 to 257)	239 (236 to 242)	237 (234 to 240)	244 (216 to 272)	244 (214 to 276)	245 (213 to 278)
Oslo	435 (395 to 476)	475 (432 to 520)	479 (433 to 525)	452 (411 to 496)	461 (430 to 493)	508 (502 to 514)	588 (581 to 595)	687 (612 to 762)	695 (613 to 781)	704 (615 to 796)
Rogaland	211 (192 to 232)	239 (218 to 262)	269 (244 to 296)	305 (278 to 334)	336 (314 to 360)	373 (369 to 377)	428 (423 to 433)	480 (430 to 534)	483 (427 to 544)	486 (427 to 550)
Troms og Finnmark	182 (165 to 200)	200 (182 to 219)	213 (193 to 234)	225 (205 to 247)	221 (207 to 237)	225 (223 to 228)	230 (227 to 232)	244 (218 to 273)	245 (216 to 276)	246 (215 to 280)
Trøndelag	307 (278 to 336)	328 (298 to 359)	352 (319 to 386)	370 (336 to 405)	378 (352 to 405)	390 (386 to 395)	423 (417 to 428)	462 (411 to 511)	465 (409 to 522)	468 (408 to 526)
Vestfold og Telemark	291 (264 to 321)	324 (294 to 355)	333 (301 to 366)	349 (317 to 382)	361 (337 to 387)	378 (374 to 383)	400 (395 to 405)	424 (377 to 474)	426 (375 to 480)	427 (372 to 485)
Vestland	408 (373 to 446)	441 (401 to 481)	474 (430 to 519)	497 (453 to 542)	517 (482 to 553)	543 (537 to 549)	585 (578 to 592)	640 (573 to 711)	644 (569 to 723)	647 (568 to 731)
Viken	524 (478 to 571)	605 (553 to 658)	742 (675 to 810)	817 (749 to 889)	879 (820 to 940)	952 (942 to 962)	1067 (1055 to 1080)	1204 (1080 to 1324)	1216 (1086 to 1358)	1228 (1092 to 1380)
Portugal	8758 (8139 to 9358)	9196 (8588 to 9845)	8901 (8275 to 9526)	10013 (9254 to 10732)	10137 (9355 to 10881)	10539 (9784 to 11300)	10791 (10028 to 11537)	10671 (9450 to 11931)	10643 (9408 to 12003)	10608 (9230 to 12049)
San Marino	15 (14 to 16)	16 (14 to 19)	18 (16 to 20)	21 (19 to 22)	24 (20 to 28)	27 (24 to 31)	31 (29 to 33)	33 (28 to 37)	33 (28 to 37)	33 (28 to 37)
Spain	27972 (26017 to 29902)	30535 (28533 to 32641)	34039 (31827 to 36458)	37438 (34695 to 40228)	38782 (36192 to 41731)	40809 (40549 to 41051)	46976 (46669 to 47279)	46003 (42071 to 49961)	45781 (41757 to 49864)	45549 (40965 to 49913)
Sweden	7038 (6546 to 7533)	7501 (7010 to 8010)	8046 (7994 to 8095)	8310 (8256 to 8368)	8588 (8527 to 8650)	8902 (8830 to 8976)	9424 (9342 to 9496)	10223 (9312 to 11128)	10300 (9327 to 11284)	10374 (9389 to 11397)
Stockholm	1079 (969 to 1189)	1199 (1077 to 1321)	1428 (1414 to 1441)	1489 (1473 to 1504)	1596 (1579 to 1613)	1765 (1744 to 1787)	1997 (1972 to 2022)	2306 (2025 to 2596)	2336 (2019 to 2673)	2366 (2040 to 2704)
Sweden except Stockholm	5959 (5533 to 6392)	6302 (5861 to 6744)	6618 (6574 to 6659)	6821 (6774 to 6870)	6992 (6940 to 7044)	7137 (7075 to 7198)	7426 (7359 to 7488)	7917 (7180 to 8663)	7964 (7163 to 8791)	8007 (7202 to 8846)
Switzerland	4718 (4403 to 5051)	5430 (5050 to 5801)	6256 (5820 to 6668)	6388 (5969 to 6826)	6866 (6418 to 7319)	7300 (6820 to 7764)	7963 (7887 to 8033)	8777 (8023 to 9566)	8851 (8016 to 9651)	8923 (8054 to 9863)
UK	50289 (47563 to 53040)	52634 (49453 to 55691)	55893 (52652 to 59288)	56334 (52744 to 59566)	57299 (53696 to 60888)	59038 (55364 to 62630)	62915 (58941 to 66829)	67250 (62548 to 71766)	67579 (63179 to 71901)	67848 (63909 to 71608)
England	40966 (38369 to 43654)	43172 (40011 to 46201)	46167 (43027 to 49503)	46776 (43145 to 49970)	47721 (44189 to 51341)	49249 (45611 to 52861)	52646 (48618 to 56630)	56654 (51965 to 61208)	56978 (52651 to 61240)	57250 (53397 to 60934)
East Midlands	3202 (2906 to 3524)	3362 (2891 to 3869)	3645 (3286 to 4030)	3839 (3465 to 4239)	3998 (3615 to 4428)	4173 (3768 to 4614)	4501 (4051 to 4985)	4853 (4187 to 5565)	4880 (4171 to 5591)	4903 (4208 to 5615)
Derby	193 (171 to 218)	206 (168 to 249)	220 (193 to 251)	218 (191 to 248)	222 (194 to 253)	230 (202 to 263)	247 (216 to 280)	267 (221 to 320)	269 (219 to 323)	270 (221 to 326)

Derbyshire	611 (544 to 679)	633 (518 to 755)	674 (591 to 757)	696 (610 to 782)	713 (626 to 802)	734 (647 to 825)	768 (673 to 863)	806 (666 to 952)	808 (664 to 961)	810 (667 to 970)
Leicester	261 (233 to 291)	273 (226 to 329)	288 (253 to 325)	284 (250 to 321)	282 (249 to 319)	281 (248 to 318)	323 (285 to 366)	374 (311 to 448)	379 (313 to 456)	384 (318 to 464)
Leicestershire	404 (362 to 452)	431 (355 to 519)	484 (425 to 547)	538 (477 to 606)	570 (503 to 644)	607 (538 to 685)	647 (571 to 731)	685 (571 to 813)	688 (561 to 826)	691 (573 to 824)
Lincolnshire	455 (407 to 511)	465 (382 to 560)	502 (443 to 567)	550 (485 to 622)	585 (518 to 663)	643 (569 to 726)	710 (625 to 806)	767 (638 to 914)	771 (635 to 922)	774 (640 to 926)
Northamptonshire	411 (368 to 460)	429 (356 to 509)	472 (418 to 533)	527 (465 to 594)	579 (512 to 652)	627 (556 to 705)	687 (607 to 776)	751 (632 to 884)	756 (630 to 895)	760 (635 to 901)
Nottingham	268 (240 to 301)	288 (237 to 346)	302 (267 to 341)	280 (248 to 316)	280 (248 to 316)	268 (238 to 303)	298 (264 to 337)	335 (280 to 400)	338 (278 to 404)	341 (281 to 410)
Nottinghamshire	576 (512 to 643)	615 (504 to 733)	676 (595 to 759)	714 (628 to 801)	734 (648 to 826)	749 (661 to 840)	783 (690 to 883)	828 (687 to 975)	831 (678 to 987)	833 (682 to 991)
Rutland	22 (20 to 25)	24 (20 to 29)	28 (24 to 31)	33 (29 to 37)	33 (29 to 37)	34 (30 to 39)	37 (33 to 42)	40 (32 to 47)	40 (32 to 48)	40 (32 to 48)
East of England	3795 (3450 to 4165)	3998 (3434 to 4585)	4406 (3979 to 4872)	4819 (4328 to 5322)	5096 (4589 to 5633)	5370 (4845 to 5925)	5809 (5219 to 6426)	6293 (5440 to 7175)	6334 (5464 to 7235)	6370 (5478 to 7264)
Bedford	103 (91 to 115)	111 (90 to 135)	124 (109 to 140)	133 (116 to 151)	135 (118 to 153)	147 (129 to 166)	157 (137 to 177)	167 (136 to 198)	168 (136 to 200)	168 (135 to 203)
Cambridgeshire	338 (302 to 378)	356 (291 to 428)	397 (351 to 448)	449 (395 to 508)	504 (445 to 570)	549 (485 to 618)	613 (539 to 693)	679 (562 to 807)	685 (564 to 819)	690 (567 to 825)
Central Bedfordshire	142 (126 to 160)	153 (124 to 185)	178 (156 to 202)	209 (183 to 238)	220 (192 to 249)	232 (203 to 265)	253 (221 to 288)	278 (227 to 333)	280 (226 to 340)	282 (229 to 341)
Essex	919 (821 to 1019)	959 (788 to 1139)	1066 (943 to 1191)	1188 (1050 to 1330)	1245 (1100 to 1393)	1307 (1156 to 1459)	1388 (1229 to 1554)	1474 (1228 to 1730)	1481 (1233 to 1751)	1488 (1229 to 1756)
Hertfordshire	756 (675 to 843)	833 (686 to 998)	925 (817 to 1040)	966 (849 to 1090)	979 (862 to 1100)	1028 (909 to 1154)	1107 (975 to 1243)	1203 (1007 to 1421)	1212 (1009 to 1436)	1220 (1012 to 1451)
Luton	128 (114 to 144)	142 (117 to 174)	160 (141 to 182)	165 (145 to 188)	172 (153 to 197)	185 (164 to 210)	201 (177 to 230)	222 (186 to 269)	224 (186 to 272)	225 (185 to 277)
Norfolk	571 (508 to 633)	579 (472 to 687)	624 (551 to 700)	697 (614 to 782)	751 (664 to 844)	796 (702 to 889)	855 (752 to 963)	910 (757 to 1070)	913 (754 to 1078)	916 (749 to 1089)
Peterborough	90 (80 to 101)	94 (76 to 114)	106 (93 to 120)	131 (115 to 148)	153 (134 to 173)	157 (138 to 177)	181 (158 to 204)	213 (175 to 254)	216 (178 to 259)	219 (179 to 263)
Southend-on-Sea	171 (152 to 194)	169 (137 to 204)	165 (145 to 188)	158 (139 to 180)	161 (141 to 184)	161 (141 to 183)	173 (151 to 197)	190 (157 to 227)	191 (155 to 230)	193 (156 to 232)
Suffolk	478 (424 to 536)	493 (400 to 590)	536 (470 to 603)	596 (521 to 672)	646 (567 to 728)	668 (585 to 752)	724 (633 to 816)	783 (644 to 925)	788 (647 to 932)	792 (647 to 940)
Thurrock	99 (88 to 111)	110 (89 to 135)	124 (108 to 140)	128 (112 to 145)	128 (112 to 146)	142 (125 to 160)	157 (137 to 178)	174 (143 to 208)	176 (143 to 211)	177 (145 to 213)
Greater London	6709 (6133 to 7324)	7259 (6316 to 8244)	7524 (6825 to 8246)	6840 (6175 to 7515)	6772 (6123 to 7459)	7238 (6562 to 7932)	8074 (7256 to 8875)	9040 (7887 to 10283)	9127 (7949 to 10374)	9205 (7987 to 10428)
Barking and Dagenham	156 (140 to 175)	167 (137 to 200)	173 (152 to 195)	163 (144 to 184)	155 (137 to 175)	164 (145 to 186)	184 (162 to 208)	214 (178 to 255)	217 (179 to 259)	220 (181 to 263)
Barnet	274 (244 to 306)	294 (243 to 353)	307 (272 to 346)	296 (260 to 333)	295 (261 to 334)	316 (279 to 355)	352 (310 to 398)	394 (328 to 467)	398 (330 to 475)	401 (332 to 479)
Bexley	182 (163 to 205)	200 (166 to 241)	218 (193 to 247)	218 (192 to 247)	218 (192 to 248)	218 (193 to 247)	231 (203 to 264)	250 (208 to 298)	252 (209 to 301)	253 (209 to 306)
Brent	238 (212 to 265)	262 (214 to 309)	277 (243 to 310)	250 (219 to 281)	239 (210 to 269)	265 (233 to 297)	307 (269 to 345)	349 (290 to 410)	353 (291 to 417)	357 (292 to 423)
Bromley	262 (234 to 293)	288 (237 to 344)	307 (271 to 347)	300 (264 to 338)	293 (257 to 331)	295 (261 to 333)	309 (270 to 351)	331 (276 to 395)	333 (275 to 398)	335 (274 to 402)
Camden	201 (180 to 223)	207 (172 to 246)	208 (184 to 233)	179 (158 to 201)	178 (157 to 201)	199 (176 to 223)	217 (191 to 244)	231 (192 to 274)	232 (193 to 276)	233 (191 to 278)
Croydon	288 (257 to 320)	316 (262 to 370)	337 (297 to 377)	322 (284 to 360)	314 (277 to 352)	332 (294 to 372)	361 (316 to 406)	399 (332 to 464)	402 (333 to 470)	405 (333 to 475)
Ealing	271 (243 to 304)	292 (243 to 346)	306 (271 to 344)	286 (253 to 323)	281 (249 to 318)	304 (269 to 343)	335 (296 to 377)	368 (309 to 435)	371 (310 to 440)	373 (309 to 445)
Enfield	239 (214 to 267)	254 (211 to 304)	267 (238 to 300)	262 (232 to 295)	259 (229 to 293)	275 (244 to 310)	309 (273 to 350)	351 (293 to 419)	355 (293 to 425)	359 (296 to 429)
Greenwich	192 (172 to 214)	204 (168 to 243)	215 (191 to 242)	215 (189 to 241)	211 (186 to 236)	216 (192 to 242)	250 (221 to 281)	296 (244 to 349)	300 (249 to 354)	304 (252 to 361)
Hackney	202 (180 to 226)	222 (183 to 265)	227 (200 to 257)	194 (170 to 219)	188 (166 to 213)	211 (185 to 237)	249 (218 to 281)	288 (238 to 342)	291 (240 to 345)	293 (241 to 350)
Hammersmith and Fulham	182 (162 to 204)	194 (157 to 232)	192 (168 to 216)	152 (132 to 171)	151 (132 to 171)	167 (146 to 188)	180 (156 to 203)	191 (158 to 227)	192 (156 to 228)	192 (157 to 229)
Haringey	217 (193 to 240)	235 (196 to 279)	243 (215 to 272)	209 (184 to 234)	205 (181 to 231)	218 (192 to 244)	251 (219 to 282)	284 (236 to 334)	287 (240 to 338)	290 (238 to 343)
Harrow	180 (160 to 200)	196 (160 to 233)	207 (183 to 232)	200 (176 to 225)	202 (178 to 228)	208 (183 to 235)	236 (208 to 267)	274 (227 to 325)	278 (230 to 329)	281 (229 to 334)
Havering	197 (176 to 220)	224 (186 to 272)	247 (219 to 279)	243 (215 to 275)	232 (205 to 263)	225 (199 to 254)	236 (208 to 269)	259 (217 to 309)	261 (217 to 313)	263 (218 to 316)
Hillingdon	197 (175 to 219)	220 (181 to 261)	238 (210 to 267)	234 (205 to 262)	233 (205 to 262)	243 (214 to 273)	270 (237 to 305)	308 (256 to 363)	311 (259 to 367)	315 (260 to 373)
Hounslow	180 (161 to 200)	193 (160 to 228)	204 (181 to 229)	201 (177 to 224)	202 (179 to 227)	213 (189 to 239)	249 (220 to 279)	292 (244 to 342)	296 (246 to 346)	299 (249 to 353)
Islington	182 (164 to 203)	201 (166 to 241)	203 (180 to 229)	166 (147 to 187)	169 (150 to 191)	177 (158 to 200)	201 (177 to 227)	230 (193 to 273)	232 (194 to 276)	234 (194 to 279)
Kensington and Chelsea	183 (163 to 204)	195 (160 to 231)	196 (173 to 220)	143 (125 to 160)	141 (124 to 159)	160 (141 to 180)	158 (139 to 178)	154 (128 to 181)	154 (128 to 183)	153 (126 to 182)
Kingston upon Thames	127 (113 to 141)	136 (111 to 161)	141 (124 to 159)	135 (118 to 151)	135 (118 to 151)	147 (129 to 165)	159 (139 to 178)	171 (141 to 201)	172 (142 to 201)	173 (142 to 205)
Lambeth	278 (246 to 311)	305 (246 to 364)	309 (271 to 349)	254 (222 to 287)	252 (221 to 285)	270 (237 to 303)	299 (261 to 338)	326 (269 to 385)	328 (268 to 387)	329 (268 to 393)
Lewisham	244 (218 to 272)	266 (217 to 317)	274 (242 to 307)	240 (211 to 270)	238 (210 to 269)	252 (223 to 283)	274 (241 to 308)	301 (251 to 355)	303 (250 to 361)	306 (251 to 364)
Merton	166 (149 to 185)	177 (146 to 210)	182 (161 to 203)	169 (148 to 190)	169 (149 to 191)	189 (167 to 212)	199 (175 to 224)	210 (174 to 247)	211 (173 to 249)	211 (173 to 250)
Newham	207 (185 to 231)	228 (189 to 274)	238 (210 to 268)	214 (188 to 242)	214 (188 to 243)	245 (215 to 277)	302 (266 to 342)	365 (304 to 434)	370 (306 to 444)	375 (311 to 451)
Redbridge	211 (188 to 235)	226 (187 to 269)	235 (208 to 265)	223 (197 to 252)	221 (195 to 250)	239 (212 to 270)	276 (244 to 313)	325 (271 to 386)	330 (274 to 389)	334 (276 to 398)
Richmond upon Thames	170 (152 to 191)	180 (148 to 213)	182 (161 to 205)	165 (146 to 186)	165 (146 to 187)	172 (153 to 195)	185 (163 to 210)	199 (166 to 234)	200 (165 to 236)	200 (165 to 238)
Southwark	234 (209 to 261)	261 (215 to 309)	264 (234 to 295)	221 (195 to 248)	224 (197 to 251)	252 (223 to 283)	283 (250 to 318)	313 (260 to 370)	315 (261 to 373)	317 (262 to 377)
Sutton	152 (136 to 170)	162 (132 to 191)	170 (150 to 191)	170 (150 to 192)	169 (148 to 191)	180 (159 to 202)	190 (167 to 215)	203 (168 to 239)	204 (169 to 241)	205 (168 to 243)
Tower Hamlets	155 (139 to 173)	169 (140 to 201)	168 (150 to 189)	145 (128 to 163)	162 (143 to 183)	196 (174 to 220)	248 (219 to 279)	298 (248 to 349)	302 (252 to 356)	306 (252 to 361)
Waltham Forest	219 (195 to 244)	230 (190 to 271)	236 (209 to 264)	219 (193 to 246)	215 (190 to 242)	220 (196 to 247)	254 (224 to 286)	299 (249 to 353)	304 (251 to 359)	308 (254 to 365)
Wandsworth	285 (256 to 317)	305 (254 to 365)	308 (274 to 344)	264 (234 to 297)	259 (231 to 291)	268 (238 to 301)	301 (267 to 338)	338 (283 to 399)	341 (286 to 403)	344 (285 to 408)
Westminster	237 (211 to 265)	251 (206 to 299)	243 (215 to 273)	190 (167 to 214)	183 (160 to 206)	200 (176 to 226)	217 (190 to 245)	231 (192 to 274)	232 (193 to 275)	233 (192 to 277)
North East England	2382 (2153 to 2630)	2521 (2143 to 2895)	2683 (2414 to 2977)	2648 (2365 to 2937)	2596 (2316 to 2892)	2548 (2279 to 2825)	2591 (2302 to 2889)	2673 (2285 to 3070)	2677 (2277 to 3078)	2678 (2271 to 3065)
County Durham	461 (410 to 517)	482 (392 to 577)	512 (450 to 579)	514 (451 to 579)	504 (441 to 570)	495 (435 to 559)	512 (448 to 579)	532 (439 to 635)	533 (439 to 636)	533 (436 to 636)
Darlington	91 (80 to 101)	93 (75 to 111)	98 (85 to 110)	99 (86 to 111)	100 (86 to 112)	98 (85 to 110)	105 (91 to 118)	113 (93 to 134)	114 (92 to 136)	114 (93 to 137)
Gateshead	204 (181 to 228)	215 (175 to 258)	226 (199 to 255)	215 (188 to 244)	203 (178 to 230)	192 (169 to 217)	199 (174 to 225)	212 (173 to 253)	212 (174 to 255)	213 (173 to 255)
Hartlepool	86 (77 to 97)	93 (76 to 112)	100 (89 to 113)	96 (84 to 109)	92 (81 to 104)	90 (80 to 103)	92 (81 to 105)	94 (78 to 112)	94 (78 to 113)	95 (78 to 114)
Middlesbrough	133 (117 to 150)	147 (118 to 179)	160 (139 to 181)	152 (131 to 173)	145 (126 to 166)	142 (123 to 161)	139 (120 to 158)	139 (114 to 167)	139 (112 to 170)	139 (112 to 169)
Newcastle upon Tyne	294 (260 to 331)	308 (250 to 371)	315 (275 to 356)	287 (250 to 325)	276 (240 to 313)	266 (232 to 302)	276 (240 to 314)	291 (238 to 348)	292 (239 to 351)	293 (239 to 353)
North Tyneside	184 (164 to 207)	197 (161 to 238)	207 (183 to 235)	200 (175 to 226)	195 (171 to 223)	192 (169 to 218)	200 (174 to 228)	213 (175 to 255)	214 (174 to 255)	214 (175 to 257)
Northumberland	262 (232 to 294)	267 (216 to 323)	282 (247 to 318)	298 (261 to 337)	306 (267 to 346)	308 (269 to 348)	316 (275 to 358)	324 (264 to 387)	324 (264 to 392)	324 (262 to 389)
Redcar and Cleveland	121 (106 to 137)	131 (105 to 159)	147 (128 to 167)	151 (131 to 173)	147 (128 to 168)	140 (122 to 160)	136 (118 to 155)	135 (109 to 162)	134 (108 to 162)	134 (108 to 162)
South Tyneside	160 (141 to 180)	171 (137 to 206)	179 (157 to 204)	164 (143 to 186)	157 (137 to 179)	154 (135 to 175)	149 (129 to 169)	148 (120 to 177)	148 (119 to 179)	148 (119 to 178)

Stockton-on-Tees	131 (116 to 147)	144 (119 to 174)	163 (144 to 184)	174 (152 to 196)	175 (153 to 198)	183 (161 to 207)	191 (167 to 217)	199 (164 to 237)	199 (164 to 240)	200 (164 to 239)
Sunderland	255 (227 to 285)	273 (224 to 328)	295 (260 to 333)	299 (262 to 338)	297 (261 to 336)	287 (251 to 324)	277 (241 to 313)	273 (227 to 325)	272 (224 to 325)	271 (224 to 326)
North West England	6404 (5838 to 7020)	6722 (5809 to 7676)	7117 (6439 to 7819)	6968 (6257 to 7668)	6852 (6172 to 7565)	6778 (6119 to 7461)	7017 (6299 to 7732)	7403 (6441 to 8458)	7431 (6461 to 8497)	7450 (6437 to 8532)
Blackburn with Darwen	136 (121 to 153)	138 (114 to 165)	142 (126 to 161)	143 (126 to 162)	138 (122 to 156)	138 (122 to 156)	147 (129 to 166)	158 (131 to 188)	159 (131 to 189)	159 (131 to 194)
Blackpool	177 (159 to 198)	163 (134 to 196)	155 (136 to 175)	150 (132 to 169)	149 (132 to 169)	143 (127 to 162)	142 (125 to 161)	145 (120 to 174)	145 (120 to 175)	146 (119 to 176)
Bolton	243 (216 to 270)	249 (204 to 295)	262 (232 to 294)	263 (231 to 296)	262 (230 to 294)	262 (232 to 293)	275 (243 to 309)	297 (248 to 349)	298 (247 to 353)	300 (248 to 358)
Bury	155 (139 to 174)	163 (132 to 196)	175 (154 to 198)	177 (156 to 200)	178 (157 to 201)	181 (160 to 204)	185 (162 to 209)	193 (160 to 231)	194 (160 to 232)	194 (158 to 235)
Cheshire East	269 (240 to 302)	284 (233 to 339)	309 (273 to 349)	327 (286 to 368)	339 (297 to 382)	351 (308 to 396)	368 (322 to 416)	387 (323 to 458)	388 (323 to 460)	389 (320 to 464)
Cheshire West and Chester	248 (221 to 278)	267 (218 to 323)	298 (262 to 336)	310 (274 to 350)	312 (275 to 354)	321 (284 to 363)	329 (289 to 373)	336 (279 to 402)	337 (278 to 403)	337 (274 to 406)
Cumbria	441 (394 to 492)	456 (373 to 545)	479 (425 to 538)	482 (425 to 543)	486 (430 to 546)	488 (433 to 551)	499 (440 to 564)	512 (425 to 604)	512 (426 to 606)	511 (421 to 609)
Halton	80 (71 to 89)	83 (68 to 100)	97 (85 to 109)	122 (107 to 137)	126 (110 to 142)	119 (104 to 133)	125 (109 to 141)	134 (111 to 159)	134 (110 to 159)	135 (109 to 162)
Knowsley	136 (120 to 152)	167 (135 to 198)	194 (170 to 219)	176 (154 to 199)	156 (136 to 176)	152 (133 to 171)	147 (127 to 166)	147 (121 to 172)	147 (120 to 173)	147 (118 to 174)
Lancashire	999 (896 to 1110)	1008 (838 to 1197)	1058 (937 to 1185)	1092 (965 to 1226)	1114 (985 to 1250)	1135 (1005 to 1273)	1168 (1032 to 1308)	1207 (1020 to 1419)	1208 (1011 to 1428)	1209 (1008 to 1437)
Liverpool	554 (495 to 619)	601 (493 to 716)	618 (547 to 694)	526 (462 to 592)	479 (423 to 540)	443 (390 to 499)	461 (405 to 521)	493 (412 to 584)	495 (409 to 587)	497 (406 to 592)
Manchester	493 (440 to 553)	533 (438 to 648)	546 (481 to 619)	466 (409 to 528)	434 (382 to 496)	420 (370 to 477)	491 (431 to 559)	579 (482 to 696)	587 (490 to 710)	595 (492 to 719)
Oldham	207 (185 to 231)	215 (176 to 257)	226 (199 to 254)	223 (196 to 251)	219 (193 to 247)	219 (194 to 247)	225 (198 to 253)	237 (197 to 281)	238 (196 to 285)	239 (195 to 286)
Rochdale	180 (161 to 201)	191 (155 to 229)	205 (181 to 232)	209 (183 to 236)	204 (180 to 231)	207 (182 to 233)	211 (185 to 239)	222 (183 to 263)	223 (183 to 266)	223 (181 to 269)
Salford	255 (228 to 283)	270 (222 to 320)	281 (248 to 315)	253 (223 to 284)	233 (205 to 261)	218 (192 to 244)	231 (204 to 261)	259 (215 to 305)	262 (215 to 309)	264 (217 to 313)
Sefton	276 (246 to 309)	292 (238 to 349)	310 (273 to 349)	302 (265 to 339)	294 (258 to 331)	285 (251 to 319)	275 (241 to 310)	272 (225 to 323)	271 (224 to 322)	270 (221 to 324)
St Helens	167 (150 to 187)	175 (146 to 209)	190 (168 to 213)	192 (169 to 216)	181 (160 to 204)	178 (157 to 200)	176 (155 to 199)	177 (148 to 209)	177 (147 to 211)	176 (145 to 211)
Stockport	254 (228 to 283)	273 (227 to 326)	296 (263 to 332)	293 (259 to 329)	289 (255 to 325)	285 (253 to 320)	283 (249 to 318)	290 (244 to 340)	290 (243 to 342)	290 (240 to 346)
Tameside	201 (180 to 225)	208 (171 to 251)	220 (194 to 249)	219 (194 to 248)	218 (193 to 246)	214 (190 to 241)	219 (191 to 248)	233 (194 to 277)	234 (192 to 282)	235 (192 to 284)
Trafford	198 (177 to 222)	216 (176 to 260)	233 (206 to 263)	224 (197 to 253)	216 (190 to 245)	210 (185 to 237)	224 (196 to 254)	246 (203 to 293)	248 (204 to 297)	249 (202 to 300)
Warrington	140 (125 to 157)	150 (125 to 181)	164 (145 to 185)	169 (148 to 192)	183 (161 to 207)	191 (168 to 215)	201 (177 to 228)	213 (178 to 256)	214 (179 to 258)	214 (178 to 260)
Wigan	268 (239 to 299)	279 (229 to 332)	302 (267 to 339)	308 (271 to 346)	306 (271 to 345)	302 (267 to 341)	316 (279 to 357)	337 (279 to 398)	338 (277 to 402)	339 (278 to 406)
Wirral	325 (289 to 363)	342 (283 to 407)	358 (315 to 404)	343 (301 to 386)	336 (295 to 379)	317 (279 to 357)	319 (279 to 361)	331 (276 to 393)	332 (275 to 394)	332 (272 to 396)
South East England	5981 (5457 to 6535)	6205 (5306 to 7158)	6712 (6055 to 7424)	7187 (6494 to 7932)	7579 (6844 to 8370)	7971 (7199 to 8793)	8569 (7706 to 9478)	9218 (7994 to 10551)	9272 (8026 to 10608)	9319 (8037 to 10702)
Bracknell Forest	46 (41 to 51)	51 (41 to 61)	63 (55 to 71)	82 (72 to 93)	97 (84 to 109)	109 (96 to 123)	113 (99 to 128)	118 (97 to 140)	118 (97 to 141)	119 (97 to 144)
Brighton and Hove	274 (244 to 306)	265 (212 to 318)	255 (224 to 288)	238 (209 to 268)	239 (210 to 271)	248 (218 to 280)	269 (235 to 304)	292 (241 to 347)	294 (239 to 351)	296 (239 to 354)
Buckinghamshire	329 (292 to 368)	363 (296 to 434)	410 (358 to 462)	440 (384 to 495)	453 (396 to 511)	476 (417 to 534)	502 (440 to 567)	533 (437 to 631)	536 (440 to 635)	538 (438 to 640)
East Sussex	436 (390 to 487)	403 (330 to 480)	398 (353 to 447)	425 (374 to 478)	466 (412 to 526)	492 (436 to 554)	525 (463 to 591)	556 (464 to 656)	558 (458 to 659)	560 (456 to 667)
Hampshire	787 (704 to 872)	837 (693 to 990)	952 (843 to 1065)	1073 (952 to 1199)	1169 (1038 to 1307)	1234 (1094 to 1380)	1312 (1159 to 1469)	1393 (1163 to 1632)	1399 (1162 to 1652)	1405 (1168 to 1655)
Isle of Wight	112 (100 to 126)	108 (88 to 132)	110 (96 to 125)	118 (103 to 134)	126 (109 to 143)	133 (116 to 151)	138 (121 to 157)	142 (118 to 171)	143 (117 to 173)	142 (116 to 173)
Kent	1087 (974 to 1210)	1103 (908 to 1301)	1174 (1041 to 1322)	1239 (1099 to 1391)	1282 (1135 to 1447)	1325 (1174 to 1491)	1451 (1281 to 1635)	1593 (1329 to 1860)	1605 (1337 to 1887)	1616 (1328 to 1908)
Medway	181 (160 to 202)	196 (161 to 237)	223 (196 to 252)	240 (210 to 271)	242 (212 to 274)	249 (219 to 281)	263 (230 to 298)	283 (236 to 338)	284 (234 to 339)	286 (233 to 344)
Milton Keynes	58 (52 to 66)	56 (46 to 69)	69 (61 to 78)	119 (104 to 135)	173 (153 to 197)	209 (185 to 237)	245 (215 to 278)	281 (234 to 335)	284 (235 to 341)	286 (236 to 347)
Oxfordshire	396 (352 to 447)	435 (354 to 530)	494 (433 to 563)	536 (469 to 611)	571 (502 to 652)	602 (528 to 687)	648 (566 to 739)	696 (577 to 838)	700 (579 to 848)	704 (578 to 855)
Portsmouth	189 (168 to 211)	190 (156 to 227)	195 (172 to 220)	192 (168 to 215)	187 (164 to 211)	187 (165 to 210)	203 (178 to 229)	221 (182 to 262)	223 (183 to 263)	224 (184 to 268)
Reading	115 (102 to 129)	124 (102 to 149)	137 (120 to 155)	137 (121 to 156)	134 (118 to 153)	143 (126 to 162)	153 (135 to 174)	165 (137 to 197)	165 (137 to 198)	166 (137 to 199)
Slough	79 (70 to 89)	90 (73 to 109)	100 (88 to 113)	101 (88 to 114)	104 (91 to 118)	119 (104 to 135)	138 (121 to 157)	160 (132 to 190)	162 (133 to 192)	164 (133 to 196)
Southampton	186 (165 to 209)	199 (161 to 239)	214 (188 to 243)	211 (184 to 239)	204 (179 to 232)	217 (192 to 246)	233 (205 to 264)	249 (204 to 298)	250 (205 to 300)	251 (206 to 303)
Surrey	856 (768 to 959)	925 (767 to 1113)	994 (881 to 1123)	1007 (889 to 1138)	1017 (898 to 1151)	1053 (933 to 1190)	1123 (990 to 1271)	1204 (1006 to 1440)	1211 (1008 to 1447)	1217 (1009 to 1458)
West Berkshire	84 (74 to 94)	91 (74 to 109)	104 (91 to 118)	120 (106 to 136)	137 (120 to 156)	144 (126 to 163)	153 (134 to 174)	163 (134 to 194)	163 (135 to 196)	164 (135 to 196)
West Sussex	589 (524 to 660)	573 (468 to 684)	597 (525 to 675)	662 (579 to 749)	706 (616 to 799)	751 (660 to 849)	803 (700 to 910)	857 (707 to 1018)	861 (708 to 1021)	865 (704 to 1034)
Windsor and Maidenhead	103 (90 to 116)	112 (91 to 136)	124 (108 to 142)	134 (116 to 153)	134 (116 to 152)	133 (116 to 150)	143 (124 to 163)	155 (127 to 187)	156 (128 to 188)	157 (128 to 190)
Wokingham	74 (65 to 82)	84 (67 to 102)	99 (87 to 113)	114 (100 to 130)	138 (121 to 157)	149 (130 to 169)	154 (134 to 174)	160 (131 to 191)	161 (131 to 192)	161 (130 to 193)
South West England	3759 (3410 to 4115)	3833 (3247 to 4412)	4082 (3659 to 4491)	4355 (3890 to 4795)	4660 (4176 to 5127)	4920 (4420 to 5409)	5262 (4685 to 5823)	5613 (4868 to 6383)	5640 (4847 to 6441)	5663 (4871 to 6459)
Bath and North East Somerset	138 (122 to 153)	146 (119 to 175)	156 (137 to 176)	161 (140 to 182)	163 (142 to 183)	168 (147 to 189)	175 (152 to 197)	180 (149 to 213)	180 (148 to 214)	180 (148 to 214)
Bournemouth	177 (158 to 197)	166 (135 to 200)	155 (136 to 175)	144 (126 to 163)	156 (137 to 177)	163 (143 to 184)	181 (158 to 205)	203 (170 to 240)	204 (168 to 244)	205 (167 to 246)
Bristol, City of	389 (345 to 433)	411 (333 to 493)	429 (376 to 483)	403 (352 to 456)	392 (343 to 441)	388 (341 to 437)	421 (366 to 478)	466 (388 to 551)	469 (384 to 557)	473 (386 to 562)
Cornwall	372 (334 to 414)	365 (300 to 436)	382 (339 to 428)	423 (374 to 474)	469 (415 to 525)	501 (445 to 562)	533 (471 to 600)	565 (475 to 662)	568 (470 to 670)	569 (470 to 676)
Devon	541 (481 to 607)	532 (428 to 638)	552 (485 to 623)	595 (520 to 672)	653 (572 to 738)	703 (615 to 791)	744 (649 to 844)	780 (644 to 925)	783 (638 to 936)	785 (640 to 940)
Dorset	279 (249 to 312)	275 (221 to 331)	293 (258 to 329)	332 (290 to 376)	363 (319 to 409)	390 (343 to 440)	413 (362 to 467)	427 (355 to 506)	427 (352 to 511)	428 (350 to 511)
Gloucestershire	400 (358 to 449)	424 (346 to 509)	467 (412 to 527)	503 (442 to 568)	532 (469 to 602)	562 (495 to 634)	594 (521 to 672)	629 (529 to 747)	632 (524 to 755)	635 (523 to 756)
North Somerset	129 (114 to 143)	129 (104 to 155)	140 (122 to 157)	161 (139 to 181)	178 (155 to 201)	188 (164 to 211)	202 (175 to 228)	216 (179 to 254)	217 (178 to 258)	218 (177 to 260)
Plymouth	210 (187 to 234)	217 (176 to 261)	235 (207 to 265)	252 (222 to 284)	252 (223 to 285)	241 (213 to 272)	254 (224 to 288)	273 (229 to 324)	274 (225 to 329)	276 (224 to 333)
Poole	105 (93 to 118)	104 (84 to 125)	109 (96 to 123)	119 (104 to 135)	133 (116 to 150)	138 (121 to 156)	147 (128 to 167)	158 (130 to 187)	159 (129 to 189)	159 (129 to 190)
Somerset	354 (316 to 396)	360 (292 to 426)	387 (340 to 436)	427 (375 to 481)	463 (407 to 521)	496 (434 to 559)	529 (462 to 598)	558 (461 to 658)	561 (457 to 665)	563 (460 to 663)
South Gloucestershire	140 (124 to 157)	154 (124 to 185)	180 (157 to 204)	201 (176 to 228)	219 (193 to 248)	244 (214 to 276)	261 (228 to 297)	279 (231 to 331)	281 (229 to 335)	282 (230 to 337)
Swindon	111 (100 to 124)	122 (100 to 146)	139 (124 to 156)	150 (133 to 169)	169 (150 to 190)	179 (159 to 200)	206 (182 to 231)	239 (202 to 281)	242 (200 to 286)	245 (202 to 290)
Torbay	125 (112 to 141)	115 (94 to 139)	111 (98 to 125)	113 (99 to 128)	121 (107 to 137)	130 (115 to 147)	132 (116 to 150)	132 (110 to 158)	132 (109 to 160)	132 (109 to 159)
Wiltshire	290 (258 to 325)	312 (252 to 376)	349 (307 to 394)	373 (328 to 421)	396 (347 to 448)	430 (378 to 484)	470 (412 to 532)	508 (425 to 601)	511 (419 to 609)	513 (418 to 613)
West Midlands	4382 (3976 to 4827)	4706 (4036 to 5428)	5127 (4622 to 5688)	5193 (4645 to 5750)	5230 (4671 to 5854)	5278 (4733 to 5847)	5571 (4972 to 6190)	5960 (5126 to 6806)	5991 (5195 to 6874)	6015 (5142 to 6895)
Birmingham	964 (860 to 1081)	1047 (867 to 1246)	1109 (981 to 1244)	1038 (913 to 1167)	1007 (884 to 1139)	985 (865 to 1110)	1063 (931 to 1197)	1178 (978 to 1387)	1188 (990 to 1407)	1197 (988 to 1424)

Coventry	277 (244 to 310)	308 (249 to 368)	338 (297 to 380)	322 (280 to 364)	305 (265 to 346)	302 (264 to 341)	315 (273 to 356)	333 (271 to 396)	335 (275 to 399)	336 (272 to 403)
Dudley	251 (223 to 282)	269 (218 to 326)	294 (260 to 333)	301 (265 to 341)	308 (270 to 350)	306 (268 to 347)	313 (274 to 355)	327 (268 to 390)	328 (269 to 393)	328 (268 to 396)
Herefordshire, County of	127 (112 to 142)	131 (106 to 157)	140 (123 to 158)	149 (131 to 168)	159 (139 to 181)	174 (154 to 197)	183 (160 to 207)	190 (155 to 226)	190 (156 to 228)	191 (155 to 228)
Sandwell	292 (258 to 326)	316 (256 to 376)	334 (292 to 376)	313 (273 to 352)	295 (256 to 332)	286 (248 to 321)	306 (265 to 345)	341 (278 to 401)	344 (281 to 407)	347 (280 to 412)
Shropshire	215 (190 to 240)	223 (181 to 269)	241 (212 to 271)	254 (223 to 286)	268 (234 to 304)	282 (248 to 318)	305 (267 to 345)	326 (268 to 387)	328 (271 to 391)	329 (269 to 396)
Solihull	141 (126 to 159)	162 (132 to 194)	189 (167 to 214)	198 (175 to 224)	200 (176 to 228)	200 (176 to 226)	206 (181 to 233)	216 (178 to 257)	217 (180 to 258)	218 (179 to 261)
Staffordshire	596 (531 to 664)	628 (513 to 748)	702 (623 to 788)	762 (671 to 856)	790 (696 to 894)	806 (714 to 909)	845 (742 to 955)	887 (736 to 1044)	889 (740 to 1049)	891 (732 to 1063)
Stoke-on-Trent	242 (215 to 272)	255 (208 to 309)	267 (235 to 302)	255 (223 to 288)	250 (219 to 284)	242 (212 to 274)	248 (216 to 281)	263 (216 to 315)	264 (217 to 318)	265 (216 to 319)
Telford and Wrekin	80 (70 to 89)	82 (66 to 97)	95 (83 to 107)	123 (107 to 138)	140 (122 to 157)	157 (137 to 177)	166 (144 to 187)	173 (142 to 203)	174 (142 to 205)	174 (141 to 207)
Walsall	225 (200 to 251)	249 (205 to 299)	275 (243 to 310)	269 (236 to 304)	263 (231 to 298)	255 (224 to 288)	268 (234 to 304)	290 (239 to 344)	292 (240 to 348)	294 (241 to 350)
Warwickshire	383 (339 to 428)	412 (331 to 495)	456 (400 to 513)	476 (417 to 535)	486 (423 to 549)	504 (440 to 566)	542 (472 to 610)	581 (474 to 686)	585 (479 to 697)	587 (474 to 703)
Wolverhampton	227 (200 to 255)	247 (201 to 298)	267 (234 to 303)	260 (227 to 294)	250 (218 to 284)	239 (210 to 270)	248 (216 to 282)	267 (219 to 318)	269 (221 to 324)	270 (219 to 325)
Worcestershire	364 (324 to 409)	378 (306 to 456)	420 (369 to 475)	474 (417 to 536)	510 (446 to 580)	540 (476 to 612)	565 (495 to 641)	587 (487 to 701)	588 (488 to 702)	589 (483 to 703)
Yorkshire and the Humber	4353 (3974 to 4763)	4566 (3939 to 5224)	4872 (4413 to 5382)	4925 (4439 to 5424)	4938 (4469 to 5457)	4972 (4490 to 5494)	5253 (4737 to 5794)	5600 (4874 to 6377)	5626 (4888 to 6436)	5647 (4882 to 6442)
Barnsley	198 (176 to 222)	211 (172 to 254)	228 (201 to 258)	227 (201 to 257)	224 (198 to 254)	219 (193 to 249)	231 (204 to 261)	249 (207 to 295)	250 (204 to 300)	251 (205 to 299)
Bradford	420 (375 to 470)	441 (361 to 533)	465 (411 to 525)	465 (410 to 526)	469 (414 to 530)	470 (415 to 530)	517 (454 to 584)	575 (478 to 684)	580 (478 to 697)	584 (480 to 703)
Calderdale	189 (168 to 211)	193 (158 to 233)	198 (174 to 224)	194 (170 to 219)	193 (169 to 219)	192 (169 to 218)	203 (178 to 230)	217 (179 to 260)	219 (180 to 264)	219 (179 to 266)
Doncaster	236 (211 to 265)	256 (210 to 308)	282 (249 to 319)	291 (256 to 330)	293 (259 to 331)	288 (255 to 326)	301 (266 to 342)	320 (265 to 380)	321 (263 to 385)	323 (264 to 386)
East Riding of Yorkshire	231 (206 to 260)	234 (192 to 284)	249 (220 to 283)	270 (237 to 305)	292 (259 to 331)	314 (277 to 354)	333 (295 to 379)	349 (288 to 417)	350 (287 to 424)	351 (285 to 423)
Kingston upon Hull, City of	244 (217 to 273)	264 (215 to 317)	284 (249 to 320)	276 (242 to 311)	265 (233 to 298)	251 (219 to 285)	255 (223 to 288)	268 (221 to 318)	269 (219 to 319)	269 (218 to 321)
Kirklees	341 (305 to 382)	351 (287 to 425)	371 (328 to 420)	378 (334 to 427)	379 (335 to 430)	388 (342 to 439)	419 (370 to 476)	454 (378 to 541)	457 (376 to 552)	459 (378 to 552)
Leeds	656 (591 to 733)	697 (579 to 831)	741 (661 to 832)	720 (640 to 810)	706 (629 to 793)	713 (636 to 804)	744 (664 to 836)	790 (665 to 935)	793 (662 to 946)	796 (657 to 952)
North East Lincolnshire	136 (122 to 151)	147 (120 to 176)	161 (143 to 181)	162 (143 to 182)	162 (144 to 181)	159 (140 to 179)	160 (141 to 180)	165 (137 to 195)	165 (136 to 197)	165 (135 to 197)
North Lincolnshire	119 (106 to 133)	129 (104 to 156)	143 (125 to 161)	151 (131 to 170)	153 (134 to 173)	153 (133 to 173)	166 (144 to 188)	181 (149 to 216)	182 (149 to 219)	183 (149 to 221)
North Yorkshire	445 (396 to 496)	448 (364 to 537)	475 (418 to 535)	508 (446 to 571)	539 (475 to 608)	568 (498 to 640)	599 (525 to 674)	625 (517 to 737)	627 (517 to 742)	628 (514 to 744)
Rotherham	204 (183 to 228)	220 (179 to 264)	244 (215 to 275)	253 (223 to 286)	255 (225 to 288)	249 (220 to 281)	257 (228 to 289)	270 (224 to 321)	271 (222 to 324)	272 (222 to 324)
Sheffield	526 (470 to 588)	549 (451 to 652)	575 (507 to 647)	551 (486 to 620)	522 (461 to 590)	512 (452 to 578)	546 (480 to 615)	587 (485 to 691)	590 (486 to 699)	593 (484 to 702)
Wakefield	267 (239 to 299)	280 (231 to 337)	304 (268 to 343)	315 (277 to 356)	315 (277 to 355)	316 (279 to 356)	326 (287 to 368)	342 (286 to 406)	343 (286 to 407)	344 (284 to 414)
York	140 (124 to 156)	144 (118 to 172)	155 (136 to 174)	164 (144 to 185)	171 (151 to 192)	180 (158 to 202)	196 (172 to 220)	208 (173 to 246)	209 (172 to 248)	210 (173 to 249)
Northern Ireland	1408 (1312 to 1500)	1460 (1349 to 1565)	1566 (1448 to 1689)	1523 (1418 to 1644)	1595 (1482 to 1713)	1703 (1573 to 1843)	1828 (1694 to 1963)	1922 (1767 to 2073)	1927 (1778 to 2076)	1930 (1796 to 2062)
Scotland	5246 (4887 to 5630)	5288 (4885 to 5684)	5366 (4960 to 5780)	5162 (4790 to 5545)	5090 (4697 to 5452)	5138 (4758 to 5514)	5356 (4949 to 5770)	5517 (4913 to 6191)	5519 (4856 to 6163)	5516 (4793 to 6278)
Wales	2669 (2481 to 2859)	2714 (2523 to 2916)	2794 (2579 to 3002)	2873 (2653 to 3090)	2893 (2672 to 3107)	2948 (2731 to 3179)	3085 (2852 to 3324)	3158 (2900 to 3394)	3156 (2930 to 3380)	3152 (2942 to 3366)
Latin America and Caribbean	141227 (136742 to 145497)	187819 (181654 to 193471)	249280 (241459 to 257030)	320068 (309851 to 330418)	390245 (377108 to 403118)	465265 (450113 to 479851)	530324 (513121 to 547083)	585435 (555393 to 612702)	590105 (557426 to 618849)	594119 (559806 to 626195)
Andean Latin America	13853 (13232 to 14465)	18093 (17111 to 19011)	23765 (22379 to 25000)	30490 (28956 to 31943)	37993 (35795 to 39984)	46289 (43402 to 49182)	53992 (51165 to 56634)	63906 (59909 to 67337)	65204 (60880 to 69018)	66133 (61436 to 70270)
Bolivia	2855 (2652 to 3052)	3516 (3047 to 3973)	4300 (3824 to 4774)	5210 (4738 to 5704)	6381 (5838 to 6902)	8289 (7670 to 8906)	10058 (9220 to 10903)	11537 (10221 to 12888)	11680 (10284 to 13026)	11797 (10327 to 13298)
Ecuador	3306 (3064 to 3529)	4447 (4079 to 4818)	6031 (5471 to 6558)	7870 (7247 to 8506)	9978 (9286 to 10602)	12542 (11597 to 13510)	15028 (14054 to 16012)	17495 (15322 to 19645)	17839 (15544 to 20118)	18065 (15505 to 20500)
Peru	7693 (7168 to 8235)	10130 (9334 to 10904)	13433 (12304 to 14471)	17410 (16173 to 18717)	21634 (19746 to 23439)	25458 (22916 to 28197)	28906 (26585 to 31179)	34874 (31924 to 37573)	35685 (32471 to 38630)	36271 (32891 to 39697)
Caribbean	17755 (17220 to 18304)	21681 (20716 to 22732)	26214 (25441 to 27046)	30760 (29744 to 31834)	35293 (33509 to 37113)	40145 (38747 to 41602)	43898 (42208 to 45615)	46891 (43946 to 50069)	47205 (44139 to 50418)	47459 (44304 to 50863)
Antigua and Barbuda	47 (44 to 50)	56 (52 to 60)	65 (60 to 69)	61 (53 to 68)	60 (56 to 65)	76 (70 to 82)	86 (80 to 93)	89 (78 to 99)	89 (78 to 100)	89 (78 to 100)
The Bahamas	80 (75 to 85)	118 (108 to 128)	170 (158 to 181)	213 (198 to 228)	257 (240 to 275)	303 (283 to 325)	354 (330 to 379)	385 (337 to 433)	387 (339 to 431)	388 (334 to 444)
Barbados	233 (216 to 248)	240 (225 to 256)	243 (225 to 258)	251 (234 to 268)	253 (236 to 271)	257 (240 to 273)	282 (263 to 299)	297 (263 to 334)	298 (258 to 337)	299 (260 to 342)
Belize	69 (64 to 73)	95 (88 to 101)	124 (116 to 133)	150 (139 to 159)	187 (174 to 201)	240 (223 to 256)	330 (308 to 351)	413 (361 to 462)	421 (366 to 475)	429 (369 to 489)
Bermuda	38 (35 to 40)	44 (41 to 47)	54 (50 to 57)	56 (52 to 59)	59 (55 to 64)	63 (59 to 67)	65 (61 to 70)	64 (58 to 70)	64 (58 to 70)	64 (57 to 70)
Cuba	5682 (5297 to 6059)	6841 (6126 to 7601)	8619 (8055 to 9217)	9939 (9213 to 10673)	10847 (9527 to 12109)	11403 (10501 to 12284)	11458 (10593 to 12375)	11362 (10098 to 12742)	11331 (9879 to 12731)	11270 (9907 to 12688)
Dominica	53 (50 to 57)	63 (59 to 67)	72 (67 to 76)	75 (70 to 81)	72 (67 to 78)	69 (64 to 74)	68 (63 to 73)	67 (59 to 76)	67 (59 to 75)	67 (58 to 76)
Dominican Republic	2330 (2164 to 2489)	3264 (3043 to 3492)	4273 (3990 to 4571)	5707 (5307 to 6119)	7151 (6510 to 7782)	8597 (7896 to 9250)	9736 (9062 to 10373)	10780 (9540 to 12165)	10899 (9429 to 12197)	11013 (9395 to 12573)
Grenada	88 (81 to 94)	93 (87 to 98)	96 (90 to 103)	95 (88 to 102)	87 (80 to 94)	104 (96 to 112)	107 (99 to 115)	104 (91 to 116)	103 (91 to 117)	103 (89 to 116)
Guyana	431 (402 to 460)	584 (545 to 623)	732 (682 to 781)	794 (740 to 847)	780 (718 to 839)	779 (719 to 842)	751 (694 to 811)	766 (680 to 852)	769 (675 to 858)	765 (670 to 859)
Haiti	3287 (3058 to 3527)	3957 (3445 to 4503)	4521 (4177 to 4875)	5121 (4705 to 5541)	6381 (5602 to 7145)	8190 (7469 to 8871)	10298 (9201 to 11434)	12387 (10361 to 14695)	12631 (10434 to 14796)	12864 (10696 to 15179)
Jamaica	1458 (1362 to 1558)	1673 (1558 to 1788)	1867 (1740 to 2006)	2207 (2028 to 2387)	2366 (2189 to 2545)	2631 (2447 to 2836)	2762 (2564 to 2973)	2799 (2472 to 3119)	2801 (2455 to 3145)	2800 (2448 to 3165)
Puerto Rico	2211 (2061 to 2363)	2424 (2267 to 2584)	2783 (2591 to 2978)	3274 (3064 to 3485)	3612 (3356 to 3875)	3880 (3616 to 4128)	3794 (3536 to 4057)	3381 (3142 to 3648)	3338 (3119 to 3569)	3294 (3048 to 3535)
Saint Kitts and Nevis	62 (51 to 73)	53 (46 to 59)	47 (44 to 50)	45 (42 to 48)	41 (38 to 45)	46 (43 to 50)	54 (47 to 60)	58 (47 to 69)	58 (47 to 69)	59 (49 to 70)
Saint Lucia	77 (72 to 82)	91 (84 to 97)	103 (96 to 110)	119 (111 to 127)	137 (126 to 146)	155 (144 to 166)	169 (158 to 181)	177 (156 to 197)	177 (155 to 199)	178 (152 to 202)
Saint Vincent and the Grenadines	76 (71 to 81)	84 (78 to 90)	89 (84 to 95)	102 (95 to 108)	110 (101 to 118)	110 (102 to 118)	111 (102 to 120)	114 (102 to 127)	114 (101 to 127)	114 (100 to 129)
Suriname	193 (180 to 207)	288 (263 to 313)	391 (362 to 420)	357 (314 to 398)	387 (338 to 432)	449 (418 to 479)	539 (494 to 582)	574 (511 to 647)	577 (510 to 643)	579 (510 to 654)
Trinidad and Tobago	667 (623 to 710)	860 (804 to 918)	967 (903 to 1029)	1089 (1014 to 1159)	1205 (1122 to 1285)	1292 (1204 to 1379)	1347 (1243 to 1449)	1393 (1231 to 1556)	1394 (1225 to 1589)	1393 (1207 to 1568)
Virgin Islands	27 (25 to 29)	33 (31 to 36)	66 (62 to 71)	99 (93 to 106)	106 (99 to 113)	111 (104 to 119)	108 (101 to 115)	90 (83 to 97)	88 (82 to 94)	86 (80 to 92)
Central Latin America	53351 (51401 to 55579)	72465 (69697 to 75551)	100093 (96253 to 104273)	132562 (127266 to 138285)	164408 (158167 to 171670)	199003 (191388 to 207839)	228501 (219832 to 238261)	250884 (240927 to 261933)	251908 (242293 to 262311)	253002 (242163 to 264759)
Colombia	11525 (10719 to 12281)	15726 (14222 to 17167)	21809 (19879 to 23667)	26851 (24076 to 29581)	32491 (29572 to 35396)	39733 (35666 to 43746)	44506 (40379 to 48960)	48034 (44412 to 51818)	48783 (44822 to 52517)	49060 (44537 to 53498)
Costa Rica	844 (785 to 902)	1243 (1138 to 1354)	1783 (1633 to 1925)	2269 (2053 to 2483)	3042 (2733 to 3357)	3904 (3637 to 4159)	4389 (4084 to 4722)	4708 (4157 to 5261)	4731 (4121 to 5303)	4748 (4175 to 5343)
El Salvador	1929 (1797 to 2059)	2603 (2420 to 2798)	3644 (3382 to 3917)	4700 (4116 to 5250)	5306 (4883 to 5726)	5862 (5240 to 6549)	6090 (5542 to 6668)	6399 (5516 to 7265)	6428 (5450 to 7372)	6450 (5430 to 7375)
Guatemala	2984 (2783 to 3197)	3929 (3549 to 4272)	4992 (4558 to 5407)	6377 (5910 to 6846)	8384 (7555 to 9162)	11077 (10238 to 11975)	13619 (12062 to 15205)	15455 (14293 to 16627)	15621 (14406 to 16897)	15767 (14360 to 17143)
Honduras	1459 (1361 to 1557)	1912 (1774 to 2052)	2507 (2280 to 2757)	3424 (3059 to 3767)	4710 (4337 to 5099)	6167 (5716 to 6661)	7988 (7309 to 8674)	9745 (8761 to 10757)	9933 (8764 to 11049)	10110 (8912 to 11286)
Mexico	27398 (25525 to 29378)	36879 (34282 to 39311)	51115 (47527 to 54484)	69677 (64797 to 74120)	85391 (79683 to 91484)	101154 (94416 to 108312)	114881 (107074 to 122403)	127622 (117627 to 137224)	128565 (119983 to 137086)	129276 (119311 to 139133)

Aguascalientes	198 (180 to 218)	260 (236 to 286)	362 (329 to 399)	541 (490 to 595)	754 (683 to 833)	988 (897 to 1088)	1216 (1104 to 1342)	1433 (1286 to 1592)	1452 (1321 to 1595)	1469 (1317 to 1633)
Baja California	248 (225 to 273)	538 (490 to 591)	917 (833 to 1008)	1233 (1121 to 1352)	1705 (1554 to 1875)	2396 (2178 to 2633)	3203 (2915 to 3526)	3805 (3424 to 4210)	3848 (3509 to 4228)	3885 (3497 to 4312)
Baja California Sur	63 (57 to 69)	85 (77 to 94)	135 (122 to 148)	222 (201 to 244)	329 (298 to 362)	445 (405 to 489)	643 (584 to 707)	797 (715 to 885)	809 (734 to 892)	820 (735 to 909)
Campeche	128 (116 to 140)	177 (161 to 193)	268 (244 to 292)	436 (395 to 476)	560 (512 to 611)	721 (654 to 789)	845 (767 to 923)	936 (843 to 1033)	942 (856 to 1029)	947 (853 to 1044)
Chiapas	956 (868 to 1051)	1277 (1159 to 1398)	1674 (1519 to 1838)	2240 (2033 to 2459)	3398 (3066 to 3734)	4085 (3703 to 4483)	4969 (4508 to 5450)	5654 (5098 to 6249)	5703 (5182 to 6253)	5747 (5163 to 6353)
Chihuahua	927 (842 to 1022)	1281 (1164 to 1407)	1706 (1545 to 1872)	2099 (1899 to 2297)	2536 (2292 to 2782)	3112 (2817 to 3425)	3422 (3098 to 3758)	3781 (3380 to 4195)	3809 (3445 to 4168)	3832 (3426 to 4251)
Coahuila	766 (691 to 845)	954 (862 to 1047)	1189 (1071 to 1306)	1615 (1456 to 1773)	2064 (1861 to 2271)	2400 (2155 to 2649)	2797 (2527 to 3077)	3168 (2817 to 3514)	3195 (2890 to 3511)	3218 (2860 to 3577)
Colima	121 (110 to 134)	173 (157 to 190)	256 (232 to 281)	360 (325 to 396)	445 (403 to 487)	539 (488 to 592)	658 (596 to 723)	741 (662 to 821)	746 (673 to 819)	750 (670 to 831)
Durango	665 (603 to 730)	807 (732 to 887)	999 (903 to 1098)	1240 (1126 to 1362)	1422 (1297 to 1561)	1533 (1395 to 1683)	1669 (1519 to 1831)	1857 (1670 to 2058)	1873 (1707 to 2056)	1888 (1697 to 2092)
Guanajuato	1421 (1289 to 1564)	1844 (1667 to 2027)	2421 (2189 to 2660)	3187 (2878 to 3503)	4131 (3750 to 4547)	4908 (4448 to 5398)	5630 (5110 to 6177)	6242 (5579 to 6919)	6283 (5701 to 6899)	6314 (5651 to 6994)
Guerrero	974 (883 to 1081)	1272 (1152 to 1399)	1710 (1545 to 1892)	2236 (2025 to 2467)	2774 (2521 to 3060)	3270 (2962 to 3617)	3539 (3196 to 3909)	3638 (3265 to 4055)	3639 (3302 to 4014)	3635 (3250 to 4063)
Hidalgo	888 (807 to 980)	1049 (957 to 1148)	1271 (1162 to 1396)	1616 (1472 to 1775)	2002 (1829 to 2204)	2371 (2160 to 2610)	2748 (2501 to 3021)	3122 (2823 to 3456)	3153 (2869 to 3461)	3178 (2872 to 3523)
Jalisco	1904 (1736 to 2086)	2586 (2357 to 2828)	3498 (3179 to 3826)	4557 (4148 to 4977)	5555 (5058 to 6079)	6582 (6014 to 7193)	7499 (6854 to 8193)	8442 (7613 to 9301)	8520 (7768 to 9283)	8582 (7741 to 9445)
México	1432 (1298 to 1575)	2020 (1831 to 2213)	4031 (3665 to 4413)	7824 (7124 to 8550)	10329 (9415 to 11272)	13181 (12028 to 14431)	15465 (14129 to 16864)	17184 (15520 to 18914)	17293 (15777 to 18881)	17374 (15679 to 19148)
Mexico City	3270 (2934 to 3570)	5062 (4558 to 5520)	7191 (6456 to 7855)	9009 (8125 to 9823)	8652 (7759 to 9504)	8882 (7989 to 9737)	8904 (7996 to 9747)	9363 (8309 to 10351)	9382 (8390 to 10286)	9365 (8300 to 10371)
Michoacán de Ocampo	1534 (1385 to 1687)	1978 (1794 to 2162)	2478 (2236 to 2716)	3059 (2759 to 3356)	3735 (3377 to 4099)	4192 (3789 to 4605)	4478 (4058 to 4901)	4835 (4338 to 5341)	4862 (4404 to 5329)	4880 (4371 to 5403)
Morelos	285 (257 to 317)	409 (368 to 453)	653 (589 to 724)	984 (883 to 1092)	1265 (1142 to 1405)	1571 (1416 to 1745)	1814 (1642 to 2006)	2000 (1793 to 2235)	2011 (1815 to 2224)	2017 (1801 to 2257)
Nayarit	308 (277 to 341)	417 (377 to 457)	581 (524 to 638)	759 (686 to 835)	866 (785 to 952)	974 (881 to 1072)	1114 (1008 to 1220)	1249 (1122 to 1381)	1259 (1139 to 1386)	1268 (1136 to 1407)
Nuevo León	787 (711 to 871)	1151 (1040 to 1270)	1788 (1617 to 1974)	2572 (2321 to 2838)	3245 (2936 to 3572)	3976 (3597 to 4400)	4749 (4312 to 5222)	5775 (5179 to 6397)	5880 (5322 to 6467)	5973 (5363 to 6636)
Oaxaca	1521 (1378 to 1665)	1831 (1663 to 1996)	2147 (1943 to 2348)	2523 (2283 to 2764)	3184 (2879 to 3490)	3662 (3317 to 4013)	3956 (3576 to 4324)	4218 (3769 to 4667)	4235 (3821 to 4645)	4244 (3790 to 4684)
Puebla	1706 (1552 to 1873)	2083 (1891 to 2286)	2684 (2438 to 2949)	3504 (3179 to 3845)	4378 (3971 to 4826)	5237 (4764 to 5763)	5974 (5443 to 6550)	6683 (6007 to 7407)	6739 (6125 to 7384)	6780 (6081 to 7508)
Querétaro	298 (271 to 327)	377 (343 to 412)	515 (468 to 566)	765 (695 to 836)	1103 (1002 to 1208)	1468 (1335 to 1606)	1871 (1698 to 2046)	2364 (2124 to 2613)	2416 (2194 to 2642)	2463 (2214 to 2732)
Quintana Roo	29 (26 to 32)	52 (47 to 57)	93 (84 to 102)	233 (212 to 256)	506 (459 to 554)	899 (815 to 987)	1340 (1213 to 1467)	1838 (1650 to 2033)	1892 (1712 to 2074)	1944 (1739 to 2152)
San Luis Potosí	904 (821 to 990)	1103 (1005 to 1203)	1366 (1242 to 1495)	1748 (1593 to 1909)	2112 (1924 to 2311)	2422 (2202 to 2648)	2656 (2419 to 2902)	2868 (2579 to 3158)	2882 (2629 to 3150)	2891 (2604 to 3194)
Sinaloa	658 (595 to 725)	899 (813 to 987)	1350 (1223 to 1484)	1928 (1749 to 2114)	2324 (2107 to 2557)	2671 (2431 to 2936)	2856 (2600 to 3138)	3076 (2766 to 3403)	3093 (2797 to 3392)	3107 (2785 to 3446)
Sonora	564 (514 to 623)	819 (744 to 900)	1167 (1059 to 1285)	1554 (1416 to 1708)	1904 (1732 to 2100)	2322 (2112 to 2549)	2710 (2472 to 2985)	2978 (2680 to 3301)	2991 (2728 to 3287)	2998 (2698 to 3317)
Tabasco	372 (337 to 407)	528 (481 to 576)	807 (733 to 885)	1127 (1022 to 1236)	1581 (1434 to 1732)	1995 (1816 to 2187)	2283 (2078 to 2493)	2443 (2191 to 2693)	2448 (2220 to 2675)	2450 (2202 to 2705)
Tamaulipas	771 (701 to 851)	1083 (985 to 1188)	1540 (1397 to 1687)	1989 (1807 to 2174)	2363 (2148 to 2598)	2857 (2597 to 3149)	3272 (2973 to 3580)	3574 (3205 to 3953)	3595 (3274 to 3939)	3610 (3247 to 3999)
Tlaxcala	300 (272 to 332)	363 (328 to 399)	447 (404 to 491)	584 (527 to 642)	804 (725 to 888)	1016 (919 to 1120)	1203 (1090 to 1323)	1360 (1214 to 1510)	1372 (1237 to 1508)	1381 (1235 to 1534)
Veracruz de Ignacio de la Llave	2149 (1937 to 2369)	2886 (2612 to 3158)	4052 (3659 to 4444)	5631 (5089 to 6178)	6594 (5948 to 7258)	7315 (6611 to 8034)	7863 (7136 to 8612)	8218 (7360 to 9103)	8224 (7444 to 9015)	8217 (7333 to 9095)
Yucatán	540 (489 to 594)	651 (590 to 714)	813 (734 to 894)	1113 (1006 to 1223)	1432 (1289 to 1577)	1735 (1567 to 1909)	2006 (1815 to 2197)	2333 (2083 to 2586)	2364 (2128 to 2594)	2390 (2128 to 2658)
Zacatecas	712 (643 to 783)	865 (785 to 951)	1006 (910 to 1102)	1193 (1081 to 1307)	1343 (1215 to 1473)	1428 (1289 to 1570)	1530 (1382 to 1680)	1647 (1467 to 1825)	1655 (1494 to 1818)	1660 (1487 to 1836)
Nicaragua	1120 (1047 to 1199)	1486 (1356 to 1617)	1958 (1814 to 2104)	2764 (2403 to 3103)	3888 (3491 to 4262)	4927 (4460 to 5403)	5735 (5167 to 6319)	6519 (5522 to 7585)	6598 (5550 to 7633)	6669 (5591 to 7775)
Panama	813 (756 to 868)	1120 (1047 to 1192)	1487 (1378 to 1583)	1878 (1750 to 2002)	2389 (2211 to 2547)	2912 (2728 to 3121)	3501 (3265 to 3745)	4153 (3653 to 4671)	4234 (3675 to 4784)	4292 (3695 to 4867)
Venezuela	5279 (4937 to 5658)	7569 (6984 to 8157)	10798 (10013 to 11575)	14622 (13584 to 15700)	18807 (17497 to 20063)	23267 (21635 to 25051)	27793 (25689 to 29914)	28249 (25005 to 31464)	27016 (23566 to 30407)	26629 (22960 to 30073)
Tropical Latin America	56268 (52582 to 60062)	75580 (70806 to 80661)	99209 (92620 to 105849)	126256 (117903 to 134723)	152552 (142133 to 163062)	179827 (167646 to 192130)	203933 (190128 to 217774)	223753 (197547 to 250094)	225788 (195842 to 254599)	227525 (196124 to 258236)
Brazil	54900 (51167 to 58671)	73764 (68962 to 78797)	96863 (90224 to 103517)	123253 (114800 to 131694)	148509 (137926 to 158977)	174681 (162292 to 186998)	197785 (183621 to 211679)	216751 (189955 to 242600)	218699 (187909 to 247252)	220356 (188145 to 250952)
Acre	122 (111 to 135)	166 (151 to 184)	223 (202 to 247)	313 (284 to 346)	419 (378 to 466)	572 (521 to 636)	766 (696 to 846)	940 (786 to 1125)	959 (791 to 1159)	977 (799 to 1196)
Alagoas	1141 (1028 to 1254)	1346 (1214 to 1478)	1672 (1505 to 1833)	2073 (1870 to 2272)	2564 (2287 to 2826)	2968 (2670 to 3256)	3339 (3006 to 3662)	3716 (3068 to 4361)	3761 (3043 to 4447)	3802 (3059 to 4552)
Amapá	40 (36 to 45)	71 (65 to 78)	118 (107 to 130)	180 (163 to 198)	277 (248 to 307)	481 (435 to 530)	684 (619 to 755)	838 (700 to 995)	854 (703 to 1020)	869 (709 to 1053)
Amazonas	549 (494 to 606)	750 (676 to 826)	989 (895 to 1093)	1452 (1309 to 1604)	2063 (1853 to 2289)	2834 (2551 to 3127)	3588 (3229 to 3969)	4218 (3466 to 5010)	4286 (3485 to 5108)	4347 (3486 to 5283)
Bahia	5105 (4635 to 5570)	6325 (5720 to 6903)	7860 (7135 to 8582)	9901 (8982 to 10801)	12074 (10881 to 13279)	13602 (12347 to 14871)	14789 (13367 to 16203)	15935 (13315 to 18744)	16067 (13255 to 18854)	16186 (13191 to 19312)
Ceará	2825 (2556 to 3088)	3506 (3188 to 3820)	4555 (4130 to 4978)	5580 (5058 to 6114)	6475 (5827 to 7127)	7746 (7017 to 8464)	8956 (8117 to 9794)	10071 (8410 to 11816)	10196 (8362 to 12011)	10315 (8370 to 12288)
Distrito Federal	58 (52 to 64)	150 (136 to 165)	547 (495 to 601)	1223 (1105 to 1349)	1617 (1453 to 1790)	2105 (1903 to 2318)	2624 (2374 to 2899)	3021 (2517 to 3592)	3060 (2515 to 3639)	3094 (2523 to 3715)
Espírito Santo	898 (810 to 988)	1226 (1108 to 1350)	1654 (1495 to 1819)	2102 (1902 to 2317)	2626 (2357 to 2912)	3191 (2885 to 3516)	3635 (3277 to 4004)	3986 (3299 to 4745)	4021 (3298 to 4771)	4051 (3283 to 4888)
Goiás	1098 (985 to 1208)	1760 (1585 to 1940)	2634 (2377 to 2897)	3425 (3070 to 3772)	4082 (3638 to 4517)	5130 (4603 to 5651)	6154 (5508 to 6795)	6896 (5624 to 8183)	6969 (5675 to 8238)	7030 (5651 to 8457)
Maranhão	1736 (1568 to 1908)	2614 (2371 to 2866)	3201 (2904 to 3510)	4161 (3766 to 4569)	5031 (4525 to 5541)	5959 (5386 to 6543)	7058 (6370 to 7751)	8236 (6850 to 9629)	8383 (6899 to 9922)	8527 (6914 to 10245)
Mato Grosso	208 (188 to 228)	367 (332 to 403)	671 (610 to 734)	1182 (1074 to 1297)	1980 (1783 to 2185)	2571 (2324 to 2821)	3108 (2808 to 3418)	3583 (2981 to 4231)	3634 (2981 to 4302)	3678 (2987 to 4417)
Mato Grosso do Sul	346 (311 to 382)	576 (520 to 634)	974 (877 to 1075)	1419 (1280 to 1562)	1791 (1603 to 1995)	2140 (1932 to 2363)	2519 (2269 to 2780)	2853 (2365 to 3406)	2887 (2331 to 3464)	2916 (2355 to 3548)
Minas Gerais	8193 (7484 to 8934)	10208 (9348 to 11172)	11958 (10936 to 13054)	13841 (12613 to 15158)	15963 (14447 to 17623)	18385 (16752 to 20136)	20255 (18448 to 22224)	21658 (18255 to 25411)	21798 (18049 to 25873)	21910 (17967 to 26304)
Pará	1192 (1078 to 1308)	1616 (1472 to 1772)	2251 (2046 to 2466)	3494 (3182 to 3836)	4866 (4375 to 5376)	6306 (5732 to 6913)	7849 (7147 to 8596)	9129 (7629 to 10749)	9264 (7646 to 10996)	9392 (7640 to 11260)
Paraíba	1807 (1632 to 1992)	2120 (1913 to 2332)	2477 (2238 to 2722)	2870 (2590 to 3160)	3244 (2895 to 3593)	3589 (3236 to 3949)	3987 (3580 to 4405)	4383 (3616 to 5204)	4427 (3610 to 5297)	4469 (3612 to 5381)
Paraná	2297 (2074 to 2538)	4454 (4049 to 4903)	7034 (6385 to 7736)	7852 (7107 to 8704)	8565 (7697 to 9500)	9816 (8906 to 10819)	10728 (9701 to 11847)	11443 (9488 to 13586)	11516 (9463 to 13737)	11568 (9377 to 14109)
Pernambuco	3575 (3255 to 3942)	4372 (3974 to 4812)	5423 (4955 to 5965)	6447 (5866 to 7109)	7267 (6567 to 8075)	8250 (7484 to 9114)	9262 (8387 to 10242)	10168 (8499 to 12086)	10262 (8432 to 12274)	10348 (8473 to 12565)
Piauí	1088 (985 to 1198)	1334 (1211 to 1470)	1754 (1593 to 1933)	2238 (2025 to 2468)	2636 (2371 to 2924)	2971 (2692 to 3264)	3320 (2993 to 3661)	3685 (3056 to 4372)	3727 (3060 to 4450)	3766 (3050 to 4558)
Rio de Janeiro	5022 (4550 to 5489)	6999 (6361 to 7629)	9329 (8477 to 10173)	11597 (10481 to 12660)	13052 (11754 to 14370)	14701 (13337 to 16066)	16412 (14842 to 17963)	17690 (14765 to 20704)	17791 (14589 to 20952)	17862 (14604 to 21243)
Rio Grande do Norte	1004 (910 to 1100)	1223 (1115 to 1338)	1604 (1461 to 1755)	2008 (1828 to 2207)	2448 (2207 to 2692)	2907 (2639 to 3170)	3346 (3039 to 3666)	3739 (3135 to 4397)	3782 (3132 to 4445)	3822 (3113 to 4572)
Rio Grande do Sul	4370 (3980 to 4789)	5639 (5131 to 6174)	6864 (6251 to 7522)	8036 (7289 to 8828)	9228 (8326 to 10164)	10366 (9435 to 11339)	10942 (9907 to 12019)	11258 (9225 to 13296)	11288 (9368 to 13380)	11300 (9278 to 13526)
Rondônia	39 (35 to 43)	72 (65 to 79)	118 (107 to 131)	497 (449 to 549)	1110 (991 to 1233)	1421 (1282 to 1569)	1625 (1468 to 1798)	1810 (1495 to 2157)	1830 (1490 to 2189)	1848 (1482 to 2238)
Roraima	19 (17 to 21)	29 (26 to 31)	42 (38 to 45)	81 (74 to 89)	205 (185 to 225)	329 (299 to 360)	464 (419 to 507)	598 (495 to 699)	613 (503 to 719)	628 (510 to 746)
Santa Catarina	1608 (1461 to 1768)	2213 (2018 to 2436)	2941 (2672 to 3237)	3713 (3375 to 4084)	4535 (4104 to 5033)	5462 (4962 to 6022)	6385 (5799 to 7052)	7159 (6039 to 8478)	7242 (5997 to 8668)	7313 (6002 to 8818)
São Paulo	9733 (8857 to 10619)	13583 (12429 to 14807)	18641 (17013 to 20350)	25839 (23596 to 28153)	31996 (29074 to 35063)	37824 (34571 to 41264)	42366 (38615 to 46316)	45613 (38604 to 53208)	45900 (38387 to 53677)	46107 (38195 to 54849)
Sergipe	677 (609 to 742)	794 (714 to 870)	939 (848 to 1028)	1176 (1057 to 1293)	1494 (1339 to 1649)	1841 (1658 to 2016)	2164 (1943 to 2373)	2431 (1995 to 2864)	2460 (2008 to 2913)	2487 (1998 to 2980)
Tocantins	151 (137 to 166)	249 (226 to 272)	388 (351 to 426)	548 (495 to 602)	903 (805 to 999)	1211 (1095 to 1328)	1461 (1318 to 1604)	1694 (1398 to 2003)	1720 (1405 to 2043)	1745 (1416 to 2090)

Paraguay	1367 (1276 to 1461)	1817 (1671 to 1961)	2345 (2158 to 2534)	3003 (2771 to 3237)	4043 (3692 to 4381)	5146 (4728 to 5584)	6148 (5390 to 6908)	7002 (5758 to 8195)	7089 (5819 to 8407)	7169 (5861 to 8461)
North Africa and Middle East	114957 (111699 to 118253)	146198 (141911 to 150452)	193584 (188131 to 199349)	258302 (251272 to 265783)	339192 (330202 to 348439)	420541 (406951 to 434269)	517741 (502626 to 533106)	606147 (586455 to 625980)	614944 (593052 to 636622)	623000 (599578 to 646200)
North Africa and Middle East	114957 (111699 to 118253)	146198 (141911 to 150452)	193584 (188131 to 199349)	258302 (251272 to 265783)	339192 (330202 to 348439)	420541 (406951 to 434269)	517741 (502626 to 533106)	606147 (586455 to 625980)	614944 (593052 to 636622)	623000 (599578 to 646200)
Afghanistan	7420 (5353 to 9249)	9085 (7460 to 10753)	11124 (9649 to 12562)	13433 (12438 to 14483)	9943 (8649 to 11276)	15936 (12763 to 18947)	21938 (16200 to 27858)	29275 (20009 to 38599)	30228 (20390 to 39369)	31222 (21632 to 40882)
Algeria	8920 (8334 to 9505)	10929 (9763 to 12066)	13708 (12473 to 14952)	18614 (17017 to 20333)	25288 (23120 to 27325)	31021 (28642 to 33456)	36253 (33430 to 39105)	42909 (36934 to 48666)	43586 (37402 to 49685)	44198 (37361 to 50961)
Bahrain	117 (109 to 125)	155 (144 to 167)	217 (201 to 234)	345 (320 to 368)	506 (470 to 544)	646 (602 to 695)	1254 (1171 to 1340)	1494 (1378 to 1596)	1514 (1409 to 1618)	1530 (1421 to 1647)
Egypt	20218 (18844 to 21516)	25917 (24223 to 27724)	34841 (31676 to 38196)	43699 (39757 to 47654)	55333 (50225 to 60508)	67264 (61466 to 72972)	84113 (75661 to 92444)	102323 (93549 to 111044)	104037 (95374 to 112630)	105627 (95745 to 115666)
Iran	16790 (15674 to 17969)	21703 (19661 to 23731)	29146 (26501 to 31695)	39953 (36295 to 43828)	57100 (51975 to 61981)	66248 (60446 to 72236)	76073 (70648 to 81525)	84358 (77386 to 92001)	84961 (76837 to 92808)	85356 (76864 to 93931)
Alborz	198 (178 to 218)	376 (323 to 430)	636 (551 to 721)	898 (772 to 1026)	1453 (1255 to 1646)	1967 (1699 to 2234)	2399 (2133 to 2662)	2901 (2532 to 3277)	2945 (2533 to 3354)	2981 (2534 to 3426)
Ardebil	451 (409 to 493)	537 (470 to 606)	667 (584 to 749)	894 (780 to 1011)	1166 (1020 to 1310)	1235 (1082 to 1393)	1288 (1155 to 1421)	1307 (1153 to 1468)	1305 (1143 to 1473)	1302 (1127 to 1483)
Bushehr	131 (119 to 145)	202 (176 to 229)	292 (256 to 331)	439 (386 to 499)	703 (621 to 791)	795 (700 to 899)	1022 (924 to 1127)	1243 (1105 to 1400)	1261 (1110 to 1423)	1276 (1111 to 1458)
Chahar Mahaal and Bakhtiari	218 (197 to 240)	267 (233 to 304)	330 (289 to 373)	477 (411 to 546)	726 (635 to 821)	822 (715 to 937)	912 (817 to 1011)	1010 (886 to 1140)	1018 (886 to 1154)	1024 (878 to 1180)
East Azarbayejan	1483 (1348 to 1631)	1794 (1568 to 2046)	2159 (1895 to 2434)	2703 (2371 to 3079)	3436 (3002 to 3887)	3519 (3081 to 3969)	3787 (3414 to 4180)	4100 (3605 to 4606)	4119 (3593 to 4656)	4129 (3564 to 4702)
Fars	591 (532 to 650)	1053 (920 to 1194)	1693 (1484 to 1908)	2451 (2137 to 2786)	3627 (3186 to 4096)	4121 (3619 to 4645)	4646 (4181 to 5128)	5056 (4470 to 5681)	5086 (4445 to 5754)	5104 (4428 to 5834)
Gilan	783 (712 to 861)	1002 (853 to 1155)	1356 (1180 to 1536)	1801 (1570 to 2047)	2280 (2006 to 2569)	2363 (2064 to 2667)	2521 (2270 to 2784)	2617 (2312 to 2938)	2619 (2289 to 2950)	2614 (2263 to 2973)
Golestan	460 (416 to 508)	522 (453 to 594)	635 (555 to 721)	915 (796 to 1045)	1344 (1174 to 1526)	1531 (1340 to 1732)	1802 (1618 to 2001)	1971 (1733 to 2222)	1978 (1718 to 2240)	1981 (1708 to 2276)
Hamadan	603 (547 to 664)	778 (674 to 883)	965 (841 to 1090)	1253 (1088 to 1427)	1672 (1468 to 1890)	1734 (1515 to 1963)	1812 (1625 to 1997)	1763 (1545 to 1978)	1753 (1523 to 1983)	1740 (1493 to 1991)
Hormozgan	190 (170 to 210)	284 (244 to 325)	402 (349 to 456)	570 (489 to 651)	913 (789 to 1033)	1205 (1045 to 1365)	1571 (1398 to 1740)	1919 (1681 to 2167)	1950 (1677 to 2215)	1976 (1682 to 2262)
Ilam	88 (79 to 97)	122 (106 to 139)	182 (158 to 206)	296 (256 to 337)	443 (389 to 500)	524 (459 to 594)	567 (510 to 626)	606 (535 to 681)	609 (532 to 689)	612 (529 to 699)
Isfahan	876 (794 to 966)	1181 (1034 to 1344)	1599 (1385 to 1826)	2427 (2121 to 2765)	3777 (3331 to 4269)	4253 (3730 to 4847)	4917 (4451 to 5443)	5356 (4756 to 6021)	5382 (4725 to 6107)	5393 (4679 to 6178)
Kerman	817 (746 to 895)	775 (681 to 877)	879 (774 to 989)	1316 (1155 to 1492)	1855 (1635 to 2089)	2283 (2015 to 2569)	2937 (2657 to 3237)	3368 (2996 to 3779)	3400 (2996 to 3828)	3423 (2991 to 3901)
Kermanshah	491 (446 to 539)	657 (572 to 743)	879 (772 to 989)	1197 (1042 to 1355)	1688 (1477 to 1901)	1880 (1645 to 2116)	1992 (1794 to 2199)	2010 (1767 to 2253)	2006 (1748 to 2263)	1998 (1726 to 2275)
Khorasan-e-Razavi	1405 (1287 to 1552)	1740 (1535 to 1980)	2163 (1914 to 2445)	3147 (2776 to 3583)	4720 (4164 to 5337)	5177 (4567 to 5877)	6091 (5517 to 6736)	6799 (6063 to 7665)	6852 (6020 to 7823)	6888 (6018 to 7886)
Khuzestan	1327 (1208 to 1451)	1473 (1287 to 1663)	1858 (1634 to 2082)	2417 (2119 to 2727)	3203 (2817 to 3600)	4100 (3615 to 4595)	4638 (4188 to 5110)	4992 (4425 to 5583)	5010 (4379 to 5643)	5017 (4342 to 5702)
Kohgiluyeh and Boyer-Ahmad	76 (69 to 83)	123 (107 to 139)	188 (164 to 212)	301 (262 to 341)	484 (423 to 545)	593 (517 to 666)	673 (604 to 741)	759 (667 to 851)	768 (670 to 866)	775 (667 to 882)
Kurdistan	526 (475 to 580)	585 (509 to 665)	678 (591 to 768)	889 (771 to 1014)	1245 (1089 to 1404)	1431 (1253 to 1617)	1529 (1369 to 1692)	1697 (1492 to 1919)	1711 (1488 to 1929)	1722 (1480 to 1970)
Lorestan	652 (589 to 718)	669 (579 to 762)	765 (667 to 865)	1113 (962 to 1267)	1550 (1348 to 1761)	1694 (1477 to 1906)	1801 (1610 to 1990)	1797 (1577 to 2026)	1793 (1544 to 2027)	1786 (1528 to 2049)
Markazi	242 (218 to 266)	465 (404 to 527)	766 (668 to 867)	943 (820 to 1072)	1197 (1049 to 1350)	1305 (1139 to 1476)	1431 (1288 to 1580)	1462 (1284 to 1638)	1460 (1272 to 1652)	1454 (1262 to 1658)
Mazandaran	1156 (1055 to 1259)	1225 (1076 to 1381)	1397 (1227 to 1561)	1895 (1657 to 2138)	2571 (2267 to 2881)	2804 (2465 to 3144)	3115 (2817 to 3416)	3485 (3080 to 3901)	3512 (3076 to 3943)	3530 (3060 to 4013)
North Khorasan	169 (153 to 187)	213 (185 to 243)	271 (237 to 309)	405 (352 to 463)	626 (546 to 713)	744 (649 to 845)	888 (796 to 988)	882 (775 to 996)	878 (764 to 1000)	873 (752 to 1003)
Qazvin	439 (400 to 485)	469 (397 to 548)	531 (439 to 633)	659 (572 to 759)	951 (836 to 1071)	1117 (974 to 1268)	1217 (1098 to 1345)	1330 (1178 to 1499)	1338 (1165 to 1524)	1343 (1164 to 1538)
Qom	104 (94 to 115)	211 (183 to 241)	372 (326 to 424)	496 (434 to 570)	734 (645 to 835)	951 (839 to 1084)	1150 (1036 to 1280)	1390 (1235 to 1578)	1412 (1237 to 1616)	1430 (1241 to 1647)
Semnan	95 (86 to 105)	157 (136 to 178)	249 (215 to 283)	344 (293 to 395)	468 (404 to 530)	545 (472 to 617)	631 (562 to 697)	754 (658 to 847)	766 (661 to 867)	776 (664 to 886)
Sistan and Baluchistan	423 (383 to 467)	442 (386 to 502)	526 (461 to 596)	858 (747 to 976)	1465 (1292 to 1652)	2029 (1776 to 2301)	2585 (2328 to 2862)	3078 (2716 to 3461)	3126 (2723 to 3550)	3167 (2738 to 3619)
South Khorasan	234 (211 to 256)	292 (254 to 330)	366 (318 to 412)	501 (435 to 567)	669 (584 to 751)	640 (559 to 723)	675 (604 to 743)	852 (751 to 956)	872 (758 to 981)	889 (765 to 1010)
Tehran	1229 (1115 to 1356)	2421 (2118 to 2748)	4229 (3718 to 4777)	5619 (4912 to 6430)	8220 (7225 to 9303)	10359 (9163 to 11670)	12245 (11070 to 13581)	14104 (12532 to 15866)	14248 (12556 to 16114)	14353 (12496 to 16407)
West Azarbayejan	573 (519 to 634)	863 (754 to 980)	1214 (1066 to 1376)	1624 (1425 to 1852)	2331 (2052 to 2645)	2686 (2369 to 3041)	3113 (2812 to 3442)	3474 (3091 to 3909)	3498 (3067 to 3964)	3513 (3045 to 4042)
Yazd	208 (188 to 230)	254 (220 to 291)	308 (266 to 354)	423 (367 to 485)	683 (596 to 780)	865 (757 to 989)	1078 (971 to 1199)	1186 (1043 to 1346)	1194 (1040 to 1365)	1198 (1035 to 1383)
Zanjan	552 (499 to 605)	549 (467 to 643)	591 (478 to 710)	680 (584 to 776)	900 (788 to 1021)	977 (847 to 1121)	1039 (932 to 1151)	1091 (959 to 1223)	1092 (950 to 1240)	1091 (942 to 1244)
Iraq	5282 (4958 to 5622)	7064 (6450 to 7661)	9730 (8733 to 10729)	13715 (12331 to 14882)	18417 (16728 to 20074)	25133 (21590 to 29077)	31802 (24192 to 38837)	39929 (29795 to 50226)	40592 (29867 to 51522)	41224 (29163 to 52061)
Jordan	460 (349 to 573)	744 (608 to 880)	1303 (1136 to 1477)	2281 (2115 to 2452)	3736 (3398 to 4091)	4819 (4385 to 5267)	7450 (6711 to 8181)	11514 (10477 to 12545)	11926 (10691 to 13029)	12325 (11066 to 13681)
Kuwait	101 (91 to 111)	281 (260 to 302)	776 (724 to 828)	1404 (1312 to 1496)	1719 (1554 to 1881)	1916 (1720 to 2108)	3004 (2774 to 3232)	4385 (3890 to 4884)	4521 (3971 to 5067)	4650 (4033 to 5277)
Lebanon	1285 (1196 to 1367)	1693 (1488 to 1903)	2197 (2047 to 2355)	2509 (2183 to 2841)	2992 (2460 to 3517)	3561 (3198 to 3966)	4176 (3809 to 4546)	5549 (4776 to 6355)	5548 (4700 to 6382)	5540 (4674 to 6394)
Libya	1068 (992 to 1140)	1413 (1281 to 1553)	1892 (1721 to 2054)	3070 (2779 to 3347)	4215 (3797 to 4648)	5093 (4592 to 5598)	6186 (5628 to 6722)	6800 (5767 to 7824)	6839 (5810 to 7906)	6871 (5809 to 7977)
Morocco	9206 (8601 to 9881)	12024 (11214 to 12857)	15703 (14526 to 16838)	20424 (18879 to 21986)	25357 (23021 to 27749)	29718 (26803 to 32630)	33627 (30433 to 36779)	36710 (33020 to 40259)	36962 (33058 to 40794)	37177 (33142 to 41310)
Oman	451 (295 to 602)	659 (484 to 833)	970 (762 to 1175)	1422 (1212 to 1640)	1984 (1808 to 2165)	2329 (2120 to 2529)	2828 (2644 to 3016)	4318 (3992 to 4638)	4514 (4226 to 4812)	4704 (4354 to 5061)
Palestine	800 (671 to 936)	908 (808 to 1011)	1113 (1015 to 1215)	1464 (1259 to 1674)	2047 (1819 to 2280)	3020 (2753 to 3295)	4124 (3774 to 4468)	4970 (4574 to 5344)	5054 (4619 to 5506)	5136 (4663 to 5613)
Qatar	26 (18 to 33)	57 (43 to 69)	131 (109 to 152)	273 (244 to 301)	445 (403 to 485)	592 (538 to 643)	1745 (1625 to 1863)	2827 (2614 to 3032)	2903 (2706 to 3100)	2977 (2746 to 3202)
Saudi Arabia	4285 (3994 to 4589)	4623 (4014 to 5230)	5924 (5357 to 6489)	9562 (8616 to 10643)	15856 (14480 to 17155)	20764 (18764 to 22785)	27945 (26051 to 30019)	36208 (31591 to 40728)	36979 (32069 to 41695)	37704 (32635 to 43017)
Sudan	5958 (5559 to 6331)	7138 (6457 to 7834)	10270 (9339 to 11184)	14496 (13279 to 15842)	20021 (18244 to 21736)	26725 (23692 to 29798)	33986 (31356 to 36811)	41901 (36302 to 47241)	42694 (36654 to 48947)	43419 (36967 to 49714)
Syria	3456 (3225 to 3694)	4768 (4433 to 5104)	6603 (6162 to 7024)	9046 (8391 to 9695)	12718 (11471 to 13900)	16729 (15088 to 18211)	22062 (19789 to 24290)	13961 (11728 to 16181)	13988 (11599 to 16346)	14030 (11466 to 16228)
Tunisia	3719 (3456 to 3972)	4301 (3920 to 4702)	5109 (4648 to 5609)	6541 (5936 to 7171)	8349 (7571 to 9146)	9841 (8931 to 10751)	10791 (9809 to 11788)	11703 (10542 to 12902)	11784 (10553 to 13034)	11843 (10595 to 13166)
Türkiye	21034 (19617 to 22415)	27350 (25464 to 29240)	35766 (33260 to 38148)	45436 (42195 to 48655)	57473 (53610 to 61149)	67054 (58221 to 75645)	74101 (68325 to 79931)	82674 (82161 to 83182)	83201 (77616 to 88893)	83612 (77066 to 89962)
United Arab Emirates	73 (60 to 86)	106 (94 to 118)	250 (231 to 270)	1073 (1002 to 1143)	1871 (1691 to 2055)	3227 (2900 to 3548)	8442 (7584 to 9325)	9504 (7985 to 10893)	9571 (8077 to 11085)	9631 (7900 to 11219)
Yemen	4277 (2743 to 5838)	5245 (3731 to 6697)	6733 (5207 to 8165)	9418 (7952 to 10828)	13634 (12345 to 14866)	18623 (17013 to 20211)	25410 (22672 to 28036)	32274 (26985 to 37491)	32969 (27627 to 38764)	33643 (28179 to 39519)
South Asia	445259 (420455 to 470882)	536596 (503889 to 569496)	684675 (645467 to 725684)	883150 (832367 to 932452)	1093413 (1028061 to 1158287)	1325086 (1245384 to 1402975)	1587521 (1492031 to 1679534)	1807416 (1654521 to 1976406)	1827971 (1652398 to 2001229)	1846571 (1668045 to 2038669)
South Asia	445259 (420455 to 470882)	536596 (503889 to 569496)	684675 (645467 to 725684)	883150 (832367 to 932452)	1093413 (1028061 to 1158287)	1325086 (1245384 to 1402975)	1587521 (1492031 to 1679534)	1807416 (1654521 to 1976406)	1827971 (1652398 to 2001229)	1846571 (1668045 to 2038669)
Bangladesh	41424 (38485 to 44153)	48142 (44528 to 51666)	65864 (59835 to 71918)	85133 (78631 to 91753)	109121 (101414 to 117222)	129406 (119819 to 138805)	147187 (136150 to 158231)	162146 (143758 to 181076)	163458 (141817 to 183801)	164636 (142940 to 185760)
Bhutan	177 (165 to 190)	233 (203 to 262)	325 (262 to 387)	463 (361 to 560)	630 (533 to 728)	645 (582 to 712)	697 (632 to 767)	760 (702 to 821)	758 (690 to 824)	757 (685 to 823)
India	360271 (335776 to 385169)	437552 (404893 to 469341)	545159 (506165 to 583815)	694398 (644854 to 742587)	853065 (788853 to 914903)	1032537 (952915 to 1109616)	1233548 (1141885 to 1324659)	1388624 (1235920 to 1556437)	1402362 (1229816 to 1567343)	1414495 (1240241 to 1601508)
Nepal	8382 (7814 to 8923)	9908 (9204 to 10649)	12090 (11197 to 12967)	15778 (14668 to 16942)	19469 (17971 to 20956)	23924 (22225 to 25547)	27622 (25604 to 29672)	30518 (26700 to 34353)	30838 (27153 to 34395)	31130 (27340 to 35331)
Pakistan	35004 (32478 to 37381)	40761 (37596 to 43774)	61237 (56396 to 66026)	87377 (80774 to 93642)	111127 (98743 to 123323)	138573 (127497 to 149808)	178466 (158773 to 198028)	225369 (208285 to 243065)	230555 (210268 to 251536)	235554 (215432 to 256773)
Azad Jammu & Kashmir	936 (819 to 1058)	986 (875 to 1102)	1420 (1247 to 1601)	1954 (1733 to 2183)	2469 (2047 to 2918)	2976 (2609 to 3367)	3457 (2797 to 4135)	4013 (3184 to 4855)	4067 (3123 to 4951)	4115 (3137 to 5089)
Balochistan	1201 (1060 to 1369)	1063 (946 to 1189)	2094 (1843 to 2364)	4251 (3778 to 4745)	5562 (4618 to 6645)	6676 (5886 to 7519)	9466 (7958 to 11115)	13508 (11967 to 15206)	13998 (12137 to 15880)	14489 (12596 to 16602)

Gilgit-Baltistan	242 (189 to 298)	277 (233 to 327)	384 (339 to 435)	564 (504 to 630)	778 (649 to 930)	1030 (831 to 1255)	1369 (997 to 1778)	1778 (1217 to 2355)	1826 (1284 to 2453)	1873 (1266 to 2458)
Islamabad Capital Territory	141 (123 to 158)	153 (129 to 178)	218 (192 to 245)	334 (298 to 371)	531 (442 to 627)	872 (769 to 979)	1459 (1221 to 1709)	2161 (1909 to 2429)	2239 (1954 to 2534)	2315 (2011 to 2646)
Khyber Pakhtunkhwa	6168 (5436 to 6936)	7040 (6341 to 7785)	9731 (8698 to 10879)	13505 (12163 to 14959)	17085 (14496 to 19764)	21570 (19312 to 24060)	28941 (24685 to 33680)	37969 (34050 to 42135)	38986 (34361 to 43802)	39977 (35122 to 45330)
Punjab	20347 (18408 to 22207)	23478 (21452 to 25351)	34457 (31354 to 37594)	47677 (43604 to 51626)	60131 (52258 to 67810)	74416 (67650 to 81304)	93503 (81579 to 105335)	115468 (105185 to 125985)	117872 (105719 to 130161)	120162 (107576 to 132817)
Sindh	5970 (5272 to 6782)	7762 (6918 to 8613)	12932 (11485 to 14499)	19092 (17119 to 21113)	24570 (20567 to 28825)	31034 (27560 to 34632)	40270 (34097 to 46758)	50472 (44946 to 56248)	51567 (45116 to 58161)	52622 (46004 to 59306)
Southeast Asia, east Asia, and Oceania	744378 (707868 to 783682)	923060 (862031 to 985603)	1144661 (1054326 to 1227131)	1394936 (1311659 to 1472700)	1689497 (1606761 to 1774023)	1855937 (1761863 to 1948976)	2014242 (1927893 to 2104034)	2162312 (2051970 to 2267618)	2174906 (2067884 to 2270838)	2185007 (2074129 to 2292836)
East Asia	553421 (518444 to 591176)	677793 (618424 to 739111)	833617 (745192 to 915605)	1007133 (925796 to 1084136)	1217440 (1136722 to 1304383)	1304755 (1217993 to 1392709)	1385491 (1301451 to 1473823)	1464290 (1356381 to 1567835)	1469471 (1369815 to 1561989)	1472775 (1369752 to 1579504)
China	535248 (499854 to 573330)	654108 (595028 to 715482)	803248 (714205 to 885788)	971250 (889206 to 1048596)	1176458 (1096739 to 1263975)	1259086 (1172263 to 1346854)	1336938 (1252377 to 1425239)	1414370 (1305491 to 1518669)	1419485 (1318719 to 1512217)	1422746 (1318759 to 1530462)
North Korea	10568 (7120 to 14165)	12873 (9550 to 16348)	15709 (12426 to 18930)	18001 (15297 to 20471)	20591 (18727 to 22437)	23404 (20852 to 25974)	25363 (23398 to 27398)	26279 (22669 to 29963)	26342 (22435 to 30041)	26392 (22353 to 30270)
Taiwan (province of China)	7605 (7558 to 7651)	10811 (10752 to 10869)	14660 (14591 to 14727)	17882 (17799 to 17964)	20391 (20277 to 20511)	22265 (22122 to 22406)	23190 (23010 to 23374)	23641 (21678 to 25466)	23645 (21519 to 25630)	23637 (21439 to 25875)
Oceania	2655 (2336 to 2964)	3291 (3083 to 3512)	4140 (3943 to 4342)	5196 (4973 to 5419)	6550 (5983 to 7092)	8346 (7949 to 8716)	10666 (10092 to 11258)	13286 (12048 to 14389)	13608 (12282 to 14959)	13928 (12507 to 15293)
American Samoa	20 (18 to 21)	21 (19 to 22)	28 (26 to 30)	33 (31 to 36)	49 (45 to 52)	58 (55 to 63)	57 (53 to 61)	51 (47 to 55)	50 (47 to 54)	50 (46 to 53)
Cook Islands	15 (14 to 16)	19 (18 to 20)	22 (20 to 23)	18 (17 to 20)	19 (17 to 20)	19 (17 to 20)	18 (17 to 20)	18 (16 to 19)	18 (16 to 19)	18 (16 to 19)
Fiji	297 (276 to 317)	408 (372 to 446)	538 (485 to 589)	657 (598 to 720)	758 (690 to 829)	816 (739 to 892)	873 (797 to 948)	917 (844 to 990)	921 (844 to 1002)	924 (839 to 1016)
Guam	61 (57 to 65)	69 (64 to 73)	87 (81 to 94)	108 (101 to 116)	137 (127 to 146)	159 (149 to 170)	164 (153 to 176)	161 (148 to 172)	160 (149 to 170)	159 (147 to 171)
Kiribati	30 (28 to 32)	37 (34 to 40)	46 (43 to 50)	62 (57 to 67)	74 (69 to 80)	87 (81 to 94)	105 (98 to 112)	118 (107 to 128)	120 (109 to 131)	121 (108 to 134)
Marshall Islands	12 (8 to 15)	17 (13 to 21)	24 (19 to 28)	34 (30 to 38)	45 (42 to 49)	53 (48 to 57)	55 (51 to 59)	56 (49 to 62)	56 (49 to 63)	56 (49 to 64)
Federated States of Micronesia	41 (39 to 44)	52 (46 to 59)	66 (53 to 80)	85 (72 to 99)	103 (94 to 114)	109 (102 to 117)	105 (98 to 112)	103 (90 to 115)	103 (89 to 117)	103 (90 to 116)
Nauru	4 (3 to 4)	5 (4 to 5)	7 (7 to 8)	9 (8 to 11)	10 (9 to 11)	11 (10 to 12)	11 (10 to 11)	11 (10 to 12)	11 (10 to 12)	11 (10 to 12)
Niue	5 (4 to 5)	5 (5 to 5)	5 (5 to 6)	4 (3 to 4)	2 (2 to 3)	2 (2 to 2)	2 (1 to 2)	2 (1 to 2)	2 (1 to 2)	2 (1 to 2)
Northern Mariana Islands	5 (4 to 5)	7 (6 to 8)	10 (9 to 10)	17 (16 to 18)	45 (42 to 48)	73 (68 to 77)	55 (51 to 59)	49 (45 to 53)	49 (45 to 52)	48 (45 to 52)
Palau	7 (7 to 8)	9 (8 to 10)	11 (10 to 12)	13 (12 to 14)	15 (14 to 17)	20 (18 to 21)	18 (17 to 20)	18 (16 to 20)	18 (16 to 20)	18 (16 to 20)
Papua New Guinea	1714 (1408 to 2008)	2072 (1870 to 2286)	2551 (2369 to 2749)	3201 (2999 to 3411)	4103 (3553 to 4637)	5518 (5145 to 5876)	7540 (7000 to 8107)	9886 (8706 to 10974)	10175 (8890 to 11495)	10462 (9096 to 11787)
Samoa	87 (81 to 93)	115 (106 to 123)	148 (137 to 159)	161 (148 to 174)	169 (157 to 182)	180 (166 to 193)	191 (176 to 205)	208 (190 to 226)	211 (192 to 232)	214 (193 to 236)
Solomon Islands	104 (97 to 111)	136 (125 to 145)	171 (160 to 182)	234 (212 to 255)	339 (309 to 371)	445 (412 to 480)	550 (507 to 591)	658 (572 to 745)	671 (571 to 767)	684 (579 to 780)
Tokelau	2 (2 to 2)	2 (2 to 2)	2 (2 to 2)	2 (1 to 2)	2 (1 to 2)	2 (1 to 2)	1 (1 to 1)	1 (1 to 1)	1 (1 to 1)	1 (1 to 2)
Tonga	50 (46 to 54)	68 (61 to 74)	87 (79 to 95)	97 (89 to 107)	99 (90 to 108)	103 (93 to 113)	106 (99 to 114)	106 (97 to 115)	106 (96 to 115)	106 (96 to 117)
Tuvalu	4 (4 to 4)	5 (5 to 6)	6 (5 to 6)	8 (8 to 9)	10 (9 to 10)	10 (9 to 11)	11 (10 to 12)	12 (11 to 14)	12 (11 to 14)	12 (11 to 14)
Vanuatu	51 (48 to 55)	65 (58 to 73)	89 (81 to 97)	119 (111 to 128)	152 (141 to 163)	194 (180 to 208)	248 (230 to 266)	300 (277 to 323)	307 (286 to 328)	313 (291 to 336)
Southeast Asia	188302 (182188 to 194573)	241976 (232753 to 251733)	306904 (295330 to 318941)	382607 (371232 to 394460)	465507 (450528 to 481579)	542836 (512516 to 573172)	618086 (598873 to 638587)	684735 (659738 to 711897)	691826 (667215 to 718646)	698305 (670177 to 727998)
Cambodia	4404 (4106 to 4715)	5868 (5370 to 6367)	7557 (6625 to 8470)	8026 (6618 to 9450)	10273 (9099 to 11507)	12533 (11531 to 13602)	14523 (13303 to 15716)	16595 (14199 to 18858)	16832 (14353 to 19152)	17044 (14539 to 19614)
Indonesia	79502 (74181 to 84930)	98372 (91366 to 105707)	123300 (113654 to 132319)	153735 (144367 to 163431)	184983 (172490 to 197569)	211679 (182890 to 240475)	241527 (225752 to 257593)	272627 (252081 to 293115)	275905 (257341 to 293507)	278914 (256923 to 300109)
Aceh	1265 (1144 to 1387)	1628 (1388 to 1894)	2110 (1801 to 2446)	2775 (2510 to 3031)	3519 (3184 to 3868)	4096 (3341 to 4880)	4551 (4121 to 4990)	5193 (4623 to 5756)	5265 (4770 to 5767)	5331 (4733 to 5902)
Bali	1562 (1405 to 1723)	1845 (1564 to 2158)	2203 (1852 to 2582)	2588 (2341 to 2838)	2906 (2617 to 3195)	3355 (2730 to 4019)	3909 (3523 to 4301)	4393 (3906 to 4887)	4437 (4009 to 4882)	4476 (3972 to 4996)
Bangka-Belitung Islands	332 (301 to 363)	415 (349 to 479)	525 (444 to 604)	680 (616 to 743)	845 (763 to 925)	1018 (834 to 1204)	1241 (1122 to 1355)	1460 (1307 to 1611)	1484 (1338 to 1626)	1505 (1342 to 1664)
Banten	1948 (1758 to 2132)	2047 (1739 to 2360)	2560 (2172 to 2962)	4169 (3776 to 4550)	8058 (7292 to 8804)	10350 (8456 to 12239)	10841 (9817 to 11844)	11888 (10640 to 13097)	12023 (10835 to 13133)	12149 (10818 to 13412)
Bengkulu	389 (353 to 429)	448 (382 to 521)	564 (482 to 658)	805 (733 to 881)	1223 (1108 to 1343)	1528 (1256 to 1826)	1755 (1594 to 1927)	2012 (1805 to 2229)	2041 (1854 to 2238)	2068 (1849 to 2290)
Gorontalo	276 (249 to 302)	370 (316 to 426)	514 (440 to 595)	609 (554 to 663)	616 (559 to 675)	801 (654 to 954)	1072 (975 to 1171)	1194 (1074 to 1318)	1202 (1093 to 1317)	1210 (1085 to 1335)
Jakarta	2517 (2282 to 2756)	3419 (2921 to 3929)	4824 (4099 to 5569)	6692 (6110 to 7289)	8558 (7771 to 9371)	9103 (7450 to 10796)	9505 (8655 to 10385)	10526 (9451 to 11598)	10638 (9667 to 11608)	10739 (9619 to 11825)
Jambi	710 (639 to 781)	860 (729 to 995)	1106 (938 to 1295)	1511 (1366 to 1659)	2080 (1877 to 2298)	2626 (2134 to 3154)	3148 (2837 to 3473)	3537 (3143 to 3934)	3575 (3226 to 3922)	3609 (3207 to 4002)
West Java	12549 (11456 to 13701)	15538 (13514 to 17602)	19742 (17106 to 22402)	24529 (22455 to 26704)	28622 (26205 to 31288)	35268 (29625 to 41563)	43514 (39879 to 47646)	48903 (44334 to 53733)	49375 (45225 to 54009)	49810 (44933 to 54975)
Central Java	13972 (12793 to 15251)	17917 (15605 to 20505)	22333 (19401 to 25445)	26582 (24482 to 28858)	29572 (27130 to 32327)	30993 (25908 to 36735)	33147 (30318 to 36248)	37216 (33709 to 41087)	37667 (34509 to 41193)	38067 (34344 to 42032)
East Java	17641 (16117 to 19181)	21687 (18823 to 24643)	26197 (22763 to 29659)	30535 (27926 to 33142)	33645 (30704 to 36672)	35618 (29743 to 41947)	38106 (34800 to 41588)	41435 (37340 to 45579)	41742 (38037 to 45480)	41988 (37782 to 46179)
West Kalimantan	1302 (1179 to 1426)	1606 (1365 to 1867)	2027 (1717 to 2341)	2645 (2405 to 2893)	3342 (3030 to 3662)	3847 (3158 to 4583)	4513 (4080 to 4964)	5419 (4830 to 6018)	5529 (5022 to 6062)	5635 (5045 to 6253)
South Kalimantan	1131 (1018 to 1245)	1361 (1139 to 1586)	1710 (1434 to 1989)	2171 (1964 to 2383)	2692 (2428 to 2960)	3292 (2639 to 3934)	3848 (3464 to 4222)	4088 (3634 to 4529)	4102 (3696 to 4491)	4113 (3650 to 4573)
Central Kalimantan	441 (398 to 483)	541 (457 to 623)	704 (596 to 807)	988 (895 to 1079)	1430 (1287 to 1570)	1829 (1474 to 2176)	2242 (2021 to 2456)	2665 (2372 to 2946)	2713 (2451 to 2967)	2757 (2455 to 3041)
East Kalimantan	455 (412 to 501)	555 (470 to 644)	735 (625 to 852)	1089 (994 to 1198)	1689 (1537 to 1865)	2331 (1912 to 2792)	3034 (2756 to 3340)	3715 (3327 to 4132)	3789 (3428 to 4162)	3859 (3432 to 4290)
North Kalimantan	68 (62 to 75)	90 (77 to 104)	130 (110 to 150)	181 (164 to 198)	242 (219 to 266)	352 (285 to 422)	525 (474 to 576)	689 (615 to 762)	707 (641 to 774)	725 (648 to 800)
Riau Islands	193 (175 to 212)	205 (175 to 237)	255 (218 to 295)	444 (404 to 486)	1010 (916 to 1109)	1404 (1156 to 1666)	1685 (1530 to 1846)	2167 (1946 to 2396)	2226 (2019 to 2437)	2282 (2036 to 2526)
Lampung	1965 (1775 to 2155)	2521 (2119 to 2904)	3475 (2942 to 4006)	4829 (4350 to 5261)	6213 (5587 to 6793)	6944 (5624 to 8278)	7735 (6987 to 8480)	9009 (8029 to 10012)	9156 (8279 to 10035)	9295 (8276 to 10267)
Maluku	542 (489 to 594)	608 (516 to 698)	726 (616 to 834)	927 (842 to 1014)	1191 (1075 to 1307)	1362 (1118 to 1623)	1565 (1405 to 1714)	1859 (1656 to 2063)	1899 (1713 to 2081)	1939 (1720 to 2142)
North Maluku	296 (267 to 326)	336 (286 to 392)	403 (339 to 470)	531 (481 to 583)	721 (651 to 796)	880 (718 to 1049)	1052 (951 to 1161)	1268 (1133 to 1407)	1295 (1172 to 1424)	1321 (1177 to 1470)
West Nusa Tenggara	1730 (1561 to 1914)	1935 (1652 to 2249)	2302 (1966 to 2680)	2872 (2597 to 3156)	3469 (3144 to 3821)	3976 (3223 to 4748)	4608 (4167 to 5083)	5269 (4701 to 5857)	5339 (4832 to 5872)	5405 (4817 to 6020)
East Nusa Tenggara	1726 (1561 to 1901)	2015 (1718 to 2336)	2398 (2033 to 2786)	2872 (2614 to 3154)	3380 (3070 to 3720)	4012 (3273 to 4828)	4790 (4347 to 5268)	5318 (4767 to 5914)	5382 (4880 to 5903)	5446 (4845 to 6063)
Papua	249 (224 to 274)	318 (269 to 370)	439 (372 to 512)	652 (591 to 716)	1055 (952 to 1166)	1747 (1421 to 2098)	2917 (2634 to 3216)	4485 (3997 to 4983)	4673 (4221 to 5133)	4858 (4314 to 5407)
West Papua	201 (182 to 221)	287 (245 to 332)	395 (337 to 458)	514 (469 to 564)	621 (563 to 684)	644 (528 to 771)	764 (694 to 838)	1096 (983 to 1215)	1143 (1033 to 1258)	1191 (1066 to 1321)
Riau	762 (688 to 841)	1005 (847 to 1166)	1367 (1147 to 1591)	1821 (1655 to 2000)	2361 (2123 to 2606)	3656 (2985 to 4389)	5551 (5009 to 6125)	6344 (5665 to 7074)	6396 (5786 to 7038)	6446 (5740 to 7158)
West Sulawesi	394 (357 to 438)	437 (371 to 506)	509 (429 to 594)	612 (557 to 675)	725 (657 to 805)	917 (750 to 1115)	1191 (1081 to 1315)	1411 (1266 to 1577)	1436 (1301 to 1586)	1460 (1303 to 1633)
South Sulawesi	3217 (2899 to 3547)	3875 (3300 to 4496)	4787 (4019 to 5615)	5815 (5262 to 6363)	6591 (5933 to 7267)	7314 (5921 to 8765)	8203 (7398 to 9039)	9196 (8140 to 10243)	9308 (8386 to 10245)	9414 (8384 to 10493)
Central Sulawesi	639 (577 to 702)	760 (644 to 882)	985 (835 to 1144)	1335 (1209 to 1465)	1760 (1592 to 1942)	2216 (1801 to 2661)	2699 (2446 to 2976)	2993 (2677 to 3322)	3022 (2726 to 3321)	3049 (2719 to 3414)
Southeast Sulawesi	480 (431 to 528)	579 (483 to 669)	727 (614 to 843)	992 (893 to 1085)	1384 (1245 to 1519)	1804 (1463 to 2143)	2261 (2034 to 2479)	2600 (2314 to 2884)	2637 (2376 to 2889)	2673 (2370 to 2956)
North Sulawesi	882 (797 to 969)	1063 (899 to 1220)	1263 (1067 to 1455)	1581 (1432 to 1731)	1961 (1769 to 2154)	2134 (1736 to 2527)	2307 (2080 to 2523)	2657 (2374 to 2929)	2697 (2433 to 2953)	2735 (2429 to 3024)
West Sumatra	1987 (1797 to 2191)	2427 (2068 to 2796)	2928 (2490 to 3385)	3573 (3242 to 3923)	4150 (3742 to 4591)	4479 (3653 to 5393)	4915 (4451 to 5421)	5594 (5000 to 6243)	5678 (5140 to 6256)	5758 (5135 to 6416)
South Sumatra	1761 (1596 to 1939)	2258 (1921 to 2595)	3004 (2552 to 3473)	4168 (3794 to 4575)	5648 (5132 to 6207)	6694 (5561 to 7968)	7616 (6949 to 8355)	8441 (7595 to 9365)	8525 (7787 to 9329)	8603 (7733 to 9577)

North Sumatra	4036 (3664 to 4418)	5174 (4391 to 5942)	6748 (5773 to 7758)	8760 (7999 to 9548)	10651 (9697 to 11654)	11853 (9879 to 14016)	13229 (12053 to 14474)	14838 (13377 to 16374)	15033 (13731 to 16441)	15220 (13645 to 16790)
Yogyakarta	1882 (1704 to 2085)	2243 (1922 to 2614)	2604 (2233 to 3048)	2887 (2626 to 3194)	3050 (2770 to 3385)	3235 (2671 to 3923)	3489 (3167 to 3868)	3748 (3375 to 4186)	3768 (3420 to 4180)	3781 (3388 to 4243)
Laos	1714 (1229 to 2192)	2135 (1673 to 2627)	2655 (2252 to 3089)	3283 (2949 to 3610)	4169 (3734 to 4576)	5388 (4852 to 5932)	6352 (5718 to 6935)	7181 (6490 to 7851)	7280 (6583 to 8009)	7378 (6610 to 8098)
Malaysia	6346 (5526 to 7124)	8336 (7819 to 8868)	10720 (9967 to 11408)	13575 (12654 to 14502)	17667 (16289 to 19001)	23817 (22249 to 25456)	28185 (26372 to 30219)	31238 (27284 to 35120)	31540 (27534 to 35744)	31812 (27220 to 35985)
Maldives	78 (72 to 83)	93 (85 to 100)	121 (110 to 130)	163 (149 to 177)	222 (207 to 237)	280 (261 to 299)	352 (320 to 385)	488 (439 to 535)	503 (451 to 553)	517 (456 to 571)
Mauritius	488 (458 to 522)	671 (616 to 726)	831 (765 to 896)	987 (899 to 1079)	1096 (1026 to 1171)	1214 (1130 to 1302)	1268 (1176 to 1365)	1274 (1111 to 1440)	1273 (1111 to 1429)	1272 (1101 to 1439)
Myanmar	19177 (17737 to 20470)	22857 (19844 to 25889)	27965 (25549 to 30436)	34363 (31449 to 37180)	40438 (36066 to 44754)	45267 (38334 to 52250)	50080 (45520 to 55060)	55296 (49461 to 60918)	55878 (49554 to 61846)	56420 (50184 to 62791)
Philippines	20308 (18951 to 21662)	28677 (26660 to 30569)	38532 (36007 to 41057)	49559 (46401 to 52615)	63008 (58854 to 67309)	79452 (73873 to 85076)	96095 (89680 to 102973)	110442 (100037 to 120023)	111909 (101474 to 123576)	113250 (100304 to 125282)
Seychelles	35 (32 to 37)	43 (40 to 46)	54 (50 to 58)	67 (61 to 72)	73 (66 to 79)	82 (75 to 88)	94 (87 to 100)	103 (91 to 116)	104 (90 to 119)	105 (91 to 121)
Sri Lanka	7832 (7330 to 8392)	10156 (9232 to 11039)	12846 (11899 to 13828)	15144 (14042 to 16258)	17131 (14920 to 19212)	18711 (16167 to 21215)	20506 (18966 to 22122)	22007 (19581 to 24309)	22147 (19506 to 24886)	22270 (19444 to 25035)
Thailand	20609 (19103 to 22014)	28137 (26187 to 30006)	35778 (33258 to 38183)	46647 (43463 to 49917)	56765 (53039 to 60702)	62519 (58478 to 66849)	67776 (63183 to 72384)	66738 (58378 to 75112)	66728 (58184 to 75657)	66683 (57543 to 75921)
Timor-Leste	451 (393 to 510)	544 (505 to 579)	548 (480 to 617)	582 (542 to 624)	781 (726 to 835)	904 (821 to 984)	1105 (1030 to 1176)	1341 (1215 to 1454)	1369 (1228 to 1507)	1398 (1249 to 1544)
Viet Nam	27086 (25244 to 28950)	35694 (31178 to 40299)	45498 (39918 to 51311)	55903 (51624 to 59893)	68227 (62742 to 73638)	80203 (74523 to 86376)	89330 (82902 to 95676)	98451 (91350 to 105193)	99393 (91320 to 106655)	100268 (92292 to 107845)
Sub-Saharan Africa	175958 (162782 to 188935)	223239 (210117 to 237168)	287066 (274829 to 299099)	372712 (361809 to 384862)	491364 (479652 to 503138)	646529 (628541 to 665655)	851661 (826746 to 877641)	1079249 (1038679 to 1120736)	1106473 (1064966 to 1150116)	1133158 (1087676 to 1180536)
Central sub-Saharan Africa	19318 (18398 to 20227)	25190 (22923 to 27472)	32767 (31172 to 34399)	41943 (39010 to 44737)	54963 (50763 to 59274)	73559 (65302 to 81308)	100132 (84899 to 114370)	130165 (105109 to 155027)	133594 (108021 to 159338)	136928 (110308 to 165520)
Angola	4371 (4077 to 4681)	5123 (4778 to 5475)	5919 (5520 to 6321)	7517 (6527 to 8459)	10276 (8379 to 12347)	14724 (12613 to 16900)	21818 (19785 to 24115)	30639 (27504 to 33667)	31678 (28382 to 34976)	32706 (29055 to 36449)
Central African Republic	1376 (1070 to 1682)	1661 (1405 to 1939)	2051 (1847 to 2255)	2310 (2092 to 2533)	2731 (2518 to 2968)	3617 (3322 to 3937)	4601 (4121 to 5098)	5285 (4446 to 6175)	5394 (4414 to 6321)	5484 (4507 to 6407)
Congo (Brazzaville)	797 (626 to 985)	1018 (865 to 1172)	1311 (1188 to 1437)	1760 (1590 to 1920)	2402 (2133 to 2653)	3155 (2795 to 3455)	4197 (3851 to 4533)	5205 (4455 to 5940)	5301 (4539 to 6106)	5392 (4589 to 6240)
DR Congo	12217 (11457 to 12980)	16709 (14444 to 18930)	22654 (21015 to 24273)	29293 (26438 to 32008)	38148 (34346 to 42288)	50183 (41939 to 58093)	66989 (51408 to 81113)	85840 (60460 to 110240)	87957 (61939 to 113160)	90018 (62963 to 117854)
Equatorial Guinea	187 (174 to 200)	208 (181 to 235)	237 (203 to 271)	296 (270 to 323)	423 (378 to 469)	654 (545 to 758)	1029 (927 to 1132)	1431 (1301 to 1566)	1473 (1323 to 1610)	1512 (1363 to 1676)
Gabon	370 (344 to 395)	470 (438 to 501)	594 (519 to 670)	767 (656 to 885)	984 (901 to 1080)	1227 (1086 to 1369)	1498 (1371 to 1623)	1765 (1580 to 1956)	1791 (1606 to 1997)	1816 (1607 to 2015)
Eastern sub-Saharan Africa	62745 (57501 to 67732)	81256 (76201 to 86523)	107258 (102522 to 112099)	142650 (138408 to 147386)	190825 (185140 to 197109)	249820 (241616 to 258731)	326757 (316665 to 336959)	406916 (389050 to 425319)	416649 (397679 to 436063)	426098 (406099 to 446710)
Burundi	2381 (1823 to 3012)	3074 (2531 to 3682)	3890 (3407 to 4379)	4412 (4069 to 4754)	5553 (5185 to 5920)	6390 (5607 to 7128)	9309 (8584 to 10045)	12337 (10652 to 13989)	12803 (10929 to 14710)	13220 (11339 to 15001)
Comoros	154 (129 to 180)	189 (169 to 208)	255 (231 to 278)	356 (331 to 380)	463 (430 to 495)	553 (505 to 601)	642 (569 to 722)	727 (604 to 852)	736 (605 to 867)	744 (612 to 882)
Djibouti	64 (48 to 79)	95 (75 to 116)	150 (127 to 172)	261 (238 to 285)	414 (369 to 460)	619 (546 to 696)	894 (831 to 962)	1195 (1044 to 1354)	1228 (1060 to 1402)	1259 (1082 to 1448)
Eritrea	1120 (791 to 1444)	1476 (1149 to 1793)	1956 (1655 to 2261)	2577 (2335 to 2815)	3406 (3034 to 3768)	3982 (3367 to 4647)	5345 (4026 to 6623)	6315 (4499 to 8089)	6456 (4675 to 8294)	6598 (4577 to 8754)
Ethiopia	17417 (12132 to 22273)	21873 (17127 to 26965)	27664 (23325 to 32010)	35019 (31956 to 38536)	50569 (46301 to 55699)	68419 (61771 to 75429)	85165 (77817 to 92200)	104414 (89393 to 119265)	106722 (91761 to 122629)	108938 (91827 to 125474)
Addis Ababa	644 (453 to 832)	823 (619 to 1046)	1094 (857 to 1349)	1460 (1273 to 1664)	2016 (1755 to 2295)	2536 (2173 to 2923)	2995 (2641 to 3376)	3389 (2700 to 4113)	3416 (2703 to 4163)	3435 (2703 to 4245)
Afar	273 (192 to 354)	246 (184 to 316)	231 (182 to 286)	308 (269 to 352)	854 (745 to 976)	1585 (1350 to 1830)	1596 (1401 to 1805)	1744 (1394 to 2115)	1764 (1401 to 2166)	1784 (1413 to 2203)
Amhara	5336 (3739 to 6862)	6697 (5148 to 8408)	8358 (6839 to 10091)	10211 (9096 to 11489)	13606 (12091 to 15383)	16761 (14654 to 19204)	19326 (17249 to 21623)	22218 (18357 to 26830)	22540 (18450 to 27387)	22863 (18513 to 27906)
Benishangul-Gumuz	265 (185 to 344)	360 (272 to 457)	443 (348 to 547)	474 (412 to 543)	491 (428 to 561)	576 (494 to 667)	802 (706 to 903)	1084 (870 to 1316)	1116 (888 to 1369)	1148 (906 to 1411)
Dire Dawa	107 (75 to 138)	152 (114 to 194)	218 (171 to 269)	266 (232 to 303)	274 (239 to 314)	303 (260 to 351)	394 (346 to 444)	488 (392 to 592)	497 (394 to 610)	505 (396 to 623)
Gambella	66 (46 to 85)	90 (68 to 114)	120 (94 to 147)	135 (118 to 154)	157 (137 to 179)	241 (206 to 276)	371 (327 to 417)	497 (397 to 601)	510 (408 to 618)	523 (414 to 638)
Harari	46 (32 to 61)	61 (45 to 78)	83 (64 to 103)	107 (93 to 123)	132 (115 to 152)	163 (138 to 189)	209 (183 to 236)	258 (203 to 316)	263 (205 to 326)	267 (207 to 332)
Oromia	5055 (3517 to 6517)	6074 (4618 to 7634)	7465 (6038 to 9038)	9962 (8799 to 11203)	16547 (14636 to 18537)	24416 (21387 to 27666)	31450 (28234 to 34761)	39716 (32860 to 46818)	40692 (33737 to 48178)	41673 (34164 to 49694)
Somali	700 (482 to 903)	900 (665 to 1143)	1140 (883 to 1395)	1580 (1366 to 1793)	2988 (2591 to 3393)	4555 (3860 to 5239)	5437 (4753 to 6089)	6673 (5349 to 8053)	6820 (5342 to 8300)	6968 (5465 to 8497)
Southern Nations, Nationalities, and Peoples	3693 (2531 to 4805)	4922 (3711 to 6284)	6567 (5264 to 7914)	8202 (7210 to 9239)	10482 (9206 to 11861)	13413 (11524 to 15324)	17670 (15570 to 19724)	22449 (18260 to 26778)	22991 (18550 to 27699)	23543 (18916 to 28498)
Tigray	1232 (853 to 1597)	1547 (1166 to 1987)	1943 (1530 to 2388)	2314 (2021 to 2630)	3022 (2632 to 3433)	3870 (3299 to 4444)	4915 (4331 to 5504)	5998 (4810 to 7233)	6114 (4915 to 7407)	6229 (4898 to 7591)
Kenya	5521 (5167 to 5879)	7879 (7278 to 8522)	11960 (11031 to 12801)	16760 (15523 to 18079)	23147 (21539 to 24874)	31115 (28770 to 33381)	40705 (37610 to 43795)	48608 (45281 to 51908)	49362 (45514 to 53270)	50062 (46231 to 54034)
Baringo	87 (78 to 97)	124 (103 to 147)	178 (157 to 200)	223 (197 to 251)	315 (280 to 355)	448 (397 to 505)	573 (509 to 645)	672 (599 to 750)	680 (600 to 771)	688 (605 to 781)
Bomet	81 (73 to 90)	123 (102 to 146)	186 (165 to 209)	244 (216 to 273)	341 (302 to 383)	511 (454 to 574)	942 (840 to 1053)	913 (820 to 1017)	899 (797 to 1011)	886 (783 to 998)
Bungoma	158 (141 to 177)	235 (194 to 281)	380 (336 to 430)	545 (480 to 617)	846 (750 to 956)	1603 (1431 to 1794)	1441 (1275 to 1625)	1749 (1563 to 1956)	1799 (1589 to 2038)	1847 (1620 to 2102)
Busia	109 (97 to 122)	147 (122 to 179)	223 (196 to 253)	331 (292 to 377)	467 (412 to 528)	604 (533 to 688)	781 (690 to 888)	936 (834 to 1051)	950 (835 to 1083)	962 (840 to 1102)
Elgeyo Marakwet	85 (76 to 95)	136 (112 to 161)	176 (155 to 197)	162 (143 to 182)	252 (222 to 282)	484 (427 to 541)	376 (330 to 421)	457 (407 to 507)	469 (411 to 527)	481 (418 to 542)
Embu	84 (75 to 94)	119 (99 to 143)	189 (166 to 214)	283 (249 to 321)	394 (348 to 447)	483 (425 to 548)	540 (476 to 611)	619 (549 to 692)	625 (547 to 708)	629 (548 to 715)
Garissa	31 (27 to 35)	41 (34 to 50)	79 (70 to 89)	137 (121 to 154)	137 (121 to 155)	330 (292 to 371)	703 (621 to 795)	911 (815 to 1015)	930 (824 to 1046)	949 (834 to 1073)
Homa Bay	261 (234 to 289)	350 (292 to 414)	465 (413 to 523)	545 (481 to 612)	680 (601 to 763)	830 (733 to 934)	999 (881 to 1125)	1145 (1017 to 1281)	1158 (1018 to 1307)	1170 (1026 to 1329)
Isiolo	19 (16 to 21)	24 (19 to 28)	34 (30 to 39)	48 (42 to 55)	74 (65 to 84)	110 (96 to 124)	156 (136 to 176)	268 (236 to 300)	282 (246 to 320)	296 (257 to 338)
Kajiado	44 (39 to 49)	60 (50 to 72)	97 (85 to 109)	167 (146 to 188)	280 (248 to 316)	453 (399 to 511)	763 (675 to 861)	1181 (1057 to 1316)	1232 (1083 to 1392)	1281 (1121 to 1450)
Kakamega	221 (199 to 244)	350 (294 to 406)	561 (502 to 622)	827 (736 to 917)	1153 (1034 to 1281)	1069 (947 to 1197)	1732 (1540 to 1934)	1930 (1731 to 2131)	1931 (1714 to 2159)	1931 (1709 to 2168)
Kericho	128 (116 to 143)	205 (172 to 242)	338 (301 to 381)	445 (397 to 502)	633 (567 to 711)	761 (679 to 856)	634 (565 to 713)	942 (848 to 1049)	989 (878 to 1118)	1035 (913 to 1173)
Kiambu	228 (204 to 254)	315 (263 to 376)	503 (446 to 567)	736 (653 to 832)	995 (887 to 1125)	1493 (1322 to 1682)	1777 (1578 to 1991)	2426 (2181 to 2708)	2505 (2220 to 2808)	2580 (2277 to 2918)
Kilifi	176 (158 to 197)	235 (195 to 280)	341 (302 to 385)	476 (420 to 538)	653 (581 to 738)	905 (801 to 1020)	1188 (1050 to 1339)	1508 (1347 to 1686)	1543 (1359 to 1747)	1576 (1391 to 1785)
Kirinyaga	110 (98 to 124)	154 (127 to 185)	230 (201 to 261)	312 (273 to 354)	412 (364 to 467)	484 (424 to 550)	554 (483 to 628)	613 (541 to 691)	617 (537 to 703)	620 (539 to 713)
Kisii	189 (170 to 211)	317 (267 to 375)	522 (463 to 585)	699 (622 to 786)	932 (831 to 1047)	1219 (1084 to 1370)	1278 (1134 to 1444)	1279 (1143 to 1432)	1276 (1127 to 1449)	1273 (1116 to 1449)
Kisumu	219 (196 to 243)	322 (269 to 378)	444 (392 to 496)	530 (467 to 597)	716 (634 to 807)	854 (754 to 960)	1005 (885 to 1131)	1183 (1050 to 1319)	1200 (1057 to 1356)	1215 (1067 to 1381)
Kitui	185 (166 to 206)	260 (217 to 309)	379 (335 to 425)	514 (455 to 578)	702 (623 to 787)	888 (786 to 999)	1056 (934 to 1189)	1189 (1062 to 1327)	1197 (1053 to 1348)	1202 (1053 to 1362)
Kwale	115 (103 to 130)	156 (127 to 189)	232 (204 to 263)	316 (277 to 360)	408 (360 to 463)	540 (474 to 615)	690 (606 to 785)	887 (788 to 997)	909 (798 to 1039)	930 (812 to 1067)
Laikipia	32 (28 to 35)	41 (34 to 49)	74 (65 to 83)	146 (129 to 165)	233 (206 to 264)	355 (313 to 400)	419 (369 to 472)	519 (464 to 579)	529 (466 to 595)	538 (471 to 610)
Lamu	14 (12 to 16)	16 (13 to 20)	26 (22 to 29)	46 (40 to 53)	63 (55 to 72)	80 (69 to 91)	107 (93 to 122)	144 (126 to 162)	148 (128 to 168)	152 (131 to 174)
Machakos	176 (157 to 196)	268 (222 to 317)	456 (403 to 512)	718 (635 to 804)	1007 (894 to 1127)	1171 (1034 to 1312)	1148 (1013 to 1290)	1449 (1288 to 1613)	1482 (1301 to 1674)	1511 (1326 to 1712)
Makueni	150 (133 to 168)	216 (178 to 256)	310 (272 to 349)	395 (346 to 445)	484 (426 to 544)	616 (539 to 695)	927 (816 to 1043)	1017 (900 to 1135)	1015 (884 to 1147)	1011 (879 to 1147)
Mandera	48 (42 to 53)	75 (62 to 89)	108 (95 to 122)	115 (101 to 131)	141 (124 to 160)	335 (294 to 381)	1155 (1018 to 1305)	932 (828 to 1045)	899 (786 to 1017)	872 (760 to 995)
Marsabit	29 (26 to 33)	32 (27 to 39)	53 (47 to 60)	105 (93 to 120)	138 (122 to 156)	195 (172 to 221)	317 (280 to 359)	468 (418 to 523)	484 (425 to 549)	500 (438 to 569)

Meru	246 (220 to 273)	368 (307 to 432)	573 (508 to 638)	815 (720 to 907)	1131 (1004 to 1262)	1433 (1267 to 1600)	1666 (1473 to 1862)	1604 (1431 to 1780)	1587 (1394 to 1783)	1567 (1375 to 1767)
Migori	158 (143 to 176)	228 (192 to 271)	324 (287 to 363)	407 (361 to 456)	544 (483 to 611)	736 (652 to 828)	968 (854 to 1087)	1162 (1035 to 1296)	1182 (1042 to 1333)	1200 (1055 to 1359)
Mombasa	145 (130 to 161)	187 (154 to 220)	273 (241 to 305)	361 (318 to 405)	494 (437 to 553)	702 (618 to 787)	982 (866 to 1101)	1201 (1072 to 1333)	1219 (1071 to 1371)	1234 (1080 to 1389)
Murang'a	221 (197 to 246)	316 (262 to 373)	479 (422 to 540)	693 (611 to 783)	886 (784 to 1002)	795 (699 to 899)	923 (811 to 1041)	1037 (918 to 1163)	1044 (912 to 1186)	1049 (913 to 1198)
Nairobi	303 (274 to 332)	363 (307 to 419)	558 (498 to 616)	961 (859 to 1059)	1431 (1284 to 1579)	2257 (2024 to 2484)	3314 (2972 to 3653)	4366 (3934 to 4777)	4467 (3987 to 4935)	4561 (4059 to 5047)
Nakuru	137 (124 to 152)	187 (157 to 220)	325 (289 to 364)	574 (513 to 643)	919 (824 to 1031)	1295 (1159 to 1452)	1661 (1493 to 1856)	2146 (1943 to 2374)	2195 (1963 to 2461)	2240 (1998 to 2517)
Nandi	108 (97 to 120)	151 (127 to 177)	231 (205 to 259)	326 (289 to 366)	469 (419 to 527)	630 (562 to 707)	786 (699 to 882)	927 (833 to 1028)	940 (833 to 1057)	952 (842 to 1071)
Narok	66 (59 to 74)	91 (76 to 109)	140 (125 to 159)	242 (215 to 274)	409 (363 to 461)	417 (370 to 470)	918 (817 to 1033)	1195 (1073 to 1332)	1213 (1078 to 1369)	1230 (1089 to 1394)
Nyamira	99 (89 to 111)	149 (124 to 178)	206 (182 to 232)	233 (206 to 264)	279 (246 to 316)	360 (315 to 408)	506 (445 to 573)	604 (537 to 677)	611 (536 to 694)	618 (538 to 703)
Nyandarua	83 (74 to 93)	119 (98 to 143)	183 (162 to 207)	250 (220 to 283)	374 (331 to 423)	509 (449 to 576)	604 (532 to 684)	624 (555 to 702)	622 (545 to 706)	620 (541 to 710)
Nyeri	172 (154 to 191)	243 (203 to 288)	370 (327 to 416)	513 (452 to 577)	634 (560 to 713)	678 (599 to 763)	702 (616 to 792)	739 (655 to 826)	740 (647 to 837)	740 (644 to 842)
Samburu	36 (32 to 40)	55 (46 to 66)	76 (67 to 86)	85 (76 to 96)	125 (111 to 141)	161 (143 to 182)	239 (211 to 269)	320 (286 to 356)	328 (291 to 371)	337 (297 to 384)
Siaya	154 (137 to 172)	235 (197 to 280)	355 (312 to 400)	469 (412 to 530)	624 (552 to 704)	774 (680 to 875)	881 (773 to 994)	1027 (909 to 1151)	1042 (913 to 1179)	1056 (918 to 1204)
Taita Taveta	57 (50 to 64)	81 (65 to 97)	119 (104 to 135)	161 (142 to 184)	229 (201 to 261)	267 (234 to 303)	298 (260 to 339)	344 (303 to 389)	349 (304 to 399)	353 (305 to 404)
Tana River	28 (25 to 31)	36 (29 to 43)	58 (51 to 67)	102 (89 to 116)	140 (123 to 159)	197 (173 to 224)	251 (220 to 286)	323 (286 to 363)	332 (291 to 378)	340 (296 to 389)
Tharaka Nithi	51 (45 to 57)	67 (54 to 80)	83 (72 to 93)	90 (79 to 102)	102 (89 to 115)	111 (97 to 125)	144 (125 to 163)	389 (344 to 435)	432 (377 to 487)	476 (412 to 540)
Trans Nzoia	55 (49 to 62)	73 (61 to 88)	139 (123 to 157)	286 (252 to 322)	436 (387 to 489)	632 (559 to 709)	853 (755 to 955)	1008 (903 to 1119)	1020 (899 to 1145)	1030 (908 to 1159)
Turkana	96 (85 to 107)	145 (118 to 173)	186 (164 to 211)	162 (142 to 184)	218 (192 to 246)	496 (435 to 559)	946 (833 to 1066)	980 (871 to 1093)	976 (858 to 1103)	972 (850 to 1105)
Uasin Gishu	89 (80 to 99)	126 (105 to 150)	213 (189 to 239)	333 (295 to 373)	484 (430 to 543)	693 (615 to 775)	961 (854 to 1075)	1221 (1096 to 1352)	1247 (1103 to 1399)	1271 (1126 to 1432)
Vihiga	148 (132 to 166)	221 (180 to 264)	285 (251 to 323)	305 (267 to 347)	376 (330 to 428)	401 (351 to 456)	571 (499 to 649)	597 (524 to 673)	593 (516 to 678)	589 (510 to 674)
Wajir	46 (41 to 51)	60 (49 to 72)	103 (90 to 116)	147 (130 to 166)	142 (124 to 160)	402 (355 to 453)	739 (647 to 833)	844 (748 to 943)	857 (753 to 966)	871 (760 to 988)
West Pokot	42 (37 to 47)	56 (46 to 66)	96 (85 to 109)	177 (157 to 201)	244 (216 to 276)	350 (310 to 397)	532 (472 to 602)	614 (548 to 690)	619 (547 to 701)	624 (551 to 708)
Madagascar	4261 (3959 to 4533)	5513 (4792 to 6206)	7088 (6397 to 7818)	9215 (8327 to 10107)	11901 (10934 to 12975)	15853 (14255 to 17520)	21430 (18884 to 24001)	27314 (25227 to 29349)	27948 (25782 to 30290)	28557 (26054 to 30956)
Malawi	2961 (2755 to 3166)	3645 (3262 to 4030)	4670 (4216 to 5094)	6530 (5991 to 7119)	9806 (8977 to 10616)	11098 (10184 to 11943)	14457 (13259 to 15630)	18564 (17263 to 19875)	19018 (17486 to 20493)	19448 (17851 to 21031)
Mozambique	6058 (5633 to 6483)	7204 (6734 to 7694)	9028 (8424 to 9667)	12319 (11419 to 13199)	13360 (11854 to 14908)	17557 (16002 to 19124)	22942 (20996 to 25022)	29491 (27024 to 31768)	30290 (27539 to 32907)	31073 (28241 to 33940)
Rwanda	2520 (2355 to 2700)	3148 (2731 to 3545)	4076 (3626 to 4529)	5399 (4967 to 5840)	7189 (6631 to 7756)	8111 (7418 to 8781)	10429 (9625 to 11268)	12730 (11382 to 14123)	13002 (11461 to 14606)	13270 (11517 to 14949)
Somalia	2435 (2257 to 2603)	2968 (2582 to 3339)	3838 (3475 to 4204)	6172 (5548 to 6760)	7939 (7317 to 8631)	10160 (8648 to 11702)	14870 (11688 to 18104)	20013 (15021 to 24975)	20804 (14979 to 26607)	21607 (15579 to 27030)
South Sudan	2579 (2313 to 2843)	3179 (2896 to 3492)	3930 (3315 to 4535)	4850 (4427 to 5248)	5877 (5193 to 6567)	7266 (6421 to 8086)	9373 (8576 to 10105)	9104 (7891 to 10365)	9413 (8108 to 10720)	9673 (8122 to 11041)
Uganda	5271 (4902 to 5620)	7365 (6861 to 7874)	10310 (9534 to 11125)	13320 (11446 to 15202)	17291 (16035 to 18565)	24260 (22162 to 26278)	32630 (29542 to 35602)	41272 (37162 to 45124)	42304 (37966 to 46634)	43315 (38655 to 48316)
Tanzania	7598 (7060 to 8092)	10302 (9402 to 11195)	13864 (12622 to 15016)	19350 (17719 to 21002)	25837 (23720 to 27939)	34303 (31481 to 37138)	44540 (41273 to 47836)	55869 (49724 to 62264)	57177 (50660 to 63868)	58448 (51504 to 65494)
Zambia	2377 (2216 to 2531)	3309 (2887 to 3704)	4528 (4185 to 4863)	6032 (5648 to 6399)	7936 (7376 to 8502)	9927 (9217 to 10623)	13761 (12923 to 14640)	18512 (16125 to 20781)	19028 (16530 to 21554)	19515 (16772 to 22271)
Southern sub-Saharan Africa	17582 (16411 to 18782)	22910 (21621 to 24332)	30790 (29166 to 32548)	40587 (36692 to 44729)	52418 (48515 to 56545)	63665 (60002 to 67287)	70861 (67130 to 74835)	78885 (72133 to 86409)	79677 (72389 to 87279)	80304 (72893 to 88169)
Botswana	403 (377 to 431)	522 (474 to 570)	676 (603 to 749)	927 (857 to 999)	1319 (1219 to 1413)	1695 (1578 to 1818)	2004 (1855 to 2154)	2328 (2075 to 2595)	2363 (2065 to 2666)	2393 (2079 to 2710)
Eswatini	257 (239 to 275)	337 (302 to 372)	443 (403 to 487)	588 (534 to 640)	806 (731 to 884)	1017 (927 to 1108)	1077 (987 to 1174)	1142 (1051 to 1230)	1150 (1055 to 1249)	1155 (1029 to 1262)
Lesotho	573 (535 to 613)	771 (696 to 840)	991 (895 to 1091)	1251 (1127 to 1368)	1533 (1395 to 1669)	1735 (1575 to 1909)	1824 (1659 to 1991)	1869 (1707 to 2031)	1874 (1703 to 2043)	1874 (1684 to 2067)
Namibia	449 (418 to 478)	575 (537 to 612)	777 (721 to 833)	1044 (909 to 1182)	1404 (1300 to 1511)	1834 (1704 to 1961)	2111 (1958 to 2268)	2377 (2090 to 2656)	2406 (2090 to 2711)	2431 (2093 to 2728)
South Africa	13152 (12211 to 14120)	16939 (15780 to 18039)	22689 (21263 to 24158)	29359 (25440 to 33287)	37013 (33156 to 40940)	45397 (41798 to 48781)	50778 (47161 to 54421)	56029 (49559 to 63222)	56505 (49419 to 63618)	56854 (49727 to 64283)
Eastern Cape	2710 (2458 to 2961)	3252 (2966 to 3539)	3681 (3344 to 4012)	4599 (3808 to 5446)	5921 (5084 to 6775)	6653 (5949 to 7307)	6492 (5850 to 7132)	6566 (5497 to 7675)	6560 (5437 to 7725)	6543 (5398 to 7724)
Free State	1041 (942 to 1148)	1456 (1328 to 1601)	1838 (1677 to 2029)	2216 (1841 to 2684)	2571 (2228 to 2977)	2787 (2507 to 3098)	2708 (2445 to 3013)	2745 (2306 to 3263)	2747 (2285 to 3283)	2741 (2264 to 3298)
Gauteng	2565 (2338 to 2801)	3331 (3046 to 3622)	4421 (4040 to 4812)	5266 (4399 to 6193)	6395 (5534 to 7298)	8782 (7917 to 9622)	11892 (10791 to 12963)	14595 (12389 to 16827)	14837 (12557 to 17148)	15044 (12644 to 17461)
KwaZulu-Natal	2521 (2295 to 2773)	3157 (2886 to 3455)	4584 (4187 to 5014)	6099 (5105 to 7239)	7683 (6661 to 8795)	9528 (8621 to 10485)	10134 (9208 to 11192)	10770 (9087 to 12647)	10835 (9131 to 12793)	10877 (9152 to 12883)
Limpopo	926 (843 to 1021)	1283 (1175 to 1408)	2303 (2113 to 2528)	3549 (2973 to 4213)	4605 (4007 to 5320)	5391 (4871 to 5939)	5391 (4900 to 5929)	5423 (4610 to 6338)	5435 (4563 to 6410)	5433 (4532 to 6419)
Mpumalanga	871 (786 to 958)	1150 (1044 to 1262)	1342 (1220 to 1469)	1750 (1445 to 2086)	2443 (2101 to 2824)	3161 (2833 to 3502)	3921 (3541 to 4328)	4765 (3980 to 5579)	4851 (4053 to 5711)	4925 (4080 to 5846)
North West	772 (694 to 851)	1087 (985 to 1193)	1656 (1497 to 1817)	2303 (1885 to 2761)	3013 (2571 to 3485)	3726 (3321 to 4118)	3513 (3142 to 3882)	3226 (2685 to 3798)	3200 (2611 to 3798)	3171 (2604 to 3768)
Northern Cape	455 (409 to 499)	573 (517 to 629)	675 (610 to 741)	790 (643 to 940)	872 (743 to 1008)	838 (744 to 930)	1085 (968 to 1201)	1464 (1210 to 1718)	1499 (1225 to 1772)	1530 (1247 to 1816)
Western Cape	1292 (1165 to 1421)	1648 (1489 to 1809)	2190 (1981 to 2406)	2787 (2273 to 3330)	3510 (2978 to 4061)	4530 (4032 to 5013)	5642 (5042 to 6226)	6476 (5376 to 7602)	6541 (5369 to 7729)	6590 (5383 to 7817)
Zimbabwe	2748 (1962 to 3486)	3766 (3021 to 4559)	5214 (4503 to 5919)	7418 (6836 to 8009)	10343 (9506 to 11135)	11985 (11064 to 12884)	13068 (11966 to 14066)	15141 (13433 to 16795)	15379 (13574 to 17250)	15595 (13802 to 17465)
Western sub-Saharan Africa	76313 (64338 to 88631)	93883 (81849 to 106690)	116252 (105118 to 127207)	147533 (138285 to 157731)	193159 (185625 to 200460)	259484 (245992 to 272509)	353910 (337835 to 369320)	463282 (438802 to 486329)	476552 (451000 to 501512)	489828 (462150 to 517876)
Benin	2122 (1598 to 2673)	2296 (1833 to 2700)	2676 (2363 to 3002)	3471 (3220 to 3730)	4851 (4464 to 5252)	6717 (6166 to 7264)	9402 (8569 to 10199)	12677 (11327 to 13995)	13087 (11650 to 14464)	13499 (11844 to 15142)
Burkina Faso	4129 (2893 to 5320)	4812 (3699 to 5969)	5897 (4948 to 6791)	7487 (6725 to 8229)	9528 (8579 to 10488)	12425 (11260 to 13669)	16801 (15232 to 18365)	21559 (20121 to 23073)	22156 (20516 to 23808)	22761 (20948 to 24636)
Cabo Verde	155 (145 to 166)	210 (196 to 225)	284 (265 to 304)	303 (283 to 325)	354 (329 to 379)	451 (420 to 482)	510 (474 to 543)	552 (485 to 619)	556 (488 to 627)	559 (487 to 634)
Cameroon	4554 (3488 to 5566)	5576 (4642 to 6467)	6715 (6001 to 7430)	8088 (7317 to 8830)	10437 (9529 to 11369)	15051 (13584 to 16615)	22054 (19771 to 24205)	29973 (25525 to 34611)	30886 (25888 to 35789)	31780 (26654 to 37155)
Chad	2418 (1636 to 3212)	2967 (2147 to 3786)	3617 (2901 to 4404)	4569 (3882 to 5255)	6026 (5507 to 6558)	8286 (7355 to 9216)	11796 (10918 to 12657)	16486 (14403 to 18779)	17108 (14741 to 19280)	17749 (15191 to 20275)
Côte d'Ivoire	3091 (2874 to 3299)	4138 (3591 to 4650)	5887 (5283 to 6468)	8341 (7545 to 9188)	12198 (11168 to 13205)	16949 (15674 to 18244)	21529 (19537 to 23348)	26612 (23970 to 29359)	27238 (24463 to 30019)	27862 (24857 to 31147)
The Gambia	258 (242 to 274)	302 (274 to 326)	458 (420 to 497)	663 (606 to 719)	982 (899 to 1067)	1351 (1241 to 1460)	1785 (1625 to 1945)	2282 (2061 to 2516)	2338 (2086 to 2606)	2394 (2109 to 2685)
Ghana	5096 (4779 to 5441)	6901 (6452 to 7349)	9008 (8399 to 9609)	11720 (10687 to 12781)	14972 (13364 to 16552)	19141 (17826 to 20370)	25378 (23675 to 27100)	32622 (28390 to 36397)	33445 (28977 to 37726)	34246 (29711 to 38854)
Guinea	2938 (2739 to 3142)	3375 (2983 to 3771)	3962 (3588 to 4326)	4662 (4264 to 5064)	5994 (5337 to 6646)	8096 (7384 to 8799)	9951 (9023 to 10935)	12739 (11452 to 14058)	13085 (11699 to 14428)	13430 (11962 to 14955)
Guinea-Bissau	536 (499 to 572)	566 (528 to 607)	641 (566 to 722)	802 (741 to 858)	1007 (934 to 1081)	1245 (1083 to 1408)	1573 (1451 to 1687)	1972 (1729 to 2227)	2019 (1746 to 2314)	2064 (1780 to 2339)
Liberia	839 (718 to 962)	1069 (990 to 1155)	1419 (1290 to 1542)	1967 (1774 to 2142)	2461 (2219 to 2727)	2848 (2521 to 3178)	4012 (3687 to 4362)	5202 (4486 to 5887)	5327 (4612 to 6035)	5459 (4607 to 6313)
Mali	3784 (3525 to 4051)	4676 (4108 to 5294)	5942 (5319 to 6584)	7258 (6558 to 7925)	8663 (7915 to 9460)	11063 (10173 to 11979)	16052 (14785 to 17301)	22408 (19554 to 25426)	23251 (19933 to 26506)	24104 (20613 to 27474)
Mauritania	647 (495 to 800)	868 (714 to 1012)	1153 (1021 to 1281)	1548 (1405 to 1681)	2055 (1888 to 2226)	2614 (2438 to 2793)	3366 (3085 to 3653)	4210 (3736 to 4659)	4304 (3839 to 4767)	4396 (3882 to 4929)
Niger	2595 (1990 to 3175)	3372 (2791 to 3958)	4484 (3960 to 4983)	5968 (5440 to 6475)	8032 (7377 to 8651)	11273 (10417 to 12122)	16439 (15127 to 17724)	23195 (20708 to 25821)	24101 (21091 to 26851)	25038 (21889 to 28031)
Nigeria	37244 (25076 to 49142)	45350 (33208 to 58083)	54810 (43596 to 65355)	68746 (59812 to 78604)	90042 (83167 to 96685)	122614 (110240 to 135425)	167723 (153244 to 182647)	218903 (196800 to 241065)	225038 (201187 to 247658)	231166 (205604 to 257994)
São Tomé and Príncipe	63 (58 to 67)	69 (64 to 74)	77 (71 to 82)	96 (88 to 103)	121 (112 to 130)	144 (133 to 155)	177 (162 to 191)	210 (187 to 234)	213 (189 to 238)	217 (191 to 243)

Senegal	2674<br>(2065 to 3343)	3537<br>(2969 to 4122)	4612<br>(4099 to 5123)	5893<br>(5372 to 6416)	7632<br>(7018 to 8238)	9931<br>(9184 to 10675)	12617<br>(11538 to 13693)	15270<br>(13625 to 17004)	15569<br>(13800 to 17239)	15860<br>(14009 to 17616)
Sierra Leone	1852<br>(1580 to 2115)	2204<br>(2019 to 2397)	2638<br>(2393 to 2891)	3286<br>(2965 to 3623)	4152<br>(3747 to 4569)	4424<br>(4008 to 4807)	6352<br>(5721 to 6995)	8382<br>(7615 to 9187)	8625<br>(7791 to 9449)	8867<br>(7944 to 9808)
Togo	1311<br>(1165 to 1467)	1590<br>(1460 to 1714)	1968<br>(1831 to 2100)	2658<br>(2479 to 2857)	3647<br>(3228 to 4067)	4854<br>(4272 to 5471)	6389<br>(5976 to 6828)	8020<br>(7029 to 9008)	8198<br>(7059 to 9224)	8371<br>(7165 to 9504)

Appendix 2 Table S5b: Population (in thousands) with 95% UIs by location, females, 1950, 1960, 1970, 1980, 1990, 2000, 2010, 2019, 2020, 2021

Location	1950	1960	1970	1980	1990	2000	2010	2019	2020	2021
Global	1263509 (1240538 to 1289164)	1519134 (1484200 to 1556025)	1844763 (1798330 to 1892408)	2225554 (2178761 to 2273987)	2647880 (2597850 to 2701944)	3026589 (2966888 to 3087055)	3458071 (3391403 to 3525442)	3857568 (3757705 to 3963237)	3896446 (3794381 to 4003170)	3931962 (3822504 to 4049401)
Central Europe, eastern Europe, and central Asia	152473 (147718 to 157152)	172175 (166617 to 177956)	190584 (185025 to 196345)	206253 (199797 to 212758)	219839 (212579 to 226682)	218314 (211011 to 225461)	216165 (208990 to 222865)	218467 (206414 to 229841)	218394 (205827 to 230499)	217944 (204997 to 230165)
Central Asia	14713 (14301 to 15115)	18440 (17852 to 19001)	24428 (23693 to 25144)	29867 (28952 to 30767)	35381 (34267 to 36466)	37907 (36014 to 39804)	41525 (38645 to 44320)	47034 (42821 to 51359)	47675 (43240 to 52289)	48279 (43310 to 53181)
Armenia	768 (714 to 821)	979 (907 to 1049)	1309 (1226 to 1397)	1625 (1503 to 1741)	1745 (1613 to 1879)	1718 (1589 to 1839)	1619 (1498 to 1742)	1567 (1376 to 1757)	1560 (1369 to 1754)	1553 (1350 to 1750)
Azerbaijan	1656 (1550 to 1766)	2064 (1910 to 2221)	2705 (2518 to 2875)	3212 (2959 to 3458)	3753 (3464 to 4021)	4235 (3938 to 4544)	4686 (4322 to 5029)	5171 (4505 to 5856)	5209 (4524 to 5899)	5240 (4532 to 5984)
Georgia	1989 (1853 to 2128)	2265 (2093 to 2425)	2541 (2362 to 2708)	2745 (2538 to 2949)	2904 (2690 to 3127)	2507 (2300 to 2712)	2105 (1908 to 2303)	1916 (1729 to 2115)	1899 (1715 to 2097)	1878 (1666 to 2085)
Kazakhstan	4014 (3748 to 4284)	5040 (4646 to 5416)	6743 (6250 to 7214)	7705 (7172 to 8281)	8448 (7791 to 9062)	7764 (7186 to 8362)	8396 (8346 to 8442)	9514 (8687 to 10305)	9644 (8752 to 10507)	9763 (8772 to 10731)
Kyrgyzstan	930 (865 to 993)	1163 (1076 to 1248)	1574 (1473 to 1681)	1906 (1763 to 2044)	2281 (2116 to 2452)	2539 (2357 to 2724)	2862 (2665 to 3065)	3368 (2937 to 3770)	3423 (2939 to 3922)	3474 (2965 to 3999)
Mongolia	403 (378 to 429)	486 (444 to 528)	641 (595 to 689)	847 (786 to 907)	1080 (1003 to 1162)	1229 (1143 to 1313)	1366 (1276 to 1462)	1631 (1512 to 1748)	1661 (1552 to 1764)	1689 (1562 to 1813)
Tajikistan	857 (802 to 913)	1090 (1007 to 1168)	1525 (1421 to 1630)	2053 (1898 to 2196)	2697 (2496 to 2909)	3176 (2970 to 3392)	3857 (3621 to 4108)	4797 (4151 to 5395)	4906 (4201 to 5586)	5012 (4343 to 5719)
Turkmenistan	655 (613 to 698)	836 (773 to 902)	1130 (1055 to 1205)	1485 (1380 to 1595)	1873 (1734 to 2014)	2124 (1849 to 2408)	2281 (2050 to 2517)	2479 (2251 to 2704)	2502 (2256 to 2756)	2523 (2261 to 2789)
Uzbekistan	3440 (3201 to 3664)	4516 (4168 to 4861)	6259 (5843 to 6703)	8290 (7717 to 8847)	10599 (9793 to 11425)	12617 (10862 to 14454)	14352 (11432 to 17188)	16590 (12518 to 20847)	16871 (12348 to 21412)	17147 (12271 to 21855)
Central Europe	46459 (45173 to 47704)	52166 (50587 to 53756)	56615 (54903 to 58407)	61261 (59264 to 63277)	63832 (61773 to 65894)	62874 (60791 to 64937)	61238 (60209 to 62212)	59658 (57330 to 61939)	59422 (57005 to 61836)	59100 (56396 to 61714)
Albania	616 (576 to 660)	821 (766 to 878)	1064 (986 to 1142)	1321 (1221 to 1418)	1604 (1488 to 1723)	1597 (1485 to 1717)	1440 (1333 to 1550)	1346 (1196 to 1495)	1337 (1172 to 1511)	1327 (1152 to 1502)
Bosnia and Herzegovina	1544 (1438 to 1650)	1813 (1677 to 1954)	1959 (1813 to 2096)	2120 (1967 to 2270)	2251 (2073 to 2432)	2033 (1784 to 2298)	1933 (1758 to 2104)	1731 (1547 to 1914)	1711 (1522 to 1902)	1688 (1483 to 1888)
Bulgaria	3578 (3327 to 3832)	3955 (3586 to 4338)	4245 (3833 to 4699)	4467 (4005 to 4884)	4400 (4040 to 4759)	4072 (3794 to 4396)	3825 (3801 to 3849)	3571 (3275 to 3889)	3541 (3226 to 3856)	3500 (3132 to 3831)
Croatia	2032 (1887 to 2182)	2177 (2011 to 2344)	2328 (2137 to 2503)	2487 (2303 to 2662)	2497 (2308 to 2692)	2369 (2200 to 2539)	2269 (2109 to 2431)	2191 (1933 to 2457)	2180 (1906 to 2433)	2167 (1892 to 2444)
Czechia	4805 (4445 to 5134)	4891 (4520 to 5251)	5052 (4726 to 5404)	5294 (4913 to 5674)	5301 (4878 to 5699)	5251 (5213 to 5289)	5340 (5301 to 5381)	5407 (4968 to 5842)	5406 (4948 to 5864)	5399 (4910 to 5896)
Hungary	4904 (4575 to 5229)	5173 (4828 to 5531)	5304 (4909 to 5680)	5475 (5133 to 5860)	5400 (5010 to 5782)	5347 (4947 to 5743)	5234 (4836 to 5616)	5074 (4466 to 5658)	5051 (4396 to 5677)	5022 (4409 to 5688)
Montenegro	215 (200 to 230)	247 (230 to 263)	275 (254 to 294)	299 (277 to 320)	314 (293 to 338)	322 (293 to 352)	320 (296 to 344)	315 (277 to 353)	314 (274 to 353)	313 (273 to 355)
North Macedonia	651 (607 to 697)	710 (656 to 763)	822 (763 to 882)	957 (883 to 1026)	991 (905 to 1084)	1024 (944 to 1107)	1060 (931 to 1184)	1076 (893 to 1264)	1075 (879 to 1283)	1072 (885 to 1275)
Poland	13088 (12168 to 13985)	15353 (14328 to 16374)	16780 (15637 to 17972)	18310 (16878 to 19839)	19584 (18029 to 21087)	19747 (18118 to 21299)	19819 (19683 to 19957)	19832 (18255 to 21345)	19796 (18165 to 21537)	19731 (17829 to 21616)
Romania	8561 (7949 to 9126)	9680 (8789 to 10599)	10585 (9580 to 11626)	11478 (10510 to 12484)	11862 (10937 to 12804)	11458 (10528 to 12422)	10601 (9817 to 11437)	9889 (8755 to 11074)	9818 (8560 to 11011)	9732 (8489 to 11055)
Serbia	3508 (3278 to 3754)	3932 (3666 to 4216)	4366 (4023 to 4712)	4733 (4384 to 5059)	4888 (4489 to 5262)	4913 (4514 to 5322)	4649 (4307 to 4986)	4524 (4050 to 5033)	4500 (3946 to 5086)	4469 (3884 to 5027)
Slovakia	1771 (1763 to 1780)	2071 (2062 to 2081)	2298 (2286 to 2309)	2531 (2518 to 2543)	2703 (2687 to 2717)	2772 (2755 to 2789)	2780 (2760 to 2801)	2785 (2546 to 3034)	2784 (2528 to 3054)	2779 (2506 to 3050)
Slovenia	780 (724 to 831)	825 (760 to 888)	892 (829 to 963)	966 (892 to 1046)	1015 (906 to 1124)	1021 (1013 to 1028)	1034 (1026 to 1043)	1045 (964 to 1130)	1043 (958 to 1135)	1042 (949 to 1133)
Eastern Europe	91301 (86692 to 95987)	101569 (96205 to 107330)	109541 (104183 to 115179)	115125 (108984 to 121581)	120627 (113592 to 127348)	117532 (110649 to 124486)	113402 (106881 to 119653)	111775 (100890 to 122490)	111297 (99650 to 122680)	110564 (98823 to 121863)
Belarus	4191 (3909 to 4463)	4659 (4308 to 5008)	5009 (4692 to 5328)	5267 (4882 to 5645)	5544 (5120 to 5964)	5414 (5012 to 5818)	5158 (4753 to 5560)	5049 (4435 to 5675)	5022 (4340 to 5686)	4983 (4286 to 5676)
Estonia	594 (591 to 596)	674 (670 to 677)	734 (730 to 738)	791 (787 to 795)	835 (830 to 840)	746 (741 to 751)	712 (707 to 718)	696 (639 to 751)	695 (640 to 753)	692 (627 to 757)
Latvia	1091 (1016 to 1165)	1186 (1097 to 1270)	1285 (1196 to 1379)	1360 (1262 to 1457)	1423 (1318 to 1529)	1286 (1191 to 1372)	1148 (1139 to 1156)	1033 (949 to 1117)	1022 (933 to 1110)	1009 (916 to 1106)
Lithuania	1319 (1227 to 1413)	1486 (1383 to 1596)	1663 (1552 to 1773)	1806 (1675 to 1939)	1937 (1795 to 2083)	1873 (1736 to 2009)	1663 (1528 to 1782)	1507 (1388 to 1632)	1490 (1362 to 1626)	1471 (1335 to 1621)
Moldova	1376 (1281 to 1469)	1631 (1508 to 1749)	1955 (1828 to 2085)	2160 (2009 to 2317)	2325 (2157 to 2496)	2188 (1984 to 2398)	2007 (1789 to 2225)	1910 (1603 to 2241)	1898 (1580 to 2199)	1883 (1558 to 2196)
Russia	61246 (57171 to 65523)	67702 (62856 to 72845)	72398 (67685 to 77555)	76014 (70501 to 81968)	80255 (73881 to 86396)	79445 (73149 to 85875)	77941 (72640 to 83232)	78037 (68533 to 87853)	77822 (67744 to 88140)	77404 (66792 to 87411)
Ukraine	21484 (19945 to 22944)	24230 (22509 to 25972)	26497 (24792 to 28139)	27727 (25523 to 29699)	28308 (26184 to 30323)	26579 (24630 to 28514)	24772 (21781 to 27821)	23543 (19108 to 27941)	23349 (19095 to 27589)	23121 (18589 to 27586)
High income	318678 (310274 to 326835)	358061 (349630 to 366096)	399467 (390161 to 408529)	434012 (423484 to 443947)	464167 (453851 to 474597)	493278 (481169 to 504619)	527759 (514721 to 539990)	552270 (537208 to 567762)	553890 (539326 to 567861)	555097 (539894 to 569929)
Australasia	5241 (4953 to 5530)	6419 (6022 to 6802)	7808 (7341 to 8271)	8961 (8424 to 9532)	10206 (9567 to 10853)	11511 (10784 to 12216)	13144 (12332 to 13934)	15124 (14138 to 16168)	15387 (14430 to 16344)	15636 (14732 to 16539)
Australia	4269 (3979 to 4552)	5209 (4807 to 5592)	6363 (5902 to 6823)	7332 (6801 to 7909)	8477 (7846 to 9097)	9545 (8818 to 10258)	10900 (10097 to 11657)	12616 (11634 to 13670)	12833 (11880 to 13769)	13048 (12142 to 13936)
New Zealand	971 (903 to 1036)	1209 (1119 to 1298)	1445 (1341 to 1547)	1629 (1511 to 1741)	1729 (1599 to 1859)	1966 (1820 to 2112)	2244 (2038 to 2465)	2508 (2315 to 2694)	2554 (2362 to 2760)	2588 (2362 to 2807)
High-income Asia Pacific	54201 (51140 to 57010)	62468 (59067 to 65904)	71690 (67719 to 75578)	81050 (76788 to 85311)	87629 (83024 to 92162)	91216 (86511 to 95992)	93971 (88861 to 98854)	94626 (89351 to 100156)	94430 (89711 to 99227)	94196 (88940 to 99669)
Brunei	28 (26 to 30)	42 (39 to 45)	63 (58 to 68)	89 (83 to 96)	122 (113 to 131)	163 (150 to 176)	191 (176 to 206)	209 (183 to 236)	211 (185 to 239)	213 (186 to 241)
Japan	43679 (40650 to 46439)	48754 (45573 to 51966)	54406 (50430 to 58217)	60601 (56456 to 64745)	63969 (59507 to 68208)	65800 (61266 to 70265)	66599 (61663 to 71198)	65839 (60886 to 70823)	65672 (61168 to 69827)	65482 (60505 to 70291)
Aichi	1784 (1615 to 1945)	2179 (1981 to 2378)	2745 (2490 to 2996)	3168 (2868 to 3452)	3391 (3077 to 3693)	3564 (3228 to 3888)	3750 (3379 to 4097)	3824 (3433 to 4218)	3826 (3456 to 4174)	3827 (3433 to 4213)
Akita	677 (612 to 741)	703 (635 to 772)	663 (601 to 730)	667 (605 to 731)	656 (592 to 718)	636 (576 to 698)	588 (530 to 646)	522 (466 to 579)	515 (466 to 566)	507 (454 to 562)
Aomori	663 (601 to 730)	746 (676 to 822)	760 (689 to 837)	806 (733 to 888)	795 (722 to 876)	789 (716 to 868)	742 (671 to 818)	673 (604 to 750)	664 (601 to 730)	655 (587 to 728)
Chiba	1126 (1013 to 1234)	1200 (1084 to 1313)	1705 (1540 to 1864)	2396 (2158 to 2620)	2798 (2521 to 3057)	2994 (2690 to 3272)	3141 (2823 to 3442)	3207 (2858 to 3529)	3210 (2890 to 3503)	3213 (2856 to 3555)
Ehime	796 (725 to 874)	793 (724 to 872)	765 (696 to 841)	805 (733 to 884)	814 (741 to 893)	803 (731 to 882)	770 (700 to 846)	720 (649 to 799)	713 (648 to 783)	706 (637 to 784)
Fukui	396 (359 to 436)	399 (360 to 440)	397 (359 to 437)	419 (379 to 461)	432 (390 to 476)	434 (391 to 479)	422 (381 to 464)	402 (360 to 447)	399 (361 to 439)	396 (355 to 441)
Fukuoka	1828 (1645 to 2000)	2080 (1875 to 2275)	2142 (1928 to 2346)	2396 (2161 to 2631)	2549 (2284 to 2802)	2667 (2400 to 2931)	2716 (2433 to 2983)	2741 (2433 to 3032)	2740 (2456 to 2999)	2737 (2440 to 3030)
Fukushima	1081 (982 to 1185)	1082 (979 to 1193)	1034 (938 to 1138)	1067 (967 to 1179)	1101 (998 to 1213)	1109 (1006 to 1225)	1061 (957 to 1168)	950 (853 to 1062)	942 (852 to 1042)	934 (836 to 1046)
Gifu	798 (723 to 878)	856 (776 to 939)	931 (841 to 1020)	1027 (931 to 1129)	1083 (975 to 1187)	1104 (1002 to 1215)	1092 (986 to 1201)	1041 (929 to 1156)	1033 (931 to 1134)	1025 (918 to 1141)
Gunma	841 (758 to 923)	835 (753 to 919)	869 (786 to 957)	958 (869 to 1053)	1013 (917 to 1112)	1041 (944 to 1146)	1036 (939 to 1137)	998 (897 to 1105)	992 (897 to 1091)	986 (883 to 1096)
Hiroshima	1088 (983 to 1189)	1144 (1036 to 1253)	1271 (1151 to 1393)	1429 (1295 to 1567)	1489 (1347 to 1627)	1510 (1364 to 1651)	1495 (1350 to 1637)	1469 (1320 to 1623)	1462 (1323 to 1600)	1455 (1303 to 1611)
Hokkaidō	2172 (1978 to 2374)	2535 (2310 to 2773)	2690 (2446 to 2934)	2895 (2640 to 3170)	2975 (2711 to 3252)	3005 (2734 to 3296)	2960 (2697 to 3245)	2821 (2546 to 3117)	2800 (2551 to 3063)	2778 (2510 to 3073)
Hyōgo	1727 (1567 to 1889)	2023 (1833 to 2219)	2417 (2188 to 2643)	2680 (2433 to 2939)	2827 (2550 to 3100)	2918 (2645 to 3197)	2958 (2668 to 3252)	2913 (2615 to 3225)	2904 (2626 to 3188)	2893 (2598 to 3206)
Ibaraki	1069 (973 to 1166)	1065 (968 to 1164)	1114 (1012 to 1216)	1311 (1196 to 1433)	1452 (1316 to 1592)	1522 (1386 to 1662)	1515 (1375 to 1657)	1464 (1320 to 1616)	1456 (1325 to 1590)	1448 (1304 to 1597)
Ishikawa	506 (460 to 555)	517 (469 to 568)	534 (484 to 586)	588 (533 to 648)	613 (558 to 673)	618 (561 to 679)	614 (554 to 676)	593 (531 to 658)	590 (533 to 649)	587 (525 to 650)
Iwate	700 (633 to 765)	759 (688 to 832)	730 (658 to 803)	750 (677 to 824)	752 (680 to 825)	748 (674 to 820)	709 (637 to 778)	646 (578 to 717)	637 (575 to 700)	629 (563 to 698)
Kagawa	498 (447 to 545)	488 (439 to 535)	488 (436 to 536)	529 (474 to 582)	542 (487 to 594)	541 (486 to 594)	522 (470 to 575)	502 (447 to 557)	500 (446 to 549)	497 (441 to 552)
Kagoshima	963 (877 to 1057)	1047 (953 to 1150)	948 (864 to 1043)	965 (880 to 1062)	974 (887 to 1071)	965 (881 to 1063)	925 (838 to 1021)	862 (779 to 960)	854 (778 to 938)	846 (761 to 941)
Kanagawa	1266 (1144 to 1384)	1724 (1565 to 1883)	2698 (2452 to 2948)	3447 (3132 to 3772)	3940 (3575 to 4306)	4238 (3848 to 4634)	4562 (4116 to 4985)	4694 (4200 to 5179)	4705 (4278 to 5136)	4714 (4231 to 5208)
Kōchi	457 (414 to 501)	451 (409 to 496)	425 (386 to 468)	444 (403 to 487)	443 (402 to 486)	437 (397 to 483)	411 (371 to 453)	375 (338 to 416)	370 (335 to 408)	366 (327 to 407)
Kumamoto	967 (877 to 1060)	985 (893 to 1081)	923 (837 to 1013)	959 (872 to 1049)	989 (898 to 1085)	999 (906 to 1096)	979 (884 to 1073)	938 (841 to 1040)	931 (843 to 1021)	924 (827 to 1028)

Kyōto	961 (869 to 1057)	1037 (940 to 1142)	1172 (1061 to 1286)	1314 (1189 to 1446)	1357 (1226 to 1494)	1384 (1250 to 1524)	1381 (1249 to 1522)	1370 (1225 to 1525)	1365 (1234 to 1500)	1359 (1215 to 1517)
Mie	774 (698 to 853)	783 (704 to 861)	819 (738 to 902)	888 (800 to 982)	941 (847 to 1034)	973 (874 to 1075)	967 (865 to 1067)	926 (825 to 1035)	920 (828 to 1020)	914 (815 to 1016)
Miyagi	853 (770 to 934)	909 (818 to 993)	951 (855 to 1042)	1077 (970 to 1178)	1163 (1050 to 1271)	1227 (1101 to 1342)	1227 (1107 to 1342)	1203 (1074 to 1332)	1198 (1079 to 1314)	1193 (1061 to 1320)
Miyazaki	569 (516 to 621)	592 (536 to 649)	566 (512 to 619)	614 (557 to 673)	630 (571 to 689)	629 (571 to 690)	613 (554 to 671)	578 (518 to 639)	574 (520 to 629)	569 (508 to 629)
Nagano	1081 (981 to 1187)	1045 (946 to 1151)	1044 (946 to 1150)	1096 (992 to 1208)	1129 (1021 to 1245)	1153 (1044 to 1268)	1126 (1020 to 1241)	1068 (959 to 1190)	1062 (964 to 1168)	1055 (944 to 1169)
Nagasaki	854 (774 to 935)	913 (828 to 1001)	842 (760 to 923)	850 (770 to 931)	843 (763 to 923)	819 (742 to 899)	775 (699 to 851)	716 (641 to 793)	708 (639 to 779)	700 (625 to 778)
Nara	403 (363 to 440)	405 (366 to 443)	489 (441 to 534)	634 (573 to 692)	724 (654 to 792)	763 (689 to 838)	748 (671 to 820)	715 (637 to 792)	711 (643 to 780)	706 (633 to 783)
Niigata	1293 (1166 to 1419)	1287 (1162 to 1411)	1240 (1118 to 1361)	1284 (1157 to 1405)	1299 (1172 to 1421)	1296 (1172 to 1419)	1248 (1122 to 1371)	1162 (1036 to 1284)	1148 (1037 to 1263)	1135 (1009 to 1258)
Ōita	663 (597 to 728)	658 (591 to 724)	629 (567 to 693)	659 (593 to 723)	665 (598 to 731)	656 (589 to 722)	642 (574 to 706)	606 (538 to 675)	600 (539 to 660)	594 (528 to 661)
Okayama	875 (798 to 959)	887 (806 to 976)	907 (825 to 996)	985 (894 to 1083)	1018 (928 to 1117)	1032 (938 to 1131)	1026 (931 to 1130)	1000 (900 to 1111)	994 (903 to 1090)	988 (890 to 1096)
Okinawa	347 (312 to 382)	429 (368 to 491)	505 (456 to 557)	575 (517 to 632)	633 (570 to 697)	679 (611 to 745)	722 (648 to 794)	755 (675 to 838)	757 (684 to 833)	758 (676 to 845)
Ōsaka	2001 (1808 to 2203)	2781 (2516 to 3061)	3868 (3507 to 4266)	4345 (3940 to 4769)	4491 (4078 to 4932)	4566 (4138 to 5017)	4626 (4197 to 5080)	4665 (4189 to 5177)	4663 (4232 to 5113)	4660 (4179 to 5165)
Saga	500 (454 to 547)	502 (455 to 550)	455 (412 to 499)	464 (421 to 508)	473 (428 to 518)	471 (426 to 516)	458 (415 to 503)	436 (391 to 480)	433 (393 to 474)	430 (386 to 478)
Saitama	1120 (1018 to 1225)	1256 (1143 to 1376)	1955 (1780 to 2138)	2733 (2494 to 2983)	3213 (2924 to 3512)	3491 (3176 to 3826)	3645 (3306 to 3988)	3734 (3372 to 4126)	3740 (3400 to 4097)	3744 (3375 to 4144)
Shiga	457 (415 to 502)	447 (406 to 491)	463 (421 to 511)	561 (508 to 617)	632 (574 to 695)	690 (626 to 759)	722 (654 to 797)	727 (655 to 807)	725 (661 to 796)	724 (652 to 805)
Shimane	478 (429 to 525)	465 (418 to 512)	415 (373 to 458)	417 (374 to 460)	416 (374 to 459)	406 (364 to 447)	382 (343 to 420)	356 (316 to 396)	353 (317 to 390)	351 (312 to 390)
Shizuoka	1296 (1172 to 1416)	1428 (1292 to 1568)	1608 (1448 to 1764)	1786 (1609 to 1962)	1897 (1709 to 2080)	1943 (1750 to 2135)	1941 (1744 to 2135)	1879 (1681 to 2086)	1869 (1680 to 2054)	1859 (1657 to 2066)
Tochigi	815 (740 to 893)	798 (724 to 873)	829 (752 to 908)	925 (839 to 1015)	991 (897 to 1088)	1026 (931 to 1126)	1022 (925 to 1124)	989 (887 to 1095)	982 (890 to 1079)	976 (875 to 1082)
Tokushima	460 (417 to 503)	447 (407 to 490)	424 (385 to 464)	438 (398 to 481)	444 (404 to 485)	440 (400 to 482)	418 (379 to 458)	386 (347 to 427)	382 (344 to 418)	377 (338 to 418)
Tōkyō	3172 (2878 to 3451)	4741 (4305 to 5189)	5705 (5200 to 6217)	5845 (5320 to 6379)	5941 (5386 to 6488)	6091 (5536 to 6657)	6664 (6032 to 7292)	7164 (6453 to 7905)	7222 (6576 to 7880)	7276 (6536 to 8025)
Tottori	317 (287 to 351)	318 (288 to 351)	306 (277 to 337)	321 (290 to 356)	327 (295 to 361)	325 (295 to 361)	312 (281 to 346)	294 (264 to 329)	292 (264 to 322)	290 (259 to 324)
Toyama	530 (481 to 581)	541 (489 to 593)	550 (497 to 602)	583 (525 to 639)	592 (536 to 649)	591 (534 to 648)	577 (520 to 631)	545 (488 to 603)	541 (488 to 594)	536 (479 to 594)
Wakayama	518 (468 to 569)	526 (475 to 579)	547 (496 to 602)	575 (521 to 632)	574 (519 to 632)	573 (517 to 632)	538 (485 to 591)	499 (447 to 554)	494 (446 to 543)	488 (436 to 545)
Yamagata	713 (645 to 781)	703 (636 to 773)	653 (588 to 718)	661 (595 to 729)	665 (596 to 734)	656 (592 to 722)	621 (560 to 685)	568 (507 to 631)	560 (506 to 618)	553 (493 to 615)
Yamaguchi	797 (718 to 871)	835 (752 to 916)	810 (731 to 888)	844 (761 to 925)	843 (759 to 925)	819 (738 to 899)	781 (702 to 858)	724 (644 to 804)	716 (643 to 785)	708 (629 to 786)
Yamanashi	427 (389 to 467)	410 (373 to 449)	404 (366 to 444)	421 (383 to 460)	442 (402 to 483)	459 (417 to 503)	448 (405 to 491)	422 (380 to 466)	418 (380 to 459)	415 (374 to 460)
South Korea	9960 (9276 to 10611)	12908 (12010 to 13855)	16208 (15073 to 17347)	19176 (17831 to 20411)	22026 (20456 to 23434)	23291 (21637 to 24837)	24745 (23013 to 26514)	25806 (23887 to 27745)	25753 (24023 to 27542)	25688 (23798 to 27459)
Singapore	533 (496 to 569)	765 (696 to 829)	1013 (943 to 1078)	1183 (1109 to 1259)	1513 (1416 to 1616)	1962 (1824 to 2095)	2436 (2267 to 2604)	2771 (2559 to 2962)	2794 (2572 to 3014)	2813 (2584 to 3043)
High-income North America	84519 (79429 to 89614)	101872 (95477 to 107837)	118141 (110937 to 125411)	130381 (121943 to 138491)	143845 (134983 to 152769)	158414 (148403 to 168209)	174120 (163585 to 184867)	186382 (173554 to 199196)	187371 (175530 to 199002)	188192 (175928 to 200230)
Canada	6924 (6445 to 7392)	9032 (8362 to 9693)	10835 (10052 to 11623)	12317 (11456 to 13196)	13806 (12763 to 14900)	15425 (14322 to 16493)	17087 (15765 to 18286)	18666 (17152 to 20128)	18829 (17439 to 20226)	18984 (17759 to 20352)
Greenland	12 (11 to 13)	16 (15 to 18)	22 (21 to 23)	23 (23 to 23)	26 (25 to 26)	26 (26 to 26)	27 (26 to 27)	27 (24 to 29)	27 (24 to 29)	27 (24 to 29)
USA	77580 (72482 to 82759)	92821 (86285 to 98808)	107280 (99931 to 114420)	118038 (109450 to 126259)	130010 (120925 to 138998)	142959 (132954 to 152874)	157003 (146191 to 167816)	167686 (154783 to 180383)	168513 (156471 to 180249)	169178 (156838 to 181462)
Alabama	1582 (1431 to 1732)	1709 (1547 to 1869)	1835 (1653 to 2018)	2043 (1841 to 2243)	2146 (1941 to 2352)	2294 (2069 to 2513)	2461 (2228 to 2693)	2573 (2294 to 2849)	2580 (2326 to 2825)	2584 (2314 to 2854)
Alaska	50 (45 to 54)	99 (90 to 108)	142 (129 to 156)	192 (174 to 211)	266 (241 to 291)	304 (276 to 333)	339 (308 to 371)	349 (313 to 386)	349 (315 to 383)	348 (313 to 384)
Arizona	378 (342 to 415)	660 (598 to 726)	939 (851 to 1034)	1402 (1264 to 1545)	1896 (1716 to 2089)	2573 (2331 to 2827)	3206 (2908 to 3531)	3608 (3223 to 4023)	3640 (3293 to 3999)	3666 (3290 to 4068)
Arkansas	969 (878 to 1061)	926 (838 to 1013)	1021 (924 to 1118)	1193 (1078 to 1304)	1242 (1125 to 1357)	1368 (1241 to 1496)	1479 (1334 to 1620)	1529 (1372 to 1683)	1531 (1387 to 1674)	1531 (1375 to 1683)
California	5410 (4932 to 5921)	8037 (7318 to 8806)	10420 (9510 to 11405)	12199 (11086 to 13375)	15190 (13871 to 16629)	17074 (15564 to 18724)	18792 (17171 to 20580)	19824 (17942 to 21890)	19888 (18128 to 21804)	19933 (18043 to 21991)
Colorado	672 (609 to 736)	901 (815 to 986)	1158 (1050 to 1267)	1479 (1340 to 1618)	1699 (1548 to 1857)	2144 (1945 to 2351)	2509 (2282 to 2741)	2869 (2582 to 3164)	2905 (2632 to 3188)	2941 (2637 to 3241)
Connecticut	1040 (945 to 1143)	1317 (1192 to 1445)	1600 (1449 to 1754)	1632 (1478 to 1789)	1722 (1567 to 1883)	1746 (1581 to 1917)	1825 (1657 to 2003)	1841 (1649 to 2044)	1839 (1663 to 2024)	1836 (1647 to 2033)
Delaware	164 (149 to 179)	230 (208 to 251)	289 (264 to 316)	311 (282 to 340)	350 (319 to 384)	403 (366 to 441)	466 (424 to 509)	509 (459 to 562)	512 (465 to 561)	515 (464 to 568)
Washington, DC	431 (390 to 476)	414 (374 to 456)	414 (375 to 456)	346 (313 to 380)	328 (298 to 361)	307 (278 to 339)	326 (296 to 359)	361 (324 to 401)	364 (329 to 400)	366 (329 to 406)
Florida	1433 (1288 to 1574)	2564 (2310 to 2805)	3646 (3281 to 4000)	5168 (4643 to 5677)	6827 (6148 to 7490)	8159 (7330 to 8938)	9662 (8723 to 10587)	10933 (9727 to 12097)	11049 (9953 to 12102)	11151 (9946 to 12282)
Georgia	1782 (1614 to 1961)	2058 (1862 to 2261)	2431 (2199 to 2683)	2861 (2579 to 3159)	3409 (3083 to 3751)	4191 (3785 to 4605)	5009 (4528 to 5503)	5500 (4933 to 6116)	5537 (5008 to 6093)	5567 (4990 to 6169)
Hawaii	228 (207 to 250)	300 (271 to 328)	383 (345 to 420)	474 (426 to 519)	556 (501 to 610)	605 (545 to 663)	677 (610 to 744)	722 (645 to 800)	725 (653 to 795)	727 (649 to 805)
Idaho	290 (262 to 318)	335 (303 to 367)	369 (332 to 405)	479 (431 to 525)	518 (467 to 567)	645 (582 to 707)	783 (708 to 858)	911 (815 to 1009)	924 (833 to 1013)	936 (836 to 1035)
Illinois	4486 (4071 to 4929)	5231 (4739 to 5742)	5868 (5304 to 6459)	5955 (5383 to 6549)	5990 (5423 to 6593)	6251 (5639 to 6892)	6499 (5876 to 7172)	6524 (5828 to 7278)	6513 (5879 to 7190)	6496 (5843 to 7193)
Indiana	2018 (1824 to 2229)	2411 (2173 to 2663)	2735 (2468 to 3022)	2855 (2571 to 3157)	2911 (2630 to 3218)	3050 (2749 to 3378)	3270 (2953 to 3600)	3436 (3071 to 3834)	3447 (3113 to 3814)	3455 (3102 to 3844)
Iowa	1333 (1216 to 1461)	1427 (1293 to 1571)	1491 (1352 to 1645)	1512 (1365 to 1672)	1458 (1324 to 1608)	1469 (1329 to 1622)	1533 (1391 to 1691)	1597 (1437 to 1775)	1602 (1455 to 1764)	1606 (1440 to 1791)
Kansas	972 (881 to 1066)	1118 (1011 to 1226)	1175 (1064 to 1287)	1222 (1101 to 1340)	1285 (1165 to 1405)	1345 (1208 to 1474)	1428 (1289 to 1561)	1475 (1317 to 1631)	1477 (1333 to 1614)	1477 (1317 to 1630)
Kentucky	1491 (1354 to 1635)	1561 (1413 to 1711)	1692 (1531 to 1855)	1890 (1708 to 2072)	1938 (1757 to 2123)	2058 (1866 to 2257)	2202 (1997 to 2414)	2277 (2042 to 2521)	2280 (2065 to 2497)	2281 (2046 to 2516)
Louisiana	1390 (1257 to 1529)	1698 (1534 to 1869)	1928 (1741 to 2121)	2191 (1969 to 2411)	2231 (2017 to 2455)	2301 (2075 to 2533)	2310 (2093 to 2535)	2358 (2104 to 2625)	2360 (2130 to 2596)	2361 (2115 to 2610)
Maine	468 (424 to 516)	500 (451 to 551)	525 (475 to 578)	586 (526 to 645)	642 (583 to 704)	648 (585 to 712)	681 (615 to 750)	693 (621 to 768)	692 (625 to 763)	691 (621 to 767)
Maryland	1199 (1087 to 1325)	1599 (1449 to 1766)	2063 (1868 to 2279)	2206 (1991 to 2438)	2511 (2279 to 2771)	2754 (2497 to 3038)	3012 (2736 to 3319)	3167 (2844 to 3531)	3176 (2873 to 3514)	3183 (2853 to 3529)
Massachusetts	2466 (2236 to 2691)	2717 (2477 to 2968)	3042 (2762 to 3329)	3046 (2763 to 3343)	3179 (2889 to 3474)	3259 (2949 to 3577)	3371 (3065 to 3689)	3594 (3224 to 3972)	3616 (3278 to 3966)	3638 (3283 to 4009)
Michigan	3226 (2939 to 3530)	4017 (3651 to 4380)	4650 (4221 to 5099)	4795 (4348 to 5250)	4871 (4435 to 5332)	5016 (4555 to 5496)	4996 (4537 to 5482)	5124 (4615 to 5671)	5136 (4682 to 5634)	5145 (4644 to 5674)
Minnesota	1509 (1366 to 1655)	1755 (1585 to 1924)	1993 (1797 to 2190)	2107 (1896 to 2316)	2272 (2058 to 2492)	2447 (2208 to 2685)	2657 (2396 to 2917)	2854 (2548 to 3160)	2871 (2591 to 3154)	2887 (2587 to 3189)
Mississippi	1118 (1012 to 1231)	1133 (1024 to 1246)	1178 (1062 to 1298)	1320 (1190 to 1455)	1369 (1239 to 1503)	1465 (1326 to 1615)	1529 (1382 to 1683)	1523 (1359 to 1695)	1519 (1370 to 1675)	1513 (1357 to 1677)
Missouri	2051 (1868 to 2254)	2256 (2057 to 2475)	2483 (2263 to 2733)	2582 (2346 to 2846)	2701 (2464 to 2969)	2841 (2576 to 3142)	3033 (2762 to 3347)	3127 (2830 to 3464)	3129 (2848 to 3444)	3128 (2812 to 3480)
Montana	287 (259 to 315)	338 (305 to 370)	358 (325 to 394)	399 (361 to 438)	411 (374 to 451)	455 (411 to 499)	490 (444 to 537)	534 (480 to 592)	539 (488 to 592)	542 (488 to 598)
Nebraska	669 (605 to 737)	726 (656 to 802)	781 (706 to 862)	813 (732 to 901)	824 (747 to 911)	861 (777 to 950)	916 (828 to 1015)	979 (876 to 1093)	985 (892 to 1084)	990 (887 to 1101)
Nevada	76 (69 to 84)	141 (127 to 154)	250 (227 to 275)	404 (365 to 445)	610 (553 to 670)	994 (898 to 1092)	1336 (1210 to 1470)	1538 (1381 to 1708)	1555 (1407 to 1710)	1570 (1410 to 1734)
New Hampshire	276 (250 to 303)	315 (287 to 346)	389 (353 to 427)	479 (434 to 527)	576 (524 to 632)	623 (566 to 684)	670 (608 to 738)	696 (628 to 774)	698 (635 to 766)	699 (631 to 775)
New Jersey	2504 (2273 to 2760)	3158 (2870 to 3461)	3799 (3463 to 4180)	3881 (3521 to 4268)	4075 (3705 to 4472)	4314 (3926 to 4742)	4494 (4085 to 4938)	4726 (4260 to 5251)	4749 (4317 to 5218)	4769 (4301 to 5276)
New Mexico	340 (307 to 373)	480 (435 to 526)	534 (482 to 586)	670 (605 to 734)	787 (712 to 861)	925 (838 to 1015)	1041 (941 to 1139)	1069 (958 to 1183)	1068 (969 to 1172)	1066 (958 to 1175)

New York	7740 (7008 to 8460)	8837 (7998 to 9661)	9758 (8844 to 10691)	9323 (8415 to 10198)	9527 (8612 to 10426)	9806 (8864 to 10728)	9925 (8955 to 10857)	10342 (9262 to 11407)	10379 (9372 to 11386)	10405 (9335 to 11474)
North Carolina	2073 (1880 to 2282)	2356 (2140 to 2580)	2673 (2425 to 2935)	3063 (2771 to 3372)	3489 (3176 to 3830)	4114 (3727 to 4526)	4918 (4464 to 5410)	5362 (4818 to 5956)	5391 (4878 to 5933)	5414 (4875 to 5976)
North Dakota	301 (272 to 331)	316 (284 to 347)	315 (284 to 347)	328 (295 to 362)	325 (294 to 358)	317 (286 to 348)	334 (302 to 368)	378 (338 to 421)	383 (346 to 422)	387 (346 to 430)
Ohio	4117 (3727 to 4517)	5040 (4544 to 5506)	5631 (5082 to 6153)	5649 (5070 to 6200)	5719 (5153 to 6265)	5768 (5194 to 6324)	5851 (5300 to 6407)	6008 (5356 to 6643)	6018 (5444 to 6588)	6024 (5387 to 6651)
Oklahoma	1132 (1023 to 1243)	1205 (1088 to 1320)	1353 (1225 to 1484)	1569 (1408 to 1725)	1644 (1487 to 1799)	1752 (1583 to 1924)	1877 (1701 to 2059)	1986 (1777 to 2190)	1995 (1798 to 2187)	2001 (1789 to 2204)
Oregon	761 (687 to 837)	907 (819 to 996)	1101 (993 to 1210)	1353 (1219 to 1486)	1479 (1340 to 1625)	1720 (1547 to 1892)	1937 (1755 to 2130)	2131 (1907 to 2366)	2148 (1942 to 2359)	2164 (1943 to 2396)
Pennsylvania	5430 (4924 to 5950)	5929 (5359 to 6489)	6284 (5677 to 6889)	6255 (5648 to 6844)	6291 (5715 to 6885)	6295 (5690 to 6885)	6510 (5903 to 7122)	6620 (5930 to 7329)	6619 (6002 to 7226)	6612 (5951 to 7296)
Rhode Island	409 (372 to 447)	447 (406 to 489)	497 (450 to 546)	502 (454 to 550)	531 (484 to 583)	539 (490 to 590)	540 (492 to 592)	562 (508 to 620)	564 (514 to 619)	567 (510 to 626)
South Carolina	1091 (987 to 1194)	1231 (1114 to 1352)	1361 (1236 to 1492)	1623 (1468 to 1780)	1836 (1676 to 2006)	2061 (1863 to 2260)	2385 (2164 to 2611)	2613 (2346 to 2895)	2630 (2390 to 2880)	2645 (2380 to 2930)
South Dakota	321 (291 to 352)	343 (311 to 375)	345 (313 to 378)	353 (319 to 388)	360 (326 to 394)	375 (340 to 411)	408 (370 to 446)	439 (394 to 485)	441 (401 to 483)	443 (397 to 487)
Tennessee	1694 (1538 to 1859)	1864 (1686 to 2047)	2090 (1890 to 2292)	2399 (2163 to 2639)	2582 (2343 to 2847)	2910 (2633 to 3200)	3259 (2958 to 3580)	3534 (3174 to 3924)	3555 (3221 to 3907)	3573 (3212 to 3957)
Texas	3923 (3549 to 4294)	4929 (4453 to 5379)	5908 (5339 to 6482)	7349 (6614 to 8055)	8806 (7973 to 9653)	10554 (9565 to 11544)	12827 (11657 to 14028)	14611 (13147 to 16131)	14764 (13411 to 16156)	14902 (13402 to 16400)
Utah	347 (313 to 379)	455 (410 to 495)	555 (502 to 605)	749 (674 to 818)	887 (802 to 968)	1118 (1006 to 1221)	1374 (1241 to 1500)	1611 (1442 to 1775)	1635 (1475 to 1786)	1659 (1483 to 1825)
Vermont	193 (175 to 213)	202 (183 to 223)	234 (211 to 259)	266 (239 to 294)	293 (266 to 323)	307 (277 to 339)	321 (291 to 355)	325 (292 to 361)	325 (293 to 359)	325 (291 to 361)
Virginia	1671 (1512 to 1846)	2027 (1830 to 2235)	2422 (2194 to 2671)	2770 (2501 to 3062)	3217 (2910 to 3549)	3632 (3285 to 4007)	4101 (3705 to 4504)	4376 (3923 to 4889)	4394 (3977 to 4844)	4407 (3962 to 4906)
Washington	1177 (1069 to 1295)	1447 (1312 to 1587)	1765 (1600 to 1939)	2113 (1915 to 2320)	2511 (2276 to 2766)	2959 (2670 to 3259)	3379 (3067 to 3710)	3829 (3442 to 4272)	3874 (3525 to 4264)	3918 (3529 to 4340)
West Virginia	1013 (918 to 1115)	965 (877 to 1057)	926 (837 to 1021)	1014 (916 to 1116)	947 (860 to 1040)	920 (834 to 1012)	925 (841 to 1019)	906 (811 to 1009)	902 (818 to 994)	897 (804 to 995)
Wisconsin	1740 (1571 to 1913)	2027 (1833 to 2223)	2308 (2077 to 2533)	2431 (2184 to 2671)	2544 (2301 to 2794)	2678 (2424 to 2938)	2856 (2580 to 3135)	2955 (2641 to 3284)	2959 (2675 to 3250)	2962 (2650 to 3277)
Wyoming	138 (125 to 152)	164 (148 to 180)	171 (154 to 188)	234 (210 to 257)	230 (208 to 255)	244 (219 to 270)	275 (248 to 302)	281 (250 to 313)	280 (253 to 309)	280 (249 to 311)
Southern Latin America	12810 (12225 to 13445)	15497 (14767 to 16220)	18242 (17357 to 19143)	21811 (20757 to 22909)	25284 (23996 to 26596)	28218 (26761 to 29734)	31343 (29750 to 33040)	34199 (31270 to 37252)	34453 (31207 to 37542)	34628 (31427 to 37946)
Argentina	8691 (8139 to 9315)	10315 (9583 to 10978)	12125 (11292 to 12932)	14588 (13612 to 15561)	16908 (15713 to 18086)	18849 (17513 to 20308)	21073 (19754 to 22501)	23056 (20190 to 26102)	23198 (20012 to 26218)	23306 (20092 to 26566)
Chile	2996 (2791 to 3193)	3887 (3620 to 4145)	4697 (4383 to 5019)	5713 (5239 to 6186)	6762 (6233 to 7309)	7668 (7027 to 8278)	8527 (7572 to 9445)	9365 (8621 to 10095)	9478 (8642 to 10347)	9547 (8683 to 10447)
Uruguay	1122 (1043 to 1198)	1294 (1182 to 1402)	1420 (1280 to 1566)	1509 (1360 to 1655)	1613 (1451 to 1789)	1699 (1540 to 1858)	1742 (1621 to 1867)	1776 (1567 to 2004)	1775 (1563 to 1998)	1773 (1555 to 2012)
Western Europe	161908 (155668 to 167776)	171804 (167129 to 176289)	183586 (178799 to 187976)	191810 (186966 to 196545)	197202 (193307 to 200803)	203920 (200000 to 207488)	215181 (211826 to 218154)	221939 (215174 to 228138)	222248 (215304 to 228878)	222444 (214716 to 229343)
Andorra	3 (3 to 3)	5 (4 to 5)	9 (8 to 10)	16 (15 to 18)	25 (25 to 26)	32 (31 to 32)	40 (40 to 41)	40 (37 to 44)	41 (37 to 45)	42 (38 to 46)
Austria	3705 (3461 to 3947)	3761 (3484 to 4039)	3942 (3656 to 4243)	3977 (3701 to 4276)	4035 (3754 to 4324)	4136 (3842 to 4434)	4294 (4258 to 4328)	4516 (4138 to 4896)	4534 (4141 to 4923)	4551 (4098 to 4956)
Belgium	4404 (4108 to 4684)	4659 (4331 to 5015)	4928 (4598 to 5262)	5030 (4645 to 5430)	5091 (4679 to 5479)	5239 (4857 to 5636)	5546 (5507 to 5588)	5798 (5350 to 6254)	5810 (5320 to 6324)	5821 (5238 to 6375)
Cyprus	249 (231 to 266)	300 (280 to 322)	325 (282 to 367)	336 (308 to 365)	387 (357 to 417)	457 (423 to 489)	564 (520 to 606)	668 (591 to 750)	678 (596 to 762)	688 (595 to 780)
Denmark	2154 (2004 to 2292)	2313 (2159 to 2475)	2485 (2309 to 2653)	2592 (2574 to 2609)	2609 (2590 to 2629)	2700 (2678 to 2722)	2793 (2769 to 2817)	2913 (2676 to 3144)	2925 (2644 to 3186)	2937 (2662 to 3217)
Finland	2103 (1954 to 2253)	2297 (2141 to 2449)	2394 (2382 to 2407)	2480 (2464 to 2496)	2580 (2563 to 2597)	2658 (2639 to 2677)	2742 (2722 to 2763)	2802 (2575 to 3034)	2802 (2563 to 3049)	2802 (2506 to 3066)
France	22195 (20663 to 23700)	24027 (22103 to 25953)	26533 (24490 to 28648)	28065 (25822 to 30219)	29617 (27503 to 31498)	30772 (28499 to 33053)	32811 (30638 to 34990)	34069 (30924 to 37275)	34144 (30916 to 37593)	34205 (30681 to 37855)
Germany	38481 (33164 to 43602)	39255 (36412 to 42070)	40808 (37921 to 43371)	41213 (38582 to 44047)	41340 (41045 to 41653)	42127 (41773 to 42498)	41636 (41277 to 42025)	42740 (39102 to 46418)	42807 (38819 to 46736)	42843 (38264 to 47163)
Greece	3981 (3717 to 4240)	4397 (4066 to 4715)	4563 (4220 to 4896)	5013 (4654 to 5388)	5267 (4875 to 5665)	5585 (5173 to 6003)	5611 (5219 to 5987)	5298 (4649 to 5888)	5268 (4579 to 5881)	5233 (4489 to 5919)
Iceland	71 (70 to 71)	86 (86 to 86)	101 (100 to 101)	113 (112 to 113)	126 (126 to 127)	140 (138 to 141)	158 (157 to 159)	170 (156 to 184)	172 (155 to 186)	173 (157 to 189)
Ireland	1494 (1398 to 1591)	1442 (1334 to 1551)	1508 (1408 to 1611)	1735 (1599 to 1868)	1807 (1673 to 1937)	1945 (1790 to 2096)	2312 (2129 to 2502)	2478 (2262 to 2702)	2488 (2253 to 2728)	2498 (2234 to 2756)
Israel	759 (707 to 810)	1066 (983 to 1144)	1510 (1393 to 1631)	1936 (1779 to 2104)	2506 (2259 to 2755)	3231 (2914 to 3577)	3956 (3628 to 4287)	4663 (4090 to 5285)	4740 (4069 to 5386)	4816 (4116 to 5546)
Italy	23983 (22328 to 25528)	25960 (23864 to 27957)	27507 (25446 to 29518)	28969 (26790 to 31013)	29231 (27178 to 31232)	29241 (27051 to 31310)	31046 (30804 to 31270)	30963 (28418 to 33361)	30833 (28053 to 33447)	30687 (27887 to 33402)
Luxembourg	152 (141 to 162)	160 (149 to 170)	174 (162 to 185)	185 (171 to 200)	194 (179 to 208)	220 (204 to 237)	253 (251 to 255)	307 (282 to 331)	314 (285 to 340)	320 (292 to 349)
Malta	174 (162 to 186)	172 (157 to 186)	168 (153 to 183)	174 (157 to 191)	188 (168 to 207)	203 (183 to 223)	213 (197 to 229)	220 (195 to 245)	221 (193 to 246)	221 (192 to 250)
Monaco	12 (11 to 13)	12 (11 to 13)	13 (12 to 14)	14 (13 to 15)	16 (15 to 17)	17 (16 to 18)	18 (16 to 20)	19 (18 to 21)	19 (18 to 21)	19 (18 to 21)
Netherlands	5038 (5010 to 5065)	5728 (5697 to 5760)	6503 (6467 to 6543)	7098 (7046 to 7145)	7548 (7493 to 7604)	8031 (7966 to 8098)	8385 (8310 to 8462)	8633 (7887 to 9366)	8651 (7918 to 9415)	8666 (7850 to 9513)
Norway	1652 (1547 to 1771)	1802 (1684 to 1926)	1953 (1820 to 2089)	2064 (1937 to 2210)	2147 (2005 to 2296)	2264 (2246 to 2283)	2436 (2416 to 2458)	2649 (2445 to 2851)	2666 (2440 to 2913)	2681 (2442 to 2951)
Agder	86 (78 to 95)	93 (84 to 102)	103 (93 to 113)	115 (104 to 126)	122 (114 to 130)	130 (129 to 132)	140 (138 to 142)	151 (135 to 169)	152 (134 to 171)	153 (134 to 173)
Innlandet	163 (148 to 179)	168 (153 to 185)	174 (158 to 191)	183 (167 to 202)	186 (174 to 200)	187 (185 to 189)	190 (188 to 192)	193 (173 to 216)	193 (170 to 218)	194 (169 to 220)
Møre og Romsdal	96 (88 to 106)	106 (96 to 116)	111 (101 to 122)	118 (107 to 129)	119 (111 to 128)	122 (120 to 123)	125 (123 to 127)	132 (118 to 147)	132 (117 to 149)	133 (116 to 150)
Nordland	109 (98 to 119)	115 (105 to 127)	118 (107 to 130)	121 (109 to 132)	120 (112 to 128)	120 (118 to 121)	118 (117 to 120)	120 (106 to 134)	120 (105 to 136)	120 (105 to 136)
Oslo	236 (214 to 258)	255 (232 to 279)	257 (233 to 282)	243 (221 to 266)	245 (228 to 262)	263 (260 to 266)	298 (294 to 301)	344 (307 to 382)	348 (307 to 391)	352 (308 to 398)
Rogaland	107 (97 to 117)	120 (109 to 131)	135 (122 to 148)	153 (139 to 168)	169 (157 to 181)	187 (185 to 189)	212 (209 to 214)	236 (211 to 263)	238 (210 to 268)	239 (210 to 270)
Troms og Finnmark	88 (80 to 97)	96 (88 to 106)	103 (94 to 114)	110 (100 to 121)	109 (102 to 116)	112 (110 to 113)	113 (112 to 115)	119 (107 to 133)	120 (105 to 135)	120 (105 to 137)
Trøndelag	154 (140 to 168)	164 (149 to 179)	176 (159 to 193)	186 (168 to 203)	190 (177 to 204)	196 (194 to 199)	210 (208 to 213)	227 (202 to 252)	228 (201 to 256)	230 (200 to 258)
Vestfold og Telemark	146 (132 to 160)	162 (146 to 177)	167 (151 to 184)	176 (160 to 193)	184 (172 to 197)	193 (190 to 195)	202 (200 to 205)	212 (188 to 237)	213 (187 to 240)	213 (186 to 242)
Vestland	208 (190 to 227)	222 (202 to 242)	238 (216 to 261)	249 (227 to 272)	259 (242 to 278)	273 (270 to 276)	291 (288 to 295)	315 (282 to 350)	316 (280 to 355)	318 (279 to 359)
Viken	260 (237 to 284)	301 (275 to 327)	371 (337 to 404)	411 (376 to 447)	444 (415 to 475)	482 (477 to 488)	537 (531 to 543)	599 (538 to 659)	605 (540 to 676)	611 (543 to 686)
Portugal	4541 (4220 to 4852)	4789 (4473 to 5127)	4668 (4340 to 4996)	5191 (4797 to 5564)	5240 (4836 to 5625)	5440 (5050 to 5833)	5618 (5221 to 6007)	5596 (4956 to 6257)	5585 (4937 to 6298)	5570 (4846 to 6326)
San Marino	8 (7 to 8)	8 (7 to 9)	9 (8 to 10)	10 (9 to 11)	12 (10 to 14)	14 (12 to 16)	16 (15 to 17)	17 (15 to 19)	17 (15 to 19)	17 (15 to 19)
Spain	14509 (13495 to 15510)	15720 (14690 to 16804)	17399 (16268 to 18635)	19070 (17673 to 20491)	19780 (18459 to 21284)	20813 (20681 to 20937)	23769 (23614 to 23923)	23489 (21481 to 25510)	23392 (21336 to 25478)	23290 (20945 to 25521)
Sweden	3533 (3286 to 3781)	3758 (3512 to 4013)	4031 (4005 to 4056)	4196 (4169 to 4225)	4349 (4318 to 4381)	4502 (4465 to 4539)	4732 (4691 to 4769)	5087 (4634 to 5537)	5121 (4637 to 5610)	5153 (4664 to 5662)
Stockholm	567 (509 to 625)	627 (563 to 691)	736 (729 to 743)	769 (761 to 777)	823 (814 to 832)	903 (892 to 914)	1009 (997 to 1022)	1153 (1012 to 1297)	1166 (1008 to 1334)	1180 (1017 to 1348)
Sweden except Stockholm	2966 (2753 to 3182)	3131 (2911 to 3351)	3295 (3273 to 3316)	3427 (3403 to 3452)	3526 (3500 to 3552)	3599 (3568 to 3630)	3723 (3689 to 3754)	3934 (3568 to 4305)	3954 (3557 to 4366)	3973 (3573 to 4390)
Switzerland	2444 (2281 to 2617)	2766 (2573 to 2955)	3173 (2952 to 3382)	3260 (3046 to 3484)	3483 (3256 to 3713)	3728 (3483 to 3965)	4035 (3996 to 4070)	4406 (4027 to 4802)	4440 (4021 to 4841)	4473 (4037 to 4944)
UK	26147 (24726 to 27579)	27198 (25554 to 28780)	28747 (27079 to 30494)	28922 (27080 to 30580)	29461 (27612 to 31305)	30258 (28378 to 32096)	32011 (29991 to 34001)	34205 (31812 to 36506)	34387 (32147 to 36585)	34543 (32538 to 36461)
England	21331 (19979 to 22730)	22313 (20680 to 23879)	23734 (22120 to 25449)	24001 (22139 to 25641)	24504 (22691 to 26363)	25197 (23336 to 27045)	26751 (24704 to 28775)	28835 (26449 to 31153)	29020 (26816 to 31191)	29182 (27218 to 31059)
East Midlands	1630 (1480 to 1794)	1702 (1463 to 1959)	1846 (1664 to 2041)	1951 (1761 to 2154)	2036 (1841 to 2255)	2121 (1915 to 2345)	2281 (2053 to 2527)	2472 (2133 to 2835)	2488 (2127 to 2851)	2503 (2148 to 2866)
Derby	100 (88 to 112)	105 (85 to 126)	112 (98 to 127)	111 (97 to 126)	114 (99 to 129)	117 (103 to 134)	125 (109 to 142)	135 (112 to 162)	136 (111 to 163)	137 (112 to 165)

Derbyshire	305 (272 to 339)	318 (260 to 379)	341 (299 to 383)	354 (310 to 397)	362 (318 to 408)	374 (329 to 420)	390 (342 to 438)	412 (341 to 487)	414 (340 to 492)	415 (342 to 497)
Leicester	137 (122 to 153)	141 (117 to 170)	147 (130 to 166)	146 (129 to 165)	146 (129 to 165)	144 (127 to 163)	164 (144 to 185)	189 (157 to 226)	191 (158 to 230)	193 (160 to 234)
Leicestershire	206 (184 to 230)	217 (179 to 261)	243 (214 to 275)	271 (241 to 306)	288 (254 to 325)	307 (272 to 346)	327 (289 to 370)	349 (291 to 414)	351 (286 to 421)	352 (292 to 421)
Lincolnshire	229 (205 to 258)	234 (193 to 283)	254 (224 to 287)	279 (246 to 316)	299 (265 to 339)	328 (290 to 371)	363 (320 to 413)	398 (331 to 474)	401 (330 to 479)	403 (334 to 483)
Northamptonshire	212 (189 to 237)	218 (182 to 259)	240 (212 to 271)	269 (237 to 303)	295 (261 to 332)	317 (281 to 357)	348 (307 to 393)	383 (322 to 450)	386 (322 to 457)	389 (325 to 460)
Nottingham	139 (124 to 156)	148 (122 to 178)	155 (137 to 174)	143 (127 to 162)	143 (126 to 161)	134 (119 to 152)	148 (131 to 167)	166 (139 to 199)	168 (138 to 200)	169 (139 to 204)
Nottinghamshire	292 (259 to 325)	309 (254 to 369)	341 (300 to 383)	362 (318 to 406)	374 (329 to 420)	383 (338 to 429)	398 (351 to 449)	420 (349 to 495)	422 (344 to 501)	423 (347 to 504)
Rutland	11 (10 to 13)	12 (10 to 14)	13 (12 to 15)	16 (14 to 18)	16 (14 to 18)	17 (15 to 19)	18 (16 to 21)	20 (16 to 24)	20 (16 to 24)	20 (16 to 24)
East of England	1953 (1776 to 2144)	2038 (1750 to 2337)	2239 (2023 to 2477)	2451 (2201 to 2707)	2596 (2338 to 2869)	2738 (2470 to 3020)	2950 (2651 to 3264)	3205 (2770 to 3654)	3228 (2785 to 3687)	3249 (2795 to 3705)
Bedford	53 (47 to 59)	56 (46 to 68)	63 (55 to 71)	67 (58 to 76)	69 (60 to 78)	74 (65 to 84)	80 (69 to 90)	86 (70 to 102)	86 (70 to 103)	87 (69 to 105)
Cambridgeshire	171 (152 to 191)	178 (146 to 215)	198 (175 to 224)	225 (198 to 254)	254 (224 to 287)	277 (244 to 312)	307 (270 to 347)	341 (283 to 406)	344 (283 to 412)	347 (285 to 415)
Central Bedfordshire	73 (64 to 82)	77 (62 to 93)	88 (77 to 101)	104 (91 to 118)	110 (96 to 124)	117 (102 to 133)	128 (111 to 145)	141 (115 to 169)	142 (115 to 172)	143 (116 to 173)
Essex	472 (422 to 524)	491 (403 to 583)	544 (482 to 608)	606 (535 to 678)	636 (562 to 712)	668 (592 to 747)	710 (629 to 795)	758 (632 to 889)	763 (635 to 901)	767 (634 to 905)
Hertfordshire	390 (348 to 435)	425 (350 to 510)	471 (416 to 529)	492 (433 to 555)	499 (439 to 561)	525 (464 to 590)	564 (497 to 634)	615 (514 to 726)	620 (516 to 734)	624 (518 to 742)
Luton	65 (58 to 73)	70 (58 to 87)	79 (70 to 90)	82 (72 to 94)	87 (77 to 99)	92 (81 to 104)	100 (88 to 114)	111 (93 to 135)	113 (94 to 137)	114 (93 to 140)
Norfolk	294 (261 to 326)	296 (242 to 352)	320 (282 to 359)	358 (316 to 402)	385 (340 to 433)	408 (360 to 456)	436 (384 to 491)	465 (386 to 547)	467 (385 to 551)	468 (383 to 557)
Peterborough	45 (40 to 51)	47 (38 to 57)	53 (47 to 60)	66 (58 to 75)	78 (68 to 88)	80 (70 to 90)	91 (80 to 103)	108 (88 to 128)	109 (90 to 131)	111 (90 to 133)
Southend-on-Sea	96 (85 to 108)	92 (74 to 111)	89 (78 to 101)	85 (74 to 96)	85 (74 to 97)	84 (74 to 95)	88 (77 to 101)	96 (79 to 115)	97 (78 to 116)	97 (79 to 117)
Suffolk	246 (218 to 276)	250 (203 to 300)	272 (238 to 306)	303 (265 to 341)	329 (289 to 371)	340 (298 to 383)	366 (320 to 413)	397 (326 to 468)	399 (328 to 473)	402 (328 to 477)
Thurrock	50 (44 to 56)	55 (44 to 67)	62 (54 to 70)	64 (56 to 73)	65 (57 to 74)	73 (64 to 82)	79 (70 to 90)	88 (72 to 105)	88 (72 to 106)	89 (73 to 107)
Greater London	3543 (3239 to 3868)	3795 (3302 to 4309)	3910 (3547 to 4285)	3546 (3201 to 3895)	3506 (3170 to 3862)	3688 (3343 to 4042)	4086 (3672 to 4492)	4603 (4016 to 5236)	4652 (4052 to 5287)	4697 (4076 to 5320)
Barking and Dagenham	79 (71 to 88)	84 (69 to 101)	88 (78 to 100)	85 (75 to 96)	80 (71 to 91)	85 (76 to 97)	95 (84 to 107)	110 (91 to 130)	111 (92 to 132)	113 (93 to 135)
Barnet	148 (132 to 165)	156 (129 to 188)	162 (143 to 182)	155 (137 to 175)	155 (137 to 175)	163 (144 to 184)	181 (159 to 205)	204 (170 to 241)	206 (171 to 246)	208 (172 to 248)
Bexley	94 (84 to 106)	103 (85 to 123)	112 (99 to 127)	112 (98 to 127)	112 (99 to 128)	113 (100 to 128)	120 (105 to 137)	130 (108 to 155)	131 (109 to 156)	132 (109 to 159)
Brent	124 (110 to 138)	135 (110 to 159)	142 (125 to 159)	128 (112 to 144)	122 (107 to 138)	134 (118 to 150)	152 (133 to 171)	174 (144 to 204)	176 (145 to 208)	178 (145 to 211)
Bromley	141 (126 to 158)	153 (126 to 183)	161 (142 to 182)	156 (137 to 176)	153 (134 to 173)	154 (136 to 173)	160 (141 to 182)	173 (144 to 206)	174 (144 to 209)	175 (144 to 211)
Camden	107 (96 to 119)	111 (92 to 132)	111 (98 to 124)	94 (83 to 105)	93 (82 to 105)	101 (90 to 114)	110 (97 to 124)	118 (98 to 140)	119 (99 to 141)	119 (98 to 143)
Croydon	156 (139 to 173)	168 (139 to 196)	176 (156 to 197)	167 (147 to 187)	163 (144 to 183)	171 (151 to 191)	186 (163 to 209)	207 (173 to 241)	209 (173 to 245)	211 (174 to 248)
Ealing	140 (126 to 157)	149 (125 to 177)	156 (139 to 176)	147 (130 to 165)	144 (128 to 163)	152 (135 to 172)	167 (148 to 188)	186 (157 to 220)	188 (157 to 223)	189 (157 to 226)
Enfield	127 (114 to 142)	133 (110 to 159)	139 (124 to 157)	136 (120 to 153)	134 (119 to 152)	142 (126 to 160)	160 (141 to 181)	183 (153 to 218)	186 (153 to 222)	188 (155 to 224)
Greenwich	100 (90 to 112)	105 (87 to 125)	111 (98 to 125)	111 (98 to 125)	110 (97 to 123)	111 (99 to 125)	126 (111 to 142)	148 (123 to 175)	151 (125 to 178)	153 (126 to 181)
Hackney	106 (94 to 118)	115 (95 to 138)	118 (103 to 133)	100 (88 to 113)	97 (86 to 110)	108 (95 to 121)	125 (110 to 142)	145 (120 to 173)	147 (122 to 175)	149 (122 to 177)
Hammersmith and Fulham	95 (84 to 107)	101 (82 to 121)	100 (87 to 112)	80 (69 to 90)	79 (69 to 90)	85 (75 to 96)	92 (80 to 104)	99 (82 to 118)	100 (81 to 119)	100 (82 to 120)
Haringey	115 (103 to 127)	124 (103 to 147)	127 (112 to 142)	108 (95 to 121)	106 (94 to 120)	111 (98 to 125)	126 (111 to 142)	143 (119 to 169)	145 (121 to 171)	147 (121 to 173)
Harrow	96 (86 to 107)	102 (84 to 122)	107 (95 to 120)	104 (91 to 117)	105 (92 to 118)	106 (93 to 120)	120 (105 to 135)	139 (115 to 165)	141 (117 to 167)	143 (117 to 170)
Havering	102 (92 to 114)	115 (95 to 139)	126 (111 to 142)	124 (110 to 140)	119 (105 to 135)	117 (103 to 132)	123 (108 to 139)	135 (113 to 161)	136 (113 to 163)	137 (113 to 165)
Hillingdon	102 (91 to 113)	112 (92 to 133)	121 (107 to 136)	118 (104 to 133)	119 (105 to 134)	125 (110 to 140)	136 (120 to 154)	154 (128 to 182)	156 (130 to 184)	158 (130 to 187)
Hounslow	94 (84 to 104)	99 (82 to 117)	104 (93 to 117)	103 (91 to 115)	104 (92 to 116)	107 (95 to 120)	124 (109 to 139)	146 (122 to 171)	148 (123 to 174)	150 (125 to 177)
Islington	95 (85 to 106)	104 (86 to 125)	105 (93 to 118)	85 (75 to 96)	88 (78 to 99)	91 (81 to 102)	102 (90 to 115)	117 (98 to 138)	118 (98 to 140)	119 (99 to 142)
Kensington and Chelsea	101 (90 to 112)	107 (88 to 127)	107 (94 to 120)	76 (67 to 86)	75 (66 to 84)	82 (73 to 92)	80 (70 to 90)	79 (66 to 93)	79 (66 to 94)	79 (65 to 94)
Kingston upon Thames	68 (60 to 76)	72 (59 to 85)	74 (65 to 83)	70 (61 to 79)	70 (61 to 78)	74 (65 to 84)	81 (71 to 91)	89 (74 to 105)	90 (74 to 105)	90 (74 to 107)
Lambeth	144 (128 to 162)	159 (128 to 189)	160 (141 to 181)	132 (115 to 149)	130 (114 to 147)	134 (118 to 150)	150 (131 to 169)	167 (138 to 198)	169 (138 to 199)	170 (138 to 203)
Lewisham	130 (115 to 144)	140 (114 to 167)	143 (127 to 161)	126 (110 to 141)	124 (109 to 140)	129 (114 to 145)	140 (123 to 157)	156 (130 to 184)	157 (130 to 187)	159 (131 to 189)
Merton	90 (80 to 100)	94 (77 to 111)	95 (84 to 107)	88 (78 to 99)	88 (77 to 99)	96 (85 to 108)	101 (88 to 113)	108 (89 to 127)	109 (90 to 128)	109 (90 to 129)
Newham	108 (96 to 120)	117 (97 to 140)	121 (107 to 137)	109 (96 to 124)	109 (96 to 124)	123 (108 to 138)	145 (128 to 164)	173 (144 to 206)	176 (145 to 211)	178 (148 to 214)
Redbridge	112 (100 to 125)	118 (98 to 141)	122 (108 to 138)	115 (102 to 130)	114 (101 to 129)	122 (108 to 138)	139 (123 to 158)	164 (137 to 195)	167 (139 to 197)	169 (140 to 202)
Richmond upon Thames	92 (82 to 103)	96 (79 to 114)	96 (85 to 109)	87 (77 to 98)	86 (76 to 98)	88 (78 to 100)	95 (84 to 108)	103 (86 to 121)	104 (86 to 122)	104 (86 to 124)
Southwark	124 (110 to 138)	137 (113 to 162)	138 (122 to 154)	115 (101 to 129)	115 (101 to 130)	127 (112 to 142)	143 (126 to 160)	161 (133 to 190)	162 (134 to 192)	164 (135 to 194)
Sutton	83 (74 to 93)	86 (71 to 102)	90 (79 to 101)	90 (79 to 101)	88 (77 to 100)	93 (82 to 104)	98 (86 to 110)	105 (87 to 124)	106 (88 to 125)	107 (88 to 127)
Tower Hamlets	77 (69 to 86)	85 (70 to 101)	85 (76 to 96)	73 (65 to 82)	82 (72 to 92)	96 (85 to 108)	120 (106 to 135)	145 (121 to 169)	147 (122 to 173)	149 (123 to 175)
Waltham Forest	116 (103 to 129)	120 (99 to 141)	123 (109 to 137)	114 (100 to 128)	111 (98 to 125)	112 (99 to 125)	127 (112 to 143)	151 (125 to 177)	153 (126 to 181)	155 (128 to 184)
Wandsworth	153 (137 to 170)	162 (134 to 193)	161 (143 to 180)	139 (123 to 156)	136 (121 to 153)	138 (122 to 155)	155 (137 to 174)	176 (148 to 208)	178 (149 to 211)	180 (149 to 214)
Westminster	124 (110 to 138)	132 (109 to 157)	128 (113 to 144)	99 (87 to 111)	95 (83 to 107)	100 (88 to 113)	106 (93 to 120)	113 (94 to 135)	114 (95 to 135)	115 (95 to 136)
North East England	1220 (1102 to 1347)	1289 (1096 to 1481)	1372 (1234 to 1522)	1359 (1213 to 1507)	1337 (1193 to 1490)	1312 (1174 to 1455)	1325 (1177 to 1477)	1367 (1169 to 1570)	1370 (1165 to 1575)	1371 (1163 to 1569)
County Durham	229 (204 to 257)	242 (196 to 289)	258 (227 to 293)	262 (230 to 295)	258 (226 to 292)	254 (224 to 287)	261 (229 to 295)	272 (224 to 325)	273 (225 to 325)	273 (223 to 326)
Darlington	47 (41 to 53)	48 (39 to 57)	50 (44 to 57)	51 (44 to 57)	52 (45 to 58)	51 (44 to 57)	54 (47 to 61)	58 (47 to 68)	58 (47 to 69)	58 (47 to 70)
Gateshead	104 (92 to 116)	110 (89 to 132)	116 (102 to 131)	111 (96 to 125)	105 (92 to 119)	99 (87 to 112)	102 (89 to 115)	108 (88 to 129)	108 (89 to 130)	109 (88 to 130)
Hartlepool	44 (39 to 49)	47 (39 to 57)	51 (45 to 58)	49 (43 to 55)	47 (42 to 54)	47 (41 to 53)	47 (42 to 54)	49 (40 to 58)	49 (40 to 58)	49 (40 to 59)
Middlesbrough	69 (61 to 78)	75 (61 to 92)	81 (71 to 92)	77 (67 to 88)	75 (65 to 85)	73 (63 to 83)	71 (61 to 81)	71 (58 to 86)	71 (58 to 87)	71 (58 to 87)
Newcastle upon Tyne	153 (136 to 173)	161 (131 to 194)	164 (143 to 185)	149 (130 to 169)	143 (124 to 162)	137 (120 to 156)	138 (120 to 157)	143 (117 to 171)	143 (118 to 172)	144 (117 to 173)
North Tyneside	97 (86 to 110)	103 (84 to 124)	108 (95 to 122)	104 (91 to 118)	102 (89 to 116)	100 (88 to 114)	104 (90 to 118)	110 (90 to 132)	111 (90 to 132)	111 (91 to 133)
Northumberland	135 (119 to 151)	136 (110 to 165)	144 (126 to 163)	153 (134 to 173)	157 (137 to 178)	158 (138 to 178)	162 (141 to 183)	167 (136 to 200)	167 (137 to 202)	167 (135 to 201)
Redcar and Cleveland	62 (54 to 70)	66 (53 to 81)	74 (65 to 84)	77 (67 to 88)	76 (66 to 86)	73 (63 to 83)	70 (61 to 80)	69 (56 to 84)	69 (56 to 84)	69 (55 to 83)
South Tyneside	82 (73 to 93)	88 (71 to 107)	92 (81 to 105)	84 (73 to 96)	81 (71 to 92)	79 (69 to 90)	77 (67 to 88)	77 (62 to 92)	77 (62 to 93)	77 (62 to 93)

Stockton-on-Tees	67 (59 to 75)	73 (60 to 88)	83 (73 to 93)	88 (77 to 100)	89 (78 to 101)	94 (82 to 106)	97 (85 to 110)	101 (84 to 121)	102 (83 to 122)	102 (84 to 122)
Sunderland	131 (117 to 147)	140 (115 to 168)	151 (133 to 170)	154 (135 to 174)	153 (134 to 173)	147 (129 to 167)	142 (124 to 161)	141 (117 to 168)	141 (116 to 168)	141 (116 to 169)
North West England	3369 (3071 to 3693)	3500 (3026 to 3996)	3678 (3328 to 4042)	3597 (3229 to 3958)	3536 (3185 to 3904)	3490 (3150 to 3841)	3572 (3206 to 3935)	3758 (3269 to 4294)	3773 (3281 to 4315)	3785 (3270 to 4335)
Blackburn with Darwen	73 (65 to 82)	72 (60 to 87)	74 (65 to 83)	74 (65 to 83)	71 (62 to 80)	70 (62 to 79)	74 (65 to 83)	79 (66 to 95)	80 (66 to 95)	80 (66 to 98)
Blackpool	97 (87 to 108)	89 (73 to 107)	84 (74 to 95)	81 (71 to 91)	79 (70 to 89)	74 (66 to 84)	72 (63 to 82)	74 (61 to 88)	74 (61 to 89)	74 (61 to 90)
Bolton	126 (113 to 141)	129 (105 to 152)	135 (119 to 151)	135 (119 to 152)	134 (118 to 151)	134 (119 to 151)	139 (123 to 157)	150 (125 to 176)	151 (125 to 179)	152 (125 to 181)
Bury	82 (74 to 92)	85 (69 to 102)	91 (80 to 103)	92 (81 to 104)	92 (81 to 104)	93 (82 to 105)	94 (83 to 107)	99 (82 to 118)	100 (82 to 119)	100 (81 to 120)
Cheshire East	140 (125 to 157)	147 (121 to 176)	160 (141 to 180)	168 (147 to 189)	174 (152 to 196)	180 (158 to 203)	188 (164 to 212)	198 (165 to 234)	199 (166 to 235)	199 (164 to 238)
Cheshire West and Chester	129 (115 to 145)	138 (112 to 166)	152 (134 to 172)	159 (140 to 179)	160 (141 to 182)	165 (146 to 187)	168 (148 to 191)	173 (144 to 207)	174 (143 to 208)	174 (142 to 210)
Cumbria	229 (205 to 256)	235 (192 to 281)	246 (218 to 276)	248 (219 to 279)	250 (221 to 281)	250 (222 to 282)	253 (223 to 286)	261 (217 to 308)	261 (217 to 309)	261 (215 to 311)
Halton	41 (36 to 46)	42 (34 to 50)	49 (43 to 55)	62 (54 to 70)	64 (56 to 72)	61 (54 to 69)	64 (56 to 72)	68 (57 to 81)	69 (57 to 81)	69 (56 to 83)
Knowsley	72 (63 to 80)	87 (70 to 103)	99 (87 to 112)	90 (79 to 102)	81 (71 to 92)	80 (70 to 90)	77 (67 to 87)	77 (64 to 91)	77 (63 to 91)	77 (62 to 92)
Lancashire	521 (467 to 579)	524 (436 to 623)	549 (486 to 615)	565 (499 to 634)	575 (509 to 645)	585 (517 to 655)	595 (525 to 666)	613 (518 to 721)	614 (514 to 726)	615 (513 to 731)
Liverpool	296 (265 to 331)	318 (260 to 379)	322 (285 to 361)	273 (240 to 308)	250 (221 to 282)	230 (203 to 260)	234 (205 to 264)	246 (205 to 291)	247 (204 to 292)	247 (202 to 295)
Manchester	256 (228 to 287)	278 (229 to 338)	281 (248 to 319)	237 (208 to 269)	222 (195 to 254)	212 (187 to 241)	244 (214 to 278)	287 (239 to 345)	291 (243 to 352)	295 (244 to 357)
Oldham	108 (97 to 121)	111 (90 to 132)	116 (102 to 130)	115 (101 to 129)	113 (99 to 127)	113 (99 to 127)	115 (101 to 129)	121 (101 to 143)	122 (100 to 146)	122 (100 to 146)
Rochdale	95 (85 to 106)	99 (80 to 118)	105 (93 to 119)	107 (94 to 121)	105 (92 to 119)	106 (93 to 119)	108 (94 to 122)	113 (94 to 135)	114 (93 to 136)	114 (93 to 138)
Salford	132 (118 to 147)	140 (115 to 165)	144 (128 to 162)	130 (114 to 146)	119 (105 to 134)	110 (97 to 123)	116 (102 to 130)	130 (108 to 153)	131 (108 to 155)	133 (109 to 157)
Sefton	151 (135 to 169)	157 (128 to 187)	163 (144 to 184)	159 (139 to 178)	155 (136 to 174)	150 (133 to 169)	144 (126 to 162)	140 (116 to 167)	140 (116 to 166)	140 (114 to 167)
St Helens	85 (76 to 95)	89 (74 to 106)	96 (85 to 108)	98 (86 to 111)	93 (82 to 105)	92 (81 to 103)	90 (79 to 101)	90 (75 to 106)	90 (75 to 107)	90 (74 to 107)
Stockport	135 (121 to 151)	143 (119 to 171)	154 (136 to 173)	152 (135 to 171)	149 (132 to 168)	148 (131 to 166)	145 (128 to 163)	148 (124 to 173)	148 (124 to 174)	148 (122 to 176)
Tameside	107 (95 to 119)	108 (89 to 130)	114 (100 to 128)	113 (100 to 128)	113 (99 to 127)	110 (98 to 124)	112 (97 to 126)	118 (98 to 141)	119 (98 to 143)	119 (97 to 144)
Trafford	106 (95 to 119)	113 (92 to 137)	120 (107 to 136)	116 (102 to 131)	112 (98 to 126)	108 (95 to 122)	114 (100 to 130)	125 (103 to 149)	126 (104 to 151)	127 (103 to 153)
Warrington	72 (65 to 81)	76 (63 to 91)	83 (73 to 93)	86 (75 to 97)	93 (82 to 105)	97 (85 to 109)	101 (89 to 115)	107 (90 to 129)	108 (90 to 130)	108 (90 to 131)
Wigan	138 (123 to 154)	142 (117 to 170)	154 (137 to 173)	158 (139 to 177)	156 (138 to 176)	154 (136 to 174)	159 (141 to 180)	168 (139 to 199)	169 (139 to 201)	170 (139 to 203)
Wirral	175 (156 to 196)	181 (150 to 216)	188 (165 to 212)	180 (158 to 203)	177 (155 to 199)	167 (147 to 188)	166 (145 to 188)	171 (143 to 202)	171 (142 to 203)	171 (141 to 204)
South East England	3162 (2886 to 3454)	3259 (2790 to 3758)	3481 (3142 to 3851)	3689 (3333 to 4071)	3886 (3509 to 4291)	4074 (3679 to 4494)	4360 (3920 to 4822)	4701 (4077 to 5380)	4732 (4096 to 5413)	4759 (4105 to 5465)
Bracknell Forest	23 (21 to 26)	25 (20 to 30)	31 (27 to 35)	40 (35 to 45)	48 (42 to 54)	54 (48 to 61)	57 (50 to 64)	60 (49 to 71)	60 (49 to 72)	60 (50 to 73)
Brighton and Hove	155 (138 to 173)	148 (119 to 178)	140 (123 to 158)	128 (112 to 144)	126 (111 to 143)	127 (112 to 144)	135 (118 to 153)	145 (120 to 173)	146 (119 to 175)	147 (119 to 176)
Buckinghamshire	171 (152 to 192)	186 (151 to 222)	207 (181 to 234)	223 (195 to 251)	230 (201 to 259)	243 (213 to 272)	255 (224 to 288)	272 (223 to 322)	274 (225 to 325)	275 (224 to 328)
East Sussex	244 (218 to 272)	224 (184 to 267)	220 (195 to 247)	231 (204 to 260)	250 (221 to 282)	259 (230 to 292)	272 (240 to 306)	286 (239 to 338)	288 (236 to 340)	289 (235 to 344)
Hampshire	407 (364 to 452)	432 (358 to 511)	486 (431 to 544)	542 (481 to 606)	592 (525 to 662)	629 (557 to 703)	669 (591 to 749)	714 (597 to 837)	718 (596 to 848)	722 (601 to 850)
Isle of Wight	60 (53 to 67)	58 (47 to 70)	58 (51 to 66)	62 (54 to 71)	66 (57 to 75)	69 (61 to 78)	71 (62 to 81)	73 (61 to 87)	73 (60 to 88)	73 (59 to 89)
Kent	571 (512 to 636)	580 (477 to 684)	613 (543 to 690)	641 (569 to 720)	662 (586 to 747)	684 (606 to 769)	742 (655 to 836)	813 (678 to 949)	819 (682 to 963)	825 (678 to 975)
Medway	93 (82 to 104)	100 (82 to 121)	113 (99 to 127)	120 (105 to 135)	122 (107 to 138)	126 (111 to 143)	133 (116 to 150)	143 (119 to 171)	144 (118 to 172)	145 (118 to 174)
Milton Keynes	29 (26 to 33)	28 (23 to 34)	35 (30 to 39)	60 (53 to 68)	88 (77 to 100)	104 (92 to 118)	124 (109 to 140)	144 (120 to 172)	146 (121 to 176)	148 (122 to 179)
Oxfordshire	203 (180 to 229)	220 (179 to 268)	246 (215 to 280)	266 (233 to 304)	287 (252 to 328)	304 (266 to 347)	327 (286 to 373)	354 (293 to 426)	356 (294 to 431)	358 (294 to 436)
Portsmouth	99 (88 to 111)	101 (83 to 121)	101 (89 to 114)	93 (82 to 105)	93 (82 to 105)	94 (83 to 106)	100 (88 to 114)	109 (89 to 128)	109 (90 to 129)	110 (90 to 132)
Reading	59 (52 to 66)	64 (52 to 77)	70 (61 to 79)	69 (61 to 79)	67 (59 to 77)	70 (62 to 80)	76 (67 to 87)	84 (70 to 100)	84 (70 to 101)	85 (70 to 102)
Slough	40 (36 to 45)	45 (36 to 54)	49 (43 to 56)	50 (44 to 57)	52 (46 to 59)	59 (51 to 67)	69 (60 to 78)	81 (67 to 96)	82 (67 to 97)	83 (67 to 99)
Southampton	95 (84 to 107)	102 (82 to 122)	109 (96 to 124)	107 (94 to 121)	104 (91 to 118)	108 (95 to 122)	115 (101 to 131)	124 (102 to 148)	125 (102 to 150)	125 (103 to 151)
Surrey	454 (407 to 508)	487 (403 to 586)	515 (457 to 582)	518 (457 to 586)	523 (461 to 592)	539 (477 to 610)	573 (505 to 648)	615 (514 to 735)	619 (515 to 739)	622 (516 to 745)
West Berkshire	43 (38 to 48)	46 (38 to 56)	53 (46 to 60)	60 (53 to 68)	69 (60 to 78)	72 (63 to 82)	77 (67 to 88)	83 (68 to 98)	83 (69 to 99)	83 (68 to 100)
West Sussex	322 (287 to 361)	311 (254 to 371)	320 (282 to 362)	351 (307 to 397)	371 (324 to 420)	391 (344 to 443)	415 (361 to 469)	442 (365 to 526)	445 (366 to 527)	447 (364 to 534)
Windsor and Maidenhead	56 (49 to 63)	59 (48 to 72)	64 (56 to 73)	68 (59 to 78)	68 (59 to 78)	67 (59 to 76)	72 (63 to 82)	79 (64 to 94)	79 (65 to 95)	80 (65 to 97)
Wokingham	38 (34 to 43)	43 (34 to 52)	50 (44 to 57)	57 (49 to 64)	69 (60 to 78)	75 (65 to 85)	78 (68 to 88)	82 (67 to 97)	82 (67 to 98)	82 (67 to 99)
South West England	1977 (1794 to 2164)	2008 (1702 to 2312)	2123 (1903 to 2336)	2251 (2011 to 2479)	2404 (2155 to 2646)	2528 (2271 to 2779)	2683 (2389 to 2969)	2865 (2484 to 3258)	2881 (2475 to 3289)	2894 (2490 to 3301)
Bath and North East Somerset	74 (66 to 83)	78 (63 to 93)	82 (72 to 92)	84 (73 to 94)	84 (74 to 95)	87 (76 to 97)	89 (78 to 100)	92 (76 to 109)	92 (76 to 110)	92 (76 to 110)
Bournemouth	101 (90 to 113)	94 (76 to 113)	87 (76 to 98)	80 (70 to 90)	84 (74 to 95)	85 (75 to 96)	91 (79 to 103)	100 (84 to 118)	101 (83 to 120)	101 (82 to 121)
Bristol, City of	204 (181 to 227)	215 (174 to 258)	222 (194 to 250)	208 (181 to 235)	202 (176 to 227)	198 (174 to 223)	212 (184 to 240)	233 (194 to 276)	235 (192 to 279)	237 (193 to 282)
Cornwall	194 (174 to 216)	191 (157 to 228)	200 (178 to 224)	220 (195 to 247)	243 (215 to 273)	259 (230 to 290)	275 (243 to 309)	294 (247 to 344)	295 (245 to 349)	297 (245 to 353)
Devon	283 (252 to 317)	279 (225 to 335)	289 (254 to 327)	310 (271 to 351)	340 (298 to 384)	364 (318 to 409)	383 (334 to 434)	402 (332 to 477)	404 (329 to 483)	405 (331 to 485)
Dorset	146 (131 to 164)	144 (116 to 174)	153 (134 to 172)	172 (150 to 195)	188 (165 to 212)	202 (178 to 227)	212 (186 to 240)	218 (182 to 259)	219 (180 to 262)	219 (179 to 262)
Gloucestershire	209 (187 to 234)	220 (179 to 264)	240 (212 to 270)	258 (227 to 291)	274 (241 to 309)	288 (254 to 325)	303 (266 to 343)	322 (271 to 382)	324 (269 to 387)	326 (268 to 388)
North Somerset	70 (61 to 78)	69 (55 to 83)	74 (64 to 83)	84 (73 to 95)	92 (80 to 104)	97 (84 to 109)	104 (90 to 117)	111 (92 to 131)	112 (92 to 133)	113 (92 to 135)
Plymouth	108 (97 to 121)	114 (92 to 136)	122 (108 to 137)	126 (111 to 142)	128 (113 to 144)	123 (109 to 139)	128 (113 to 145)	138 (116 to 163)	138 (113 to 166)	139 (113 to 168)
Poole	56 (50 to 63)	55 (45 to 67)	58 (51 to 65)	62 (54 to 71)	69 (61 to 78)	72 (63 to 82)	75 (65 to 86)	79 (65 to 94)	79 (65 to 95)	80 (65 to 95)
Somerset	186 (166 to 208)	187 (152 to 222)	200 (175 to 225)	219 (192 to 247)	238 (209 to 268)	255 (223 to 288)	271 (237 to 306)	287 (237 to 339)	289 (235 to 342)	290 (237 to 342)
South Gloucestershire	71 (63 to 80)	78 (63 to 94)	91 (79 to 103)	102 (89 to 115)	111 (97 to 125)	123 (108 to 140)	132 (115 to 150)	141 (117 to 167)	142 (116 to 169)	142 (116 to 170)
Swindon	56 (51 to 62)	62 (50 to 74)	70 (62 to 79)	76 (68 to 86)	86 (76 to 96)	90 (80 to 101)	103 (91 to 116)	120 (101 to 141)	121 (100 to 143)	123 (101 to 146)
Torbay	69 (62 to 78)	64 (52 to 77)	62 (55 to 70)	62 (54 to 70)	65 (57 to 74)	68 (60 to 77)	68 (60 to 78)	69 (57 to 82)	69 (57 to 83)	69 (57 to 83)
Wiltshire	149 (132 to 167)	159 (129 to 191)	176 (155 to 199)	188 (165 to 213)	201 (176 to 227)	218 (192 to 245)	238 (209 to 269)	258 (216 to 306)	260 (213 to 310)	261 (213 to 312)
West Midlands	2226 (2020 to 2452)	2373 (2034 to 2738)	2586 (2331 to 2869)	2633 (2355 to 2916)	2665 (2380 to 2983)	2691 (2413 to 2981)	2823 (2520 to 3138)	3023 (2600 to 3453)	3041 (2637 to 3490)	3056 (2612 to 3503)
Birmingham	492 (439 to 552)	531 (439 to 632)	561 (496 to 629)	528 (465 to 594)	515 (453 to 583)	504 (442 to 568)	540 (473 to 608)	599 (497 to 705)	605 (504 to 716)	610 (503 to 725)

Coventry	138 (122 to 155)	153 (124 to 183)	168 (148 to 190)	162 (141 to 183)	155 (135 to 176)	152 (132 to 171)	158 (137 to 179)	169 (138 to 202)	171 (140 to 203)	172 (139 to 206)
Dudley	128 (114 to 143)	135 (110 to 164)	148 (131 to 168)	153 (134 to 173)	156 (137 to 178)	156 (137 to 176)	159 (139 to 180)	167 (137 to 199)	167 (137 to 200)	168 (137 to 202)
Herefordshire, County of	66 (58 to 74)	67 (54 to 80)	71 (63 to 80)	75 (66 to 85)	81 (71 to 92)	89 (79 to 101)	93 (81 to 105)	96 (79 to 114)	96 (79 to 115)	97 (78 to 116)
Sandwell	149 (131 to 166)	158 (128 to 188)	168 (147 to 189)	160 (139 to 179)	151 (131 to 170)	147 (128 to 165)	156 (135 to 176)	172 (140 to 203)	174 (142 to 206)	175 (142 to 208)
Shropshire	109 (97 to 122)	113 (92 to 136)	122 (107 to 137)	128 (112 to 145)	136 (119 to 155)	143 (125 to 161)	154 (135 to 174)	165 (136 to 196)	166 (137 to 198)	167 (136 to 201)
Solihull	73 (65 to 83)	83 (68 to 100)	96 (85 to 109)	101 (89 to 114)	103 (90 to 117)	103 (91 to 116)	106 (93 to 120)	111 (92 to 132)	112 (93 to 133)	112 (92 to 134)
Staffordshire	296 (264 to 330)	314 (257 to 374)	353 (313 to 396)	384 (338 to 432)	400 (352 to 452)	410 (363 to 463)	427 (375 to 483)	448 (372 to 528)	450 (374 to 530)	451 (370 to 537)
Stoke-on-Trent	124 (110 to 139)	130 (106 to 157)	136 (120 to 154)	130 (114 to 147)	128 (112 to 145)	124 (109 to 140)	125 (109 to 141)	131 (108 to 157)	131 (108 to 158)	132 (107 to 159)
Telford and Wrekin	39 (34 to 44)	40 (32 to 48)	47 (41 to 53)	62 (54 to 70)	71 (62 to 80)	80 (70 to 90)	84 (73 to 94)	88 (72 to 103)	88 (72 to 104)	88 (71 to 105)
Walsall	114 (102 to 128)	125 (103 to 150)	138 (122 to 156)	136 (120 to 154)	134 (117 to 152)	131 (115 to 148)	136 (119 to 155)	147 (121 to 175)	148 (122 to 177)	149 (122 to 178)
Warwickshire	195 (172 to 217)	208 (167 to 250)	230 (202 to 258)	241 (211 to 271)	246 (215 to 279)	257 (224 to 288)	274 (239 to 309)	295 (240 to 348)	297 (243 to 354)	298 (241 to 357)
Wolverhampton	115 (101 to 129)	124 (100 to 149)	134 (117 to 152)	131 (115 to 149)	128 (111 to 145)	121 (106 to 137)	125 (109 to 143)	135 (111 to 161)	136 (112 to 164)	137 (111 to 165)
Worcestershire	189 (168 to 212)	193 (156 to 233)	214 (188 to 242)	242 (213 to 273)	260 (228 to 296)	275 (243 to 312)	286 (251 to 325)	299 (248 to 358)	300 (249 to 359)	301 (247 to 359)
Yorkshire and the Humber	2251 (2055 to 2464)	2348 (2025 to 2687)	2498 (2262 to 2759)	2526 (2277 to 2782)	2538 (2297 to 2805)	2555 (2307 to 2823)	2671 (2409 to 2946)	2841 (2473 to 3235)	2855 (2481 to 3267)	2867 (2479 to 3271)
Barnsley	98 (88 to 110)	106 (86 to 127)	115 (101 to 130)	115 (102 to 130)	115 (101 to 130)	112 (99 to 128)	117 (104 to 133)	127 (105 to 150)	127 (104 to 153)	128 (105 to 153)
Bradford	224 (200 to 250)	230 (188 to 278)	240 (212 to 271)	239 (210 to 270)	242 (214 to 273)	242 (214 to 273)	263 (231 to 297)	291 (242 to 346)	294 (242 to 353)	296 (243 to 356)
Calderdale	101 (90 to 113)	102 (83 to 123)	104 (91 to 117)	101 (88 to 114)	100 (88 to 113)	99 (88 to 112)	104 (91 to 118)	111 (92 to 133)	112 (92 to 135)	112 (92 to 136)
Doncaster	117 (105 to 132)	127 (104 to 154)	141 (125 to 160)	147 (129 to 166)	149 (132 to 169)	147 (130 to 167)	153 (135 to 173)	162 (134 to 192)	162 (133 to 195)	163 (133 to 195)
East Riding of Yorkshire	121 (108 to 137)	122 (100 to 147)	129 (114 to 146)	139 (122 to 157)	150 (133 to 170)	161 (142 to 182)	170 (151 to 194)	179 (148 to 214)	180 (147 to 218)	181 (147 to 218)
Kingston upon Hull, City of	128 (114 to 143)	138 (112 to 165)	147 (129 to 166)	142 (124 to 160)	136 (120 to 153)	127 (111 to 144)	127 (111 to 144)	134 (111 to 159)	135 (110 to 160)	135 (109 to 161)
Kirklees	178 (159 to 200)	181 (148 to 220)	191 (169 to 216)	195 (172 to 220)	195 (172 to 221)	199 (176 to 226)	213 (187 to 241)	229 (191 to 273)	231 (190 to 279)	232 (191 to 279)
Leeds	342 (308 to 382)	361 (300 to 431)	382 (341 to 429)	372 (330 to 418)	364 (324 to 409)	369 (328 to 416)	380 (339 to 427)	402 (338 to 475)	403 (337 to 481)	405 (334 to 484)
North East Lincolnshire	72 (64 to 80)	77 (62 to 92)	83 (74 to 93)	83 (73 to 93)	83 (74 to 93)	82 (72 to 92)	81 (72 to 92)	84 (70 to 99)	84 (69 to 100)	84 (69 to 100)
North Lincolnshire	58 (52 to 65)	63 (51 to 77)	71 (62 to 80)	76 (66 to 86)	78 (68 to 88)	78 (68 to 88)	84 (73 to 95)	92 (75 to 109)	92 (75 to 111)	93 (76 to 112)
North Yorkshire	232 (207 to 259)	232 (189 to 278)	244 (215 to 275)	261 (229 to 293)	277 (244 to 312)	291 (255 to 328)	304 (267 to 342)	316 (261 to 373)	317 (262 to 375)	318 (260 to 377)
Rotherham	101 (91 to 113)	110 (89 to 132)	123 (108 to 139)	129 (114 to 146)	130 (115 to 147)	128 (113 to 144)	131 (116 to 148)	137 (114 to 163)	138 (113 to 165)	138 (113 to 165)
Sheffield	271 (242 to 303)	283 (232 to 336)	295 (261 to 332)	284 (250 to 320)	269 (237 to 304)	262 (231 to 296)	277 (243 to 311)	297 (246 to 350)	299 (246 to 354)	301 (245 to 356)
Wakefield	133 (119 to 149)	141 (116 to 169)	154 (136 to 174)	160 (141 to 181)	161 (142 to 181)	162 (143 to 183)	166 (146 to 187)	174 (146 to 207)	175 (146 to 208)	176 (145 to 211)
York	74 (66 to 83)	76 (62 to 91)	80 (71 to 91)	85 (75 to 96)	89 (78 to 100)	93 (82 to 105)	100 (88 to 113)	106 (88 to 125)	107 (88 to 127)	107 (88 to 127)
Northern Ireland	721 (672 to 768)	748 (691 to 802)	797 (737 to 859)	775 (722 to 837)	816 (758 to 876)	872 (805 to 944)	931 (863 to 1000)	976 (897 to 1053)	978 (903 to 1054)	980 (912 to 1047)
Scotland	2732 (2546 to 2933)	2751 (2541 to 2957)	2783 (2572 to 2997)	2669 (2476 to 2867)	2649 (2444 to 2837)	2667 (2470 to 2863)	2759 (2549 to 2972)	2826 (2516 to 3171)	2826 (2486 to 3156)	2824 (2453 to 3214)
Wales	1363 (1267 to 1460)	1386 (1288 to 1489)	1434 (1323 to 1540)	1476 (1363 to 1588)	1492 (1378 to 1602)	1521 (1409 to 1641)	1570 (1452 to 1692)	1568 (1440 to 1685)	1563 (1451 to 1674)	1558 (1454 to 1664)
Latin America and Caribbean	70760 (68523 to 72903)	94103 (91014 to 96931)	125036 (121099 to 128932)	160980 (155855 to 166180)	197331 (190692 to 203843)	235947 (228251 to 243380)	269419 (260654 to 277923)	298215 (282873 to 312164)	300748 (284042 to 315445)	303018 (285443 to 319452)
Andean Latin America	7005 (6691 to 7313)	9089 (8594 to 9551)	11873 (11180 to 12489)	15269 (14501 to 15995)	19113 (18009 to 20113)	23227 (21782 to 24676)	27006 (25598 to 28323)	31899 (29898 to 33609)	32529 (30377 to 34429)	33008 (30666 to 35074)
Bolivia	1451 (1348 to 1551)	1781 (1543 to 2012)	2174 (1933 to 2413)	2632 (2394 to 2882)	3225 (2951 to 3489)	4163 (3852 to 4473)	5031 (4612 to 5454)	5764 (5106 to 6439)	5837 (5139 to 6509)	5900 (5165 to 6651)
Ecuador	1667 (1545 to 1779)	2225 (2040 to 2410)	3010 (2730 to 3273)	3940 (3628 to 4258)	5016 (4668 to 5330)	6309 (5834 to 6796)	7572 (7082 to 8068)	8801 (7708 to 9882)	8968 (7814 to 10114)	9085 (7797 to 10309)
Peru	3887 (3622 to 4161)	5084 (4684 to 5472)	6690 (6127 to 7206)	8697 (8079 to 9349)	10871 (9922 to 11778)	12755 (11481 to 14127)	14403 (13247 to 15535)	17334 (15868 to 18676)	17724 (16128 to 19187)	18023 (16344 to 19725)
Caribbean	8905 (8640 to 9176)	10866 (10381 to 11388)	13144 (12758 to 13563)	15449 (14937 to 15985)	17871 (16979 to 18789)	20321 (19614 to 21050)	22167 (21318 to 23036)	23686 (22192 to 25304)	23850 (22296 to 25483)	23991 (22389 to 25728)
Antigua and Barbuda	25 (24 to 27)	30 (28 to 32)	34 (32 to 37)	31 (27 to 35)	31 (29 to 33)	40 (37 to 44)	45 (42 to 48)	46 (40 to 51)	46 (40 to 51)	46 (40 to 51)
The Bahamas	43 (40 to 46)	61 (56 to 67)	86 (80 to 91)	109 (102 to 117)	131 (122 to 140)	156 (145 to 167)	182 (170 to 195)	199 (174 to 224)	200 (175 to 223)	201 (173 to 230)
Barbados	131 (121 to 139)	131 (122 to 139)	129 (120 to 137)	132 (123 to 141)	132 (123 to 141)	133 (125 to 142)	146 (136 to 155)	154 (136 to 173)	154 (134 to 174)	155 (135 to 177)
Belize	35 (33 to 37)	48 (44 to 51)	62 (58 to 66)	74 (69 to 79)	92 (86 to 99)	119 (111 to 127)	165 (154 to 176)	207 (182 to 232)	212 (184 to 239)	216 (186 to 246)
Bermuda	19 (18 to 21)	22 (21 to 24)	27 (25 to 28)	28 (26 to 30)	31 (28 to 33)	33 (31 to 35)	34 (32 to 36)	33 (30 to 36)	33 (30 to 36)	33 (30 to 36)
Cuba	2779 (2591 to 2963)	3317 (2971 to 3686)	4200 (3925 to 4492)	4904 (4546 to 5266)	5388 (4733 to 6015)	5683 (5233 to 6122)	5733 (5300 to 6191)	5708 (5073 to 6401)	5696 (4966 to 6400)	5672 (4986 to 6385)
Dominica	28 (27 to 30)	33 (31 to 36)	38 (35 to 40)	38 (36 to 41)	36 (34 to 39)	35 (32 to 37)	34 (31 to 36)	33 (29 to 37)	33 (29 to 37)	33 (29 to 37)
Dominican Republic	1151 (1069 to 1229)	1619 (1509 to 1732)	2135 (1994 to 2285)	2834 (2635 to 3038)	3648 (3321 to 3970)	4336 (3983 to 4665)	4845 (4510 to 5162)	5359 (4742 to 6047)	5419 (4688 to 6064)	5477 (4672 to 6253)
Grenada	49 (45 to 52)	50 (47 to 53)	51 (48 to 55)	49 (46 to 53)	44 (41 to 48)	53 (49 to 57)	53 (49 to 57)	51 (44 to 57)	50 (44 to 57)	50 (43 to 56)
Guyana	217 (202 to 232)	293 (273 to 312)	367 (342 to 392)	400 (372 to 426)	393 (362 to 424)	389 (359 to 421)	377 (348 to 406)	387 (343 to 430)	389 (341 to 434)	388 (339 to 435)
Haiti	1690 (1572 to 1813)	2047 (1782 to 2330)	2335 (2157 to 2518)	2631 (2417 to 2846)	3280 (2879 to 3672)	4214 (3843 to 4564)	5275 (4713 to 5857)	6318 (5285 to 7496)	6443 (5322 to 7547)	6562 (5456 to 7743)
Jamaica	751 (701 to 802)	868 (808 to 928)	954 (890 to 1025)	1122 (1031 to 1214)	1204 (1115 to 1296)	1336 (1243 to 1440)	1394 (1294 to 1501)	1410 (1246 to 1572)	1411 (1237 to 1584)	1410 (1233 to 1594)
Puerto Rico	1103 (1028 to 1179)	1222 (1142 to 1302)	1415 (1318 to 1515)	1677 (1570 to 1785)	1860 (1728 to 1995)	2009 (1873 to 2138)	1974 (1840 to 2111)	1773 (1648 to 1914)	1752 (1637 to 1873)	1730 (1601 to 1856)
Saint Kitts and Nevis	34 (28 to 40)	29 (25 to 32)	25 (23 to 27)	23 (22 to 25)	21 (20 to 23)	23 (22 to 25)	27 (24 to 30)	29 (23 to 35)	29 (23 to 35)	29 (24 to 35)
Saint Lucia	41 (38 to 43)	48 (44 to 51)	54 (51 to 58)	62 (58 to 66)	70 (65 to 75)	79 (73 to 85)	85 (79 to 91)	88 (78 to 99)	89 (78 to 99)	89 (76 to 101)
Saint Vincent and the Grenadines	41 (38 to 43)	44 (41 to 48)	47 (44 to 50)	52 (49 to 56)	55 (51 to 59)	54 (51 to 58)	55 (50 to 59)	56 (50 to 62)	56 (49 to 62)	56 (49 to 63)
Suriname	97 (91 to 104)	145 (132 to 157)	195 (181 to 209)	180 (158 to 200)	192 (168 to 215)	223 (208 to 238)	269 (247 to 290)	290 (258 to 327)	292 (258 to 325)	293 (258 to 331)
Trinidad and Tobago	335 (313 to 357)	432 (404 to 461)	489 (456 to 520)	545 (508 to 581)	602 (561 to 642)	643 (600 to 687)	671 (619 to 721)	694 (614 to 775)	695 (611 to 792)	696 (602 to 783)
Virgin Islands	14 (13 to 15)	17 (16 to 18)	33 (31 to 36)	52 (48 to 55)	55 (51 to 58)	58 (54 to 62)	56 (52 to 60)	46 (43 to 50)	45 (42 to 48)	44 (41 to 47)
Central Latin America	26804 (25821 to 27928)	36278 (34890 to 37828)	50223 (48301 to 52327)	66807 (64133 to 69693)	83227 (80068 to 86905)	101288 (97399 to 105803)	116493 (112077 to 121477)	128303 (123212 to 133954)	128963 (124042 to 134275)	129666 (124096 to 135688)
Colombia	5795 (5390 to 6176)	7957 (7197 to 8687)	11069 (10089 to 12012)	13579 (12176 to 14960)	16437 (14960 to 17907)	20201 (18133 to 22241)	22686 (20582 to 24956)	24505 (22657 to 26435)	24860 (22842 to 26763)	25015 (22709 to 27278)
Costa Rica	423 (393 to 452)	620 (568 to 676)	888 (814 to 959)	1132 (1024 to 1238)	1519 (1365 to 1677)	1954 (1820 to 2082)	2233 (2078 to 2402)	2428 (2144 to 2713)	2443 (2128 to 2738)	2455 (2159 to 2763)
El Salvador	975 (908 to 1041)	1319 (1227 to 1418)	1834 (1703 to 1972)	2362 (2069 to 2639)	2716 (2499 to 2931)	3049 (2726 to 3406)	3209 (2920 to 3513)	3396 (2927 to 3855)	3413 (2894 to 3915)	3427 (2885 to 3918)
Guatemala	1473 (1374 to 1578)	1938 (1751 to 2107)	2485 (2268 to 2691)	3206 (2971 to 3442)	4255 (3834 to 4649)	5647 (5220 to 6105)	6987 (6188 to 7800)	7959 (7360 to 8562)	8047 (7421 to 8705)	8129 (7403 to 8838)
Honduras	727 (678 to 776)	958 (889 to 1028)	1261 (1147 to 1387)	1726 (1542 to 1899)	2369 (2182 to 2565)	3108 (2881 to 3357)	4067 (3722 to 4417)	5005 (4499 to 5524)	5104 (4504 to 5678)	5200 (4584 to 5805)
Mexico	13849 (12902 to 14850)	18462 (17163 to 19680)	25567 (23773 to 27252)	35163 (32700 to 37405)	43338 (40441 to 46431)	51651 (48210 to 55306)	58716 (54726 to 62560)	65228 (60119 to 70135)	65755 (61365 to 70113)	66185 (61083 to 71231)

Aguascalientes	100 (91 to 111)	131 (119 to 144)	183 (166 to 201)	275 (249 to 302)	386 (349 to 427)	509 (462 to 560)	624 (566 to 688)	731 (656 to 813)	741 (675 to 814)	750 (673 to 834)
Baja California	120 (109 to 132)	267 (243 to 293)	459 (417 to 505)	624 (567 to 684)	849 (774 to 933)	1186 (1078 to 1304)	1586 (1443 to 1745)	1882 (1693 to 2082)	1904 (1736 to 2092)	1925 (1732 to 2136)
Baja California Sur	31 (28 to 34)	42 (38 to 46)	66 (59 to 72)	109 (98 to 120)	161 (146 to 177)	218 (198 to 239)	314 (285 to 345)	391 (351 to 434)	397 (360 to 438)	403 (361 to 447)
Campeche	63 (58 to 69)	87 (79 to 95)	133 (121 to 145)	217 (197 to 237)	278 (254 to 303)	361 (327 to 394)	426 (387 to 465)	474 (427 to 523)	478 (434 to 522)	481 (433 to 530)
Chiapas	474 (430 to 521)	630 (571 to 689)	826 (749 to 906)	1107 (1005 to 1215)	1694 (1529 to 1862)	2058 (1865 to 2259)	2529 (2294 to 2774)	2888 (2604 to 3192)	2916 (2649 to 3197)	2941 (2642 to 3251)
Chihuahua	461 (419 to 509)	634 (576 to 696)	847 (767 to 929)	1057 (957 to 1157)	1274 (1152 to 1398)	1559 (1411 to 1716)	1720 (1557 to 1888)	1904 (1702 to 2113)	1920 (1736 to 2101)	1933 (1729 to 2145)
Coahuila	383 (346 to 423)	472 (427 to 518)	589 (530 to 646)	810 (731 to 890)	1036 (934 to 1140)	1207 (1084 to 1332)	1407 (1271 to 1548)	1591 (1415 to 1765)	1605 (1452 to 1764)	1618 (1438 to 1798)
Colima	62 (56 to 68)	87 (79 to 95)	127 (115 to 140)	180 (163 to 198)	223 (203 to 245)	272 (247 to 299)	331 (300 to 364)	375 (334 to 415)	378 (341 to 415)	380 (340 to 421)
Durango	328 (298 to 360)	396 (359 to 435)	491 (443 to 539)	620 (563 to 681)	719 (656 to 789)	780 (710 to 857)	847 (771 to 929)	939 (845 to 1041)	948 (863 to 1040)	955 (858 to 1058)
Guanajuato	715 (649 to 787)	921 (833 to 1012)	1205 (1090 to 1325)	1610 (1454 to 1770)	2125 (1930 to 2340)	2552 (2313 to 2807)	2916 (2646 to 3199)	3206 (2866 to 3554)	3228 (2928 to 3544)	3244 (2903 to 3594)
Guerrero	492 (446 to 545)	635 (575 to 698)	855 (772 to 945)	1122 (1016 to 1238)	1413 (1284 to 1558)	1682 (1523 to 1861)	1820 (1643 to 2010)	1888 (1694 to 2105)	1892 (1717 to 2087)	1894 (1694 to 2117)
Hidalgo	446 (405 to 492)	522 (476 to 572)	632 (577 to 694)	804 (733 to 884)	1013 (926 to 1115)	1220 (1112 to 1343)	1421 (1294 to 1563)	1619 (1463 to 1792)	1636 (1489 to 1796)	1651 (1491 to 1830)
Jalisco	975 (888 to 1068)	1306 (1189 to 1428)	1762 (1602 to 1927)	2327 (2118 to 2541)	2857 (2602 to 3127)	3378 (3087 to 3691)	3821 (3492 to 4174)	4288 (3867 to 4725)	4330 (3948 to 4717)	4364 (3936 to 4804)
México	714 (647 to 785)	997 (904 to 1092)	1998 (1817 to 2188)	3931 (3579 to 4297)	5228 (4765 to 5705)	6721 (6132 to 7359)	7919 (7235 to 8636)	8816 (7961 to 9703)	8882 (8103 to 9697)	8935 (8063 to 9849)
Mexico City	1741 (1562 to 1901)	2635 (2374 to 2873)	3712 (3334 to 4054)	4679 (4220 to 5100)	4497 (4033 to 4939)	4625 (4160 to 5071)	4638 (4165 to 5077)	4868 (4321 to 5381)	4883 (4367 to 5353)	4883 (4328 to 5407)
Michoacán de Ocampo	773 (698 to 850)	986 (894 to 1079)	1234 (1113 to 1352)	1548 (1396 to 1698)	1919 (1735 to 2107)	2175 (1966 to 2389)	2312 (2095 to 2530)	2482 (2227 to 2742)	2497 (2262 to 2736)	2507 (2246 to 2776)
Morelos	143 (129 to 159)	204 (184 to 226)	327 (295 to 362)	496 (445 to 551)	646 (583 to 717)	811 (731 to 901)	937 (848 to 1036)	1033 (926 to 1154)	1040 (938 to 1150)	1045 (933 to 1169)
Nayarit	154 (138 to 170)	206 (186 to 226)	286 (258 to 314)	377 (341 to 415)	434 (393 to 477)	490 (443 to 539)	558 (506 to 611)	629 (565 to 695)	635 (574 to 698)	640 (573 to 710)
Nuevo León	393 (355 to 435)	572 (517 to 631)	889 (804 to 981)	1289 (1163 to 1423)	1625 (1470 to 1789)	1993 (1803 to 2205)	2378 (2159 to 2615)	2884 (2585 to 3194)	2936 (2657 to 3229)	2984 (2679 to 3316)
Oaxaca	770 (698 to 843)	919 (835 to 1002)	1079 (976 to 1180)	1268 (1147 to 1389)	1620 (1465 to 1776)	1891 (1712 to 2072)	2059 (1862 to 2251)	2199 (1965 to 2433)	2209 (1993 to 2423)	2216 (1979 to 2445)
Puebla	868 (789 to 953)	1046 (950 to 1149)	1345 (1223 to 1478)	1774 (1609 to 1947)	2239 (2031 to 2468)	2706 (2461 to 2978)	3105 (2829 to 3404)	3469 (3118 to 3845)	3499 (3180 to 3834)	3523 (3160 to 3902)
Querétaro	149 (135 to 164)	187 (171 to 205)	256 (233 to 282)	383 (348 to 419)	559 (508 to 613)	754 (686 to 825)	962 (873 to 1052)	1207 (1084 to 1334)	1233 (1120 to 1349)	1257 (1130 to 1395)
Quintana Roo	14 (12 to 15)	24 (22 to 27)	45 (41 to 49)	113 (103 to 124)	244 (222 to 267)	438 (397 to 481)	659 (597 to 722)	910 (816 to 1006)	937 (848 to 1027)	964 (862 to 1067)
San Luis Potosí	452 (410 to 495)	546 (497 to 595)	676 (614 to 739)	875 (797 to 956)	1069 (974 to 1169)	1238 (1126 to 1353)	1360 (1239 to 1486)	1470 (1322 to 1619)	1478 (1348 to 1616)	1485 (1337 to 1640)
Sinaloa	328 (297 to 361)	441 (400 to 485)	661 (598 to 726)	955 (866 to 1047)	1160 (1051 to 1276)	1337 (1216 to 1469)	1435 (1306 to 1576)	1554 (1397 to 1719)	1564 (1414 to 1716)	1573 (1410 to 1744)
Sonora	281 (256 to 310)	405 (368 to 445)	579 (525 to 637)	772 (704 to 849)	946 (861 to 1044)	1156 (1051 to 1269)	1345 (1227 to 1482)	1485 (1336 to 1646)	1493 (1362 to 1641)	1500 (1350 to 1659)
Tabasco	185 (168 to 203)	261 (237 to 285)	398 (361 to 436)	559 (507 to 613)	789 (716 to 865)	1007 (916 to 1104)	1160 (1055 to 1267)	1246 (1118 to 1374)	1250 (1134 to 1366)	1253 (1126 to 1383)
Tamaulipas	383 (348 to 422)	539 (490 to 591)	771 (699 to 844)	1005 (913 to 1098)	1193 (1084 to 1311)	1443 (1311 to 1590)	1653 (1502 to 1809)	1812 (1625 to 2004)	1824 (1661 to 1998)	1833 (1649 to 2030)
Tlaxcala	150 (135 to 165)	180 (162 to 197)	220 (199 to 242)	292 (264 to 322)	406 (367 to 449)	519 (469 to 572)	620 (562 to 682)	700 (625 to 778)	707 (637 to 777)	712 (637 to 791)
Veracruz de Ignacio de la Llave	1080 (973 to 1190)	1437 (1300 to 1573)	2011 (1816 to 2206)	2823 (2551 to 3097)	3327 (3001 to 3663)	3752 (3391 to 4121)	4056 (3681 to 4443)	4261 (3816 to 4719)	4269 (3864 to 4679)	4271 (3812 to 4727)
Yucatán	268 (242 to 294)	321 (291 to 352)	405 (366 to 446)	558 (504 to 613)	721 (649 to 795)	877 (792 to 965)	1016 (919 to 1113)	1185 (1058 to 1313)	1201 (1081 to 1318)	1216 (1082 to 1351)
Zacatecas	355 (321 to 390)	429 (389 to 471)	502 (454 to 550)	603 (546 to 661)	685 (620 to 752)	736 (665 to 810)	784 (708 to 861)	842 (750 to 933)	847 (765 to 931)	851 (762 to 941)
Nicaragua	569 (532 to 608)	751 (685 to 818)	994 (921 to 1068)	1405 (1222 to 1578)	1980 (1778 to 2170)	2496 (2260 to 2737)	2903 (2616 to 3199)	3298 (2794 to 3837)	3339 (2808 to 3862)	3376 (2831 to 3936)
Panama	401 (373 to 428)	551 (515 to 587)	733 (679 to 780)	929 (865 to 990)	1179 (1091 to 1258)	1442 (1351 to 1545)	1739 (1622 to 1860)	2060 (1812 to 2317)	2099 (1822 to 2372)	2129 (1833 to 2414)
Venezuela	2592 (2425 to 2779)	3720 (3433 to 4009)	5391 (4999 to 5779)	7304 (6786 to 7843)	9433 (8776 to 10063)	11739 (10915 to 12639)	13954 (12897 to 15019)	14424 (12768 to 16066)	13902 (12127 to 15646)	13750 (11856 to 15528)
Tropical Latin America	28046 (26211 to 29937)	37870 (35478 to 40414)	49796 (46490 to 53128)	63456 (59257 to 67713)	77121 (71850 to 82436)	91112 (84933 to 97347)	103752 (96718 to 110803)	114327 (100913 to 127791)	115407 (100093 to 130155)	116354 (100287 to 132067)
Brazil	27348 (25488 to 29226)	36946 (34541 to 39467)	48614 (45282 to 51954)	61960 (57711 to 66204)	75112 (69759 to 80406)	88562 (82280 to 94806)	100713 (93500 to 107788)	110864 (97158 to 124085)	111900 (96146 to 126510)	112806 (96316 to 128468)
Acre	55 (50 to 61)	78 (71 to 86)	107 (97 to 119)	152 (138 to 168)	205 (185 to 228)	284 (258 to 315)	381 (346 to 421)	469 (393 to 562)	479 (395 to 579)	488 (399 to 597)
Alagoas	590 (531 to 648)	693 (625 to 761)	853 (768 to 936)	1057 (954 to 1159)	1312 (1170 to 1446)	1516 (1363 to 1663)	1717 (1546 to 1883)	1925 (1589 to 2259)	1948 (1577 to 2304)	1970 (1586 to 2359)
Amapá	19 (17 to 21)	35 (32 to 38)	59 (53 to 65)	90 (82 to 99)	138 (124 to 153)	239 (217 to 264)	342 (309 to 377)	421 (351 to 500)	429 (353 to 513)	437 (357 to 530)
Amazonas	266 (240 to 294)	365 (329 to 403)	490 (443 to 541)	718 (647 to 793)	1027 (922 to 1140)	1407 (1266 to 1552)	1779 (1601 to 1968)	2101 (1726 to 2495)	2136 (1737 to 2546)	2168 (1738 to 2634)
Bahia	2608 (2368 to 2845)	3240 (2930 to 3536)	4013 (3643 to 4381)	5006 (4541 to 5460)	6118 (5513 to 6728)	6863 (6229 to 7504)	7516 (6793 to 8234)	8172 (6829 to 9612)	8246 (6803 to 9676)	8314 (6776 to 9919)
Ceará	1438 (1302 to 1572)	1801 (1637 to 1962)	2329 (2112 to 2545)	2861 (2594 to 3134)	3331 (2998 to 3665)	3957 (3585 to 4324)	4578 (4149 to 5006)	5176 (4322 to 6071)	5241 (4298 to 6173)	5304 (4304 to 6319)
Distrito Federal	21 (19 to 23)	56 (51 to 62)	271 (246 to 298)	627 (567 to 692)	839 (754 to 929)	1096 (990 to 1206)	1366 (1236 to 1509)	1574 (1311 to 1871)	1594 (1310 to 1895)	1612 (1315 to 1936)
Espírito Santo	439 (396 to 483)	604 (546 to 665)	819 (741 to 901)	1042 (943 to 1148)	1311 (1177 to 1453)	1606 (1452 to 1770)	1840 (1658 to 2027)	2026 (1677 to 2411)	2045 (1677 to 2426)	2061 (1670 to 2487)
Goiás	544 (488 to 599)	862 (777 to 950)	1285 (1159 to 1413)	1684 (1509 to 1855)	2032 (1811 to 2248)	2572 (2307 to 2832)	3092 (2768 to 3414)	3481 (2838 to 4131)	3520 (2866 to 4160)	3553 (2856 to 4272)
Maranhão	866 (782 to 952)	1294 (1174 to 1419)	1601 (1453 to 1756)	2092 (1893 to 2297)	2538 (2283 to 2795)	2985 (2698 to 3277)	3537 (3192 to 3884)	4157 (3458 to 4861)	4234 (3484 to 5012)	4310 (3495 to 5179)
Mato Grosso	99 (90 to 109)	174 (158 to 191)	316 (288 to 346)	564 (512 to 619)	955 (860 to 1054)	1248 (1128 to 1370)	1518 (1372 to 1670)	1763 (1467 to 2083)	1790 (1468 to 2119)	1813 (1473 to 2177)
Mato Grosso do Sul	166 (150 to 183)	279 (252 to 307)	469 (423 to 518)	686 (619 to 756)	885 (792 to 986)	1067 (964 to 1178)	1262 (1136 to 1393)	1436 (1190 to 1714)	1454 (1174 to 1744)	1469 (1187 to 1788)
Minas Gerais	4091 (3737 to 4461)	5113 (4682 to 5596)	6003 (5490 to 6552)	6938 (6322 to 7598)	8048 (7285 to 8884)	9277 (8455 to 10162)	10271 (9354 to 11270)	11041 (9307 to 12952)	11117 (9205 to 13199)	11180 (9167 to 13420)
Pará	591 (534 to 648)	802 (730 to 878)	1118 (1017 to 1225)	1722 (1568 to 1891)	2407 (2164 to 2660)	3112 (2828 to 3411)	3887 (3539 to 4257)	4554 (3805 to 5363)	4624 (3817 to 5489)	4692 (3816 to 5626)
Paraíba	926 (837 to 1021)	1098 (991 to 1208)	1288 (1164 to 1415)	1487 (1342 to 1637)	1675 (1495 to 1856)	1843 (1662 to 2028)	2050 (1841 to 2265)	2263 (1867 to 2687)	2286 (1863 to 2736)	2308 (1865 to 2779)
Paraná	1109 (1001 to 1225)	2148 (1952 to 2364)	3421 (3105 to 3763)	3884 (3516 to 4306)	4293 (3858 to 4761)	4945 (4486 to 5450)	5446 (4925 to 6015)	5847 (4848 to 6943)	5888 (4837 to 7025)	5920 (4798 to 7219)
Pernambuco	1835 (1671 to 2023)	2264 (2058 to 2492)	2804 (2562 to 3083)	3328 (3028 to 3670)	3752 (3390 to 4169)	4253 (3858 to 4698)	4793 (4341 to 5301)	5282 (4416 to 6278)	5332 (4383 to 6378)	5378 (4404 to 6529)
Piauí	547 (496 to 603)	676 (614 to 745)	890 (808 to 980)	1141 (1033 to 1258)	1346 (1211 to 1493)	1508 (1366 to 1657)	1690 (1523 to 1864)	1885 (1563 to 2236)	1907 (1565 to 2277)	1928 (1561 to 2334)
Rio de Janeiro	2493 (2259 to 2726)	3532 (3210 to 3850)	4746 (4313 to 5175)	5920 (5350 to 6461)	6741 (6072 to 7422)	7643 (6936 to 8353)	8565 (7748 to 9374)	9239 (7712 to 10814)	9294 (7619 to 10940)	9333 (7630 to 11096)
Rio Grande do Norte	511 (464 to 561)	629 (573 to 688)	823 (750 to 901)	1027 (935 to 1129)	1255 (1131 to 1380)	1481 (1344 to 1615)	1706 (1549 to 1868)	1915 (1606 to 2251)	1938 (1604 to 2278)	1958 (1596 to 2343)
Rio Grande do Sul	2161 (1968 to 2368)	2820 (2565 to 3086)	3442 (3134 to 3771)	4050 (3674 to 4449)	4682 (4226 to 5157)	5277 (4803 to 5772)	5605 (5076 to 6157)	5795 (4750 to 6845)	5813 (4823 to 6892)	5822 (4781 to 6966)
Rondônia	17 (15 to 19)	32 (29 to 35)	59 (53 to 65)	235 (212 to 259)	535 (478 to 594)	691 (624 to 763)	796 (719 to 881)	895 (739 to 1066)	906 (737 to 1083)	916 (734 to 1109)
Roraima	9 (8 to 10)	14 (12 to 15)	20 (18 to 22)	39 (35 to 43)	93 (83 to 101)	160 (145 to 175)	228 (206 to 249)	293 (242 to 342)	300 (246 to 352)	308 (250 to 365)
Santa Catarina	786 (714 to 864)	1091 (995 to 1201)	1456 (1323 to 1603)	1839 (1671 to 2022)	2261 (2045 to 2509)	2737 (2486 to 3017)	3212 (2917 to 3548)	3612 (3047 to 4277)	3655 (3026 to 4376)	3693 (3029 to 4452)
São Paulo	4731 (4304 to 5163)	6713 (6143 to 7317)	9260 (8450 to 10114)	12902 (11782 to 14057)	16134 (14656 to 17682)	19266 (17610 to 21018)	21709 (19790 to 23736)	23442 (19846 to 27341)	23596 (19735 to 27590)	23716 (19649 to 28219)
Sergipe	354 (319 to 388)	414 (372 to 453)	484 (437 to 529)	601 (540 to 661)	761 (682 to 840)	937 (844 to 1026)	1110 (997 to 1218)	1259 (1034 to 1484)	1275 (1041 to 1509)	1290 (1036 to 1546)
Tocantins	74 (67 to 81)	120 (109 to 131)	189 (171 to 207)	267 (242 to 294)	439 (392 to 486)	591 (534 to 648)	718 (648 to 788)	841 (694 to 994)	854 (698 to 1015)	868 (704 to 1039)

Paraguay	698 (652 to 746)	924 (850 to 997)	1182 (1087 to 1277)	1496 (1381 to 1613)	2009 (1835 to 2177)	2550 (2343 to 2767)	3040 (2665 to 3415)	3463 (2848 to 4053)	3507 (2879 to 4159)	3548 (2901 to 4188)
North Africa and Middle East	55809 (54235 to 57412)	70804 (68739 to 72918)	94537 (91896 to 97360)	126011 (122554 to 129680)	165457 (161062 to 170006)	204937 (198295 to 211600)	249995 (242754 to 257578)	291512 (281957 to 301169)	295710 (285104 to 306271)	299630 (288268 to 310877)
North Africa and Middle East	55809 (54235 to 57412)	70804 (68739 to 72918)	94537 (91896 to 97360)	126011 (122554 to 129680)	165457 (161062 to 170006)	204937 (198295 to 211600)	249995 (242754 to 257578)	291512 (281957 to 301169)	295710 (285104 to 306271)	299630 (288268 to 310877)
Afghanistan	3459 (2496 to 4312)	4296 (3527 to 5084)	5311 (4607 to 5998)	6506 (6024 to 7015)	5095 (4431 to 5777)	7937 (6357 to 9437)	10810 (7983 to 13726)	14329 (9793 to 18892)	14787 (9974 to 19258)	15265 (10576 to 19988)
Algeria	4410 (4120 to 4699)	5434 (4854 to 5999)	6807 (6194 to 7425)	9244 (8450 to 10097)	12485 (11414 to 13491)	15328 (14153 to 16532)	17888 (16495 to 19295)	21118 (18177 to 23951)	21451 (18408 to 24453)	21759 (18393 to 25088)
Bahrain	49 (46 to 53)	71 (66 to 76)	100 (93 to 108)	145 (134 to 155)	213 (198 to 229)	276 (257 to 297)	472 (441 to 505)	554 (511 to 591)	560 (521 to 599)	567 (527 to 610)
Egypt	10174 (9482 to 10826)	12911 (12067 to 13811)	17172 (15612 to 18826)	21357 (19430 to 23290)	26974 (24484 to 29497)	32864 (30032 to 35653)	40879 (36771 to 44927)	49383 (45148 to 53592)	50189 (46010 to 54334)	50951 (46184 to 55793)
Iran	8084 (7547 to 8652)	10586 (9590 to 11575)	14136 (12853 to 15372)	19440 (17660 to 21325)	27992 (25480 to 30385)	32502 (29656 to 35440)	37575 (34896 to 40268)	41492 (38063 to 45251)	41769 (37775 to 45627)	41965 (37790 to 46181)
Alborz	86 (77 to 94)	176 (151 to 201)	306 (265 to 346)	432 (372 to 494)	703 (607 to 796)	946 (817 to 1075)	1173 (1044 to 1302)	1421 (1240 to 1605)	1443 (1241 to 1643)	1461 (1242 to 1679)
Ardebil	212 (192 to 232)	259 (226 to 292)	322 (282 to 362)	431 (376 to 488)	570 (498 to 640)	614 (538 to 692)	637 (571 to 703)	638 (563 to 717)	637 (557 to 719)	635 (550 to 723)
Bushehr	62 (56 to 69)	98 (86 to 111)	141 (124 to 160)	215 (189 to 244)	347 (306 to 390)	387 (341 to 437)	468 (423 to 516)	587 (522 to 661)	598 (526 to 675)	608 (529 to 694)
Chahar Mahaal and Bakhtiari	104 (94 to 114)	129 (112 to 146)	160 (140 to 181)	233 (201 to 267)	355 (311 to 402)	408 (355 to 465)	453 (406 to 503)	496 (435 to 560)	499 (434 to 566)	502 (431 to 578)
East Azarbayejan	714 (649 to 786)	869 (759 to 992)	1041 (913 to 1173)	1315 (1154 to 1498)	1688 (1475 to 1910)	1729 (1514 to 1951)	1866 (1682 to 2060)	2007 (1765 to 2255)	2015 (1758 to 2278)	2019 (1743 to 2299)
Fars	289 (260 to 317)	515 (450 to 584)	819 (718 to 923)	1192 (1040 to 1355)	1776 (1560 to 2006)	2021 (1775 to 2279)	2300 (2069 to 2538)	2482 (2195 to 2789)	2494 (2180 to 2822)	2502 (2170 to 2860)
Gilan	397 (361 to 436)	520 (443 to 599)	694 (604 to 786)	894 (779 to 1015)	1134 (997 to 1277)	1186 (1036 to 1339)	1267 (1141 to 1399)	1299 (1147 to 1458)	1298 (1135 to 1462)	1295 (1121 to 1473)
Golestan	216 (195 to 238)	253 (219 to 287)	313 (274 to 356)	454 (395 to 518)	668 (583 to 759)	772 (675 to 873)	903 (811 to 1003)	981 (863 to 1106)	985 (855 to 1115)	986 (851 to 1133)
Hamadan	298 (270 to 328)	380 (329 to 431)	464 (404 to 524)	600 (520 to 683)	812 (712 to 917)	860 (751 to 973)	898 (805 to 990)	865 (758 to 971)	860 (747 to 973)	853 (732 to 976)
Hormozgan	94 (85 to 105)	141 (121 to 161)	195 (169 to 221)	278 (238 to 317)	446 (385 to 505)	580 (503 to 657)	769 (684 to 852)	935 (820 to 1057)	950 (817 to 1079)	962 (819 to 1101)
Ilam	43 (39 to 47)	58 (50 to 66)	86 (75 to 97)	143 (124 to 162)	215 (189 to 243)	257 (225 to 291)	280 (252 to 309)	298 (263 to 335)	300 (262 to 339)	301 (260 to 344)
Isfahan	416 (377 to 459)	575 (504 to 654)	774 (671 to 884)	1169 (1021 to 1332)	1828 (1612 to 2066)	2067 (1813 to 2356)	2412 (2183 to 2670)	2633 (2337 to 2959)	2645 (2323 to 3002)	2653 (2302 to 3039)
Kerman	398 (363 to 436)	384 (337 to 434)	435 (384 to 490)	651 (571 to 738)	914 (806 to 1030)	1119 (988 to 1259)	1452 (1314 to 1600)	1635 (1454 to 1834)	1647 (1451 to 1854)	1655 (1446 to 1886)
Kermanshah	235 (213 to 258)	313 (272 to 354)	416 (365 to 468)	577 (502 to 653)	817 (715 to 920)	913 (799 to 1028)	983 (885 to 1085)	991 (872 to 1111)	989 (862 to 1116)	986 (852 to 1122)
Khorasan-e-Razavi	678 (621 to 748)	856 (755 to 974)	1062 (940 to 1200)	1550 (1368 to 1765)	2334 (2060 to 2640)	2579 (2275 to 2927)	3040 (2753 to 3361)	3359 (2995 to 3787)	3382 (2971 to 3861)	3398 (2969 to 3890)
Khuzestan	667 (607 to 730)	727 (635 to 821)	897 (789 to 1005)	1172 (1027 to 1322)	1572 (1382 to 1766)	1998 (1762 to 2240)	2290 (2068 to 2523)	2455 (2176 to 2746)	2463 (2152 to 2773)	2465 (2134 to 2802)
Kohgiluyeh and Boyer-Ahmad	37 (33 to 40)	59 (52 to 67)	91 (79 to 102)	146 (127 to 165)	237 (207 to 267)	292 (255 to 329)	336 (302 to 370)	371 (326 to 416)	374 (326 to 422)	377 (325 to 429)
Kurdistan	253 (229 to 279)	280 (243 to 318)	322 (280 to 364)	429 (372 to 489)	610 (534 to 688)	703 (616 to 794)	760 (680 to 840)	833 (732 to 942)	839 (730 to 946)	844 (725 to 965)
Lorestan	318 (287 to 351)	324 (280 to 368)	366 (319 to 414)	537 (464 to 612)	757 (658 to 860)	829 (723 to 933)	893 (798 to 987)	888 (779 to 1001)	885 (763 to 1001)	882 (755 to 1012)
Markazi	108 (97 to 119)	221 (192 to 251)	368 (321 to 416)	456 (396 to 518)	593 (520 to 669)	650 (567 to 735)	706 (635 to 779)	719 (632 to 806)	718 (626 to 812)	716 (621 to 816)
Mazandaran	557 (509 to 607)	602 (528 to 678)	693 (609 to 775)	944 (825 to 1065)	1282 (1130 to 1437)	1402 (1233 to 1572)	1549 (1401 to 1699)	1728 (1527 to 1934)	1741 (1525 to 1955)	1751 (1518 to 1990)
North Khorasan	82 (74 to 91)	105 (92 to 120)	134 (116 to 152)	199 (173 to 227)	310 (271 to 353)	376 (329 to 428)	446 (400 to 496)	436 (384 to 493)	434 (378 to 494)	431 (372 to 495)
Qazvin	208 (189 to 229)	231 (196 to 270)	260 (215 to 310)	321 (279 to 370)	466 (409 to 524)	546 (476 to 620)	598 (540 to 662)	650 (575 to 732)	653 (569 to 744)	655 (568 to 751)
Qom	48 (44 to 53)	100 (87 to 115)	174 (153 to 198)	233 (204 to 268)	357 (314 to 406)	462 (408 to 527)	562 (506 to 626)	681 (605 to 773)	692 (606 to 792)	701 (609 to 808)
Semnan	44 (40 to 48)	77 (67 to 87)	122 (106 to 139)	168 (143 to 193)	228 (197 to 257)	264 (228 to 298)	311 (277 to 343)	370 (323 to 416)	376 (324 to 425)	380 (326 to 434)
Sistan and Baluchistan	201 (182 to 221)	216 (188 to 245)	257 (225 to 291)	421 (367 to 479)	724 (638 to 816)	994 (870 to 1127)	1286 (1158 to 1424)	1516 (1337 to 1704)	1537 (1339 to 1746)	1556 (1345 to 1778)
South Khorasan	109 (99 to 120)	142 (123 to 160)	179 (156 to 201)	247 (214 to 279)	329 (288 to 370)	315 (275 to 356)	335 (300 to 369)	418 (369 to 469)	427 (371 to 480)	435 (375 to 494)
Tehran	576 (523 to 636)	1166 (1020 to 1323)	2021 (1776 to 2283)	2707 (2366 to 3098)	4001 (3517 to 4529)	5010 (4431 to 5644)	6023 (5445 to 6681)	6983 (6205 to 7855)	7057 (6219 to 7980)	7115 (6195 to 8132)
West Azarbayejan	278 (252 to 308)	417 (365 to 474)	581 (510 to 659)	792 (695 to 903)	1147 (1010 to 1302)	1319 (1163 to 1493)	1539 (1390 to 1701)	1705 (1517 to 1919)	1716 (1505 to 1944)	1723 (1493 to 1982)
Yazd	106 (96 to 117)	131 (113 to 150)	155 (134 to 179)	206 (179 to 237)	331 (289 to 378)	416 (365 to 476)	520 (469 to 579)	575 (506 to 653)	579 (505 to 662)	582 (502 to 671)
Zanjan	250 (226 to 275)	263 (224 to 308)	286 (231 to 343)	330 (283 to 376)	443 (388 to 503)	487 (423 to 560)	518 (465 to 574)	537 (472 to 602)	538 (468 to 610)	537 (463 to 612)
Iraq	2636 (2474 to 2805)	3479 (3177 to 3774)	4741 (4256 to 5228)	6612 (5945 to 7174)	8933 (8114 to 9736)	12051 (10352 to 13942)	15334 (11665 to 18726)	19220 (14342 to 24177)	19548 (14383 to 24811)	19864 (14052 to 25085)
Jordan	221 (168 to 275)	362 (296 to 429)	634 (553 to 719)	1100 (1020 to 1182)	1781 (1620 to 1951)	2326 (2116 to 2542)	3550 (3198 to 3899)	5362 (4879 to 5842)	5546 (4972 to 6059)	5725 (5141 to 6356)
Kuwait	34 (31 to 38)	104 (96 to 112)	336 (313 to 359)	602 (562 to 641)	736 (666 to 806)	774 (695 to 852)	1276 (1179 to 1373)	2038 (1807 to 2269)	2117 (1859 to 2373)	2194 (1903 to 2489)
Lebanon	615 (572 to 654)	827 (727 to 929)	1079 (1005 to 1156)	1261 (1097 to 1428)	1508 (1240 to 1773)	1784 (1602 to 1987)	2111 (1925 to 2298)	2761 (2376 to 3161)	2768 (2345 to 3184)	2773 (2339 to 3200)
Libya	507 (471 to 541)	675 (612 to 741)	916 (833 to 994)	1441 (1304 to 1571)	2016 (1816 to 2223)	2482 (2238 to 2728)	2976 (2707 to 3234)	3295 (2794 to 3791)	3317 (2818 to 3834)	3336 (2821 to 3873)
Morocco	4470 (4176 to 4798)	5987 (5584 to 6402)	7841 (7253 to 8408)	10194 (9423 to 10973)	12706 (11536 to 13905)	15014 (13541 to 16485)	16897 (15291 to 18480)	18242 (16409 to 20006)	18355 (16416 to 20258)	18454 (16451 to 20506)
Oman	221 (145 to 296)	301 (221 to 381)	409 (322 to 496)	575 (490 to 663)	816 (743 to 890)	1016 (925 to 1104)	1184 (1107 to 1262)	1677 (1550 to 1801)	1743 (1631 to 1858)	1807 (1673 to 1944)
Palestine	391 (328 to 457)	458 (408 to 510)	568 (518 to 620)	739 (635 to 845)	1019 (906 to 1135)	1481 (1350 to 1616)	2032 (1860 to 2202)	2438 (2244 to 2622)	2479 (2266 to 2701)	2518 (2287 to 2753)
Qatar	13 (9 to 16)	25 (19 to 30)	50 (41 to 58)	93 (83 to 102)	148 (134 to 161)	208 (189 to 226)	431 (401 to 460)	784 (725 to 840)	831 (774 to 887)	877 (809 to 944)
Saudi Arabia	1699 (1584 to 1820)	2028 (1761 to 2294)	2753 (2490 to 3016)	4399 (3964 to 4896)	6991 (6384 to 7564)	9248 (8357 to 10148)	12045 (11228 to 12938)	15104 (13178 to 16990)	15397 (13352 to 17361)	15675 (13567 to 17884)
Sudan	2717 (2535 to 2887)	2733 (2472 to 3000)	4834 (4396 to 5264)	7210 (6604 to 7879)	9913 (9033 to 10762)	13202 (11704 to 14721)	16743 (15448 to 18135)	20600 (17847 to 23225)	20994 (18024 to 24069)	21362 (18187 to 24459)
Syria	1687 (1574 to 1803)	2327 (2164 to 2491)	3218 (3003 to 3423)	4422 (4102 to 4740)	6216 (5607 to 6794)	8154 (7354 to 8876)	10640 (9544 to 11715)	7246 (6087 to 8398)	7263 (6022 to 8487)	7286 (5955 to 8427)
Tunisia	1874 (1742 to 2002)	2131 (1942 to 2330)	2506 (2280 to 2751)	3220 (2922 to 3531)	4118 (3734 to 4510)	4888 (4436 to 5340)	5408 (4916 to 5908)	5873 (5291 to 6475)	5915 (5297 to 6543)	5947 (5321 to 6612)
Türkiye	10415 (9714 to 11100)	13389 (12465 to 14314)	17600 (16367 to 18773)	22352 (20757 to 23936)	28357 (26451 to 30171)	33116 (28754 to 37359)	36850 (33978 to 39750)	41155 (40899 to 41407)	41442 (38660 to 44277)	41679 (38416 to 44845)
United Arab Emirates	36 (29 to 43)	46 (41 to 51)	90 (83 to 97)	331 (309 to 353)	661 (597 to 726)	1053 (946 to 1157)	2190 (1967 to 2419)	2616 (2198 to 2999)	2652 (2238 to 3072)	2685 (2203 to 3128)
Yemen	2089 (1340 to 2852)	2618 (1862 to 3342)	3396 (2626 to 4118)	4710 (3977 to 5415)	6688 (6055 to 7292)	9095 (8308 to 9870)	12497 (11151 to 13789)	15957 (13341 to 18536)	16313 (13670 to 19180)	16661 (13955 to 19571)
South Asia	215080 (203063 to 227502)	258929 (243109 to 274852)	328103 (309296 to 347865)	424408 (399992 to 448167)	525104 (493779 to 556174)	638207 (599880 to 675670)	772590 (726348 to 817213)	885181 (810901 to 967955)	895781 (810286 to 980473)	905595 (818357 to 999629)
South Asia	215080 (203063 to 227502)	258929 (243109 to 274852)	328103 (309296 to 347865)	424408 (399992 to 448167)	525104 (493779 to 556174)	638207 (599880 to 675670)	772590 (726348 to 817213)	885181 (810901 to 967955)	895781 (810286 to 980473)	905595 (818357 to 999629)
Bangladesh	19727 (18327 to 21026)	23034 (21305 to 24720)	31421 (28545 to 34309)	41094 (37955 to 44290)	53027 (49281 to 56963)	62741 (58093 to 67298)	73215 (67725 to 78708)	82114 (72802 to 91700)	82914 (71937 to 93233)	83658 (72633 to 94392)
Bhutan	89 (83 to 95)	117 (102 to 132)	162 (130 to 193)	224 (175 to 271)	297 (251 to 343)	306 (276 to 337)	331 (300 to 365)	364 (336 to 393)	364 (332 to 396)	364 (330 to 396)
India	174886 (162995 to 186972)	211980 (196157 to 227380)	262292 (243531 to 280890)	334314 (310461 to 357514)	409192 (378391 to 438854)	496689 (458388 to 533767)	598354 (553891 to 642549)	676896 (602459 to 758698)	683905 (599757 to 764363)	690260 (605225 to 781520)
Nepal	4229 (3942 to 4502)	5012 (4656 to 5386)	5979 (5538 to 6413)	7700 (7159 to 8268)	9709 (8962 to 10450)	11938 (11090 to 12748)	14150 (13116 to 15200)	15951 (13956 to 17956)	16144 (14215 to 18006)	16324 (14337 to 18527)
Pakistan	16150 (14984 to 17246)	18786 (17328 to 20175)	28249 (26016 to 30458)	41075 (37971 to 44020)	52880 (46987 to 58683)	66533 (61215 to 71927)	86540 (76991 to 96026)	109855 (101528 to 118481)	112454 (102559 to 122688)	114989 (105166 to 125348)
Azad Jammu & Kashmir	452 (395 to 510)	486 (432 to 544)	680 (597 to 767)	940 (834 to 1050)	1214 (1007 to 1436)	1478 (1296 to 1672)	1720 (1392 to 2057)	1999 (1586 to 2419)	2027 (1556 to 2466)	2052 (1564 to 2538)
Balochistan	536 (473 to 612)	473 (421 to 529)	957 (842 to 1079)	1955 (1737 to 2182)	2554 (2121 to 3053)	3106 (2738 to 3499)	4468 (3756 to 5251)	6390 (5660 to 7197)	6625 (5745 to 7519)	6861 (5963 to 7862)

Gilgit-Baltistan	108 (84 to 133)	132 (111 to 156)	190 (168 to 215)	275 (245 to 307)	373 (311 to 446)	489 (395 to 596)	648 (471 to 841)	839 (574 to 1112)	862 (606 to 1158)	885 (598 to 1161)
Islamabad Capital Territory	59 (52 to 66)	66 (56 to 77)	96 (85 to 109)	152 (135 to 168)	243 (203 to 287)	402 (354 to 451)	685 (573 to 803)	1025 (905 to 1152)	1063 (928 to 1203)	1100 (956 to 1258)
Khyber Pakhtunkhwa	2949 (2600 to 3316)	3319 (2989 to 3670)	4840 (4328 to 5407)	6491 (5847 to 7189)	8145 (6909 to 9426)	10531 (9427 to 11748)	14251 (12160 to 16577)	18661 (16741 to 20708)	19161 (16883 to 21527)	19653 (17264 to 22278)
Punjab	9407 (8510 to 10265)	10864 (9930 to 11729)	15600 (14183 to 17039)	22374 (20460 to 24228)	28833 (25062 to 32512)	35910 (32643 to 39233)	45601 (39801 to 51354)	56739 (51712 to 61906)	57966 (51996 to 63999)	59152 (52973 to 65342)
Sindh	2638 (2329 to 2999)	3446 (3069 to 3826)	5885 (5226 to 6591)	8889 (7971 to 9832)	11517 (9630 to 13528)	14618 (12982 to 16318)	19167 (16219 to 22266)	24202 (21542 to 26998)	24750 (21644 to 27930)	25286 (22099 to 28528)
Southeast Asia, east Asia, and Oceania	363456 (345657 to 382336)	453521 (423754 to 483925)	562823 (519022 to 603074)	685923 (645203 to 723775)	827915 (787913 to 868816)	910367 (864595 to 955557)	992709 (950456 to 1036478)	1064545 (1010420 to 1115901)	1070492 (1018103 to 1117455)	1075374 (1021275 to 1128212)
East Asia	268098 (251234 to 286442)	330344 (301442 to 360266)	406285 (363344 to 446112)	490754 (451202 to 528176)	590382 (551358 to 632449)	633468 (591403 to 676197)	678101 (637053 to 721298)	716068 (663414 to 766611)	718379 (669774 to 763600)	719864 (669637 to 771932)
China	258777 (241665 to 277189)	318189 (289449 to 348044)	390979 (347640 to 431153)	472621 (432693 to 510261)	569618 (531023 to 611991)	610430 (568354 to 652972)	653622 (612310 to 696776)	690902 (637753 to 741831)	693179 (644014 to 738461)	694642 (643907 to 747206)
North Korea	5610 (3780 to 7520)	6873 (5099 to 8729)	8335 (6593 to 10044)	9577 (8138 to 10891)	10910 (9922 to 11888)	12160 (10834 to 13495)	12942 (11939 to 13980)	13250 (11430 to 15108)	13267 (11300 to 15130)	13280 (11247 to 15231)
Taiwan (province of China)	3710 (3687 to 3733)	5282 (5253 to 5310)	6971 (6938 to 7003)	8556 (8516 to 8595)	9853 (9798 to 9911)	10878 (10808 to 10947)	11537 (11447 to 11628)	11915 (10926 to 12835)	11932 (10859 to 12934)	11942 (10831 to 13073)
Oceania	1227 (1080 to 1370)	1563 (1464 to 1667)	1991 (1896 to 2088)	2497 (2390 to 2603)	3158 (2886 to 3418)	4038 (3847 to 4217)	5150 (4874 to 5435)	6424 (5828 to 6956)	6581 (5942 to 7230)	6738 (6055 to 7394)
American Samoa	10 (9 to 10)	10 (10 to 11)	14 (13 to 15)	16 (15 to 18)	24 (22 to 25)	29 (27 to 31)	28 (26 to 30)	25 (23 to 27)	25 (23 to 26)	24 (22 to 26)
Cook Islands	7 (7 to 8)	9 (9 to 10)	10 (10 to 11)	9 (8 to 9)	9 (8 to 10)	9 (8 to 10)	9 (9 to 10)	9 (8 to 10)	9 (8 to 10)	9 (8 to 10)
Fiji	142 (132 to 151)	198 (181 to 217)	265 (239 to 290)	325 (296 to 356)	373 (340 to 408)	400 (362 to 437)	428 (391 to 465)	452 (416 to 488)	454 (416 to 494)	456 (414 to 501)
Guam	20 (18 to 21)	29 (27 to 30)	39 (36 to 42)	52 (48 to 55)	64 (59 to 68)	78 (73 to 83)	80 (75 to 86)	79 (73 to 85)	79 (74 to 84)	79 (72 to 84)
Kiribati	15 (14 to 16)	19 (17 to 21)	24 (22 to 25)	31 (29 to 34)	37 (35 to 40)	44 (41 to 47)	53 (50 to 57)	60 (55 to 65)	61 (55 to 67)	62 (55 to 69)
Marshall Islands	5 (4 to 7)	8 (6 to 10)	12 (9 to 14)	17 (15 to 19)	22 (20 to 24)	26 (24 to 28)	27 (25 to 29)	27 (24 to 30)	27 (24 to 31)	27 (24 to 31)
Federated States of Micronesia	20 (18 to 21)	25 (22 to 28)	32 (26 to 38)	41 (35 to 48)	50 (46 to 56)	54 (50 to 57)	52 (48 to 55)	50 (44 to 56)	50 (44 to 57)	50 (44 to 57)
Nauru	1 (1 to 1)	1 (1 to 2)	3 (3 to 3)	4 (4 to 5)	5 (5 to 5)	5 (5 to 6)	5 (5 to 6)	5 (5 to 6)	5 (5 to 6)	5 (5 to 6)
Niue	2 (2 to 3)	3 (2 to 3)	3 (2 to 3)	2 (2 to 2)	1 (1 to 1)	1 (1 to 1)	1 (1 to 1)	1 (1 to 1)	1 (1 to 1)	1 (1 to 1)
Northern Mariana Islands	2 (2 to 2)	3 (3 to 4)	5 (4 to 5)	8 (7 to 9)	21 (20 to 23)	39 (36 to 42)	27 (25 to 28)	23 (22 to 25)	23 (21 to 25)	23 (21 to 25)
Palau	4 (3 to 4)	5 (4 to 5)	5 (5 to 6)	6 (6 to 6)	7 (7 to 8)	9 (8 to 10)	9 (8 to 9)	8 (7 to 9)	8 (7 to 9)	8 (7 to 9)
Papua New Guinea	791 (650 to 927)	980 (884 to 1081)	1224 (1137 to 1319)	1526 (1430 to 1626)	1970 (1706 to 2226)	2657 (2477 to 2829)	3623 (3364 to 3896)	4757 (4189 to 5280)	4896 (4278 to 5532)	5036 (4379 to 5674)
Samoa	42 (39 to 45)	56 (52 to 60)	72 (66 to 77)	78 (72 to 84)	80 (75 to 86)	86 (80 to 92)	92 (85 to 99)	101 (92 to 110)	102 (93 to 112)	104 (94 to 114)
Solomon Islands	48 (45 to 51)	64 (59 to 68)	81 (75 to 86)	113 (102 to 123)	163 (149 to 178)	215 (199 to 232)	268 (247 to 288)	322 (280 to 365)	328 (280 to 375)	335 (283 to 382)
Tokelau	1 (1 to 1)	1 (1 to 1)	1 (1 to 1)	1 (1 to 1)	1 (1 to 1)	1 (1 to 1)	1 (1 to 1)	1 (1 to 1)	1 (1 to 1)	1 (1 to 1)
Tonga	25 (23 to 26)	33 (30 to 36)	42 (39 to 46)	48 (44 to 52)	49 (45 to 54)	51 (46 to 55)	53 (49 to 56)	53 (49 to 58)	53 (48 to 58)	53 (48 to 58)
Tuvalu	2 (2 to 2)	3 (3 to 3)	3 (3 to 3)	4 (4 to 5)	5 (5 to 5)	5 (5 to 5)	5 (5 to 6)	6 (5 to 6)	6 (5 to 7)	6 (5 to 7)
Vanuatu	22 (20 to 23)	30 (26 to 33)	42 (38 to 45)	56 (52 to 60)	74 (69 to 79)	95 (88 to 102)	122 (113 to 131)	148 (137 to 160)	152 (142 to 162)	155 (144 to 167)
Southeast Asia	94132 (91049 to 97278)	121613 (116973 to 126572)	154547 (148696 to 160659)	192673 (186901 to 198652)	234375 (226843 to 242479)	272861 (257712 to 288116)	309458 (299904 to 319648)	342053 (329559 to 355578)	345533 (333099 to 358954)	348773 (334876 to 363654)
Cambodia	2195 (2046 to 2350)	2934 (2685 to 3183)	3731 (3271 to 4181)	4310 (3554 to 5075)	5397 (4780 to 6046)	6463 (5946 to 7013)	7439 (6814 to 8050)	8456 (7236 to 9610)	8572 (7309 to 9753)	8676 (7401 to 9984)
Indonesia	39969 (37293 to 42698)	49806 (46259 to 53520)	62312 (57437 to 66870)	77239 (72533 to 82111)	92684 (86425 to 98990)	105889 (91488 to 120294)	119900 (112070 to 127876)	134647 (124500 to 144766)	136231 (127065 to 144922)	137716 (126857 to 148181)
Aceh	640 (579 to 702)	821 (699 to 955)	1054 (899 to 1221)	1374 (1243 to 1502)	1750 (1583 to 1923)	2069 (1687 to 2465)	2276 (2061 to 2497)	2586 (2303 to 2867)	2622 (2376 to 2872)	2656 (2359 to 2941)
Bali	784 (705 to 864)	933 (791 to 1091)	1116 (938 to 1308)	1302 (1178 to 1428)	1456 (1311 to 1601)	1672 (1360 to 2002)	1941 (1749 to 2135)	2180 (1939 to 2425)	2201 (1988 to 2422)	2220 (1970 to 2478)
Bangka-Belitung Islands	164 (148 to 179)	205 (173 to 238)	258 (218 to 297)	333 (302 to 364)	416 (375 to 454)	497 (407 to 588)	597 (540 to 652)	706 (632 to 779)	718 (648 to 787)	730 (651 to 807)
Banten	938 (846 to 1026)	1009 (858 to 1163)	1275 (1083 to 1476)	2071 (1876 to 2260)	3989 (3610 to 4360)	5070 (4143 to 5995)	5286 (4786 to 5775)	5809 (5199 to 6400)	5881 (5300 to 6424)	5949 (5298 to 6568)
Bengkulu	199 (180 to 219)	228 (194 to 265)	283 (242 to 331)	397 (362 to 435)	595 (539 to 653)	747 (614 to 892)	858 (779 to 943)	979 (878 to 1085)	993 (902 to 1089)	1006 (900 to 1114)
Gorontalo	150 (136 to 164)	200 (171 to 231)	272 (233 to 315)	308 (280 to 335)	304 (275 to 332)	401 (327 to 477)	532 (484 to 581)	588 (529 to 649)	592 (538 to 648)	596 (534 to 657)
Jakarta	1272 (1153 to 1393)	1714 (1464 to 1970)	2392 (2032 to 2762)	3301 (3013 to 3595)	4237 (3847 to 4640)	4472 (3660 to 5305)	4697 (4278 to 5133)	5204 (4672 to 5733)	5258 (4778 to 5738)	5307 (4753 to 5843)
Jambi	340 (307 to 375)	416 (353 to 482)	536 (455 to 628)	733 (663 to 805)	1016 (917 to 1122)	1290 (1048 to 1549)	1535 (1384 to 1693)	1726 (1534 to 1919)	1746 (1575 to 1915)	1764 (1568 to 1956)
West Java	6082 (5549 to 6641)	7809 (6789 to 8845)	10004 (8669 to 11352)	12327 (11284 to 13419)	14295 (13086 to 15626)	17524 (14718 to 20658)	21360 (19570 to 23387)	24034 (21785 to 26408)	24282 (22239 to 26562)	24518 (22117 to 27060)
Central Java	7174 (6567 to 7826)	9171 (7988 to 10493)	11379 (9889 to 12962)	13499 (12432 to 14653)	14957 (13724 to 16351)	15653 (13089 to 18557)	16676 (15255 to 18233)	18531 (16788 to 20457)	18732 (17159 to 20483)	18913 (17061 to 20881)
East Java	8964 (8191 to 9747)	11042 (9585 to 12546)	13356 (11607 to 15117)	15574 (14247 to 16902)	17152 (15656 to 18692)	18075 (15101 to 21286)	19282 (17615 to 21036)	20773 (18723 to 22847)	20897 (19045 to 22766)	20997 (18896 to 23091)
West Kalimantan	664 (601 to 727)	807 (685 to 938)	1007 (853 to 1162)	1302 (1184 to 1424)	1638 (1484 to 1795)	1894 (1555 to 2256)	2200 (1989 to 2420)	2626 (2340 to 2916)	2679 (2433 to 2937)	2731 (2445 to 3031)
South Kalimantan	574 (516 to 632)	700 (586 to 815)	873 (732 to 1016)	1093 (989 to 1200)	1349 (1216 to 1483)	1642 (1317 to 1962)	1902 (1713 to 2087)	2014 (1791 to 2232)	2022 (1822 to 2214)	2029 (1801 to 2256)
Central Kalimantan	212 (192 to 233)	263 (222 to 303)	344 (291 to 394)	480 (435 to 524)	690 (622 to 758)	882 (711 to 1049)	1070 (965 to 1172)	1275 (1135 to 1410)	1300 (1174 to 1421)	1323 (1178 to 1459)
East Kalimantan	210 (191 to 232)	260 (220 to 302)	347 (295 to 401)	516 (471 to 568)	801 (729 to 884)	1110 (911 to 1330)	1445 (1313 to 1591)	1772 (1587 to 1971)	1809 (1636 to 1986)	1844 (1640 to 2050)
North Kalimantan	31 (28 to 34)	42 (36 to 49)	62 (53 to 71)	86 (78 to 94)	114 (103 to 125)	167 (135 to 200)	246 (222 to 270)	324 (289 to 358)	333 (301 to 364)	342 (305 to 377)
Riau Islands	91 (82 to 100)	97 (83 to 112)	121 (104 to 140)	215 (195 to 235)	486 (441 to 534)	671 (552 to 796)	820 (744 to 898)	1058 (950 to 1170)	1086 (986 to 1190)	1114 (994 to 1234)
Lampung	892 (805 to 978)	1191 (1001 to 1372)	1669 (1412 to 1925)	2332 (2100 to 2540)	3024 (2719 to 3307)	3386 (2742 to 4038)	3753 (3390 to 4115)	4376 (3899 to 4864)	4451 (4023 to 4878)	4522 (4027 to 4996)
Maluku	275 (248 to 302)	309 (262 to 355)	363 (308 to 417)	456 (414 to 499)	587 (530 to 644)	678 (557 to 808)	770 (691 to 843)	916 (816 to 1016)	936 (844 to 1026)	957 (849 to 1057)
North Maluku	150 (135 to 165)	169 (143 to 197)	198 (167 to 231)	257 (233 to 283)	351 (317 to 387)	434 (354 to 518)	513 (464 to 567)	616 (551 to 684)	630 (570 to 693)	643 (573 to 716)
West Nusa Tenggara	867 (782 to 959)	984 (840 to 1143)	1165 (995 to 1357)	1447 (1309 to 1591)	1775 (1609 to 1955)	2072 (1679 to 2474)	2369 (2143 to 2614)	2643 (2358 to 2938)	2672 (2419 to 2940)	2701 (2407 to 3008)
East Nusa Tenggara	868 (785 to 956)	1020 (869 to 1182)	1205 (1022 to 1400)	1437 (1308 to 1578)	1701 (1545 to 1872)	2043 (1667 to 2459)	2404 (2182 to 2644)	2656 (2381 to 2954)	2689 (2438 to 2949)	2723 (2423 to 3032)
Papua	125 (113 to 138)	157 (133 to 183)	214 (182 to 250)	312 (283 to 343)	497 (448 to 549)	823 (669 to 988)	1373 (1240 to 1514)	2100 (1872 to 2333)	2187 (1975 to 2402)	2272 (2018 to 2529)
West Papua	103 (93 to 113)	140 (119 to 162)	188 (161 to 218)	245 (223 to 268)	299 (271 to 329)	308 (252 to 369)	358 (325 to 393)	515 (462 to 571)	538 (487 to 592)	562 (503 to 623)
Riau	358 (323 to 395)	489 (412 to 568)	673 (564 to 783)	893 (811 to 981)	1155 (1039 to 1275)	1802 (1472 to 2164)	2692 (2428 to 2970)	3078 (2749 to 3432)	3108 (2811 to 3420)	3137 (2794 to 3484)
West Sulawesi	217 (196 to 241)	236 (200 to 273)	267 (225 to 312)	311 (283 to 343)	366 (332 to 407)	467 (382 to 568)	593 (538 to 655)	694 (622 to 775)	705 (639 to 780)	717 (640 to 802)
South Sulawesi	1679 (1513 to 1851)	2016 (1716 to 2339)	2481 (2083 to 2910)	2982 (2699 to 3264)	3369 (3033 to 3714)	3776 (3058 to 4525)	4199 (3787 to 4627)	4643 (4111 to 5172)	4694 (4229 to 5166)	4742 (4223 to 5285)
Central Sulawesi	299 (270 to 328)	368 (312 to 427)	481 (408 to 559)	649 (588 to 713)	858 (776 to 946)	1091 (886 to 1310)	1316 (1193 to 1452)	1450 (1297 to 1610)	1464 (1321 to 1609)	1478 (1318 to 1655)
Southeast Sulawesi	269 (241 to 295)	314 (262 to 363)	382 (322 to 442)	505 (454 to 552)	696 (626 to 763)	912 (740 to 1084)	1127 (1013 to 1235)	1280 (1139 to 1420)	1298 (1169 to 1421)	1315 (1166 to 1454)
North Sulawesi	456 (412 to 501)	537 (454 to 617)	627 (530 to 723)	776 (703 to 850)	964 (870 to 1059)	1046 (851 to 1239)	1128 (1017 to 1234)	1295 (1157 to 1428)	1314 (1185 to 1439)	1332 (1184 to 1473)
West Sumatra	1069 (967 to 1179)	1276 (1087 to 1471)	1514 (1287 to 1750)	1827 (1657 to 2006)	2116 (1908 to 2341)	2291 (1868 to 2758)	2477 (2244 to 2733)	2782 (2487 to 3106)	2821 (2554 to 3109)	2859 (2550 to 3185)
South Sumatra	869 (787 to 956)	1118 (951 to 1286)	1488 (1263 to 1720)	2064 (1879 to 2266)	2810 (2553 to 3088)	3321 (2759 to 3953)	3733 (3405 to 4095)	4127 (3714 to 4578)	4170 (3809 to 4564)	4211 (3785 to 4688)

North Sumatra	1973 (1790 to 2159)	2588 (2196 to 2972)	3377 (2889 to 3883)	4366 (3987 to 4758)	5329 (4852 to 5831)	5978 (4982 to 7069)	6612 (6023 to 7234)	7402 (6673 to 8168)	7503 (6853 to 8207)	7602 (6815 to 8385)
Yogyakarta	1013 (917 to 1122)	1176 (1008 to 1370)	1340 (1150 to 1568)	1468 (1335 to 1623)	1543 (1401 to 1712)	1626 (1343 to 1972)	1760 (1597 to 1951)	1890 (1703 to 2112)	1899 (1724 to 2106)	1905 (1707 to 2137)
Laos	854 (612 to 1092)	1080 (846 to 1329)	1356 (1150 to 1577)	1683 (1512 to 1850)	2122 (1901 to 2329)	2706 (2437 to 2979)	3170 (2854 to 3461)	3571 (3227 to 3904)	3619 (3272 to 3981)	3667 (3285 to 4025)
Malaysia	3029 (2638 to 3401)	4061 (3809 to 4320)	5314 (4941 to 5655)	6753 (6295 to 7214)	8751 (8068 to 9412)	11679 (10910 to 12483)	13670 (12791 to 14657)	15078 (13169 to 16951)	15221 (13287 to 17250)	15354 (13137 to 17367)
Maldives	36 (33 to 38)	43 (39 to 47)	57 (52 to 61)	78 (71 to 84)	108 (101 to 116)	138 (128 to 147)	165 (150 to 181)	193 (174 to 212)	196 (176 to 216)	199 (175 to 219)
Mauritius	244 (229 to 261)	334 (307 to 361)	414 (381 to 446)	495 (451 to 541)	548 (513 to 586)	612 (569 to 656)	640 (594 to 690)	645 (562 to 729)	645 (563 to 723)	644 (558 to 729)
Myanmar	9441 (8732 to 10078)	11430 (9923 to 12947)	14043 (12829 to 15283)	17255 (15792 to 18670)	20512 (18295 to 22702)	23265 (19702 to 26854)	25863 (23508 to 28435)	28600 (25583 to 31508)	28903 (25632 to 31990)	29188 (25962 to 32485)
Philippines	10096 (9421 to 10769)	14206 (13206 to 15143)	19228 (17968 to 20489)	24603 (23036 to 26120)	31263 (29202 to 33397)	39313 (36553 to 42096)	47428 (44262 to 50823)	54443 (49314 to 59166)	55162 (50019 to 60913)	55832 (49450 to 61764)
Seychelles	18 (17 to 19)	22 (21 to 24)	27 (25 to 29)	33 (30 to 36)	36 (33 to 40)	41 (37 to 44)	45 (42 to 48)	49 (43 to 55)	49 (43 to 56)	50 (43 to 57)
Sri Lanka	3675 (3440 to 3938)	4852 (4411 to 5275)	6221 (5763 to 6697)	7413 (6874 to 7958)	8519 (7420 to 9554)	9487 (8198 to 10757)	10540 (9748 to 11371)	11379 (10124 to 12569)	11455 (10089 to 12872)	11524 (10061 to 12954)
Thailand	10168 (9424 to 10861)	13966 (12998 to 14894)	17912 (16650 to 19116)	23401 (21804 to 25042)	28618 (26739 to 30602)	31699 (29650 to 33894)	34483 (32146 to 36827)	34447 (30132 to 38769)	34479 (30064 to 39092)	34494 (29766 to 39272)
Timor-Leste	222 (194 to 251)	262 (243 to 279)	263 (231 to 296)	285 (265 to 305)	378 (351 to 404)	443 (402 to 482)	541 (504 to 575)	662 (599 to 718)	676 (606 to 744)	691 (617 to 763)
Viet Nam	14049 (13094 to 15015)	18421 (16090 to 20797)	23418 (20546 to 26409)	28835 (26628 to 30893)	35098 (32277 to 37882)	40730 (37846 to 43865)	45125 (41878 to 48330)	49406 (45843 to 52789)	49844 (45795 to 53486)	50253 (46256 to 54051)
Sub-Saharan Africa	87253 (80702 to 93702)	111542 (105116 to 118402)	144212 (138147 to 150133)	187966 (182546 to 194027)	248068 (242182 to 253972)	325539 (316495 to 334988)	429435 (417007 to 442424)	547379 (526866 to 568199)	561431 (540722 to 583717)	575303 (552193 to 599028)
Central sub-Saharan Africa	9138 (8705 to 9572)	12491 (11374 to 13618)	16595 (15766 to 17427)	21226 (19741 to 22634)	27722 (25594 to 29893)	37006 (32861 to 40882)	50199 (42633 to 57279)	65171 (52780 to 77514)	66888 (54228 to 79652)	68571 (55349 to 82689)
Angola	2202 (2054 to 2358)	2506 (2337 to 2678)	2829 (2638 to 3021)	3631 (3153 to 4086)	5095 (4155 to 6122)	7468 (6398 to 8572)	11203 (10159 to 12383)	15774 (14160 to 17332)	16309 (14612 to 18007)	16841 (14961 to 18768)
Central African Republic	699 (543 to 854)	863 (730 to 1007)	1071 (964 to 1177)	1191 (1079 to 1306)	1387 (1279 to 1508)	1820 (1672 to 1981)	2315 (2074 to 2565)	2688 (2261 to 3140)	2745 (2246 to 3216)	2794 (2296 to 3264)
Congo (Brazzaville)	401 (315 to 495)	524 (445 to 603)	680 (616 to 745)	906 (819 to 988)	1227 (1090 to 1355)	1604 (1421 to 1757)	2118 (1943 to 2287)	2617 (2240 to 2986)	2665 (2282 to 3070)	2711 (2308 to 3138)
DR Congo	5547 (5202 to 5893)	8247 (7129 to 9343)	11588 (10750 to 12416)	14955 (13497 to 16341)	19297 (17374 to 21392)	25161 (21027 to 29126)	33303 (25557 to 40324)	42521 (29948 to 54607)	43566 (30679 to 56049)	44591 (31189 to 58380)
Equatorial Guinea	92 (85 to 98)	102 (89 to 116)	119 (102 to 136)	152 (138 to 165)	219 (196 to 243)	331 (276 to 384)	492 (444 to 542)	658 (598 to 719)	675 (606 to 738)	691 (623 to 766)
Gabon	198 (184 to 211)	248 (231 to 265)	308 (269 to 348)	391 (335 to 451)	497 (455 to 545)	622 (551 to 694)	767 (702 to 831)	914 (818 to 1012)	928 (832 to 1035)	942 (834 to 1045)
Eastern sub-Saharan Africa	31700 (29071 to 34191)	41083 (38518 to 43743)	54087 (51696 to 56561)	71986 (69846 to 74355)	96168 (93319 to 99315)	125829 (121695 to 130225)	164449 (159436 to 169566)	205082 (196202 to 214298)	209995 (200557 to 219664)	214810 (204817 to 224995)
Burundi	1237 (948 to 1566)	1560 (1284 to 1869)	1950 (1708 to 2195)	2275 (2098 to 2451)	2851 (2662 to 3040)	3283 (2881 to 3662)	4684 (4319 to 5054)	6169 (5327 to 6995)	6395 (5459 to 7348)	6600 (5661 to 7489)
Comoros	78 (65 to 91)	96 (86 to 106)	129 (117 to 141)	178 (166 to 190)	233 (216 to 249)	279 (255 to 303)	323 (286 to 363)	364 (303 to 427)	369 (303 to 434)	373 (306 to 442)
Djibouti	32 (24 to 40)	48 (38 to 58)	75 (63 to 86)	126 (115 to 137)	196 (174 to 217)	290 (255 to 326)	414 (385 to 446)	551 (481 to 624)	566 (488 to 646)	580 (499 to 668)
Eritrea	564 (398 to 727)	737 (574 to 895)	974 (824 to 1125)	1286 (1165 to 1404)	1704 (1518 to 1885)	1984 (1677 to 2315)	2644 (1992 to 3276)	3110 (2215 to 3983)	3178 (2301 to 4083)	3247 (2253 to 4308)
Ethiopia	8731 (6081 to 11165)	11050 (8653 to 13623)	14079 (11871 to 16291)	17873 (16310 to 19668)	25229 (23100 to 27788)	33849 (30560 to 37317)	42168 (38530 to 45651)	51794 (44300 to 59104)	52905 (45488 to 60791)	54032 (45545 to 62234)
Addis Ababa	304 (213 to 392)	404 (304 to 514)	559 (438 to 689)	772 (673 to 881)	1045 (910 to 1190)	1307 (1120 to 1507)	1576 (1390 to 1777)	1801 (1436 to 2186)	1817 (1439 to 2214)	1830 (1441 to 2262)
Afar	150 (105 to 194)	126 (94 to 161)	115 (91 to 142)	149 (130 to 170)	389 (339 to 444)	698 (595 to 806)	713 (625 to 805)	795 (636 to 965)	806 (640 to 989)	818 (648 to 1009)
Amhara	2643 (1850 to 3399)	3313 (2546 to 4164)	4130 (3376 to 4995)	5065 (4509 to 5698)	6726 (5974 to 7607)	8339 (7286 to 9550)	9622 (8585 to 10767)	11073 (9146 to 13357)	11238 (9200 to 13658)	11406 (9237 to 13921)
Benishangul-Gumuz	146 (102 to 189)	192 (145 to 244)	231 (181 to 284)	241 (210 to 276)	243 (212 to 278)	284 (243 to 328)	394 (347 to 444)	532 (427 to 646)	548 (436 to 672)	564 (445 to 692)
Dire Dawa	54 (38 to 70)	81 (60 to 103)	118 (92 to 145)	141 (123 to 161)	138 (120 to 158)	150 (129 to 174)	196 (172 to 221)	244 (195 to 295)	248 (197 to 305)	252 (198 to 312)
Gambella	45 (31 to 58)	64 (48 to 81)	89 (70 to 110)	98 (85 to 111)	88 (76 to 100)	113 (97 to 129)	181 (159 to 204)	249 (199 to 301)	257 (205 to 310)	264 (209 to 322)
Harari	25 (17 to 32)	33 (25 to 43)	45 (35 to 56)	57 (49 to 65)	67 (58 to 77)	81 (68 to 94)	103 (90 to 117)	127 (100 to 156)	130 (101 to 161)	132 (102 to 164)
Oromia	2602 (1810 to 3352)	3142 (2392 to 3949)	3889 (3148 to 4707)	5177 (4575 to 5821)	8334 (7374 to 9335)	12144 (10649 to 13758)	15579 (13983 to 17219)	19630 (16234 to 23133)	20114 (16699 to 23810)	20605 (16883 to 24580)
Somali	395 (272 to 510)	479 (354 to 608)	592 (459 to 725)	806 (697 to 915)	1409 (1222 to 1601)	2033 (1721 to 2339)	2440 (2133 to 2734)	3047 (2442 to 3680)	3121 (2443 to 3801)	3197 (2507 to 3899)
Southern Nations, Nationalities, and Peoples	1768 (1211 to 2301)	2451 (1848 to 3133)	3339 (2680 to 4023)	4197 (3688 to 4725)	5260 (4619 to 5950)	6730 (5783 to 7690)	8869 (7816 to 9899)	11262 (9164 to 13423)	11536 (9316 to 13891)	11817 (9502 to 14303)
Tigray	599 (415 to 777)	765 (577 to 982)	973 (766 to 1195)	1170 (1022 to 1330)	1530 (1333 to 1737)	1971 (1681 to 2264)	2496 (2199 to 2794)	3032 (2434 to 3659)	3090 (2485 to 3738)	3148 (2477 to 3837)
Kenya	2790 (2611 to 2970)	3993 (3688 to 4319)	5963 (5500 to 6382)	8422 (7801 to 9085)	11652 (10843 to 12521)	15687 (14504 to 16829)	20440 (18886 to 21992)	24442 (22769 to 26101)	24832 (22896 to 26797)	25200 (23272 to 27199)
Baringo	44 (40 to 49)	64 (53 to 76)	89 (79 to 101)	109 (97 to 123)	159 (141 to 179)	224 (199 to 253)	285 (253 to 321)	331 (295 to 370)	335 (296 to 380)	339 (298 to 385)
Bomet	41 (37 to 45)	61 (51 to 73)	91 (80 to 102)	118 (105 to 133)	168 (149 to 189)	255 (227 to 286)	474 (423 to 530)	459 (412 to 511)	452 (400 to 508)	446 (394 to 502)
Bungoma	84 (75 to 94)	123 (101 to 146)	192 (170 to 217)	276 (244 to 313)	432 (383 to 488)	823 (735 to 922)	737 (652 to 832)	895 (800 to 1002)	921 (814 to 1044)	946 (830 to 1076)
Busia	61 (54 to 68)	82 (68 to 99)	117 (103 to 133)	172 (152 to 196)	248 (219 to 281)	318 (281 to 362)	407 (359 to 462)	486 (433 to 546)	494 (435 to 563)	501 (437 to 574)
Elgeyo Marakwet	47 (42 to 52)	74 (61 to 87)	90 (79 to 101)	80 (71 to 90)	126 (111 to 141)	241 (213 to 270)	189 (166 to 212)	227 (202 to 252)	233 (204 to 262)	239 (207 to 269)
Embu	44 (39 to 49)	62 (51 to 74)	96 (84 to 108)	143 (126 to 162)	201 (178 to 228)	247 (217 to 280)	273 (240 to 308)	308 (273 to 344)	310 (272 to 352)	312 (272 to 355)
Garissa	17 (15 to 19)	22 (18 to 26)	39 (34 to 44)	62 (55 to 70)	66 (58 to 74)	156 (138 to 176)	325 (287 to 367)	413 (369 to 460)	421 (373 to 475)	430 (378 to 487)
Homa Bay	138 (124 to 153)	188 (157 to 222)	242 (215 to 272)	282 (249 to 317)	358 (317 to 402)	433 (383 to 488)	520 (459 to 586)	598 (531 to 669)	605 (532 to 683)	612 (536 to 695)
Isiolo	9 (8 to 10)	12 (10 to 14)	17 (15 to 19)	23 (20 to 26)	36 (32 to 41)	55 (48 to 62)	76 (66 to 85)	128 (113 to 143)	135 (117 to 153)	141 (122 to 161)
Kajiado	20 (18 to 23)	28 (23 to 34)	46 (40 to 52)	80 (70 to 90)	136 (121 to 154)	226 (199 to 255)	380 (336 to 429)	591 (529 to 659)	617 (543 to 697)	643 (563 to 728)
Kakamega	122 (110 to 135)	191 (160 to 221)	294 (263 to 325)	426 (379 to 472)	604 (542 to 670)	556 (493 to 623)	895 (796 to 1000)	997 (895 to 1101)	999 (886 to 1116)	999 (885 to 1121)
Kericho	55 (50 to 62)	94 (79 to 112)	158 (141 to 178)	214 (191 to 241)	312 (279 to 351)	380 (339 to 427)	314 (279 to 352)	470 (423 to 523)	494 (438 to 558)	518 (457 to 587)
Kiambu	106 (95 to 118)	152 (127 to 182)	246 (218 to 278)	367 (325 to 415)	493 (439 to 558)	744 (659 to 839)	897 (797 to 1006)	1233 (1109 to 1376)	1274 (1130 to 1429)	1314 (1160 to 1486)
Kilifi	90 (81 to 101)	119 (98 to 141)	172 (152 to 193)	245 (217 to 276)	338 (301 to 382)	468 (415 to 528)	612 (541 to 690)	774 (692 to 865)	791 (697 to 896)	808 (714 to 916)
Kirinyaga	57 (51 to 64)	81 (67 to 97)	119 (105 to 135)	159 (139 to 180)	207 (183 to 235)	242 (212 to 275)	279 (244 to 317)	308 (272 to 347)	310 (270 to 353)	311 (271 to 358)
Kisii	97 (88 to 108)	160 (135 to 190)	259 (230 to 290)	358 (319 to 402)	485 (432 to 544)	632 (562 to 710)	667 (592 to 753)	664 (594 to 744)	663 (586 to 752)	661 (580 to 753)
Kisumu	110 (98 to 122)	165 (138 to 194)	222 (196 to 249)	263 (232 to 297)	363 (321 to 409)	437 (386 to 492)	514 (452 to 578)	607 (539 to 678)	617 (543 to 697)	625 (549 to 711)
Kitui	103 (93 to 115)	142 (118 to 168)	200 (177 to 224)	268 (237 to 301)	368 (327 to 413)	468 (414 to 526)	552 (489 to 621)	611 (545 to 681)	613 (539 to 691)	615 (539 to 697)
Kwale	62 (56 to 70)	83 (68 to 100)	118 (104 to 134)	159 (139 to 181)	210 (185 to 237)	277 (243 to 315)	353 (310 to 402)	450 (400 to 506)	461 (405 to 527)	472 (412 to 541)
Laikipia	13 (12 to 15)	18 (15 to 22)	34 (30 to 38)	70 (62 to 79)	117 (103 to 133)	179 (157 to 201)	210 (185 to 237)	258 (230 to 288)	263 (232 to 296)	267 (234 to 303)
Lamu	8 (7 to 9)	9 (7 to 11)	13 (11 to 15)	22 (19 to 25)	29 (25 to 33)	38 (33 to 44)	51 (44 to 58)	67 (59 to 76)	69 (60 to 79)	71 (61 to 82)
Machakos	88 (79 to 98)	136 (113 to 161)	230 (203 to 258)	366 (324 to 410)	519 (460 to 580)	599 (529 to 671)	578 (510 to 650)	722 (641 to 803)	738 (647 to 833)	752 (659 to 852)
Makueni	79 (70 to 88)	112 (93 to 133)	157 (138 to 177)	199 (174 to 224)	249 (219 to 280)	318 (278 to 358)	474 (417 to 533)	510 (451 to 569)	508 (443 to 574)	505 (439 to 573)
Mandera	25 (22 to 28)	37 (31 to 44)	50 (44 to 57)	55 (48 to 62)	68 (60 to 77)	158 (139 to 180)	527 (464 to 596)	457 (406 to 512)	445 (389 to 503)	435 (380 to 495)
Marsabit	14 (12 to 16)	15 (13 to 18)	26 (23 to 29)	51 (45 to 58)	68 (60 to 77)	97 (86 to 110)	152 (134 to 173)	219 (195 to 245)	226 (199 to 257)	233 (204 to 266)

Meru	134 (120 to 148)	196 (163 to 230)	292 (259 to 325)	406 (359 to 452)	576 (511 to 643)	727 (642 to 812)	840 (743 to 939)	805 (717 to 893)	796 (699 to 894)	786 (690 to 886)
Migori	91 (82 to 100)	127 (106 to 150)	171 (152 to 192)	213 (188 to 239)	286 (254 to 321)	381 (337 to 428)	498 (439 to 560)	601 (536 to 670)	612 (539 to 690)	622 (547 to 704)
Mombasa	60 (53 to 66)	81 (66 to 96)	118 (104 to 132)	155 (137 to 175)	219 (193 to 246)	323 (284 to 362)	474 (417 to 531)	594 (530 to 659)	604 (530 to 679)	613 (536 to 690)
Murang'a	130 (116 to 145)	181 (150 to 213)	257 (227 to 291)	351 (310 to 397)	458 (405 to 518)	412 (362 to 466)	473 (416 to 533)	519 (459 to 582)	521 (456 to 593)	523 (455 to 597)
Nairobi	111 (100 to 122)	131 (111 to 152)	245 (218 to 270)	521 (466 to 574)	621 (557 to 687)	1053 (944 to 1160)	1623 (1456 to 1791)	2191 (1973 to 2399)	2246 (2006 to 2483)	2299 (2047 to 2546)
Nakuru	59 (53 to 65)	87 (73 to 102)	154 (137 to 173)	273 (244 to 306)	455 (408 to 510)	645 (577 to 724)	829 (745 to 926)	1074 (972 to 1188)	1099 (982 to 1232)	1122 (1001 to 1262)
Nandi	52 (47 to 58)	77 (65 to 90)	116 (103 to 130)	158 (140 to 177)	233 (208 to 262)	314 (281 to 353)	394 (350 to 441)	464 (417 to 515)	470 (417 to 529)	477 (421 to 537)
Narok	33 (29 to 37)	45 (38 to 54)	69 (61 to 78)	120 (106 to 136)	205 (181 to 231)	207 (184 to 234)	455 (405 to 512)	594 (533 to 662)	604 (537 to 681)	613 (542 to 695)
Nyamira	52 (46 to 58)	76 (63 to 90)	102 (90 to 114)	117 (104 to 133)	143 (126 to 162)	186 (163 to 210)	262 (231 to 297)	312 (278 to 350)	316 (278 to 359)	320 (279 to 364)
Nyandarua	37 (33 to 42)	56 (46 to 67)	89 (78 to 100)	125 (110 to 142)	190 (168 to 215)	257 (226 to 291)	308 (271 to 349)	315 (280 to 354)	314 (274 to 356)	312 (272 to 358)
Nyeri	89 (80 to 99)	122 (102 to 145)	185 (164 to 208)	263 (232 to 295)	325 (287 to 365)	347 (306 to 390)	357 (313 to 402)	371 (329 to 415)	372 (325 to 420)	371 (323 to 423)
Samburu	19 (17 to 21)	28 (23 to 33)	39 (34 to 44)	43 (38 to 49)	63 (55 to 71)	84 (75 to 95)	119 (105 to 134)	157 (141 to 176)	162 (143 to 183)	166 (146 to 189)
Siaya	85 (76 to 95)	127 (107 to 151)	184 (161 to 207)	241 (212 to 272)	329 (291 to 371)	411 (361 to 465)	464 (407 to 524)	537 (475 to 602)	545 (477 to 616)	552 (480 to 629)
Taita Taveta	27 (24 to 31)	39 (32 to 47)	58 (51 to 66)	81 (71 to 92)	117 (103 to 133)	133 (117 to 151)	145 (127 to 165)	168 (148 to 189)	170 (148 to 195)	172 (149 to 197)
Tana River	14 (12 to 16)	18 (15 to 22)	29 (25 to 33)	50 (44 to 57)	68 (60 to 78)	97 (85 to 111)	125 (110 to 143)	160 (142 to 180)	164 (144 to 187)	168 (147 to 193)
Tharaka Nithi	28 (25 to 32)	36 (29 to 43)	43 (37 to 48)	44 (39 to 50)	52 (46 to 59)	57 (50 to 64)	75 (65 to 84)	196 (173 to 219)	217 (189 to 244)	238 (206 to 270)
Trans Nzoia	21 (19 to 24)	31 (26 to 37)	63 (56 to 72)	138 (121 to 155)	215 (191 to 241)	319 (282 to 358)	428 (379 to 480)	508 (454 to 563)	514 (453 to 577)	519 (458 to 584)
Turkana	44 (39 to 50)	68 (55 to 81)	87 (76 to 98)	80 (70 to 90)	114 (100 to 128)	251 (220 to 283)	453 (399 to 511)	471 (419 to 526)	470 (413 to 532)	469 (410 to 534)
Uasin Gishu	37 (34 to 42)	57 (47 to 68)	100 (89 to 112)	159 (141 to 179)	234 (209 to 263)	338 (300 to 378)	478 (424 to 535)	610 (548 to 676)	624 (552 to 700)	636 (564 to 716)
Vihiga	86 (76 to 96)	122 (100 to 146)	151 (133 to 170)	157 (138 to 179)	199 (174 to 226)	212 (185 to 241)	300 (262 to 341)	308 (270 to 347)	306 (266 to 349)	303 (263 to 347)
Wajir	22 (20 to 25)	29 (23 to 34)	48 (42 to 54)	69 (61 to 78)	68 (60 to 77)	186 (164 to 209)	334 (292 to 377)	394 (348 to 440)	402 (353 to 452)	410 (358 to 465)
West Pokot	22 (20 to 25)	29 (24 to 34)	48 (42 to 54)	88 (78 to 100)	122 (108 to 138)	176 (155 to 199)	268 (237 to 303)	309 (276 to 347)	312 (275 to 353)	314 (277 to 356)
Madagascar	2096 (1947 to 2229)	2721 (2365 to 3063)	3520 (3176 to 3882)	4603 (4160 to 5049)	5963 (5479 to 6501)	7975 (7171 to 8813)	10824 (9538 to 12122)	13832 (12775 to 14862)	14156 (13059 to 15342)	14471 (13202 to 15686)
Malawi	1539 (1432 to 1646)	1923 (1721 to 2126)	2436 (2199 to 2657)	3367 (3089 to 3671)	4982 (4561 to 5394)	5652 (5186 to 6082)	7400 (6786 to 8000)	9519 (8852 to 10191)	9752 (8966 to 10508)	9975 (9156 to 10787)
Mozambique	3148 (2927 to 3369)	3716 (3473 to 3969)	4642 (4331 to 4970)	6306 (5845 to 6756)	6967 (6181 to 7774)	9110 (8303 to 9923)	11882 (10875 to 12960)	15295 (14015 to 16476)	15710 (14283 to 17067)	16119 (14650 to 17606)
Rwanda	1341 (1253 to 1437)	1650 (1431 to 1858)	2121 (1887 to 2357)	2771 (2549 to 2997)	3683 (3396 to 3973)	4246 (3883 to 4597)	5391 (4975 to 5825)	6544 (5851 to 7260)	6679 (5887 to 7502)	6812 (5912 to 7673)
Somalia	1123 (1041 to 1200)	1379 (1200 to 1551)	1788 (1619 to 1959)	2866 (2577 to 3139)	3800 (3502 to 4132)	4946 (4210 to 5696)	7250 (5699 to 8827)	9784 (7343 to 12209)	10166 (7320 to 13002)	10557 (7611 to 13206)
South Sudan	1288 (1155 to 1419)	1563 (1424 to 1717)	1889 (1593 to 2180)	2282 (2083 to 2469)	2767 (2445 to 3092)	3472 (3068 to 3864)	4524 (4139 to 4877)	4542 (3937 to 5171)	4692 (4042 to 5344)	4822 (4049 to 5504)
Uganda	2576 (2396 to 2747)	3662 (3412 to 3916)	5114 (4729 to 5518)	6644 (5709 to 7582)	8777 (8139 to 9423)	12401 (11328 to 13432)	16617 (15044 to 18130)	20934 (18850 to 22888)	21450 (19251 to 23646)	21959 (19597 to 24494)
Tanzania	3959 (3679 to 4217)	5305 (4842 to 5765)	7079 (6445 to 7668)	9874 (9042 to 10717)	13260 (12173 to 14338)	17519 (16078 to 18967)	22794 (21122 to 24481)	28681 (25526 to 31964)	29360 (26014 to 32796)	30026 (26459 to 33646)
Zambia	1184 (1103 to 1260)	1661 (1449 to 1859)	2303 (2129 to 2474)	3074 (2879 to 3261)	4036 (3751 to 4324)	5033 (4673 to 5385)	6962 (6538 to 7406)	9344 (8139 to 10489)	9603 (8343 to 10878)	9852 (8467 to 11243)
Southern sub-Saharan Africa	8680 (8104 to 9269)	11442 (10800 to 12149)	15624 (14803 to 16510)	20780 (18791 to 22887)	26996 (24988 to 29117)	33043 (31146 to 34923)	36479 (34560 to 38520)	40380 (36947 to 44219)	40776 (37057 to 44670)	41102 (37324 to 45119)
Botswana	198 (185 to 211)	263 (238 to 287)	351 (313 to 389)	489 (452 to 526)	688 (636 to 737)	875 (815 to 939)	1025 (950 to 1103)	1179 (1051 to 1315)	1196 (1045 to 1349)	1211 (1052 to 1371)
Eswatini	132 (123 to 141)	174 (156 to 192)	233 (212 to 256)	311 (283 to 339)	423 (384 to 464)	535 (488 to 583)	562 (515 to 613)	584 (537 to 629)	587 (538 to 637)	589 (525 to 643)
Lesotho	336 (314 to 359)	437 (394 to 476)	561 (506 to 617)	705 (636 to 771)	836 (761 to 910)	928 (842 to 1021)	957 (870 to 1044)	964 (881 to 1048)	965 (878 to 1052)	965 (867 to 1064)
Namibia	215 (200 to 229)	282 (264 to 301)	382 (354 to 409)	522 (454 to 591)	717 (664 to 772)	941 (875 to 1006)	1085 (1006 to 1165)	1224 (1076 to 1368)	1239 (1077 to 1396)	1253 (1079 to 1406)
South Africa	6436 (5975 to 6909)	8401 (7826 to 8946)	11467 (10746 to 12209)	14980 (12980 to 16984)	19057 (17071 to 21079)	23598 (21727 to 25357)	26098 (24239 to 27970)	28578 (25278 to 32247)	28812 (25199 to 32439)	28991 (25357 to 32779)
Eastern Cape	1481 (1344 to 1616)	1783 (1628 to 1940)	2040 (1856 to 2222)	2542 (2106 to 3001)	3212 (2760 to 3675)	3569 (3192 to 3918)	3437 (3099 to 3776)	3454 (2892 to 4037)	3452 (2861 to 4065)	3444 (2842 to 4064)
Free State	511 (465 to 564)	691 (630 to 759)	881 (803 to 972)	1070 (889 to 1294)	1270 (1101 to 1470)	1438 (1294 to 1598)	1399 (1264 to 1556)	1406 (1182 to 1672)	1407 (1171 to 1681)	1404 (1159 to 1690)
Gauteng	1052 (956 to 1152)	1481 (1350 to 1613)	2053 (1872 to 2238)	2476 (2067 to 2923)	3070 (2654 to 3511)	4351 (3920 to 4772)	5898 (5347 to 6431)	7233 (6128 to 8342)	7357 (6217 to 8508)	7466 (6273 to 8676)
KwaZulu-Natal	1279 (1163 to 1406)	1623 (1485 to 1777)	2406 (2199 to 2635)	3189 (2674 to 3780)	4027 (3495 to 4612)	5052 (4573 to 5558)	5320 (4835 to 5872)	5613 (4739 to 6591)	5643 (4756 to 6659)	5663 (4768 to 6709)
Limpopo	499 (454 to 549)	695 (636 to 763)	1287 (1182 to 1411)	1954 (1639 to 2320)	2494 (2169 to 2879)	2921 (2640 to 3219)	2876 (2615 to 3164)	2851 (2424 to 3333)	2853 (2396 to 3363)	2848 (2375 to 3365)
Mpumalanga	420 (379 to 462)	559 (508 to 613)	635 (577 to 695)	857 (708 to 1022)	1253 (1078 to 1448)	1640 (1470 to 1816)	2004 (1810 to 2213)	2417 (2018 to 2828)	2459 (2055 to 2894)	2496 (2070 to 2962)
North West	362 (325 to 400)	501 (453 to 550)	774 (700 to 851)	1122 (918 to 1347)	1521 (1297 to 1758)	1869 (1664 to 2066)	1732 (1549 to 1914)	1593 (1327 to 1879)	1582 (1291 to 1879)	1570 (1289 to 1866)
Northern Cape	213 (191 to 234)	271 (244 to 297)	326 (294 to 357)	390 (318 to 464)	438 (374 to 507)	428 (380 to 475)	551 (491 to 610)	738 (610 to 867)	756 (618 to 894)	772 (629 to 917)
Western Cape	619 (558 to 682)	796 (719 to 873)	1066 (965 to 1171)	1379 (1124 to 1648)	1772 (1502 to 2048)	2330 (2074 to 2578)	2882 (2576 to 3182)	3272 (2714 to 3838)	3303 (2712 to 3904)	3328 (2722 to 3944)
Zimbabwe	1363 (973 to 1730)	1885 (1512 to 2282)	2631 (2272 to 2986)	3773 (3477 to 4074)	5274 (4848 to 5678)	6165 (5691 to 6628)	6752 (6183 to 7268)	7851 (6965 to 8708)	7976 (7040 to 8947)	8093 (7162 to 9063)
Western sub-Saharan Africa	37736 (31832 to 43818)	46527 (40640 to 52779)	57907 (52431 to 63232)	73973 (69392 to 79049)	97182 (93433 to 100839)	129661 (123019 to 136102)	178308 (170246 to 186074)	236746 (224136 to 248657)	243772 (230465 to 256697)	250821 (236440 to 265302)
Benin	1123 (846 to 1415)	1217 (971 to 1431)	1417 (1251 to 1589)	1812 (1681 to 1947)	2488 (2289 to 2694)	3445 (3162 to 3726)	4805 (4379 to 5212)	6443 (5757 to 7113)	6648 (5918 to 7347)	6854 (6014 to 7688)
Burkina Faso	1916 (1342 to 2469)	2368 (1821 to 2938)	3002 (2519 to 3457)	3859 (3467 to 4242)	4919 (4429 to 5415)	6412 (5811 to 7054)	8657 (7848 to 9463)	11122 (10380 to 11903)	11432 (10585 to 12284)	11747 (10811 to 12714)
Cabo Verde	85 (79 to 91)	112 (104 to 119)	147 (137 to 157)	163 (152 to 175)	186 (173 to 199)	232 (217 to 249)	257 (239 to 274)	274 (241 to 307)	276 (242 to 311)	277 (242 to 314)
Cameroon	2313 (1771 to 2827)	2831 (2357 to 3283)	3430 (3065 to 3795)	4108 (3717 to 4485)	5293 (4833 to 5766)	7618 (6875 to 8409)	11091 (9942 to 12172)	15011 (12783 to 17333)	15464 (12962 to 17920)	15912 (13346 to 18604)
Chad	1215 (822 to 1614)	1507 (1091 to 1923)	1853 (1487 to 2257)	2351 (1998 to 2704)	3100 (2833 to 3373)	4237 (3761 to 4713)	5971 (5526 to 6406)	8292 (7244 to 9445)	8602 (7412 to 9694)	8923 (7637 to 10192)
Côte d'Ivoire	1413 (1314 to 1508)	1947 (1690 to 2188)	2819 (2530 to 3097)	4046 (3660 to 4457)	5960 (5457 to 6452)	8289 (7665 to 8922)	10441 (9475 to 11323)	12867 (11590 to 14195)	13170 (11828 to 14515)	13474 (12021 to 15063)
The Gambia	120 (113 to 128)	147 (133 to 158)	225 (206 to 244)	332 (303 to 360)	491 (450 to 534)	681 (625 to 735)	907 (826 to 988)	1158 (1046 to 1277)	1187 (1058 to 1323)	1215 (1071 to 1363)
Ghana	2499 (2344 to 2668)	3427 (3204 to 3650)	4523 (4217 to 4824)	5913 (5392 to 6448)	7541 (6731 to 8337)	9645 (8982 to 10264)	12945 (12076 to 13823)	16734 (14563 to 18671)	17161 (14868 to 19357)	17577 (15249 to 19942)
Guinea	1430 (1334 to 1529)	1669 (1476 to 1865)	1992 (1804 to 2175)	2374 (2172 to 2579)	3071 (2734 to 3405)	4156 (3790 to 4516)	5138 (4659 to 5647)	6557 (5894 to 7235)	6732 (6019 to 7424)	6909 (6154 to 7694)
Guinea-Bissau	269 (250 to 286)	282 (262 to 302)	330 (292 to 372)	417 (386 to 446)	519 (482 to 557)	639 (556 to 722)	807 (744 to 865)	1010 (885 to 1140)	1033 (894 to 1185)	1057 (911 to 1197)
Liberia	408 (349 to 468)	531 (492 to 574)	708 (644 to 769)	974 (879 to 1061)	1228 (1108 to 1361)	1428 (1264 to 1594)	1992 (1831 to 2166)	2565 (2212 to 2903)	2627 (2274 to 2975)	2691 (2270 to 3111)
Mali	1903 (1773 to 2038)	2372 (2084 to 2686)	3027 (2710 to 3354)	3706 (3349 to 4047)	4401 (4021 to 4806)	5576 (5128 to 6038)	8071 (7434 to 8699)	11264 (9829 to 12781)	11687 (10019 to 13323)	12118 (10363 to 13813)
Mauritania	330 (252 to 408)	442 (364 to 516)	586 (519 to 651)	784 (712 to 851)	1036 (952 to 1123)	1321 (1232 to 1411)	1707 (1565 to 1853)	2139 (1898 to 2366)	2187 (1951 to 2422)	2234 (1973 to 2505)
Niger	1367 (1048 to 1672)	1741 (1440 to 2043)	2288 (2021 to 2543)	3024 (2756 to 3281)	4047 (3717 to 4359)	5642 (5214 to 6067)	8260 (7601 to 8906)	11671 (10420 to 12992)	12127 (10612 to 13511)	12599 (11014 to 14105)
Nigeria	18361 (12362 to 24226)	22178 (16240 to 28405)	26851 (21357 to 32017)	34038 (29615 to 38919)	44948 (41516 to 48264)	60443 (54343 to 66758)	84336 (77055 to 91840)	113607 (102136 to 125109)	117051 (104645 to 128816)	120493 (107169 to 134477)
São Tomé and Príncipe	25 (23 to 27)	30 (28 to 33)	38 (35 to 40)	48 (44 to 52)	61 (57 to 66)	73 (67 to 78)	89 (82 to 96)	105 (93 to 117)	106 (94 to 118)	108 (95 to 121)

Senegal	1345 (1038 to 1681)	1772 (1487 to 2065)	2316 (2058 to 2572)	2998 (2733 to 3264)	3910 (3596 to 4220)	5045 (4665 to 5423)	6332 (5790 to 6872)	7607 (6788 to 8471)	7753 (6872 to 8585)	7896 (6974 to 8770)
Sierra Leone	932 (795 to 1064)	1115 (1021 to 1212)	1326 (1203 to 1453)	1652 (1491 to 1821)	2111 (1905 to 2323)	2292 (2077 to 2491)	3238 (2916 to 3566)	4227 (3840 to 4633)	4346 (3926 to 4761)	4465 (4001 to 4939)
Togo	679 (604 to 760)	836 (767 to 901)	1027 (956 to 1096)	1369 (1277 to 1471)	1868 (1654 to 2084)	2484 (2186 to 2799)	3262 (3051 to 3486)	4089 (3584 to 4593)	4180 (3599 to 4703)	4268 (3653 to 4846)

Appendix 2 Table S5c: Population (in thousands) with 95% UIs by location, males, 1950, 1960, 1970, 1980, 1990, 2000, 2010, 2019, 2020, 2021

Location	1950	1960	1970	1980	1990	2000	2010	2019	2020	2021
Global	1261161 (1236901 to 1287513)	1519714 (1482829 to 1557910)	1855824 (1807146 to 1905676)	2244932 (2195910 to 2295574)	2685743 (2633235 to 2743089)	3072189 (3009463 to 3135654)	3492685 (3423799 to 3563243)	3887760 (3784809 to 3997322)	3925918 (3820202 to 4037387)	3959391 (3843846 to 4082398)
Central Europe, eastern Europe, and central Asia	127120 (123322 to 130824)	148480 (143800 to 153222)	169202 (164428 to 174104)	186075 (180625 to 191664)	201055 (194643 to 207125)	199082 (192567 to 205330)	196392 (189951 to 202380)	200097 (189407 to 210239)	200169 (188831 to 210921)	199888 (188281 to 210911)
Central Asia	13194 (12825 to 13553)	16835 (16296 to 17349)	22934 (22248 to 23602)	28336 (27480 to 29176)	33931 (32848 to 34975)	36492 (34628 to 38333)	40392 (37534 to 43159)	46233 (42029 to 50537)	46905 (42456 to 51472)	47529 (42618 to 52441)
Armenia	682 (635 to 730)	903 (837 to 968)	1255 (1175 to 1339)	1547 (1430 to 1657)	1675 (1549 to 1804)	1600 (1480 to 1713)	1495 (1383 to 1608)	1458 (1280 to 1635)	1451 (1273 to 1631)	1443 (1255 to 1626)
Azerbaijan	1468 (1374 to 1565)	1879 (1739 to 2022)	2556 (2380 to 2717)	3060 (2819 to 3295)	3574 (3299 to 3830)	4049 (3766 to 4345)	4611 (4252 to 4947)	5184 (4516 to 5871)	5228 (4540 to 5920)	5261 (4550 to 6009)
Georgia	1692 (1576 to 1810)	1949 (1801 to 2086)	2253 (2094 to 2402)	2425 (2242 to 2605)	2619 (2426 to 2820)	2223 (2040 to 2405)	1890 (1713 to 2068)	1758 (1586 to 1940)	1745 (1576 to 1927)	1729 (1534 to 1920)
Kazakhstan	3598 (3360 to 3840)	4572 (4216 to 4914)	6258 (5801 to 6695)	7173 (6677 to 7710)	7944 (7326 to 8521)	7207 (6671 to 7763)	7809 (7762 to 7851)	8942 (8165 to 9686)	9073 (8235 to 9886)	9192 (8259 to 10104)
Kyrgyzstan	817 (760 to 872)	1043 (965 to 1120)	1446 (1353 to 1544)	1803 (1667 to 1934)	2182 (2024 to 2345)	2475 (2298 to 2655)	2791 (2599 to 2990)	3286 (2865 to 3678)	3339 (2867 to 3827)	3389 (2892 to 3901)
Mongolia	402 (377 to 428)	483 (441 to 525)	639 (593 to 687)	851 (790 to 912)	1078 (1001 to 1159)	1216 (1131 to 1299)	1349 (1260 to 1444)	1592 (1477 to 1707)	1620 (1514 to 1721)	1647 (1523 to 1767)
Tajikistan	806 (753 to 858)	1037 (959 to 1112)	1479 (1378 to 1580)	2009 (1857 to 2150)	2672 (2473 to 2882)	3188 (2981 to 3404)	3933 (3692 to 4189)	4927 (4263 to 5540)	5040 (4316 to 5738)	5148 (4462 to 5875)
Turkmenistan	605 (566 to 644)	782 (722 to 843)	1098 (1025 to 1172)	1438 (1336 to 1545)	1826 (1690 to 1964)	2138 (1861 to 2424)	2353 (2114 to 2596)	2587 (2348 to 2822)	2612 (2355 to 2877)	2635 (2361 to 2913)
Uzbekistan	3124 (2907 to 3327)	4185 (3862 to 4504)	5948 (5553 to 6370)	8031 (7475 to 8570)	10360 (9572 to 11168)	12396 (10671 to 14201)	14161 (11280 to 16960)	16499 (12449 to 20733)	16796 (12292 to 21316)	17086 (12227 to 21776)
Central Europe	42745 (41599 to 43898)	49255 (47775 to 50753)	54299 (52653 to 56000)	59158 (57253 to 61120)	61260 (59325 to 63256)	59582 (57623 to 61532)	57931 (56952 to 58876)	56714 (54533 to 58872)	56489 (54193 to 58755)	56164 (53601 to 58631)
Albania	649 (607 to 696)	869 (811 to 930)	1128 (1045 to 1211)	1413 (1307 to 1518)	1701 (1578 to 1828)	1596 (1485 to 1716)	1447 (1339 to 1557)	1363 (1211 to 1514)	1352 (1186 to 1529)	1341 (1164 to 1518)
Bosnia and Herzegovina	1430 (1332 to 1528)	1682 (1556 to 1813)	1887 (1747 to 2019)	2100 (1949 to 2250)	2248 (2070 to 2428)	1944 (1705 to 2197)	1869 (1700 to 2035)	1660 (1483 to 1835)	1639 (1457 to 1821)	1614 (1418 to 1806)
Bulgaria	3562 (3313 to 3815)	3948 (3580 to 4331)	4240 (3829 to 4693)	4455 (3994 to 4870)	4280 (3929 to 4628)	3872 (3608 to 4180)	3610 (3587 to 3633)	3366 (3087 to 3667)	3332 (3037 to 3629)	3287 (2942 to 3598)
Croatia	1865 (1732 to 2003)	2010 (1856 to 2163)	2188 (2009 to 2353)	2345 (2172 to 2511)	2364 (2185 to 2549)	2205 (2048 to 2364)	2116 (1967 to 2267)	2062 (1819 to 2313)	2053 (1796 to 2292)	2042 (1783 to 2304)
Czechia	4309 (3986 to 4604)	4595 (4246 to 4933)	4741 (4434 to 5071)	4977 (4619 to 5334)	4993 (4594 to 5368)	4978 (4942 to 5015)	5134 (5096 to 5174)	5240 (4814 to 5661)	5241 (4797 to 5685)	5233 (4759 to 5715)
Hungary	4513 (4210 to 4812)	4817 (4496 to 5151)	4988 (4617 to 5342)	5142 (4820 to 5503)	4995 (4634 to 5348)	4856 (4493 to 5216)	4728 (4368 to 5073)	4615 (4062 to 5147)	4599 (4003 to 5169)	4575 (4017 to 5181)
Montenegro	197 (183 to 210)	233 (217 to 249)	264 (244 to 282)	294 (273 to 314)	312 (290 to 336)	315 (287 to 344)	312 (288 to 335)	308 (271 to 345)	307 (267 to 345)	305 (267 to 346)
North Macedonia	669 (624 to 717)	726 (670 to 780)	845 (785 to 907)	987 (911 to 1058)	1002 (915 to 1096)	1035 (954 to 1119)	1082 (950 to 1209)	1109 (920 to 1302)	1108 (906 to 1322)	1104 (912 to 1313)
Poland	11460 (10654 to 12245)	14079 (13139 to 15014)	15790 (14715 to 16912)	17415 (16053 to 18870)	18589 (17113 to 20016)	18576 (17043 to 20036)	18563 (18436 to 18693)	18613 (17133 to 20032)	18576 (17046 to 20210)	18507 (16723 to 20275)
Romania	8025 (7452 to 8555)	9235 (8384 to 10111)	10228 (9256 to 11233)	11199 (10255 to 12181)	11518 (10619 to 12432)	10959 (10069 to 11881)	10086 (9340 to 10881)	9374 (8299 to 10497)	9299 (8108 to 10428)	9207 (8031 to 10459)
Serbia	3339 (3121 to 3574)	3818 (3560 to 4094)	4312 (3973 to 4653)	4674 (4329 to 4996)	4741 (4354 to 5104)	4756 (4370 to 5152)	4465 (4137 to 4789)	4491 (4020 to 4996)	4476 (3924 to 5058)	4451 (3868 to 5006)
Slovakia	1659 (1651 to 1667)	2000 (1991 to 2010)	2241 (2230 to 2252)	2449 (2436 to 2461)	2580 (2566 to 2594)	2618 (2602 to 2634)	2630 (2611 to 2650)	2654 (2426 to 2891)	2655 (2411 to 2912)	2651 (2390 to 2909)
Slovenia	693 (644 to 739)	755 (695 to 812)	829 (771 to 896)	911 (842 to 988)	958 (855 to 1060)	972 (965 to 979)	1007 (999 to 1015)	1029 (950 to 1113)	1029 (945 to 1120)	1028 (937 to 1118)
Eastern Europe	71182 (67596 to 74847)	82390 (78035 to 87063)	91969 (87450 to 96697)	98580 (93330 to 104100)	105864 (99692 to 111762)	103008 (96973 to 109101)	98068 (92443 to 103465)	97150 (87670 to 106483)	96775 (86650 to 106697)	96195 (85984 to 106043)
Belarus	3208 (2992 to 3416)	3761 (3478 to 4043)	4269 (3999 to 4541)	4579 (4244 to 4907)	4901 (4526 to 5272)	4801 (4445 to 5159)	4497 (4144 to 4847)	4401 (3865 to 4946)	4377 (3783 to 4955)	4341 (3734 to 4945)
Estonia	437 (435 to 439)	531 (529 to 534)	619 (616 to 622)	682 (678 to 685)	733 (729 to 738)	648 (644 to 652)	620 (615 to 625)	619 (568 to 667)	619 (570 to 671)	619 (561 to 676)
Latvia	806 (751 to 861)	935 (864 to 1001)	1080 (1005 to 1160)	1161 (1077 to 1244)	1236 (1145 to 1328)	1098 (1016 to 1171)	968 (961 to 975)	879 (808 to 951)	871 (796 to 947)	861 (782 to 944)
Lithuania	1086 (1010 to 1164)	1266 (1178 to 1360)	1468 (1370 to 1565)	1613 (1497 to 1732)	1737 (1610 to 1868)	1647 (1525 to 1766)	1428 (1312 to 1530)	1287 (1186 to 1394)	1273 (1164 to 1389)	1257 (1141 to 1385)
Moldova	1141 (1062 to 1218)	1410 (1303 to 1511)	1710 (1598 to 1824)	1941 (1806 to 2083)	2122 (1969 to 2278)	2008 (1821 to 2201)	1840 (1640 to 2039)	1740 (1460 to 2041)	1727 (1438 to 2001)	1711 (1415 to 1995)
Russia	48070 (44872 to 51426)	55034 (51095 to 59215)	60891 (56927 to 65229)	65248 (60517 to 70359)	70730 (65112 to 76141)	69767 (64238 to 75414)	67409 (62823 to 71984)	67902 (59633 to 76443)	67752 (58978 to 76735)	67449 (58202 to 76169)
Ukraine	16434 (15257 to 17551)	19453 (18071 to 20851)	21932 (20521 to 23291)	23355 (21499 to 25016)	24406 (22575 to 26143)	23039 (21350 to 24717)	21306 (18733 to 23928)	20322 (16493 to 24118)	20155 (16483 to 23816)	19957 (16044 to 23810)
High income	304621 (296583 to 312226)	343221 (335203 to 351074)	382067 (373026 to 390689)	414978 (404924 to 424440)	444850 (434965 to 454798)	474747 (463051 to 485605)	508952 (496376 to 520842)	533936 (519286 to 548979)	535513 (521496 to 549177)	536569 (521958 to 550919)
Australasia	5384 (5087 to 5681)	6560 (6154 to 6953)	7898 (7425 to 8366)	8931 (8396 to 9501)	10071 (9439 to 10710)	11238 (10527 to 11928)	12832 (12034 to 13605)	14807 (13843 to 15829)	15076 (14141 to 16010)	15326 (14436 to 16211)
Australia	4402 (4102 to 4693)	5335 (4923 to 5727)	6448 (5980 to 6914)	7315 (6785 to 7891)	8382 (7758 to 8996)	9340 (8629 to 10038)	10677 (9889 to 11418)	12328 (11369 to 13359)	12537 (11607 to 13452)	12744 (11859 to 13612)
New Zealand	982 (913 to 1048)	1225 (1134 to 1315)	1450 (1346 to 1552)	1617 (1499 to 1727)	1688 (1561 to 1815)	1898 (1757 to 2040)	2155 (1957 to 2367)	2479 (2288 to 2663)	2539 (2347 to 2744)	2582 (2356 to 2800)
High-income Asia Pacific	52872 (49895 to 55599)	61066 (57781 to 64375)	70116 (66283 to 73884)	79523 (75374 to 83706)	85751 (81237 to 90160)	88920 (84387 to 93538)	90805 (85946 to 95495)	91743 (86733 to 97088)	91514 (87008 to 96162)	91250 (86238 to 96494)
Brunei	34 (32 to 36)	45 (42 to 49)	72 (67 to 77)	103 (95 to 110)	137 (127 to 147)	169 (156 to 182)	204 (188 to 219)	232 (203 to 261)	235 (206 to 266)	238 (208 to 269)
Japan	42040 (39125 to 44697)	47146 (44070 to 50253)	52595 (48751 to 56279)	58866 (54840 to 62891)	61860 (57545 to 65959)	63188 (58835 to 67476)	63387 (58689 to 67764)	62569 (57862 to 67306)	62405 (58125 to 66353)	62215 (57486 to 66784)
Aichi	1685 (1526 to 1837)	2106 (1914 to 2299)	2755 (2498 to 3007)	3181 (2881 to 3466)	3419 (3102 to 3723)	3573 (3236 to 3898)	3761 (3390 to 4108)	3820 (3430 to 4213)	3819 (3450 to 4165)	3816 (3421 to 4203)
Akita	661 (598 to 724)	658 (594 to 722)	609 (552 to 670)	618 (561 to 677)	599 (541 to 656)	577 (522 to 633)	523 (472 to 574)	468 (418 to 519)	462 (418 to 508)	456 (408 to 505)
Aomori	651 (591 to 717)	709 (642 to 781)	704 (638 to 775)	754 (686 to 830)	722 (656 to 796)	718 (652 to 791)	662 (599 to 730)	601 (540 to 670)	595 (539 to 655)	589 (527 to 654)
Chiba	1058 (953 to 1160)	1155 (1043 to 1264)	1734 (1566 to 1895)	2434 (2193 to 2661)	2853 (2571 to 3116)	3028 (2721 to 3308)	3127 (2810 to 3425)	3170 (2825 to 3488)	3171 (2854 to 3460)	3171 (2819 to 3508)
Ehime	759 (691 to 833)	736 (672 to 810)	689 (627 to 758)	736 (670 to 809)	734 (668 to 805)	719 (655 to 790)	688 (624 to 755)	651 (587 to 723)	646 (588 to 711)	641 (579 to 713)
Fukui	373 (338 to 410)	368 (332 to 405)	366 (332 to 404)	394 (356 to 433)	410 (371 to 452)	411 (370 to 453)	396 (358 to 435)	384 (344 to 426)	382 (346 to 420)	379 (340 to 423)
Fukuoka	1783 (1605 to 1951)	1987 (1791 to 2174)	1982 (1783 to 2170)	2248 (2027 to 2467)	2348 (2104 to 2580)	2430 (2187 to 2669)	2437 (2184 to 2678)	2470 (2192 to 2733)	2471 (2215 to 2706)	2471 (2202 to 2737)
Fukushima	1032 (938 to 1132)	1007 (911 to 1110)	962 (872 to 1059)	1015 (919 to 1122)	1048 (950 to 1155)	1061 (961 to 1171)	1005 (906 to 1106)	931 (836 to 1041)	922 (833 to 1020)	912 (816 to 1021)
Gifu	777 (703 to 854)	812 (737 to 892)	871 (787 to 954)	977 (885 to 1074)	1027 (925 to 1126)	1043 (947 to 1148)	1028 (928 to 1130)	984 (878 to 1093)	978 (881 to 1073)	972 (870 to 1081)
Gunma	797 (718 to 875)	777 (701 to 856)	828 (749 to 912)	930 (843 to 1022)	994 (899 to 1091)	1017 (922 to 1120)	1008 (913 to 1106)	979 (881 to 1085)	976 (882 to 1073)	972 (870 to 1079)
Hiroshima	1038 (938 to 1135)	1080 (978 to 1183)	1217 (1102 to 1335)	1367 (1239 to 1499)	1413 (1277 to 1543)	1418 (1281 to 1551)	1399 (1263 to 1532)	1388 (1247 to 1533)	1381 (1250 to 1511)	1375 (1231 to 1522)
Hokkaidō	2217 (2020 to 2423)	2594 (2365 to 2838)	2619 (2382 to 2857)	2799 (2552 to 3063)	2783 (2536 to 3043)	2761 (2511 to 3026)	2670 (2432 to 2928)	2528 (2281 to 2795)	2511 (2287 to 2748)	2493 (2253 to 2756)
Hyōgo	1661 (1506 to 1816)	1957 (1773 to 2148)	2353 (2130 to 2574)	2565 (2328 to 2813)	2664 (2404 to 2923)	2722 (2467 to 2984)	2724 (2457 to 2995)	2655 (2382 to 2939)	2644 (2391 to 2903)	2632 (2362 to 2916)
Ibaraki	1016 (925 to 1109)	1021 (929 to 1116)	1081 (983 to 1181)	1302 (1188 to 1423)	1450 (1315 to 1590)	1518 (1382 to 1658)	1509 (1370 to 1651)	1461 (1318 to 1613)	1455 (1325 to 1589)	1449 (1305 to 1598)
Ishikawa	471 (428 to 516)	474 (430 to 521)	493 (447 to 541)	556 (503 to 612)	575 (523 to 631)	581 (528 to 639)	575 (518 to 633)	563 (503 to 624)	561 (507 to 616)	558 (500 to 619)
Iwate	681 (616 to 744)	717 (649 to 785)	676 (609 to 744)	706 (638 to 776)	697 (630 to 765)	696 (627 to 762)	650 (584 to 714)	603 (539 to 669)	594 (536 to 653)	587 (525 to 651)
Kagawa	468 (420 to 512)	448 (403 to 491)	442 (395 to 486)	492 (441 to 541)	503 (452 to 551)	502 (451 to 552)	483 (435 to 531)	473 (420 to 524)	469 (418 to 515)	465 (413 to 516)
Kagoshima	897 (816 to 984)	956 (870 to 1049)	827 (754 to 910)	860 (784 to 948)	863 (786 to 949)	856 (782 to 943)	816 (739 to 901)	769 (694 to 855)	763 (695 to 839)	757 (681 to 843)
Kanagawa	1274 (1152 to 1393)	1782 (1616 to 1947)	2883 (2620 to 3150)	3607 (3277 to 3945)	4165 (3779 to 4551)	4376 (3974 to 4785)	4622 (4172 to 5048)	4660 (4167 to 5141)	4662 (4238 to 5090)	4662 (4185 to 5148)
Kōchi	436 (395 to 478)	420 (381 to 462)	382 (347 to 421)	406 (368 to 446)	397 (360 to 435)	391 (355 to 432)	366 (330 to 403)	338 (304 to 375)	334 (302 to 368)	330 (296 to 368)
Kumamoto	905 (820 to 991)	905 (821 to 993)	820 (743 to 900)	872 (792 to 953)	890 (807 to 976)	897 (813 to 984)	872 (787 to 956)	845 (757 to 936)	840 (761 to 921)	835 (747 to 928)

Kyōto	911 (824 to 1002)	993 (900 to 1093)	1129 (1022 to 1239)	1266 (1146 to 1395)	1290 (1166 to 1421)	1295 (1169 to 1426)	1275 (1154 to 1407)	1258 (1126 to 1401)	1253 (1133 to 1378)	1247 (1115 to 1392)
Mie	722 (651 to 795)	732 (659 to 806)	762 (686 to 839)	838 (755 to 927)	890 (801 to 978)	920 (826 to 1017)	920 (823 to 1015)	885 (789 to 989)	881 (793 to 977)	876 (782 to 974)
Miyagi	846 (764 to 926)	865 (779 to 946)	911 (819 to 998)	1049 (945 to 1148)	1126 (1017 to 1231)	1182 (1060 to 1293)	1159 (1045 to 1267)	1150 (1027 to 1274)	1144 (1030 to 1254)	1138 (1013 to 1259)
Miyazaki	548 (497 to 598)	563 (510 to 617)	512 (463 to 560)	564 (511 to 618)	565 (512 to 619)	564 (512 to 618)	546 (493 to 597)	517 (463 to 572)	514 (465 to 563)	510 (455 to 565)
Nagano	1023 (929 to 1124)	974 (882 to 1073)	960 (870 to 1058)	1032 (934 to 1138)	1073 (970 to 1183)	1102 (997 to 1212)	1071 (970 to 1180)	1025 (920 to 1142)	1019 (925 to 1122)	1014 (906 to 1123)
Nagasaki	833 (755 to 912)	875 (793 to 959)	768 (694 to 842)	777 (704 to 852)	755 (683 to 826)	728 (659 to 799)	682 (615 to 749)	636 (569 to 705)	630 (568 to 693)	623 (556 to 693)
Nara	377 (339 to 411)	390 (352 to 427)	461 (416 to 504)	600 (543 to 656)	678 (612 to 740)	704 (635 to 773)	676 (606 to 741)	640 (570 to 709)	635 (575 to 697)	630 (565 to 699)
Niigata	1221 (1101 to 1339)	1204 (1087 to 1319)	1170 (1054 to 1284)	1222 (1102 to 1338)	1229 (1108 to 1345)	1226 (1109 to 1344)	1174 (1055 to 1290)	1100 (981 to 1216)	1089 (983 to 1197)	1078 (958 to 1194)
Ōita	619 (557 to 679)	603 (542 to 663)	555 (500 to 611)	597 (537 to 655)	598 (538 to 658)	587 (527 to 646)	576 (515 to 634)	548 (487 to 610)	543 (488 to 597)	538 (478 to 599)
Okayama	823 (750 to 901)	814 (739 to 895)	839 (763 to 921)	927 (842 to 1019)	948 (865 to 1041)	955 (868 to 1047)	948 (860 to 1043)	929 (836 to 1033)	924 (840 to 1013)	920 (828 to 1020)
Okinawa	305 (274 to 336)	369 (317 to 423)	464 (418 to 511)	557 (502 to 613)	609 (549 to 671)	657 (592 to 722)	699 (627 to 769)	734 (656 to 815)	737 (665 to 811)	739 (659 to 823)
Ōsaka	1943 (1755 to 2138)	2815 (2547 to 3098)	3906 (3542 to 4305)	4289 (3890 to 4707)	4375 (3974 to 4804)	4374 (3964 to 4806)	4344 (3942 to 4769)	4313 (3873 to 4786)	4306 (3907 to 4721)	4296 (3854 to 4761)
Saga	467 (424 to 511)	457 (415 to 501)	404 (365 to 443)	421 (381 to 461)	425 (385 to 466)	424 (383 to 464)	410 (371 to 449)	395 (355 to 435)	393 (356 to 430)	390 (350 to 433)
Saitama	1072 (974 to 1173)	1230 (1119 to 1348)	1998 (1819 to 2186)	2800 (2556 to 3056)	3306 (3010 to 3612)	3560 (3240 to 3900)	3686 (3344 to 4032)	3711 (3353 to 4099)	3712 (3375 to 4065)	3711 (3345 to 4106)
Shiga	422 (382 to 463)	412 (374 to 452)	422 (383 to 465)	541 (490 to 596)	613 (557 to 675)	676 (613 to 744)	705 (639 to 778)	710 (640 to 789)	709 (646 to 778)	708 (637 to 787)
Shimane	455 (408 to 499)	442 (398 to 486)	378 (339 to 416)	387 (347 to 427)	383 (344 to 422)	372 (334 to 409)	351 (315 to 386)	335 (297 to 372)	332 (298 to 367)	329 (293 to 366)
Shizuoka	1237 (1119 to 1353)	1382 (1251 to 1517)	1555 (1400 to 1705)	1736 (1563 to 1906)	1850 (1666 to 2029)	1895 (1707 to 2082)	1889 (1696 to 2077)	1831 (1638 to 2033)	1823 (1640 to 2003)	1815 (1618 to 2017)
Tochigi	768 (697 to 842)	745 (676 to 815)	789 (716 to 865)	906 (823 to 995)	984 (891 to 1081)	1016 (921 to 1114)	1014 (917 to 1115)	988 (886 to 1094)	982 (890 to 1079)	977 (876 to 1083)
Tokushima	437 (396 to 478)	417 (379 to 457)	387 (351 to 423)	406 (368 to 445)	405 (369 to 443)	400 (363 to 438)	379 (344 to 415)	353 (317 to 391)	350 (316 to 383)	346 (310 to 383)
Tōkyō	3238 (2939 to 3522)	5070 (4605 to 5542)	5919 (5400 to 6452)	5954 (5420 to 6496)	6016 (5456 to 6567)	6091 (5536 to 6654)	6556 (5936 to 7176)	6945 (6257 to 7666)	6988 (6362 to 7623)	7027 (6311 to 7751)
Tottori	297 (268 to 328)	293 (265 to 323)	277 (250 to 305)	297 (269 to 329)	302 (273 to 334)	299 (271 to 332)	286 (258 to 317)	272 (244 to 304)	270 (244 to 298)	268 (239 to 300)
Toyama	498 (451 to 545)	510 (461 to 559)	505 (456 to 553)	546 (492 to 598)	551 (498 to 603)	551 (498 to 604)	538 (485 to 589)	514 (460 to 569)	511 (462 to 562)	508 (454 to 563)
Wakayama	486 (440 to 535)	495 (447 to 545)	516 (467 to 568)	536 (486 to 590)	523 (473 to 576)	518 (467 to 571)	479 (433 to 527)	446 (399 to 496)	442 (400 to 486)	438 (391 to 489)
Yamagata	676 (613 to 741)	645 (584 to 709)	603 (544 to 664)	621 (559 to 685)	622 (558 to 687)	615 (555 to 677)	575 (518 to 634)	532 (475 to 592)	527 (476 to 581)	521 (465 to 579)
Yamaguchi	777 (700 to 849)	795 (716 to 872)	737 (666 to 809)	777 (700 to 851)	762 (686 to 836)	738 (665 to 810)	700 (629 to 769)	655 (583 to 728)	648 (583 to 712)	642 (571 to 713)
Yamanashi	402 (366 to 440)	387 (352 to 424)	376 (340 to 413)	401 (365 to 439)	428 (389 to 468)	446 (405 to 489)	431 (390 to 472)	408 (368 to 452)	405 (368 to 444)	402 (362 to 446)
South Korea	10186 (9486 to 10851)	13035 (12129 to 13991)	16387 (15239 to 17538)	19322 (17967 to 20566)	22220 (20636 to 23640)	23495 (21828 to 25056)	24573 (22854 to 26330)	26044 (24108 to 28001)	25969 (24225 to 27773)	25883 (23979 to 27668)
Singapore	612 (569 to 653)	839 (764 to 910)	1062 (989 to 1131)	1232 (1155 to 1312)	1534 (1436 to 1638)	2067 (1921 to 2206)	2642 (2459 to 2824)	2897 (2675 to 3097)	2904 (2674 to 3134)	2914 (2677 to 3152)
High-income North America	84322 (79252 to 89391)	100125 (93899 to 105965)	113061 (106180 to 119972)	124000 (115981 to 131677)	137564 (129099 to 146083)	153012 (143341 to 162473)	168804 (158584 to 179229)	180477 (168064 to 192877)	181336 (169880 to 192585)	181982 (170134 to 193620)
Canada	7099 (6607 to 7579)	9266 (8579 to 9944)	10894 (10107 to 11687)	12154 (11304 to 13021)	13446 (12430 to 14512)	14877 (13813 to 15907)	16463 (15189 to 17617)	18135 (16664 to 19556)	18315 (16963 to 19674)	18485 (17292 to 19816)
Greenland	11 (11 to 12)	17 (16 to 18)	24 (23 to 26)	27 (27 to 27)	30 (30 to 30)	30 (30 to 30)	30 (30 to 30)	29 (27 to 32)	29 (27 to 32)	29 (27 to 32)
USA	77209 (72136 to 82364)	90840 (84443 to 96698)	102140 (95143 to 108938)	111817 (103681 to 119604)	124084 (115413 to 132662)	138102 (128436 to 147680)	152308 (141820 to 162797)	162310 (149820 to 174599)	162988 (151341 to 174339)	163465 (151542 to 175334)
Alabama	1536 (1390 to 1681)	1637 (1482 to 1791)	1718 (1547 to 1888)	1895 (1708 to 2081)	1981 (1792 to 2171)	2147 (1937 to 2351)	2329 (2108 to 2548)	2438 (2174 to 2700)	2444 (2204 to 2676)	2446 (2190 to 2701)
Alaska	80 (73 to 88)	133 (121 to 145)	171 (156 to 188)	217 (197 to 238)	297 (269 to 325)	326 (296 to 358)	369 (335 to 404)	384 (344 to 424)	384 (347 to 421)	383 (344 to 422)
Arizona	390 (353 to 428)	674 (611 to 741)	913 (827 to 1004)	1362 (1228 to 1501)	1858 (1681 to 2048)	2571 (2329 to 2826)	3176 (2881 to 3498)	3515 (3140 to 3919)	3538 (3201 to 3887)	3554 (3190 to 3945)
Arkansas	969 (878 to 1062)	904 (818 to 989)	965 (873 to 1056)	1118 (1010 to 1222)	1160 (1051 to 1268)	1308 (1187 to 1431)	1431 (1290 to 1567)	1480 (1329 to 1630)	1482 (1343 to 1620)	1481 (1330 to 1628)
California	5458 (4977 to 5974)	8062 (7343 to 8833)	10136 (9251 to 11093)	11901 (10819 to 13046)	15269 (13950 to 16714)	17011 (15508 to 18653)	18619 (17015 to 20389)	19563 (17707 to 21602)	19608 (17876 to 21497)	19628 (17770 to 21650)
Colorado	683 (619 to 748)	895 (810 to 980)	1133 (1028 to 1240)	1461 (1324 to 1599)	1673 (1525 to 1830)	2181 (1978 to 2391)	2528 (2300 to 2762)	2847 (2563 to 3140)	2879 (2608 to 3160)	2909 (2608 to 3207)
Connecticut	1018 (925 to 1118)	1279 (1157 to 1403)	1513 (1370 to 1658)	1524 (1379 to 1670)	1623 (1477 to 1775)	1645 (1489 to 1806)	1737 (1576 to 1906)	1763 (1579 to 1957)	1761 (1593 to 1939)	1759 (1578 to 1947)
Delaware	162 (147 to 177)	227 (206 to 249)	276 (252 to 302)	291 (264 to 318)	331 (301 to 362)	381 (347 to 418)	439 (399 to 480)	477 (430 to 527)	480 (436 to 525)	481 (434 to 531)
Washington, DC	387 (350 to 427)	369 (334 to 406)	361 (327 to 397)	299 (270 to 329)	287 (260 to 316)	274 (248 to 303)	293 (266 to 323)	324 (291 to 360)	327 (296 to 359)	329 (295 to 365)
Florida	1406 (1264 to 1545)	2505 (2256 to 2740)	3414 (3073 to 3745)	4781 (4295 to 5250)	6431 (5792 to 7054)	7798 (7006 to 8540)	9268 (8368 to 10154)	10422 (9271 to 11530)	10519 (9476 to 11523)	10599 (9455 to 11674)
Georgia	1729 (1566 to 1903)	1984 (1794 to 2179)	2305 (2084 to 2543)	2683 (2418 to 2963)	3226 (2918 to 3549)	4067 (3674 to 4469)	4793 (4332 to 5266)	5166 (4633 to 5744)	5189 (4694 to 5712)	5205 (4667 to 5768)
Hawaii	277 (250 to 303)	349 (315 to 381)	415 (374 to 455)	499 (449 to 546)	577 (521 to 633)	613 (552 to 672)	681 (614 to 749)	725 (648 to 803)	727 (655 to 798)	729 (651 to 807)
Idaho	310 (281 to 340)	348 (314 to 380)	369 (332 to 405)	479 (431 to 525)	514 (464 to 563)	650 (586 to 712)	788 (712 to 864)	912 (815 to 1010)	924 (833 to 1013)	935 (835 to 1033)
Illinois	4447 (4036 to 4887)	5091 (4611 to 5589)	5551 (5018 to 6112)	5615 (5076 to 6177)	5680 (5141 to 6253)	6015 (5425 to 6633)	6274 (5672 to 6925)	6288 (5617 to 7014)	6273 (5663 to 6926)	6251 (5624 to 6923)
Indiana	2015 (1820 to 2225)	2363 (2129 to 2610)	2610 (2356 to 2883)	2700 (2432 to 2986)	2750 (2484 to 3041)	2945 (2654 to 3261)	3177 (2870 to 3498)	3331 (2977 to 3717)	3339 (3016 to 3696)	3344 (3003 to 3721)
Iowa	1344 (1225 to 1472)	1397 (1265 to 1538)	1416 (1284 to 1562)	1434 (1294 to 1585)	1374 (1248 to 1516)	1419 (1284 to 1567)	1509 (1369 to 1664)	1582 (1424 to 1758)	1587 (1441 to 1747)	1590 (1426 to 1774)
Kansas	982 (890 to 1077)	1111 (1005 to 1218)	1136 (1028 to 1244)	1175 (1059 to 1288)	1240 (1124 to 1357)	1317 (1183 to 1443)	1411 (1273 to 1542)	1458 (1302 to 1612)	1459 (1317 to 1595)	1458 (1300 to 1609)
Kentucky	1506 (1367 to 1651)	1552 (1406 to 1701)	1636 (1480 to 1794)	1809 (1635 to 1983)	1827 (1656 to 2002)	1974 (1790 to 2164)	2139 (1940 to 2345)	2221 (1992 to 2458)	2224 (2014 to 2435)	2223 (1995 to 2453)
Louisiana	1354 (1225 to 1489)	1637 (1480 to 1802)	1835 (1657 to 2018)	2069 (1859 to 2277)	2077 (1878 to 2286)	2166 (1953 to 2384)	2221 (2012 to 2438)	2289 (2043 to 2548)	2291 (2068 to 2520)	2291 (2053 to 2533)
Maine	467 (423 to 515)	493 (444 to 543)	501 (453 to 552)	555 (498 to 611)	610 (554 to 670)	616 (557 to 678)	654 (591 to 721)	670 (601 to 743)	670 (605 to 738)	669 (600 to 741)
Maryland	1199 (1087 to 1324)	1577 (1430 to 1742)	1978 (1791 to 2186)	2077 (1875 to 2296)	2370 (2151 to 2614)	2581 (2340 to 2846)	2830 (2570 to 3117)	2988 (2683 to 3331)	2997 (2710 to 3315)	3003 (2691 to 3331)
Massachusetts	2336 (2118 to 2548)	2558 (2331 to 2794)	2795 (2538 to 3060)	2777 (2520 to 3048)	2945 (2676 to 3221)	3041 (2751 to 3338)	3168 (2881 to 3468)	3385 (3036 to 3741)	3406 (3088 to 3735)	3426 (3091 to 3776)
Michigan	3302 (3008 to 3613)	3988 (3625 to 4349)	4483 (4070 to 4915)	4572 (4146 to 5006)	4613 (4200 to 5047)	4838 (4394 to 5303)	4826 (4381 to 5294)	4923 (4434 to 5449)	4930 (4494 to 5409)	4932 (4453 to 5439)
Minnesota	1540 (1395 to 1689)	1739 (1571 to 1906)	1921 (1733 to 2112)	2030 (1827 to 2231)	2194 (1987 to 2406)	2406 (2171 to 2640)	2626 (2368 to 2882)	2820 (2518 to 3123)	2836 (2559 to 3115)	2850 (2554 to 3148)
Mississippi	1101 (996 to 1212)	1098 (993 to 1209)	1111 (1002 to 1224)	1229 (1108 to 1354)	1261 (1141 to 1384)	1372 (1241 to 1512)	1449 (1310 to 1595)	1443 (1288 to 1607)	1438 (1297 to 1586)	1431 (1283 to 1586)
Missouri	1994 (1818 to 2193)	2168 (1975 to 2378)	2325 (2119 to 2559)	2400 (2180 to 2646)	2519 (2298 to 2769)	2698 (2446 to 2984)	2922 (2661 to 3225)	3025 (2737 to 3351)	3026 (2753 to 3330)	3023 (2717 to 3363)
Montana	317 (287 to 349)	353 (319 to 387)	359 (326 to 394)	398 (360 to 438)	404 (369 to 444)	453 (409 to 497)	495 (449 to 542)	544 (489 to 603)	548 (496 to 602)	551 (496 to 608)
Nebraska	684 (618 to 754)	719 (650 to 795)	749 (677 to 827)	777 (699 to 860)	787 (713 to 869)	840 (758 to 926)	905 (819 to 1003)	971 (869 to 1084)	977 (885 to 1076)	982 (880 to 1092)
Nevada	87 (79 to 96)	152 (137 to 167)	258 (235 to 283)	415 (375 to 457)	634 (575 to 696)	1035 (935 to 1137)	1366 (1237 to 1503)	1545 (1387 to 1716)	1559 (1410 to 1714)	1570 (1409 to 1734)
New Hampshire	270 (244 to 296)	307 (280 to 336)	373 (339 to 410)	457 (414 to 502)	555 (505 to 609)	604 (549 to 663)	654 (594 to 721)	679 (613 to 755)	680 (619 to 747)	681 (615 to 755)
New Jersey	2453 (2227 to 2704)	3056 (2779 to 3351)	3571 (3255 to 3928)	3589 (3257 to 3947)	3824 (3476 to 4195)	4079 (3712 to 4483)	4277 (3887 to 4699)	4510 (4064 to 5011)	4531 (4119 to 4979)	4549 (4102 to 5033)
New Mexico	357 (322 to 392)	493 (447 to 540)	521 (470 to 572)	652 (589 to 714)	764 (691 to 835)	898 (813 to 985)	1020 (922 to 1115)	1051 (942 to 1162)	1049 (952 to 1151)	1046 (941 to 1153)

New York	7443 (6738 to 8138)	8354 (7560 to 9133)	8962 (8122 to 9820)	8461 (7639 to 9257)	8805 (7956 to 9637)	9155 (8273 to 10018)	9339 (8426 to 10219)	9745 (8726 to 10750)	9777 (8829 to 10728)	9797 (8789 to 10805)
North Carolina	2059 (1867 to 2266)	2314 (2102 to 2535)	2574 (2335 to 2825)	2897 (2620 to 3189)	3297 (3000 to 3619)	3968 (3593 to 4364)	4698 (4265 to 5168)	5048 (4536 to 5608)	5066 (4583 to 5576)	5077 (4571 to 5604)
North Dakota	331 (299 to 364)	332 (299 to 365)	321 (289 to 354)	333 (300 to 367)	324 (293 to 356)	316 (285 to 348)	343 (310 to 377)	391 (350 to 436)	396 (358 to 436)	401 (358 to 445)
Ohio	4056 (3671 to 4449)	4895 (4413 to 5347)	5319 (4800 to 5812)	5293 (4751 to 5808)	5335 (4807 to 5846)	5461 (4917 to 5988)	5600 (5072 to 6133)	5764 (5138 to 6373)	5772 (5221 to 6319)	5773 (5162 to 6374)
Oklahoma	1139 (1029 to 1250)	1181 (1067 to 1294)	1291 (1168 to 1415)	1500 (1346 to 1649)	1565 (1415 to 1712)	1698 (1535 to 1864)	1844 (1671 to 2023)	1960 (1753 to 2161)	1968 (1774 to 2159)	1973 (1764 to 2173)
Oregon	791 (714 to 870)	904 (816 to 993)	1060 (956 to 1166)	1316 (1185 to 1444)	1435 (1300 to 1576)	1697 (1526 to 1866)	1903 (1725 to 2093)	2079 (1861 to 2309)	2093 (1893 to 2299)	2106 (1891 to 2332)
Pennsylvania	5315 (4820 to 5823)	5665 (5120 to 6199)	5833 (5270 to 6395)	5765 (5207 to 6309)	5811 (5278 to 6358)	5898 (5331 to 6449)	6211 (5630 to 6797)	6369 (5704 to 7051)	6370 (5776 to 6956)	6364 (5728 to 7023)
Rhode Island	401 (365 to 439)	434 (395 to 475)	480 (435 to 526)	459 (415 to 503)	492 (448 to 540)	500 (454 to 548)	506 (462 to 555)	529 (479 to 584)	532 (484 to 583)	534 (481 to 590)
South Carolina	1061 (960 to 1162)	1211 (1096 to 1330)	1318 (1197 to 1445)	1542 (1394 to 1690)	1729 (1578 to 1889)	1953 (1765 to 2142)	2266 (2055 to 2481)	2485 (2231 to 2753)	2500 (2272 to 2738)	2512 (2260 to 2782)
South Dakota	346 (314 to 379)	354 (321 to 387)	341 (309 to 374)	345 (311 to 379)	350 (317 to 383)	371 (336 to 406)	410 (372 to 448)	444 (399 to 491)	446 (406 to 489)	448 (402 to 493)
Tennessee	1658 (1506 to 1819)	1792 (1621 to 1968)	1965 (1777 to 2154)	2245 (2023 to 2469)	2408 (2184 to 2654)	2770 (2506 to 3044)	3110 (2823 to 3415)	3349 (3007 to 3719)	3365 (3049 to 3698)	3376 (3035 to 3738)
Texas	3968 (3588 to 4343)	4881 (4409 to 5326)	5691 (5142 to 6243)	7129 (6418 to 7814)	8577 (7769 to 9403)	10433 (9458 to 11411)	12663 (11509 to 13847)	14320 (12887 to 15810)	14454 (13129 to 15816)	14566 (13099 to 16030)
Utah	356 (321 to 388)	457 (412 to 498)	543 (491 to 593)	738 (665 to 807)	878 (794 to 958)	1125 (1013 to 1229)	1390 (1256 to 1518)	1624 (1453 to 1789)	1647 (1486 to 1799)	1670 (1493 to 1837)
Vermont	192 (174 to 212)	197 (178 to 217)	224 (203 to 248)	253 (228 to 280)	282 (256 to 310)	296 (267 to 327)	313 (283 to 345)	318 (285 to 353)	318 (287 to 351)	317 (284 to 353)
Virginia	1719 (1556 to 1899)	2039 (1841 to 2247)	2373 (2150 to 2617)	2667 (2408 to 2949)	3106 (2809 to 3425)	3508 (3173 to 3871)	3965 (3582 to 4355)	4224 (3787 to 4719)	4239 (3838 to 4673)	4249 (3820 to 4731)
Washington	1257 (1141 to 1383)	1476 (1337 to 1619)	1749 (1586 to 1922)	2090 (1894 to 2294)	2479 (2247 to 2731)	2942 (2655 to 3241)	3363 (3052 to 3693)	3802 (3418 to 4241)	3845 (3499 to 4232)	3885 (3499 to 4305)
West Virginia	1027 (931 to 1130)	941 (855 to 1031)	873 (789 to 962)	957 (864 to 1052)	879 (798 to 965)	874 (792 to 962)	903 (820 to 994)	892 (798 to 993)	887 (804 to 978)	881 (790 to 978)
Wisconsin	1773 (1601 to 1950)	2018 (1825 to 2213)	2232 (2009 to 2450)	2340 (2102 to 2570)	2445 (2211 to 2685)	2621 (2373 to 2876)	2822 (2550 to 3098)	2929 (2618 to 3255)	2933 (2650 to 3221)	2934 (2624 to 3247)
Wyoming	158 (143 to 175)	174 (157 to 191)	173 (155 to 191)	246 (220 to 271)	232 (209 to 256)	248 (223 to 274)	287 (258 to 315)	297 (265 to 331)	297 (268 to 328)	296 (264 to 330)
Southern Latin America	12903 (12305 to 13543)	15367 (14641 to 16087)	17874 (17004 to 18762)	21119 (20095 to 22181)	24254 (23019 to 25513)	27023 (25626 to 28481)	29888 (28365 to 31502)	32644 (29848 to 35552)	32906 (29821 to 35850)	33066 (30016 to 36232)
Argentina	8874 (8310 to 9511)	10336 (9603 to 11001)	11985 (11163 to 12784)	14173 (13225 to 15118)	16203 (15058 to 17332)	17949 (16677 to 19338)	20038 (18784 to 21396)	21950 (19221 to 24849)	22086 (19053 to 24962)	22181 (19123 to 25284)
Chile	2896 (2698 to 3087)	3740 (3483 to 3988)	4500 (4199 to 4809)	5500 (5044 to 5955)	6523 (6013 to 7050)	7475 (6850 to 8069)	8232 (7310 to 9118)	9055 (8336 to 9762)	9183 (8373 to 10024)	9251 (8413 to 10123)
Uruguay	1131 (1052 to 1208)	1289 (1178 to 1397)	1387 (1250 to 1529)	1445 (1302 to 1586)	1527 (1373 to 1693)	1598 (1449 to 1747)	1617 (1505 to 1734)	1636 (1443 to 1846)	1635 (1440 to 1840)	1632 (1431 to 1852)
Western Europe	149140 (143796 to 154225)	160102 (155850 to 164198)	173118 (168658 to 177193)	181405 (176860 to 185844)	187210 (183490 to 190633)	194554 (190829 to 197936)	206623 (203425 to 209427)	214266 (207695 to 220280)	214681 (207908 to 221069)	214945 (207489 to 221618)
Andorra	3 (3 to 3)	5 (4 to 5)	10 (9 to 11)	18 (17 to 20)	29 (29 to 29)	34 (34 to 34)	44 (43 to 44)	43 (39 to 46)	43 (39 to 47)	44 (40 to 49)
Austria	3205 (2994 to 3414)	3280 (3039 to 3523)	3484 (3231 to 3749)	3561 (3314 to 3829)	3733 (3473 to 4001)	3882 (3606 to 4162)	4077 (4042 to 4108)	4385 (4017 to 4754)	4409 (4027 to 4787)	4432 (3991 to 4827)
Belgium	4212 (3929 to 4480)	4465 (4151 to 4806)	4720 (4404 to 5040)	4803 (4436 to 5185)	4888 (4492 to 5261)	5017 (4651 to 5397)	5329 (5291 to 5369)	5617 (5183 to 6059)	5634 (5159 to 6132)	5648 (5083 to 6186)
Cyprus	240 (223 to 256)	291 (271 to 312)	325 (282 to 367)	333 (305 to 362)	391 (361 to 422)	462 (428 to 494)	558 (515 to 600)	652 (577 to 732)	661 (581 to 742)	670 (579 to 759)
Denmark	2119 (1971 to 2255)	2275 (2123 to 2435)	2447 (2274 to 2612)	2525 (2508 to 2542)	2535 (2516 to 2554)	2635 (2614 to 2657)	2742 (2718 to 2765)	2887 (2652 to 3116)	2902 (2622 to 3160)	2915 (2643 to 3193)
Finland	1924 (1788 to 2062)	2136 (1991 to 2277)	2238 (2227 to 2251)	2321 (2306 to 2336)	2430 (2414 to 2446)	2531 (2513 to 2550)	2641 (2621 to 2661)	2729 (2508 to 2955)	2732 (2499 to 2973)	2734 (2445 to 2992)
France	20949 (19503 to 22369)	22761 (20939 to 24585)	25368 (23415 to 27390)	26900 (24750 to 28964)	28155 (26145 to 29943)	29136 (26984 to 31297)	30941 (28892 to 32996)	32074 (29113 to 35092)	32137 (29099 to 35383)	32185 (28868 to 35619)
Germany	32887 (28342 to 37263)	34116 (31645 to 36562)	36643 (34051 to 38944)	37476 (35084 to 40054)	38601 (38326 to 38894)	40188 (39851 to 40542)	40004 (39659 to 40378)	42216 (38623 to 45848)	42395 (38445 to 46286)	42528 (37983 to 46816)
Greece	3793 (3542 to 4041)	4194 (3878 to 4498)	4372 (4043 to 4691)	4834 (4488 to 5196)	5122 (4740 to 5509)	5504 (5098 to 5916)	5462 (5081 to 5829)	5019 (4404 to 5578)	4983 (4331 to 5563)	4942 (4239 to 5590)
Iceland	70 (70 to 71)	88 (87 to 88)	103 (103 to 104)	115 (114 to 115)	127 (127 to 128)	140 (139 to 141)	161 (159 to 162)	174 (160 to 189)	176 (159 to 191)	178 (161 to 195)
Ireland	1556 (1456 to 1657)	1461 (1352 to 1572)	1525 (1424 to 1629)	1756 (1619 to 1891)	1795 (1662 to 1924)	1922 (1769 to 2072)	2286 (2105 to 2474)	2426 (2215 to 2646)	2435 (2205 to 2670)	2444 (2186 to 2696)
Israel	798 (744 to 852)	1103 (1017 to 1183)	1531 (1412 to 1655)	1939 (1782 to 2108)	2455 (2213 to 2699)	3159 (2848 to 3497)	3890 (3567 to 4215)	4621 (4052 to 5237)	4700 (4035 to 5341)	4778 (4084 to 5502)
Italy	22722 (21154 to 24185)	24939 (22925 to 26857)	26357 (24382 to 28285)	27452 (25387 to 29389)	27567 (25631 to 29454)	27418 (25365 to 29358)	29280 (29052 to 29492)	29333 (26922 to 31605)	29240 (26603 to 31719)	29125 (26468 to 31702)
Luxembourg	150 (140 to 161)	156 (145 to 166)	167 (156 to 178)	176 (163 to 190)	187 (172 to 200)	214 (198 to 230)	250 (248 to 252)	311 (286 to 335)	318 (289 to 345)	324 (297 to 354)
Malta	160 (149 to 171)	158 (145 to 172)	155 (141 to 170)	166 (150 to 183)	183 (164 to 202)	199 (180 to 219)	211 (195 to 227)	220 (195 to 245)	221 (193 to 246)	221 (192 to 250)
Monaco	9 (9 to 10)	10 (9 to 10)	11 (10 to 12)	12 (11 to 13)	14 (14 to 15)	16 (15 to 17)	17 (16 to 19)	18 (17 to 20)	18 (17 to 20)	18 (17 to 20)
Netherlands	4999 (4971 to 5025)	5689 (5657 to 5720)	6475 (6439 to 6514)	6994 (6943 to 7041)	7374 (7321 to 7429)	7858 (7794 to 7923)	8208 (8134 to 8283)	8503 (7769 to 9225)	8525 (7803 to 9279)	8544 (7739 to 9380)
Norway	1626 (1522 to 1743)	1790 (1672 to 1913)	1932 (1800 to 2066)	2026 (1900 to 2168)	2099 (1960 to 2243)	2216 (2198 to 2235)	2428 (2408 to 2451)	2697 (2489 to 2902)	2717 (2487 to 2970)	2737 (2492 to 3012)
Agder	86 (78 to 95)	93 (85 to 102)	102 (93 to 113)	113 (102 to 124)	120 (111 to 128)	128 (126 to 129)	139 (138 to 141)	154 (137 to 172)	155 (136 to 175)	156 (136 to 177)
Innlandet	170 (155 to 187)	175 (159 to 192)	178 (162 to 196)	184 (167 to 203)	184 (172 to 197)	183 (181 to 185)	187 (184 to 189)	194 (173 to 217)	194 (171 to 220)	195 (170 to 221)
Møre og Romsdal	95 (87 to 104)	107 (97 to 117)	113 (102 to 124)	118 (108 to 129)	119 (111 to 128)	122 (121 to 123)	127 (125 to 128)	137 (123 to 153)	138 (122 to 155)	138 (121 to 157)
Nordland	113 (102 to 124)	122 (110 to 134)	123 (112 to 136)	124 (112 to 136)	121 (113 to 129)	119 (118 to 121)	119 (117 to 120)	124 (110 to 138)	124 (109 to 140)	124 (109 to 141)
Oslo	199 (181 to 218)	220 (200 to 241)	221 (200 to 243)	210 (190 to 230)	217 (202 to 232)	245 (242 to 248)	291 (287 to 294)	343 (305 to 380)	347 (306 to 390)	352 (307 to 398)
Rogaland	105 (95 to 115)	119 (109 to 131)	134 (121 to 147)	152 (138 to 166)	168 (156 to 180)	186 (184 to 188)	217 (214 to 219)	244 (219 to 272)	246 (217 to 277)	247 (217 to 280)
Troms og Finnmark	94 (85 to 103)	103 (94 to 113)	110 (99 to 121)	115 (105 to 126)	113 (105 to 120)	114 (112 to 115)	116 (115 to 118)	125 (111 to 139)	125 (111 to 141)	126 (110 to 143)
Trøndelag	153 (139 to 167)	165 (149 to 180)	177 (160 to 193)	184 (168 to 202)	188 (175 to 201)	194 (191 to 196)	212 (210 to 215)	234 (208 to 260)	236 (208 to 265)	238 (208 to 268)
Vestfold og Telemark	146 (132 to 160)	163 (147 to 178)	166 (150 to 182)	172 (156 to 189)	177 (166 to 190)	185 (183 to 188)	198 (195 to 200)	212 (188 to 237)	213 (188 to 240)	214 (187 to 243)
Vestland	201 (183 to 219)	219 (199 to 239)	236 (214 to 259)	247 (226 to 270)	258 (240 to 276)	270 (267 to 273)	294 (290 to 297)	325 (291 to 361)	327 (290 to 367)	329 (289 to 372)
Viken	264 (241 to 288)	304 (278 to 331)	371 (338 to 405)	406 (372 to 442)	435 (406 to 465)	469 (464 to 475)	530 (524 to 536)	604 (542 to 665)	611 (545 to 682)	617 (549 to 694)
Portugal	4217 (3919 to 4506)	4407 (4116 to 4717)	4233 (3935 to 4530)	4822 (4457 to 5169)	4897 (4519 to 5256)	5099 (4733 to 5467)	5173 (4807 to 5530)	5074 (4494 to 5673)	5058 (4471 to 5704)	5038 (4384 to 5723)
San Marino	8 (7 to 8)	8 (7 to 9)	9 (8 to 10)	10 (9 to 11)	12 (10 to 14)	13 (12 to 15)	15 (14 to 16)	16 (14 to 18)	16 (14 to 18)	16 (14 to 18)
Spain	13463 (12522 to 14392)	14815 (13843 to 15837)	16641 (15559 to 17823)	18368 (17022 to 19737)	19003 (17734 to 20447)	19996 (19869 to 20115)	23206 (23055 to 23356)	22514 (20590 to 24451)	22389 (20422 to 24386)	22260 (20019 to 24392)
Sweden	3505 (3260 to 3751)	3743 (3498 to 3997)	4015 (3989 to 4039)	4114 (4087 to 4143)	4239 (4209 to 4269)	4401 (4365 to 4437)	4691 (4650 to 4727)	5136 (4679 to 5591)	5179 (4690 to 5674)	5220 (4725 to 5735)
Stockholm	512 (459 to 564)	572 (514 to 630)	692 (686 to 699)	720 (712 to 727)	773 (764 to 781)	863 (852 to 873)	988 (975 to 1000)	1154 (1013 to 1298)	1170 (1011 to 1339)	1186 (1023 to 1356)
Sweden except Stockholm	2993 (2780 to 3210)	3171 (2950 to 3393)	3323 (3301 to 3343)	3394 (3371 to 3419)	3466 (3440 to 3491)	3538 (3507 to 3568)	3704 (3670 to 3734)	3983 (3612 to 4357)	4009 (3606 to 4426)	4034 (3628 to 4456)
Switzerland	2274 (2122 to 2435)	2664 (2477 to 2846)	3083 (2868 to 3286)	3128 (2923 to 3342)	3383 (3162 to 3606)	3572 (3338 to 3799)	3928 (3891 to 3963)	4371 (3995 to 4764)	4412 (3996 to 4810)	4450 (4017 to 4919)
UK	24142 (22837 to 25461)	25436 (23899 to 26912)	27146 (25573 to 28795)	27412 (25663 to 28987)	27838 (26084 to 29583)	28780 (26986 to 30534)	30904 (28949 to 32828)	33045 (30737 to 35260)	33192 (31032 to 35316)	33305 (31373 to 35148)
England	19635 (18391 to 20924)	20858 (19332 to 22322)	22433 (20907 to 24054)	22774 (21007 to 24329)	23217 (21498 to 24978)	24051 (22275 to 25815)	25896 (23914 to 27855)	27819 (25516 to 30054)	27958 (25835 to 30050)	28069 (26180 to 29875)
East Midlands	1571 (1427 to 1730)	1660 (1427 to 1909)	1798 (1622 to 1988)	1889 (1705 to 2085)	1962 (1774 to 2173)	2052 (1853 to 2269)	2220 (1998 to 2458)	2381 (2054 to 2730)	2392 (2044 to 2740)	2400 (2060 to 2750)
Derby	93 (83 to 105)	101 (83 to 122)	109 (95 to 124)	107 (94 to 122)	109 (95 to 124)	113 (99 to 129)	122 (107 to 139)	132 (109 to 158)	133 (108 to 159)	133 (109 to 161)

Derbyshire	306 (272 to 340)	315 (258 to 376)	333 (292 to 374)	343 (300 to 384)	350 (307 to 394)	361 (318 to 405)	378 (331 to 425)	394 (325 to 465)	394 (324 to 469)	395 (325 to 473)
Leicester	124 (111 to 138)	132 (109 to 159)	140 (123 to 159)	138 (122 to 156)	136 (121 to 154)	137 (121 to 155)	160 (141 to 181)	186 (155 to 222)	188 (156 to 227)	191 (158 to 230)
Leicestershire	198 (178 to 222)	214 (176 to 257)	240 (211 to 272)	266 (237 to 300)	282 (249 to 318)	300 (266 to 338)	320 (282 to 361)	336 (280 to 399)	338 (275 to 405)	338 (281 to 404)
Lincolnshire	226 (202 to 254)	230 (189 to 278)	248 (219 to 280)	271 (239 to 307)	286 (253 to 324)	315 (278 to 355)	346 (305 to 394)	369 (307 to 440)	370 (305 to 443)	371 (306 to 444)
Northamptonshire	200 (179 to 223)	210 (175 to 250)	232 (205 to 262)	258 (228 to 291)	284 (251 to 320)	309 (274 to 348)	339 (300 to 383)	368 (310 to 433)	370 (309 to 438)	372 (310 to 440)
Nottingham	129 (116 to 145)	140 (115 to 168)	148 (131 to 167)	137 (121 to 154)	137 (121 to 155)	134 (119 to 152)	150 (133 to 170)	169 (141 to 202)	170 (140 to 203)	172 (141 to 207)
Nottinghamshire	284 (253 to 317)	305 (251 to 364)	335 (295 to 376)	352 (309 to 395)	361 (318 to 406)	366 (323 to 411)	385 (339 to 434)	408 (338 to 480)	409 (334 to 486)	410 (335 to 487)
Rutland	11 (10 to 12)	12 (10 to 15)	14 (13 to 16)	17 (15 to 19)	17 (15 to 19)	18 (15 to 20)	19 (17 to 22)	20 (16 to 24)	20 (16 to 24)	20 (16 to 24)
East of England	1842 (1674 to 2022)	1959 (1684 to 2248)	2166 (1956 to 2395)	2368 (2127 to 2615)	2500 (2251 to 2763)	2632 (2375 to 2905)	2858 (2568 to 3162)	3088 (2669 to 3521)	3106 (2679 to 3548)	3121 (2684 to 3559)
Bedford	50 (44 to 56)	55 (44 to 66)	62 (54 to 70)	66 (57 to 75)	66 (58 to 75)	72 (64 to 82)	77 (67 to 87)	81 (66 to 96)	81 (66 to 97)	82 (65 to 98)
Cambridgeshire	168 (149 to 187)	177 (145 to 213)	199 (176 to 224)	224 (197 to 254)	251 (221 to 283)	272 (240 to 307)	306 (269 to 345)	338 (280 to 402)	340 (280 to 407)	343 (282 to 410)
Central Bedfordshire	70 (62 to 78)	76 (62 to 92)	89 (78 to 102)	105 (92 to 120)	110 (96 to 125)	115 (101 to 132)	126 (109 to 143)	137 (112 to 164)	138 (111 to 167)	139 (112 to 168)
Essex	447 (399 to 496)	468 (385 to 556)	522 (462 to 583)	582 (514 to 652)	609 (538 to 681)	638 (565 to 713)	678 (600 to 759)	716 (596 to 840)	719 (598 to 850)	721 (595 to 851)
Hertfordshire	366 (327 to 408)	407 (336 to 488)	455 (402 to 511)	474 (417 to 535)	480 (423 to 540)	503 (444 to 564)	543 (478 to 609)	589 (492 to 695)	592 (493 to 702)	596 (495 to 709)
Luton	63 (56 to 71)	71 (59 to 88)	81 (71 to 92)	82 (73 to 94)	86 (76 to 98)	93 (82 to 106)	101 (89 to 116)	110 (92 to 134)	111 (92 to 135)	112 (92 to 137)
Norfolk	277 (247 to 308)	282 (230 to 335)	304 (268 to 341)	339 (299 to 380)	366 (324 to 411)	388 (342 to 433)	419 (368 to 472)	445 (370 to 524)	447 (369 to 527)	448 (366 to 532)
Peterborough	45 (40 to 50)	47 (38 to 57)	53 (46 to 60)	65 (57 to 74)	75 (66 to 85)	77 (68 to 87)	89 (78 to 101)	106 (87 to 126)	107 (88 to 128)	108 (89 to 130)
Southend-on-Sea	76 (67 to 86)	77 (62 to 93)	76 (67 to 87)	74 (65 to 84)	76 (67 to 87)	77 (68 to 87)	84 (74 to 96)	94 (78 to 112)	95 (76 to 114)	95 (77 to 114)
Suffolk	232 (206 to 260)	243 (197 to 290)	264 (232 to 297)	294 (257 to 331)	317 (278 to 357)	328 (287 to 369)	358 (313 to 403)	387 (318 to 456)	389 (319 to 460)	390 (319 to 463)
Thurrock	49 (44 to 55)	56 (45 to 68)	62 (54 to 70)	63 (55 to 72)	63 (55 to 71)	69 (61 to 78)	77 (68 to 88)	86 (71 to 103)	87 (71 to 105)	88 (72 to 106)
Greater London	3166 (2894 to 3456)	3465 (3014 to 3936)	3614 (3278 to 3961)	3295 (2975 to 3620)	3266 (2953 to 3597)	3550 (3218 to 3890)	3988 (3585 to 4384)	4437 (3871 to 5047)	4475 (3897 to 5087)	4508 (3911 to 5107)
Barking and Dagenham	77 (69 to 86)	83 (68 to 99)	84 (74 to 95)	78 (69 to 89)	75 (66 to 85)	79 (70 to 89)	89 (79 to 101)	105 (87 to 124)	106 (88 to 126)	107 (88 to 128)
Barnet	126 (112 to 140)	138 (114 to 165)	145 (128 to 163)	140 (123 to 158)	141 (124 to 159)	152 (135 to 171)	171 (150 to 193)	190 (158 to 226)	192 (159 to 229)	193 (160 to 231)
Bexley	88 (79 to 99)	98 (81 to 118)	106 (94 to 121)	106 (93 to 120)	106 (93 to 120)	105 (93 to 119)	111 (98 to 127)	120 (100 to 143)	121 (101 to 145)	122 (100 to 147)
Brent	114 (102 to 127)	127 (104 to 150)	135 (119 to 151)	122 (107 to 137)	117 (102 to 131)	132 (116 to 147)	155 (135 to 174)	175 (145 to 206)	177 (146 to 209)	179 (146 to 213)
Bromley	121 (108 to 135)	135 (111 to 162)	147 (129 to 166)	144 (127 to 162)	140 (123 to 159)	141 (125 to 160)	148 (130 to 168)	158 (132 to 188)	159 (131 to 190)	159 (130 to 191)
Camden	94 (84 to 104)	96 (79 to 114)	97 (86 to 109)	85 (75 to 96)	85 (75 to 96)	97 (86 to 109)	106 (94 to 120)	113 (94 to 134)	113 (94 to 135)	114 (93 to 136)
Croydon	132 (118 to 147)	148 (123 to 174)	161 (142 to 180)	155 (137 to 173)	151 (134 to 170)	162 (143 to 181)	175 (153 to 197)	191 (159 to 223)	193 (159 to 225)	194 (159 to 227)
Ealing	131 (118 to 147)	142 (119 to 169)	149 (133 to 168)	140 (123 to 157)	137 (121 to 155)	152 (134 to 171)	168 (148 to 189)	182 (153 to 215)	183 (153 to 217)	184 (152 to 219)
Enfield	112 (100 to 125)	121 (100 to 145)	128 (114 to 144)	126 (111 to 142)	124 (110 to 141)	133 (118 to 150)	149 (132 to 169)	168 (140 to 200)	170 (140 to 203)	171 (141 to 205)
Greenwich	92 (82 to 103)	99 (81 to 118)	104 (92 to 117)	103 (91 to 116)	101 (89 to 113)	104 (93 to 117)	124 (109 to 139)	147 (122 to 174)	149 (124 to 176)	152 (125 to 180)
Hackney	96 (86 to 108)	107 (88 to 128)	110 (96 to 124)	94 (82 to 106)	91 (80 to 102)	103 (91 to 116)	124 (108 to 140)	142 (118 to 169)	144 (119 to 171)	145 (119 to 173)
Hammersmith and Fulham	87 (77 to 97)	93 (75 to 111)	92 (81 to 104)	72 (63 to 82)	72 (63 to 81)	82 (72 to 92)	88 (76 to 99)	92 (76 to 109)	92 (75 to 109)	92 (75 to 110)
Haringey	102 (91 to 113)	111 (93 to 132)	117 (103 to 130)	101 (89 to 113)	99 (87 to 111)	107 (94 to 119)	124 (109 to 140)	140 (117 to 165)	142 (118 to 167)	143 (117 to 169)
Harrow	83 (74 to 93)	93 (76 to 111)	99 (88 to 112)	96 (85 to 109)	97 (86 to 110)	102 (89 to 115)	117 (103 to 132)	135 (112 to 160)	137 (113 to 161)	138 (112 to 164)
Havering	95 (85 to 106)	110 (91 to 133)	121 (108 to 137)	119 (105 to 134)	113 (100 to 128)	108 (96 to 122)	114 (100 to 129)	124 (104 to 149)	125 (104 to 150)	126 (104 to 152)
Hillingdon	95 (85 to 106)	107 (88 to 128)	117 (103 to 131)	115 (101 to 129)	114 (100 to 128)	119 (104 to 133)	134 (118 to 151)	154 (128 to 181)	155 (129 to 183)	157 (130 to 186)
Hounslow	86 (77 to 96)	94 (78 to 111)	100 (89 to 112)	98 (86 to 109)	99 (88 to 111)	106 (94 to 119)	125 (110 to 140)	146 (122 to 171)	147 (123 to 173)	149 (124 to 176)
Islington	87 (78 to 97)	97 (80 to 116)	99 (88 to 111)	81 (72 to 91)	82 (72 to 92)	87 (77 to 98)	99 (87 to 112)	113 (95 to 134)	115 (95 to 136)	115 (96 to 138)
Kensington and Chelsea	82 (73 to 91)	88 (72 to 104)	89 (79 to 100)	66 (58 to 75)	66 (58 to 75)	78 (69 to 87)	78 (69 to 88)	75 (63 to 88)	75 (62 to 89)	75 (61 to 89)
Kingston upon Thames	59 (53 to 66)	64 (52 to 76)	67 (59 to 75)	64 (56 to 72)	65 (57 to 73)	73 (64 to 81)	78 (68 to 87)	82 (68 to 96)	82 (68 to 96)	82 (68 to 98)
Lambeth	134 (118 to 149)	146 (118 to 175)	149 (131 to 168)	122 (107 to 138)	122 (107 to 138)	136 (120 to 153)	150 (131 to 169)	159 (131 to 187)	159 (130 to 188)	159 (130 to 190)
Lewisham	115 (102 to 128)	126 (103 to 150)	131 (116 to 147)	114 (100 to 129)	114 (100 to 129)	124 (109 to 139)	134 (118 to 151)	145 (121 to 171)	146 (120 to 174)	147 (121 to 175)
Merton	76 (68 to 85)	83 (68 to 99)	86 (76 to 97)	80 (70 to 90)	81 (72 to 92)	93 (82 to 105)	98 (86 to 111)	102 (84 to 120)	102 (84 to 121)	102 (84 to 121)
Newham	99 (89 to 111)	112 (92 to 134)	117 (103 to 132)	105 (92 to 118)	105 (92 to 119)	123 (108 to 138)	157 (138 to 178)	192 (160 to 228)	195 (161 to 234)	197 (164 to 237)
Redbridge	98 (88 to 110)	107 (89 to 128)	113 (100 to 127)	107 (95 to 121)	107 (94 to 121)	117 (104 to 133)	137 (121 to 155)	161 (134 to 191)	163 (135 to 192)	165 (136 to 197)
Richmond upon Thames	78 (70 to 88)	83 (69 to 99)	86 (76 to 97)	78 (69 to 88)	79 (69 to 89)	84 (74 to 95)	90 (80 to 102)	96 (80 to 113)	96 (79 to 113)	96 (79 to 114)
Southwark	110 (98 to 123)	124 (103 to 147)	127 (112 to 141)	106 (93 to 119)	108 (95 to 122)	125 (111 to 141)	141 (124 to 158)	152 (126 to 180)	153 (127 to 181)	154 (127 to 182)
Sutton	69 (62 to 77)	75 (62 to 89)	80 (71 to 90)	81 (71 to 91)	81 (71 to 91)	88 (77 to 98)	92 (81 to 104)	97 (80 to 115)	98 (81 to 116)	98 (81 to 116)
Tower Hamlets	78 (70 to 87)	84 (69 to 100)	83 (74 to 93)	72 (64 to 81)	81 (71 to 91)	100 (89 to 112)	128 (113 to 144)	153 (128 to 180)	155 (129 to 183)	157 (130 to 186)
Waltham Forest	103 (92 to 115)	110 (91 to 130)	114 (100 to 127)	105 (93 to 118)	104 (91 to 117)	109 (97 to 122)	127 (112 to 143)	149 (124 to 175)	151 (125 to 178)	153 (126 to 181)
Wandsworth	132 (118 to 147)	144 (119 to 172)	147 (131 to 164)	126 (112 to 141)	123 (110 to 139)	130 (115 to 146)	146 (129 to 164)	161 (135 to 191)	163 (136 to 192)	164 (136 to 195)
Westminster	113 (101 to 126)	119 (98 to 141)	115 (102 to 129)	91 (80 to 103)	88 (77 to 99)	100 (88 to 113)	111 (97 to 125)	117 (97 to 139)	118 (98 to 140)	118 (97 to 140)
North East England	1162 (1051 to 1283)	1231 (1047 to 1414)	1311 (1180 to 1454)	1289 (1151 to 1430)	1258 (1123 to 1402)	1235 (1105 to 1369)	1266 (1125 to 1412)	1306 (1117 to 1500)	1307 (1112 to 1503)	1307 (1108 to 1496)
County Durham	232 (207 to 260)	241 (196 to 288)	253 (223 to 287)	252 (221 to 284)	245 (215 to 278)	241 (212 to 272)	250 (219 to 283)	260 (214 to 310)	260 (214 to 310)	260 (213 to 310)
Darlington	44 (38 to 49)	45 (37 to 54)	47 (41 to 53)	48 (42 to 54)	48 (42 to 54)	47 (41 to 53)	51 (44 to 58)	55 (45 to 66)	56 (45 to 67)	56 (46 to 67)
Gateshead	100 (89 to 112)	105 (85 to 127)	110 (97 to 125)	105 (91 to 118)	98 (86 to 111)	93 (82 to 105)	97 (85 to 110)	104 (85 to 124)	104 (85 to 125)	104 (85 to 125)
Hartlepool	42 (38 to 48)	45 (37 to 55)	49 (43 to 56)	47 (41 to 53)	44 (39 to 51)	44 (39 to 50)	45 (39 to 51)	46 (38 to 55)	46 (38 to 55)	46 (38 to 55)
Middlesbrough	64 (56 to 72)	72 (58 to 87)	78 (68 to 89)	75 (65 to 85)	71 (61 to 81)	69 (60 to 78)	68 (59 to 77)	68 (55 to 82)	68 (55 to 83)	68 (55 to 82)
Newcastle upon Tyne	141 (124 to 158)	147 (119 to 177)	151 (132 to 171)	138 (120 to 156)	133 (115 to 151)	129 (113 to 146)	138 (120 to 157)	148 (121 to 177)	149 (122 to 178)	149 (122 to 180)
North Tyneside	87 (77 to 98)	94 (77 to 114)	100 (88 to 113)	96 (84 to 108)	93 (81 to 106)	92 (81 to 105)	96 (84 to 110)	103 (84 to 123)	103 (84 to 123)	103 (84 to 124)
Northumberland	128 (113 to 143)	131 (106 to 158)	138 (121 to 155)	145 (127 to 164)	149 (130 to 169)	150 (131 to 170)	154 (134 to 175)	157 (128 to 188)	157 (128 to 189)	156 (126 to 188)
Redcar and Cleveland	59 (52 to 67)	65 (52 to 78)	73 (63 to 83)	74 (64 to 85)	71 (62 to 82)	68 (59 to 77)	66 (57 to 75)	65 (53 to 79)	65 (53 to 79)	65 (52 to 78)
South Tyneside	77 (69 to 87)	83 (66 to 100)	87 (76 to 99)	80 (69 to 91)	76 (66 to 87)	75 (65 to 85)	72 (63 to 82)	71 (57 to 85)	71 (57 to 86)	71 (57 to 85)

Stockton-on-Tees	64 (57 to 72)	71 (59 to 86)	81 (71 to 91)	85 (75 to 96)	85 (75 to 97)	89 (78 to 101)	94 (82 to 106)	98 (81 to 116)	98 (80 to 117)	98 (80 to 117)
Sunderland	123 (110 to 138)	133 (109 to 160)	144 (127 to 162)	145 (127 to 164)	144 (126 to 163)	139 (122 to 157)	135 (117 to 152)	132 (110 to 157)	131 (108 to 157)	131 (108 to 157)
North West England	3035 (2768 to 3327)	3222 (2784 to 3680)	3439 (3111 to 3779)	3372 (3028 to 3710)	3316 (2986 to 3661)	3289 (2969 to 3620)	3445 (3093 to 3796)	3645 (3172 to 4164)	3657 (3180 to 4182)	3665 (3167 to 4197)
Blackburn with Darwen	63 (56 to 71)	66 (54 to 79)	68 (60 to 77)	69 (61 to 78)	67 (59 to 76)	68 (60 to 77)	73 (64 to 83)	78 (65 to 93)	79 (65 to 94)	79 (65 to 96)
Blackpool	80 (72 to 90)	74 (61 to 89)	71 (62 to 80)	69 (61 to 78)	70 (62 to 80)	69 (61 to 78)	70 (61 to 79)	71 (59 to 85)	71 (59 to 86)	71 (58 to 86)
Bolton	116 (104 to 130)	121 (99 to 143)	127 (113 to 143)	128 (112 to 144)	128 (112 to 143)	127 (113 to 143)	136 (120 to 153)	147 (123 to 172)	147 (122 to 174)	148 (122 to 177)
Bury	73 (65 to 82)	78 (63 to 94)	84 (74 to 95)	86 (75 to 97)	86 (76 to 98)	88 (78 to 99)	90 (79 to 103)	94 (78 to 112)	94 (78 to 113)	94 (77 to 114)
Cheshire East	129 (115 to 145)	137 (112 to 164)	150 (132 to 169)	159 (139 to 179)	165 (145 to 186)	171 (150 to 193)	181 (158 to 204)	189 (158 to 224)	189 (158 to 224)	190 (156 to 226)
Cheshire West and Chester	119 (106 to 134)	130 (106 to 157)	145 (128 to 164)	152 (134 to 171)	152 (134 to 172)	156 (138 to 177)	160 (141 to 182)	163 (135 to 195)	163 (135 to 195)	163 (133 to 197)
Cumbria	211 (189 to 236)	221 (181 to 264)	233 (207 to 262)	234 (207 to 264)	236 (209 to 266)	238 (211 to 269)	246 (216 to 277)	251 (209 to 296)	251 (209 to 297)	250 (206 to 298)
Halton	39 (35 to 44)	41 (34 to 50)	48 (42 to 54)	60 (52 to 67)	61 (54 to 69)	57 (51 to 64)	61 (53 to 69)	65 (54 to 77)	66 (54 to 78)	66 (53 to 79)
[truncated: 96,208 more chars]
